# From serum metabolites to the gut: revealing metabolic clues to susceptibility to subtypes of Crohn’s disease and ulcerative colitis

**DOI:** 10.3389/fendo.2024.1375896

**Published:** 2024-08-08

**Authors:** Fan Li, Zhaodi Wang, Tongyu Tang, Qi Zhao, Zhi Wang, Xiaoping Han, Zifeng Xu, Yu Chang, Hongyan Li, Sileng Hu, Chanjiao Yu, Shiyu Chang, Yue Liu, Yuqin Li

**Affiliations:** ^1^ Department of Gastroenterology, The First Hospital of Jilin University, Changchun, China; ^2^ Norman Bethune Health Science Center, Jilin University, Changchun, China

**Keywords:** Crohn’s disease, inflammatory bowel disease, ulcerative colitis, metabolite, metabolic pathway, Mendelian randomization

## Abstract

**Background and aims:**

Inflammatory bowel disease (IBD) is a common chronic inflammatory bowel disease characterized by diarrhea and abdominal pain. Recently human metabolites have been found to help explain the underlying biological mechanisms of diseases of the intestinal system, so we aimed to assess the causal relationship between human blood metabolites and susceptibility to IBD subtypes.

**Methods:**

We selected a genome-wide association study (GWAS) of 275 metabolites as the exposure factor, and the GWAS dataset of 10 IBD subtypes as the outcome, followed by univariate and multivariate analyses using a two-sample Mendelian randomization study (MR) to study the causal relationship between exposure and outcome, respectively. A series of sensitivity analyses were also performed to ensure the robustness of the results.

**Results:**

A total of 107 metabolites were found to be causally associated on univariate analysis after correcting for false discovery rate (FDR), and a total of 9 metabolites were found to be significantly causally associated on subsequent multivariate and sensitivity analyses. In addition we found causal associations between 7 metabolite pathways and 6 IBD subtypes.

**Conclusion:**

Our study confirms that blood metabolites and certain metabolic pathways are causally associated with the development of IBD subtypes and their parenteral manifestations. The exploration of the mechanisms of novel blood metabolites on IBD may provide new therapeutic ideas for IBD patients.

## 1 Introduction

Ulcerative colitis (UC) and Crohn’s disease (CD), collectively referred to as inflammatory bowel disease, are a group of chronic, recurrent autoimmune diseases. The interaction of genetic and environmental factors that influence the immune response leads to inflammatory bowel disease (1).The most common symptoms of CD or UC include diarrhea, abdominal pain, bloody stools, and weight loss. UC involvement is primarily in the colorectum, and CD can involve the entire GI tract, but primarily in the ileum. The differences between CD and UC subtypes at different sites have been debated. Dulai et al. on the basis of the differences in CD subtypes (2), suggested that a distinction should be made between ileal dominant CD and isolated colonic CD at the time of diagnosis (3). At the World Congress of Gastroenterology in Montreal in 2006, significant differences were found in patients with UC involving the rectum, left colon, and total colon based on the natural history of the disease, response to medications, risk of neoplasia, and serologic and genetic markers in patients with UC (4–6), based on which the Montreal UC Classification was popularized to differentiate between the Static severity (4). Like the subtypes at different sites, primary sclerosing cholangitis associated UC (UC-PSC) (7), CD and UC associated spondyloarthritis (CD-SpA, UC-SpA) (8) also have distinct clinical, cellular and microbiological features. These extraintestinal manifestations of UC and CD also warrant further investigation into their pathogenesis. In recent years, metabolomics has been extensively studied in patients with IBD. Single biomarker approaches cannot be considered ideal for clinical application in IBD with complex mechanisms. Metabolomics, by measuring hundreds of metabolites in biological samples, allows for the characterization of potential mechanisms specific to different disease subtypes (9).A study by Schicho et al. found that energy metabolites such as methanol, mannose, and formic acid (10). were the metabolites with the most significant increase in serum and plasma of patients with IBD, and other studies support the observation of altered energy metabolism (11, 12),, including metabolites involved in amino acid cycling and TCA cycling (13). However, relatively few metabolomics studies have been conducted for the different subtypes of UC and CD.

Mendelian randomization mimics the random grouping of individuals at birth by identifying different single-nucleotide polymorphisms and identifying the causal relationship between exposure and outcome at the gene level. Since genetic differences accompany individuals throughout their lives from birth, Mendelian randomization studies effectively eliminate the effects of general confounding factors such as age, social status, and economic level, and allow for a clear direction of causality.

In this study, we explored the metabolite phenotypes responsible for the pathogenesis of 10 IBD subtypes and extraintestinal manifestations through a two-sample MR study using the 275 blood metabolite GWAS dataset. The findings of this research will guide further investigations into the diagnostic and prognostic implications of blood metabolites for IBD subtypes.

## 2 Methods

### 2.1 Data sources

We used summary data for multiple cohorts of the study. Metabolite data were derived from a genome-wide association study of 275 blood metabolites in 7,822 adults from 2 European population studies determined in 2014 by shin et al. Hundreds of associations and their metabolic contexts reported in this study define a system-wide molecular readout atlas of human gene activity measured *in vivo* (14). We selected these metabolites into nine subclasses of lipids, fatty acids, and carbohydrates, as defined in the Kyoto Encyclopedia of Genes and Genomes (KEGG) database. The outcome data comes from FinnGen, a database that collects and analyzes genomic and health data from 500,000 Finnish Biobank participants and provides novel medical and treatment-related insights (15).It provided a GWAS dataset of UC and CD stratified diagnoses and comorbidities for us to choose from, which contained a sample size of 373,819 individuals, with a total of 15,779 patients with UC and CD. The GWAS dataset associated with gut microbiota metabolomic pathways was published by Lopera-Maya et al. in 2022. The study was based on a multidisciplinary prospective cohort study of a population residing in northern Netherlands, evaluating the impact of various exposures and lifestyles on gut microbiota composition among 167,729 individuals. This study included data from 7738 participants, encompassing 205 gut microbiota-associated metabolic pathways.

Databases of exposures and outcomes were derived from European populations and included both males and females to avoid population stratification bias (16). Details of the dataset information and stratification are shown in **Table 1**, and comprehensive dataset information is provided in **Supplementary Table 1**.

**Table 1:** Inclusion information and stratification details of the dataset.

Datasets	ICD-10 encoding	Montreal classification	Case	Sample Size	Year	Authors	Gender	Population	NSNP
Crohn’s disease of small intestine	K50.0	L1+part of L4	2004	361931	2023	FinnGen	Males and Females	European	20167370
Crohn’s disease of colon	K50.1	L2	1581	361508	2023	FinnGen	Males and Females	European	20167370
Crohn’s disease of ileocolon	K50.2	L3	2098	362025	2023	FinnGen	Males and Females	European	20167370
Arthropathy in Crohn disease	M07.4*K50.9		273	373819	2023	FinnGen	Males and Females	European	20167370
Ulcerative proctitis	K51.2	E1	1773	361700	2023	FinnGen	Males and Females	European	20167370
Ulcerative rectosigmoiditis	K51.3		2487	362414	2023	FinnGen	Males and Females	European	20167370
Left-sided ulcerative colitis	K51.5	E2	4085	364012	2023	FinnGen	Males and Females	European	20167370
Ulcerative pancolitis	K51.0	E3	933	360860	2023	FinnGen	Males and Females	European	20167370
Ulcerative colitis with PSC	K83.0*K51		209	364784	2023	FinnGen	Males and Females	European	20167370
Arthropathy in ulcerative colitis	M07.5*K51.9		336	373882	2023	FinnGen	Males and Females	European	20167370
Serum level of 275 metabolites	–	–	7822	7822	2014	Shin et al.	Males and Females	European	2546774
Gut microbiota pathway of 205	–	–	7738	7738	2022	Lopera-Maya et al.	Males and Females	European	5566712

ICD, International Classification of Diseases; PSC, Primary Sclerosing Cholangitis; SNP, Single Nucleotide Polymorphism.

### 2.2 Research approach

In this study, we used two-sample MR (TSMR) to investigate the causal relationship between blood metabolites and IBD subtypes and their parenteral manifestations. We stratified the IBD subtypes by combining the Montreal typing and The International Classification of Diseases (ICD)-10 classification methods, details of which are shown in **Table 1**.We investigated single nucleotide polymorphisms (SNPs) as instrumental variables (IVs) and performed Univariate Mendelian randomization (UVMR) analyses using inverse variance weighting (IVW), MR-Egger regression, weighted median method, weighted mode method, and MR-RAPS method after screening qualified IVs, and conducted sensitivity analyses such as the MR-Egger intercept test, Cochran’s Q test, and the leave-one-out test on the results to ensure that the results were robust.

### 2.3 Selection of instrumental variables

In this study, adhering to the three basic assumptions of association, independence, and exclusivity, the following steps were performed to screen the IVs: first, SNPs significantly associated with exposure were extracted at the genome-wide significance level (threshold alpha = 5 × 10^ -8), and in order to obtain enough SNPs, we lowered the threshold to alpha = 1 × 10^-5 for batches that could not be extracted. Then, the criterion of r2 < 0.01 and kb = 10000 was set to remove SNPs with chained disequilibrium. The F-statistic is an indicator of the degree of association based on regression analysis, and in the instrumental variable analysis method, instrumental variables with an F-statistic of < 10 are considered to be invalid weak IVs. We calculated the association F-value of each SNP with exposure and removed weak instrumental variables.

**Figure 1:**
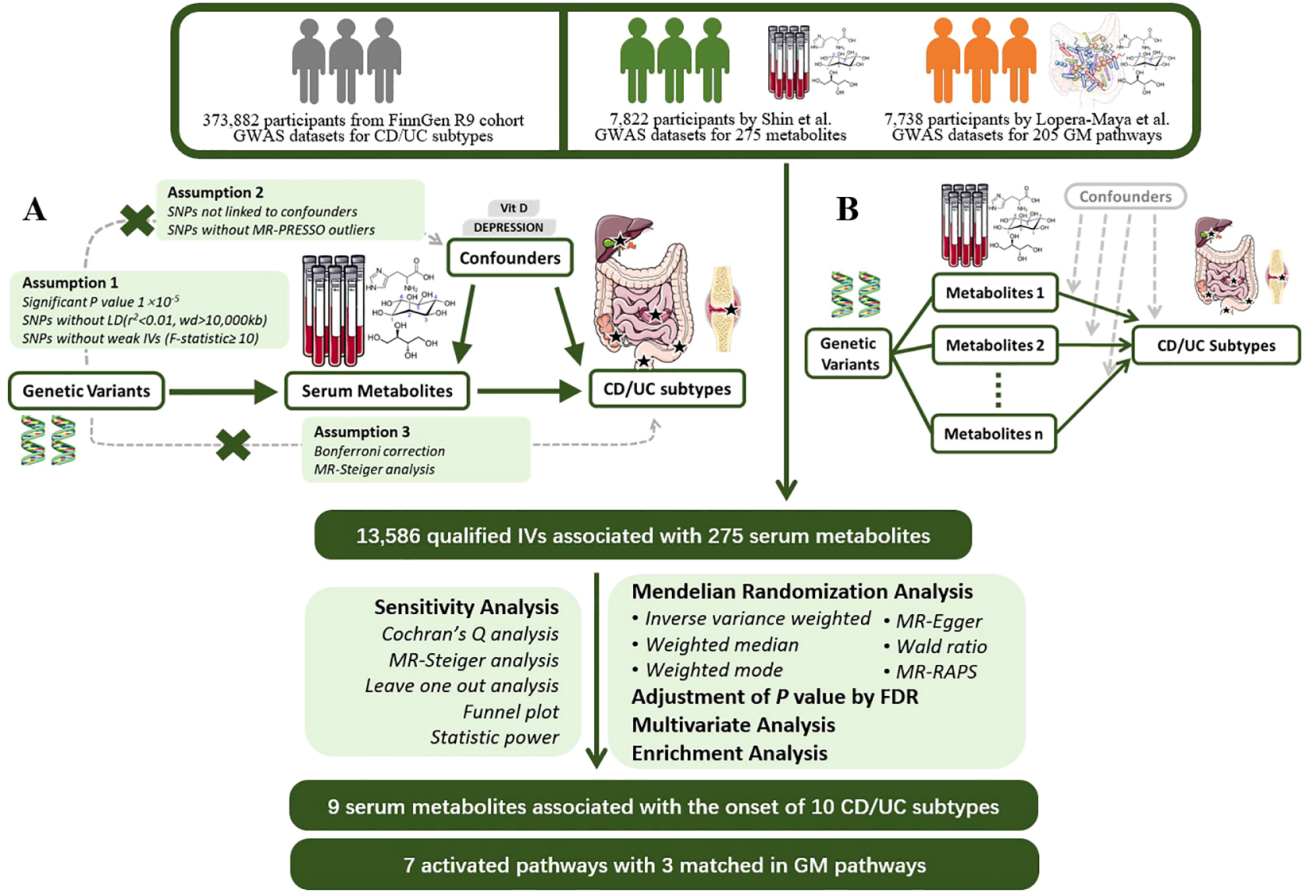
MR Study Design for the Association between Plasma Metabolites and IBD Subtypes This figure illustrates the workflow of Mendelian randomization analysis in this study. **(A)** Mendelian randomization three hypotheses and experimental principles; **(B)** Multivariable Mendelian randomization experimental principles. GWAS, Genome-Wide Association Study; CD, Crohn’s Disease; UC, Ulcerative Colitis; MR, Mendelian Randomization; PRESSO, Pleiotropy RESidual Sum and Outlier; LD, Linkage Disequilibrium; IV, Instrumental Variable; RAPS, Robust Adjusted Profile Score; FDR, False Discovery Rate.

We next used PhenoScanner searches for each SNP to exclude SNPs associated with confounding factors such as serum vitamin D levels, depression, and other confounding factors to avoid the influence of confounding factors on the results (17).Next, we applied MR-PRESSO to detect hetero-SNPs and correct their horizontal pleiotropy. We also analyzed the direction of causal estimation by MR- Steiger to remove all SNPs incorrectly (18).Finally we removed SNPs directly associated with outcome according to Bonferroni correction (P<0.05/n, n refers to the number of remaining SNPs).The flowchart and directed acyclic diagram consisting of MR research hypotheses are shown in **Figure 1**.

### 2.4 Univariate MR analysis

In this study on the relationship between blood metabolites and IBD subtypes, including their extraintestinal manifestations, we employed five methods: Inverse Variance Weighted (IVW) method, MR-Egger regression, Weighted Median, Weighted Mode, and MR-Robust Adjusted Profile Score (PAPS).

The flowchart and directed acyclic graph composed of the MR study hypotheses are shown in **Figure 1**. IVW is characterized by a regression that does not take into account the presence of an intercept term and is fitted with the inverse of the ending variance (the quadratic of se) as weights, and its estimation can be obtained by calculating the slope of a weighted linear regression (19) (20). When the instrumental variables satisfy the three hypotheses, the IVW approach provides a robust estimate of the causal relationship between exposure and outcome and will be preferentially used for assessment (21).The MR-Egger method is similar to the IVW method except that the regression model includes an intercept term. We preferred this method to be used for assessment when there is multiple validity in the data. The weighted median and weighted mode methods are based on the majority validity assumption and the plurality validity assumption, respectively, to calculate the causal effect (21, 22).

Effective IV exceeds 50%, the median of the weighted median ratio estimates will converge to the true causal effect. At less than 50%, when no larger group of invalid instrumental variables with the same ratio estimand exists, weighted mode can be used to determine the true causal effect. In the presence of heterogeneity among SNPs, the weighted median and IVW methods were required to jointly support the conclusion of significance.

The MR-RAPS method is a common modeling approach that is based on estimating causal effects based on the multiplicity of effects obeying a normal distribution centered on zero with positional variance and using a probability profile likelihood function.

In the analysis we corrected the p-values of the MR results with false discovery rate (FDR) method and inferred the causality using the corrected p-value < 0.05 as the criterion.

### 2.5 Sensitivity analysis

Therefore, in the current study we performed the Q-test for IVW and MR-Egger to evaluate the heterogeneity between IVs by calculating the weighted sum of the squared distances between the variant-specific estimates and the overall estimates, and concluded that heterogeneity existed in SNPs with a Q-test P-value < 0.05. We utilize the MR-Steiger model to estimate horizontal multinomials based on their intercepts to ensure the robustness of the results (23). The Instrument Strength Independent of Direct Effect (InSIDE) assumption and the NO Measurement Error (NOME) hypothesis that need to be satisfied for MR-Egger regression. We constructed a funnel plot and calculated the I2 statistic to ensure the validity of these assumptions. When I2 < 90% and the primary analytical method is MR-Egger, a correction for causal estimates is required (24) (25).Finally, we calculated the statistical power and conducted leave-one-out sensitivity analyses using individual SNPs (26).

### 2.6 Multivariate MR analysis

After univariate MR, we performed multivariate analyses of significant metabolites using the same parameters to find independently significant plasma markers by IVW, MR-Egger, weighted median, and LASSO regression methods. Heterogeneity and pleiotropy were also analyzed by sensitivity analysis.

### 2.7 Metabolic pathway analysis

Existing metabolite sets were utilized, culminating in metabolic pathway analysis based on KEGG databases using Metabo Analyst 5.0 (https://www.metaboanalyst.ca/), a user-friendly online tool for streamlining metabolomics data analysis.

### 2.8 Visualization and statistical software

In this study we used scatter plots, regression plots, forest plots, and leave-one-out forest plots to present the study findings, as detailed in **Supplementary Figures 1**-**4**. The expression of the overall results is demonstrated by means of circular heat maps and forest plots. Several figures were partly generated using Servier Medical Art (smart.servier.com), provided by Servier, licensed under a Creative Commons Attribution 3.0 unported license. Statistical analyses and visualizations were performed in this study using R (version 4.1.2; R Foundation for Statistical Computing, Vienna, Austria), with the application of the “TwoSampleMR”, “MR-PRESSO”, “mr. raps”, “forestploter” packages and some basic R packages. Calculation codes are provided in **Supplementary File 1**.

## 3 Result

### 3.1 Selection of instrumental variables

**Figure 2:**
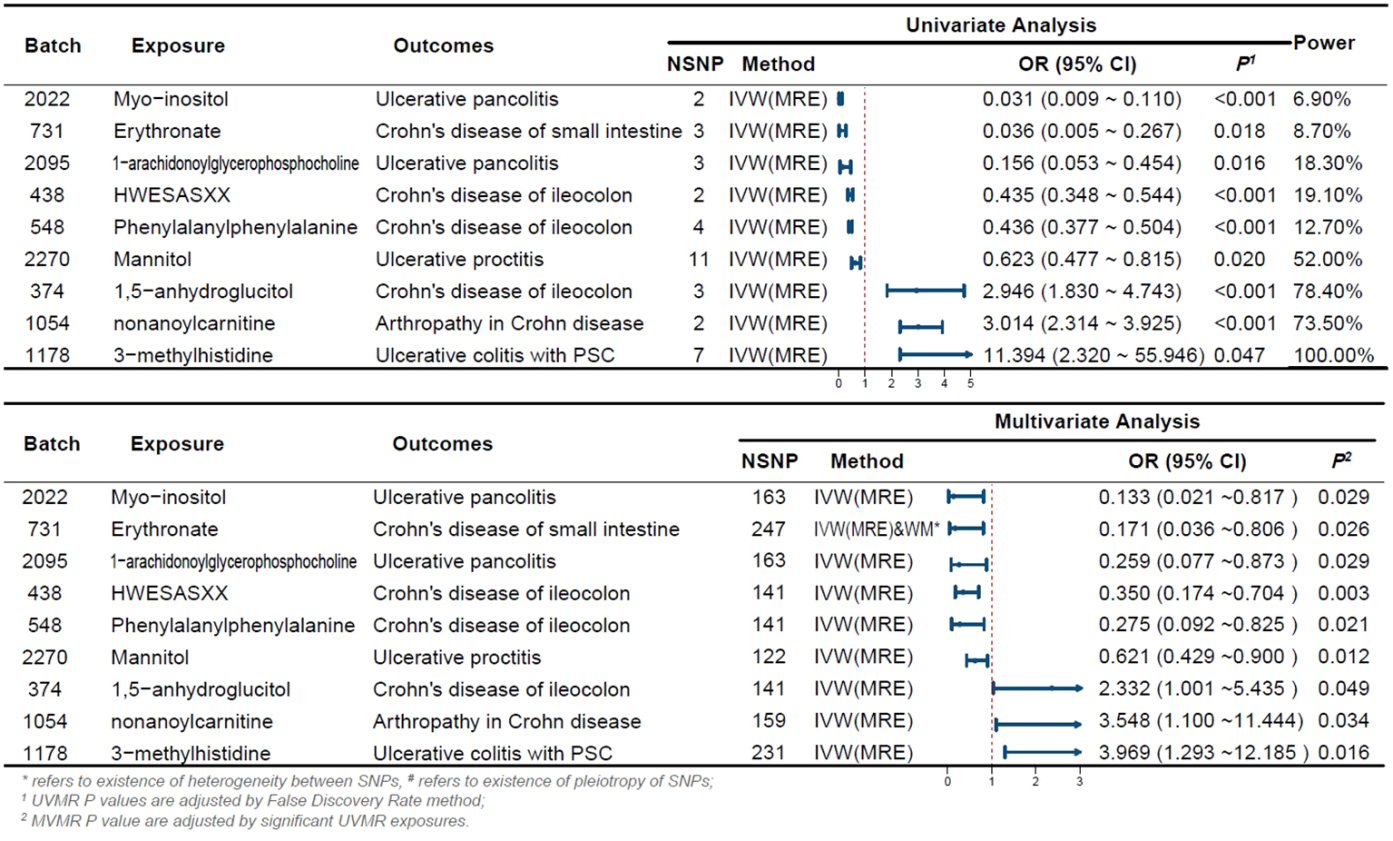
Forest Plot of Significant Univariate and Multivariate MR Analyses The forest plot illustrates the results of univariable and multivariable MR analysis. *indicates the existence of heterogeneity between SNPs, ^#^ indicates the existence of pleiotropy of SNPs; ^1^ UVMR P-values are adjusted by the False Discovery Rate method; ^2^ MVMR P-values are adjusted by significant UVMR exposures. OR, odds ratio; CI, confidence interval; PSC, primary sclerosing cholangitis; UVMR, univariable MR; MVMR, multivariable MR; NSNP, number of SNPs.

Initially, we screened a total of 16,522 SNPs associated with 275 plasma metabolites and did not find any weak variable instruments, 289 SNPs were missing from the endpoint database and were deleted, 2,027 SNPs were ambiguous SNPs, palindromic SNPs were deleted when merging the datasets, and 467 SNPs were associated with confounders such as serum vitamin D levels and depression after PhenoScanner searching, and these were deleted if they did not meet the independence assumption. The MR-PRESSO test identified 42 SNPs with horizontal pleiotropy; 111 SNPs directly associated with outcome were removed after bonferroni correction. 13,586 eligible SNPs were finally included in the study, with the number of SNPs included in significant results depicted in **Figure 2**.

### 3.2 Causal effects of blood metabolites on 9 subtypes of IBD

We summarize the details of the MR study in the **Supplementary Information**.

**Figure 3:**
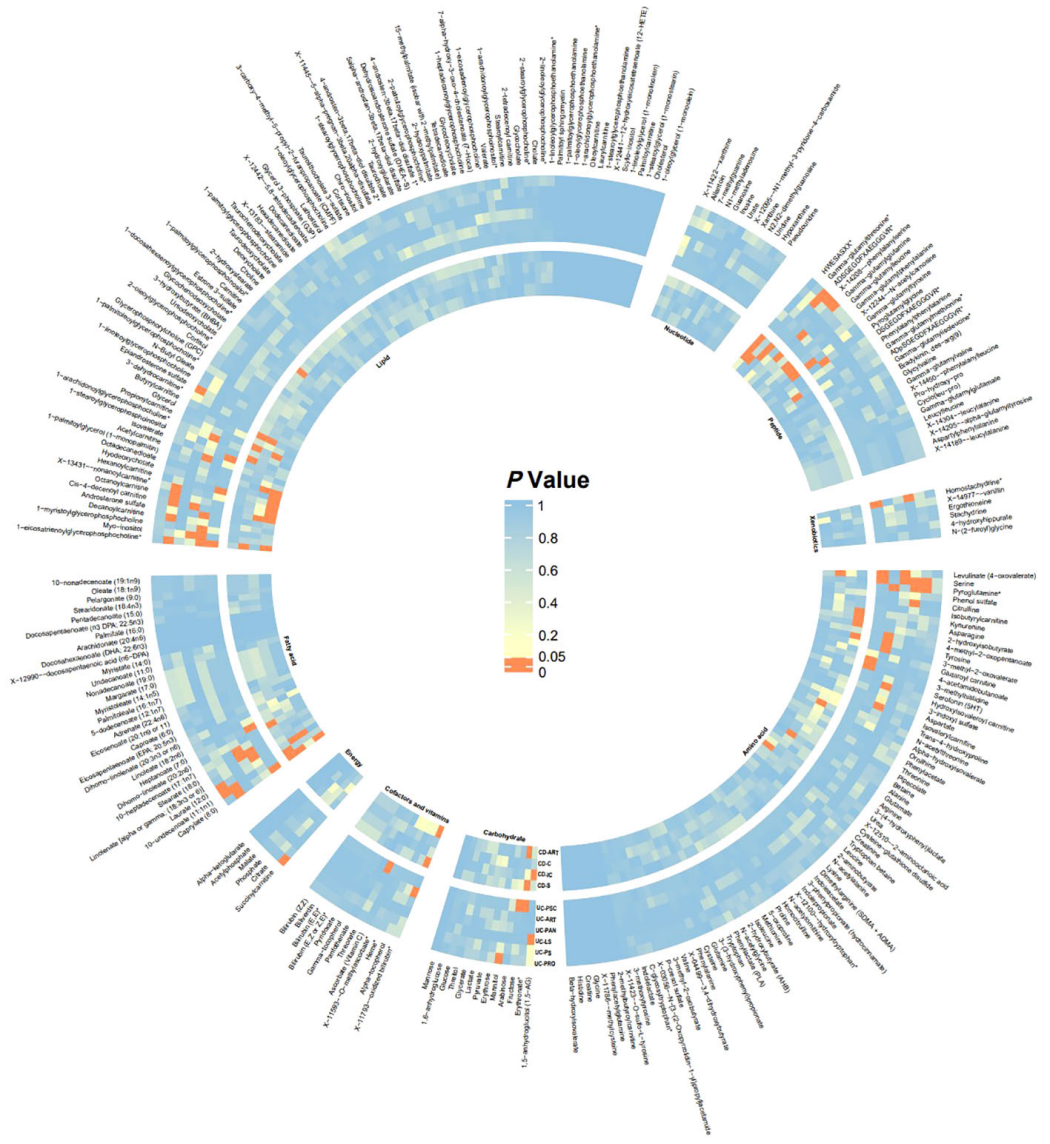
Heatmap of Significance in UVMR Analysis The heatmap displays the significance P-values of Mendelian randomization across different batches, with significant batches shown in red. From inner to outer circles are CD-ART, CD-C, CD-IC, CD-S, UC-PSC, UC-ART, UC-PAN, UC-LS, UC-PS, UC-PRO. Starting from the top left and proceeding clockwise, the groups are Lipid, Nucleotide, Peptide, Xenobiotics, Amino acid, Carbohydrate, Cofactors and vitamins, Energy, Fatty acid. CD-ART, Arthropathy in Crohn disease; CD-C, Crohn’s disease of colon; CD-IC, Crohn’s disease of ileocolon; CD-S, Crohn’s disease of small intestine; UC-PSC, Ulcerative colitis with PSC; UC-ART, Arthropathy in ulcerative colitis; UC-PAN, Ulcerative pancolitis; UC-LS, Left-sided ulcerative colitis; UC-PS, Ulcerative rectosigmoiditis; UC-PRO, Ulcerative proctitis.

In this study, 275 plasma metabolites were included in the analysis. The number of SNPs per type of gut flora ranged from 1 to 31, and details of the IVs for 150 metabolites are listed in the **Supplementary Table**. By univariate and multivariate MR analyses, we found that 9 metabolites had significant causal effects on IBD subtypes, of which 6 metabolites were protective against different IBD subtypes and three metabolites promoted the development of different IBD subtypes (as illustrated in **Figure 2**). The results of MR analysis are depicted in the circular heatmap (**Figure 3**), with detailed MR analysis results and the SNPs included therein provided in **Supplementary Tables 2**, **3**, while information on confounding-related SNPs is available in **Supplementary Table 4**.

We estimated the causal associations of these 275 metabolites with 10 IBD subtypes and their extraintestinal manifestations using MR analysis, and a total of 107 associations were identified in univariate analyses, which involved 62 metabolites. Subsequent multivariate analyses were performed, and 9 metabolites were observed to have independent effects on outcome. This includes 2 metabolites from the peptide metabolism pathway, 3 metabolites from the carbohydrate pathway, 3 metabolites from the lipid metabolism pathway, and 1 metabolite from the amino acid metabolism pathway. Protection against CD subtypes was observed with three metabolites; specifically, Erythronate reduced the risk of small intestinal Crohn’s disease; HWESASXX and Phenylalanylphenylalanine reduced the risk of Crohn’s disease of ileocolon. The development of CD subtypes is promoted by two metabolites; 1,5-anhydroglucitol (1,5-AG) increases the risk of Crohn’s disease of ileocolon, and nonanoylcarnitine increases the risk of arthritis in Crohn’s disease. Against UC subtypes, three metabolites showed protective effects; 1-arachidonoylglycerophosphocholine and Myo-inositol reduced the risk of ulcerative pancolitis; and mannitol reduced the risk of ulcerative proctitis. A metabolite was found to promote the pathogenesis of UC subtypes, with 3-methylhistidine increasing the risk of ulcerative colitis with PSC.

Although the IVW approach is highly effective in inferring causal relationships between exposures and disease outcomes, it is known to be susceptible to weak instrumental variable bias. We therefore conducted sensitivity and multivariate analyses of the above data to assess whether the results were robust. With the exception of heterogeneity in the causal relationship between erythritol and small intestinal Crohn’s disease (however, both the IVW method and the WM method results support a significant causal relationship), no evidence of heterogeneity or pleiotropy was found for the above significant results (as shown in **Table 2**). Detailed sensitivity analysis results are provided in **Supplementary Tables 5**-**7**.

**Table 2:** Sensitivity analysis results.

Batch	Exposure	Outcomes	Univariate Analysis			Multivariate Analysis		
			*P_pleiotropy_*	*P_heterogenity_*	Casual Direction	*P_pleiotropy_*	*P_heterogenity_*	*P_WM_*
2022	Myo-inositol	Ulcerative pancolitis	-	-	TRUE	0.917	0.126	0.009
731	Erythronate	Crohn’s disease of small intestine	0.596	0.727	TRUE	0.811	0.082	0.041
2095	1-arachidonoylglycerophosphocholine	Ulcerative pancolitis	0.894	0.610	TRUE	0.677	0.344	0.026
438	HWESASXX	Crohn’s disease of ileocolon	-	-	TRUE	0.443	0.025	0.011
548	Phenylalanylphenylalanine	Crohn’s disease of ileocolon	0.980	0.998	TRUE	0.917	0.126	0.013
2270	Mannitol	Ulcerative proctitis	0.187	0.899	TRUE	0.568	0.191	0.013
374	1,5-anhydroglucitol	Crohn’s disease of ileocolon	0.785	0.885	TRUE	0.811	0.082	0.154
1054	nonanoylcarnitine	Arthropathy in Crohn disease	-	-	TRUE	0.917	0.126	0.006
1178	3-methylhistidine	Ulcerative colitis with PSC	0.979	0.419	TRUE	0.265	0.871	0.173

TRUE Casual Direction indicates that the MR result passed the MR-Steiger forward causality test. Batches with less than 3 SNPs are not available for Cochran’s Q test and MR-Egger intercept analysis. WM, weighted median method; PSC, Primary Sclerosing Cholangitis.

### 3.3 Metabolic pathway analysis

We also identified seven important metabolic pathways associated with IBD subtypes in the present study (**Table 3**). 3-Methylhistidine was involved in the histidine metabolic pathway (P=0. 010). Among the metabolites that passed through the UMR only, those involved in histidine metabolism, valine, leucine, and isoleucine biosynthesis, arginine biosynthesis, aminoacyl-tRNA biosynthesis, alanine, aspartate, and glutamate metabolism, phenylalanine, tyrosine, and tryptophan metabolism, phenylalanine, tyrosine, and tryptophan biosynthesis, and unsaturated fatty acid pathway biosynthesis (P<0. 05). In the metabolic pathways of gut microbiota, we identified matches with L-glutamate degradation V, super pathway of L-isoleucine biosynthesis I, and super pathway of arginine and polyamine biosynthesis (**Table 3**, **Supplementary Table 8**).

**Table 3:** Key metabolic pathways involved in the pathogenesis of IBD subtypes.

Pathway	Serum				*Gut Microbiota*		
	Both in MVMR and UVMR	Significant in UVMR	*Outcomes*	*P_Enrichment_*	*Matched pathway*	*Outcomes*	*P_MR_*
**Histidine metabolism**	3-Methylhistidine	L-Glutamic acid	UC-PSC, CD-C	0.010			
**Valine, leucine and isoleucine biosynthesis**	-	3-Methyl-2-oxovaleric acid, Ketoleucine	UC-ART	0.002	Super pathway of L-isoleucine biosynthesis I	UC-ART	0.002
**Arginine biosynthesis**	-	L-Glutamic acid, Citrulline	CD-C, UC-LS	0.007	Super pathway of arginine and polyamine biosynthesis	UC-LS	0.004
**Aminoacyl-tRNA biosynthesis**	-	L-Asparagine, L-Tyrosine, L-Glutamic acid	CD-C, CD-S	0.010			
**Alanine, aspartate and glutamate metabolism**	-	L-Asparagine, L-Glutamic acid	CD-C, CD-S	0.029	L-glutamate degradation V	CD-C	0.039
**Phenylalanine, tyrosine and tryptophan biosynthesis**	-	L-Tyrosine	CD-C	0.038			
**Biosynthesis of unsaturated fatty acids**	-	Stearic acid, Alpha-Linolenic acid	UC-ART	0.046			

CD-ART, Arthropathy in Crohn disease; CD-C, Crohn’s disease of colon; CD-IC, Crohn’s disease of ileocolon; CD-S, Crohn’s disease of small intestine; UC-PSC, Ulcerative colitis with PSC; UC-ART, Arthropathy in ulcerative colitis; UC-PAN, Ulcerative pancolitis; UC-LS, Left-sided ulcerative colitis; UC-PS, Ulcerative rectosigmoiditis; UC-PRO, Ulcerative proctitis, UVMR, univariate MR; MVMR, multivariate MR.

## 4 Discussion

In recent years, it has been found that IBD can occur in combination with a variety of metabolic diseases, and metabolomic studies have continued to identify metabolites and metabolic pathways associated with intestinal inflammation and IBD (11, 27), and have become the focus of in-depth research. Blood is the most commonly used sample source for metabolomics identification because it contains a large number of detectable metabolites and can be easily obtained in large sample sizes to help screen for circulating biomarkers of IBD risk (28). Our study confirms the existence of an subtypes-specific metabolic profile in IBD and identifies key metabolites and metabolic pathways associated with IBD subtypes pathogenesis and its associated phenotypes.

**Table 4** summarizes the changes in metabolite levels in the bodies of patients with IBD from previous studies, as well as the effects of certain metabolites on IBD patients. Our previous studies have identified different risk factors for intestinal flora in different subtypes of CD or UC (29), and recent studies of CD subtypes have identified metabolomic differences. A mouse experiment by Baur et al. identified metabolomic differences in Crohn’s disease mice with different sites of involvement (30). Whereas Schwärzler et al. found a higher inflammatory profile in patients with ileal CD rather than isolated colonic CD (31).Serum levels of sphingolipid metabolites such as S1P (Sphingosine 1 phosphate) are higher in CD patients with ileocecal involvement compared to colonic disease (32).Serum anti-Brewer’s yeast antibodies better point to patients with ileal Crohn’s disease (33).. The high abundance of adherent-invasive E. coli (AIEC) possessed by patients with ileal CD compared to colonic CD may differentiate the metabolomics of different subtypes of CD patients through its ability to activate the expression of innate immune/pro-inflammatory genes (34, 35). However, stratification studies of UC subtypes remain limited. Previous stratification studies have led us to stratify the subtypes and extraintestinal manifestations of UC and CD, combining multiple metabolites with different CD/UC subtypes and obtaining many reliable results.

**Table 4:** Evidence of the association between metabolites and IBD onset in previous literature.

Metabolites	Impact on IBD	PMID
**Alanine, glutamine, histidine, leucine, phenylalanine, tyrosine, valine**	Decreased levels of the aforementioned amino acids in the serum of CD and UC patients.	38156773
**Omega-3 and omega-6 polyunsaturated fatty acids**	Decreased levels of the aforementioned substances in the serum of CD patients. Decreased levels of ω-3 polyunsaturated fatty acids in the serum of UC patients.	37008284
**Tetracosanoic acid, phosphatidylcholine (PC), lysophosphatidylcholine (LPC), sphingomyelin (SM), glycerides**	Decreased levels of the aforementioned substances in the serum of colonic CD and UC patients. The levels of arachidonoyl ethanolamide, palmitoyl ethanolamide, branched fatty acid esters of hydroxy fatty acids (FAHFA), and three isomers of hexadecanoic acid (palmitoyl stearin, stearoyl palmitin, and stearin olein) were higher in colonic CD patients than in UC patients.	31368421
**2-Arachidonoylglycerol**	Increased levels of these substances can lead to a significant reduction in colitis and associated systemic and central inflammation.	21551239
**Inositol and its phosphates**	The aforementioned substances can inhibit inflammatory responses and carcinogenic effects in IBD.	33374769
**1,5-Anhydroglucitol (1,5-AG), 1,5-Anhydroglucitol-6-phosphate (1,5-AG6P)**	Toxic accumulation of the aforementioned substances can lead to reduced neutrophil counts, impaired neutrophil function, and a significant propensity for developing inflammatory bowel disease (IBD).	36507137

In the amino acid metabolic pathway we identified a causal relationship between one metabolite and IBD. Amino acids are essential constituents of the human body, both for protein synthesis and through catabolism in important life activities of the body. It has been noted that the abundance of genes for the metabolism and biosynthesis of almost all amino acids is decreased in IBD patients (36). 3-Methylhistidine (3-MH) is a histidine derivative produced by degradation of several tissues, especially skeletal muscle (37). Histidine can affect acute and chronic inflammation and modulate key events in the immune response by producing histamine through decarboxylation reactions (38). It has been shown that histidine supplementation inhibits oxidative stress in intestinal epithelial cells thereby reducing damage to the gut as well as exerting anti-inflammatory effects by inhibiting TNF-α-induced IL-8 secretion (39). In addition, it has been found that dietary histidine ameliorates colitis by modulating NF-κB activation as well as inhibiting the production of pro-inflammatory cytokines by macrophages in an IL-10-deficient cellular metastasis model of Crohn’s disease (40). Decreased plasma histidine can increase the risk of recurrence in patients with ulcerative colitis in remission (41). However, our study discovered a positive causal relationship between high levels of 3-MH and the onset of ulcerative colitis with PSC, the mechanisms of which remain unknown and warrant further investigation.

Previous studies have demonstrated a strong association between foodborne bioactive peptides and the development of IBD (42).It can prevent and treat colitis by regulating four mechanisms: inflammatory cytokines, inflammatory pathways, intestinal epithelial barrier and intestinal flora balance (43). Phenylalanylphenylalanine is a peptide substance product resulting from the incomplete catabolism of proteolytic metabolism, which is strongly associated with a variety of diseases: one study found a positive correlation between Phenylalanylphenylalanine and the development of pancreatic ductal adenocarcinoma pancreatic ductal adenocarcinoma (PDAC) (44); It increases in lung cancer and decreases in tuberculosis, and can be used as a potential diagnostic marker to differentiate between lung cancer and tuberculosis (45). Phenylalanylphenylalanine and HWESASXX were also found to be causally associated with Crohn’s disease of ileocolon. However, the mechanisms by which such metabolites affect IBD are not yet fully understood and further experimental explorations are needed.

Furthermore, there is a close relationship between lipid metabolism and IBD, with various fatty acids and lipid metabolites attenuating the expression of the TNFα gene during the pathology of IBD, such as oleic acid, w-3 polyunsaturated fatty acids, arachidonic acid, and prostaglandins derived from phosphatidylcholine (46). Macrophages play a role in the pathogenesis of IBD through the cPLA2α/COX-1 pathway, which has been identified to have anti-inflammatory, immune-modulatory, intestinal microbiota-regulating, and barrier-maintaining effects (47). The existence of a causal relationship between inositol and ulcerative proctitis may be due to the following reasons, Inositol and inositol phosphates have been shown to have a variety of health benefits such as anticancer, antidiabetic, antioxidant and anti-inflammatory (48). Specifically, inositol hexakisphosphate (IP6) reduces cell necrosis and pro-inflammatory cytokine mRNA release at sites of inflammation (49, 50), and myoIns likewise have the ability to downregulate inflammation and cytokine release (51). We hypothesized that inositol and phosphatidylinositol could protect against ulcerative proctitis by decreasing the local response to intestinal mucosal inflammation. Our study found a causal relationship between nonanoylcarnitine (nonanoylcarnitine) and IBD and related extraintestinal manifestations, and acylcarnitines were found to serve as an alternative energy source for oxidative metabolism in a study examining carnitine and its derivatives in relation to alterations in IBD flora. Acetylcarnitine dietary supplementation increases carnitine levels in the gut and promotes the recovery of health in rodent models of enteropathogenic Escherichia coli infection (52). In the mouse experiments conducted by Lemons et al., it was found that consuming animal products rich in carnitine and acylcarnitines is associated with an increased risk of IBD (52). The mechanism of action here is not fully understood and still needs to be further explored.

Previous studies have indicated that 1,5-anhydroglucitol (1,5-AG) is a carbohydrate-like metabolite whose enzymatic side reaction produces 1,5-anhydroglucitol-6-phosphate (1,5-AG6P). 1,5-AG6P is a hexokinase inhibitor whose accumulation in cells inhibits the phosphorylation of glucose thereby affecting the glycolytic process. Neutrophils can suppress intestinal inflammation in IBD patients by modulating immune responses, oxidative stress, and generating pro-inflammatory cytokines, chemokines, and calprotectin (53). And since glycolysis is its only source of energy, an increase in 1,5-AG in the body may have unfavorable consequences for patients with Crohn’s disease of ileocolon through mechanisms that have not yet been clarified. Our study did confirm a causal relationship between 1,5-AG and the development of Crohn’s disease of ileocolon. Mannose, a monosaccharide *in vivo*, has been shown in mouse experiments to ameliorate colitis by strengthening tight junction proteins, inhibit mitochondrial dysfunction during inflammation by enhancing lysosomal integrity, and limit the release of histone B for the purpose of maintaining homeostasis in the intestinal epithelium (54). Mannitol is a monosaccharide derivative, and in an *in vitro* assay conducted by Yanjun Guo et al. it was found that mannitol can induce vasorelaxation through hypertonicity as well as SKCa (Small-conductance Ca2+-activated K+ channels) and IKCa (Intermediate-conductance) mediated EDH (Endothelium dependent hyperpolarization); leading to vasorelaxation, which may play a key physiological role in enhancing postprandial small resistance vascular blood flow and thus intestinal perfusion (55). In the current study we found that mannitol reduced the risk of ulcerative proctitis, and we speculate that it may be related to the above mechanism, but further experimental verification is needed. In addition, we found that Erythronate (erythritol) as well as mannitol are causally related to IBD, but the exact mechanism is still unclear and needs to be further explored. It is noteworthy that both amino acid-related metabolites, peptide metabolites, lipid metabolites, and carbohydrate metabolites share a common pathway to exert a protective effect against IBD, i.e., modulation of different pro-inflammatory factors and inhibition of the body’s inflammatory response to exert a protective effect on the intestinal mucosa.

In the present study we also identified seven metabolic pathways associated with the 6 IBD subtypes and their extraintestinal manifestations, namely valine, leucine and isoleucine biosynthesis, arginine biosynthesis, histidine metabolism, aminoacyl-tRNA biosynthesis, alanine, aspartate and glutamate metabolism, phenylalanine, tyrosine and tryptophan biosynthesis and unsaturated fatty acid biosynthesis. Overlapping metabolic pathways between various IBD subtypes have also been identified. The arginine synthesis pathway is significantly associated with left-sided ulcerative colitis and Crohn’s disease of the large intestine. This pathway involves metabolites related to glutamate and arginine. Glutamate has been shown in previous models of inflammation to improve intestinal barrier function, alleviate inflammation, and inhibit protein degradation via the corticotropin-releasing hormone (CRH)/CRH receptor 1, toll-like receptor (TLR) 4, and nucleotide-binding oligo-structural domain protein (NOD)/NF-κB, as well as the mammalian target of rapamycin (mTOR) signaling pathways, which can exert a protective effect against IBD (56). The polyamine pathway of spermidine metabolism produces putrescine, spermidine and spermine that stimulate colonic epithelial cell growth and regulate epithelial cell apoptosis, with anti-apoptotic and pro-apoptotic effects (57). Histidine metabolism is regulated for inflammation as previously described. The biosynthesis of valine, leucine, and isoleucine is an important part of the branched-chain amino acids and may act as a modulator of intestinal development, nutrient transport, and immune-related functions, thereby improving intestinal health (58).

Epidemiologic evidence on the effect of polyunsaturated fatty acids (PUFA) on inflammatory bowel disease (IBD) is conflicting (59). In our study, we found that alpha-linolenic acid involved in this metabolic pathway had a mitigating effect on arthropathy associated with ulcerative colitis, but the results were not sufficiently reliable and need to be verified by further studies. Pathways for histidine metabolism, aminoacyl-tRNA biosynthesis, alanine, aspartate, and glutamate metabolism, as well as biosynthesis with phenylalanine, tyrosine, and tryptophan are also present in a wide range of IBD outcomes involving glutamate, tyrosine, and aspartate.

This MR study has several strengths. First, to our knowledge, this is the first MR study to systematically assess the causal role of human blood metabolites in IBD subtypes and their parenteral manifestations. Second, we underwent rigorous instrumental variable screening and MR design, performed several sensitivity analyses and multivariate MR analyses to ensure robust results and explored independent blood metabolic markers. Finally, the dataset planning is also a major highlight, as we included datasets with European populations to minimize the error of results due to population bias. The large sample of the dataset also overcomes the sampling error brought about by the random effect. However, our study has some limitations. For example, some batches of SNP data were still pleiotropic after MR-PRESSO, and the conclusions obtained by MR-Egger may not be sufficiently robust. Second, the study population was focused on Europeans, so the conclusions cannot be generalized to larger populations for the time being. Third, since the ratio estimation method assumes linear causality, the present study cannot exclude that blood metabolites have a nonlinear relationship on outcome. Fourthly, due to the lack of relevant datasets, we stratified only by the sites of Crohn’s disease, without stratifying by severity (stricturing, penetrating, perianal disease). Lastly, this study was confined to etiological exploration and did not extend to investigating the post-onset details of metabolites and IBD. Future research should follow the findings of this study to conduct prospective cohort studies with repeated measures, and dynamically monitor targeted levels of serum metabolites, in association with symptoms, CRP levels, etc., during active or remission phases, to further delineate the diagnostic and prognostic significance of specific blood metabolites.

## 5 Conclusion

Our study suggests that blood metabolites may influence the pathogenesis of inflammatory bowel disease (IBD) in a causal manner, specifically, 9 metabolites, including Erythronate, Myo-inositol, and Mannitol, may be biomarkers used in public health for screening and prevention of 10 IBD subtypes, as well as potential molecules for the study of the pathogenesis of IBD. Exploration of the Mechanisms of Novel Blood Metabolites in IBD may Provide New Diagnostic Insights for Patients with IBD.

## Data availability statement

The original contributions presented in the study are included in the article/**Supplementary Material**. Further inquiries can be directed to the corresponding author.

## Author contributions

FL: Writing – original draft. ZDW: Writing – original draft. TT: Writing – review & editing. QZ: Writing – review & editing. ZW: Writing – review & editing. XH: Writing – review & editing. ZX: Writing – review & editing. YC: Writing – review & editing. HL: Writing – review & editing. SH: Writing – review & editing. CY: Writing – review & editing. SC: Writing – review & editing. YL: Writing – review & editing. YL: Writing – review & editing.

## Supplementary material

Supplementary Table 1 Dataset information included in MR studies.

Supplementary Table 2 MR results for all batches.

Supplementary Table 3 SNP information included in MR studies.

Supplementary Table 4 SNP information related to confounding factors.

Supplementary Table 5 MR-Steiger test results for SNPs.

Supplementary Table 6 Cochrane Q-test results for all batches.

Supplementary Table 7 Results of pleiotropic tests for all batches.

Supplementary Table 8 MR results for GM pathway on IBD subtypes.

Supplementary File Calculation codes for mendelian randomization analysis.

Supplementary Figure 1 Forest Plots.

Supplementary Figure 2 Scatter Plots.

Supplementary Figure 3 Leave-one-out Plots.

Supplementary Figure 4 Funnel Plots.
