# Supplementary material for: From serum metabolites to the gut: revealing metabolic clues to susceptibility to subtypes of Crohn’s disease and ulcerative colitis
Source: Front Endocrinol (Lausanne). 2024 Aug 8;15:1375896. doi: 10.3389/fendo.2024.1375896 (PMC11338916; doi:10.3389/fendo.2024.1375896)
Supplement: Supplementary Figure 2 — Scatter Plots. [file DataSheet_2.pdf]

Batch 2 : Tryptophan on Crohn's disease of colon

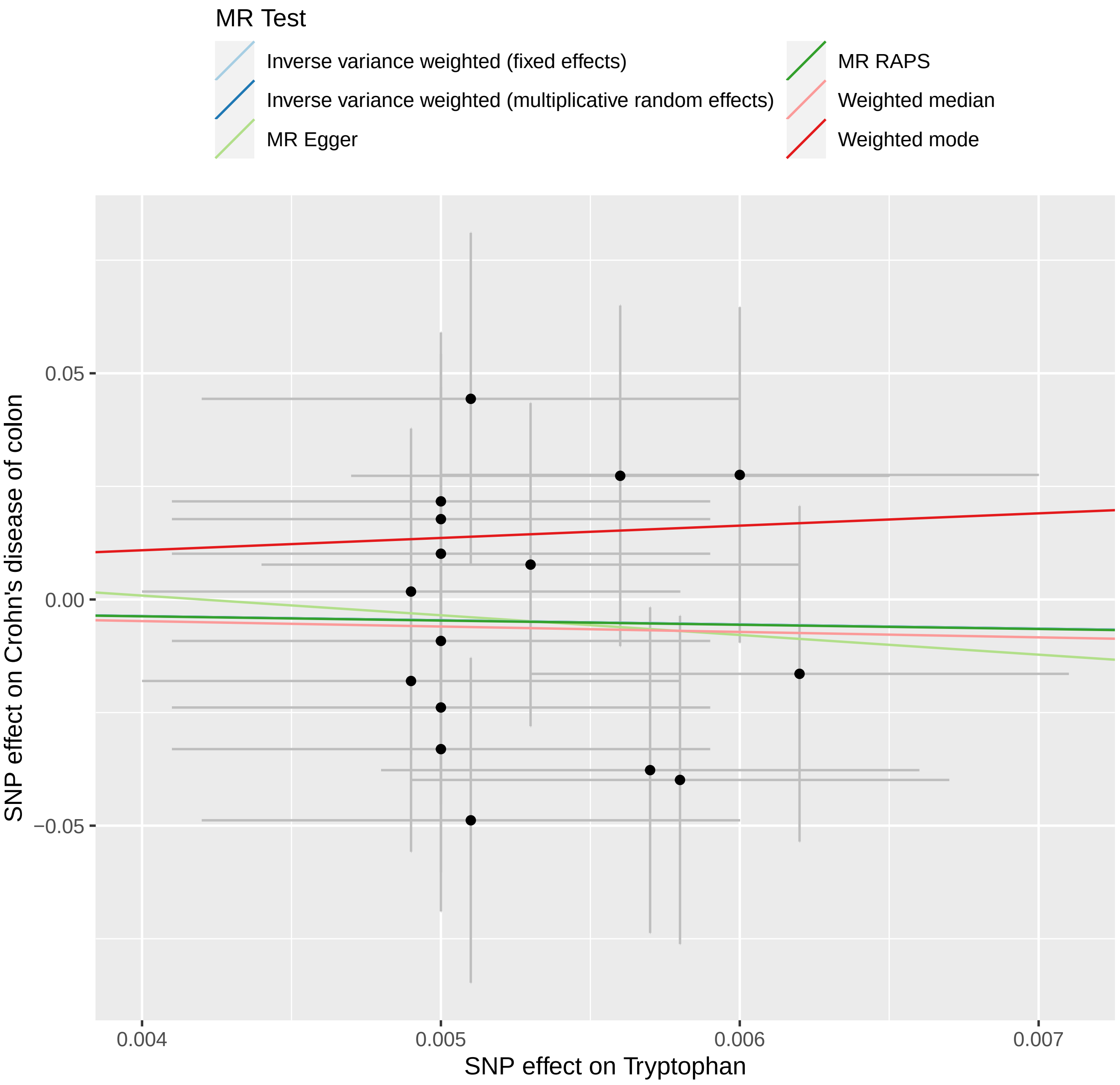

Batch 4 : Leucine on Crohn's disease of colon

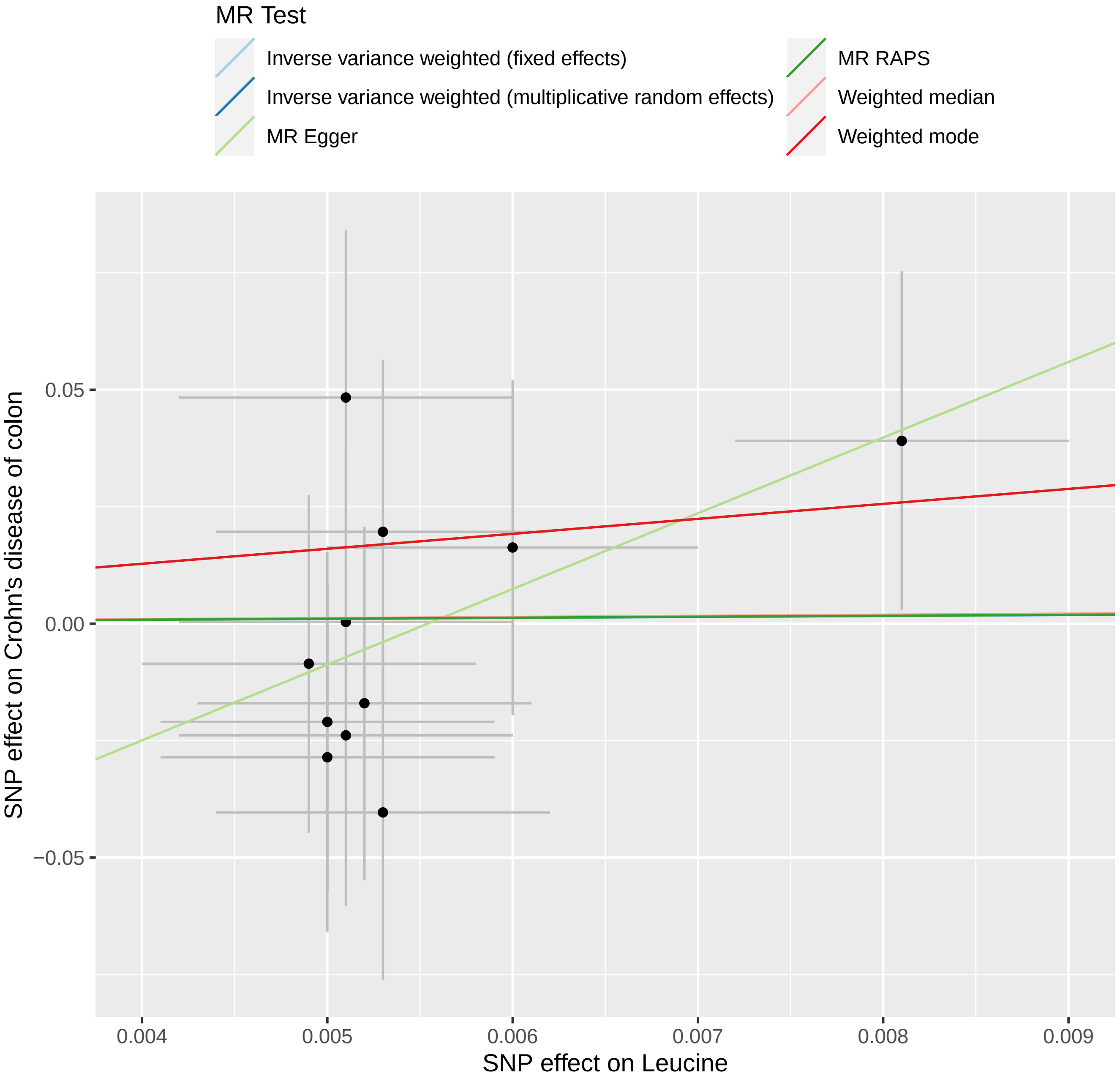

Batch 8 : Lactate on Crohn's disease of colon

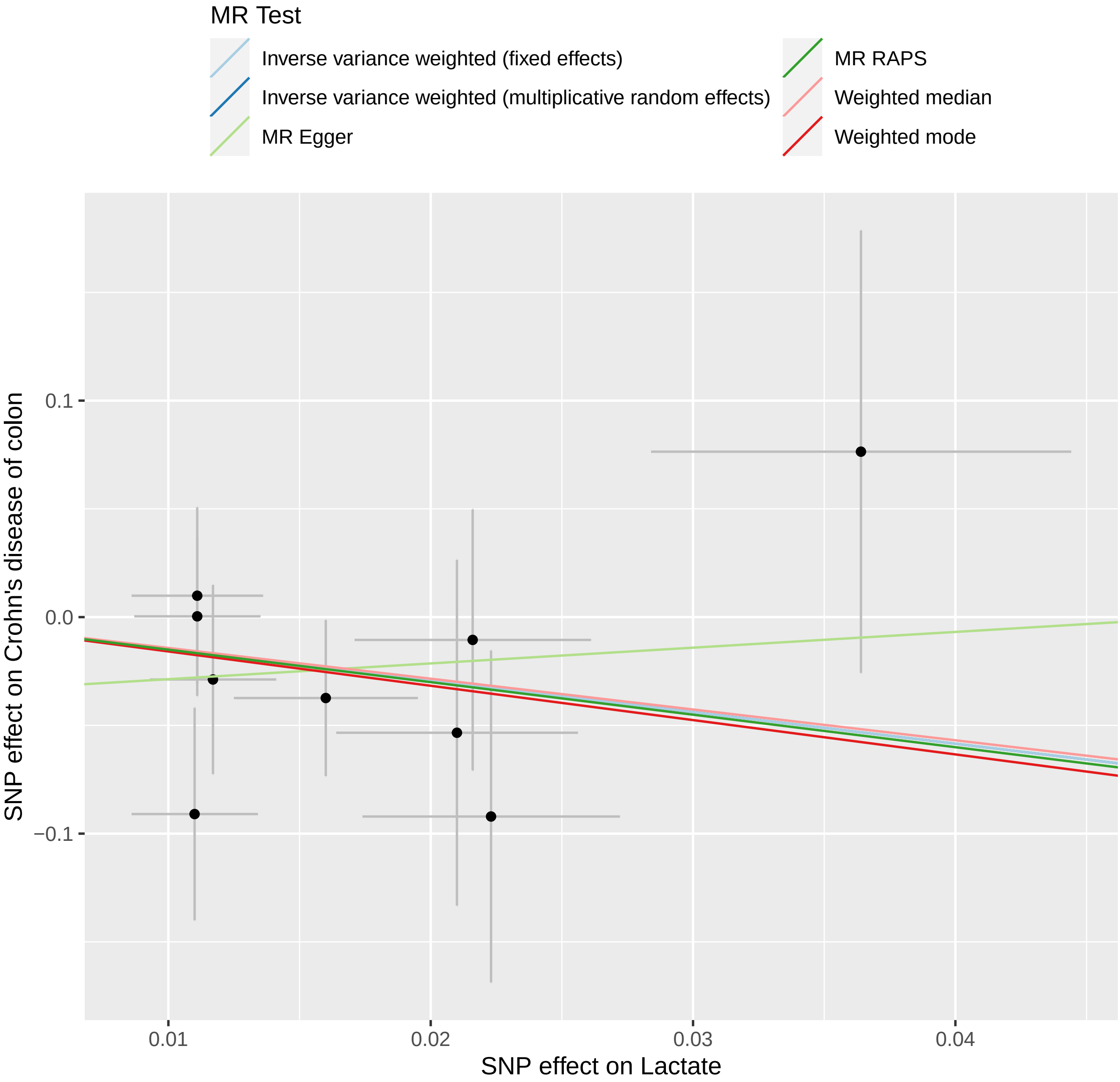

Batch 9 : 3−hydroxybutyrate (BHBA) on Crohn's disease of colon

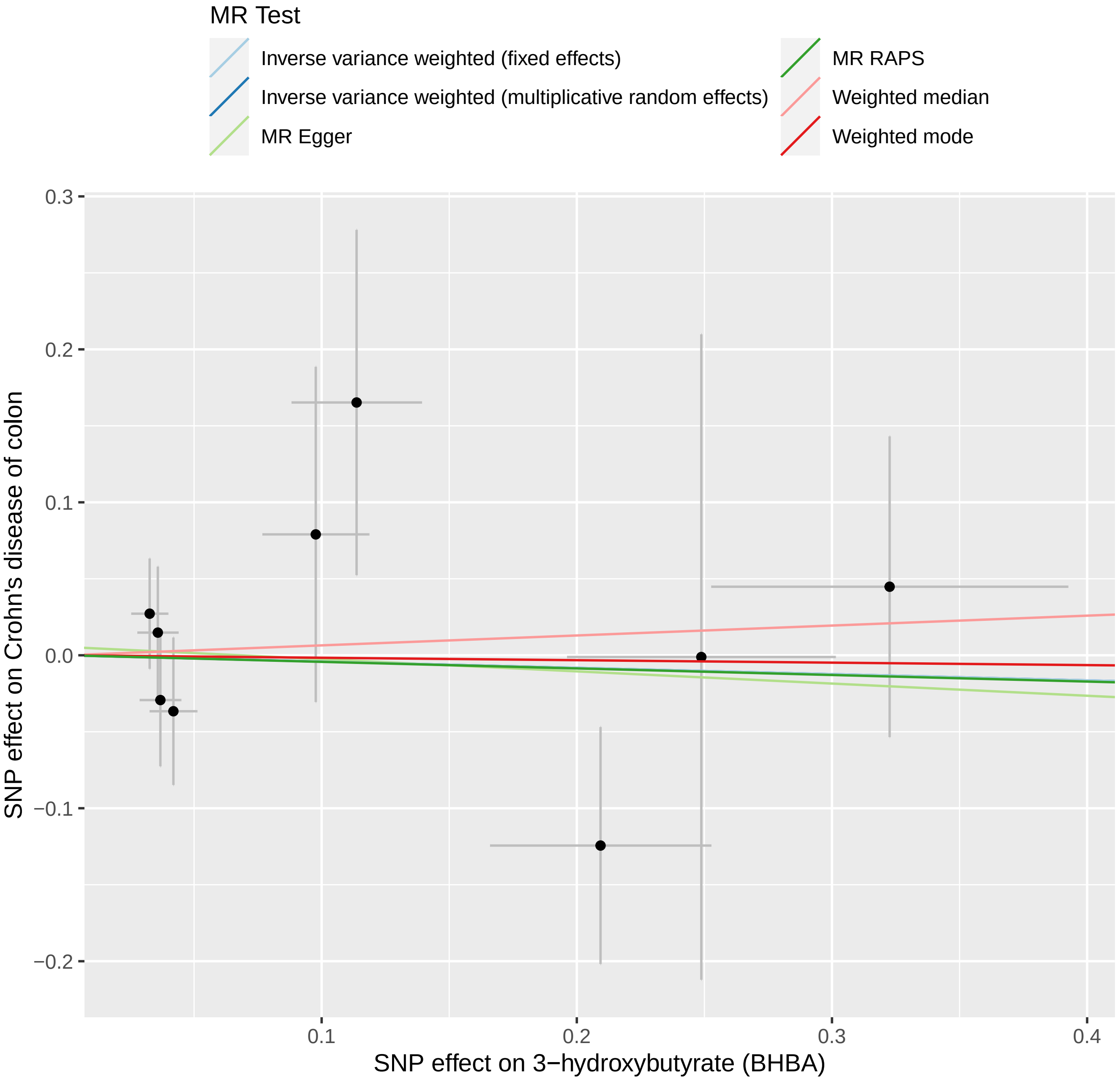

Batch 10 : Arabinose on Crohn's disease of colon

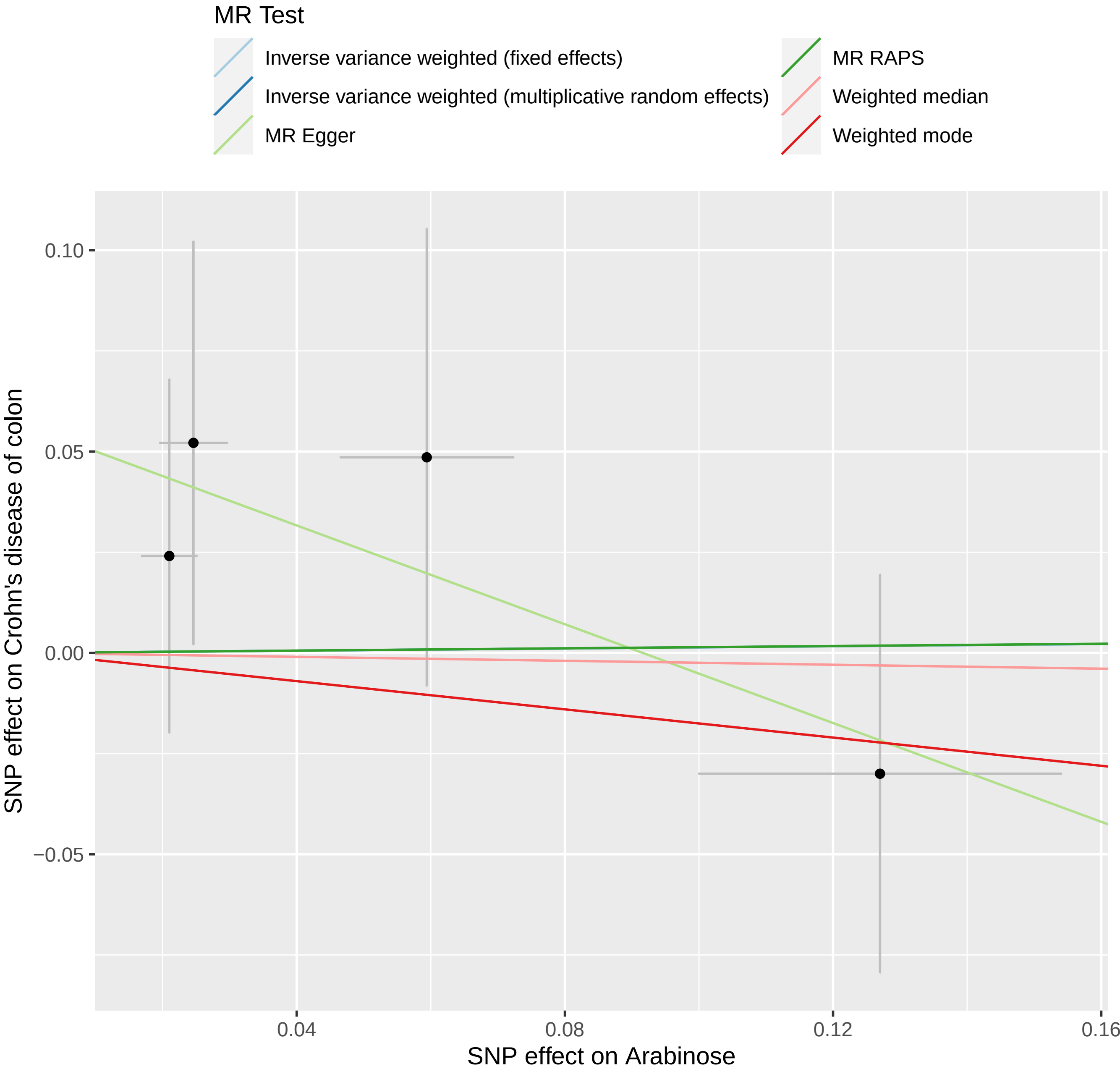

Batch 11 : Fructose on Crohn's disease of colon

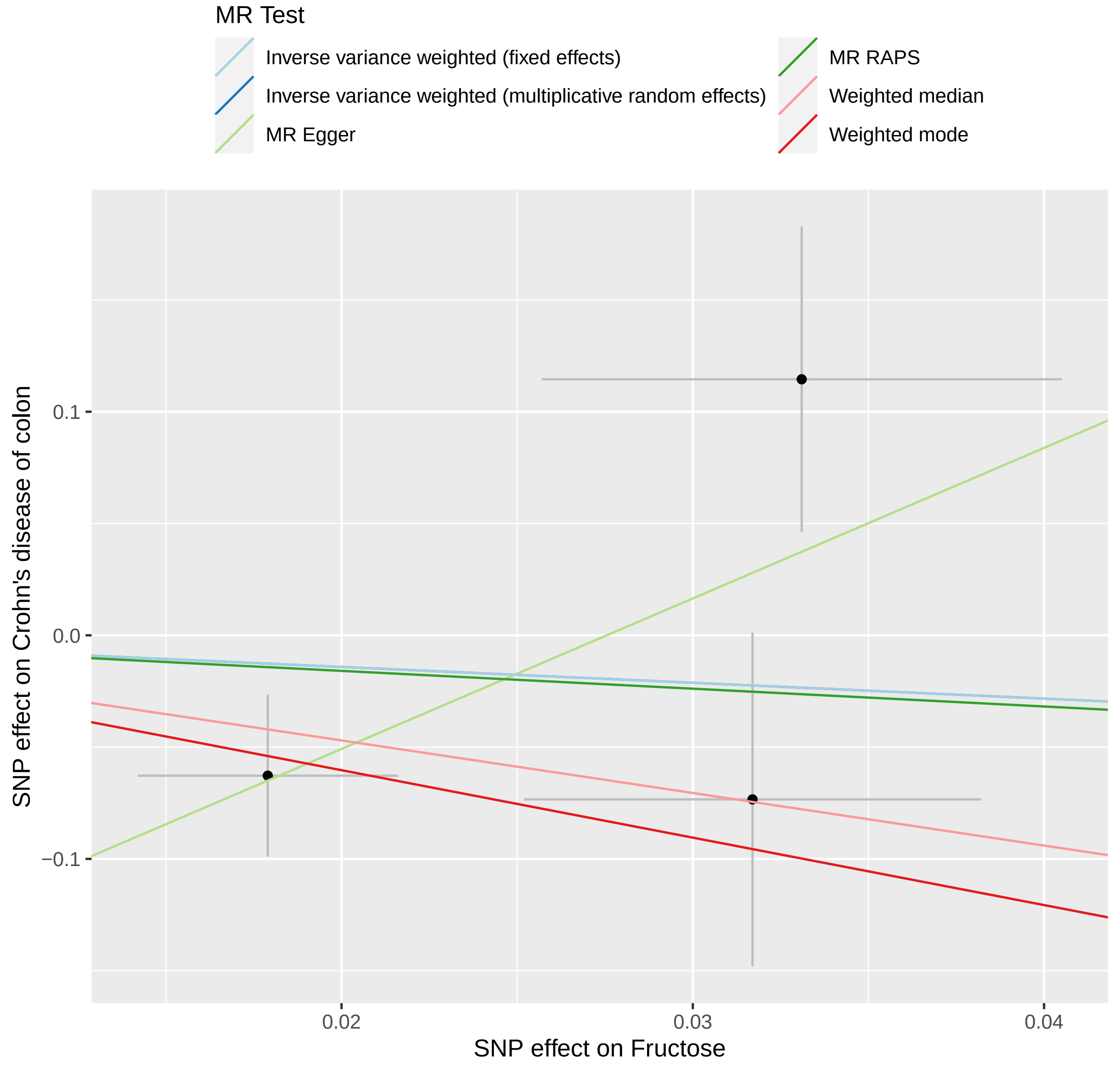

Batch 13 : Pyruvate on Crohn's disease of colon

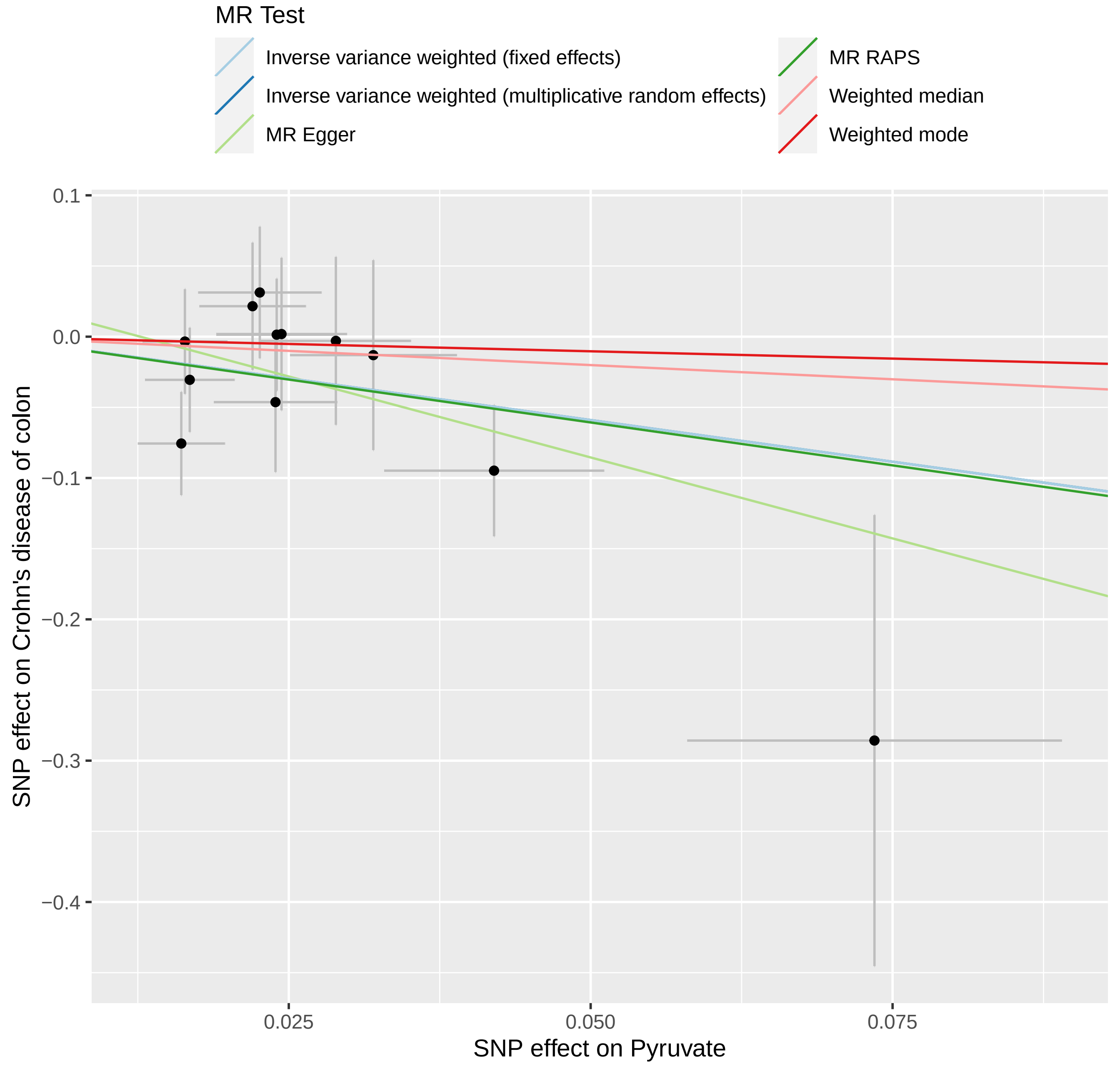

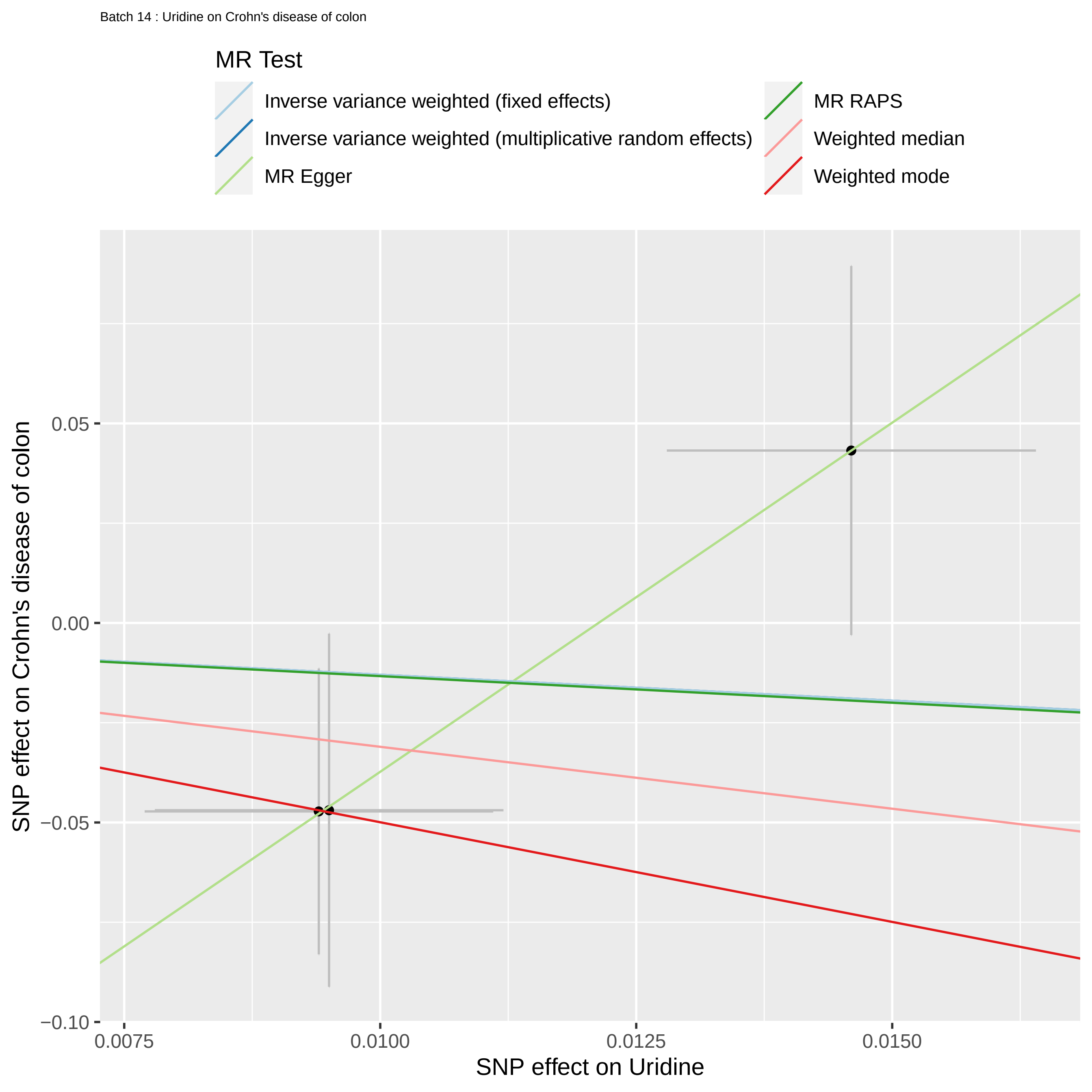
Batch 14 : Uridine on Crohn's disease of colon
MR Test
Inverse variance weighted (fixed effects)
Inverse variance weighted (multiplicative random effects)
MR Egger
MR RAPS
Weighted median
Weighted mode
SNP effect on Crohn's disease of colon
0.05
0.00
−0.05
−0.10
0.0075
0.0100
0.0125
0.0150
SNP effect on Uridine

Batch 15 : Linoleate (18:2n6) on Crohn's disease of colon

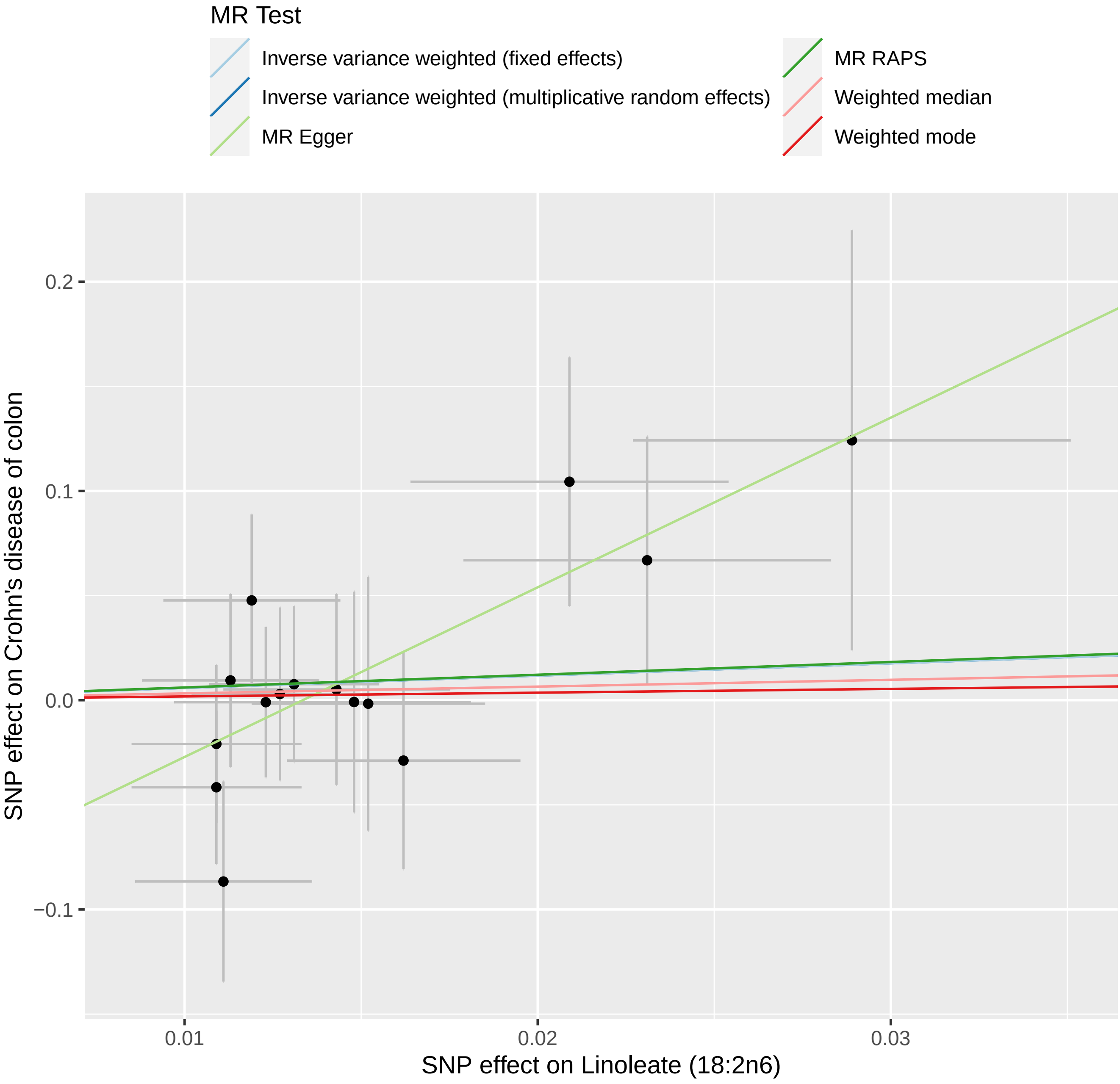

Batch 16 : Allantoin on Crohn's disease of colon

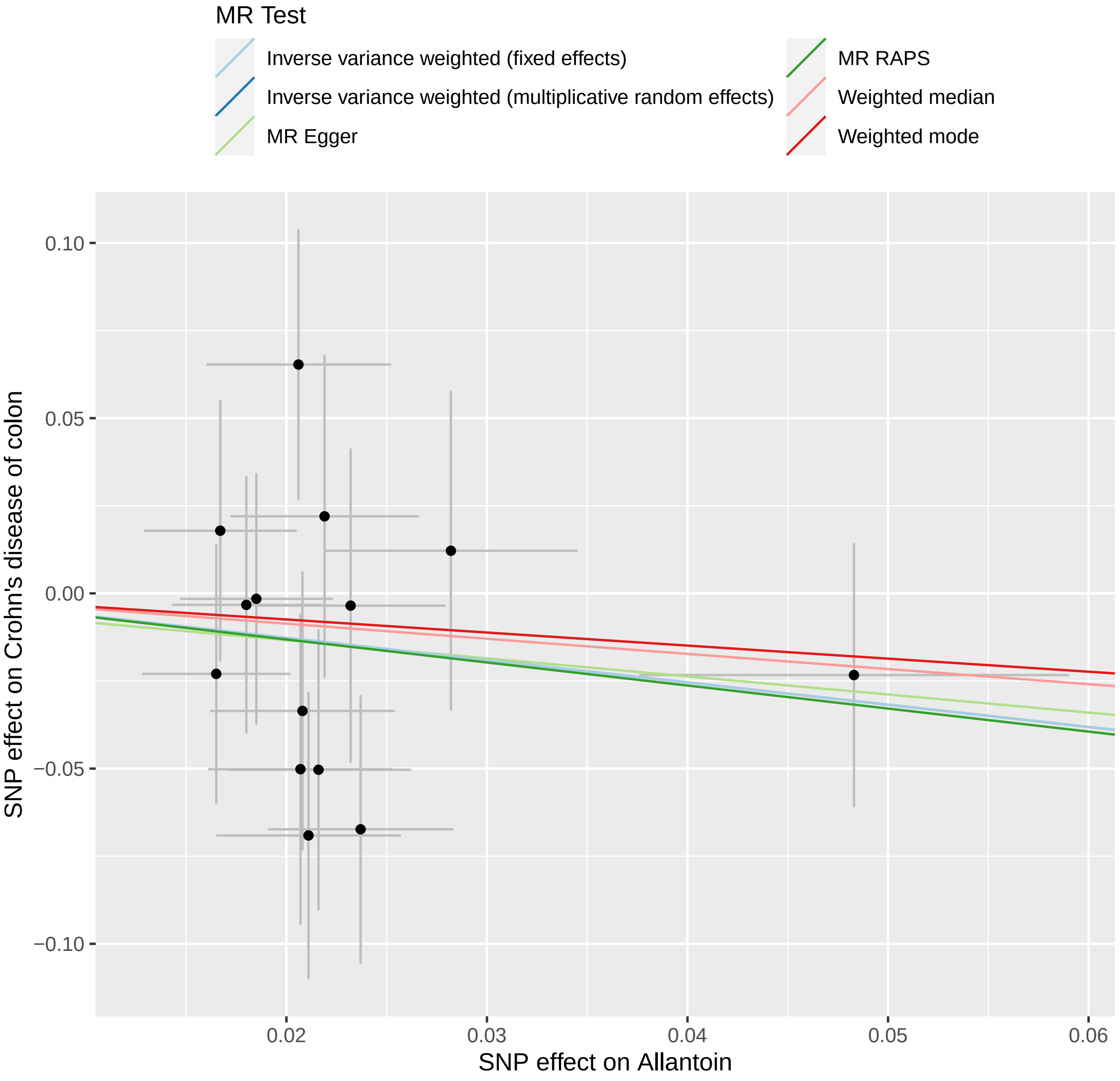

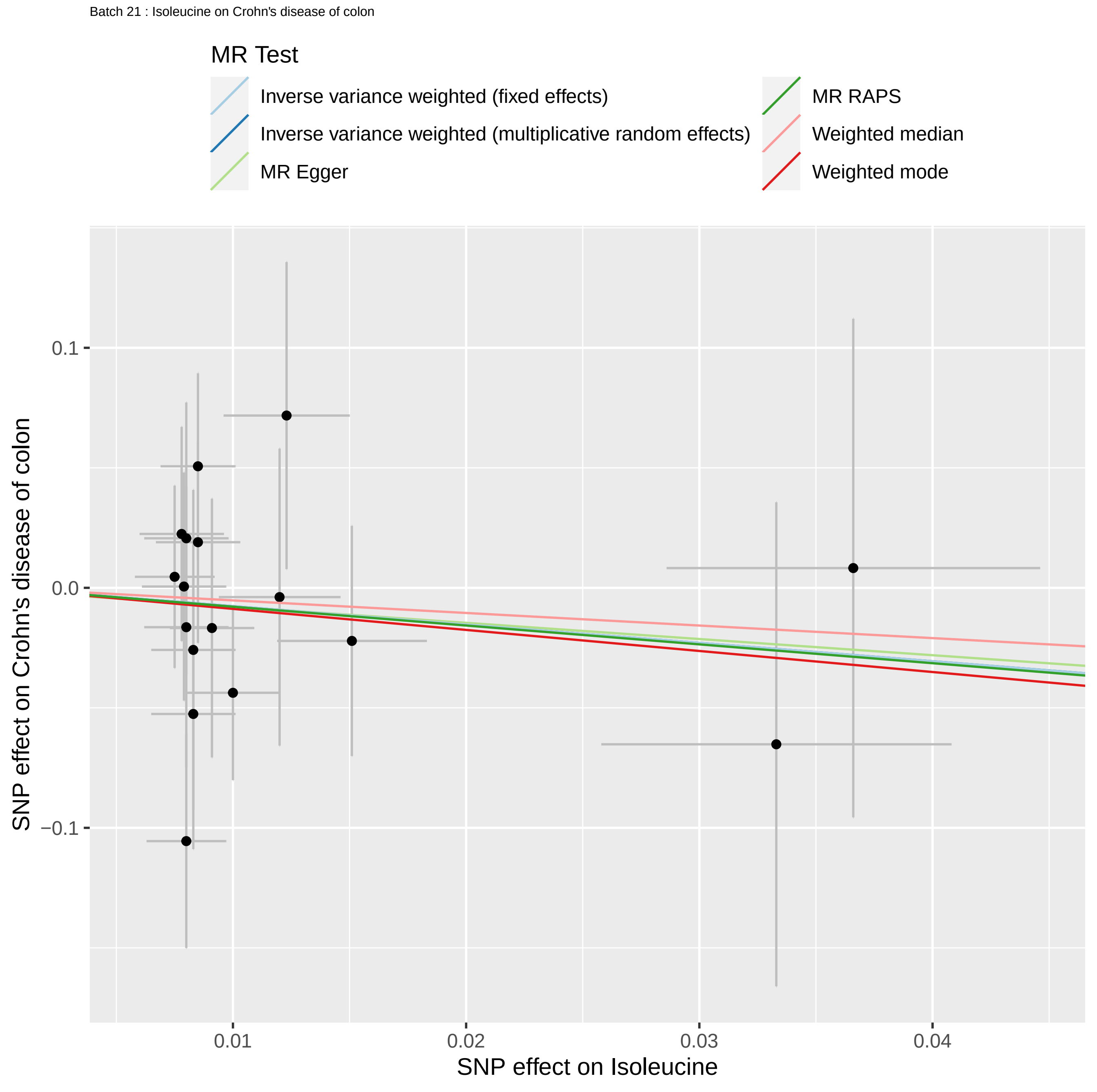
Batch 21 : Isoleucine on Crohn's disease of colon
MR Test
Inverse variance weighted (fixed effects)
Inverse variance weighted (multiplicative random effects)
MR Egger
MR RAPS
Weighted median
Weighted mode
SNP effect on Crohn's disease of colon
0.1
0.0
−0.1
0.01
0.02
0.03
0.04
SNP effect on Isoleucine

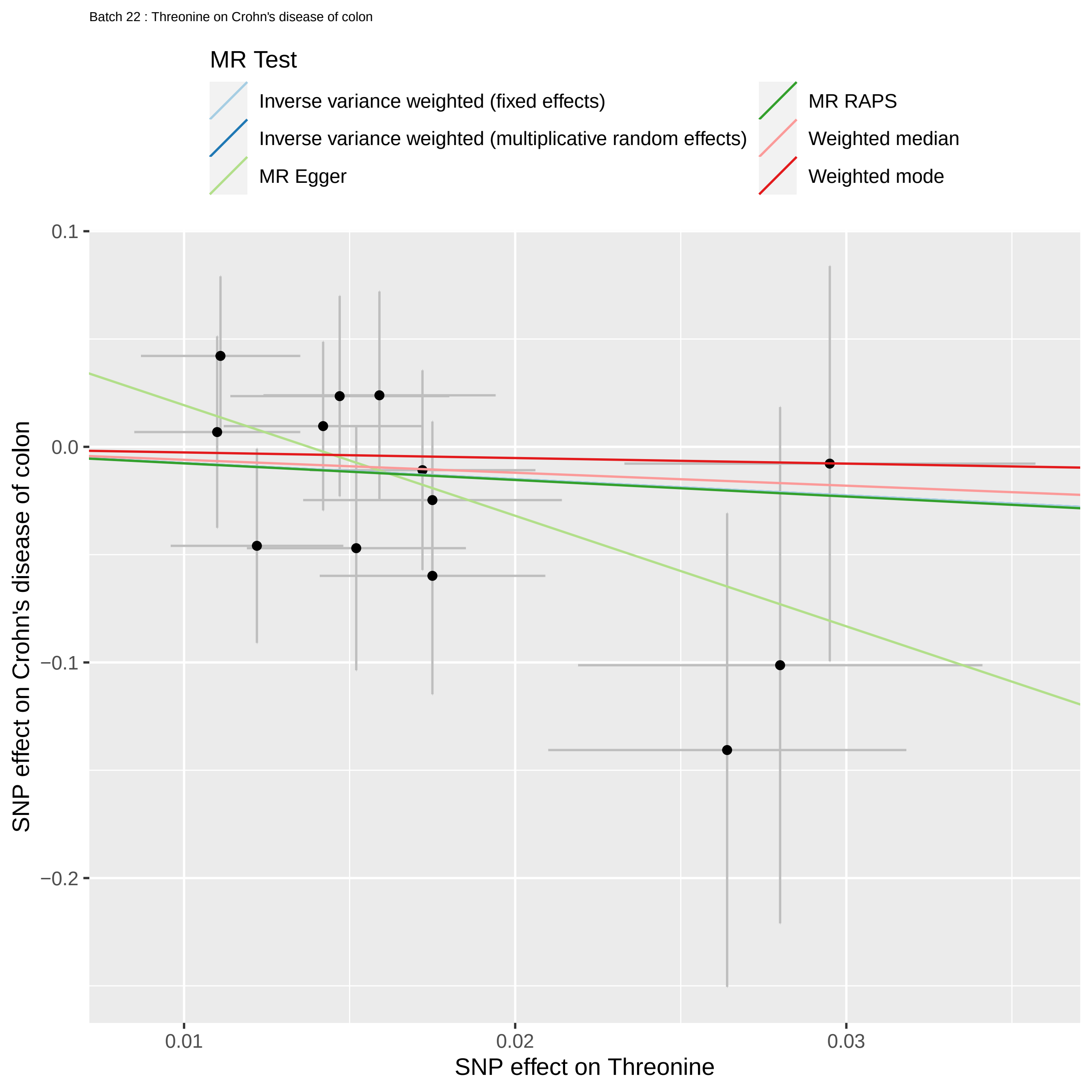

Batch 22 : Threonine on Crohn's disease of colon
MR Test
Inverse variance weighted (fixed effects)
Inverse variance weighted (multiplicative random effects)
MR Egger
MR RAPS
Weighted median
Weighted mode
0.1
0.0
−0.1
−0.2
SNP effect on Crohn's disease of colon
0.01
0.02
0.03
SNP effect on Threonine

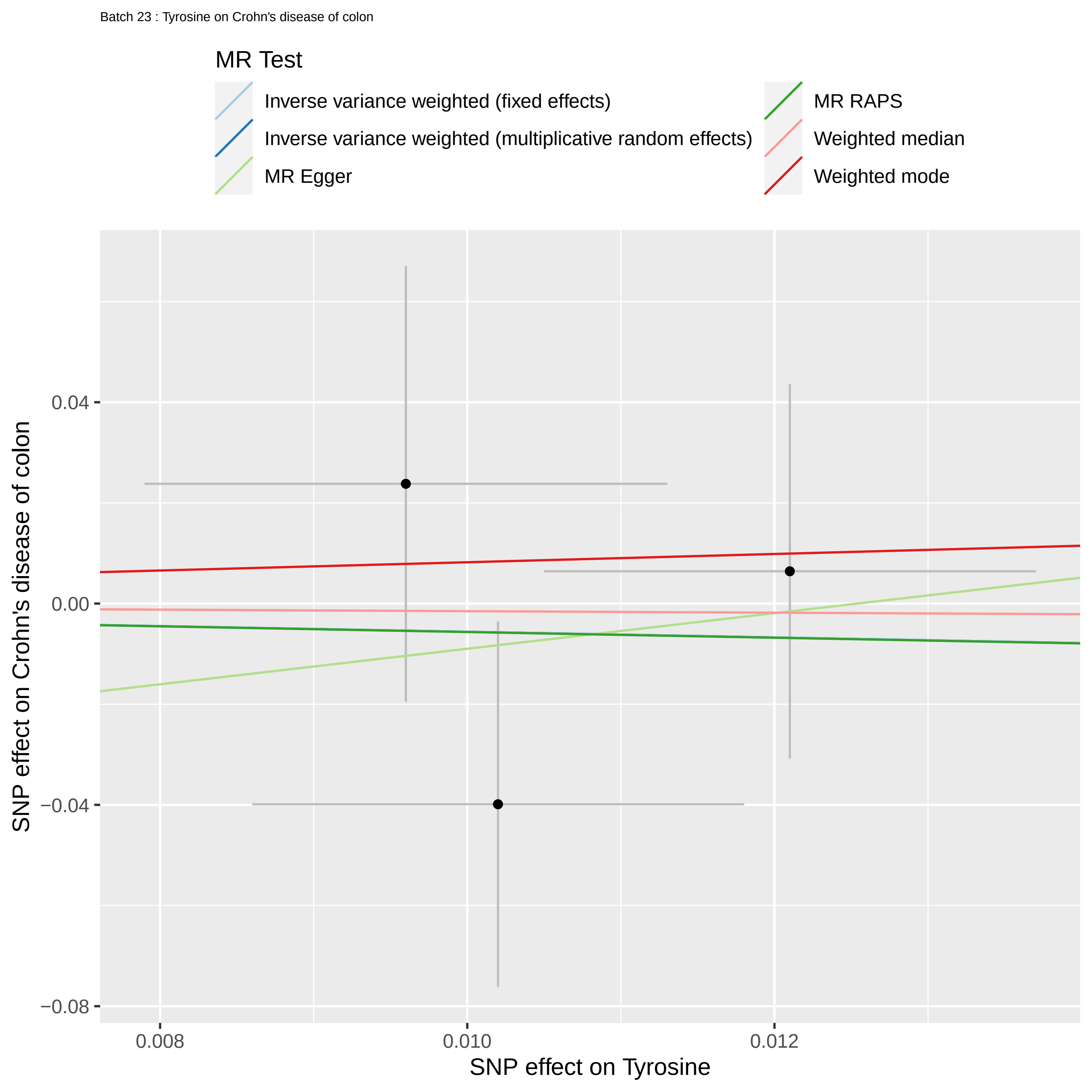
Batch 23 : Tyrosine on Crohn's disease of colon
MR Test
Inverse variance weighted (fixed effects)
Inverse variance weighted (multiplicative random effects)
MR Egger
MR RAPS
Weighted median
Weighted mode
0.04
0.00
−0.04
−0.08
SNP effect on Crohn's disease of colon
0.008
0.010
0.012
SNP effect on Tyrosine

Batch 29 : Stearate (18:0) on Crohn's disease of colon

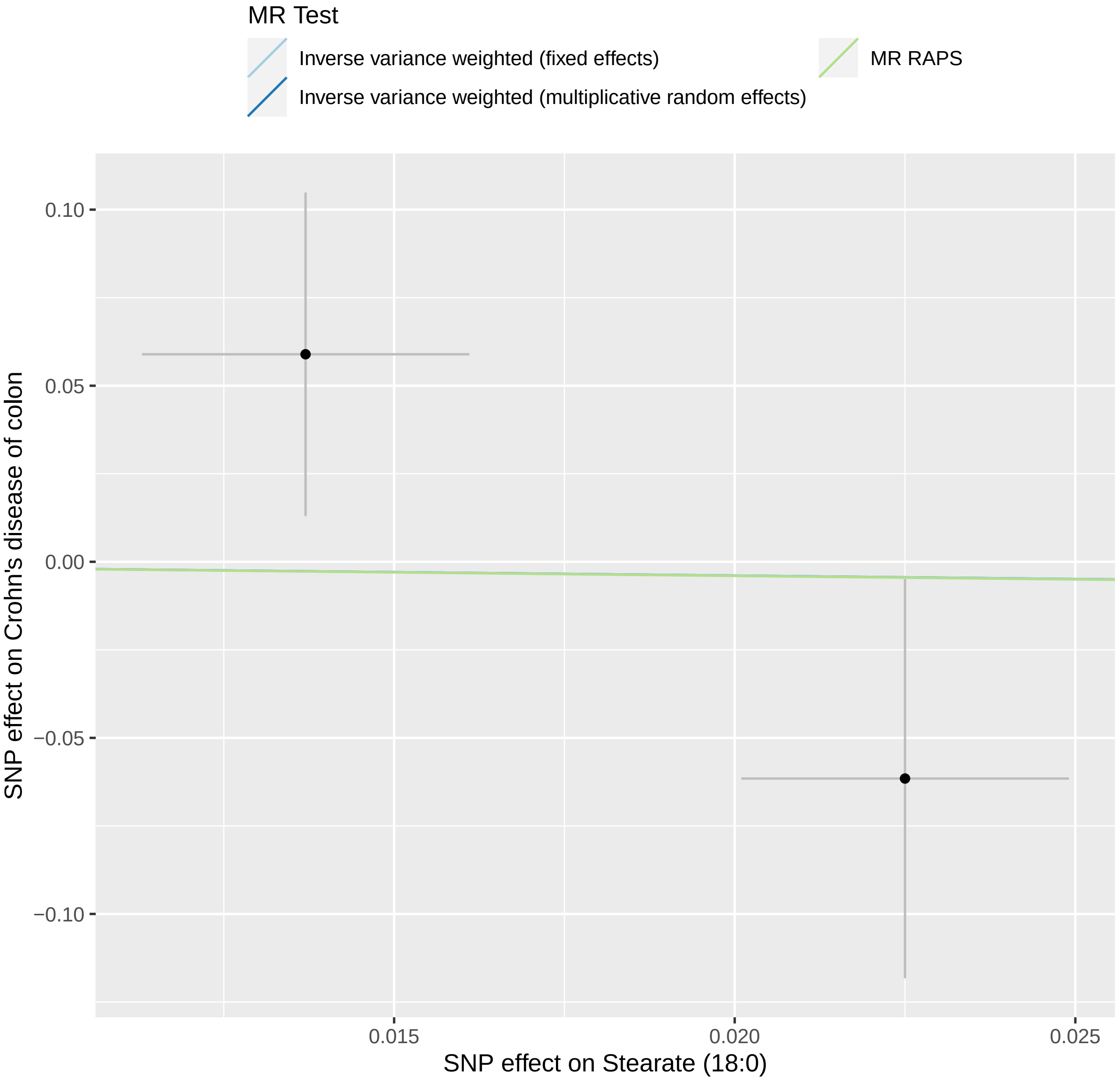

Batch 32 : Myristate (14:0) on Crohn's disease of colon

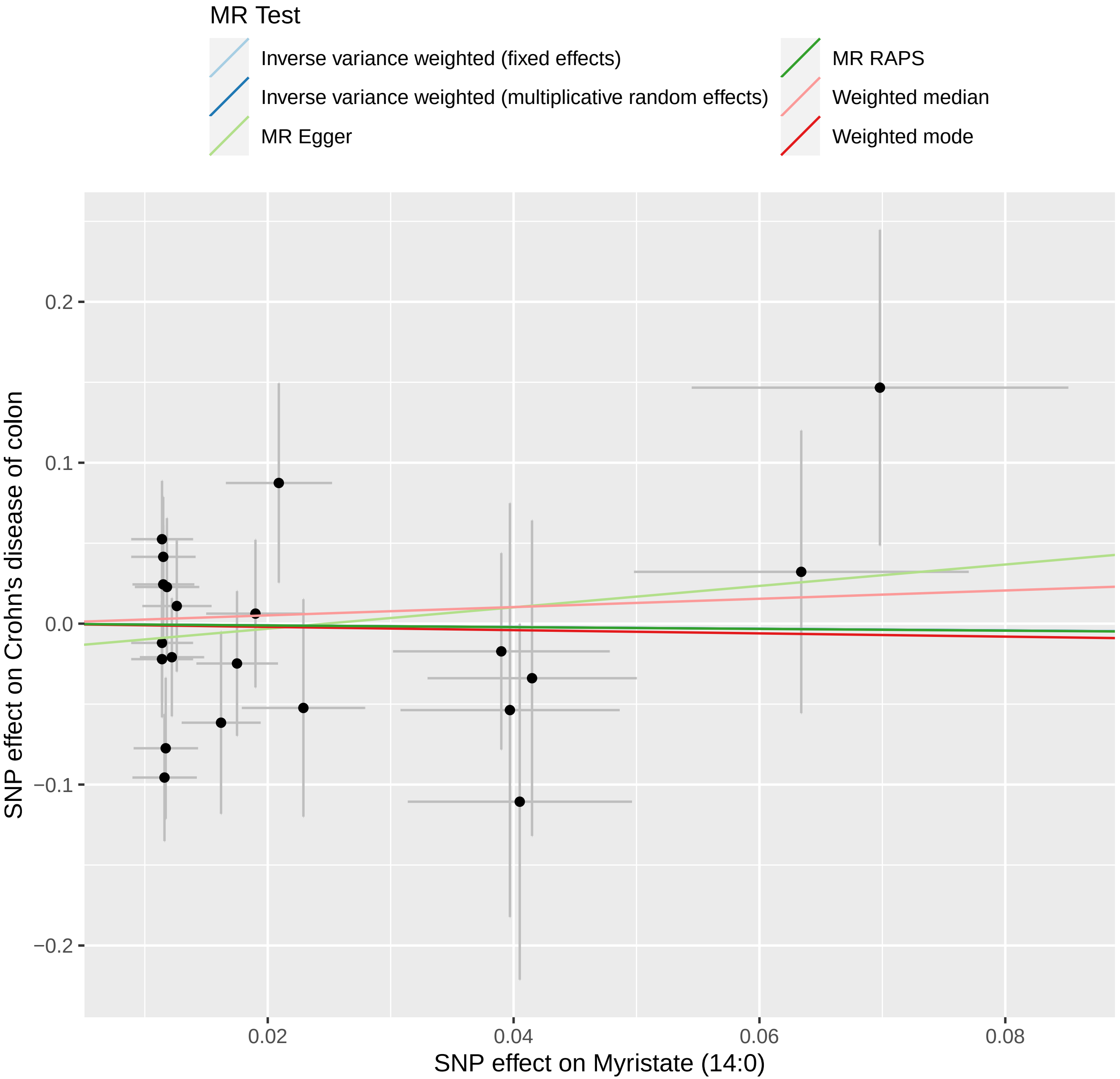

Batch 34 : Ornithine on Crohn's disease of colon

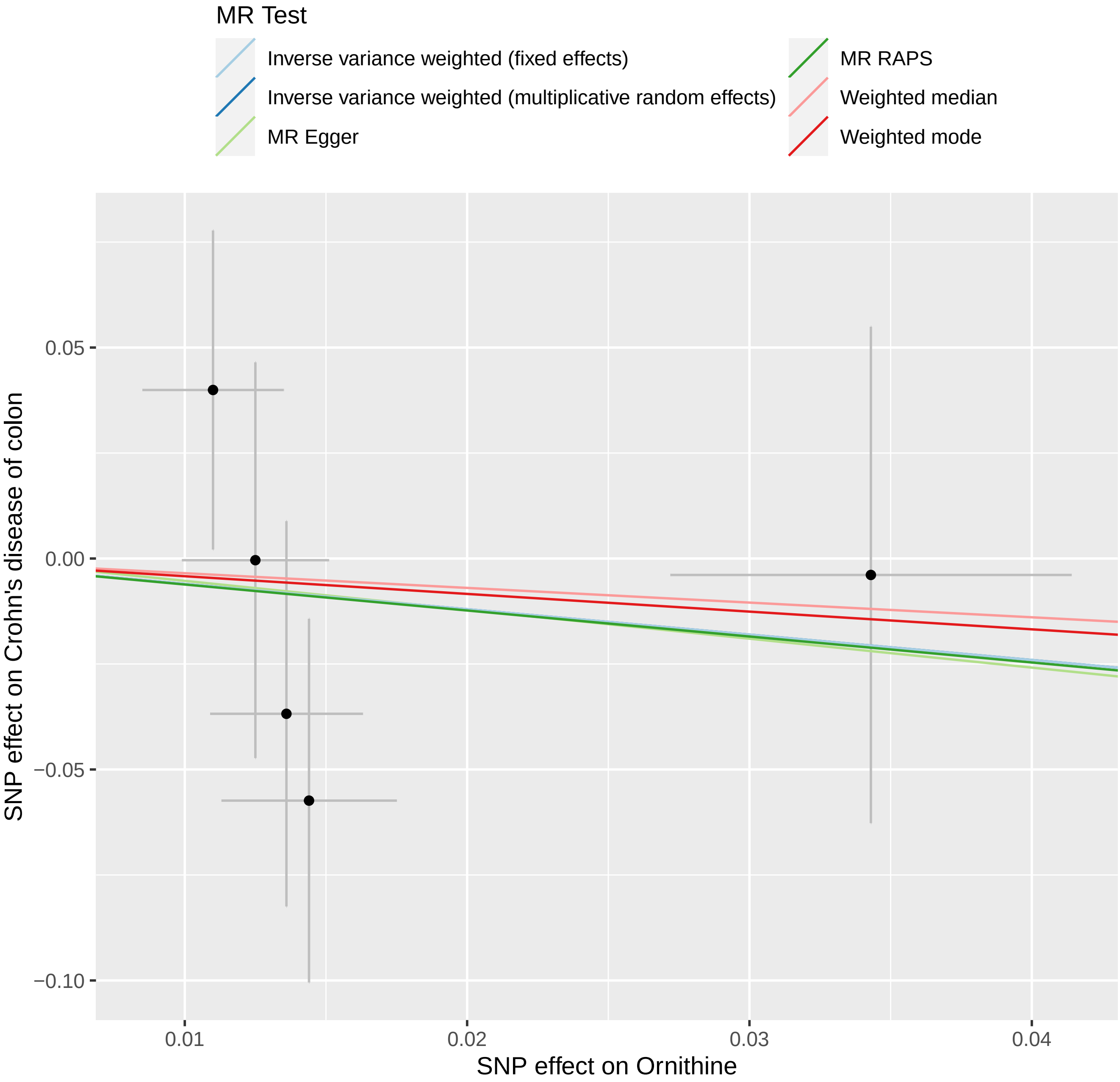

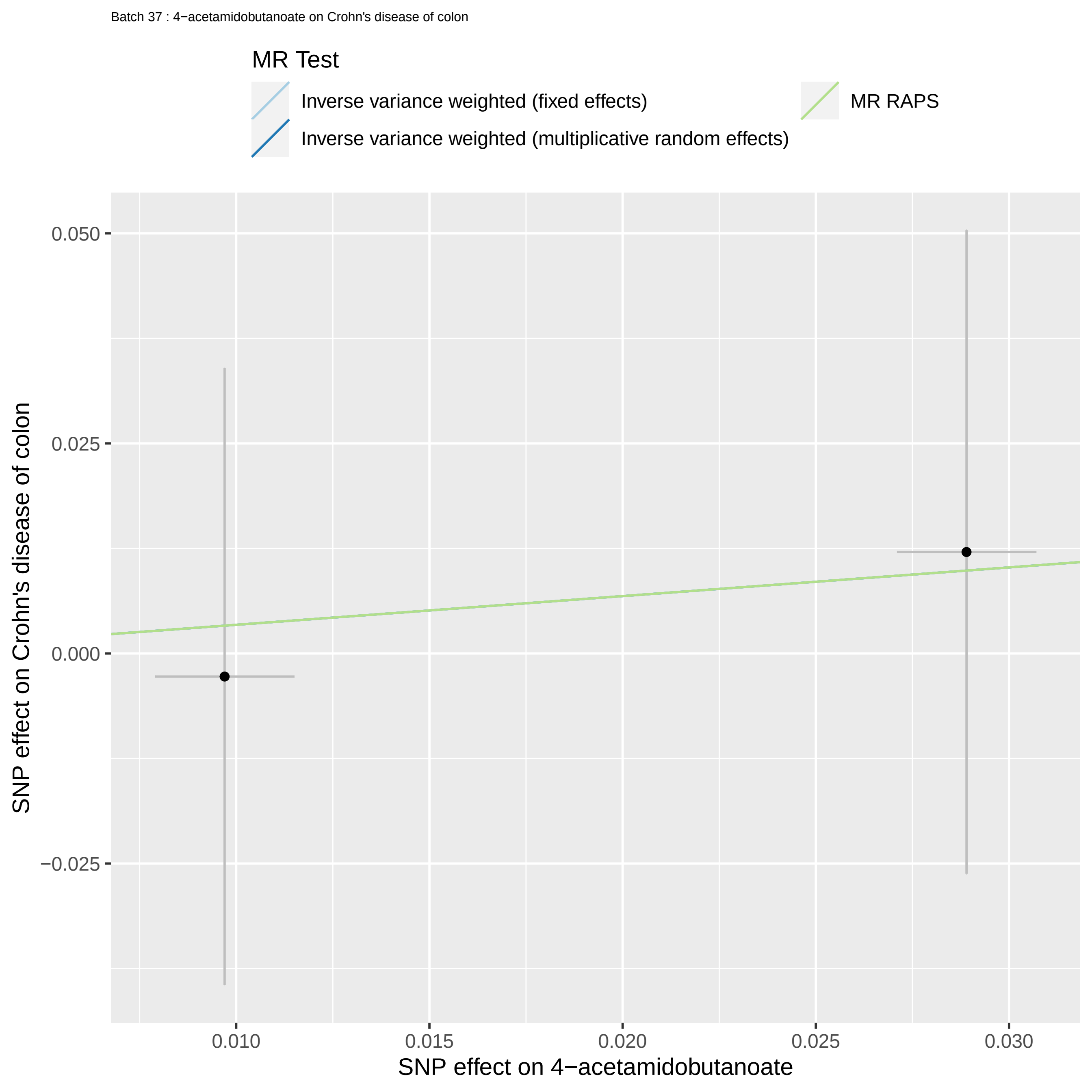
Batch 37 : 4−acetamidobutanoate on Crohn's disease of colon
MR Test
Inverse variance weighted (fixed effects)
Inverse variance weighted (multiplicative random effects)
MR RAPS
0.050
0.025
0.000
−0.025
SNP effect on Crohn's disease of colon
0.010
0.015
0.020
0.025
0.030
SNP effect on 4−acetamidobutanoate

Batch 38 : Alpha−tocopherol on Crohn's disease of colon

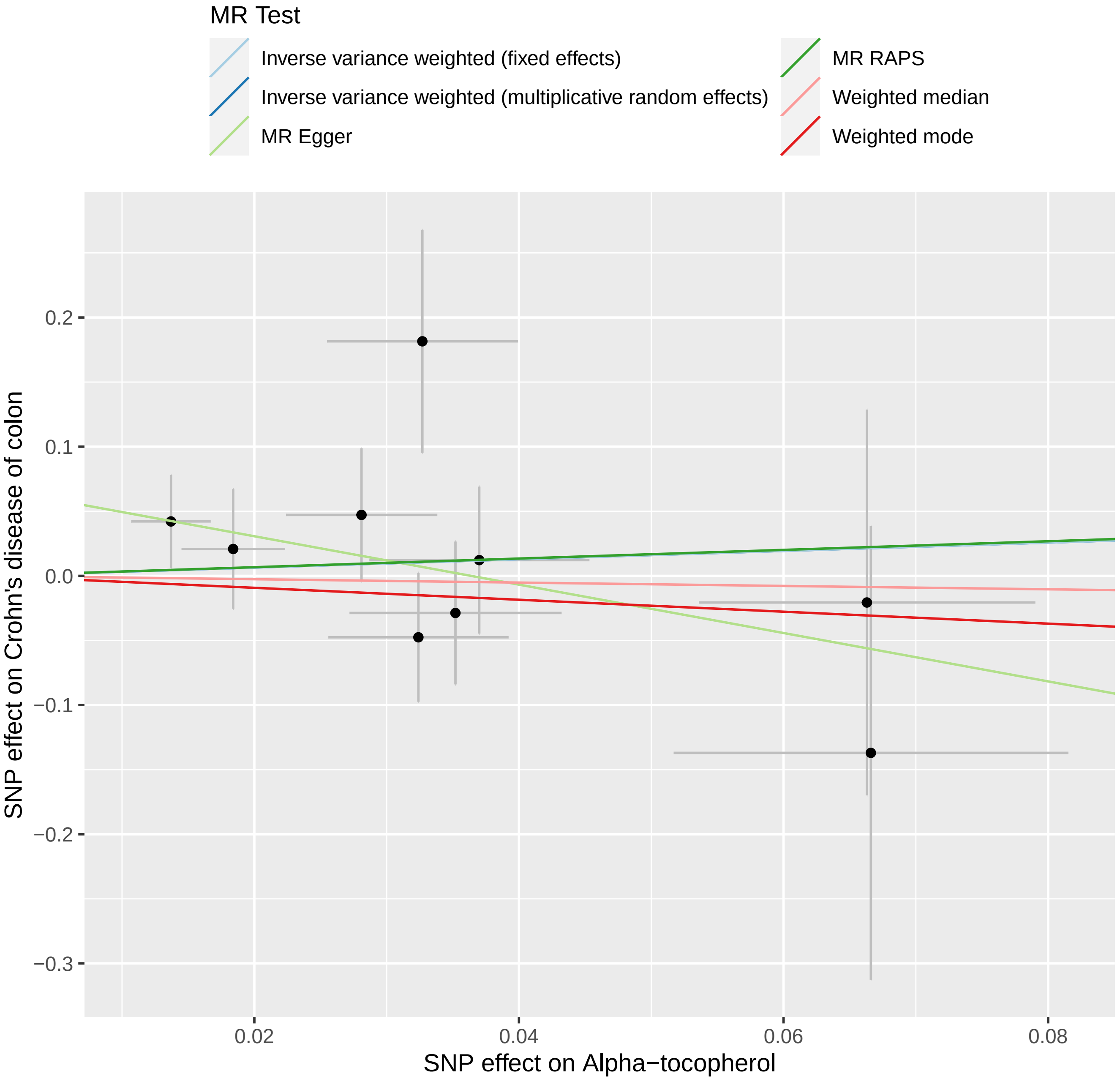

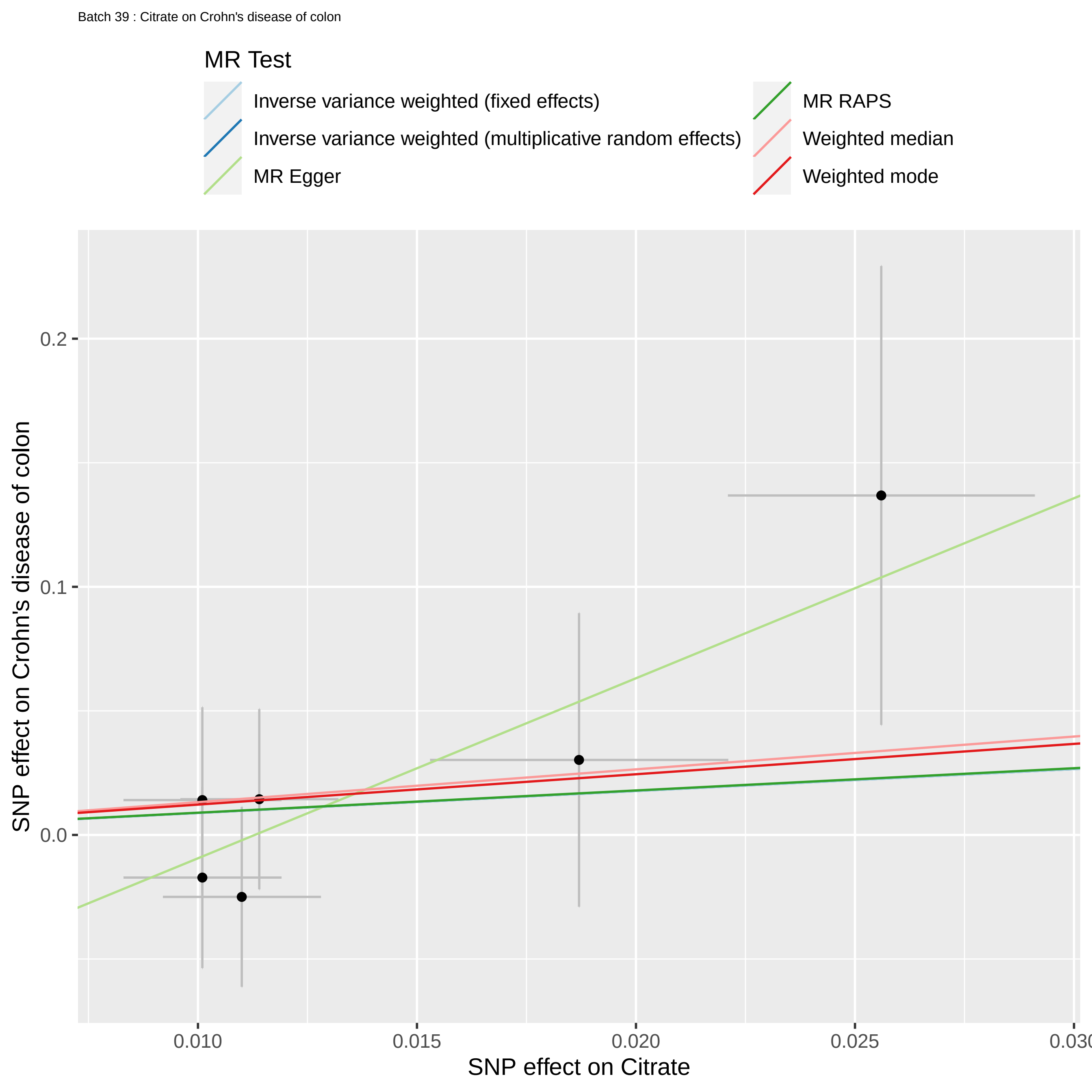

Batch 39 : Citrate on Crohn's disease of colon
MR Test
Inverse variance weighted (fixed effects)
Inverse variance weighted (multiplicative random effects)
MR Egger
MR RAPS
Weighted median
Weighted mode
0.2
0.1
0.0
SNP effect on Crohn's disease of colon
0.010
0.015
0.020
0.025
0.030
SNP effect on Citrate

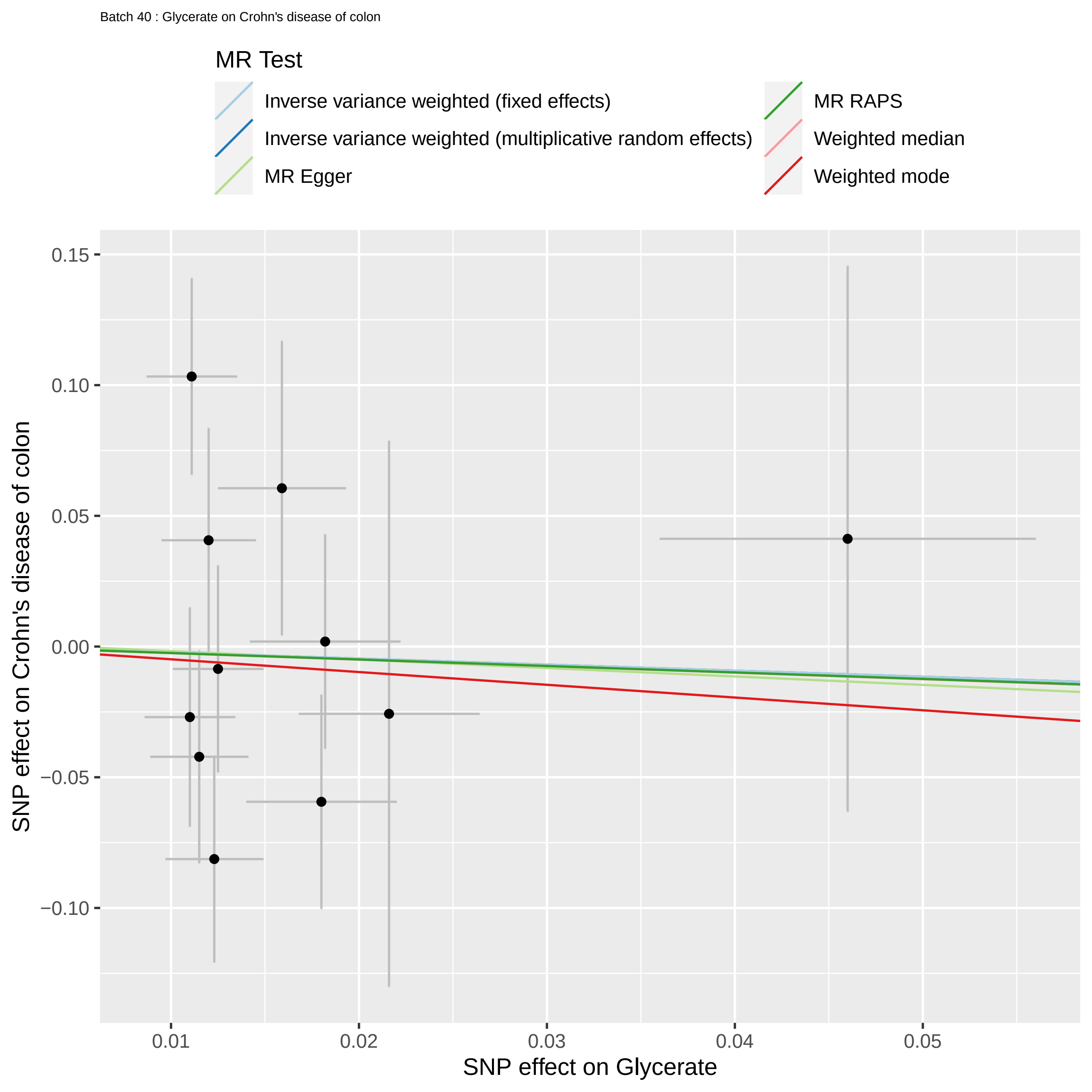
Batch 40 : Glycerate on Crohn's disease of colon
MR Test
Inverse variance weighted (fixed effects)
Inverse variance weighted (multiplicative random effects)
MR Egger
MR RAPS
Weighted median
Weighted mode
0.15
0.10
0.05
0.00
−0.05
−0.10
SNP effect on Crohn's disease of colon
0.01
0.02
0.03
0.04
0.05
SNP effect on Glycerate

Batch 41 : Guanosine on Crohn's disease of colon

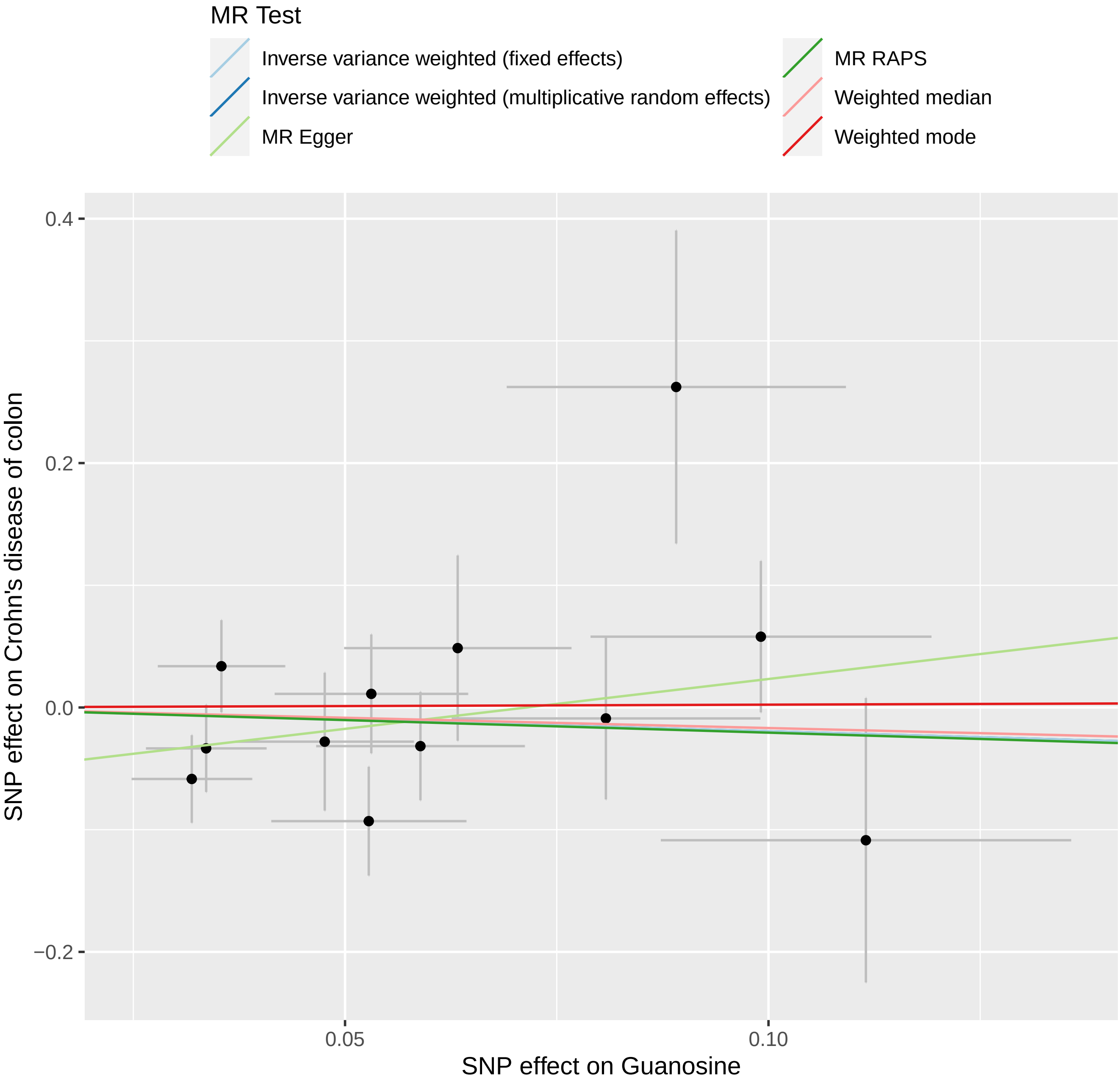

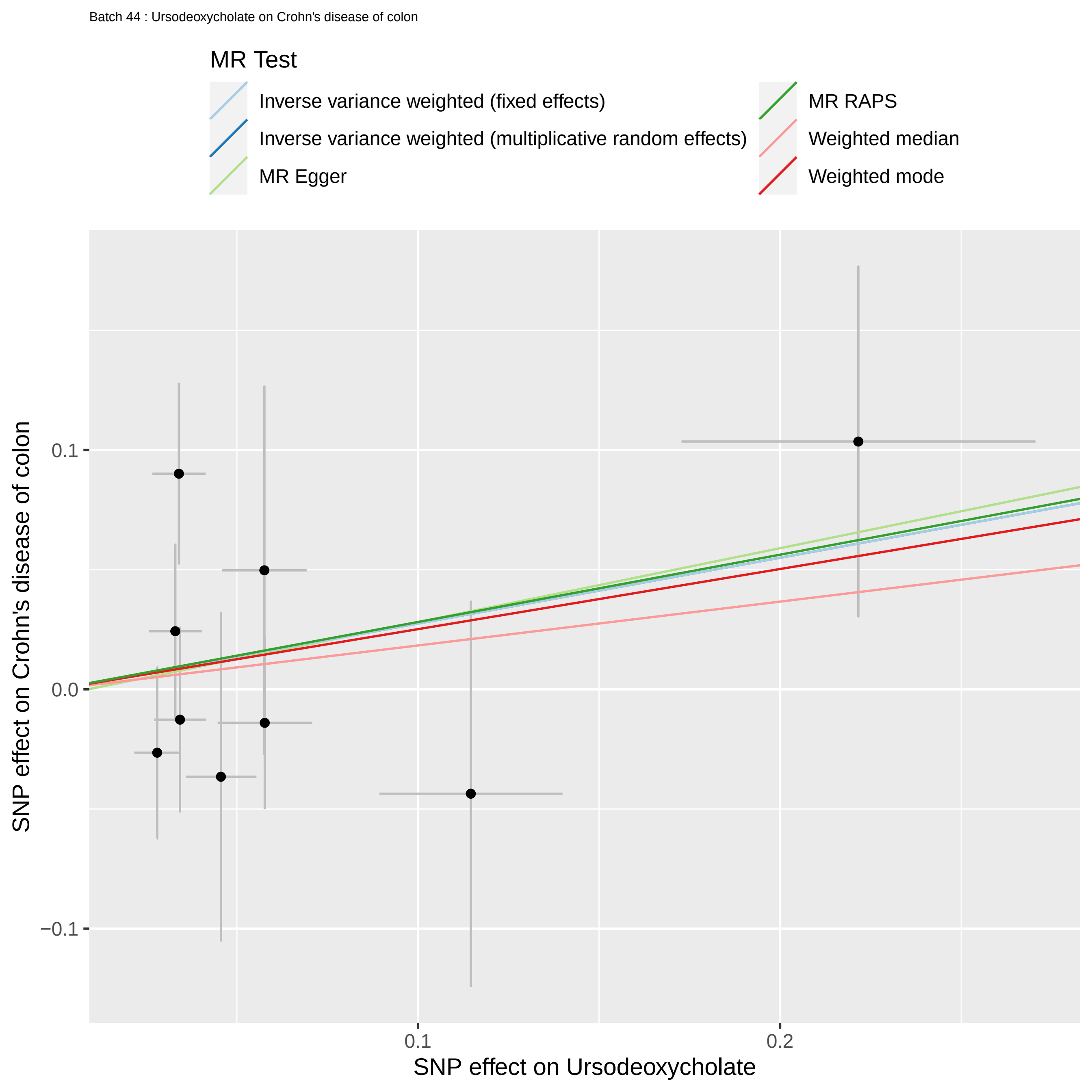

Batch 44 : Ursodeoxycholate on Crohn's disease of colon
MR Test
Inverse variance weighted (fixed effects)
Inverse variance weighted (multiplicative random effects)
MR Egger
MR RAPS
Weighted median
Weighted mode
0.1
0.0
−0.1
SNP effect on Crohn's disease of colon
0.1
0.2
SNP effect on Ursodeoxycholate

Batch 45 : Arginine on Crohn's disease of colon

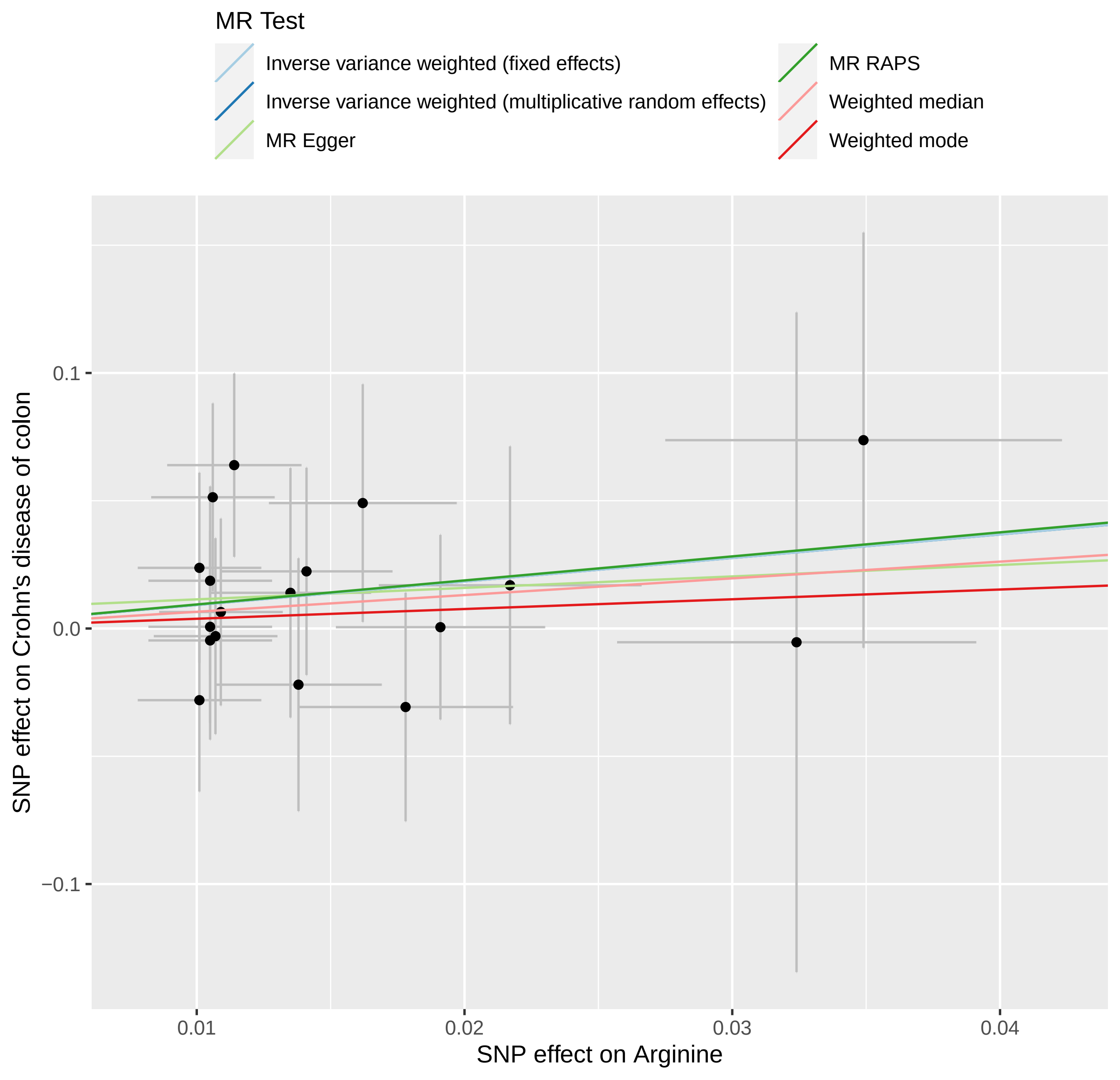

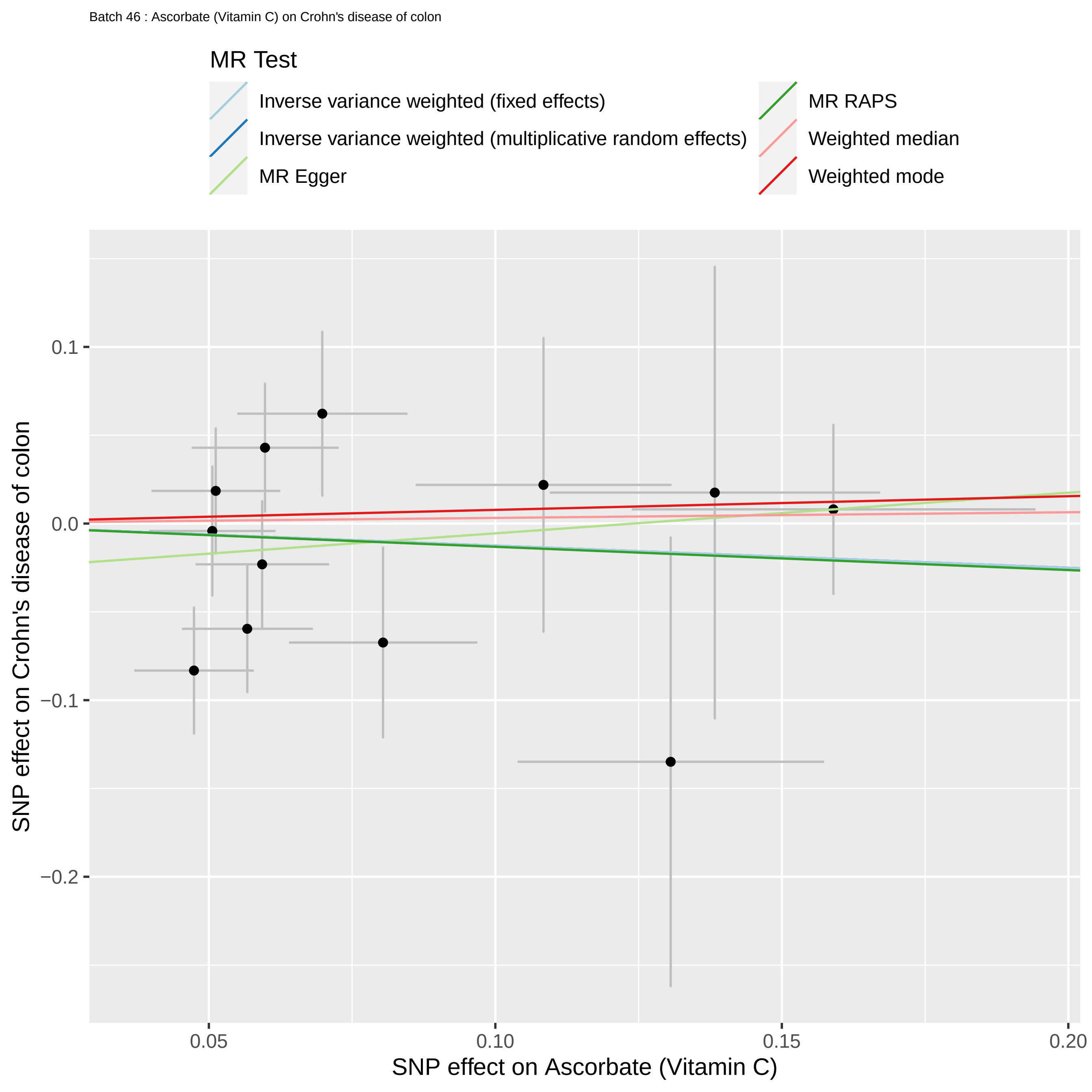
Batch 46 : Ascorbate (Vitamin C) on Crohn's disease of colon
MR Test
Inverse variance weighted (fixed effects)
Inverse variance weighted (multiplicative random effects)
MR Egger
MR RAPS
Weighted median
Weighted mode
0.1
0.0
−0.1
−0.2
0.05
0.10
0.15
0.20
SNP effect on Crohn's disease of colon
SNP effect on Ascorbate (Vitamin C)

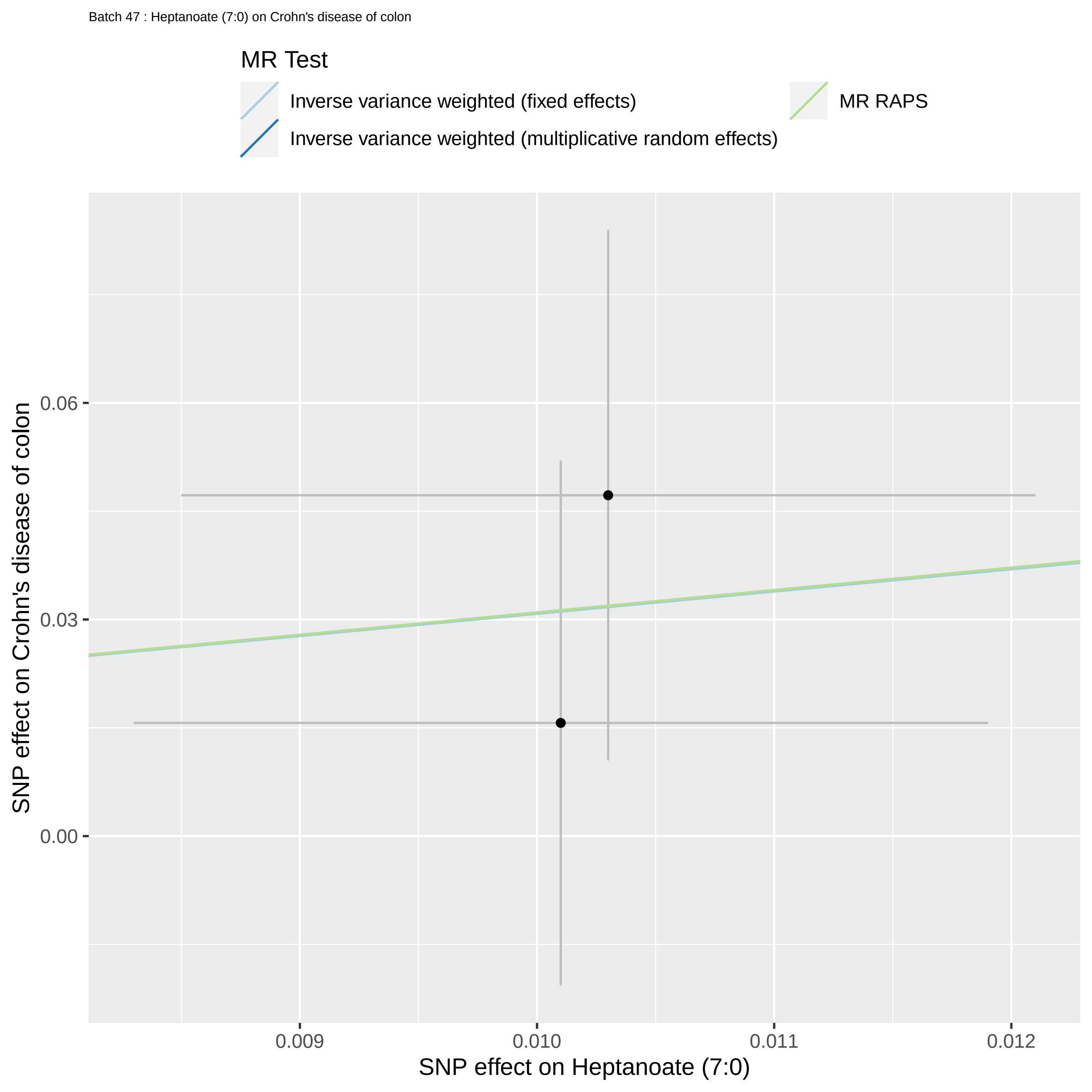
Batch 47 : Heptanoate (7:0) on Crohn's disease of colon
MR Test
Inverse variance weighted (fixed effects)
Inverse variance weighted (multiplicative random effects)
MR RAPS
0.06
0.03
0.00
0.009
0.010
0.011
0.012
SNP effect on Heptanoate (7:0)
SNP effect on Crohn's disease of colon

Batch 48 : Laurate (12:0) on Crohn's disease of colon

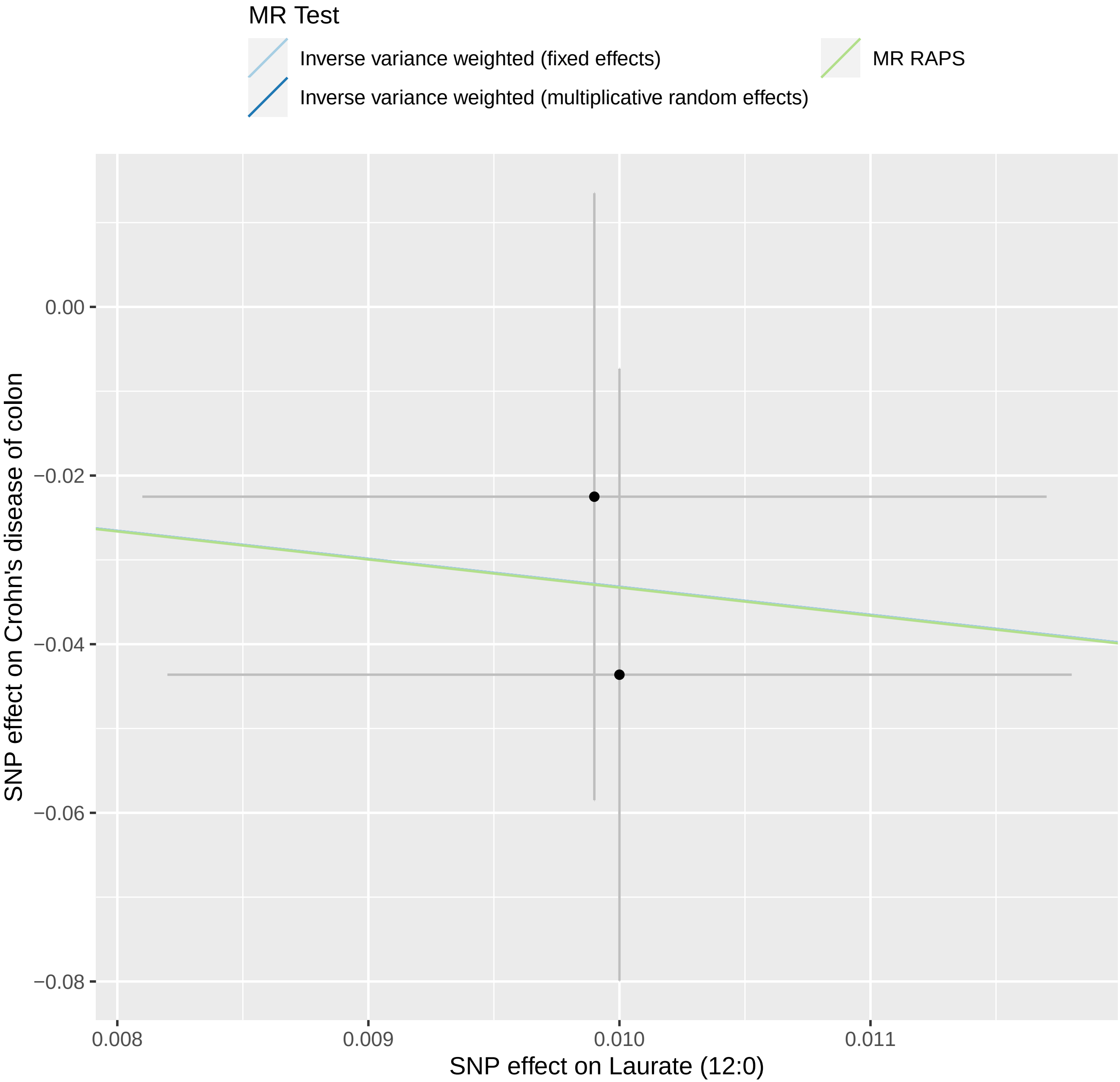

Batch 50 : Urea on Crohn's disease of colon

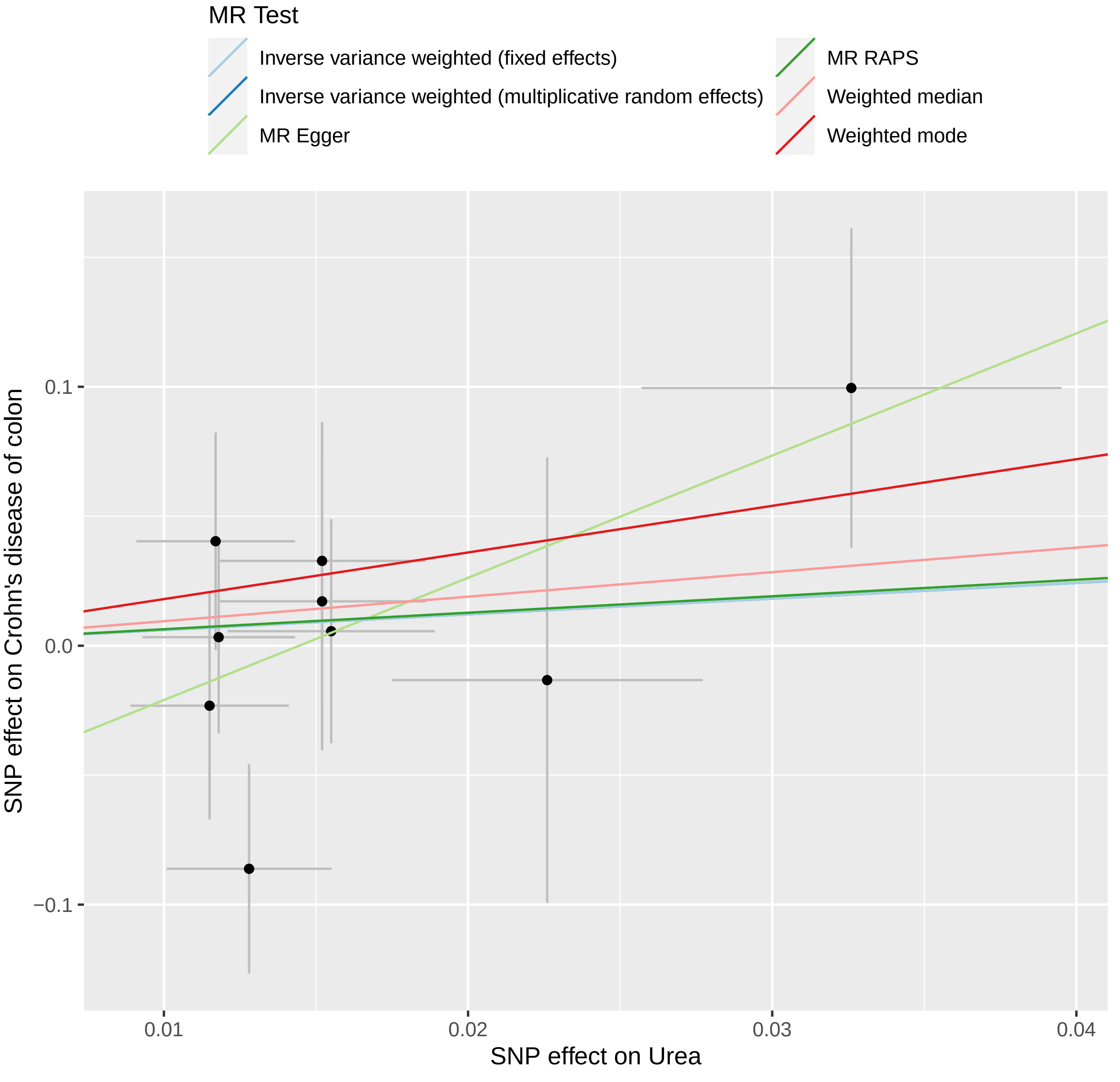

Batch 51 : Cortisol on Crohn's disease of colon

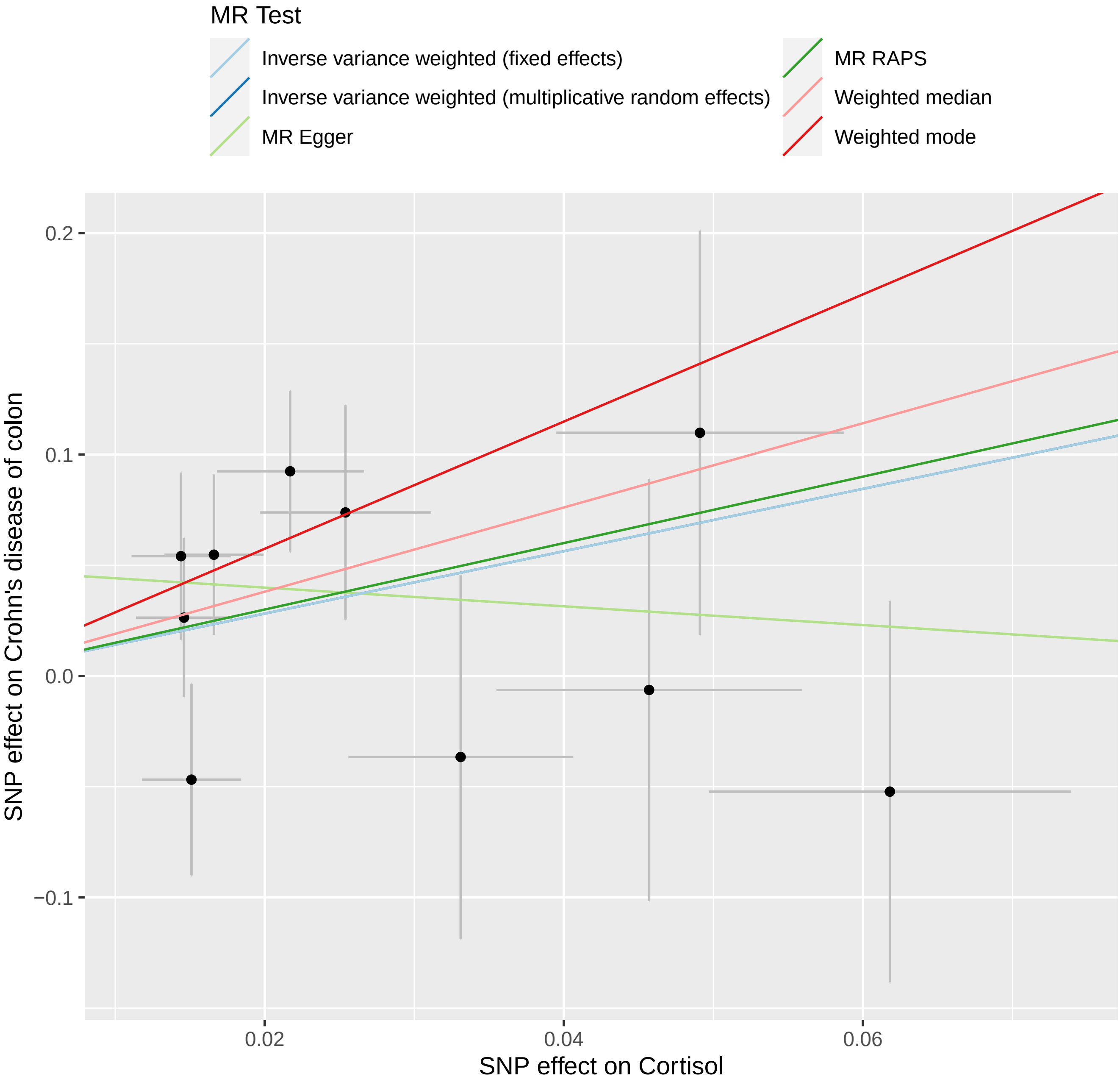

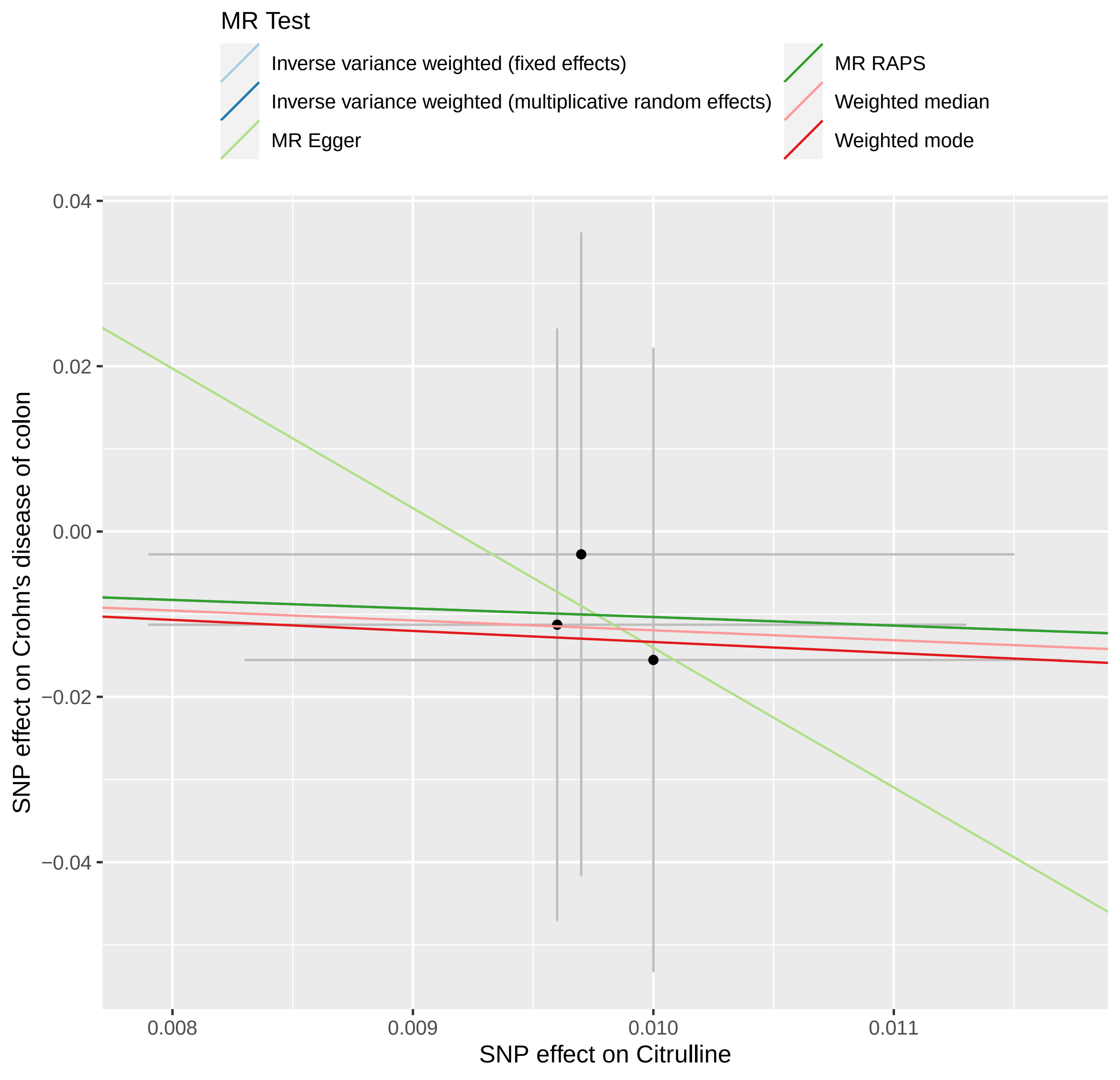
MR Test
Inverse variance weighted (fixed effects)
Inverse variance weighted (multiplicative random effects)
MR Egger
MR RAPS
Weighted median
Weighted mode
0.04
0.02
0.00
−0.02
−0.04
0.008
0.009
0.010
0.011
SNP effect on Citrulline
SNP effect on Crohn's disease of colon

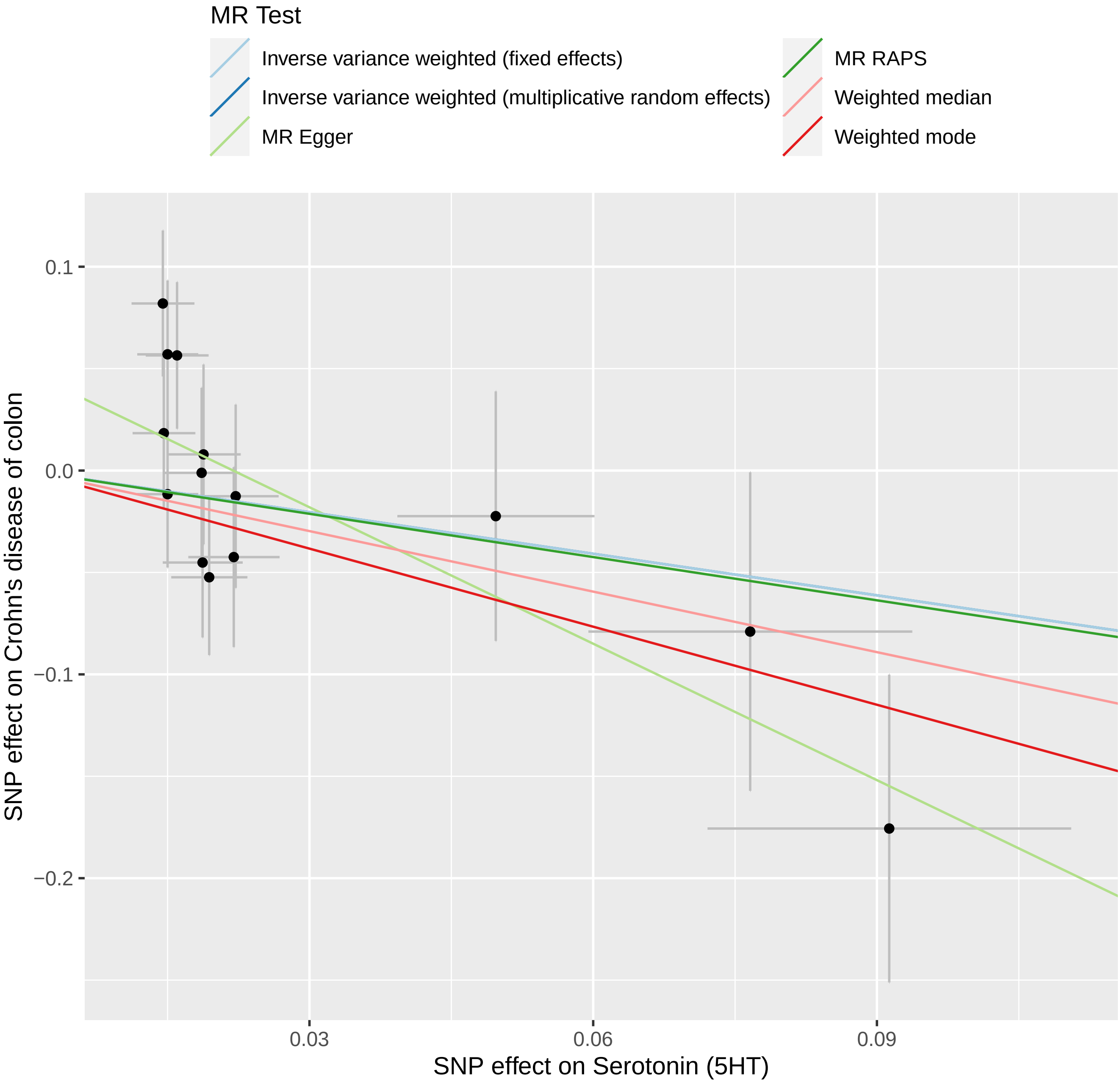
MR Test
Inverse variance weighted (fixed effects)
Inverse variance weighted (multiplicative random effects)
MR Egger
MR RAPS
Weighted median
Weighted mode
0.1
0.0
−0.1
−0.2
SNP effect on Crohn's disease of colon
0.03
0.06
0.09
SNP effect on Serotonin (5HT)

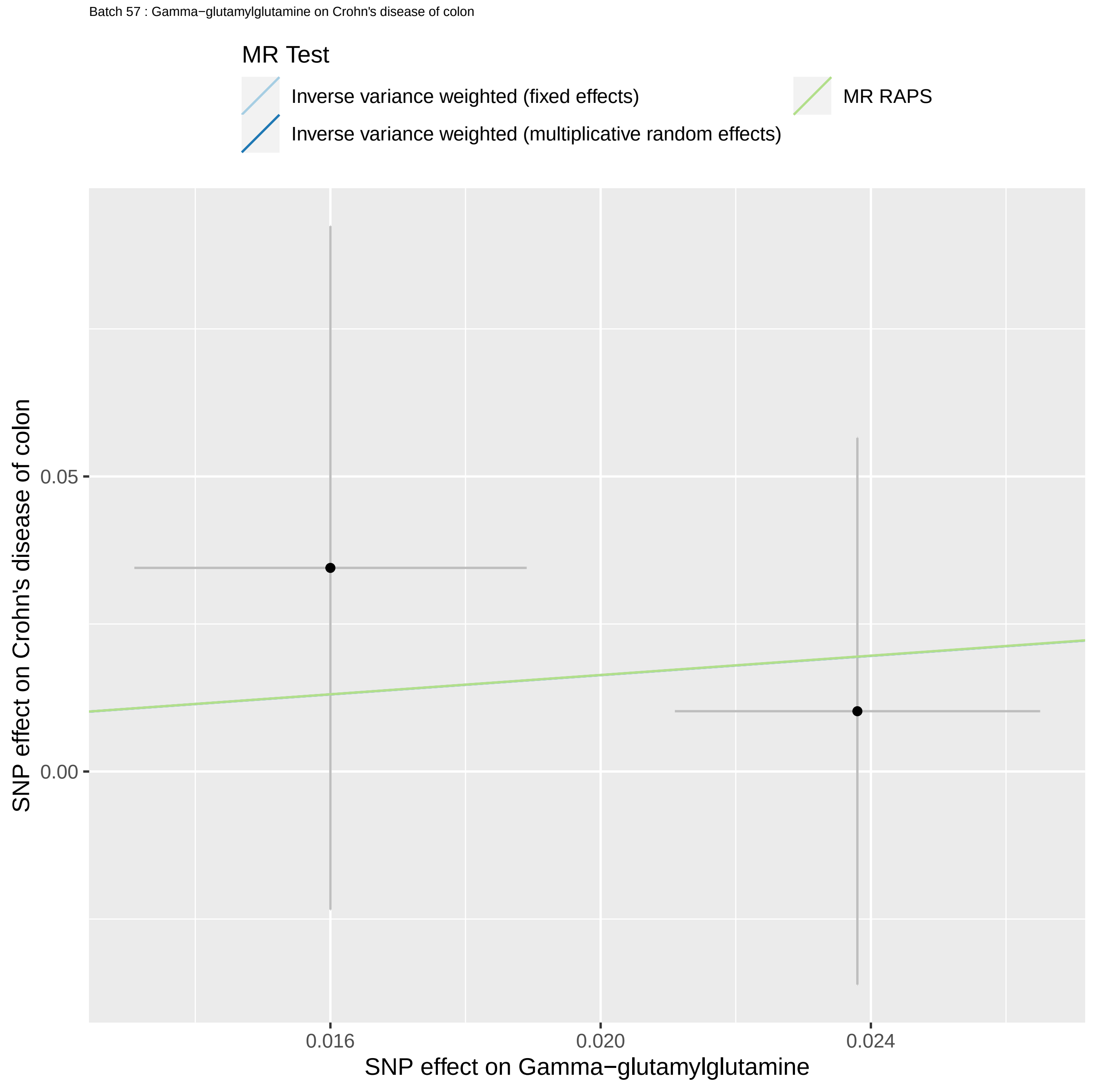
Batch 57 : Gamma−glutamylglutamine on Crohn's disease of colon
MR Test
Inverse variance weighted (fixed effects)
Inverse variance weighted (multiplicative random effects)
MR RAPS
0.05
0.00
0.016
0.020
0.024
SNP effect on Crohn's disease of colon
SNP effect on Gamma−glutamylglutamine

Batch 58 : Gamma−glutamyltyrosine on Crohn's disease of colon

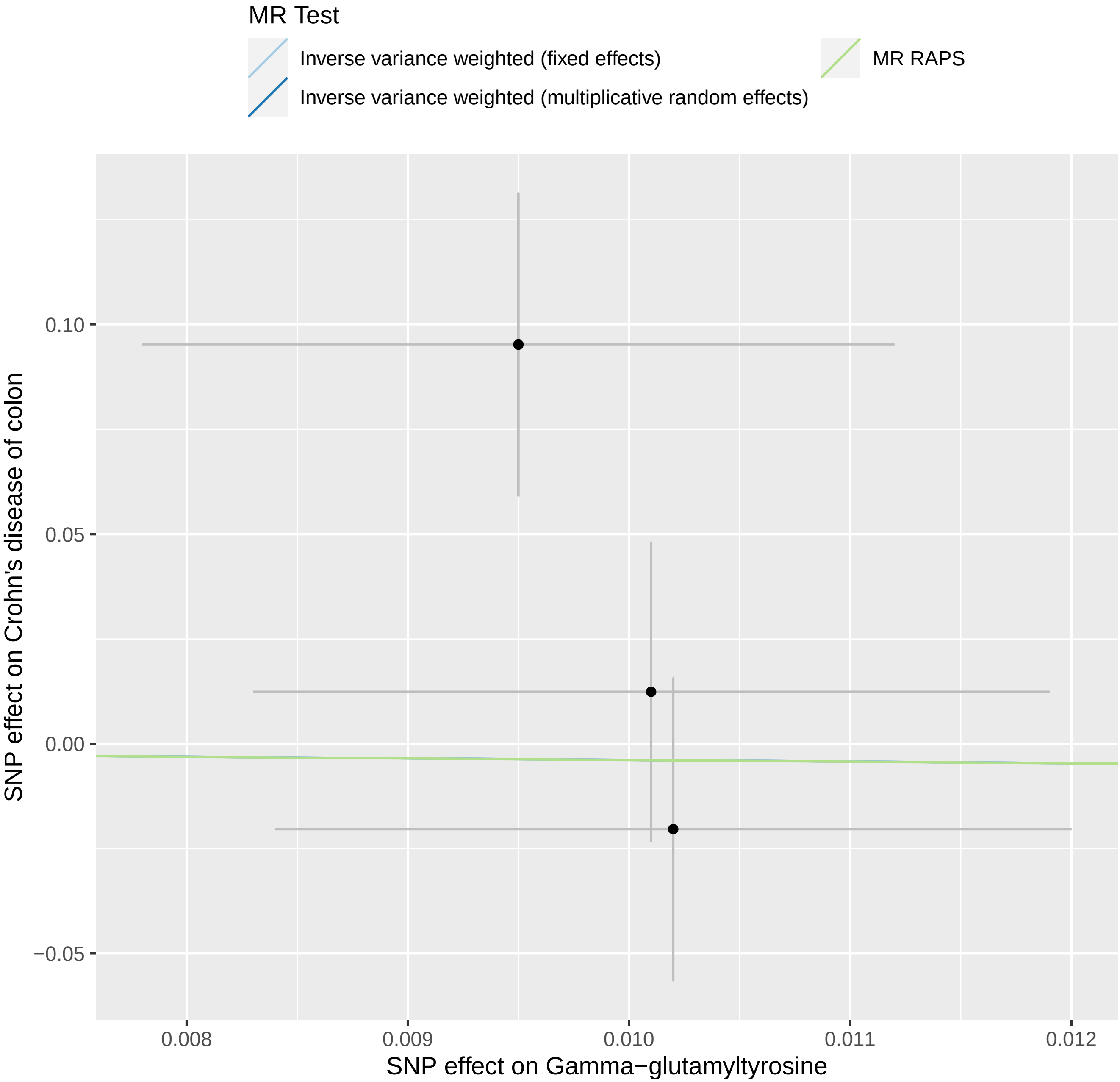

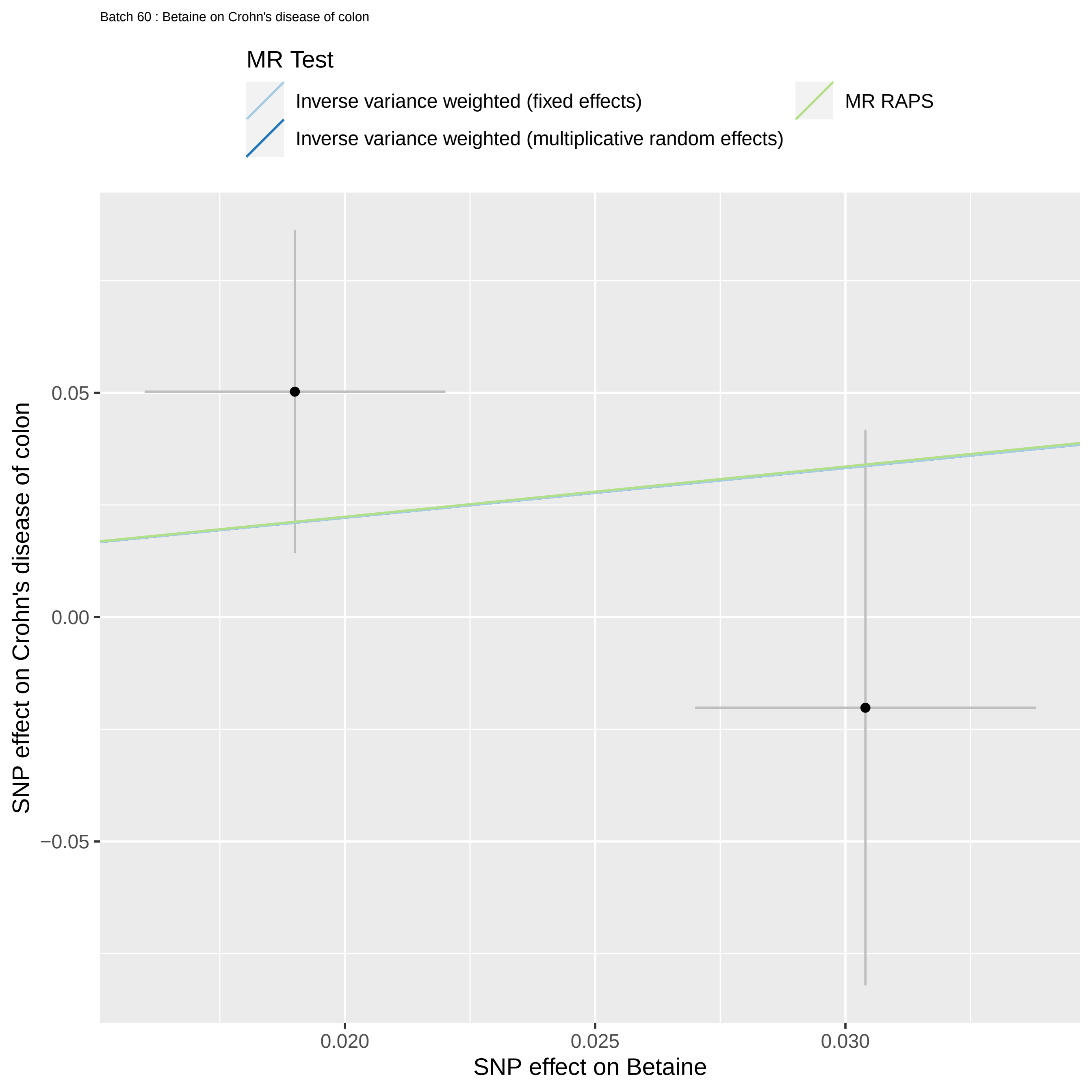
Batch 60 : Betaine on Crohn's disease of colon
MR Test
Inverse variance weighted (fixed effects)
Inverse variance weighted (multiplicative random effects)
MR RAPS
0.05
0.00
−0.05
0.020
0.025
0.030
SNP effect on Betaine
SNP effect on Crohn's disease of colon

Batch 61 : Xanthine on Crohn's disease of colon

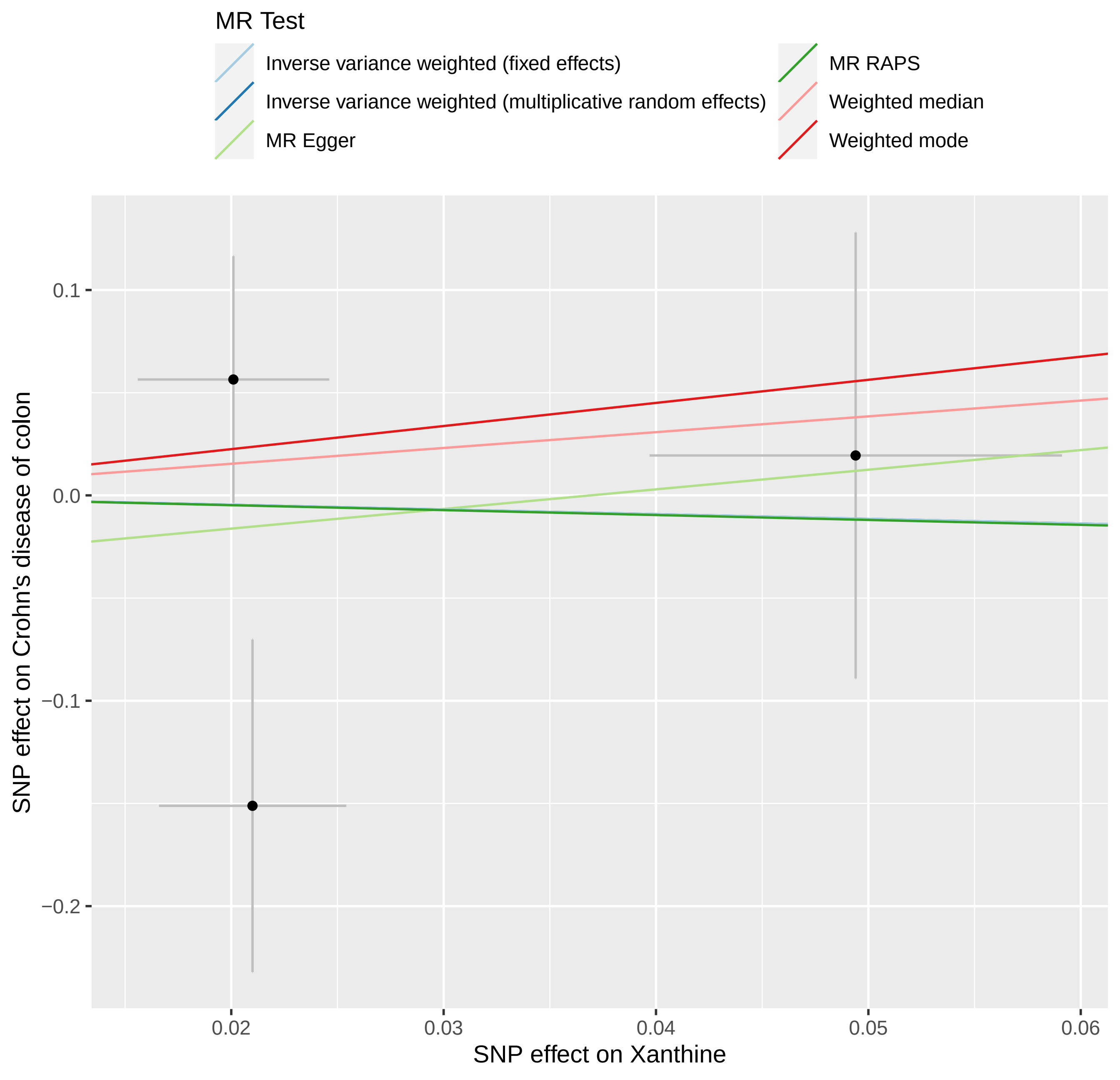

Batch 62 : Phosphate on Crohn's disease of colon

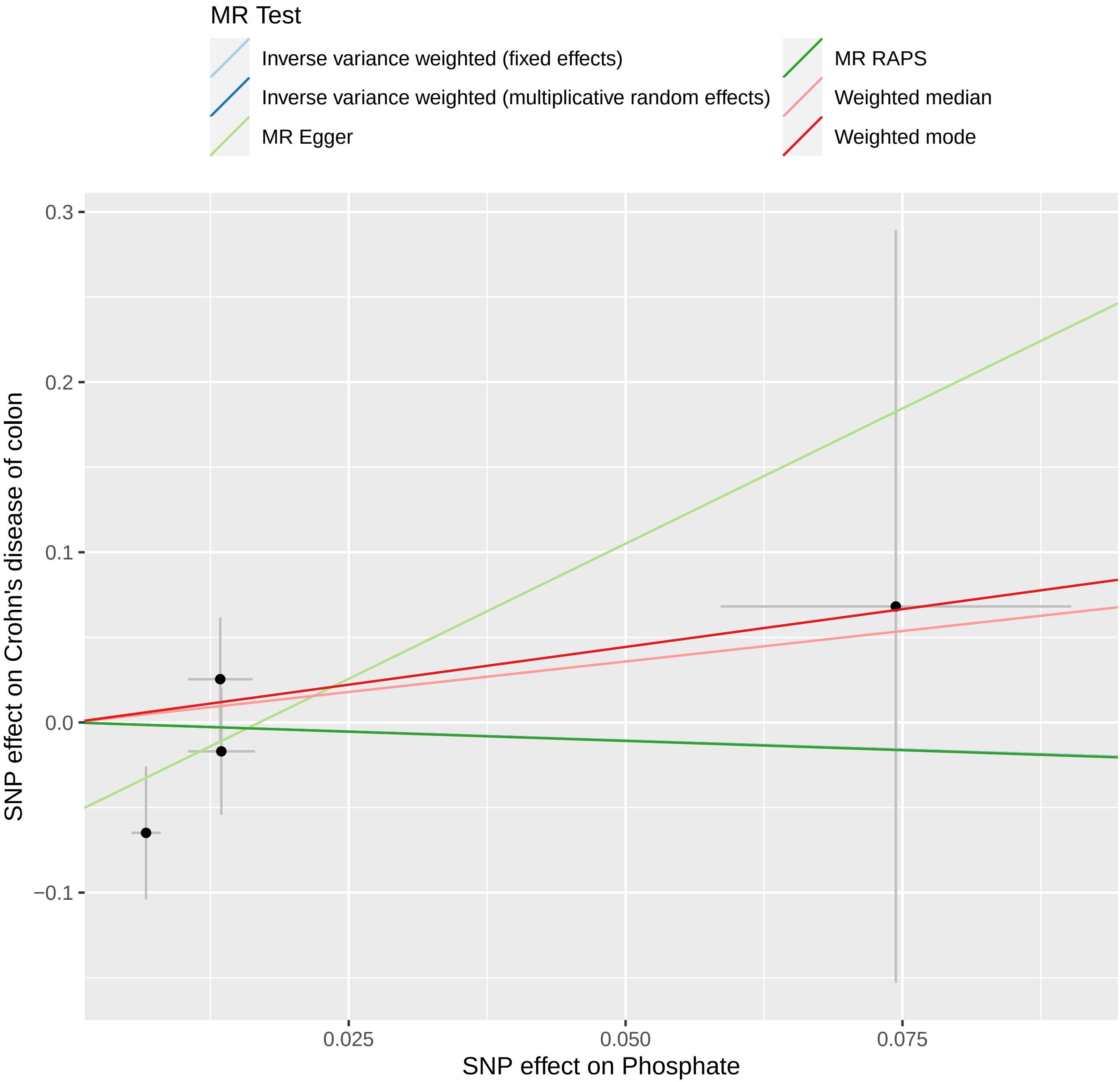

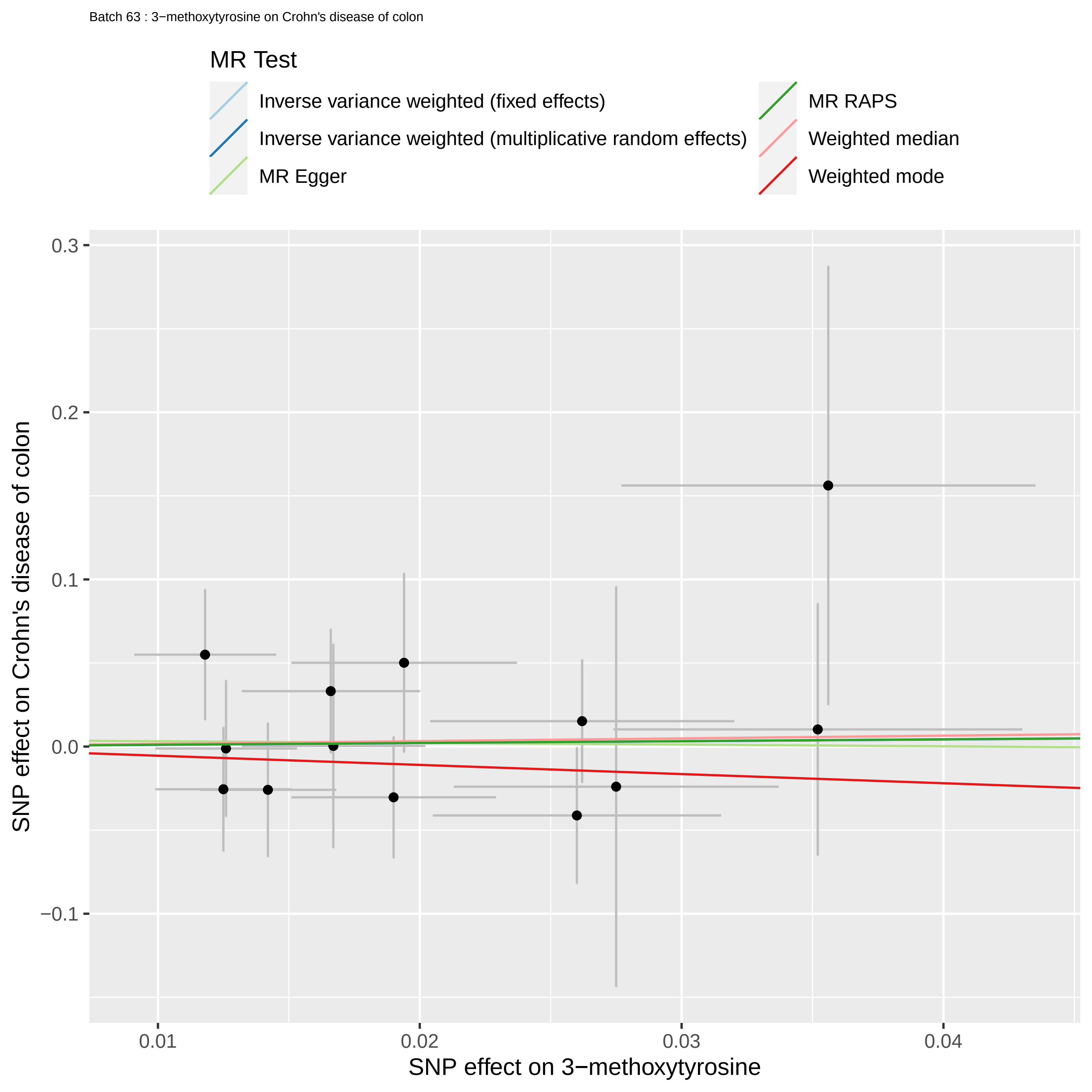

Batch 63 : 3−methoxytyrosine on Crohn's disease of colon
MR Test
Inverse variance weighted (fixed effects)
Inverse variance weighted (multiplicative random effects)
MR Egger
MR RAPS
Weighted median
Weighted mode
SNP effect on Crohn's disease of colon
0.3
0.2
0.1
0.0
−0.1
0.01
0.02
0.03
0.04
SNP effect on 3−methoxytyrosine

Batch 65 : Undecanoate (11:0) on Crohn's disease of colon

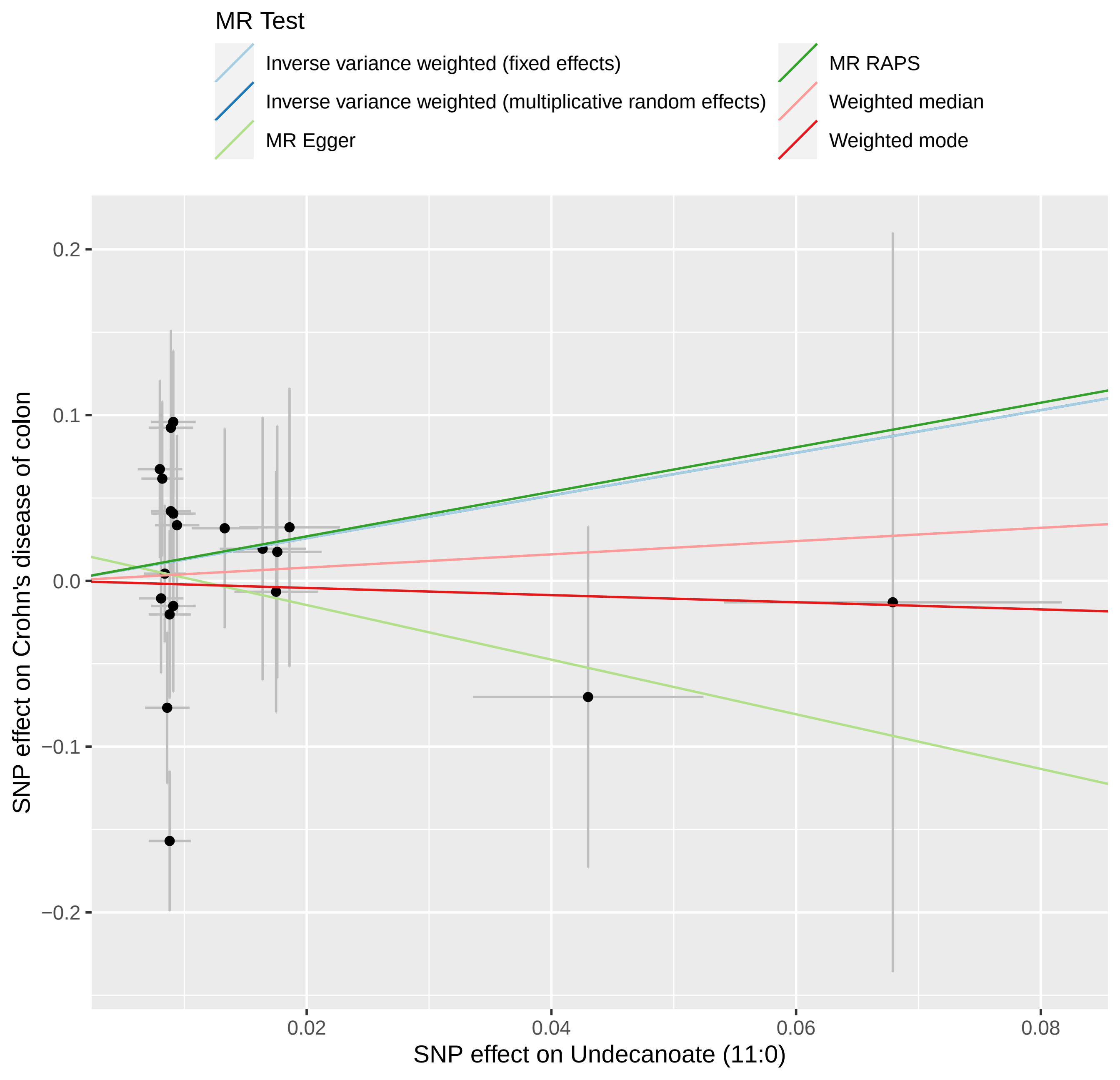

Batch 67 : Taurodeoxycholate on Crohn's disease of colon

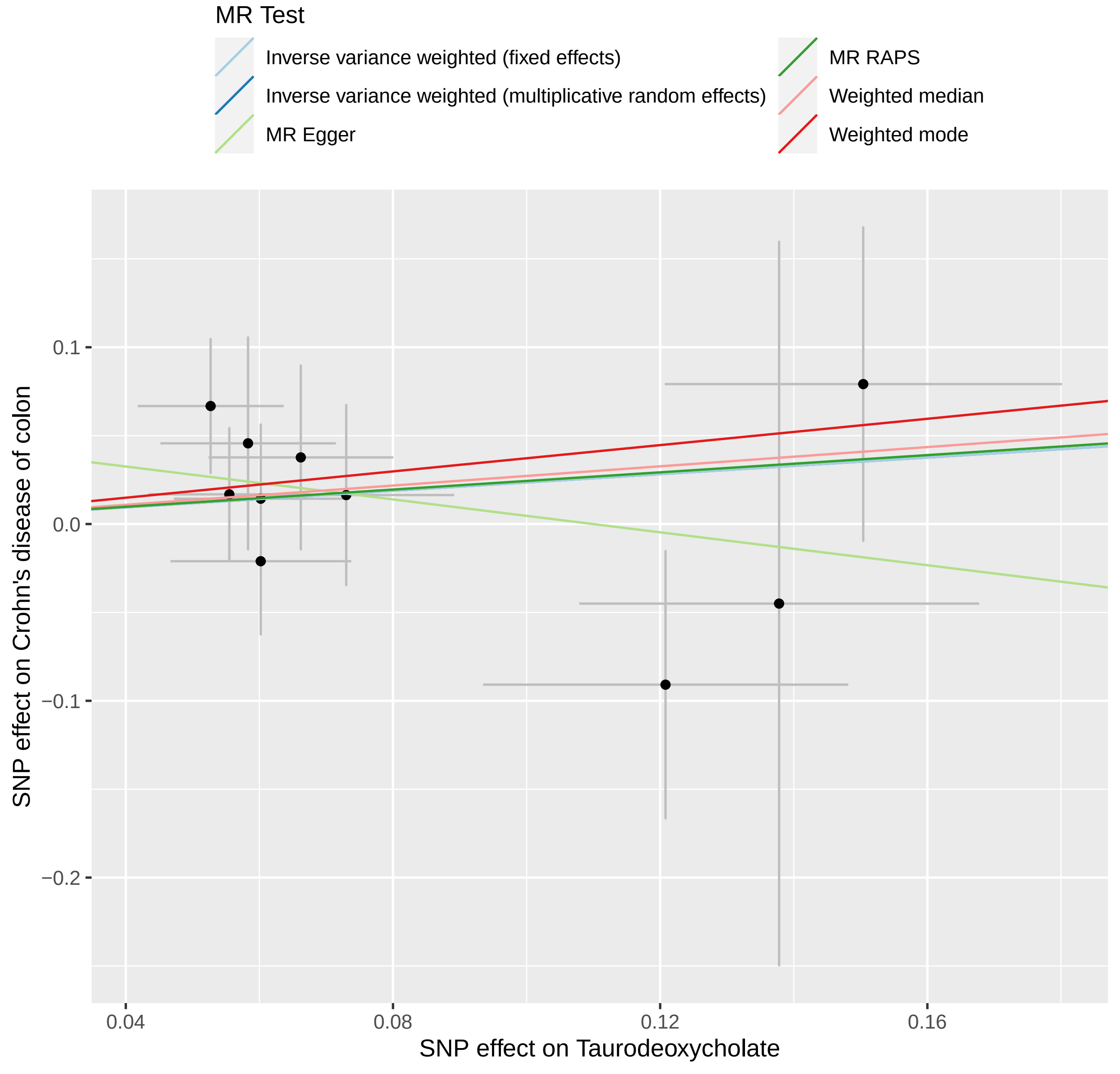

Batch 68 : Glycerol on Crohn's disease of colon

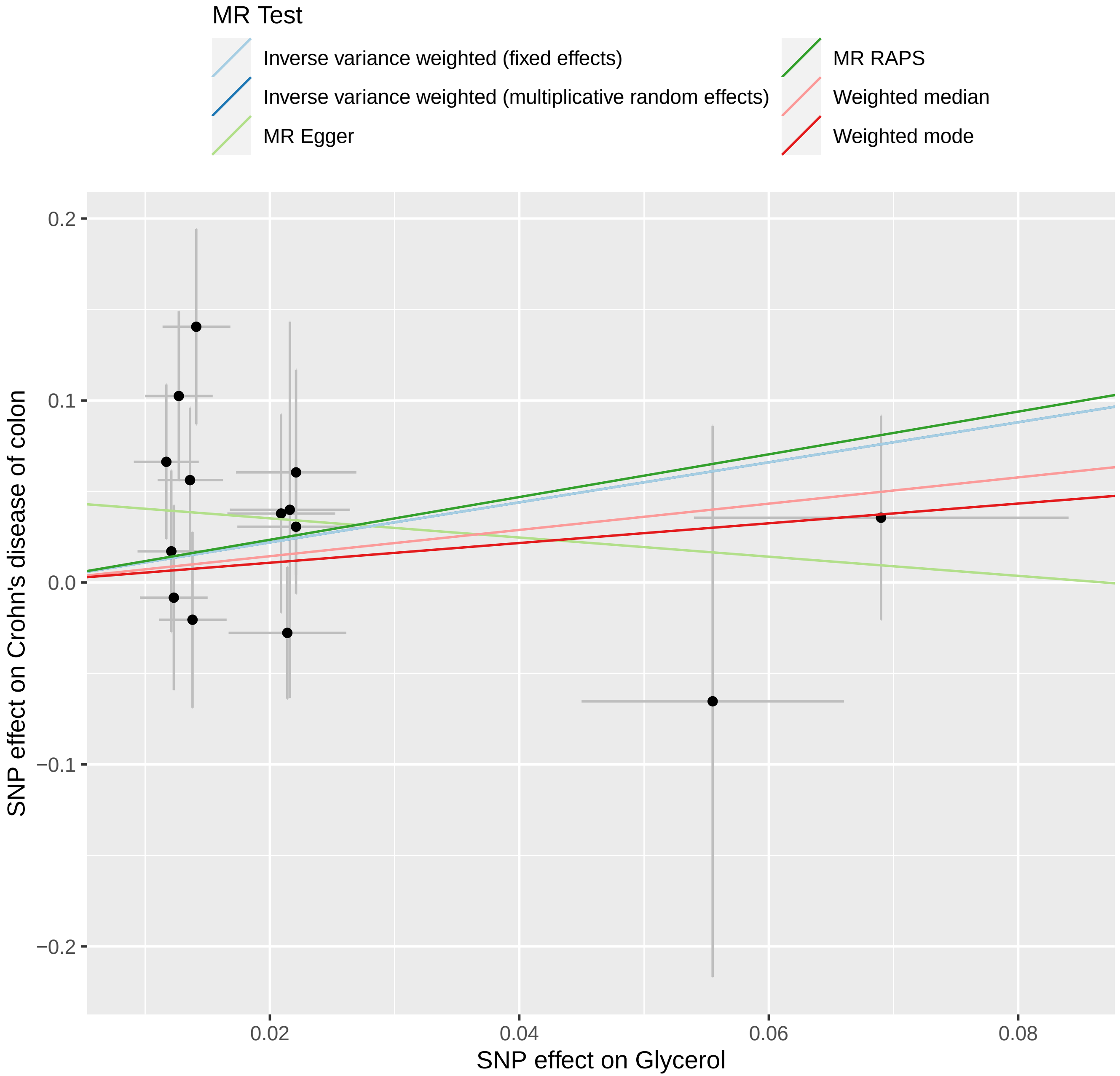

Batch 69 : Kynurenine on Crohn's disease of colon

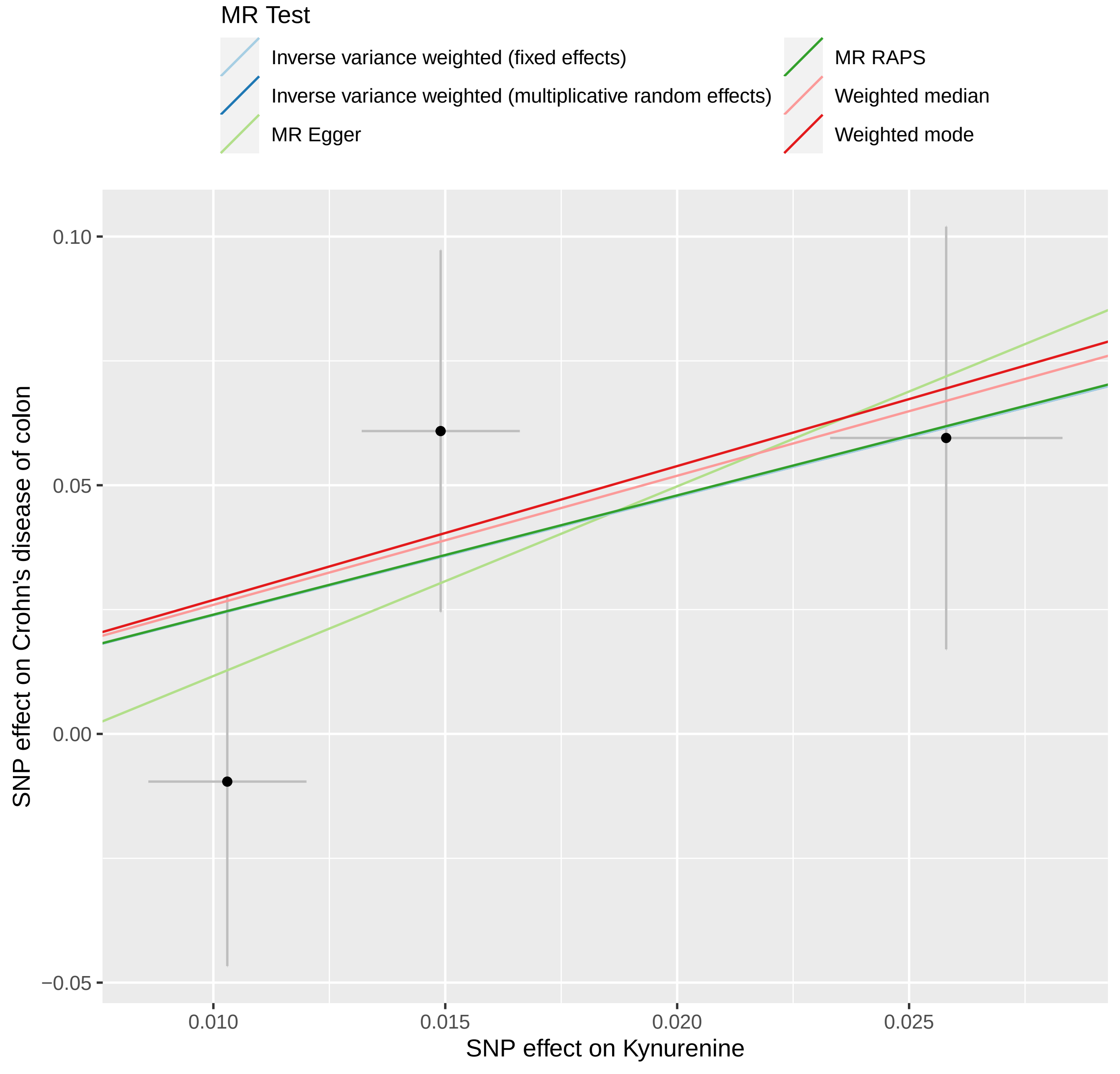

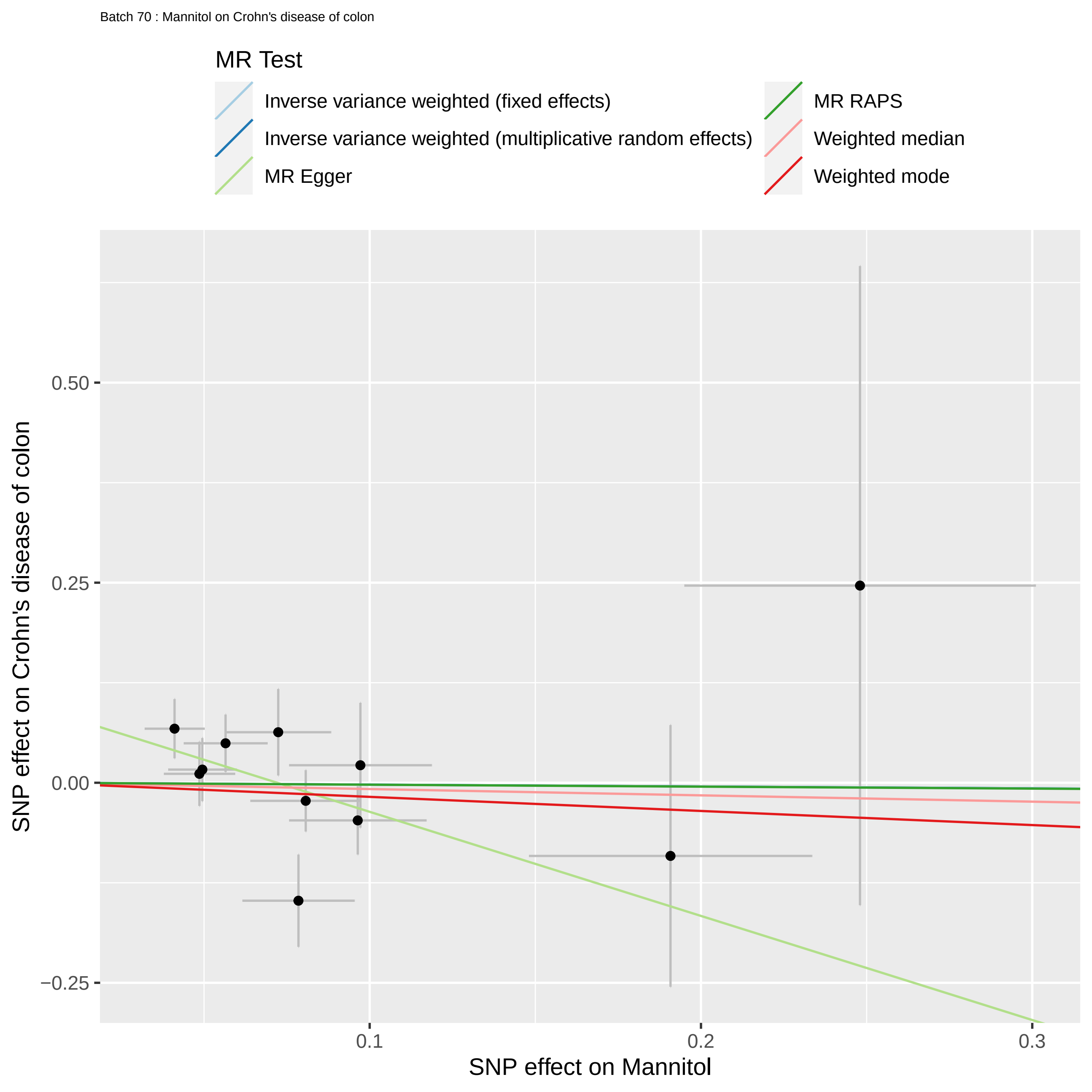

Batch 70 : Mannitol on Crohn's disease of colon
MR Test
Inverse variance weighted (fixed effects)
Inverse variance weighted (multiplicative random effects)
MR Egger
MR RAPS
Weighted median
Weighted mode
SNP effect on Crohn's disease of colon
0.50
0.25
0.00
−0.25
0.1
0.2
0.3
SNP effect on Mannitol

Batch 71 : Glycerol 3−phosphate (G3P) on Crohn's disease of colon

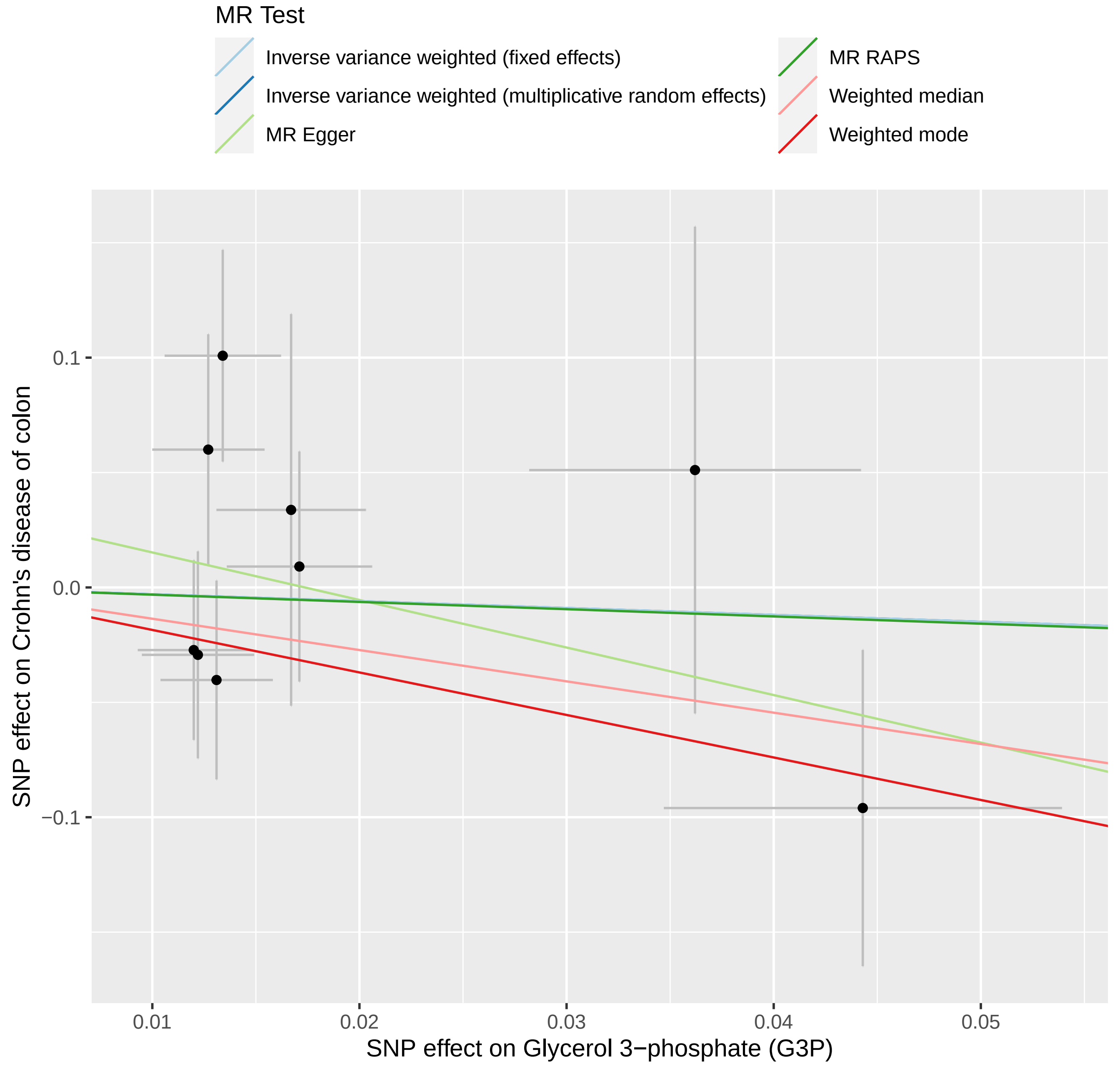

Batch 73 : Carnitine on Crohn's disease of colon

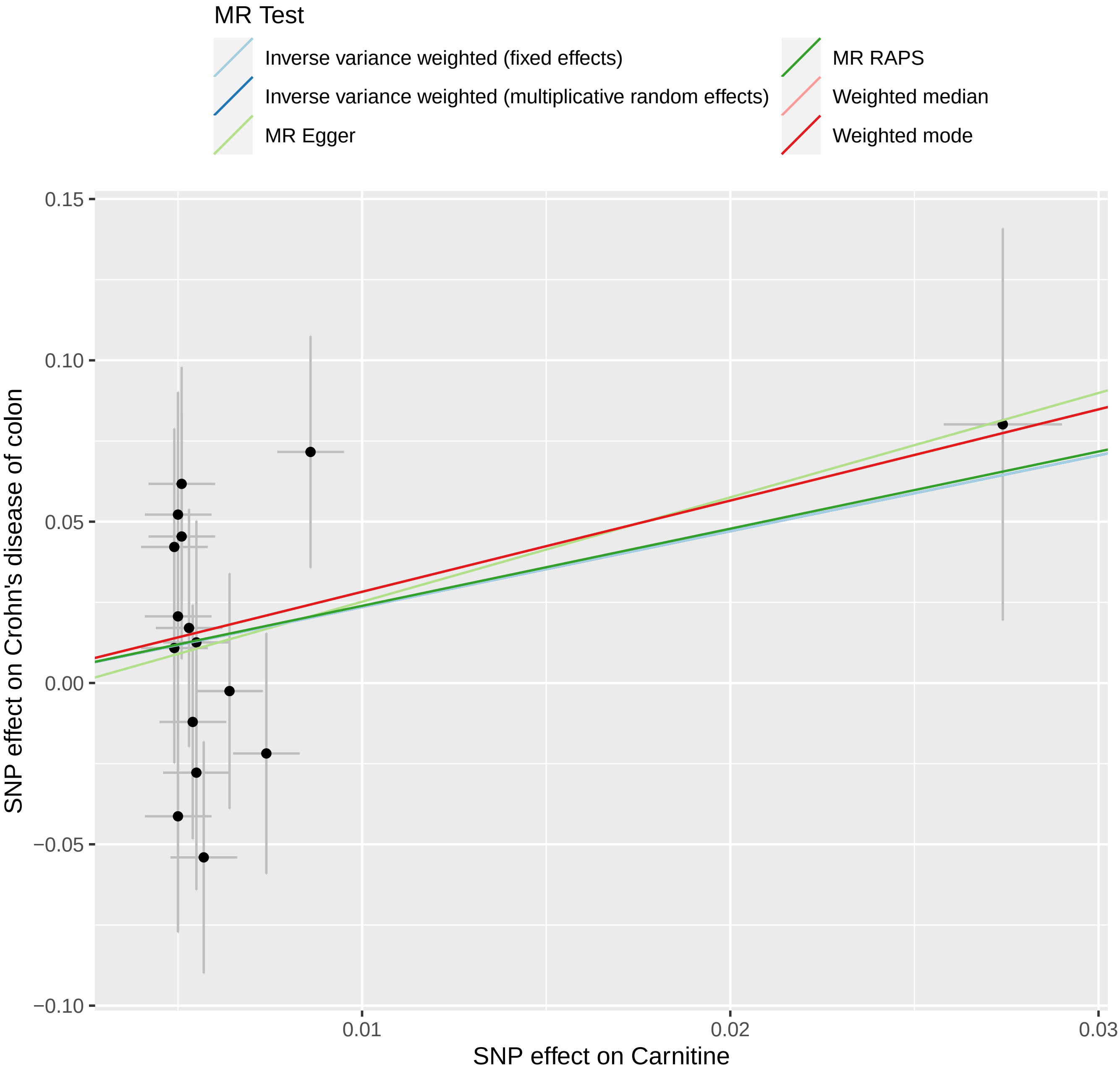

Batch 74 : Choline on Crohn's disease of colon

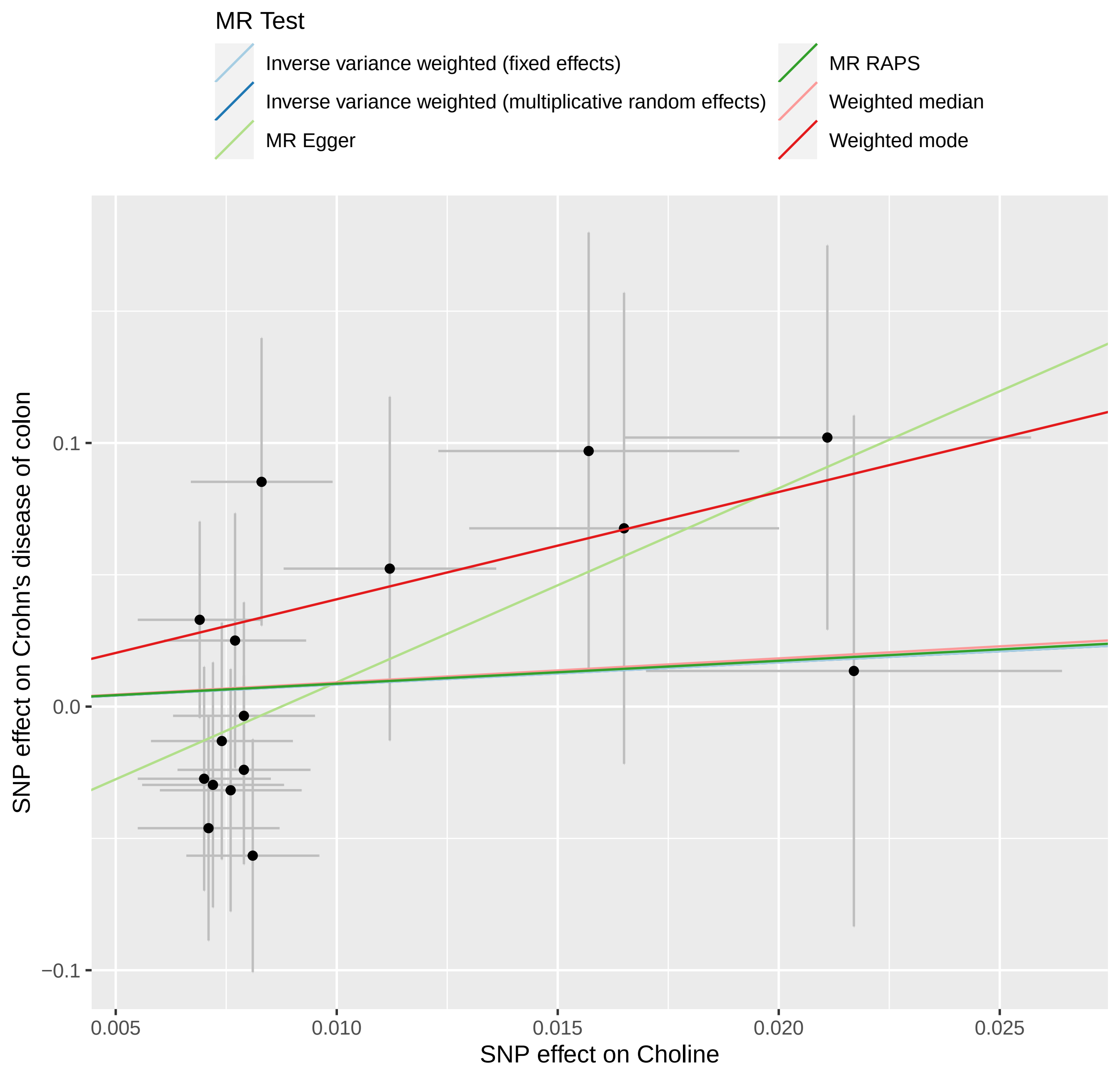

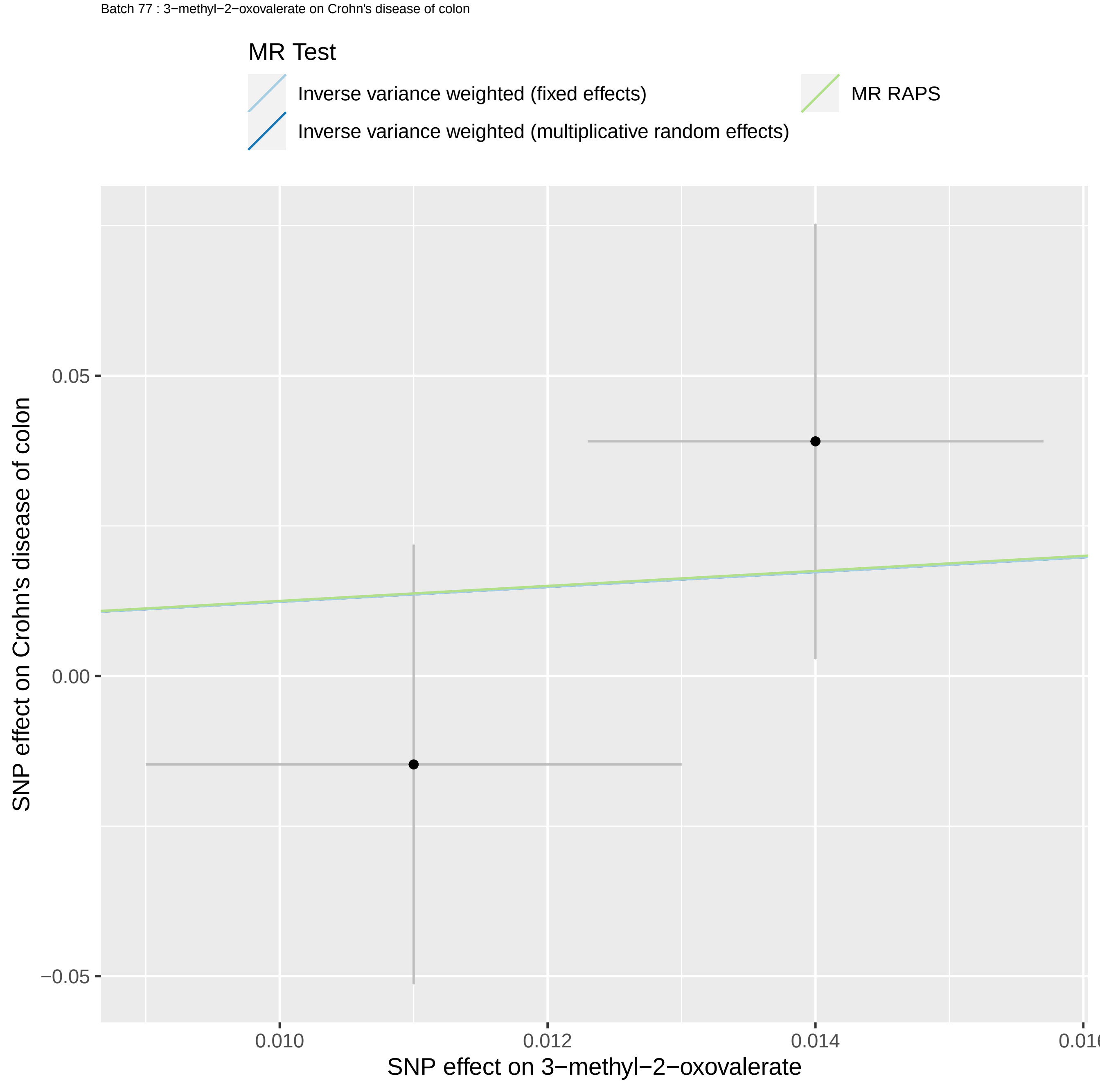

Batch 77 : 3−methyl−2−oxovalerate on Crohn's disease of colon
MR Test
Inverse variance weighted (fixed effects)
Inverse variance weighted (multiplicative random effects)
MR RAPS
SNP effect on Crohn's disease of colon
0.05
0.00
−0.05
0.010
0.012
0.014
SNP effect on 3−methyl−2−oxovalerate

Batch 78 : 3−methylhistidine on Crohn's disease of colon

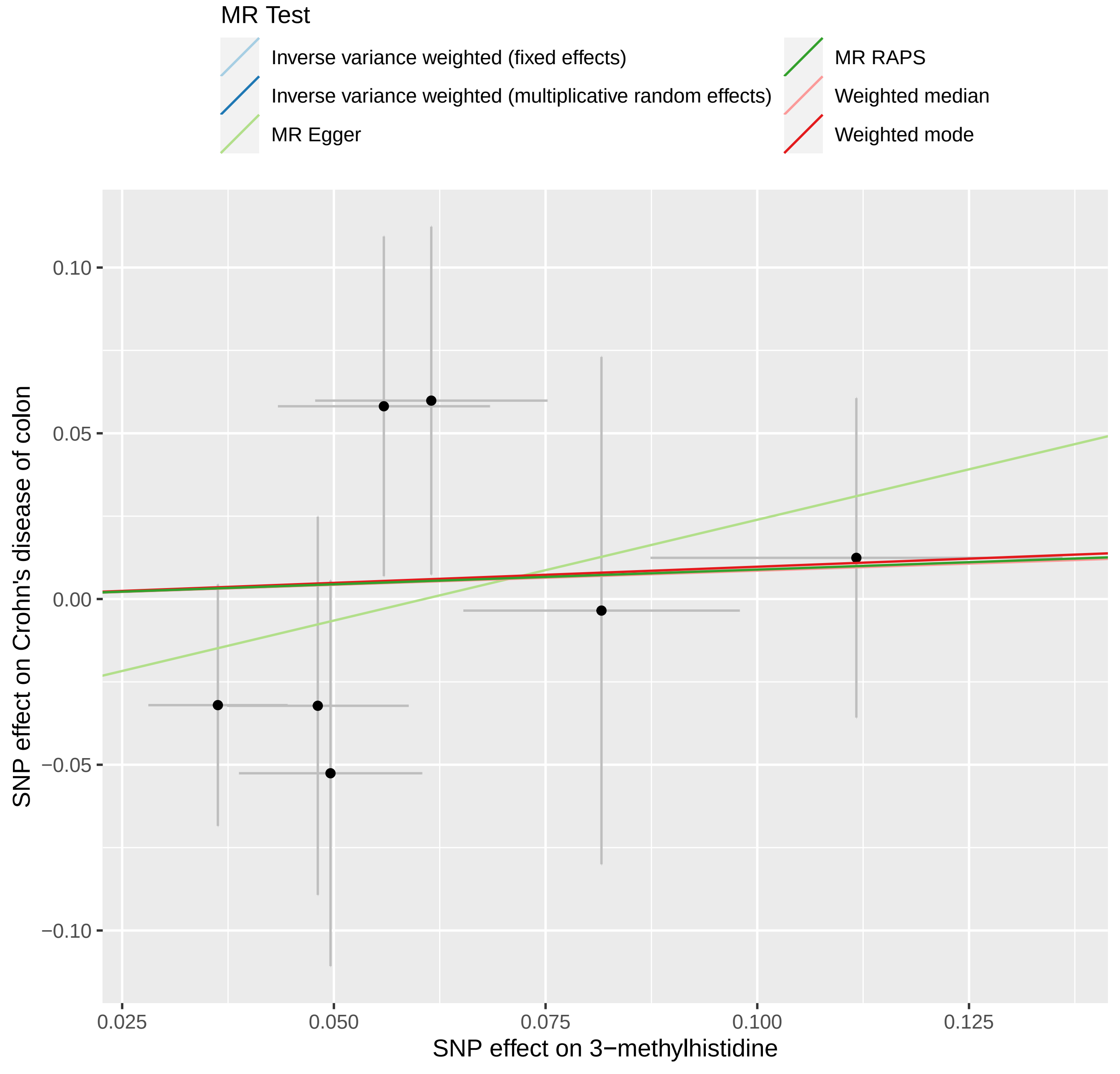

Batch 80 : Phenylacetate on Crohn's disease of colon

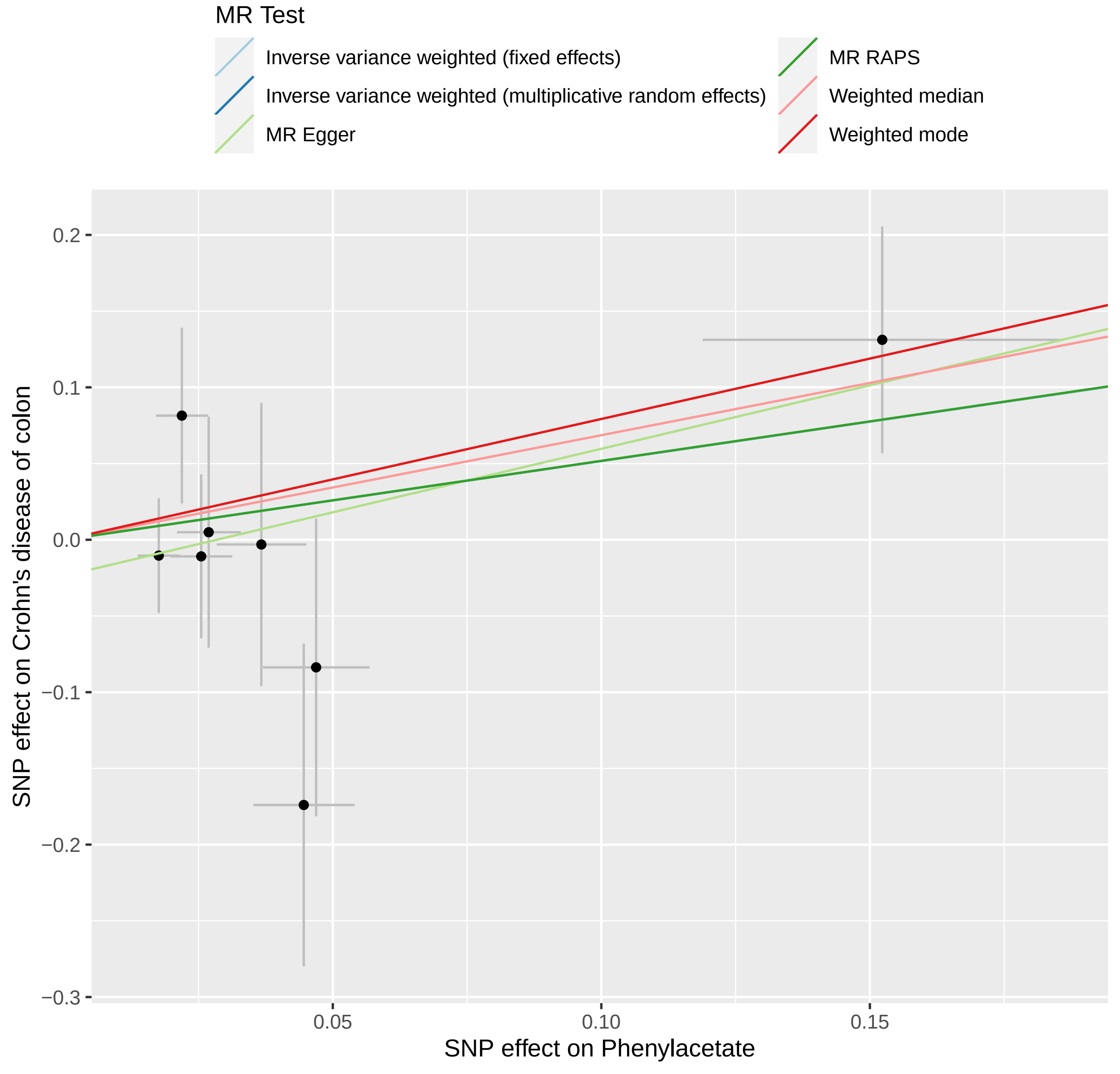

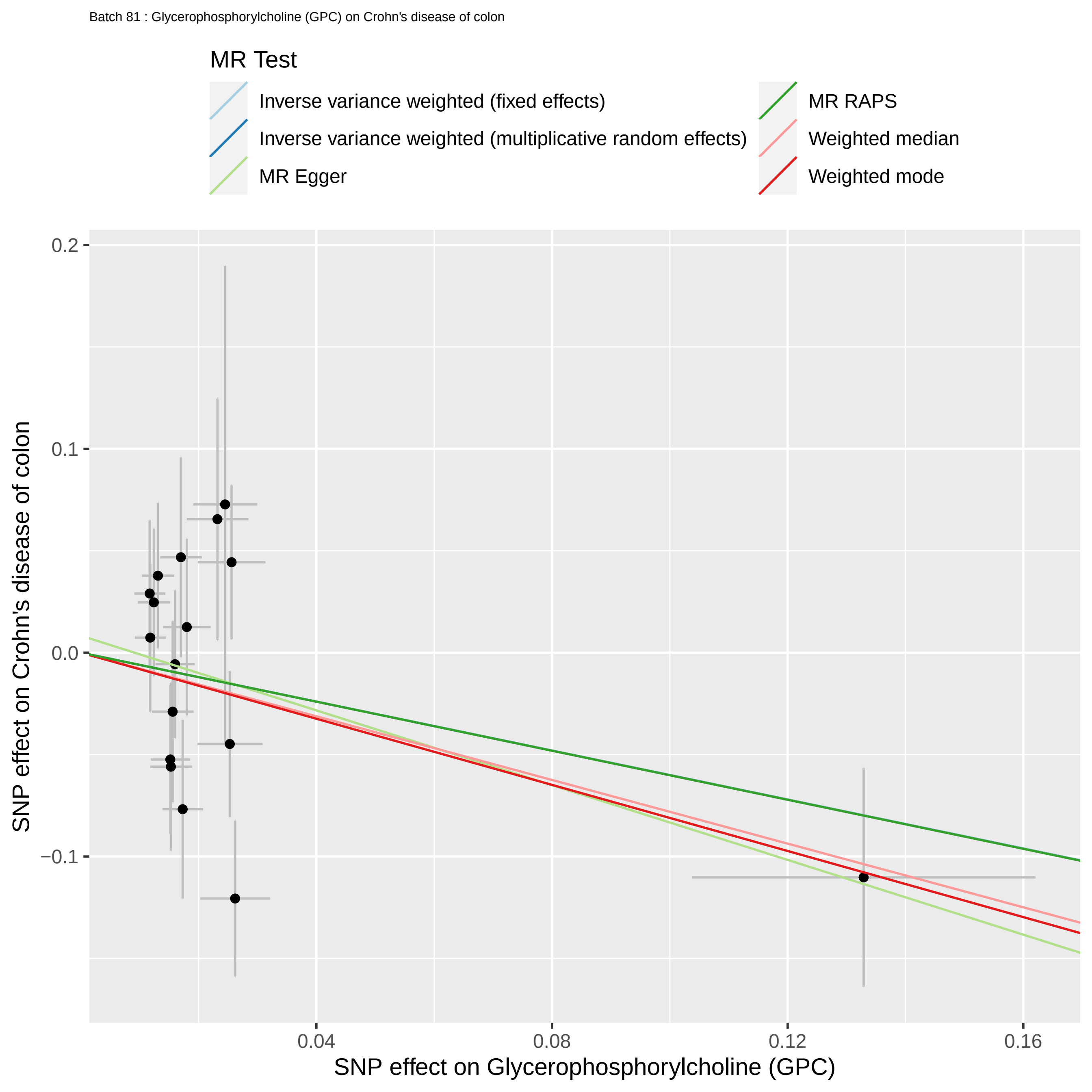

Batch 81 : Glycerophosphorylcholine (GPC) on Crohn's disease of colon
MR Test
Inverse variance weighted (fixed effects)
Inverse variance weighted (multiplicative random effects)
MR Egger
MR RAPS
Weighted median
Weighted mode
0.2
0.1
0.0
−0.1
0.04
0.08
0.12
0.16
SNP effect on Crohn's disease of colon
SNP effect on Glycerophosphorylcholine (GPC)

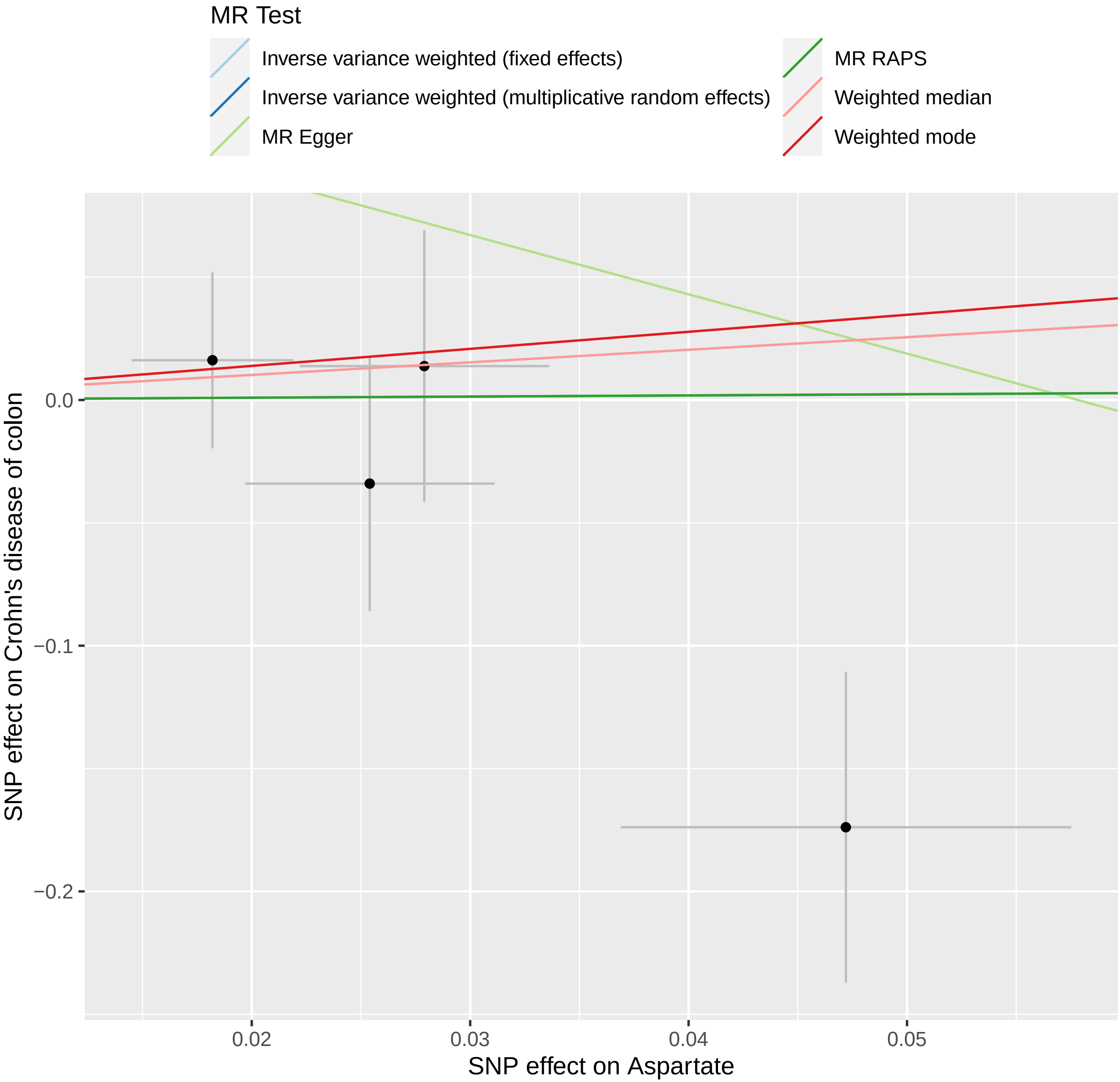
MR Test
Inverse variance weighted (fixed effects)
Inverse variance weighted (multiplicative random effects)
MR Egger
MR RAPS
Weighted median
Weighted mode
0.0
−0.1
−0.2
0.02
0.03
0.04
0.05
SNP effect on Aspartate
SNP effect on Crohn's disease of colon

Batch 84 : Dihomo−linoleate (20:2n6) on Crohn's disease of colon

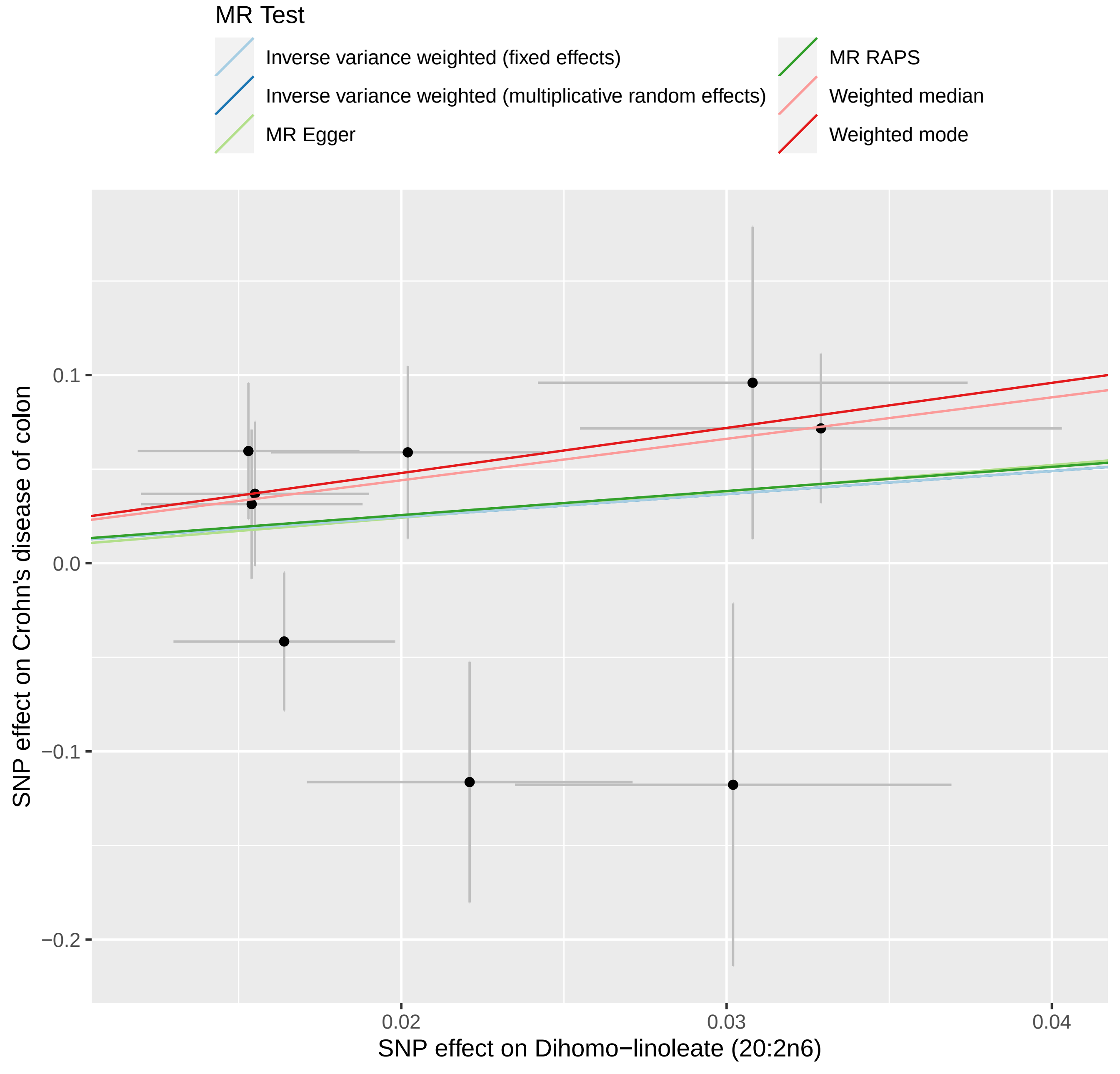

Batch 85 : 2−hydroxystearate on Crohn's disease of colon

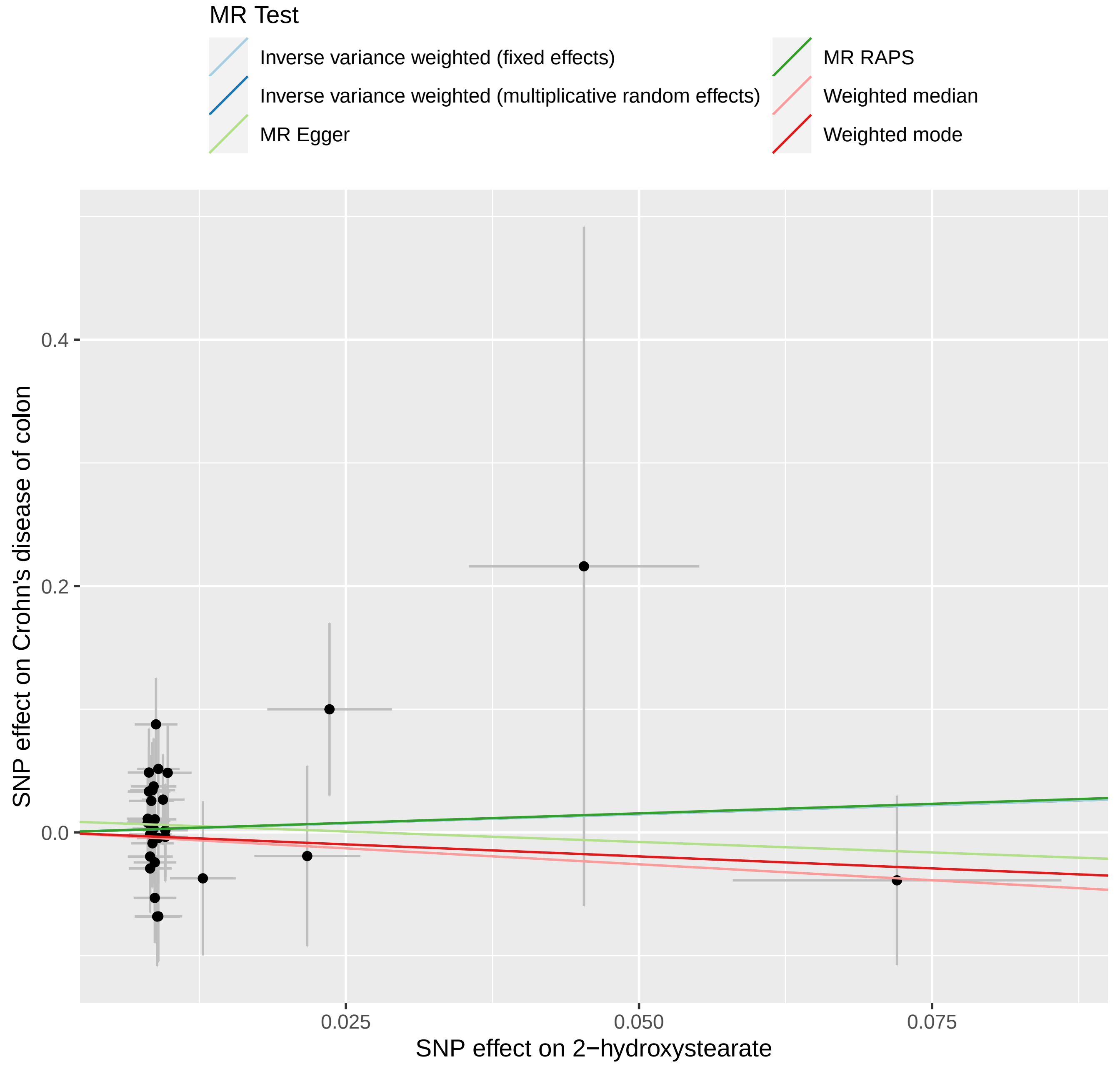

Batch 87 : Glycylvaline on Crohn's disease of colon

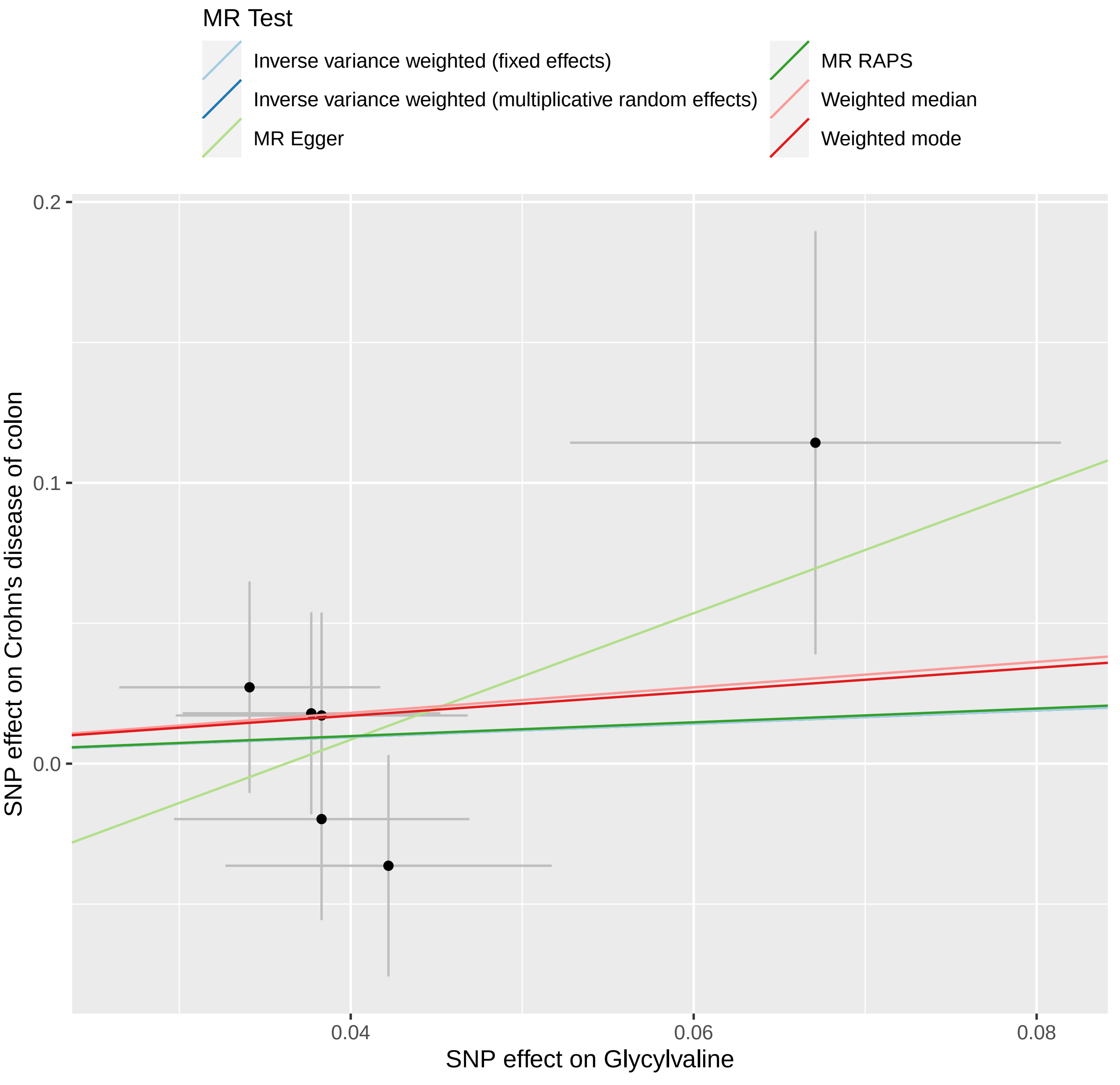

Batch 88 : Gamma−glutamylleucine on Crohn's disease of colon

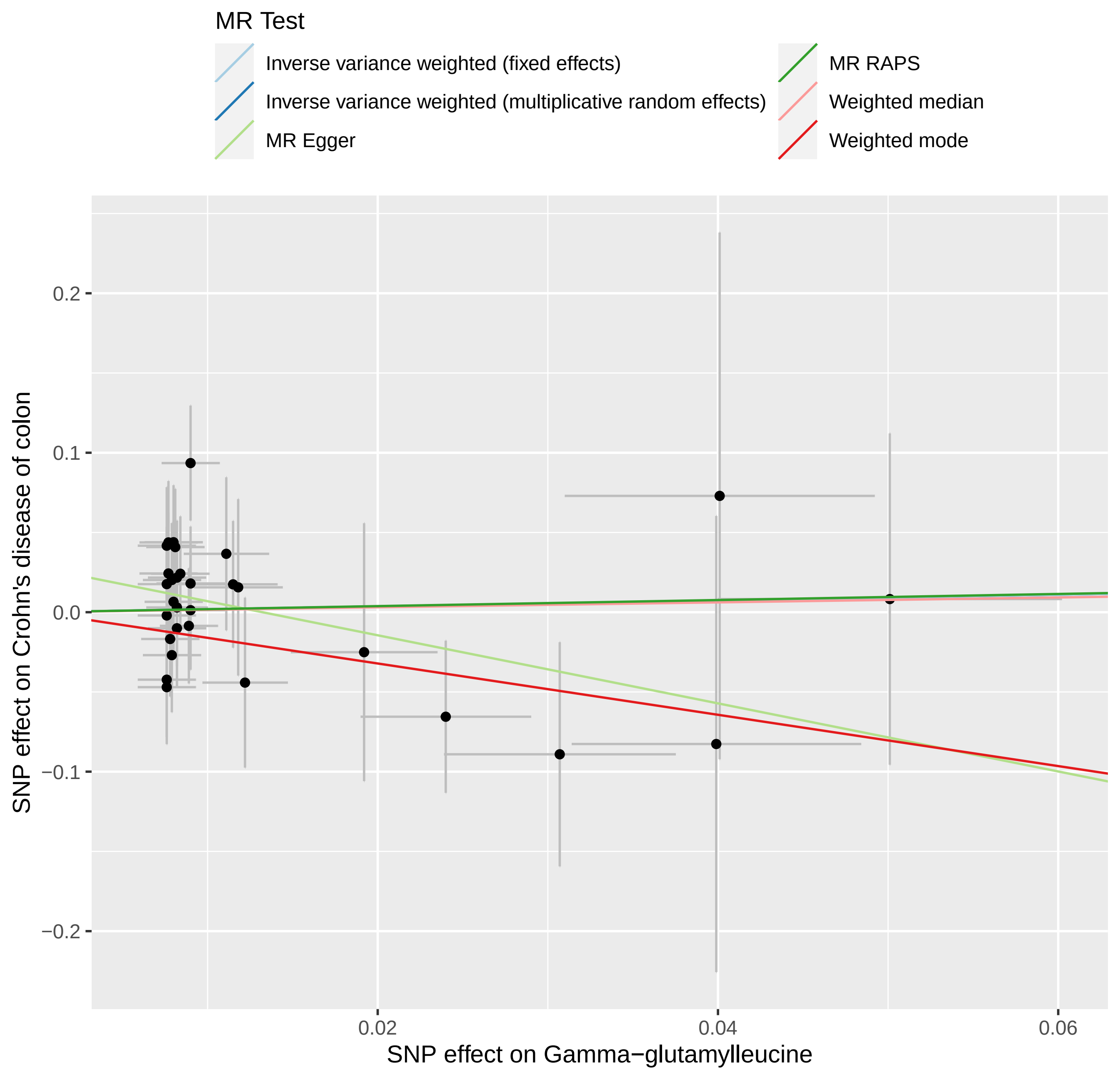

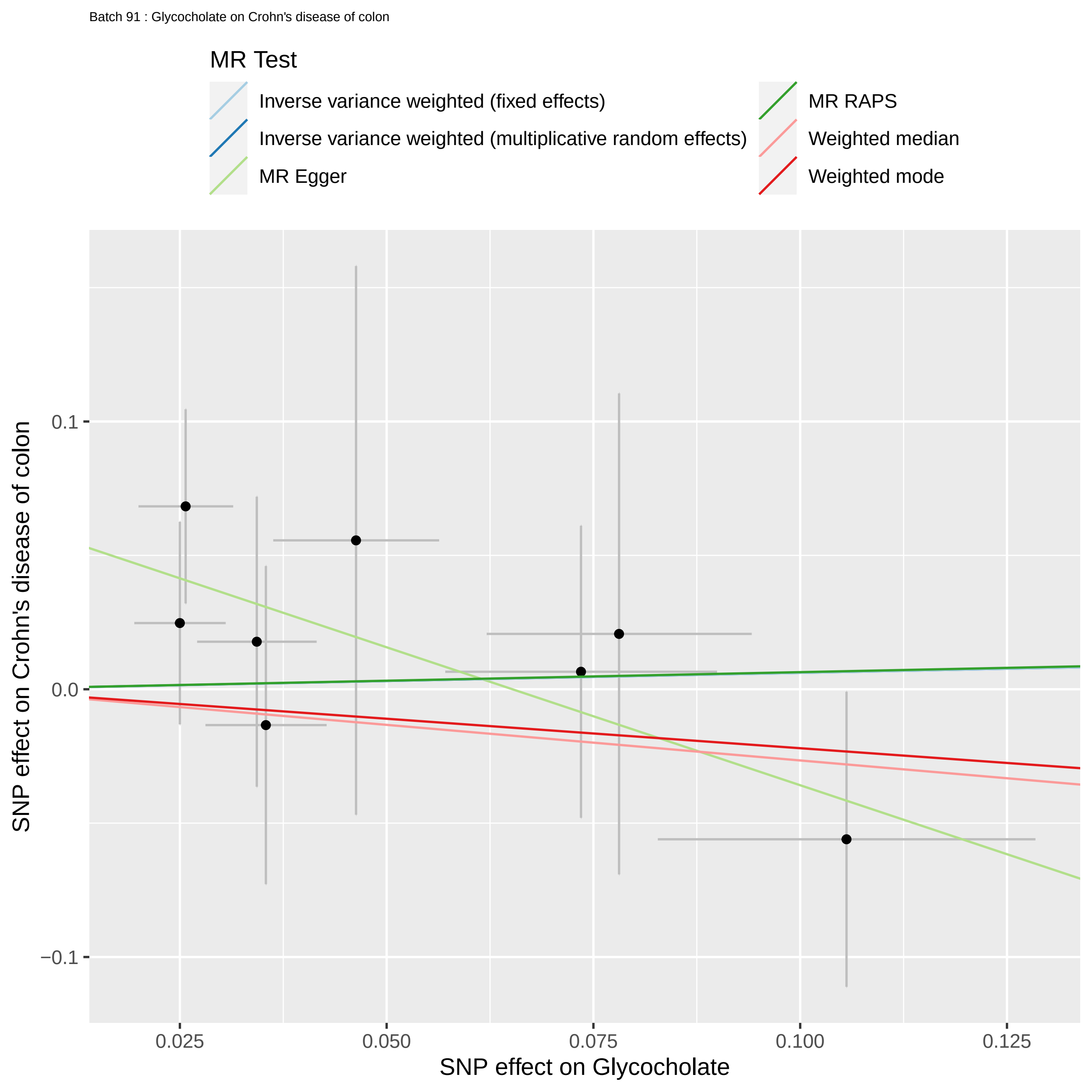
Batch 91 : Glycocholate on Crohn's disease of colon
MR Test
Inverse variance weighted (fixed effects)
Inverse variance weighted (multiplicative random effects)
MR Egger
MR RAPS
Weighted median
Weighted mode
SNP effect on Crohn's disease of colon
0.1
0.0
−0.1
0.025
0.050
0.075
0.100
0.125
SNP effect on Glycocholate

Batch 92 : Glycodeoxycholate on Crohn's disease of colon

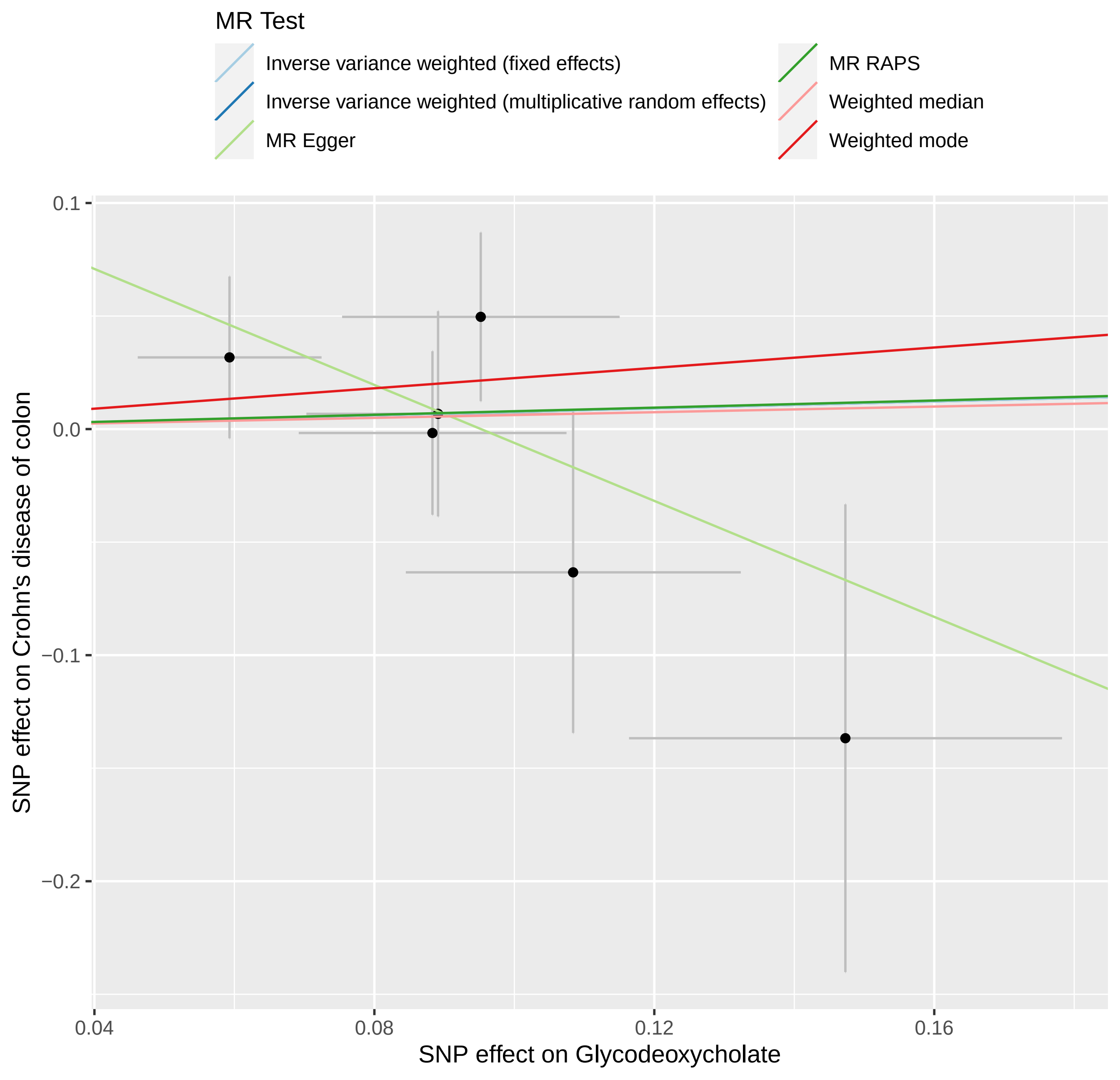

Batch 93 : Taurochenodeoxycholate on Crohn's disease of colon

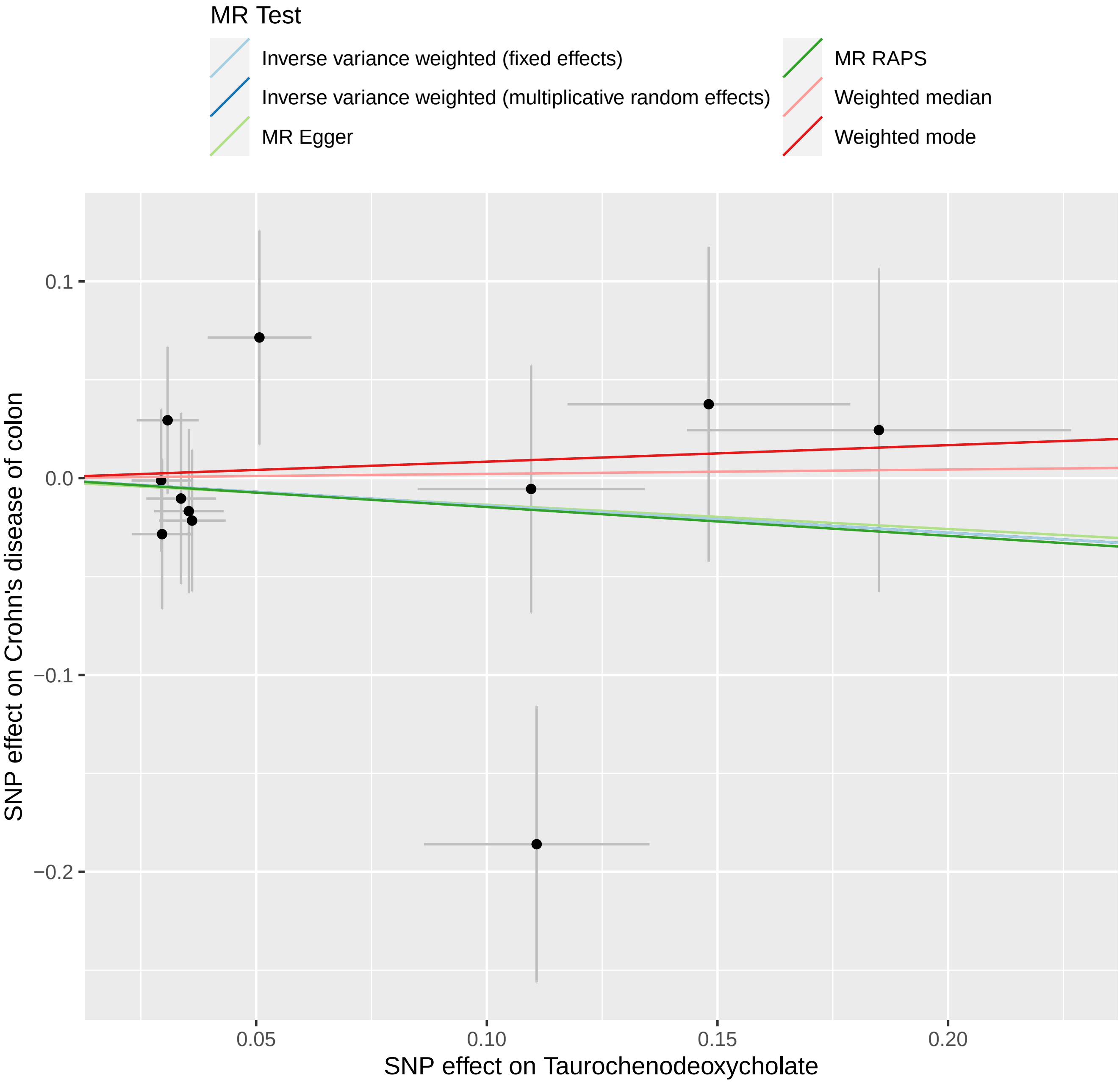

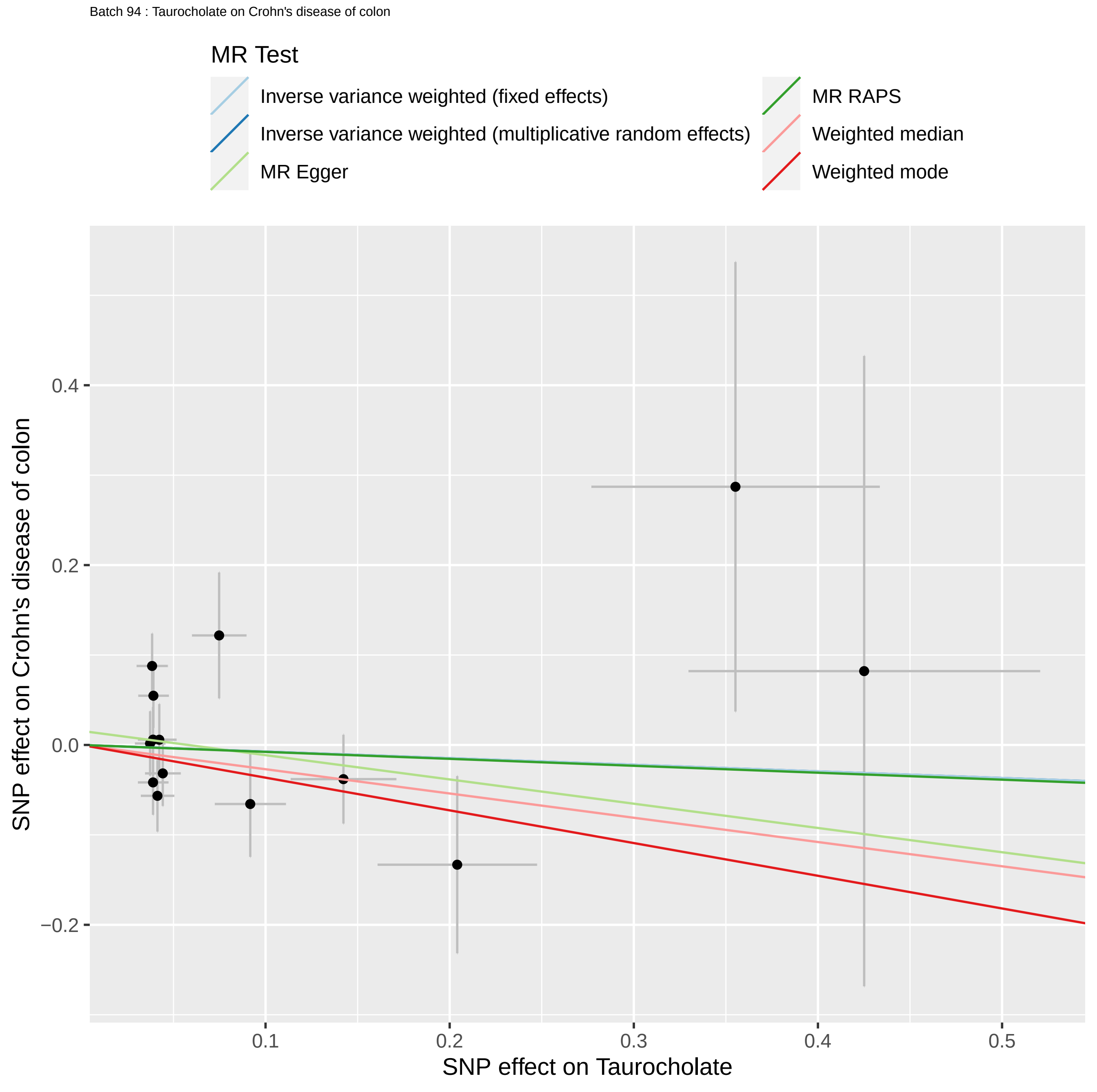
Batch 94 : Taurocholate on Crohn's disease of colon
MR Test
Inverse variance weighted (fixed effects)
Inverse variance weighted (multiplicative random effects)
MR Egger
MR RAPS
Weighted median
Weighted mode
0.4
0.2
0.0
−0.2
0.1
0.2
0.3
0.4
0.5
SNP effect on Taurocholate
SNP effect on Crohn's disease of colon

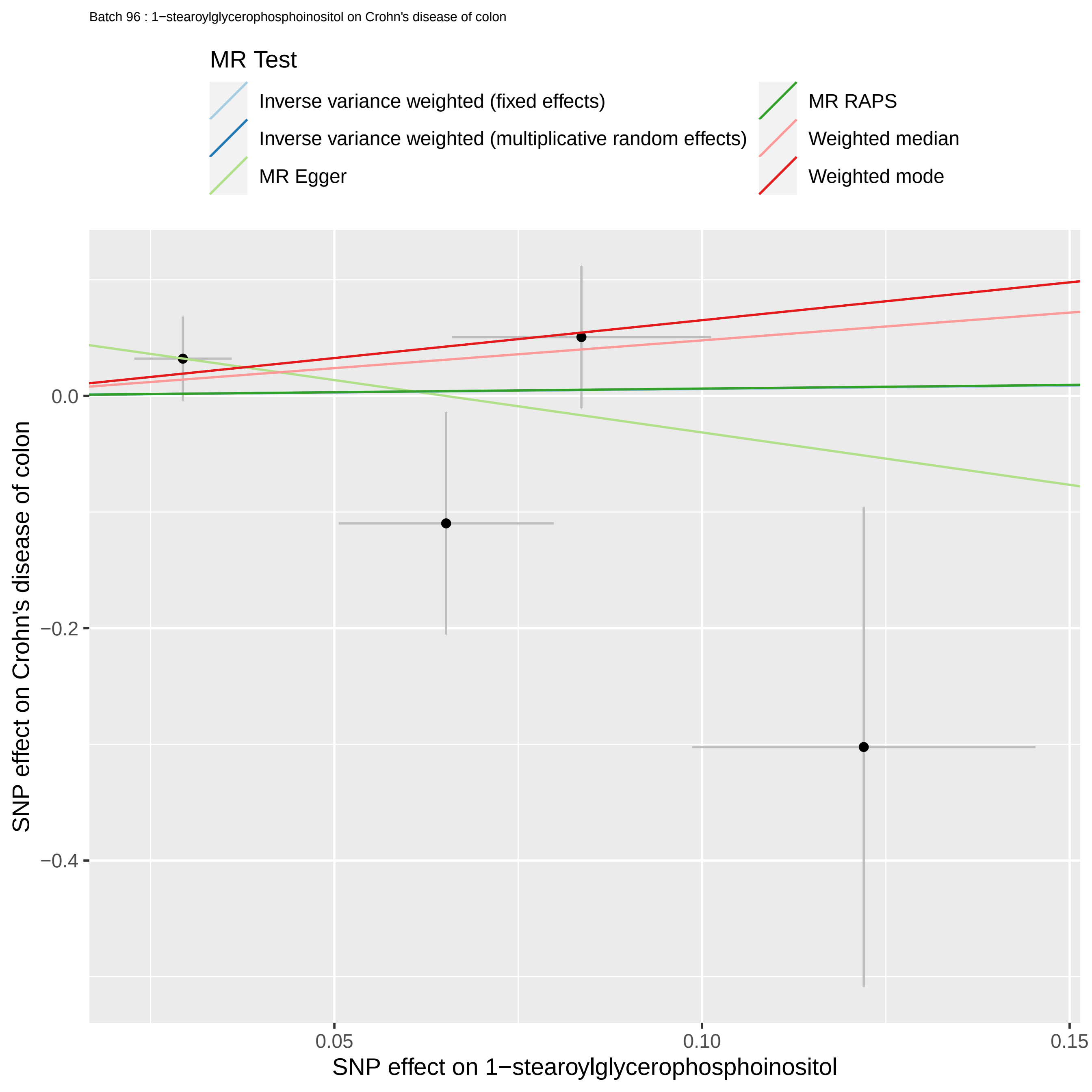
Batch 96 : 1−stearoylglycerophosphoinositol on Crohn's disease of colon
MR Test
Inverse variance weighted (fixed effects)
Inverse variance weighted (multiplicative random effects)
MR Egger
MR RAPS
Weighted median
Weighted mode
0.0
−0.2
−0.4
0.05
0.10
0.15
SNP effect on Crohn's disease of colon
SNP effect on 1−stearoylglycerophosphoinositol

Batch 97 : Myo−inositol on Crohn's disease of colon

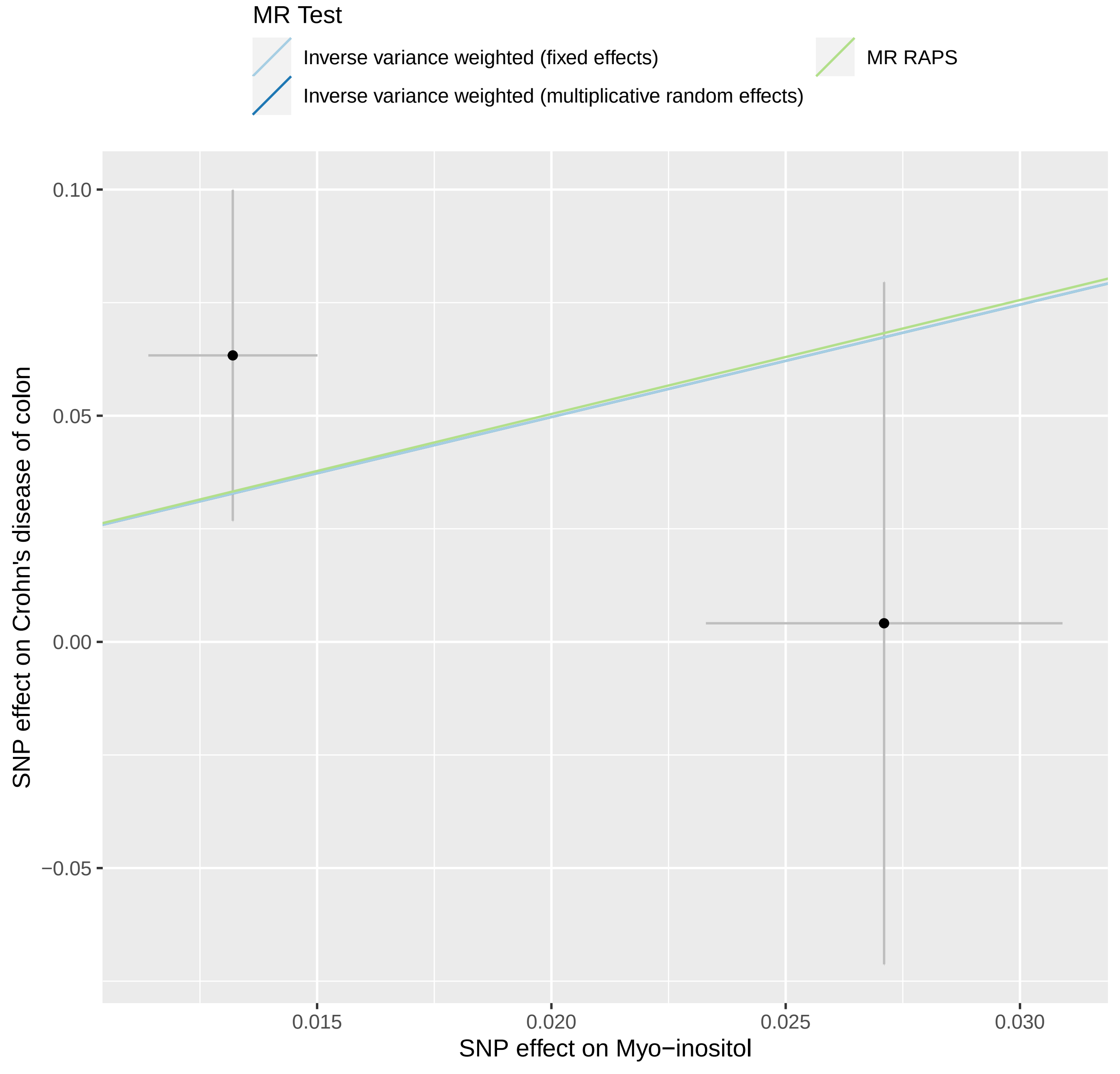

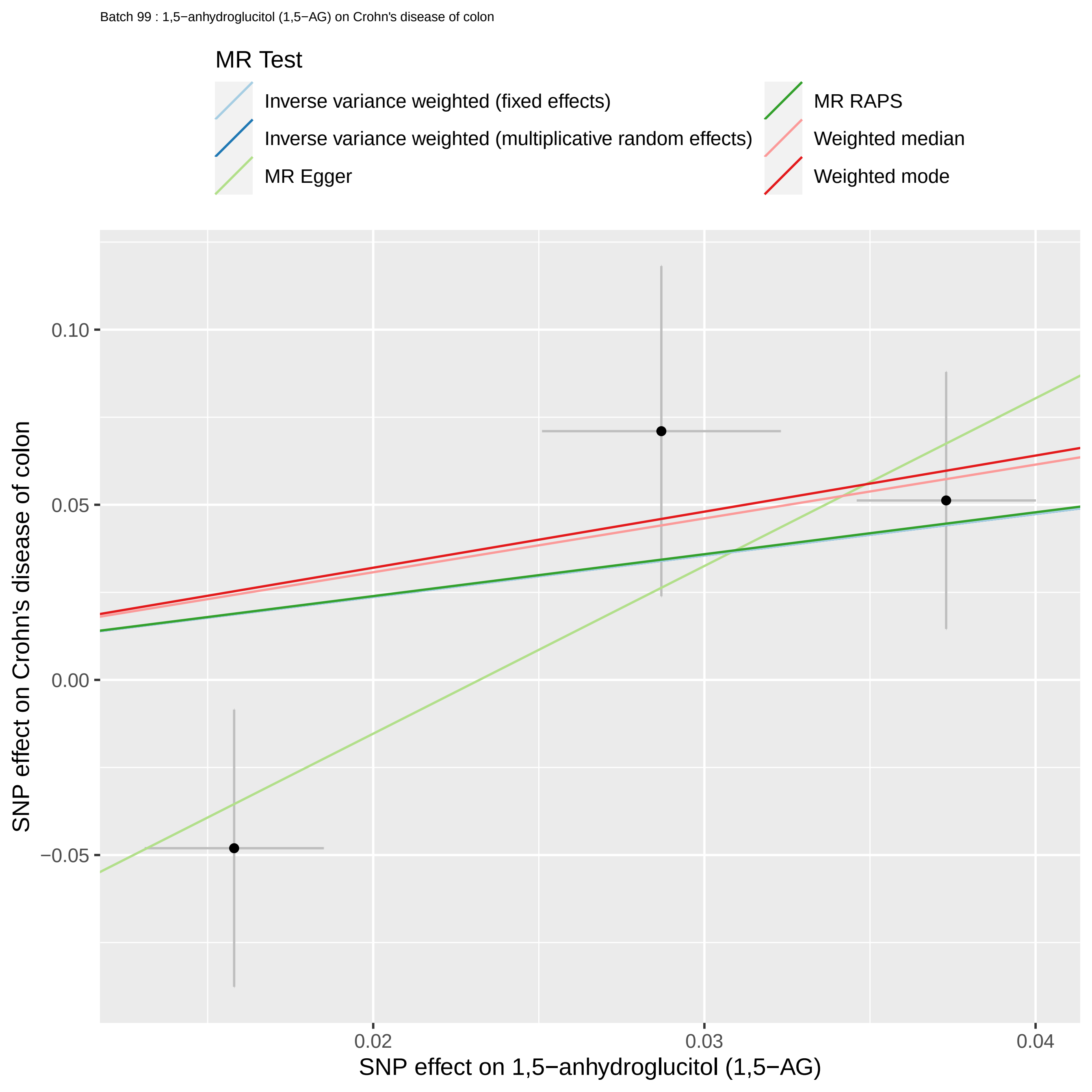

Batch 99 : 1,5−anhydroglucitol (1,5−AG) on Crohn's disease of colon
MR Test
Inverse variance weighted (fixed effects)
Inverse variance weighted (multiplicative random effects)
MR Egger
MR RAPS
Weighted median
Weighted mode
0.10
0.05
0.00
−0.05
0.02
0.03
0.04
SNP effect on Crohn's disease of colon
SNP effect on 1,5−anhydroglucitol (1,5−AG)

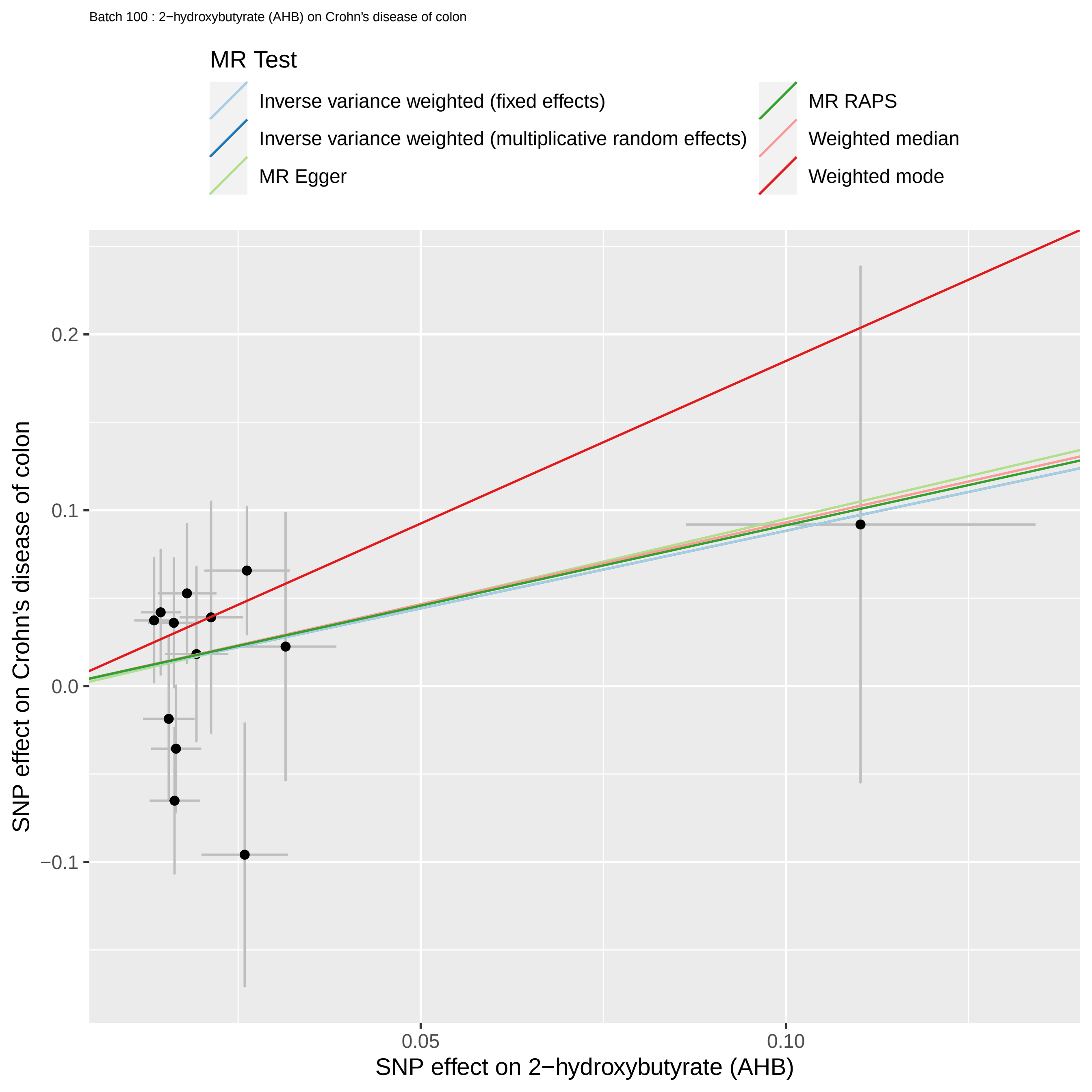
Batch 100 : 2−hydroxybutyrate (AHB) on Crohn's disease of colon
MR Test
Inverse variance weighted (fixed effects)
Inverse variance weighted (multiplicative random effects)
MR Egger
MR RAPS
Weighted median
Weighted mode
0.2
0.1
0.0
−0.1
0.05
0.10
SNP effect on Crohn's disease of colon
SNP effect on 2−hydroxybutyrate (AHB)

Batch 103 : 1−palmitoylglycerol (1−monopalmitin) on Crohn's disease of colon

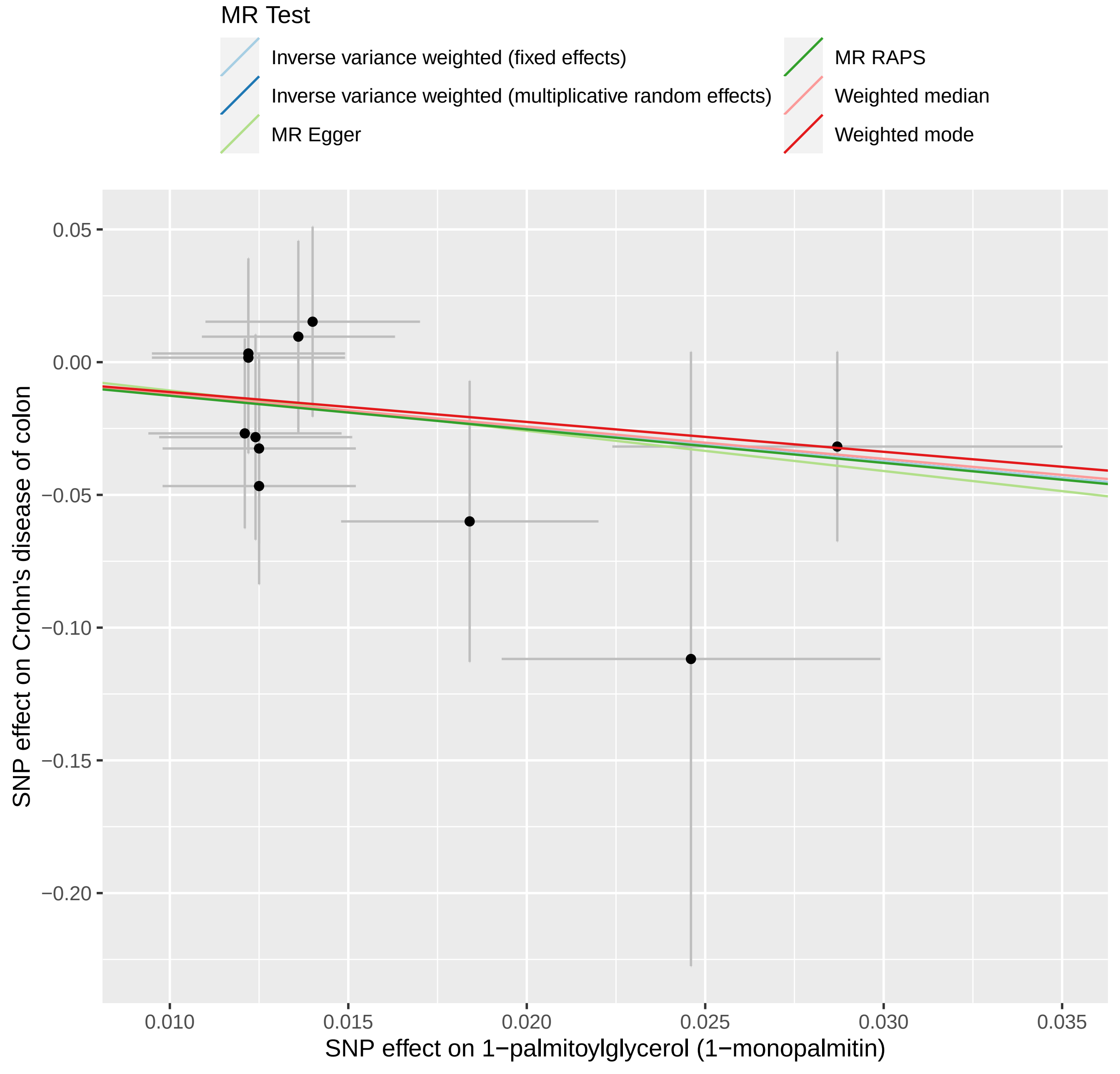

Batch 106 : 2−hydroxyisobutyrate on Crohn's disease of colon

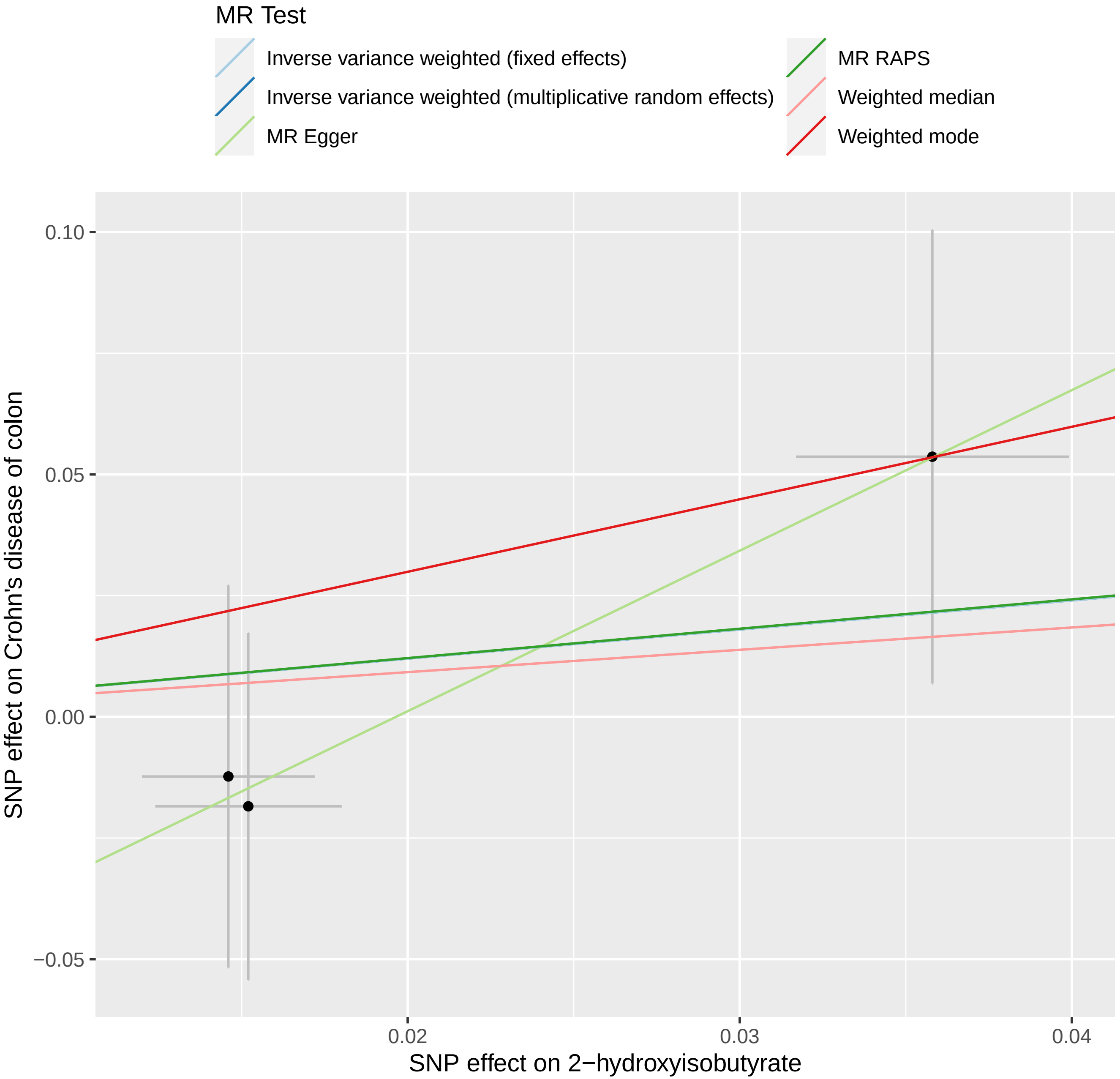

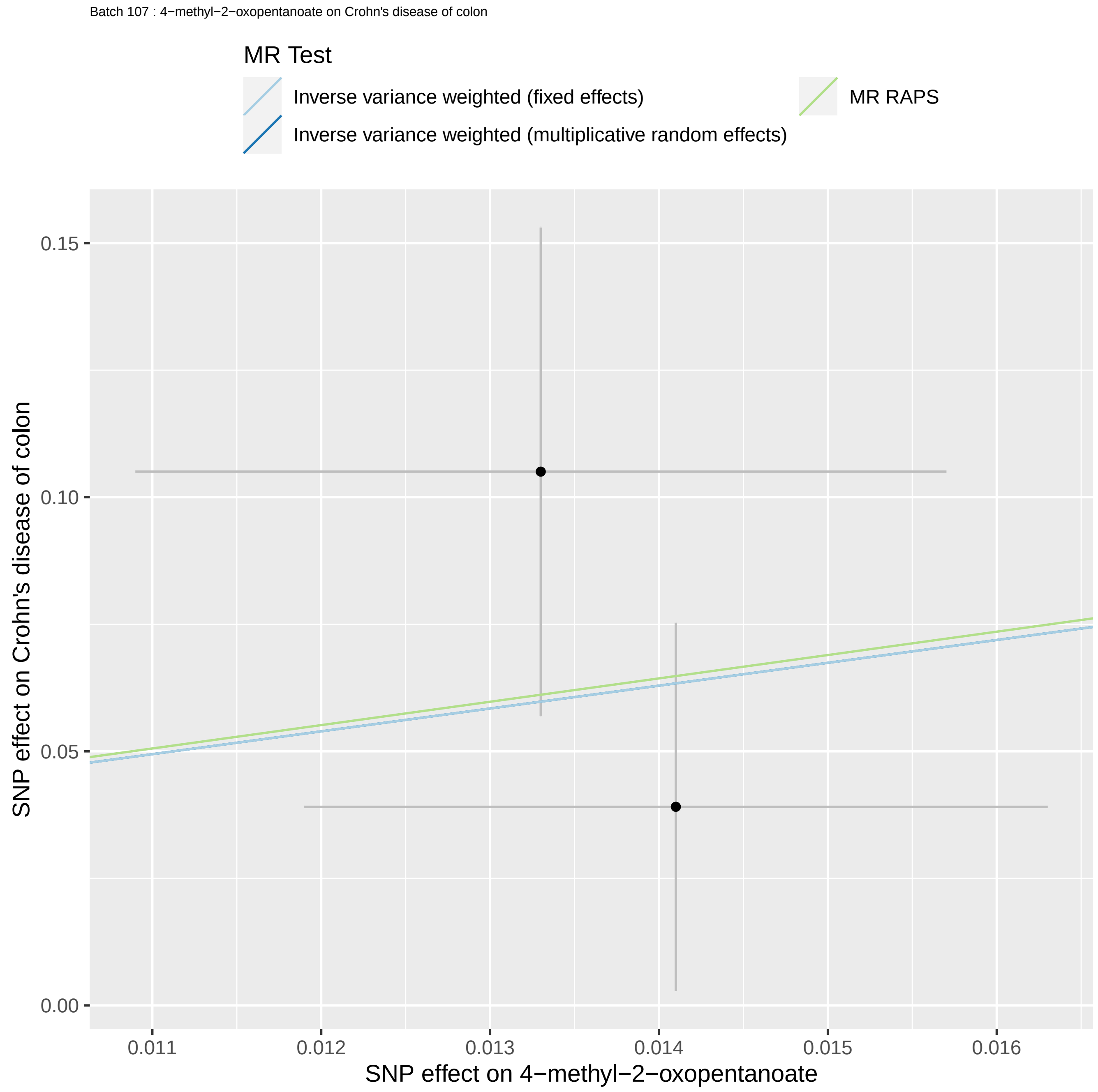
Batch 107 : 4−methyl−2−oxopentanoate on Crohn's disease of colon
MR Test
Inverse variance weighted (fixed effects)
Inverse variance weighted (multiplicative random effects)
MR RAPS
SNP effect on Crohn's disease of colon
0.15
0.10
0.05
0.00
0.011
0.012
0.013
0.014
0.015
0.016
SNP effect on 4−methyl−2−oxopentanoate

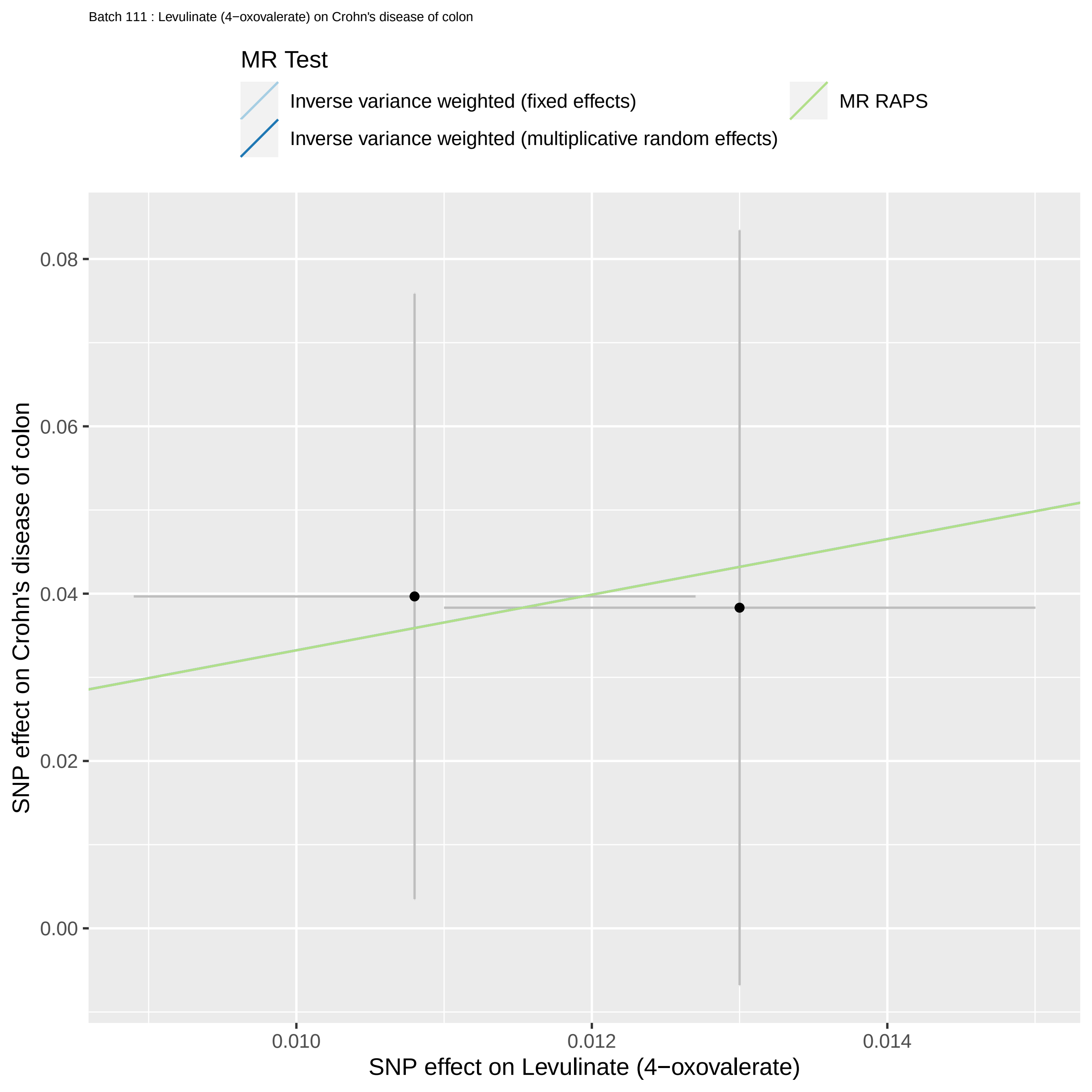
Batch 111 : Levulinate (4−oxovalerate) on Crohn's disease of colon
MR Test
Inverse variance weighted (fixed effects)
Inverse variance weighted (multiplicative random effects)
MR RAPS
0.08
0.06
0.04
0.02
0.00
0.010
0.012
0.014
SNP effect on Crohn's disease of colon
SNP effect on Levulinate (4−oxovalerate)

Batch 113 : Cholate on Crohn's disease of colon

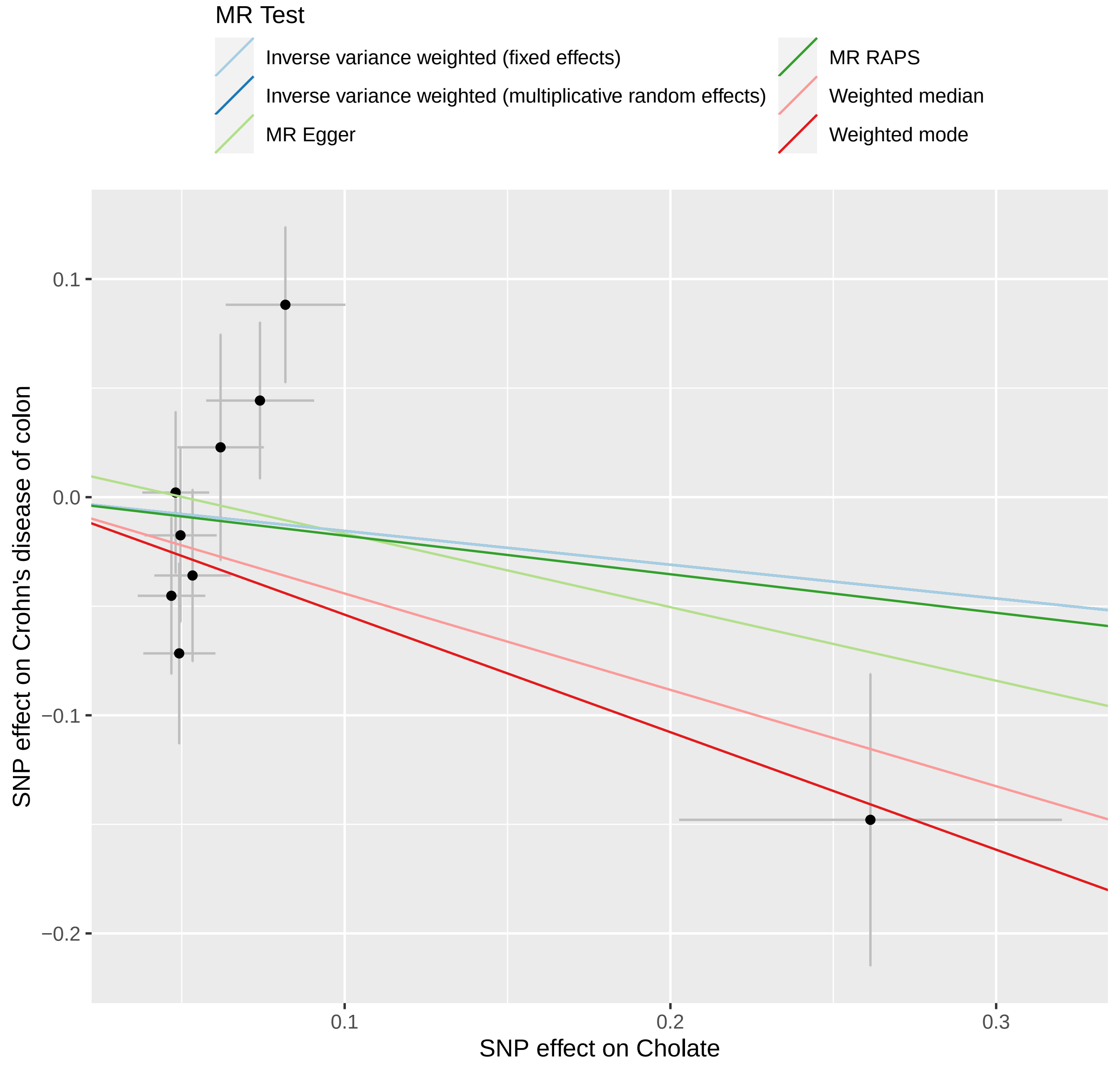

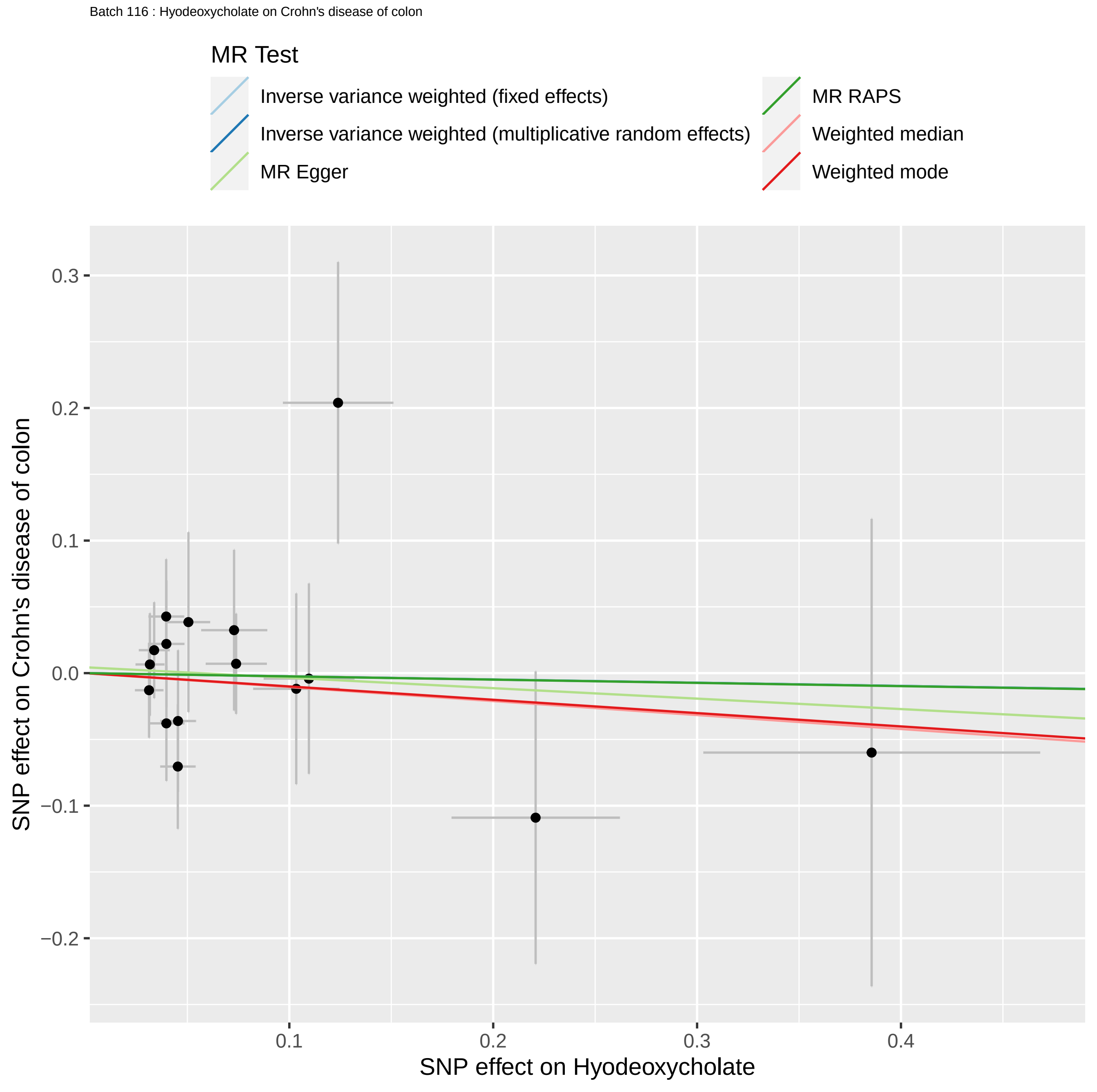

Batch 116 : Hyodeoxycholate on Crohn's disease of colon
MR Test
Inverse variance weighted (fixed effects)
Inverse variance weighted (multiplicative random effects)
MR Egger
MR RAPS
Weighted median
Weighted mode
0.3
0.2
0.1
0.0
−0.1
−0.2
0.1
0.2
0.3
0.4
SNP effect on Crohn's disease of colon
SNP effect on Hyodeoxycholate

Batch 117 : 3−indoxyl sulfate on Crohn's disease of colon

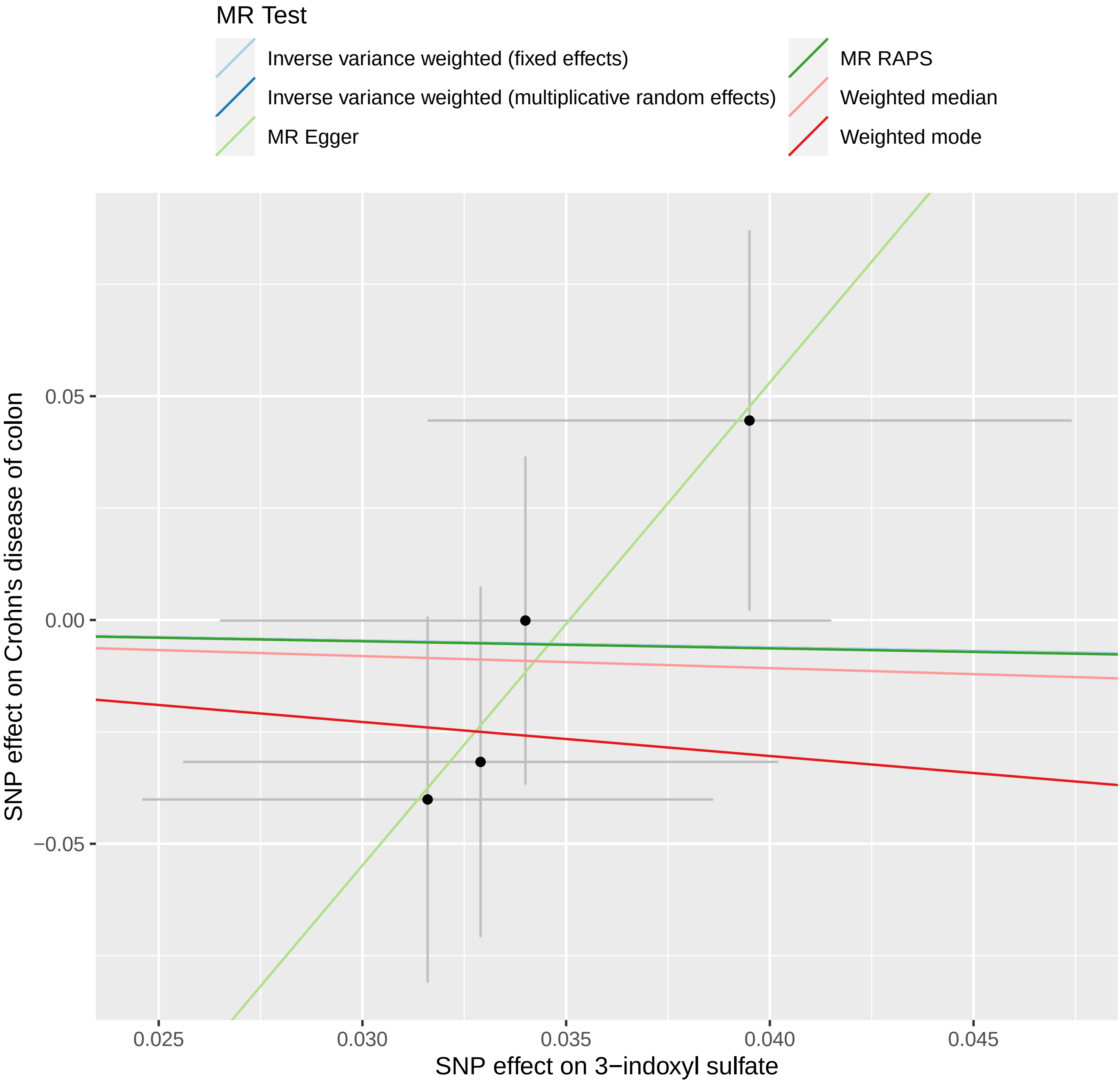

Batch 121 : Erythrose on Crohn's disease of colon

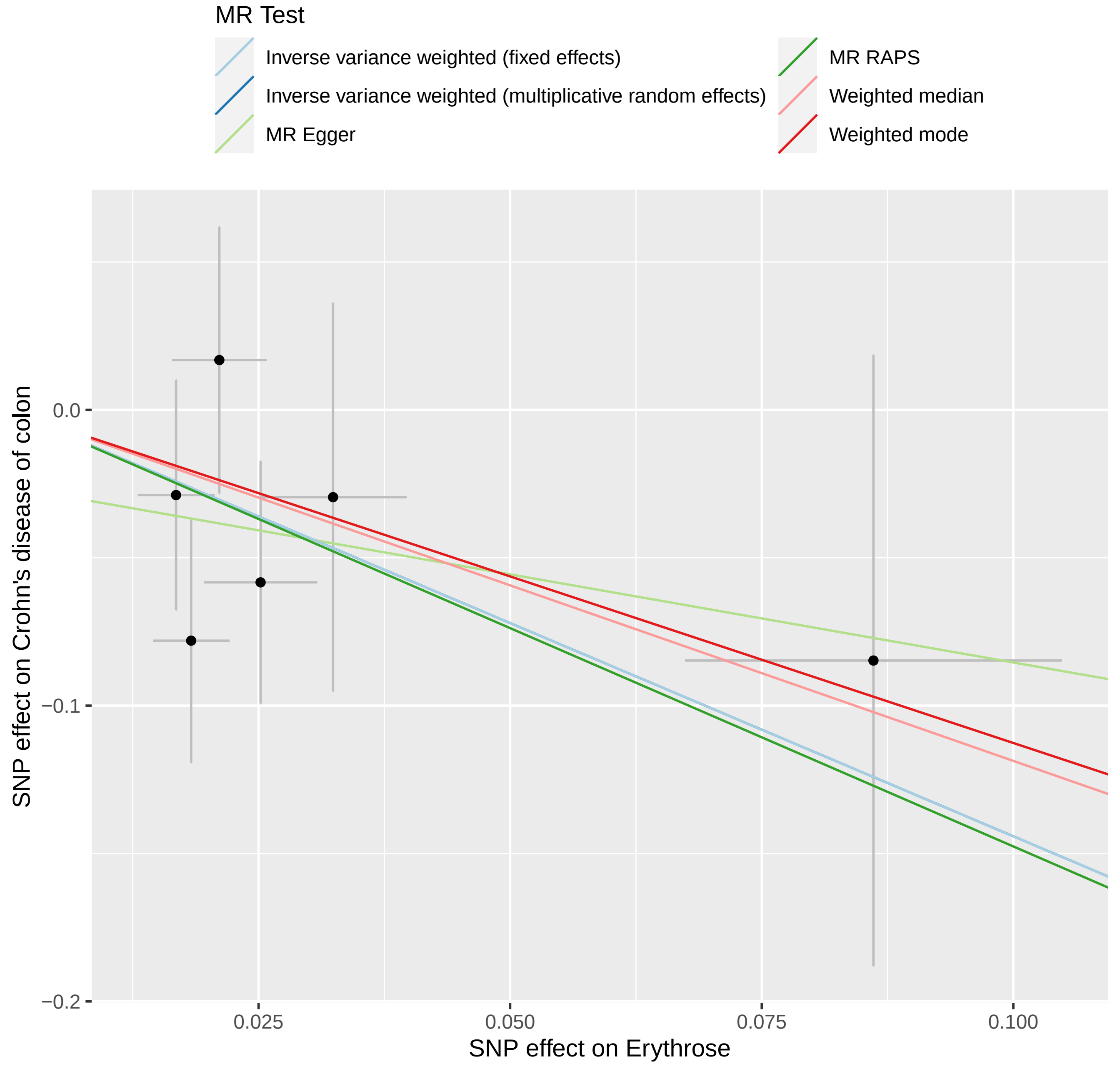

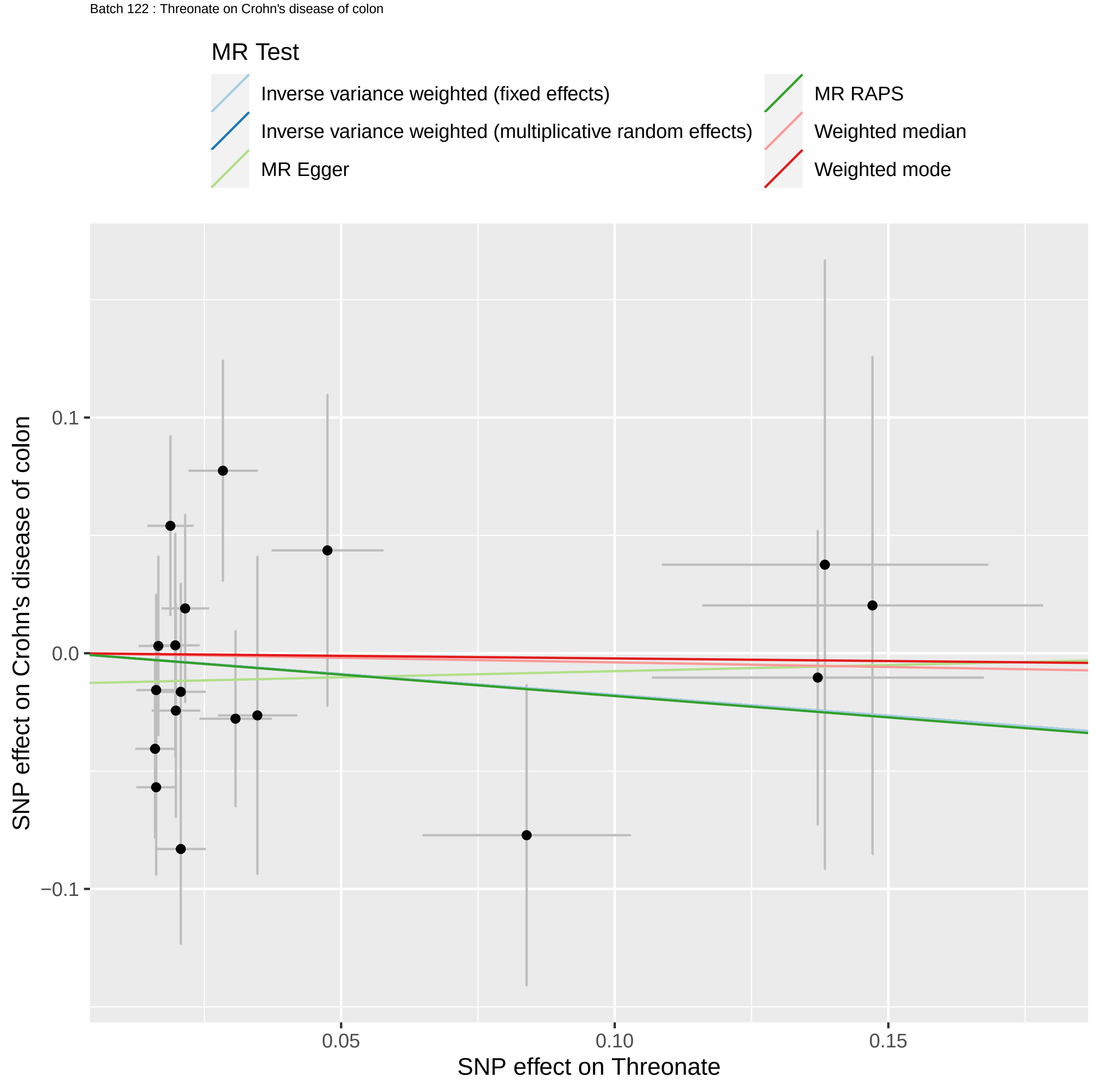

Batch 122 : Threonate on Crohn's disease of colon
MR Test
Inverse variance weighted (fixed effects)
Inverse variance weighted (multiplicative random effects)
MR Egger
MR RAPS
Weighted median
Weighted mode
SNP effect on Crohn's disease of colon
0.1
0.0
−0.1
0.05
0.10
0.15
SNP effect on Threonate

Batch 123 : Cysteine on Crohn's disease of colon

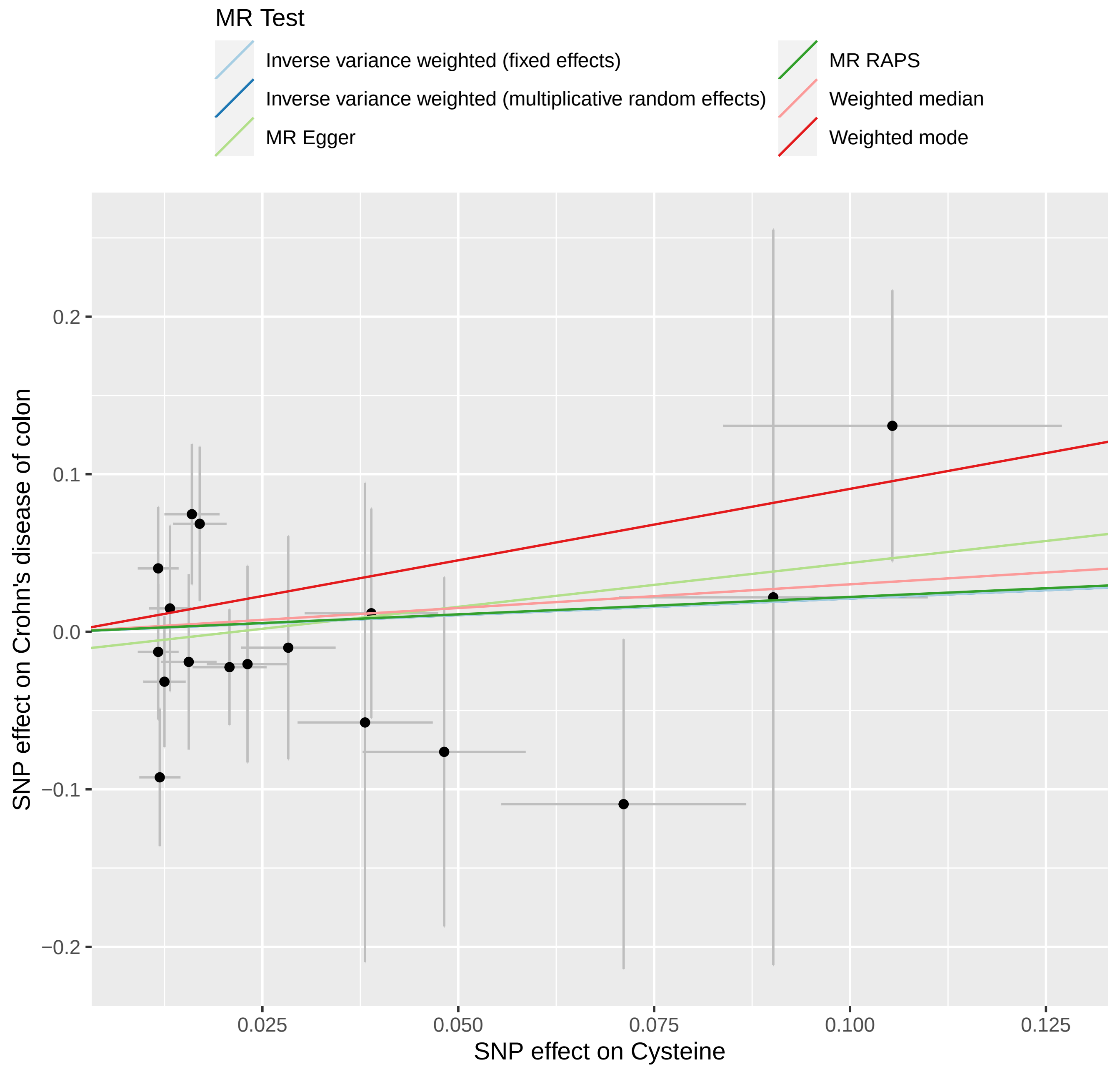

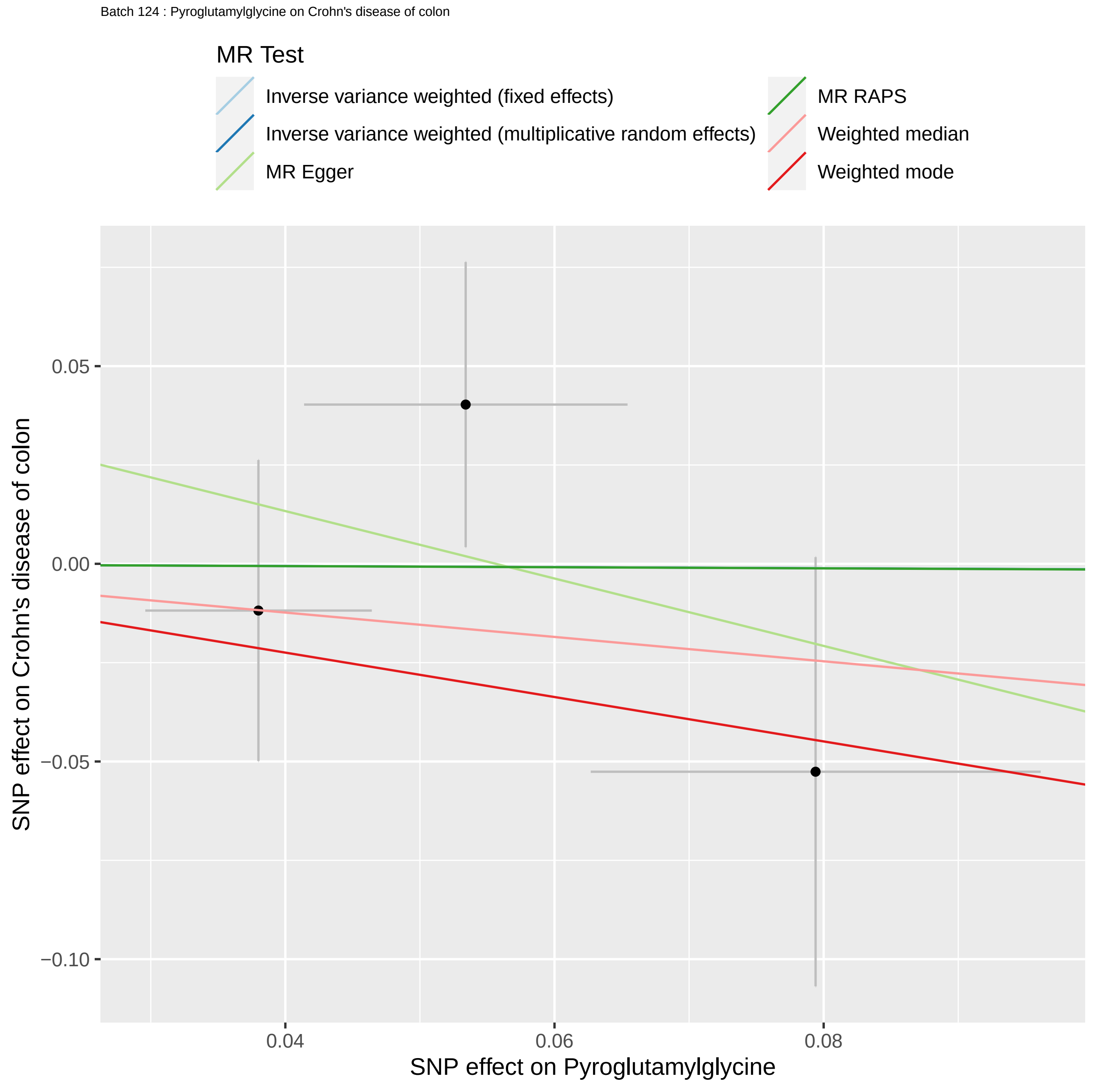
Batch 124 : Pyroglutamylglycine on Crohn's disease of colon
MR Test
Inverse variance weighted (fixed effects)
Inverse variance weighted (multiplicative random effects)
MR Egger
MR RAPS
Weighted median
Weighted mode
0.05
0.00
−0.05
−0.10
0.04
0.06
0.08
SNP effect on Crohn's disease of colon
SNP effect on Pyroglutamylglycine

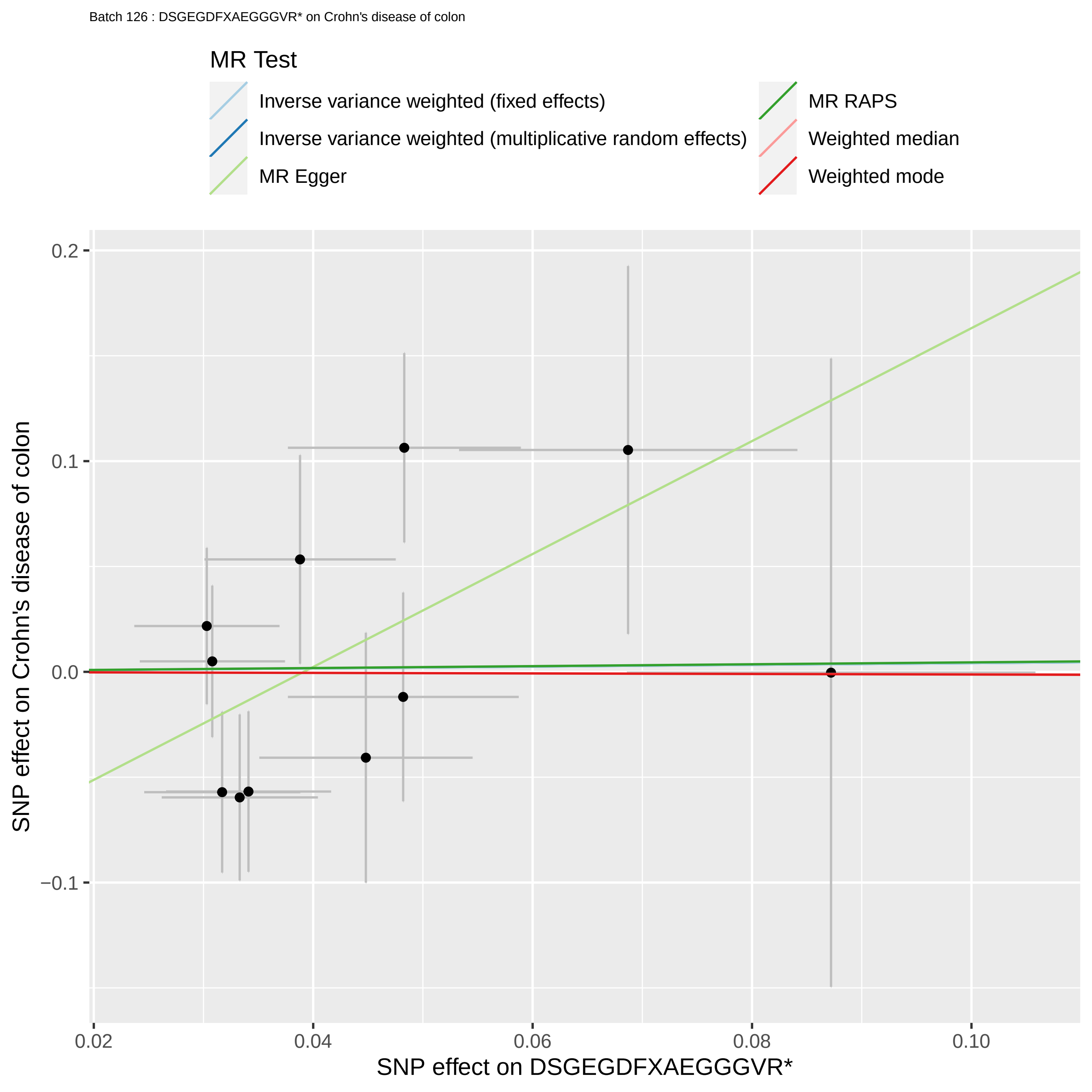
Batch 126 : DSGEGDFXAEGGGVR* on Crohn's disease of colon
MR Test
Inverse variance weighted (fixed effects)
Inverse variance weighted (multiplicative random effects)
MR Egger
MR RAPS
Weighted median
Weighted mode
0.2
0.1
0.0
−0.1
0.02
0.04
0.06
0.08
0.10
SNP effect on Crohn's disease of colon
SNP effect on DSGEGDFXAEGGGVR*

Batch 127 : Pyridoxate on Crohn's disease of colon

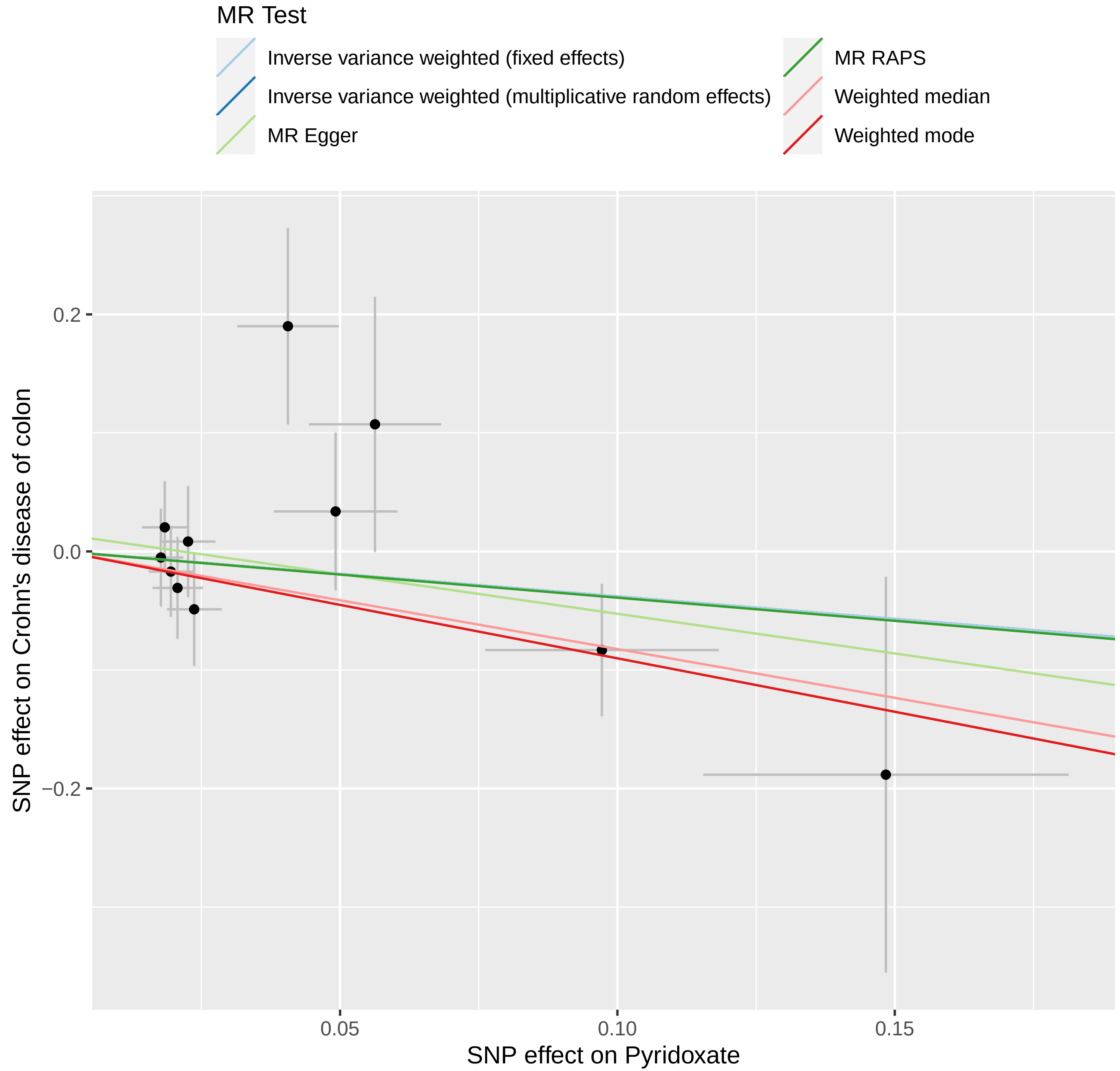

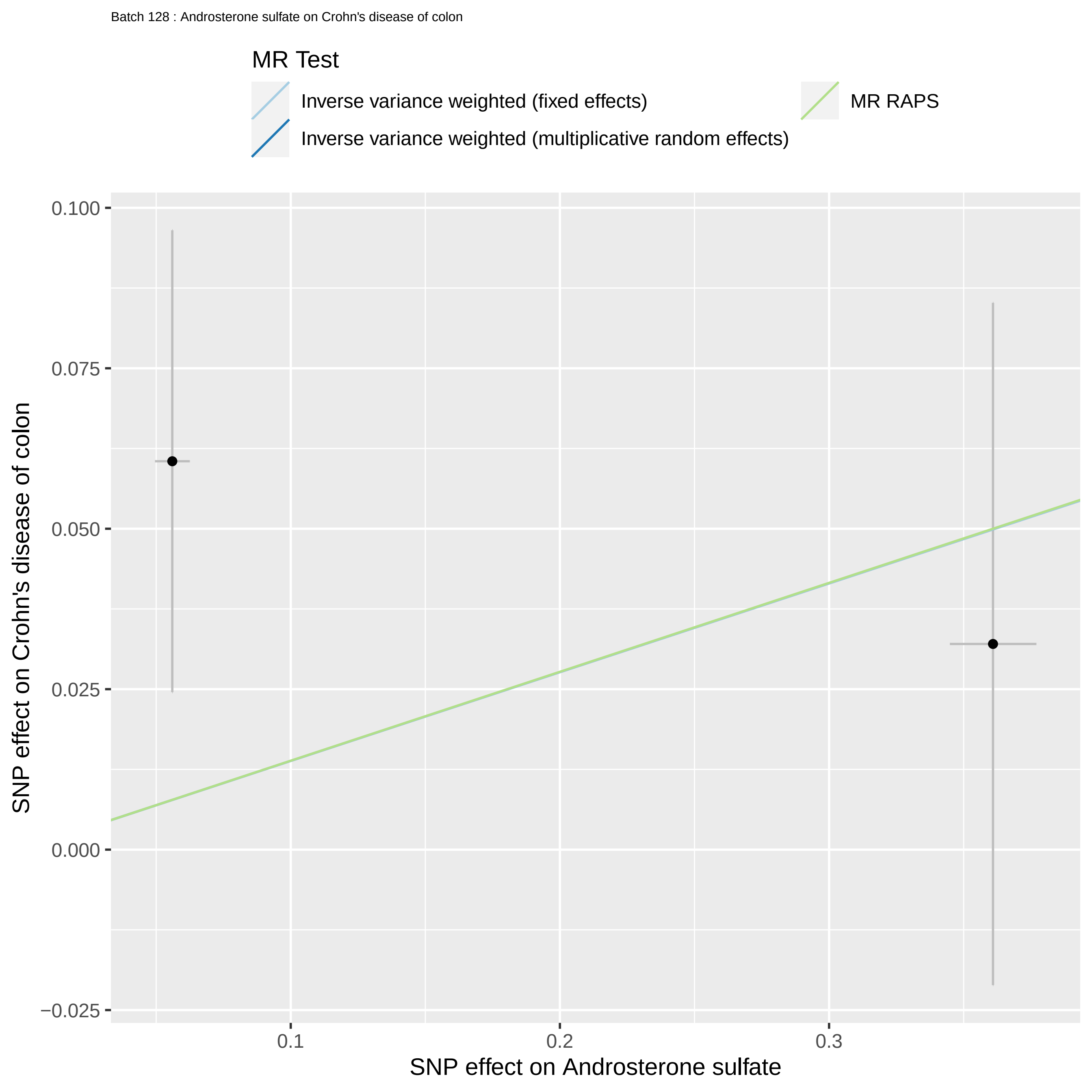

Batch 128 : Androsterone sulfate on Crohn's disease of colon
MR Test
Inverse variance weighted (fixed effects)
Inverse variance weighted (multiplicative random effects)
MR RAPS
0.100
0.075
0.050
0.025
0.000
−0.025
SNP effect on Crohn's disease of colon
0.1
0.2
0.3
SNP effect on Androsterone sulfate

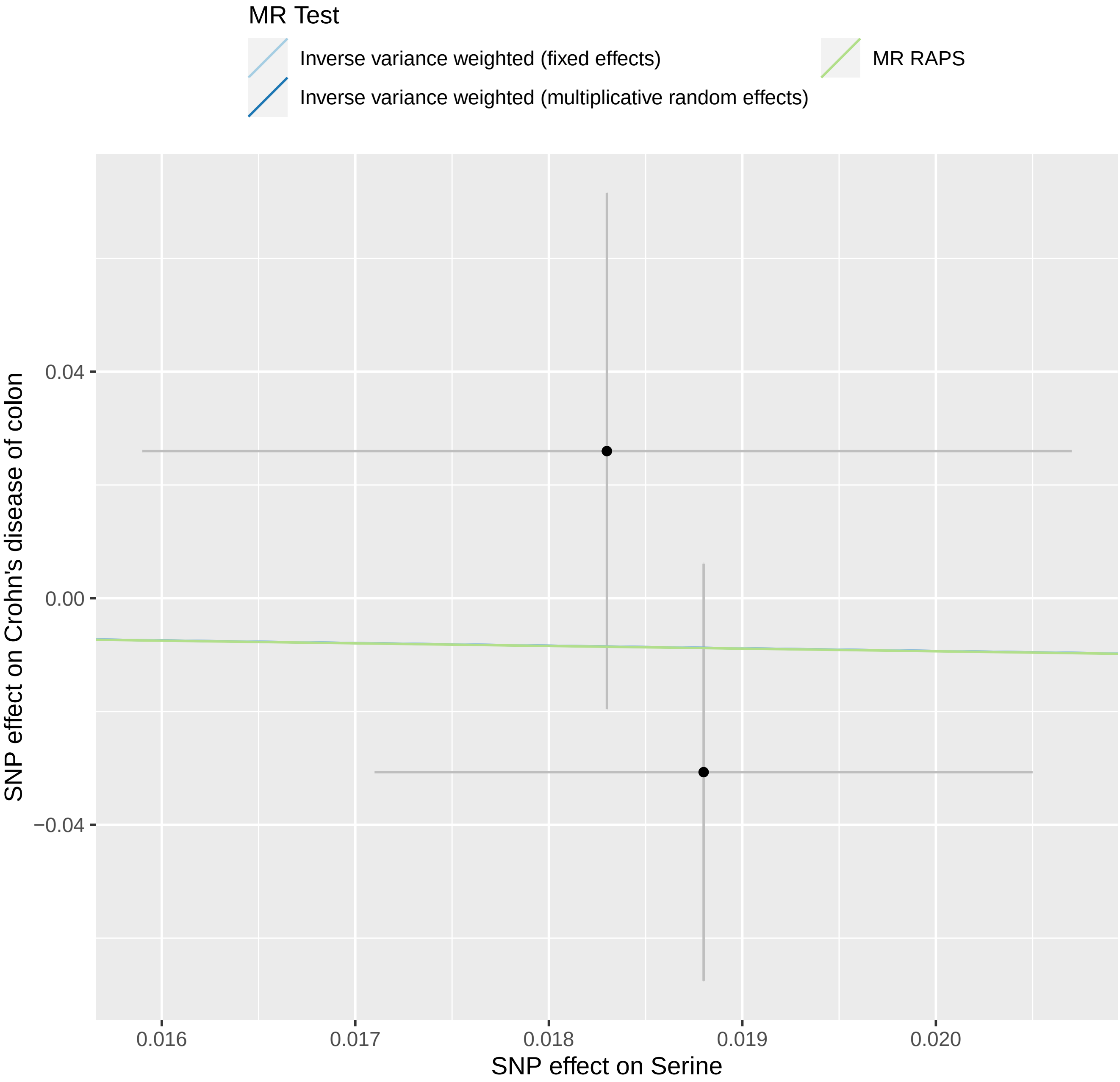
MR Test
Inverse variance weighted (fixed effects)
Inverse variance weighted (multiplicative random effects)
MR RAPS
0.04
0.00
−0.04
0.016
0.017
0.018
0.019
0.020
SNP effect on Crohn's disease of colon
SNP effect on Serine

Batch 133 : Trans−4−hydroxyproline on Crohn's disease of colon

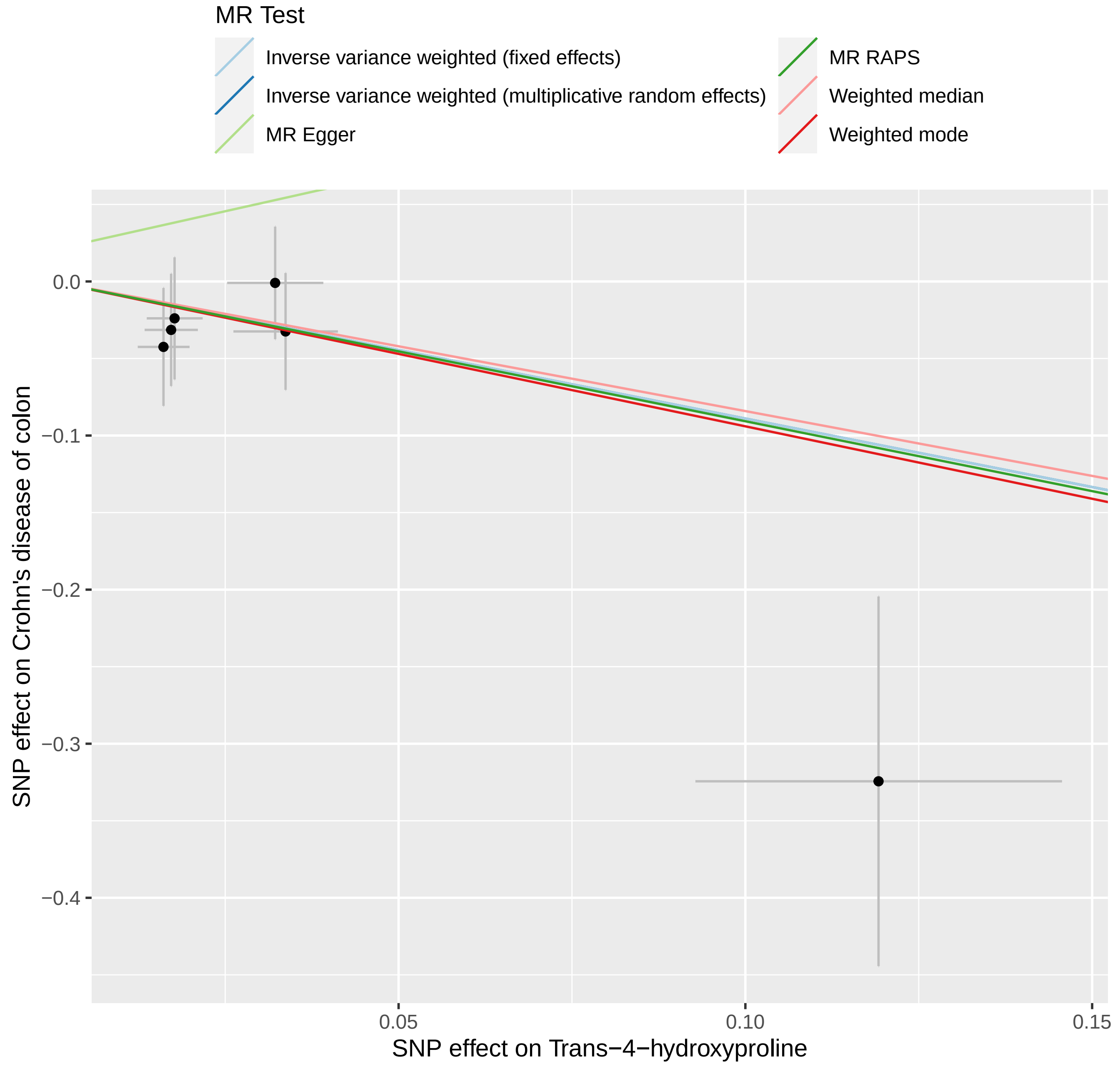

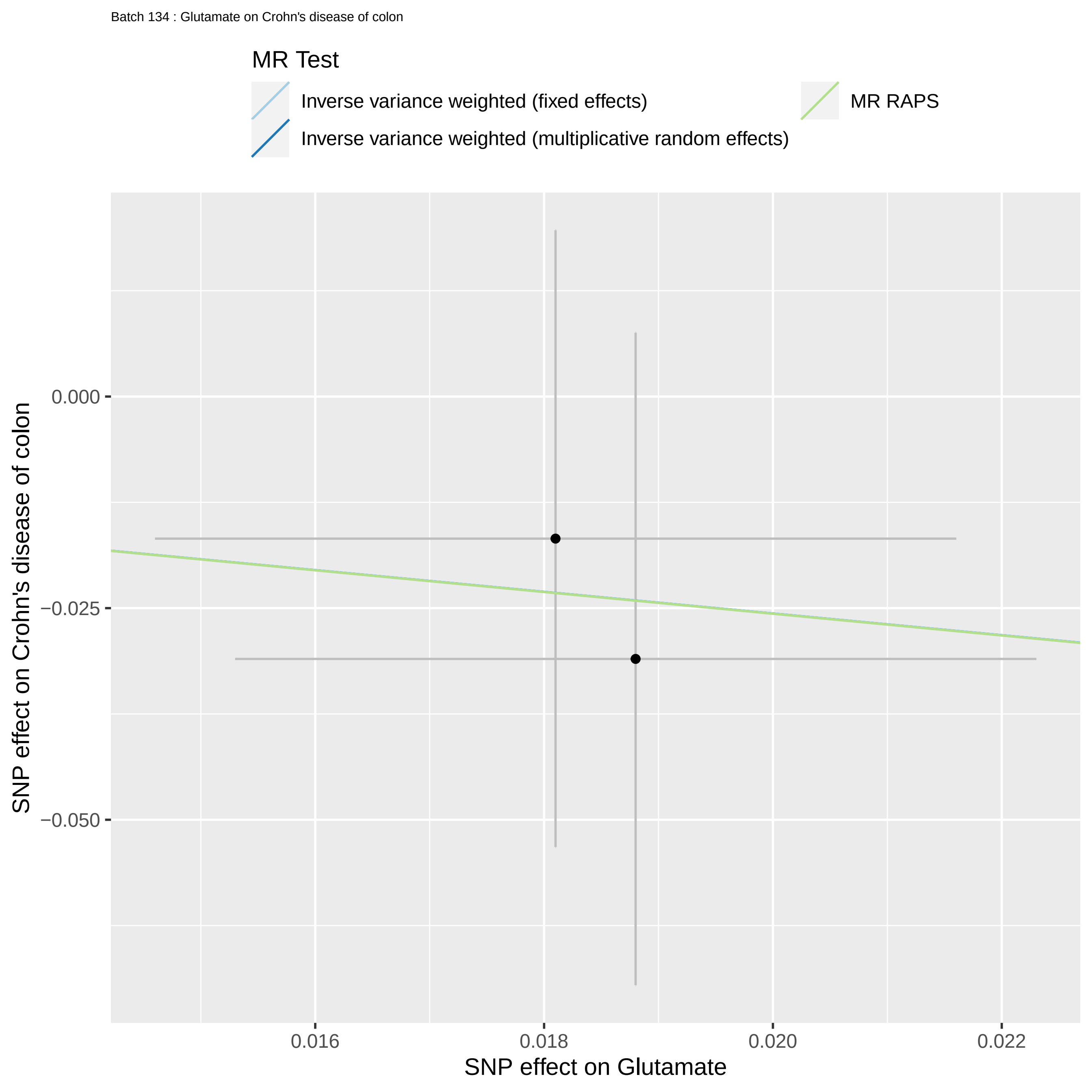
Batch 134 : Glutamate on Crohn's disease of colon
MR Test
Inverse variance weighted (fixed effects)
Inverse variance weighted (multiplicative random effects)
MR RAPS
0.000
−0.025
−0.050
0.016
0.018
0.020
0.022
SNP effect on Crohn's disease of colon
SNP effect on Glutamate

Batch 135 : Hexanoylcarnitine on Crohn's disease of colon

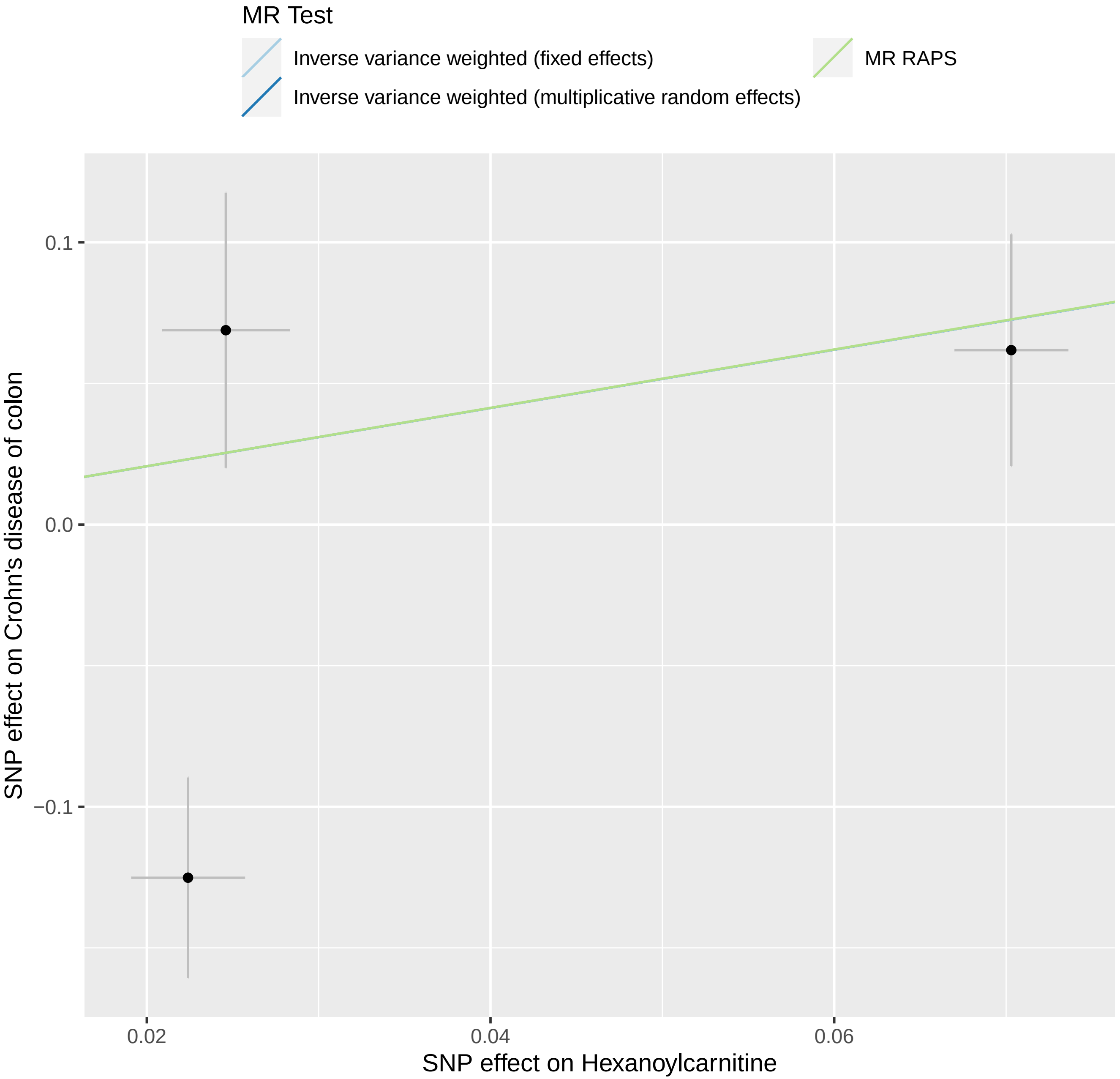

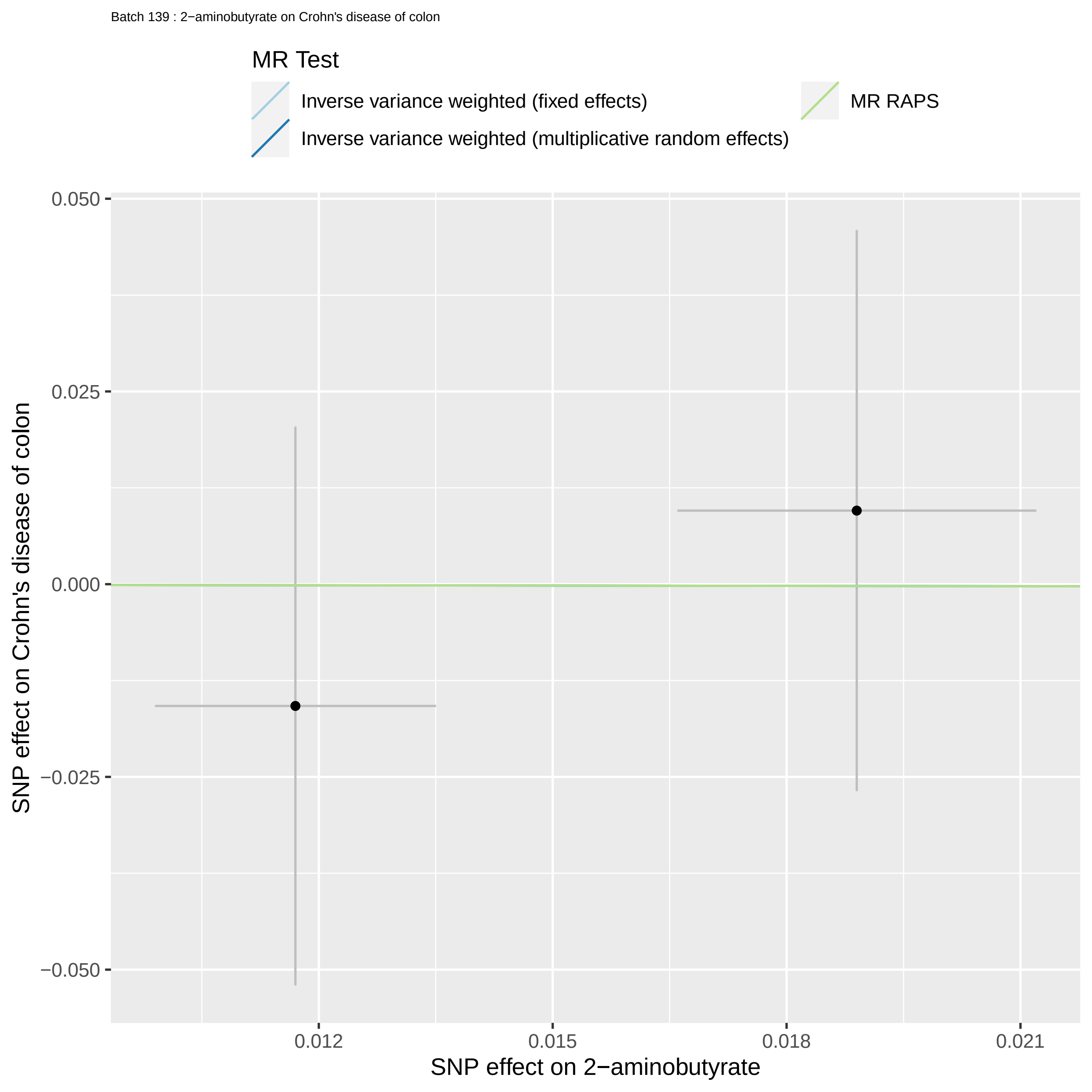

Batch 139 : 2−aminobutyrate on Crohn's disease of colon
MR Test
Inverse variance weighted (fixed effects)
Inverse variance weighted (multiplicative random effects)
MR RAPS
0.050
0.025
0.000
−0.025
−0.050
SNP effect on Crohn's disease of colon
0.012
0.015
0.018
0.021
SNP effect on 2−aminobutyrate

Batch 144 : Butyrylcarnitine on Crohn's disease of colon

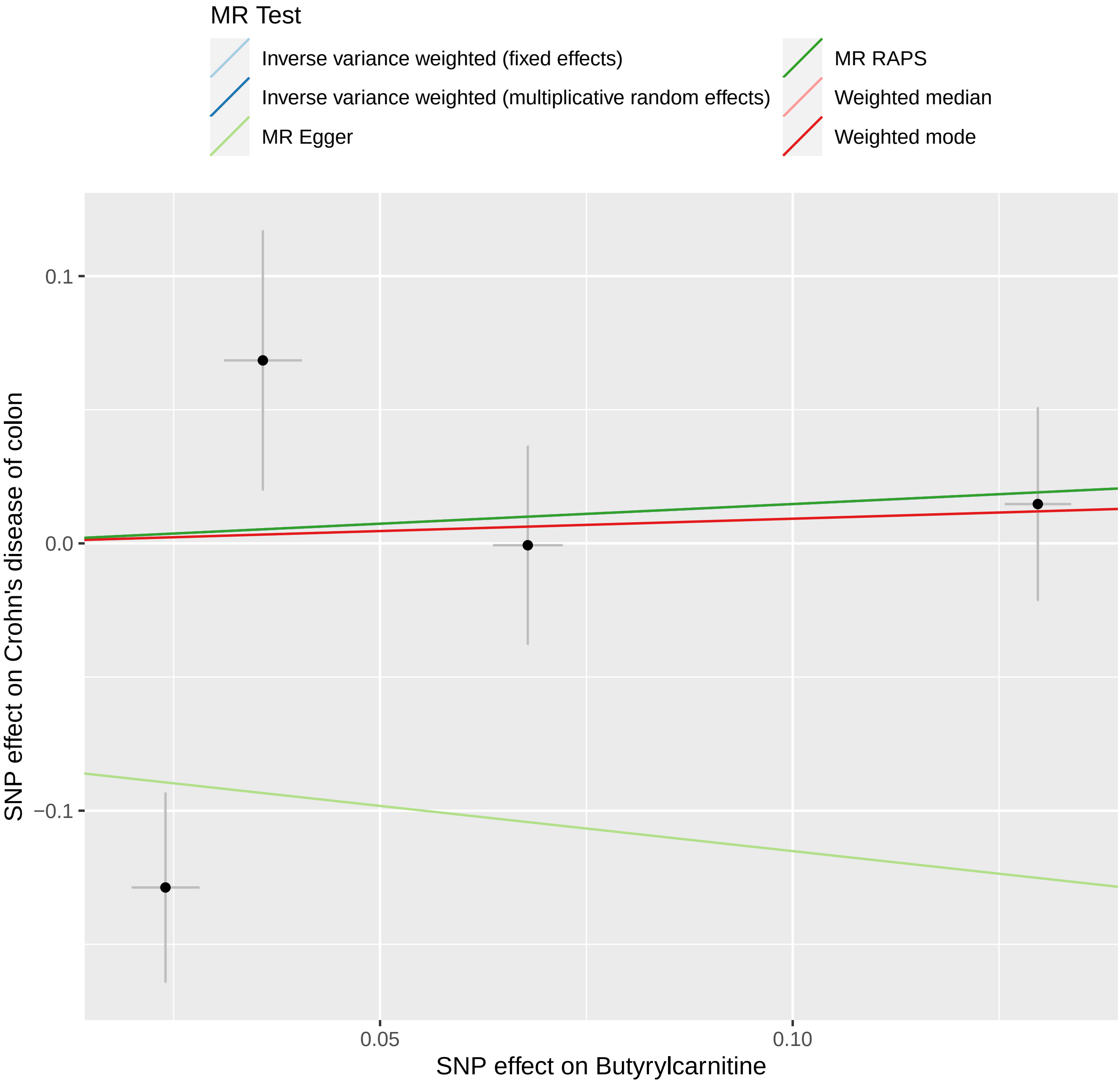

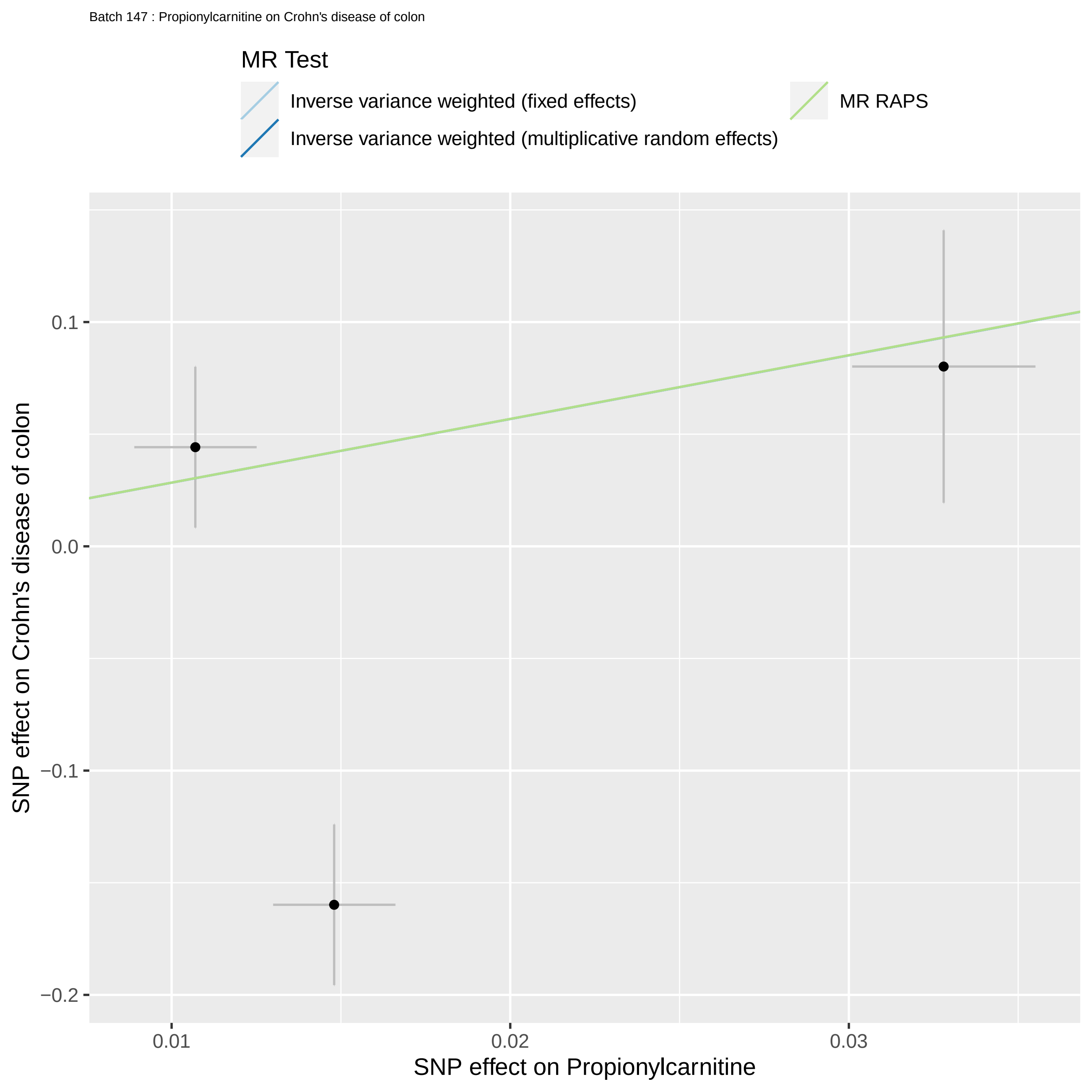
Batch 147 : Propionylcarnitine on Crohn's disease of colon
MR Test
Inverse variance weighted (fixed effects)
Inverse variance weighted (multiplicative random effects)
MR RAPS
SNP effect on Crohn's disease of colon
0.1
0.0
−0.1
−0.2
0.01
0.02
0.03
SNP effect on Propionylcarnitine

Batch 148 : Caproate (6:0) on Crohn's disease of colon

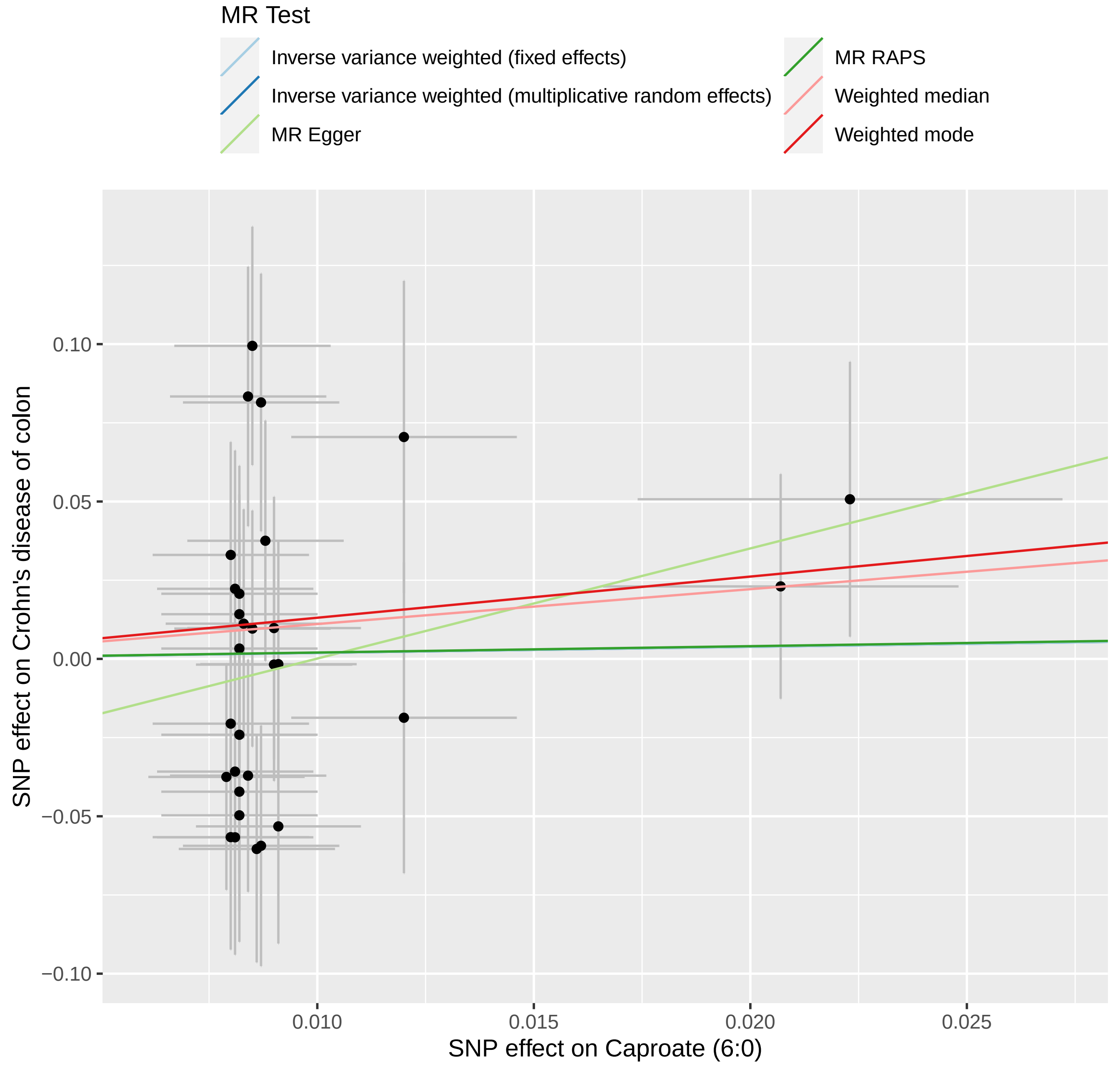

Batch 149 : Caprylate (8:0) on Crohn's disease of colon

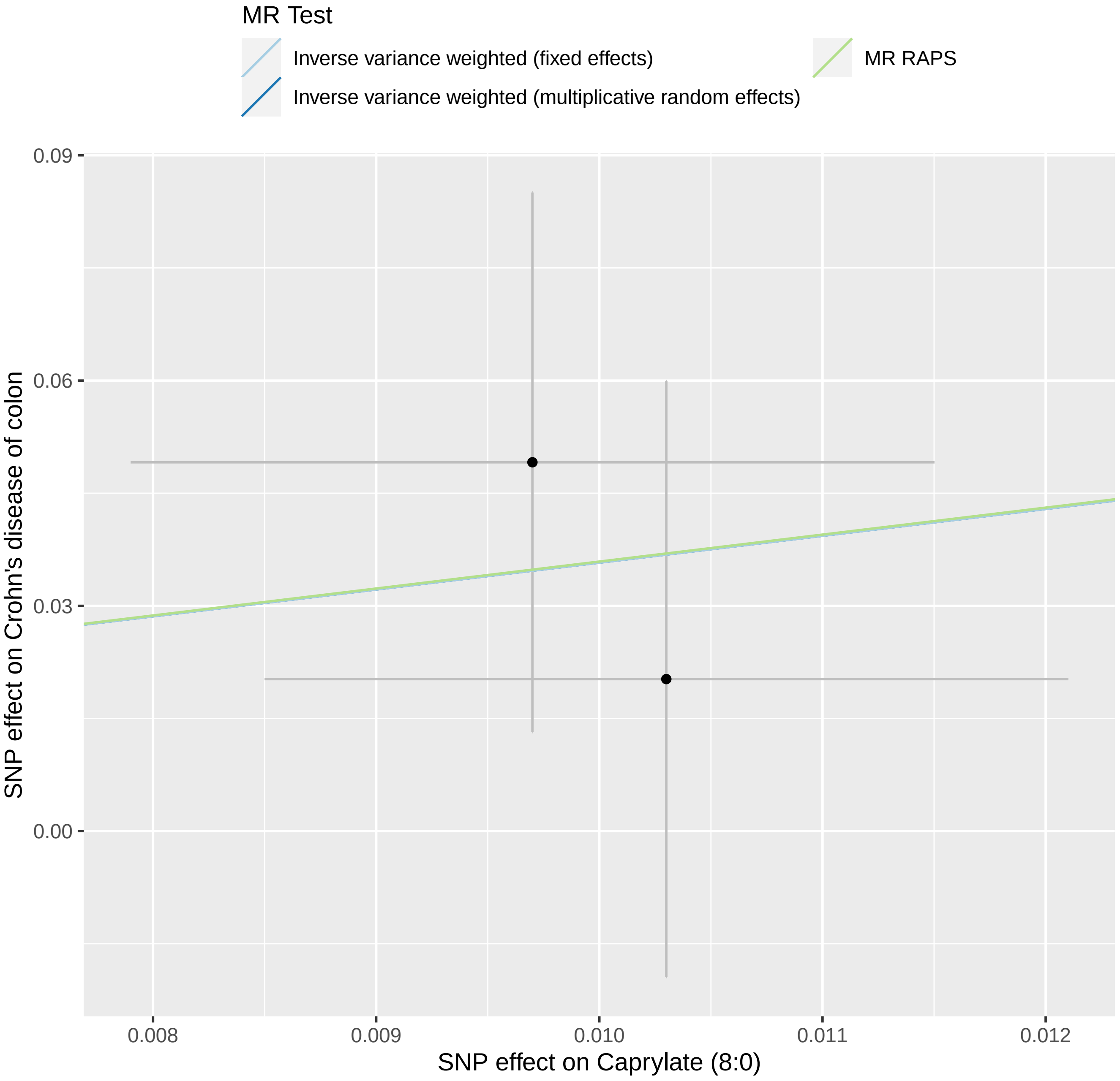

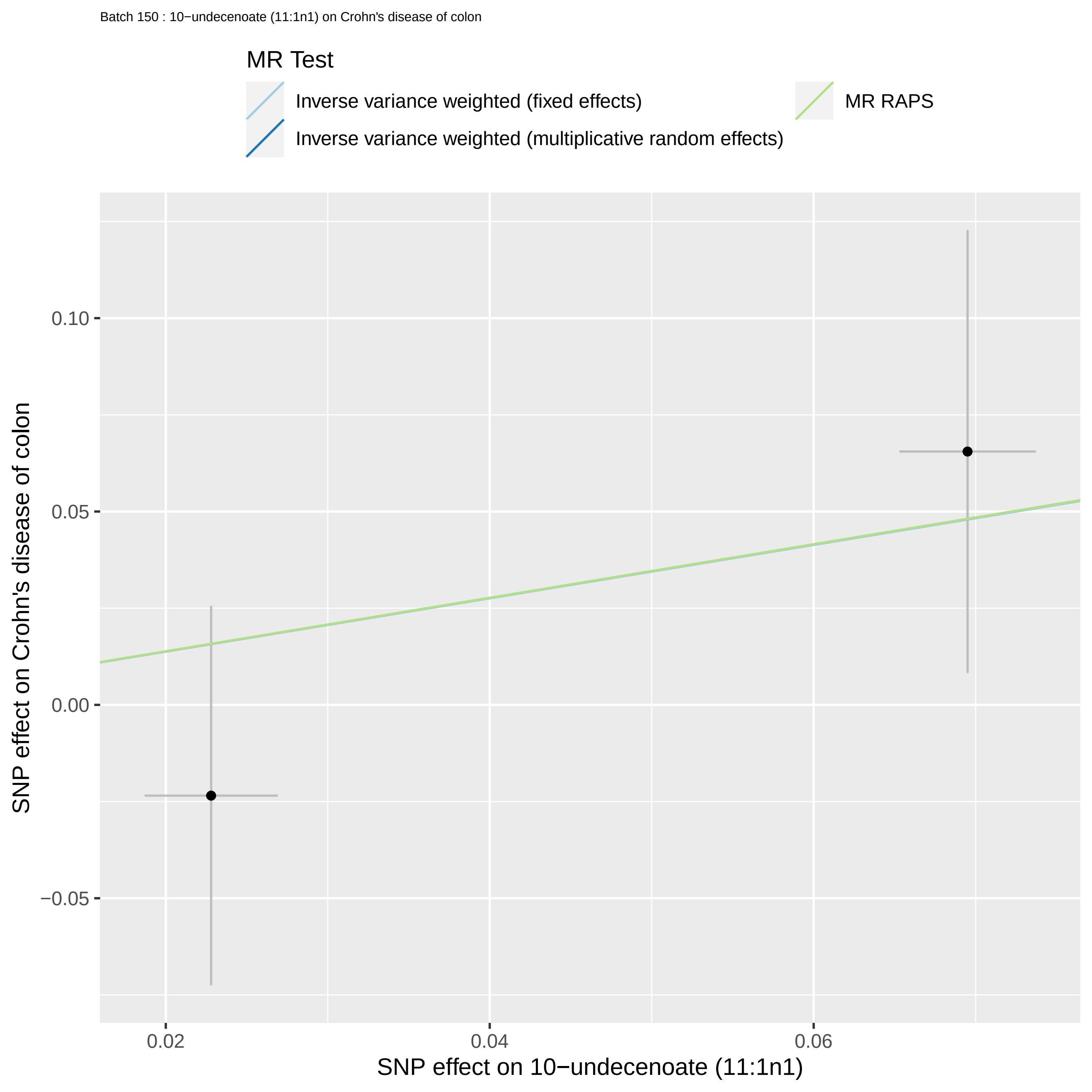
Batch 150 : 10−undecenoate (11:1n1) on Crohn's disease of colon
MR Test
Inverse variance weighted (fixed effects)
Inverse variance weighted (multiplicative random effects)
MR RAPS
0.10
0.05
0.00
−0.05
0.02
0.04
0.06
SNP effect on Crohn's disease of colon
SNP effect on 10−undecenoate (11:1n1)

Batch 152 : Phenol sulfate on Crohn's disease of colon

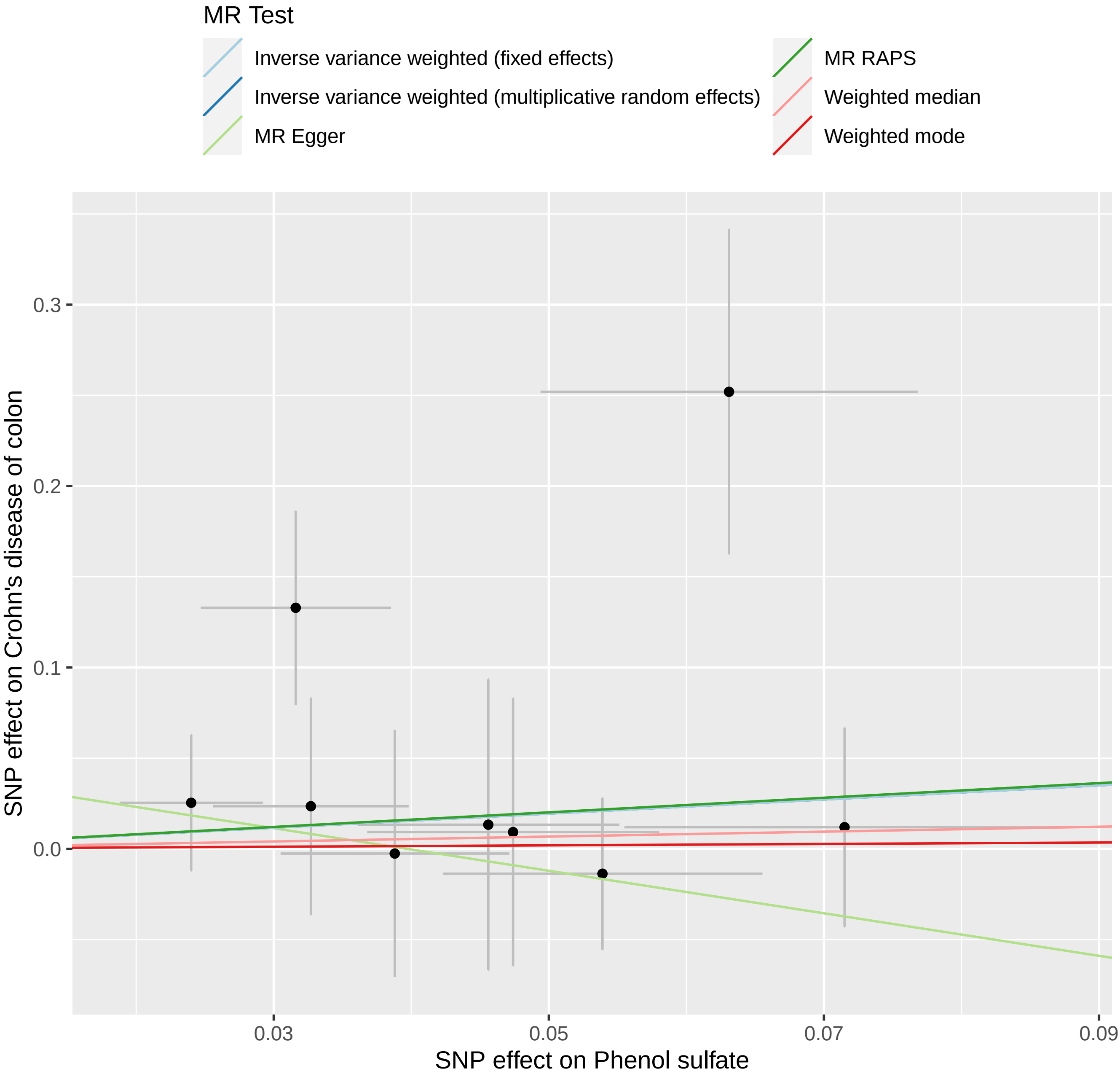

Batch 154 : Heme* on Crohn's disease of colon

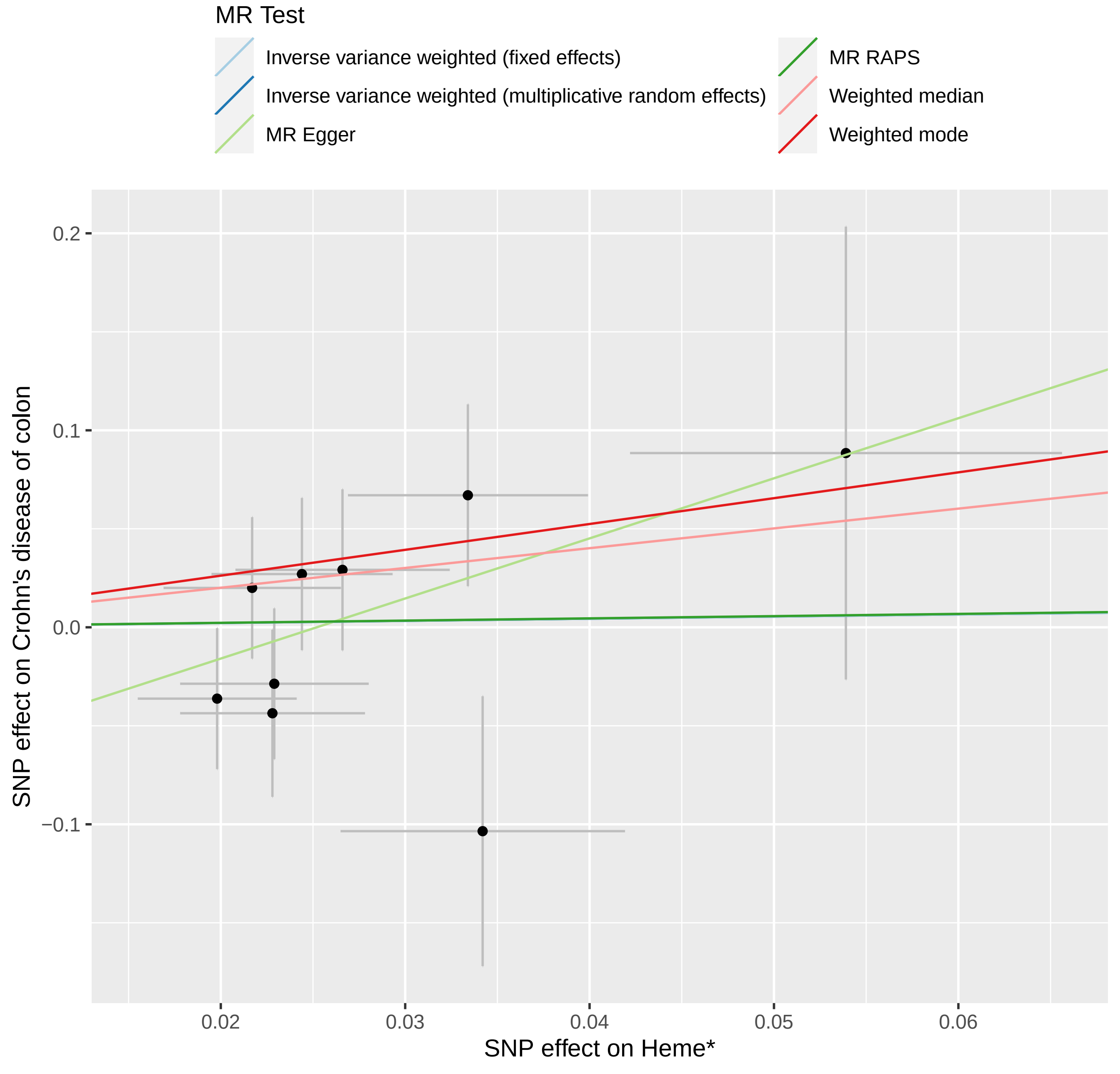

Batch 157 : Pyroglutamine* on Crohn's disease of colon

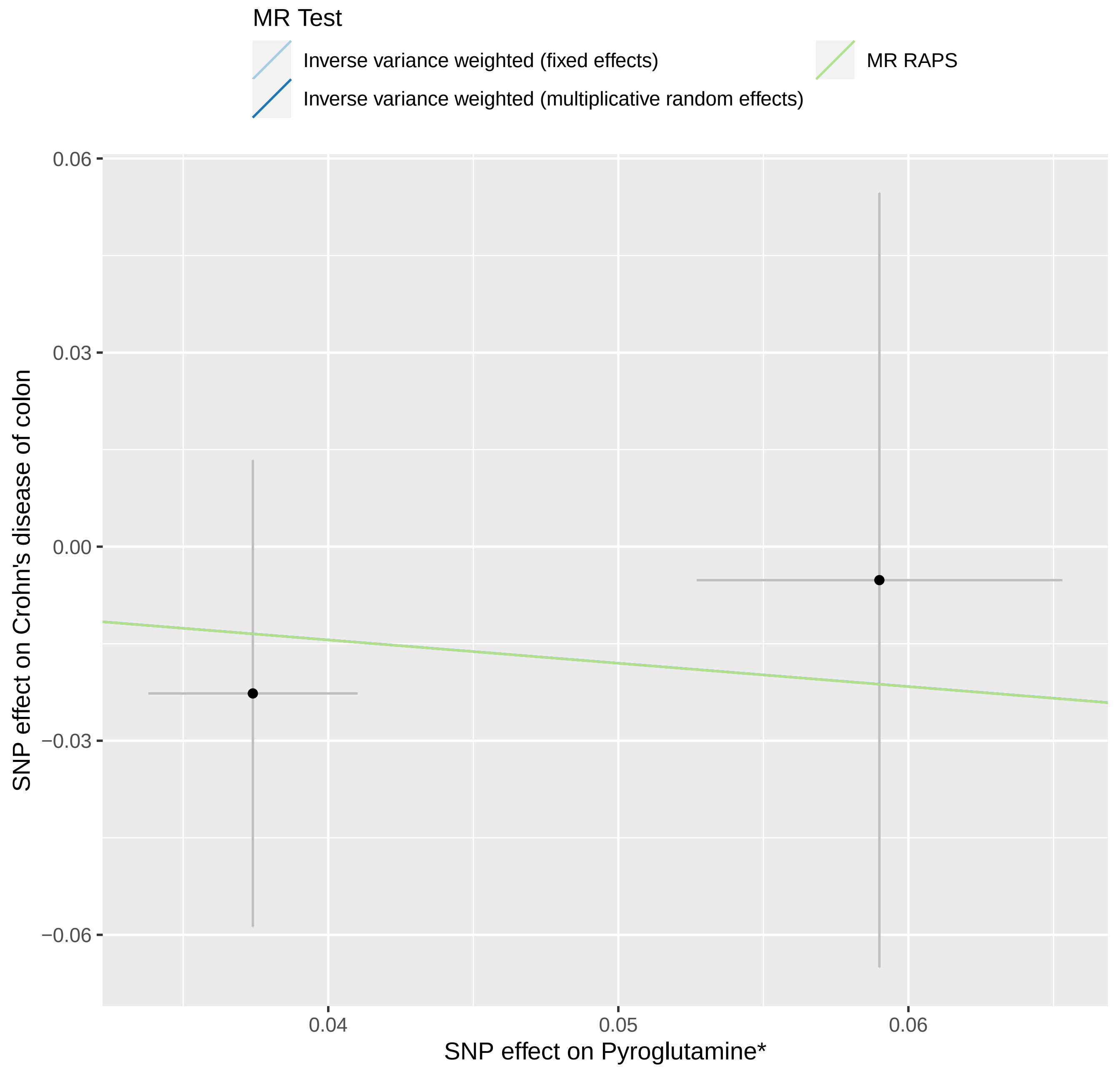

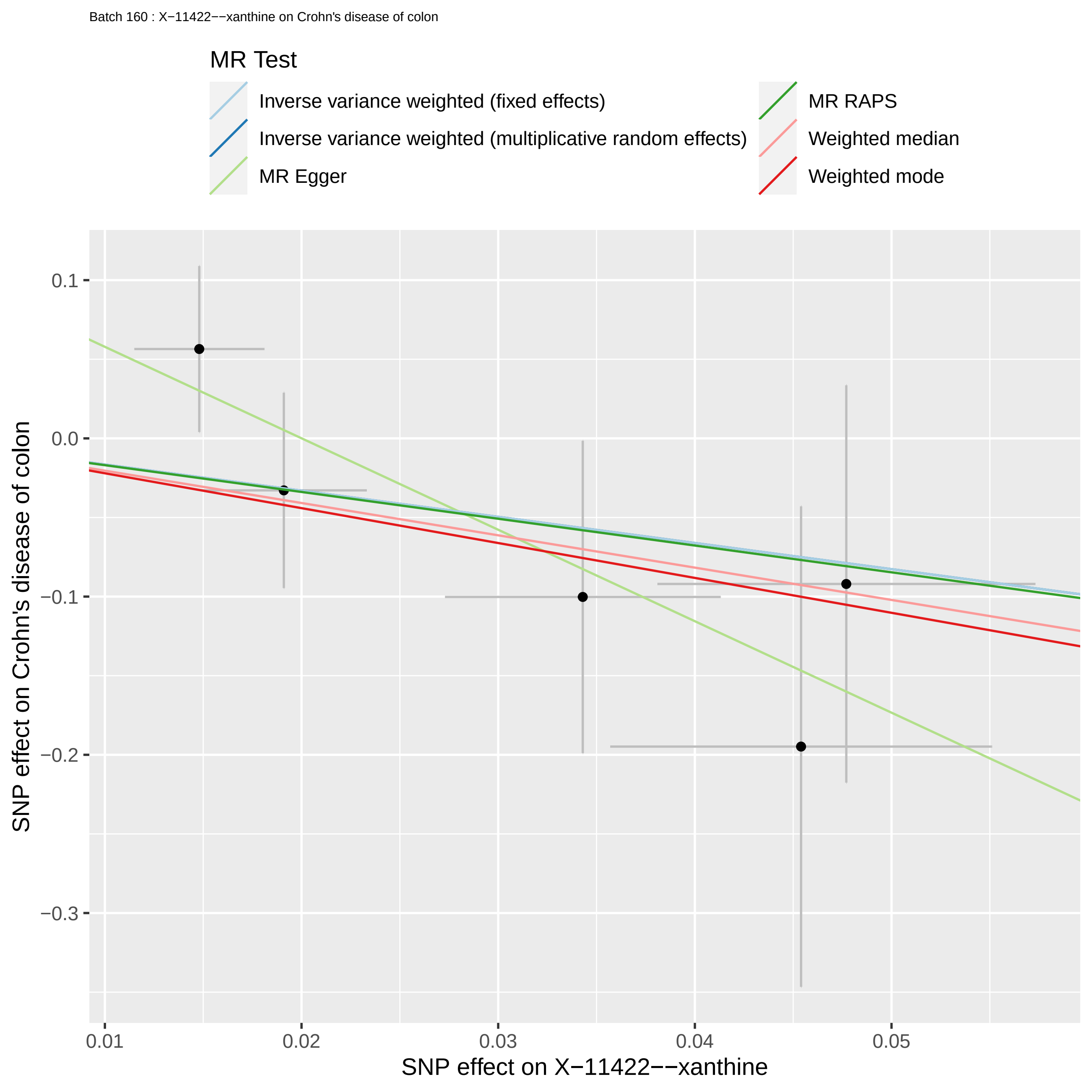
Batch 160 : X−11422−−xanthine on Crohn's disease of colon
MR Test
Inverse variance weighted (fixed effects)
Inverse variance weighted (multiplicative random effects)
MR Egger
MR RAPS
Weighted median
Weighted mode
SNP effect on Crohn's disease of colon
0.1
0.0
−0.1
−0.2
−0.3
0.01
0.02
0.03
0.04
0.05
SNP effect on X−11422−−xanthine

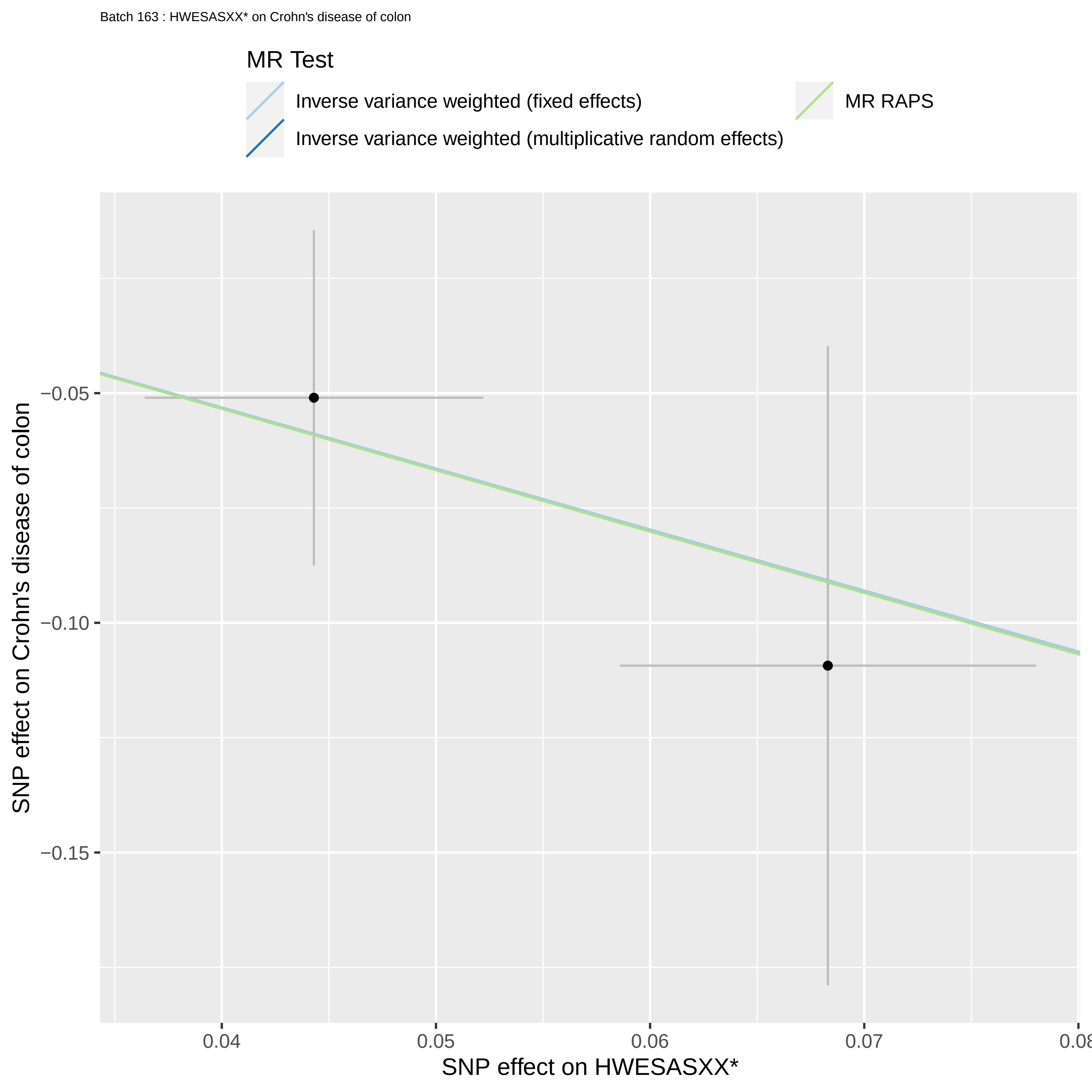
Batch 163 : HWESASXX* on Crohn's disease of colon
MR Test
Inverse variance weighted (fixed effects)
Inverse variance weighted (multiplicative random effects)
MR RAPS
SNP effect on Crohn's disease of colon
−0.05
−0.10
−0.15
0.04
0.05
0.06
0.07
0.08
SNP effect on HWESASXX*

Batch 164 : X−11593−−O−methylascorbate* on Crohn's disease of colon

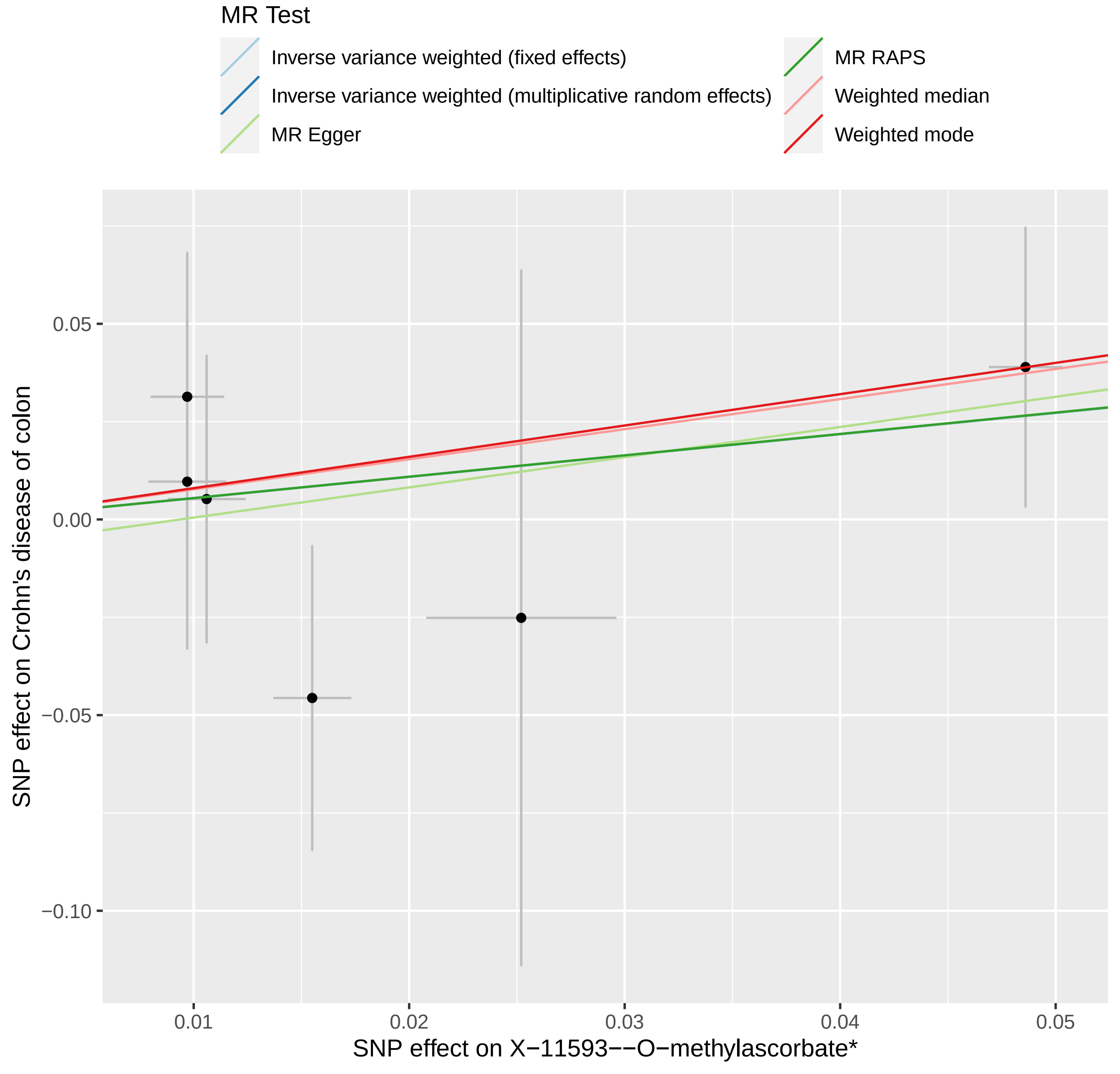

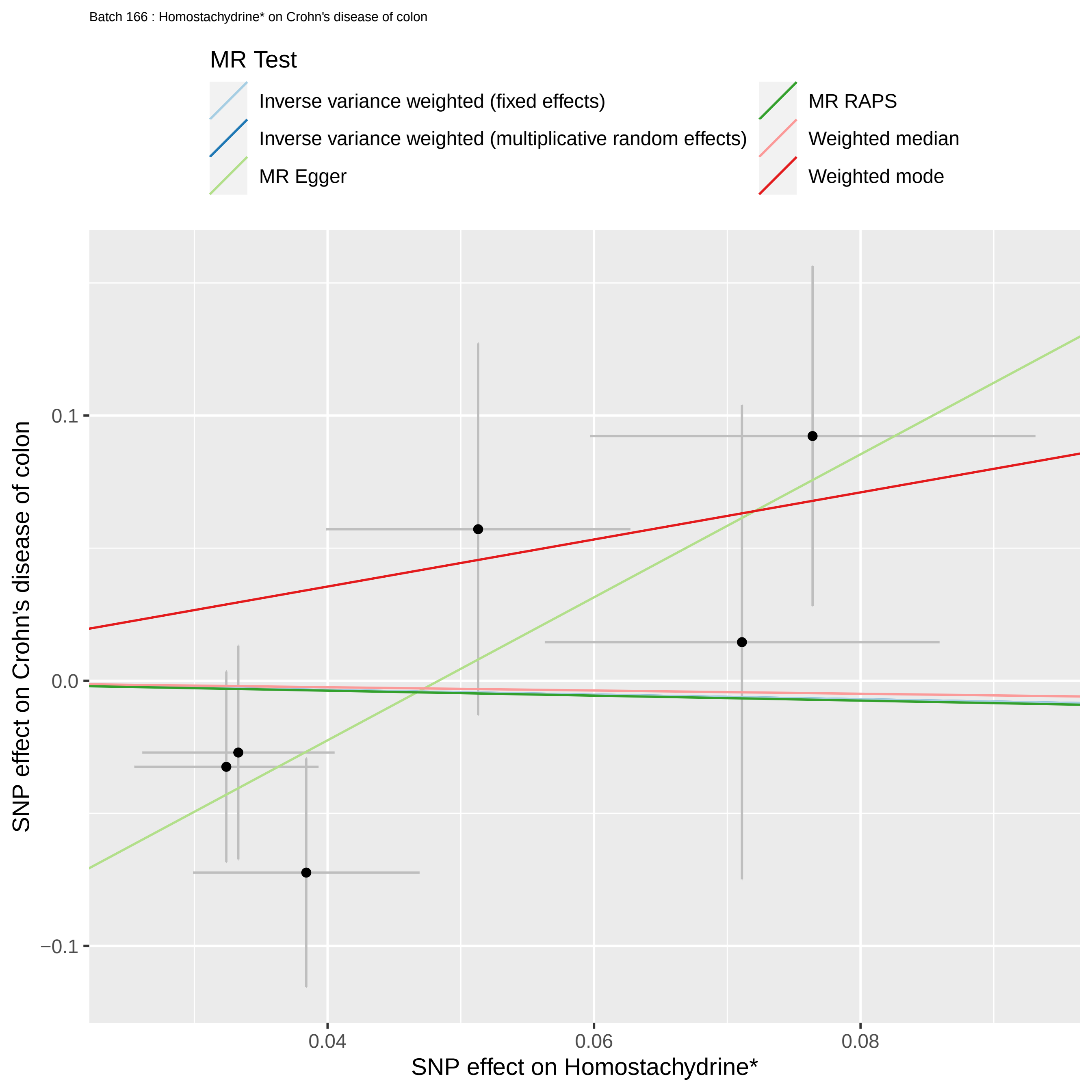

Batch 166 : Homostachydrine* on Crohn's disease of colon
MR Test
Inverse variance weighted (fixed effects)
Inverse variance weighted (multiplicative random effects)
MR Egger
MR RAPS
Weighted median
Weighted mode
0.1
0.0
−0.1
0.04
0.06
0.08
SNP effect on Crohn's disease of colon
SNP effect on Homostachydrine*

Batch 167 : ADSGEGDFXAEGGGVR* on Crohn's disease of colon

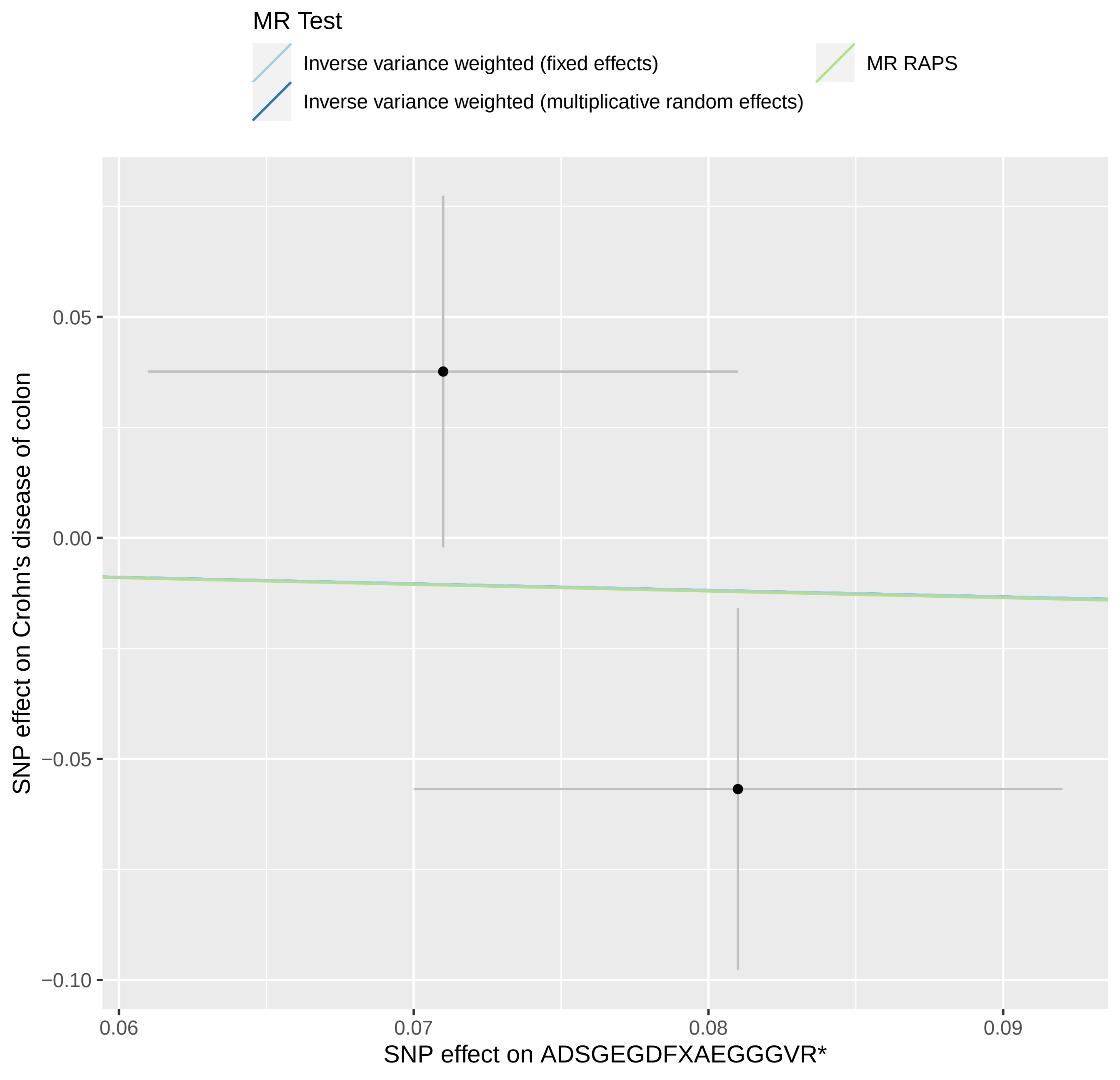

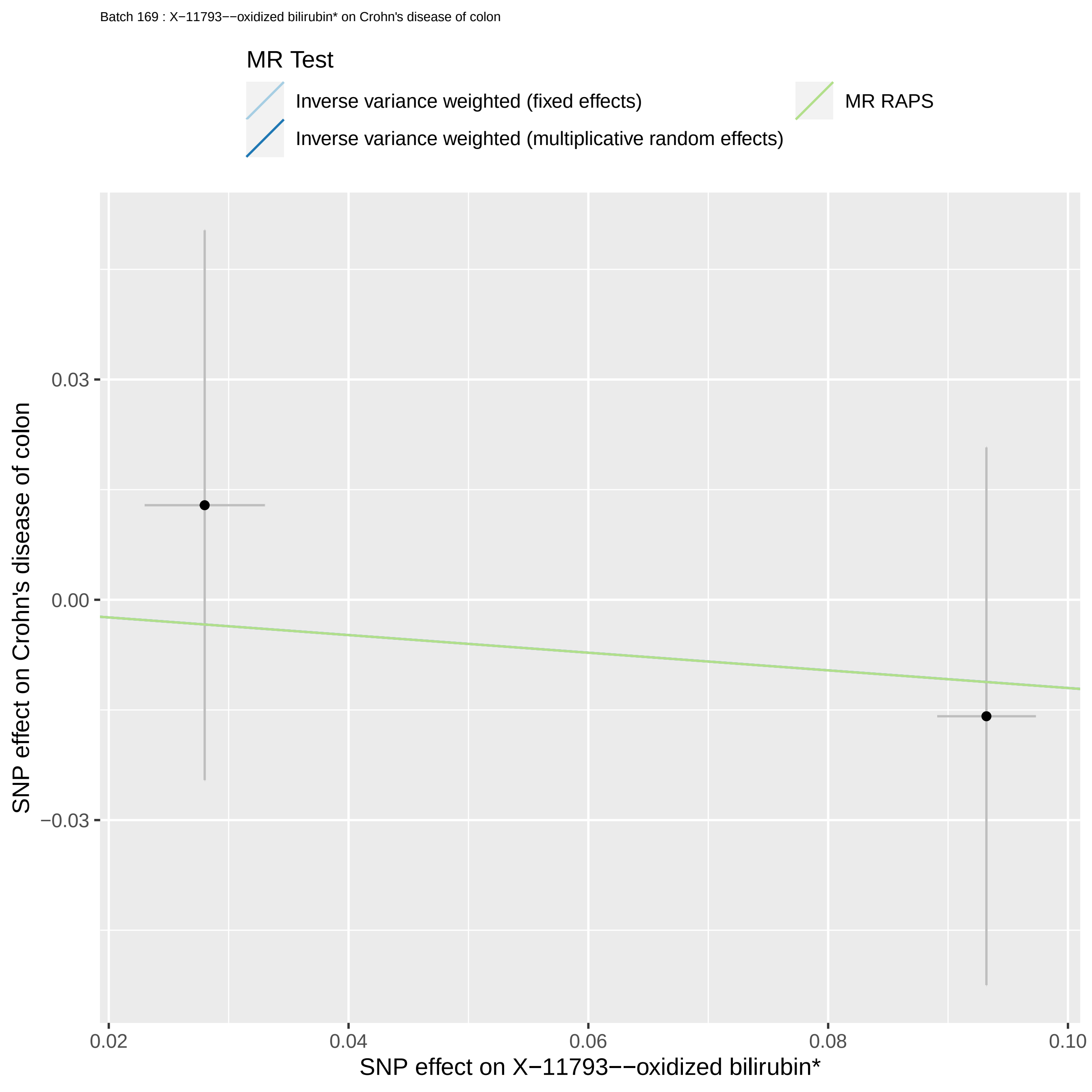

Batch 169 : X−11793−−oxidized bilirubin* on Crohn's disease of colon
MR Test
Inverse variance weighted (fixed effects)
Inverse variance weighted (multiplicative random effects)
MR RAPS
SNP effect on Crohn's disease of colon
0.03
0.00
−0.03
0.02
0.04
0.06
0.08
0.10
SNP effect on X−11793−−oxidized bilirubin*

Batch 170 : 1−arachidonoylglycerophosphocholine* on Crohn's disease of colon

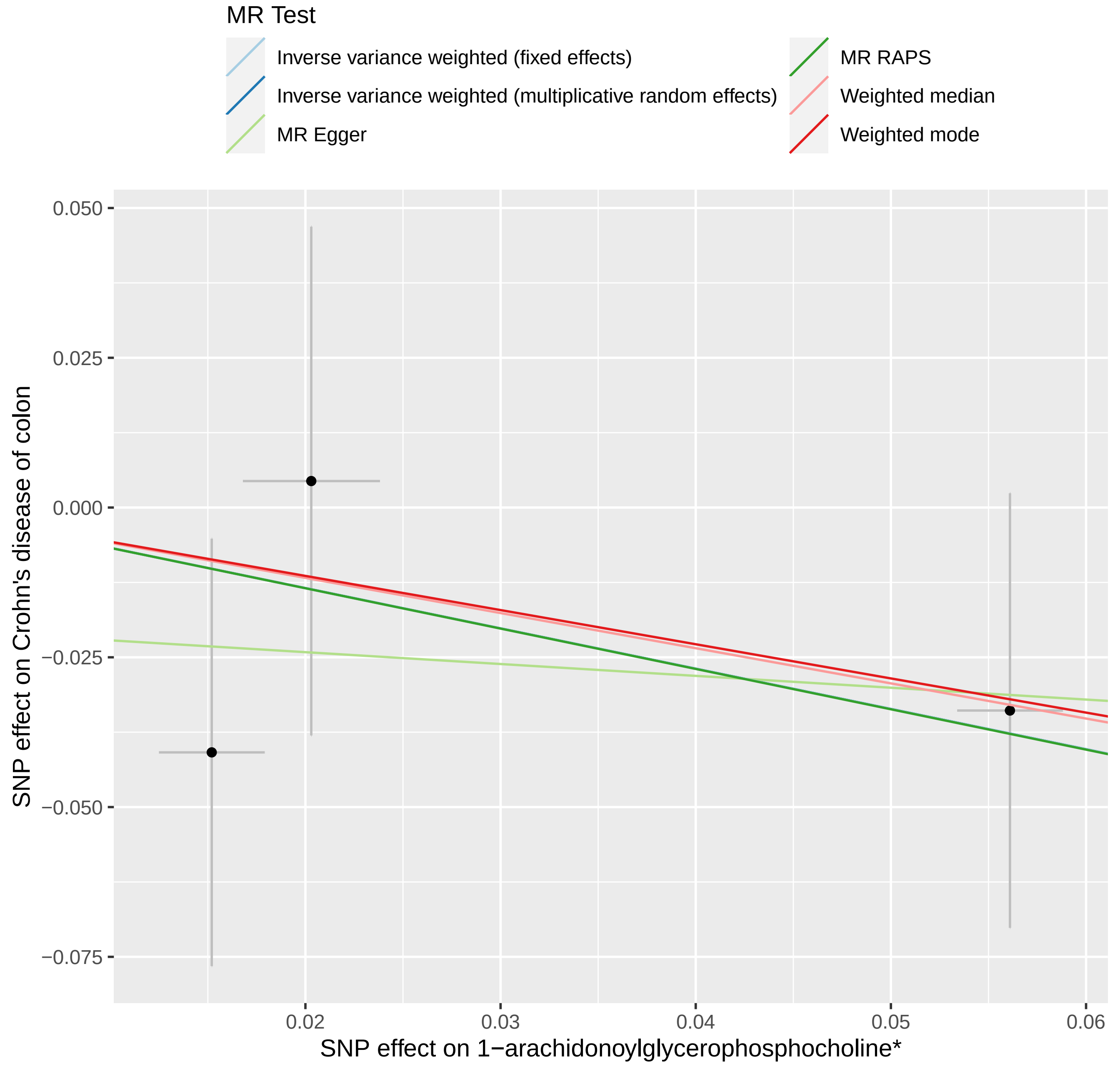

Batch 172 : Gamma−glutamylmethionine* on Crohn's disease of colon

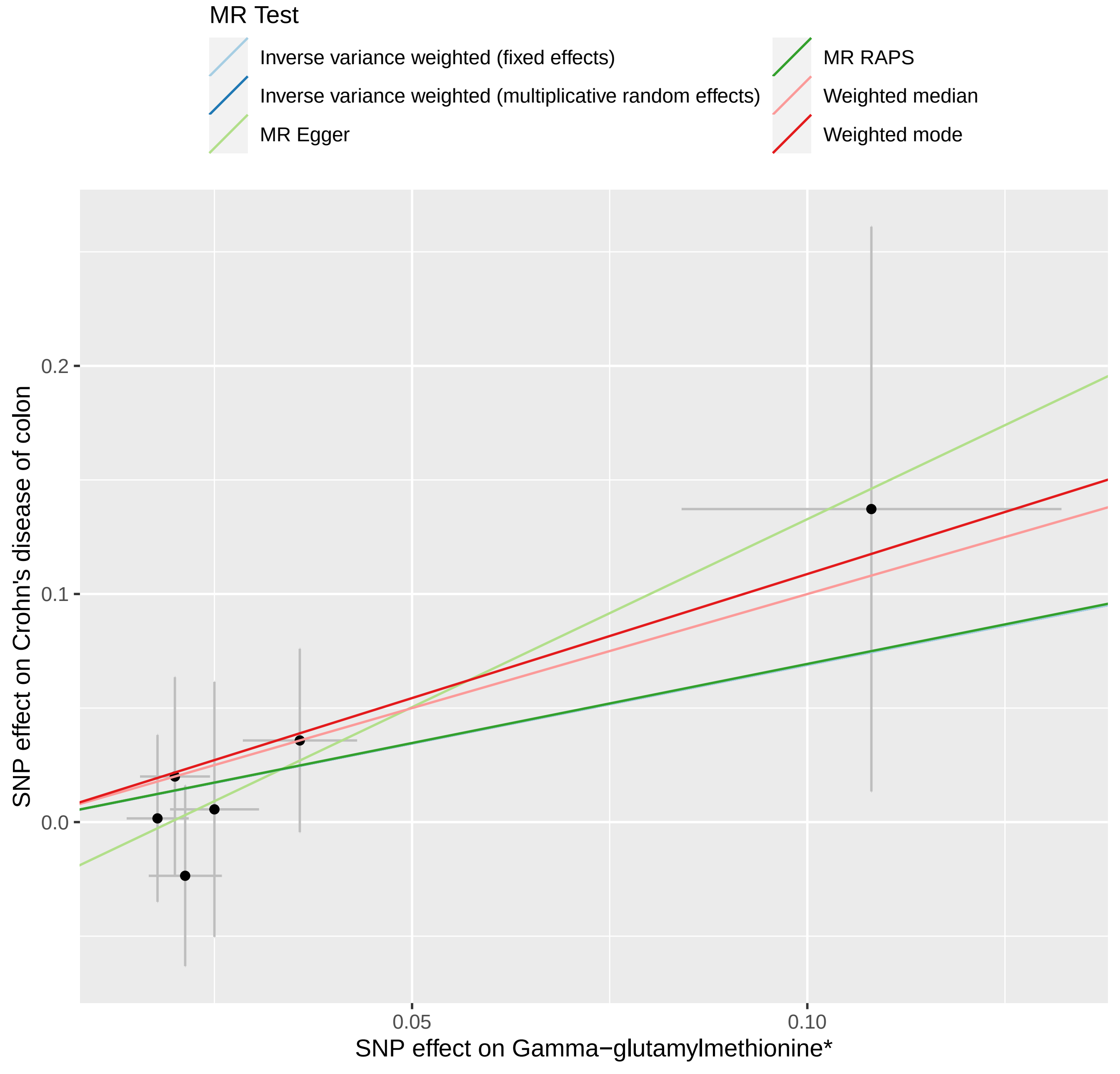

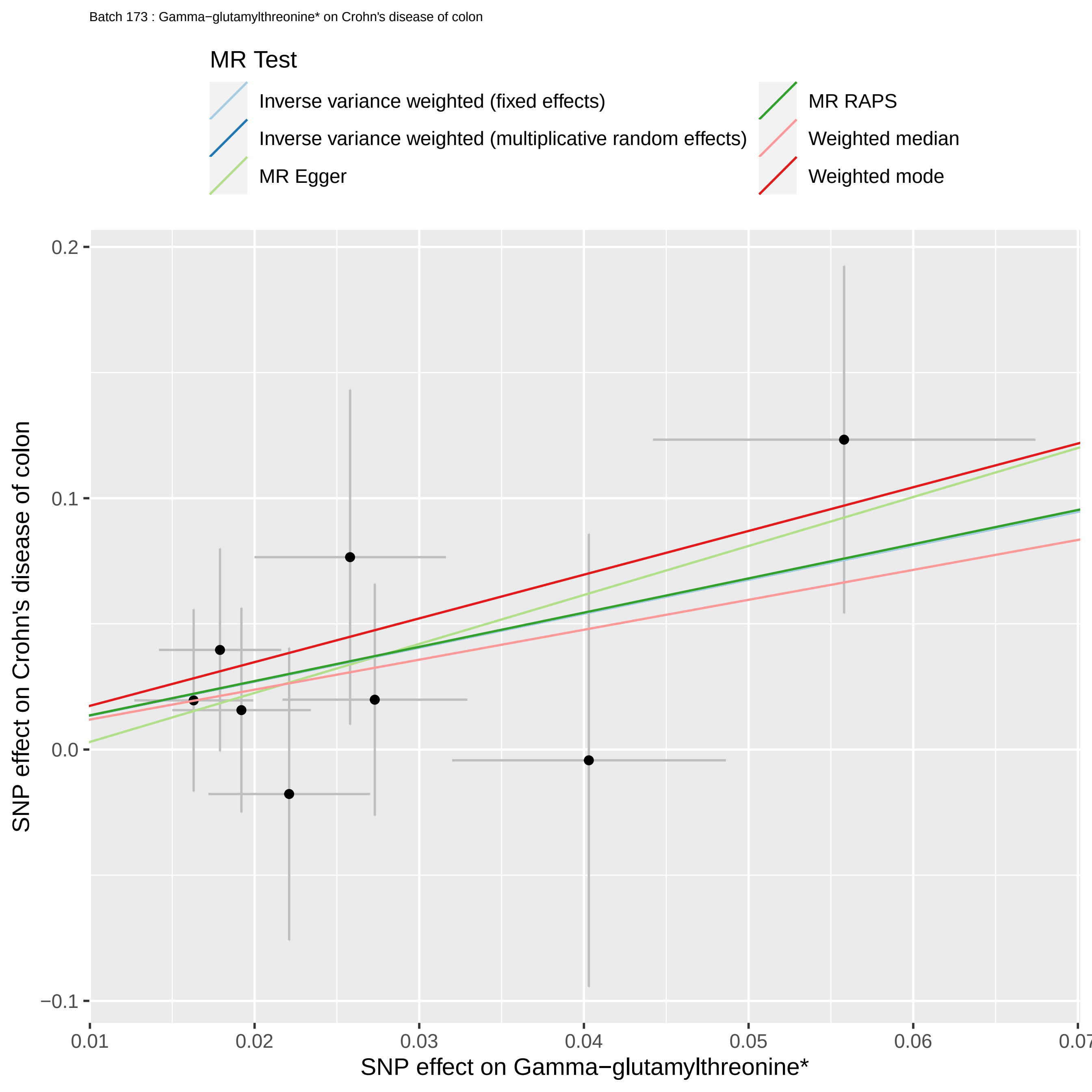
Batch 173 : Gamma−glutamylthreonine* on Crohn's disease of colon
MR Test
Inverse variance weighted (fixed effects)
Inverse variance weighted (multiplicative random effects)
MR Egger
MR RAPS
Weighted median
Weighted mode
0.2
0.1
0.0
−0.1
0.01
0.02
0.03
0.04
0.05
0.06
0.07
SNP effect on Crohn's disease of colon
SNP effect on Gamma−glutamylthreonine*

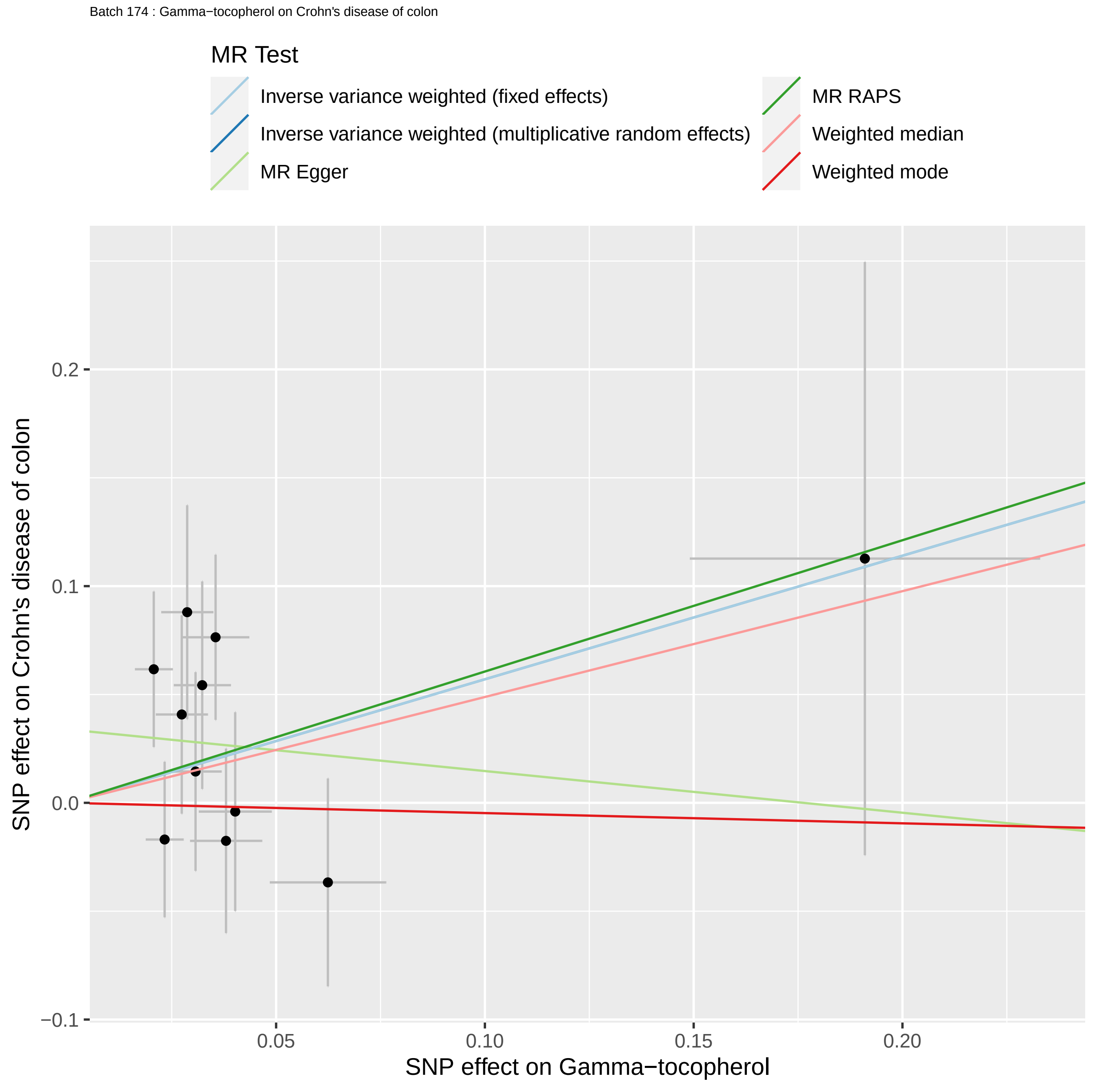

Batch 174 : Gamma−tocopherol on Crohn's disease of colon
MR Test
Inverse variance weighted (fixed effects)
Inverse variance weighted (multiplicative random effects)
MR Egger
MR RAPS
Weighted median
Weighted mode
SNP effect on Crohn's disease of colon
0.2
0.1
0.0
−0.1
0.05
0.10
0.15
0.20
SNP effect on Gamma−tocopherol

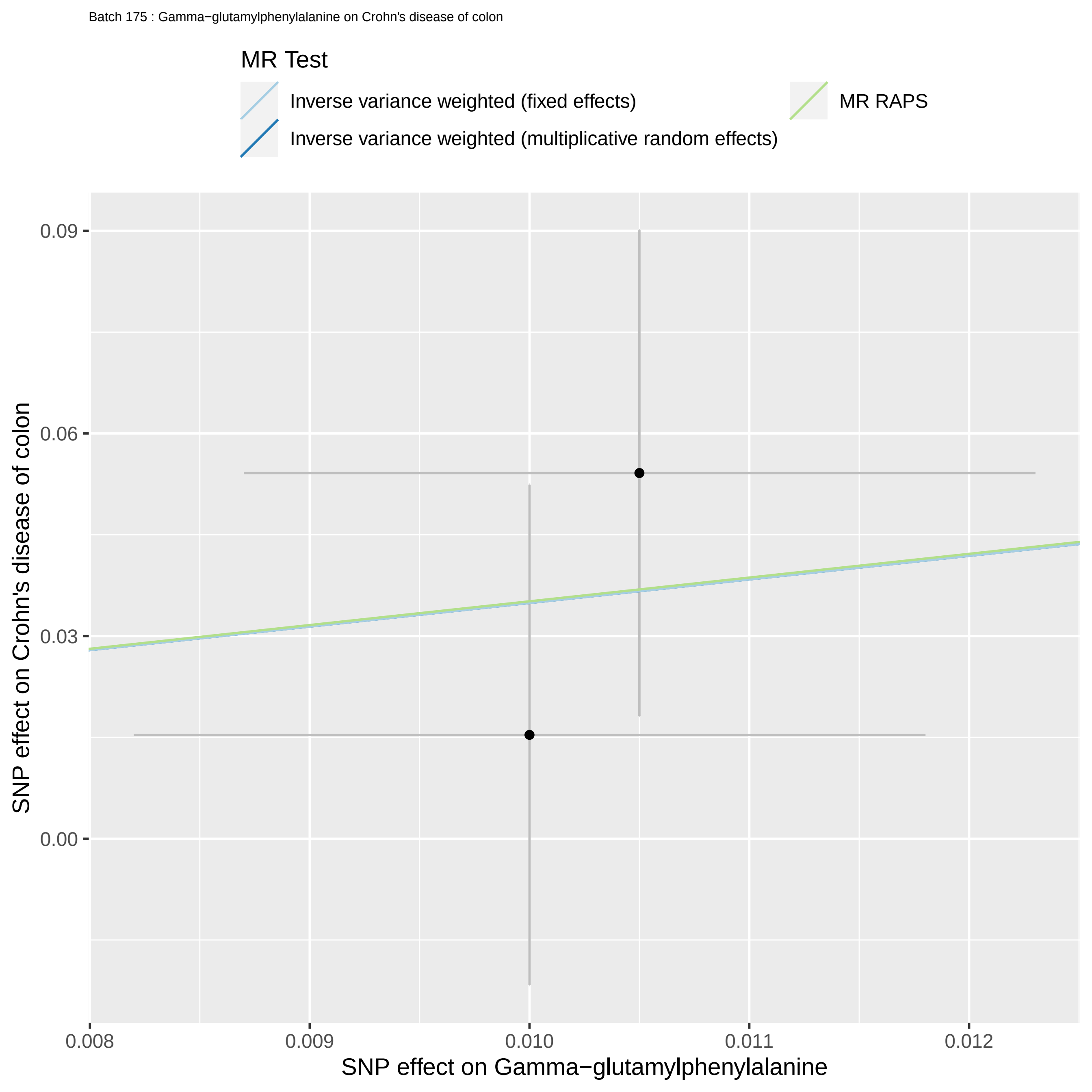

Batch 175 : Gamma−glutamylphenylalanine on Crohn's disease of colon
MR Test
Inverse variance weighted (fixed effects)
Inverse variance weighted (multiplicative random effects)
MR RAPS
0.09
0.06
0.03
0.00
SNP effect on Crohn's disease of colon
0.008
0.009
0.010
0.011
0.012
SNP effect on Gamma−glutamylphenylalanine

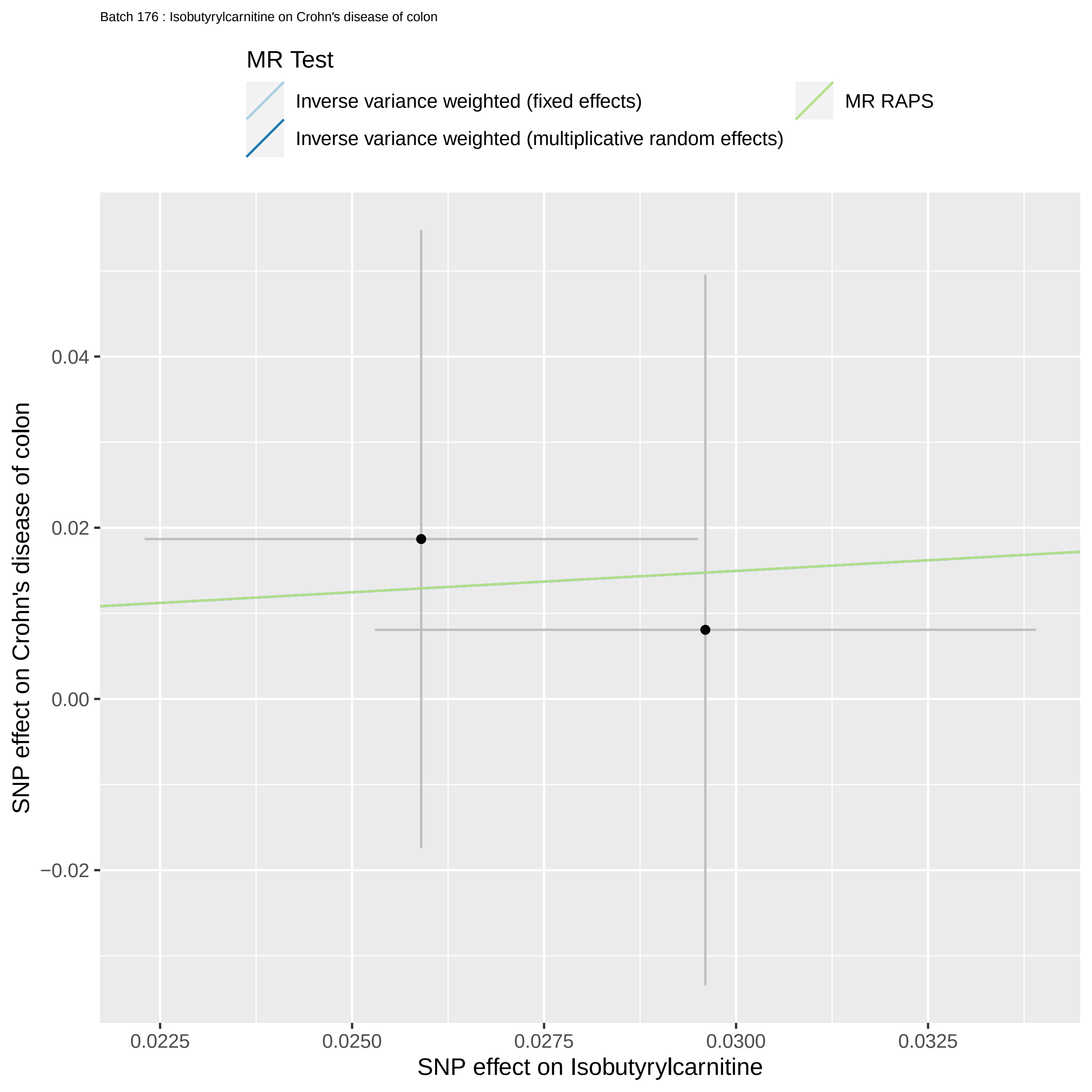
Batch 176 : Isobutyrylcarnitine on Crohn's disease of colon
MR Test
Inverse variance weighted (fixed effects)
Inverse variance weighted (multiplicative random effects)
MR RAPS
0.04
0.02
0.00
−0.02
SNP effect on Crohn's disease of colon
0.0225
0.0250
0.0275
0.0300
0.0325
SNP effect on Isobutyrylcarnitine

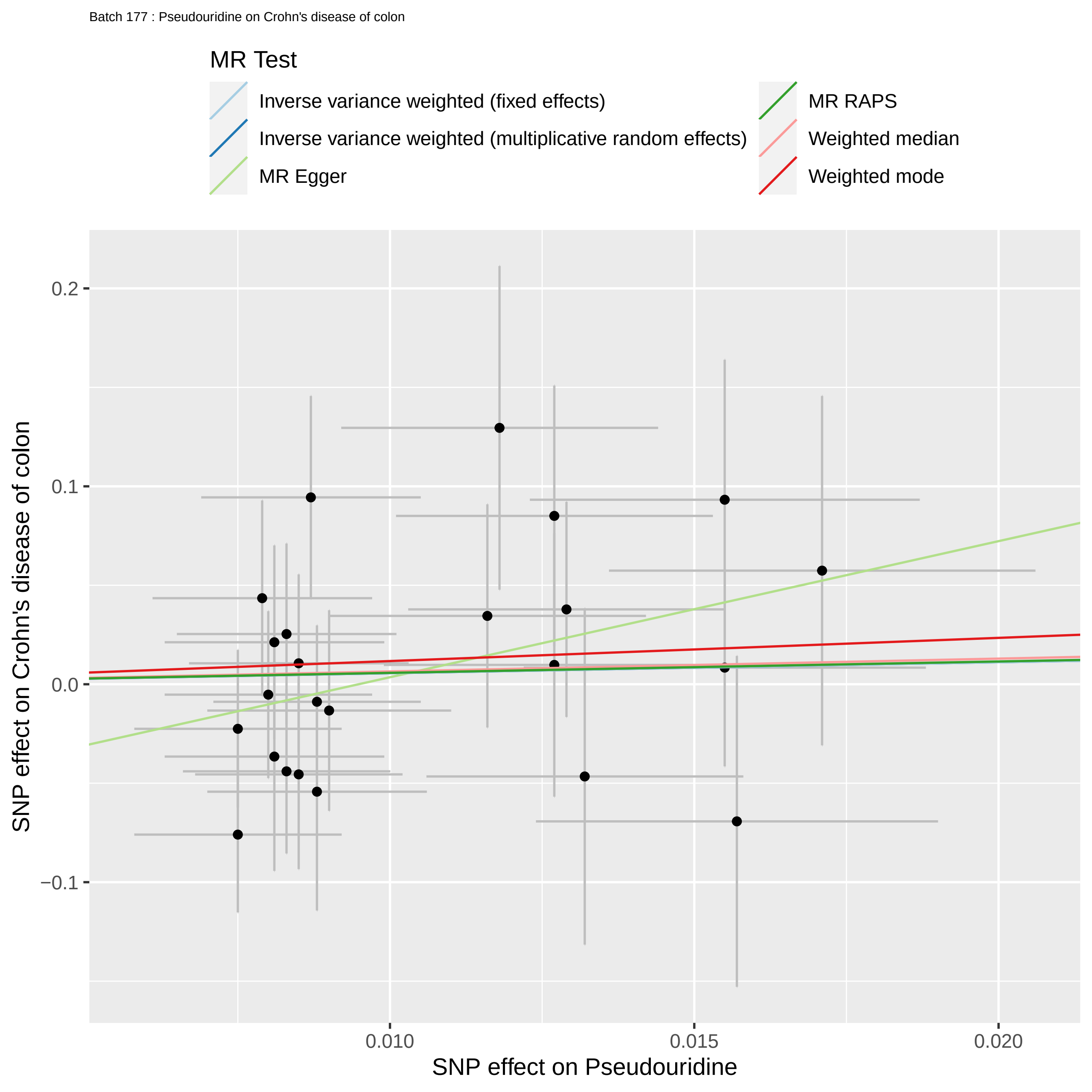
Batch 177 : Pseudouridine on Crohn's disease of colon
MR Test
Inverse variance weighted (fixed effects)
Inverse variance weighted (multiplicative random effects)
MR Egger
MR RAPS
Weighted median
Weighted mode
0.2
0.1
0.0
−0.1
0.010
0.015
0.020
SNP effect on Pseudouridine
SNP effect on Crohn's disease of colon

Batch 178 : Valerate on Crohn's disease of colon

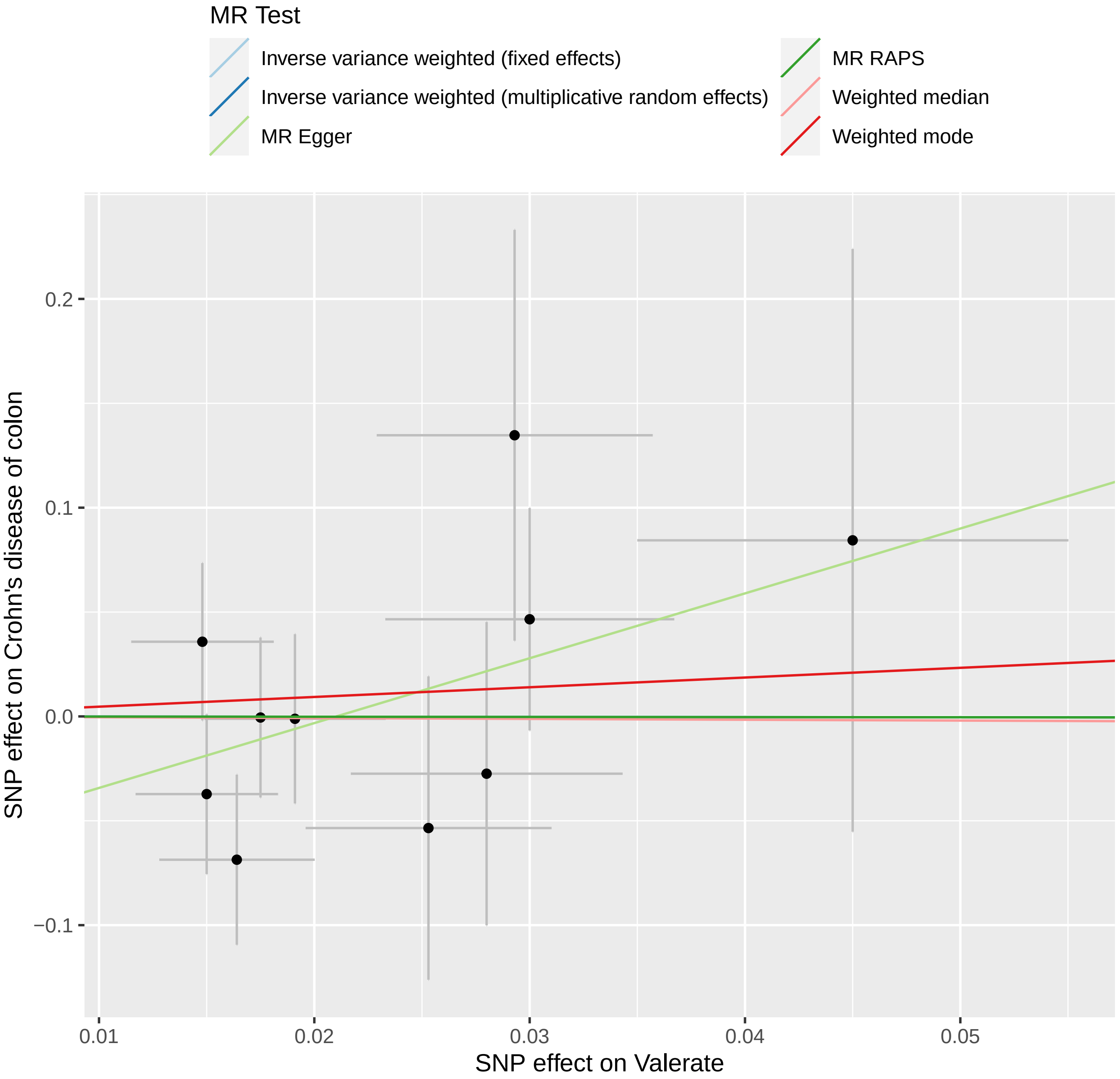

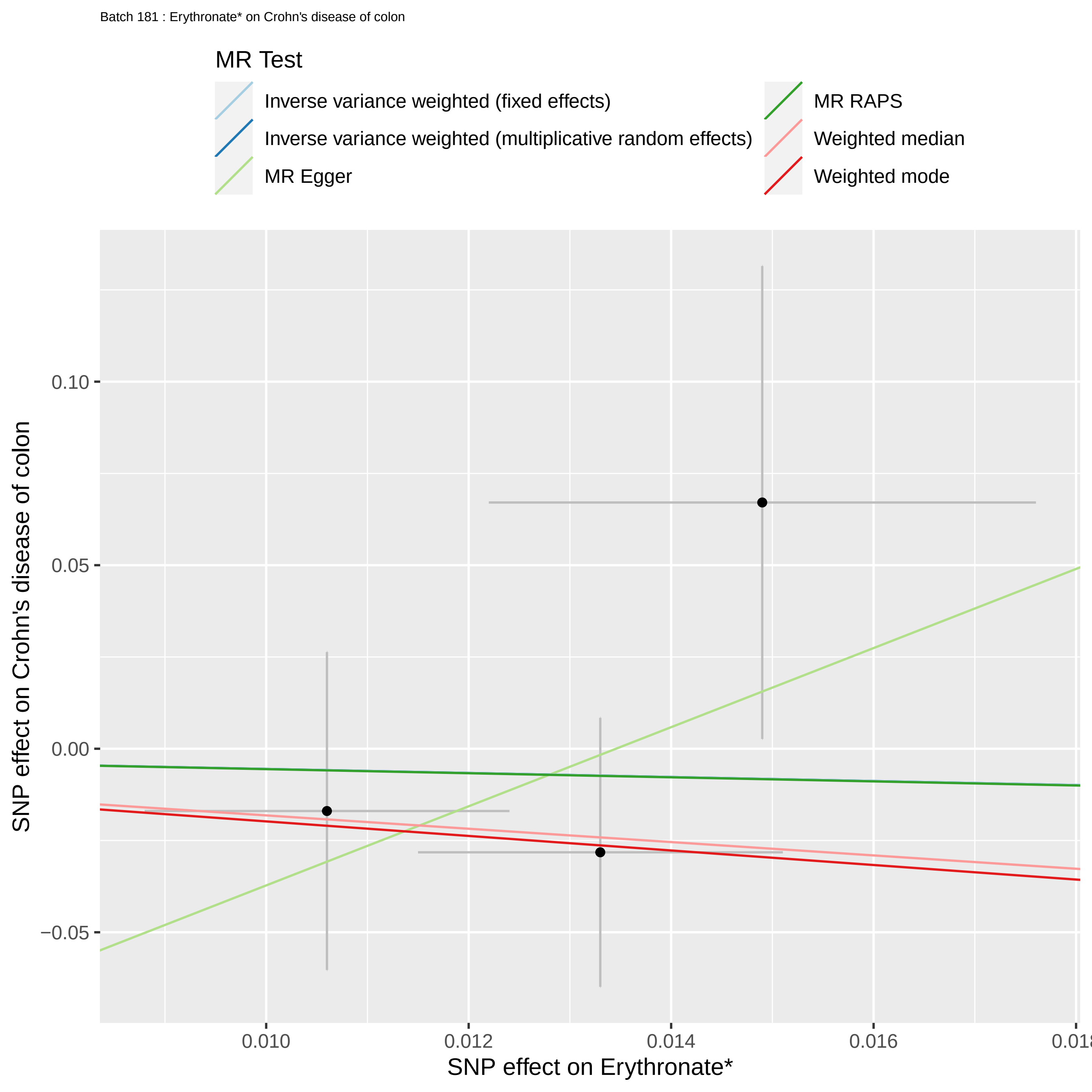

Batch 181 : Erythronate* on Crohn's disease of colon
MR Test
Inverse variance weighted (fixed effects)
Inverse variance weighted (multiplicative random effects)
MR Egger
MR RAPS
Weighted median
Weighted mode
0.10
0.05
0.00
−0.05
0.010
0.012
0.014
0.016
0.01
SNP effect on Erythronate*
SNP effect on Crohn's disease of colon

Batch 182 : Lathosterol on Crohn's disease of colon

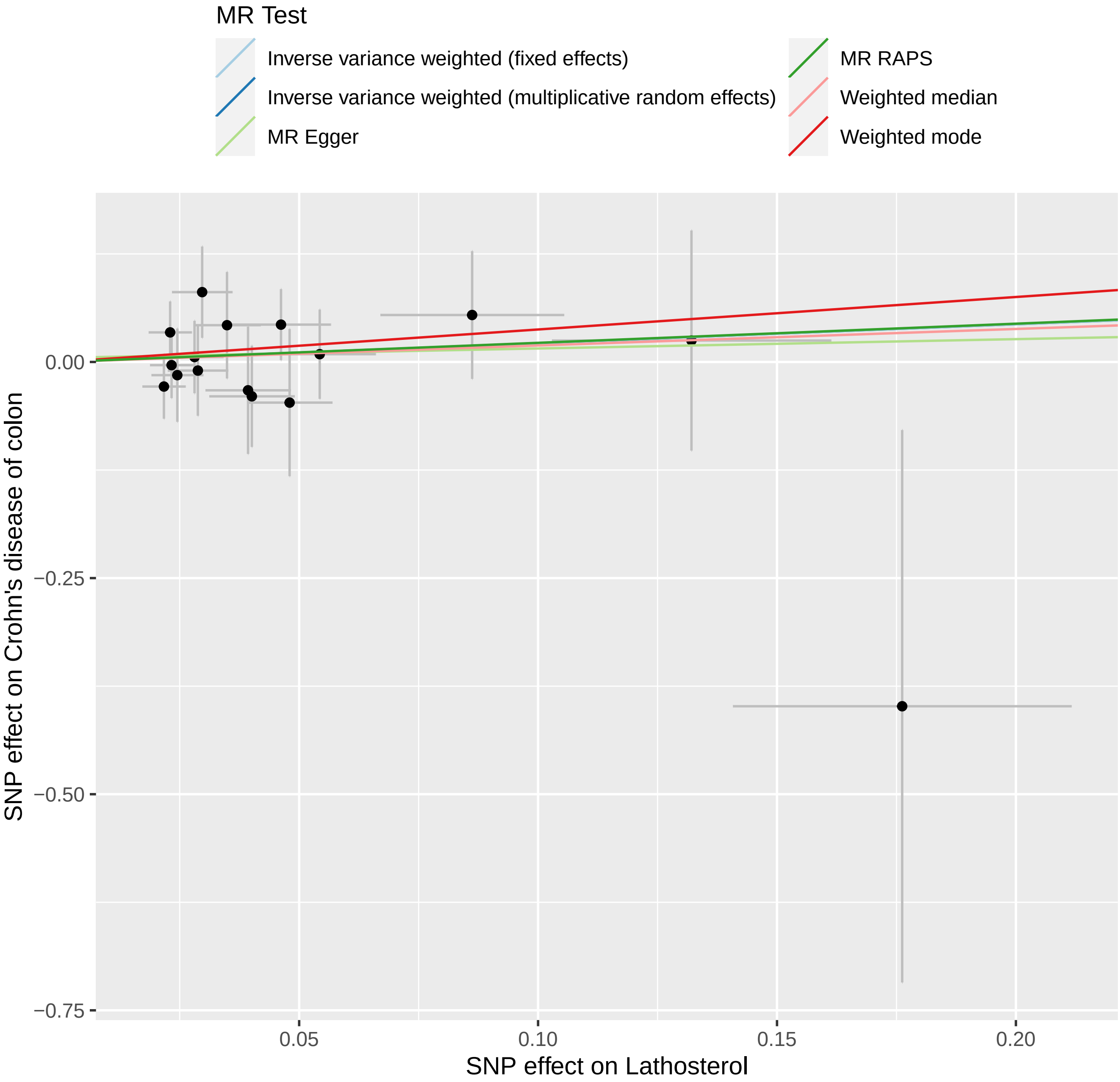

Batch 185 : Eicosenoate (20:1n9 or 11) on Crohn's disease of colon

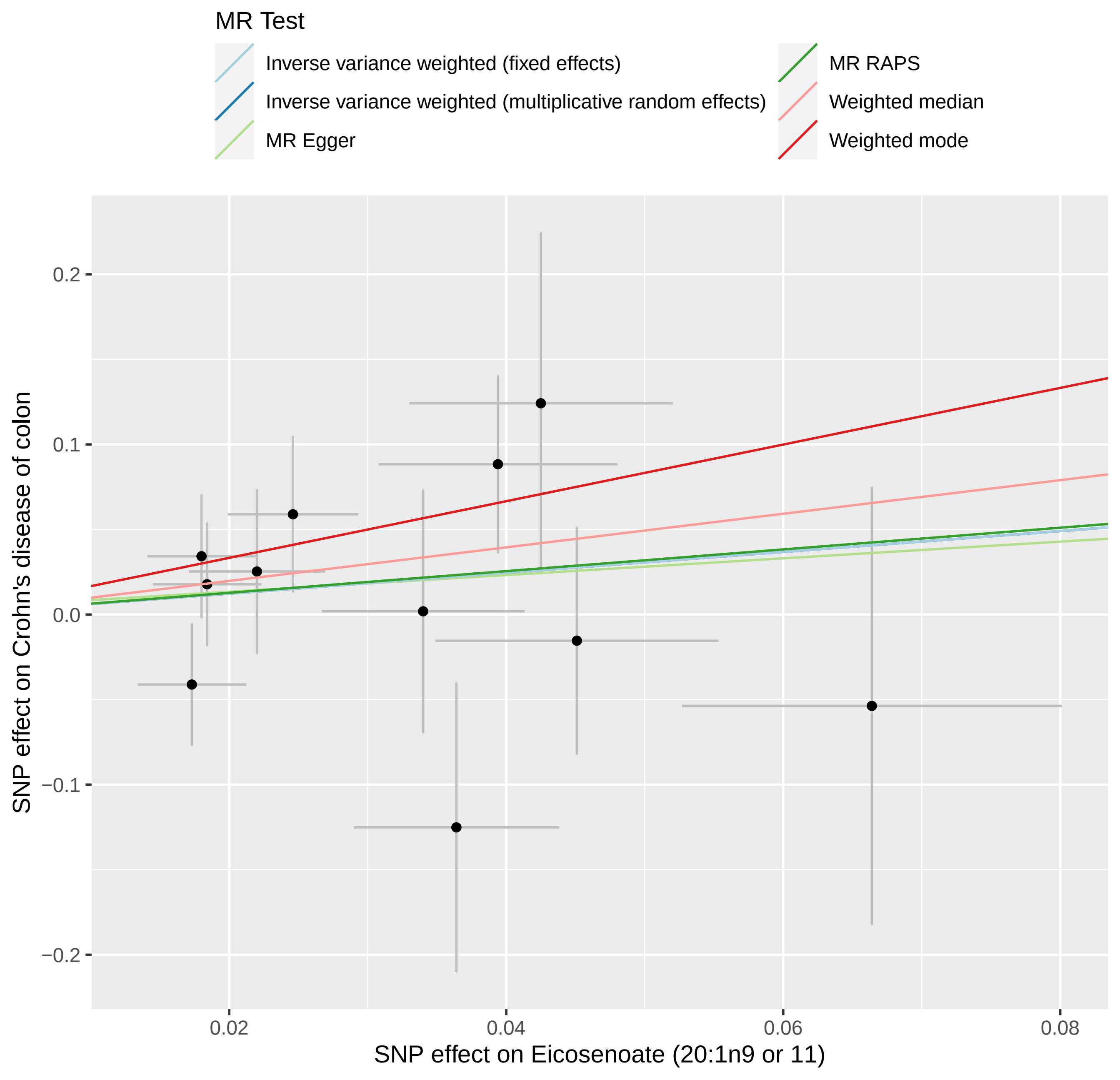

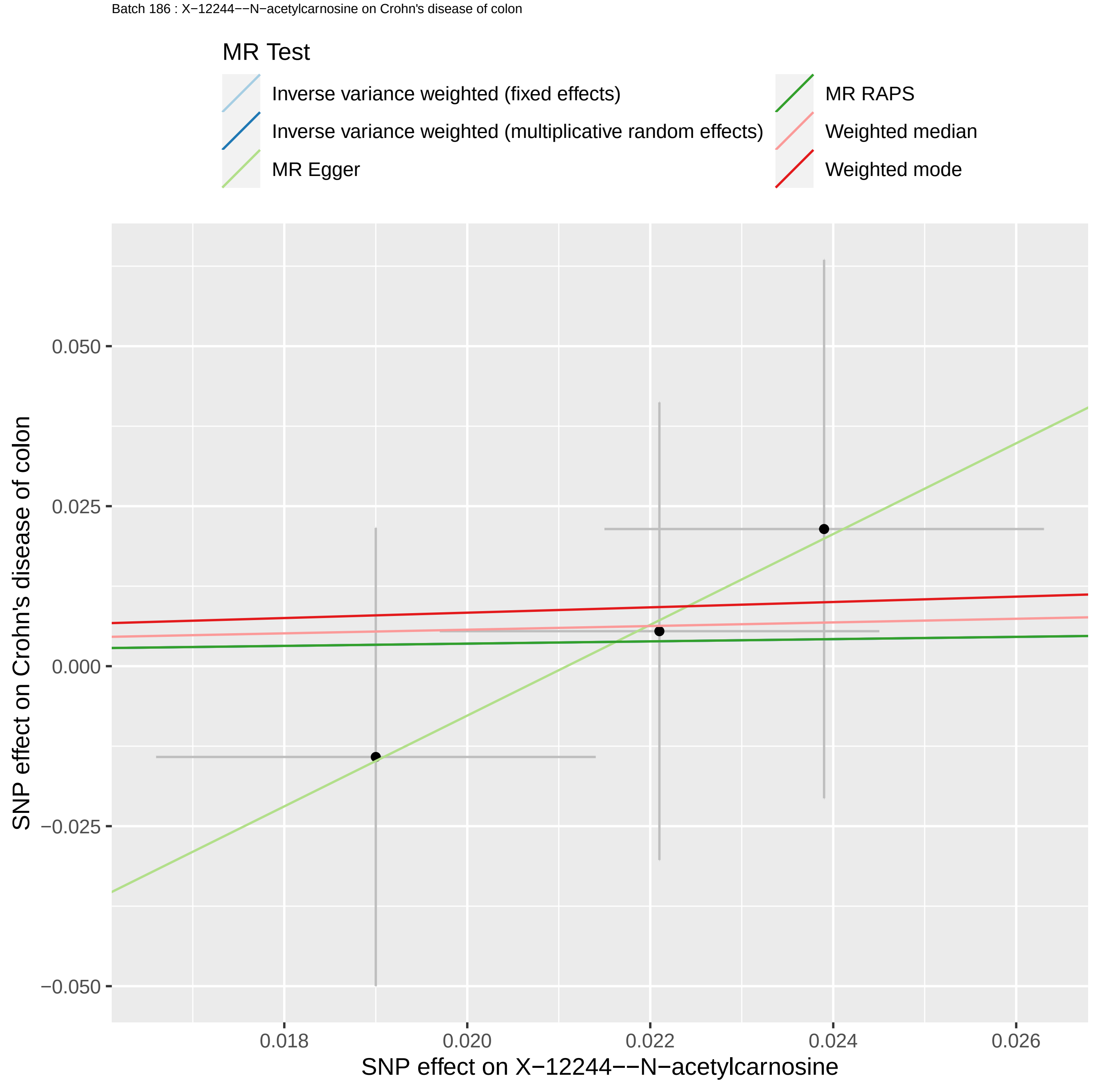
Batch 186 : X−12244−−N−acetylcarnosine on Crohn's disease of colon
MR Test
Inverse variance weighted (fixed effects)
Inverse variance weighted (multiplicative random effects)
MR Egger
MR RAPS
Weighted median
Weighted mode
0.050
0.025
0.000
−0.025
−0.050
0.018
0.020
0.022
0.024
0.026
SNP effect on Crohn's disease of colon
SNP effect on X−12244−−N−acetylcarnosine

Batch 188 : 1−eicosatrienoylglycerophosphocholine* on Crohn's disease of colon

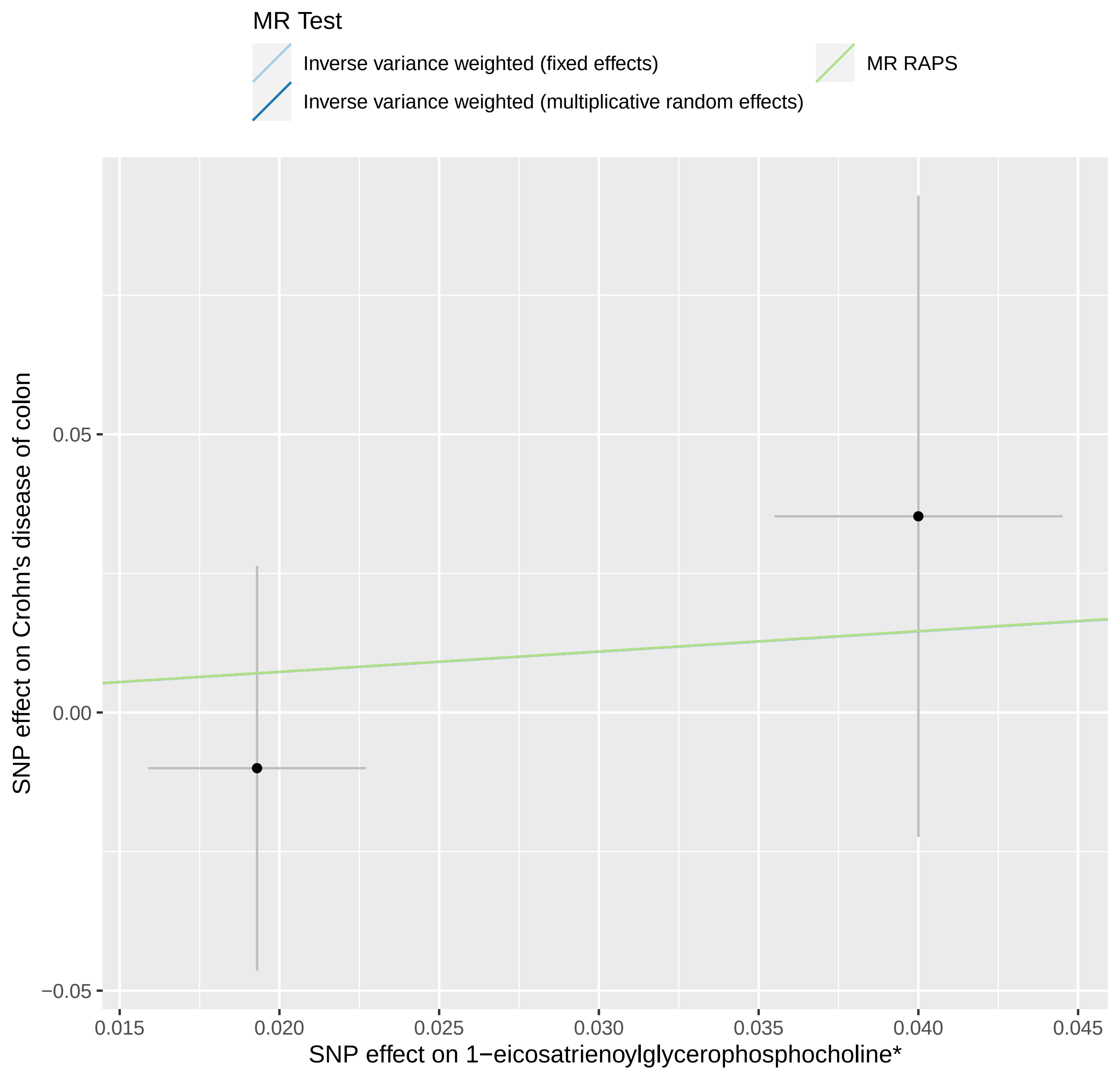

Batch 189 : 1−docosahexaenoylglycerophosphocholine* on Crohn's disease of colon

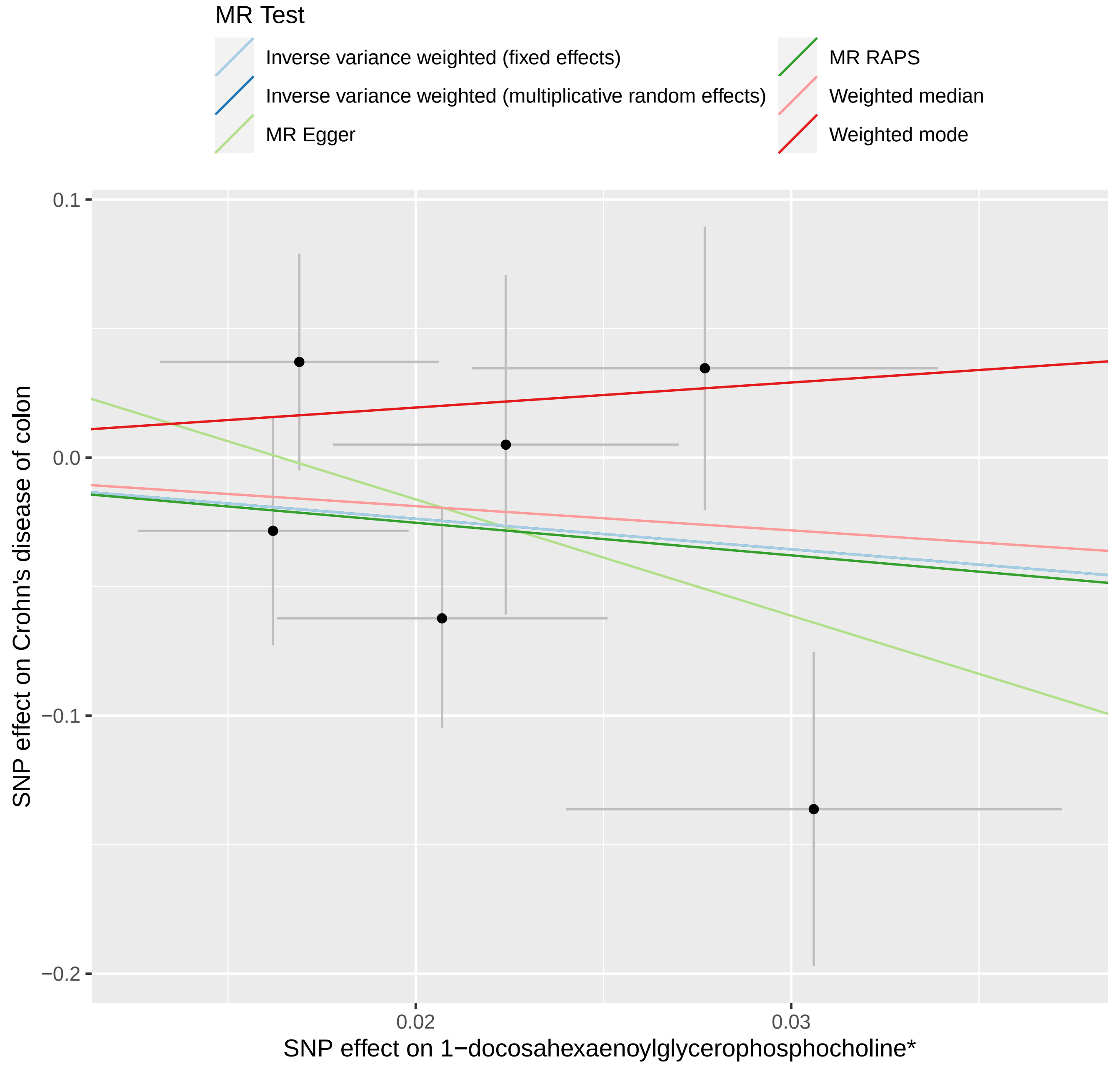

Batch 193 : Octanoylcarnitine on Crohn's disease of colon

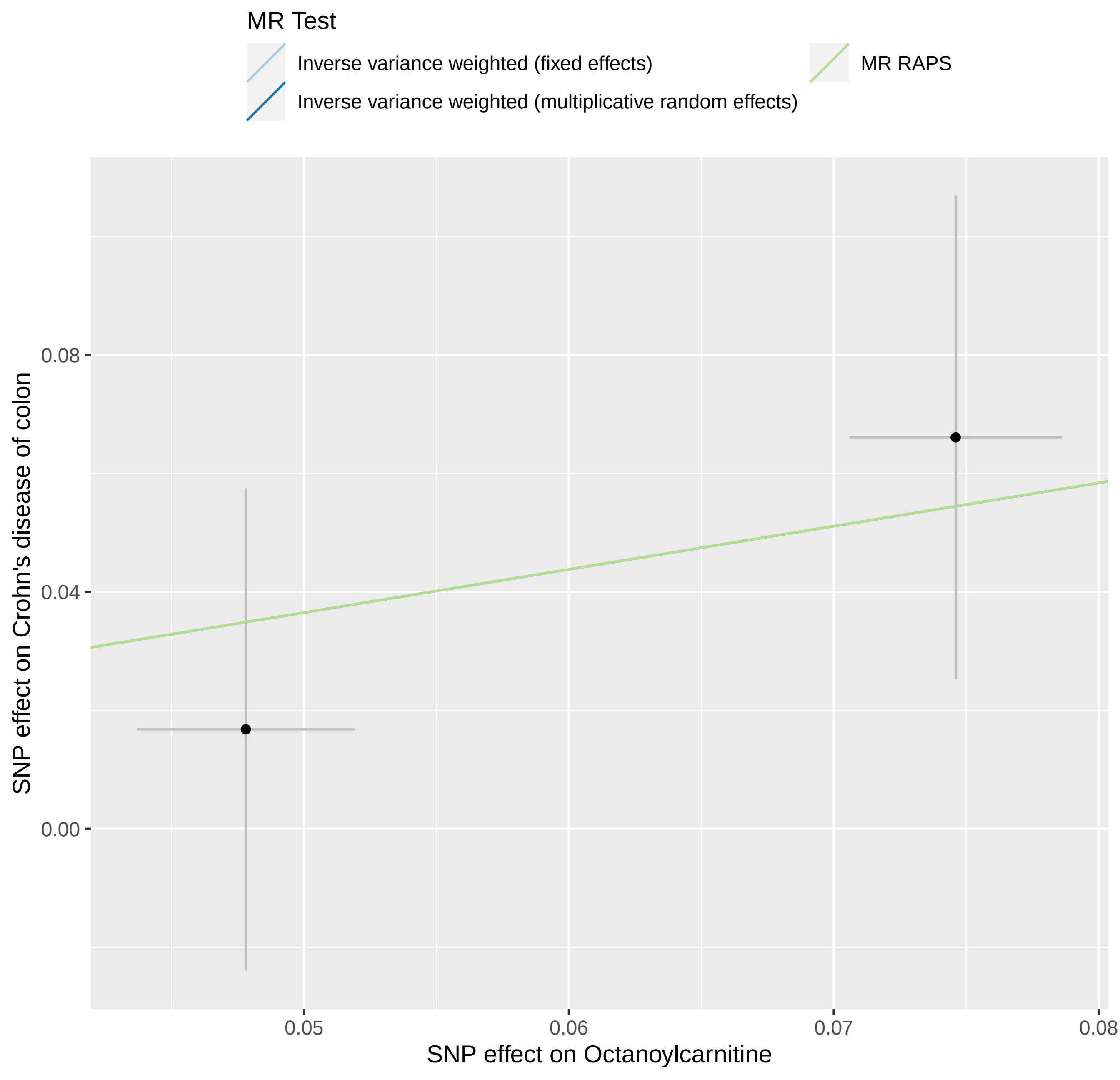

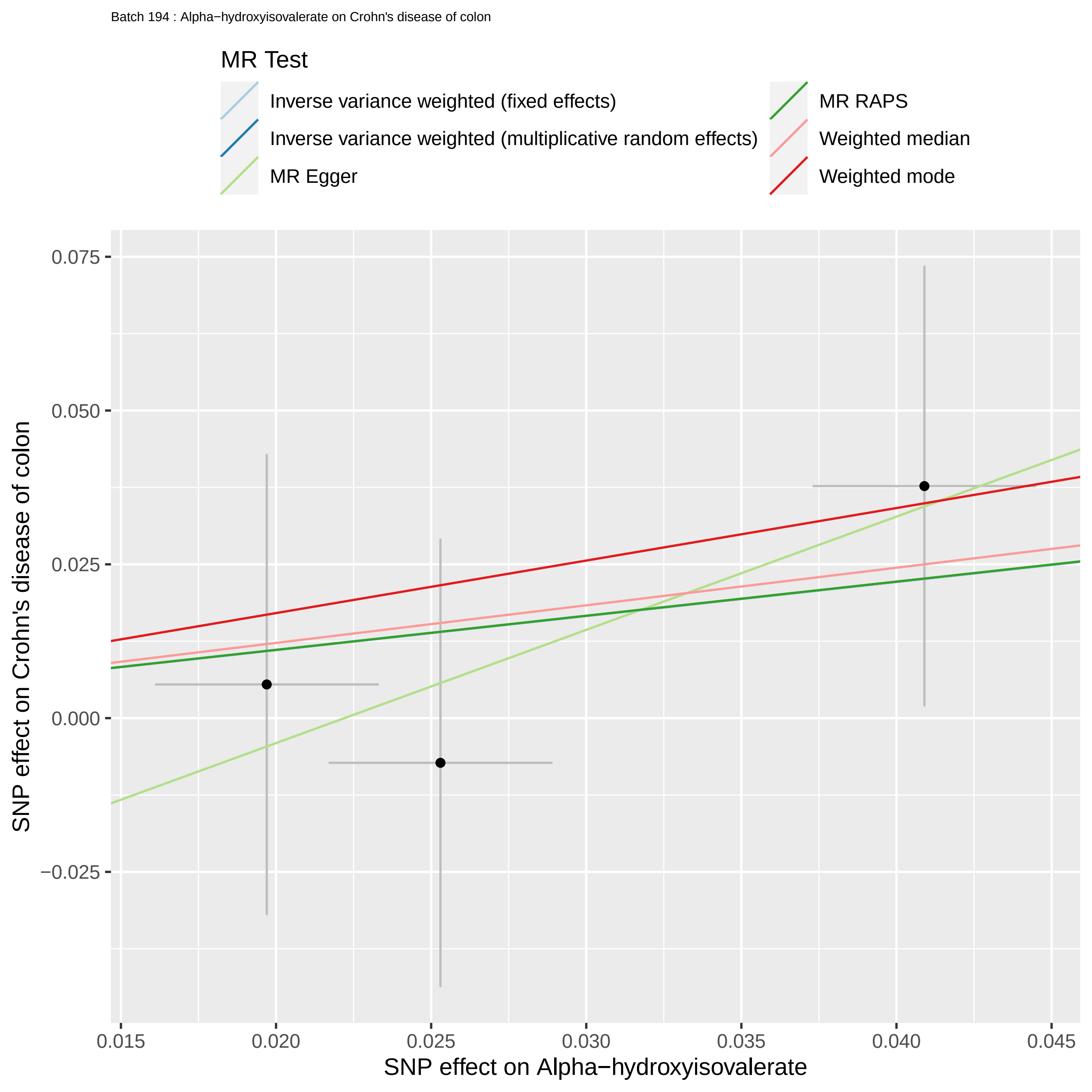

Batch 194 : Alpha−hydroxyisovalerate on Crohn's disease of colon
MR Test
Inverse variance weighted (fixed effects)
Inverse variance weighted (multiplicative random effects)
MR Egger
MR RAPS
Weighted median
Weighted mode
0.075
0.050
0.025
0.000
−0.025
0.015
0.020
0.025
0.030
0.035
0.040
0.045
SNP effect on Crohn's disease of colon
SNP effect on Alpha−hydroxyisovalerate

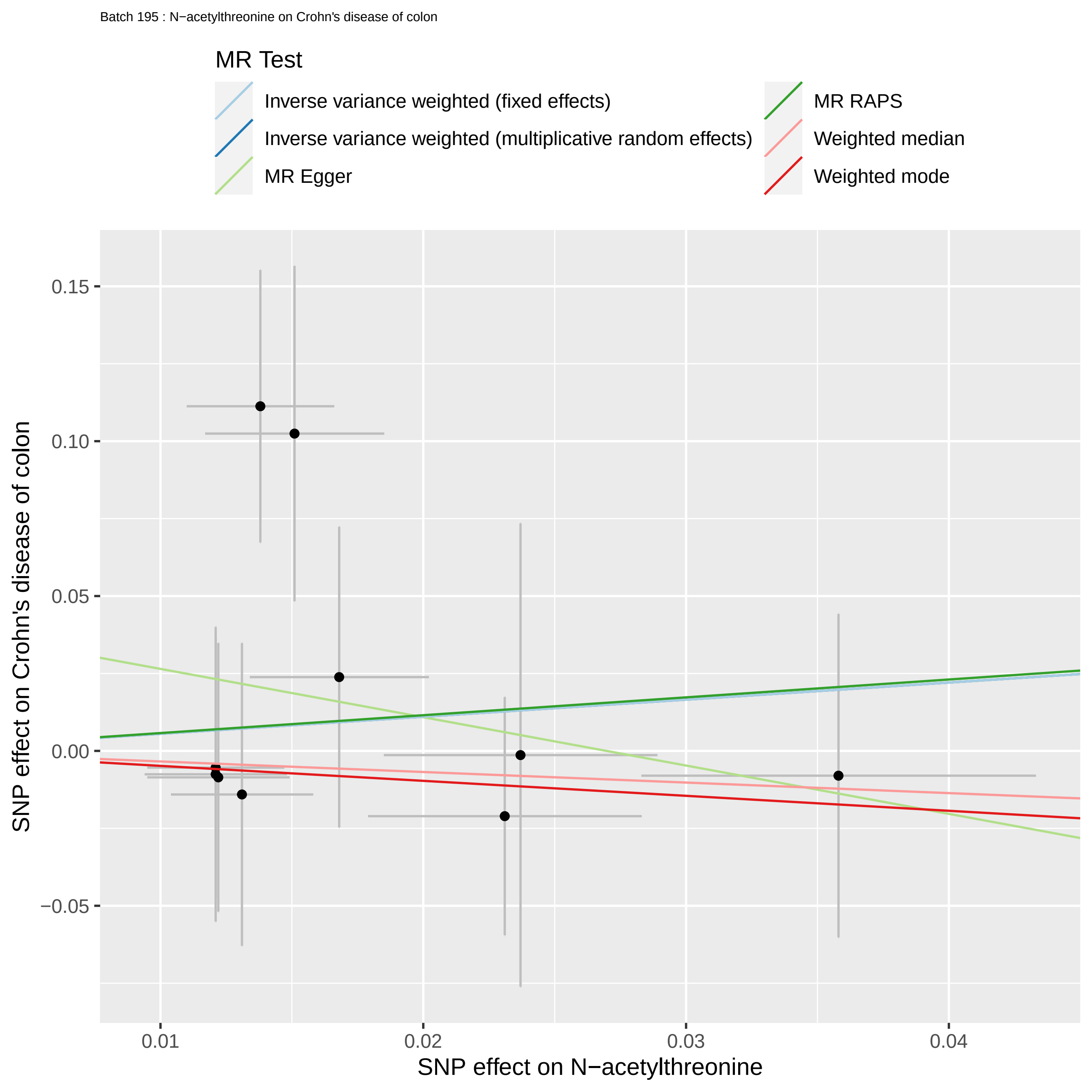

Batch 195 : N−acetylthreonine on Crohn's disease of colon
MR Test
Inverse variance weighted (fixed effects)
Inverse variance weighted (multiplicative random effects)
MR Egger
MR RAPS
Weighted median
Weighted mode
SNP effect on Crohn's disease of colon
0.15
0.10
0.05
0.00
−0.05
0.01
0.02
0.03
0.04
SNP effect on N−acetylthreonine

Batch 196 : Decanoylcarnitine on Crohn's disease of colon

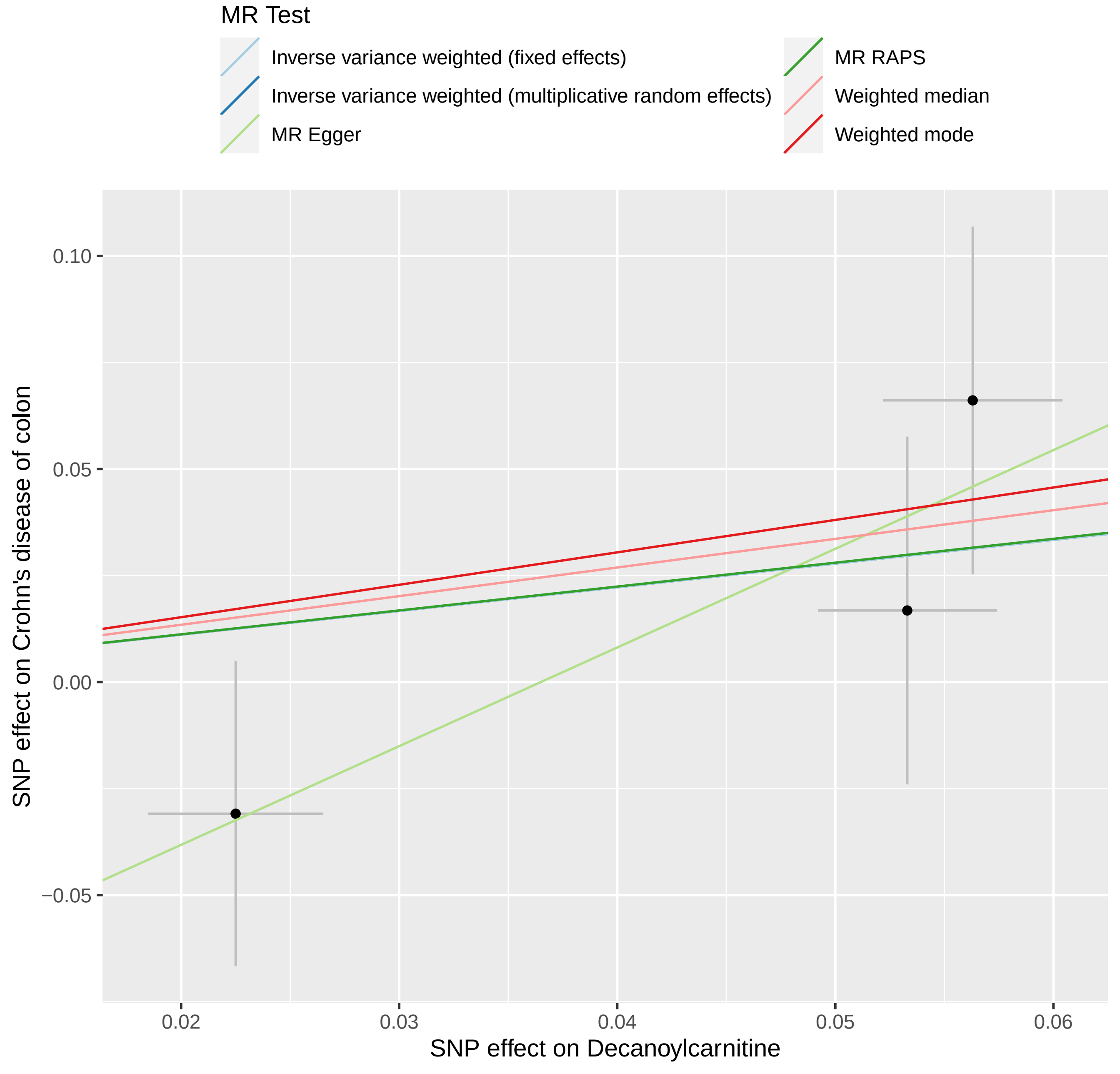

Batch 197 : 1−palmitoylglycerophosphocholine on Crohn's disease of colon

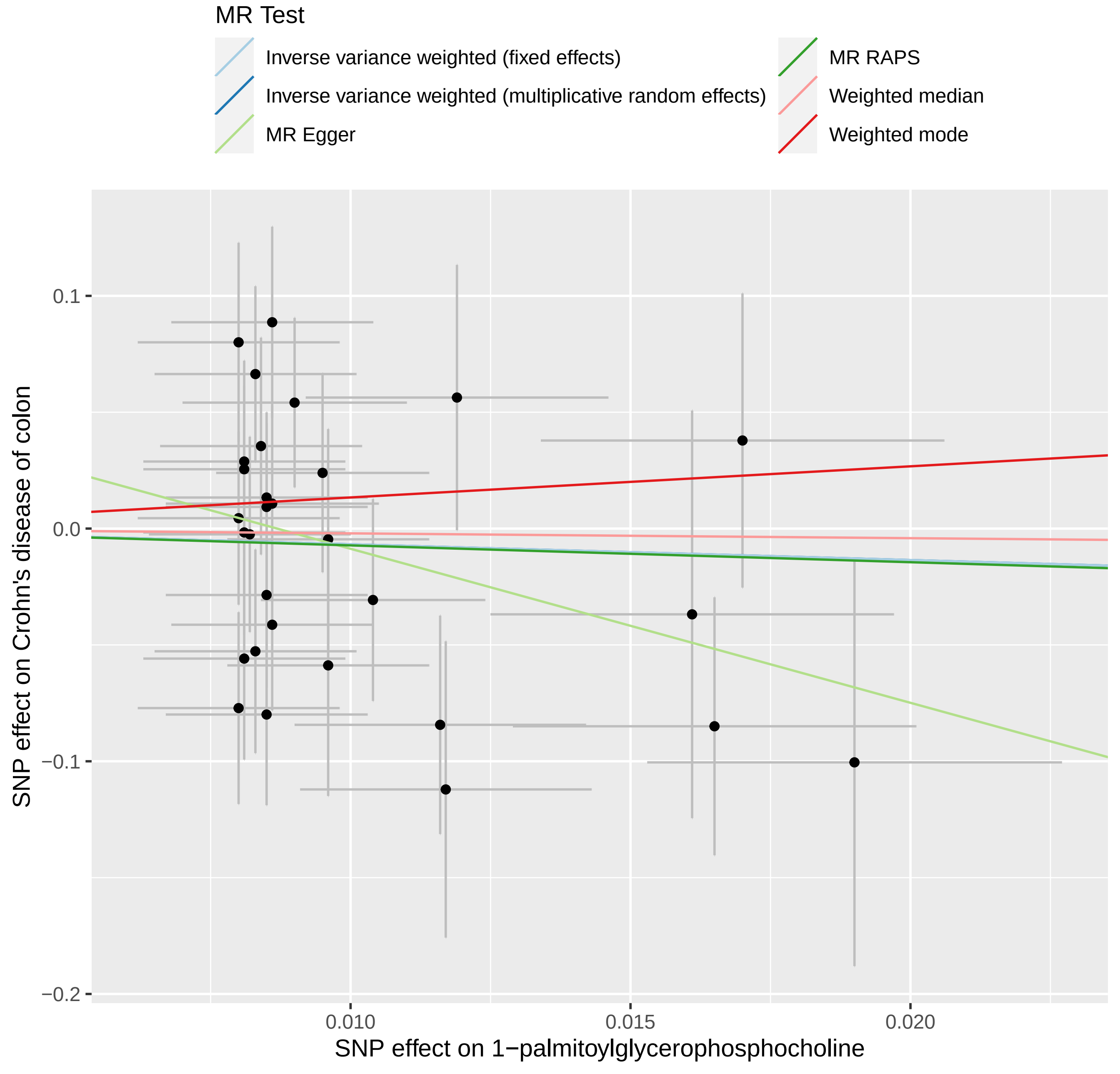

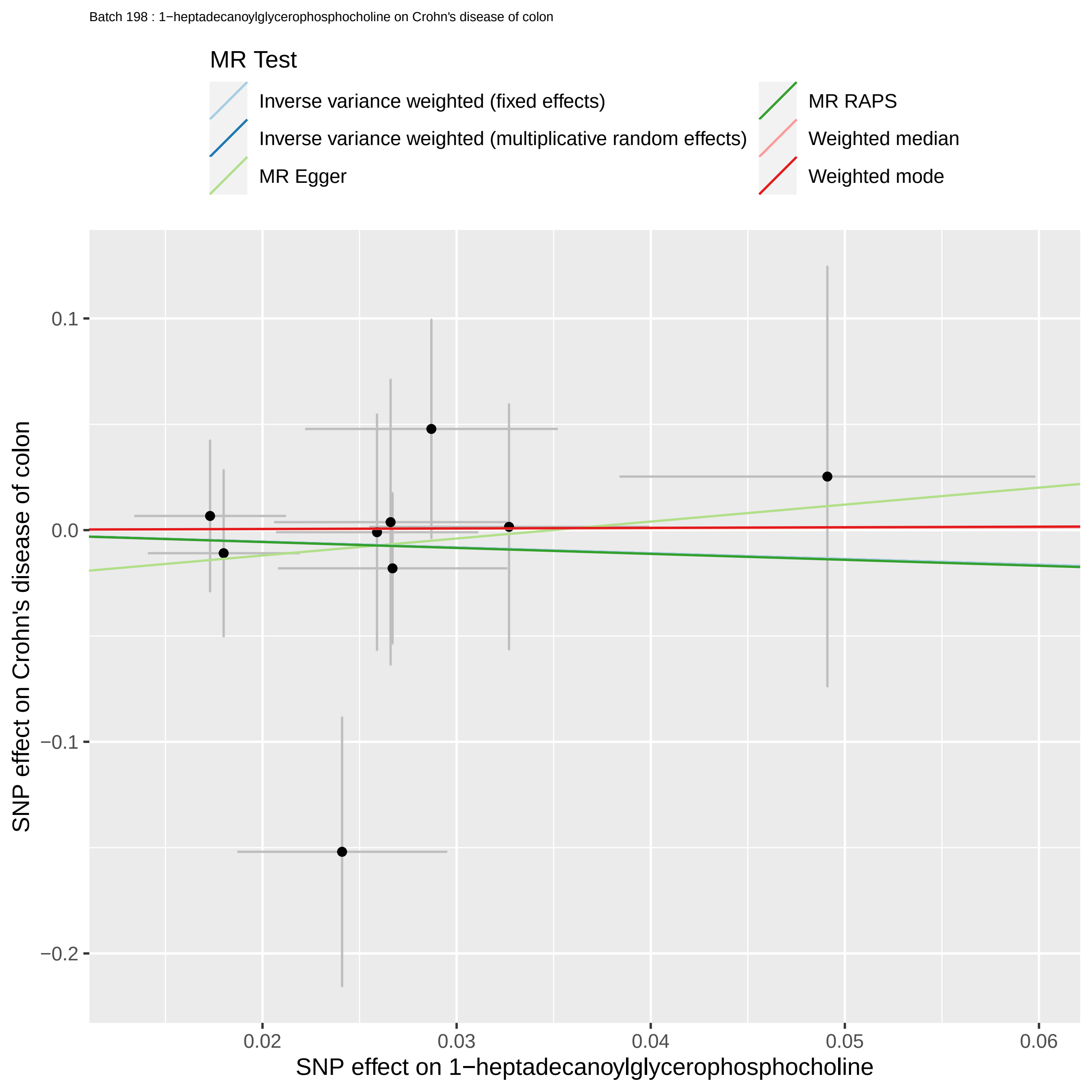
Batch 198 : 1−heptadecanoylglycerophosphocholine on Crohn's disease of colon
MR Test
Inverse variance weighted (fixed effects)
Inverse variance weighted (multiplicative random effects)
MR Egger
MR RAPS
Weighted median
Weighted mode
0.1
0.0
−0.1
−0.2
0.02
0.03
0.04
0.05
0.06
SNP effect on Crohn's disease of colon
SNP effect on 1−heptadecanoylglycerophosphocholine

Batch 199 : 1−oleoylglycerophosphocholine on Crohn's disease of colon

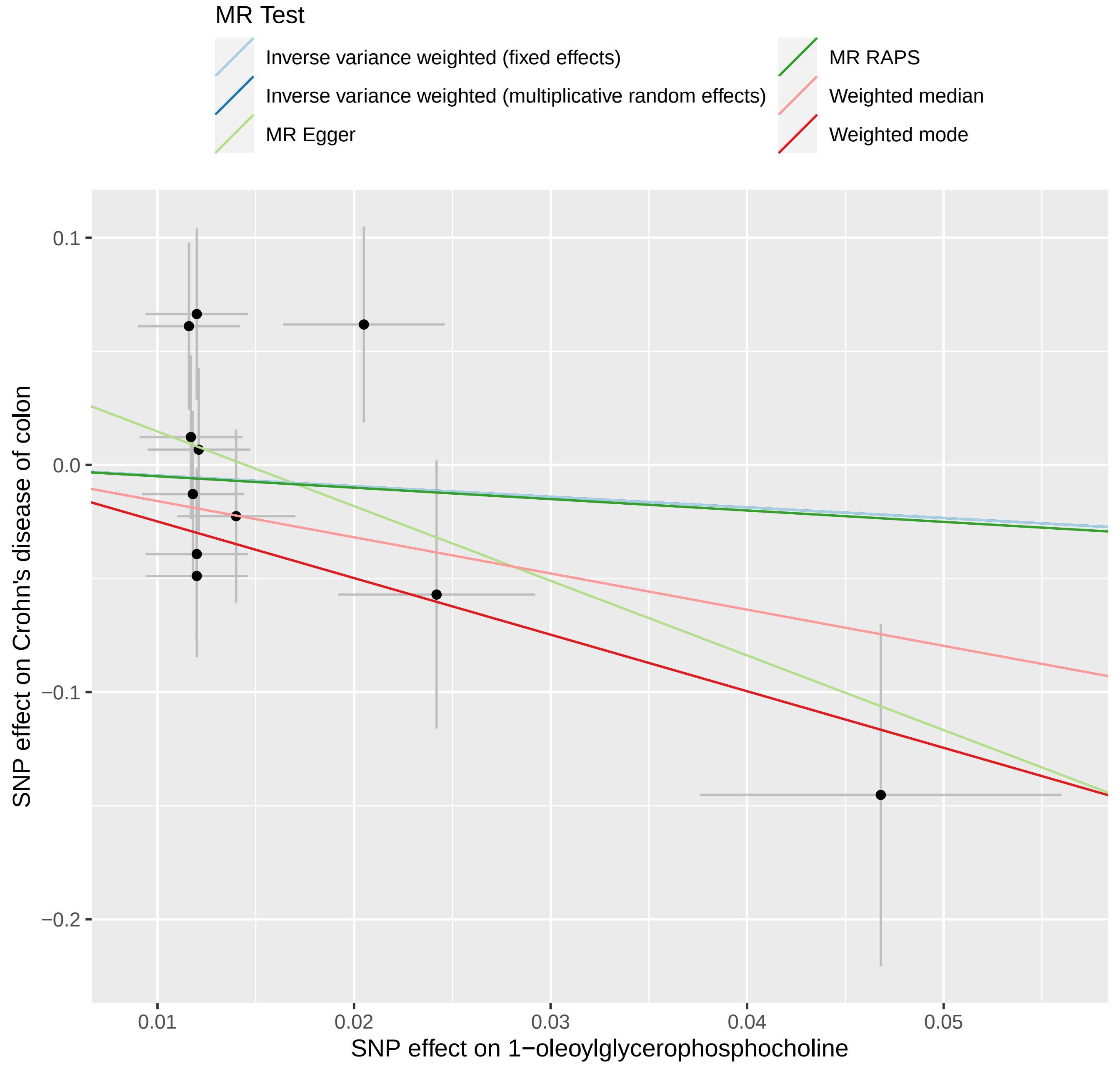

Batch 200 : 1−stearoylglycerophosphocholine on Crohn's disease of colon

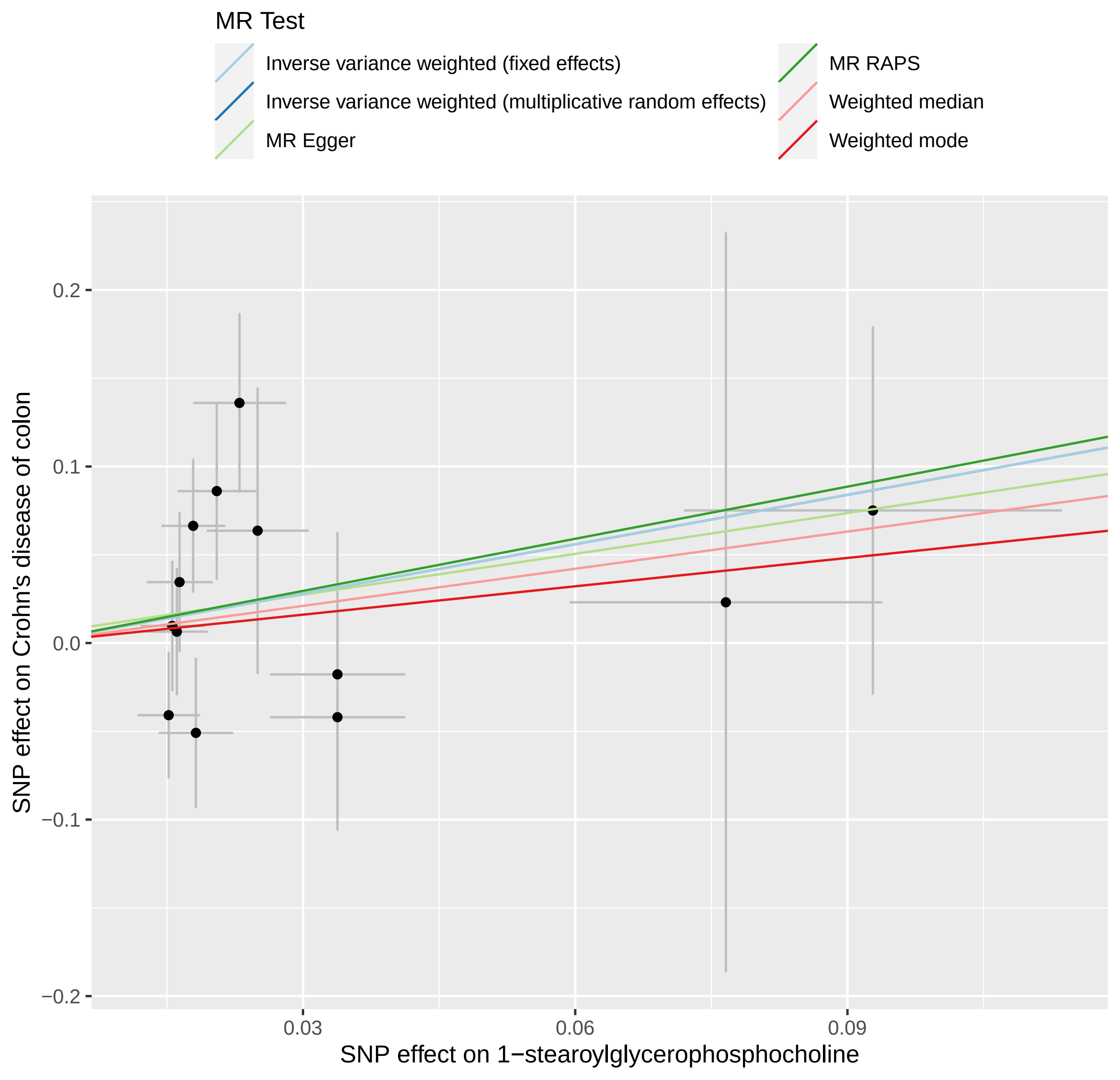

Batch 203 : 10−heptadecenoate (17:1n7) on Crohn's disease of colon

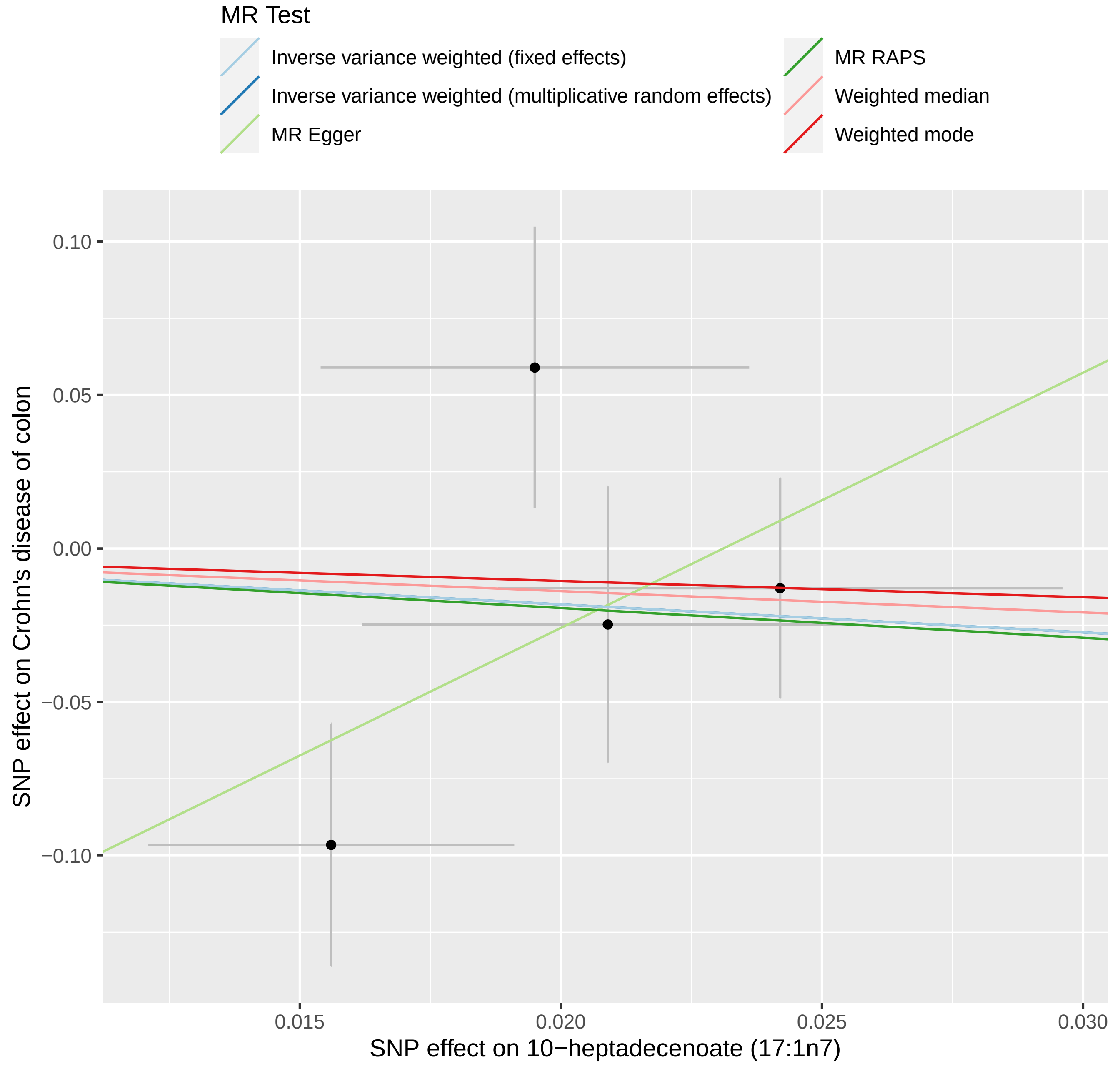

Batch 205 : Epiandrosterone sulfate on Crohn's disease of colon

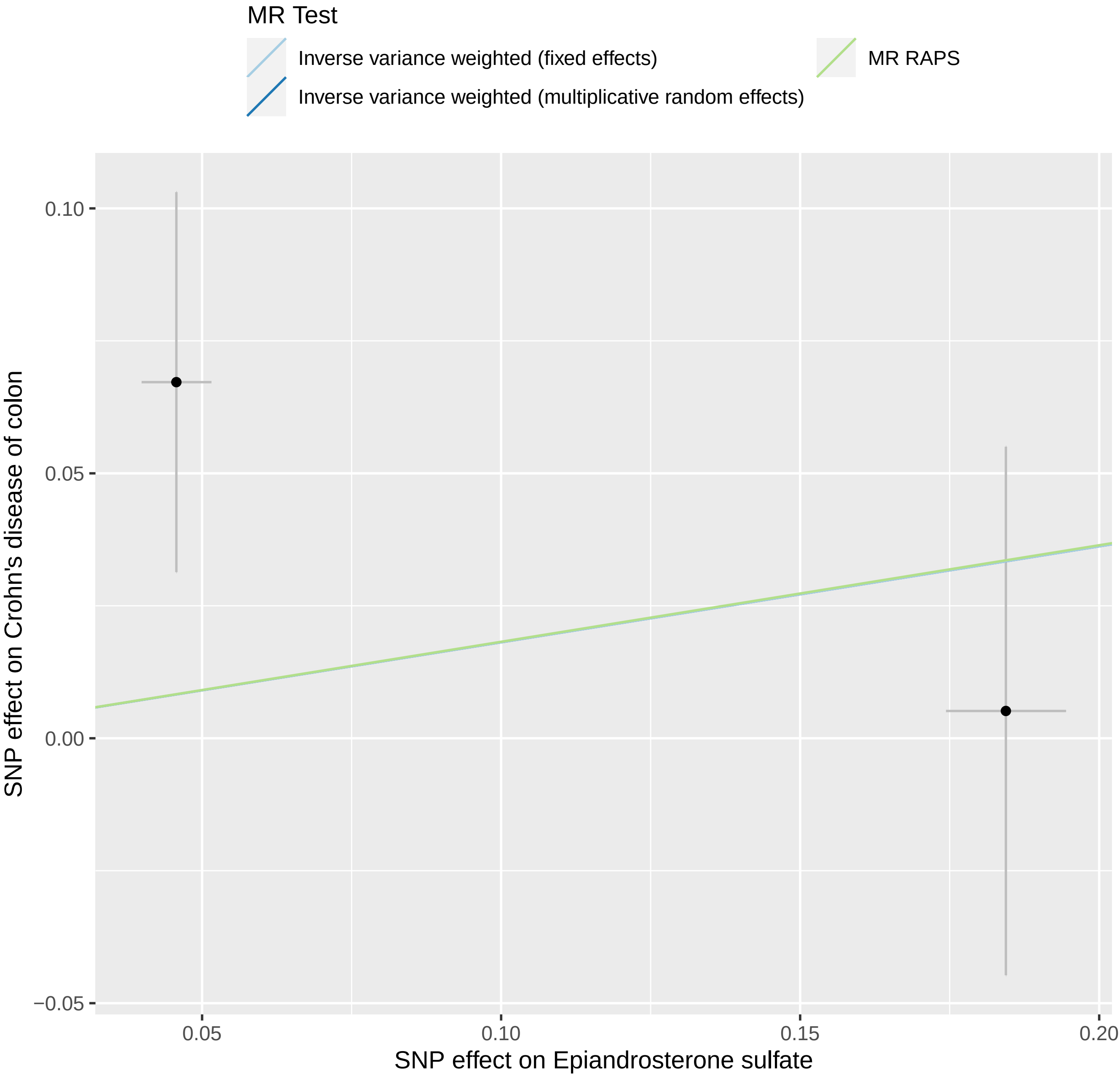

Batch 206 : Linolenate [alpha or gamma; (18:3n3 or 6)] on Crohn's disease of colon

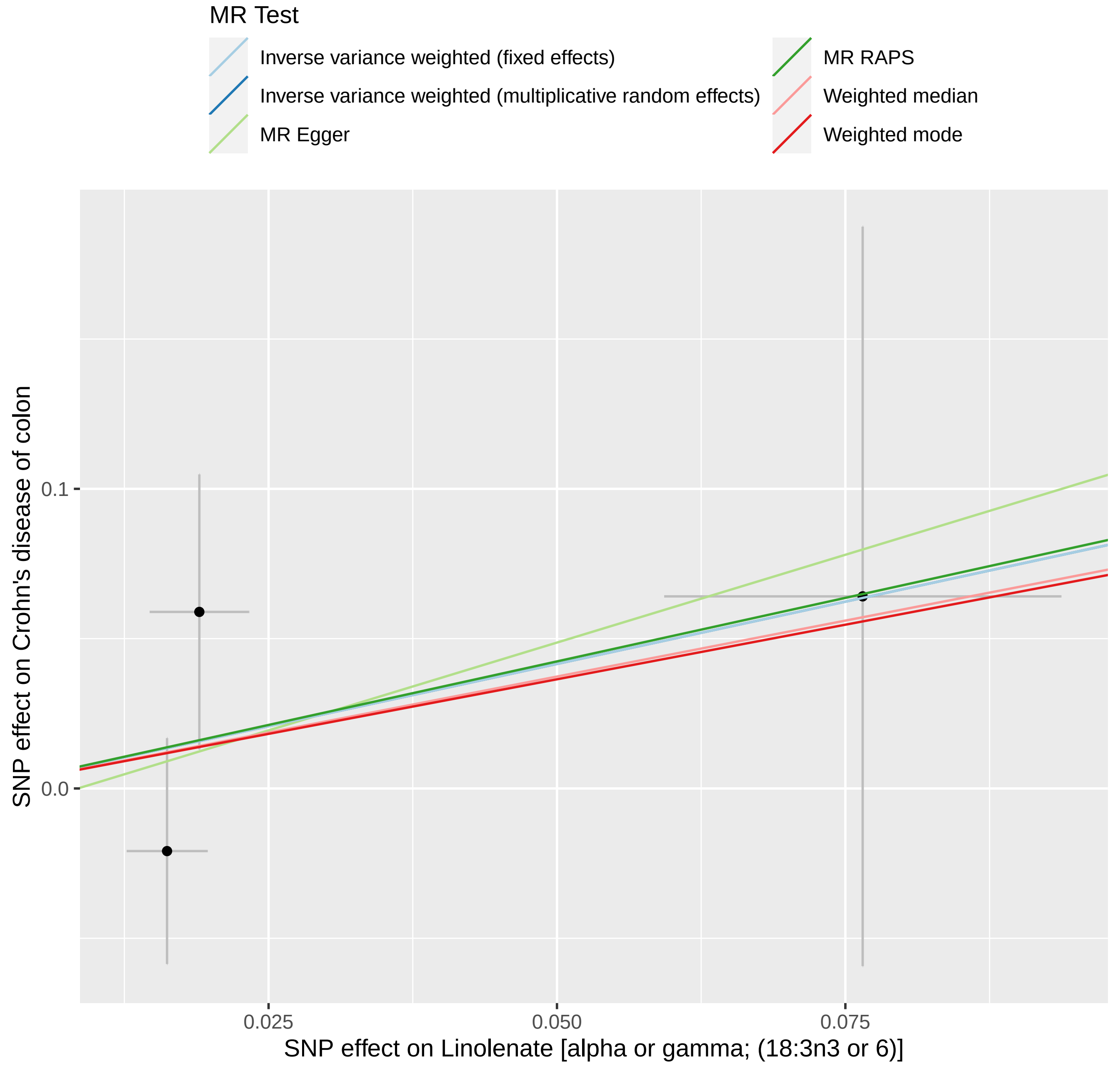

Batch 207 : X−12510−−2−aminooctanoic acid on Crohn's disease of colon

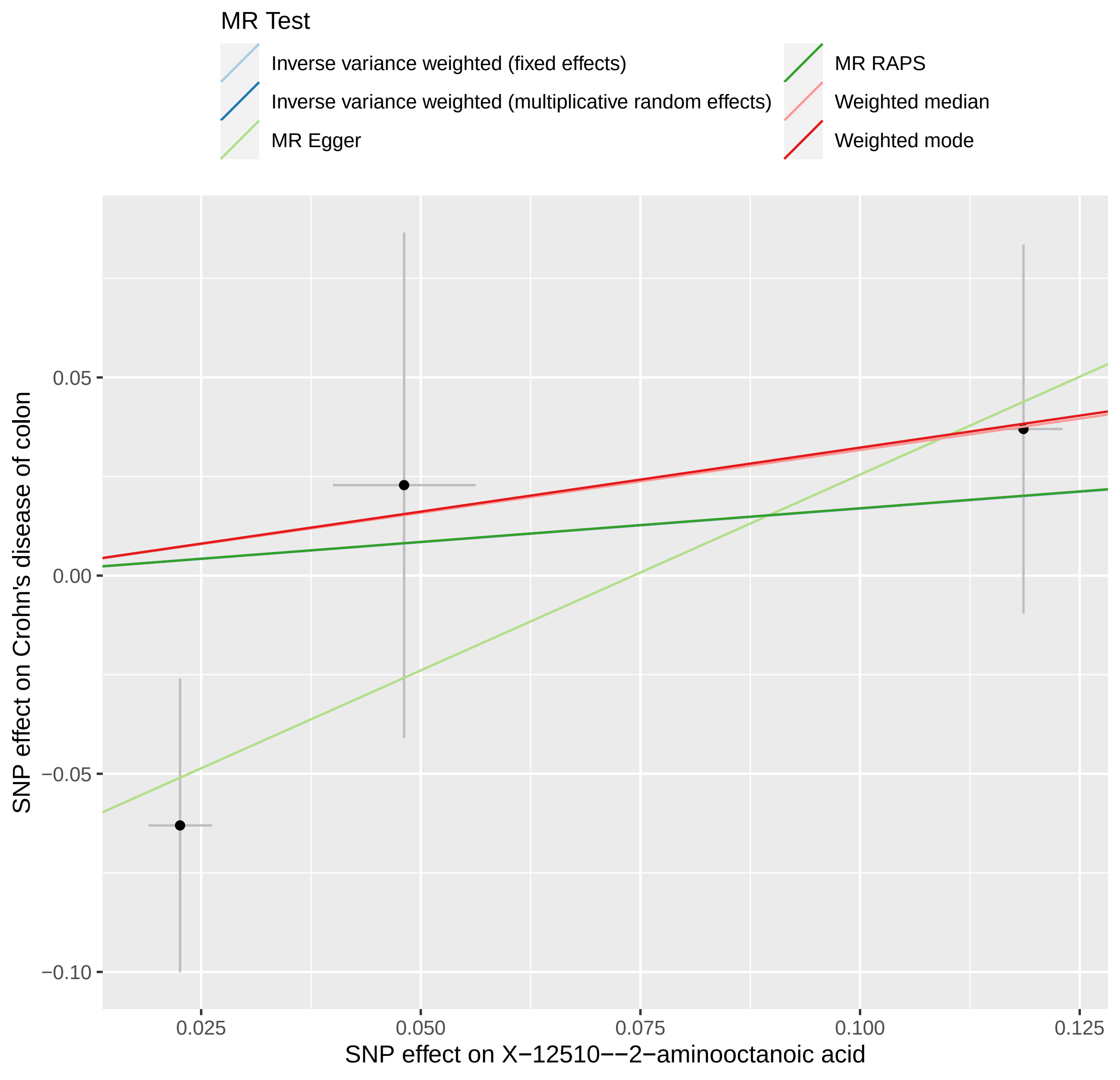

Batch 210 : Asparagine on Crohn's disease of colon

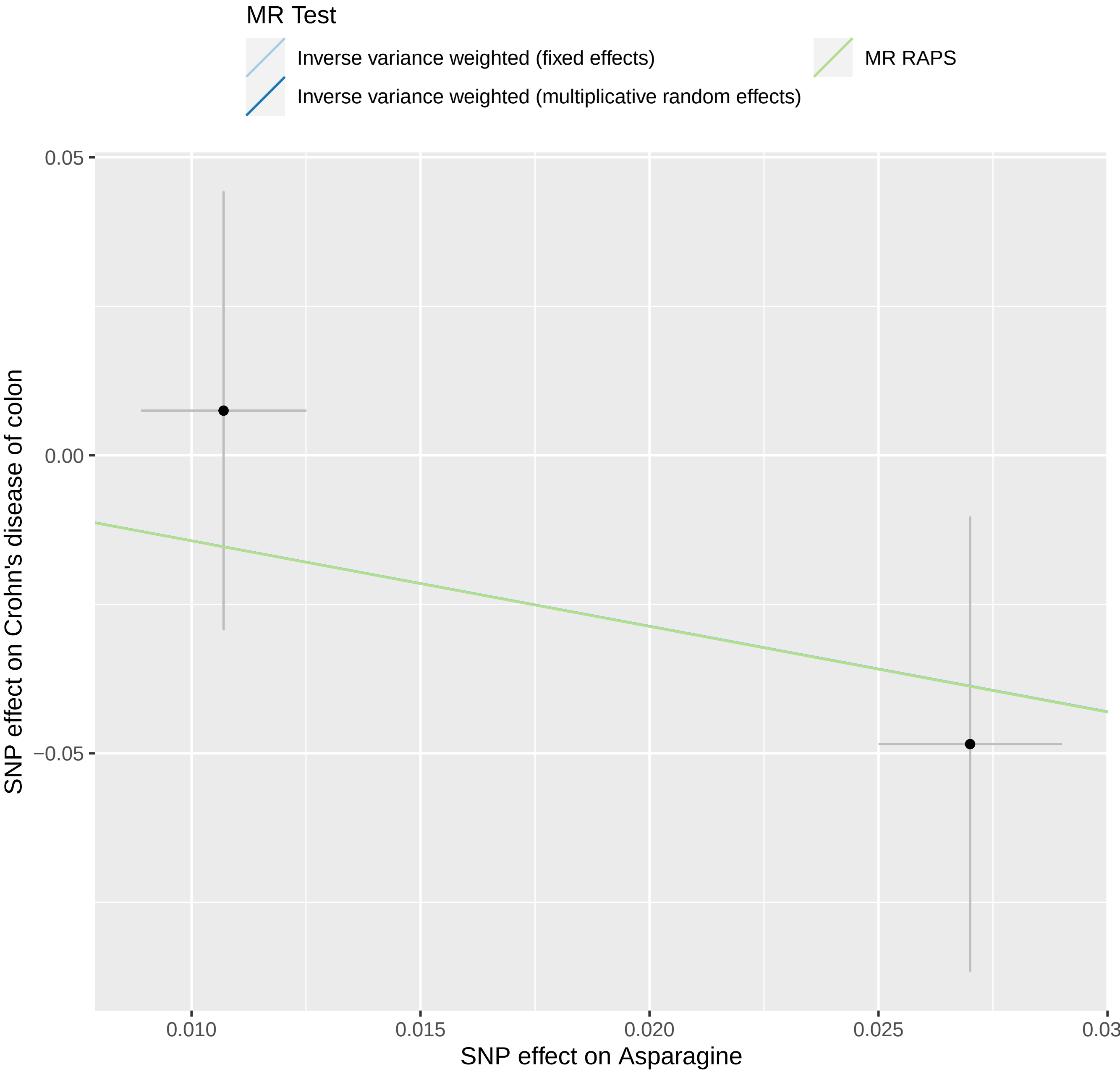

Batch 211 : Stachydrine on Crohn's disease of colon

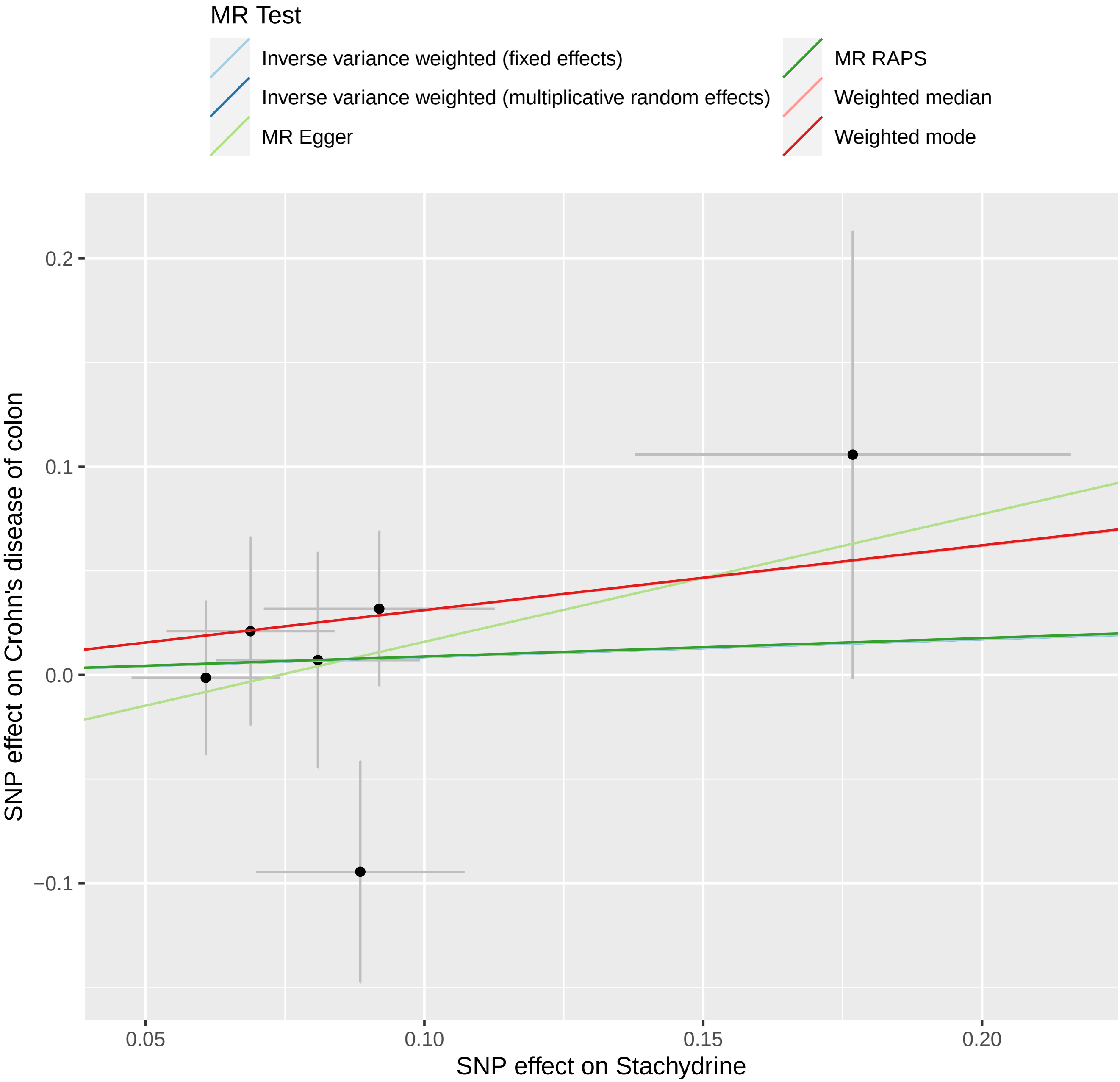

Batch 212 : Isovalerylcarnitine on Crohn's disease of colon

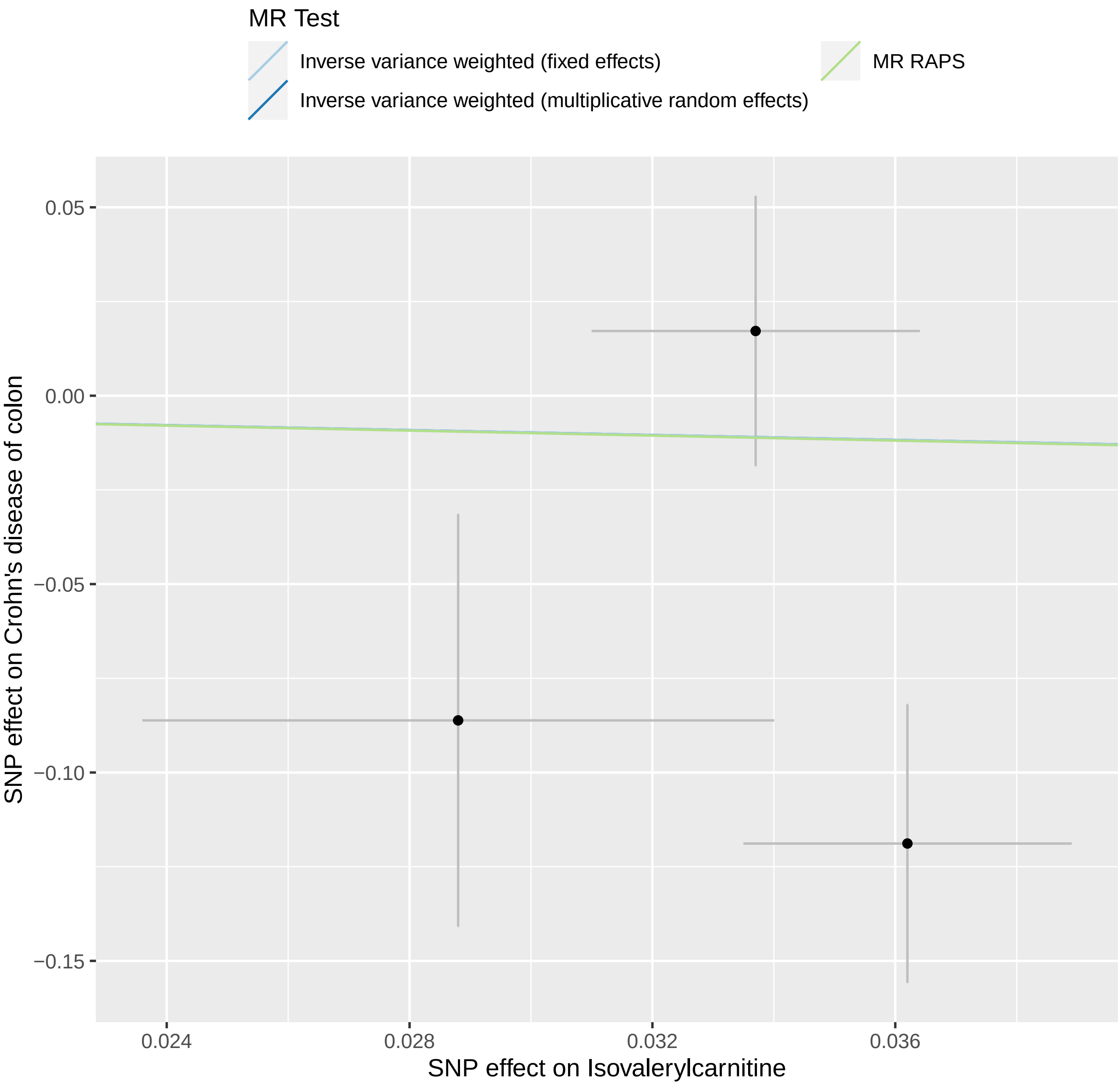

Batch 215 : 1−linoleoylglycerophosphocholine on Crohn's disease of colon

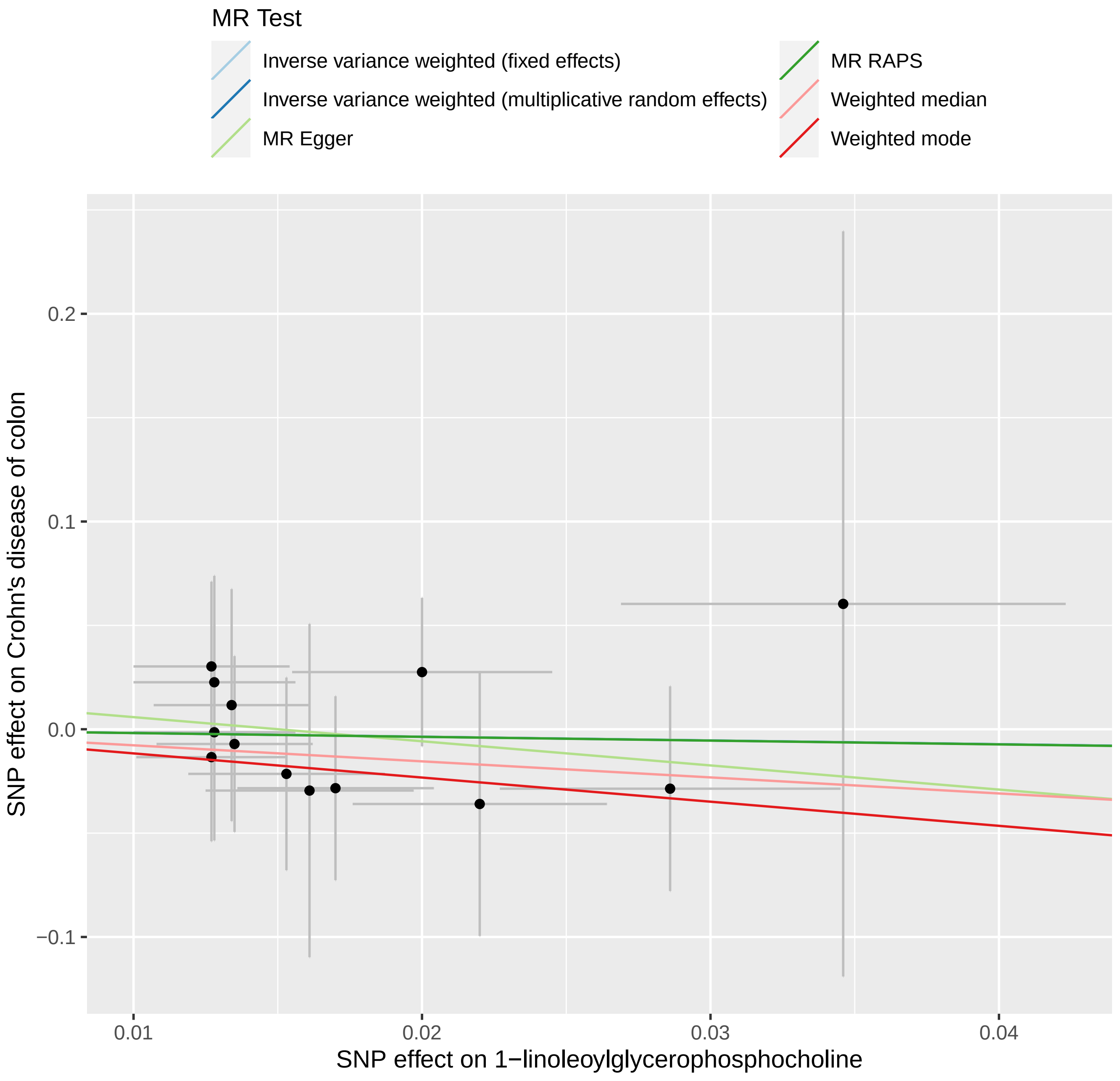

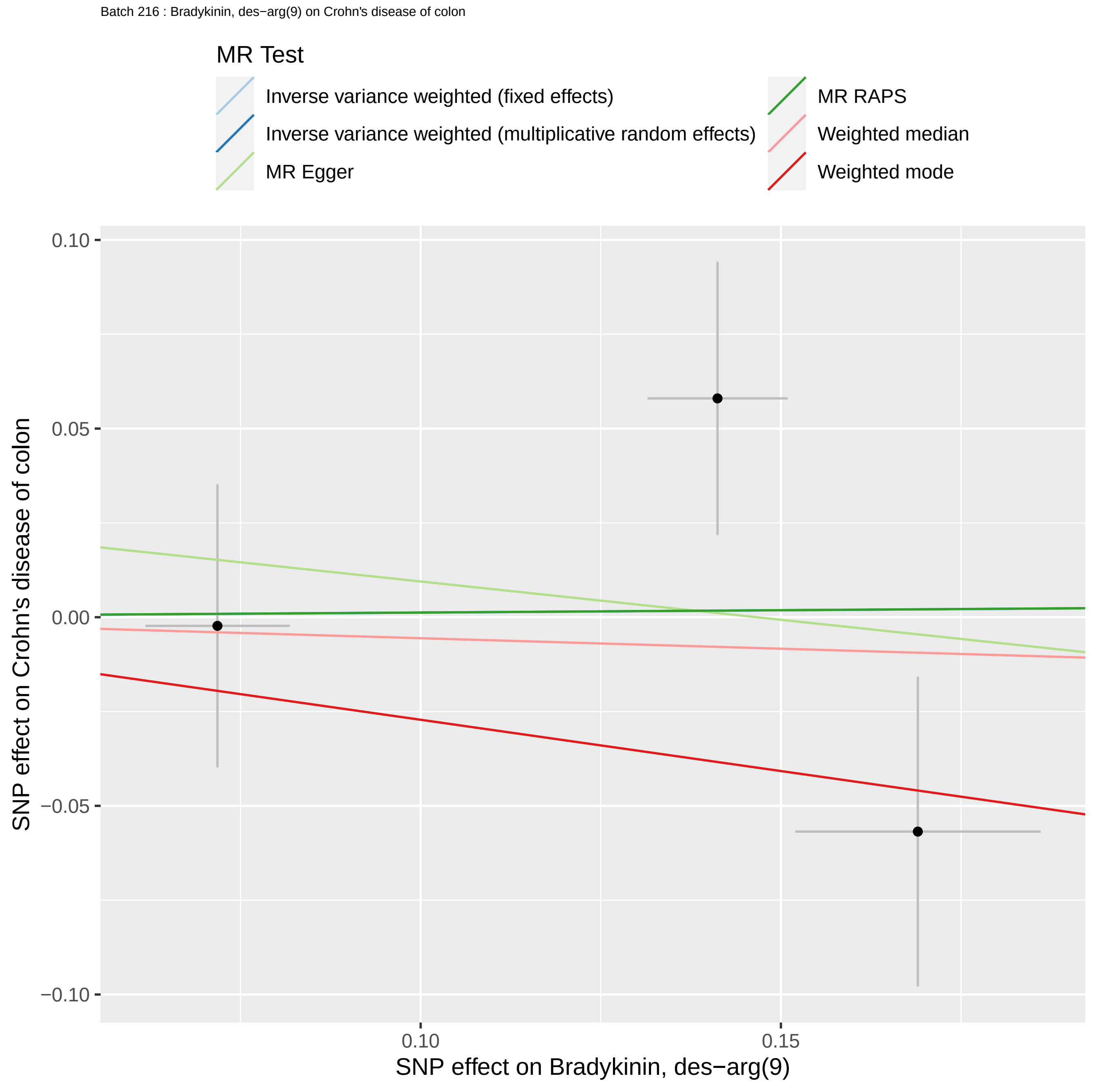
Batch 216 : Bradykinin, des−arg(9) on Crohn's disease of colon
MR Test
Inverse variance weighted (fixed effects)
Inverse variance weighted (multiplicative random effects)
MR Egger
MR RAPS
Weighted median
Weighted mode
0.10
0.05
0.00
−0.05
−0.10
0.10
0.15
SNP effect on Crohn's disease of colon
SNP effect on Bradykinin, des−arg(9)

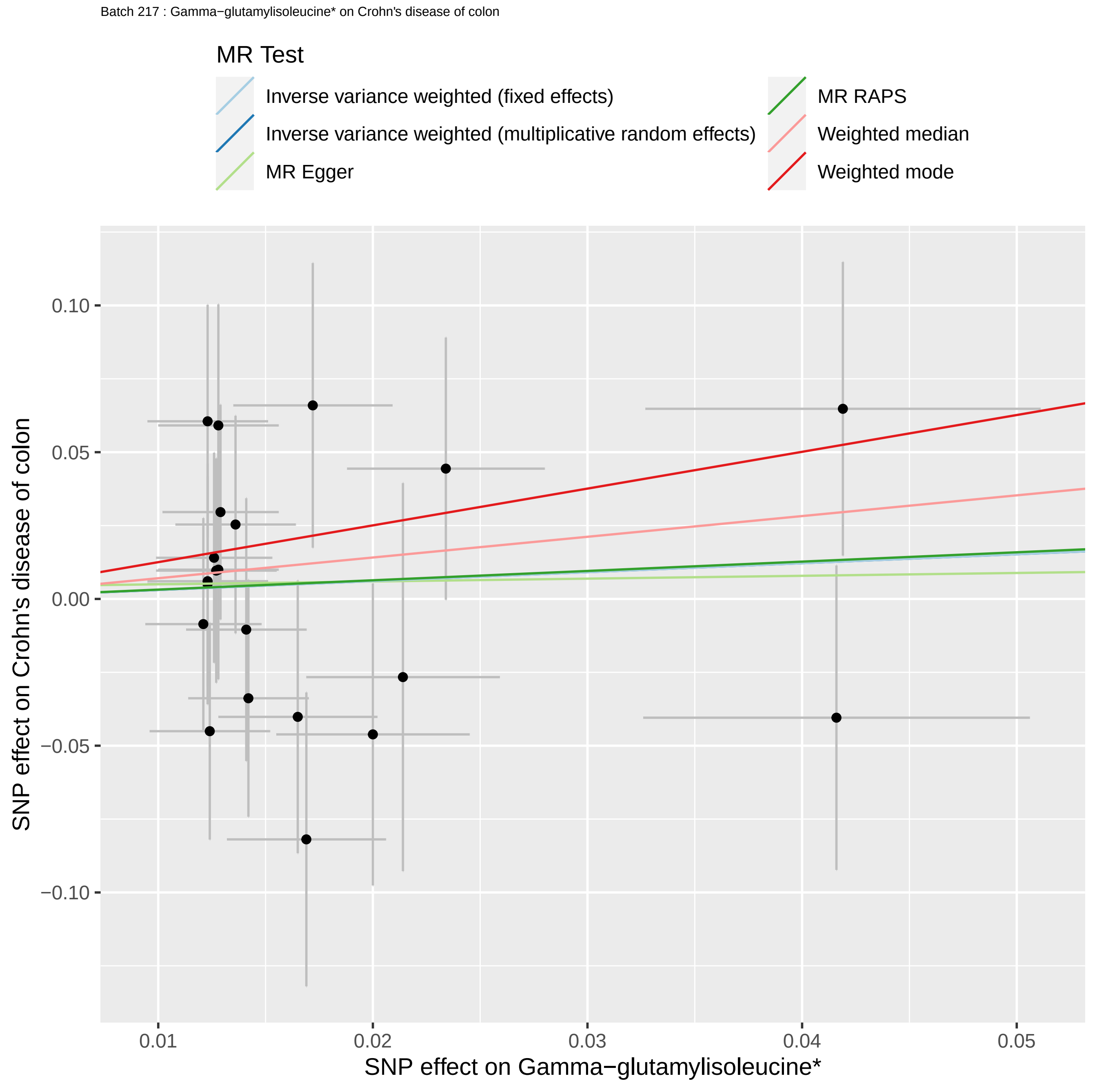
Batch 217 : Gamma−glutamylisoleucine* on Crohn's disease of colon
MR Test
Inverse variance weighted (fixed effects)
Inverse variance weighted (multiplicative random effects)
MR Egger
MR RAPS
Weighted median
Weighted mode
SNP effect on Crohn's disease of colon
0.10
0.05
0.00
−0.05
−0.10
0.01
0.02
0.03
0.04
0.05
SNP effect on Gamma−glutamylisoleucine*

Batch 220 : Isovalerate on Crohn's disease of colon

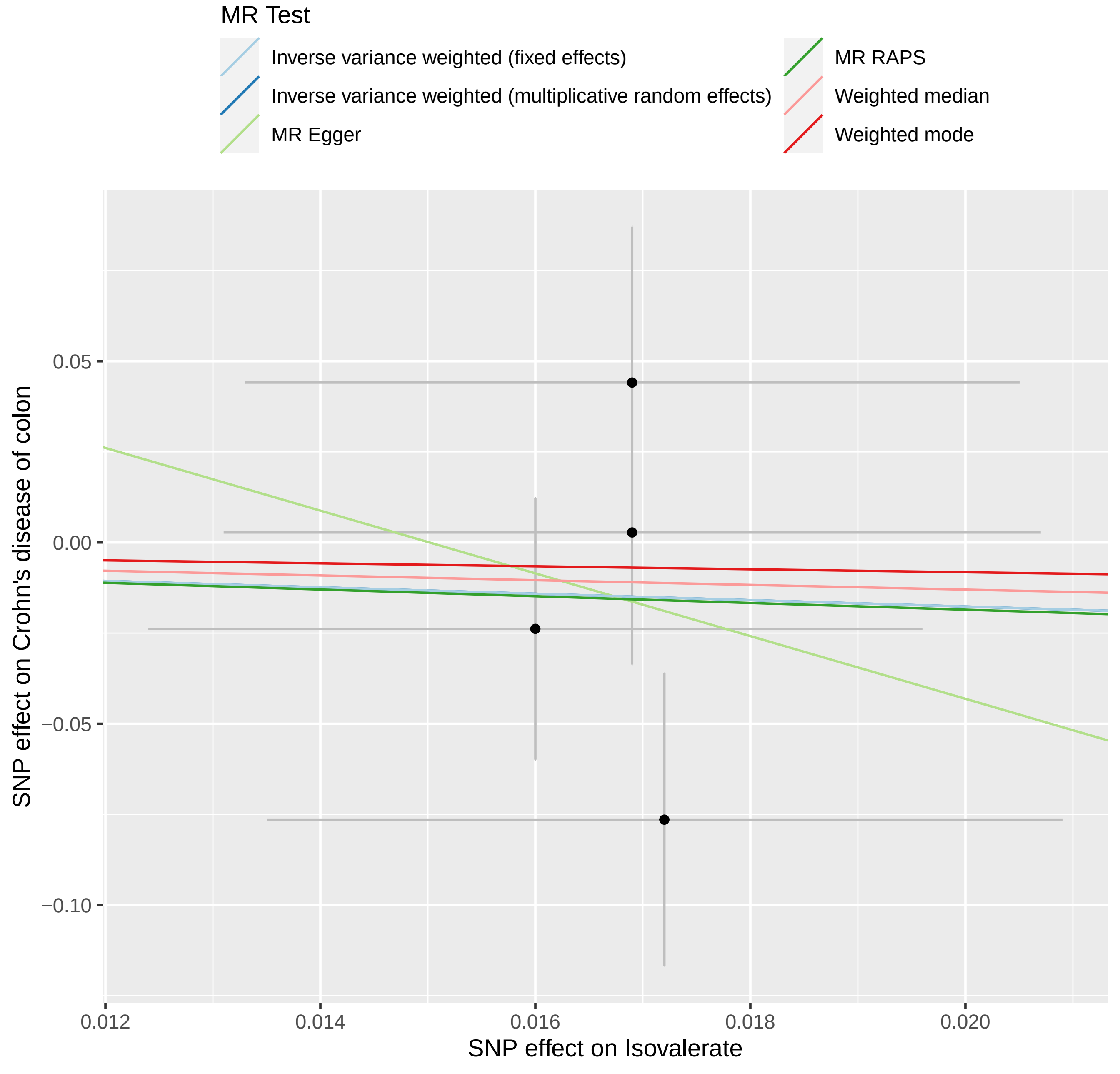

Batch 221 : X−13183−−stearamide on Crohn's disease of colon

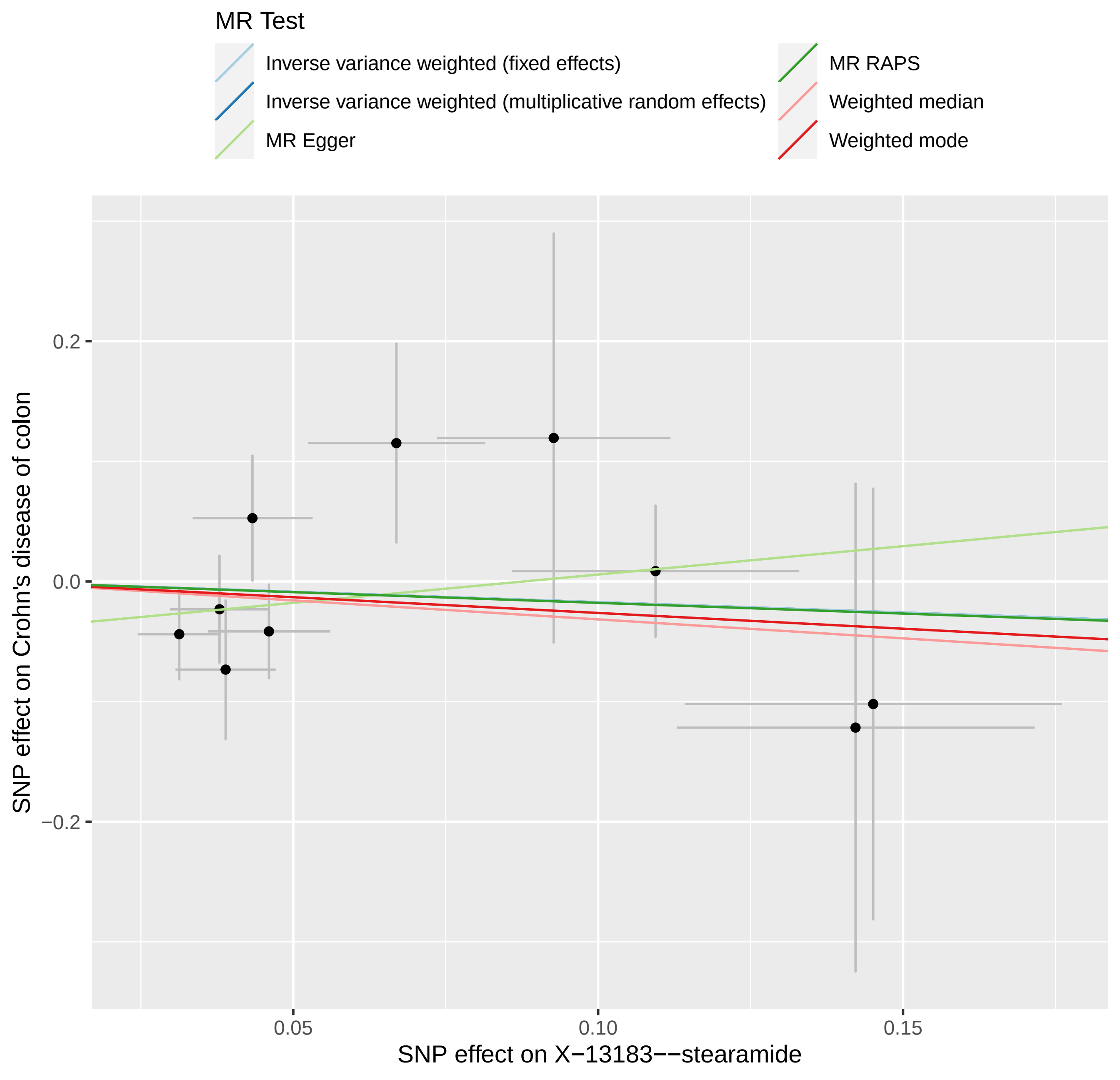

Batch 222 : 7−methylguanine on Crohn's disease of colon

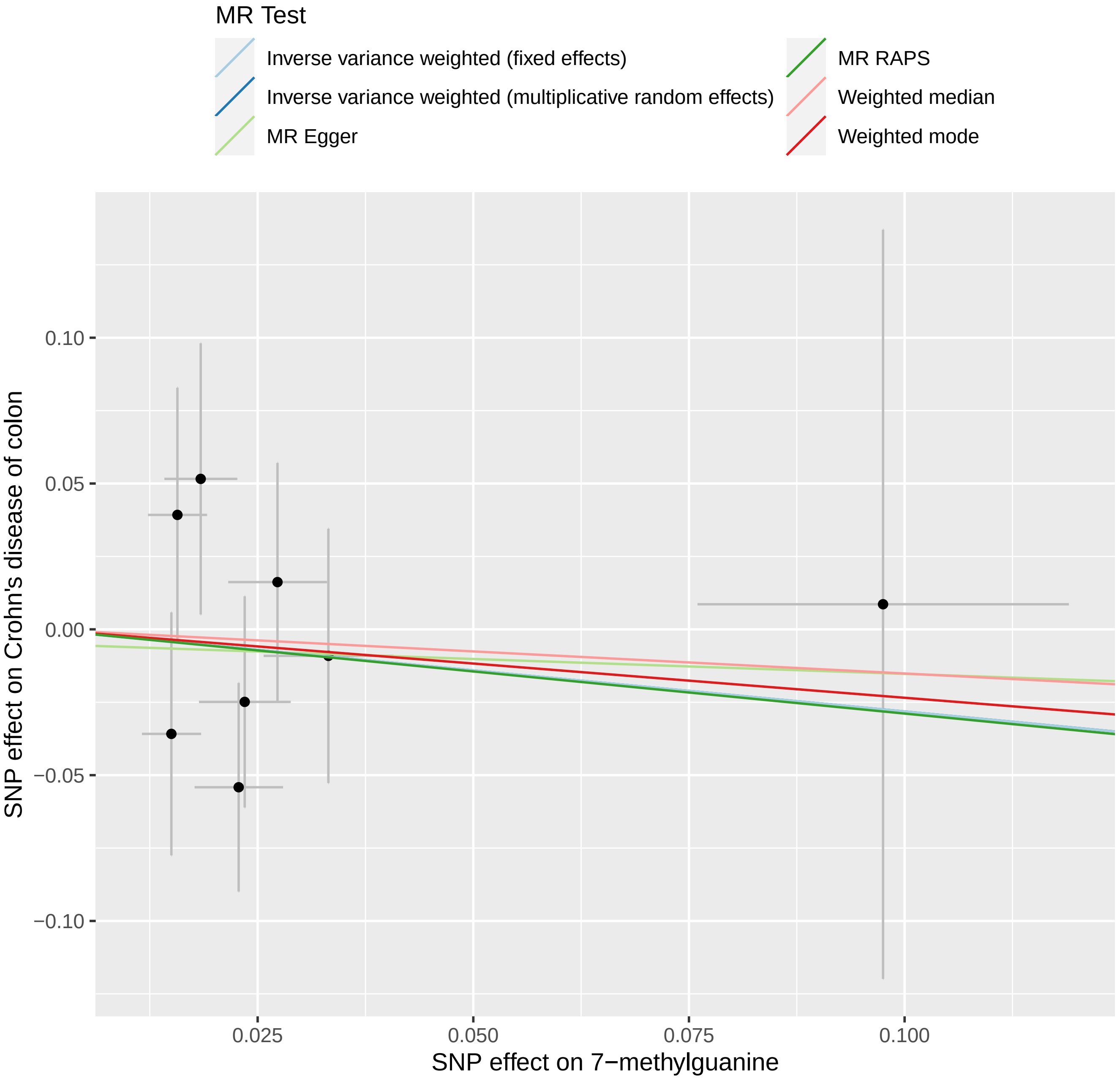

Batch 229 : X−13431−−nonanoylcarnitine* on Crohn's disease of colon

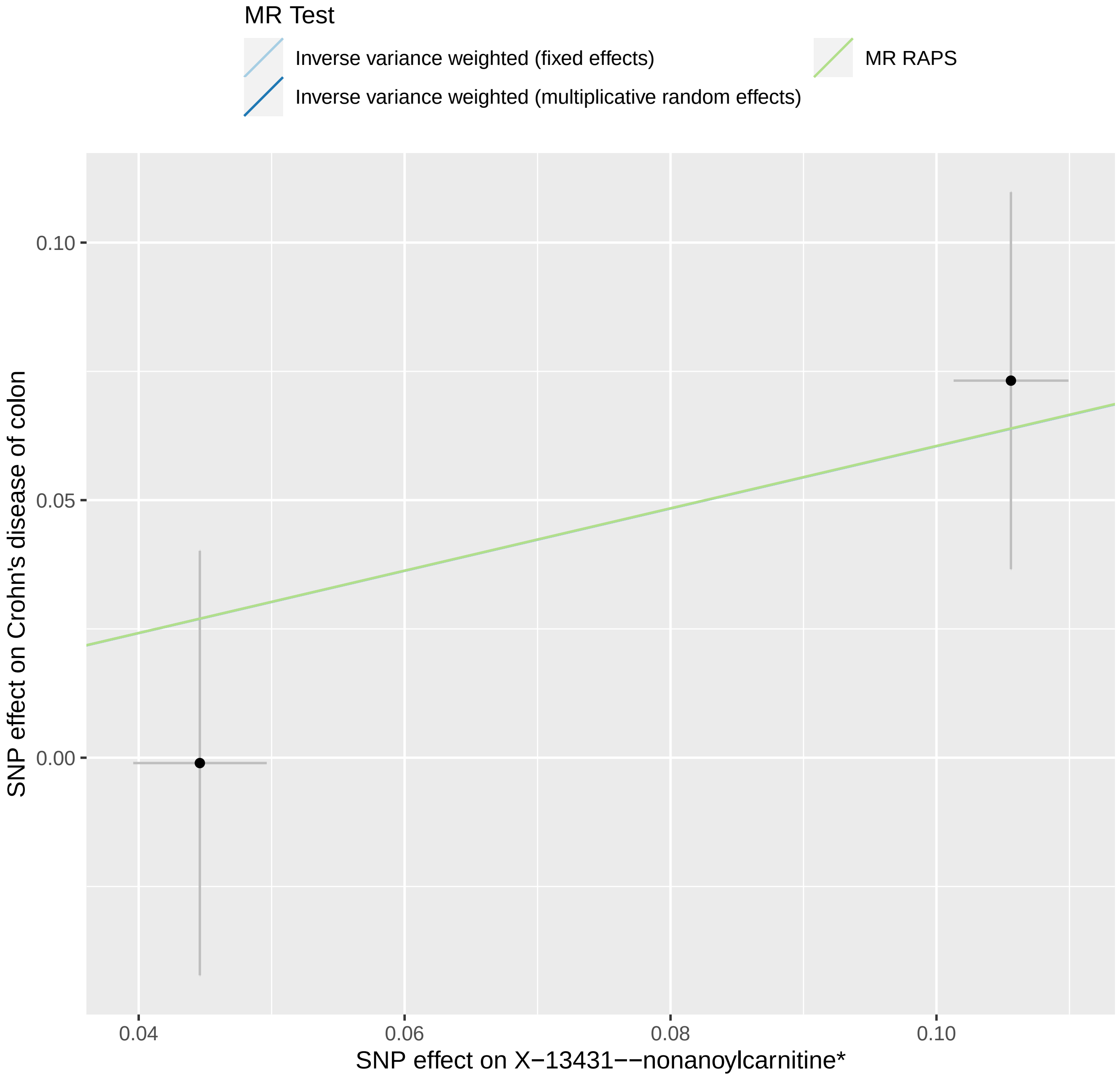

Batch 230 : 2−palmitoylglycerophosphocholine* on Crohn's disease of colon

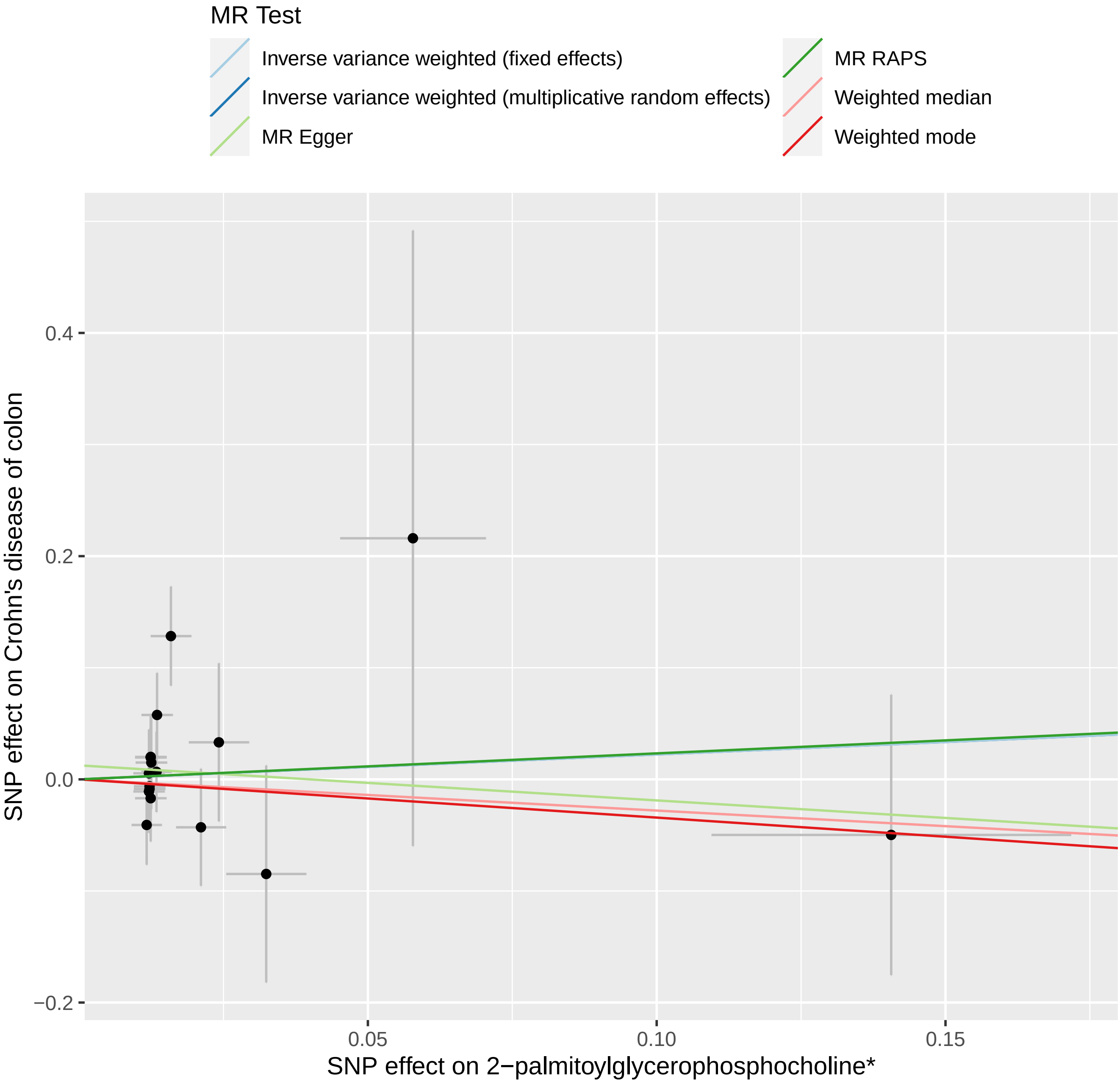

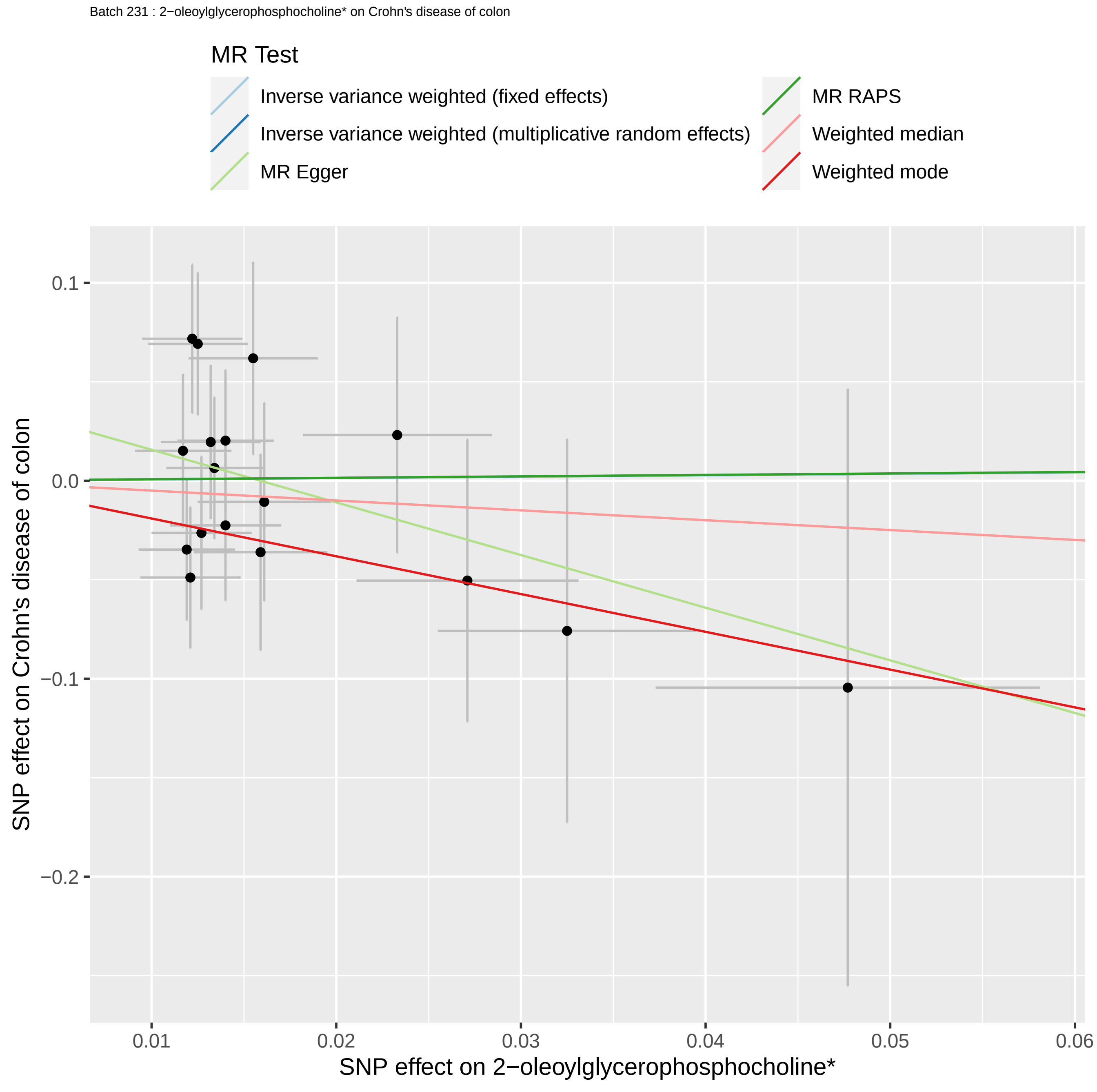
Batch 231 : 2−oleoylglycerophosphocholine* on Crohn's disease of colon
MR Test
Inverse variance weighted (fixed effects)
Inverse variance weighted (multiplicative random effects)
MR Egger
MR RAPS
Weighted median
Weighted mode
0.1
0.0
−0.1
−0.2
0.01
0.02
0.03
0.04
0.05
0.06
SNP effect on Crohn's disease of colon
SNP effect on 2−oleoylglycerophosphocholine*

Batch 234 : 1−palmitoylglycerophosphoinositol* on Crohn's disease of colon

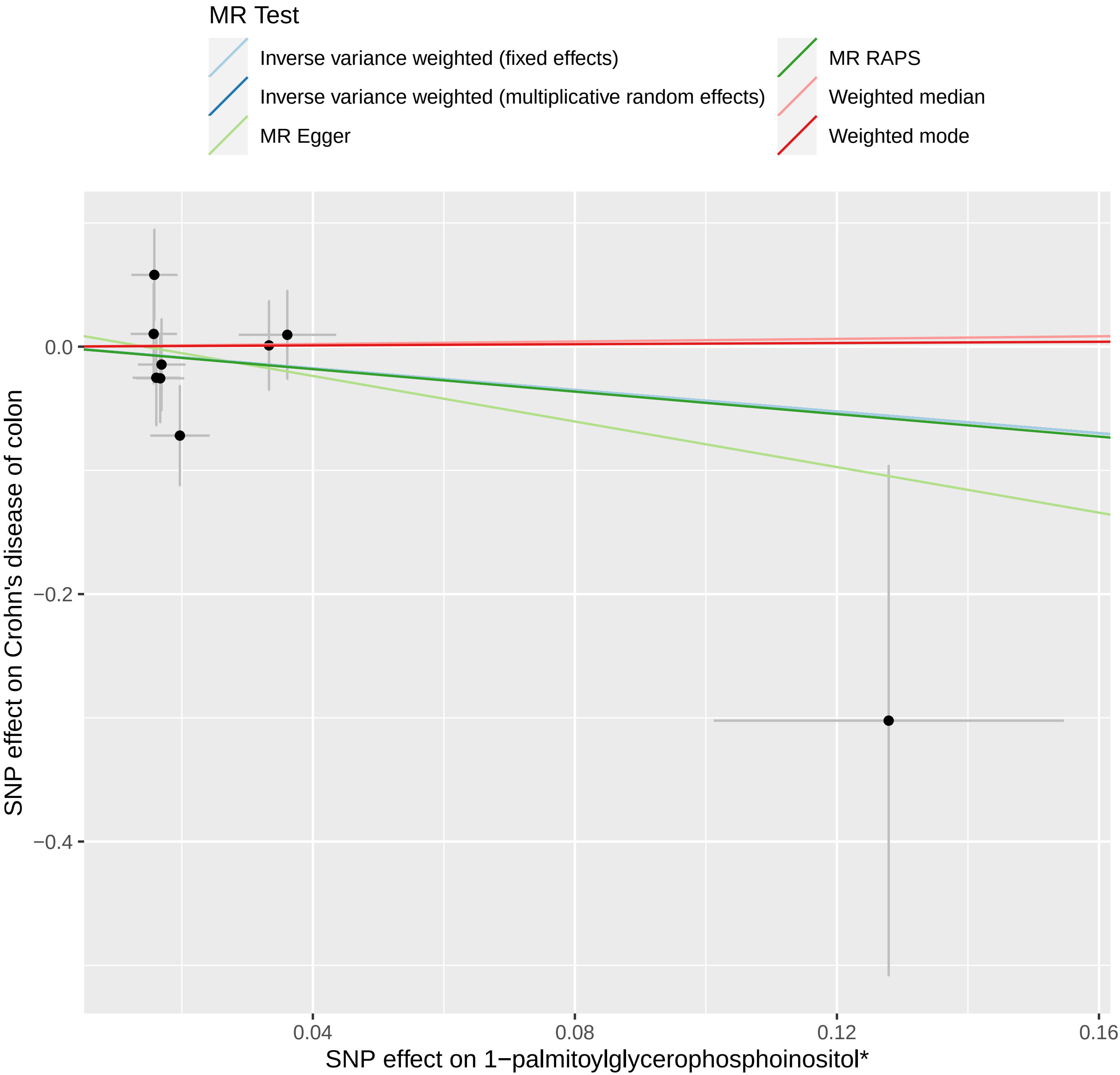

Batch 237 : Glutaroyl carnitine on Crohn's disease of colon

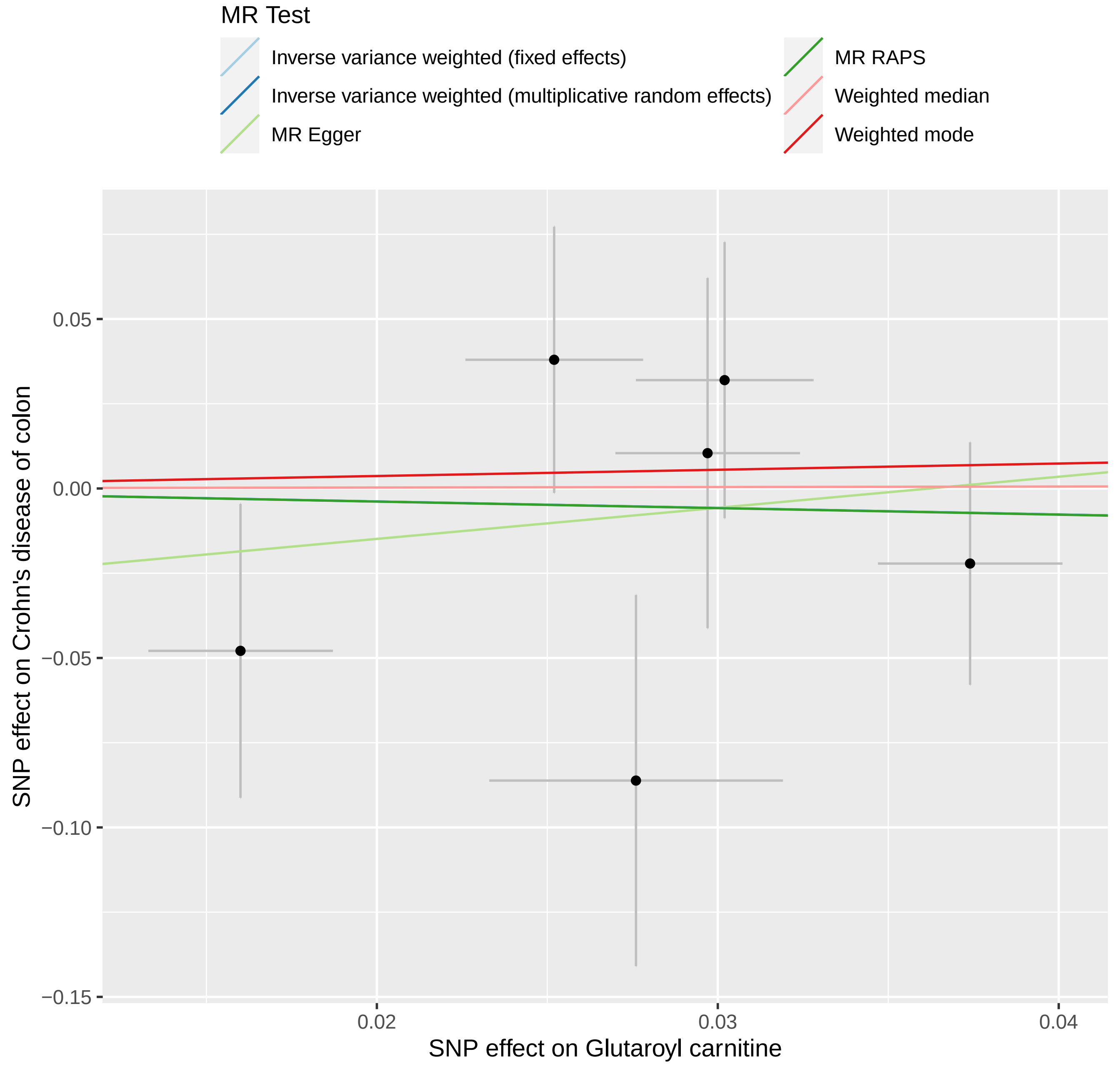

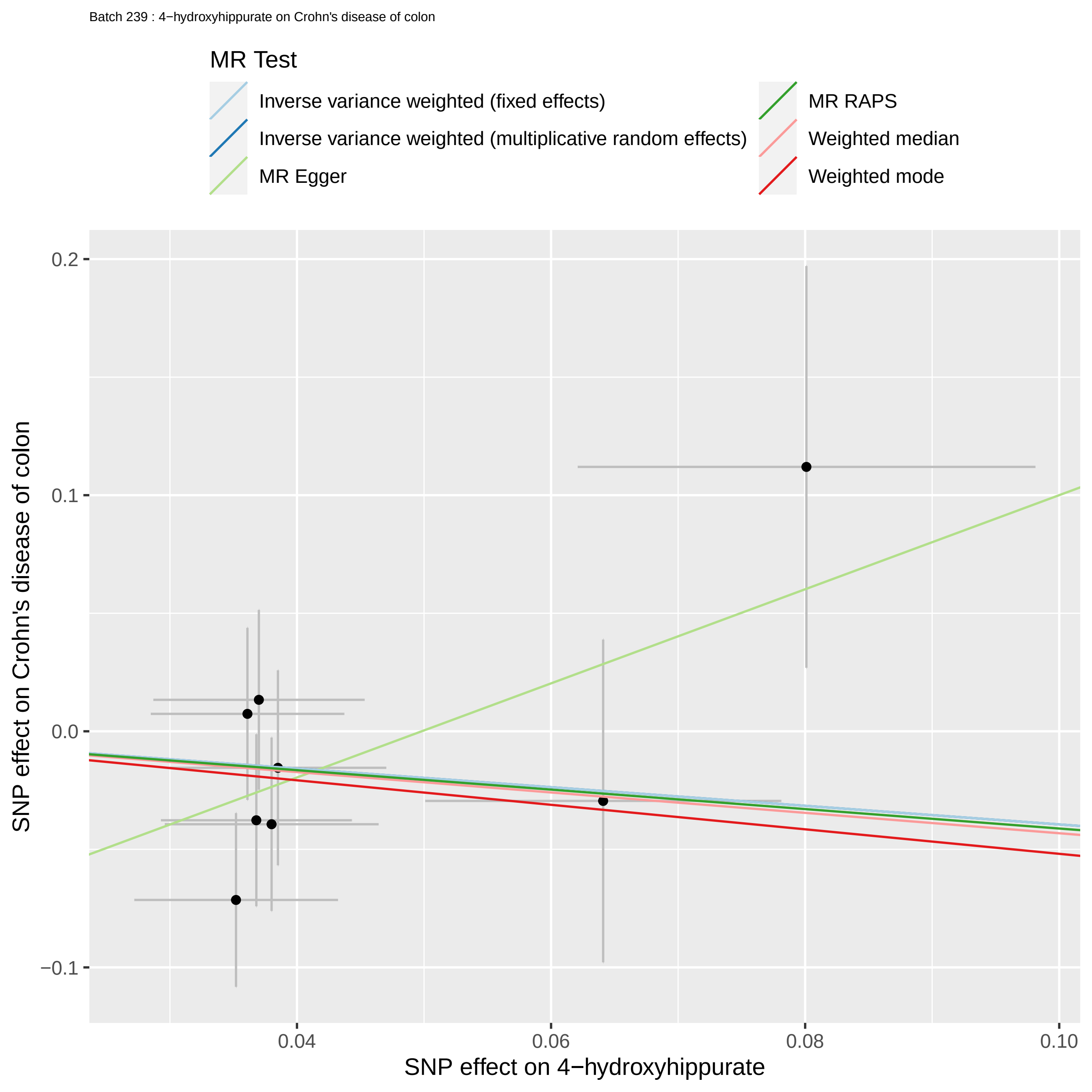
Batch 239 : 4−hydroxyhippurate on Crohn's disease of colon
MR Test
Inverse variance weighted (fixed effects)
Inverse variance weighted (multiplicative random effects)
MR Egger
MR RAPS
Weighted median
Weighted mode
0.2
0.1
0.0
−0.1
0.04
0.06
0.08
0.10
SNP effect on Crohn's disease of colon
SNP effect on 4−hydroxyhippurate

Batch 240 : 1−myristoylglycerophosphocholine on Crohn's disease of colon

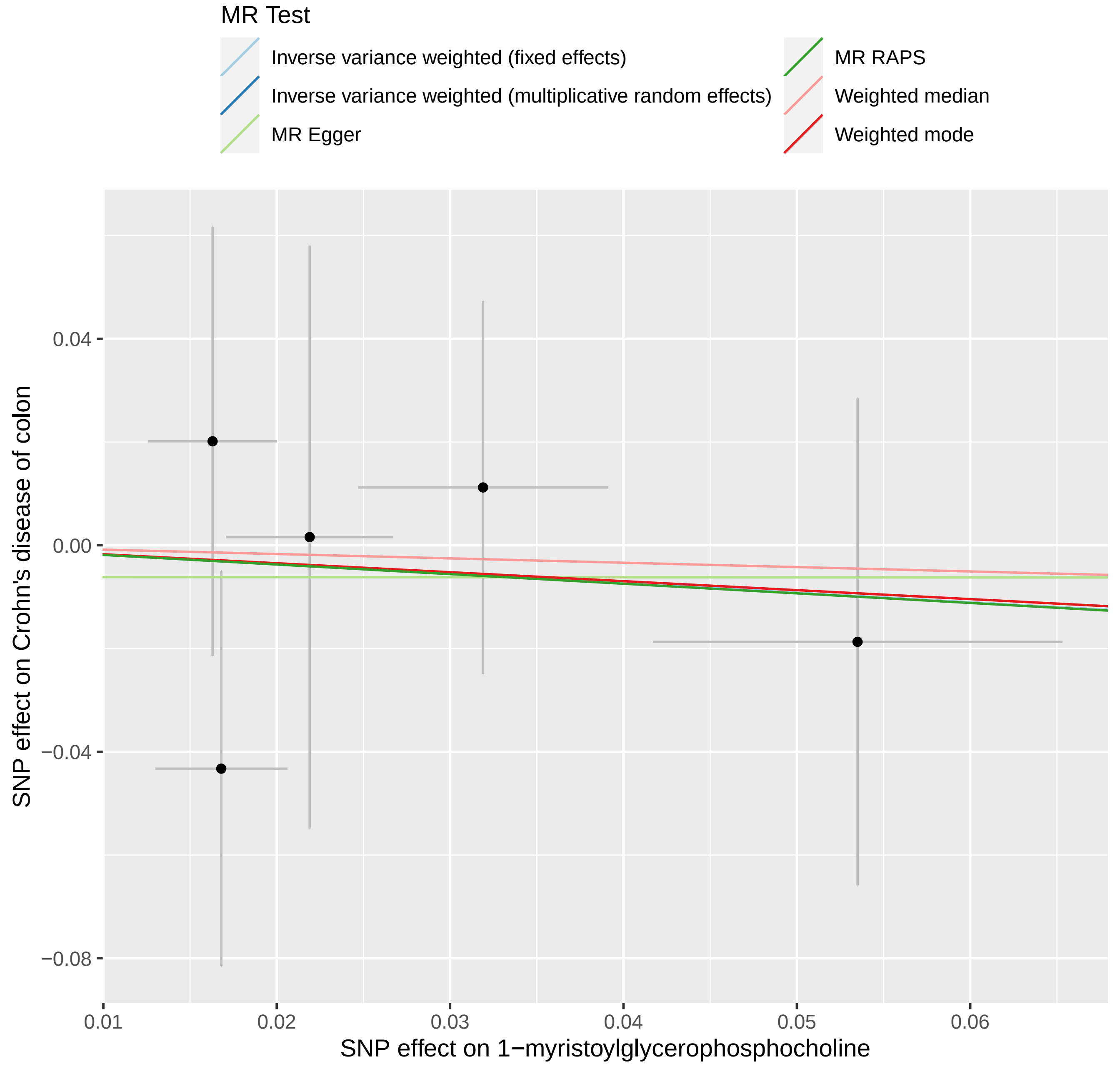

Batch 243 : 3−(3−hydroxyphenyl)propionate on Crohn's disease of colon

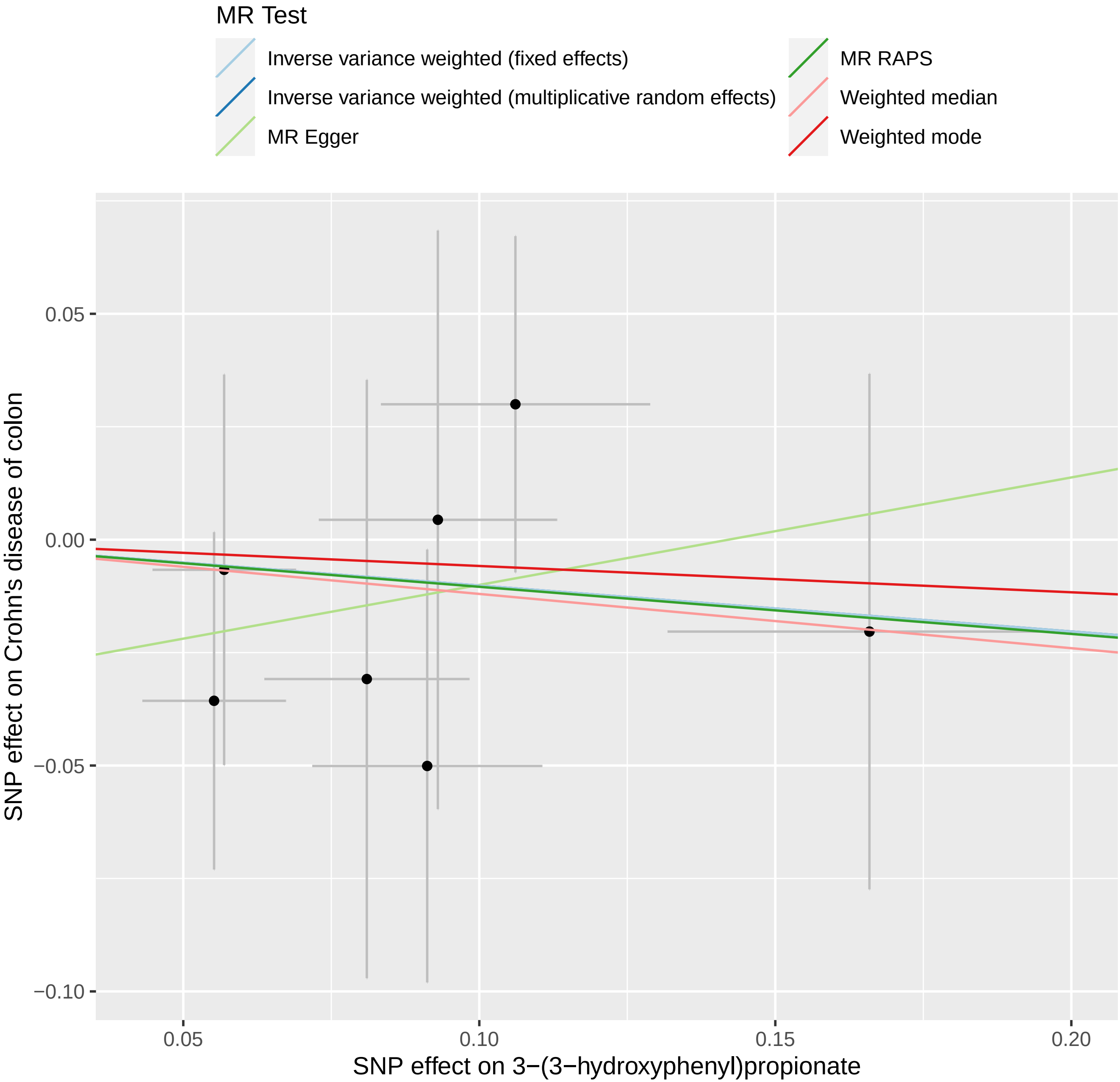

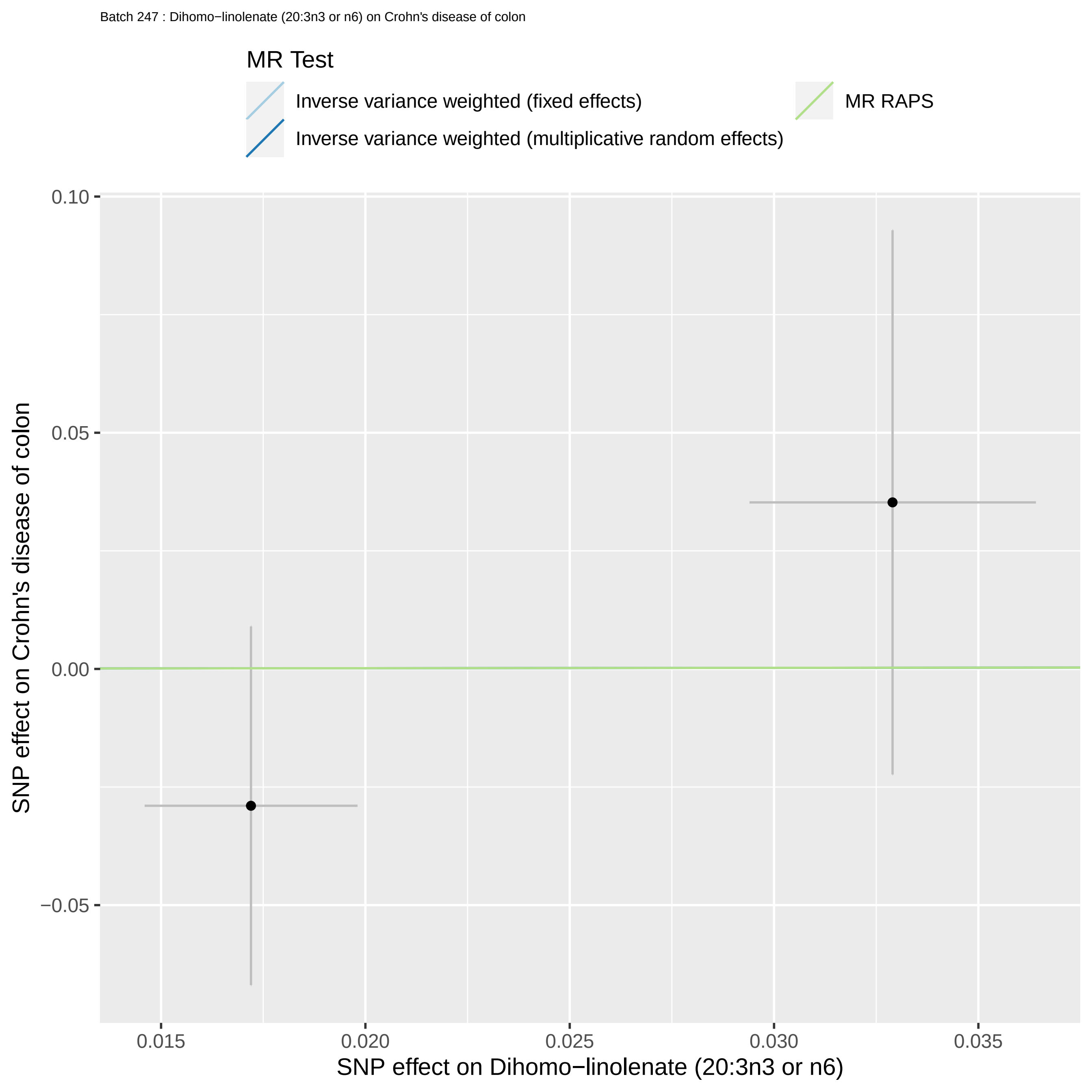

Batch 247 : Dihomo−linolenate (20:3n3 or n6) on Crohn's disease of colon
MR Test
Inverse variance weighted (fixed effects)
Inverse variance weighted (multiplicative random effects)
MR RAPS
0.10
0.05
0.00
−0.05
0.015
0.020
0.025
0.030
0.035
SNP effect on Crohn's disease of colon
SNP effect on Dihomo−linolenate (20:3n3 or n6)

Batch 248 : Threitol on Crohn's disease of colon

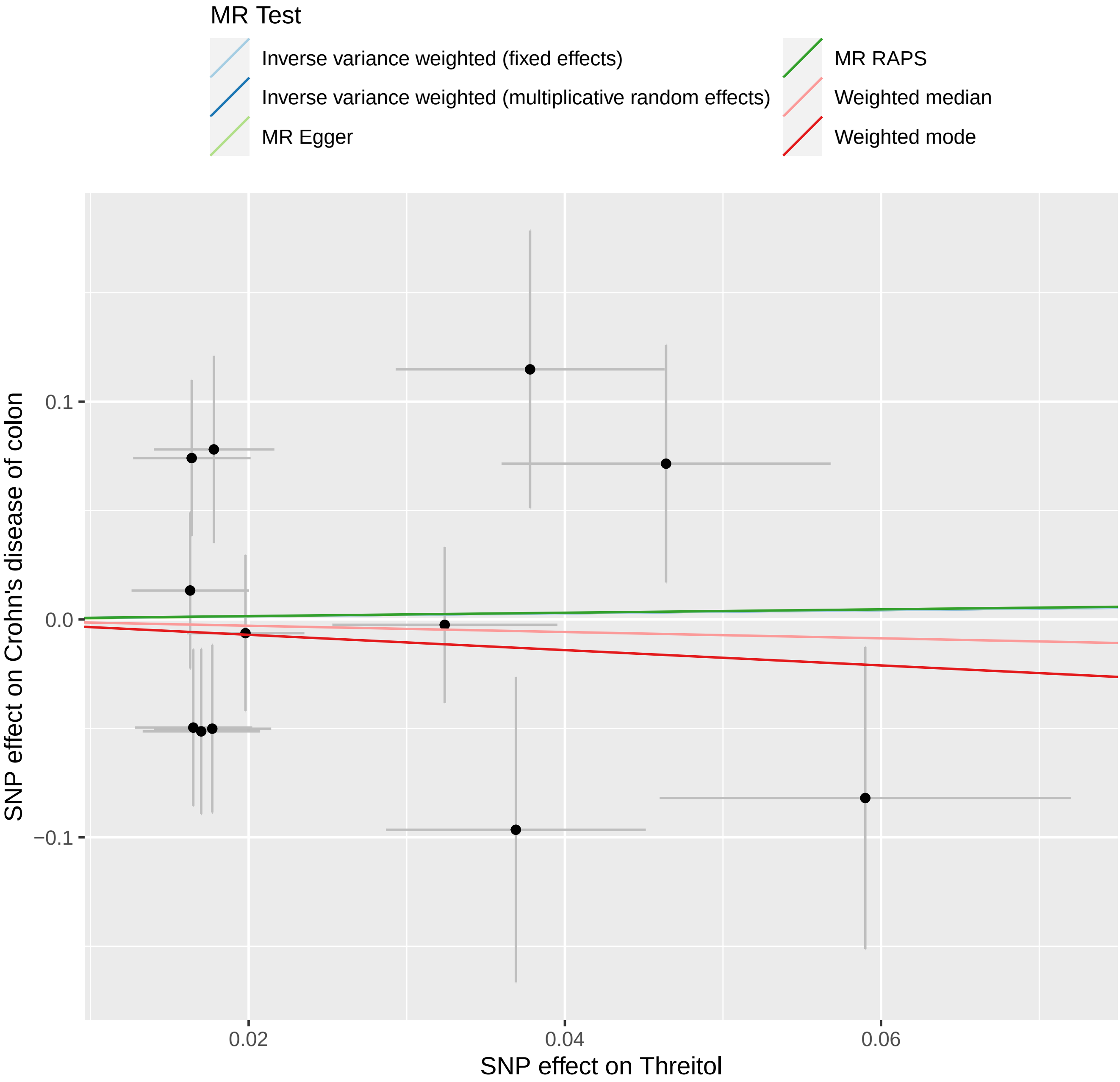

Batch 249 : P−cresol sulfate on Crohn's disease of colon

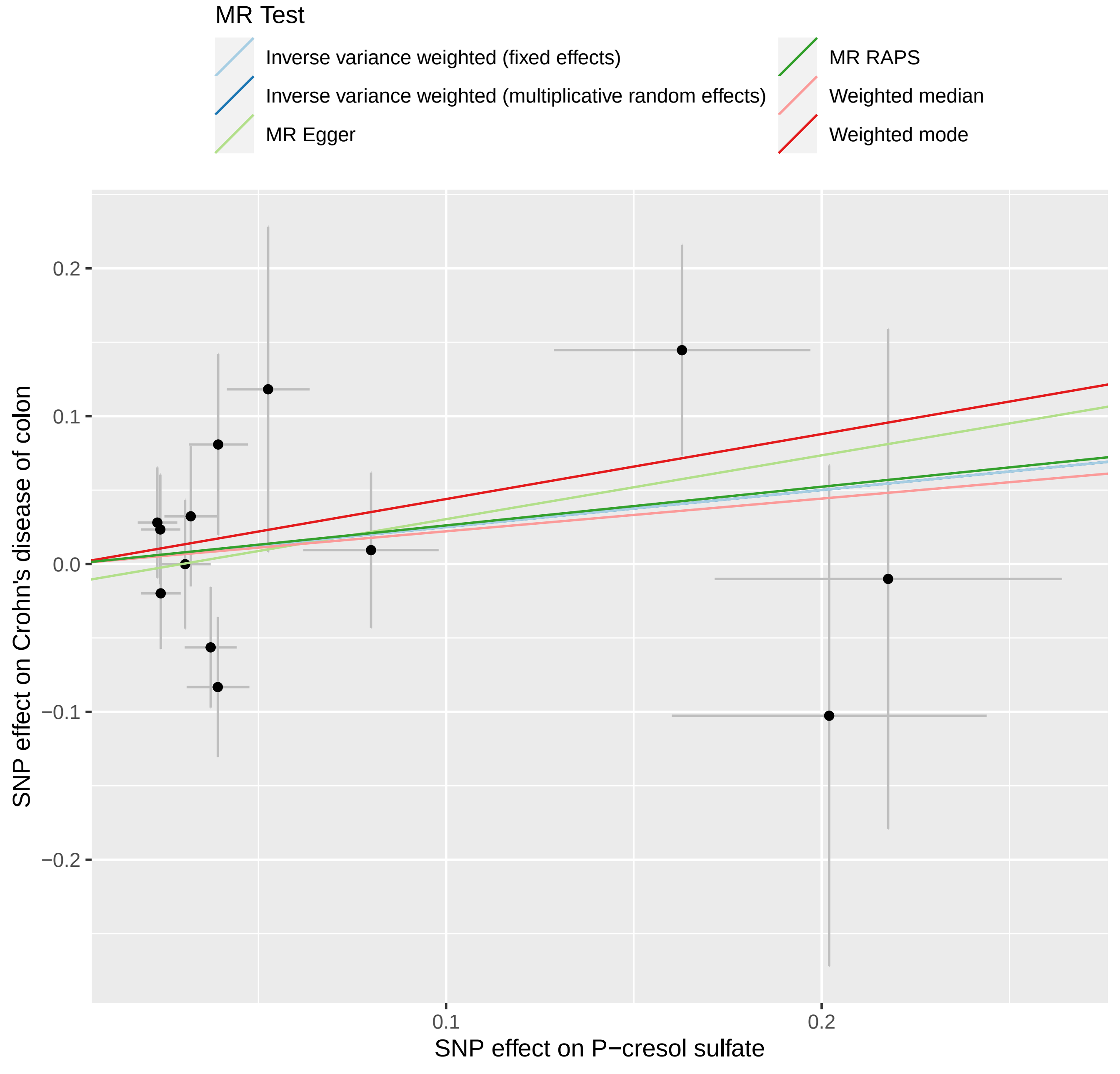

Batch 252 : X−14208−−phenylalanylserine on Crohn's disease of colon

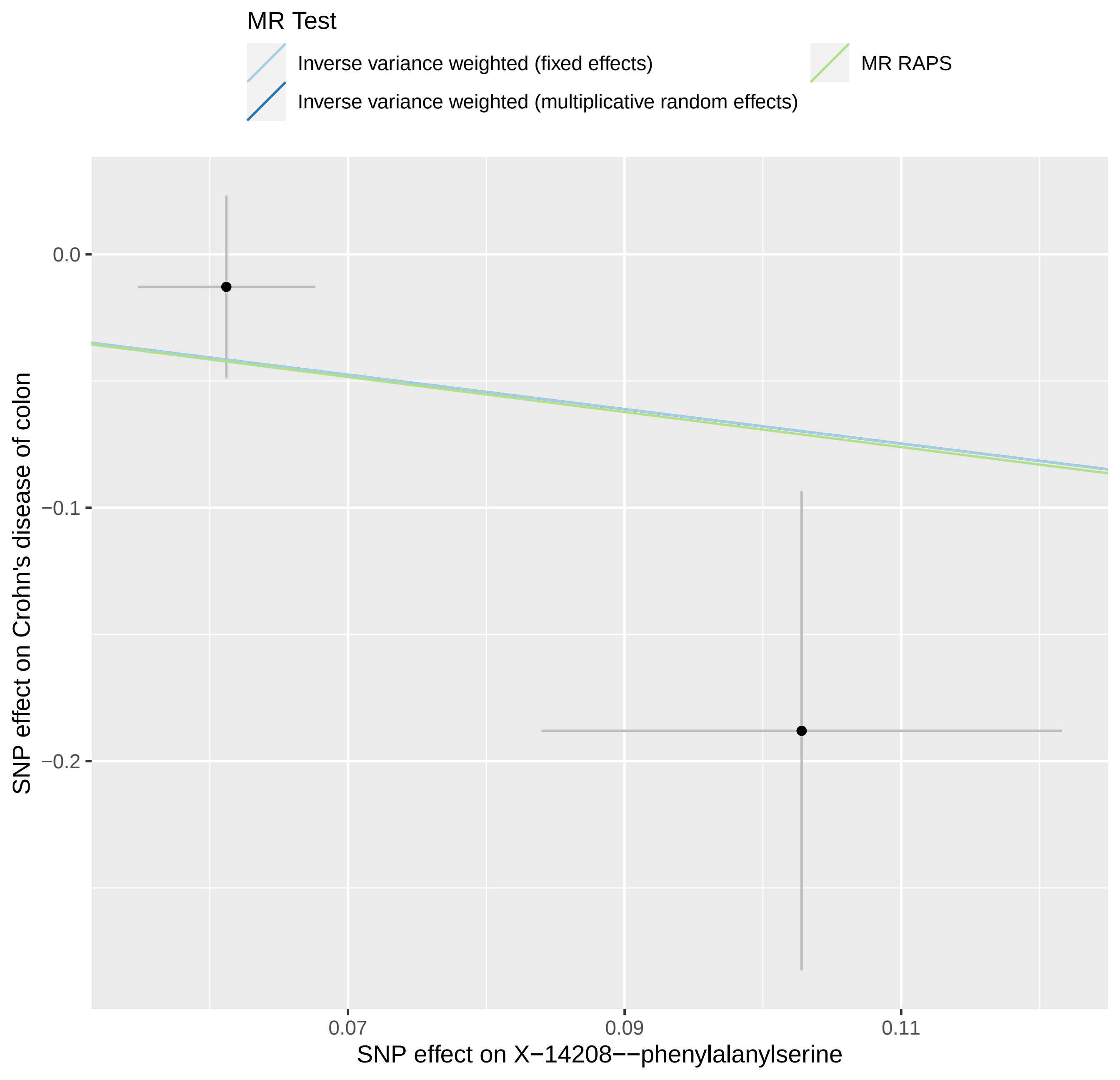

Batch 254 : X−14450−−phenylalanylleucine on Crohn's disease of colon

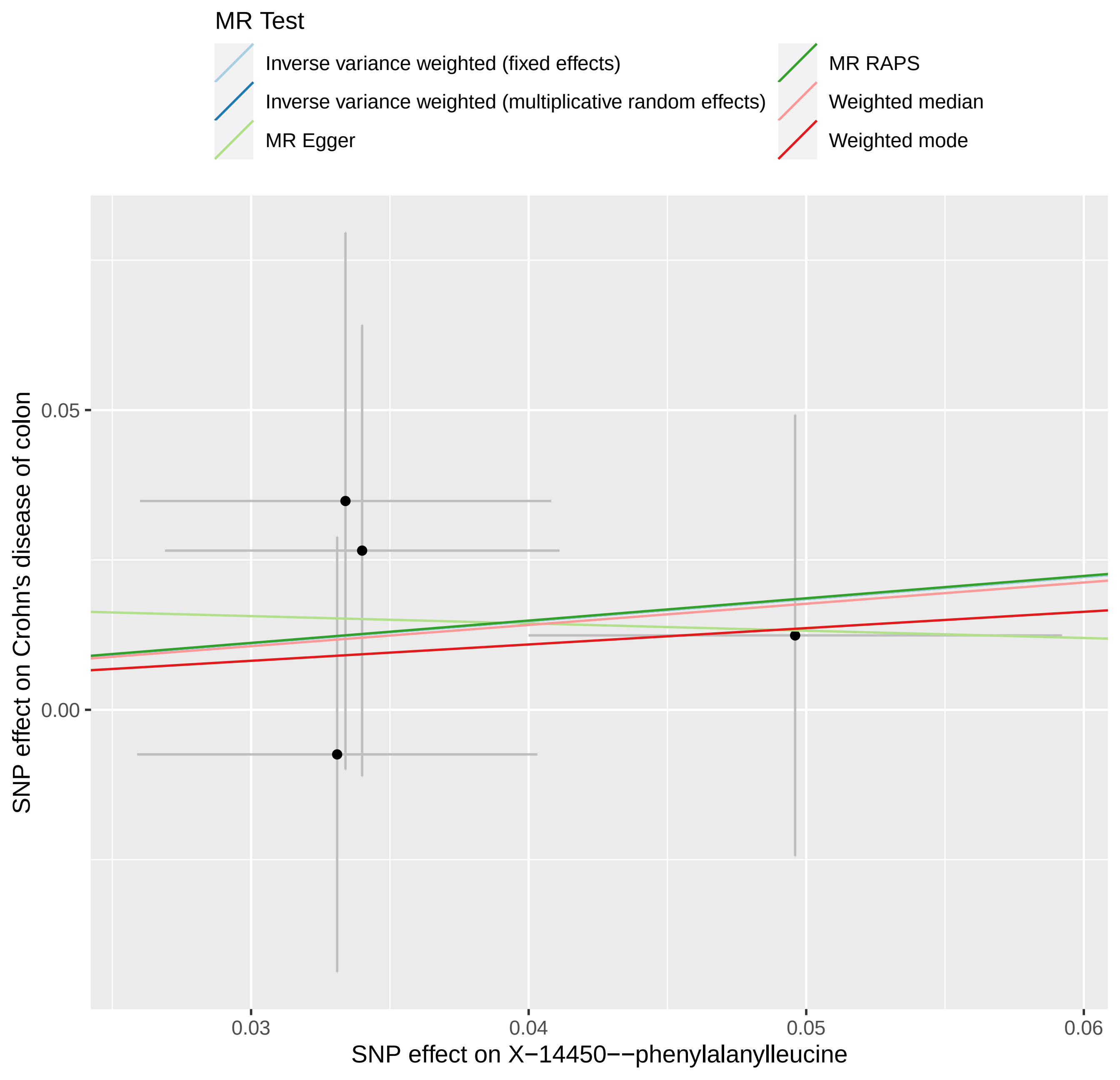

Batch 255 : Gamma−glutamylglutamate on Crohn's disease of colon

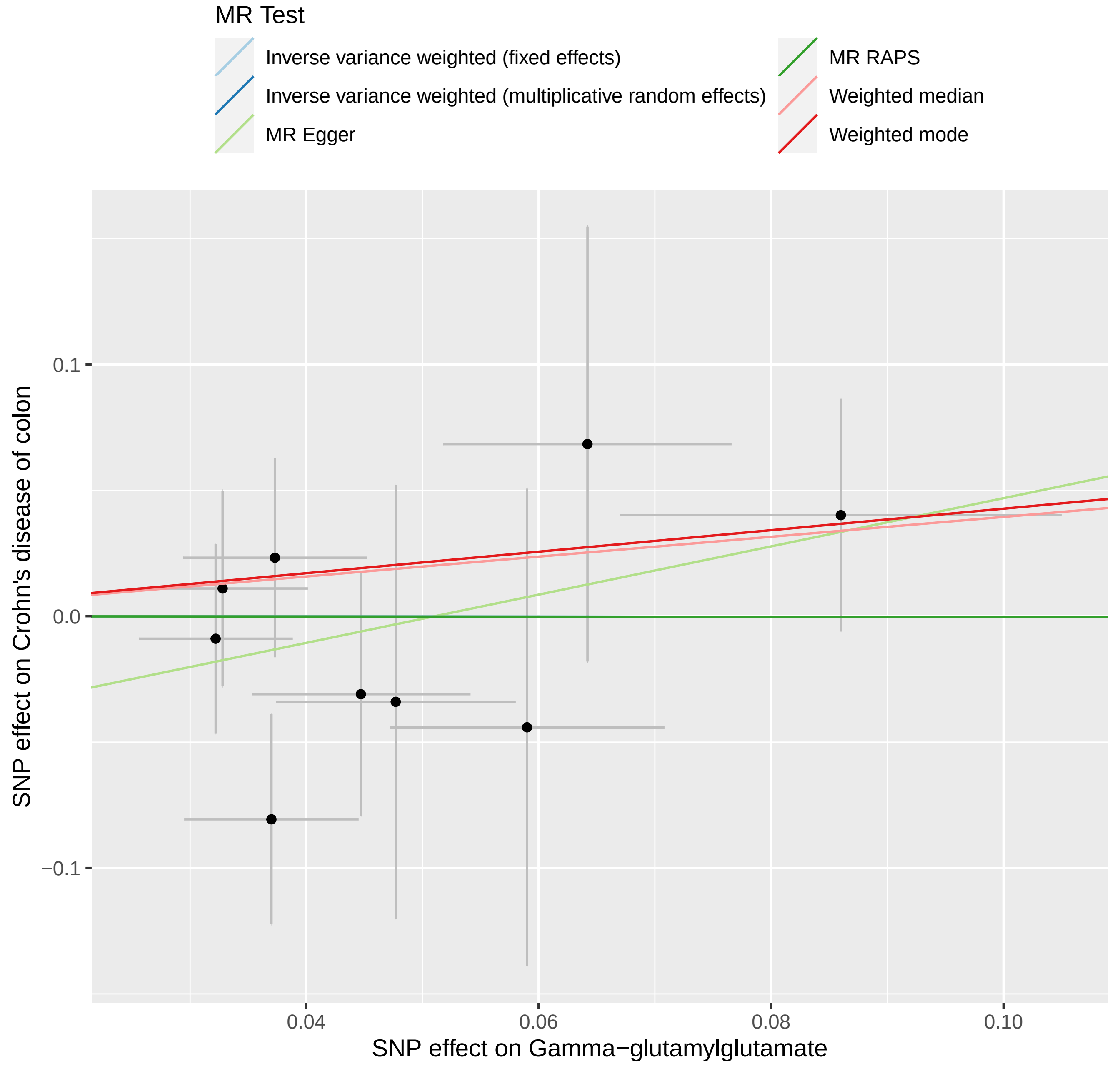

Batch 256 : Octadecanedioate on Crohn's disease of colon

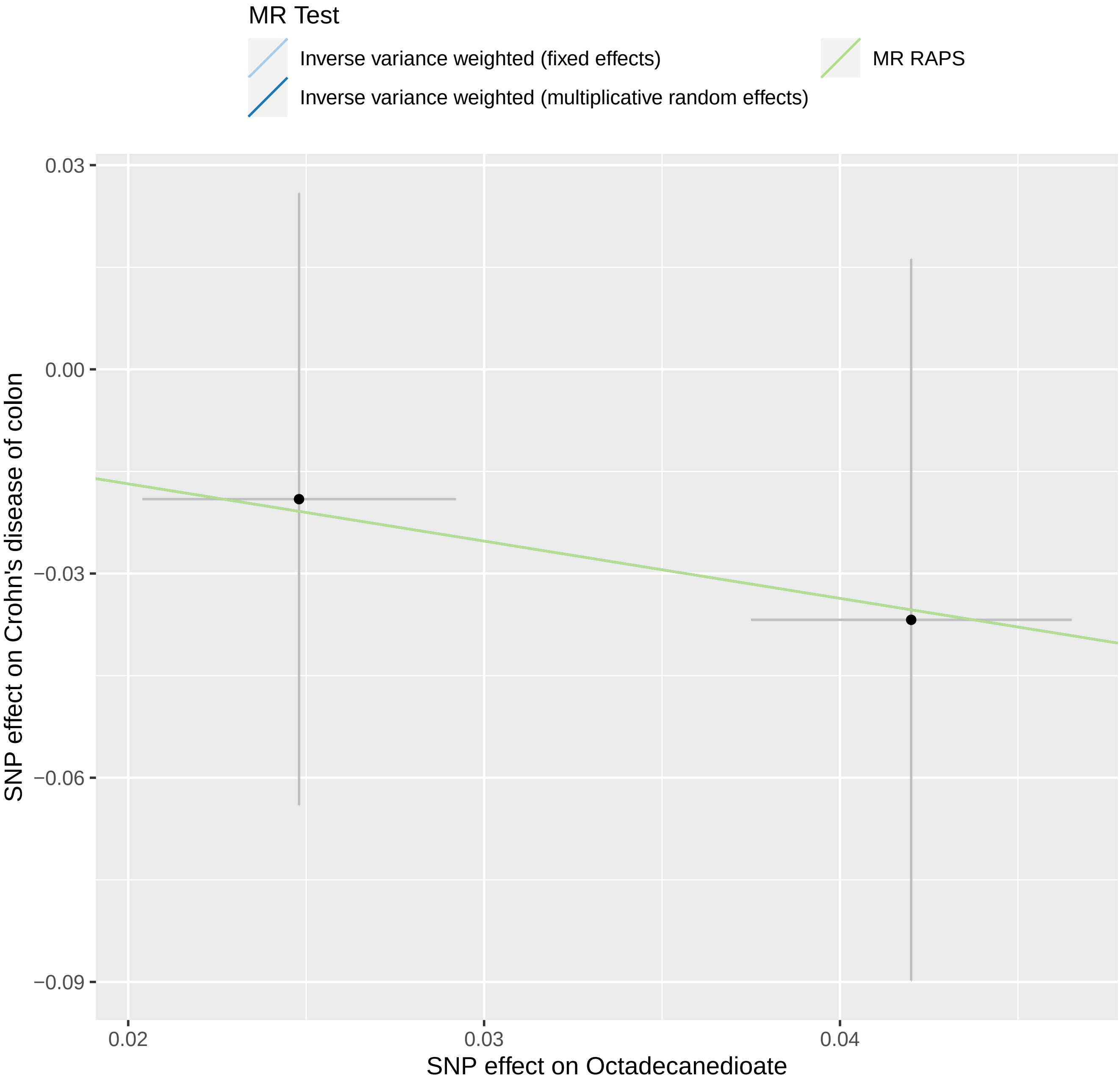

Batch 257 : Leucylleucine on Crohn's disease of colon

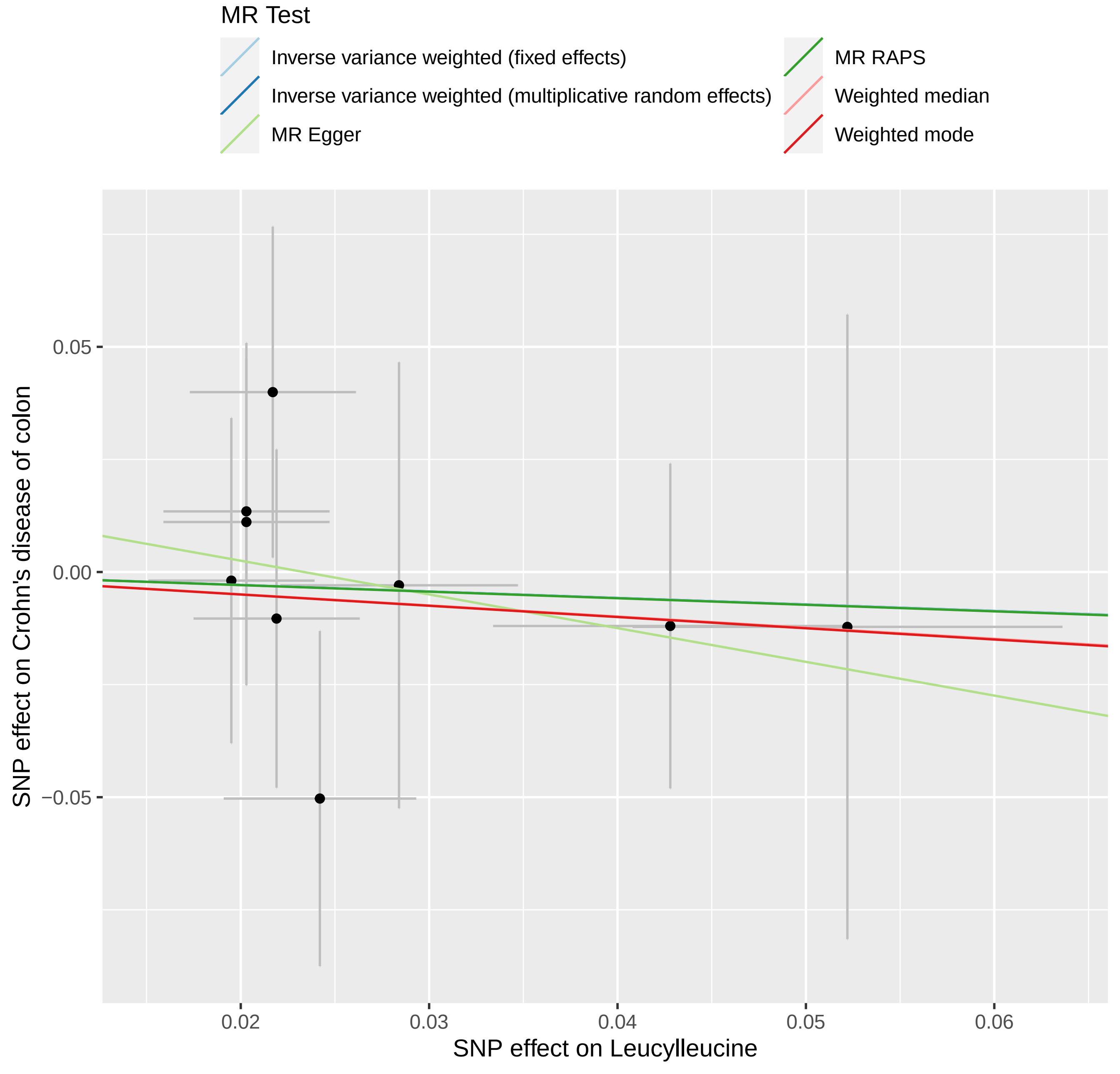

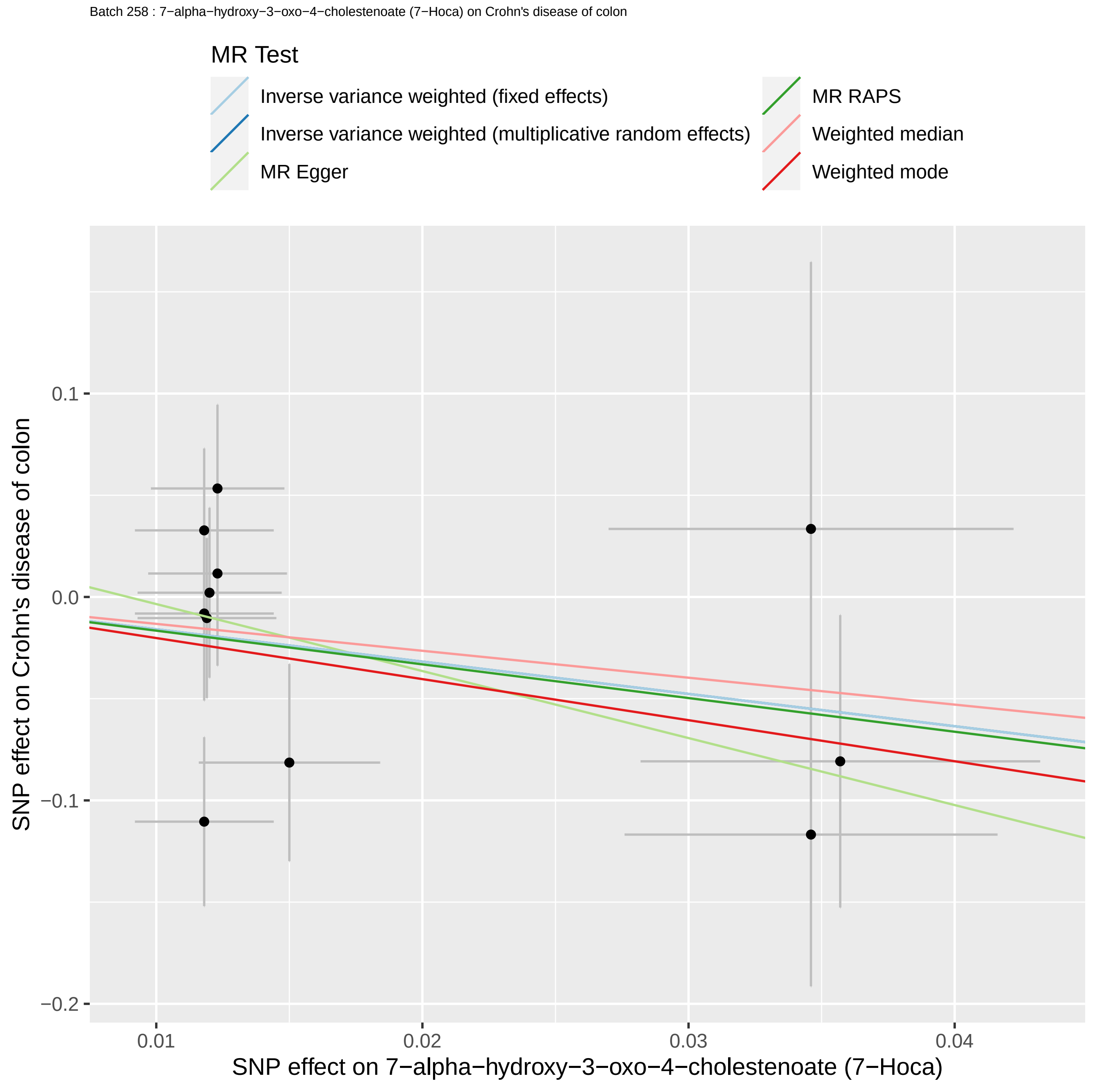
Batch 258 : 7−alpha−hydroxy−3−oxo−4−cholestenoate (7−Hoca) on Crohn's disease of colon
MR Test
Inverse variance weighted (fixed effects)
Inverse variance weighted (multiplicative random effects)
MR Egger
MR RAPS
Weighted median
Weighted mode
0.1
0.0
−0.1
−0.2
0.01
0.02
0.03
0.04
SNP effect on Crohn's disease of colon
SNP effect on 7−alpha−hydroxy−3−oxo−4−cholestenoate (7−Hoca)

Batch 259 : N−Butyl Oleate on Crohn's disease of colon

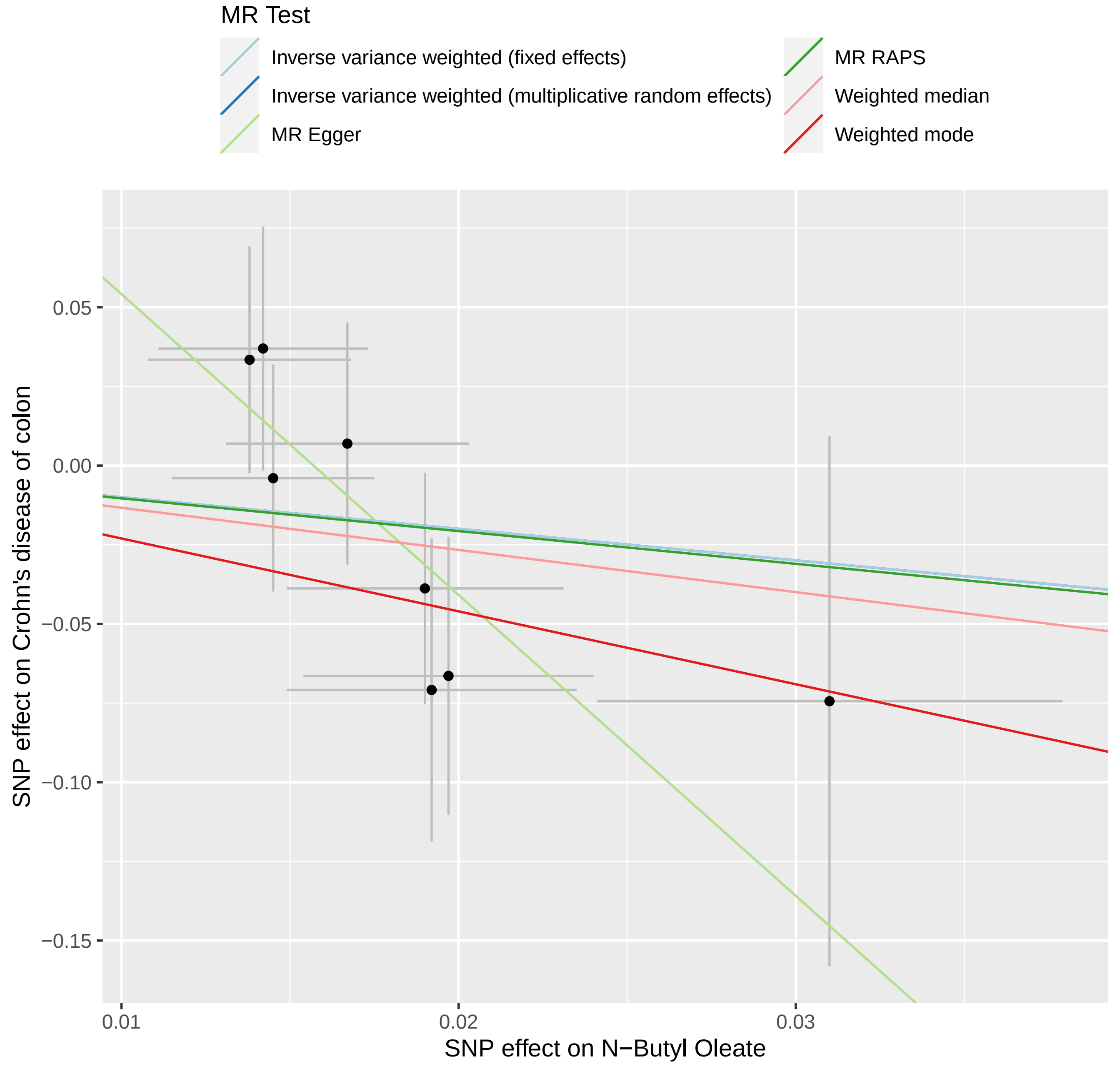

Batch 261 : Taurolithocholate 3−sulfate on Crohn's disease of colon

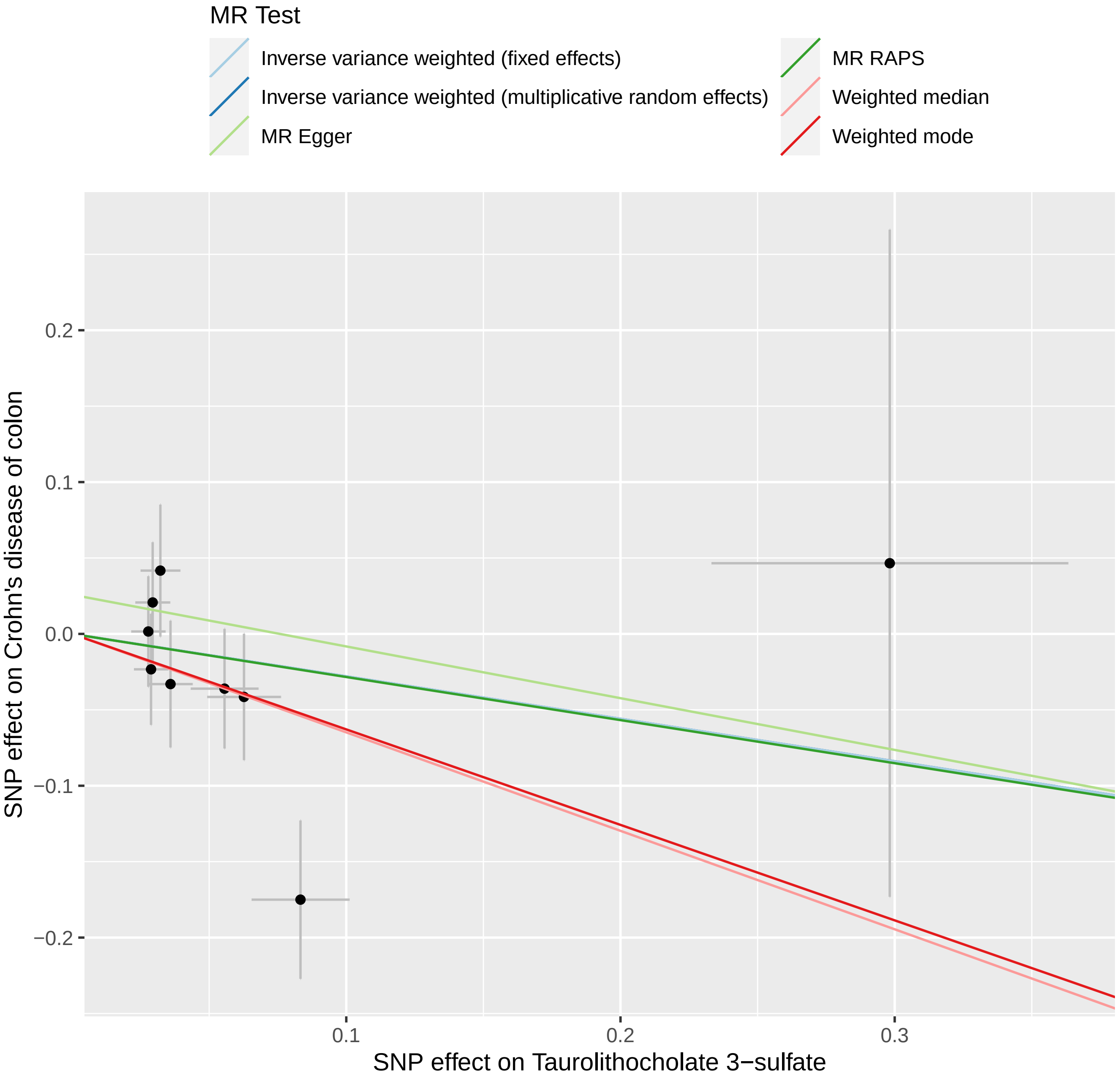

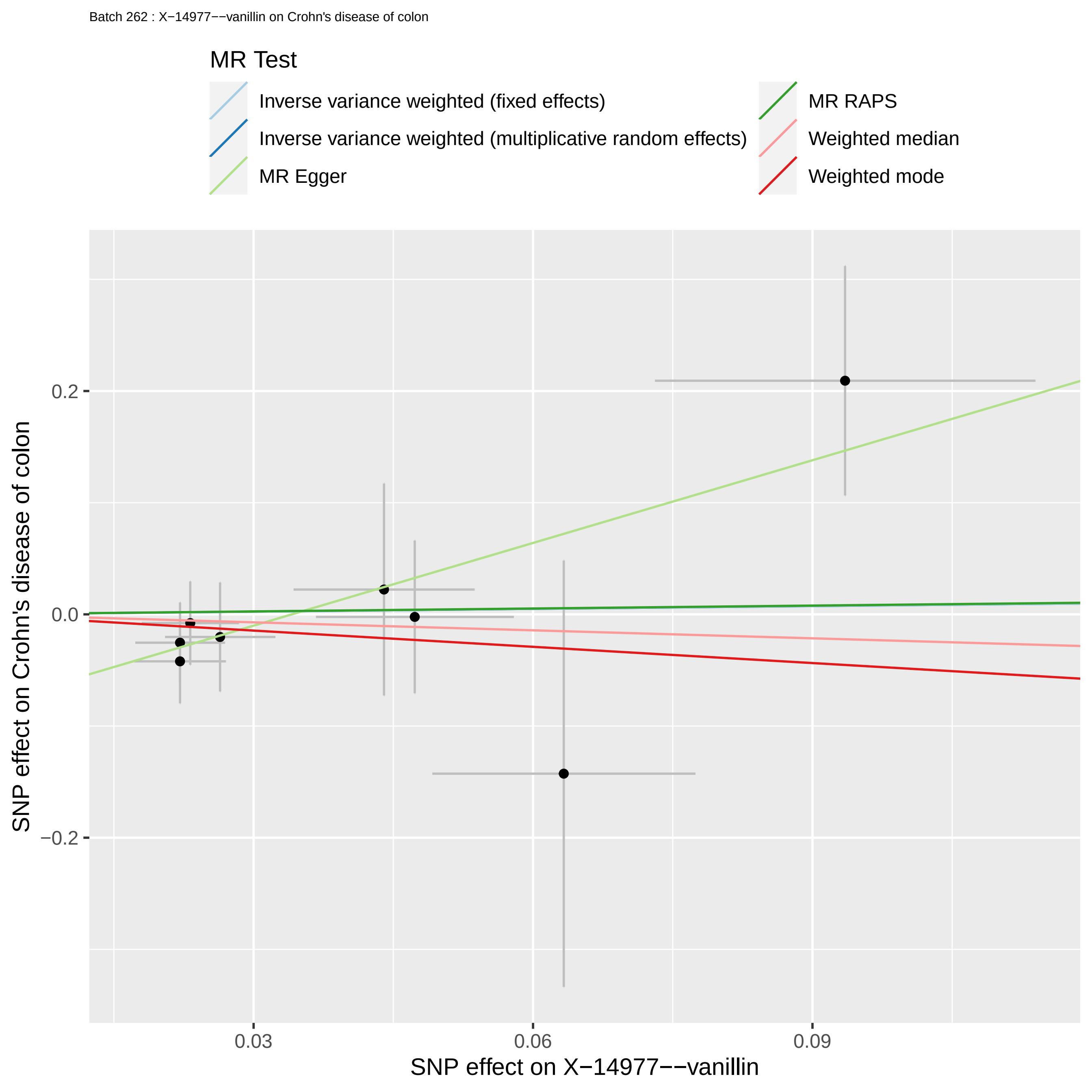

Batch 262 : X−14977−−vanillin on Crohn's disease of colon
MR Test
Inverse variance weighted (fixed effects)
Inverse variance weighted (multiplicative random effects)
MR Egger
MR RAPS
Weighted median
Weighted mode
0.2
0.0
−0.2
0.03
0.06
0.09
SNP effect on Crohn's disease of colon
SNP effect on X−14977−−vanillin

Batch 263 : Succinylcarnitine on Crohn's disease of colon

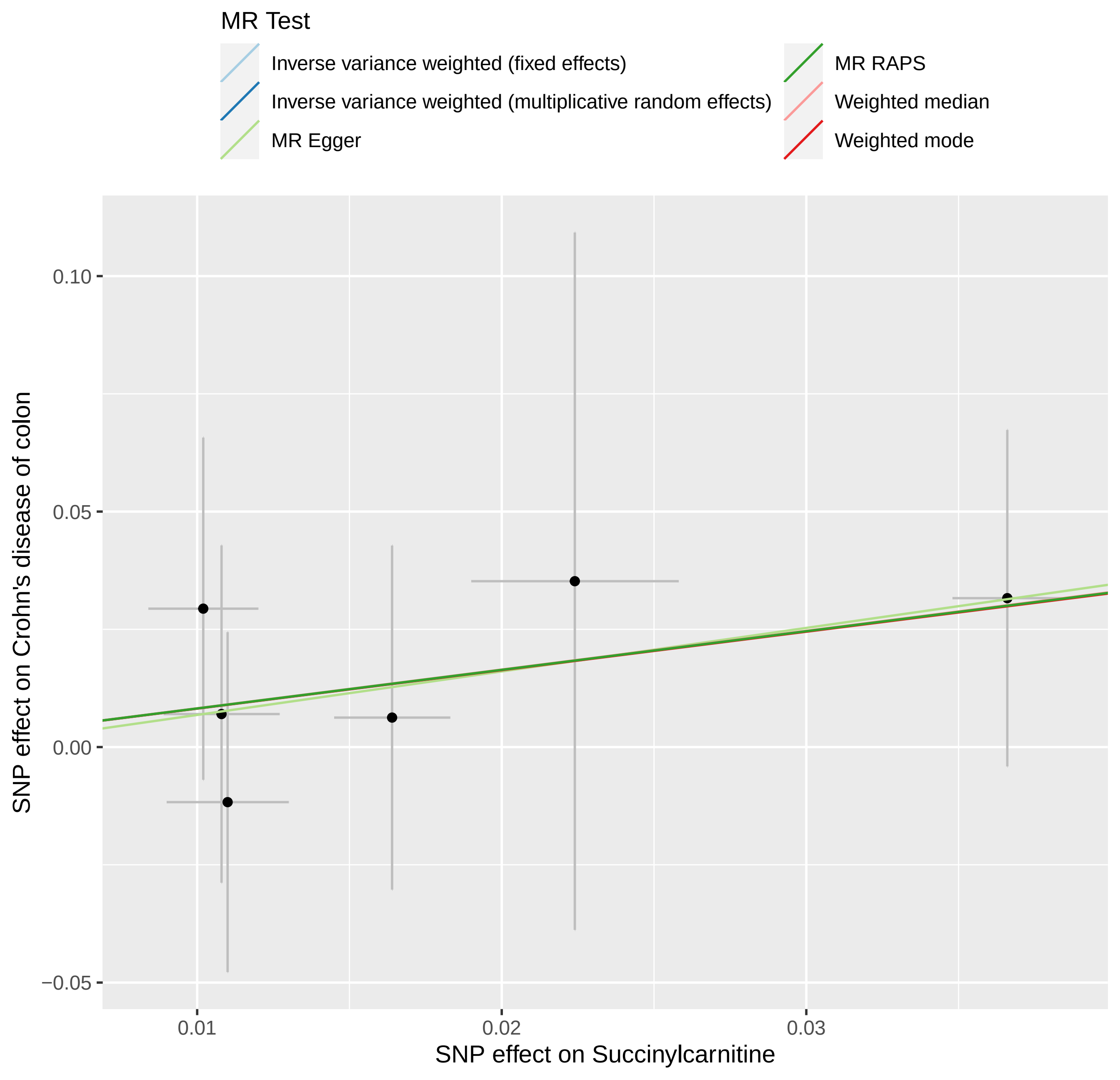

Batch 265 : Cyclo(leu−pro) on Crohn's disease of colon

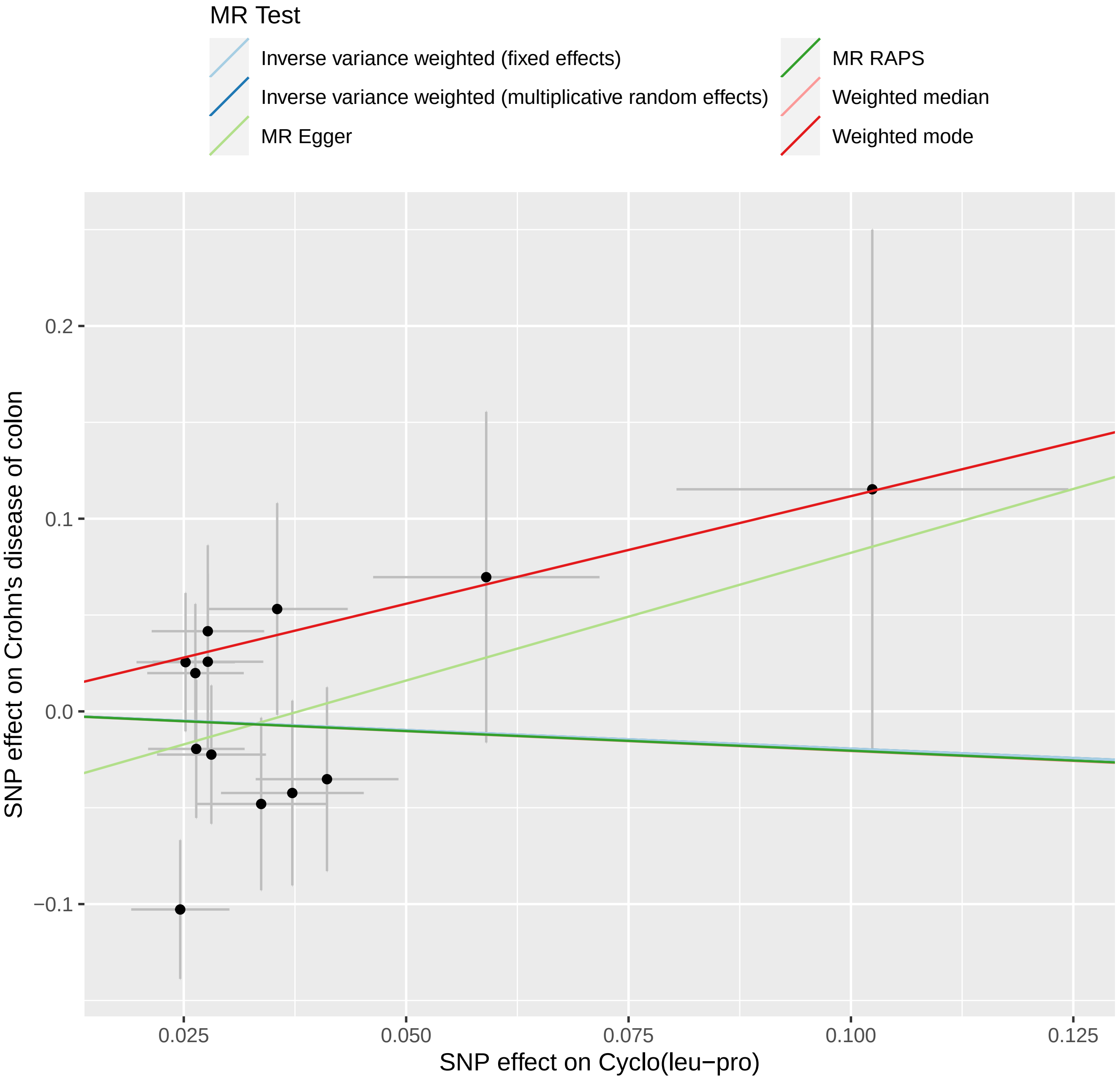

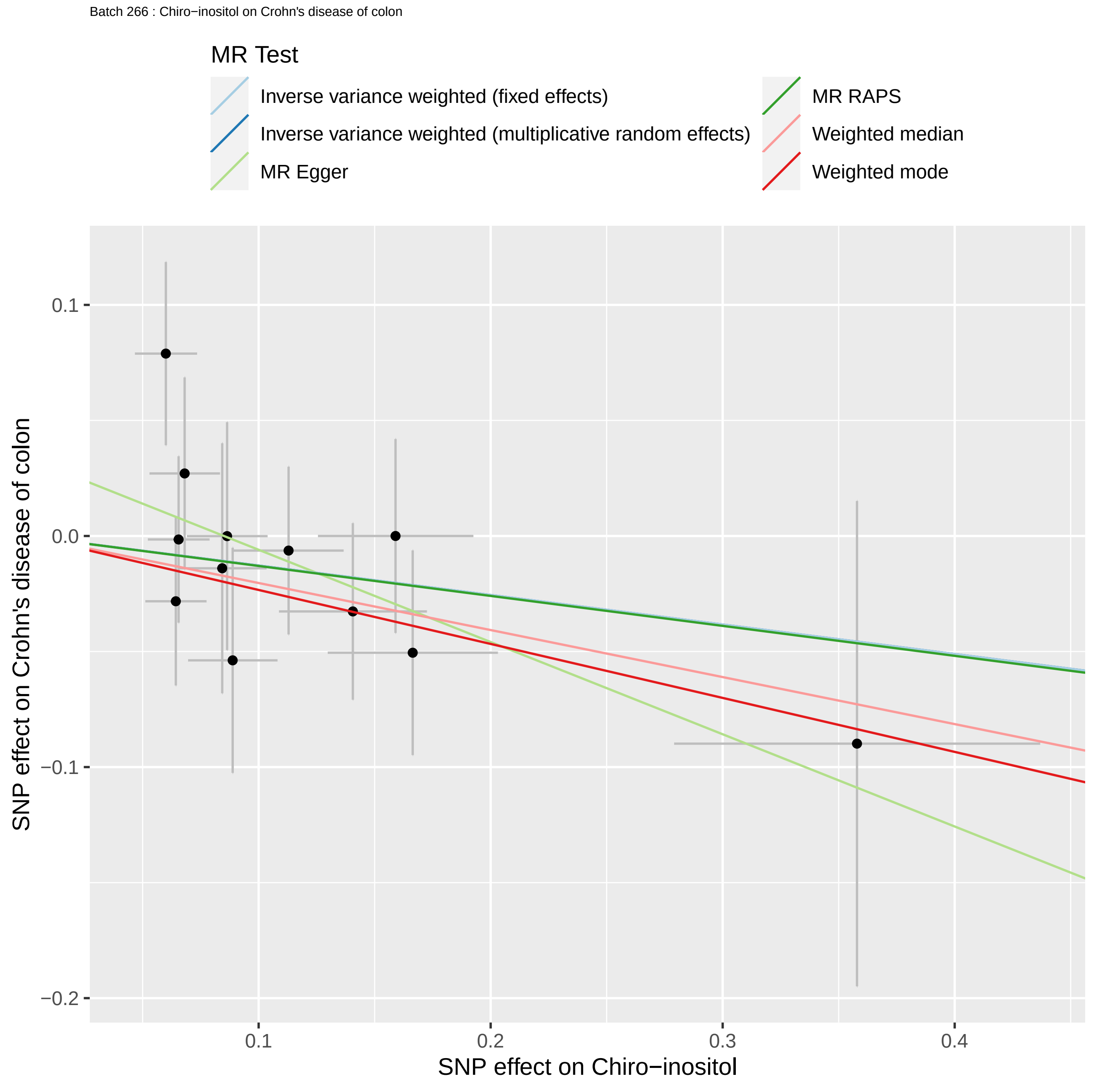

Batch 266 : Chiro−inositol on Crohn's disease of colon
MR Test
Inverse variance weighted (fixed effects)
Inverse variance weighted (multiplicative random effects)
MR Egger
MR RAPS
Weighted median
Weighted mode
0.1
0.0
−0.1
−0.2
0.1
0.2
0.3
0.4
SNP effect on Crohn's disease of colon
SNP effect on Chiro−inositol

Batch 270 : 2−hydroxyglutarate on Crohn's disease of colon

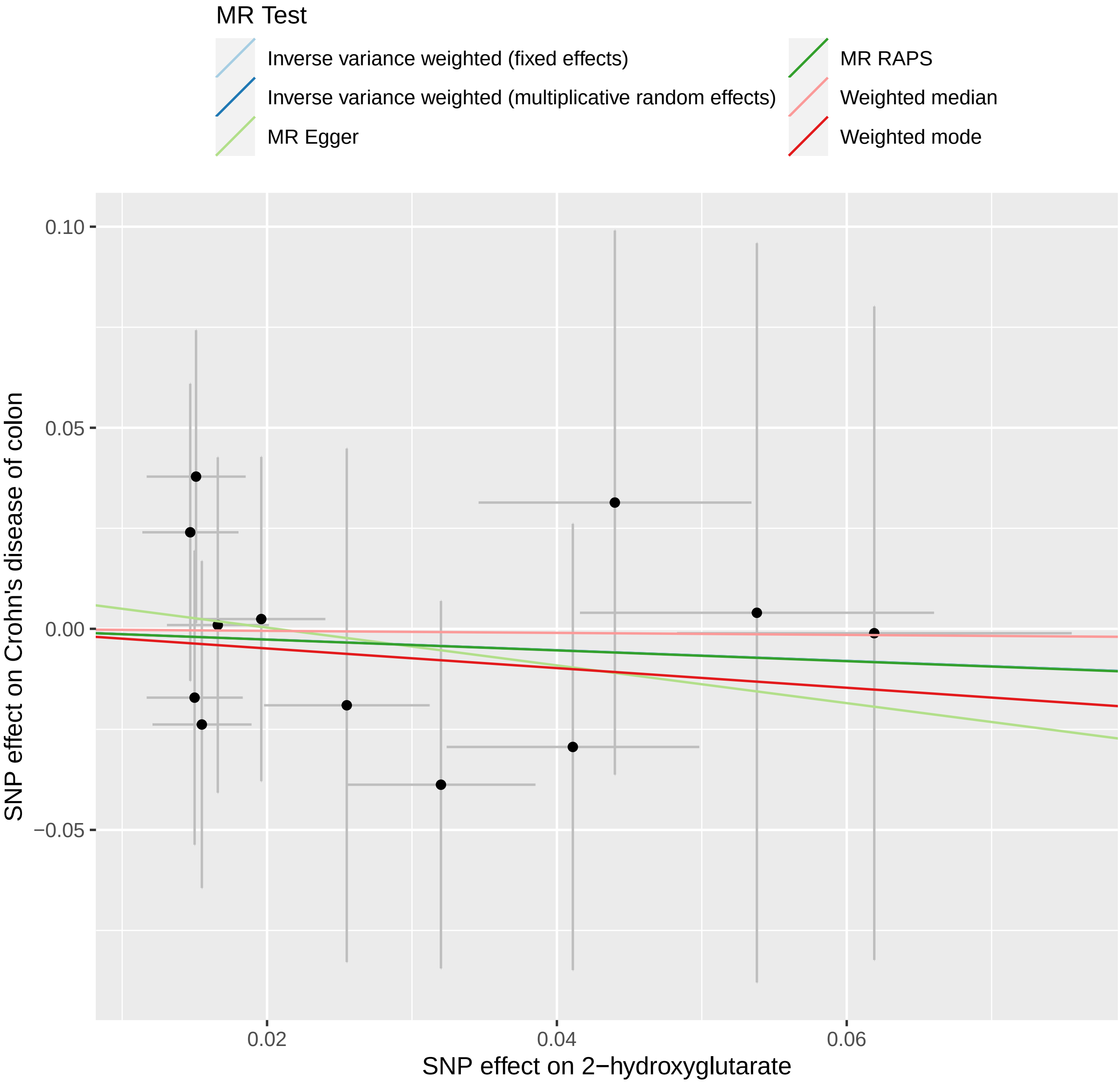

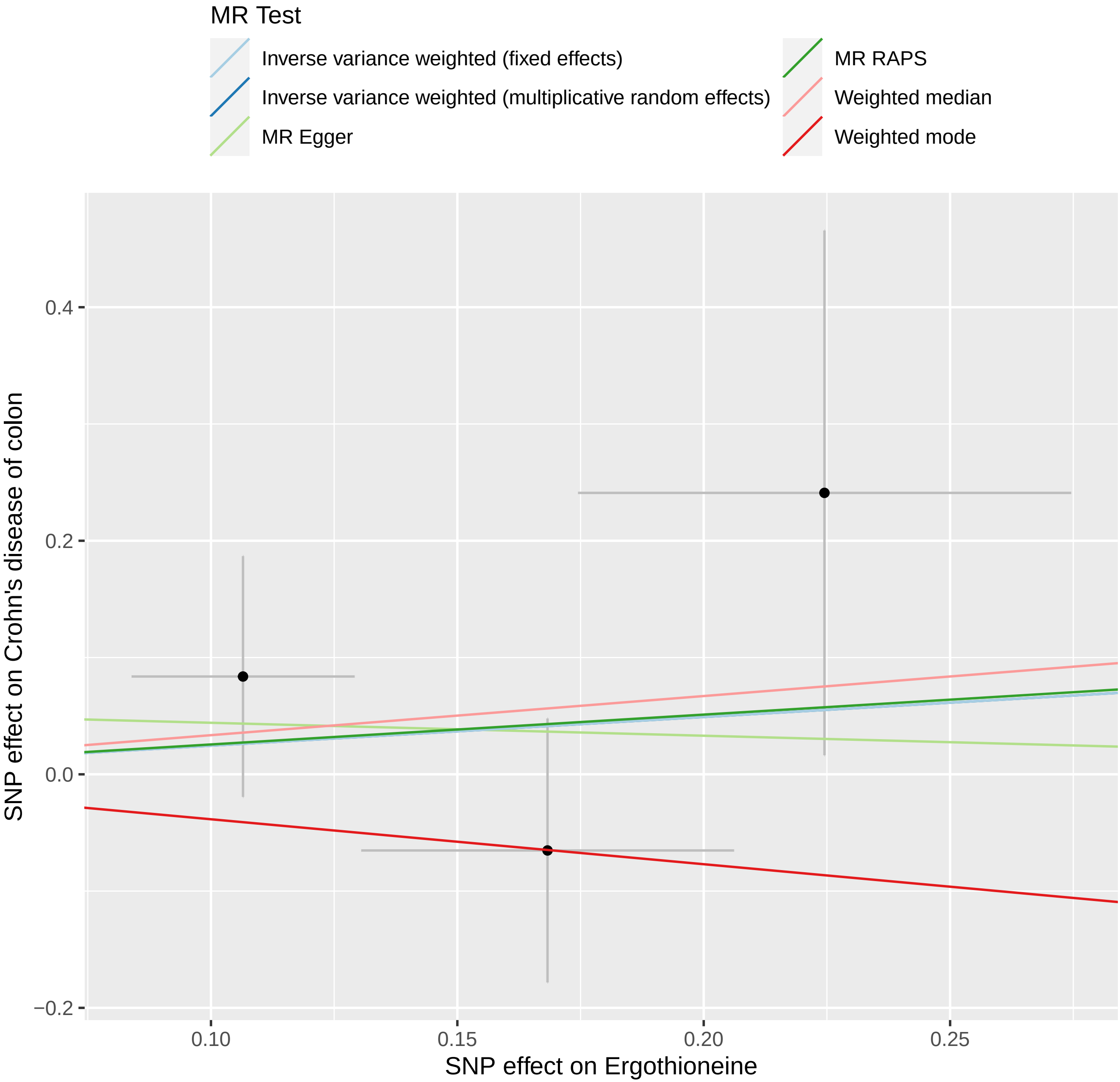
MR Test
Inverse variance weighted (fixed effects)
Inverse variance weighted (multiplicative random effects)
MR Egger
MR RAPS
Weighted median
Weighted mode
0.4
0.2
0.0
−0.2
0.10
0.15
0.20
0.25
SNP effect on Crohn's disease of colon
SNP effect on Ergothioneine

Batch 273 : Phenylalanylphenylalanine on Crohn's disease of colon

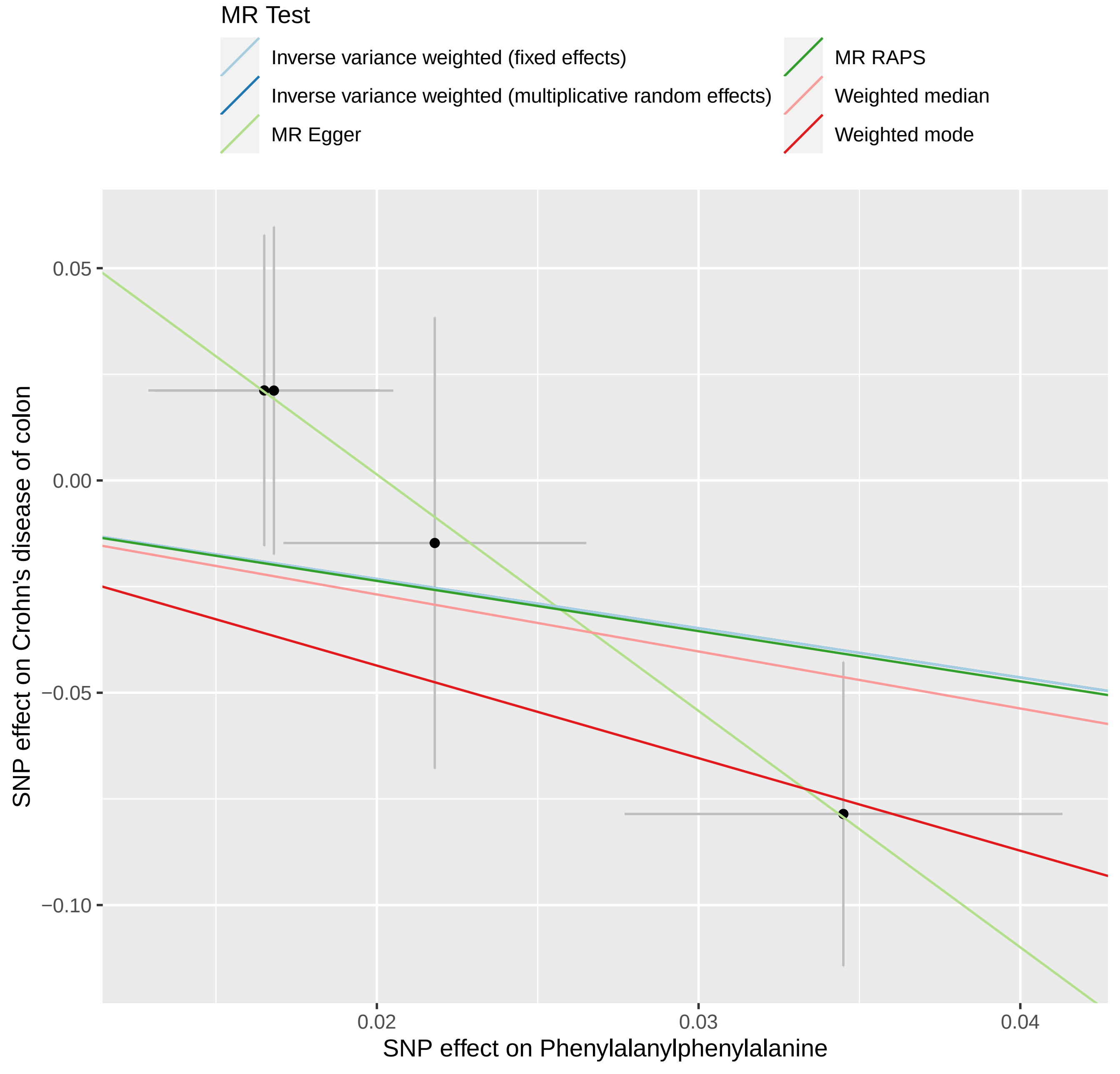

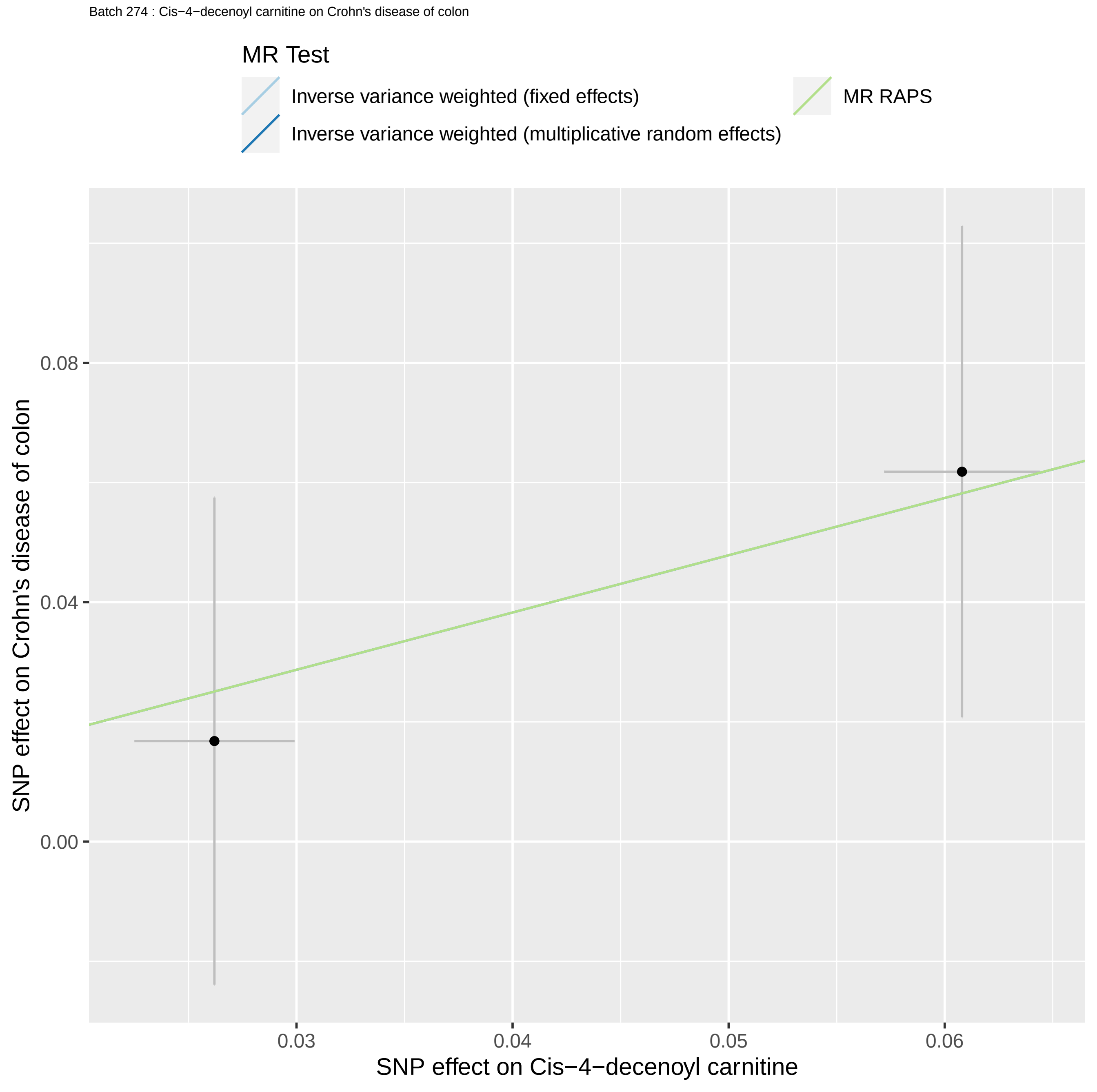

Batch 274 : Cis−4−decenoyl carnitine on Crohn's disease of colon
MR Test
Inverse variance weighted (fixed effects)
Inverse variance weighted (multiplicative random effects)
MR RAPS
0.08
0.04
0.00
SNP effect on Crohn's disease of colon
0.03
0.04
0.05
0.06
SNP effect on Cis−4−decenoyl carnitine

Batch 275 : 15−methylpalmitate (isobar with 2−methylpalmitate) on Crohn's disease of colon

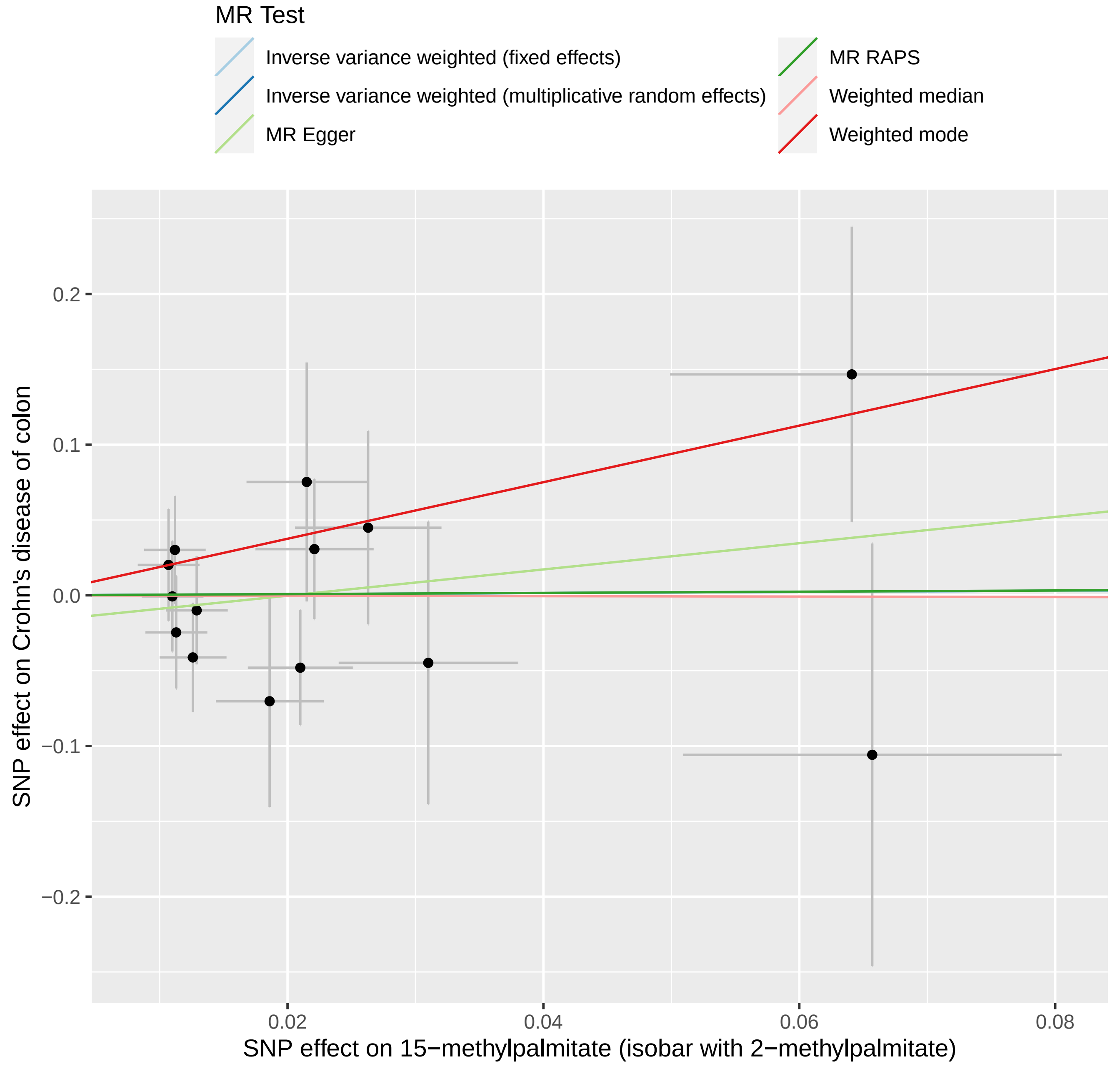

Batch 277 : Tryptophan on Crohn's disease of ileocolon

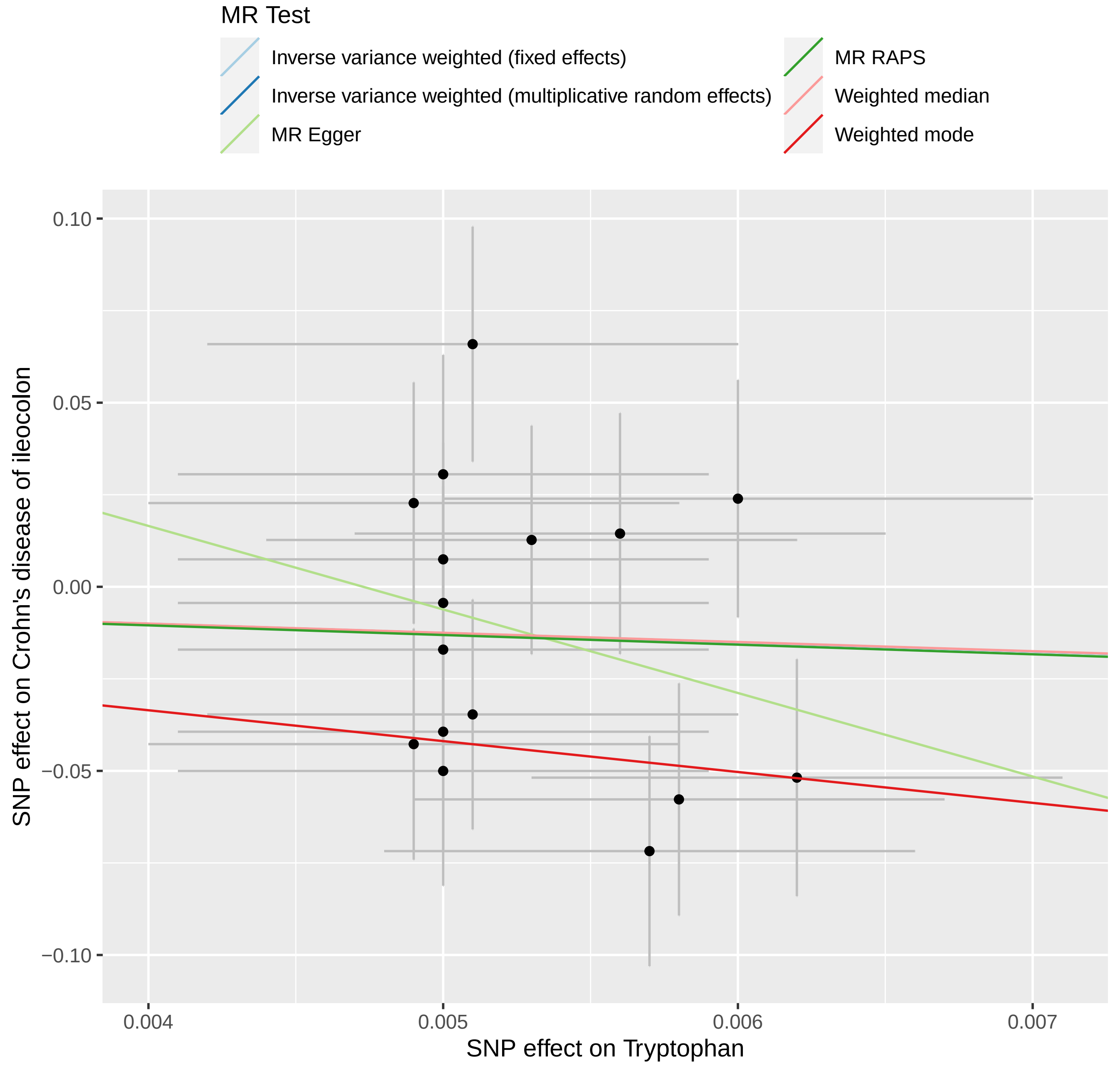

Batch 279 : Leucine on Crohn's disease of ileocolon

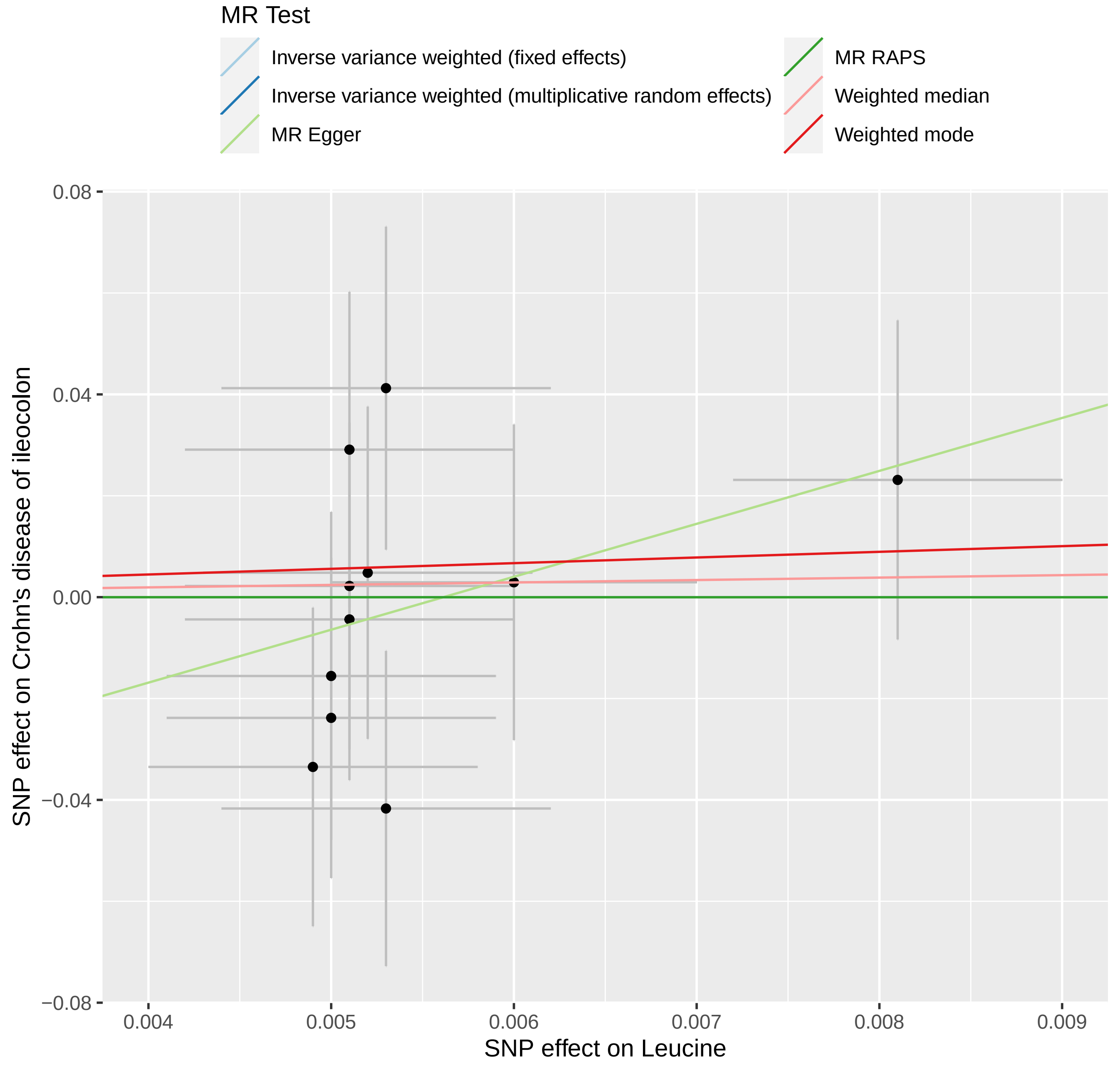

Batch 283 : Lactate on Crohn's disease of ileocolon

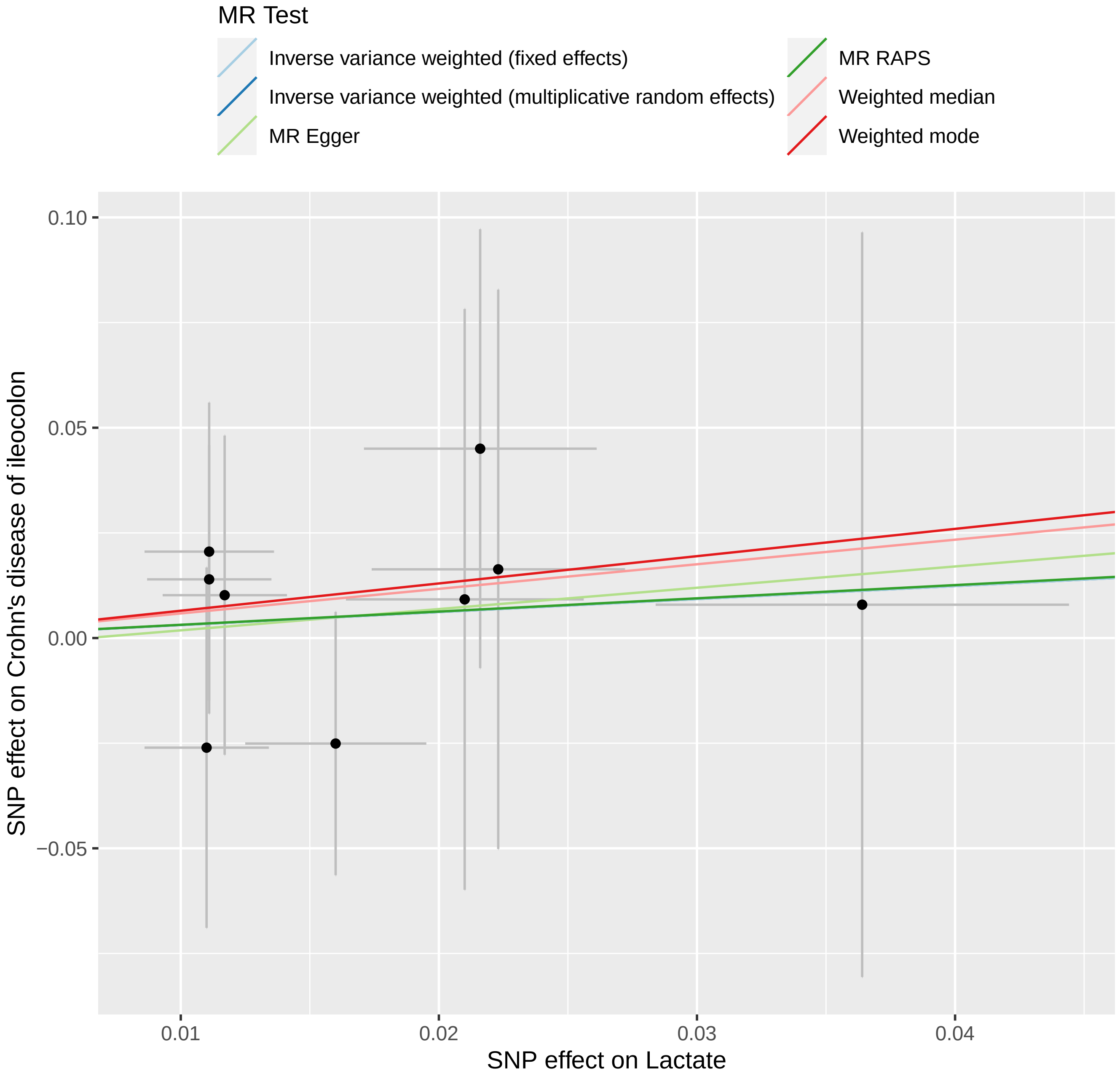

Batch 284 : 3−hydroxybutyrate (BHBA) on Crohn's disease of ileocolon

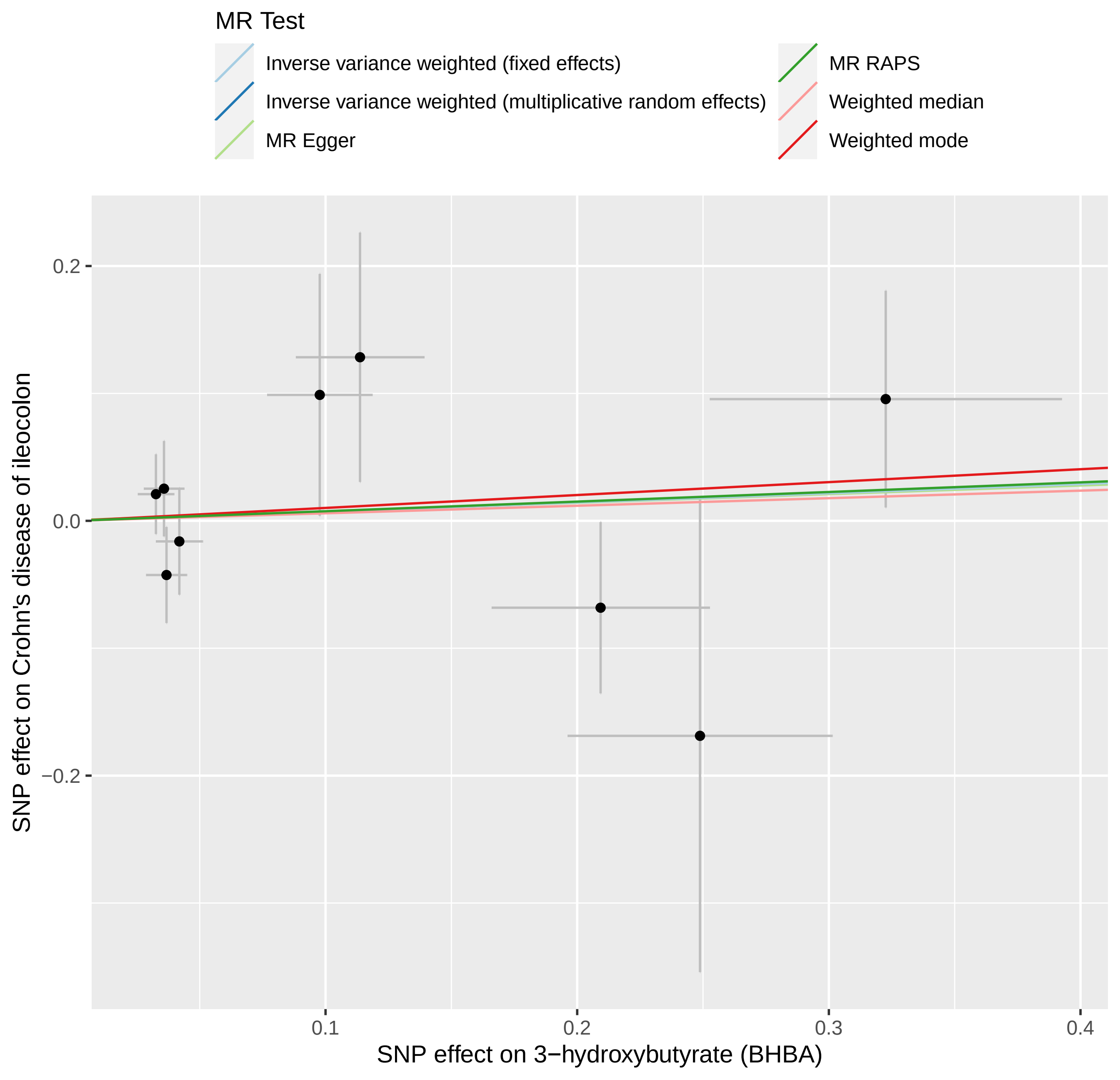

Batch 285 : Arabinose on Crohn's disease of ileocolon

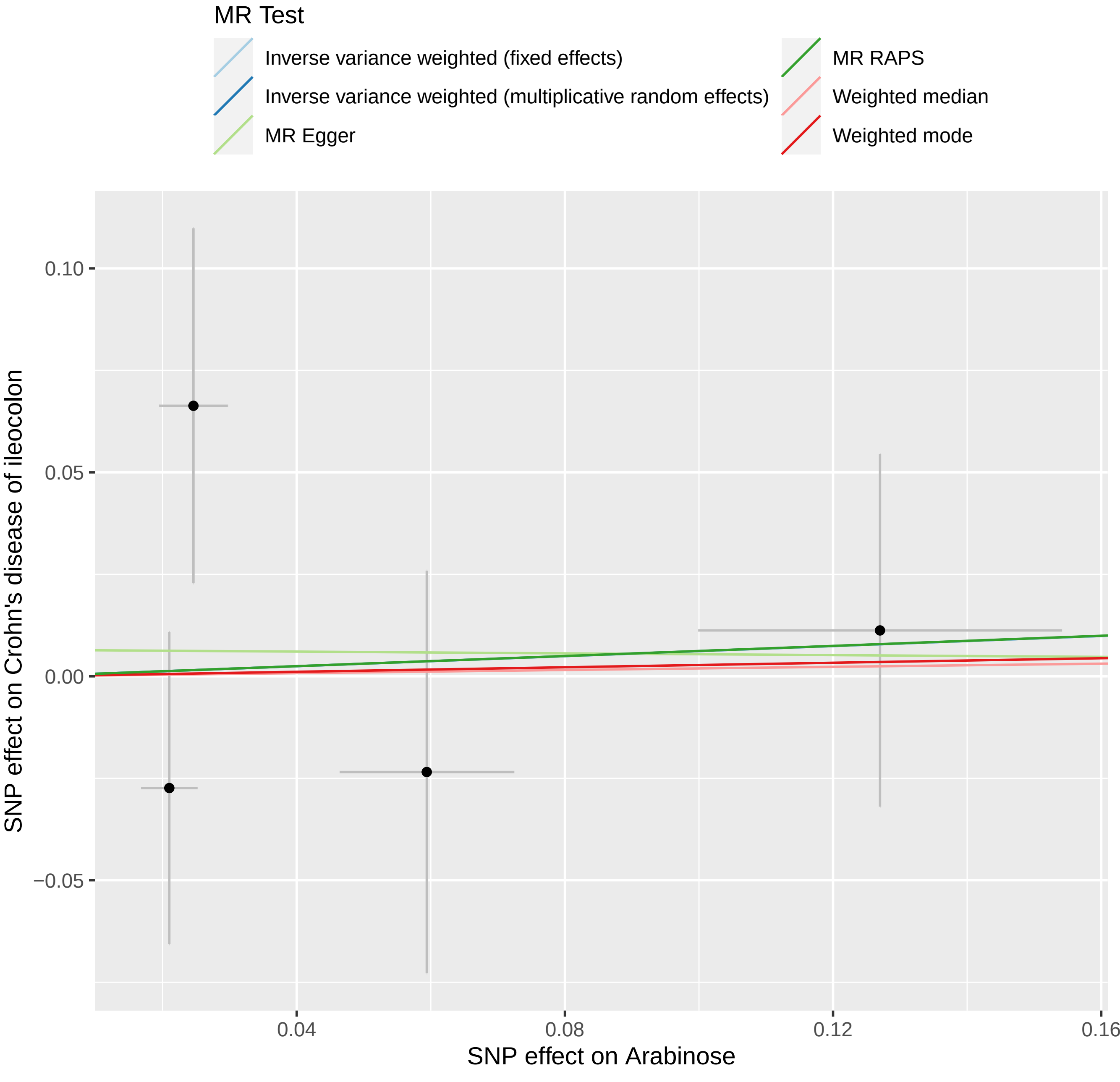

Batch 286 : Fructose on Crohn's disease of ileocolon

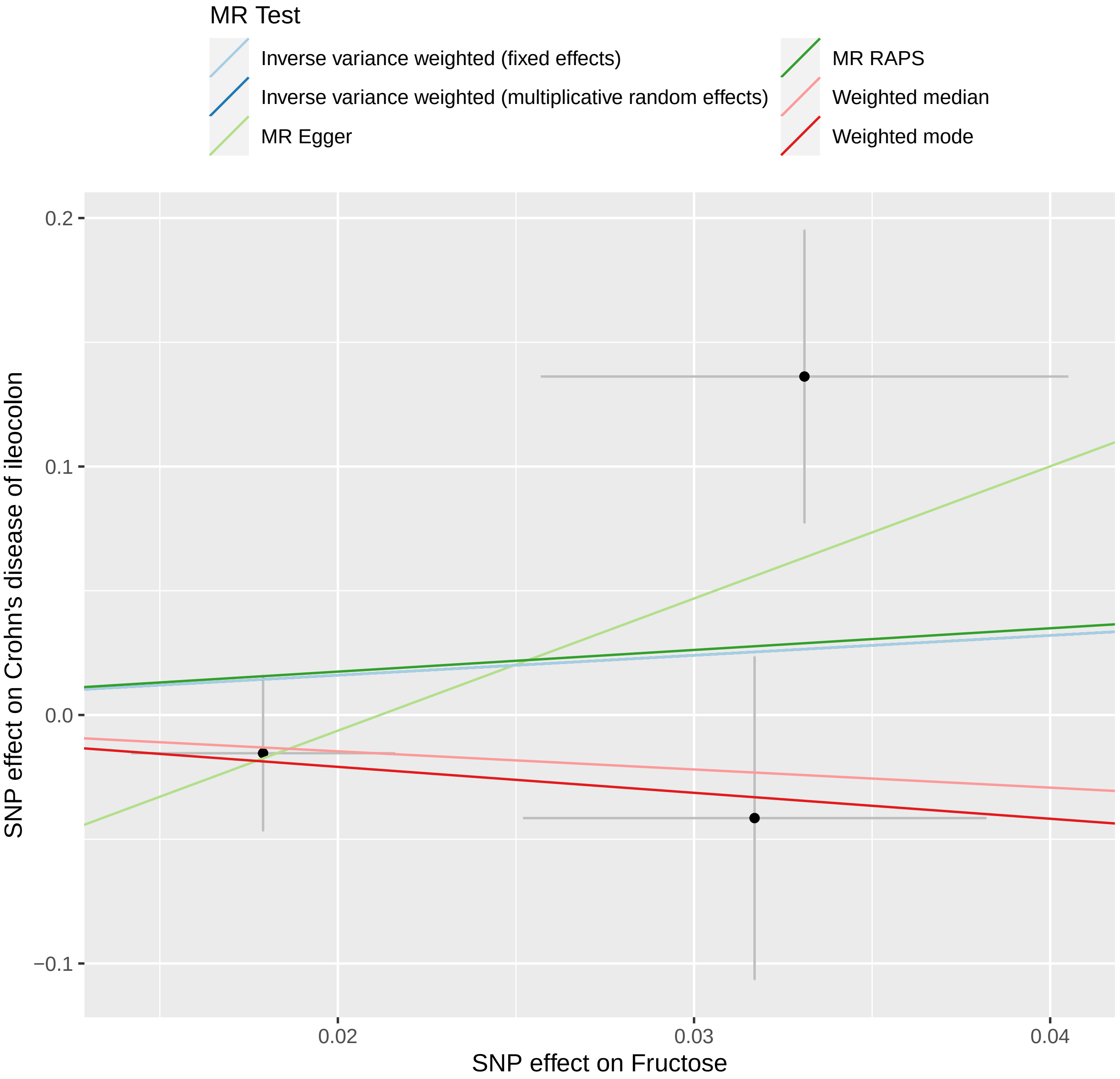

Batch 288 : Pyruvate on Crohn's disease of ileocolon

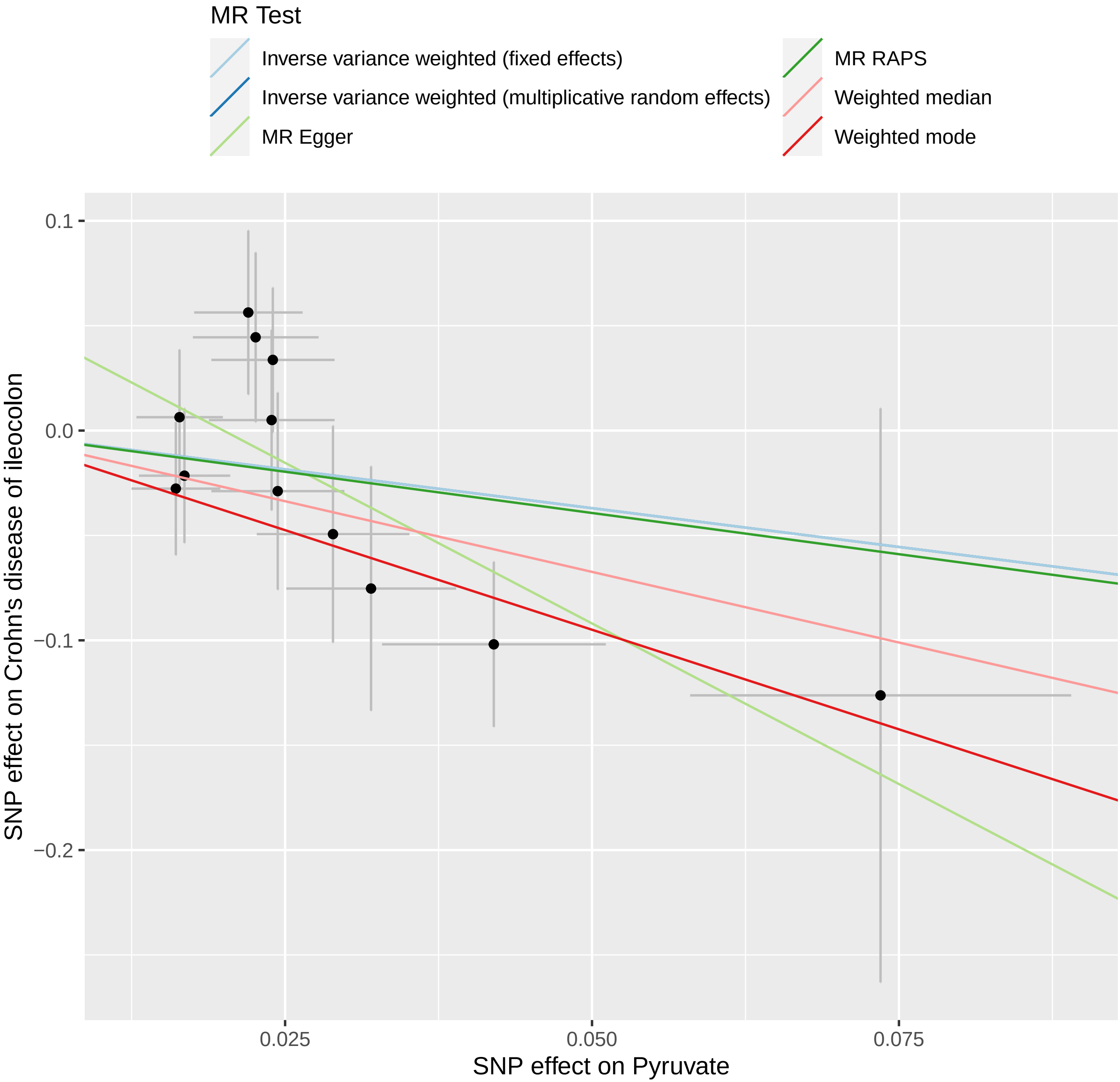

Batch 289 : Uridine on Crohn's disease of ileocolon

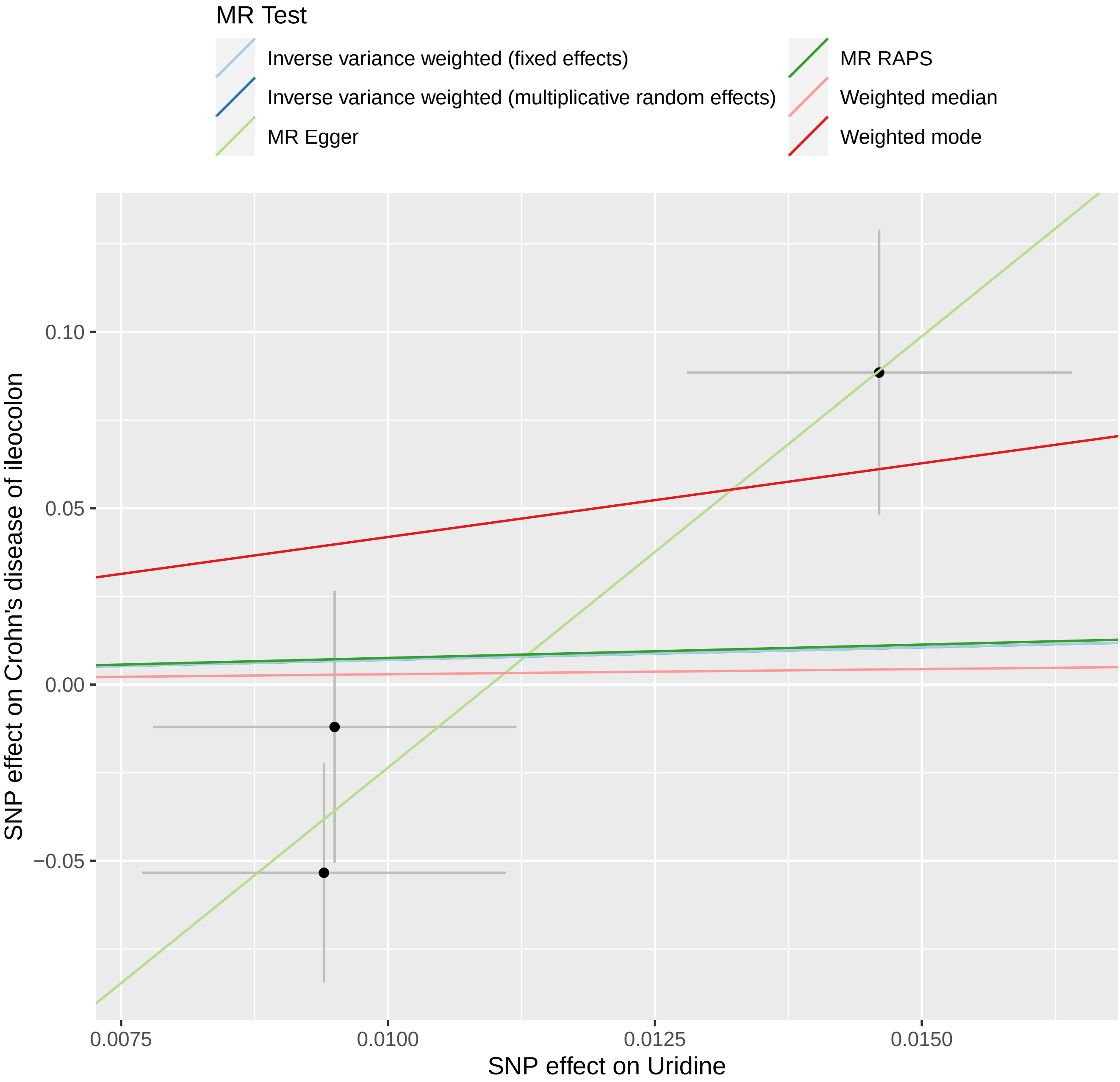

Batch 290 : Linoleate (18:2n6) on Crohn's disease of ileocolon

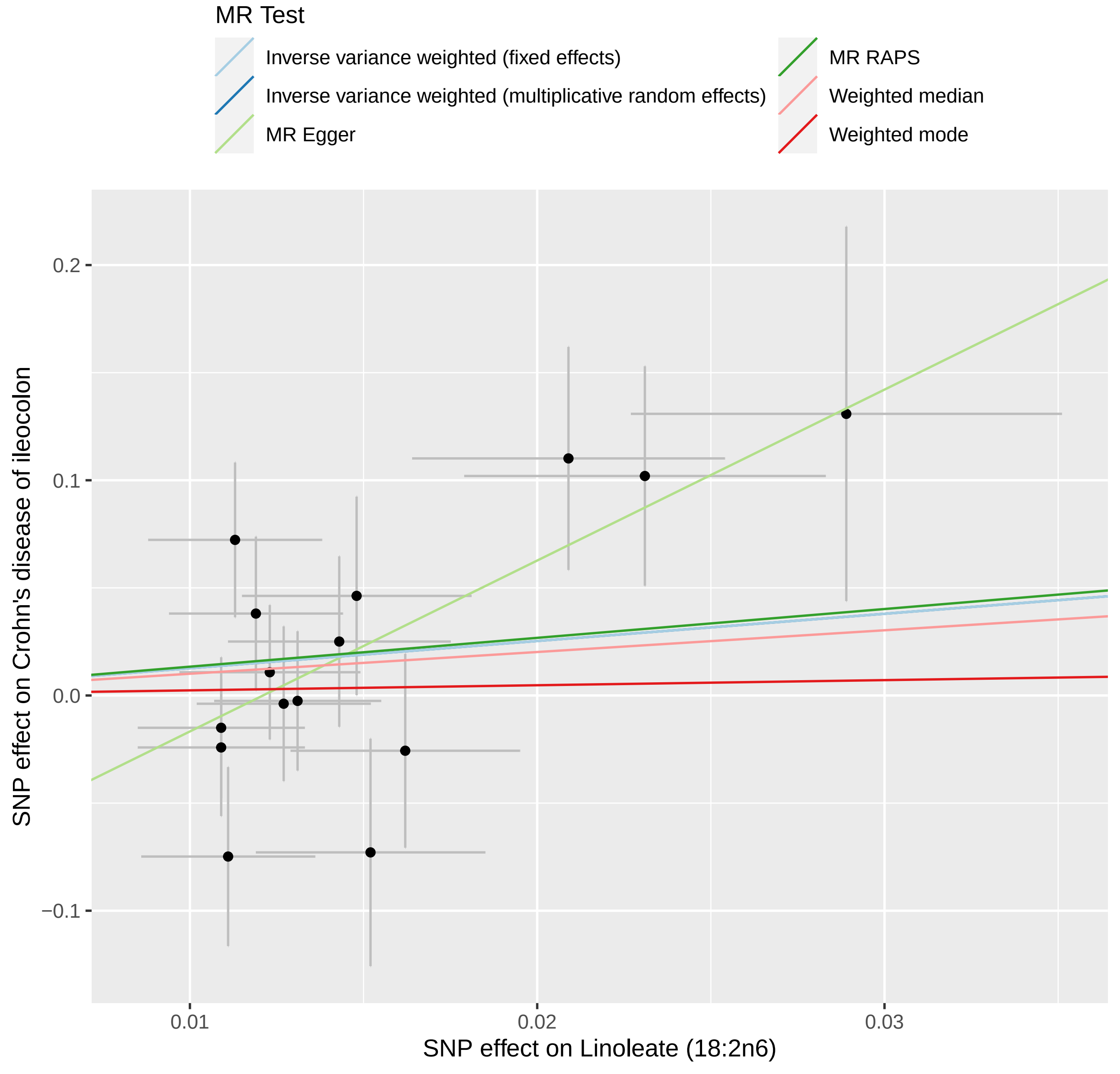

Batch 291 : Allantoin on Crohn's disease of ileocolon

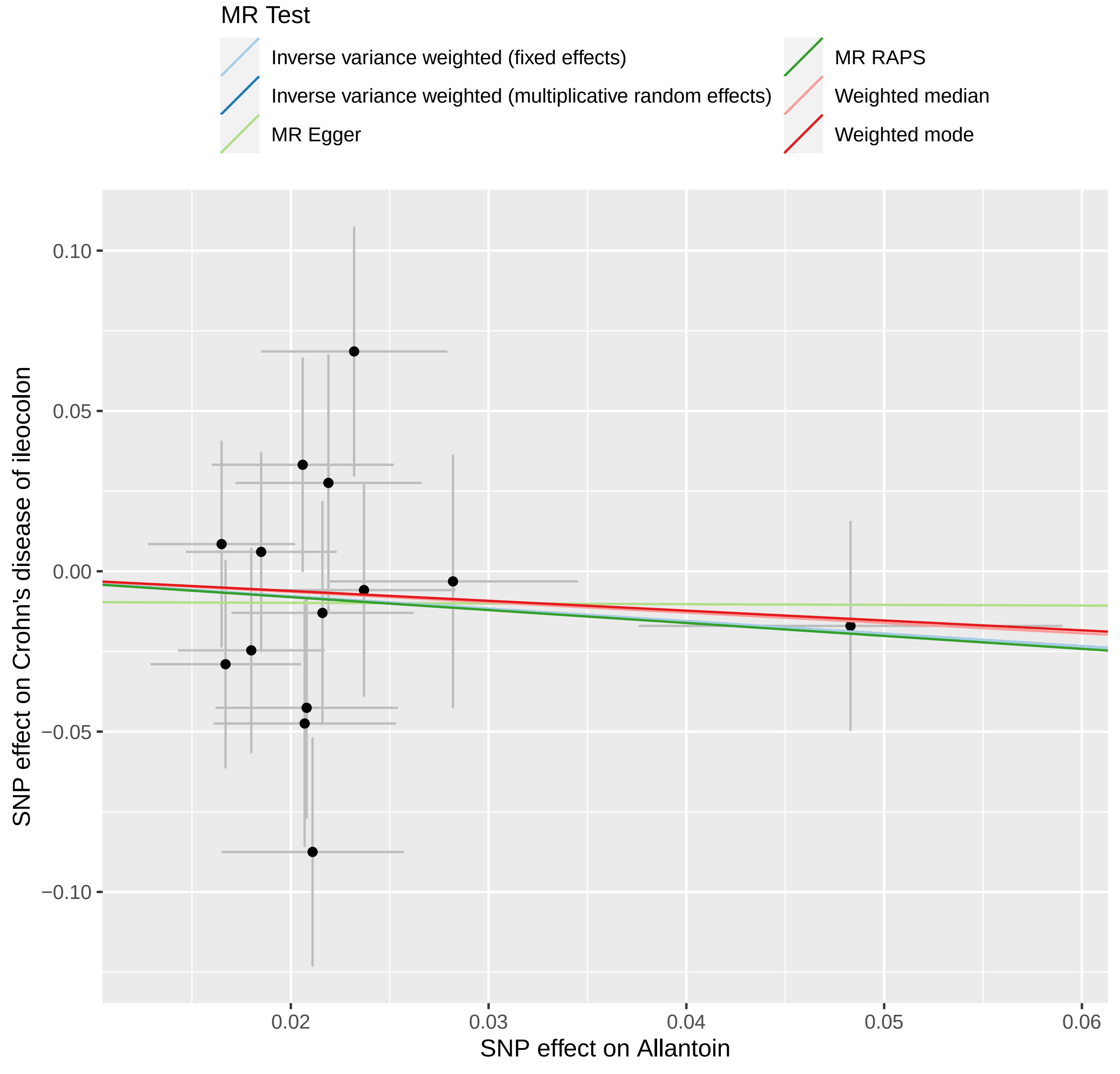

Batch 296 : Isoleucine on Crohn's disease of ileocolon

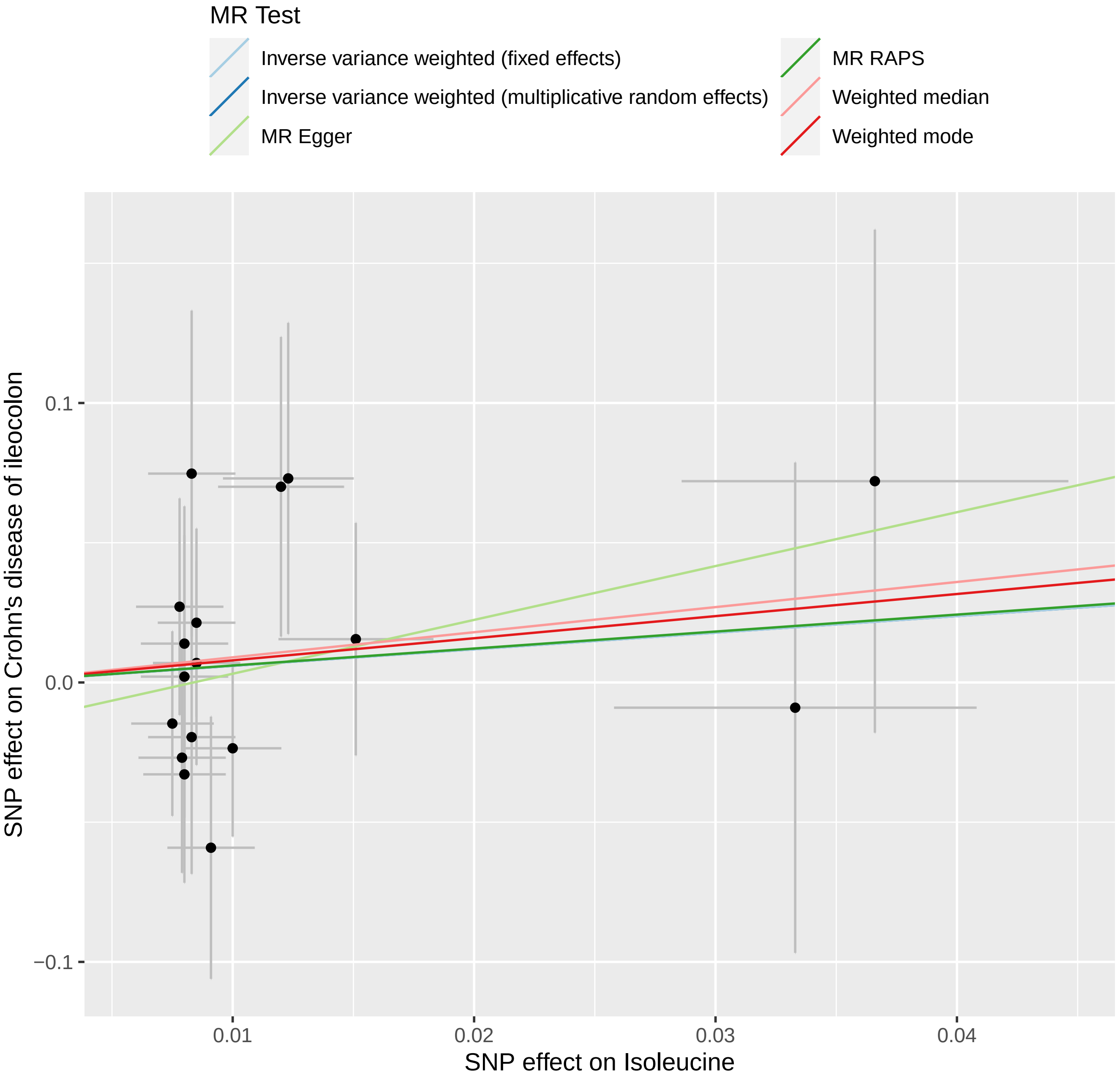

Batch 297 : Threonine on Crohn's disease of ileocolon

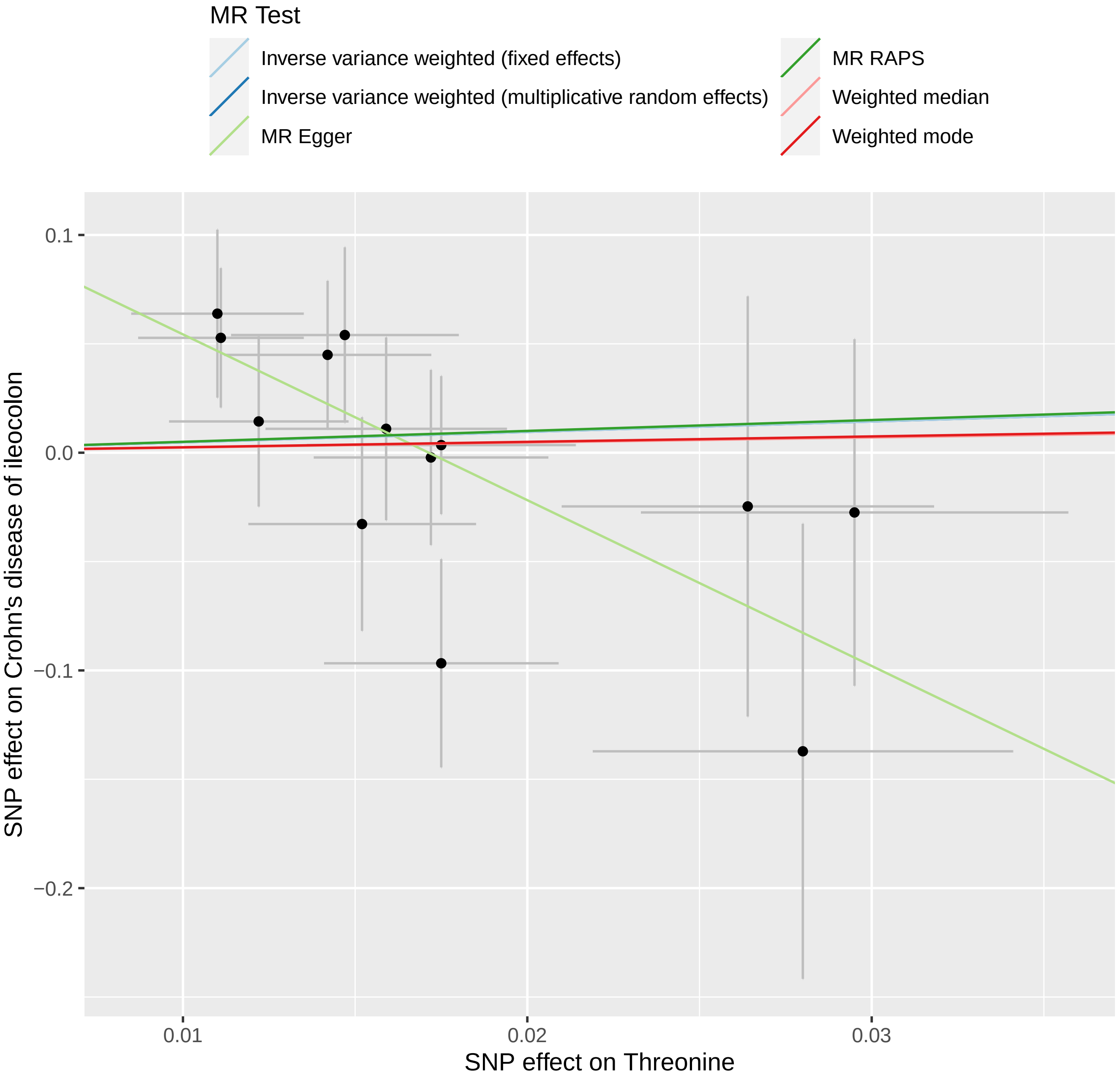

Batch 298 : Tyrosine on Crohn's disease of ileocolon

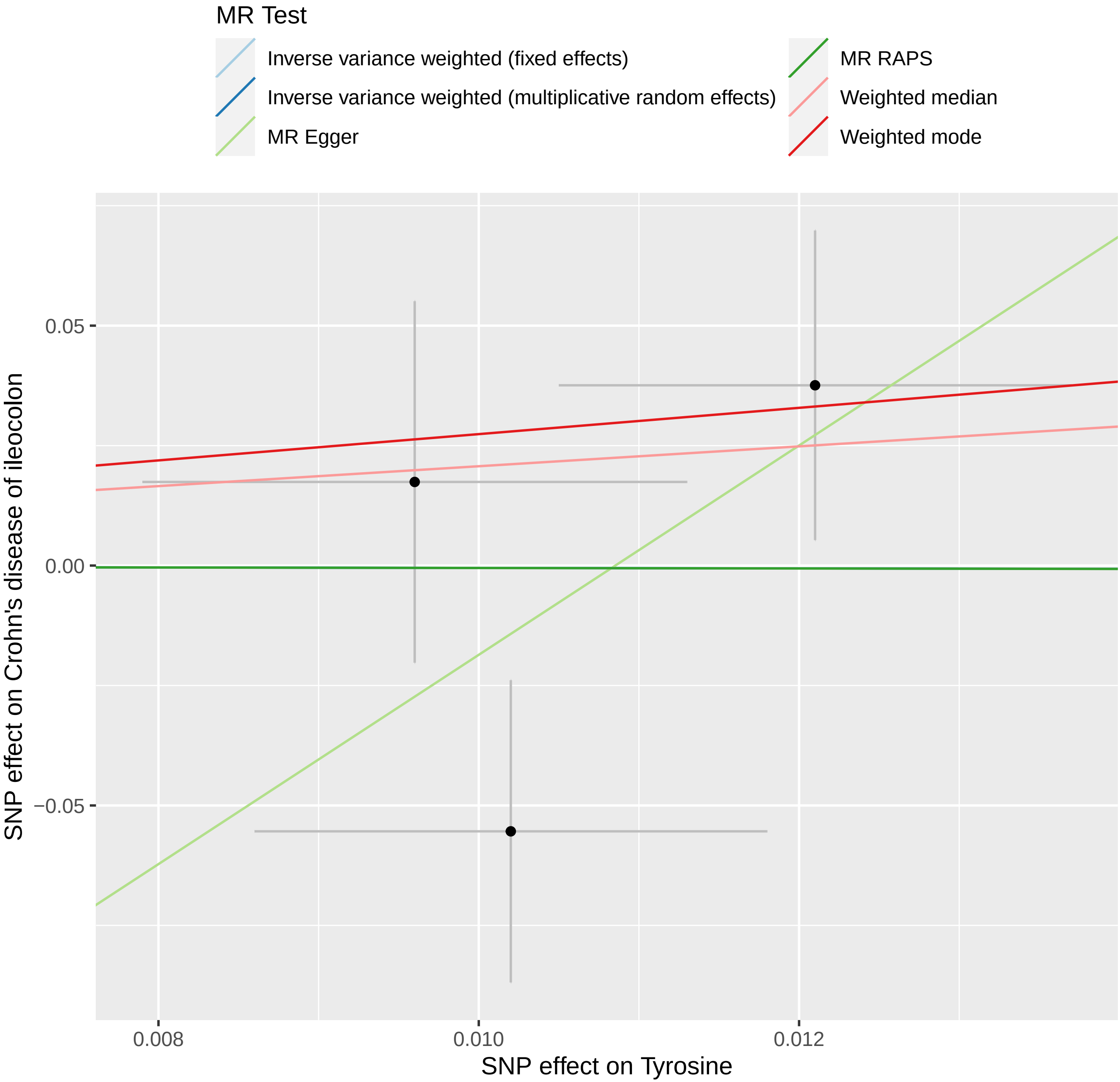

Batch 304 : Stearate (18:0) on Crohn's disease of ileocolon

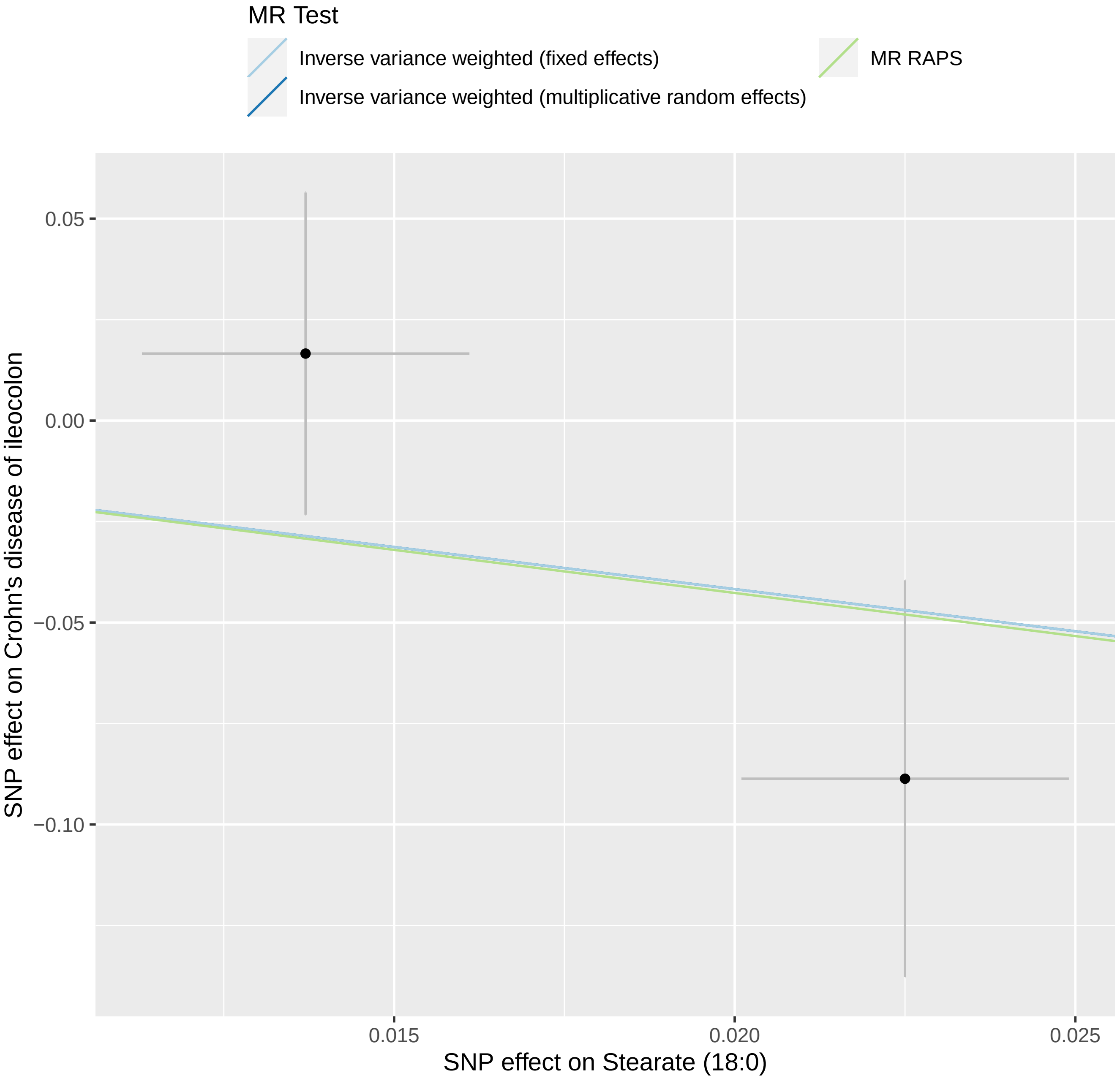

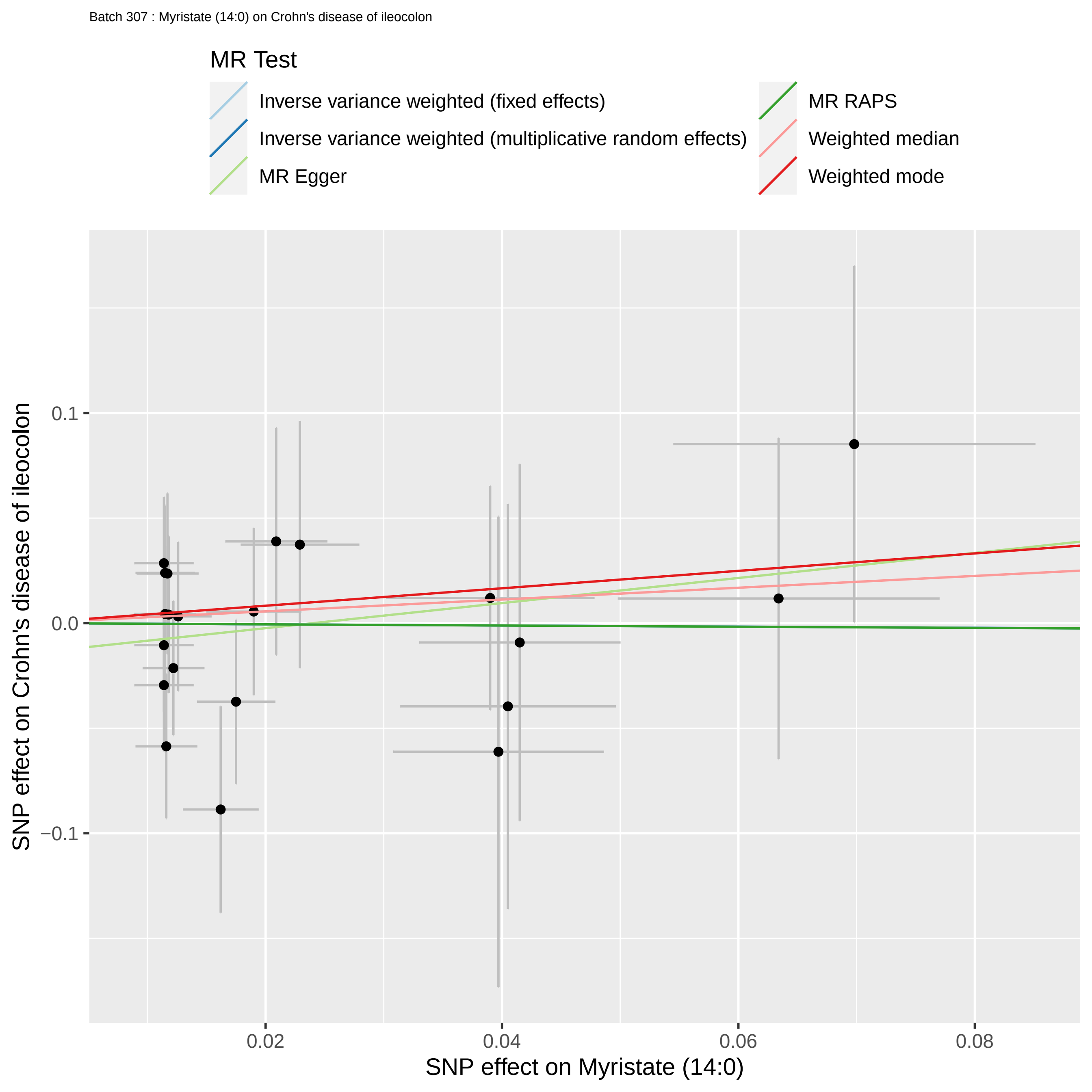
Batch 307 : Myristate (14:0) on Crohn's disease of ileocolon
MR Test
Inverse variance weighted (fixed effects)
Inverse variance weighted (multiplicative random effects)
MR Egger
MR RAPS
Weighted median
Weighted mode
0.1
0.0
−0.1
0.02
0.04
0.06
0.08
SNP effect on Myristate (14:0)
SNP effect on Crohn's disease of ileocolon

Batch 309 : Ornithine on Crohn's disease of ileocolon

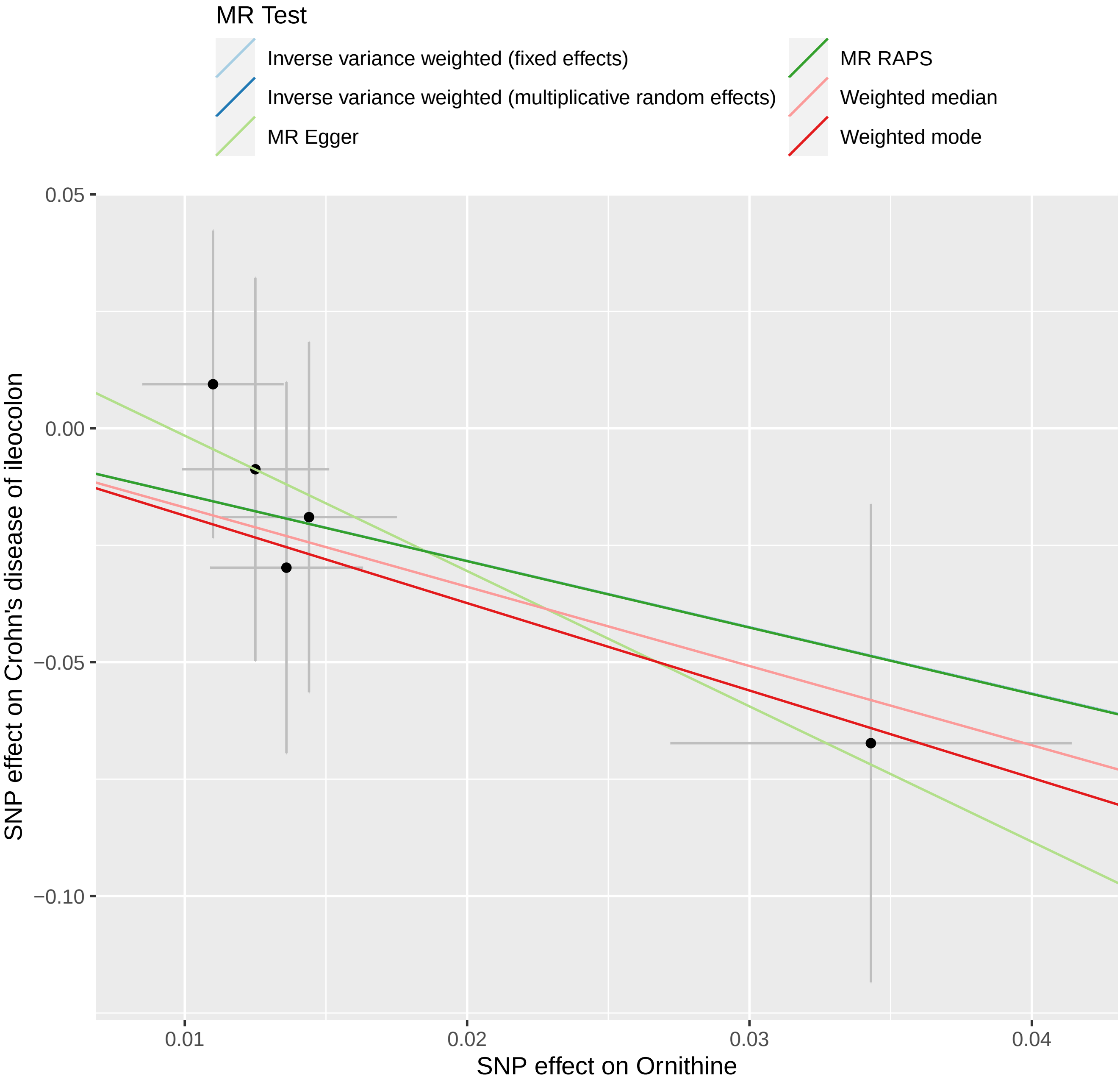

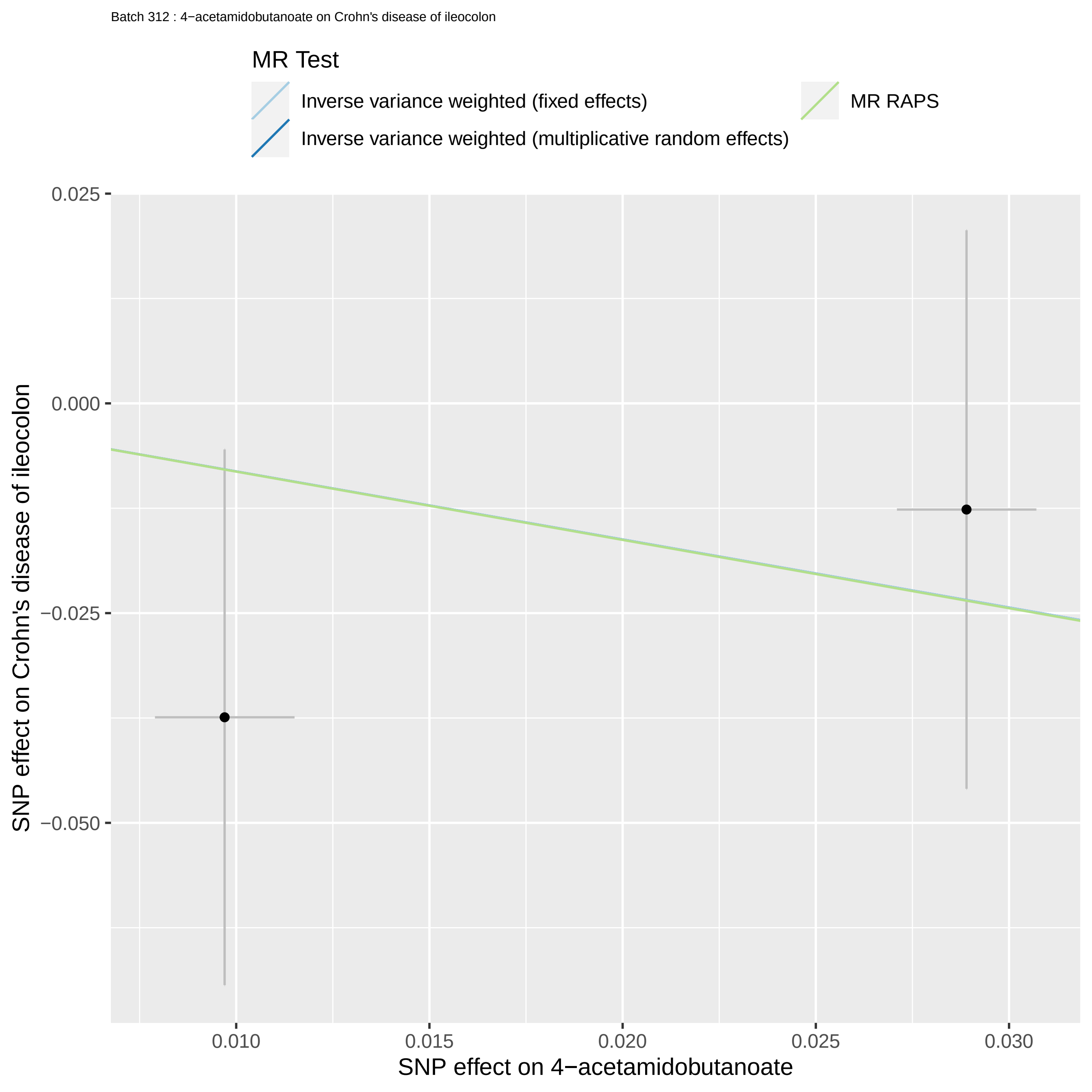

Batch 312 : 4−acetamidobutanoate on Crohn's disease of ileocolon
MR Test
Inverse variance weighted (fixed effects)
Inverse variance weighted (multiplicative random effects)
MR RAPS
0.025
0.000
−0.025
−0.050
0.010
0.015
0.020
0.025
0.030
SNP effect on Crohn's disease of ileocolon
SNP effect on 4−acetamidobutanoate

Batch 313 : Alpha−tocopherol on Crohn's disease of ileocolon

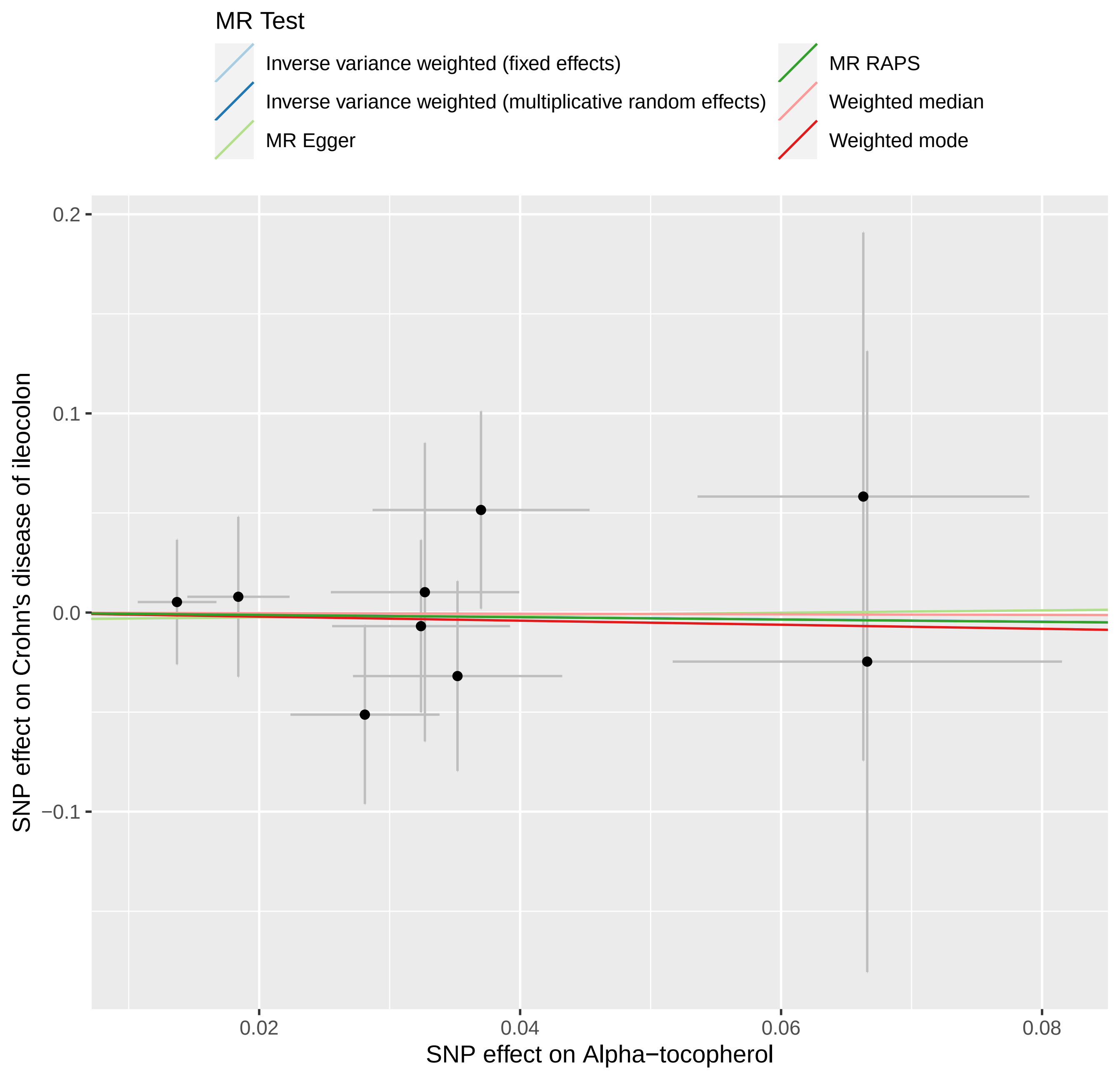

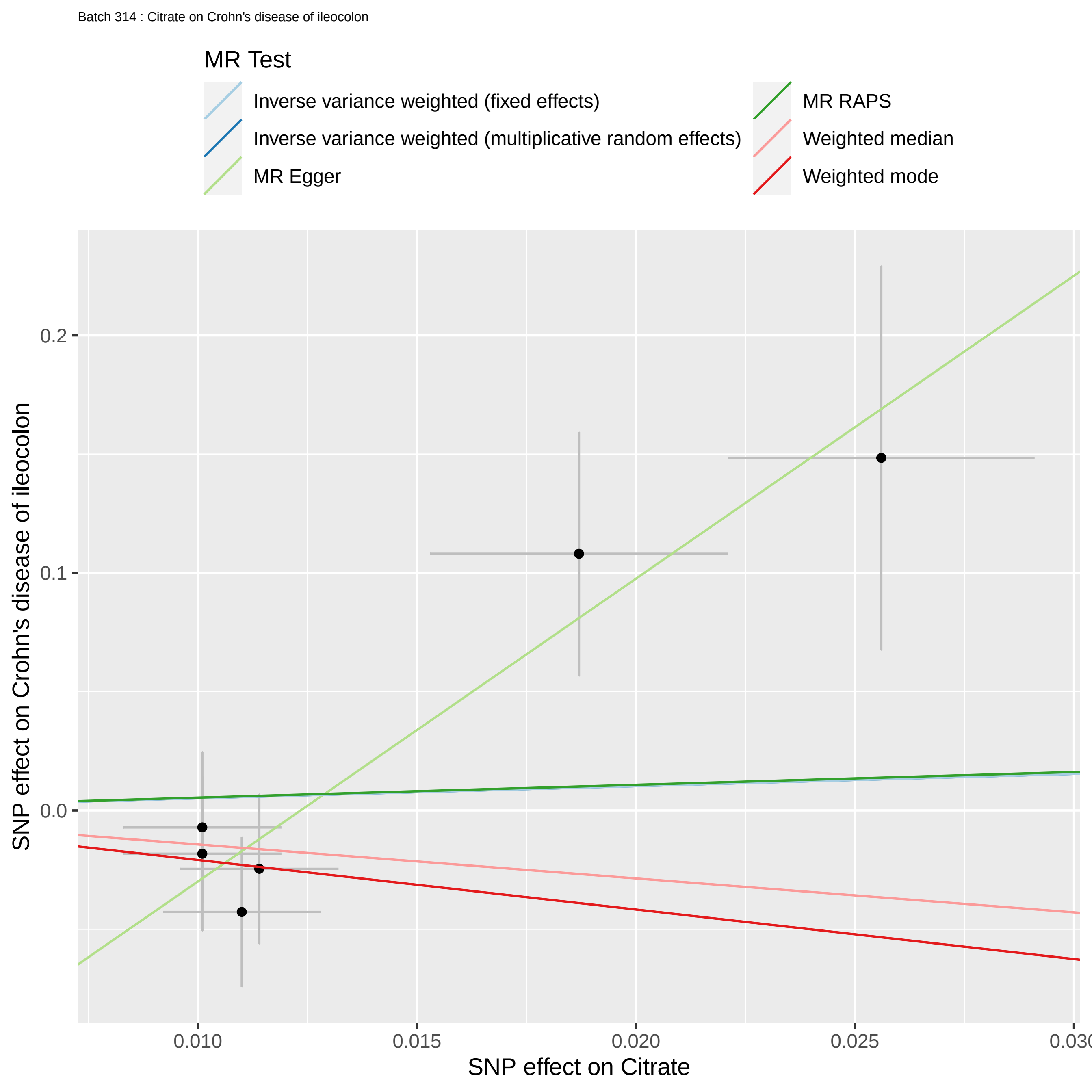
Batch 314 : Citrate on Crohn's disease of ileocolon
MR Test
Inverse variance weighted (fixed effects)
Inverse variance weighted (multiplicative random effects)
MR Egger
MR RAPS
Weighted median
Weighted mode
0.2
0.1
0.0
0.010
0.015
0.020
0.025
0.030
SNP effect on Citrate
SNP effect on Crohn's disease of ileocolon

Batch 315 : Glycerate on Crohn's disease of ileocolon

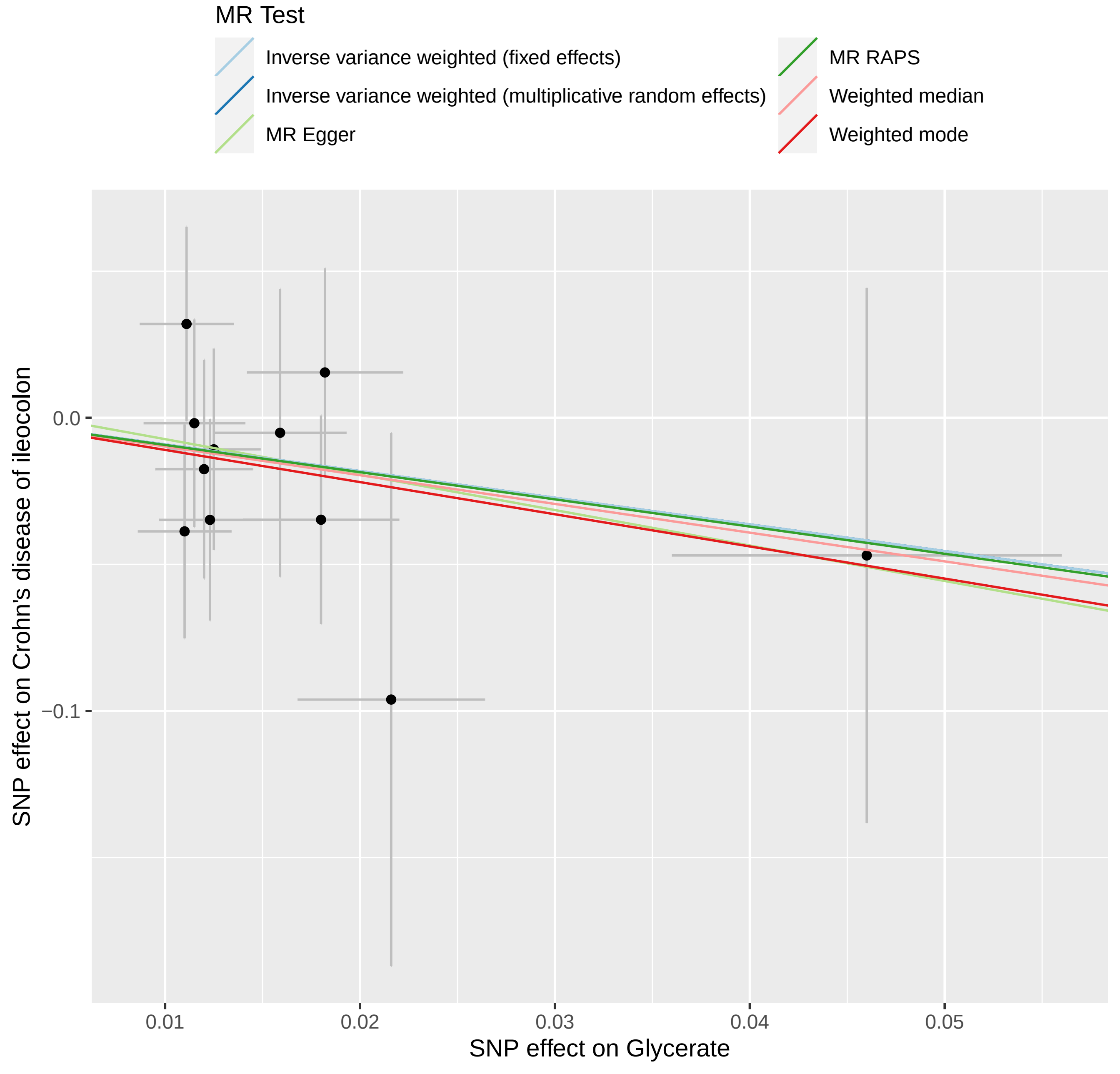

Batch 316 : Guanosine on Crohn's disease of ileocolon

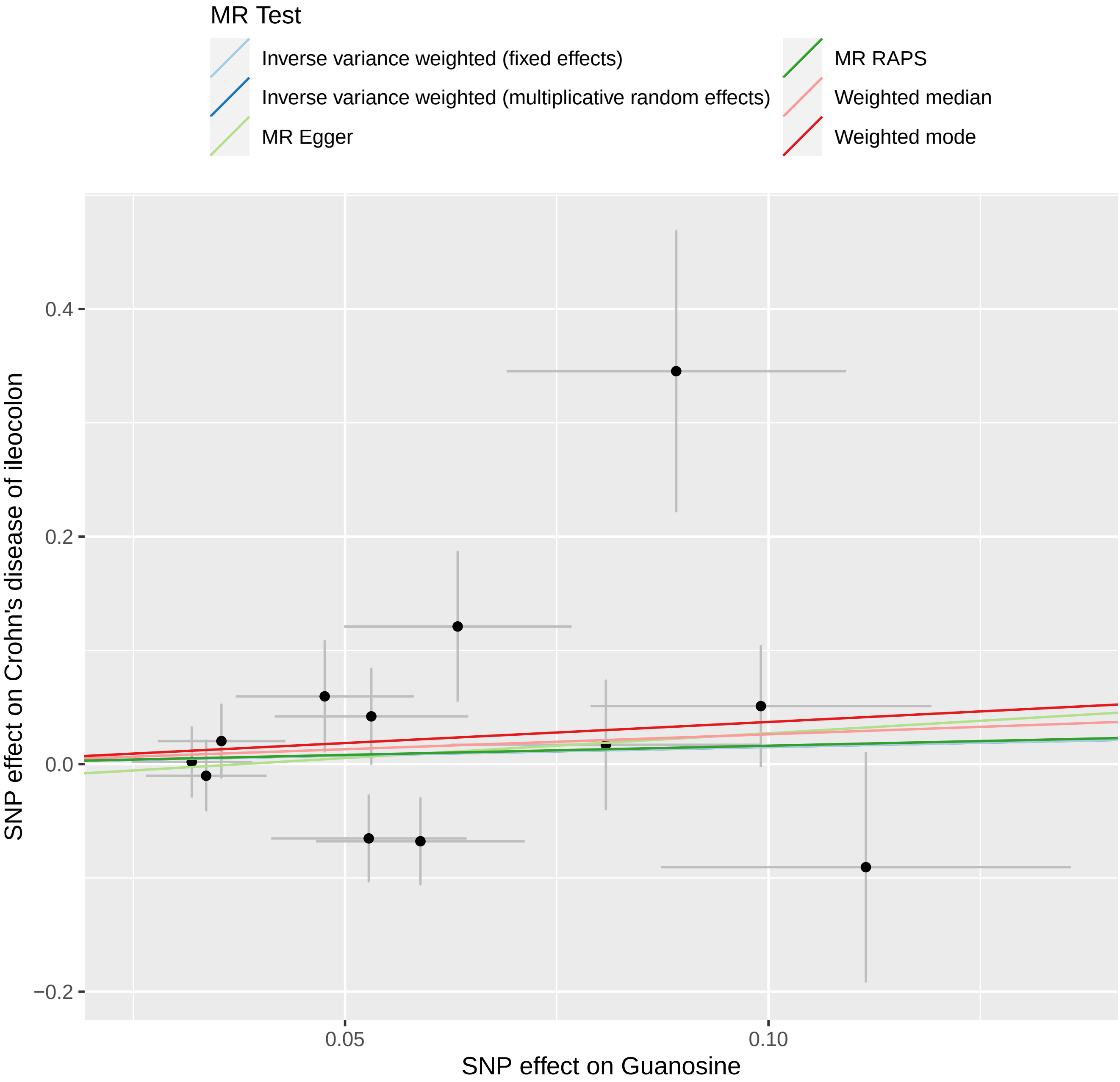

Batch 319 : Ursodeoxycholate on Crohn's disease of ileocolon

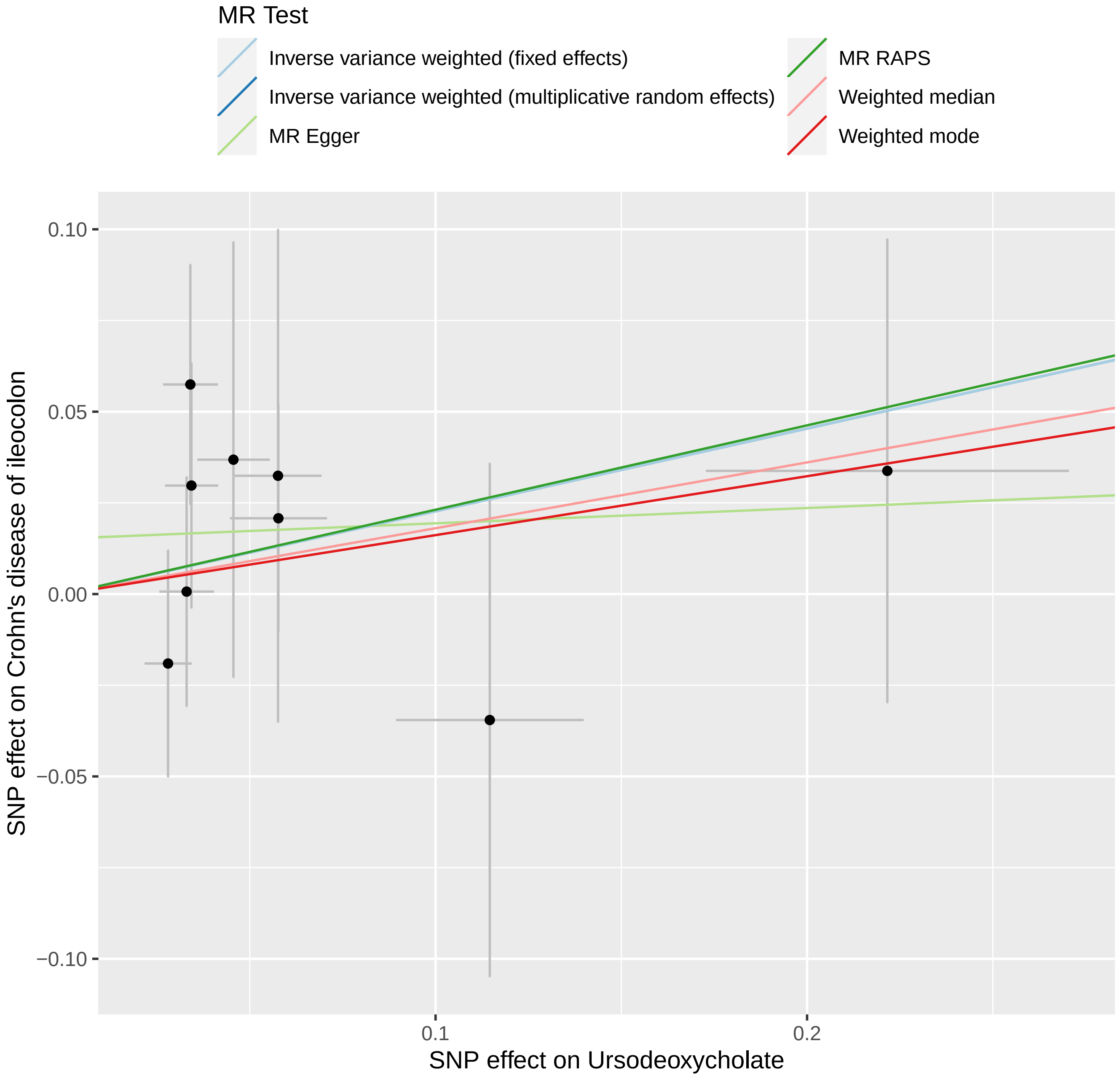

Batch 320 : Arginine on Crohn's disease of ileocolon

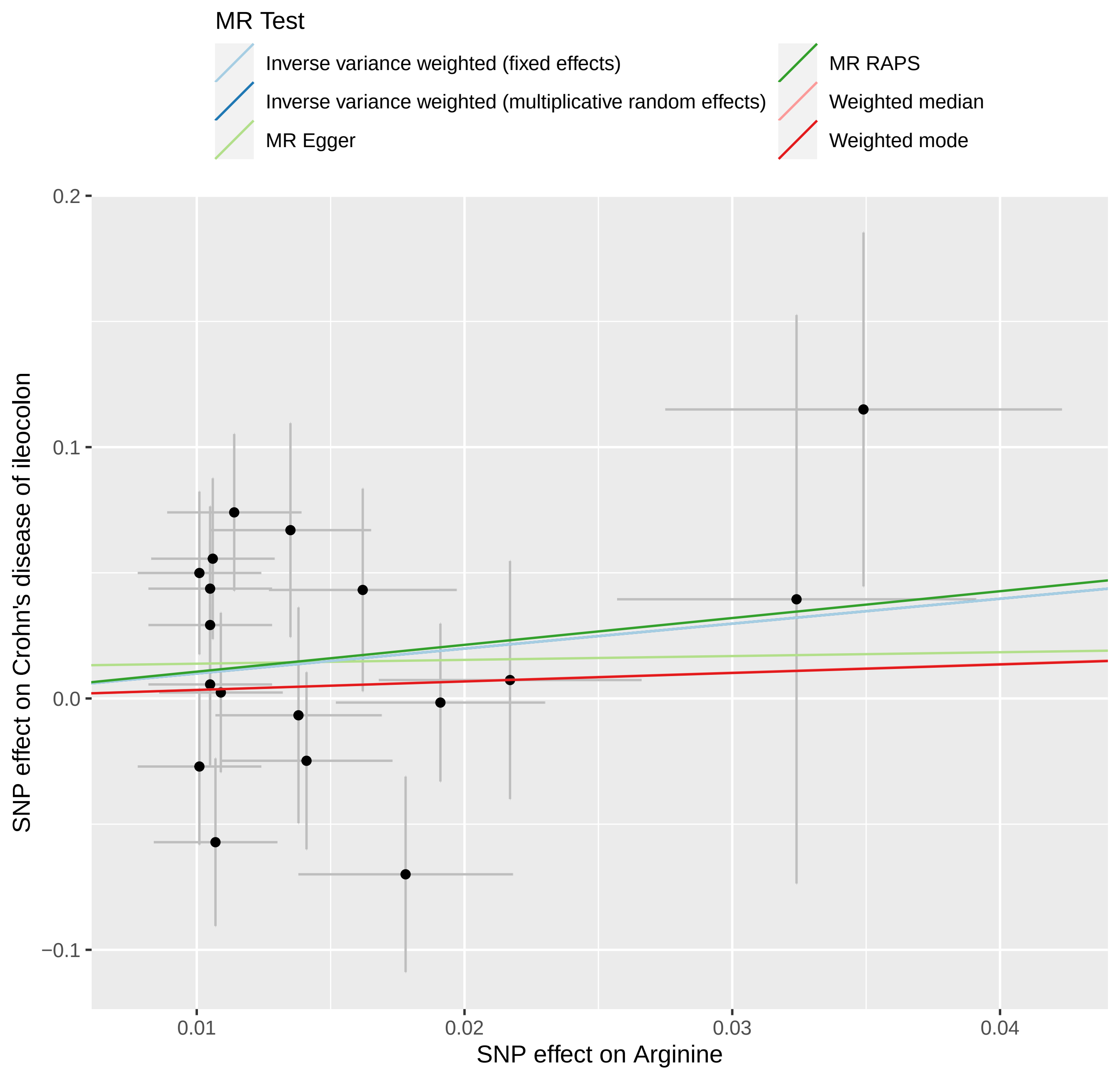

Batch 321 : Ascorbate (Vitamin C) on Crohn's disease of ileocolon

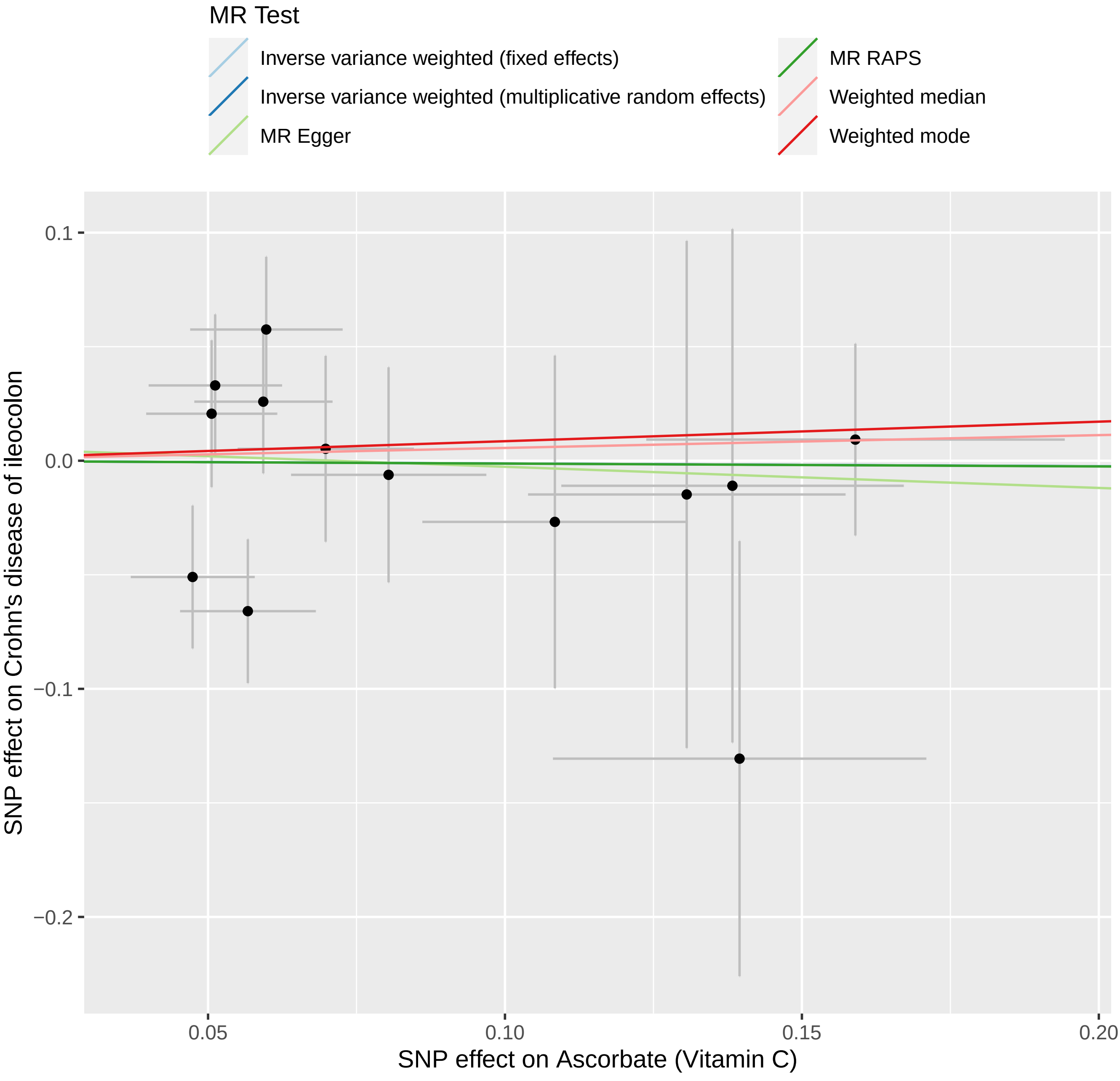

Batch 322 : Heptanoate (7:0) on Crohn's disease of ileocolon

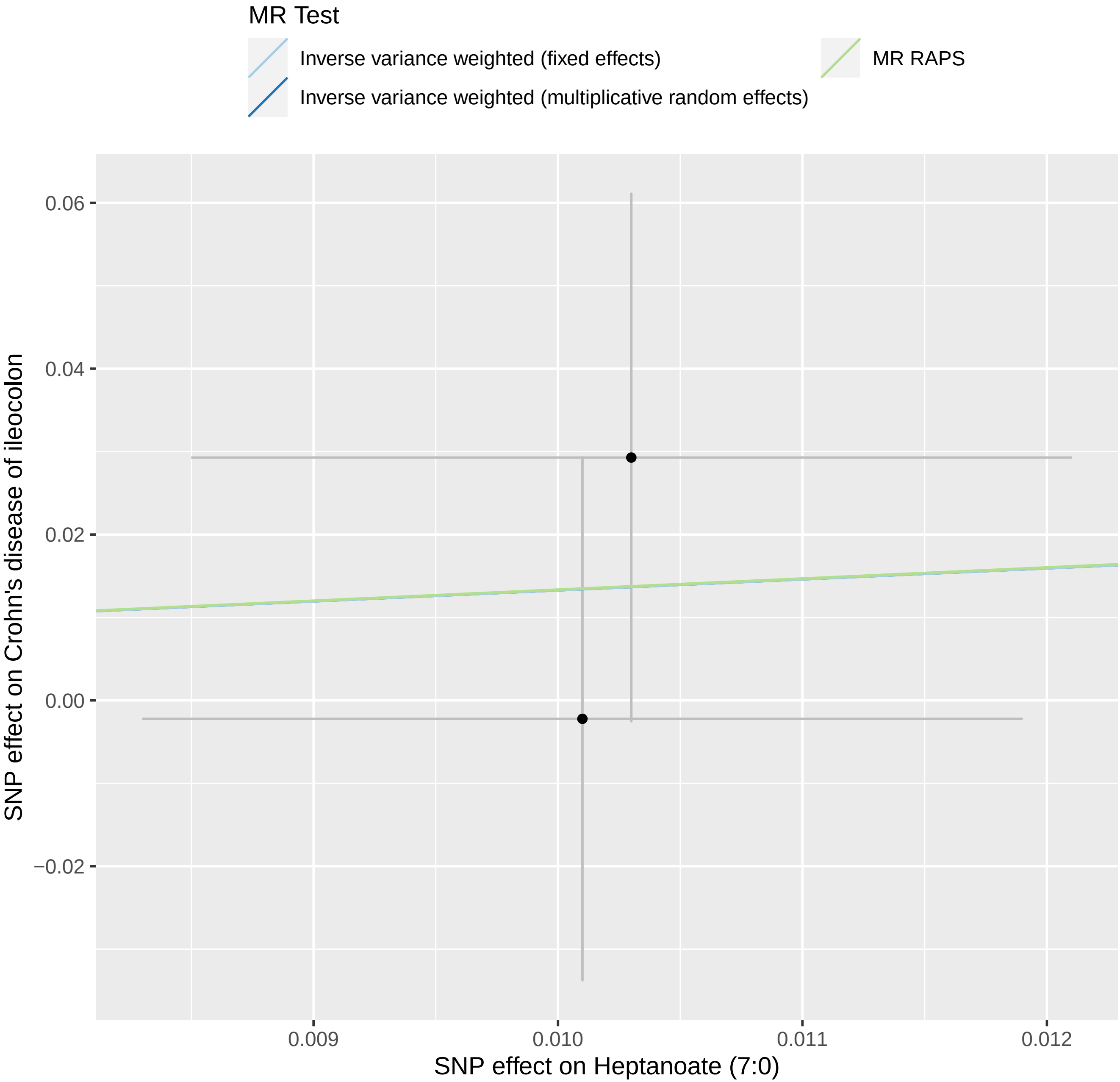

Batch 323 : Laurate (12:0) on Crohn's disease of ileocolon

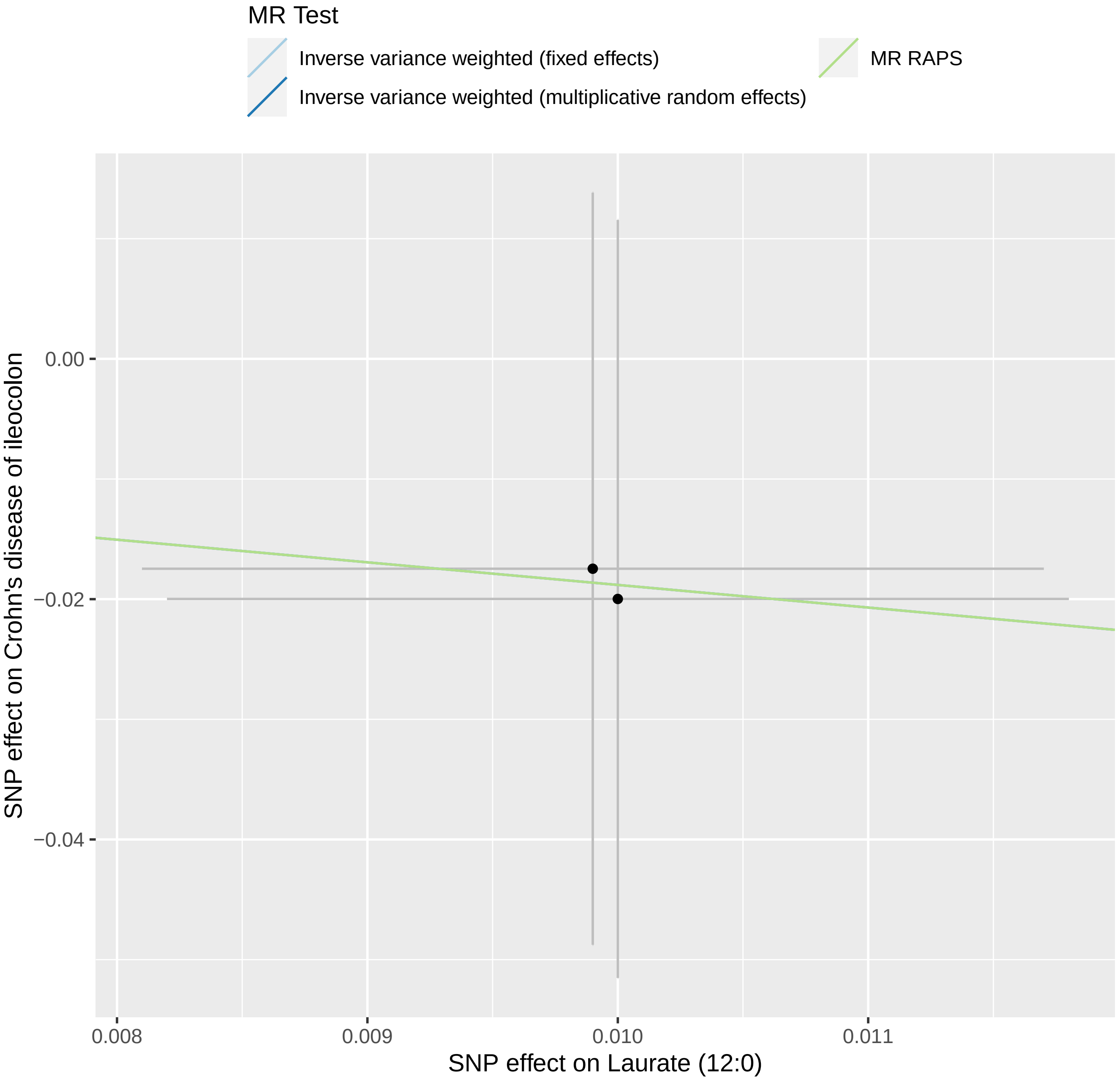

Batch 325 : Urea on Crohn's disease of ileocolon

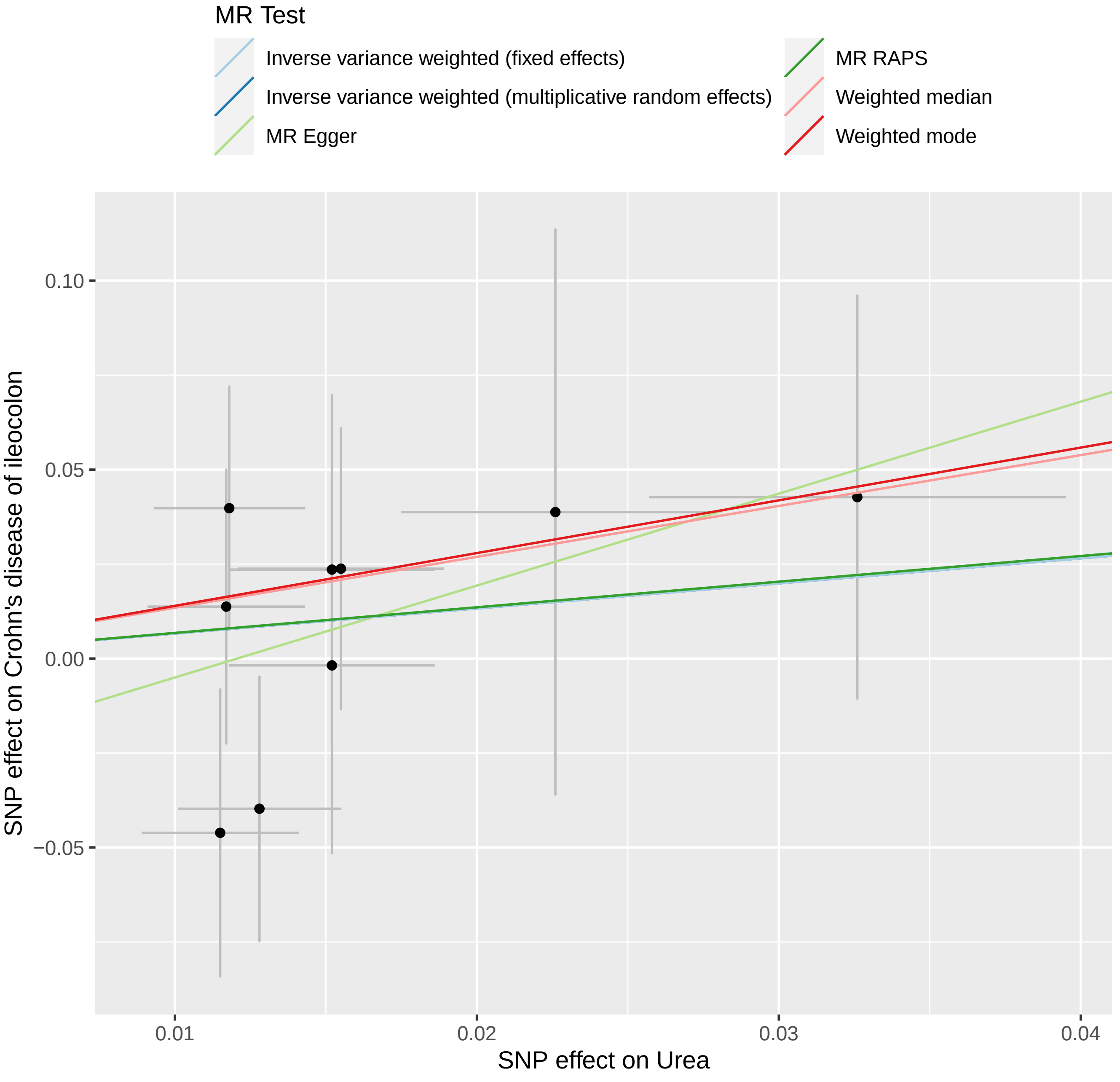

Batch 326 : Cortisol on Crohn's disease of ileocolon

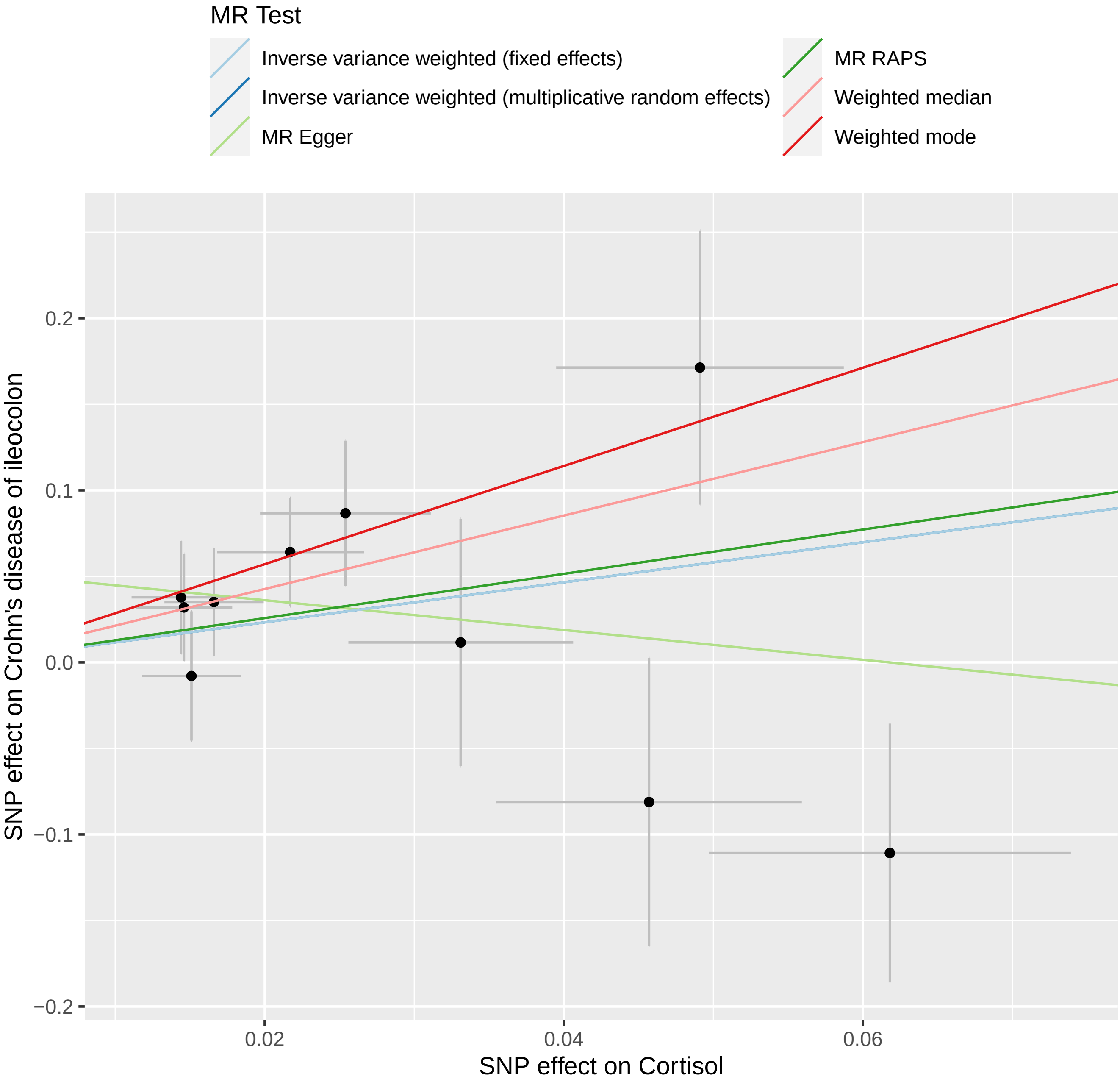

Batch 329 : Citrulline on Crohn's disease of ileocolon

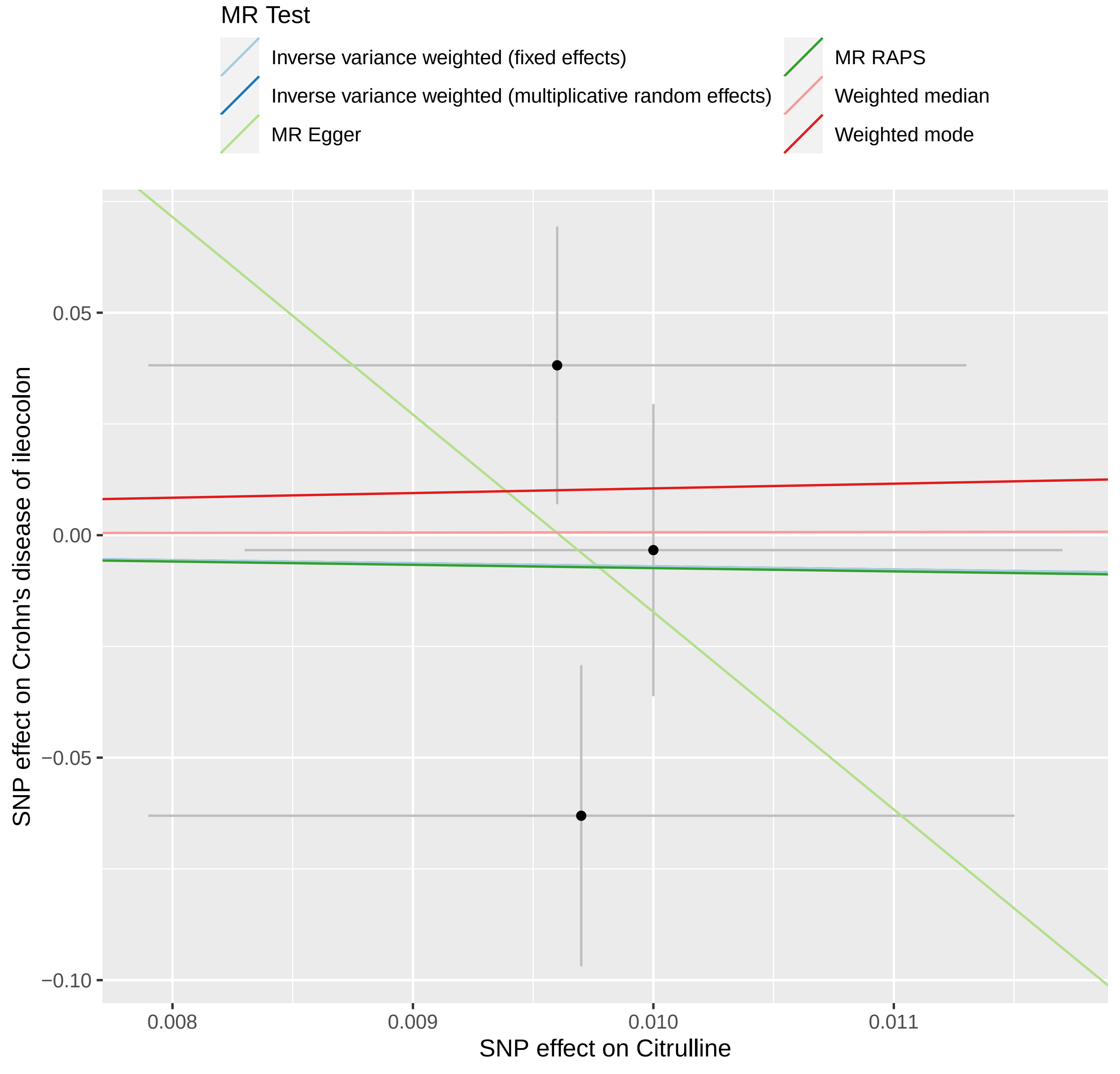

Batch 331 : Serotonin (5HT) on Crohn's disease of ileocolon

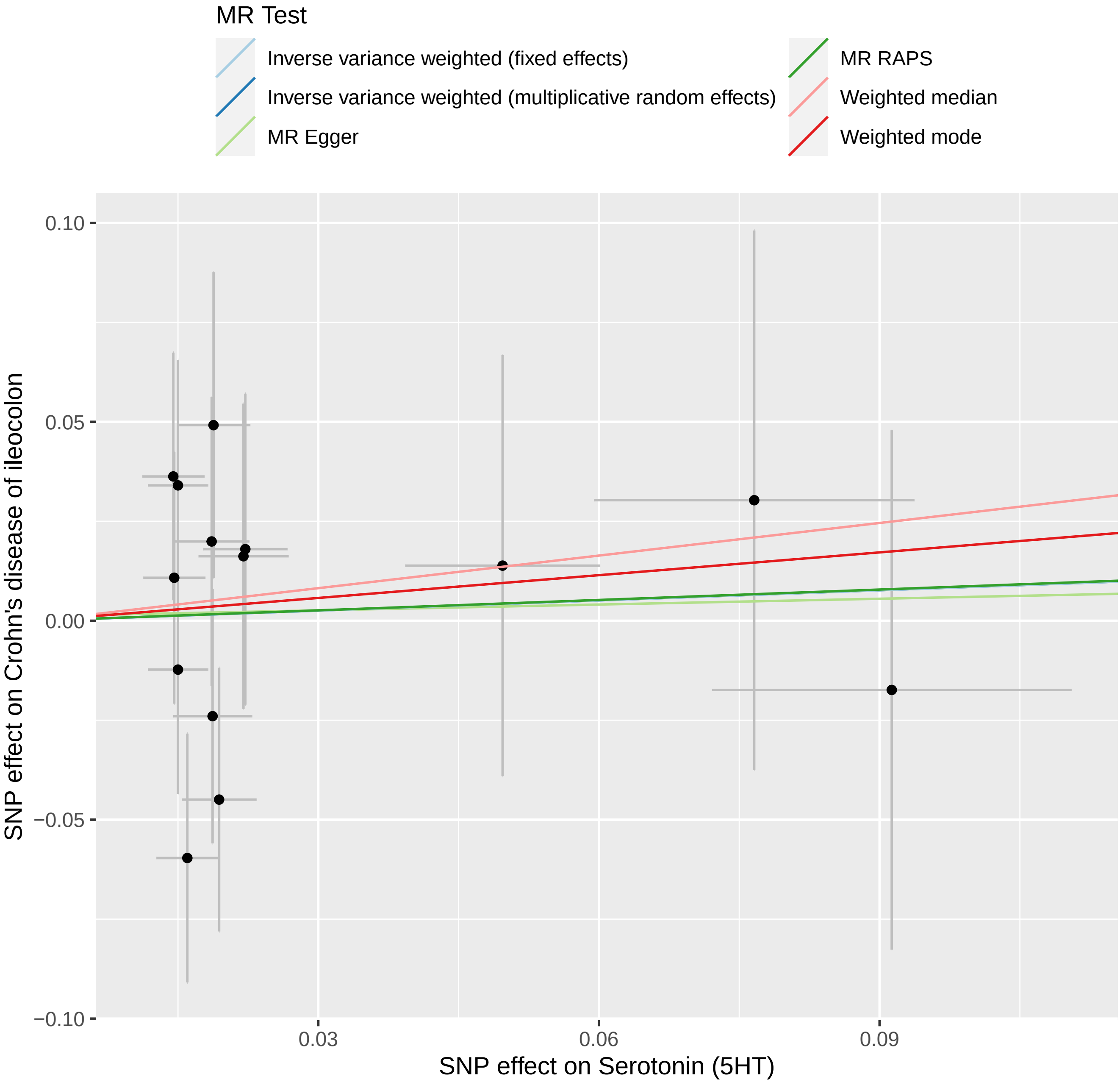

Batch 332 : Gamma−glutamylglutamine on Crohn's disease of ileocolon

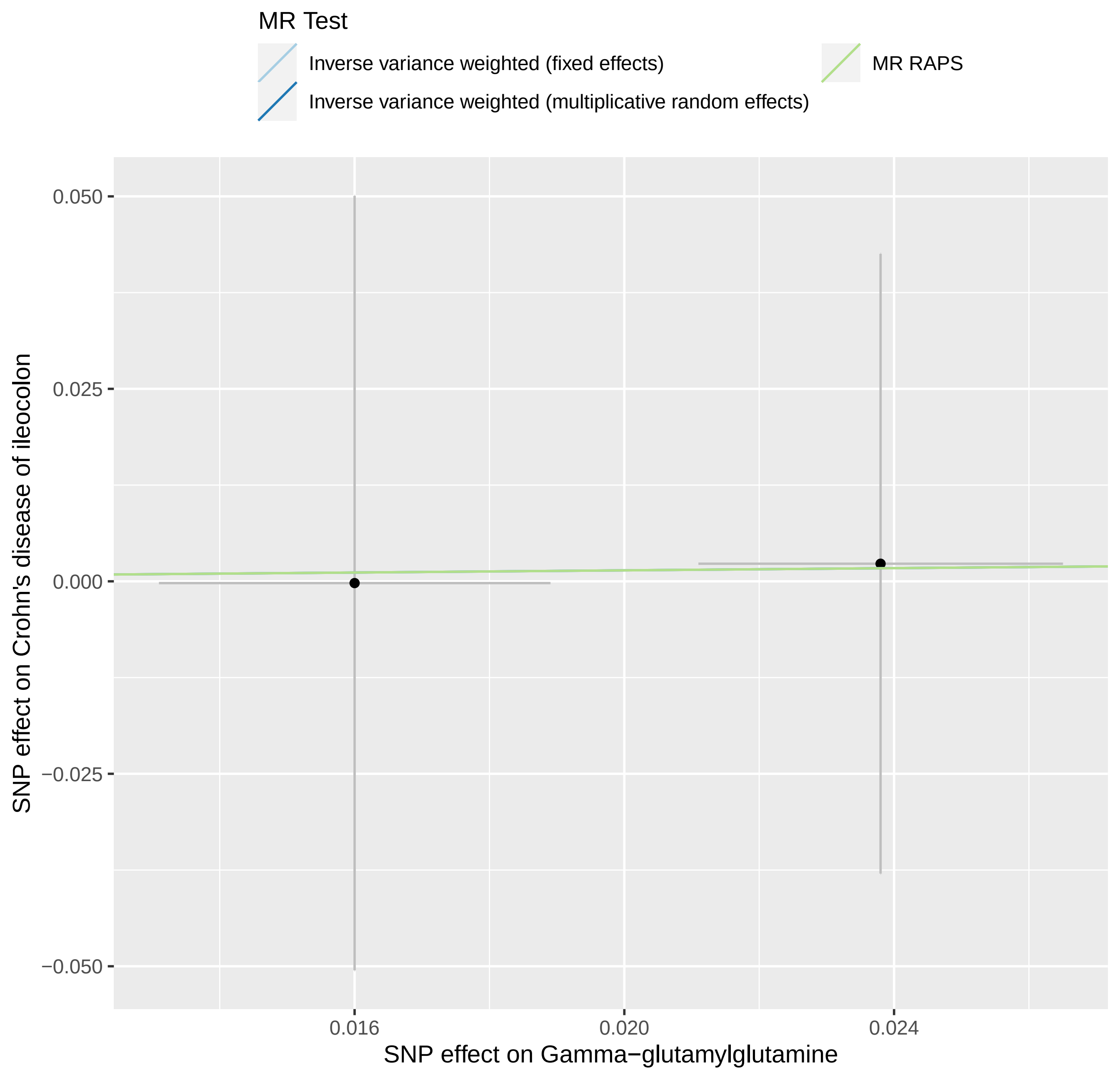

Batch 333 : Gamma−glutamyltyrosine on Crohn's disease of ileocolon

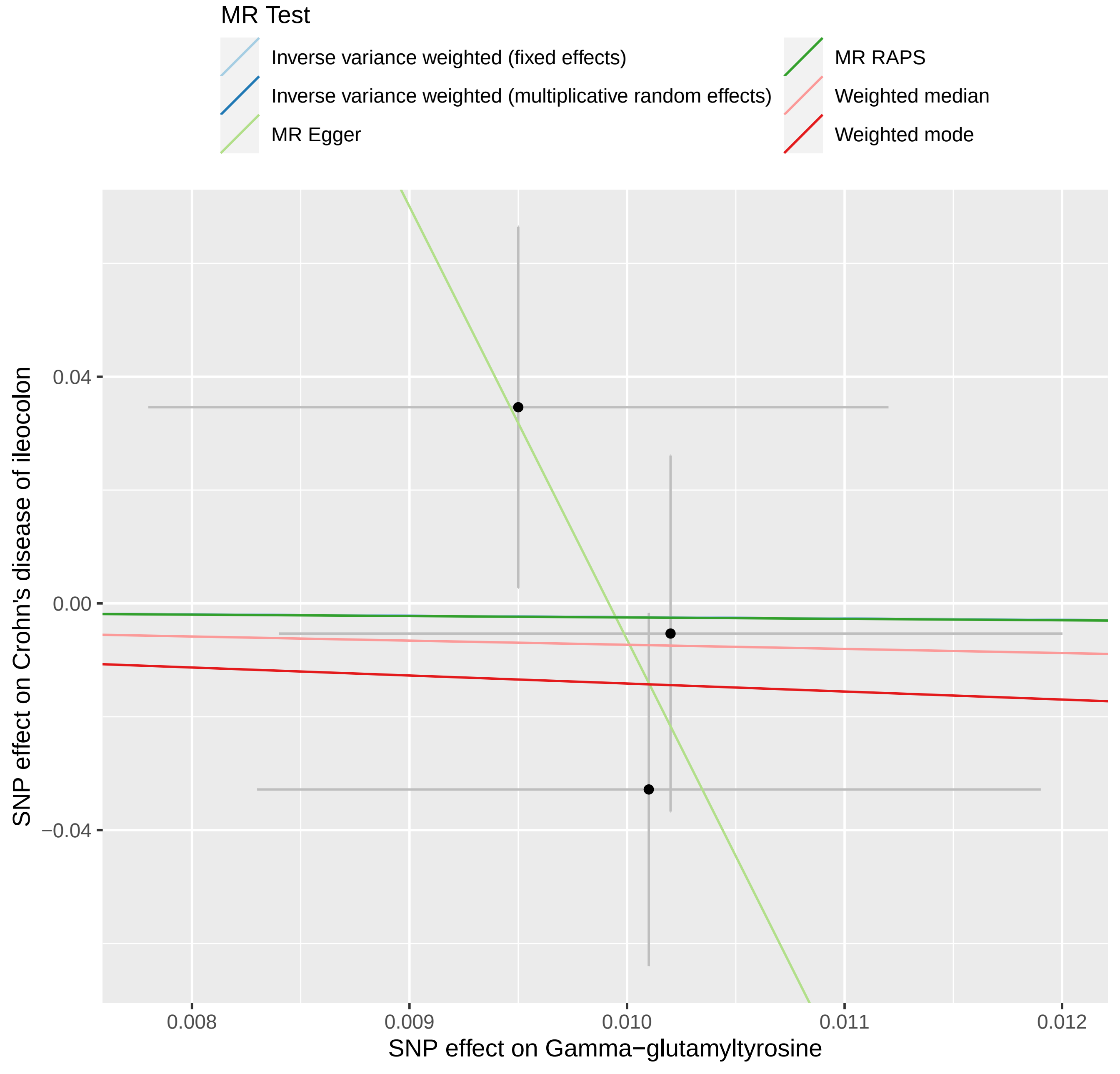

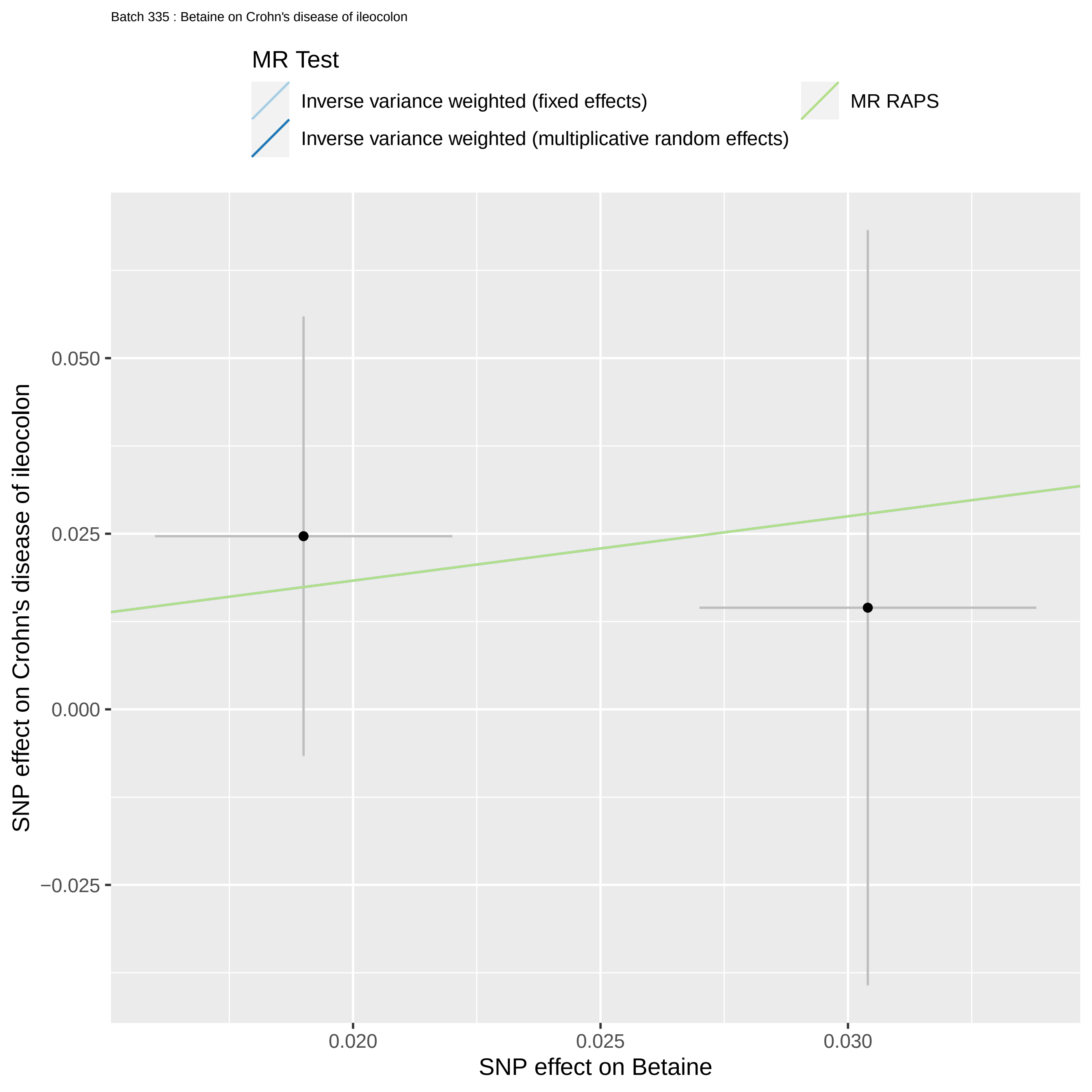
Batch 335 : Betaine on Crohn's disease of ileocolon
MR Test
Inverse variance weighted (fixed effects)
Inverse variance weighted (multiplicative random effects)
MR RAPS
0.050
0.025
0.000
−0.025
0.020
0.025
0.030
SNP effect on Crohn's disease of ileocolon
SNP effect on Betaine

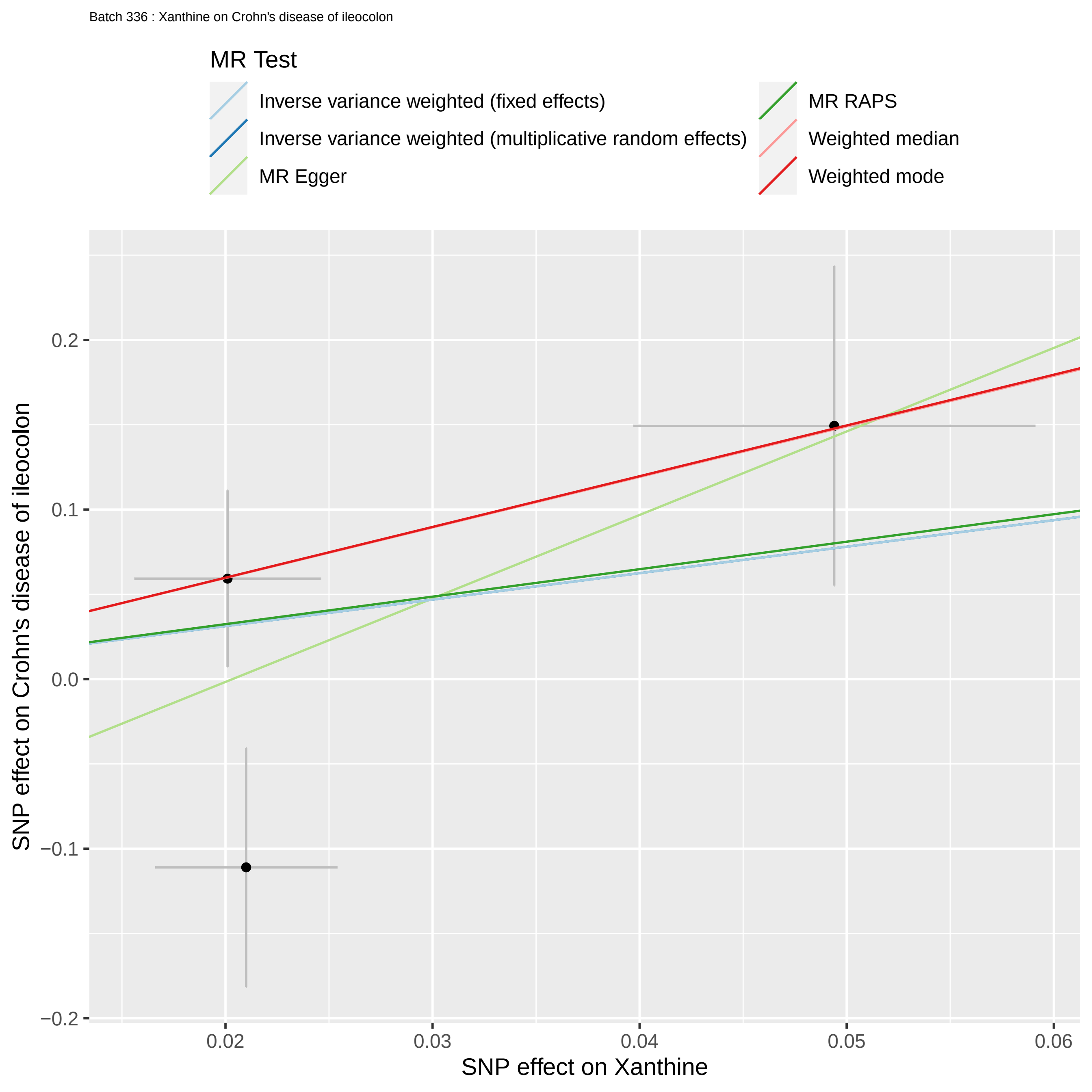
Batch 336 : Xanthine on Crohn's disease of ileocolon
MR Test
Inverse variance weighted (fixed effects)
Inverse variance weighted (multiplicative random effects)
MR Egger
MR RAPS
Weighted median
Weighted mode
SNP effect on Crohn's disease of ileocolon
0.2
0.1
0.0
−0.1
−0.2
0.02
0.03
0.04
0.05
0.06
SNP effect on Xanthine

Batch 337 : Phosphate on Crohn's disease of ileocolon

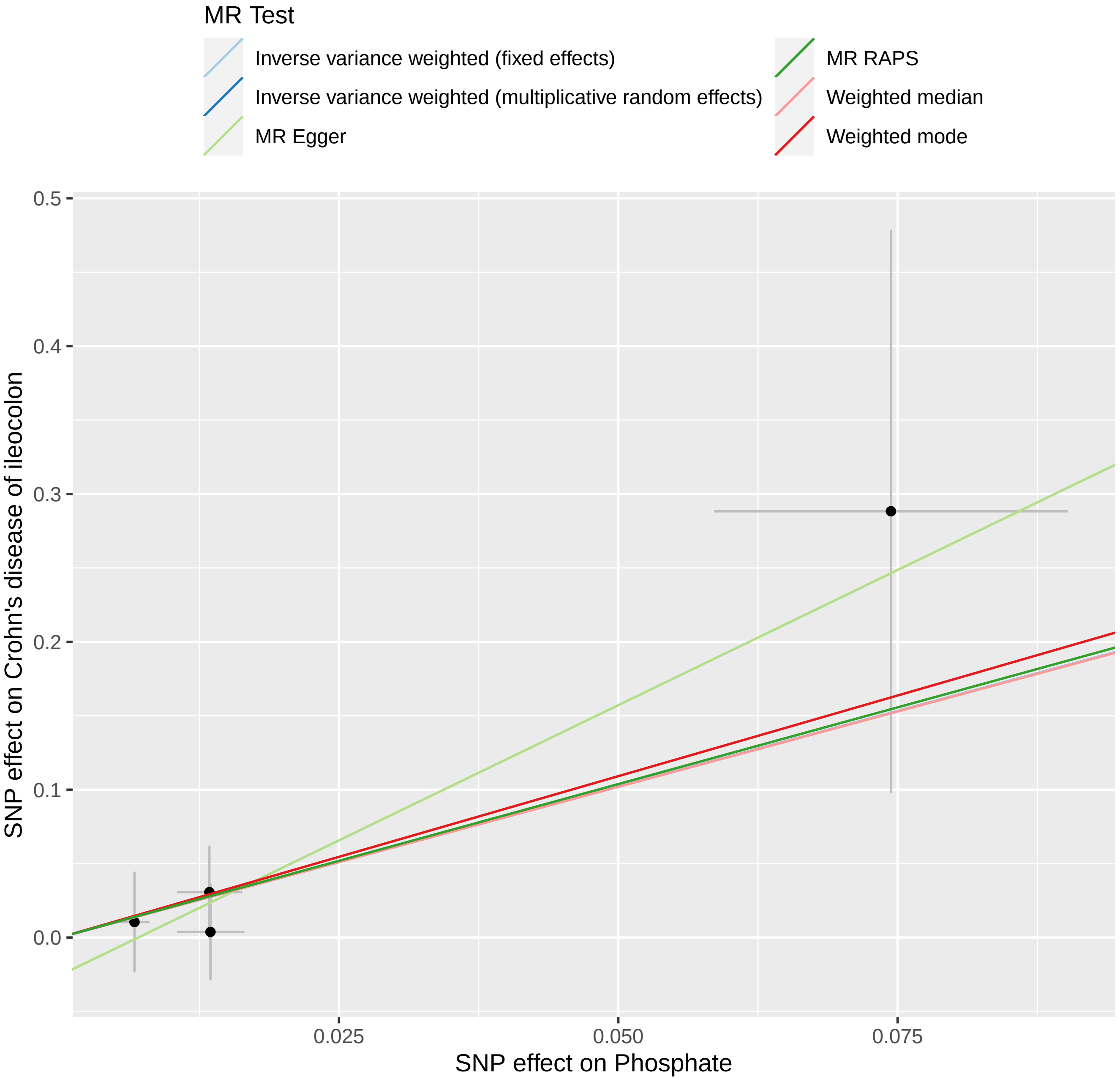

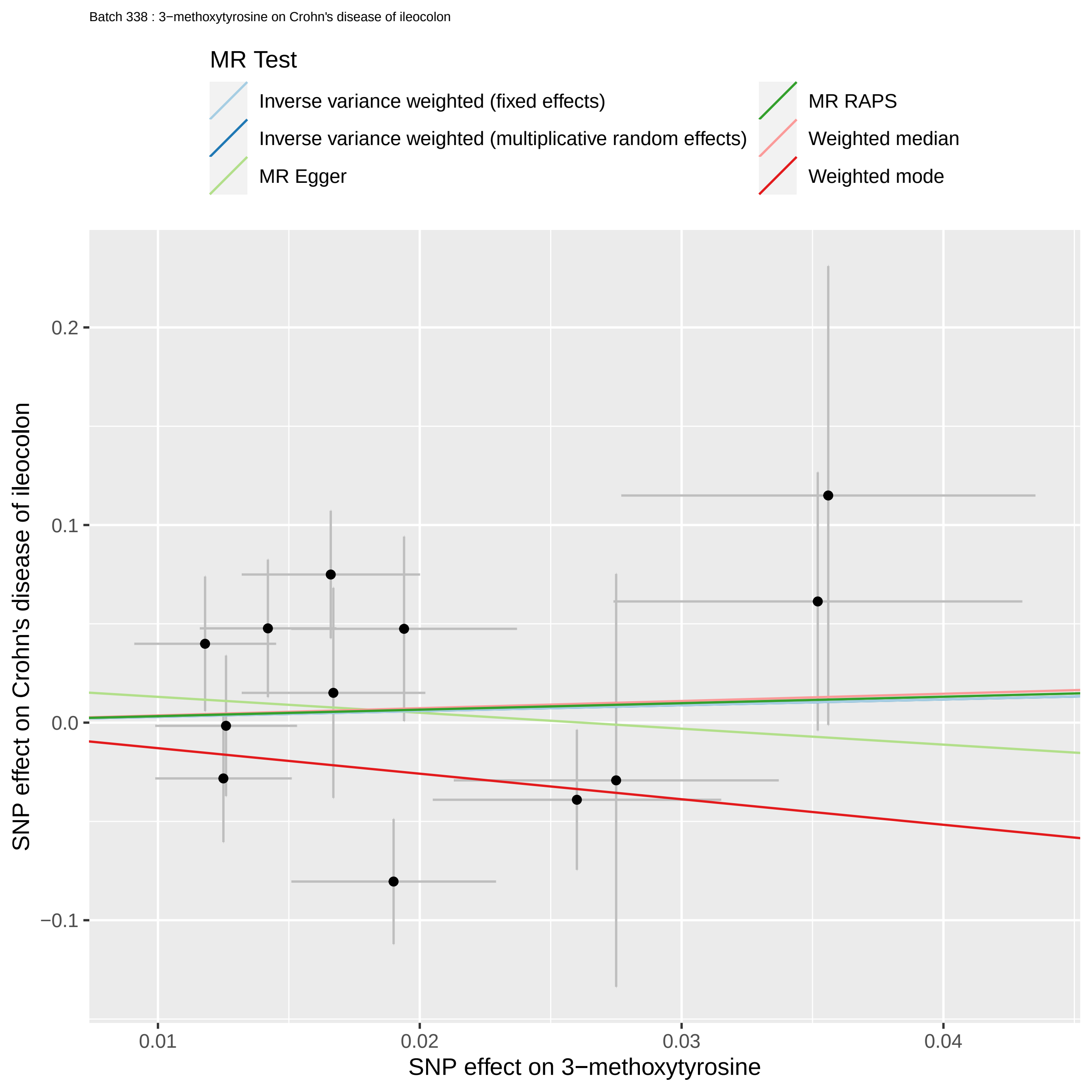
Batch 338 : 3−methoxytyrosine on Crohn's disease of ileocolon
MR Test
Inverse variance weighted (fixed effects)
Inverse variance weighted (multiplicative random effects)
MR Egger
MR RAPS
Weighted median
Weighted mode
SNP effect on Crohn's disease of ileocolon
0.2
0.1
0.0
−0.1
0.01
0.02
0.03
0.04
SNP effect on 3−methoxytyrosine

Batch 340 : Undecanoate (11:0) on Crohn's disease of ileocolon

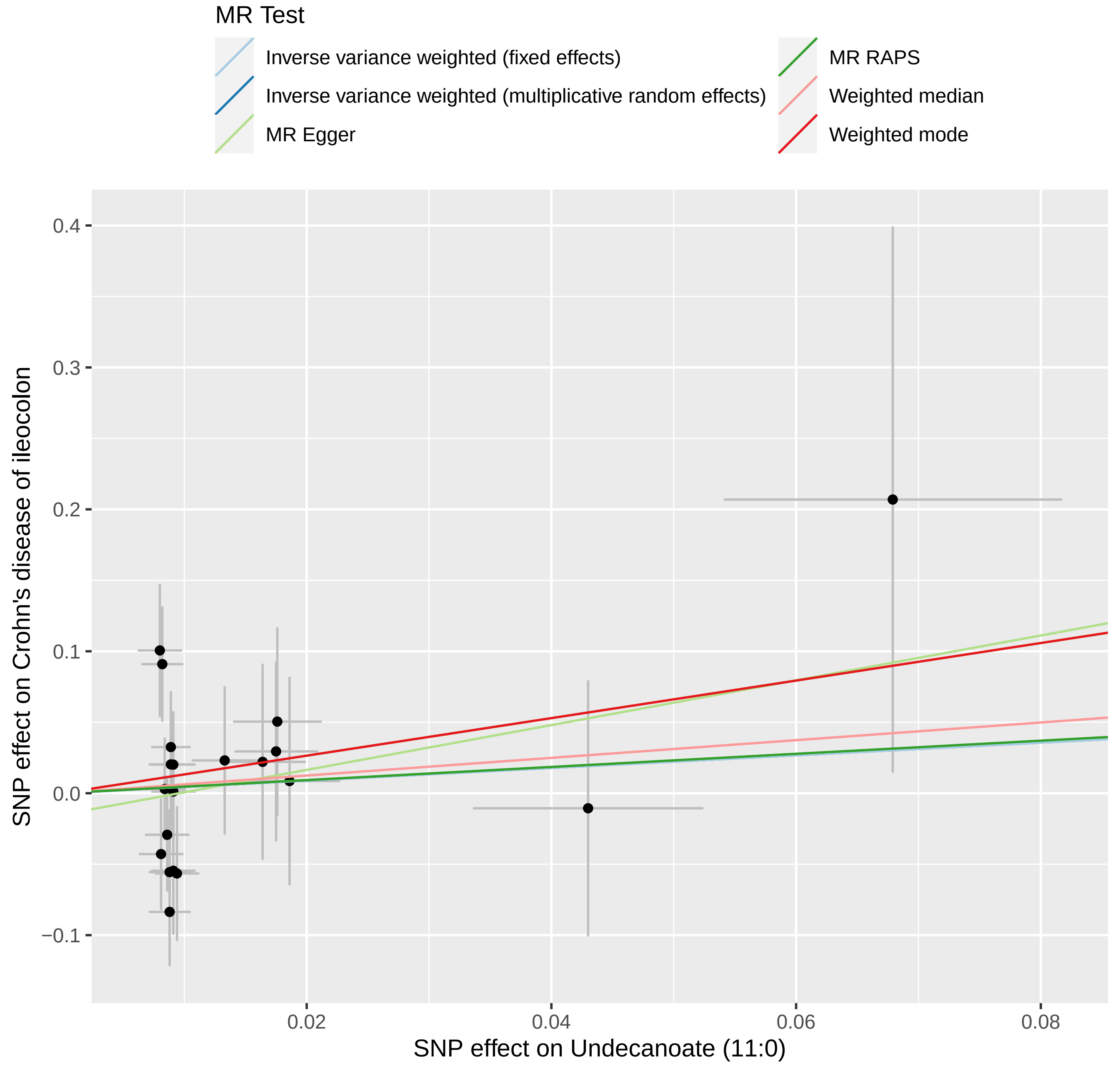

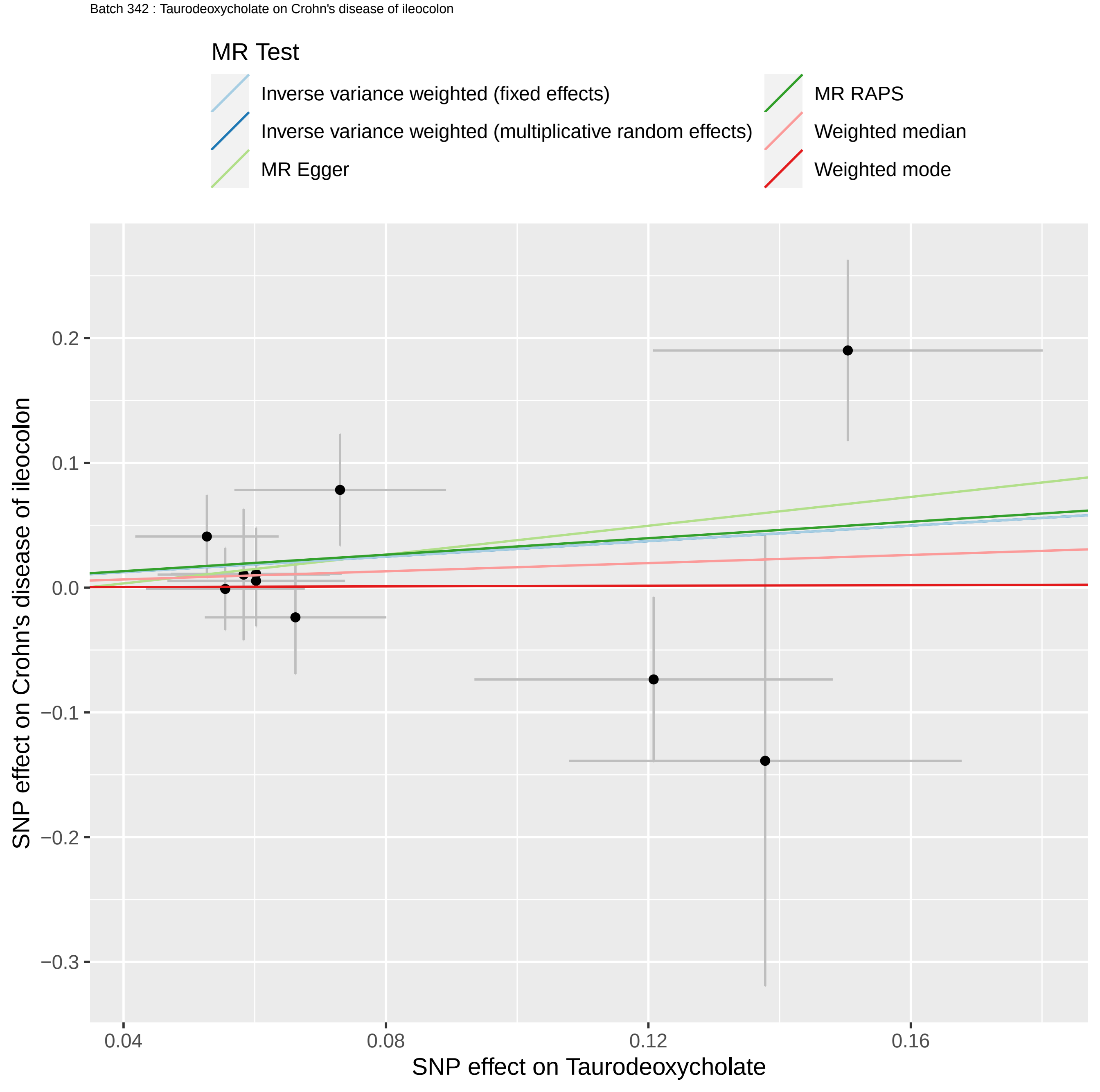

Batch 342 : Taurodeoxycholate on Crohn's disease of ileocolon
MR Test
Inverse variance weighted (fixed effects)
Inverse variance weighted (multiplicative random effects)
MR Egger
MR RAPS
Weighted median
Weighted mode
0.2
0.1
0.0
−0.1
−0.2
−0.3
0.04
0.08
0.12
0.16
SNP effect on Crohn's disease of ileocolon
SNP effect on Taurodeoxycholate

Batch 343 : Glycerol on Crohn's disease of ileocolon

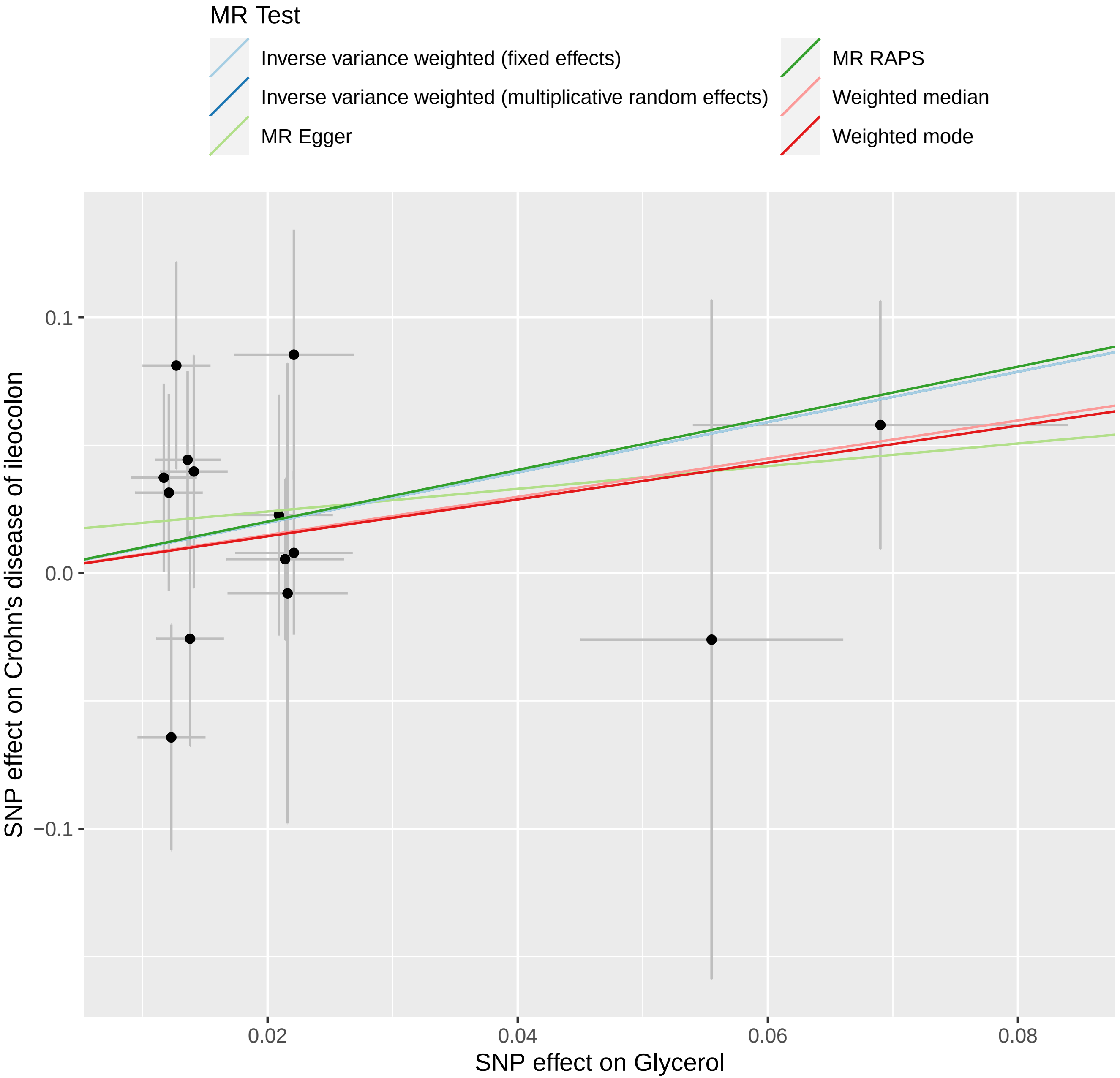

Batch 344 : Kynurenine on Crohn's disease of ileocolon

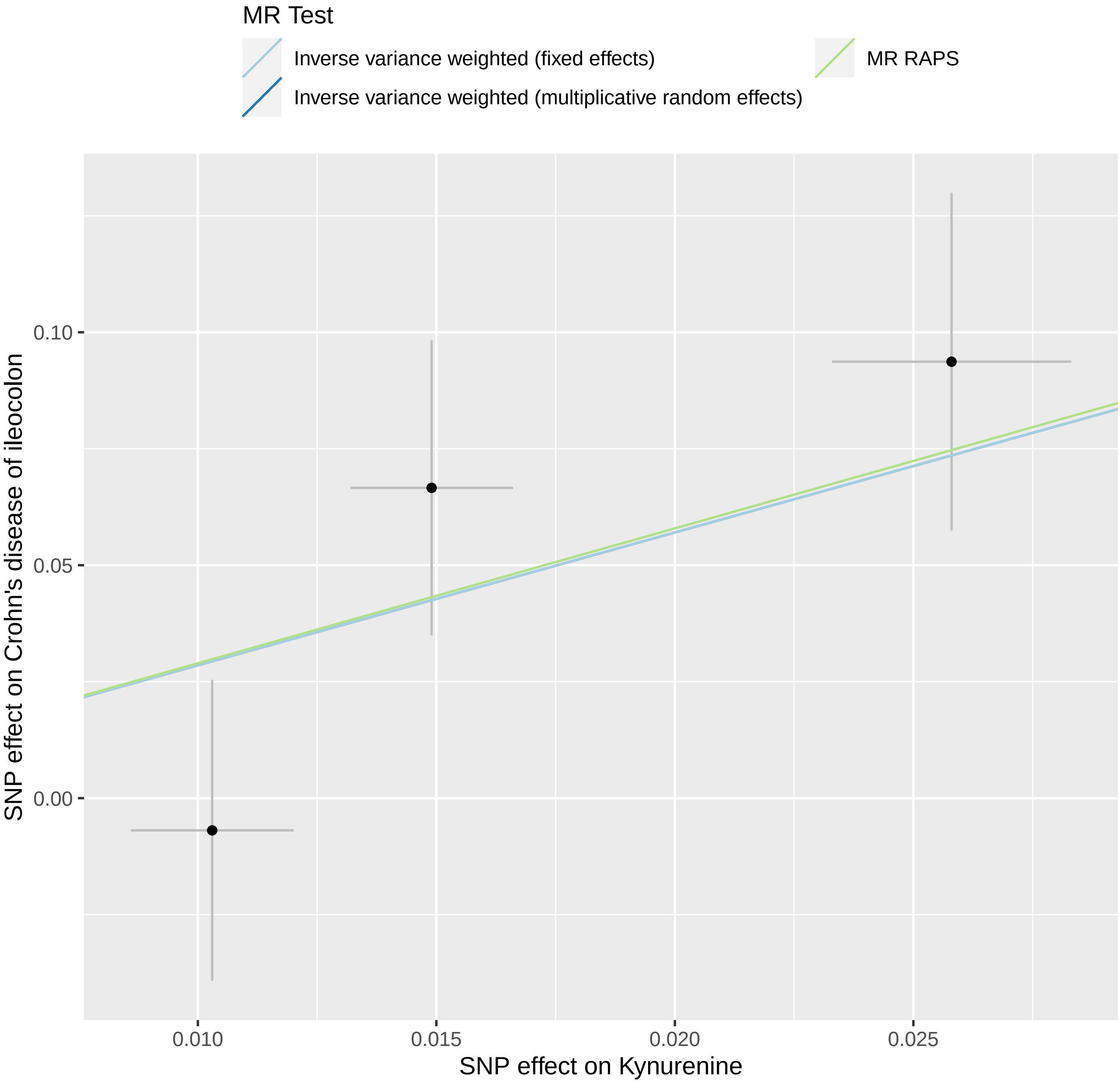

Batch 345 : Mannitol on Crohn's disease of ileocolon

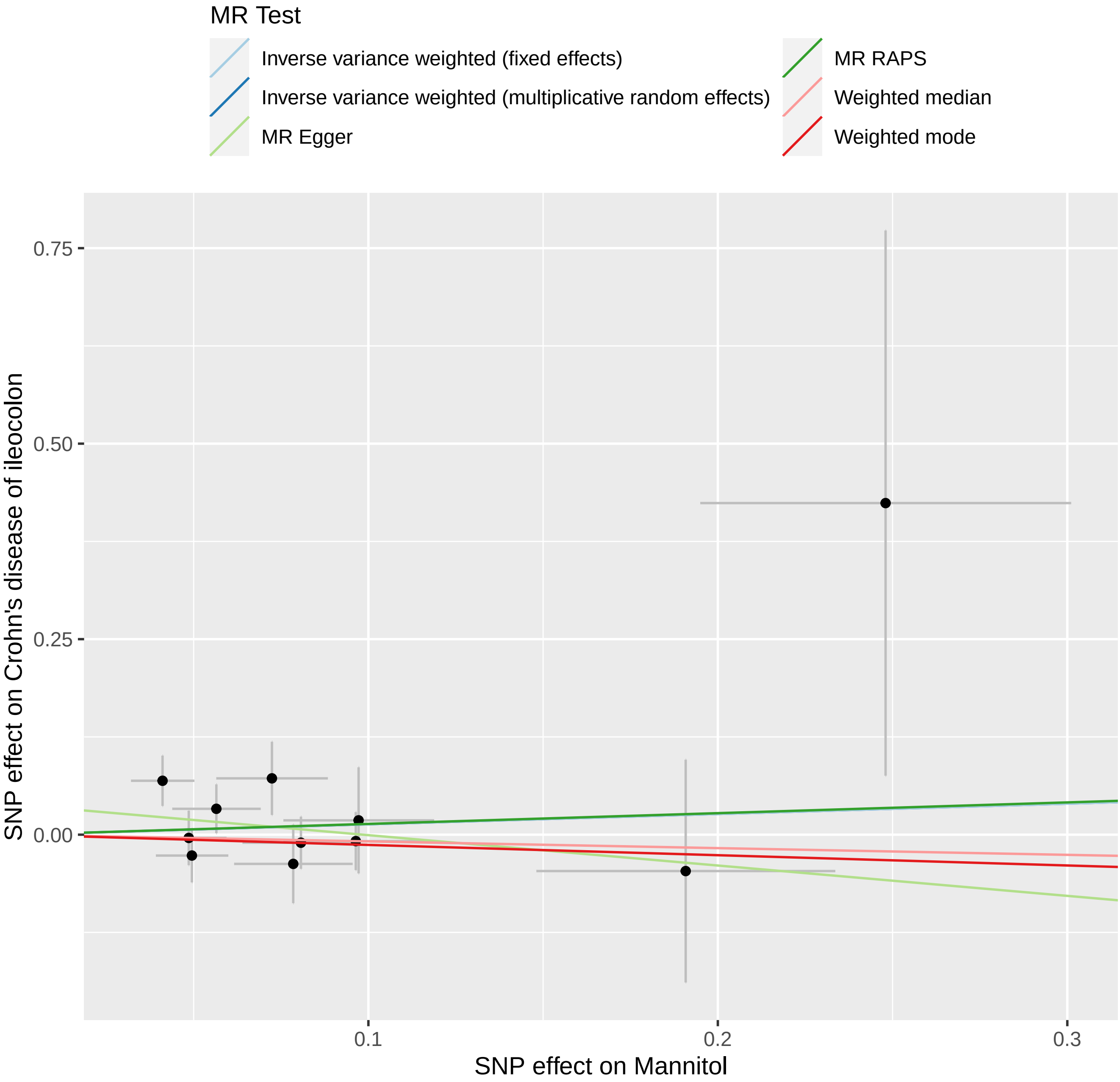

Batch 346 : Glycerol 3−phosphate (G3P) on Crohn's disease of ileocolon

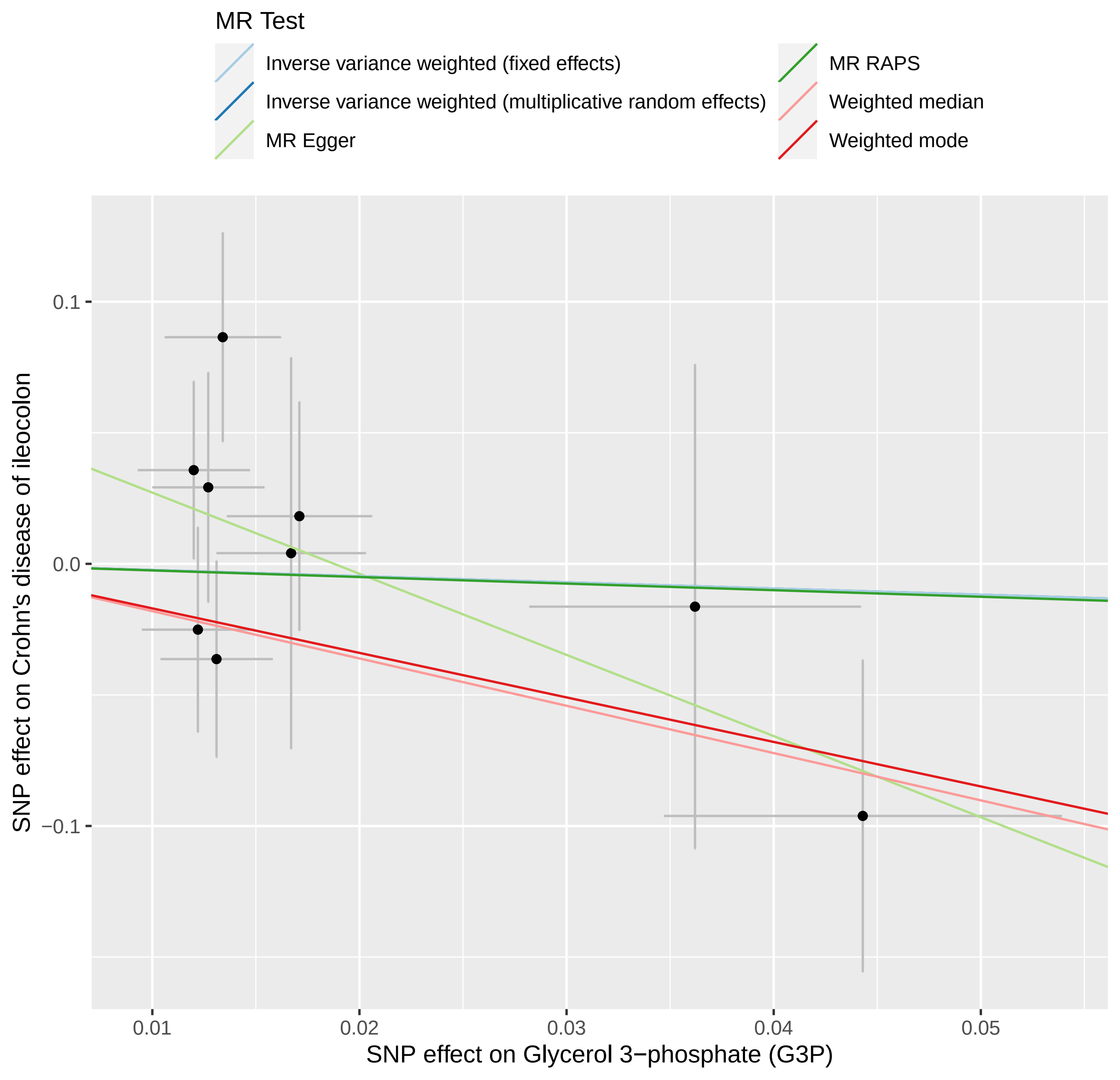

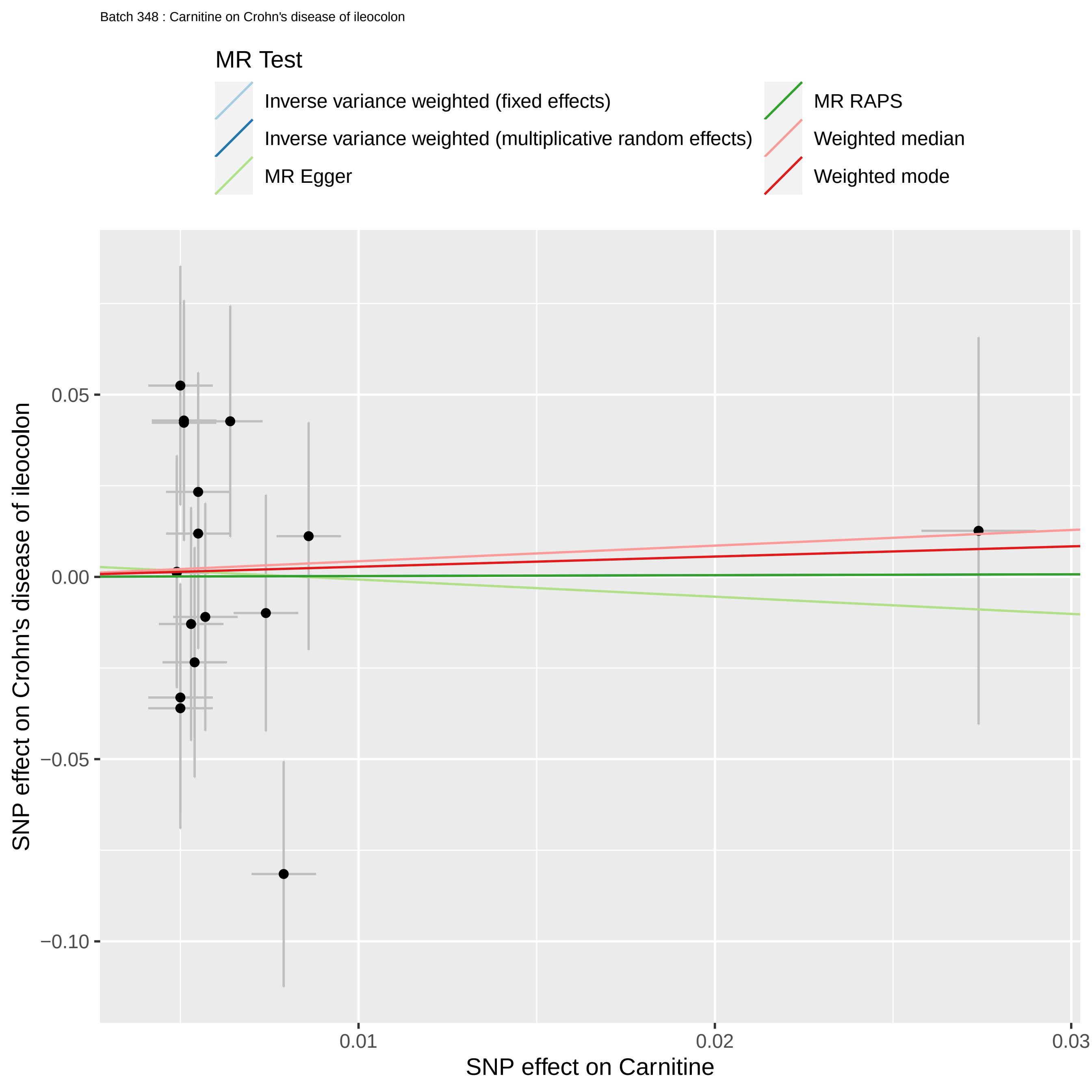
Batch 348 : Carnitine on Crohn's disease of ileocolon
MR Test
Inverse variance weighted (fixed effects)
Inverse variance weighted (multiplicative random effects)
MR Egger
MR RAPS
Weighted median
Weighted mode
SNP effect on Crohn's disease of ileocolon
0.05
0.00
−0.05
−0.10
0.01
0.02
0.03
SNP effect on Carnitine

Batch 349 : Choline on Crohn's disease of ileocolon

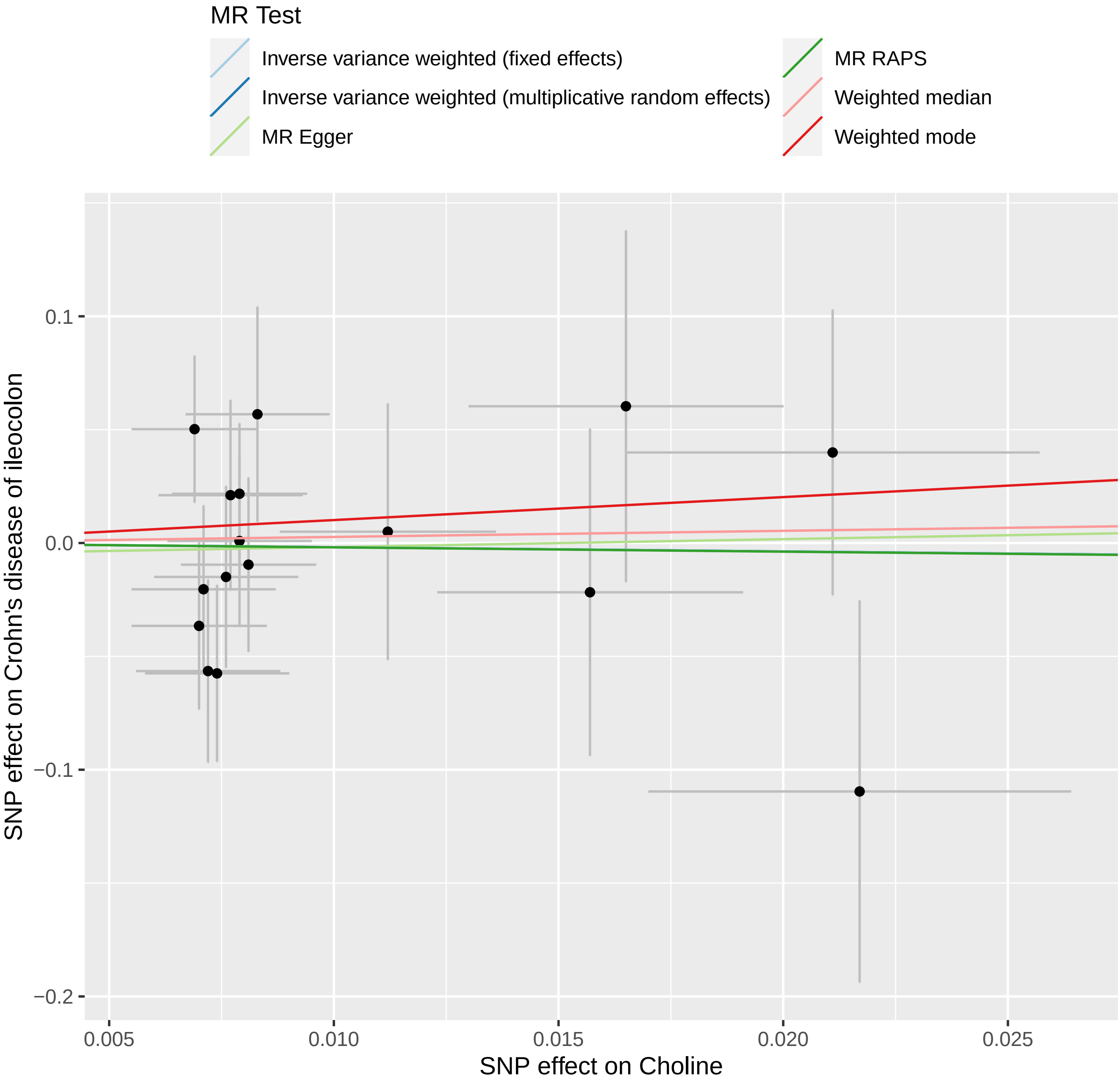

Batch 352 : 3−methyl−2−oxovalerate on Crohn's disease of ileocolon

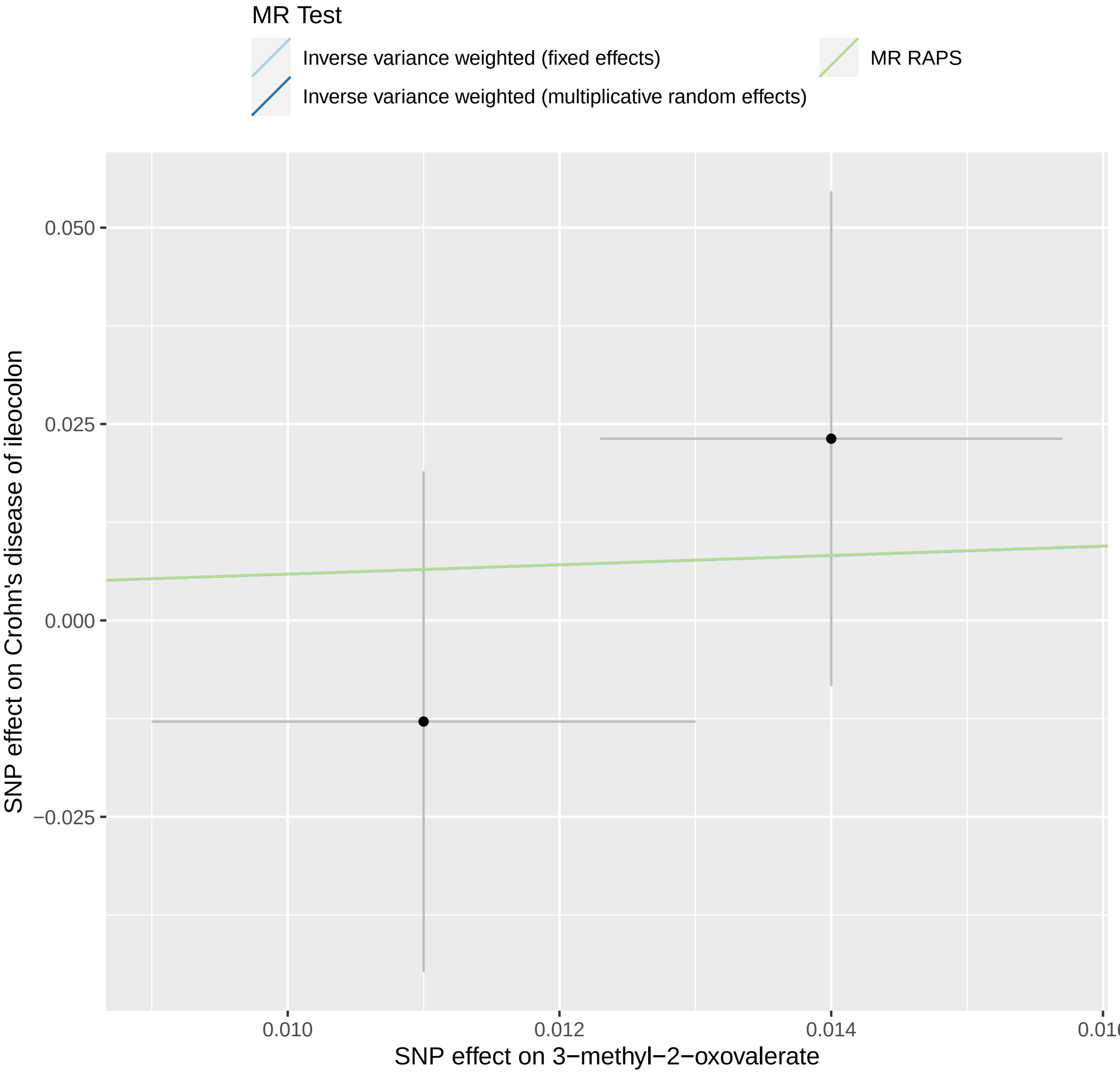

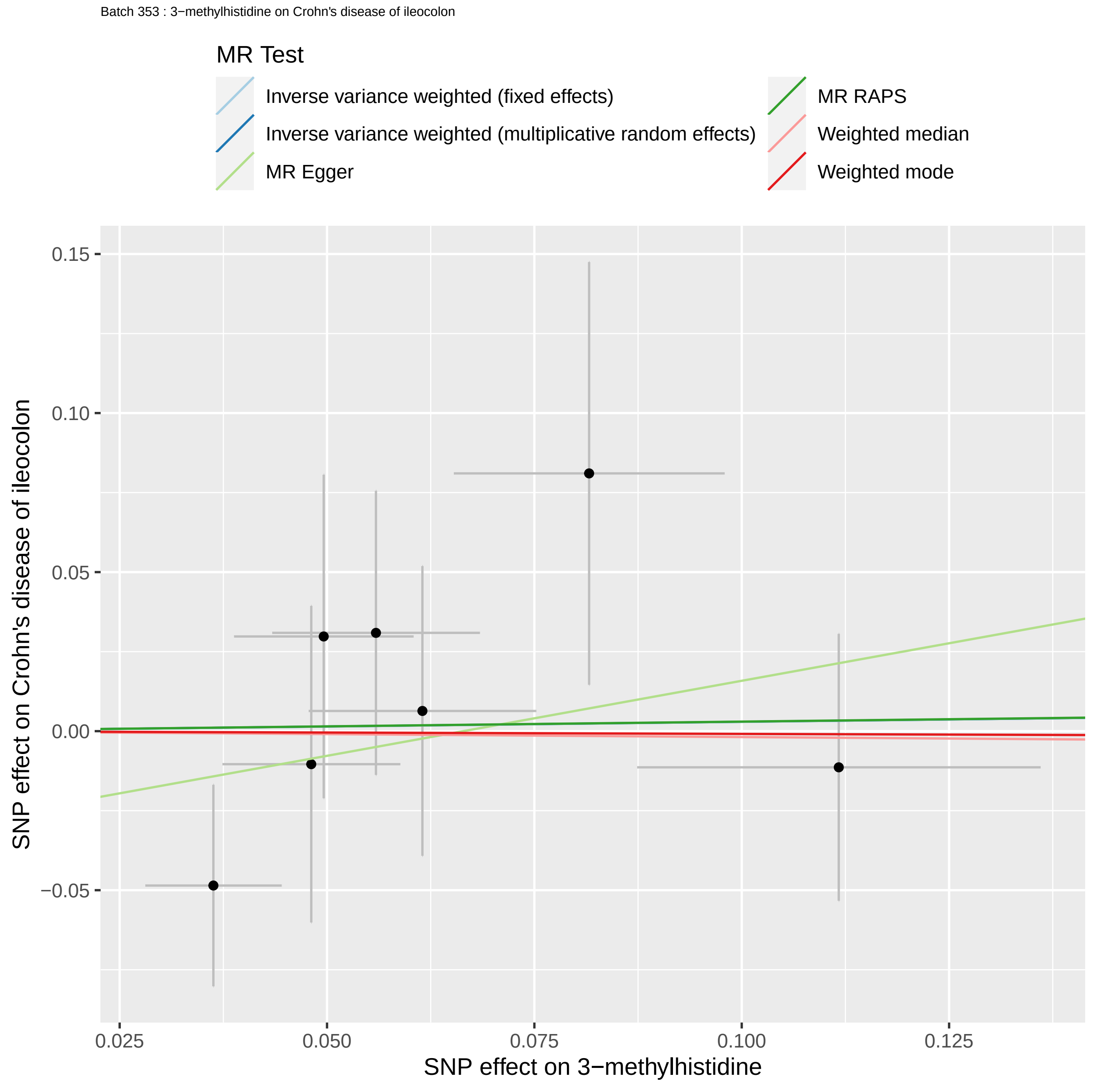
Batch 353 : 3−methylhistidine on Crohn's disease of ileocolon
MR Test
Inverse variance weighted (fixed effects)
Inverse variance weighted (multiplicative random effects)
MR Egger
MR RAPS
Weighted median
Weighted mode
0.15
0.10
0.05
0.00
−0.05
SNP effect on Crohn's disease of ileocolon
0.025
0.050
0.075
0.100
0.125
SNP effect on 3−methylhistidine

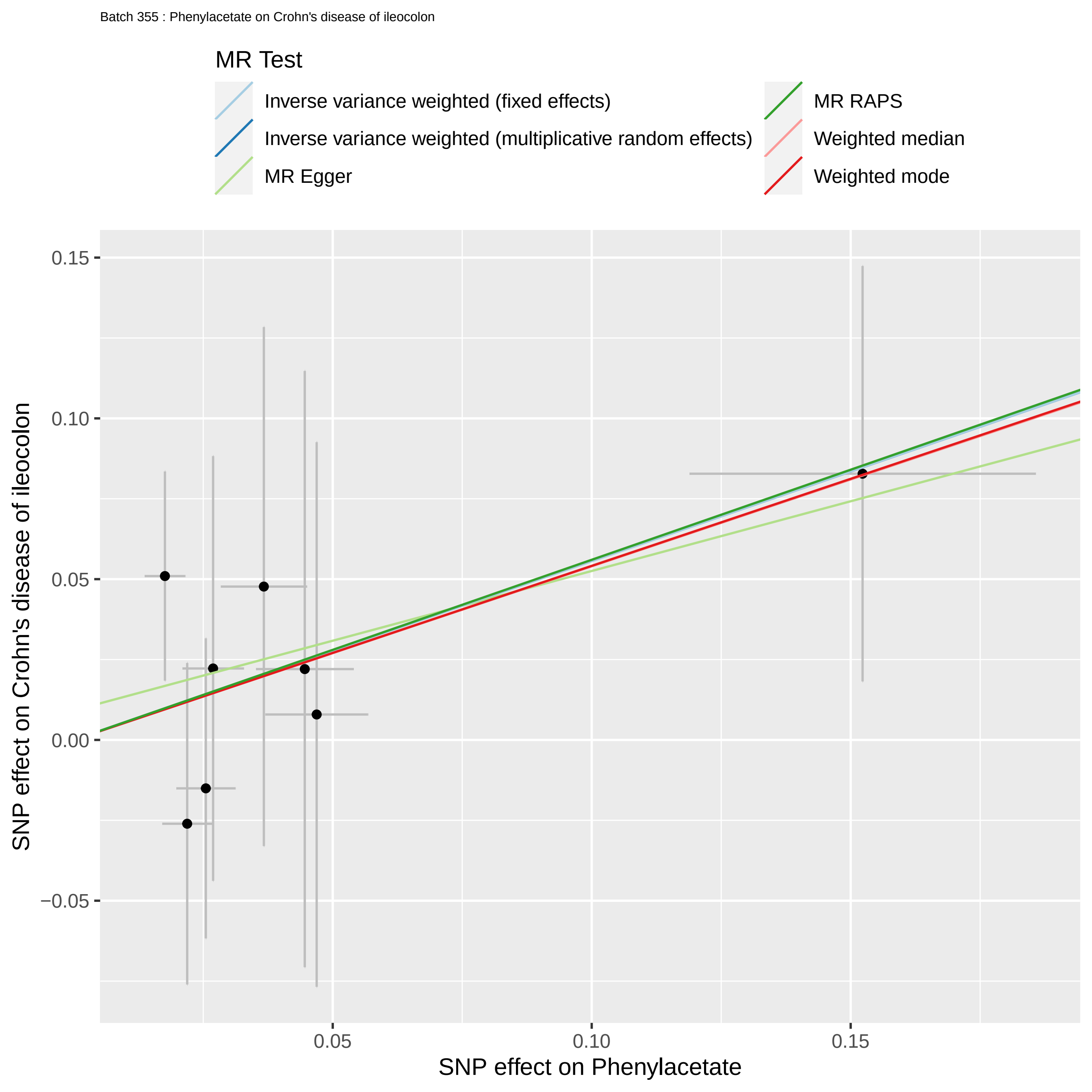

Batch 355 : Phenylacetate on Crohn's disease of ileocolon
MR Test
Inverse variance weighted (fixed effects)
Inverse variance weighted (multiplicative random effects)
MR Egger
MR RAPS
Weighted median
Weighted mode
0.15
0.10
0.05
0.00
−0.05
0.05
0.10
0.15
SNP effect on Crohn's disease of ileocolon
SNP effect on Phenylacetate

Batch 356 : Glycerophosphorylcholine (GPC) on Crohn's disease of ileocolon

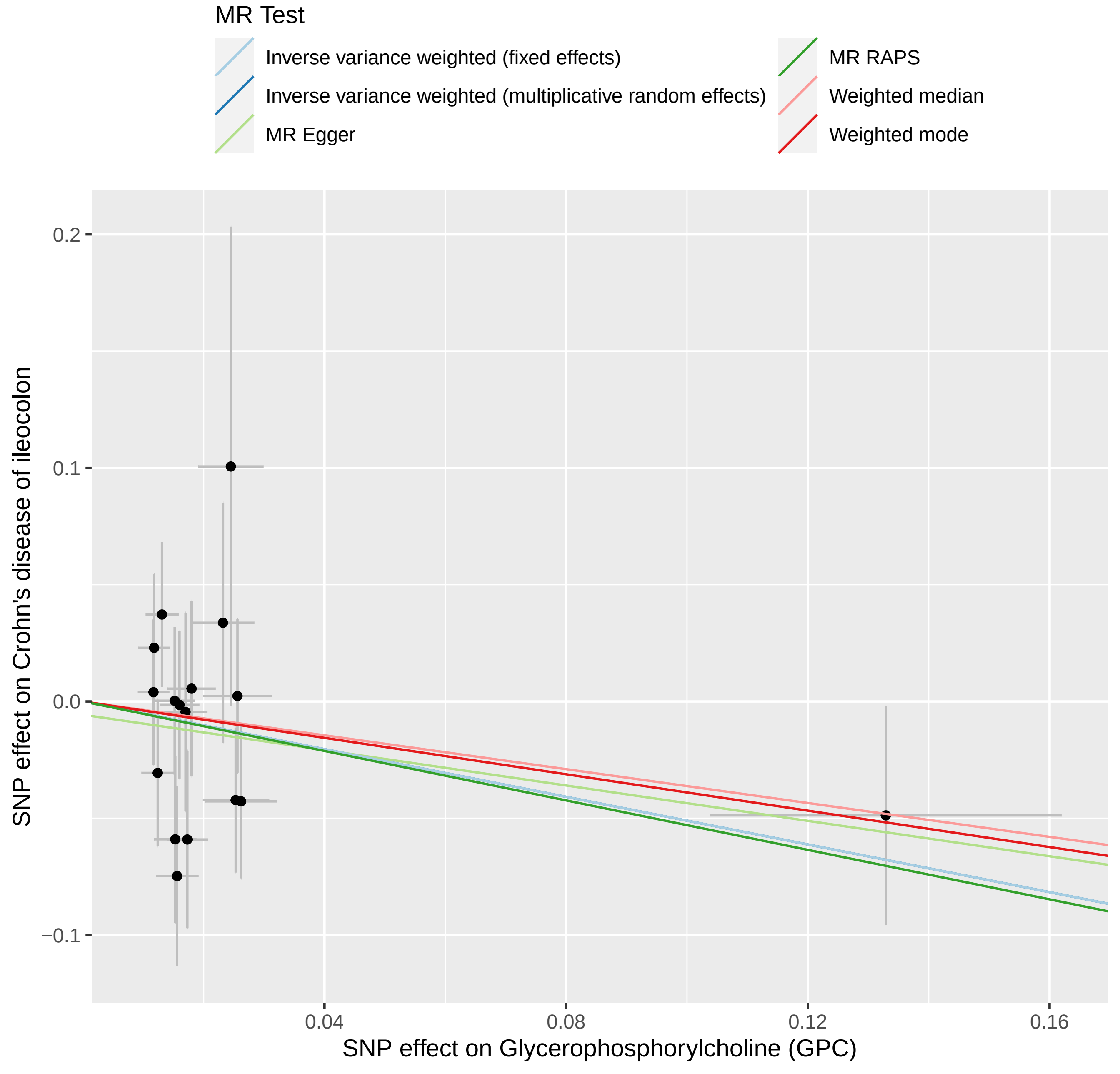

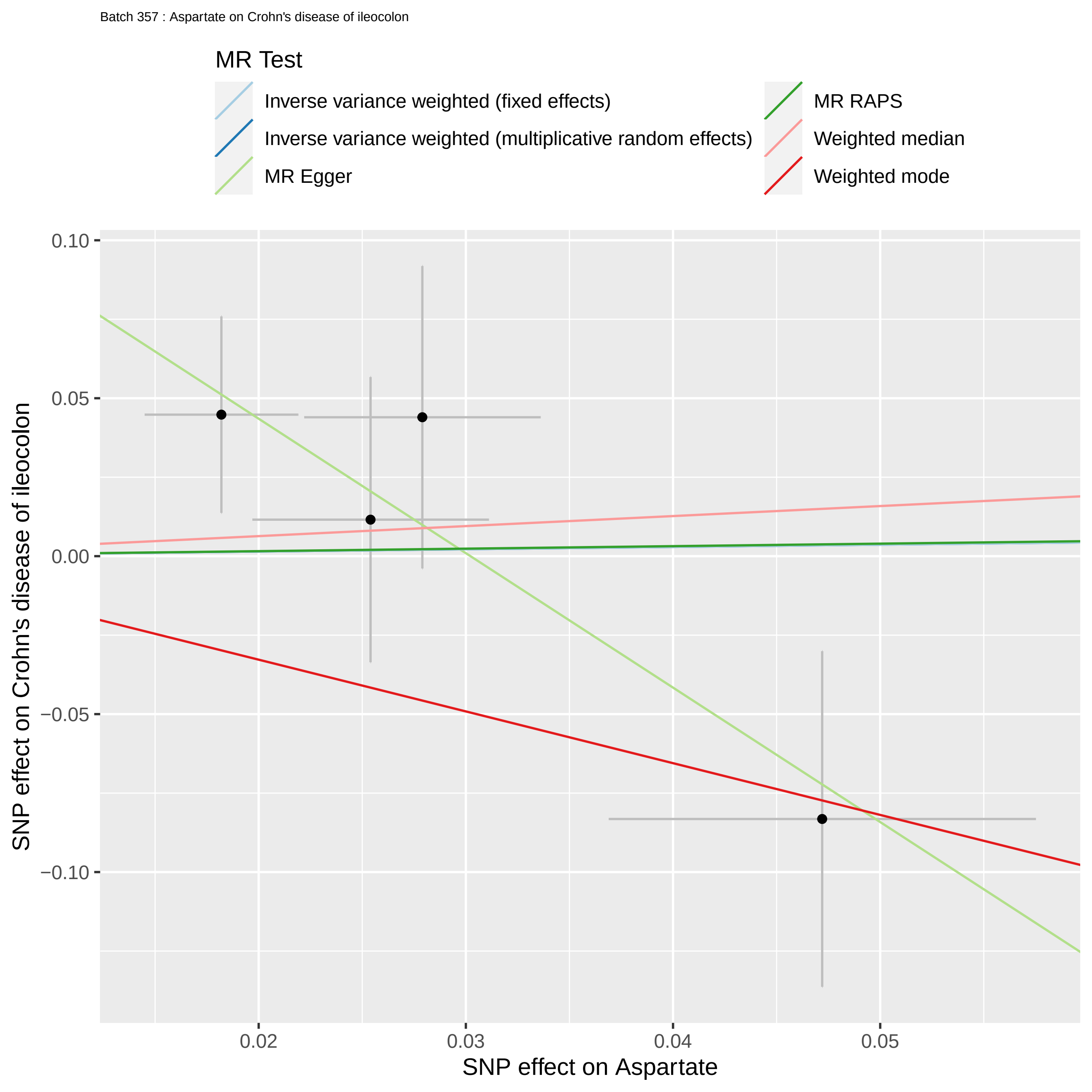

Batch 357 : Aspartate on Crohn's disease of ileocolon
MR Test
Inverse variance weighted (fixed effects)
Inverse variance weighted (multiplicative random effects)
MR Egger
MR RAPS
Weighted median
Weighted mode
0.10
0.05
0.00
−0.05
−0.10
0.02
0.03
0.04
0.05
SNP effect on Crohn's disease of ileocolon
SNP effect on Aspartate

Batch 359 : Dihomo−linoleate (20:2n6) on Crohn's disease of ileocolon

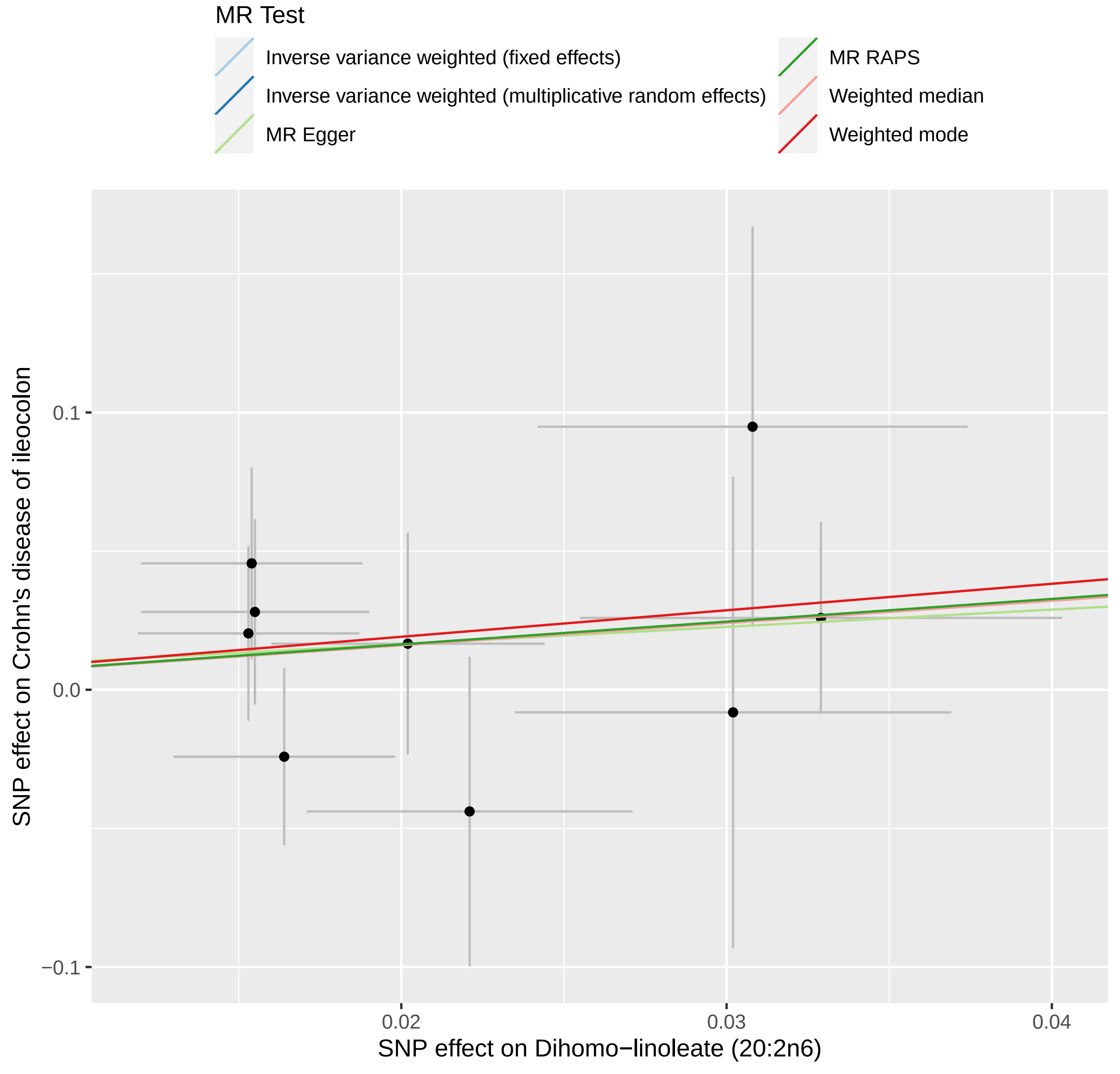

Batch 360 : 2−hydroxystearate on Crohn's disease of ileocolon

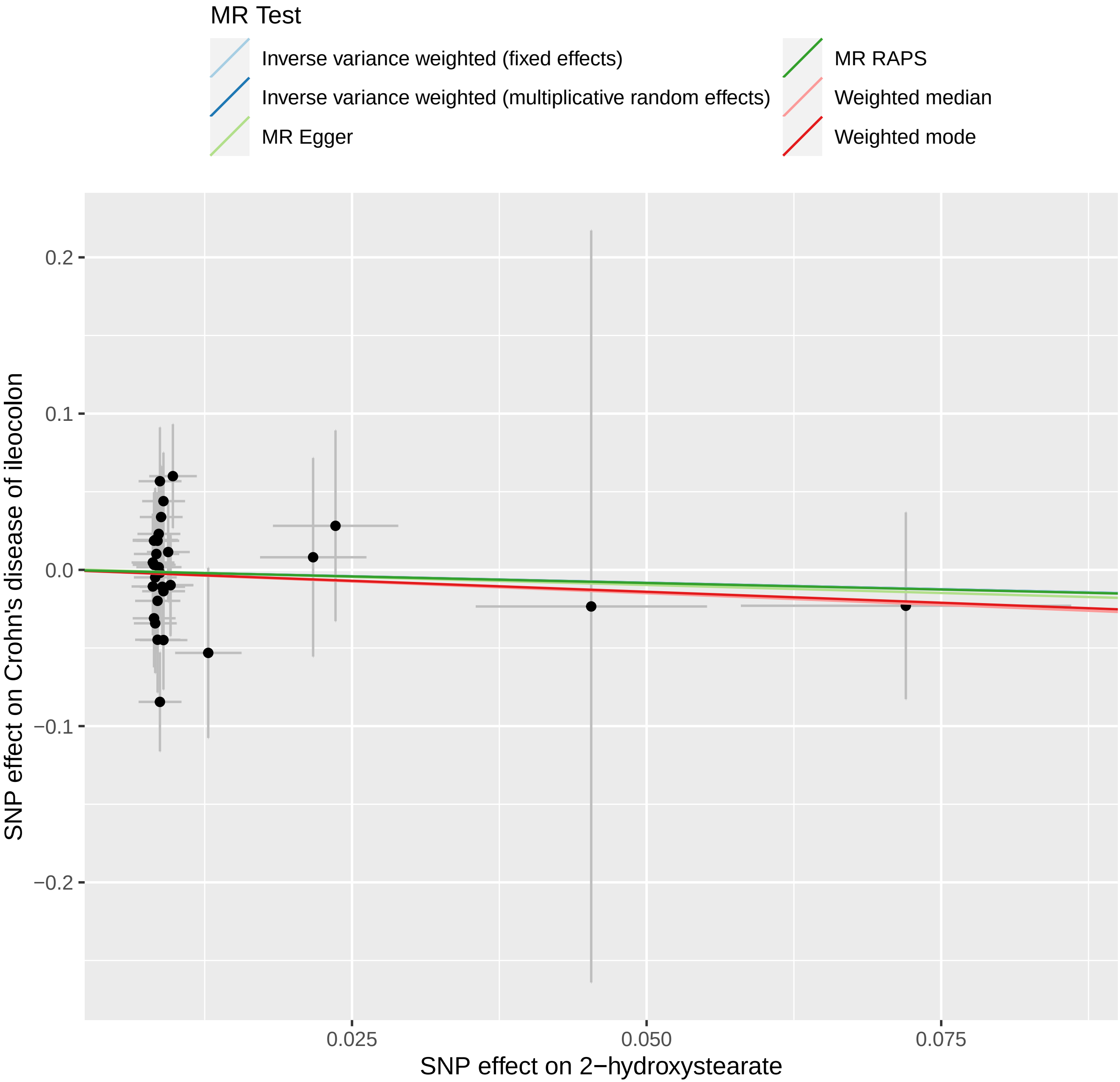

Batch 362 : Glycylvaline on Crohn's disease of ileocolon

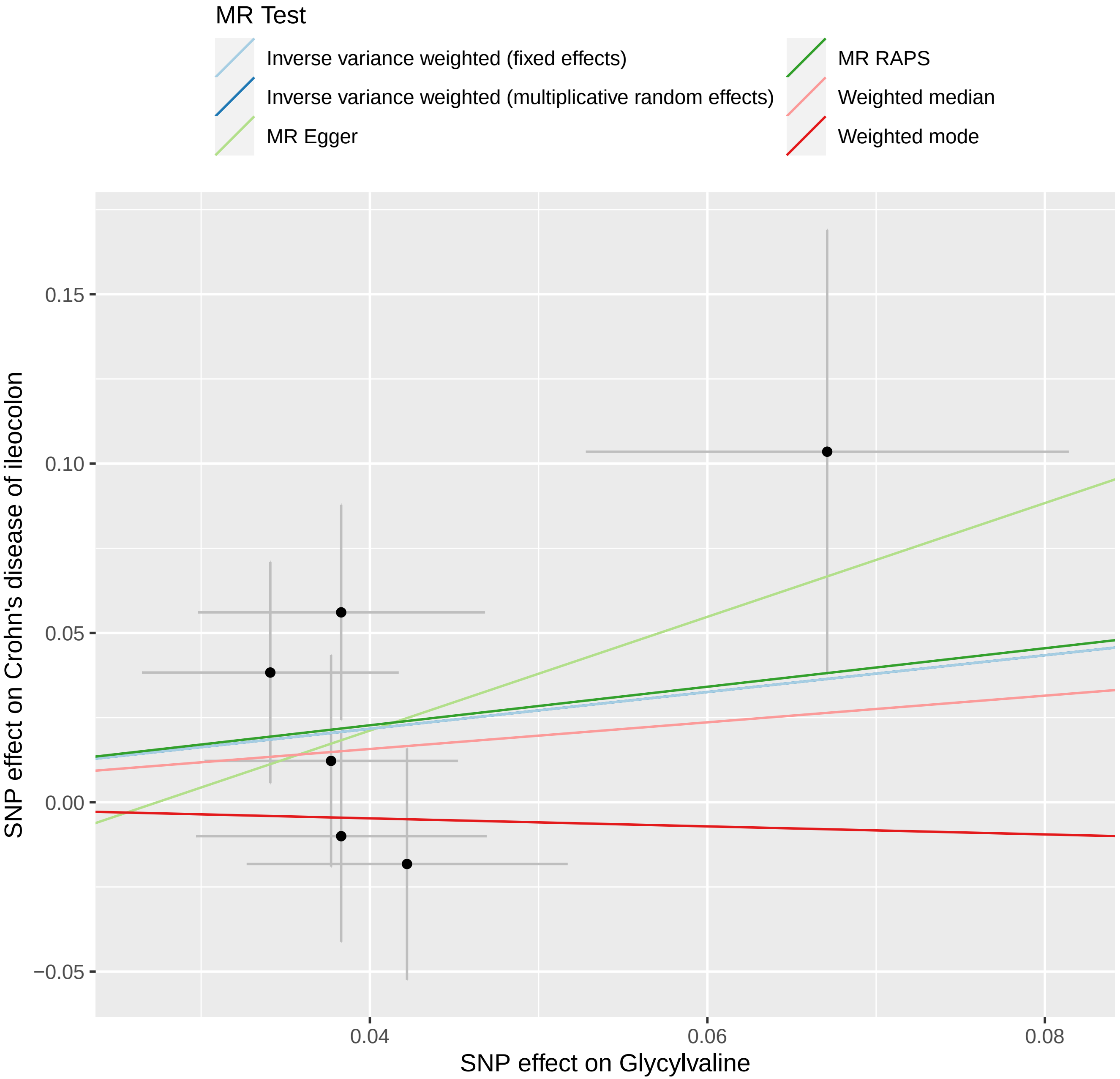

Batch 363 : Gamma−glutamylleucine on Crohn's disease of ileocolon

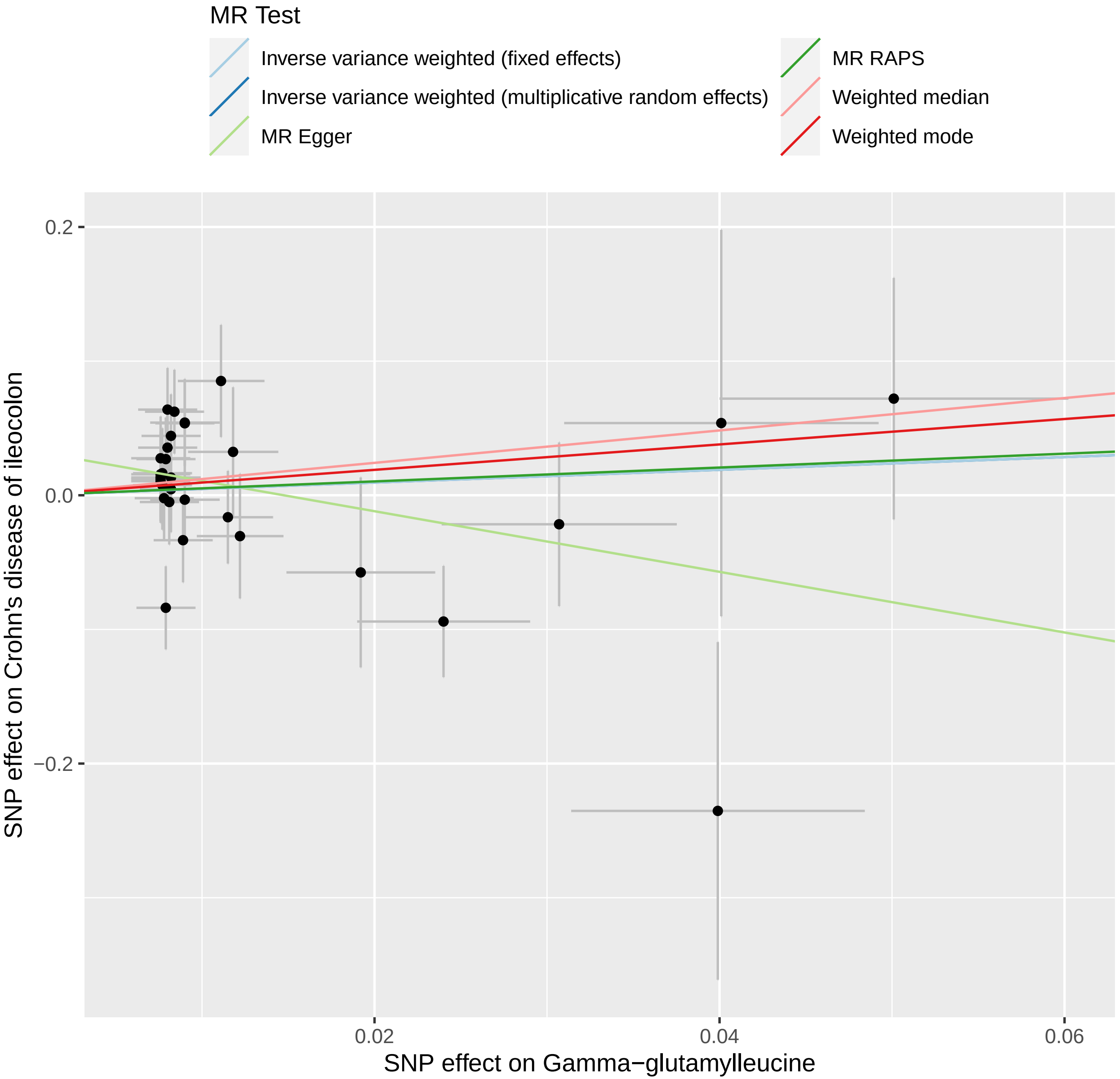

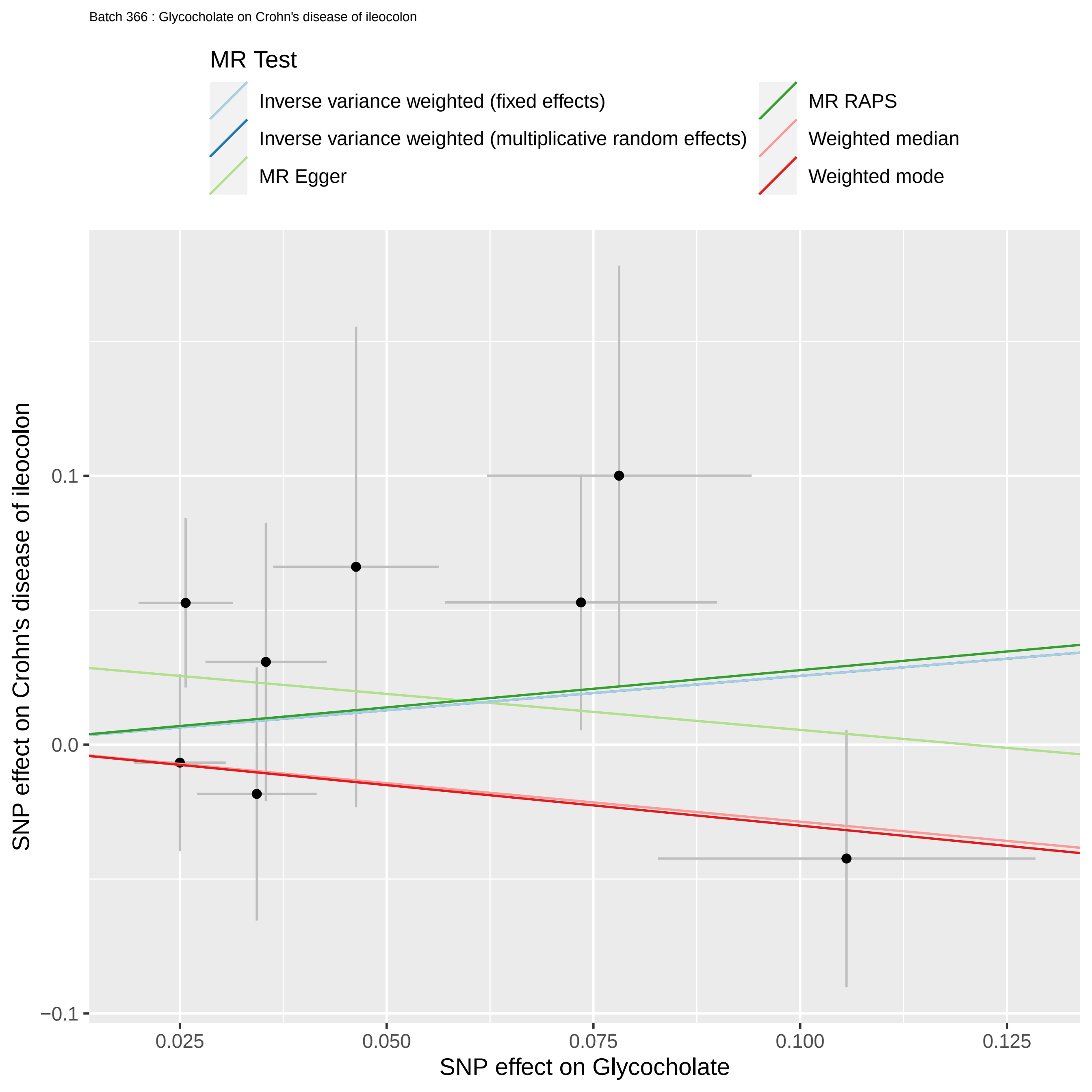

Batch 366 : Glycocholate on Crohn's disease of ileocolon
MR Test
Inverse variance weighted (fixed effects)
Inverse variance weighted (multiplicative random effects)
MR Egger
MR RAPS
Weighted median
Weighted mode
SNP effect on Crohn's disease of ileocolon
0.1
0.0
−0.1
0.025
0.050
0.075
0.100
0.125
SNP effect on Glycocholate

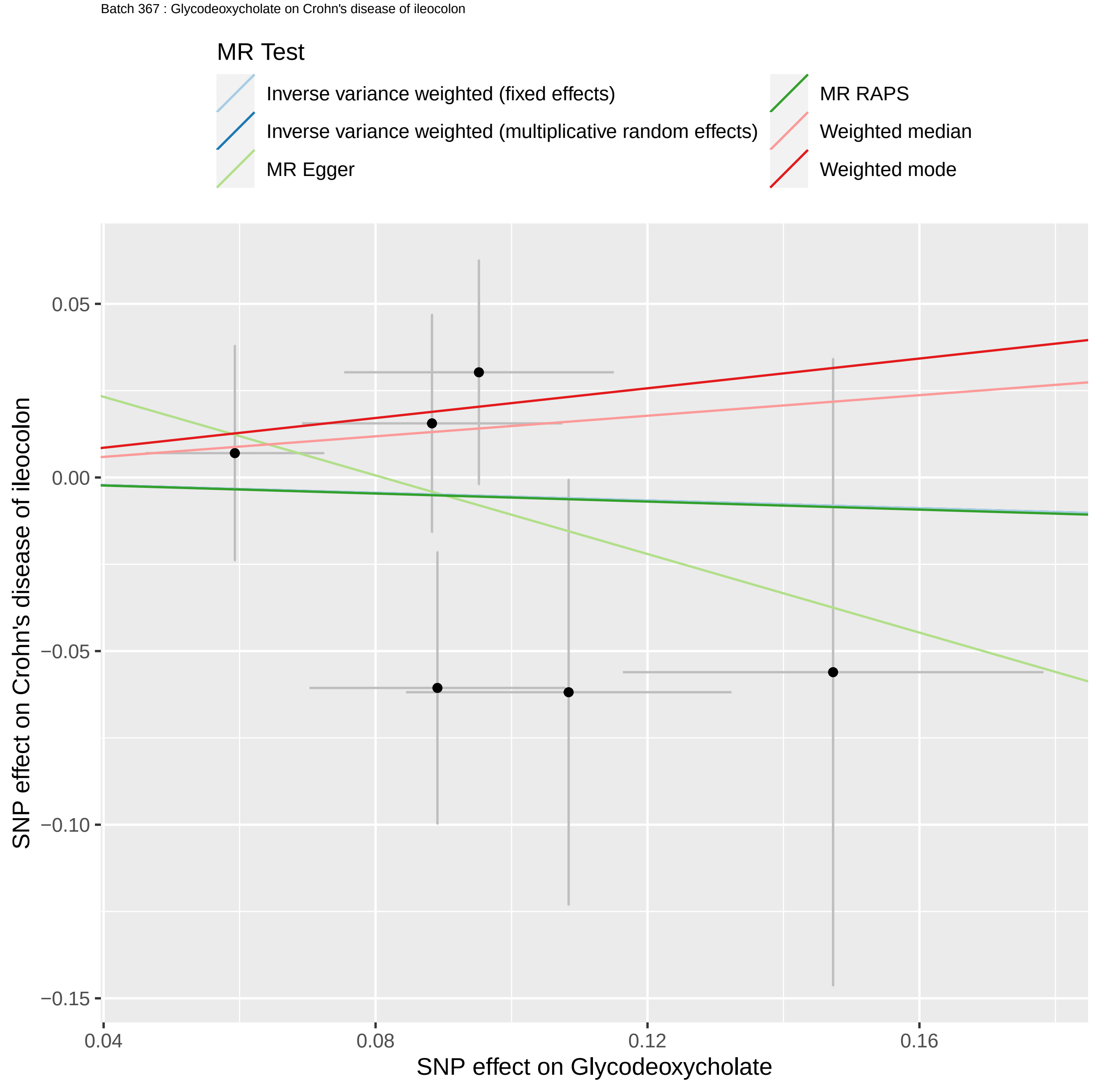

Batch 367 : Glycodeoxycholate on Crohn's disease of ileocolon
MR Test
Inverse variance weighted (fixed effects)
Inverse variance weighted (multiplicative random effects)
MR Egger
MR RAPS
Weighted median
Weighted mode
0.05
0.00
−0.05
−0.10
−0.15
0.04
0.08
0.12
0.16
SNP effect on Glycodeoxycholate
SNP effect on Crohn's disease of ileocolon

Batch 368 : Taurochenodeoxycholate on Crohn's disease of ileocolon

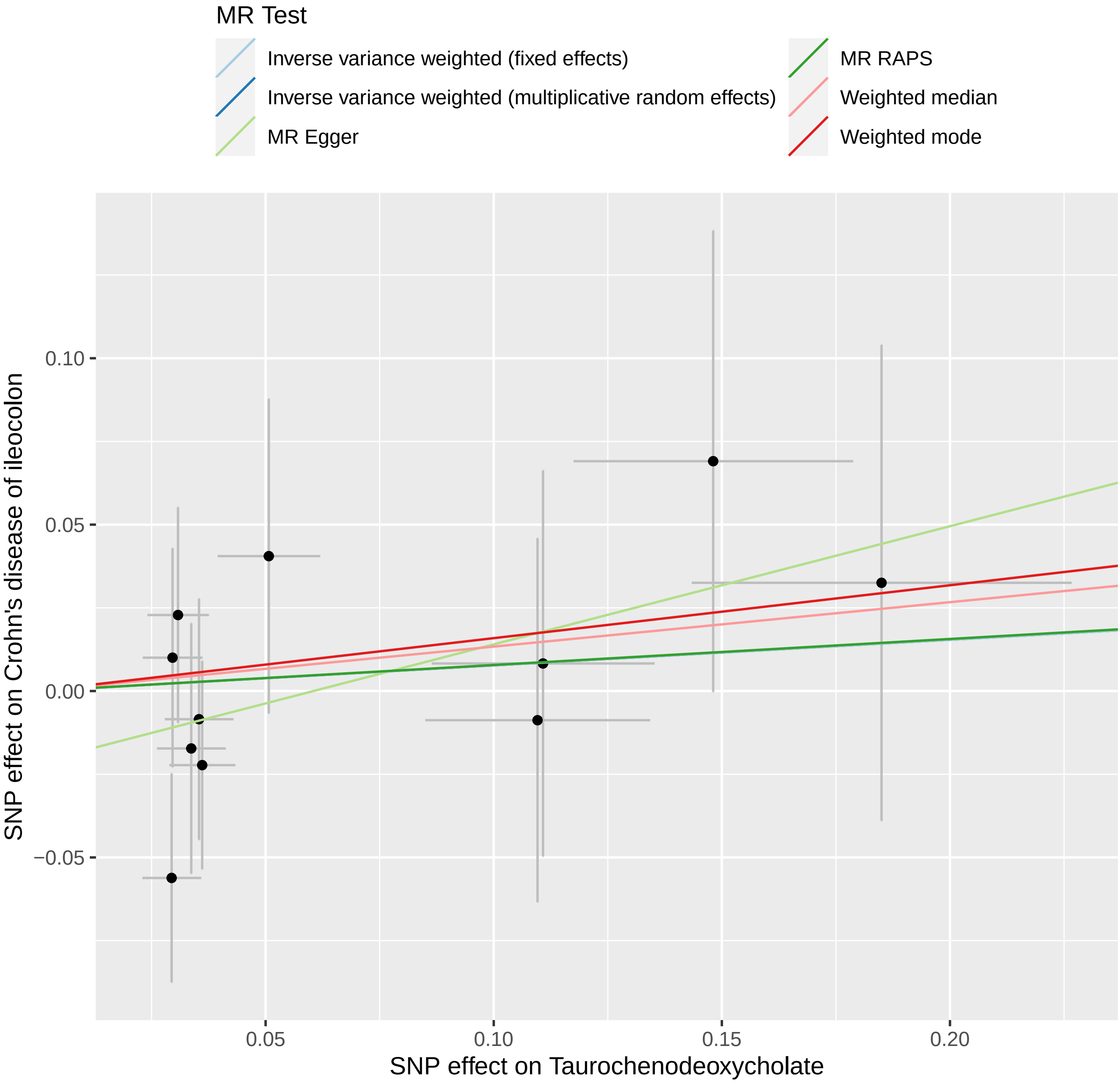

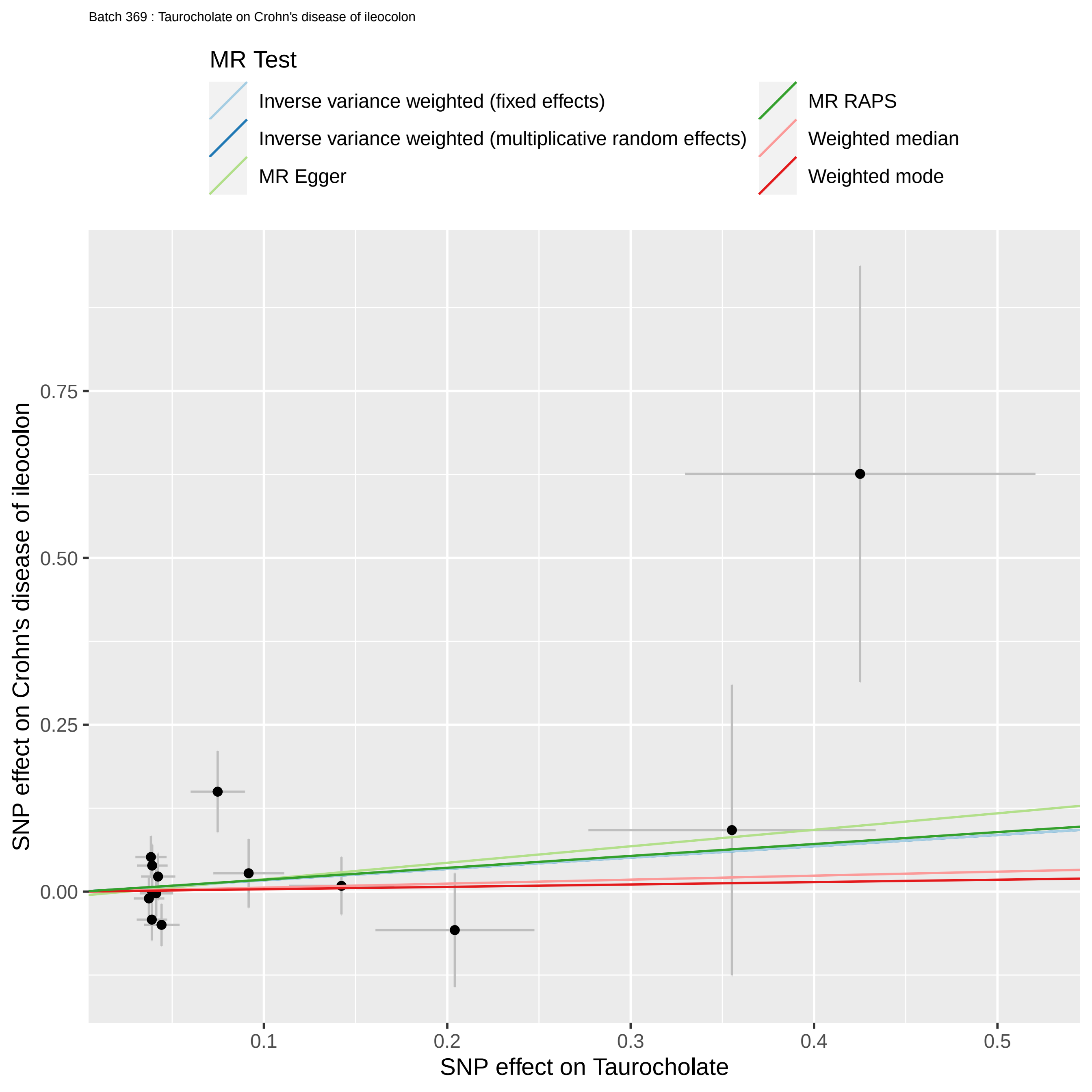

Batch 369 : Taurocholate on Crohn's disease of ileocolon
MR Test
Inverse variance weighted (fixed effects)
Inverse variance weighted (multiplicative random effects)
MR Egger
MR RAPS
Weighted median
Weighted mode
0.75
0.50
0.25
0.00
0.1
0.2
0.3
0.4
0.5
SNP effect on Crohn's disease of ileocolon
SNP effect on Taurocholate

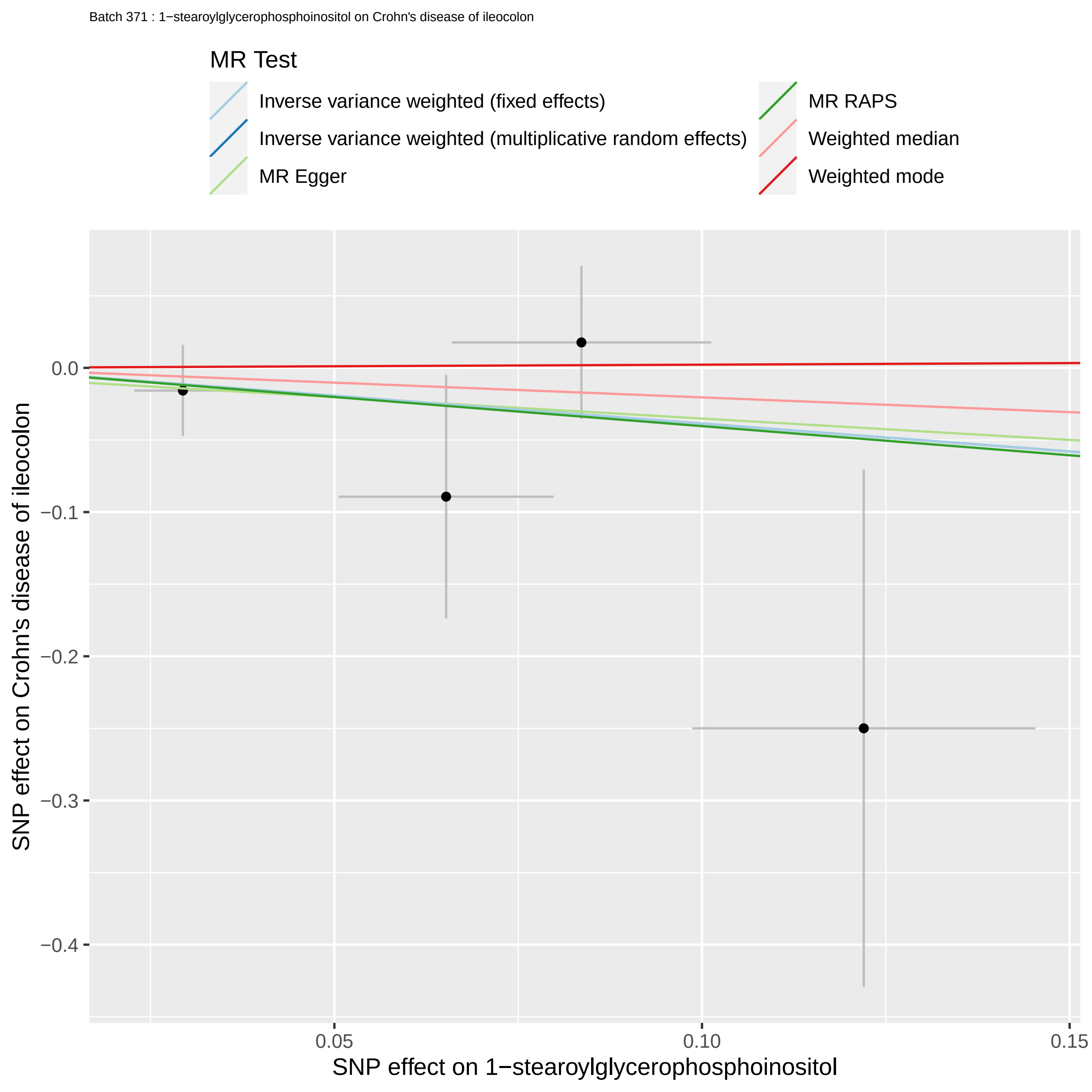

Batch 371 : 1−stearoylglycerophosphoinositol on Crohn's disease of ileocolon
MR Test
Inverse variance weighted (fixed effects)
Inverse variance weighted (multiplicative random effects)
MR Egger
MR RAPS
Weighted median
Weighted mode
0.0
−0.1
−0.2
−0.3
−0.4
0.05
0.10
0.15
SNP effect on Crohn's disease of ileocolon
SNP effect on 1−stearoylglycerophosphoinositol

Batch 372 : Myo−inositol on Crohn's disease of ileocolon

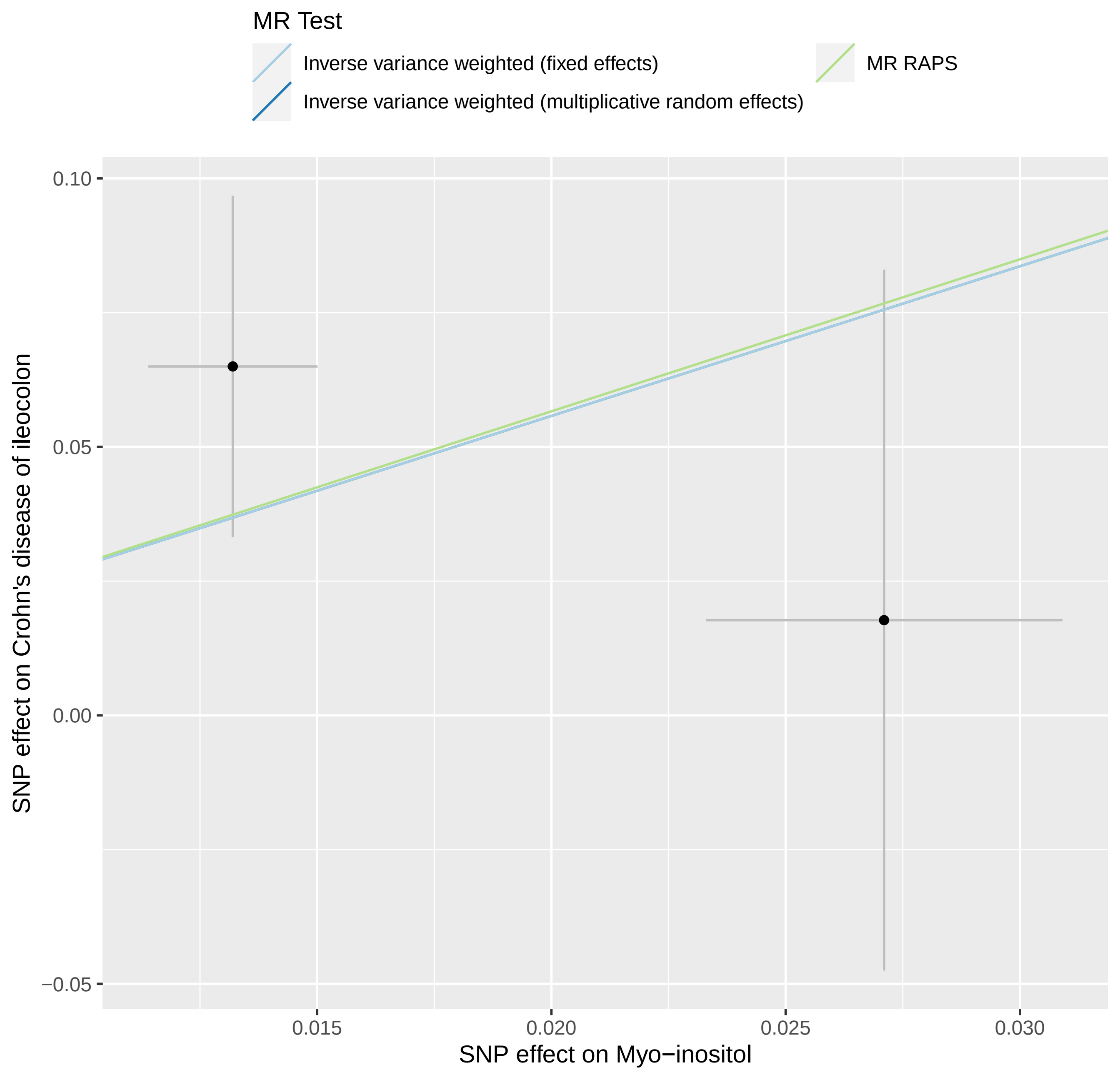

Batch 374 : 1,5−anhydroglucitol (1,5−AG) on Crohn's disease of ileocolon

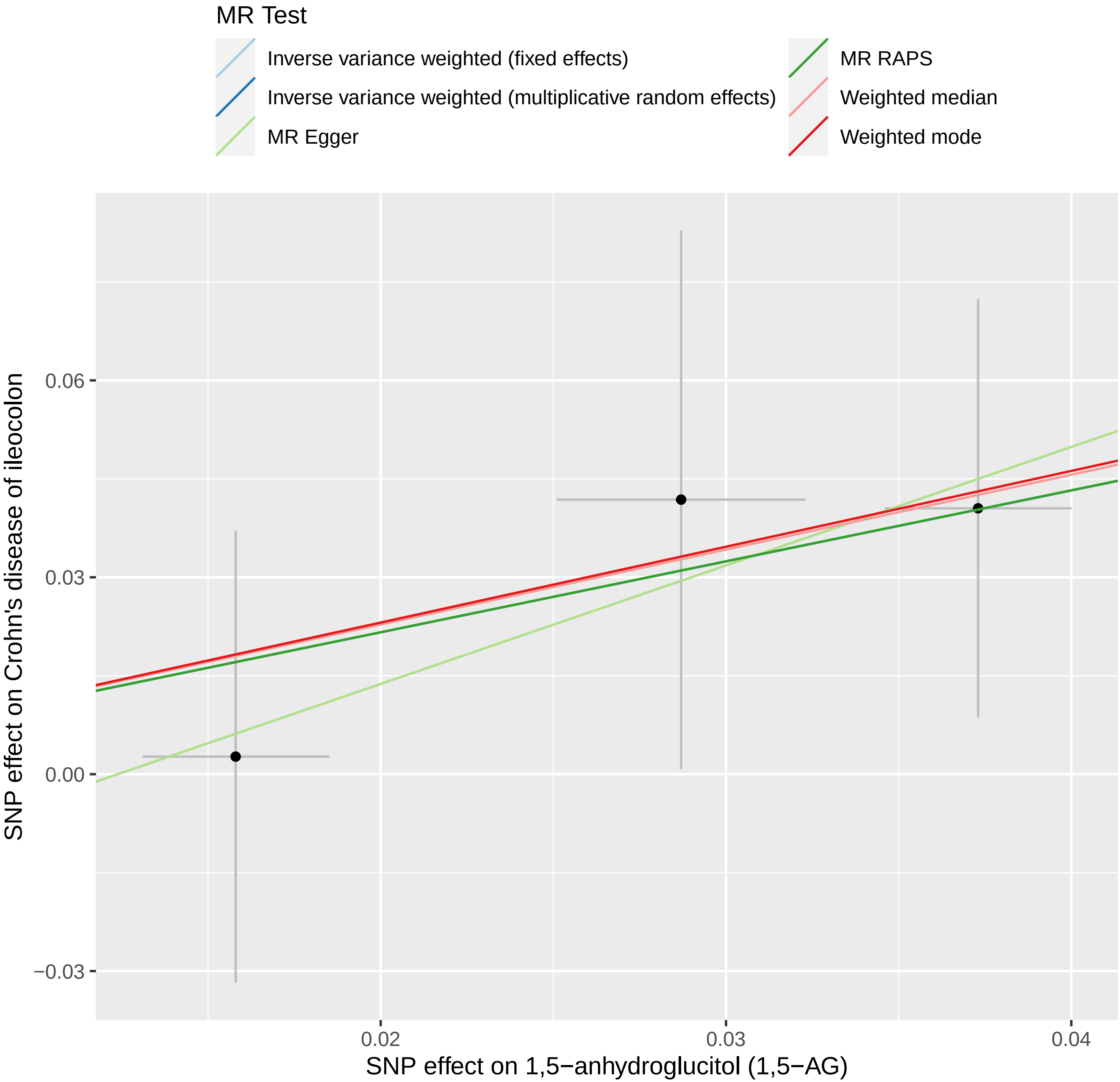

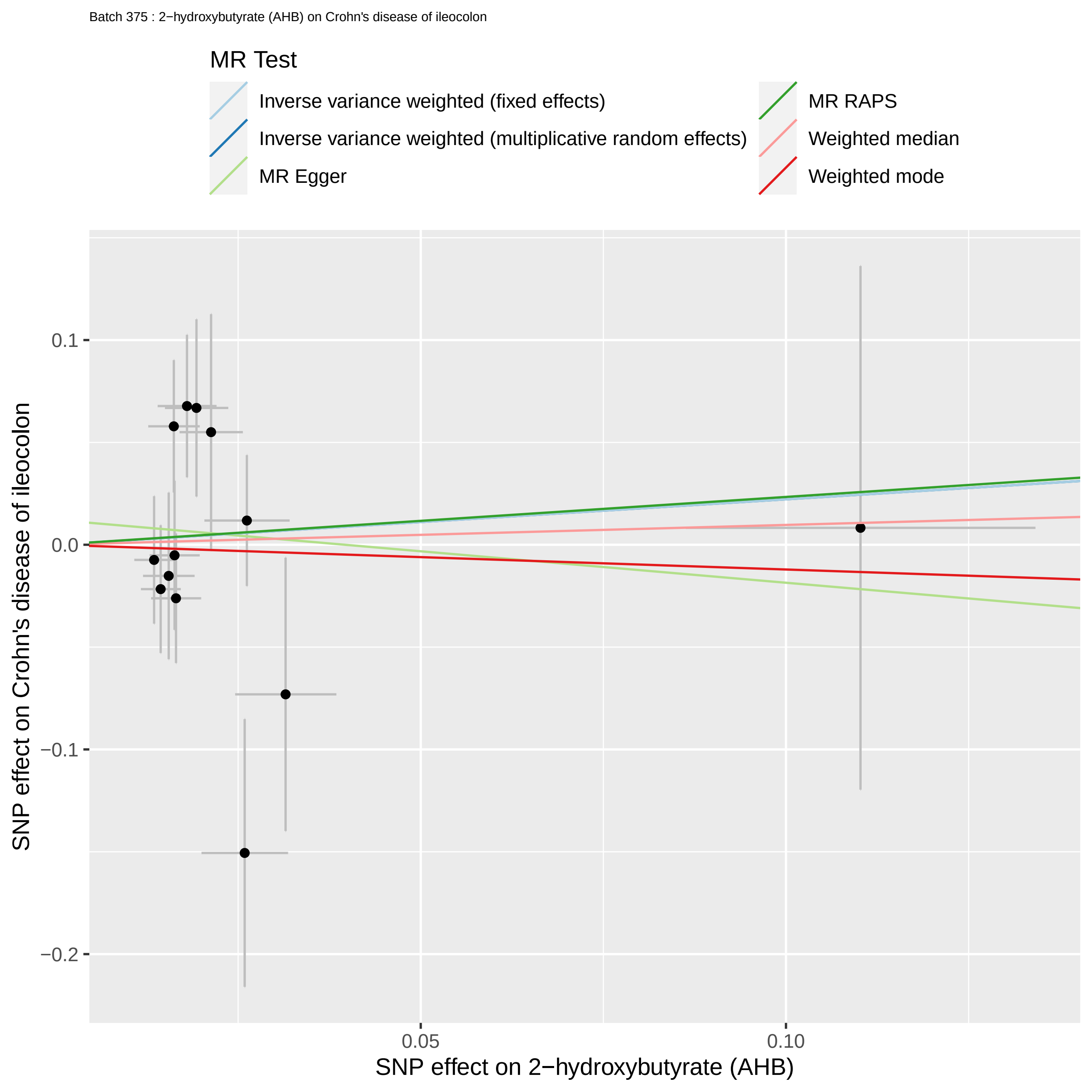
Batch 375 : 2−hydroxybutyrate (AHB) on Crohn's disease of ileocolon
MR Test
Inverse variance weighted (fixed effects)
Inverse variance weighted (multiplicative random effects)
MR Egger
MR RAPS
Weighted median
Weighted mode
0.1
0.0
−0.1
−0.2
0.05
0.10
SNP effect on 2−hydroxybutyrate (AHB)
SNP effect on Crohn's disease of ileocolon

Batch 378 : 1−palmitoylglycerol (1−monopalmitin) on Crohn's disease of ileocolon

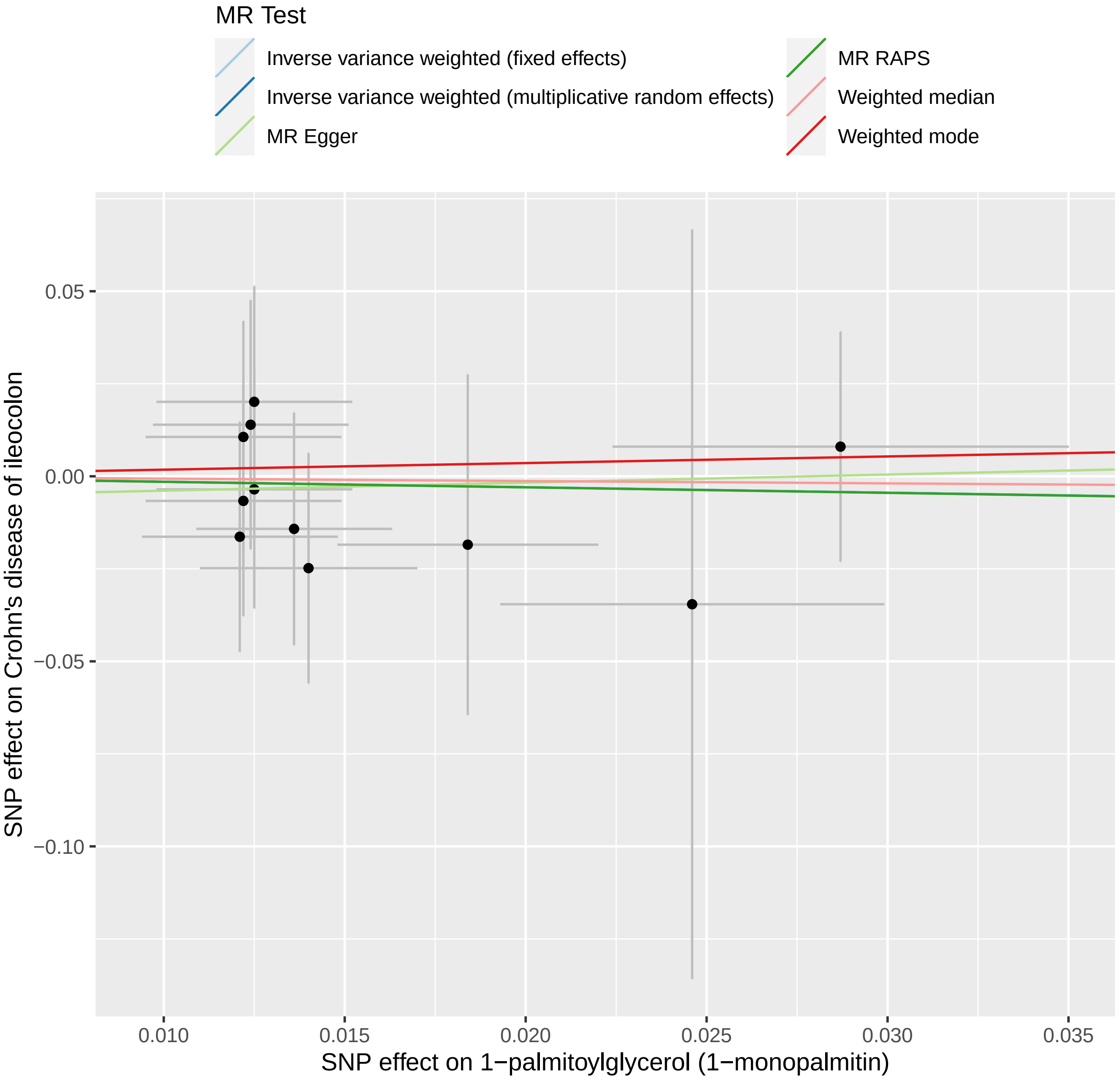

Batch 381 : 2−hydroxyisobutyrate on Crohn's disease of ileocolon

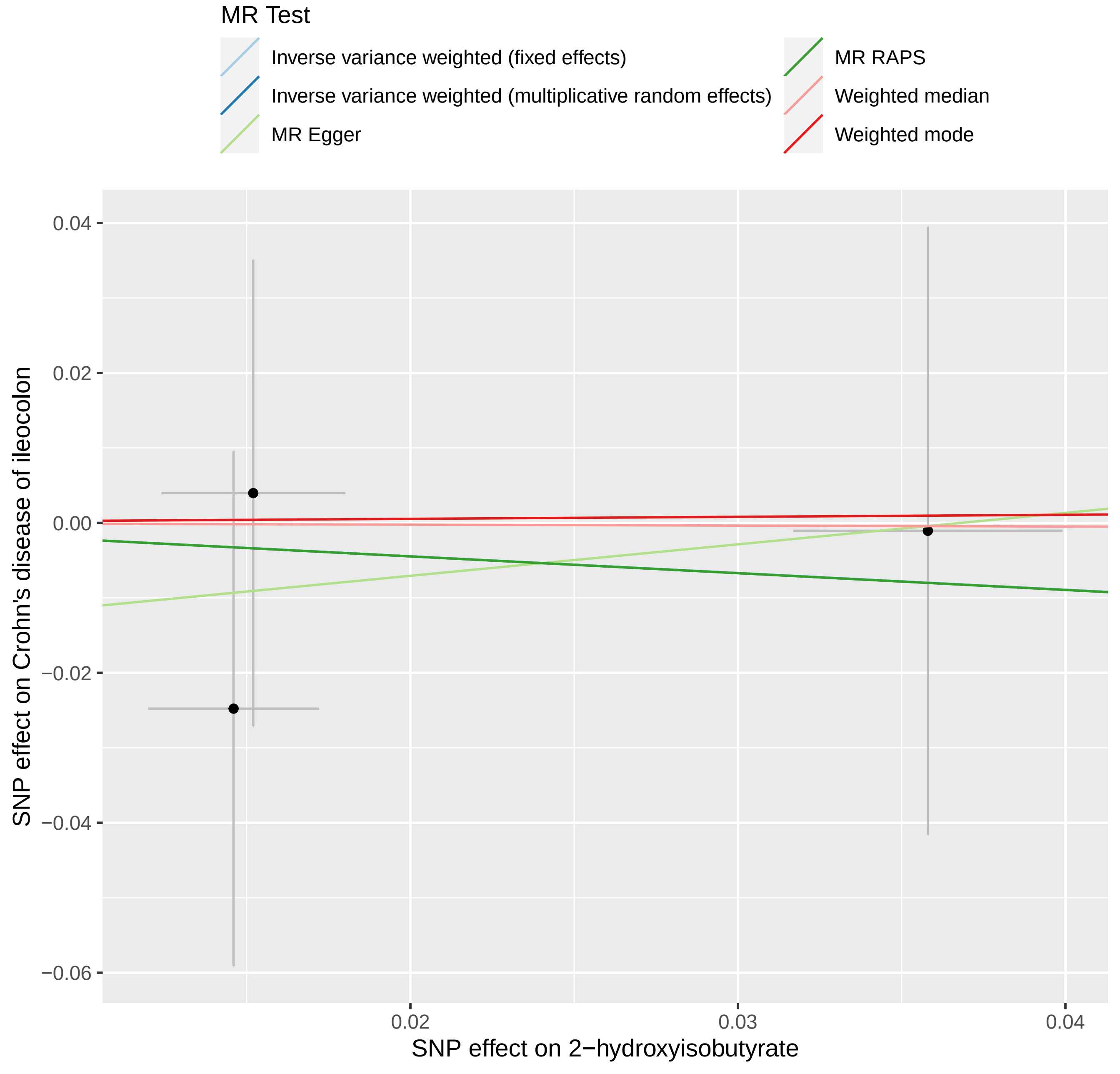

Batch 382 : 4−methyl−2−oxopentanoate on Crohn's disease of ileocolon

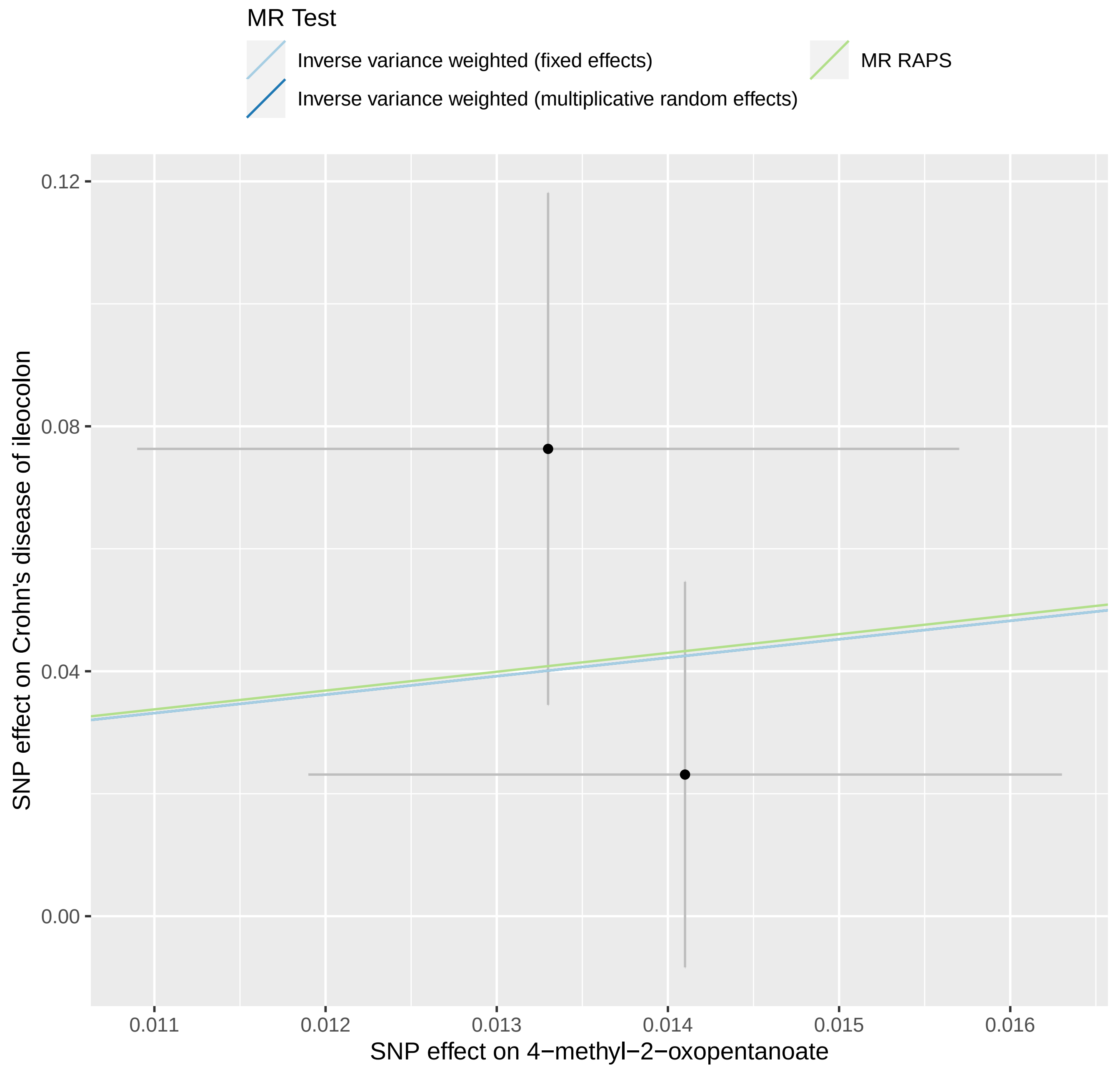

Batch 386 : Levulinate (4−oxovalerate) on Crohn's disease of ileocolon

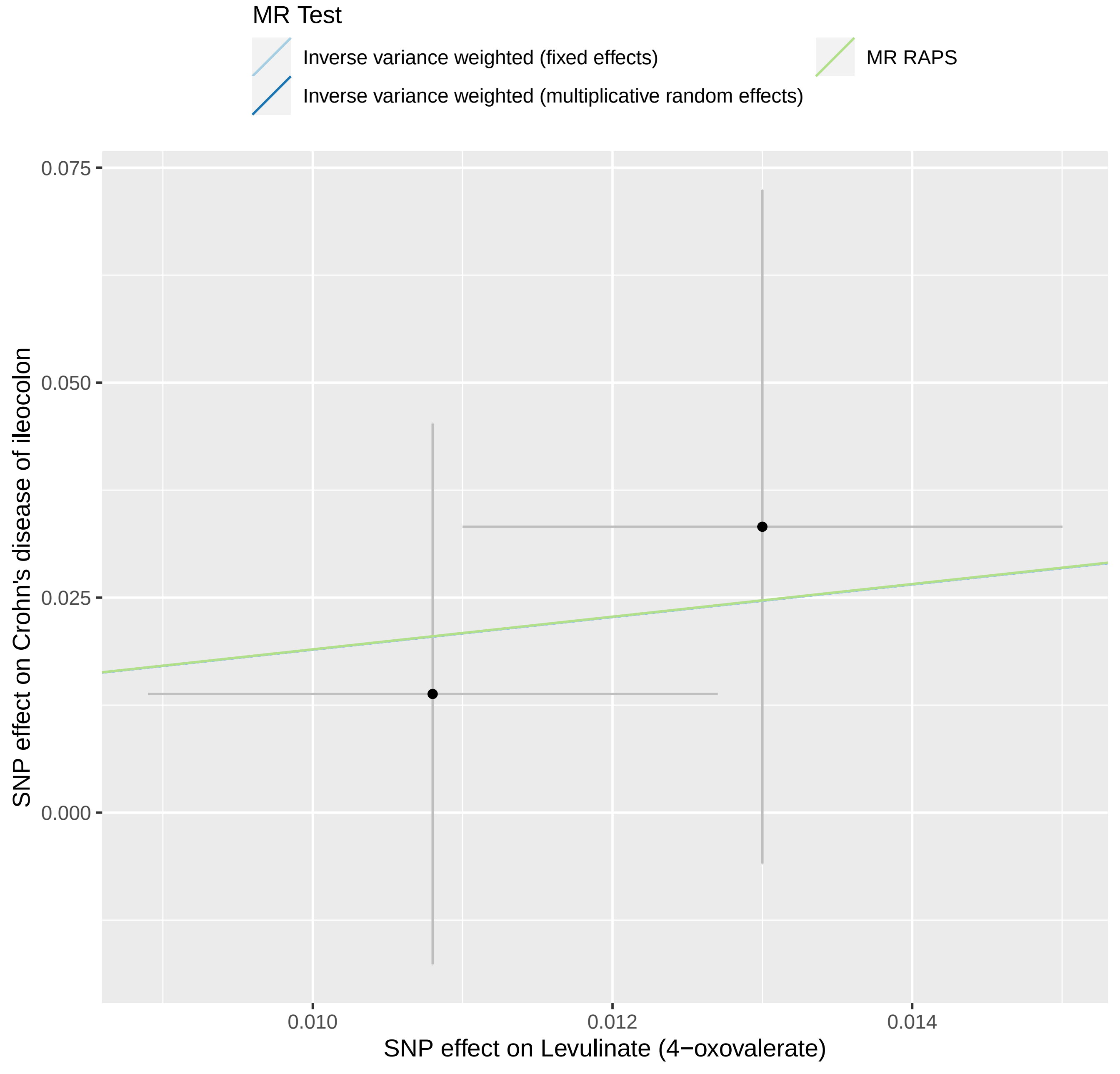

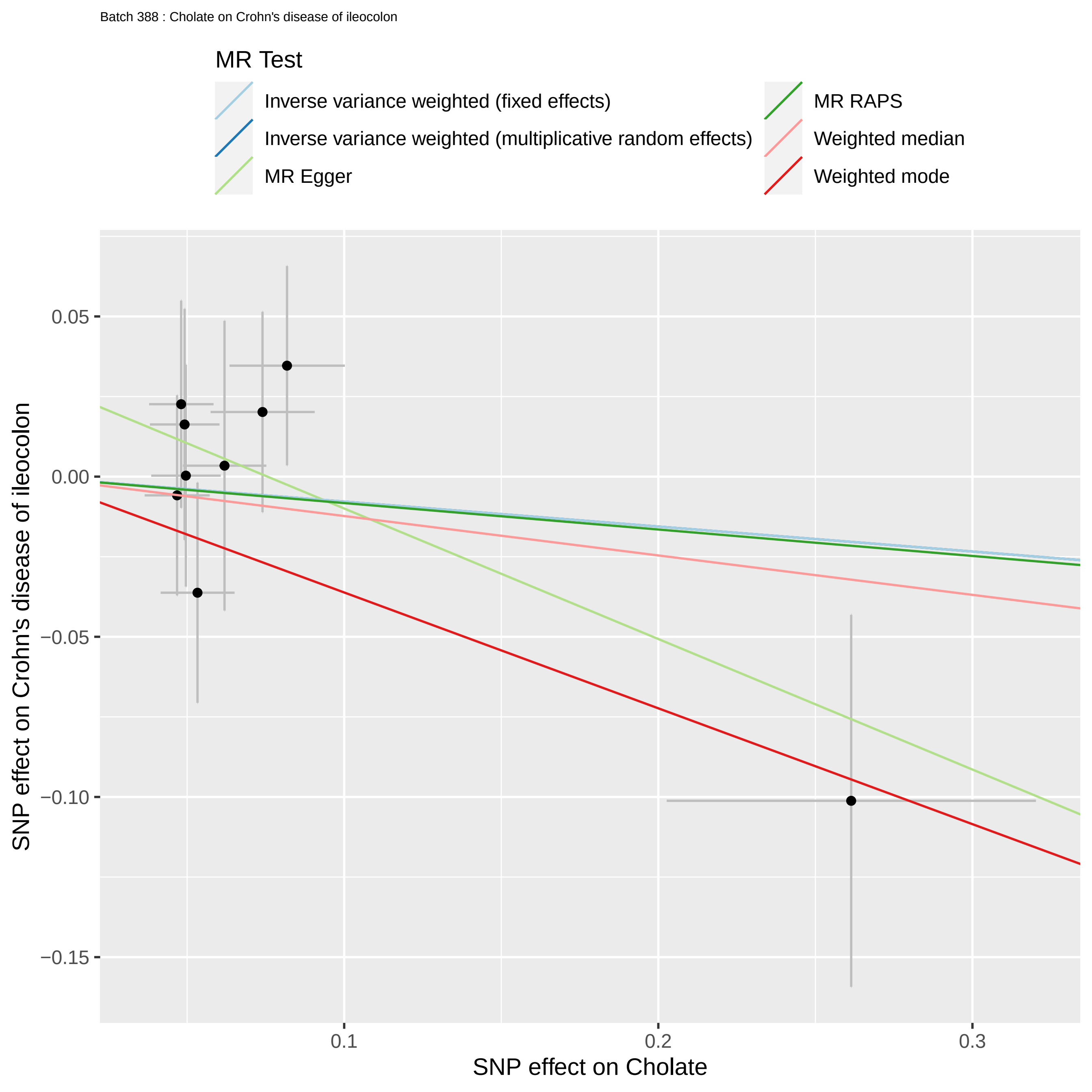

Batch 388 : Cholate on Crohn's disease of ileocolon
MR Test
Inverse variance weighted (fixed effects)
Inverse variance weighted (multiplicative random effects)
MR Egger
MR RAPS
Weighted median
Weighted mode
0.05
0.00
−0.05
−0.10
−0.15
0.1
0.2
0.3
SNP effect on Crohn's disease of ileocolon
SNP effect on Cholate

Batch 391 : Hyodeoxycholate on Crohn's disease of ileocolon

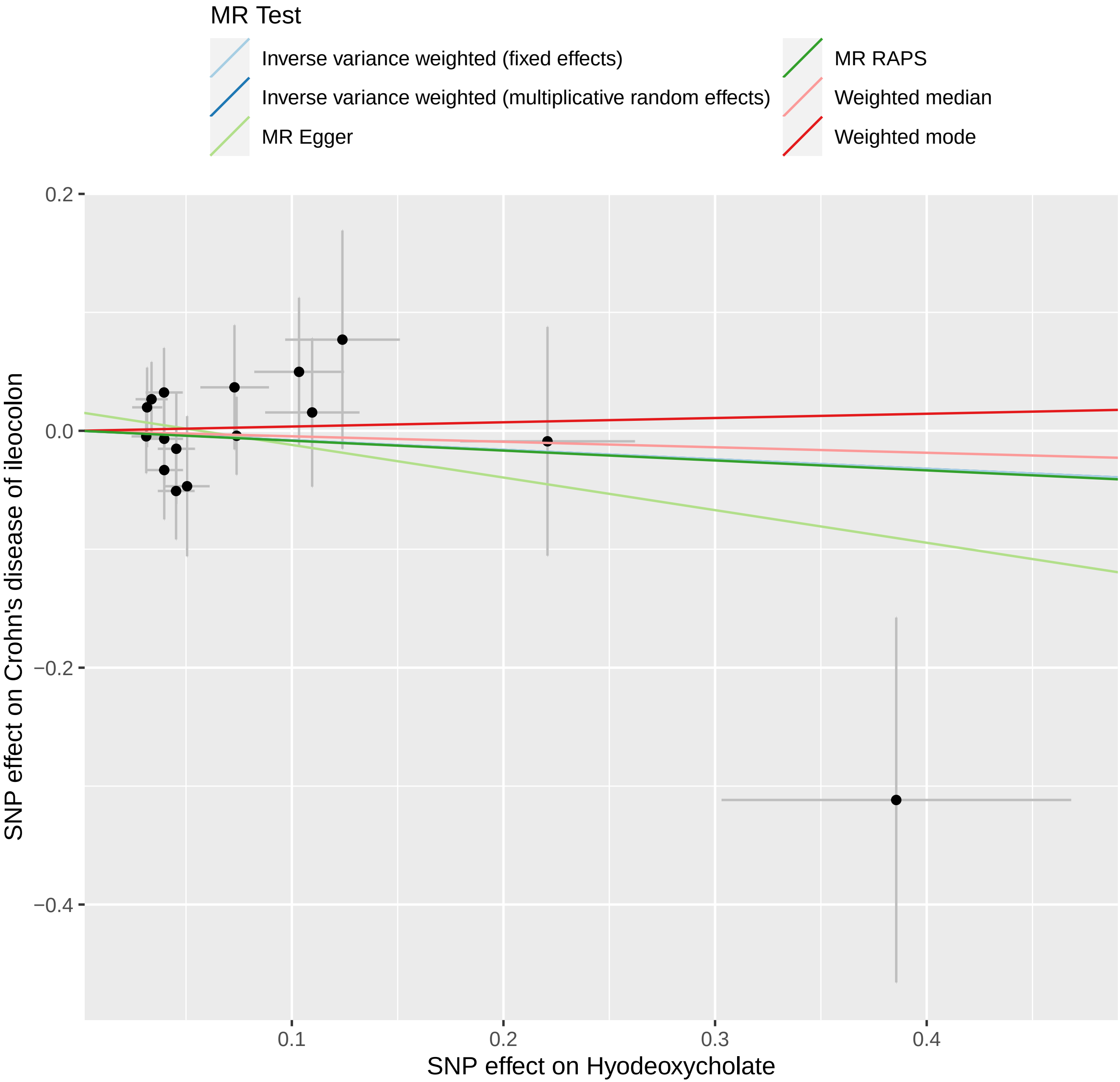

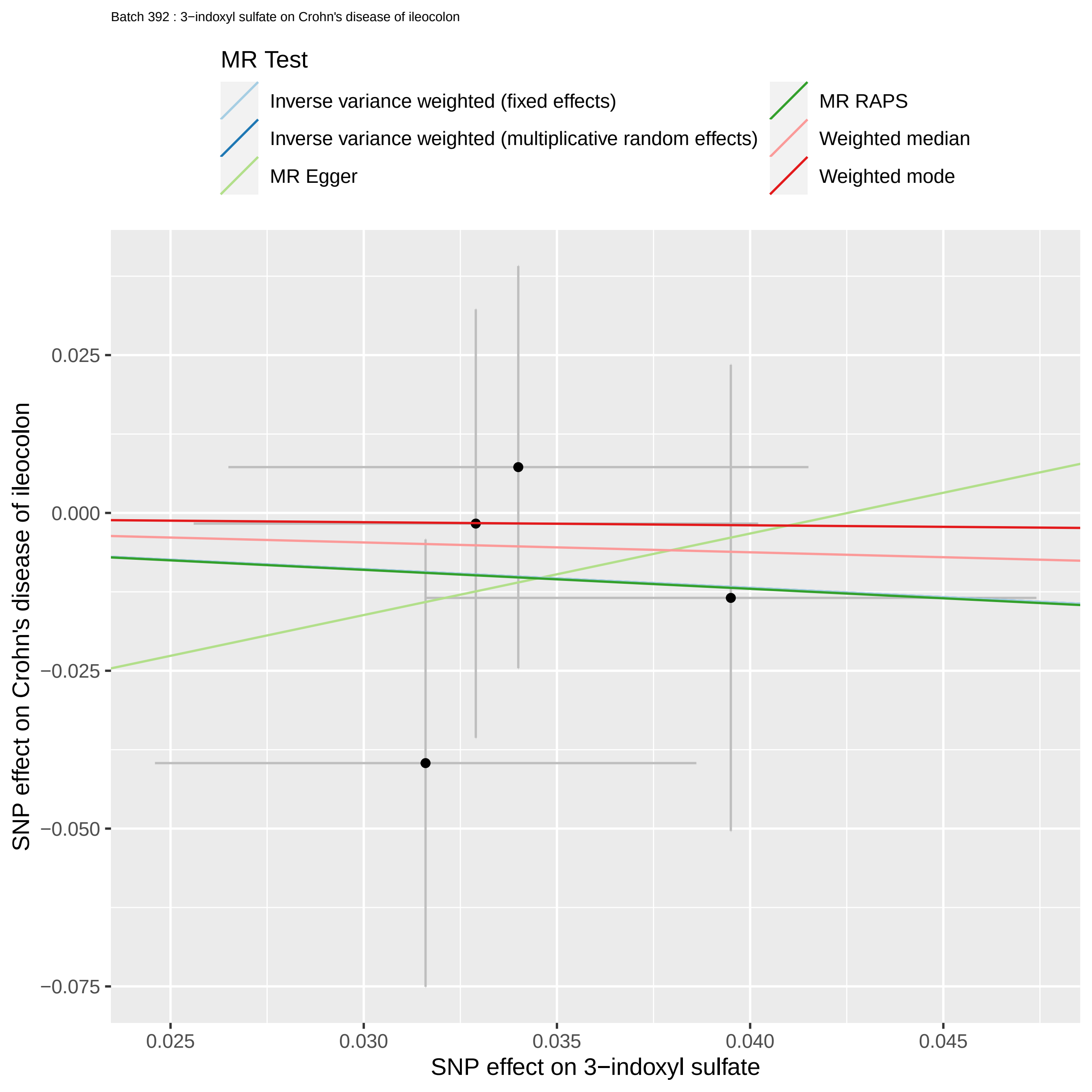
Batch 392 : 3−indoxyl sulfate on Crohn's disease of ileocolon
MR Test
Inverse variance weighted (fixed effects)
Inverse variance weighted (multiplicative random effects)
MR Egger
MR RAPS
Weighted median
Weighted mode
SNP effect on Crohn's disease of ileocolon
0.025
0.000
−0.025
−0.050
−0.075
0.025
0.030
0.035
0.040
0.045
SNP effect on 3−indoxyl sulfate

Batch 396 : Erythrose on Crohn's disease of ileocolon

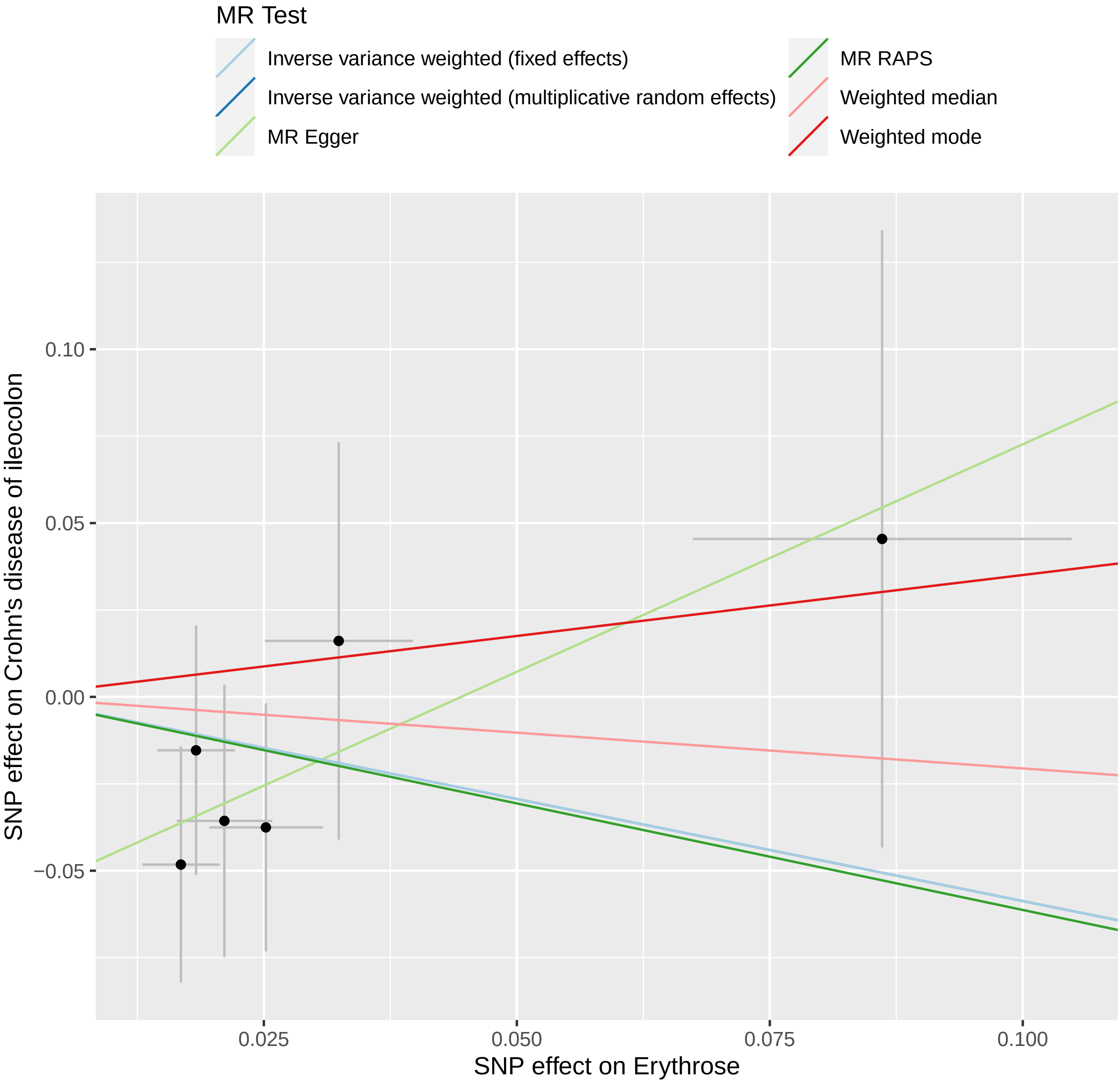

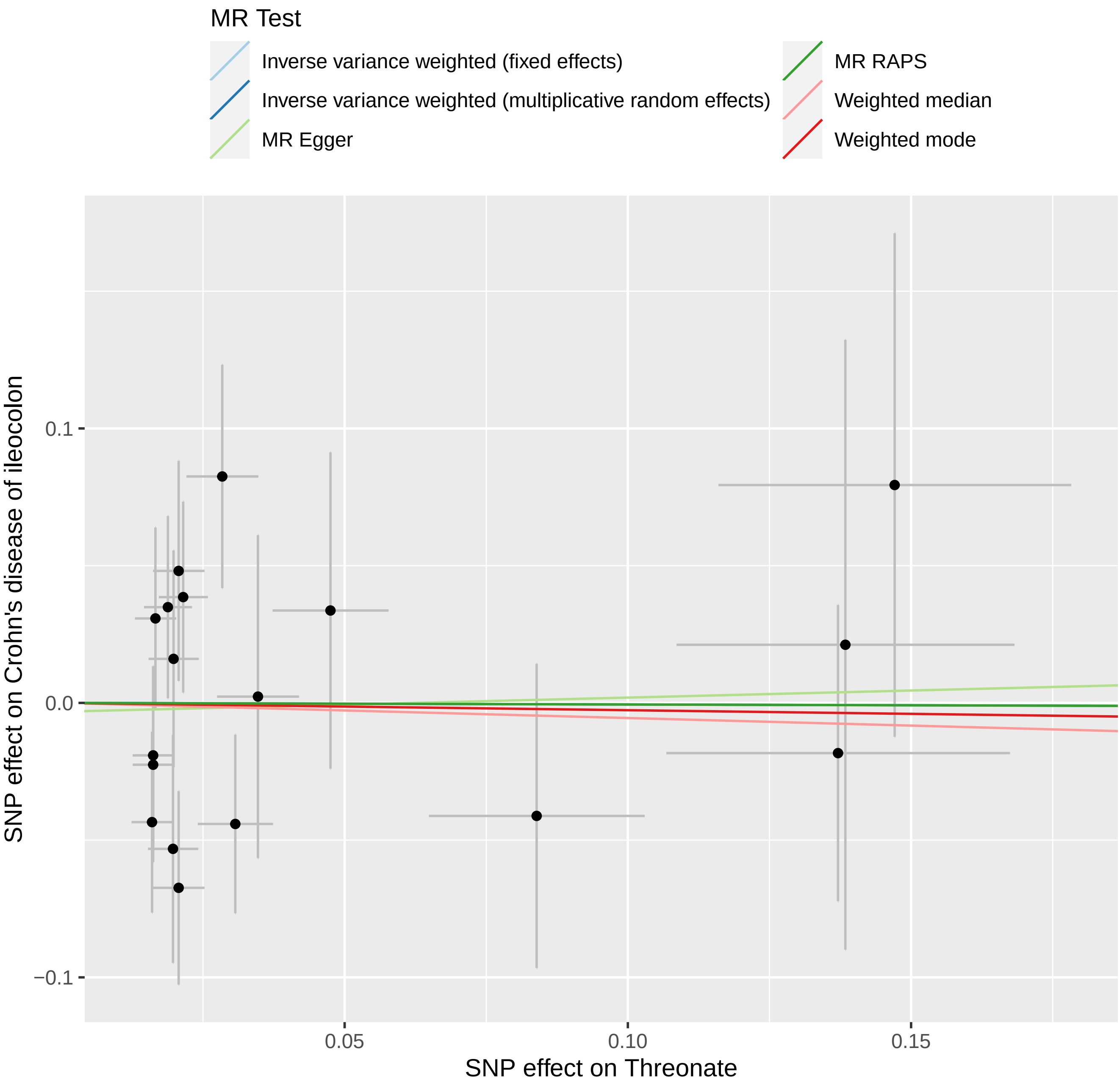
MR Test
Inverse variance weighted (fixed effects)
Inverse variance weighted (multiplicative random effects)
MR Egger
MR RAPS
Weighted median
Weighted mode
0.1
0.0
−0.1
0.05
0.10
0.15
SNP effect on Crohn's disease of ileocolon
SNP effect on Threonate

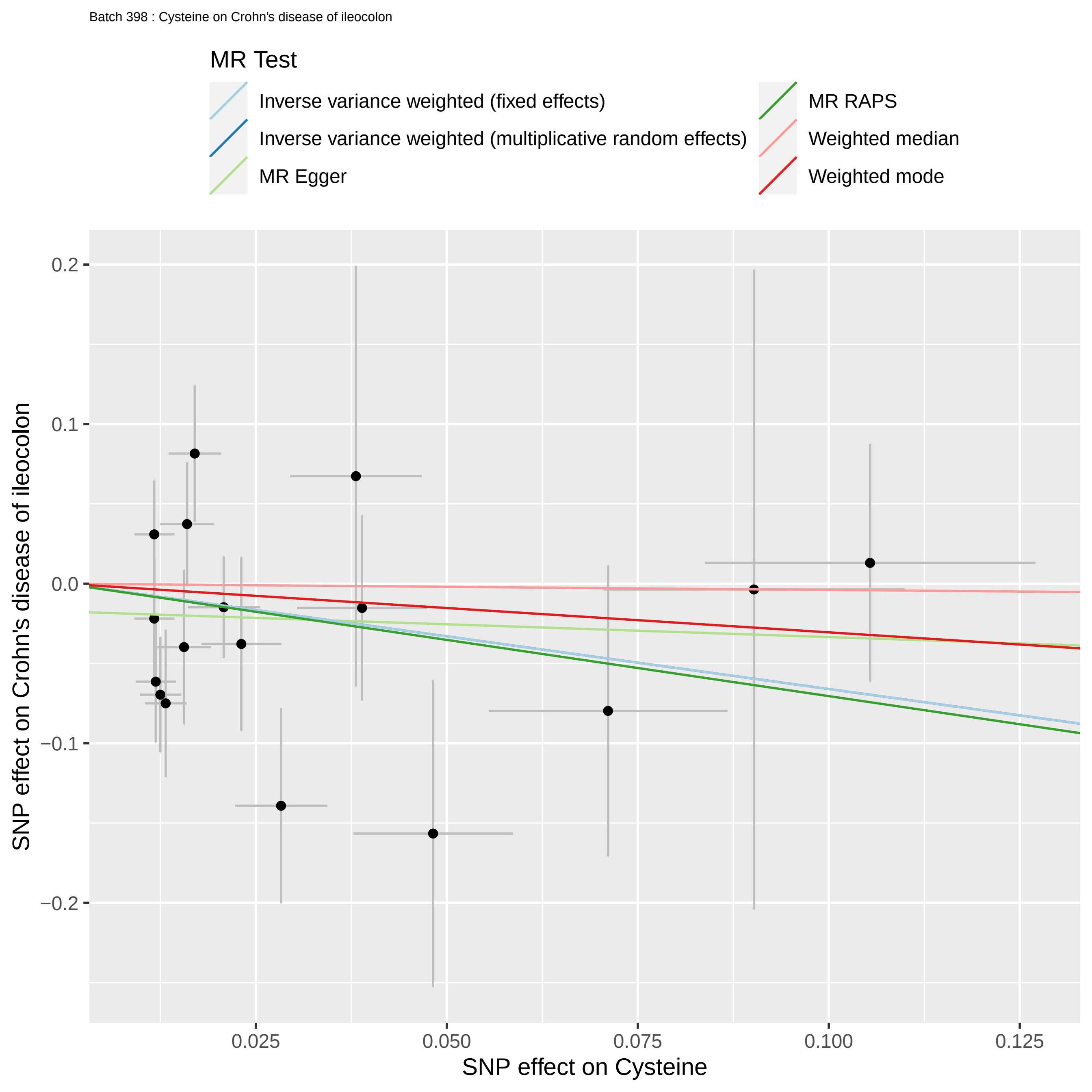

Batch 398 : Cysteine on Crohn's disease of ileocolon
MR Test
Inverse variance weighted (fixed effects)
Inverse variance weighted (multiplicative random effects)
MR Egger
MR RAPS
Weighted median
Weighted mode
SNP effect on Crohn's disease of ileocolon
0.2
0.1
0.0
−0.1
−0.2
0.025
0.050
0.075
0.100
0.125
SNP effect on Cysteine

Batch 399 : Pyroglutamylglycine on Crohn's disease of ileocolon

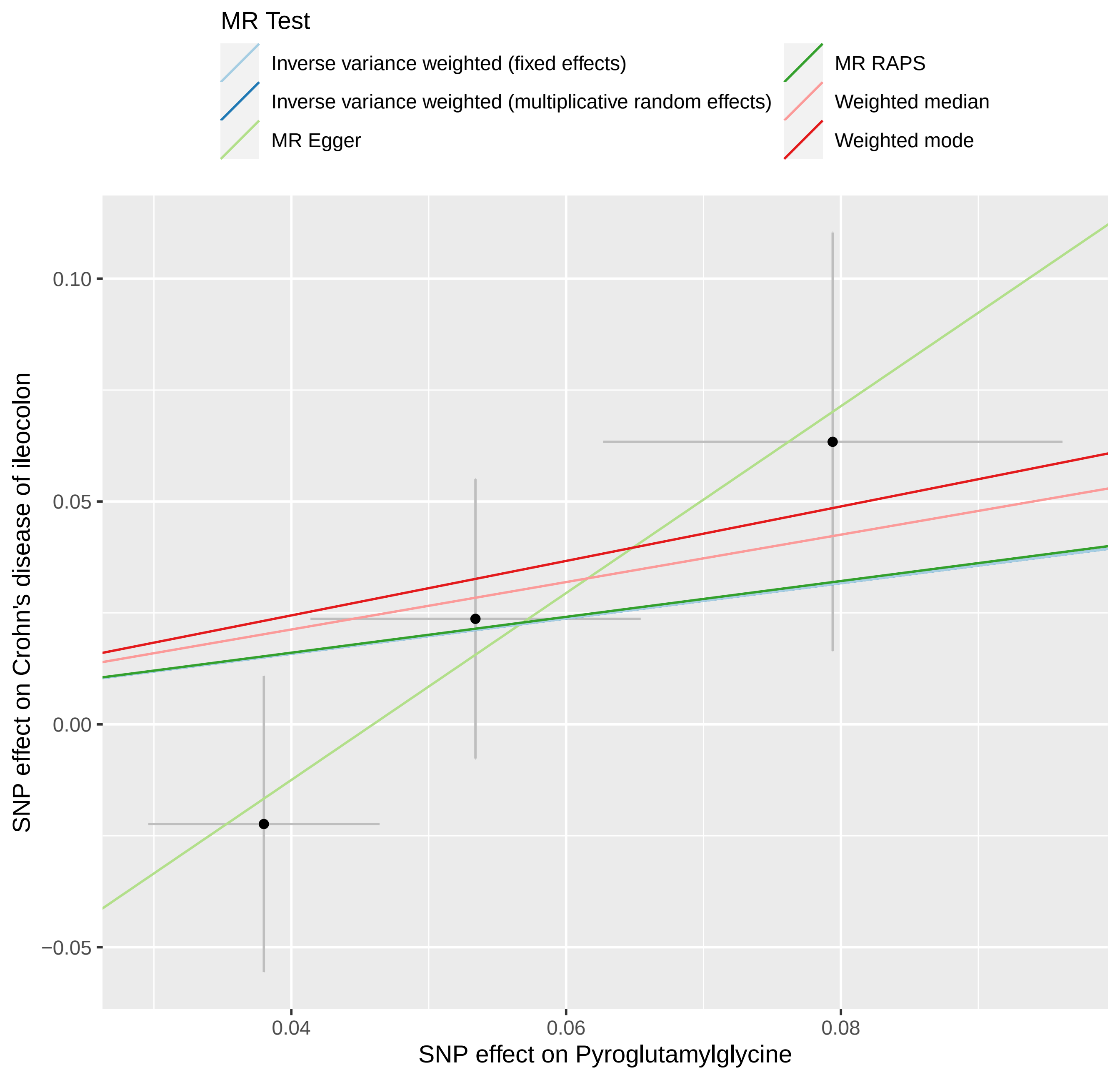

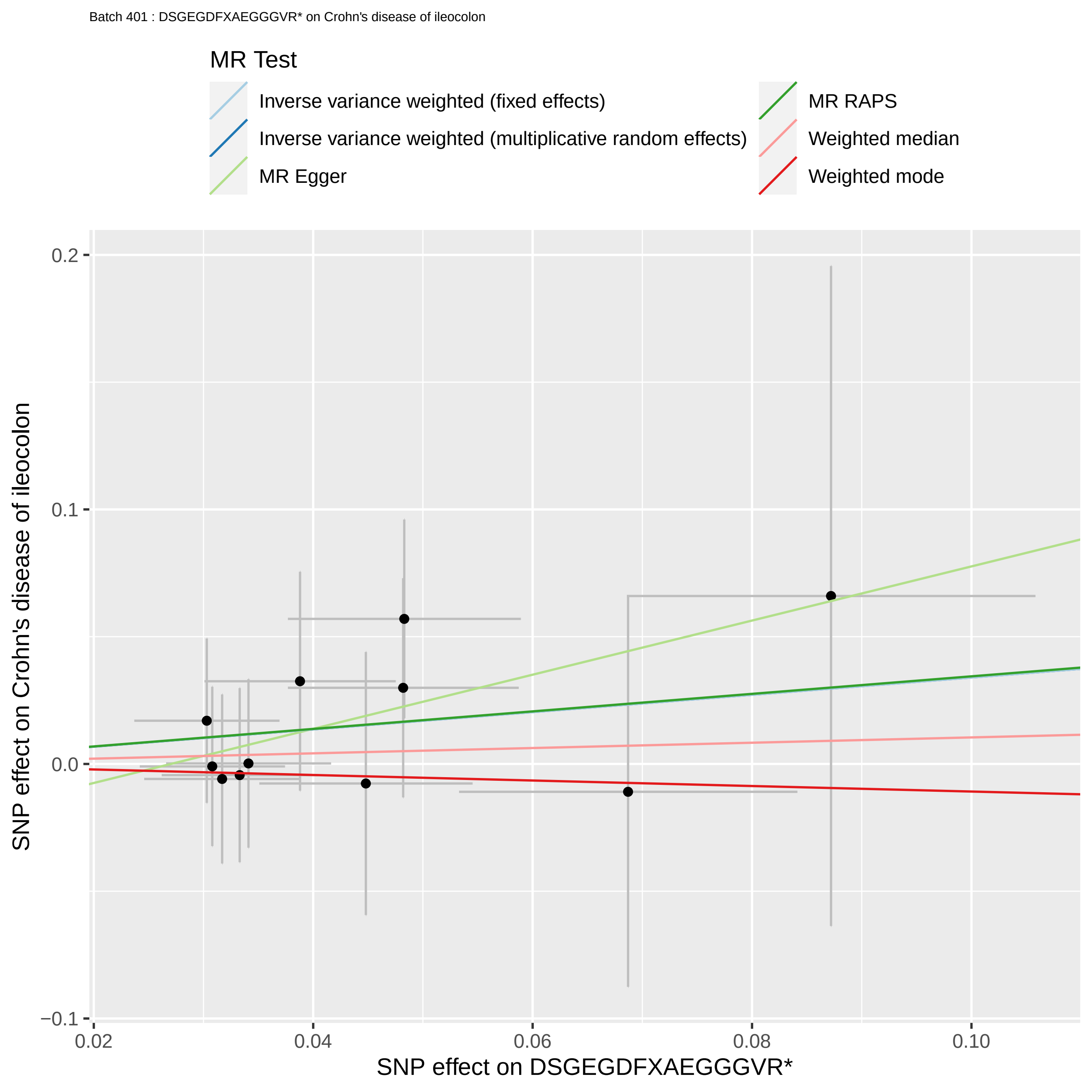

Batch 401 : DSGEGDFXAEGGGVR* on Crohn's disease of ileocolon
MR Test
Inverse variance weighted (fixed effects)
Inverse variance weighted (multiplicative random effects)
MR Egger
MR RAPS
Weighted median
Weighted mode
SNP effect on Crohn's disease of ileocolon
0.2
0.1
0.0
−0.1
0.02
0.04
0.06
0.08
0.10
SNP effect on DSGEGDFXAEGGGVR*

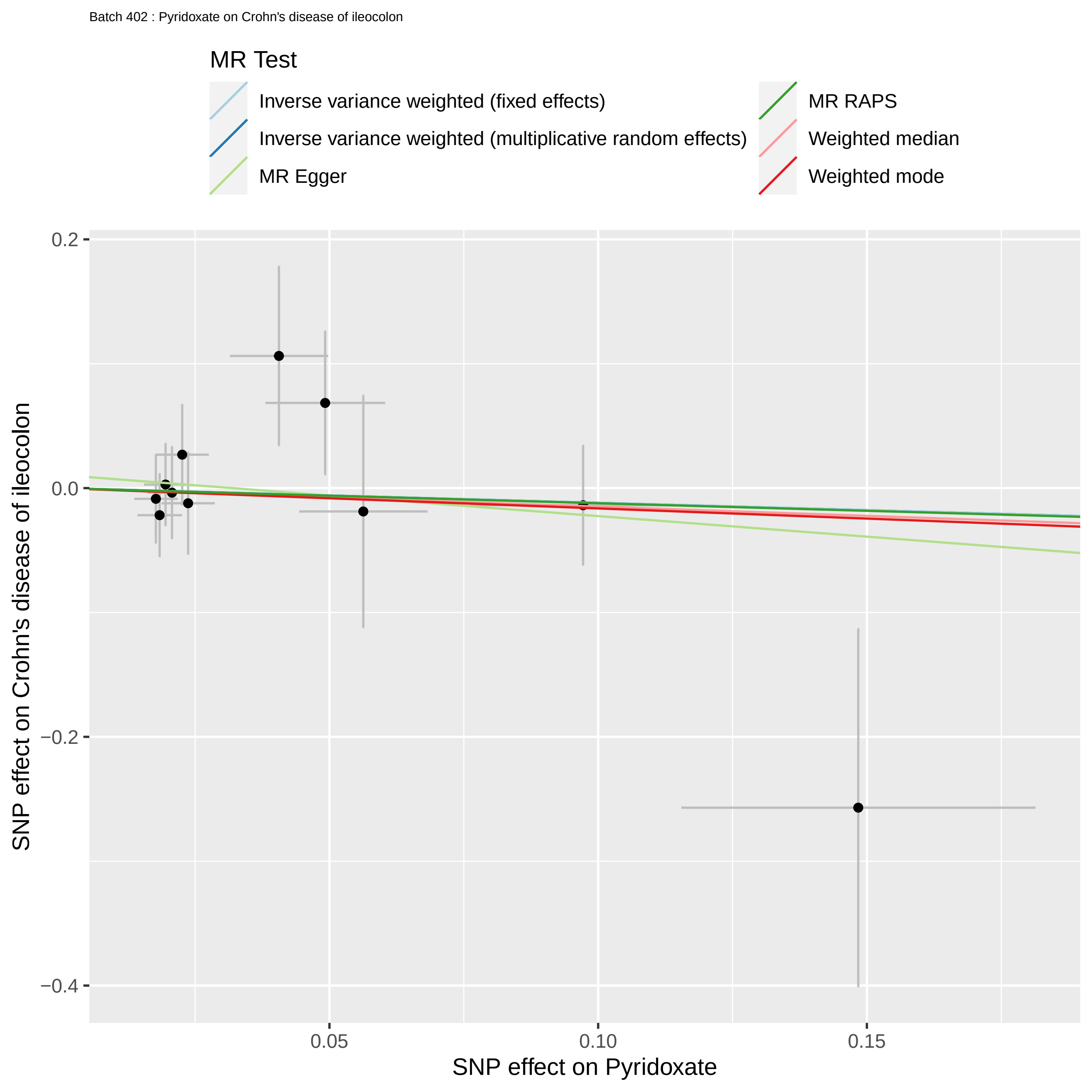
Batch 402 : Pyridoxate on Crohn's disease of ileocolon
MR Test
Inverse variance weighted (fixed effects)
Inverse variance weighted (multiplicative random effects)
MR Egger
MR RAPS
Weighted median
Weighted mode
0.2
0.0
−0.2
−0.4
0.05
0.10
0.15
SNP effect on Pyridoxate
SNP effect on Crohn's disease of ileocolon

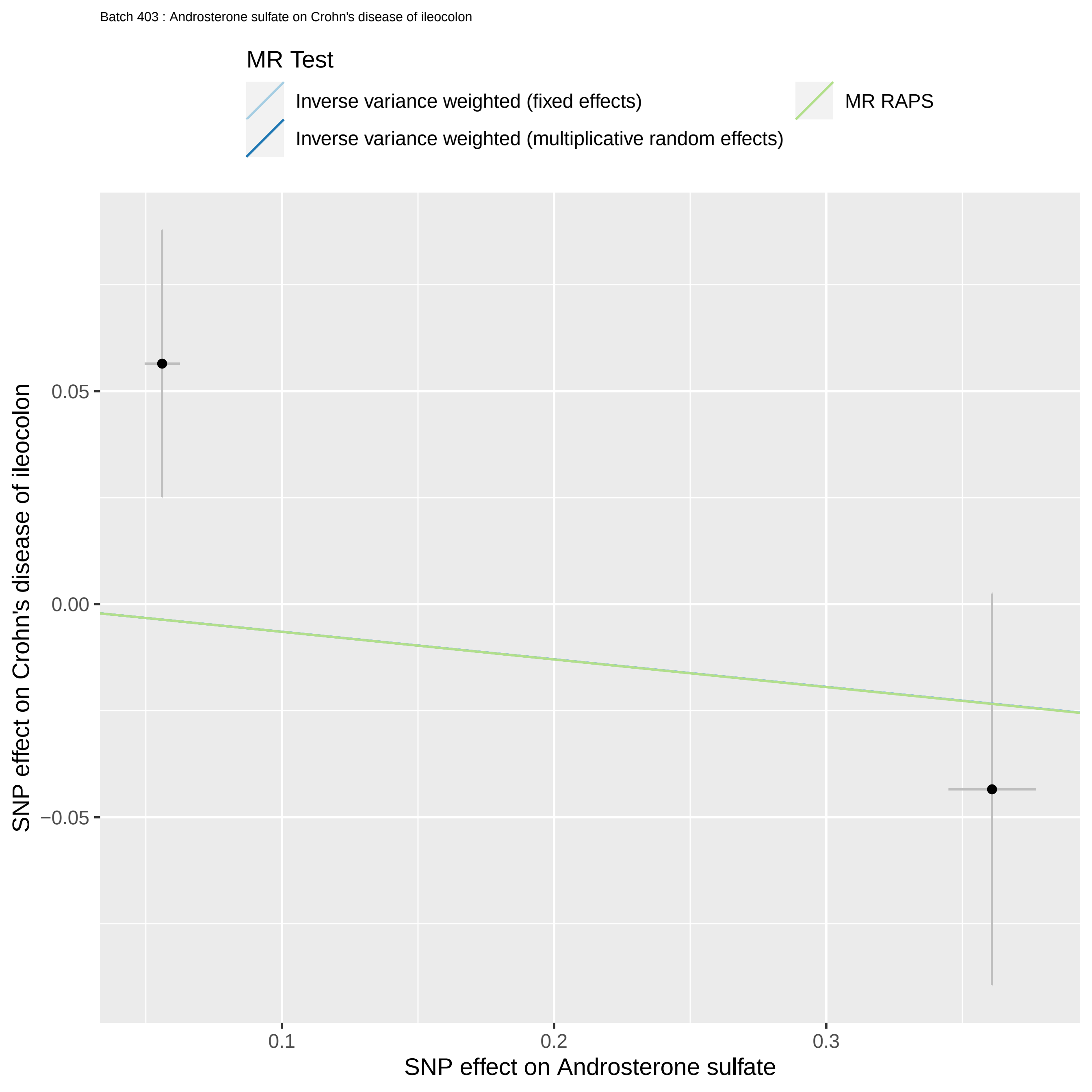

Batch 403 : Androsterone sulfate on Crohn's disease of ileocolon
MR Test
Inverse variance weighted (fixed effects)
Inverse variance weighted (multiplicative random effects)
MR RAPS
0.05
0.00
−0.05
0.1
0.2
0.3
SNP effect on Androsterone sulfate
SNP effect on Crohn's disease of ileocolon

Batch 408 : Trans−4−hydroxyproline on Crohn's disease of ileocolon

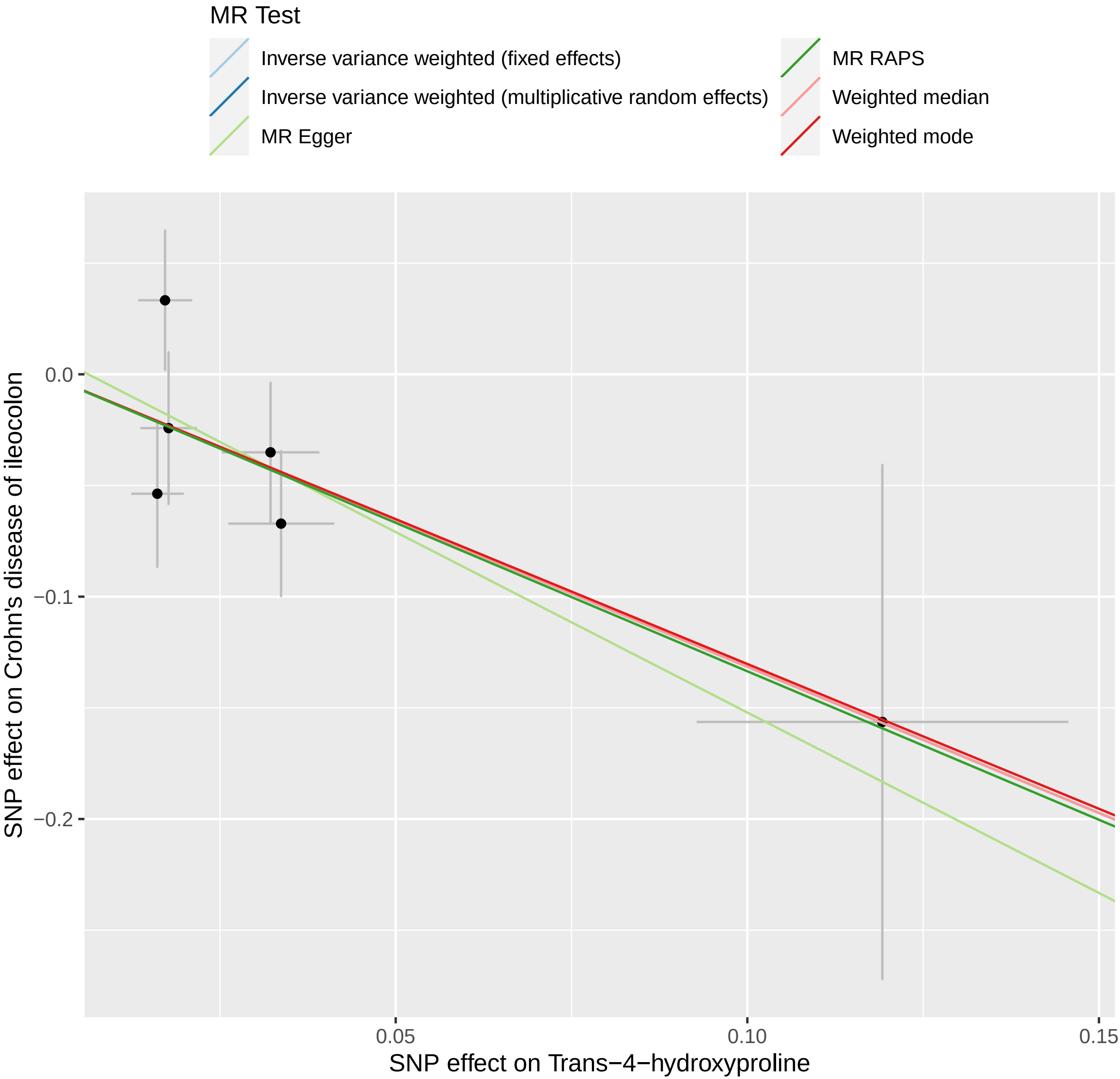

Batch 410 : Hexanoylcarnitine on Crohn's disease of ileocolon

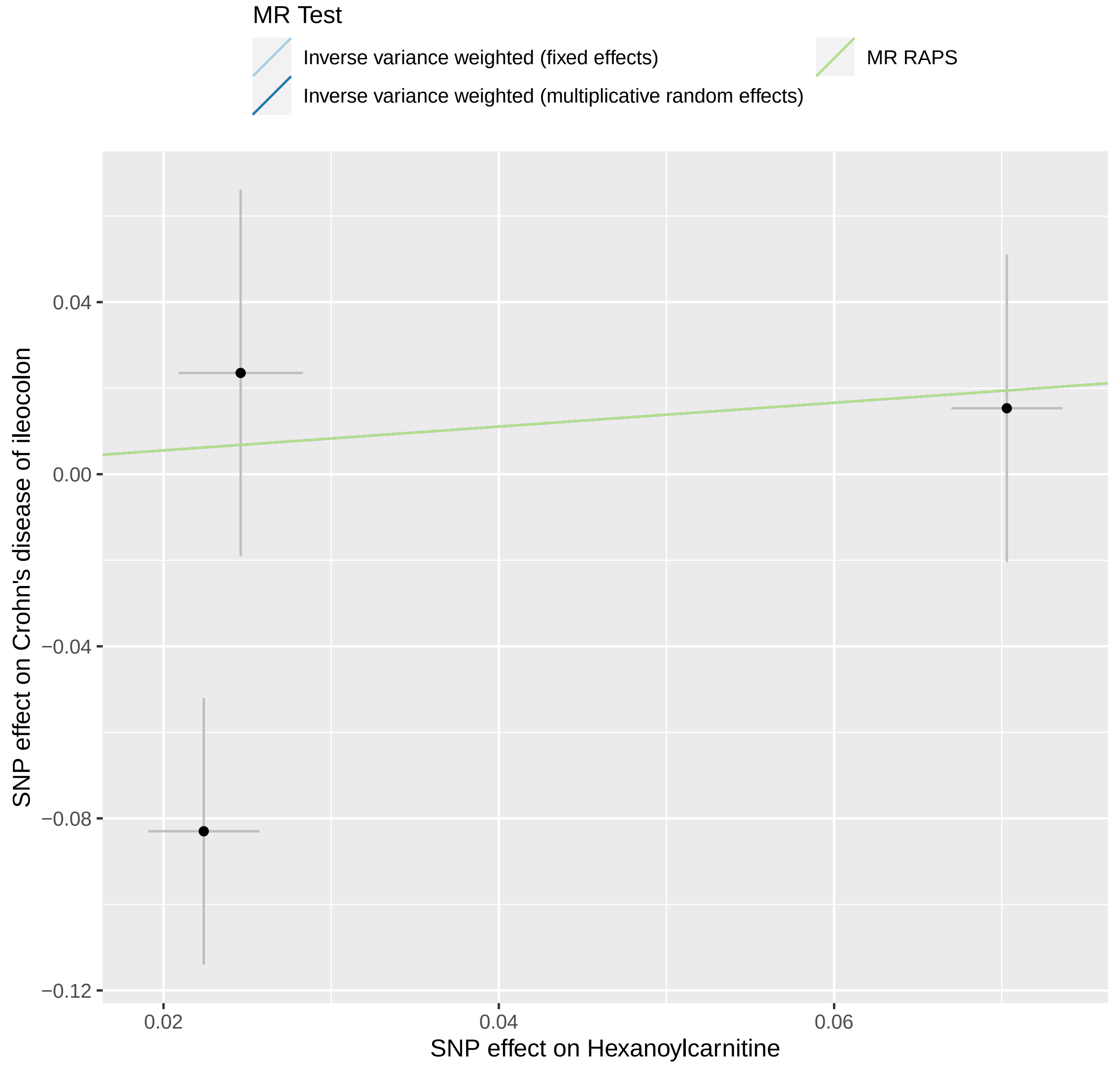

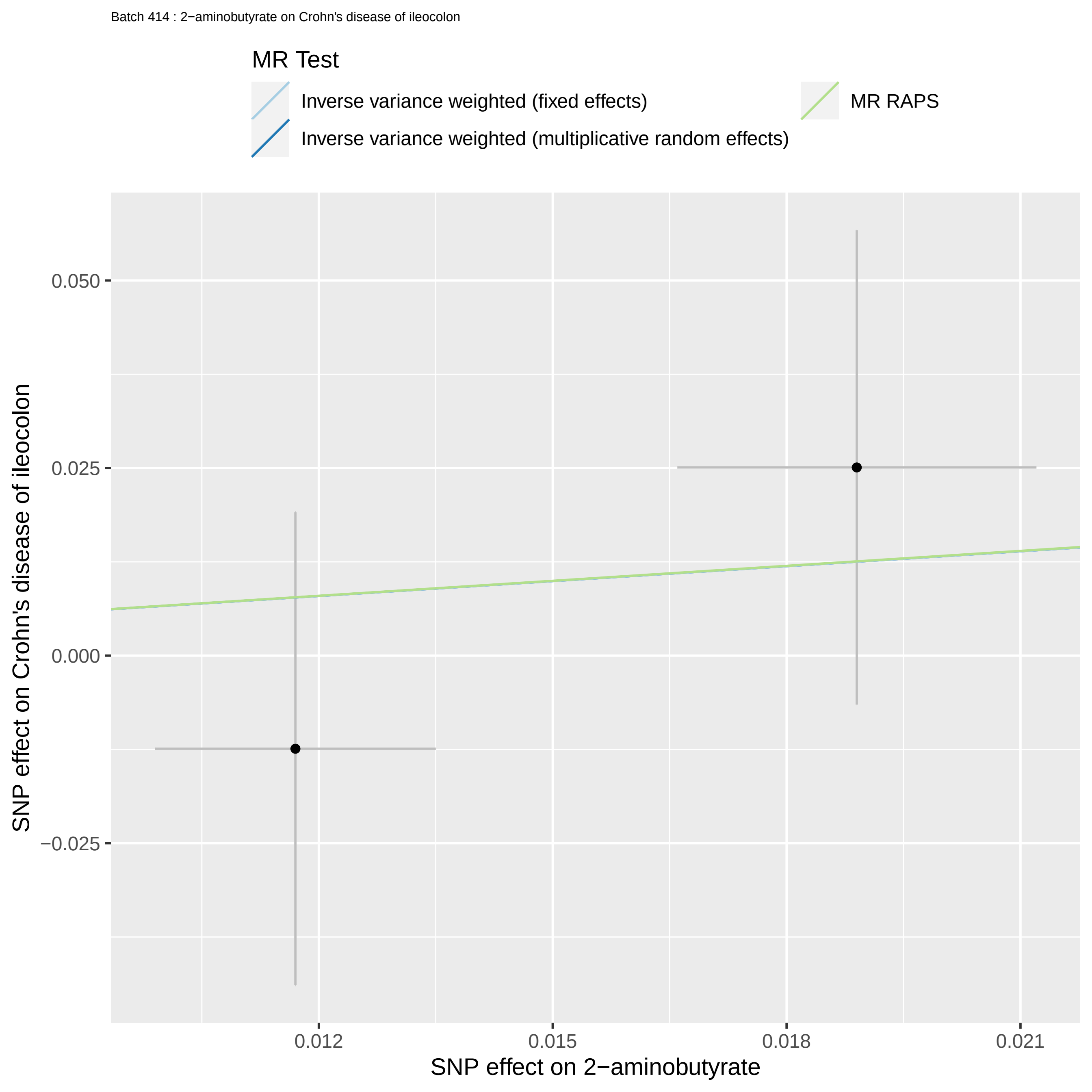
Batch 414 : 2−aminobutyrate on Crohn's disease of ileocolon
MR Test
Inverse variance weighted (fixed effects)
Inverse variance weighted (multiplicative random effects)
MR RAPS
0.050
0.025
0.000
−0.025
0.012
0.015
0.018
0.021
SNP effect on Crohn's disease of ileocolon
SNP effect on 2−aminobutyrate

Batch 419 : Butyrylcarnitine on Crohn's disease of ileocolon

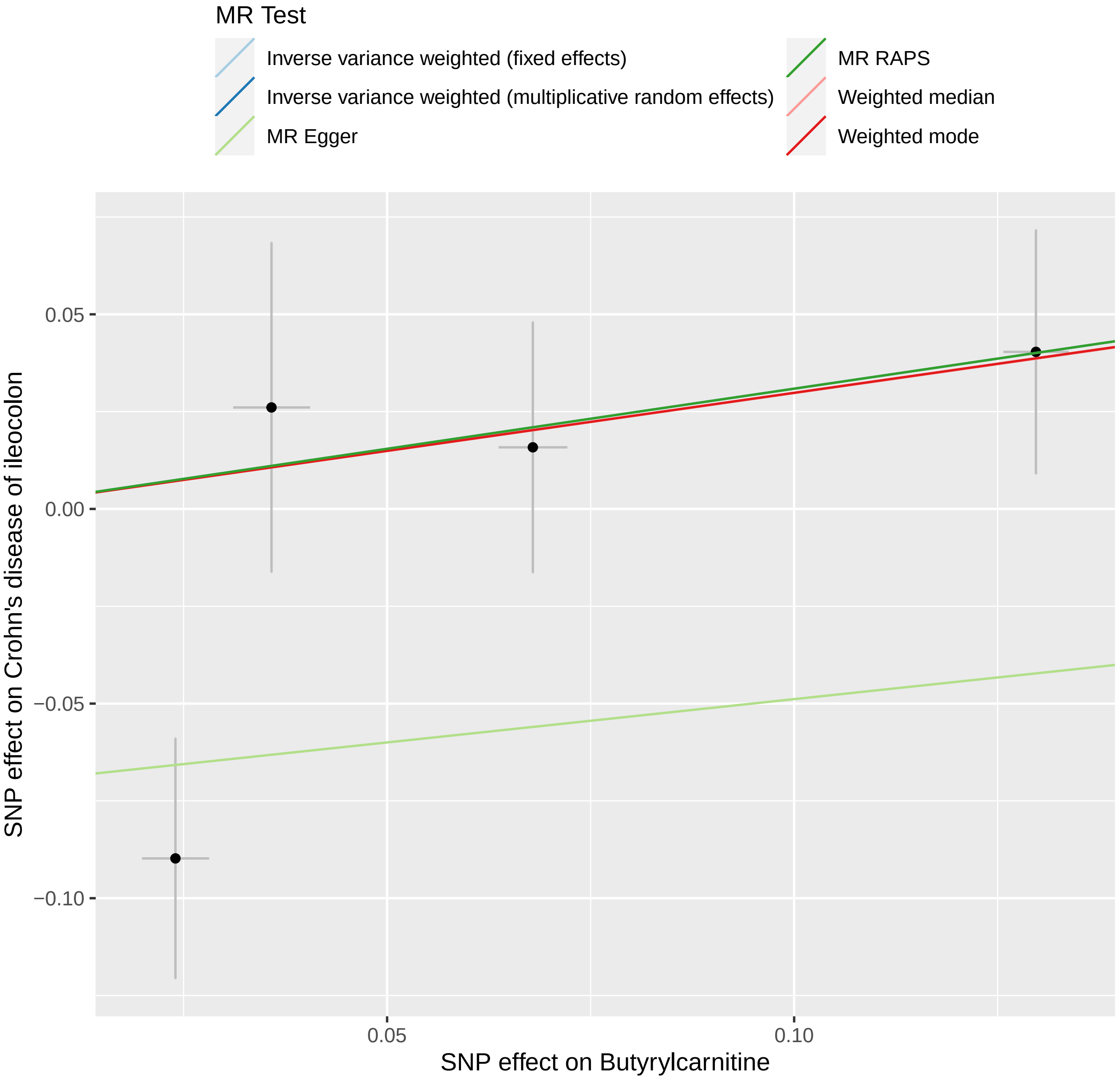

Batch 422 : Propionylcarnitine on Crohn's disease of ileocolon

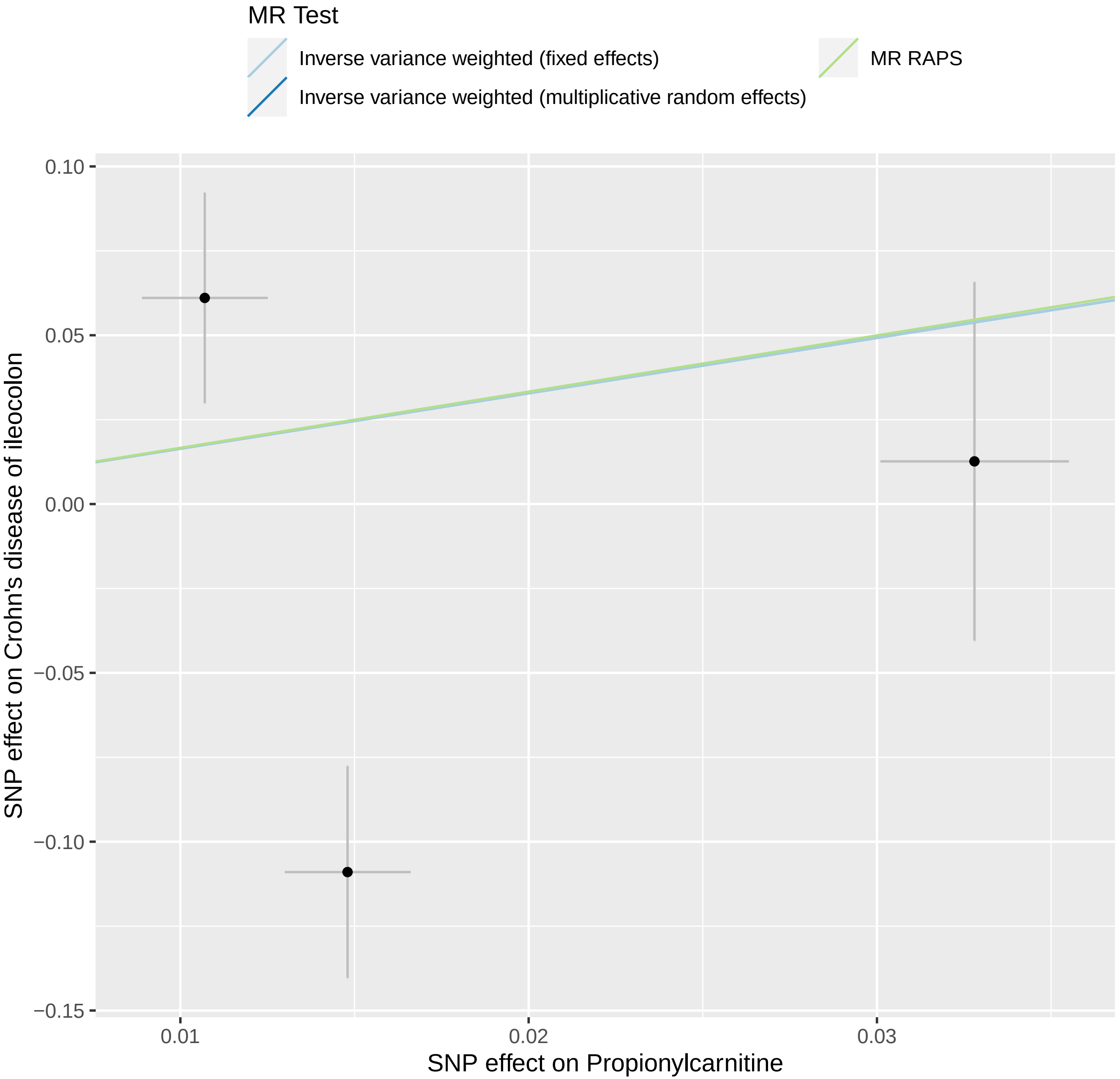

Batch 423 : Caproate (6:0) on Crohn's disease of ileocolon

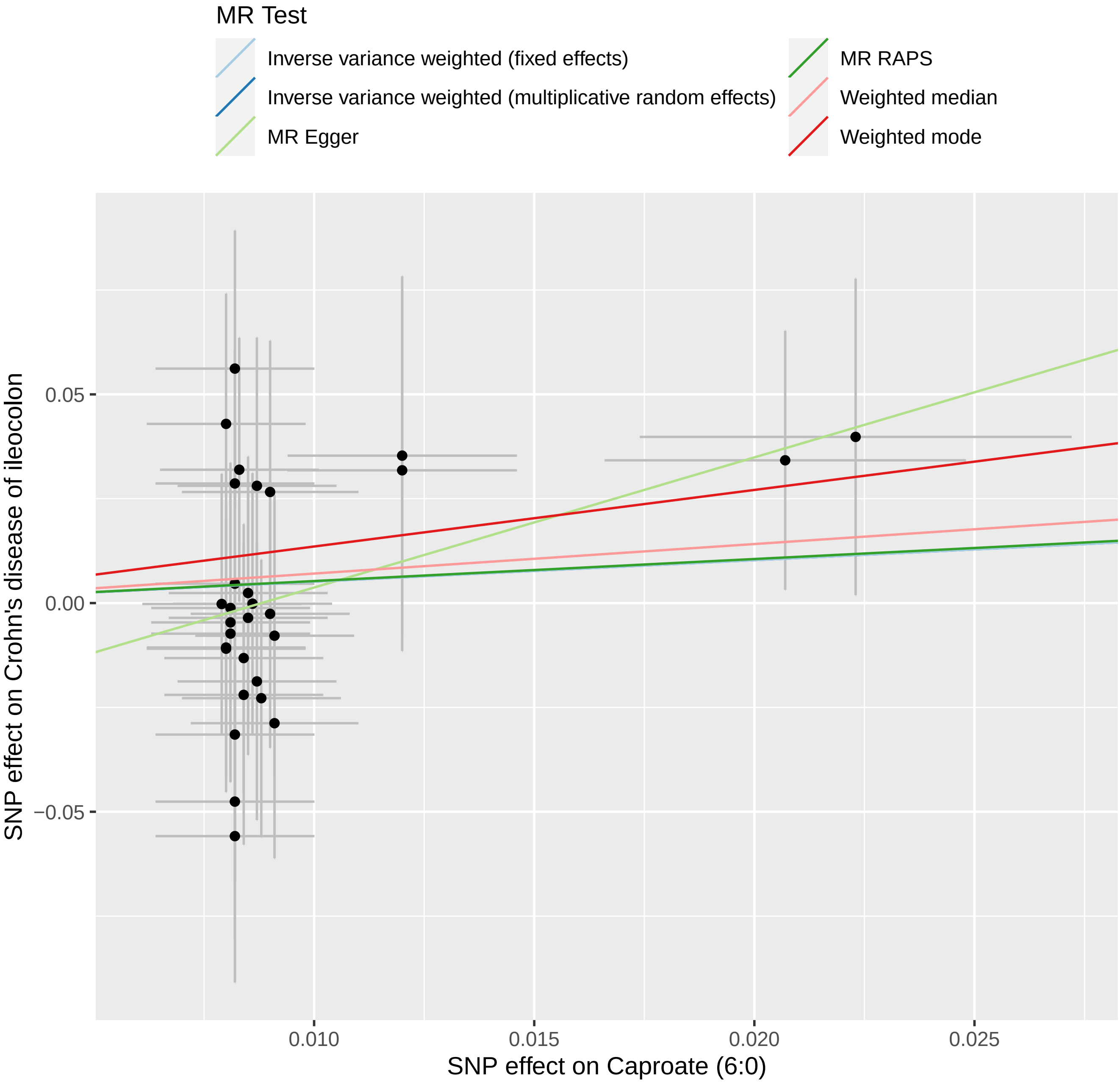

Batch 424 : Caprylate (8:0) on Crohn's disease of ileocolon

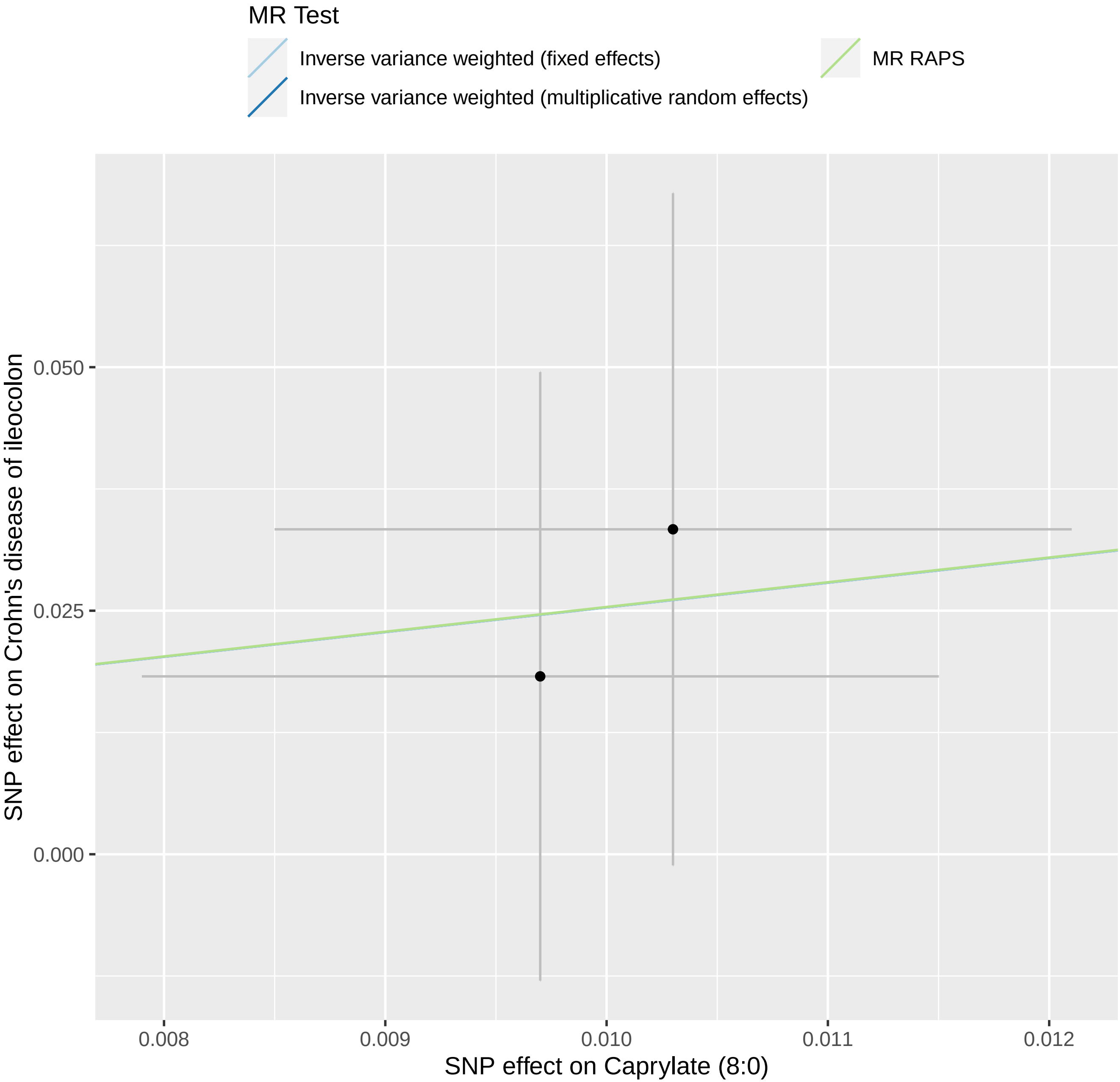

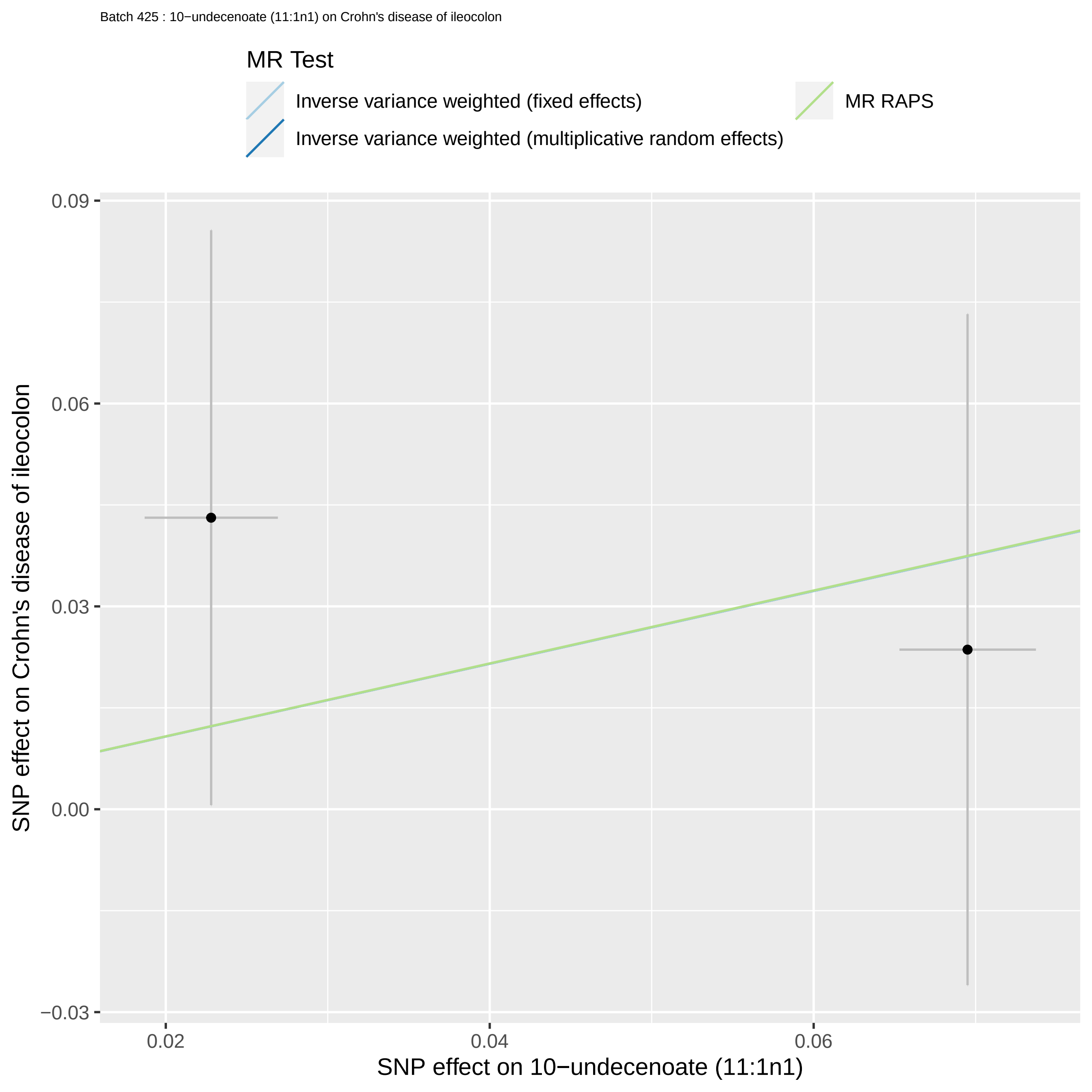

Batch 425 : 10−undecenoate (11:1n1) on Crohn's disease of ileocolon
MR Test
Inverse variance weighted (fixed effects)
Inverse variance weighted (multiplicative random effects)
MR RAPS
0.09
0.06
0.03
0.00
−0.03
0.02
0.04
0.06
SNP effect on Crohn's disease of ileocolon
SNP effect on 10−undecenoate (11:1n1)

Batch 427 : Phenol sulfate on Crohn's disease of ileocolon

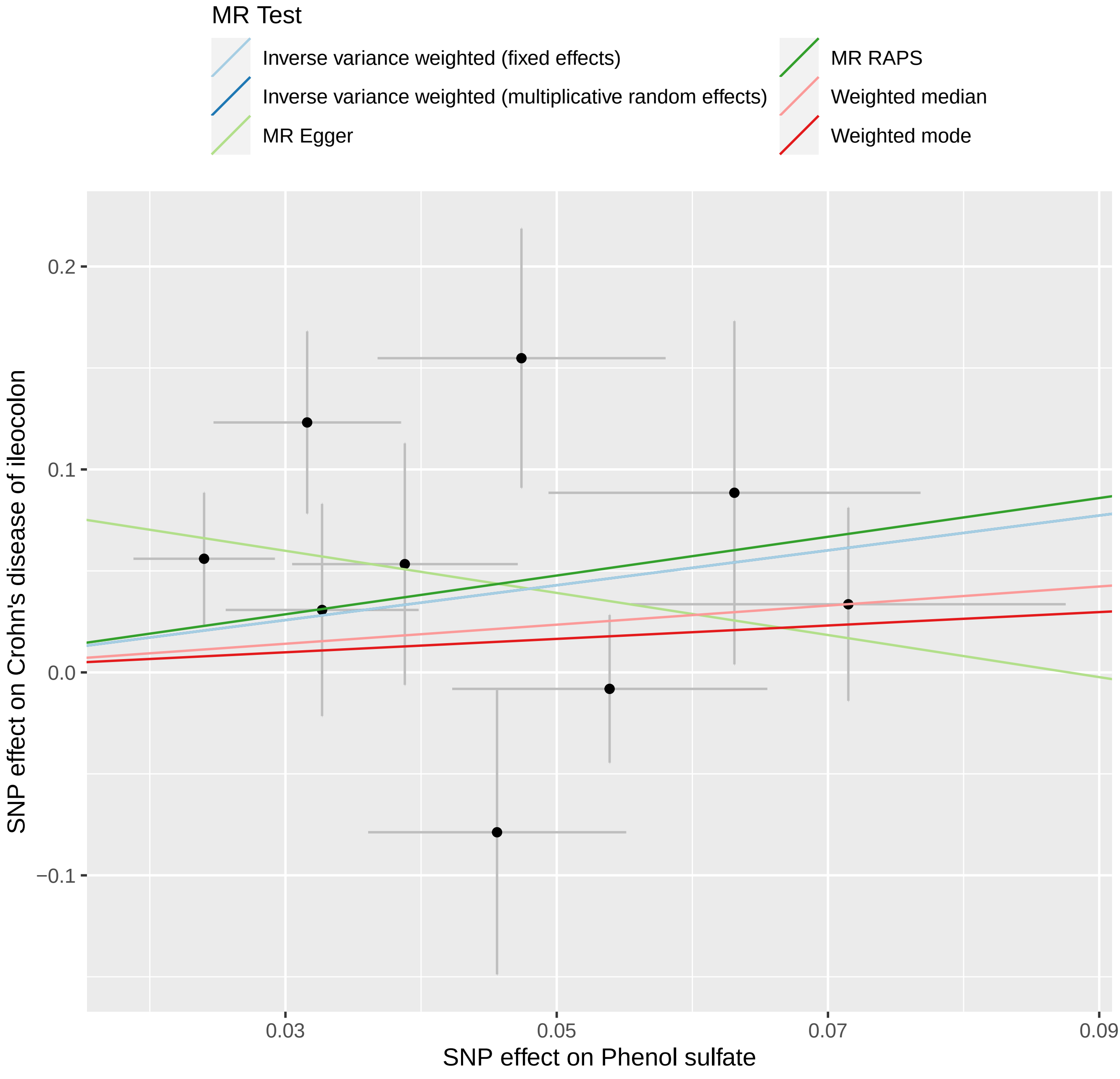

Batch 429 : Heme* on Crohn's disease of ileocolon

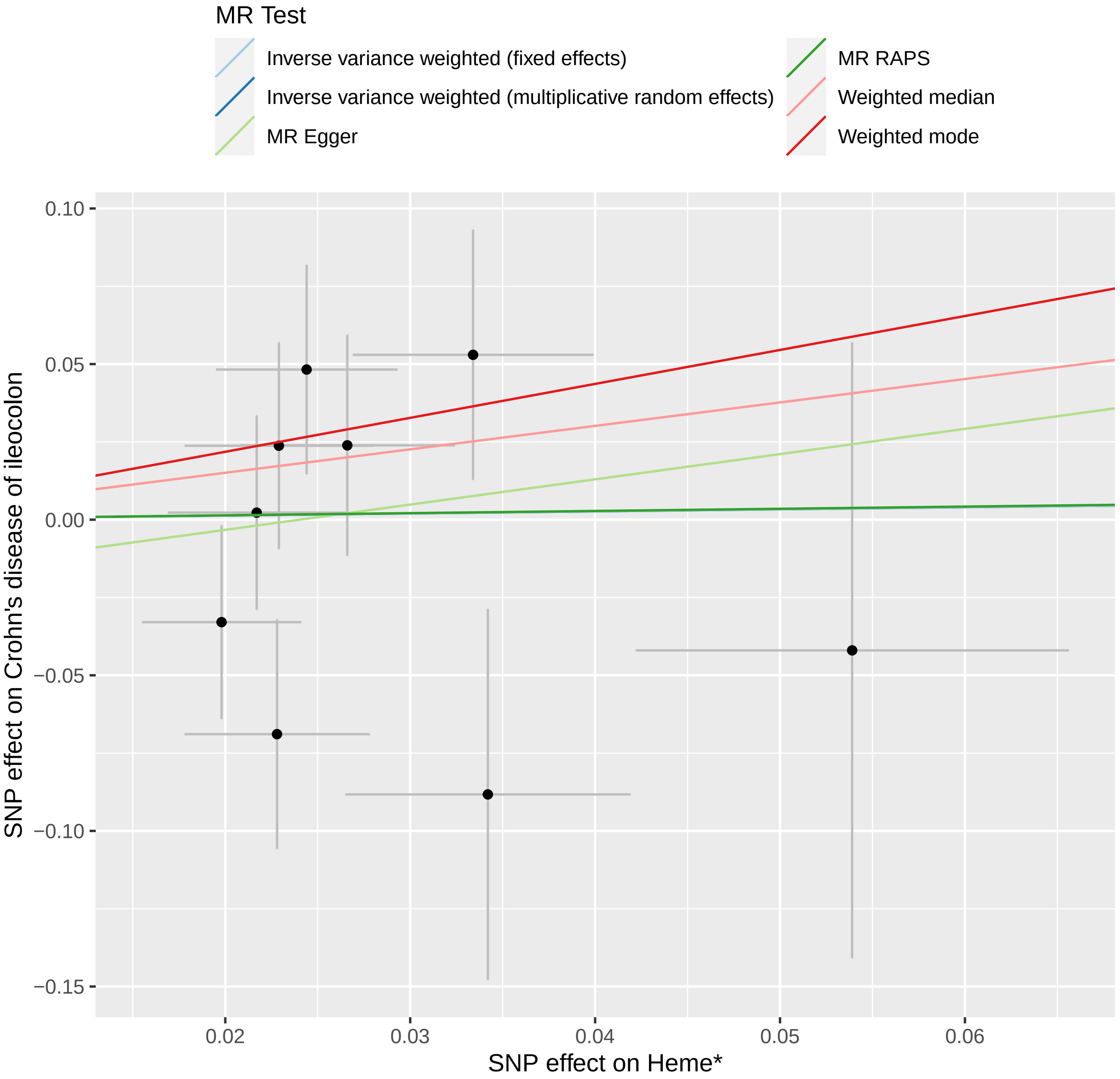

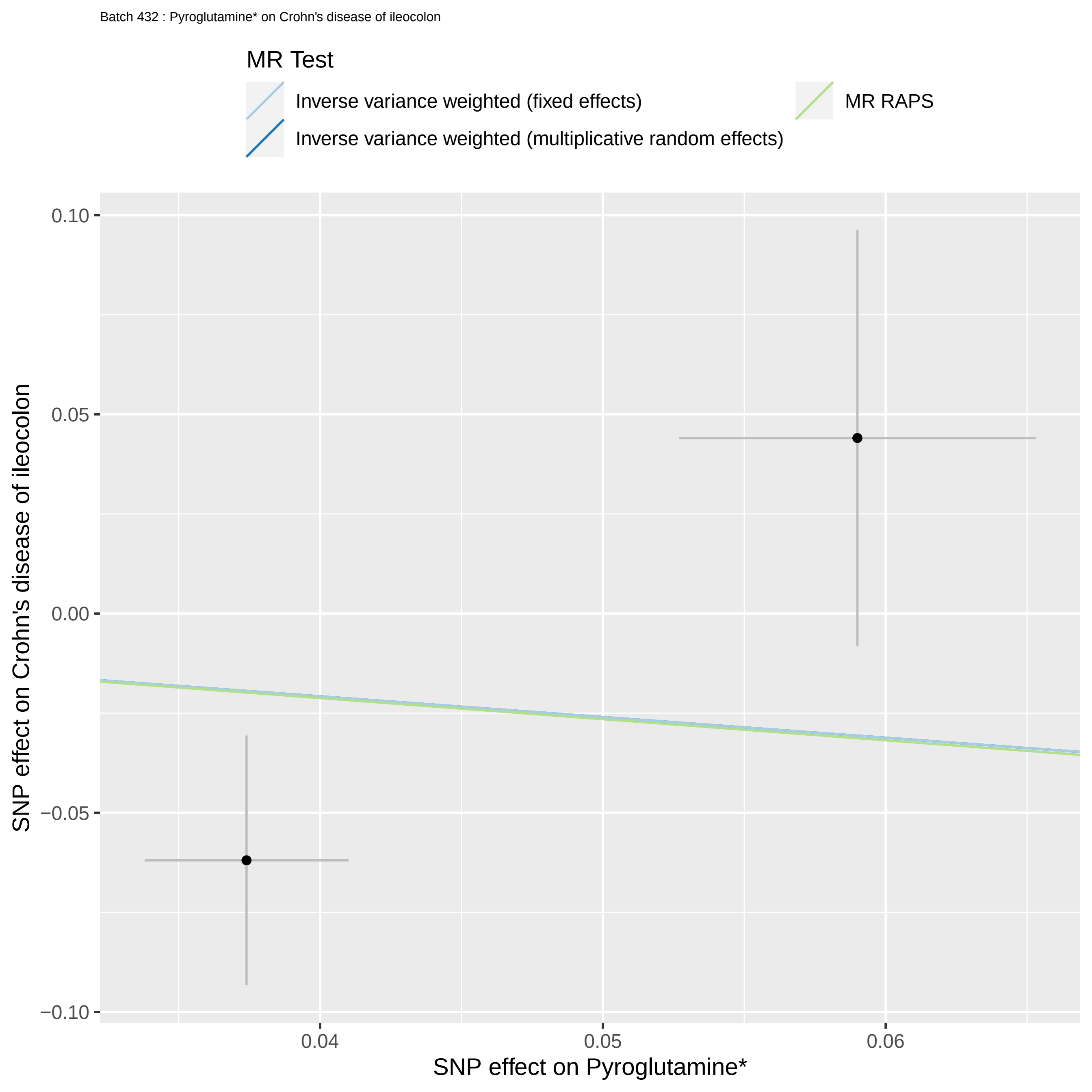

Batch 432 : Pyroglutamine* on Crohn's disease of ileocolon
MR Test
Inverse variance weighted (fixed effects)
Inverse variance weighted (multiplicative random effects)
MR RAPS
0.10
0.05
0.00
−0.05
−0.10
0.04
0.05
0.06
SNP effect on Crohn's disease of ileocolon
SNP effect on Pyroglutamine*

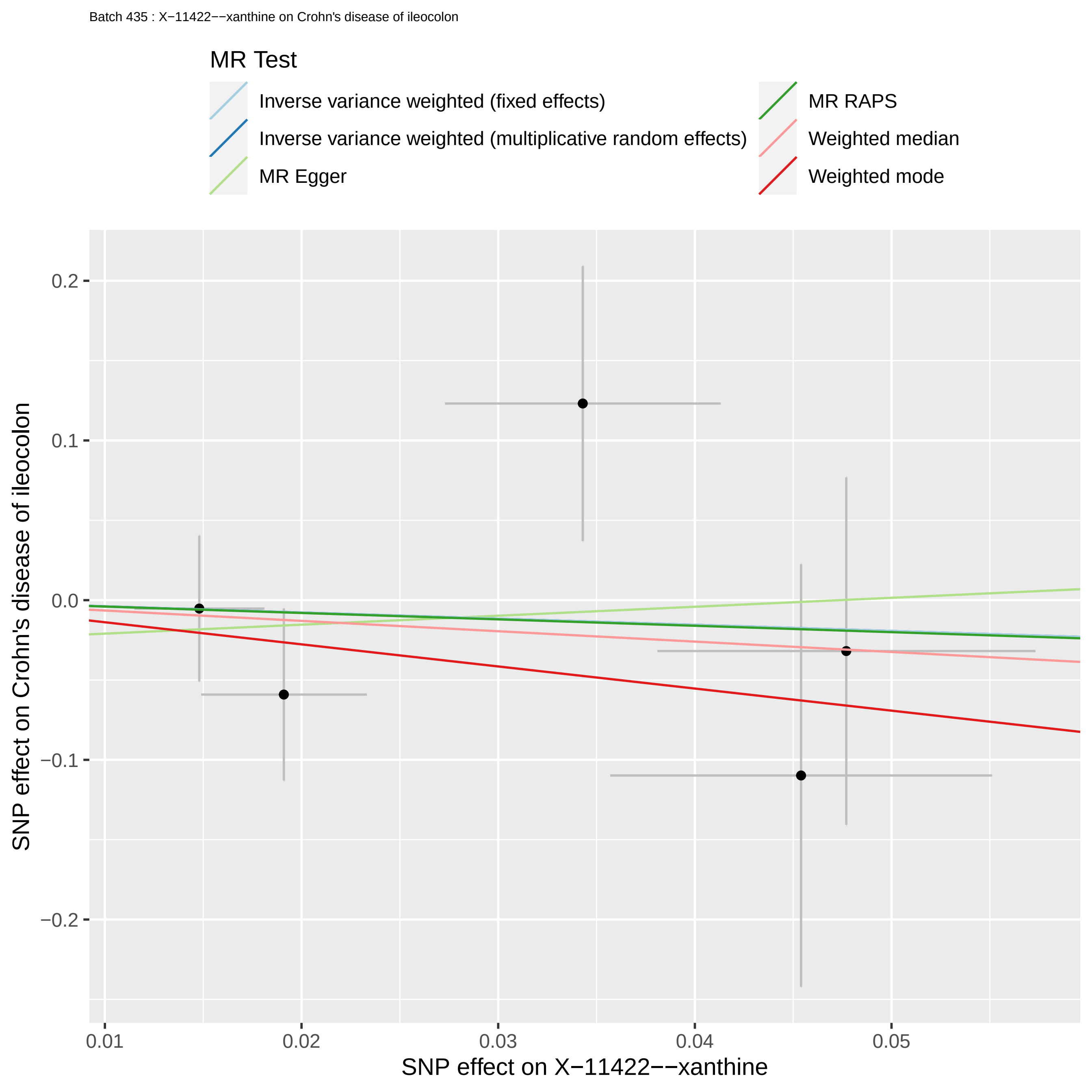

Batch 435 : X−11422−−xanthine on Crohn's disease of ileocolon
MR Test
Inverse variance weighted (fixed effects)
Inverse variance weighted (multiplicative random effects)
MR Egger
MR RAPS
Weighted median
Weighted mode
0.2
0.1
0.0
−0.1
−0.2
SNP effect on Crohn's disease of ileocolon
0.01
0.02
0.03
0.04
0.05
SNP effect on X−11422−−xanthine

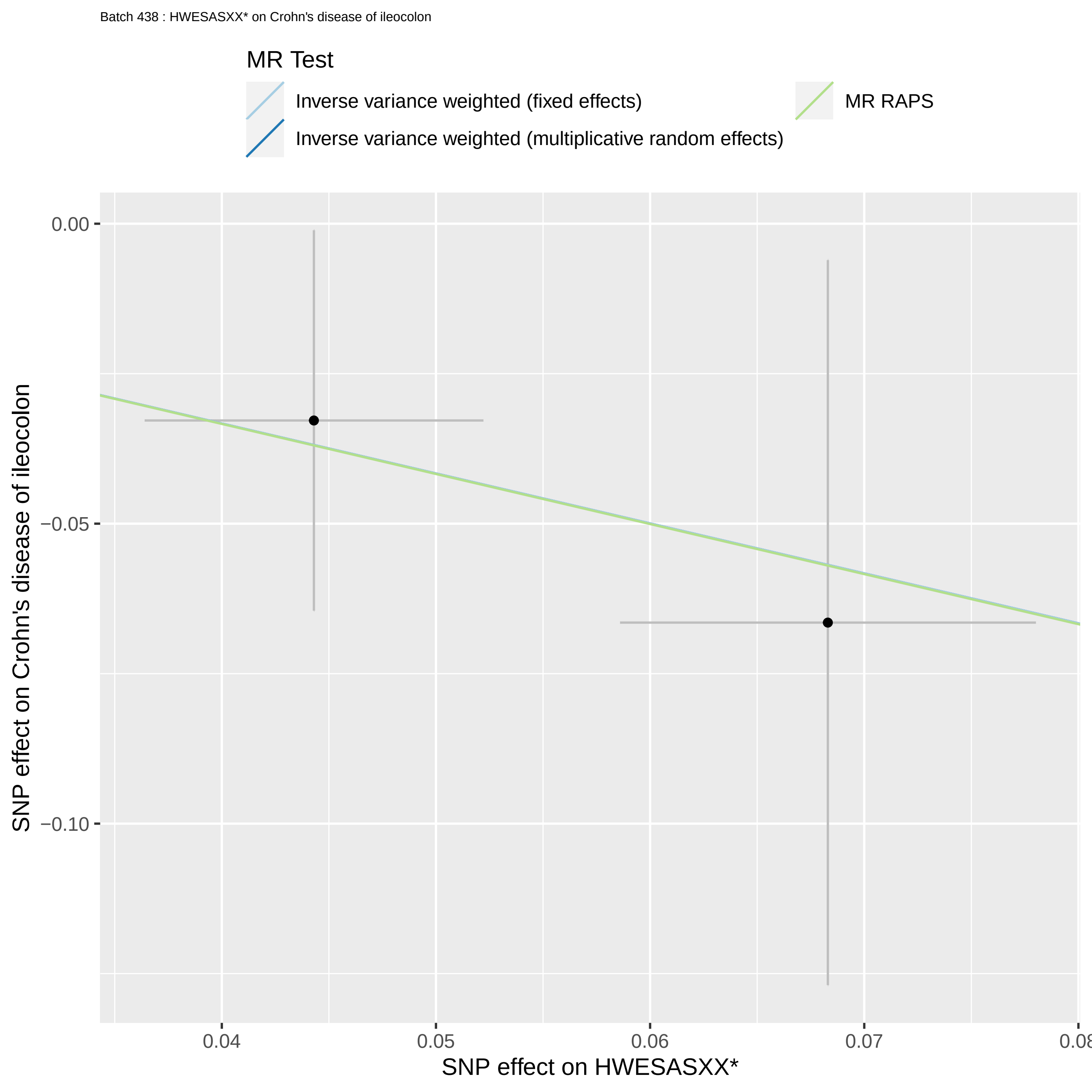
Batch 438 : HWESASXX* on Crohn's disease of ileocolon
MR Test
Inverse variance weighted (fixed effects)
Inverse variance weighted (multiplicative random effects)
MR RAPS
0.00
−0.05
−0.10
SNP effect on Crohn's disease of ileocolon
0.04
0.05
0.06
0.07
0.08
SNP effect on HWESASXX*

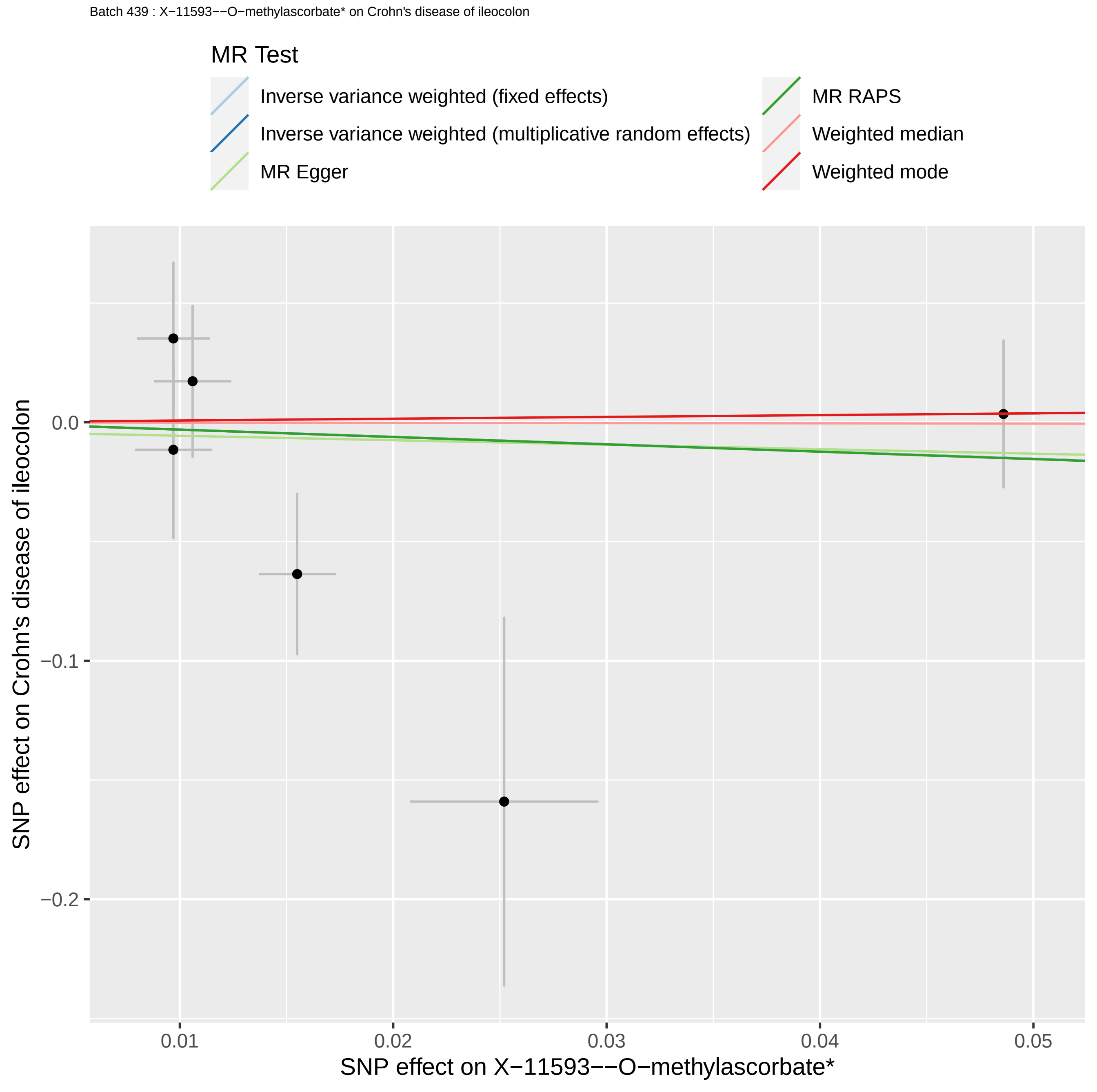
Batch 439 : X−11593−−O−methylascorbate* on Crohn's disease of ileocolon
MR Test
Inverse variance weighted (fixed effects)
Inverse variance weighted (multiplicative random effects)
MR Egger
MR RAPS
Weighted median
Weighted mode
SNP effect on Crohn's disease of ileocolon
0.0
−0.1
−0.2
0.01
0.02
0.03
0.04
0.05
SNP effect on X−11593−−O−methylascorbate*

Batch 441 : Homostachydrine* on Crohn's disease of ileocolon

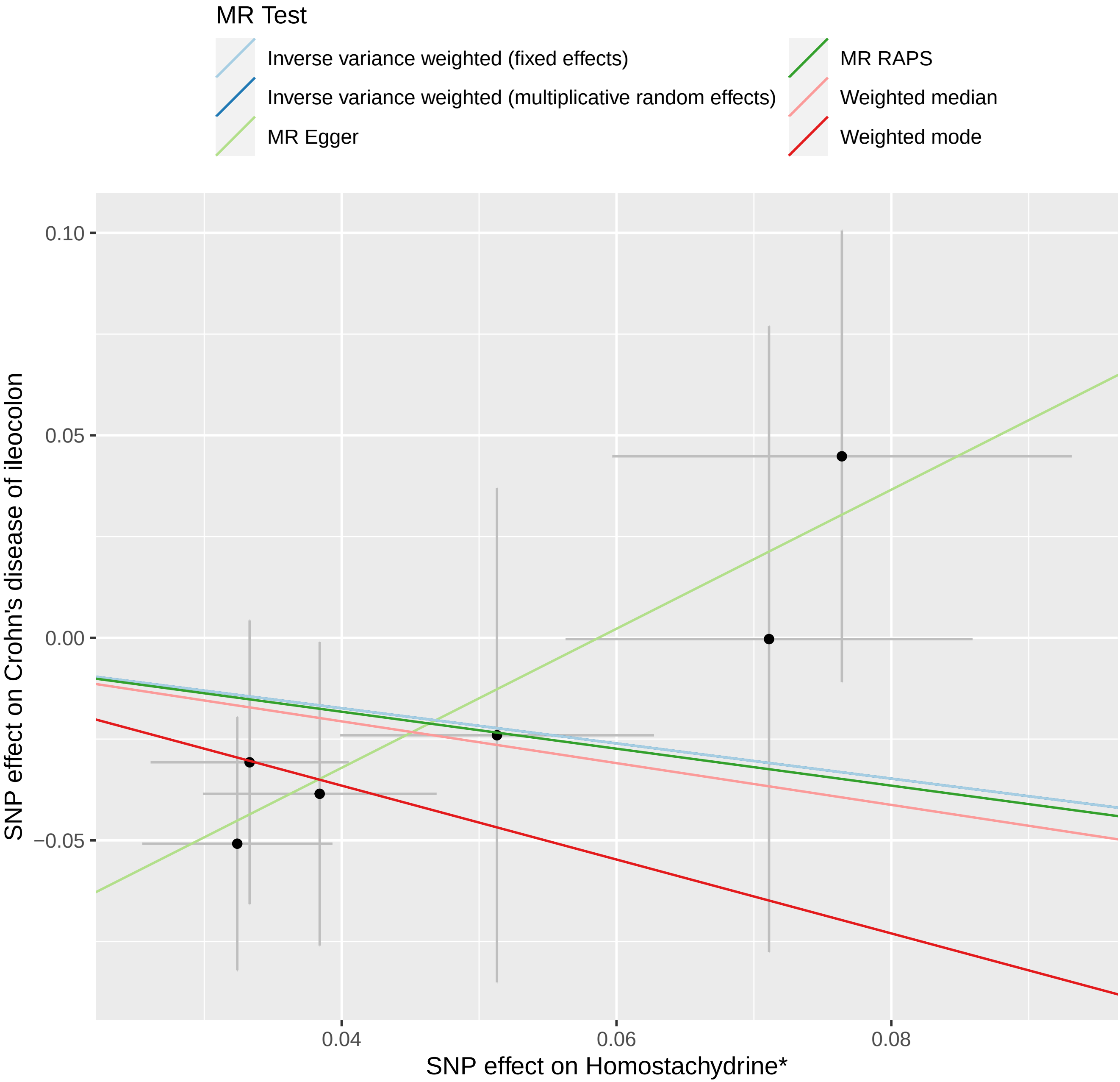

Batch 442 : ADSGEGDFXAEGGGVR* on Crohn's disease of ileocolon

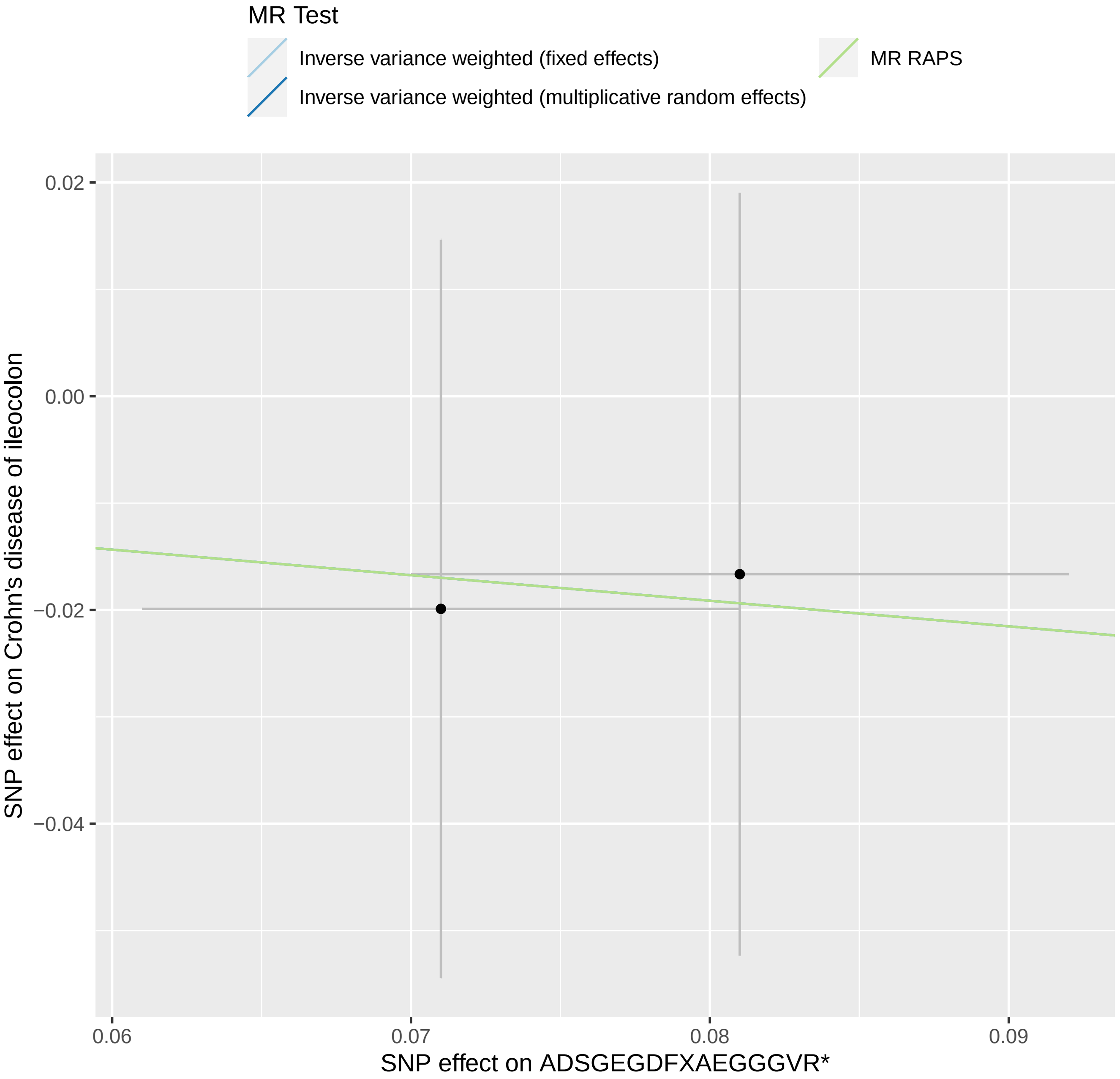

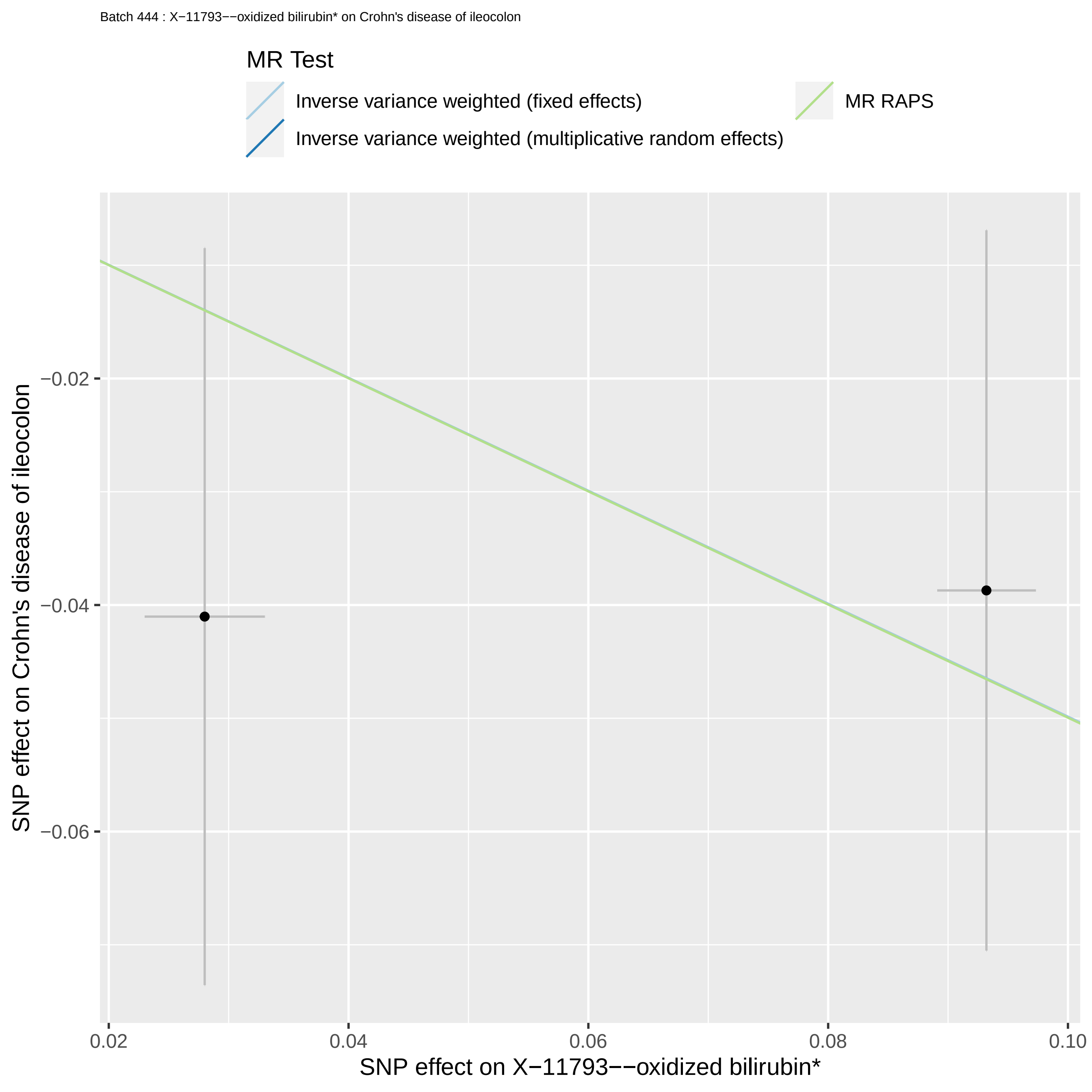

Batch 444 : X−11793−−oxidized bilirubin* on Crohn's disease of ileocolon
MR Test
Inverse variance weighted (fixed effects)
Inverse variance weighted (multiplicative random effects)
MR RAPS
−0.02
−0.04
−0.06
0.02
0.04
0.06
0.08
0.10
SNP effect on Crohn's disease of ileocolon
SNP effect on X−11793−−oxidized bilirubin*

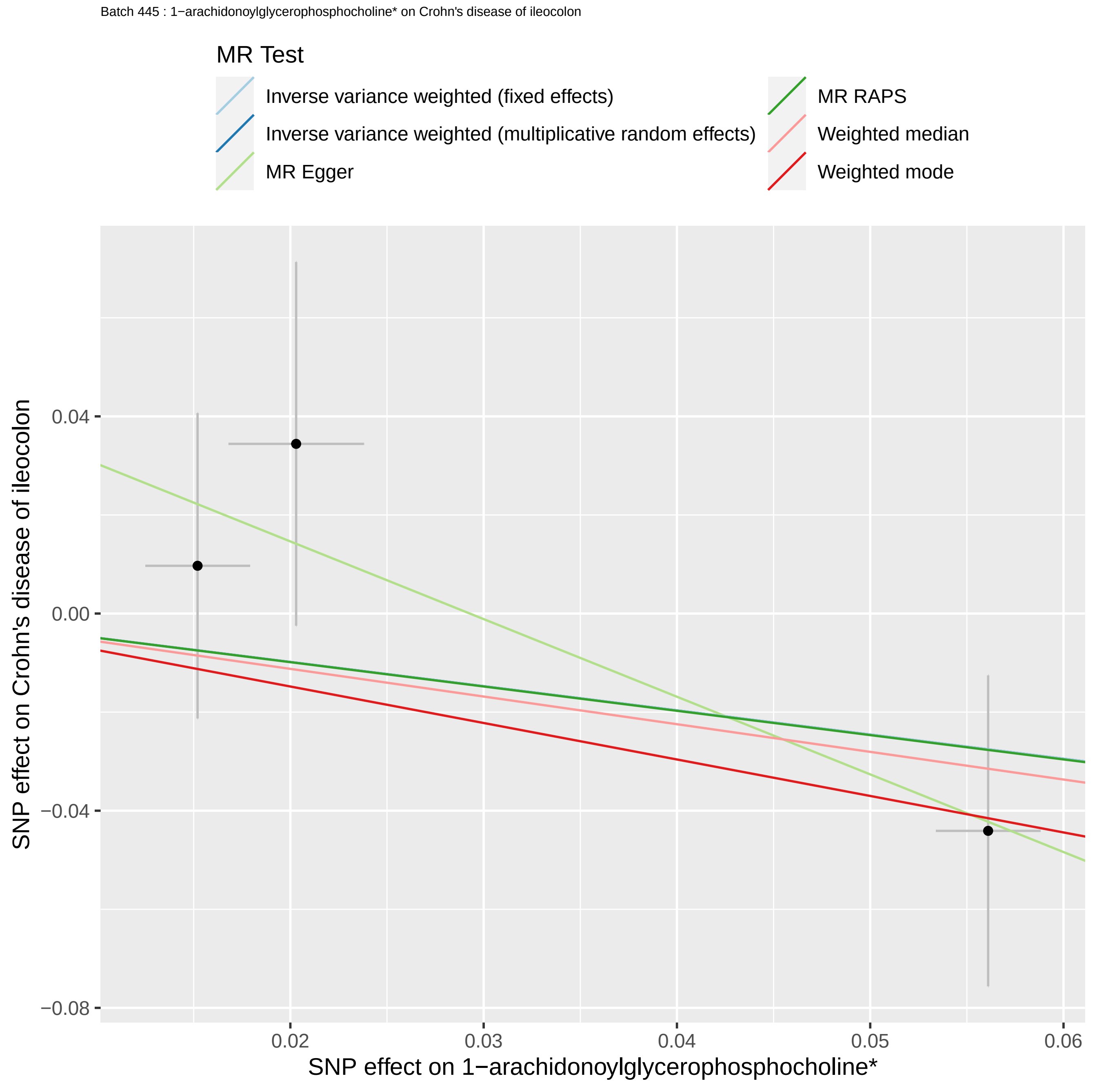
Batch 445 : 1−arachidonoylglycerophosphocholine* on Crohn's disease of ileocolon
MR Test
Inverse variance weighted (fixed effects)
Inverse variance weighted (multiplicative random effects)
MR Egger
MR RAPS
Weighted median
Weighted mode
0.04
0.00
−0.04
−0.08
0.02
0.03
0.04
0.05
0.06
SNP effect on Crohn's disease of ileocolon
SNP effect on 1−arachidonoylglycerophosphocholine*

Batch 447 : Gamma−glutamylmethionine* on Crohn's disease of ileocolon

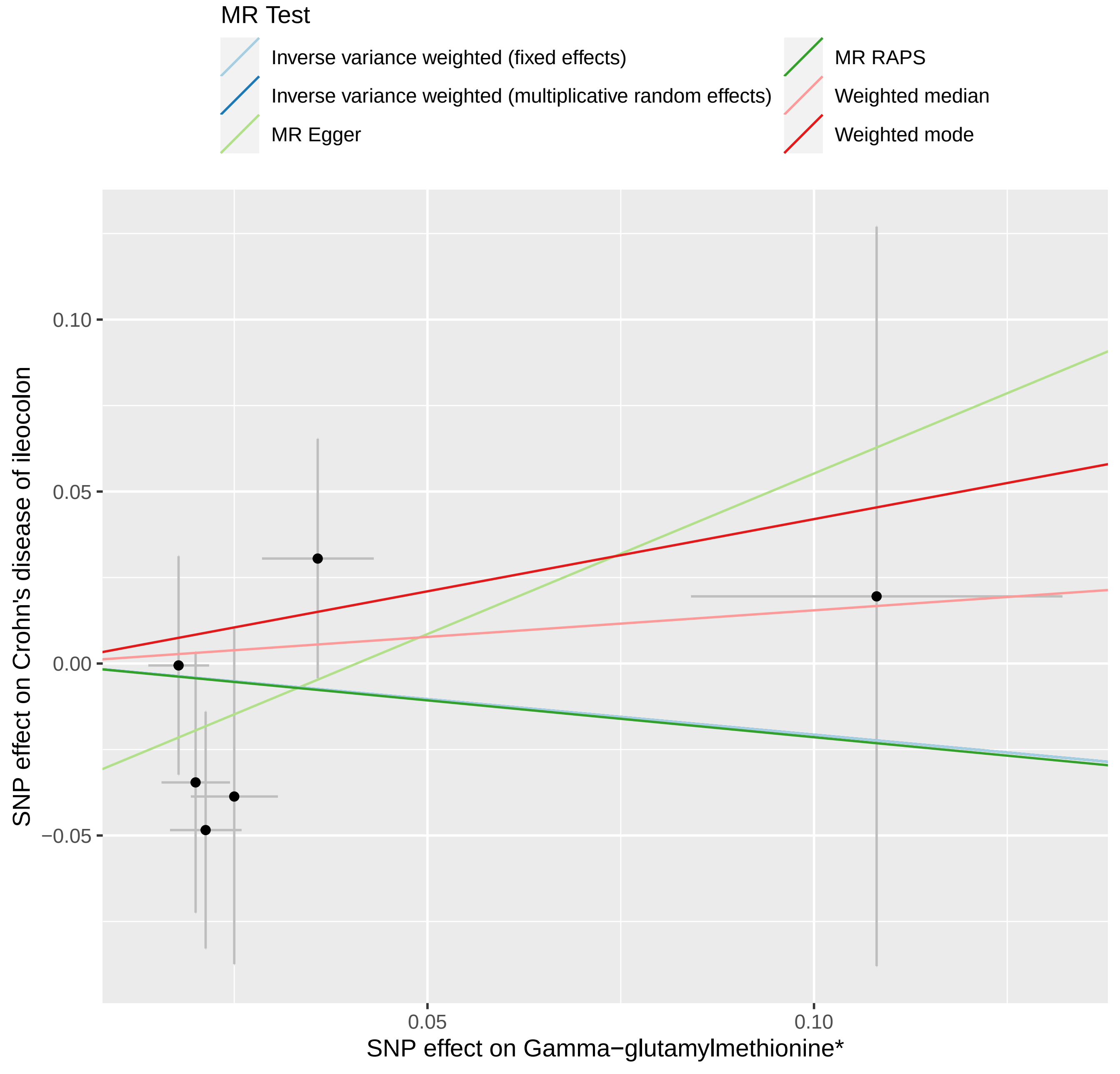

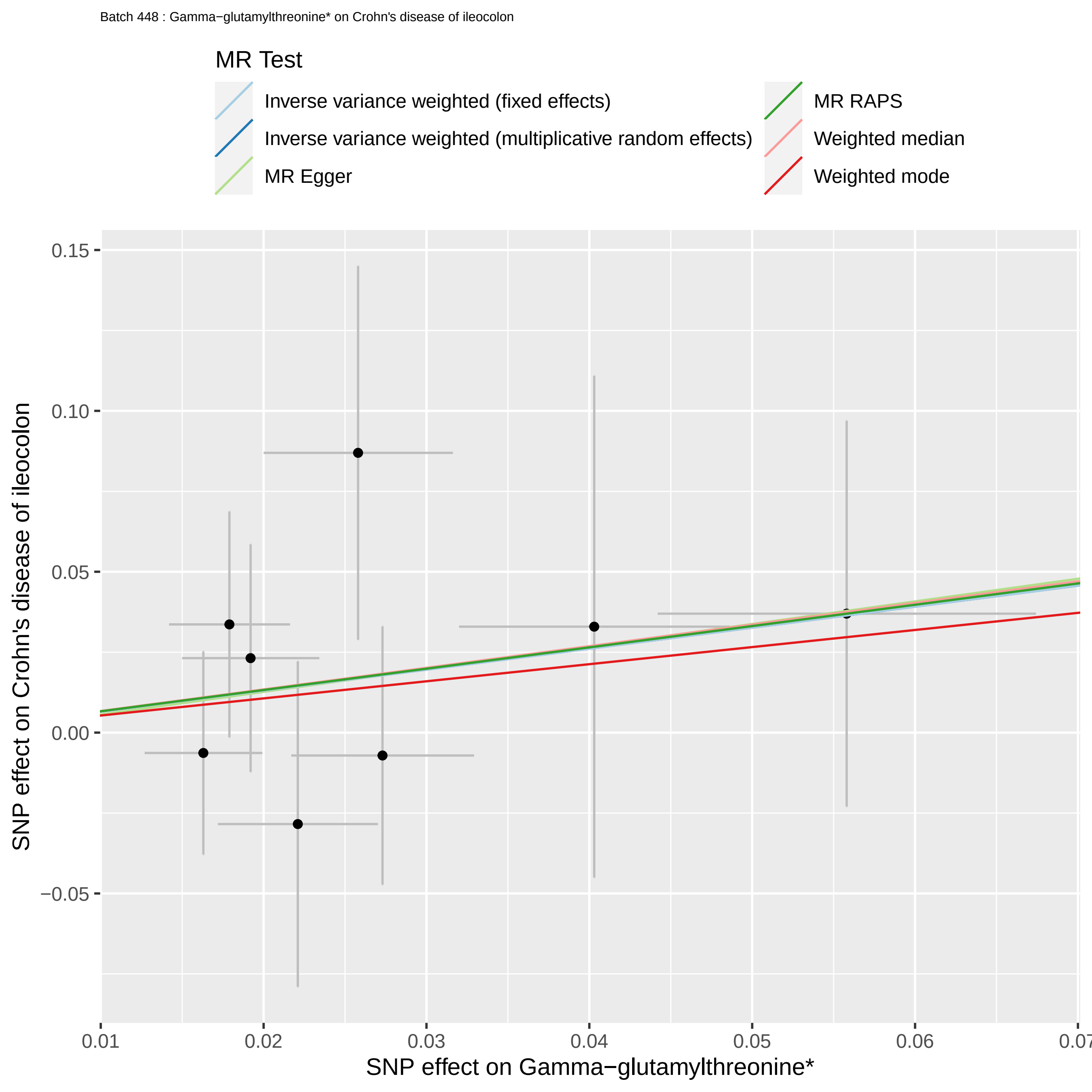

Batch 448 : Gamma−glutamylthreonine* on Crohn's disease of ileocolon
MR Test
Inverse variance weighted (fixed effects)
Inverse variance weighted (multiplicative random effects)
MR Egger
MR RAPS
Weighted median
Weighted mode
0.15
0.10
0.05
0.00
−0.05
0.01
0.02
0.03
0.04
0.05
0.06
0.07
SNP effect on Crohn's disease of ileocolon
SNP effect on Gamma−glutamylthreonine*

Batch 449 : Gamma−tocopherol on Crohn's disease of ileocolon

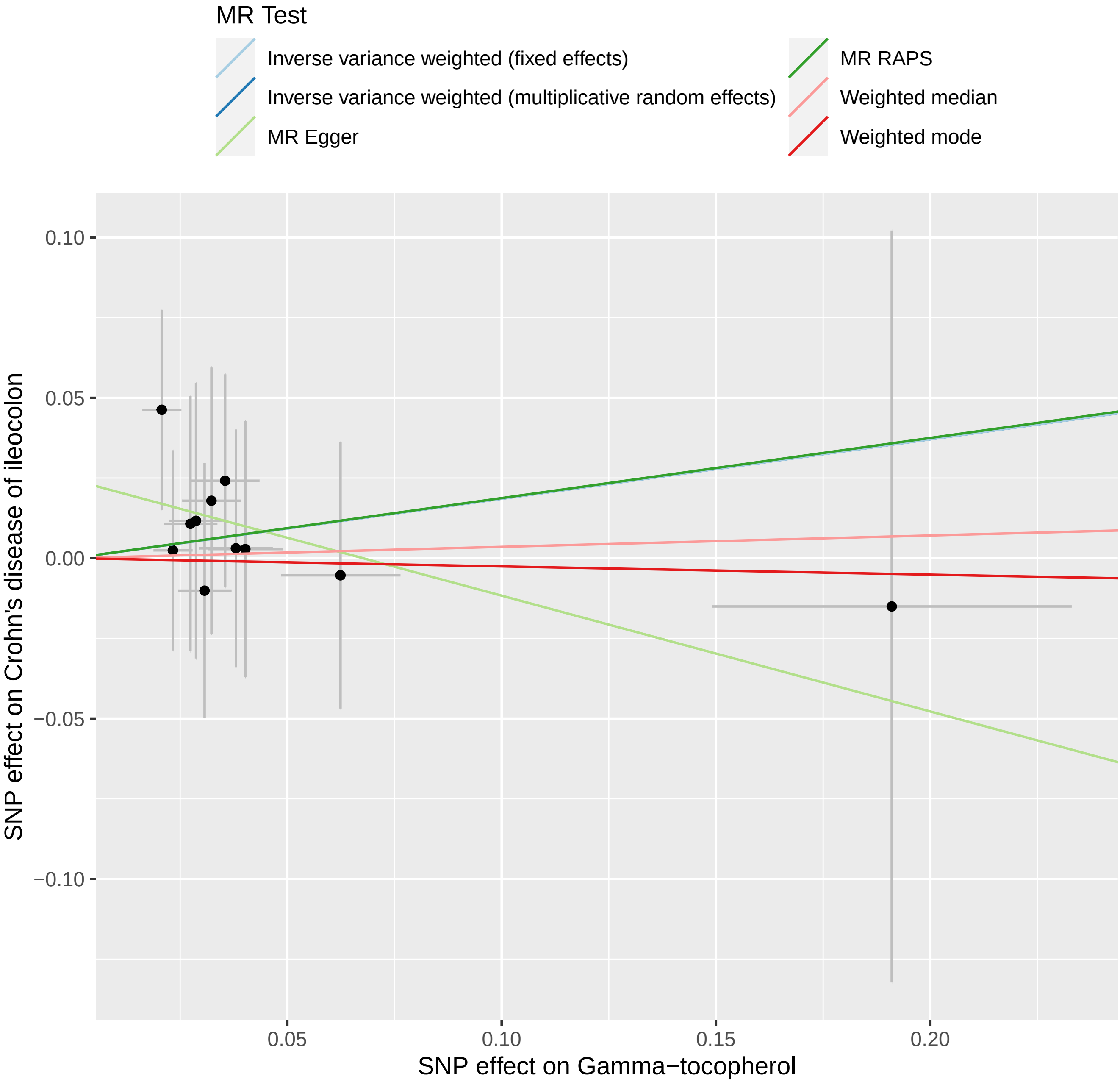

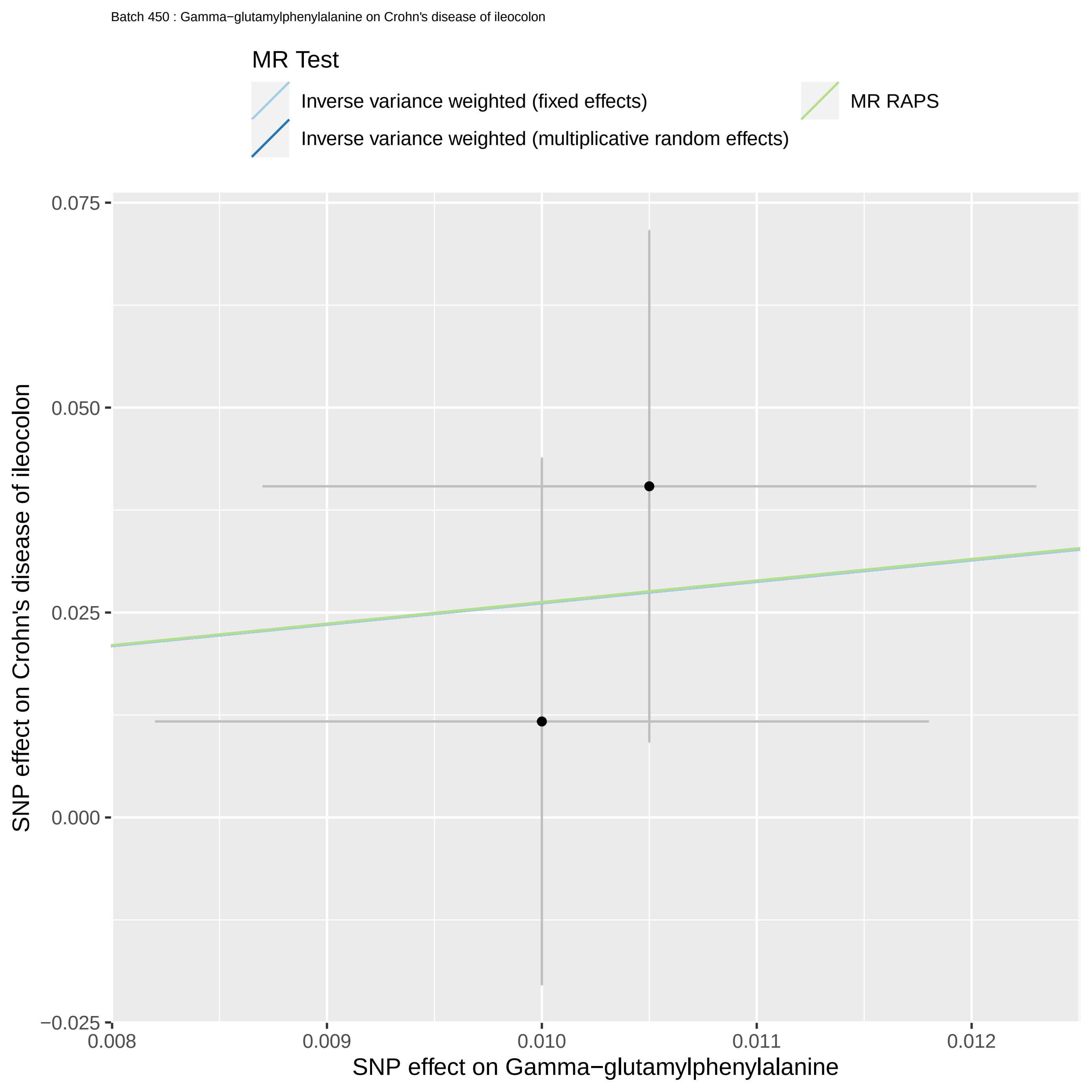

Batch 450 : Gamma−glutamylphenylalanine on Crohn's disease of ileocolon
MR Test
Inverse variance weighted (fixed effects)
Inverse variance weighted (multiplicative random effects)
MR RAPS
0.075
0.050
0.025
0.000
−0.025
0.008
0.009
0.010
0.011
0.012
SNP effect on Crohn's disease of ileocolon
SNP effect on Gamma−glutamylphenylalanine

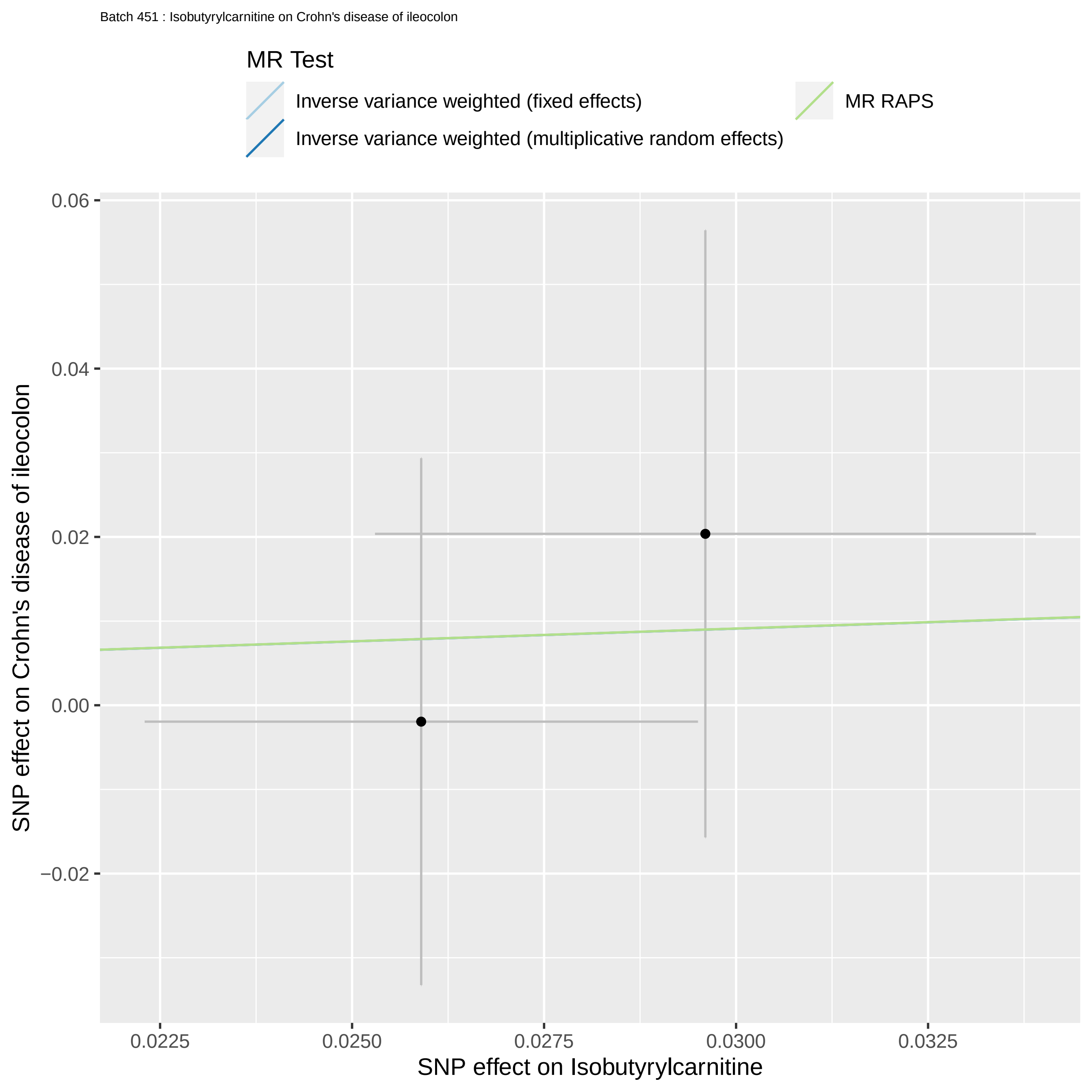
Batch 451 : Isobutyrylcarnitine on Crohn's disease of ileocolon
MR Test
Inverse variance weighted (fixed effects)
Inverse variance weighted (multiplicative random effects)
MR RAPS
0.06
0.04
0.02
0.00
−0.02
0.0225
0.0250
0.0275
0.0300
0.0325
SNP effect on Crohn's disease of ileocolon
SNP effect on Isobutyrylcarnitine

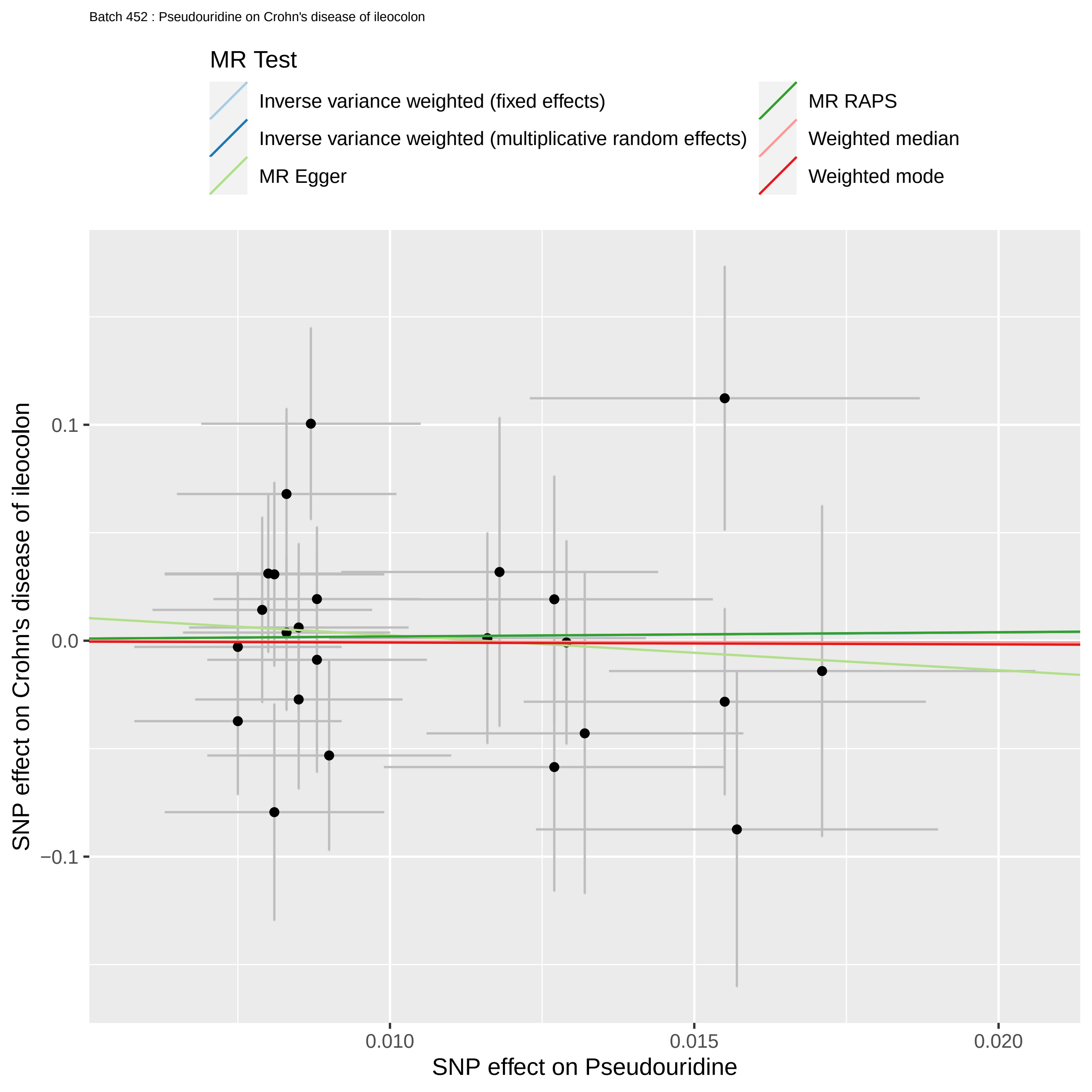
Batch 452 : Pseudouridine on Crohn's disease of ileocolon
MR Test
Inverse variance weighted (fixed effects)
Inverse variance weighted (multiplicative random effects)
MR Egger
MR RAPS
Weighted median
Weighted mode
SNP effect on Crohn's disease of ileocolon
0.1
0.0
−0.1
0.010
0.015
0.020
SNP effect on Pseudouridine

Batch 453 : Valerate on Crohn's disease of ileocolon

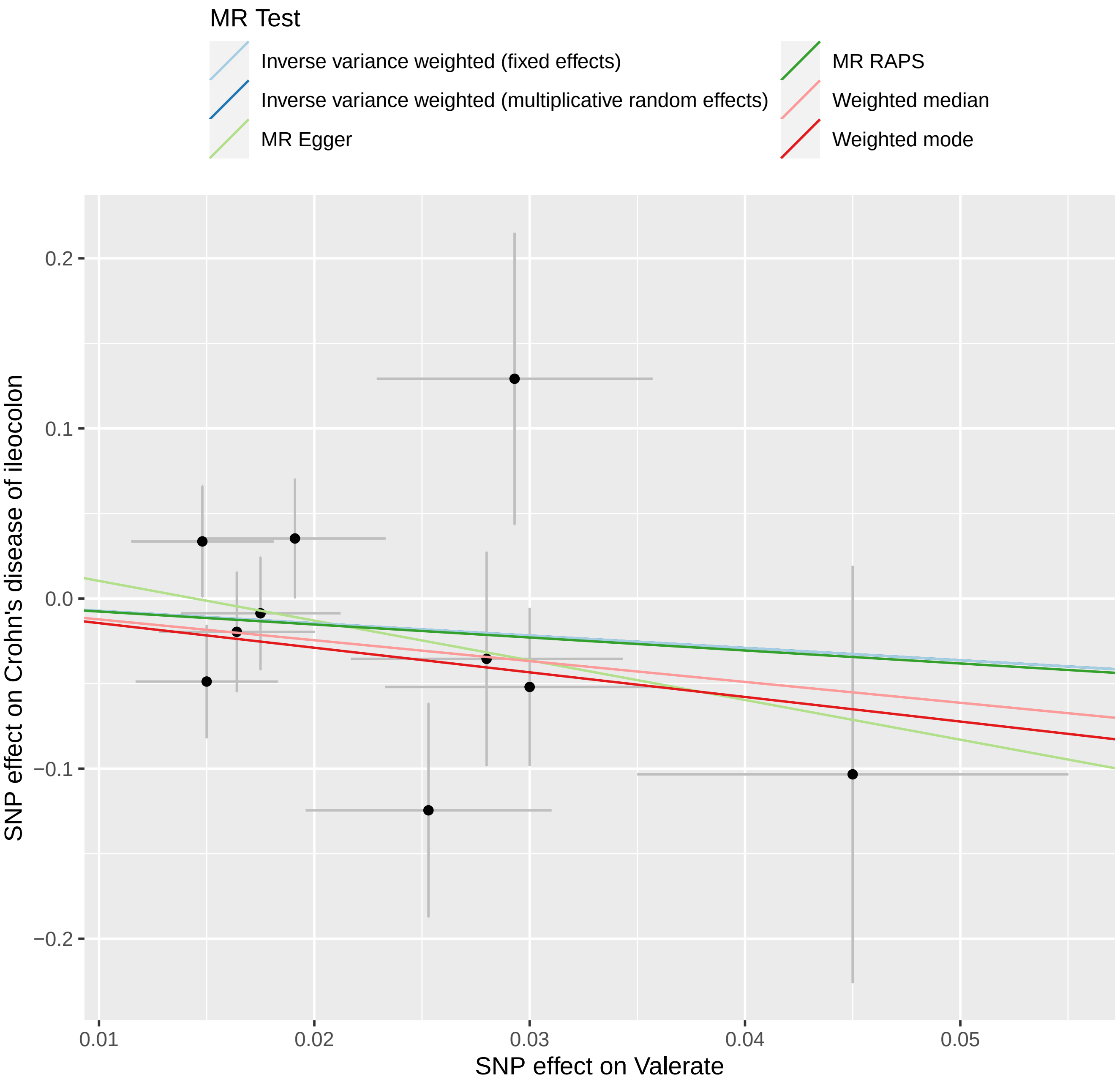

Batch 456 : Erythronate* on Crohn's disease of ileocolon

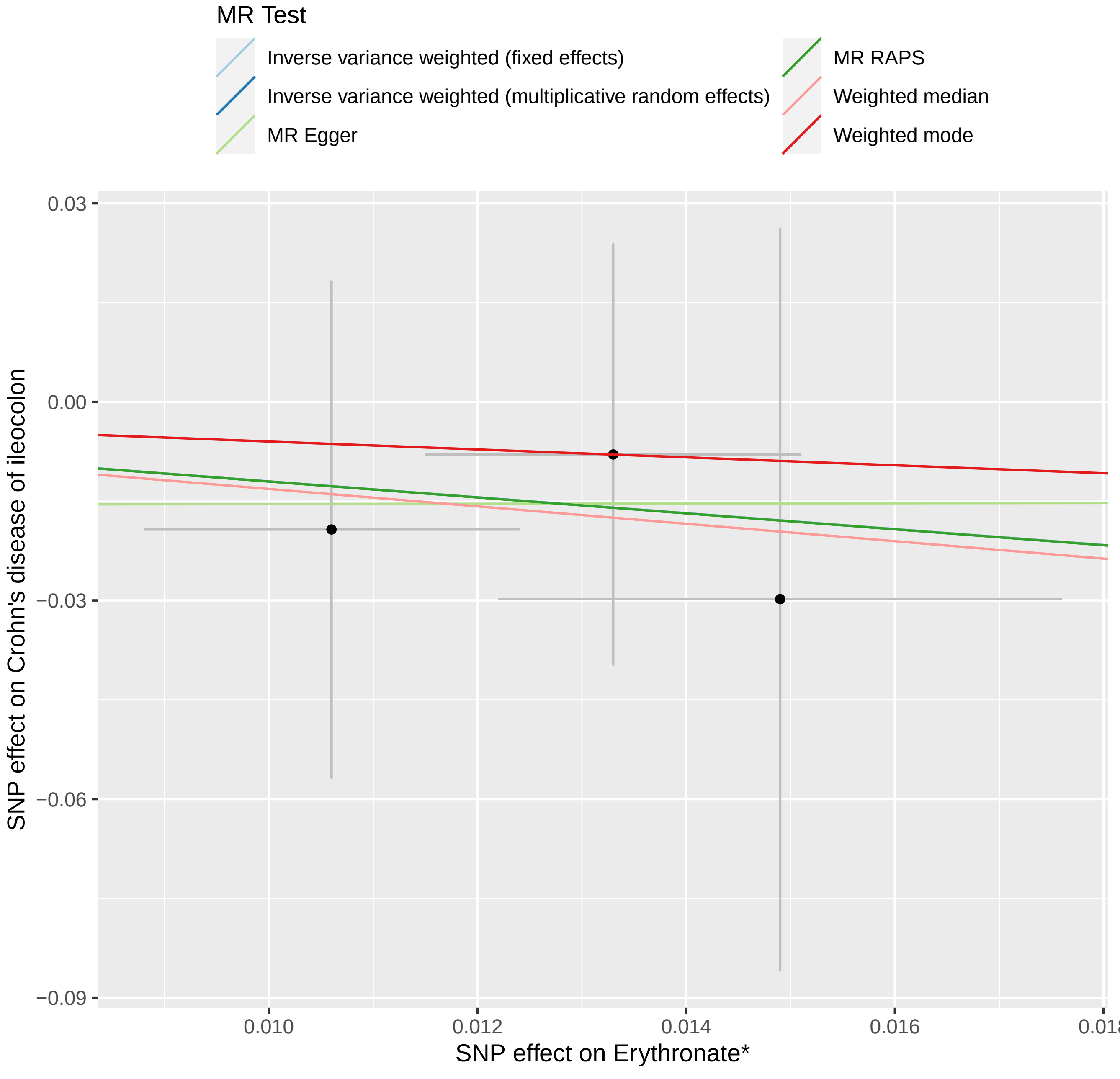

Batch 457 : Lathosterol on Crohn's disease of ileocolon

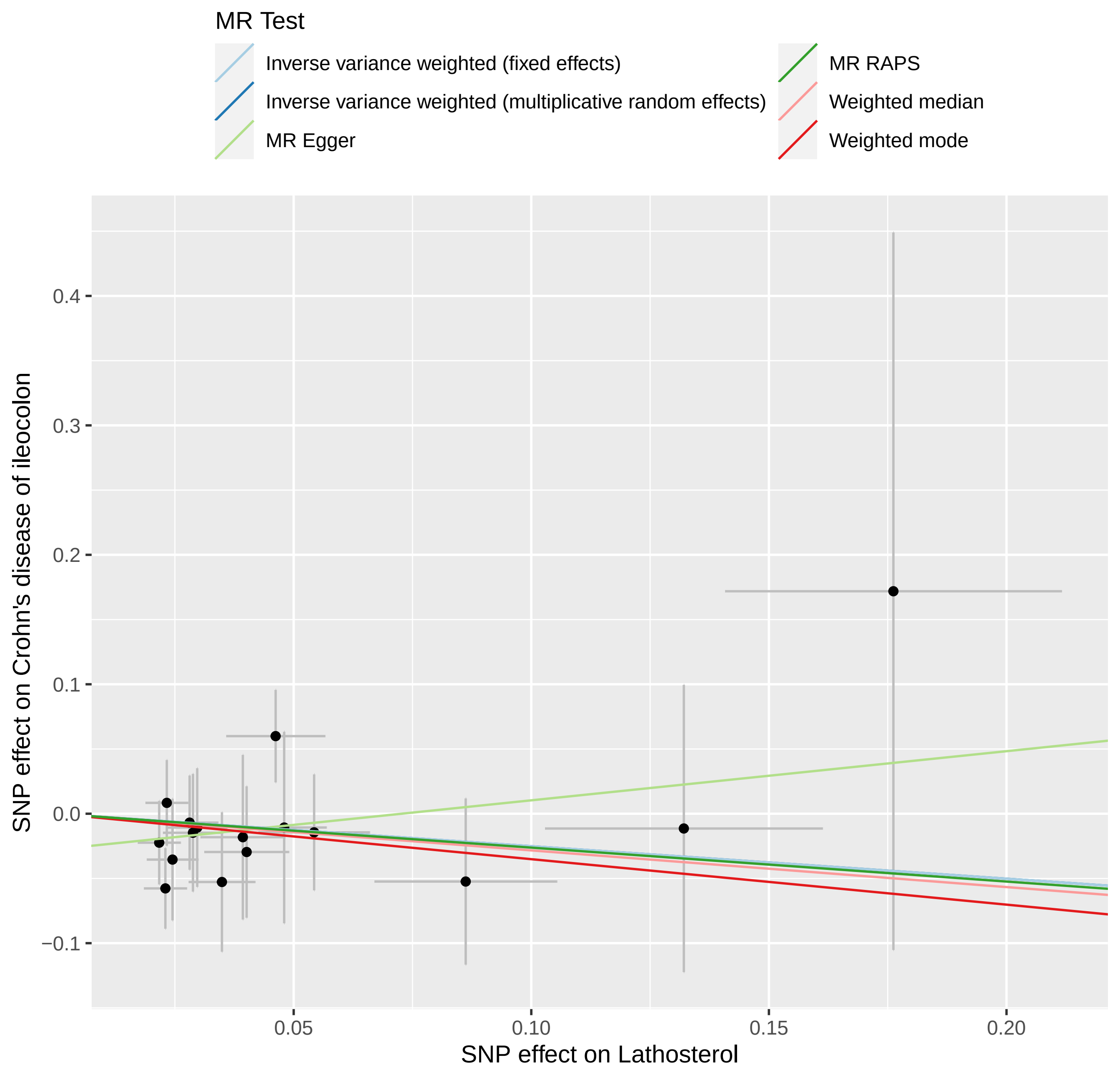

Batch 460 : Eicosenoate (20:1n9 or 11) on Crohn's disease of ileocolon

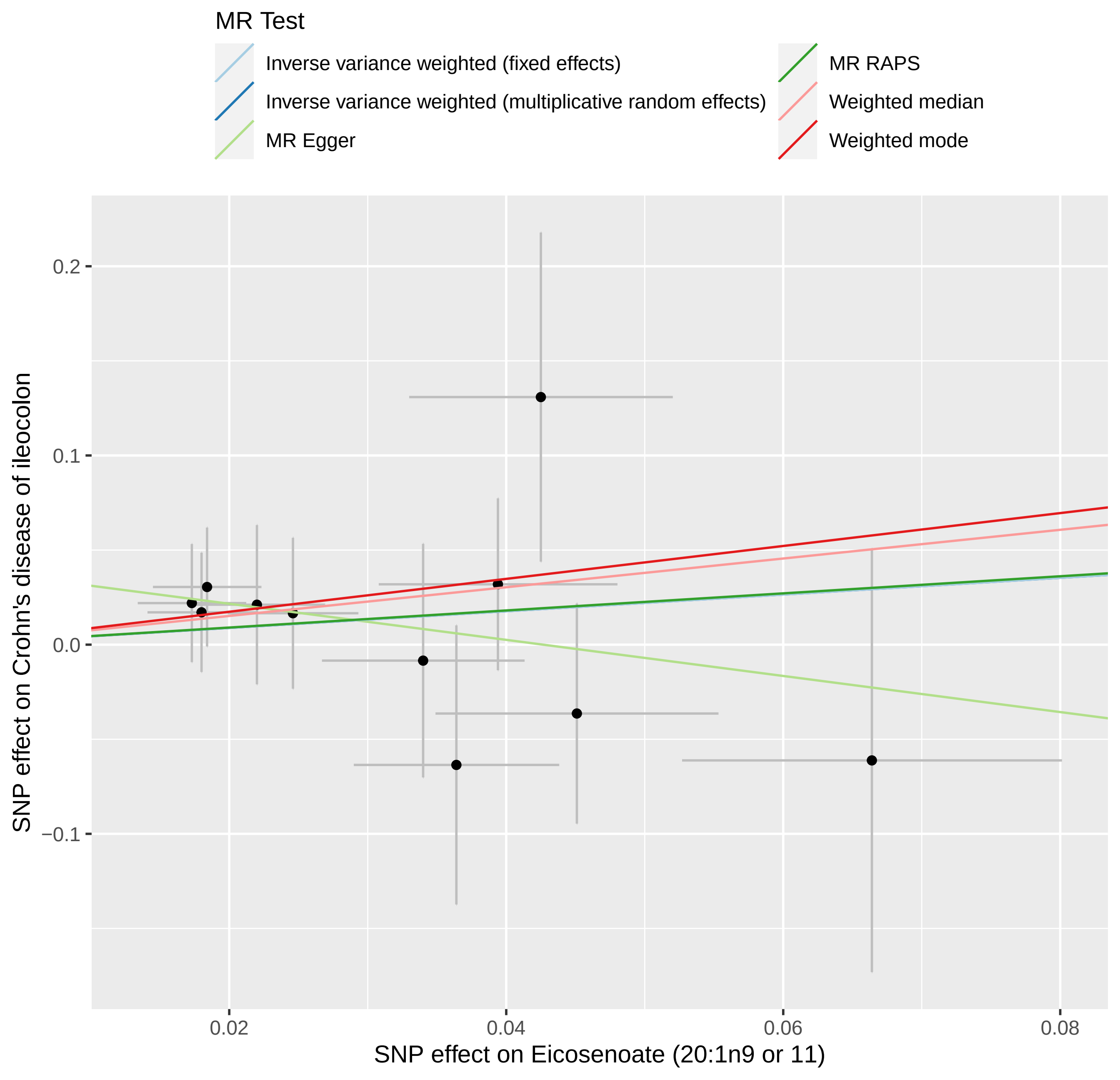

Batch 461 : X−12244−−N−acetylcarnosine on Crohn's disease of ileocolon

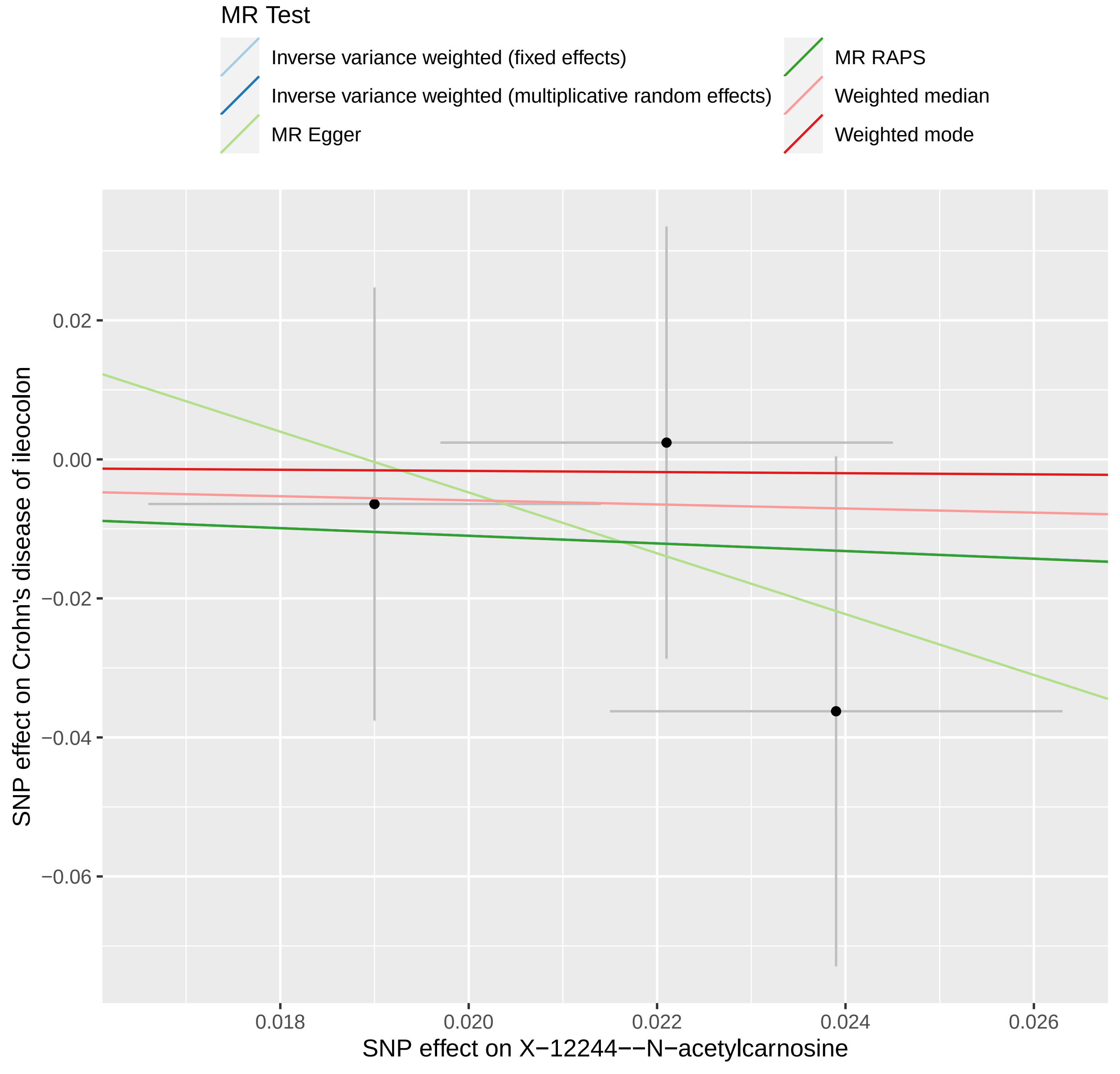

Batch 463 : 1−eicosatrienoylglycerophosphocholine* on Crohn's disease of ileocolon

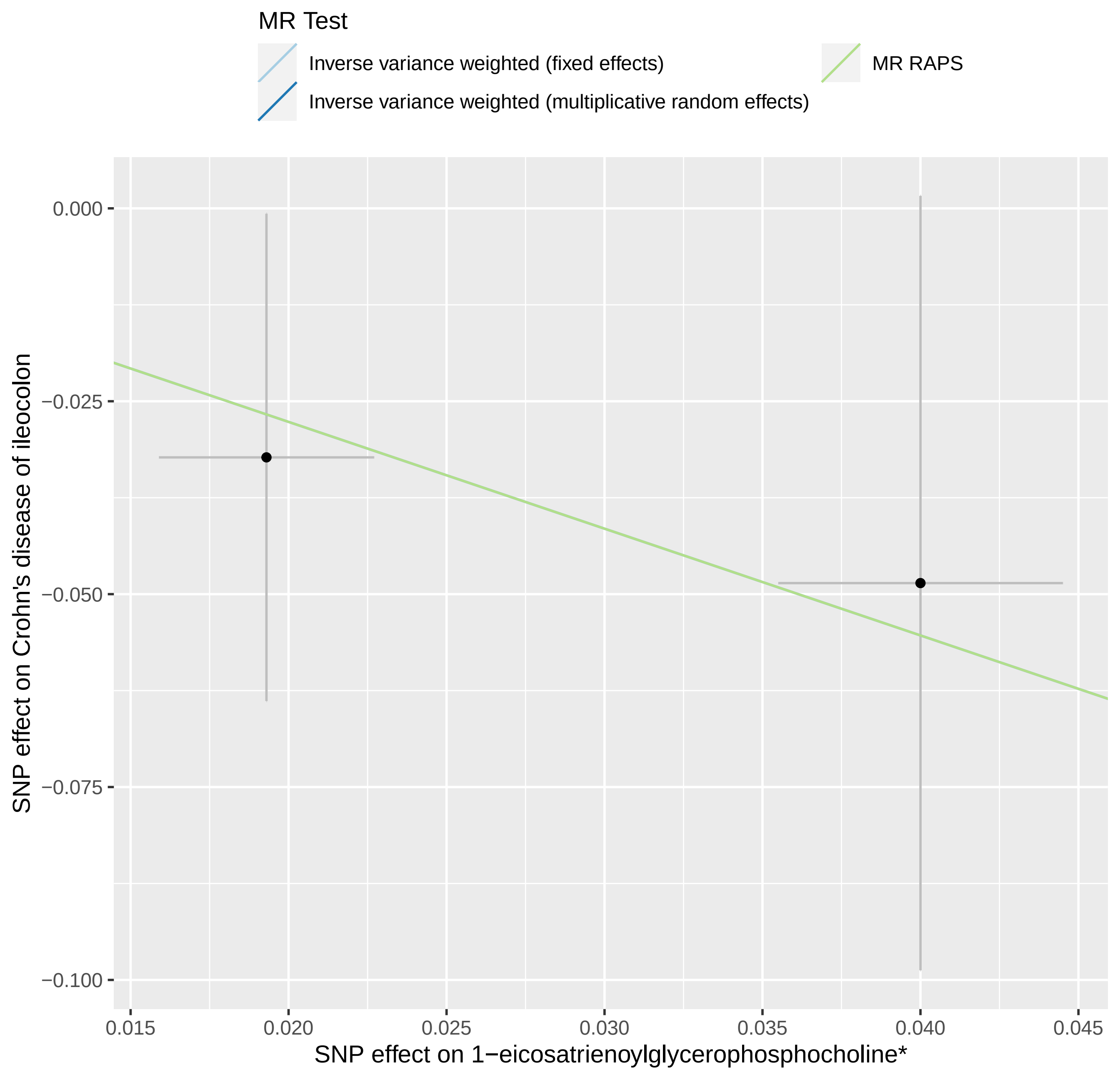

Batch 464 : 1−docosahexaenoylglycerophosphocholine* on Crohn's disease of ileocolon

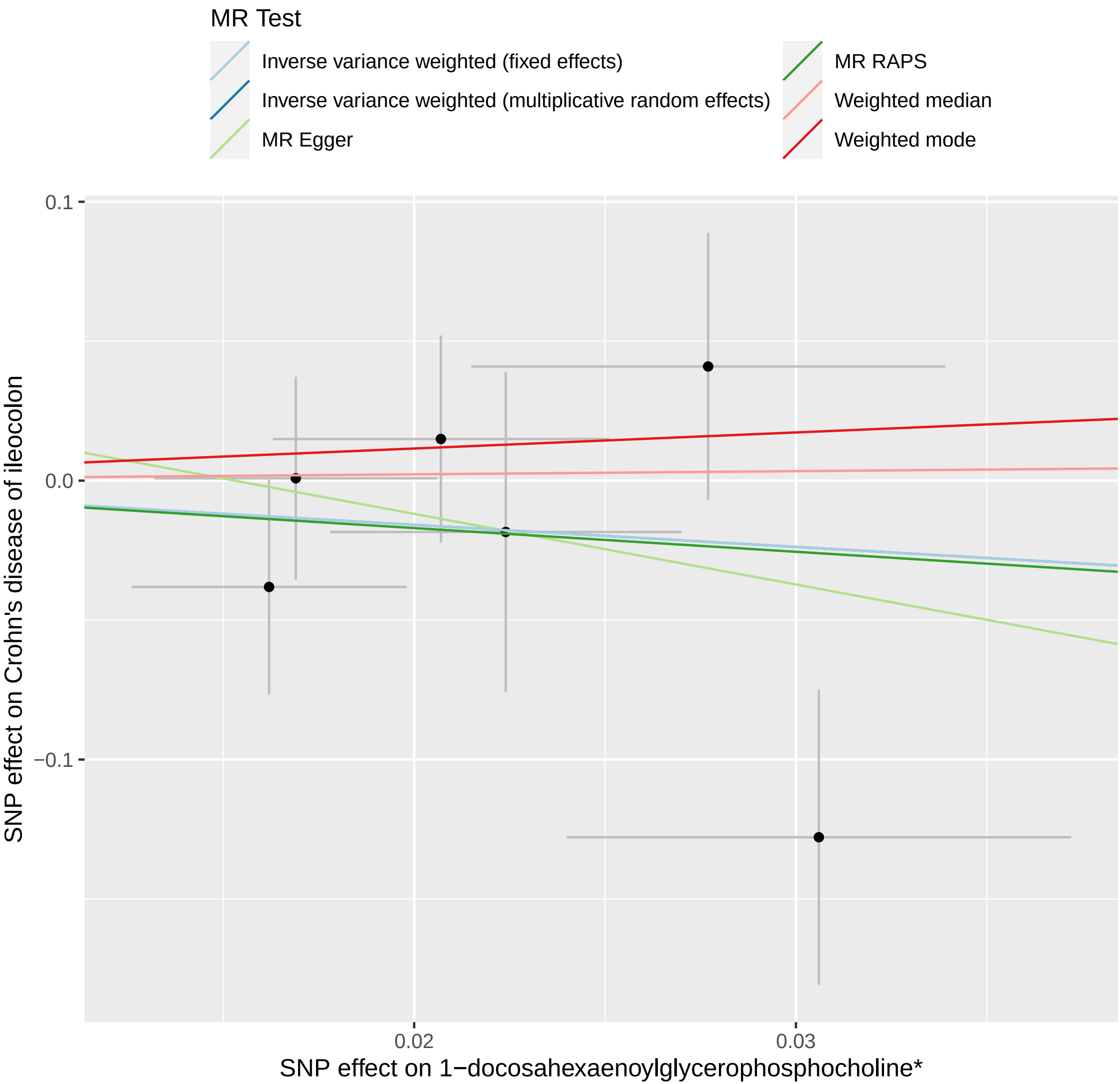

Batch 468 : Octanoylcarnitine on Crohn's disease of ileocolon

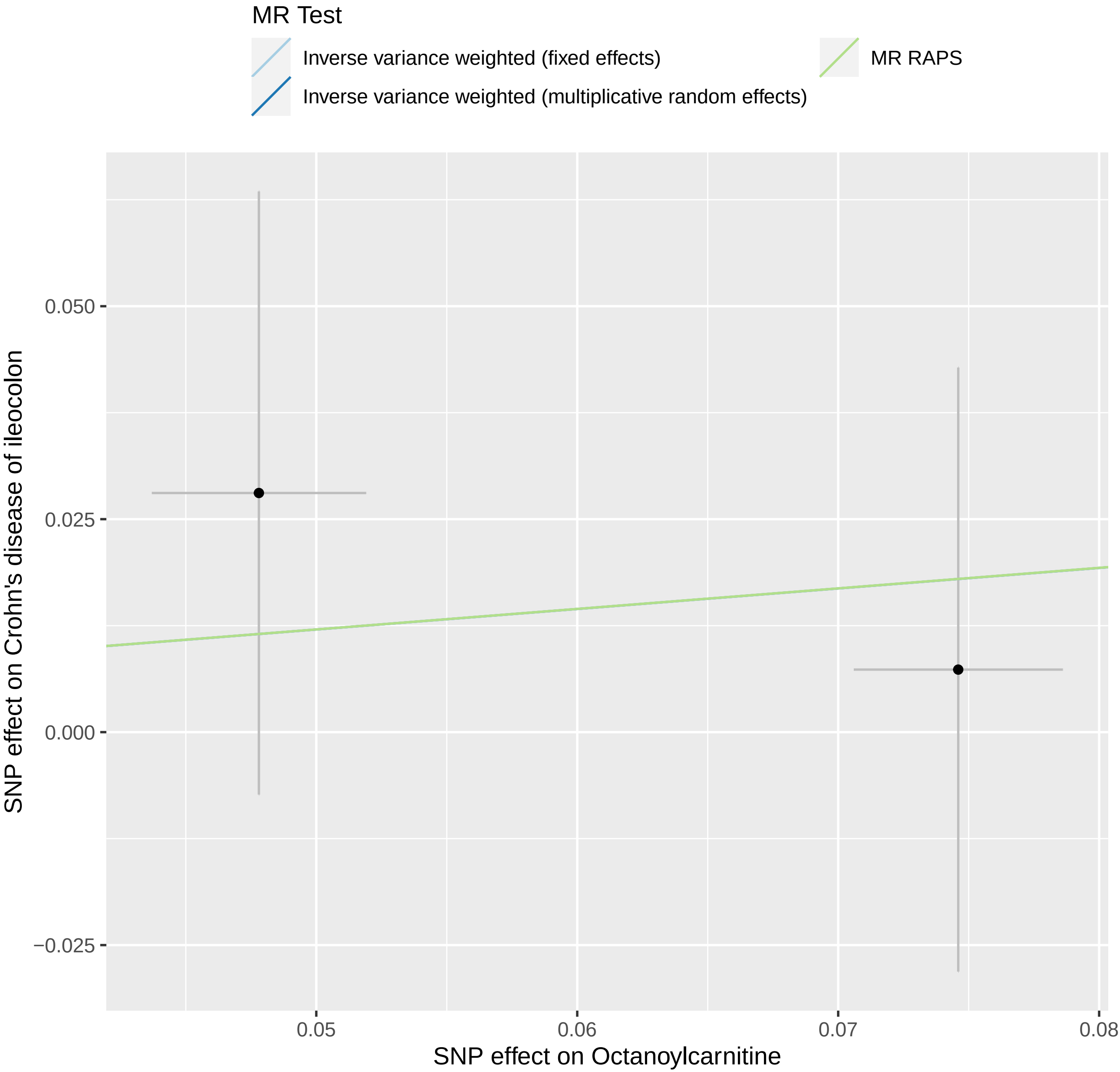

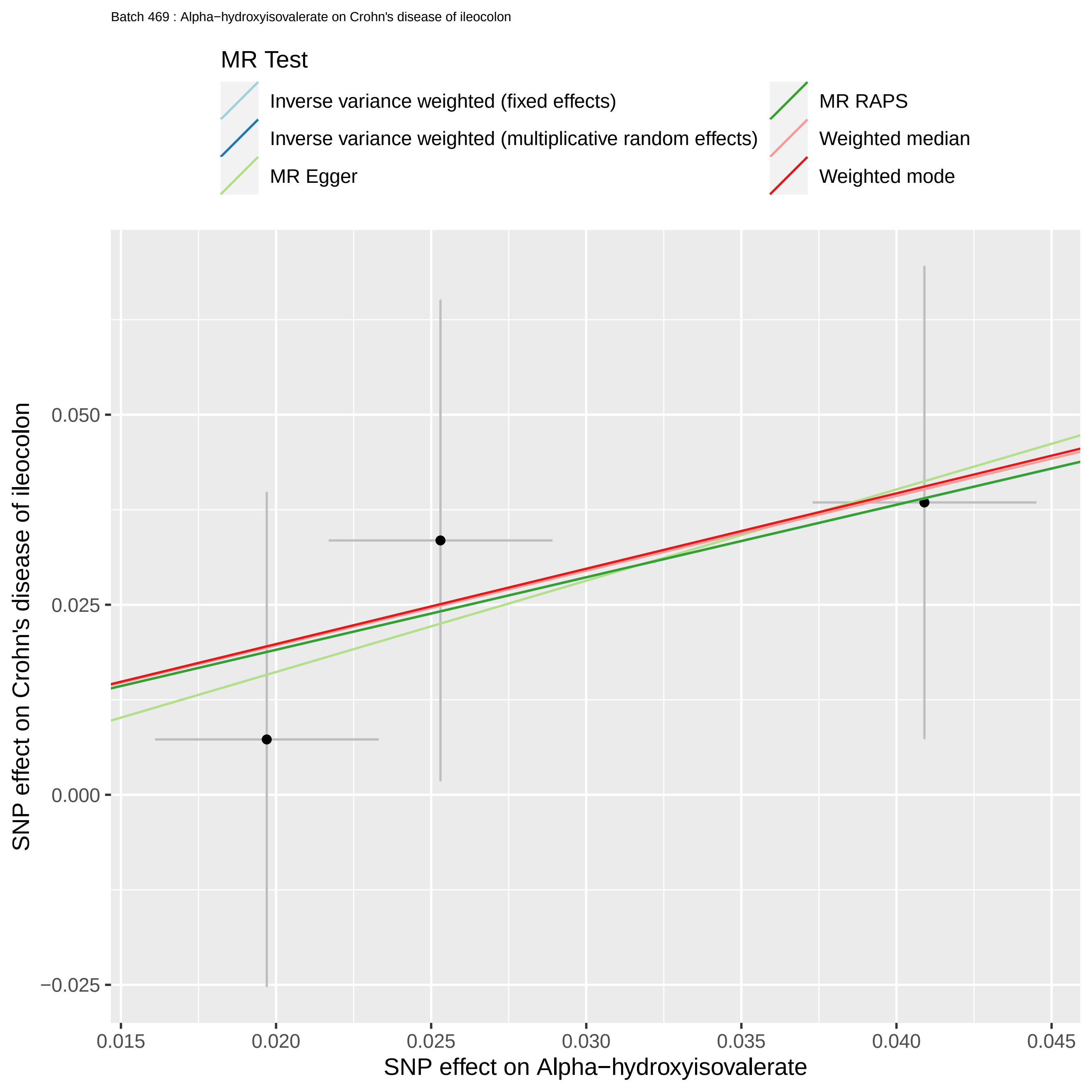
Batch 469 : Alpha−hydroxyisovalerate on Crohn's disease of ileocolon
MR Test
Inverse variance weighted (fixed effects)
Inverse variance weighted (multiplicative random effects)
MR Egger
MR RAPS
Weighted median
Weighted mode
SNP effect on Crohn's disease of ileocolon
0.050
0.025
0.000
−0.025
0.015
0.020
0.025
0.030
0.035
0.040
0.045
SNP effect on Alpha−hydroxyisovalerate

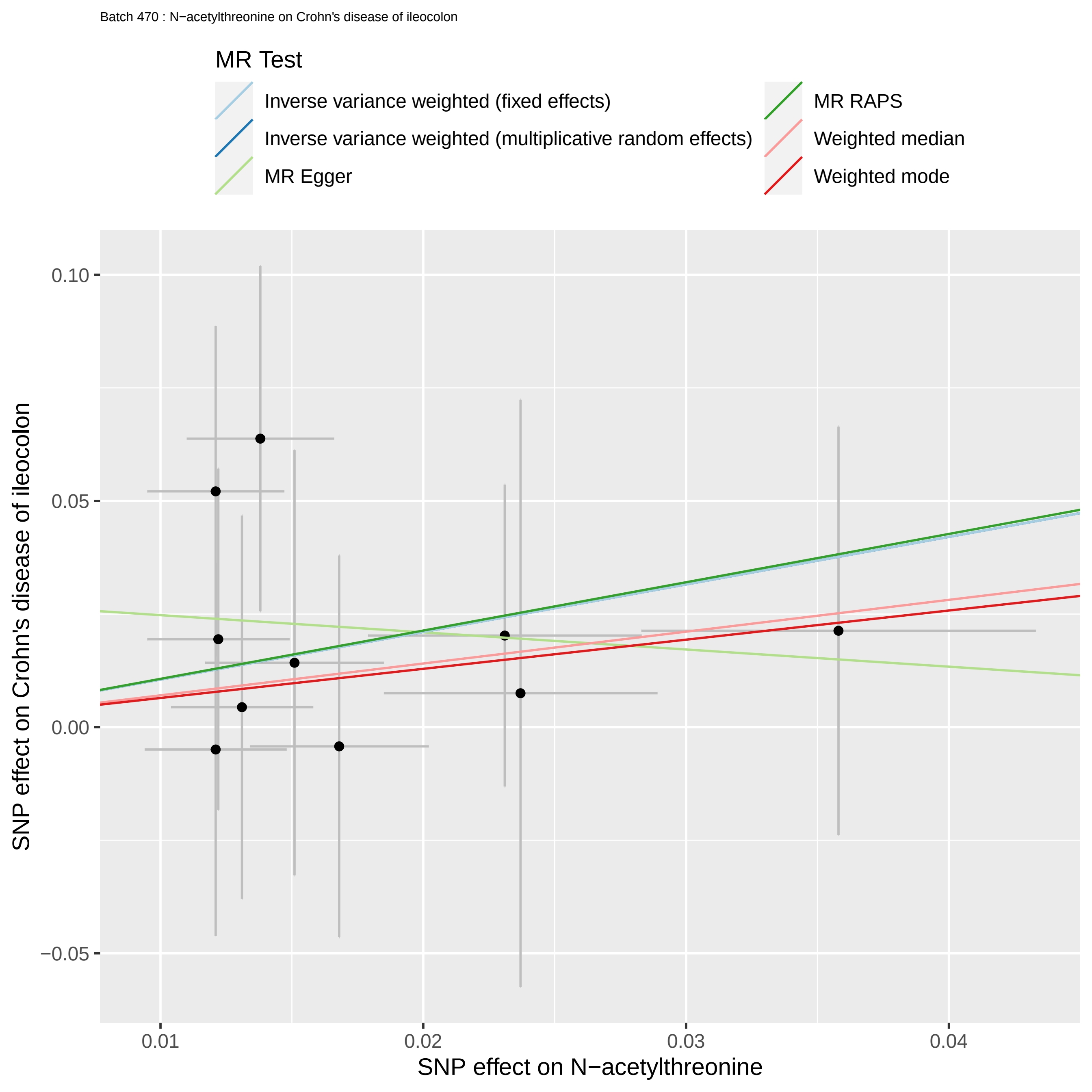

Batch 470 : N−acetylthreonine on Crohn's disease of ileocolon
MR Test
Inverse variance weighted (fixed effects)
Inverse variance weighted (multiplicative random effects)
MR Egger
MR RAPS
Weighted median
Weighted mode
0.10
0.05
0.00
−0.05
0.01
0.02
0.03
0.04
SNP effect on Crohn's disease of ileocolon
SNP effect on N−acetylthreonine

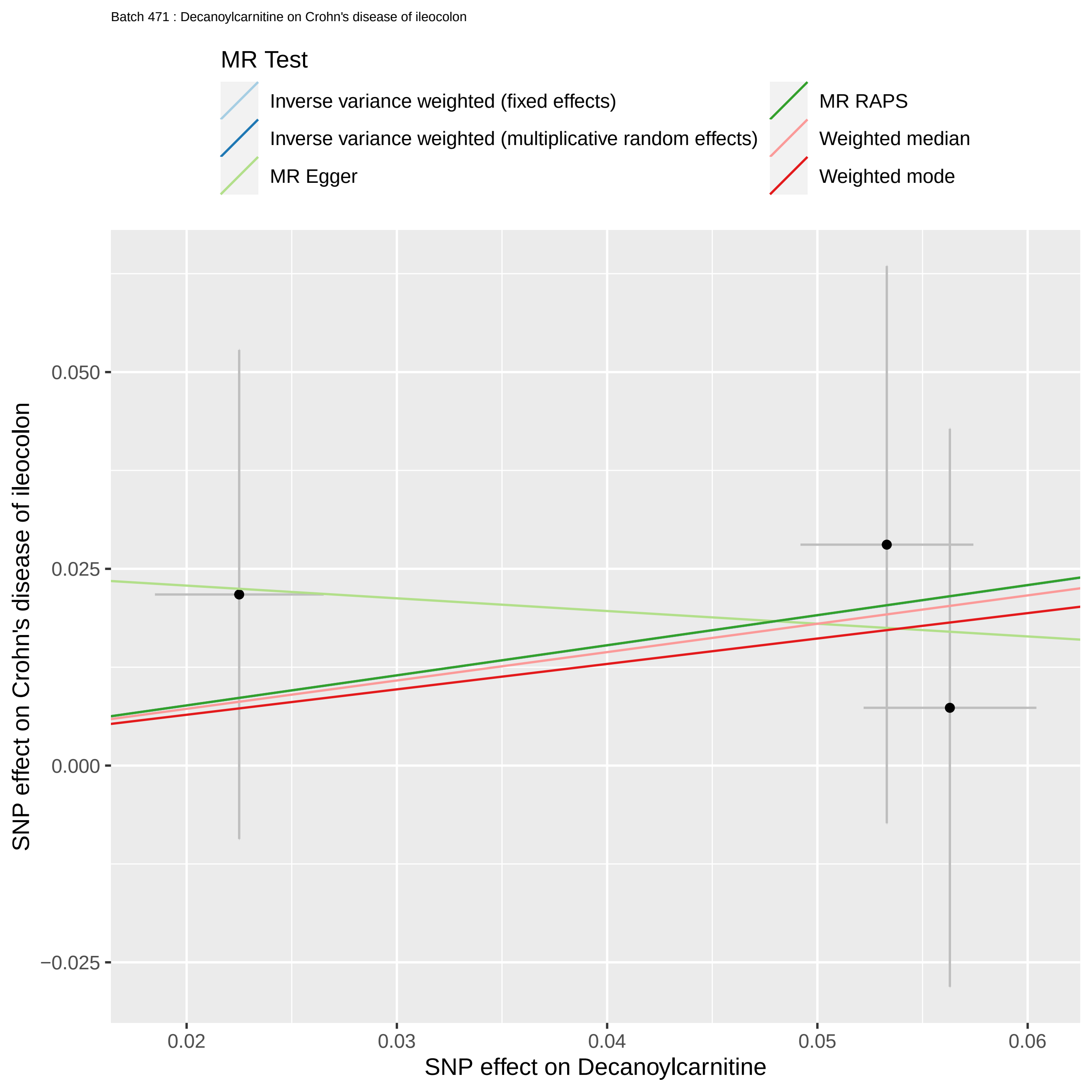
Batch 471 : Decanoylcarnitine on Crohn's disease of ileocolon
MR Test
Inverse variance weighted (fixed effects)
Inverse variance weighted (multiplicative random effects)
MR Egger
MR RAPS
Weighted median
Weighted mode
0.050
0.025
0.000
−0.025
SNP effect on Crohn's disease of ileocolon
0.02
0.03
0.04
0.05
0.06
SNP effect on Decanoylcarnitine

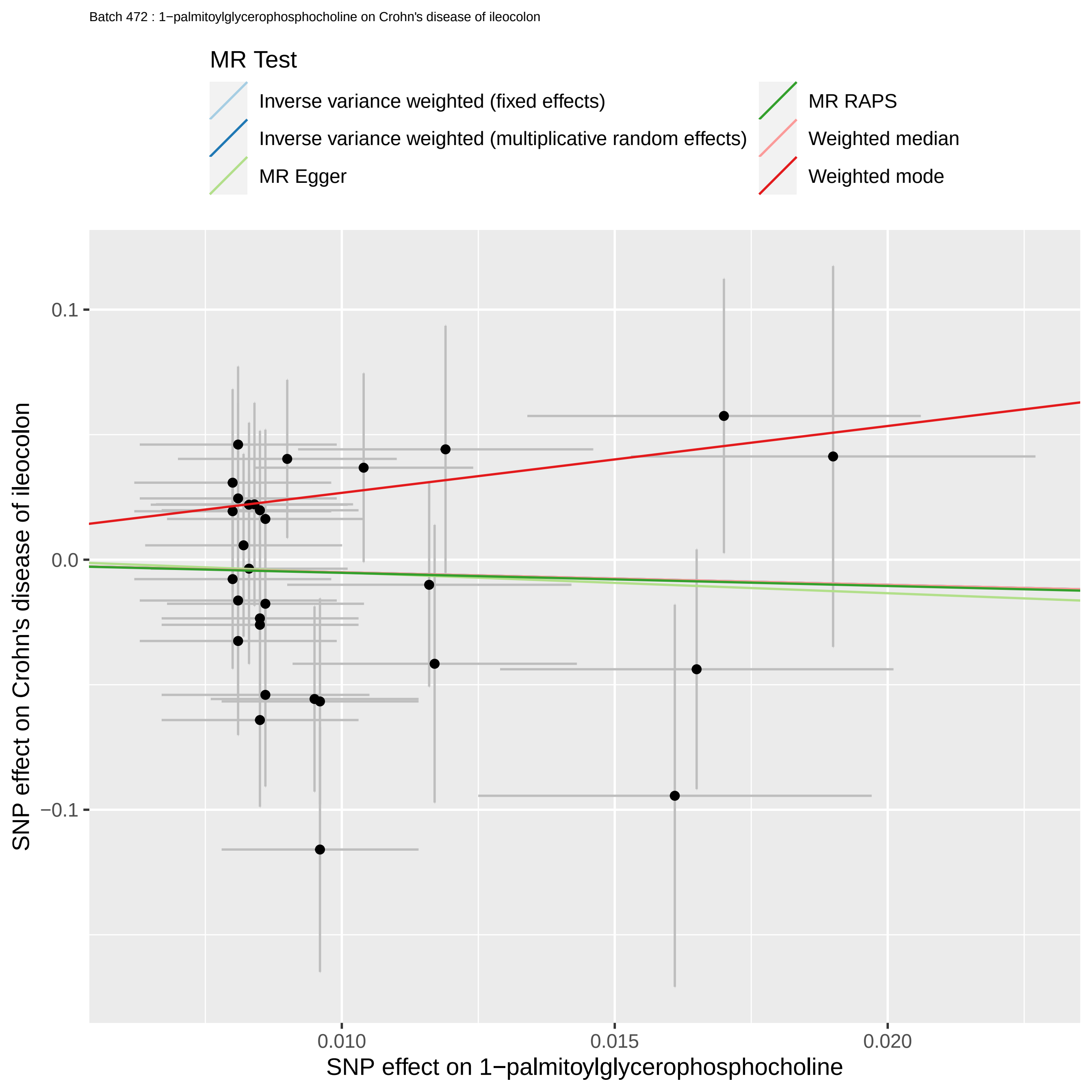
Batch 472 : 1−palmitoylglycerophosphocholine on Crohn's disease of ileocolon
MR Test
Inverse variance weighted (fixed effects)
Inverse variance weighted (multiplicative random effects)
MR Egger
MR RAPS
Weighted median
Weighted mode
0.1
0.0
−0.1
0.010
0.015
0.020
SNP effect on Crohn's disease of ileocolon
SNP effect on 1−palmitoylglycerophosphocholine

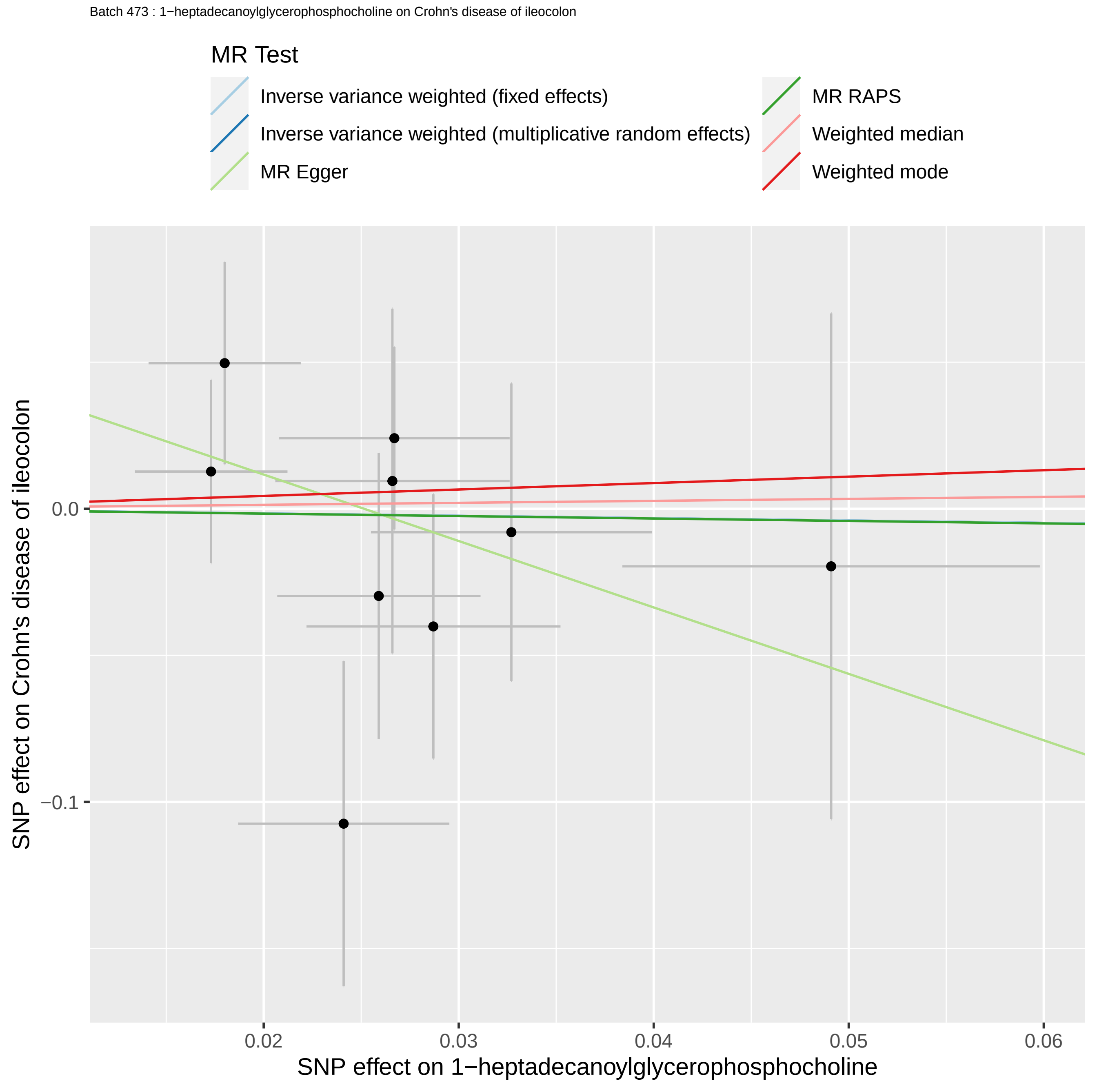

Batch 473 : 1−heptadecanoylglycerophosphocholine on Crohn's disease of ileocolon
MR Test
Inverse variance weighted (fixed effects)
Inverse variance weighted (multiplicative random effects)
MR Egger
MR RAPS
Weighted median
Weighted mode
0.0
−0.1
0.02
0.03
0.04
0.05
0.06
SNP effect on Crohn's disease of ileocolon
SNP effect on 1−heptadecanoylglycerophosphocholine

Batch 474 : 1−oleoylglycerophosphocholine on Crohn's disease of ileocolon

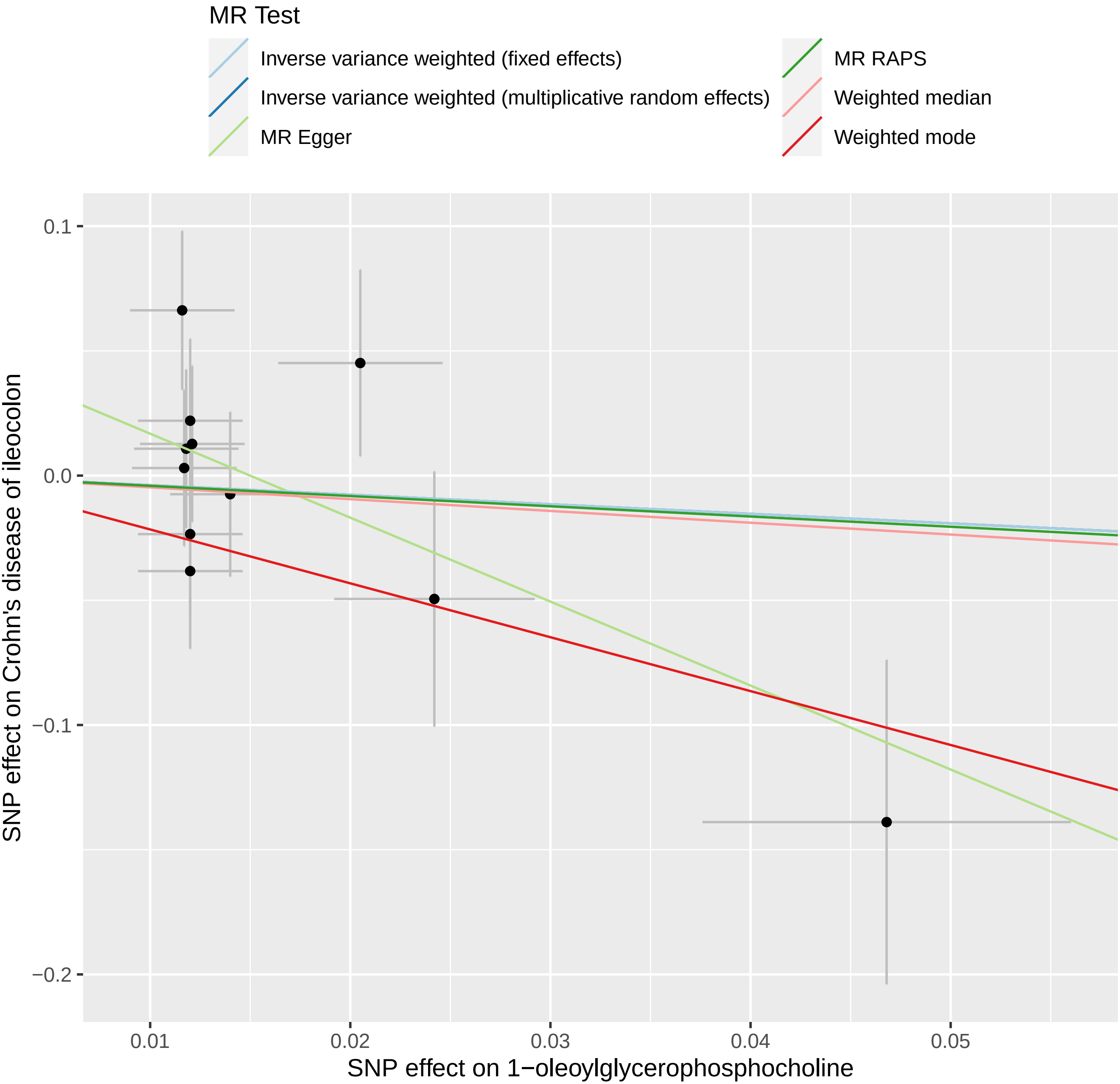

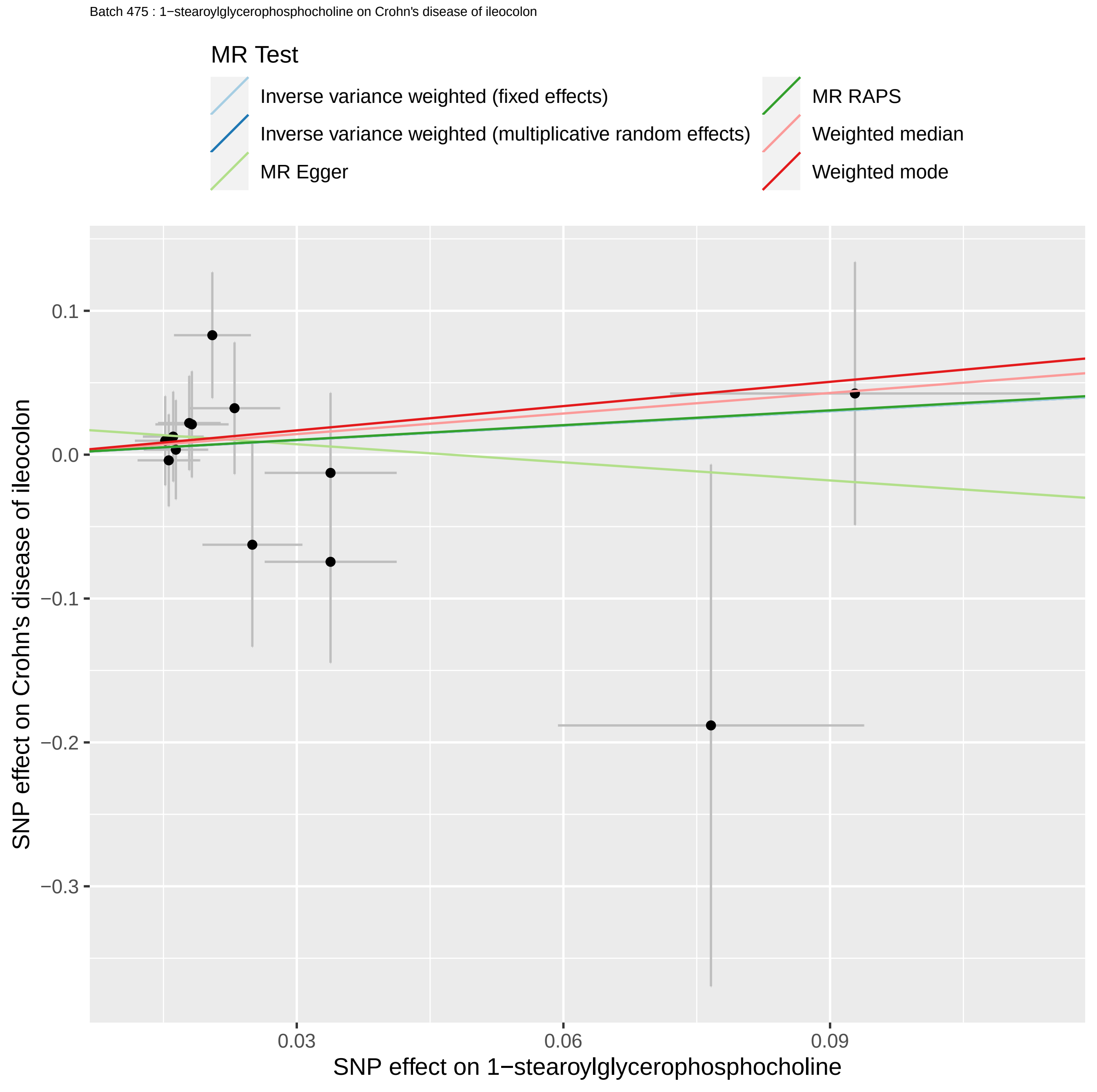
Batch 475 : 1−stearoylglycerophosphocholine on Crohn's disease of ileocolon
MR Test
Inverse variance weighted (fixed effects)
Inverse variance weighted (multiplicative random effects)
MR Egger
MR RAPS
Weighted median
Weighted mode
0.1
0.0
−0.1
−0.2
−0.3
0.03
0.06
0.09
SNP effect on Crohn's disease of ileocolon
SNP effect on 1−stearoylglycerophosphocholine

Batch 478 : 10−heptadecenoate (17:1n7) on Crohn's disease of ileocolon

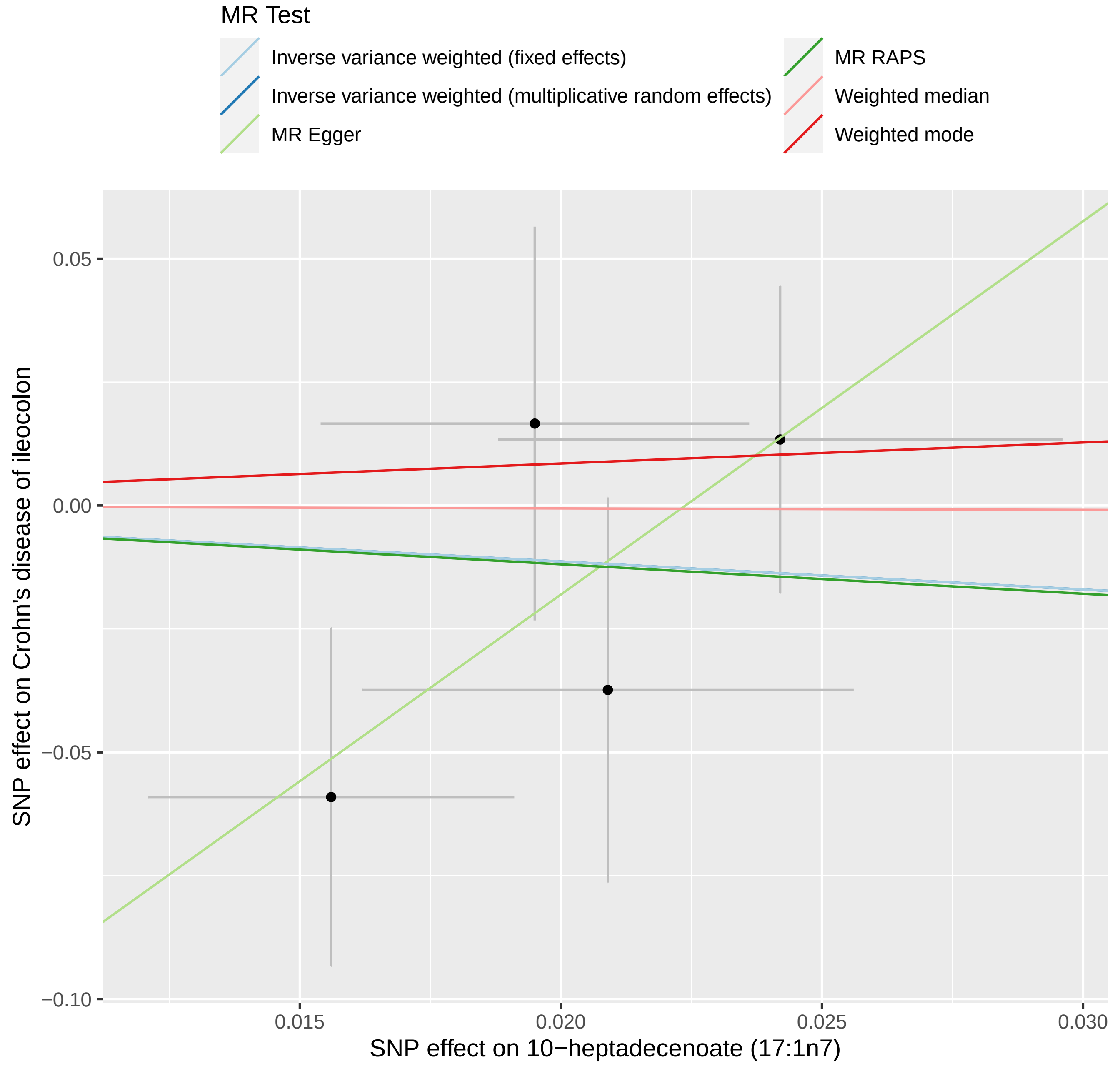

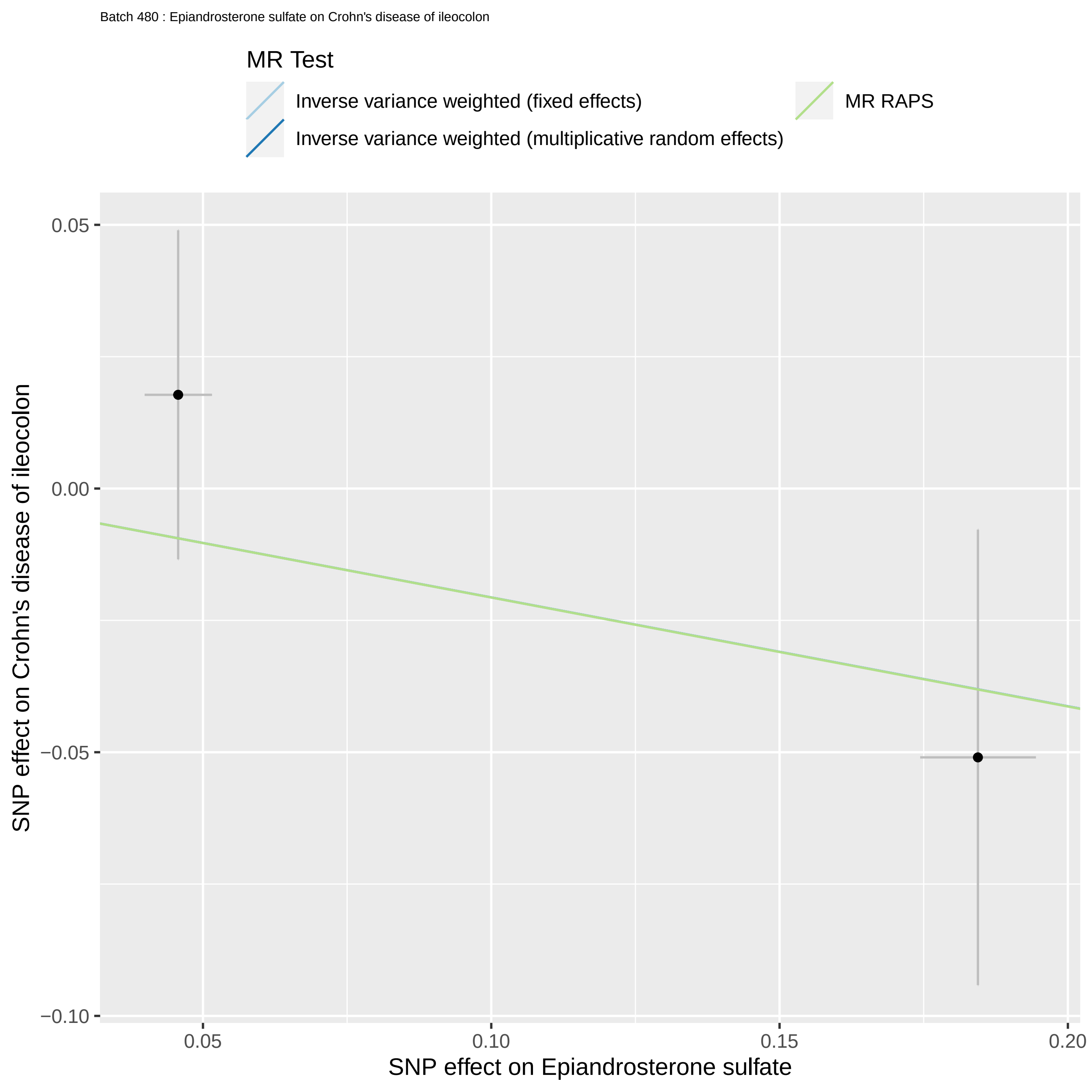

Batch 480 : Epiandrosterone sulfate on Crohn's disease of ileocolon
MR Test
Inverse variance weighted (fixed effects)
Inverse variance weighted (multiplicative random effects)
MR RAPS
0.05
0.00
−0.05
−0.10
0.05
0.10
0.15
0.20
SNP effect on Epiandrosterone sulfate
SNP effect on Crohn's disease of ileocolon

Batch 481 : Linolenate [alpha or gamma; (18:3n3 or 6)] on Crohn's disease of ileocolon

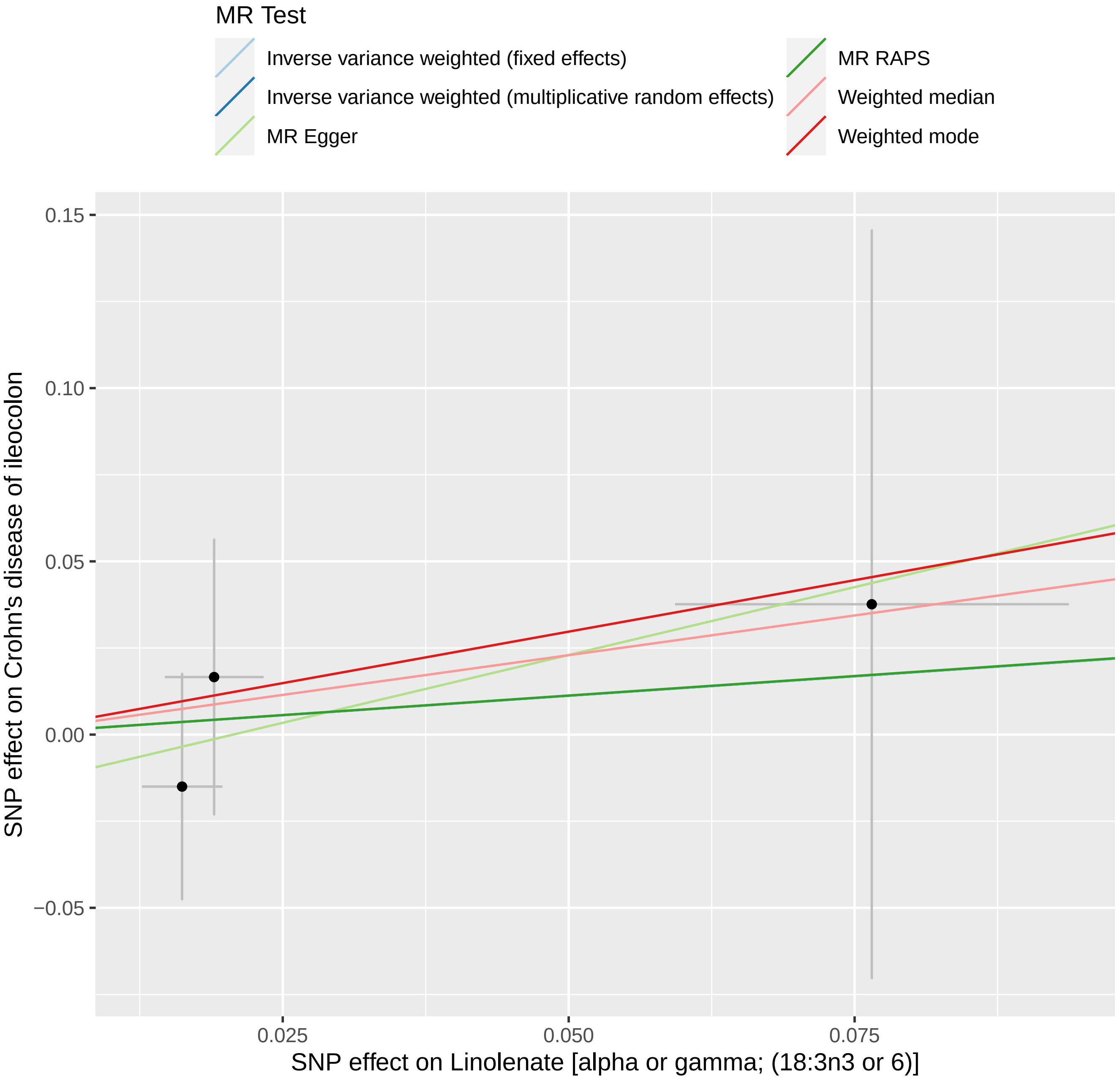

Batch 482 : X−12510−−2−aminooctanoic acid on Crohn's disease of ileocolon

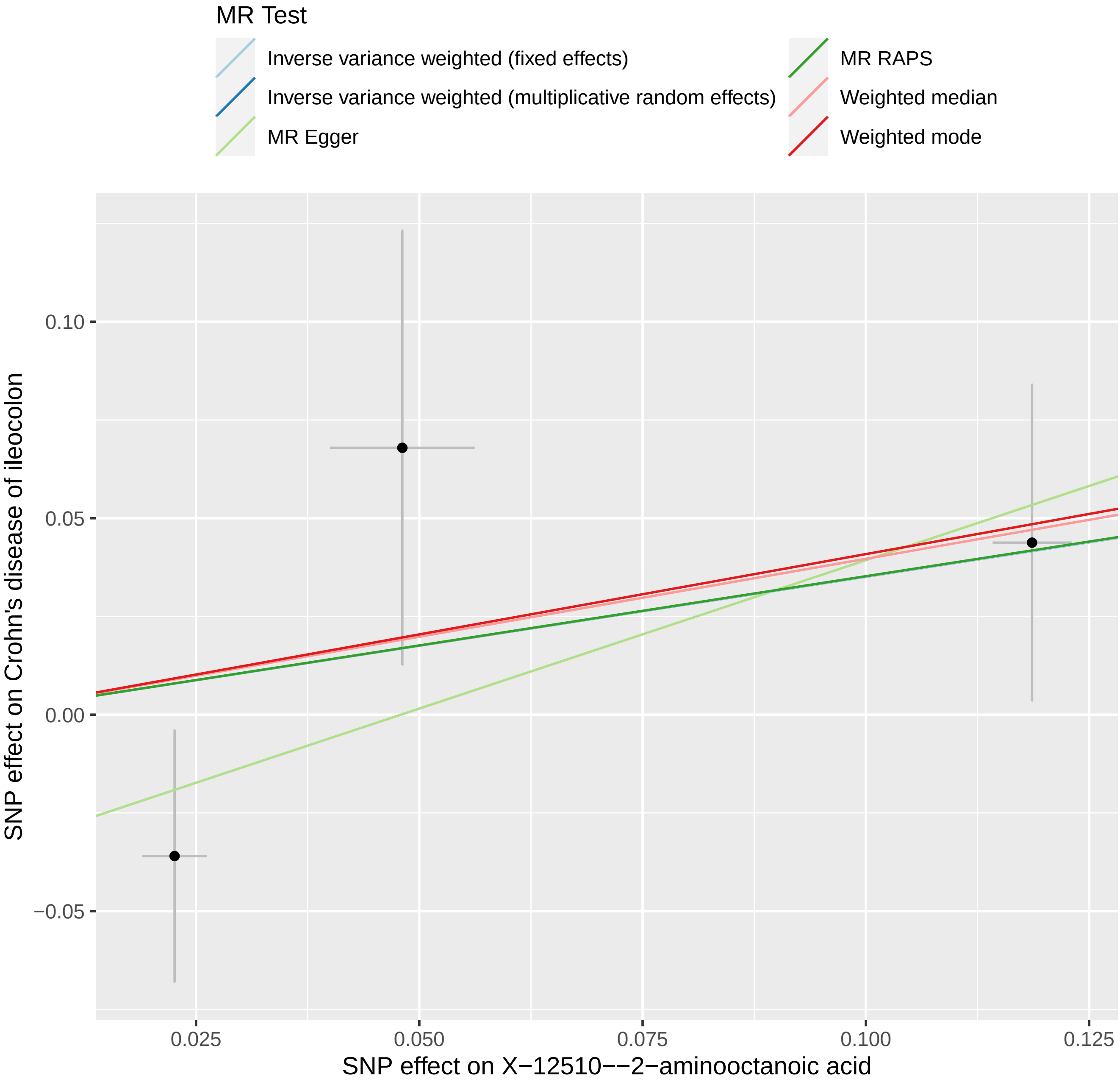

Batch 485 : Asparagine on Crohn's disease of ileocolon

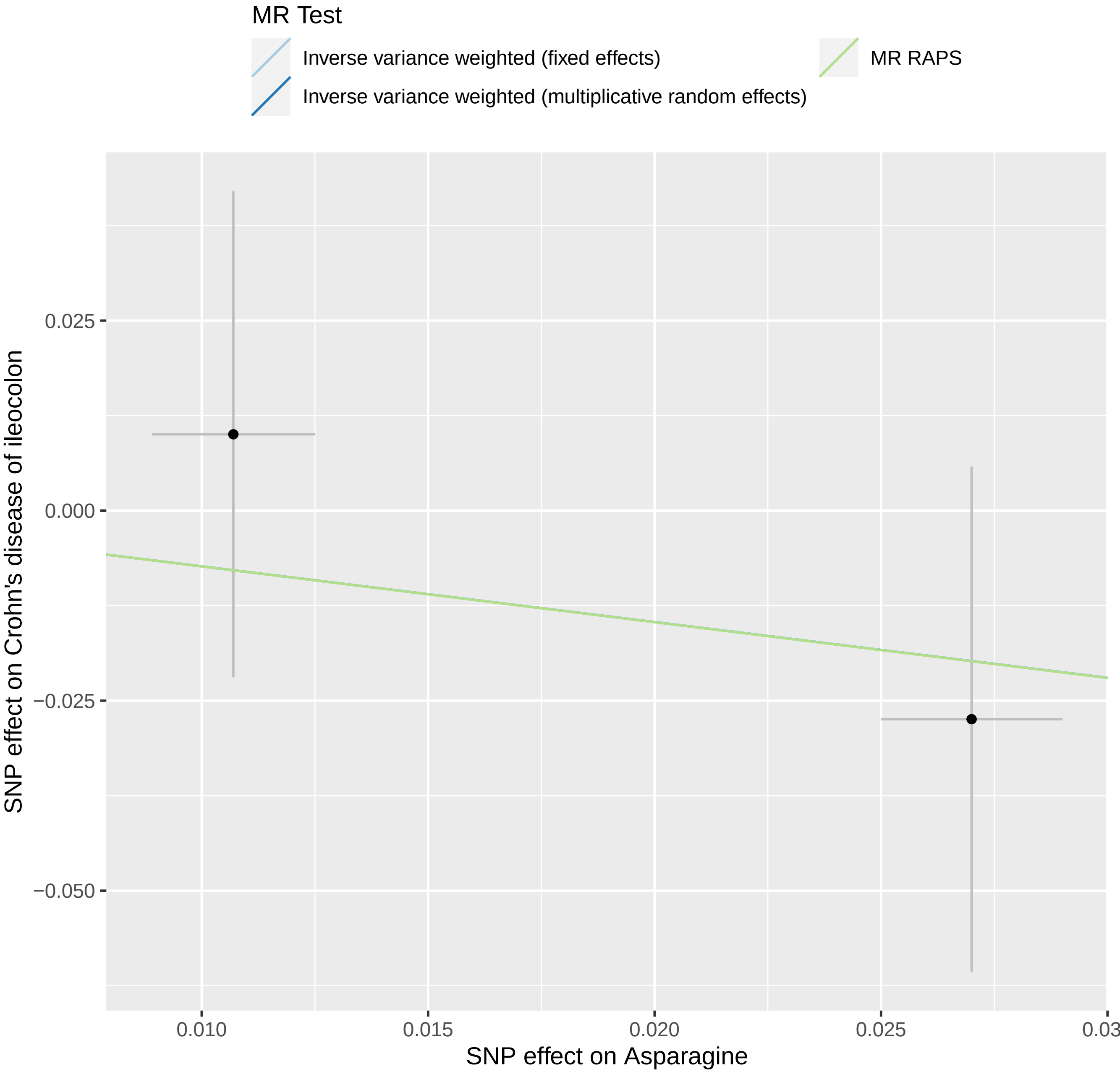

Batch 486 : Stachydrine on Crohn's disease of ileocolon

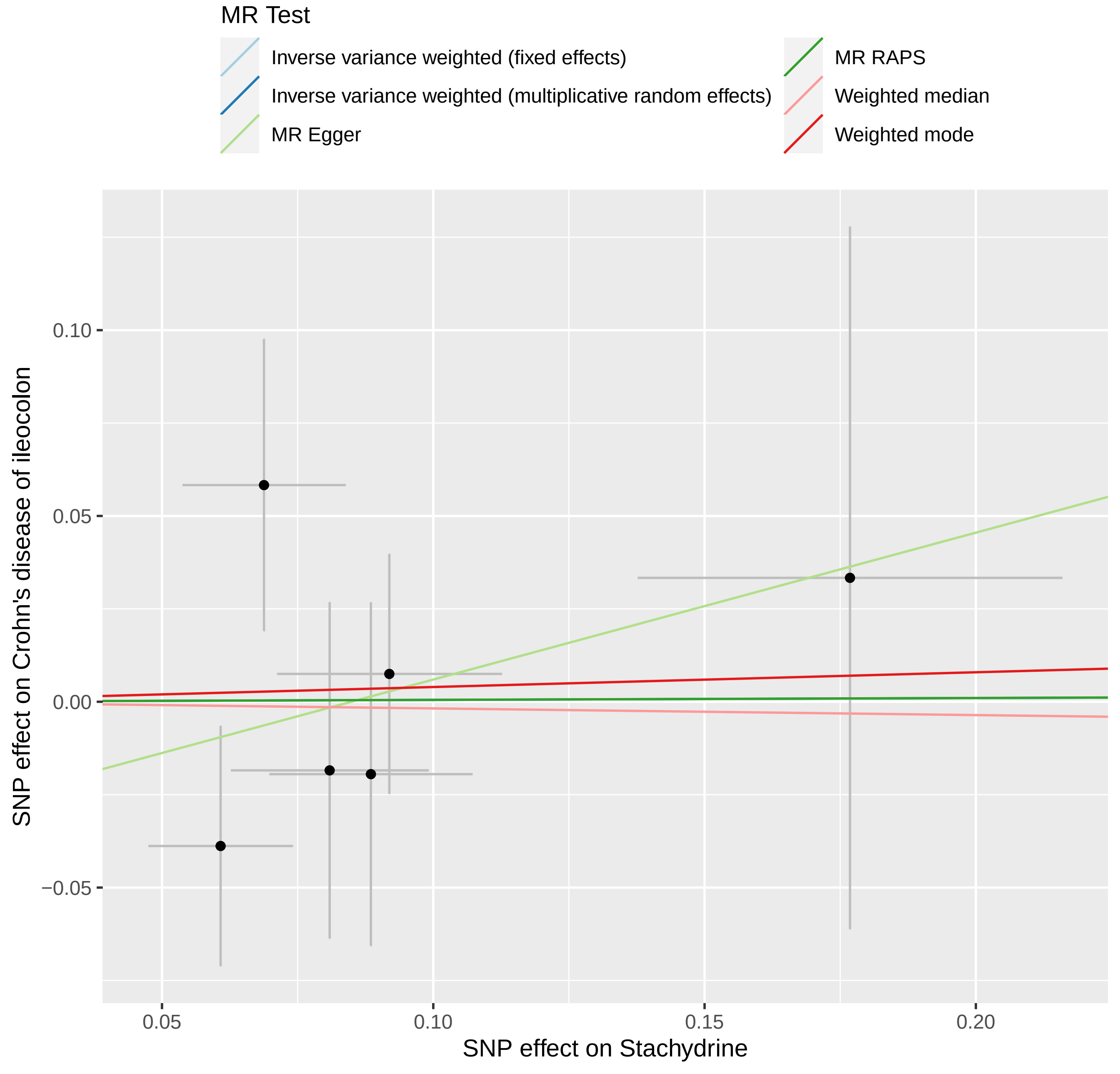

Batch 487 : Isovalerylcarnitine on Crohn's disease of ileocolon

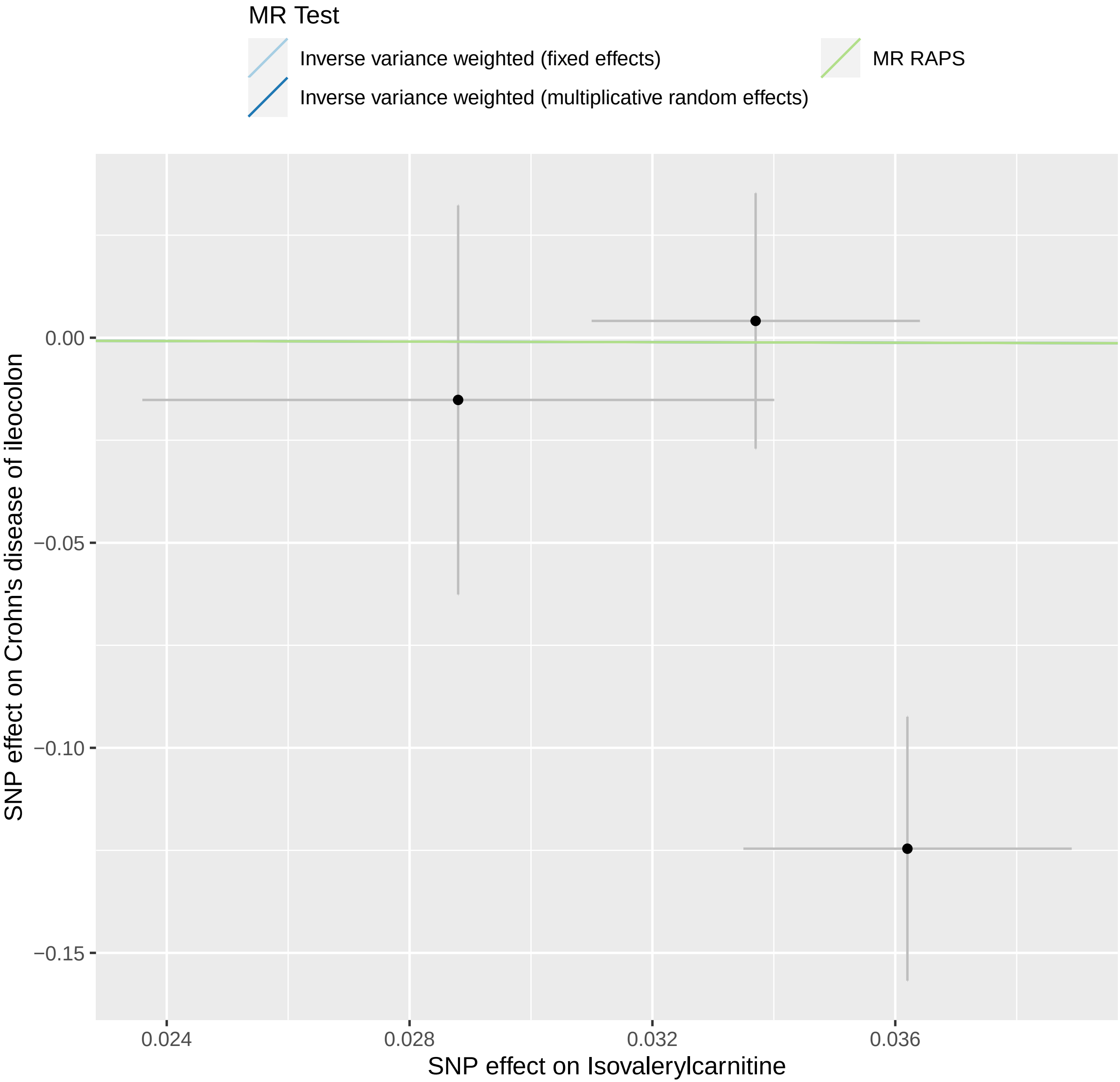

Batch 490 : 1−linoleoylglycerophosphocholine on Crohn's disease of ileocolon

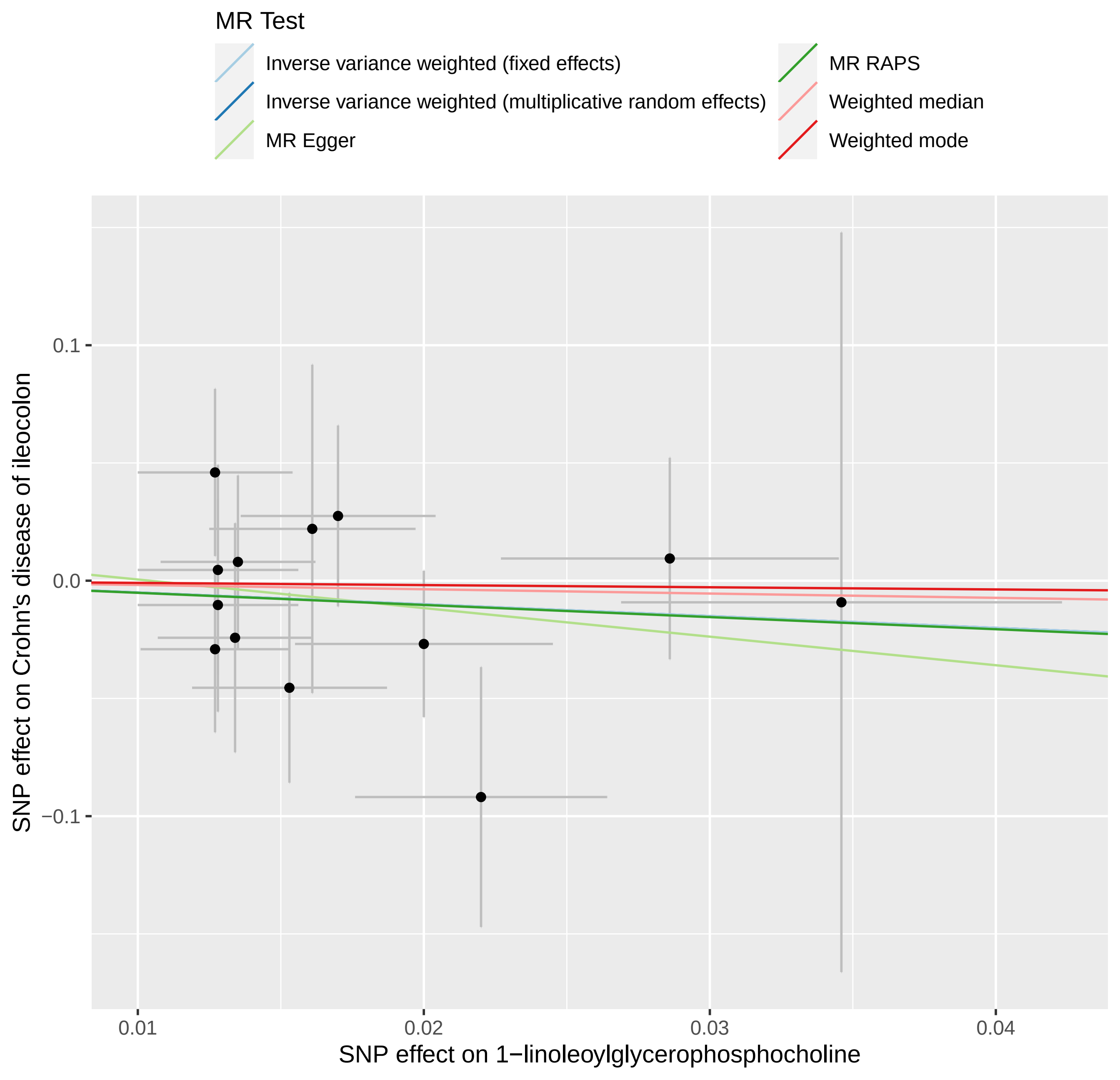

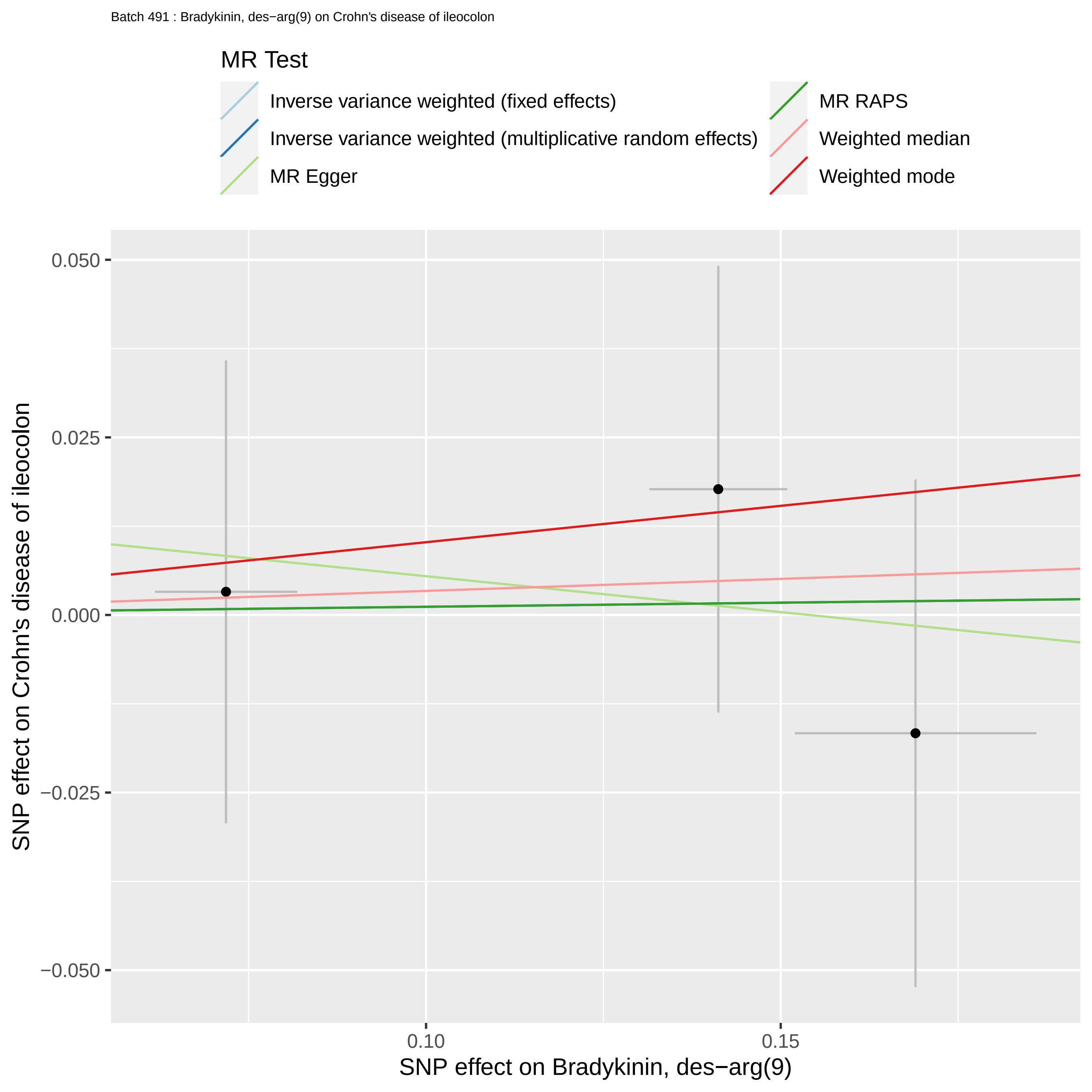

Batch 491 : Bradykinin, des−arg(9) on Crohn's disease of ileocolon
MR Test
Inverse variance weighted (fixed effects)
Inverse variance weighted (multiplicative random effects)
MR Egger
MR RAPS
Weighted median
Weighted mode
0.050
0.025
0.000
−0.025
−0.050
0.10
0.15
SNP effect on Bradykinin, des−arg(9)
SNP effect on Crohn's disease of ileocolon

Batch 492 : Gamma−glutamylisoleucine* on Crohn's disease of ileocolon

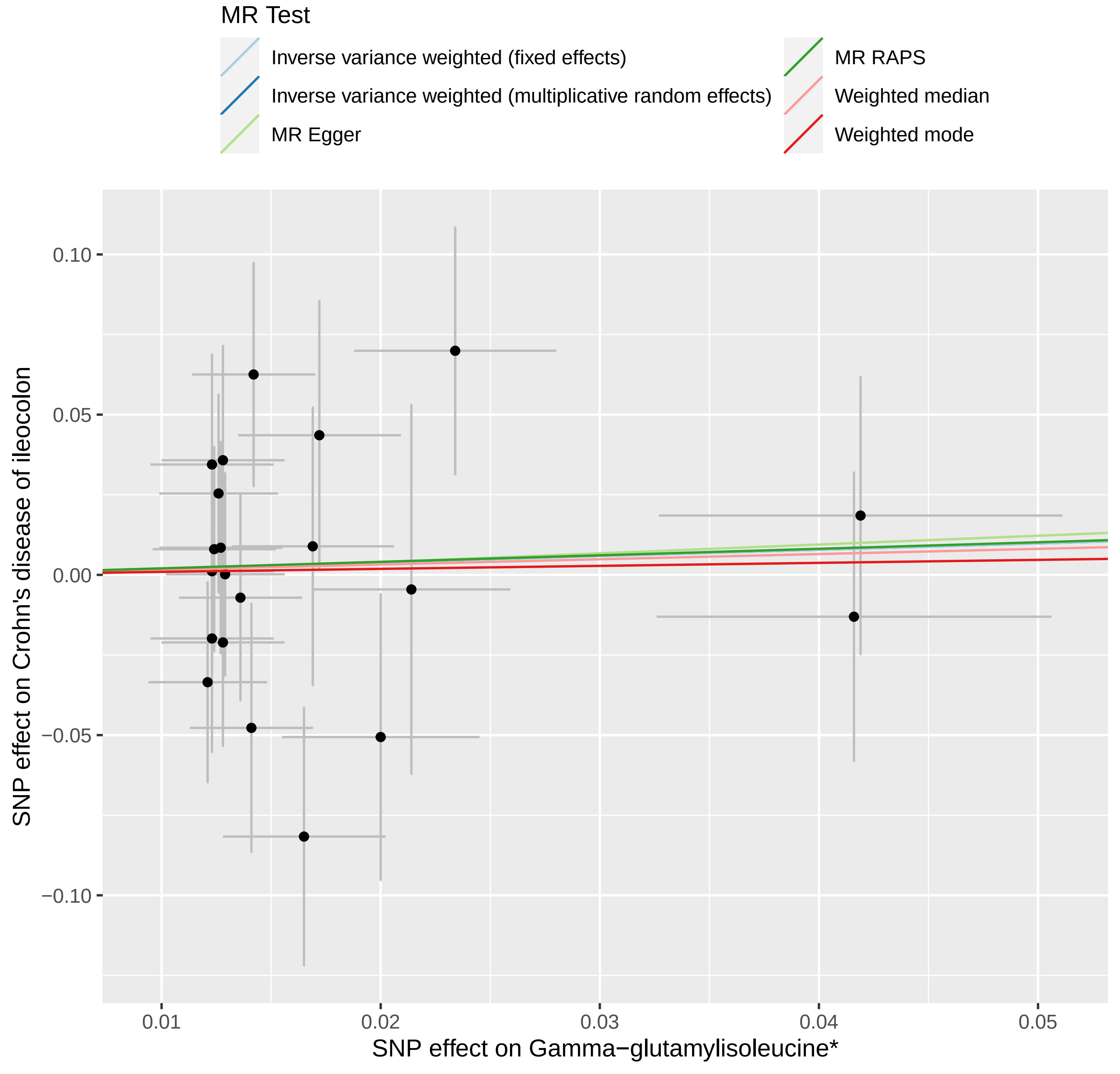

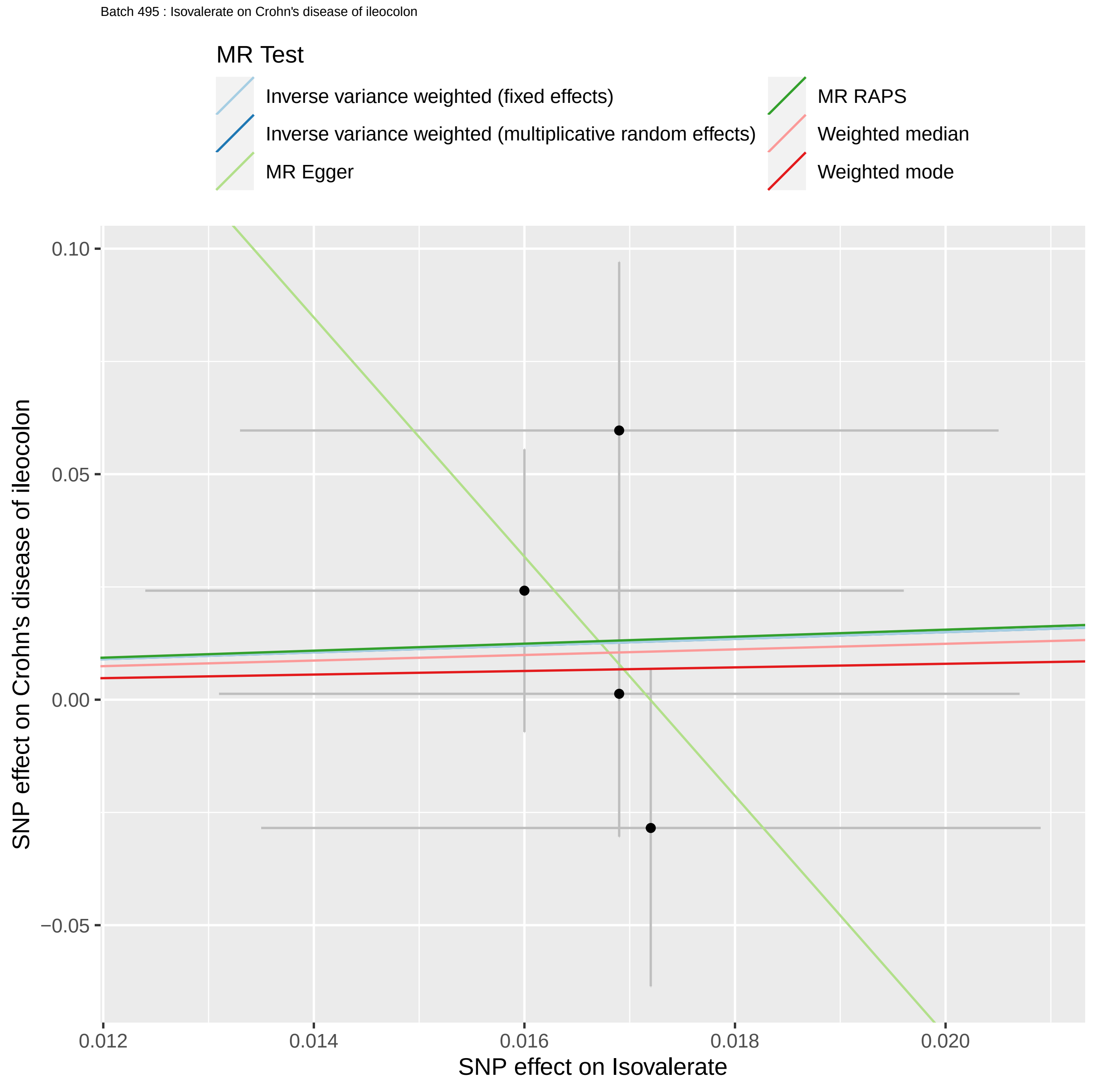

Batch 495 : Isovalerate on Crohn's disease of ileocolon
MR Test
Inverse variance weighted (fixed effects)
Inverse variance weighted (multiplicative random effects)
MR Egger
MR RAPS
Weighted median
Weighted mode
0.10
0.05
0.00
−0.05
0.012
0.014
0.016
0.018
0.020
SNP effect on Crohn's disease of ileocolon
SNP effect on Isovalerate

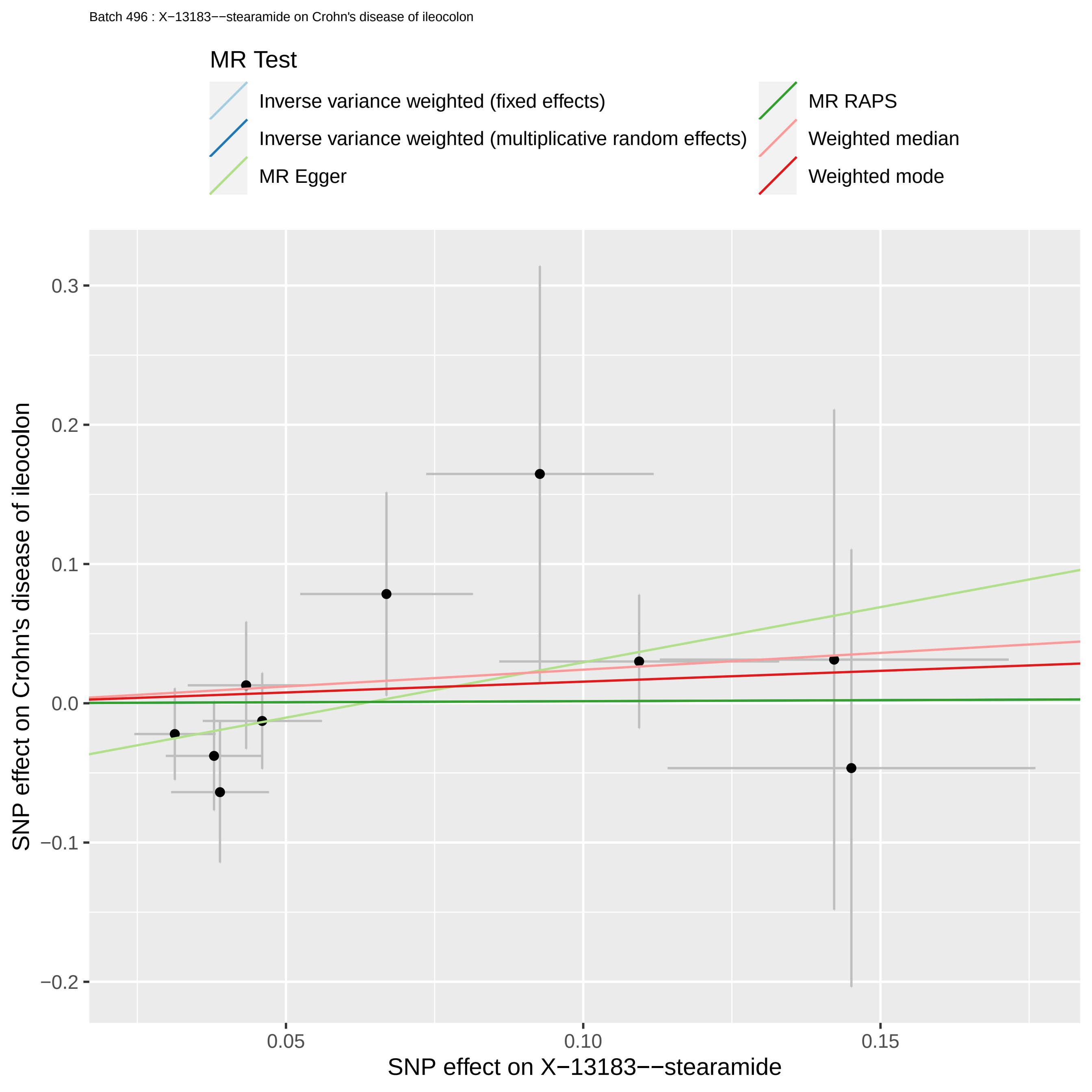
Batch 496 : X−13183−−stearamide on Crohn's disease of ileocolon
MR Test
Inverse variance weighted (fixed effects)
Inverse variance weighted (multiplicative random effects)
MR Egger
MR RAPS
Weighted median
Weighted mode
SNP effect on Crohn's disease of ileocolon
0.3
0.2
0.1
0.0
−0.1
−0.2
0.05
0.10
0.15
SNP effect on X−13183−−stearamide

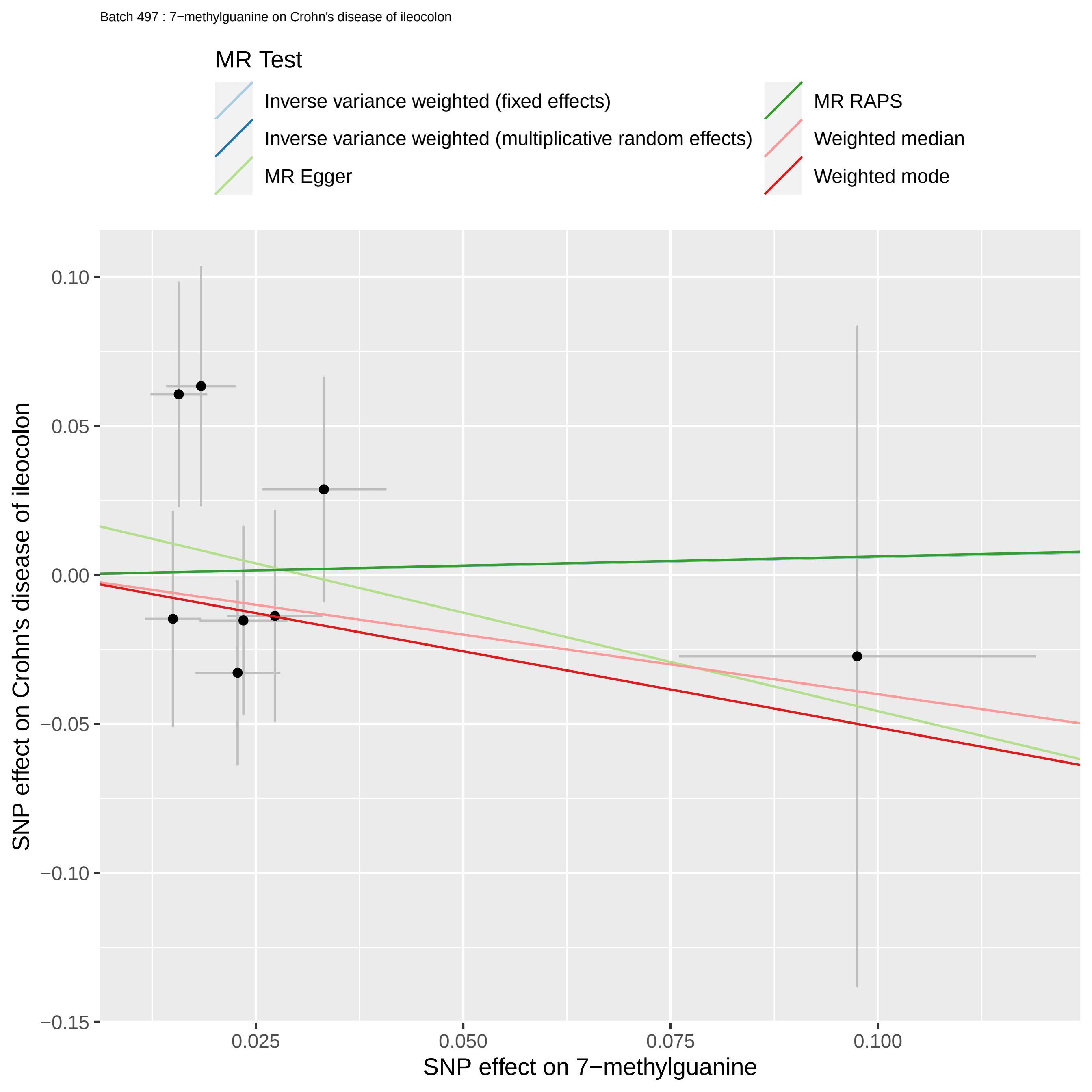
Batch 497 : 7−methylguanine on Crohn's disease of ileocolon
MR Test
Inverse variance weighted (fixed effects)
Inverse variance weighted (multiplicative random effects)
MR Egger
MR RAPS
Weighted median
Weighted mode
0.10
0.05
0.00
−0.05
−0.10
−0.15
0.025
0.050
0.075
0.100
SNP effect on Crohn's disease of ileocolon
SNP effect on 7−methylguanine

Batch 505 : 2−palmitoylglycerophosphocholine* on Crohn's disease of ileocolon

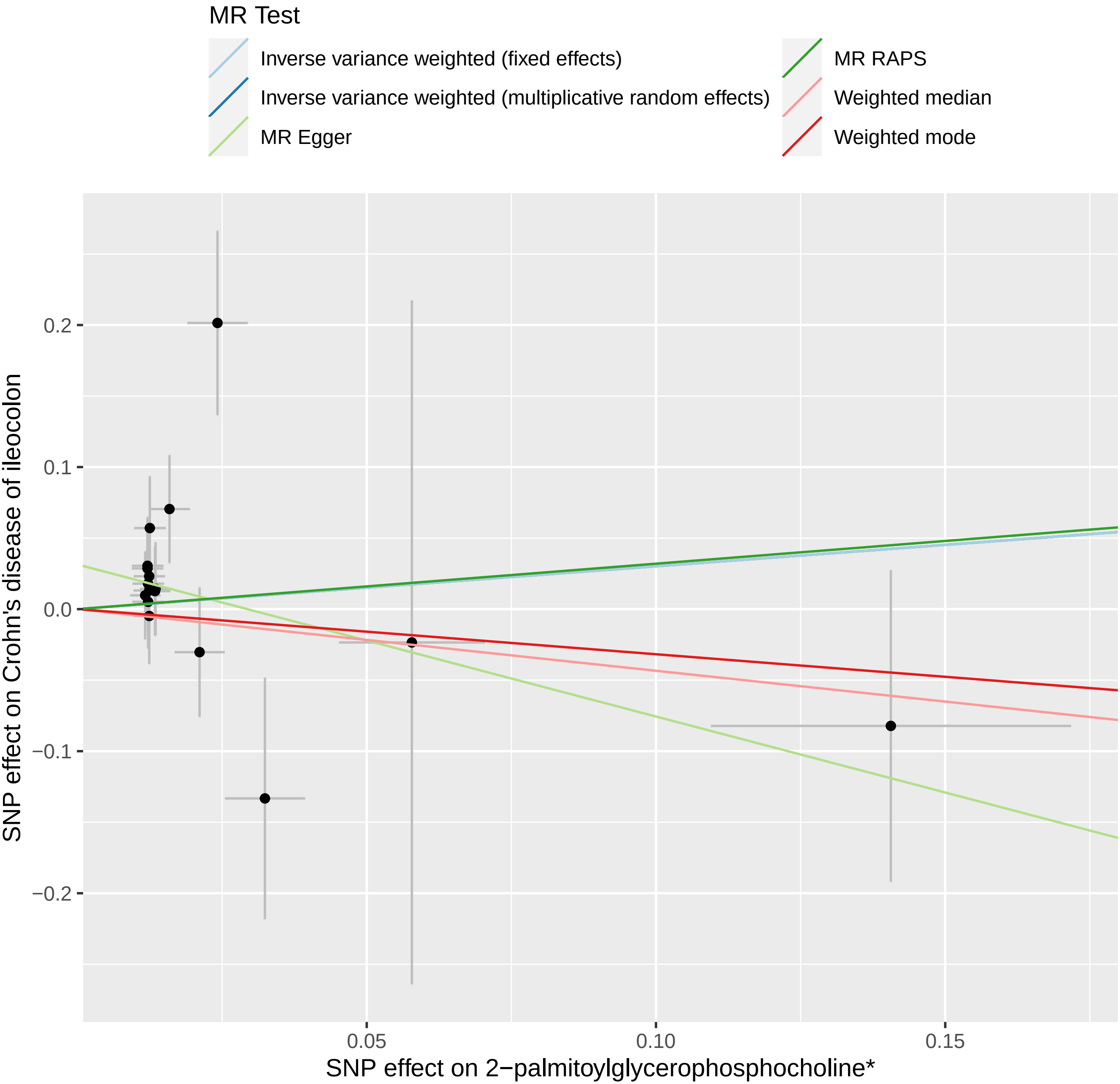

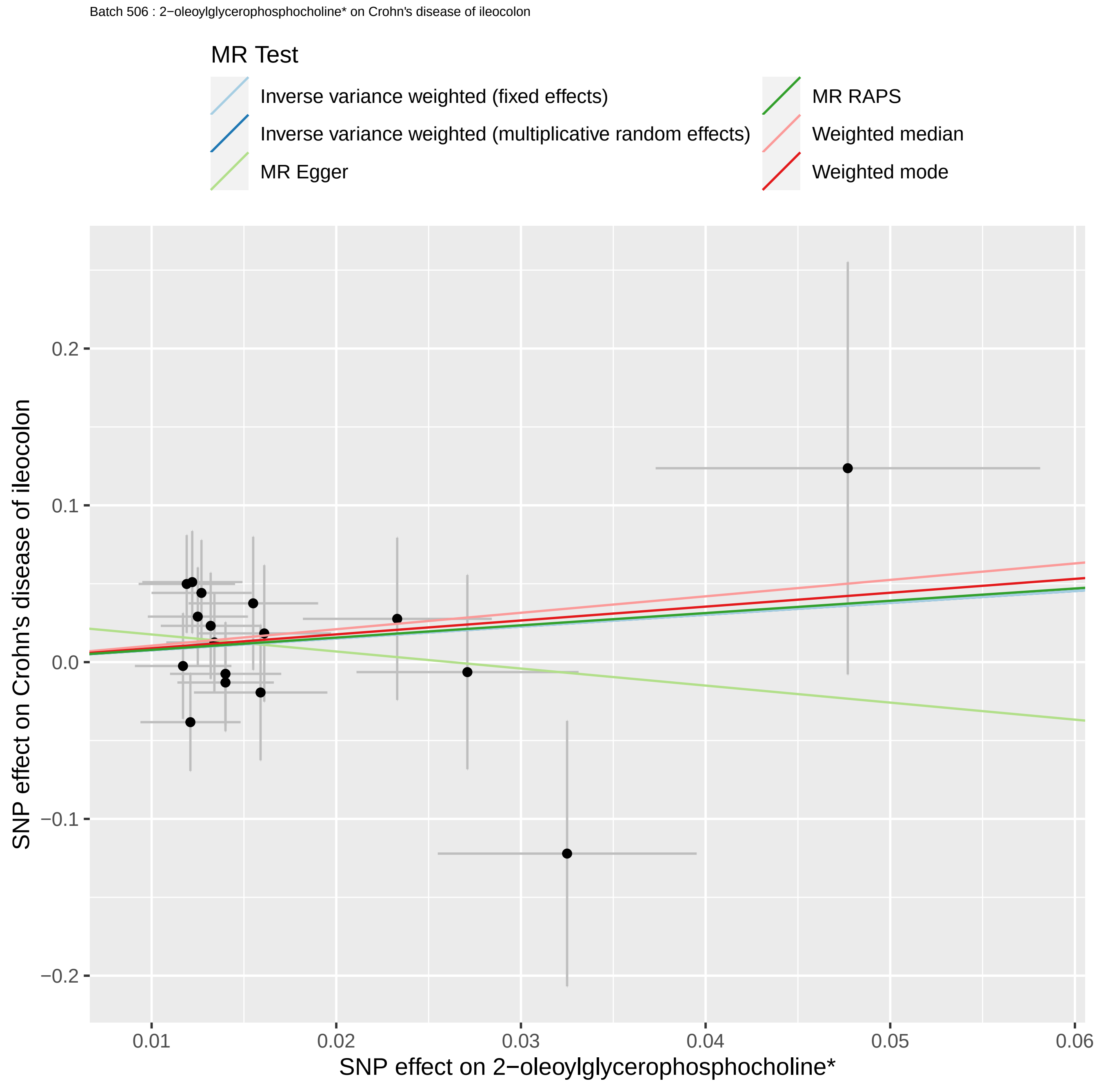

Batch 506 : 2−oleoylglycerophosphocholine* on Crohn's disease of ileocolon
MR Test
Inverse variance weighted (fixed effects)
Inverse variance weighted (multiplicative random effects)
MR Egger
MR RAPS
Weighted median
Weighted mode
0.2
0.1
0.0
−0.1
−0.2
0.01
0.02
0.03
0.04
0.05
0.06
SNP effect on Crohn's disease of ileocolon
SNP effect on 2−oleoylglycerophosphocholine*

Batch 509 : 1−palmitoylglycerophosphoinositol* on Crohn's disease of ileocolon

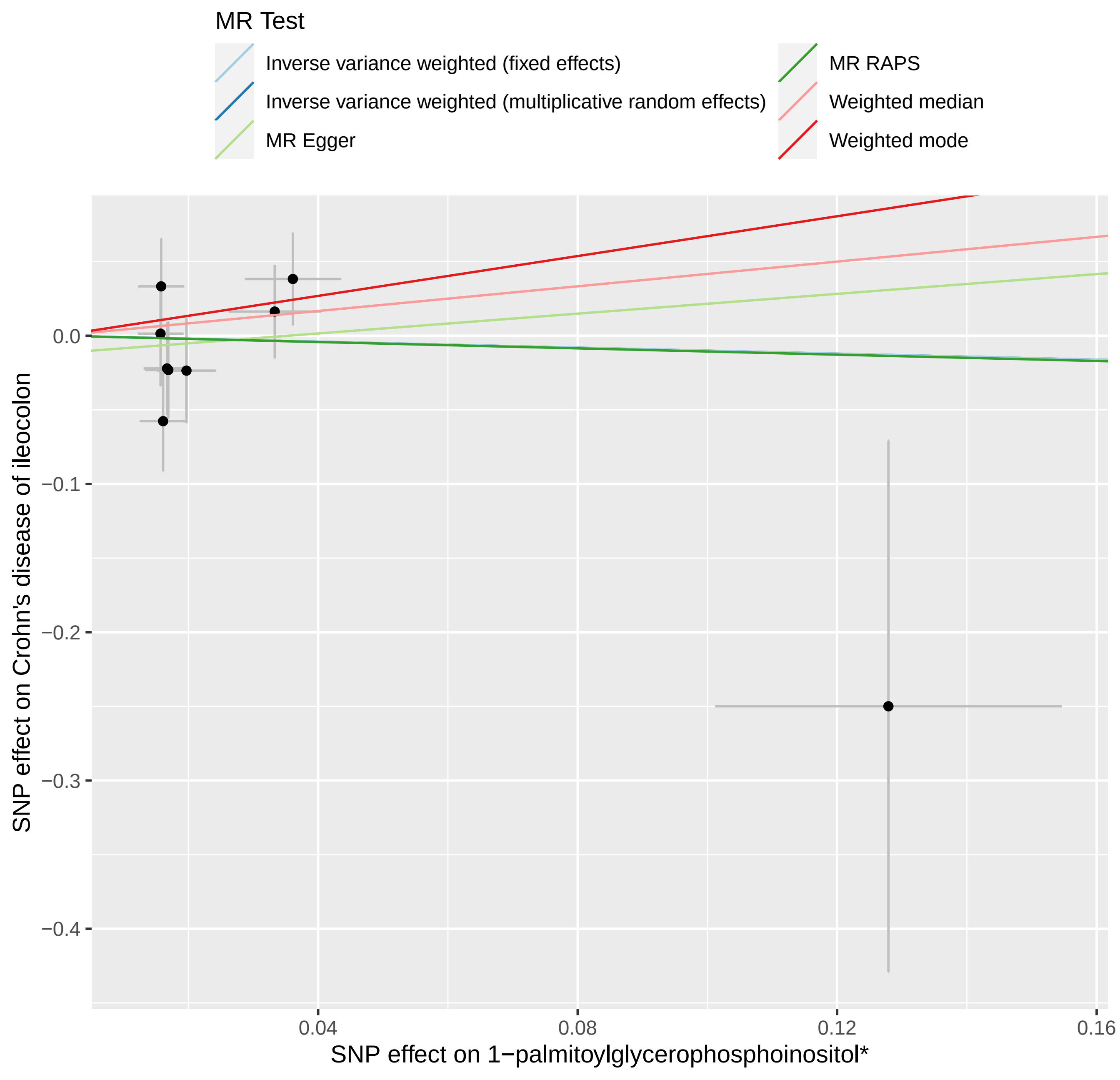

Batch 511 : Hydroxyisovaleroyl carnitine on Crohn's disease of ileocolon

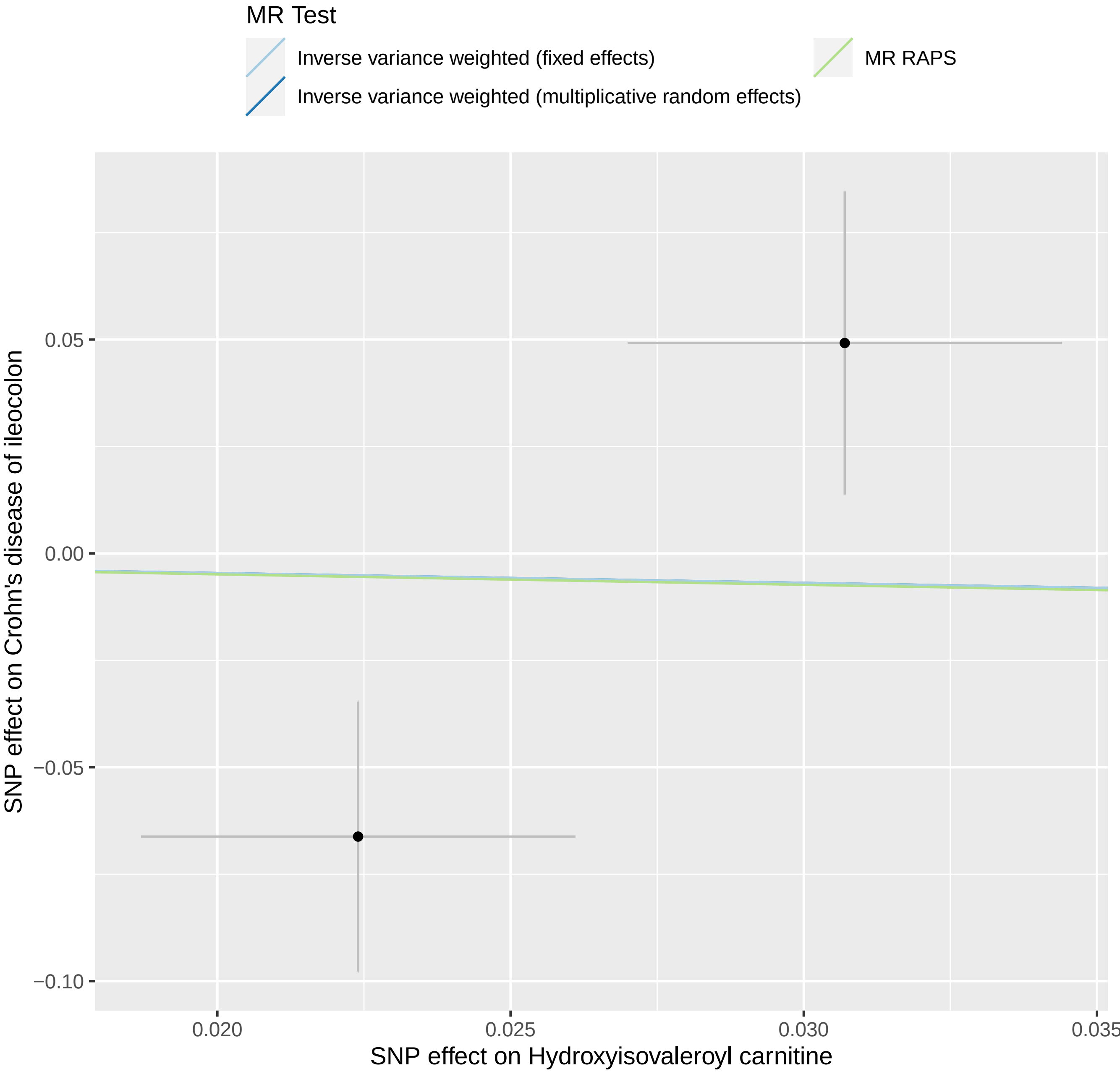

Batch 512 : Glutaroyl carnitine on Crohn's disease of ileocolon

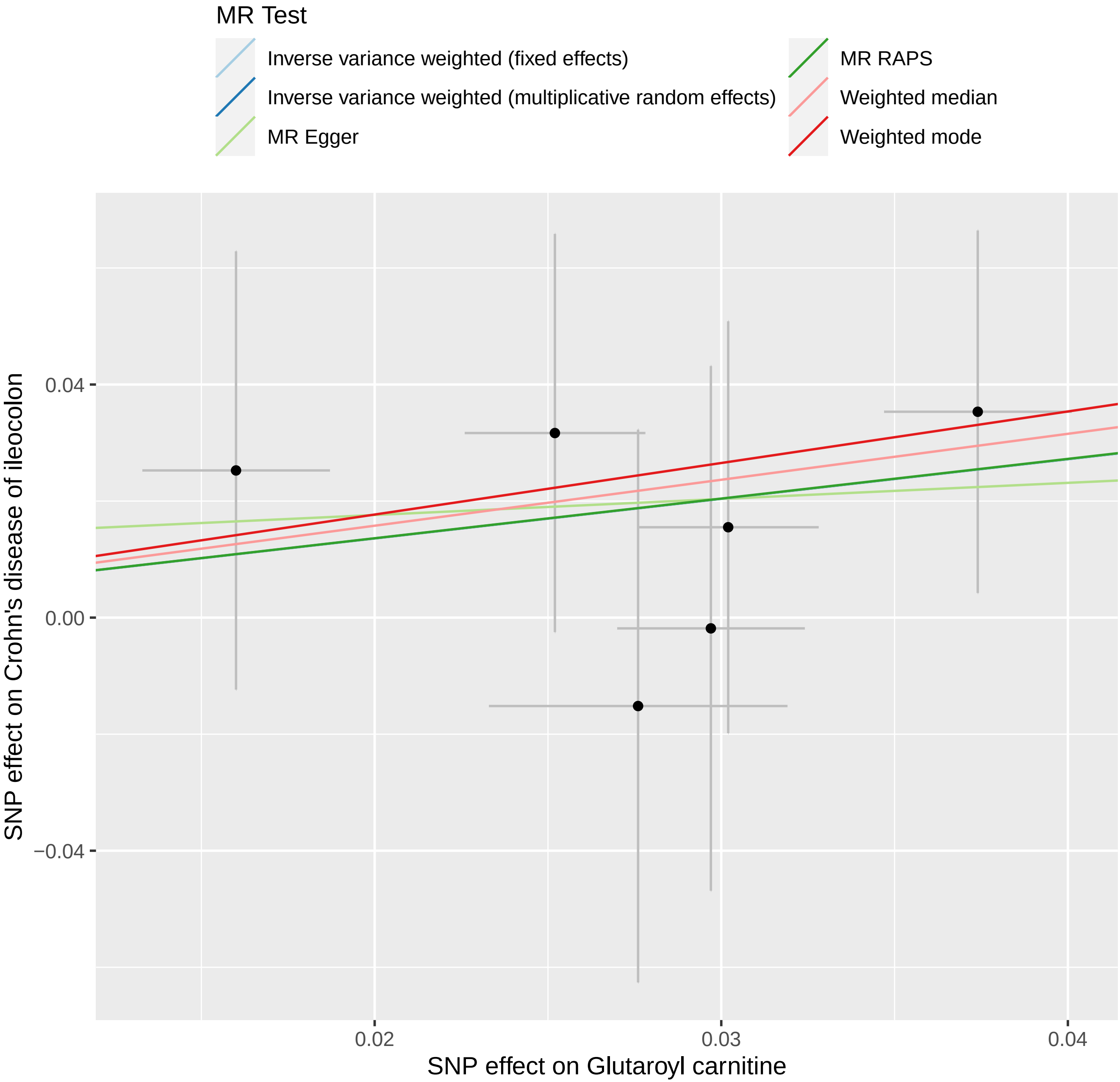

Batch 514 : 4−hydroxyhippurate on Crohn's disease of ileocolon

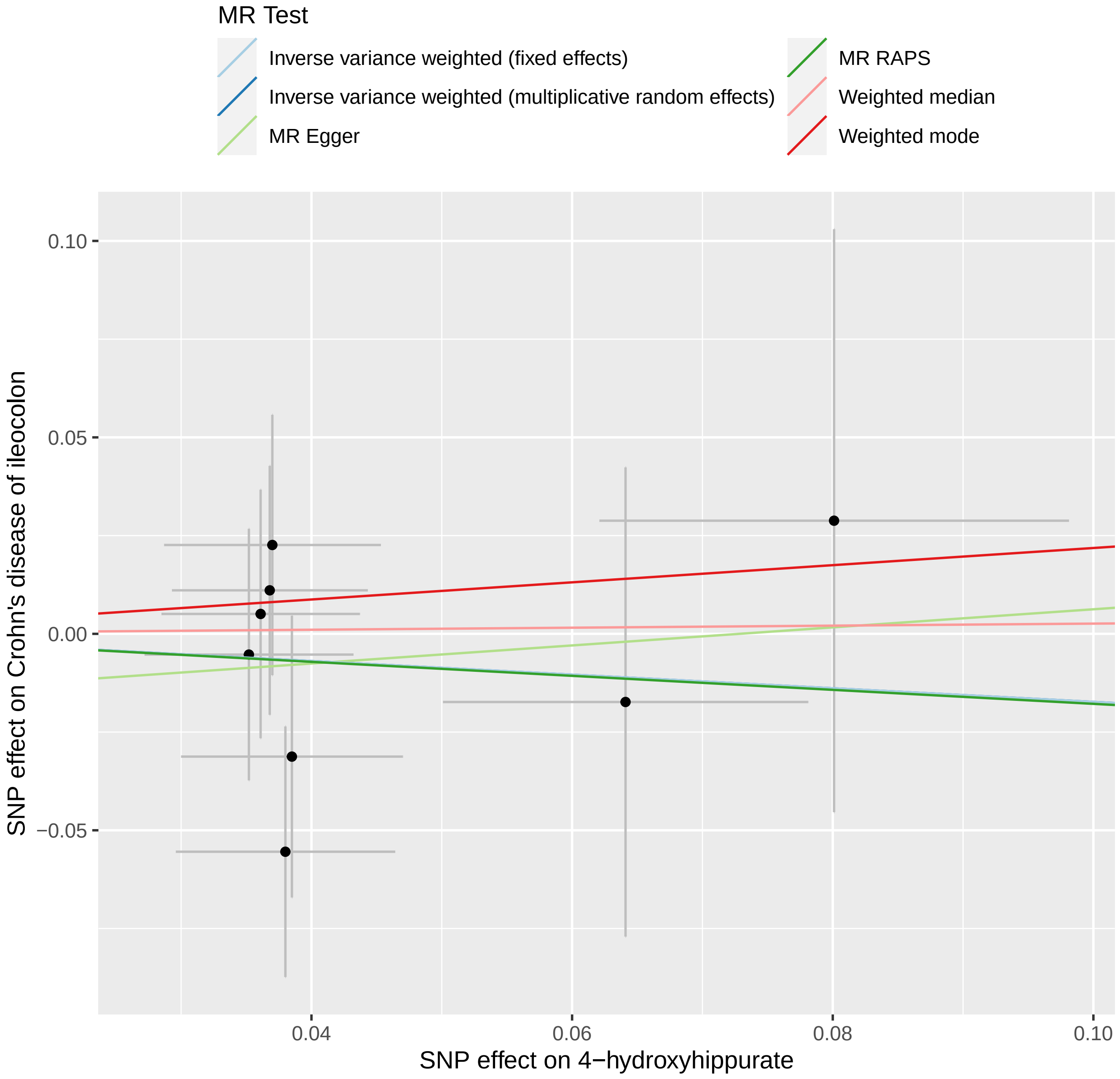

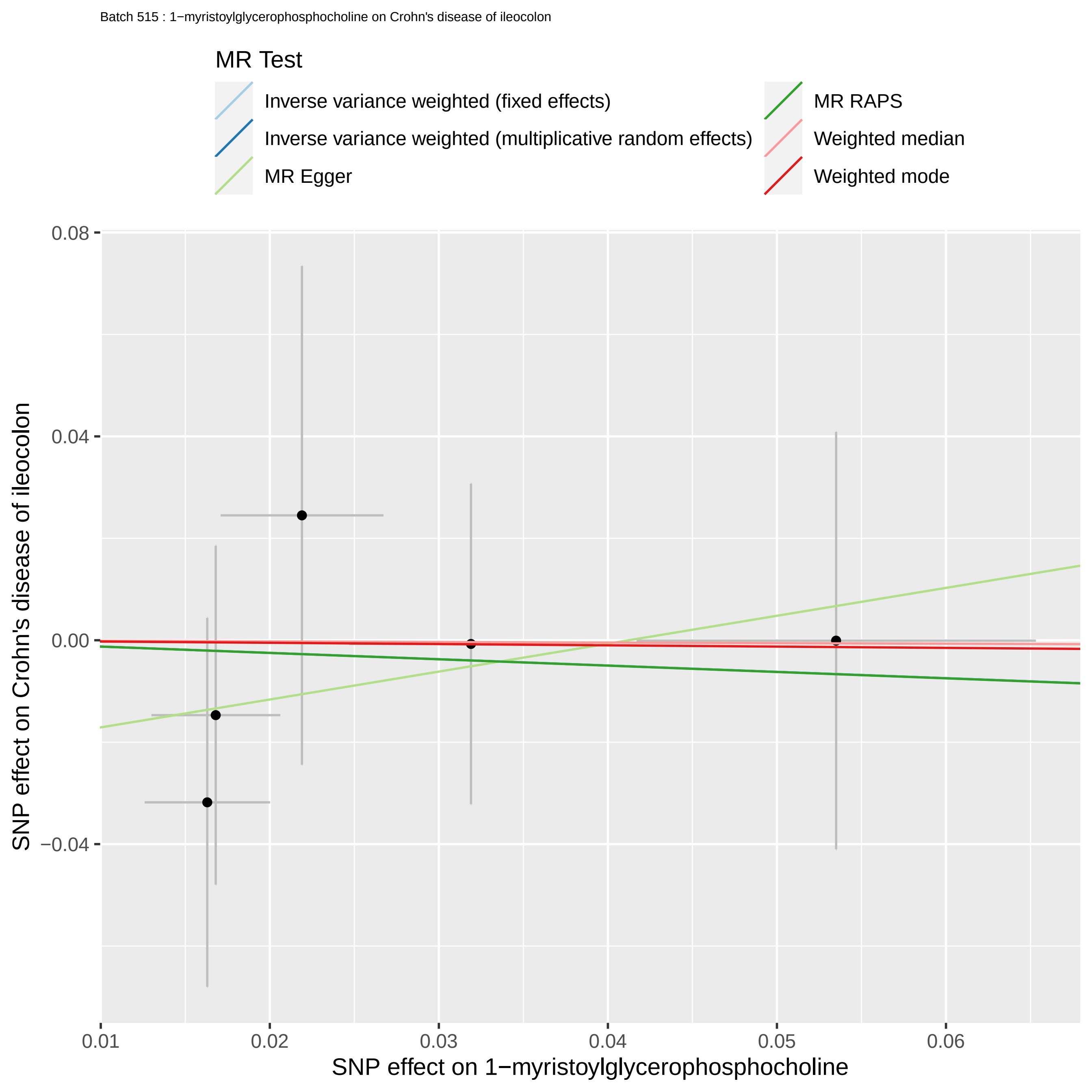

Batch 515 : 1−myristoylglycerophosphocholine on Crohn's disease of ileocolon
MR Test
Inverse variance weighted (fixed effects)
Inverse variance weighted (multiplicative random effects)
MR Egger
MR RAPS
Weighted median
Weighted mode
0.08
0.04
0.00
−0.04
0.01
0.02
0.03
0.04
0.05
0.06
SNP effect on Crohn's disease of ileocolon
SNP effect on 1−myristoylglycerophosphocholine

Batch 518 : 3−(3−hydroxyphenyl)propionate on Crohn's disease of ileocolon

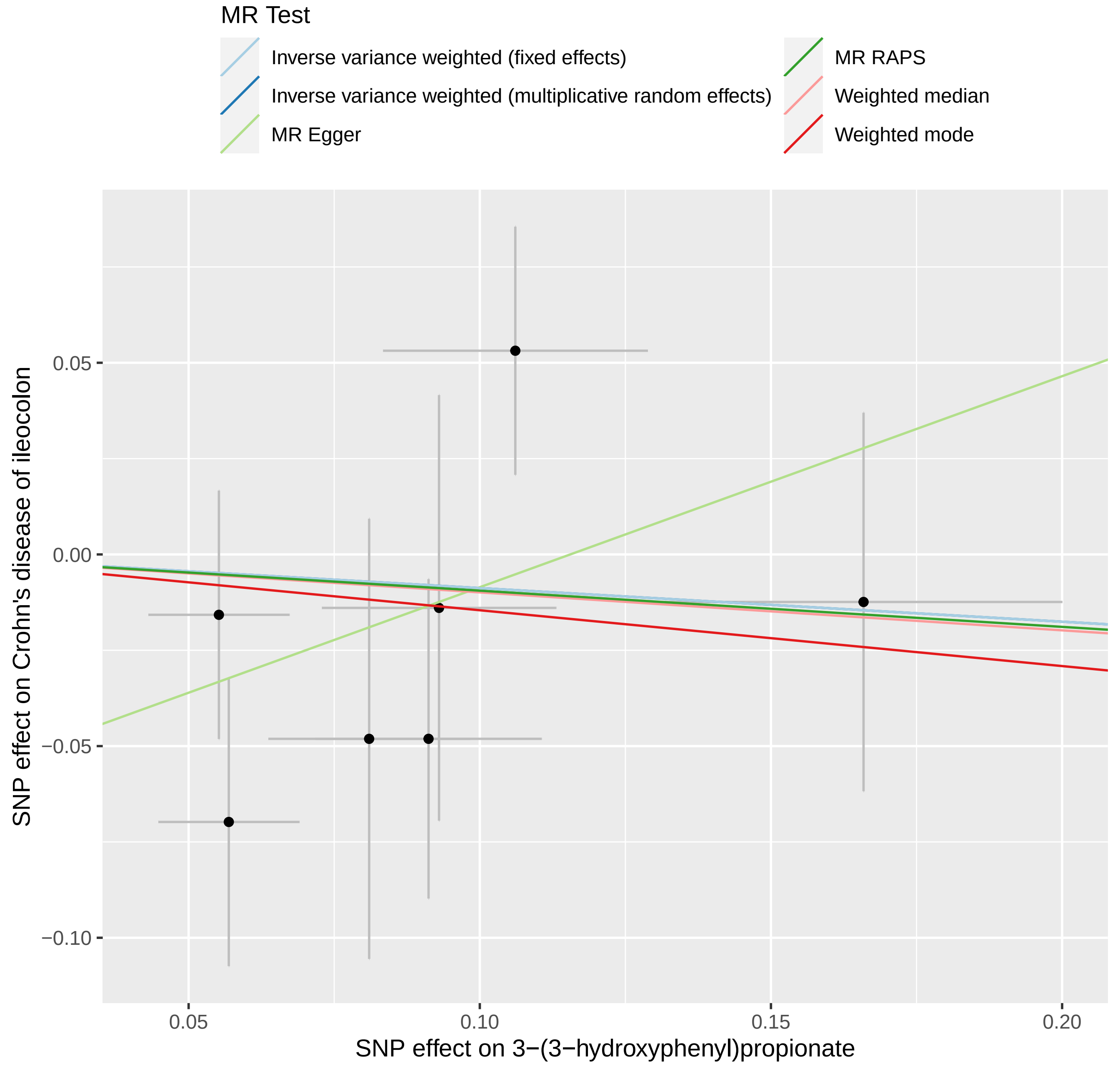

Batch 522 : Dihomo−linolenate (20:3n3 or n6) on Crohn's disease of ileocolon

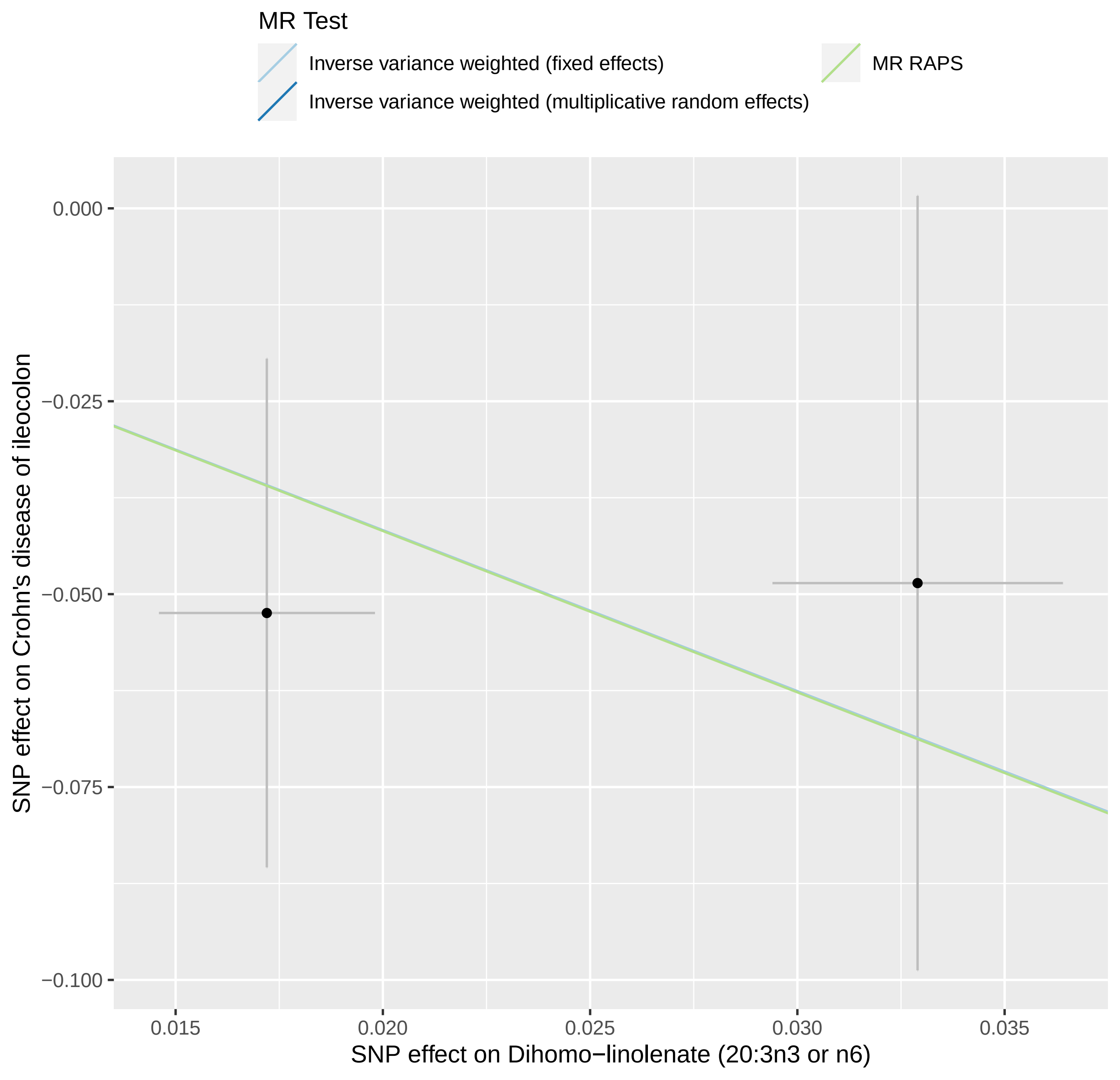

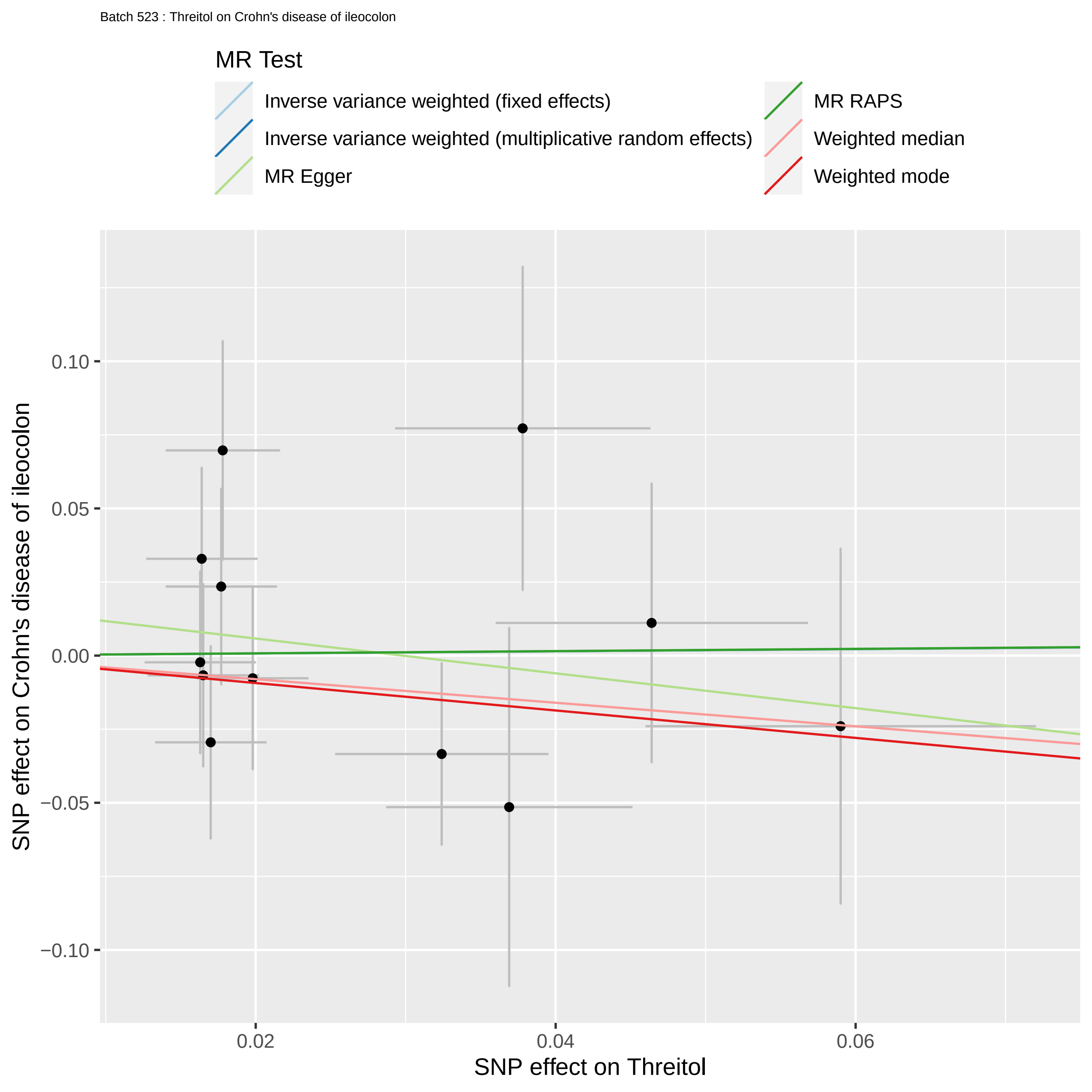

Batch 523 : Threitol on Crohn's disease of ileocolon
MR Test
Inverse variance weighted (fixed effects)
Inverse variance weighted (multiplicative random effects)
MR Egger
MR RAPS
Weighted median
Weighted mode
0.10
0.05
0.00
−0.05
−0.10
SNP effect on Crohn's disease of ileocolon
0.02
0.04
0.06
SNP effect on Threitol

Batch 524 : P−cresol sulfate on Crohn's disease of ileocolon

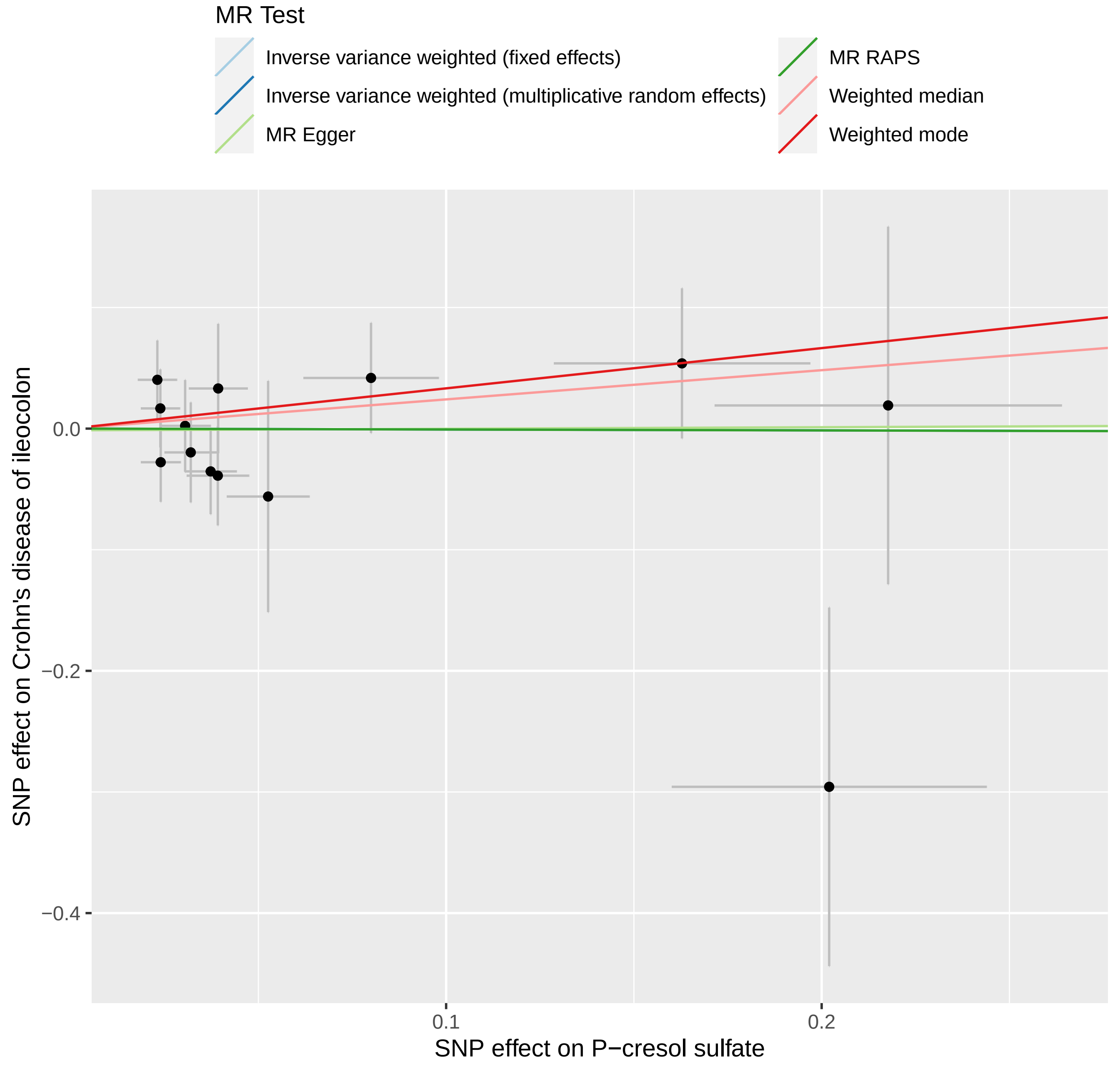

Batch 527 : X−14208−−phenylalanylserine on Crohn's disease of ileocolon

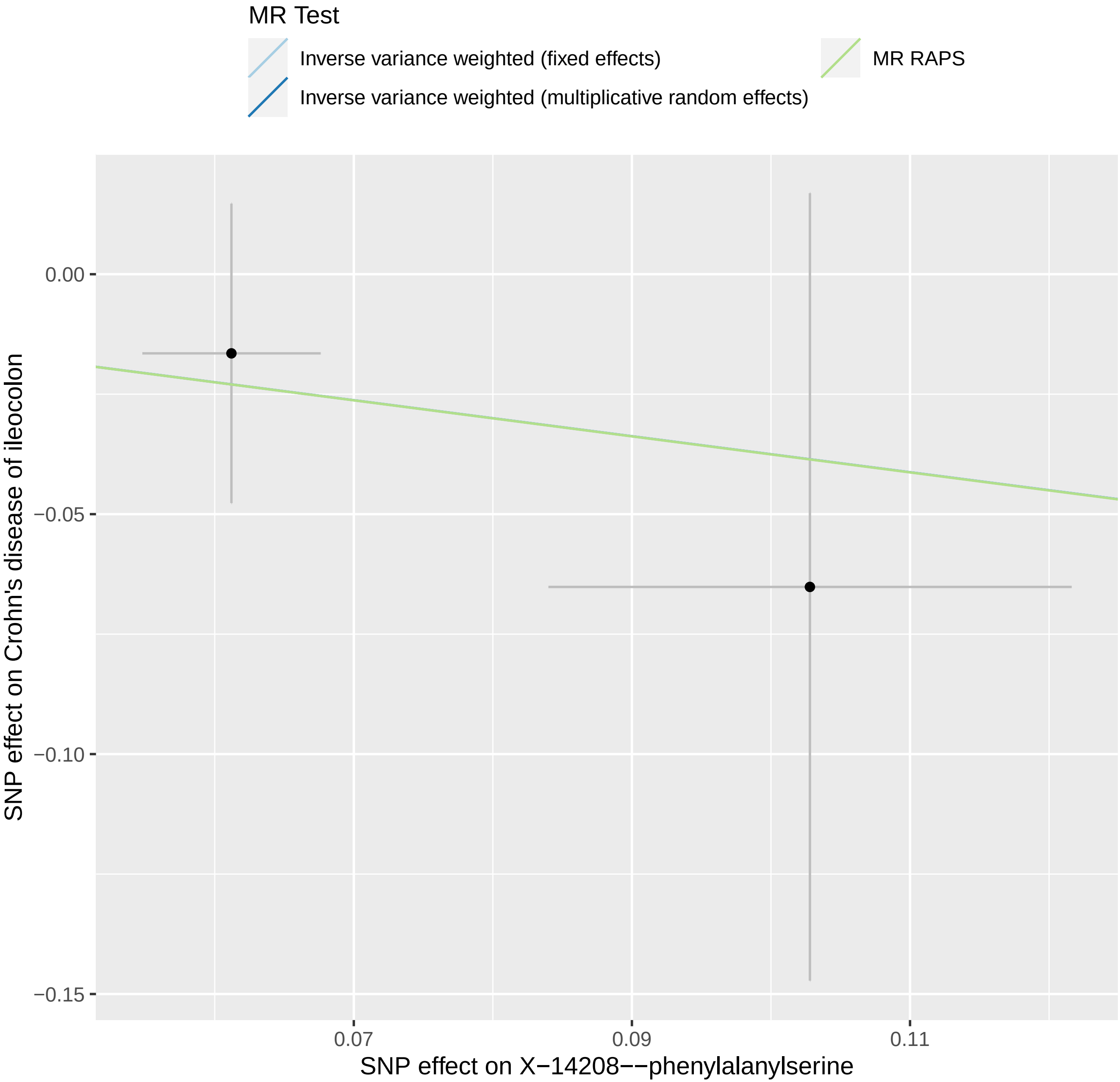

Batch 529 : X−14450−−phenylalanylleucine on Crohn's disease of ileocolon

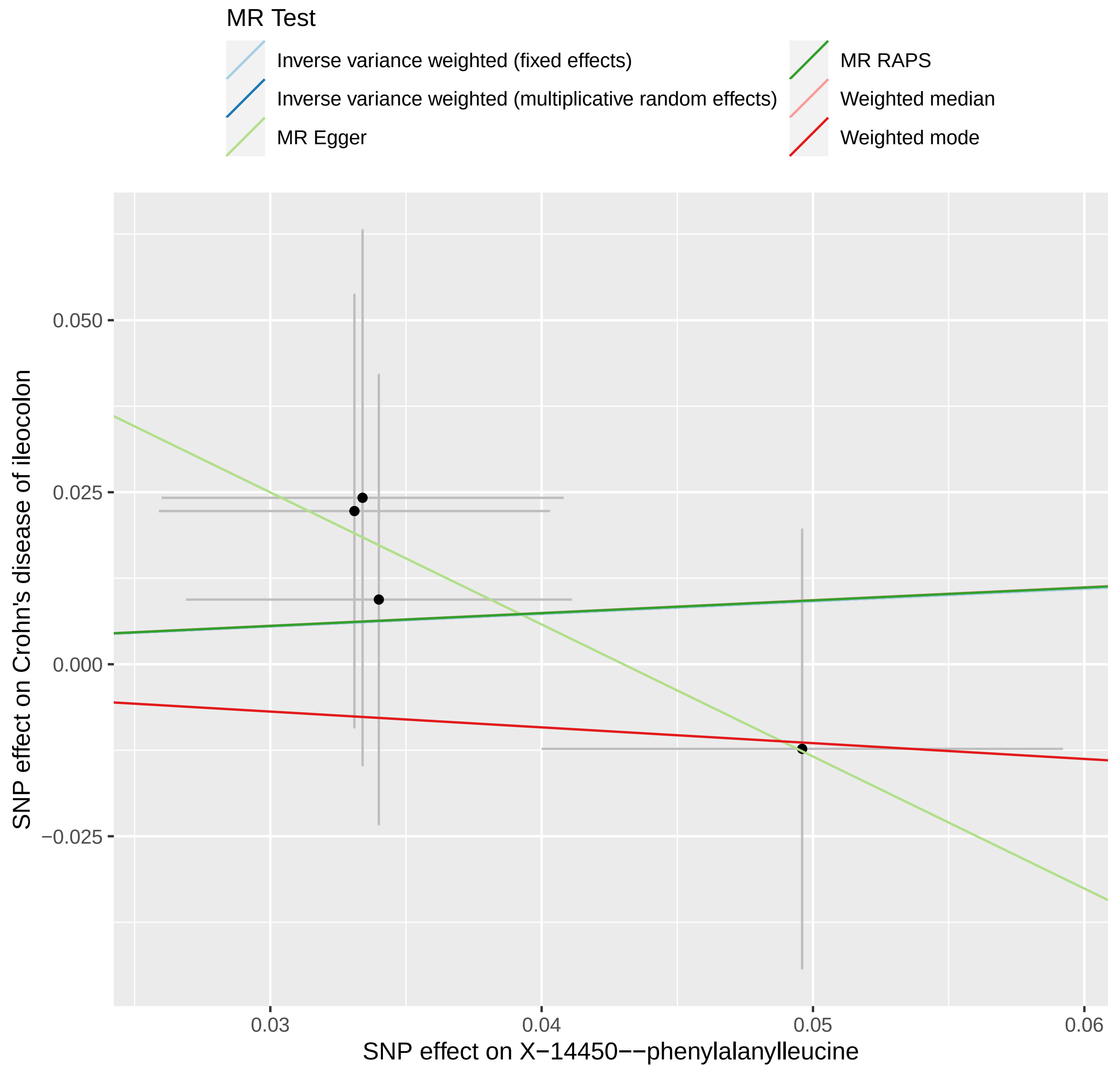

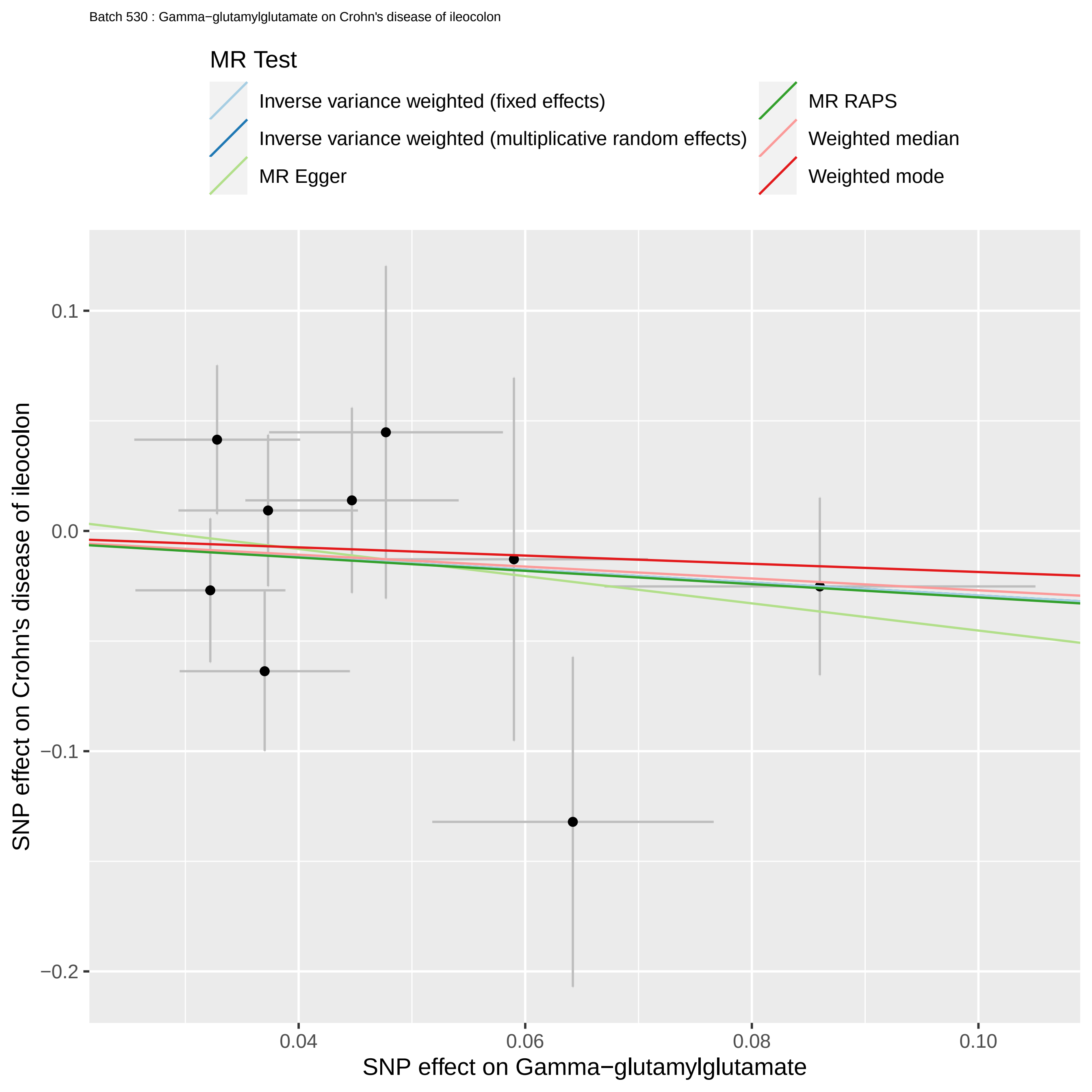
Batch 530 : Gamma−glutamylglutamate on Crohn's disease of ileocolon
MR Test
Inverse variance weighted (fixed effects)
Inverse variance weighted (multiplicative random effects)
MR Egger
MR RAPS
Weighted median
Weighted mode
SNP effect on Crohn's disease of ileocolon
SNP effect on Gamma−glutamylglutamate
0.1
0.0
−0.1
−0.2
0.04
0.06
0.08
0.10

Batch 531 : Octadecanedioate on Crohn's disease of ileocolon

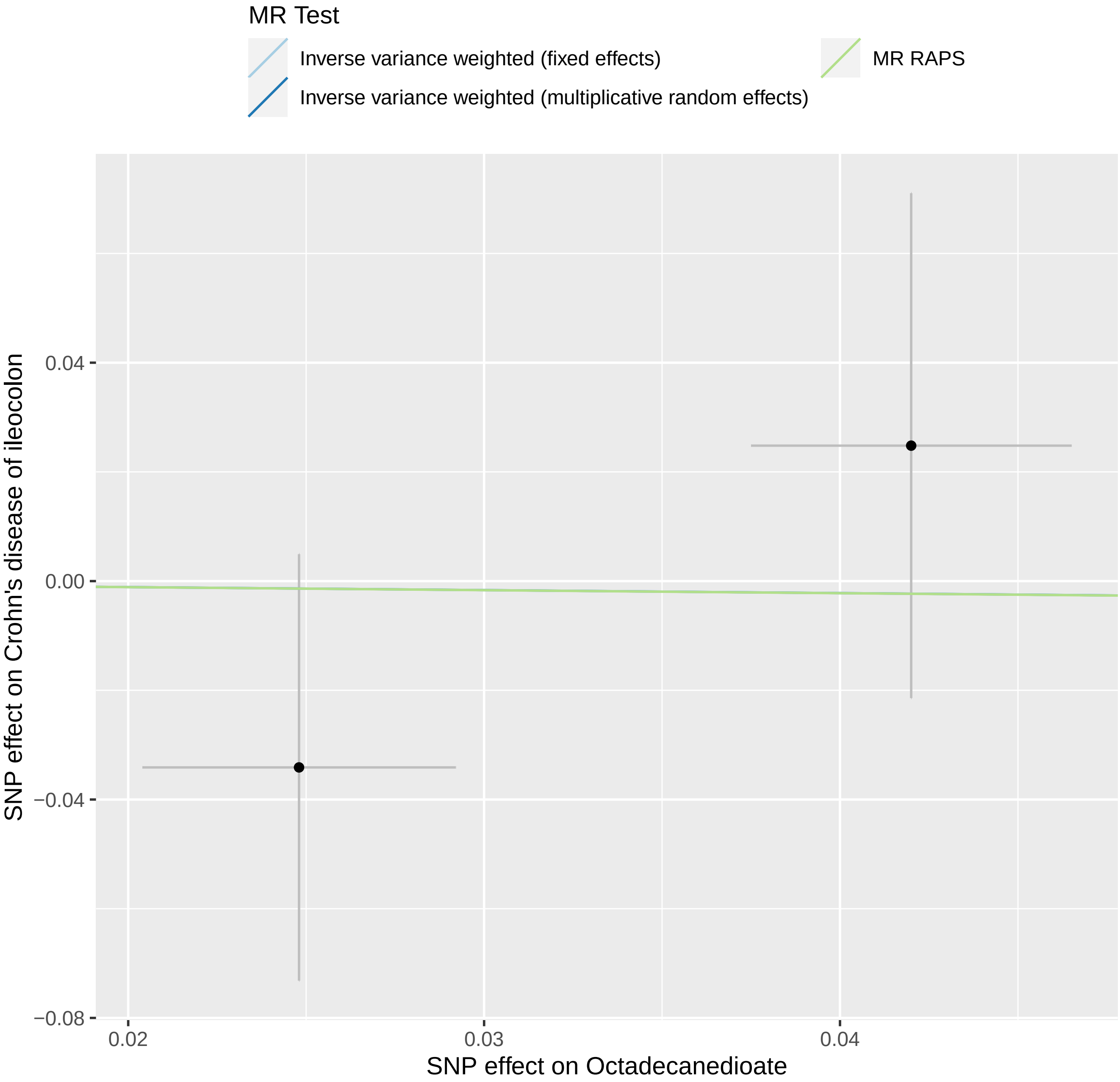

Batch 532 : Leucylleucine on Crohn's disease of ileocolon

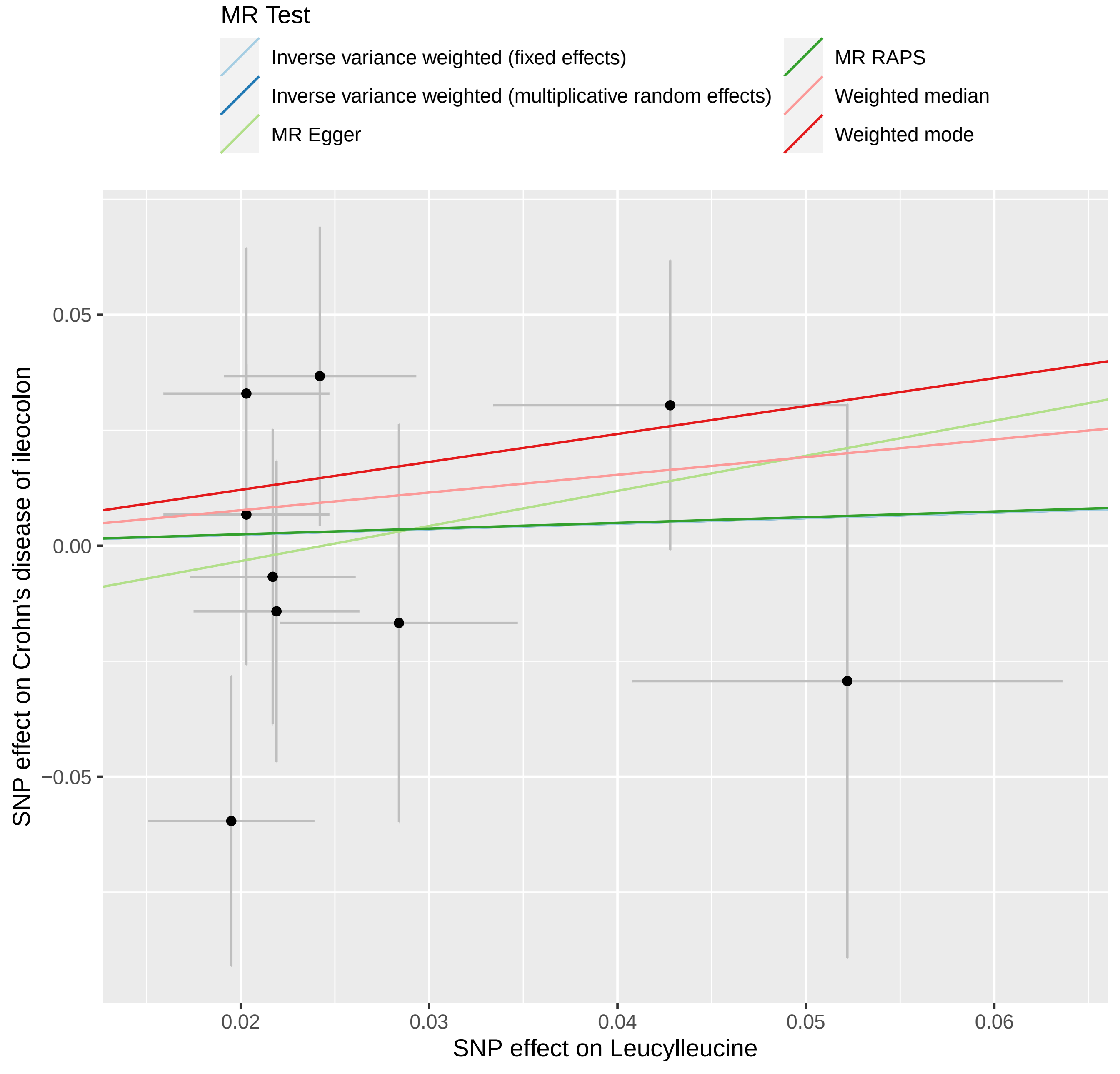

Batch 533 : 7−alpha−hydroxy−3−oxo−4−cholestenoate (7−Hoca) on Crohn's disease of ileocolon

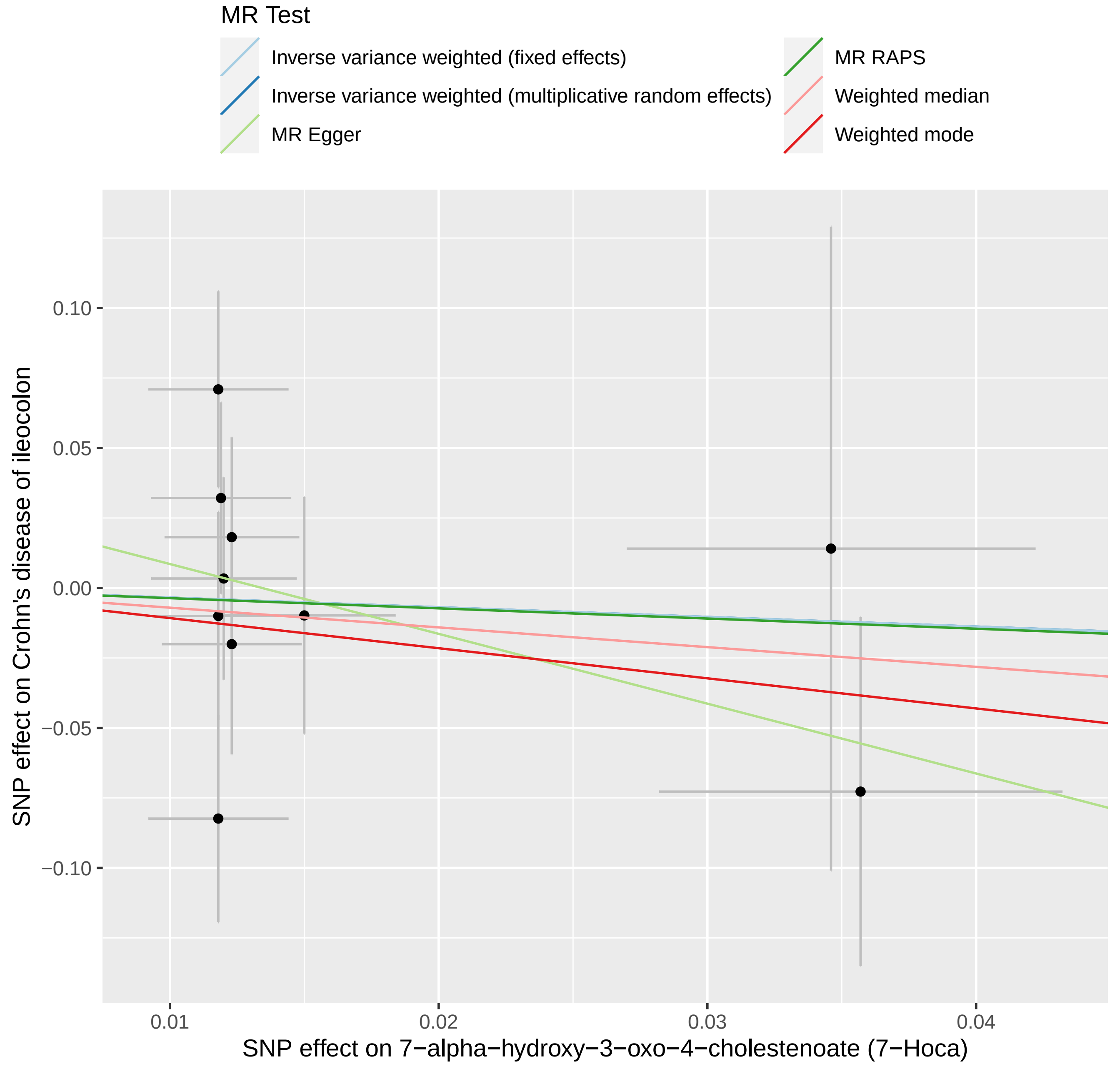

Batch 534 : N−Butyl Oleate on Crohn's disease of ileocolon

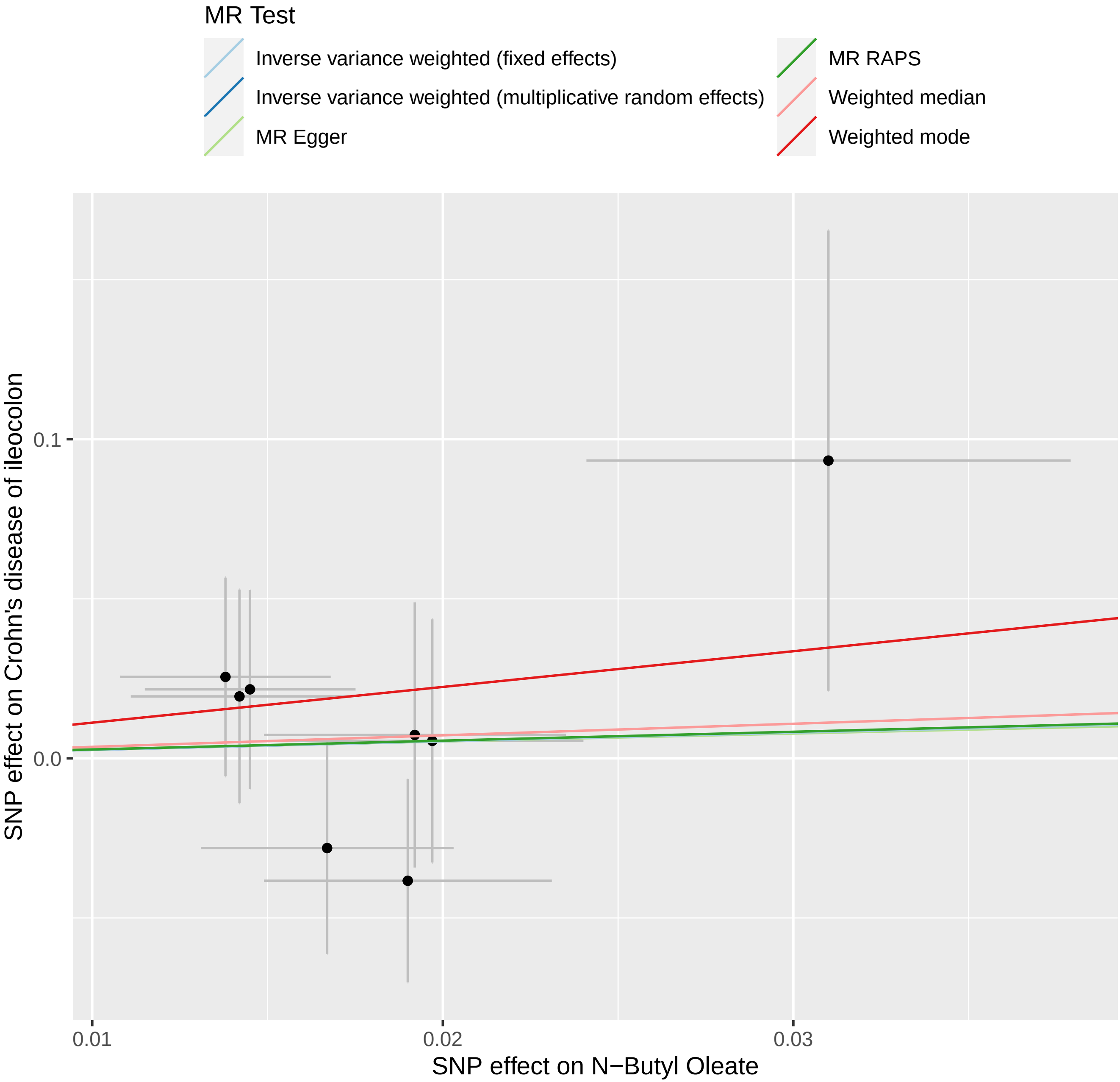

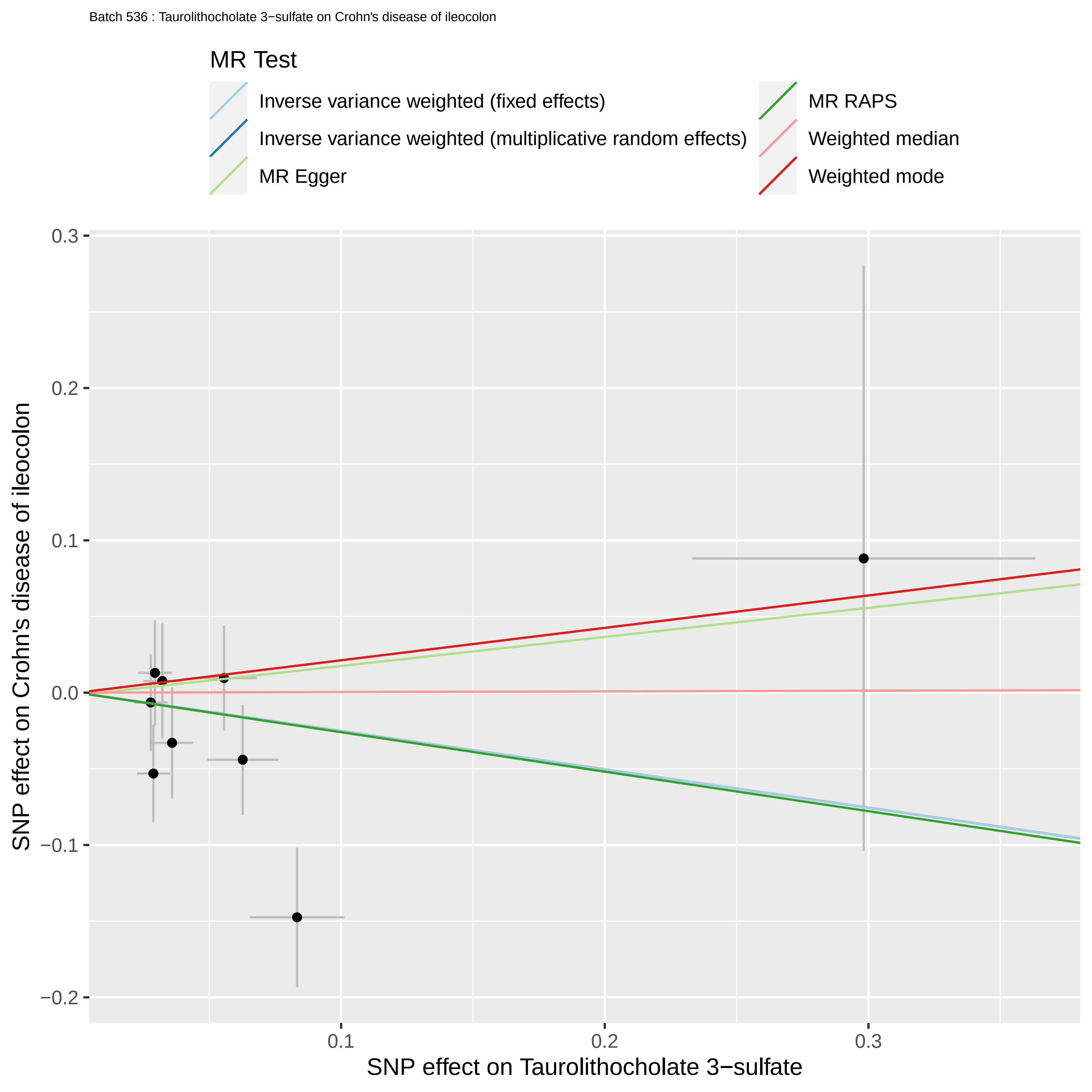
Batch 536 : Taurolithocholate 3−sulfate on Crohn's disease of ileocolon
MR Test
Inverse variance weighted (fixed effects)
Inverse variance weighted (multiplicative random effects)
MR Egger
MR RAPS
Weighted median
Weighted mode
0.3
0.2
0.1
0.0
−0.1
−0.2
0.1
0.2
0.3
SNP effect on Crohn's disease of ileocolon
SNP effect on Taurolithocholate 3−sulfate

Batch 537 : X−14977−−vanillin on Crohn's disease of ileocolon

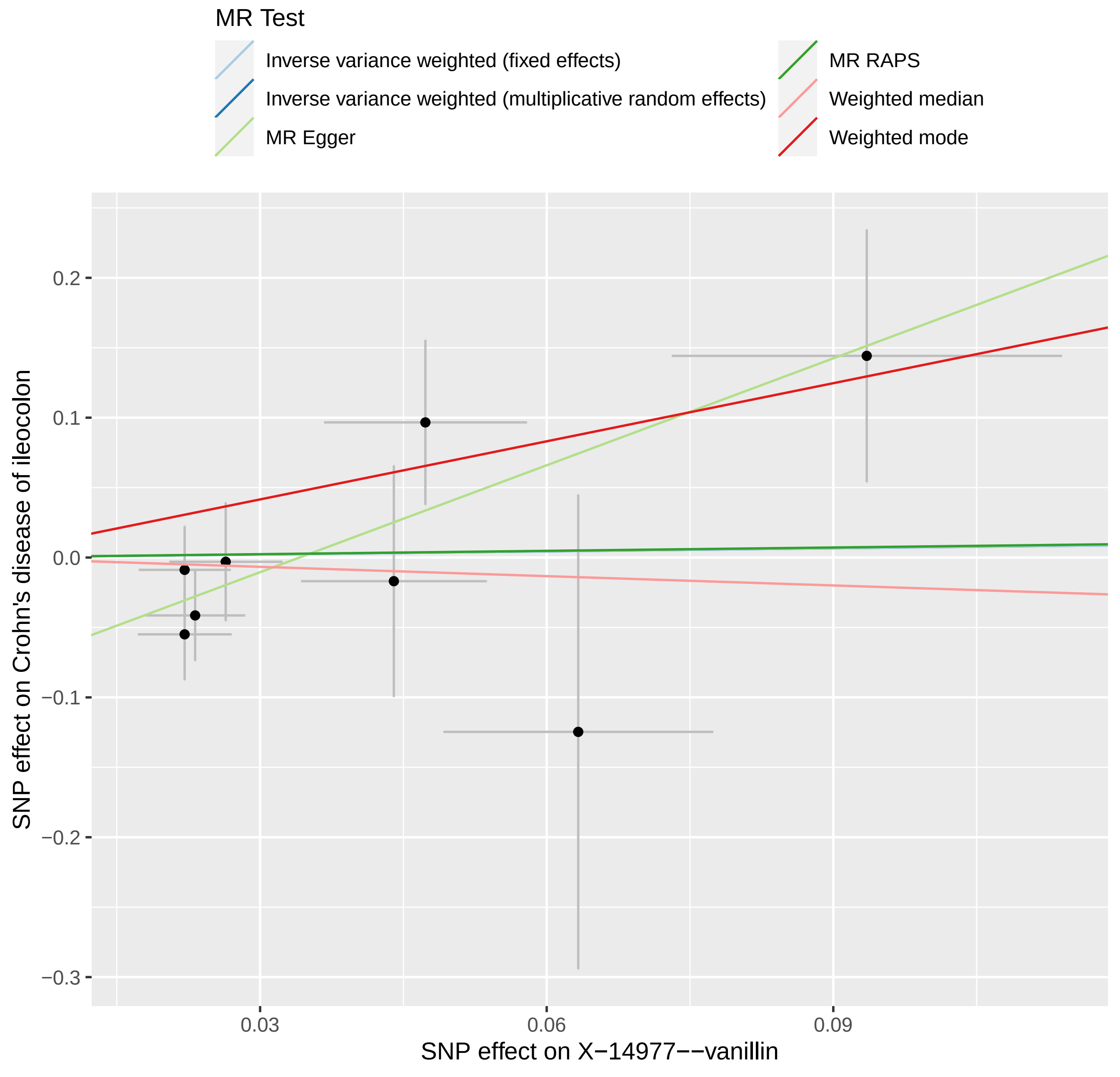

Batch 538 : Succinylcarnitine on Crohn's disease of ileocolon

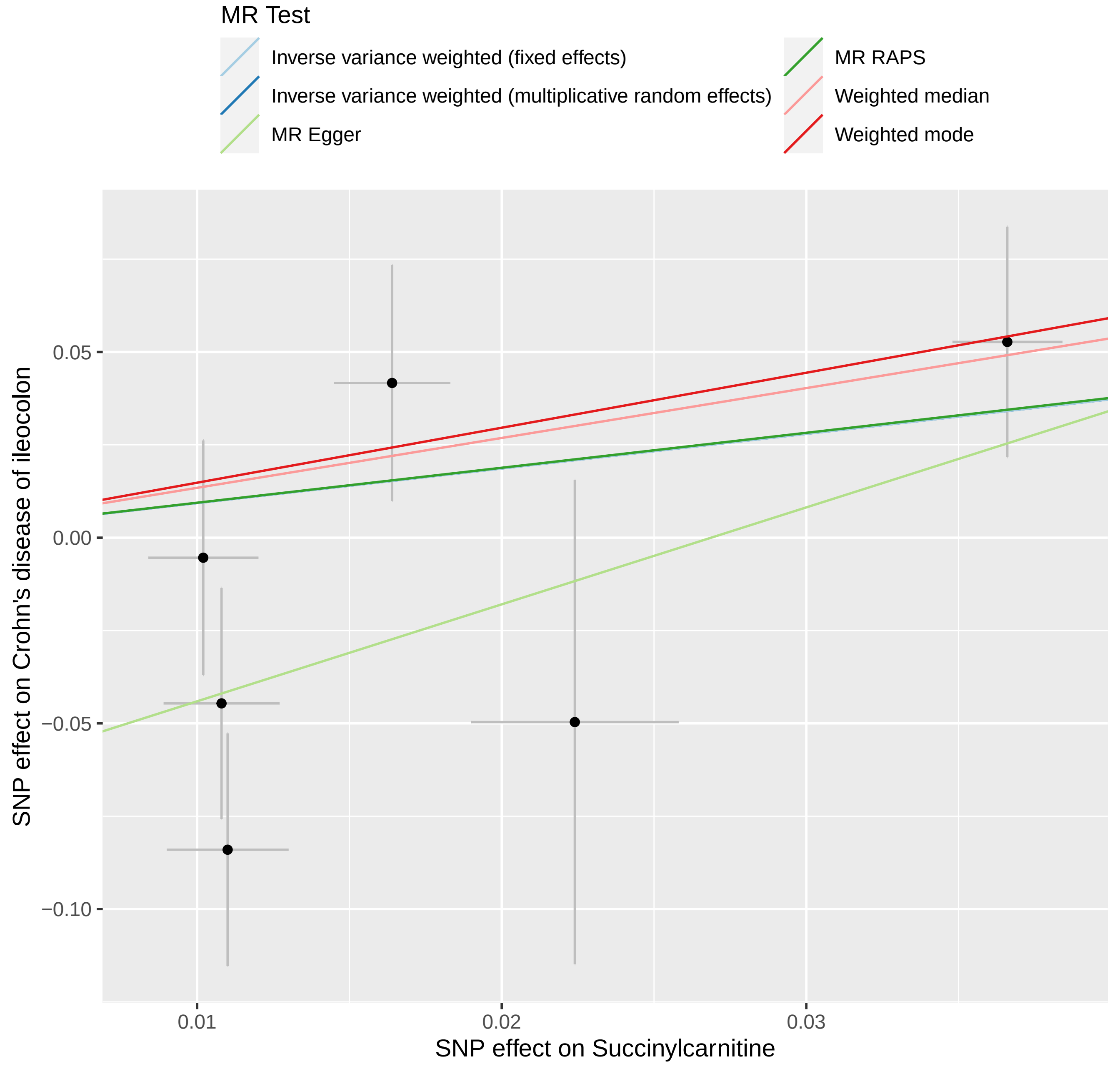

Batch 540 : Cyclo(leu−pro) on Crohn's disease of ileocolon

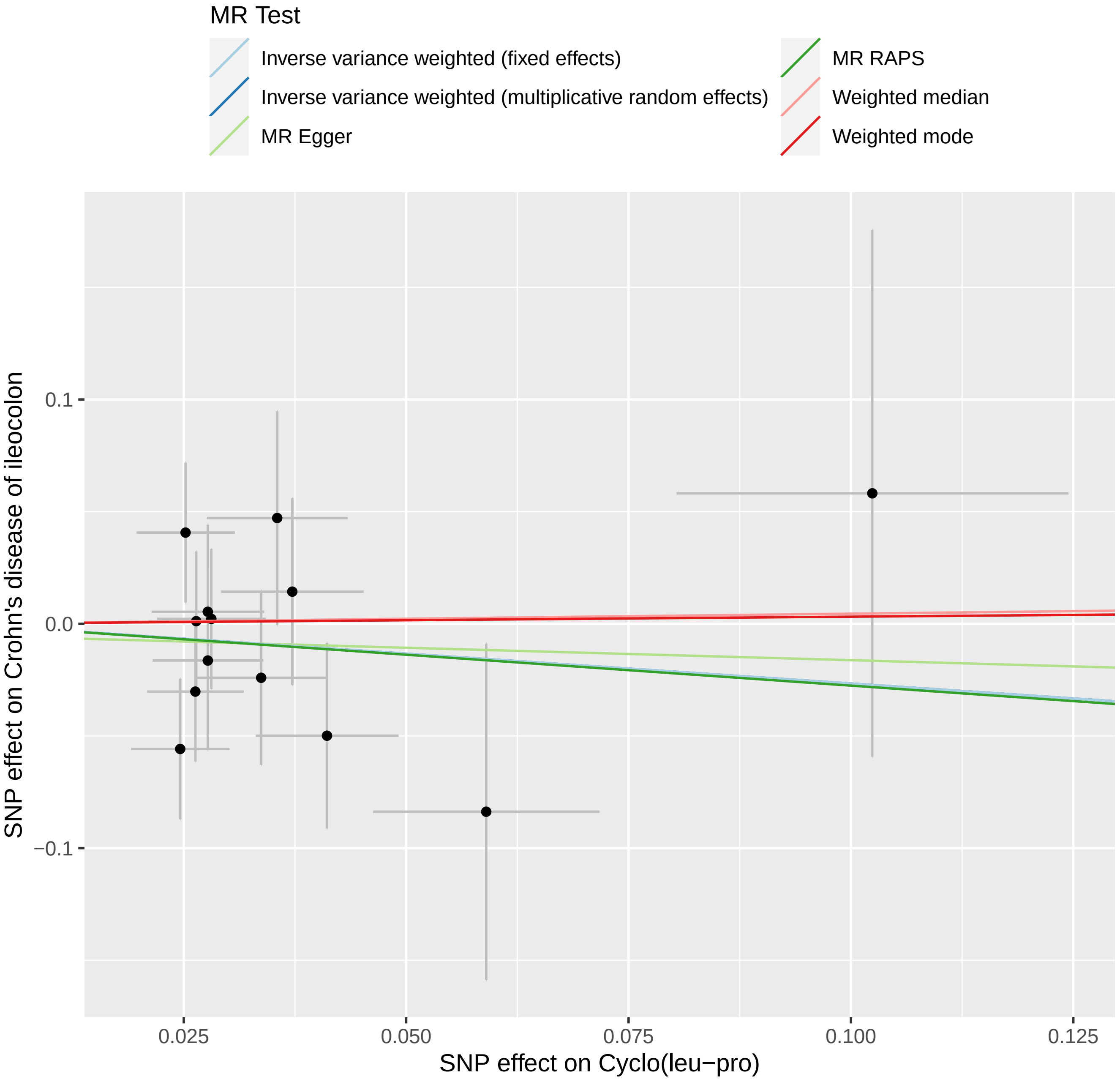

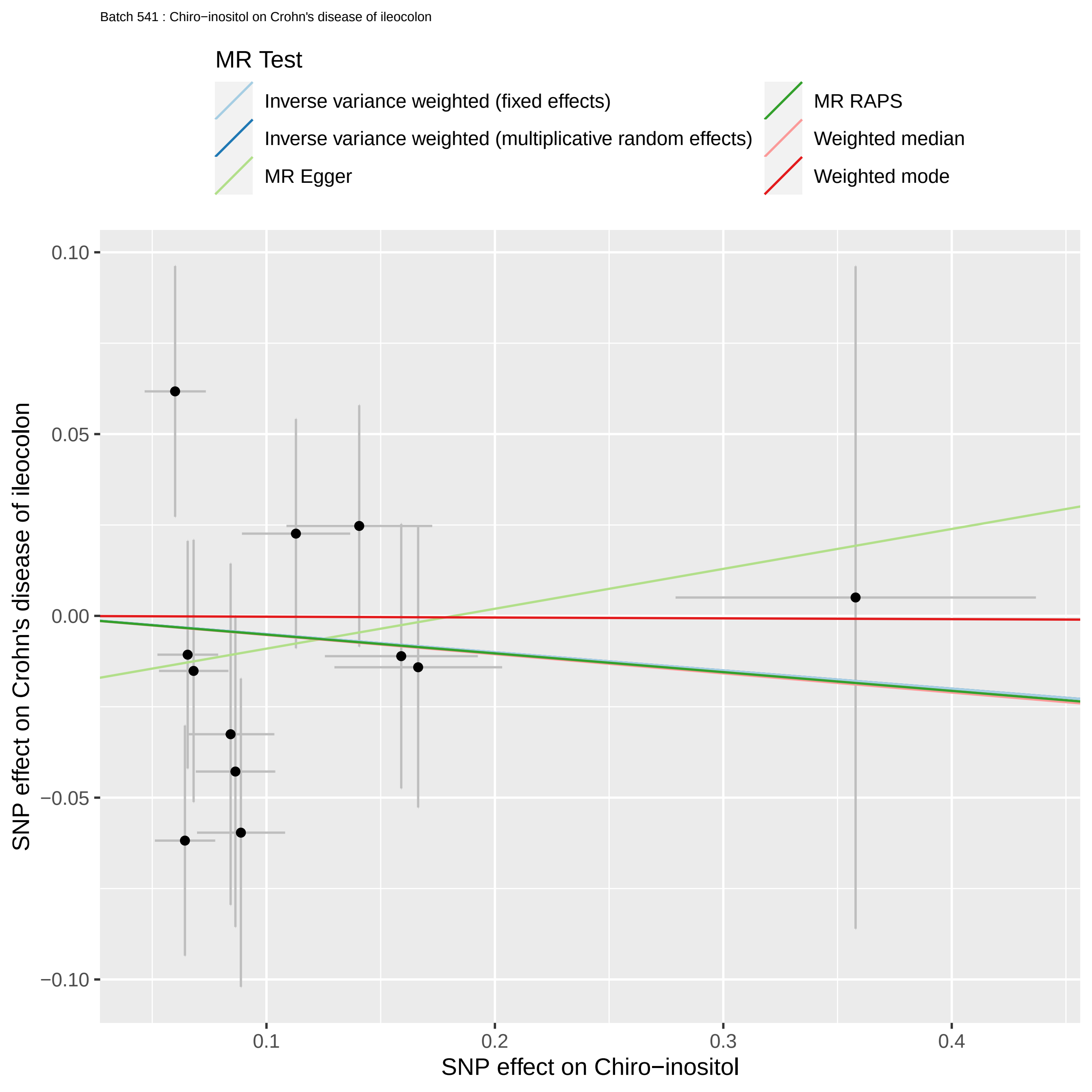

Batch 541 : Chiro−inositol on Crohn's disease of ileocolon
MR Test
Inverse variance weighted (fixed effects)
Inverse variance weighted (multiplicative random effects)
MR Egger
MR RAPS
Weighted median
Weighted mode
0.10
0.05
0.00
−0.05
−0.10
0.1
0.2
0.3
0.4
SNP effect on Crohn's disease of ileocolon
SNP effect on Chiro−inositol

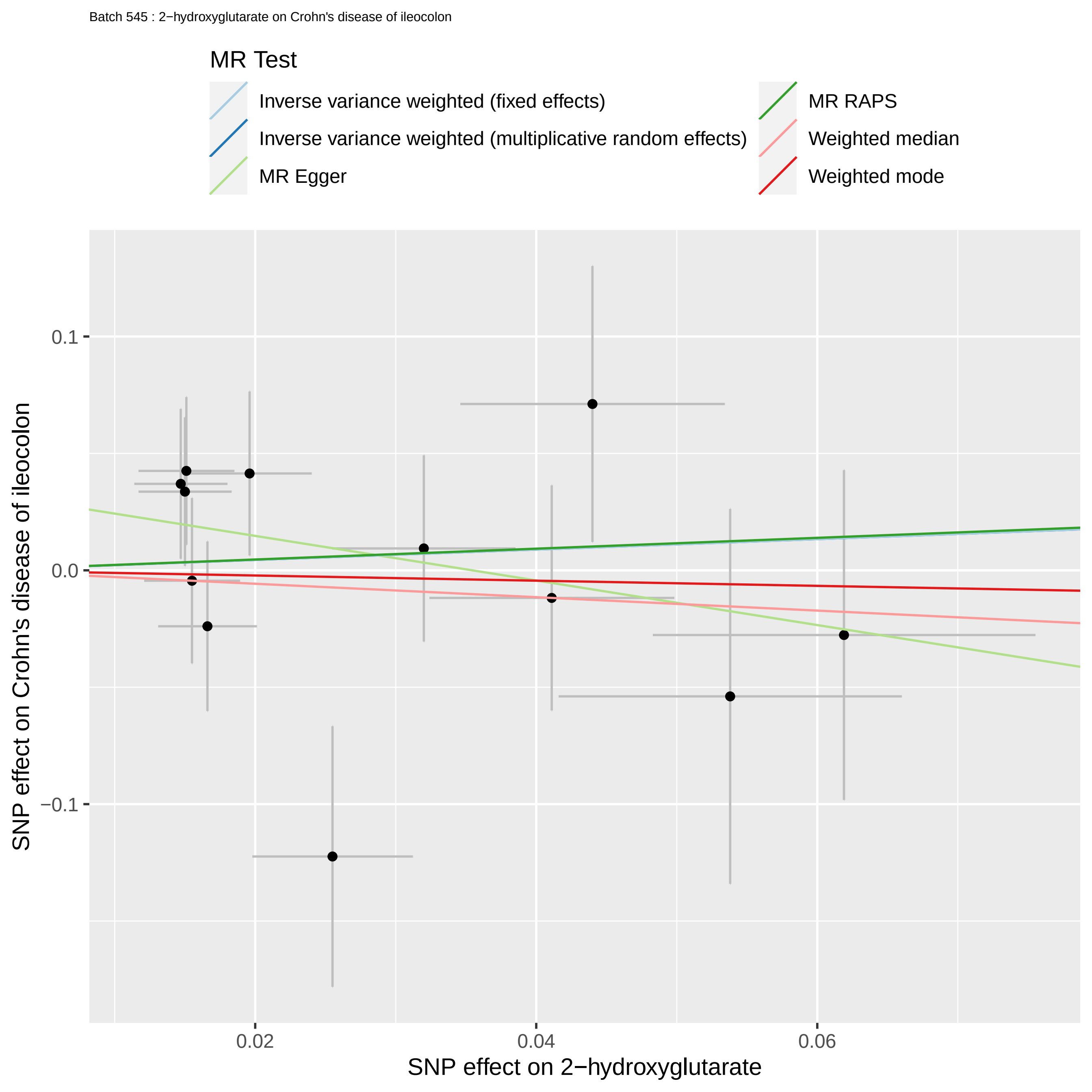

Batch 545 : 2−hydroxyglutarate on Crohn's disease of ileocolon
MR Test
Inverse variance weighted (fixed effects)
Inverse variance weighted (multiplicative random effects)
MR Egger
MR RAPS
Weighted median
Weighted mode
0.1
0.0
−0.1
0.02
0.04
0.06
SNP effect on Crohn's disease of ileocolon
SNP effect on 2−hydroxyglutarate

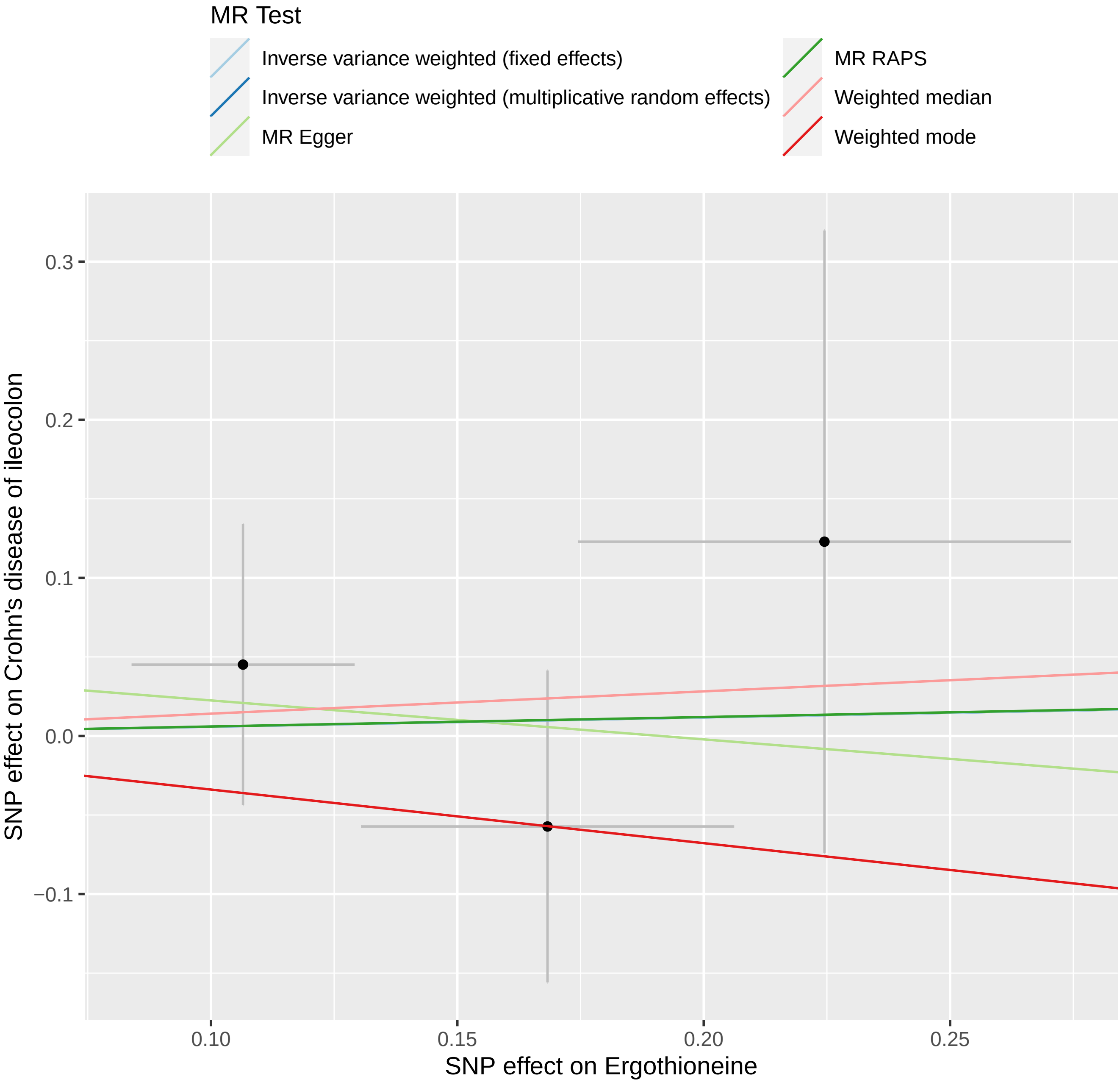
MR Test
Inverse variance weighted (fixed effects)
Inverse variance weighted (multiplicative random effects)
MR Egger
MR RAPS
Weighted median
Weighted mode
0.3
0.2
0.1
0.0
−0.1
0.10
0.15
0.20
0.25
SNP effect on Crohn's disease of ileocolon
SNP effect on Ergothioneine

Batch 548 : Phenylalanylphenylalanine on Crohn's disease of ileocolon

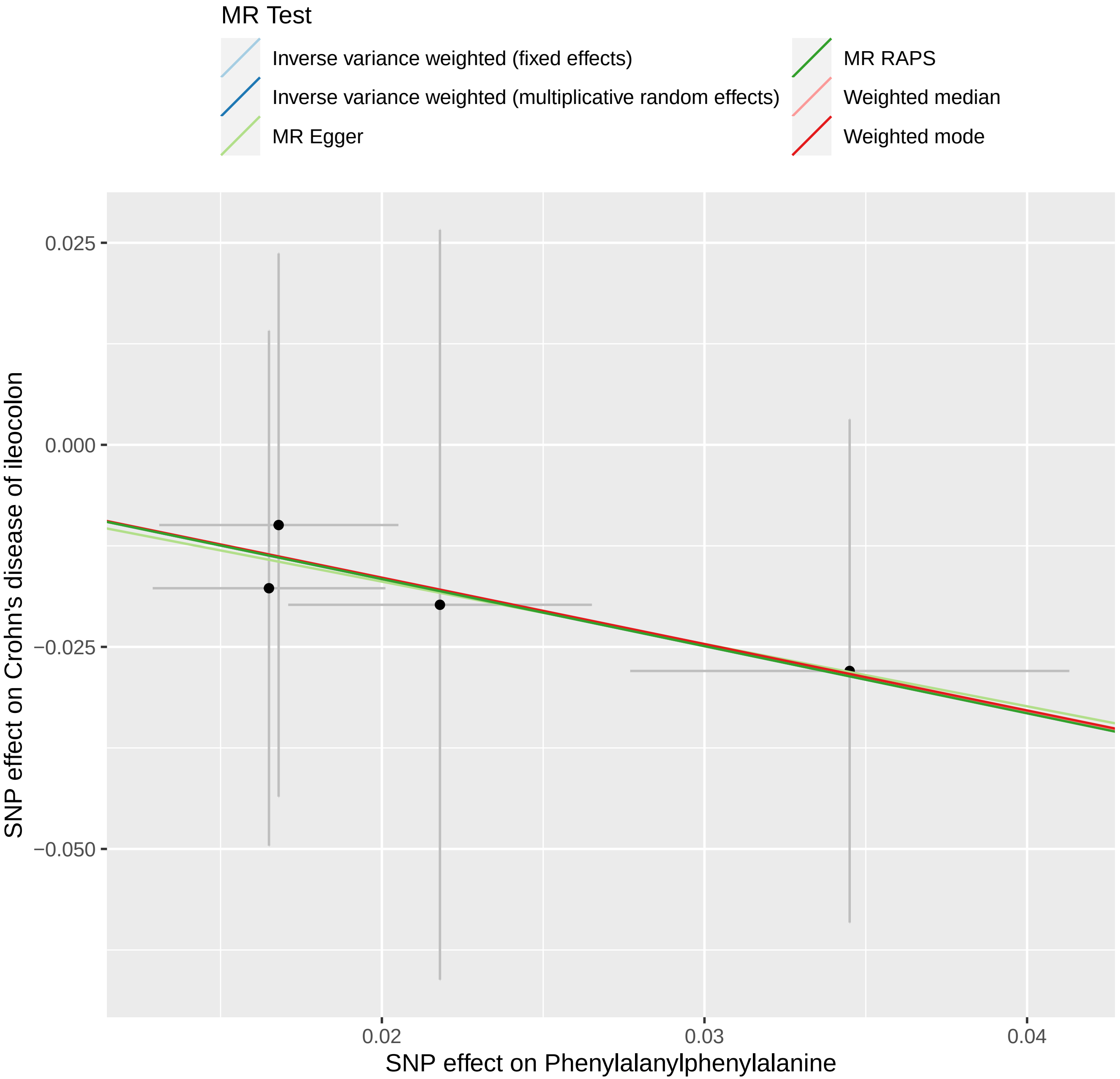

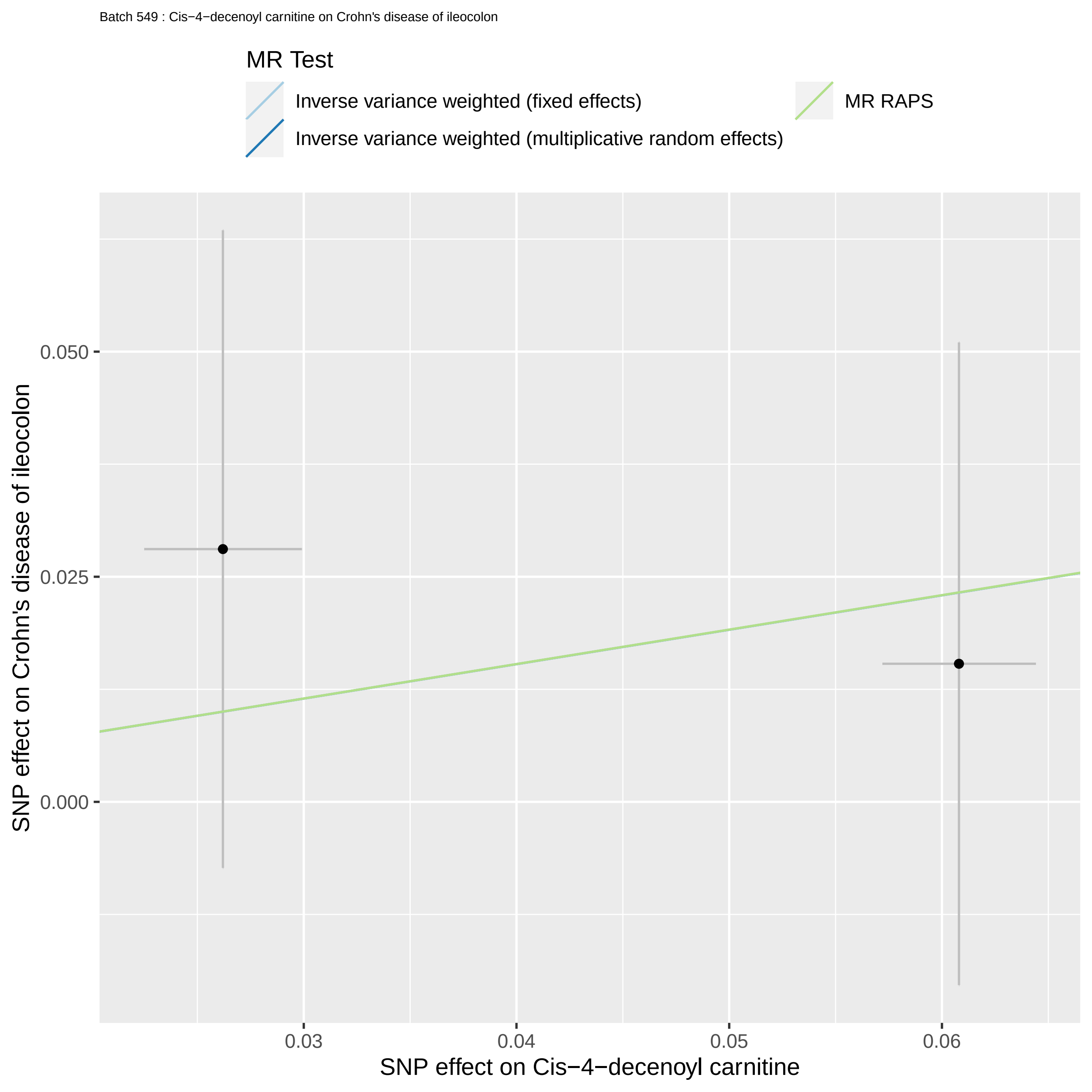
Batch 549 : Cis−4−decenoyl carnitine on Crohn's disease of ileocolon
MR Test
Inverse variance weighted (fixed effects)
Inverse variance weighted (multiplicative random effects)
MR RAPS
0.050
0.025
0.000
0.03
0.04
0.05
0.06
SNP effect on Crohn's disease of ileocolon
SNP effect on Cis−4−decenoyl carnitine

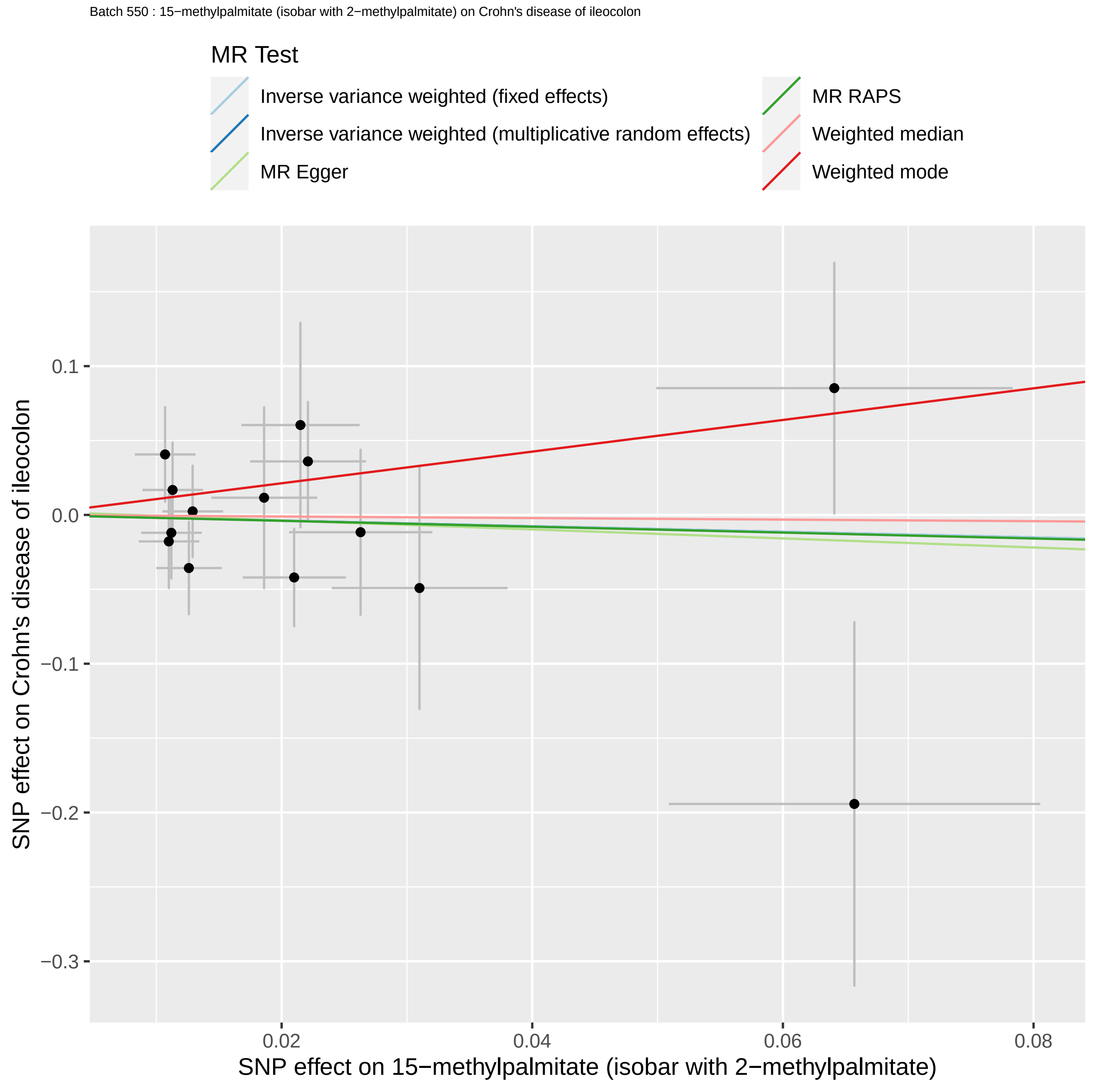

Batch 550 : 15−methylpalmitate (isobar with 2−methylpalmitate) on Crohn's disease of ileocolon
MR Test
Inverse variance weighted (fixed effects)
Inverse variance weighted (multiplicative random effects)
MR Egger
MR RAPS
Weighted median
Weighted mode
0.1
0.0
−0.1
−0.2
−0.3
0.02
0.04
0.06
0.08
SNP effect on Crohn's disease of ileocolon
SNP effect on 15−methylpalmitate (isobar with 2−methylpalmitate)

Batch 552 : Tryptophan on Crohn's disease of small intestine

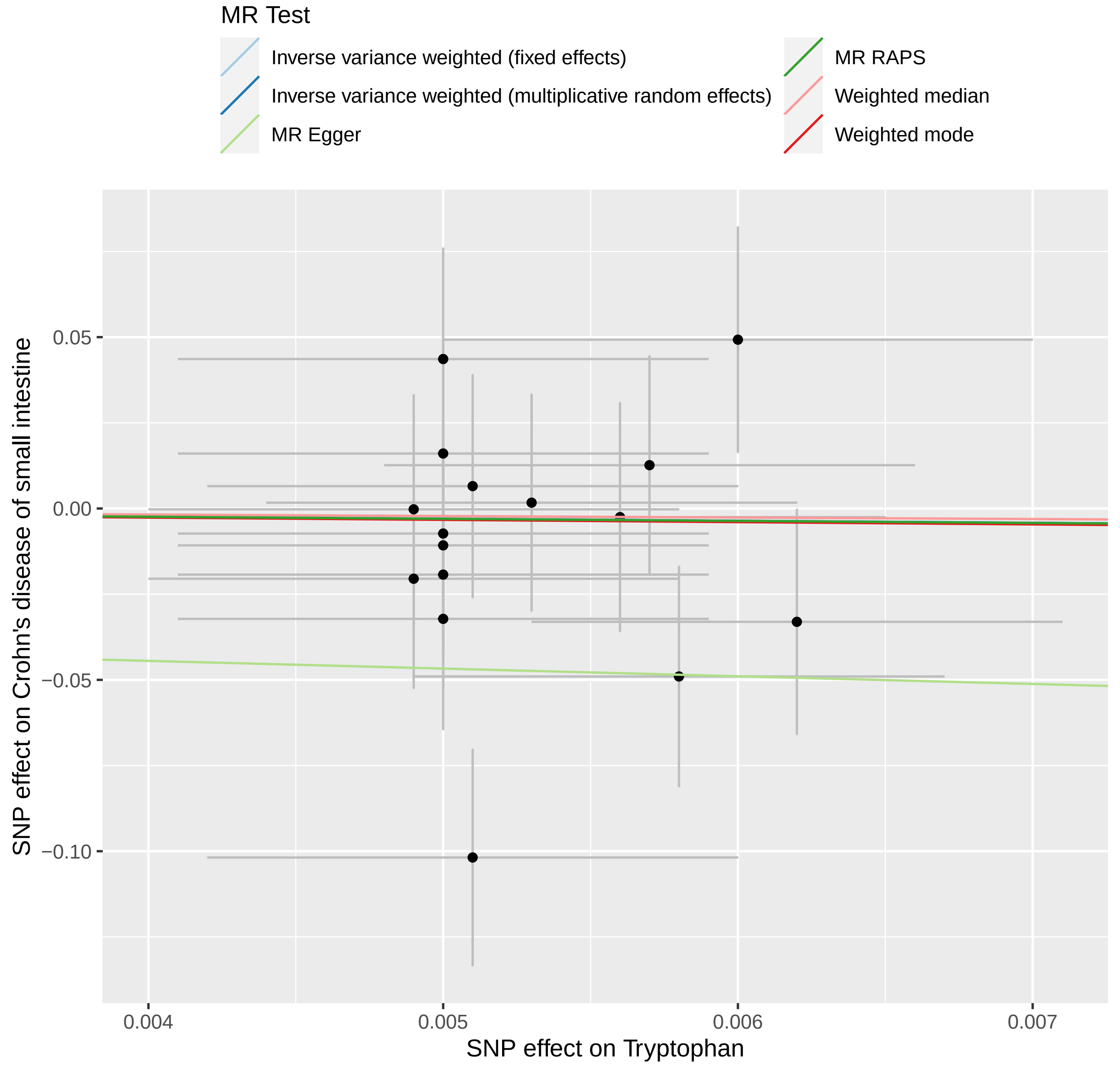

Batch 554 : Leucine on Crohn's disease of small intestine

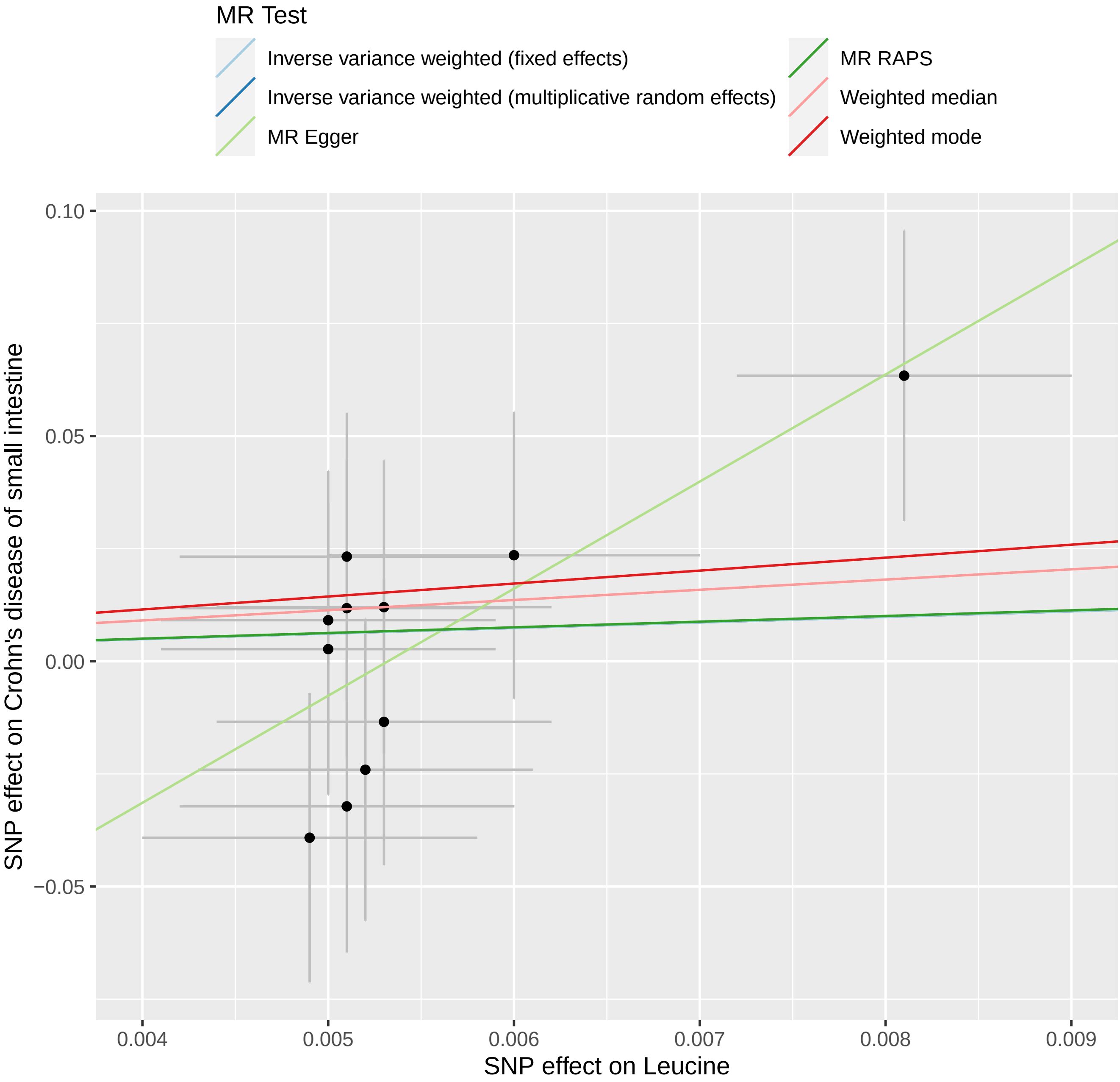

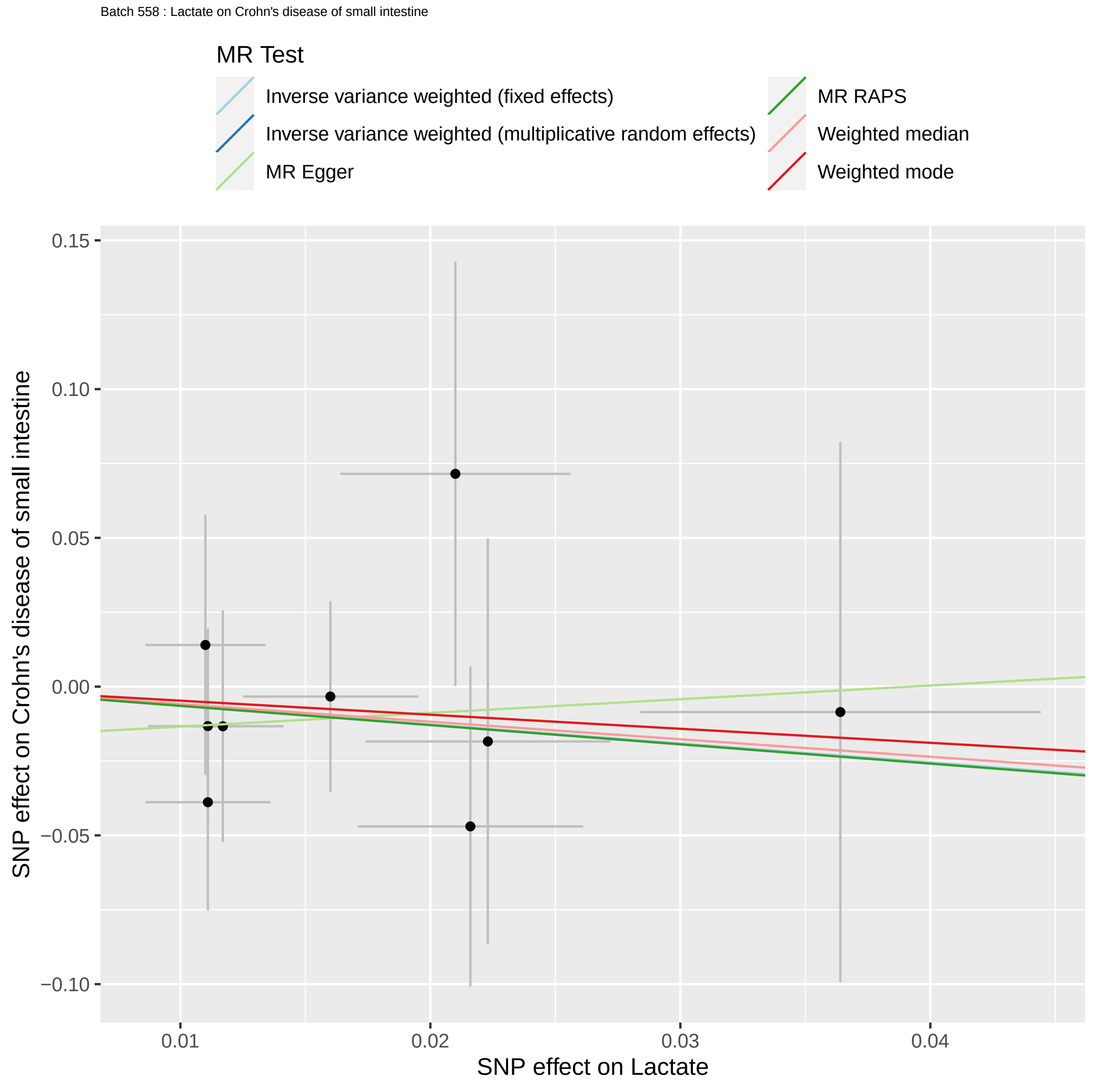
Batch 558 : Lactate on Crohn's disease of small intestine
MR Test
Inverse variance weighted (fixed effects)
Inverse variance weighted (multiplicative random effects)
MR Egger
MR RAPS
Weighted median
Weighted mode
SNP effect on Crohn's disease of small intestine
0.15
0.10
0.05
0.00
−0.05
−0.10
0.01
0.02
0.03
0.04
SNP effect on Lactate

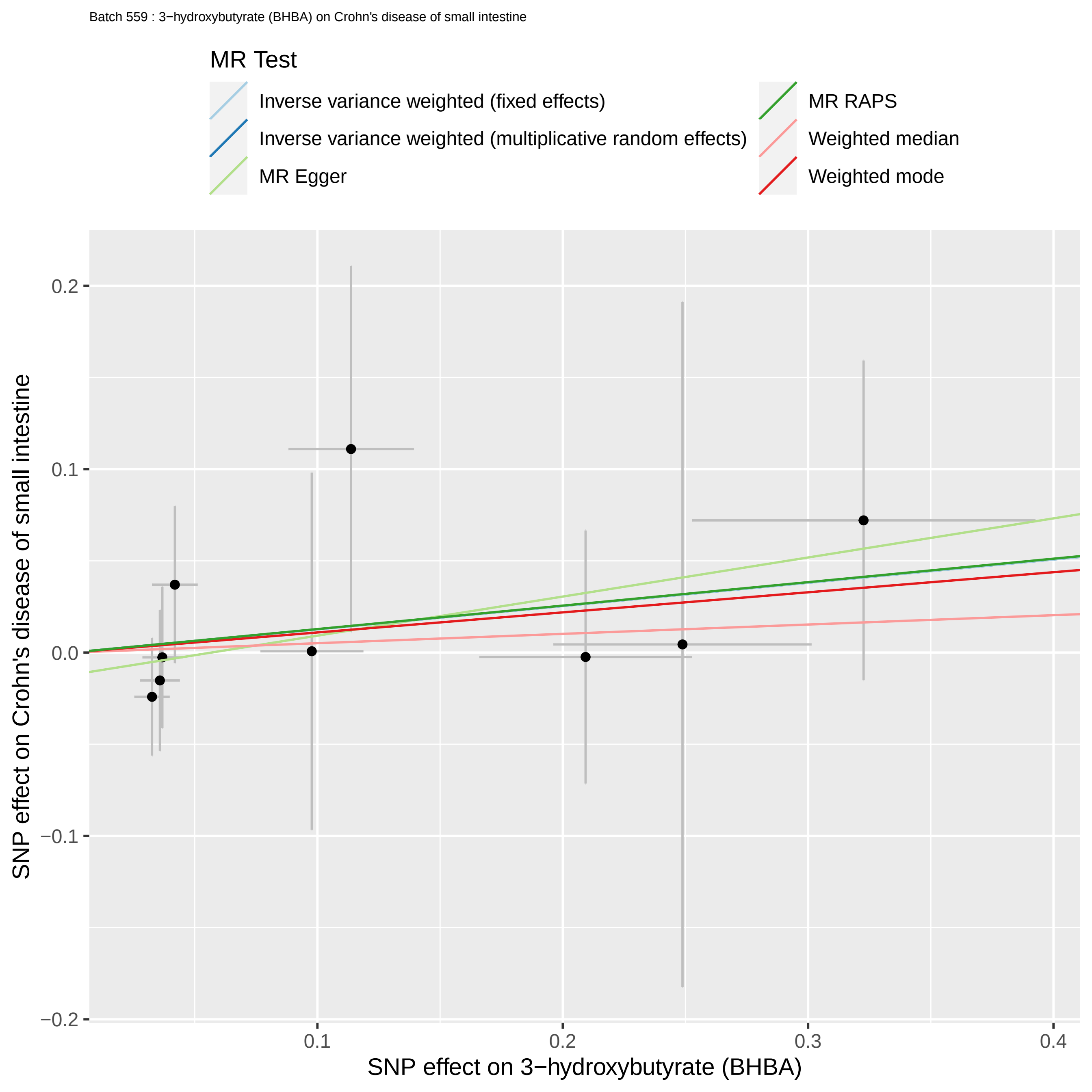
Batch 559 : 3−hydroxybutyrate (BHBA) on Crohn's disease of small intestine
MR Test
Inverse variance weighted (fixed effects)
Inverse variance weighted (multiplicative random effects)
MR Egger
MR RAPS
Weighted median
Weighted mode
SNP effect on Crohn's disease of small intestine
0.2
0.1
0.0
−0.1
−0.2
0.1
0.2
0.3
0.4
SNP effect on 3−hydroxybutyrate (BHBA)

Batch 560 : Arabinose on Crohn's disease of small intestine

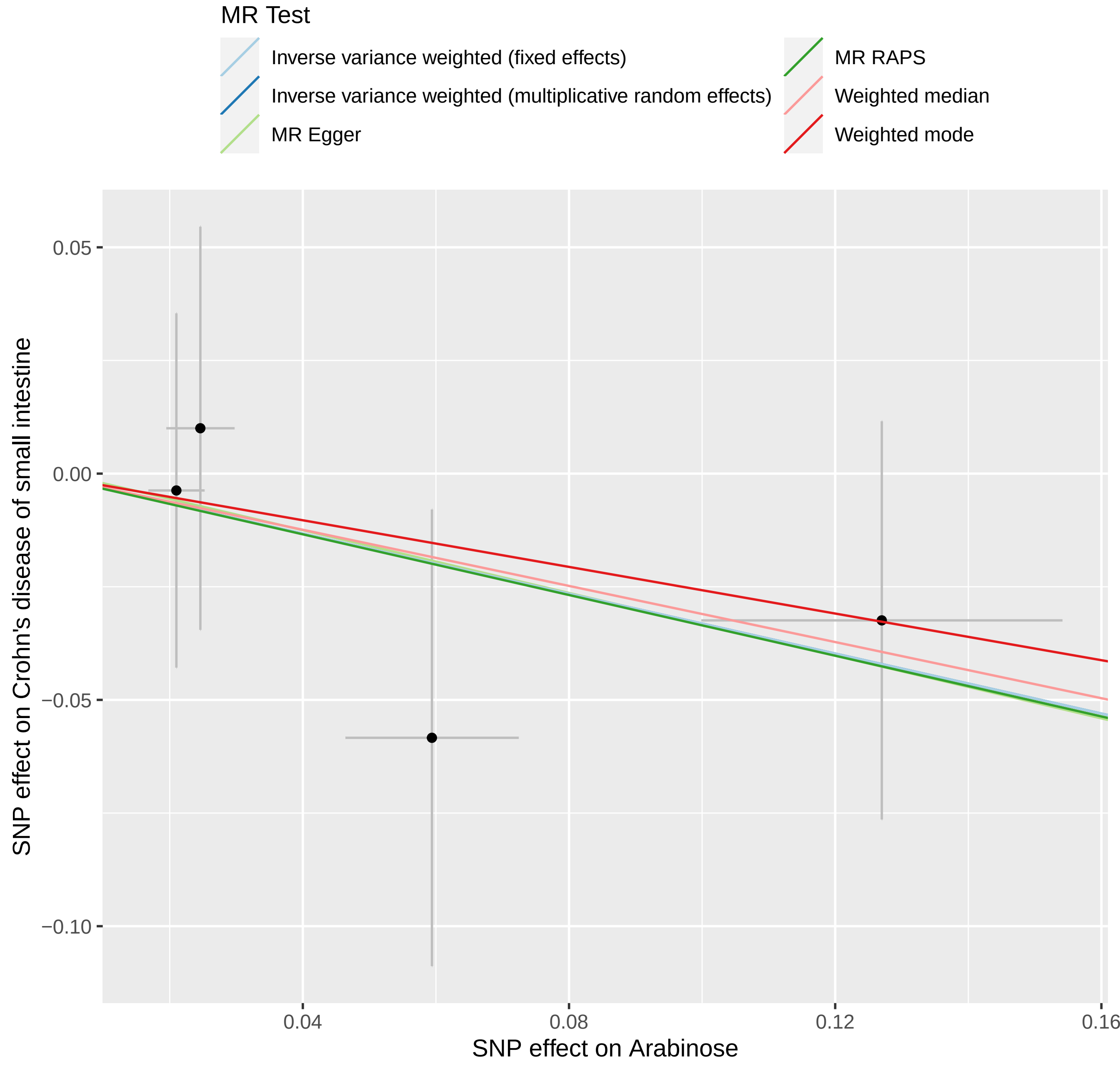

Batch 561 : Fructose on Crohn's disease of small intestine

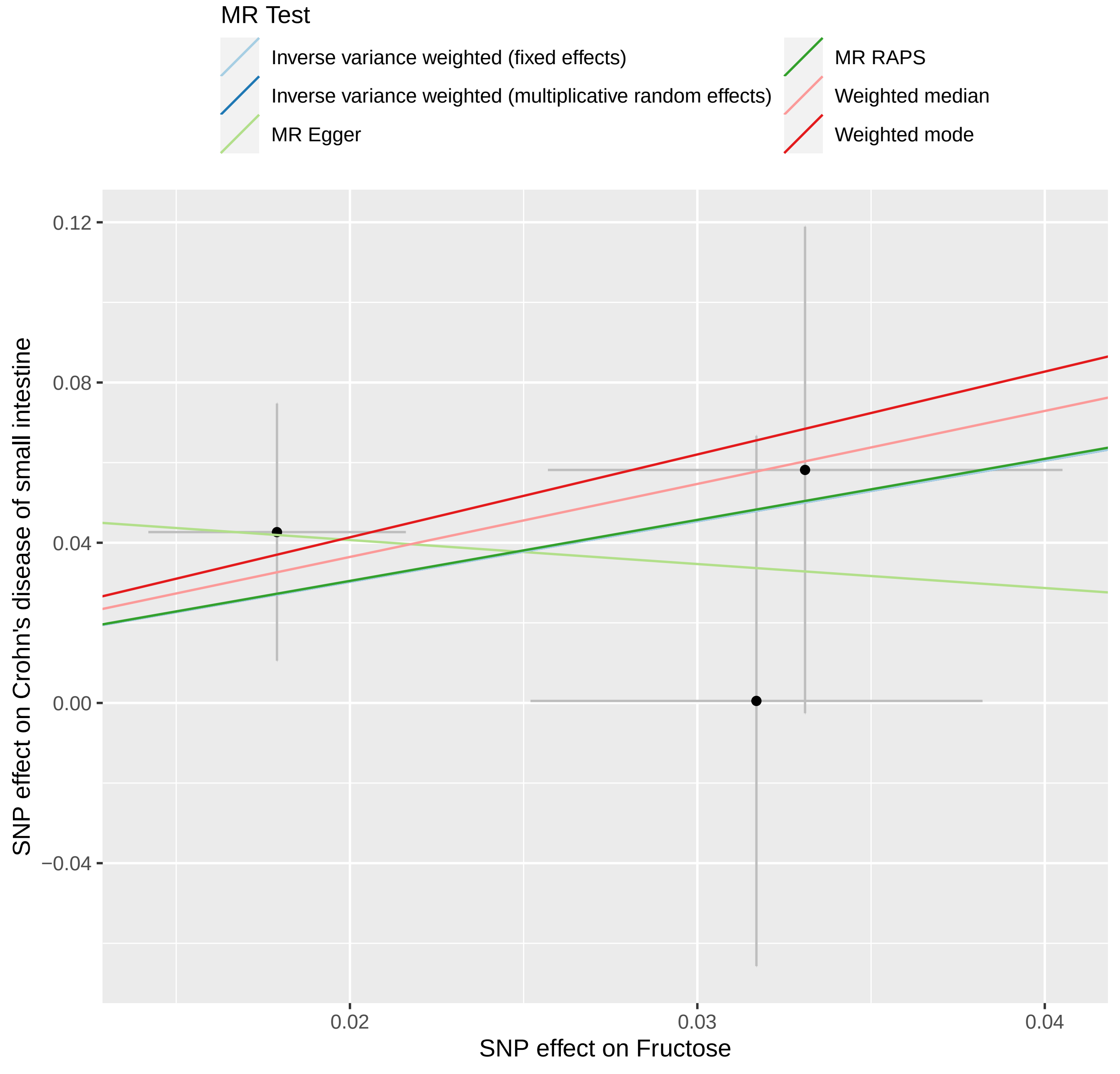

Batch 563 : Pyruvate on Crohn's disease of small intestine

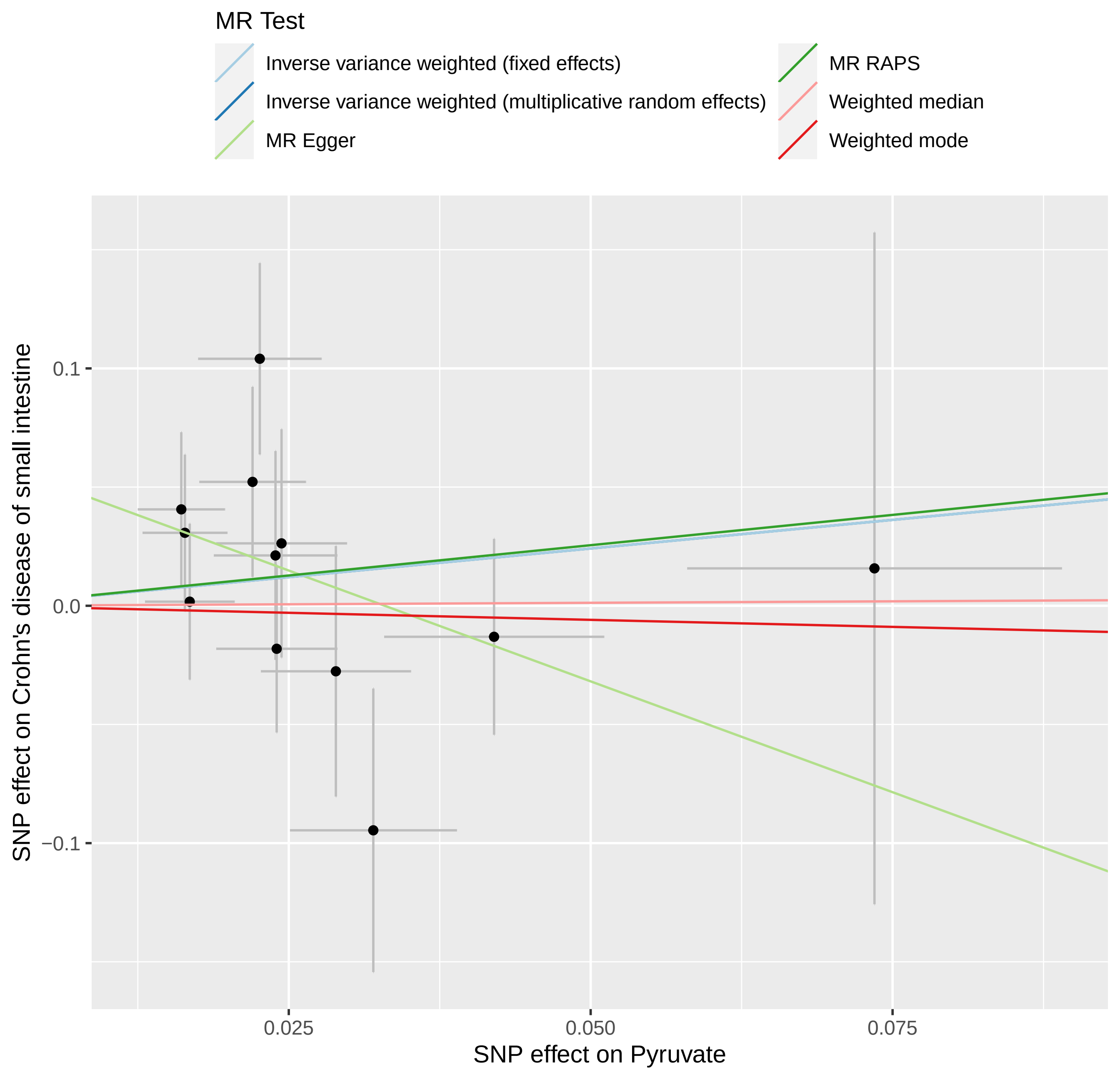

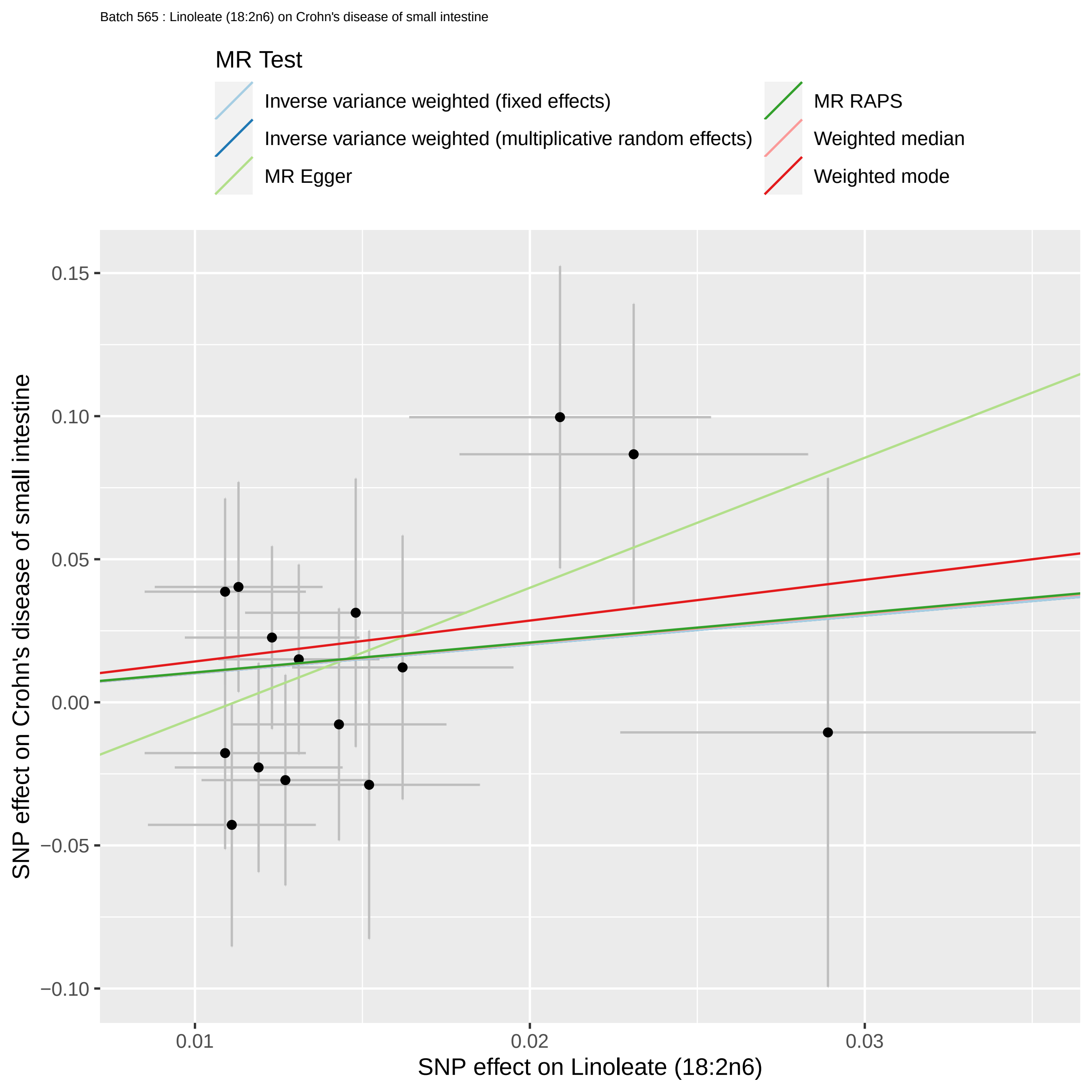

Batch 565 : Linoleate (18:2n6) on Crohn's disease of small intestine
MR Test
Inverse variance weighted (fixed effects)
Inverse variance weighted (multiplicative random effects)
MR Egger
MR RAPS
Weighted median
Weighted mode
0.15
0.10
0.05
0.00
−0.05
−0.10
0.01
0.02
0.03
SNP effect on Crohn's disease of small intestine
SNP effect on Linoleate (18:2n6)

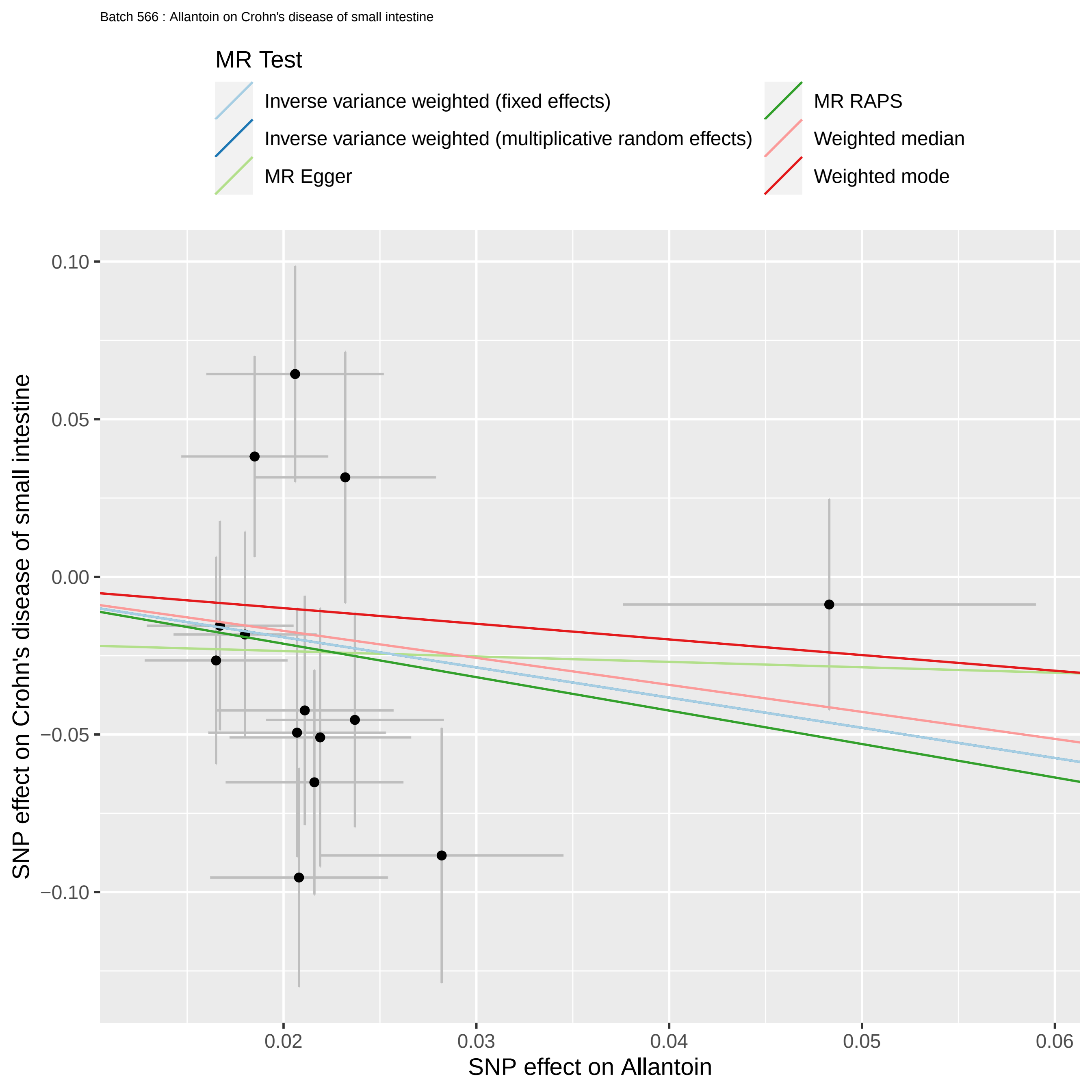

Batch 566 : Allantoin on Crohn's disease of small intestine
MR Test
Inverse variance weighted (fixed effects)
Inverse variance weighted (multiplicative random effects)
MR Egger
MR RAPS
Weighted median
Weighted mode
0.10
0.05
0.00
−0.05
−0.10
0.02
0.03
0.04
0.05
0.06
SNP effect on Crohn's disease of small intestine
SNP effect on Allantoin

Batch 571 : Isoleucine on Crohn's disease of small intestine

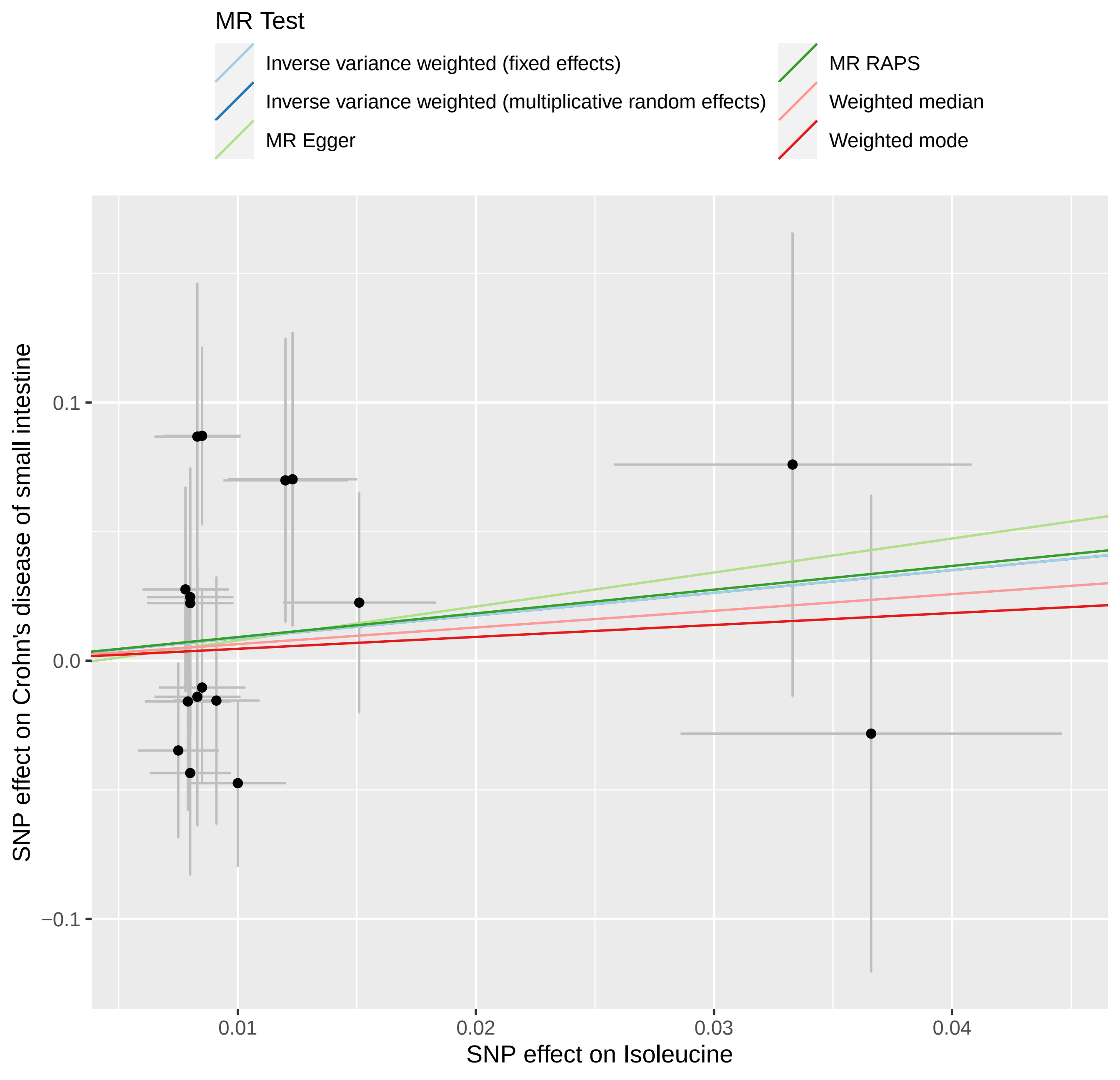

Batch 572 : Threonine on Crohn's disease of small intestine

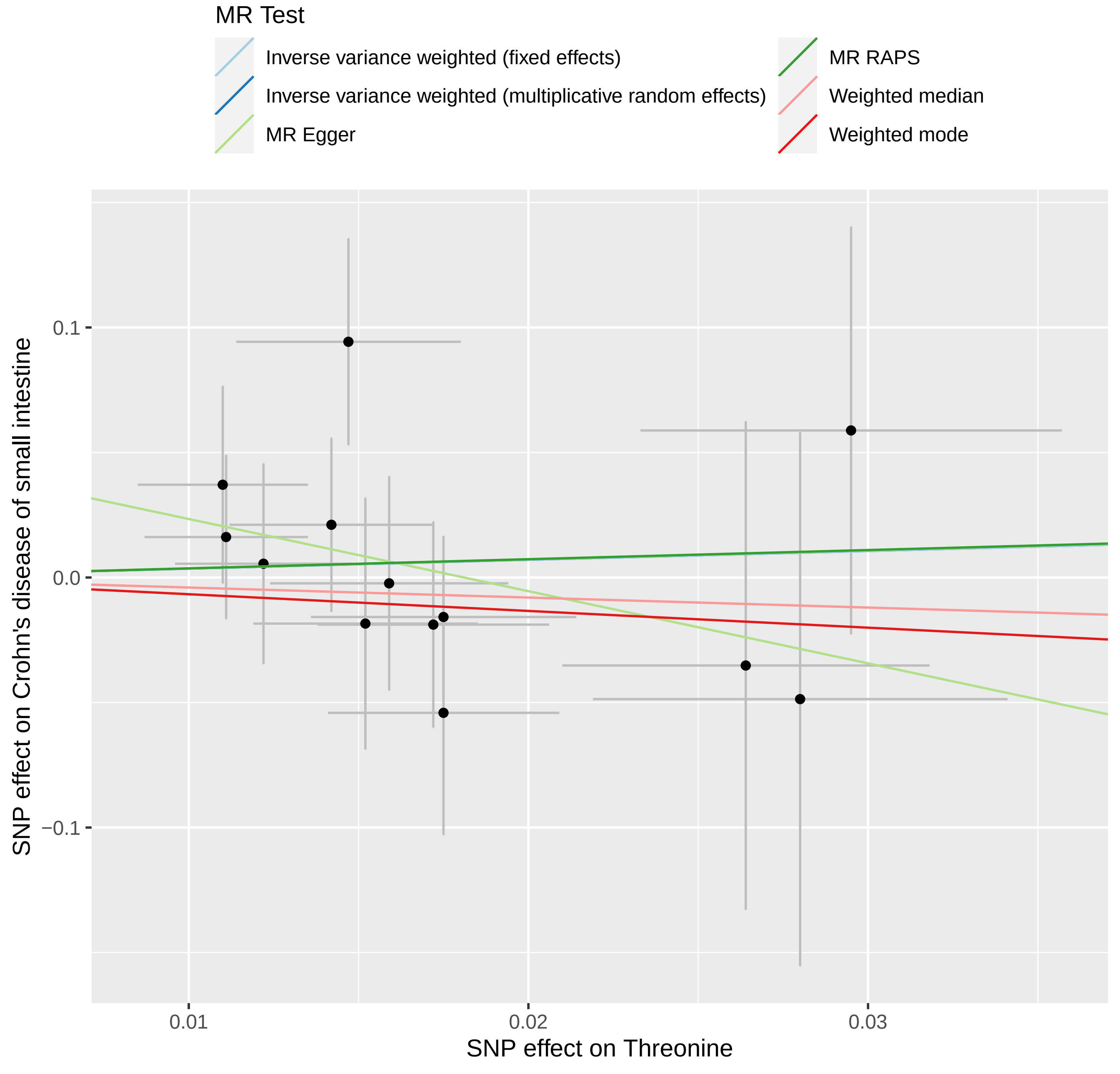

Batch 573 : Tyrosine on Crohn's disease of small intestine

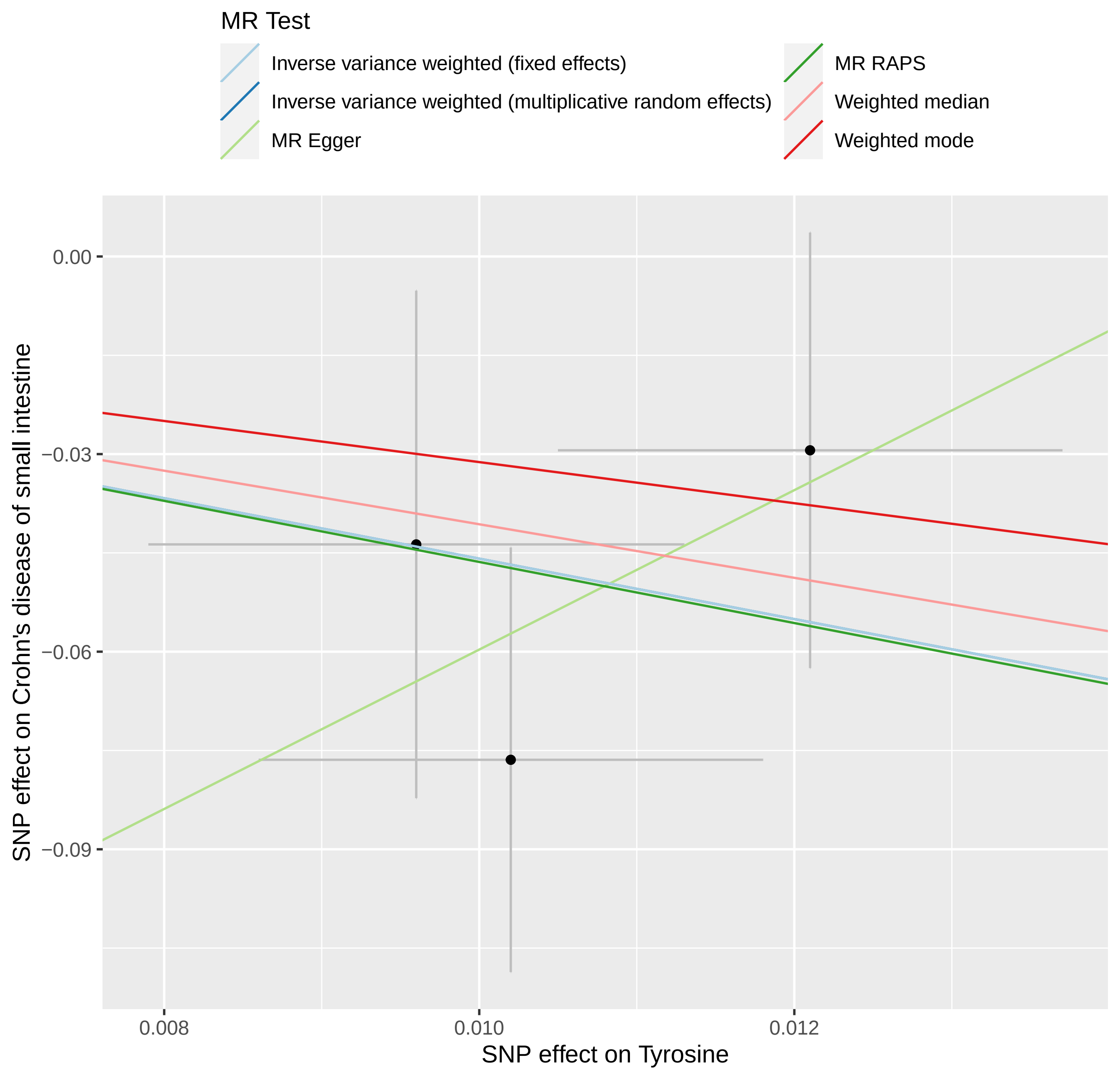

Batch 579 : Stearate (18:0) on Crohn's disease of small intestine

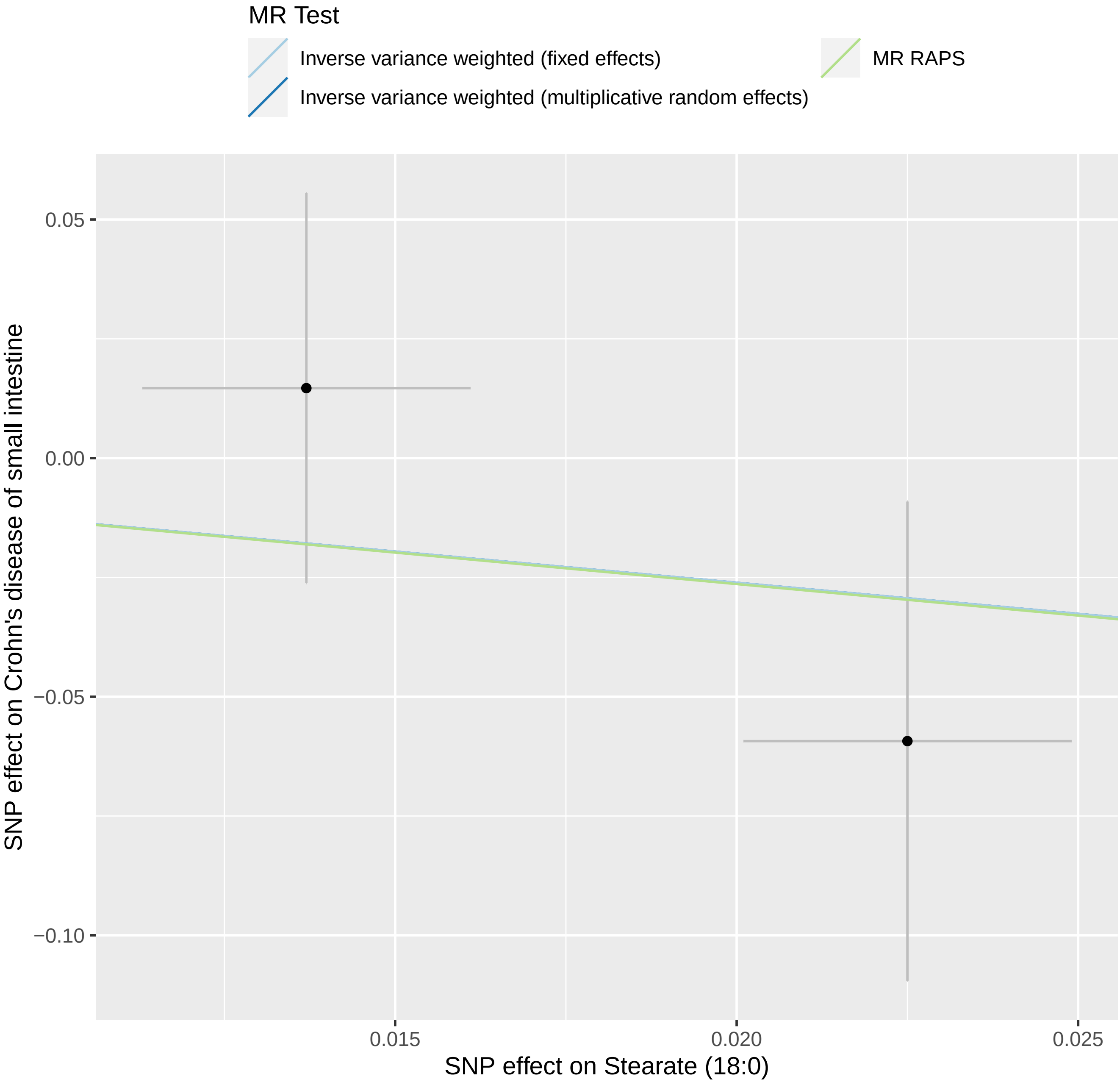

Batch 582 : Myristate (14:0) on Crohn's disease of small intestine

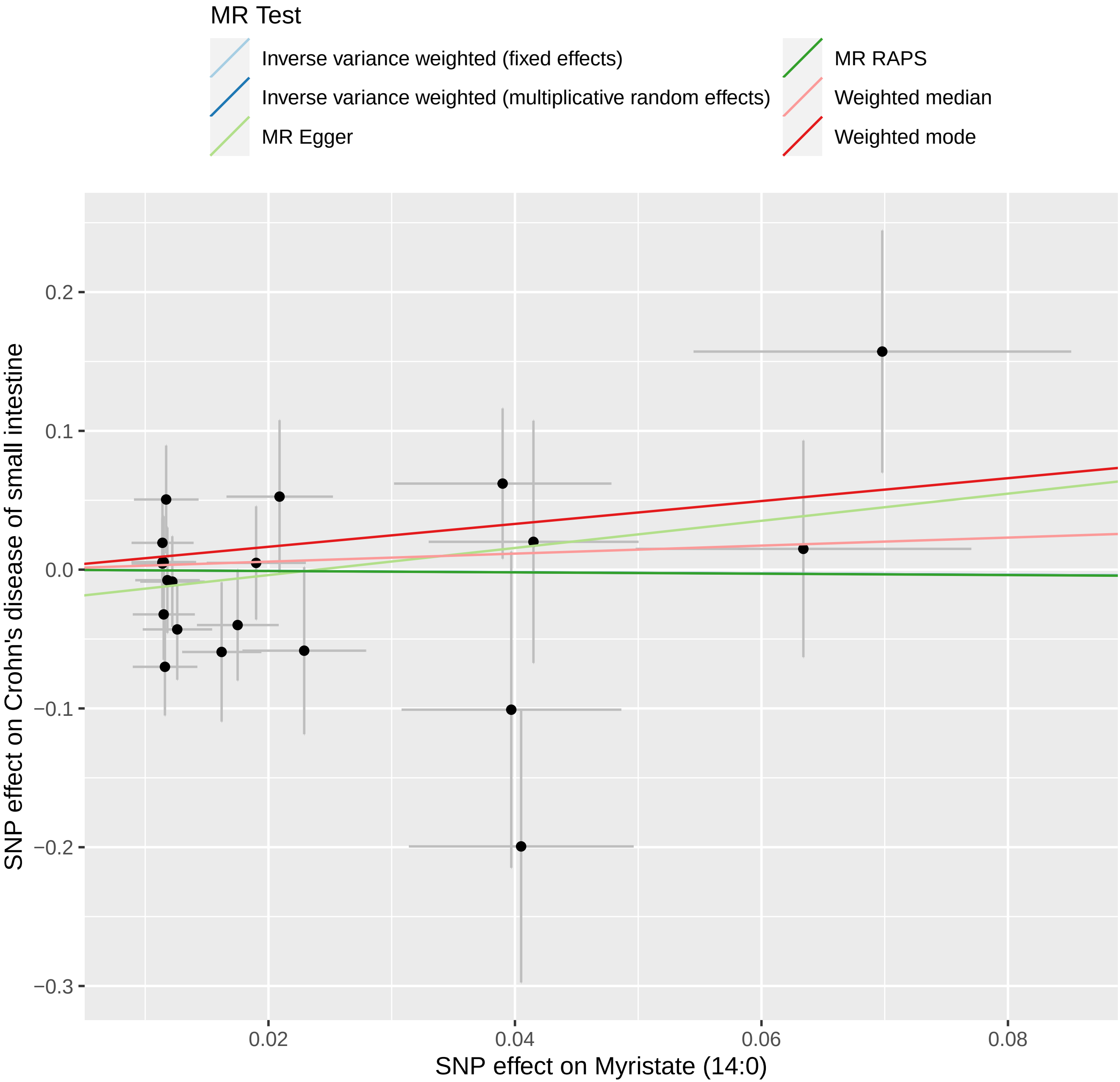

Batch 584 : Ornithine on Crohn's disease of small intestine

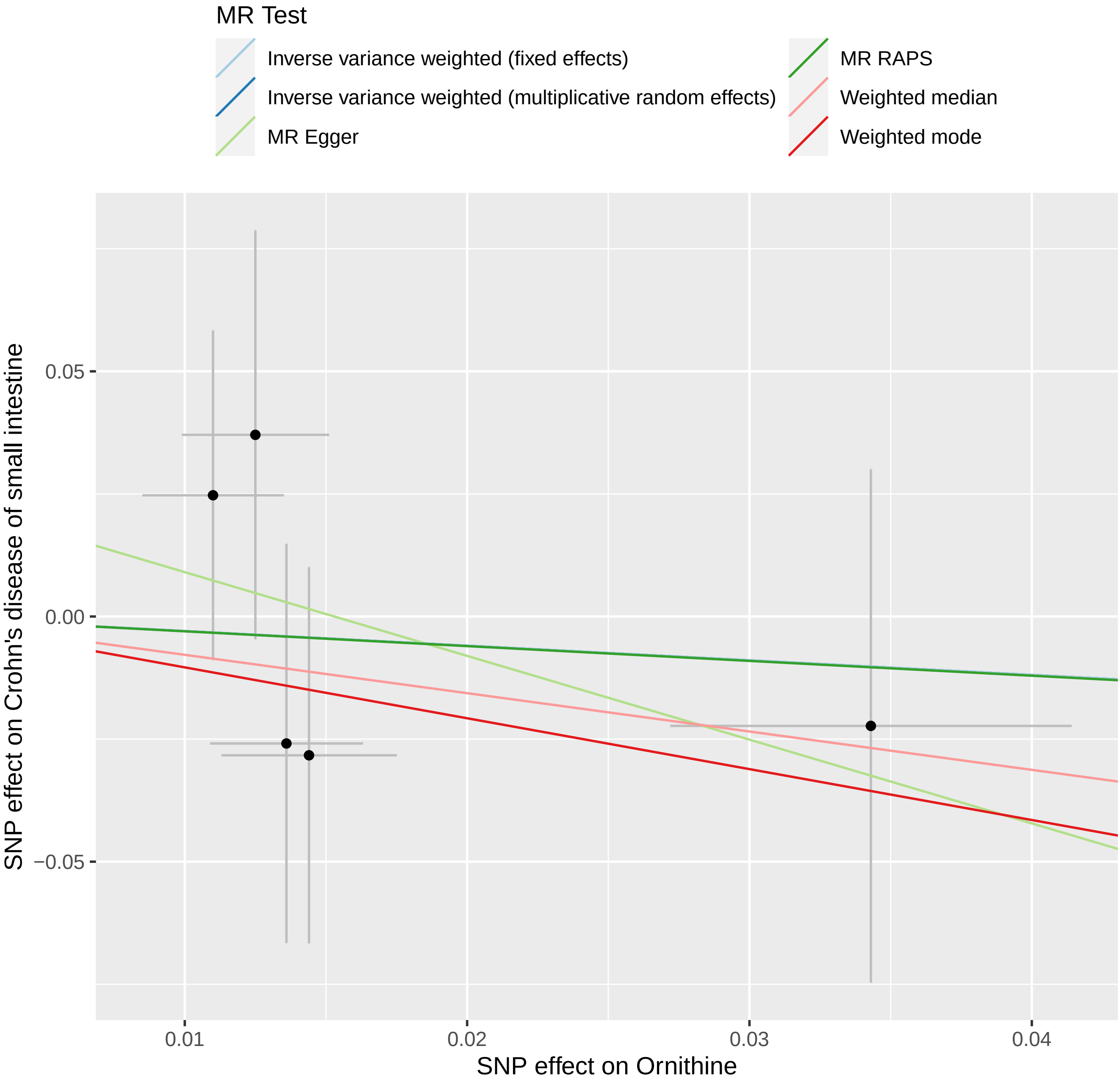

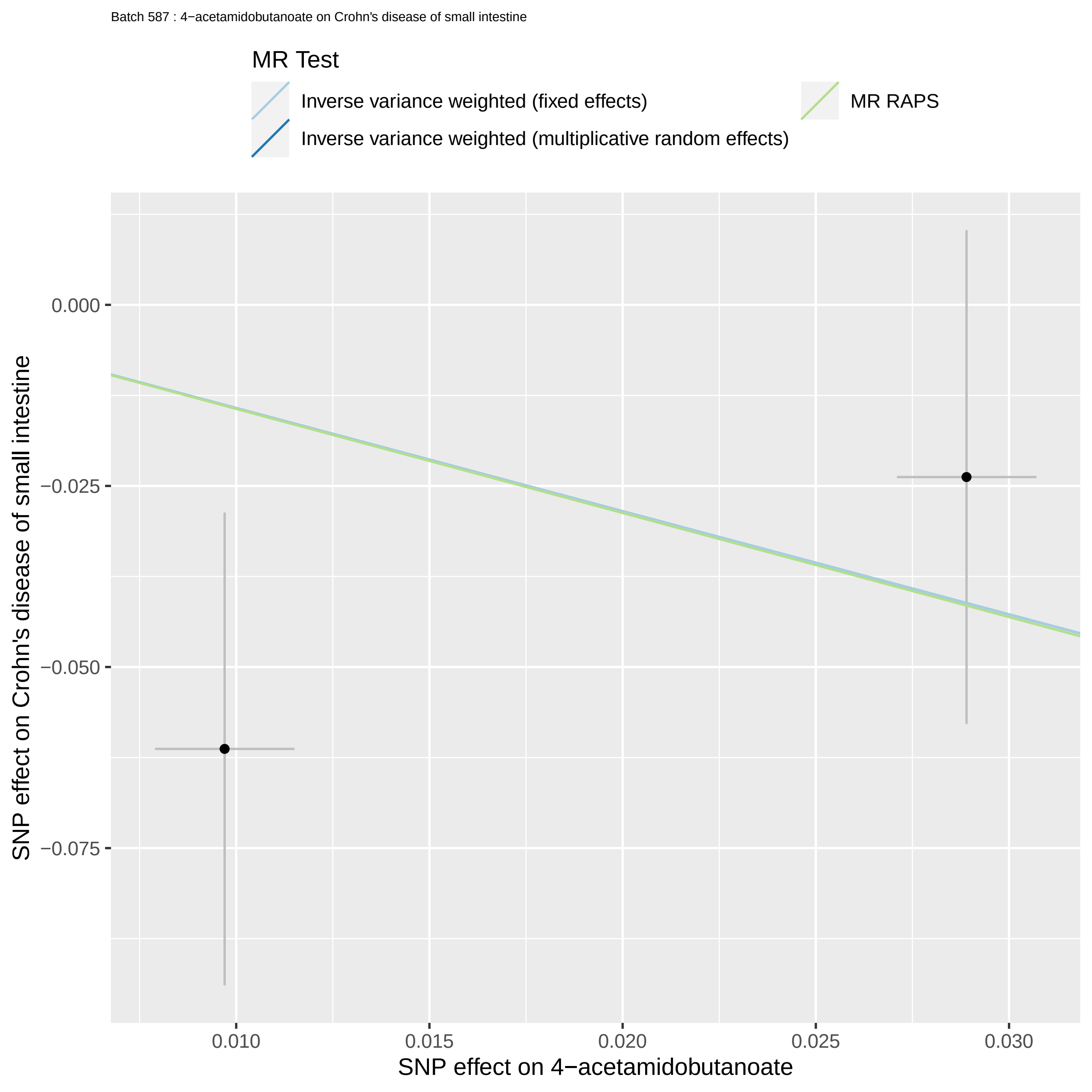
Batch 587 : 4−acetamidobutanoate on Crohn's disease of small intestine
MR Test
Inverse variance weighted (fixed effects)
Inverse variance weighted (multiplicative random effects)
MR RAPS
0.000
−0.025
−0.050
−0.075
SNP effect on Crohn's disease of small intestine
0.010
0.015
0.020
0.025
0.030
SNP effect on 4−acetamidobutanoate

Batch 588 : Alpha−tocopherol on Crohn's disease of small intestine

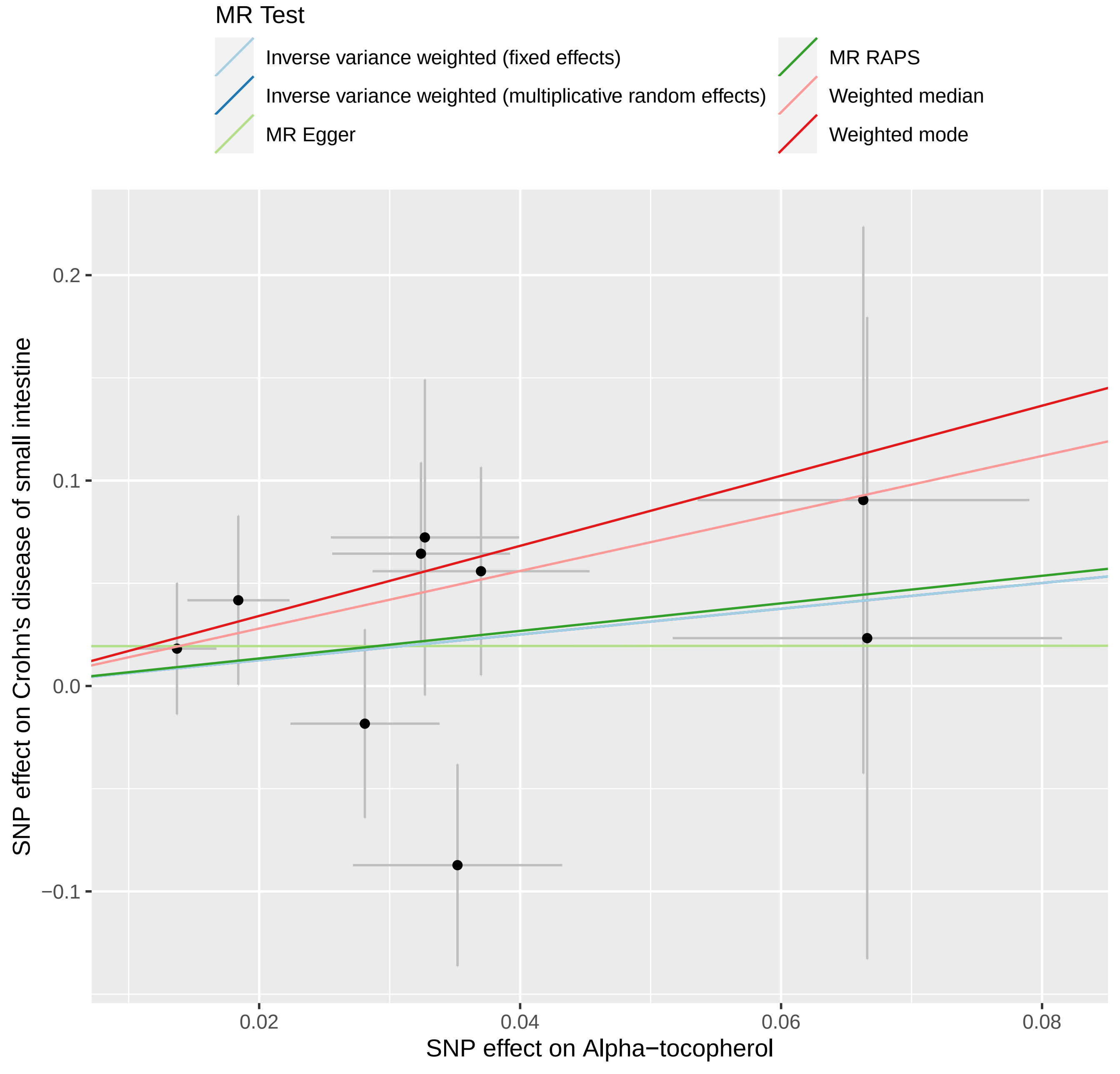

Batch 589 : Citrate on Crohn's disease of small intestine

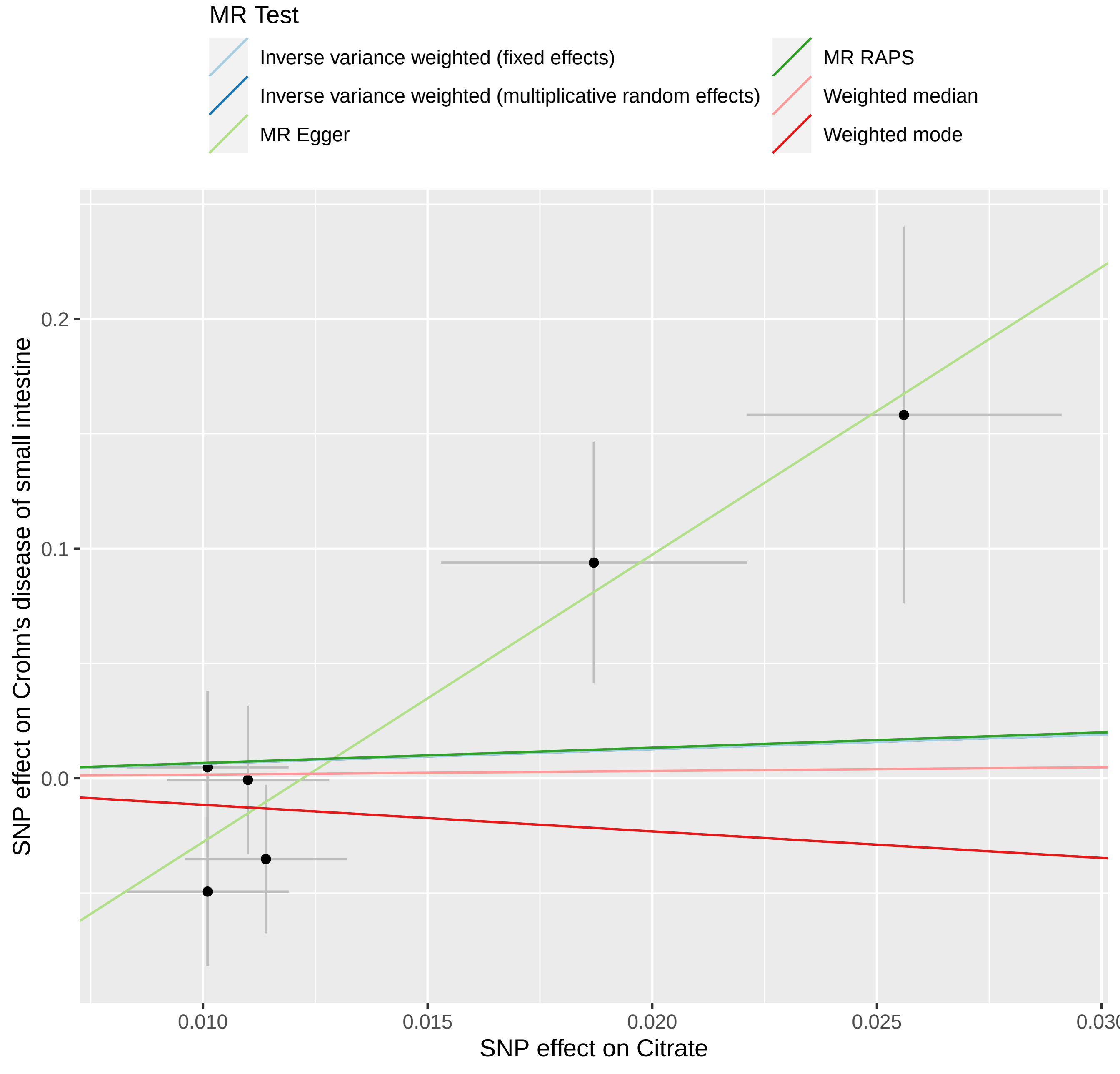

Batch 590 : Glycerate on Crohn's disease of small intestine

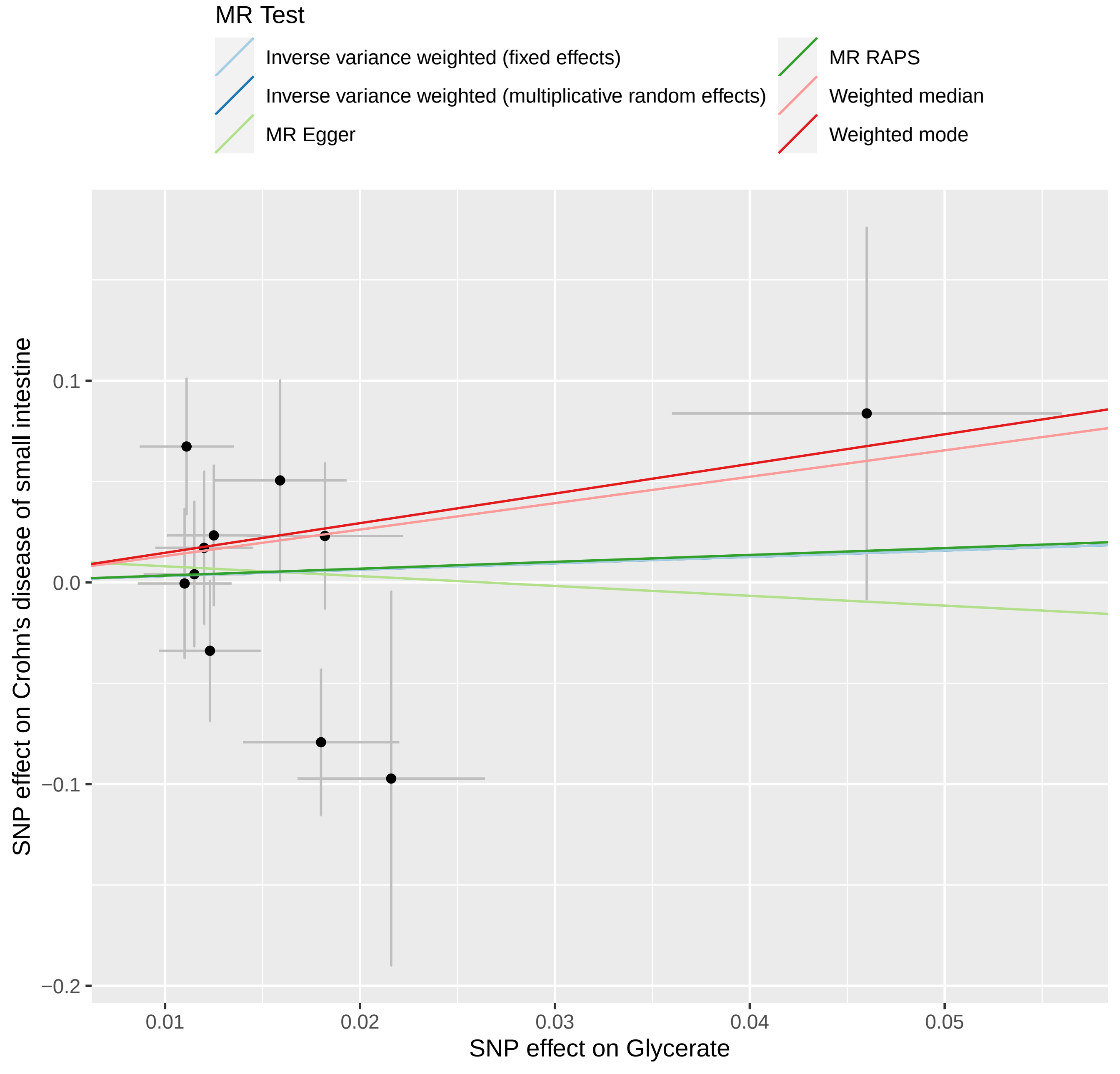

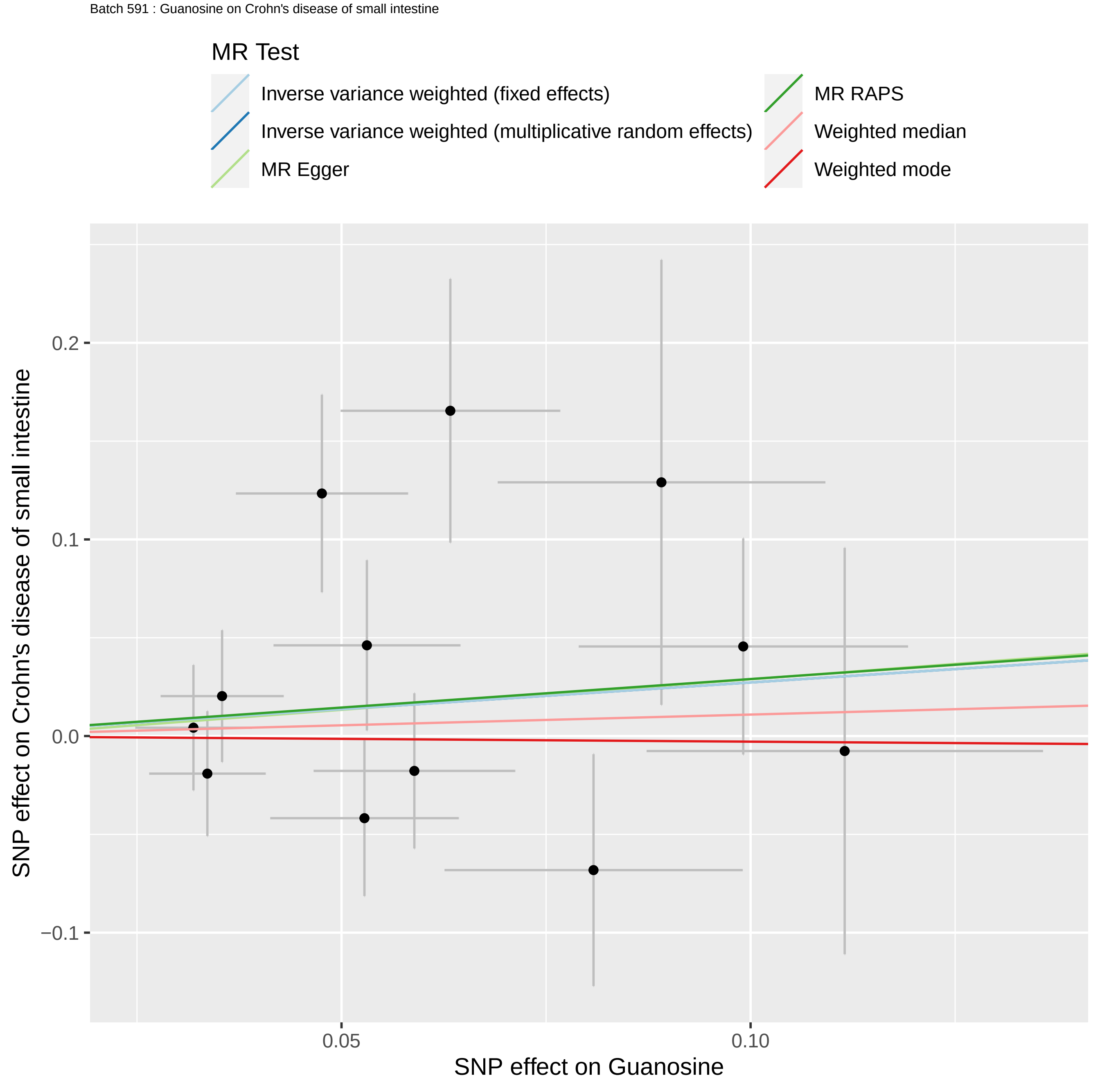

Batch 591 : Guanosine on Crohn's disease of small intestine
MR Test
Inverse variance weighted (fixed effects)
Inverse variance weighted (multiplicative random effects)
MR Egger
MR RAPS
Weighted median
Weighted mode
0.2
0.1
0.0
−0.1
0.05
0.10
SNP effect on Crohn's disease of small intestine
SNP effect on Guanosine

Batch 594 : Ursodeoxycholate on Crohn's disease of small intestine

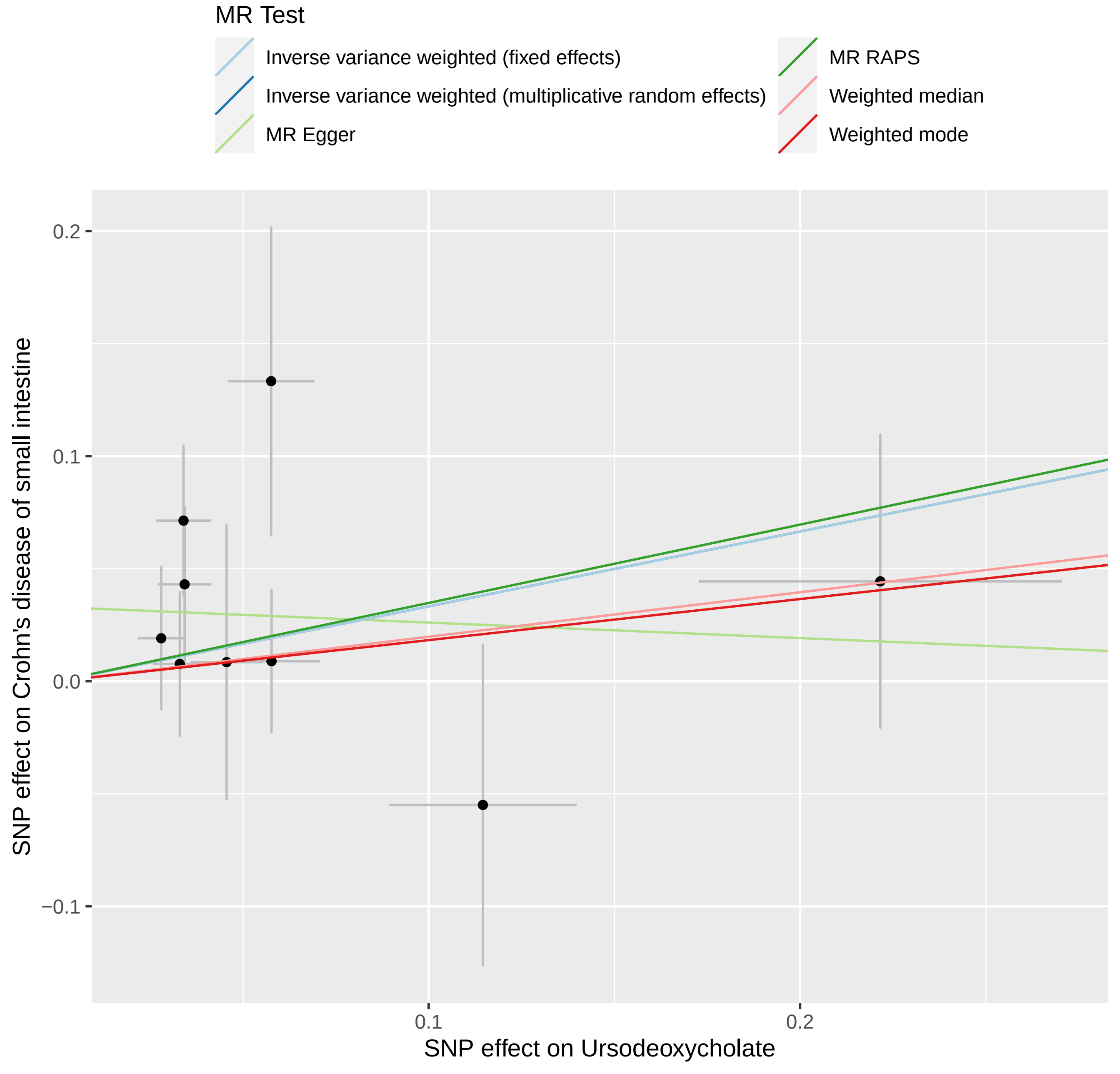

Batch 595 : Arginine on Crohn's disease of small intestine

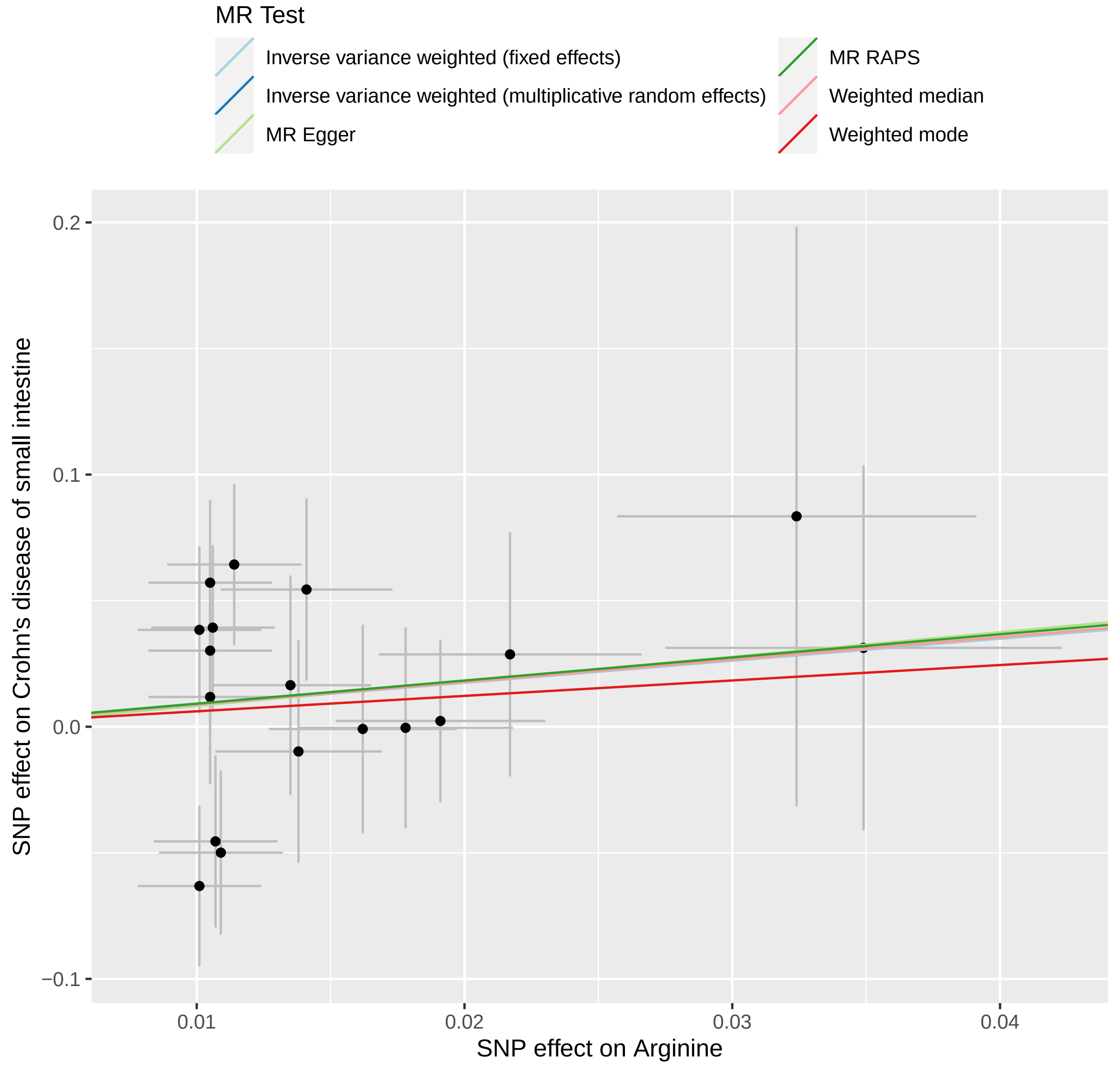

Batch 596 : Ascorbate (Vitamin C) on Crohn's disease of small intestine

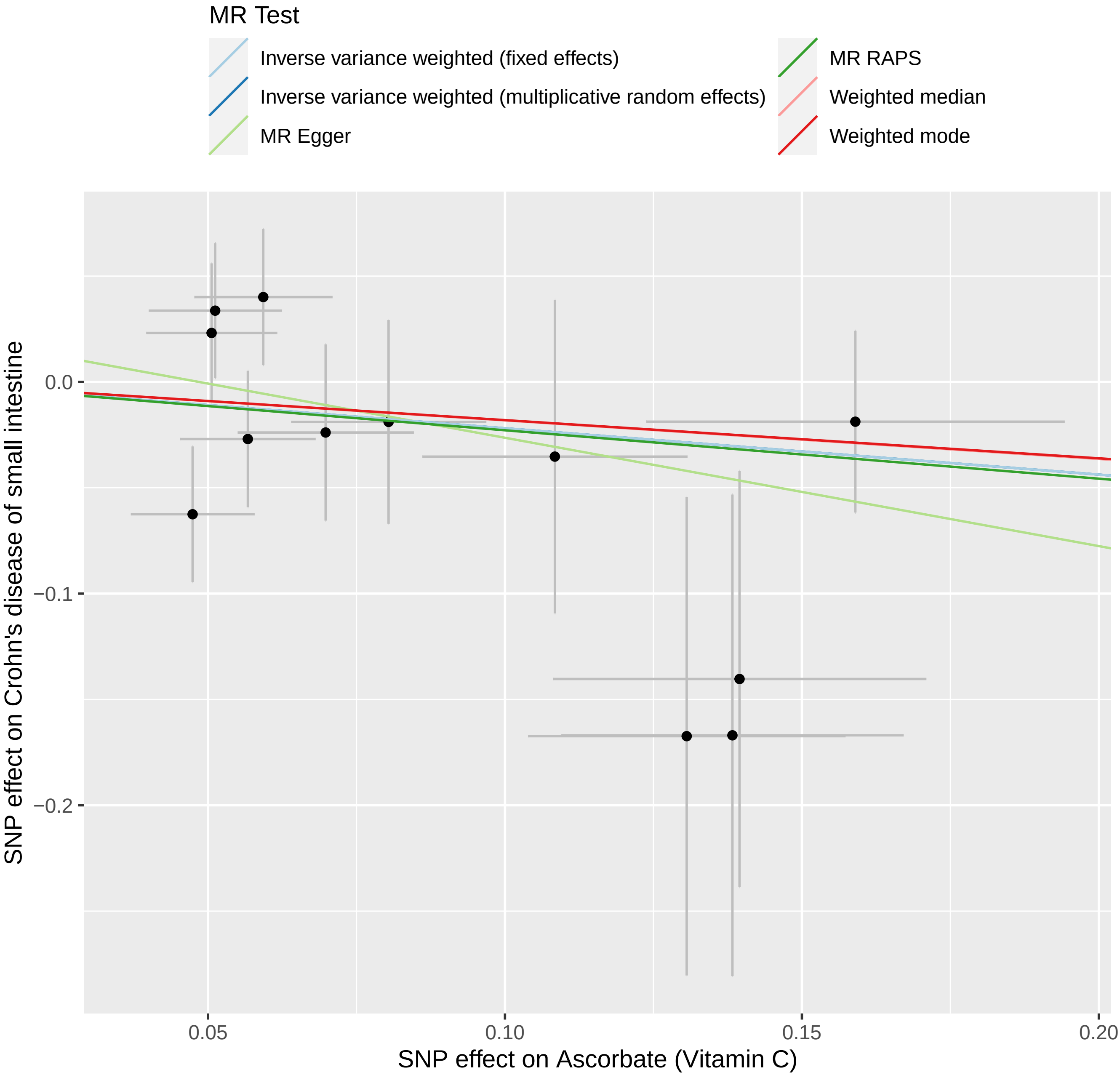

Batch 597 : Heptanoate (7:0) on Crohn's disease of small intestine

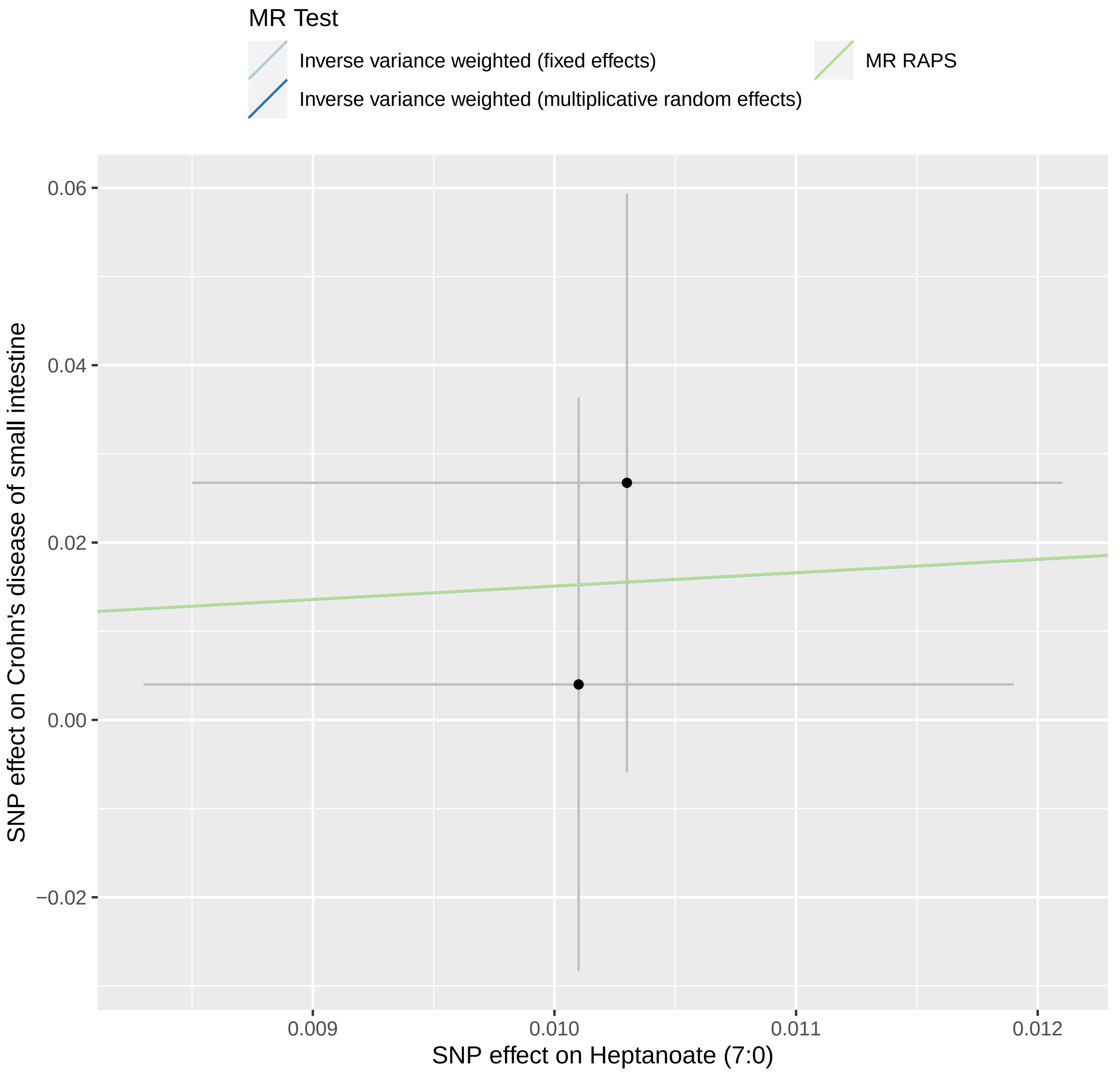

Batch 598 : Laurate (12:0) on Crohn's disease of small intestine

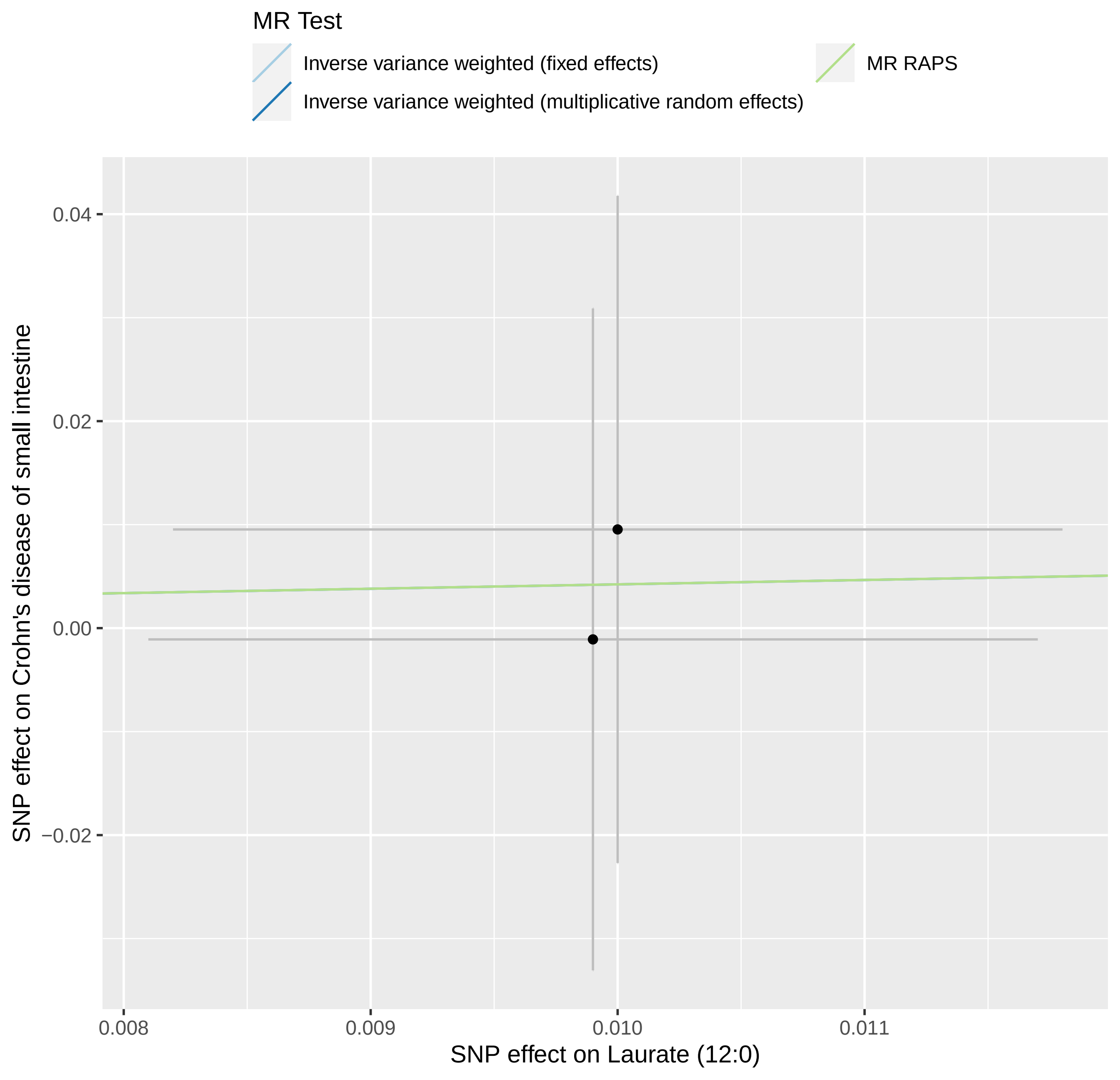

Batch 600 : Urea on Crohn's disease of small intestine

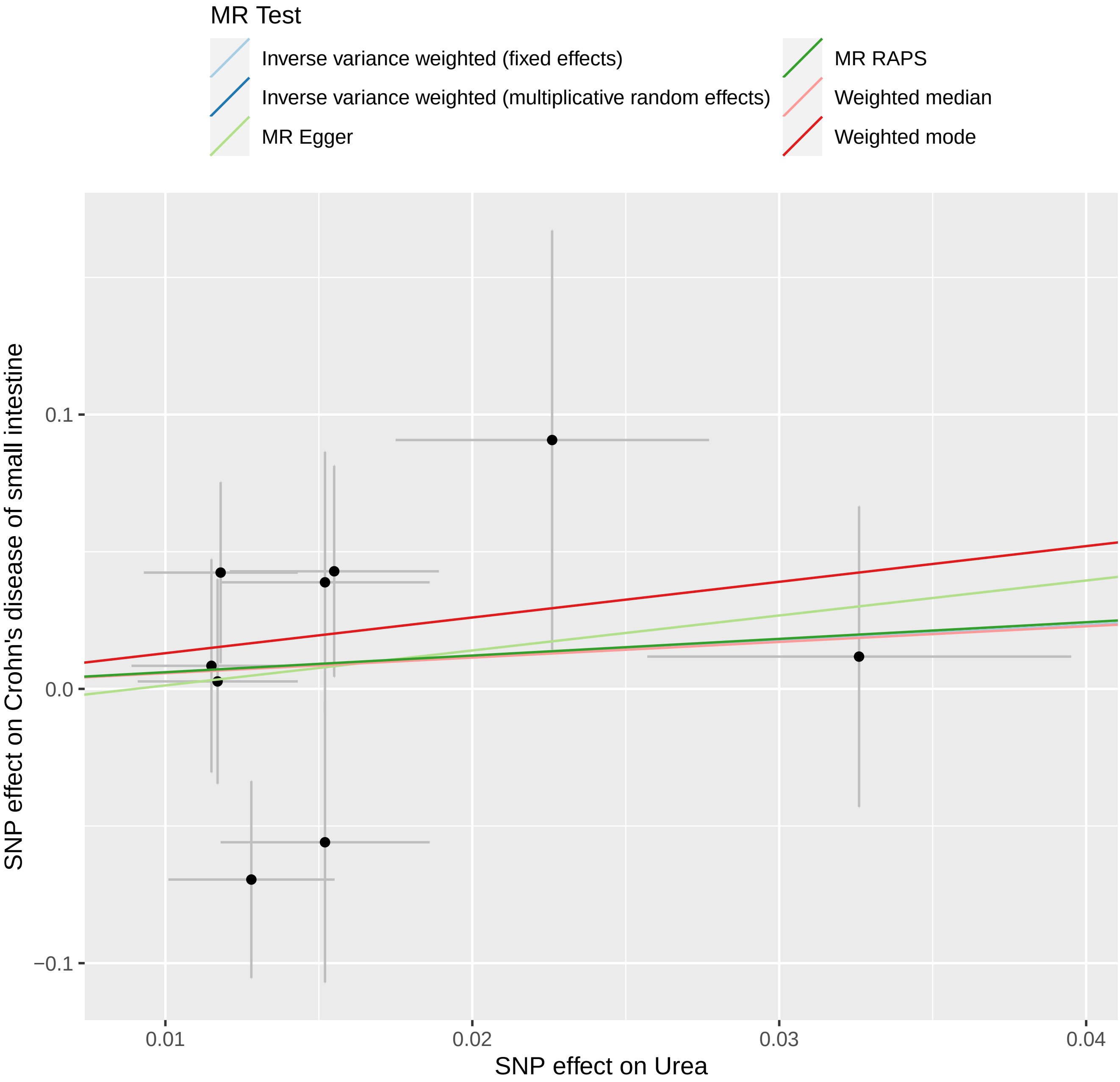

Batch 601 : Cortisol on Crohn's disease of small intestine

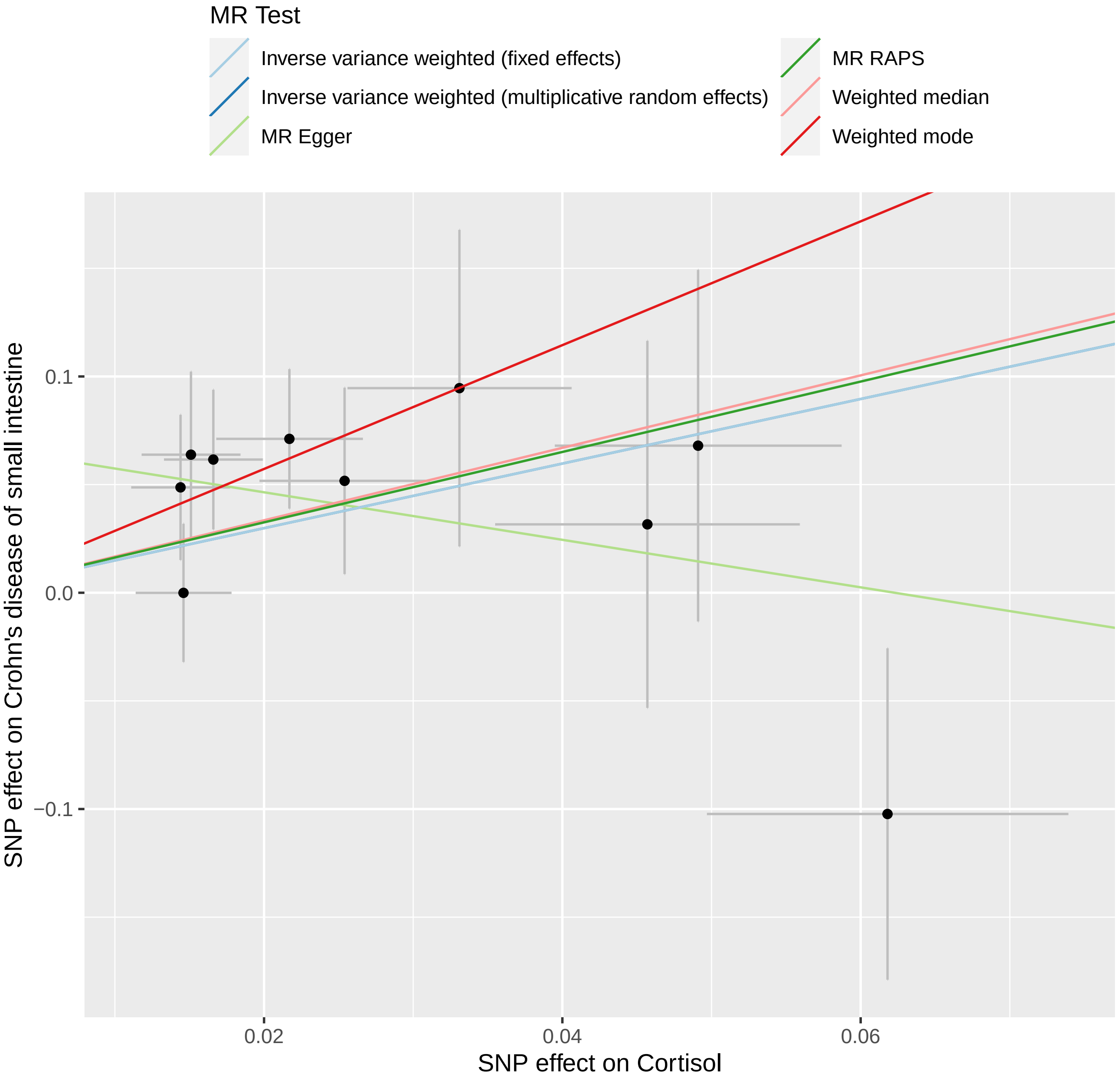

Batch 604 : Citrulline on Crohn's disease of small intestine

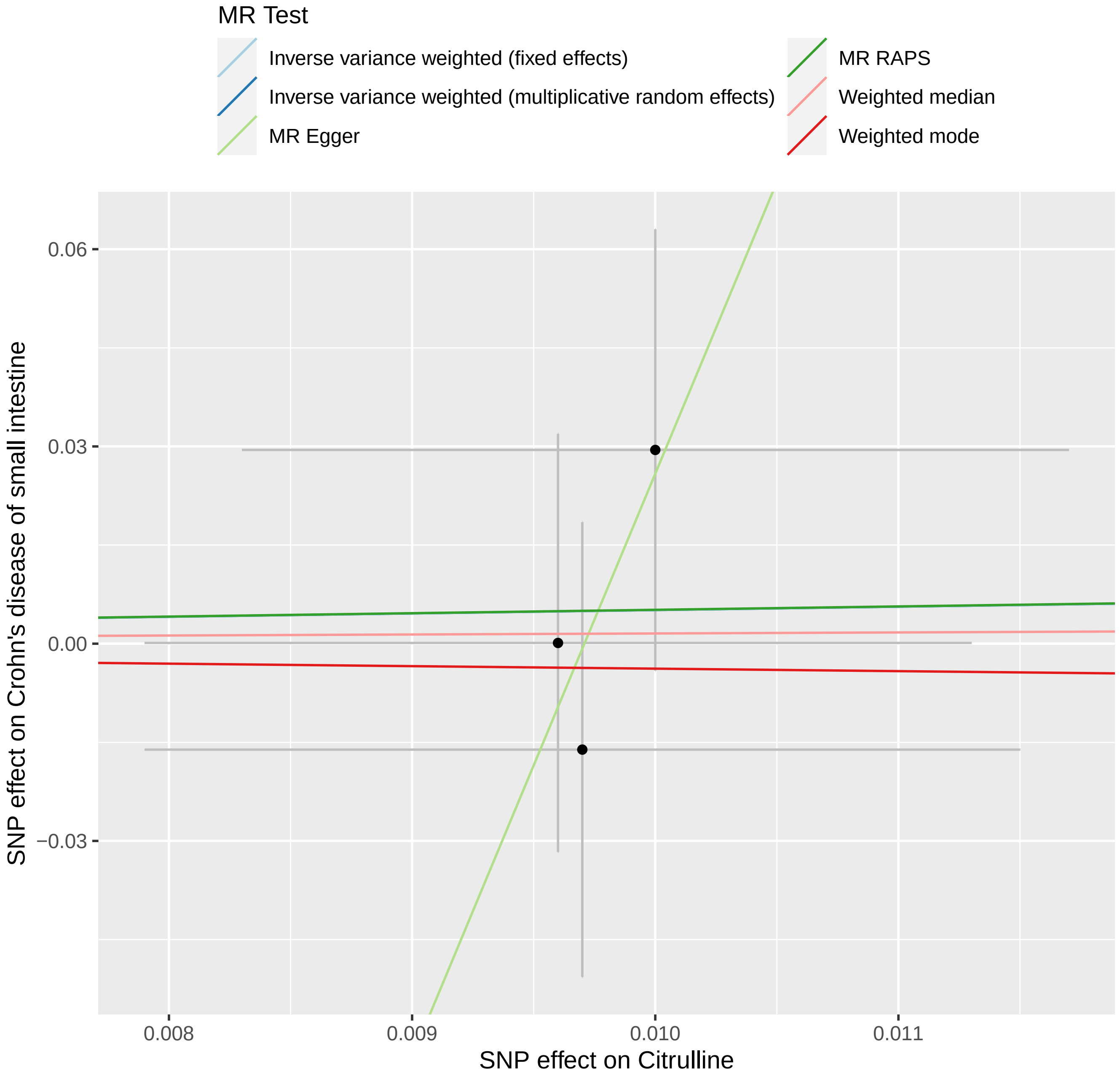

Batch 606 : Serotonin (5HT) on Crohn's disease of small intestine

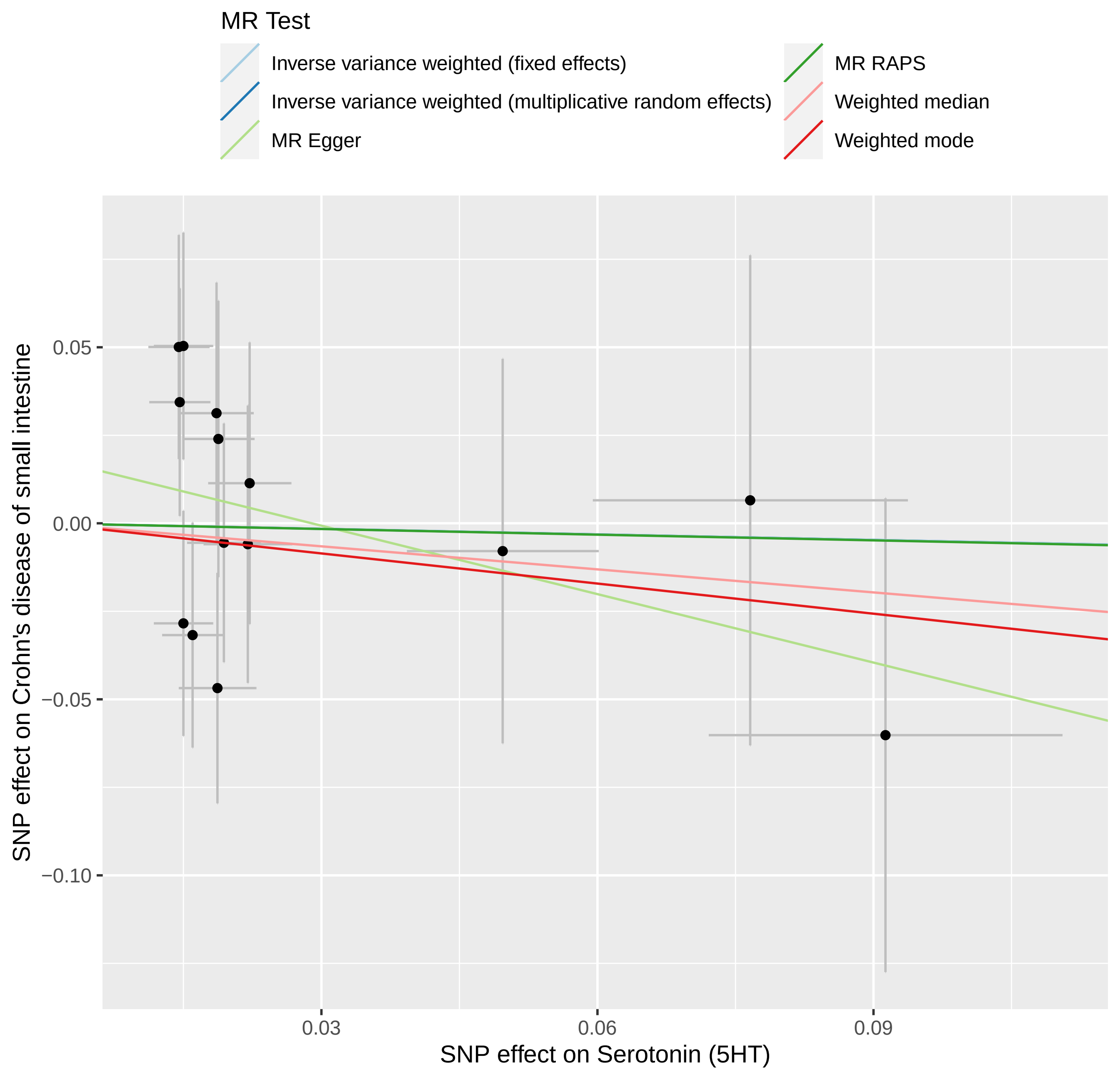

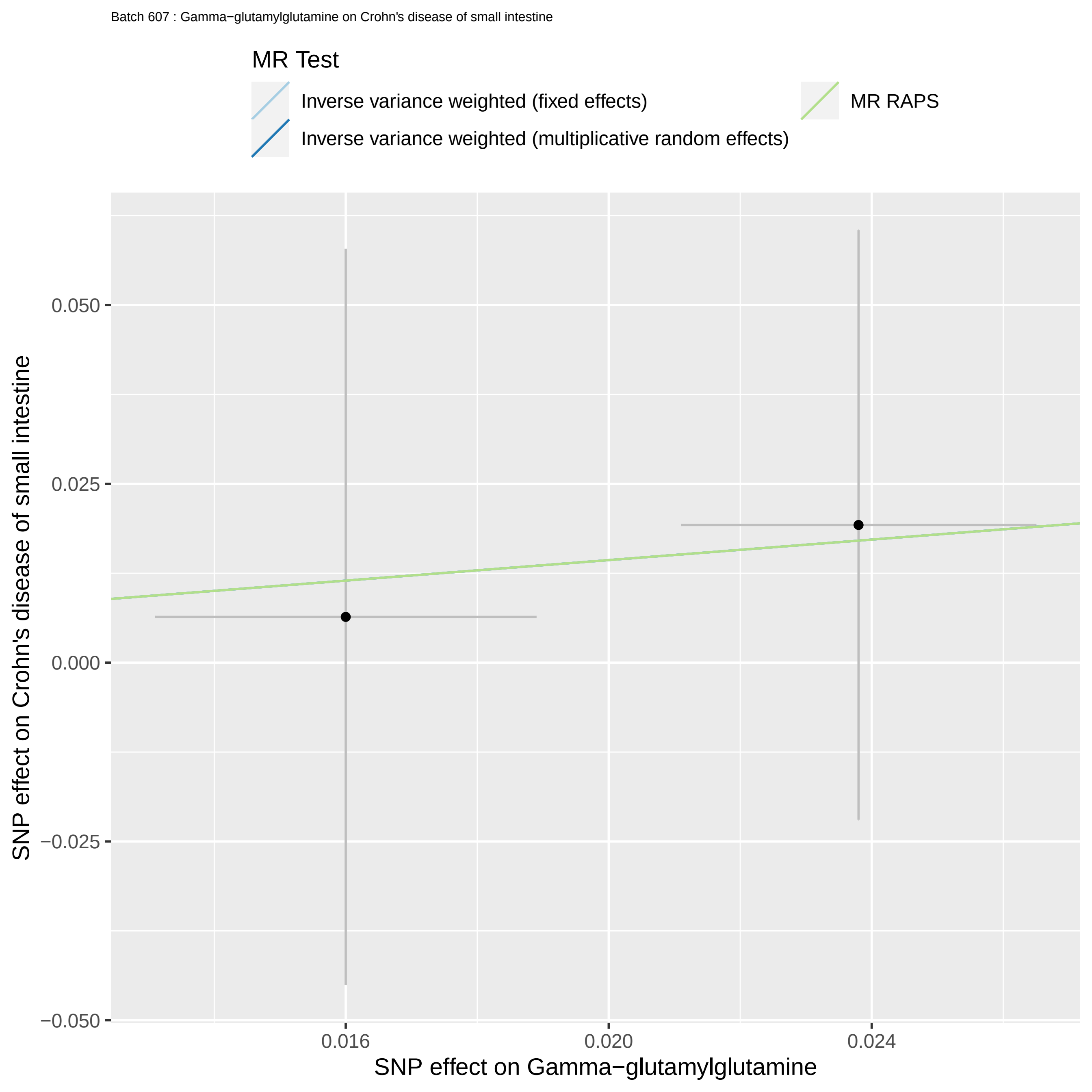

Batch 607 : Gamma−glutamylglutamine on Crohn's disease of small intestine
MR Test
Inverse variance weighted (fixed effects)
Inverse variance weighted (multiplicative random effects)
MR RAPS
0.050
0.025
0.000
−0.025
−0.050
0.016
0.020
0.024
SNP effect on Crohn's disease of small intestine
SNP effect on Gamma−glutamylglutamine

Batch 608 : Gamma−glutamyltyrosine on Crohn's disease of small intestine

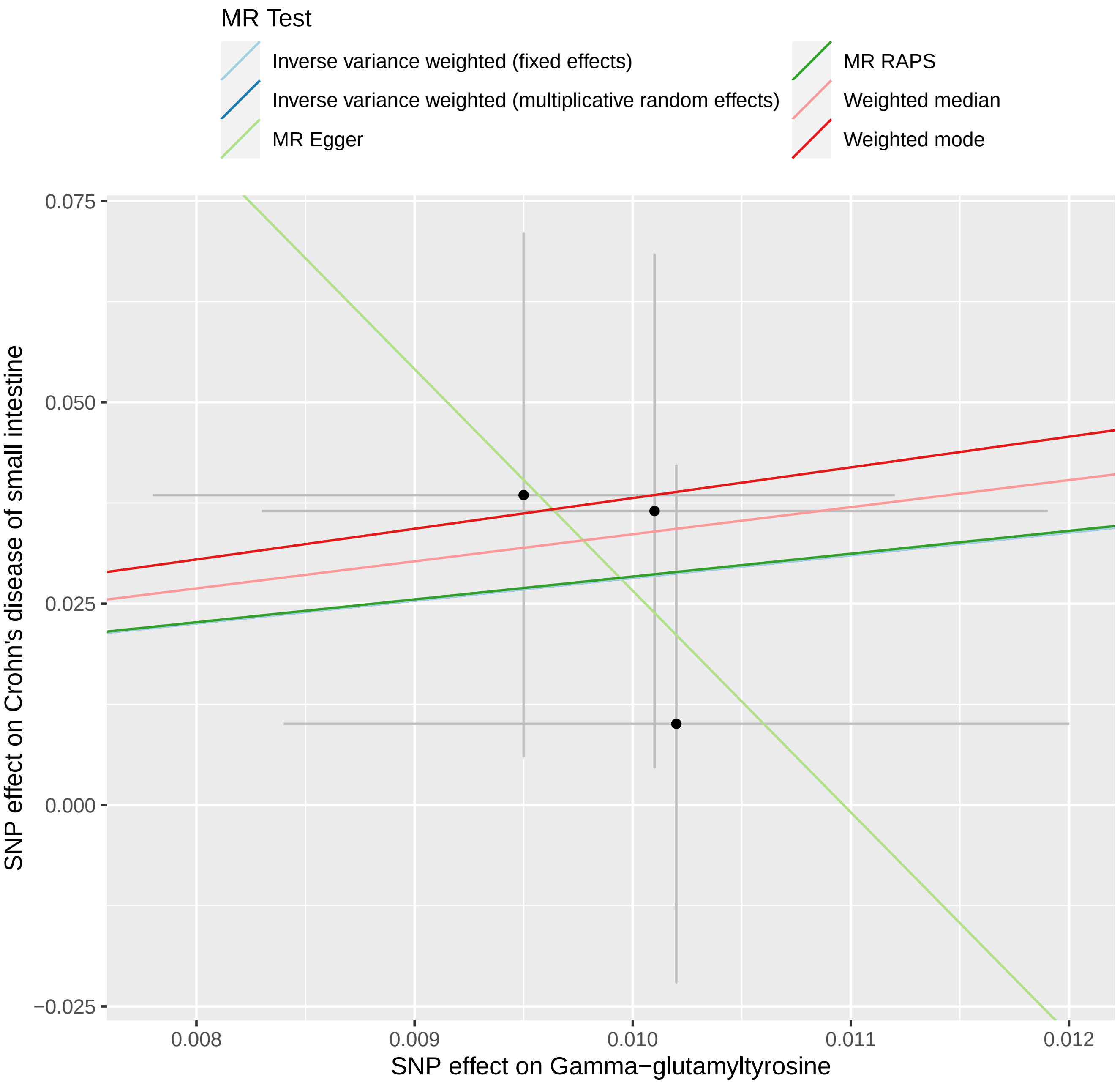

Batch 610 : Betaine on Crohn's disease of small intestine

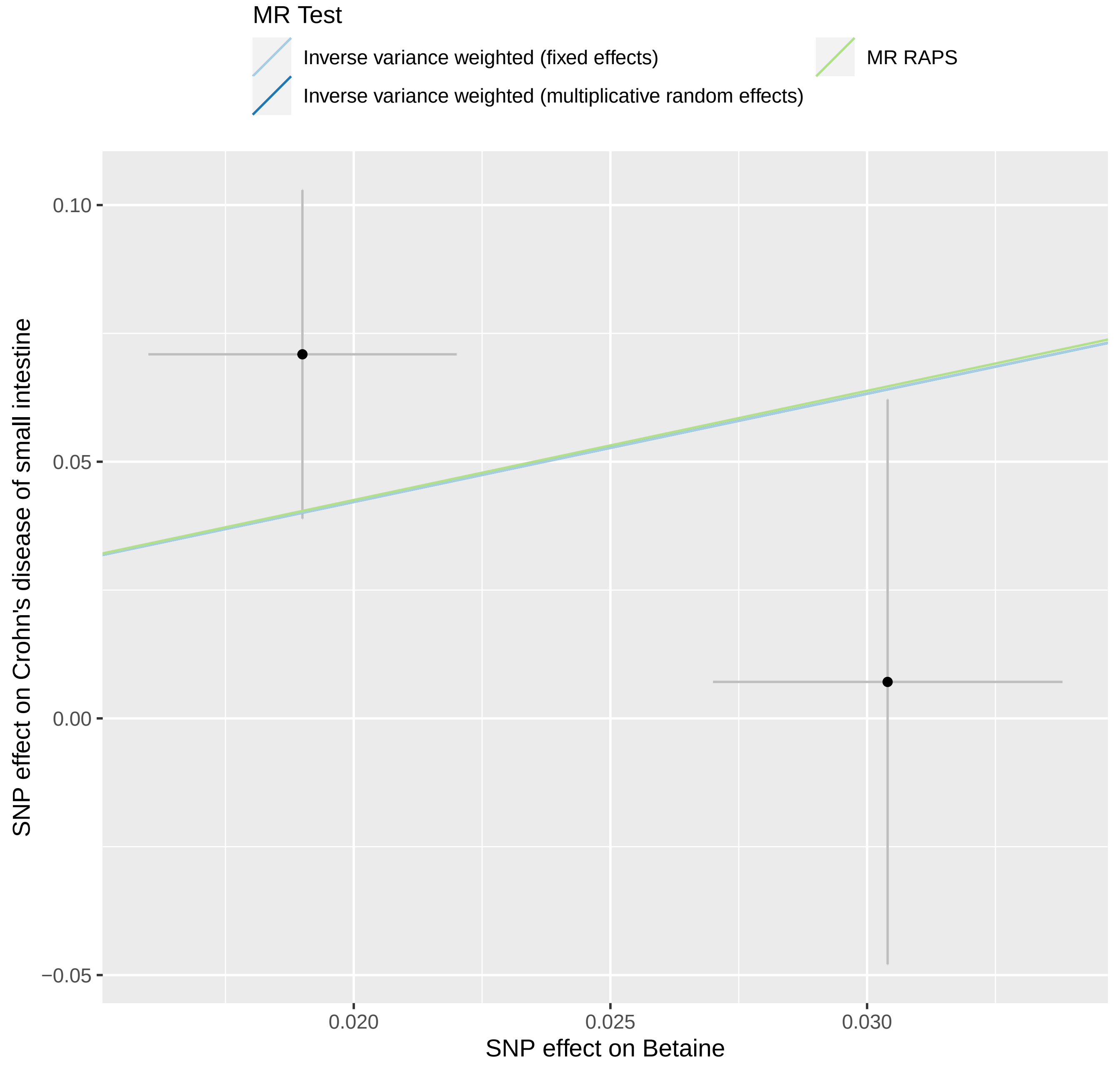

Batch 611 : Xanthine on Crohn's disease of small intestine

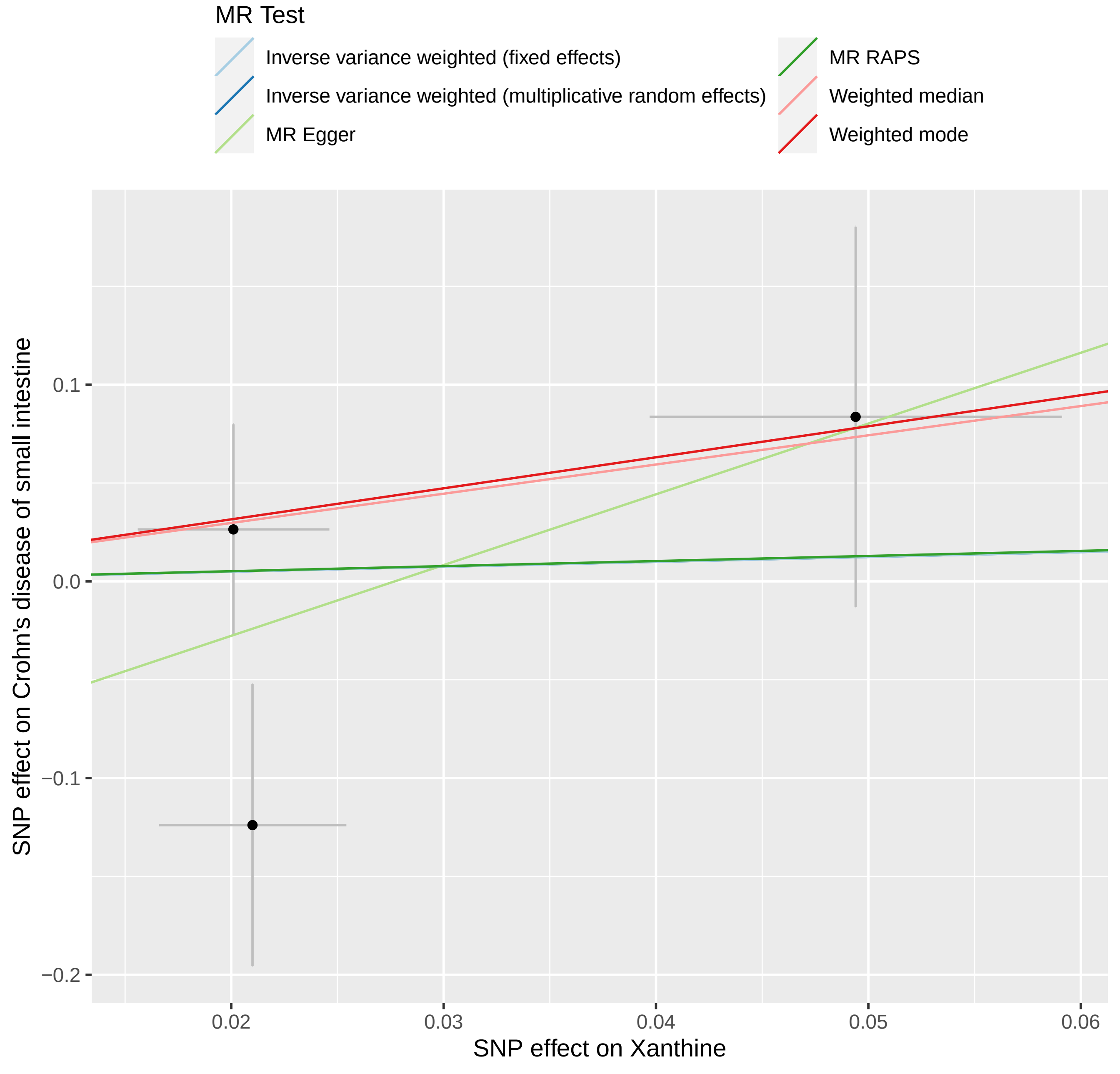

Batch 612 : Phosphate on Crohn's disease of small intestine

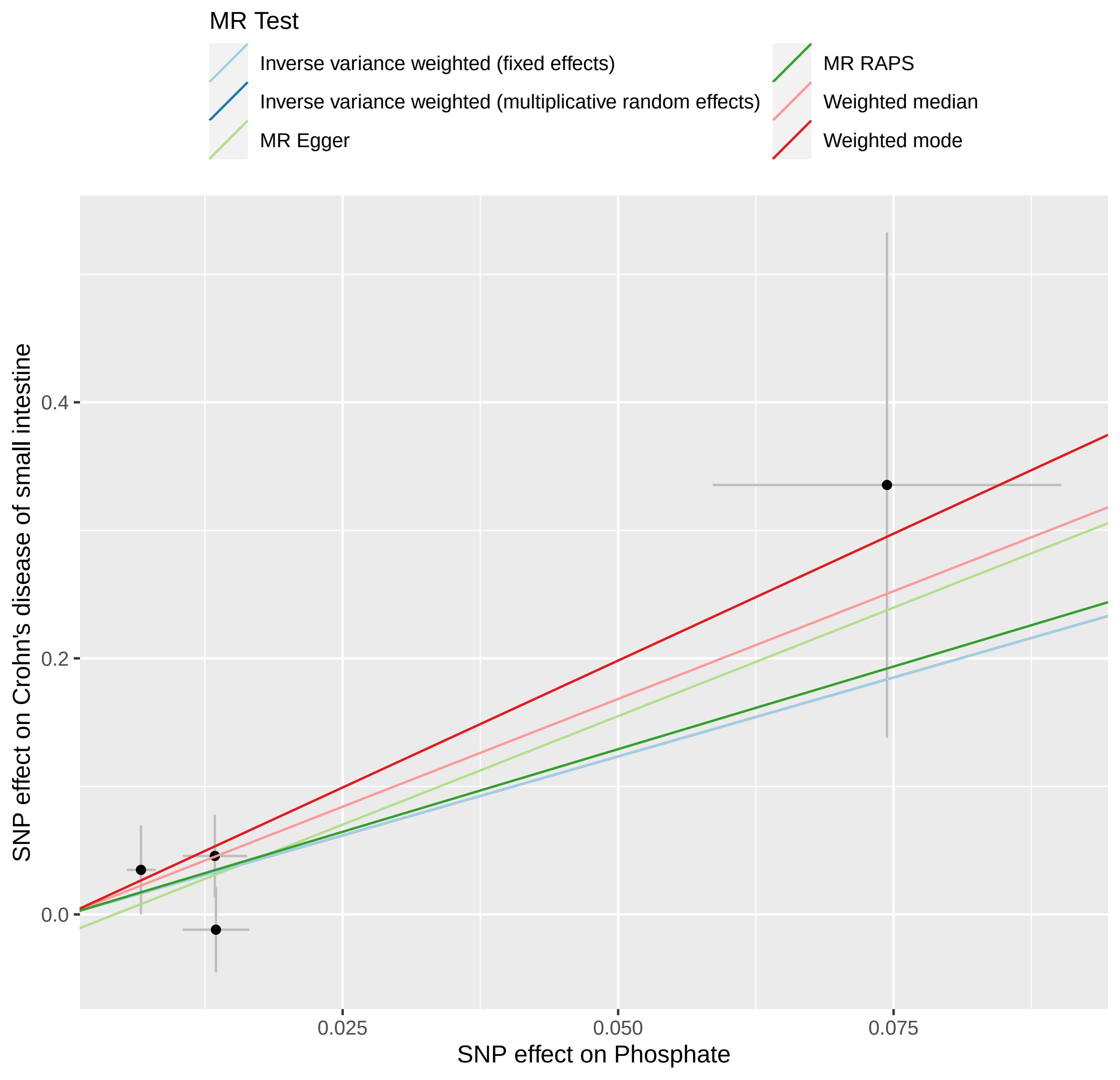

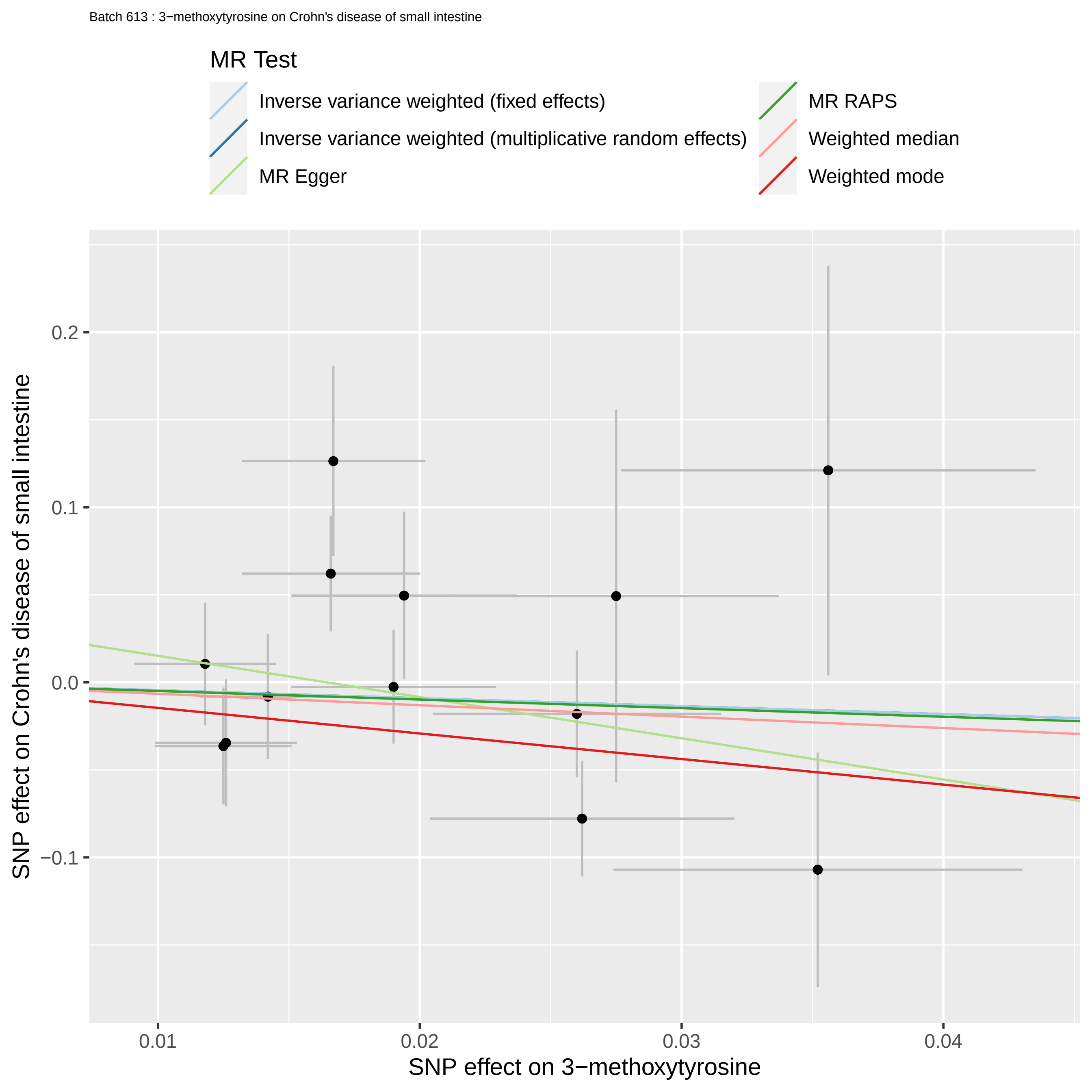

Batch 613 : 3−methoxytyrosine on Crohn's disease of small intestine
MR Test
Inverse variance weighted (fixed effects)
Inverse variance weighted (multiplicative random effects)
MR Egger
MR RAPS
Weighted median
Weighted mode
SNP effect on Crohn's disease of small intestine
0.2
0.1
0.0
−0.1
0.01
0.02
0.03
0.04
SNP effect on 3−methoxytyrosine

Batch 615 : Undecanoate (11:0) on Crohn's disease of small intestine

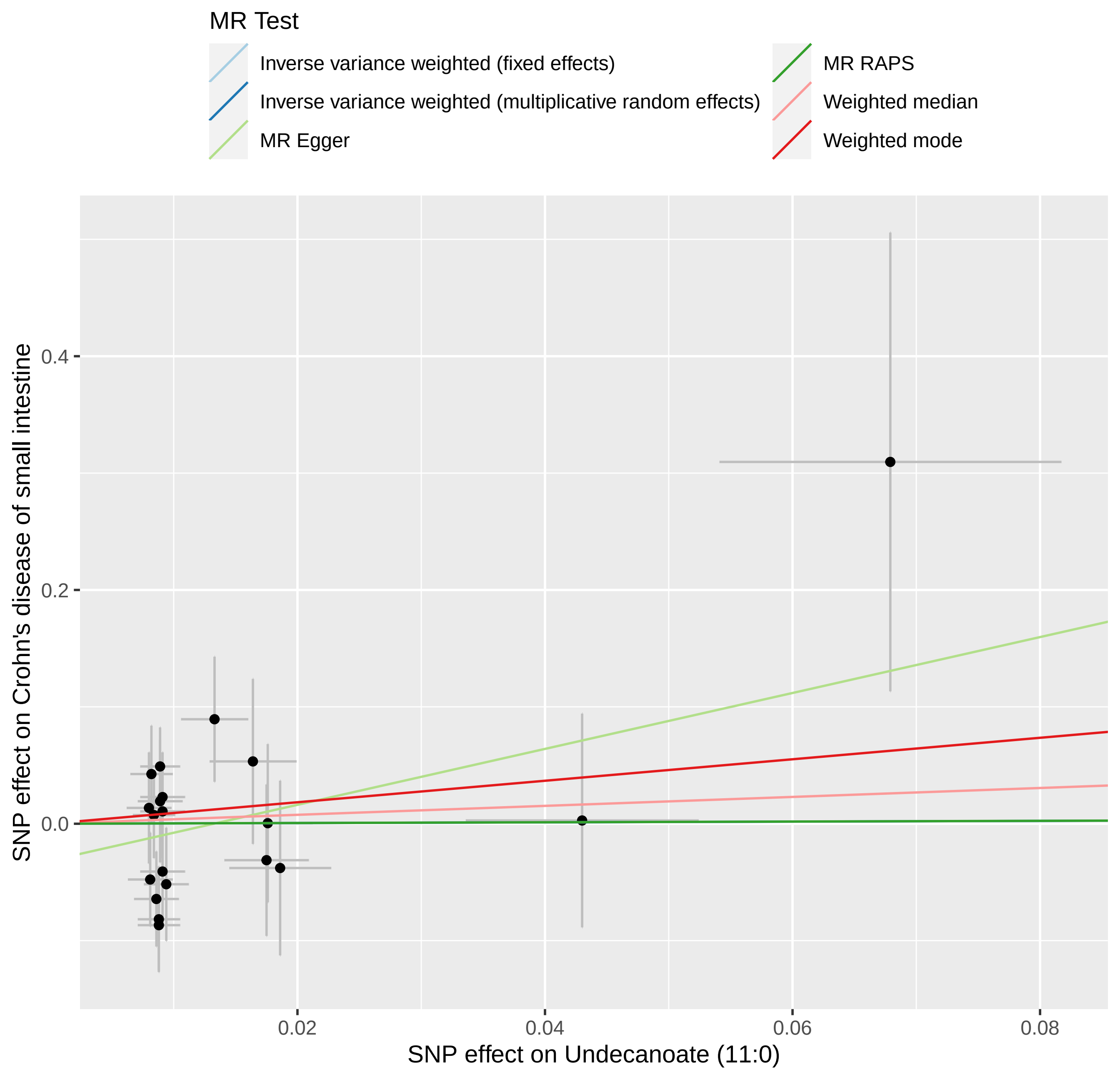

Batch 617 : Taurodeoxycholate on Crohn's disease of small intestine

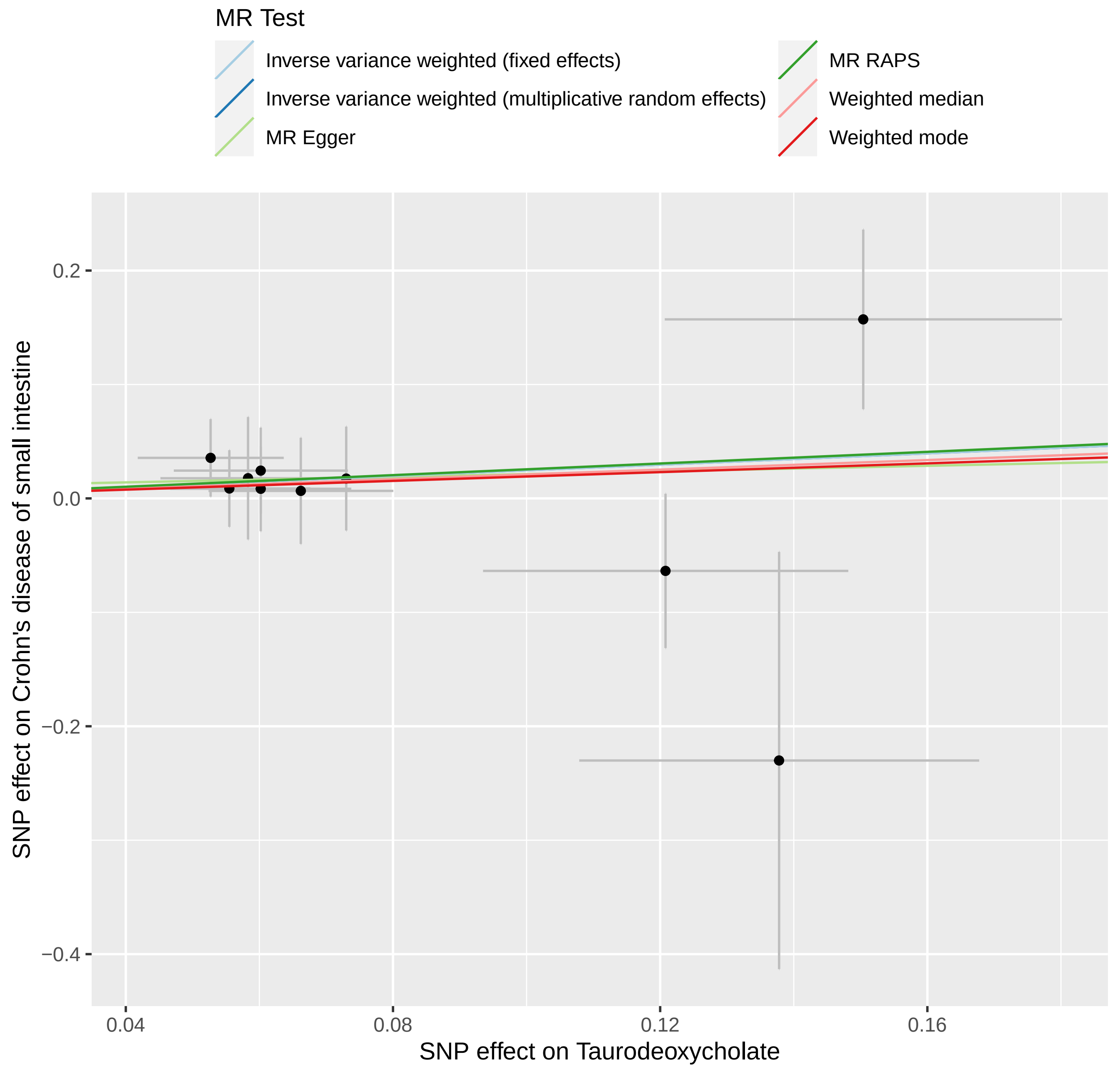

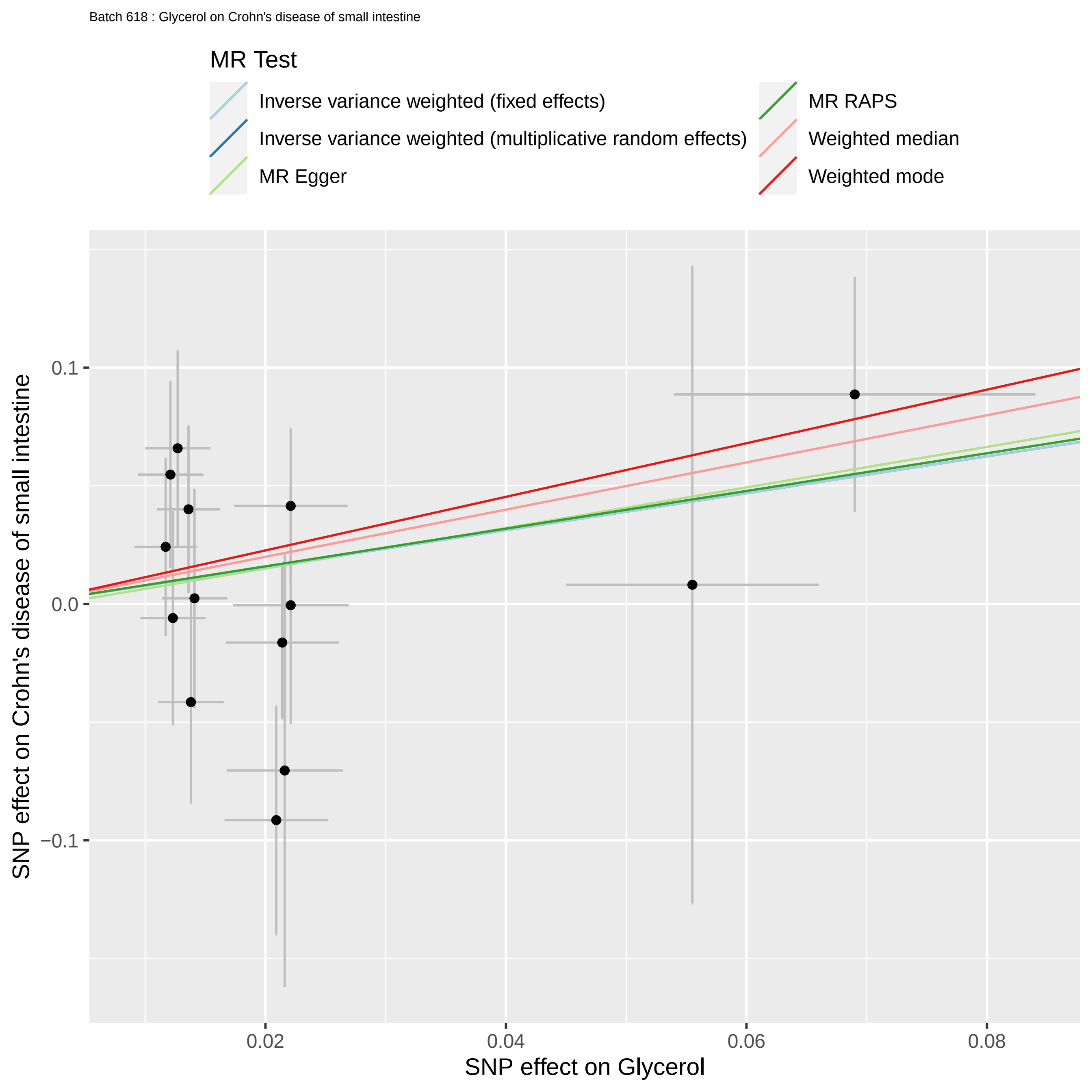
Batch 618 : Glycerol on Crohn's disease of small intestine
MR Test
Inverse variance weighted (fixed effects)
Inverse variance weighted (multiplicative random effects)
MR Egger
MR RAPS
Weighted median
Weighted mode
0.1
0.0
−0.1
0.02
0.04
0.06
0.08
SNP effect on Crohn's disease of small intestine
SNP effect on Glycerol

Batch 619 : Kynurenine on Crohn's disease of small intestine

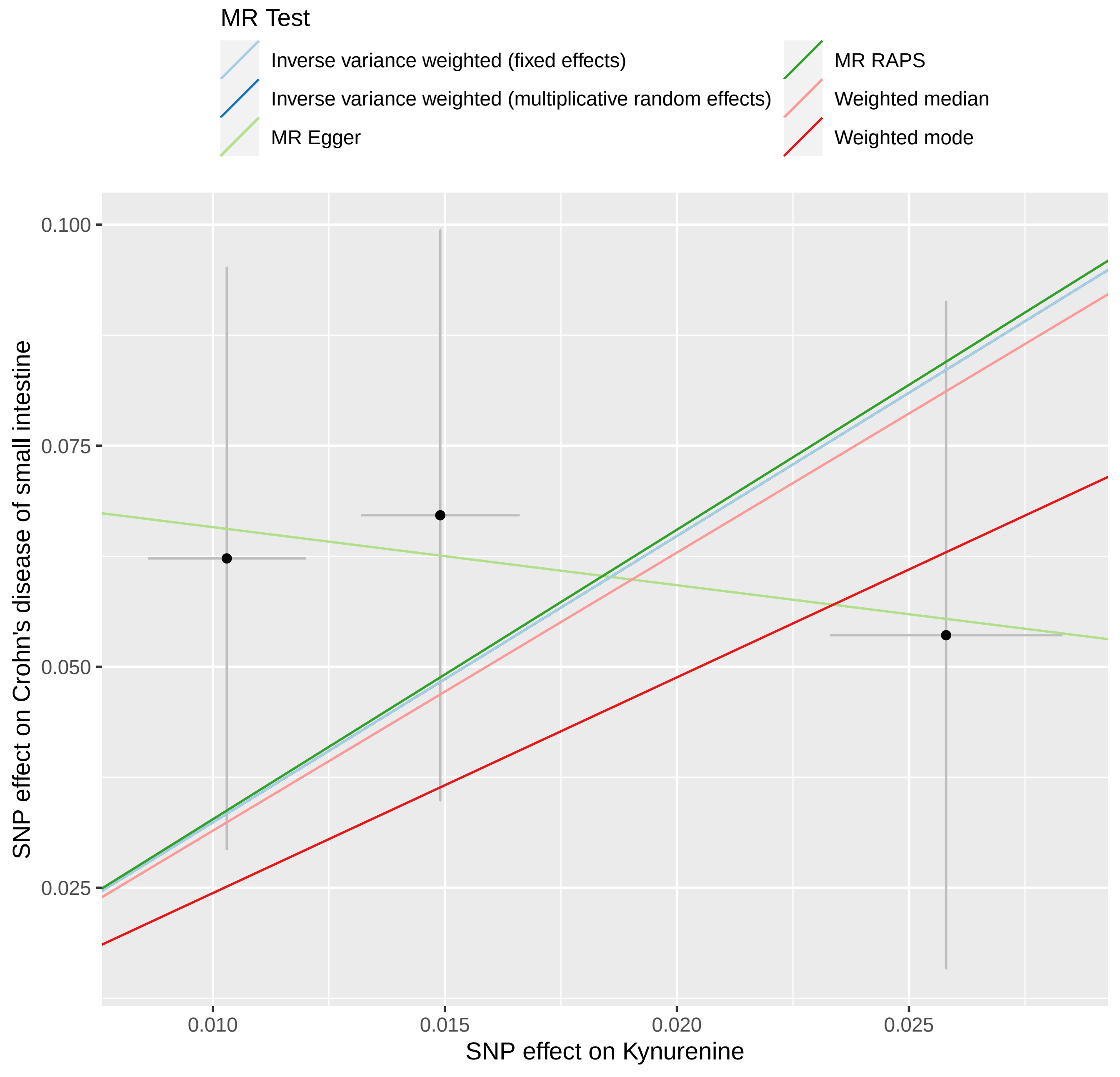

Batch 620 : Mannitol on Crohn's disease of small intestine

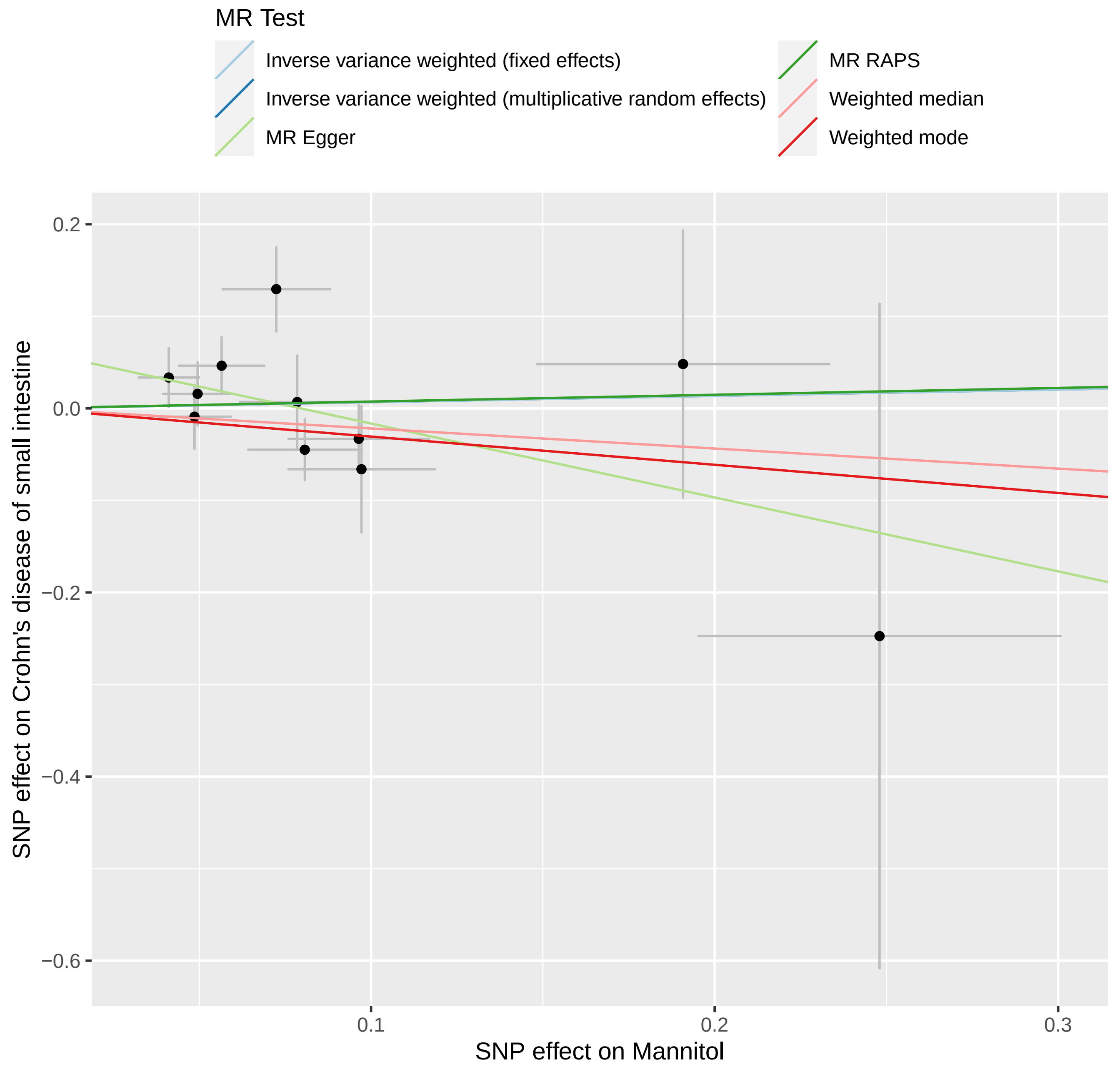

Batch 621 : Glycerol 3−phosphate (G3P) on Crohn's disease of small intestine

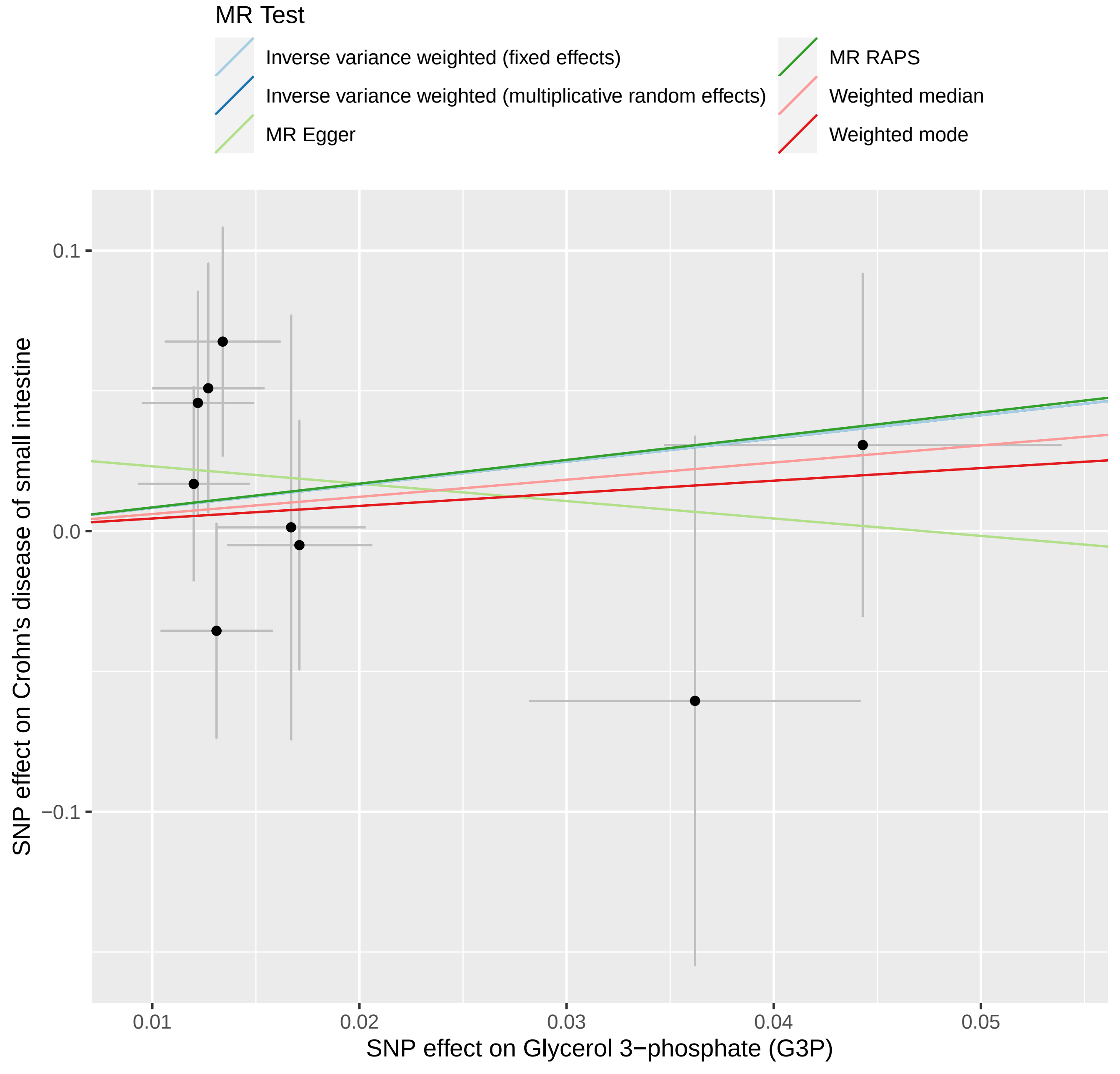

Batch 623 : Carnitine on Crohn's disease of small intestine

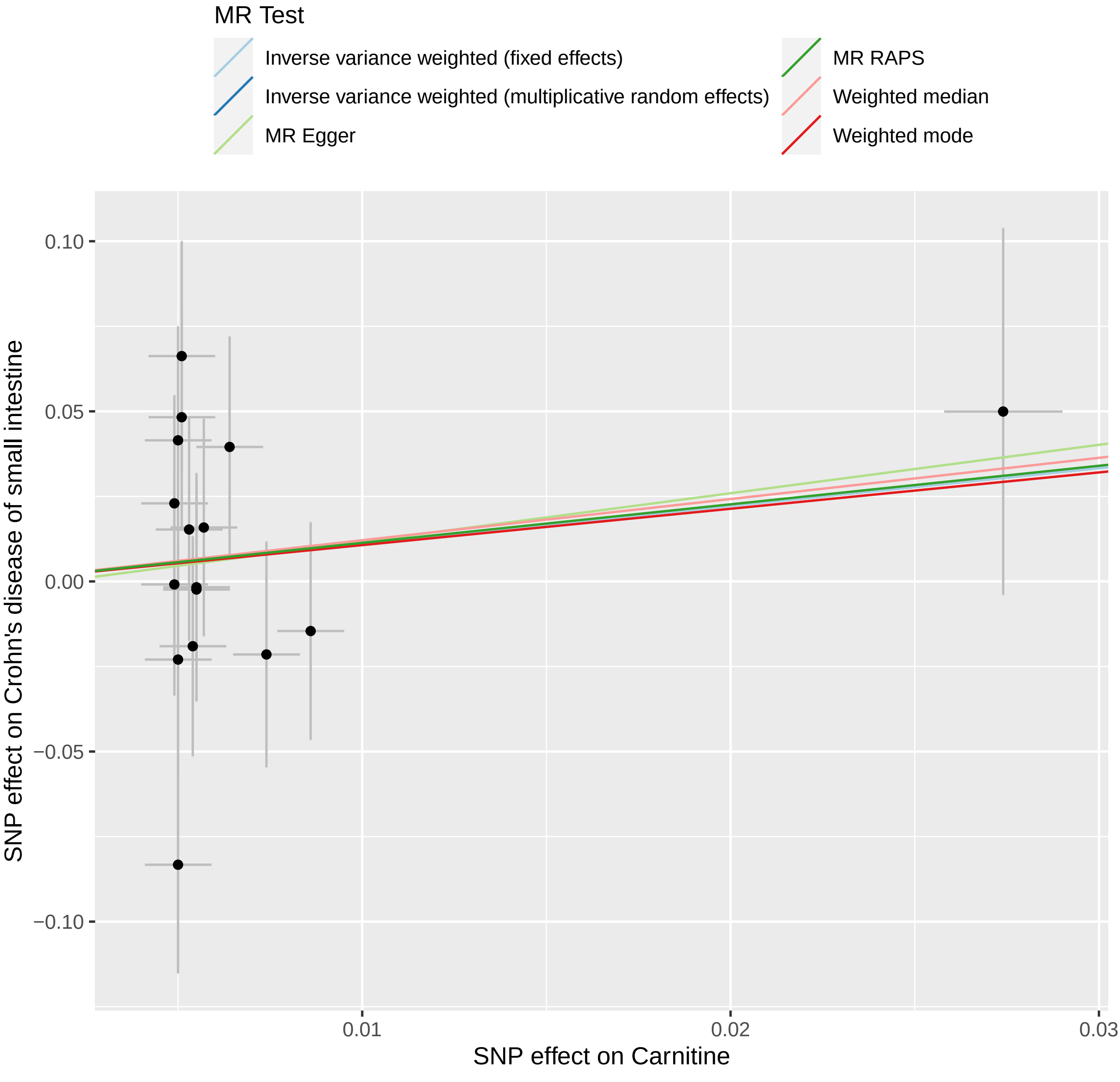

Batch 624 : Choline on Crohn's disease of small intestine

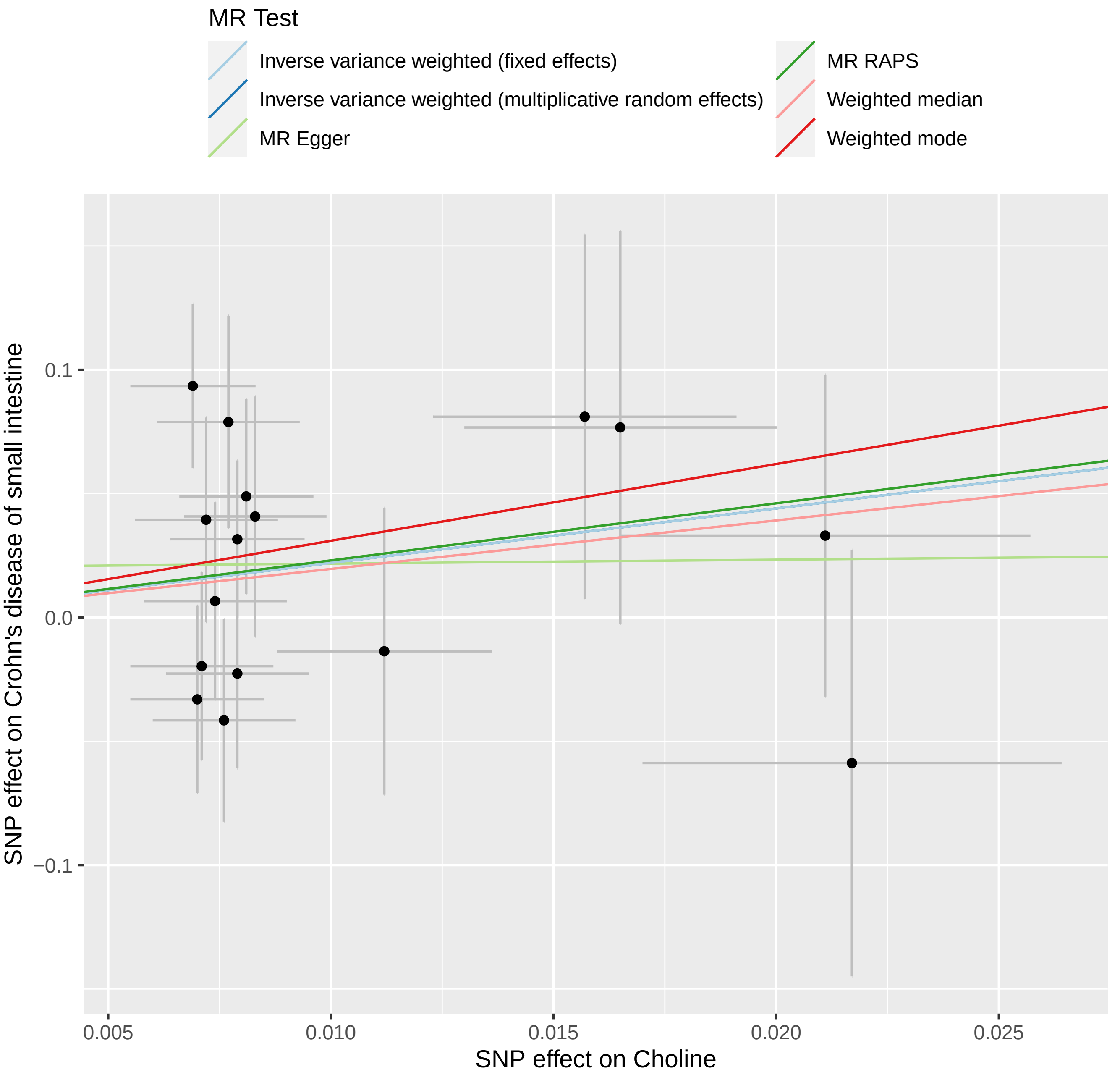

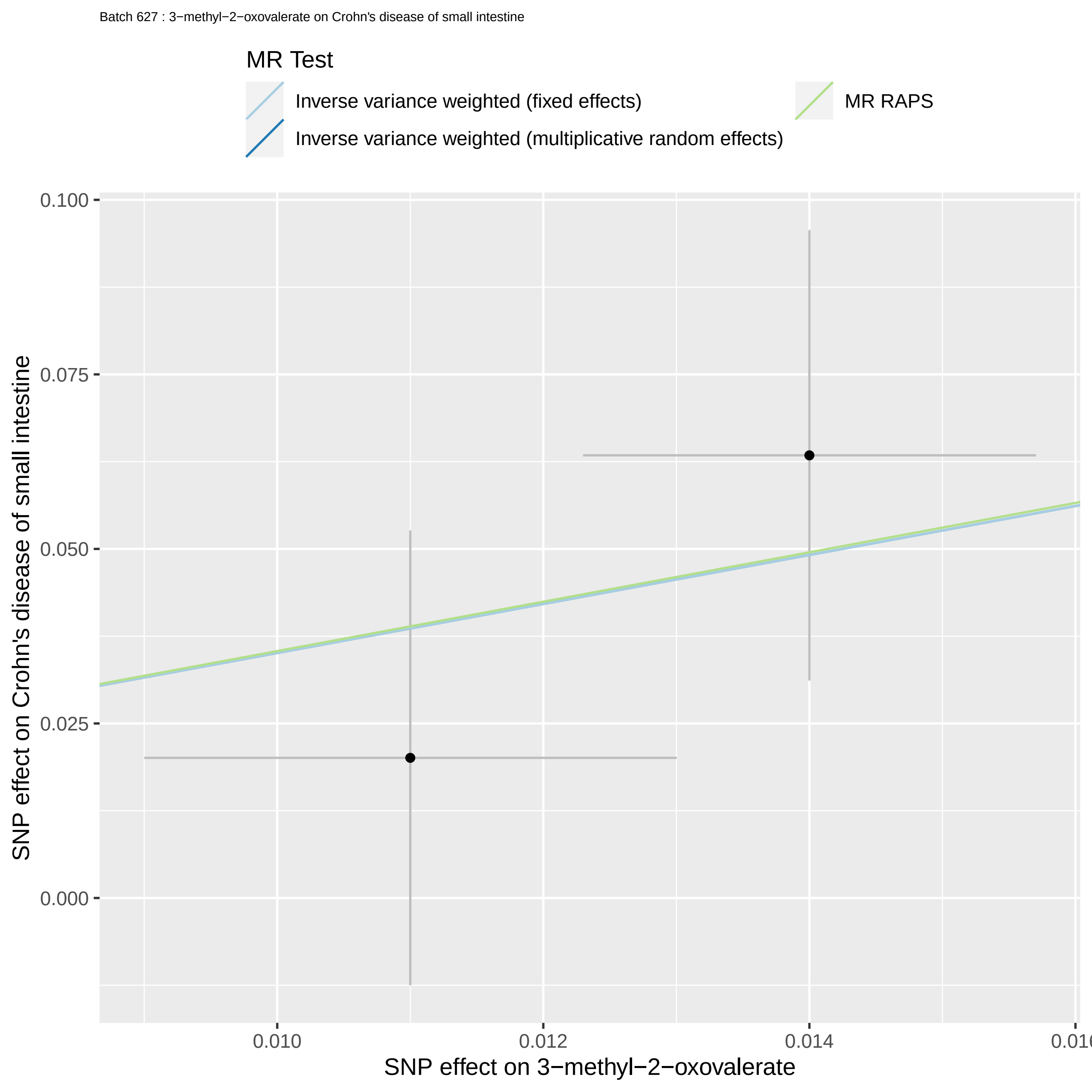

Batch 627 : 3−methyl−2−oxovalerate on Crohn's disease of small intestine
MR Test
Inverse variance weighted (fixed effects)
Inverse variance weighted (multiplicative random effects)
MR RAPS
SNP effect on Crohn's disease of small intestine
0.100
0.075
0.050
0.025
0.000
0.010
0.012
0.014
SNP effect on 3−methyl−2−oxovalerate

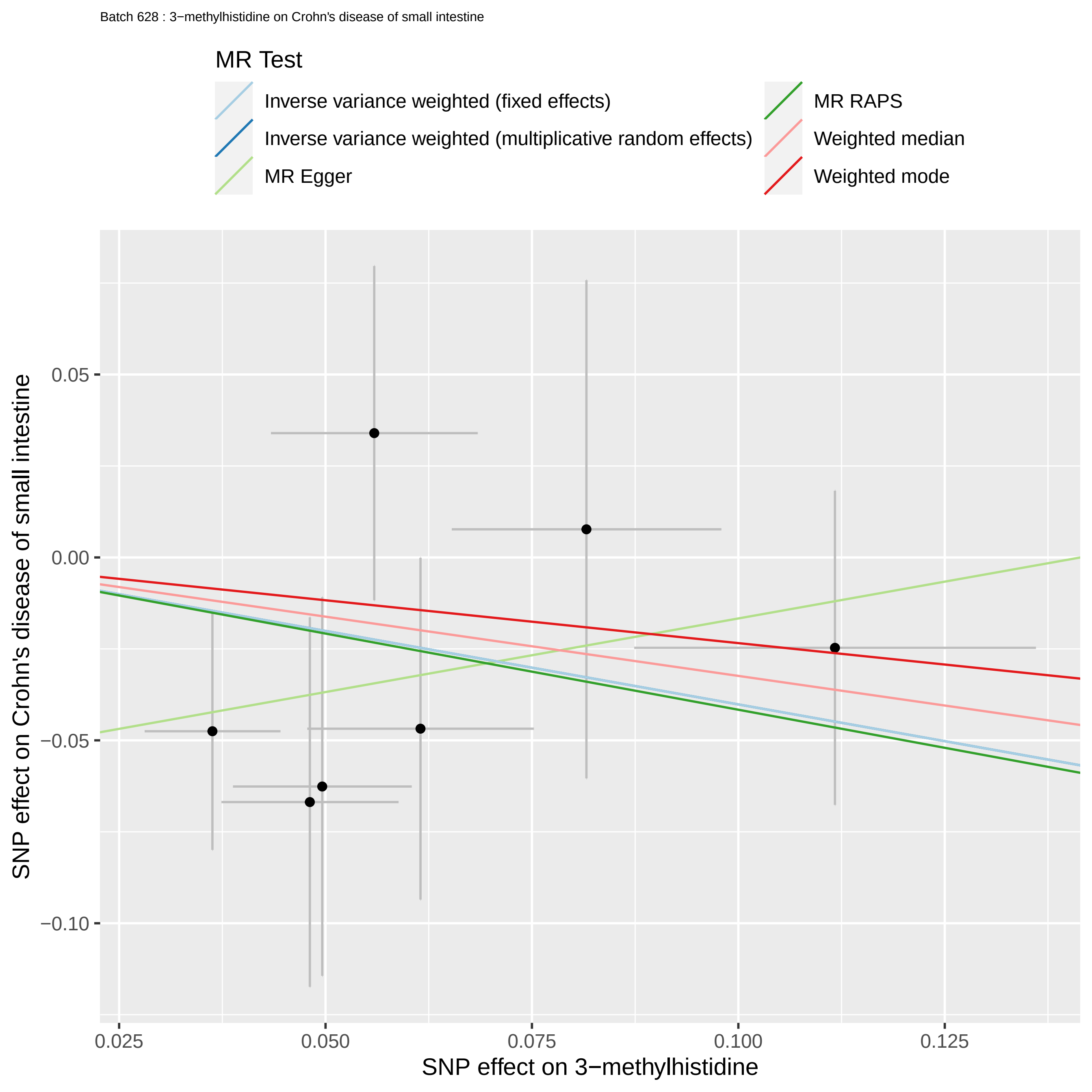
Batch 628 : 3−methylhistidine on Crohn's disease of small intestine
MR Test
Inverse variance weighted (fixed effects)
Inverse variance weighted (multiplicative random effects)
MR Egger
MR RAPS
Weighted median
Weighted mode
0.05
0.00
−0.05
−0.10
0.025
0.050
0.075
0.100
0.125
SNP effect on Crohn's disease of small intestine
SNP effect on 3−methylhistidine

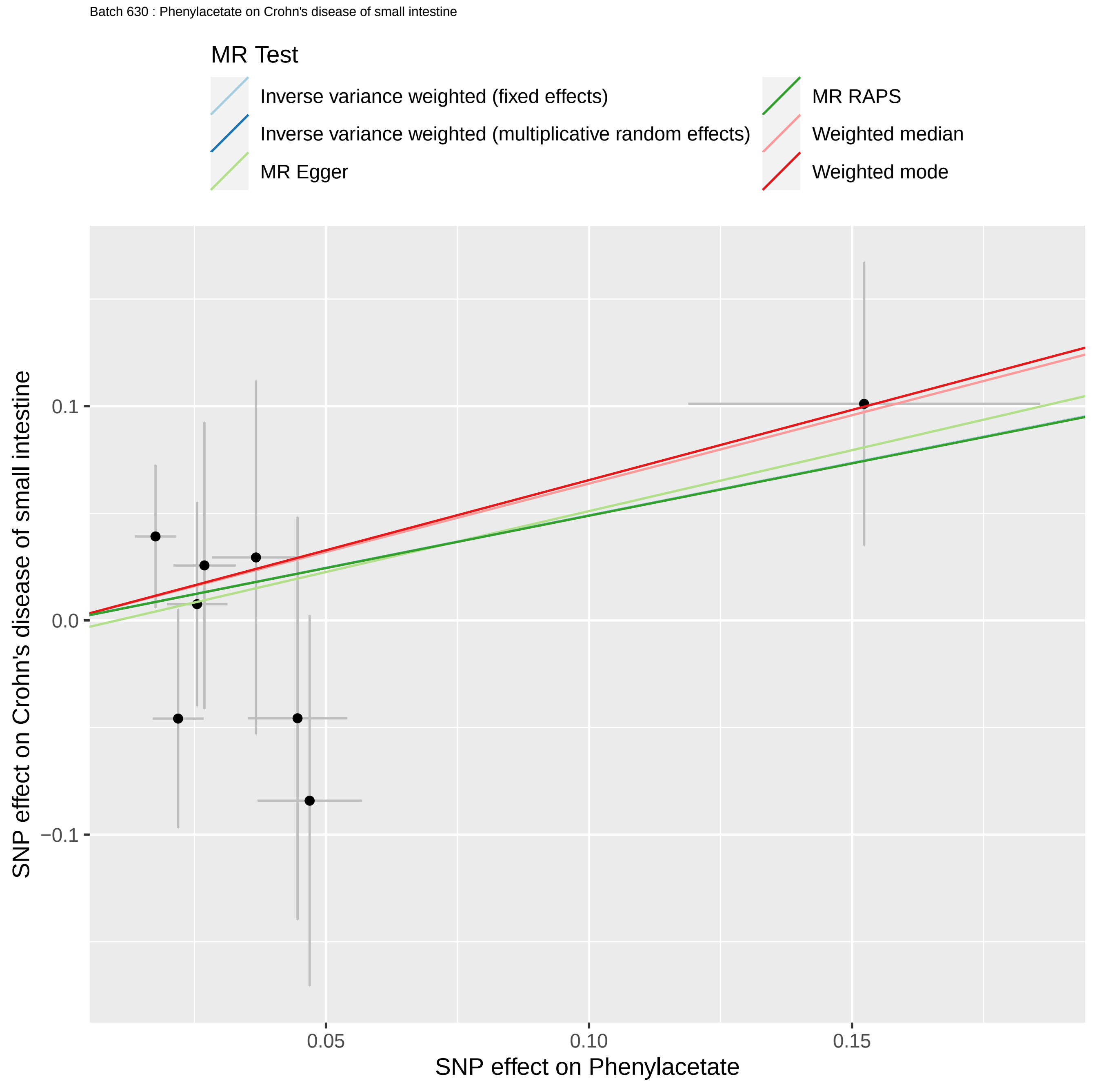

Batch 630 : Phenylacetate on Crohn's disease of small intestine
MR Test
Inverse variance weighted (fixed effects)
Inverse variance weighted (multiplicative random effects)
MR Egger
MR RAPS
Weighted median
Weighted mode
0.1
0.0
−0.1
0.05
0.10
0.15
SNP effect on Crohn's disease of small intestine
SNP effect on Phenylacetate

Batch 631 : Glycerophosphorylcholine (GPC) on Crohn's disease of small intestine

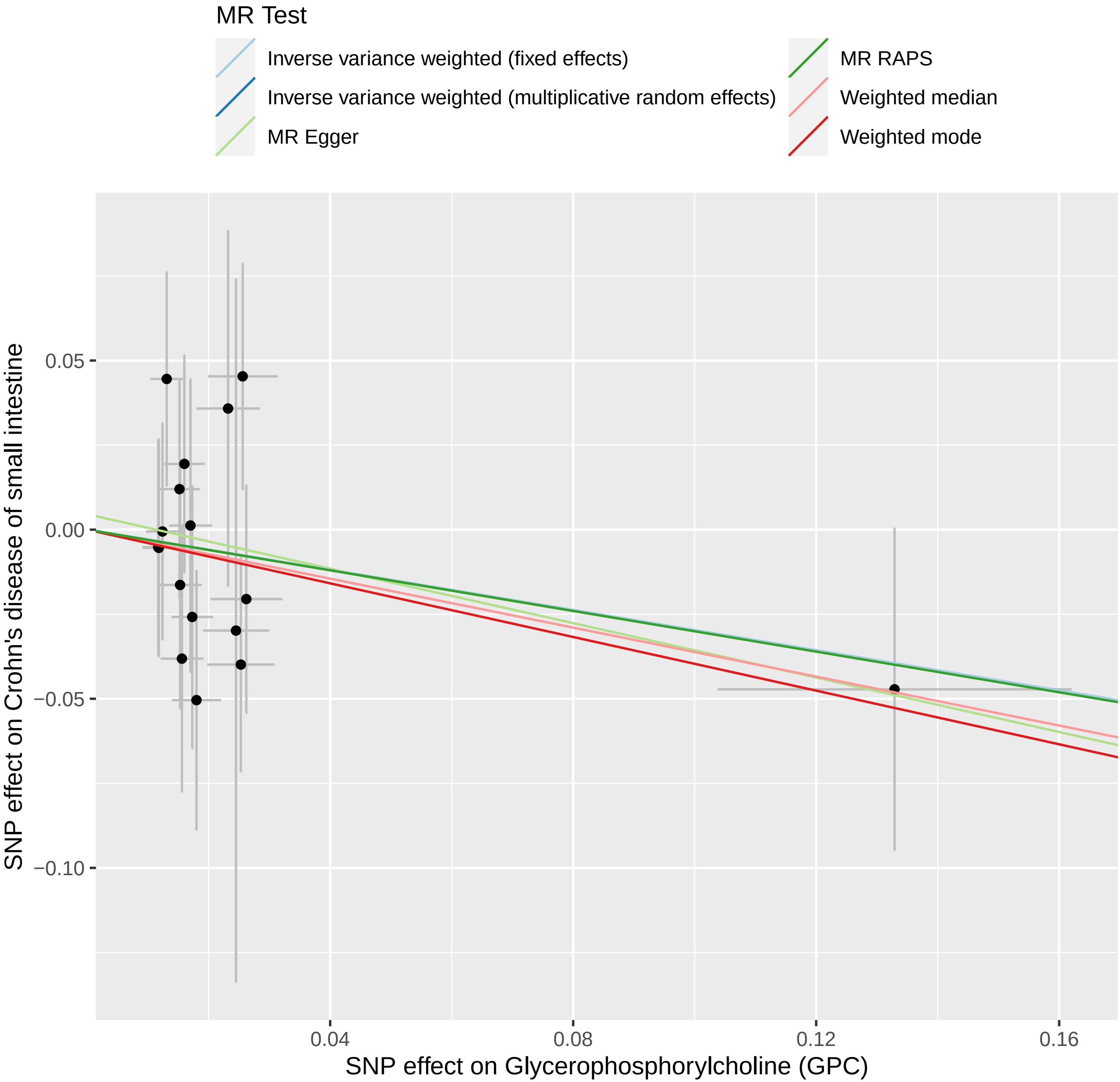

Batch 632 : Aspartate on Crohn's disease of small intestine

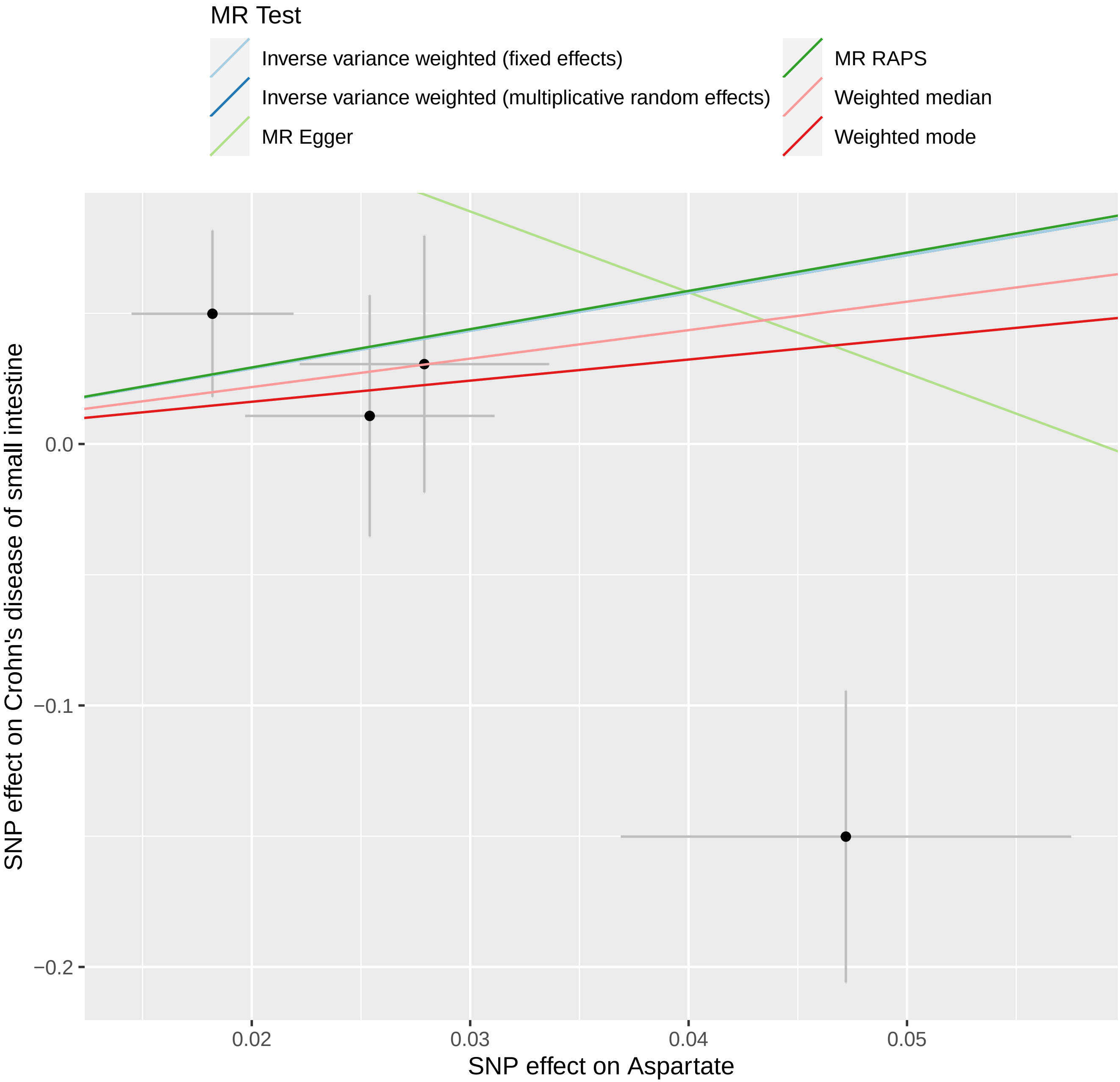

Batch 634 : Dihomo−linoleate (20:2n6) on Crohn's disease of small intestine

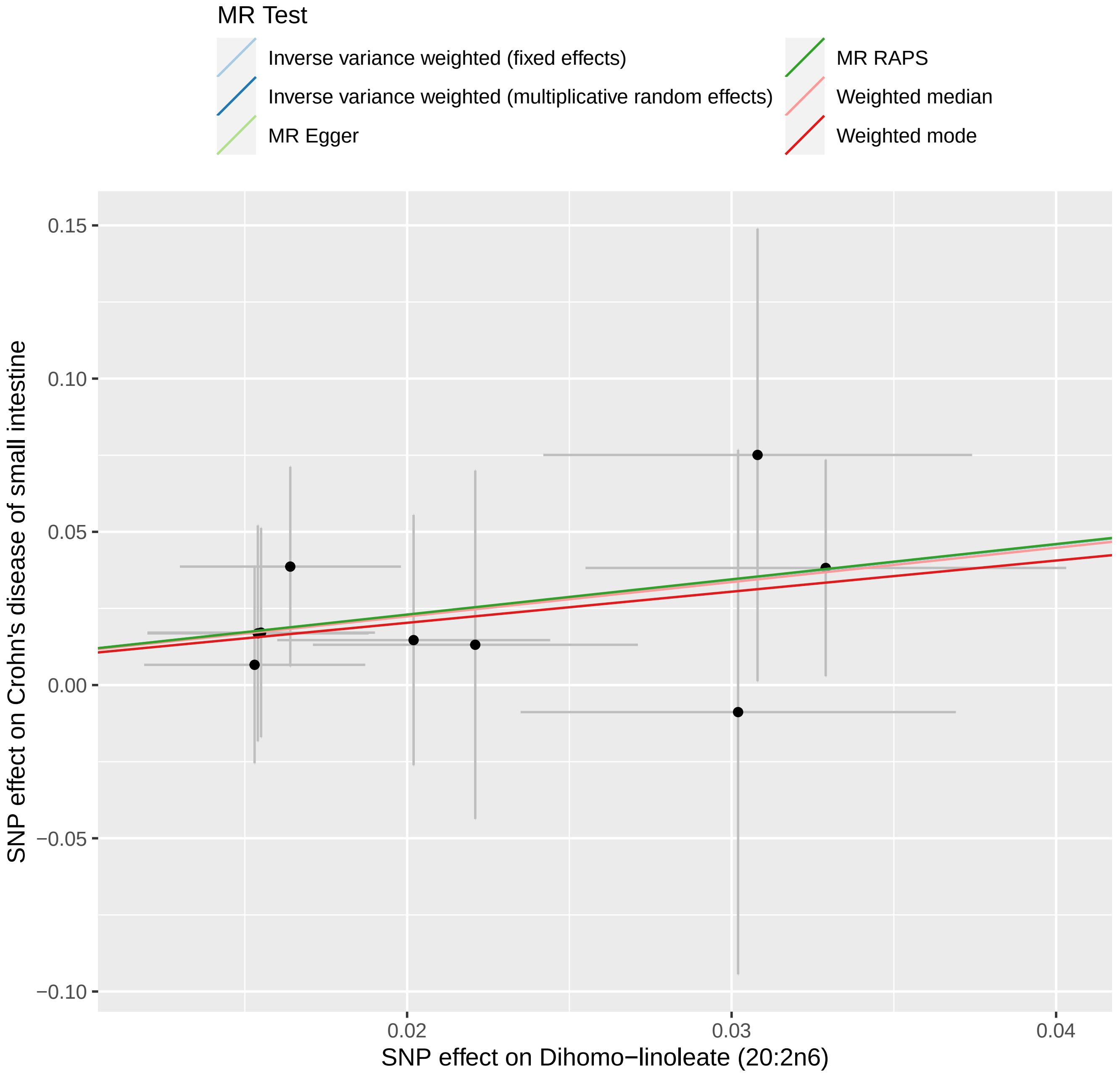

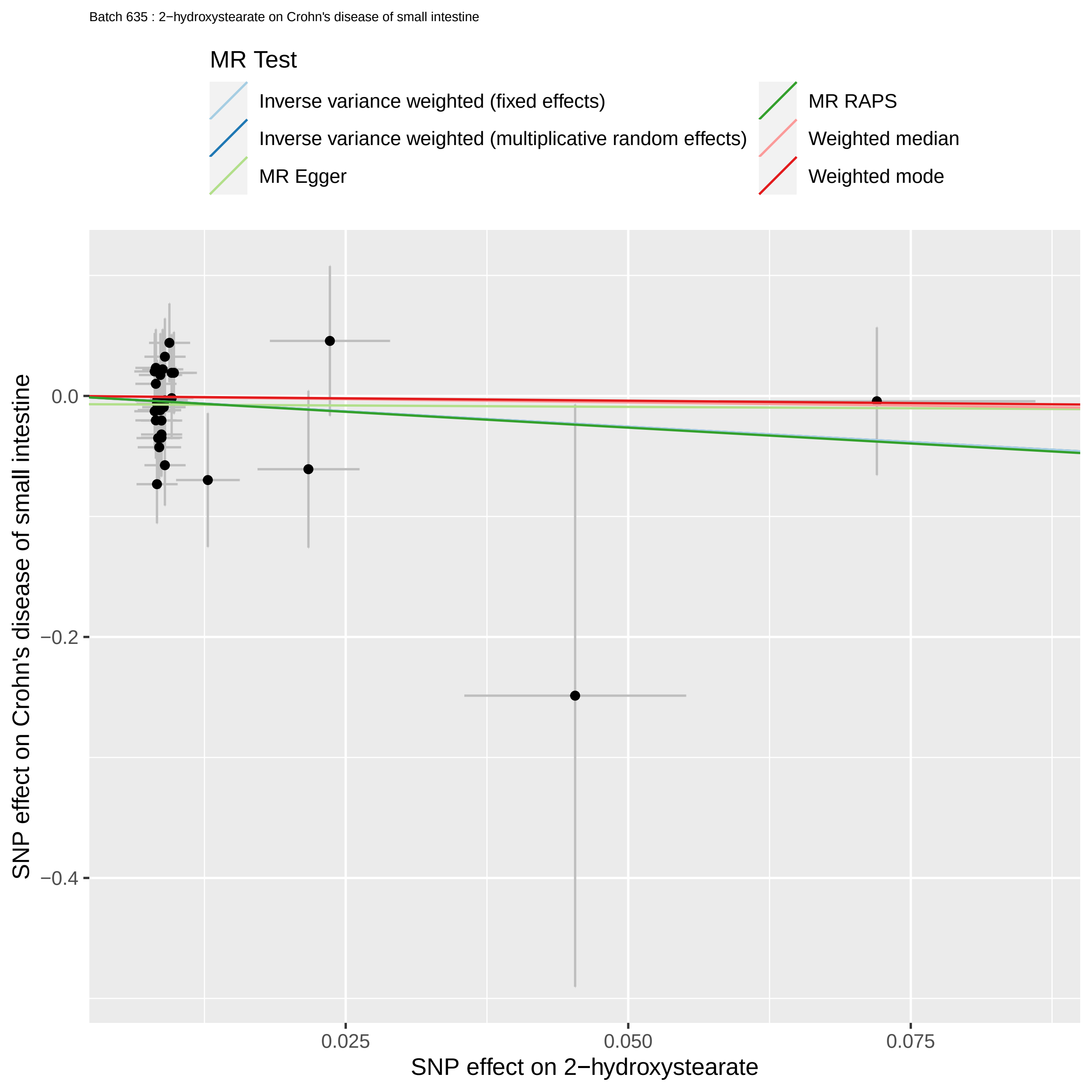
Batch 635 : 2−hydroxystearate on Crohn's disease of small intestine
MR Test
Inverse variance weighted (fixed effects)
Inverse variance weighted (multiplicative random effects)
MR Egger
MR RAPS
Weighted median
Weighted mode
SNP effect on Crohn's disease of small intestine
0.0
−0.2
−0.4
0.025
0.050
0.075
SNP effect on 2−hydroxystearate

Batch 637 : Glycylvaline on Crohn's disease of small intestine

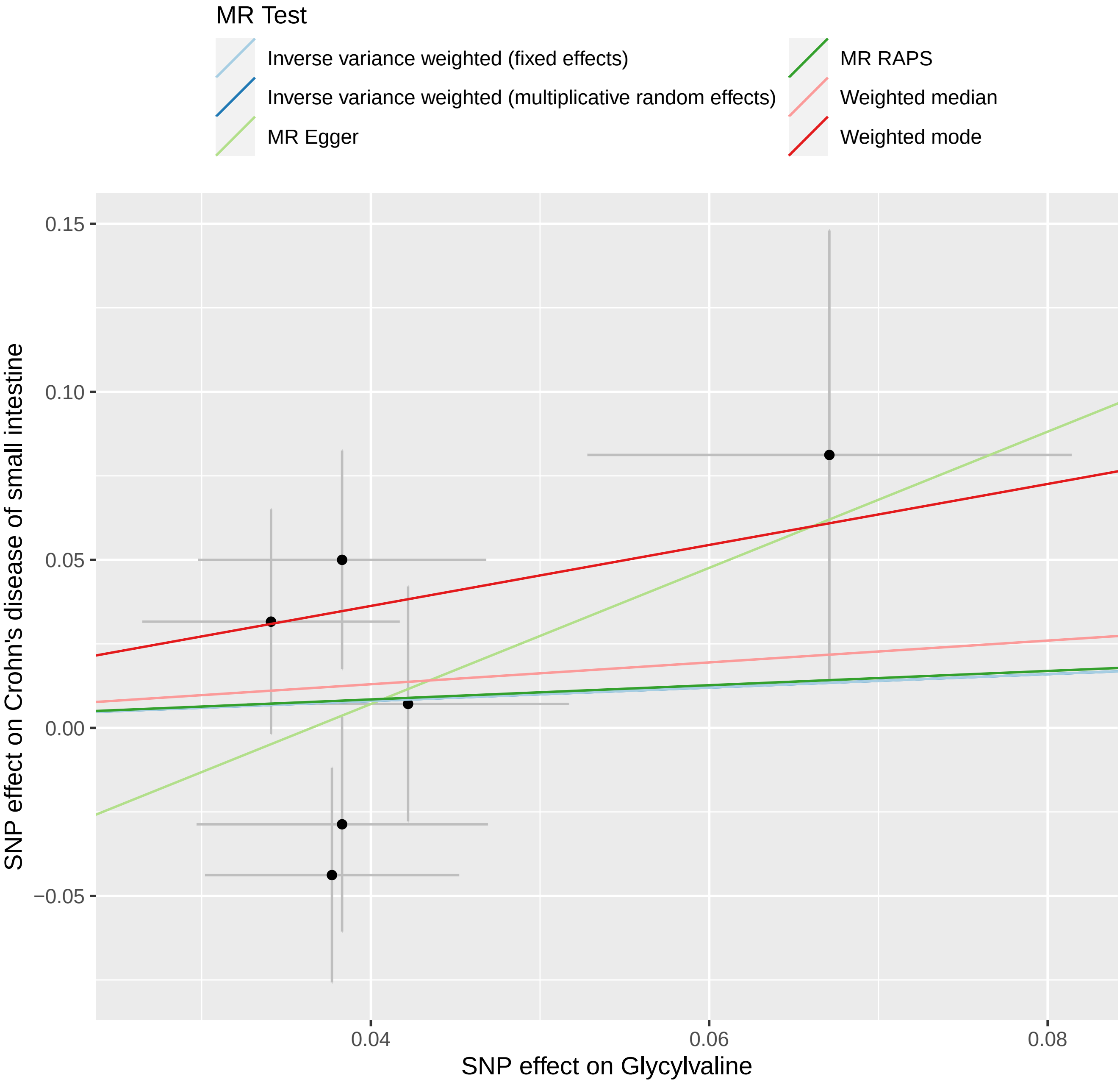

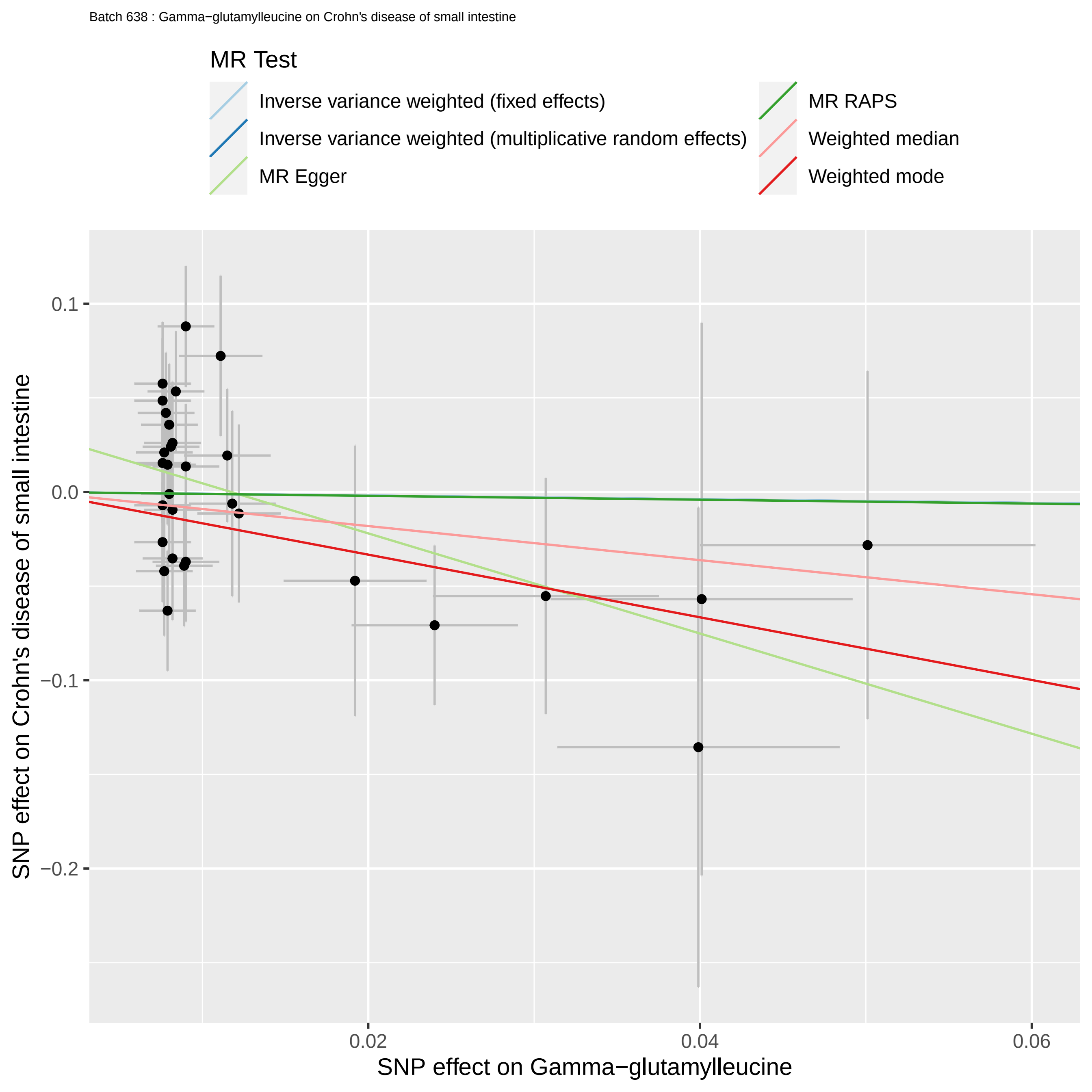

Batch 638 : Gamma−glutamylleucine on Crohn's disease of small intestine
MR Test
Inverse variance weighted (fixed effects)
Inverse variance weighted (multiplicative random effects)
MR Egger
MR RAPS
Weighted median
Weighted mode
0.1
0.0
−0.1
−0.2
0.02
0.04
0.06
SNP effect on Crohn's disease of small intestine
SNP effect on Gamma−glutamylleucine

Batch 641 : Glycocholate on Crohn's disease of small intestine

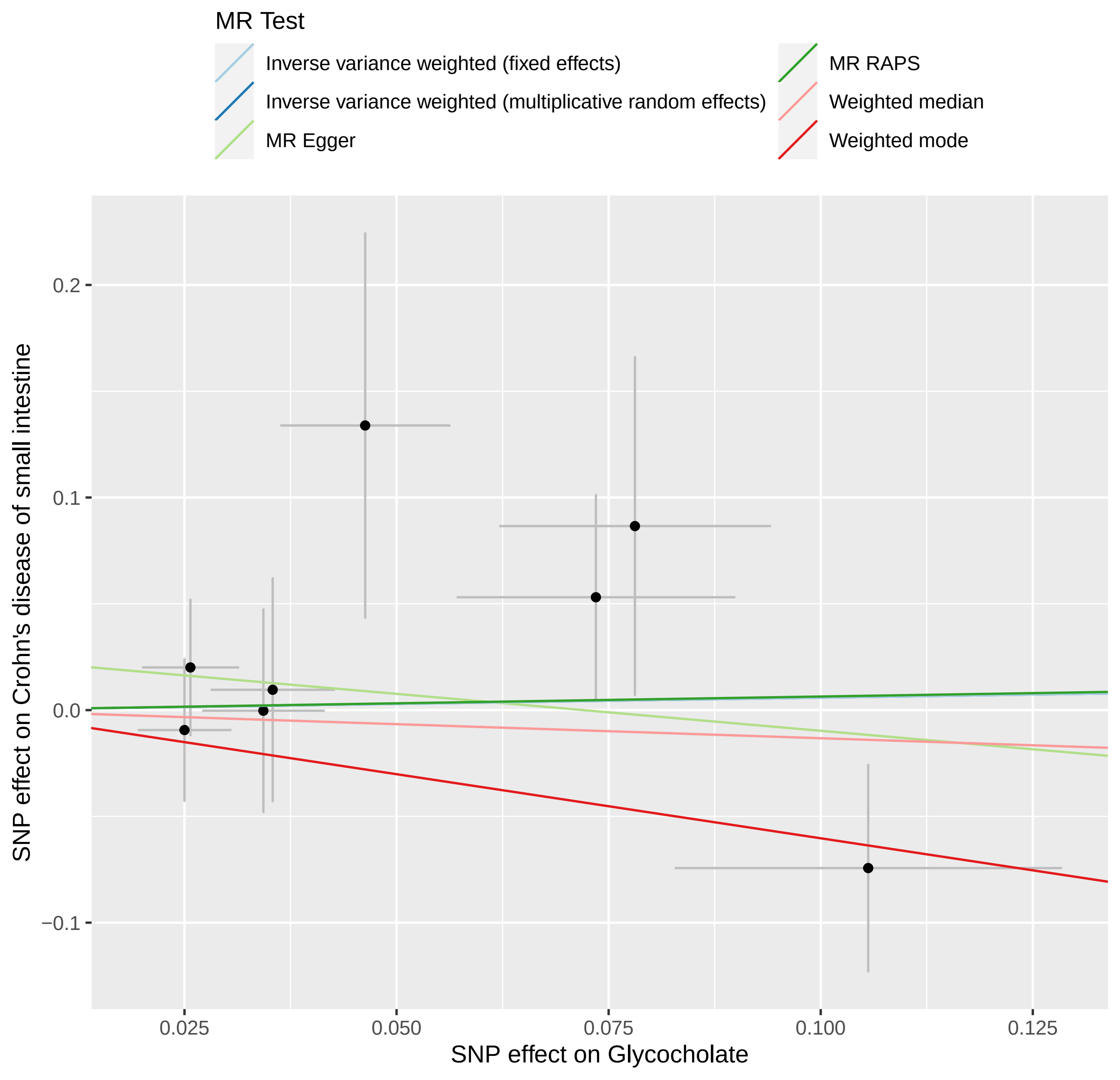

Batch 642 : Glycodeoxycholate on Crohn's disease of small intestine

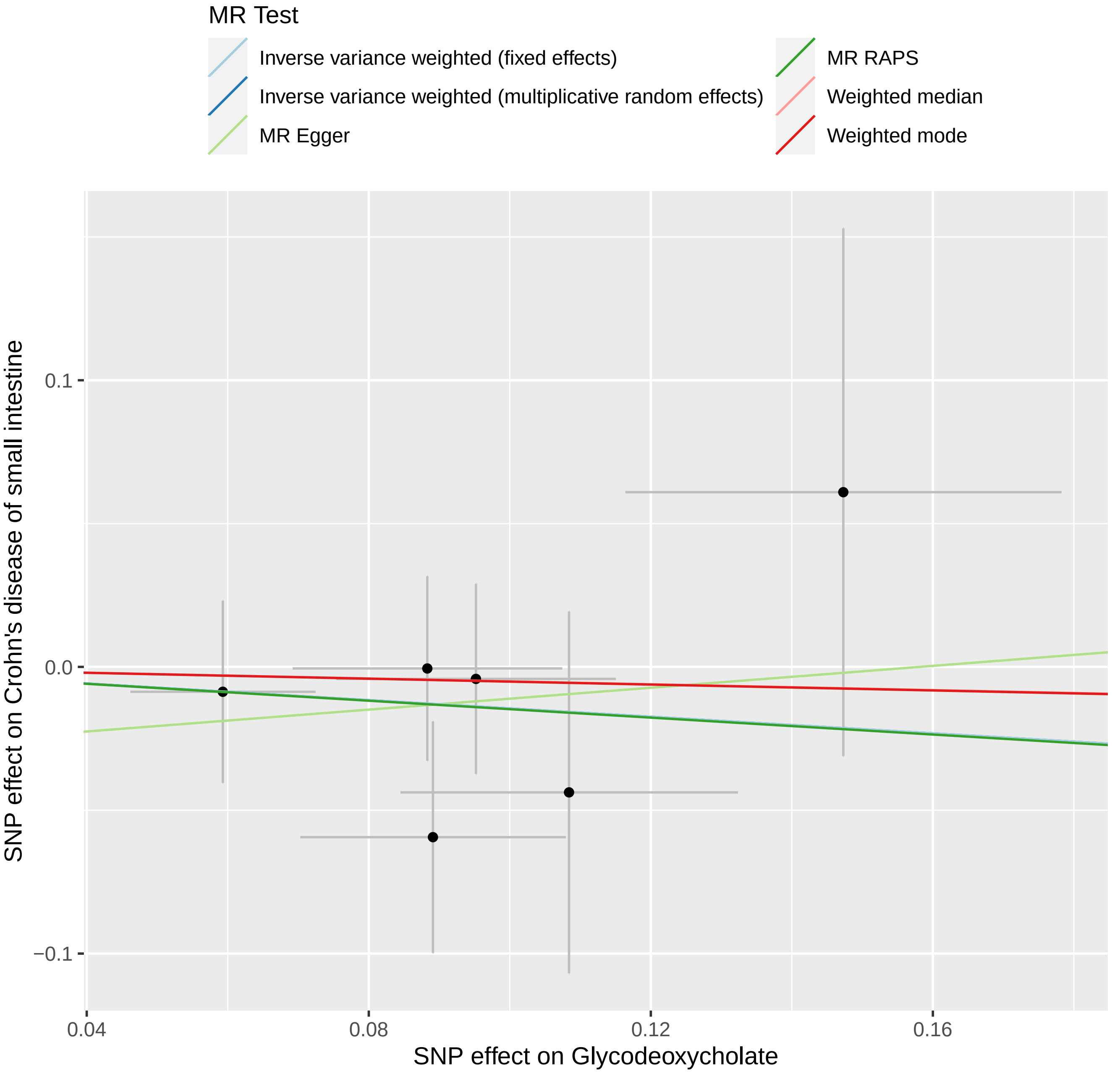

Batch 643 : Taurochenodeoxycholate on Crohn's disease of small intestine

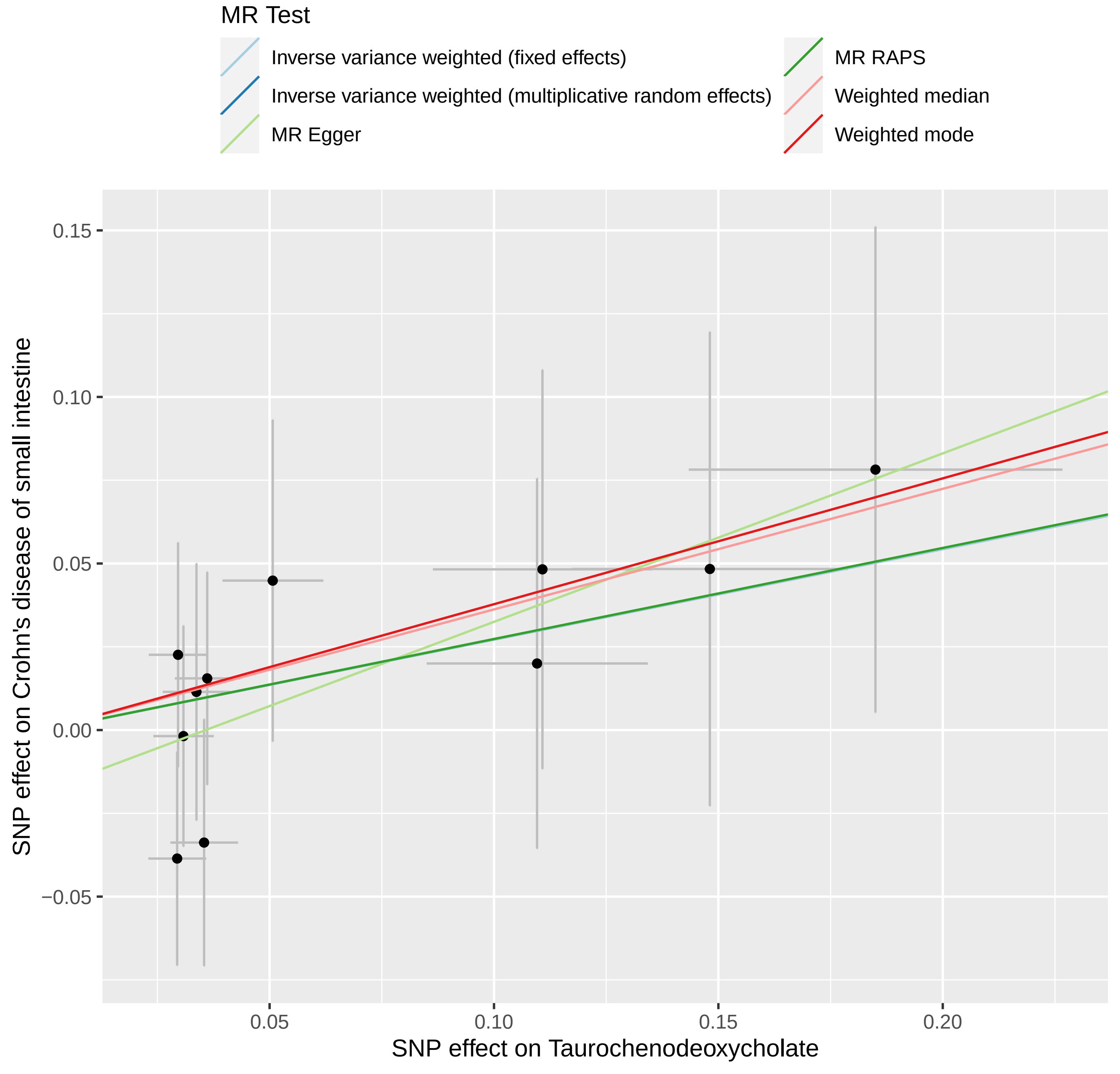

Batch 644 : Taurocholate on Crohn's disease of small intestine

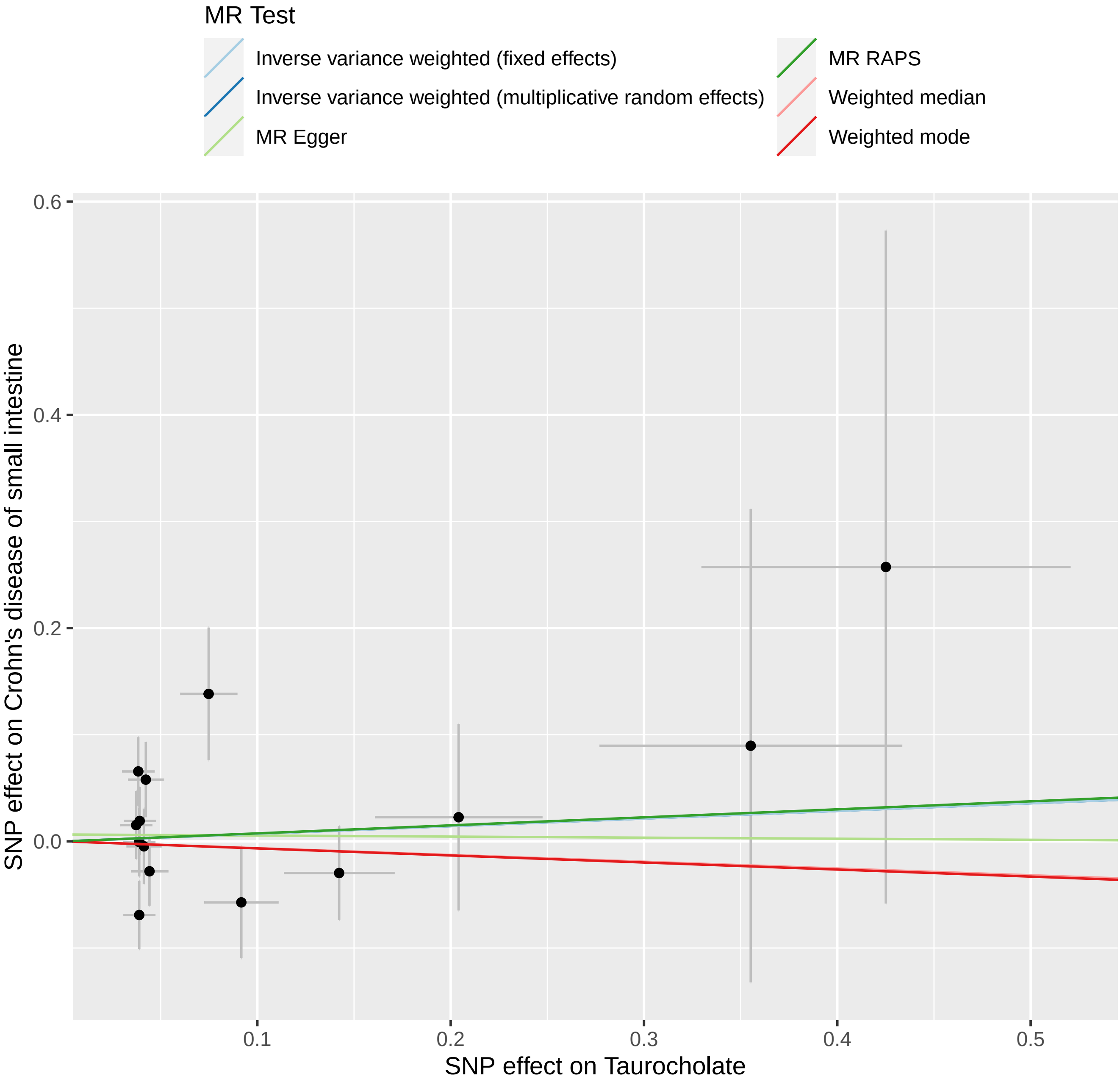

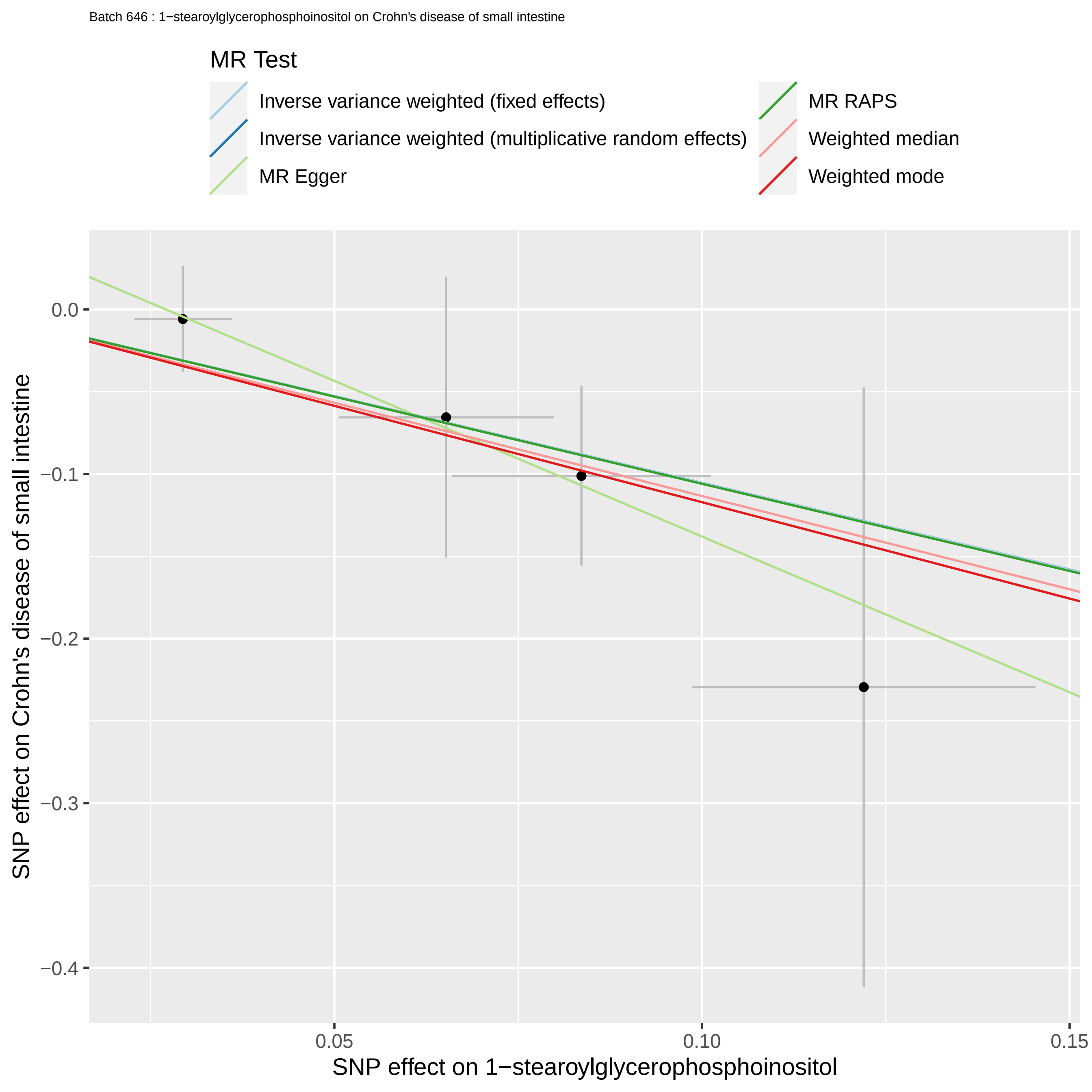

Batch 646 : 1−stearoylglycerophosphoinositol on Crohn's disease of small intestine
MR Test
Inverse variance weighted (fixed effects)
Inverse variance weighted (multiplicative random effects)
MR Egger
MR RAPS
Weighted median
Weighted mode
SNP effect on Crohn's disease of small intestine
0.0
−0.1
−0.2
−0.3
−0.4
0.05
0.10
0.15
SNP effect on 1−stearoylglycerophosphoinositol

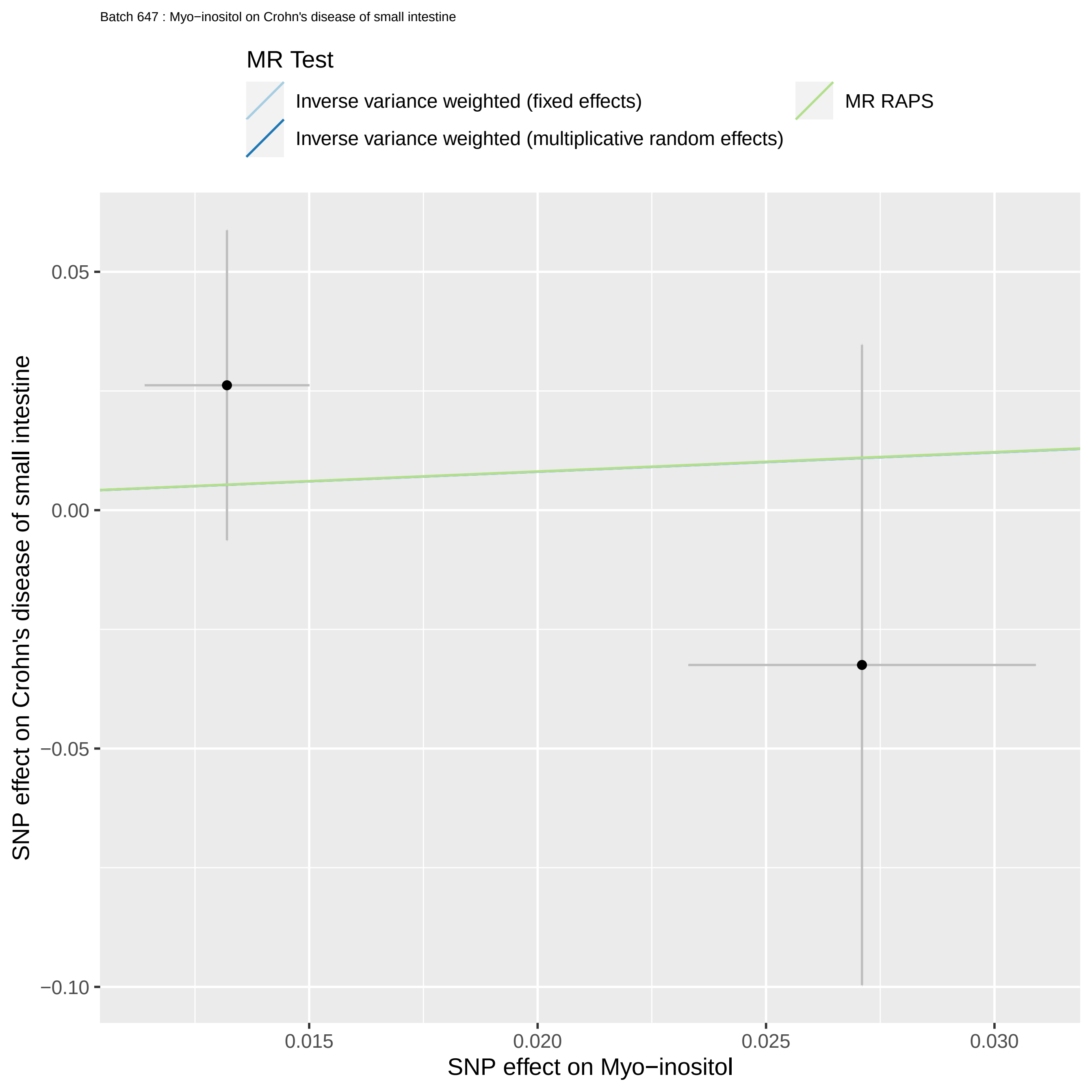
Batch 647 : Myo−inositol on Crohn's disease of small intestine
MR Test
Inverse variance weighted (fixed effects)
Inverse variance weighted (multiplicative random effects)
MR RAPS
0.05
0.00
−0.05
−0.10
0.015
0.020
0.025
0.030
SNP effect on Crohn's disease of small intestine
SNP effect on Myo−inositol

Batch 649 : 1,5−anhydroglucitol (1,5−AG) on Crohn's disease of small intestine

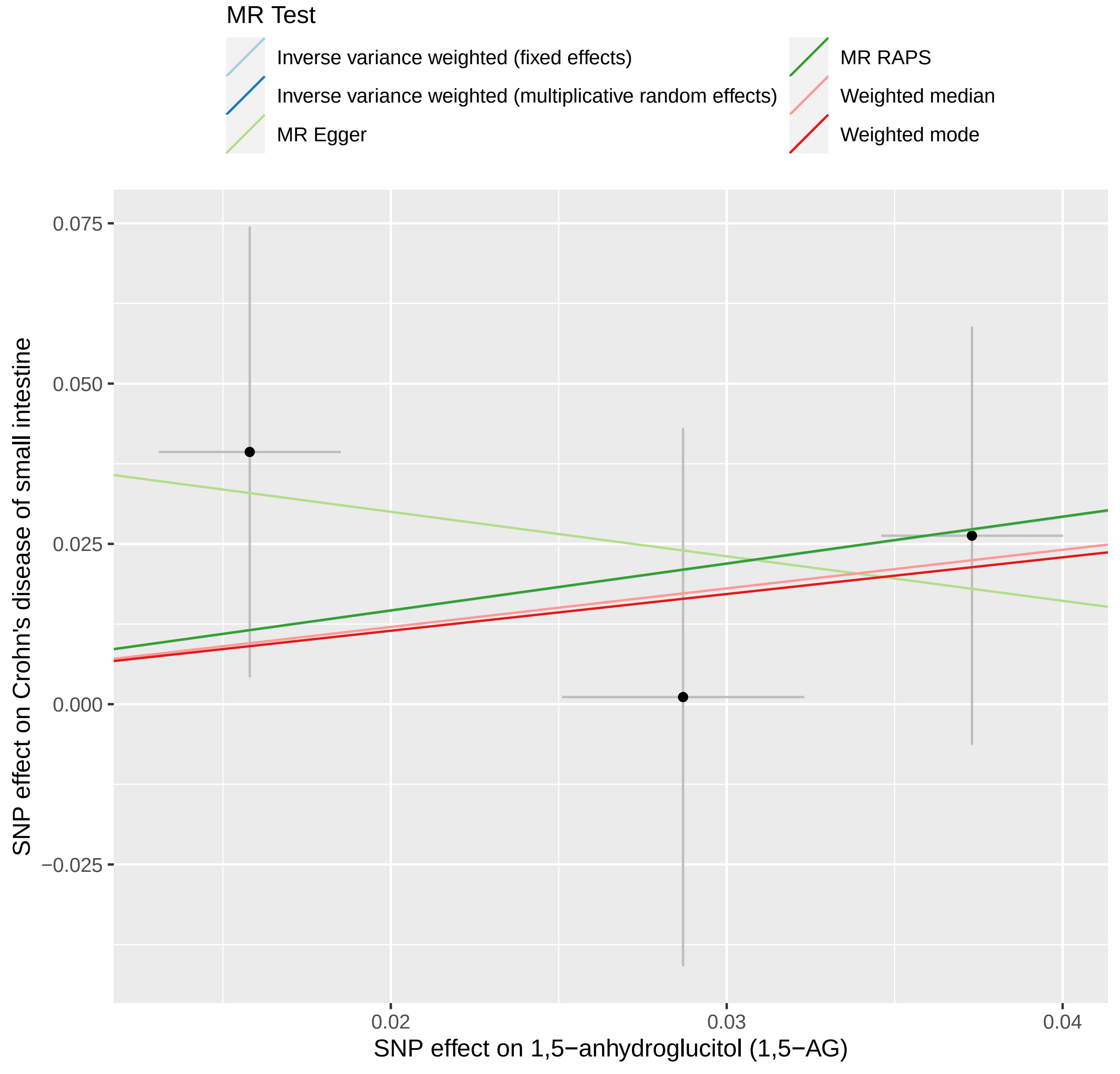

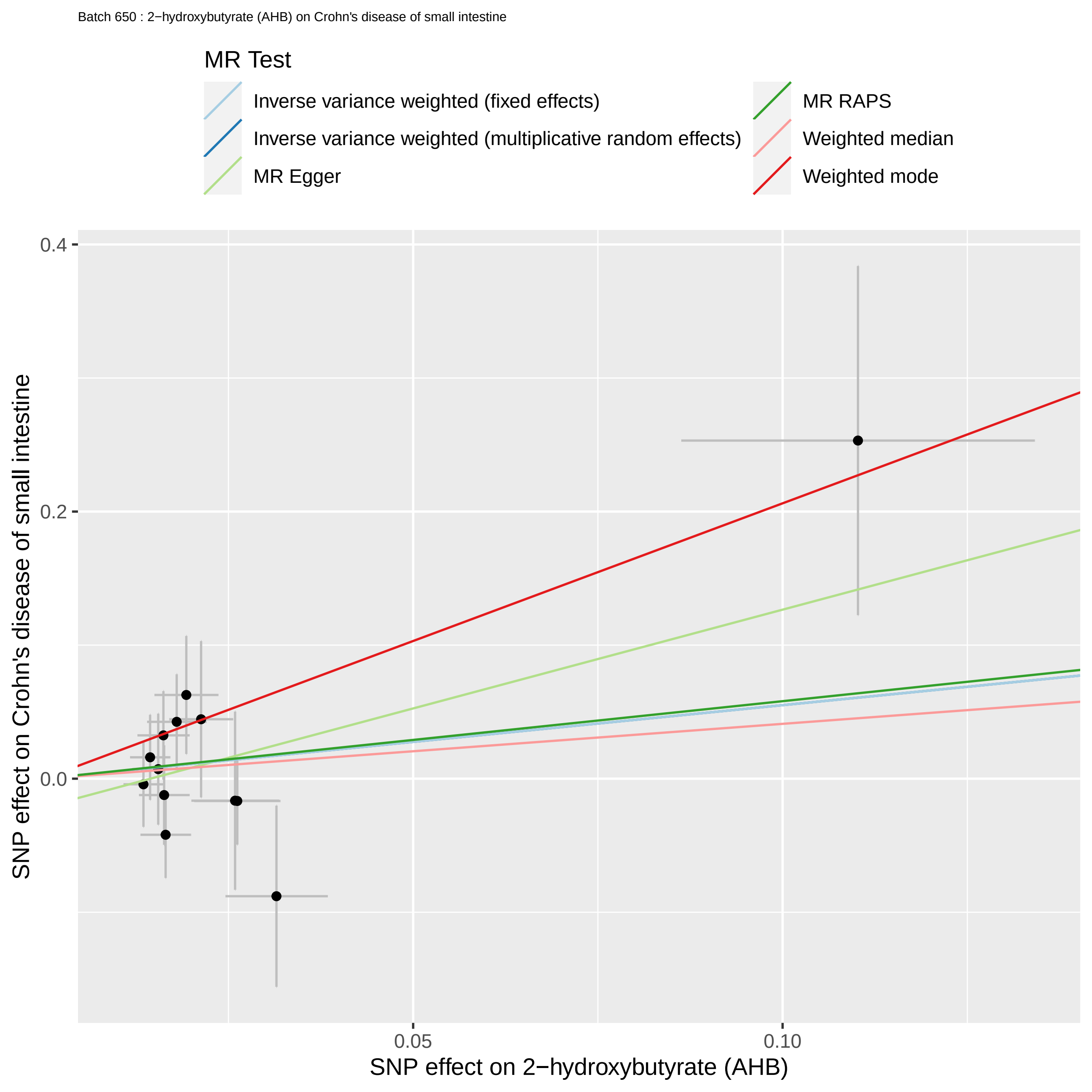

Batch 650 : 2−hydroxybutyrate (AHB) on Crohn's disease of small intestine
MR Test
Inverse variance weighted (fixed effects)
Inverse variance weighted (multiplicative random effects)
MR Egger
MR RAPS
Weighted median
Weighted mode
0.4
0.2
0.0
0.05
0.10
SNP effect on Crohn's disease of small intestine
SNP effect on 2−hydroxybutyrate (AHB)

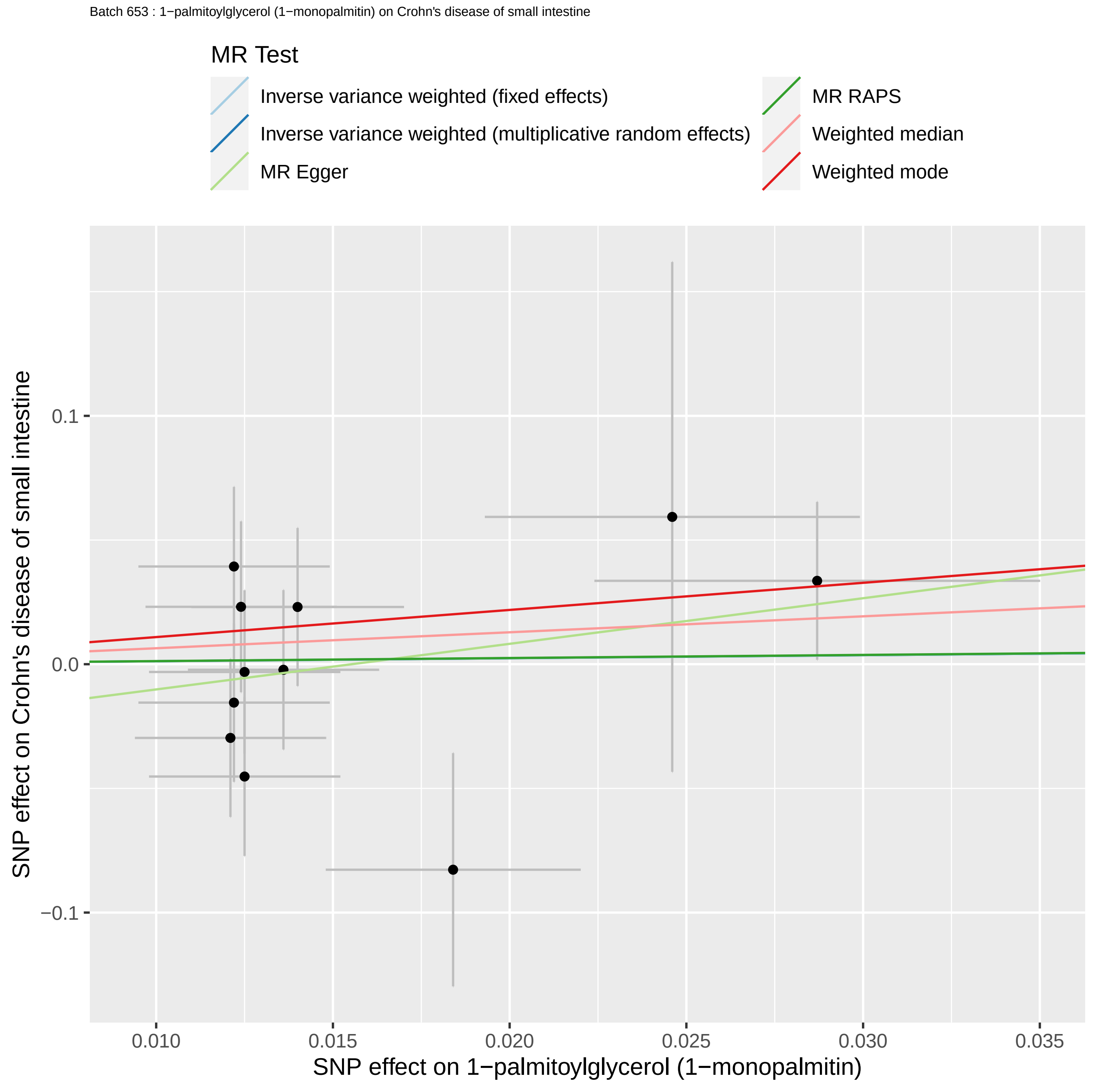

Batch 653 : 1−palmitoylglycerol (1−monopalmitin) on Crohn's disease of small intestine
MR Test
Inverse variance weighted (fixed effects)
Inverse variance weighted (multiplicative random effects)
MR Egger
MR RAPS
Weighted median
Weighted mode
0.1
0.0
−0.1
0.010
0.015
0.020
0.025
0.030
0.035
SNP effect on Crohn's disease of small intestine
SNP effect on 1−palmitoylglycerol (1−monopalmitin)

Batch 656 : 2−hydroxyisobutyrate on Crohn's disease of small intestine

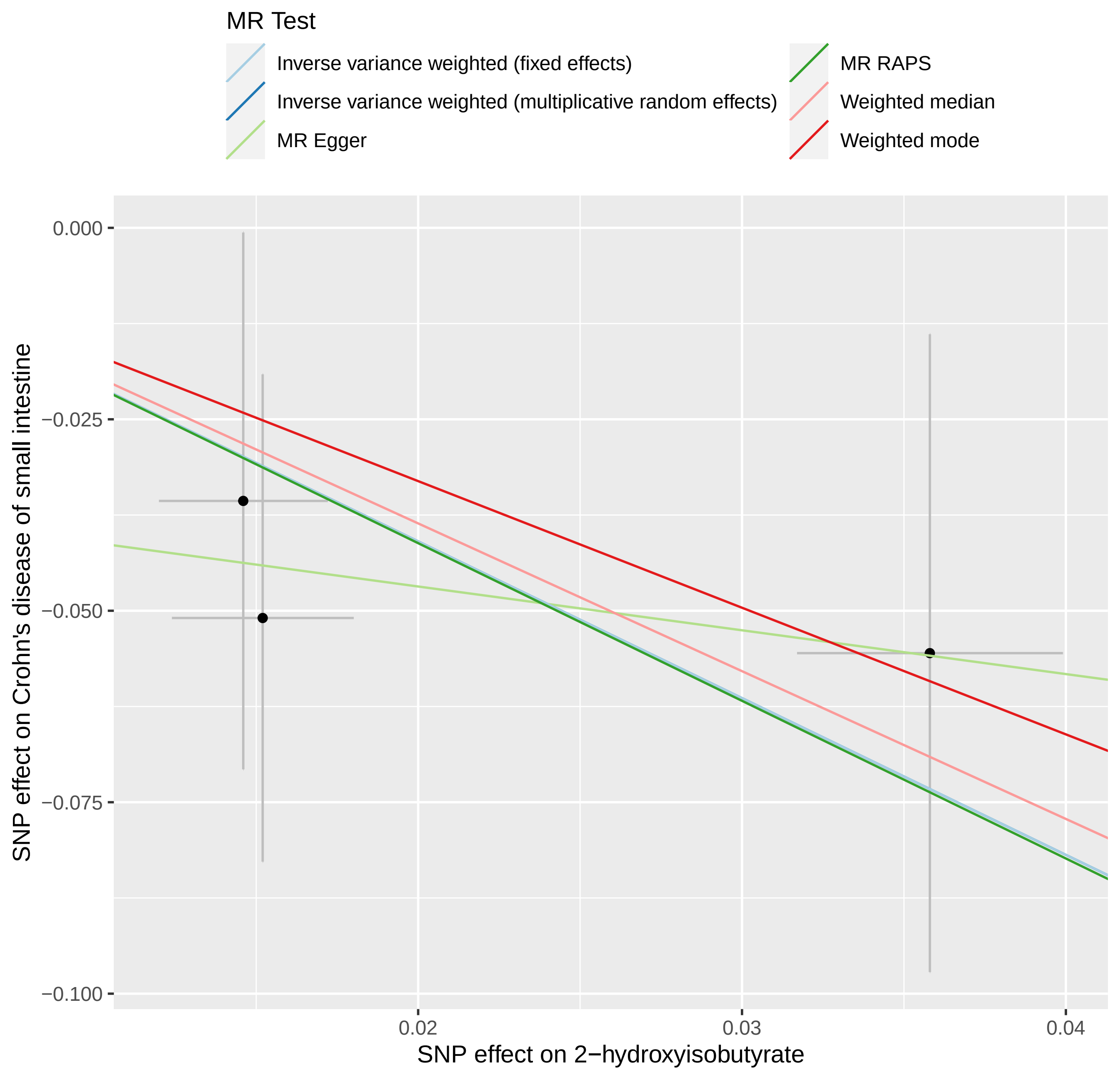

Batch 657 : 4−methyl−2−oxopentanoate on Crohn's disease of small intestine

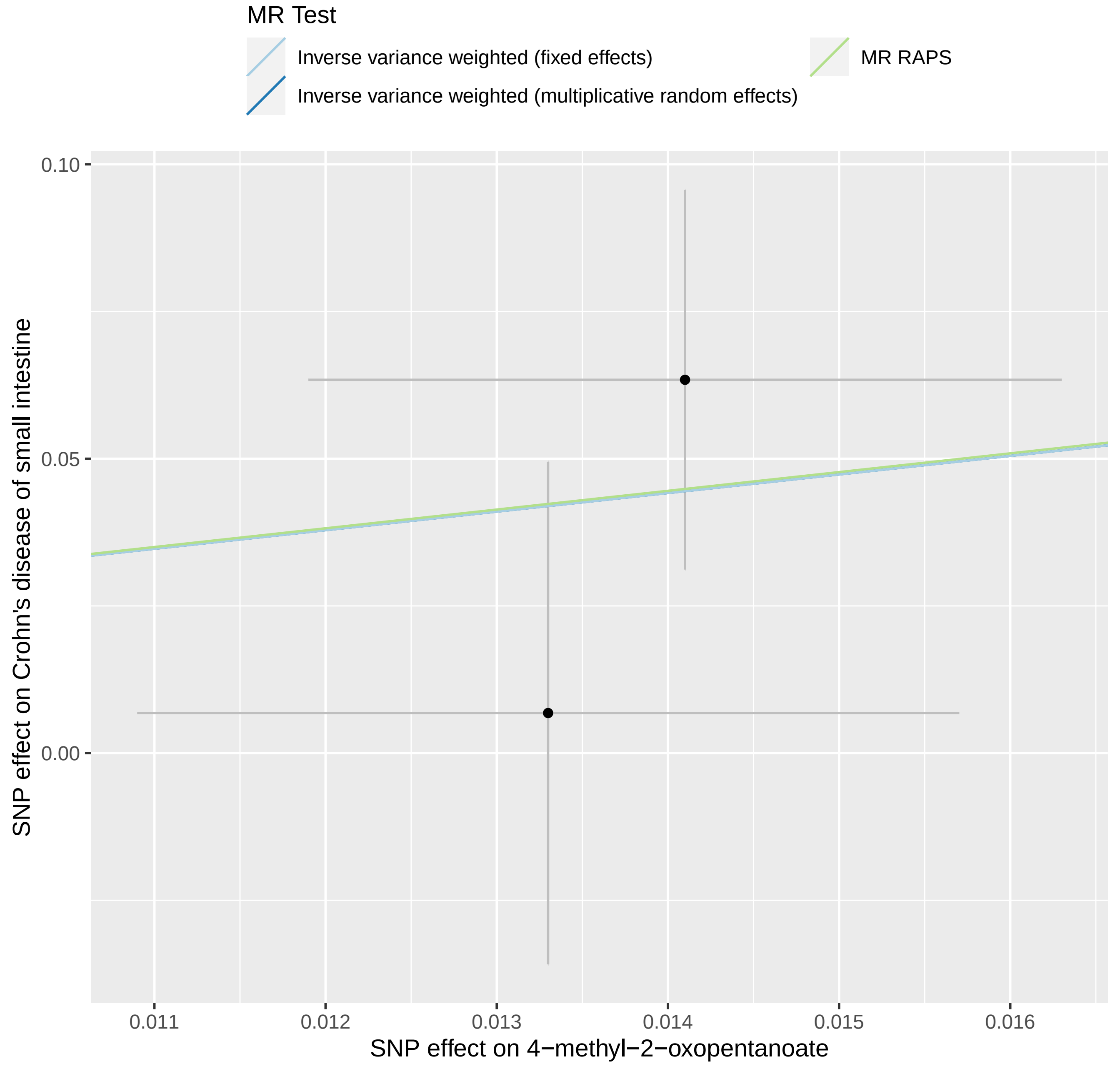

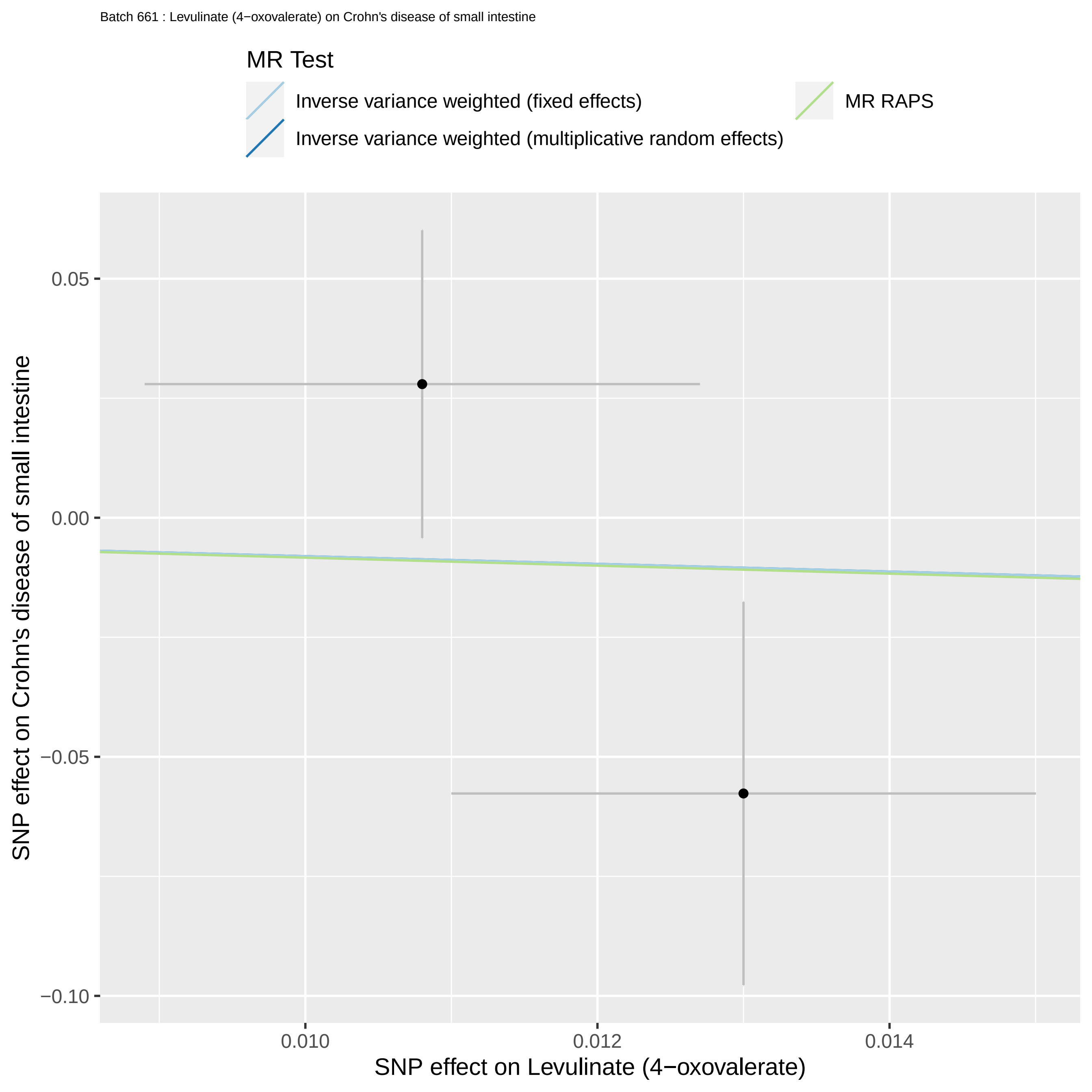

Batch 661 : Levulinate (4−oxovalerate) on Crohn's disease of small intestine
MR Test
Inverse variance weighted (fixed effects)
Inverse variance weighted (multiplicative random effects)
MR RAPS
0.05
0.00
−0.05
−0.10
0.010
0.012
0.014
SNP effect on Levulinate (4−oxovalerate)
SNP effect on Crohn's disease of small intestine

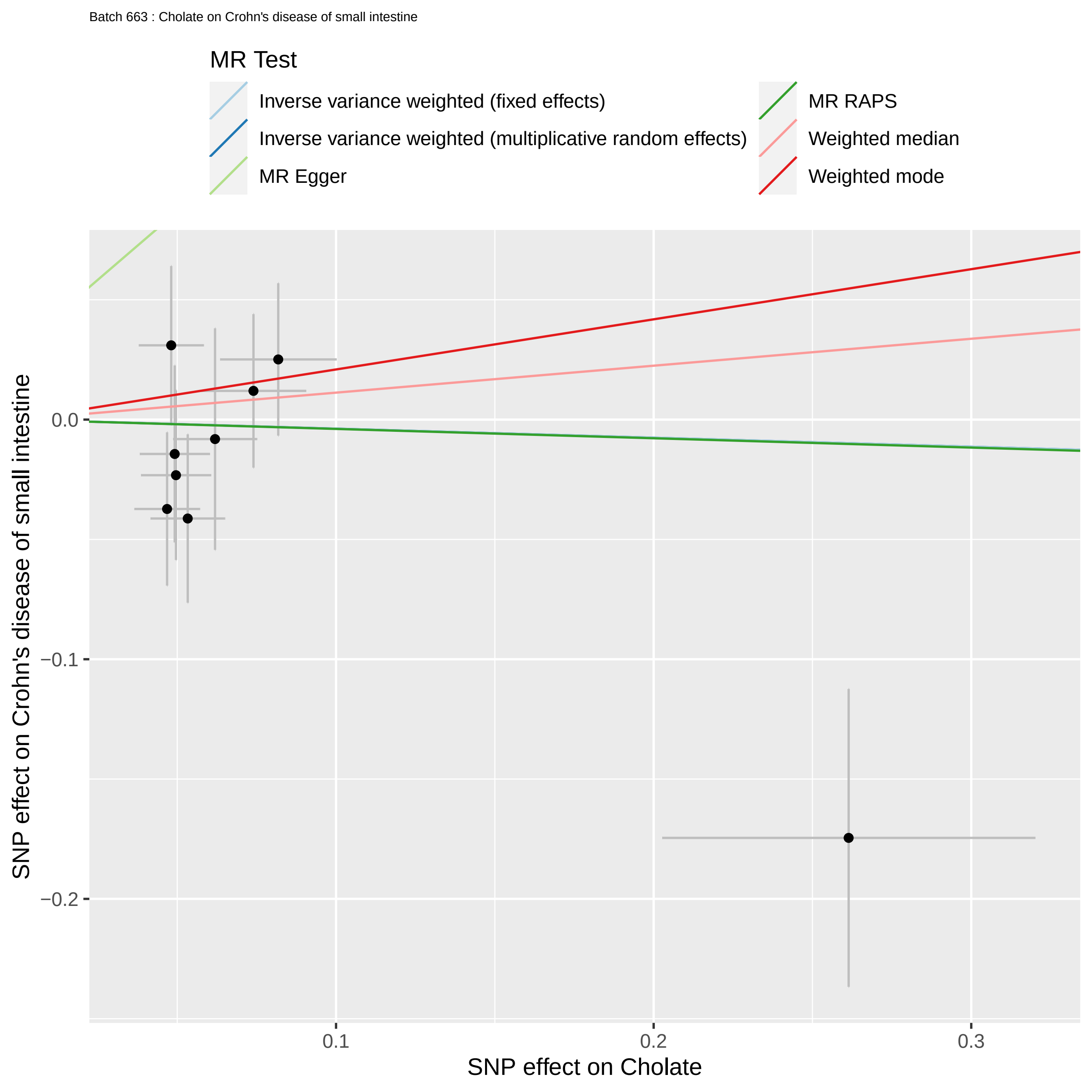

Batch 663 : Cholate on Crohn's disease of small intestine
MR Test
Inverse variance weighted (fixed effects)
Inverse variance weighted (multiplicative random effects)
MR Egger
MR RAPS
Weighted median
Weighted mode
SNP effect on Crohn's disease of small intestine
0.0
−0.1
−0.2
0.1
0.2
0.3
SNP effect on Cholate

Batch 666 : Hyodeoxycholate on Crohn's disease of small intestine

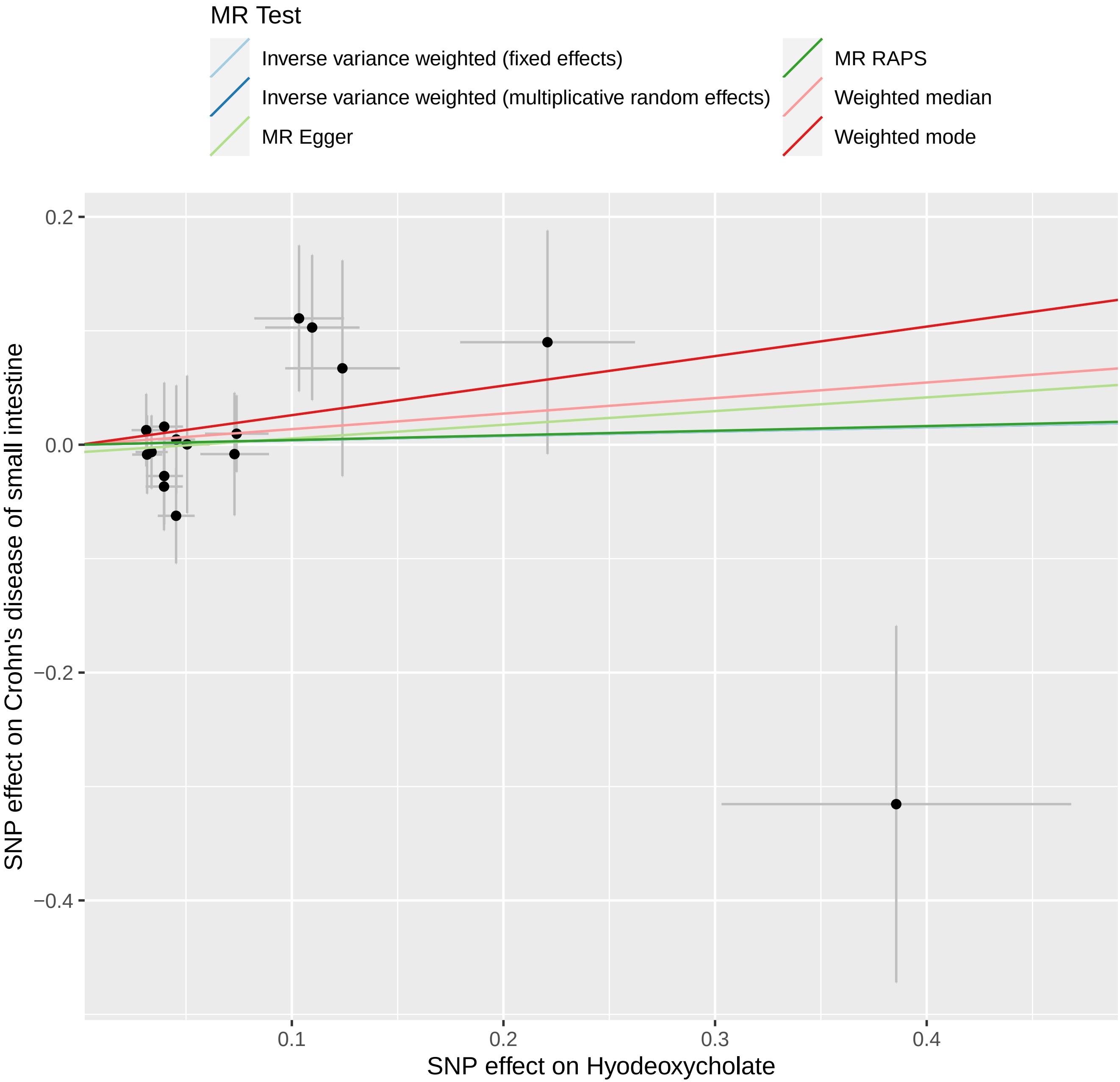

Batch 667 : 3−indoxyl sulfate on Crohn's disease of small intestine

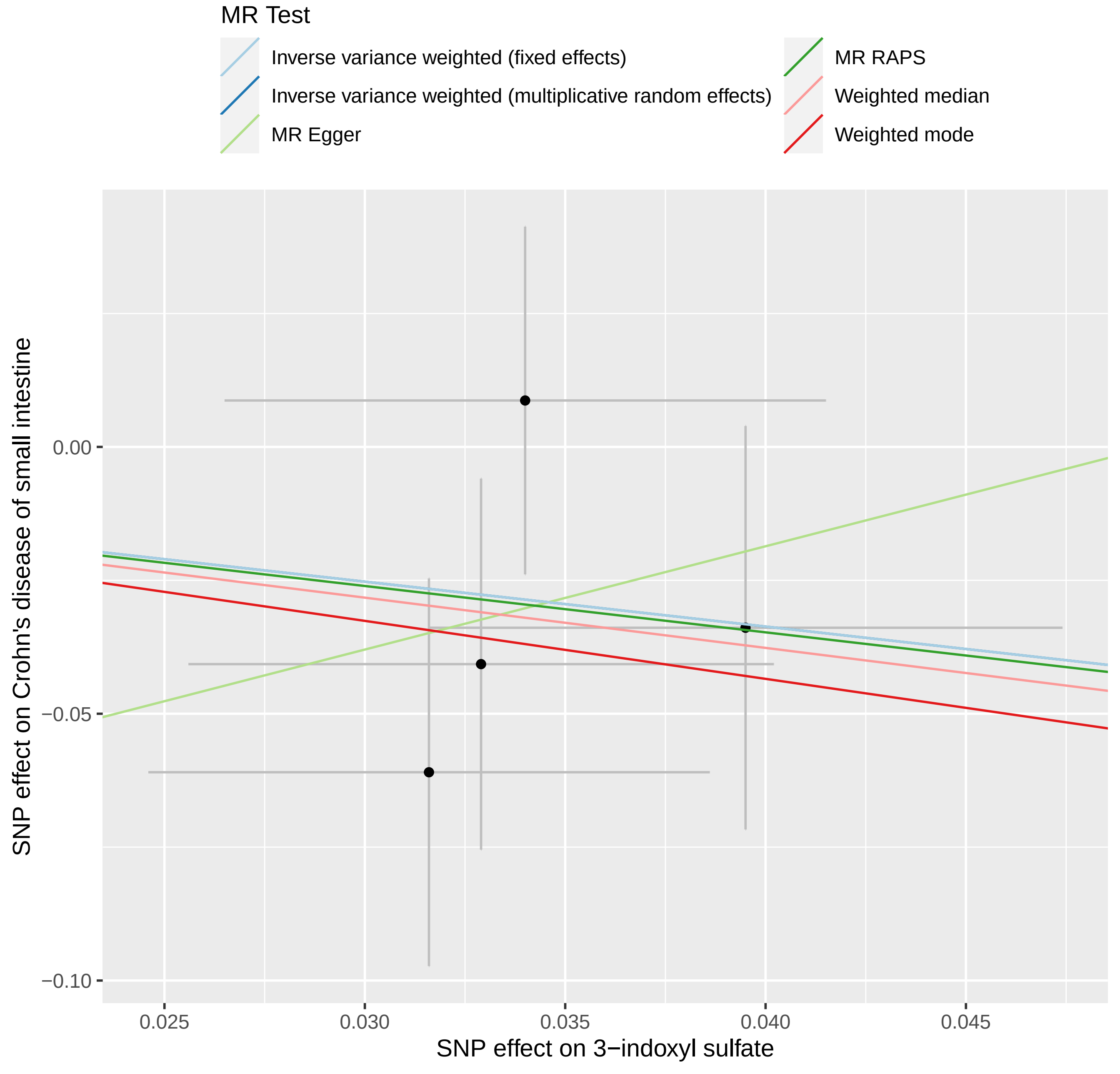

Batch 671 : Erythrose on Crohn's disease of small intestine

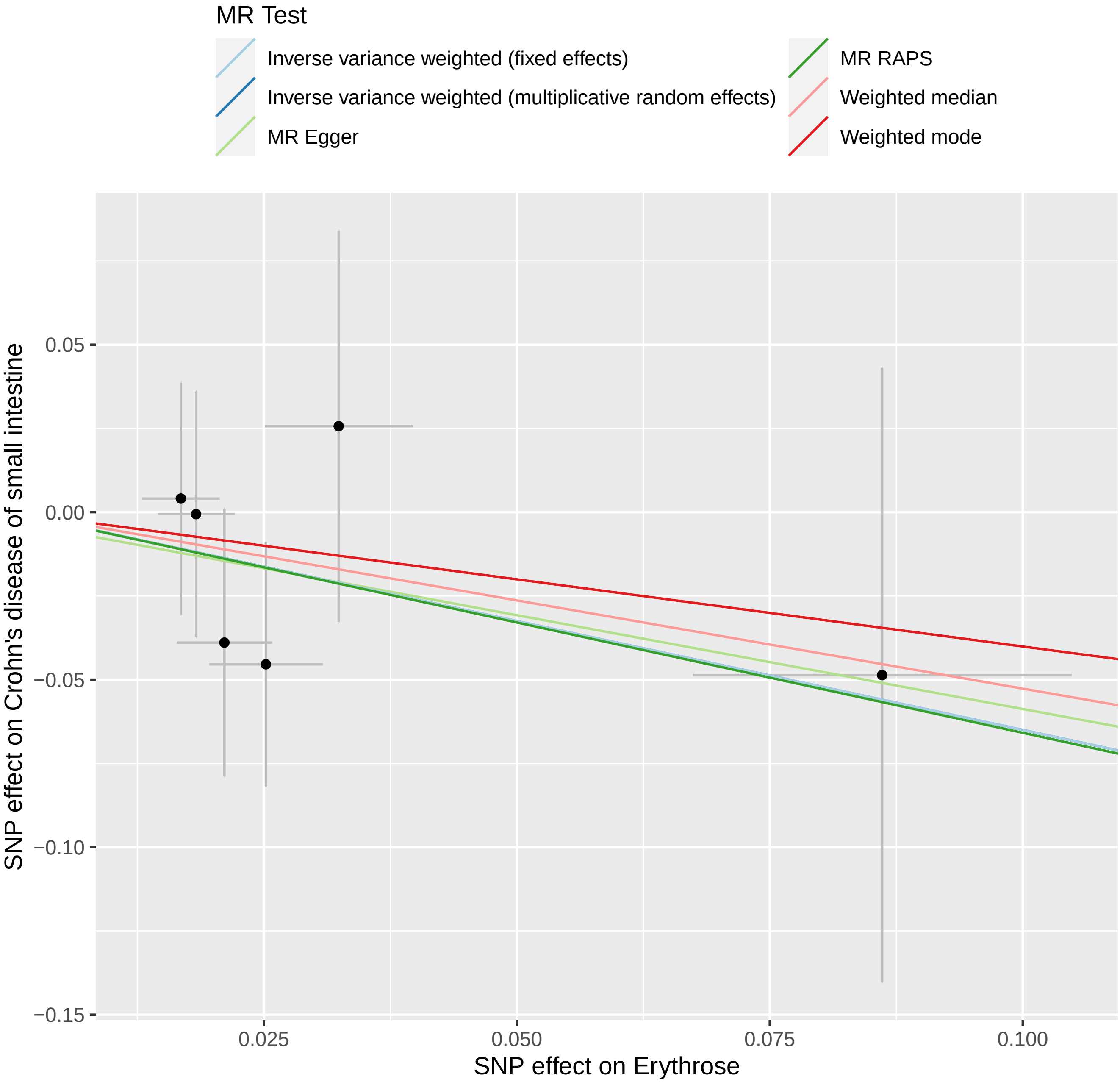

Batch 672 : Threonate on Crohn's disease of small intestine

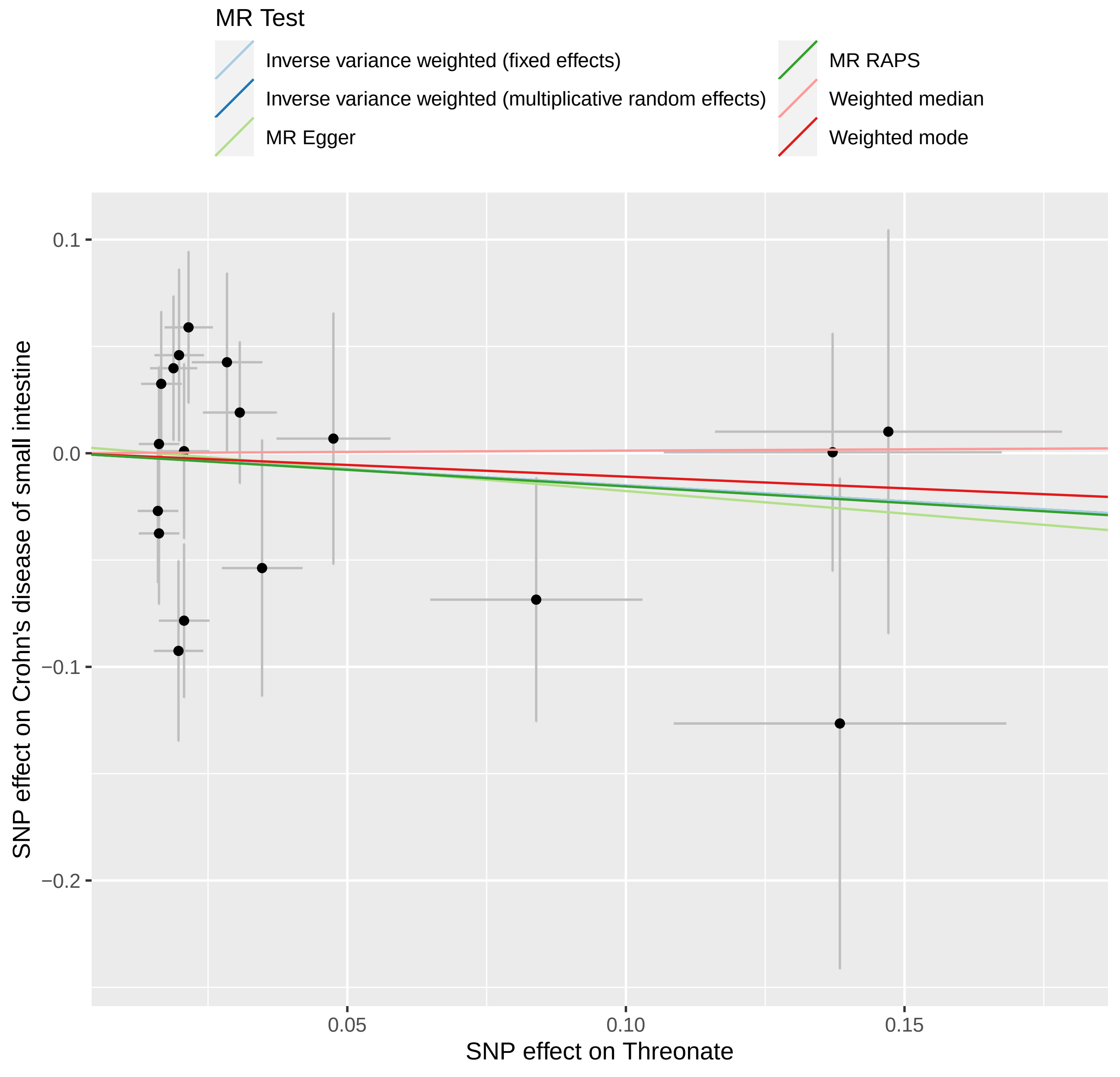

Batch 673 : Cysteine on Crohn's disease of small intestine

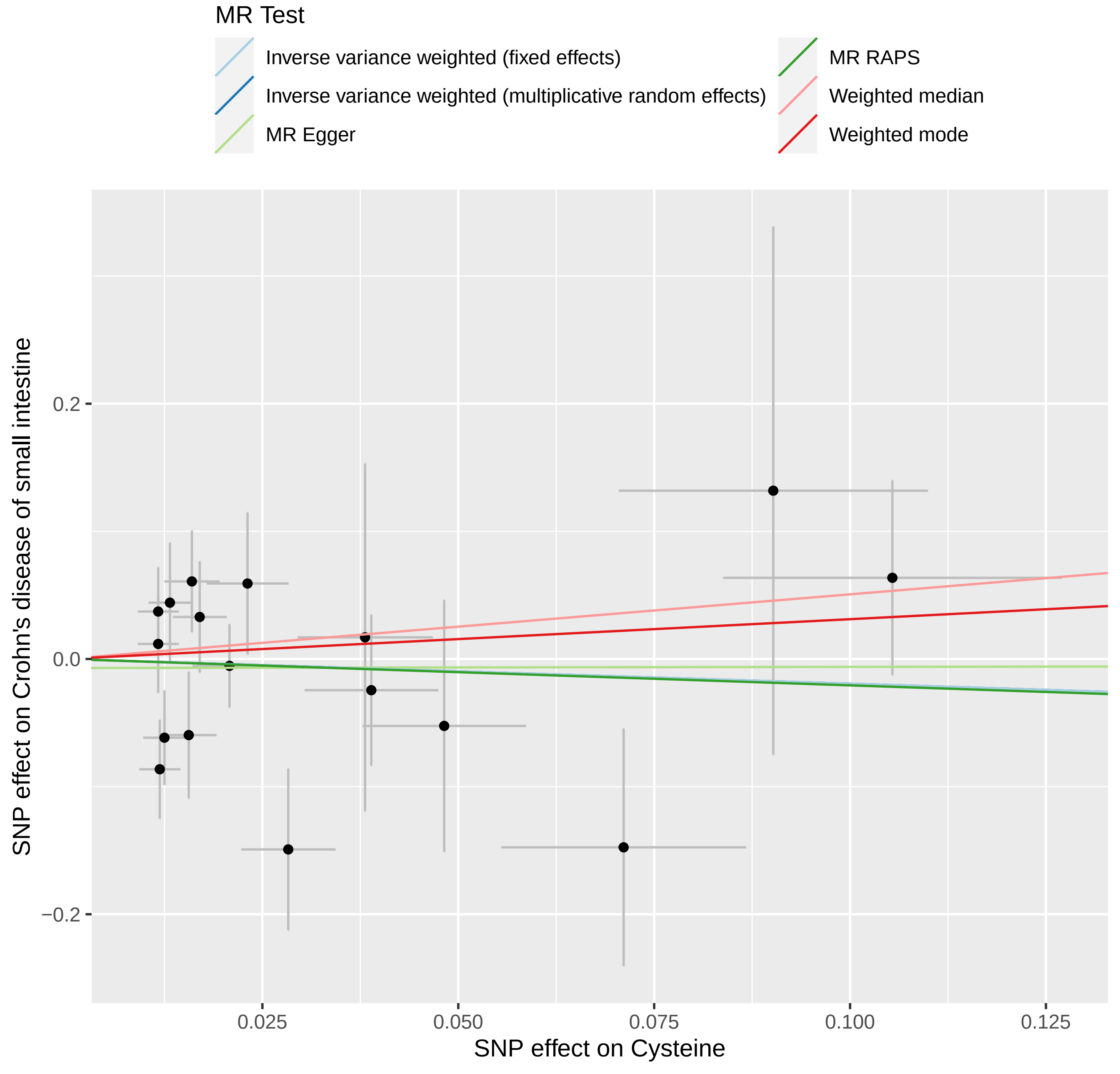

Batch 674 : Pyroglutamylglycine on Crohn's disease of small intestine

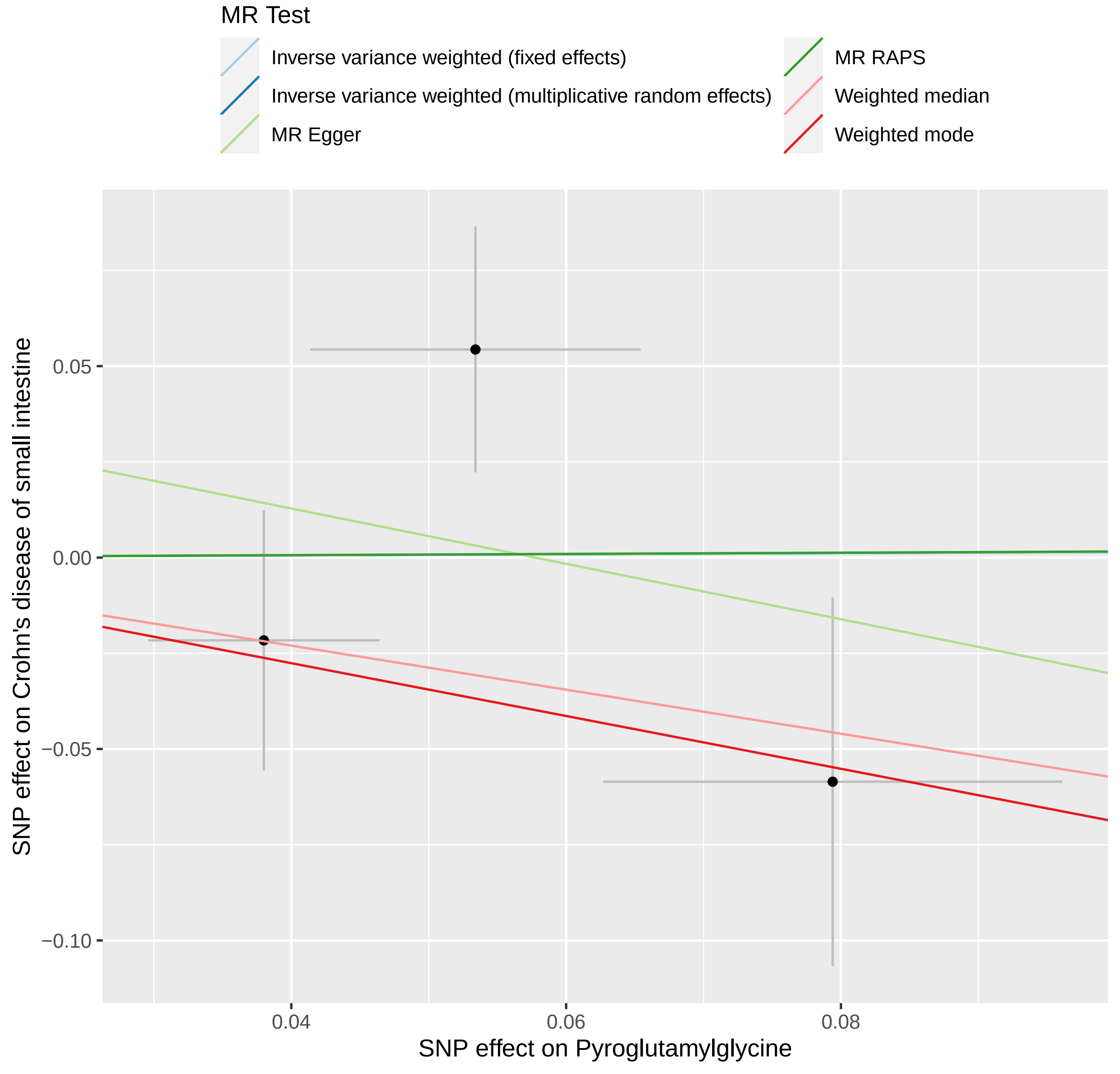

Batch 676 : DSGEGDFXAEGGGVR* on Crohn's disease of small intestine

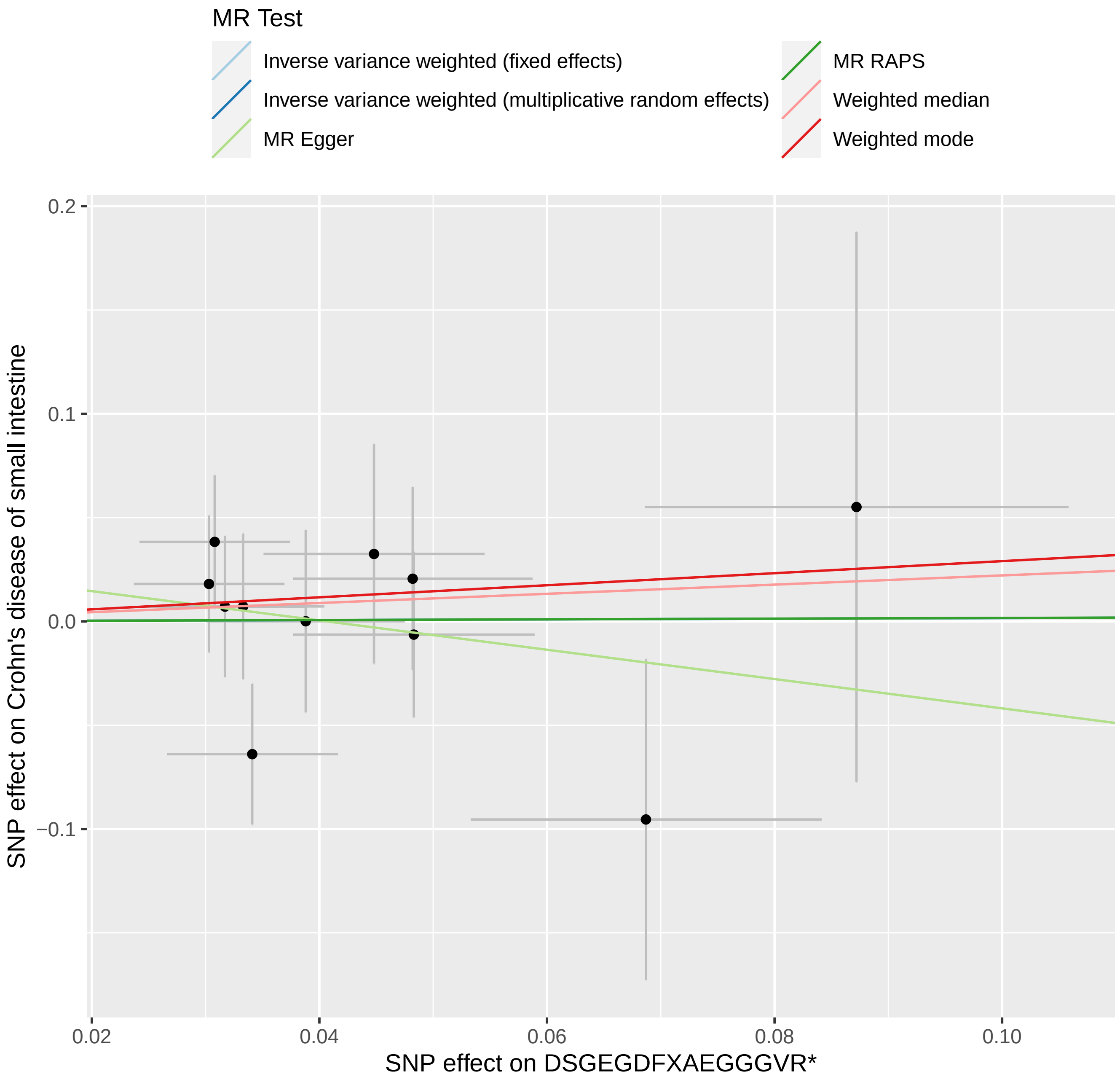

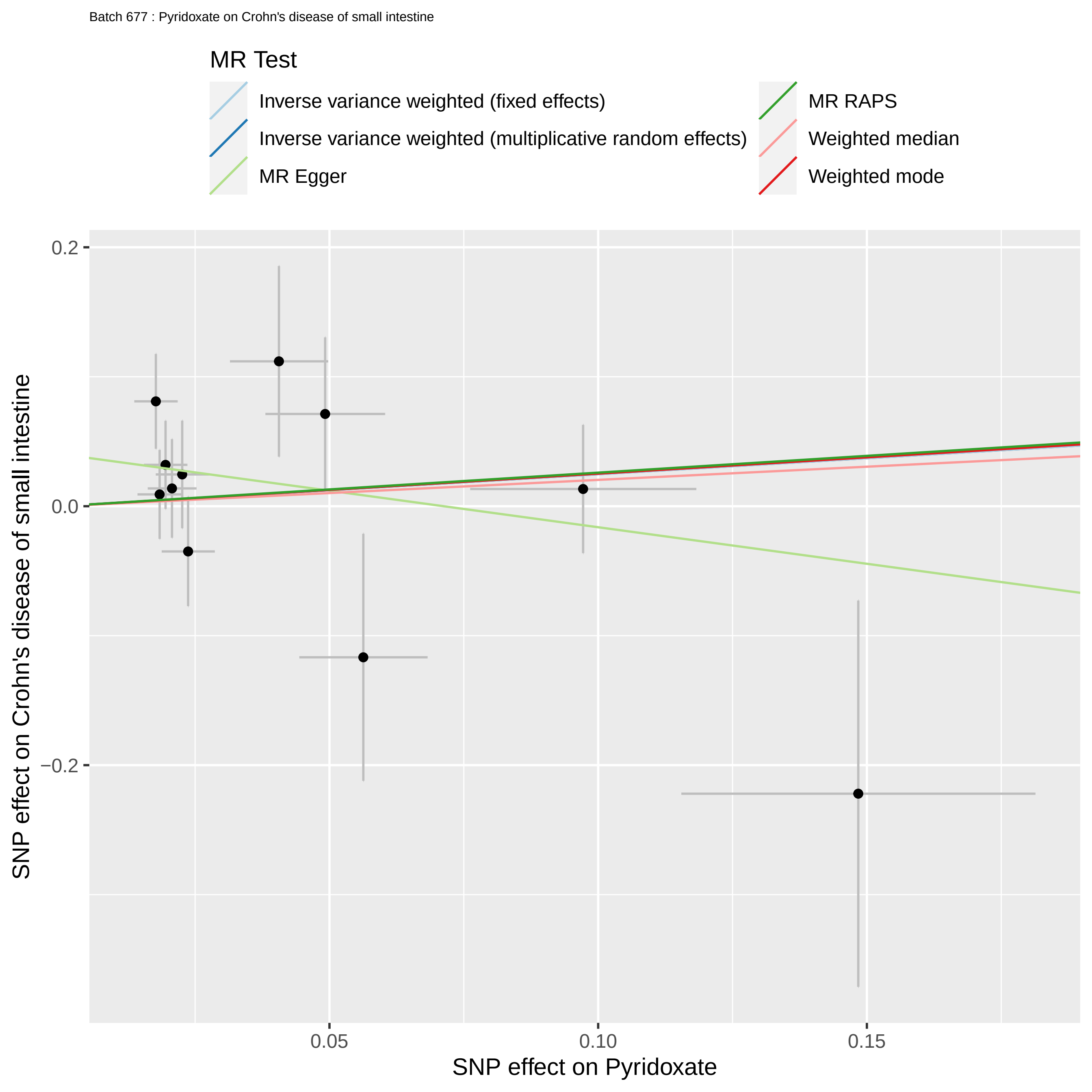
Batch 677 : Pyridoxate on Crohn's disease of small intestine
MR Test
Inverse variance weighted (fixed effects)
Inverse variance weighted (multiplicative random effects)
MR Egger
MR RAPS
Weighted median
Weighted mode
0.2
0.0
−0.2
0.05
0.10
0.15
SNP effect on Pyridoxate
SNP effect on Crohn's disease of small intestine

Batch 678 : Androsterone sulfate on Crohn's disease of small intestine

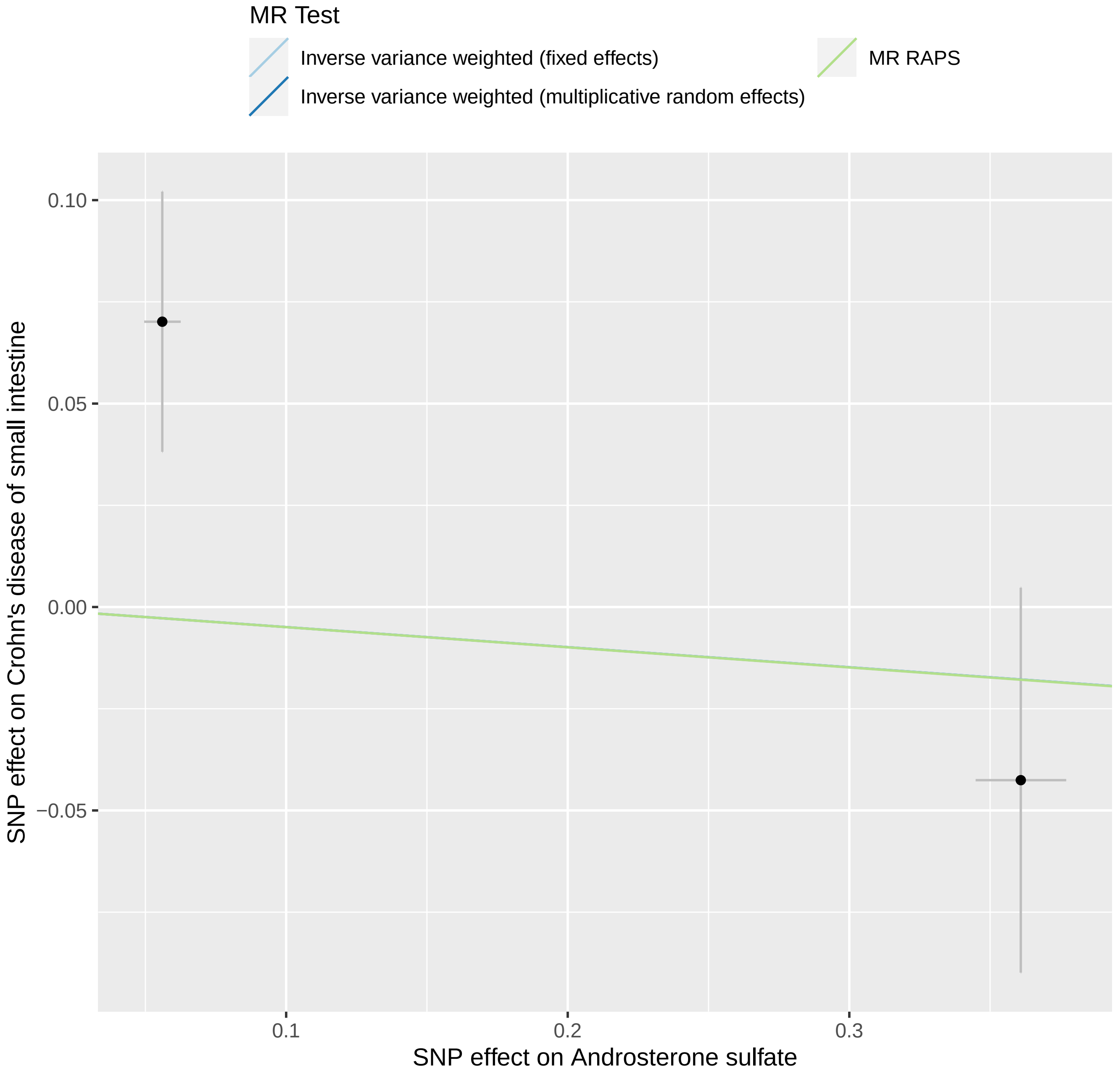

Batch 682 : Serine on Crohn's disease of small intestine

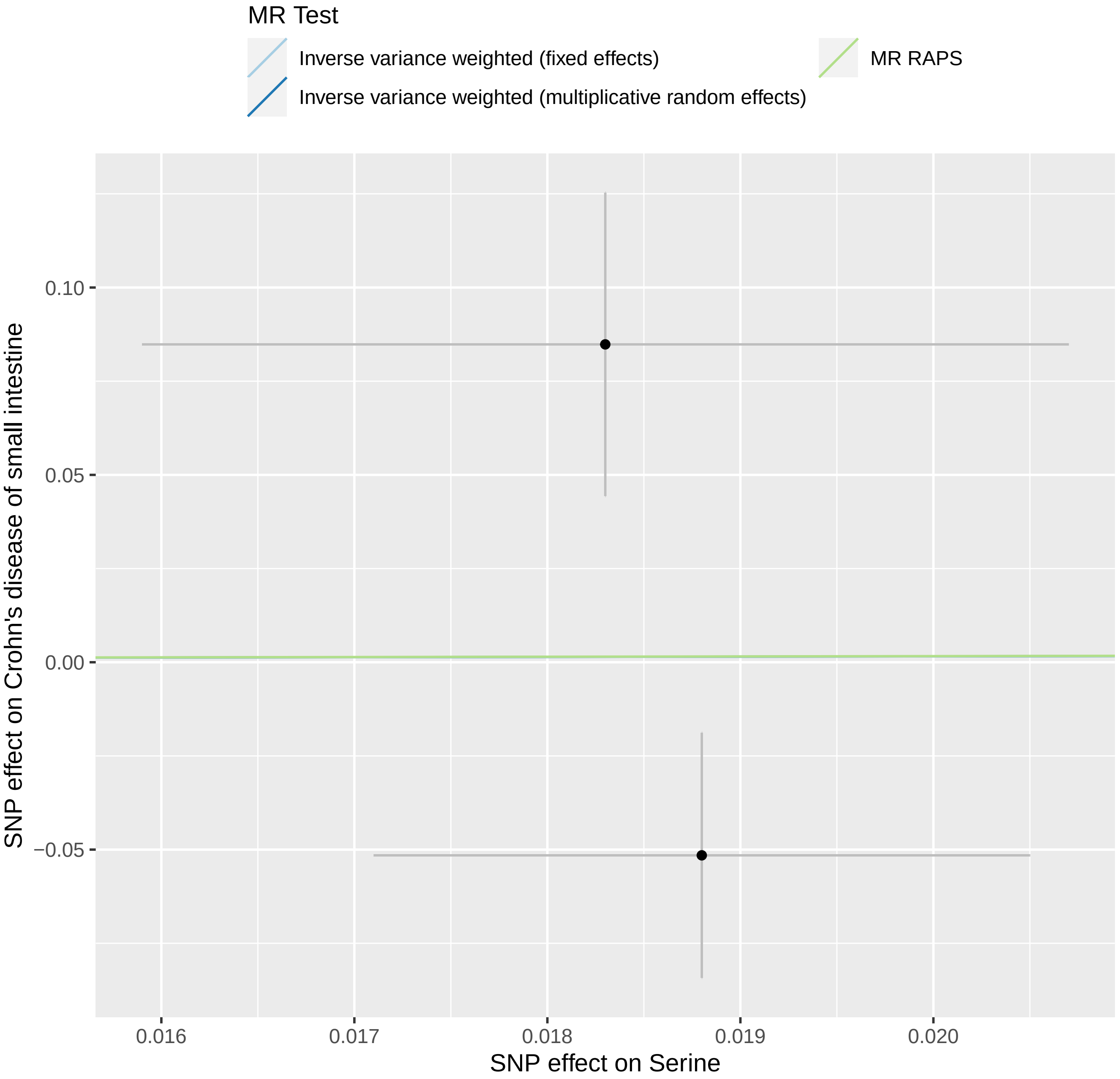

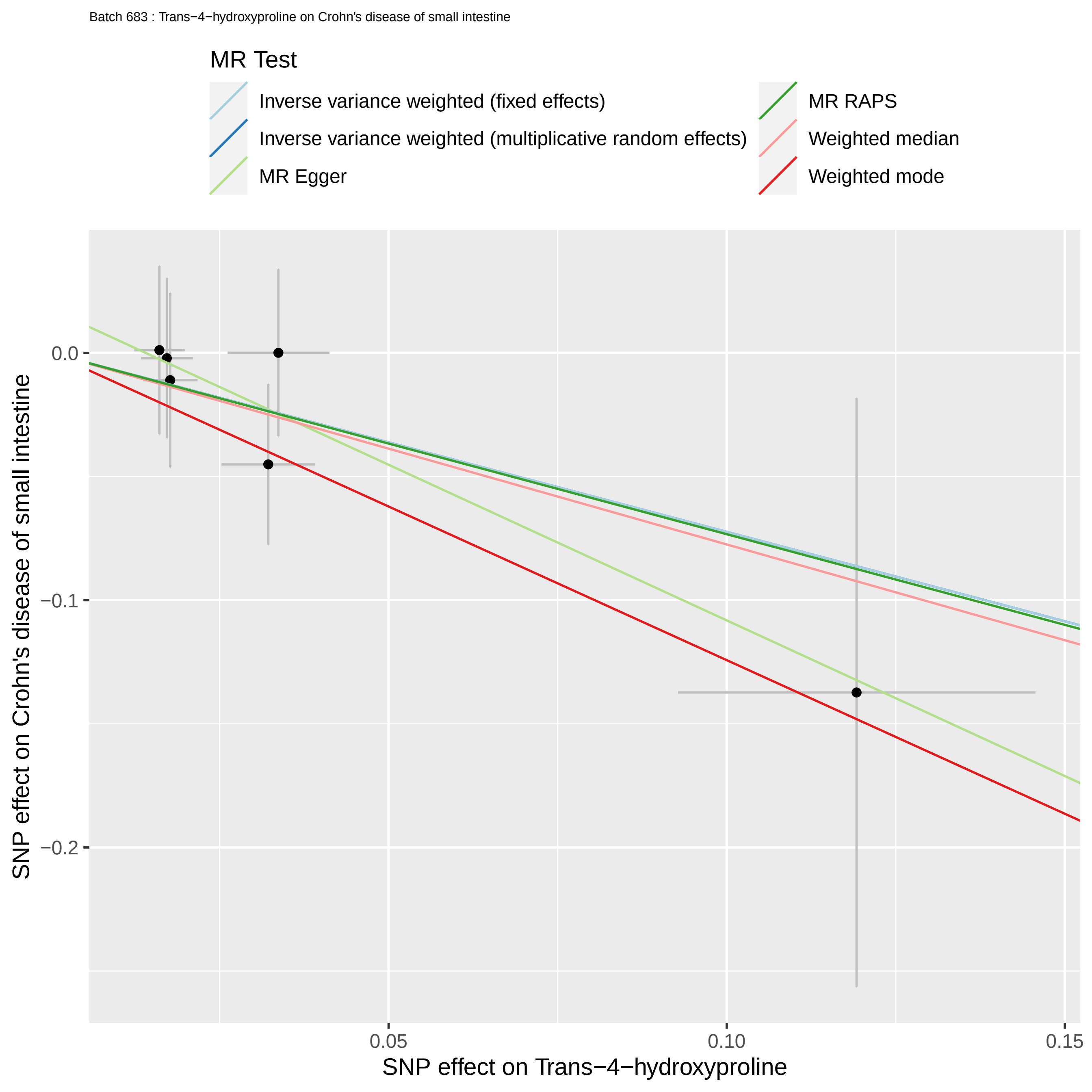

Batch 683 : Trans−4−hydroxyproline on Crohn's disease of small intestine
MR Test
Inverse variance weighted (fixed effects)
Inverse variance weighted (multiplicative random effects)
MR Egger
MR RAPS
Weighted median
Weighted mode
0.0
−0.1
−0.2
0.05
0.10
0.15
SNP effect on Crohn's disease of small intestine
SNP effect on Trans−4−hydroxyproline

Batch 685 : Hexanoylcarnitine on Crohn's disease of small intestine

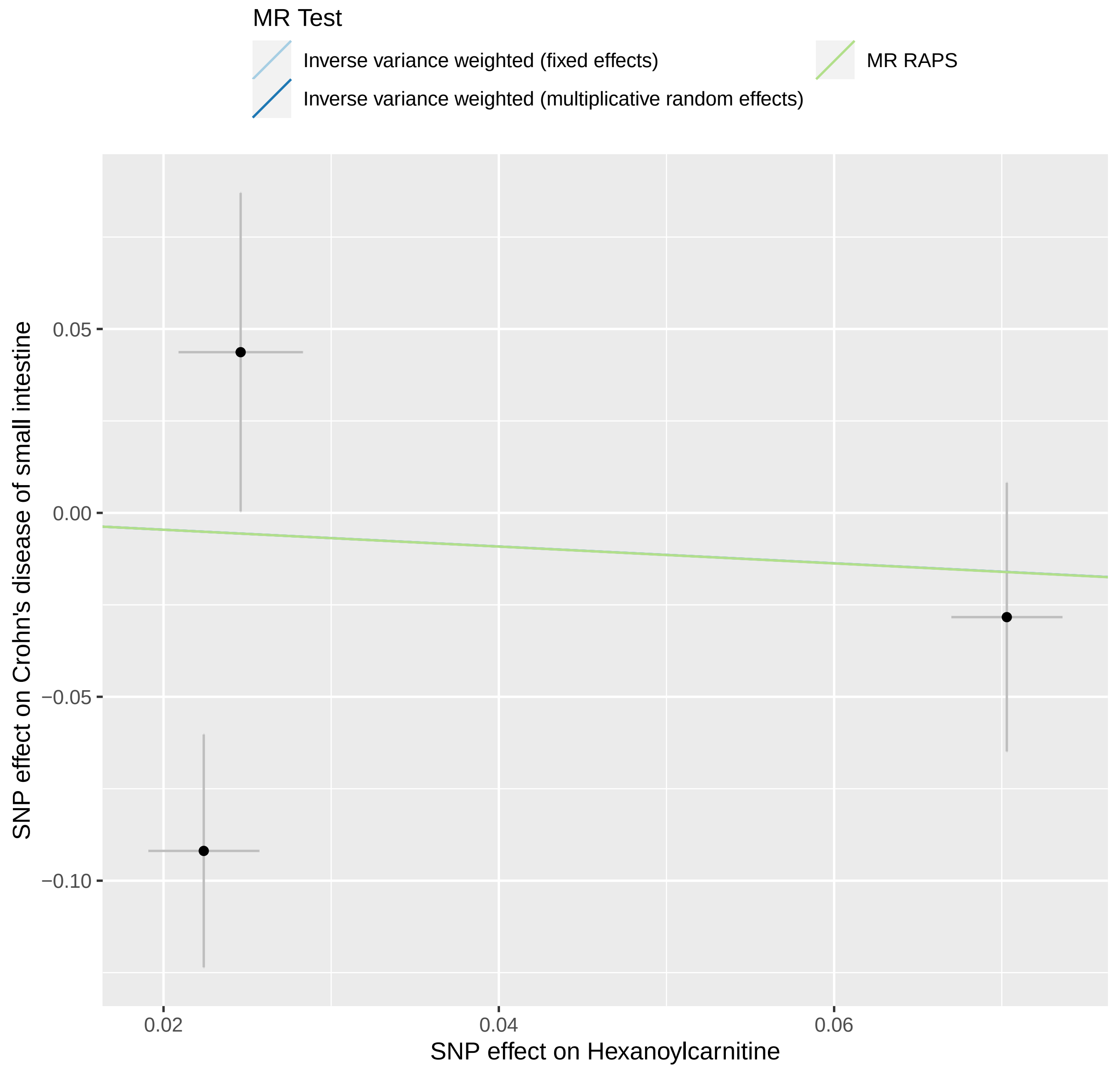

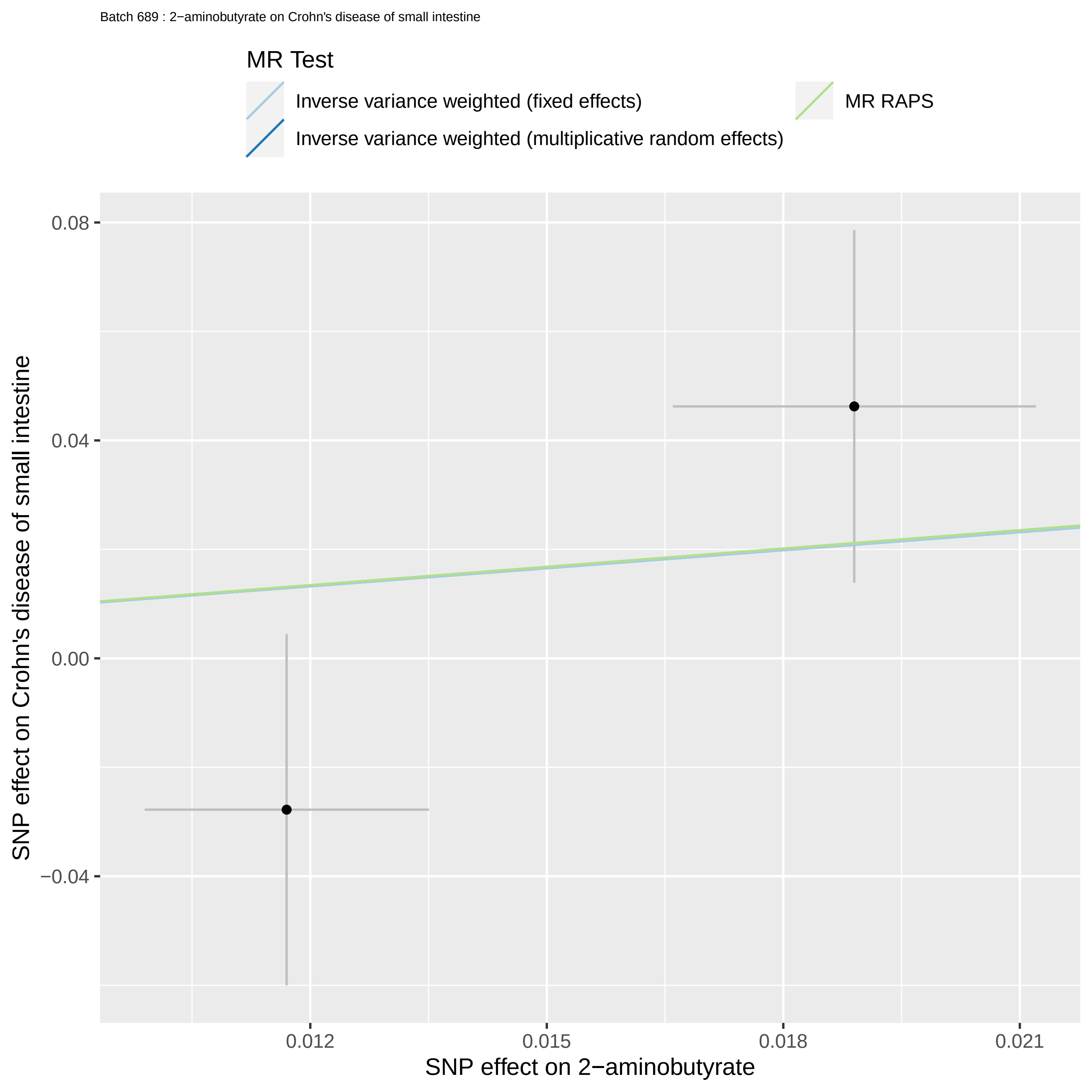
Batch 689 : 2−aminobutyrate on Crohn's disease of small intestine
MR Test
Inverse variance weighted (fixed effects)
Inverse variance weighted (multiplicative random effects)
MR RAPS
0.08
0.04
0.00
−0.04
0.012
0.015
0.018
0.021
SNP effect on 2−aminobutyrate
SNP effect on Crohn's disease of small intestine

Batch 694 : Butyrylcarnitine on Crohn's disease of small intestine

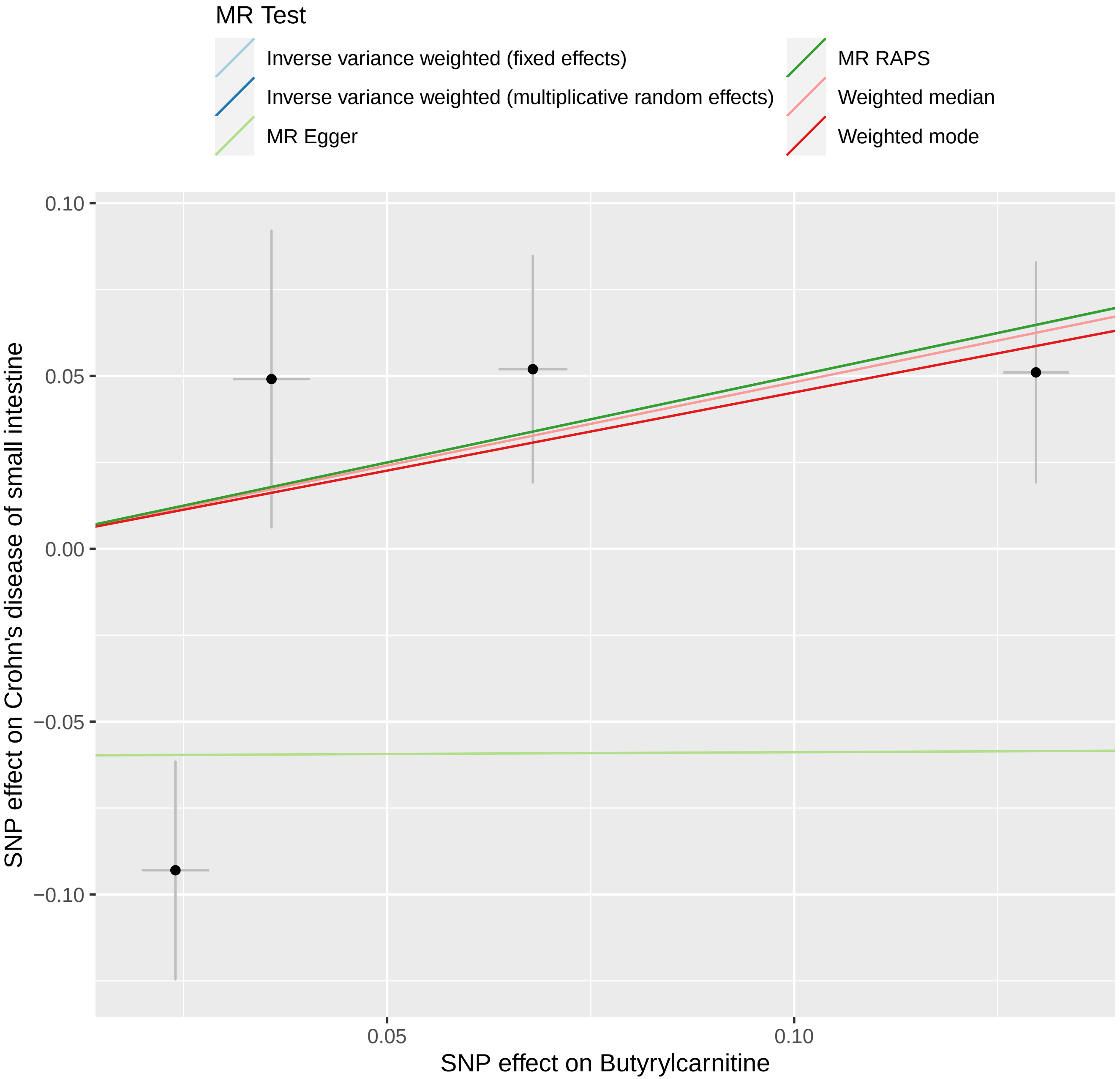

Batch 697 : Propionylcarnitine on Crohn's disease of small intestine

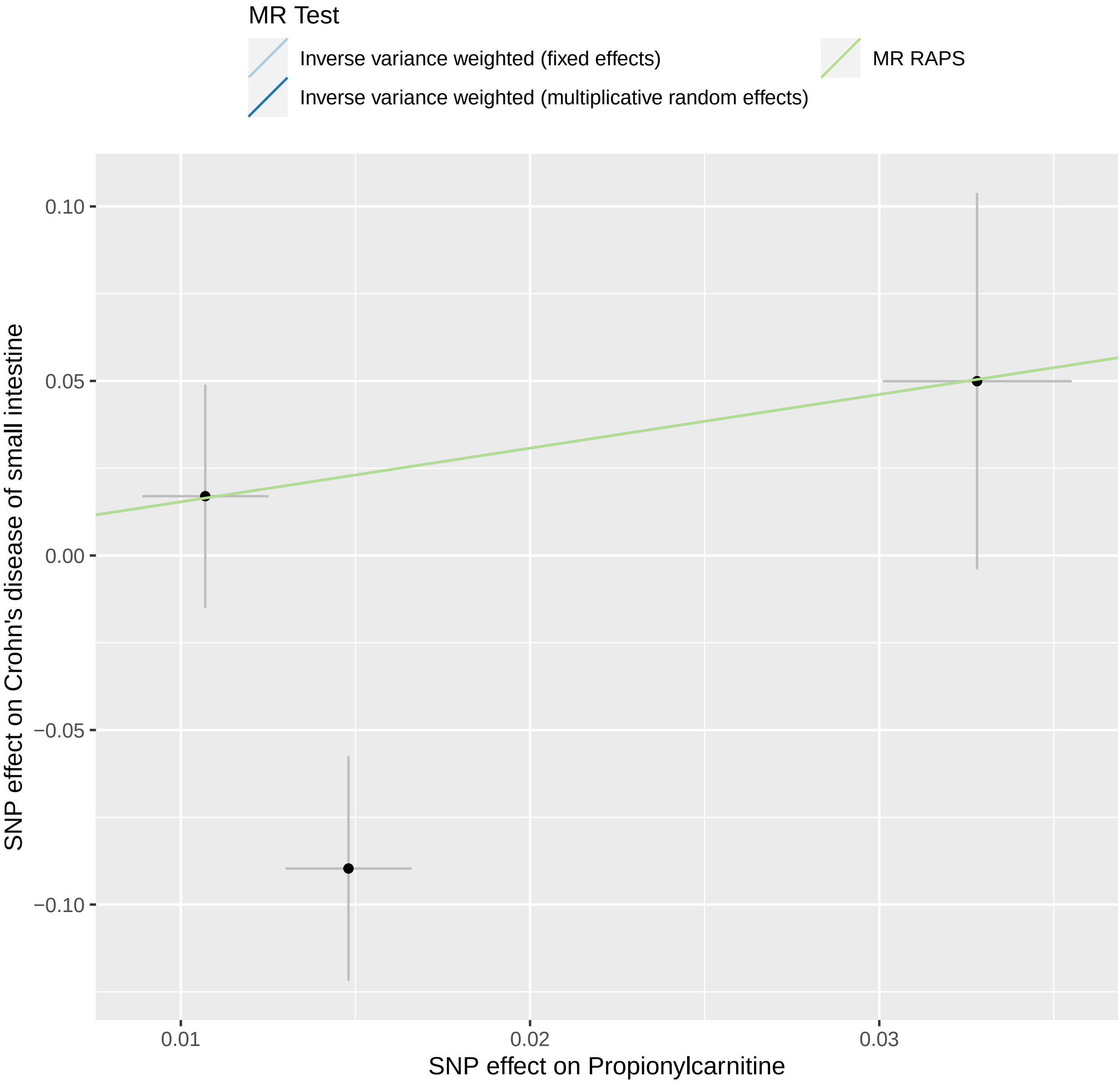

Batch 698 : Caproate (6:0) on Crohn's disease of small intestine

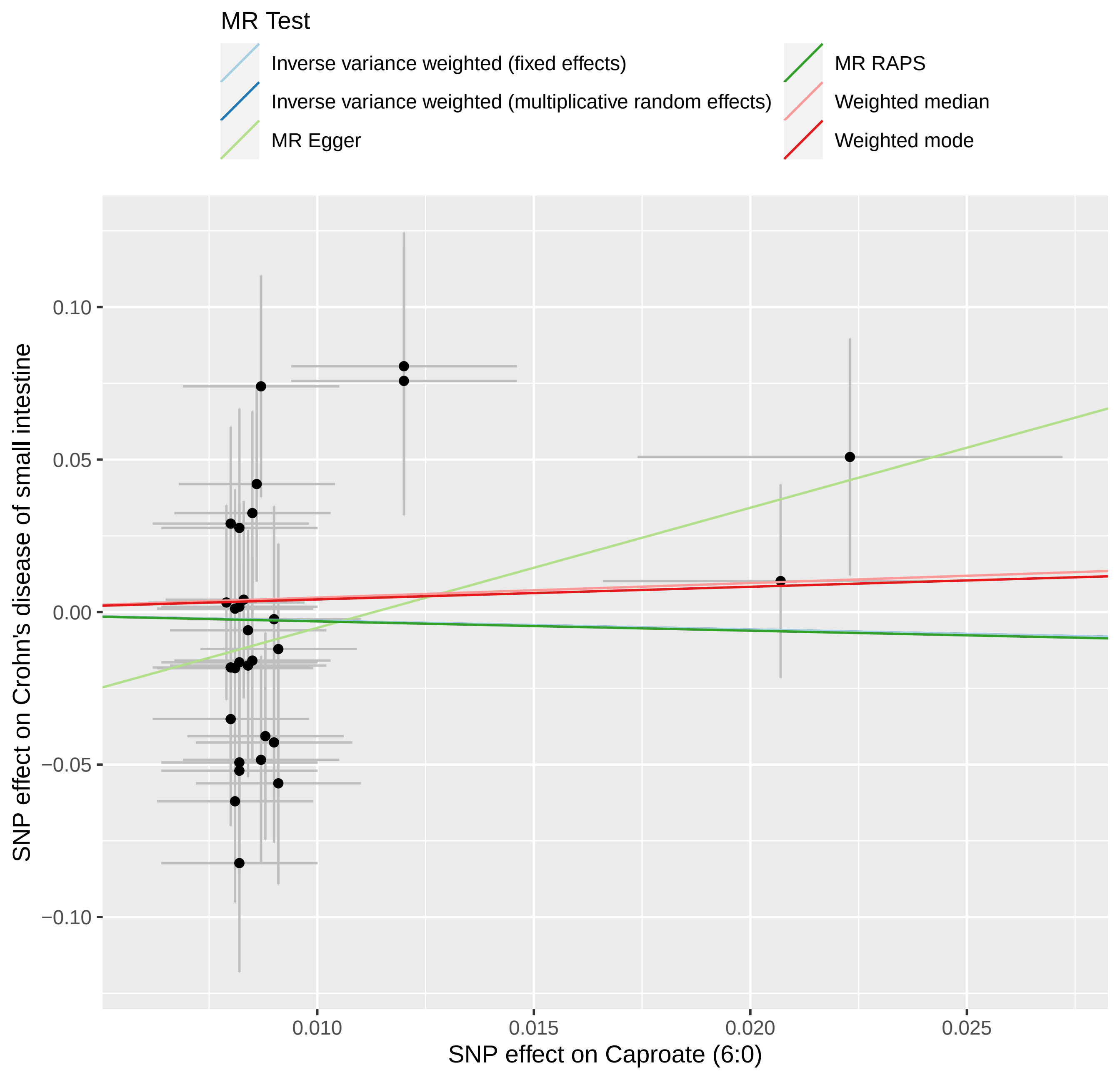

Batch 699 : Caprylate (8:0) on Crohn's disease of small intestine

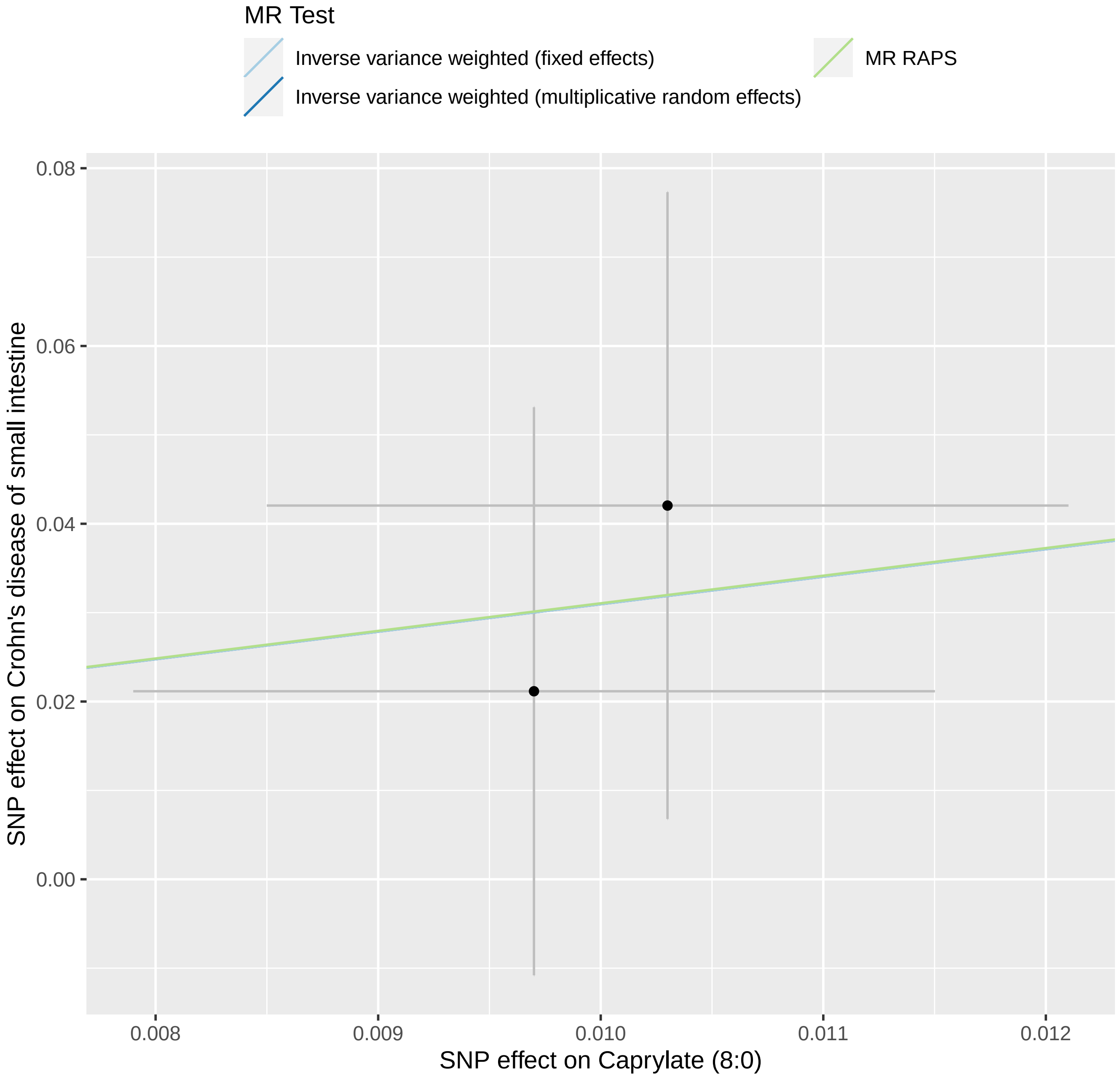

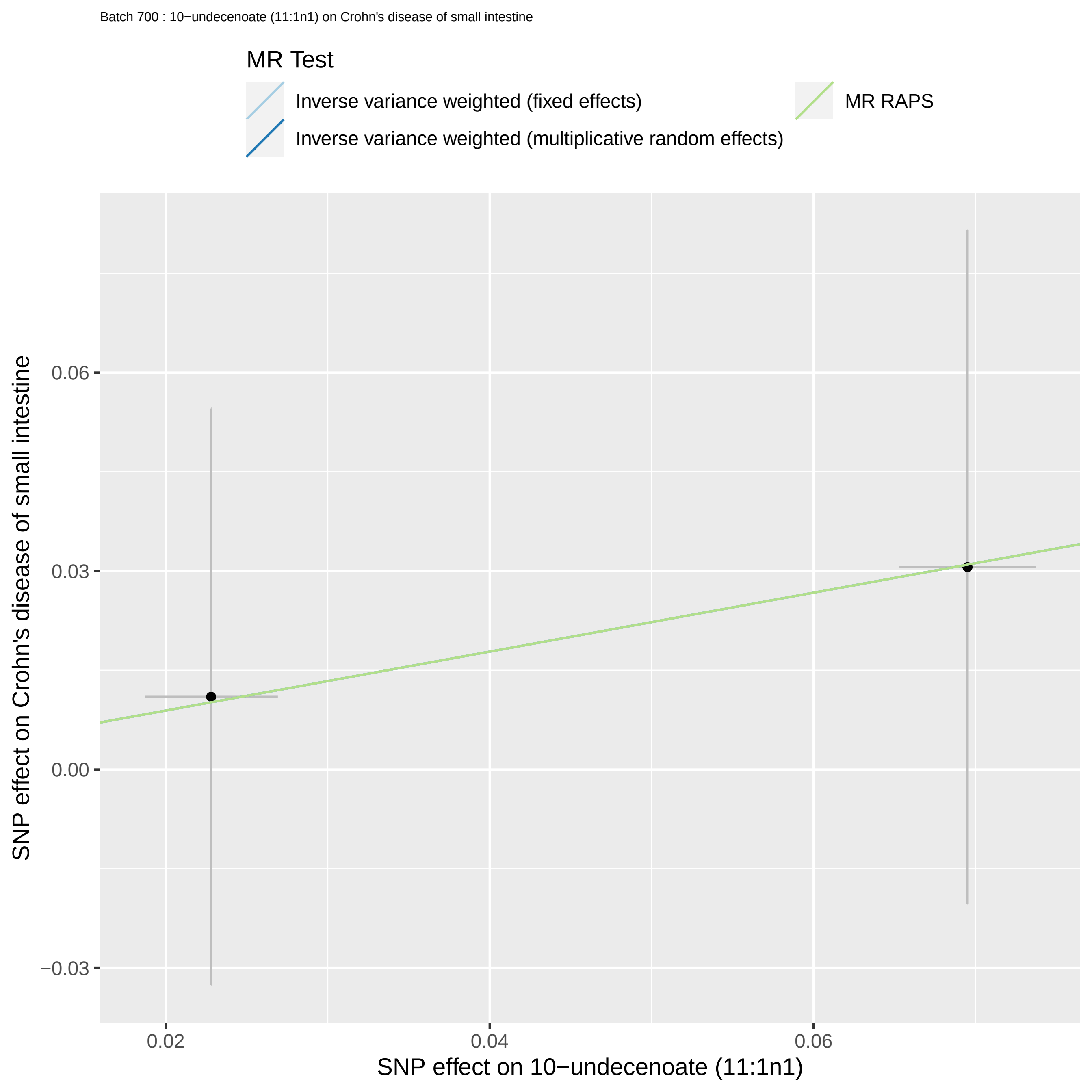
Batch 700 : 10−undecenoate (11:1n1) on Crohn's disease of small intestine
MR Test
Inverse variance weighted (fixed effects)
Inverse variance weighted (multiplicative random effects)
MR RAPS
SNP effect on Crohn's disease of small intestine
0.06
0.03
0.00
−0.03
0.02
0.04
0.06
SNP effect on 10−undecenoate (11:1n1)

Batch 702 : Phenol sulfate on Crohn's disease of small intestine

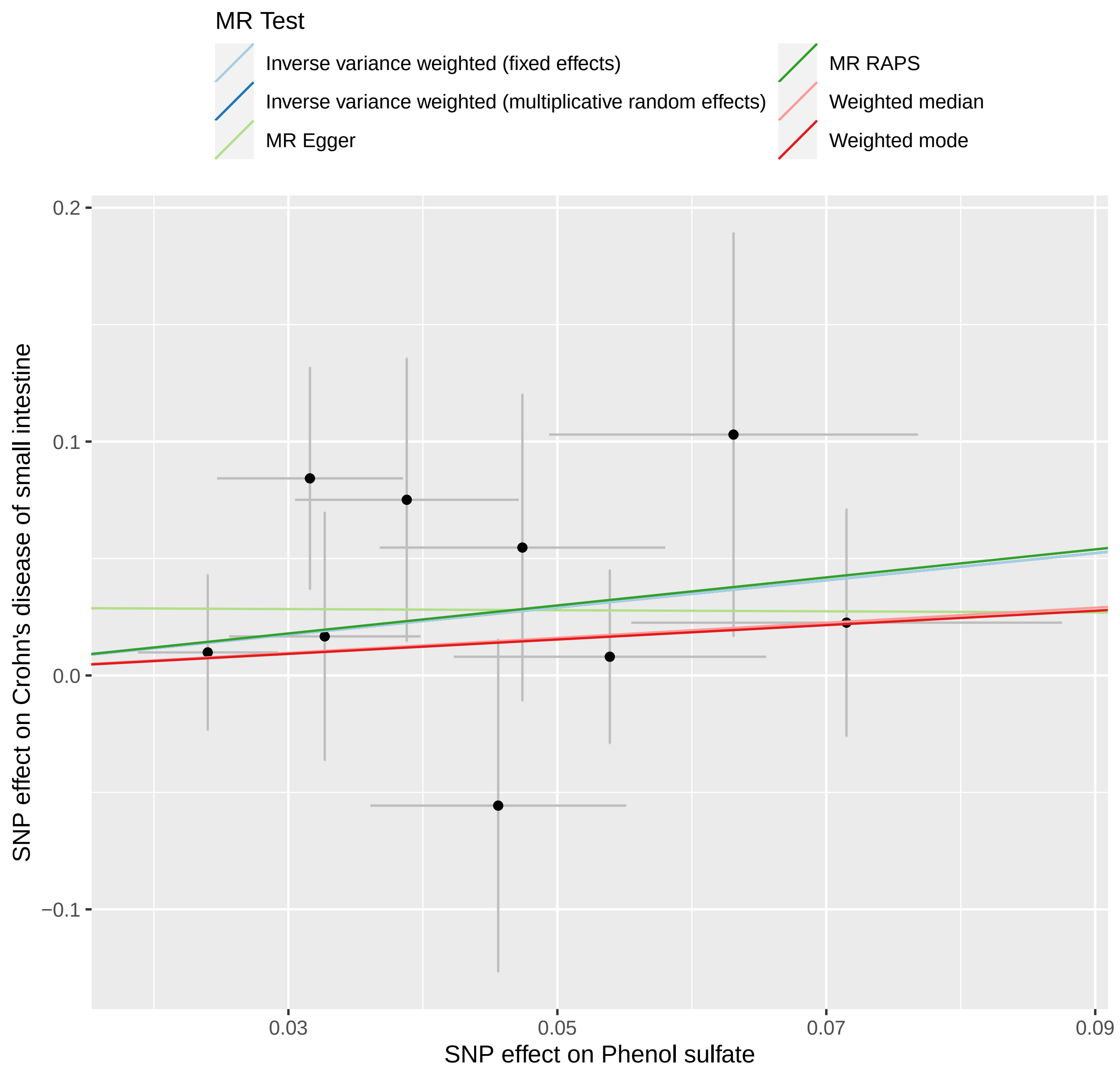

Batch 704 : Heme* on Crohn's disease of small intestine

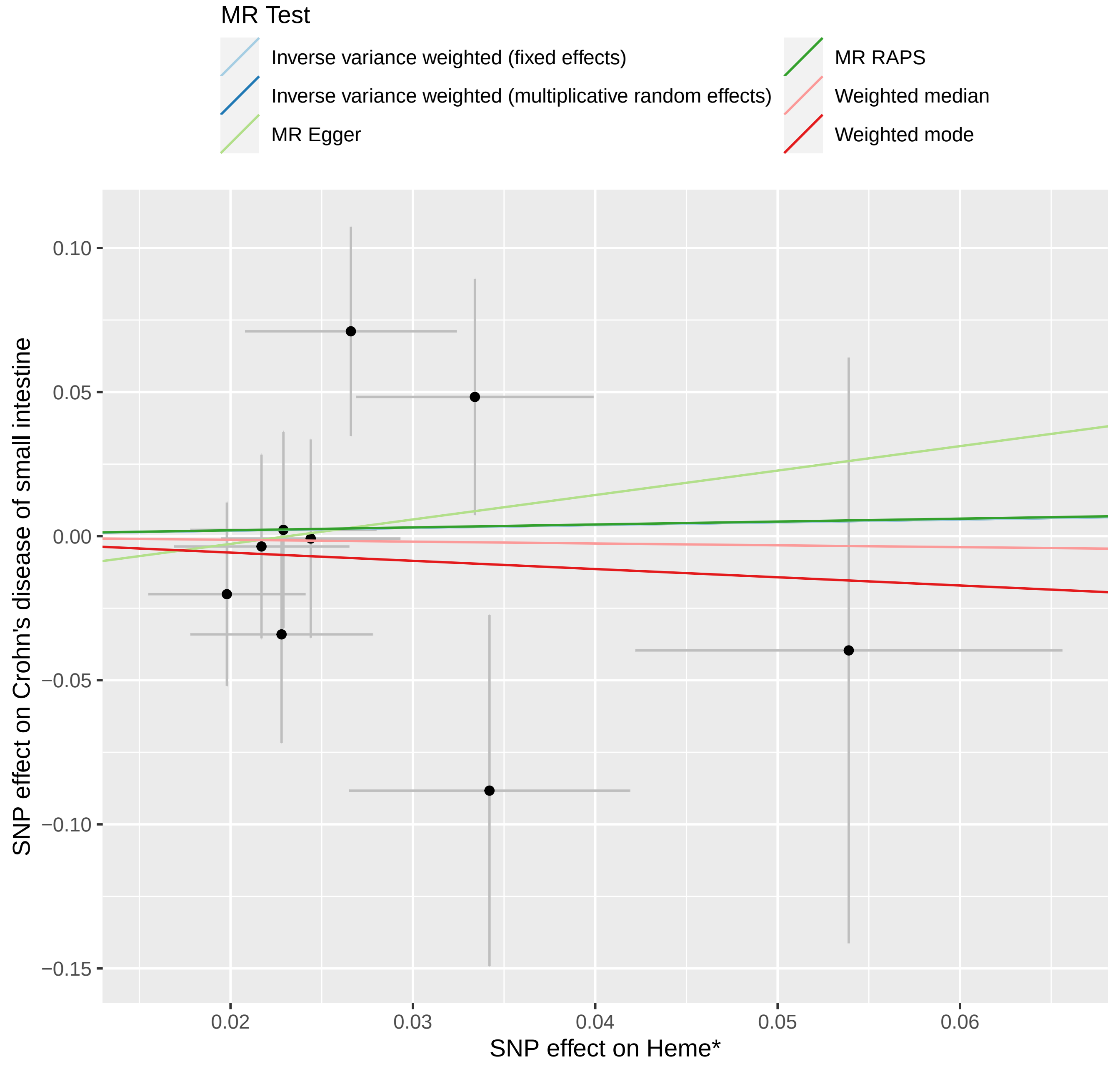

Batch 707 : Pyroglutamine* on Crohn's disease of small intestine

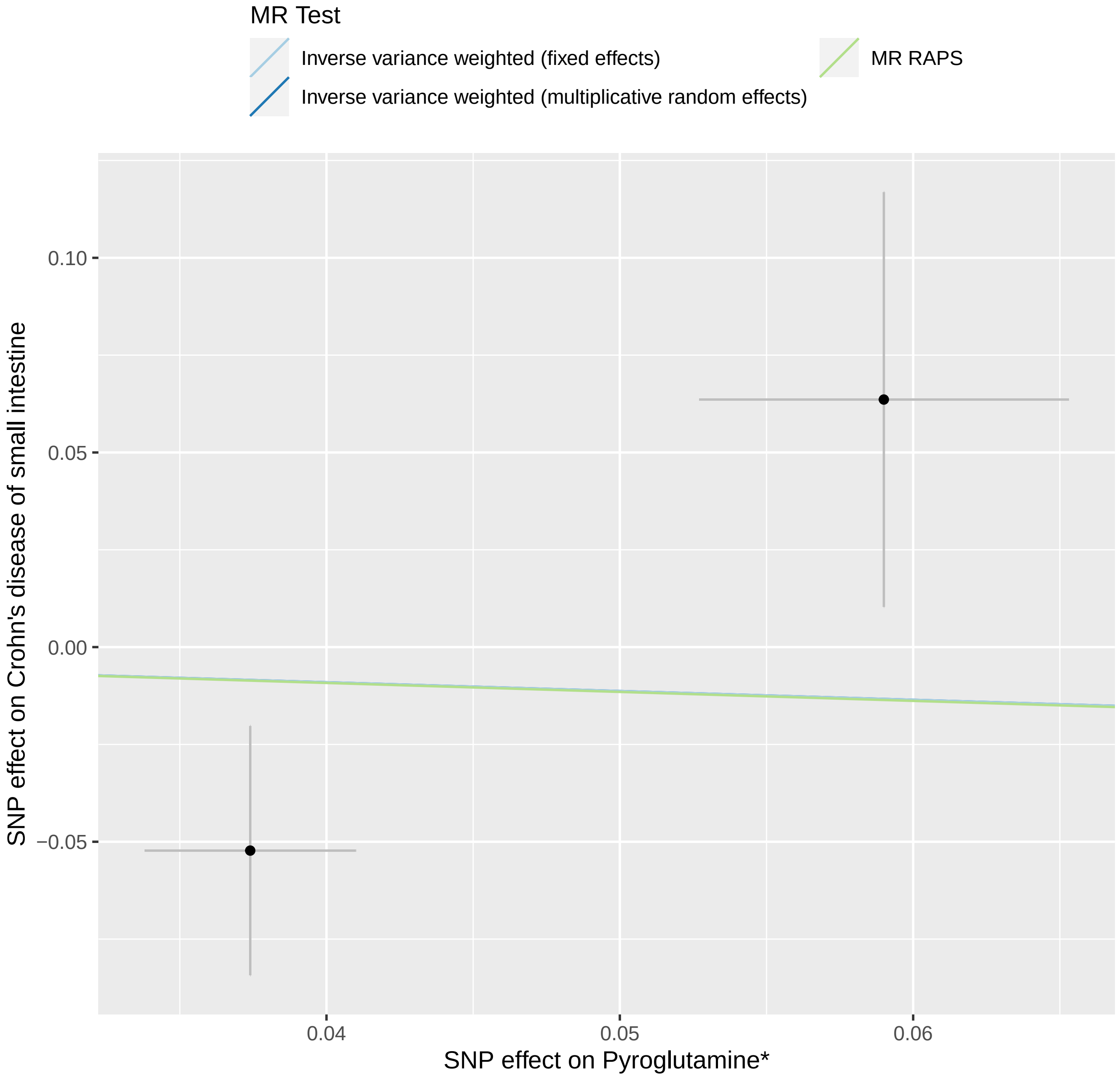

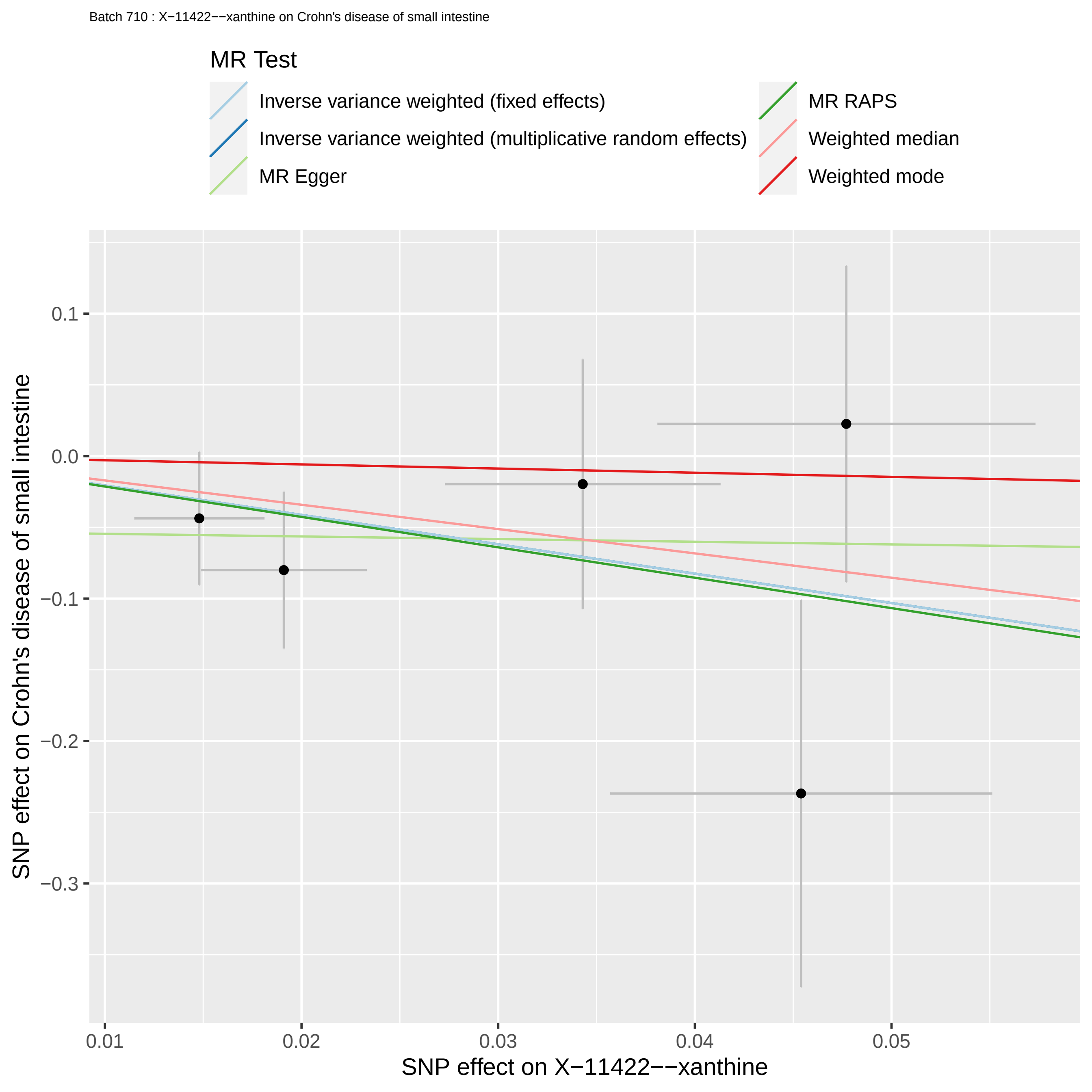

Batch 710 : X−11422−−xanthine on Crohn's disease of small intestine
MR Test
Inverse variance weighted (fixed effects)
Inverse variance weighted (multiplicative random effects)
MR Egger
MR RAPS
Weighted median
Weighted mode
0.1
0.0
−0.1
−0.2
−0.3
SNP effect on Crohn's disease of small intestine
0.01
0.02
0.03
0.04
0.05
SNP effect on X−11422−−xanthine

Batch 713 : HWESASXX* on Crohn's disease of small intestine

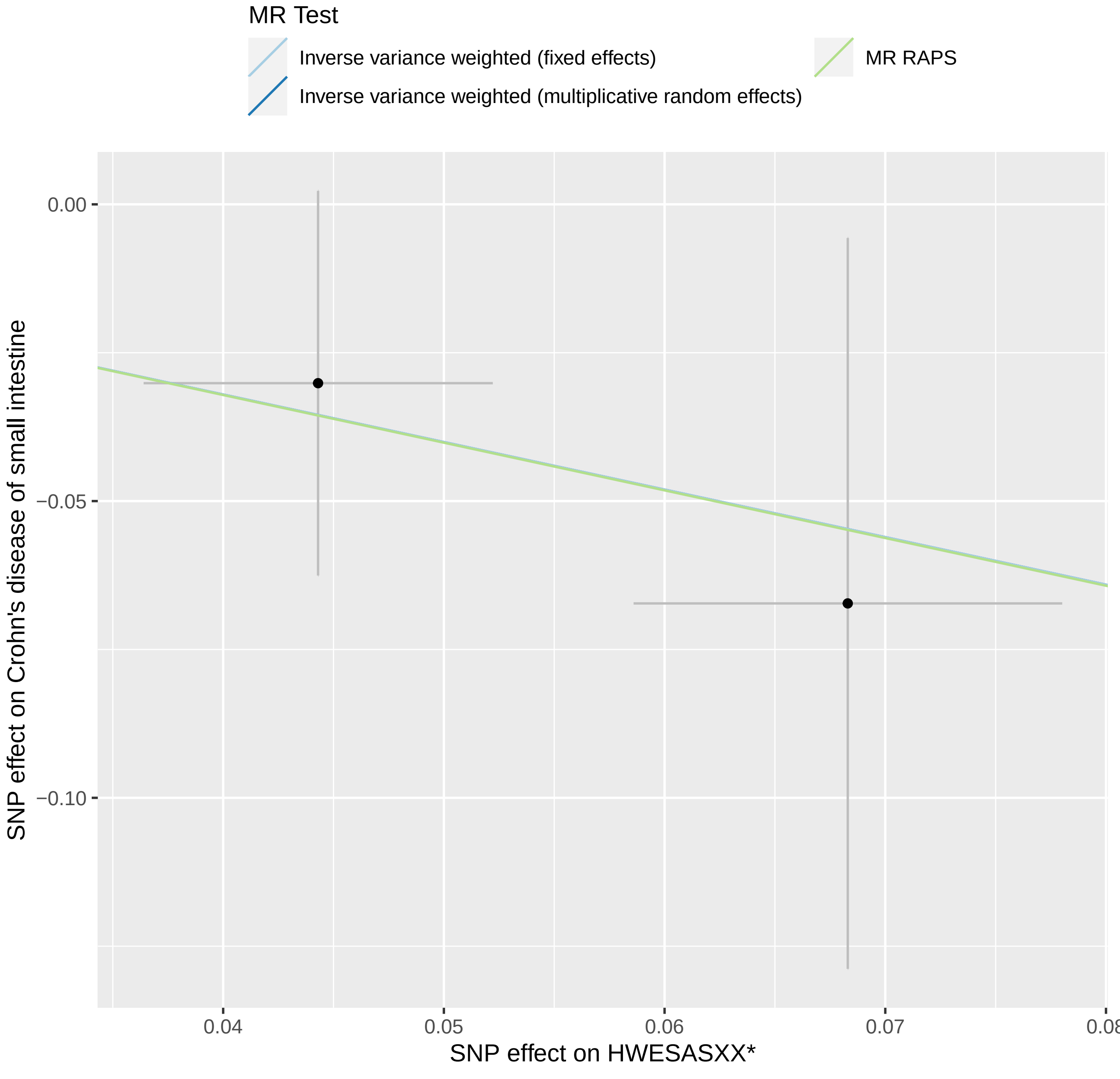

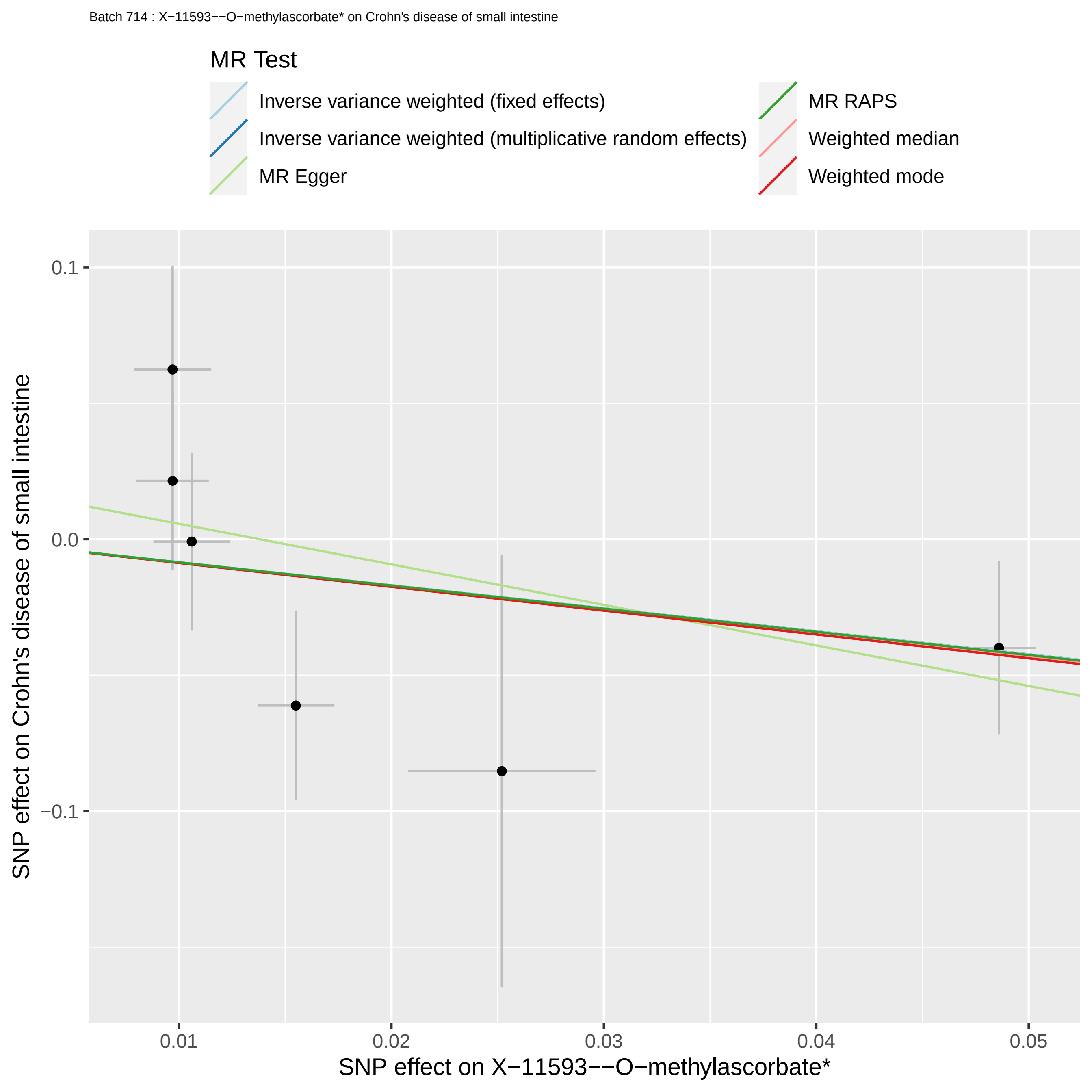

Batch 714 : X−11593−−O−methylascorbate* on Crohn's disease of small intestine
MR Test
Inverse variance weighted (fixed effects)
Inverse variance weighted (multiplicative random effects)
MR Egger
MR RAPS
Weighted median
Weighted mode
0.1
0.0
−0.1
0.01
0.02
0.03
0.04
0.05
SNP effect on Crohn's disease of small intestine
SNP effect on X−11593−−O−methylascorbate*

Batch 716 : Homostachydrine* on Crohn's disease of small intestine

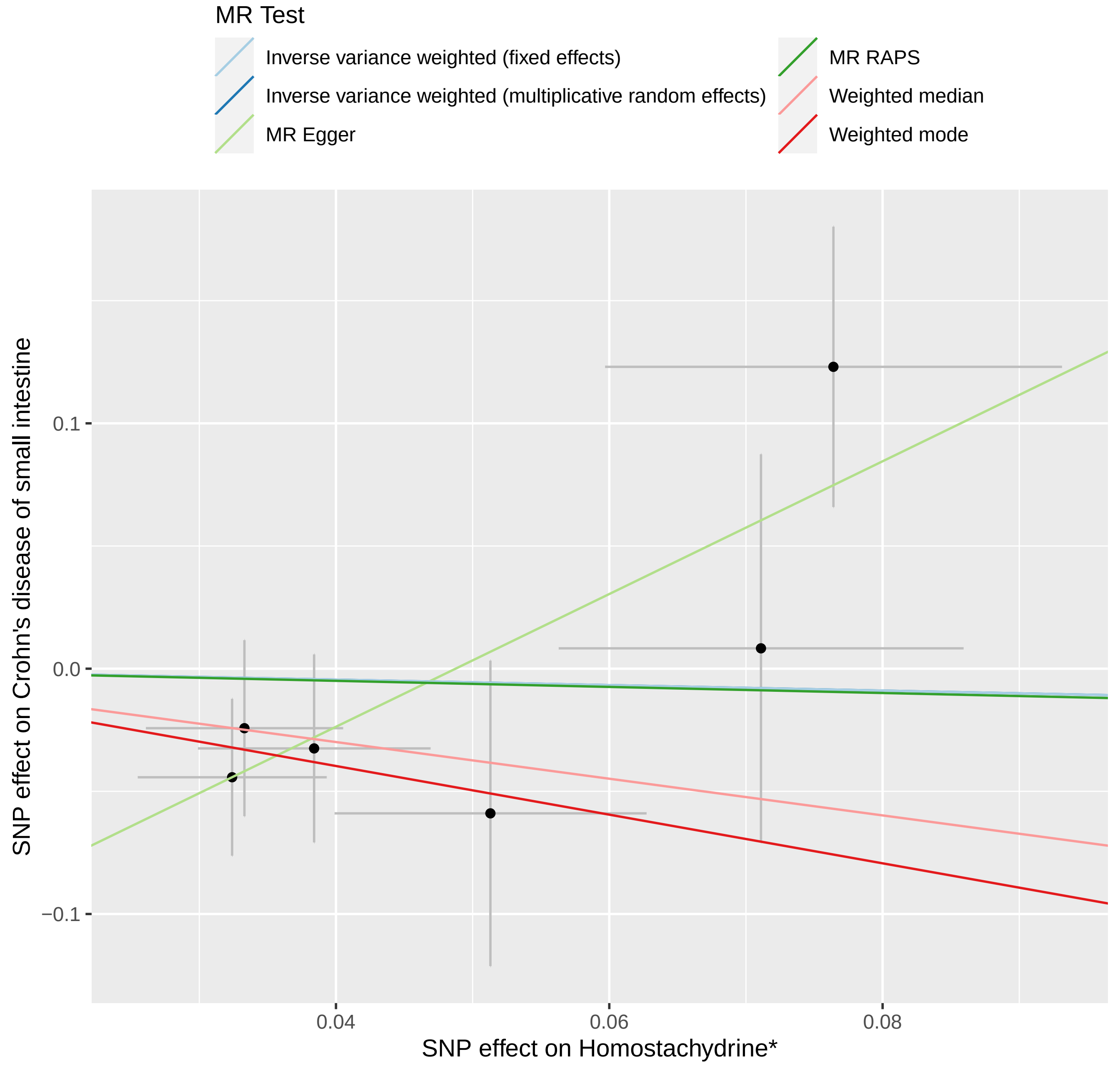

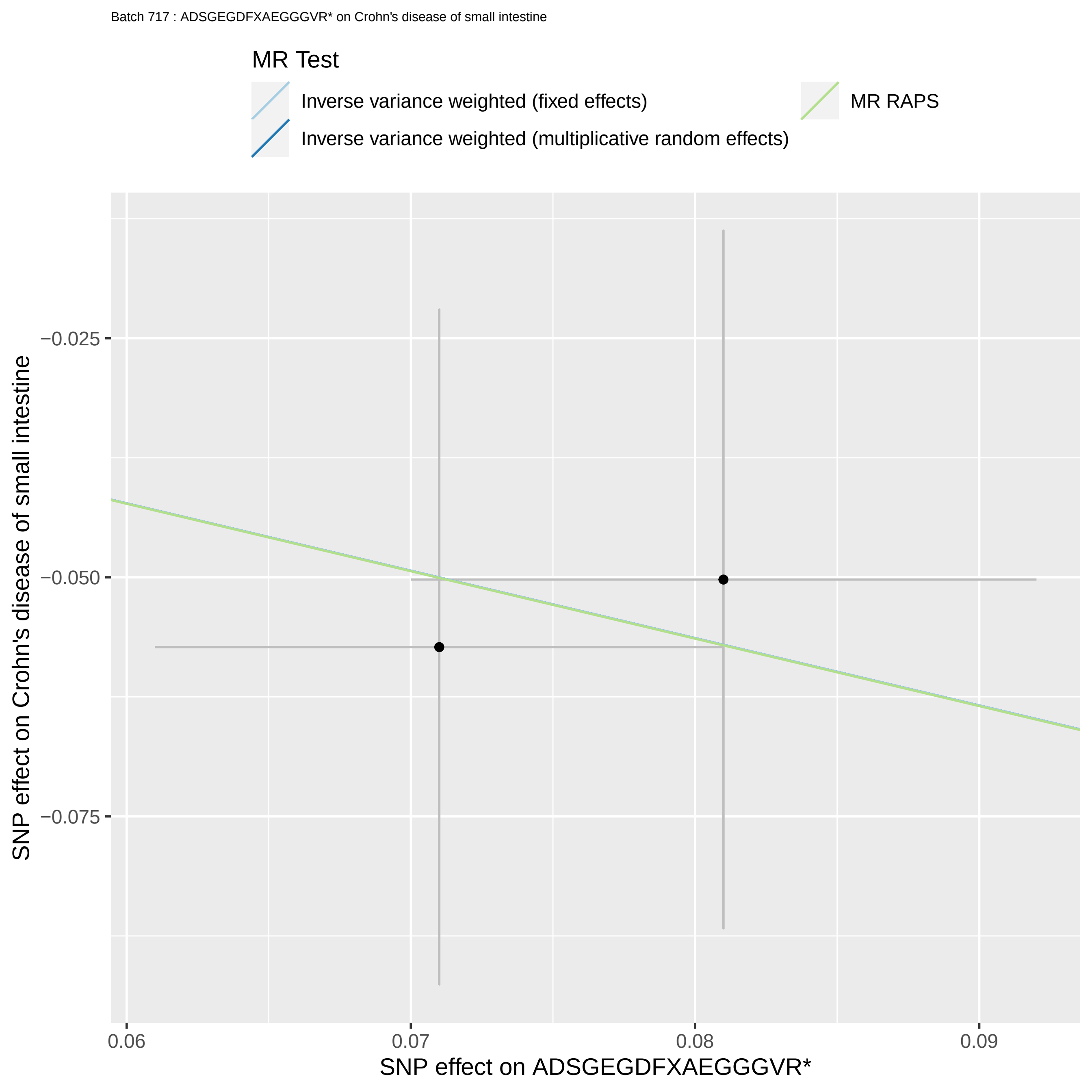
Batch 717 : ADSGEGDFXAEGGGVR* on Crohn's disease of small intestine
MR Test
Inverse variance weighted (fixed effects)
Inverse variance weighted (multiplicative random effects)
MR RAPS
−0.025
−0.050
−0.075
0.06
0.07
0.08
0.09
SNP effect on Crohn's disease of small intestine
SNP effect on ADSGEGDFXAEGGGVR*

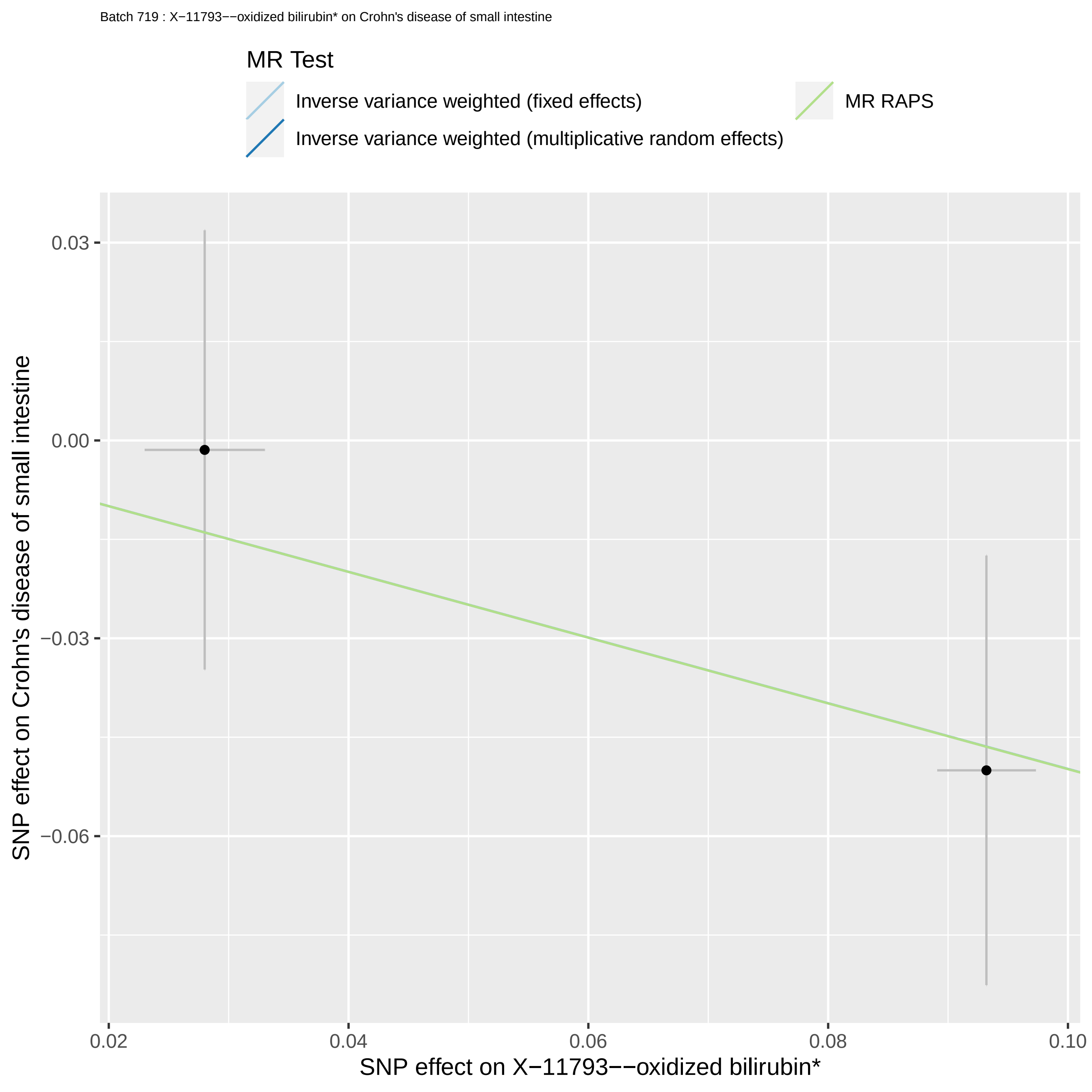
Batch 719 : X−11793−−oxidized bilirubin* on Crohn's disease of small intestine
MR Test
Inverse variance weighted (fixed effects)
Inverse variance weighted (multiplicative random effects)
MR RAPS
0.03
0.00
−0.03
−0.06
0.02
0.04
0.06
0.08
0.10
SNP effect on Crohn's disease of small intestine
SNP effect on X−11793−−oxidized bilirubin*

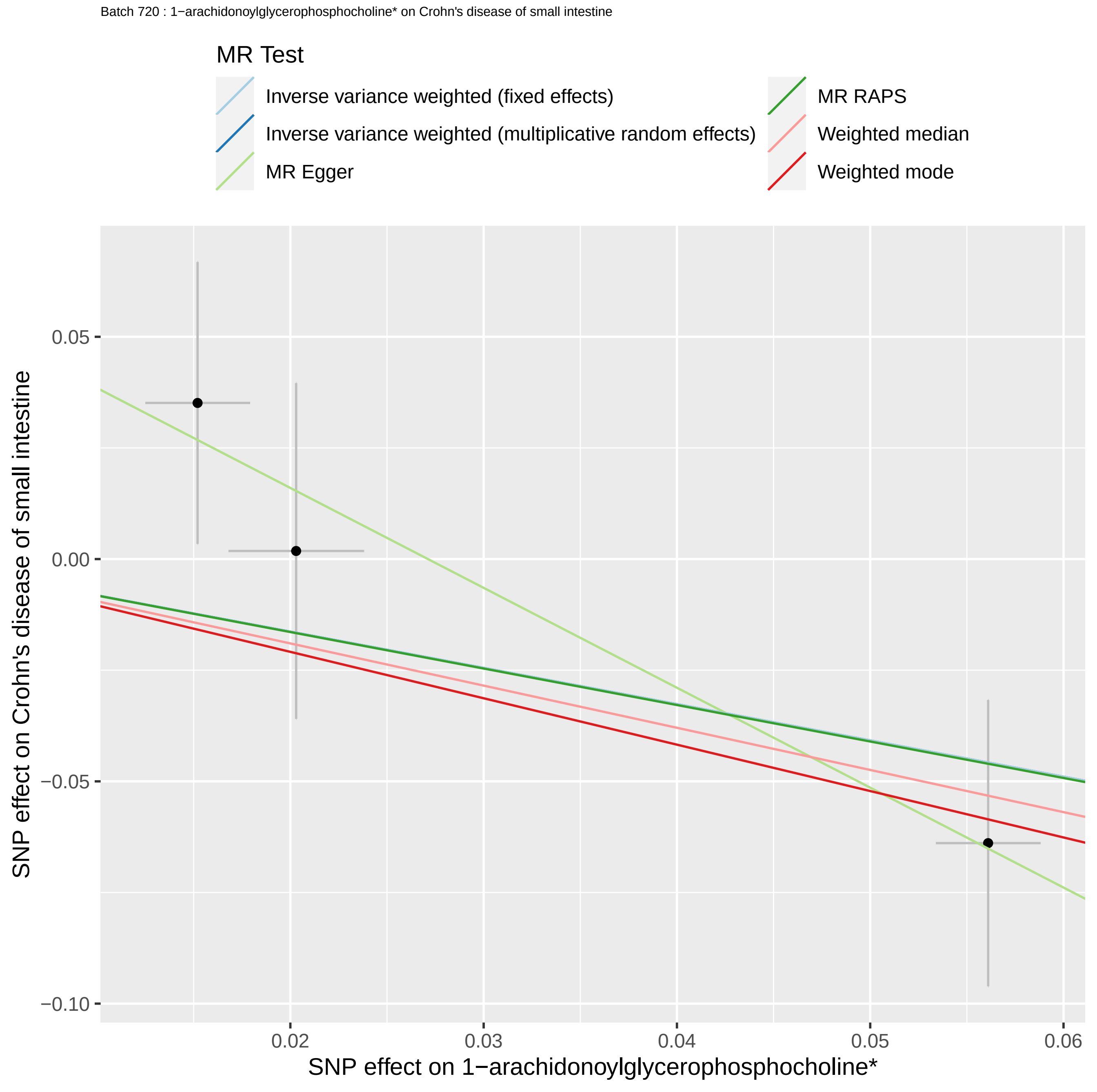

Batch 720 : 1−arachidonoylglycerophosphocholine* on Crohn's disease of small intestine
MR Test
Inverse variance weighted (fixed effects)
Inverse variance weighted (multiplicative random effects)
MR Egger
MR RAPS
Weighted median
Weighted mode
0.05
0.00
−0.05
−0.10
0.02
0.03
0.04
0.05
0.06
SNP effect on Crohn's disease of small intestine
SNP effect on 1−arachidonoylglycerophosphocholine*

Batch 722 : Gamma−glutamylmethionine* on Crohn's disease of small intestine

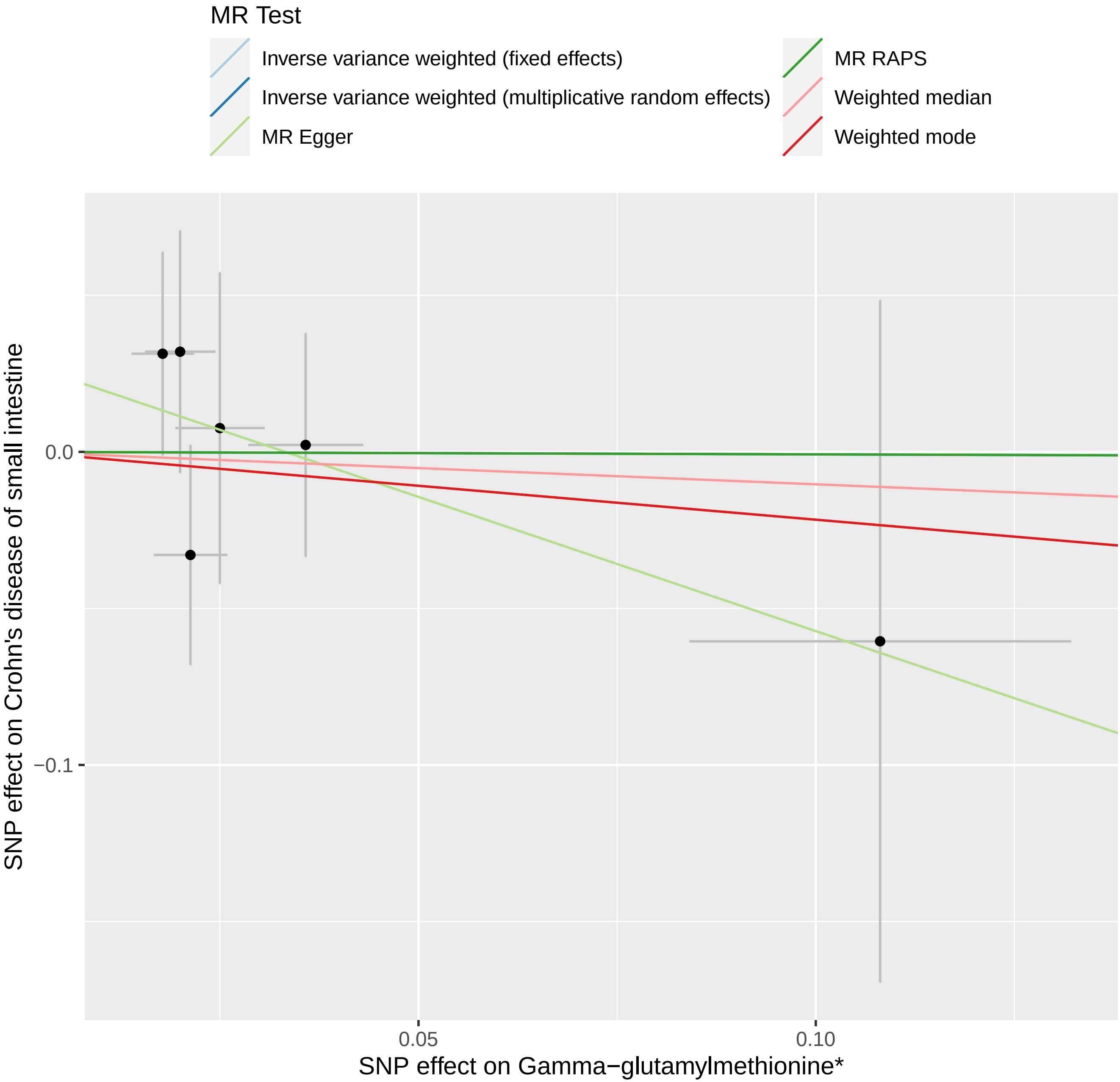

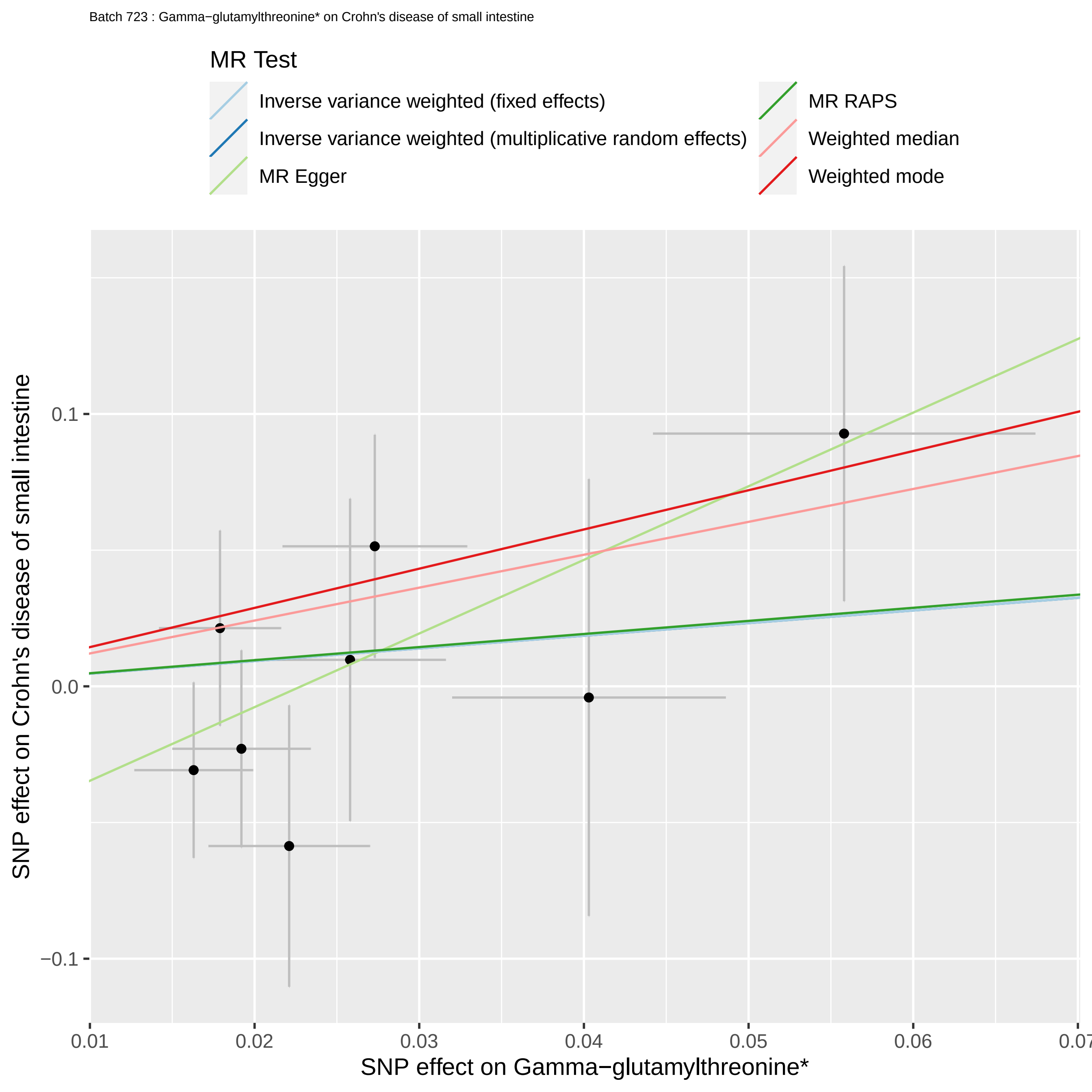
Batch 723 : Gamma−glutamylthreonine* on Crohn's disease of small intestine
MR Test
Inverse variance weighted (fixed effects)
Inverse variance weighted (multiplicative random effects)
MR Egger
MR RAPS
Weighted median
Weighted mode
0.1
0.0
−0.1
0.01
0.02
0.03
0.04
0.05
0.06
0.07
SNP effect on Crohn's disease of small intestine
SNP effect on Gamma−glutamylthreonine*

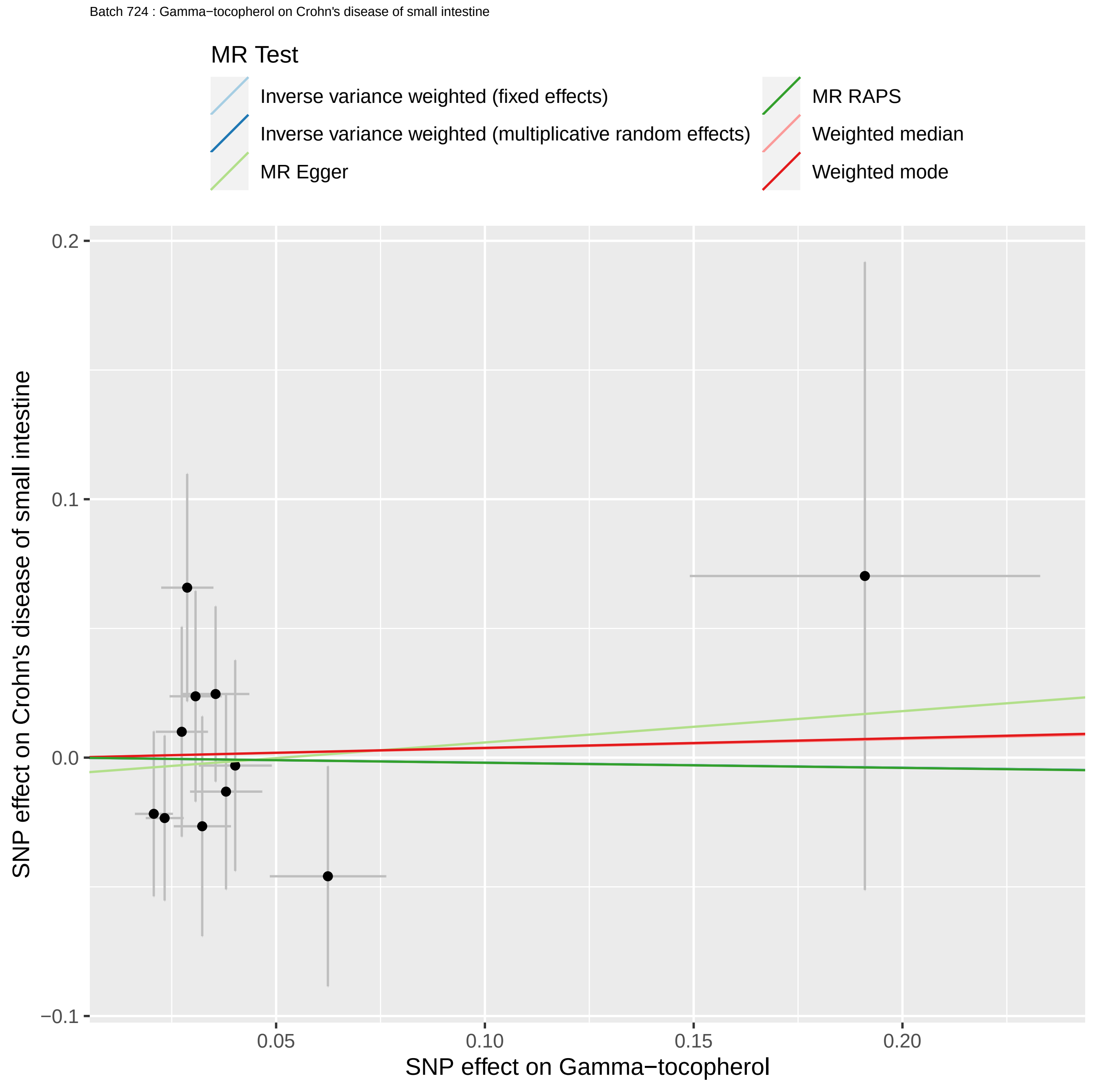

Batch 724 : Gamma−tocopherol on Crohn's disease of small intestine
MR Test
Inverse variance weighted (fixed effects)
Inverse variance weighted (multiplicative random effects)
MR Egger
MR RAPS
Weighted median
Weighted mode
SNP effect on Crohn's disease of small intestine
SNP effect on Gamma−tocopherol
0.2
0.1
0.0
−0.1
0.05
0.10
0.15
0.20

Batch 725 : Gamma−glutamylphenylalanine on Crohn's disease of small intestine

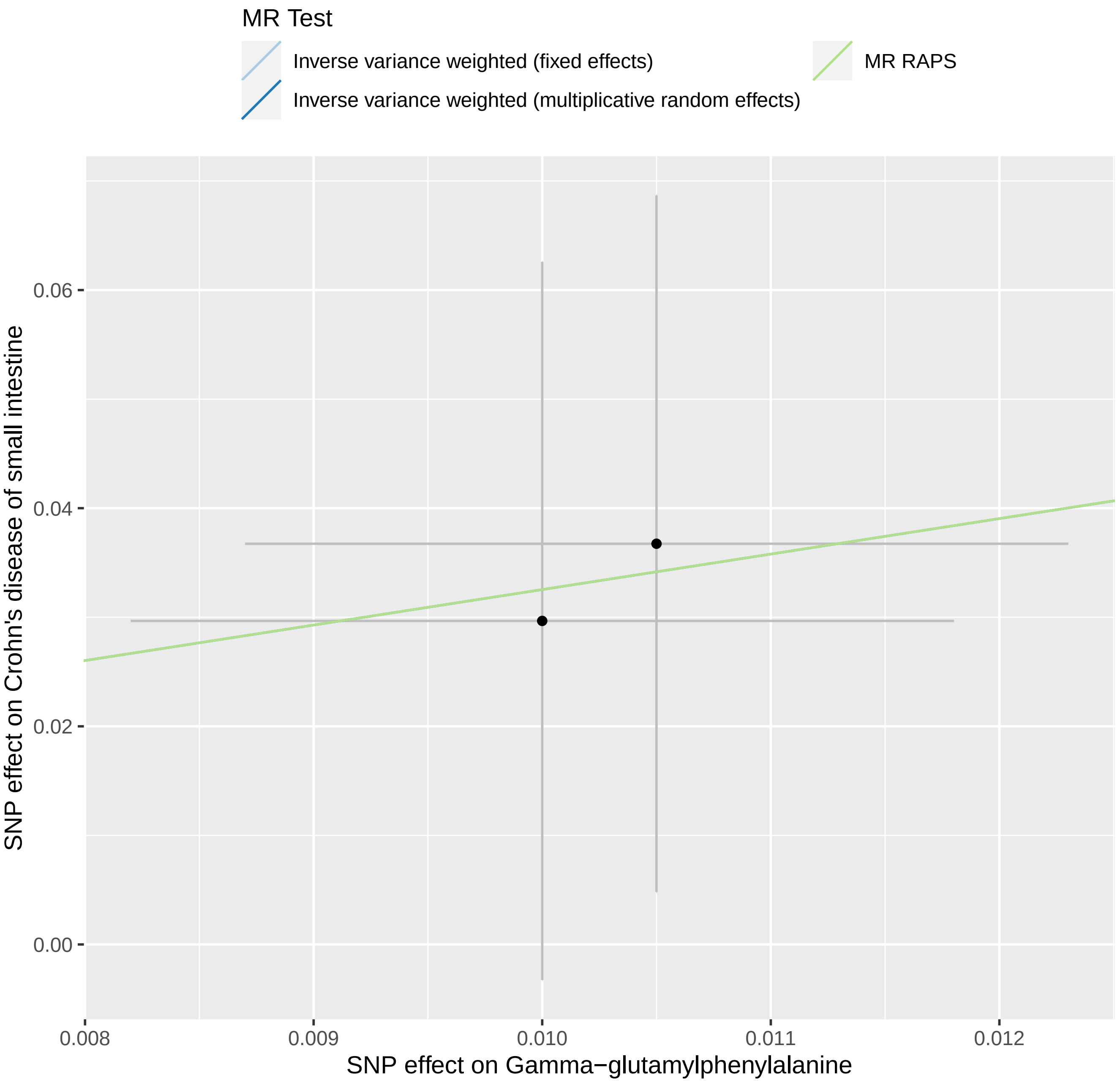

Batch 726 : Isobutyrylcarnitine on Crohn's disease of small intestine

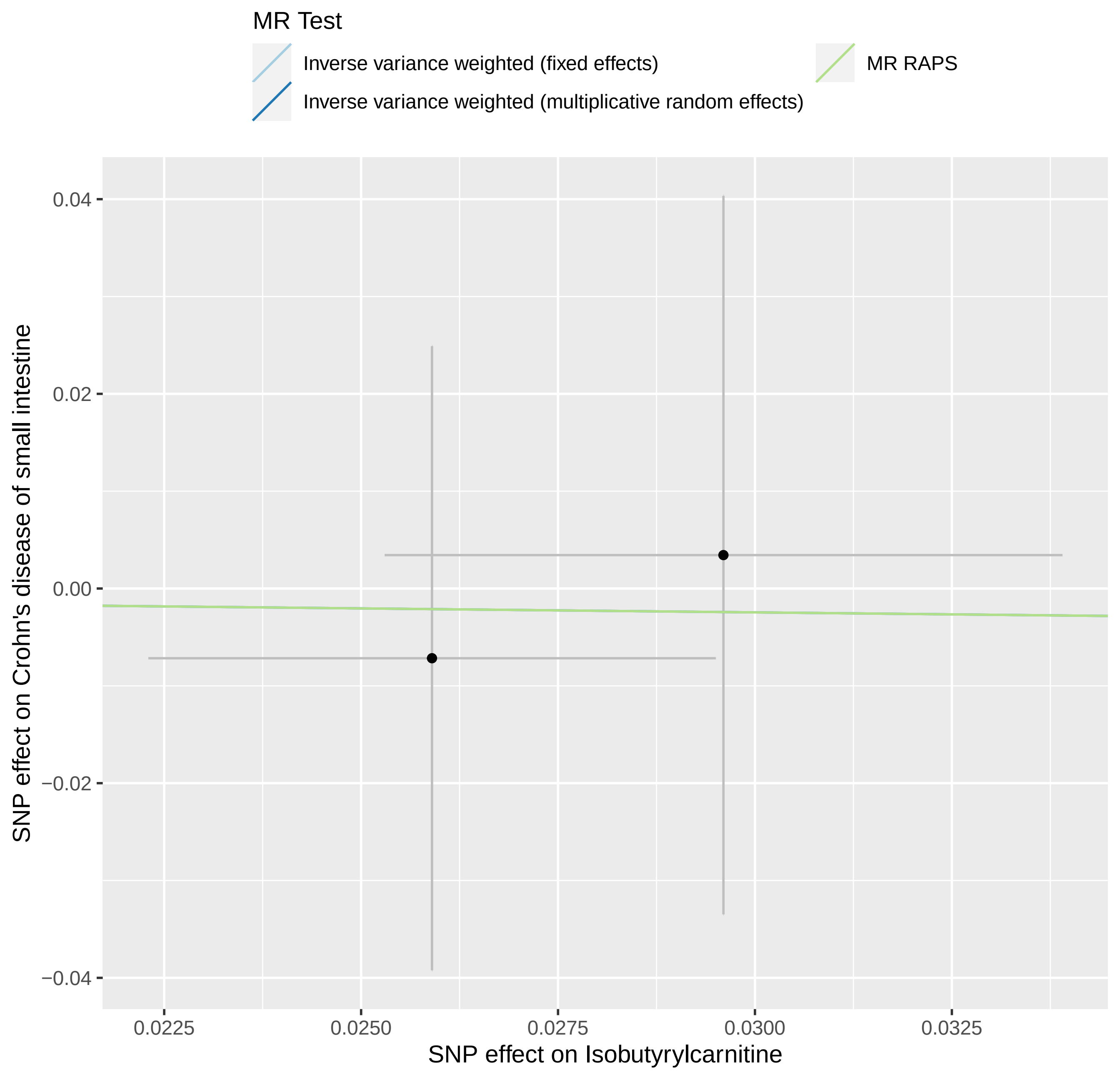

Batch 727 : Pseudouridine on Crohn's disease of small intestine

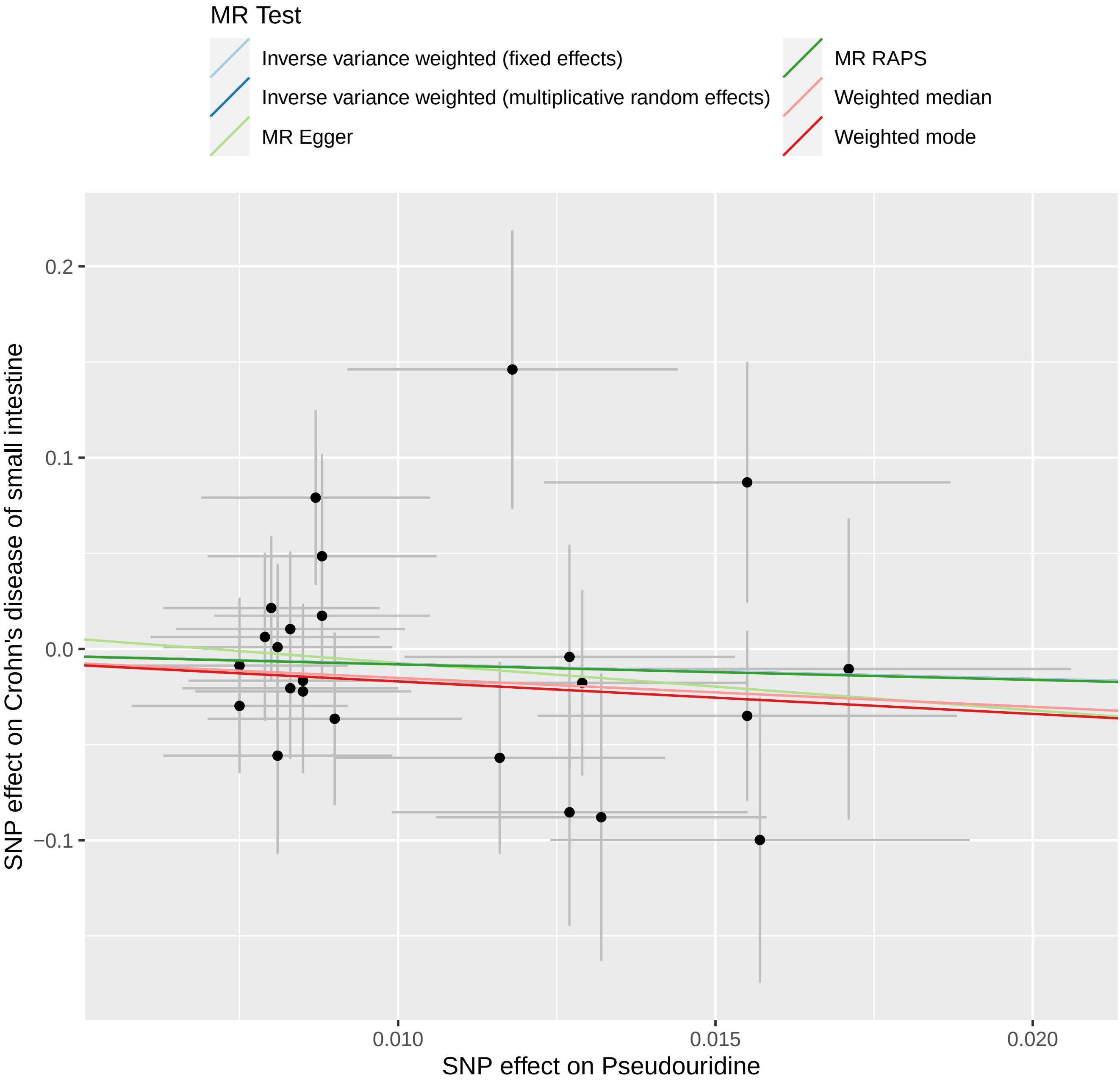

Batch 728 : Valerate on Crohn's disease of small intestine

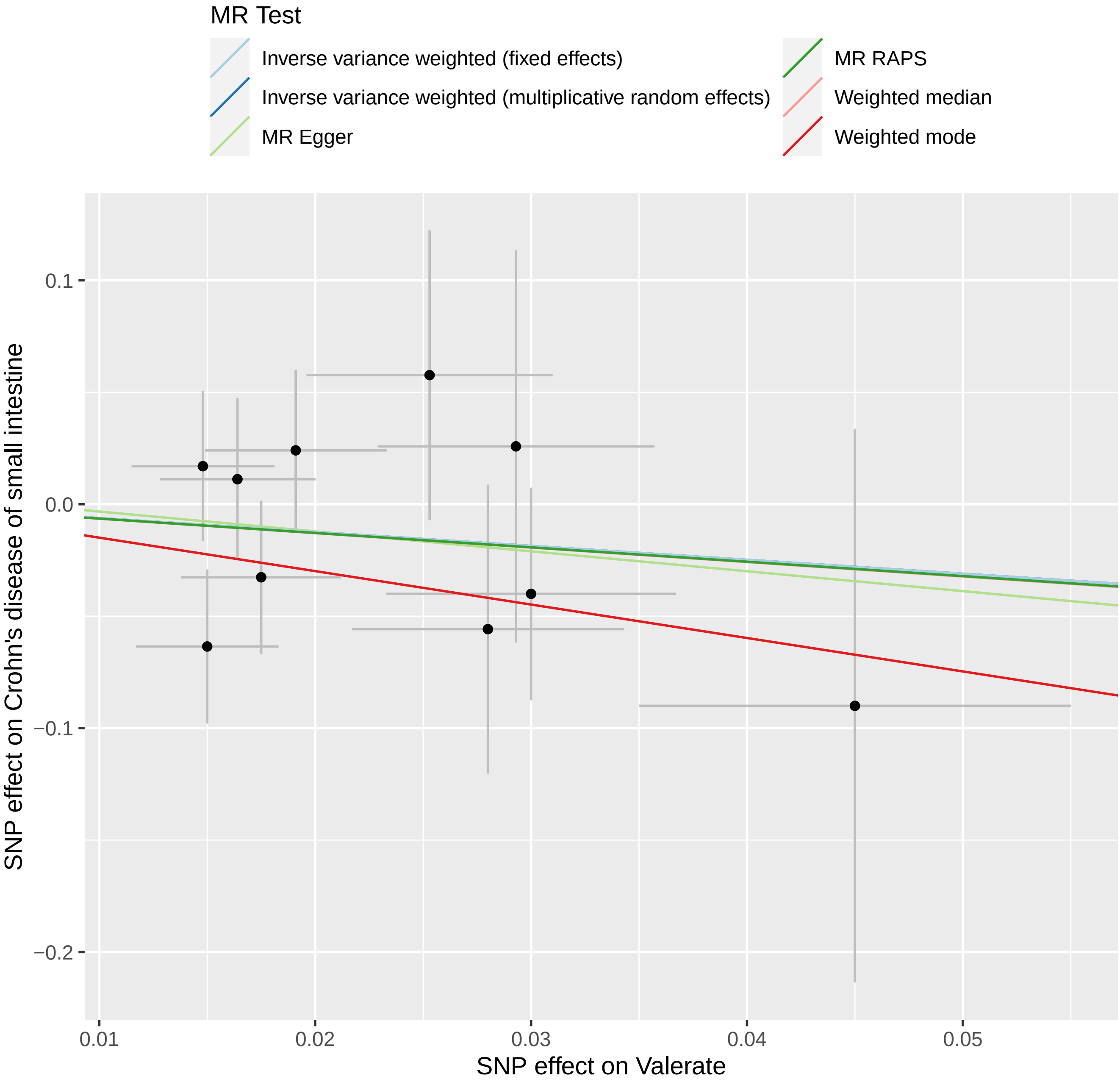

Batch 731 : Erythronate* on Crohn's disease of small intestine

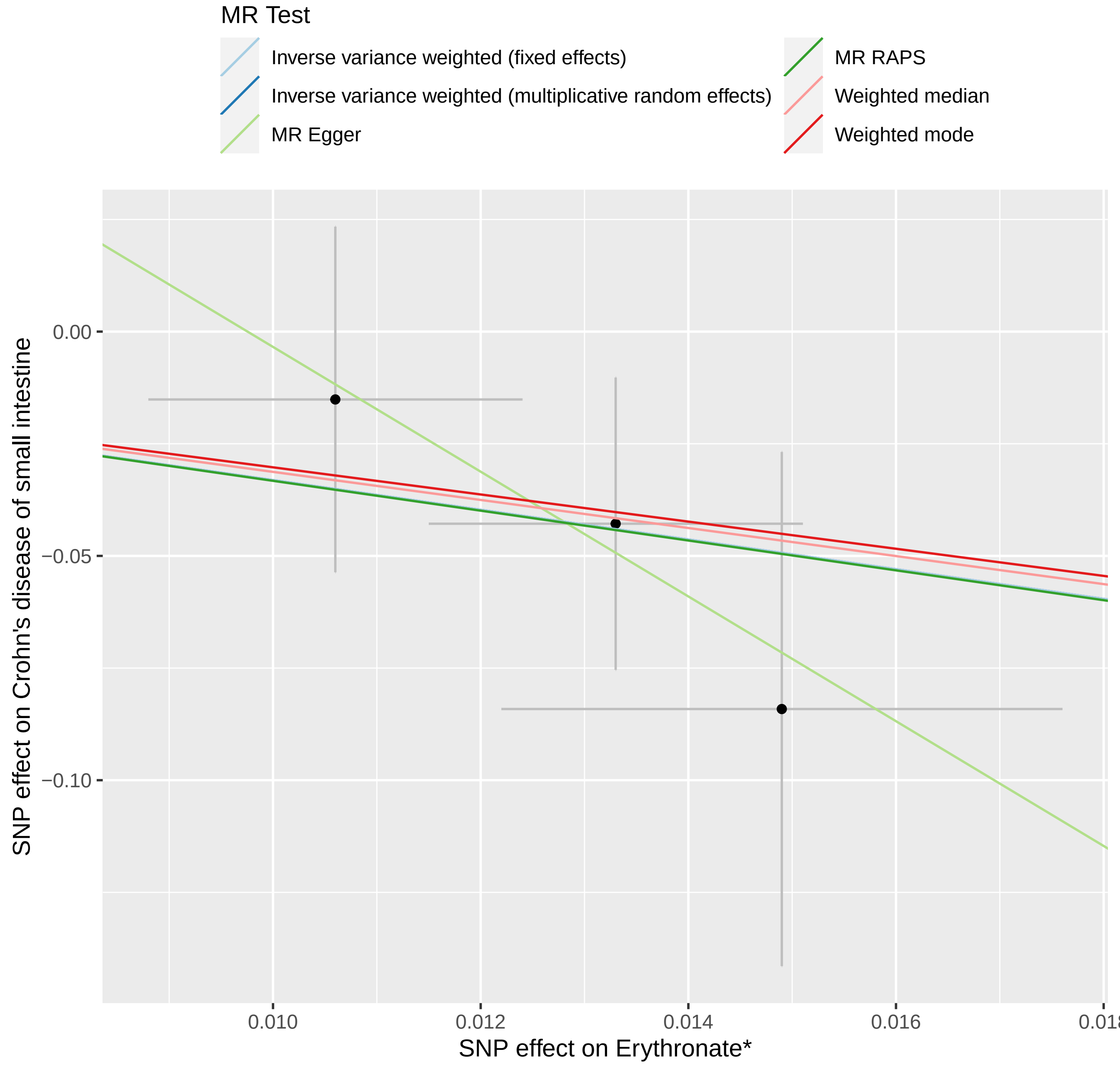

Batch 732 : Lathosterol on Crohn's disease of small intestine

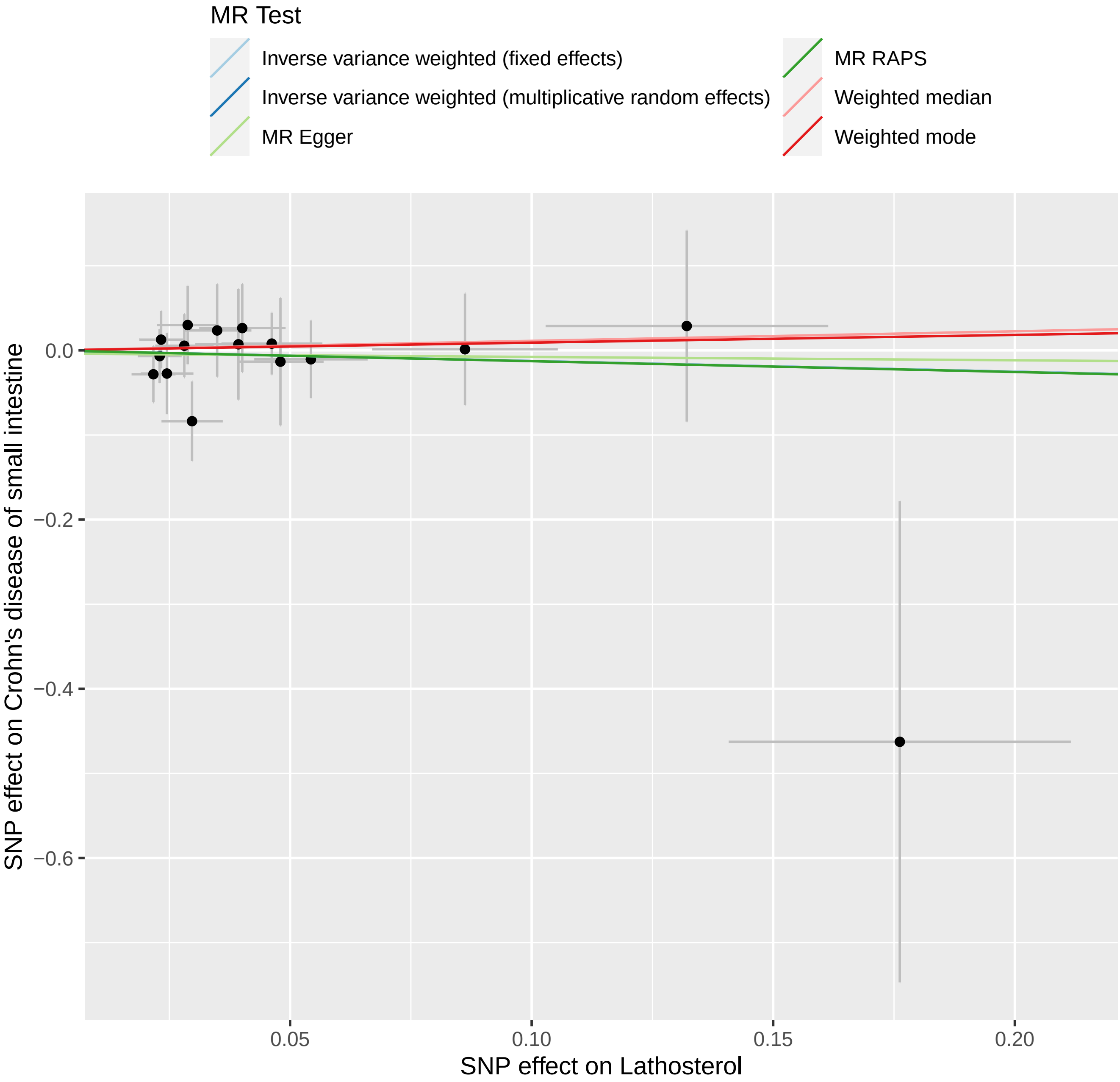

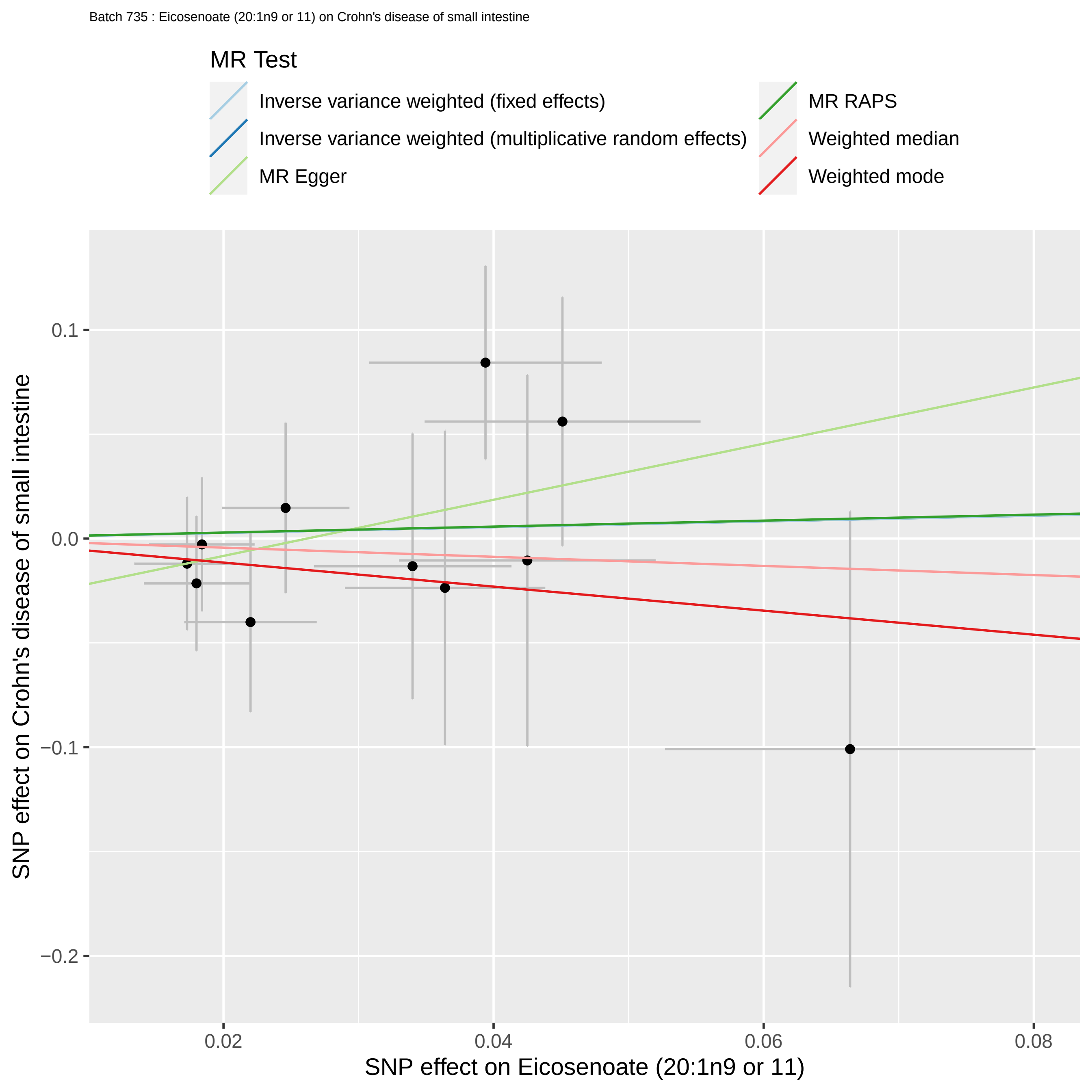

Batch 735 : Eicosenoate (20:1n9 or 11) on Crohn's disease of small intestine
MR Test
Inverse variance weighted (fixed effects)
Inverse variance weighted (multiplicative random effects)
MR Egger
MR RAPS
Weighted median
Weighted mode
0.1
0.0
−0.1
−0.2
0.02
0.04
0.06
0.08
SNP effect on Crohn's disease of small intestine
SNP effect on Eicosenoate (20:1n9 or 11)

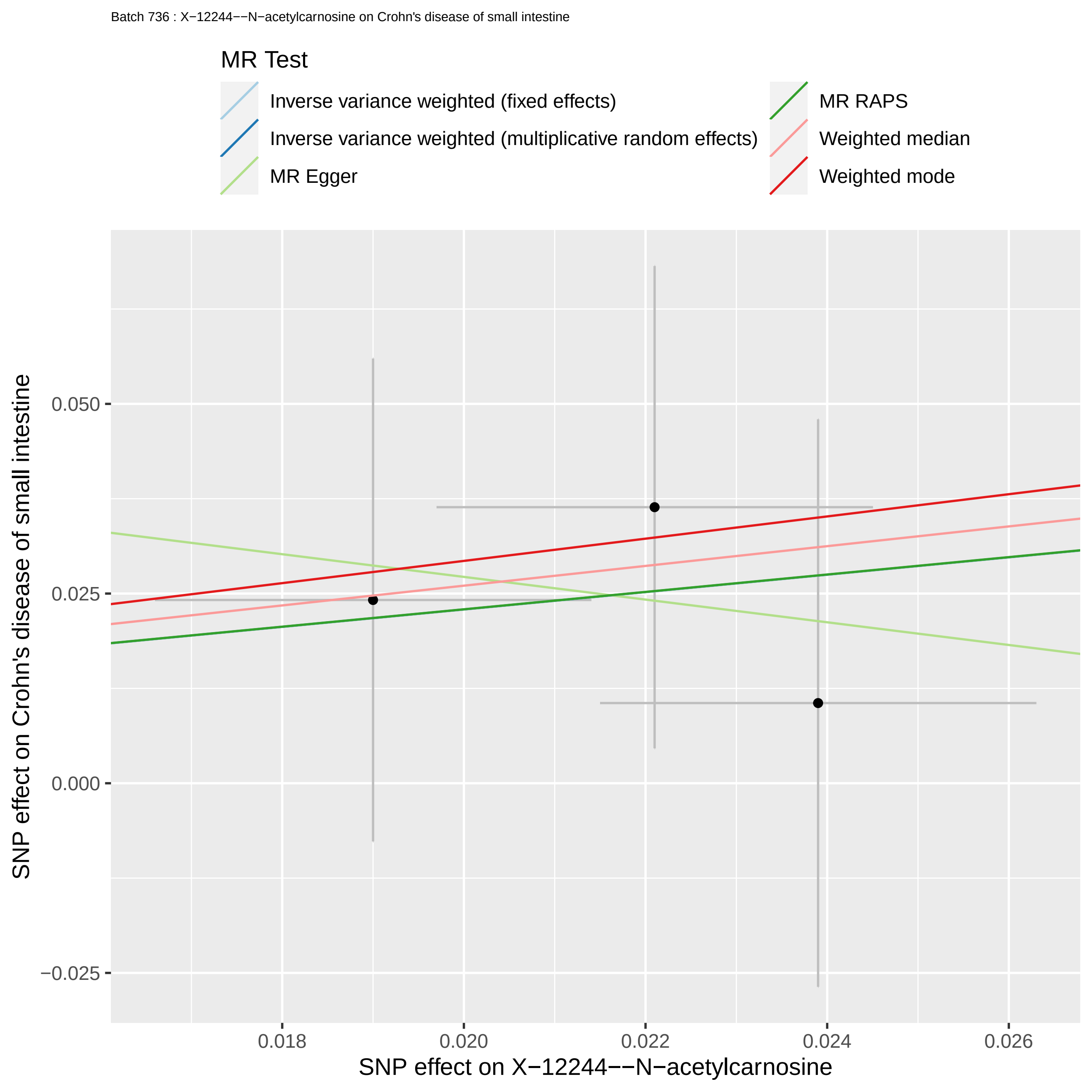
Batch 736 : X−12244−−N−acetylcarnosine on Crohn's disease of small intestine
MR Test
Inverse variance weighted (fixed effects)
Inverse variance weighted (multiplicative random effects)
MR Egger
MR RAPS
Weighted median
Weighted mode
0.050
0.025
0.000
−0.025
0.018
0.020
0.022
0.024
0.026
SNP effect on Crohn's disease of small intestine
SNP effect on X−12244−−N−acetylcarnosine

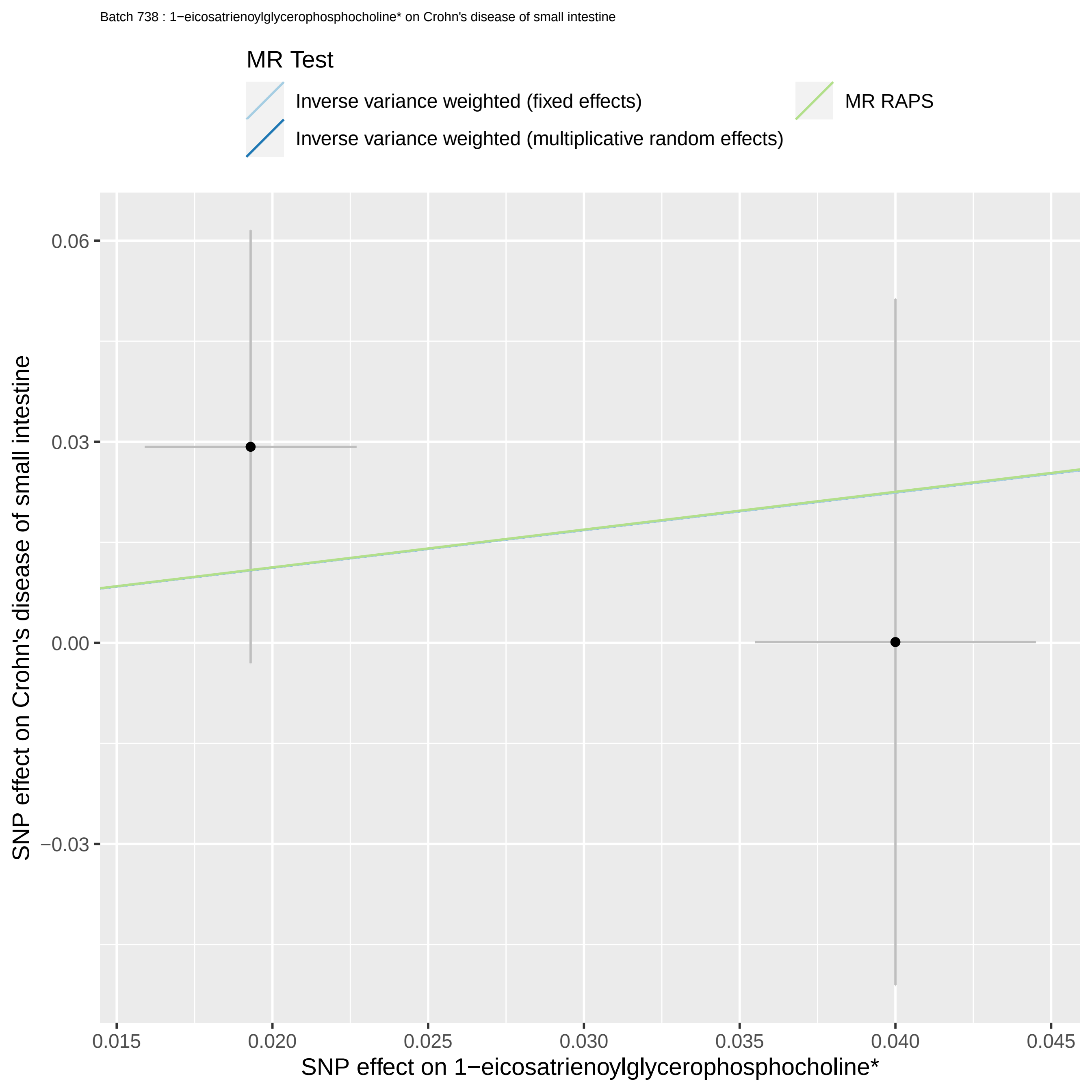
Batch 738 : 1−eicosatrienoylglycerophosphocholine* on Crohn's disease of small intestine
MR Test
Inverse variance weighted (fixed effects)
Inverse variance weighted (multiplicative random effects)
MR RAPS
0.06
0.03
0.00
−0.03
0.015
0.020
0.025
0.030
0.035
0.040
0.045
SNP effect on Crohn's disease of small intestine
SNP effect on 1−eicosatrienoylglycerophosphocholine*

Batch 739 : 1−docosahexaenoylglycerophosphocholine* on Crohn's disease of small intestine

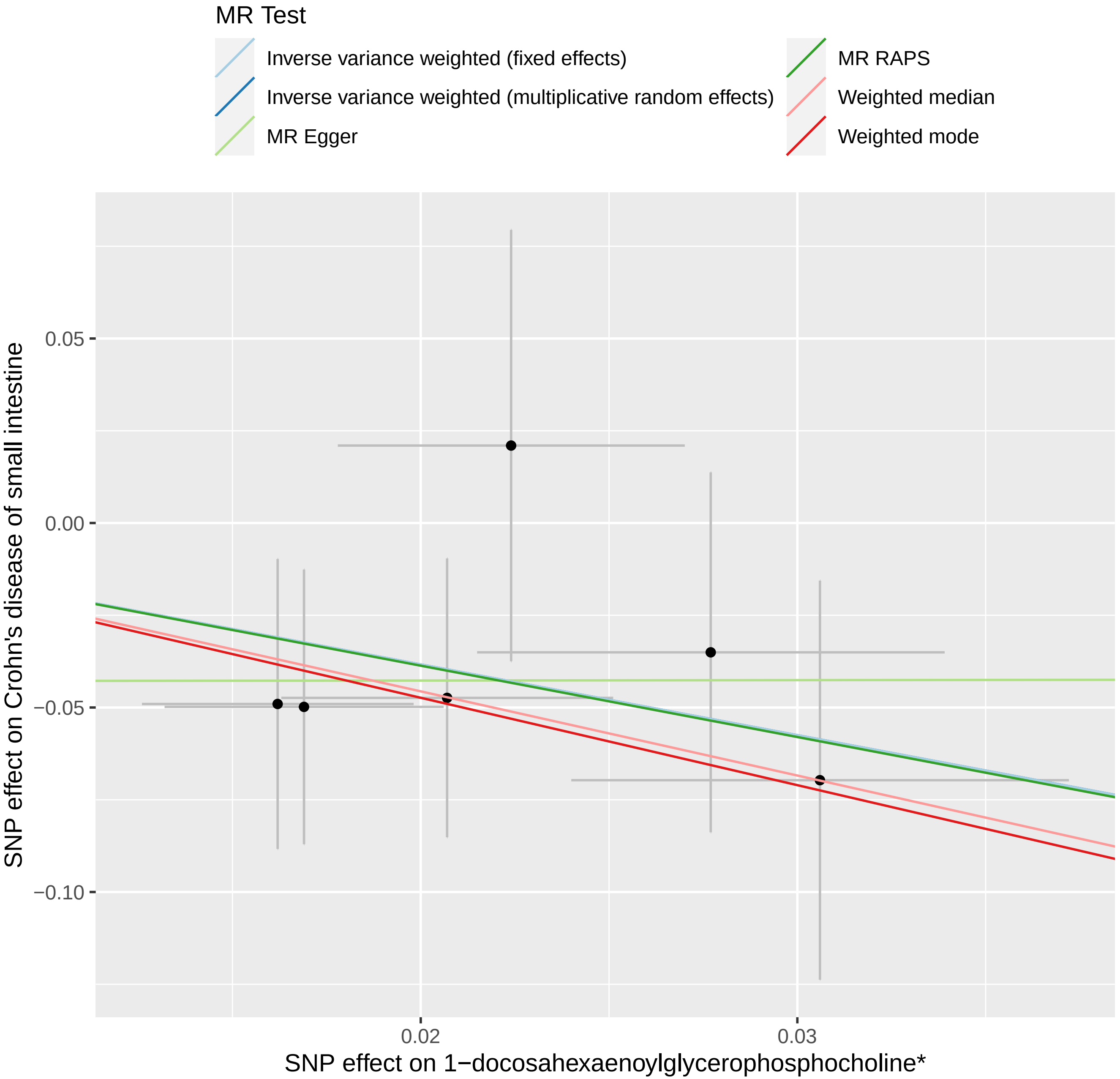

Batch 743 : Octanoylcarnitine on Crohn's disease of small intestine

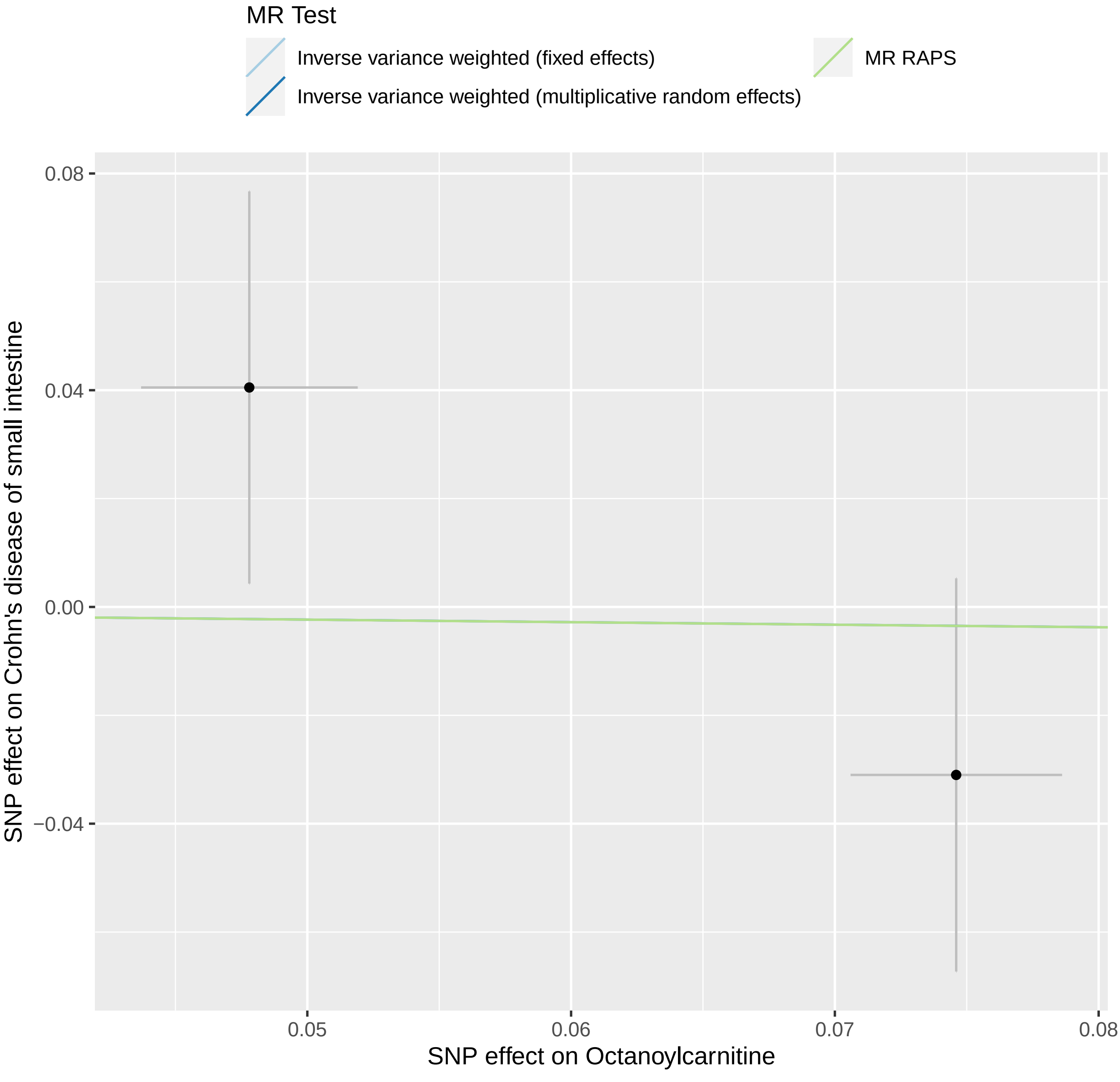

Batch 744 : Alpha−hydroxyisovalerate on Crohn's disease of small intestine

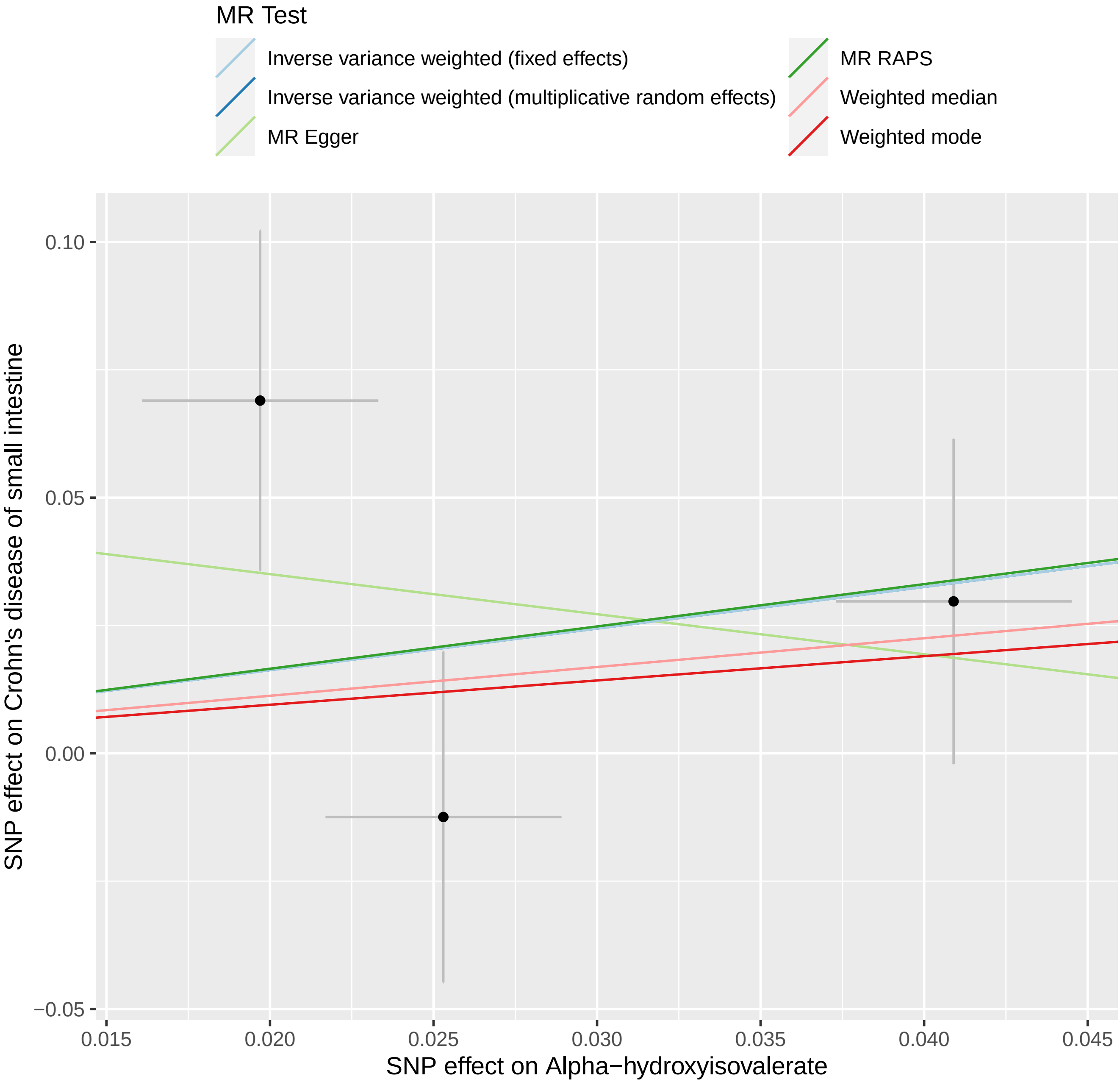

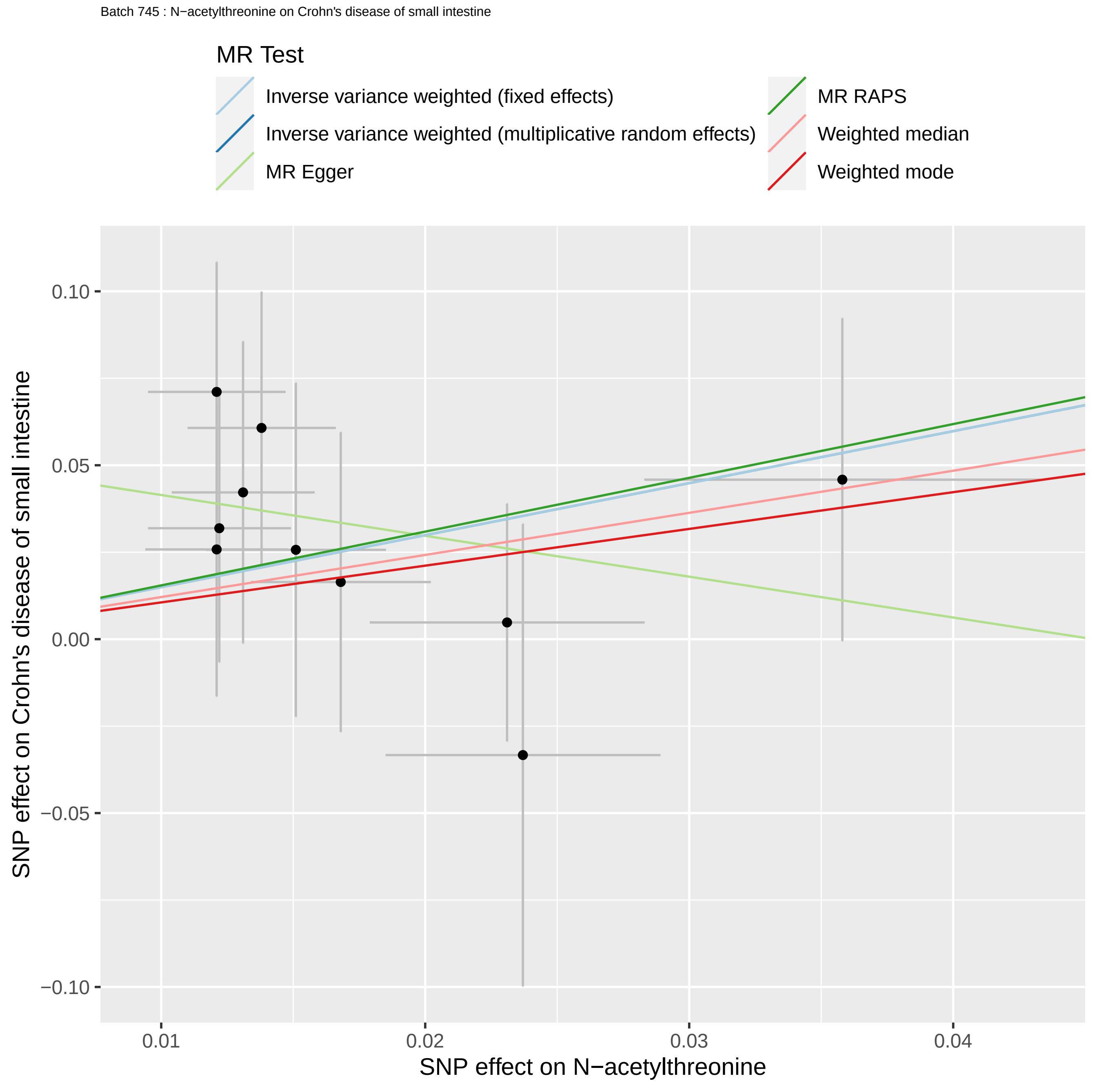

Batch 745 : N−acetylthreonine on Crohn's disease of small intestine
MR Test
Inverse variance weighted (fixed effects)
Inverse variance weighted (multiplicative random effects)
MR Egger
MR RAPS
Weighted median
Weighted mode
0.10
0.05
0.00
−0.05
−0.10
0.01
0.02
0.03
0.04
SNP effect on Crohn's disease of small intestine
SNP effect on N−acetylthreonine

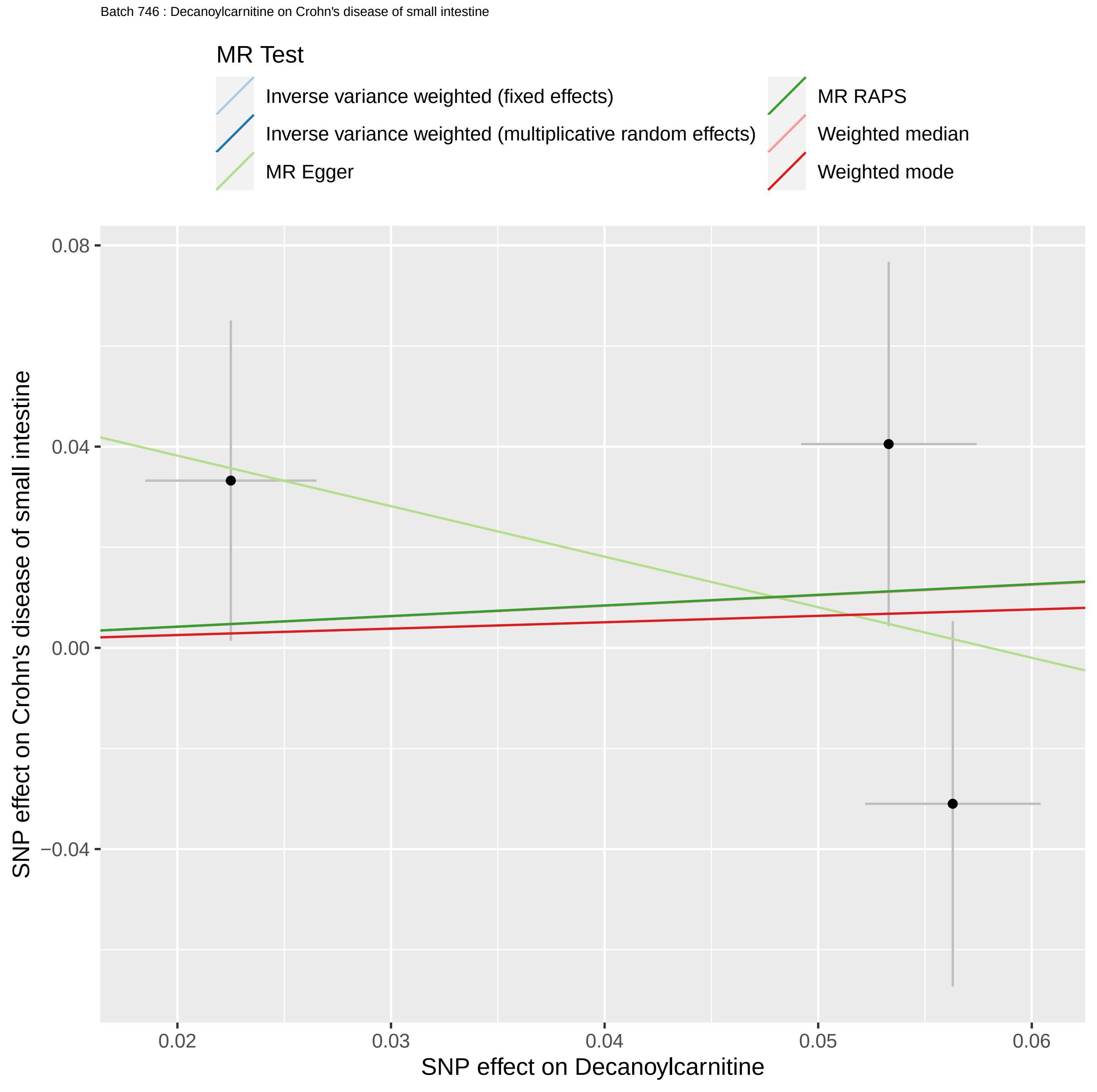
Batch 746 : Decanoylcarnitine on Crohn's disease of small intestine
MR Test
Inverse variance weighted (fixed effects)
Inverse variance weighted (multiplicative random effects)
MR Egger
MR RAPS
Weighted median
Weighted mode
0.08
0.04
0.00
−0.04
SNP effect on Crohn's disease of small intestine
0.02
0.03
0.04
0.05
0.06
SNP effect on Decanoylcarnitine

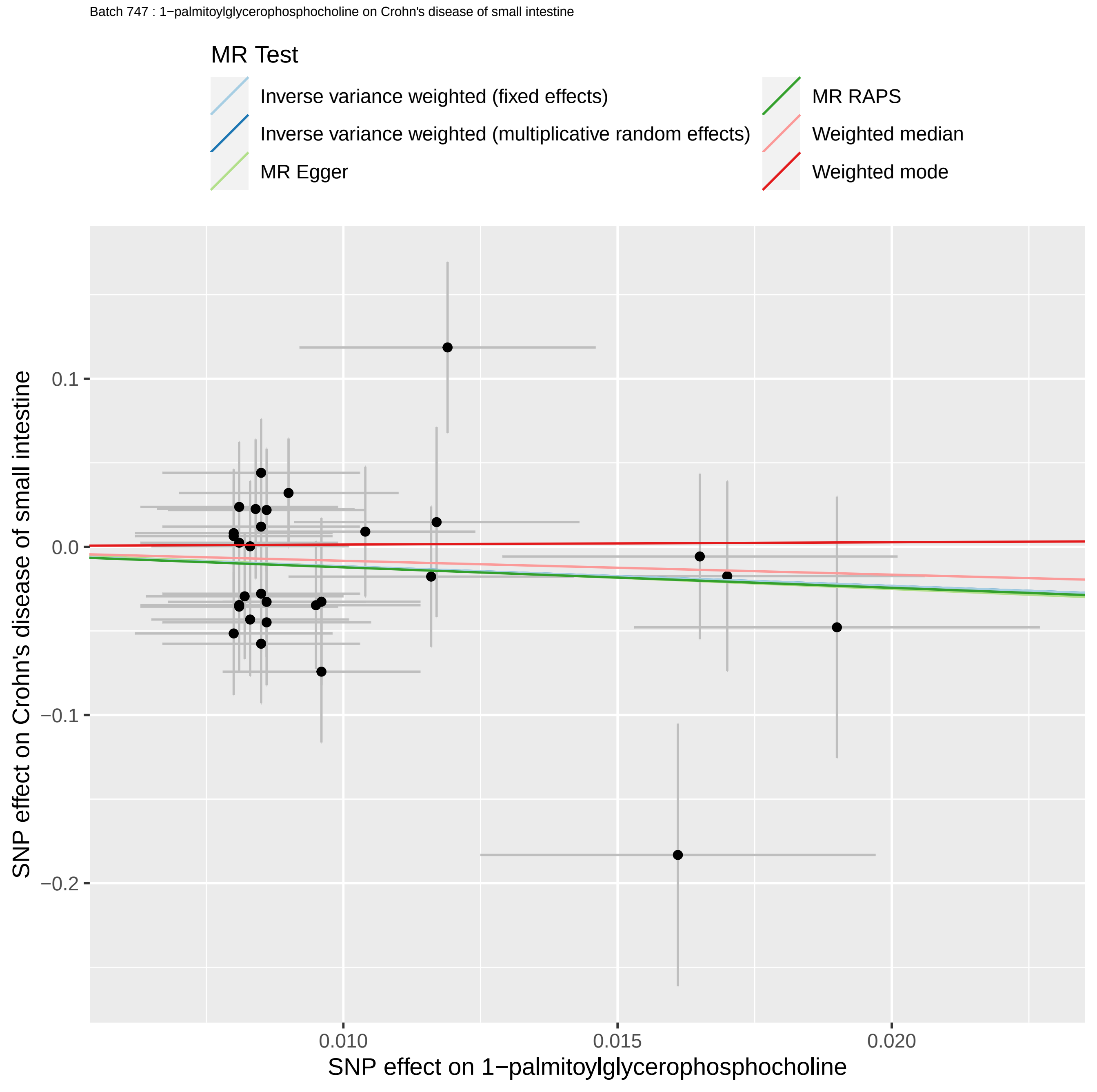

Batch 747 : 1−palmitoylglycerophosphocholine on Crohn's disease of small intestine
MR Test
Inverse variance weighted (fixed effects)
Inverse variance weighted (multiplicative random effects)
MR Egger
MR RAPS
Weighted median
Weighted mode
SNP effect on Crohn's disease of small intestine
0.1
0.0
−0.1
−0.2
0.010
0.015
0.020
SNP effect on 1−palmitoylglycerophosphocholine

Batch 748 : 1−heptadecanoylglycerophosphocholine on Crohn's disease of small intestine

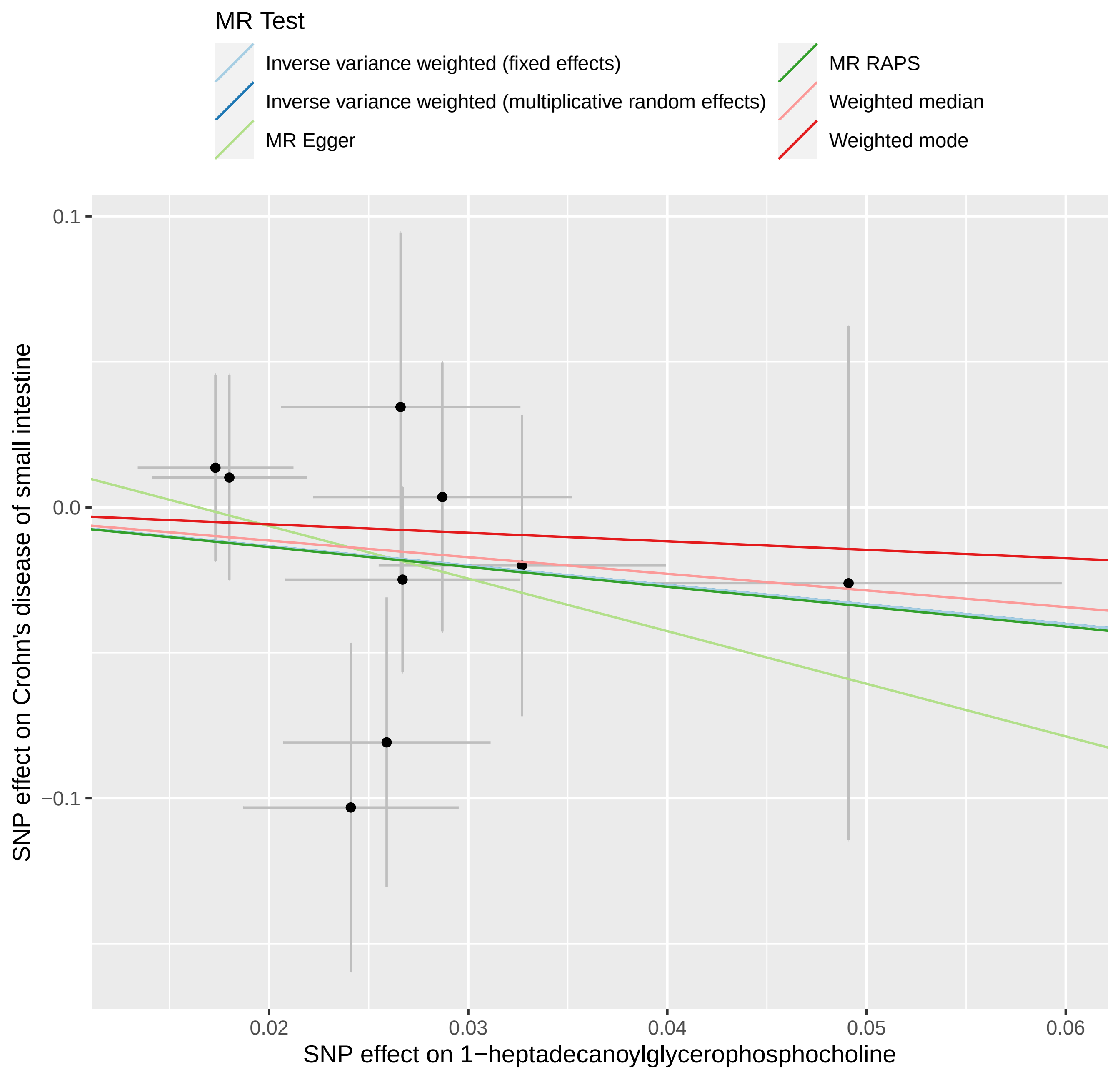

Batch 749 : 1−oleoylglycerophosphocholine on Crohn's disease of small intestine

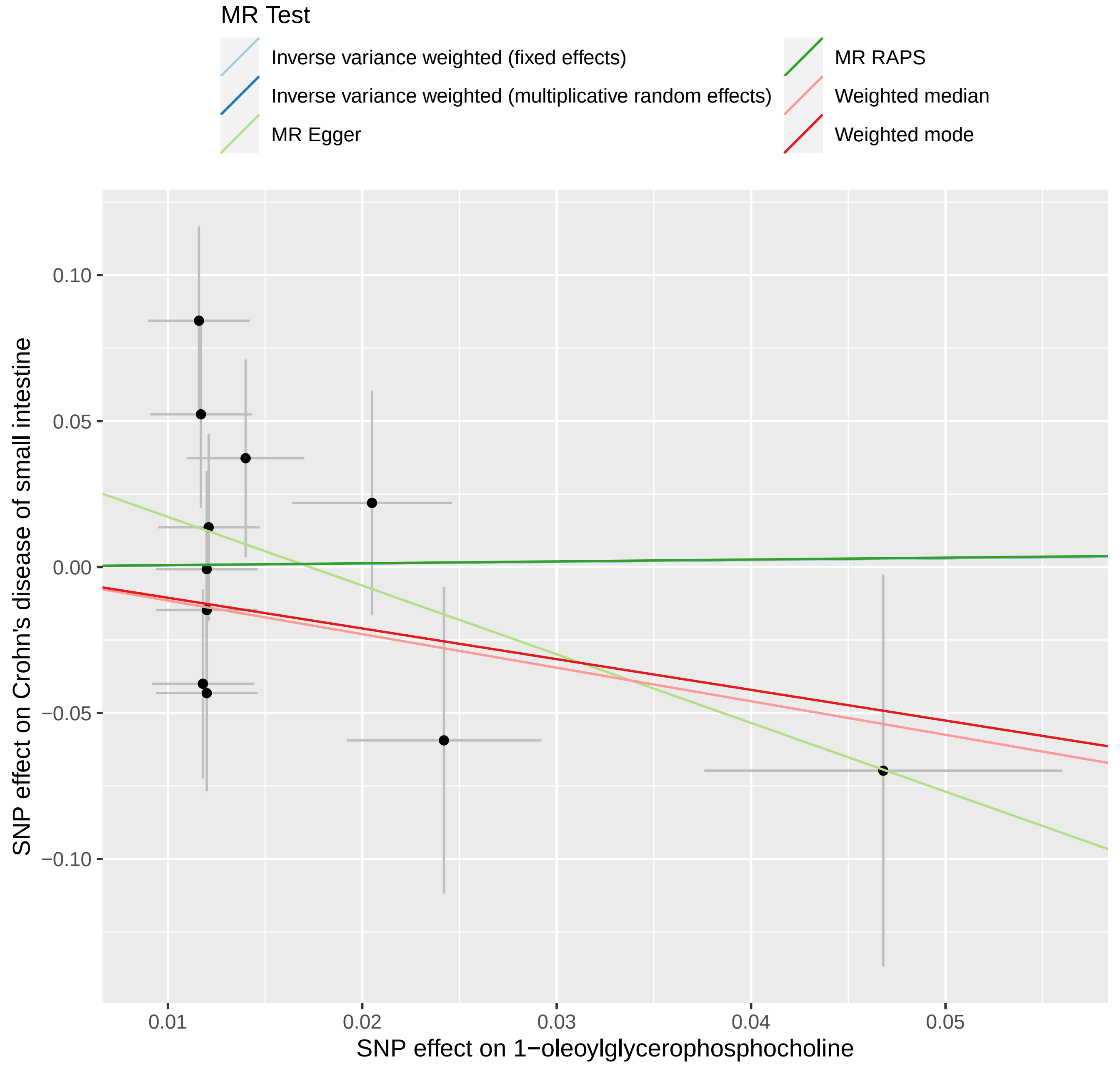

Batch 750 : 1−stearoylglycerophosphocholine on Crohn's disease of small intestine

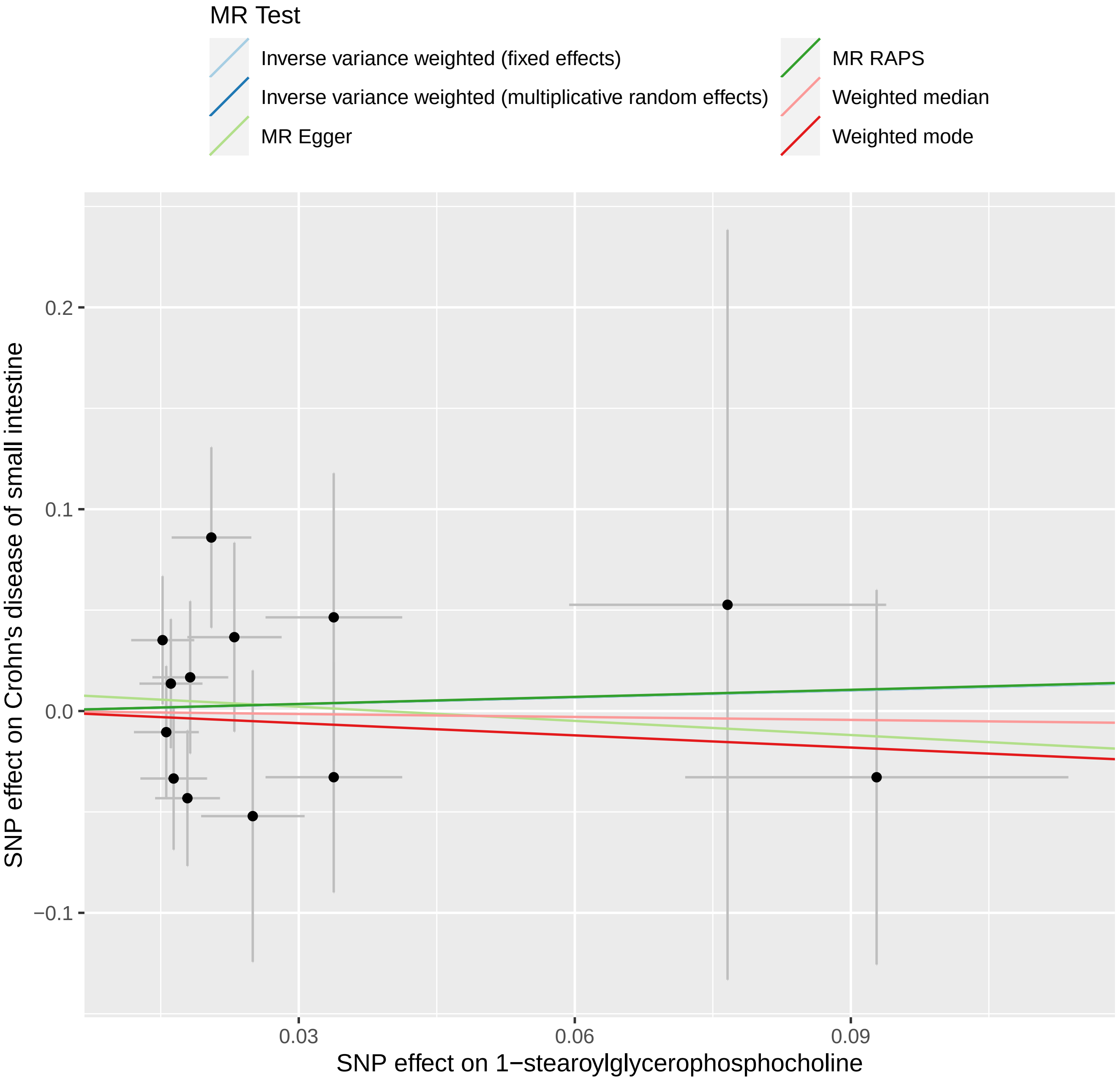

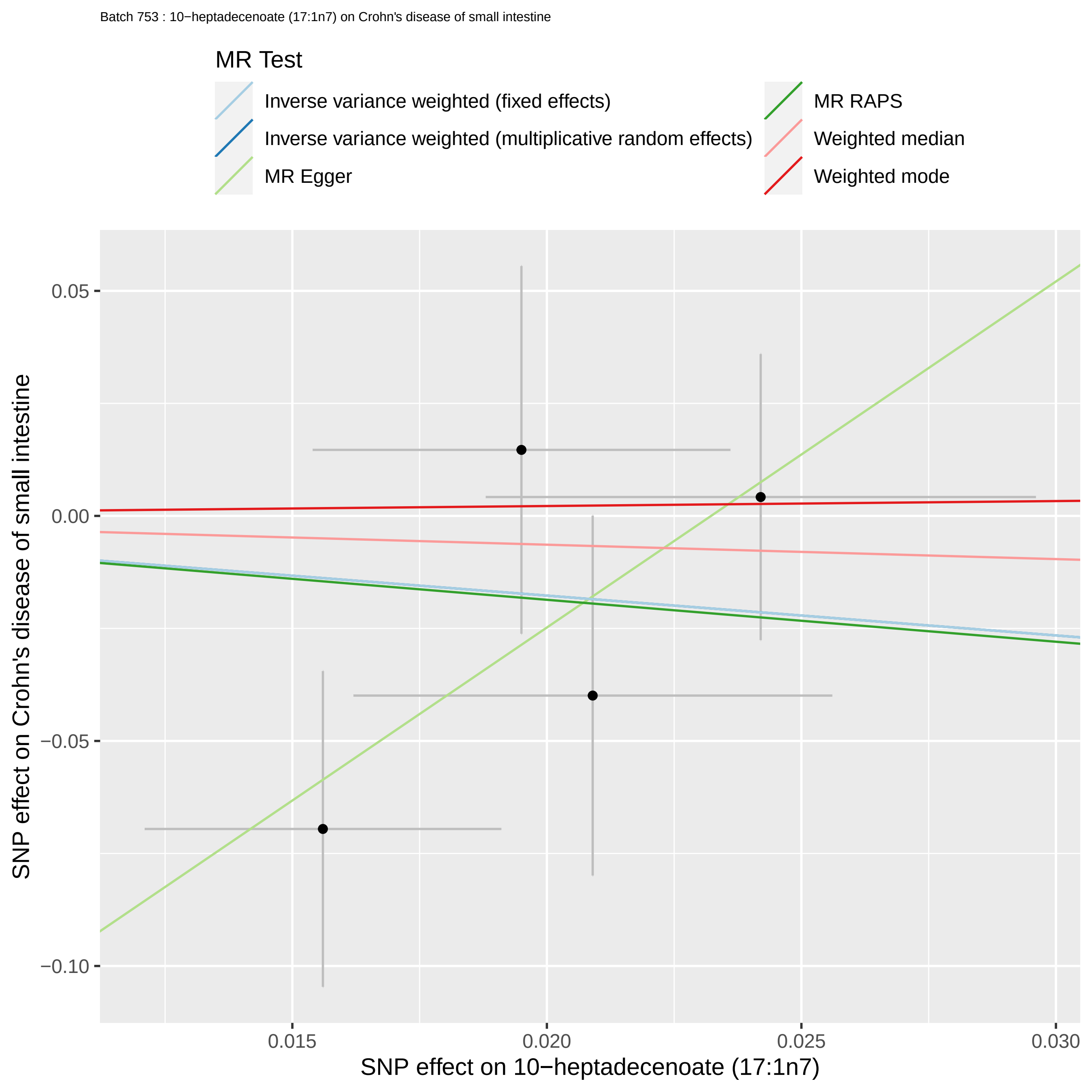

Batch 753 : 10−heptadecenoate (17:1n7) on Crohn's disease of small intestine
MR Test
Inverse variance weighted (fixed effects)
Inverse variance weighted (multiplicative random effects)
MR Egger
MR RAPS
Weighted median
Weighted mode
0.05
0.00
−0.05
−0.10
0.015
0.020
0.025
0.030
SNP effect on 10−heptadecenoate (17:1n7)
SNP effect on Crohn's disease of small intestine

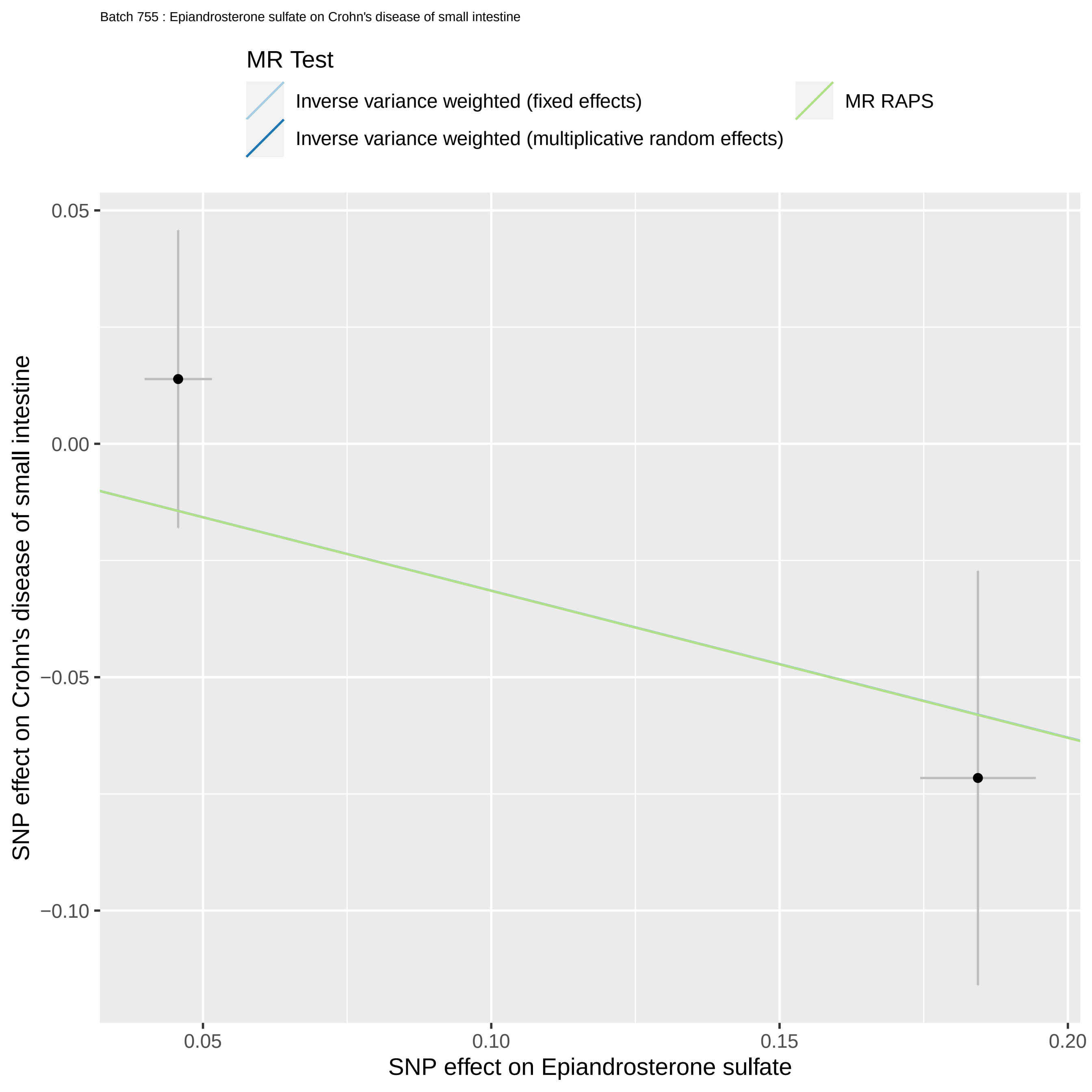

Batch 755 : Epiandrosterone sulfate on Crohn's disease of small intestine
MR Test
Inverse variance weighted (fixed effects)
Inverse variance weighted (multiplicative random effects)
MR RAPS
0.05
0.00
−0.05
−0.10
0.05
0.10
0.15
0.20
SNP effect on Epiandrosterone sulfate
SNP effect on Crohn's disease of small intestine

Batch 756 : Linolenate [alpha or gamma; (18:3n3 or 6)] on Crohn's disease of small intestine

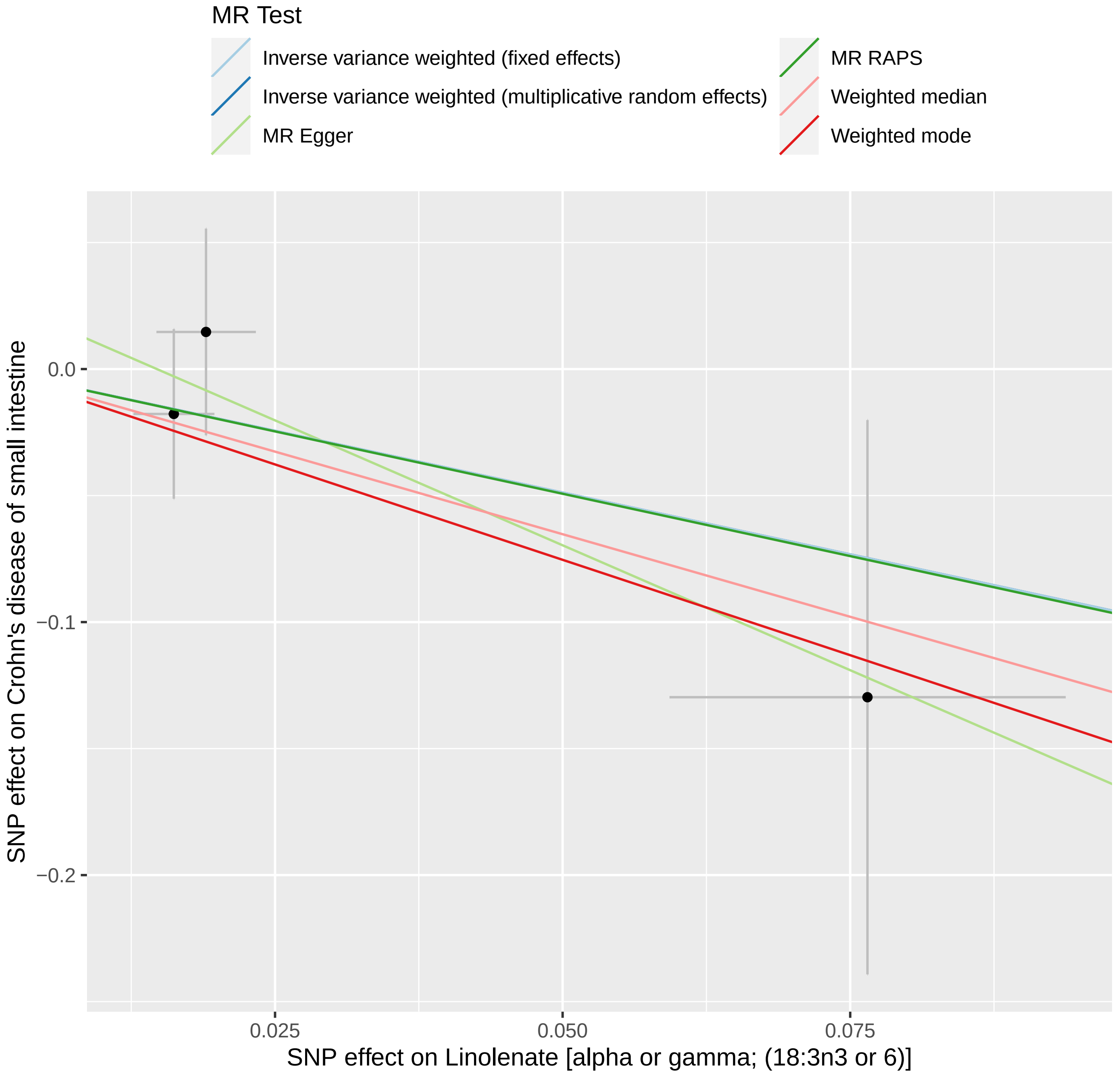

Batch 757 : X−12510−−2−aminooctanoic acid on Crohn's disease of small intestine

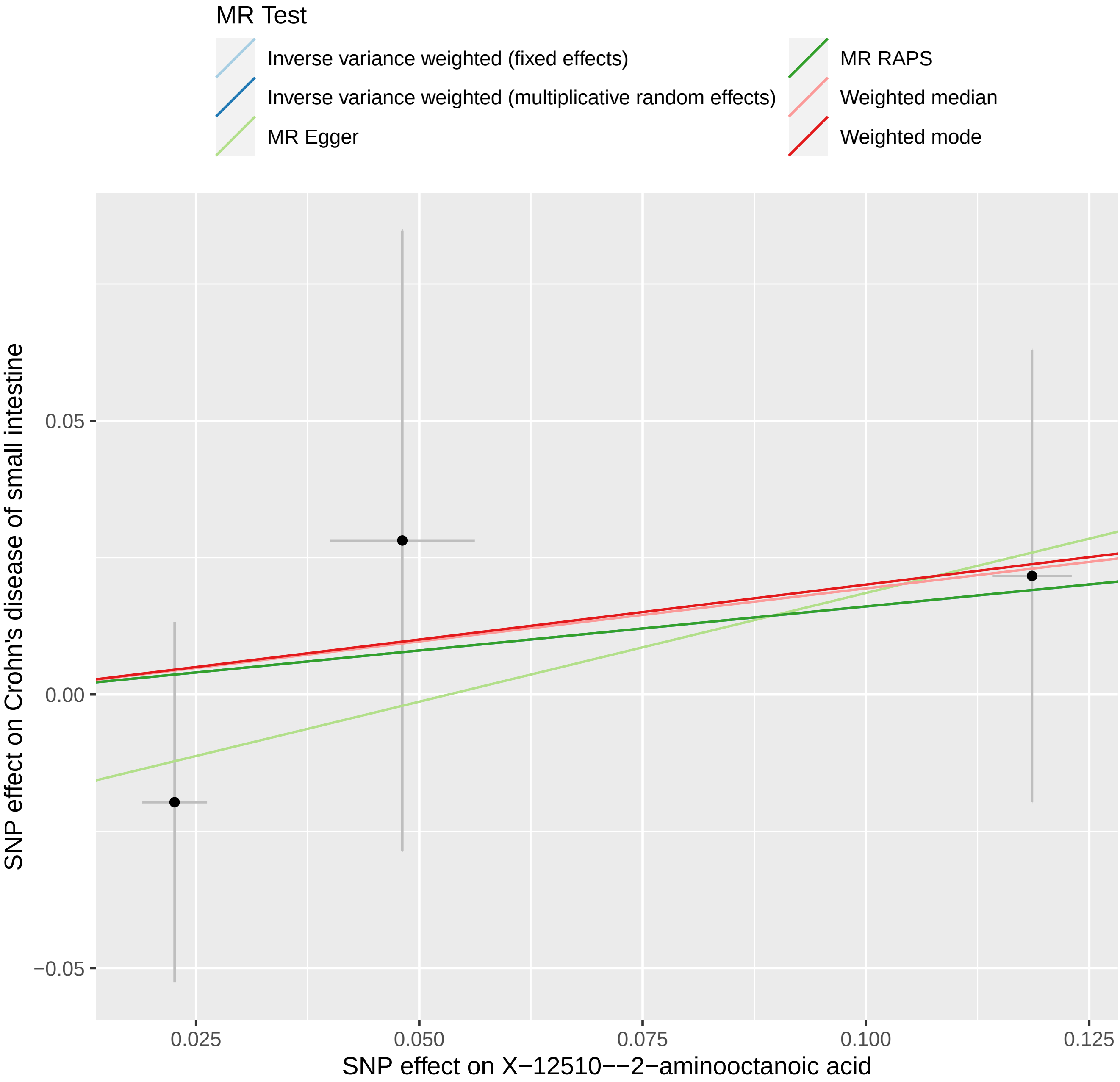

Batch 760 : Asparagine on Crohn's disease of small intestine

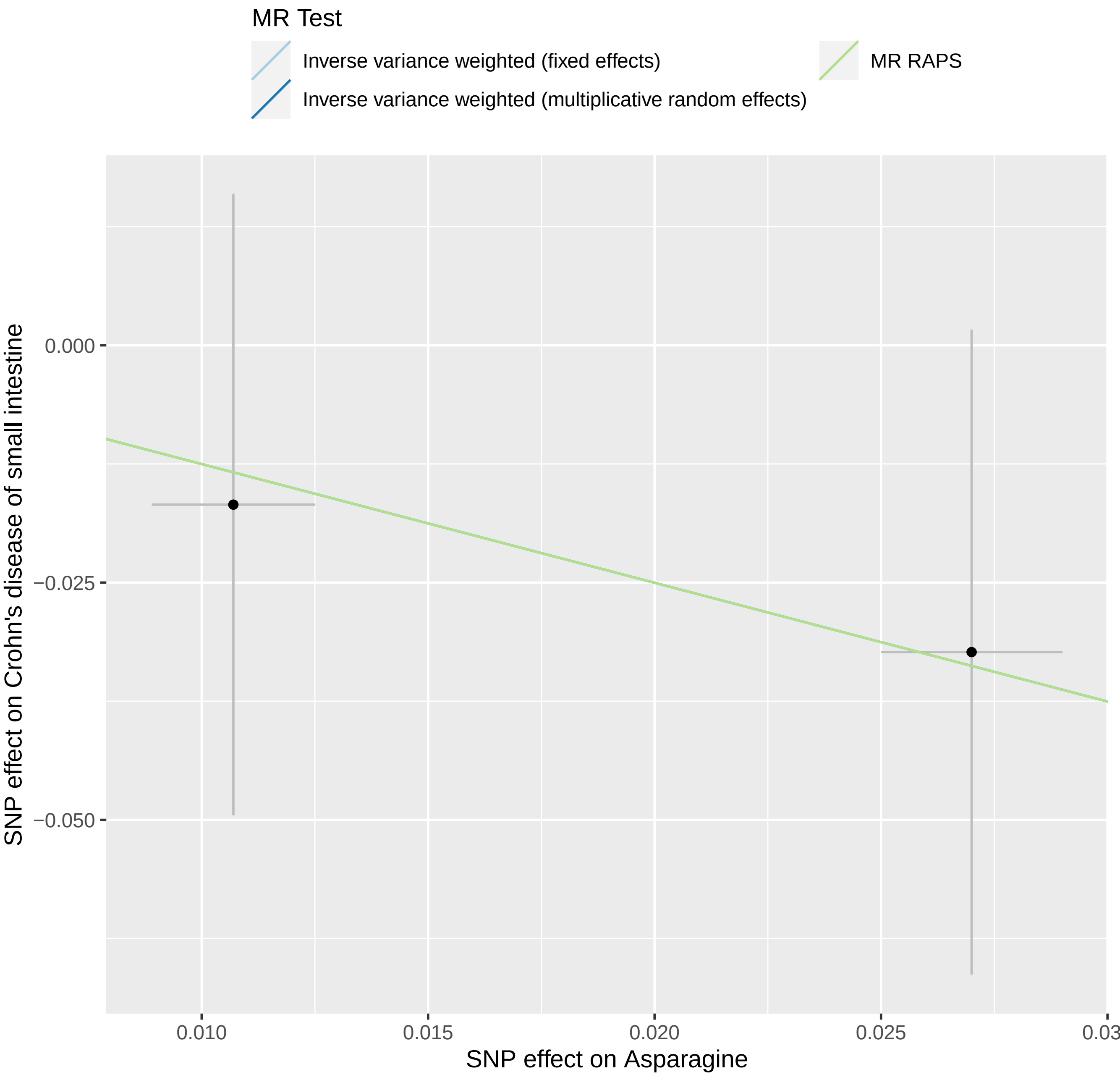

Batch 761 : Stachydrine on Crohn's disease of small intestine

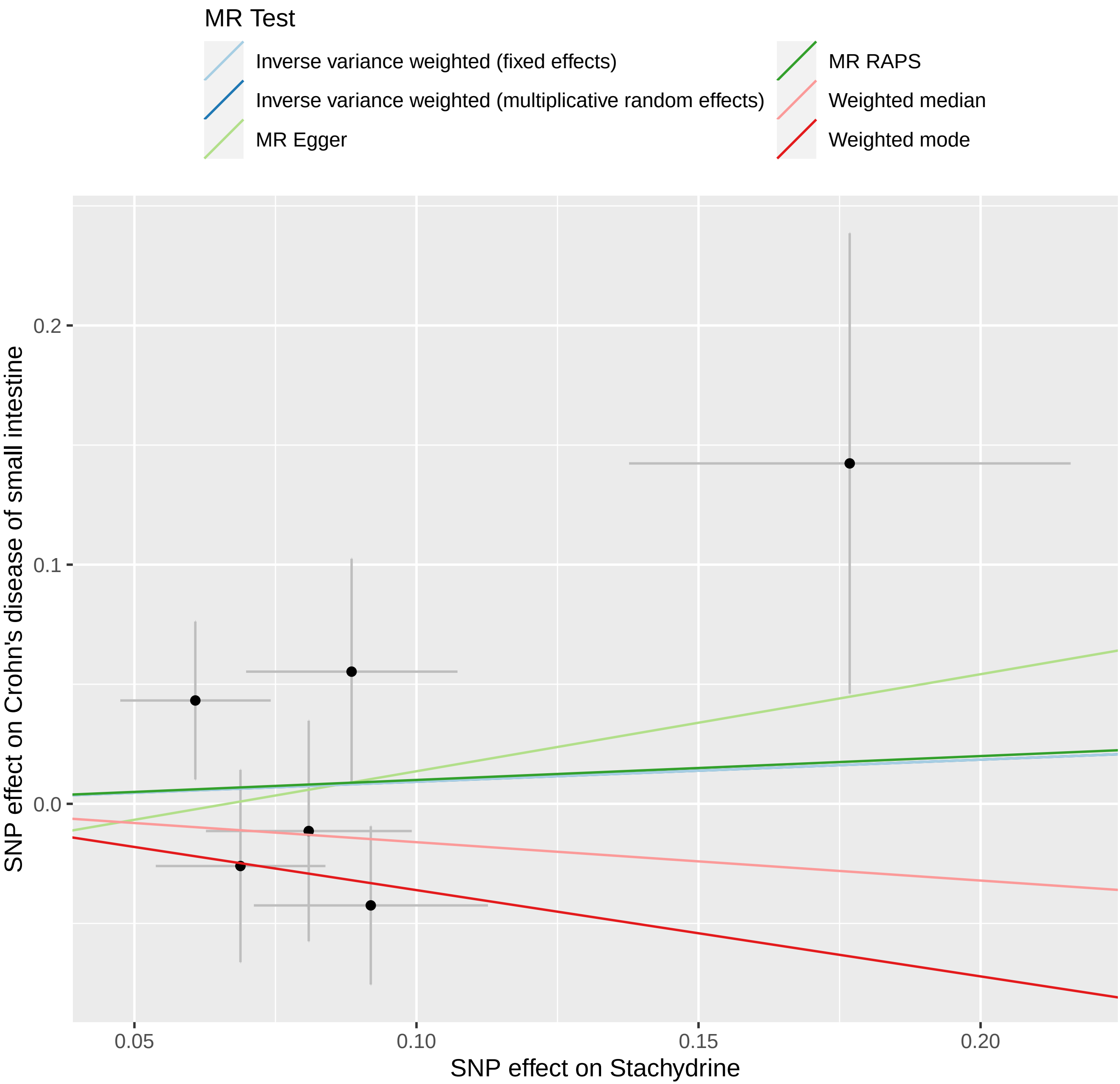

Batch 762 : Isovalerylcarnitine on Crohn's disease of small intestine

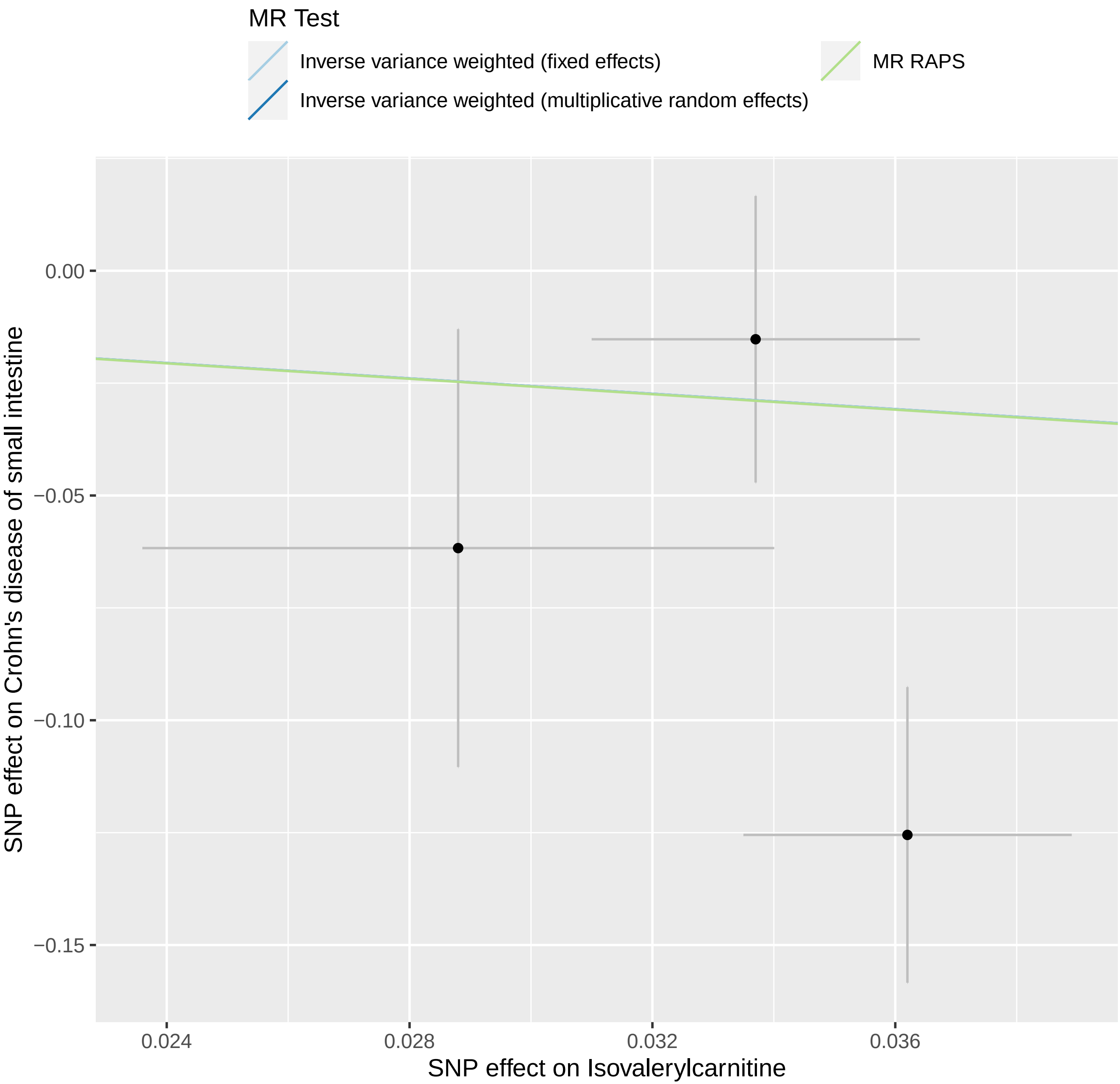

Batch 765 : 1−linoleoylglycerophosphocholine on Crohn's disease of small intestine

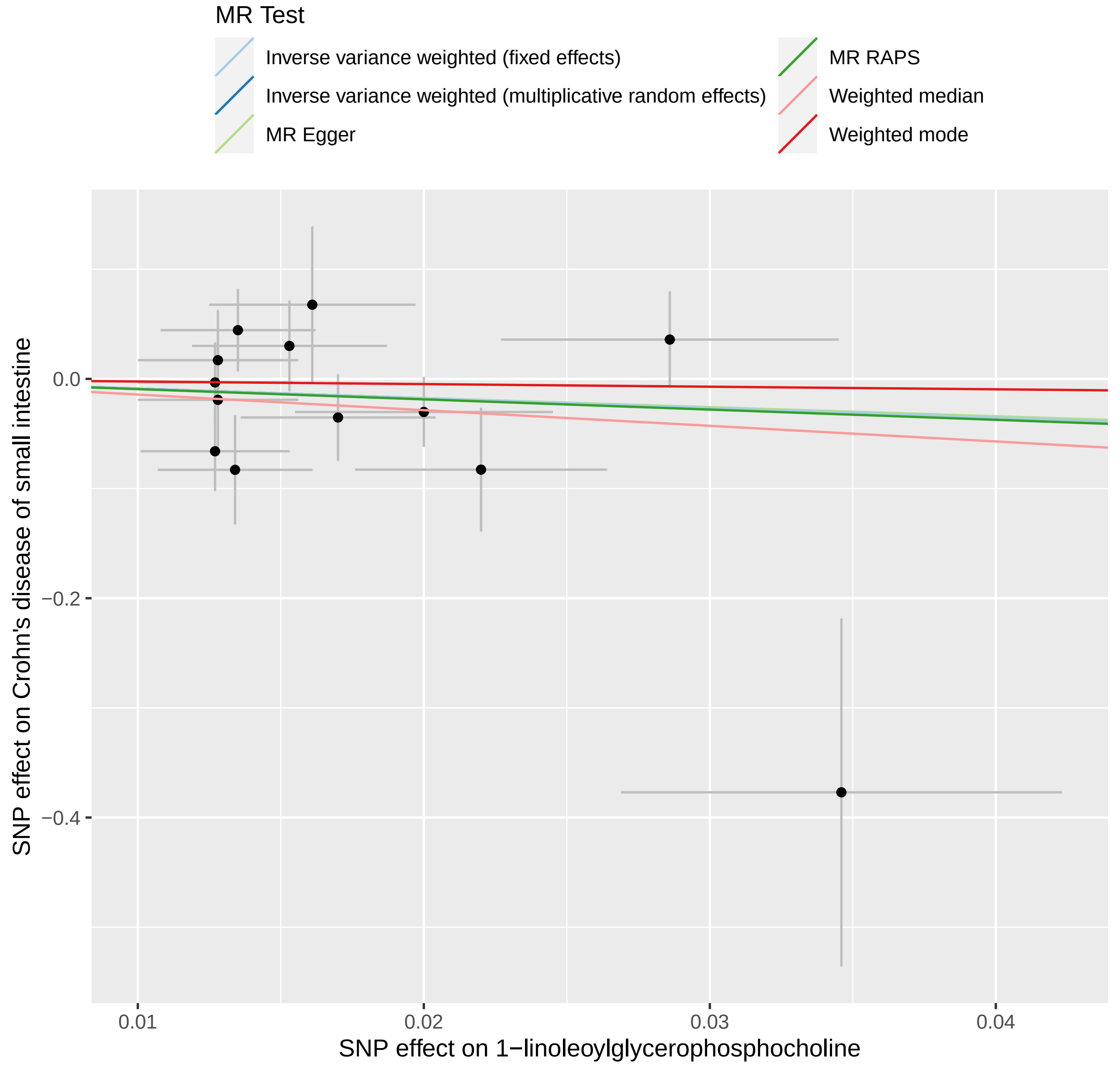

Batch 766 : Bradykinin, des−arg(9) on Crohn's disease of small intestine

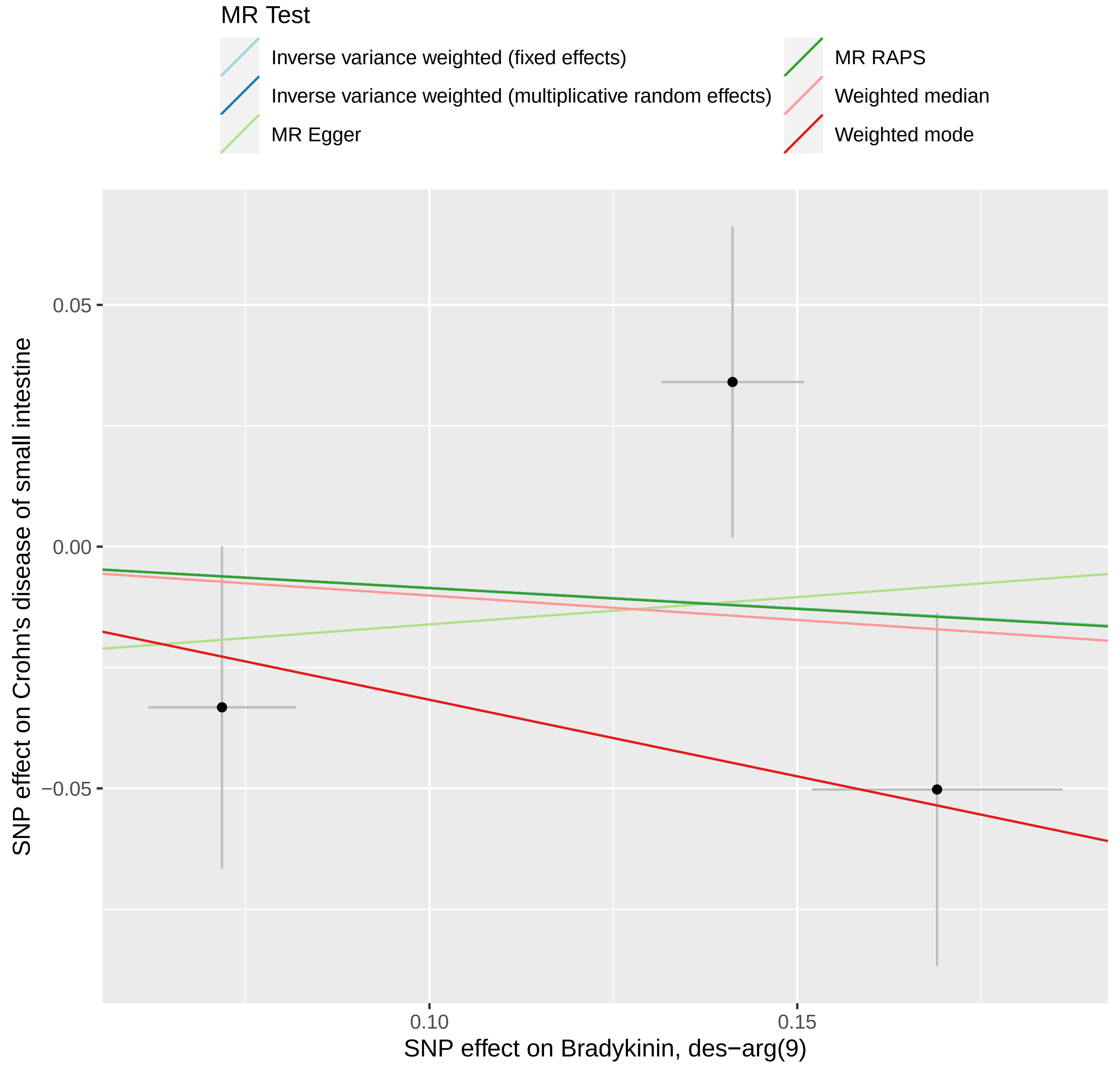

Batch 767 : Gamma−glutamylisoleucine* on Crohn's disease of small intestine

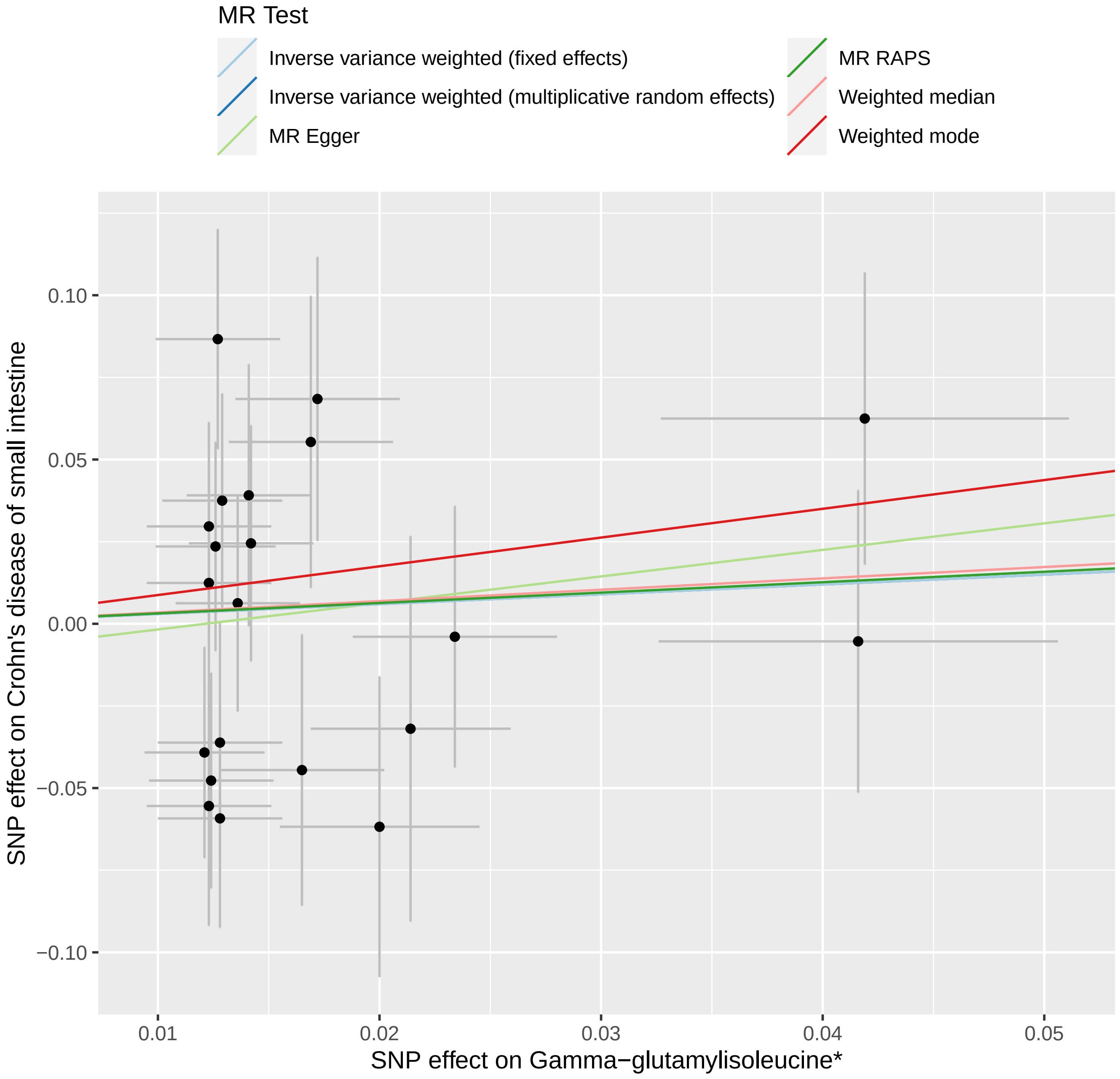

Batch 770 : Isovalerate on Crohn's disease of small intestine

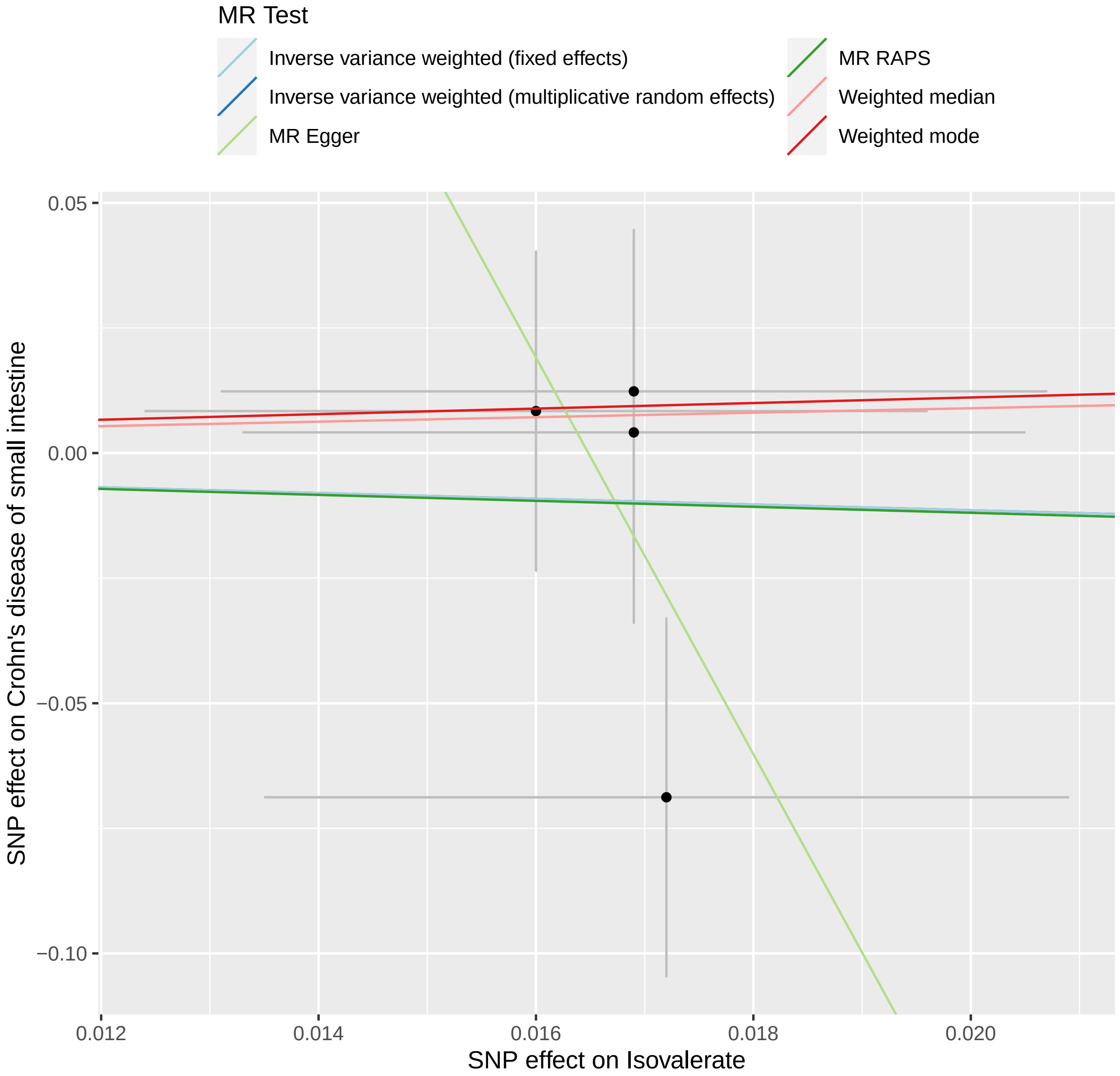

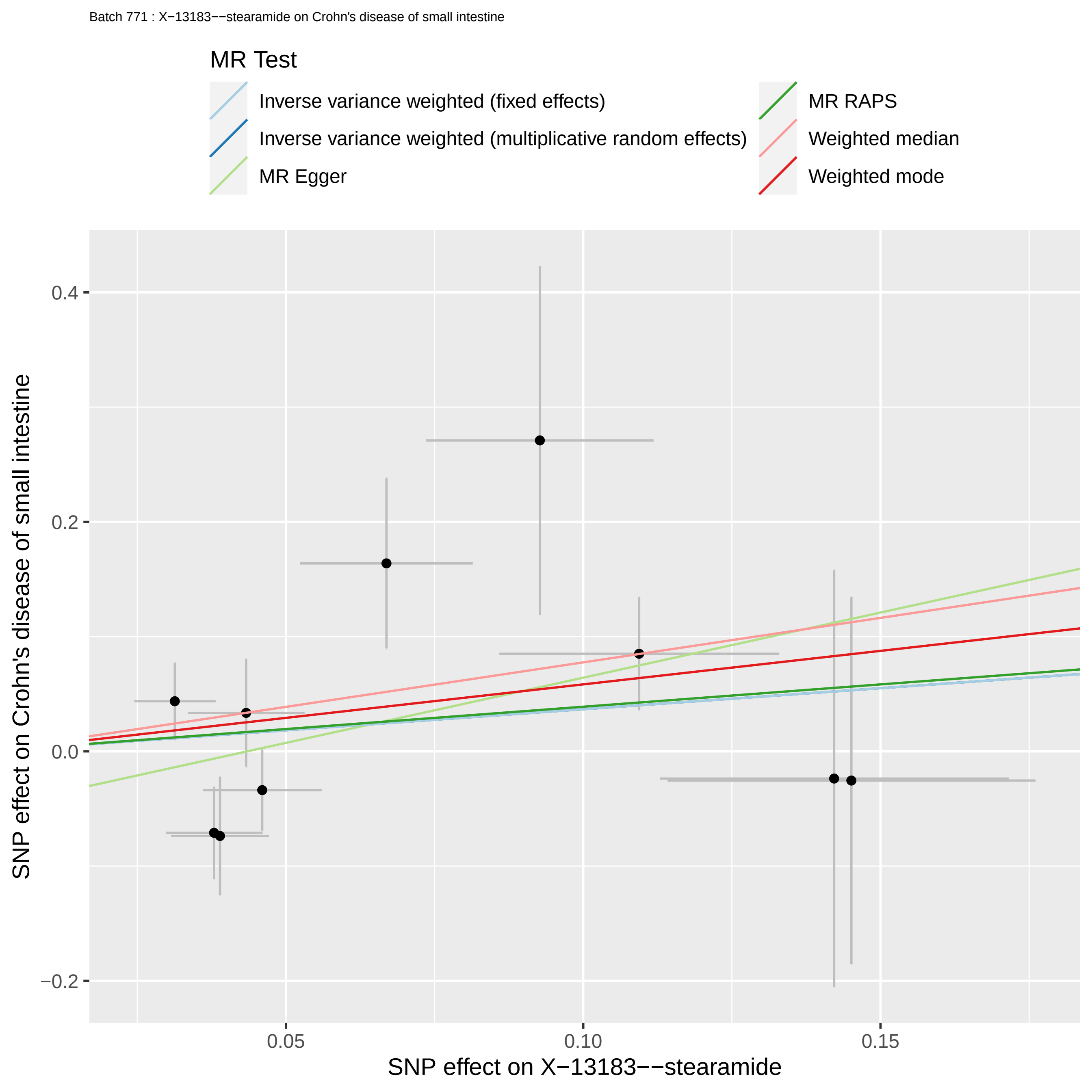
Batch 771 : X−13183−−stearamide on Crohn's disease of small intestine
MR Test
Inverse variance weighted (fixed effects)
Inverse variance weighted (multiplicative random effects)
MR Egger
MR RAPS
Weighted median
Weighted mode
0.4
0.2
0.0
−0.2
0.05
0.10
0.15
SNP effect on Crohn's disease of small intestine
SNP effect on X−13183−−stearamide

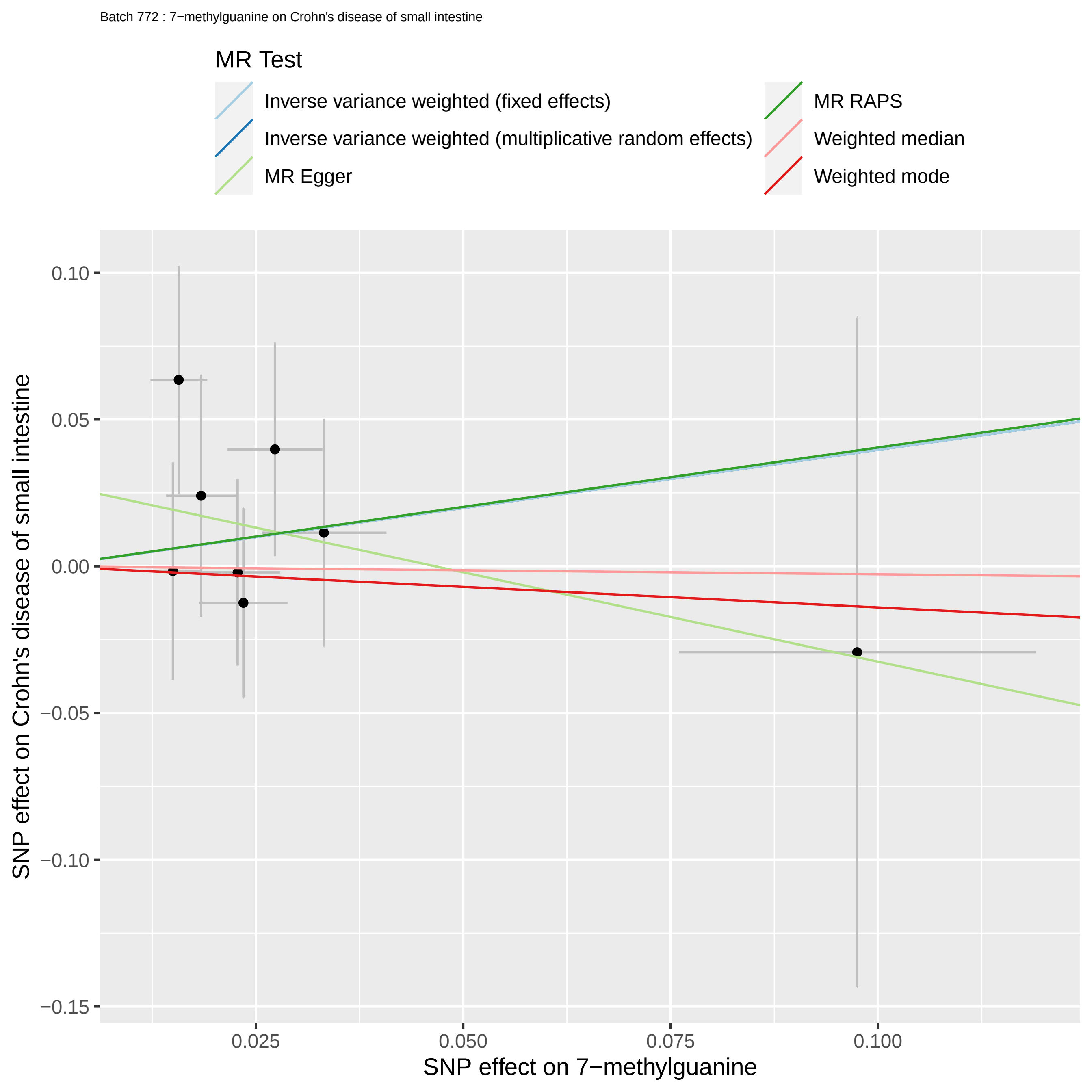
Batch 772 : 7−methylguanine on Crohn's disease of small intestine
MR Test
Inverse variance weighted (fixed effects)
Inverse variance weighted (multiplicative random effects)
MR Egger
MR RAPS
Weighted median
Weighted mode
0.10
0.05
0.00
−0.05
−0.10
−0.15
0.025
0.050
0.075
0.100
SNP effect on Crohn's disease of small intestine
SNP effect on 7−methylguanine

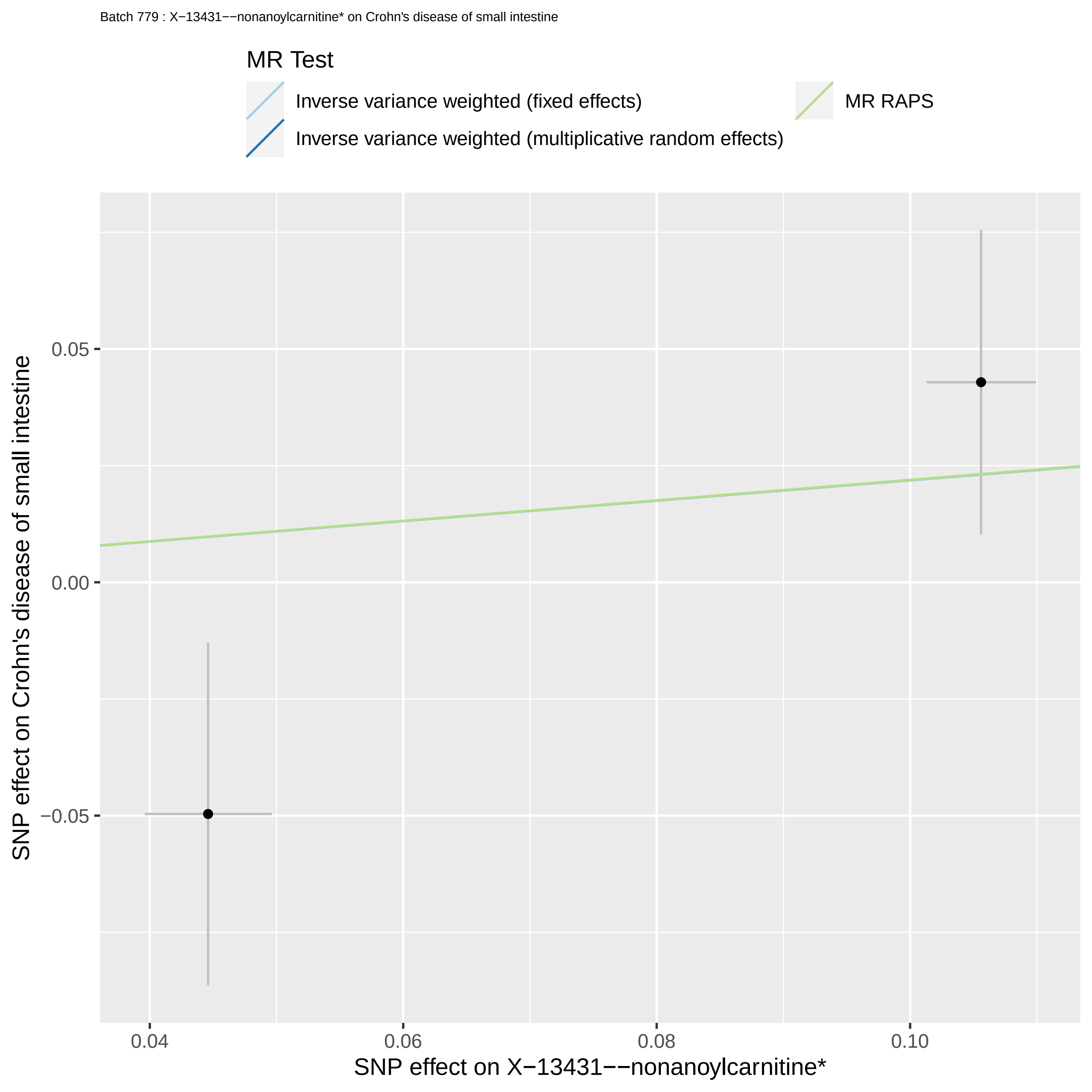
Batch 779 : X−13431−−nonanoylcarnitine* on Crohn's disease of small intestine
MR Test
Inverse variance weighted (fixed effects)
Inverse variance weighted (multiplicative random effects)
MR RAPS
0.05
0.00
−0.05
0.04
0.06
0.08
0.10
SNP effect on Crohn's disease of small intestine
SNP effect on X−13431−−nonanoylcarnitine*

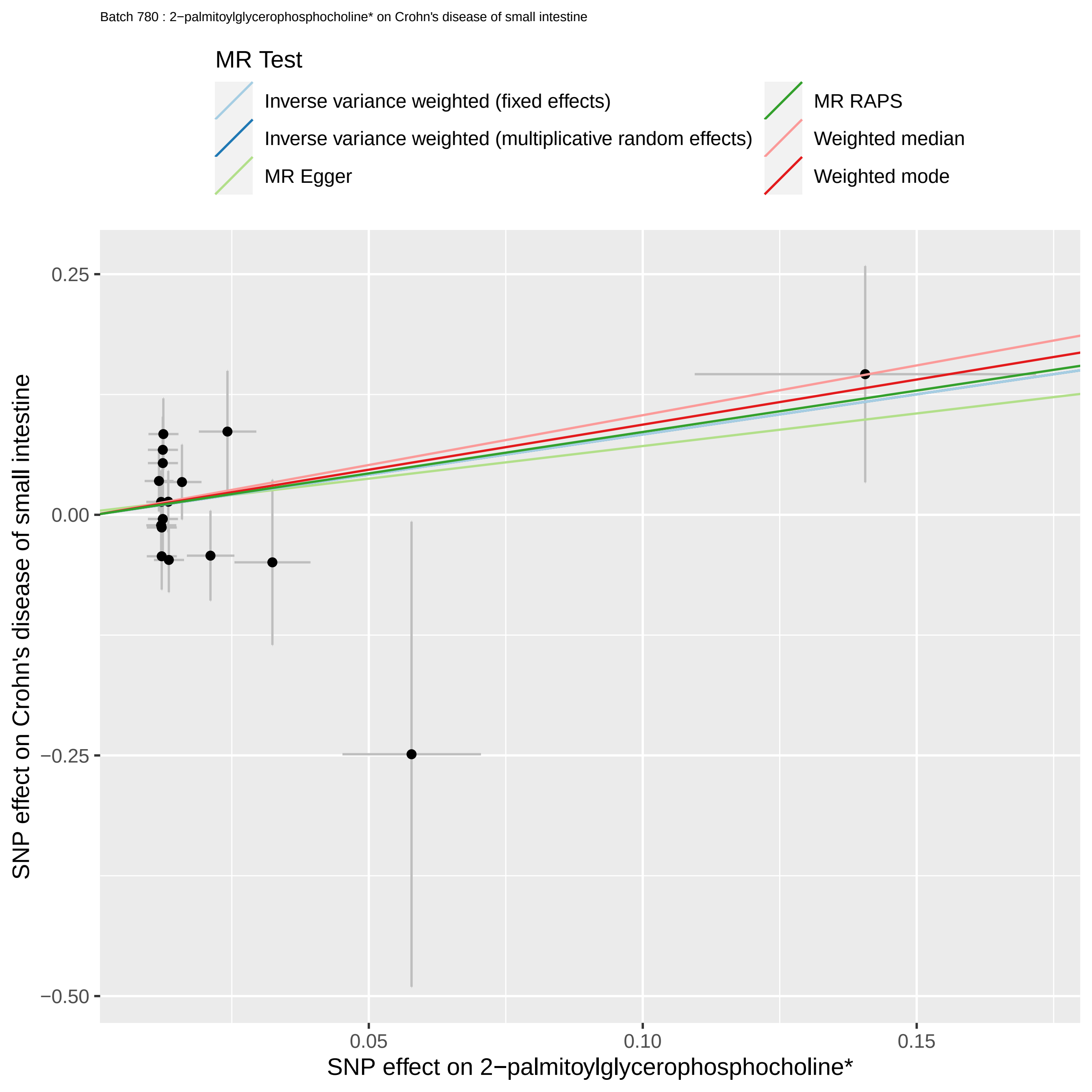

Batch 780 : 2−palmitoylglycerophosphocholine* on Crohn's disease of small intestine
MR Test
Inverse variance weighted (fixed effects)
Inverse variance weighted (multiplicative random effects)
MR Egger
MR RAPS
Weighted median
Weighted mode
0.25
0.00
−0.25
−0.50
0.05
0.10
0.15
SNP effect on Crohn's disease of small intestine
SNP effect on 2−palmitoylglycerophosphocholine*

Batch 781 : 2−oleoylglycerophosphocholine* on Crohn's disease of small intestine

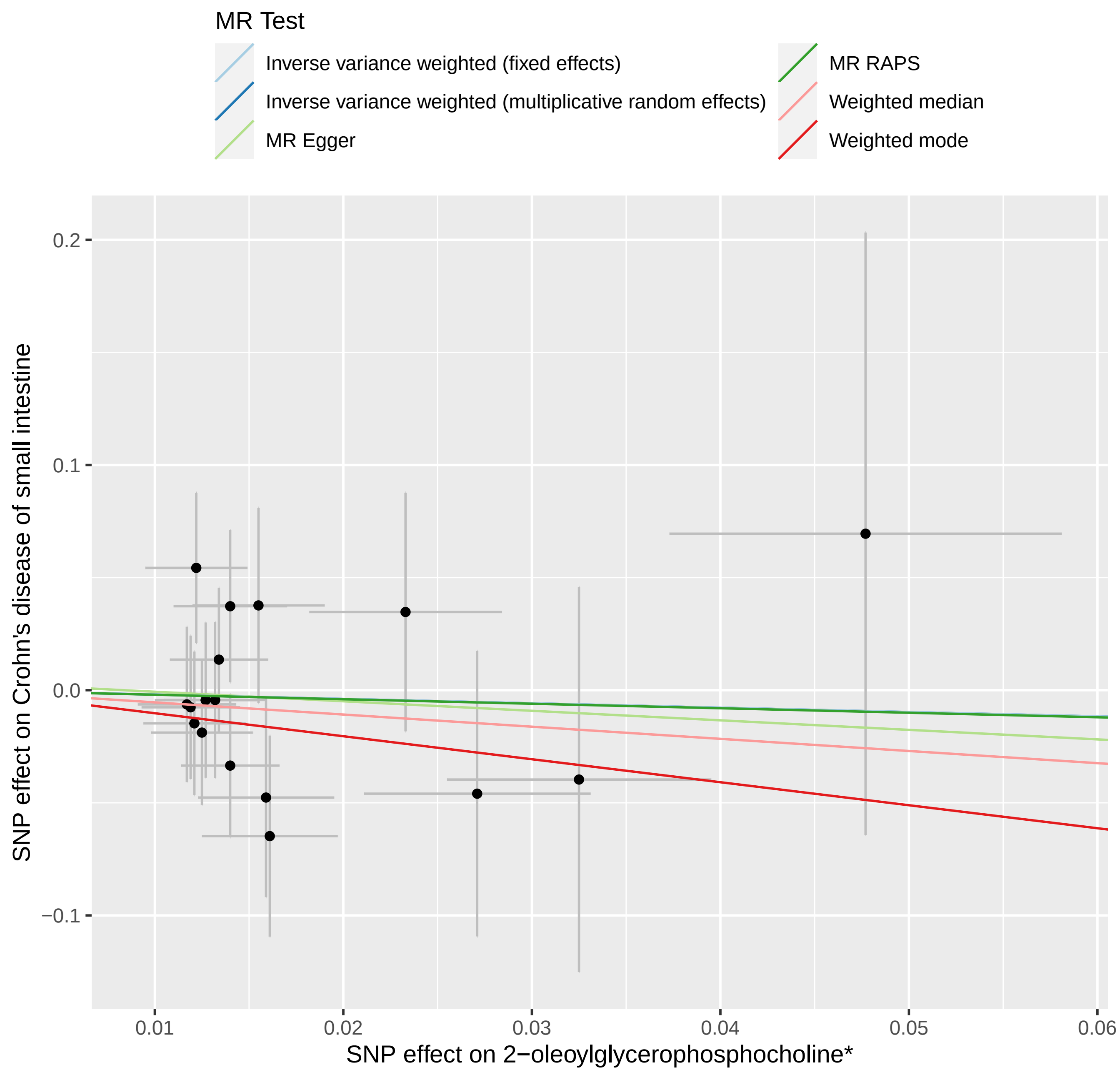

Batch 784 : 1−palmitoylglycerophosphoinositol* on Crohn's disease of small intestine

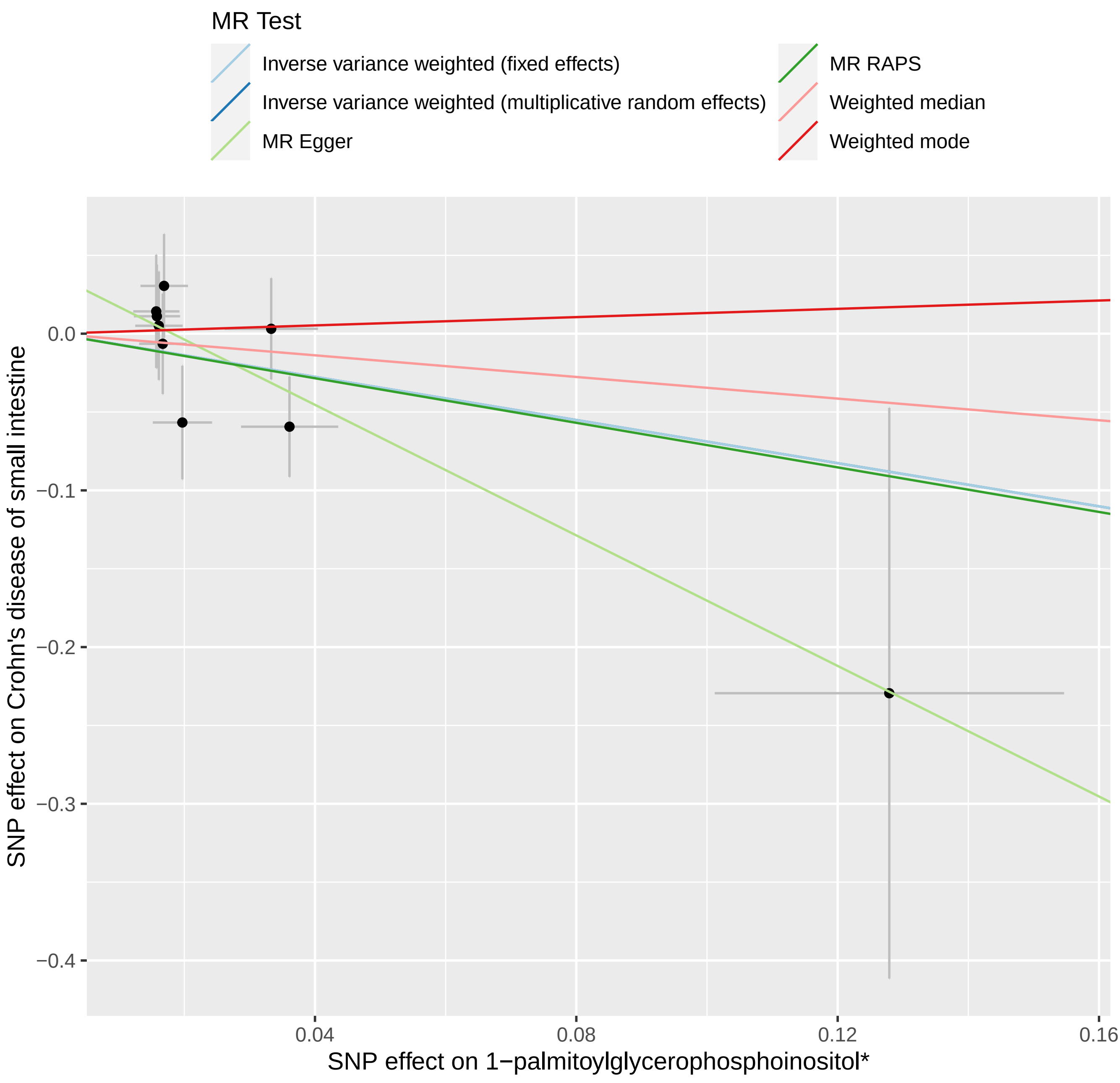

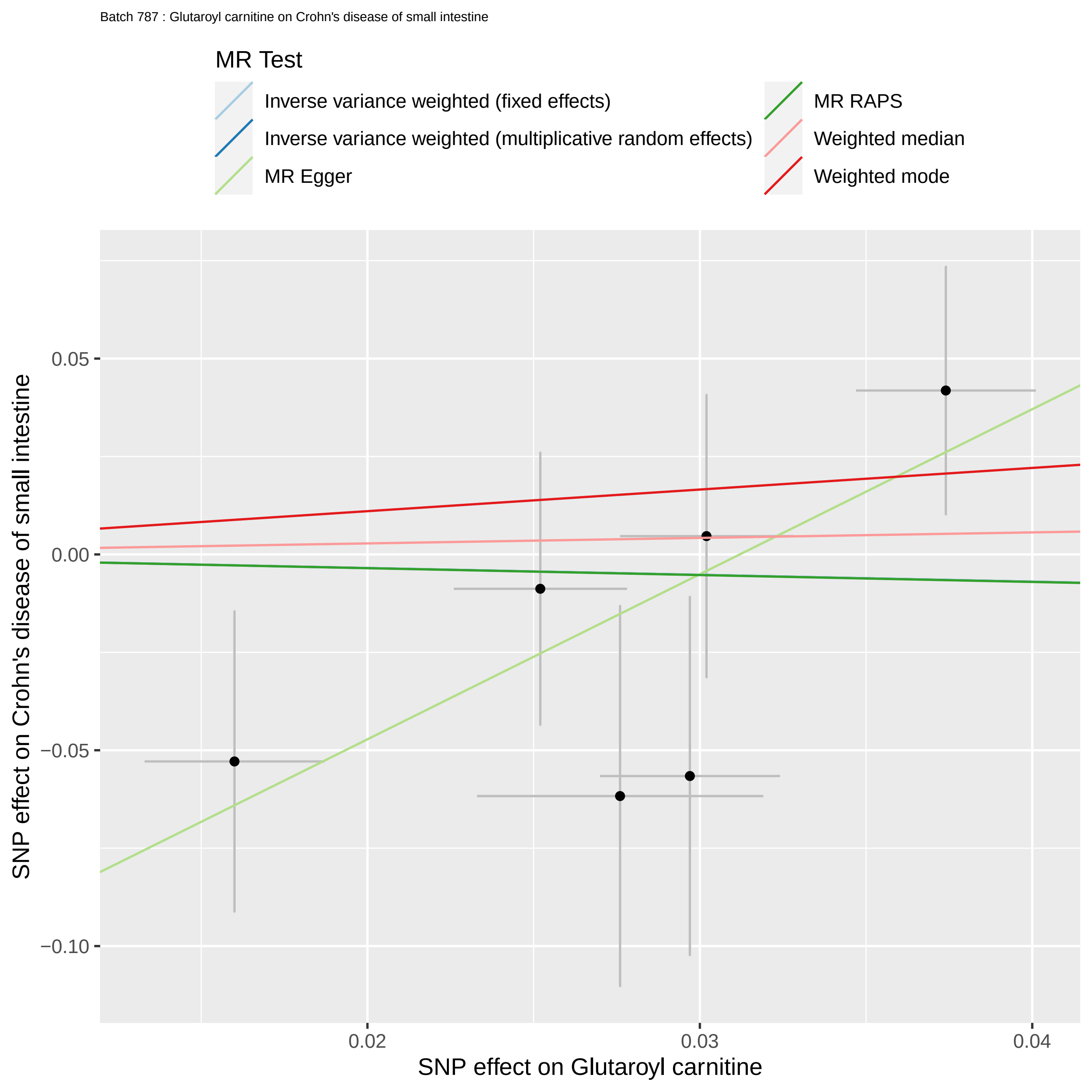

Batch 787 : Glutaroyl carnitine on Crohn's disease of small intestine
MR Test
Inverse variance weighted (fixed effects)
Inverse variance weighted (multiplicative random effects)
MR Egger
MR RAPS
Weighted median
Weighted mode
0.05
0.00
−0.05
−0.10
0.02
0.03
0.04
SNP effect on Crohn's disease of small intestine
SNP effect on Glutaroyl carnitine

Batch 789 : 4−hydroxyhippurate on Crohn's disease of small intestine

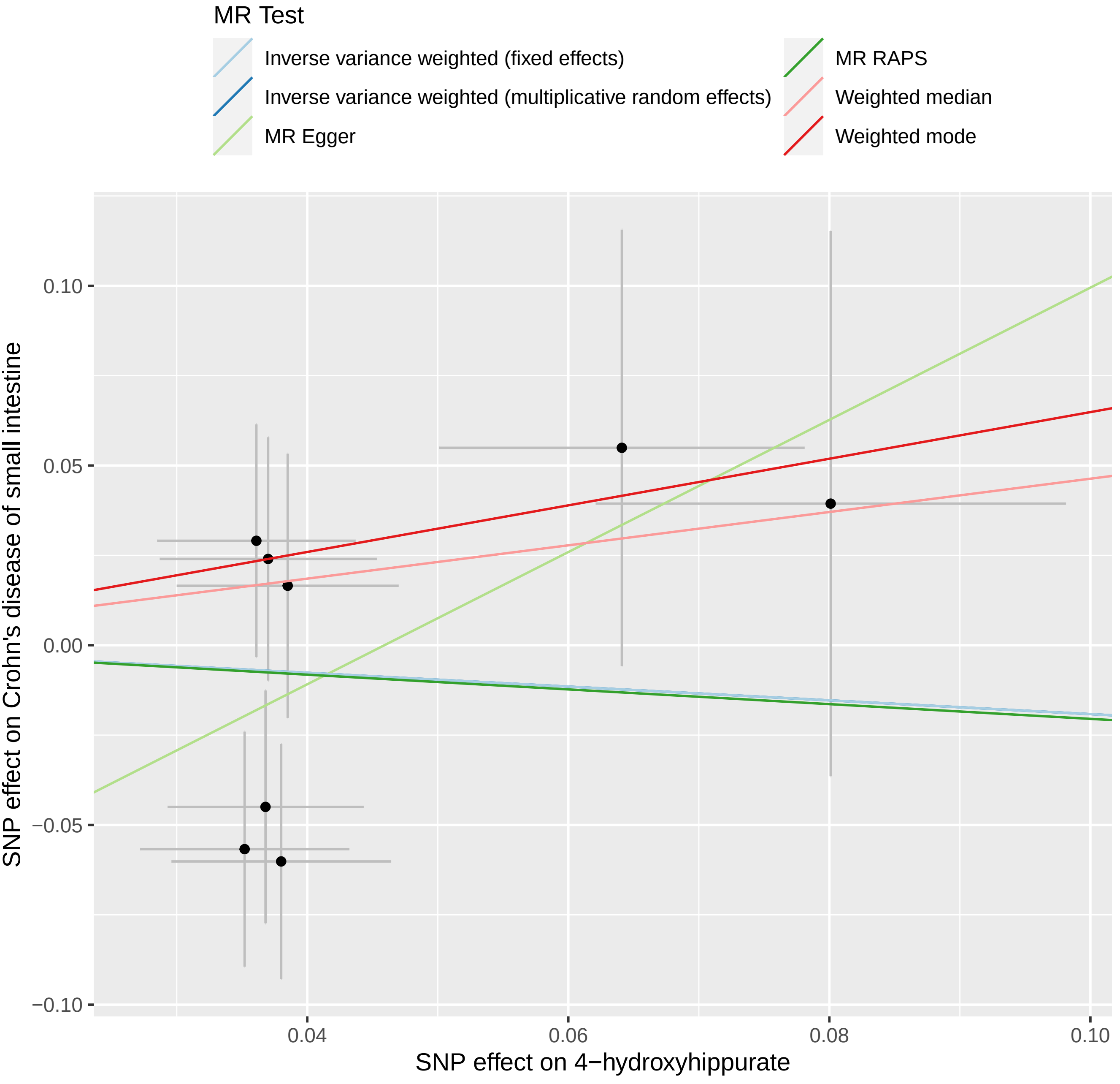

Batch 790 : 1−myristoylglycerophosphocholine on Crohn's disease of small intestine

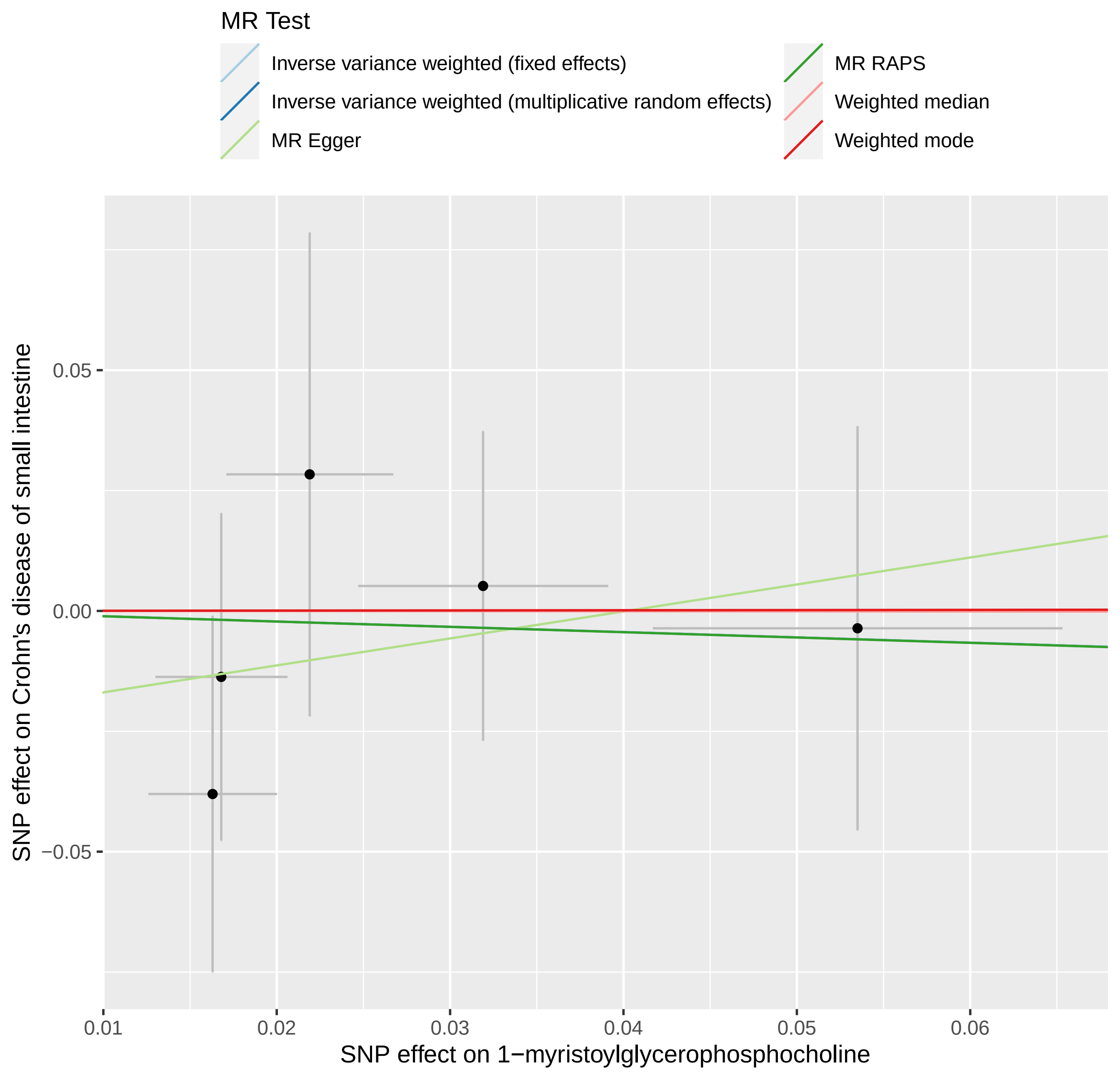

Batch 793 : 3−(3−hydroxyphenyl)propionate on Crohn's disease of small intestine

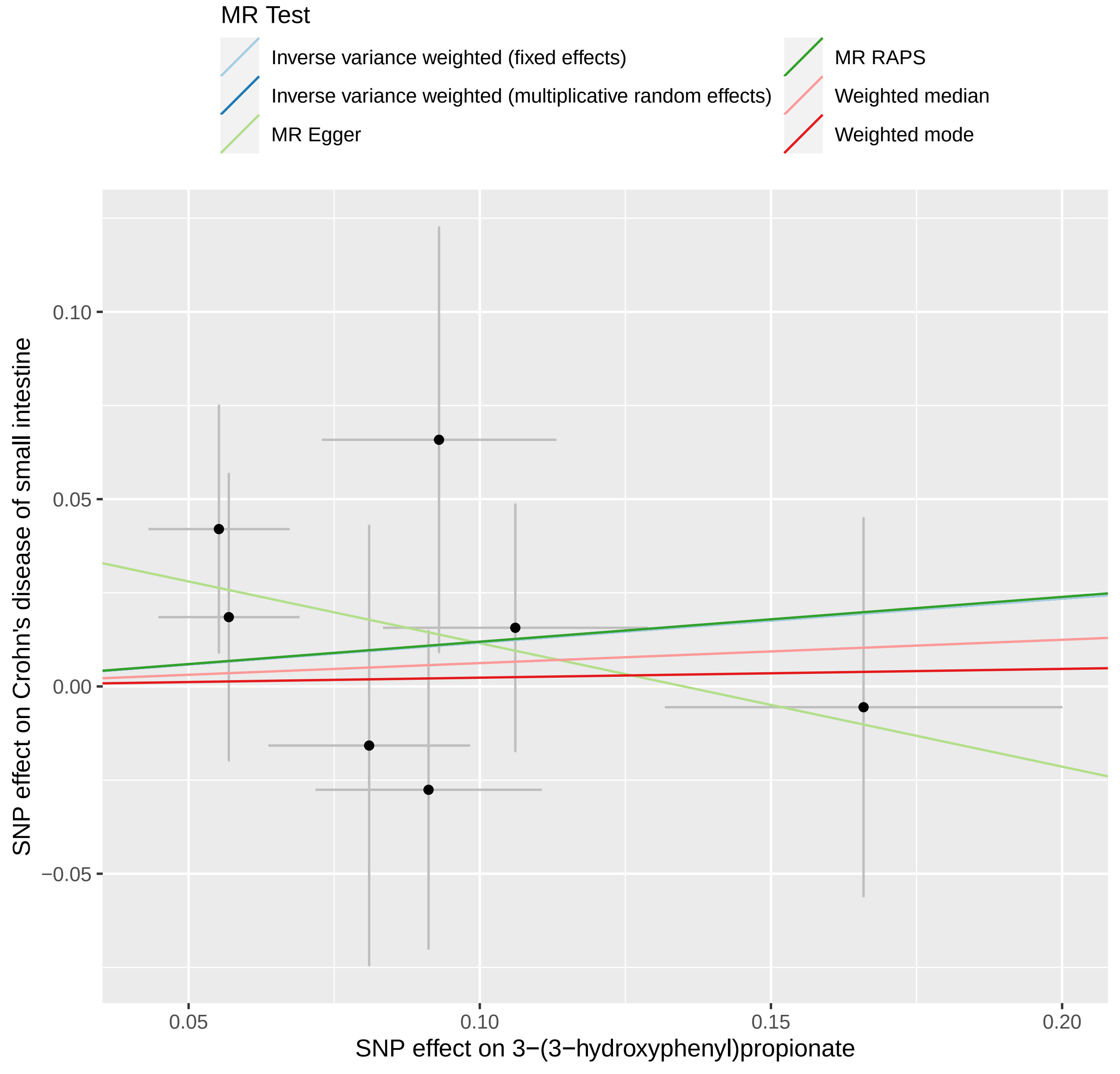

Batch 797 : Dihomo−linolenate (20:3n3 or n6) on Crohn's disease of small intestine

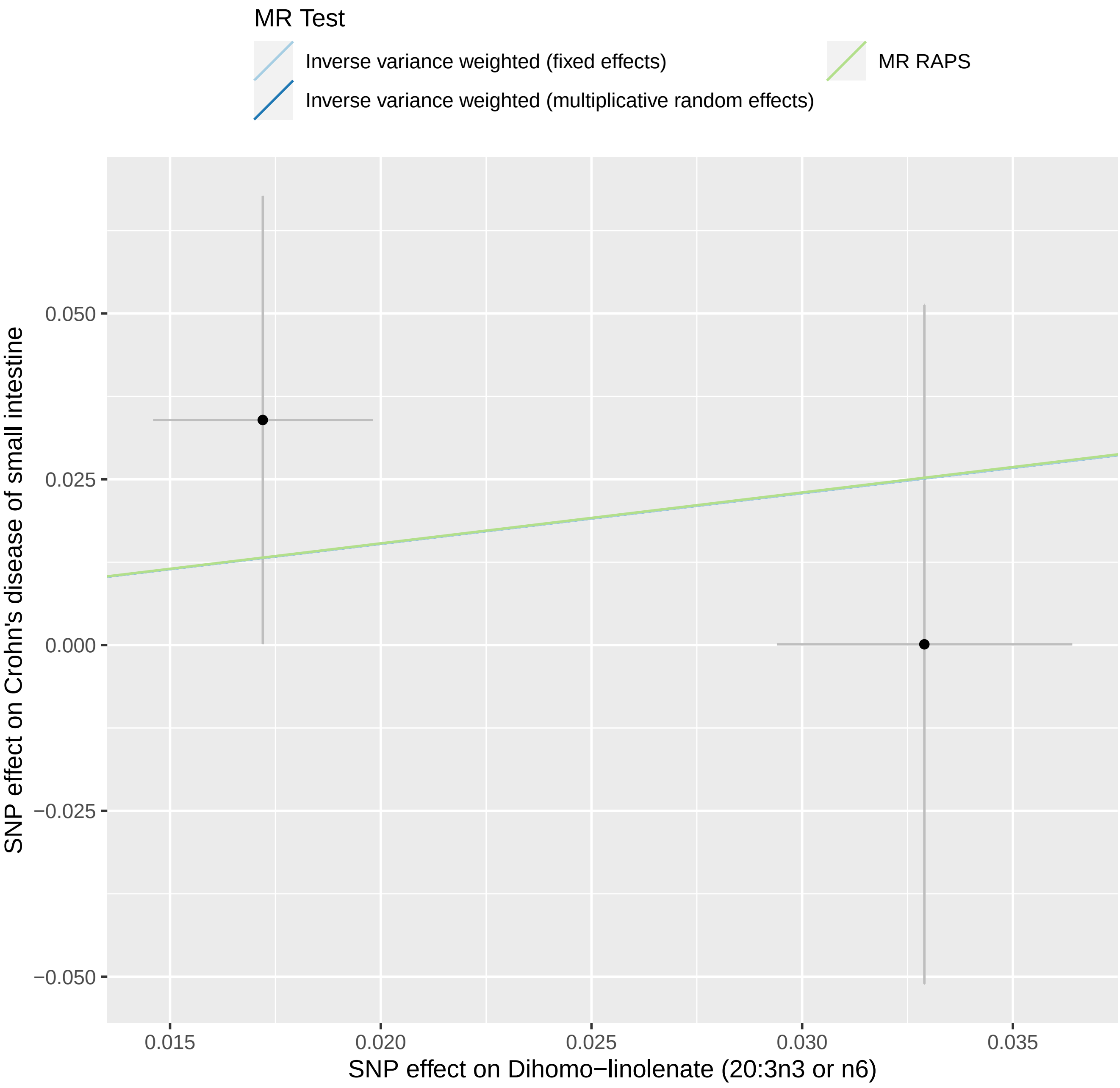

Batch 798 : Threitol on Crohn's disease of small intestine

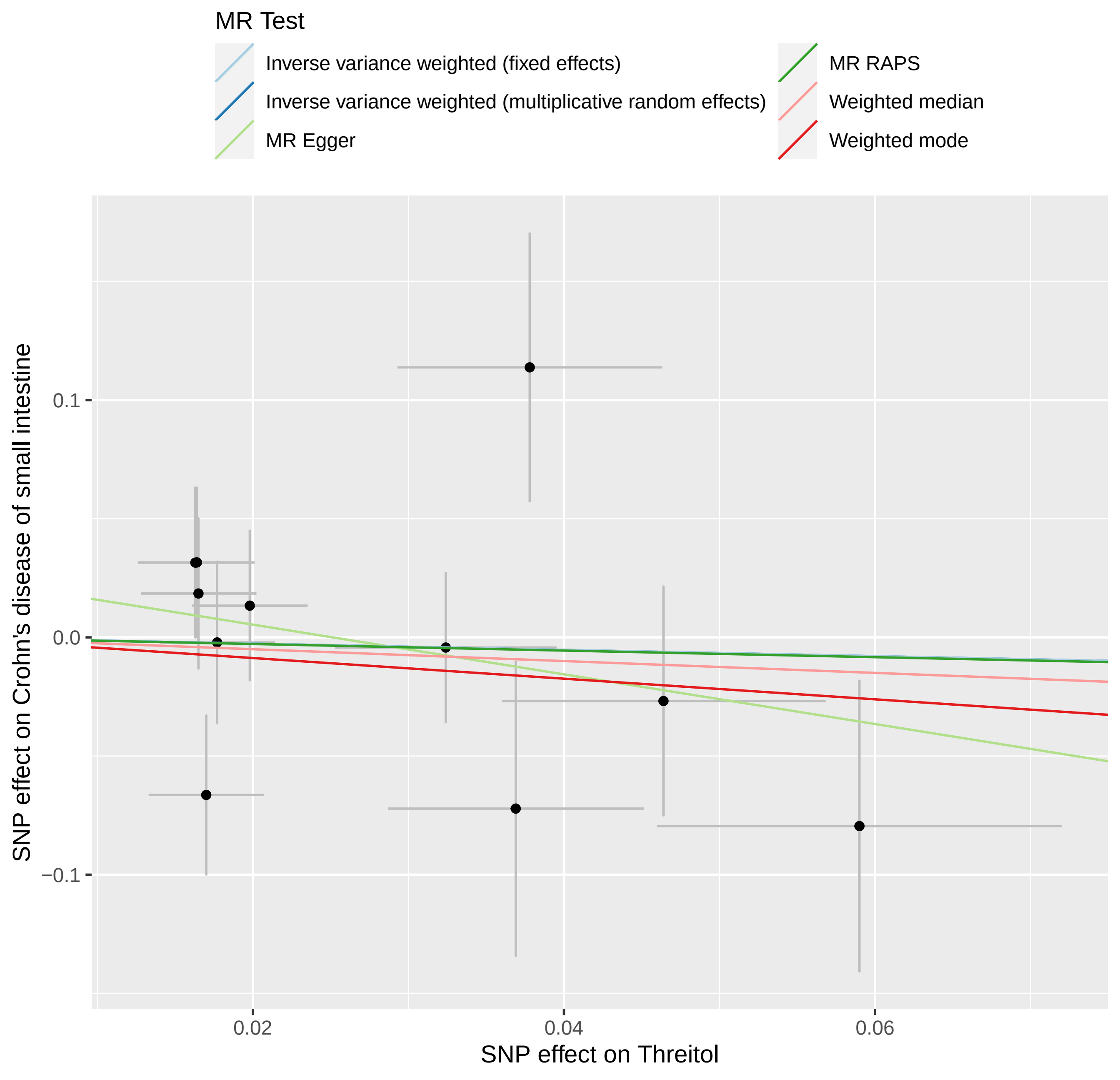

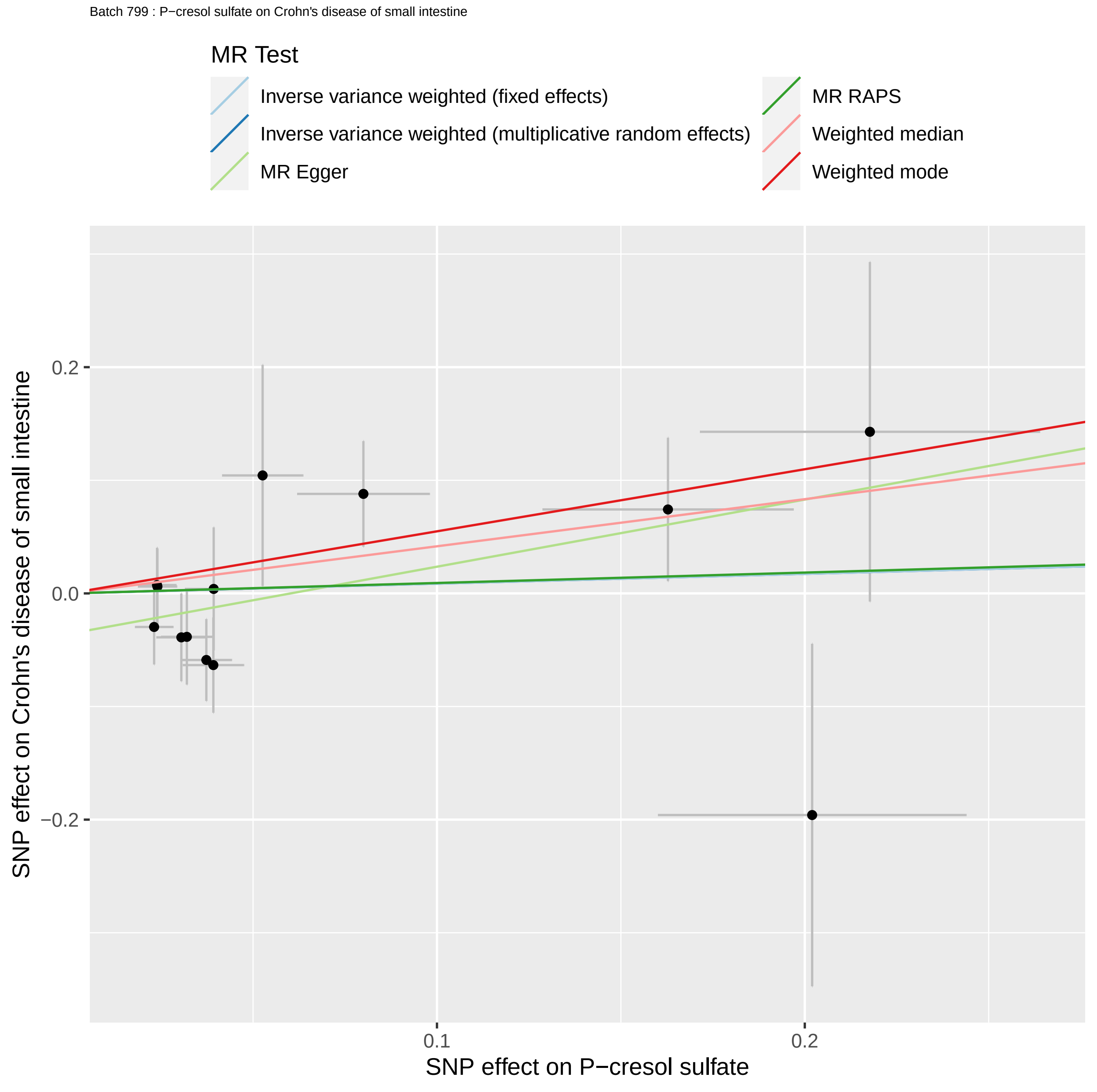

Batch 799 : P−cresol sulfate on Crohn's disease of small intestine
MR Test
Inverse variance weighted (fixed effects)
Inverse variance weighted (multiplicative random effects)
MR Egger
MR RAPS
Weighted median
Weighted mode
0.2
0.0
−0.2
0.1
0.2
SNP effect on Crohn's disease of small intestine
SNP effect on P−cresol sulfate

Batch 802 : X−14208−−phenylalanylserine on Crohn's disease of small intestine

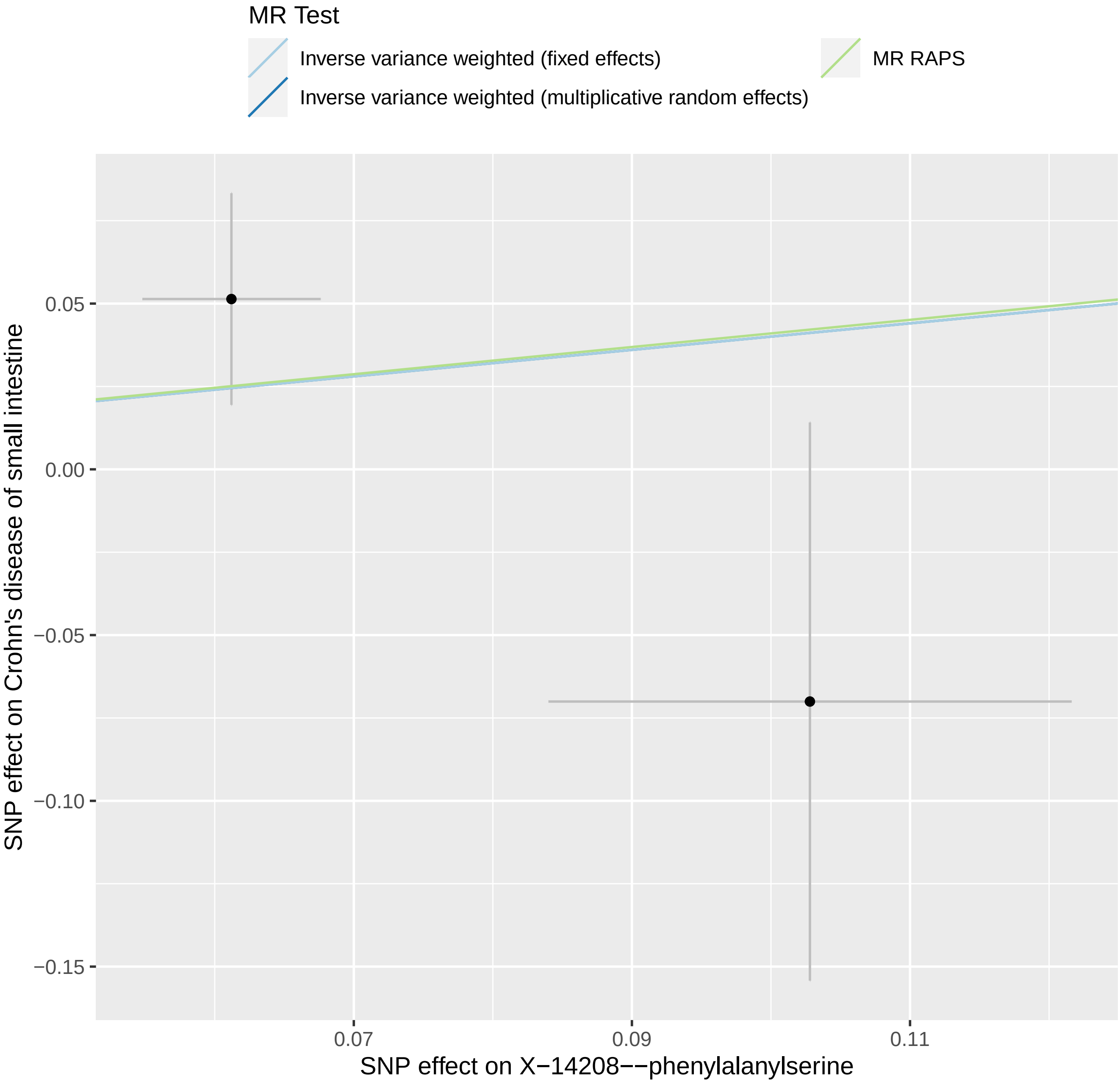

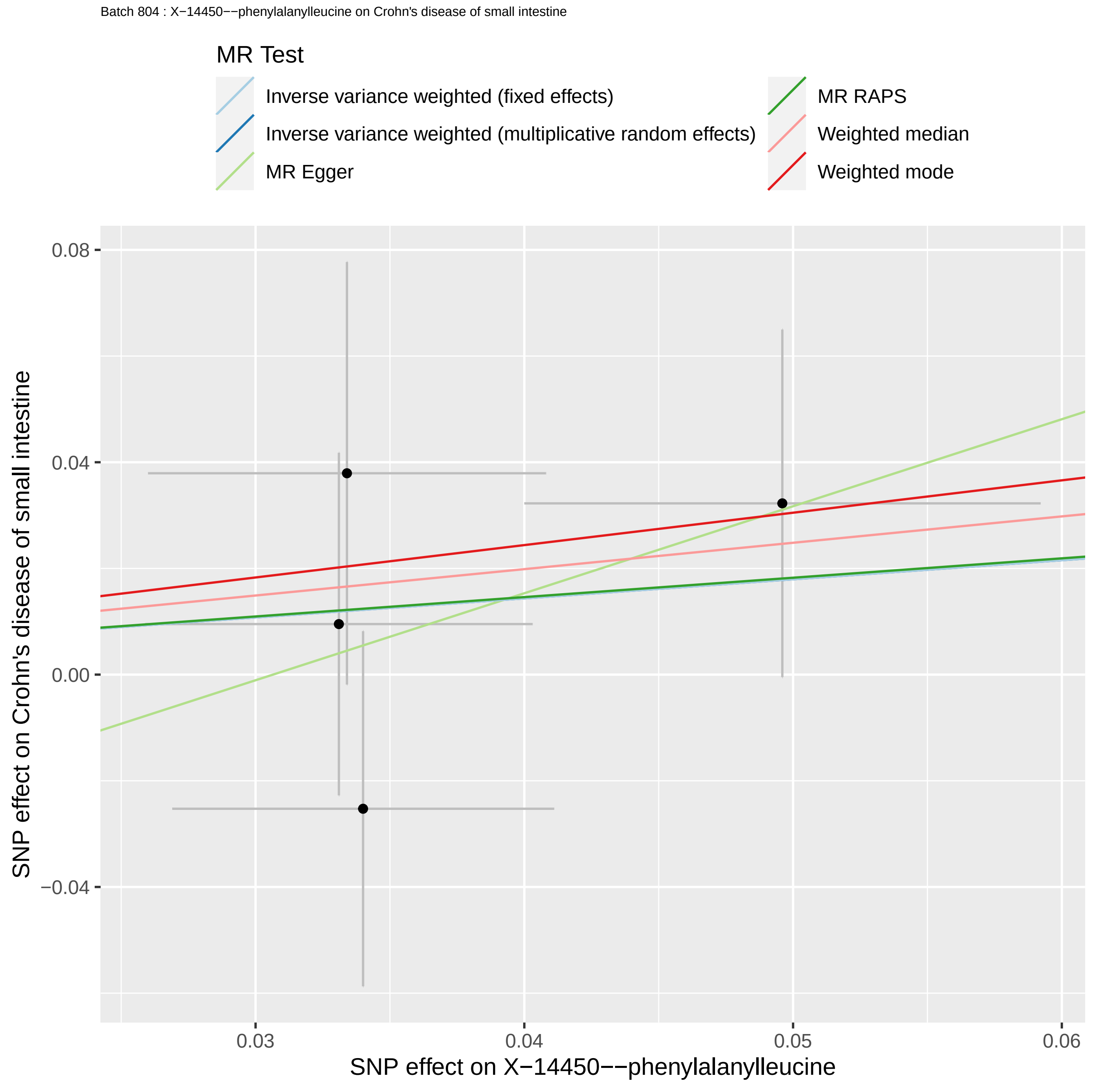

Batch 804 : X−14450−−phenylalanylleucine on Crohn's disease of small intestine
MR Test
Inverse variance weighted (fixed effects)
Inverse variance weighted (multiplicative random effects)
MR Egger
MR RAPS
Weighted median
Weighted mode
0.08
0.04
0.00
−0.04
0.03
0.04
0.05
0.06
SNP effect on Crohn's disease of small intestine
SNP effect on X−14450−−phenylalanylleucine

Batch 805 : Gamma−glutamylglutamate on Crohn's disease of small intestine

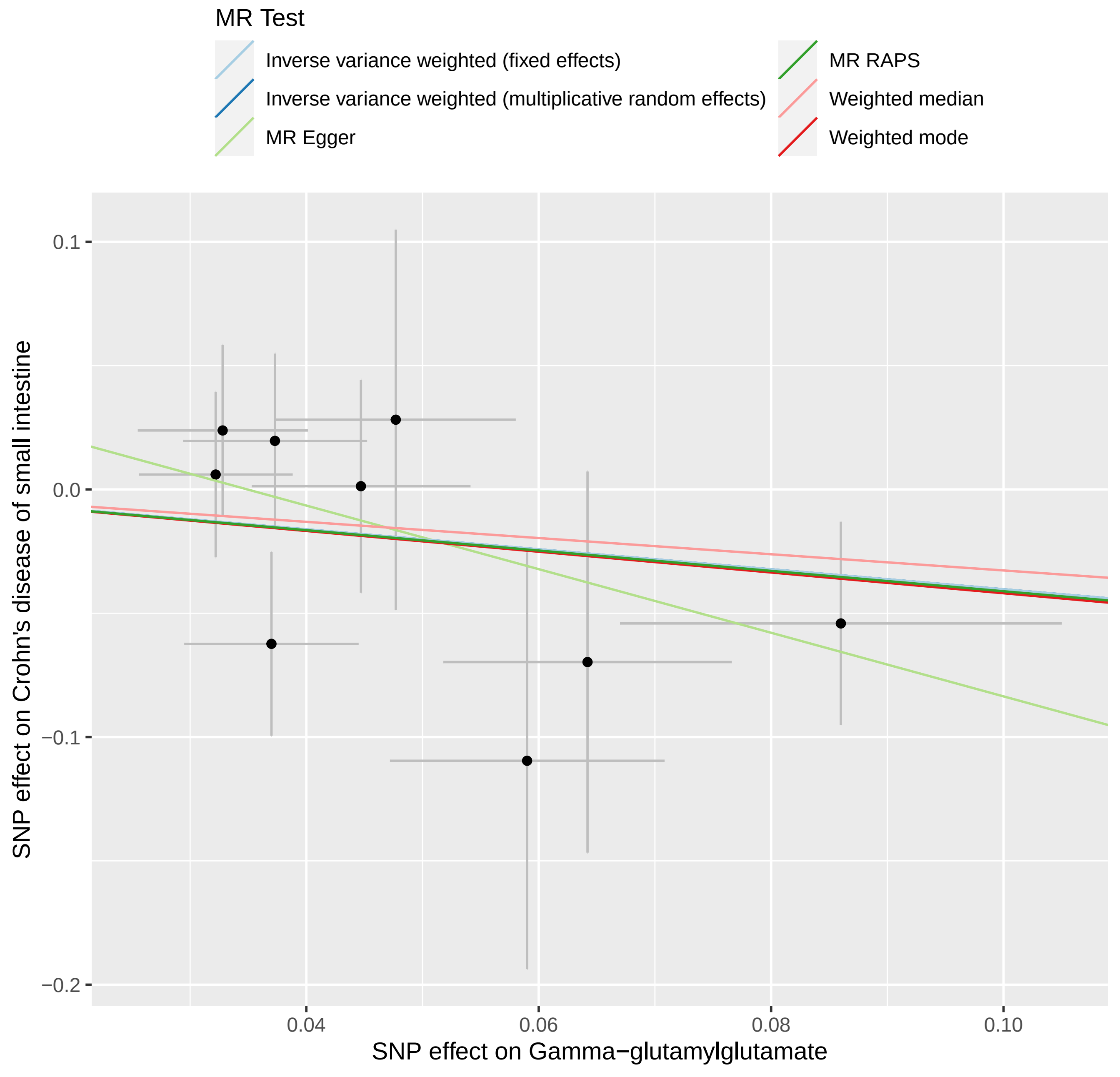

Batch 806 : Octadecanedioate on Crohn's disease of small intestine

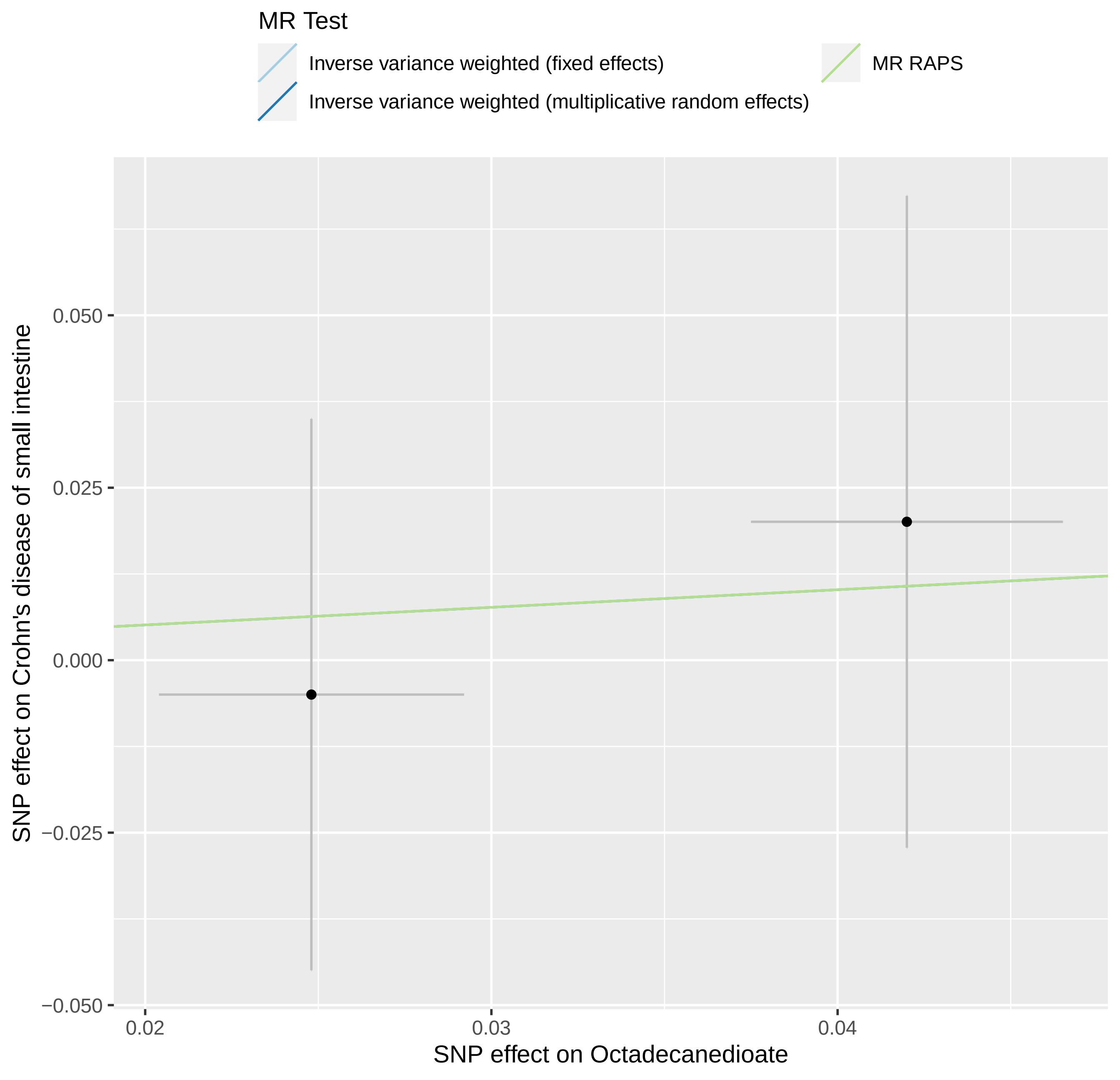

Batch 807 : Leucylleucine on Crohn's disease of small intestine

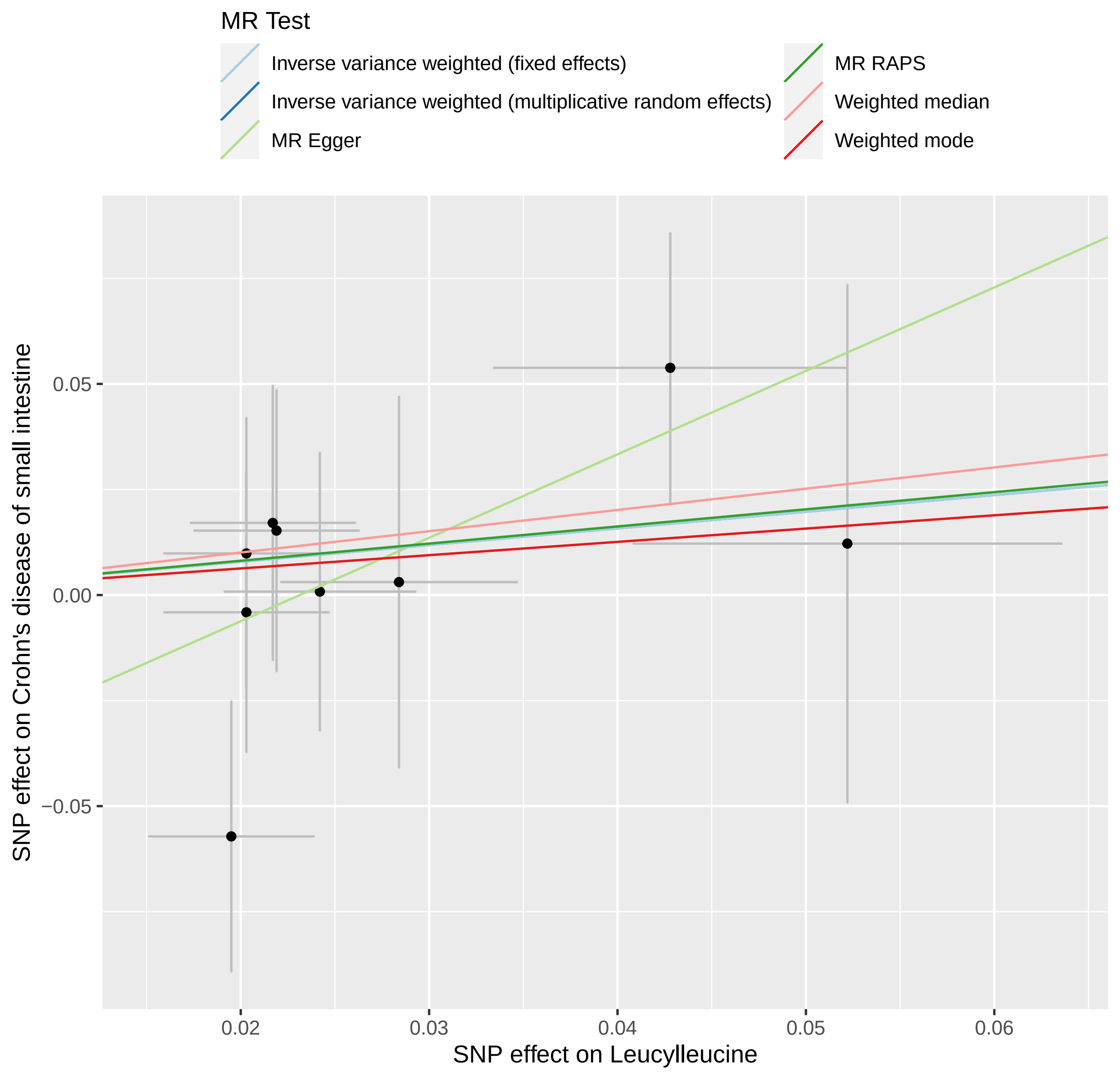

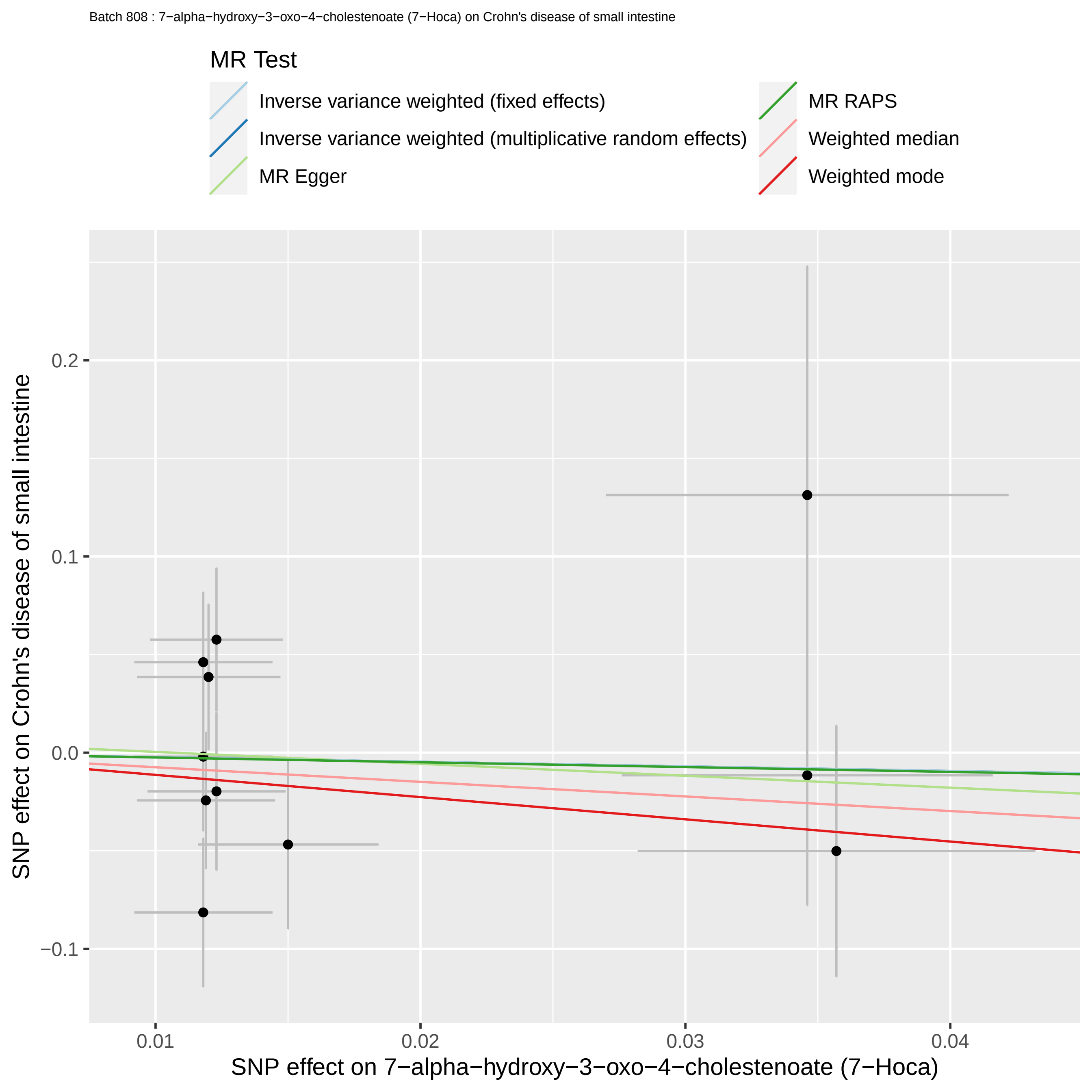

Batch 808 : 7−alpha−hydroxy−3−oxo−4−cholestenoate (7−Hoca) on Crohn's disease of small intestine
MR Test
Inverse variance weighted (fixed effects)
Inverse variance weighted (multiplicative random effects)
MR Egger
MR RAPS
Weighted median
Weighted mode
SNP effect on Crohn's disease of small intestine
0.2
0.1
0.0
−0.1
0.01
0.02
0.03
0.04
SNP effect on 7−alpha−hydroxy−3−oxo−4−cholestenoate (7−Hoca)

Batch 809 : N−Butyl Oleate on Crohn's disease of small intestine

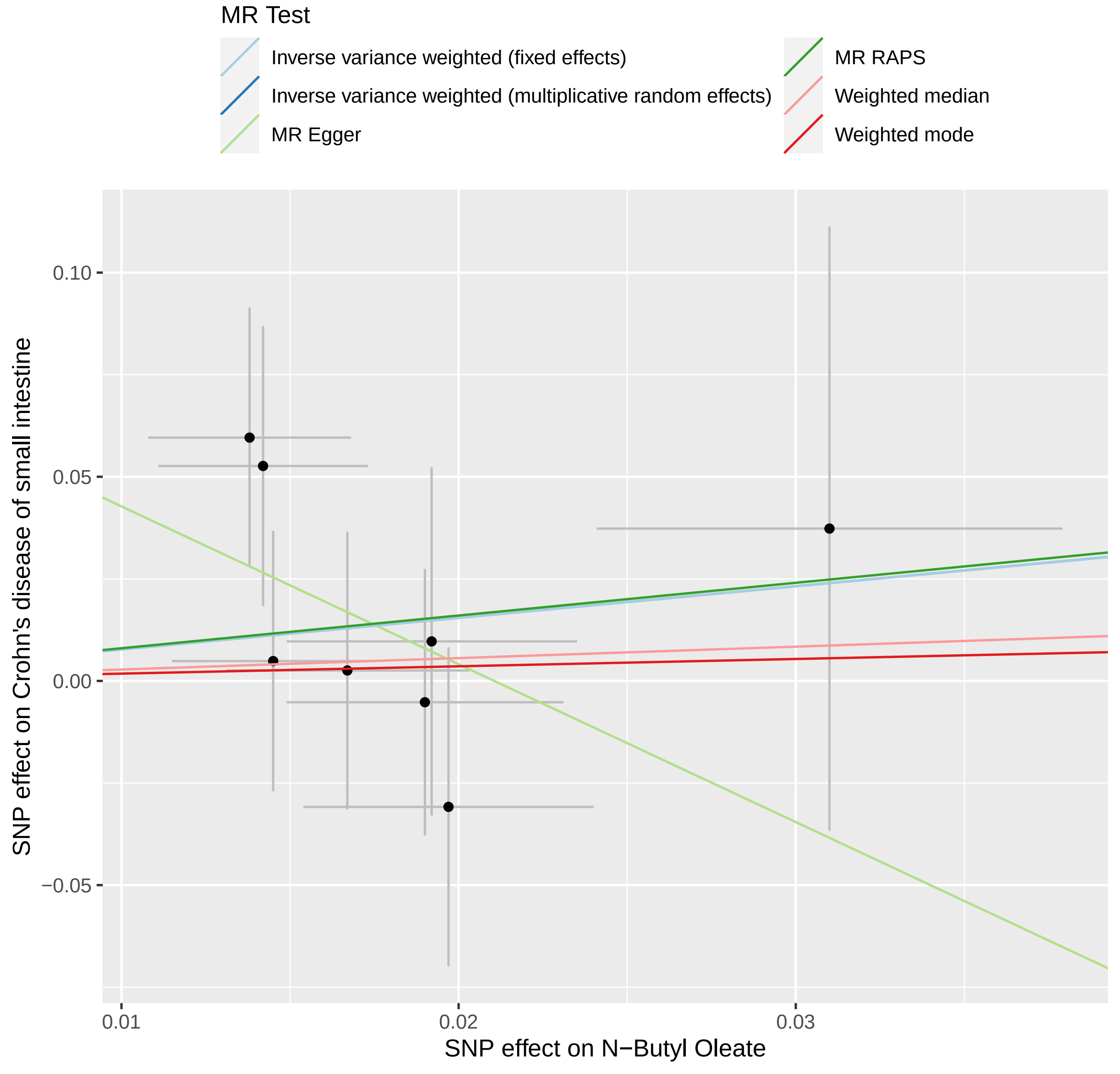

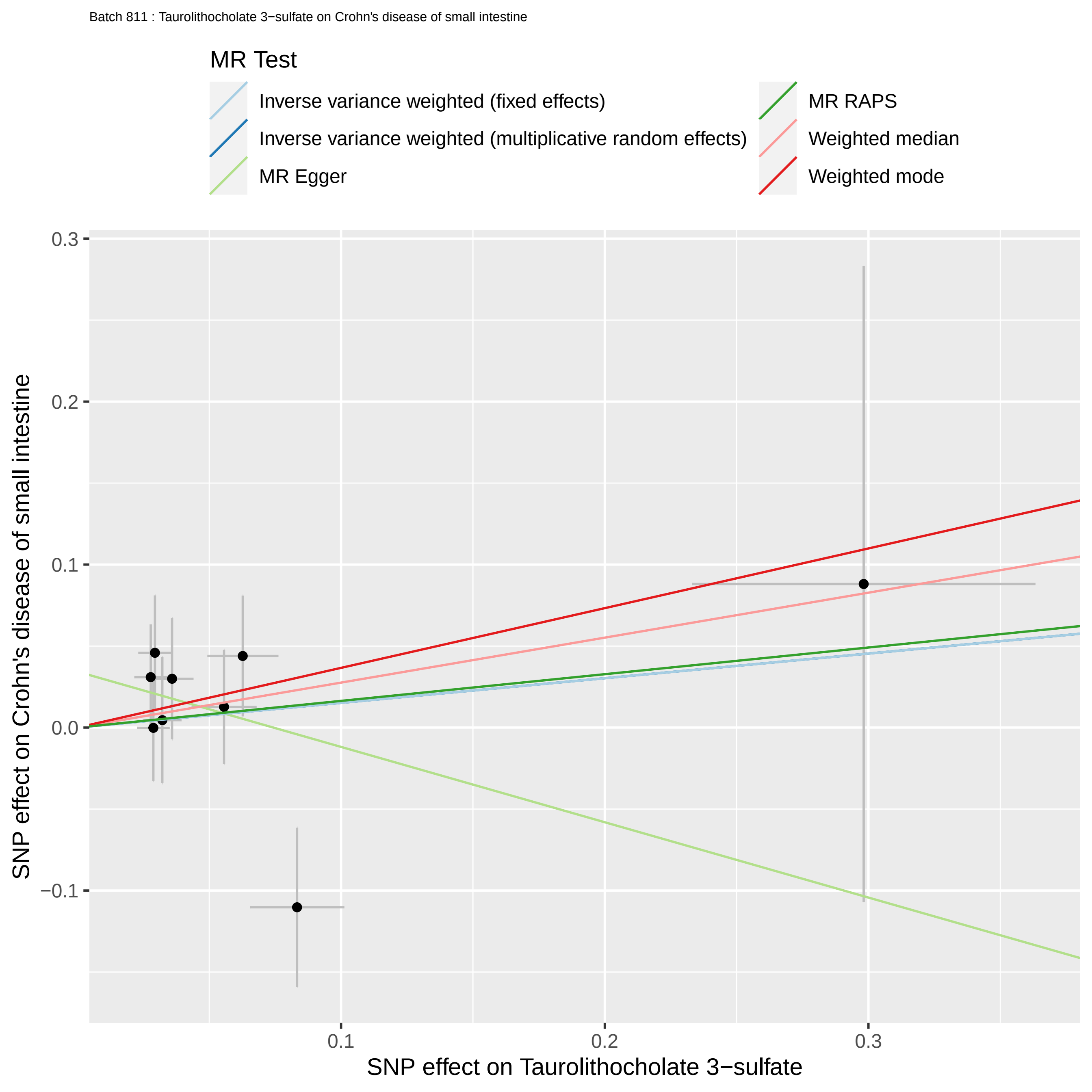
Batch 811 : Taurolithocholate 3−sulfate on Crohn's disease of small intestine
MR Test
Inverse variance weighted (fixed effects)
Inverse variance weighted (multiplicative random effects)
MR Egger
MR RAPS
Weighted median
Weighted mode
0.3
0.2
0.1
0.0
−0.1
0.1
0.2
0.3
SNP effect on Crohn's disease of small intestine
SNP effect on Taurolithocholate 3−sulfate

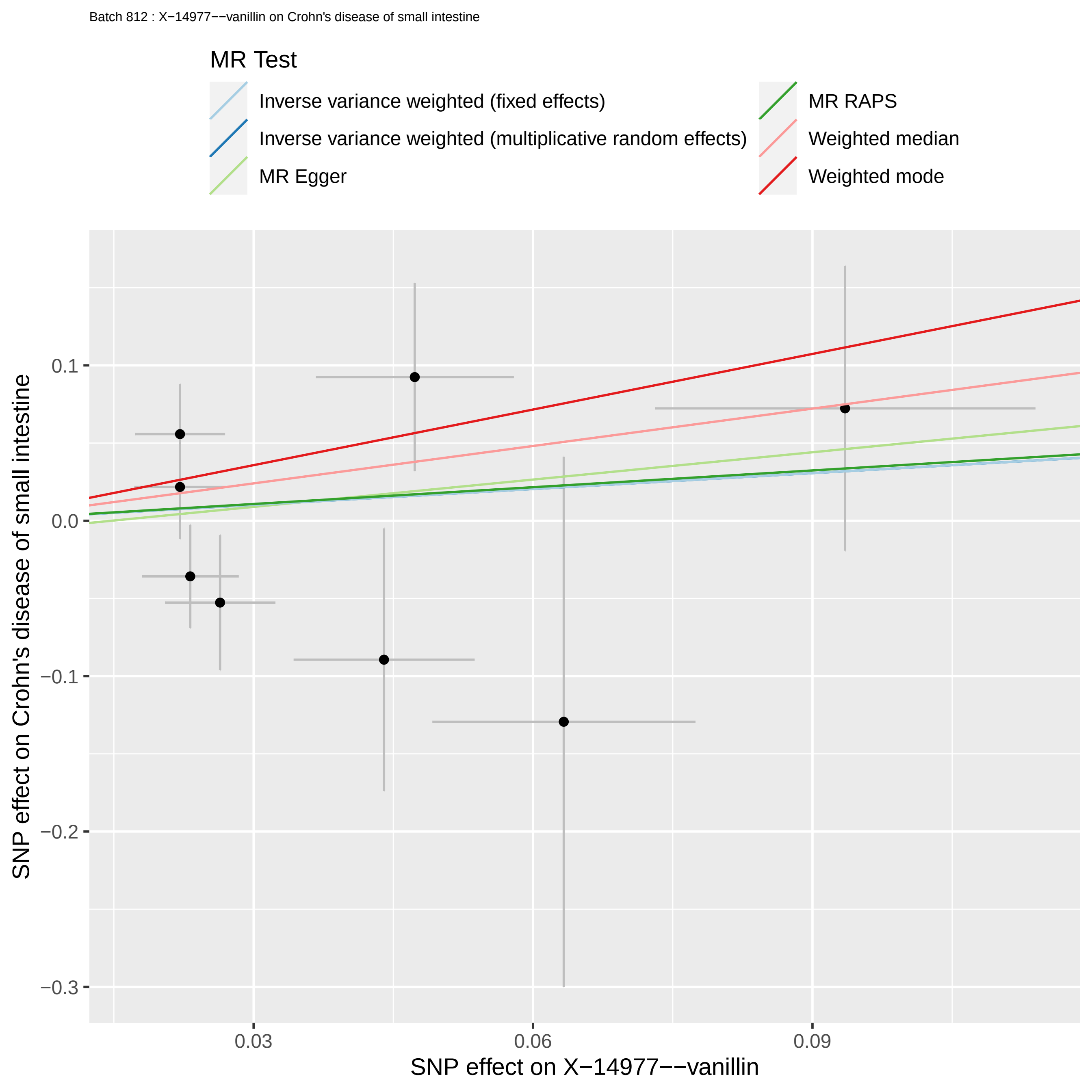

Batch 812 : X−14977−−vanillin on Crohn's disease of small intestine
MR Test
Inverse variance weighted (fixed effects)
Inverse variance weighted (multiplicative random effects)
MR Egger
MR RAPS
Weighted median
Weighted mode
0.1
0.0
−0.1
−0.2
−0.3
0.03
0.06
0.09
SNP effect on Crohn's disease of small intestine
SNP effect on X−14977−−vanillin

Batch 813 : Succinylcarnitine on Crohn's disease of small intestine

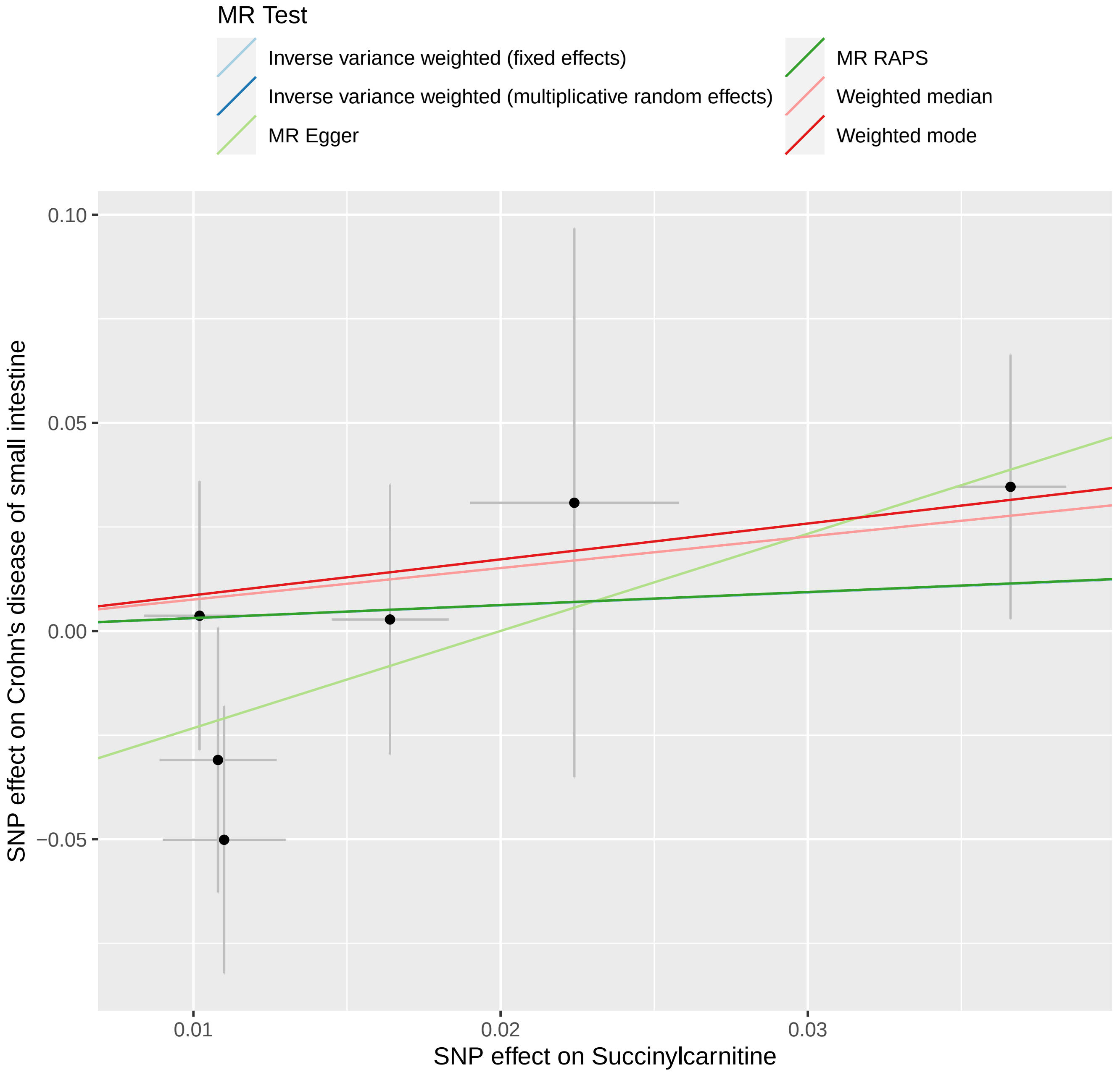

Batch 815 : Cyclo(leu−pro) on Crohn's disease of small intestine

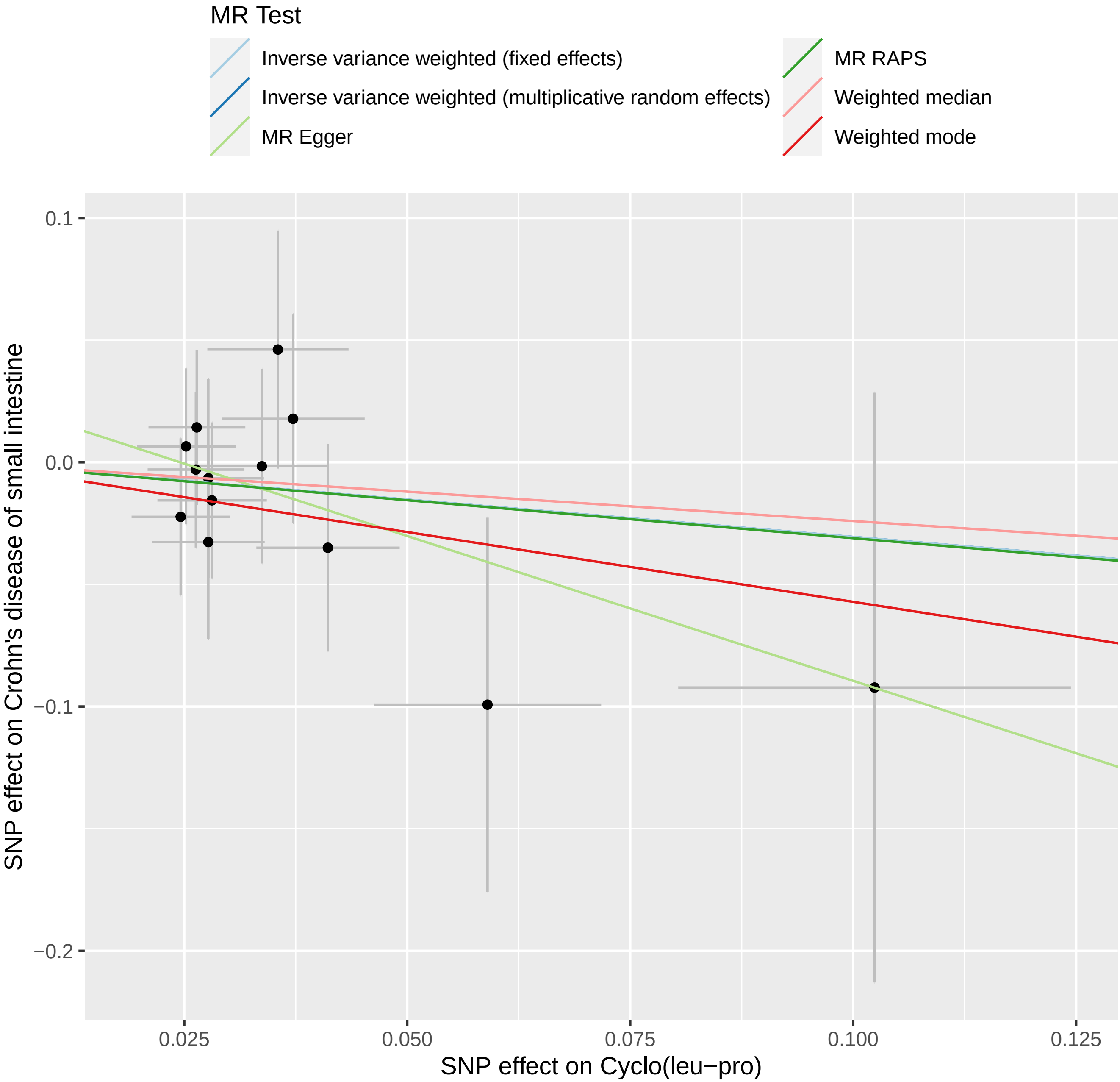

Batch 816 : Chiro−inositol on Crohn's disease of small intestine

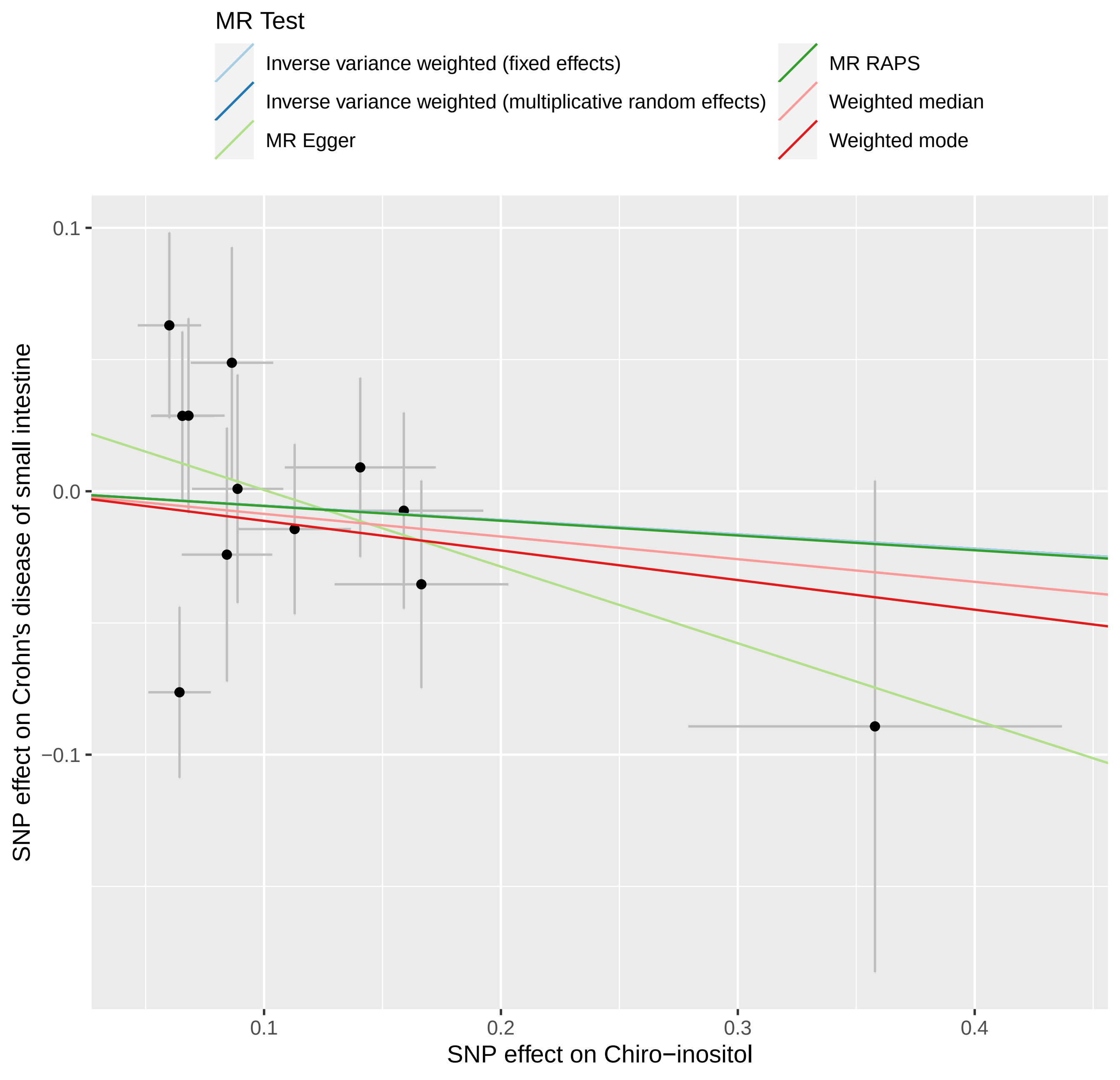

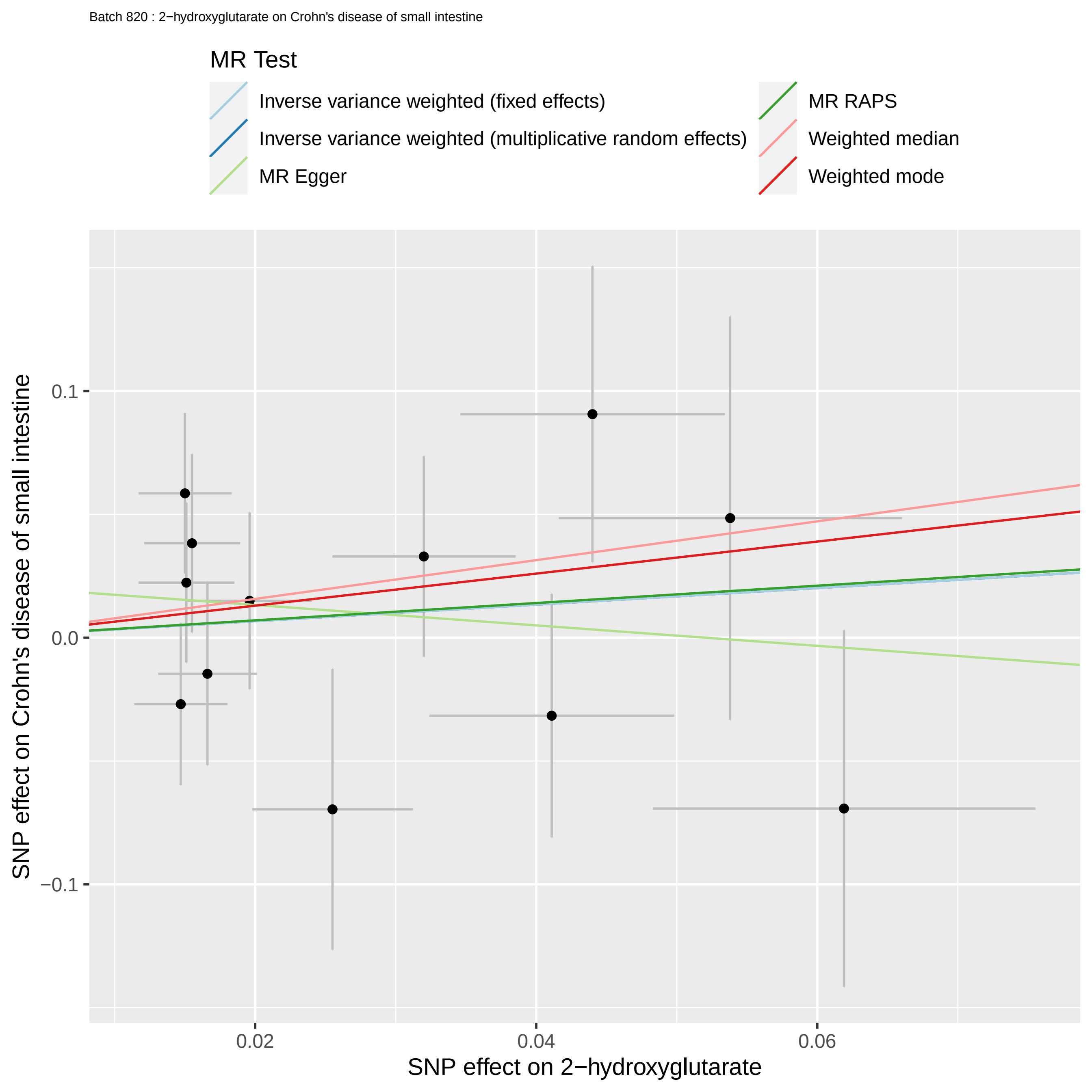
Batch 820 : 2−hydroxyglutarate on Crohn's disease of small intestine
MR Test
Inverse variance weighted (fixed effects)
Inverse variance weighted (multiplicative random effects)
MR Egger
MR RAPS
Weighted median
Weighted mode
SNP effect on Crohn's disease of small intestine
0.1
0.0
−0.1
0.02
0.04
0.06
SNP effect on 2−hydroxyglutarate

Batch 821 : Ergothioneine on Crohn's disease of small intestine

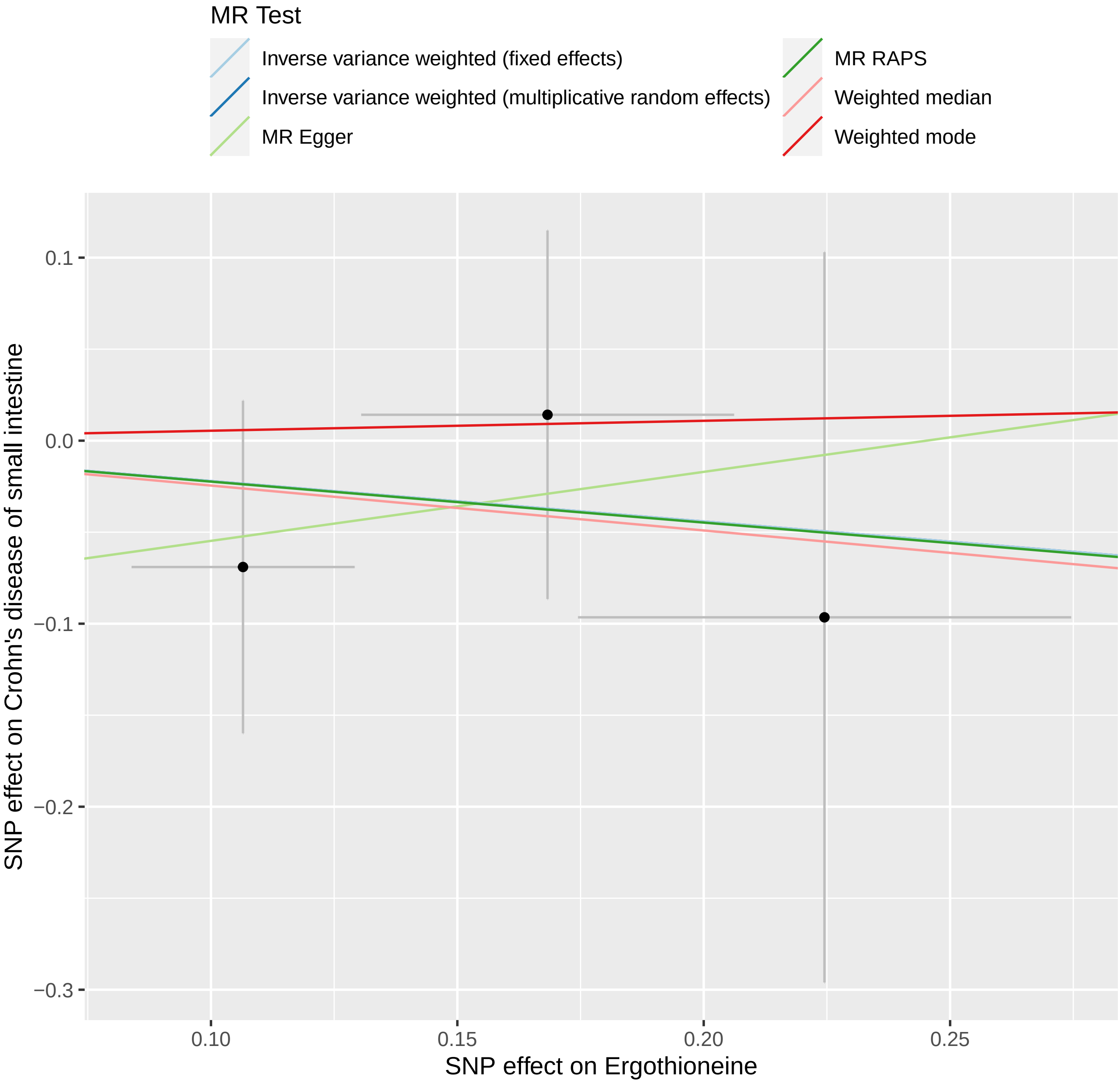

Batch 823 : Phenylalanylphenylalanine on Crohn's disease of small intestine

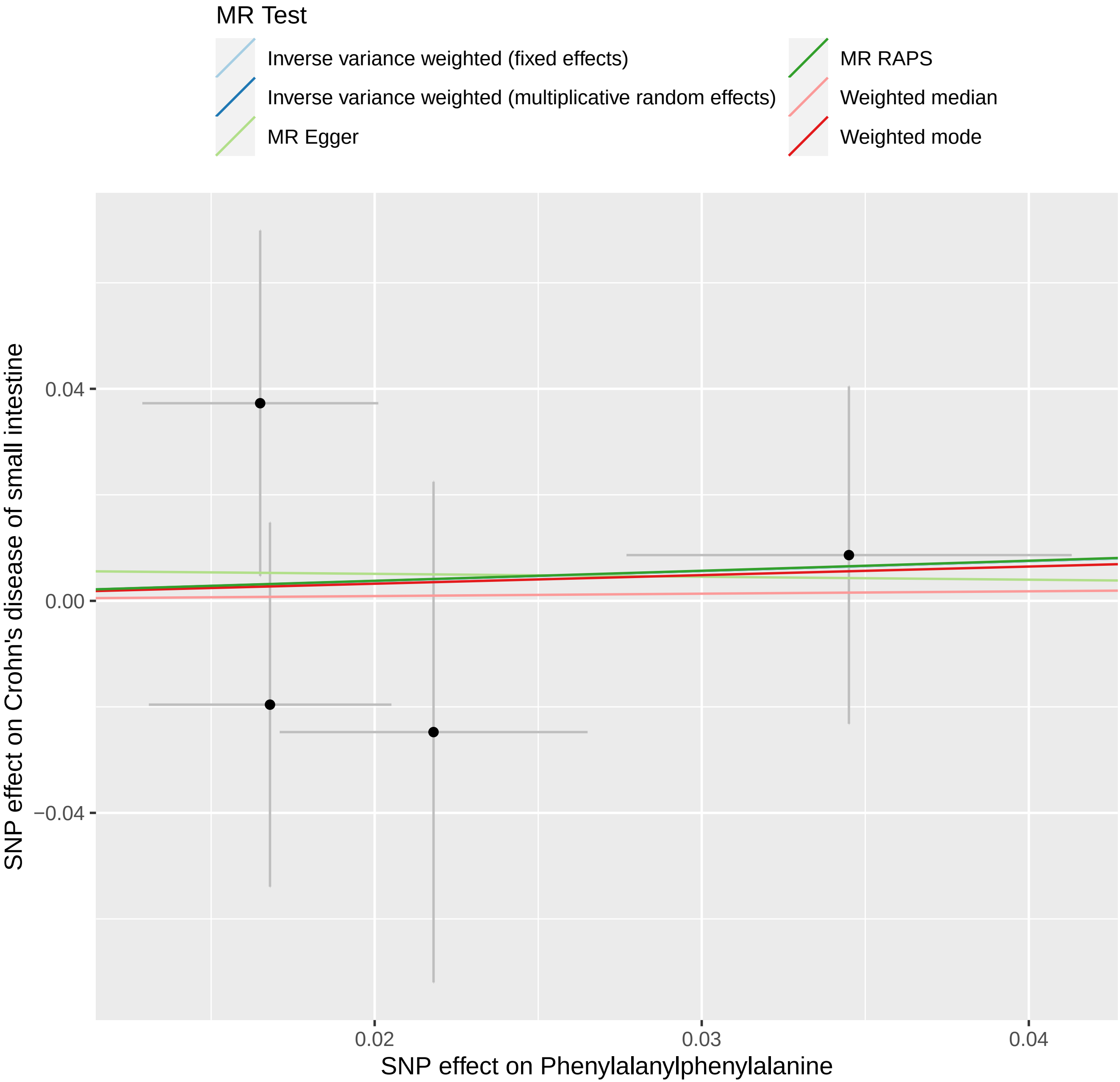

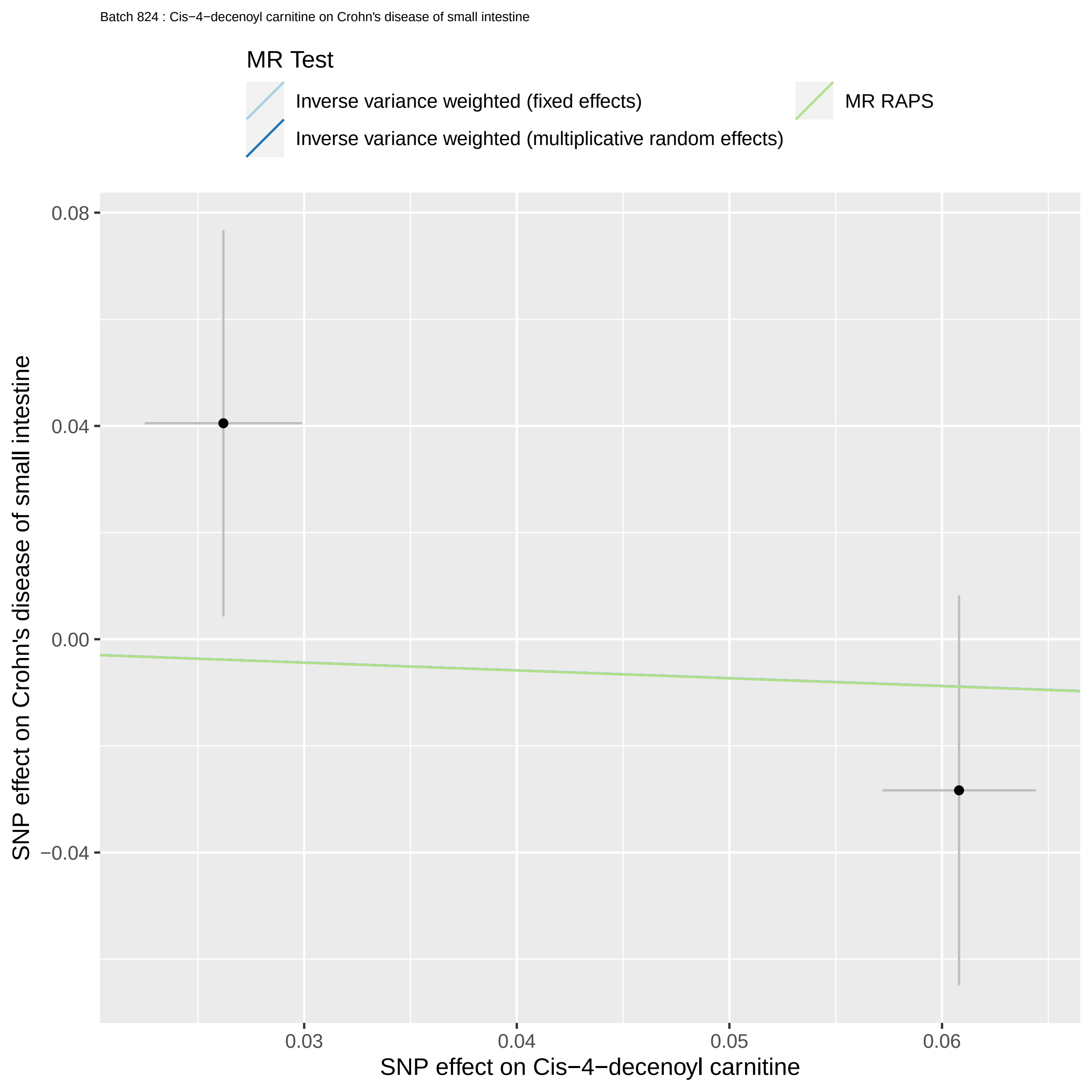

Batch 824 : Cis−4−decenoyl carnitine on Crohn's disease of small intestine
MR Test
Inverse variance weighted (fixed effects)
Inverse variance weighted (multiplicative random effects)
MR RAPS
0.08
0.04
0.00
−0.04
0.03
0.04
0.05
0.06
SNP effect on Crohn's disease of small intestine
SNP effect on Cis−4−decenoyl carnitine

Batch 825 : 15−methylpalmitate (isobar with 2−methylpalmitate) on Crohn's disease of small intestine

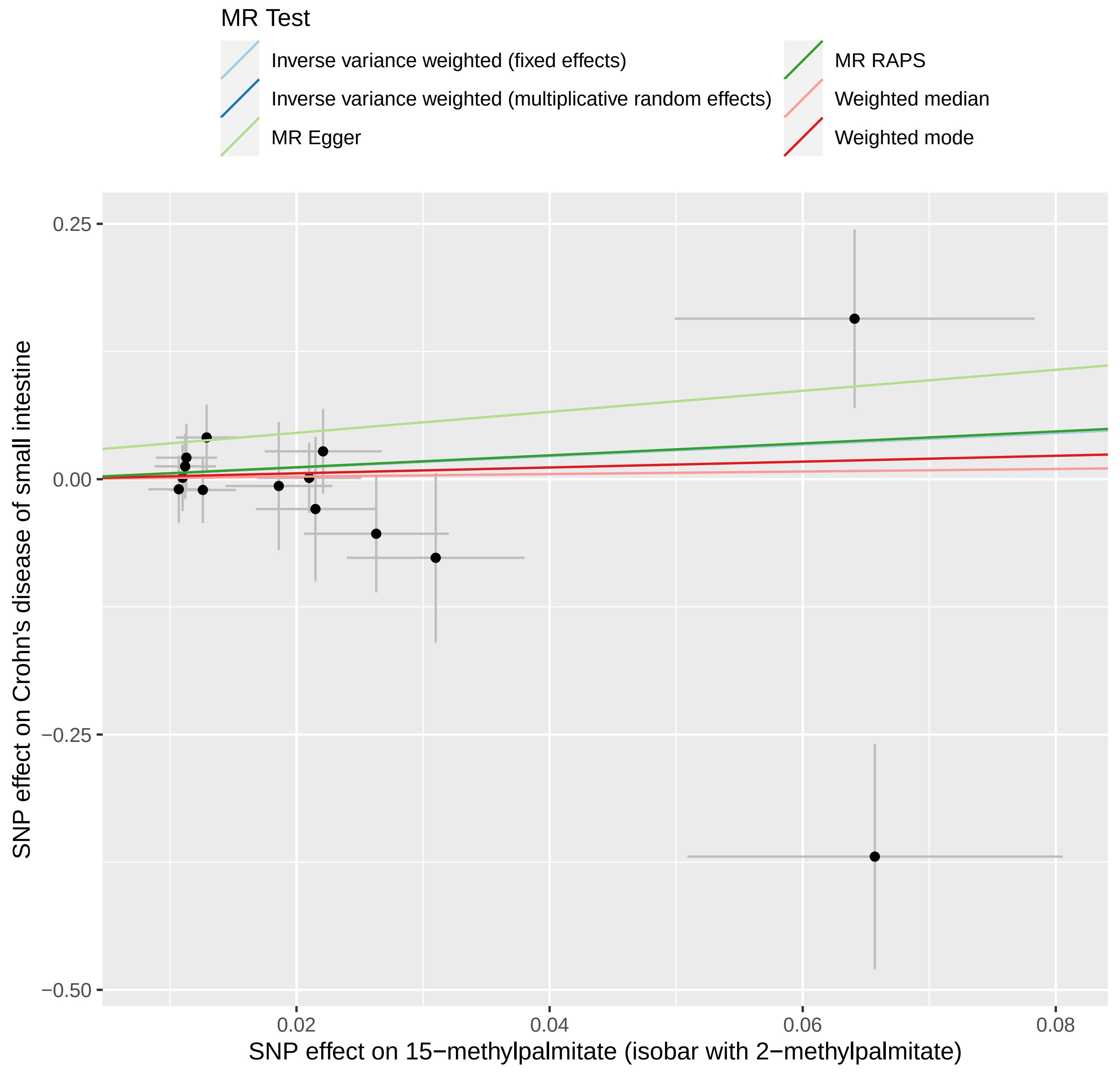

Batch 827 : Tryptophan on Arthropathy in Crohn disease

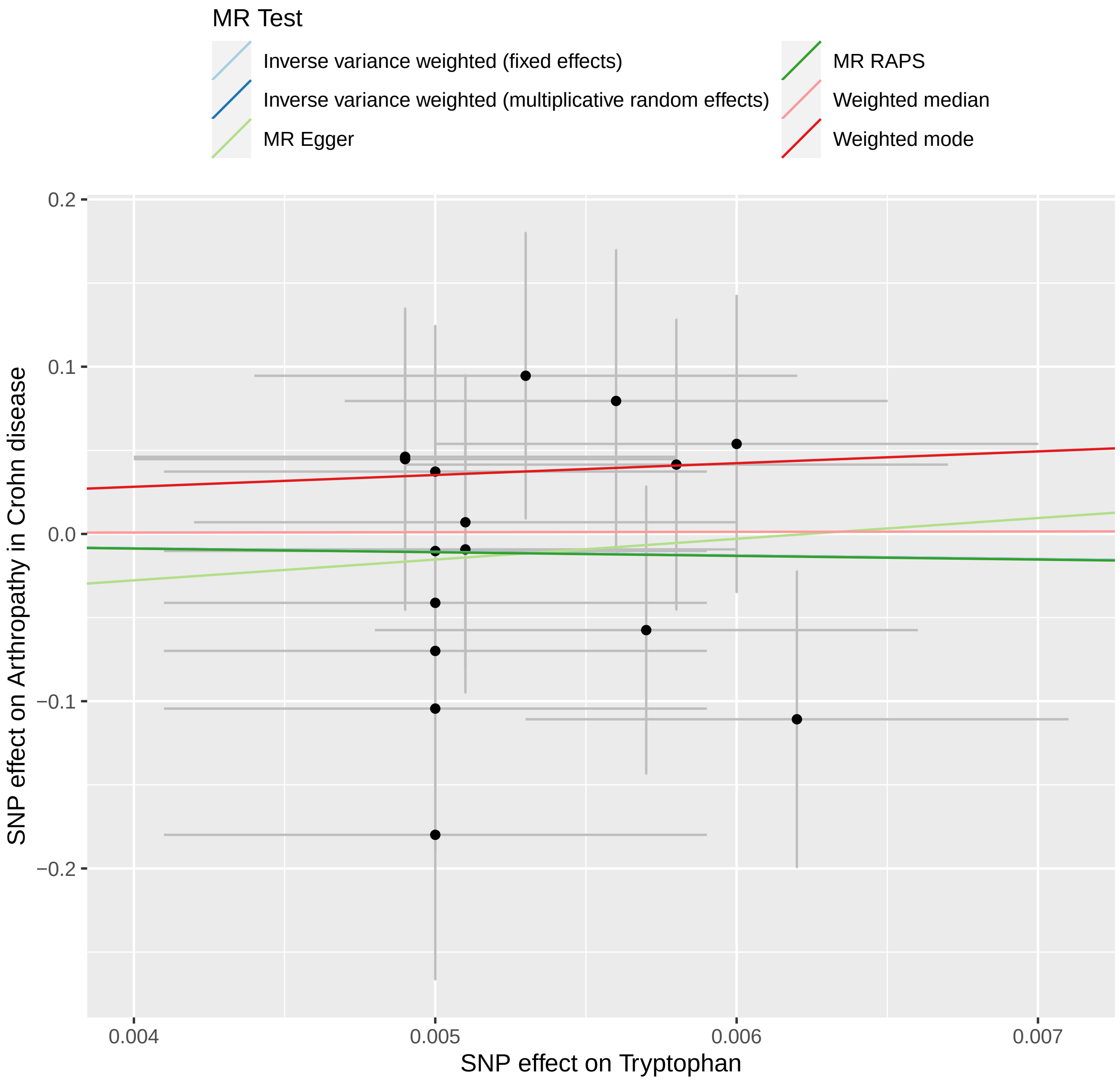

Batch 829 : Leucine on Arthropathy in Crohn disease

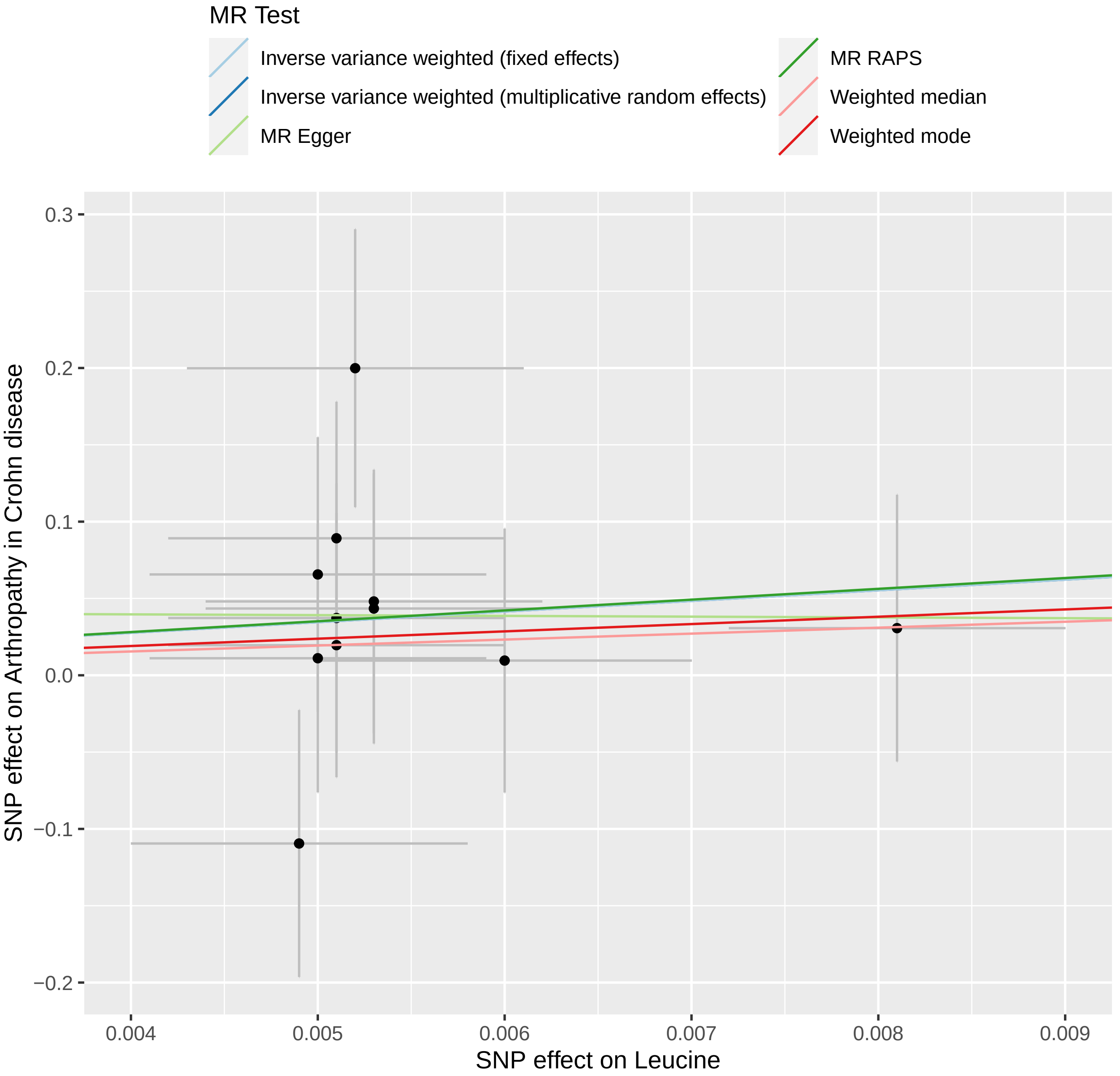

Batch 833 : Lactate on Arthropathy in Crohn disease

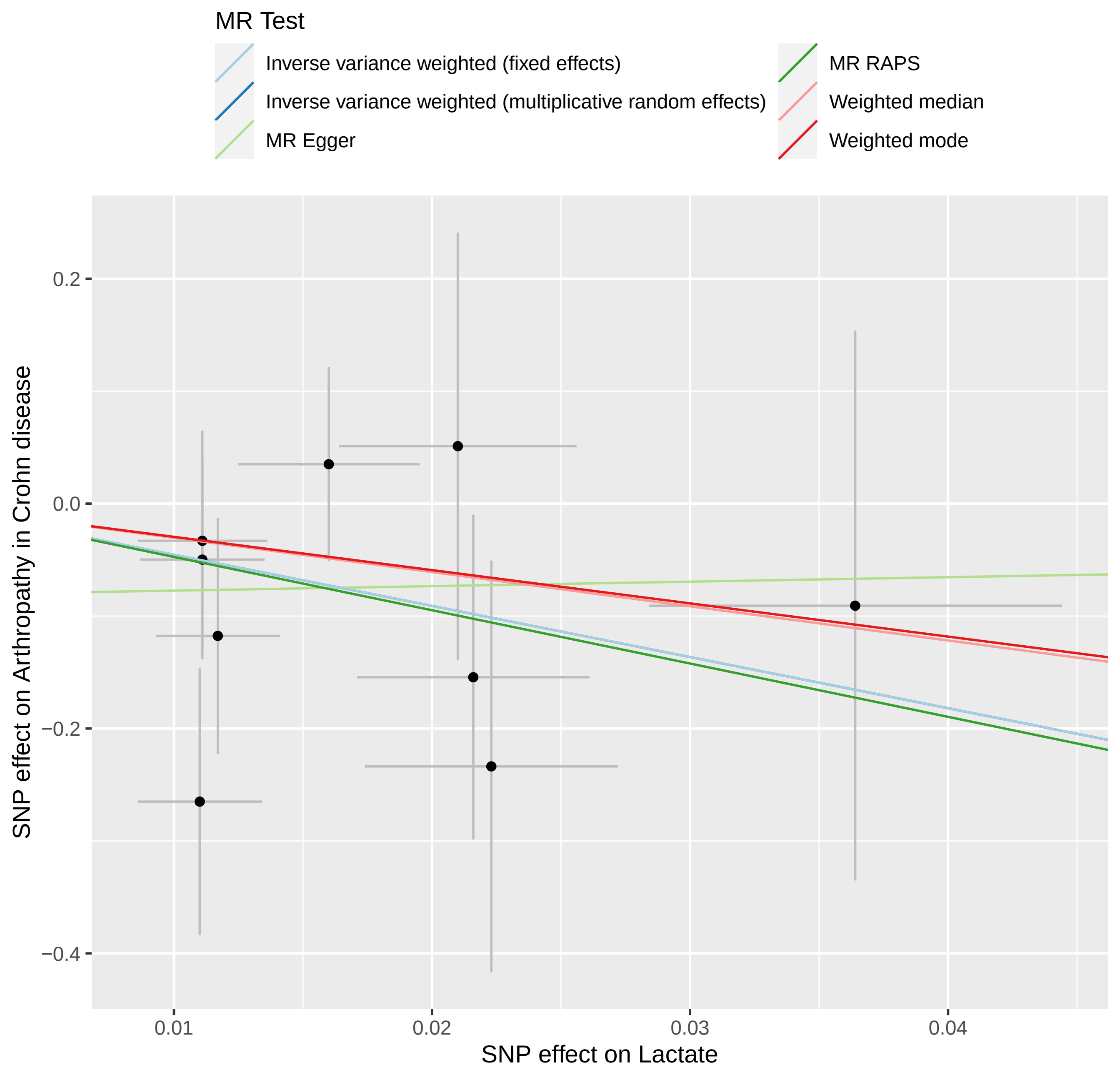

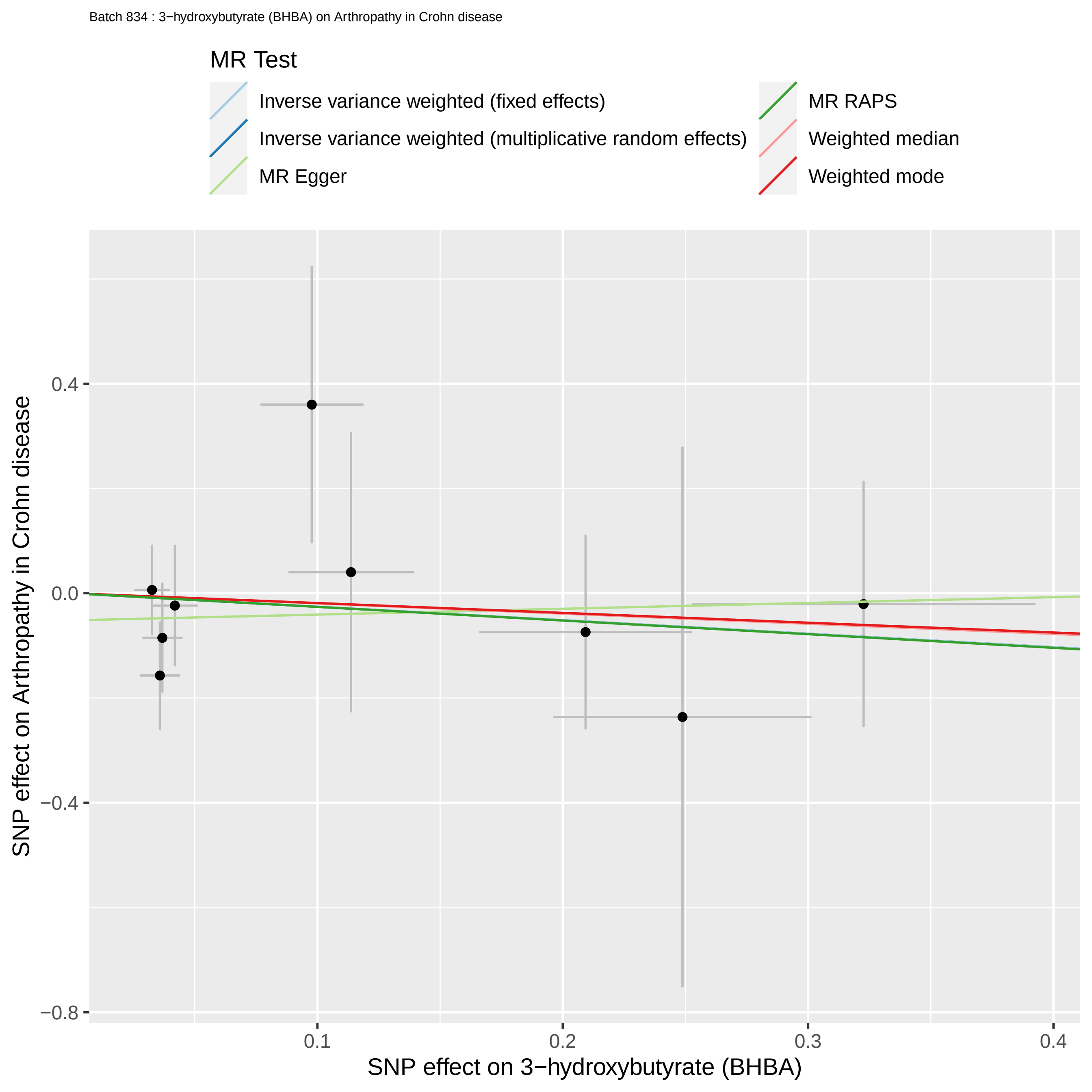
Batch 834 : 3−hydroxybutyrate (BHBA) on Arthropathy in Crohn disease
MR Test
Inverse variance weighted (fixed effects)
Inverse variance weighted (multiplicative random effects)
MR Egger
MR RAPS
Weighted median
Weighted mode
0.4
0.0
−0.4
−0.8
0.1
0.2
0.3
0.4
SNP effect on Arthropathy in Crohn disease
SNP effect on 3−hydroxybutyrate (BHBA)

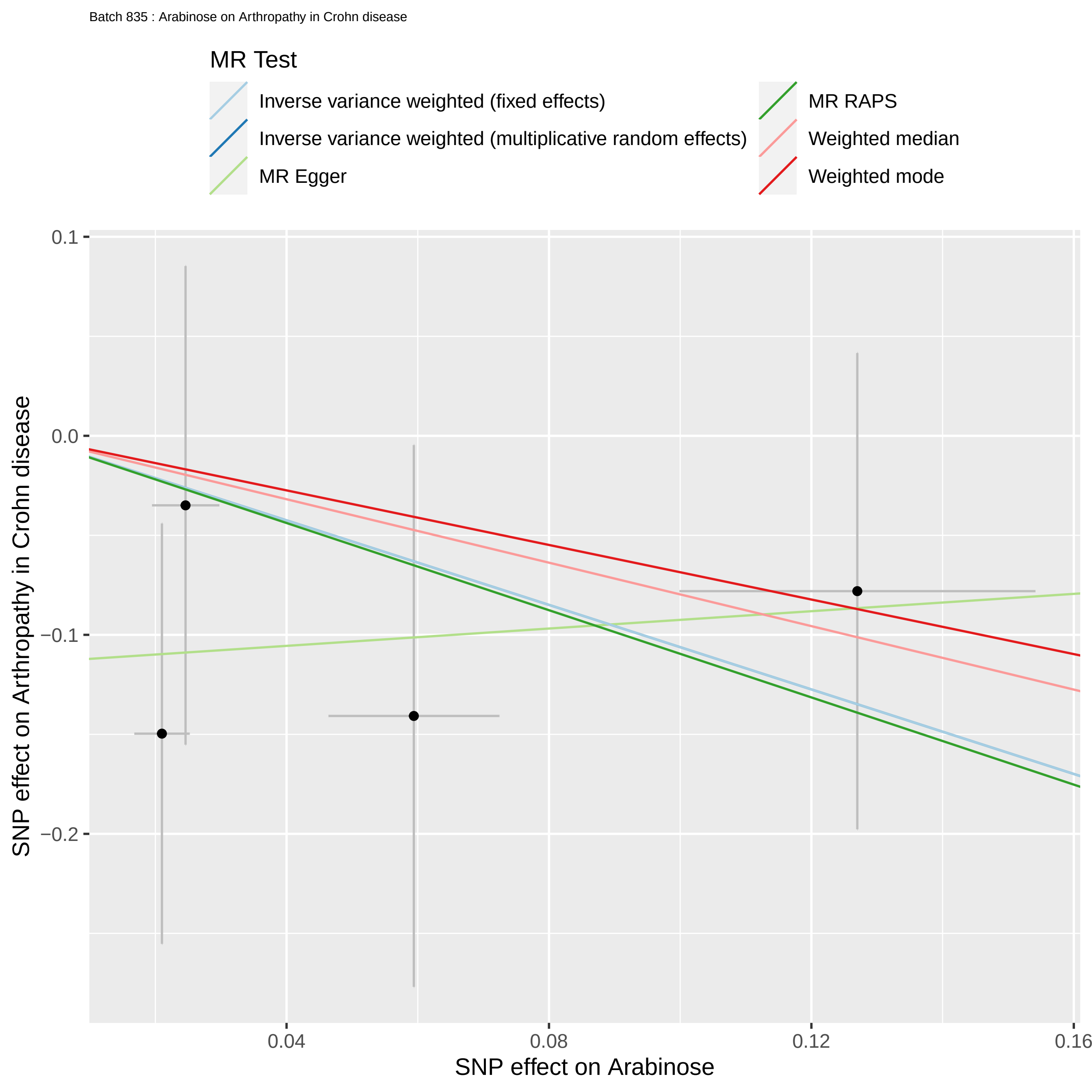

Batch 835 : Arabinose on Arthropathy in Crohn disease
MR Test
Inverse variance weighted (fixed effects)
Inverse variance weighted (multiplicative random effects)
MR Egger
MR RAPS
Weighted median
Weighted mode
0.1
0.0
−0.1
−0.2
0.04
0.08
0.12
0.16
SNP effect on Arthropathy in Crohn disease
SNP effect on Arabinose

Batch 836 : Fructose on Arthropathy in Crohn disease

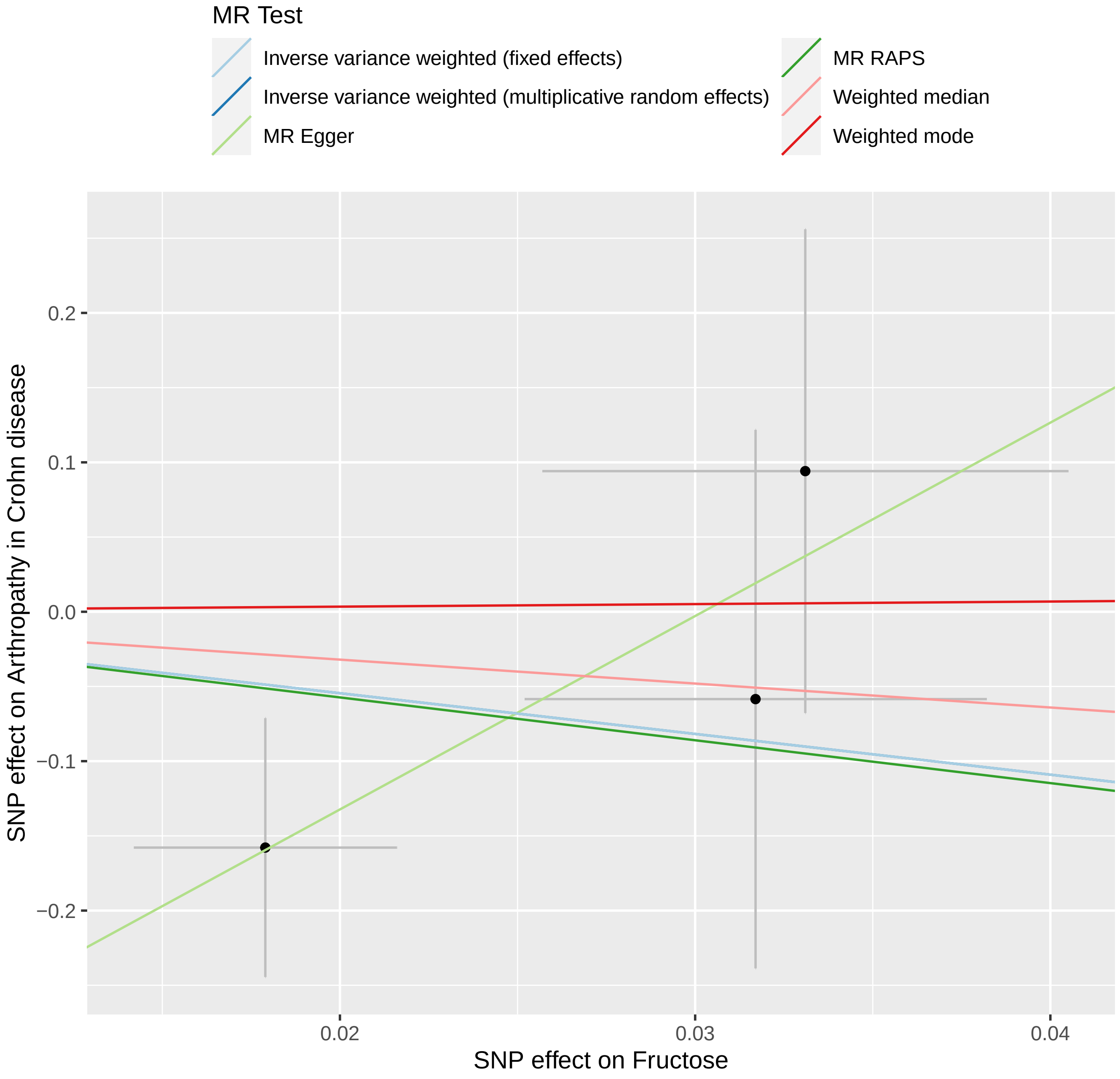

Batch 838 : Pyruvate on Arthropathy in Crohn disease

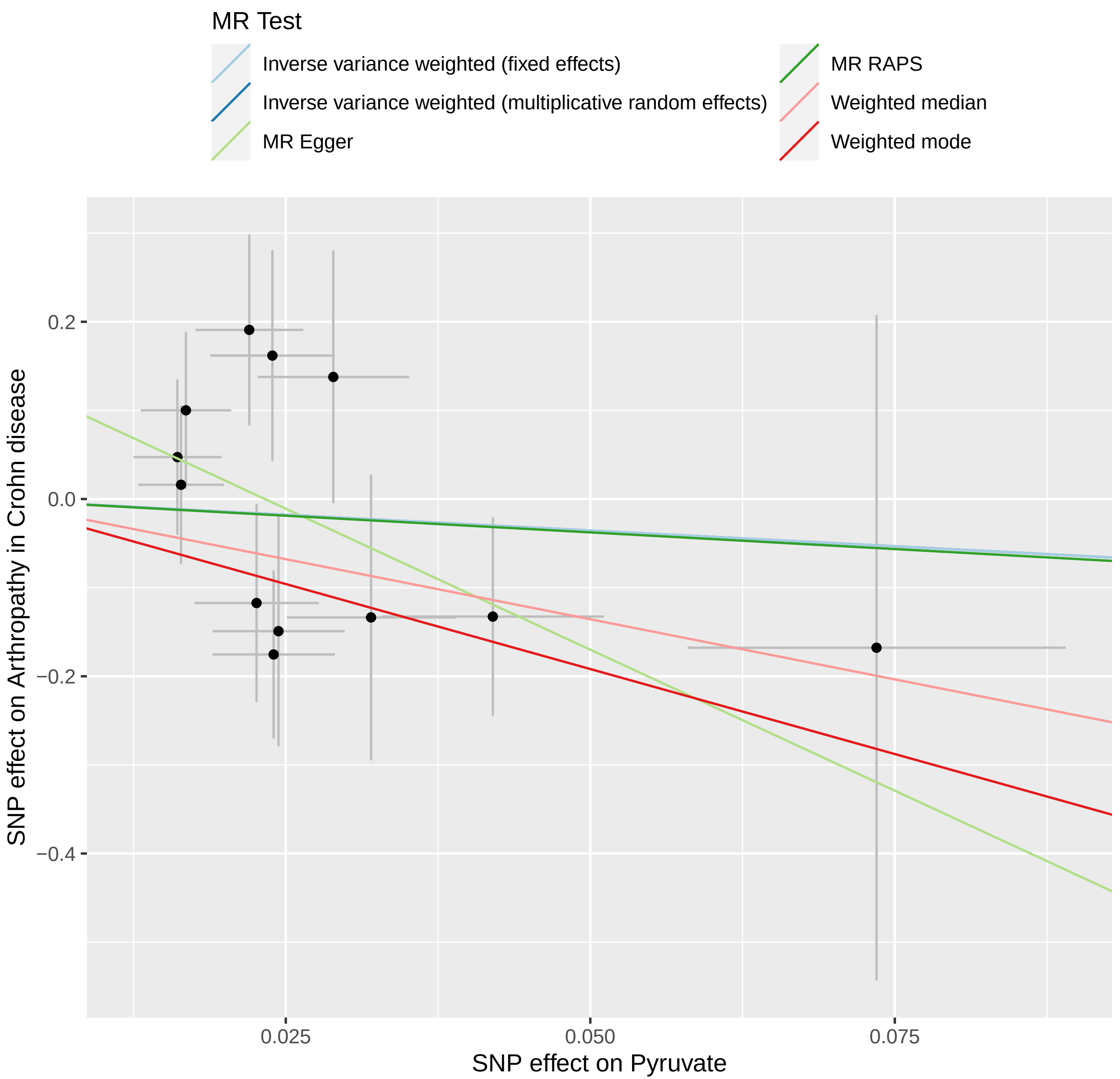

Batch 839 : Uridine on Arthropathy in Crohn disease

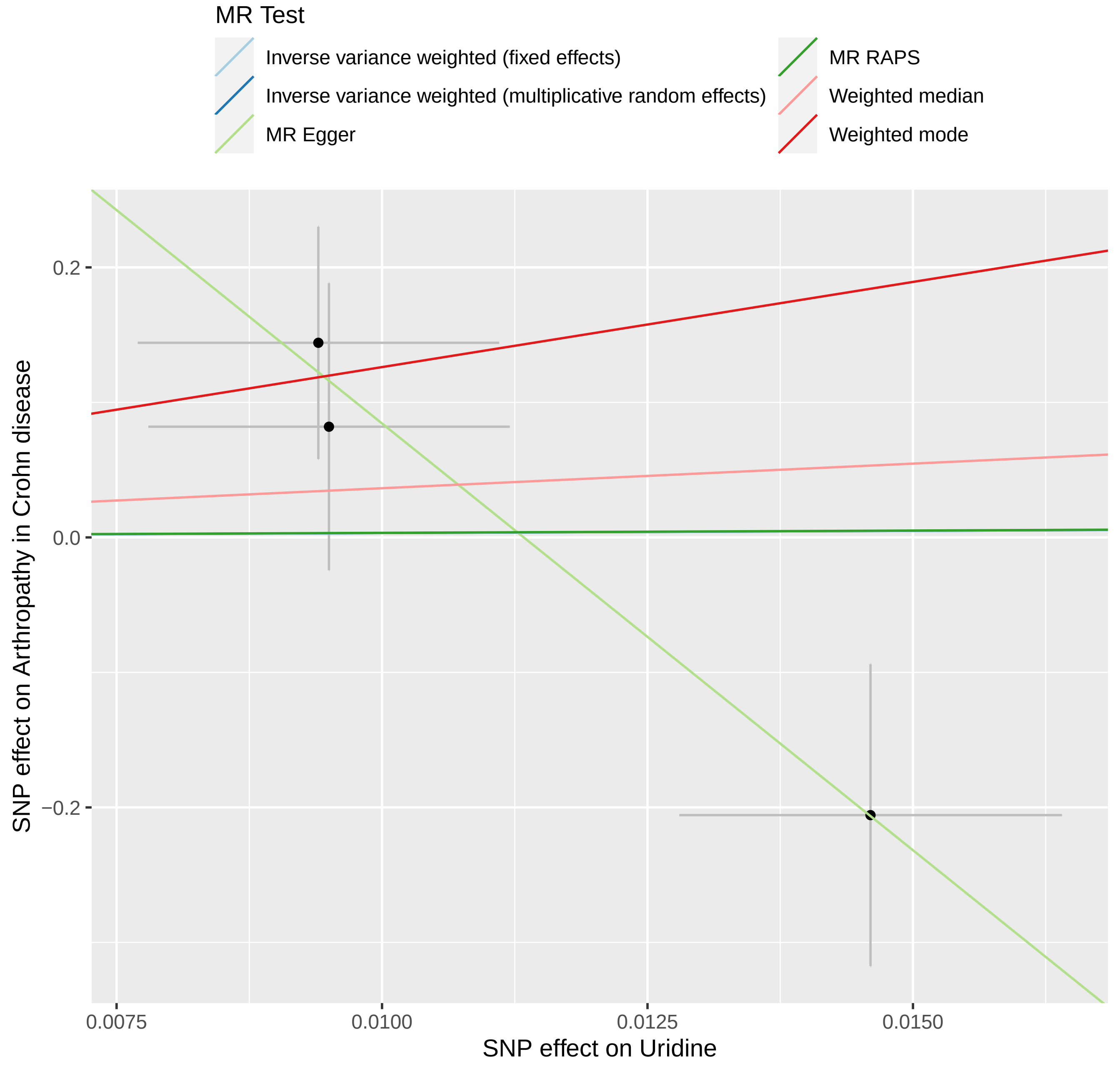

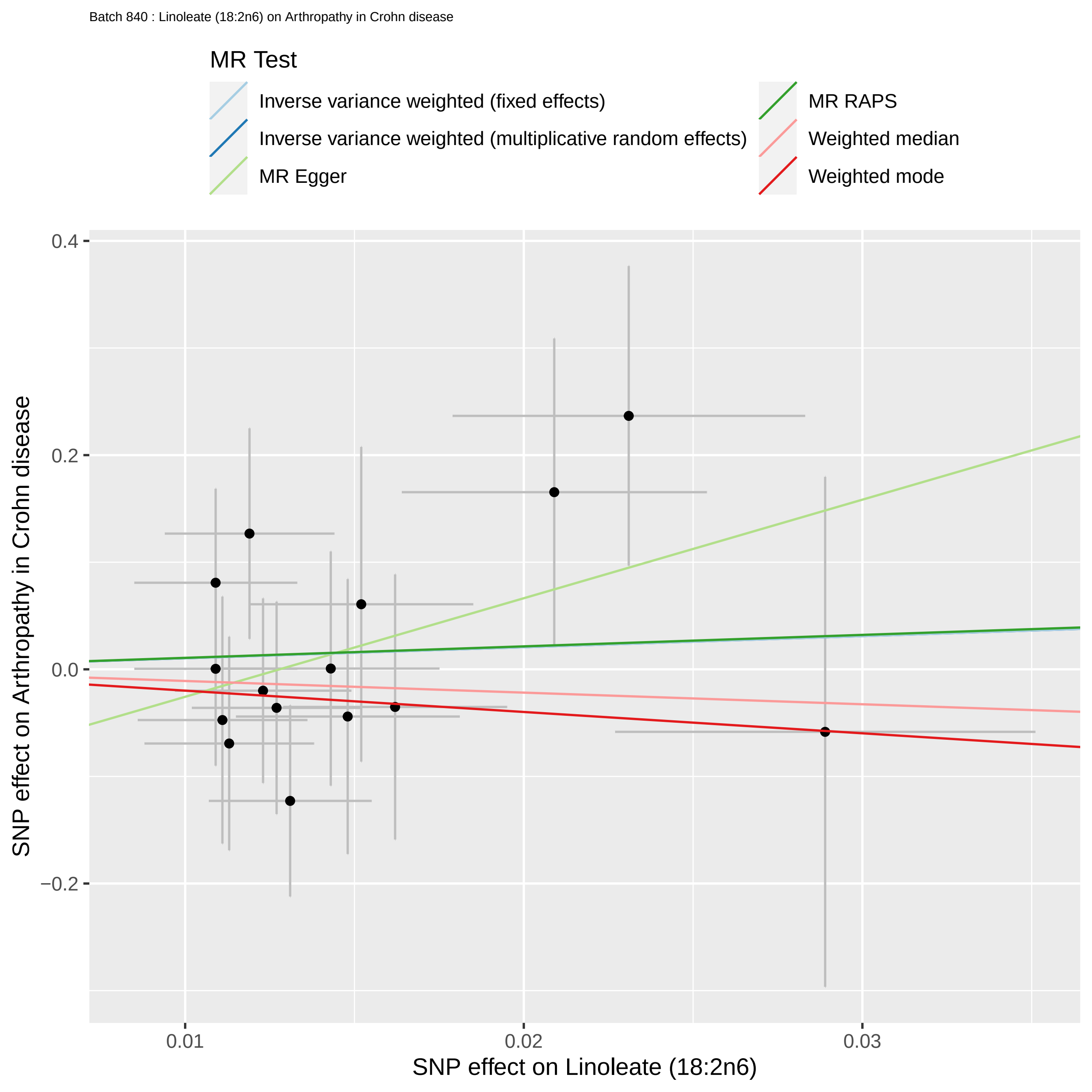

Batch 840 : Linoleate (18:2n6) on Arthropathy in Crohn disease
MR Test
Inverse variance weighted (fixed effects)
Inverse variance weighted (multiplicative random effects)
MR Egger
MR RAPS
Weighted median
Weighted mode
0.4
0.2
0.0
−0.2
0.01
0.02
0.03
SNP effect on Arthropathy in Crohn disease
SNP effect on Linoleate (18:2n6)

Batch 841 : Allantoin on Arthropathy in Crohn disease

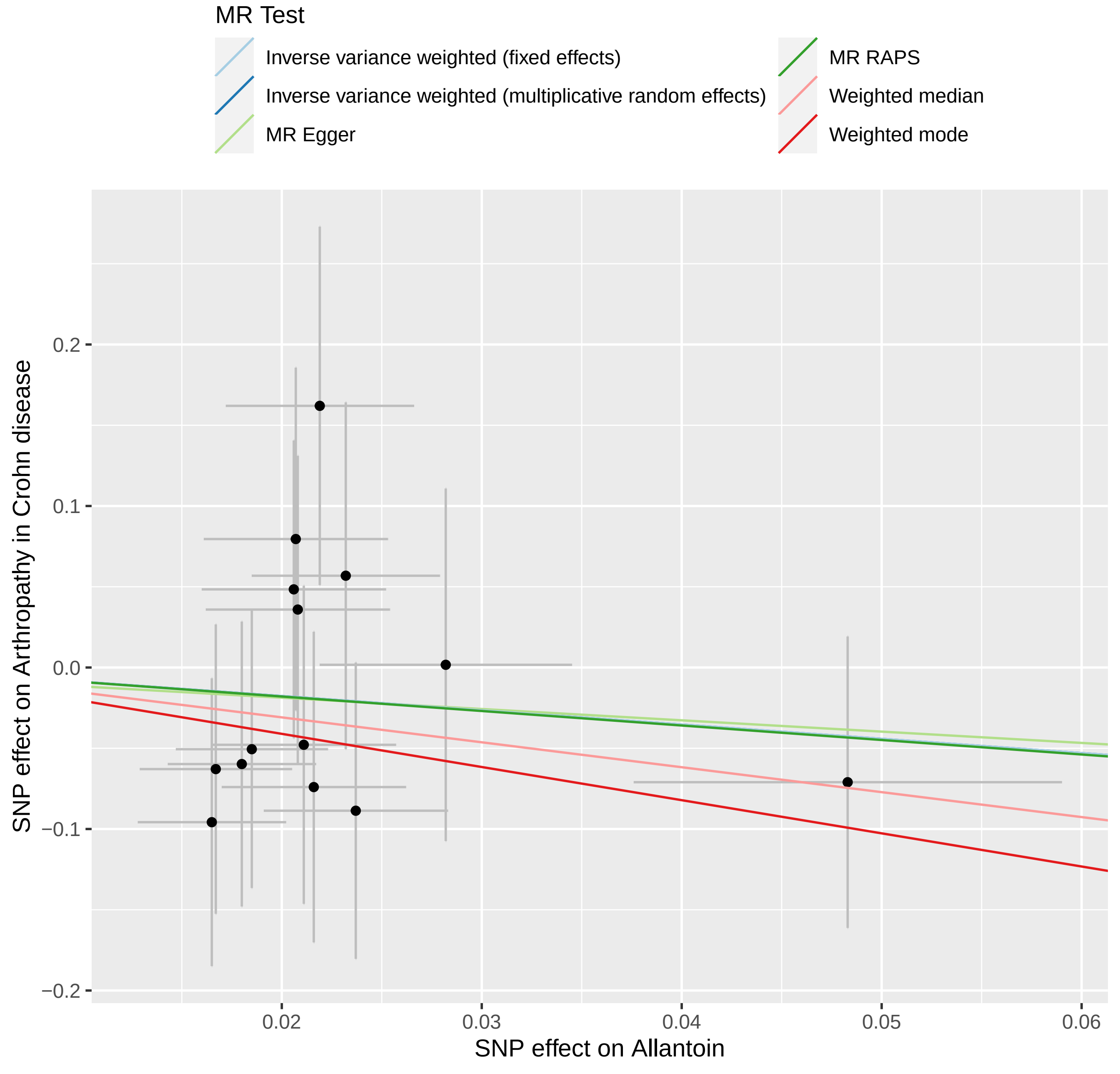

Batch 846 : Isoleucine on Arthropathy in Crohn disease

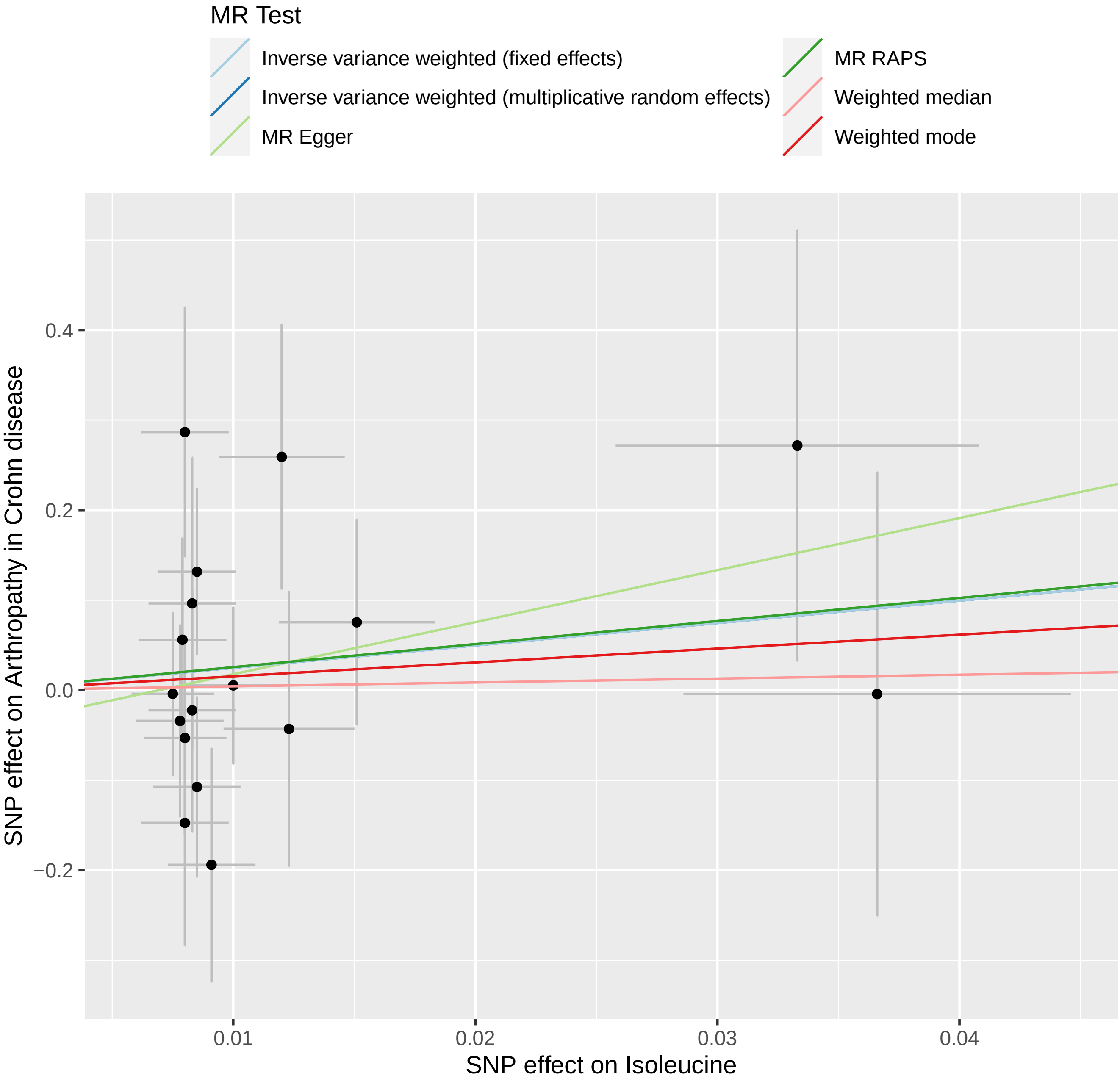

Batch 847 : Threonine on Arthropathy in Crohn disease

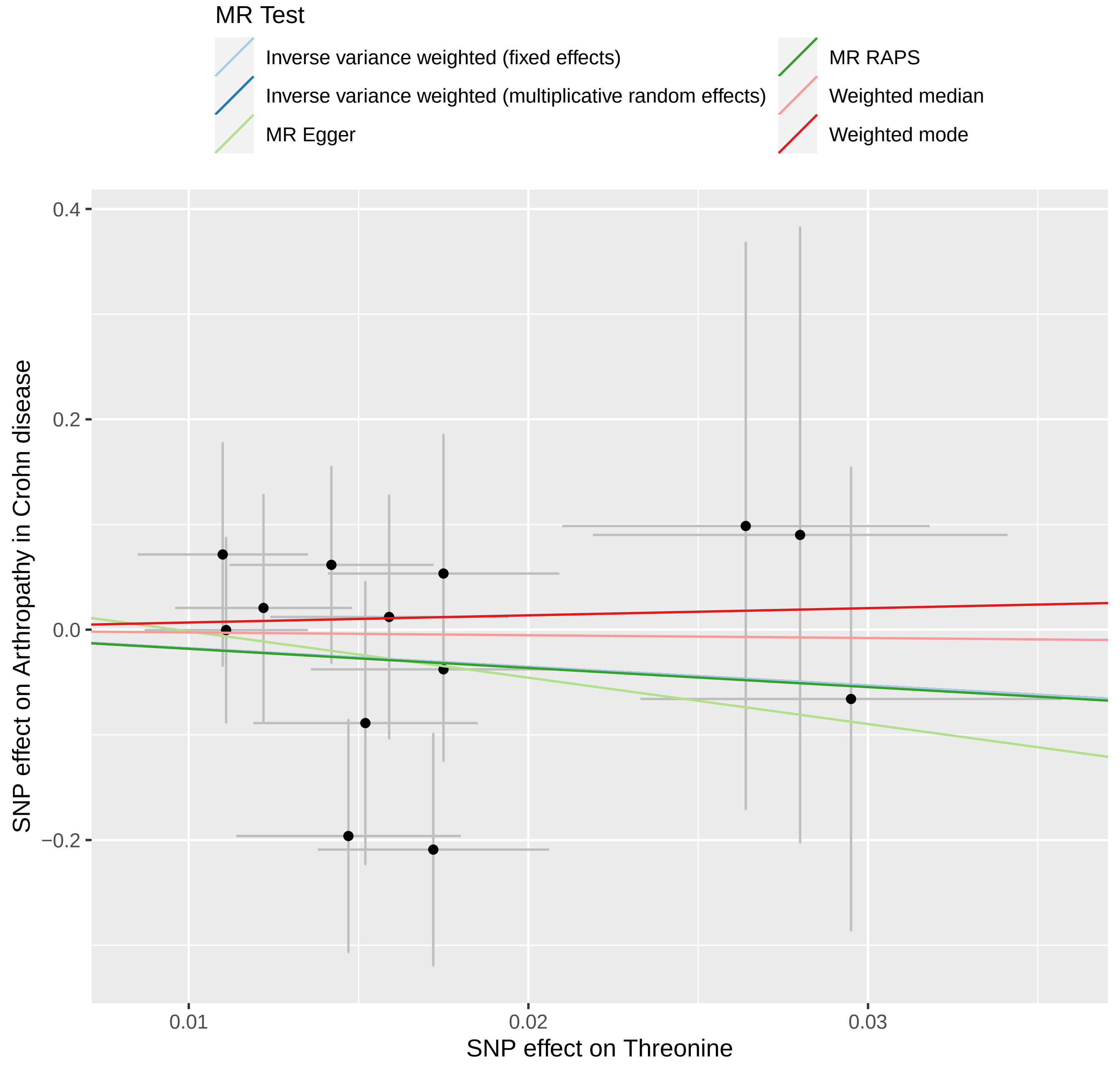

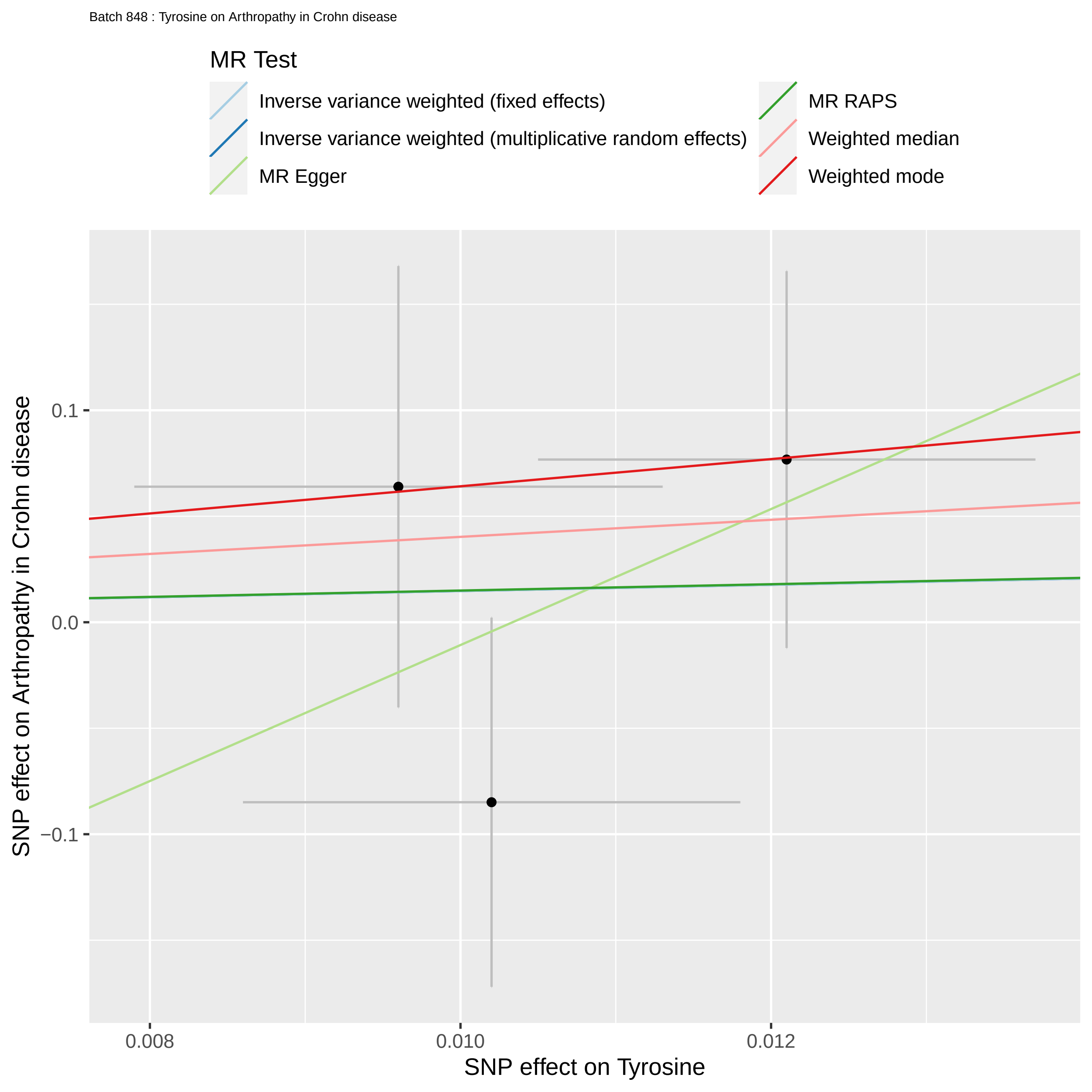
Batch 848 : Tyrosine on Arthropathy in Crohn disease
MR Test
Inverse variance weighted (fixed effects)
Inverse variance weighted (multiplicative random effects)
MR Egger
MR RAPS
Weighted median
Weighted mode
0.1
0.0
−0.1
0.008
0.010
0.012
SNP effect on Arthropathy in Crohn disease
SNP effect on Tyrosine

Batch 854 : Stearate (18:0) on Arthropathy in Crohn disease

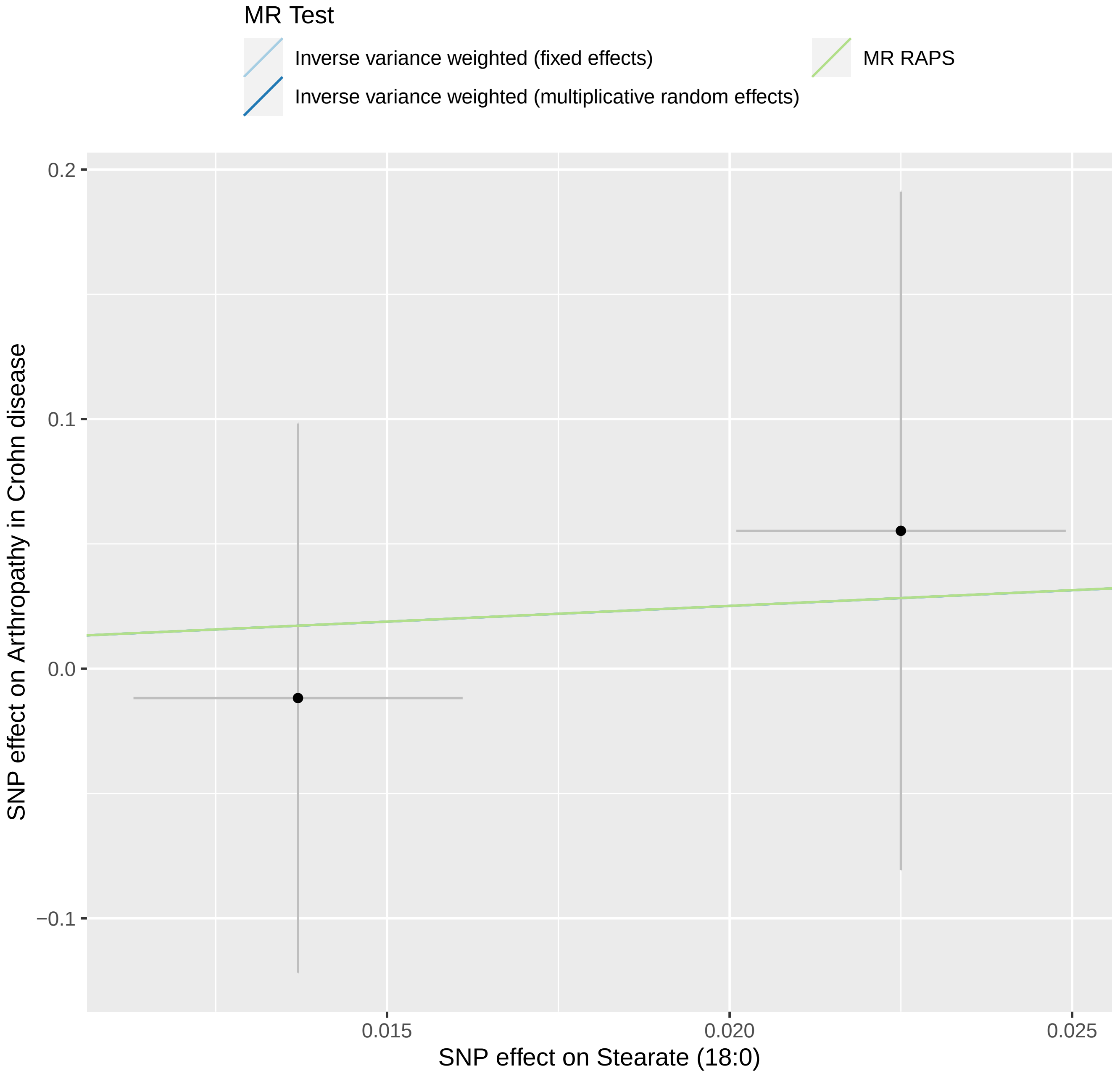

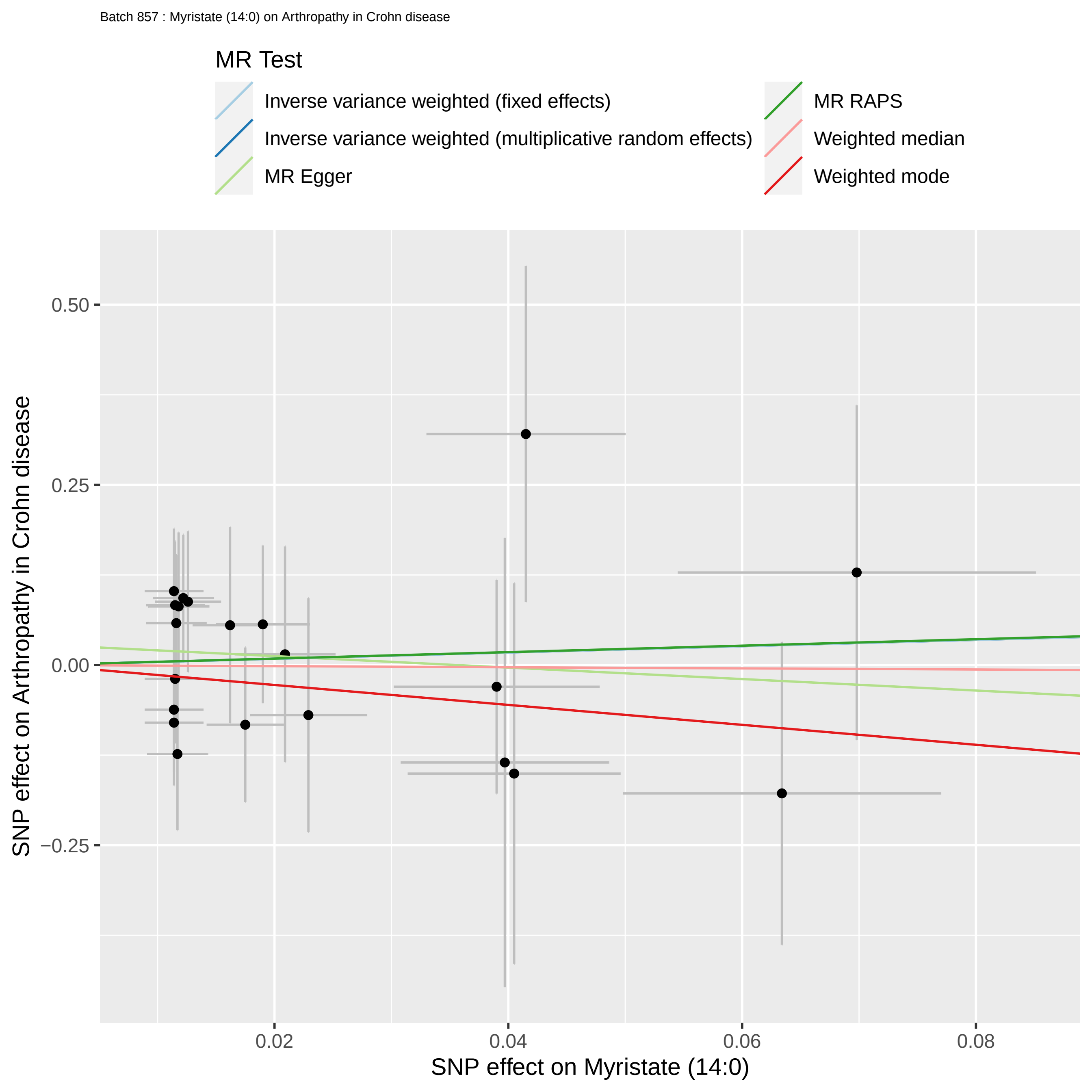

Batch 857 : Myristate (14:0) on Arthropathy in Crohn disease
MR Test
Inverse variance weighted (fixed effects)
Inverse variance weighted (multiplicative random effects)
MR Egger
MR RAPS
Weighted median
Weighted mode
0.50
0.25
0.00
−0.25
0.02
0.04
0.06
0.08
SNP effect on Arthropathy in Crohn disease
SNP effect on Myristate (14:0)

Batch 859 : Ornithine on Arthropathy in Crohn disease

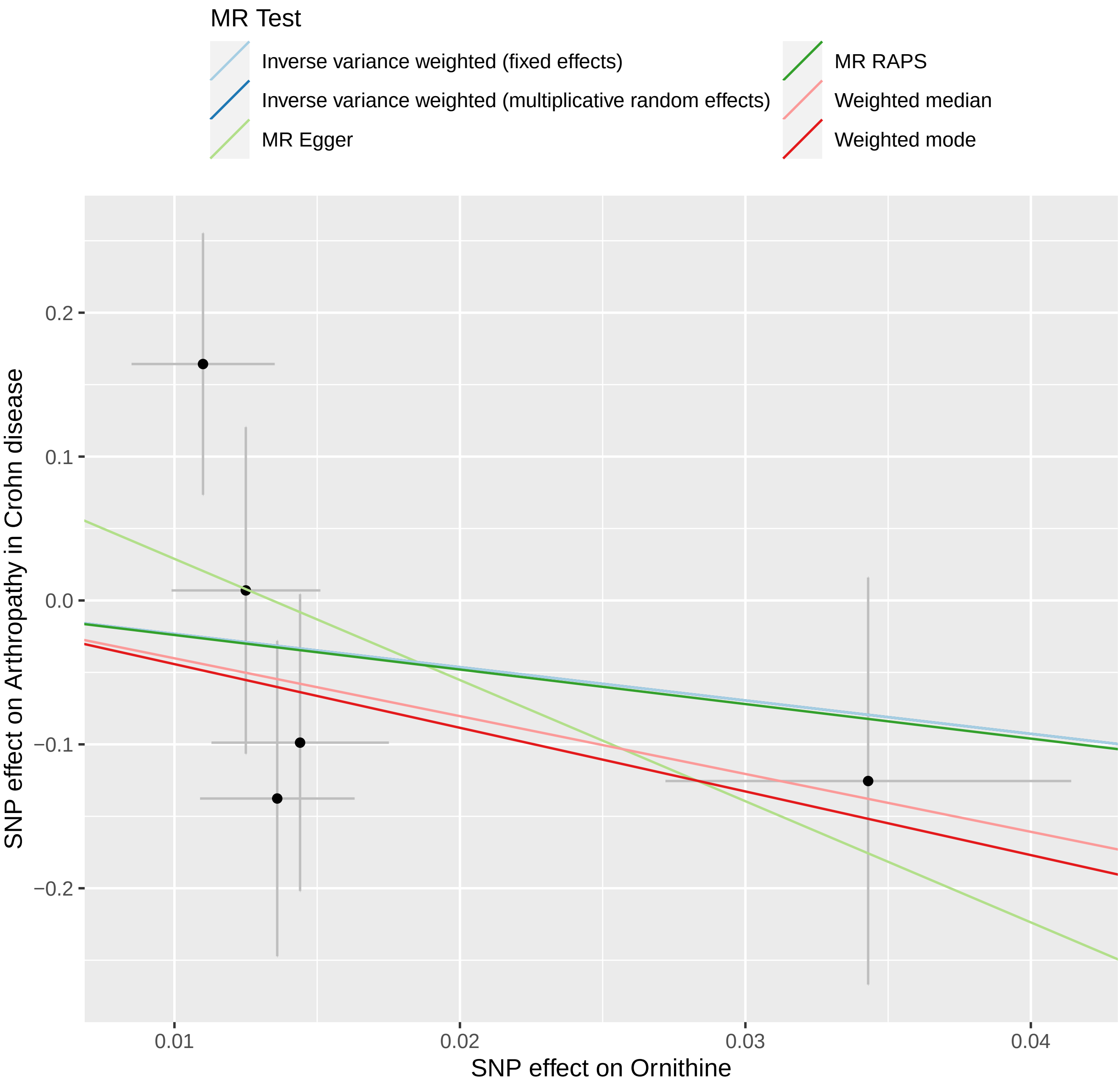

Batch 862 : 4−acetamidobutanoate on Arthropathy in Crohn disease

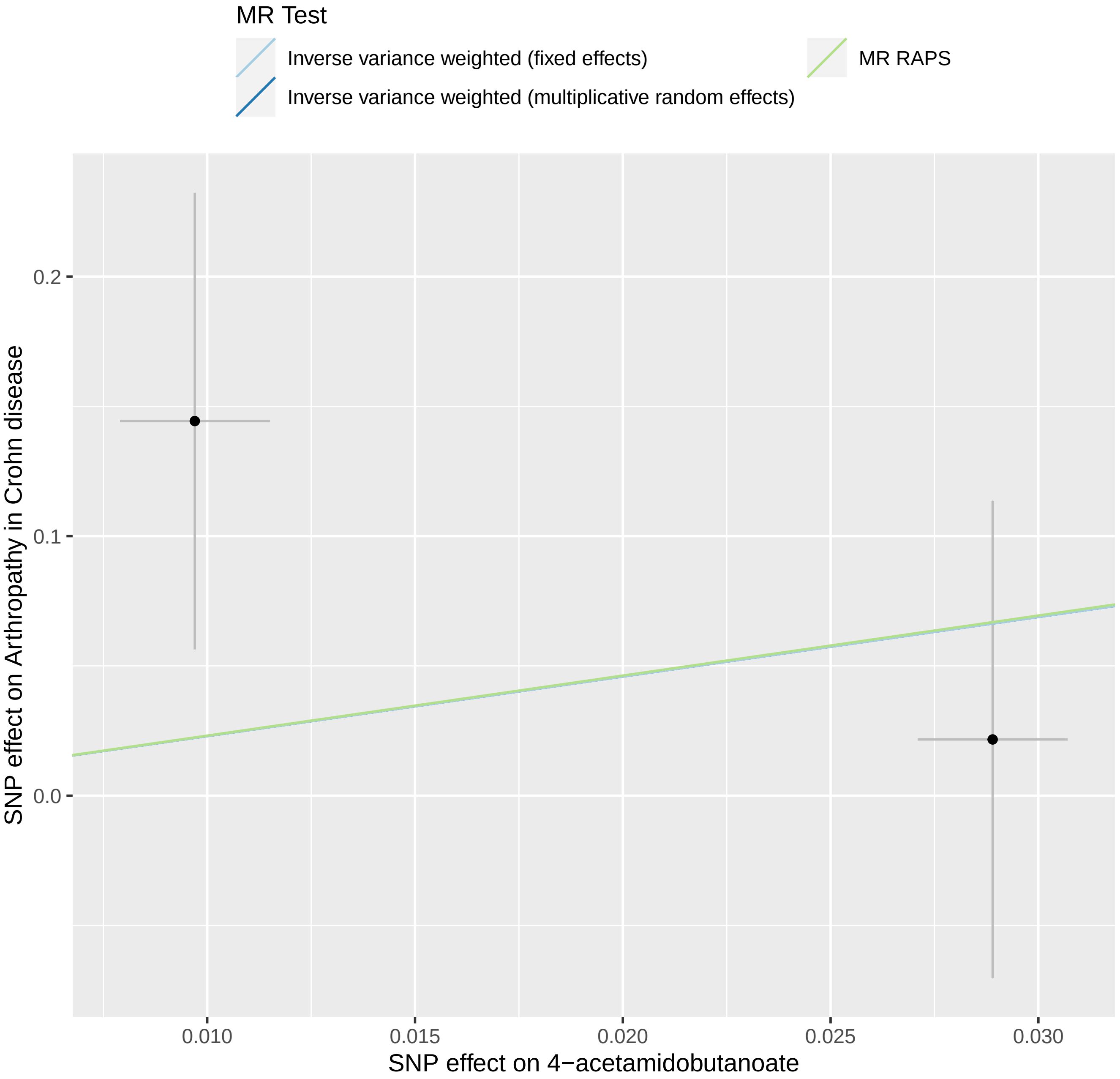

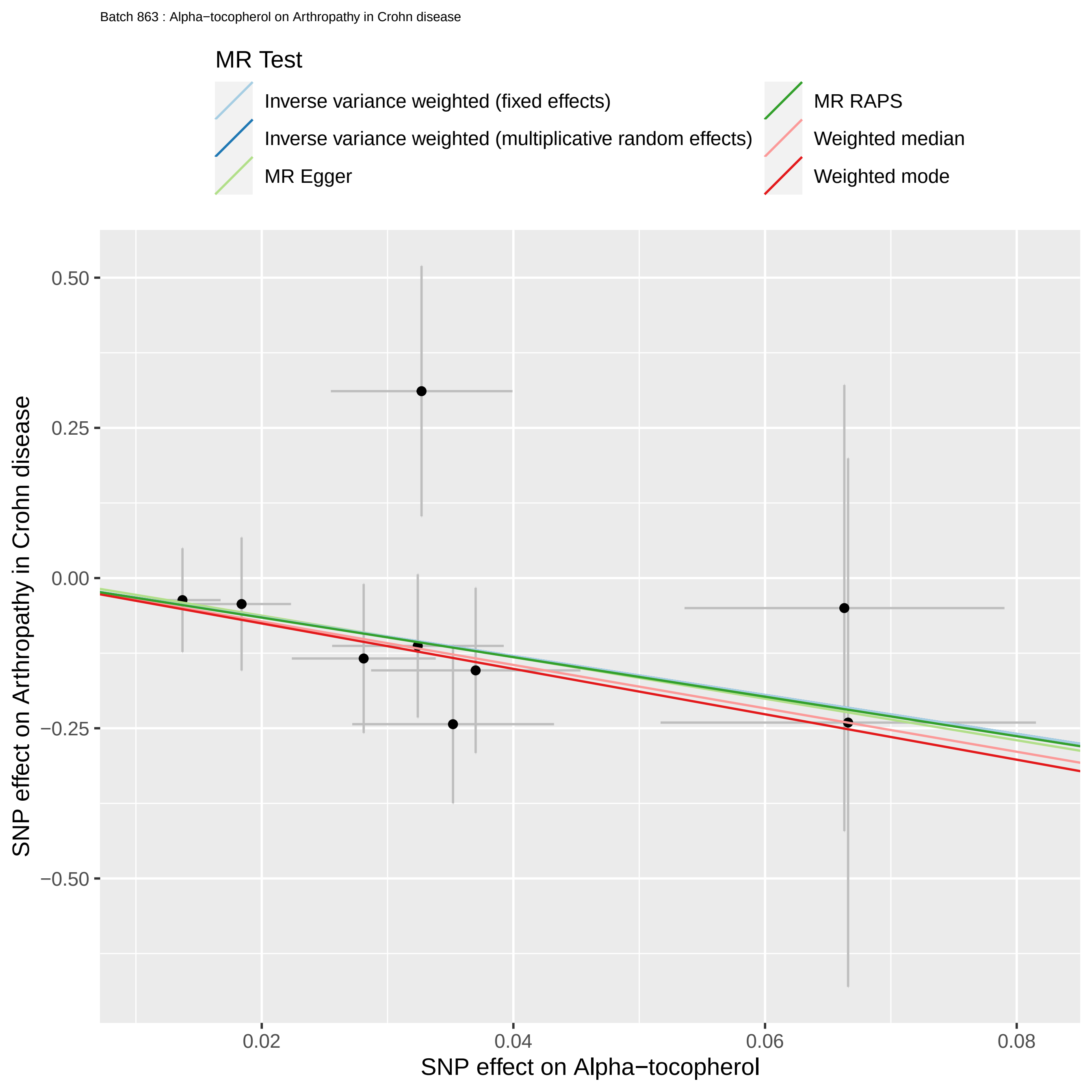
Batch 863 : Alpha−tocopherol on Arthropathy in Crohn disease
MR Test
Inverse variance weighted (fixed effects)
Inverse variance weighted (multiplicative random effects)
MR Egger
MR RAPS
Weighted median
Weighted mode
0.50
0.25
0.00
−0.25
−0.50
SNP effect on Arthropathy in Crohn disease
0.02
0.04
0.06
0.08
SNP effect on Alpha−tocopherol

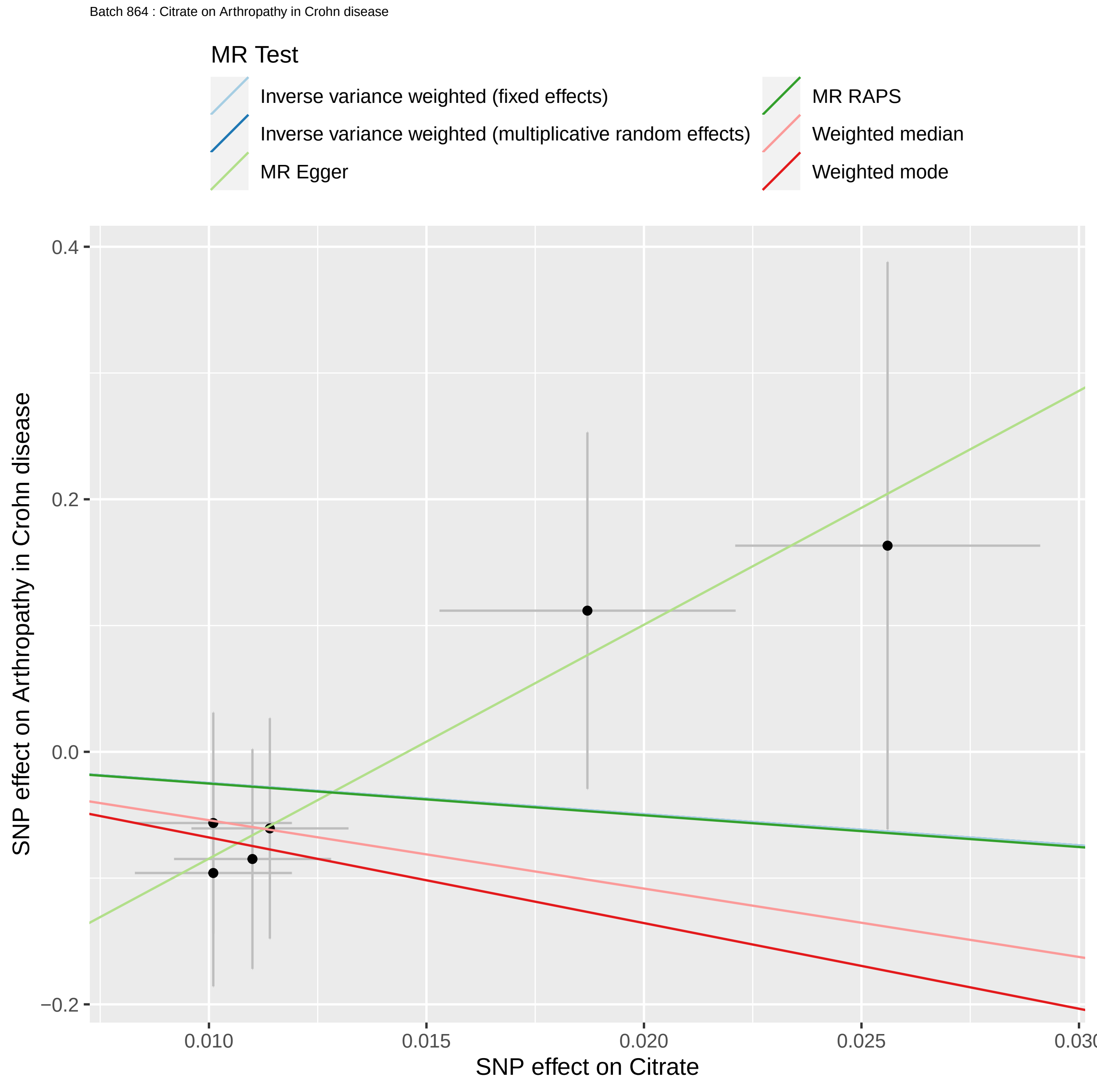

Batch 864 : Citrate on Arthropathy in Crohn disease
MR Test
Inverse variance weighted (fixed effects)
Inverse variance weighted (multiplicative random effects)
MR Egger
MR RAPS
Weighted median
Weighted mode
0.4
0.2
0.0
−0.2
0.010
0.015
0.020
0.025
0.030
SNP effect on Arthropathy in Crohn disease
SNP effect on Citrate

Batch 865 : Glycerate on Arthropathy in Crohn disease

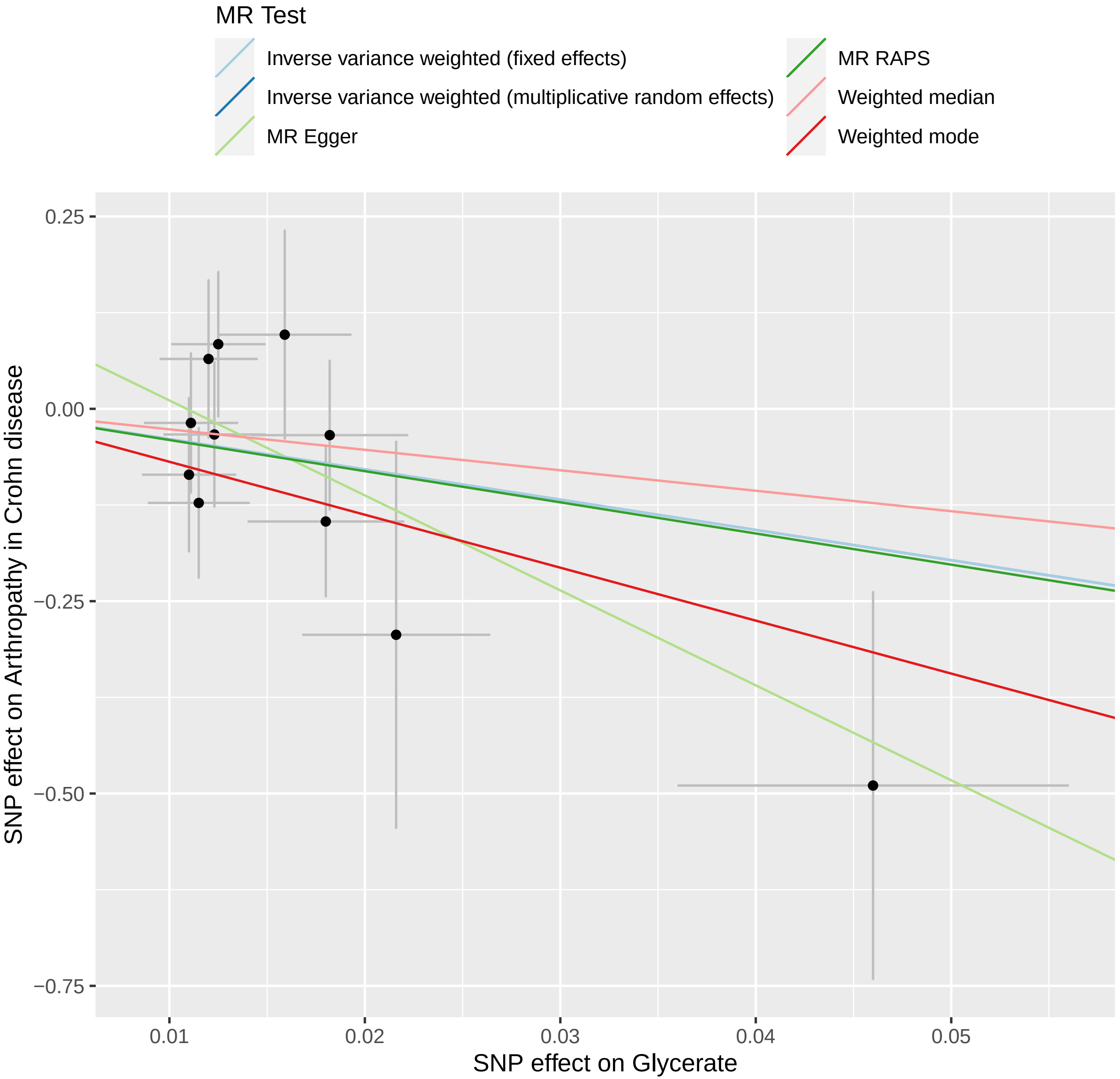

Batch 866 : Guanosine on Arthropathy in Crohn disease

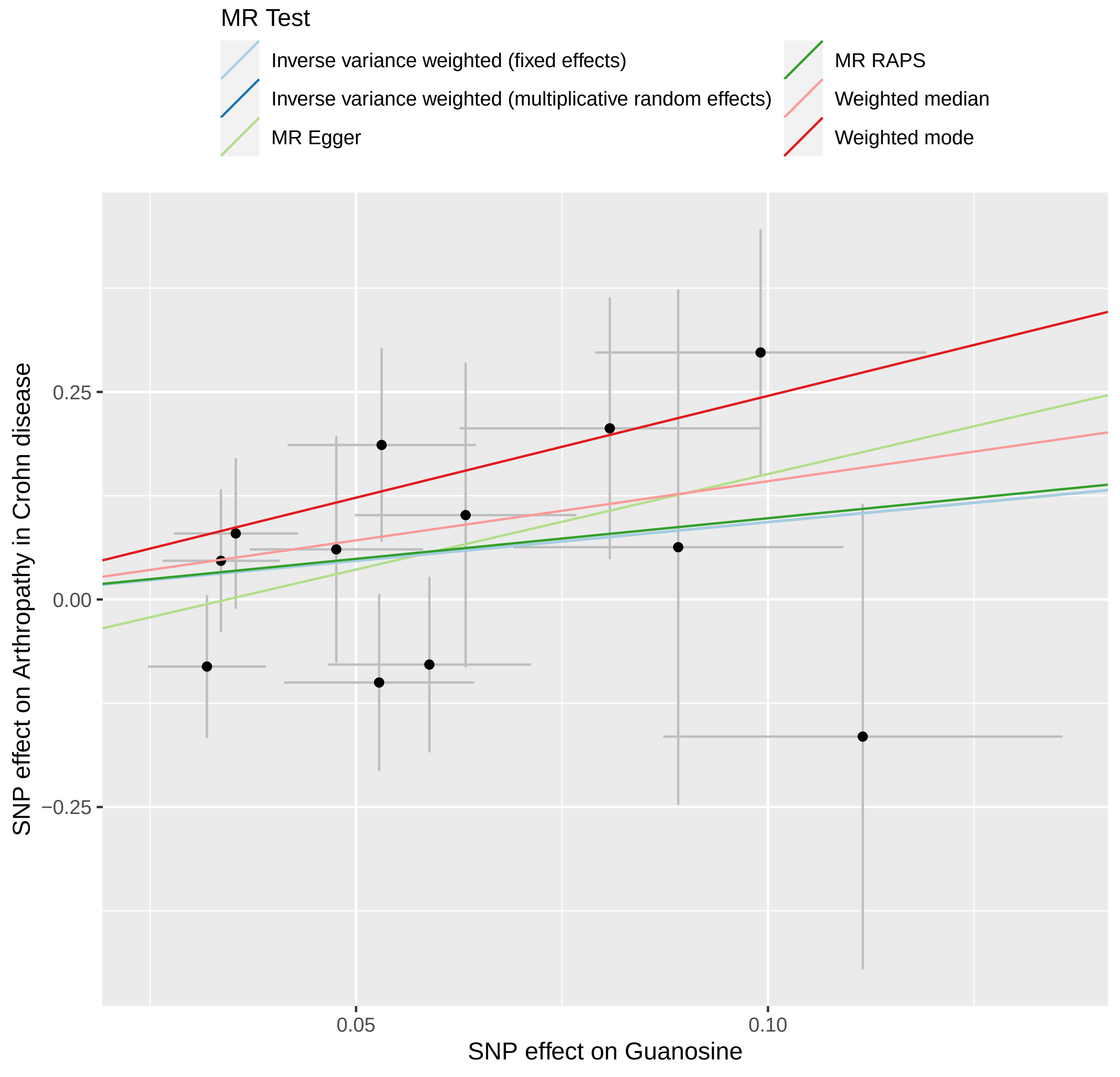

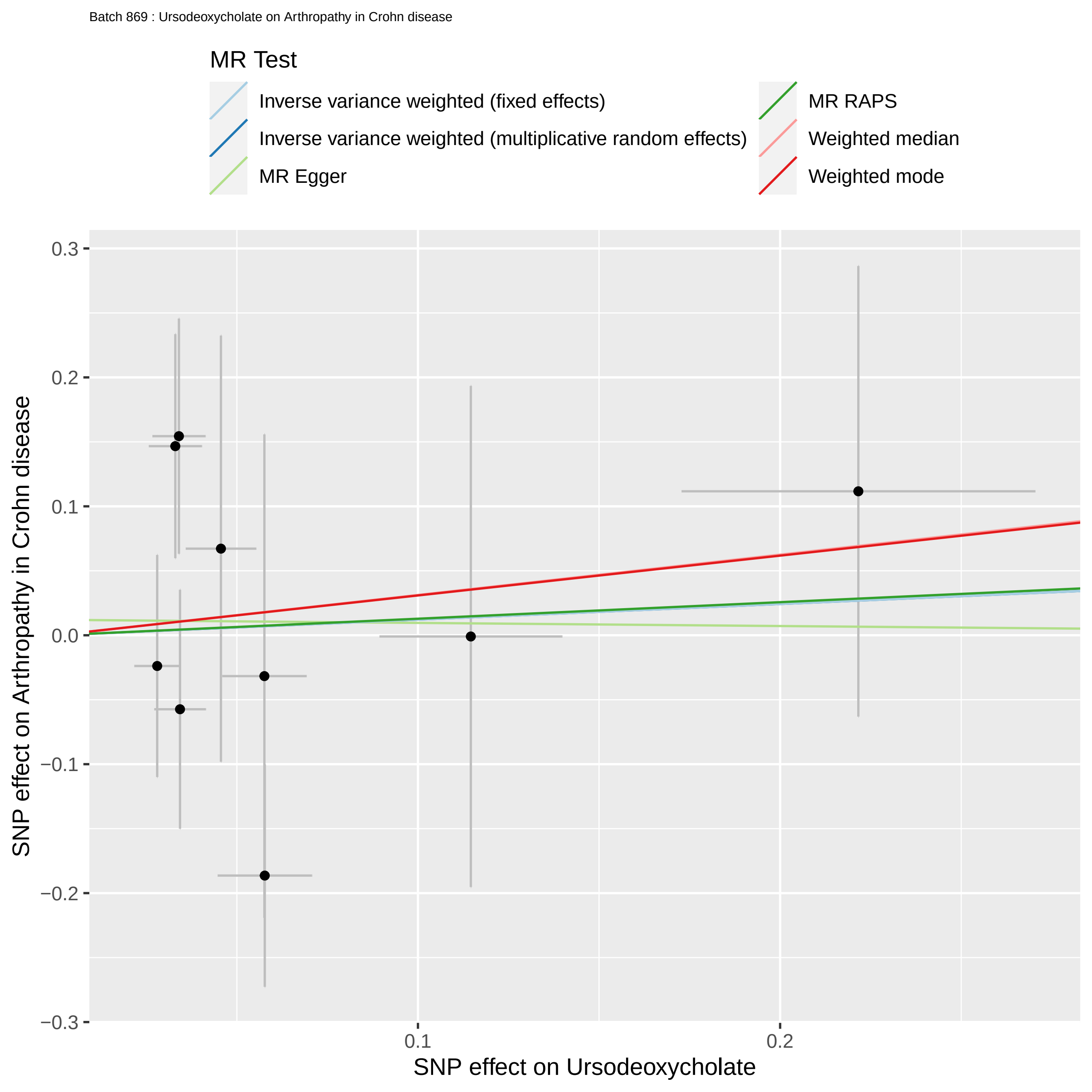
Batch 869 : Ursodeoxycholate on Arthropathy in Crohn disease
MR Test
Inverse variance weighted (fixed effects)
Inverse variance weighted (multiplicative random effects)
MR Egger
MR RAPS
Weighted median
Weighted mode
0.3
0.2
0.1
0.0
−0.1
−0.2
−0.3
0.1
0.2
SNP effect on Arthropathy in Crohn disease
SNP effect on Ursodeoxycholate

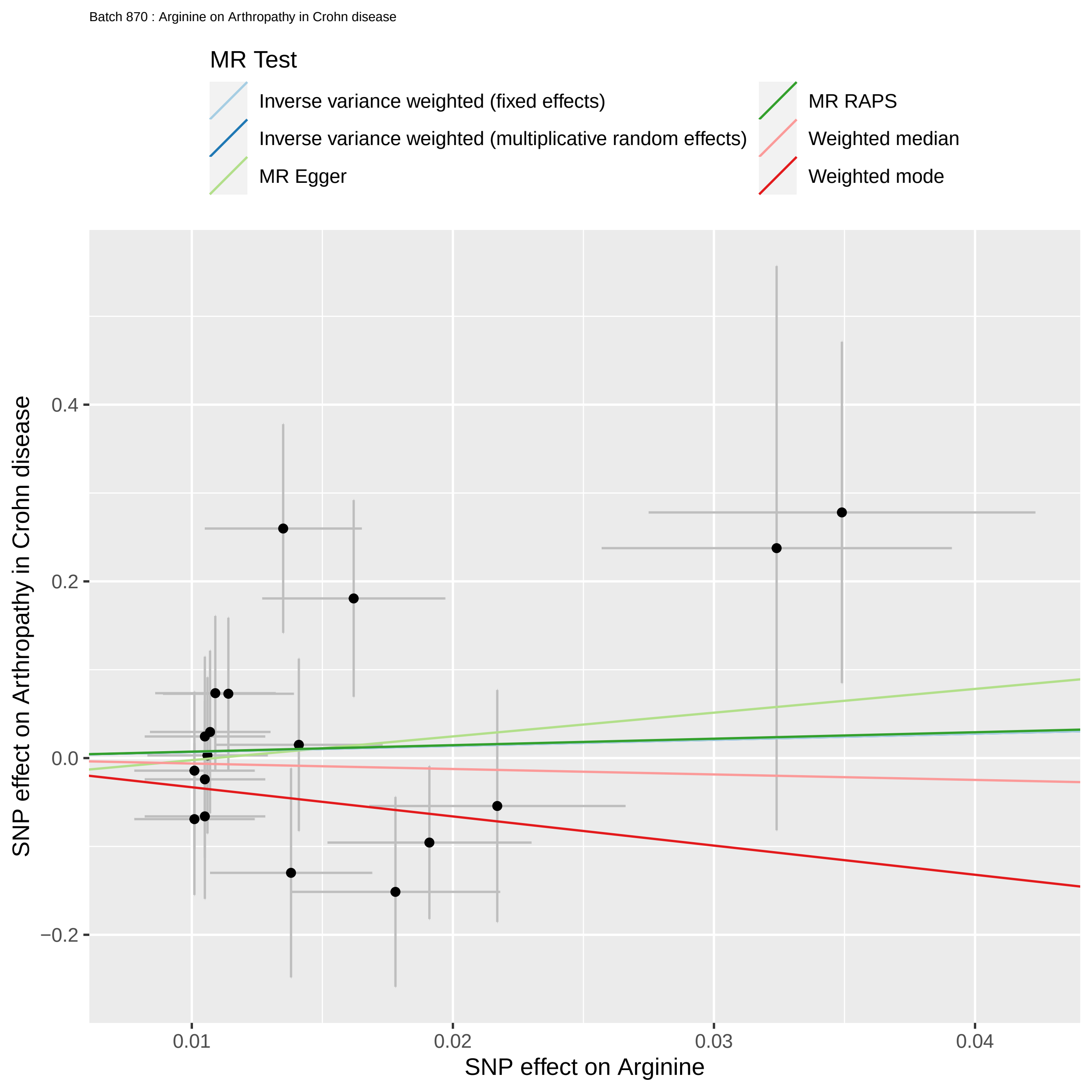
Batch 870 : Arginine on Arthropathy in Crohn disease
MR Test
Inverse variance weighted (fixed effects)
Inverse variance weighted (multiplicative random effects)
MR Egger
MR RAPS
Weighted median
Weighted mode
0.4
0.2
0.0
−0.2
0.01
0.02
0.03
0.04
SNP effect on Arthropathy in Crohn disease
SNP effect on Arginine

Batch 871 : Ascorbate (Vitamin C) on Arthropathy in Crohn disease

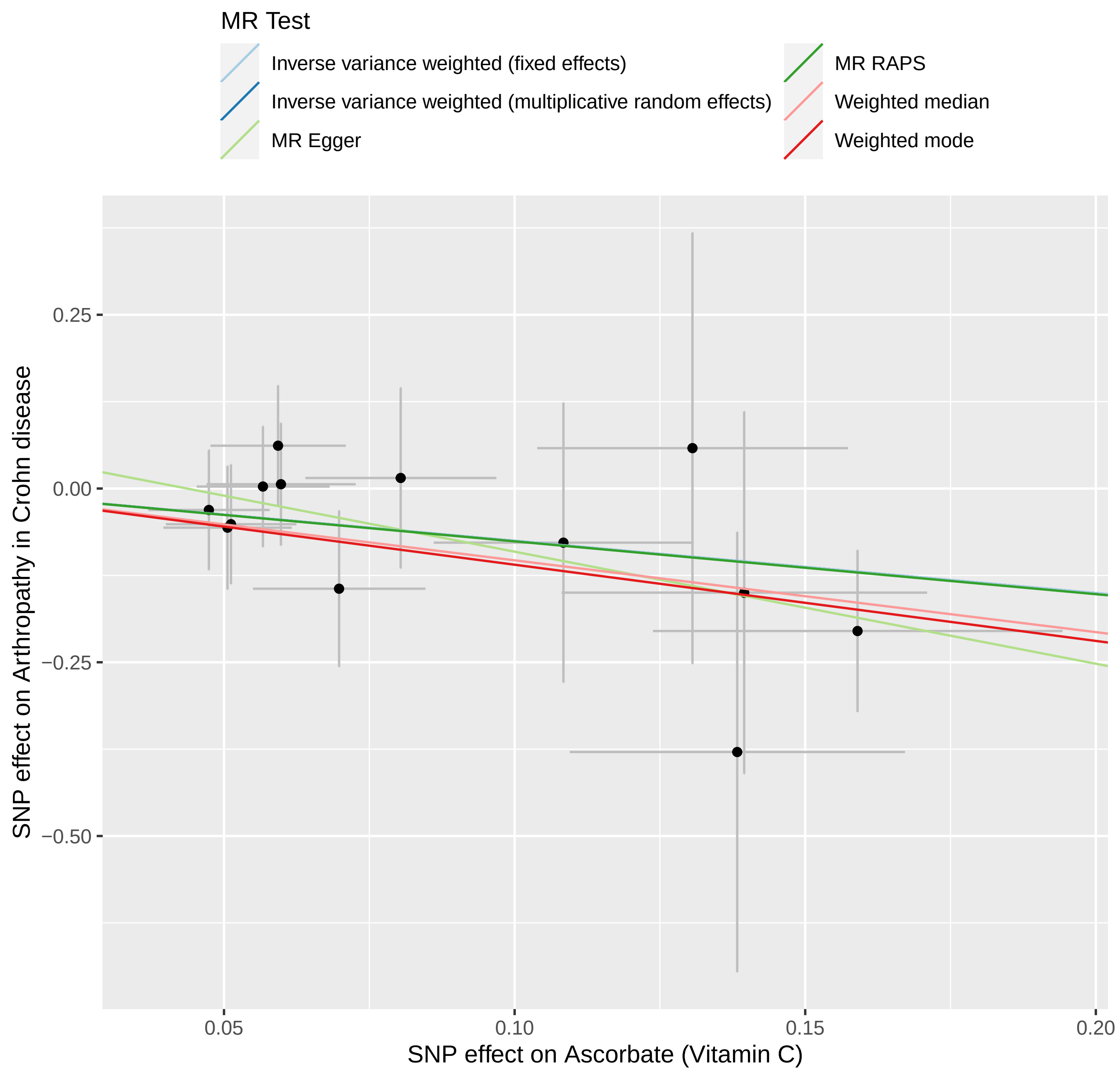

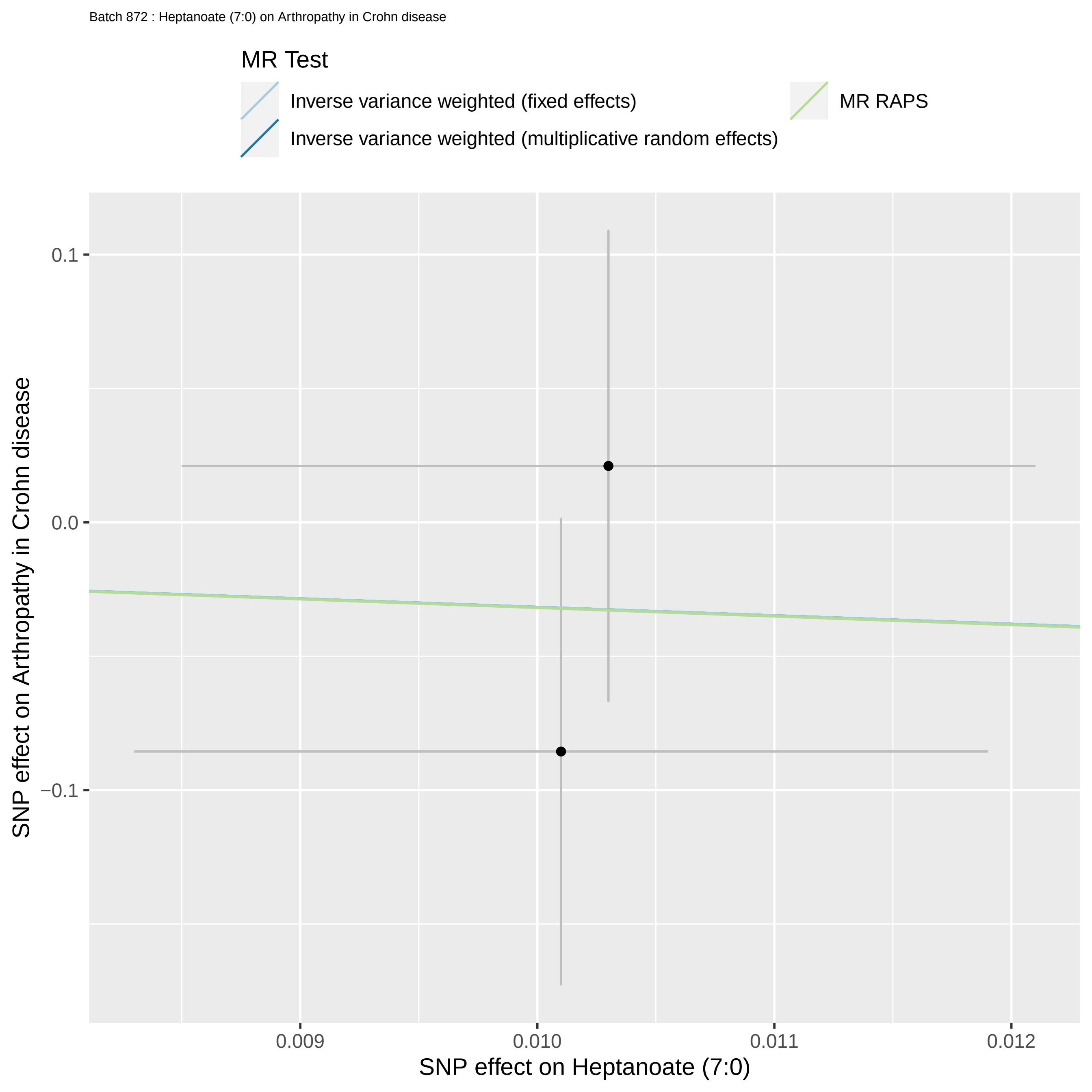

Batch 872 : Heptanoate (7:0) on Arthropathy in Crohn disease
MR Test
Inverse variance weighted (fixed effects)
Inverse variance weighted (multiplicative random effects)
MR RAPS
0.1
0.0
−0.1
SNP effect on Arthropathy in Crohn disease
0.009
0.010
0.011
0.012
SNP effect on Heptanoate (7:0)

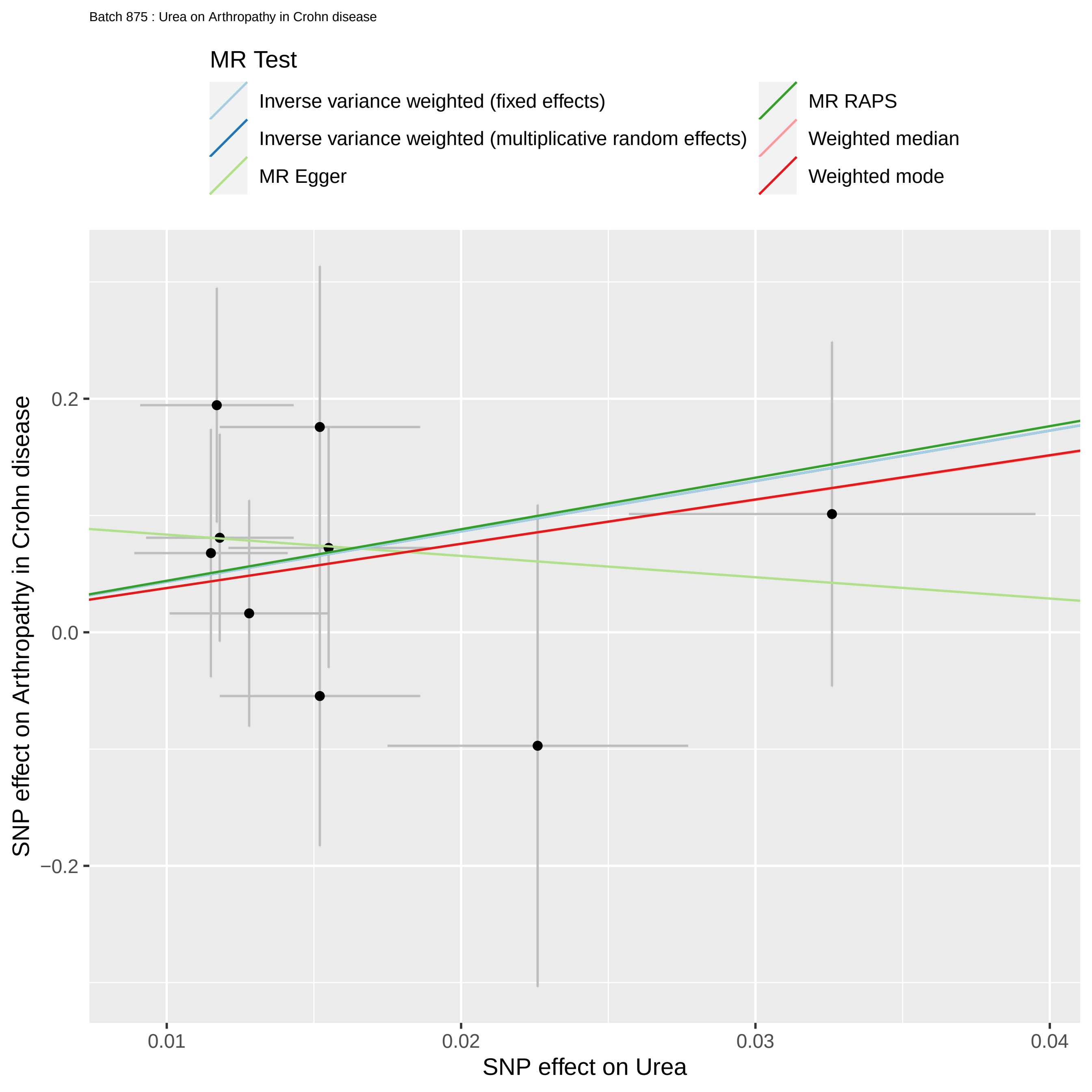
Batch 875 : Urea on Arthropathy in Crohn disease
MR Test
Inverse variance weighted (fixed effects)
Inverse variance weighted (multiplicative random effects)
MR Egger
MR RAPS
Weighted median
Weighted mode
0.2
0.0
−0.2
0.01
0.02
0.03
0.04
SNP effect on Arthropathy in Crohn disease
SNP effect on Urea

Batch 876 : Cortisol on Arthropathy in Crohn disease

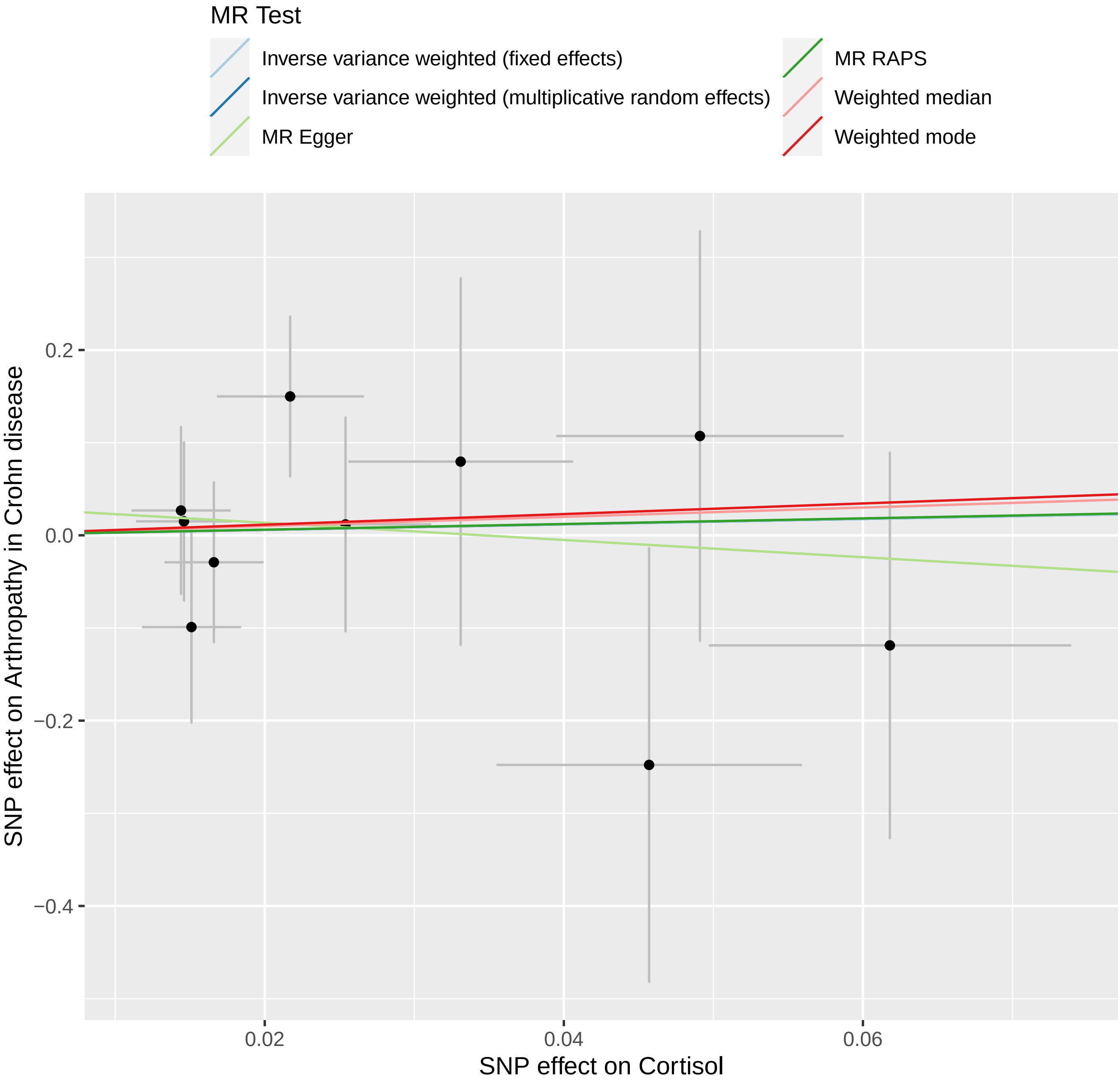

Batch 879 : Citrulline on Arthropathy in Crohn disease

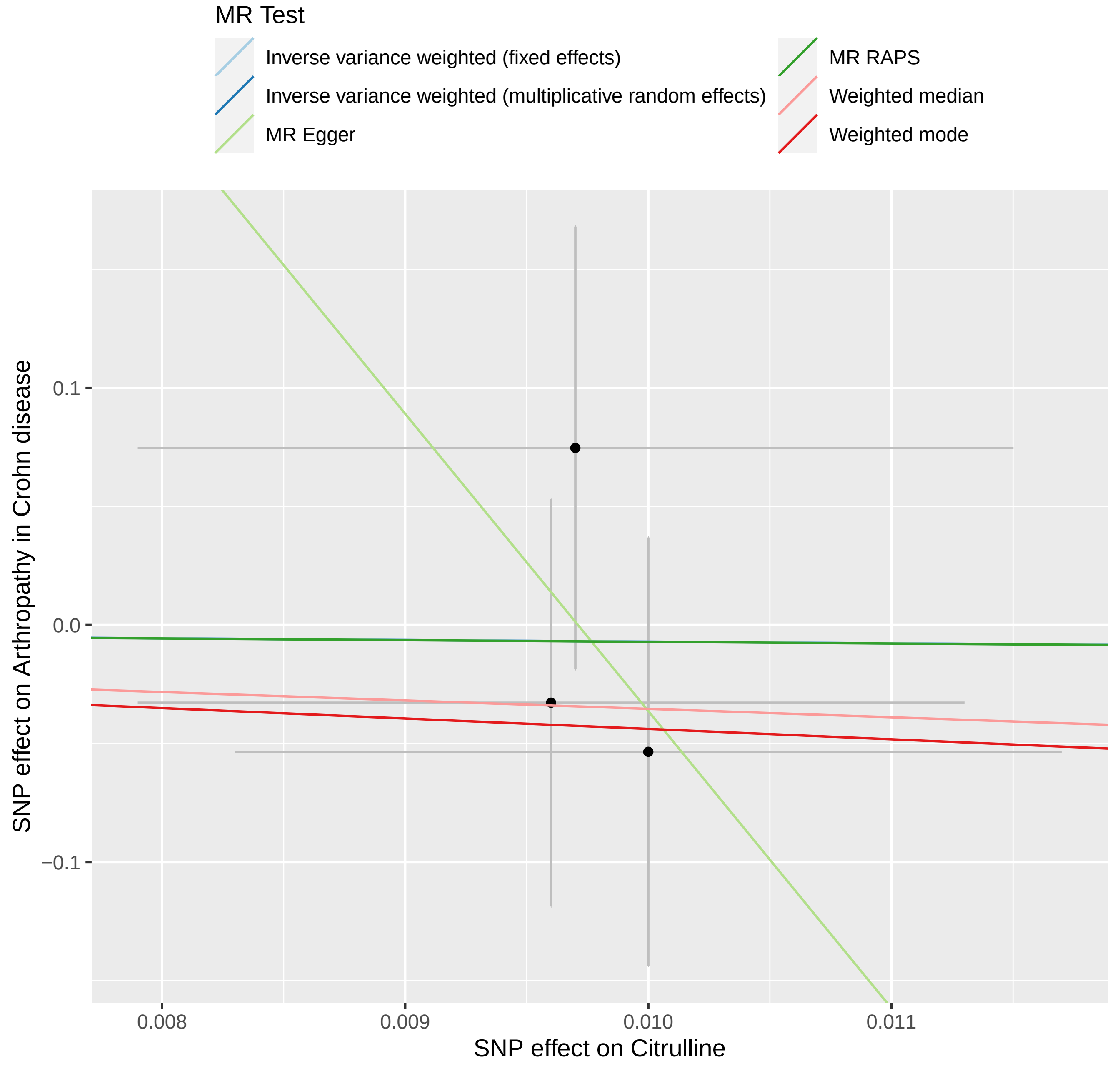

Batch 881 : Serotonin (5HT) on Arthropathy in Crohn disease

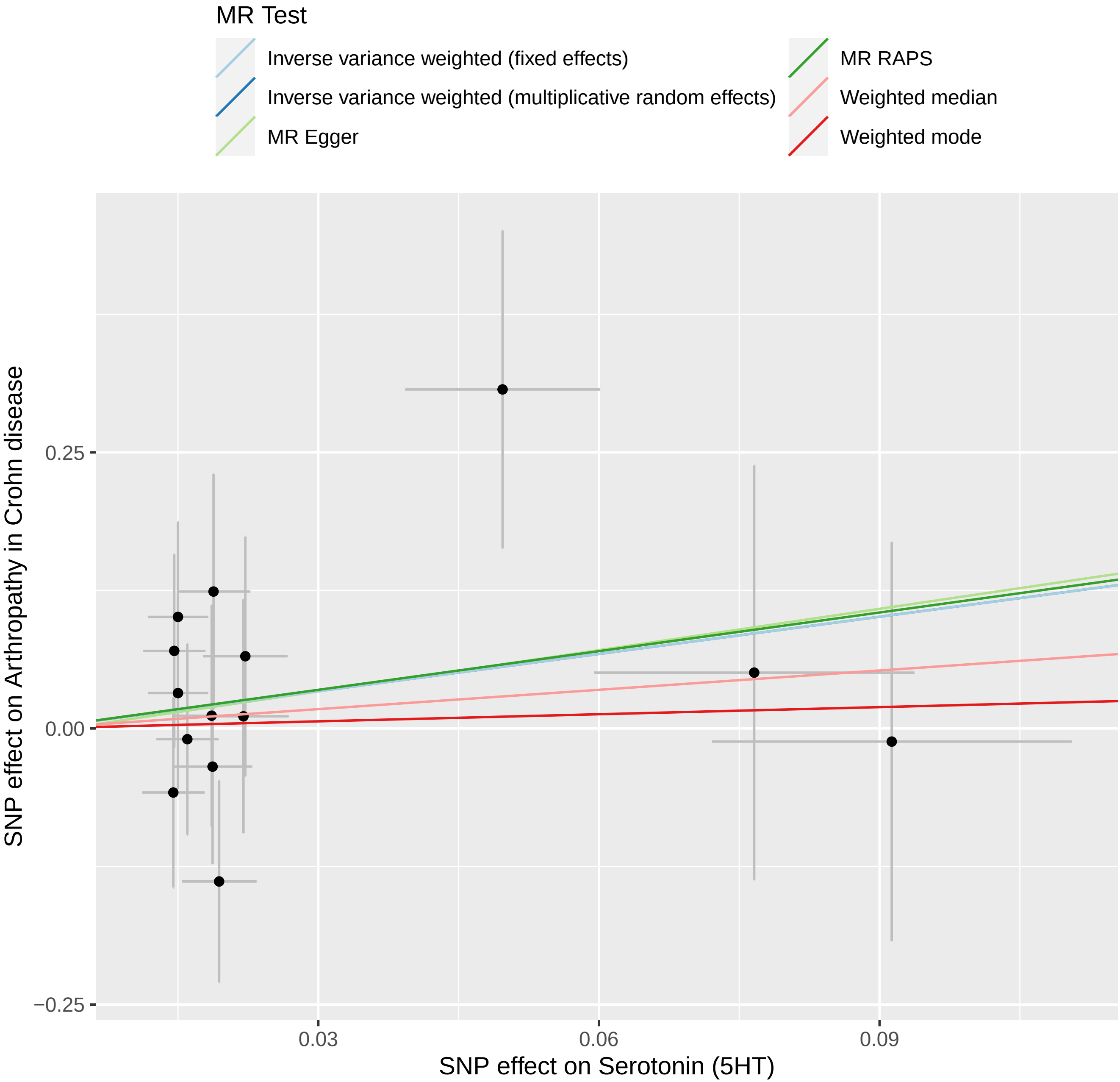

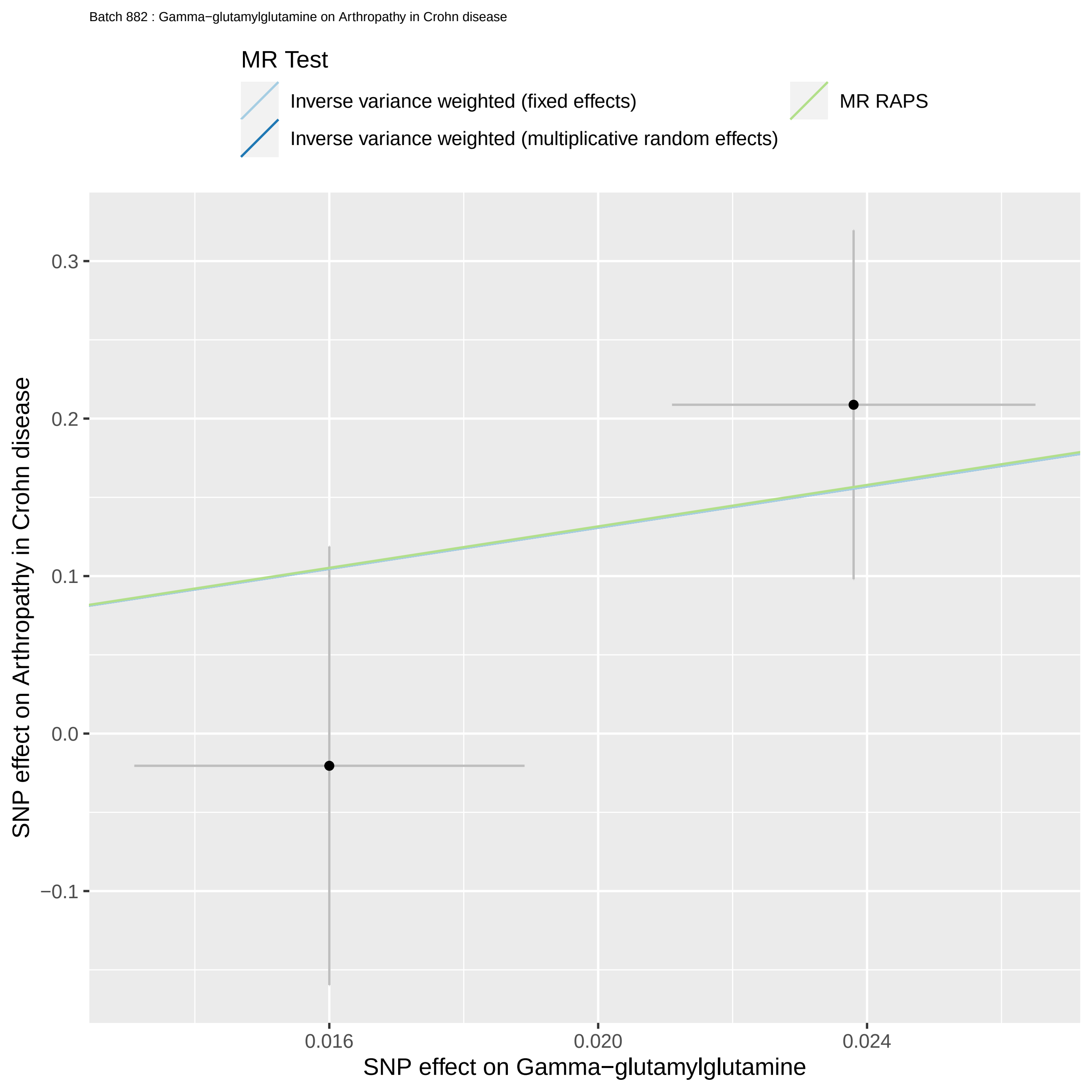
Batch 882 : Gamma−glutamylglutamine on Arthropathy in Crohn disease
MR Test
Inverse variance weighted (fixed effects)
Inverse variance weighted (multiplicative random effects)
MR RAPS
0.3
0.2
0.1
0.0
−0.1
0.016
0.020
0.024
SNP effect on Arthropathy in Crohn disease
SNP effect on Gamma−glutamylglutamine

Batch 883 : Gamma−glutamyltyrosine on Arthropathy in Crohn disease

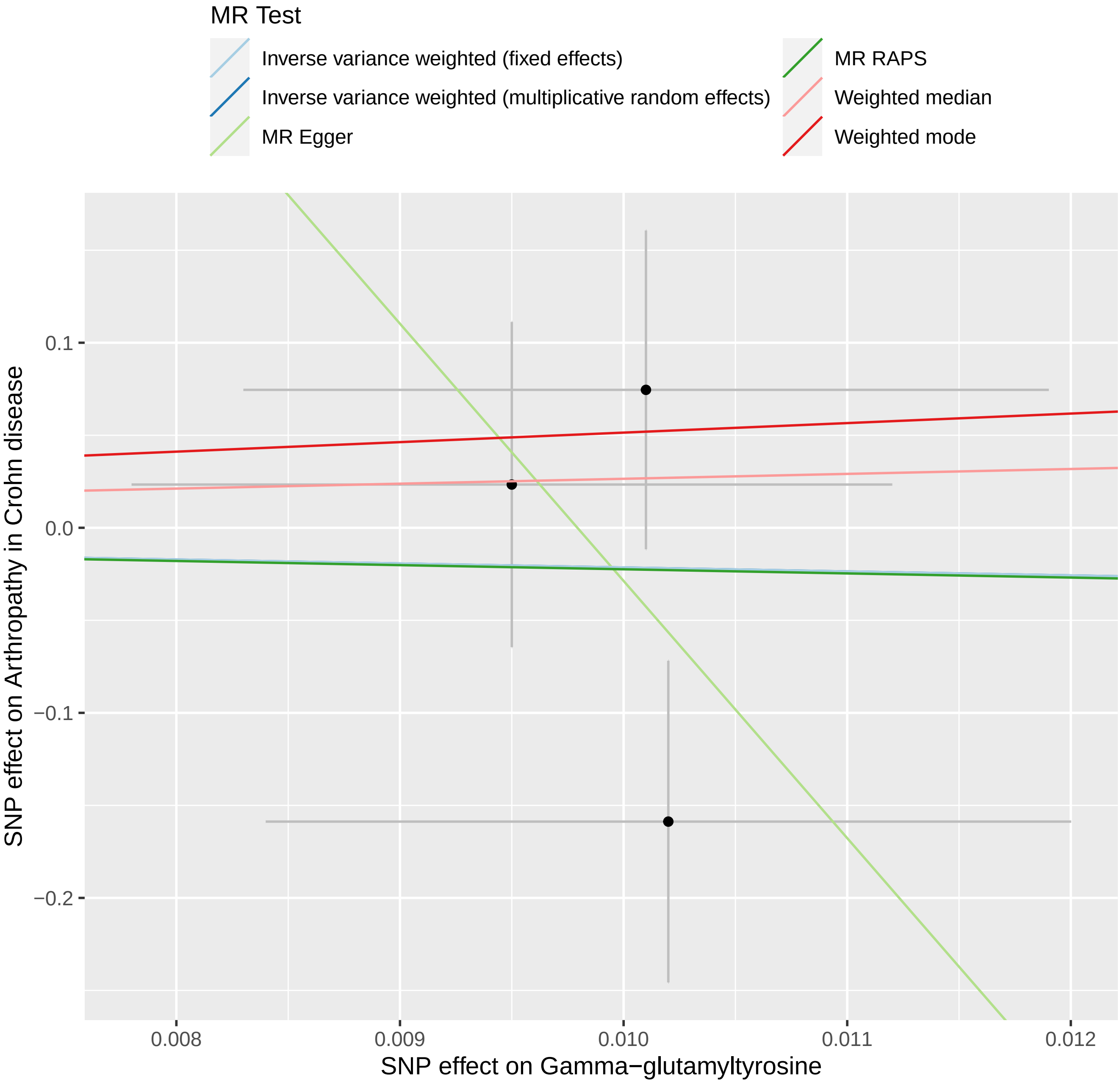

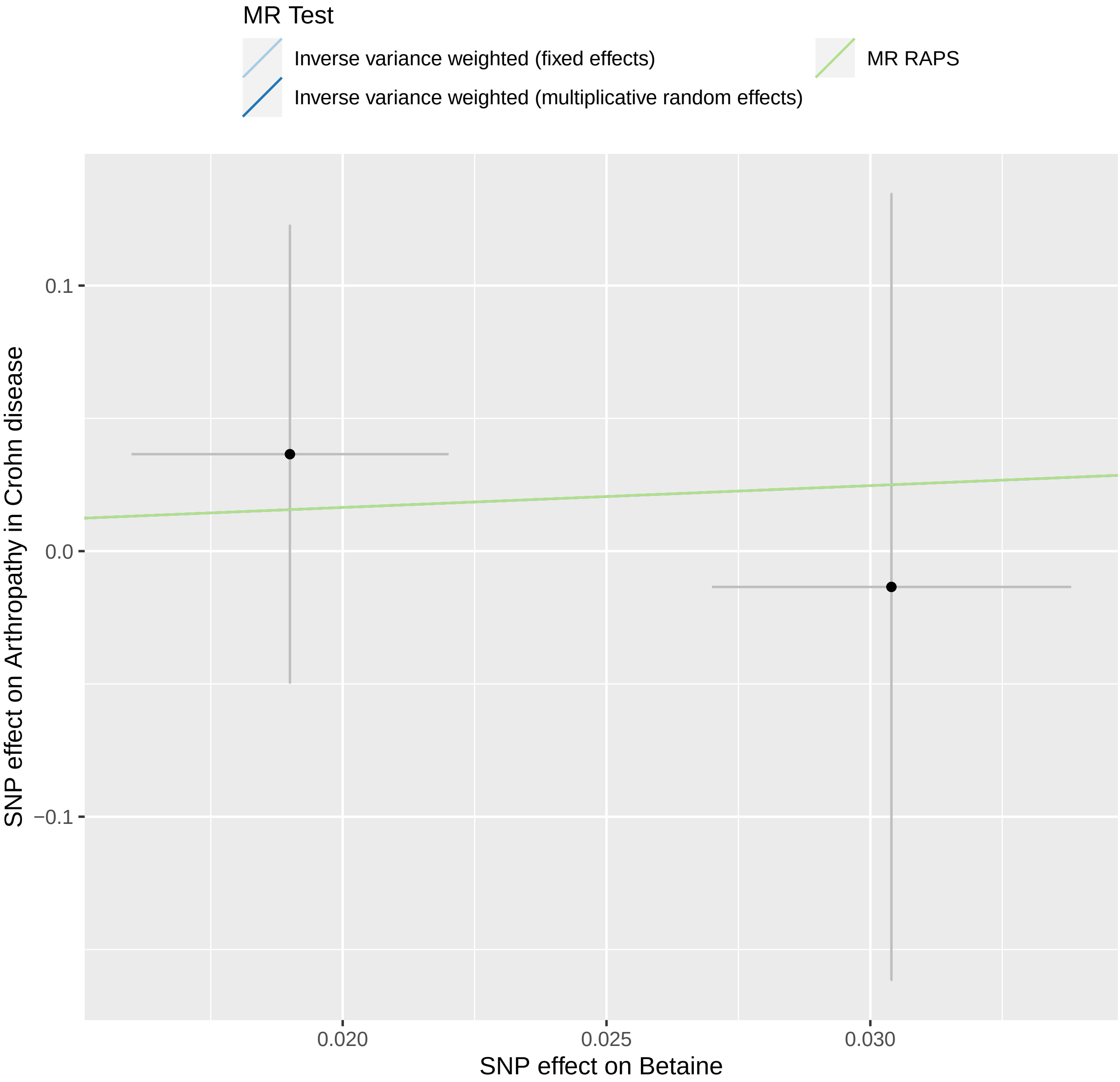
MR Test
Inverse variance weighted (fixed effects)
Inverse variance weighted (multiplicative random effects)
MR RAPS
0.1
0.0
−0.1
0.020
0.025
0.030
SNP effect on Arthropathy in Crohn disease
SNP effect on Betaine

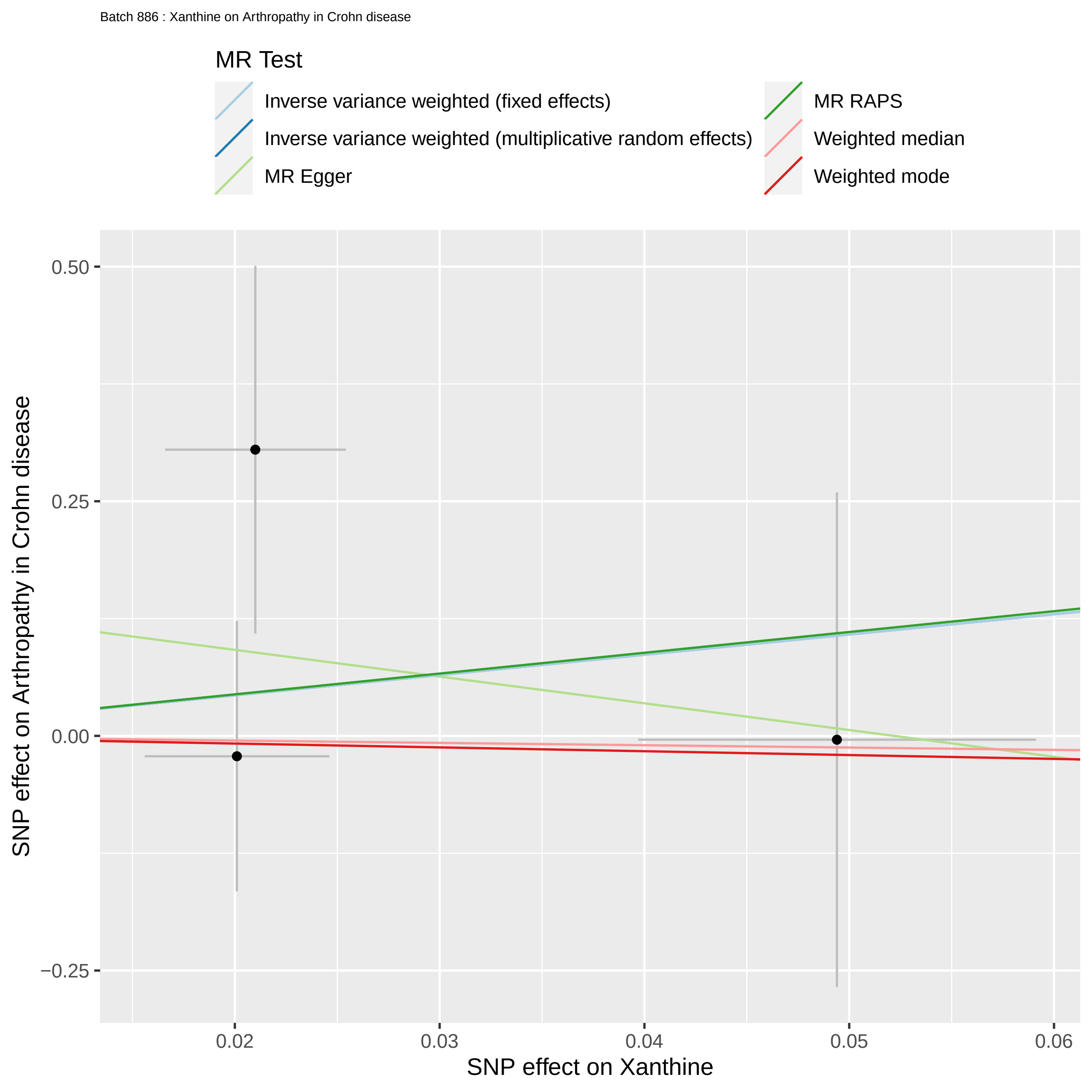

Batch 886 : Xanthine on Arthropathy in Crohn disease
MR Test
Inverse variance weighted (fixed effects)
Inverse variance weighted (multiplicative random effects)
MR Egger
MR RAPS
Weighted median
Weighted mode
0.50
0.25
0.00
−0.25
0.02
0.03
0.04
0.05
0.06
SNP effect on Arthropathy in Crohn disease
SNP effect on Xanthine

Batch 887 : Phosphate on Arthropathy in Crohn disease

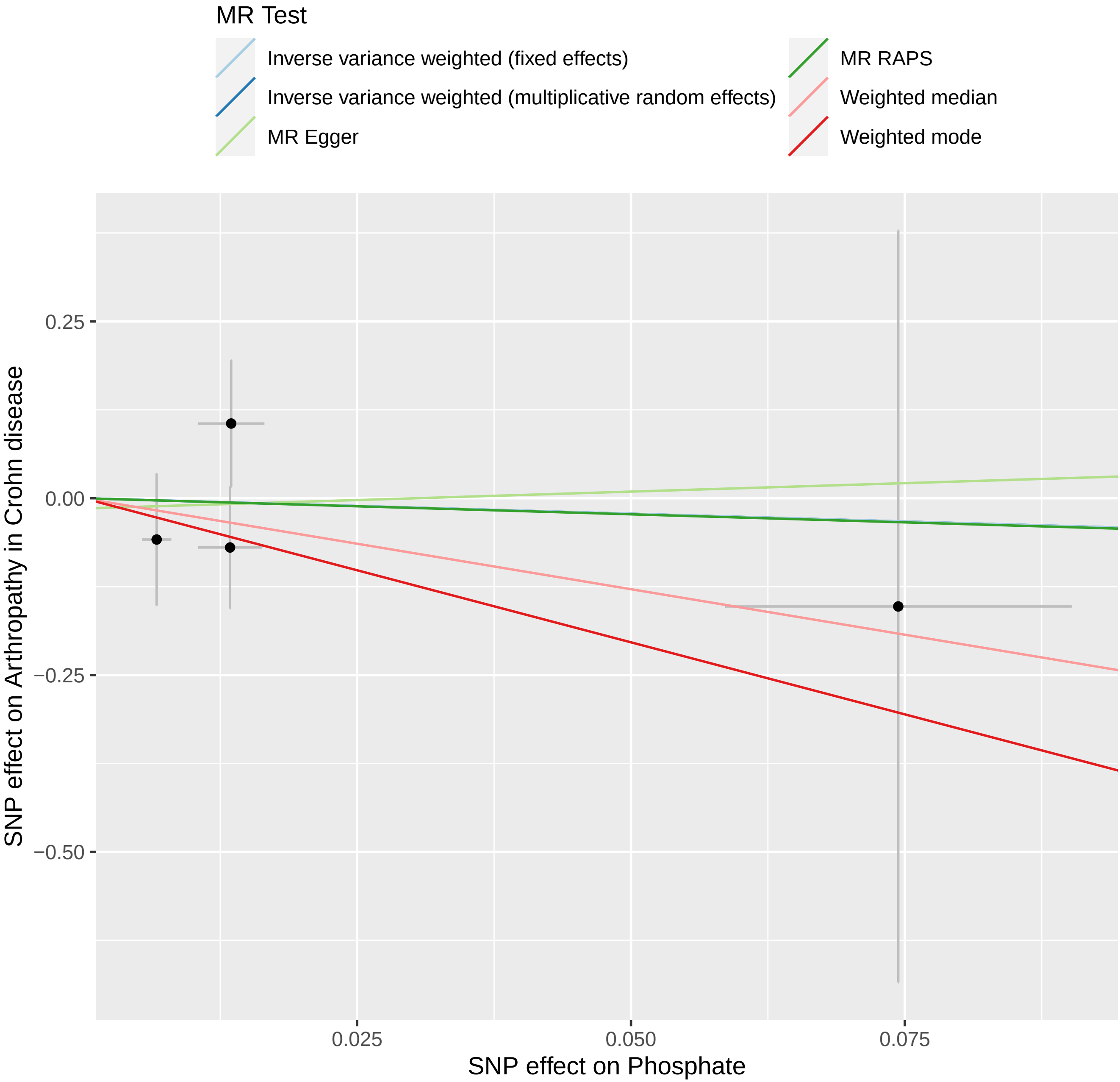

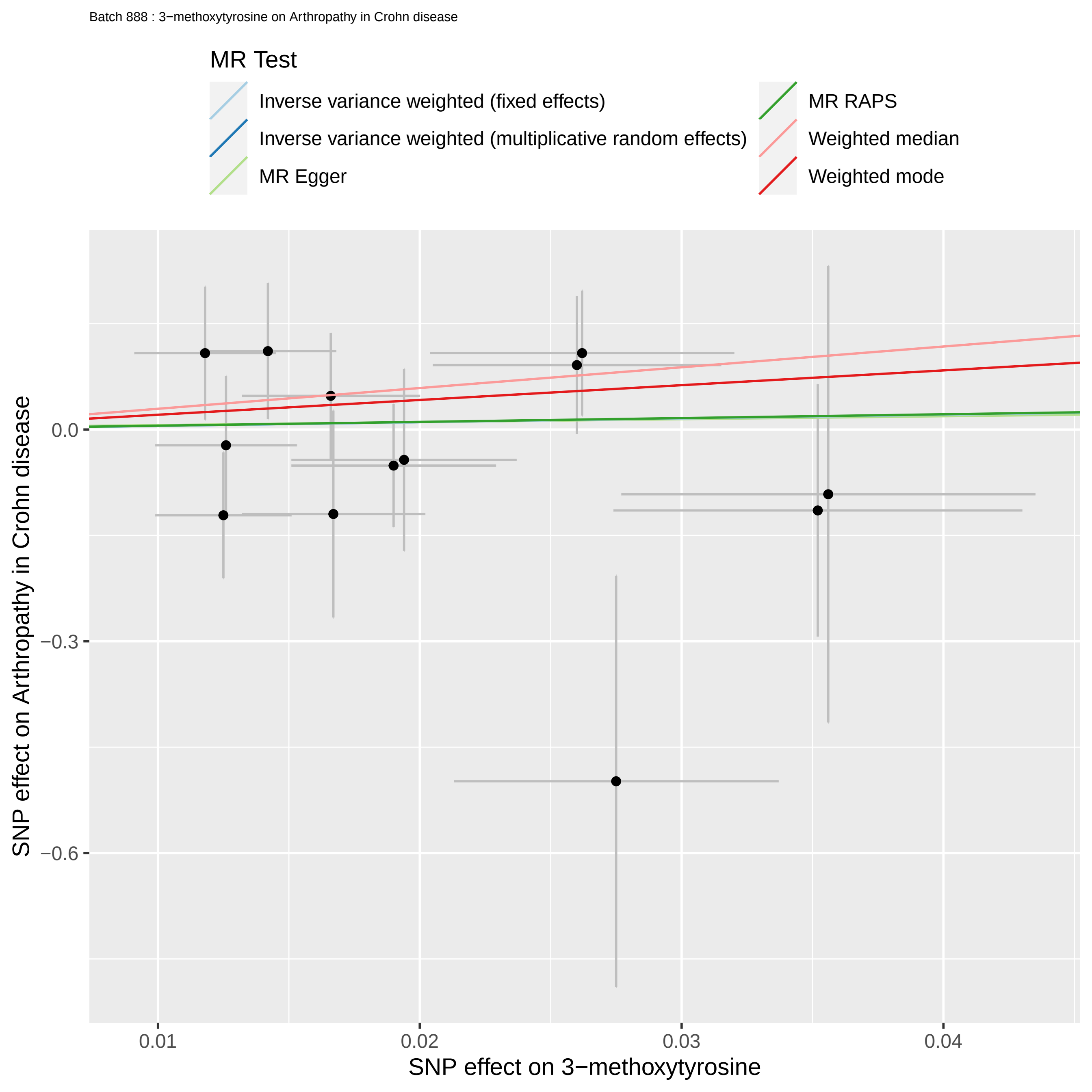

Batch 888 : 3−methoxytyrosine on Arthropathy in Crohn disease
MR Test
Inverse variance weighted (fixed effects)
Inverse variance weighted (multiplicative random effects)
MR Egger
MR RAPS
Weighted median
Weighted mode
SNP effect on Arthropathy in Crohn disease
0.0
−0.3
−0.6
0.01
0.02
0.03
0.04
SNP effect on 3−methoxytyrosine

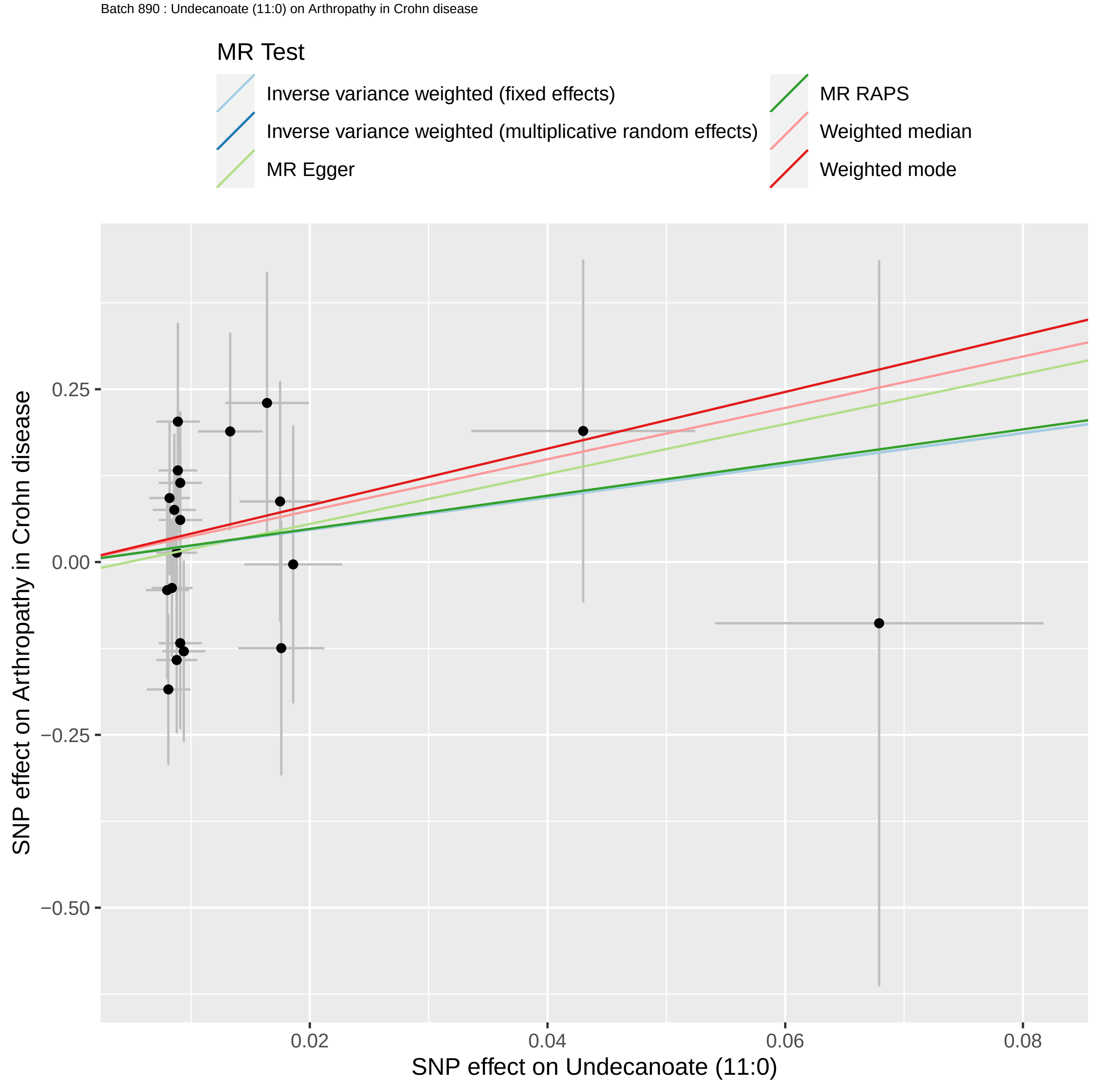

Batch 890 : Undecanoate (11:0) on Arthropathy in Crohn disease
MR Test
Inverse variance weighted (fixed effects)
Inverse variance weighted (multiplicative random effects)
MR Egger
MR RAPS
Weighted median
Weighted mode
0.25
0.00
−0.25
−0.50
0.02
0.04
0.06
0.08
SNP effect on Arthropathy in Crohn disease
SNP effect on Undecanoate (11:0)

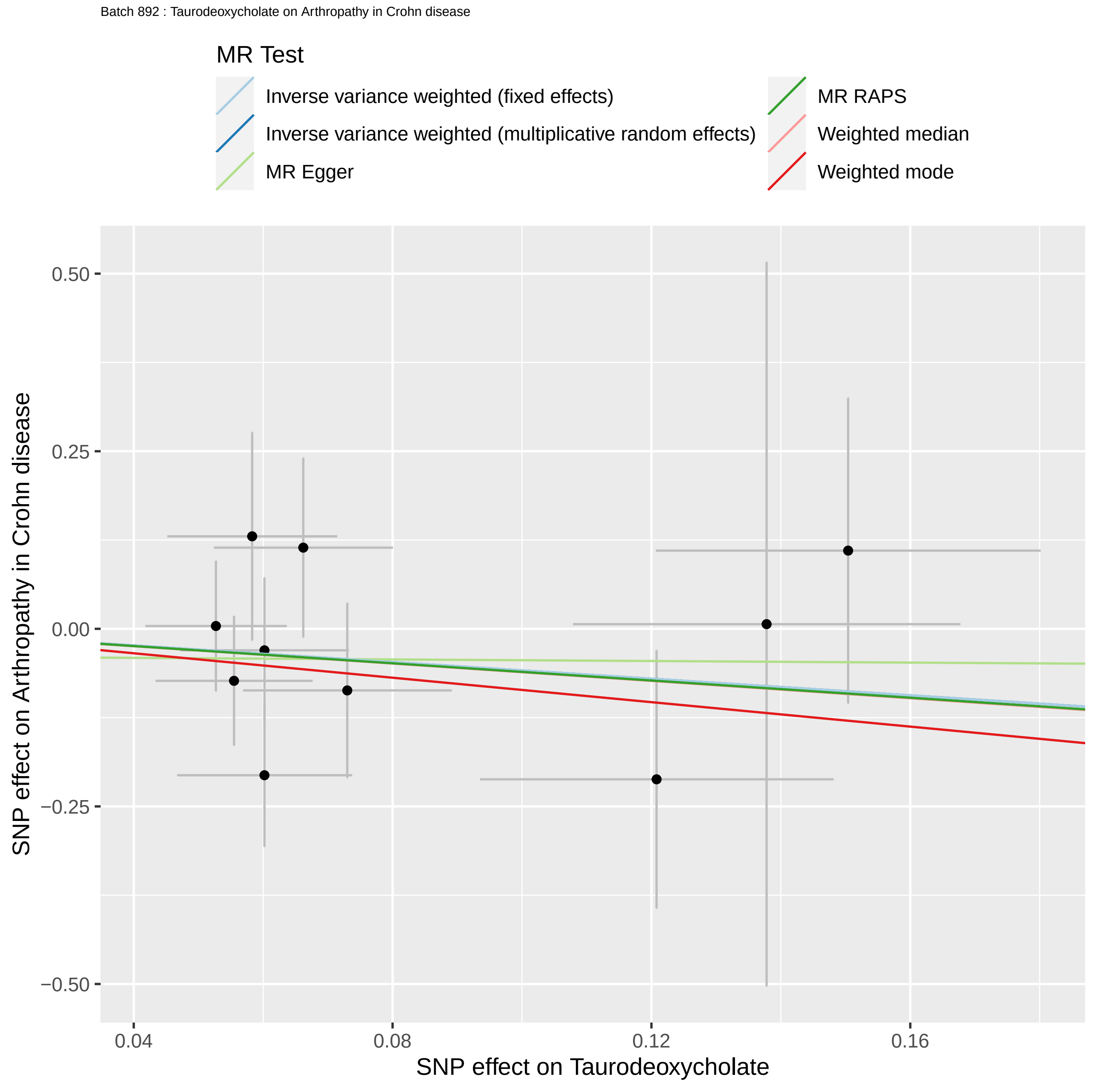

Batch 892 : Taurodeoxycholate on Arthropathy in Crohn disease
MR Test
Inverse variance weighted (fixed effects)
Inverse variance weighted (multiplicative random effects)
MR Egger
MR RAPS
Weighted median
Weighted mode
0.50
0.25
0.00
−0.25
−0.50
SNP effect on Arthropathy in Crohn disease
0.04
0.08
0.12
0.16
SNP effect on Taurodeoxycholate

Batch 893 : Glycerol on Arthropathy in Crohn disease

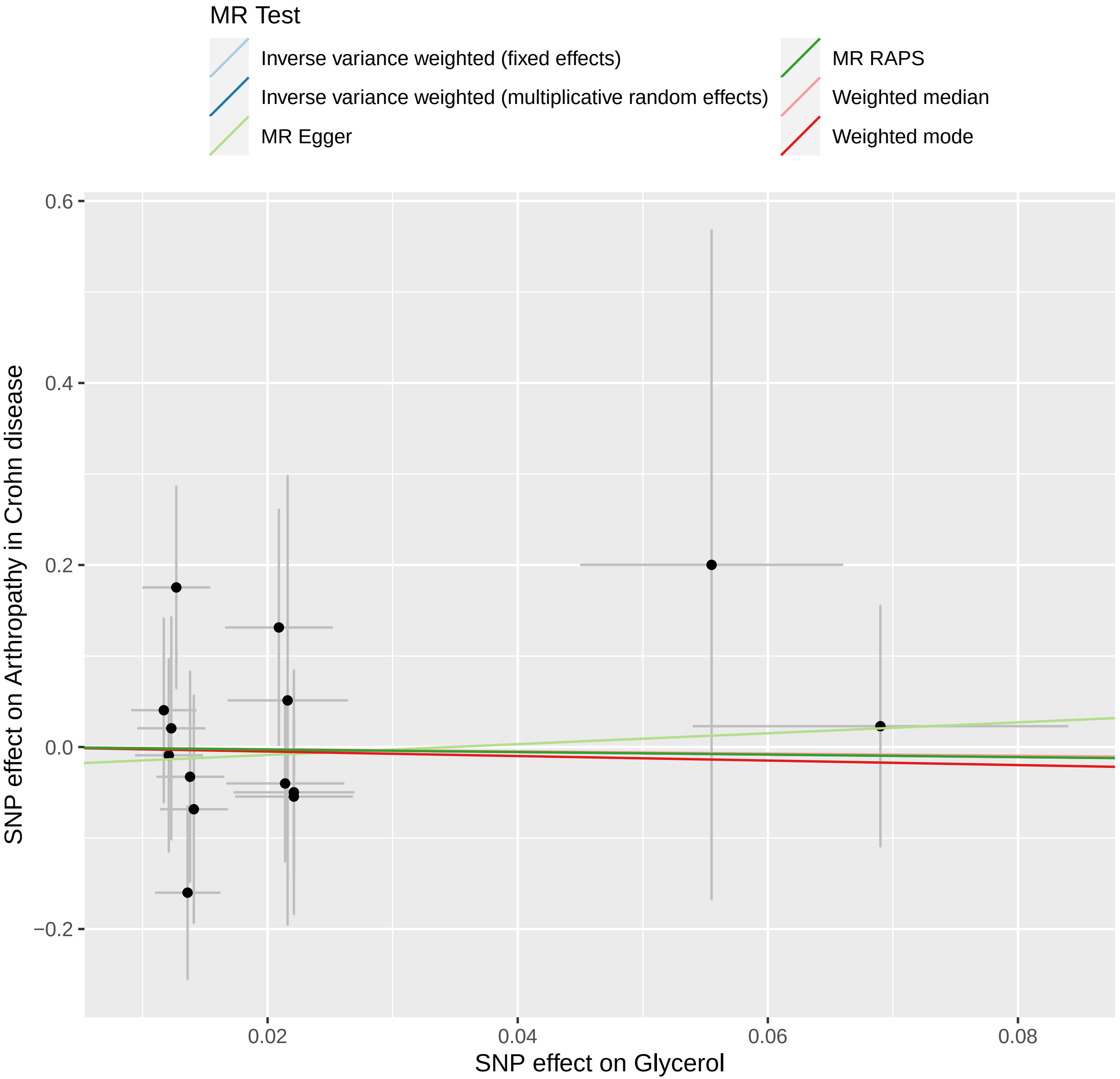

Batch 894 : Kynurenine on Arthropathy in Crohn disease

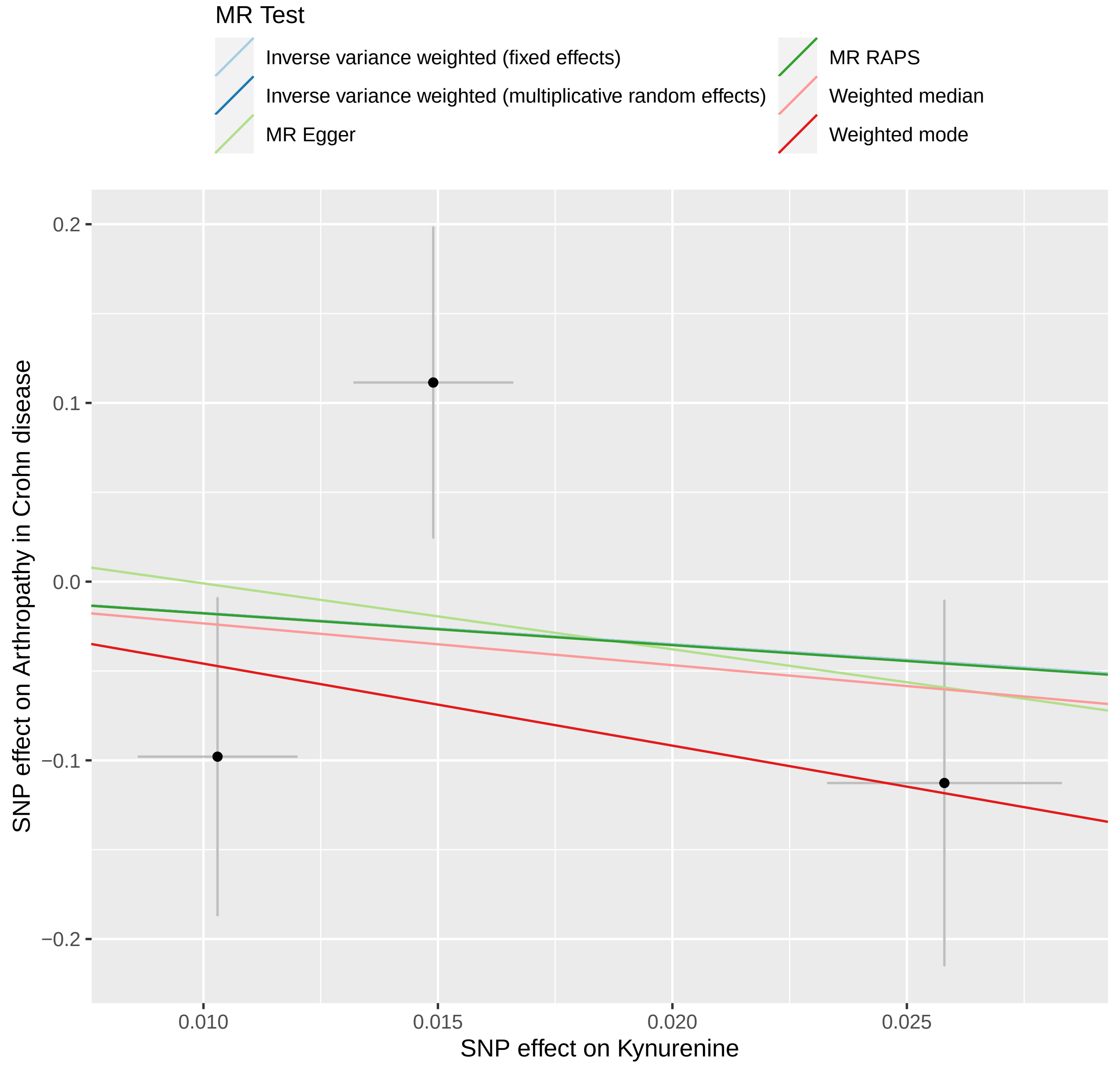

Batch 895 : Mannitol on Arthropathy in Crohn disease

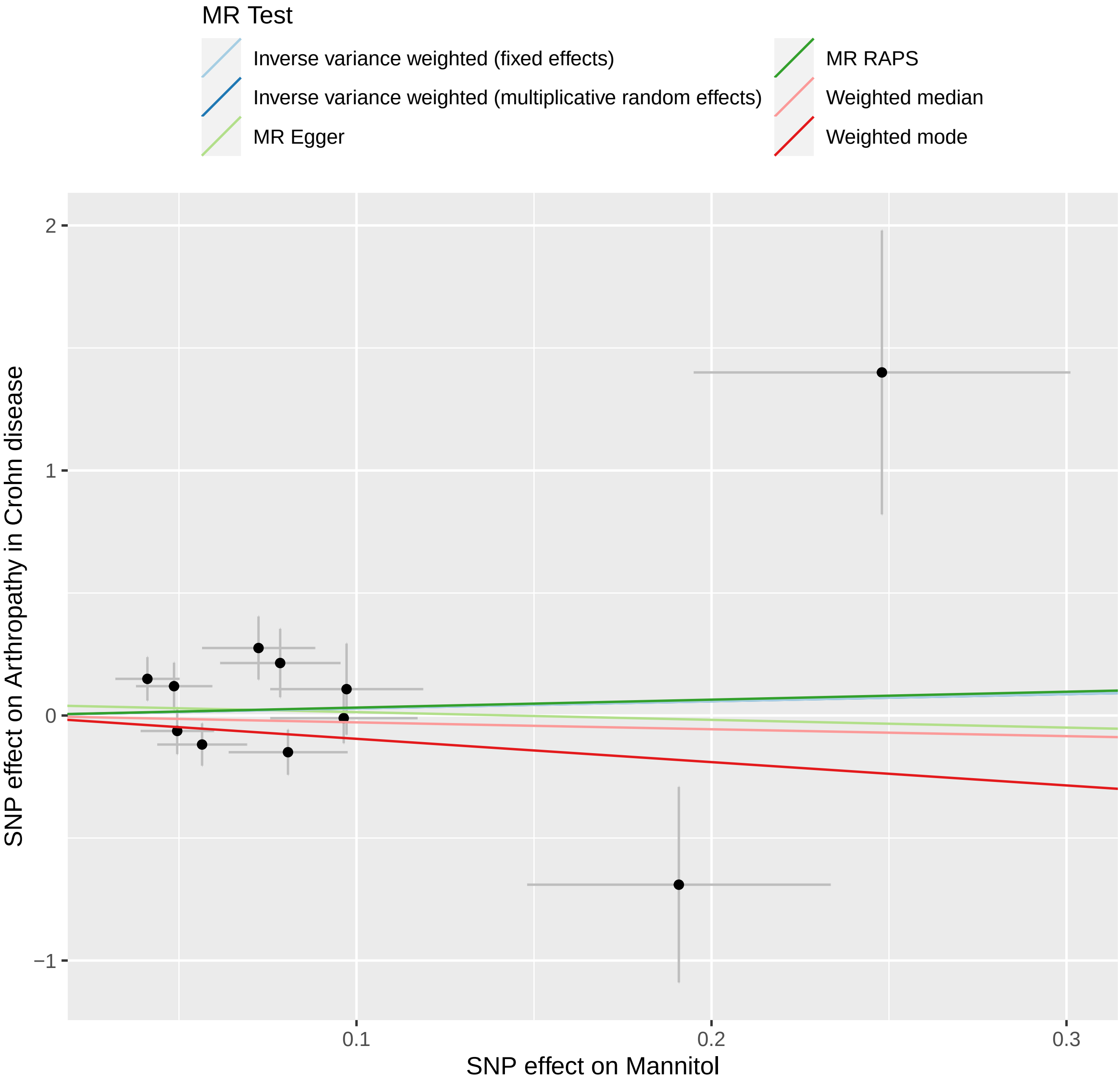

Batch 896 : Glycerol 3−phosphate (G3P) on Arthropathy in Crohn disease

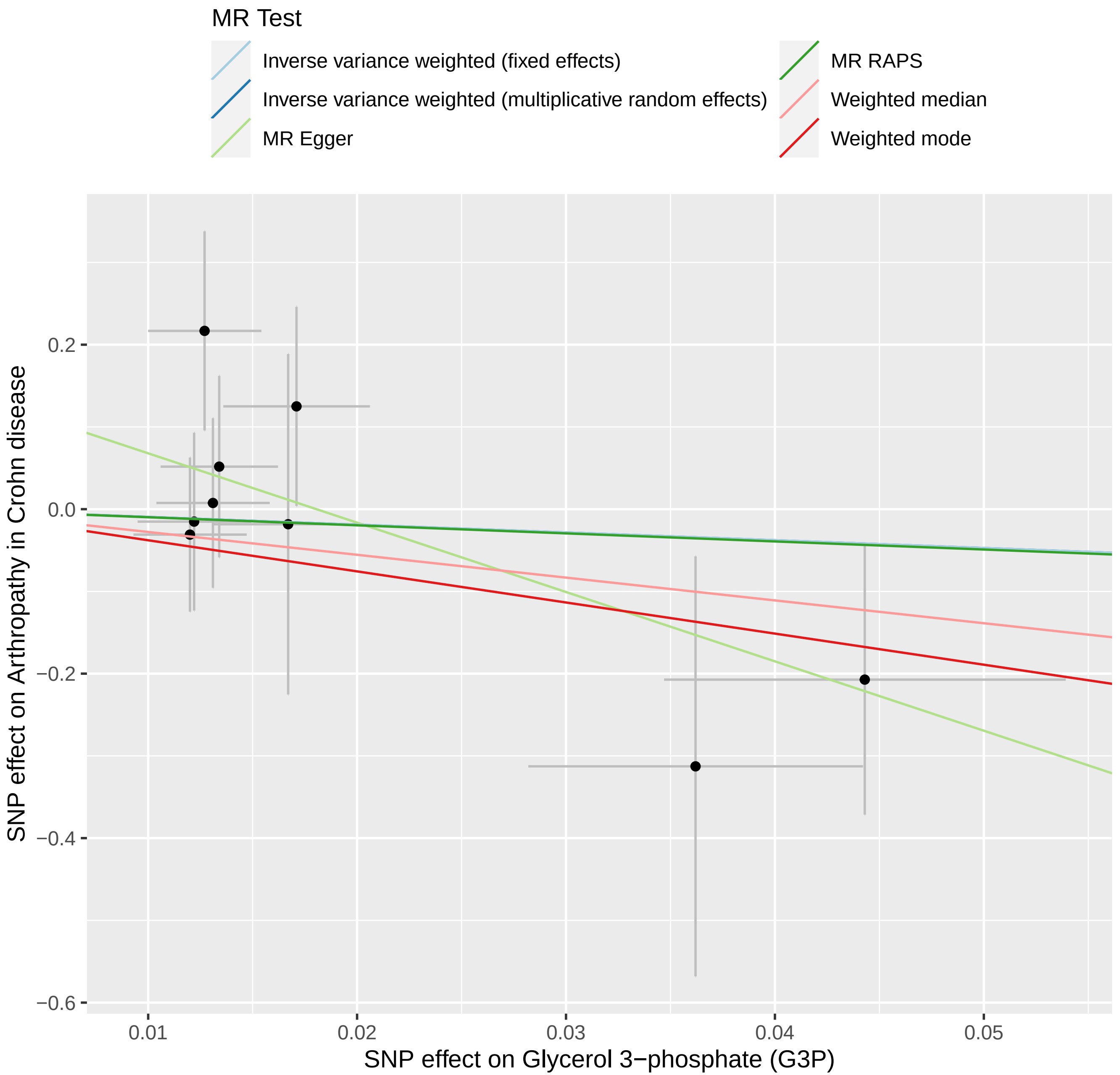

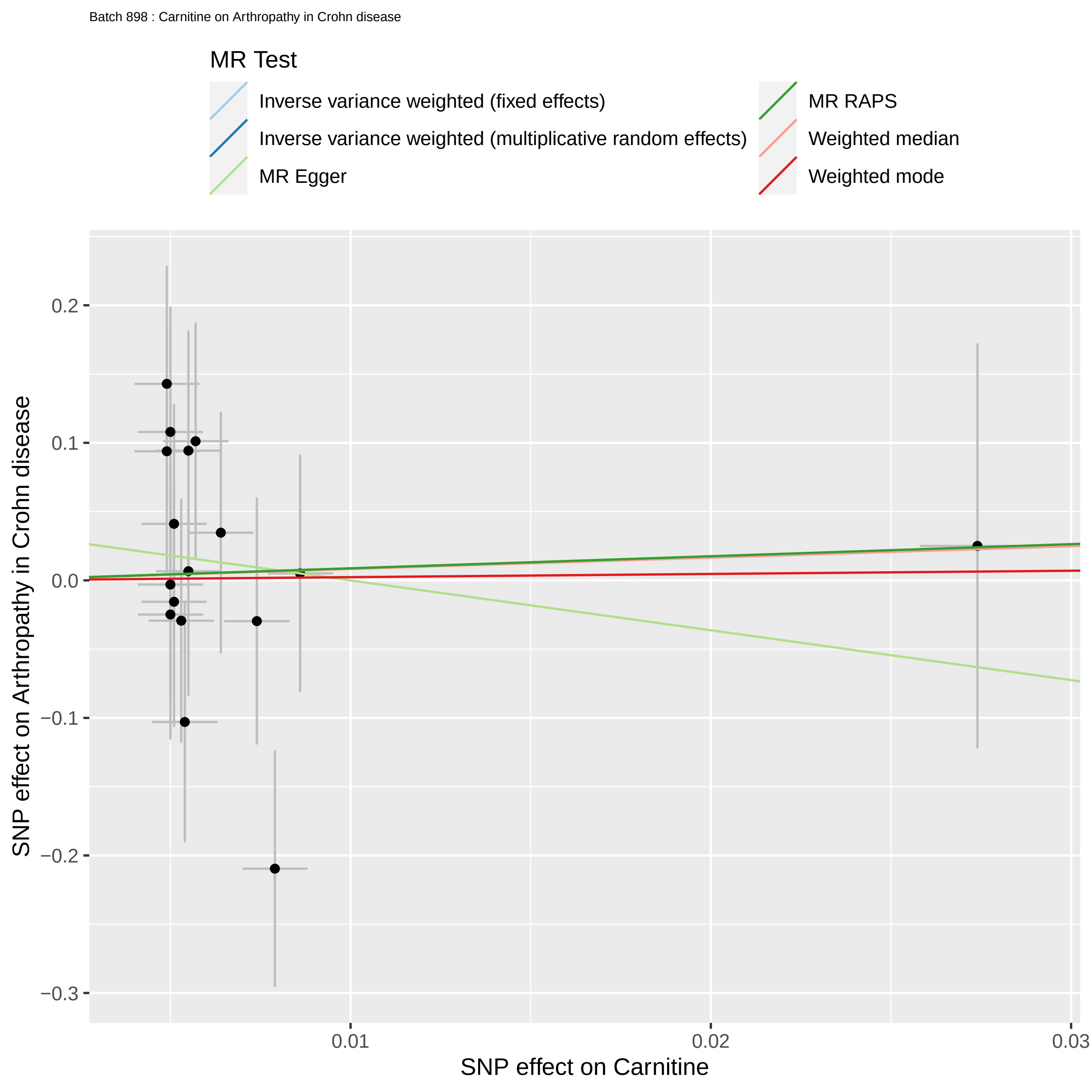
Batch 898 : Carnitine on Arthropathy in Crohn disease
MR Test
Inverse variance weighted (fixed effects)
Inverse variance weighted (multiplicative random effects)
MR Egger
MR RAPS
Weighted median
Weighted mode
SNP effect on Arthropathy in Crohn disease
0.2
0.1
0.0
−0.1
−0.2
−0.3
0.01
0.02
0.03
SNP effect on Carnitine

Batch 899 : Choline on Arthropathy in Crohn disease

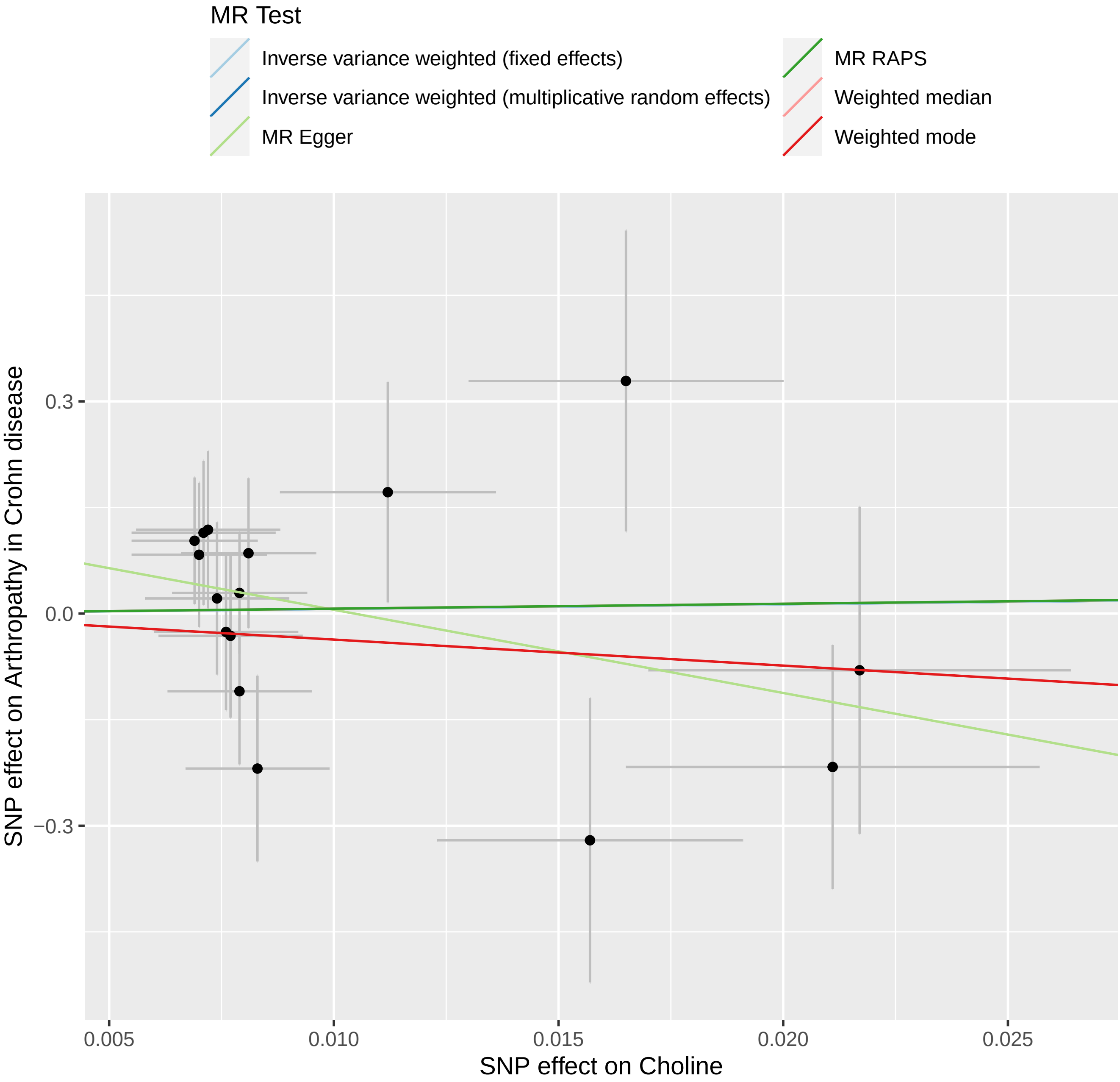

Batch 902 : 3−methyl−2−oxovalerate on Arthropathy in Crohn disease

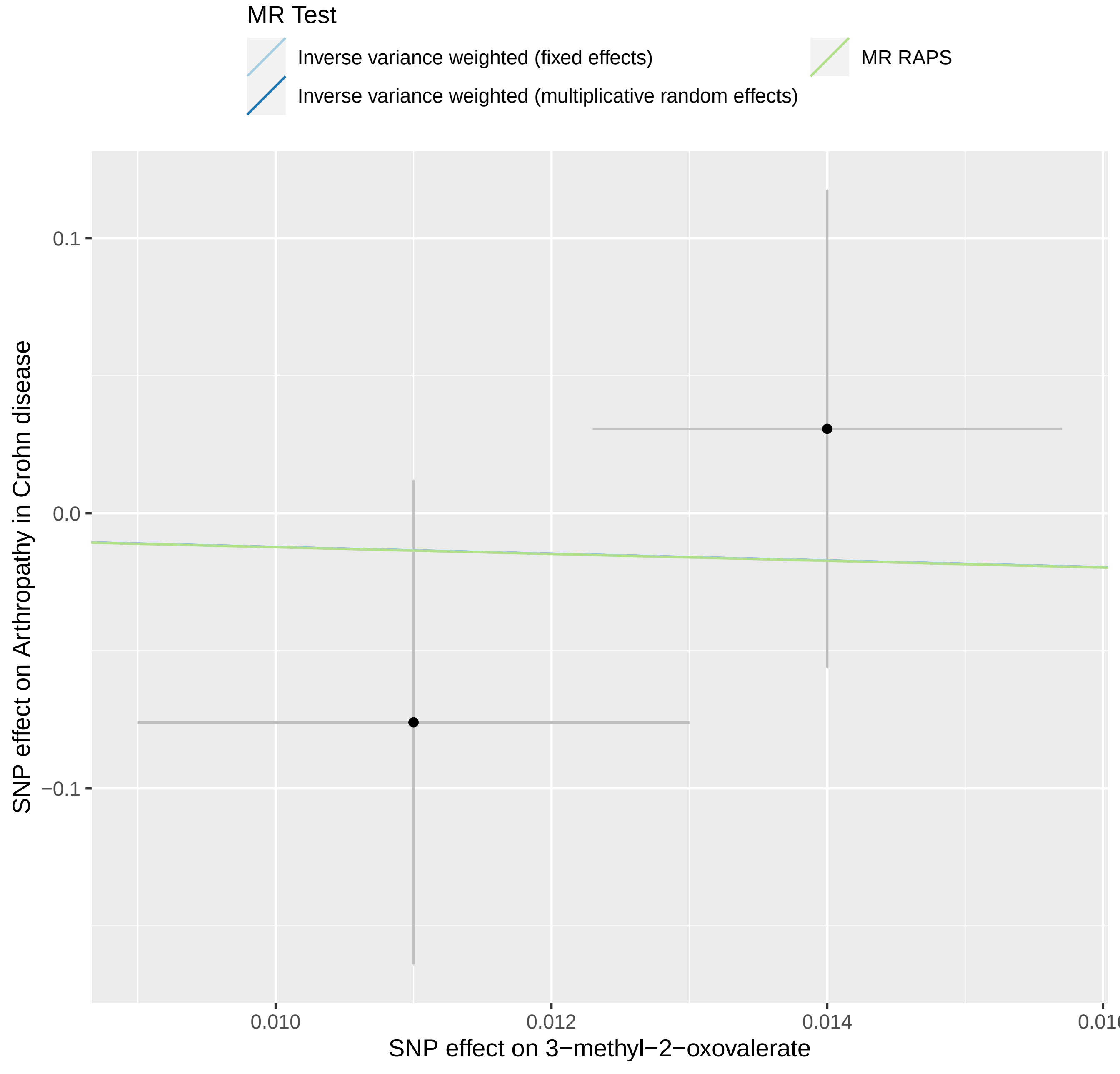

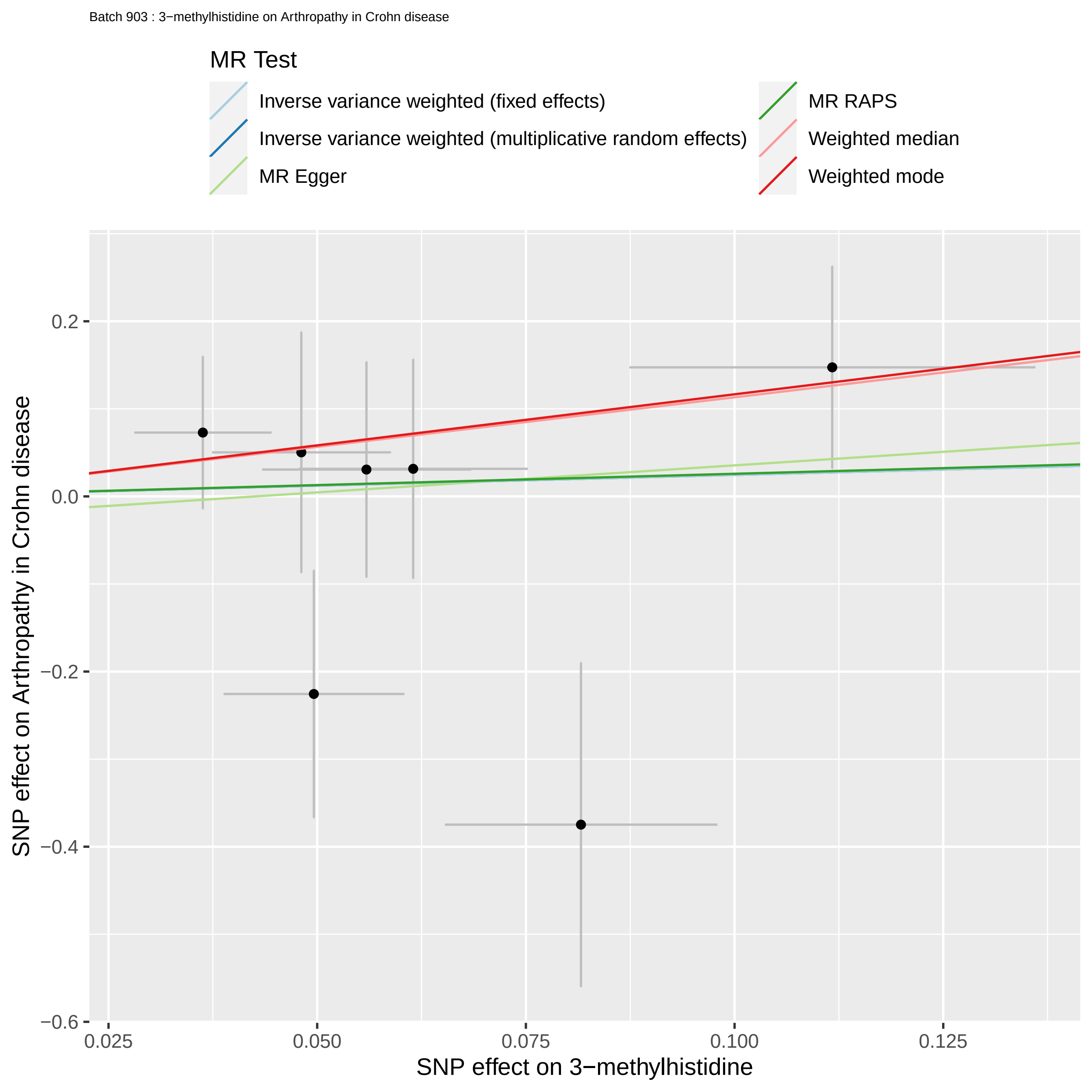
Batch 903 : 3−methylhistidine on Arthropathy in Crohn disease
MR Test
Inverse variance weighted (fixed effects)
Inverse variance weighted (multiplicative random effects)
MR Egger
MR RAPS
Weighted median
Weighted mode
SNP effect on Arthropathy in Crohn disease
0.2
0.0
−0.2
−0.4
−0.6
0.025
0.050
0.075
0.100
0.125
SNP effect on 3−methylhistidine

Batch 905 : Phenylacetate on Arthropathy in Crohn disease

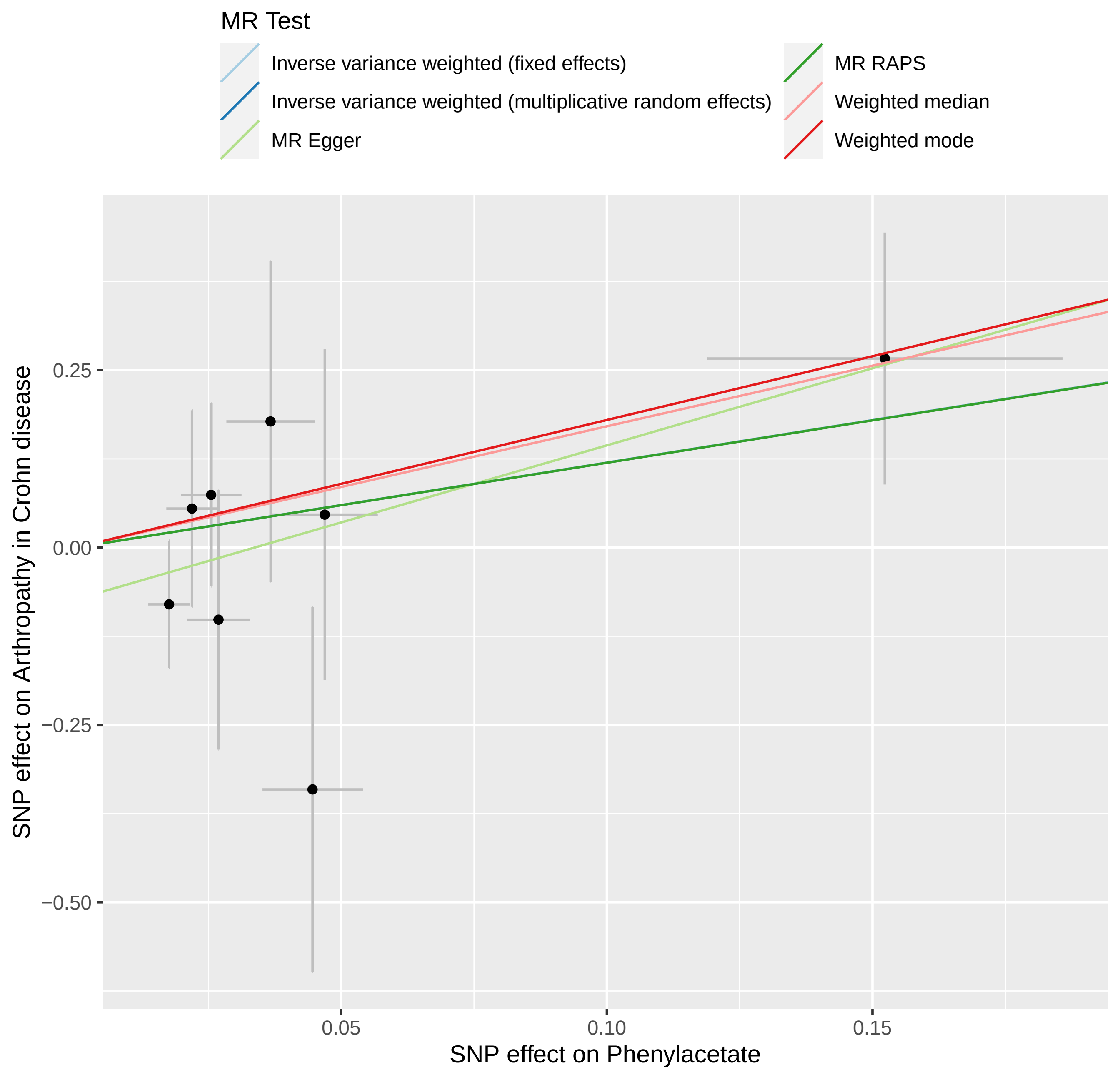

Batch 906 : Glycerophosphorylcholine (GPC) on Arthropathy in Crohn disease

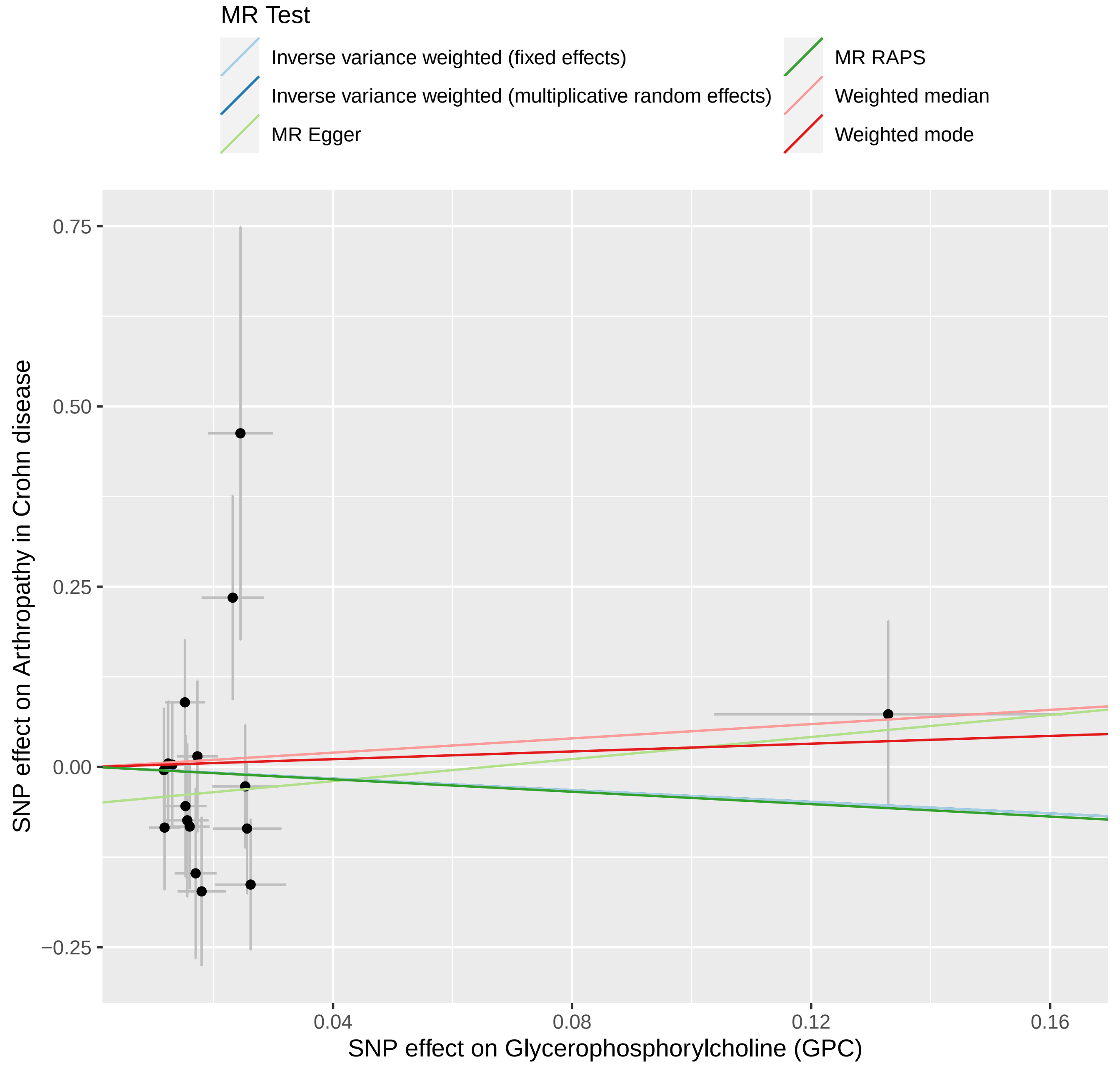

Batch 907 : Aspartate on Arthropathy in Crohn disease

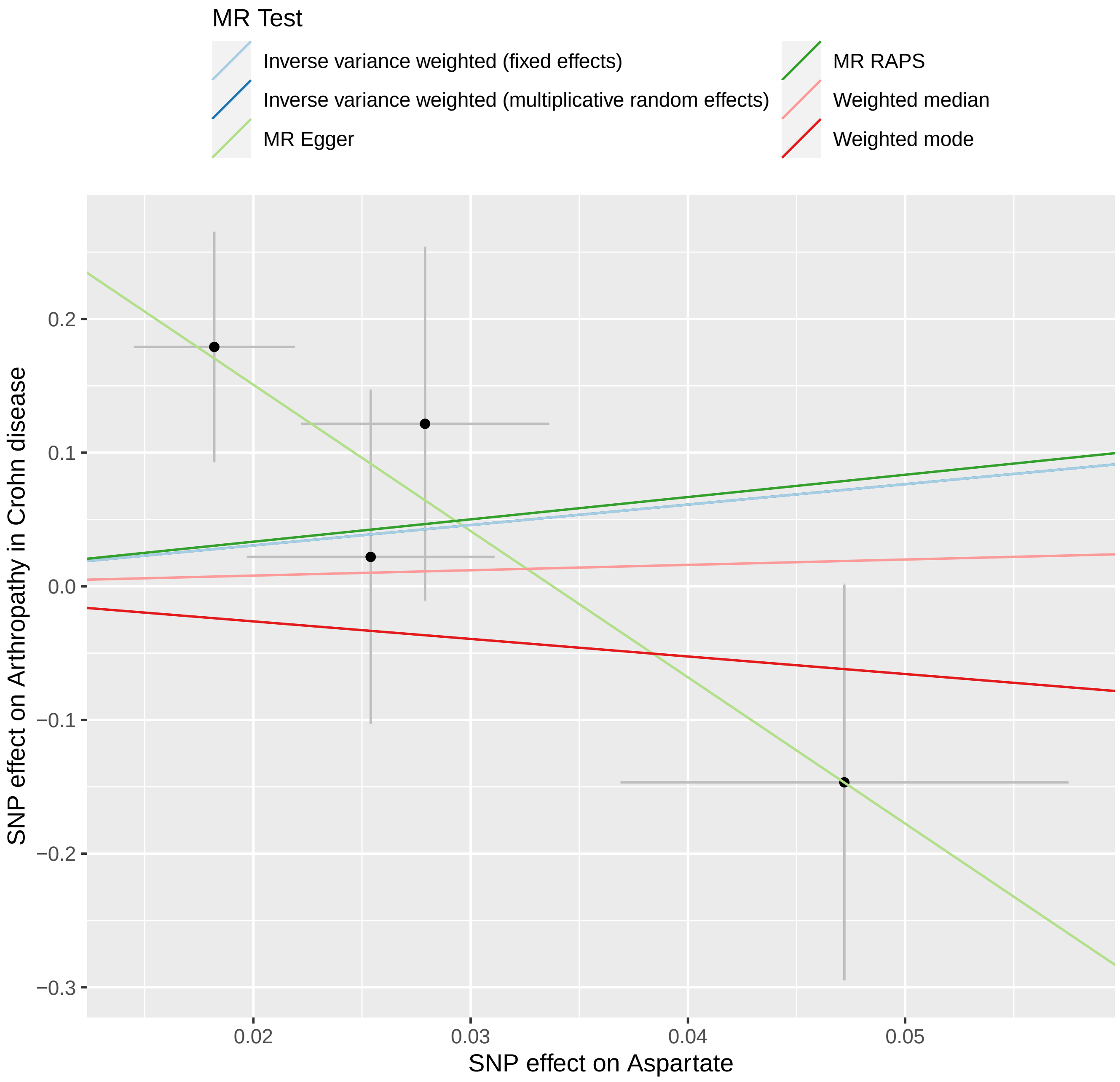

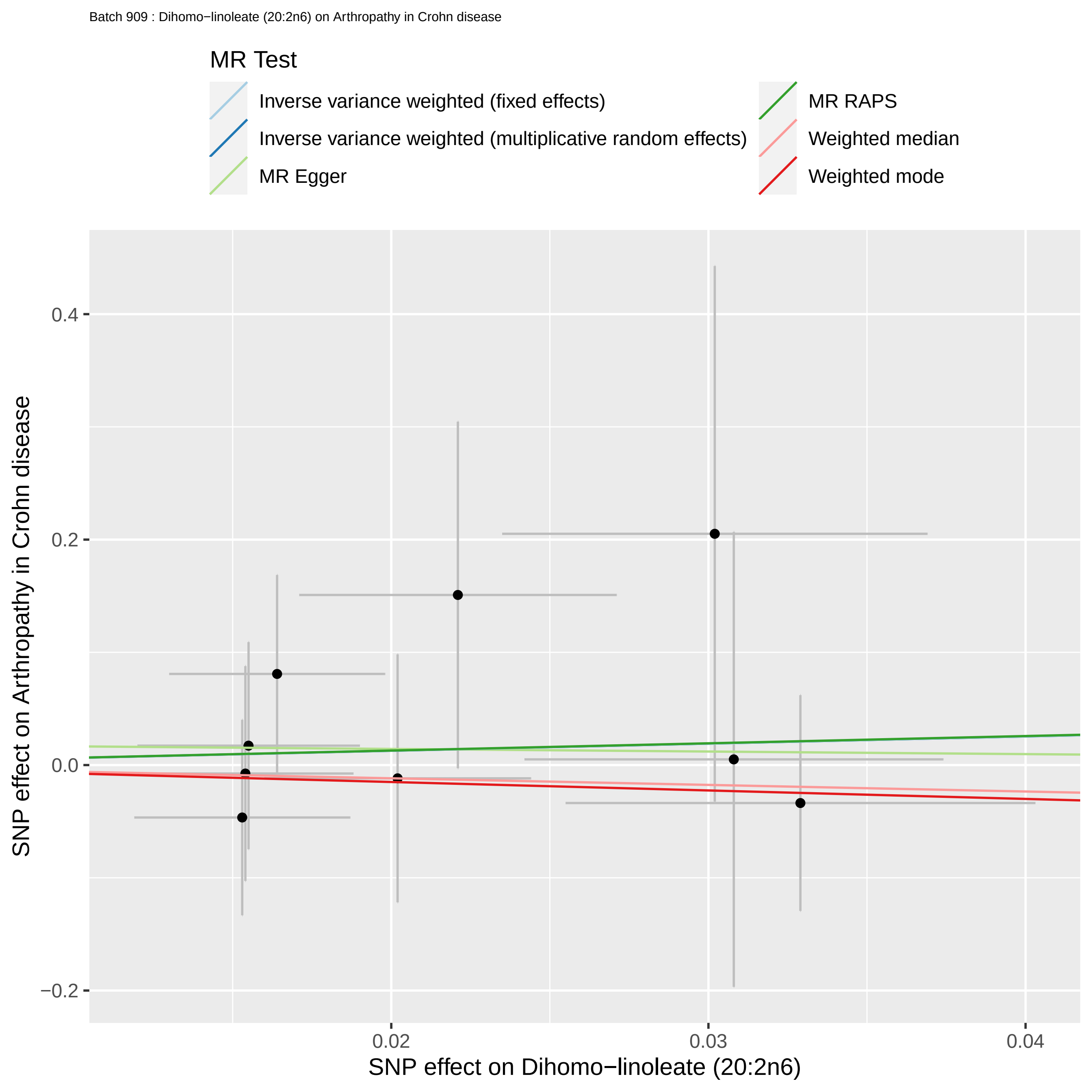
Batch 909 : Dihomo−linoleate (20:2n6) on Arthropathy in Crohn disease
MR Test
Inverse variance weighted (fixed effects)
Inverse variance weighted (multiplicative random effects)
MR Egger
MR RAPS
Weighted median
Weighted mode
0.4
0.2
0.0
−0.2
0.02
0.03
0.04
SNP effect on Arthropathy in Crohn disease
SNP effect on Dihomo−linoleate (20:2n6)

Batch 910 : 2−hydroxystearate on Arthropathy in Crohn disease

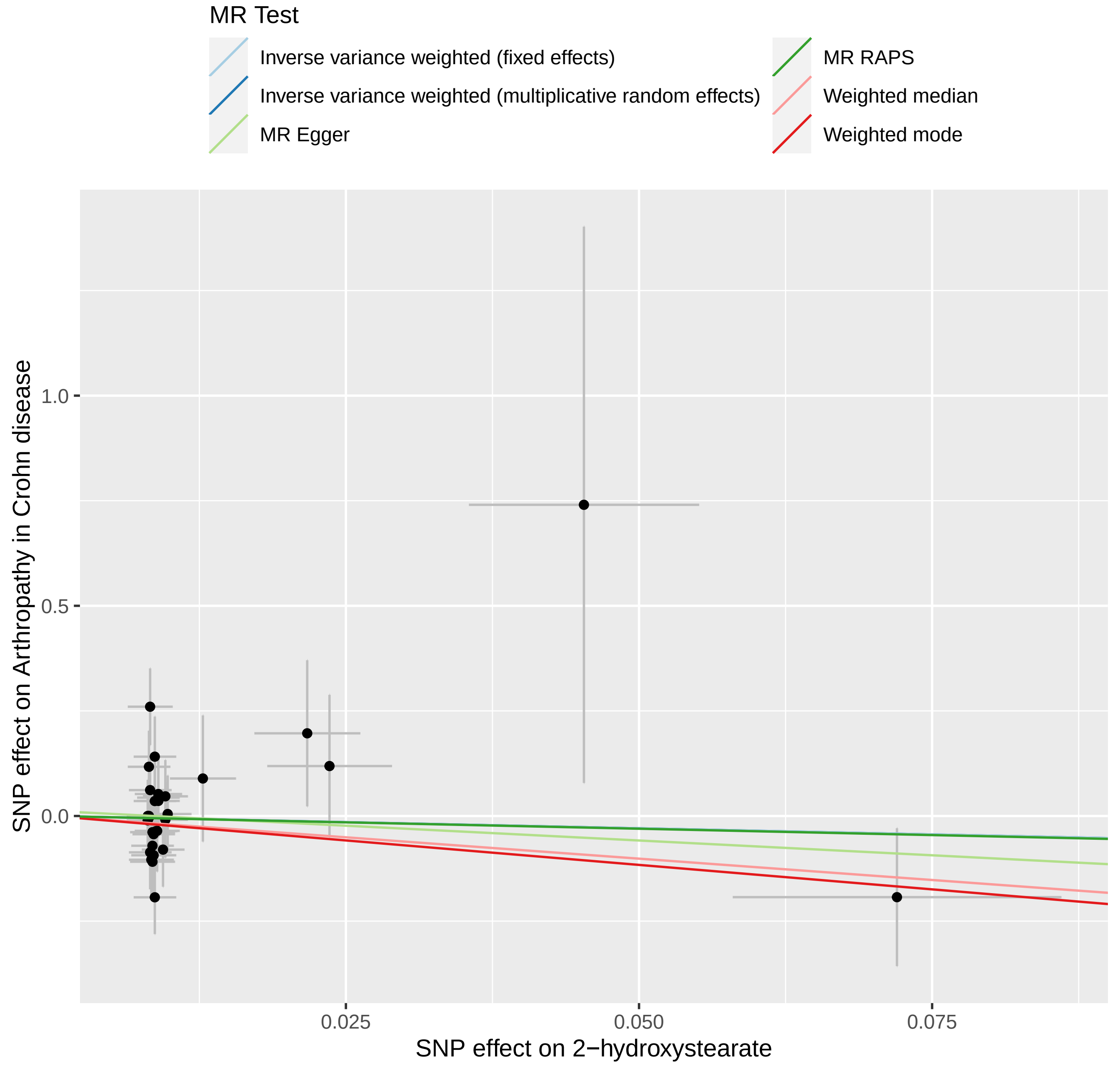

Batch 912 : Glycylvaline on Arthropathy in Crohn disease

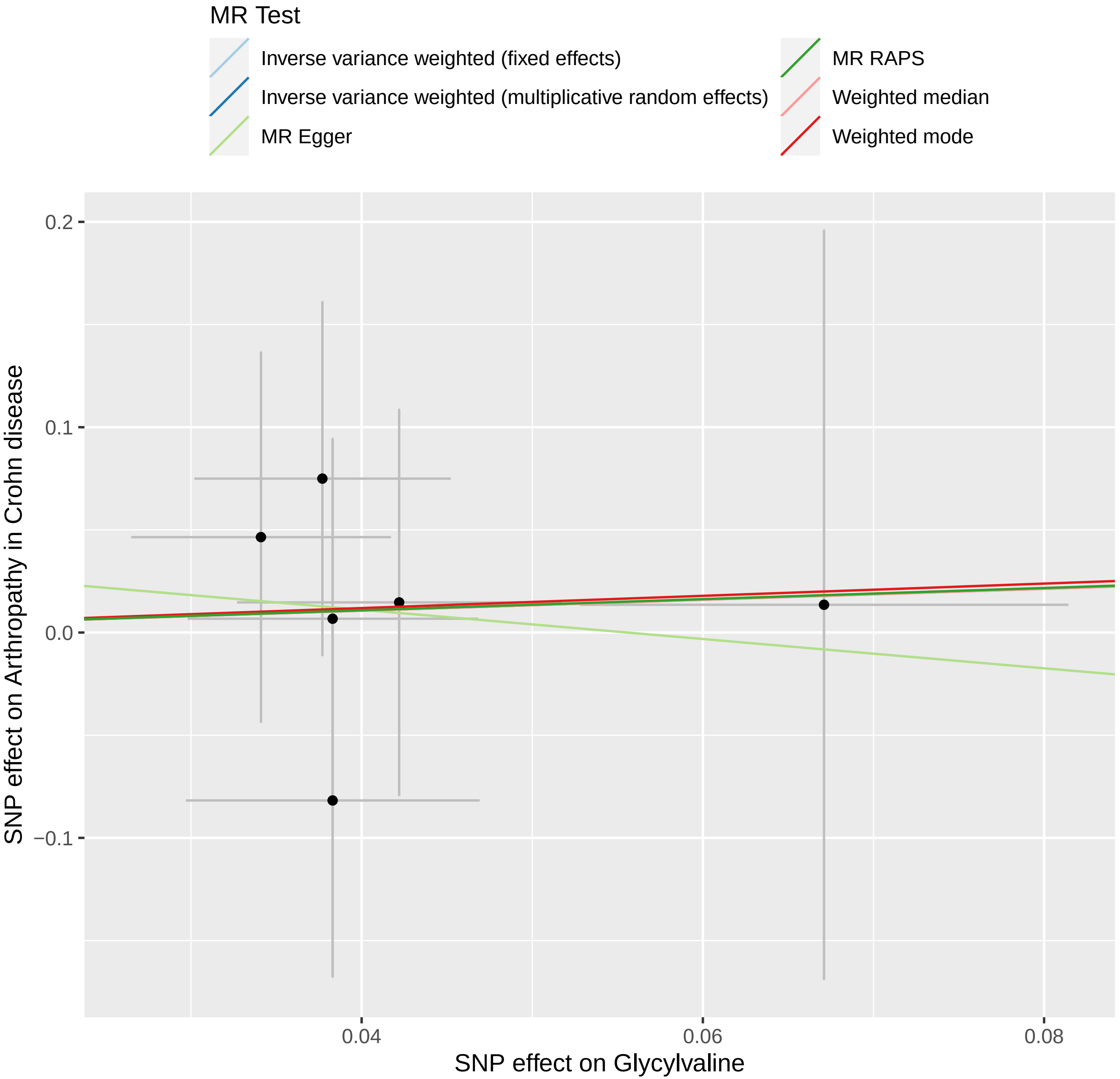

Batch 913 : Gamma−glutamylleucine on Arthropathy in Crohn disease

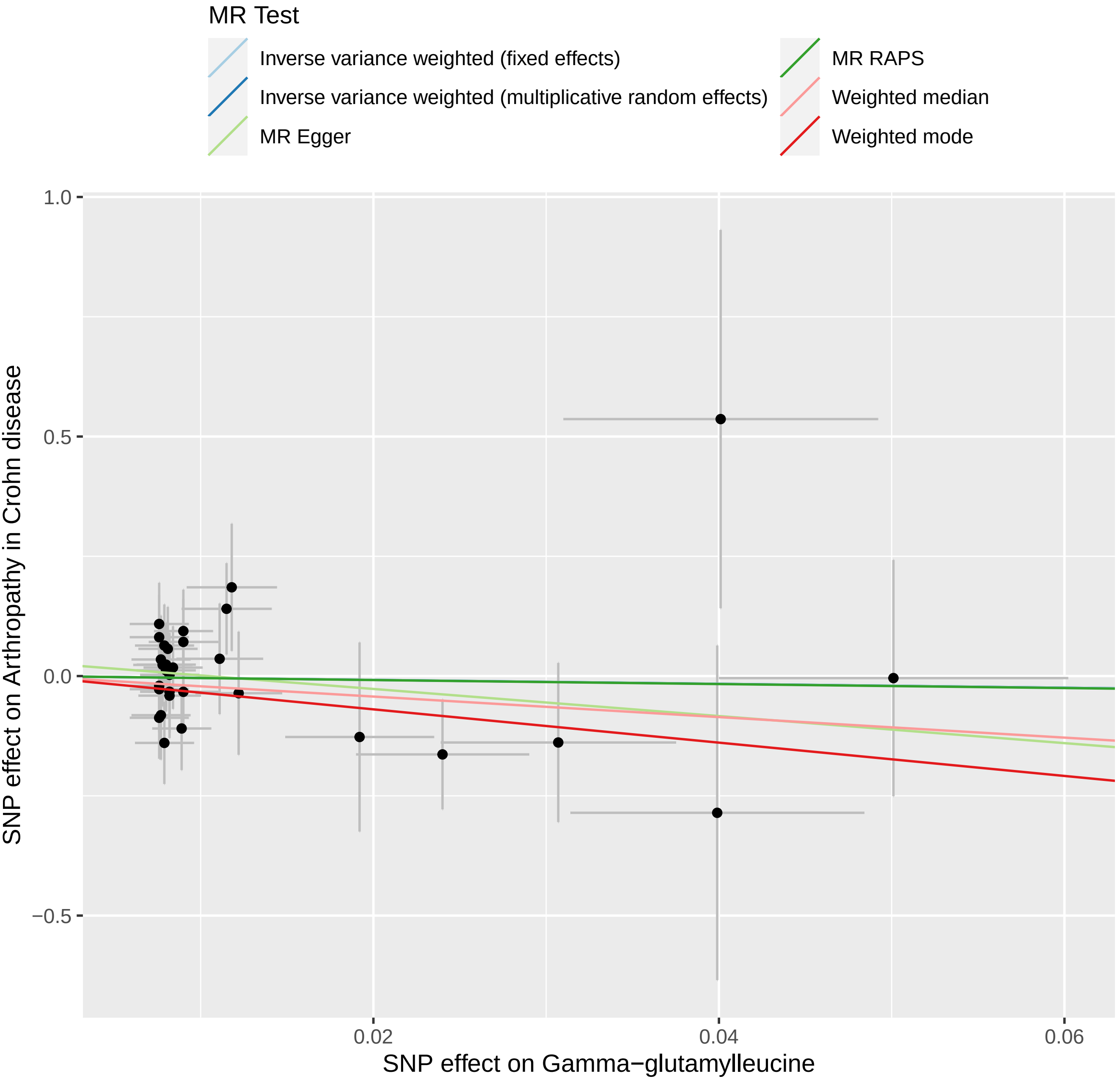

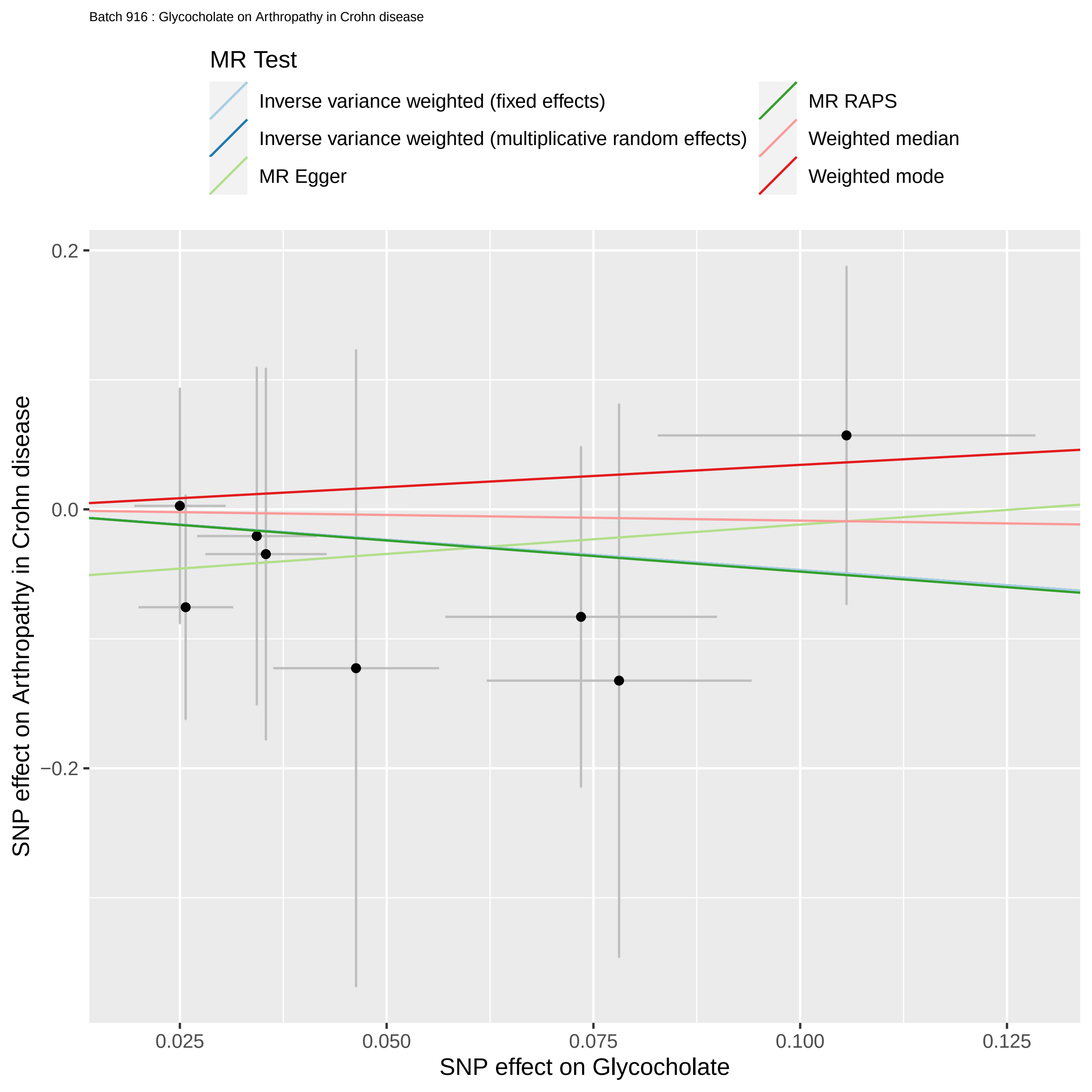
Batch 916 : Glycocholate on Arthropathy in Crohn disease
MR Test
Inverse variance weighted (fixed effects)
Inverse variance weighted (multiplicative random effects)
MR Egger
MR RAPS
Weighted median
Weighted mode
0.2
0.0
−0.2
0.025
0.050
0.075
0.100
0.125
SNP effect on Arthropathy in Crohn disease
SNP effect on Glycocholate

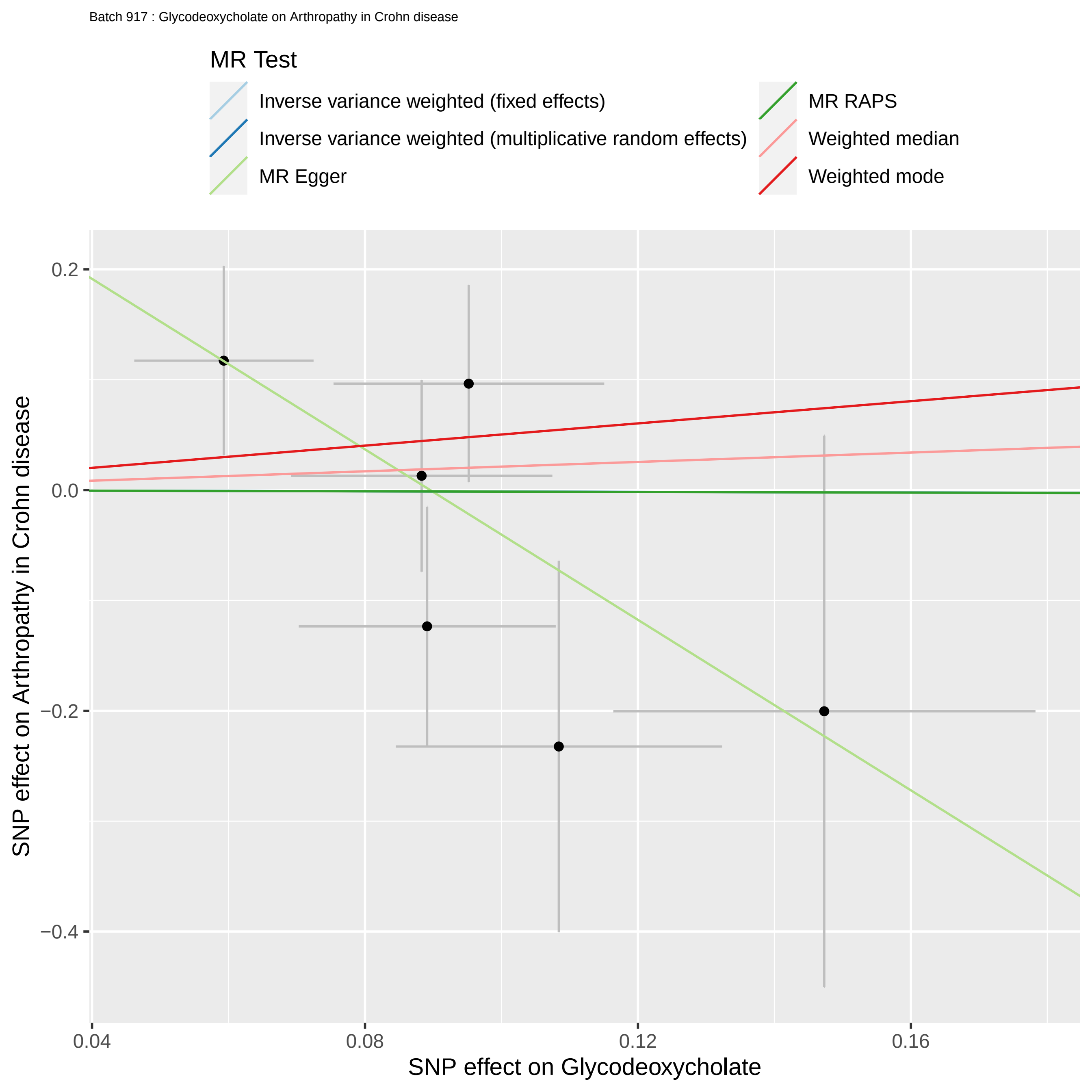

Batch 917 : Glycodeoxycholate on Arthropathy in Crohn disease
MR Test
Inverse variance weighted (fixed effects)
Inverse variance weighted (multiplicative random effects)
MR Egger
MR RAPS
Weighted median
Weighted mode
0.2
0.0
−0.2
−0.4
0.04
0.08
0.12
0.16
SNP effect on Glycodeoxycholate
SNP effect on Arthropathy in Crohn disease

Batch 918 : Taurochenodeoxycholate on Arthropathy in Crohn disease

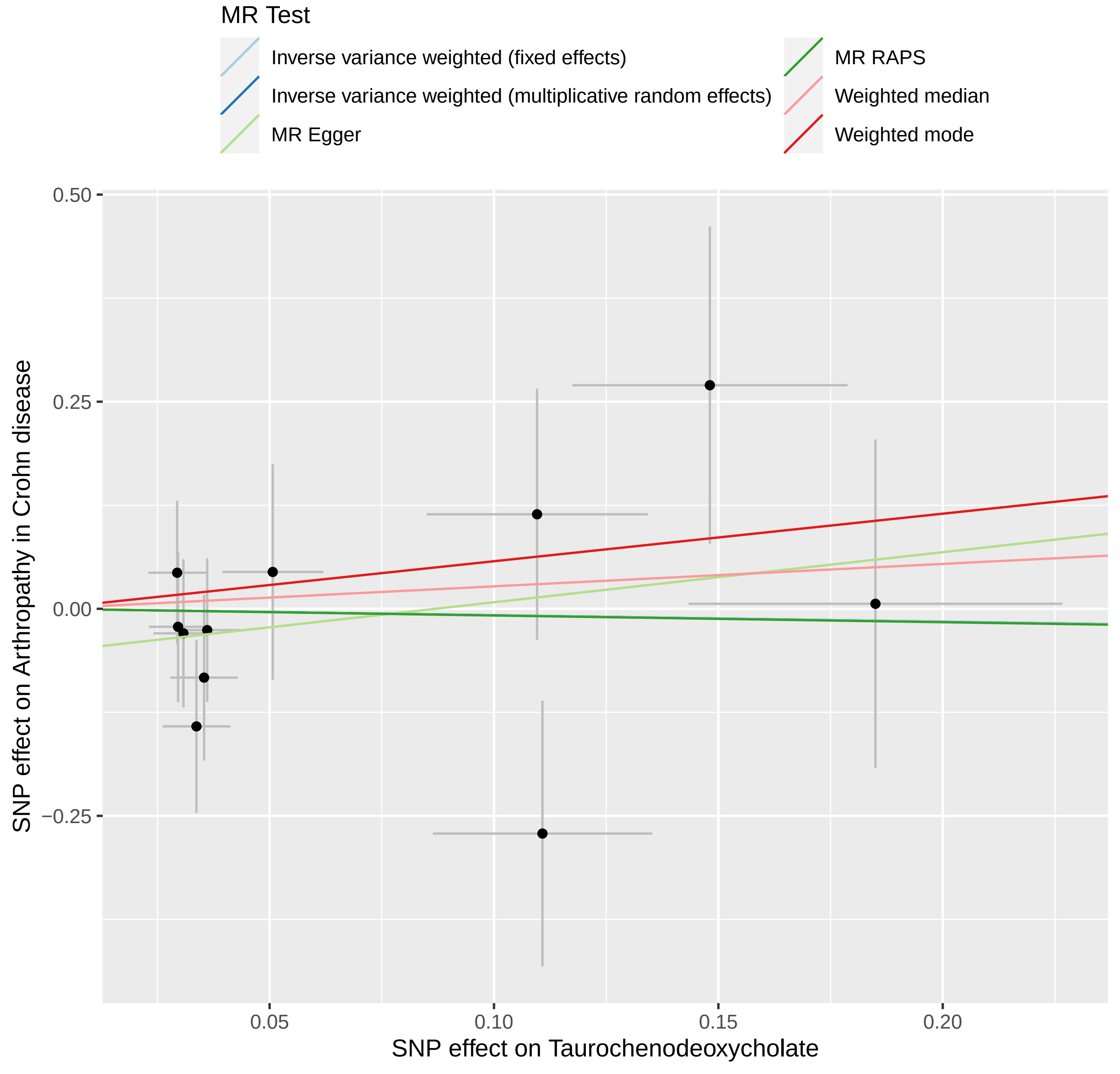

Batch 919 : Taurocholate on Arthropathy in Crohn disease

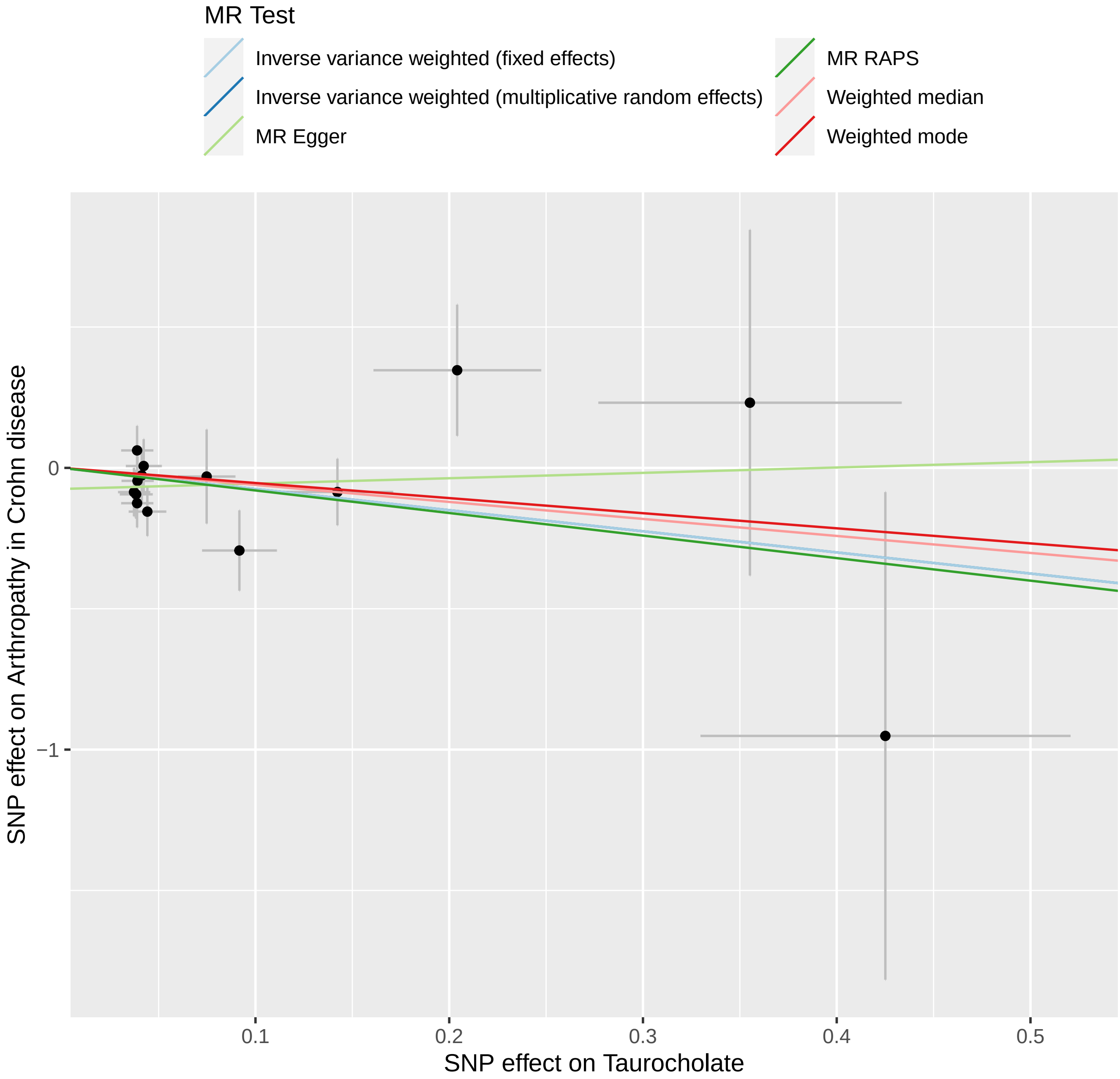

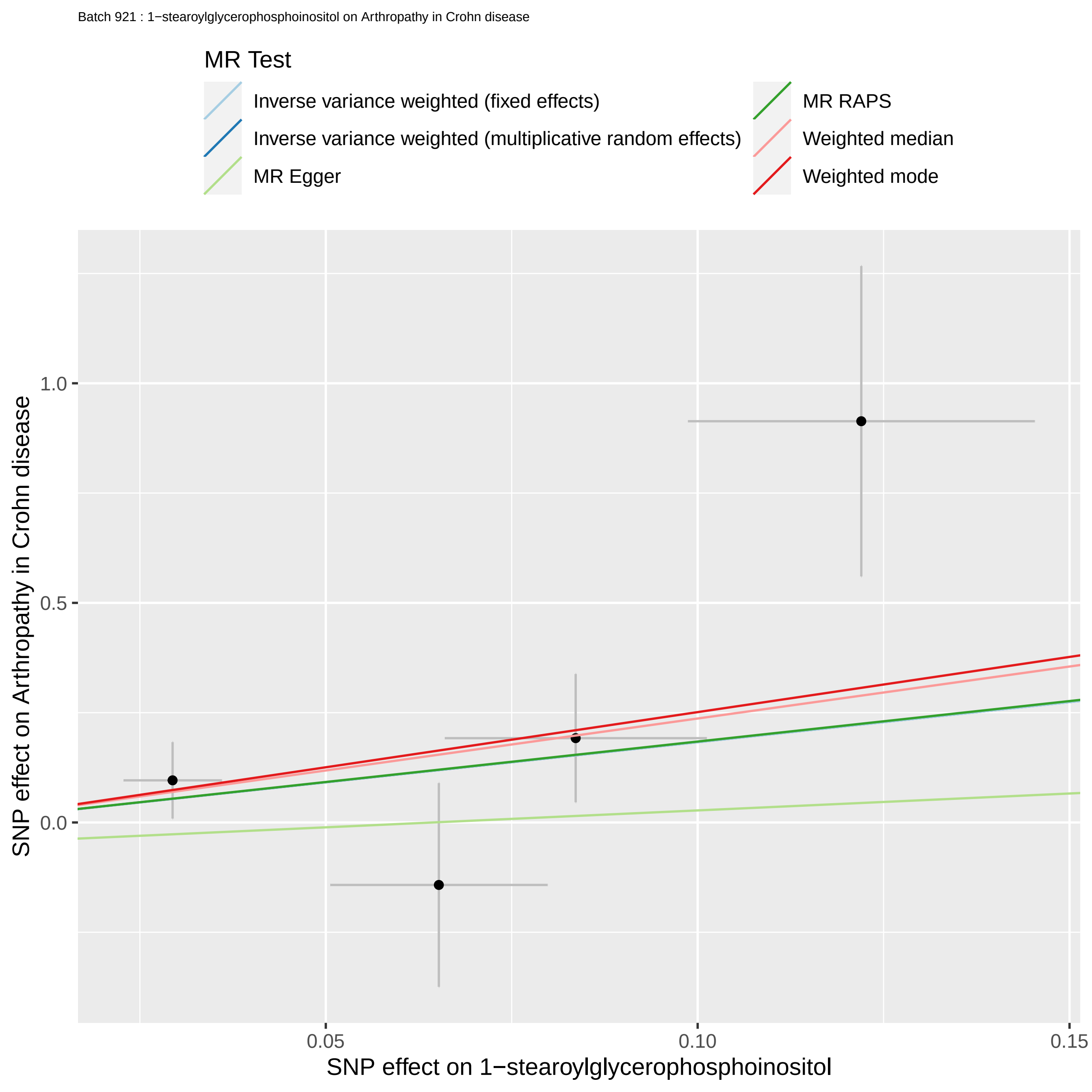

Batch 921 : 1−stearoylglycerophosphoinositol on Arthropathy in Crohn disease
MR Test
Inverse variance weighted (fixed effects)
Inverse variance weighted (multiplicative random effects)
MR Egger
MR RAPS
Weighted median
Weighted mode
SNP effect on Arthropathy in Crohn disease
1.0
0.5
0.0
0.05
0.10
0.15
SNP effect on 1−stearoylglycerophosphoinositol

Batch 922 : Myo−inositol on Arthropathy in Crohn disease

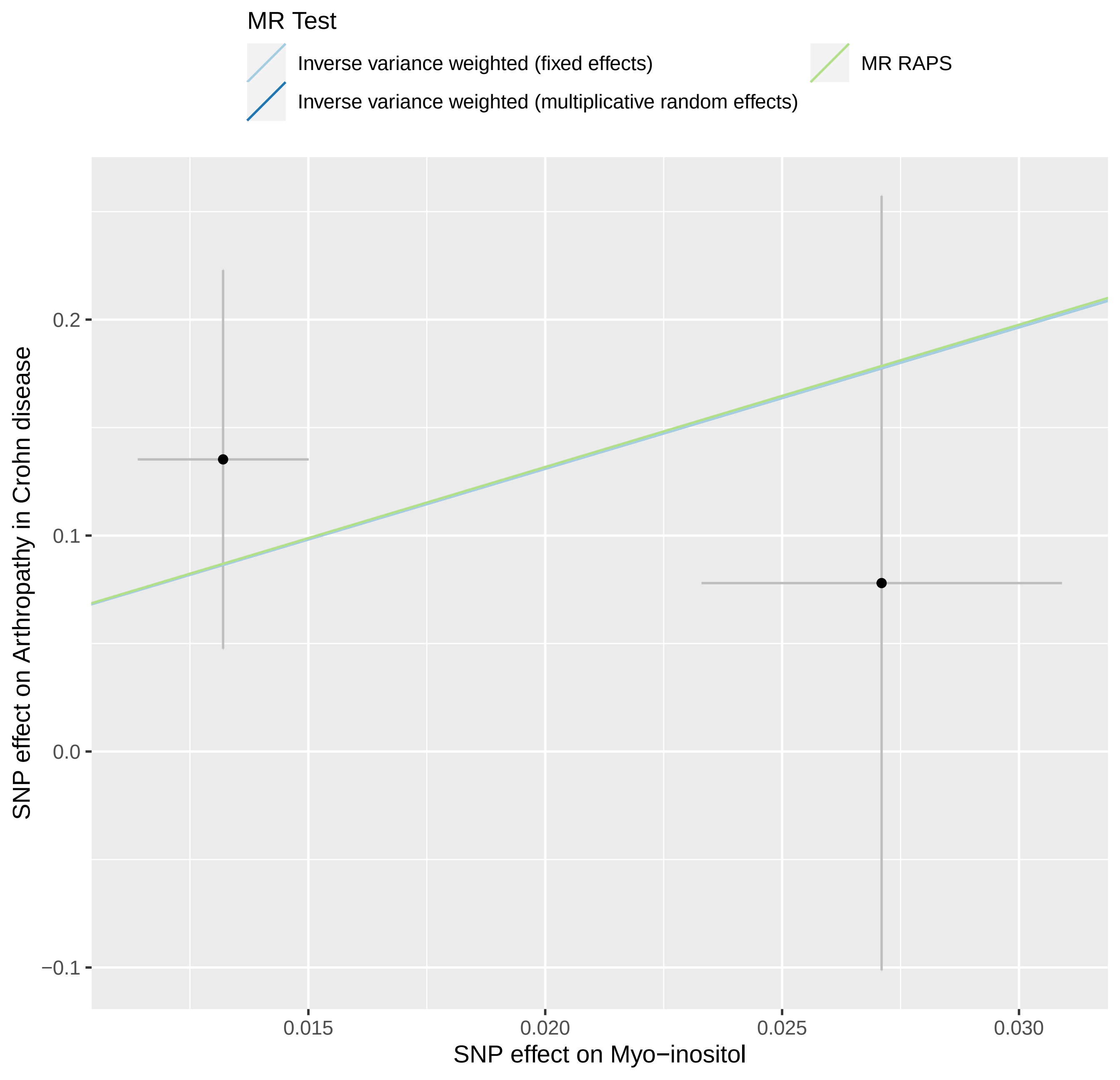

Batch 924 : 1,5−anhydroglucitol (1,5−AG) on Arthropathy in Crohn disease

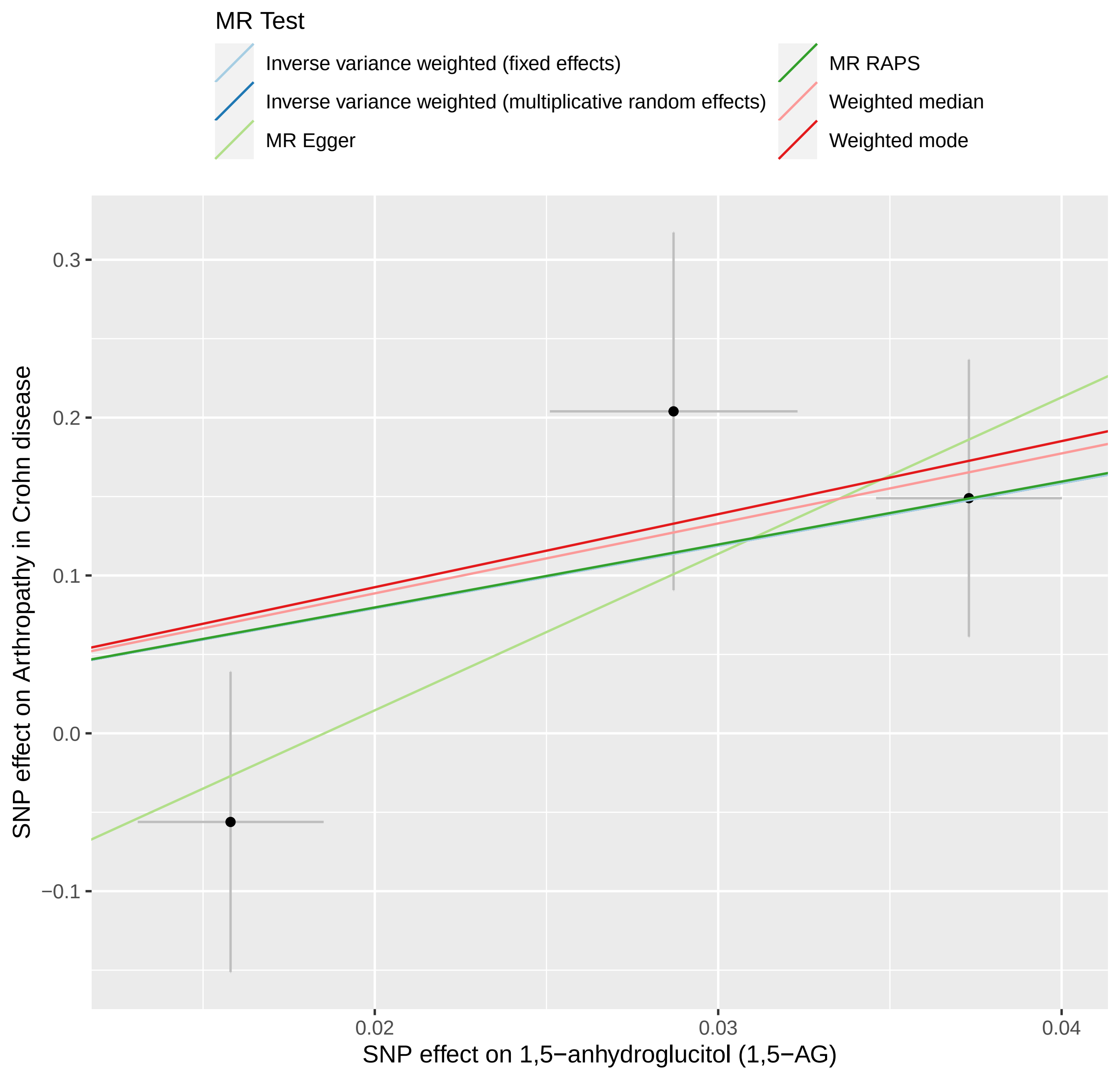

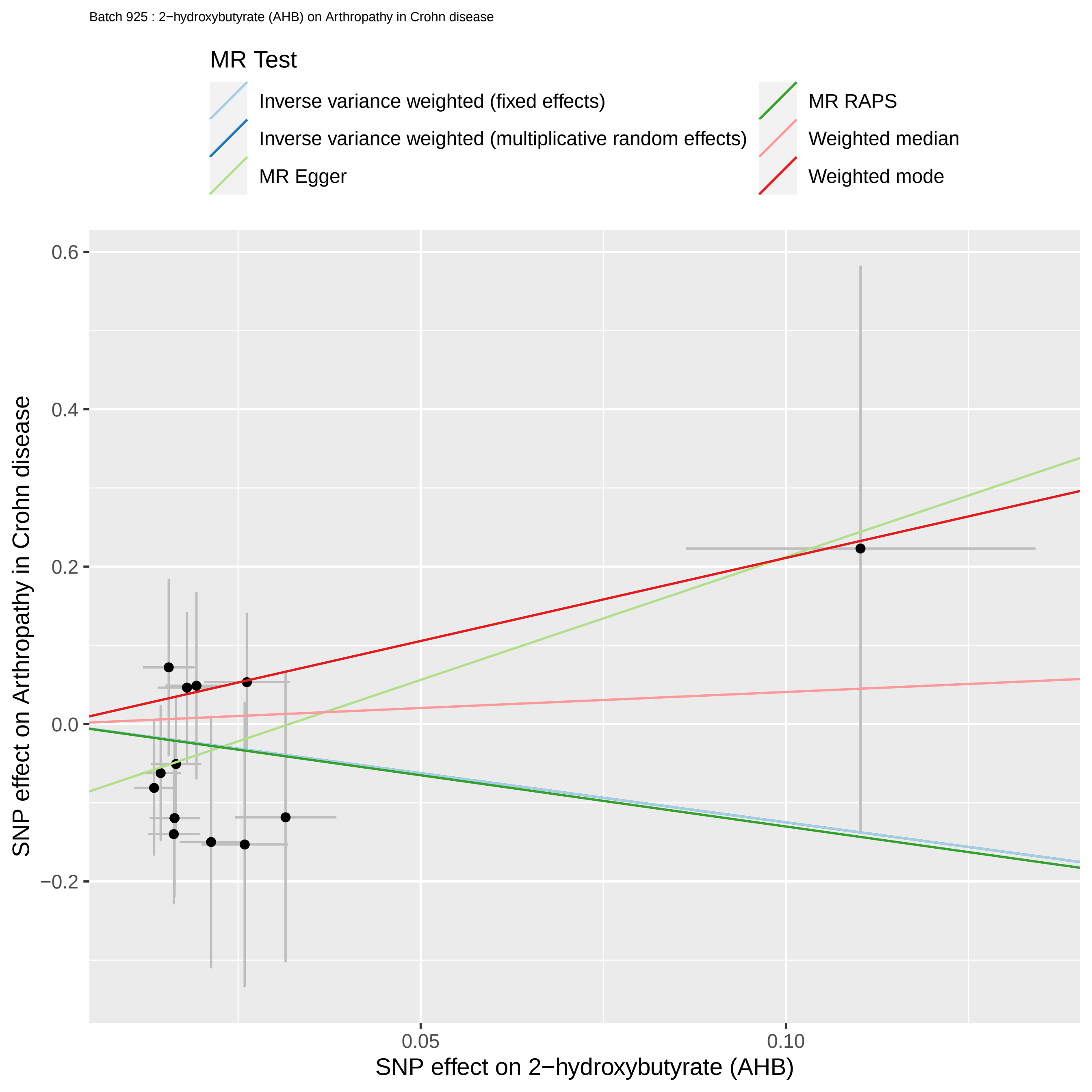
Batch 925 : 2−hydroxybutyrate (AHB) on Arthropathy in Crohn disease
MR Test
Inverse variance weighted (fixed effects)
Inverse variance weighted (multiplicative random effects)
MR Egger
MR RAPS
Weighted median
Weighted mode
0.6
0.4
0.2
0.0
−0.2
0.05
0.10
SNP effect on Arthropathy in Crohn disease
SNP effect on 2−hydroxybutyrate (AHB)

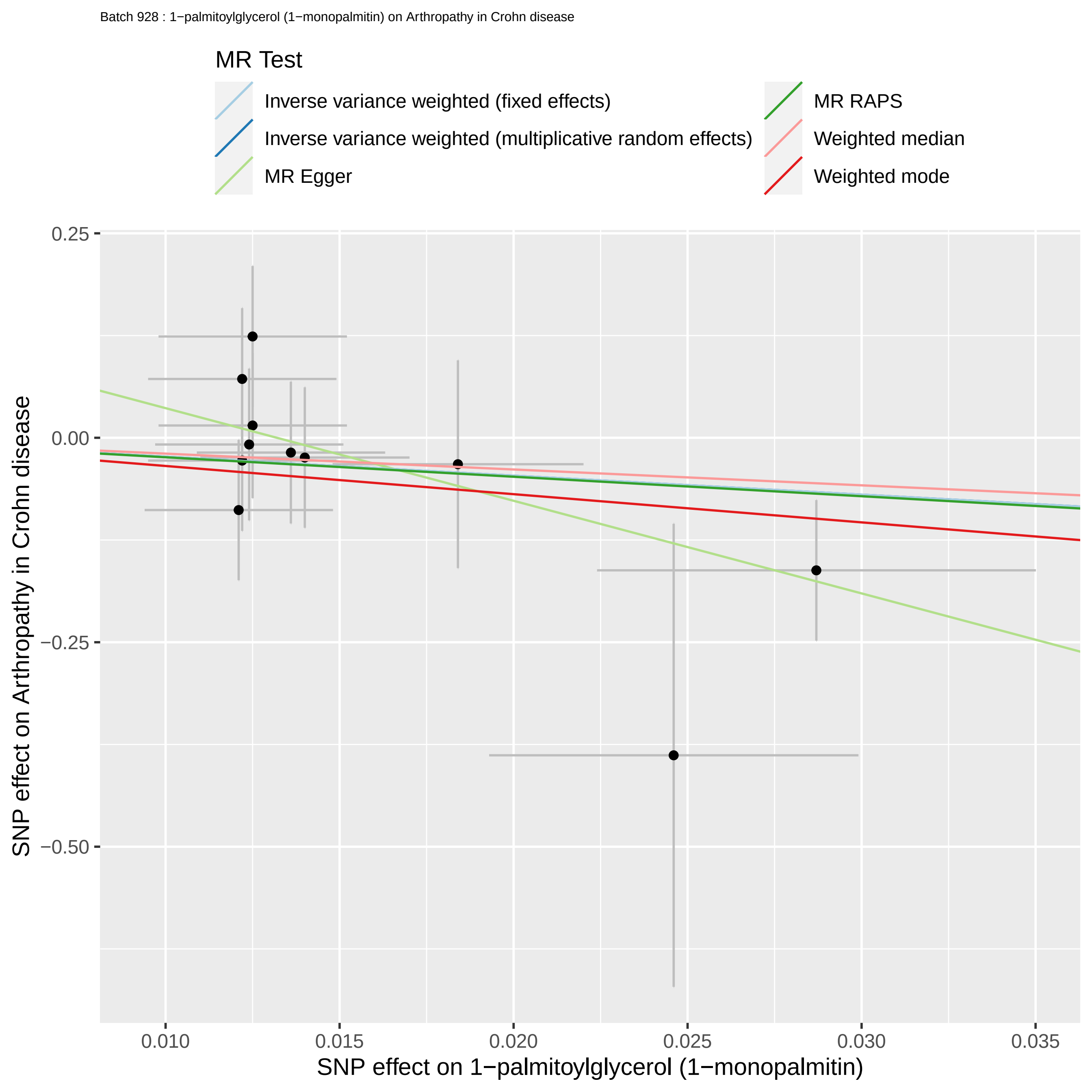
Batch 928 : 1−palmitoylglycerol (1−monopalmitin) on Arthropathy in Crohn disease
MR Test
Inverse variance weighted (fixed effects)
Inverse variance weighted (multiplicative random effects)
MR Egger
MR RAPS
Weighted median
Weighted mode
0.25
0.00
−0.25
−0.50
0.010
0.015
0.020
0.025
0.030
0.035
SNP effect on Arthropathy in Crohn disease
SNP effect on 1−palmitoylglycerol (1−monopalmitin)

Batch 931 : 2−hydroxyisobutyrate on Arthropathy in Crohn disease

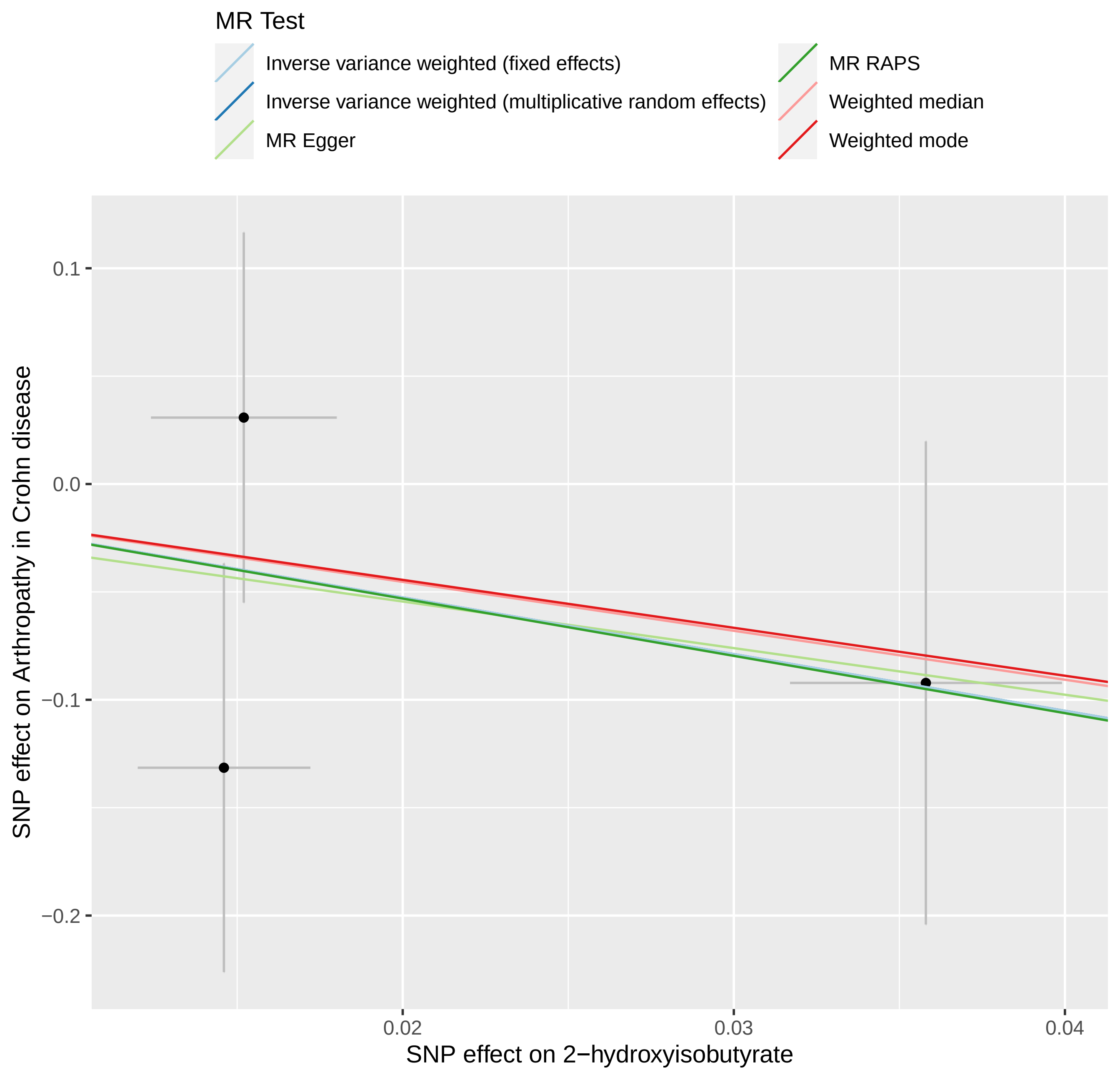

Batch 932 : 4−methyl−2−oxopentanoate on Arthropathy in Crohn disease

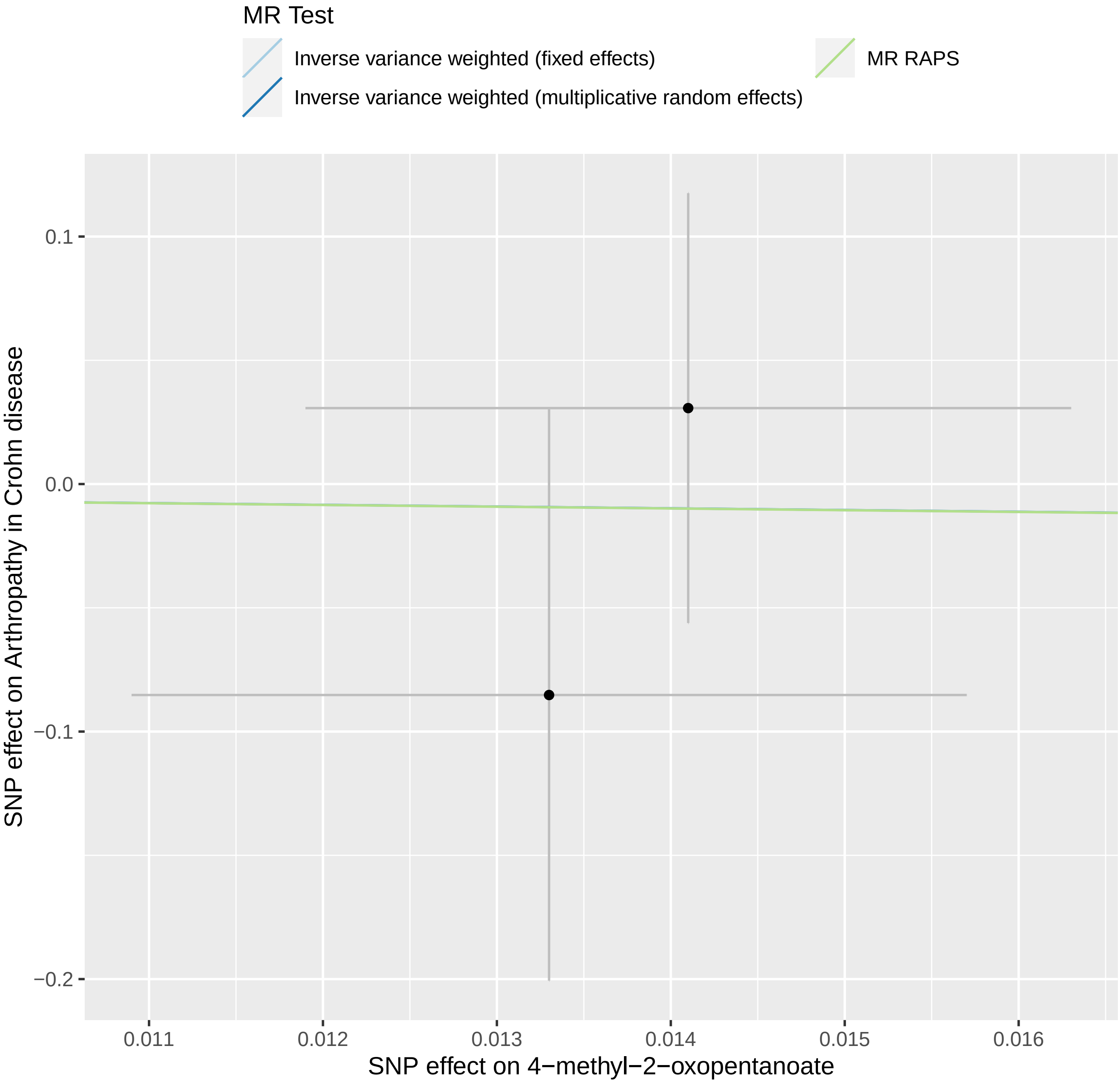

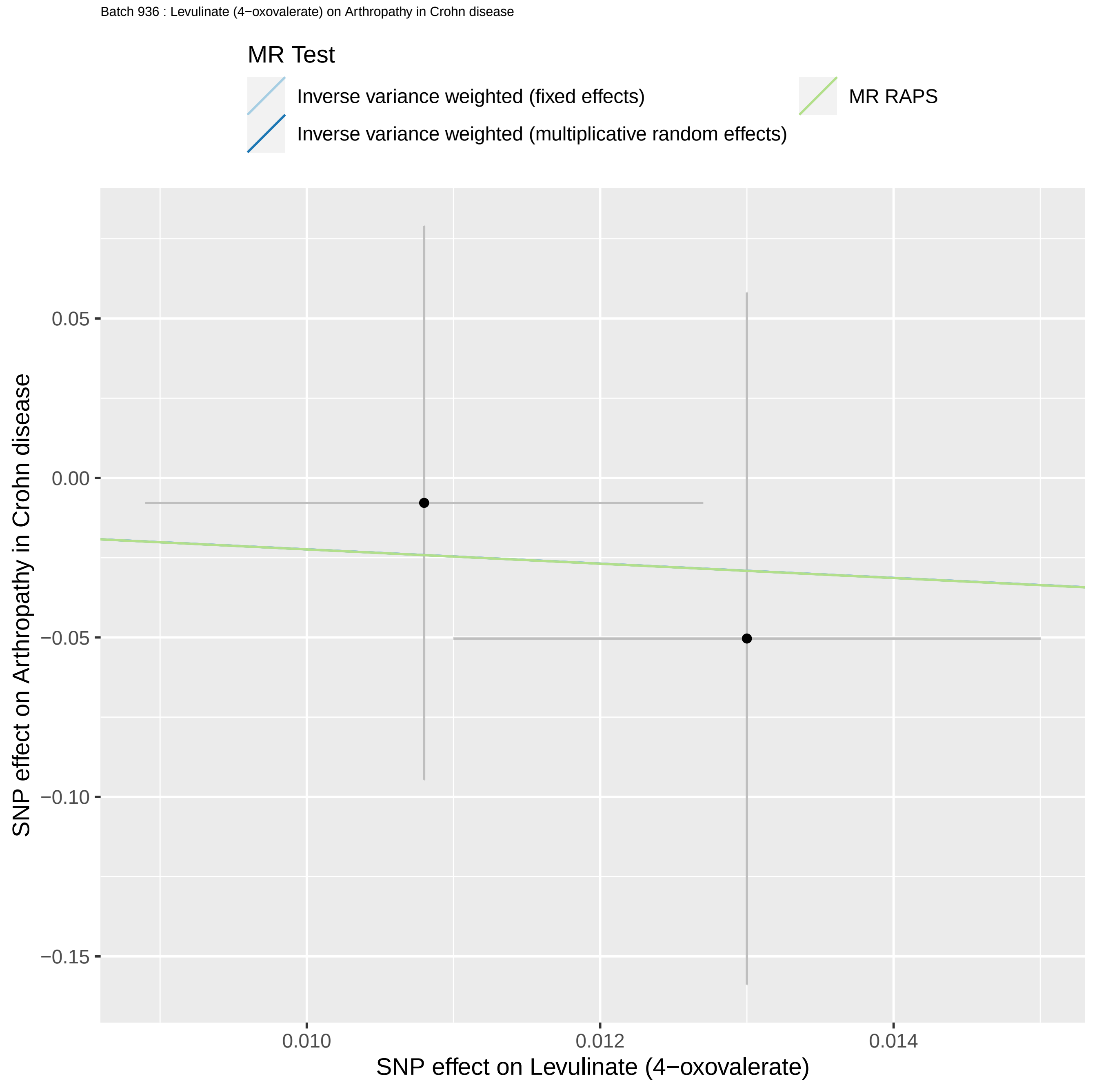
Batch 936 : Levulinate (4−oxovalerate) on Arthropathy in Crohn disease
MR Test
Inverse variance weighted (fixed effects)
Inverse variance weighted (multiplicative random effects)
MR RAPS
SNP effect on Arthropathy in Crohn disease
0.05
0.00
−0.05
−0.10
−0.15
0.010
0.012
0.014
SNP effect on Levulinate (4−oxovalerate)

Batch 938 : Cholate on Arthropathy in Crohn disease

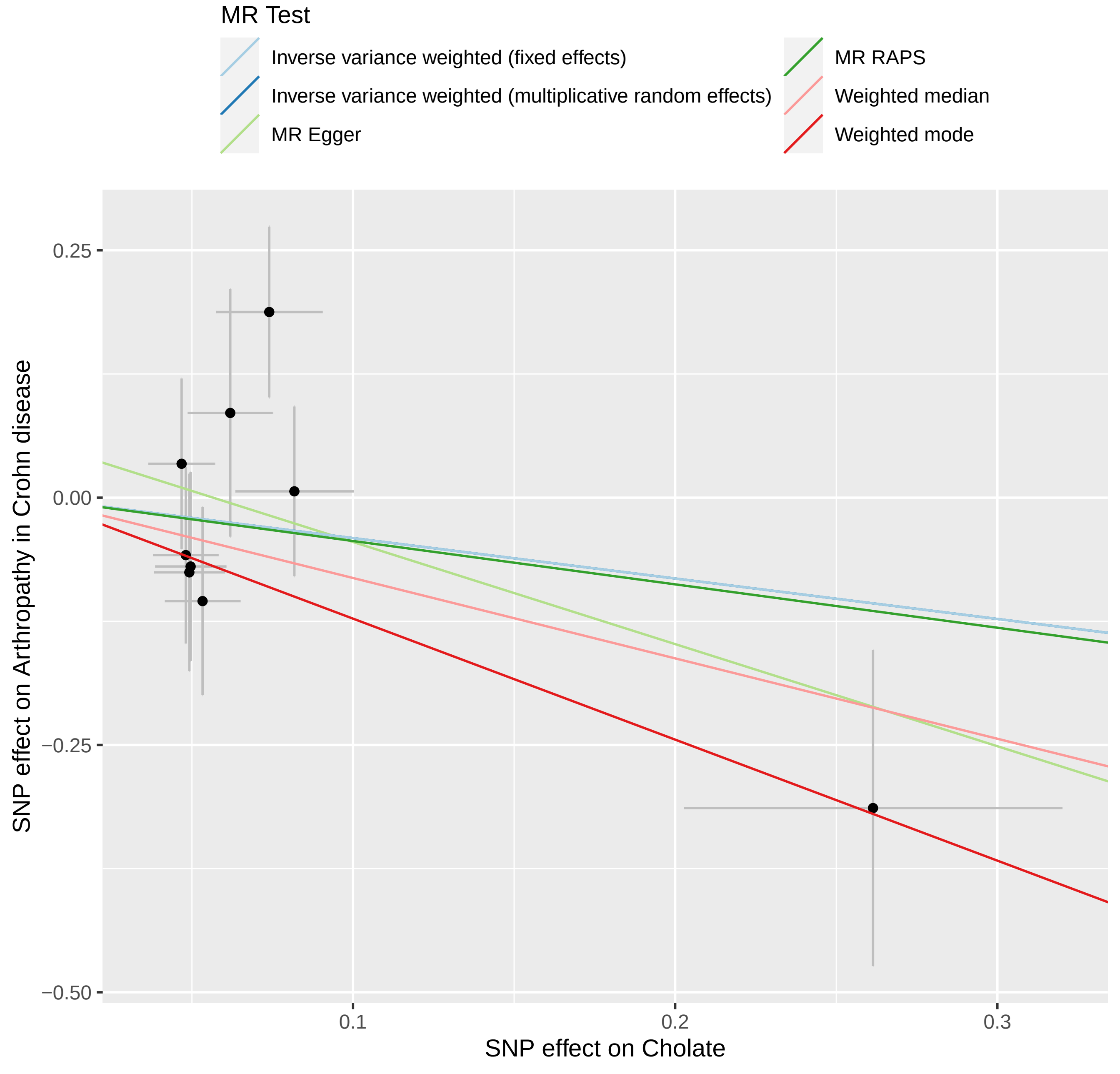

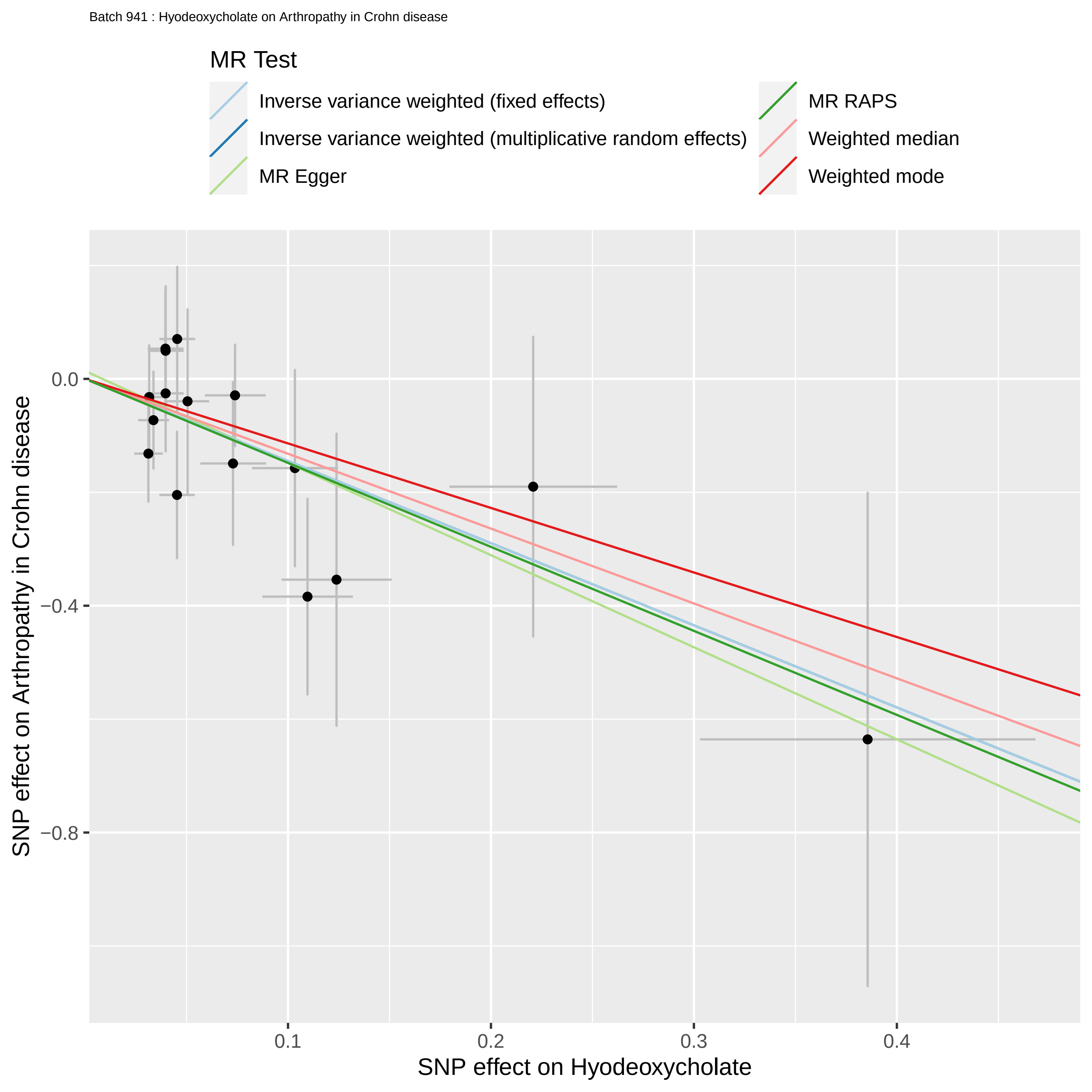
Batch 941 : Hyodeoxycholate on Arthropathy in Crohn disease
MR Test
Inverse variance weighted (fixed effects)
Inverse variance weighted (multiplicative random effects)
MR Egger
MR RAPS
Weighted median
Weighted mode
SNP effect on Arthropathy in Crohn disease
0.0
−0.4
−0.8
0.1
0.2
0.3
0.4
SNP effect on Hyodeoxycholate

Batch 942 : 3−indoxyl sulfate on Arthropathy in Crohn disease

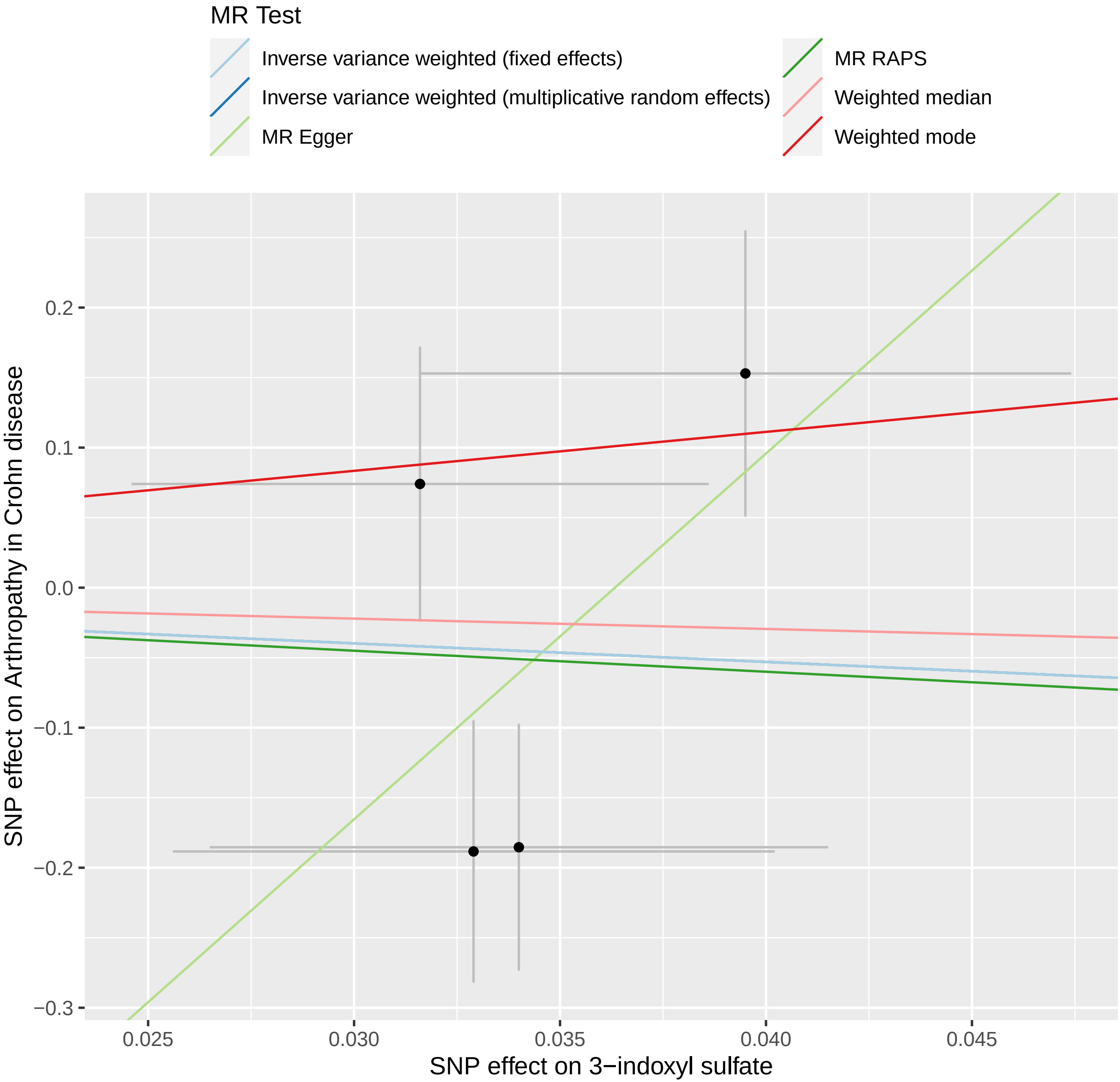

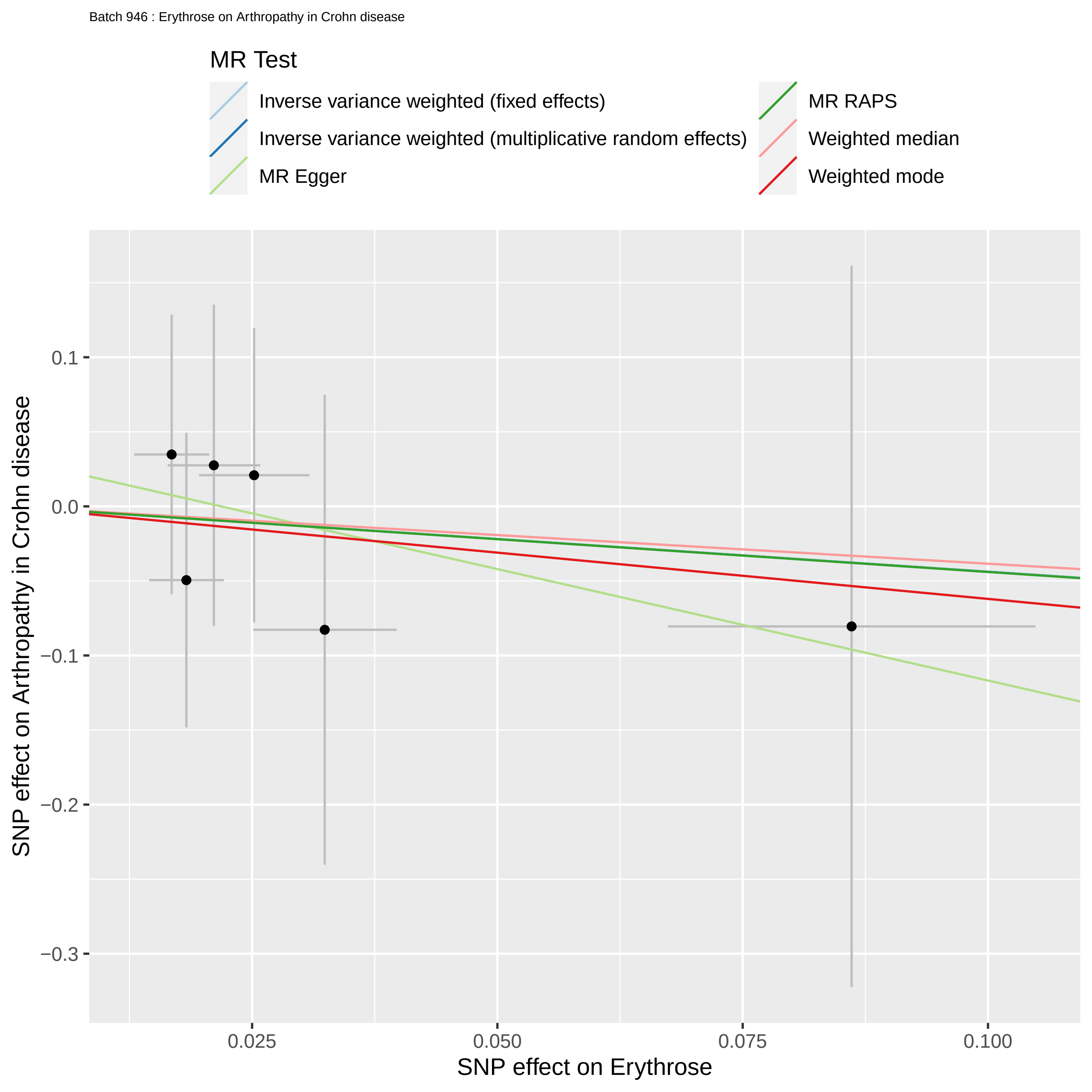

Batch 946 : Erythrose on Arthropathy in Crohn disease
MR Test
Inverse variance weighted (fixed effects)
Inverse variance weighted (multiplicative random effects)
MR Egger
MR RAPS
Weighted median
Weighted mode
0.1
0.0
−0.1
−0.2
−0.3
0.025
0.050
0.075
0.100
SNP effect on Arthropathy in Crohn disease
SNP effect on Erythrose

Batch 947 : Threonate on Arthropathy in Crohn disease

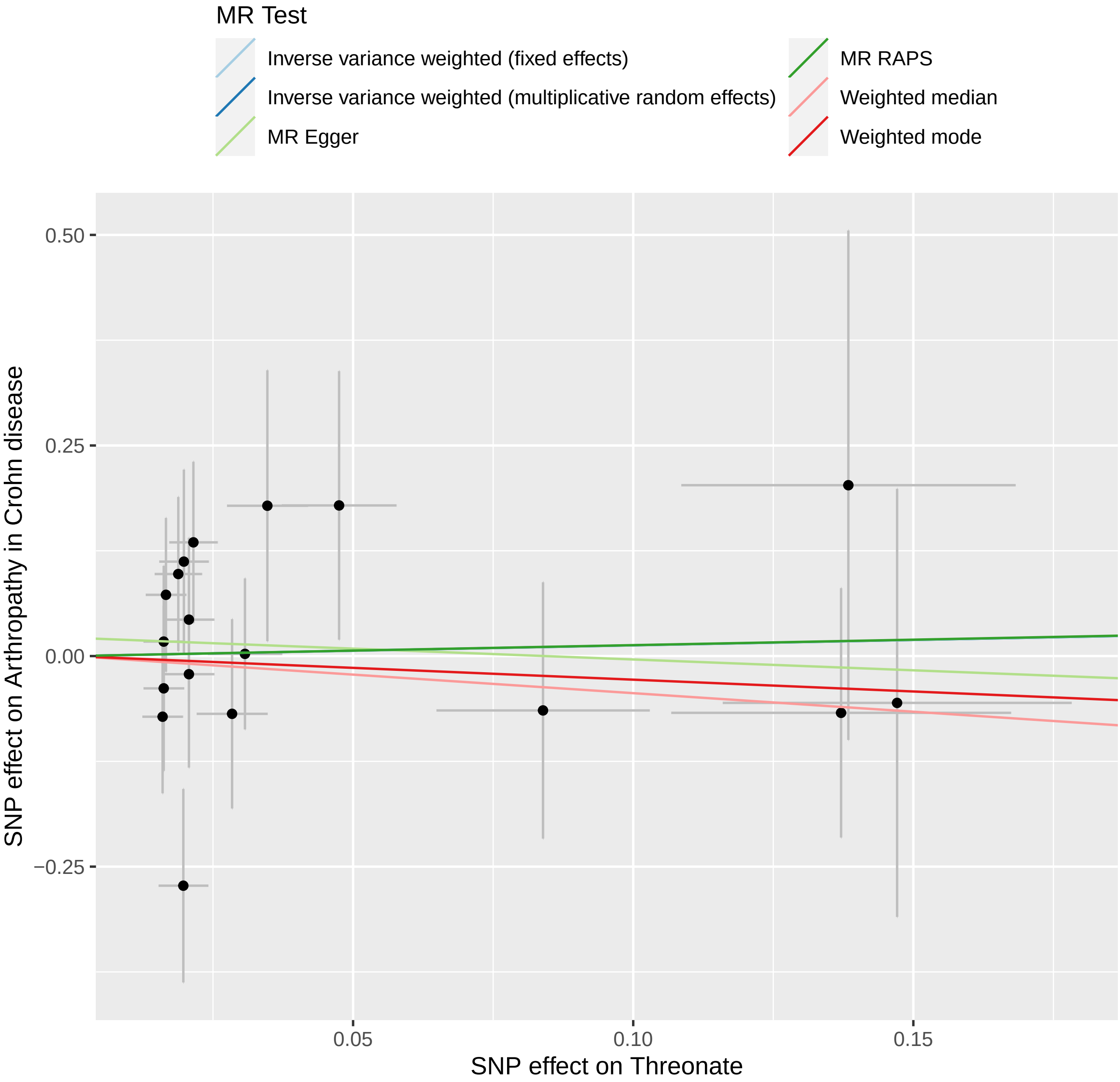

Batch 948 : Cysteine on Arthropathy in Crohn disease

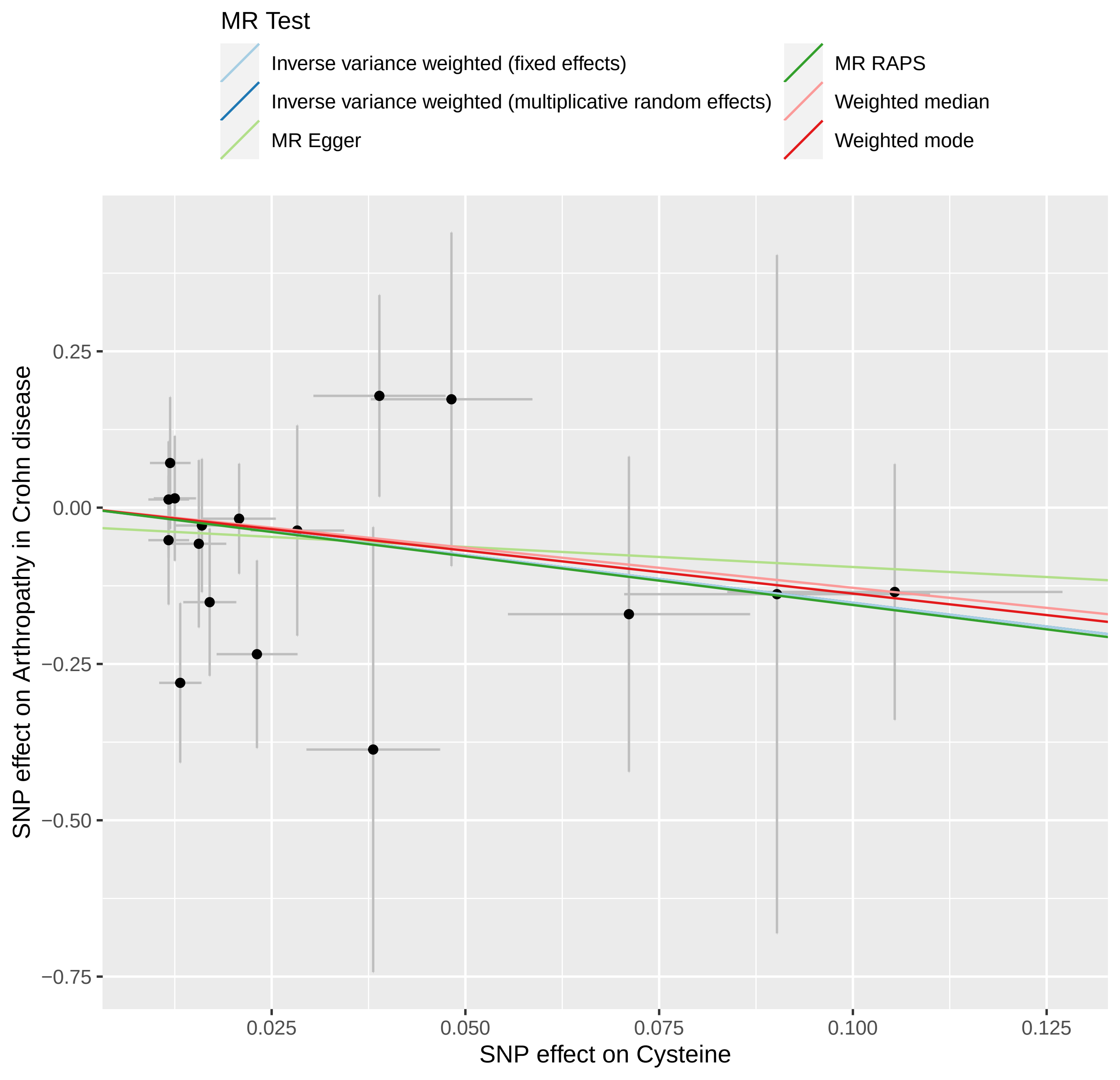

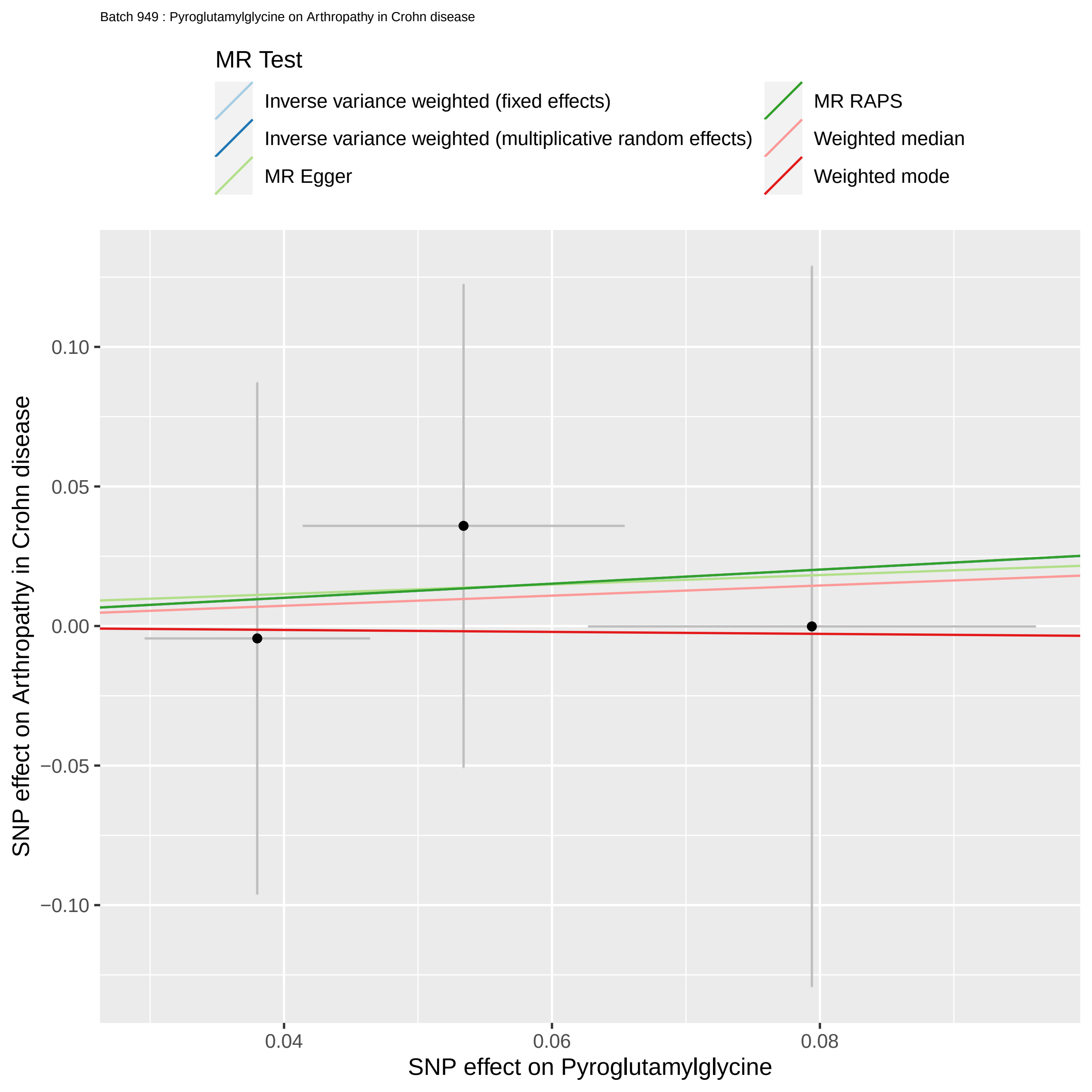

Batch 949 : Pyroglutamylglycine on Arthropathy in Crohn disease
MR Test
Inverse variance weighted (fixed effects)
Inverse variance weighted (multiplicative random effects)
MR Egger
MR RAPS
Weighted median
Weighted mode
0.10
0.05
0.00
−0.05
−0.10
0.04
0.06
0.08
SNP effect on Arthropathy in Crohn disease
SNP effect on Pyroglutamylglycine

Batch 951 : DSGEGDFXAEGGGVR* on Arthropathy in Crohn disease

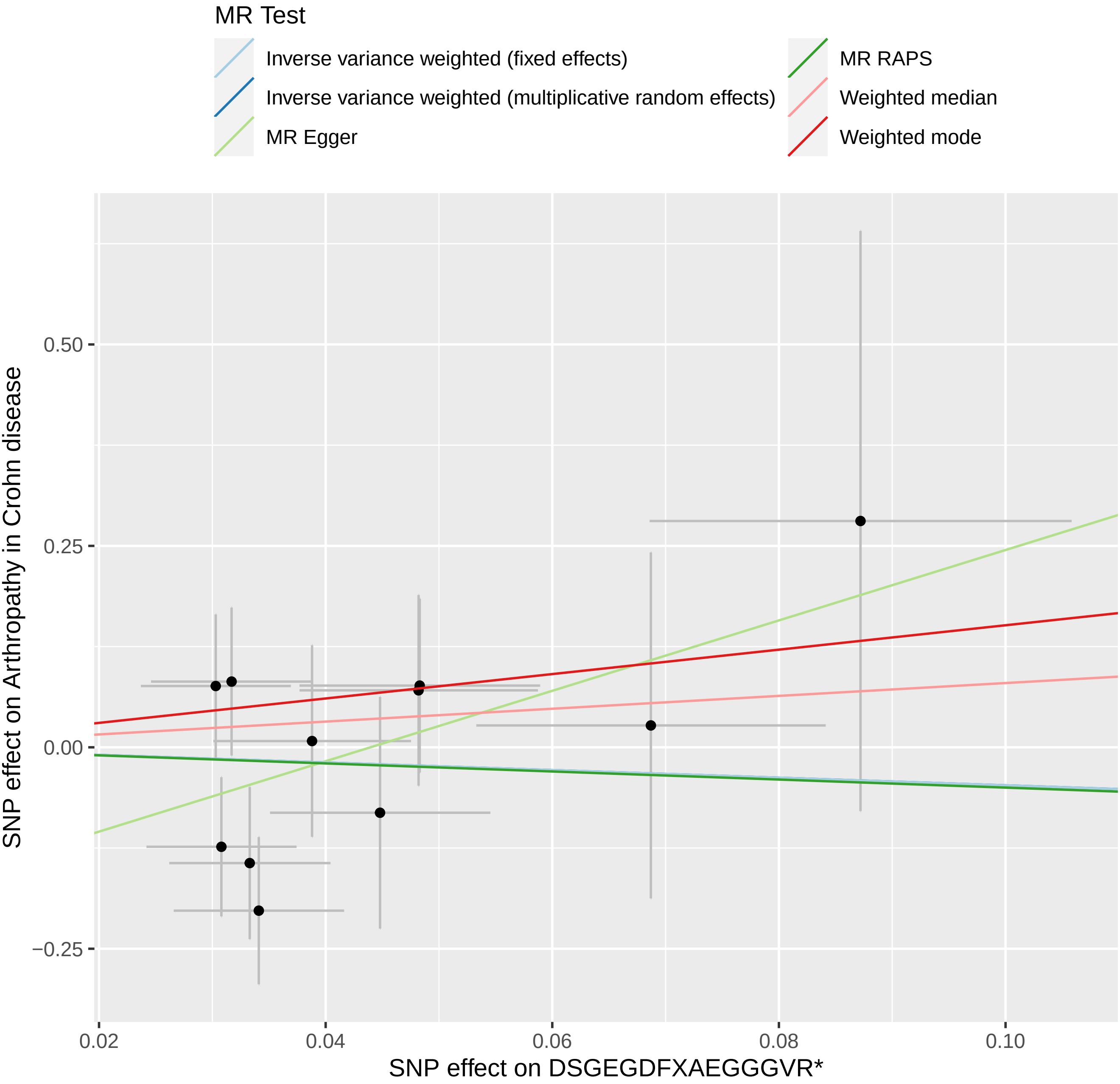

Batch 952 : Pyridoxate on Arthropathy in Crohn disease

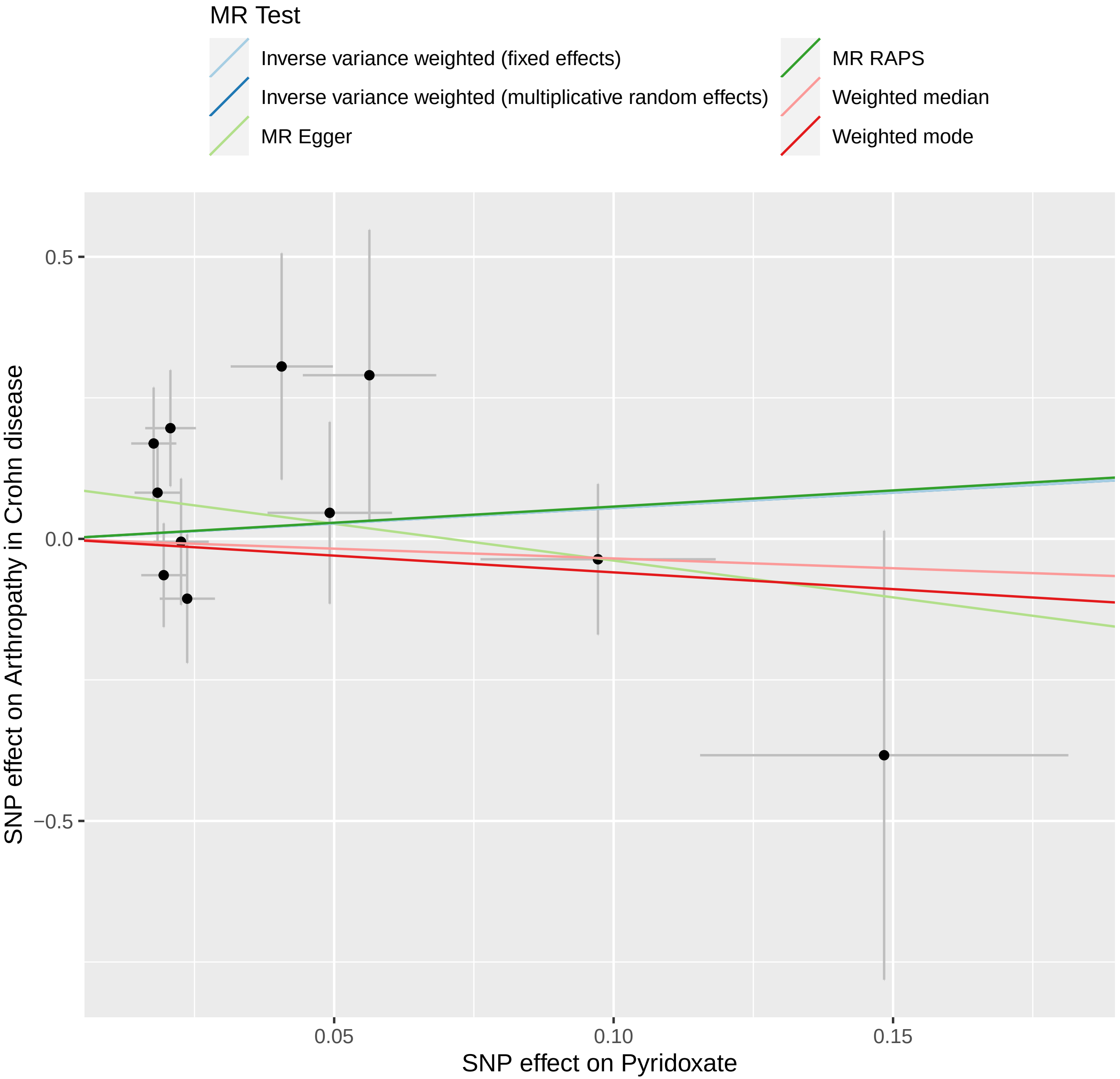

Batch 953 : Androsterone sulfate on Arthropathy in Crohn disease

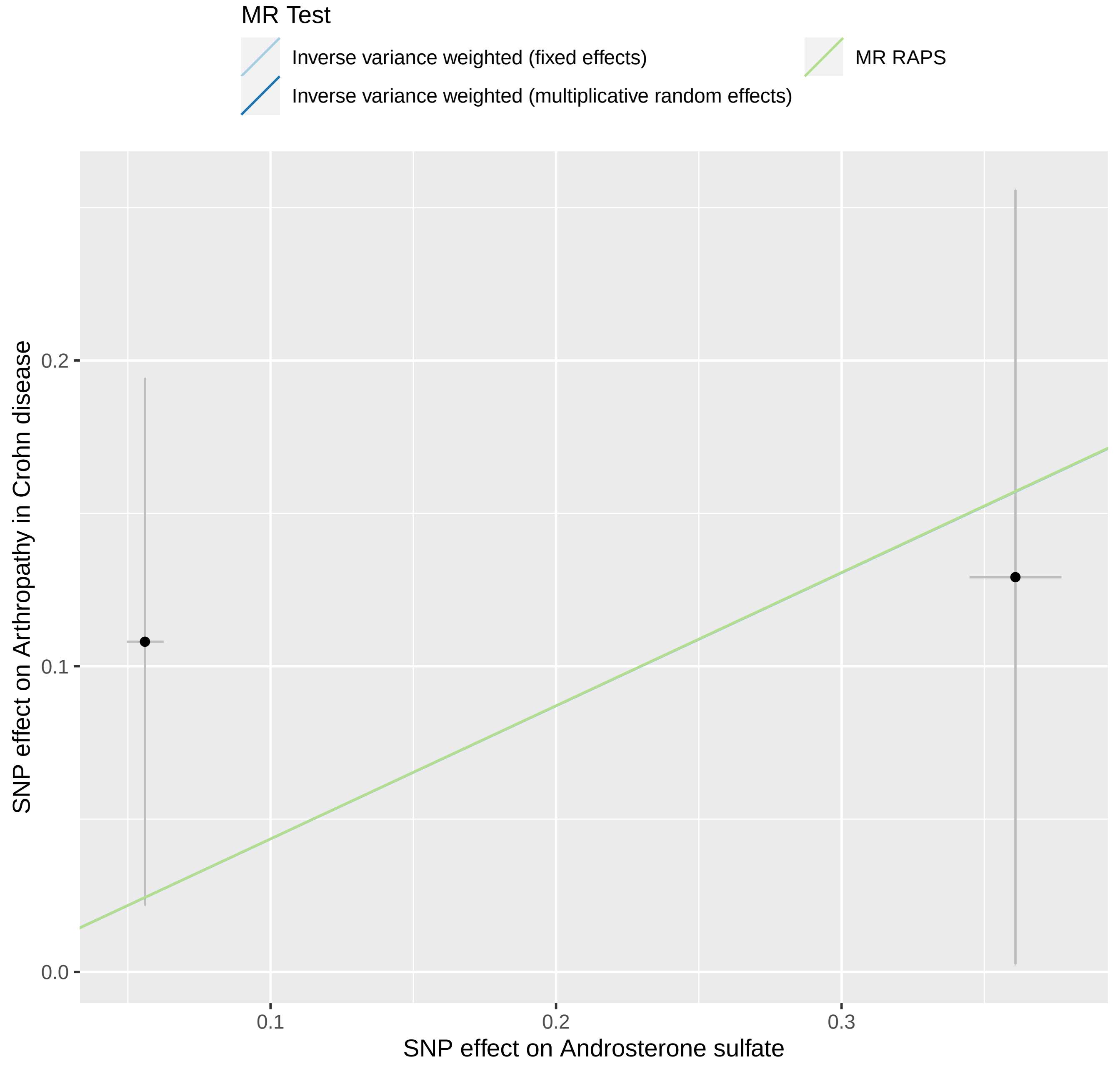

Batch 957 : Serine on Arthropathy in Crohn disease

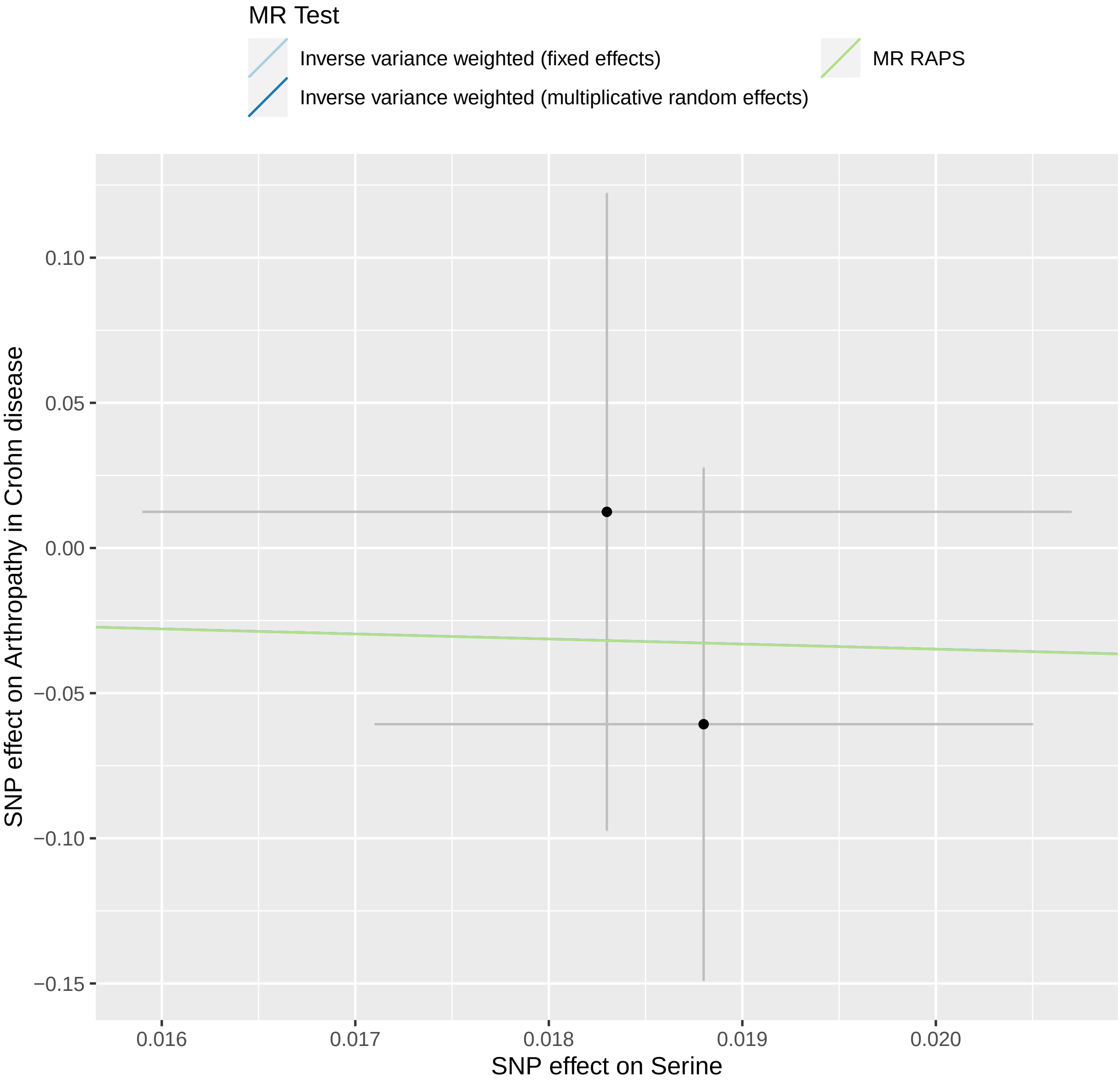

Batch 958 : Trans−4−hydroxyproline on Arthropathy in Crohn disease

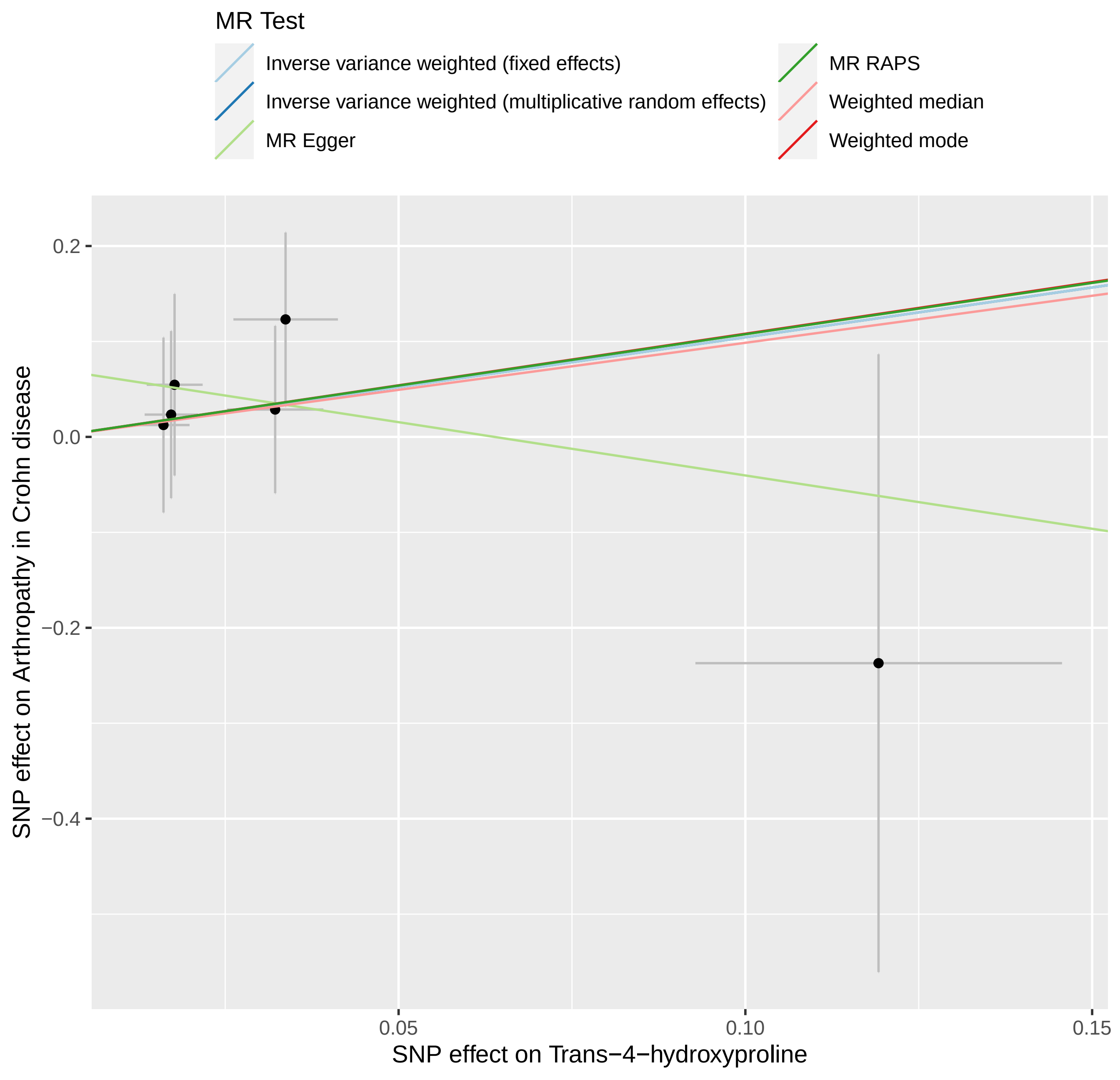

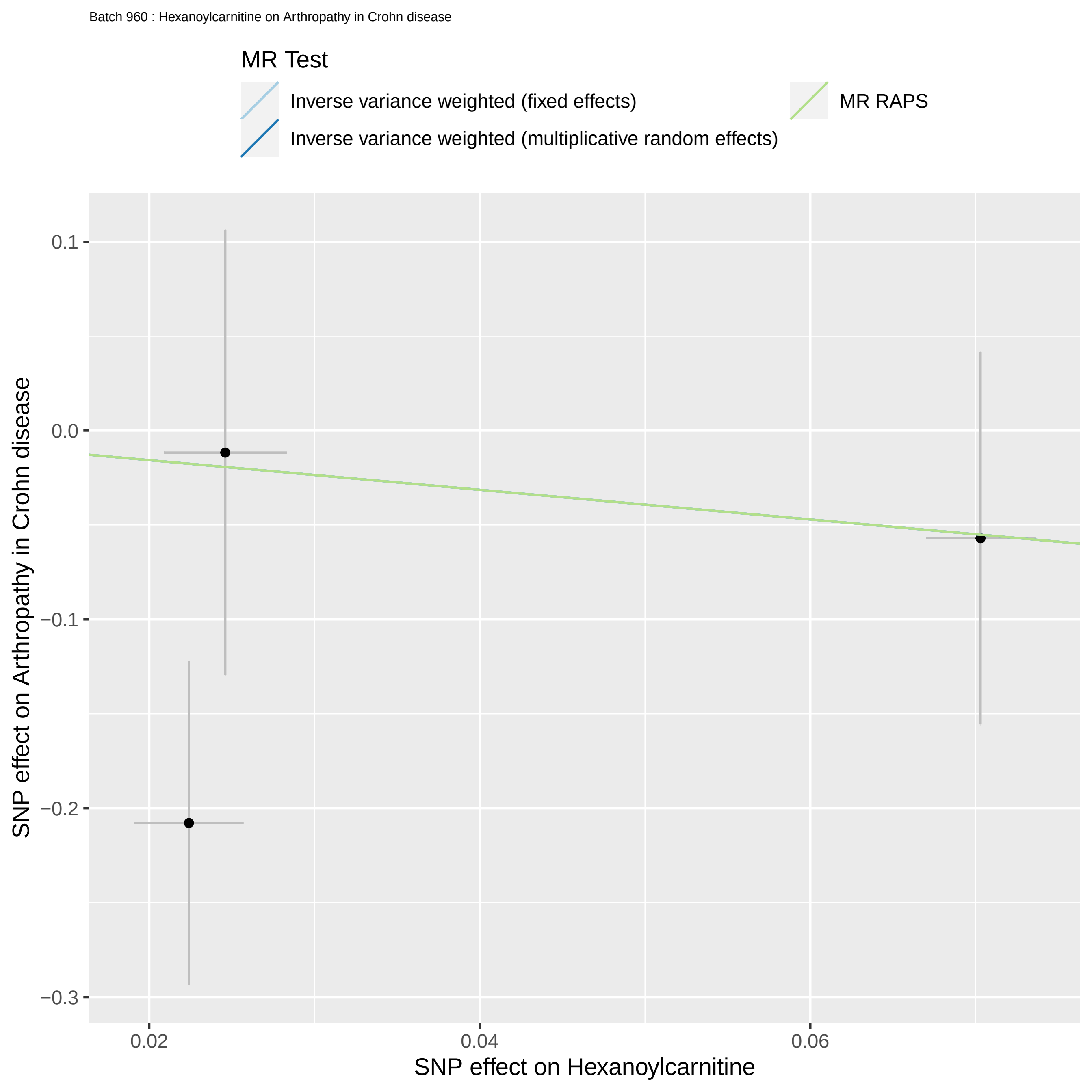

Batch 960 : Hexanoylcarnitine on Arthropathy in Crohn disease
MR Test
Inverse variance weighted (fixed effects)
Inverse variance weighted (multiplicative random effects)
MR RAPS
0.1
0.0
−0.1
−0.2
−0.3
0.02
0.04
0.06
SNP effect on Arthropathy in Crohn disease
SNP effect on Hexanoylcarnitine

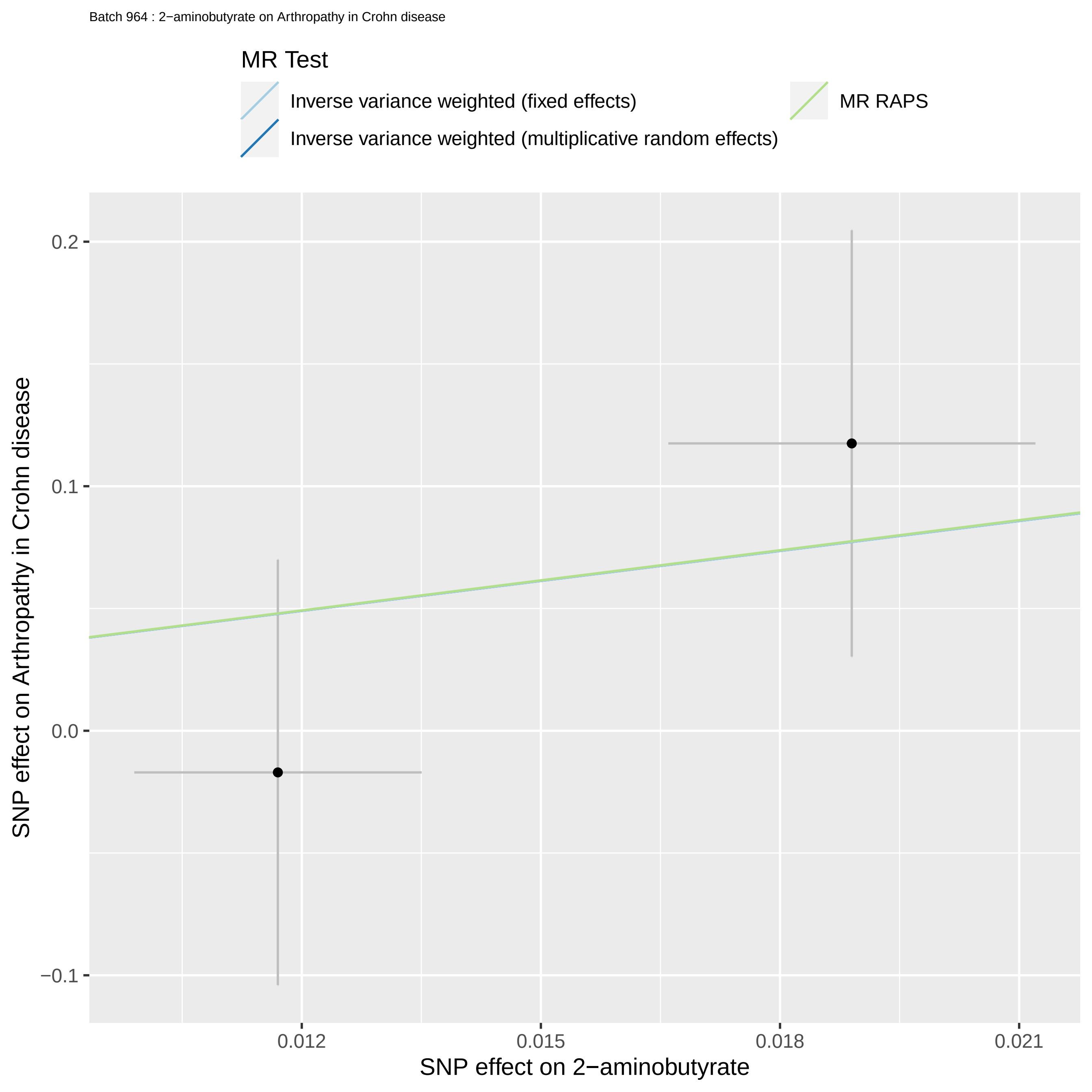

Batch 964 : 2−aminobutyrate on Arthropathy in Crohn disease
MR Test
Inverse variance weighted (fixed effects)
Inverse variance weighted (multiplicative random effects)
MR RAPS
SNP effect on Arthropathy in Crohn disease
0.2
0.1
0.0
−0.1
0.012
0.015
0.018
0.021
SNP effect on 2−aminobutyrate

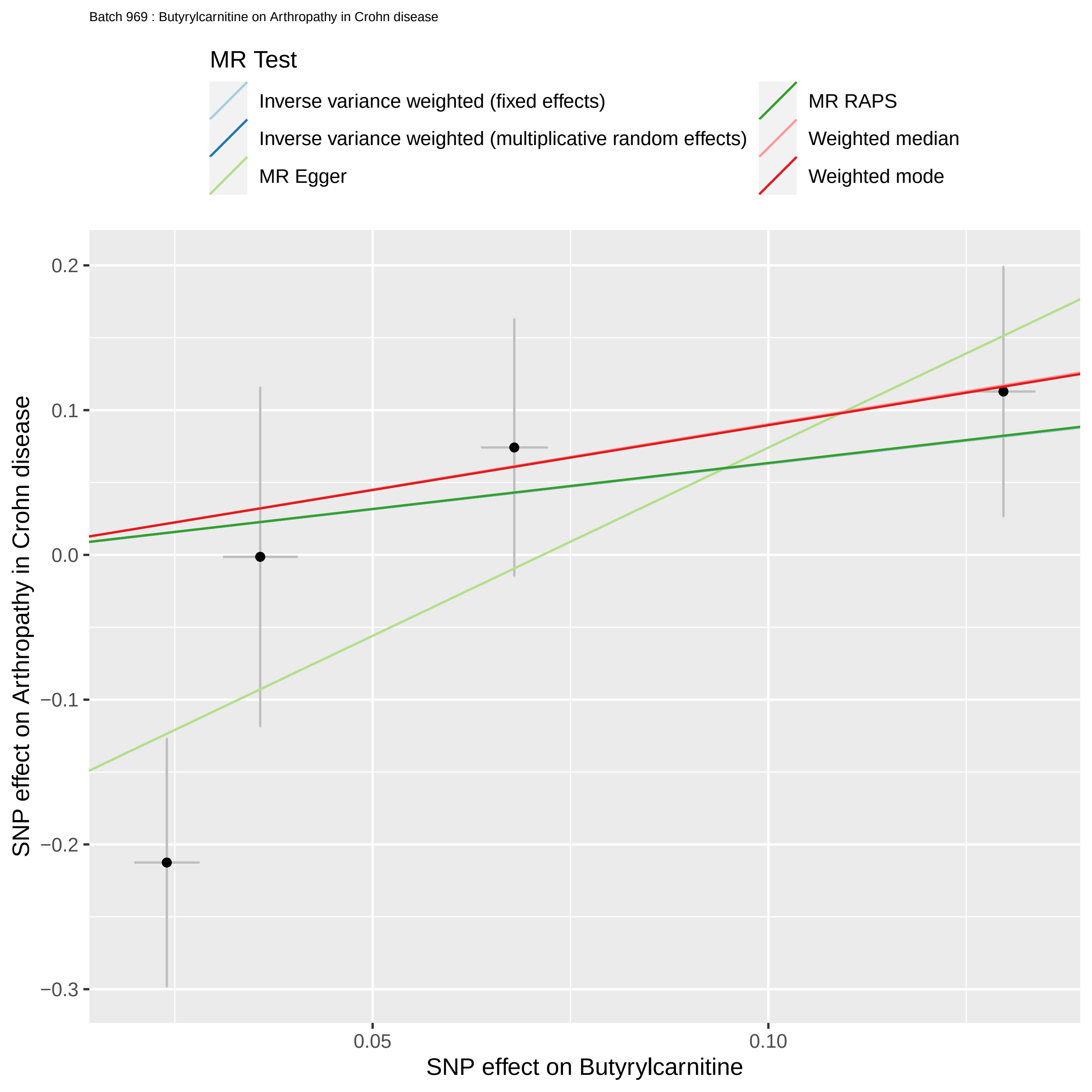

Batch 969 : Butyrylcarnitine on Arthropathy in Crohn disease
MR Test
Inverse variance weighted (fixed effects)
Inverse variance weighted (multiplicative random effects)
MR Egger
MR RAPS
Weighted median
Weighted mode
0.2
0.1
0.0
−0.1
−0.2
−0.3
SNP effect on Arthropathy in Crohn disease
0.05
0.10
SNP effect on Butyrylcarnitine

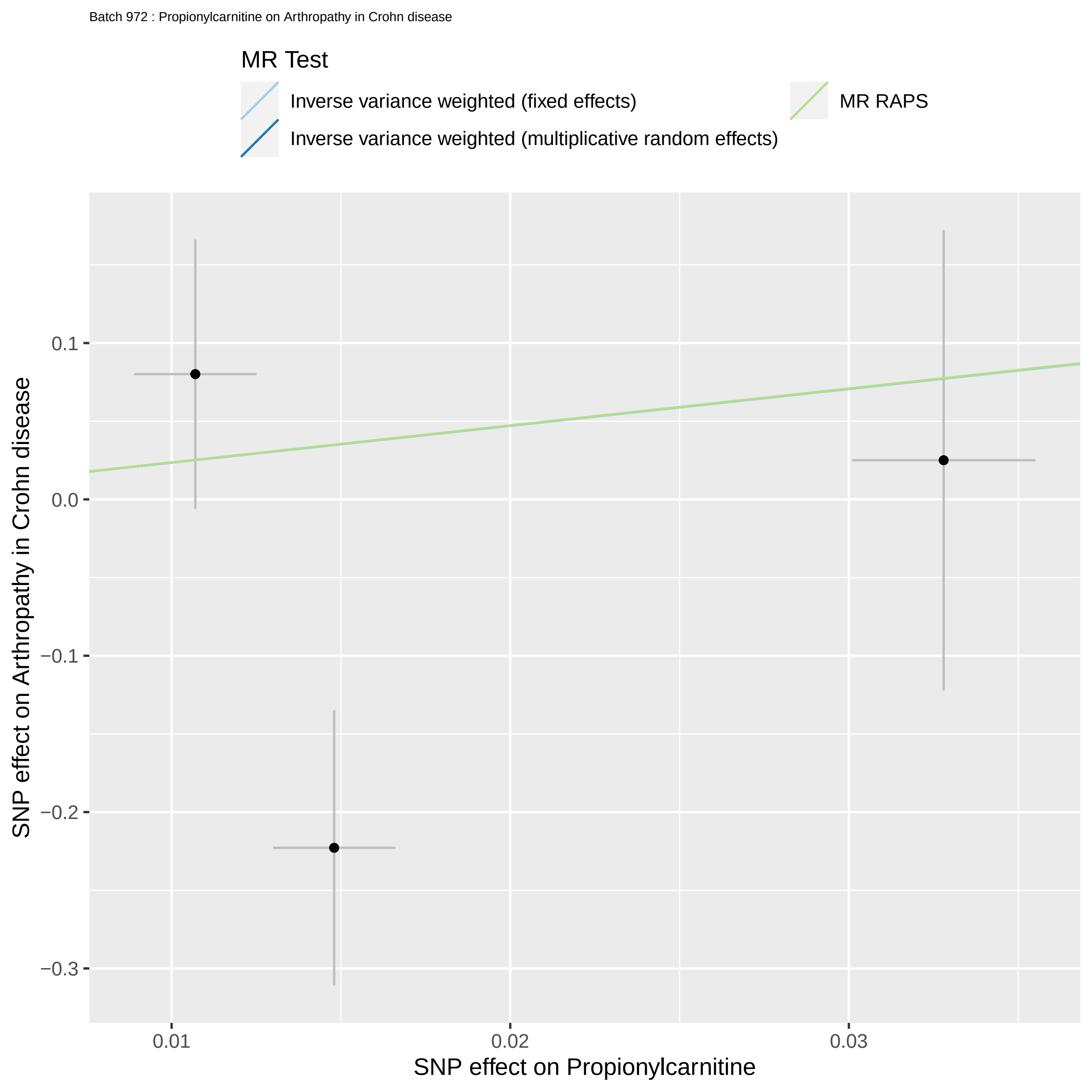
Batch 972 : Propionylcarnitine on Arthropathy in Crohn disease
MR Test
Inverse variance weighted (fixed effects)
Inverse variance weighted (multiplicative random effects)
MR RAPS
SNP effect on Arthropathy in Crohn disease
0.1
0.0
−0.1
−0.2
−0.3
0.01
0.02
0.03
SNP effect on Propionylcarnitine

Batch 973 : Caproate (6:0) on Arthropathy in Crohn disease

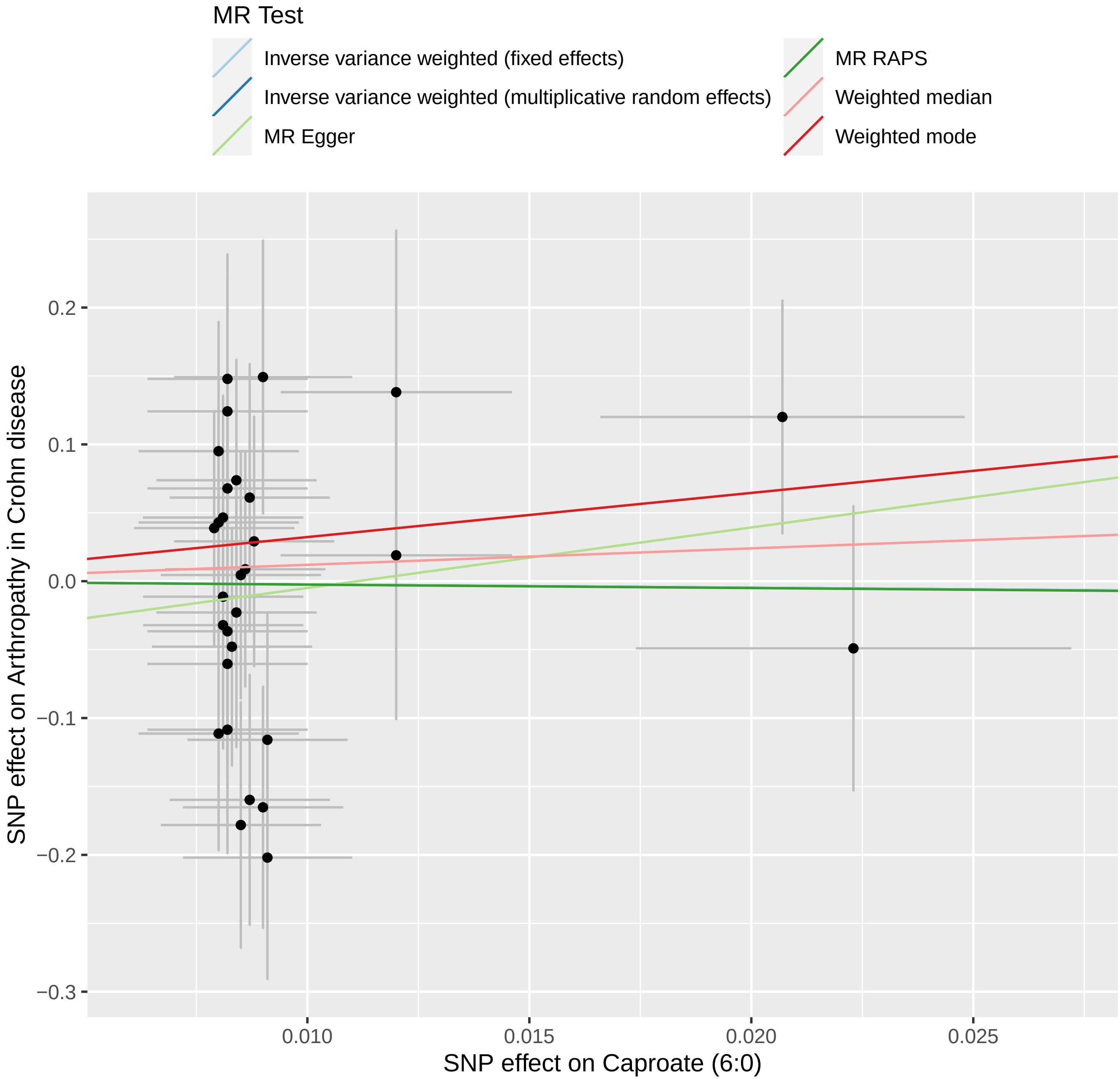

Batch 974 : Caprylate (8:0) on Arthropathy in Crohn disease

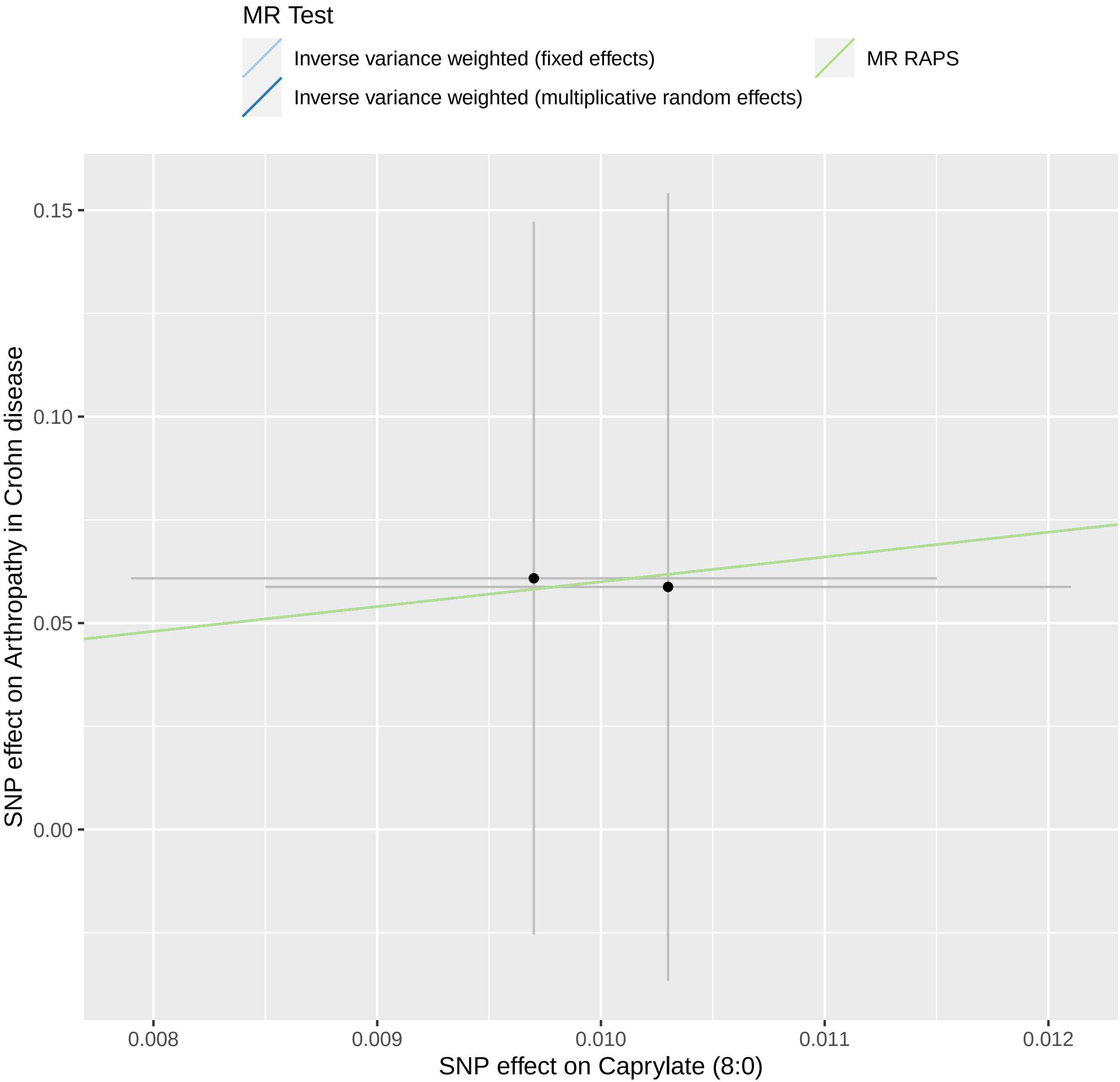

Batch 975 : 10−undecenoate (11:1n1) on Arthropathy in Crohn disease

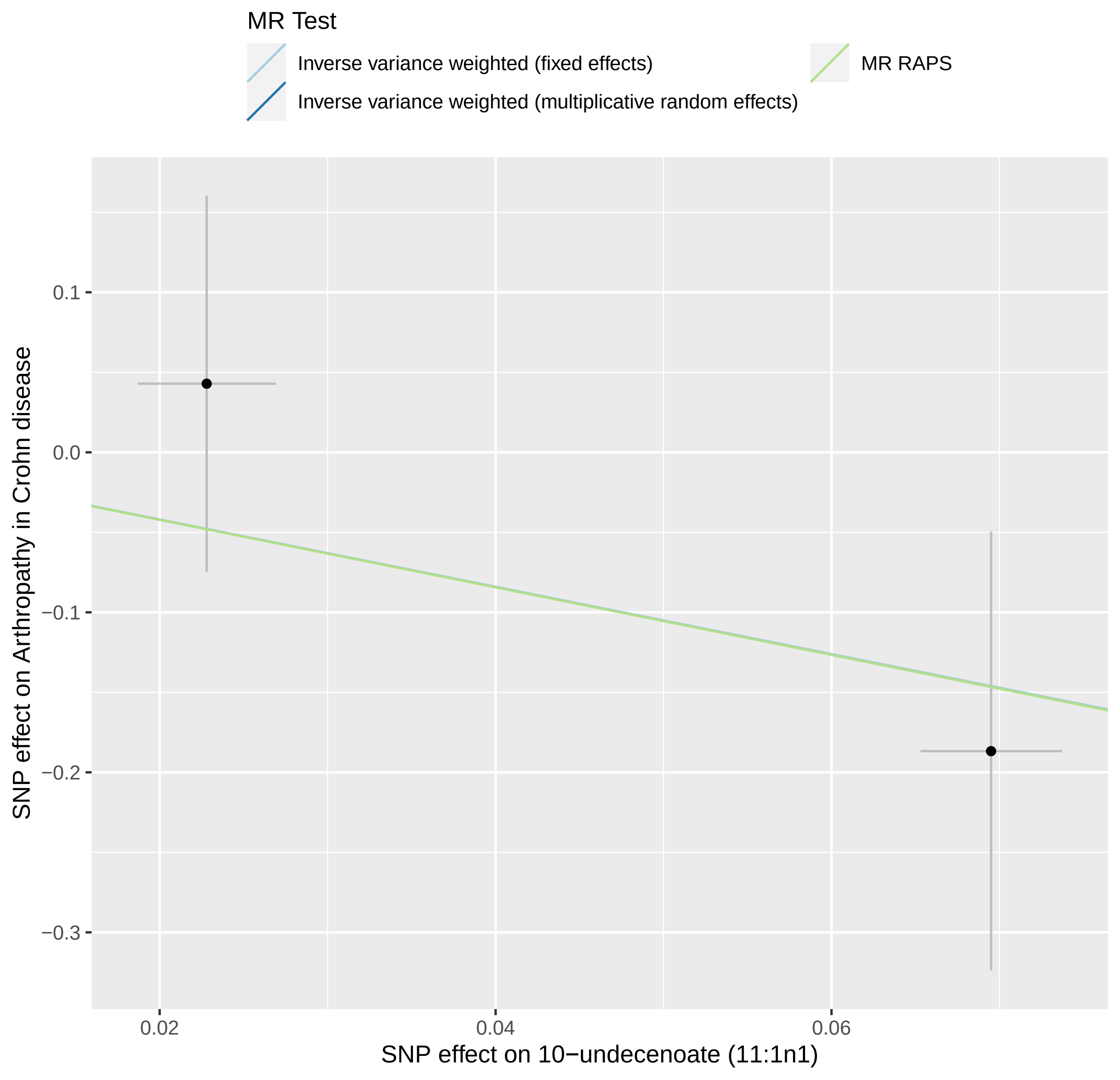

Batch 977 : Phenol sulfate on Arthropathy in Crohn disease

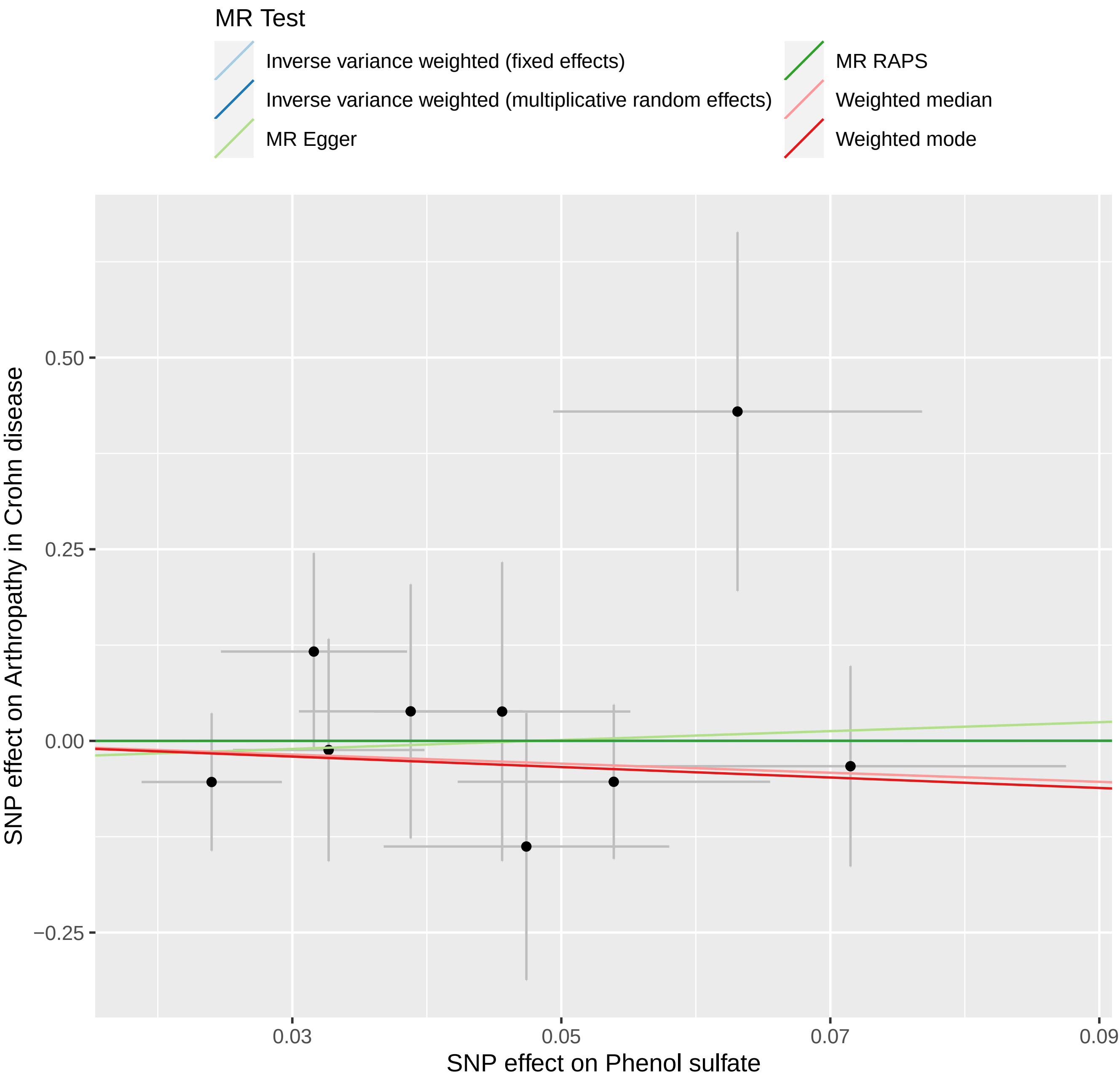

Batch 979 : Heme* on Arthropathy in Crohn disease

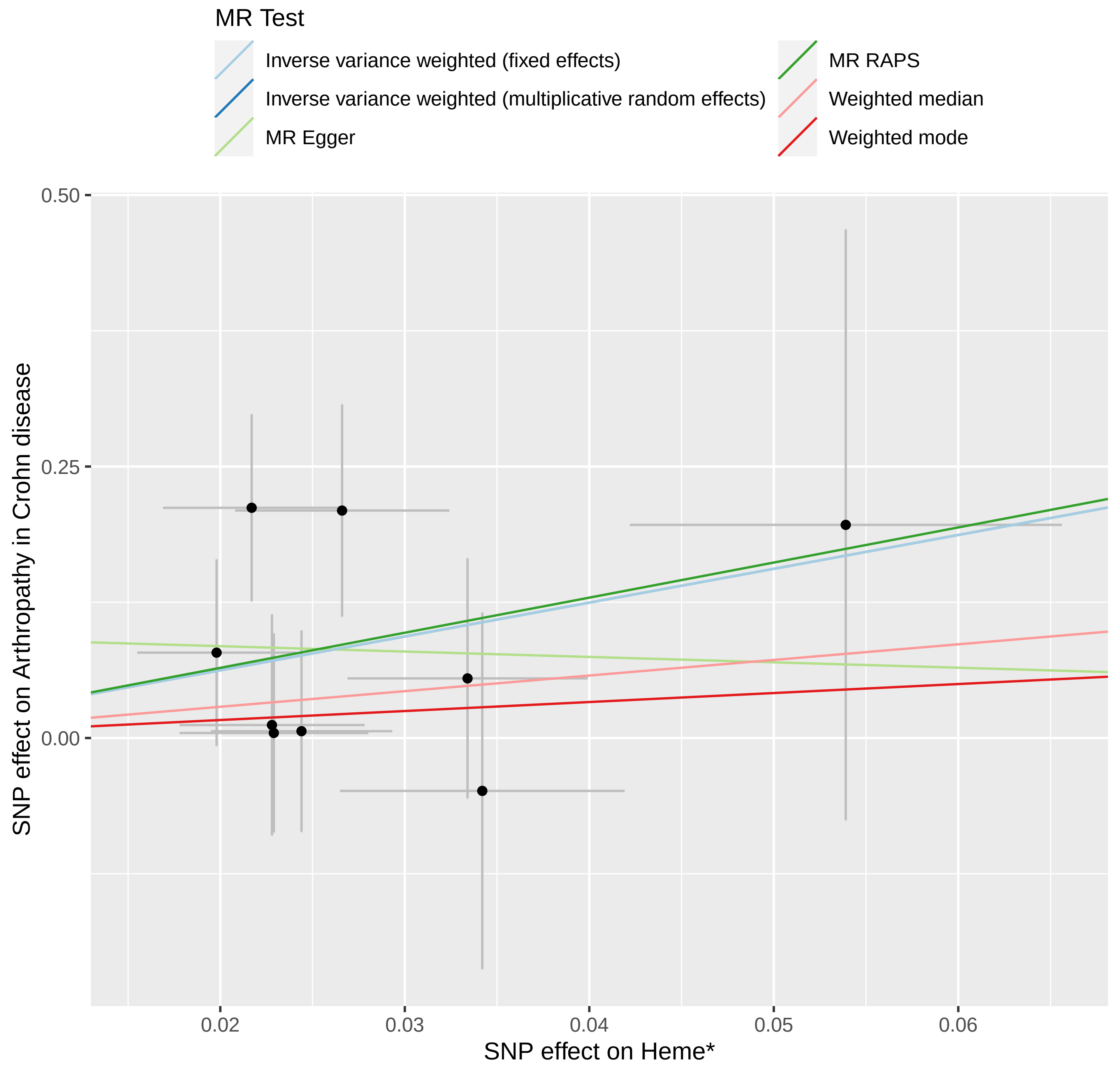

Batch 982 : Pyroglutamine* on Arthropathy in Crohn disease

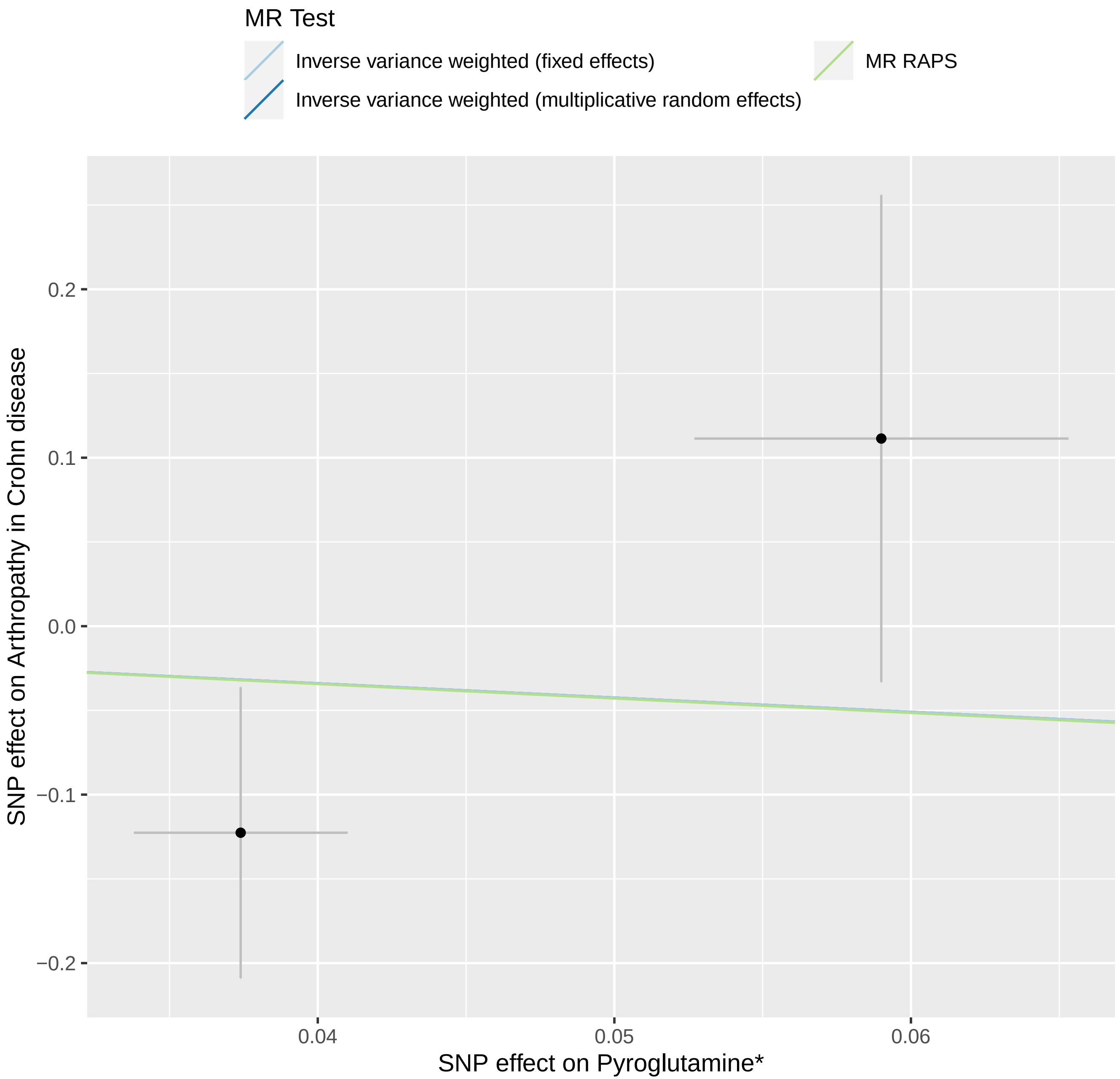

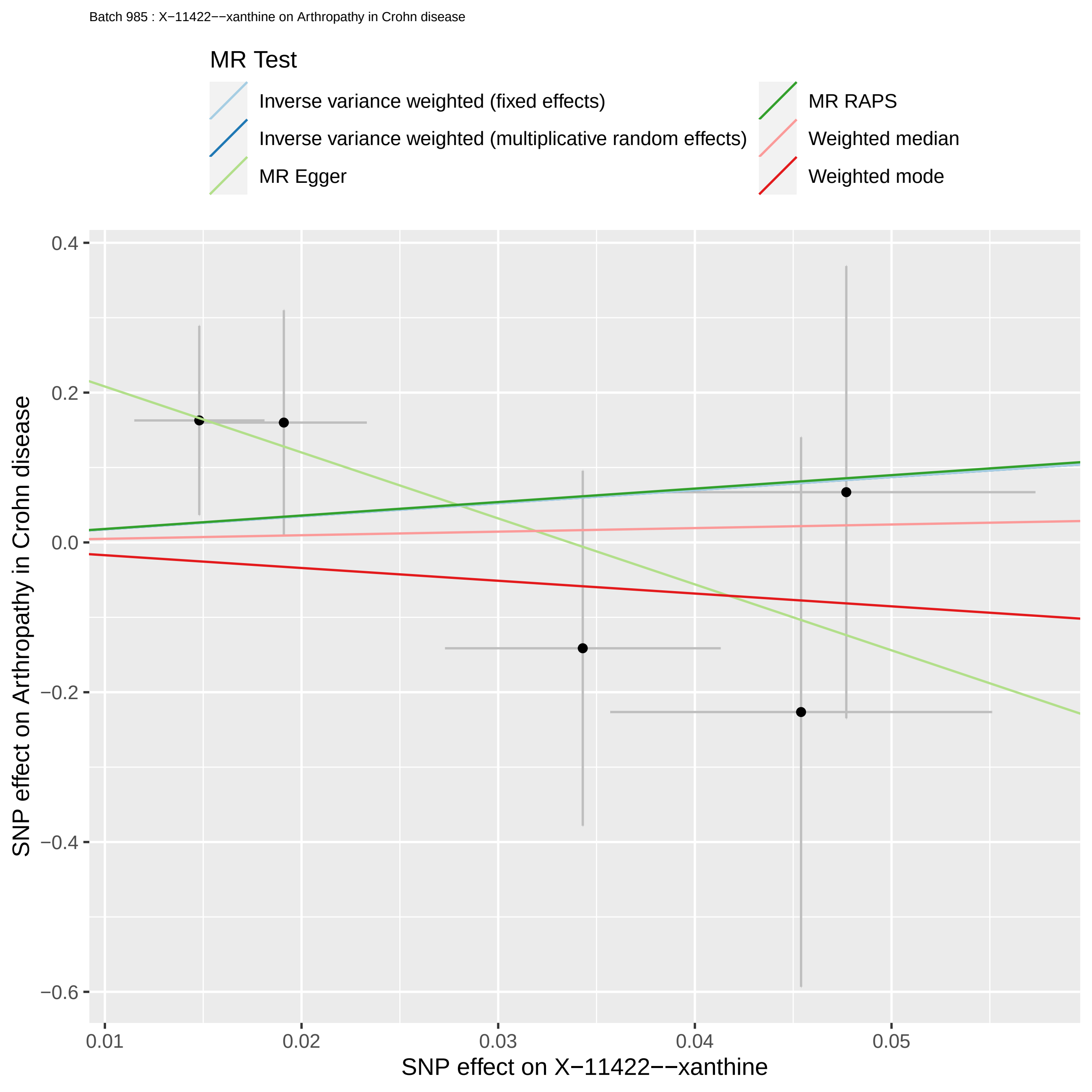
Batch 985 : X−11422−−xanthine on Arthropathy in Crohn disease
MR Test
Inverse variance weighted (fixed effects)
Inverse variance weighted (multiplicative random effects)
MR Egger
MR RAPS
Weighted median
Weighted mode
0.4
0.2
0.0
−0.2
−0.4
−0.6
SNP effect on Arthropathy in Crohn disease
0.01
0.02
0.03
0.04
0.05
SNP effect on X−11422−−xanthine

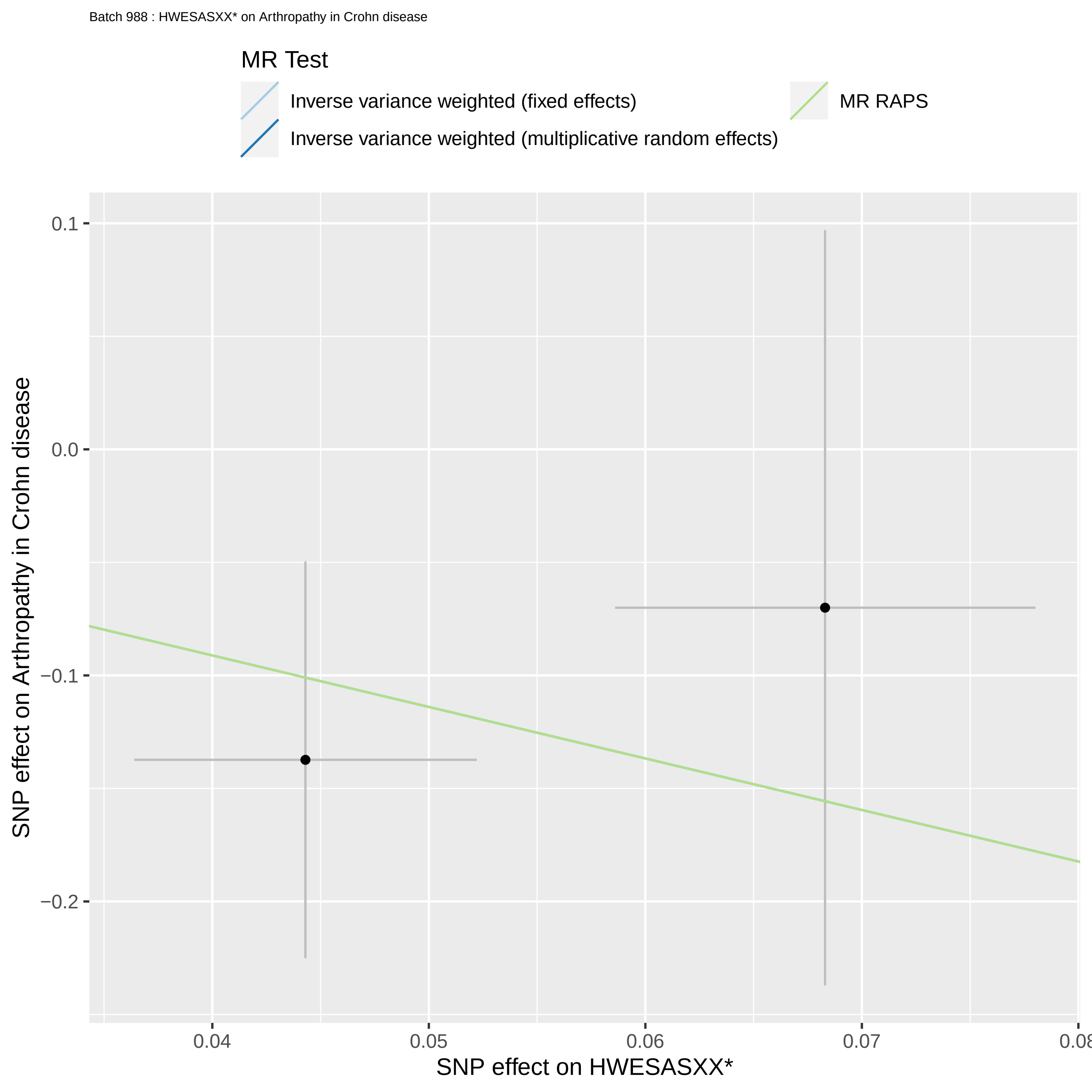

Batch 988 : HWESASXX* on Arthropathy in Crohn disease
MR Test
Inverse variance weighted (fixed effects)
Inverse variance weighted (multiplicative random effects)
MR RAPS
0.1
0.0
−0.1
−0.2
0.04
0.05
0.06
0.07
0.08
SNP effect on Arthropathy in Crohn disease
SNP effect on HWESASXX*

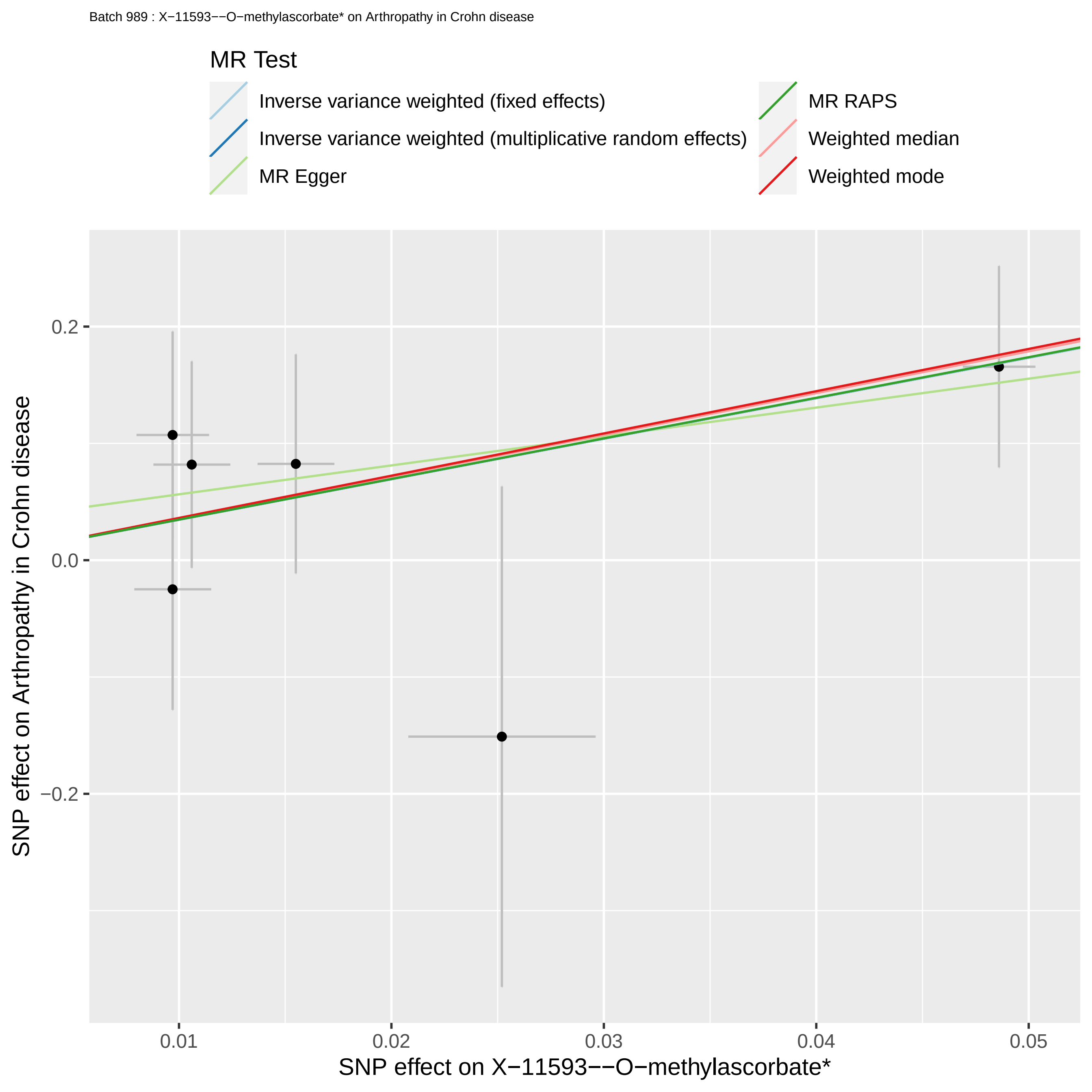
Batch 989 : X−11593−−O−methylascorbate* on Arthropathy in Crohn disease
MR Test
Inverse variance weighted (fixed effects)
Inverse variance weighted (multiplicative random effects)
MR Egger
MR RAPS
Weighted median
Weighted mode
0.2
0.0
−0.2
0.01
0.02
0.03
0.04
0.05
SNP effect on Arthropathy in Crohn disease
SNP effect on X−11593−−O−methylascorbate*

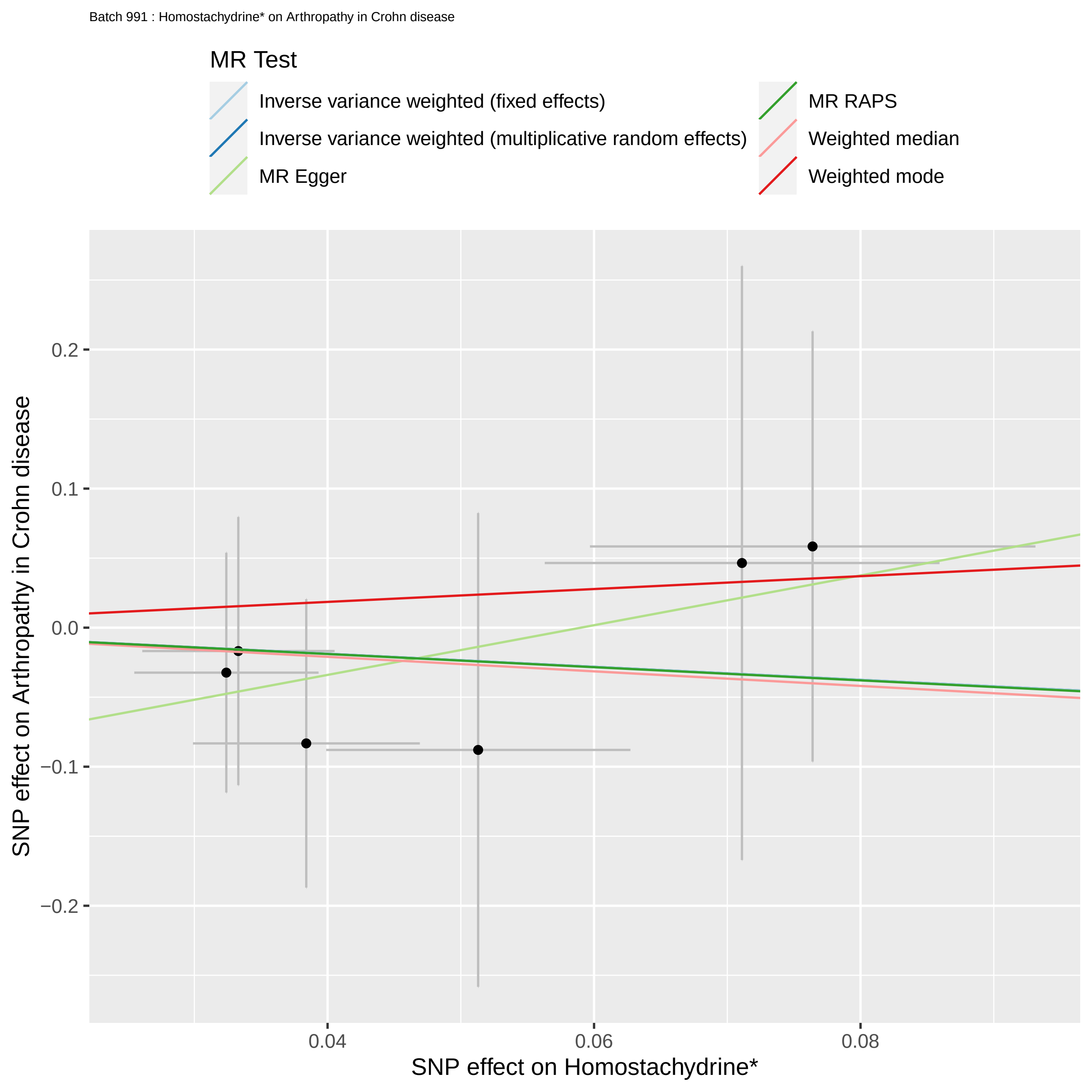
Batch 991 : Homostachydrine* on Arthropathy in Crohn disease
MR Test
Inverse variance weighted (fixed effects)
Inverse variance weighted (multiplicative random effects)
MR Egger
MR RAPS
Weighted median
Weighted mode
SNP effect on Arthropathy in Crohn disease
0.2
0.1
0.0
−0.1
−0.2
0.04
0.06
0.08
SNP effect on Homostachydrine*

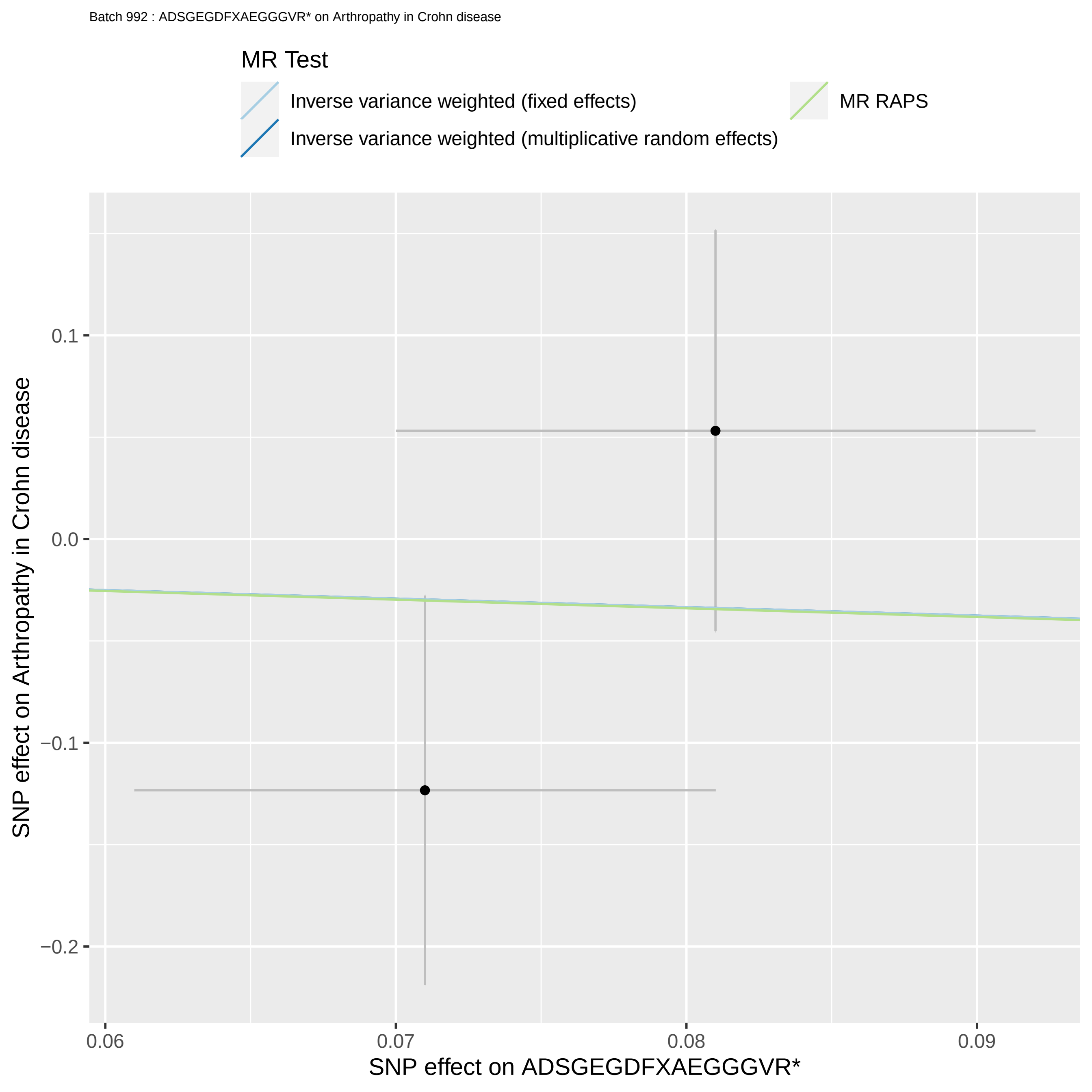
Batch 992 : ADSGEGDFXAEGGGVR* on Arthropathy in Crohn disease
MR Test
Inverse variance weighted (fixed effects)
Inverse variance weighted (multiplicative random effects)
MR RAPS
SNP effect on Arthropathy in Crohn disease
0.1
0.0
−0.1
−0.2
0.06
0.07
0.08
0.09
SNP effect on ADSGEGDFXAEGGGVR*

Batch 994 : X−11793−−oxidized bilirubin* on Arthropathy in Crohn disease

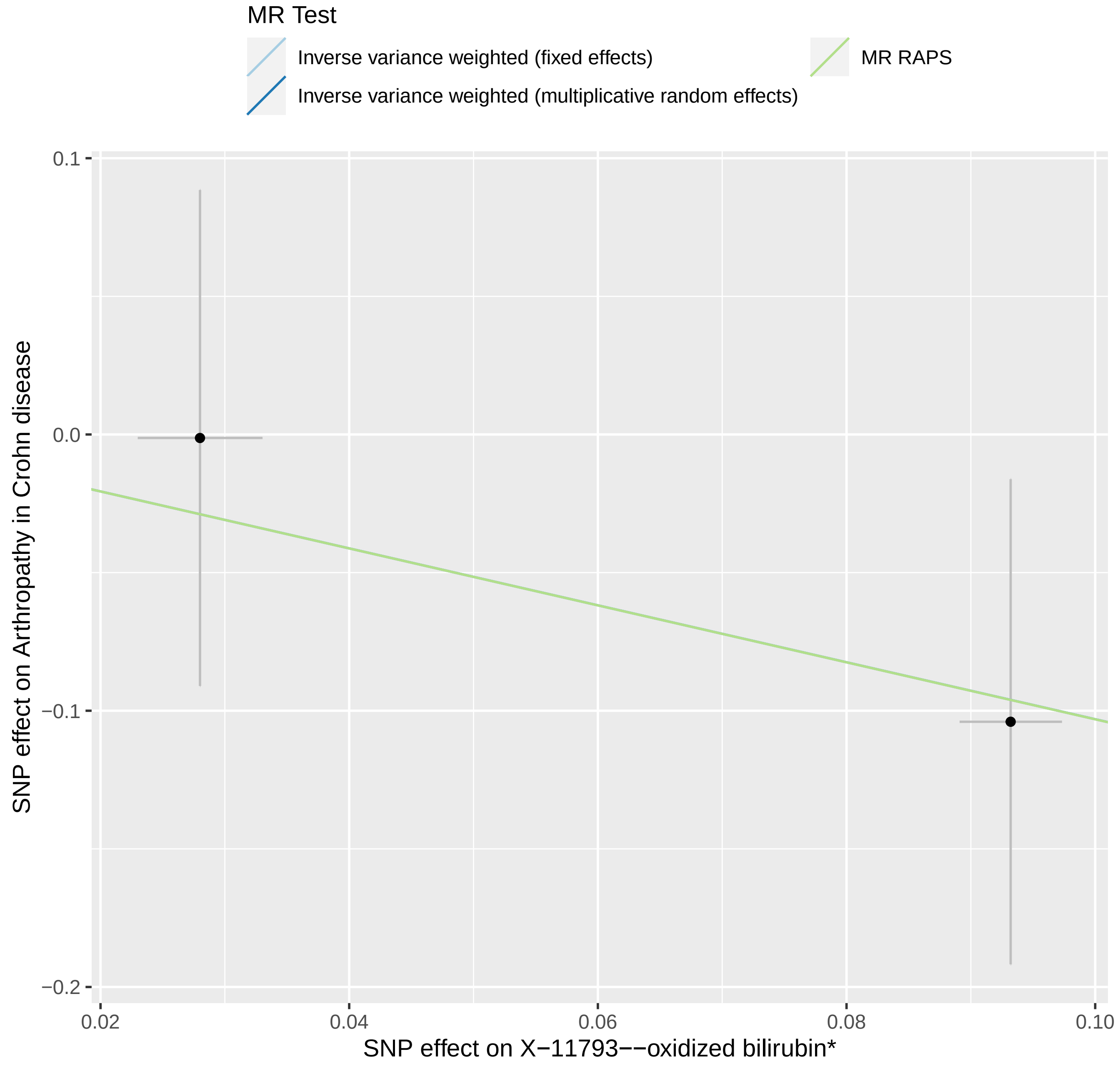

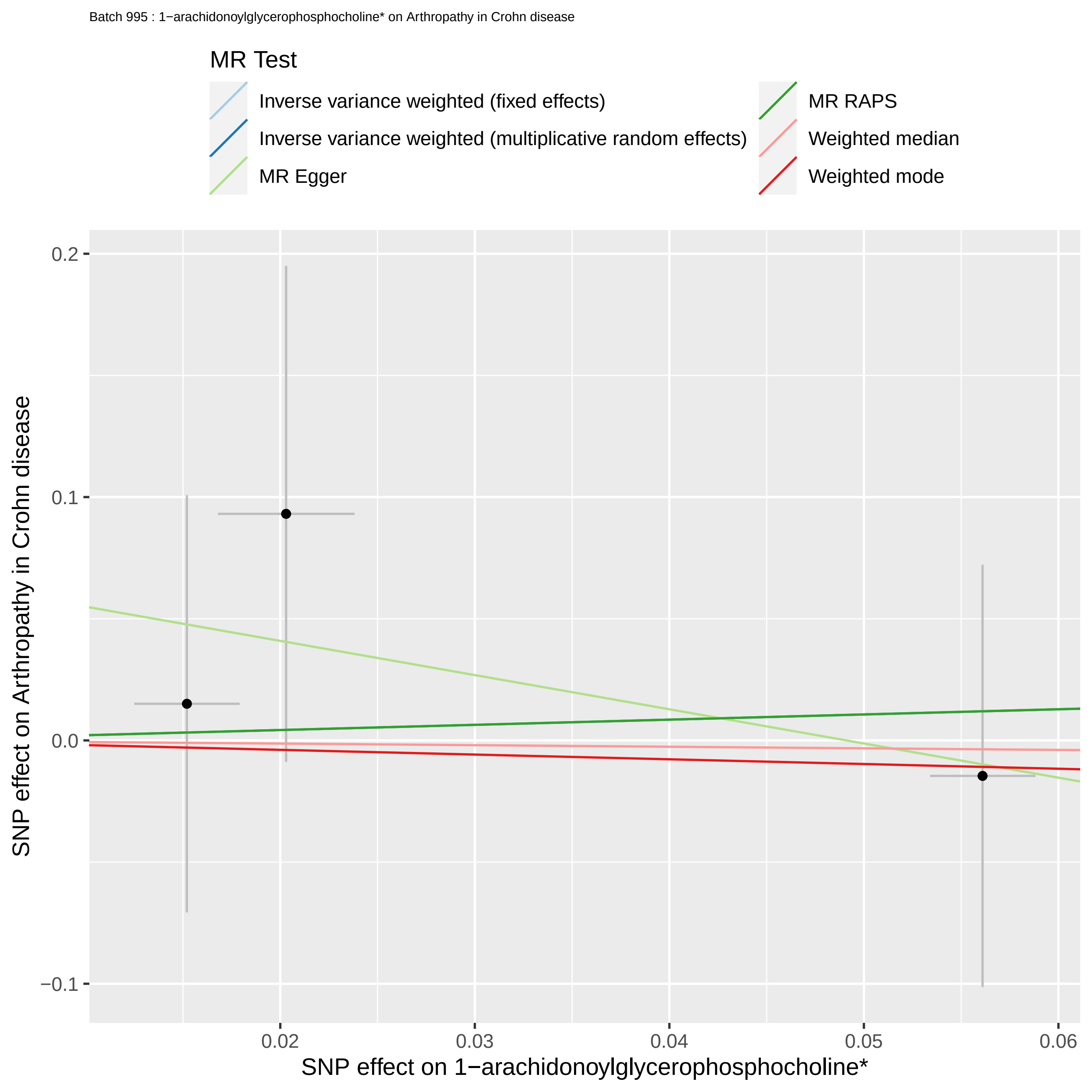

Batch 995 : 1−arachidonoylglycerophosphocholine* on Arthropathy in Crohn disease
MR Test
Inverse variance weighted (fixed effects)
Inverse variance weighted (multiplicative random effects)
MR Egger
MR RAPS
Weighted median
Weighted mode
SNP effect on Arthropathy in Crohn disease
0.2
0.1
0.0
−0.1
0.02
0.03
0.04
0.05
0.06
SNP effect on 1−arachidonoylglycerophosphocholine*

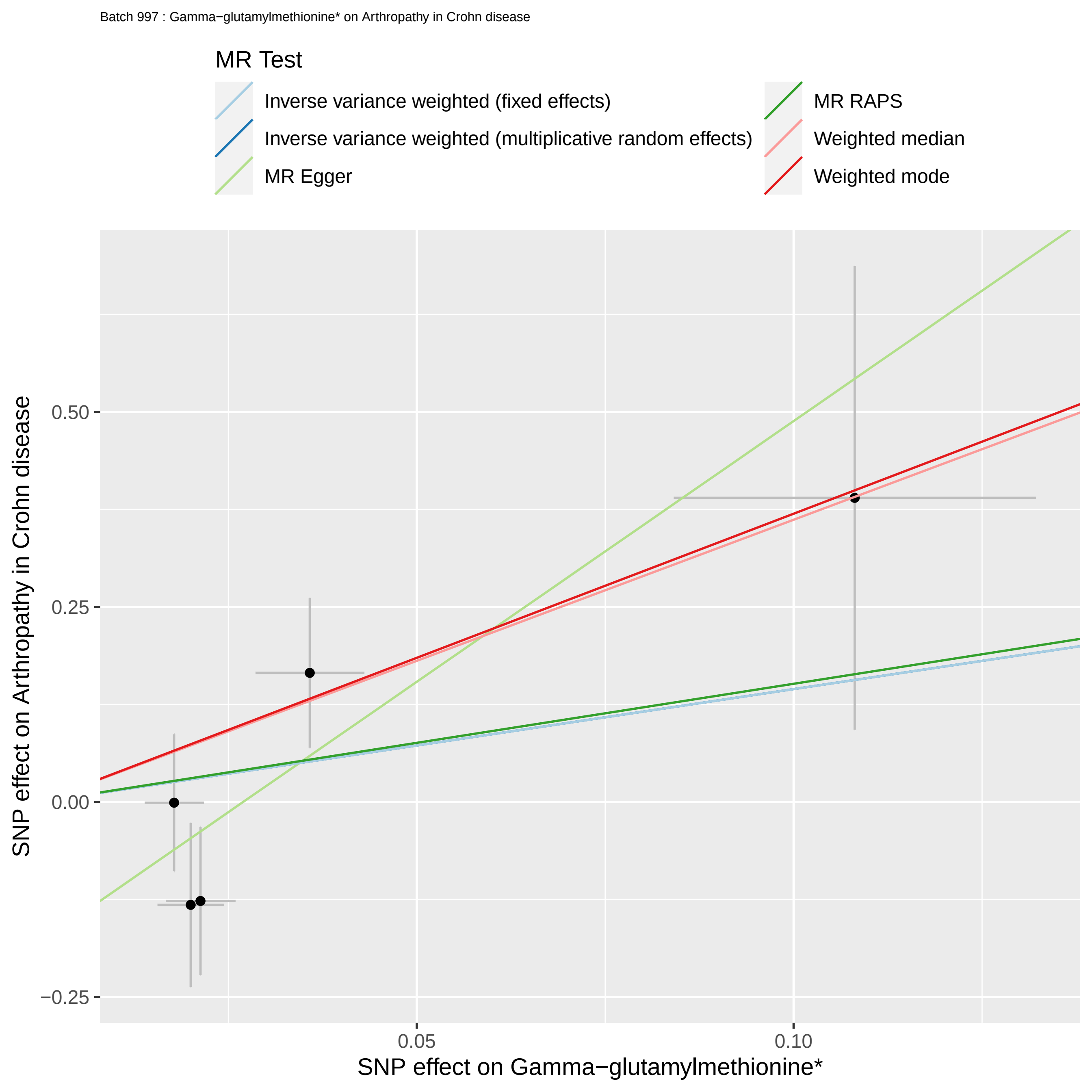

Batch 997 : Gamma−glutamylmethionine* on Arthropathy in Crohn disease
MR Test
Inverse variance weighted (fixed effects)
Inverse variance weighted (multiplicative random effects)
MR Egger
MR RAPS
Weighted median
Weighted mode
SNP effect on Arthropathy in Crohn disease
0.50
0.25
0.00
−0.25
0.05
0.10
SNP effect on Gamma−glutamylmethionine*

Batch 998 : Gamma−glutamylthreonine* on Arthropathy in Crohn disease

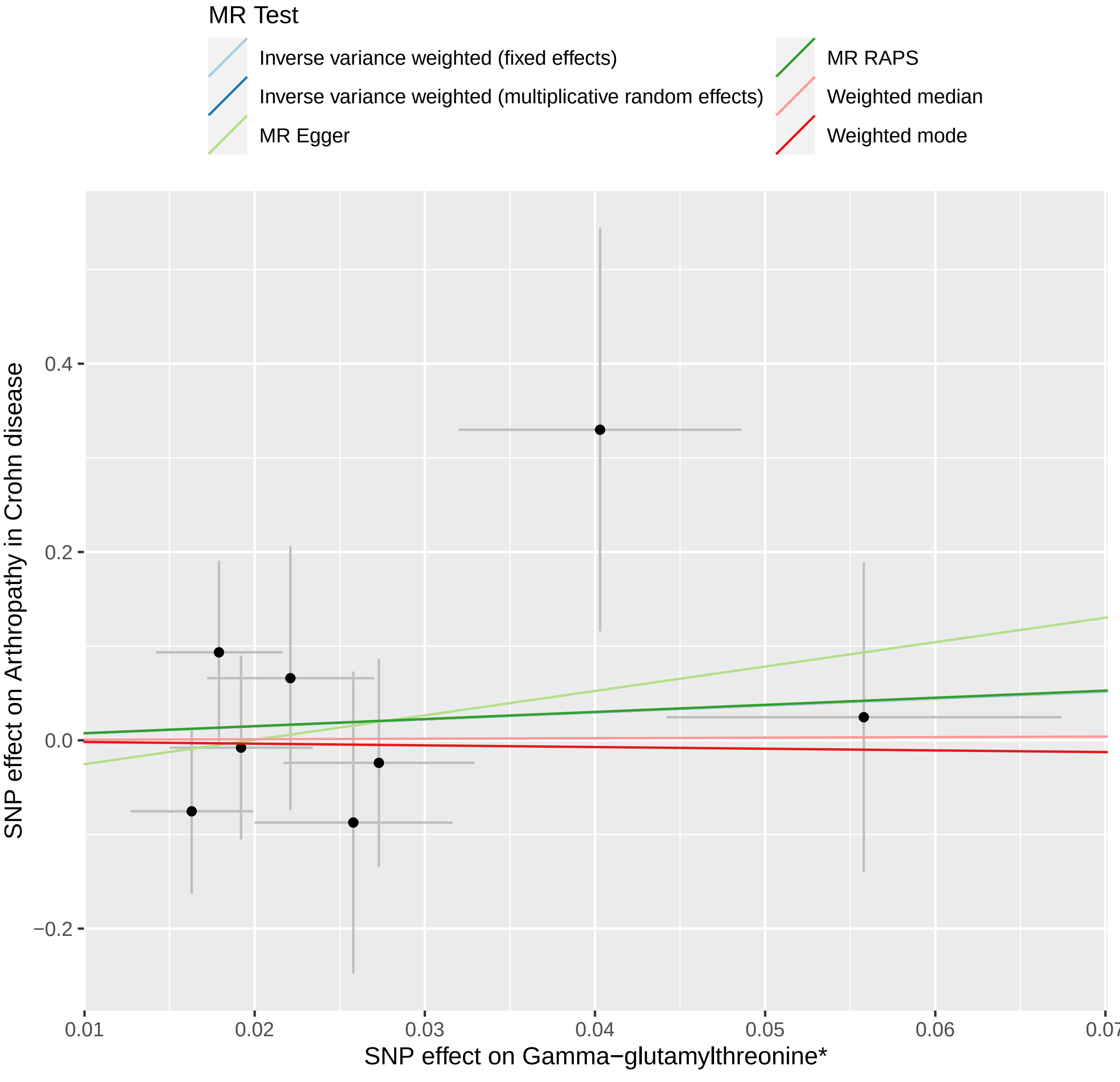

Batch 999 : Gamma−tocopherol on Arthropathy in Crohn disease

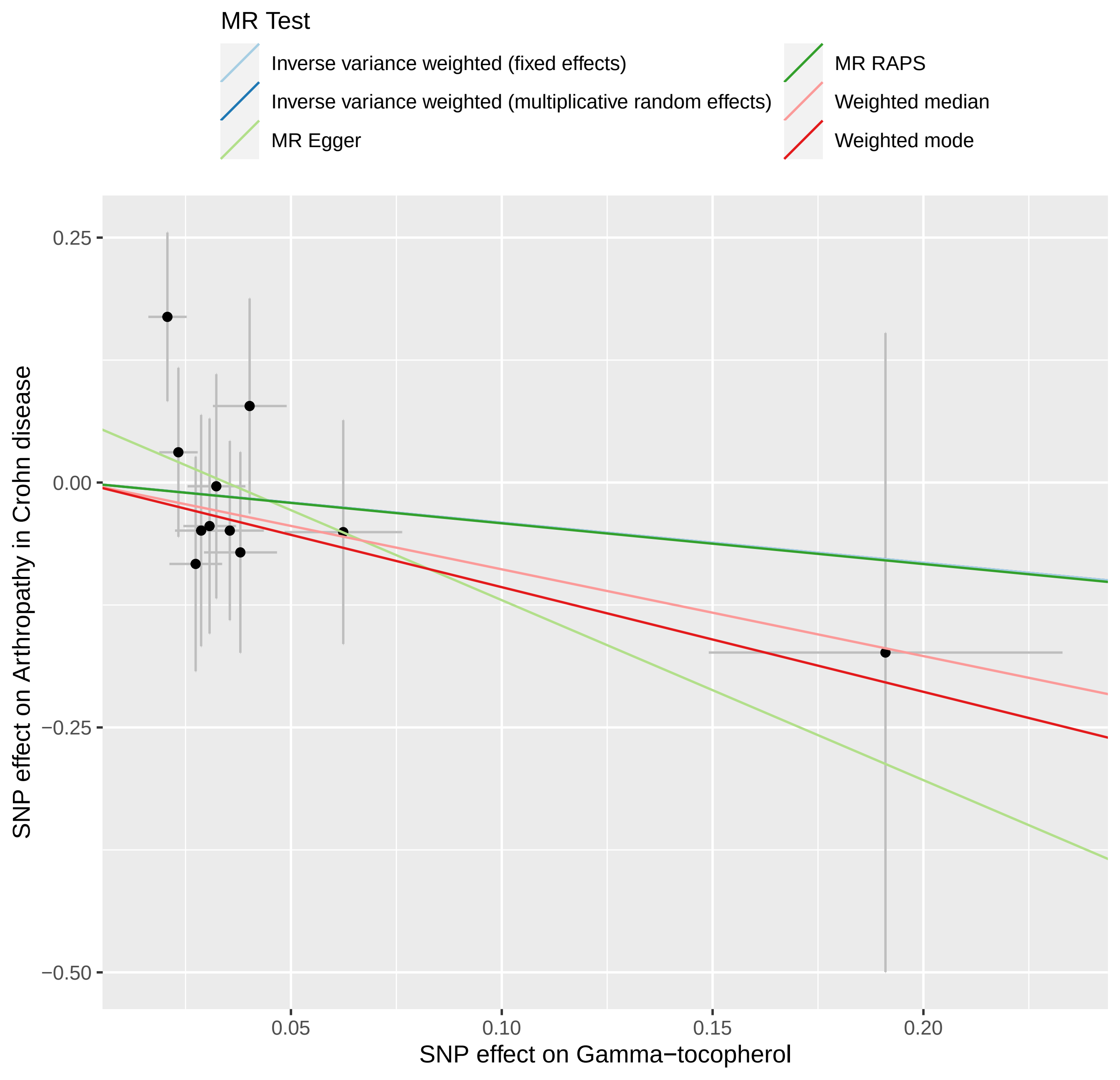

Batch 1000 : Gamma−glutamylphenylalanine on Arthropathy in Crohn disease

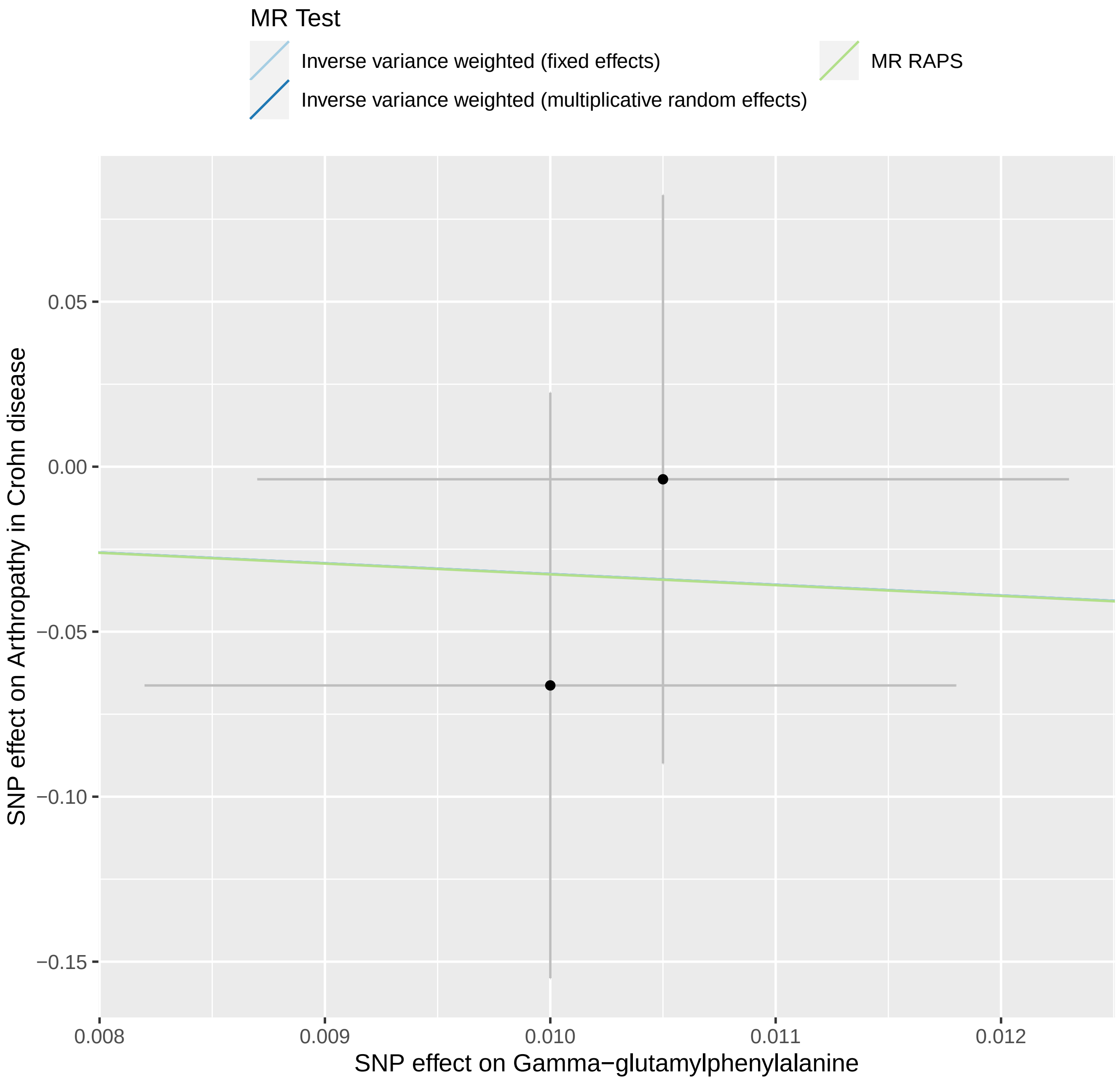

Batch 1002 : Pseudouridine on Arthropathy in Crohn disease

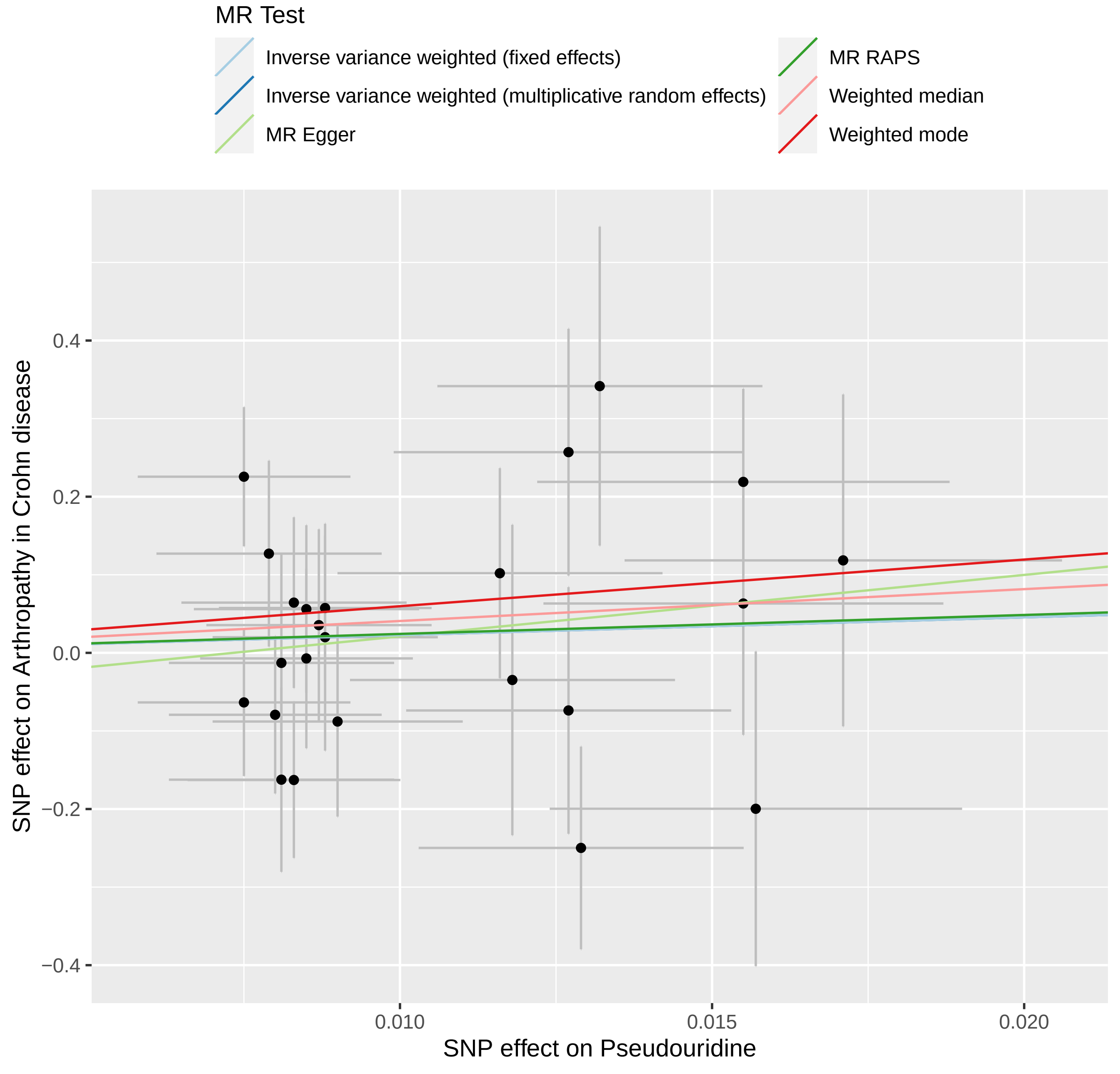

Batch 1003 : Valerate on Arthropathy in Crohn disease

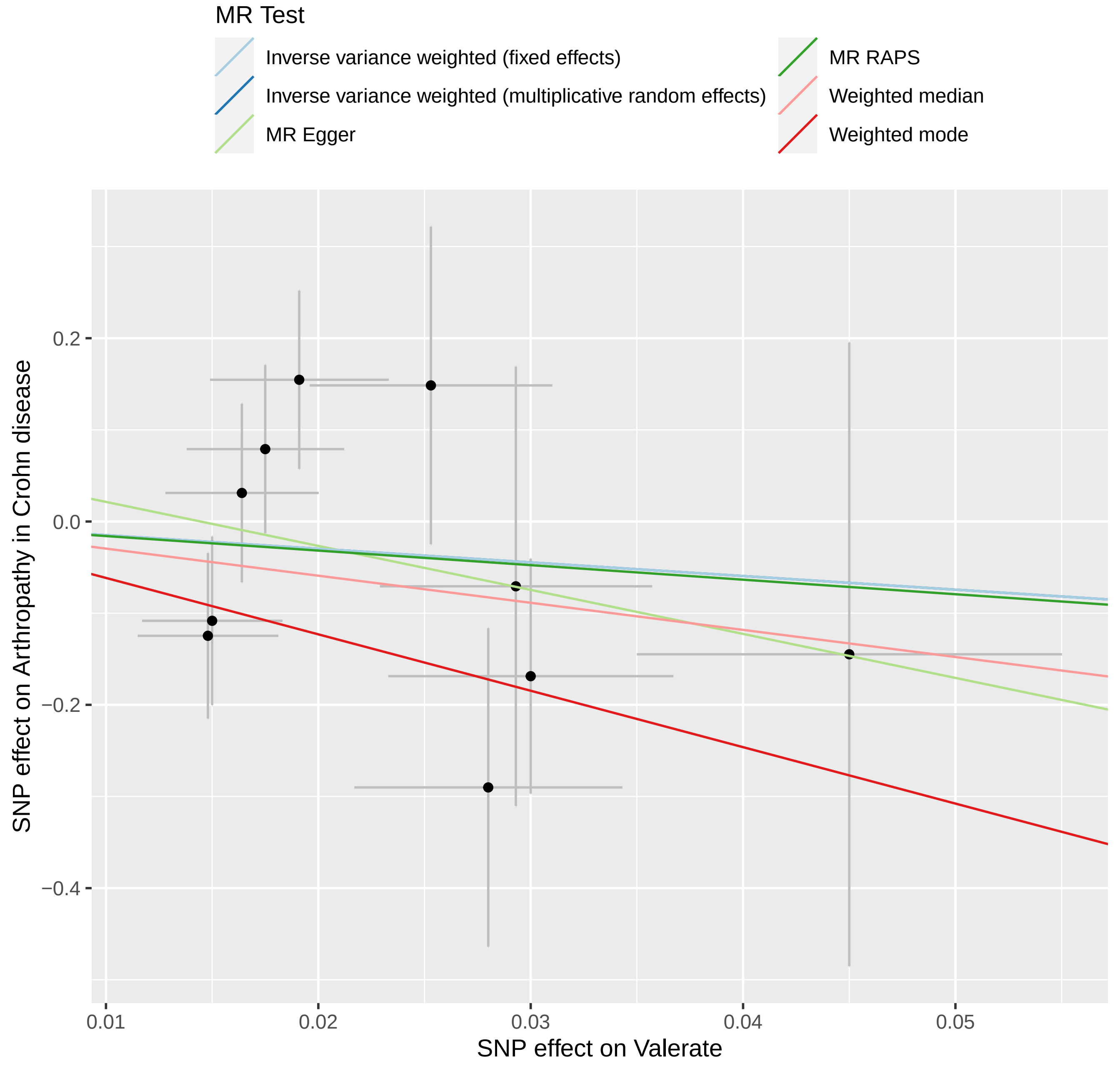

Batch 1006 : Erythronate* on Arthropathy in Crohn disease

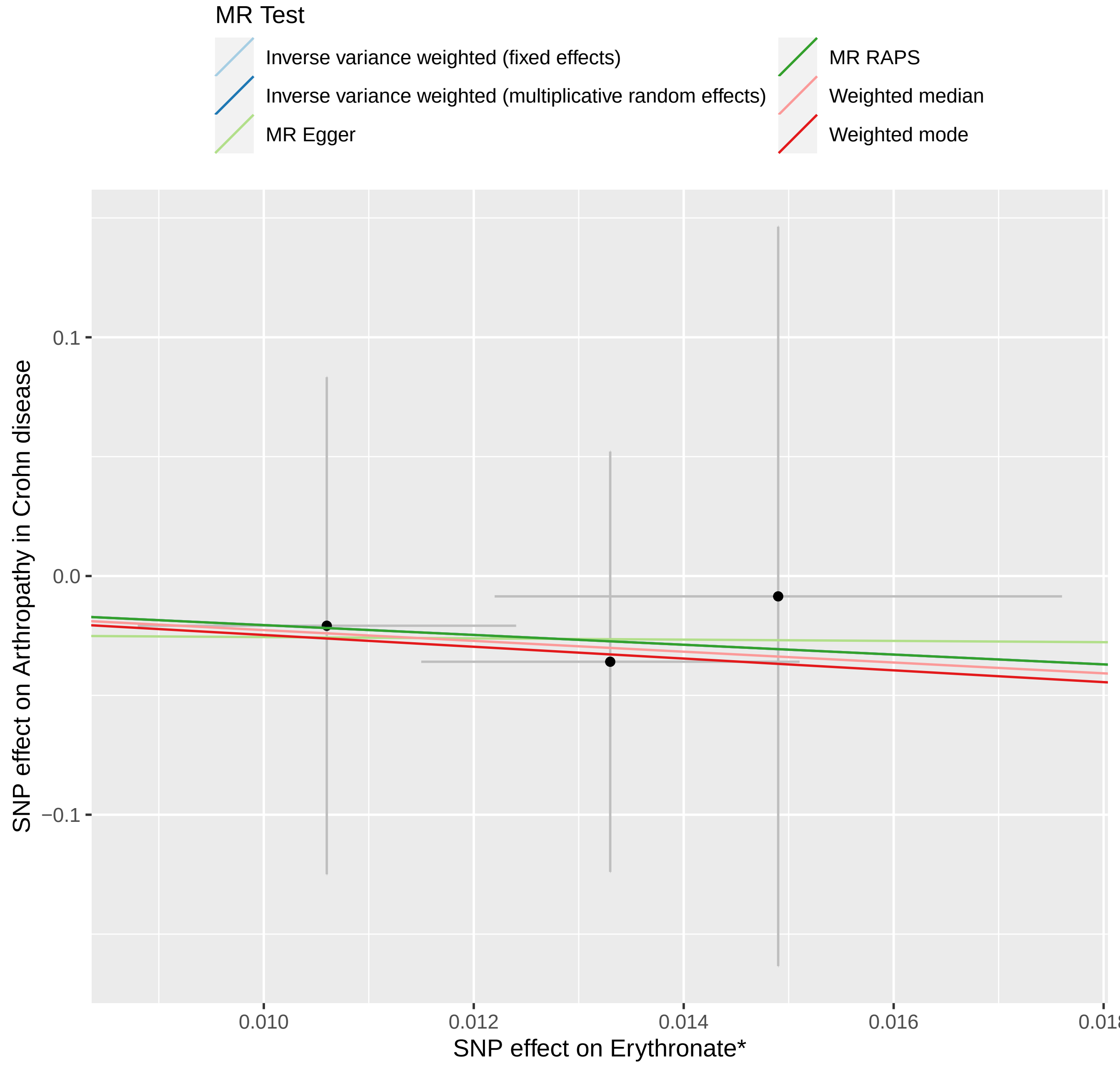

Batch 1007 : Lathosterol on Arthropathy in Crohn disease

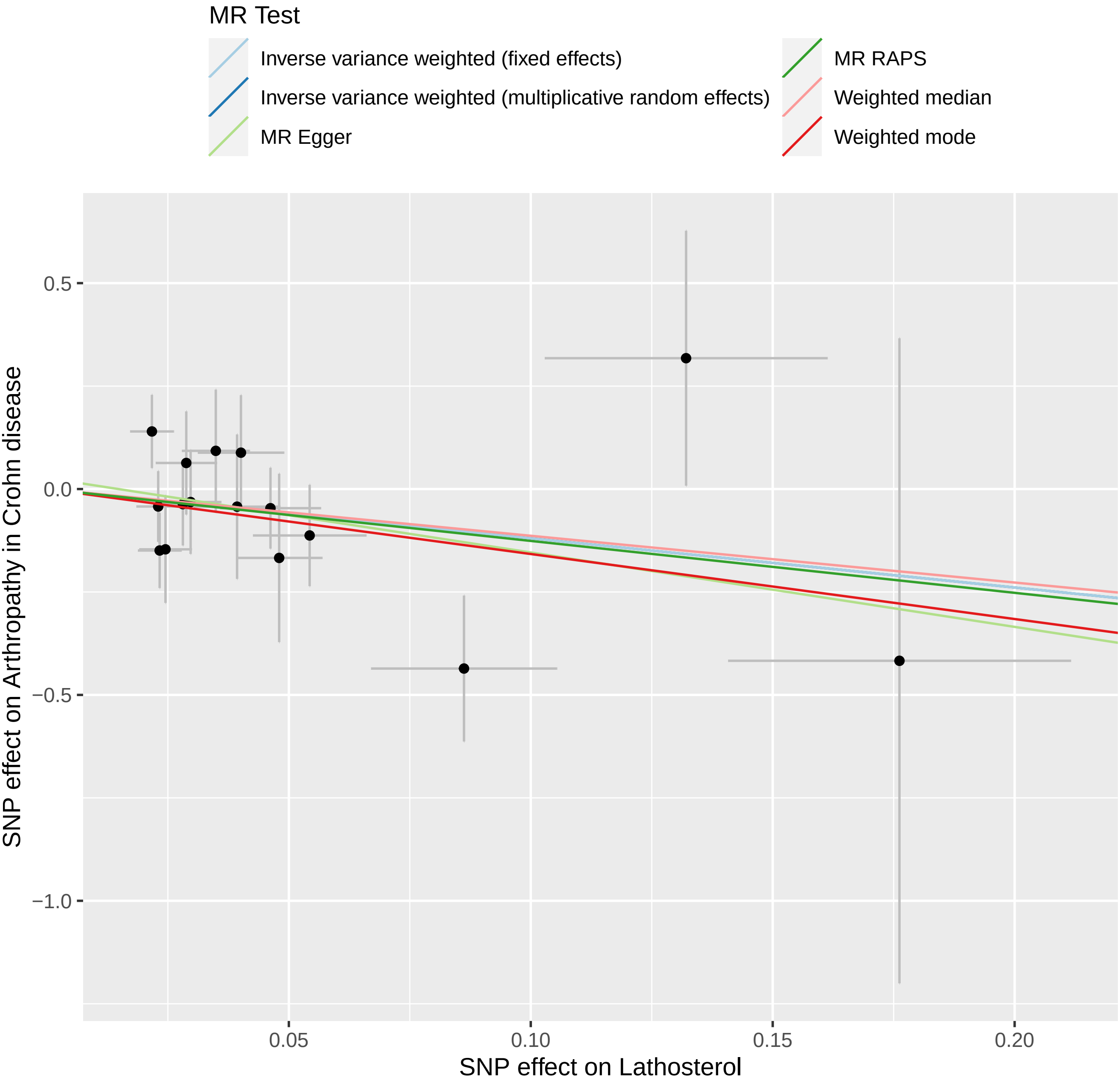

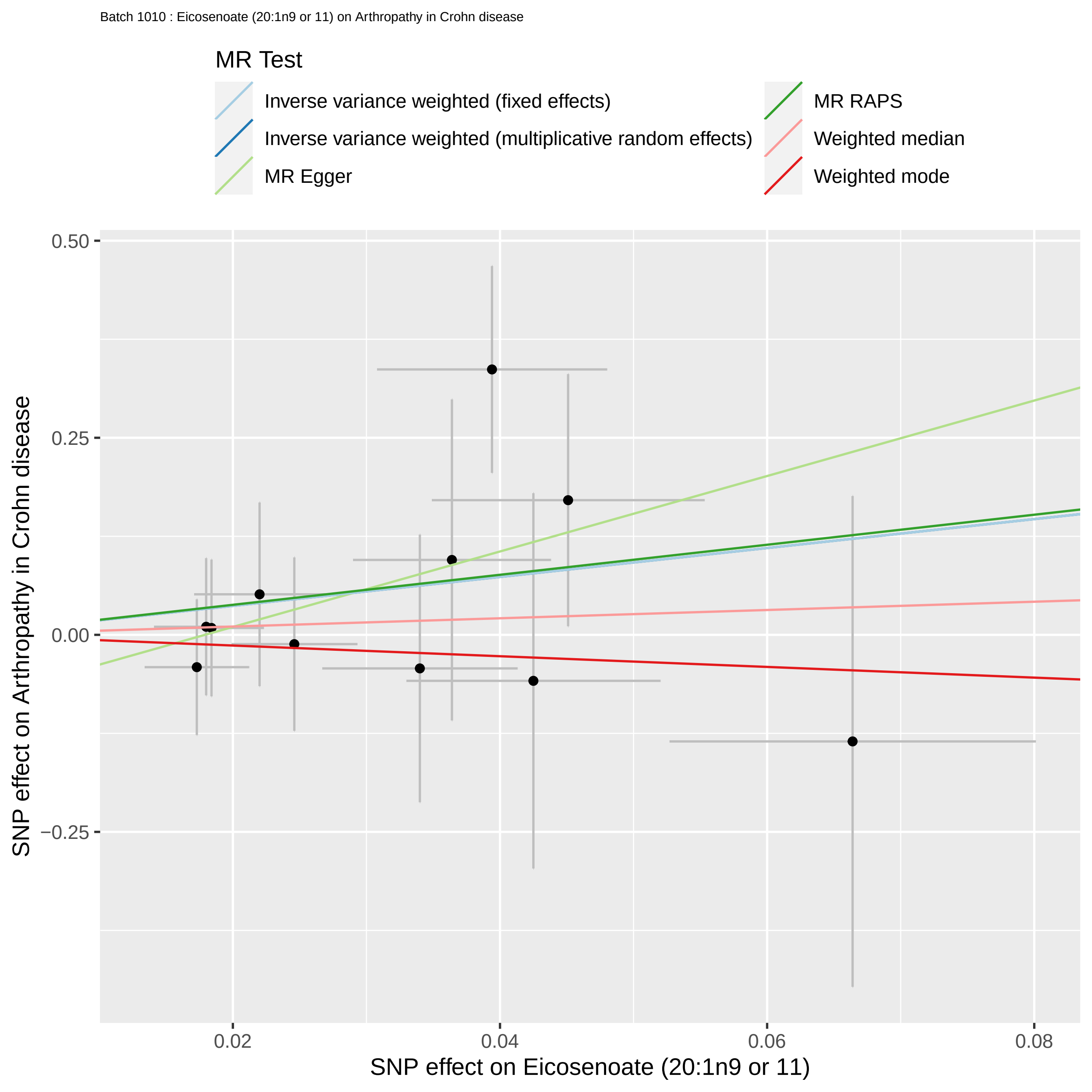

Batch 1010 : Eicosenoate (20:1n9 or 11) on Arthropathy in Crohn disease
MR Test
Inverse variance weighted (fixed effects)
Inverse variance weighted (multiplicative random effects)
MR Egger
MR RAPS
Weighted median
Weighted mode
0.50
0.25
0.00
−0.25
0.02
0.04
0.06
0.08
SNP effect on Arthropathy in Crohn disease
SNP effect on Eicosenoate (20:1n9 or 11)

Batch 1011 : X−12244−−N−acetylcarnosine on Arthropathy in Crohn disease

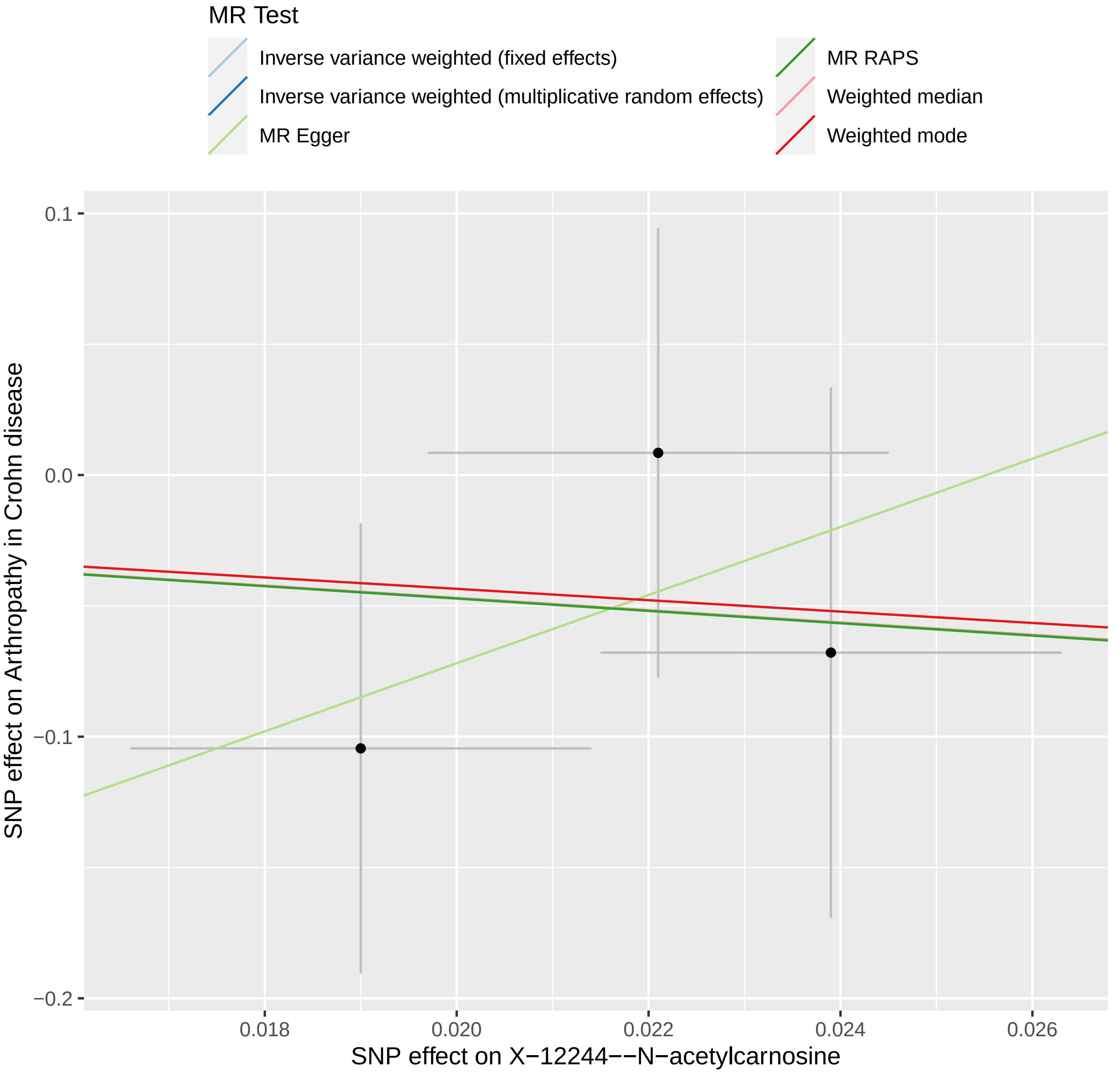

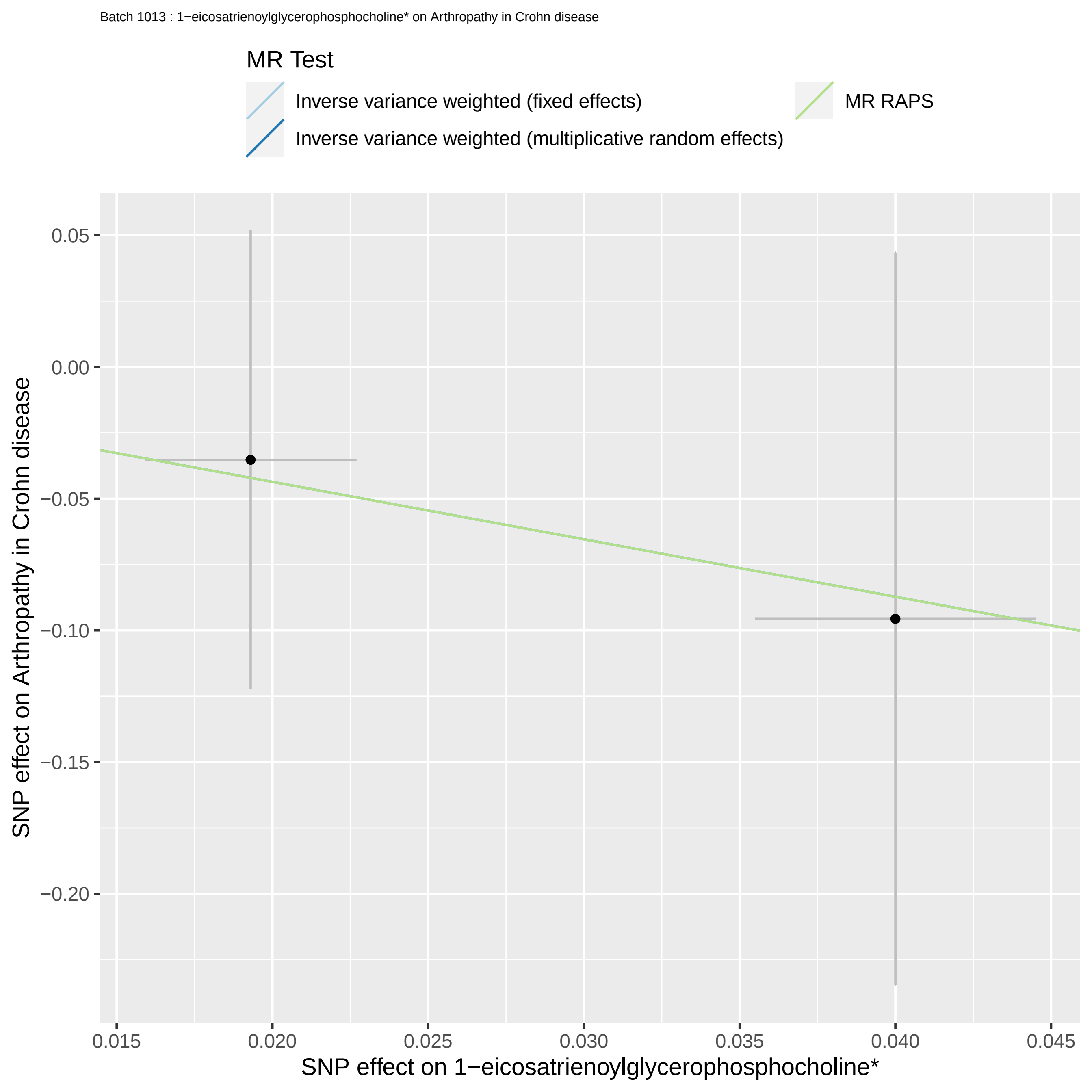
Batch 1013 : 1−eicosatrienoylglycerophosphocholine* on Arthropathy in Crohn disease
MR Test
Inverse variance weighted (fixed effects)
Inverse variance weighted (multiplicative random effects)
MR RAPS
0.05
0.00
−0.05
−0.10
−0.15
−0.20
SNP effect on Arthropathy in Crohn disease
0.015
0.020
0.025
0.030
0.035
0.040
0.045
SNP effect on 1−eicosatrienoylglycerophosphocholine*

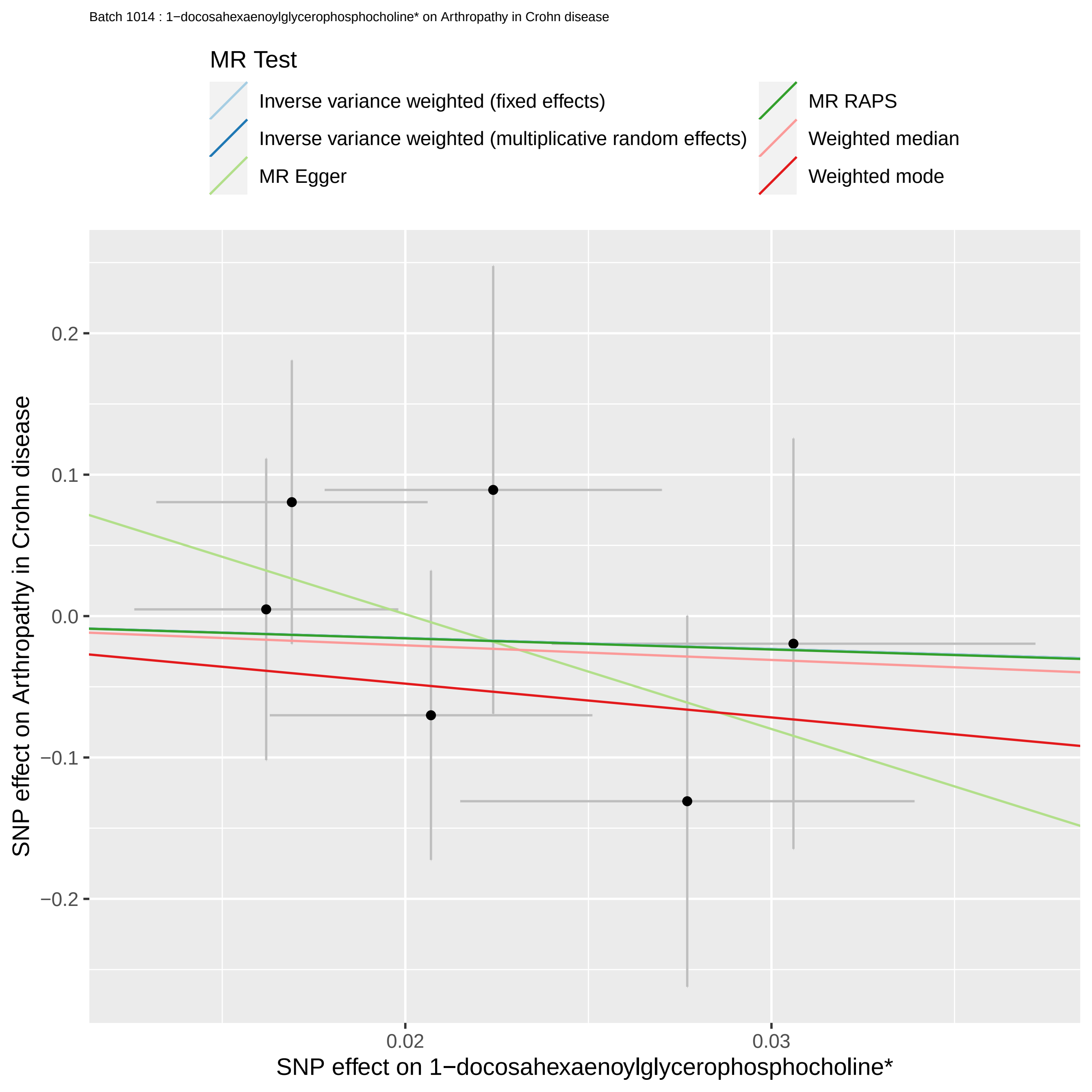
Batch 1014 : 1−docosahexaenoylglycerophosphocholine* on Arthropathy in Crohn disease
MR Test
Inverse variance weighted (fixed effects)
Inverse variance weighted (multiplicative random effects)
MR Egger
MR RAPS
Weighted median
Weighted mode
0.2
0.1
0.0
−0.1
−0.2
0.02
0.03
SNP effect on Arthropathy in Crohn disease
SNP effect on 1−docosahexaenoylglycerophosphocholine*

Batch 1018 : Octanoylcarnitine on Arthropathy in Crohn disease

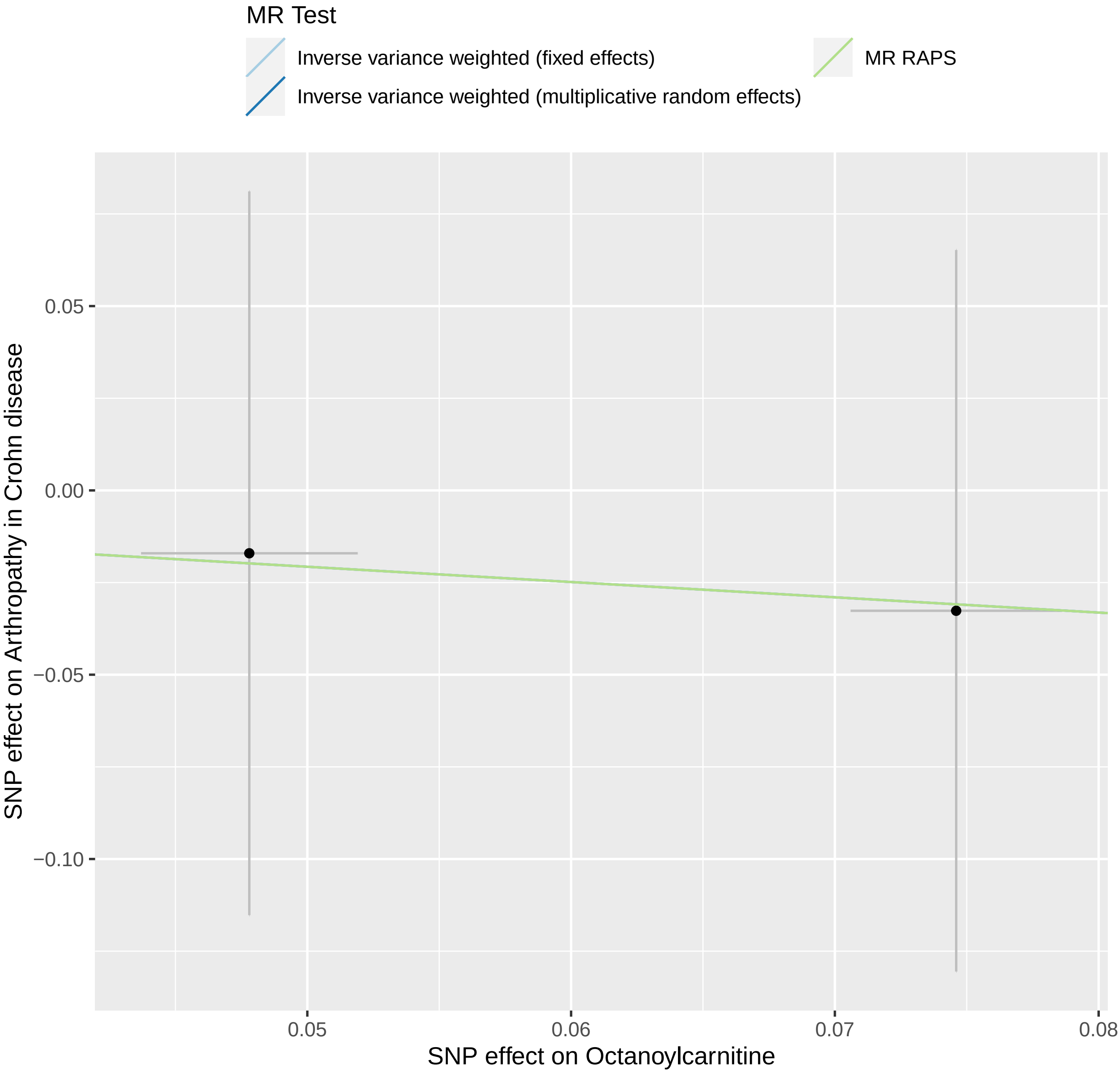

Batch 1019 : Alpha−hydroxyisovalerate on Arthropathy in Crohn disease

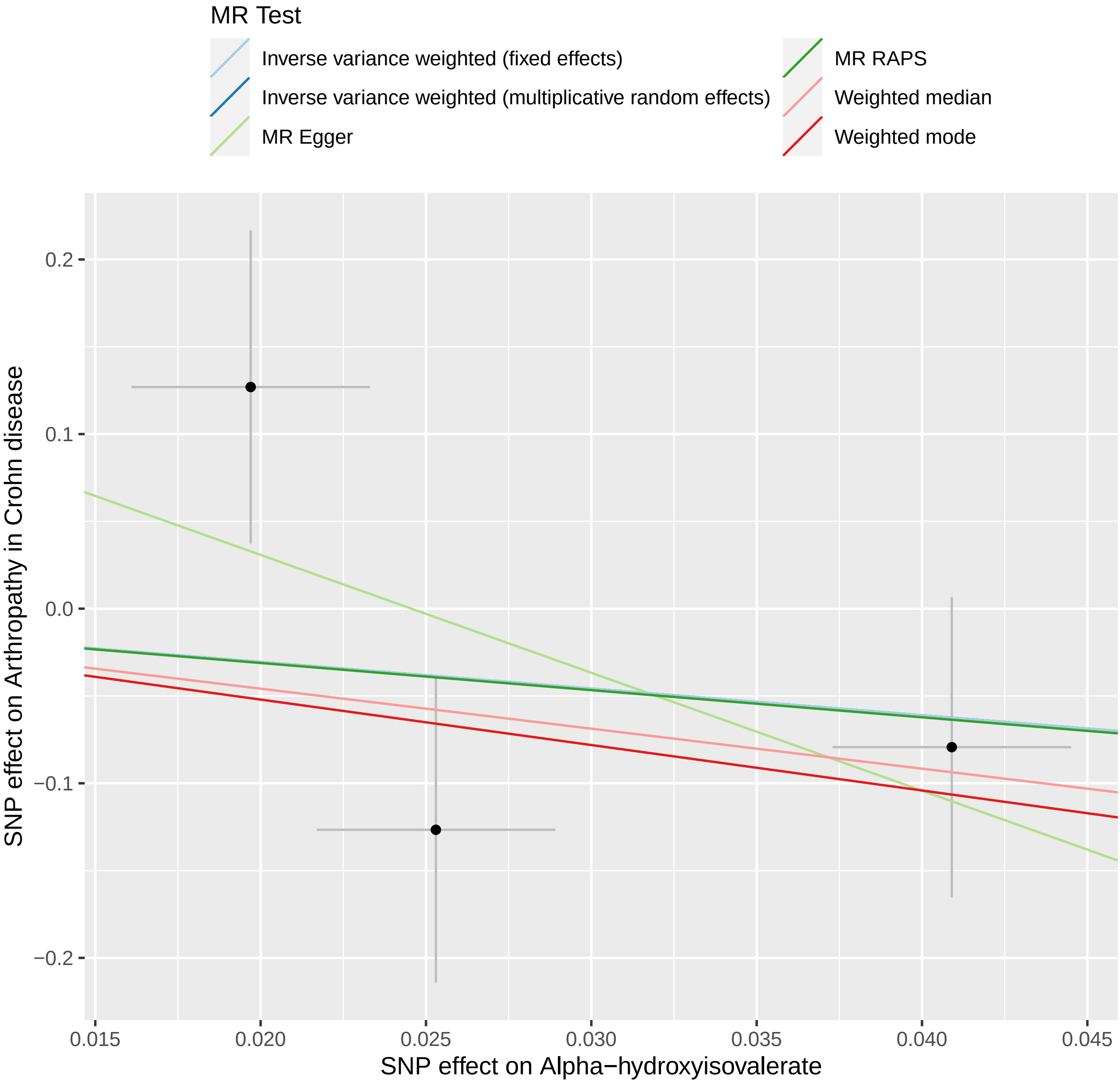

Batch 1020 : N−acetylthreonine on Arthropathy in Crohn disease

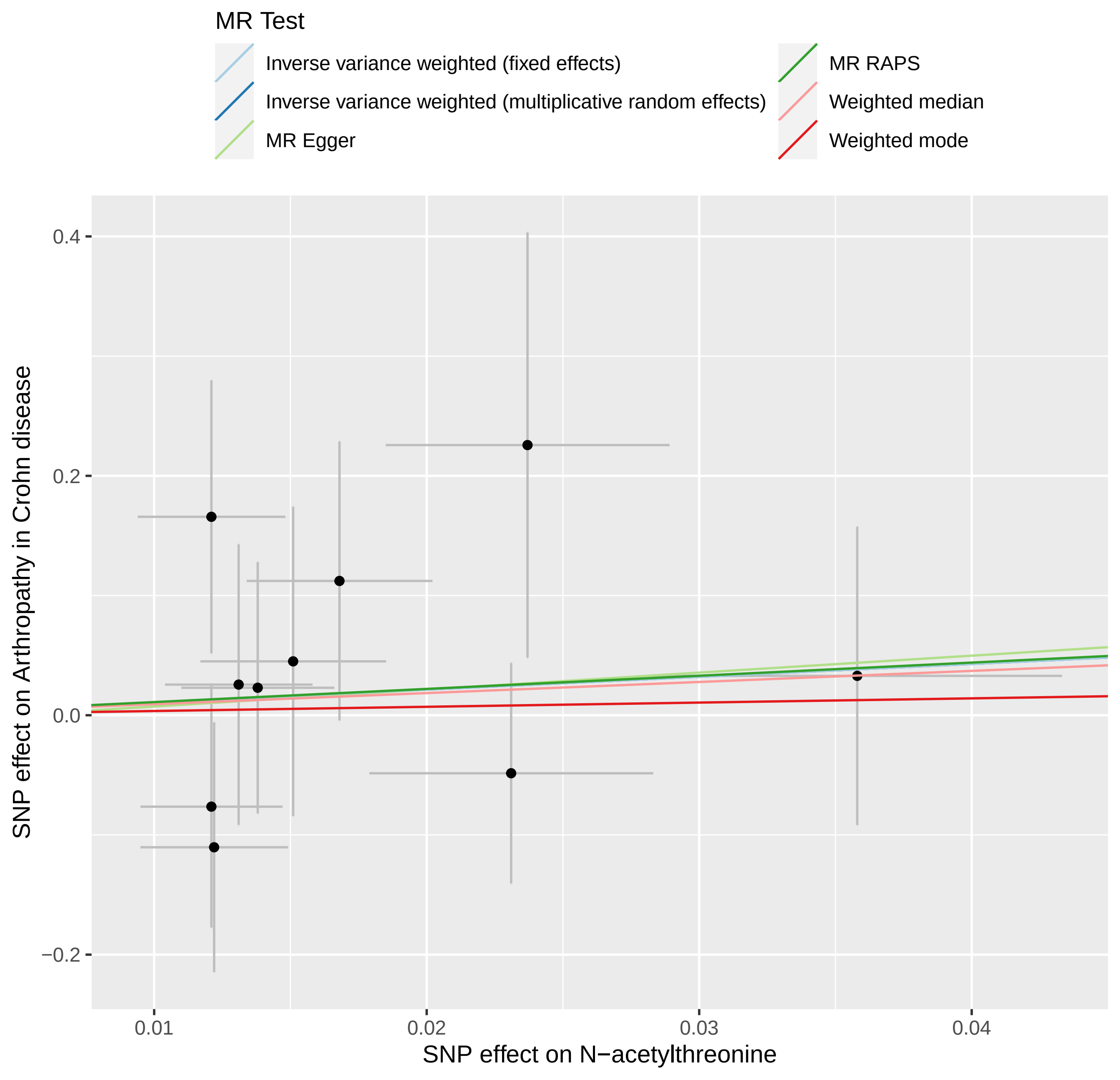

Batch 1021 : Decanoylcarnitine on Arthropathy in Crohn disease

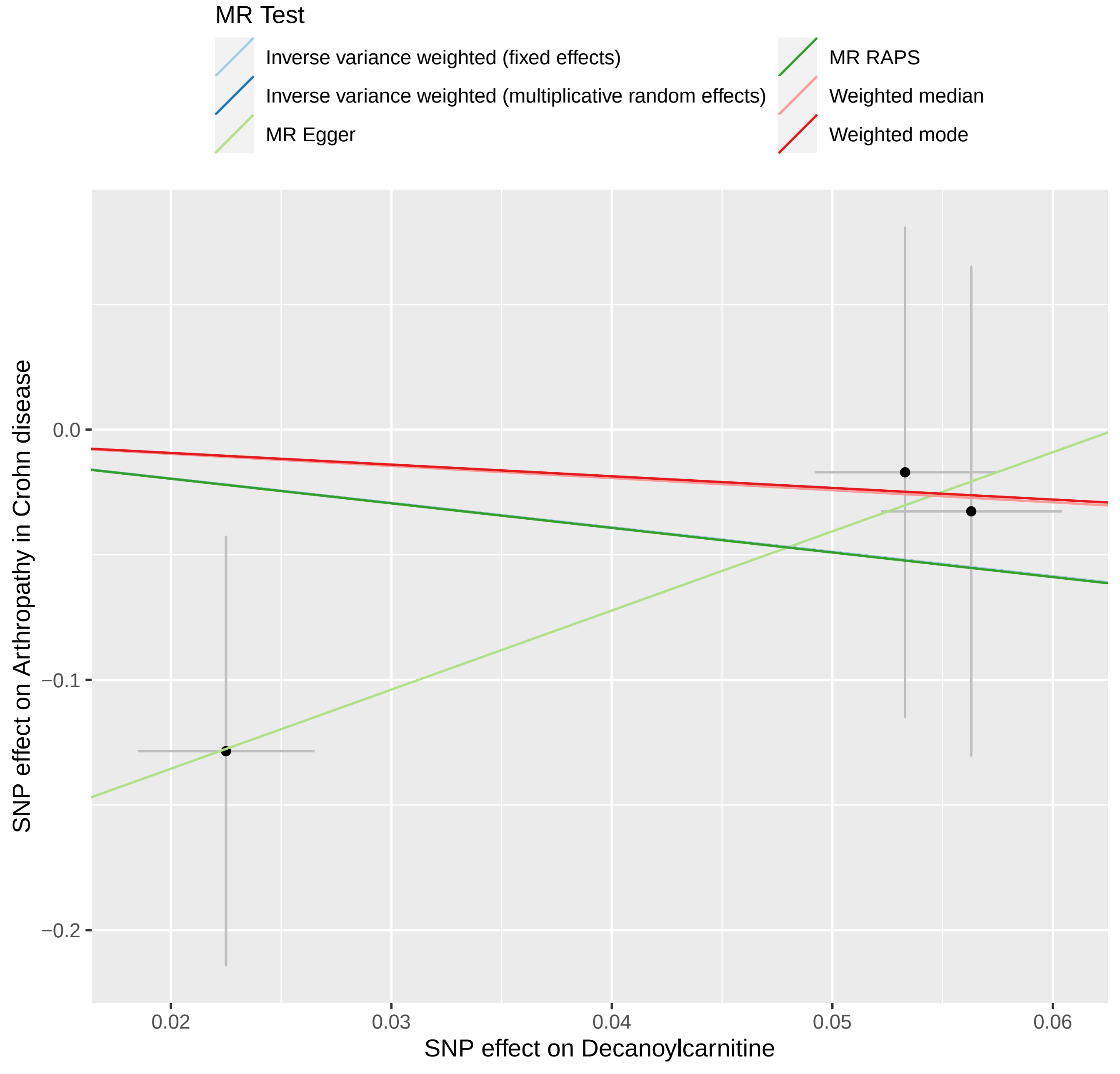

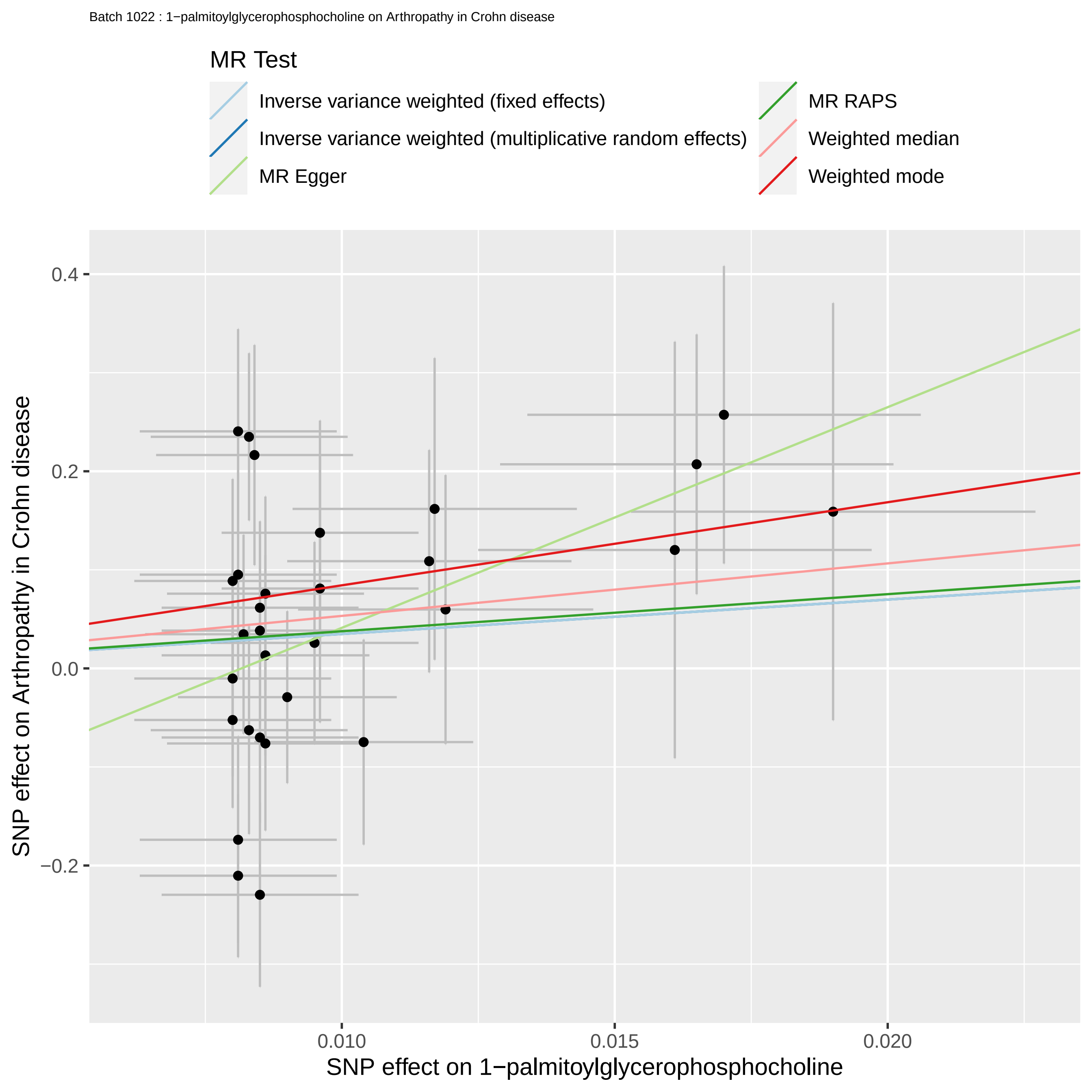
Batch 1022 : 1−palmitoylglycerophosphocholine on Arthropathy in Crohn disease
MR Test
Inverse variance weighted (fixed effects)
Inverse variance weighted (multiplicative random effects)
MR Egger
MR RAPS
Weighted median
Weighted mode
0.4
0.2
0.0
−0.2
0.010
0.015
0.020
SNP effect on Arthropathy in Crohn disease
SNP effect on 1−palmitoylglycerophosphocholine

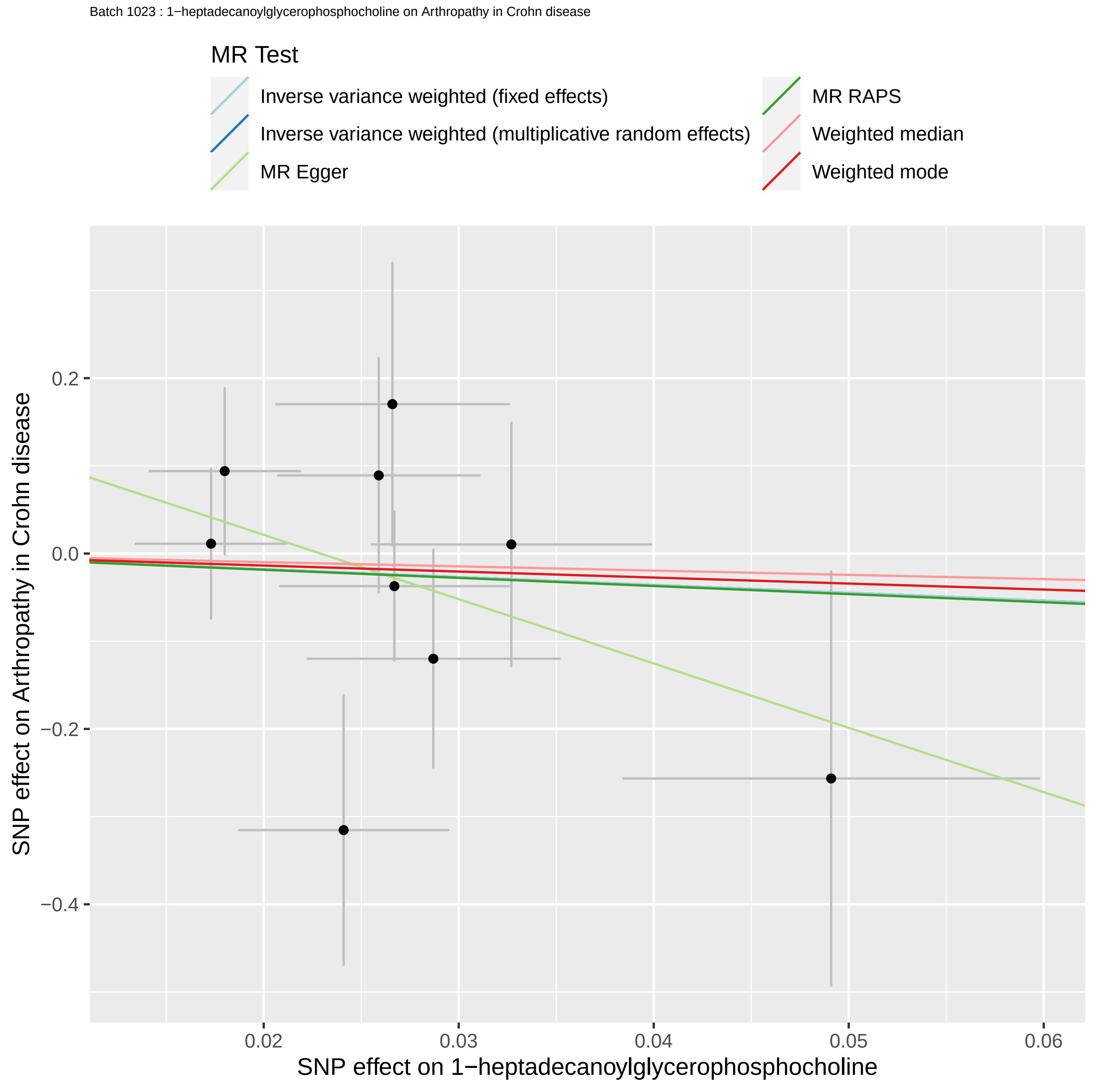
Batch 1023 : 1−heptadecanoylglycerophosphocholine on Arthropathy in Crohn disease
MR Test
Inverse variance weighted (fixed effects)
Inverse variance weighted (multiplicative random effects)
MR Egger
MR RAPS
Weighted median
Weighted mode
SNP effect on Arthropathy in Crohn disease
0.2
0.0
−0.2
−0.4
0.02
0.03
0.04
0.05
0.06
SNP effect on 1−heptadecanoylglycerophosphocholine

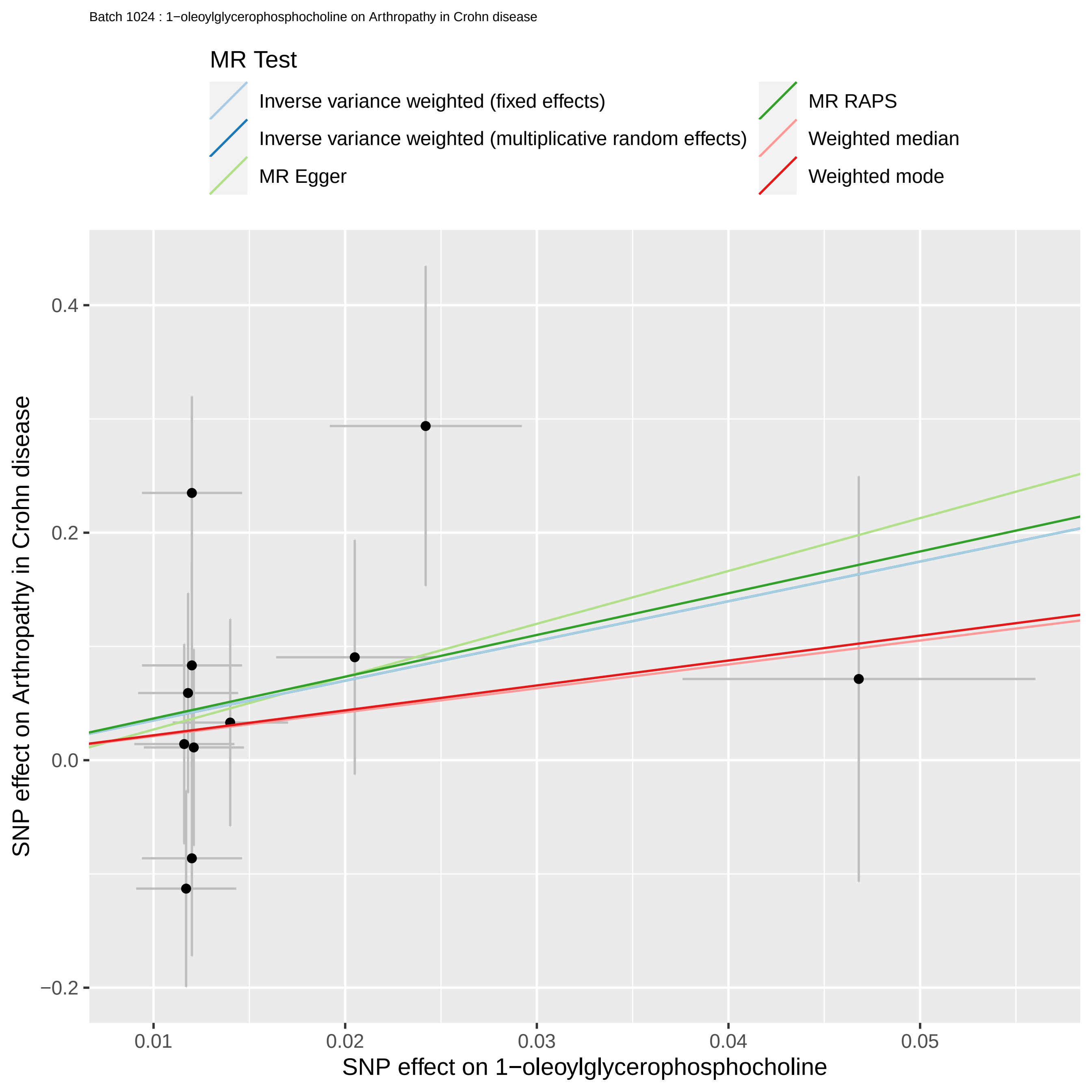
Batch 1024 : 1−oleoylglycerophosphocholine on Arthropathy in Crohn disease
MR Test
Inverse variance weighted (fixed effects)
Inverse variance weighted (multiplicative random effects)
MR Egger
MR RAPS
Weighted median
Weighted mode
SNP effect on Arthropathy in Crohn disease
0.4
0.2
0.0
−0.2
0.01
0.02
0.03
0.04
0.05
SNP effect on 1−oleoylglycerophosphocholine

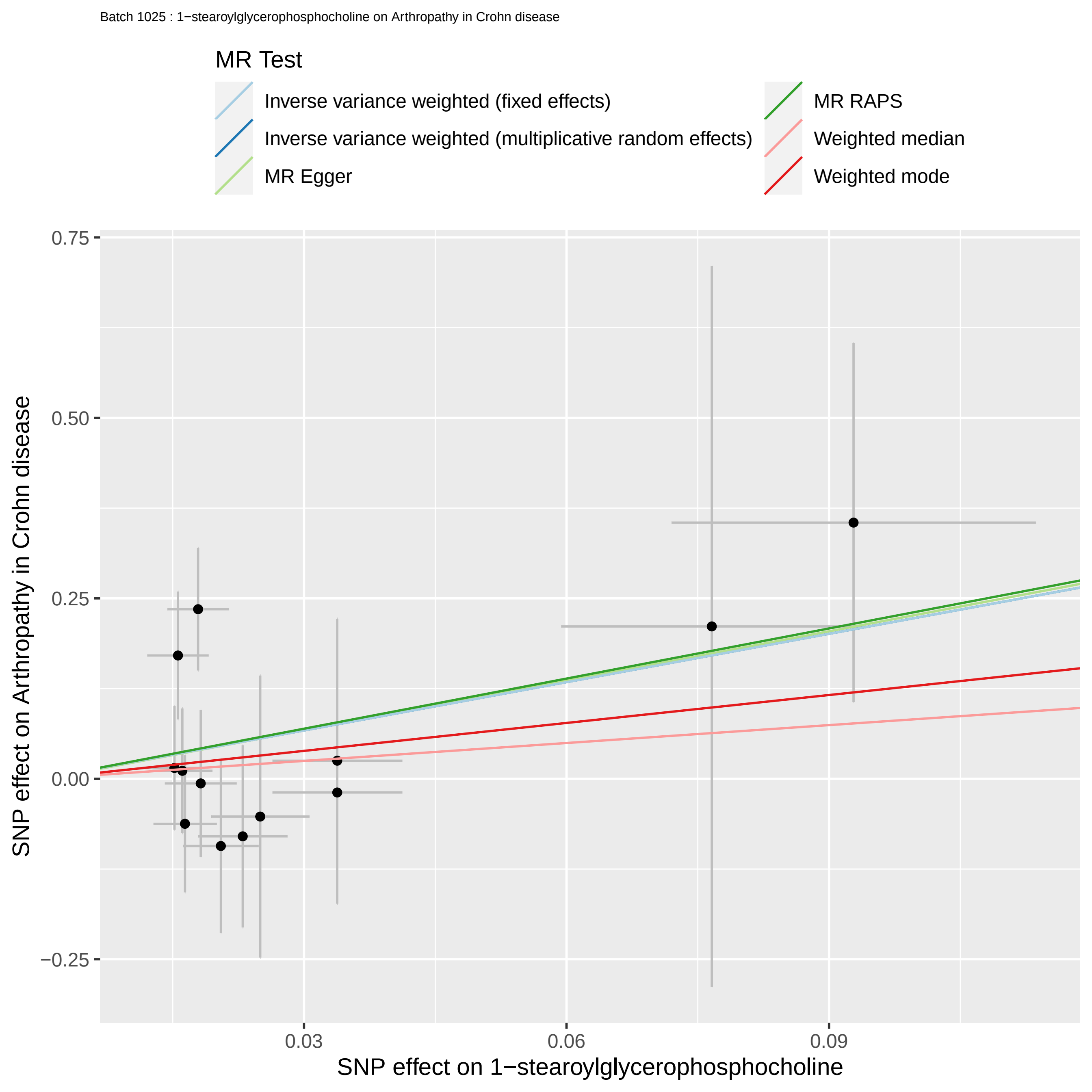

Batch 1025 : 1−stearoylglycerophosphocholine on Arthropathy in Crohn disease
MR Test
Inverse variance weighted (fixed effects)
Inverse variance weighted (multiplicative random effects)
MR Egger
MR RAPS
Weighted median
Weighted mode
0.75
0.50
0.25
0.00
−0.25
0.03
0.06
0.09
SNP effect on Arthropathy in Crohn disease
SNP effect on 1−stearoylglycerophosphocholine

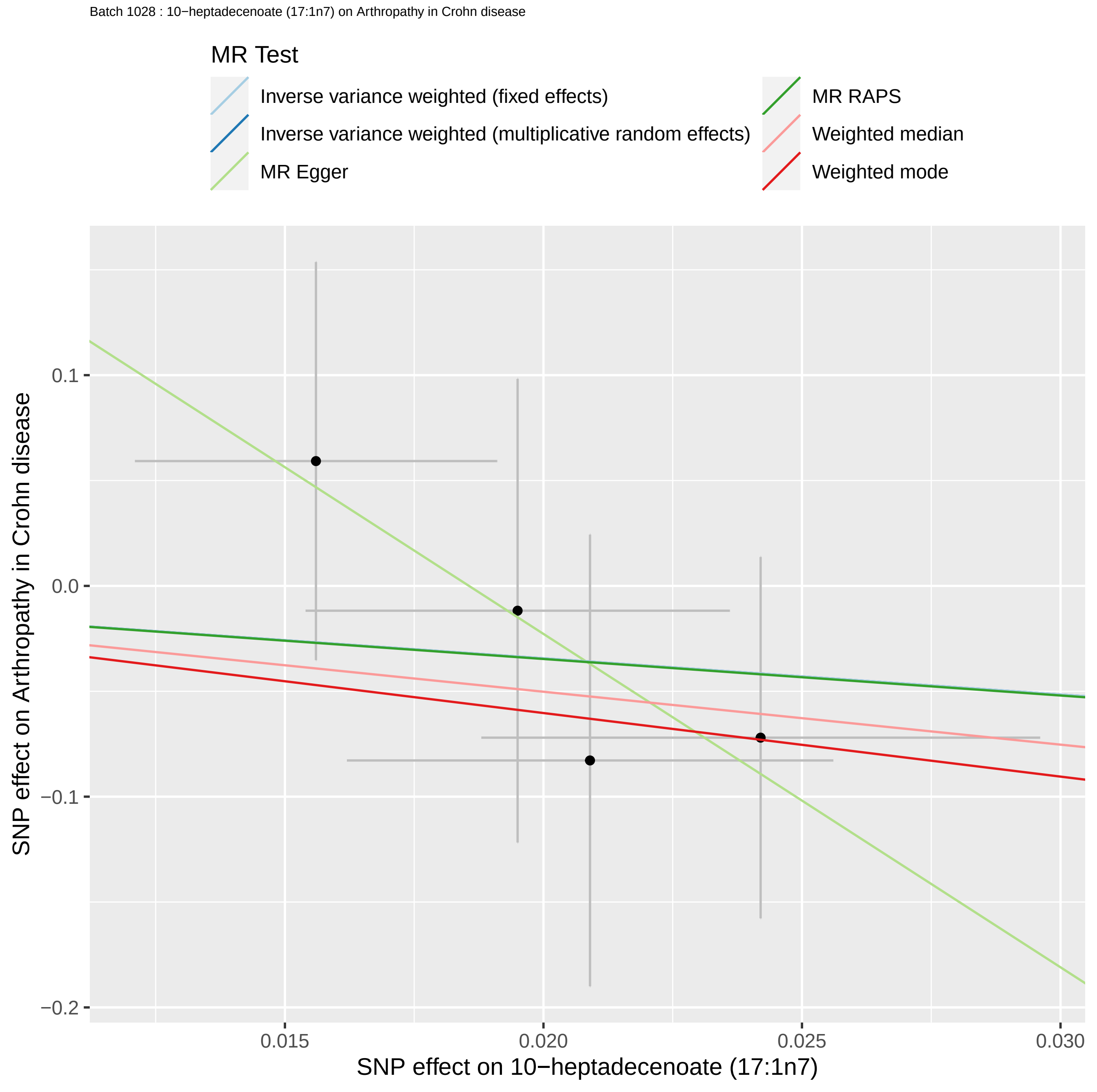
Batch 1028 : 10−heptadecenoate (17:1n7) on Arthropathy in Crohn disease
MR Test
Inverse variance weighted (fixed effects)
Inverse variance weighted (multiplicative random effects)
MR Egger
MR RAPS
Weighted median
Weighted mode
0.1
0.0
−0.1
−0.2
0.015
0.020
0.025
0.030
SNP effect on Arthropathy in Crohn disease
SNP effect on 10−heptadecenoate (17:1n7)

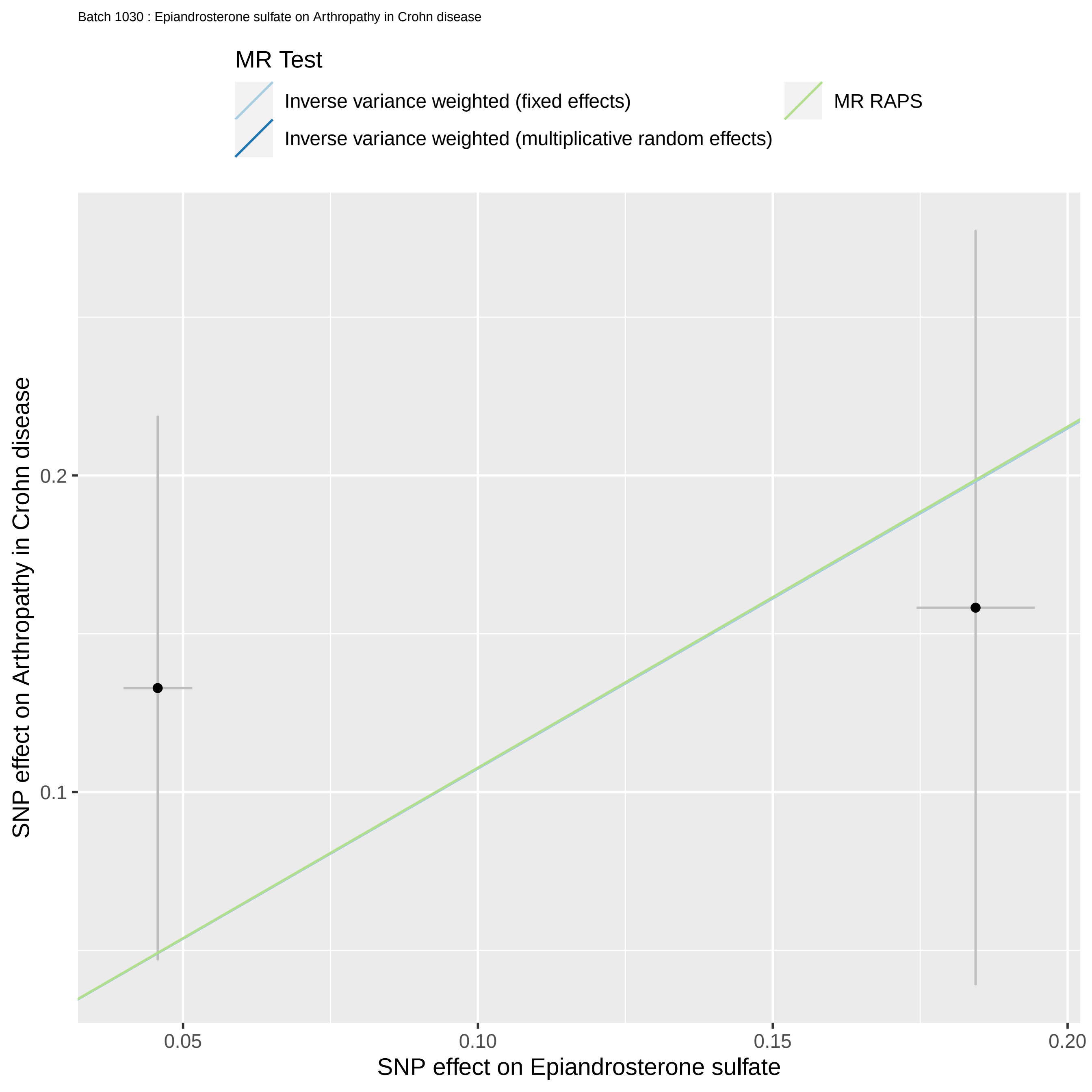
Batch 1030 : Epiandrosterone sulfate on Arthropathy in Crohn disease
MR Test
Inverse variance weighted (fixed effects)
Inverse variance weighted (multiplicative random effects)
MR RAPS
SNP effect on Arthropathy in Crohn disease
0.2
0.1
0.05
0.10
0.15
0.20
SNP effect on Epiandrosterone sulfate

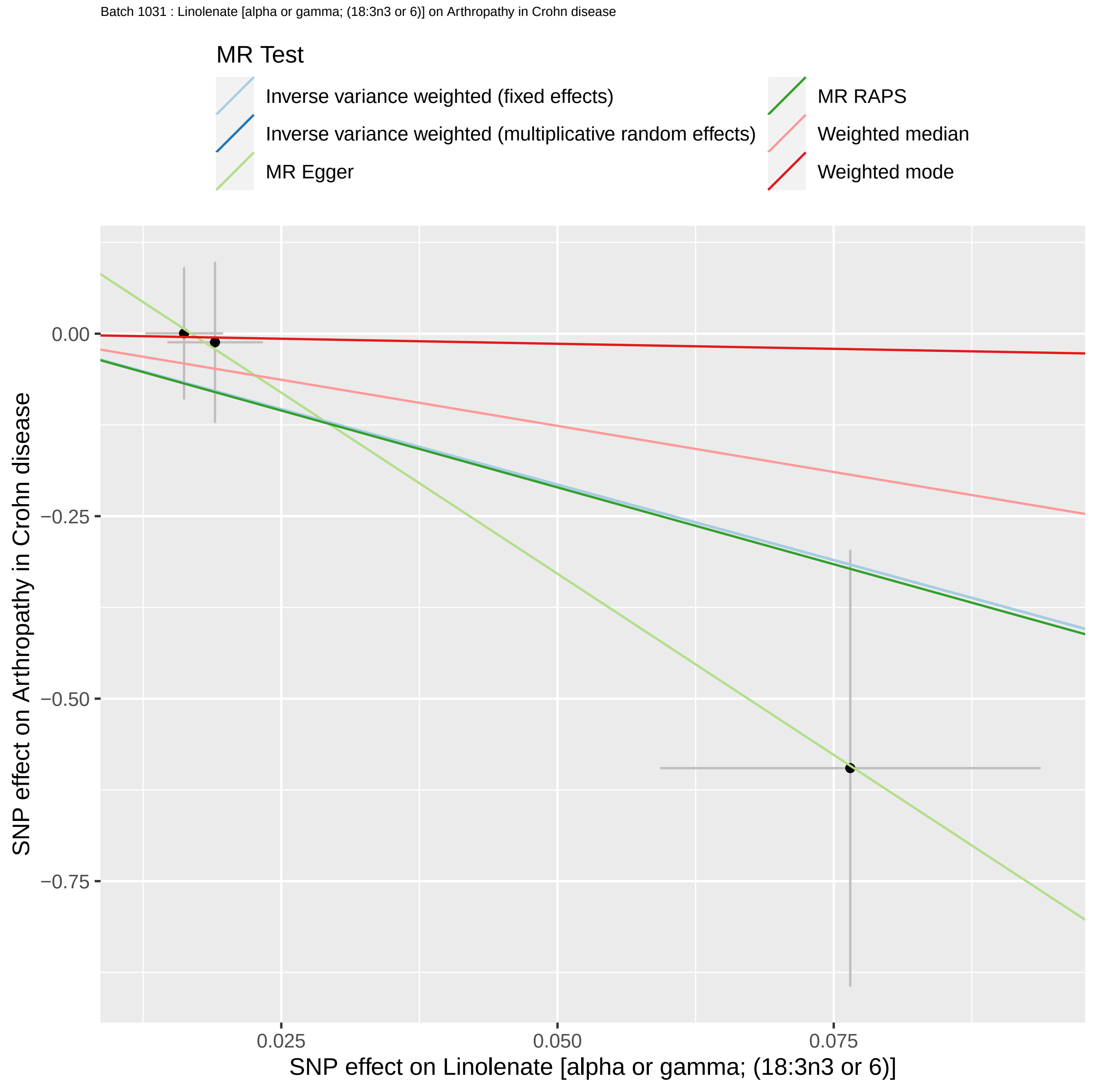
Batch 1031 : Linolenate [alpha or gamma; (18:3n3 or 6)] on Arthropathy in Crohn disease
MR Test
Inverse variance weighted (fixed effects)
Inverse variance weighted (multiplicative random effects)
MR Egger
MR RAPS
Weighted median
Weighted mode
0.00
−0.25
−0.50
−0.75
0.025
0.050
0.075
SNP effect on Arthropathy in Crohn disease
SNP effect on Linolenate [alpha or gamma; (18:3n3 or 6)]

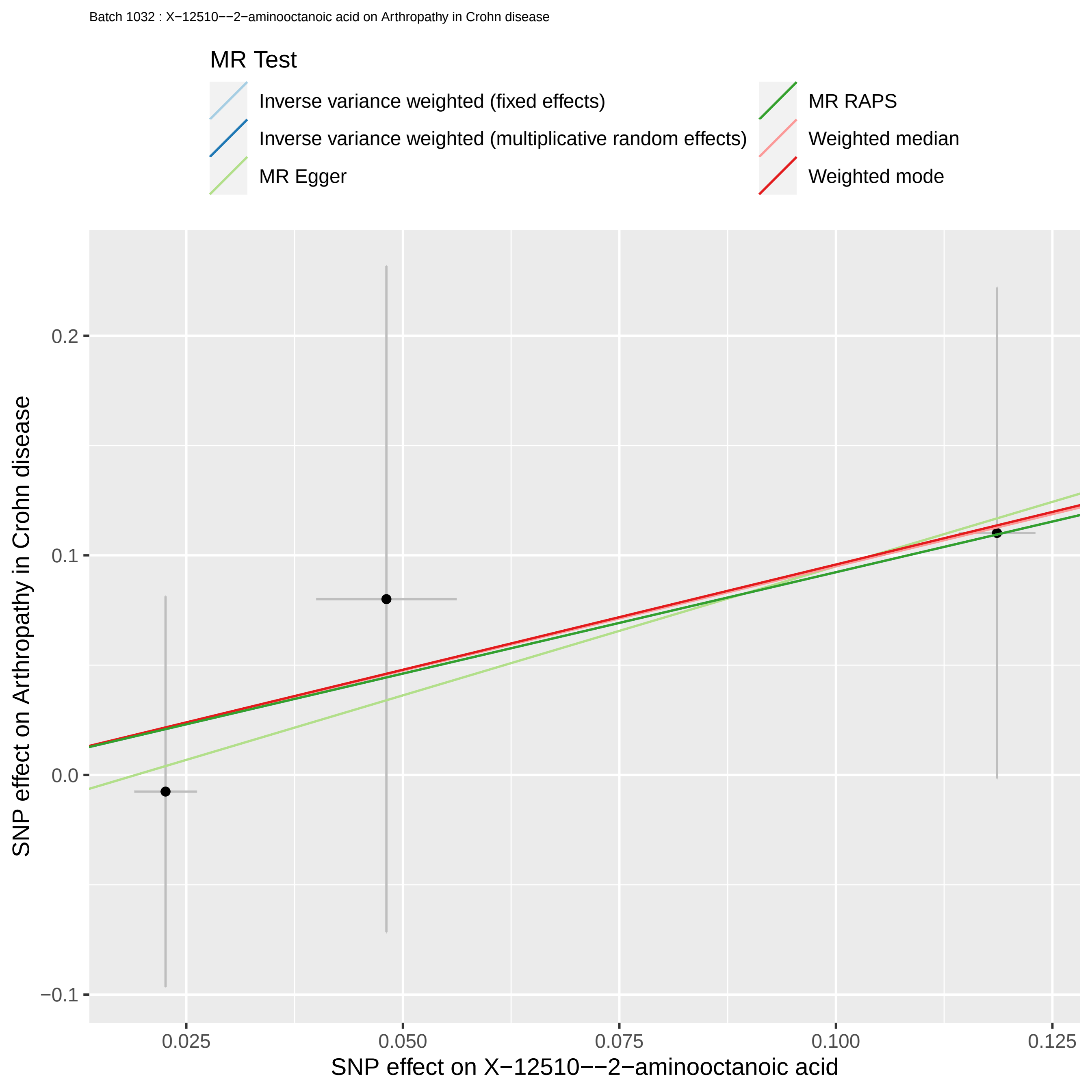
Batch 1032 : X−12510−−2−aminooctanoic acid on Arthropathy in Crohn disease
MR Test
Inverse variance weighted (fixed effects)
Inverse variance weighted (multiplicative random effects)
MR Egger
MR RAPS
Weighted median
Weighted mode
SNP effect on Arthropathy in Crohn disease
SNP effect on X−12510−−2−aminooctanoic acid
0.2
0.1
0.0
−0.1
0.025
0.050
0.075
0.100
0.125

Batch 1035 : Asparagine on Arthropathy in Crohn disease

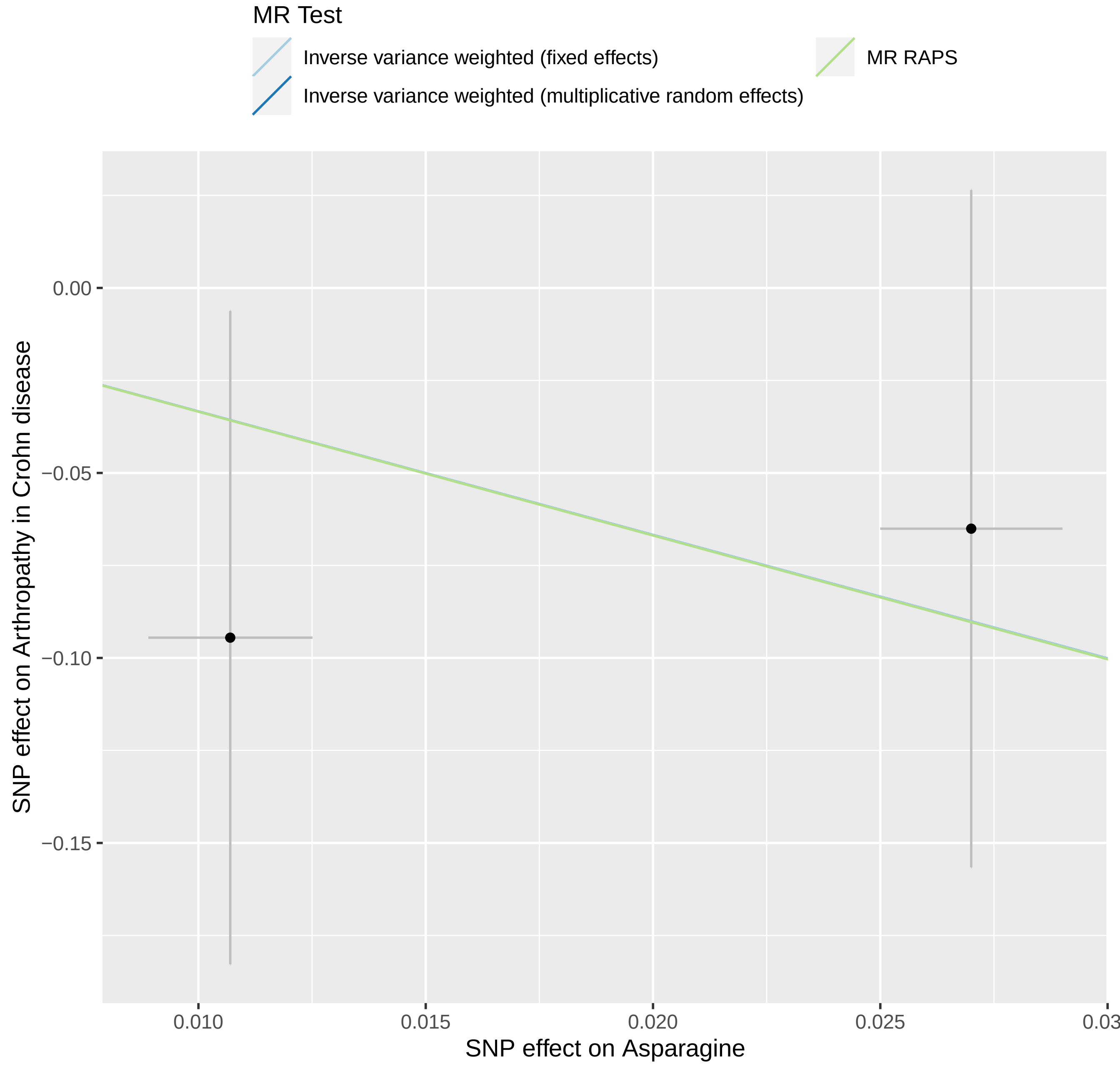

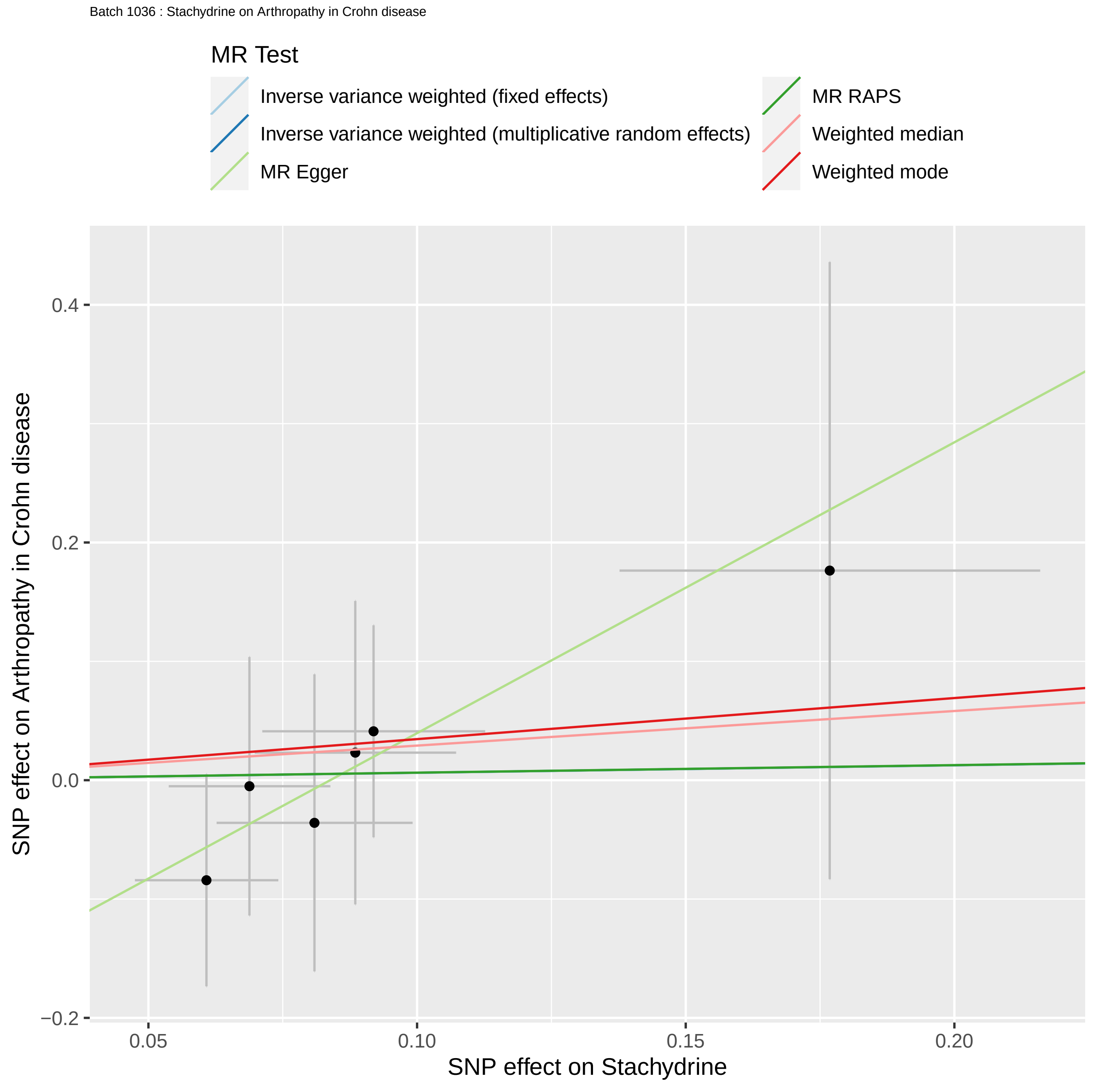

Batch 1036 : Stachydrine on Arthropathy in Crohn disease
MR Test
Inverse variance weighted (fixed effects)
Inverse variance weighted (multiplicative random effects)
MR Egger
MR RAPS
Weighted median
Weighted mode
0.4
0.2
0.0
−0.2
0.05
0.10
0.15
0.20
SNP effect on Arthropathy in Crohn disease
SNP effect on Stachydrine

Batch 1037 : Isovalerylcarnitine on Arthropathy in Crohn disease

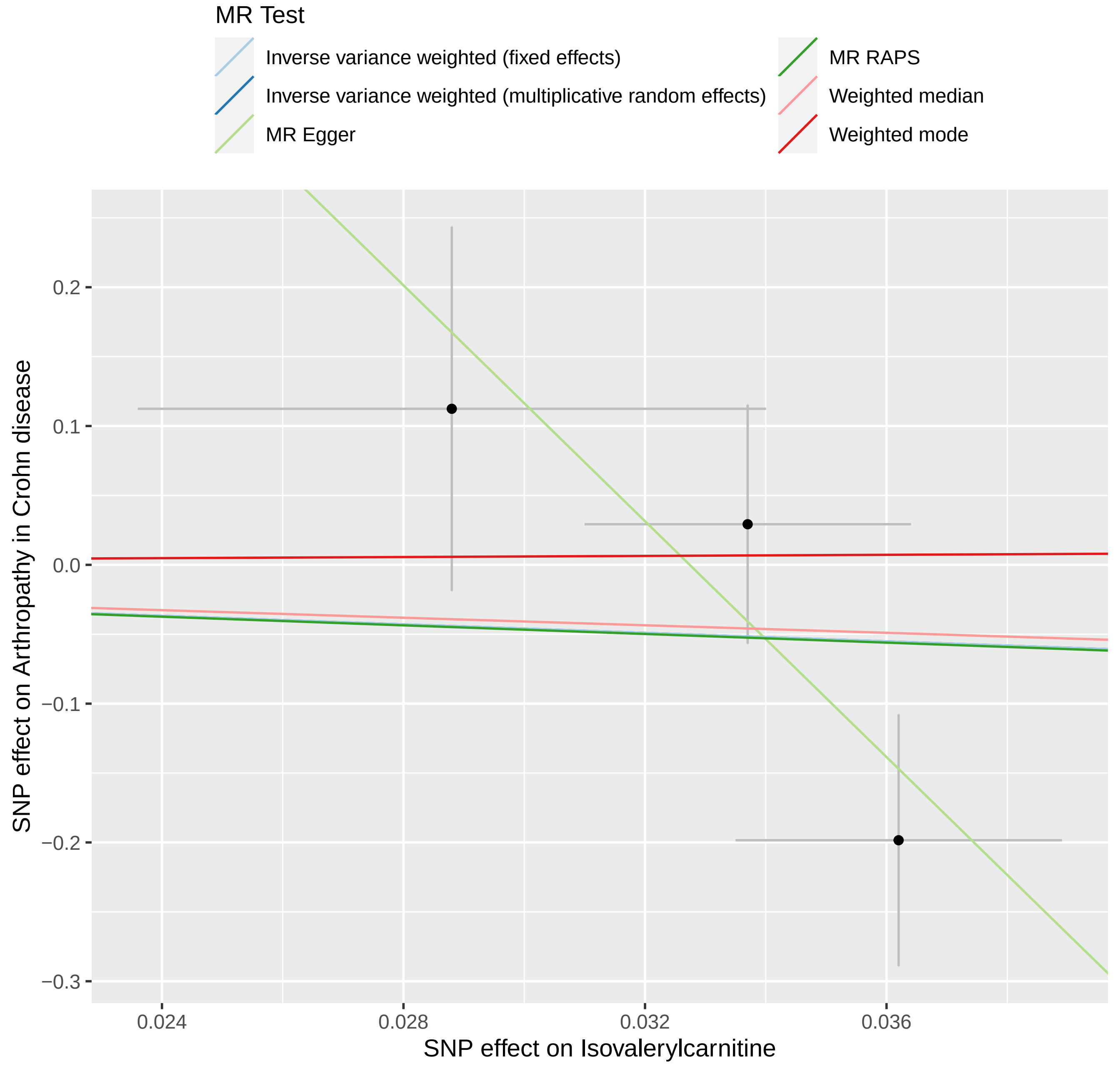

Batch 1040 : 1−linoleoylglycerophosphocholine on Arthropathy in Crohn disease

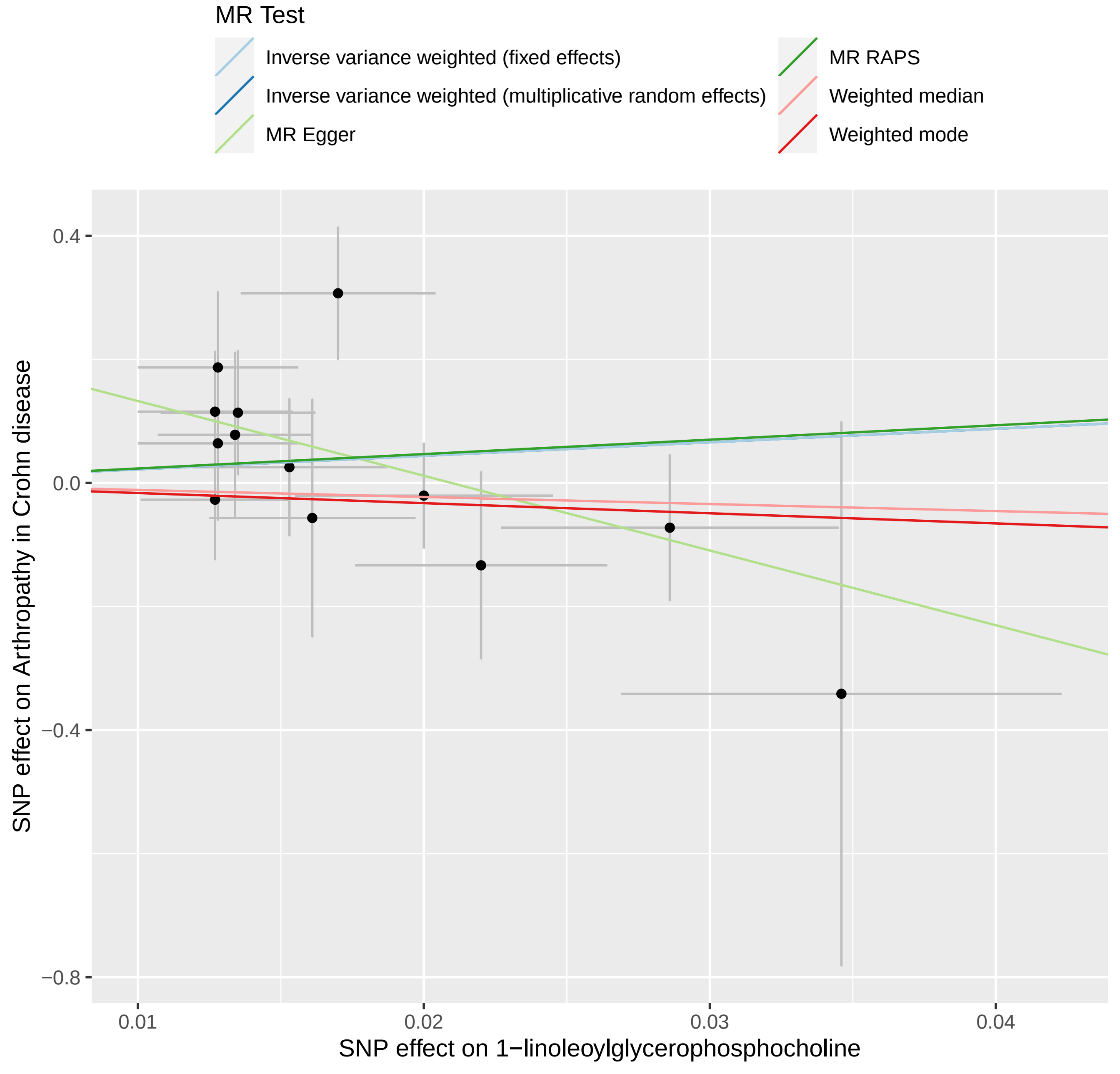

Batch 1041 : Bradykinin, des−arg(9) on Arthropathy in Crohn disease

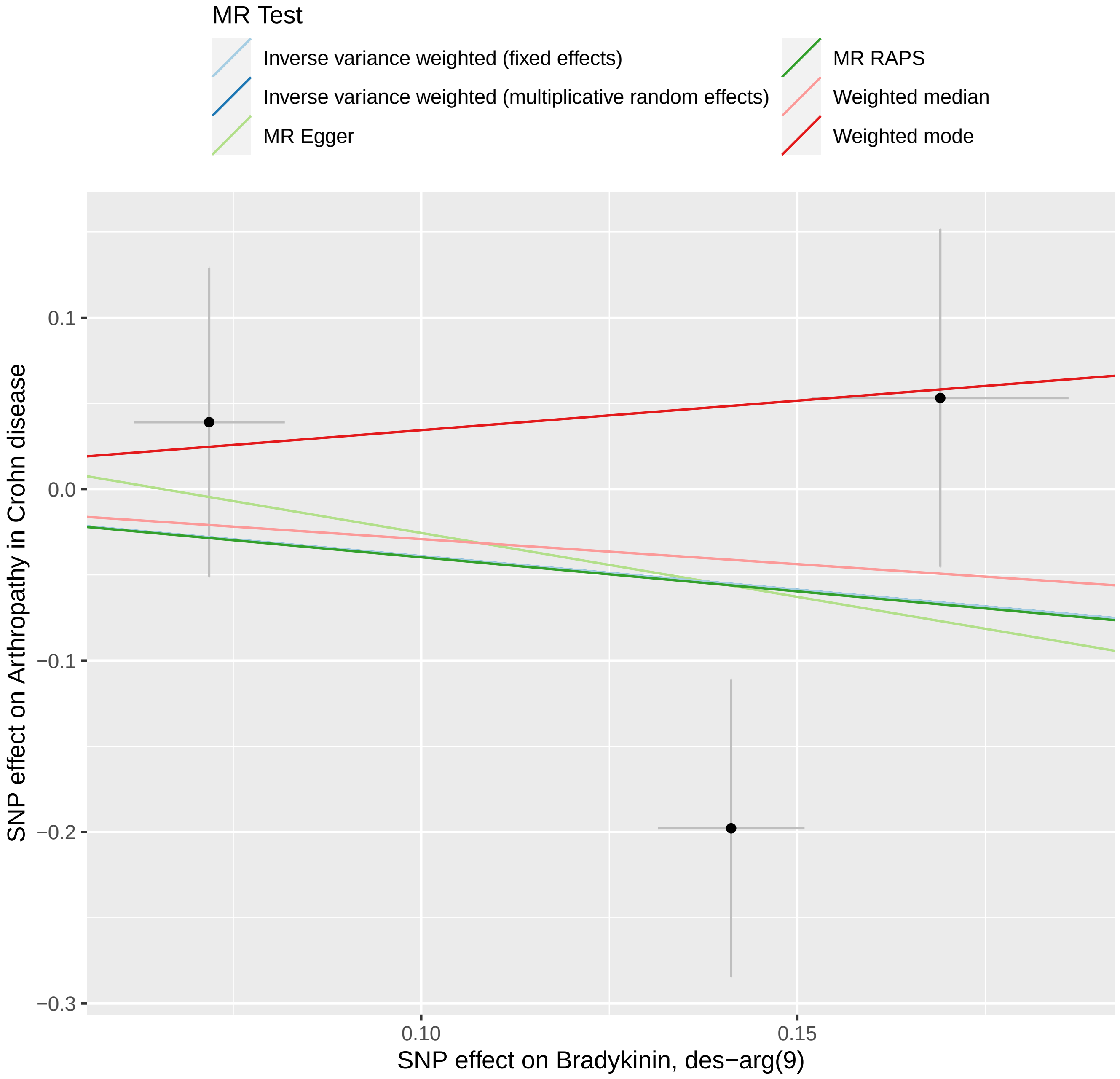

Batch 1042 : Gamma−glutamylisoleucine* on Arthropathy in Crohn disease

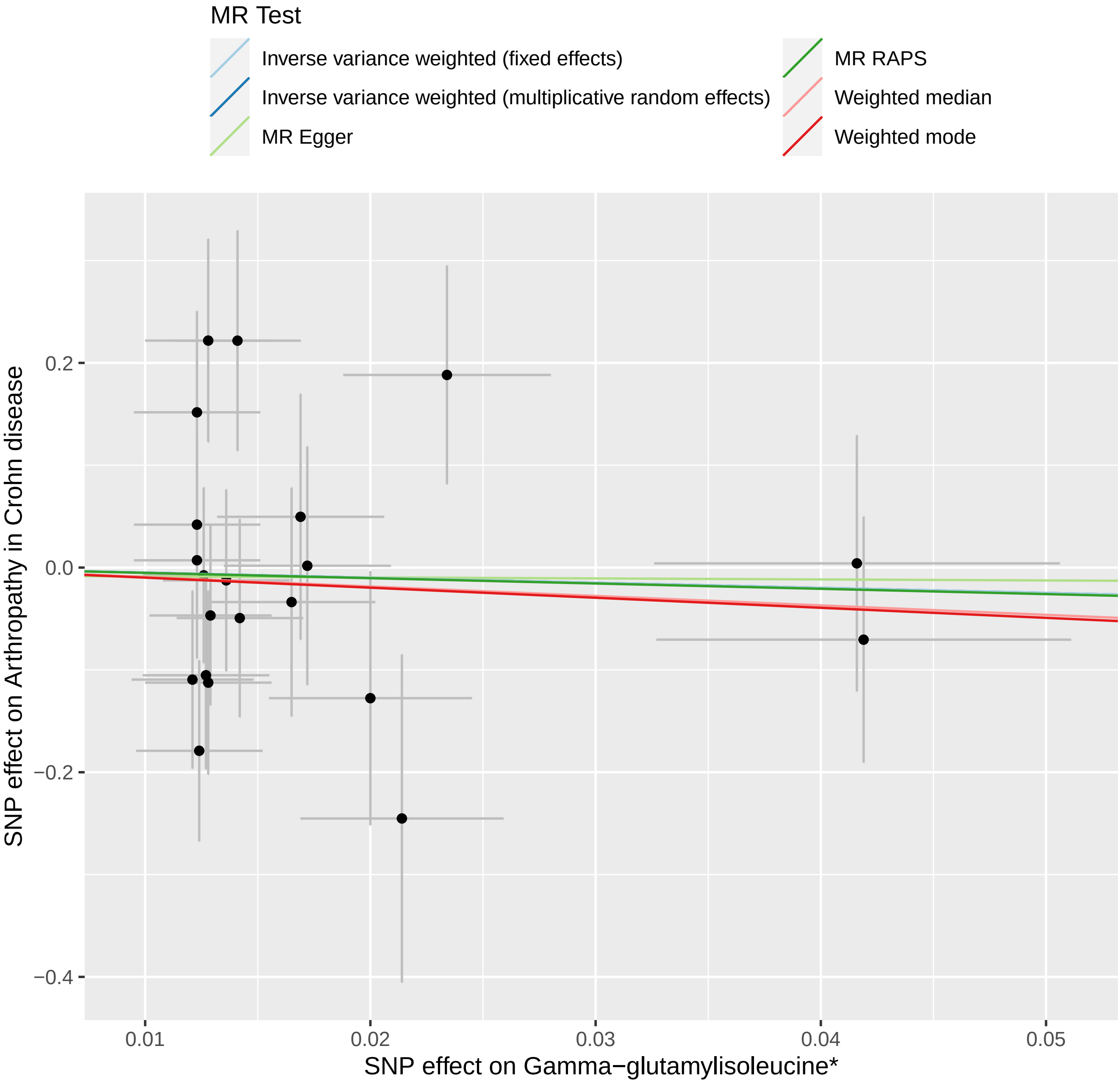

Batch 1045 : Isovalerate on Arthropathy in Crohn disease

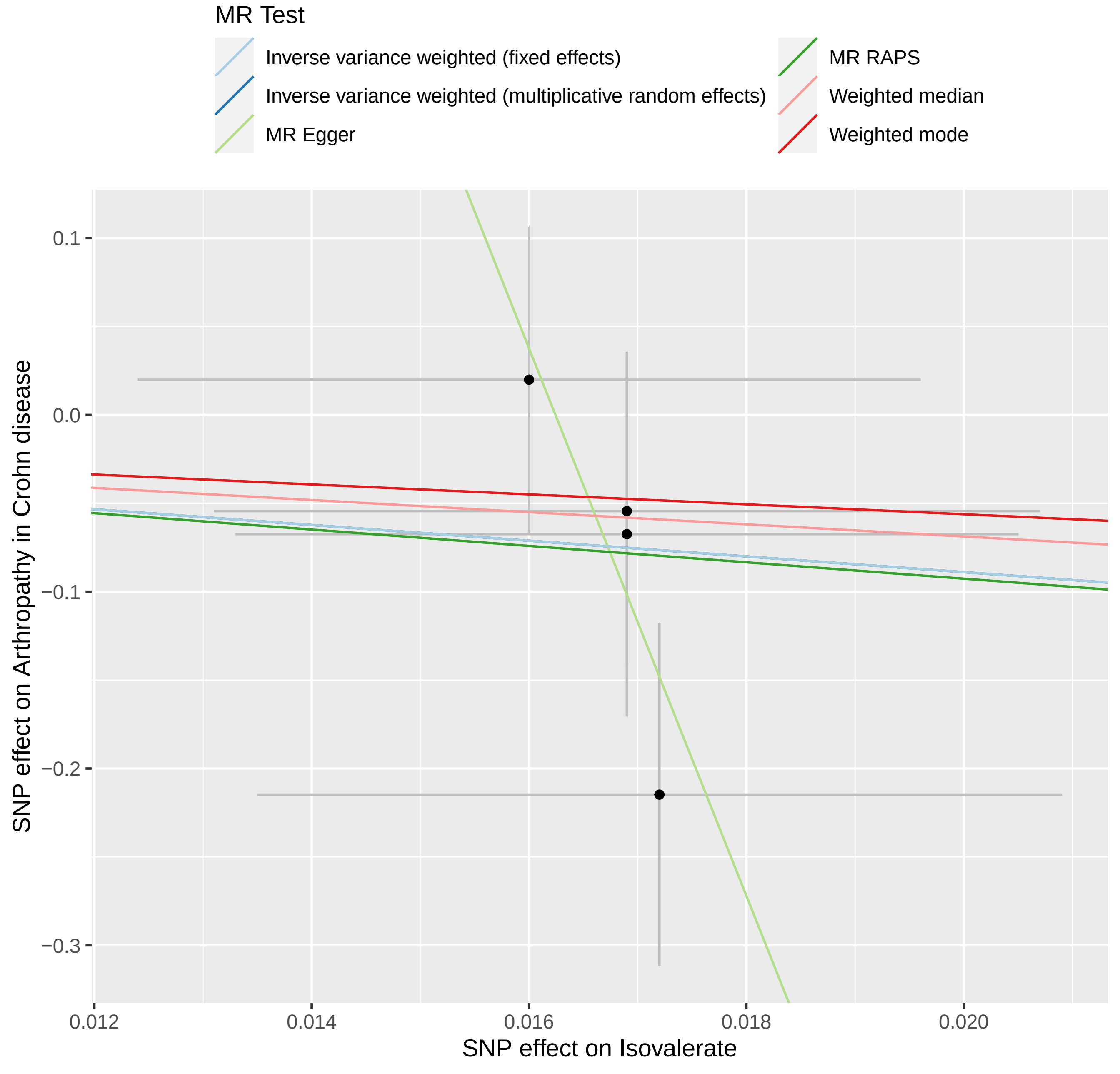

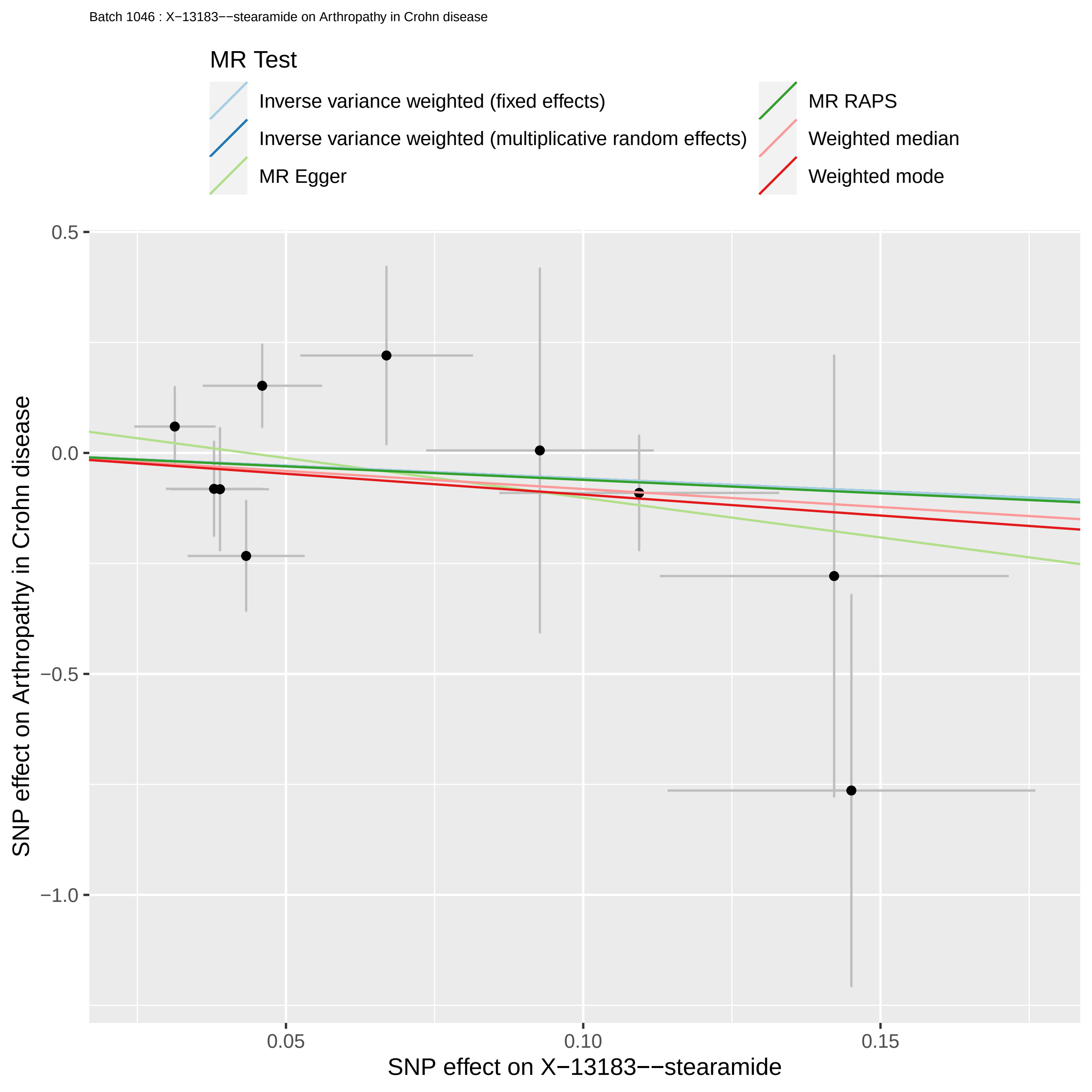

Batch 1046 : X−13183−−stearamide on Arthropathy in Crohn disease
MR Test
Inverse variance weighted (fixed effects)
Inverse variance weighted (multiplicative random effects)
MR Egger
MR RAPS
Weighted median
Weighted mode
0.5
0.0
−0.5
−1.0
0.05
0.10
0.15
SNP effect on Arthropathy in Crohn disease
SNP effect on X−13183−−stearamide

Batch 1047 : 7−methylguanine on Arthropathy in Crohn disease

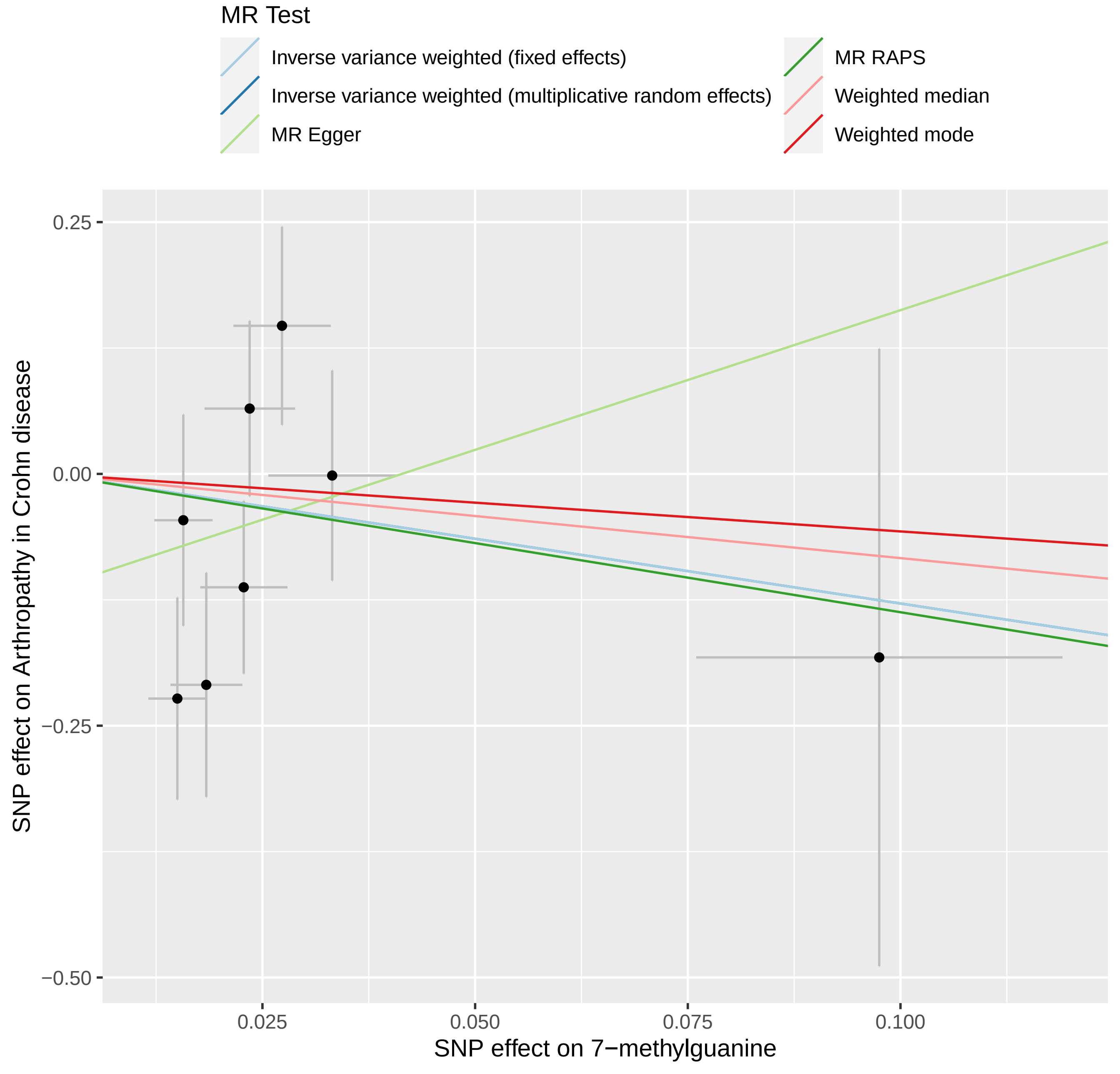

Batch 1054 : X−13431−−nonanoylcarnitine* on Arthropathy in Crohn disease

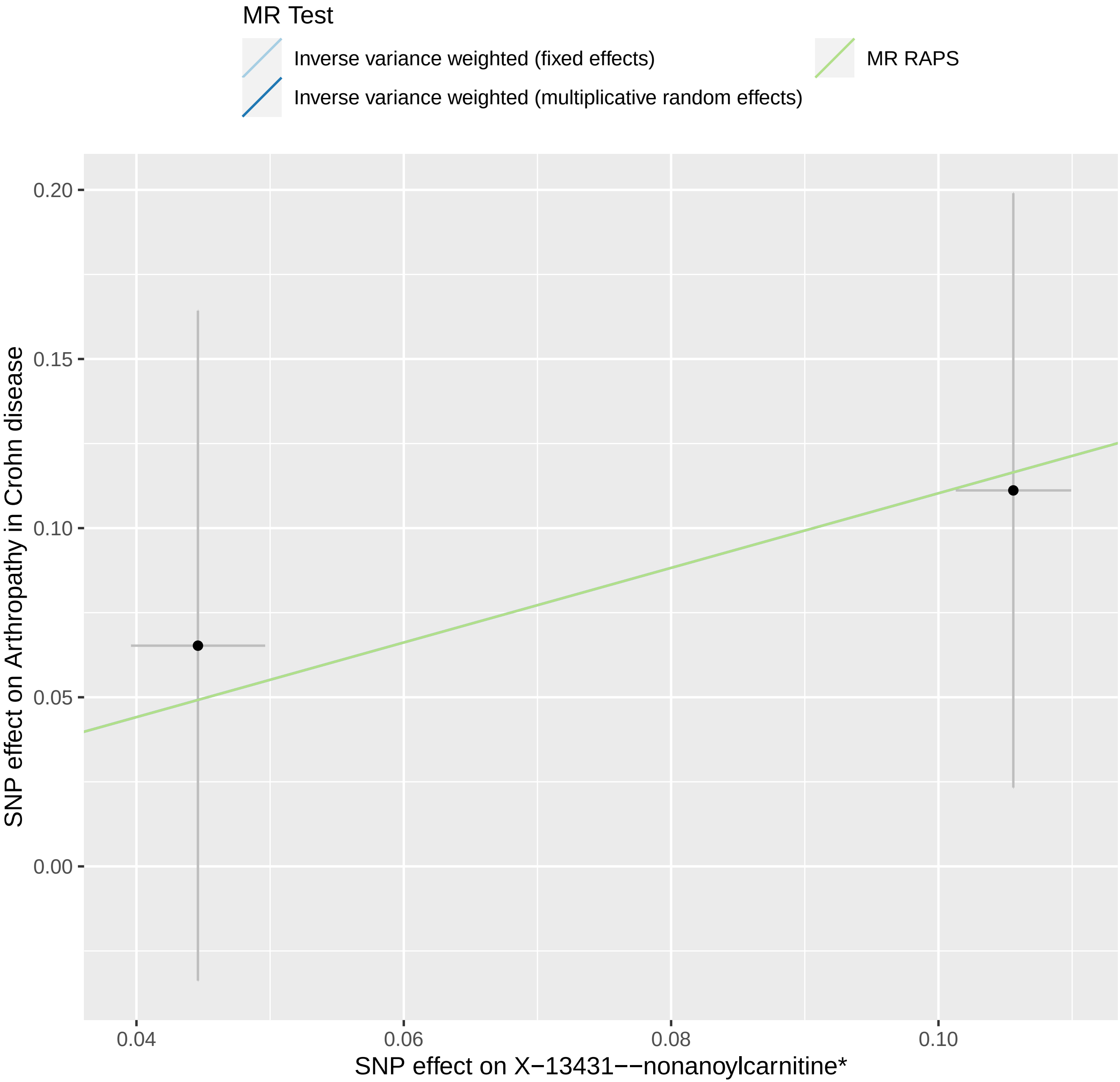

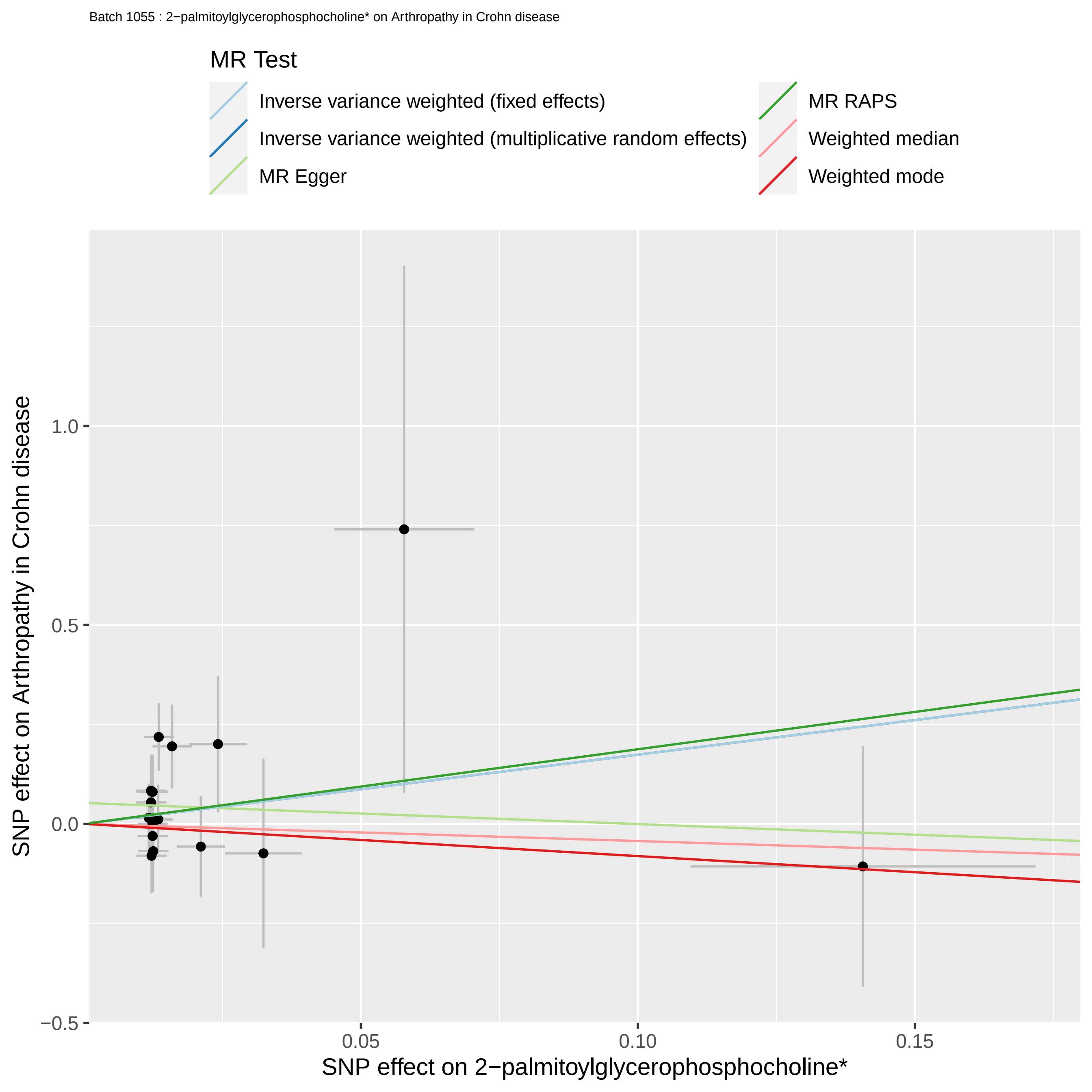
Batch 1055 : 2−palmitoylglycerophosphocholine* on Arthropathy in Crohn disease
MR Test
Inverse variance weighted (fixed effects)
Inverse variance weighted (multiplicative random effects)
MR Egger
MR RAPS
Weighted median
Weighted mode
SNP effect on Arthropathy in Crohn disease
1.0
0.5
0.0
−0.5
0.05
0.10
0.15
SNP effect on 2−palmitoylglycerophosphocholine*

Batch 1056 : 2−oleoylglycerophosphocholine* on Arthropathy in Crohn disease

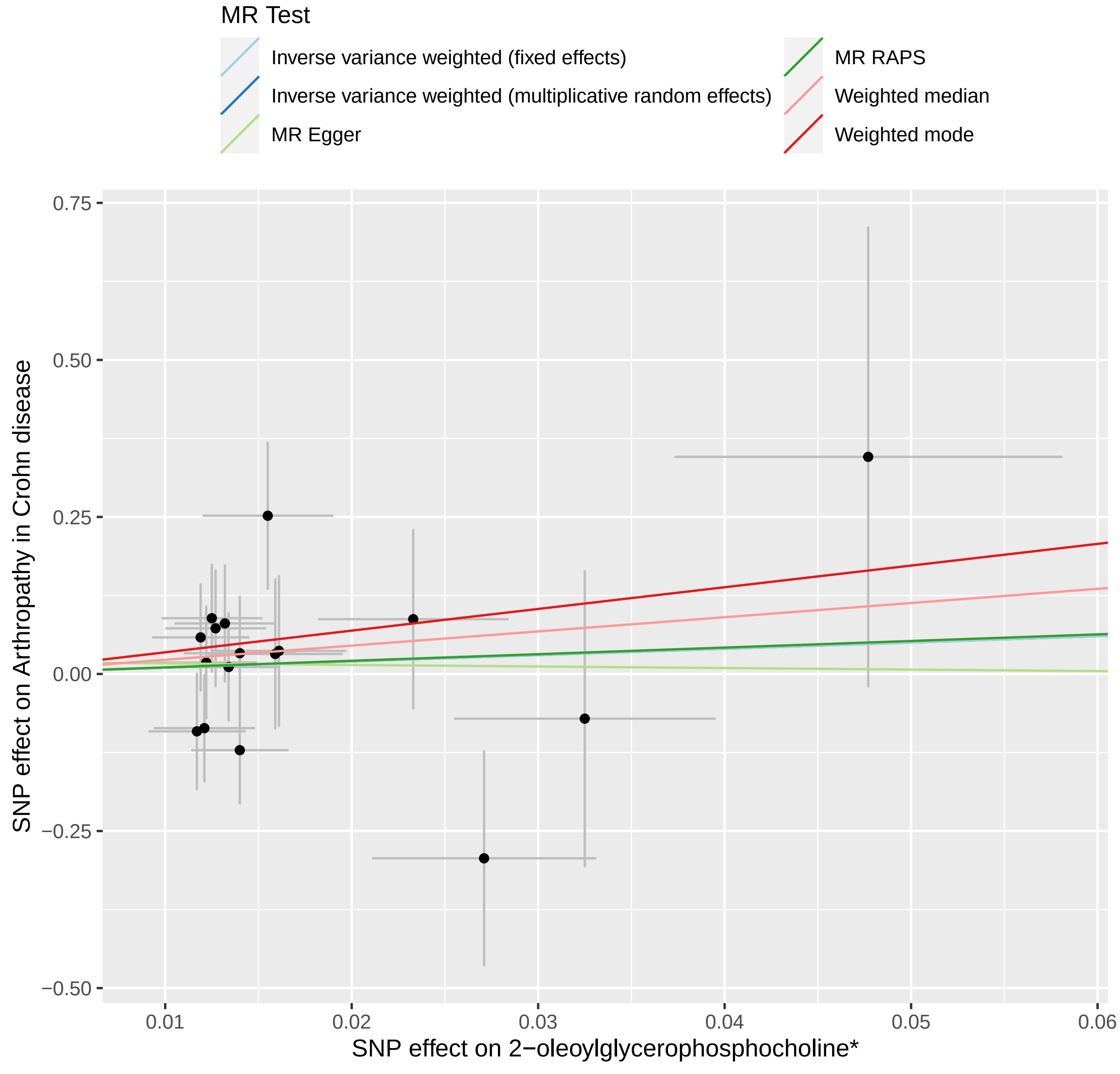

Batch 1059 : 1−palmitoylglycerophosphoinositol* on Arthropathy in Crohn disease

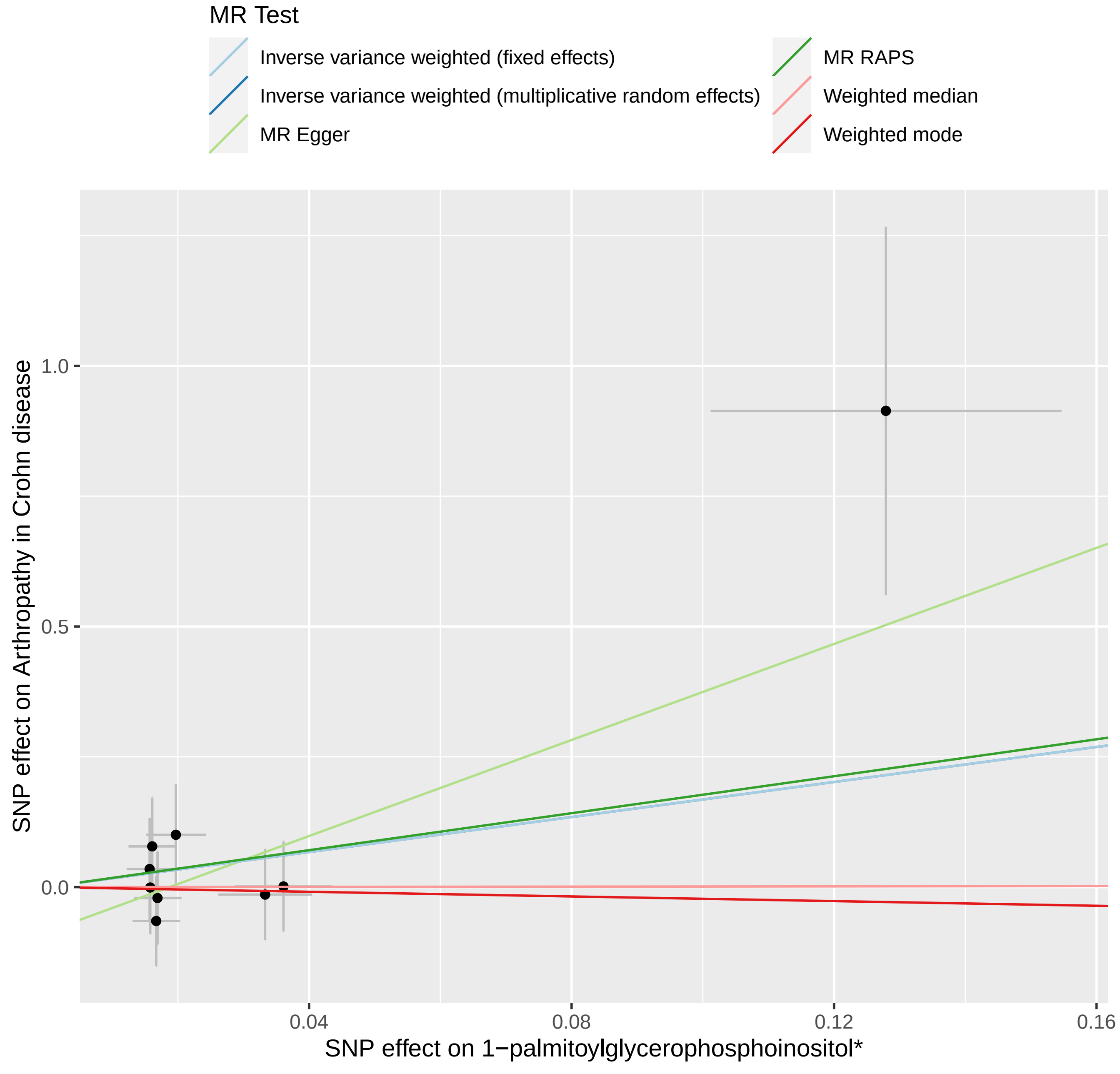

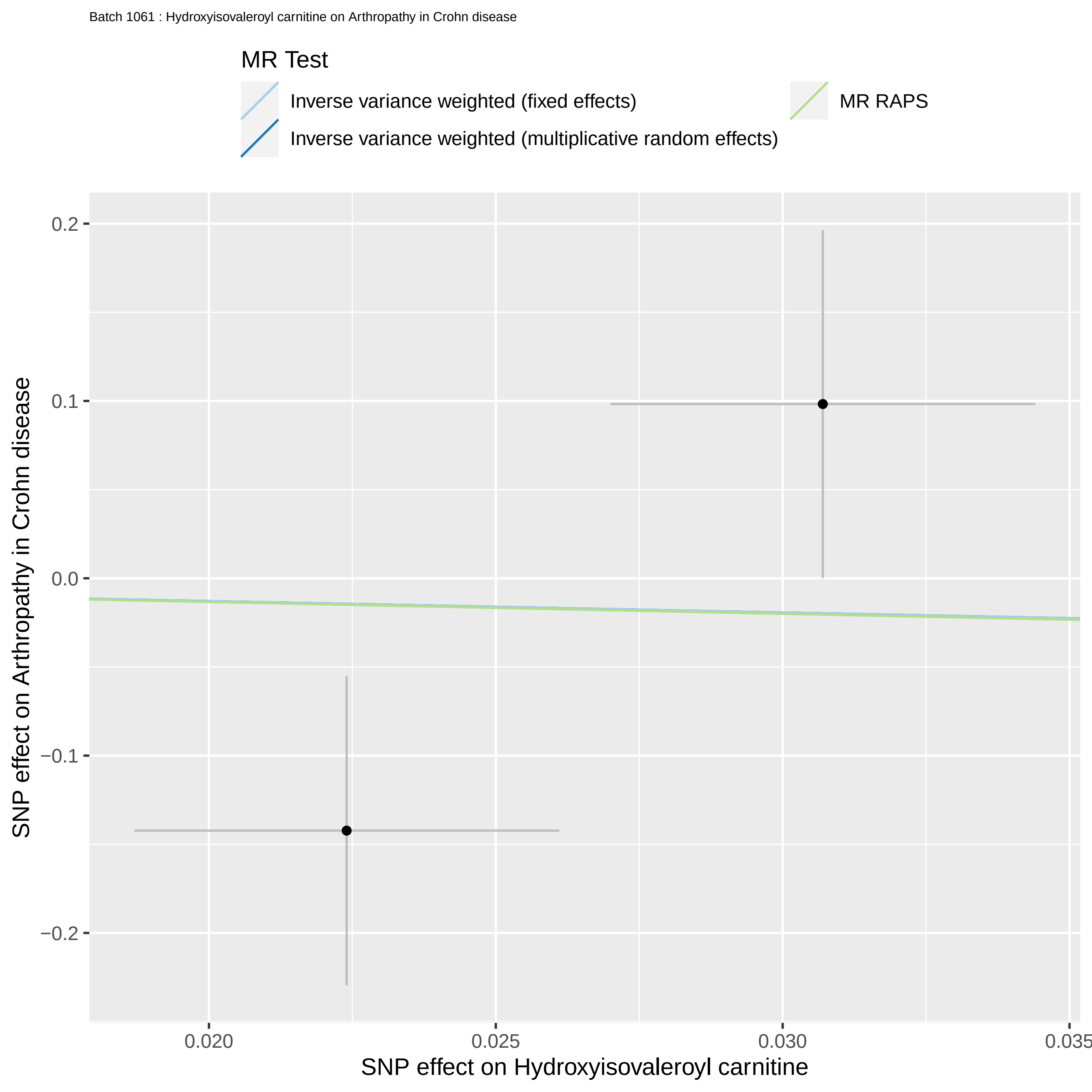

Batch 1061 : Hydroxyisovaleroyl carnitine on Arthropathy in Crohn disease
MR Test
Inverse variance weighted (fixed effects)
Inverse variance weighted (multiplicative random effects)
MR RAPS
0.2
0.1
0.0
−0.1
−0.2
0.020
0.025
0.030
0.035
SNP effect on Arthropathy in Crohn disease
SNP effect on Hydroxyisovaleroyl carnitine

Batch 1062 : Glutaroyl carnitine on Arthropathy in Crohn disease

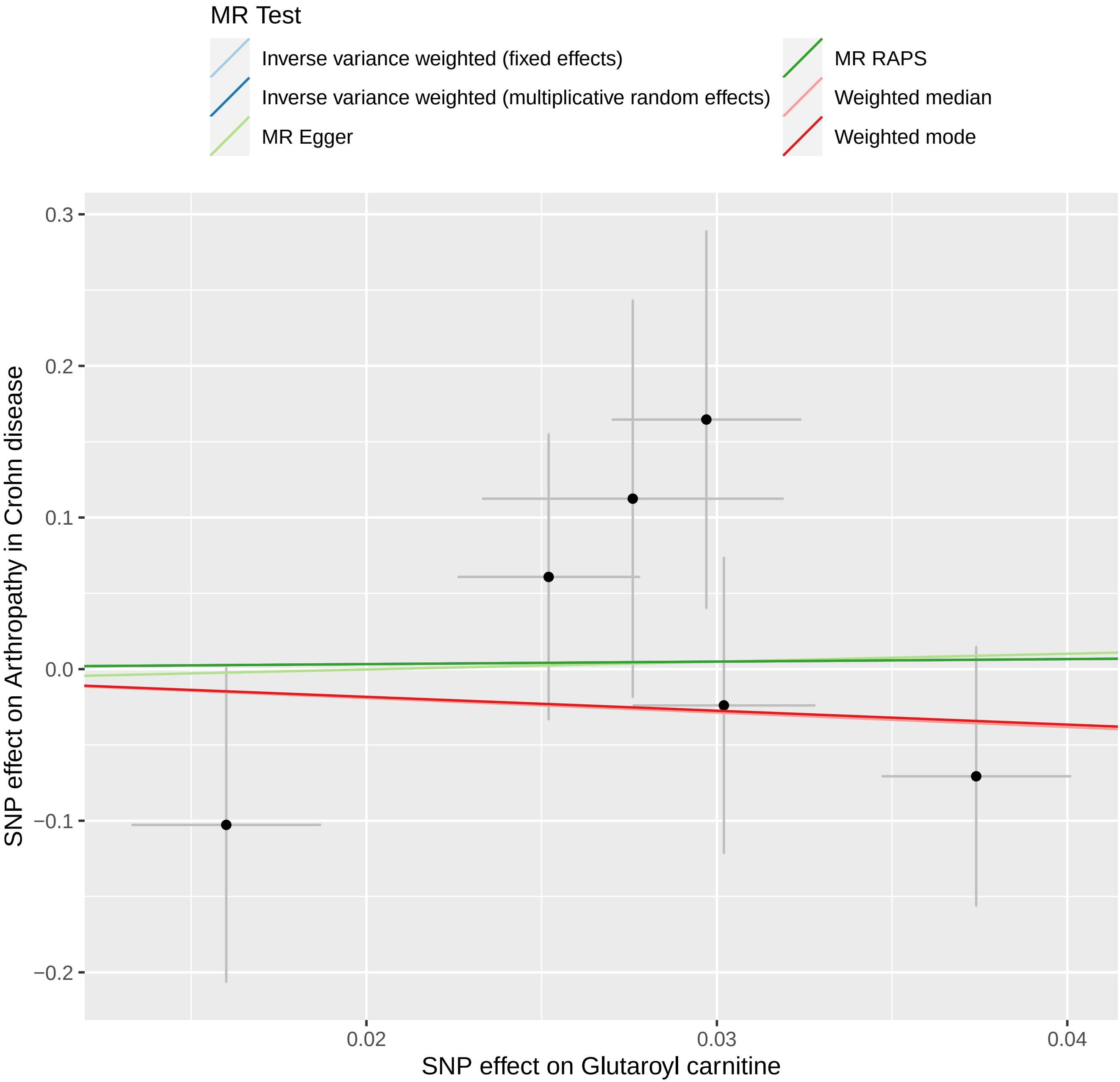

Batch 1064 : 4−hydroxyhippurate on Arthropathy in Crohn disease

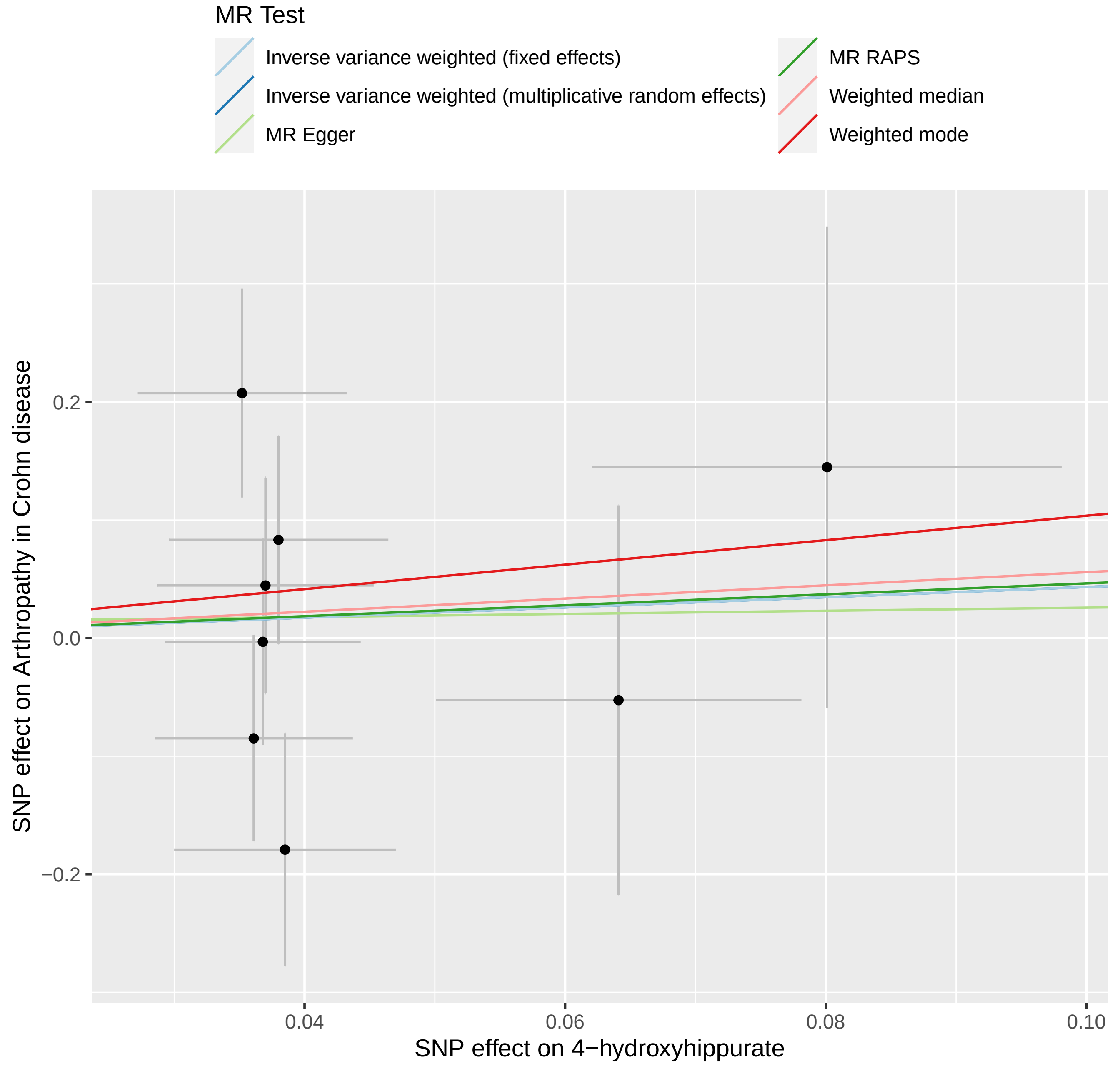

Batch 1065 : 1−myristoylglycerophosphocholine on Arthropathy in Crohn disease

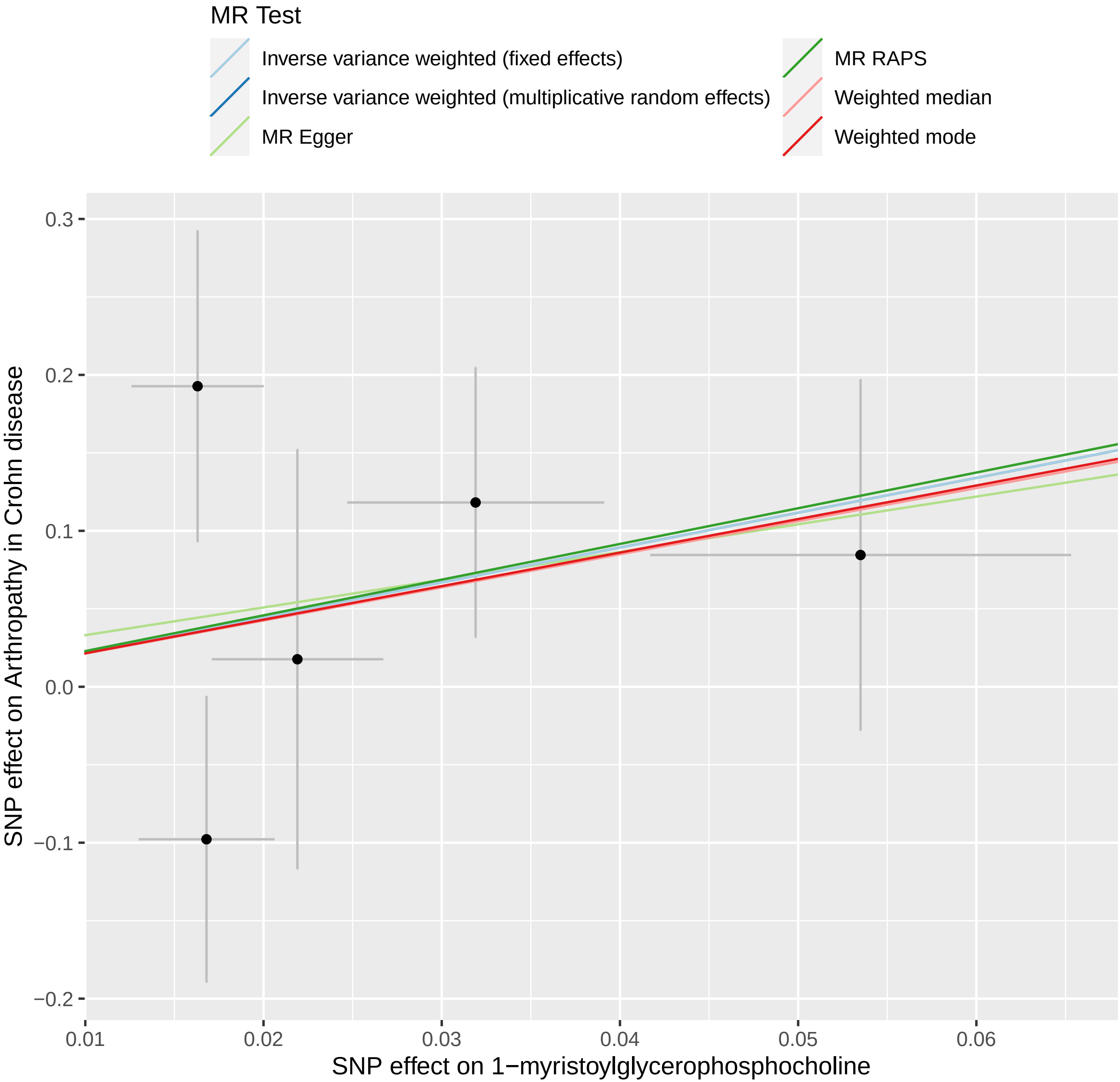

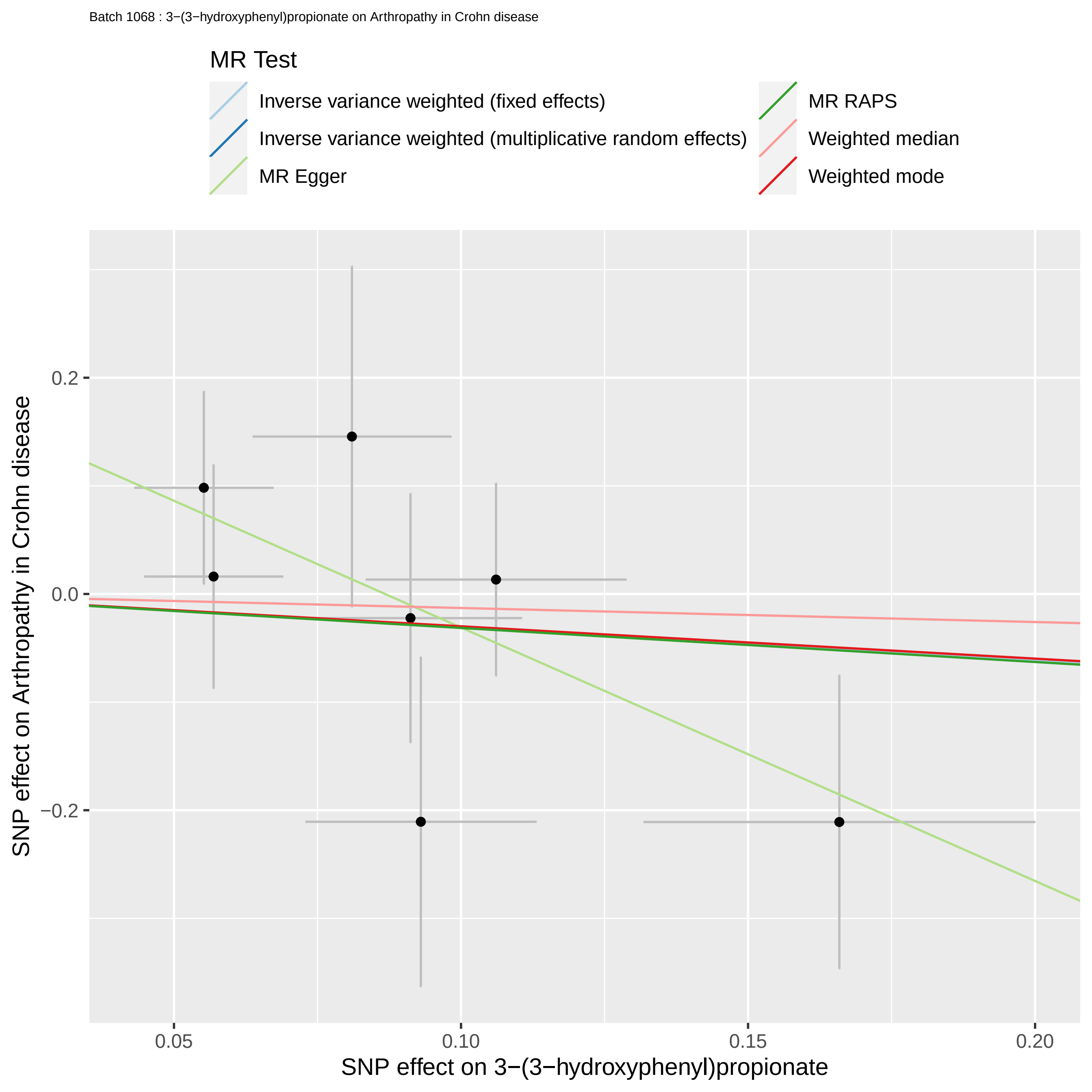
Batch 1068 : 3−(3−hydroxyphenyl)propionate on Arthropathy in Crohn disease
MR Test
Inverse variance weighted (fixed effects)
Inverse variance weighted (multiplicative random effects)
MR Egger
MR RAPS
Weighted median
Weighted mode
0.2
0.0
−0.2
0.05
0.10
0.15
0.20
SNP effect on Arthropathy in Crohn disease
SNP effect on 3−(3−hydroxyphenyl)propionate

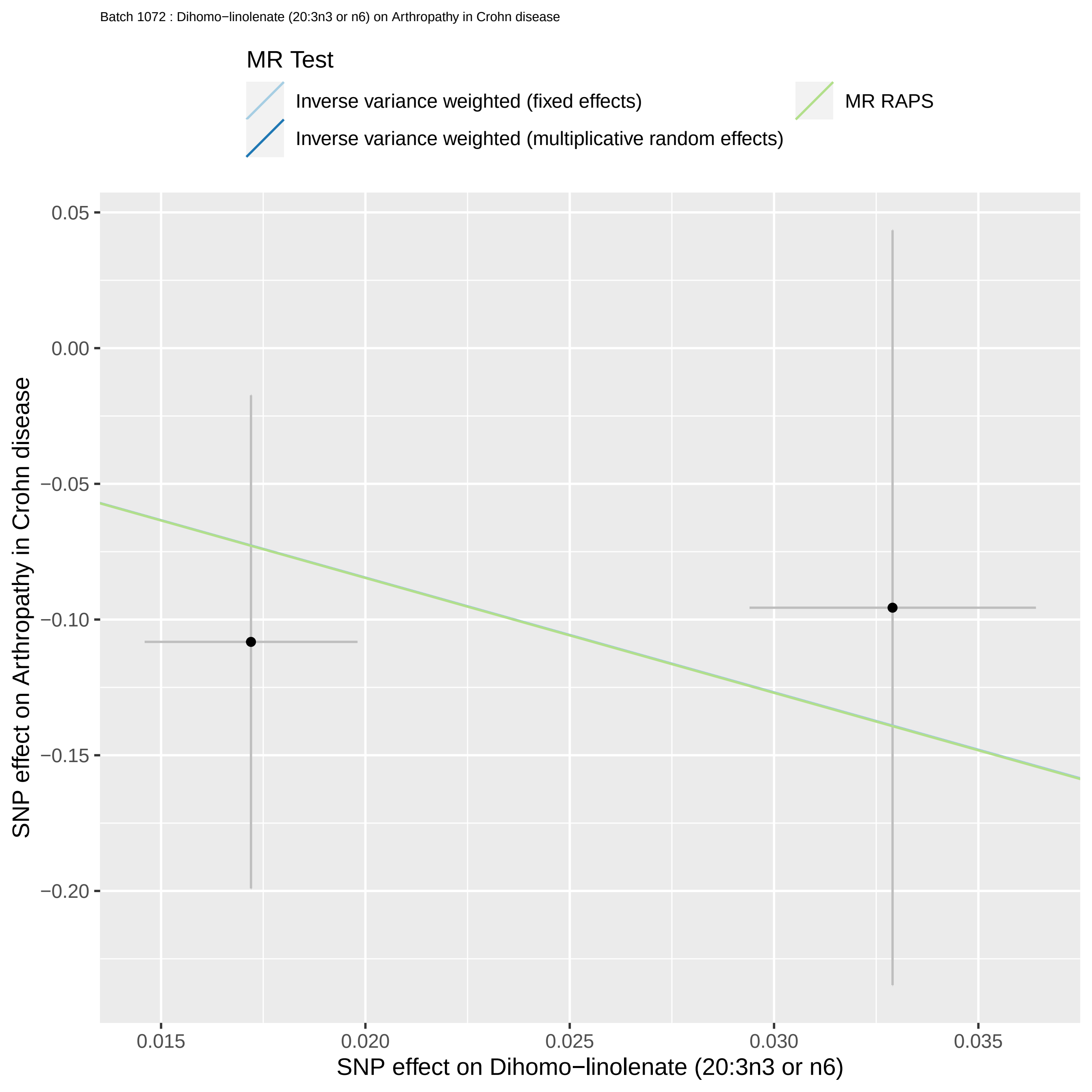

Batch 1072 : Dihomo−linolenate (20:3n3 or n6) on Arthropathy in Crohn disease
MR Test
Inverse variance weighted (fixed effects)
Inverse variance weighted (multiplicative random effects)
MR RAPS
0.05
0.00
−0.05
−0.10
−0.15
−0.20
0.015
0.020
0.025
0.030
0.035
SNP effect on Arthropathy in Crohn disease
SNP effect on Dihomo−linolenate (20:3n3 or n6)

Batch 1073 : Threitol on Arthropathy in Crohn disease

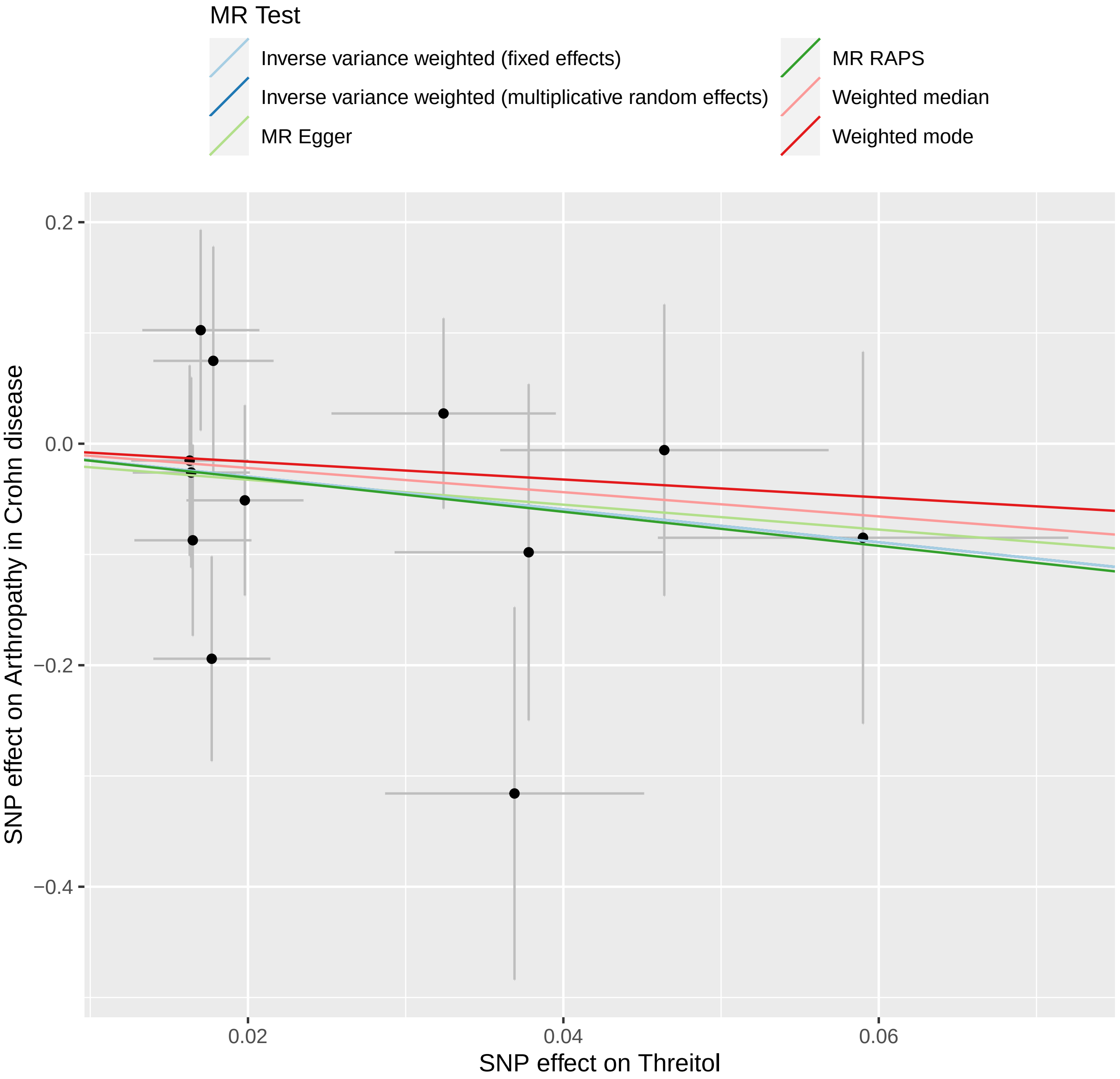

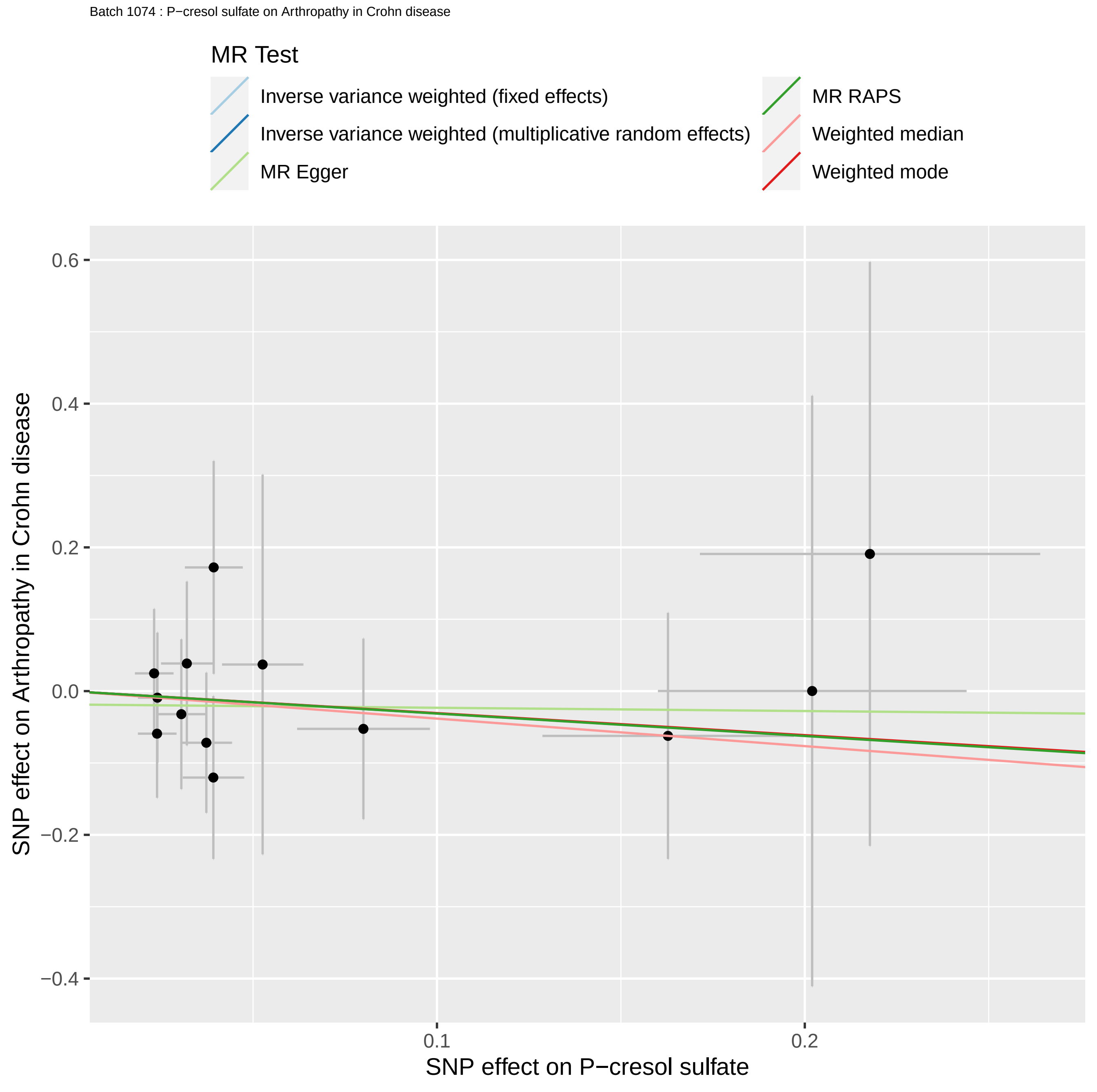
Batch 1074 : P−cresol sulfate on Arthropathy in Crohn disease
MR Test
Inverse variance weighted (fixed effects)
Inverse variance weighted (multiplicative random effects)
MR Egger
MR RAPS
Weighted median
Weighted mode
0.6
0.4
0.2
0.0
−0.2
−0.4
SNP effect on Arthropathy in Crohn disease
0.1
0.2
SNP effect on P−cresol sulfate

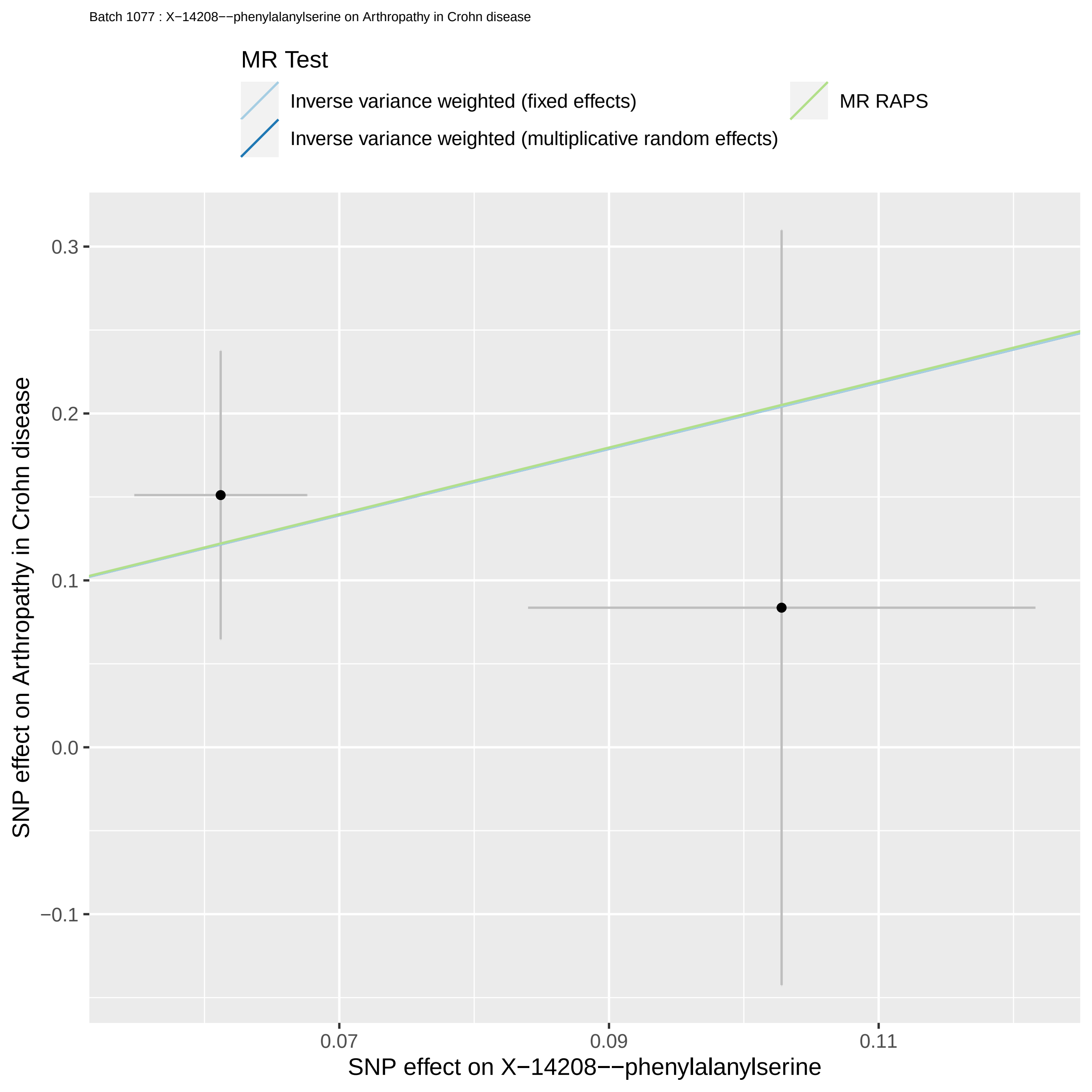
Batch 1077 : X−14208−−phenylalanylserine on Arthropathy in Crohn disease
MR Test
Inverse variance weighted (fixed effects)
Inverse variance weighted (multiplicative random effects)
MR RAPS
0.3
0.2
0.1
0.0
−0.1
SNP effect on Arthropathy in Crohn disease
0.07
0.09
0.11
SNP effect on X−14208−−phenylalanylserine

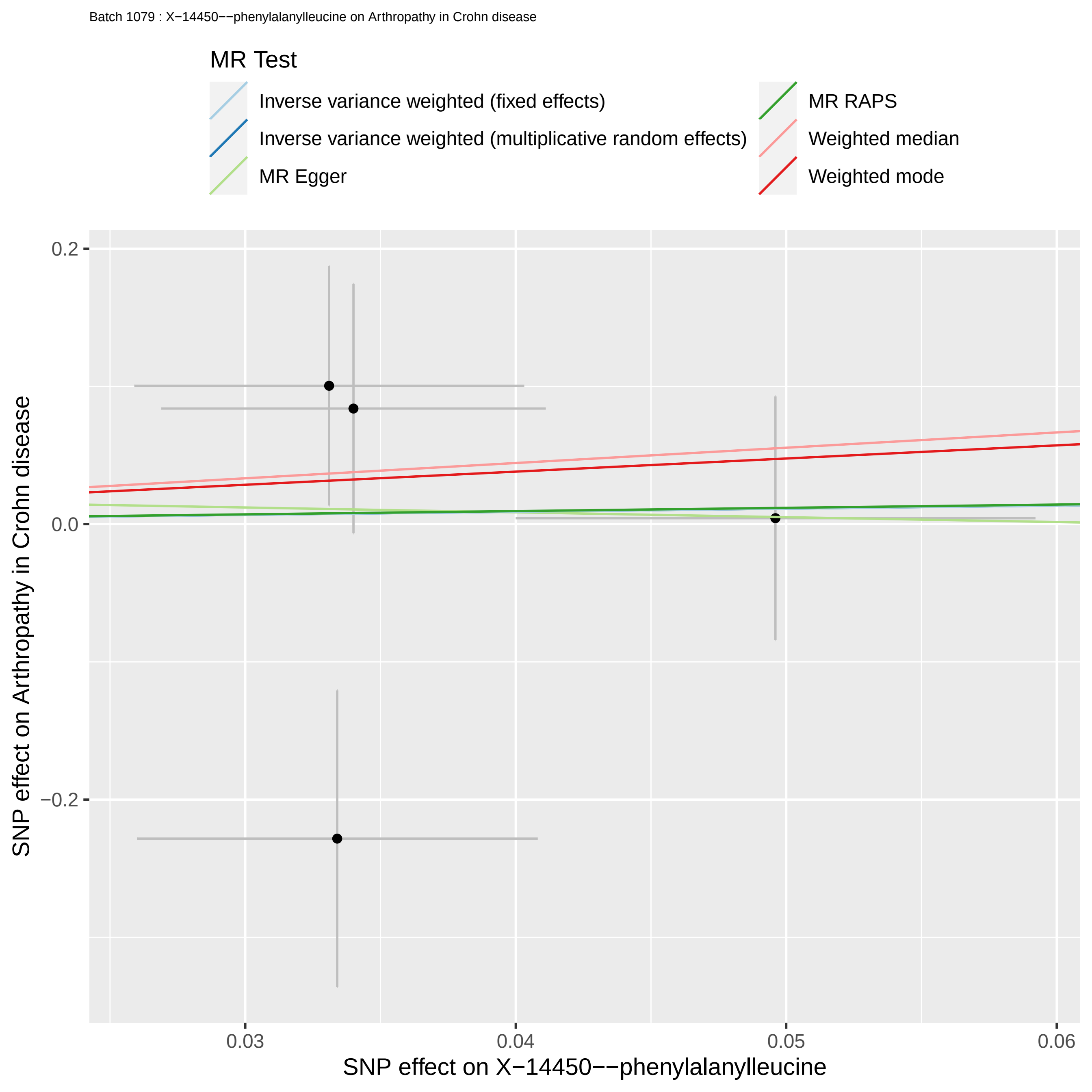

Batch 1079 : X−14450−−phenylalanylleucine on Arthropathy in Crohn disease
MR Test
Inverse variance weighted (fixed effects)
Inverse variance weighted (multiplicative random effects)
MR Egger
MR RAPS
Weighted median
Weighted mode
0.2
0.0
−0.2
0.03
0.04
0.05
0.06
SNP effect on X−14450−−phenylalanylleucine
SNP effect on Arthropathy in Crohn disease

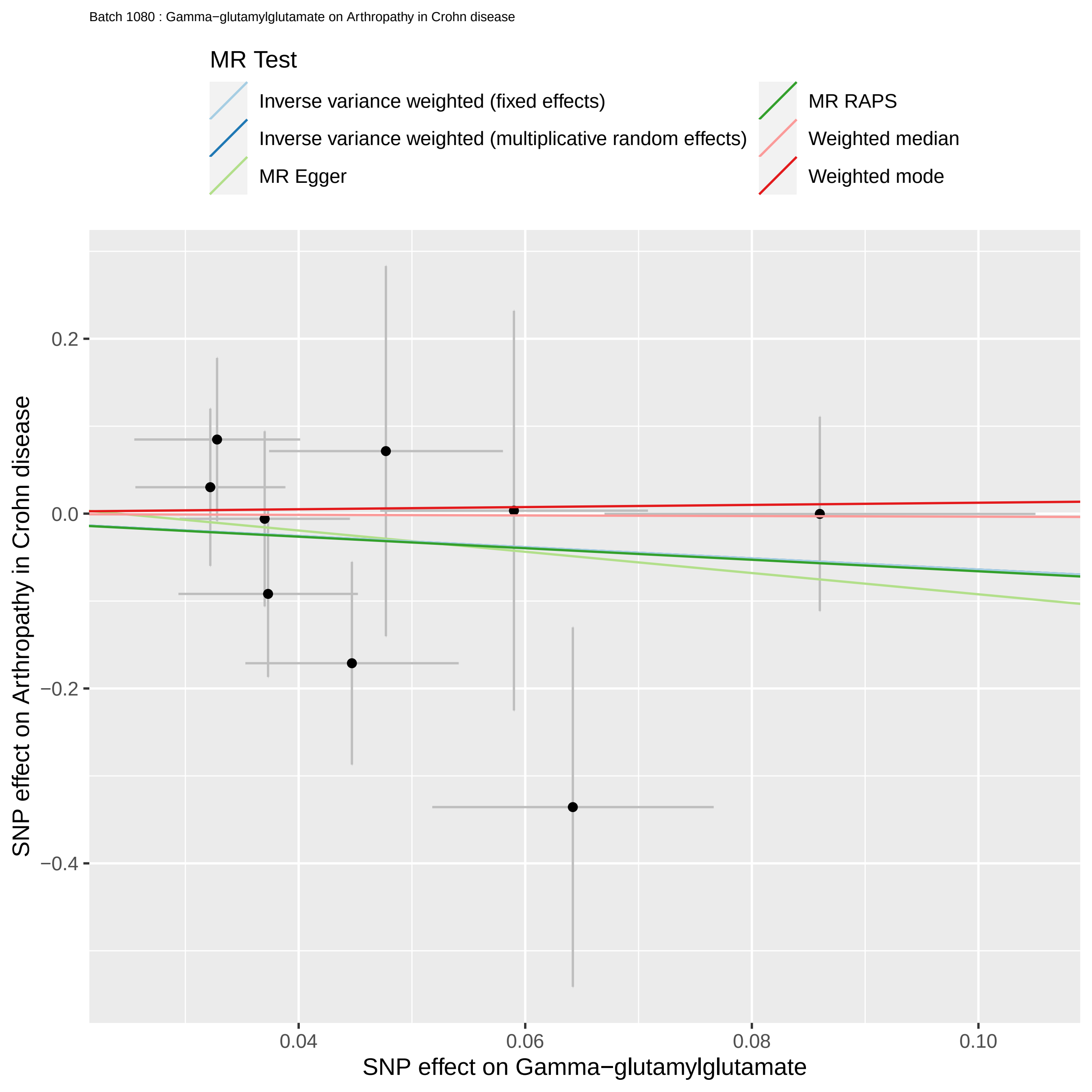
Batch 1080 : Gamma−glutamylglutamate on Arthropathy in Crohn disease
MR Test
Inverse variance weighted (fixed effects)
Inverse variance weighted (multiplicative random effects)
MR Egger
MR RAPS
Weighted median
Weighted mode
0.2
0.0
−0.2
−0.4
0.04
0.06
0.08
0.10
SNP effect on Arthropathy in Crohn disease
SNP effect on Gamma−glutamylglutamate

Batch 1081 : Octadecanedioate on Arthropathy in Crohn disease

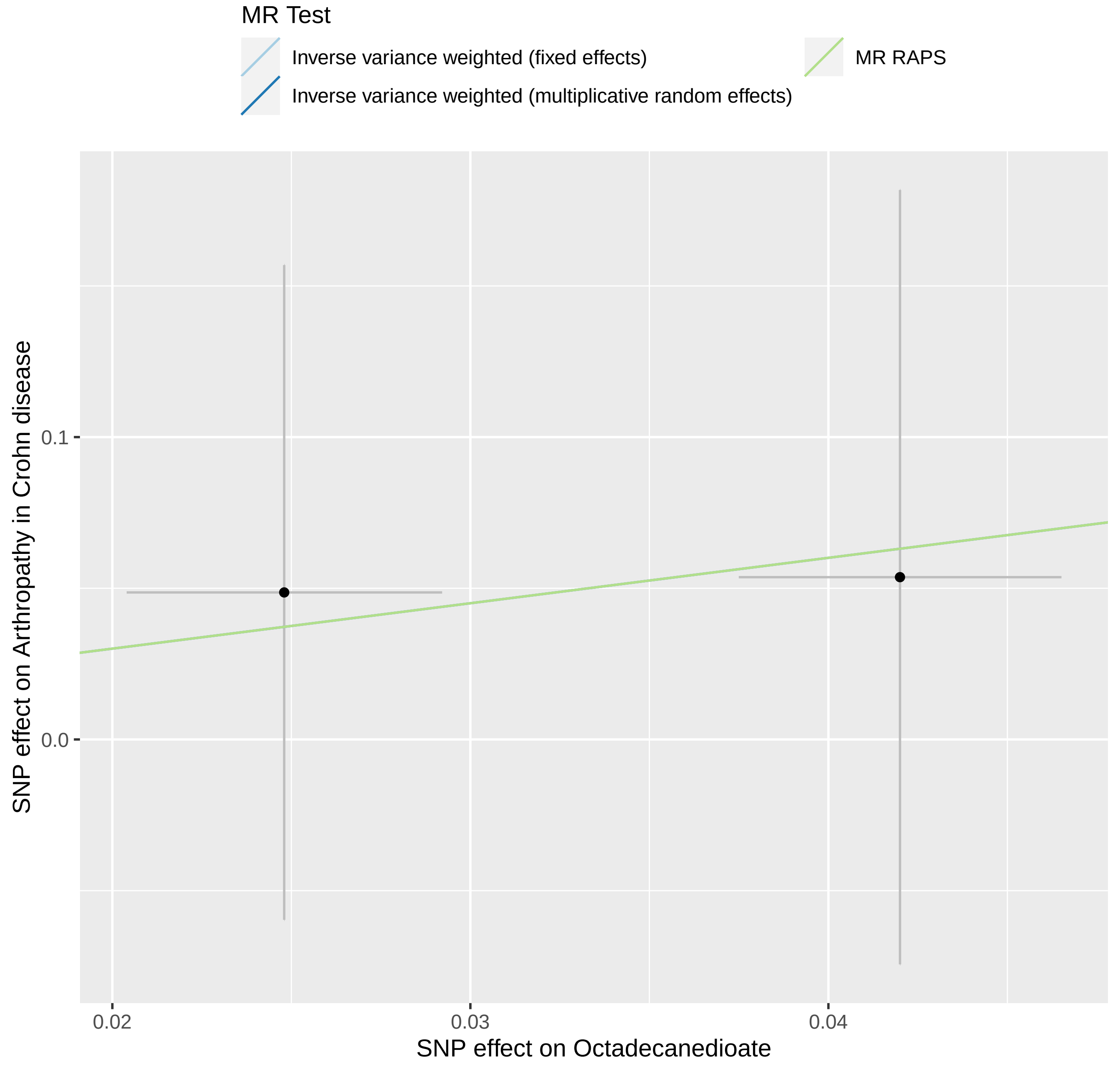

Batch 1082 : Leucylleucine on Arthropathy in Crohn disease

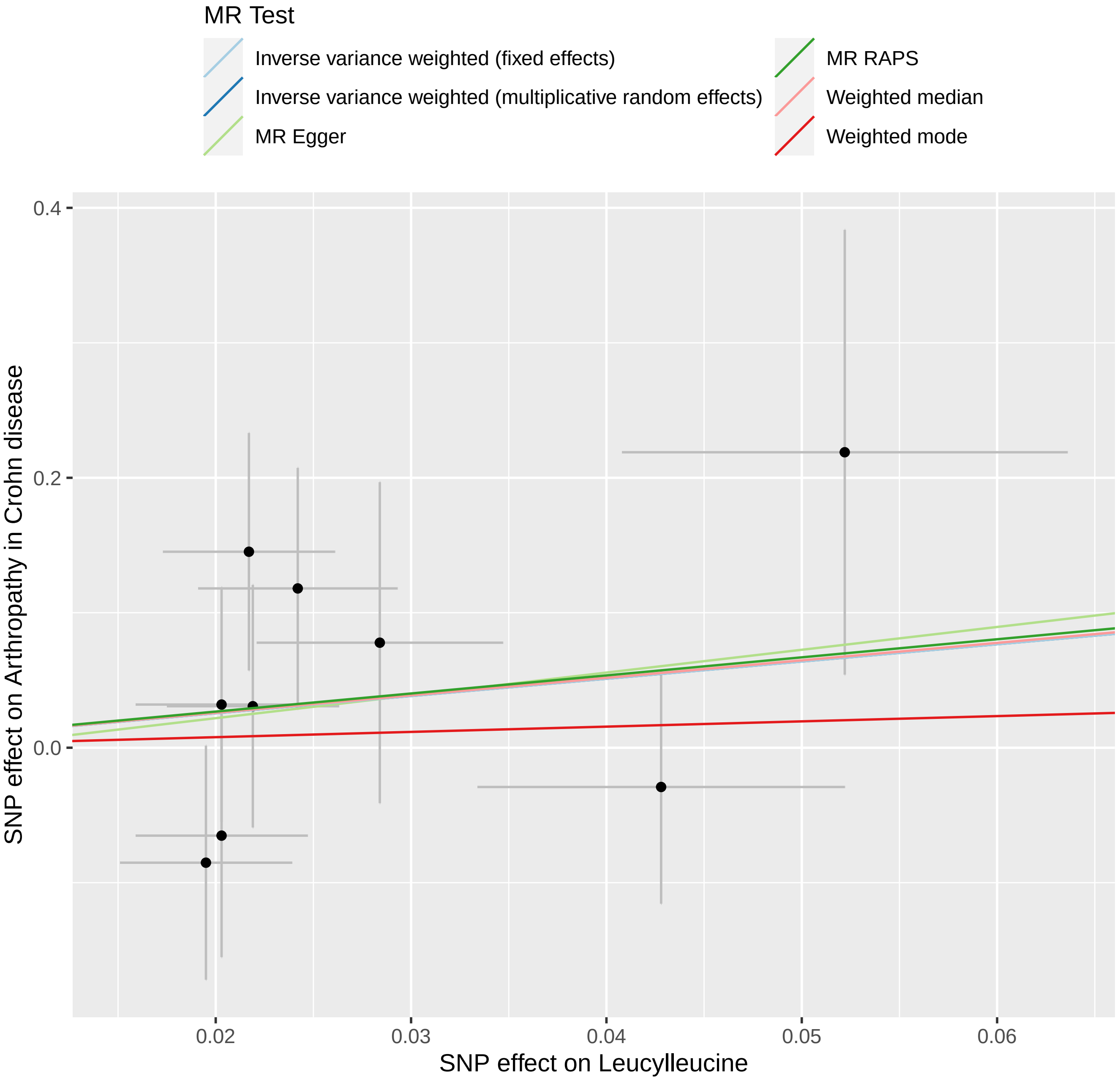

Batch 1083 : 7−alpha−hydroxy−3−oxo−4−cholestenoate (7−Hoca) on Arthropathy in Crohn disease

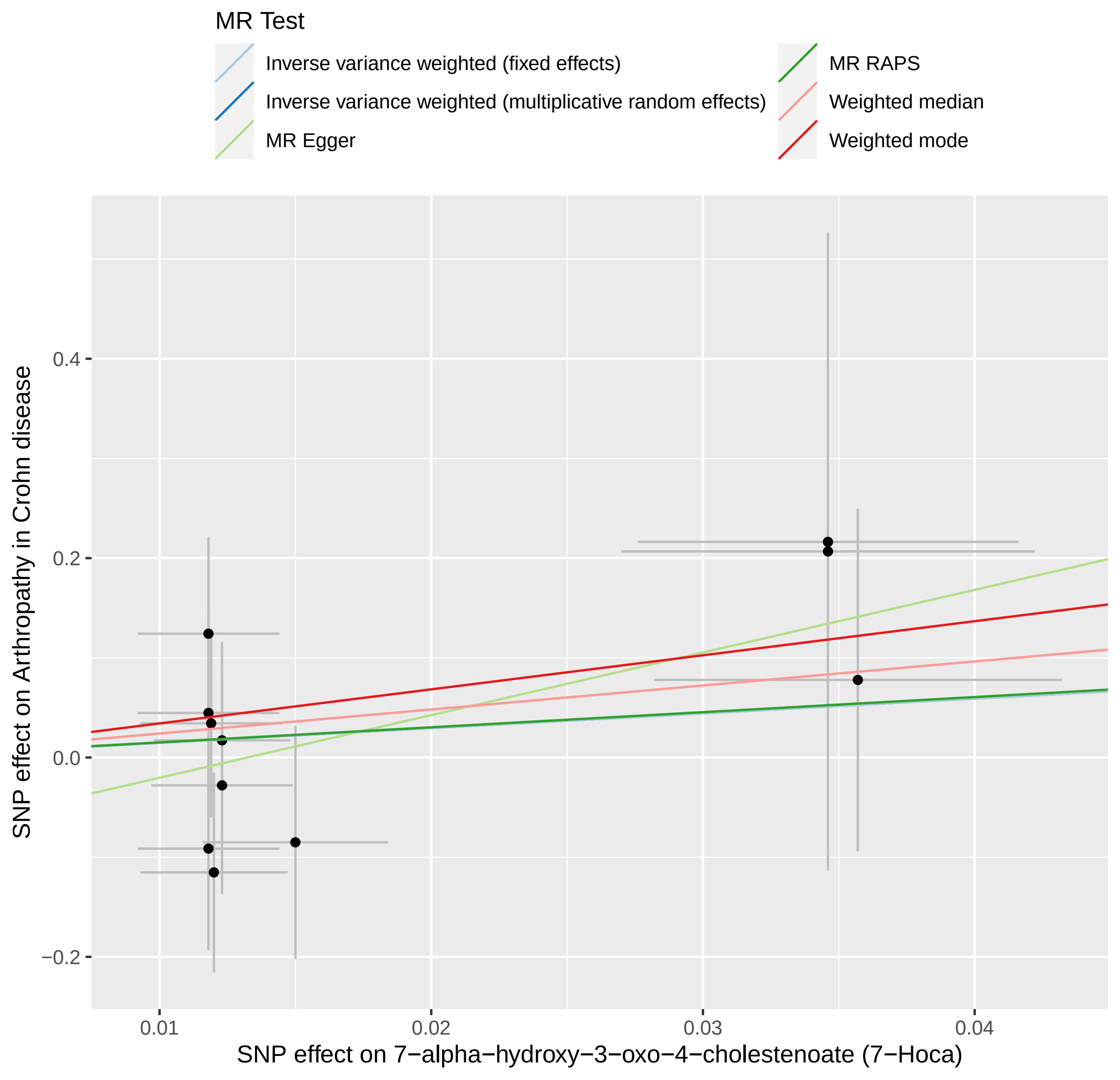

Batch 1084 : N−Butyl Oleate on Arthropathy in Crohn disease

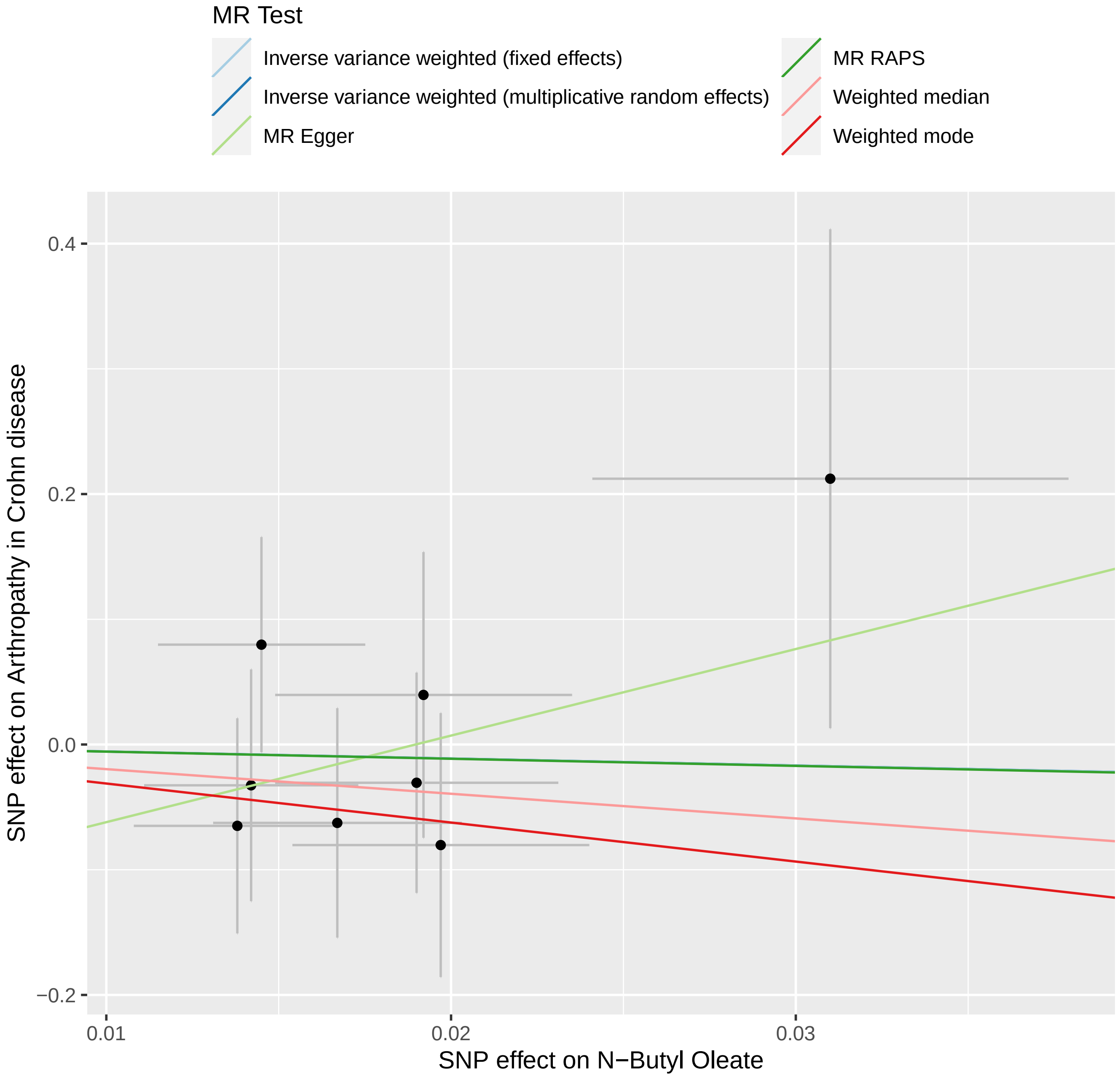

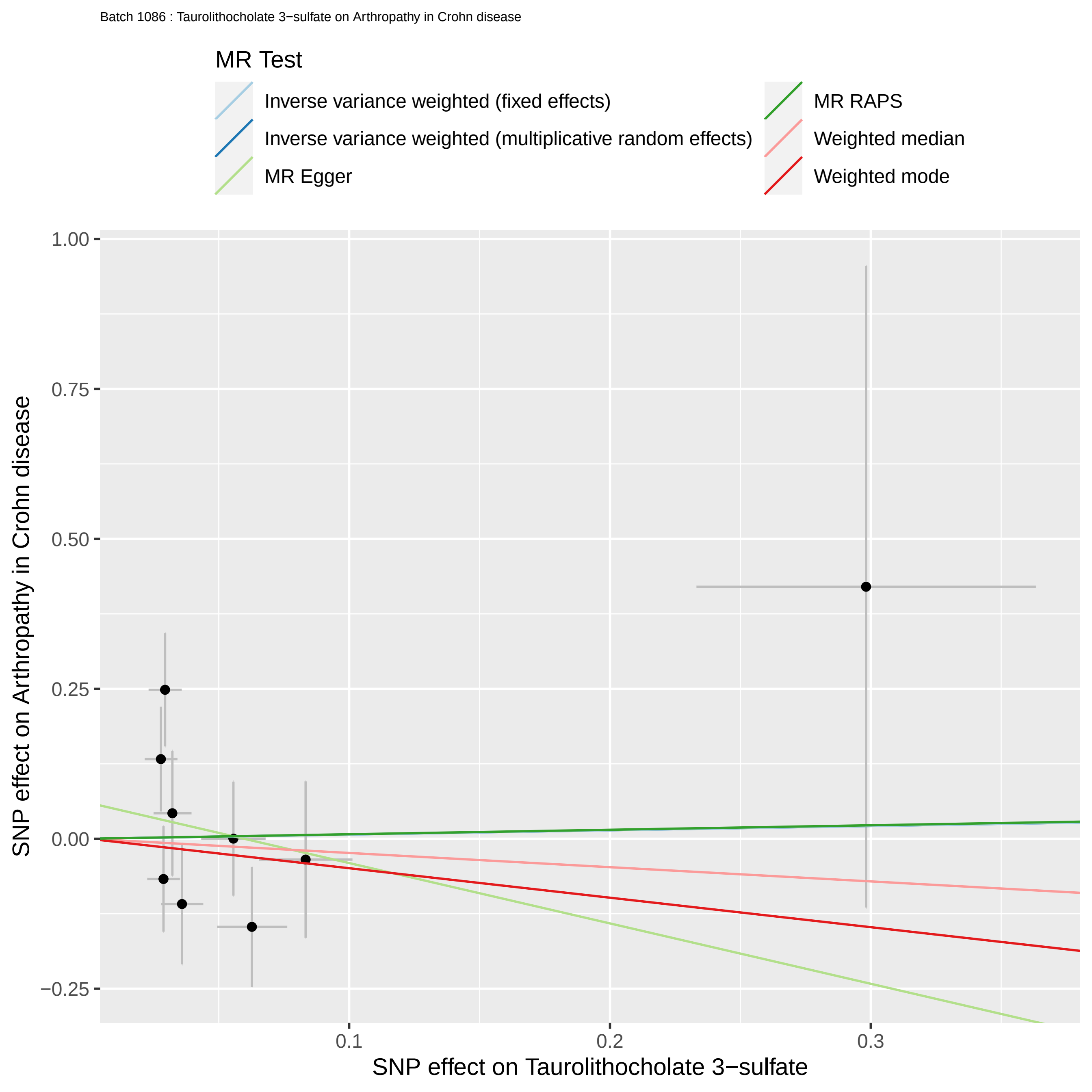

Batch 1086 : Taurolithocholate 3−sulfate on Arthropathy in Crohn disease
MR Test
Inverse variance weighted (fixed effects)
Inverse variance weighted (multiplicative random effects)
MR Egger
MR RAPS
Weighted median
Weighted mode
SNP effect on Arthropathy in Crohn disease
1.00
0.75
0.50
0.25
0.00
−0.25
0.1
0.2
0.3
SNP effect on Taurolithocholate 3−sulfate

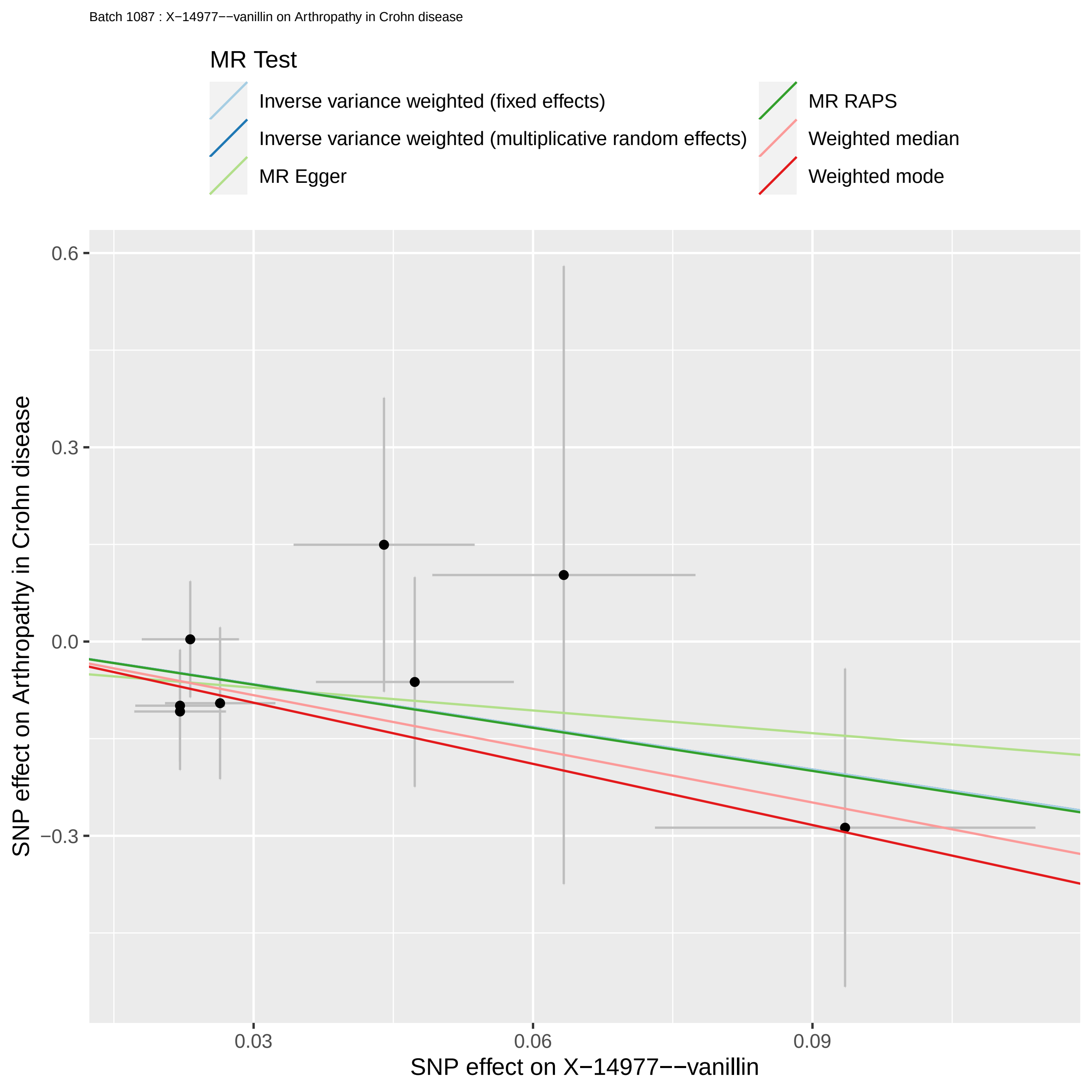
Batch 1087 : X−14977−−vanillin on Arthropathy in Crohn disease
MR Test
Inverse variance weighted (fixed effects)
Inverse variance weighted (multiplicative random effects)
MR Egger
MR RAPS
Weighted median
Weighted mode
0.6
0.3
0.0
−0.3
0.03
0.06
0.09
SNP effect on Arthropathy in Crohn disease
SNP effect on X−14977−−vanillin

Batch 1088 : Succinylcarnitine on Arthropathy in Crohn disease

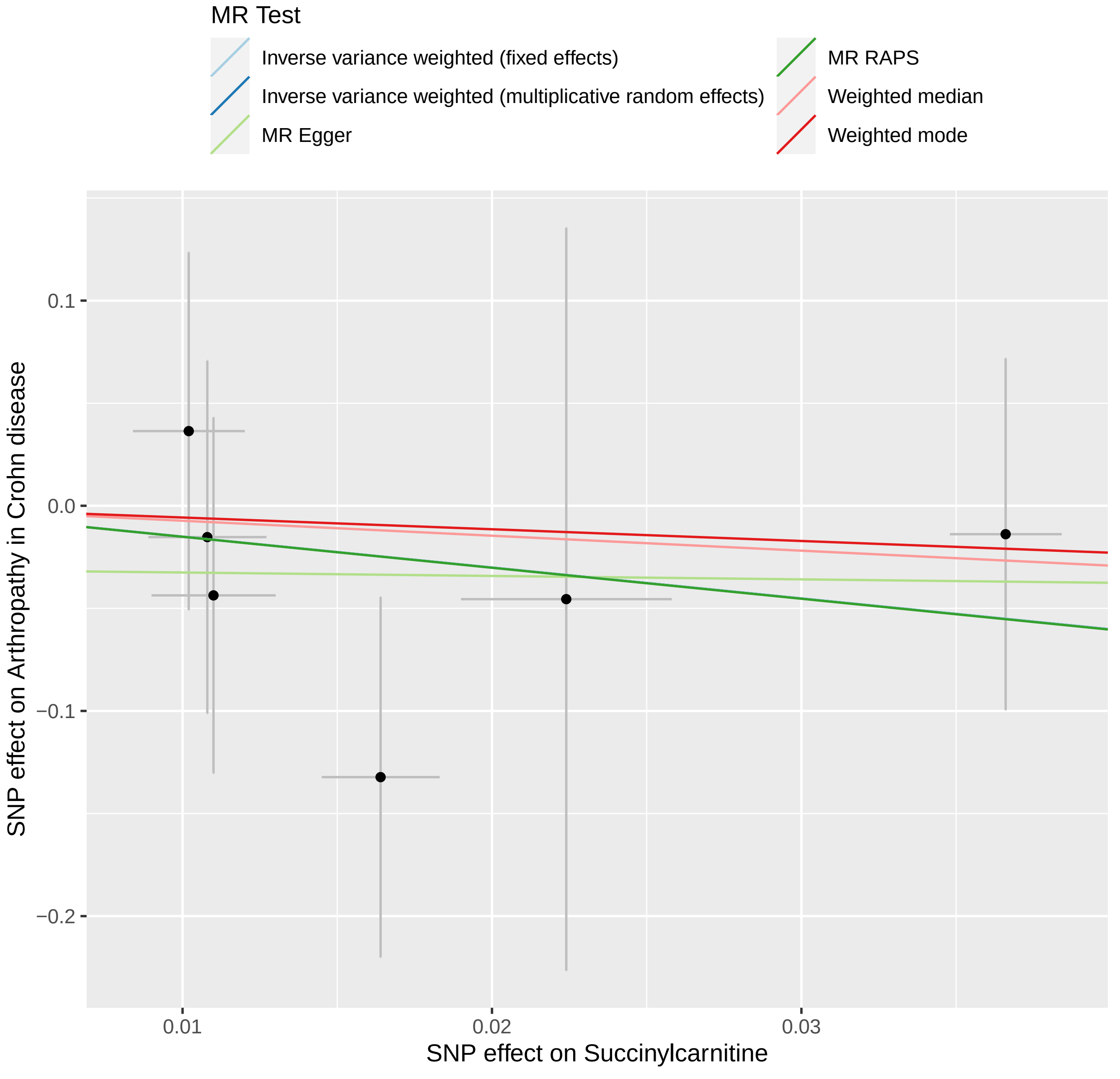

Batch 1090 : Cyclo(leu−pro) on Arthropathy in Crohn disease

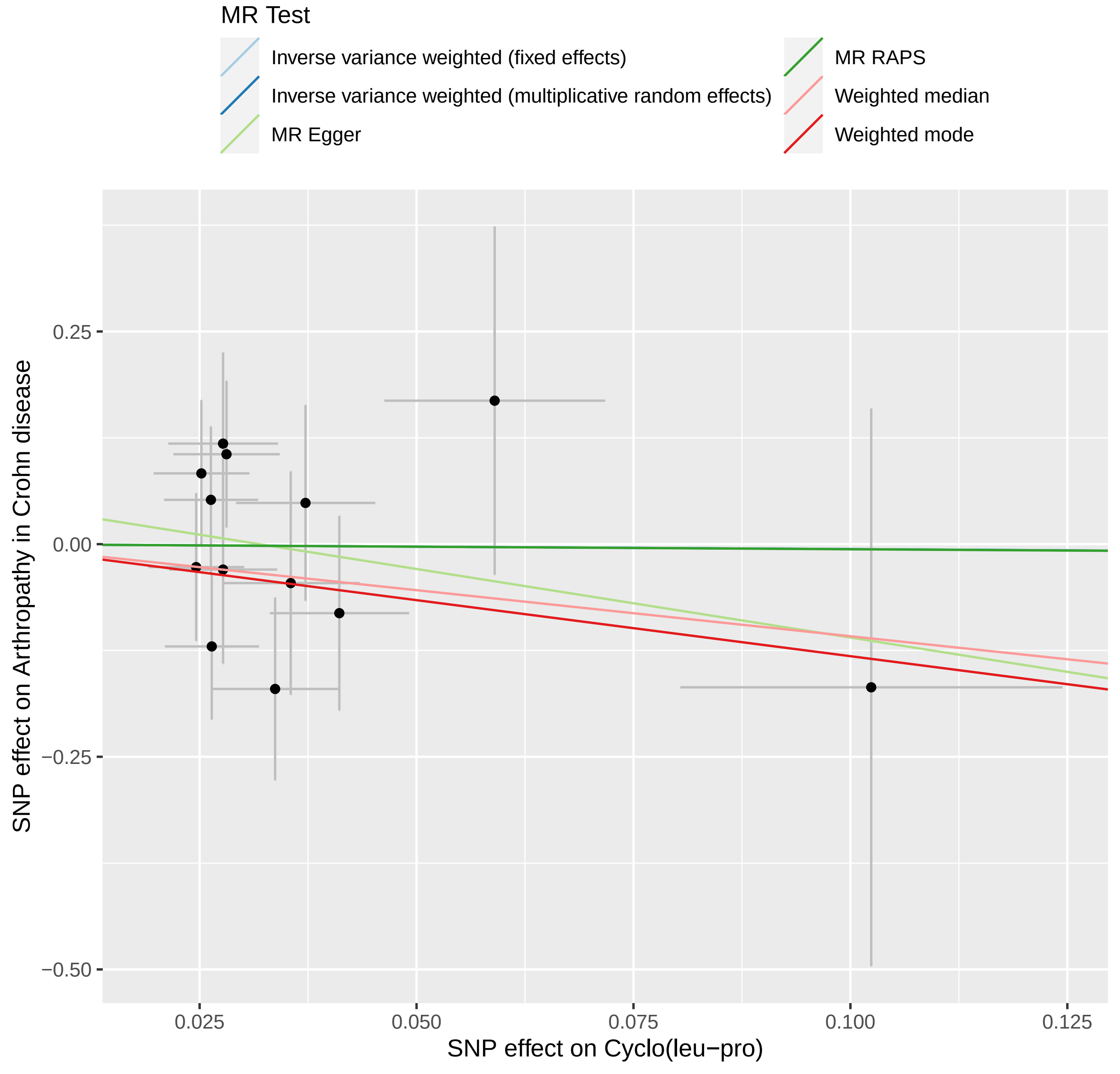

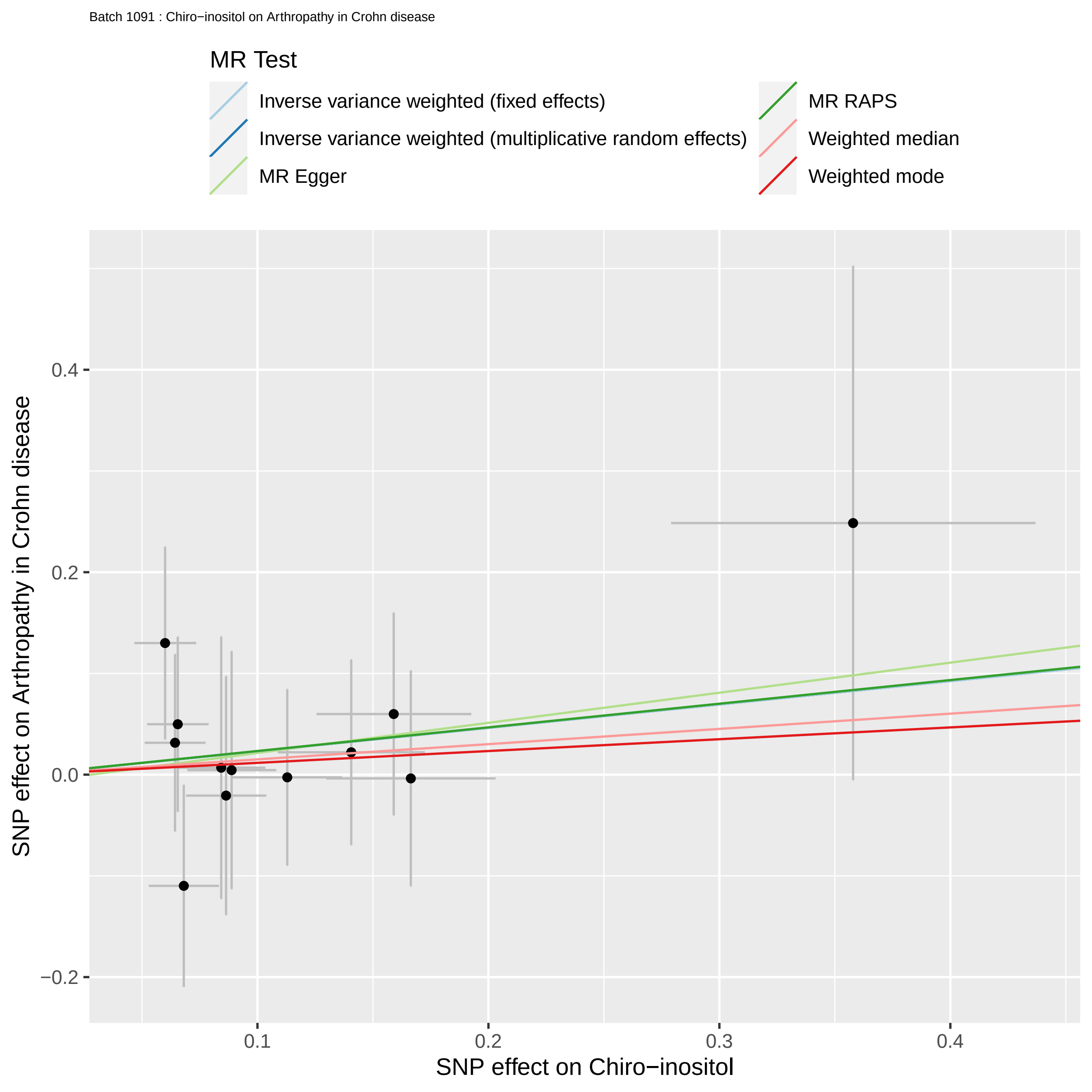

Batch 1091 : Chiro−inositol on Arthropathy in Crohn disease
MR Test
Inverse variance weighted (fixed effects)
Inverse variance weighted (multiplicative random effects)
MR Egger
MR RAPS
Weighted median
Weighted mode
0.4
0.2
0.0
−0.2
0.1
0.2
0.3
0.4
SNP effect on Arthropathy in Crohn disease
SNP effect on Chiro−inositol

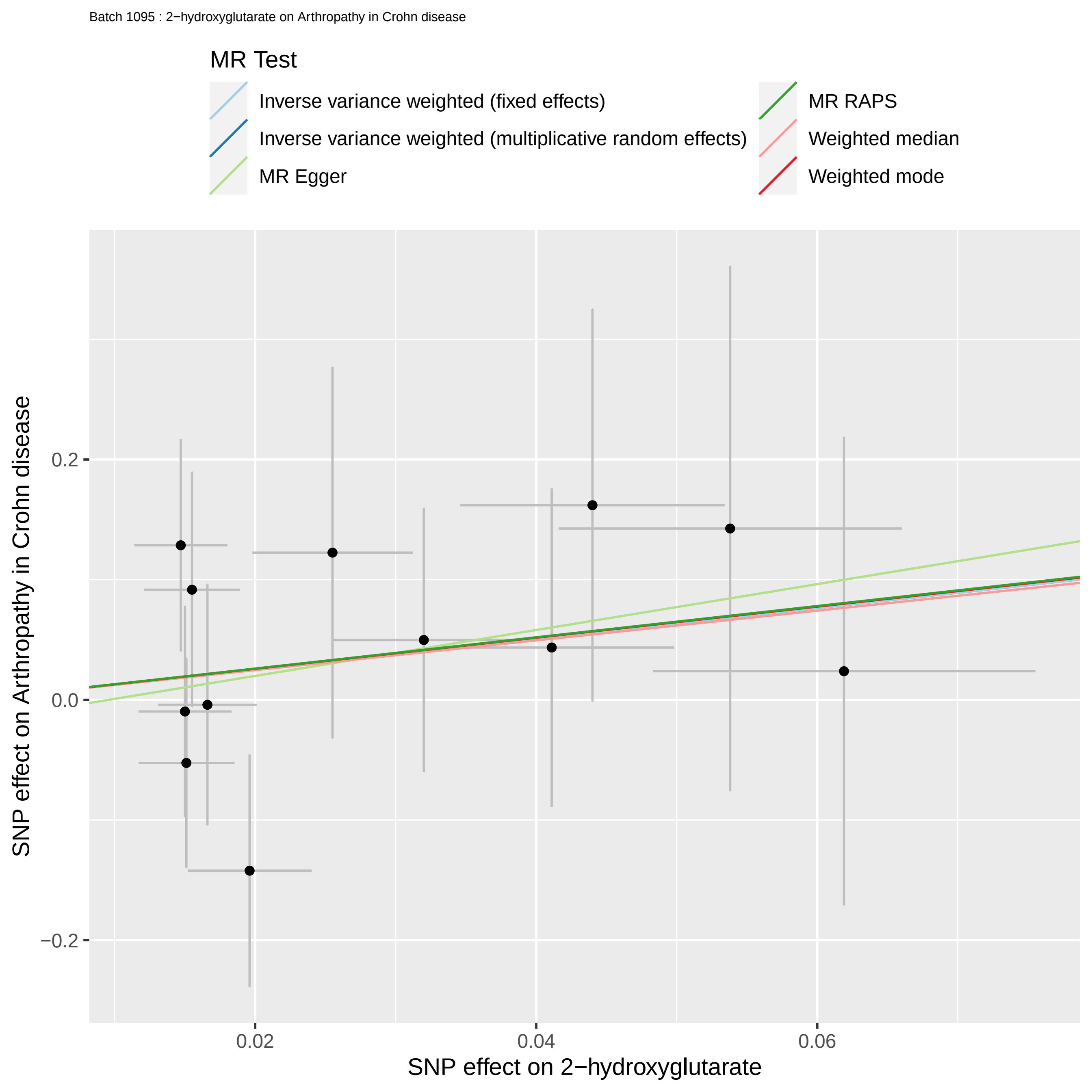

Batch 1095 : 2−hydroxyglutarate on Arthropathy in Crohn disease
MR Test
Inverse variance weighted (fixed effects)
Inverse variance weighted (multiplicative random effects)
MR Egger
MR RAPS
Weighted median
Weighted mode
0.2
0.0
−0.2
0.02
0.04
0.06
SNP effect on Arthropathy in Crohn disease
SNP effect on 2−hydroxyglutarate

Batch 1096 : Ergothioneine on Arthropathy in Crohn disease

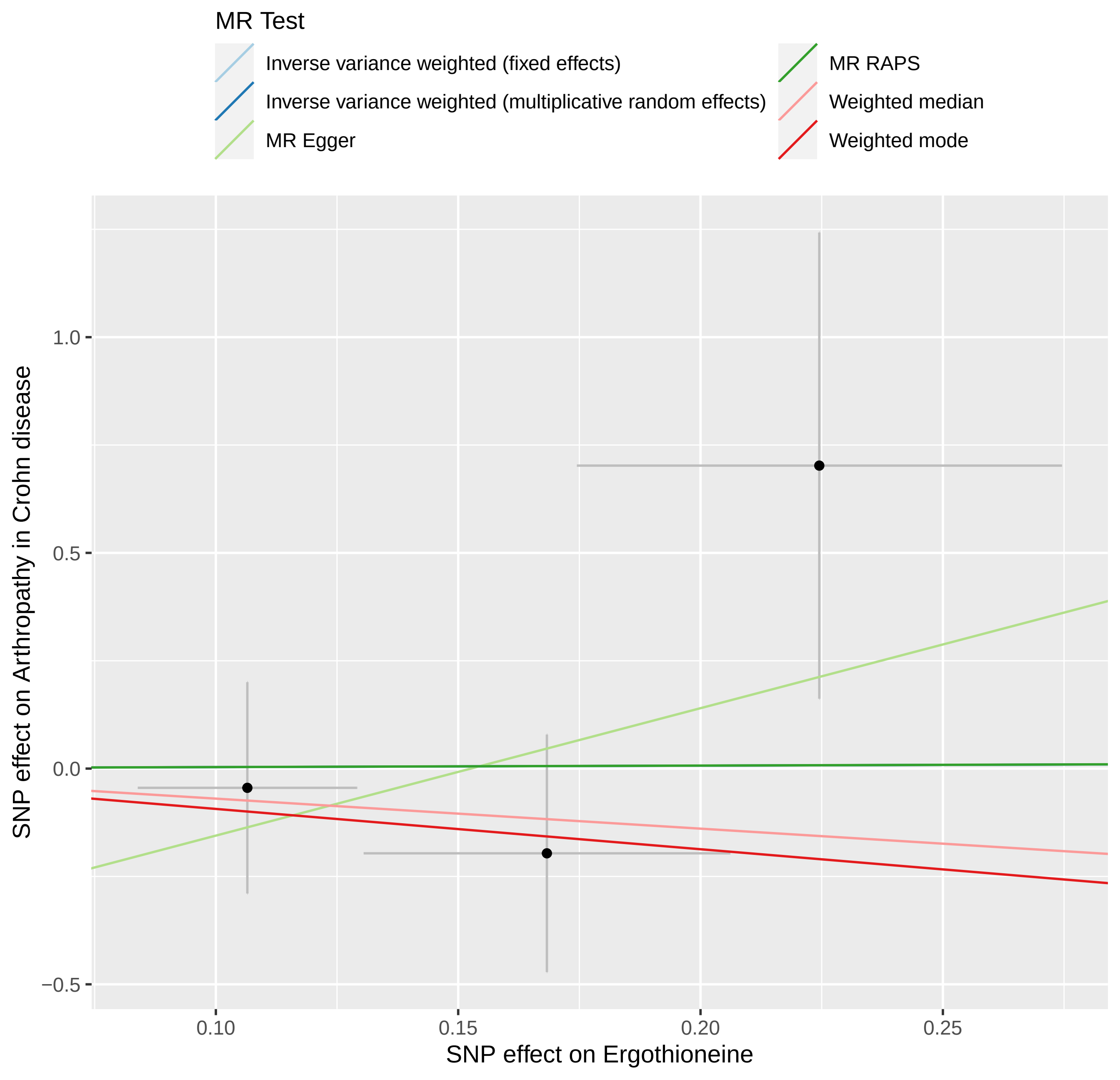

Batch 1098 : Phenylalanylphenylalanine on Arthropathy in Crohn disease

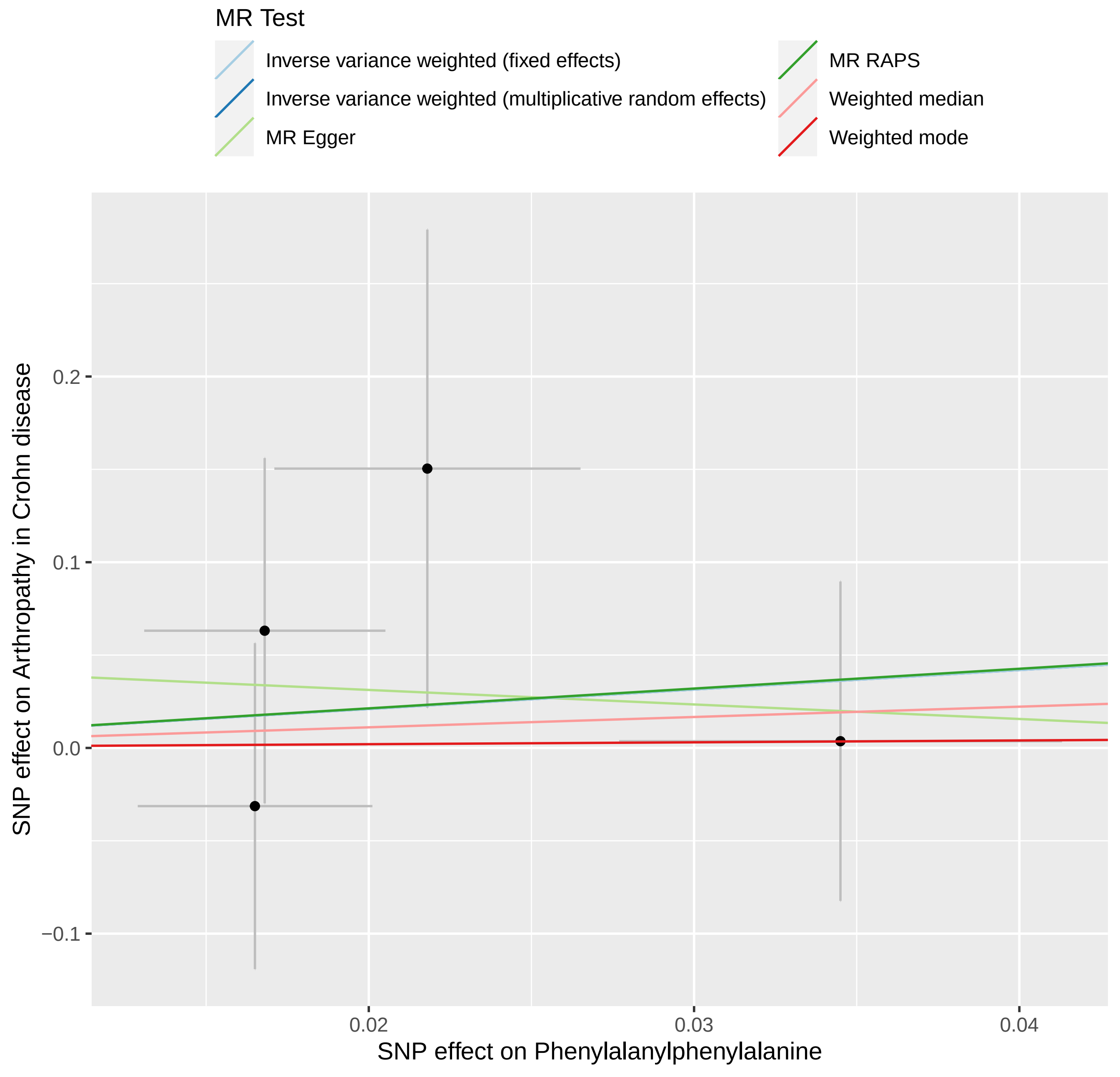

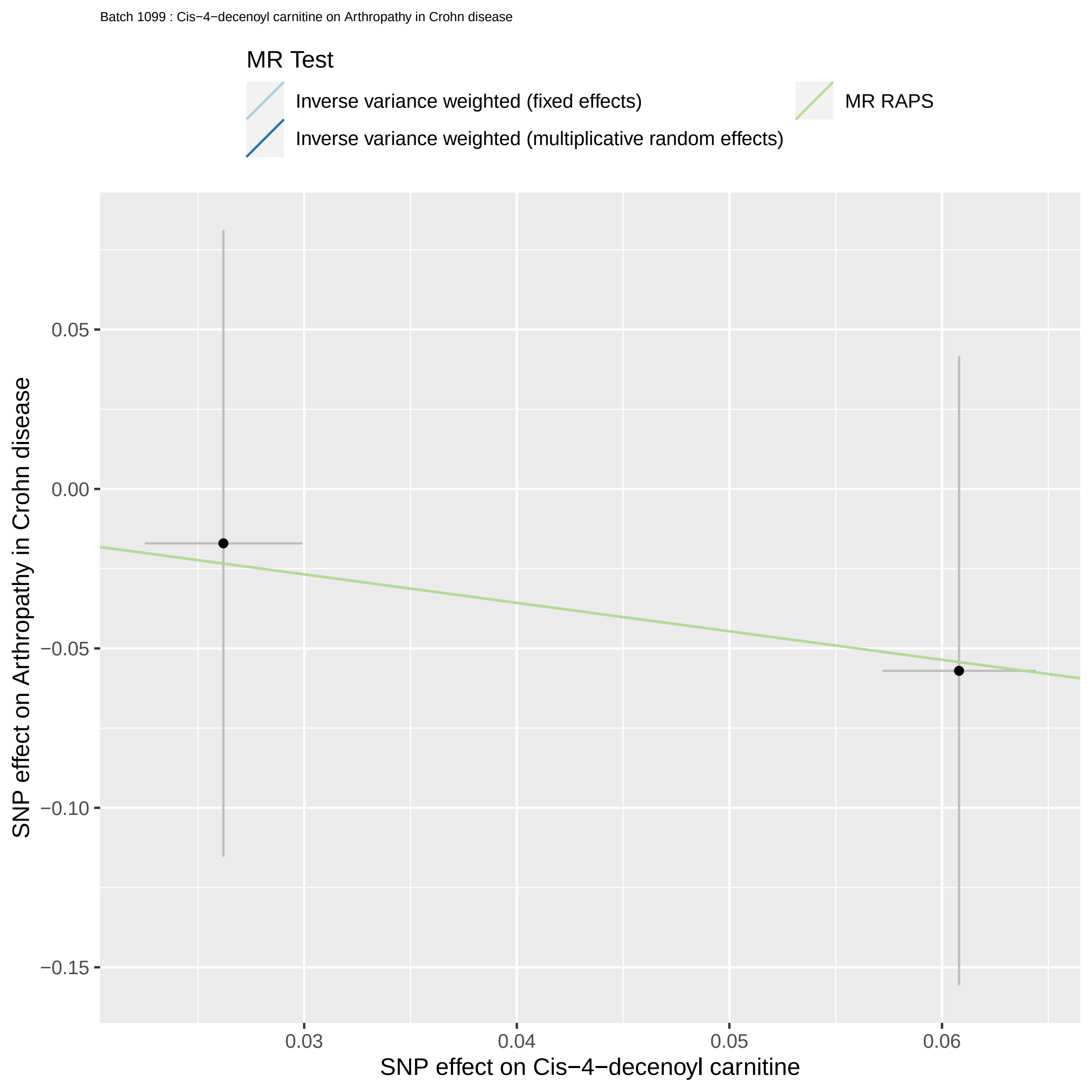

Batch 1099 : Cis−4−decenoyl carnitine on Arthropathy in Crohn disease
MR Test
Inverse variance weighted (fixed effects)
Inverse variance weighted (multiplicative random effects)
MR RAPS
0.05
0.00
−0.05
−0.10
−0.15
0.03
0.04
0.05
0.06
SNP effect on Arthropathy in Crohn disease
SNP effect on Cis−4−decenoyl carnitine

Batch 1100 : 15−methylpalmitate (isobar with 2−methylpalmitate) on Arthropathy in Crohn disease

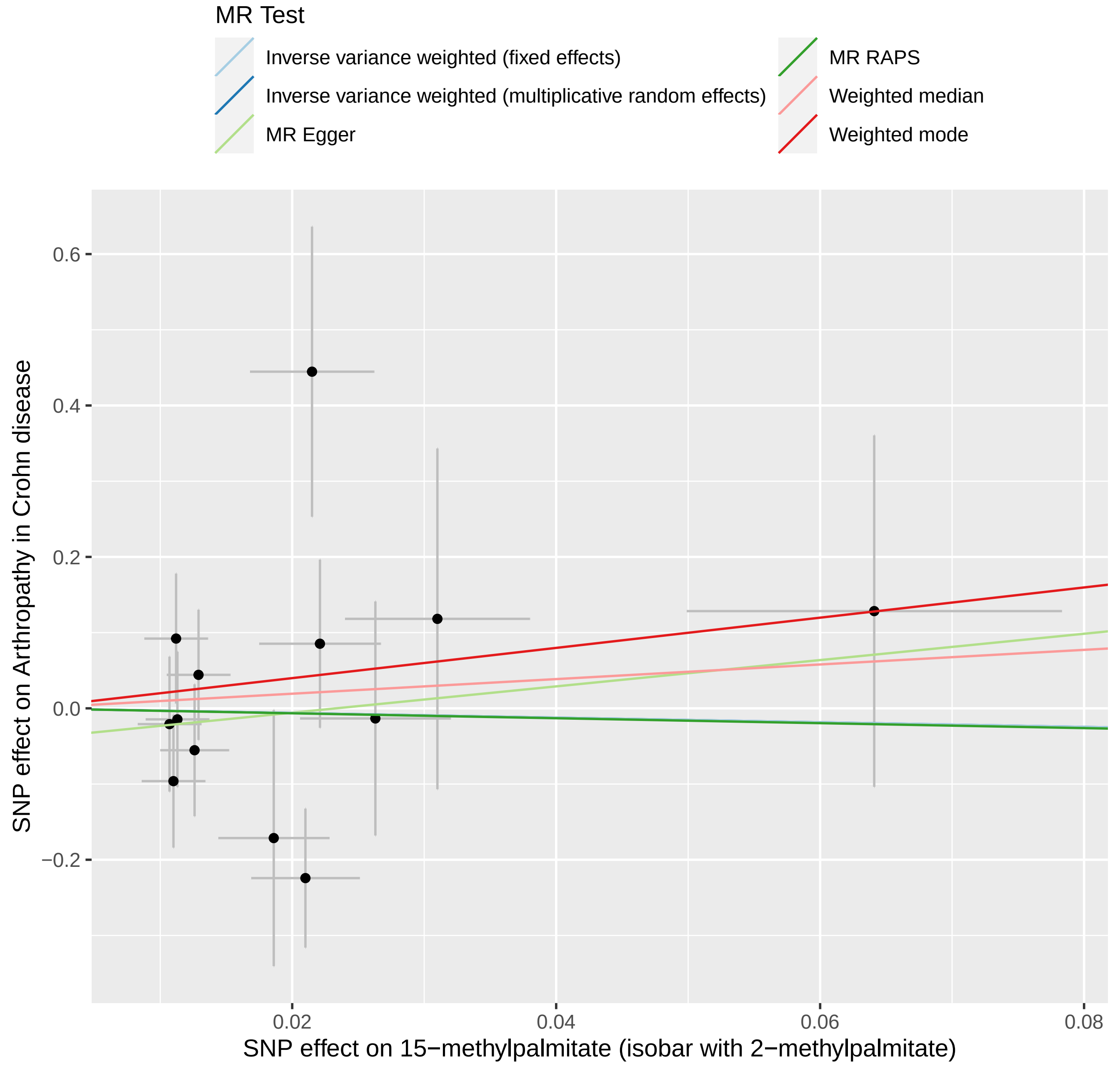

Batch 1102 : Tryptophan on Ulcerative colitis with PSC

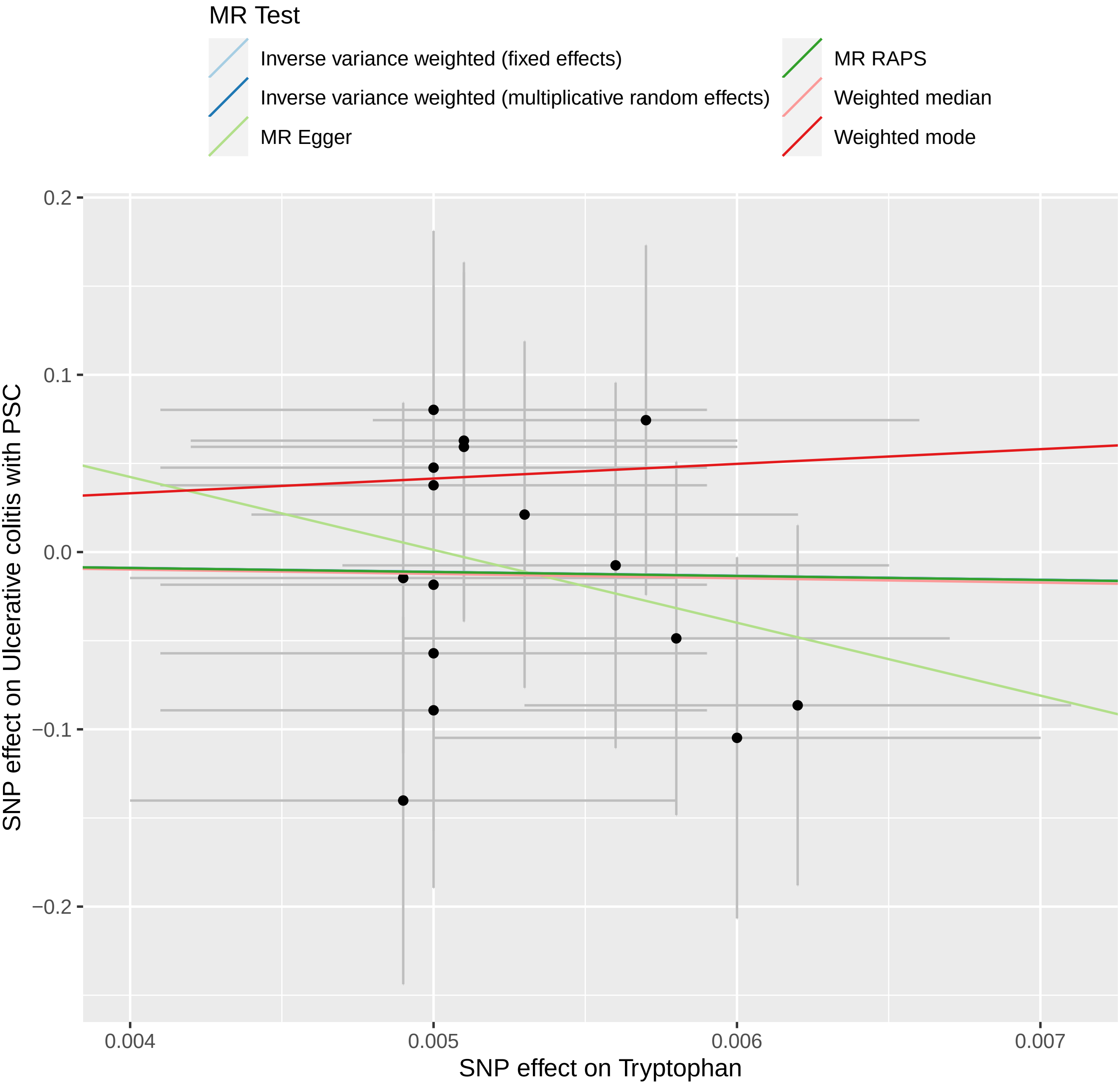

Batch 1104 : Leucine on Ulcerative colitis with PSC

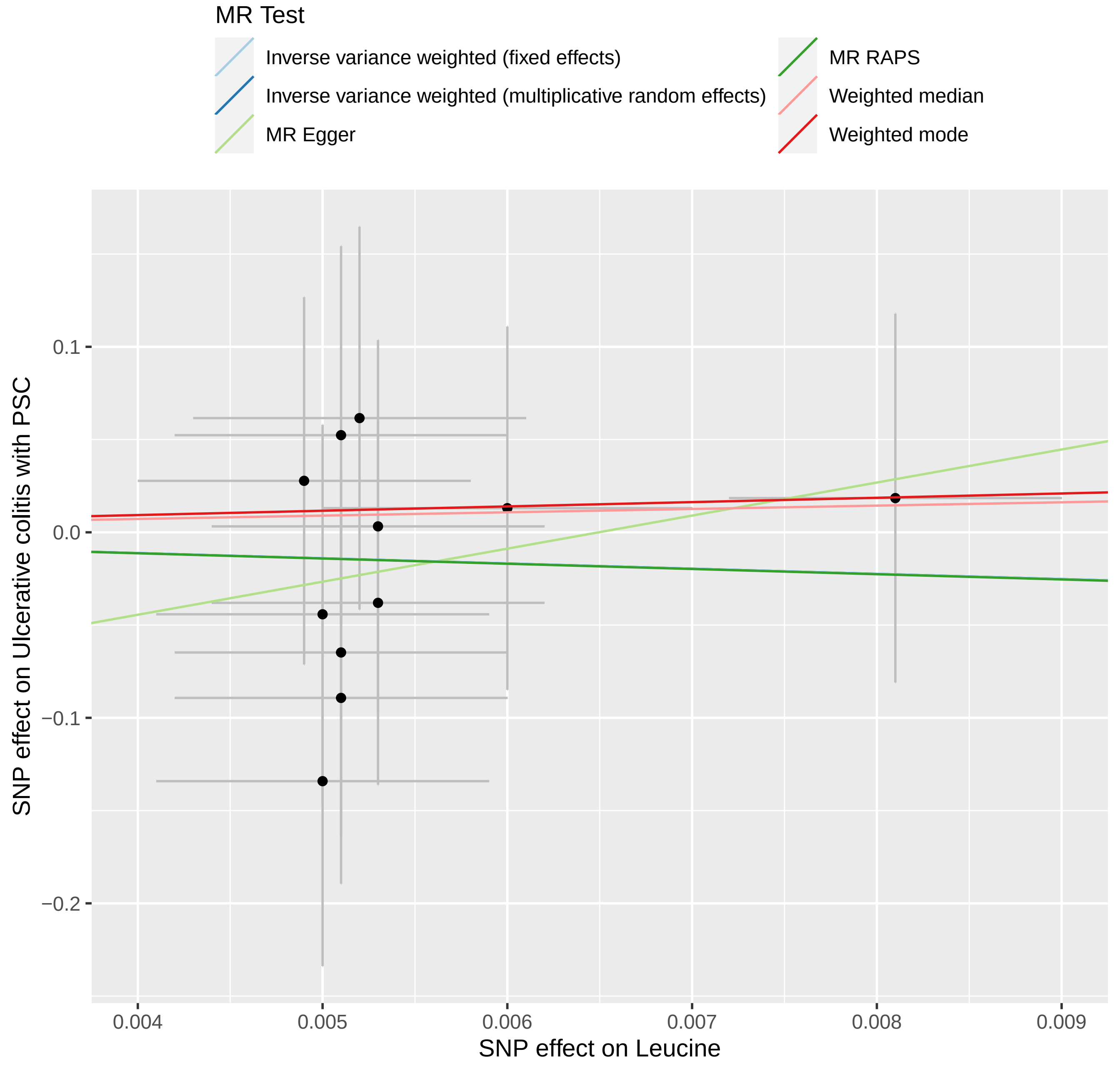

Batch 1108 : Lactate on Ulcerative colitis with PSC

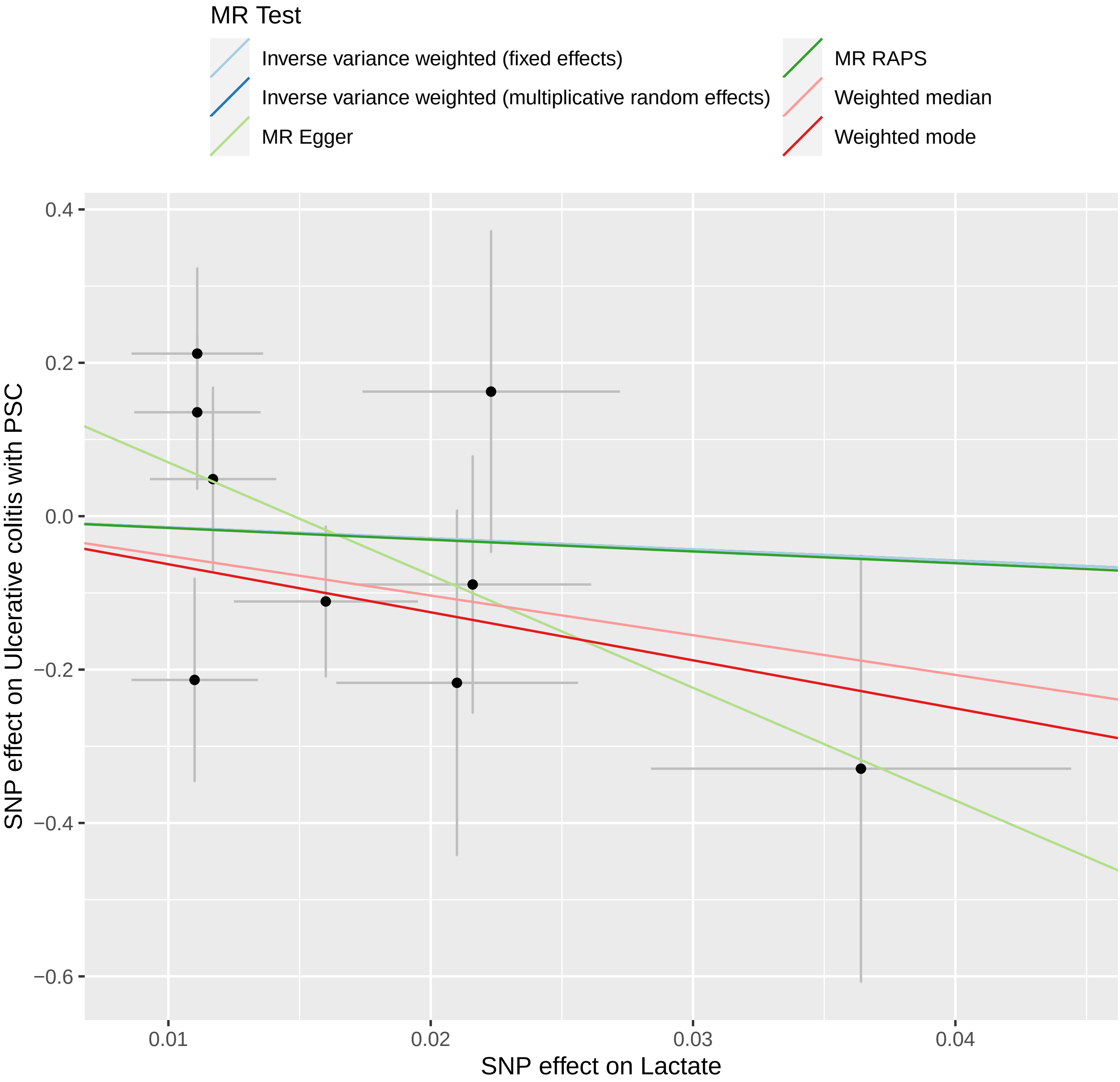

Batch 1109 : 3−hydroxybutyrate (BHBA) on Ulcerative colitis with PSC

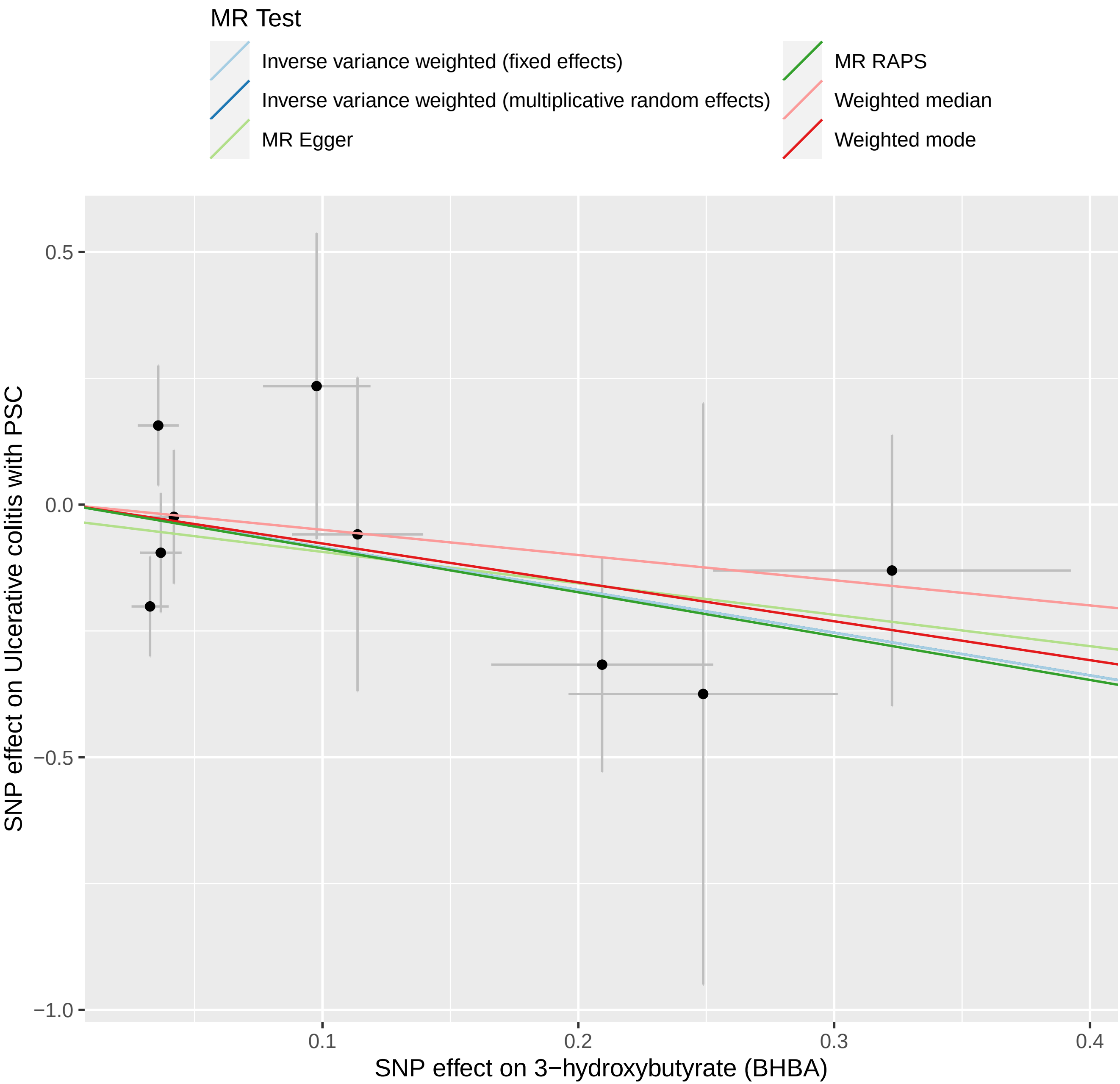

Batch 1110 : Arabinose on Ulcerative colitis with PSC

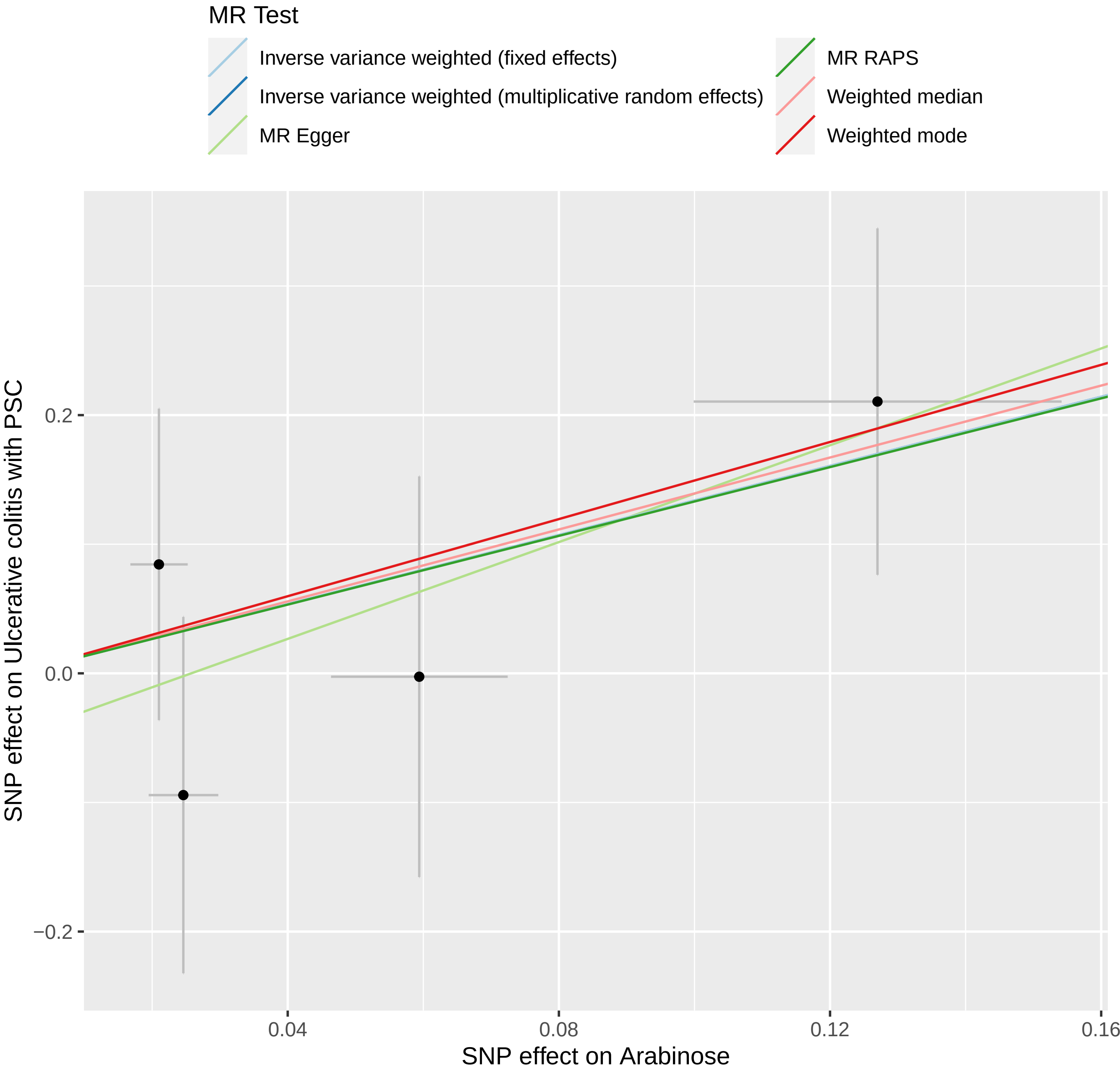

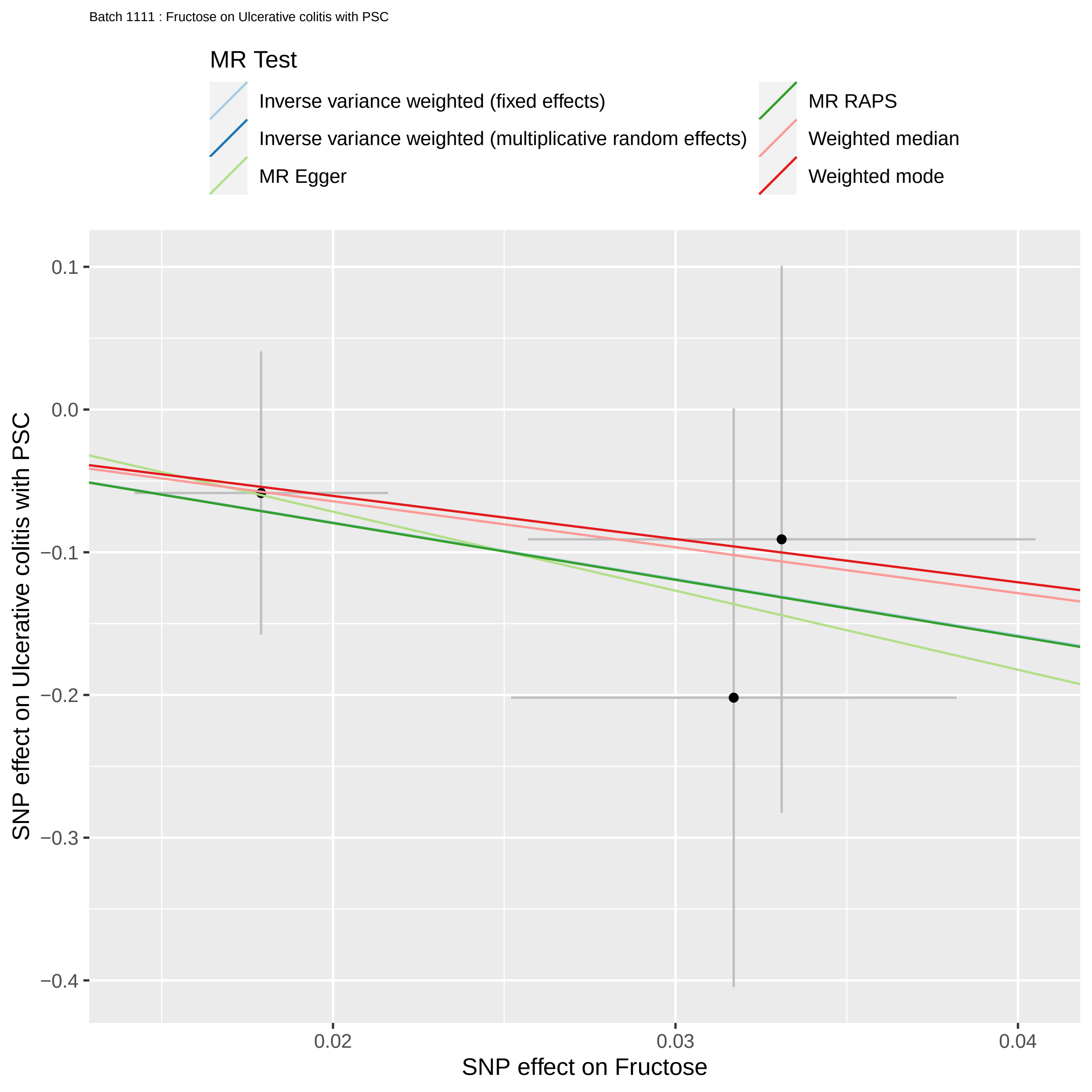
Batch 1111 : Fructose on Ulcerative colitis with PSC
MR Test
Inverse variance weighted (fixed effects)
Inverse variance weighted (multiplicative random effects)
MR Egger
MR RAPS
Weighted median
Weighted mode
0.1
0.0
−0.1
−0.2
−0.3
−0.4
SNP effect on Ulcerative colitis with PSC
0.02
0.03
0.04
SNP effect on Fructose

Batch 1113 : Pyruvate on Ulcerative colitis with PSC

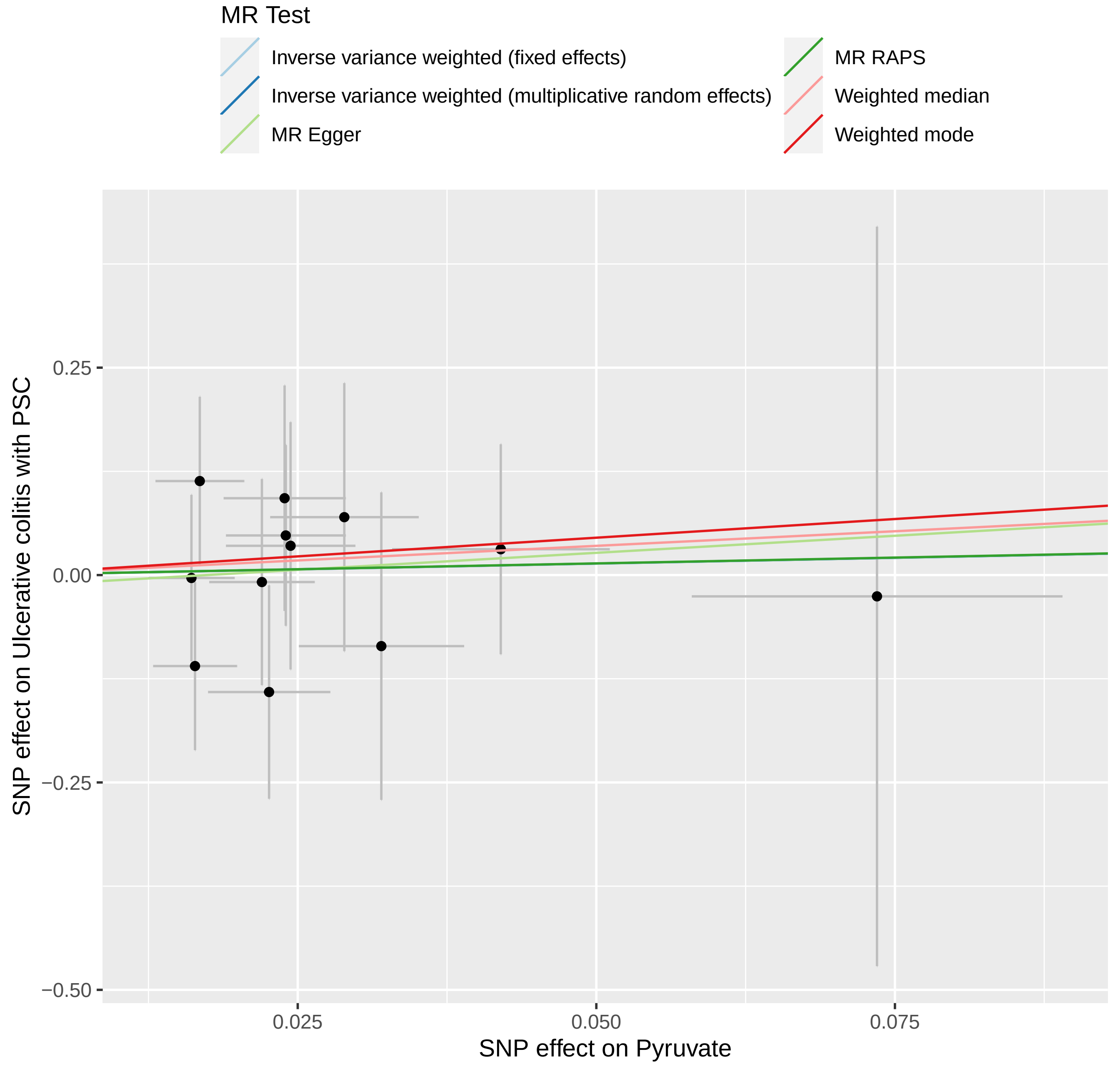

Batch 1114 : Uridine on Ulcerative colitis with PSC

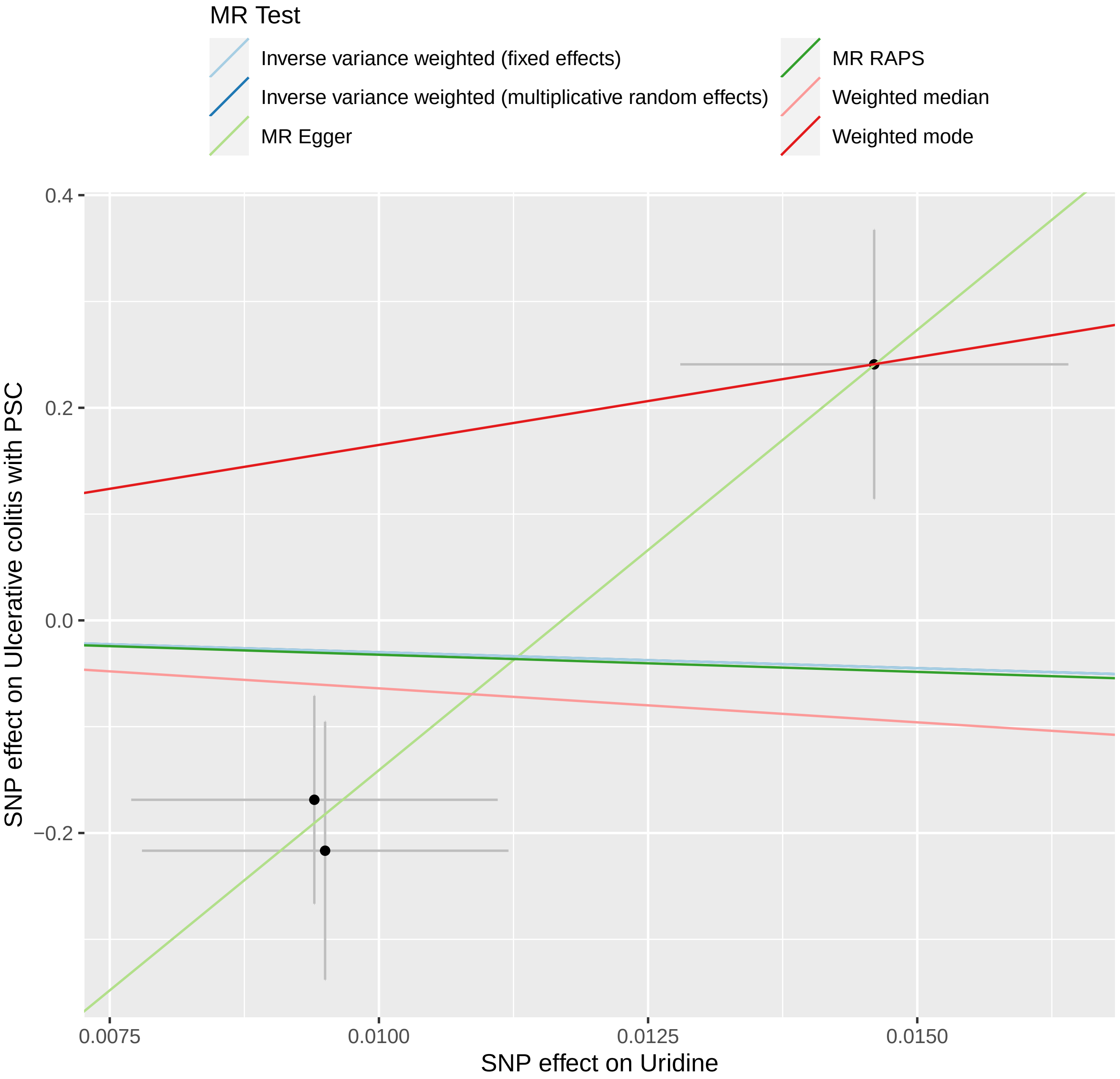

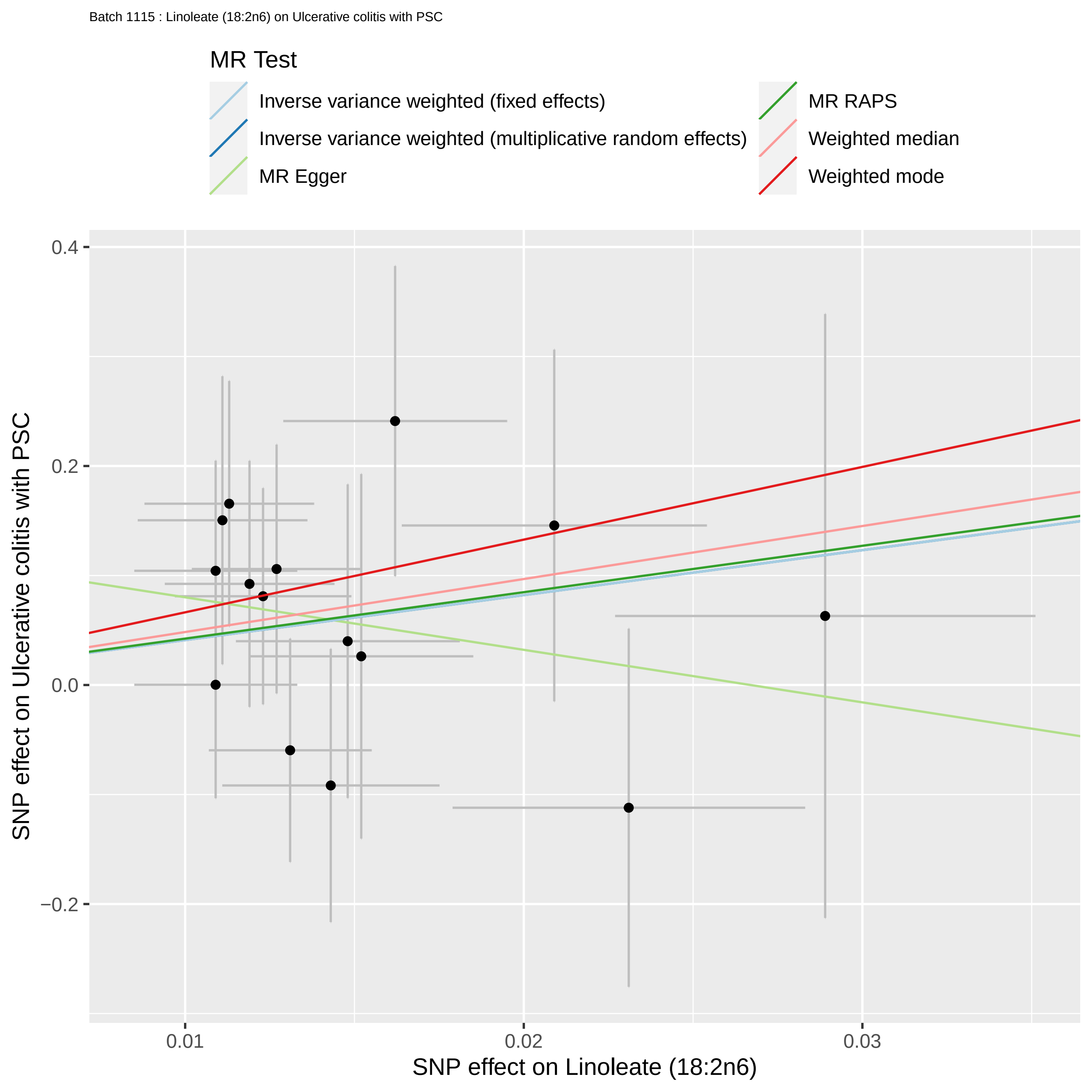
Batch 1115 : Linoleate (18:2n6) on Ulcerative colitis with PSC
MR Test
Inverse variance weighted (fixed effects)
Inverse variance weighted (multiplicative random effects)
MR Egger
MR RAPS
Weighted median
Weighted mode
0.4
0.2
0.0
−0.2
0.01
0.02
0.03
SNP effect on Ulcerative colitis with PSC
SNP effect on Linoleate (18:2n6)

Batch 1116 : Allantoin on Ulcerative colitis with PSC

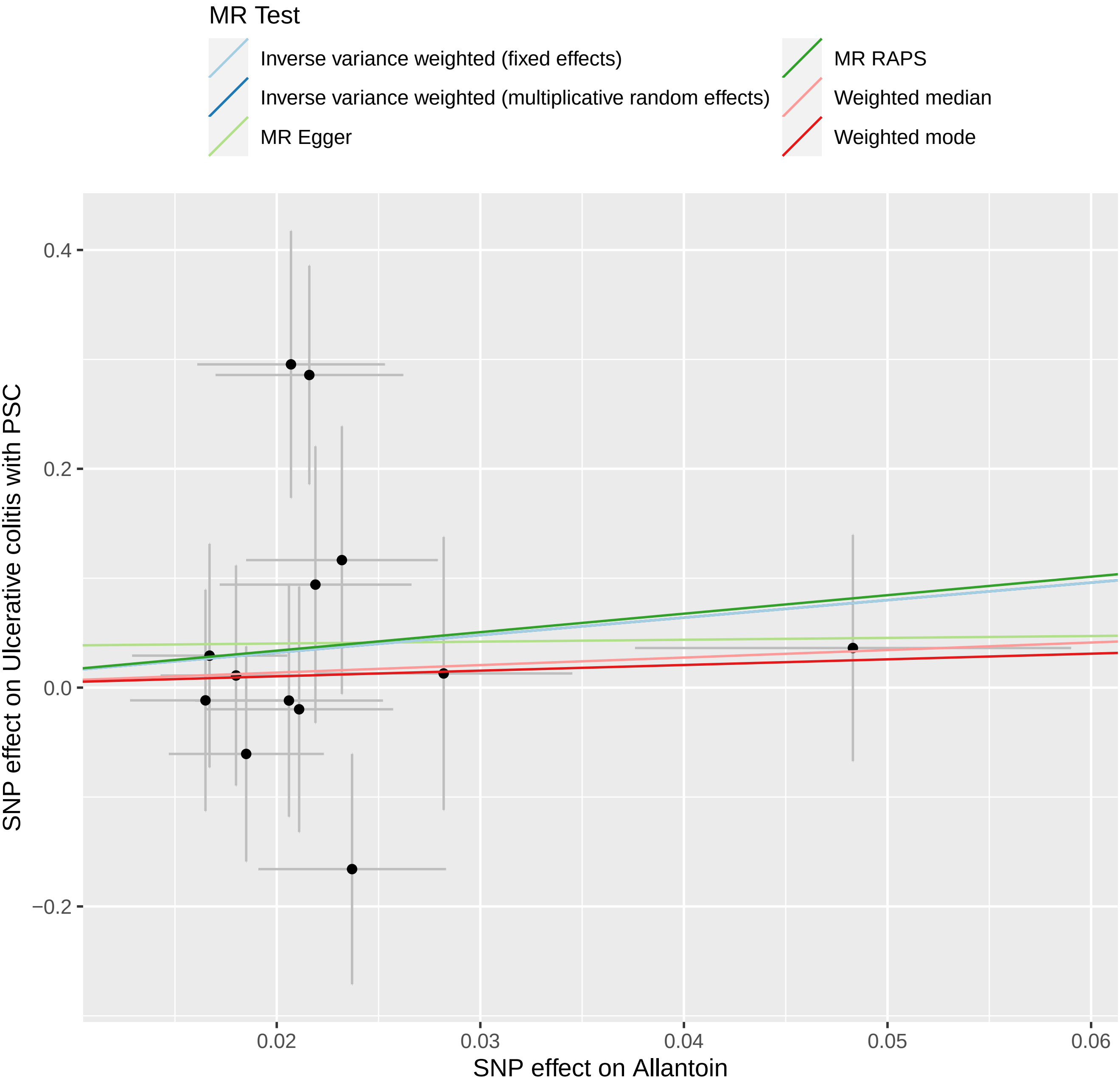

Batch 1121 : Isoleucine on Ulcerative colitis with PSC

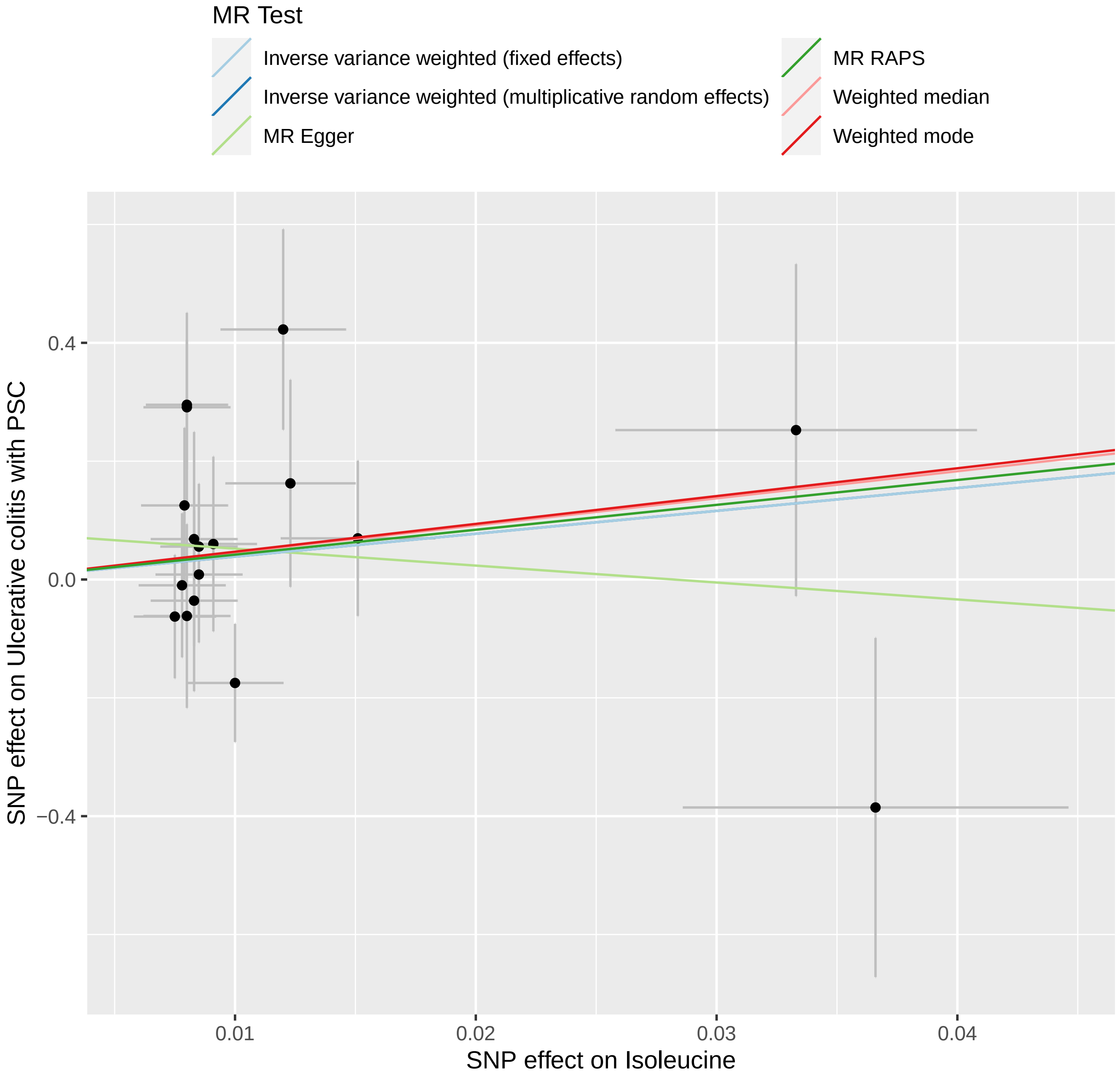

Batch 1122 : Threonine on Ulcerative colitis with PSC

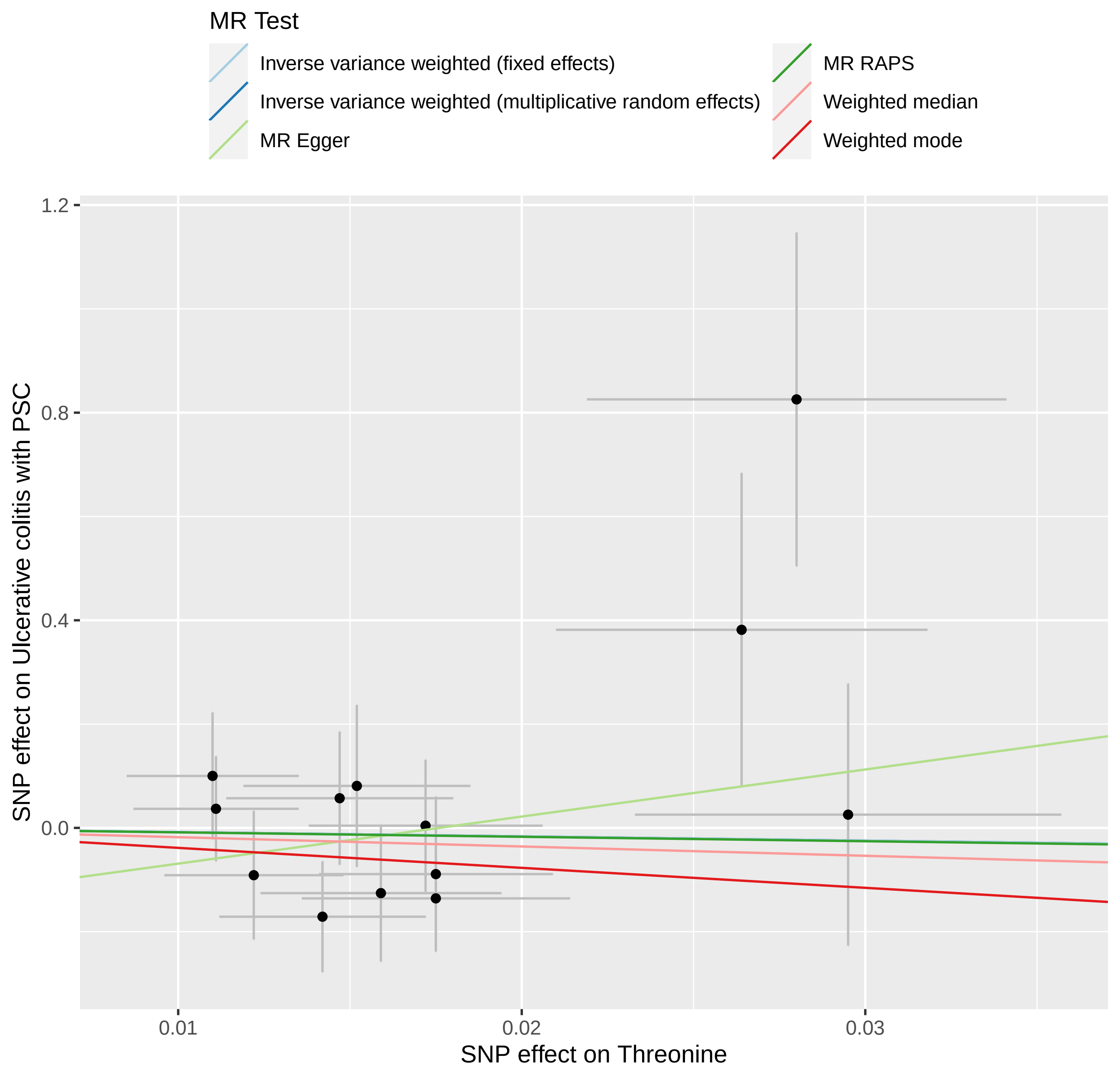

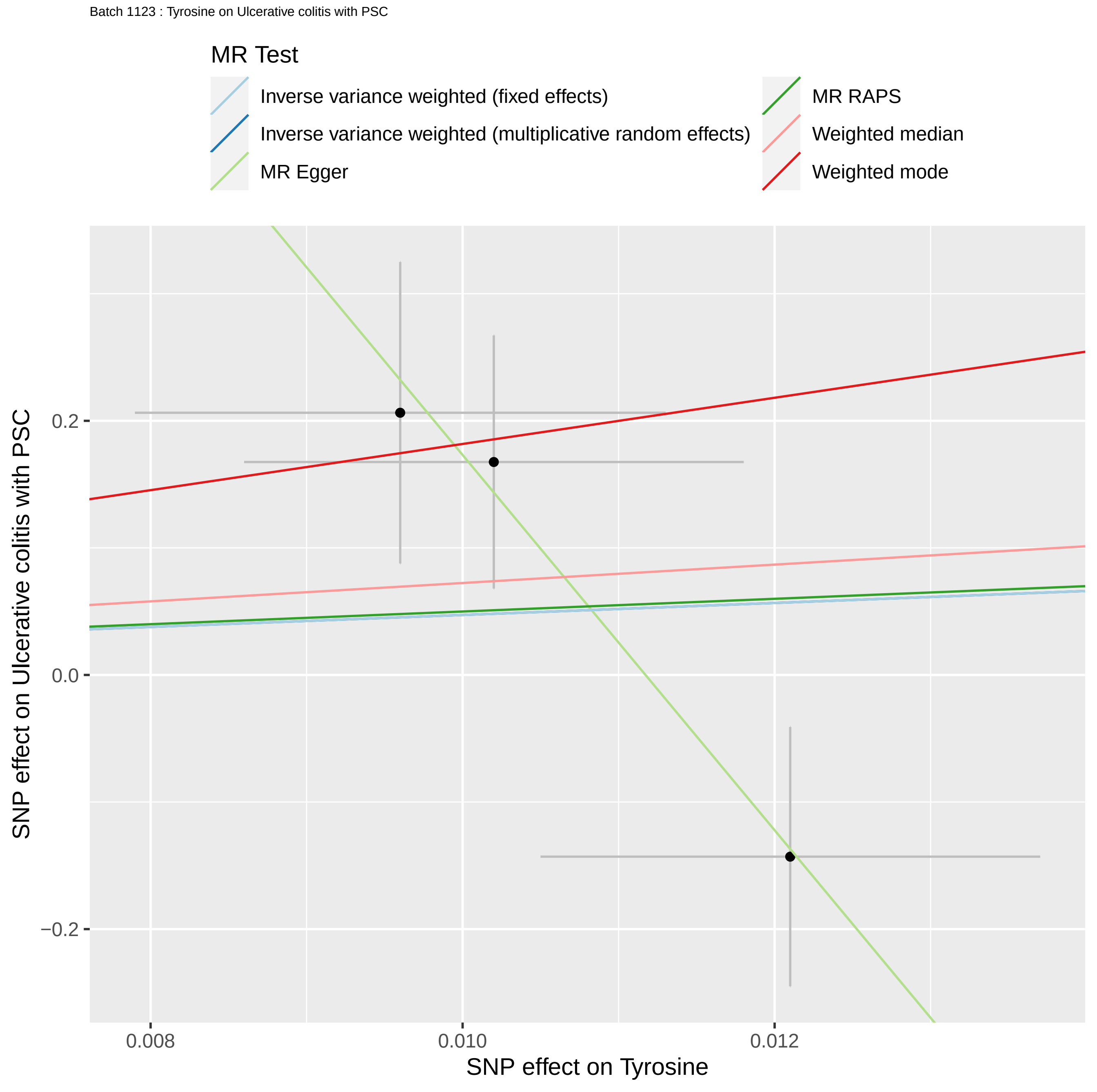

Batch 1123 : Tyrosine on Ulcerative colitis with PSC
MR Test
Inverse variance weighted (fixed effects)
Inverse variance weighted (multiplicative random effects)
MR Egger
MR RAPS
Weighted median
Weighted mode
0.2
0.0
−0.2
0.008
0.010
0.012
SNP effect on Tyrosine
SNP effect on Ulcerative colitis with PSC

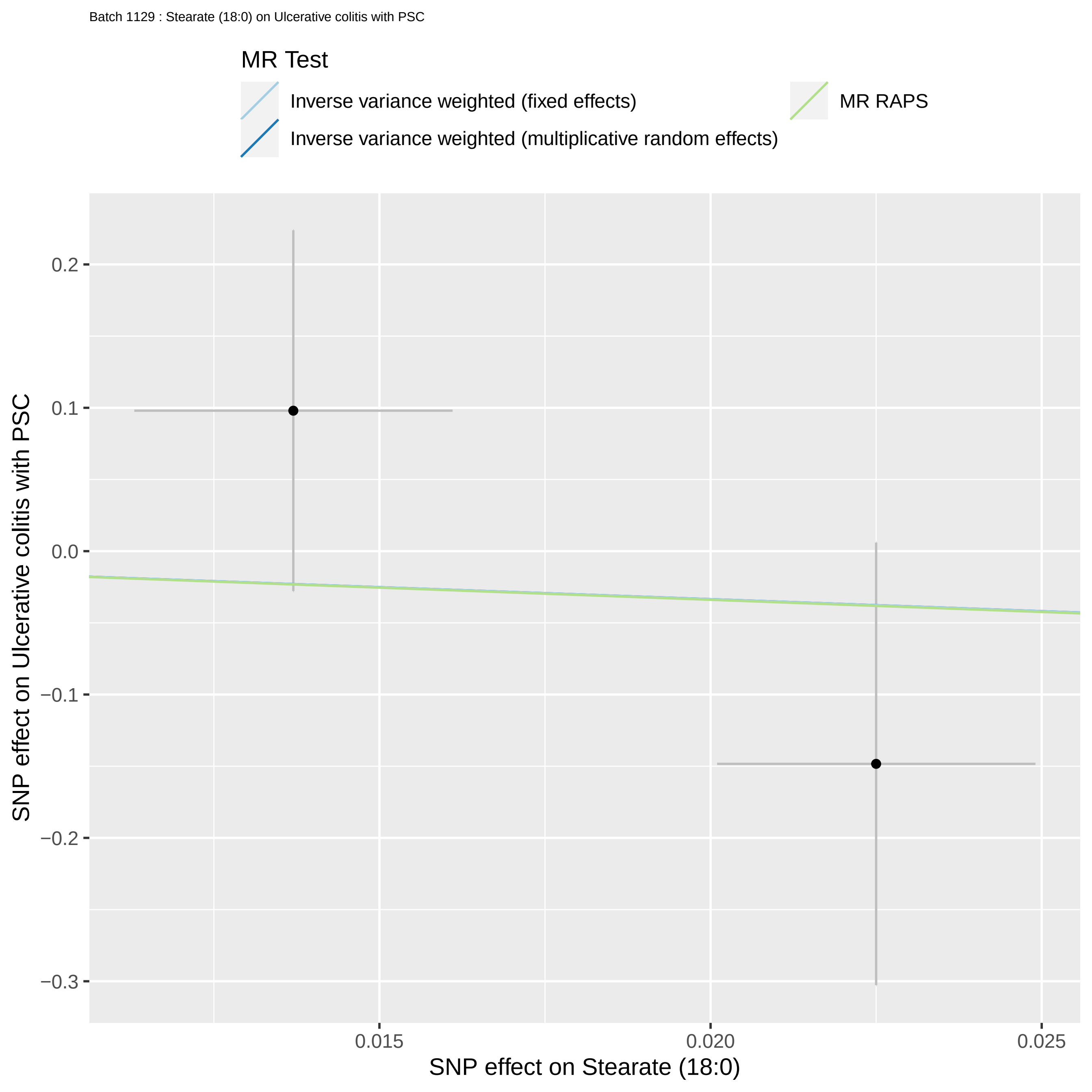

Batch 1129 : Stearate (18:0) on Ulcerative colitis with PSC
MR Test
Inverse variance weighted (fixed effects)
Inverse variance weighted (multiplicative random effects)
MR RAPS
0.2
0.1
0.0
−0.1
−0.2
−0.3
SNP effect on Ulcerative colitis with PSC
0.015
0.020
0.025
SNP effect on Stearate (18:0)

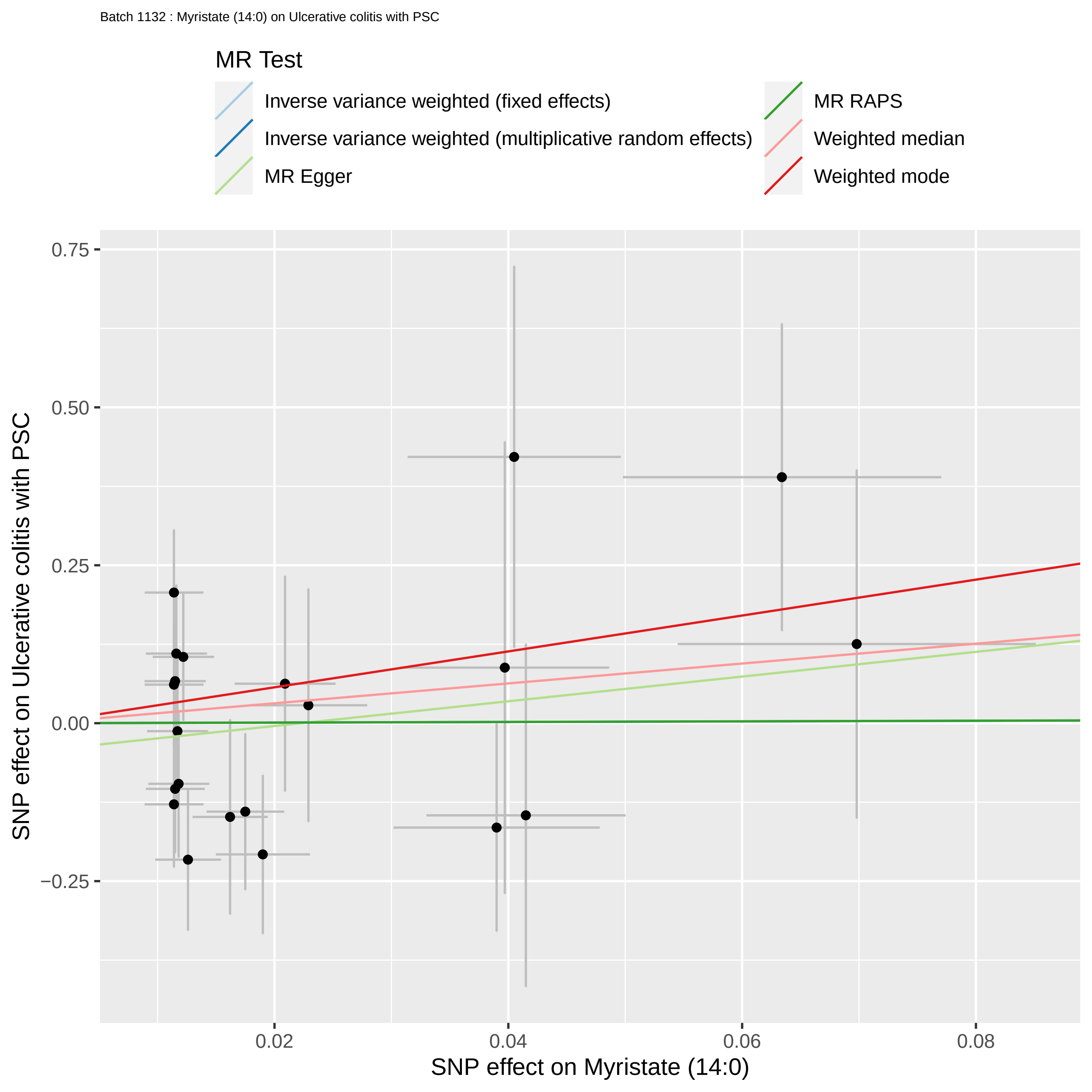

Batch 1132 : Myristate (14:0) on Ulcerative colitis with PSC
MR Test
Inverse variance weighted (fixed effects)
Inverse variance weighted (multiplicative random effects)
MR Egger
MR RAPS
Weighted median
Weighted mode
0.75
0.50
0.25
0.00
−0.25
0.02
0.04
0.06
0.08
SNP effect on Ulcerative colitis with PSC
SNP effect on Myristate (14:0)

Batch 1134 : Ornithine on Ulcerative colitis with PSC

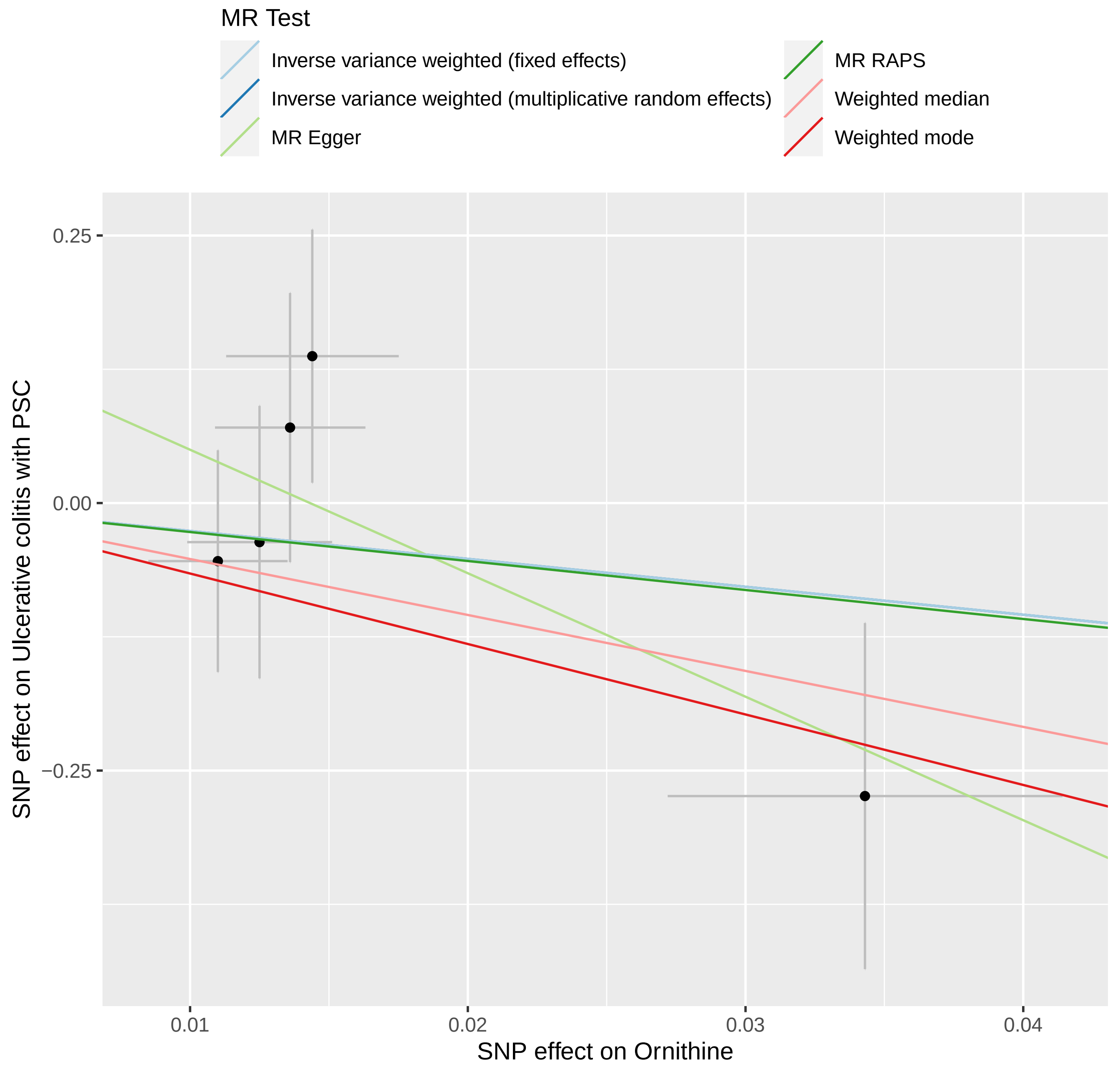

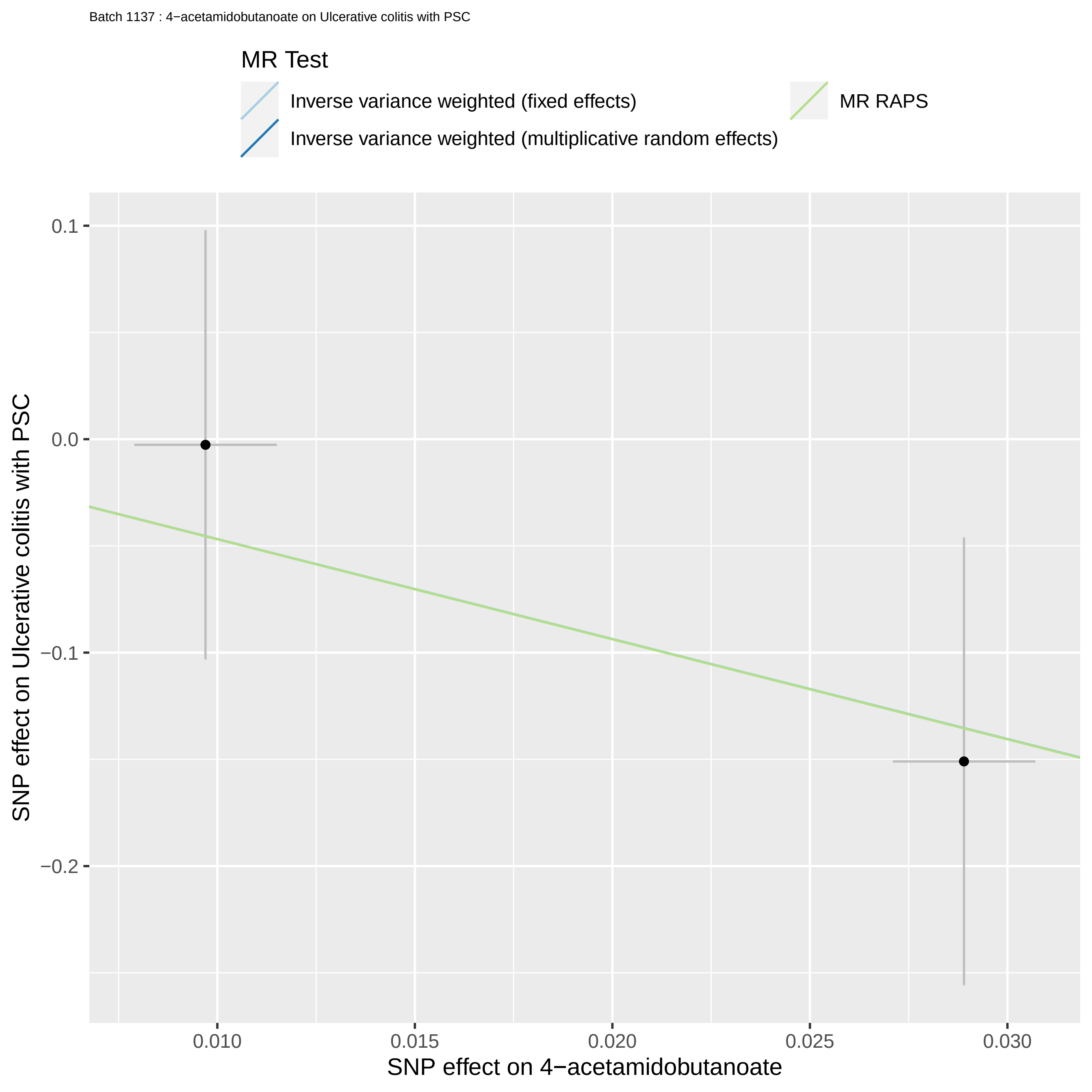

Batch 1137 : 4−acetamidobutanoate on Ulcerative colitis with PSC
MR Test
Inverse variance weighted (fixed effects)
Inverse variance weighted (multiplicative random effects)
MR RAPS
0.1
0.0
−0.1
−0.2
SNP effect on Ulcerative colitis with PSC
0.010
0.015
0.020
0.025
0.030
SNP effect on 4−acetamidobutanoate

Batch 1138 : Alpha−tocopherol on Ulcerative colitis with PSC

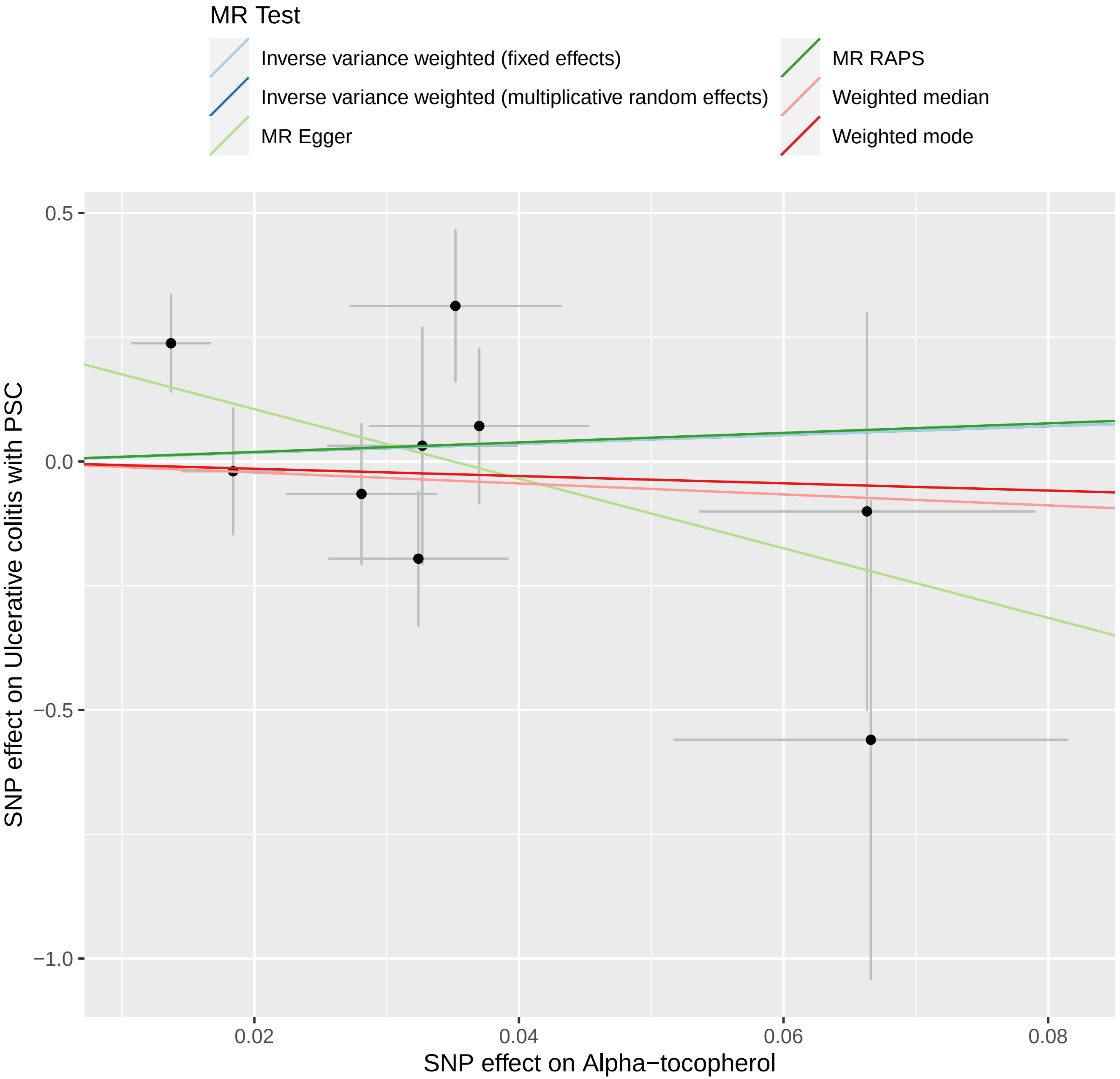

Batch 1139 : Citrate on Ulcerative colitis with PSC

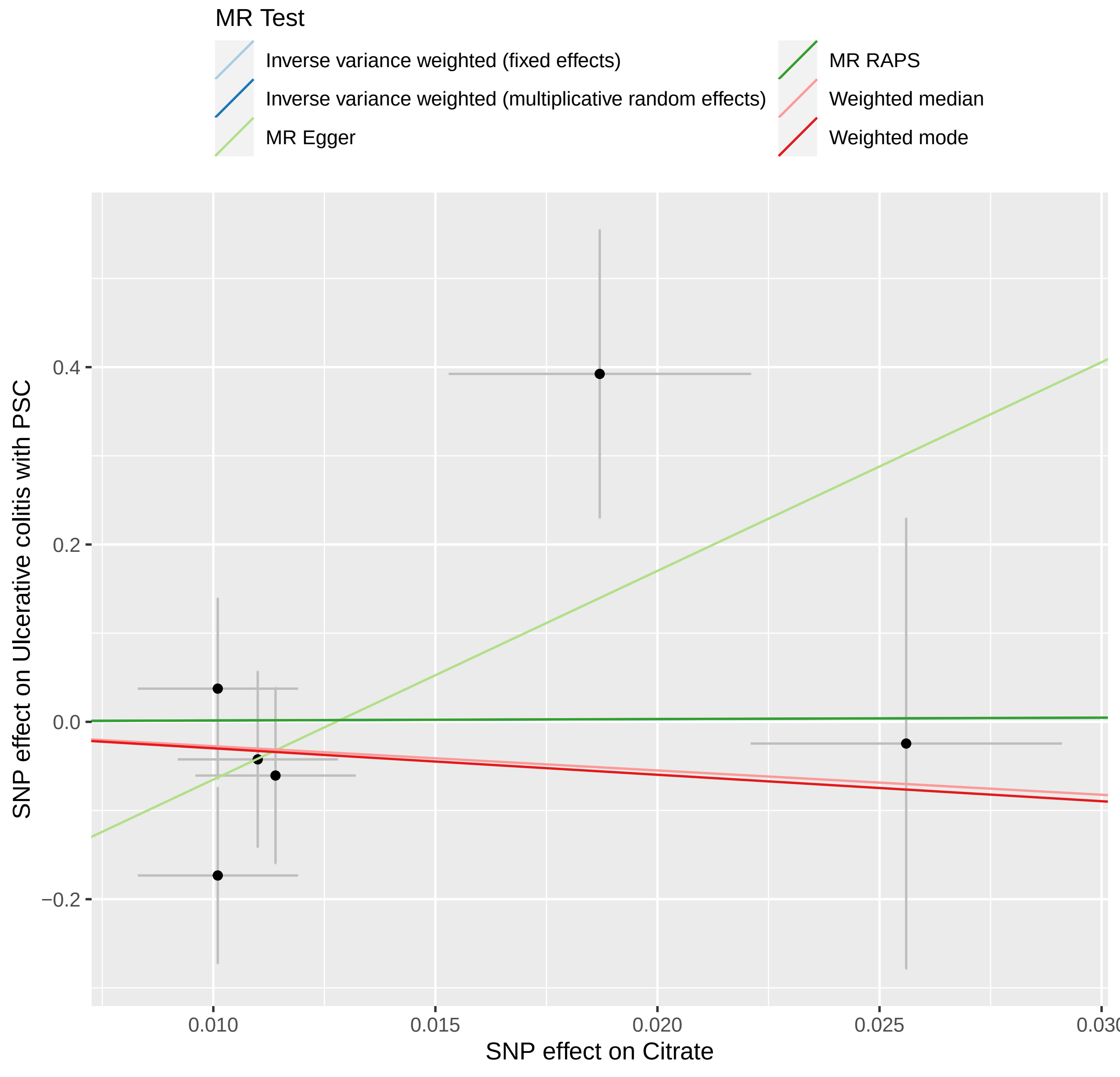

Batch 1140 : Glycerate on Ulcerative colitis with PSC

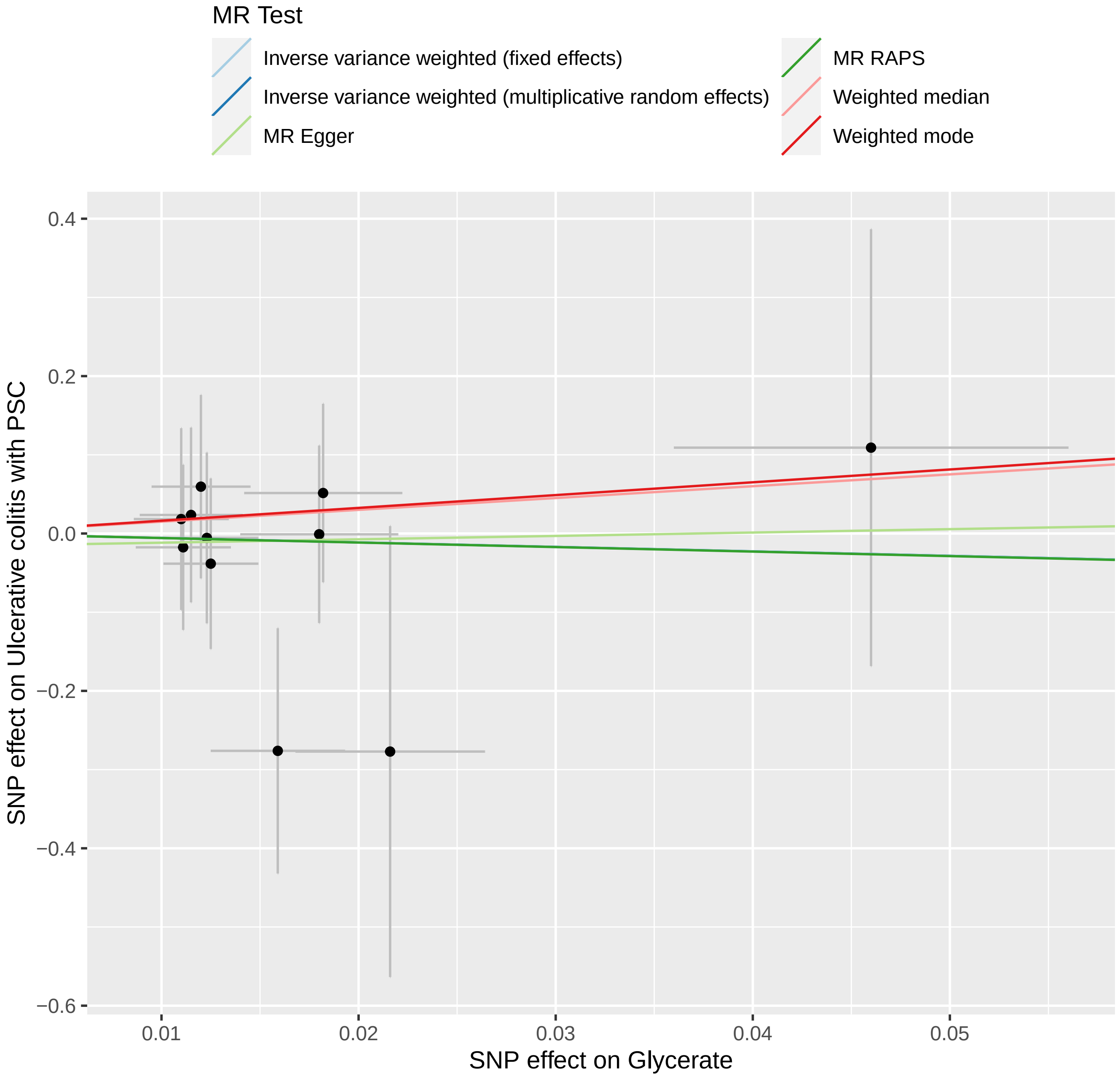

Batch 1141 : Guanosine on Ulcerative colitis with PSC

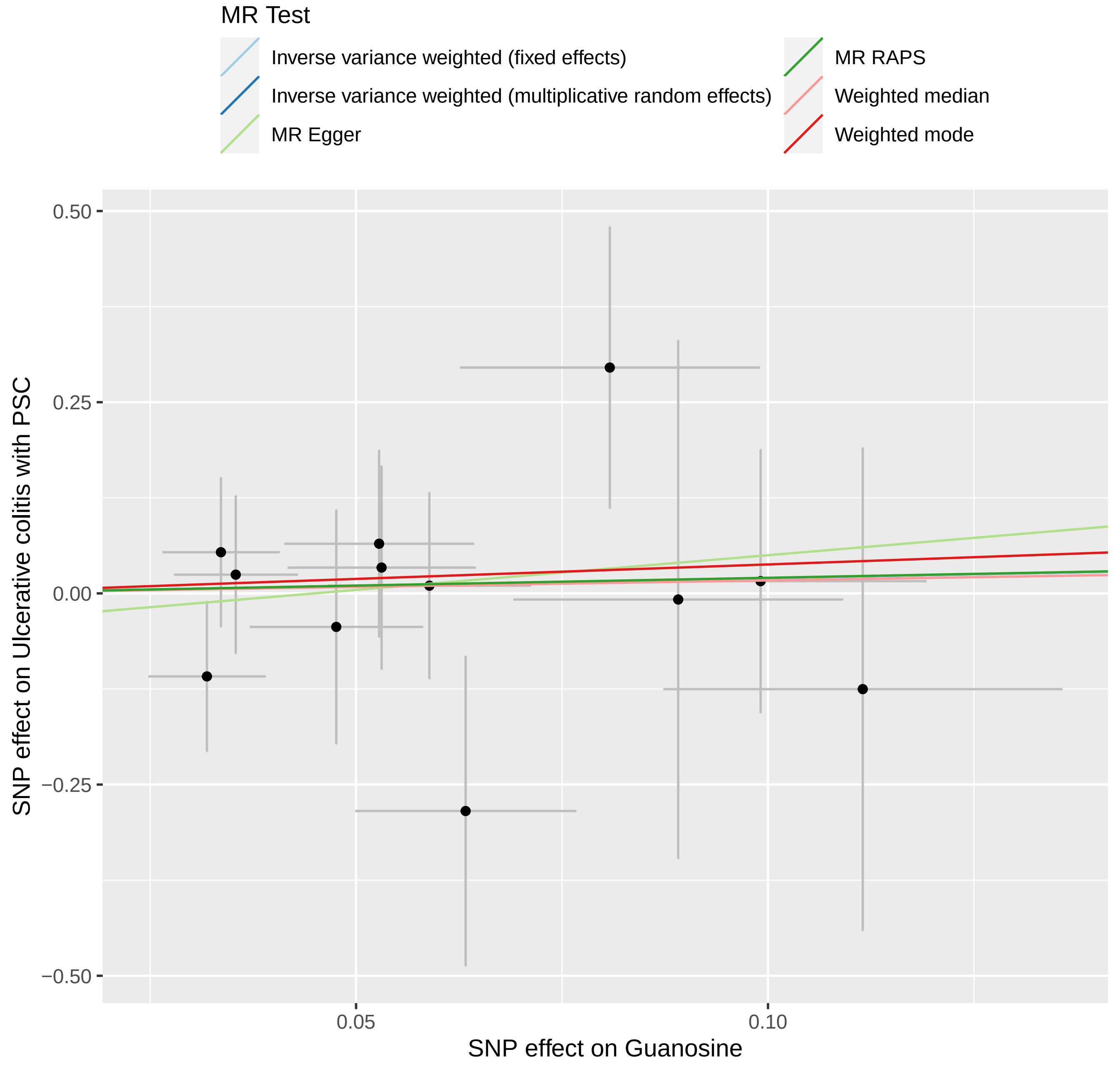

Batch 1144 : Ursodeoxycholate on Ulcerative colitis with PSC

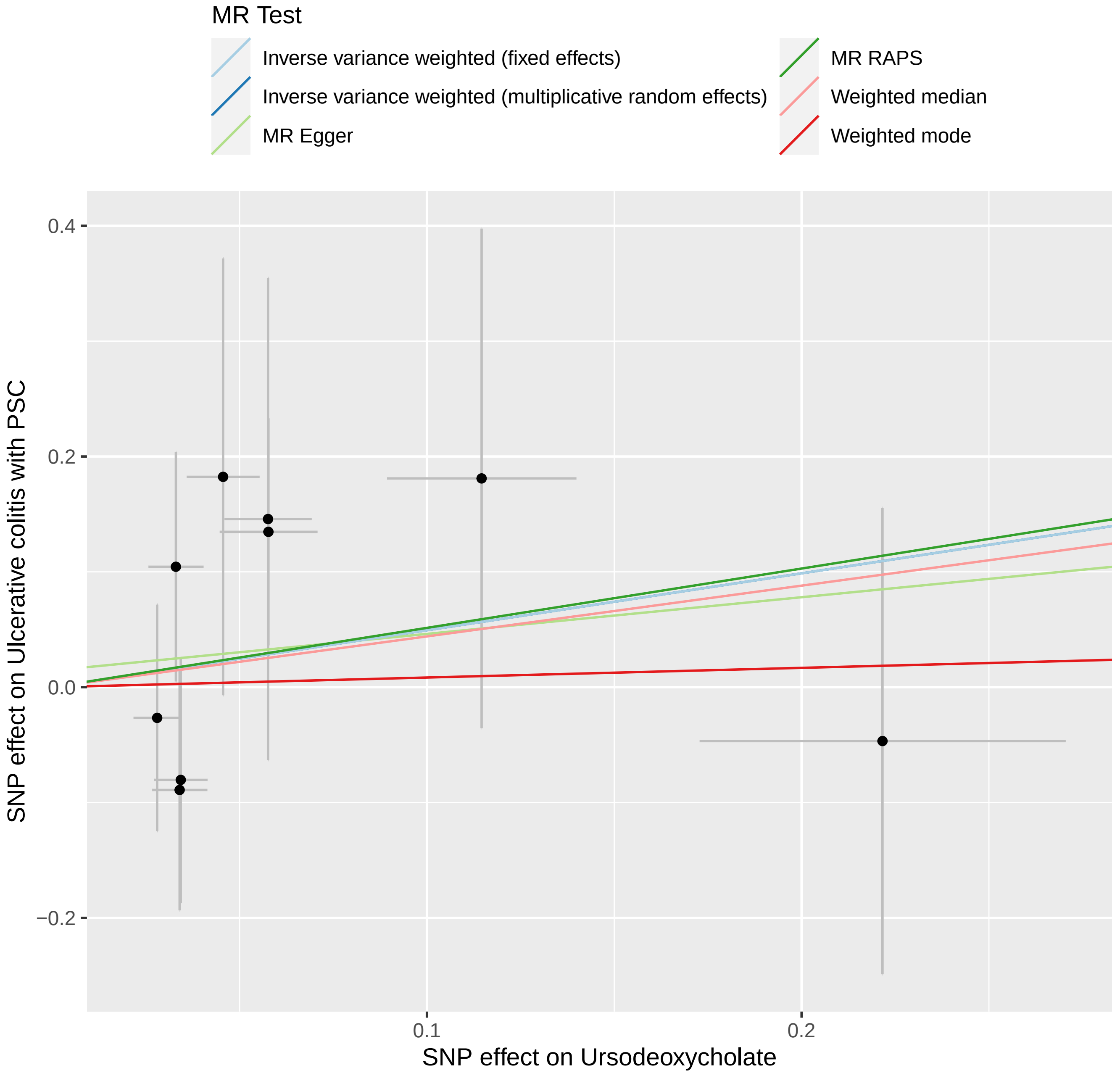

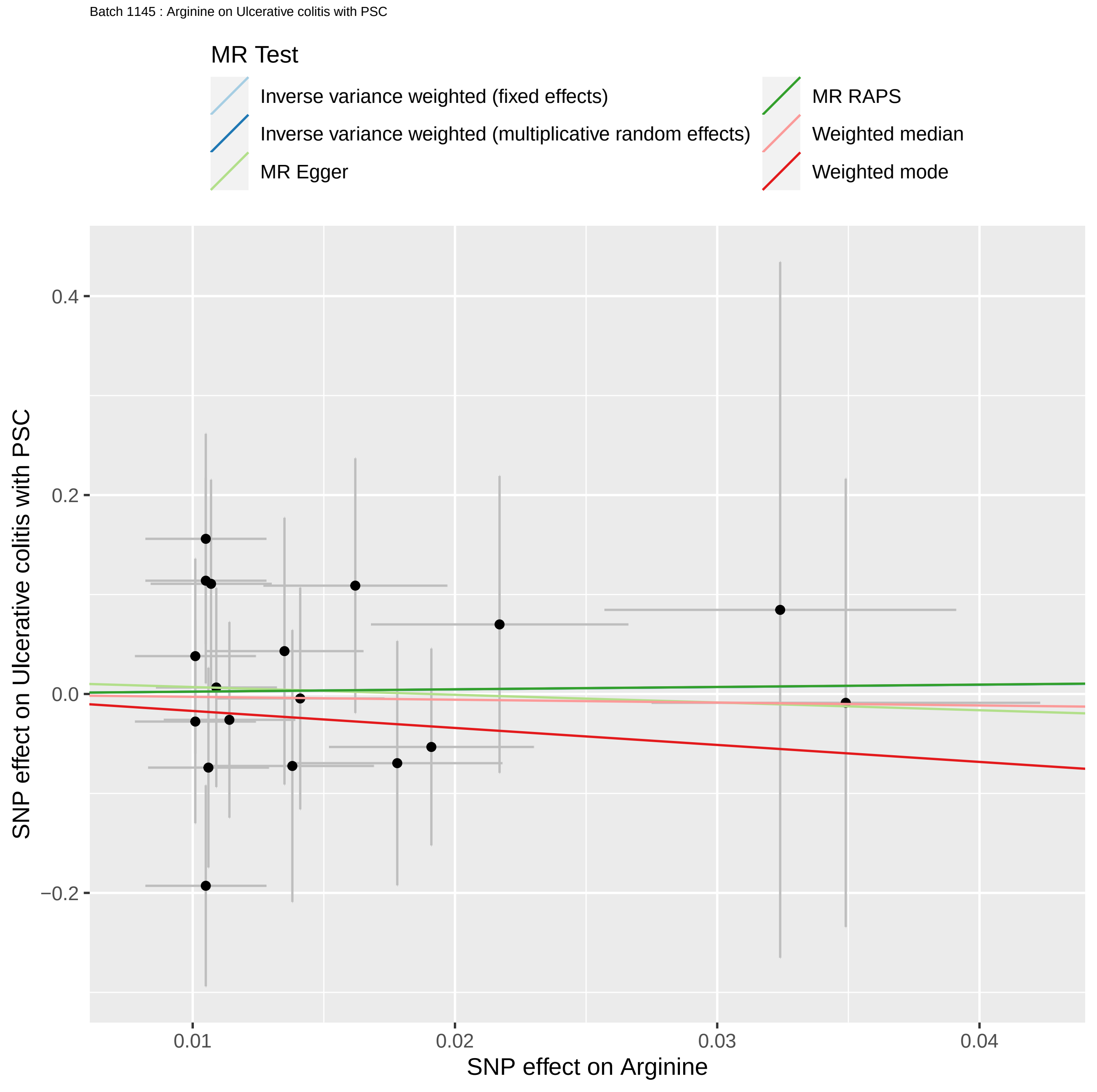
Batch 1145 : Arginine on Ulcerative colitis with PSC
MR Test
Inverse variance weighted (fixed effects)
Inverse variance weighted (multiplicative random effects)
MR Egger
MR RAPS
Weighted median
Weighted mode
0.4
0.2
0.0
−0.2
0.01
0.02
0.03
0.04
SNP effect on Ulcerative colitis with PSC
SNP effect on Arginine

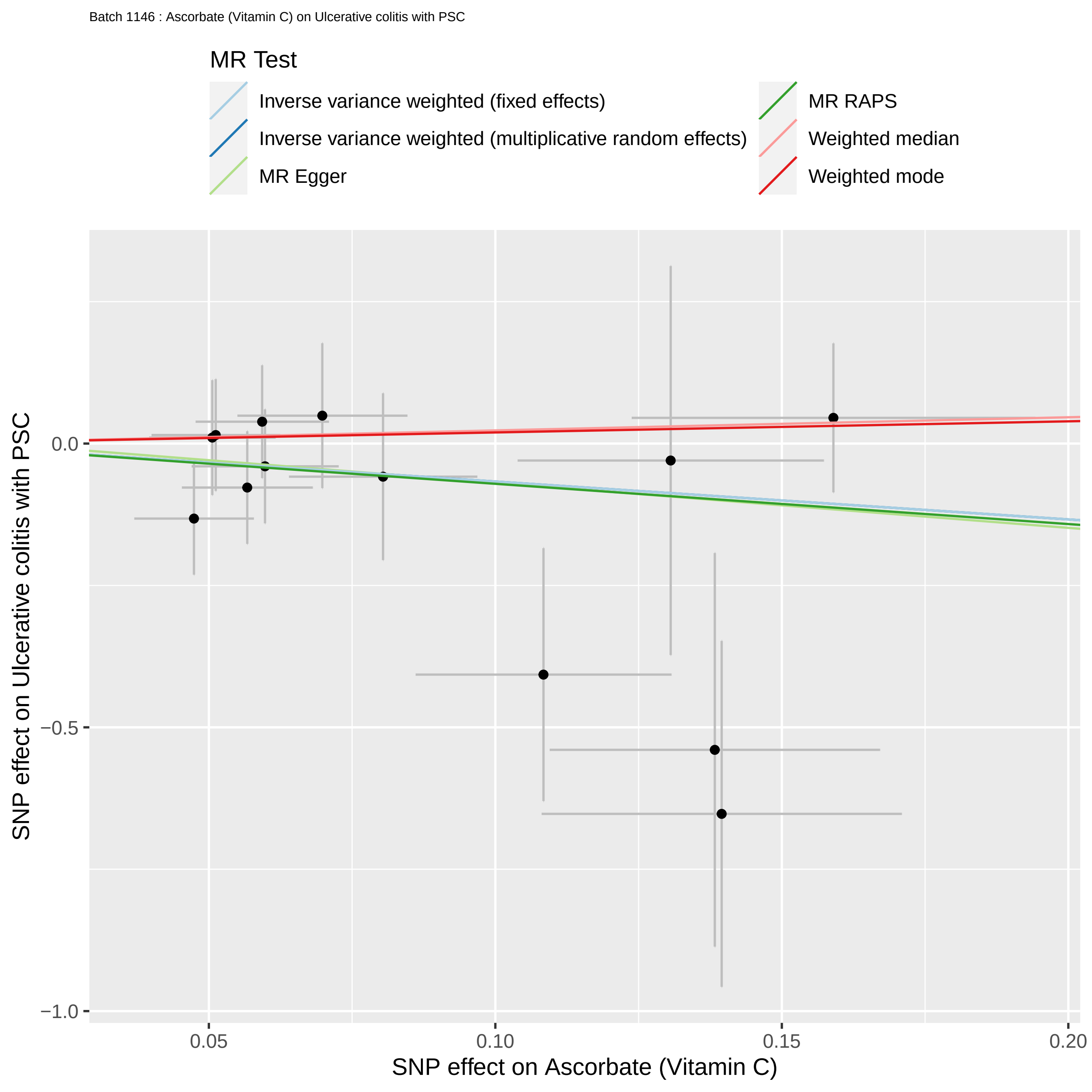
Batch 1146 : Ascorbate (Vitamin C) on Ulcerative colitis with PSC
MR Test
Inverse variance weighted (fixed effects)
Inverse variance weighted (multiplicative random effects)
MR Egger
MR RAPS
Weighted median
Weighted mode
0.0
−0.5
−1.0
0.05
0.10
0.15
0.20
SNP effect on Ulcerative colitis with PSC
SNP effect on Ascorbate (Vitamin C)

Batch 1147 : Heptanoate (7:0) on Ulcerative colitis with PSC

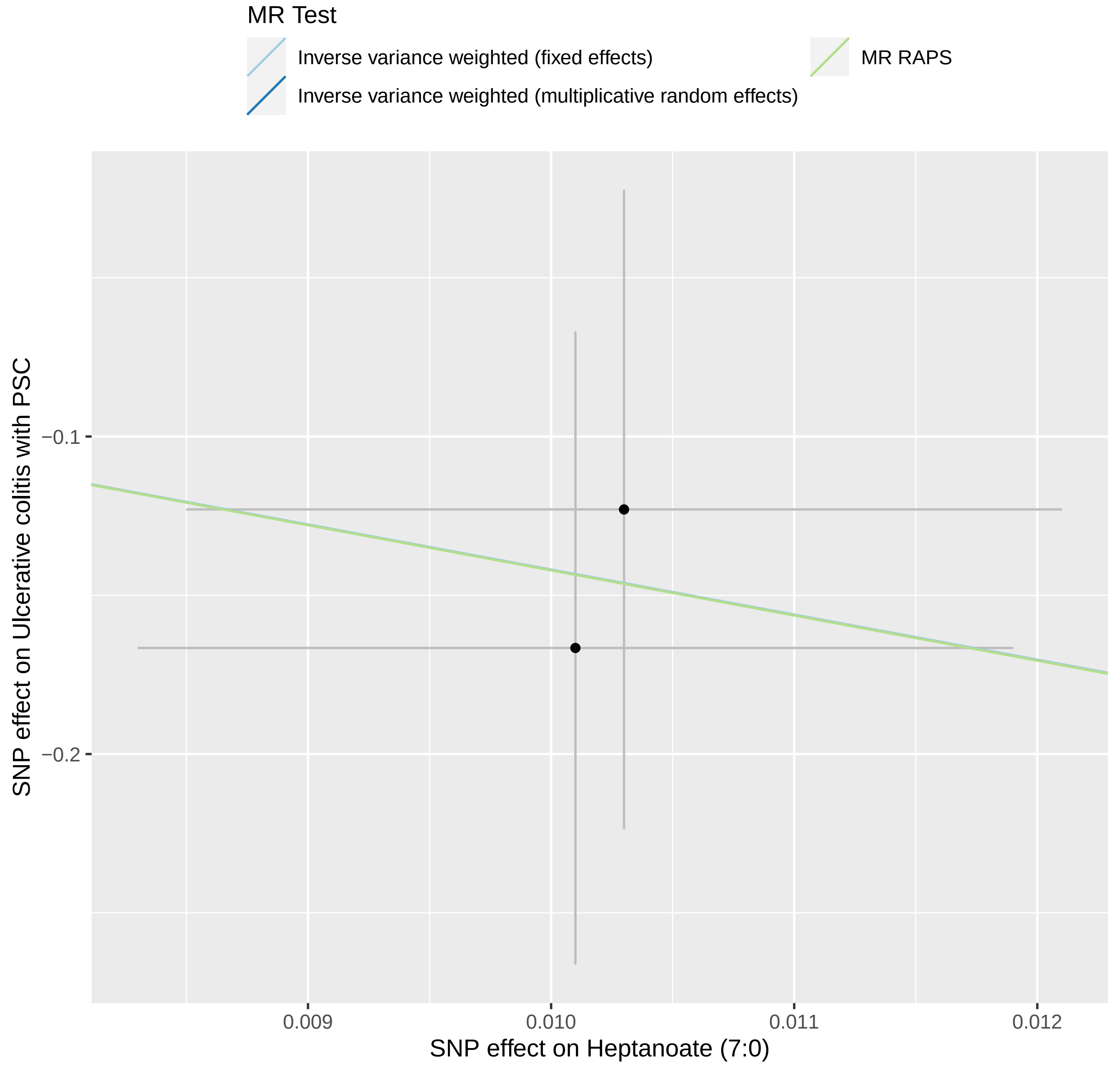

Batch 1148 : Laurate (12:0) on Ulcerative colitis with PSC

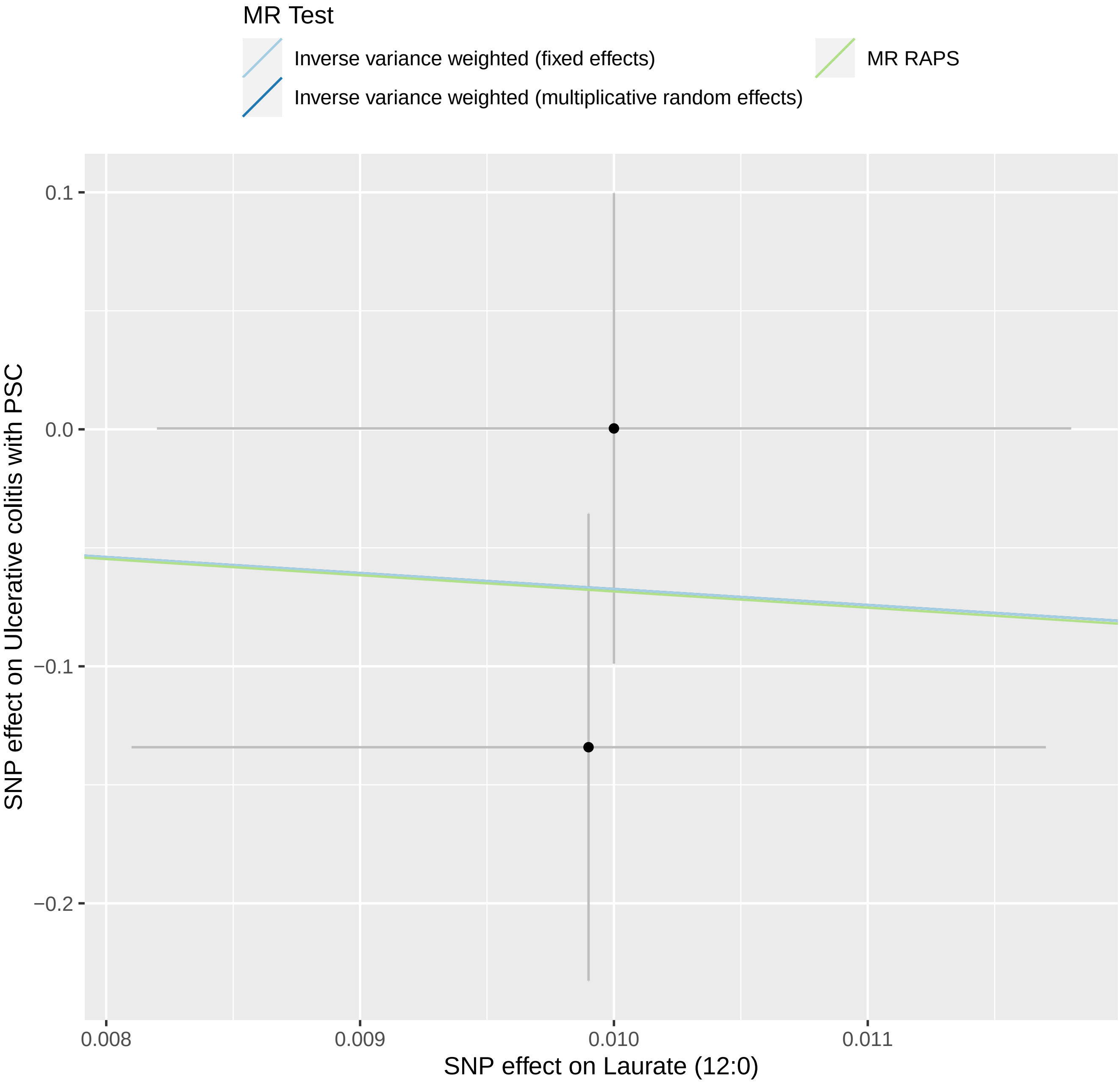

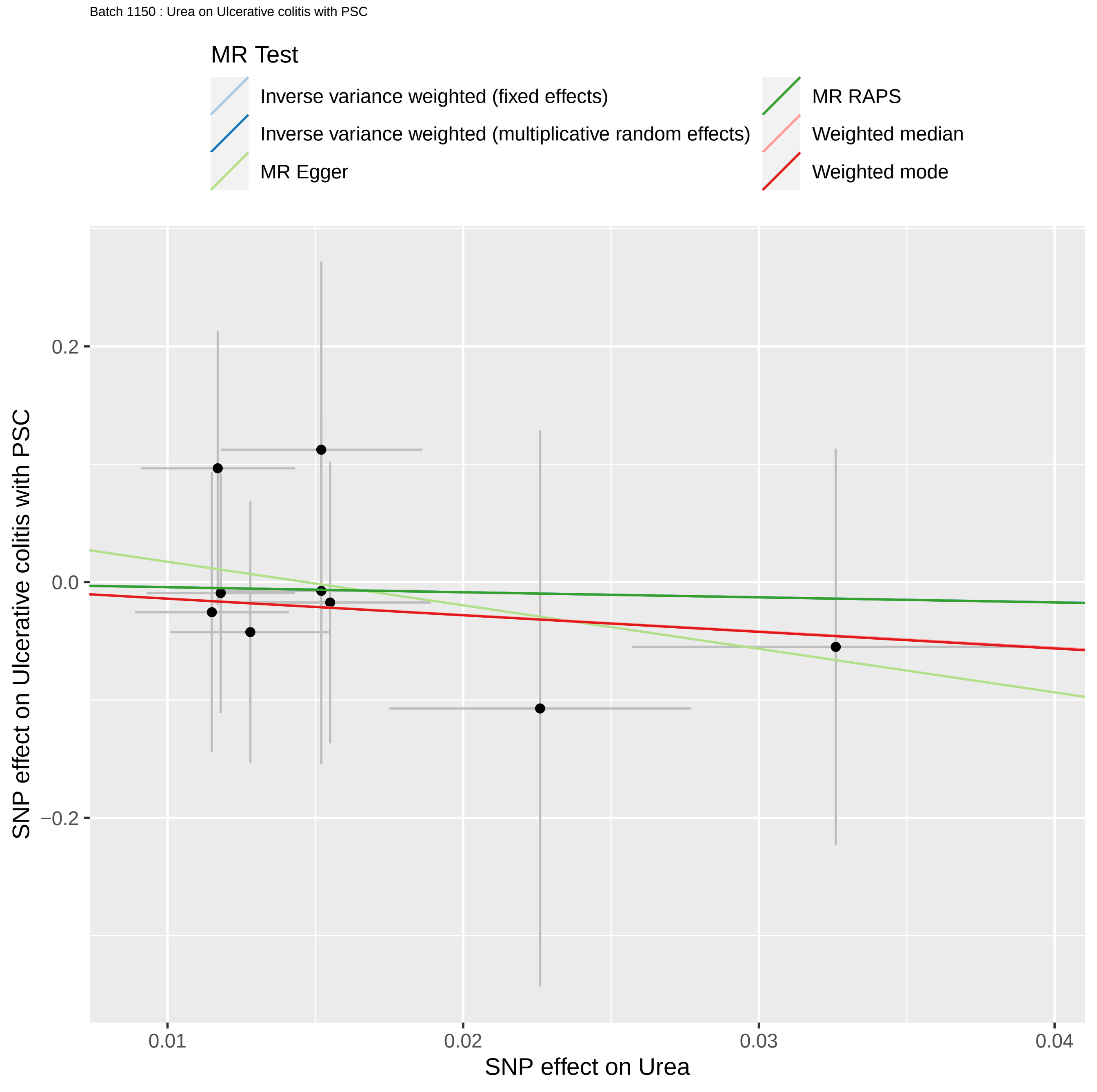

Batch 1150 : Urea on Ulcerative colitis with PSC
MR Test
Inverse variance weighted (fixed effects)
Inverse variance weighted (multiplicative random effects)
MR Egger
MR RAPS
Weighted median
Weighted mode
0.2
0.0
−0.2
0.01
0.02
0.03
0.04
SNP effect on Ulcerative colitis with PSC
SNP effect on Urea

Batch 1151 : Cortisol on Ulcerative colitis with PSC

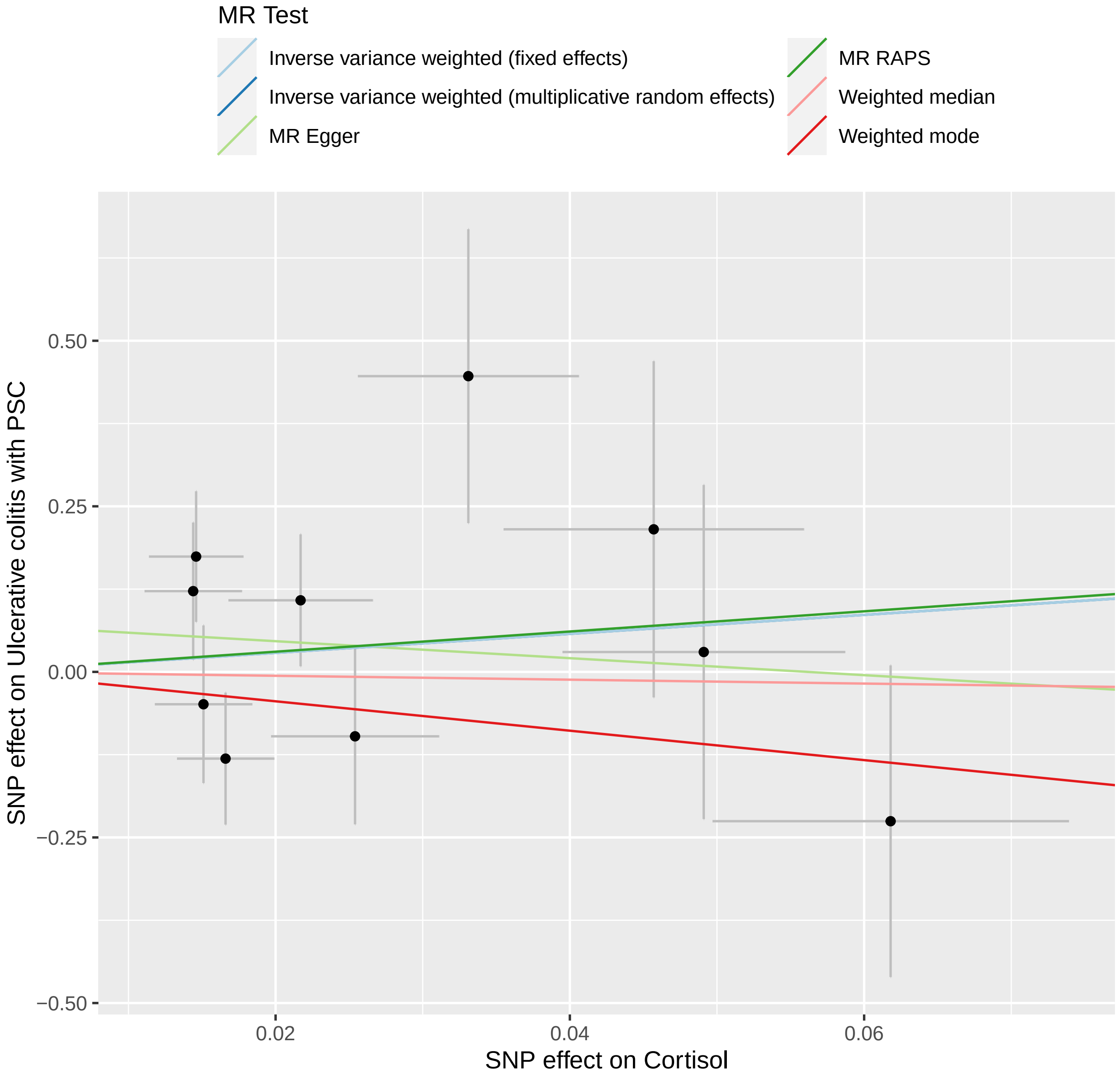

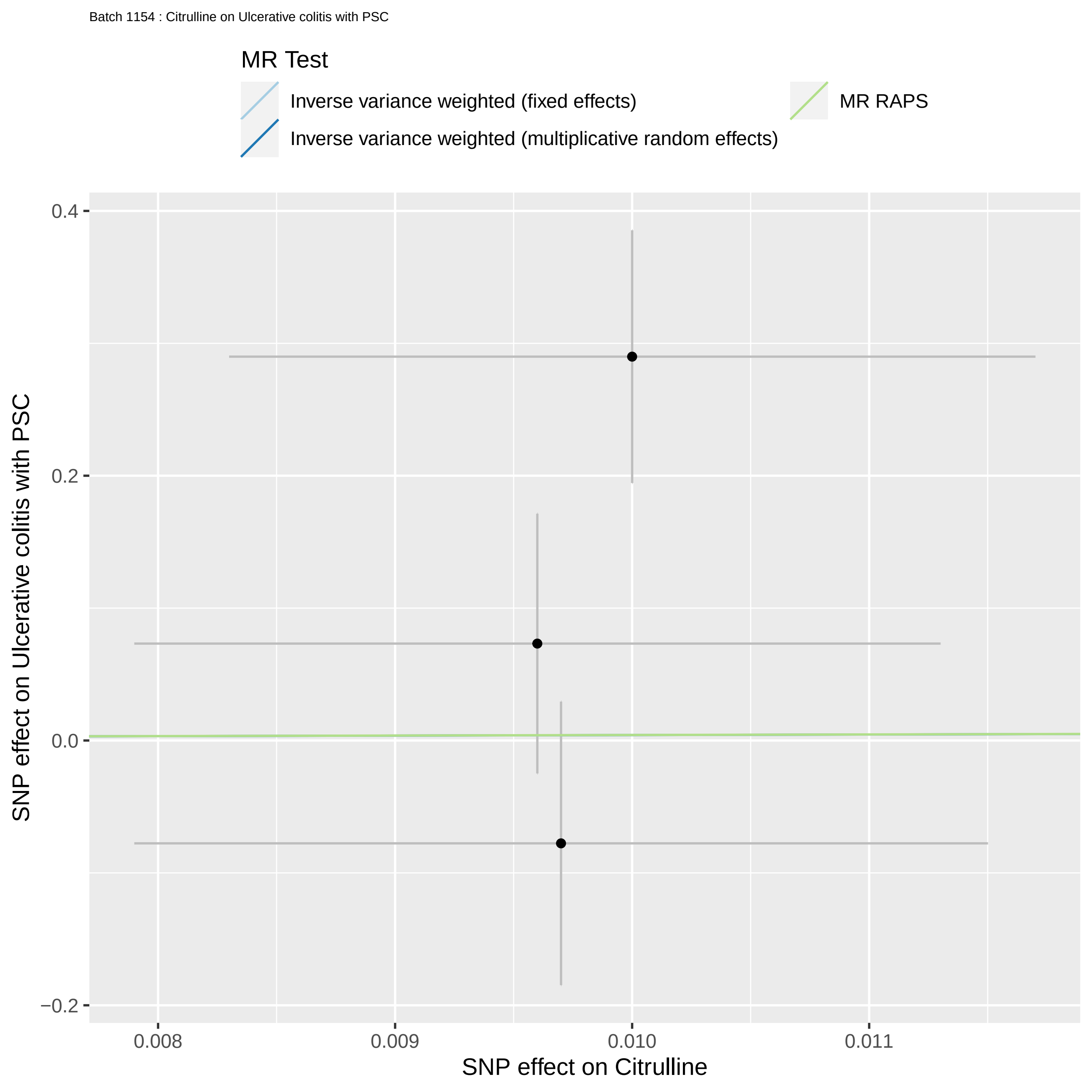
Batch 1154 : Citrulline on Ulcerative colitis with PSC
MR Test
Inverse variance weighted (fixed effects)
Inverse variance weighted (multiplicative random effects)
MR RAPS
SNP effect on Ulcerative colitis with PSC
0.4
0.2
0.0
−0.2
0.008
0.009
0.010
0.011
SNP effect on Citrulline

Batch 1156 : Serotonin (5HT) on Ulcerative colitis with PSC

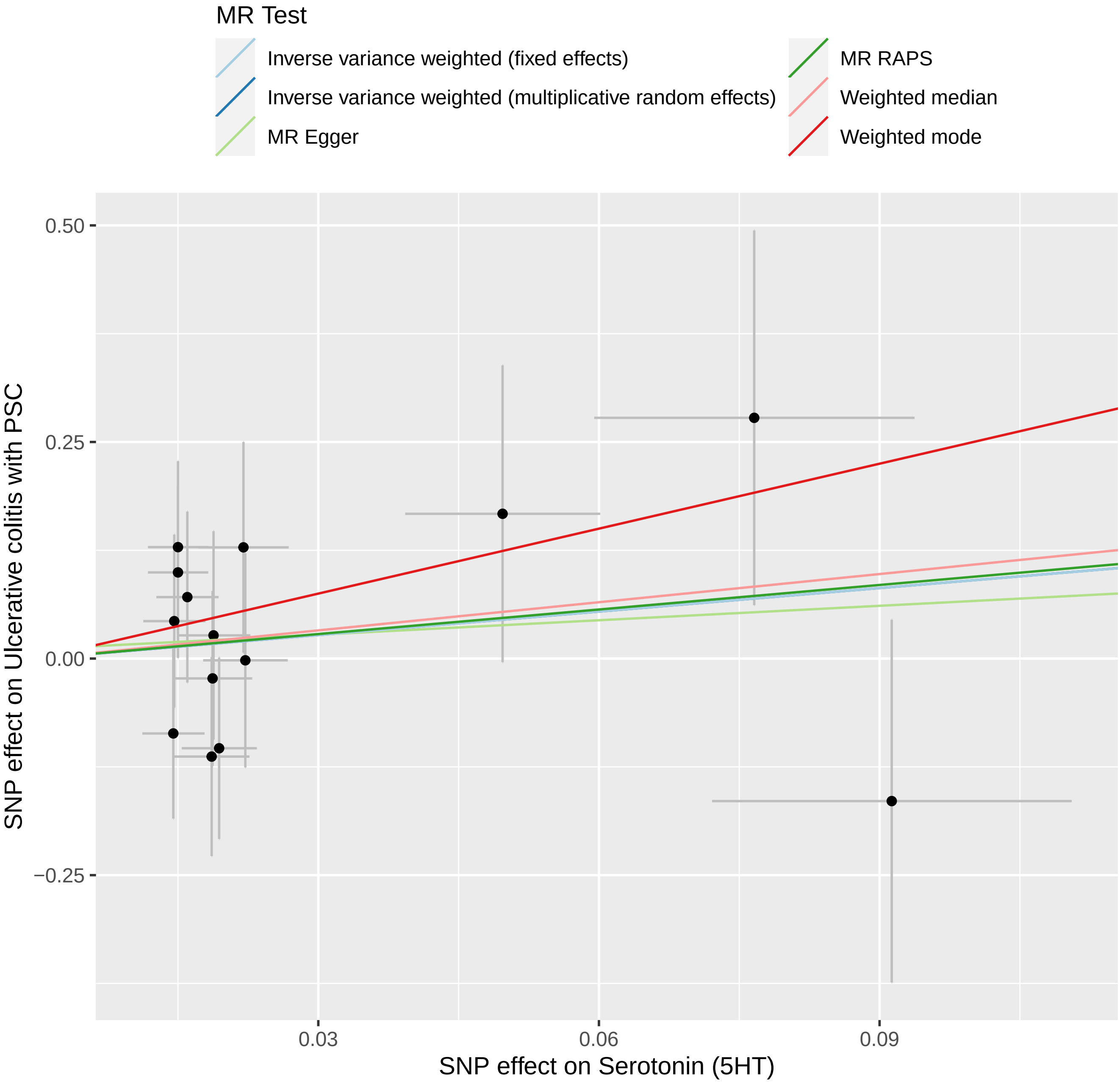

Batch 1157 : Gamma−glutamylglutamine on Ulcerative colitis with PSC

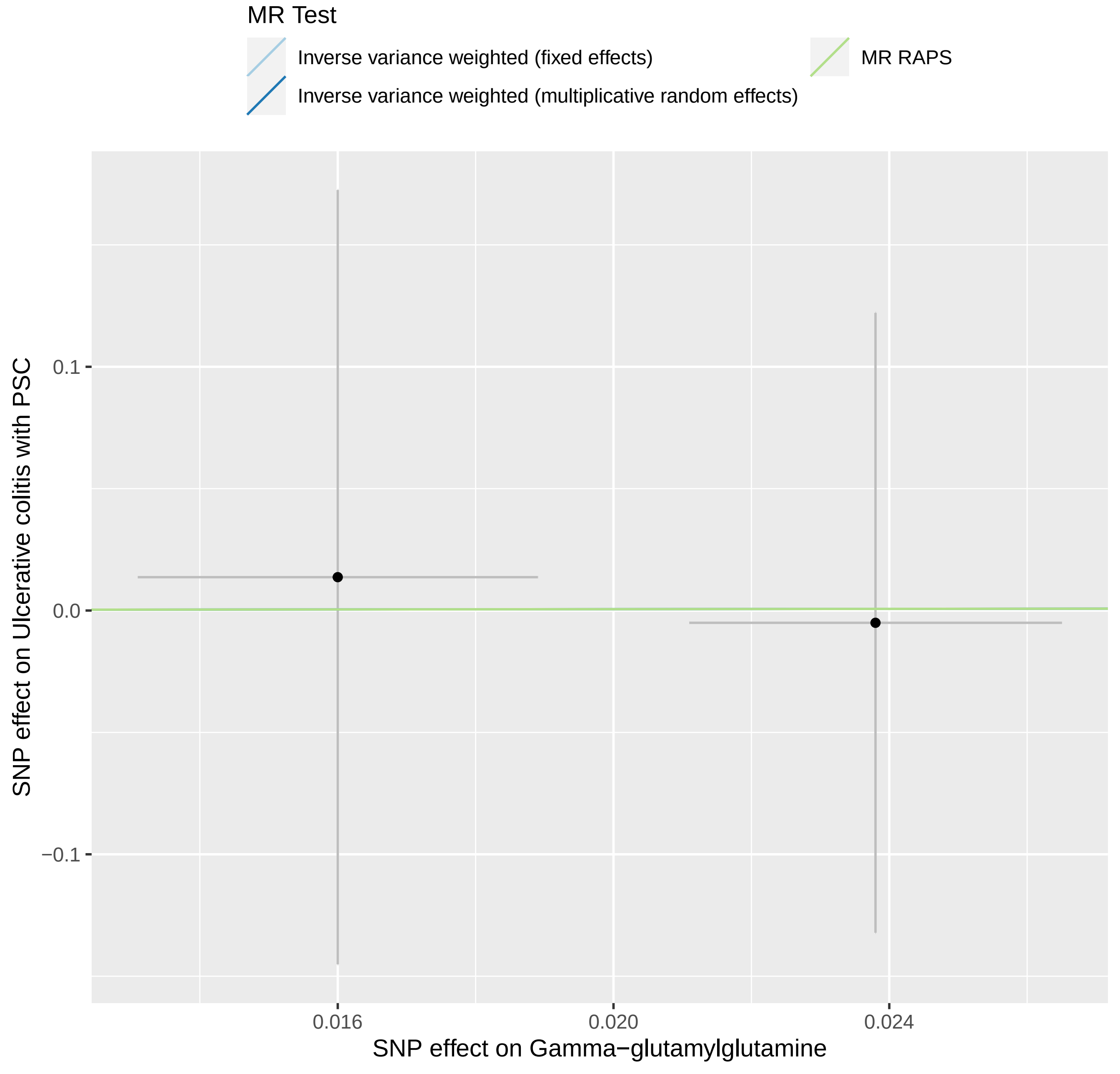

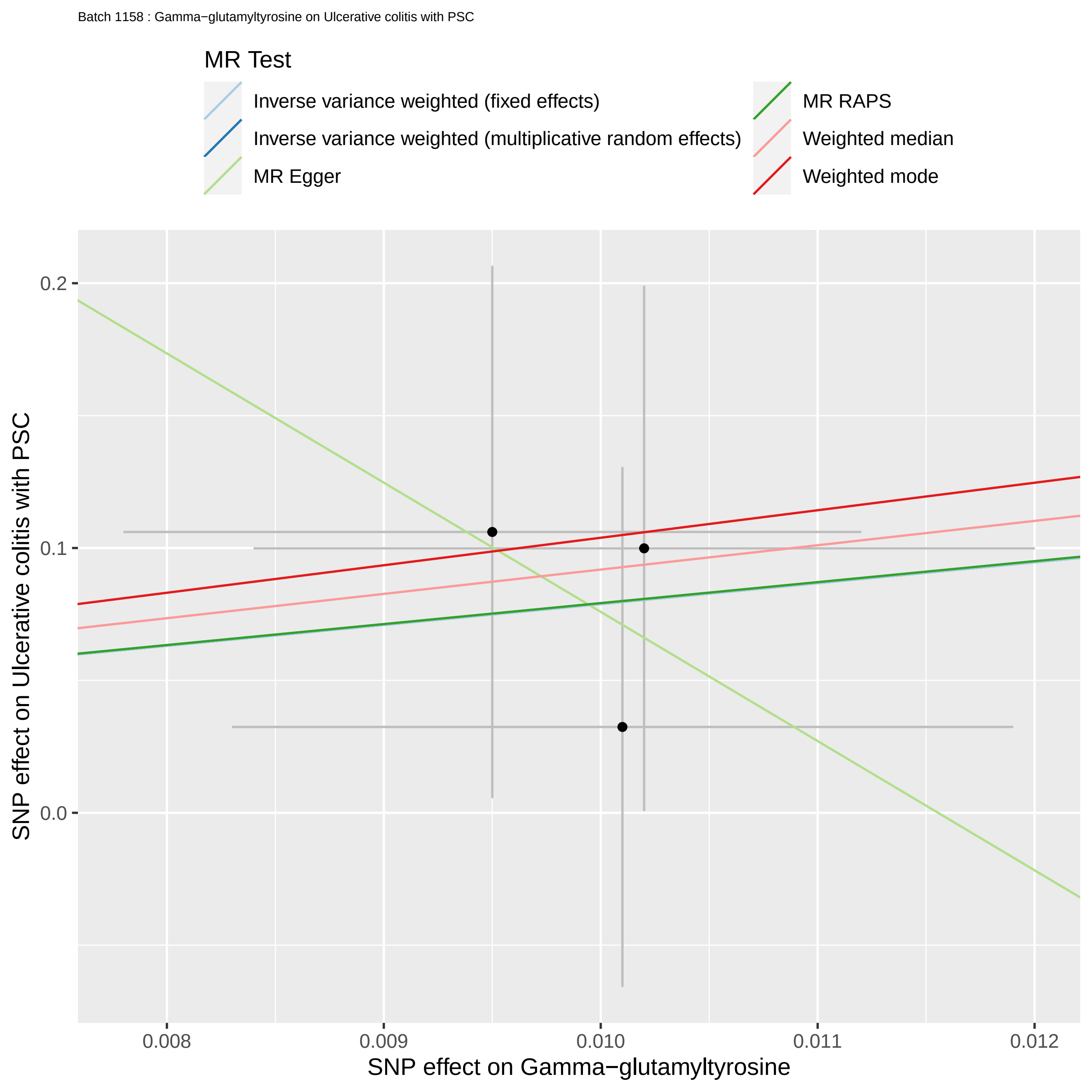
Batch 1158 : Gamma−glutamyltyrosine on Ulcerative colitis with PSC
MR Test
Inverse variance weighted (fixed effects)
Inverse variance weighted (multiplicative random effects)
MR Egger
MR RAPS
Weighted median
Weighted mode
0.2
0.1
0.0
0.008
0.009
0.010
0.011
0.012
SNP effect on Ulcerative colitis with PSC
SNP effect on Gamma−glutamyltyrosine

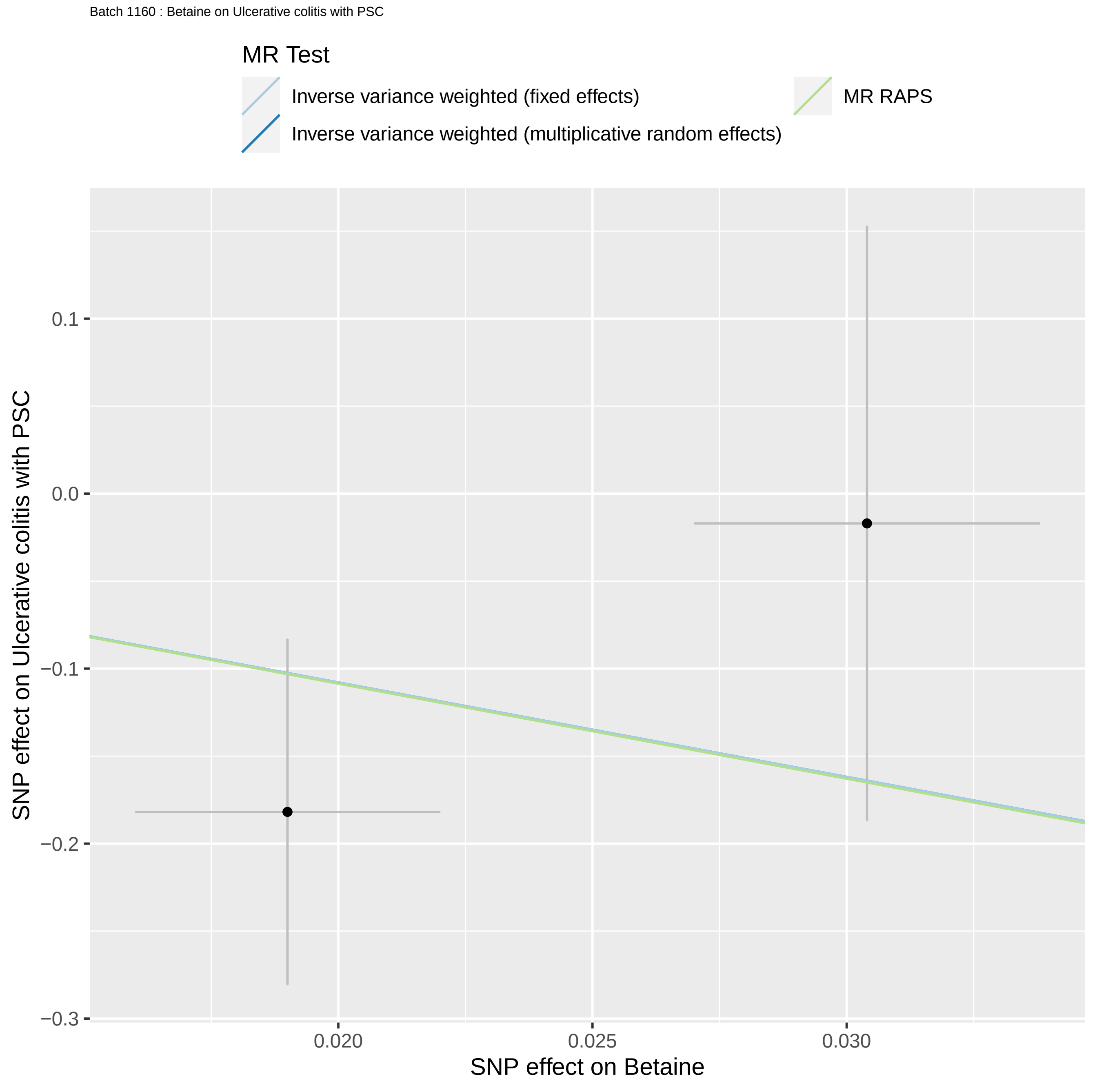

Batch 1160 : Betaine on Ulcerative colitis with PSC
MR Test
Inverse variance weighted (fixed effects)
Inverse variance weighted (multiplicative random effects)
MR RAPS
0.1
0.0
−0.1
−0.2
−0.3
SNP effect on Ulcerative colitis with PSC
0.020
0.025
0.030
SNP effect on Betaine

Batch 1161 : Xanthine on Ulcerative colitis with PSC

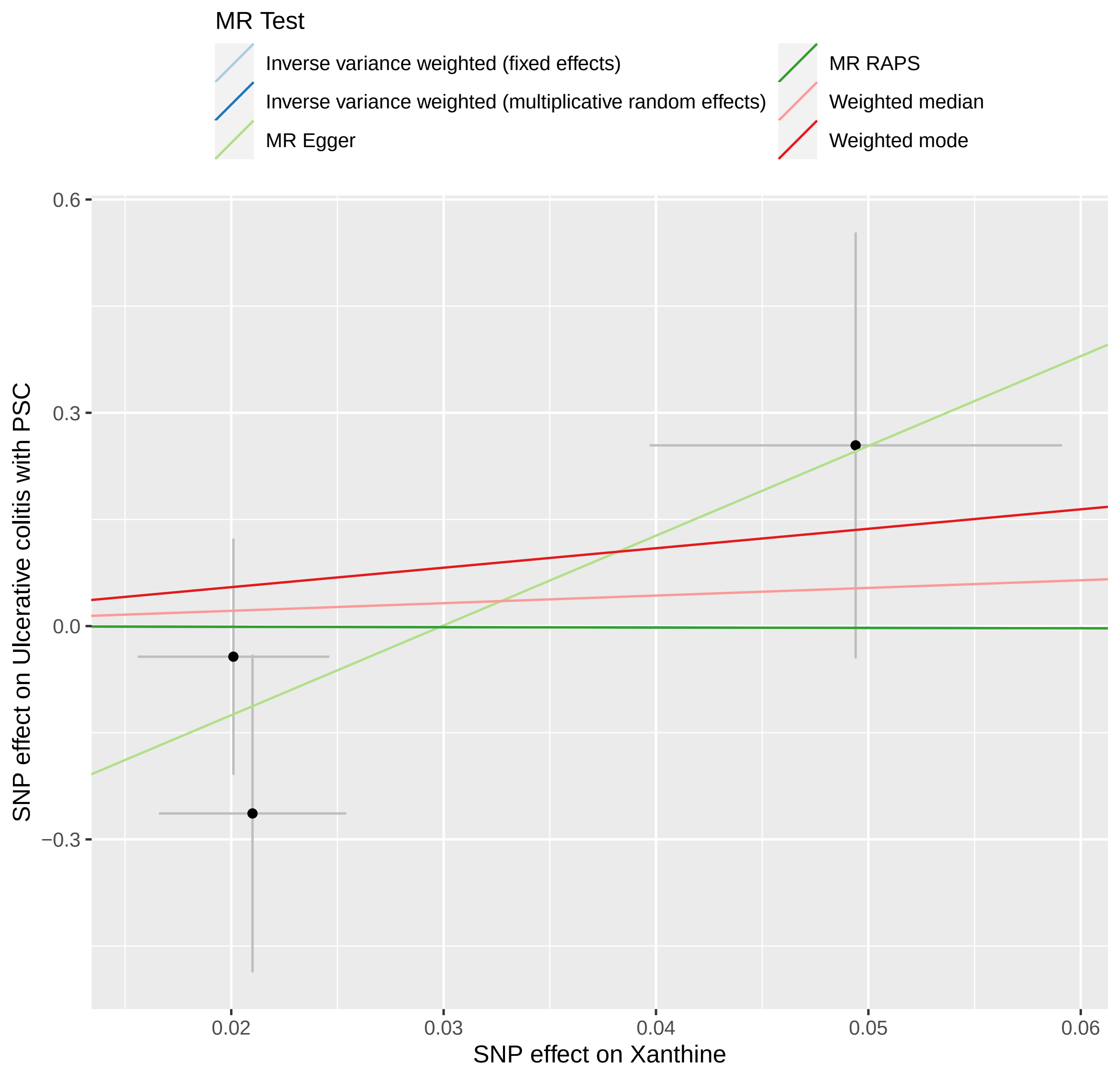

Batch 1162 : Phosphate on Ulcerative colitis with PSC

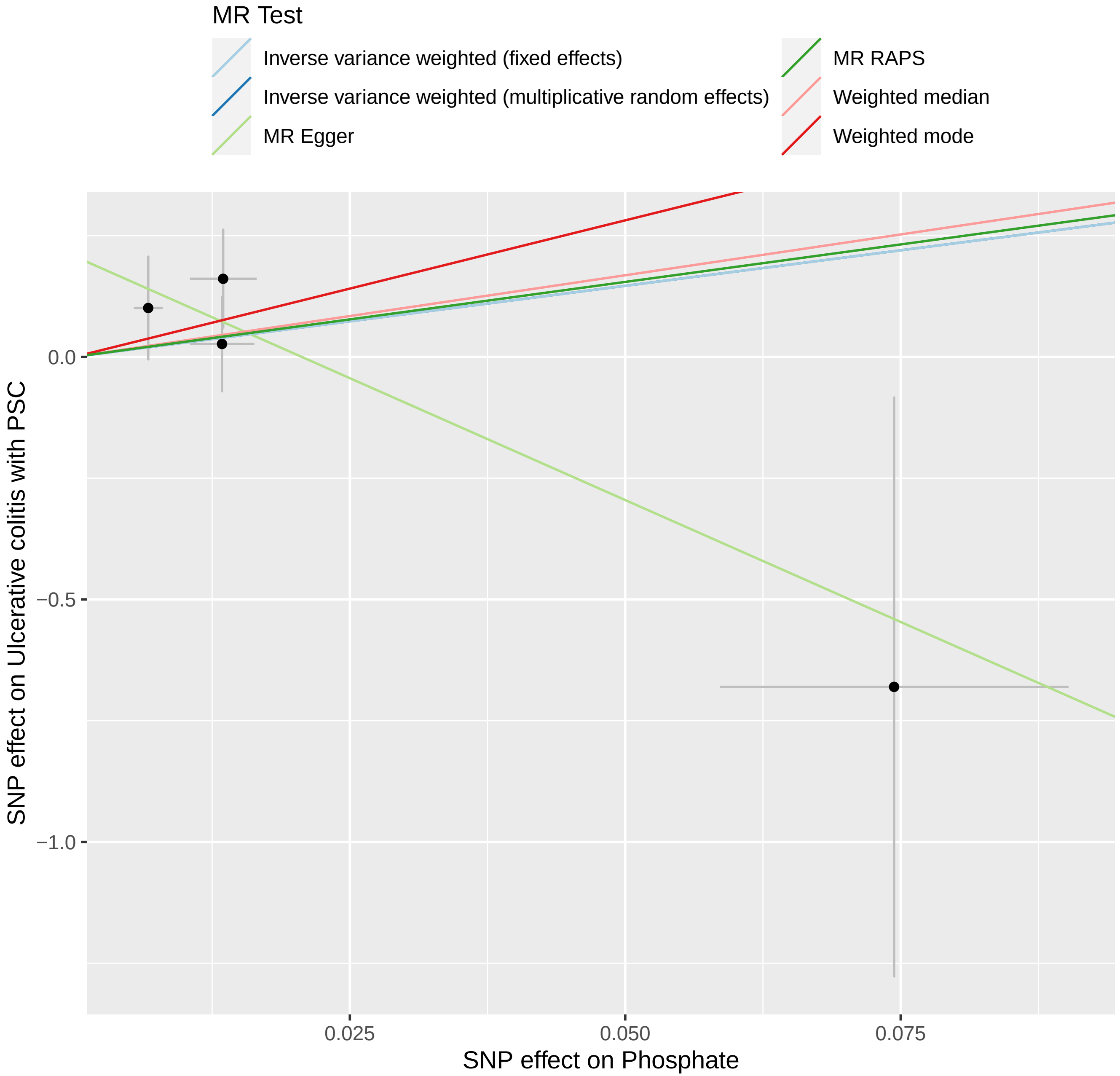

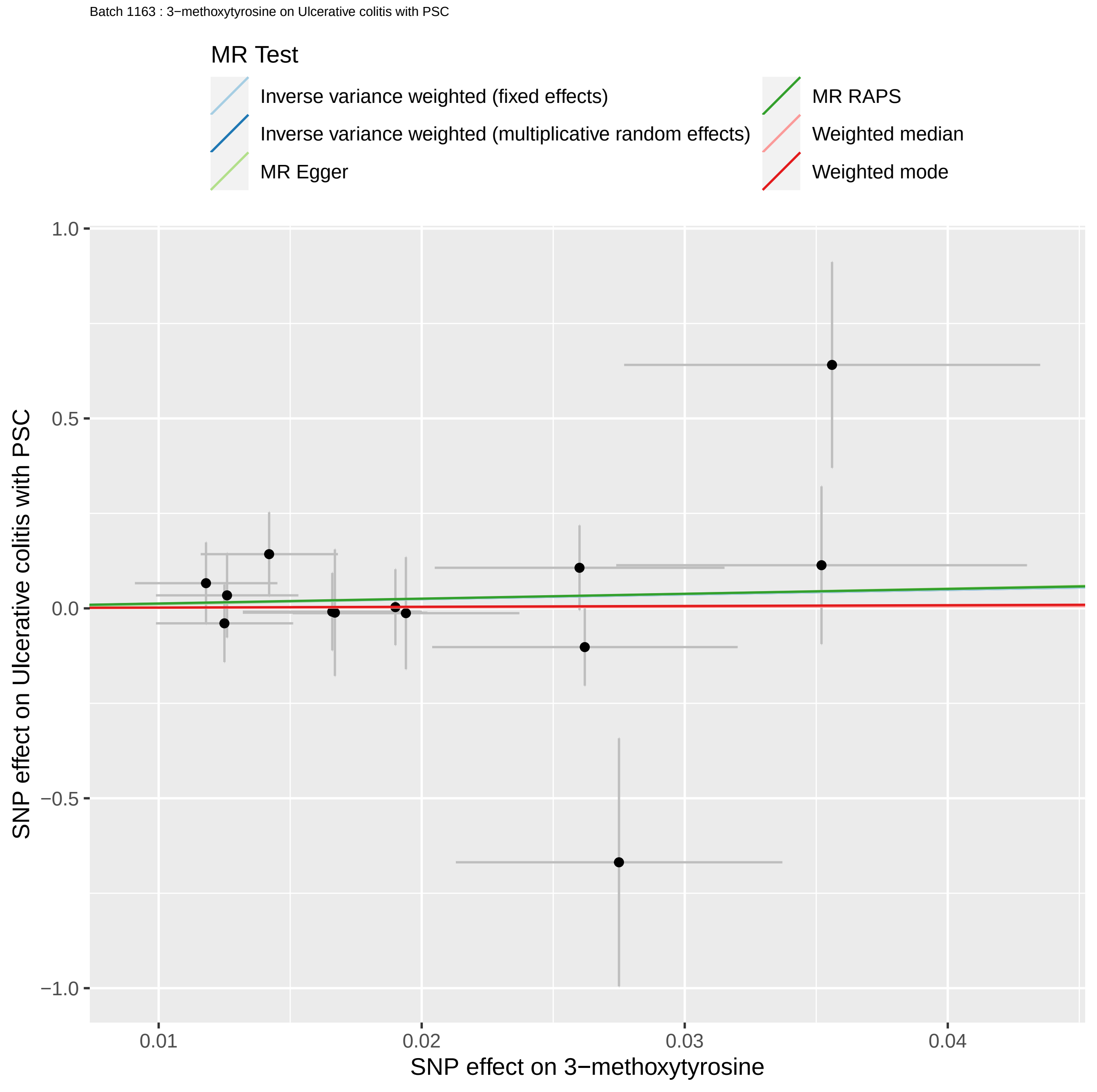
Batch 1163 : 3−methoxytyrosine on Ulcerative colitis with PSC
MR Test
Inverse variance weighted (fixed effects)
Inverse variance weighted (multiplicative random effects)
MR Egger
MR RAPS
Weighted median
Weighted mode
1.0
0.5
0.0
−0.5
−1.0
SNP effect on Ulcerative colitis with PSC
0.01
0.02
0.03
0.04
SNP effect on 3−methoxytyrosine

Batch 1165 : Undecanoate (11:0) on Ulcerative colitis with PSC

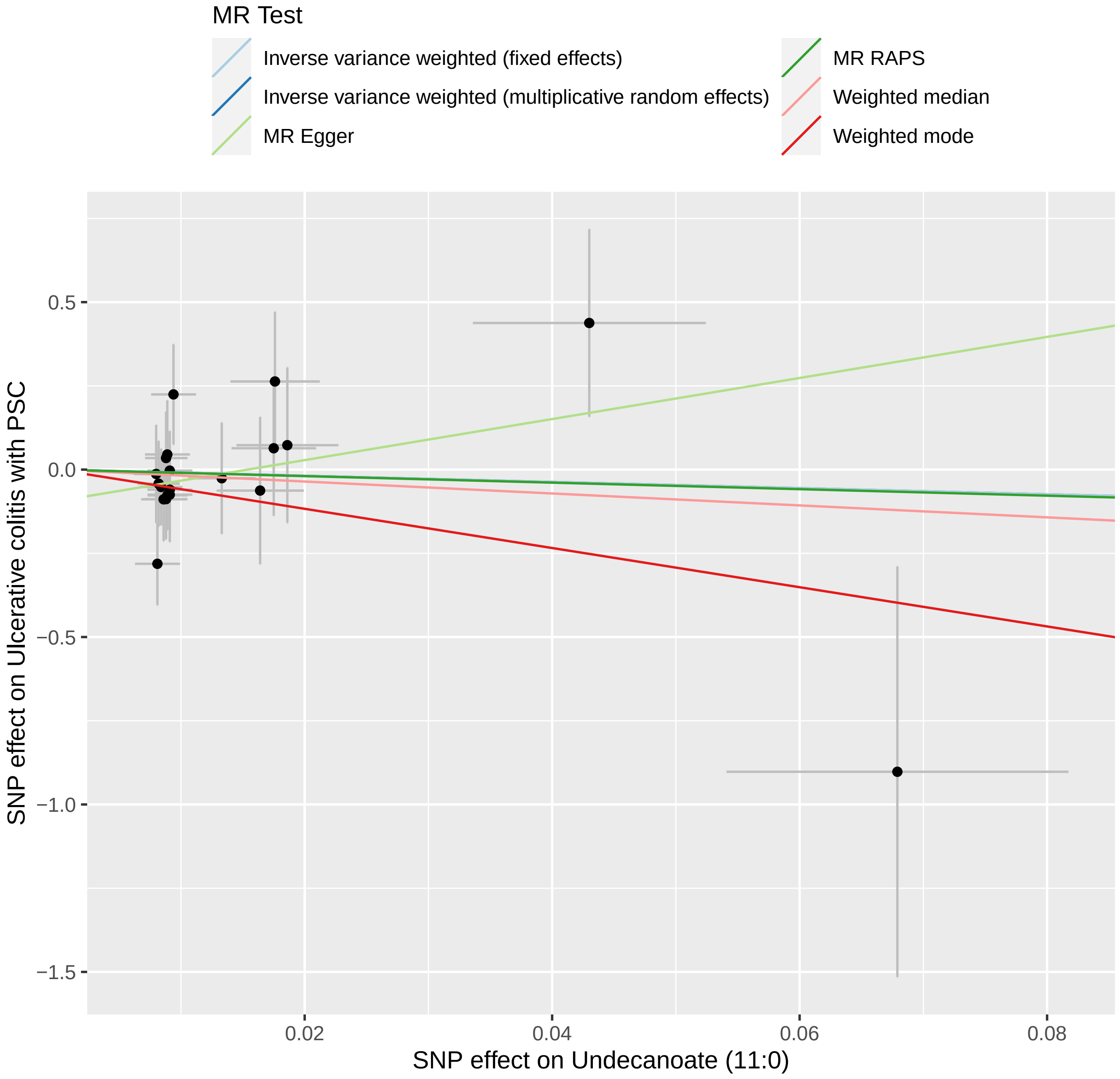

Batch 1167 : Taurodeoxycholate on Ulcerative colitis with PSC

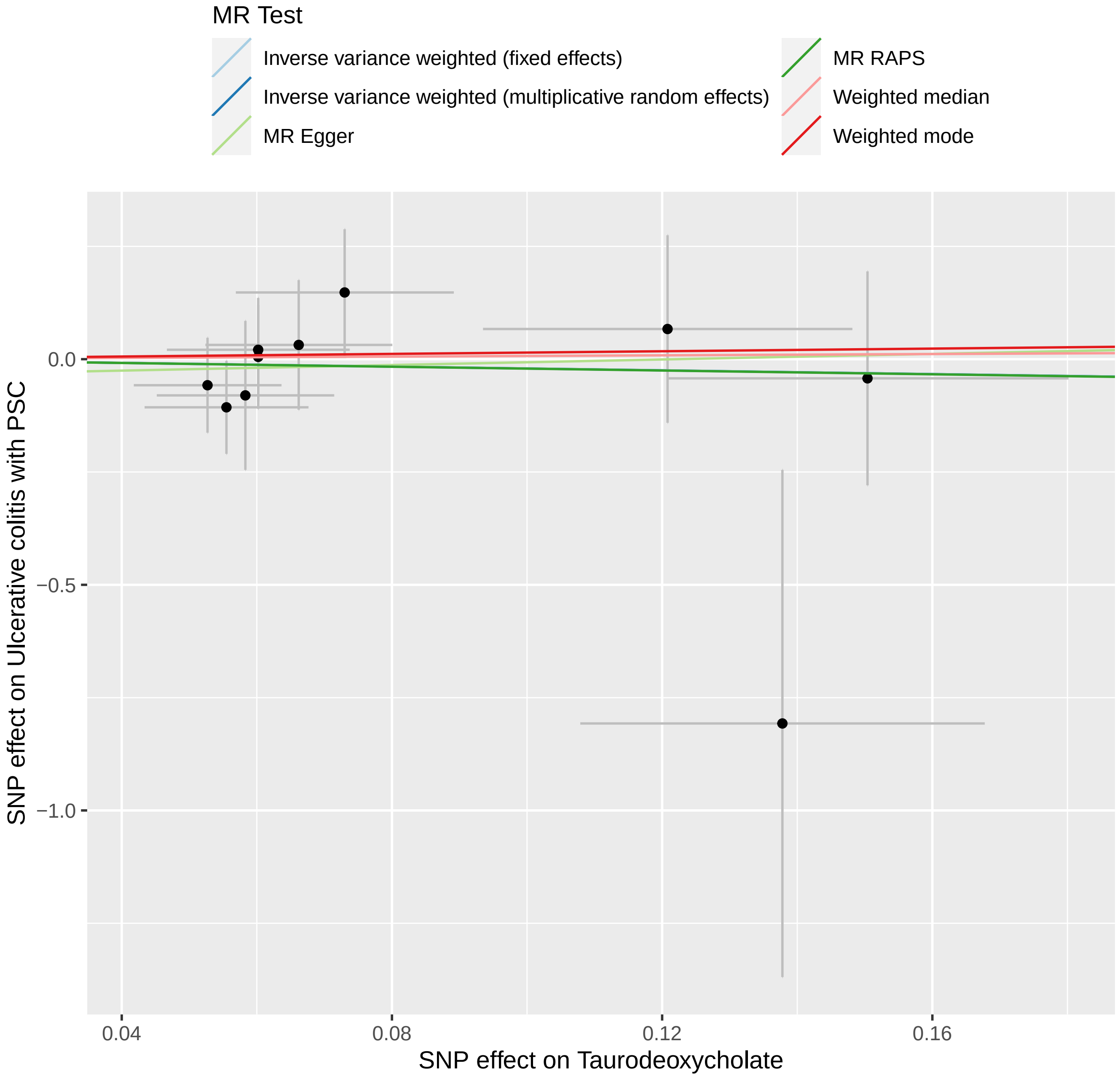

Batch 1168 : Glycerol on Ulcerative colitis with PSC

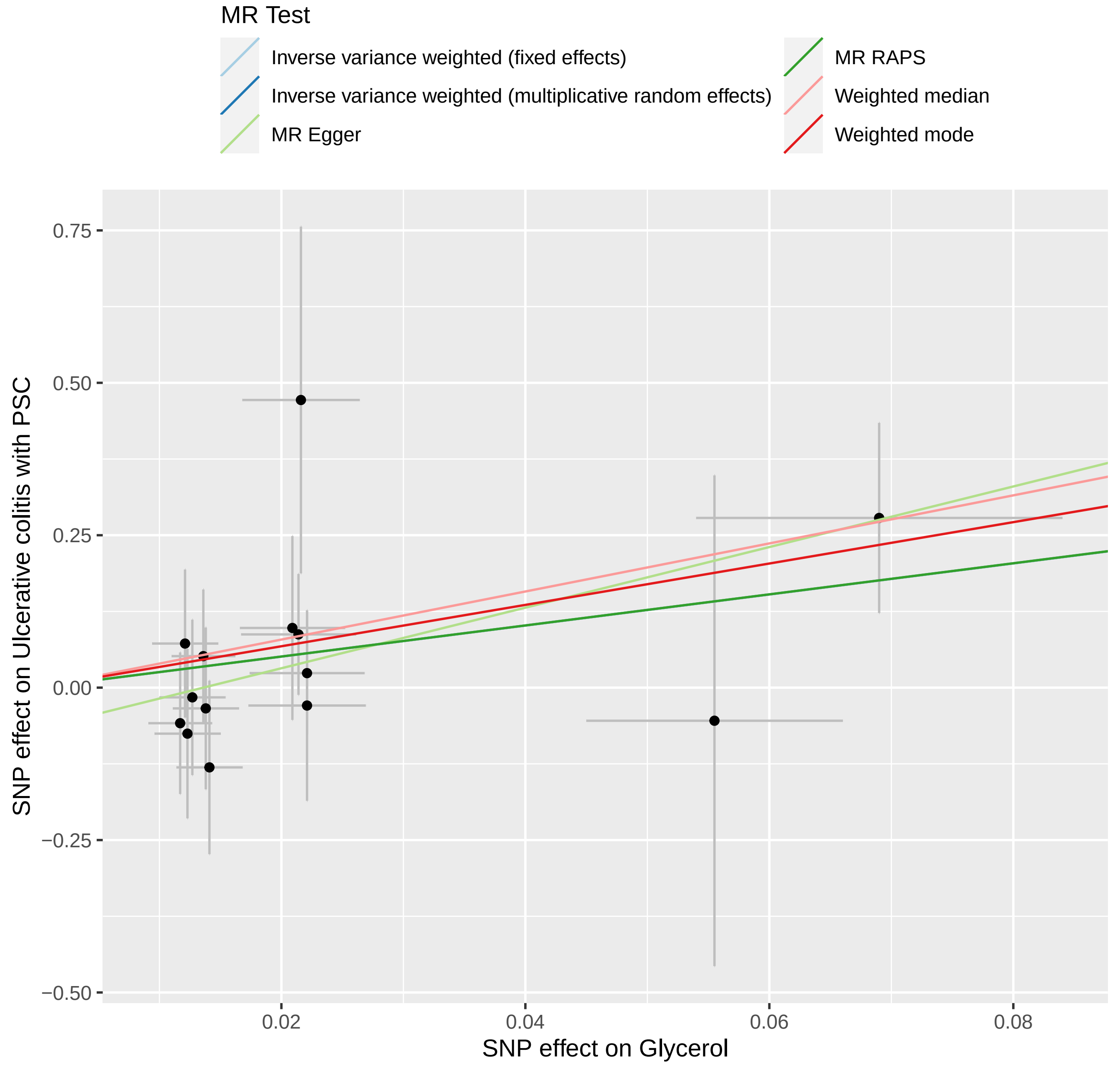

Batch 1169 : Kynurenine on Ulcerative colitis with PSC

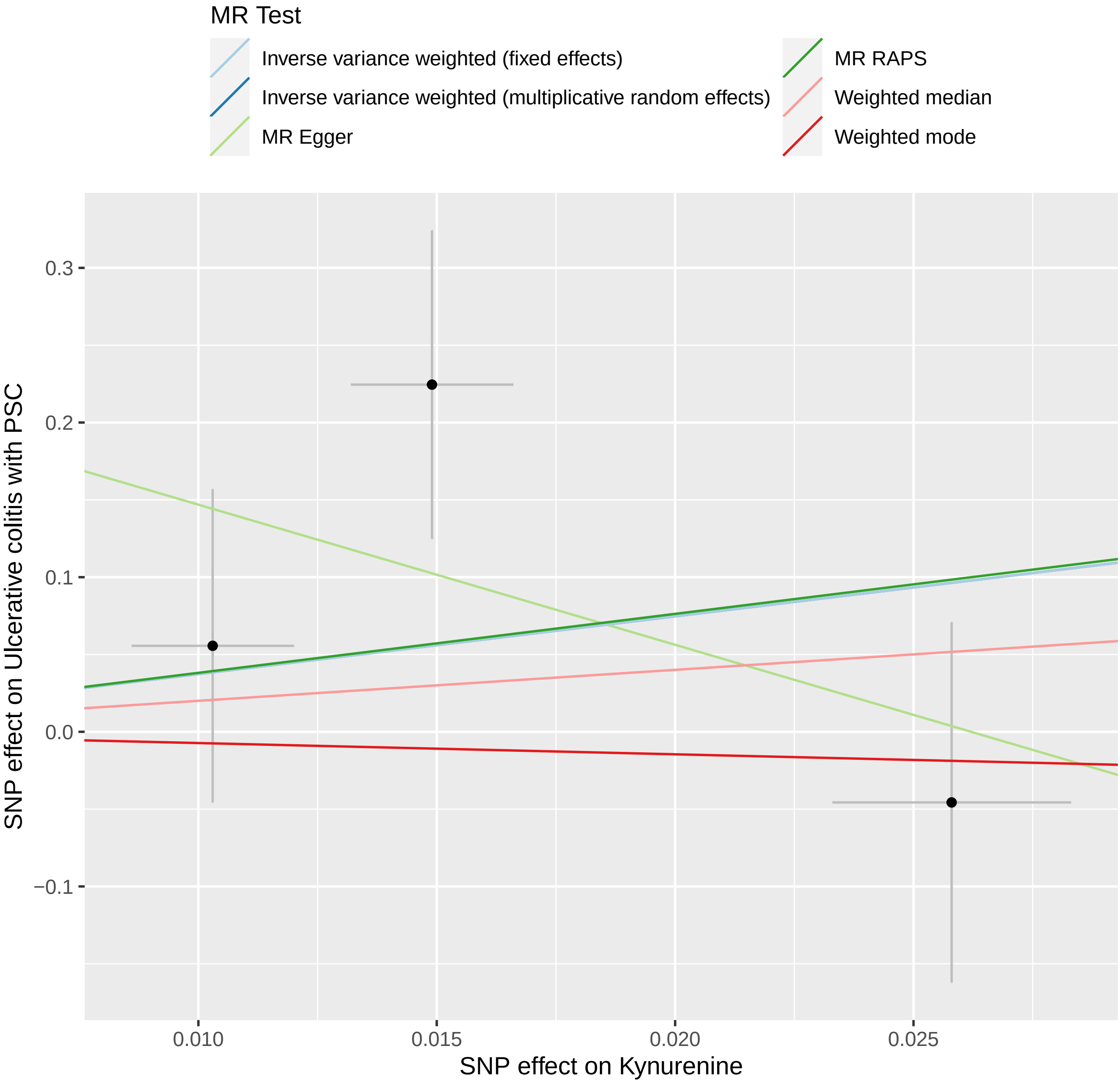

Batch 1170 : Mannitol on Ulcerative colitis with PSC

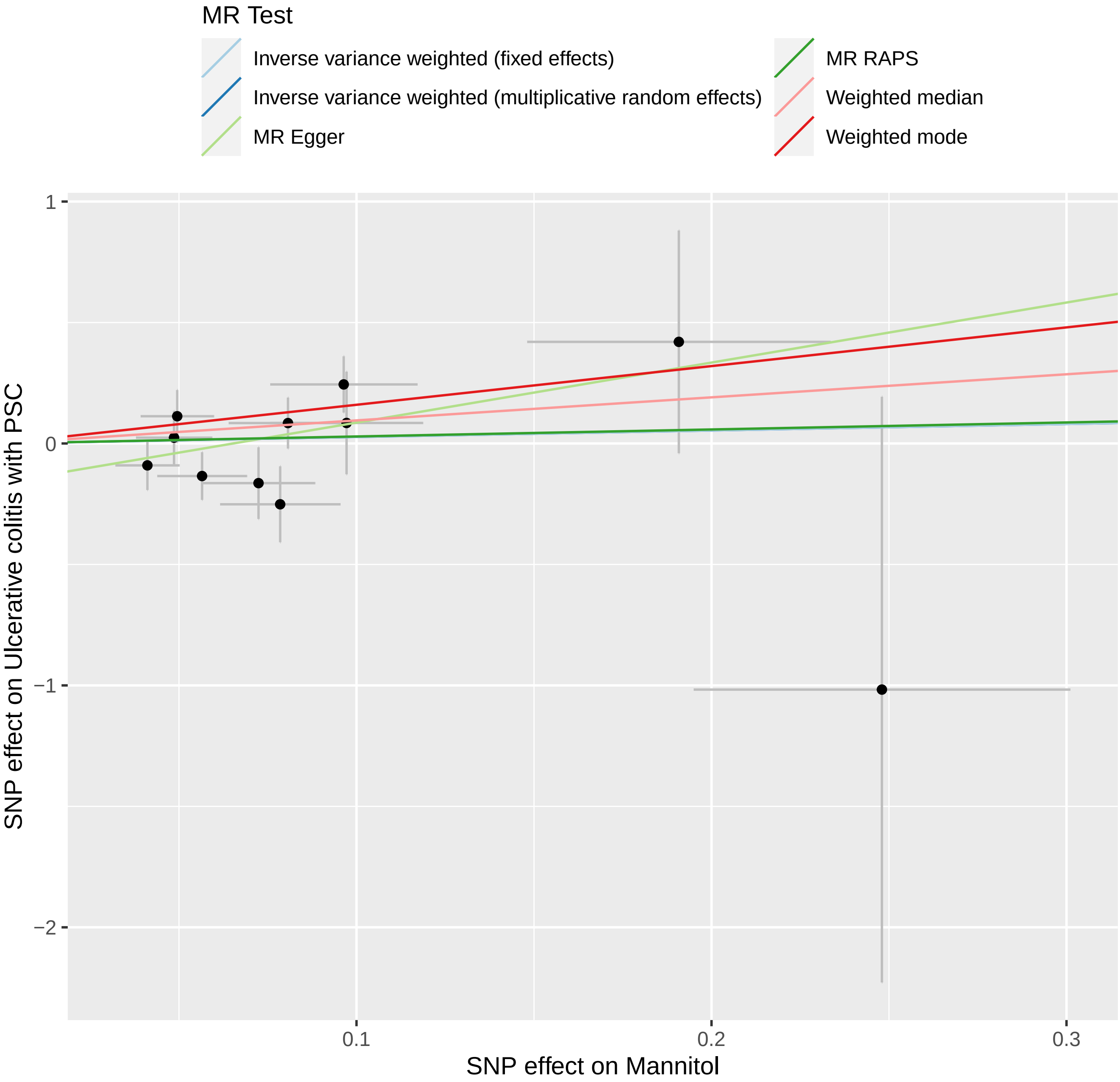

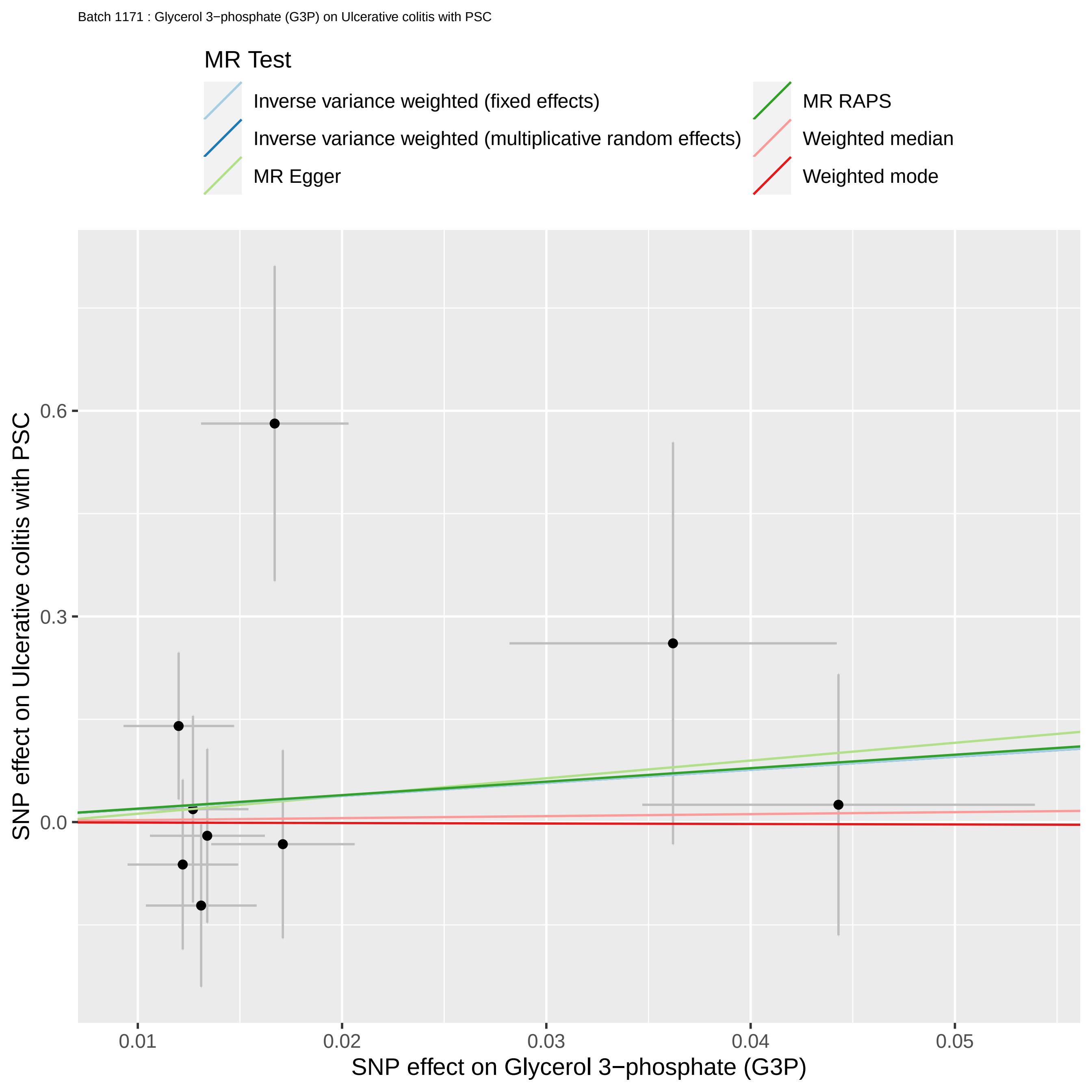

Batch 1171 : Glycerol 3−phosphate (G3P) on Ulcerative colitis with PSC
MR Test
Inverse variance weighted (fixed effects)
Inverse variance weighted (multiplicative random effects)
MR Egger
MR RAPS
Weighted median
Weighted mode
0.6
0.3
0.0
0.01
0.02
0.03
0.04
0.05
SNP effect on Glycerol 3−phosphate (G3P)
SNP effect on Ulcerative colitis with PSC

Batch 1173 : Carnitine on Ulcerative colitis with PSC

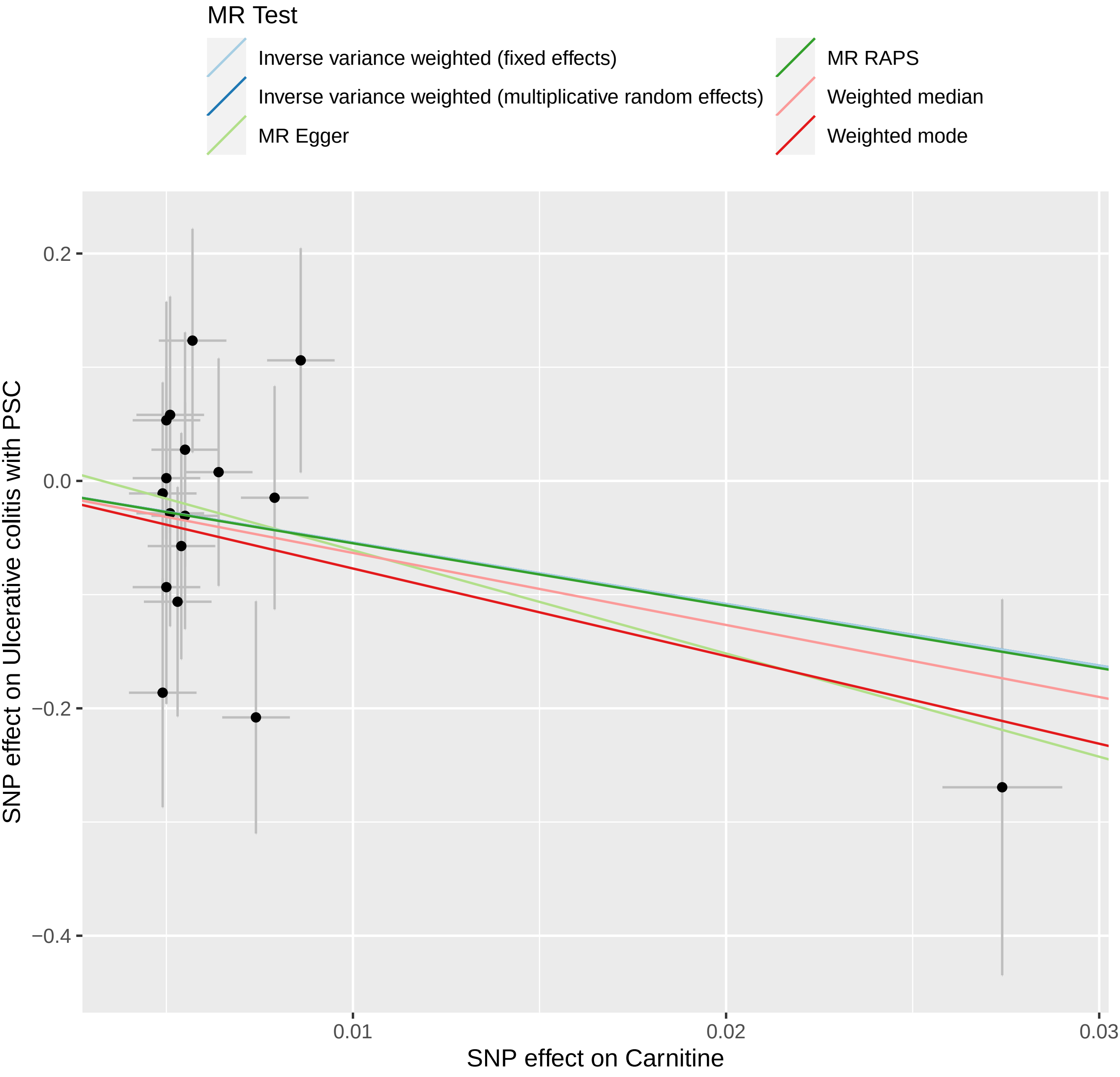

Batch 1174 : Choline on Ulcerative colitis with PSC

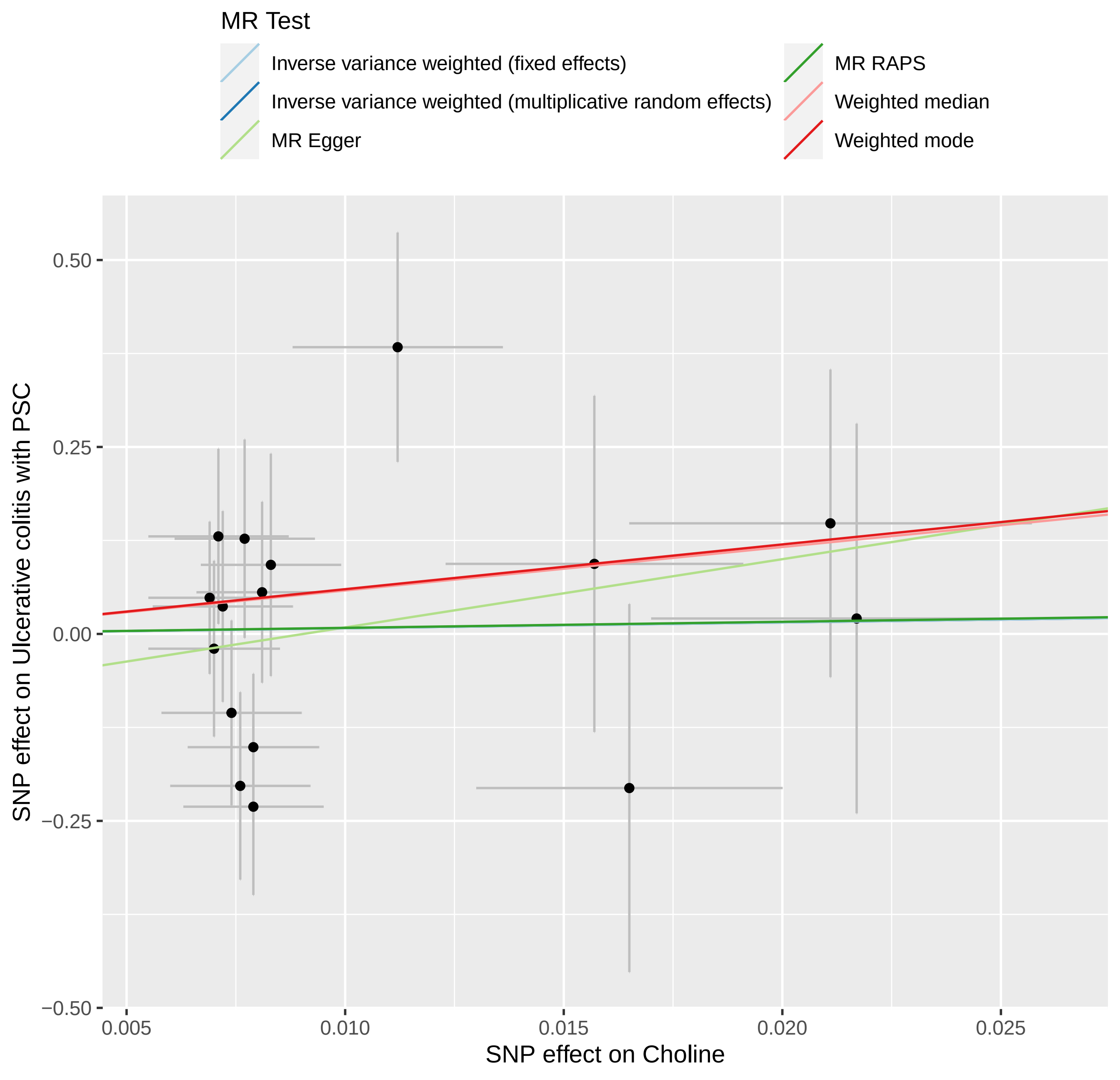

Batch 1177 : 3−methyl−2−oxovalerate on Ulcerative colitis with PSC

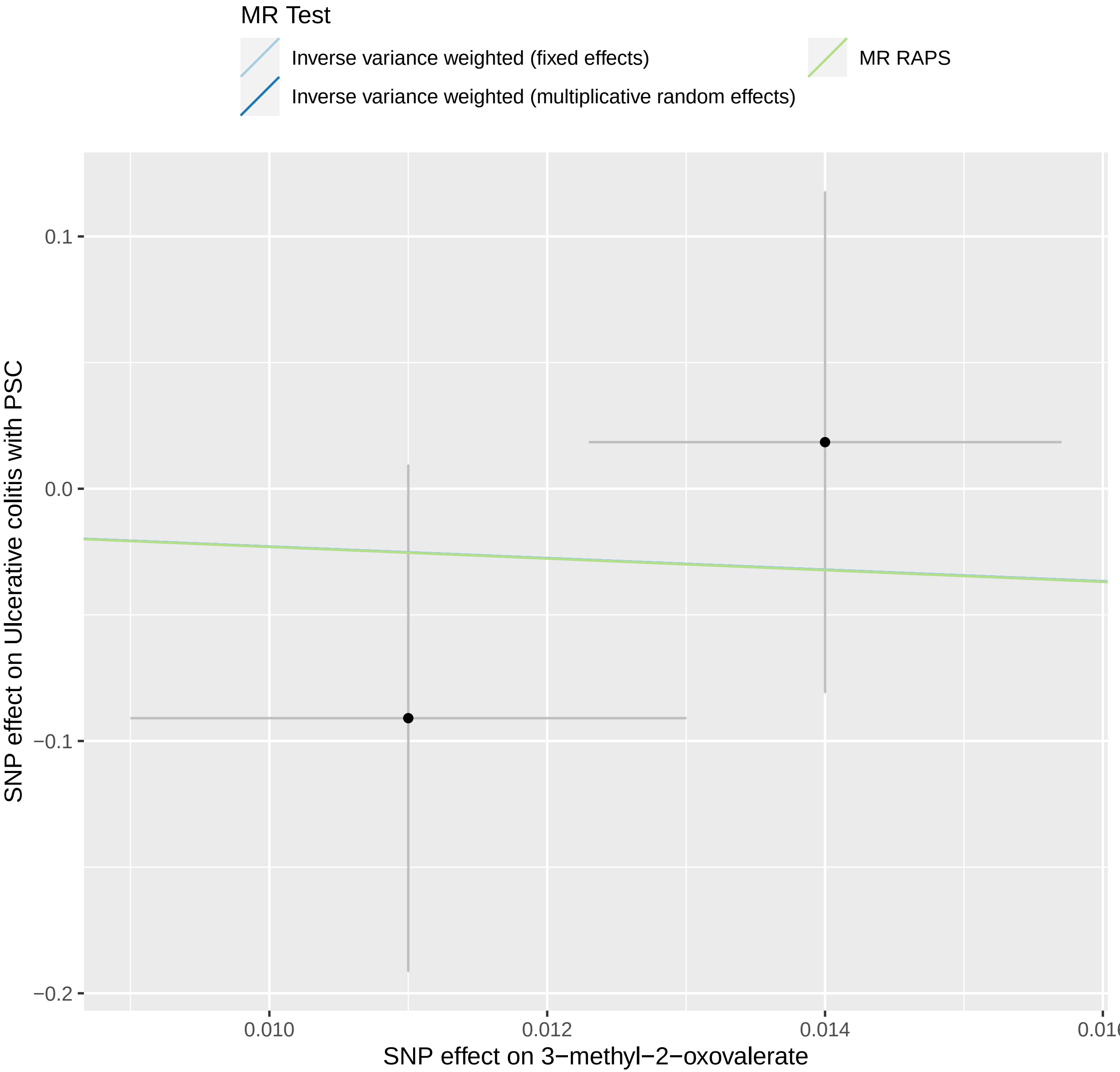

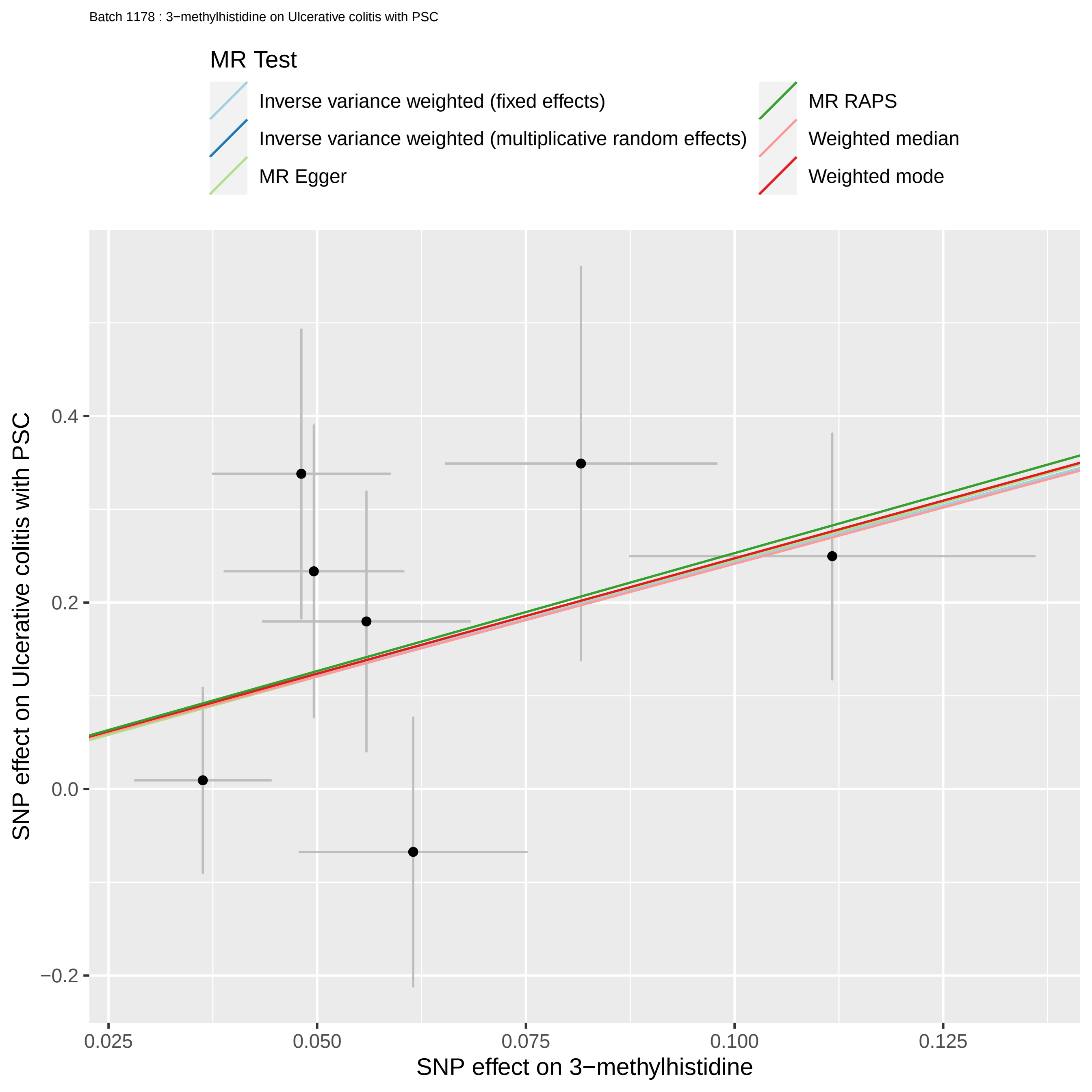

Batch 1178 : 3−methylhistidine on Ulcerative colitis with PSC
MR Test
Inverse variance weighted (fixed effects)
Inverse variance weighted (multiplicative random effects)
MR Egger
MR RAPS
Weighted median
Weighted mode
SNP effect on Ulcerative colitis with PSC
0.4
0.2
0.0
−0.2
0.025
0.050
0.075
0.100
0.125
SNP effect on 3−methylhistidine

Batch 1180 : Phenylacetate on Ulcerative colitis with PSC

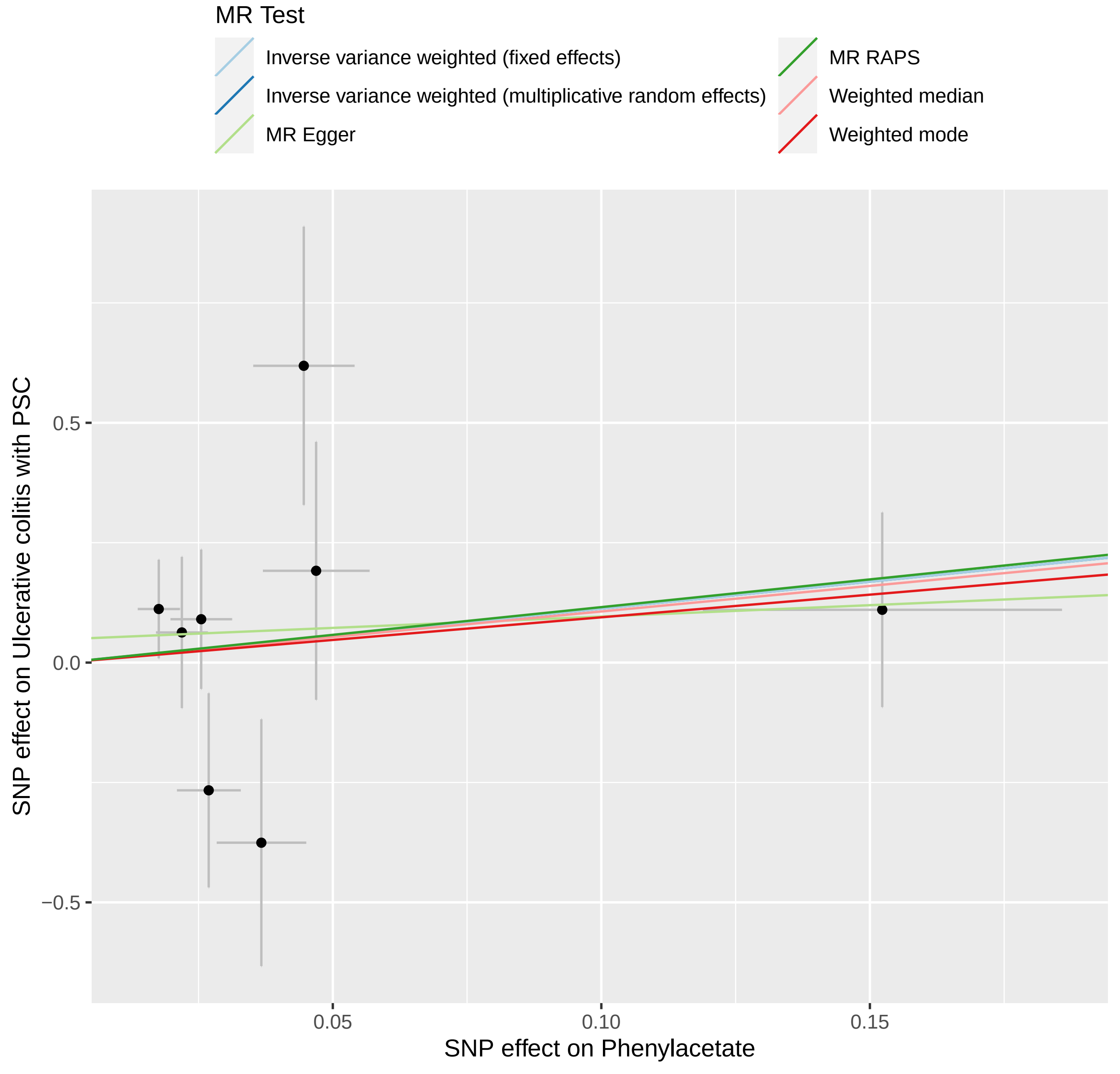

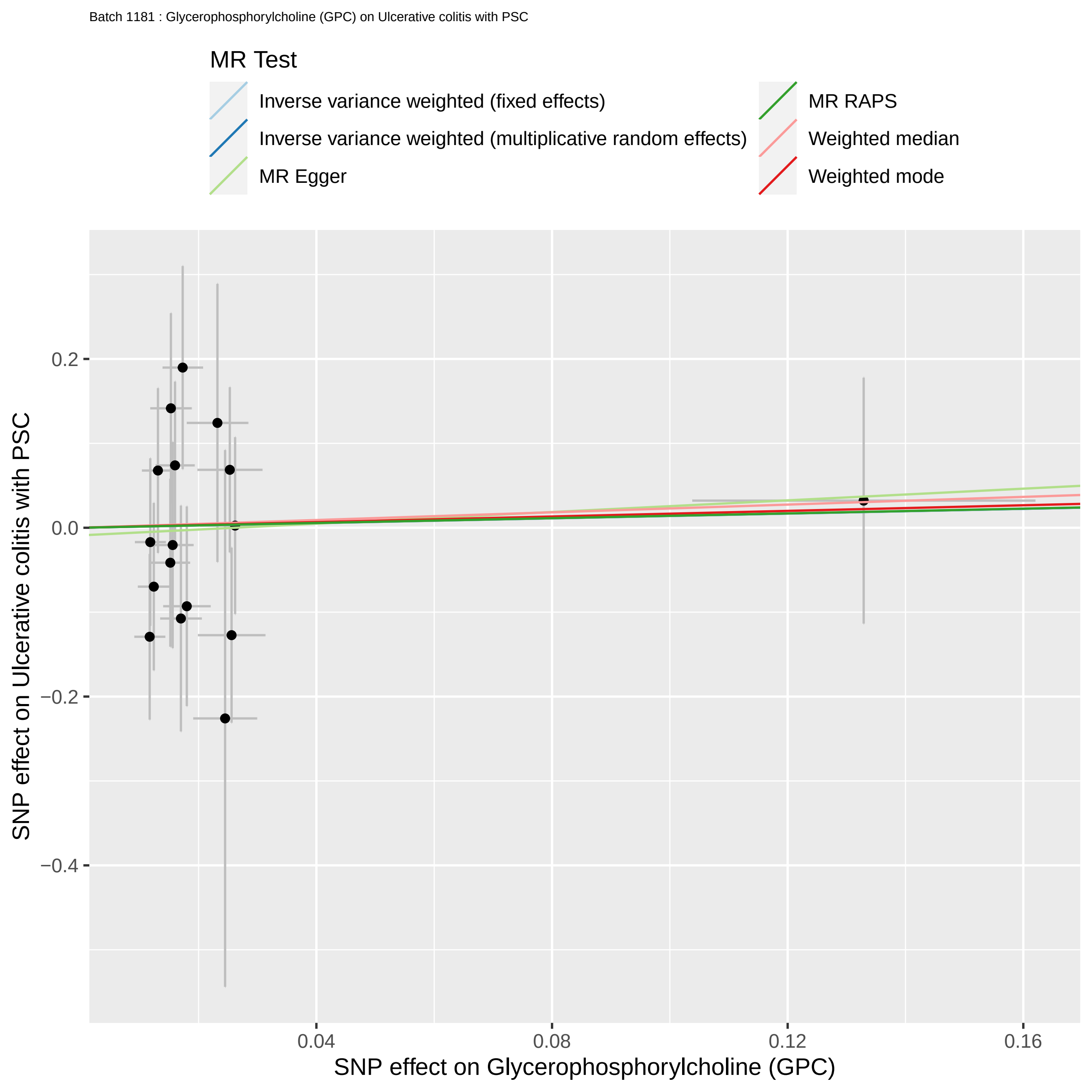

Batch 1181 : Glycerophosphorylcholine (GPC) on Ulcerative colitis with PSC
MR Test
Inverse variance weighted (fixed effects)
Inverse variance weighted (multiplicative random effects)
MR Egger
MR RAPS
Weighted median
Weighted mode
0.2
0.0
−0.2
−0.4
0.04
0.08
0.12
0.16
SNP effect on Ulcerative colitis with PSC
SNP effect on Glycerophosphorylcholine (GPC)

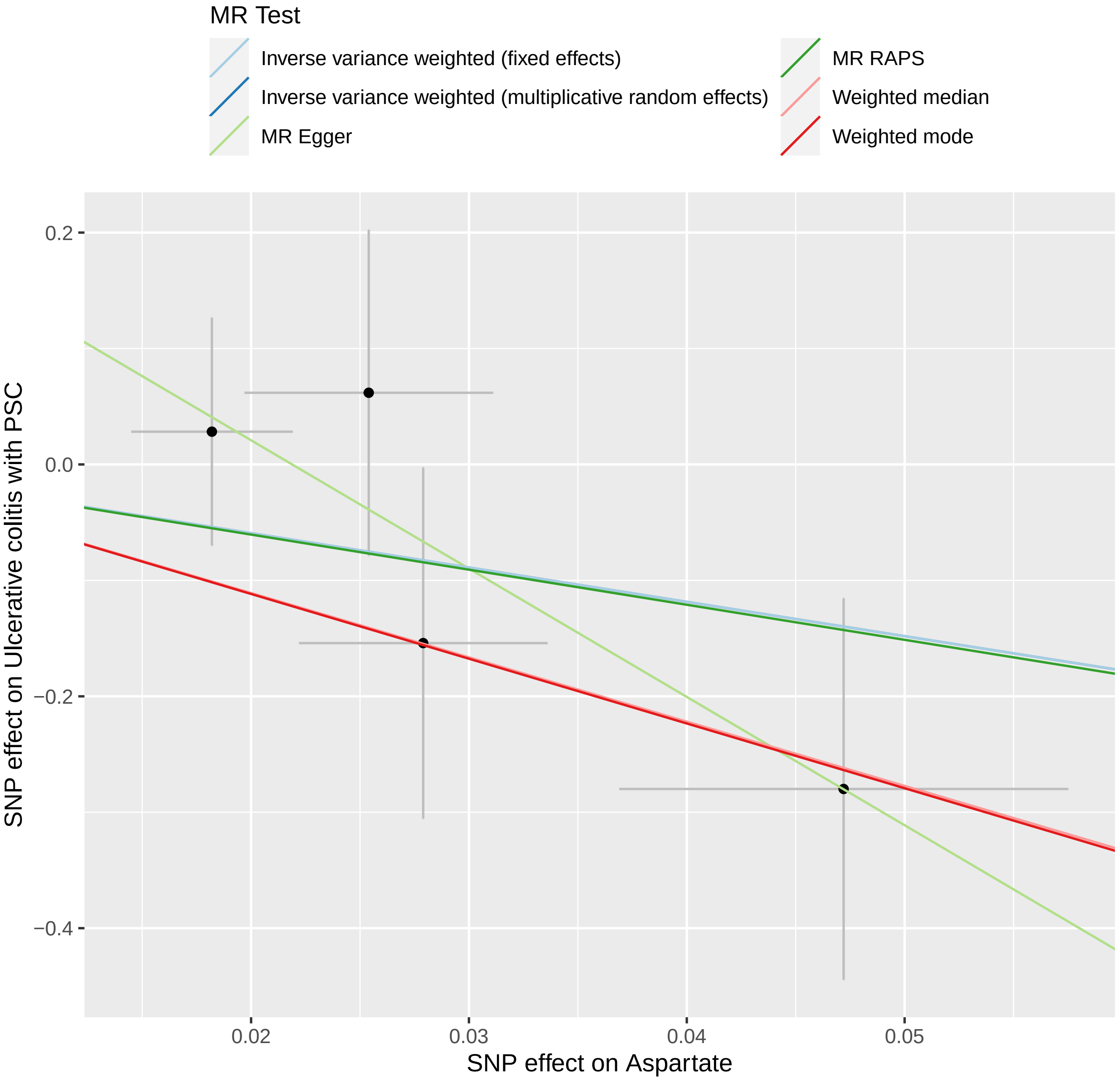
MR Test
Inverse variance weighted (fixed effects)
Inverse variance weighted (multiplicative random effects)
MR Egger
MR RAPS
Weighted median
Weighted mode
SNP effect on Ulcerative colitis with PSC
0.2
0.0
−0.2
−0.4
0.02
0.03
0.04
0.05
SNP effect on Aspartate

Batch 1184 : Dihomo−linoleate (20:2n6) on Ulcerative colitis with PSC

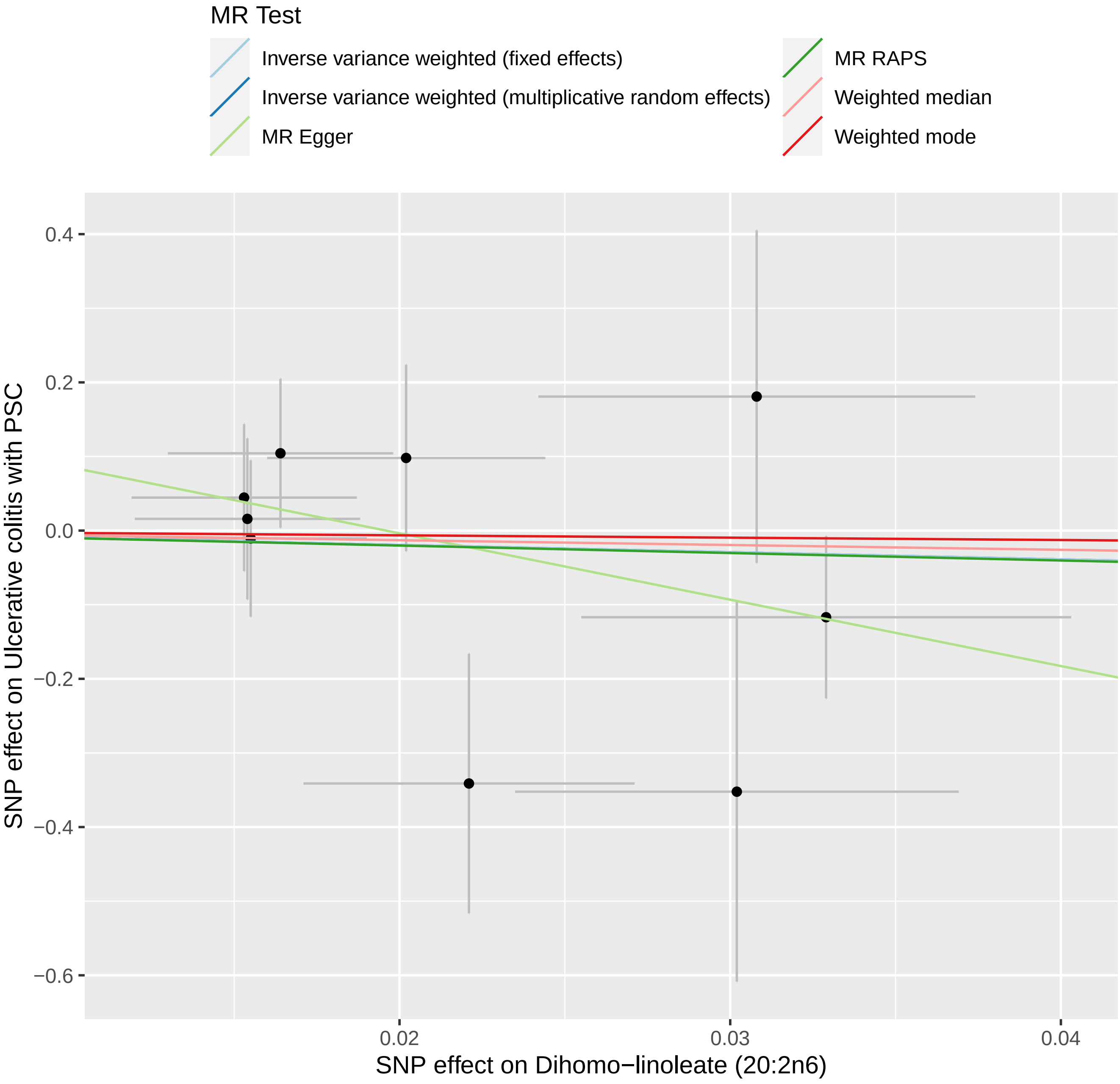

Batch 1185 : 2−hydroxystearate on Ulcerative colitis with PSC

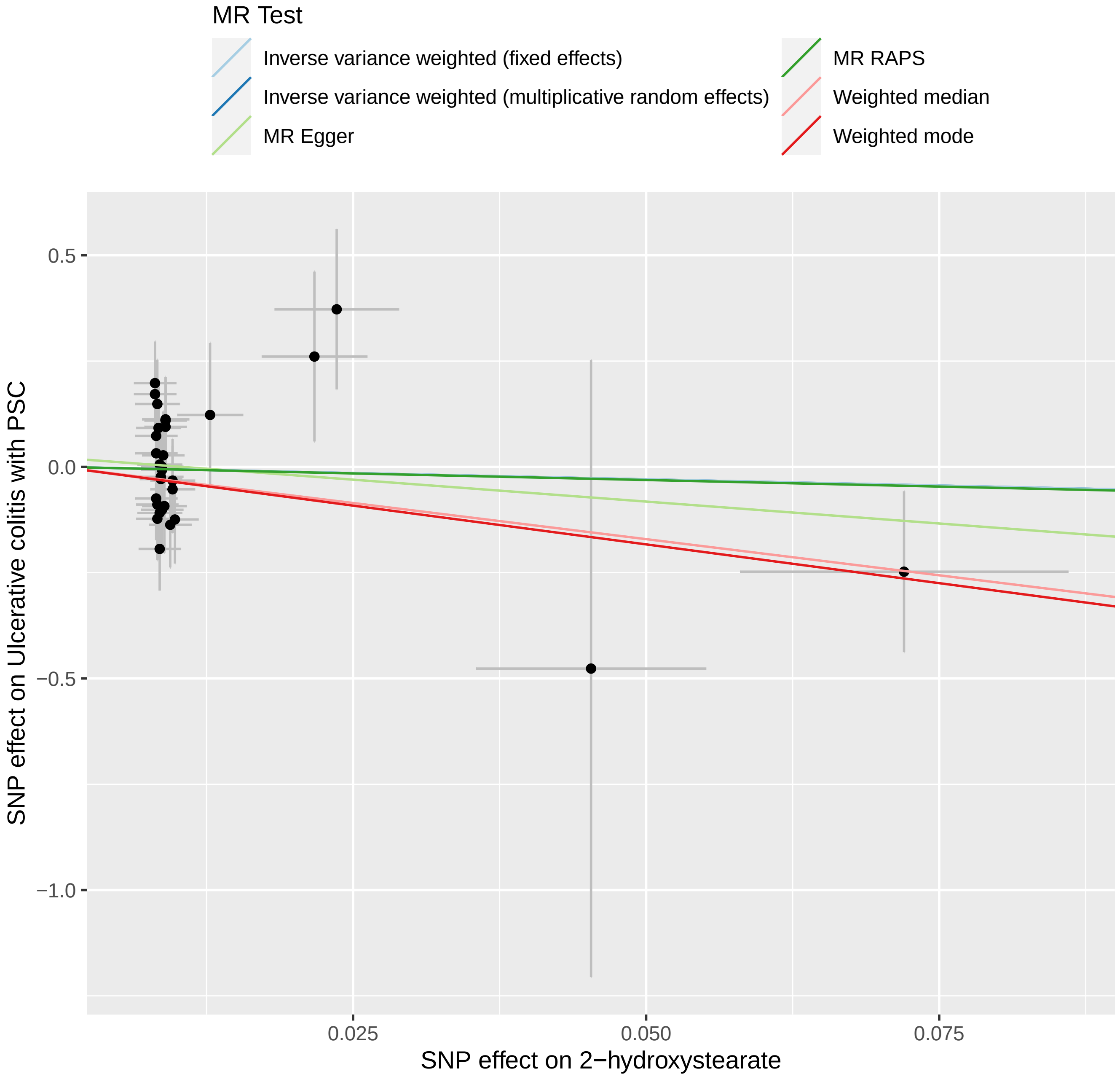

Batch 1187 : Glycylvaline on Ulcerative colitis with PSC

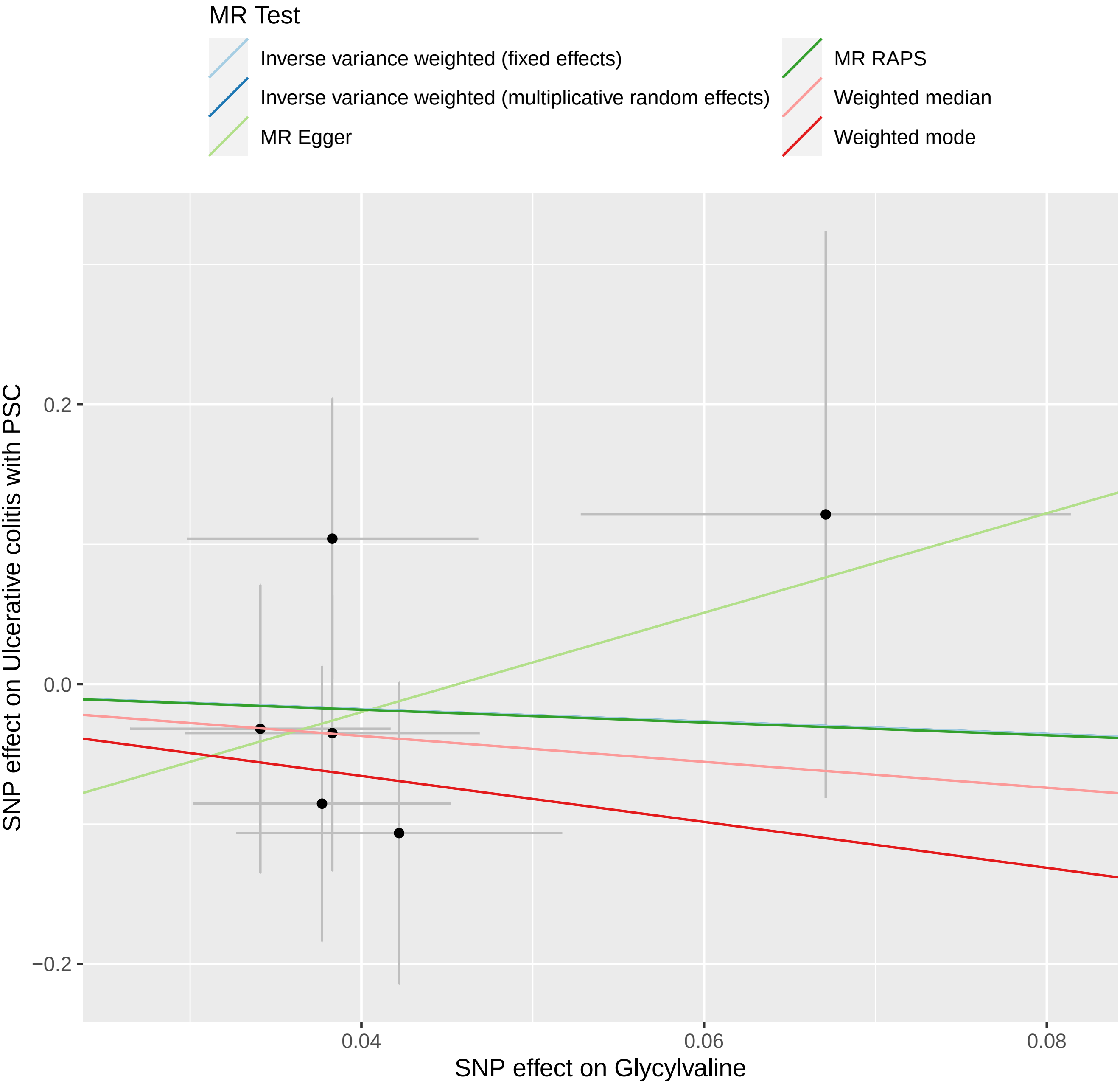

Batch 1188 : Gamma−glutamylleucine on Ulcerative colitis with PSC

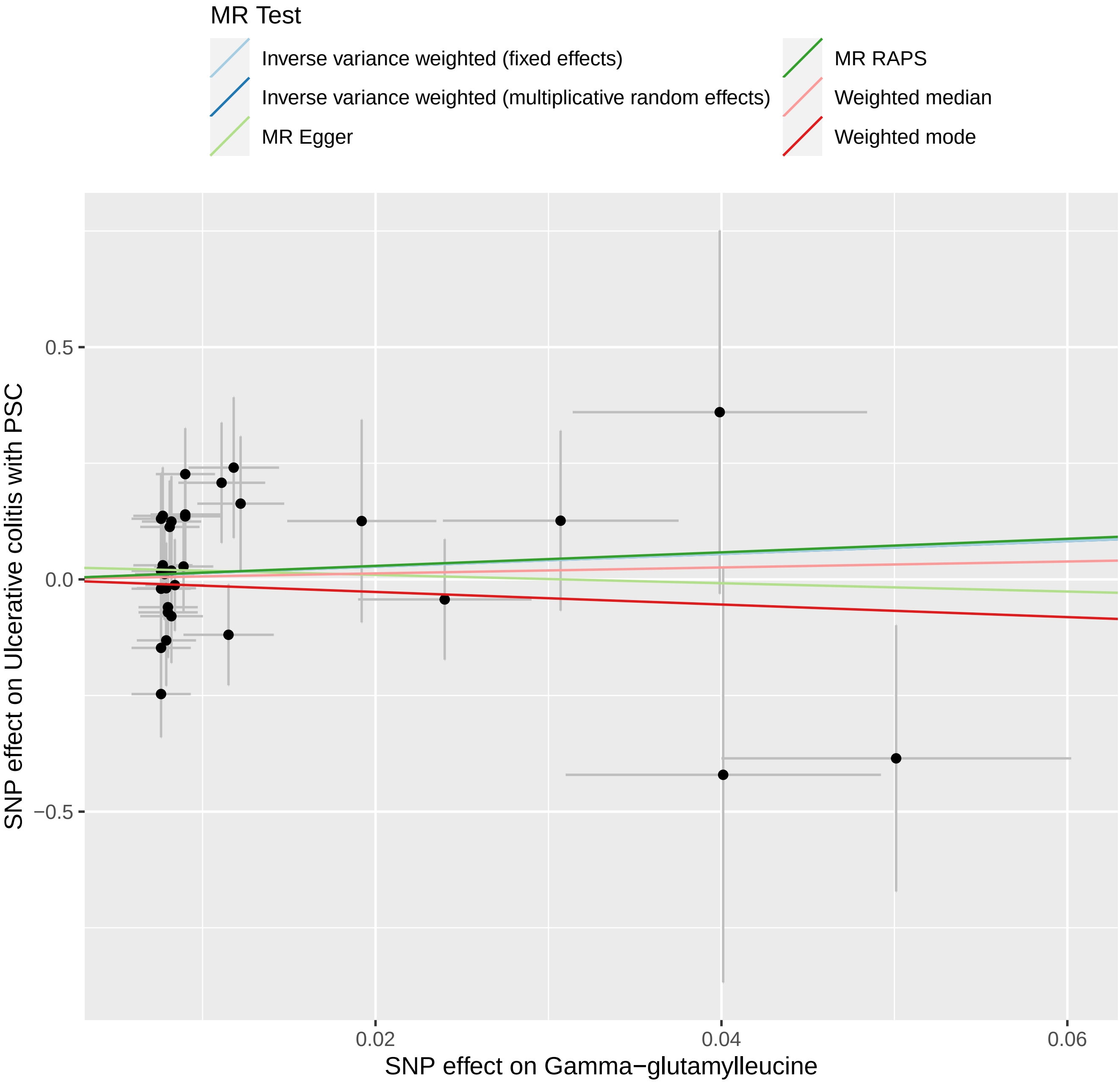

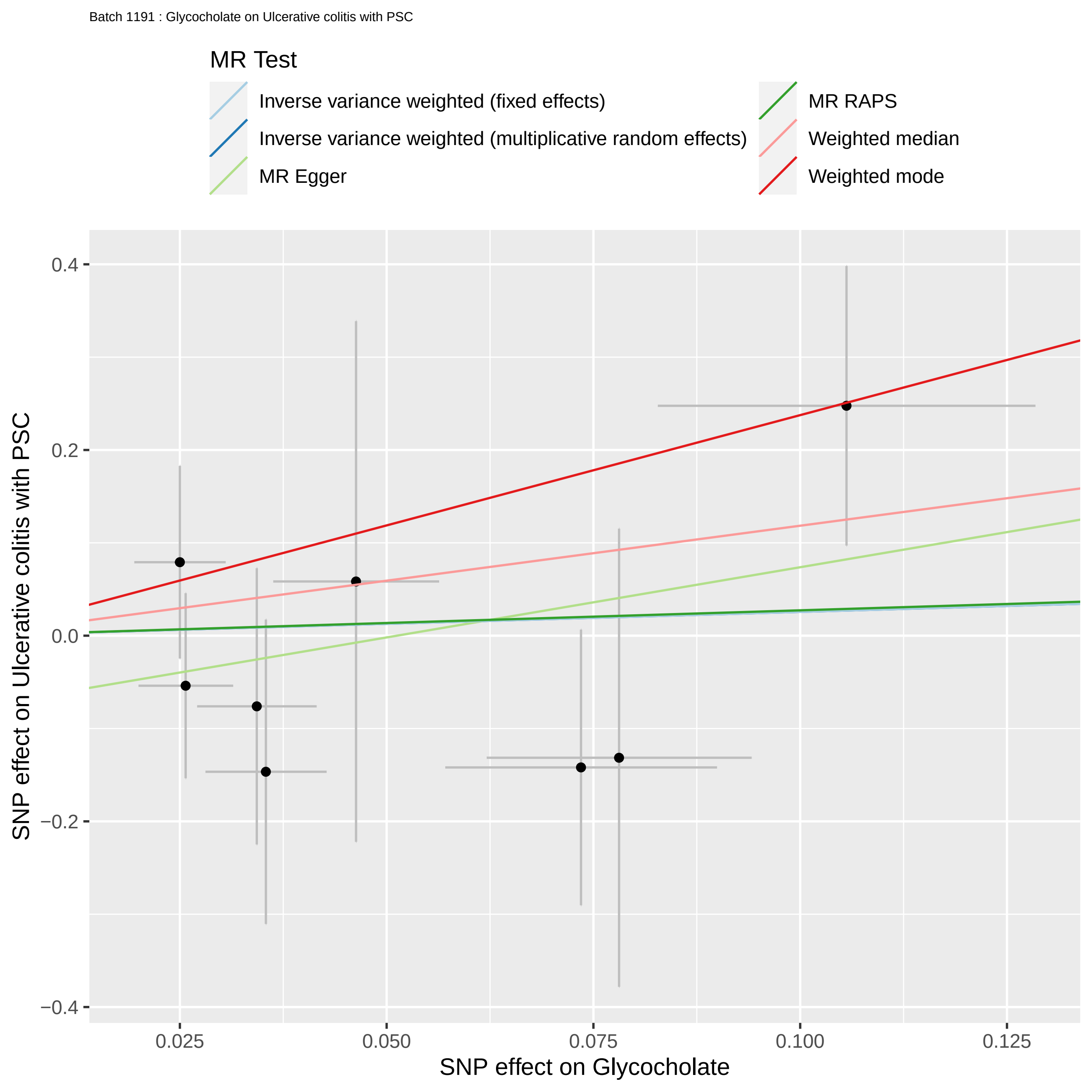

Batch 1191 : Glycocholate on Ulcerative colitis with PSC
MR Test
Inverse variance weighted (fixed effects)
Inverse variance weighted (multiplicative random effects)
MR Egger
MR RAPS
Weighted median
Weighted mode
0.4
0.2
0.0
−0.2
−0.4
0.025
0.050
0.075
0.100
0.125
SNP effect on Ulcerative colitis with PSC
SNP effect on Glycocholate

Batch 1192 : Glycodeoxycholate on Ulcerative colitis with PSC

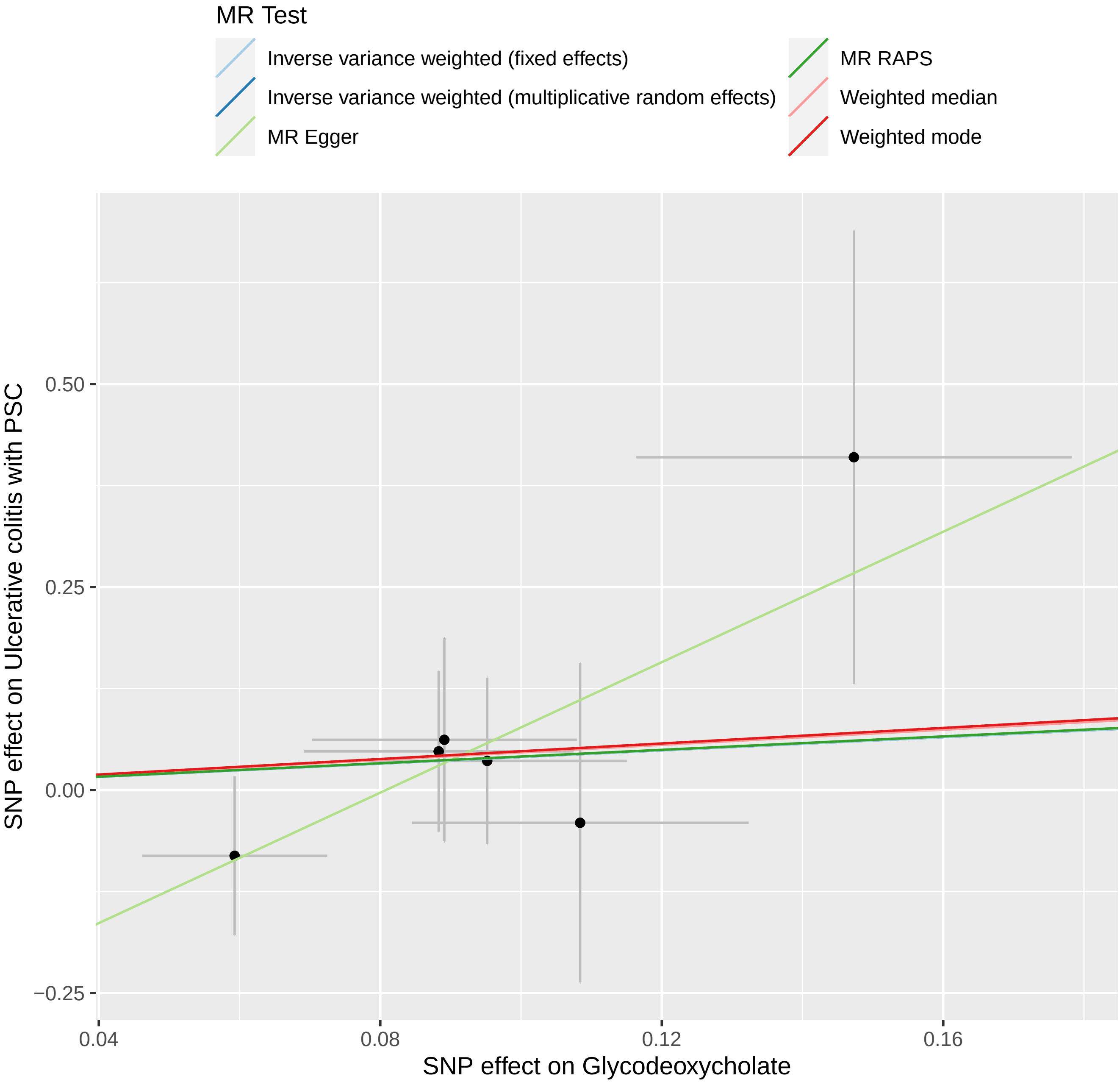

Batch 1193 : Taurochenodeoxycholate on Ulcerative colitis with PSC

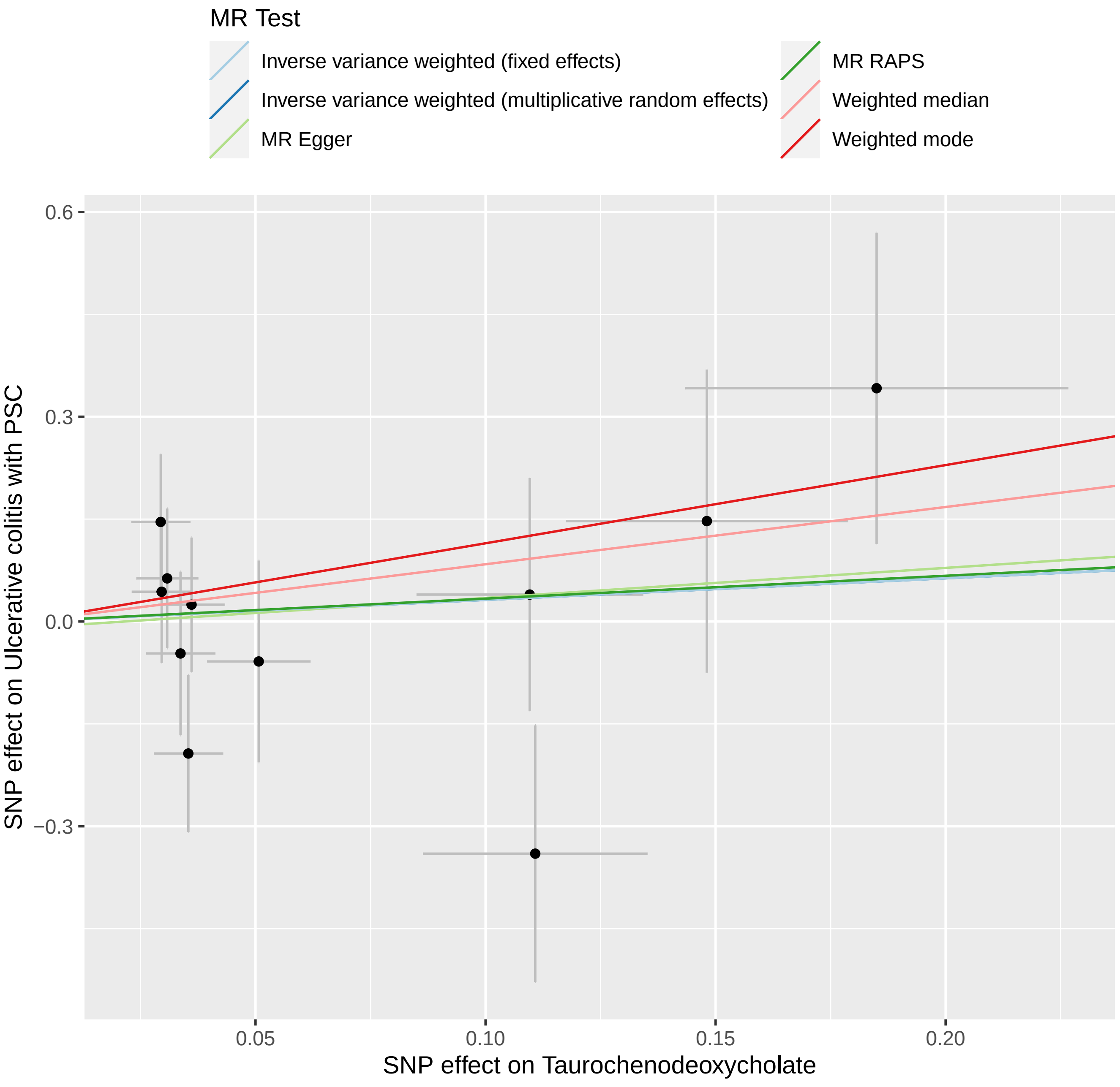

Batch 1194 : Taurocholate on Ulcerative colitis with PSC

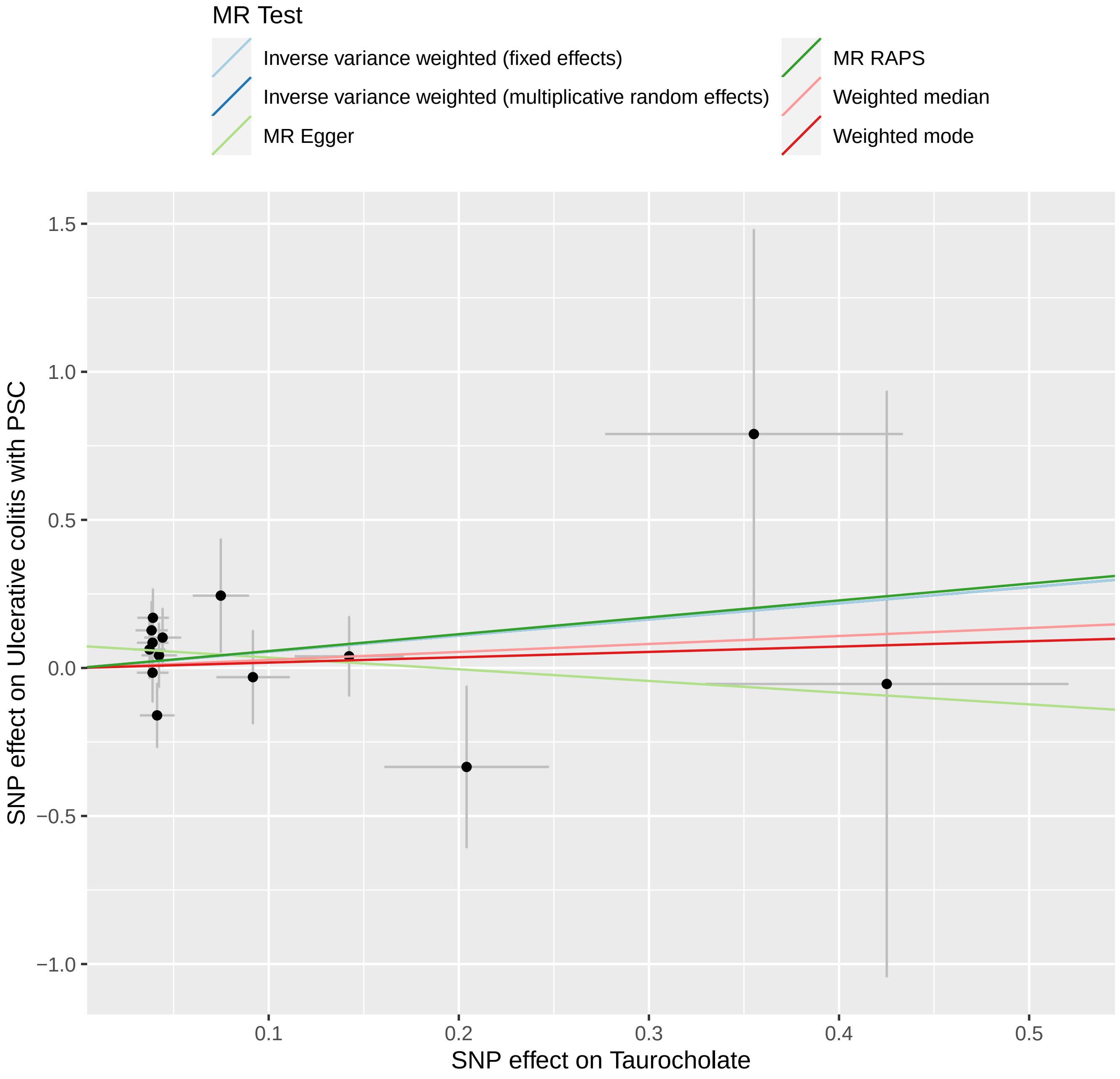

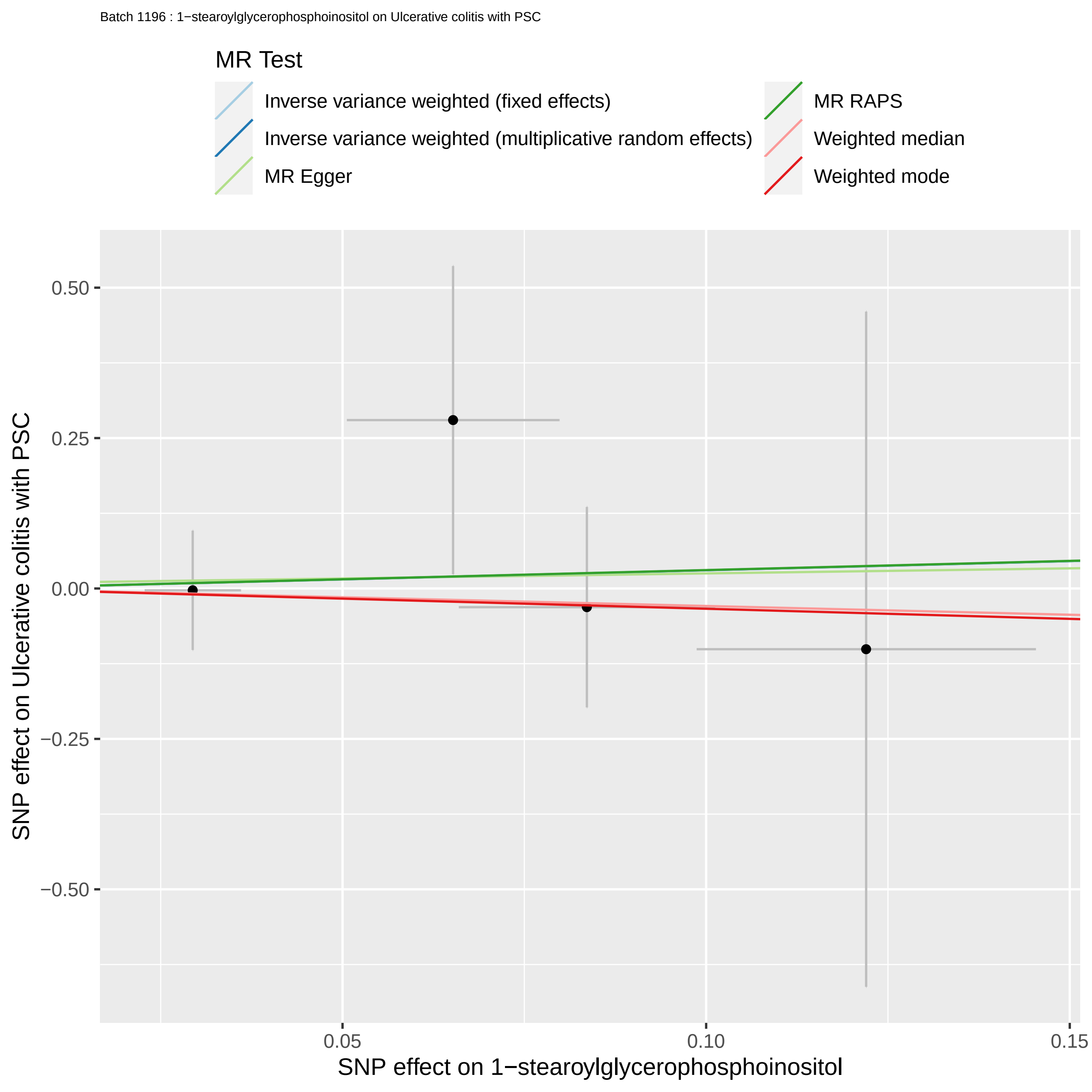
Batch 1196 : 1−stearoylglycerophosphoinositol on Ulcerative colitis with PSC
MR Test
Inverse variance weighted (fixed effects)
Inverse variance weighted (multiplicative random effects)
MR Egger
MR RAPS
Weighted median
Weighted mode
0.50
0.25
0.00
−0.25
−0.50
0.05
0.10
0.15
SNP effect on 1−stearoylglycerophosphoinositol
SNP effect on Ulcerative colitis with PSC

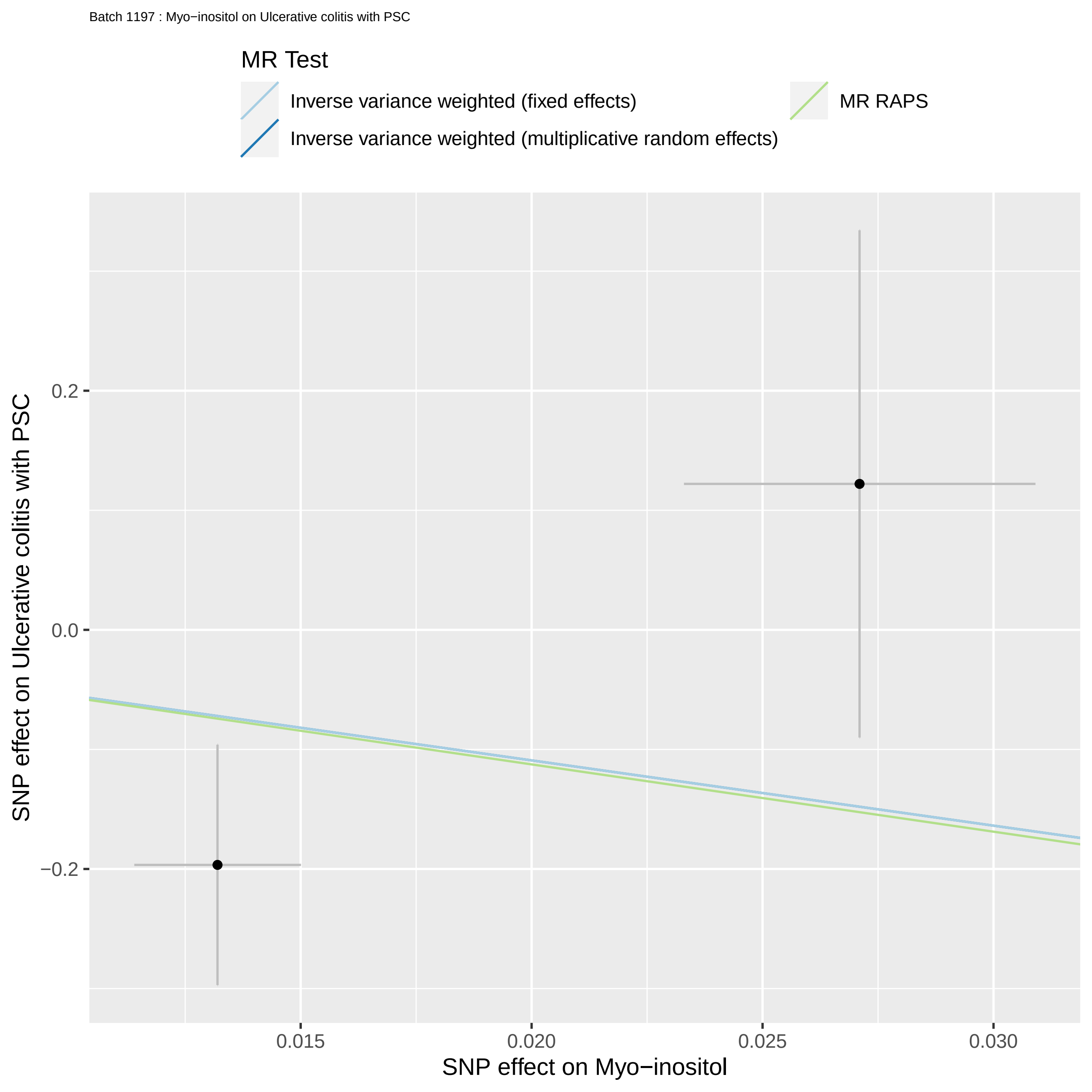
Batch 1197 : Myo−inositol on Ulcerative colitis with PSC
MR Test
Inverse variance weighted (fixed effects)
Inverse variance weighted (multiplicative random effects)
MR RAPS
0.2
0.0
−0.2
SNP effect on Ulcerative colitis with PSC
0.015
0.020
0.025
0.030
SNP effect on Myo−inositol

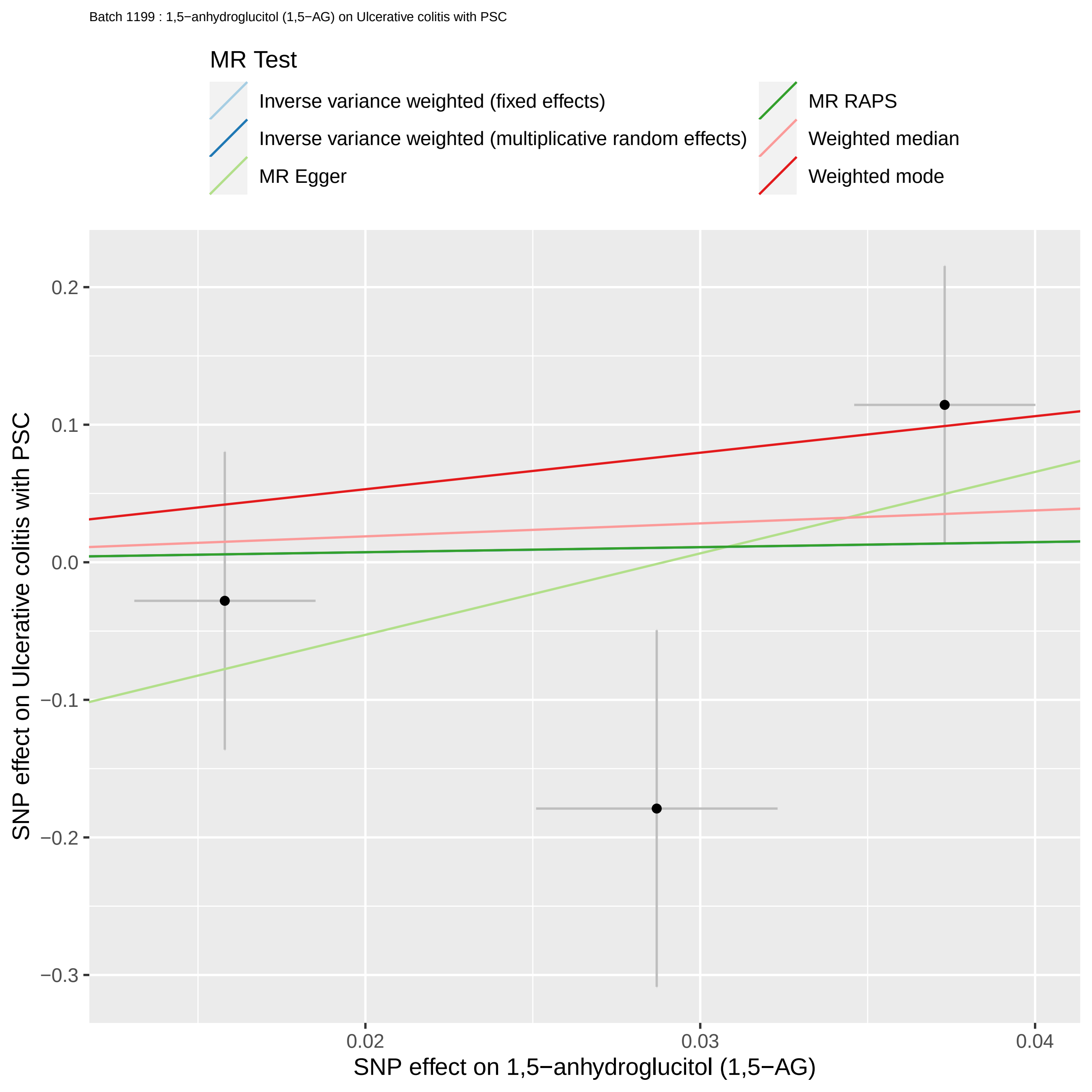
Batch 1199 : 1,5−anhydroglucitol (1,5−AG) on Ulcerative colitis with PSC
MR Test
Inverse variance weighted (fixed effects)
Inverse variance weighted (multiplicative random effects)
MR Egger
MR RAPS
Weighted median
Weighted mode
0.2
0.1
0.0
−0.1
−0.2
−0.3
0.02
0.03
0.04
SNP effect on Ulcerative colitis with PSC
SNP effect on 1,5−anhydroglucitol (1,5−AG)

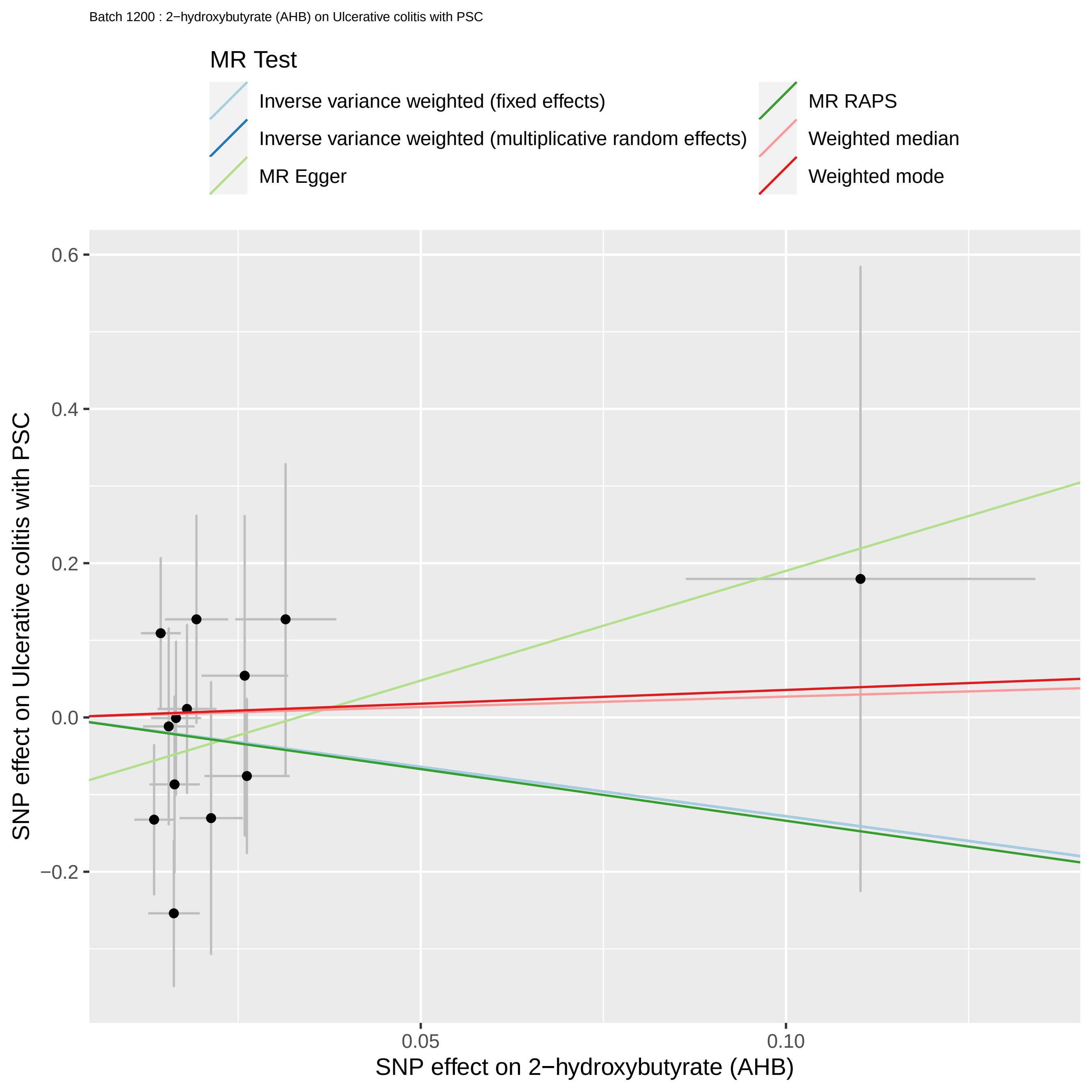

Batch 1200 : 2−hydroxybutyrate (AHB) on Ulcerative colitis with PSC
MR Test
Inverse variance weighted (fixed effects)
Inverse variance weighted (multiplicative random effects)
MR Egger
MR RAPS
Weighted median
Weighted mode
0.6
0.4
0.2
0.0
−0.2
0.05
0.10
SNP effect on Ulcerative colitis with PSC
SNP effect on 2−hydroxybutyrate (AHB)

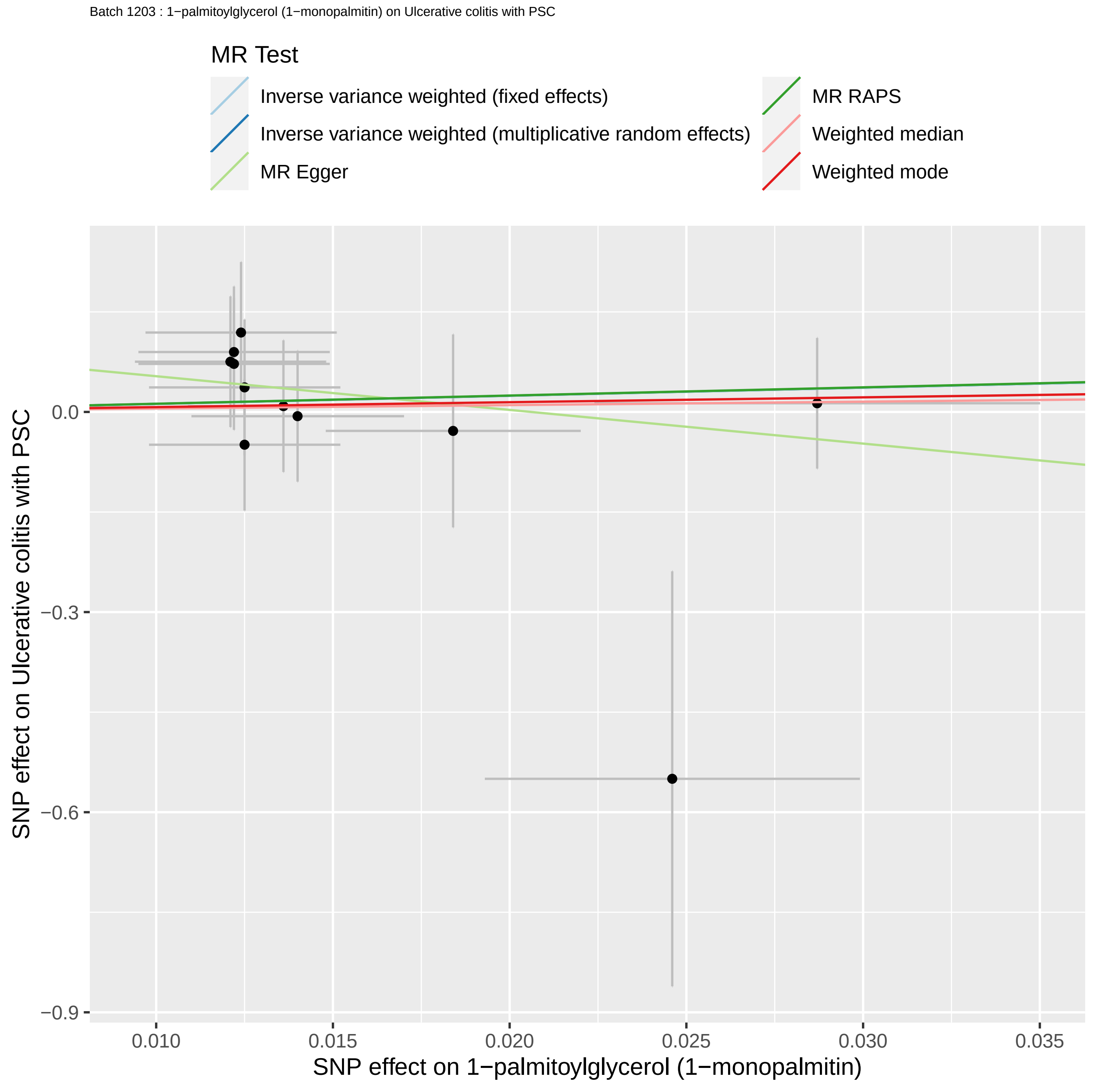
Batch 1203 : 1−palmitoylglycerol (1−monopalmitin) on Ulcerative colitis with PSC
MR Test
Inverse variance weighted (fixed effects)
Inverse variance weighted (multiplicative random effects)
MR Egger
MR RAPS
Weighted median
Weighted mode
0.0
−0.3
−0.6
−0.9
0.010
0.015
0.020
0.025
0.030
0.035
SNP effect on 1−palmitoylglycerol (1−monopalmitin)
SNP effect on Ulcerative colitis with PSC

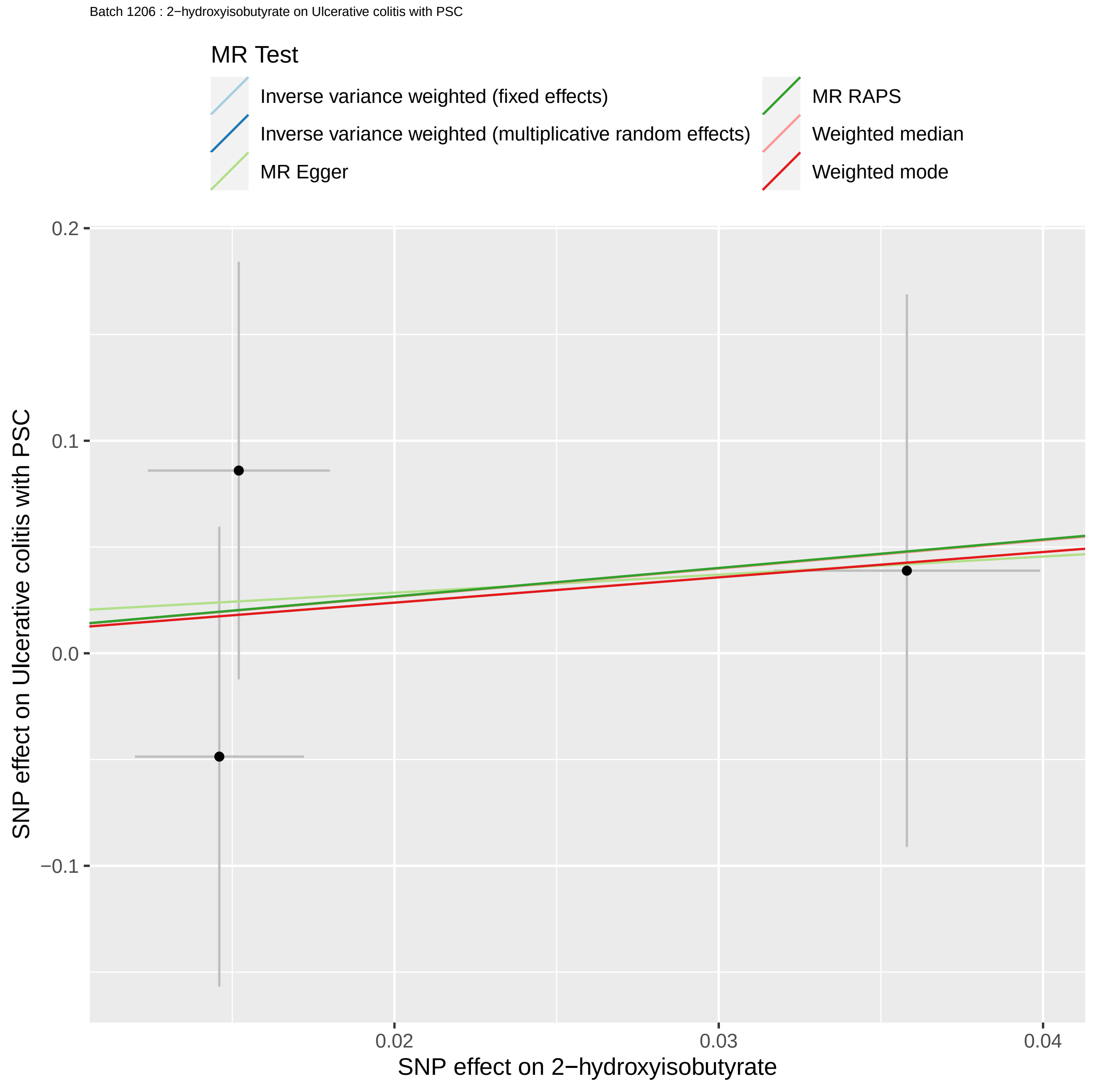

Batch 1206 : 2−hydroxyisobutyrate on Ulcerative colitis with PSC
MR Test
Inverse variance weighted (fixed effects)
Inverse variance weighted (multiplicative random effects)
MR Egger
MR RAPS
Weighted median
Weighted mode
0.2
0.1
0.0
−0.1
0.02
0.03
0.04
SNP effect on Ulcerative colitis with PSC
SNP effect on 2−hydroxyisobutyrate

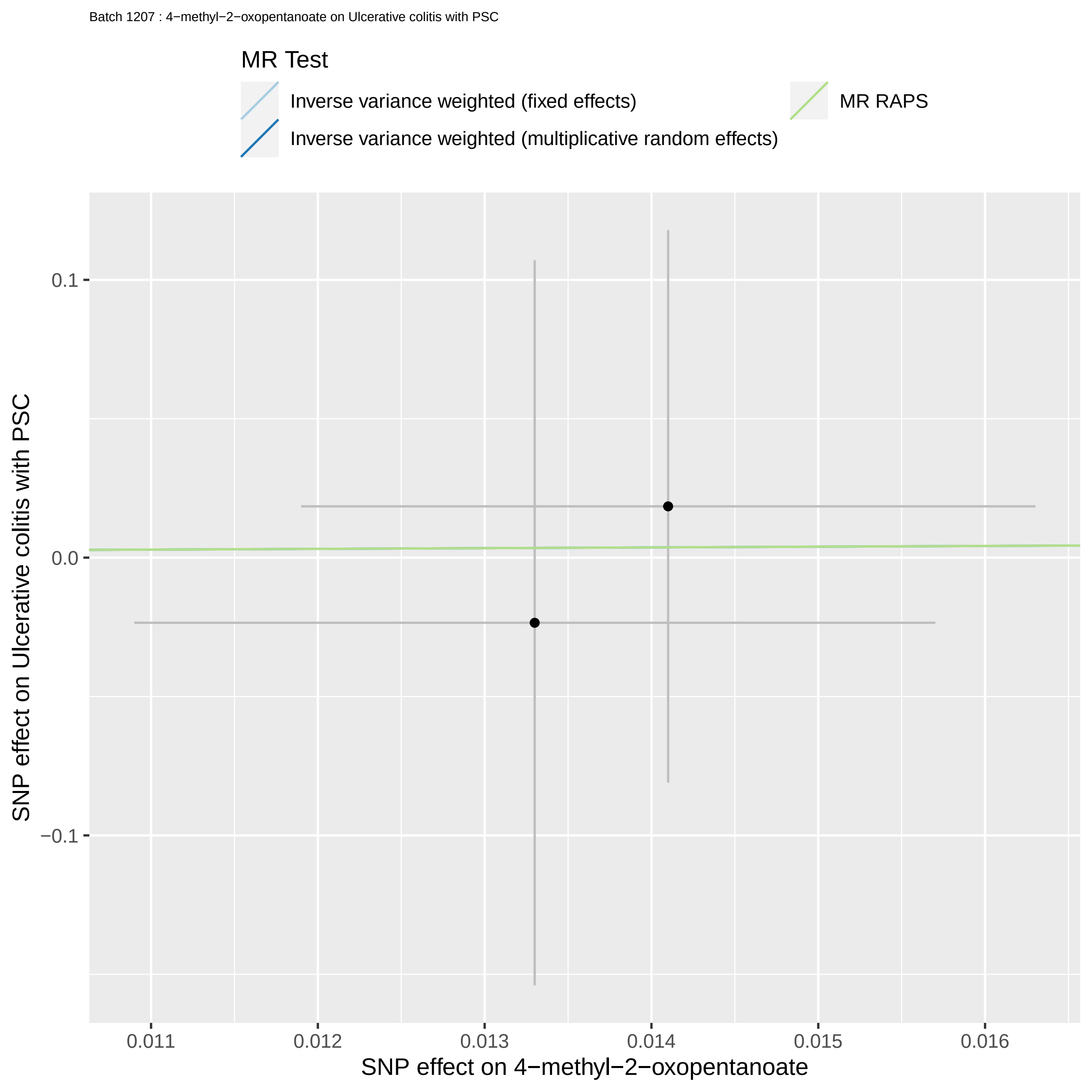
Batch 1207 : 4−methyl−2−oxopentanoate on Ulcerative colitis with PSC
MR Test
Inverse variance weighted (fixed effects)
Inverse variance weighted (multiplicative random effects)
MR RAPS
SNP effect on Ulcerative colitis with PSC
0.1
0.0
−0.1
0.011
0.012
0.013
0.014
0.015
0.016
SNP effect on 4−methyl−2−oxopentanoate

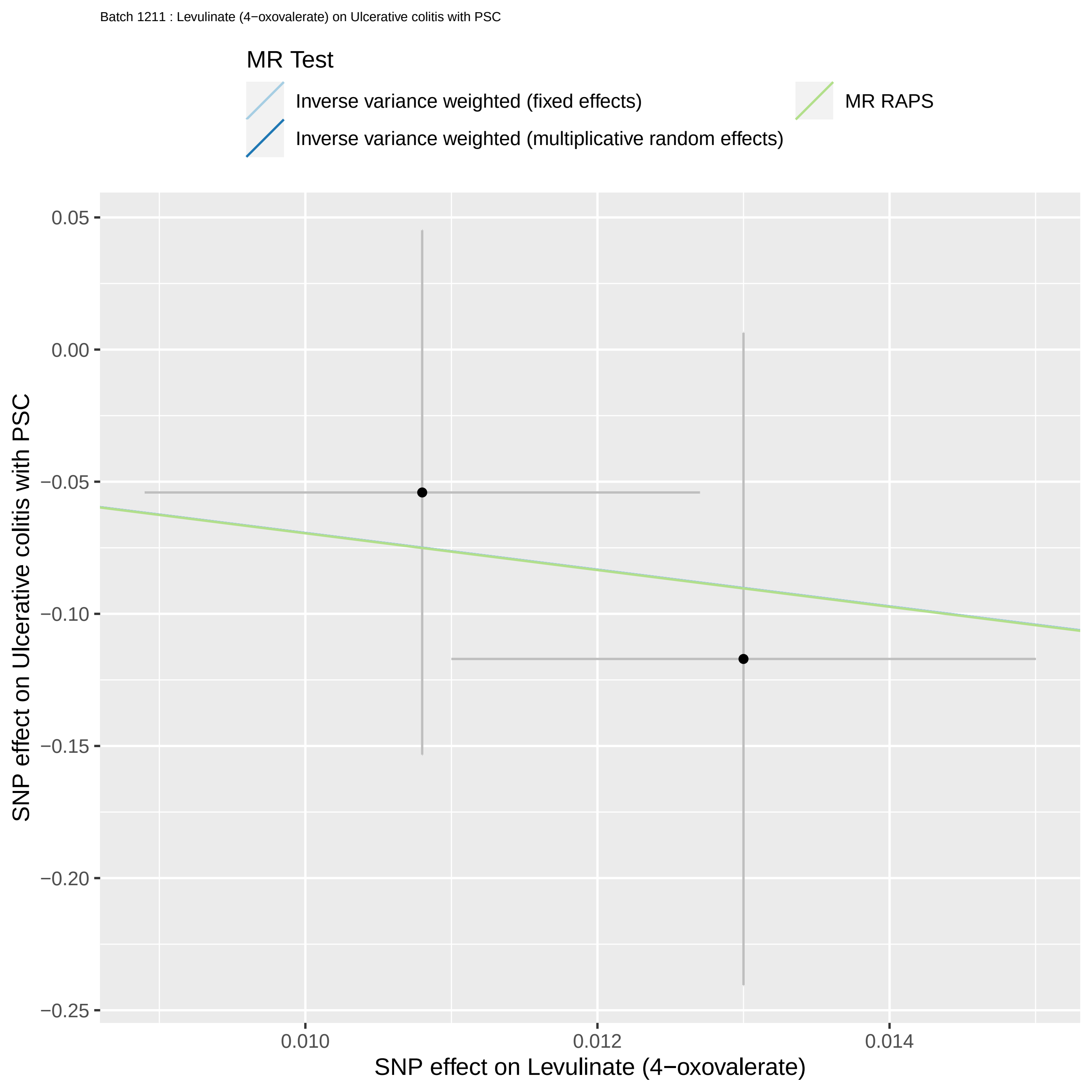

Batch 1211 : Levulinate (4−oxovalerate) on Ulcerative colitis with PSC
MR Test
Inverse variance weighted (fixed effects)
Inverse variance weighted (multiplicative random effects)
MR RAPS
0.05
0.00
−0.05
−0.10
−0.15
−0.20
−0.25
0.010
0.012
0.014
SNP effect on Levulinate (4−oxovalerate)
SNP effect on Ulcerative colitis with PSC

Batch 1213 : Cholate on Ulcerative colitis with PSC

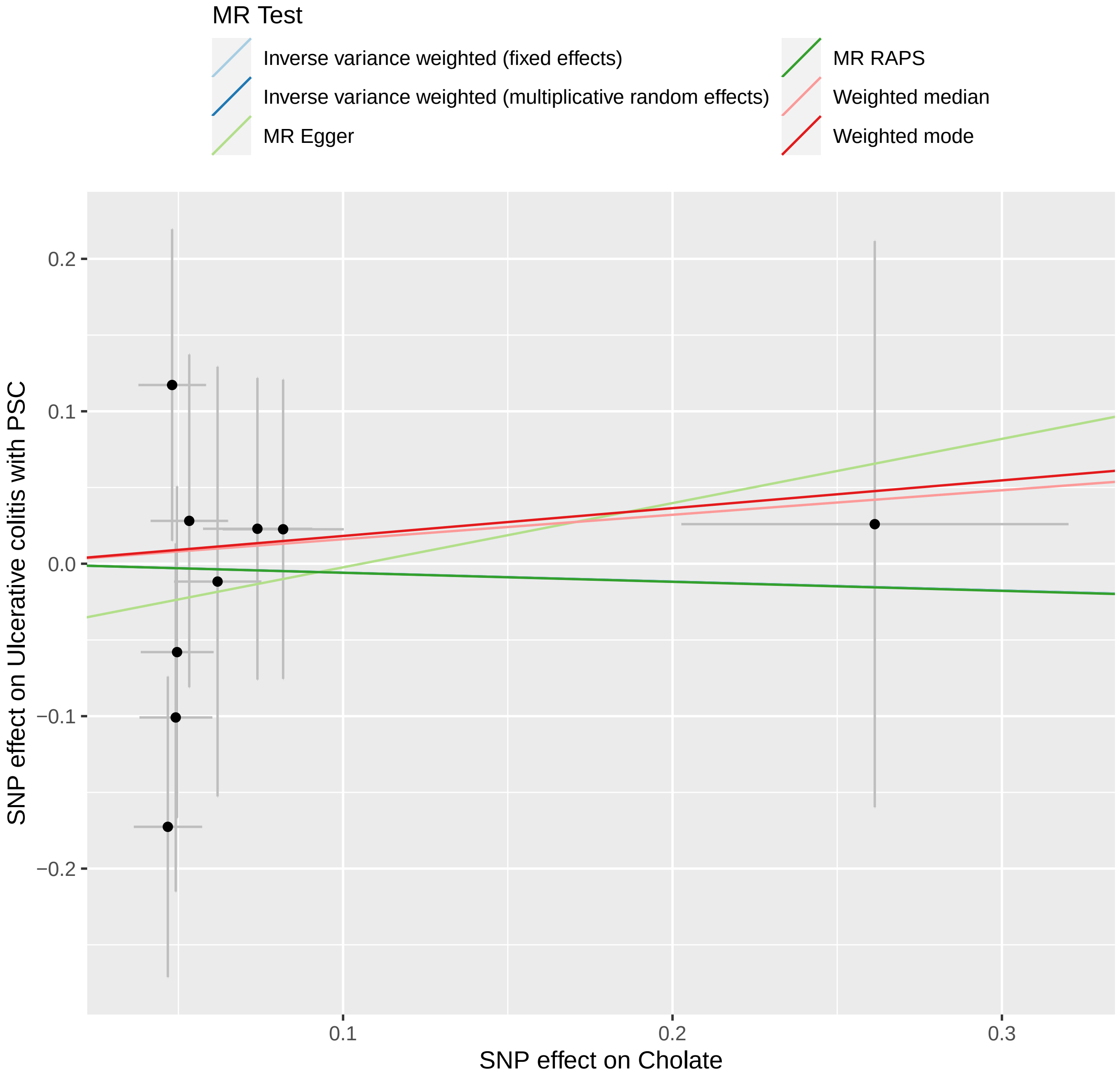

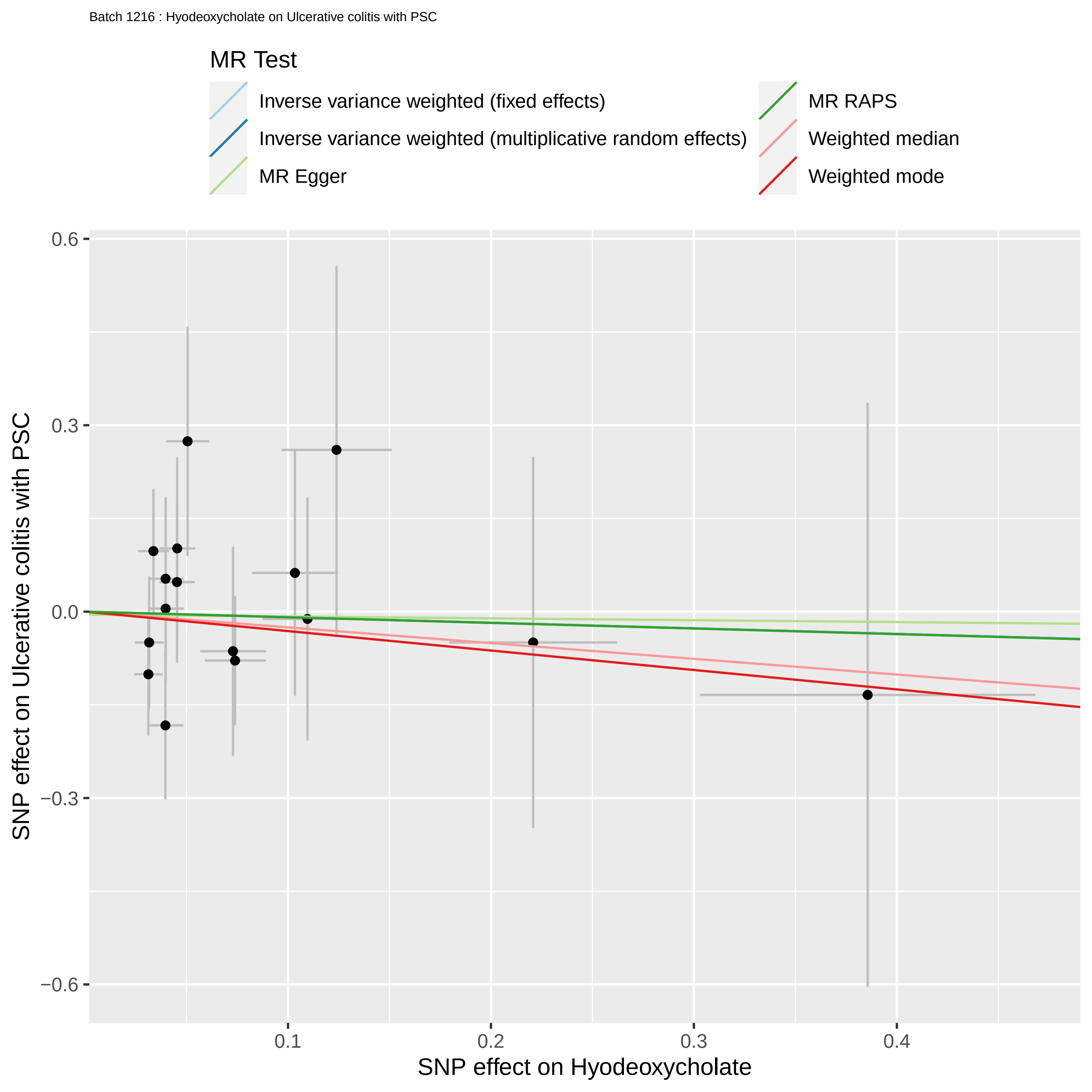
Batch 1216 : Hyodeoxycholate on Ulcerative colitis with PSC
MR Test
Inverse variance weighted (fixed effects)
Inverse variance weighted (multiplicative random effects)
MR Egger
MR RAPS
Weighted median
Weighted mode
0.6
0.3
0.0
−0.3
−0.6
0.1
0.2
0.3
0.4
SNP effect on Ulcerative colitis with PSC
SNP effect on Hyodeoxycholate

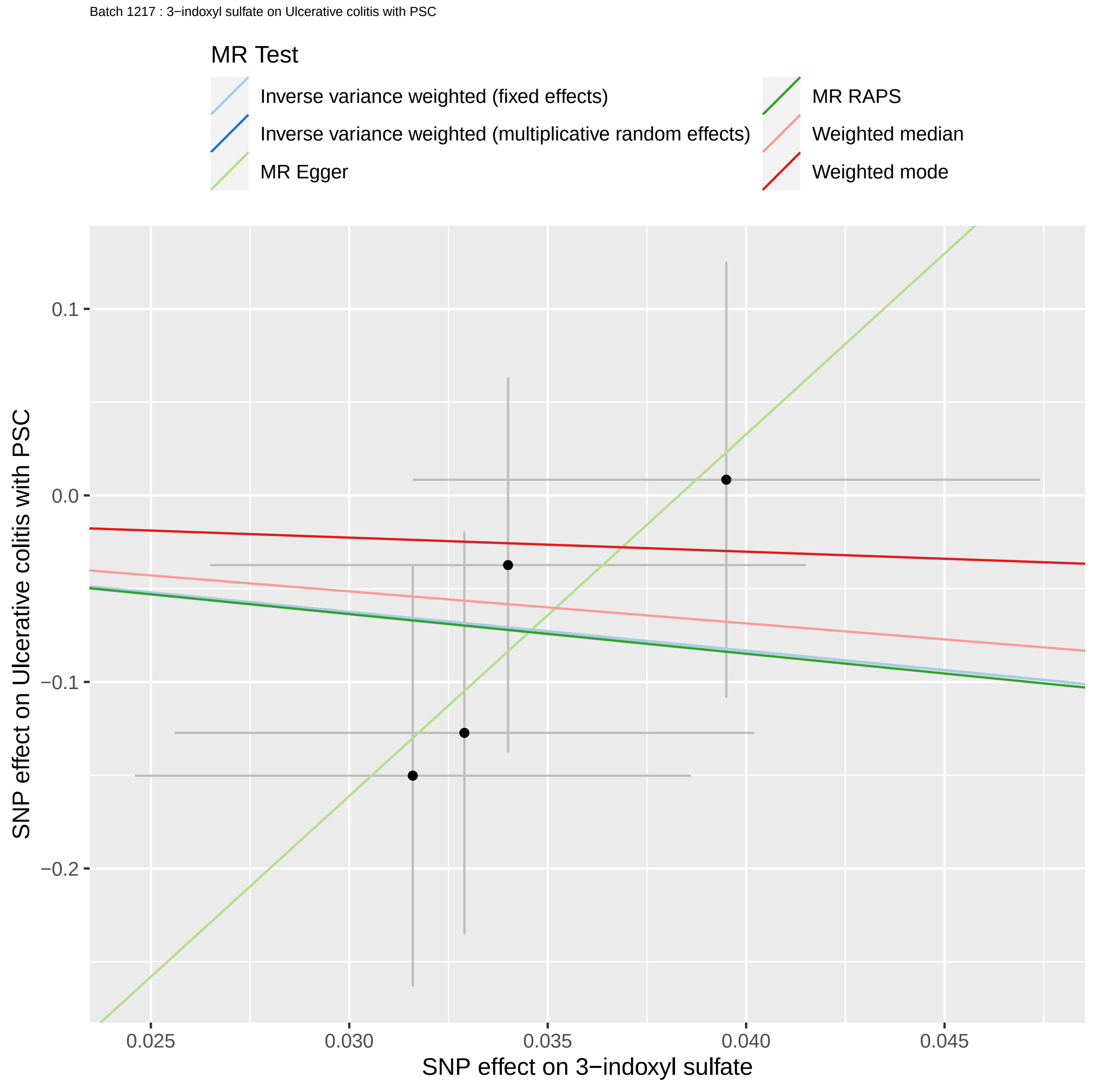

Batch 1217 : 3−indoxyl sulfate on Ulcerative colitis with PSC
MR Test
Inverse variance weighted (fixed effects)
Inverse variance weighted (multiplicative random effects)
MR Egger
MR RAPS
Weighted median
Weighted mode
SNP effect on Ulcerative colitis with PSC
0.1
0.0
−0.1
−0.2
0.025
0.030
0.035
0.040
0.045
SNP effect on 3−indoxyl sulfate

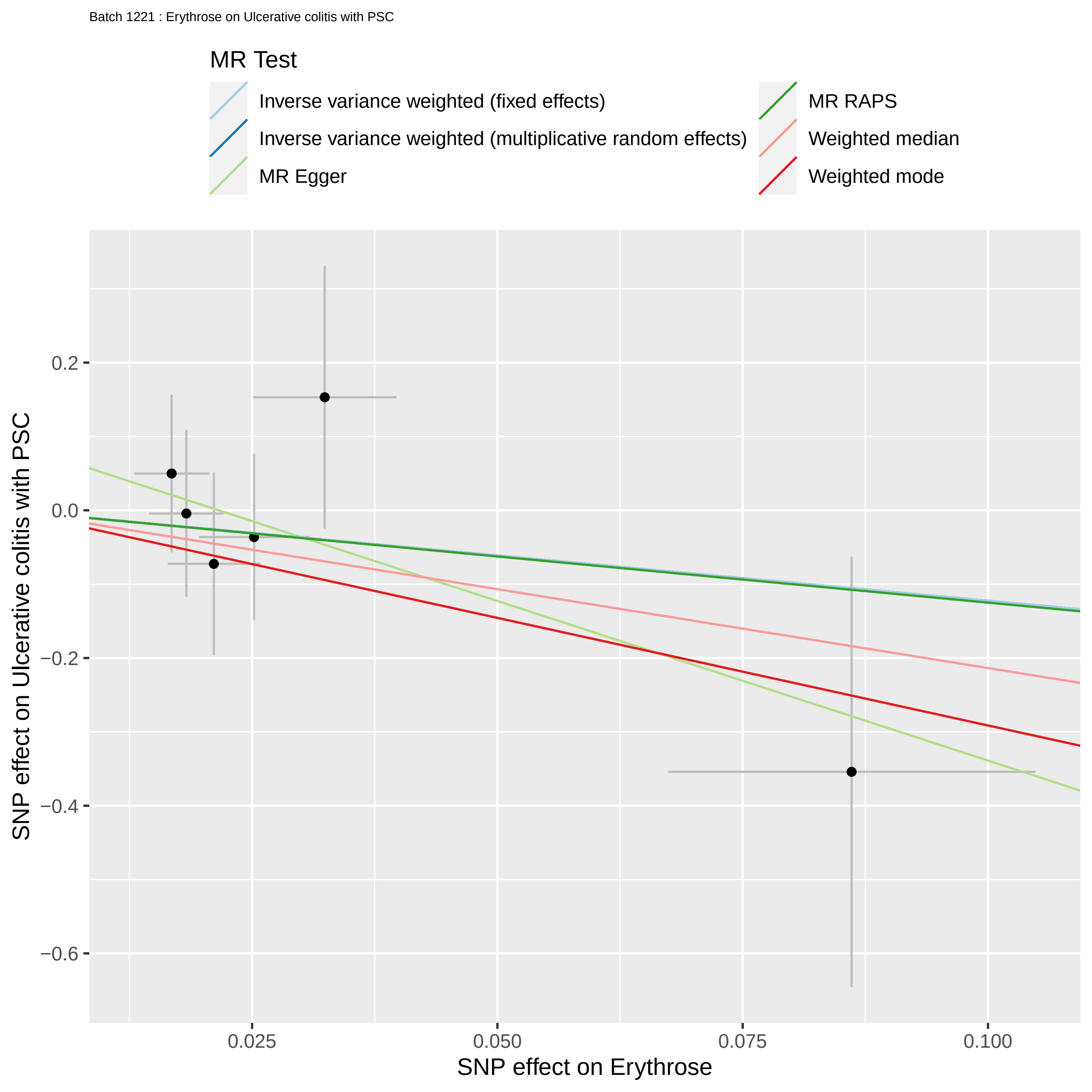
Batch 1221 : Erythrose on Ulcerative colitis with PSC
MR Test
Inverse variance weighted (fixed effects)
Inverse variance weighted (multiplicative random effects)
MR Egger
MR RAPS
Weighted median
Weighted mode
0.2
0.0
−0.2
−0.4
−0.6
0.025
0.050
0.075
0.100
SNP effect on Ulcerative colitis with PSC
SNP effect on Erythrose

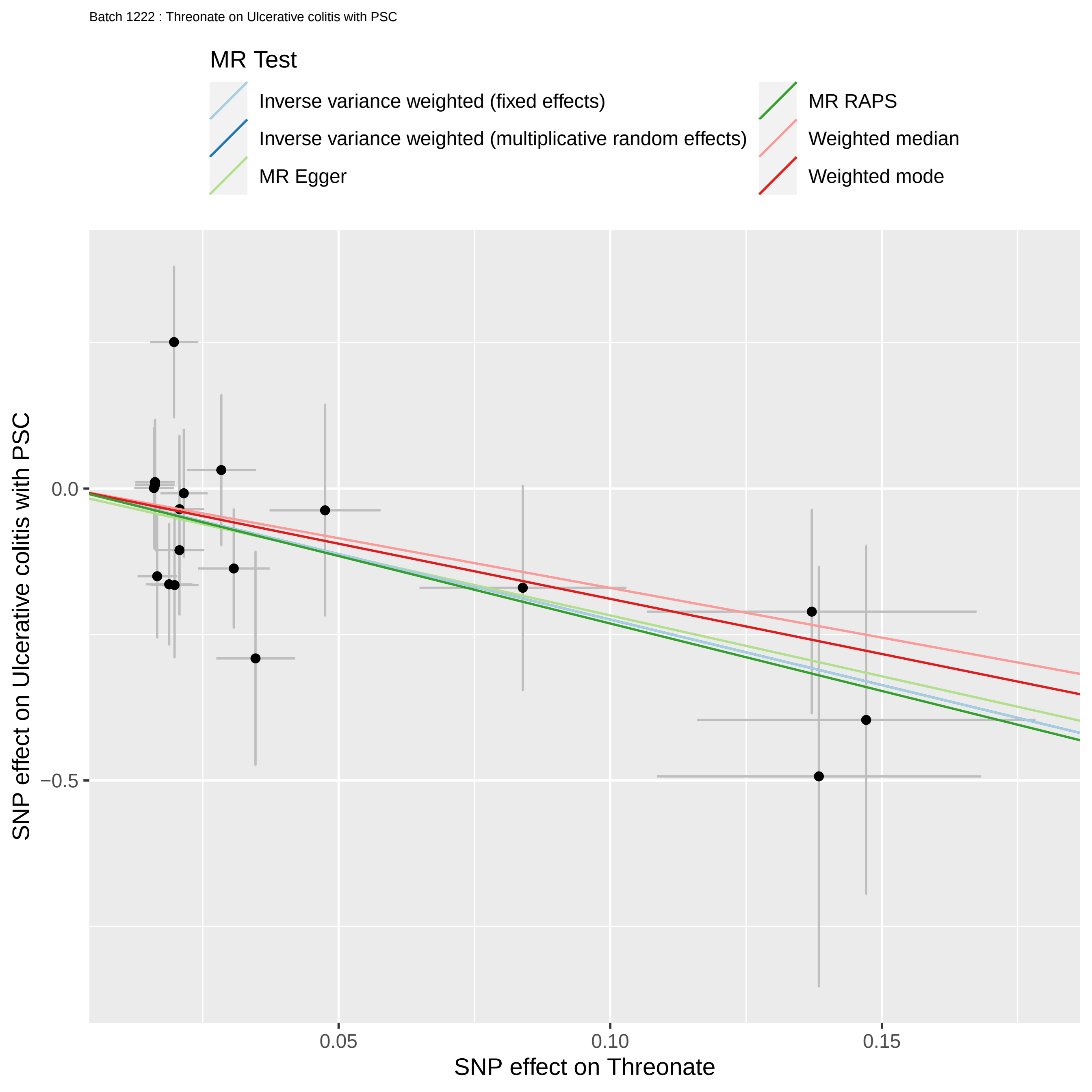

Batch 1222 : Threonate on Ulcerative colitis with PSC
MR Test
Inverse variance weighted (fixed effects)
Inverse variance weighted (multiplicative random effects)
MR Egger
MR RAPS
Weighted median
Weighted mode
SNP effect on Ulcerative colitis with PSC
0.0
−0.5
0.05
0.10
0.15
SNP effect on Threonate

Batch 1223 : Cysteine on Ulcerative colitis with PSC

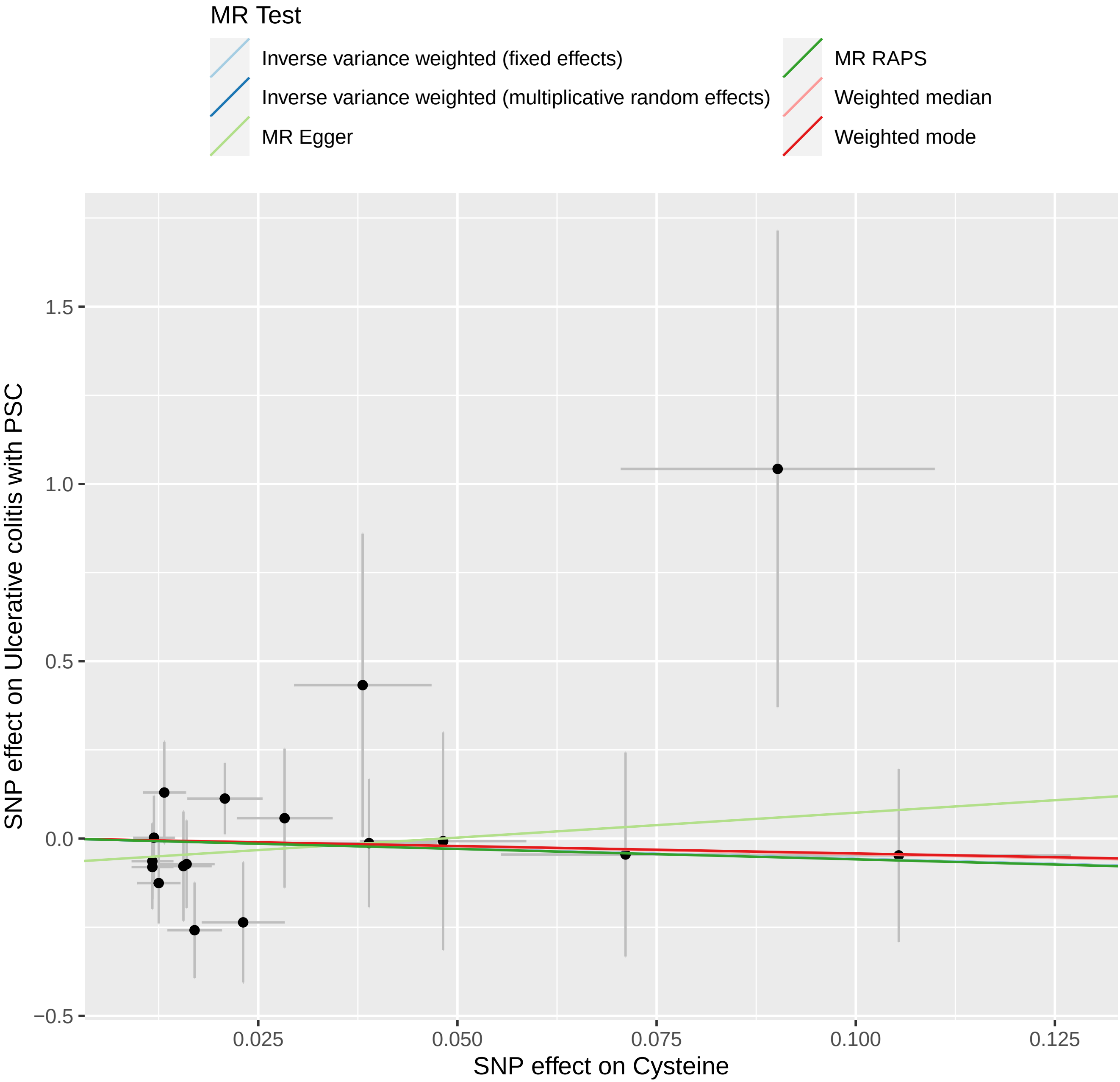

Batch 1224 : Pyroglutamylglycine on Ulcerative colitis with PSC

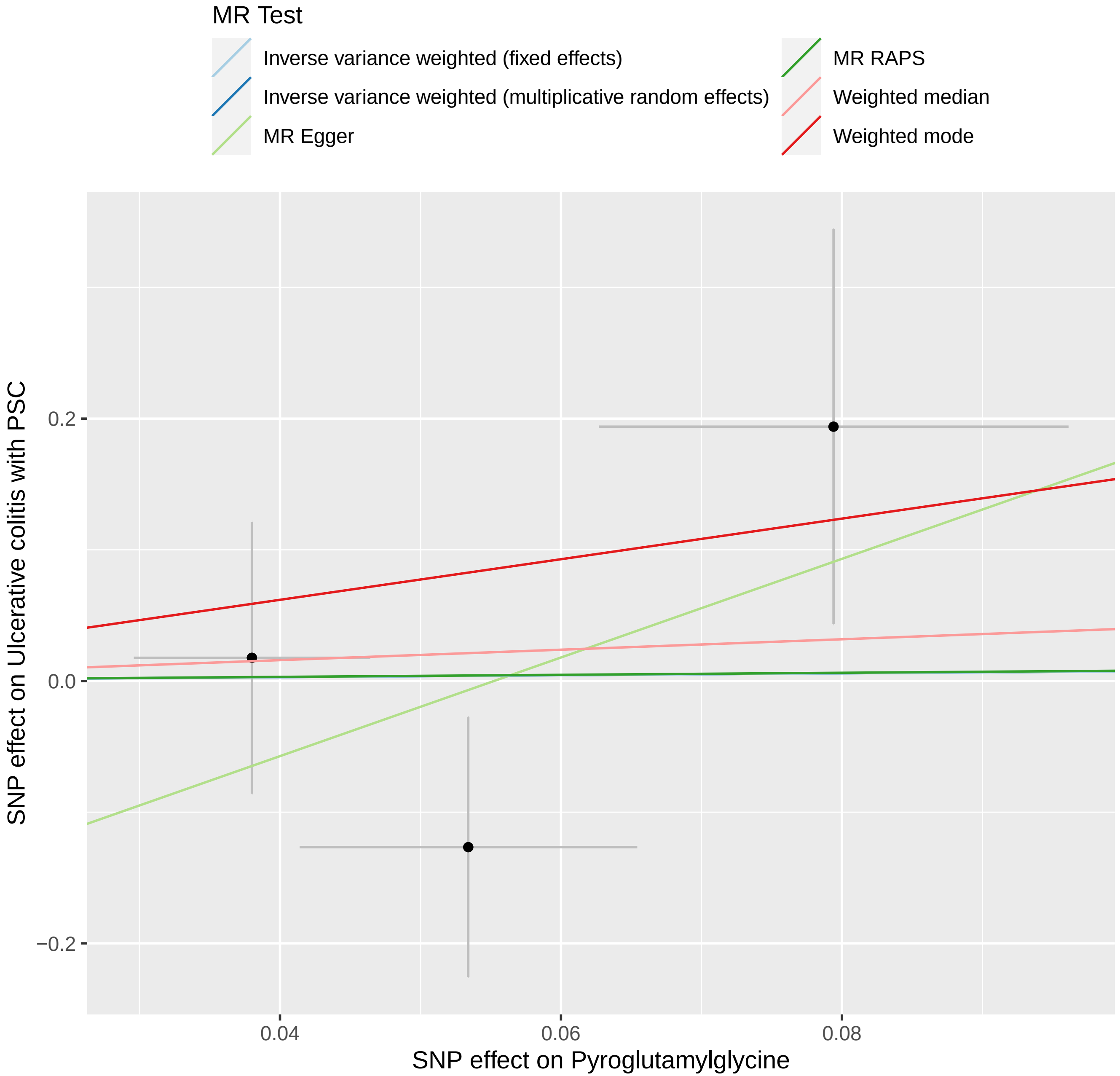

Batch 1226 : DSGEGDFXAEGGGVR* on Ulcerative colitis with PSC

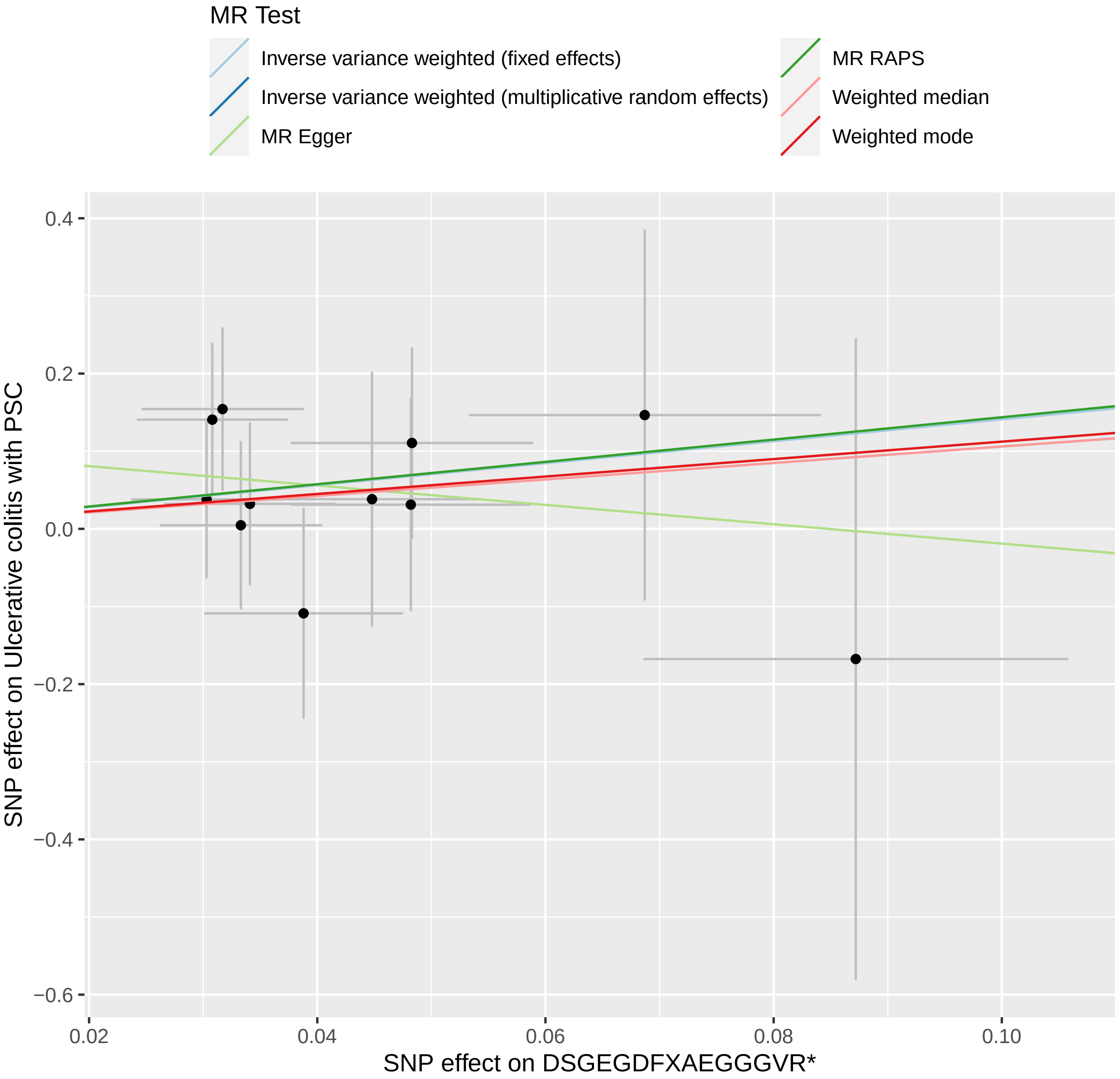

Batch 1227 : Pyridoxate on Ulcerative colitis with PSC

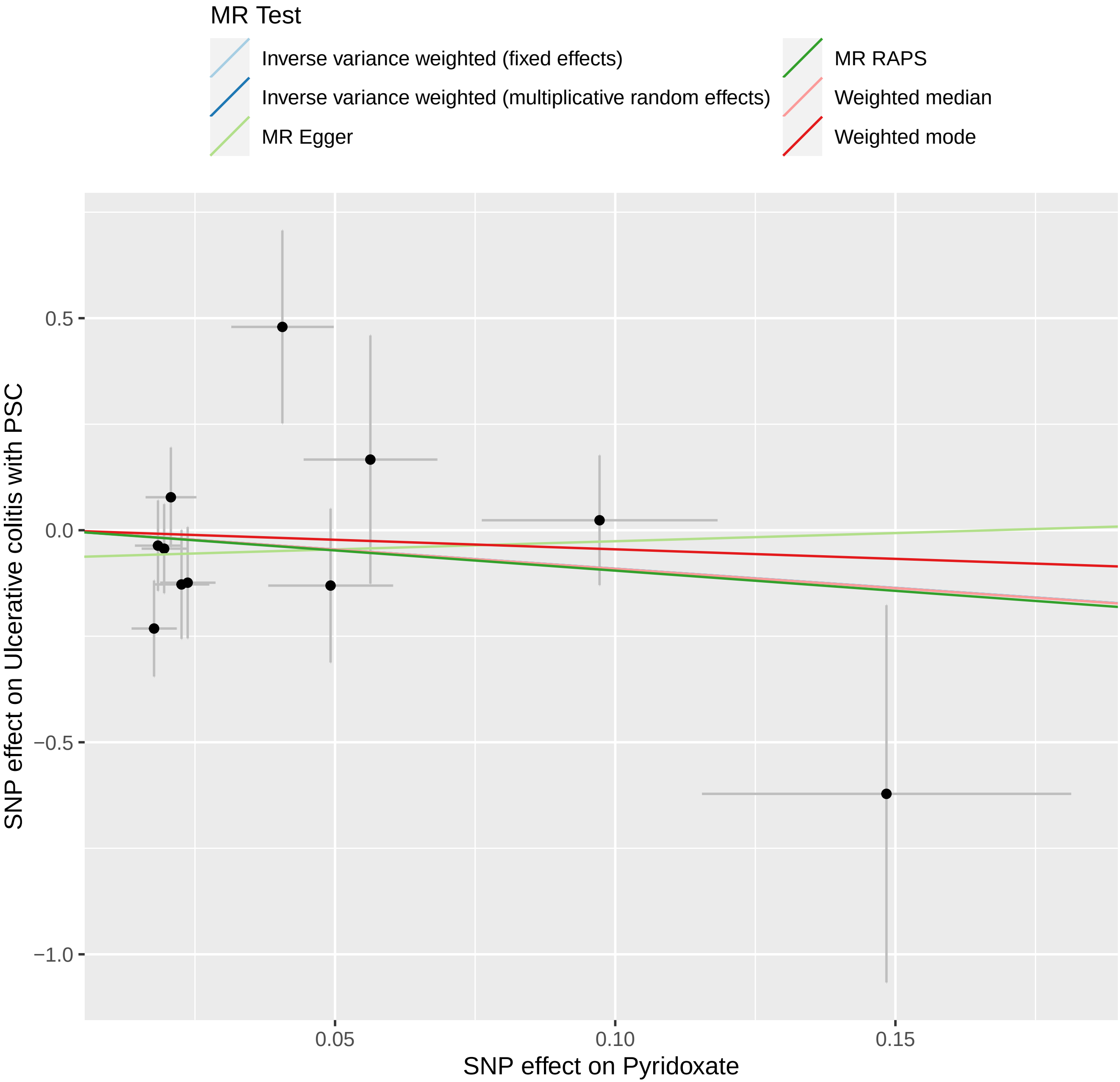

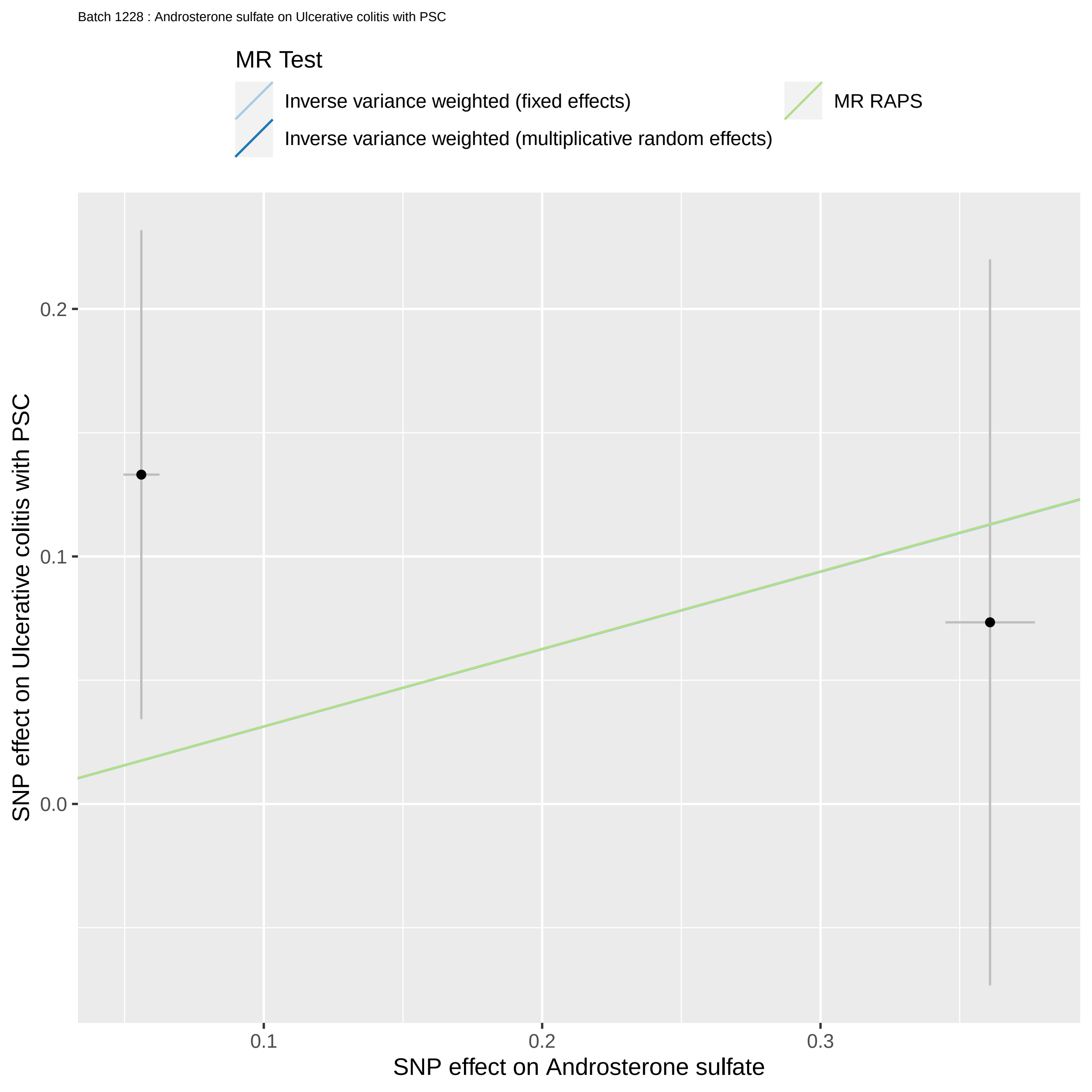
Batch 1228 : Androsterone sulfate on Ulcerative colitis with PSC
MR Test
Inverse variance weighted (fixed effects)
Inverse variance weighted (multiplicative random effects)
MR RAPS
SNP effect on Ulcerative colitis with PSC
0.2
0.1
0.0
0.1
0.2
0.3
SNP effect on Androsterone sulfate

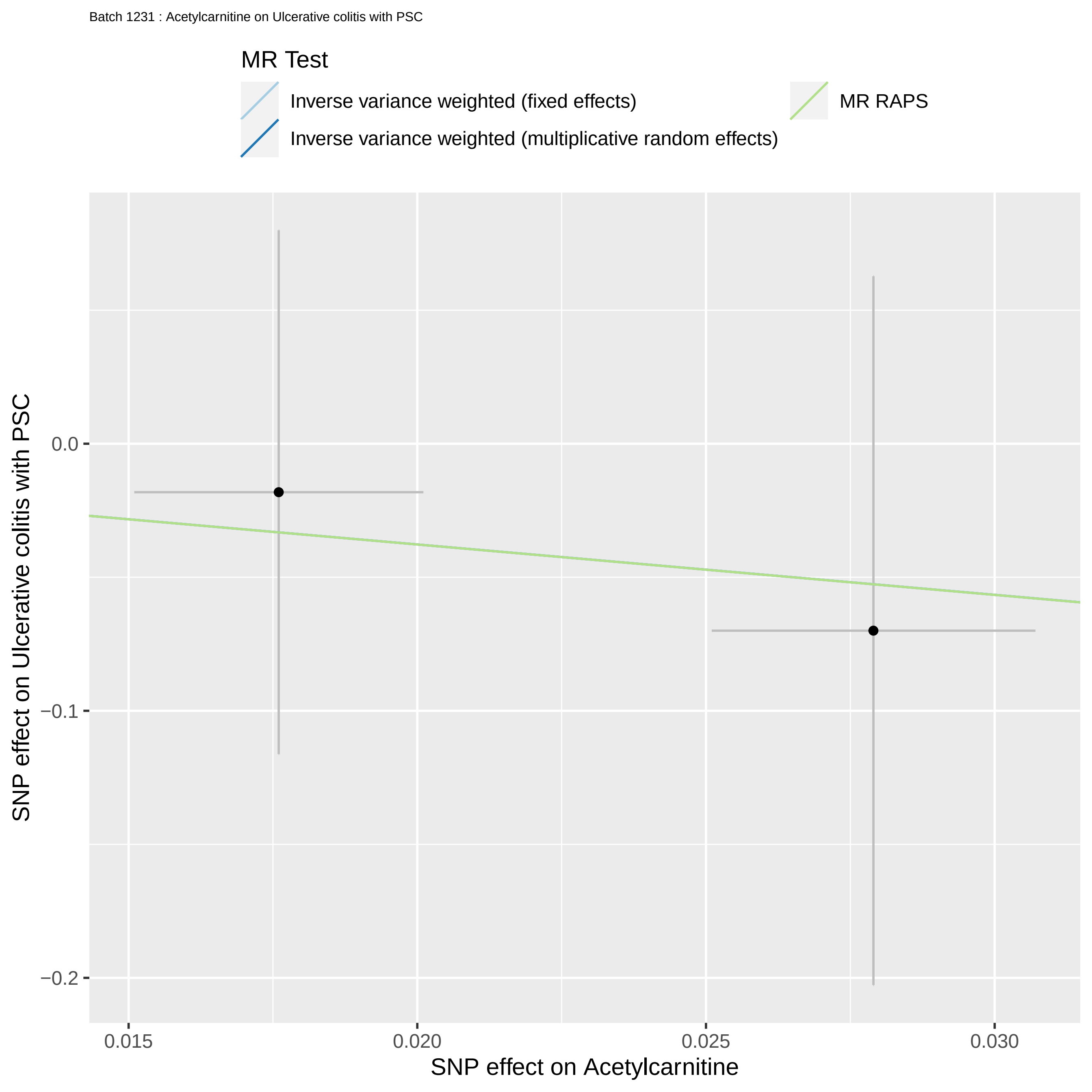

Batch 1231 : Acetylcarnitine on Ulcerative colitis with PSC
MR Test
Inverse variance weighted (fixed effects)
Inverse variance weighted (multiplicative random effects)
MR RAPS
SNP effect on Ulcerative colitis with PSC
0.0
−0.1
−0.2
0.015
0.020
0.025
0.030
SNP effect on Acetylcarnitine

Batch 1232 : Serine on Ulcerative colitis with PSC

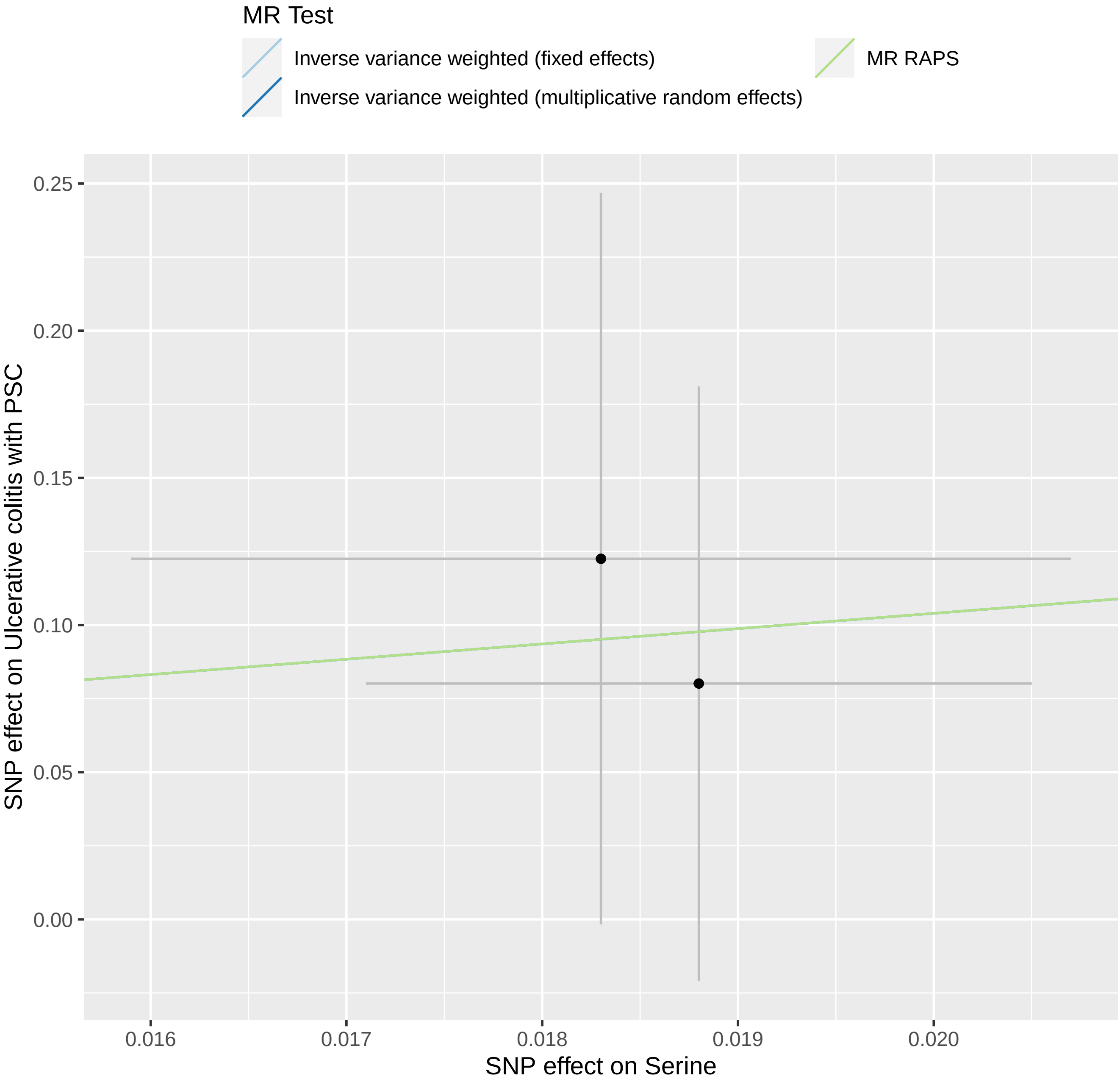

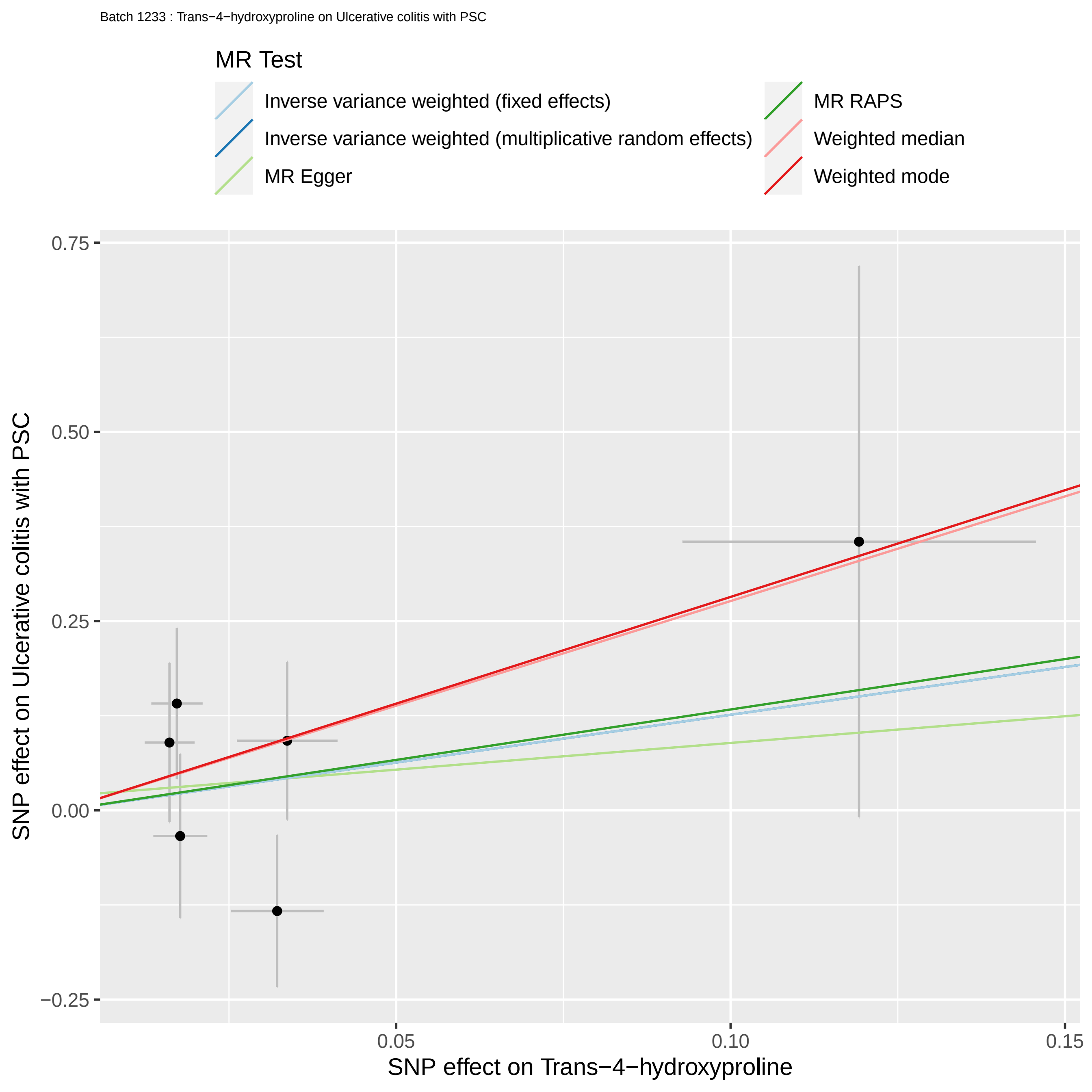
Batch 1233 : Trans−4−hydroxyproline on Ulcerative colitis with PSC
MR Test
Inverse variance weighted (fixed effects)
Inverse variance weighted (multiplicative random effects)
MR Egger
MR RAPS
Weighted median
Weighted mode
0.75
0.50
0.25
0.00
−0.25
0.05
0.10
0.15
SNP effect on Ulcerative colitis with PSC
SNP effect on Trans−4−hydroxyproline

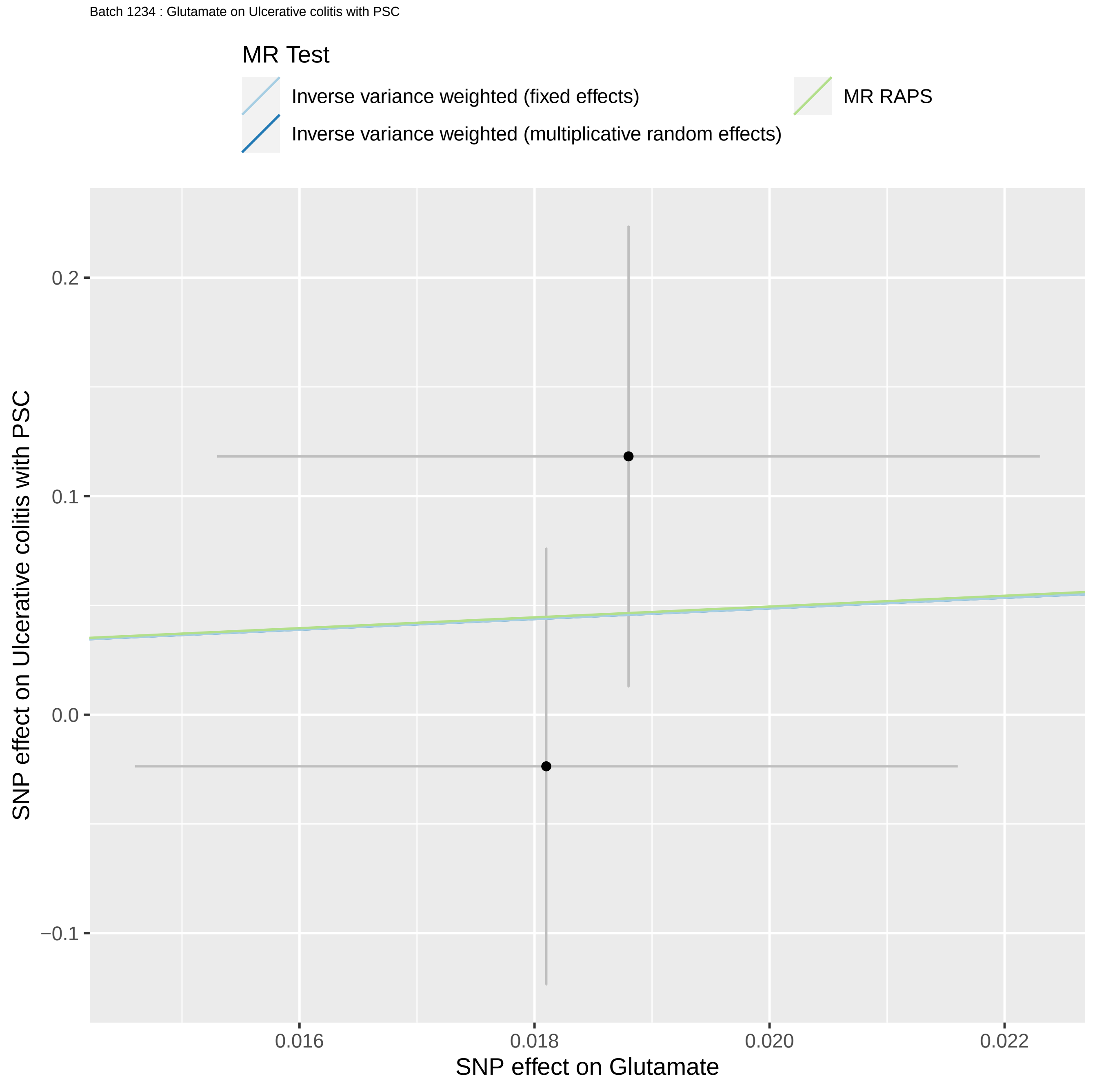
Batch 1234 : Glutamate on Ulcerative colitis with PSC
MR Test
Inverse variance weighted (fixed effects)
Inverse variance weighted (multiplicative random effects)
MR RAPS
0.2
0.1
0.0
−0.1
SNP effect on Ulcerative colitis with PSC
0.016
0.018
0.020
0.022
SNP effect on Glutamate

Batch 1235 : Hexanoylcarnitine on Ulcerative colitis with PSC

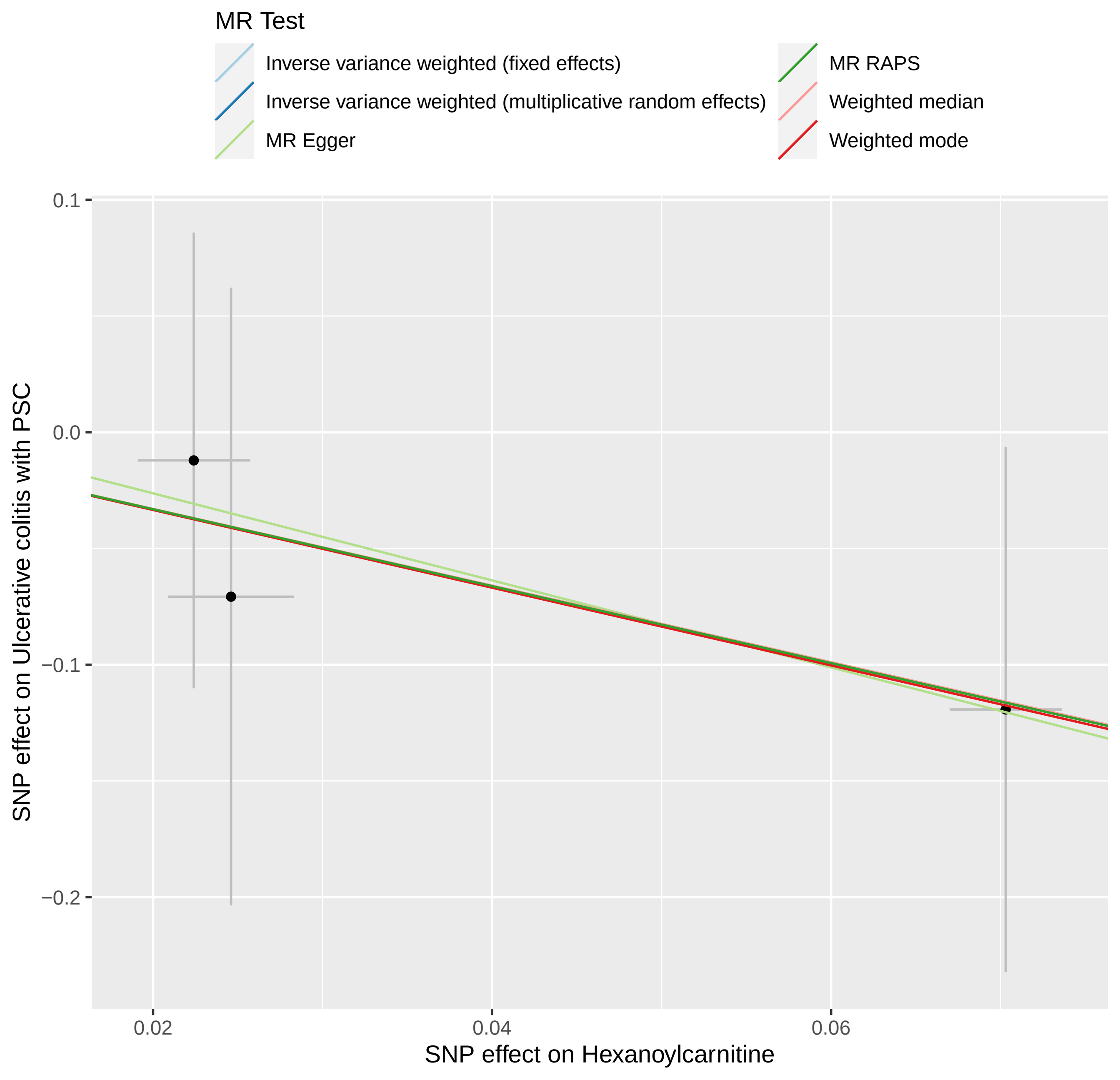

Batch 1239 : 2−aminobutyrate on Ulcerative colitis with PSC

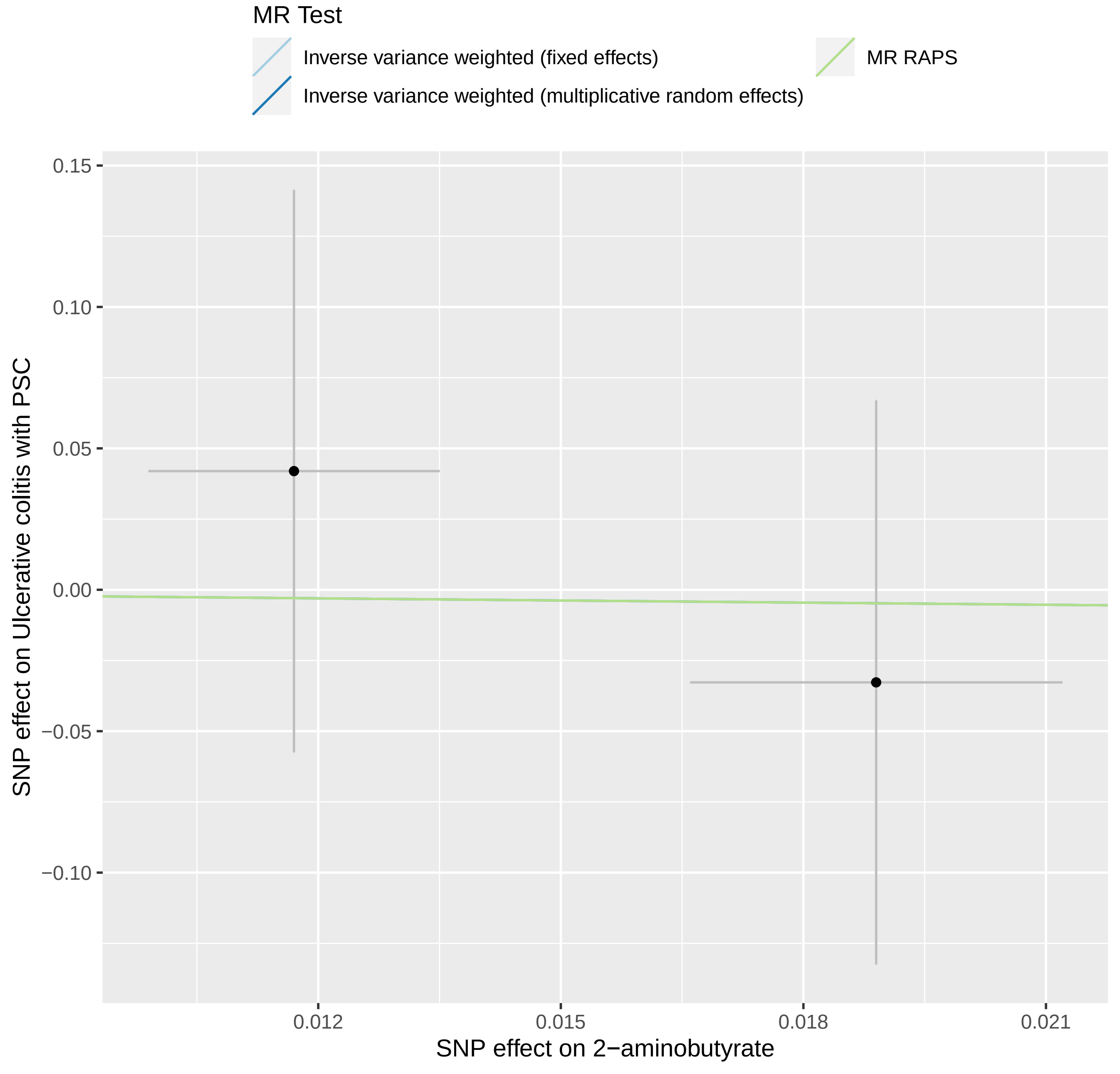

Batch 1244 : Butyrylcarnitine on Ulcerative colitis with PSC

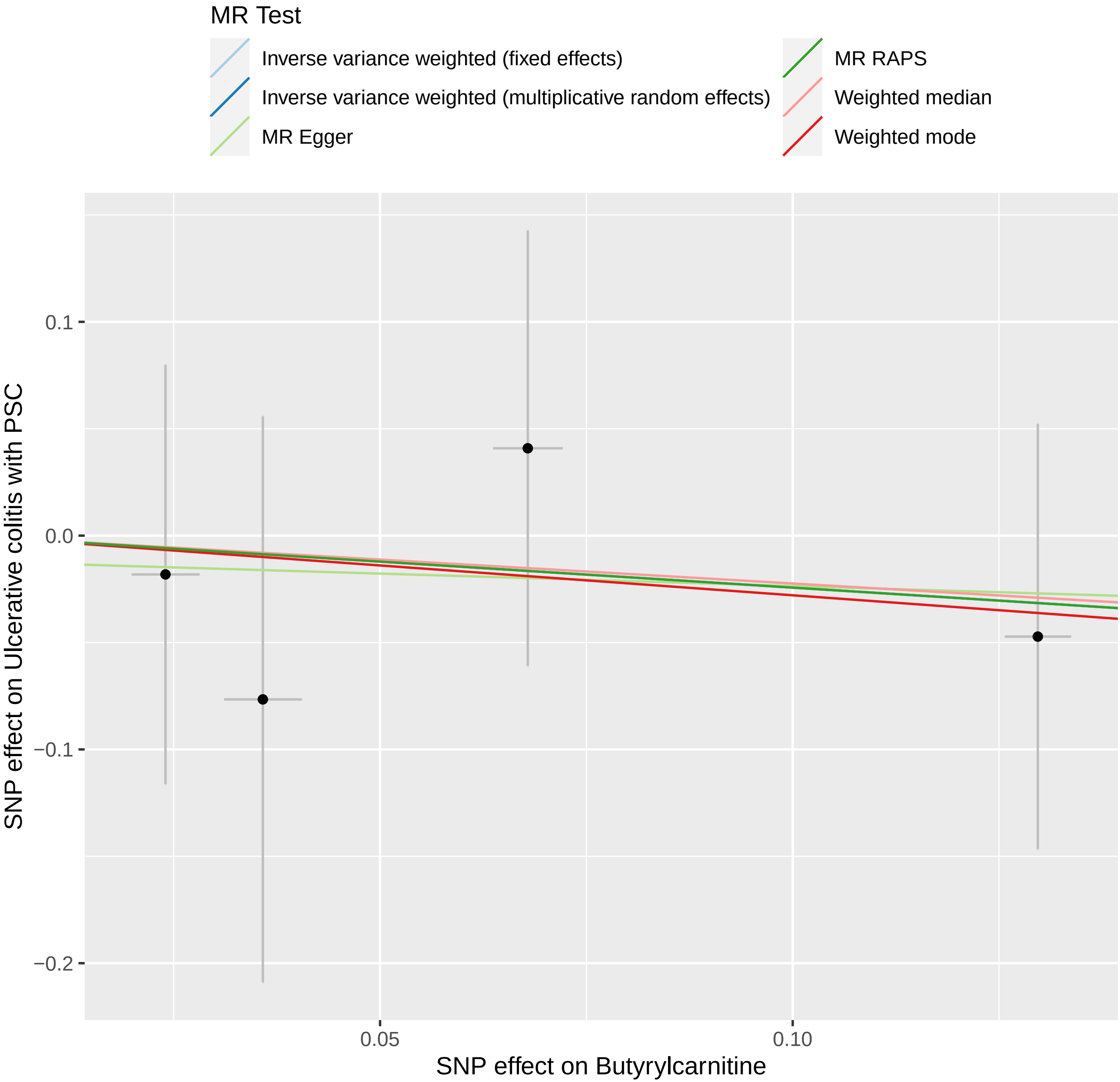

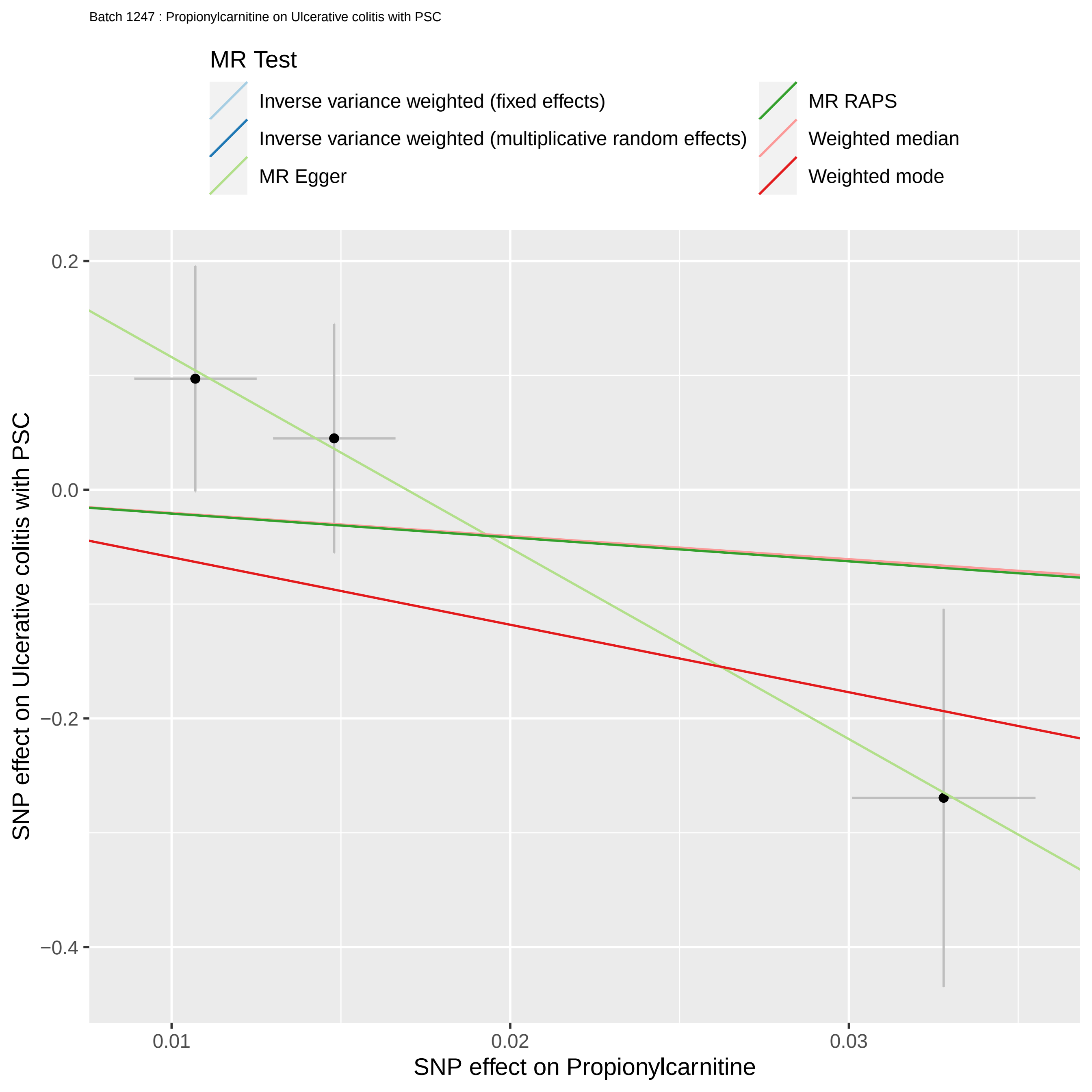
Batch 1247 : Propionylcarnitine on Ulcerative colitis with PSC
MR Test
Inverse variance weighted (fixed effects)
Inverse variance weighted (multiplicative random effects)
MR Egger
MR RAPS
Weighted median
Weighted mode
0.2
0.0
−0.2
−0.4
0.01
0.02
0.03
SNP effect on Ulcerative colitis with PSC
SNP effect on Propionylcarnitine

Batch 1248 : Caproate (6:0) on Ulcerative colitis with PSC

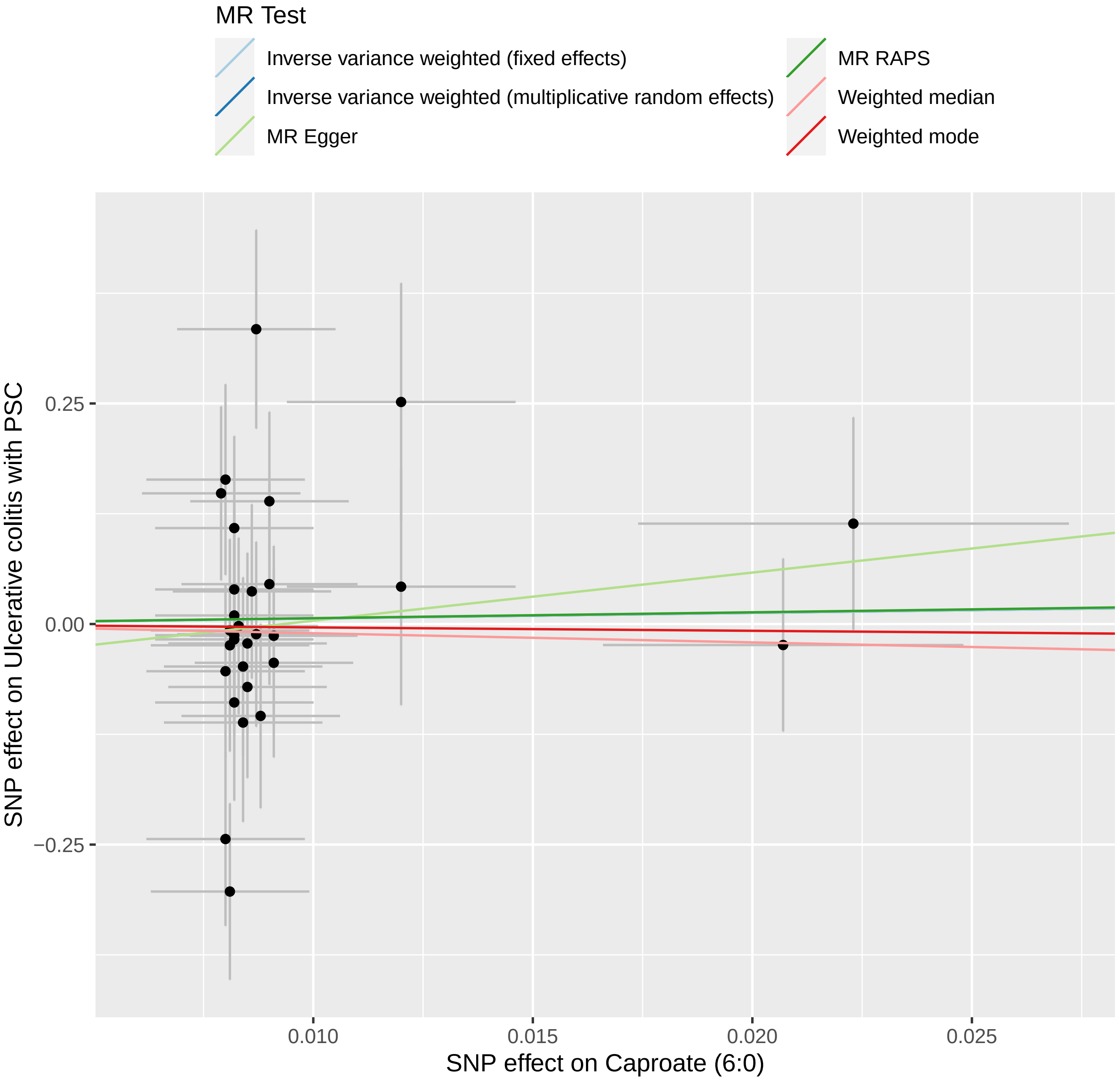

Batch 1249 : Caprylate (8:0) on Ulcerative colitis with PSC

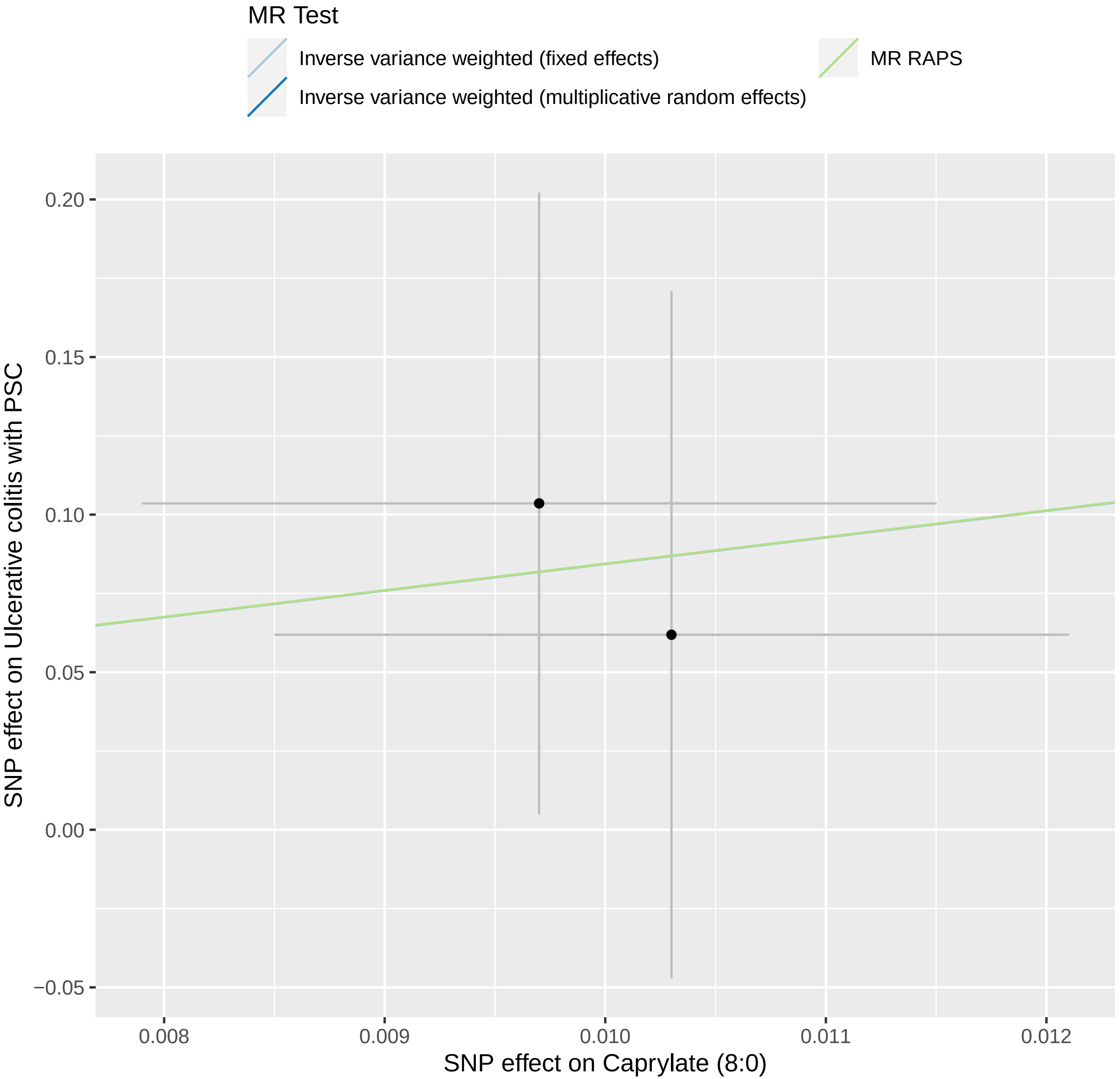

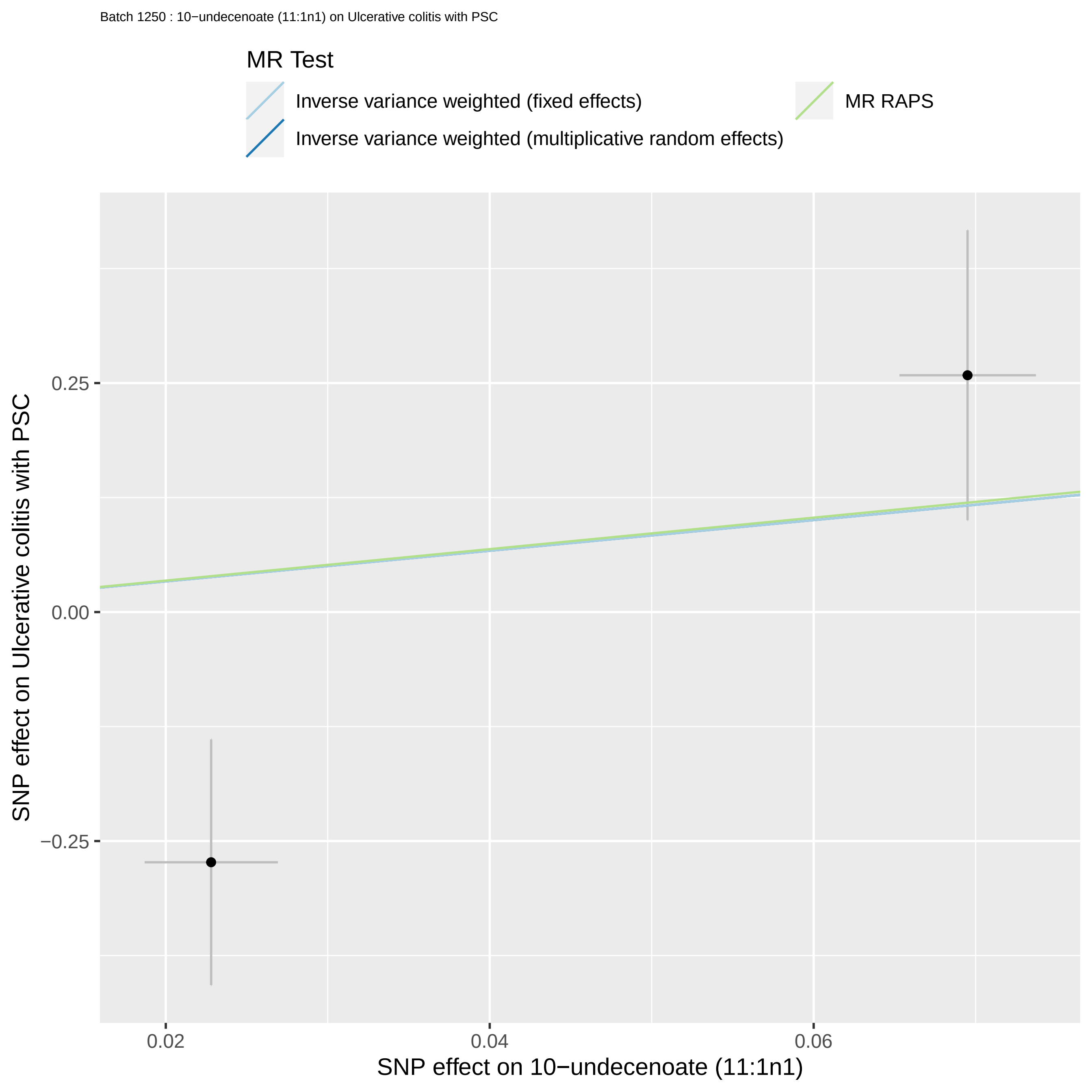
Batch 1250 : 10−undecenoate (11:1n1) on Ulcerative colitis with PSC
MR Test
Inverse variance weighted (fixed effects)
Inverse variance weighted (multiplicative random effects)
MR RAPS
SNP effect on Ulcerative colitis with PSC
0.25
0.00
−0.25
0.02
0.04
0.06
SNP effect on 10−undecenoate (11:1n1)

Batch 1252 : Phenol sulfate on Ulcerative colitis with PSC

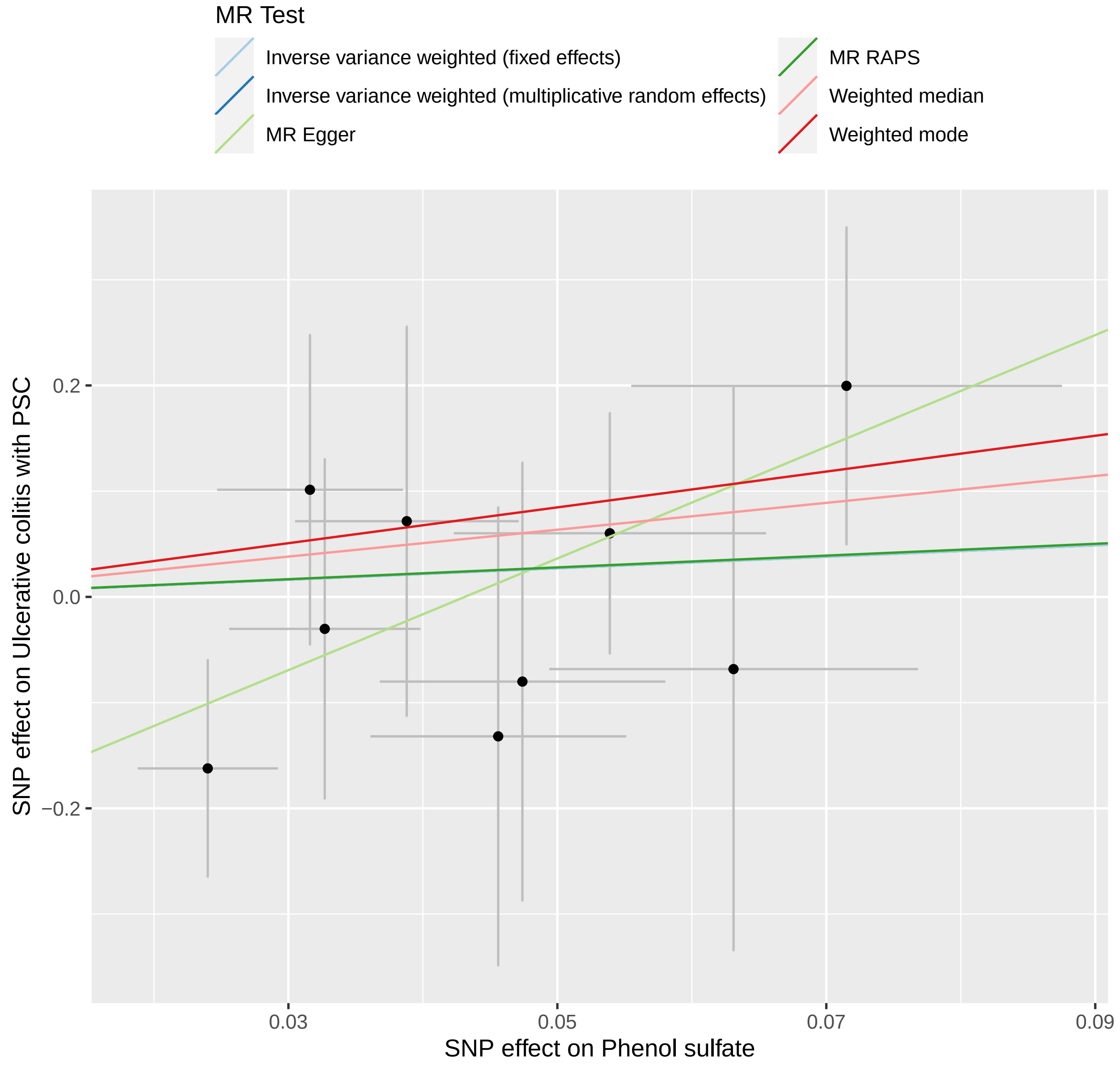

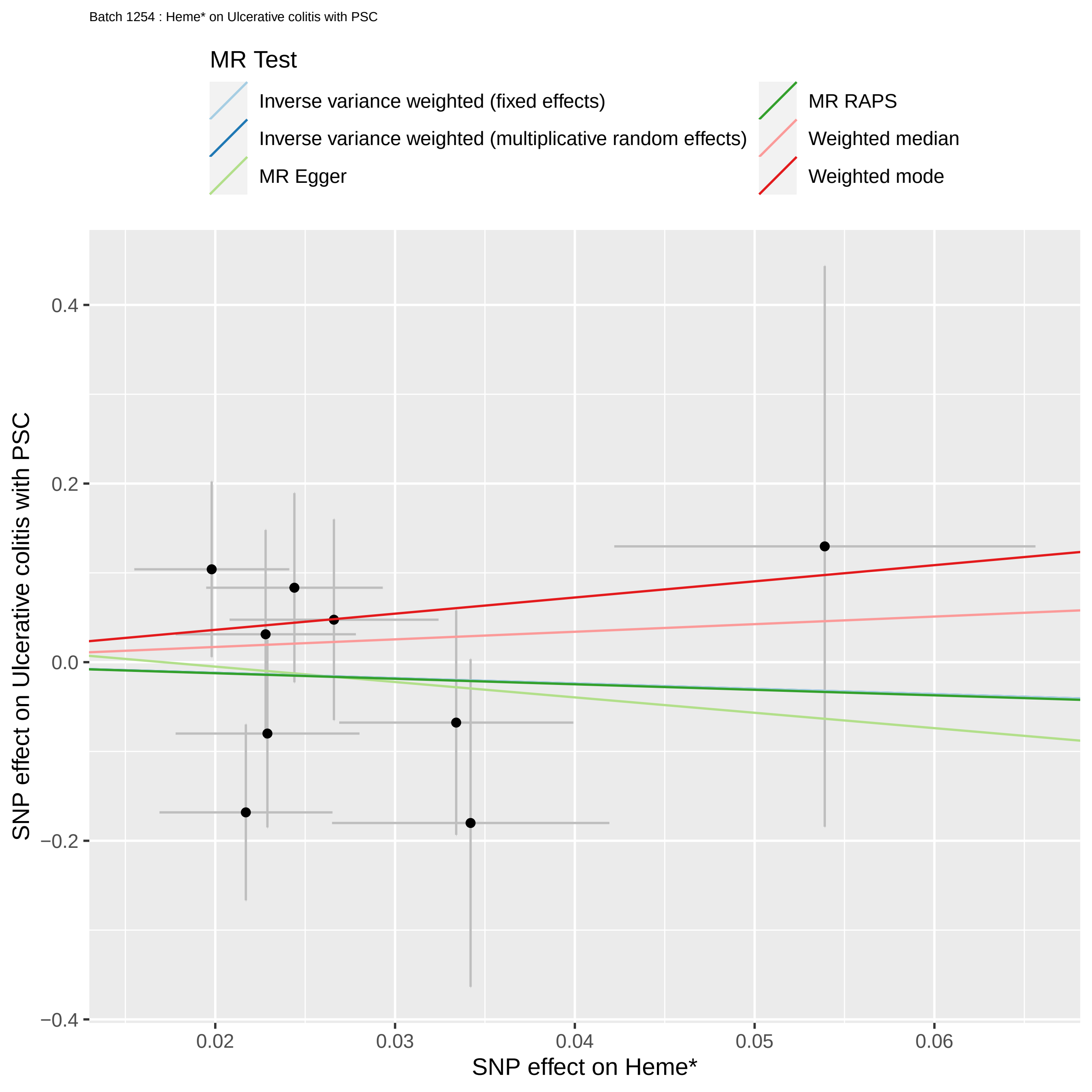
Batch 1254 : Heme* on Ulcerative colitis with PSC
MR Test
Inverse variance weighted (fixed effects)
Inverse variance weighted (multiplicative random effects)
MR Egger
MR RAPS
Weighted median
Weighted mode
0.4
0.2
0.0
−0.2
−0.4
0.02
0.03
0.04
0.05
0.06
SNP effect on Ulcerative colitis with PSC
SNP effect on Heme*

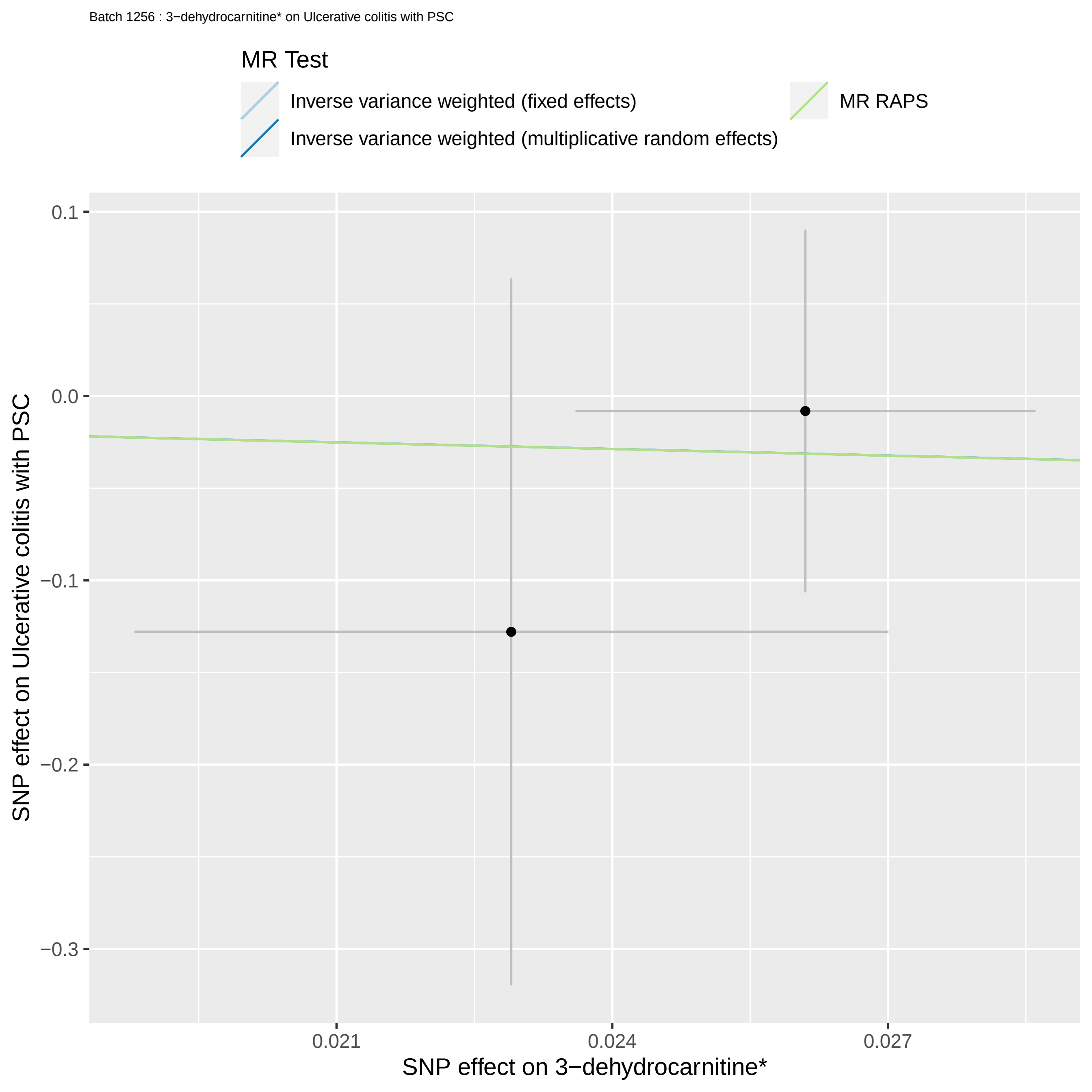
Batch 1256 : 3−dehydrocarnitine* on Ulcerative colitis with PSC
MR Test
Inverse variance weighted (fixed effects)
Inverse variance weighted (multiplicative random effects)
MR RAPS
0.1
0.0
−0.1
−0.2
−0.3
0.021
0.024
0.027
SNP effect on 3−dehydrocarnitine*
SNP effect on Ulcerative colitis with PSC

Batch 1257 : Pyroglutamine* on Ulcerative colitis with PSC

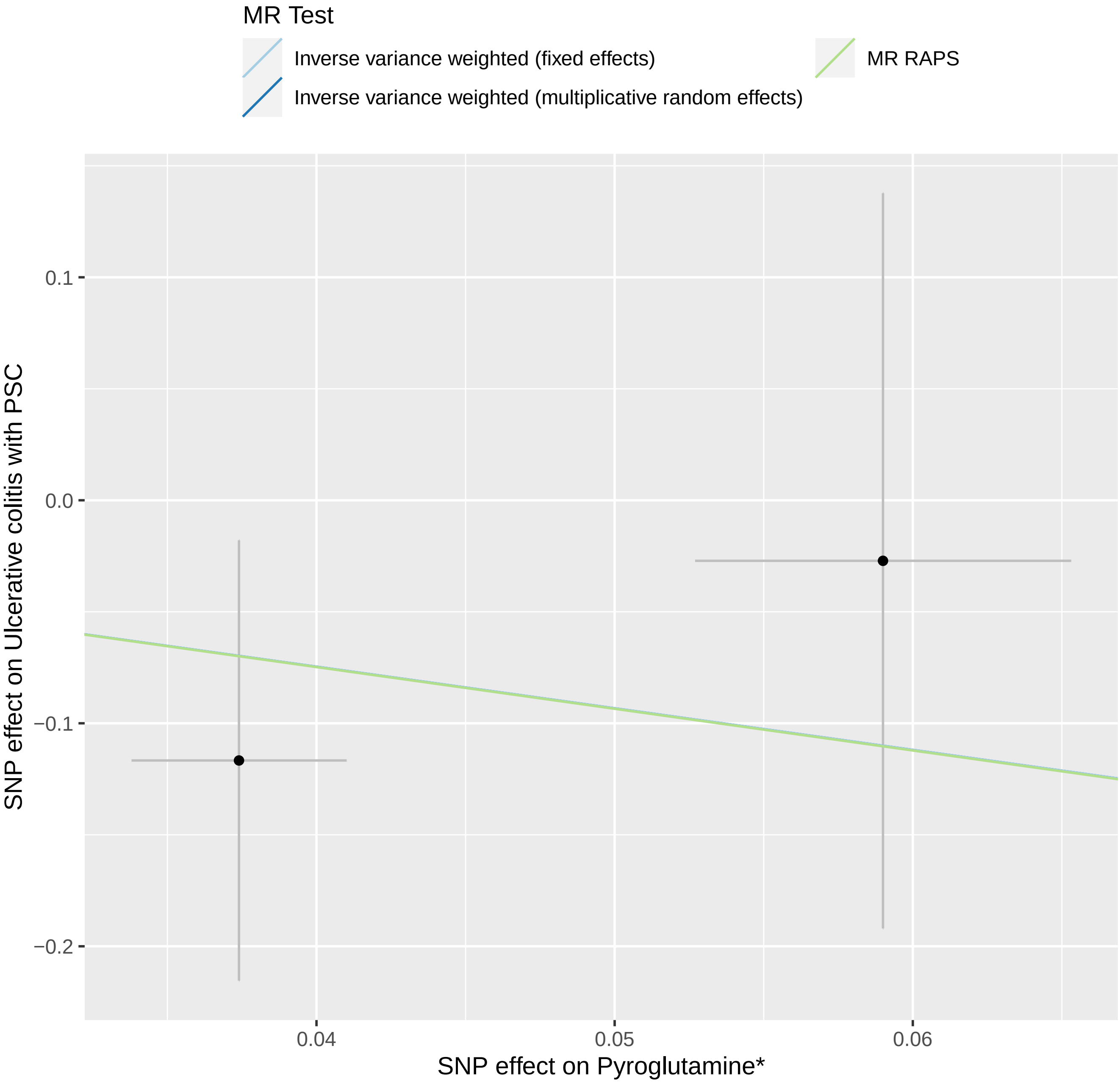

Batch 1260 : X−11422−−xanthine on Ulcerative colitis with PSC

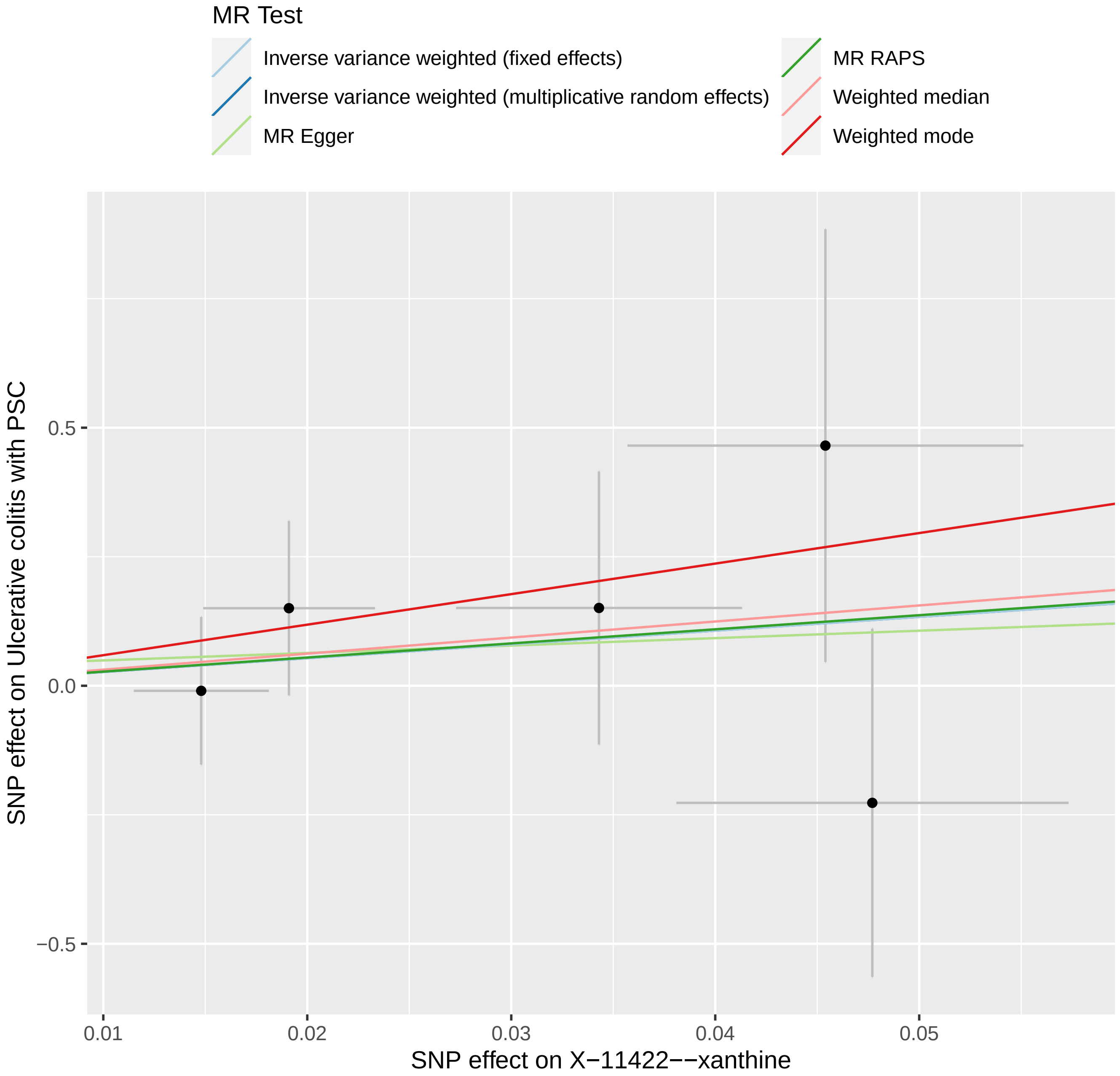

Batch 1263 : HWESASXX* on Ulcerative colitis with PSC

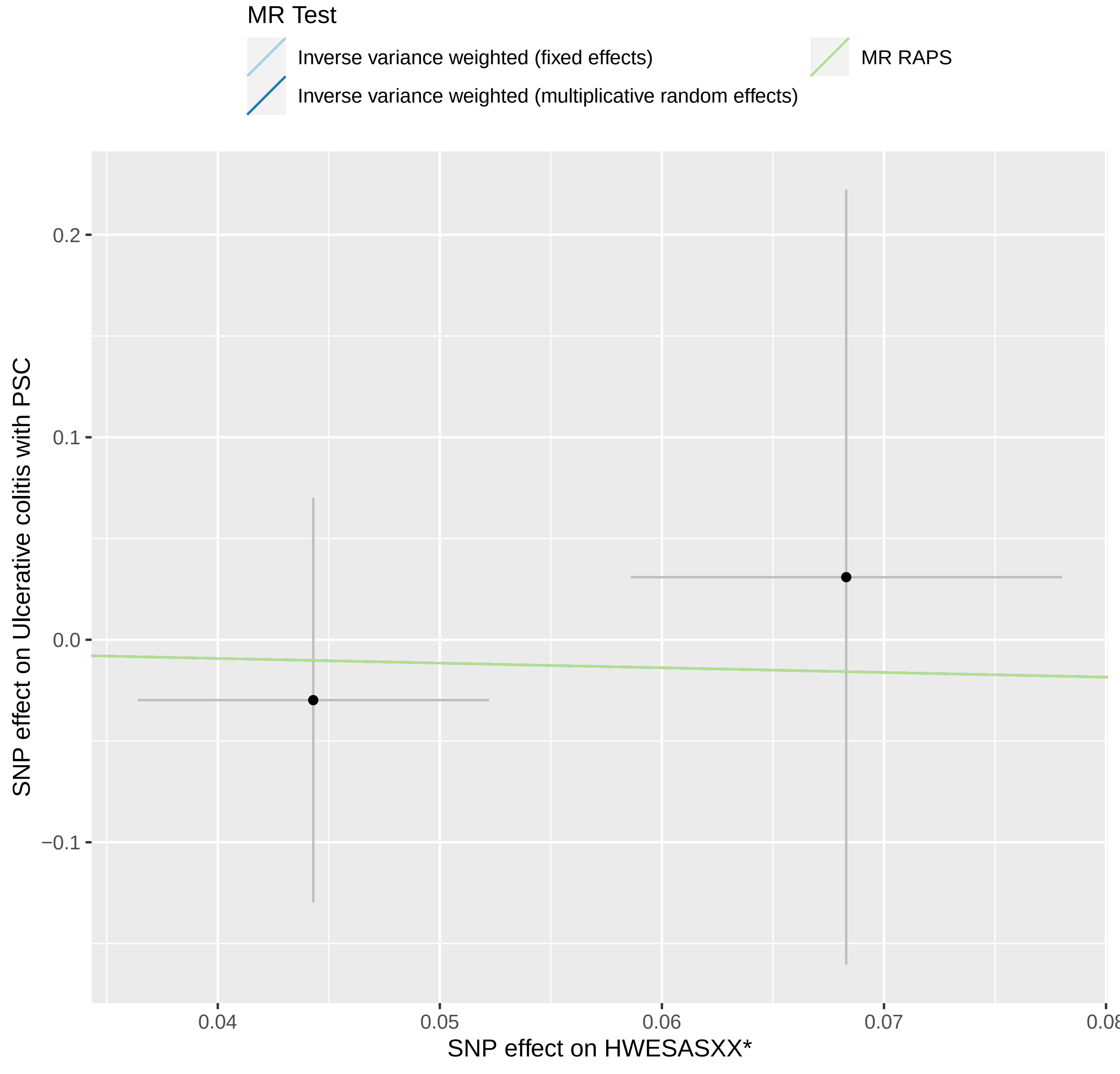

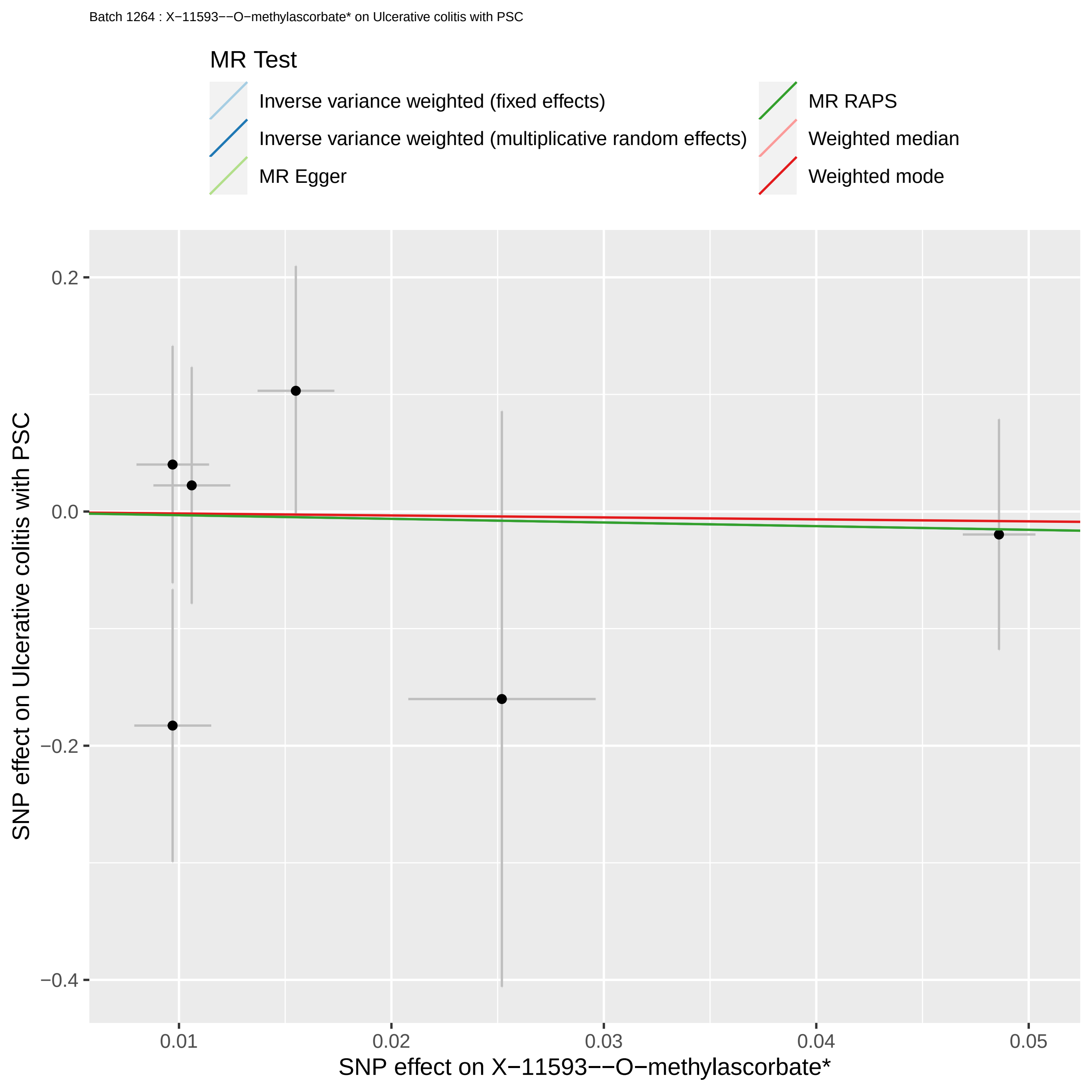
Batch 1264 : X−11593−−O−methylascorbate* on Ulcerative colitis with PSC
MR Test
Inverse variance weighted (fixed effects)
Inverse variance weighted (multiplicative random effects)
MR Egger
MR RAPS
Weighted median
Weighted mode
0.2
0.0
−0.2
−0.4
0.01
0.02
0.03
0.04
0.05
SNP effect on Ulcerative colitis with PSC
SNP effect on X−11593−−O−methylascorbate*

Batch 1266 : Homostachydrine* on Ulcerative colitis with PSC

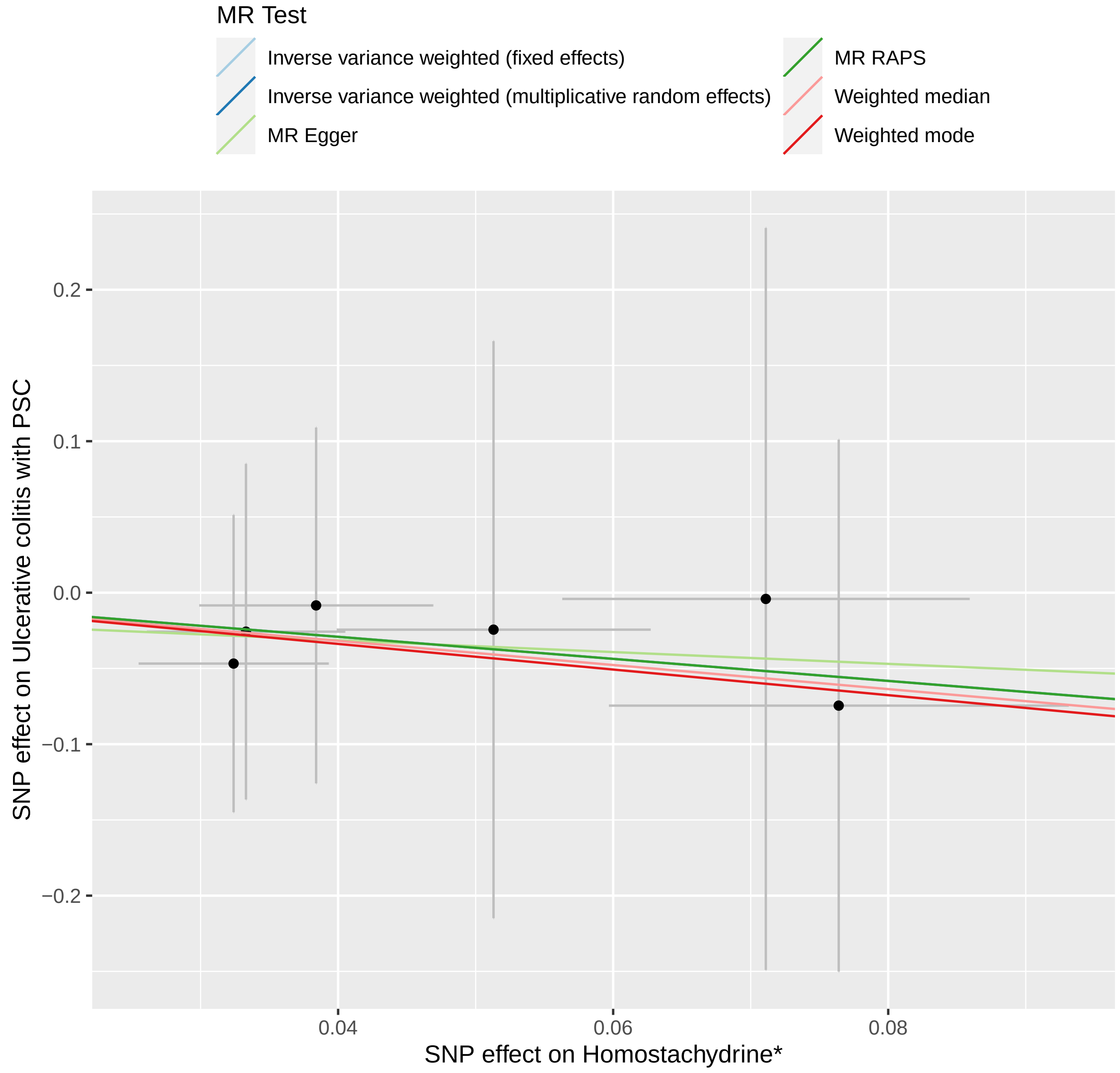

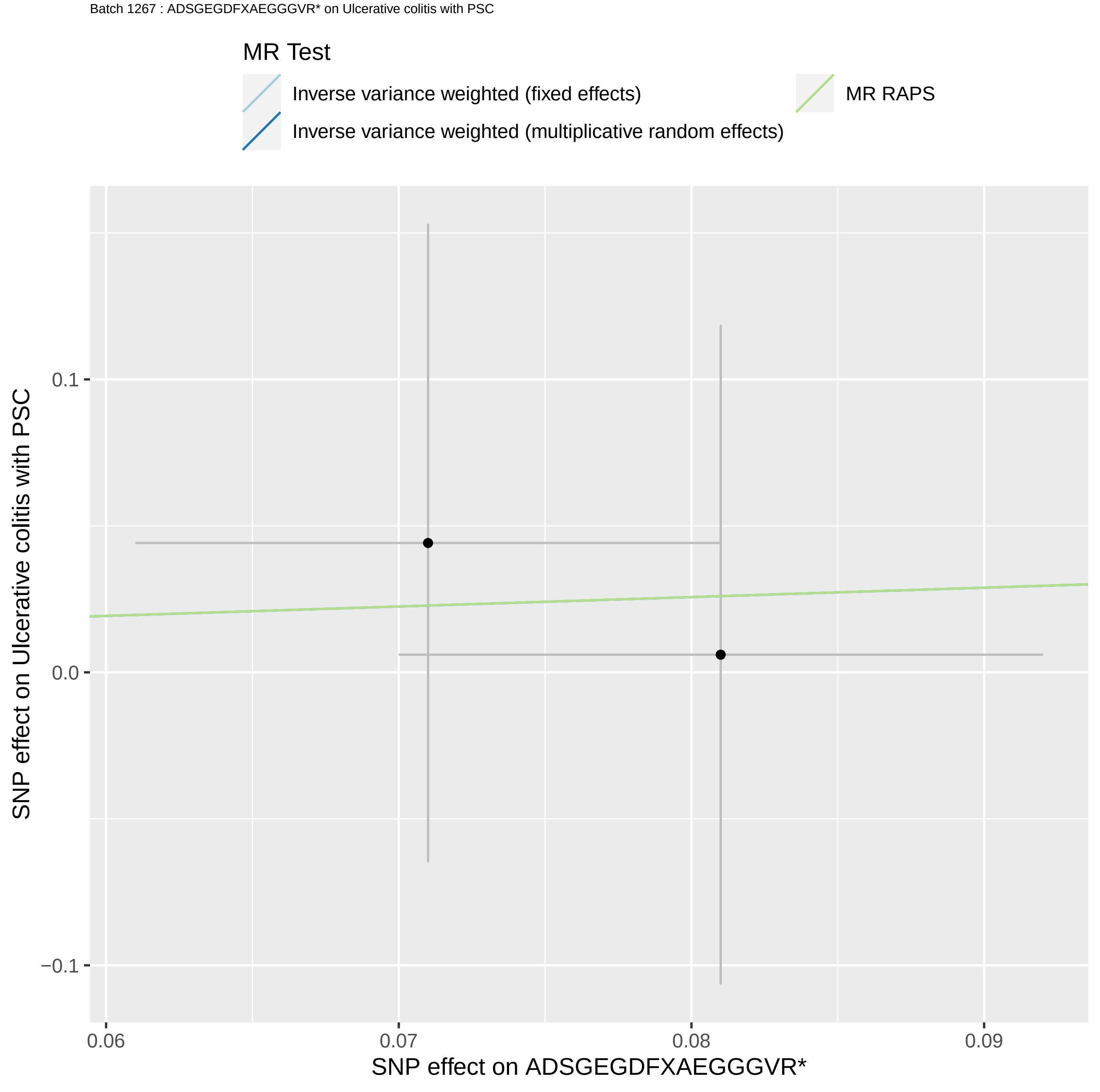
Batch 1267 : ADSGEGDFXAEGGGVR* on Ulcerative colitis with PSC
MR Test
Inverse variance weighted (fixed effects)
Inverse variance weighted (multiplicative random effects)
MR RAPS
SNP effect on Ulcerative colitis with PSC
0.1
0.0
−0.1
0.06
0.07
0.08
0.09
SNP effect on ADSGEGDFXAEGGGVR*

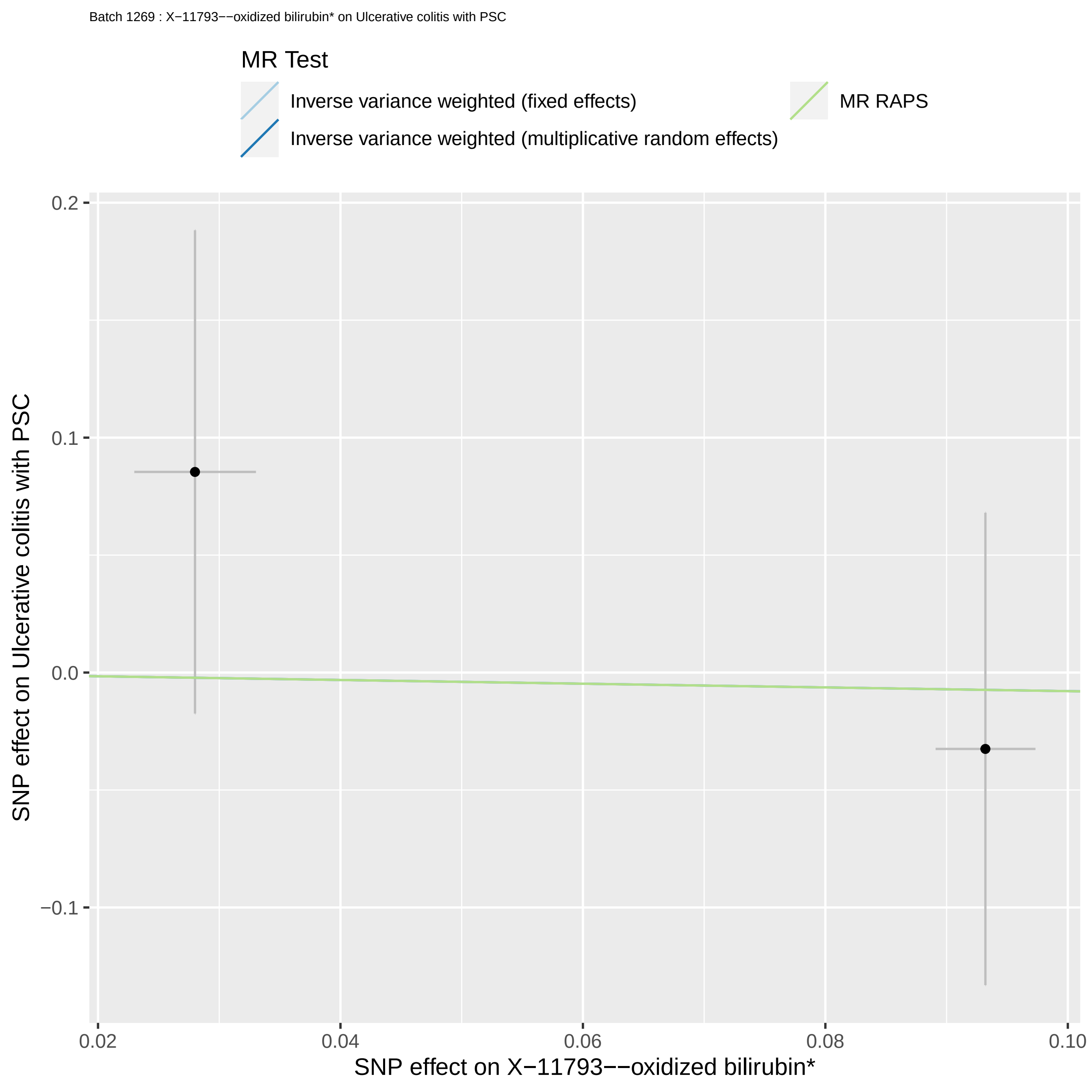
Batch 1269 : X−11793−−oxidized bilirubin* on Ulcerative colitis with PSC
MR Test
Inverse variance weighted (fixed effects)
Inverse variance weighted (multiplicative random effects)
MR RAPS
0.2
0.1
0.0
−0.1
0.02
0.04
0.06
0.08
0.10
SNP effect on Ulcerative colitis with PSC
SNP effect on X−11793−−oxidized bilirubin*

Batch 1270 : 1−arachidonoylglycerophosphocholine* on Ulcerative colitis with PSC

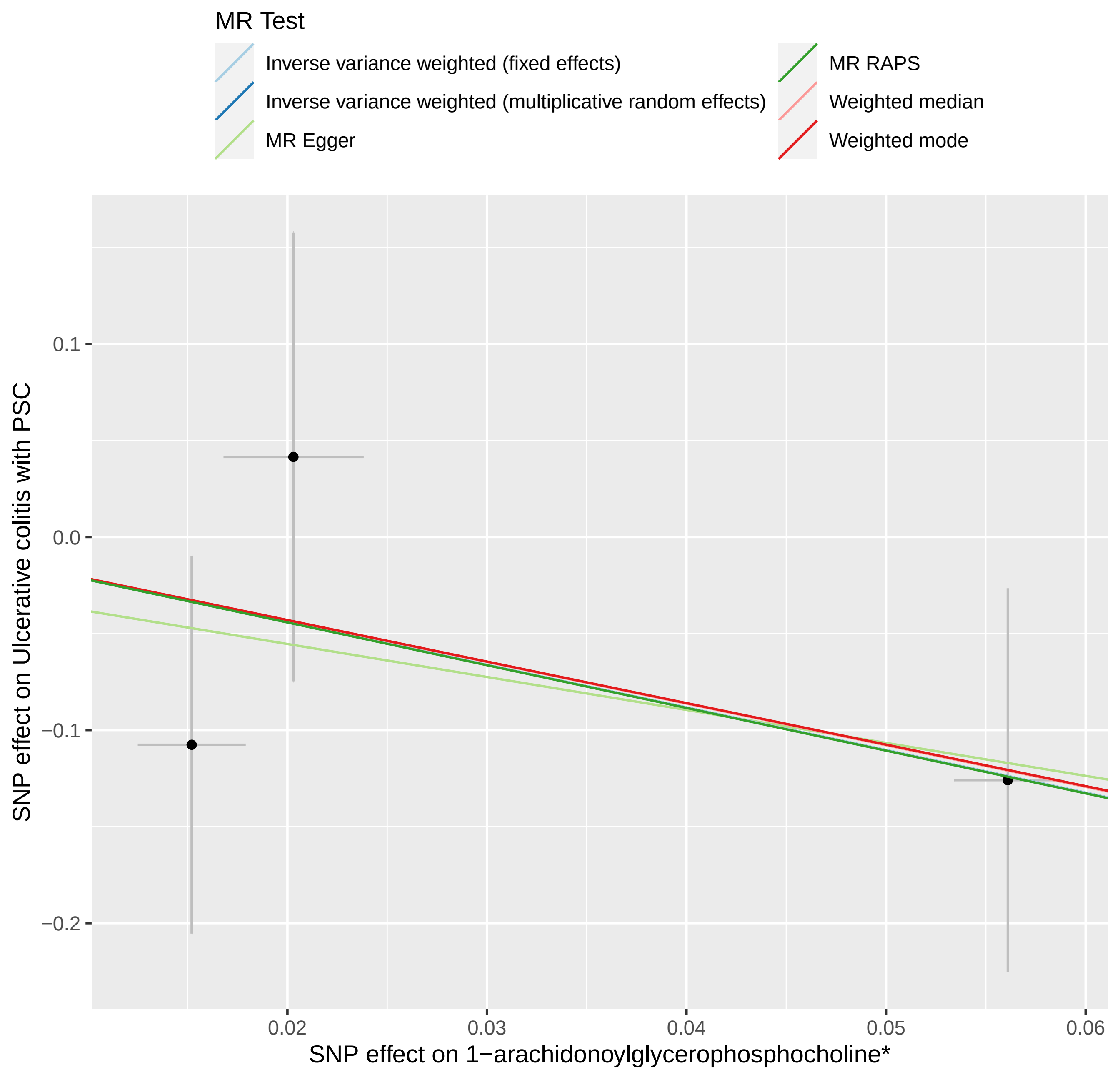

Batch 1272 : Gamma−glutamylmethionine* on Ulcerative colitis with PSC

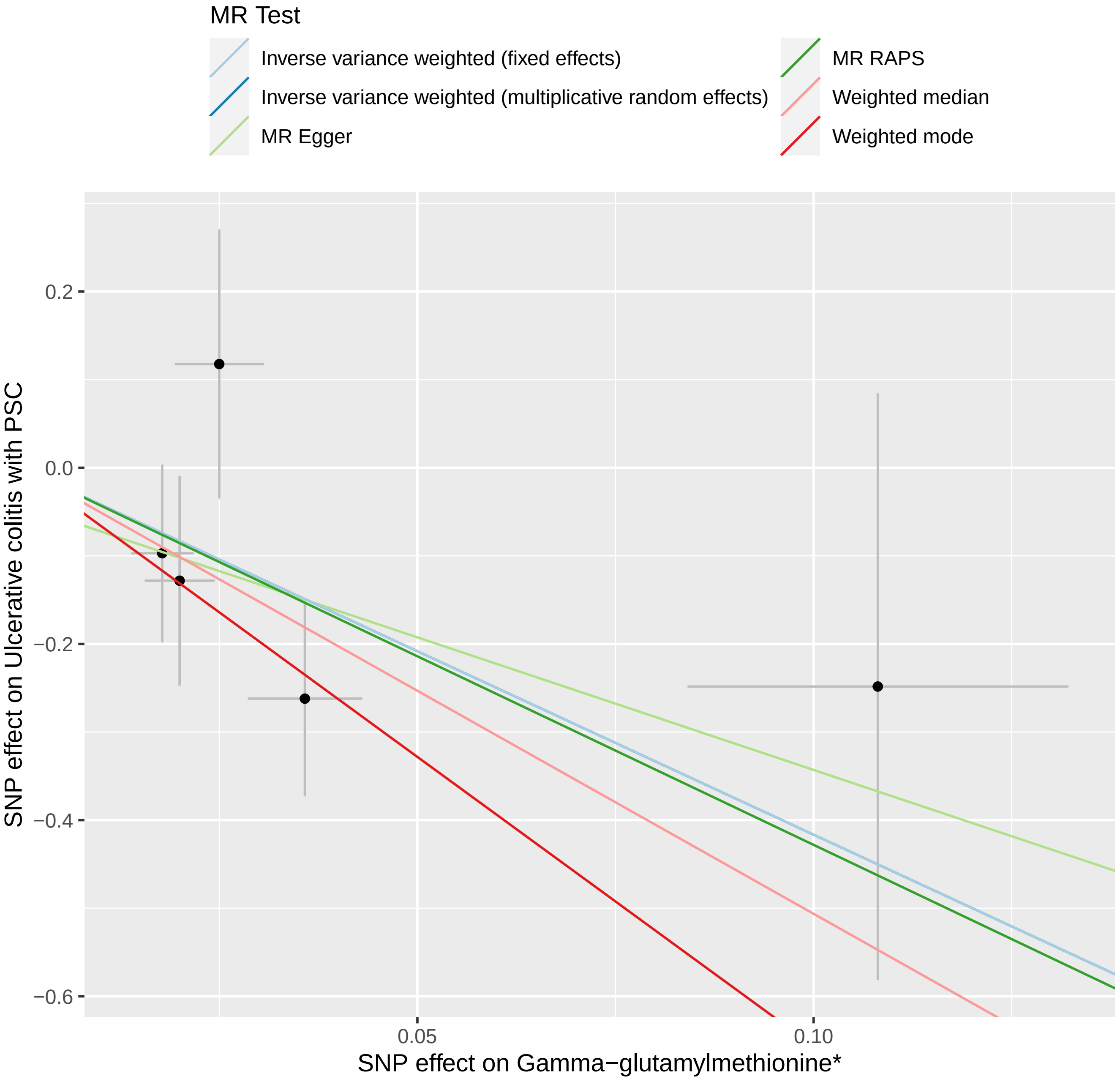

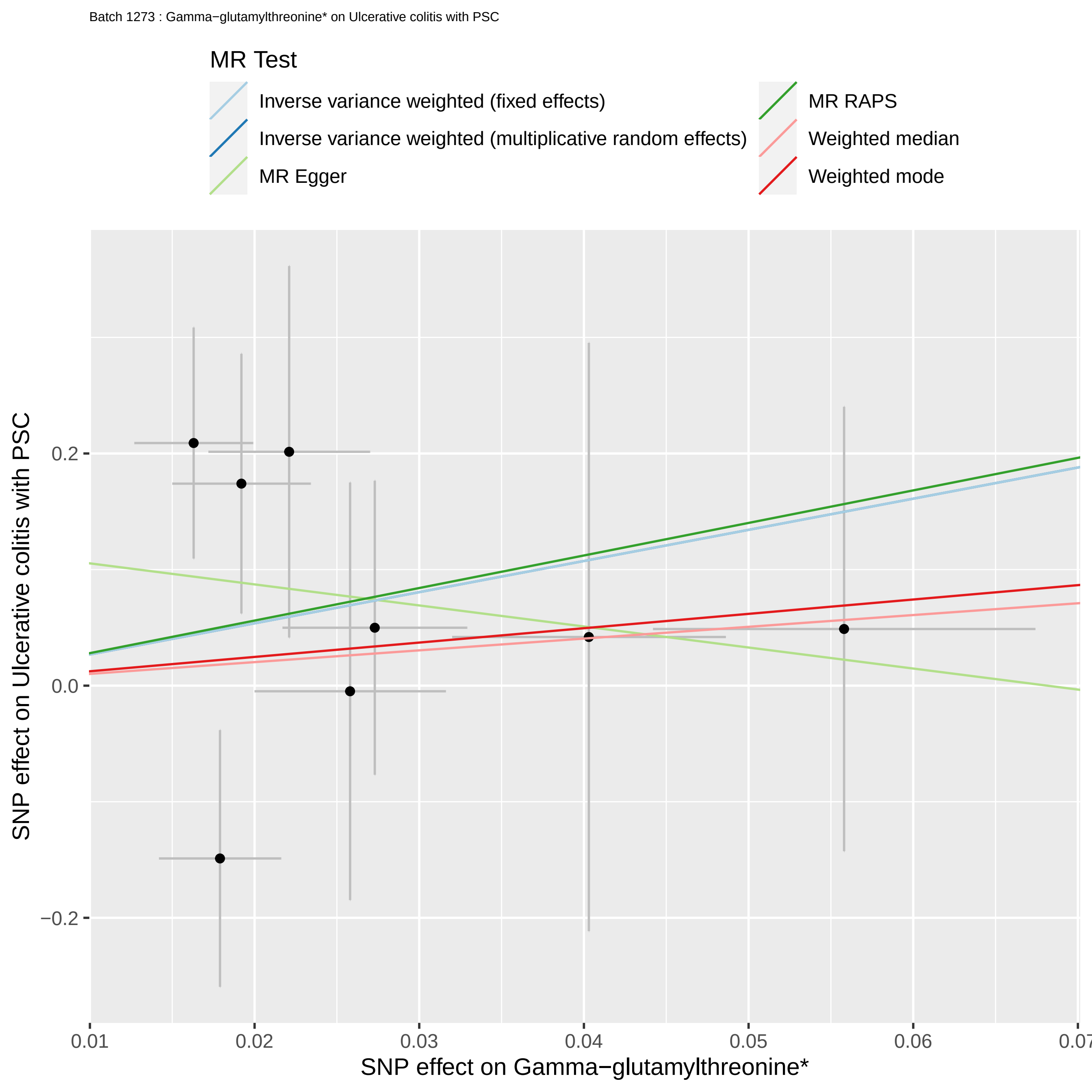

Batch 1273 : Gamma−glutamylthreonine* on Ulcerative colitis with PSC
MR Test
Inverse variance weighted (fixed effects)
Inverse variance weighted (multiplicative random effects)
MR Egger
MR RAPS
Weighted median
Weighted mode
0.2
0.0
−0.2
0.01
0.02
0.03
0.04
0.05
0.06
0.07
SNP effect on Ulcerative colitis with PSC
SNP effect on Gamma−glutamylthreonine*

Batch 1274 : Gamma−tocopherol on Ulcerative colitis with PSC

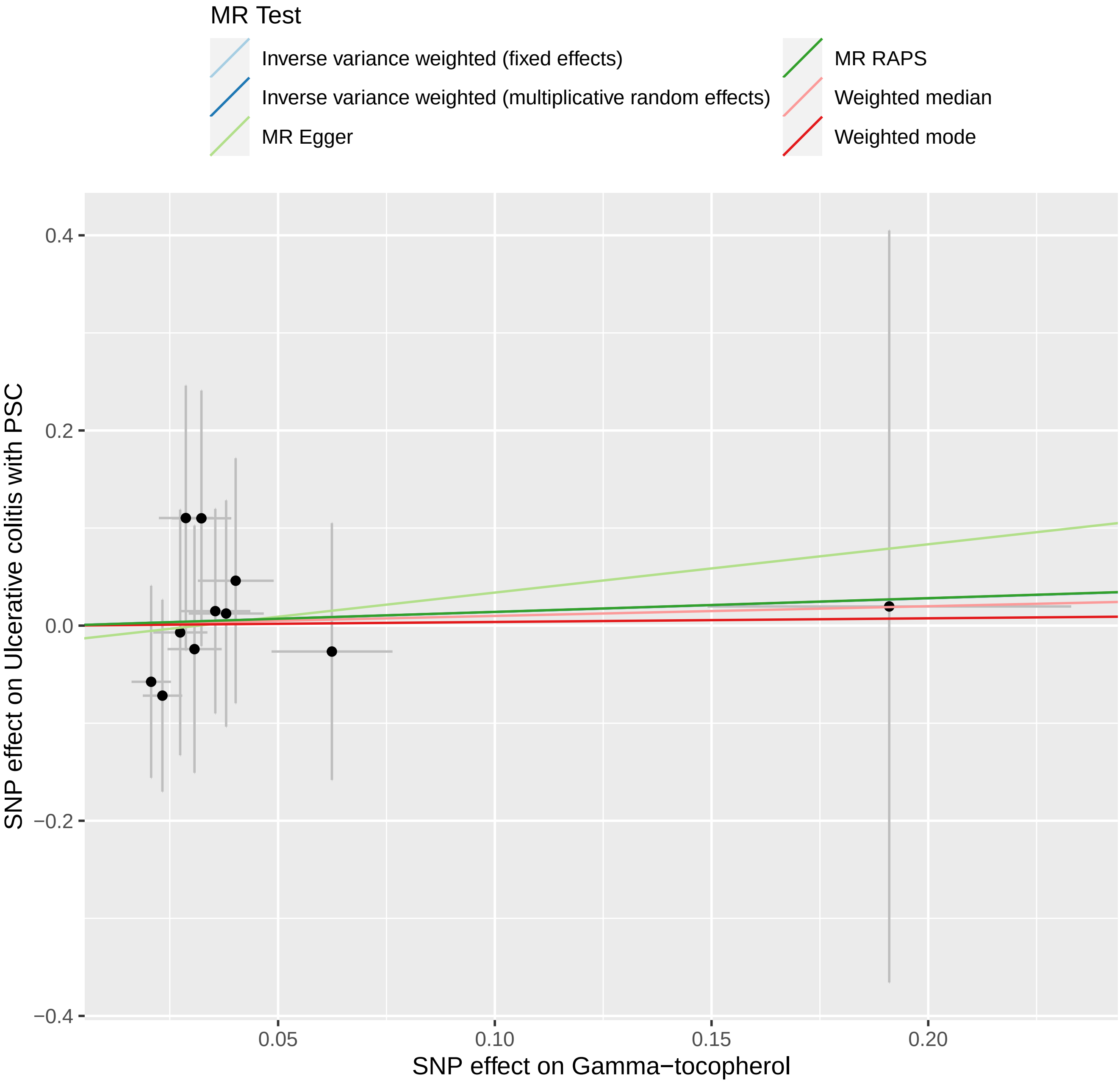

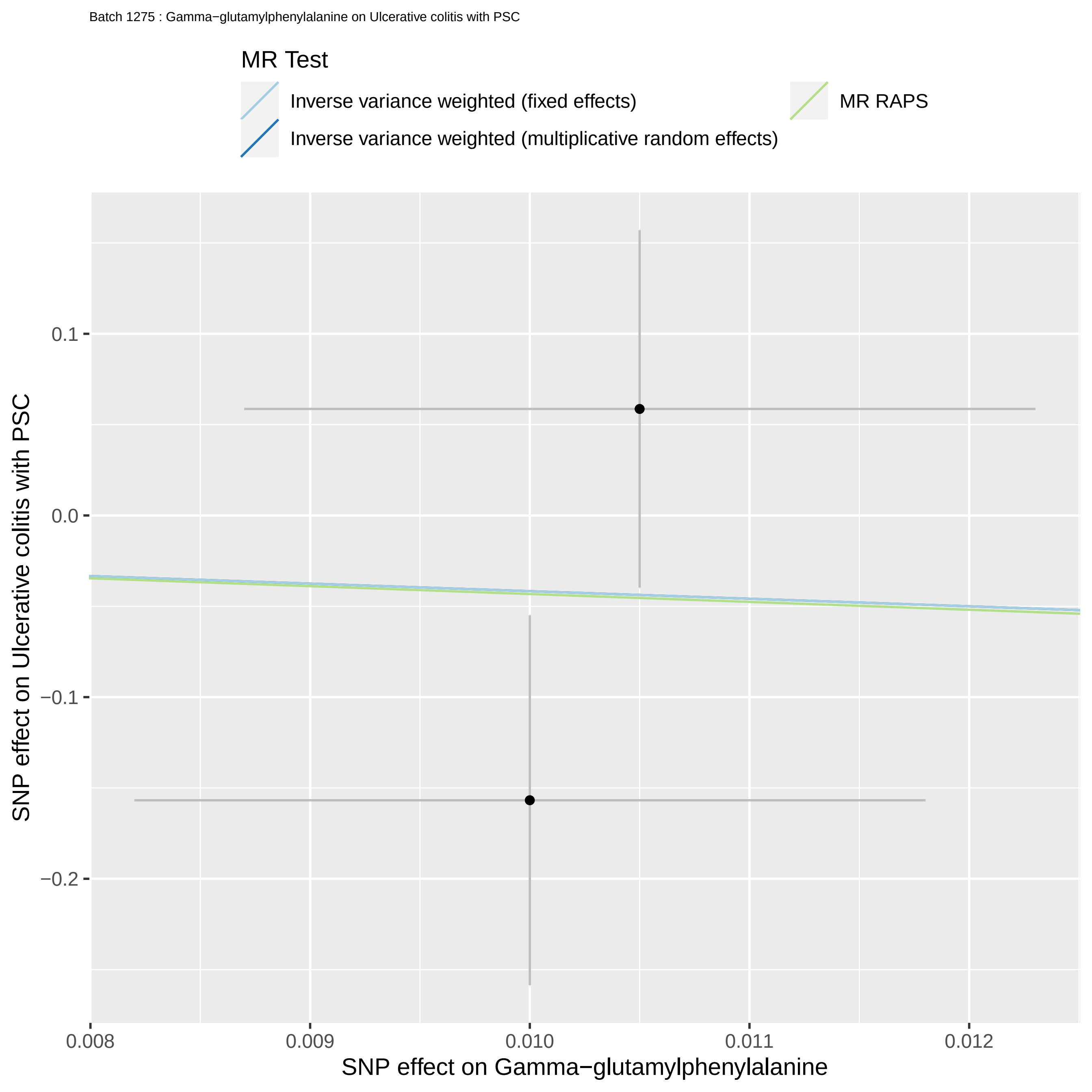
Batch 1275 : Gamma−glutamylphenylalanine on Ulcerative colitis with PSC
MR Test
Inverse variance weighted (fixed effects)
Inverse variance weighted (multiplicative random effects)
MR RAPS
0.1
0.0
−0.1
−0.2
SNP effect on Ulcerative colitis with PSC
0.008
0.009
0.010
0.011
0.012
SNP effect on Gamma−glutamylphenylalanine

Batch 1276 : Isobutyrylcarnitine on Ulcerative colitis with PSC

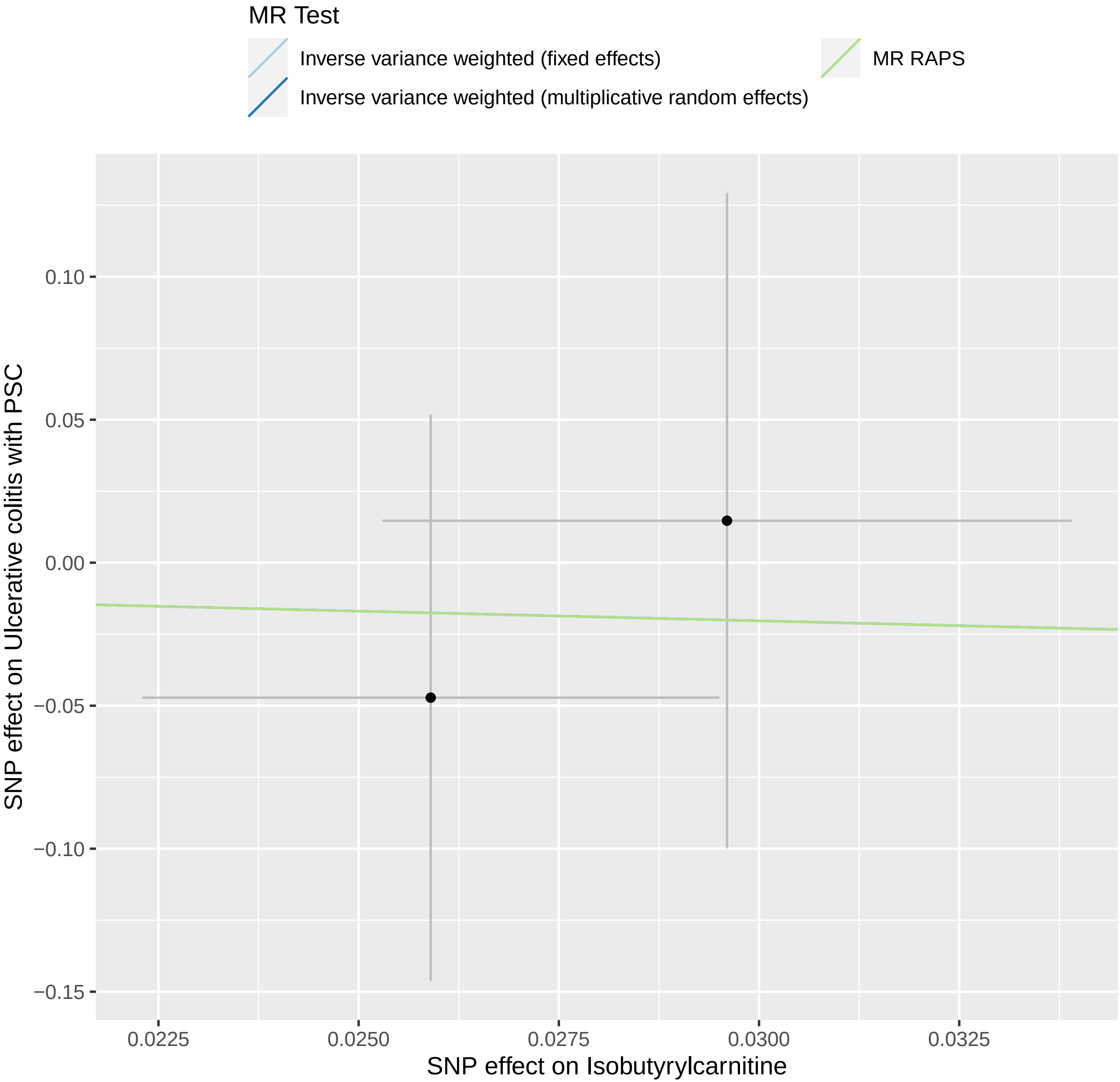

Batch 1277 : Pseudouridine on Ulcerative colitis with PSC

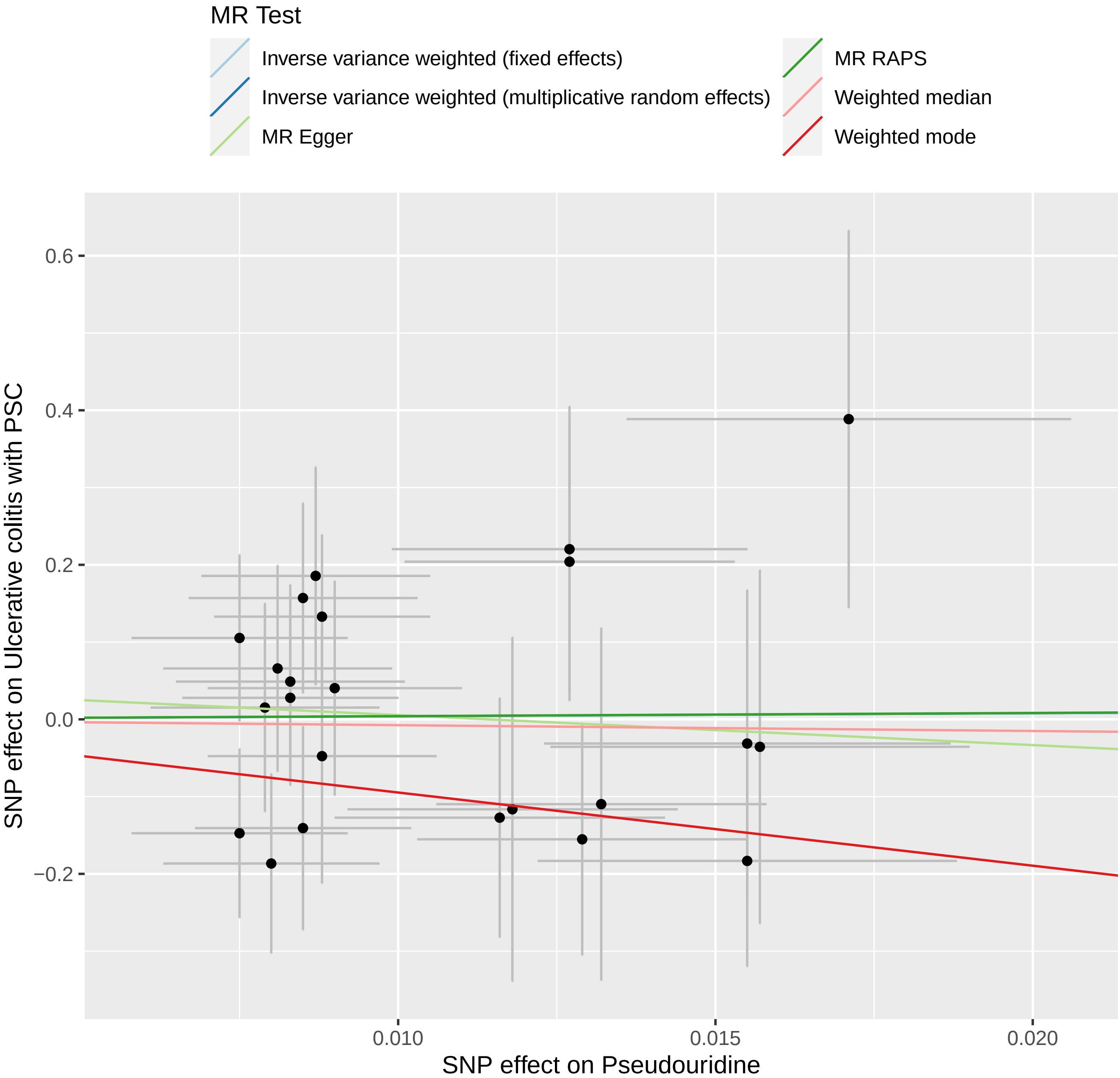

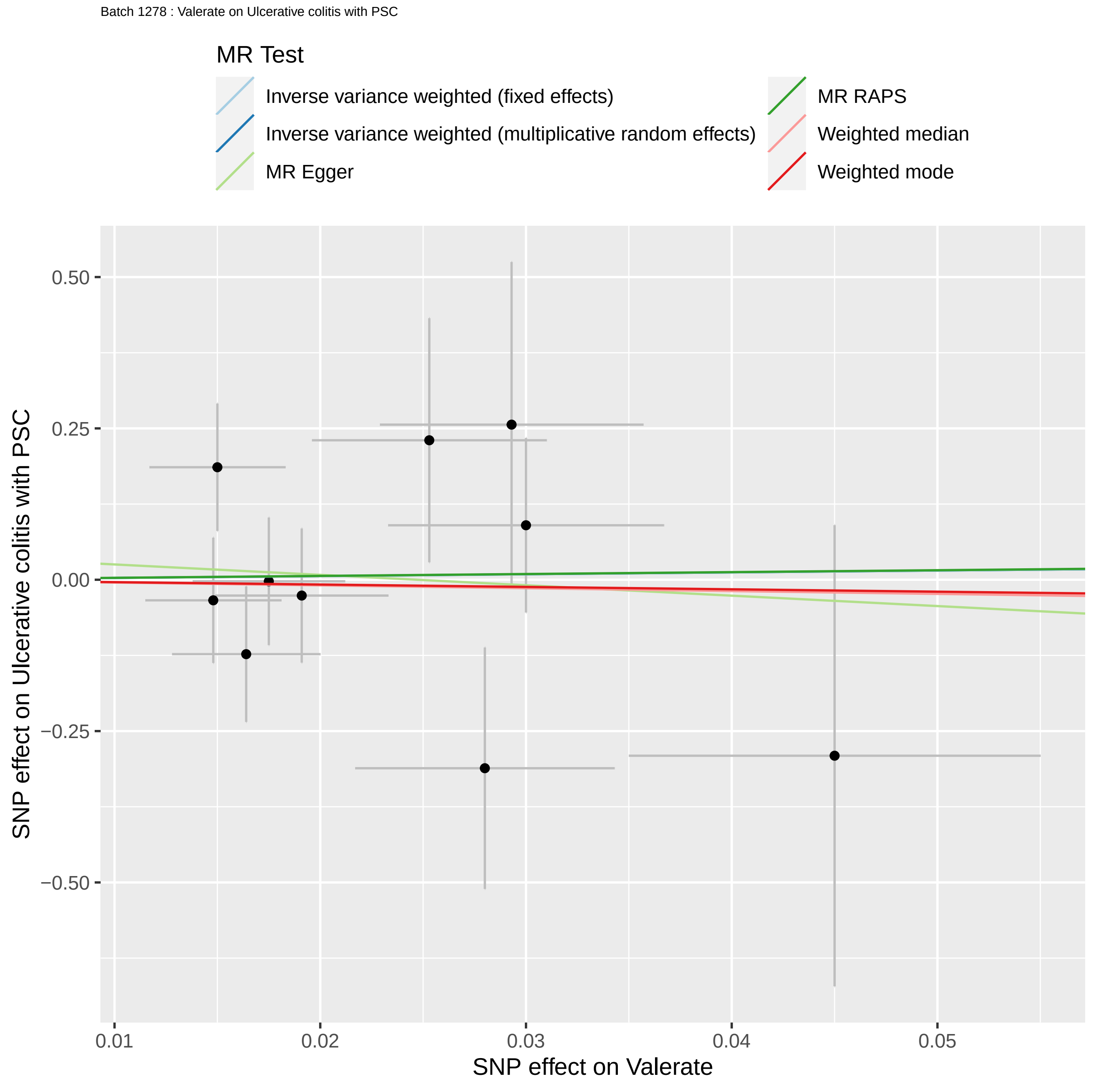
Batch 1278 : Valerate on Ulcerative colitis with PSC
MR Test
Inverse variance weighted (fixed effects)
Inverse variance weighted (multiplicative random effects)
MR Egger
MR RAPS
Weighted median
Weighted mode
0.50
0.25
0.00
−0.25
−0.50
0.01
0.02
0.03
0.04
0.05
SNP effect on Valerate
SNP effect on Ulcerative colitis with PSC

Batch 1281 : Erythronate* on Ulcerative colitis with PSC

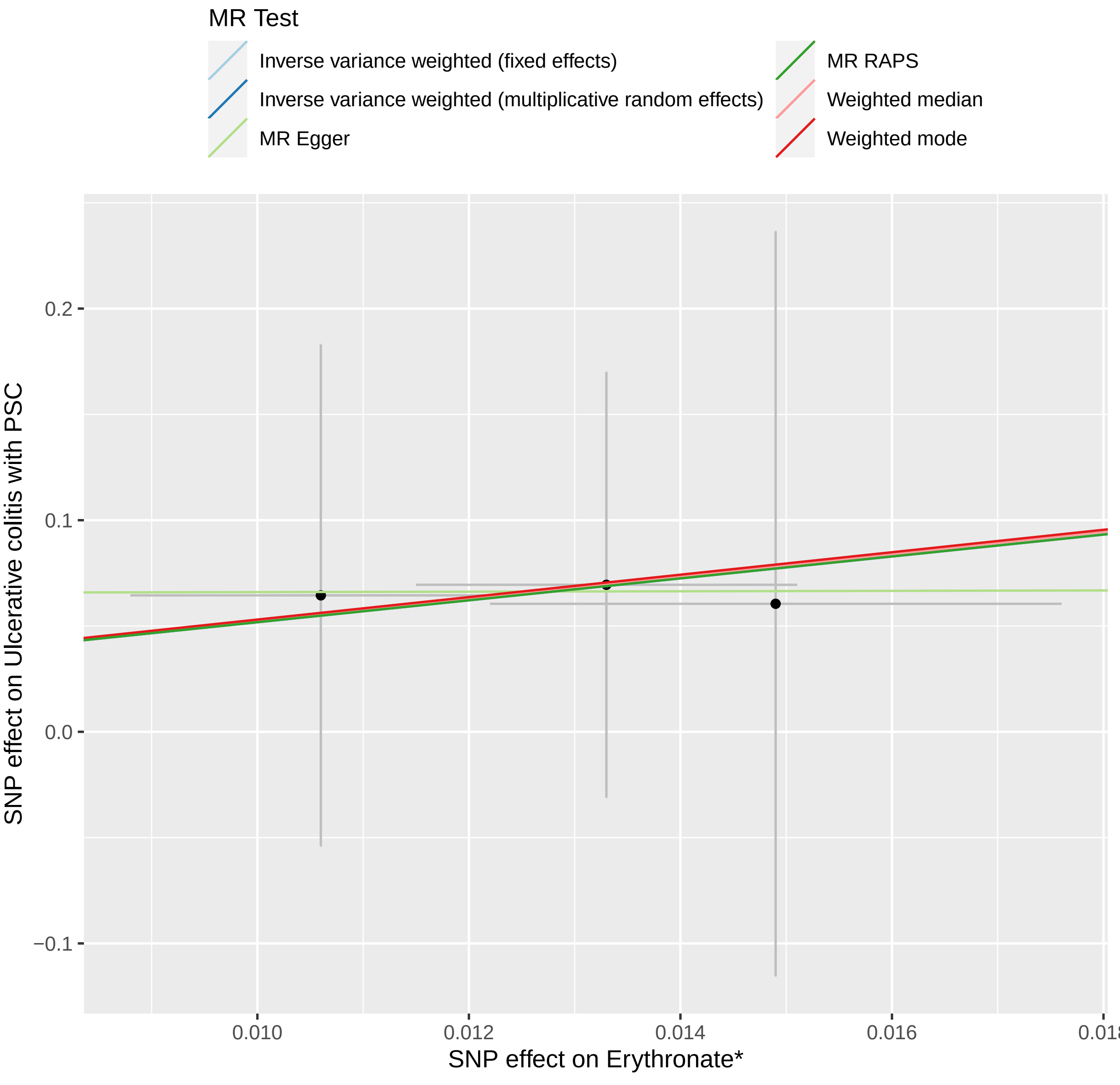

Batch 1282 : Lathosterol on Ulcerative colitis with PSC

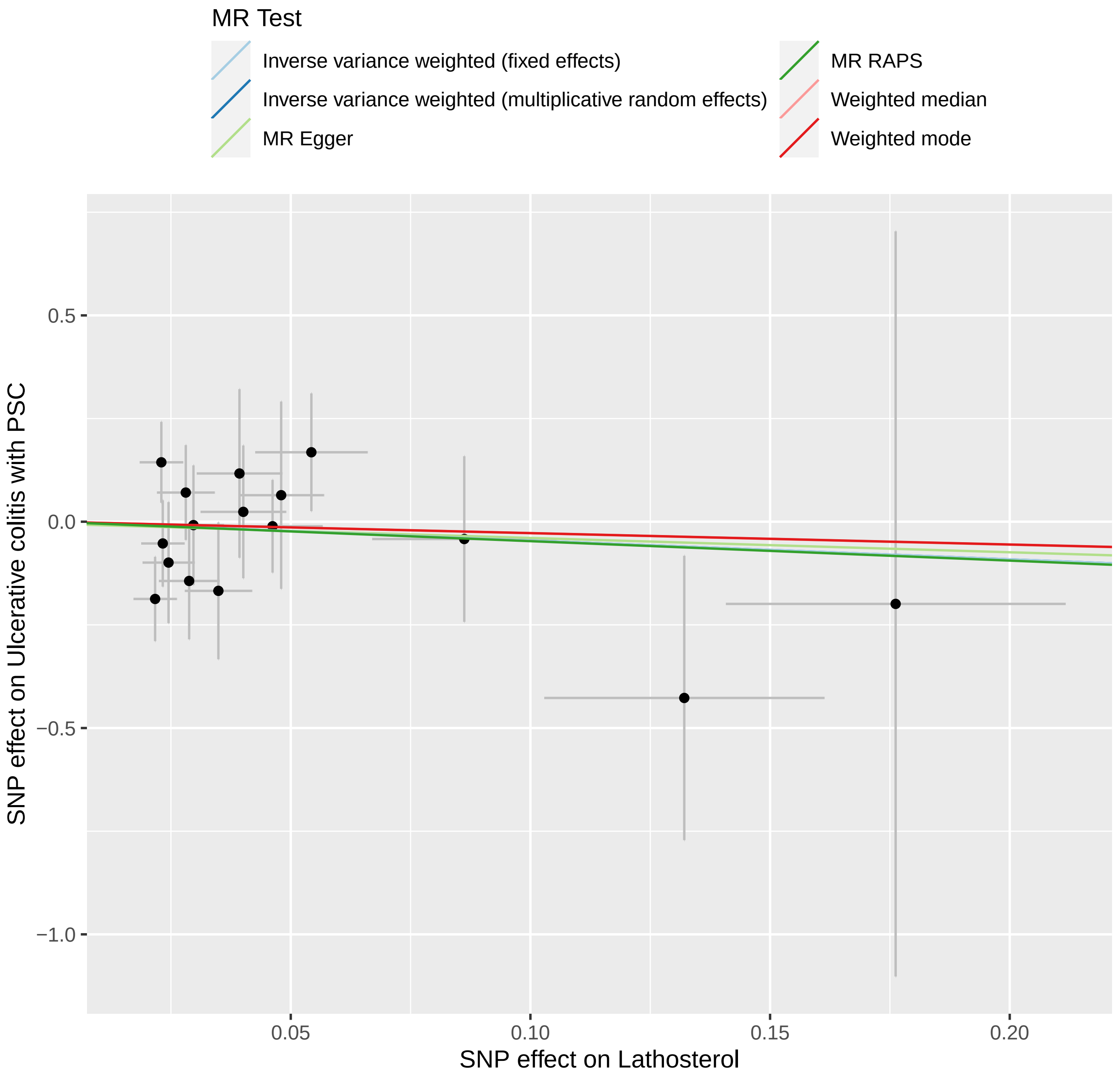

Batch 1285 : Eicosenoate (20:1n9 or 11) on Ulcerative colitis with PSC

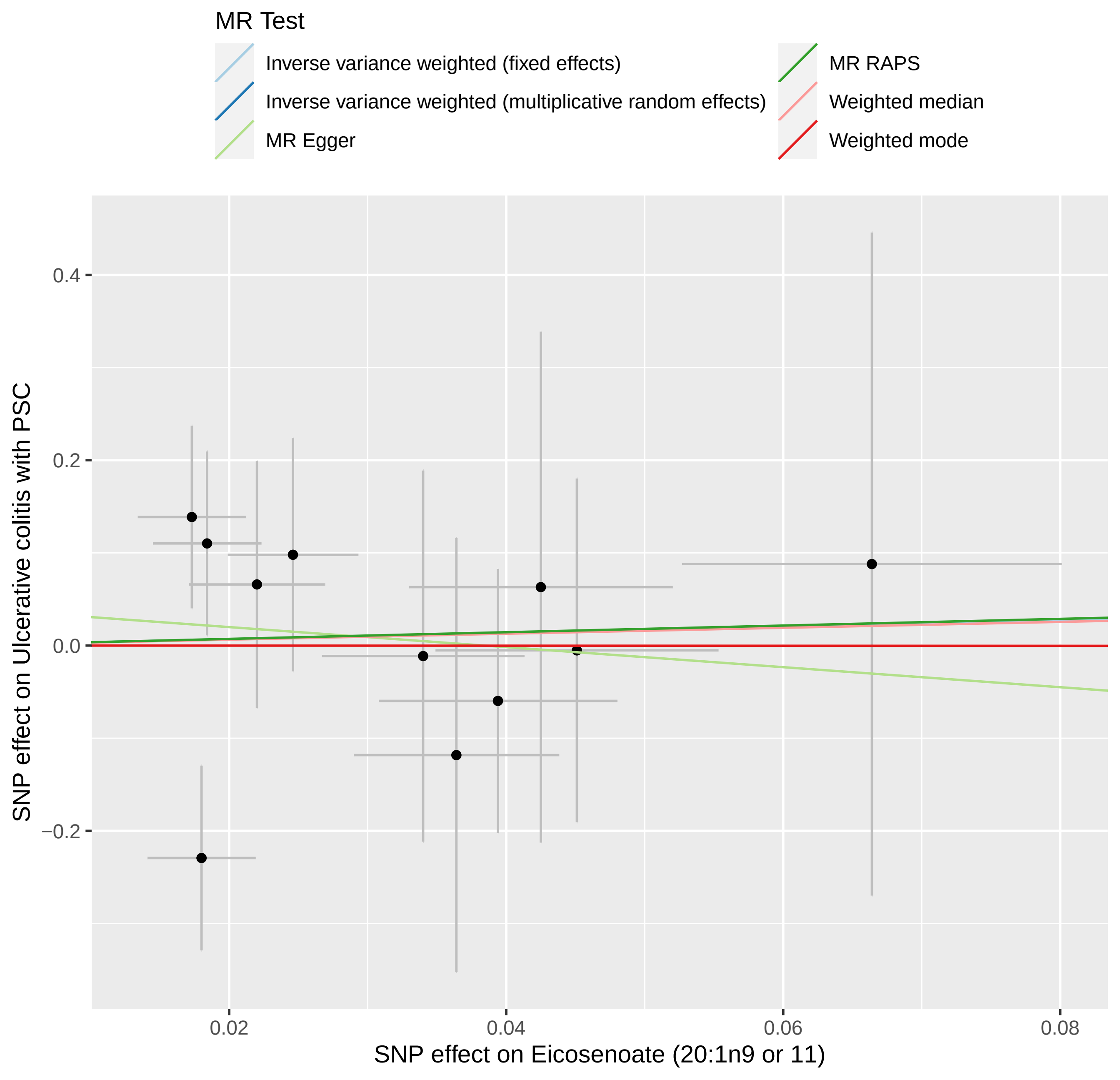

Batch 1286 : X−12244−−N−acetylcarnosine on Ulcerative colitis with PSC

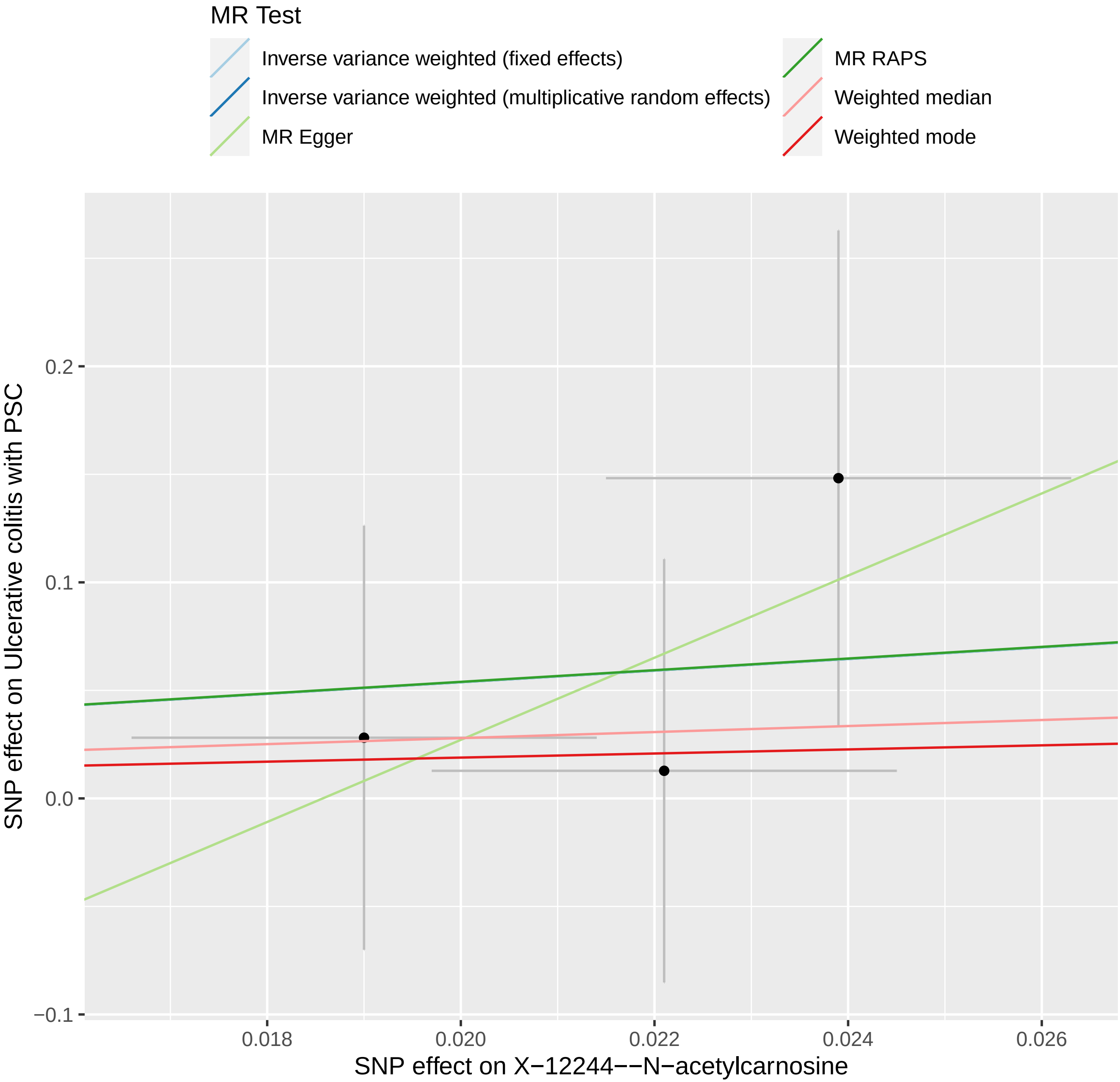

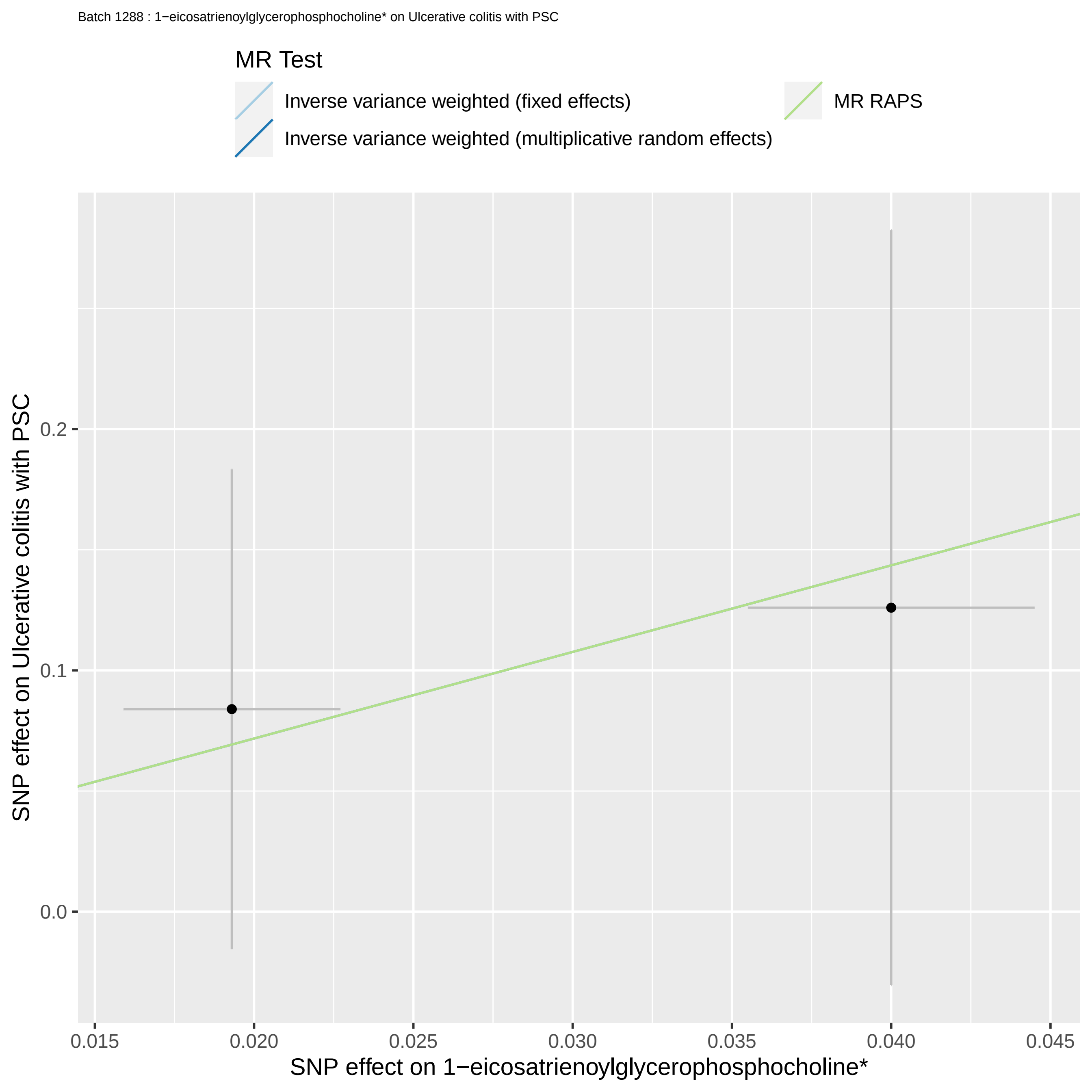
Batch 1288 : 1−eicosatrienoylglycerophosphocholine* on Ulcerative colitis with PSC
MR Test
Inverse variance weighted (fixed effects)
Inverse variance weighted (multiplicative random effects)
MR RAPS
SNP effect on Ulcerative colitis with PSC
0.2
0.1
0.0
0.015
0.020
0.025
0.030
0.035
0.040
0.045
SNP effect on 1−eicosatrienoylglycerophosphocholine*

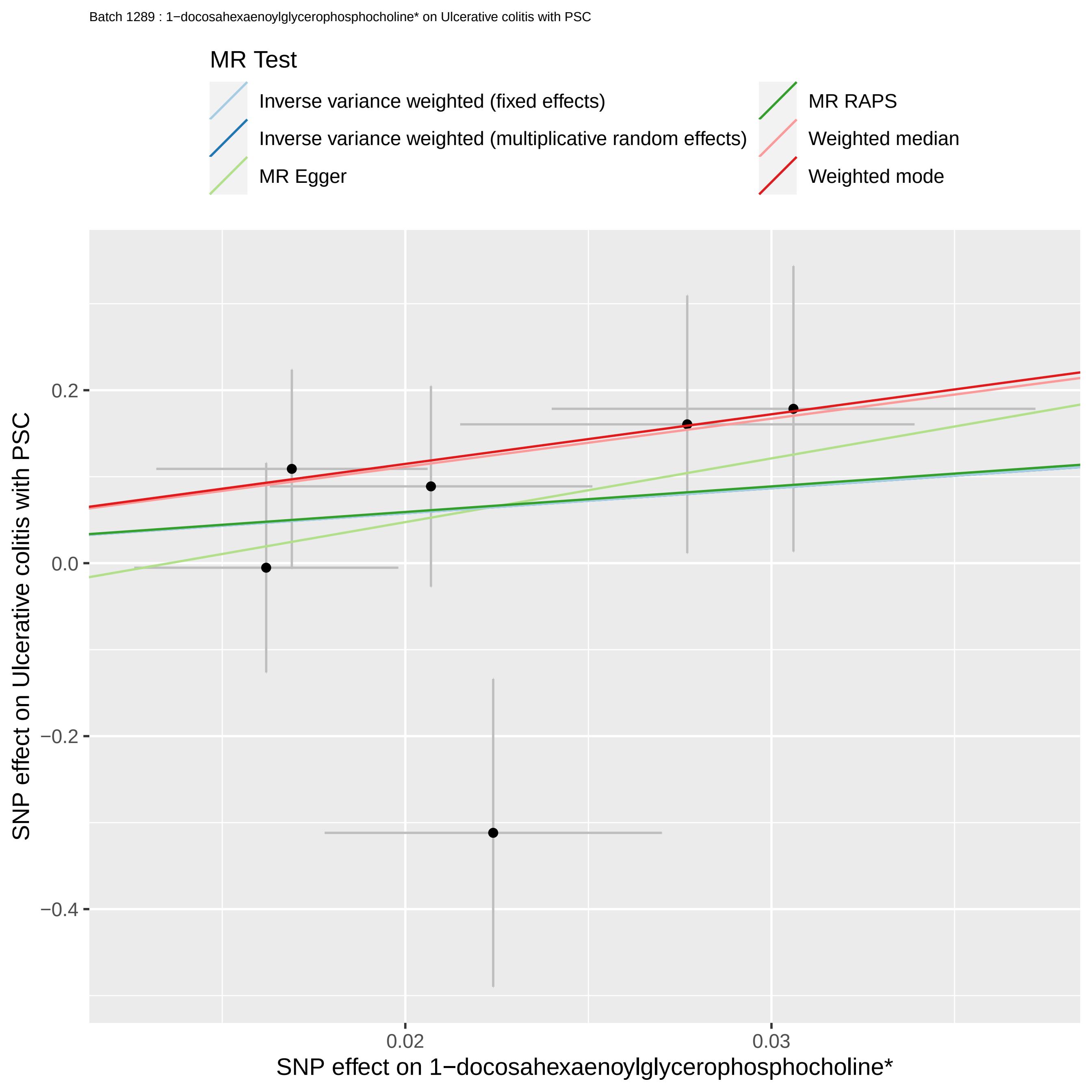
Batch 1289 : 1−docosahexaenoylglycerophosphocholine* on Ulcerative colitis with PSC
MR Test
Inverse variance weighted (fixed effects)
Inverse variance weighted (multiplicative random effects)
MR Egger
MR RAPS
Weighted median
Weighted mode
SNP effect on Ulcerative colitis with PSC
0.2
0.0
−0.2
−0.4
0.02
0.03
SNP effect on 1−docosahexaenoylglycerophosphocholine*

Batch 1293 : Octanoylcarnitine on Ulcerative colitis with PSC

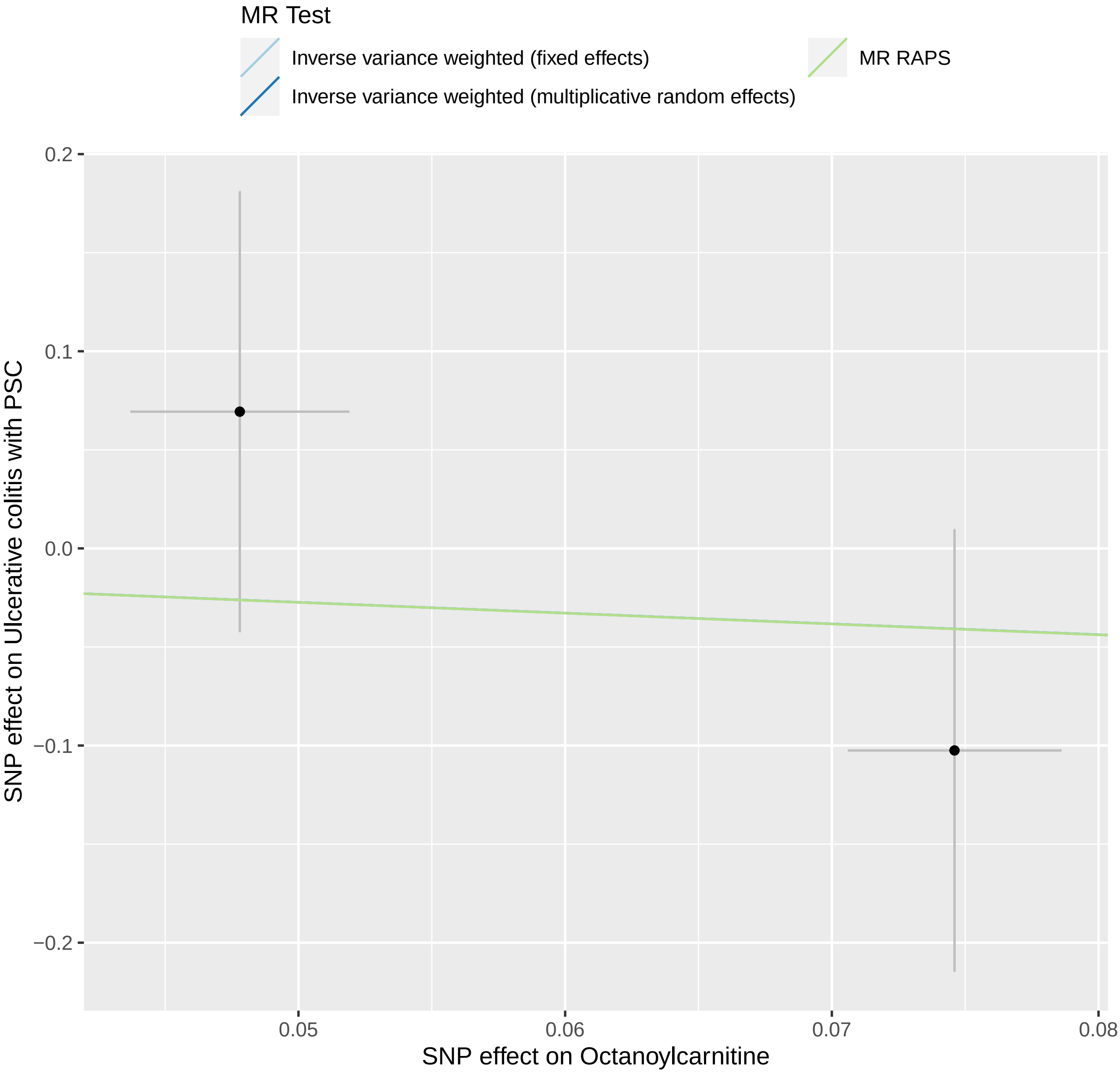

Batch 1294 : Alpha−hydroxyisovalerate on Ulcerative colitis with PSC

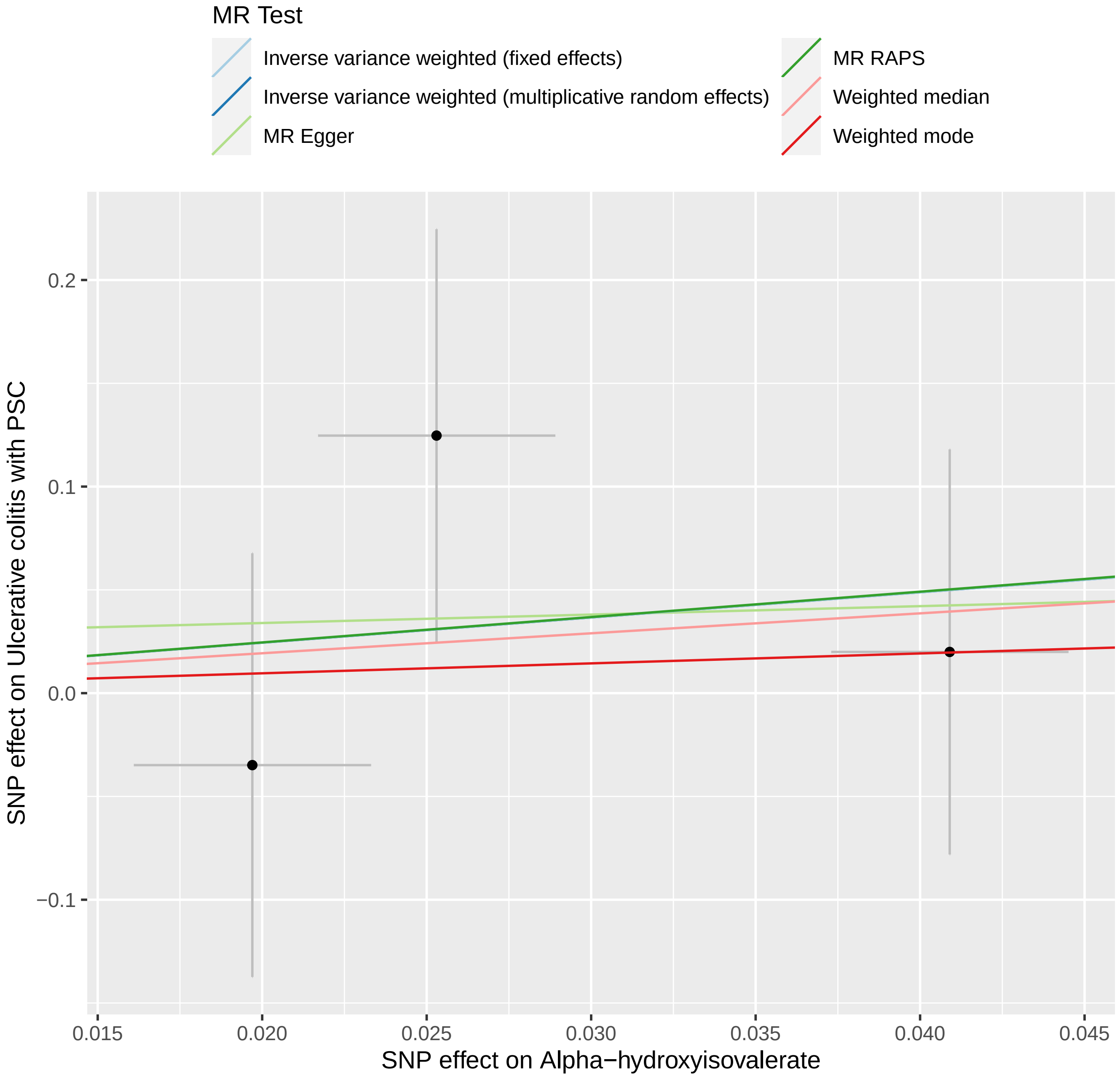

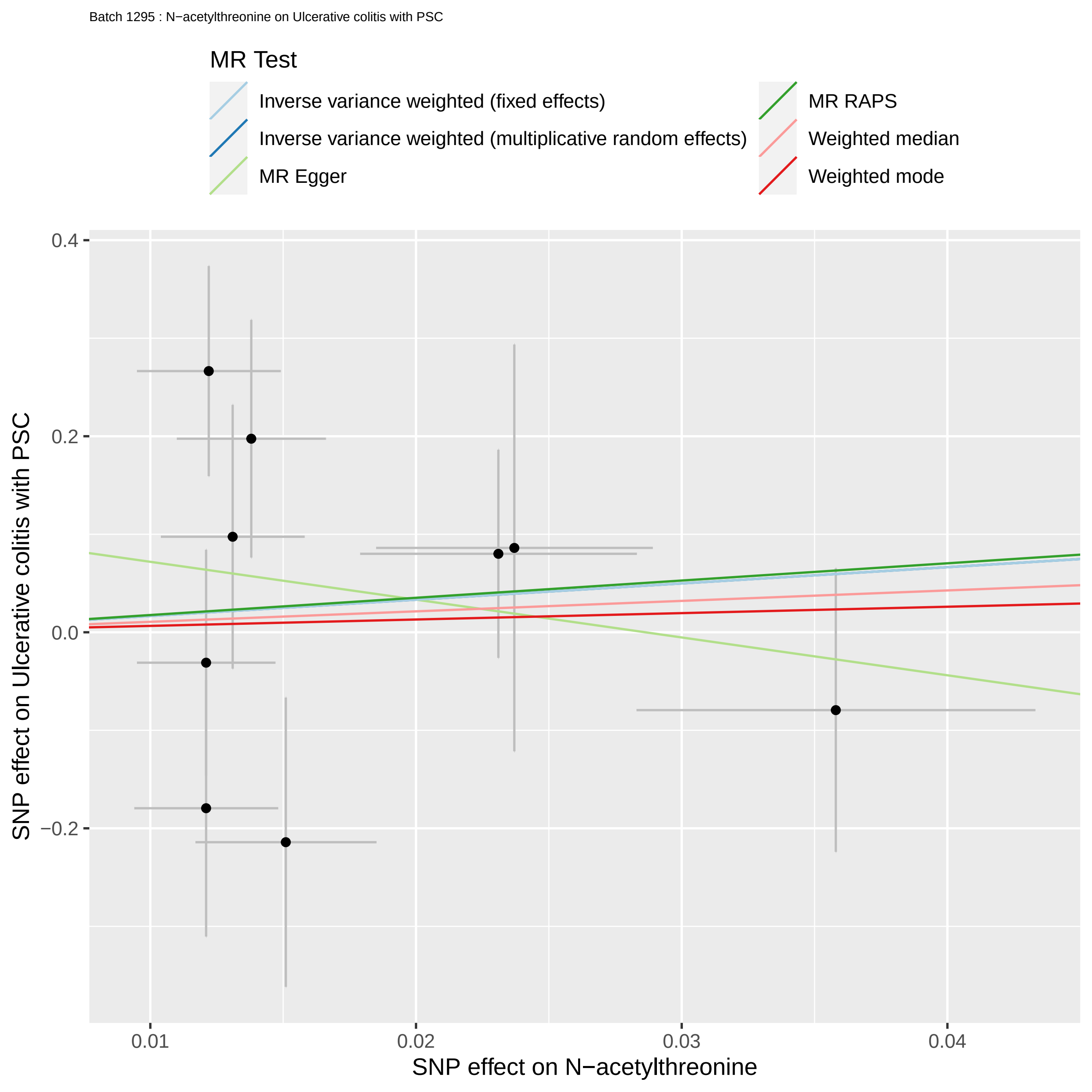

Batch 1295 : N−acetylthreonine on Ulcerative colitis with PSC
MR Test
Inverse variance weighted (fixed effects)
Inverse variance weighted (multiplicative random effects)
MR Egger
MR RAPS
Weighted median
Weighted mode
0.4
0.2
0.0
−0.2
0.01
0.02
0.03
0.04
SNP effect on Ulcerative colitis with PSC
SNP effect on N−acetylthreonine

Batch 1296 : Decanoylcarnitine on Ulcerative colitis with PSC

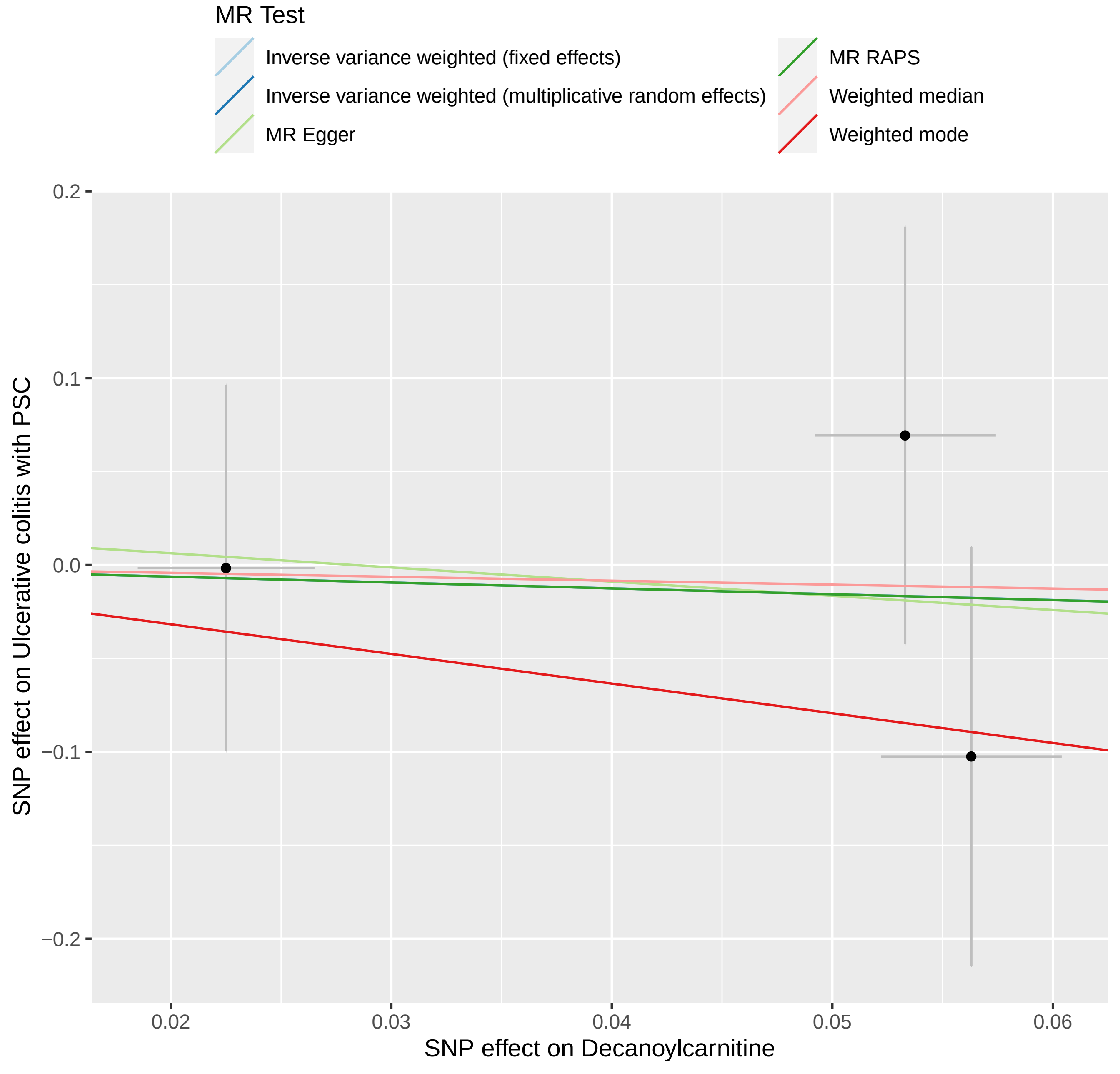

Batch 1297 : 1−palmitoylglycerophosphocholine on Ulcerative colitis with PSC

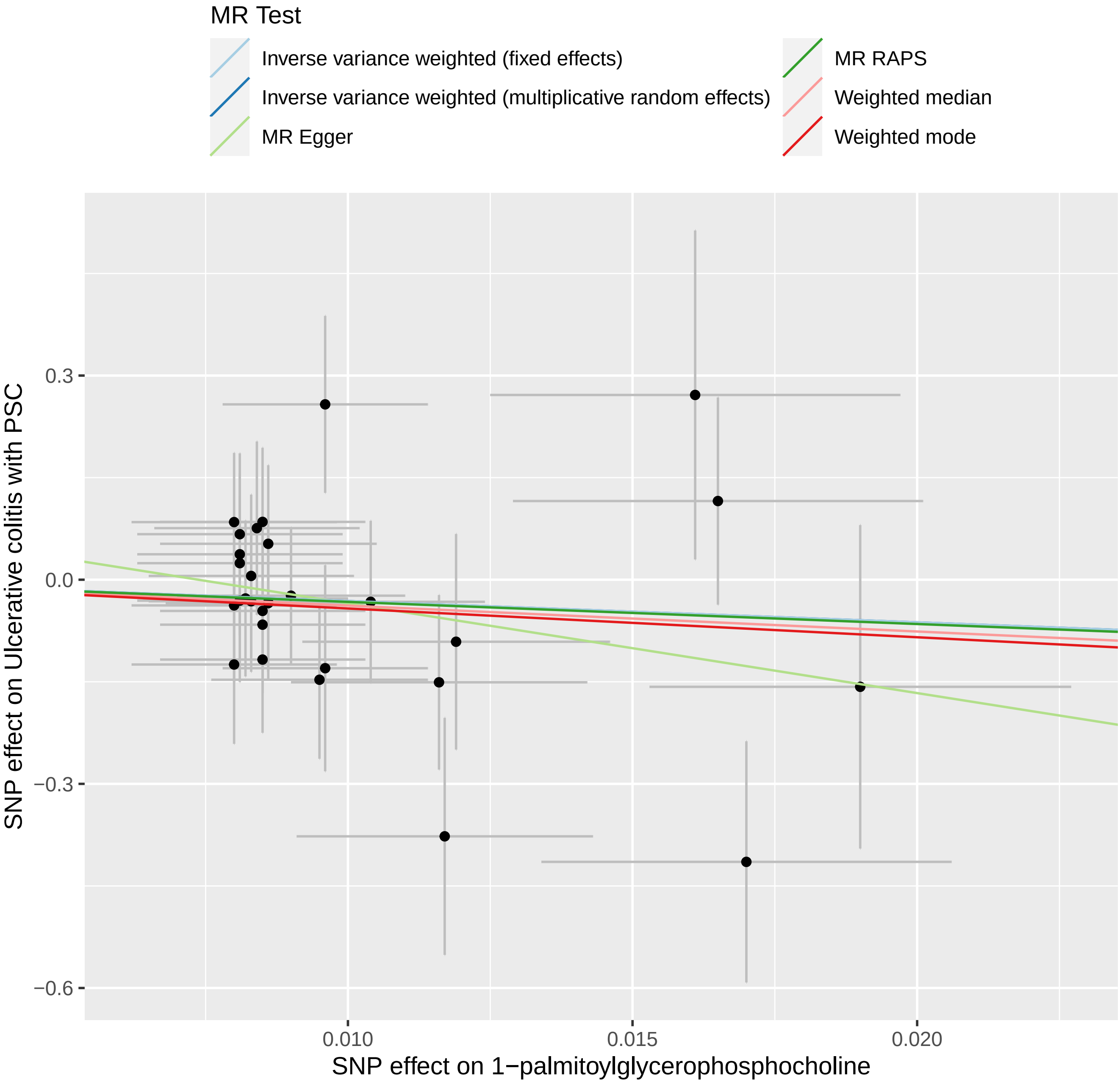

Batch 1298 : 1−heptadecanoylglycerophosphocholine on Ulcerative colitis with PSC

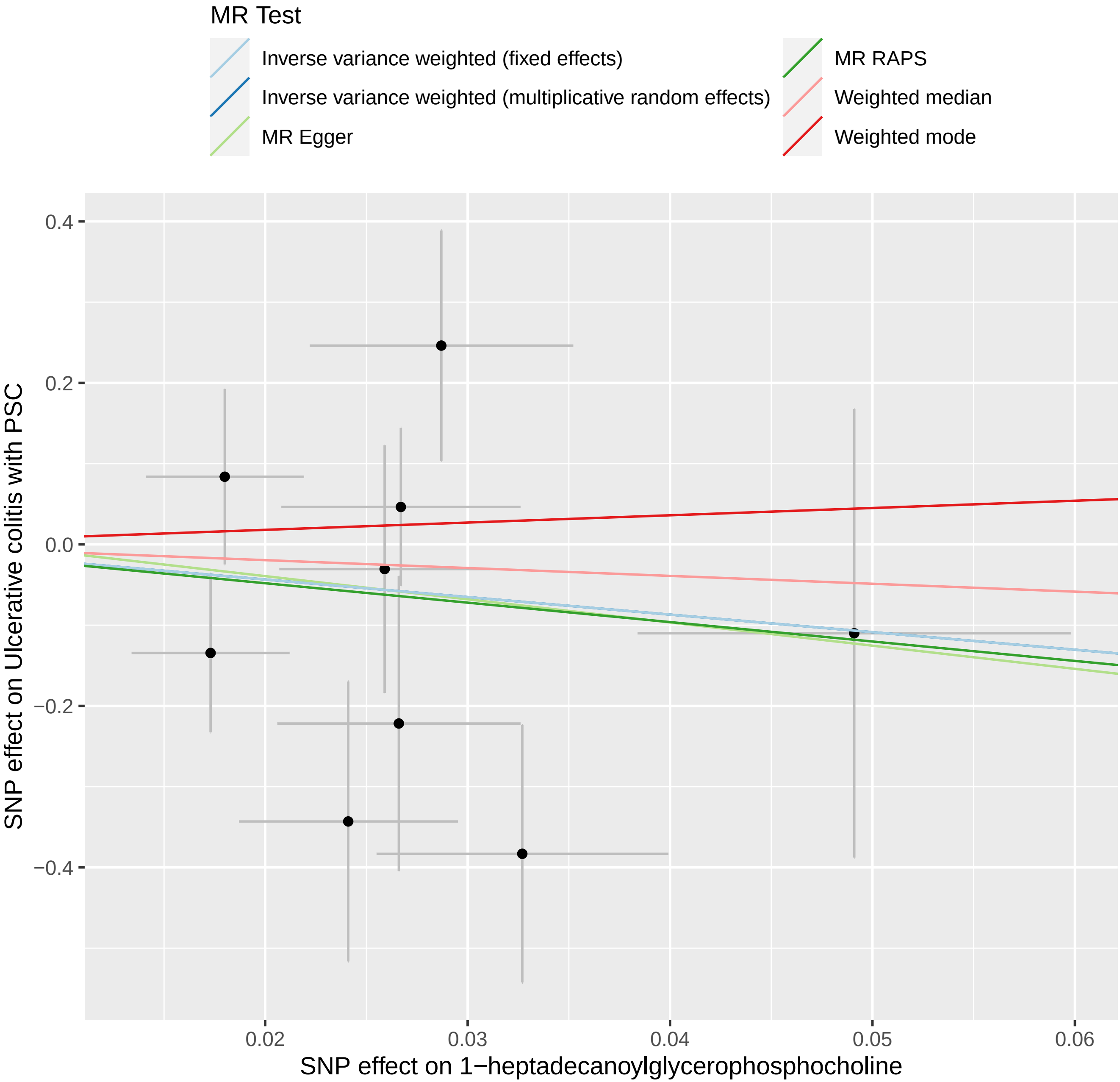

Batch 1299 : 1−oleoylglycerophosphocholine on Ulcerative colitis with PSC

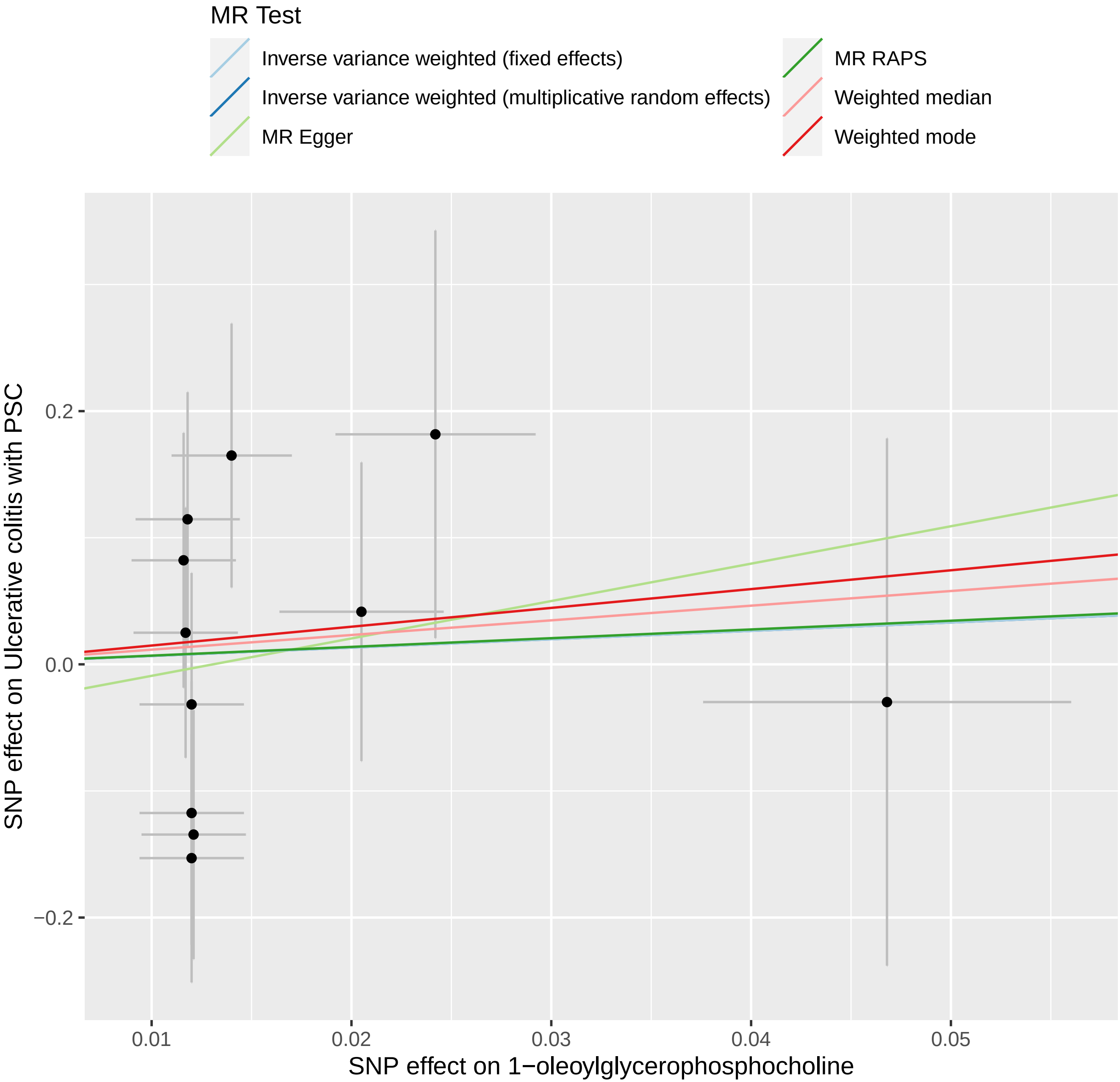

Batch 1300 : 1−stearoylglycerophosphocholine on Ulcerative colitis with PSC

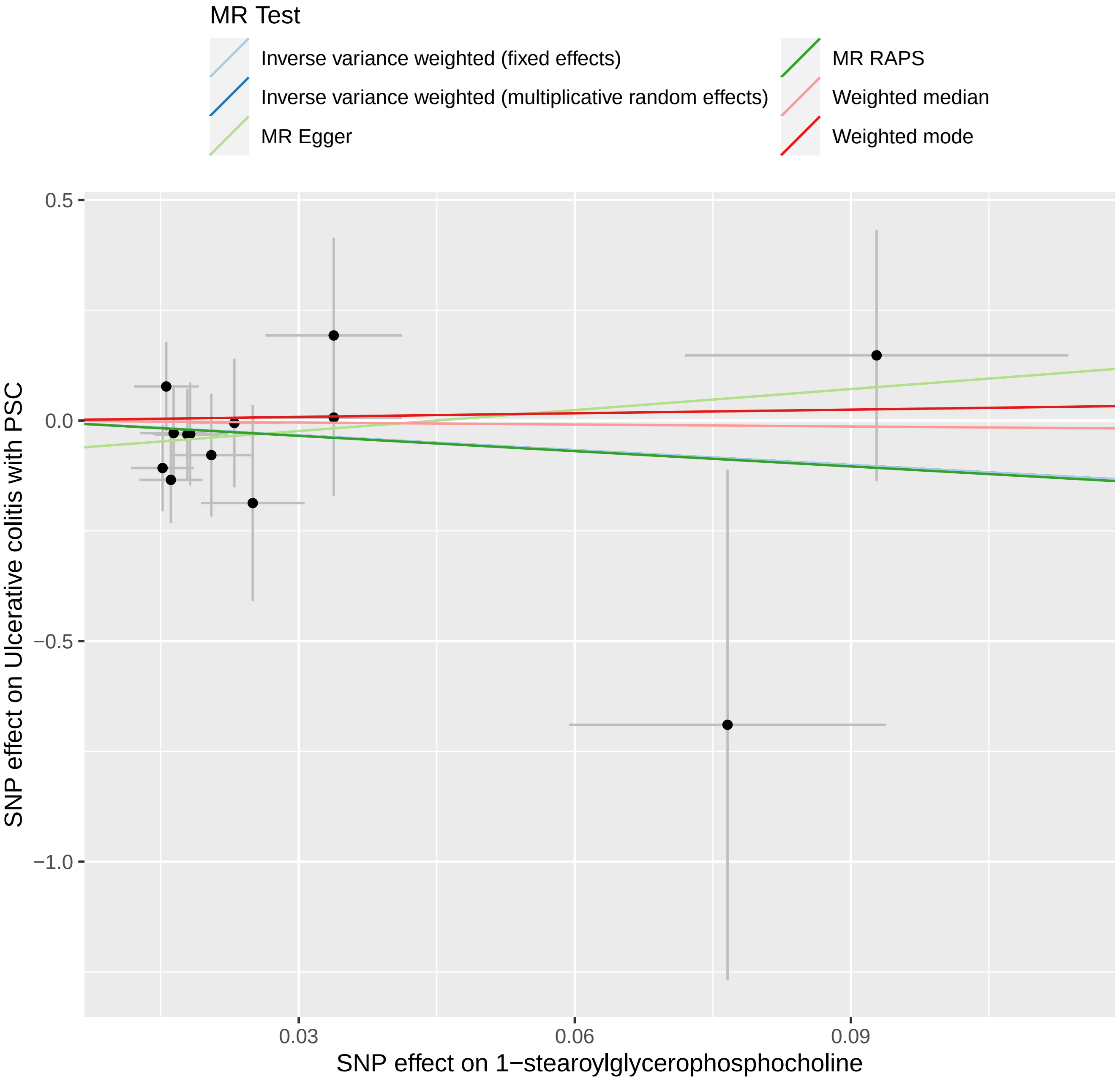

Batch 1303 : 10−heptadecenoate (17:1n7) on Ulcerative colitis with PSC

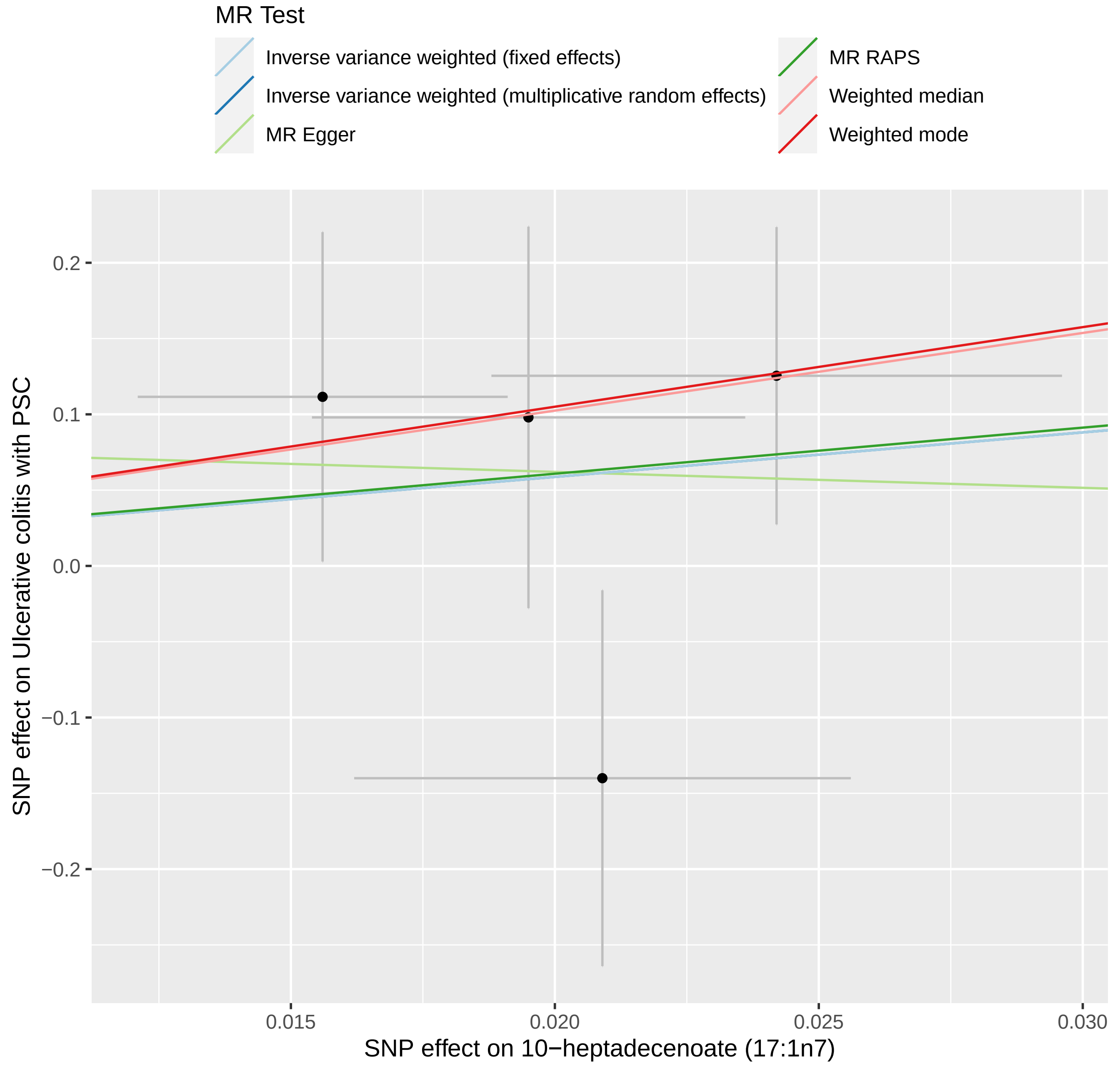

Batch 1305 : Epiandrosterone sulfate on Ulcerative colitis with PSC

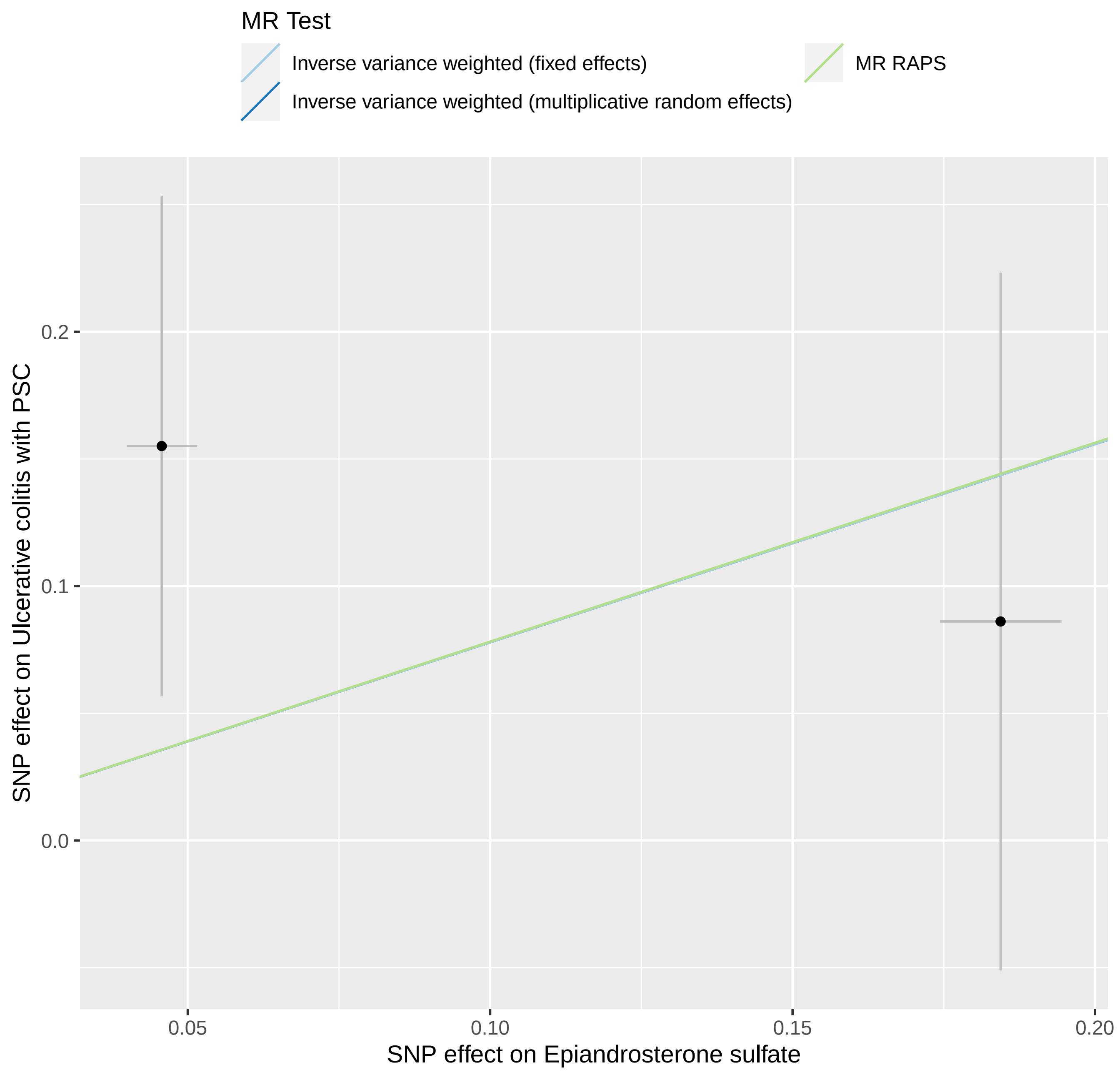

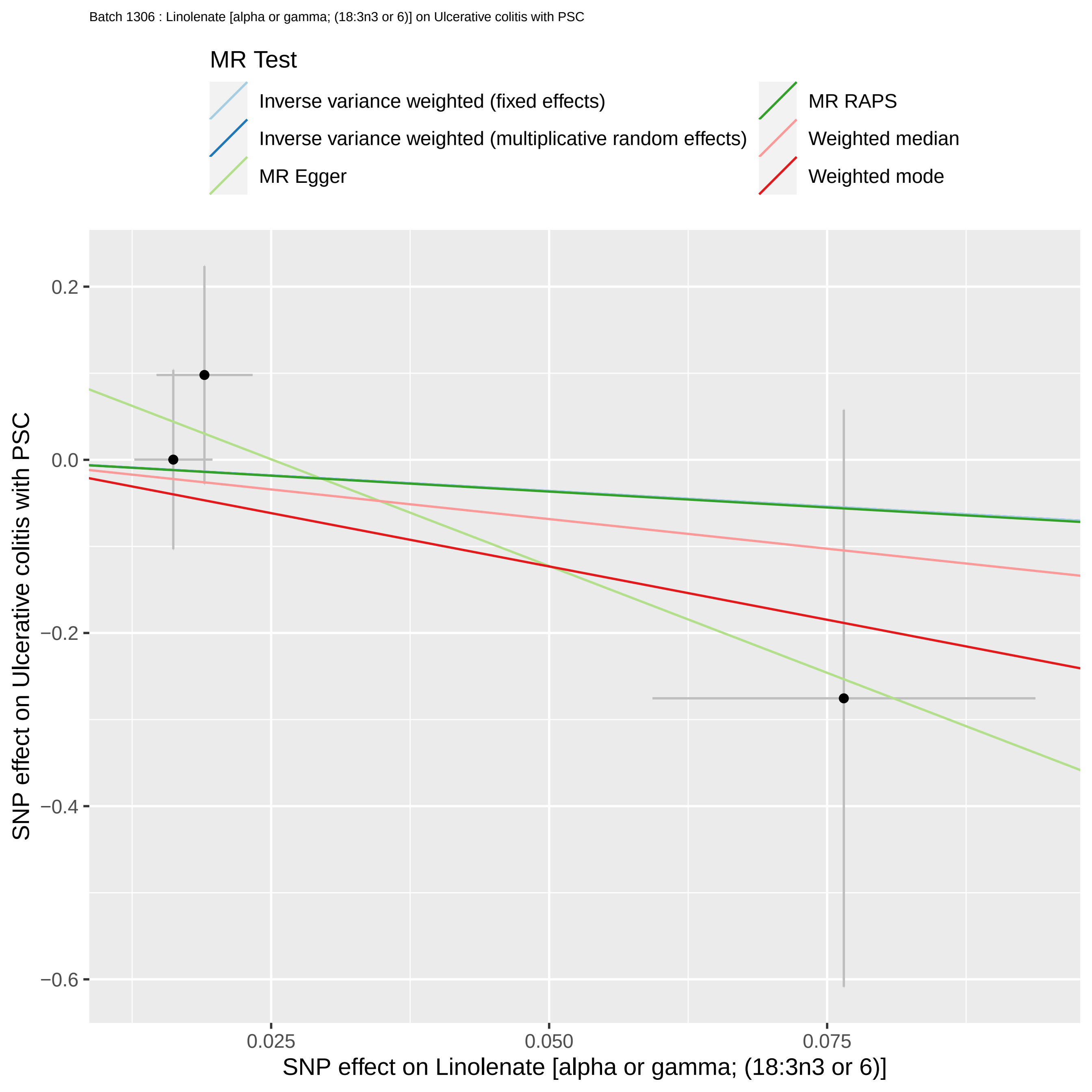
Batch 1306 : Linolenate [alpha or gamma; (18:3n3 or 6)] on Ulcerative colitis with PSC
MR Test
Inverse variance weighted (fixed effects)
Inverse variance weighted (multiplicative random effects)
MR Egger
MR RAPS
Weighted median
Weighted mode
0.2
0.0
−0.2
−0.4
−0.6
0.025
0.050
0.075
SNP effect on Ulcerative colitis with PSC
SNP effect on Linolenate [alpha or gamma; (18:3n3 or 6)]

Batch 1307 : X−12510−−2−aminooctanoic acid on Ulcerative colitis with PSC

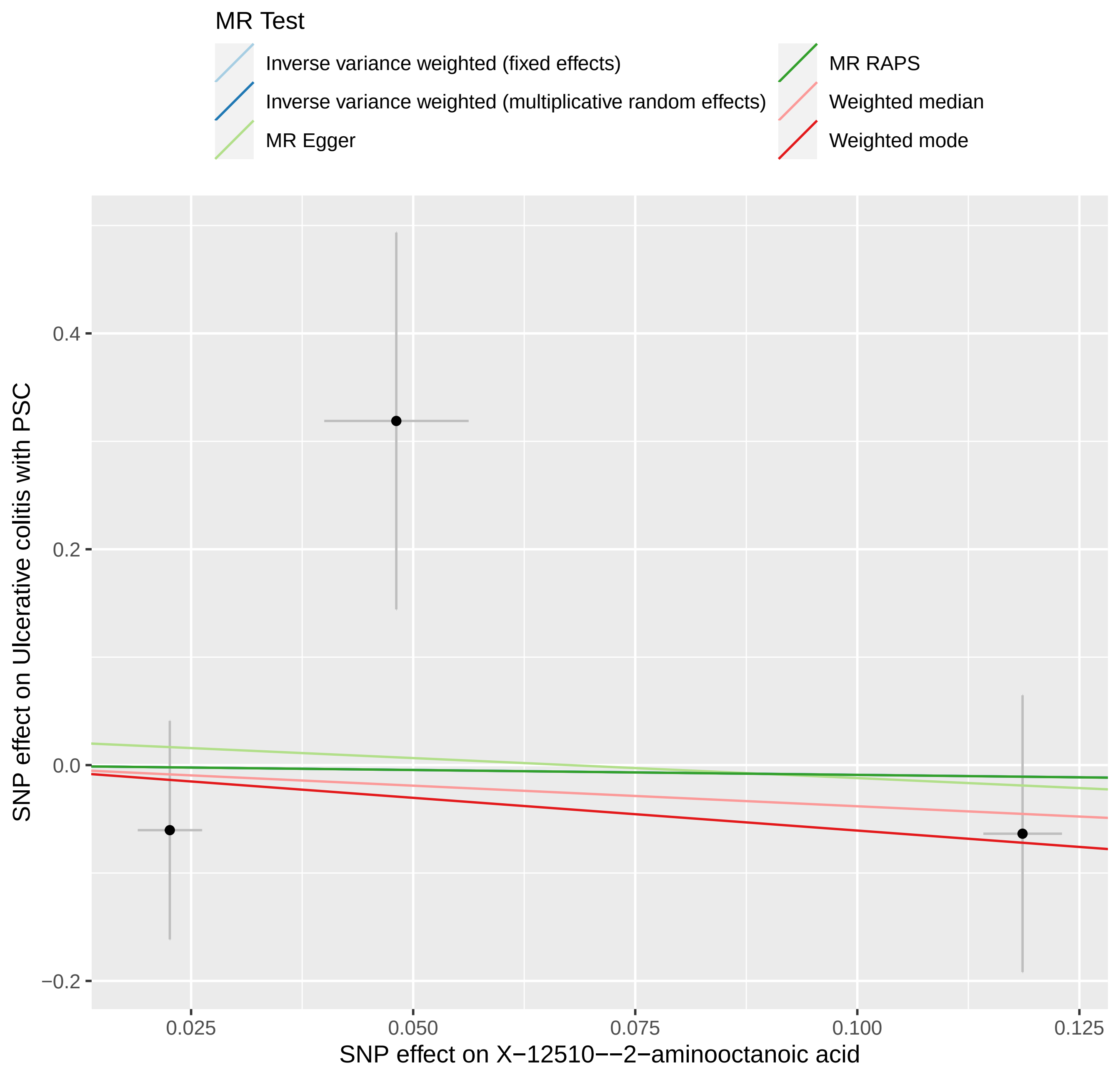

Batch 1310 : Asparagine on Ulcerative colitis with PSC

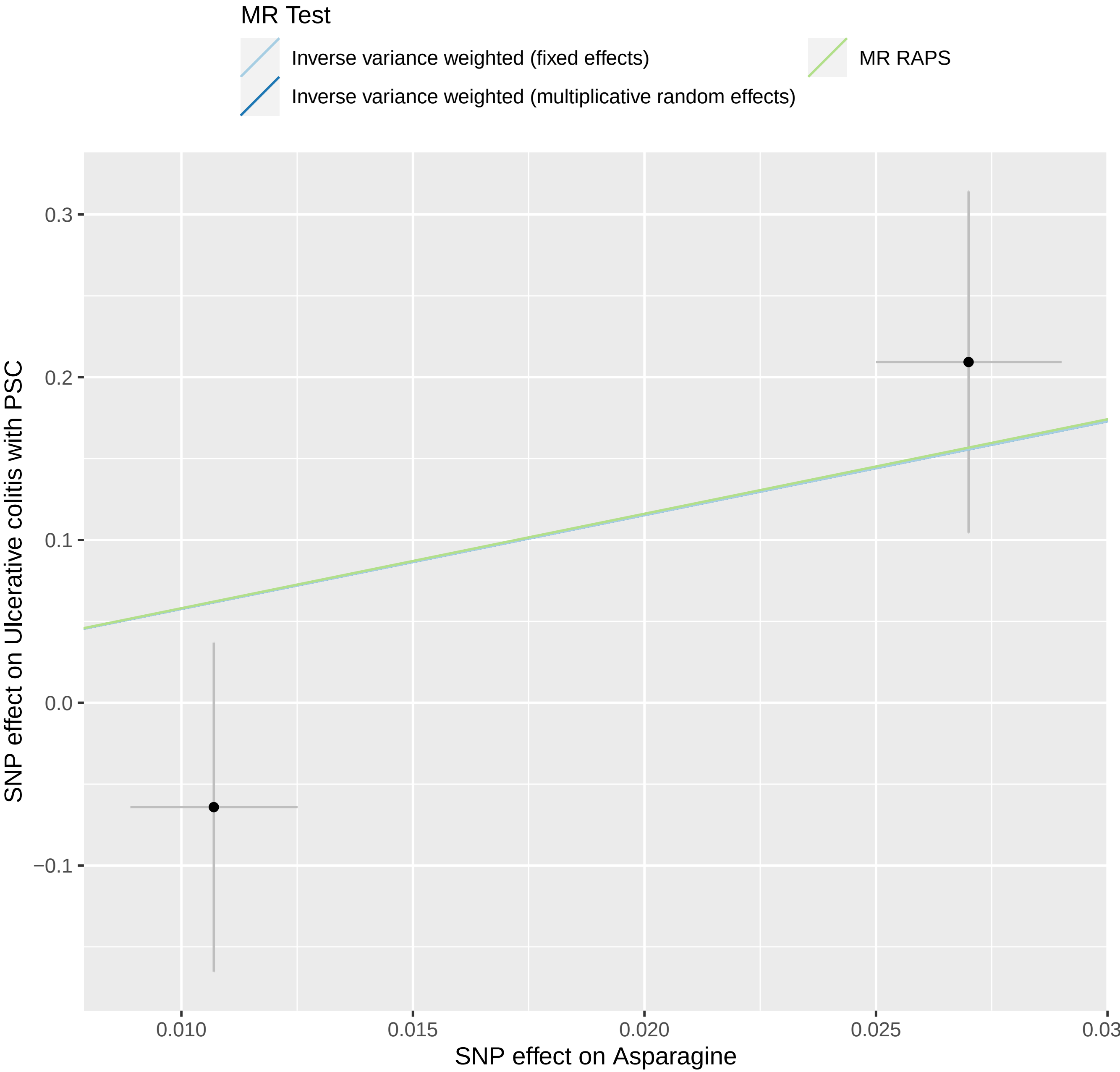

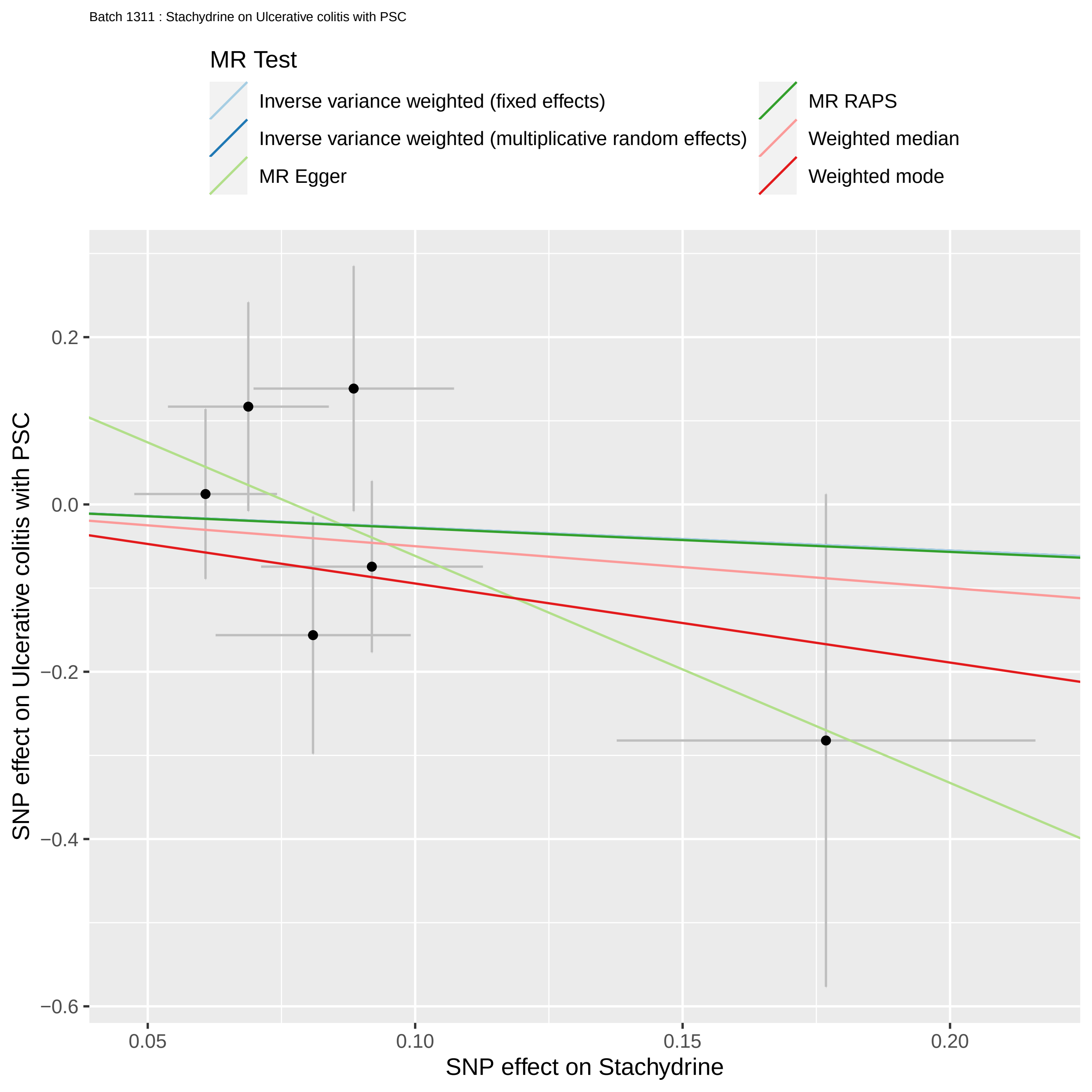

Batch 1311 : Stachydrine on Ulcerative colitis with PSC
MR Test
Inverse variance weighted (fixed effects)
Inverse variance weighted (multiplicative random effects)
MR Egger
MR RAPS
Weighted median
Weighted mode
0.2
0.0
−0.2
−0.4
−0.6
0.05
0.10
0.15
0.20
SNP effect on Stachydrine
SNP effect on Ulcerative colitis with PSC

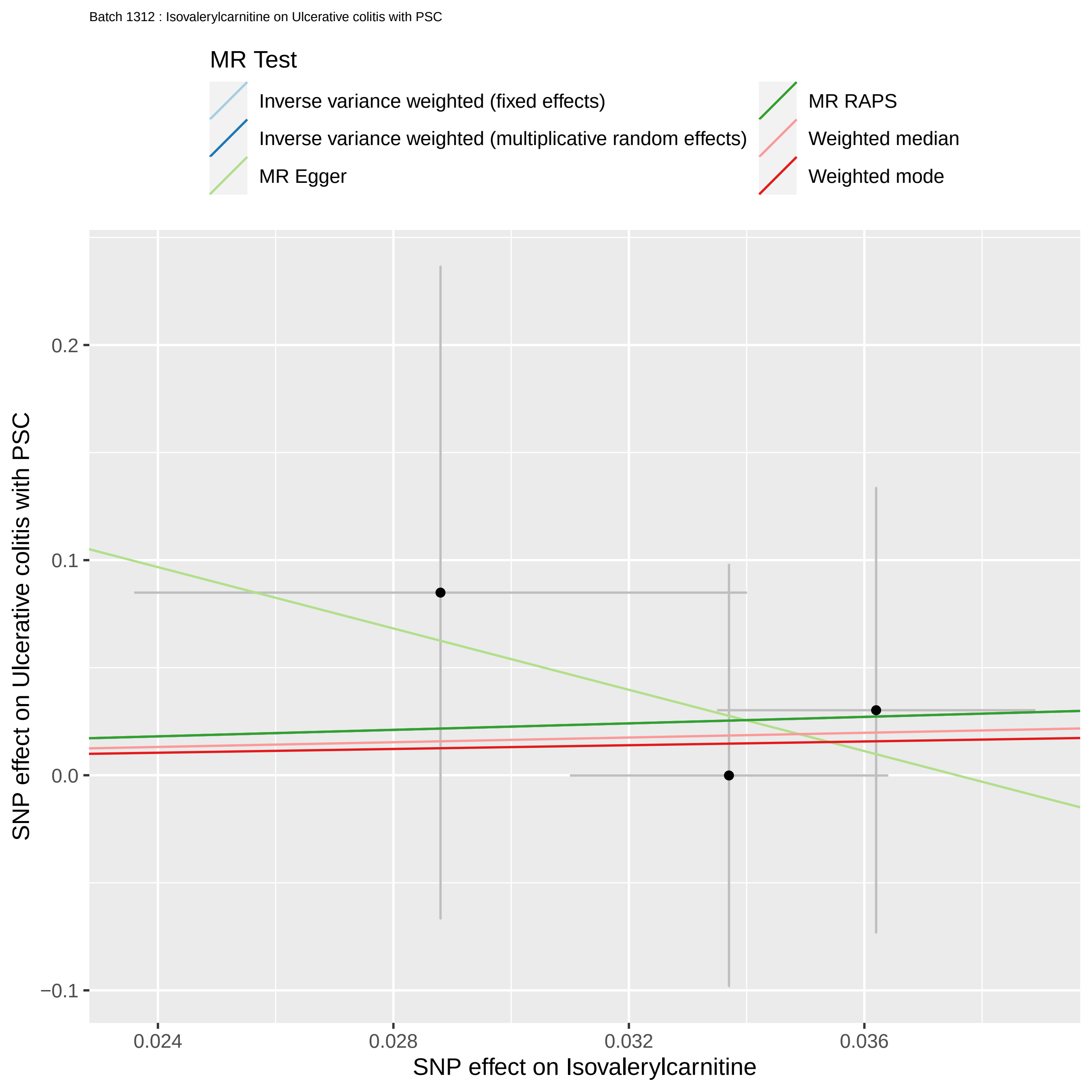

Batch 1312 : Isovalerylcarnitine on Ulcerative colitis with PSC
MR Test
Inverse variance weighted (fixed effects)
Inverse variance weighted (multiplicative random effects)
MR Egger
MR RAPS
Weighted median
Weighted mode
0.2
0.1
0.0
−0.1
0.024
0.028
0.032
0.036
SNP effect on Isovalerylcarnitine
SNP effect on Ulcerative colitis with PSC

Batch 1315 : 1−linoleoylglycerophosphocholine on Ulcerative colitis with PSC

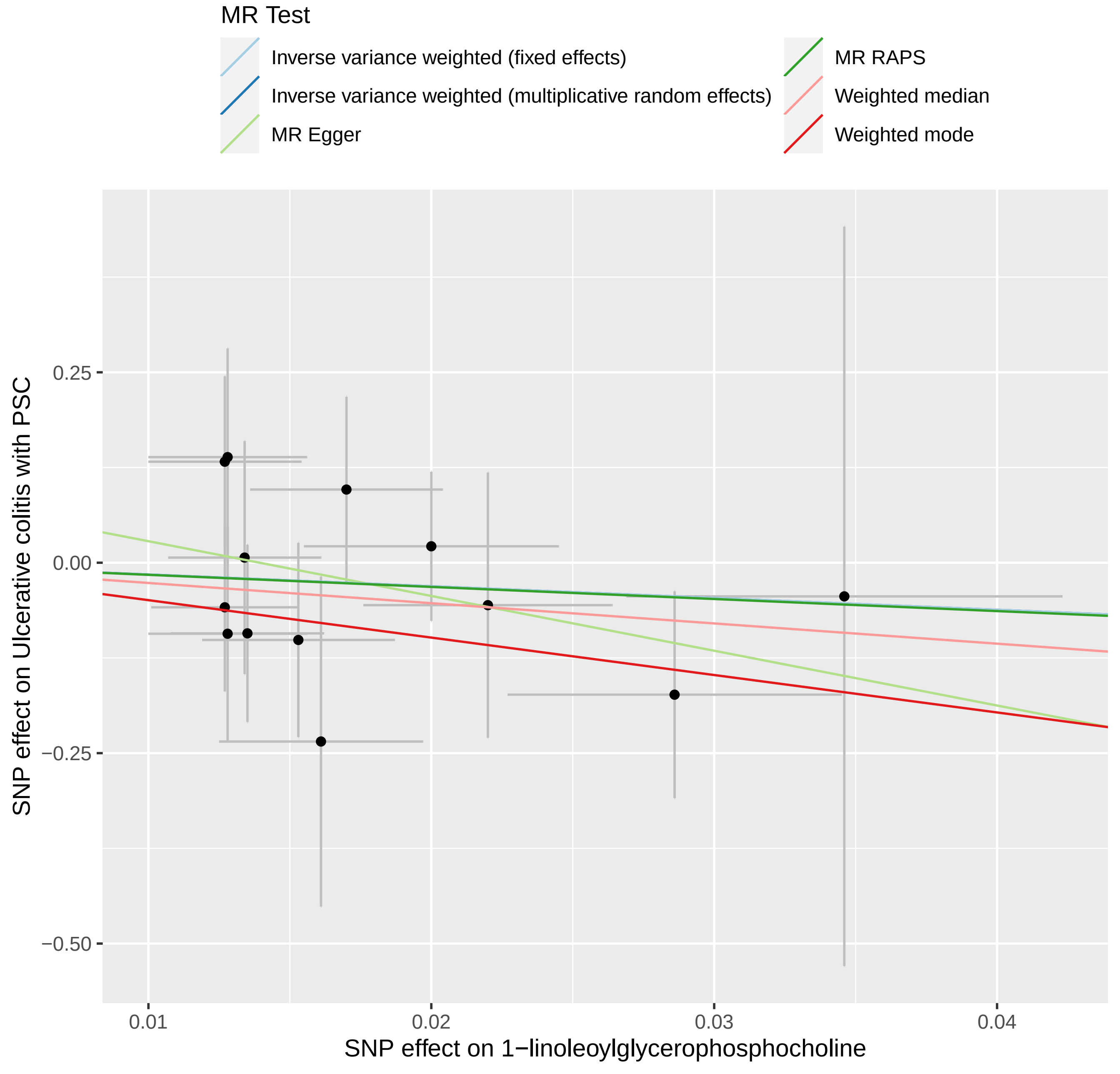

Batch 1316 : Bradykinin, des−arg(9) on Ulcerative colitis with PSC

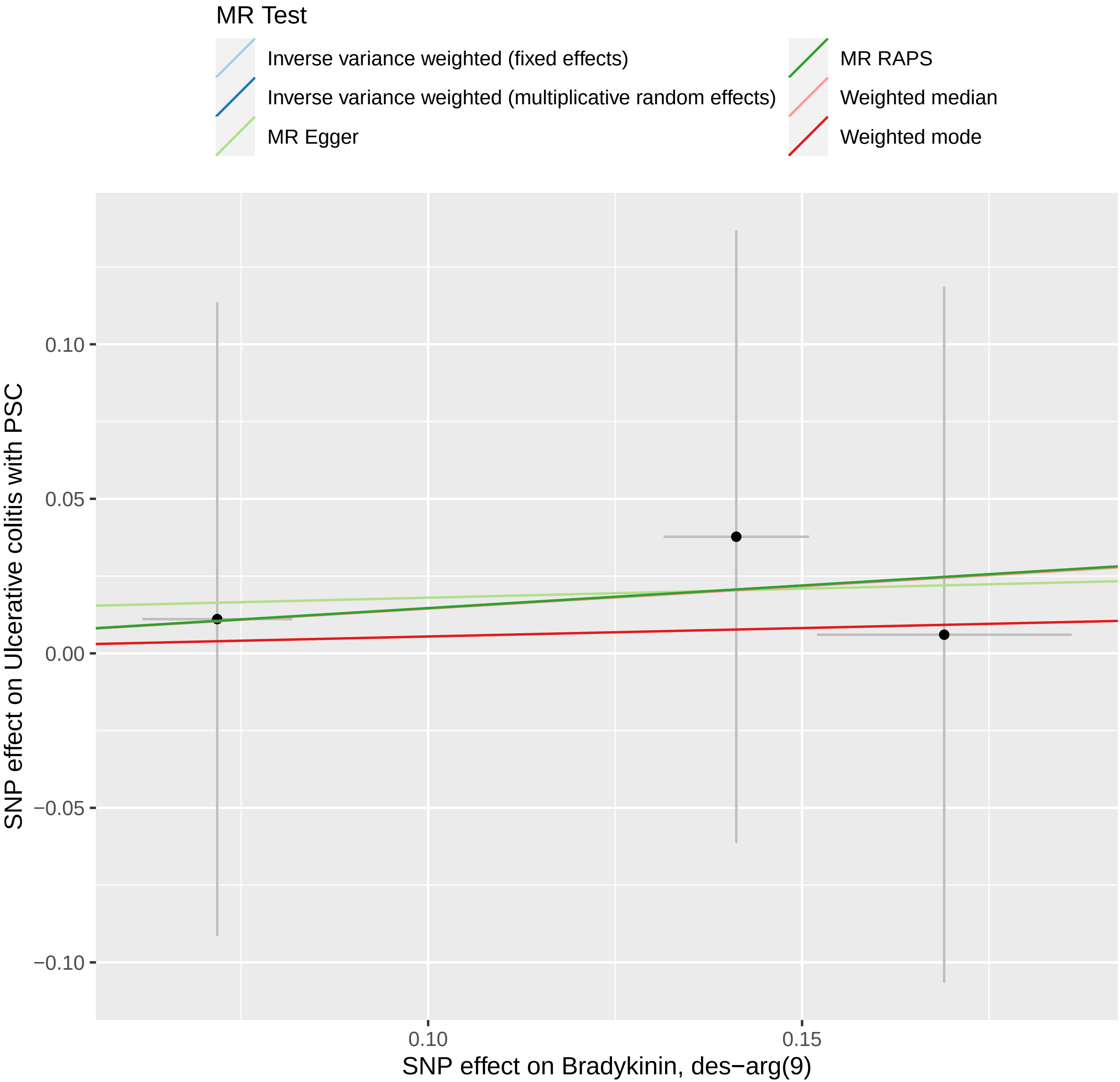

Batch 1317 : Gamma−glutamylisoleucine* on Ulcerative colitis with PSC

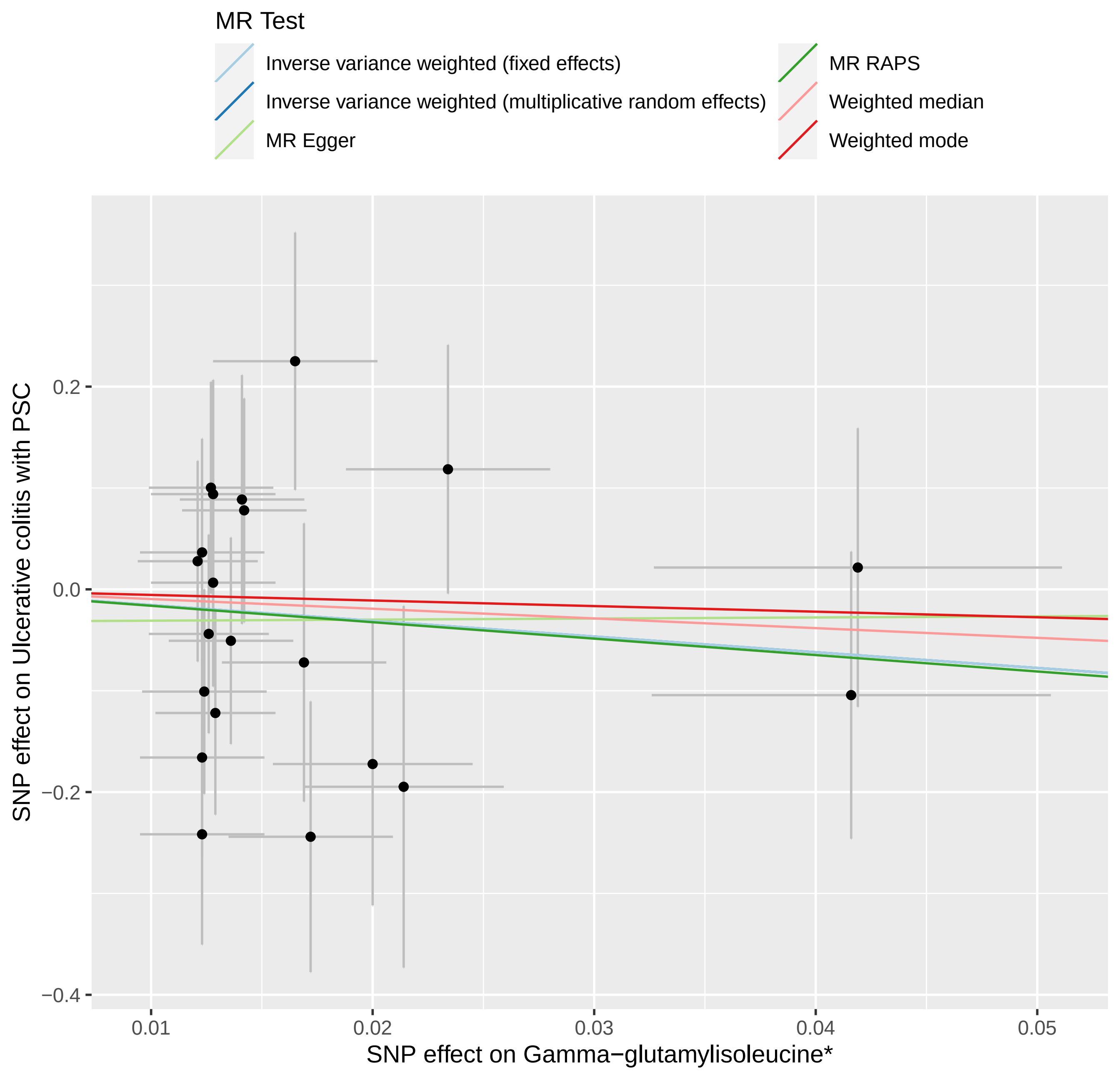

Batch 1320 : Isovalerate on Ulcerative colitis with PSC

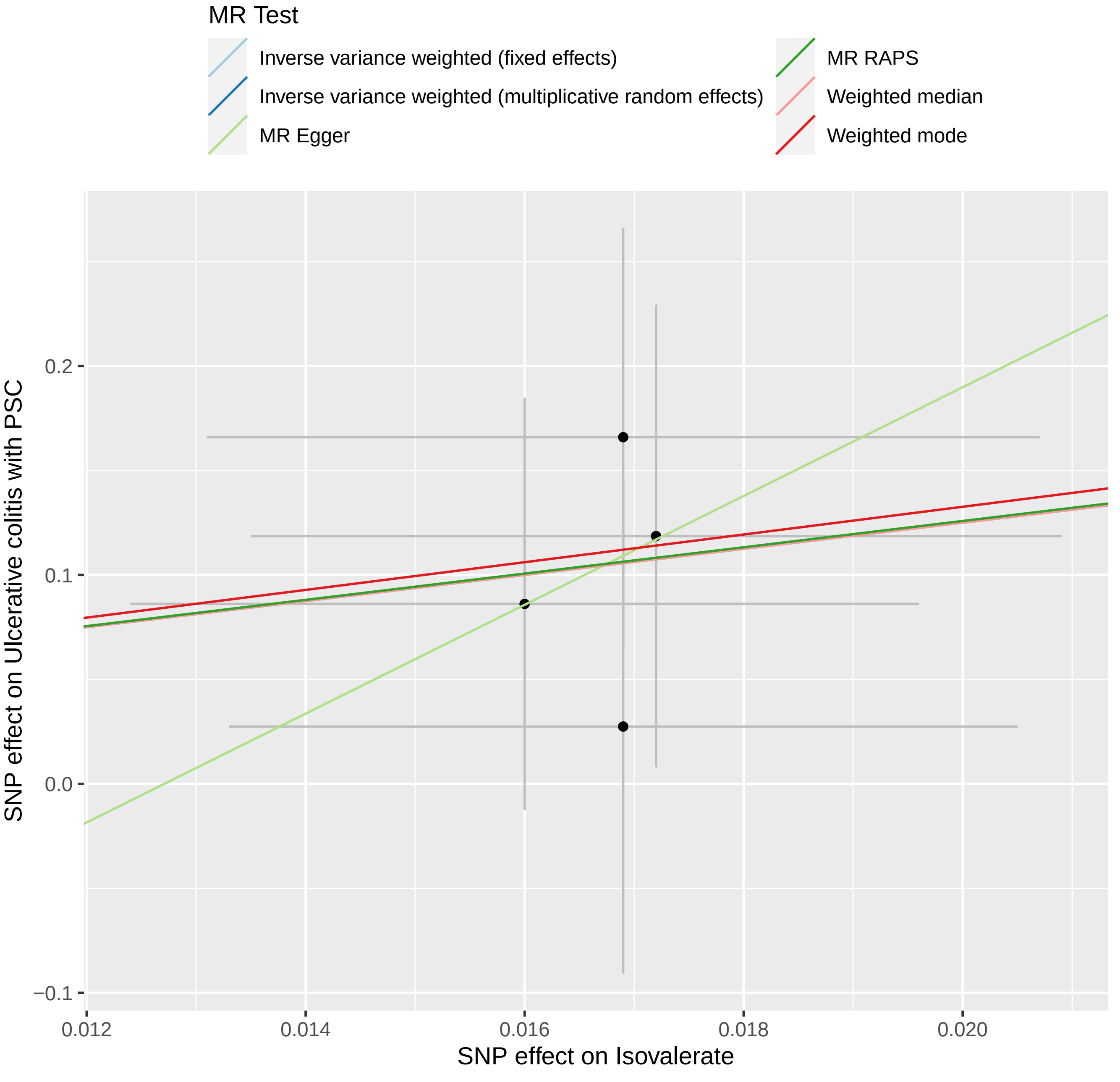

Batch 1321 : X−13183−−stearamide on Ulcerative colitis with PSC

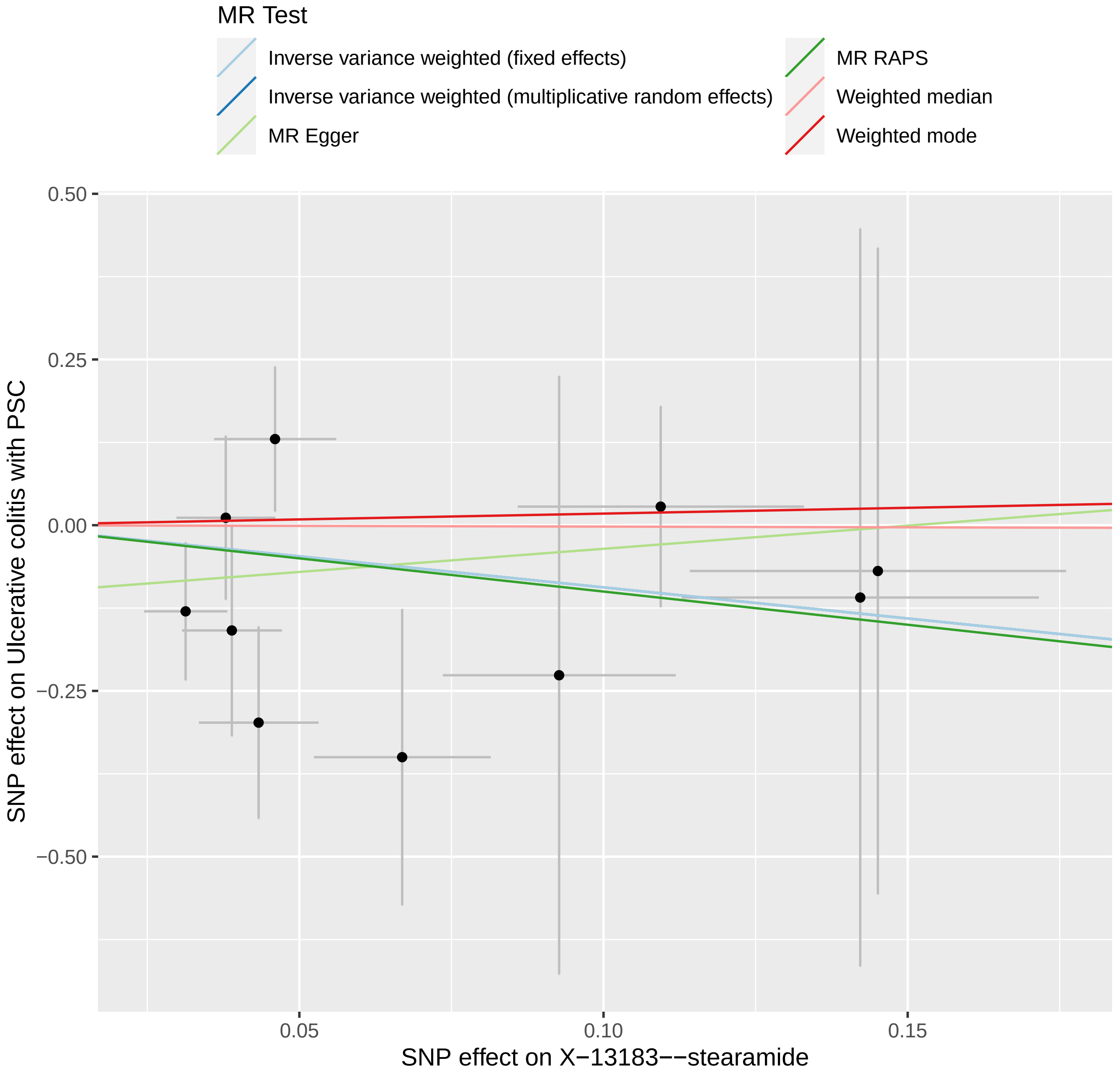

Batch 1322 : 7−methylguanine on Ulcerative colitis with PSC

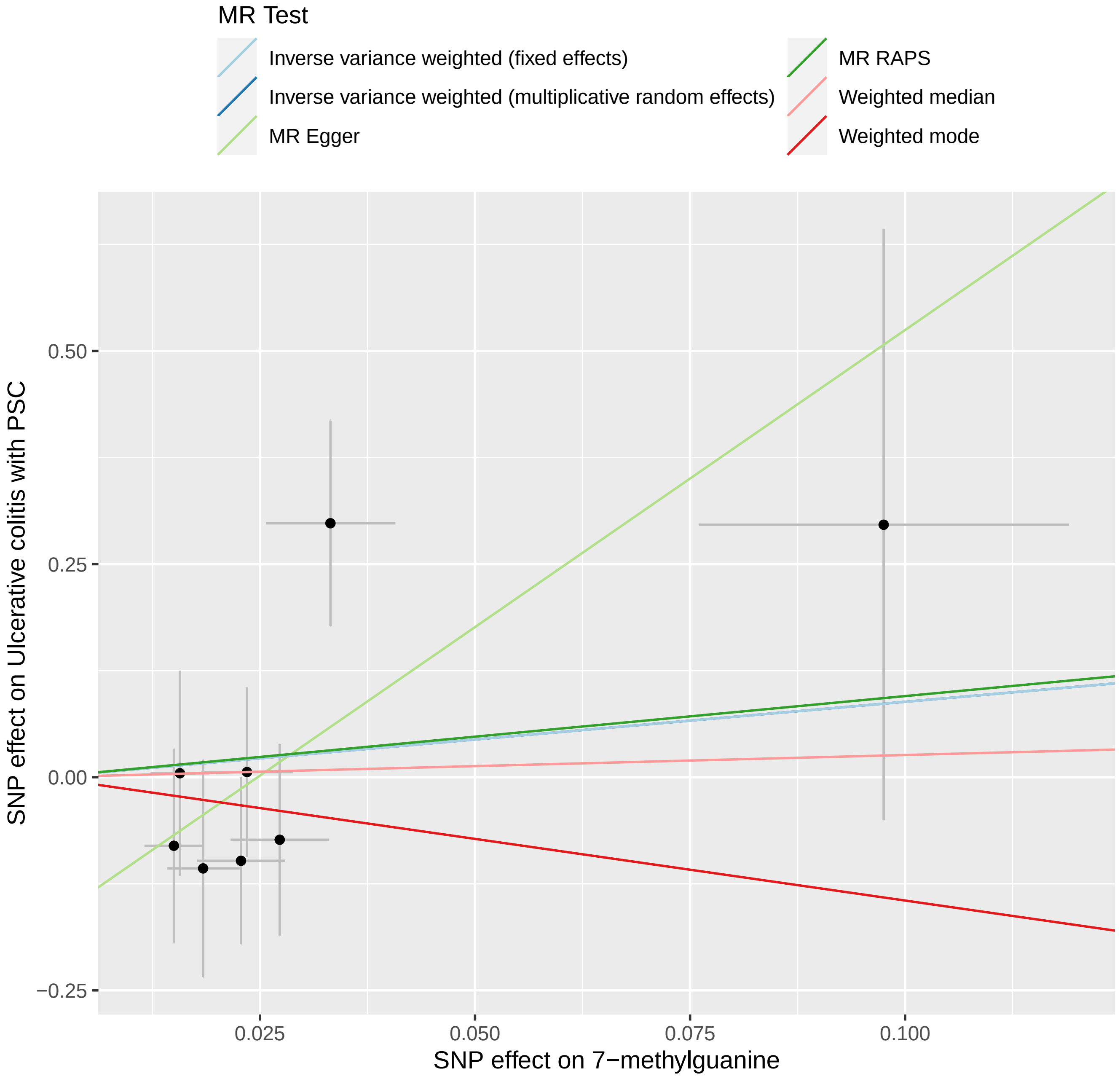

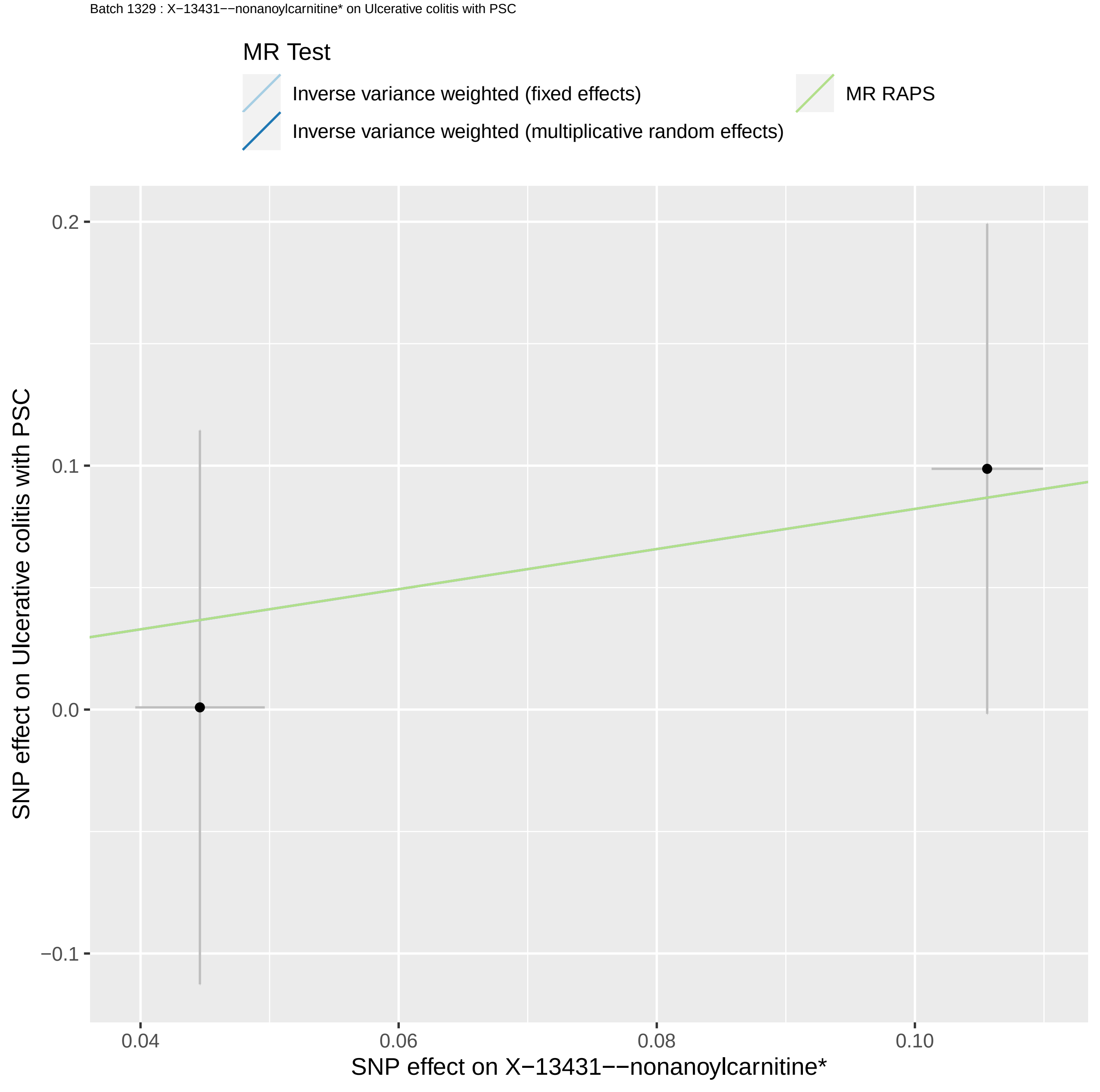

Batch 1329 : X−13431−−nonanoylcarnitine* on Ulcerative colitis with PSC
MR Test
Inverse variance weighted (fixed effects)
Inverse variance weighted (multiplicative random effects)
MR RAPS
SNP effect on Ulcerative colitis with PSC
0.2
0.1
0.0
−0.1
0.04
0.06
0.08
0.10
SNP effect on X−13431−−nonanoylcarnitine*

Batch 1330 : 2−palmitoylglycerophosphocholine* on Ulcerative colitis with PSC

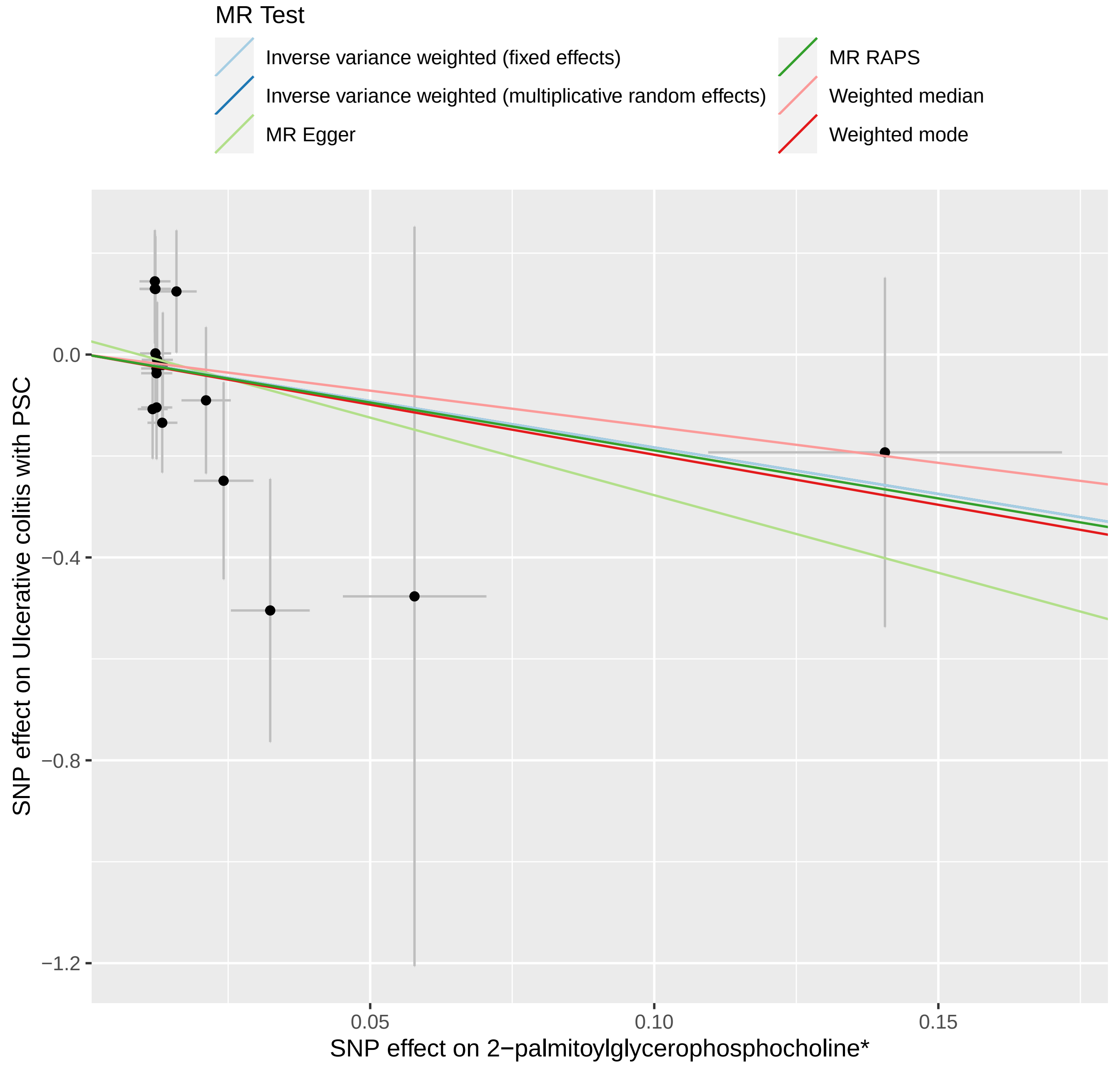

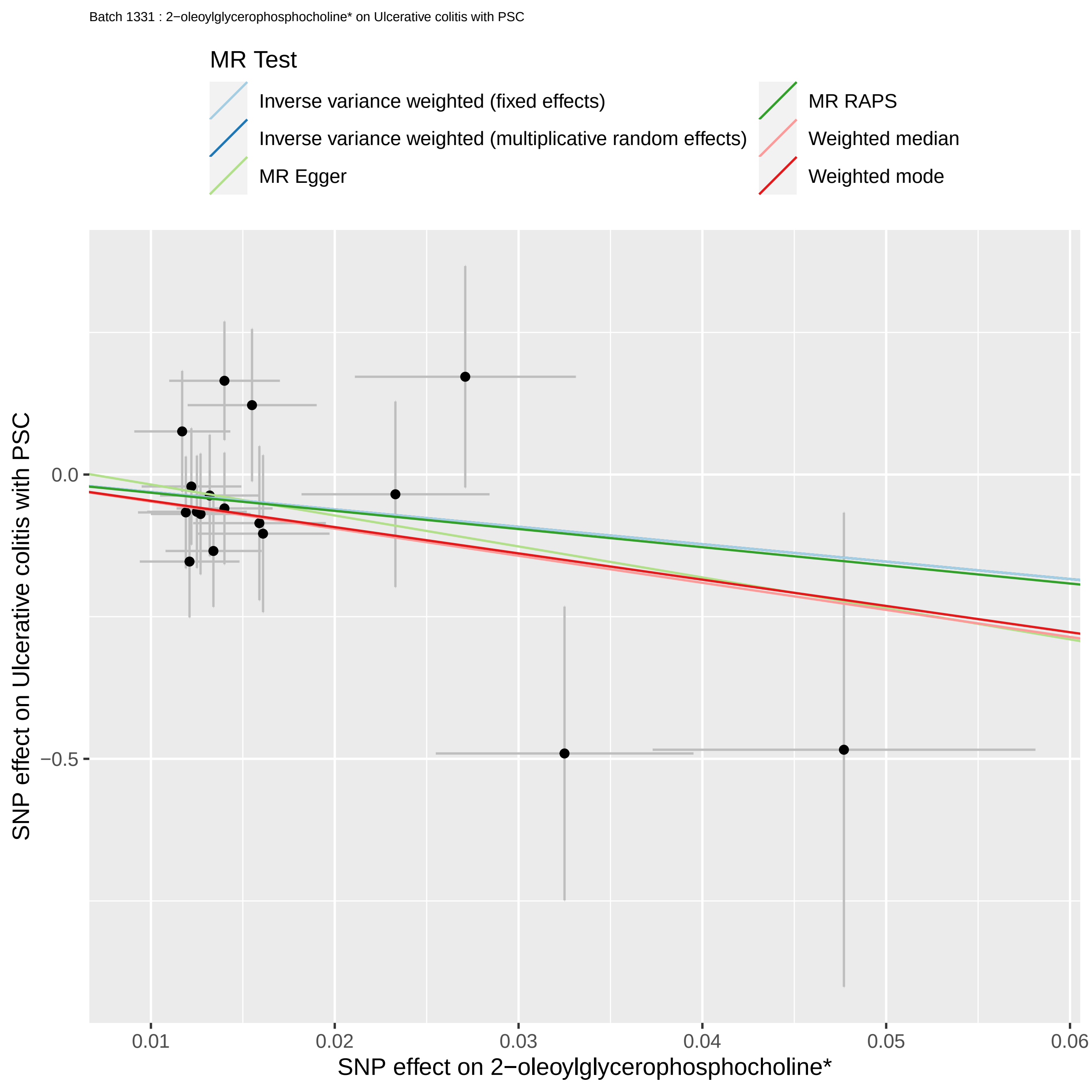

Batch 1331 : 2−oleoylglycerophosphocholine* on Ulcerative colitis with PSC
MR Test
Inverse variance weighted (fixed effects)
Inverse variance weighted (multiplicative random effects)
MR Egger
MR RAPS
Weighted median
Weighted mode
0.0
−0.5
0.01
0.02
0.03
0.04
0.05
0.06
SNP effect on 2−oleoylglycerophosphocholine*
SNP effect on Ulcerative colitis with PSC

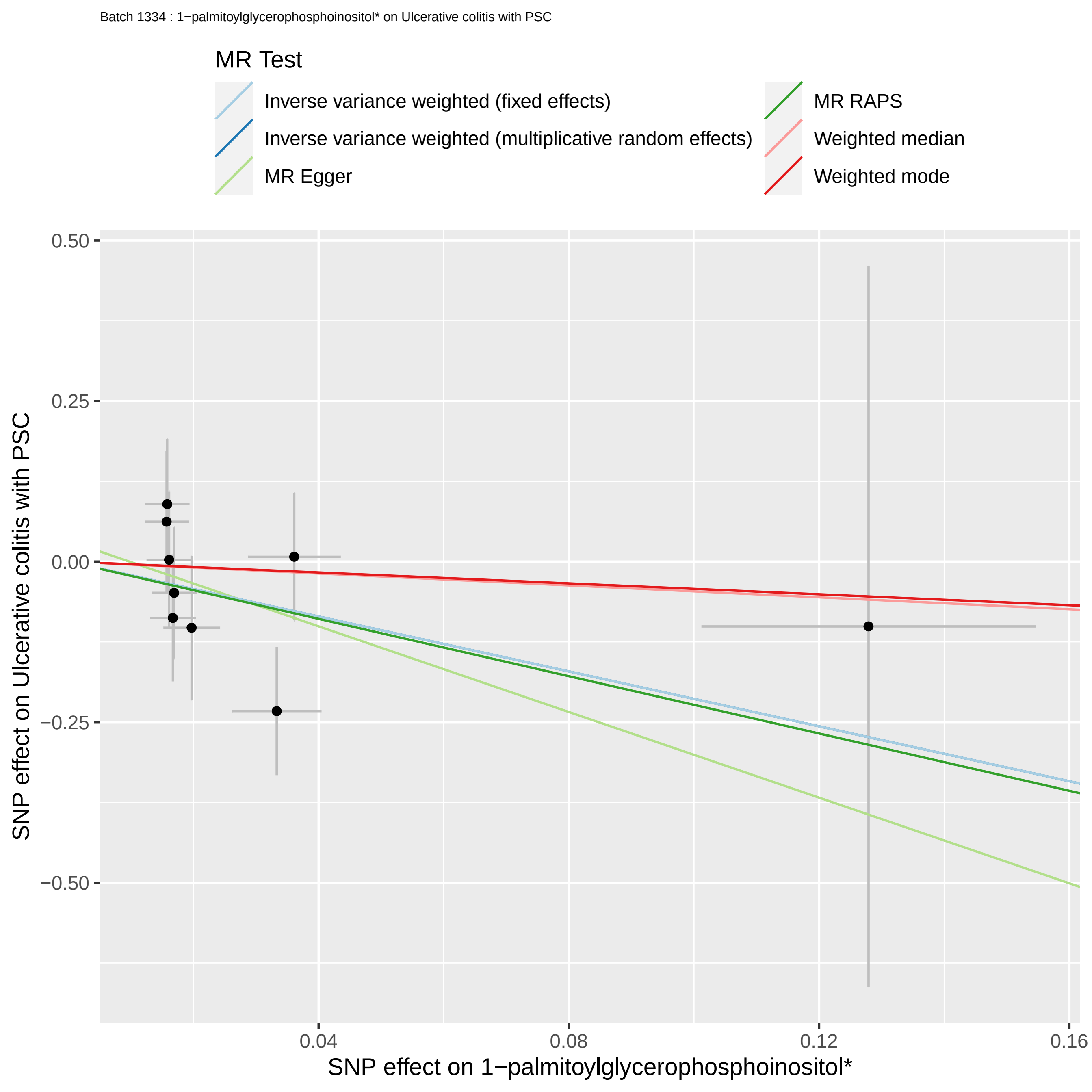
Batch 1334 : 1−palmitoylglycerophosphoinositol* on Ulcerative colitis with PSC
MR Test
Inverse variance weighted (fixed effects)
Inverse variance weighted (multiplicative random effects)
MR Egger
MR RAPS
Weighted median
Weighted mode
0.50
0.25
0.00
−0.25
−0.50
0.04
0.08
0.12
0.16
SNP effect on Ulcerative colitis with PSC
SNP effect on 1−palmitoylglycerophosphoinositol*

Batch 1336 : Hydroxyisovaleroyl carnitine on Ulcerative colitis with PSC

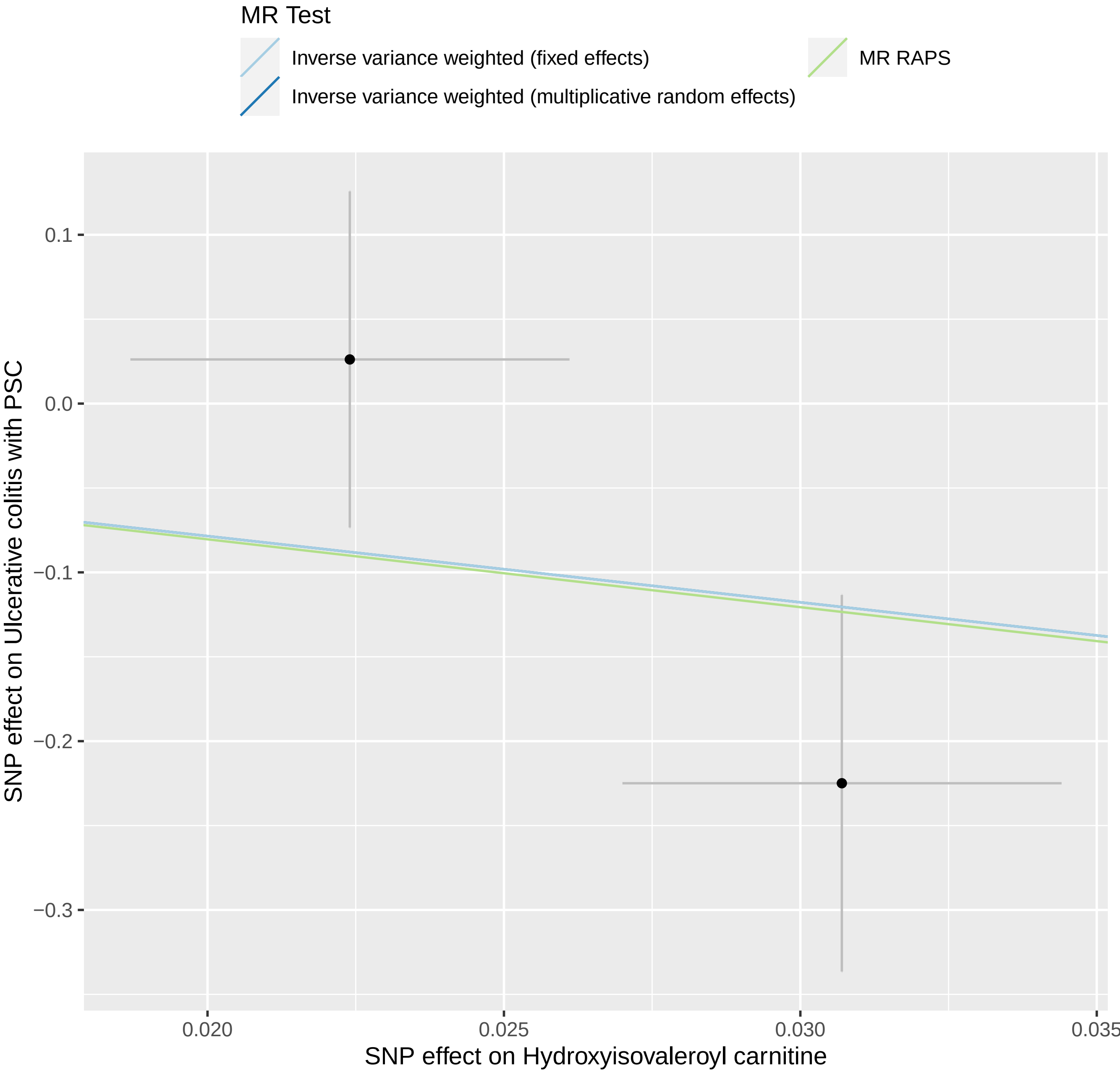

Batch 1337 : Glutaroyl carnitine on Ulcerative colitis with PSC

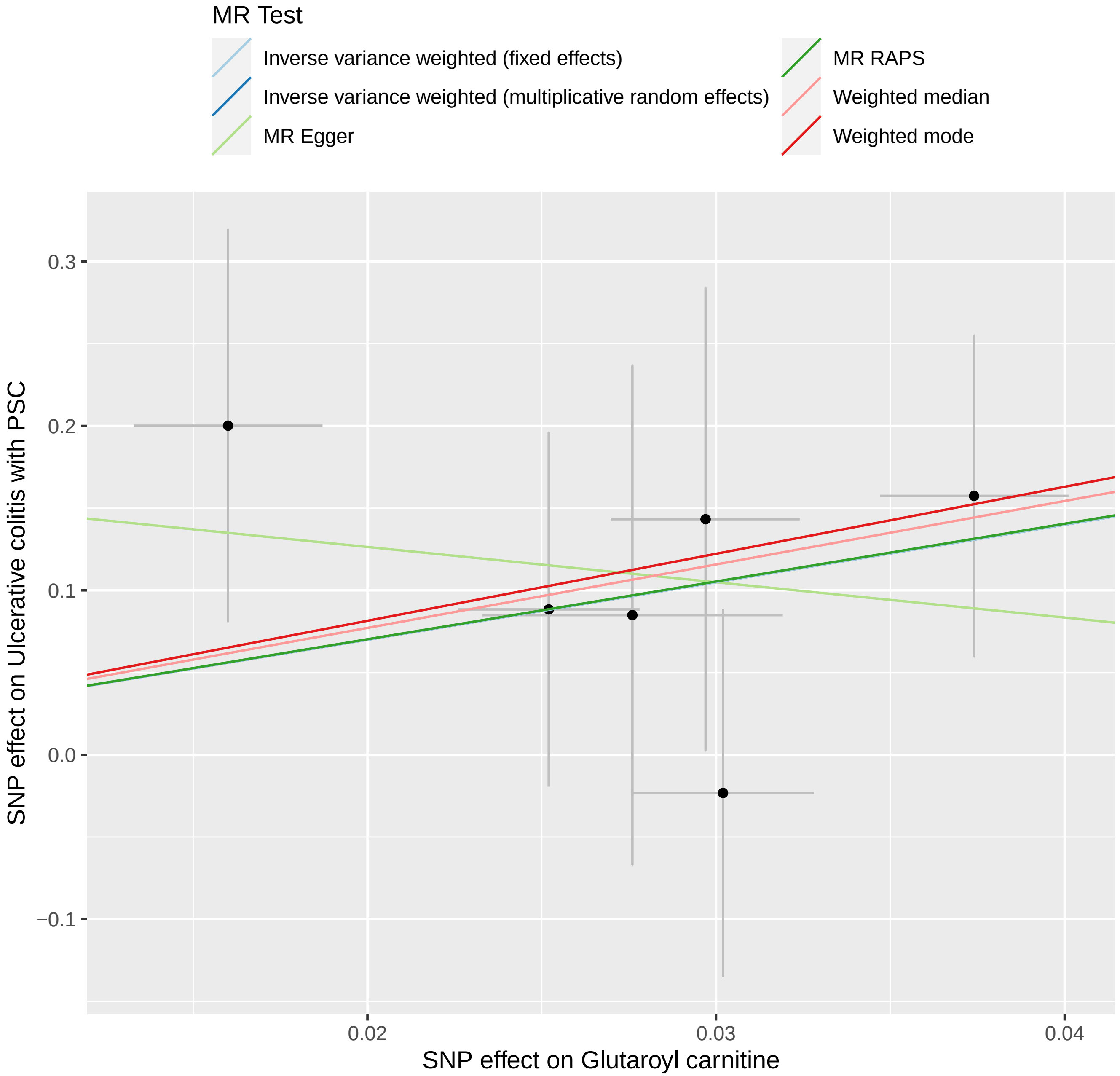

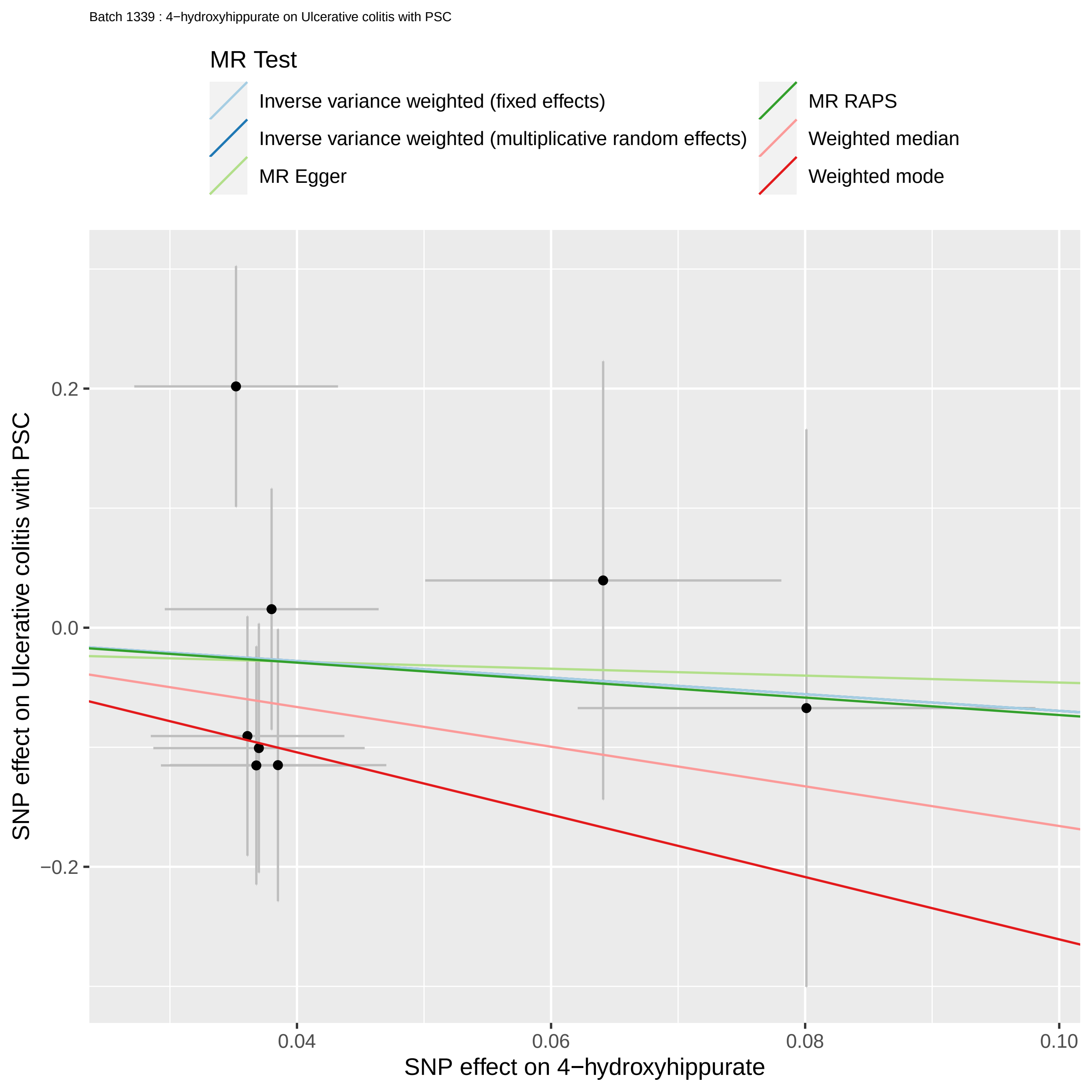
Batch 1339 : 4−hydroxyhippurate on Ulcerative colitis with PSC
MR Test
Inverse variance weighted (fixed effects)
Inverse variance weighted (multiplicative random effects)
MR Egger
MR RAPS
Weighted median
Weighted mode
SNP effect on Ulcerative colitis with PSC
0.2
0.0
−0.2
0.04
0.06
0.08
0.10
SNP effect on 4−hydroxyhippurate

Batch 1340 : 1−myristoylglycerophosphocholine on Ulcerative colitis with PSC

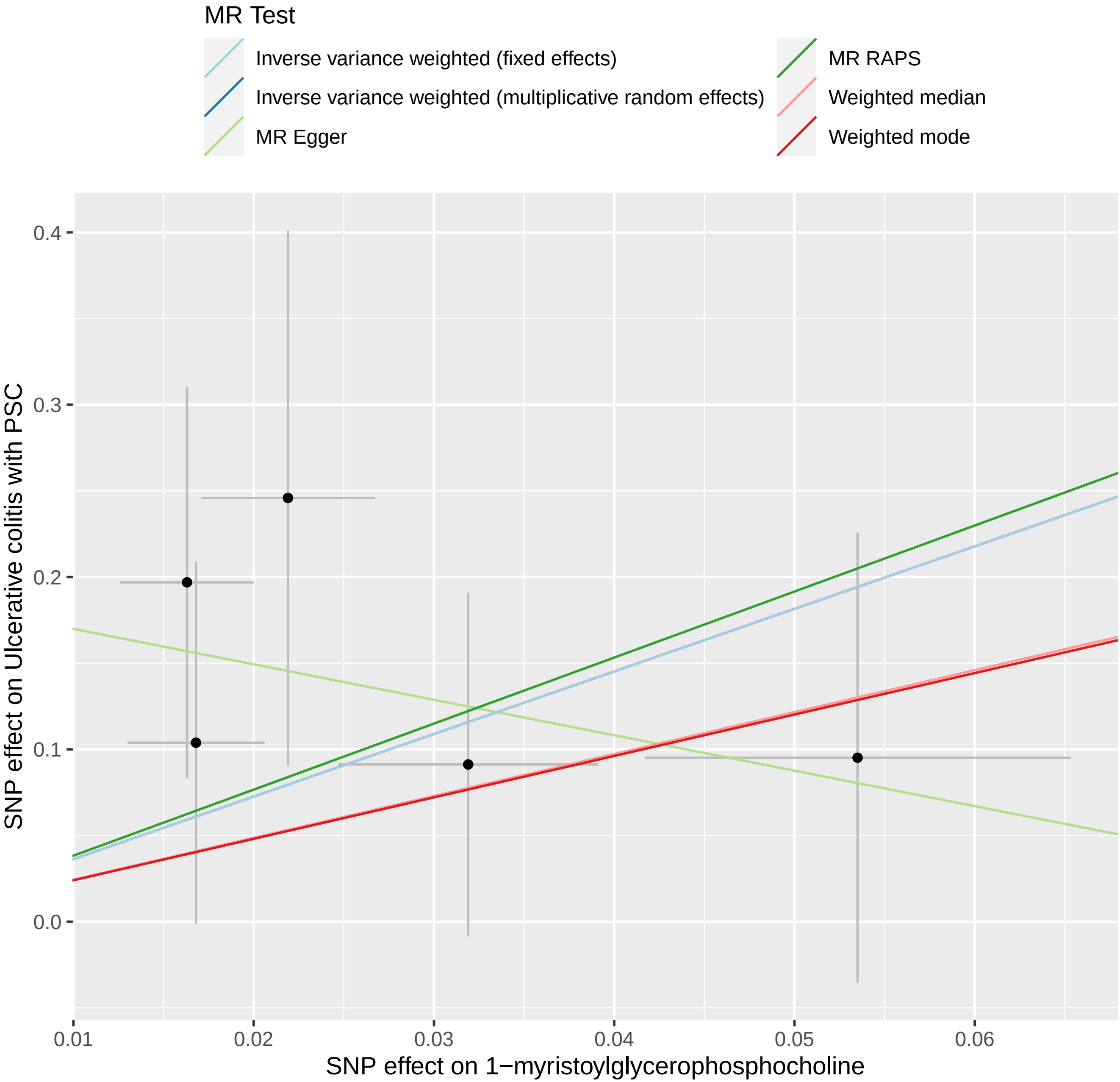

Batch 1343 : 3−(3−hydroxyphenyl)propionate on Ulcerative colitis with PSC

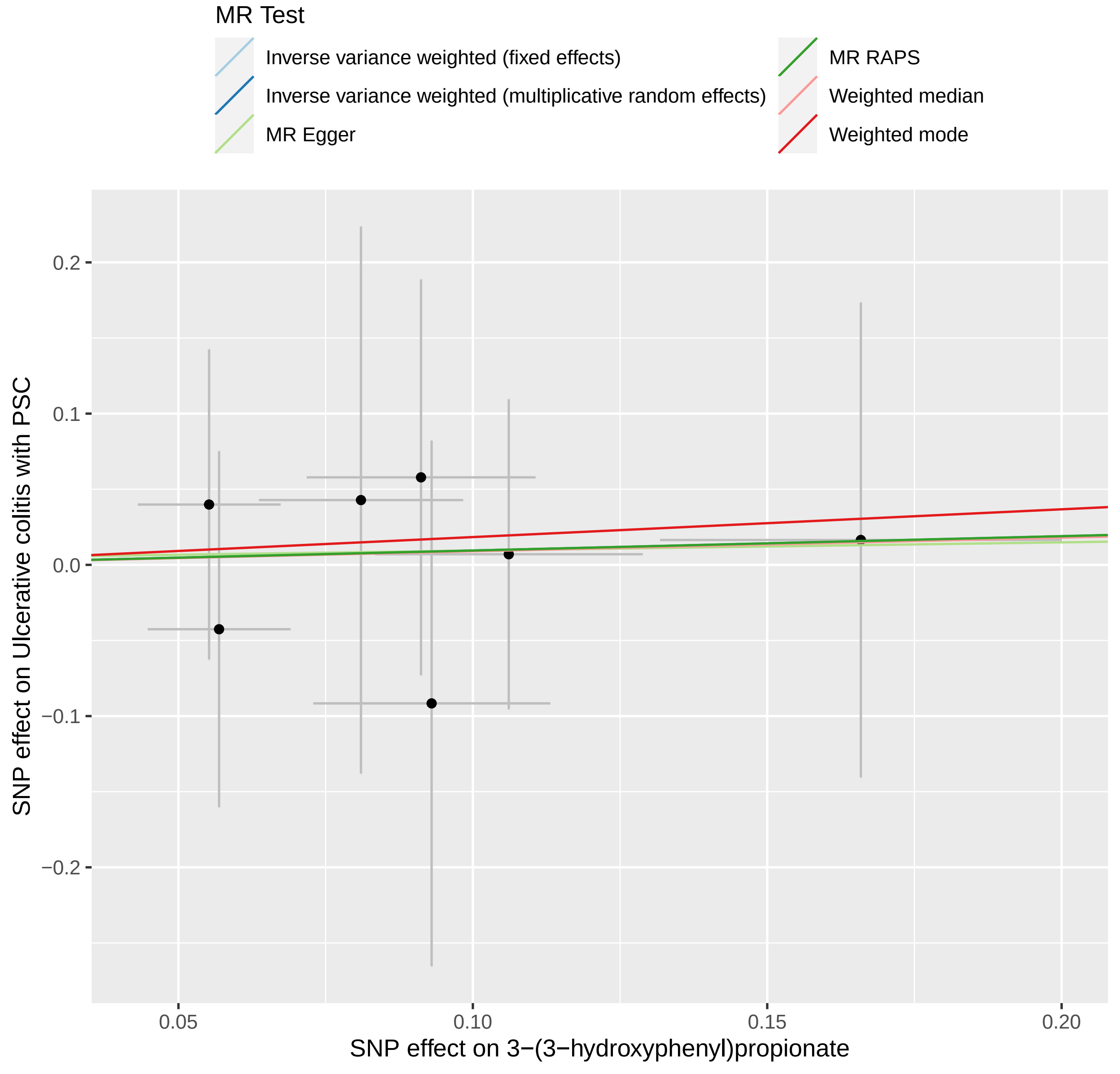

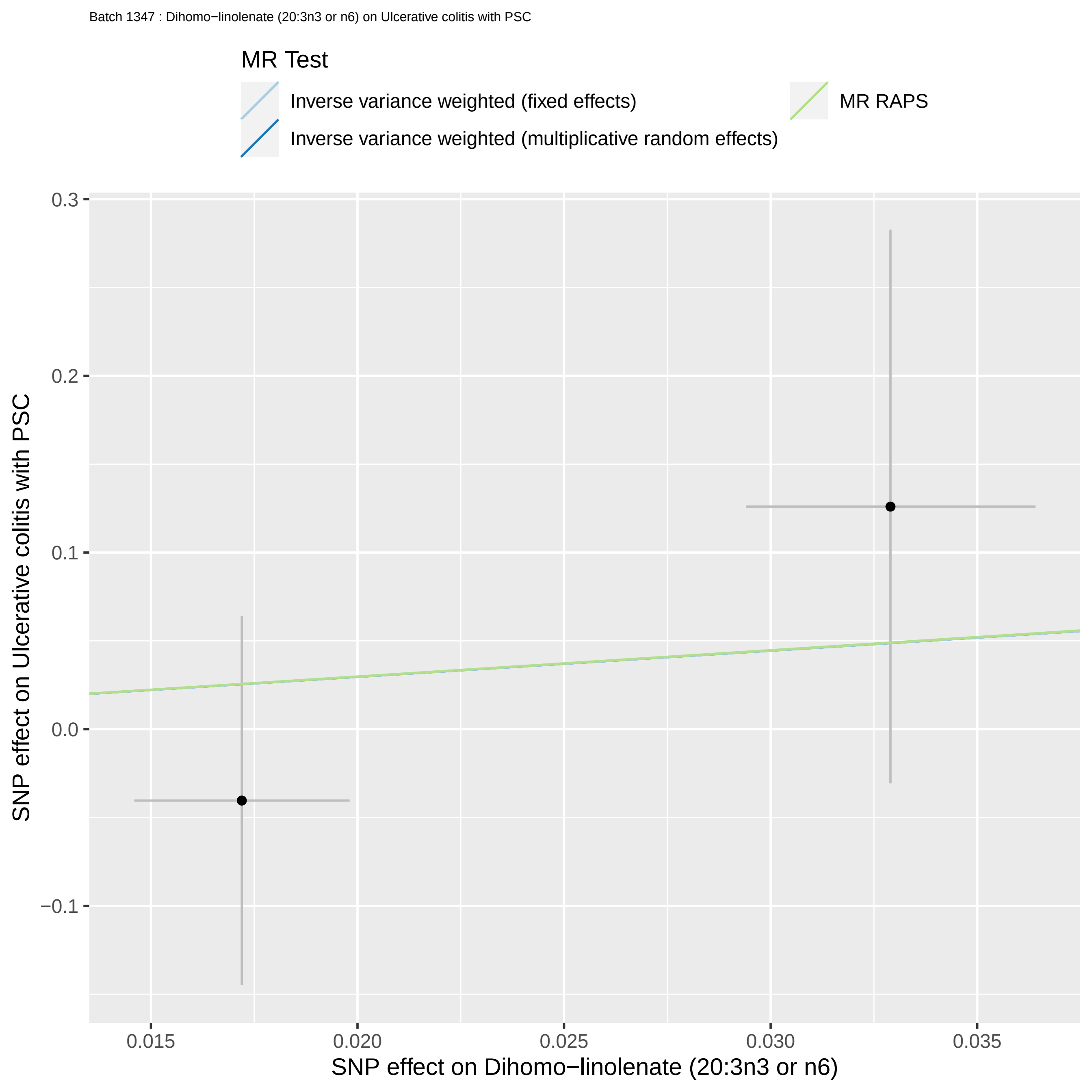
Batch 1347 : Dihomo−linolenate (20:3n3 or n6) on Ulcerative colitis with PSC
MR Test
Inverse variance weighted (fixed effects)
Inverse variance weighted (multiplicative random effects)
MR RAPS
SNP effect on Ulcerative colitis with PSC
0.3
0.2
0.1
0.0
−0.1
0.015
0.020
0.025
0.030
0.035
SNP effect on Dihomo−linolenate (20:3n3 or n6)

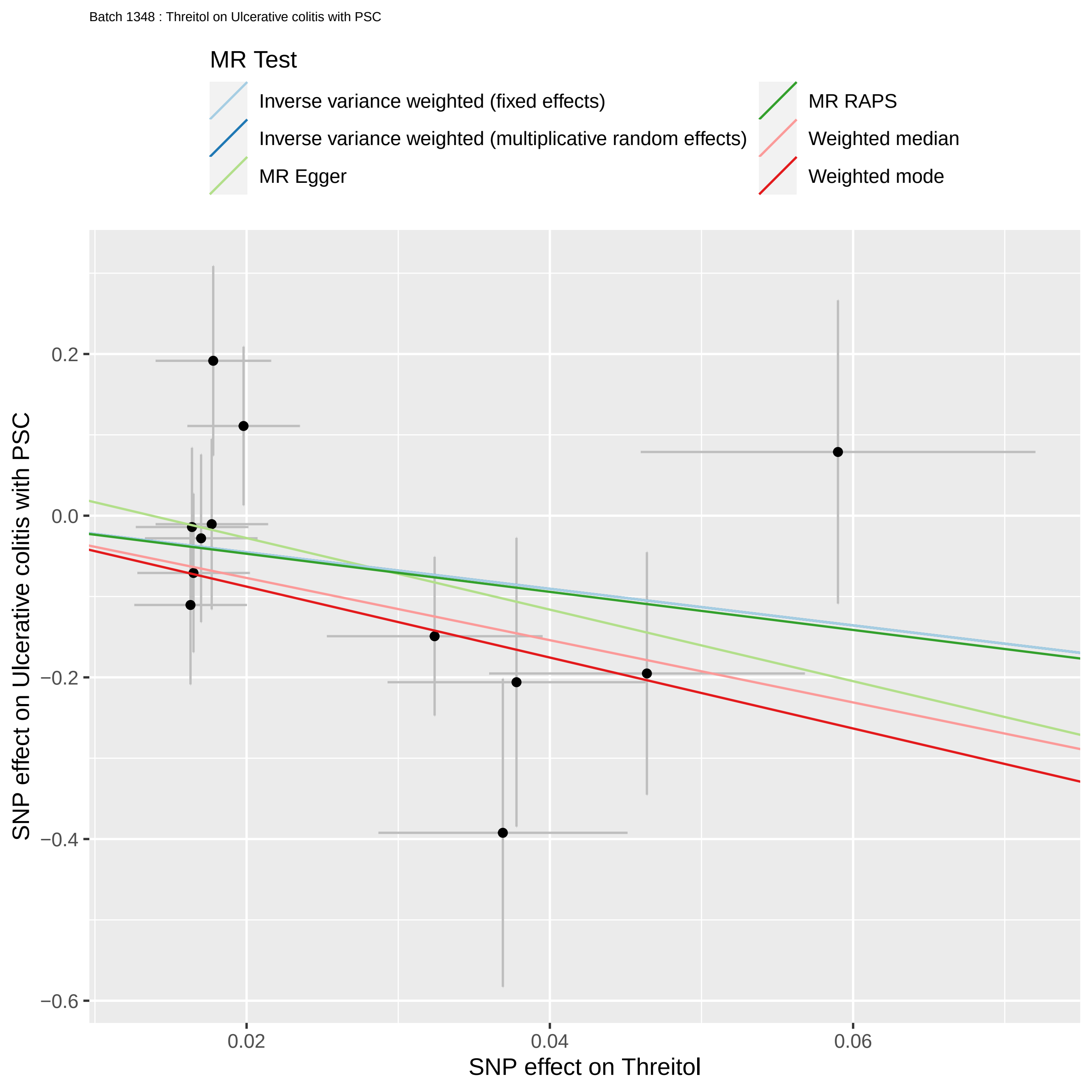

Batch 1348 : Threitol on Ulcerative colitis with PSC
MR Test
Inverse variance weighted (fixed effects)
Inverse variance weighted (multiplicative random effects)
MR Egger
MR RAPS
Weighted median
Weighted mode
0.2
0.0
−0.2
−0.4
−0.6
0.02
0.04
0.06
SNP effect on Ulcerative colitis with PSC
SNP effect on Threitol

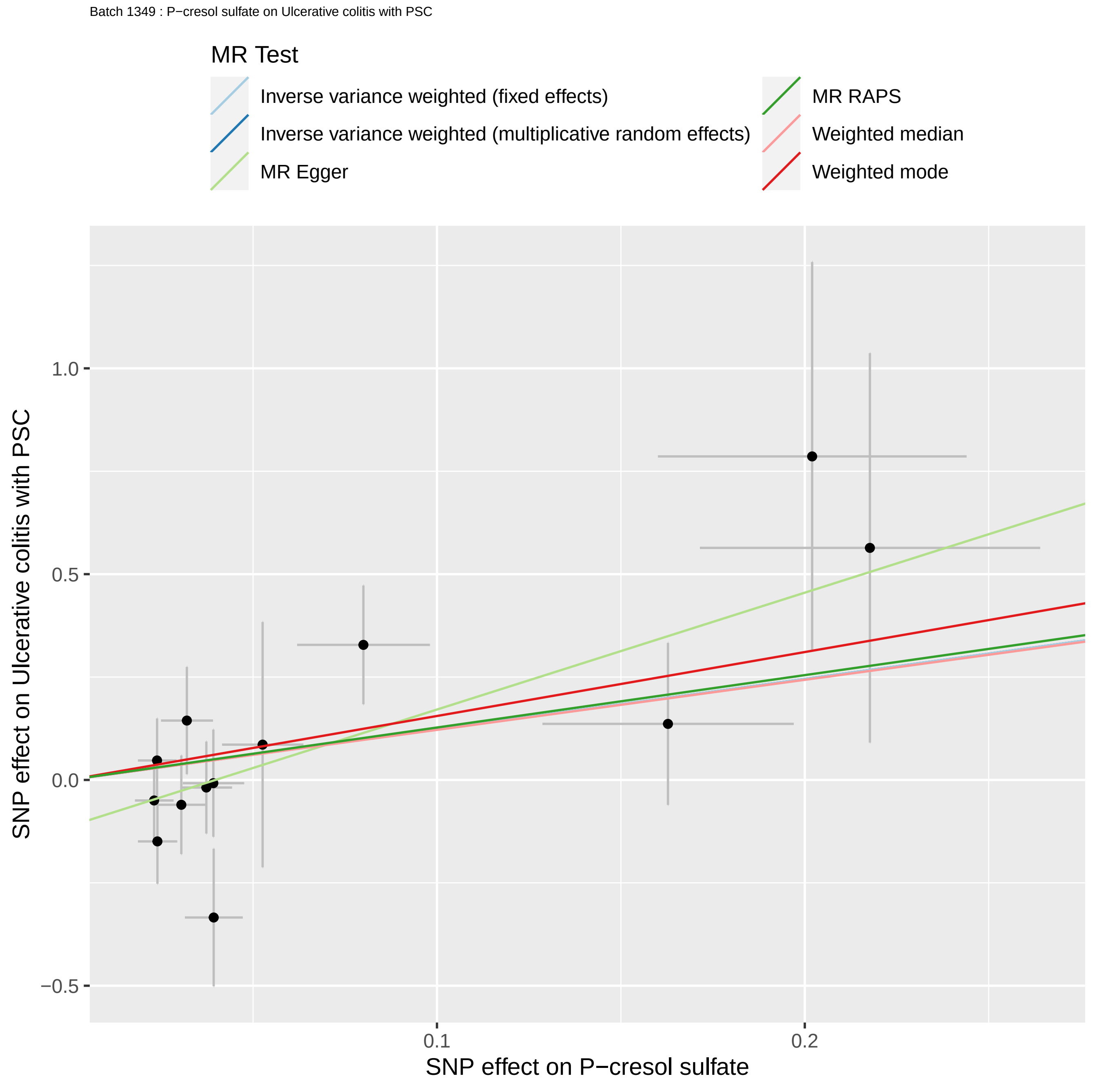
Batch 1349 : P−cresol sulfate on Ulcerative colitis with PSC
MR Test
Inverse variance weighted (fixed effects)
Inverse variance weighted (multiplicative random effects)
MR Egger
MR RAPS
Weighted median
Weighted mode
SNP effect on Ulcerative colitis with PSC
1.0
0.5
0.0
−0.5
0.1
0.2
SNP effect on P−cresol sulfate

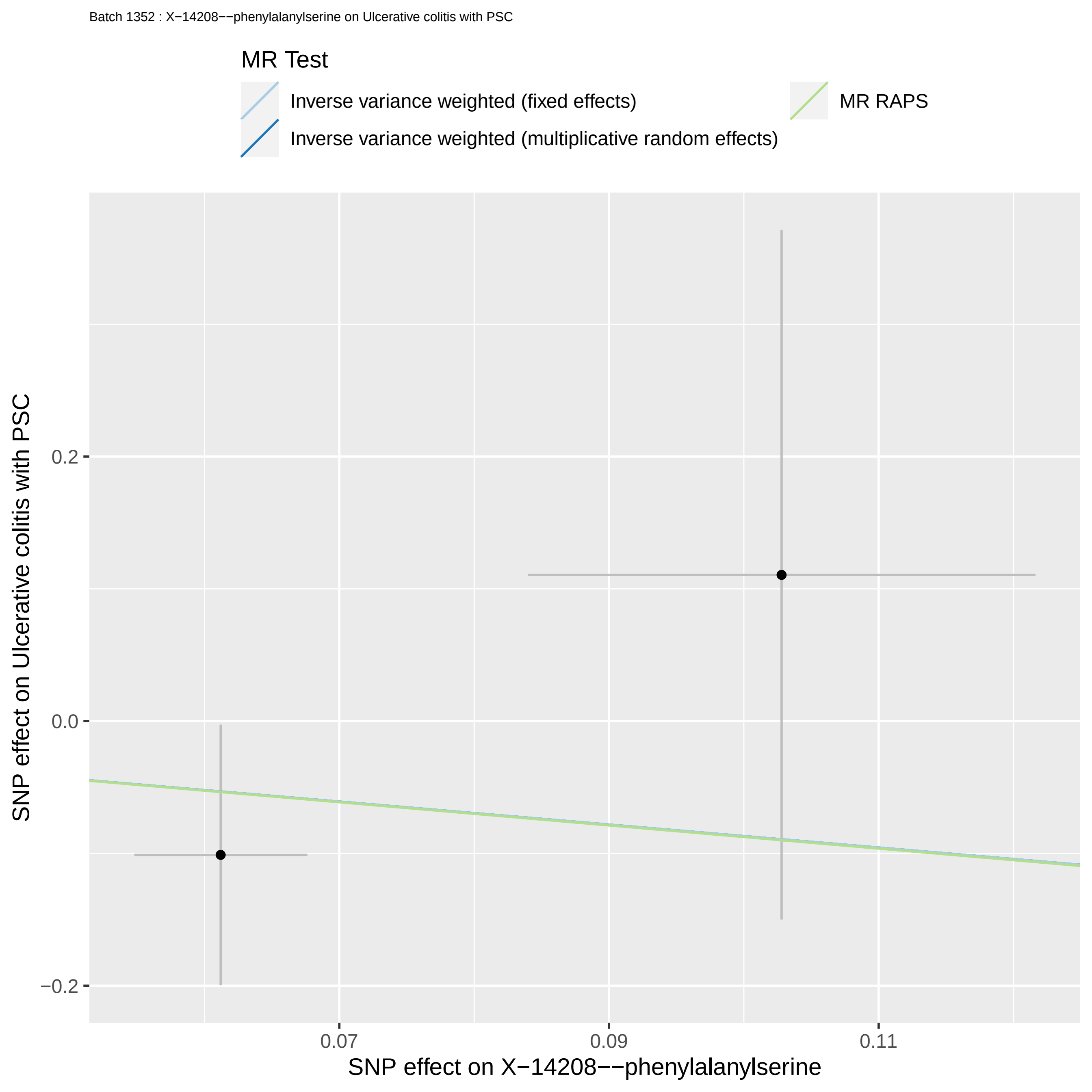
Batch 1352 : X−14208−−phenylalanylserine on Ulcerative colitis with PSC
MR Test
Inverse variance weighted (fixed effects)
Inverse variance weighted (multiplicative random effects)
MR RAPS
SNP effect on Ulcerative colitis with PSC
0.2
0.0
−0.2
0.07
0.09
0.11
SNP effect on X−14208−−phenylalanylserine

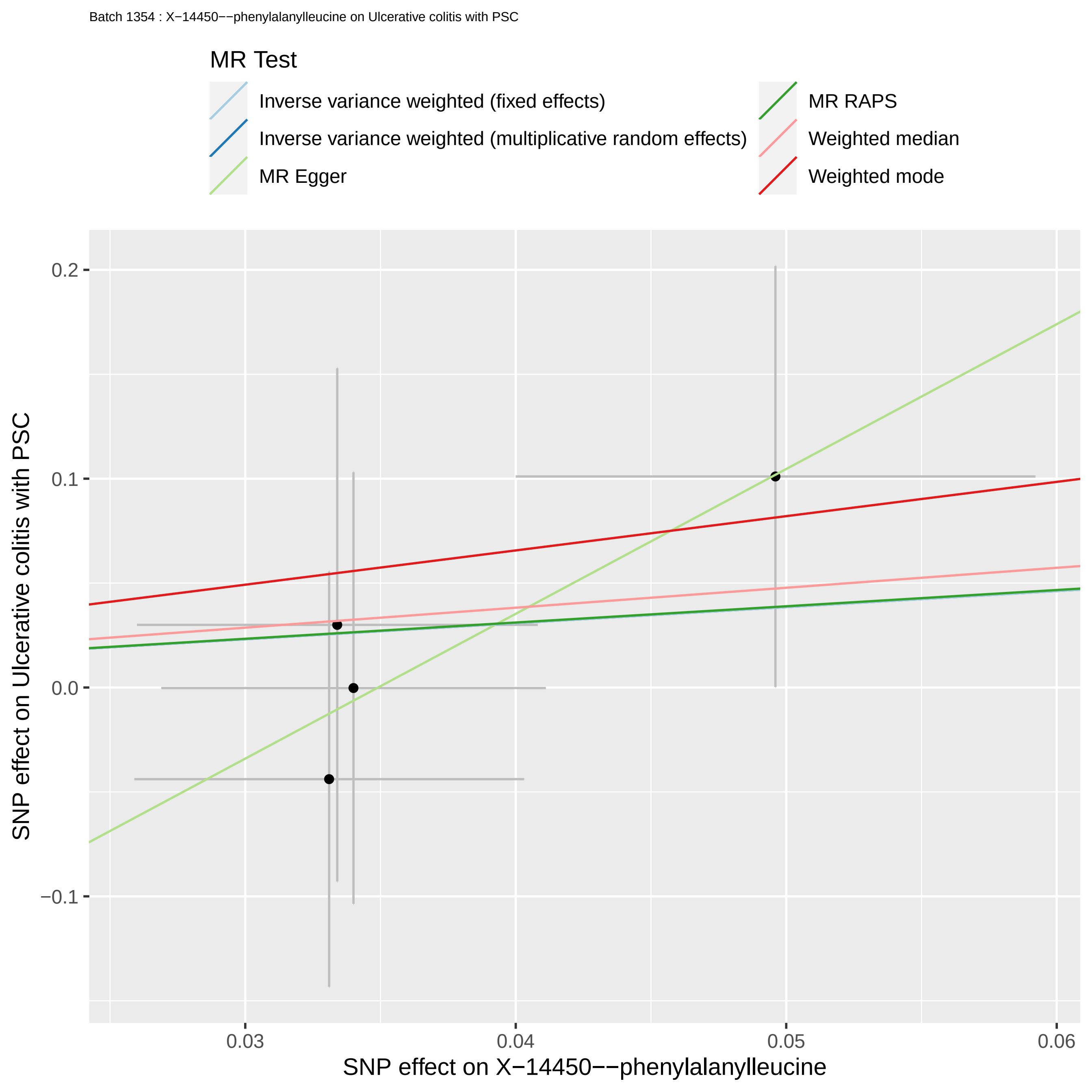
Batch 1354 : X−14450−−phenylalanylleucine on Ulcerative colitis with PSC
MR Test
Inverse variance weighted (fixed effects)
Inverse variance weighted (multiplicative random effects)
MR Egger
MR RAPS
Weighted median
Weighted mode
0.2
0.1
0.0
−0.1
0.03
0.04
0.05
0.06
SNP effect on Ulcerative colitis with PSC
SNP effect on X−14450−−phenylalanylleucine

Batch 1355 : Gamma−glutamylglutamate on Ulcerative colitis with PSC

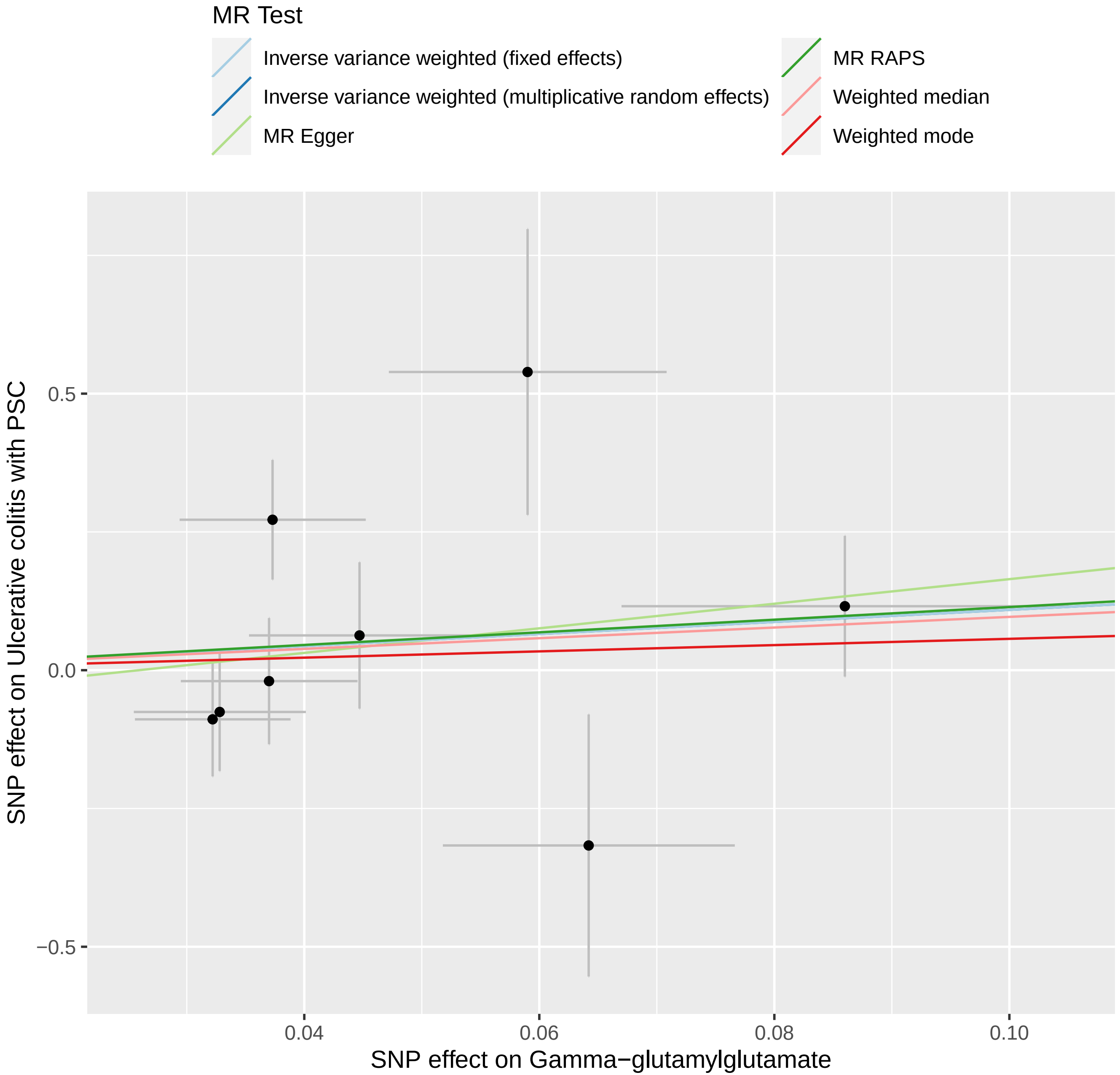

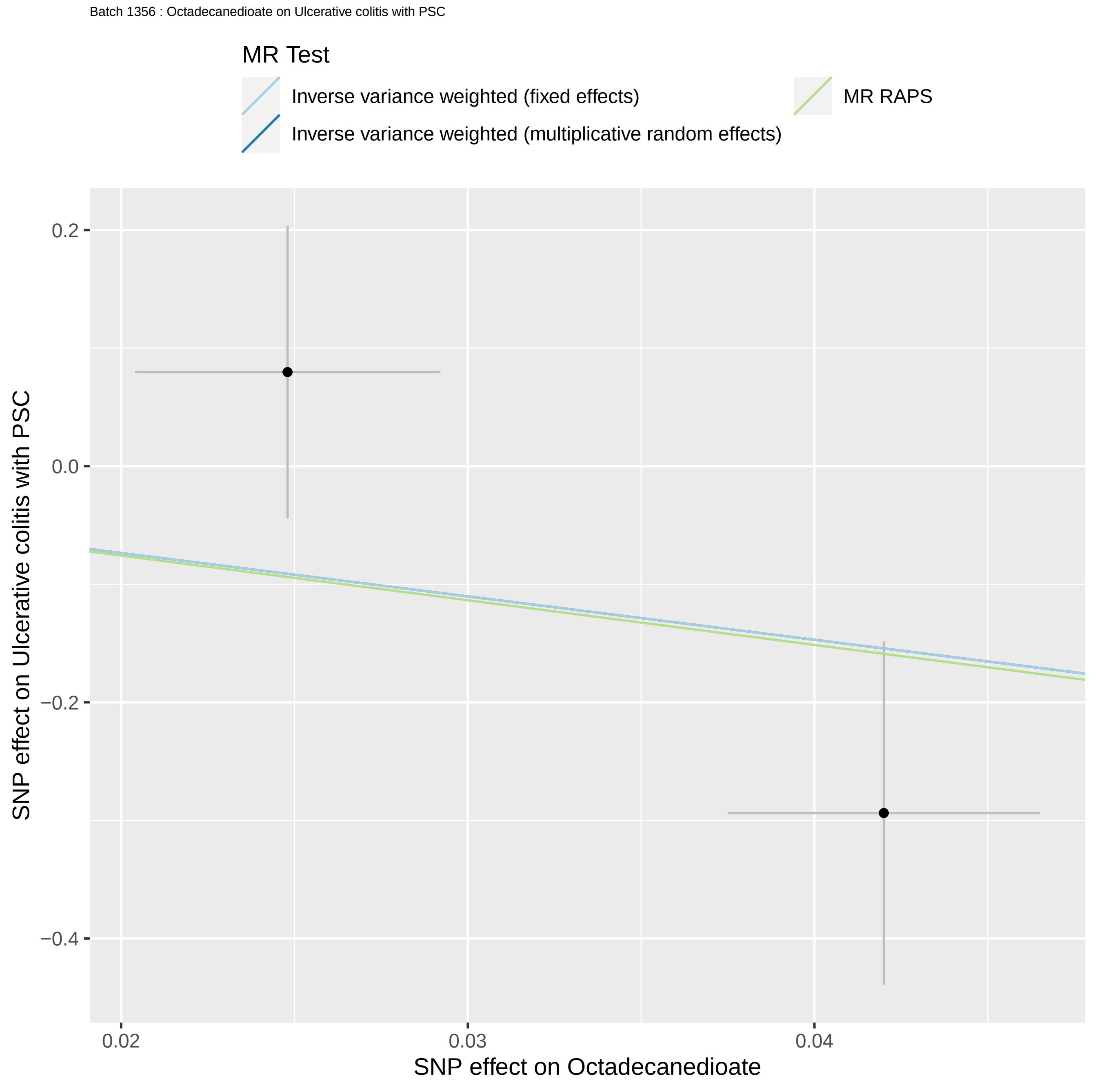
Batch 1356 : Octadecanedioate on Ulcerative colitis with PSC
MR Test
Inverse variance weighted (fixed effects)
Inverse variance weighted (multiplicative random effects)
MR RAPS
0.2
0.0
−0.2
−0.4
SNP effect on Ulcerative colitis with PSC
0.02
0.03
0.04
SNP effect on Octadecanedioate

Batch 1357 : Leucylleucine on Ulcerative colitis with PSC

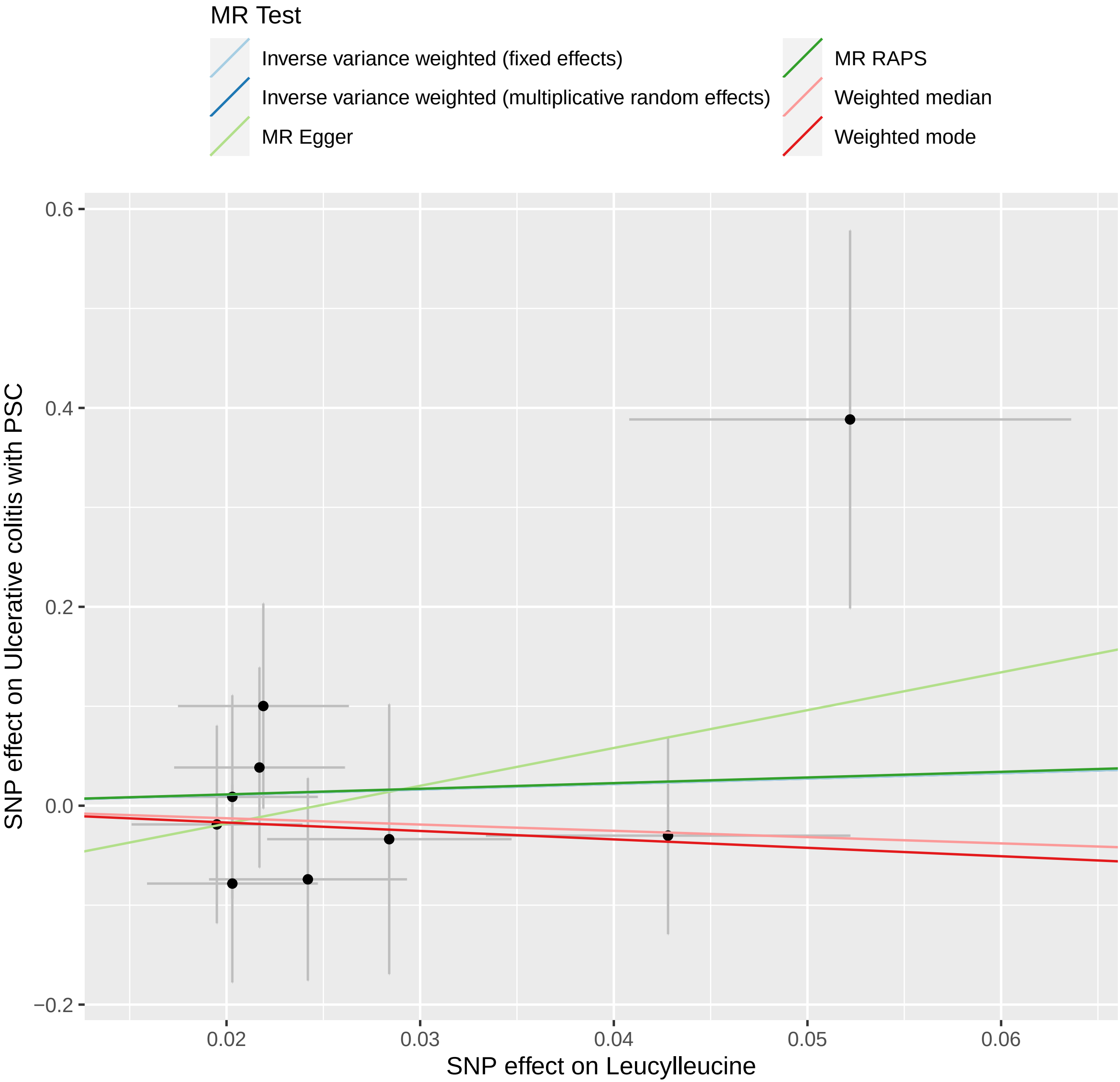

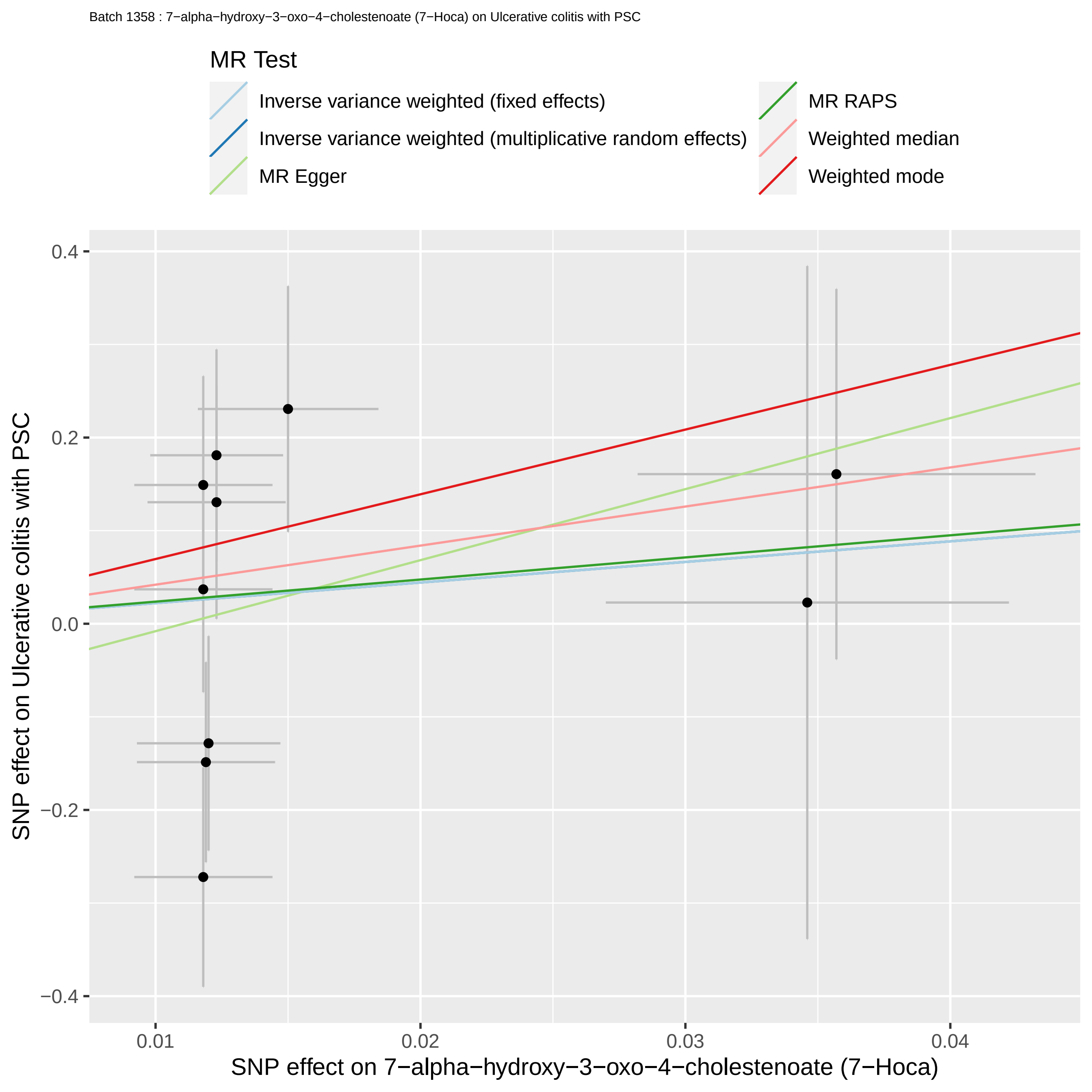

Batch 1358 : 7−alpha−hydroxy−3−oxo−4−cholestenoate (7−Hoca) on Ulcerative colitis with PSC
MR Test
Inverse variance weighted (fixed effects)
Inverse variance weighted (multiplicative random effects)
MR Egger
MR RAPS
Weighted median
Weighted mode
0.4
0.2
0.0
−0.2
−0.4
0.01
0.02
0.03
0.04
SNP effect on Ulcerative colitis with PSC
SNP effect on 7−alpha−hydroxy−3−oxo−4−cholestenoate (7−Hoca)

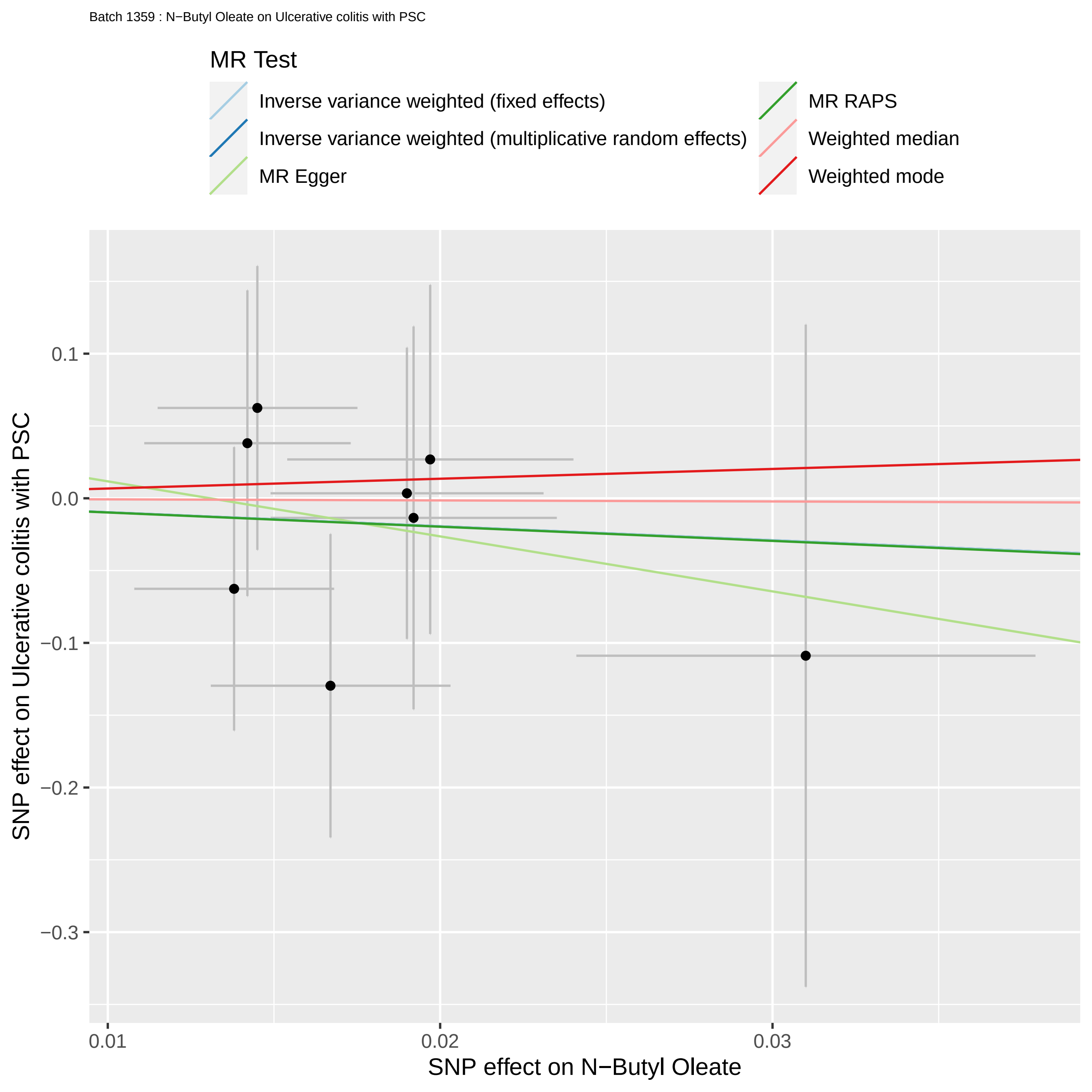

Batch 1359 : N−Butyl Oleate on Ulcerative colitis with PSC
MR Test
Inverse variance weighted (fixed effects)
Inverse variance weighted (multiplicative random effects)
MR Egger
MR RAPS
Weighted median
Weighted mode
0.1
0.0
−0.1
−0.2
−0.3
0.01
0.02
0.03
SNP effect on Ulcerative colitis with PSC
SNP effect on N−Butyl Oleate

Batch 1361 : Taurolithocholate 3−sulfate on Ulcerative colitis with PSC

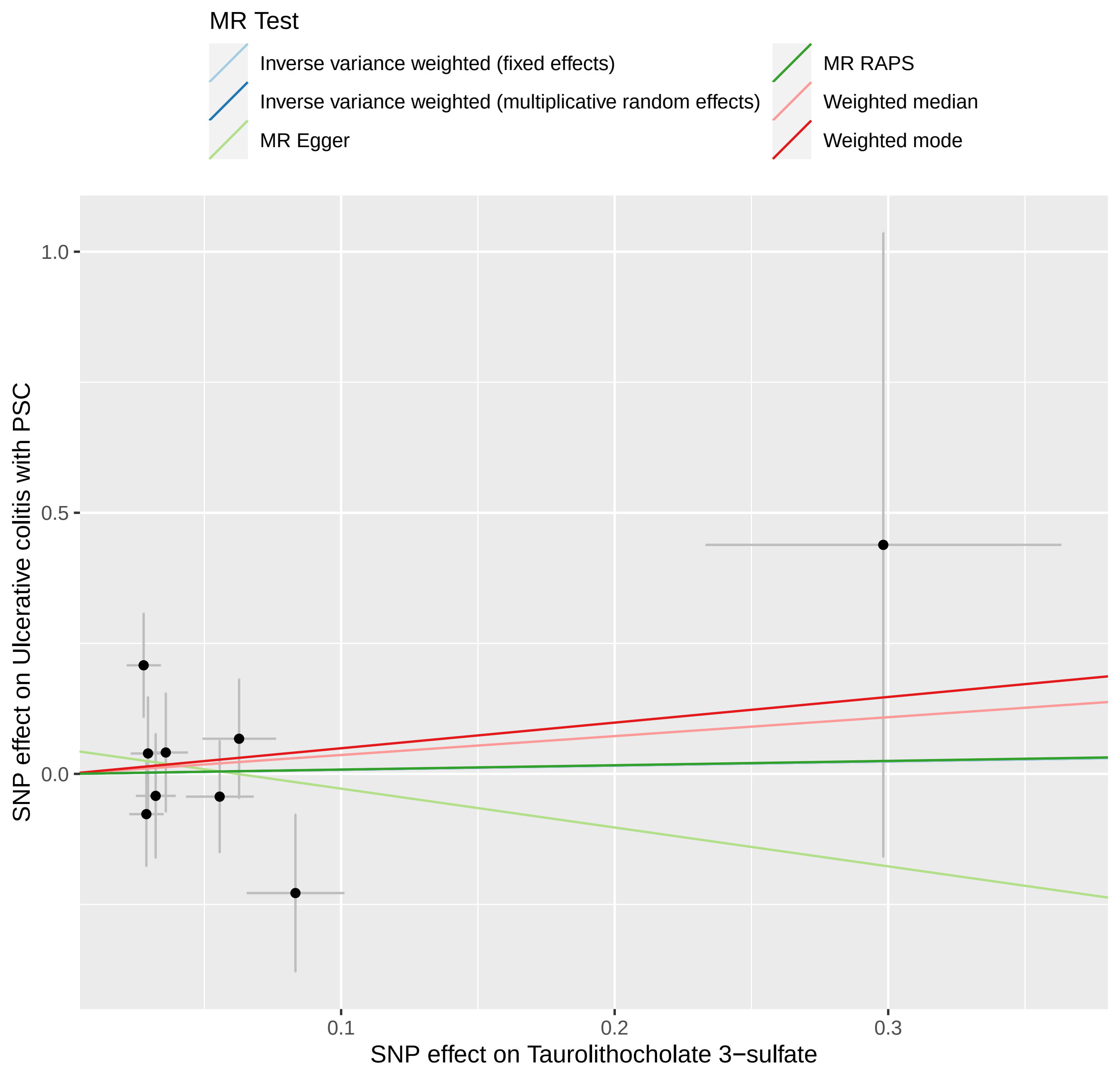

Batch 1362 : X−14977−−vanillin on Ulcerative colitis with PSC

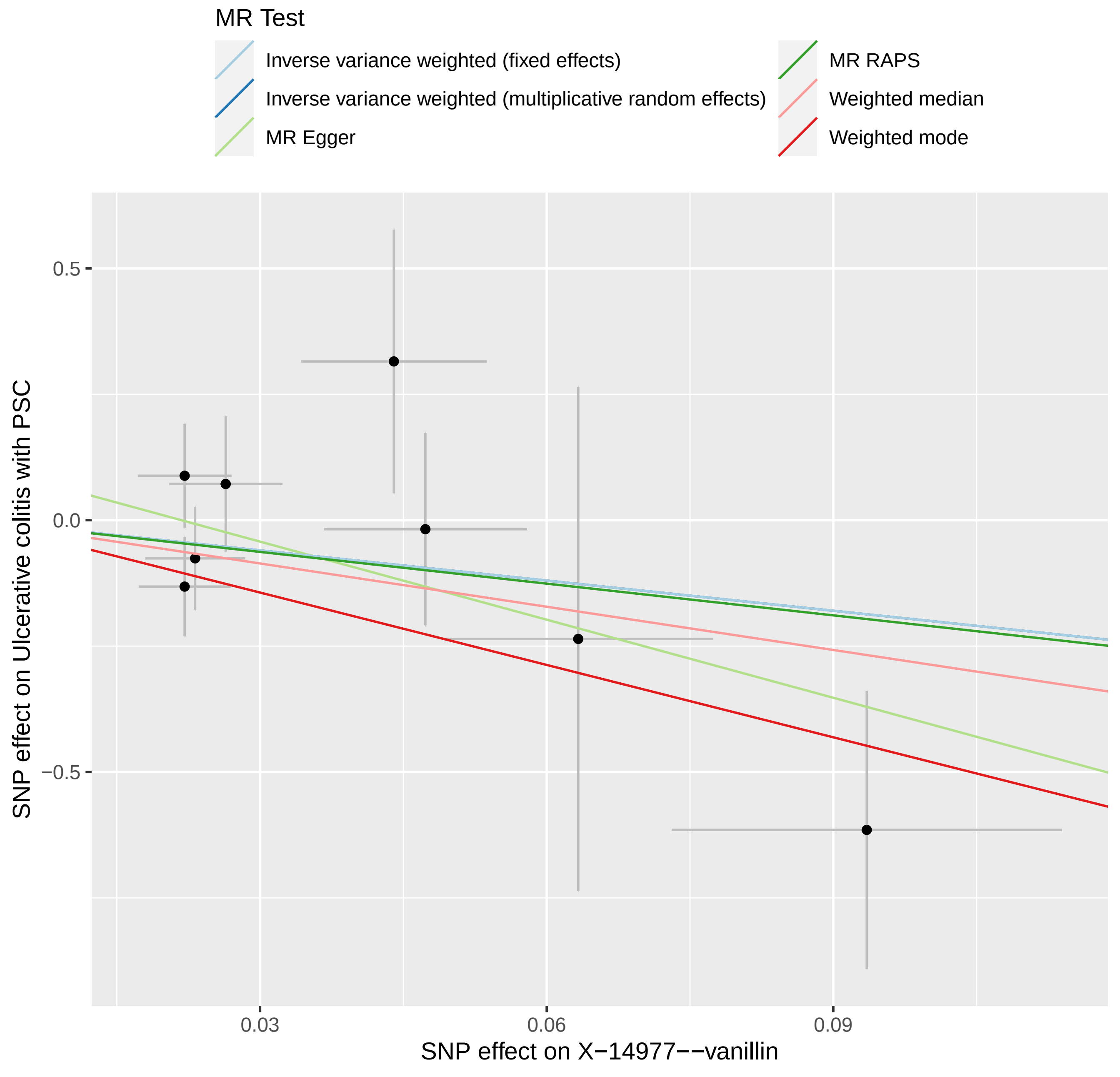

Batch 1363 : Succinylcarnitine on Ulcerative colitis with PSC

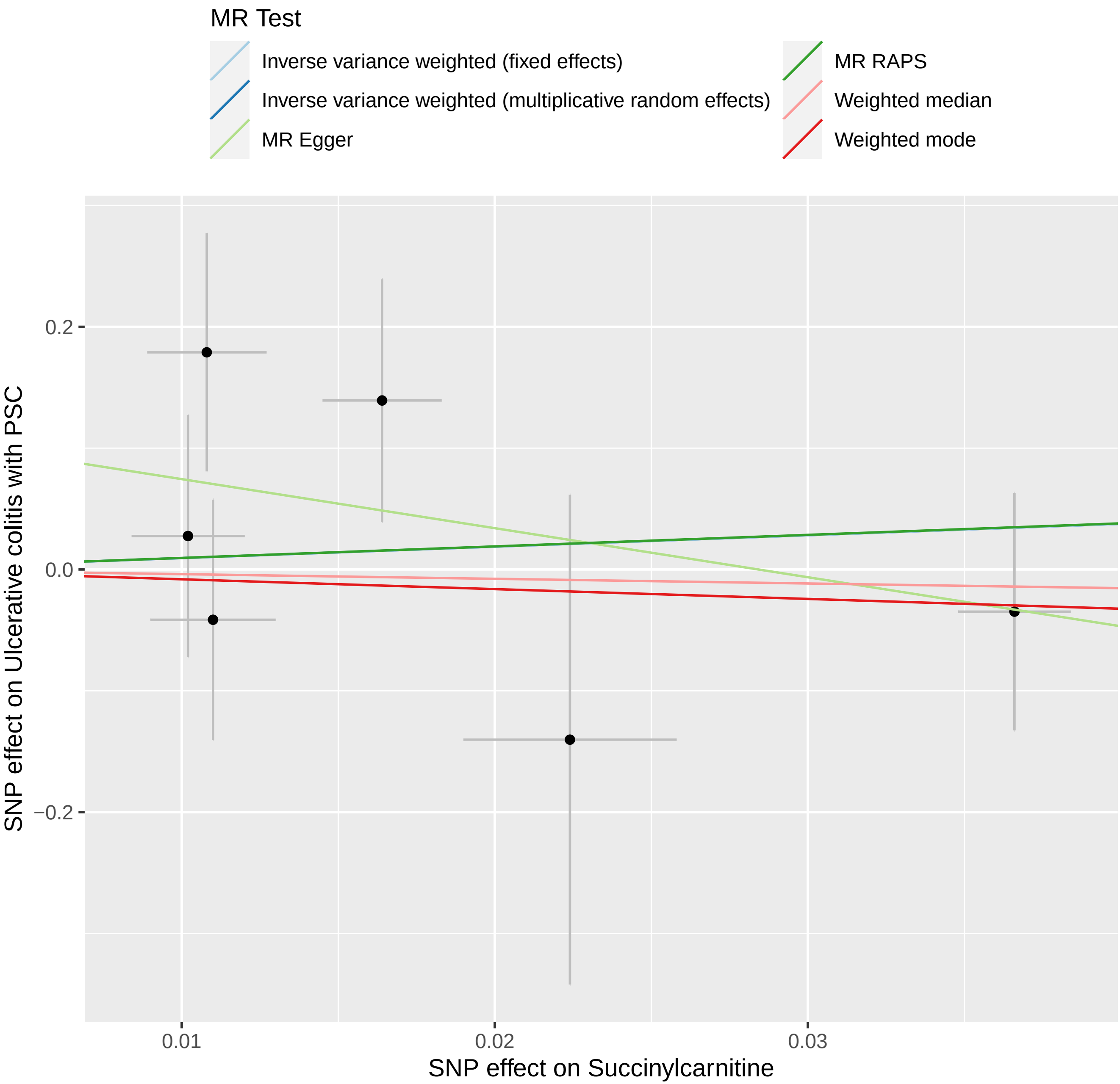

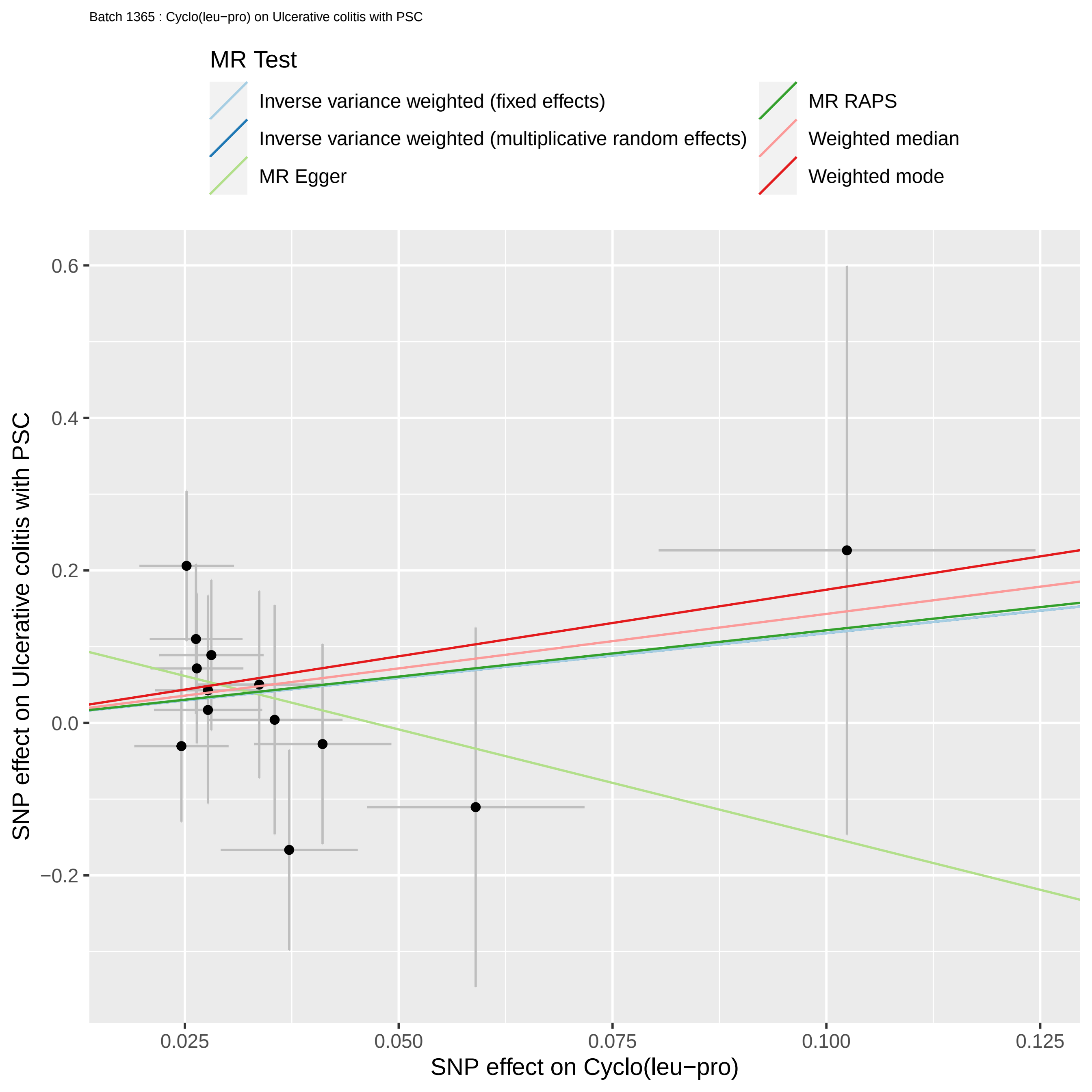

Batch 1365 : Cyclo(leu−pro) on Ulcerative colitis with PSC
MR Test
Inverse variance weighted (fixed effects)
Inverse variance weighted (multiplicative random effects)
MR Egger
MR RAPS
Weighted median
Weighted mode
0.6
0.4
0.2
0.0
−0.2
0.025
0.050
0.075
0.100
0.125
SNP effect on Ulcerative colitis with PSC
SNP effect on Cyclo(leu−pro)

Batch 1366 : Chiro−inositol on Ulcerative colitis with PSC

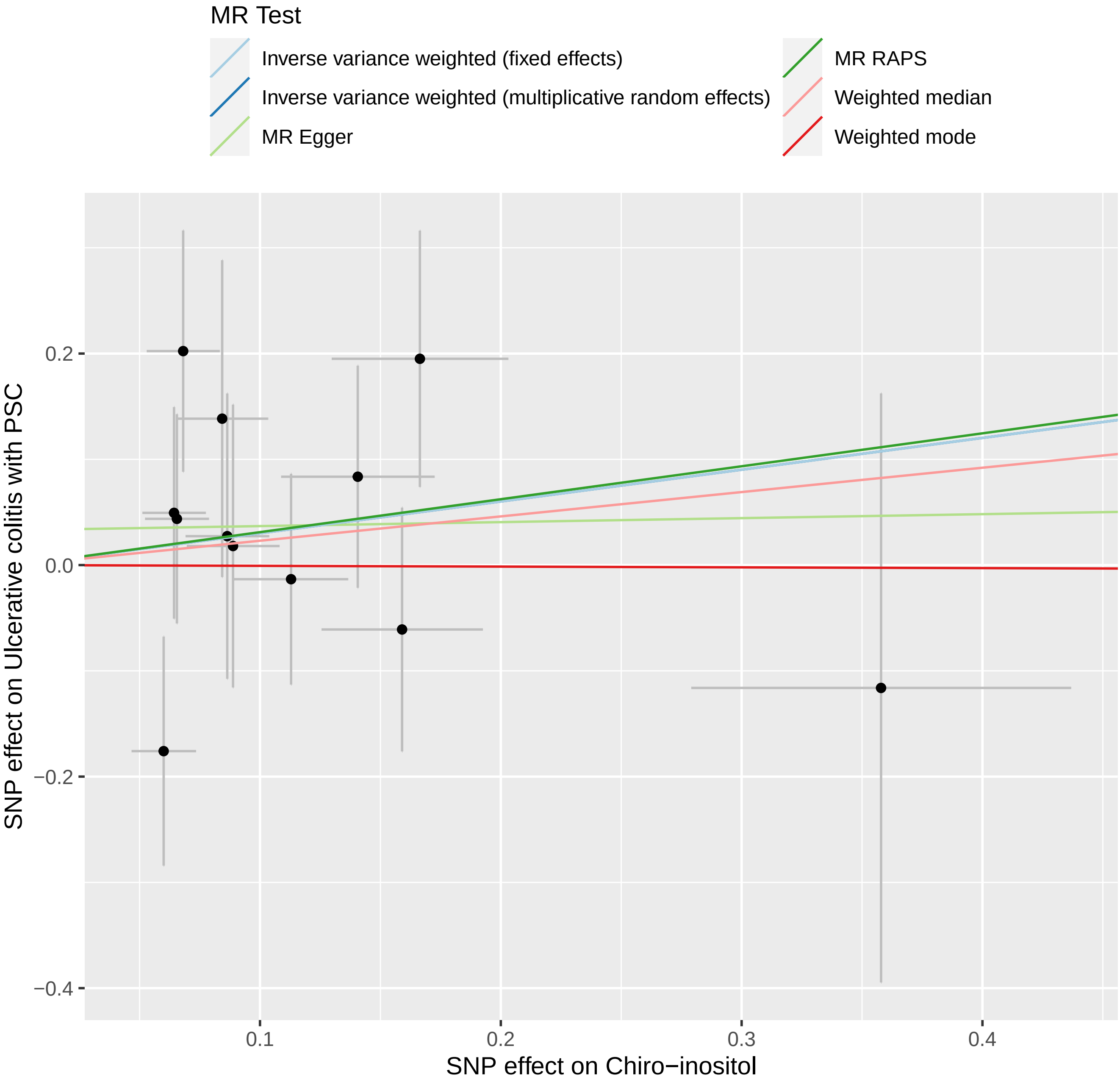

Batch 1370 : 2−hydroxyglutarate on Ulcerative colitis with PSC

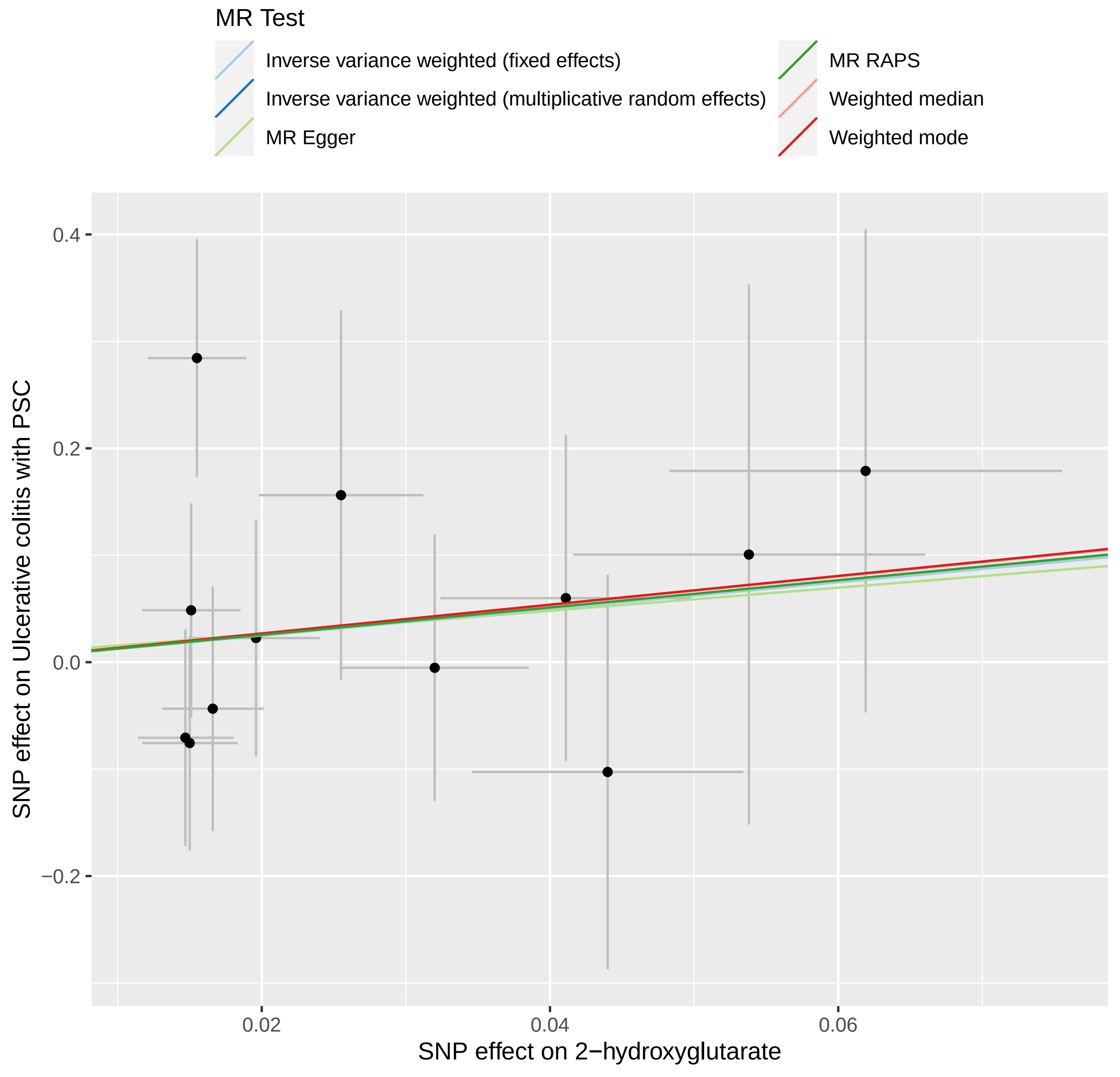

Batch 1371 : Ergothioneine on Ulcerative colitis with PSC

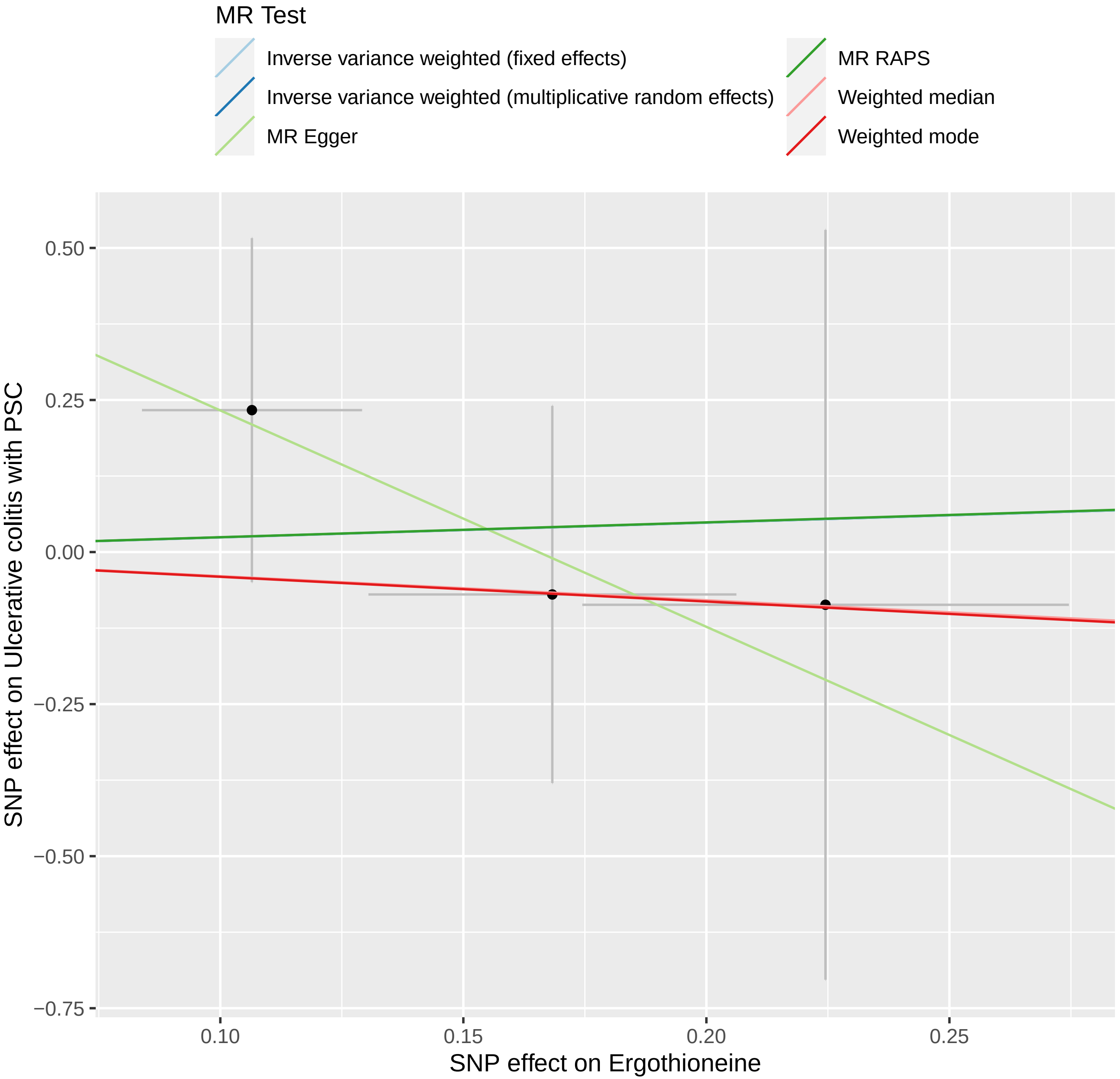

Batch 1373 : Phenylalanylphenylalanine on Ulcerative colitis with PSC

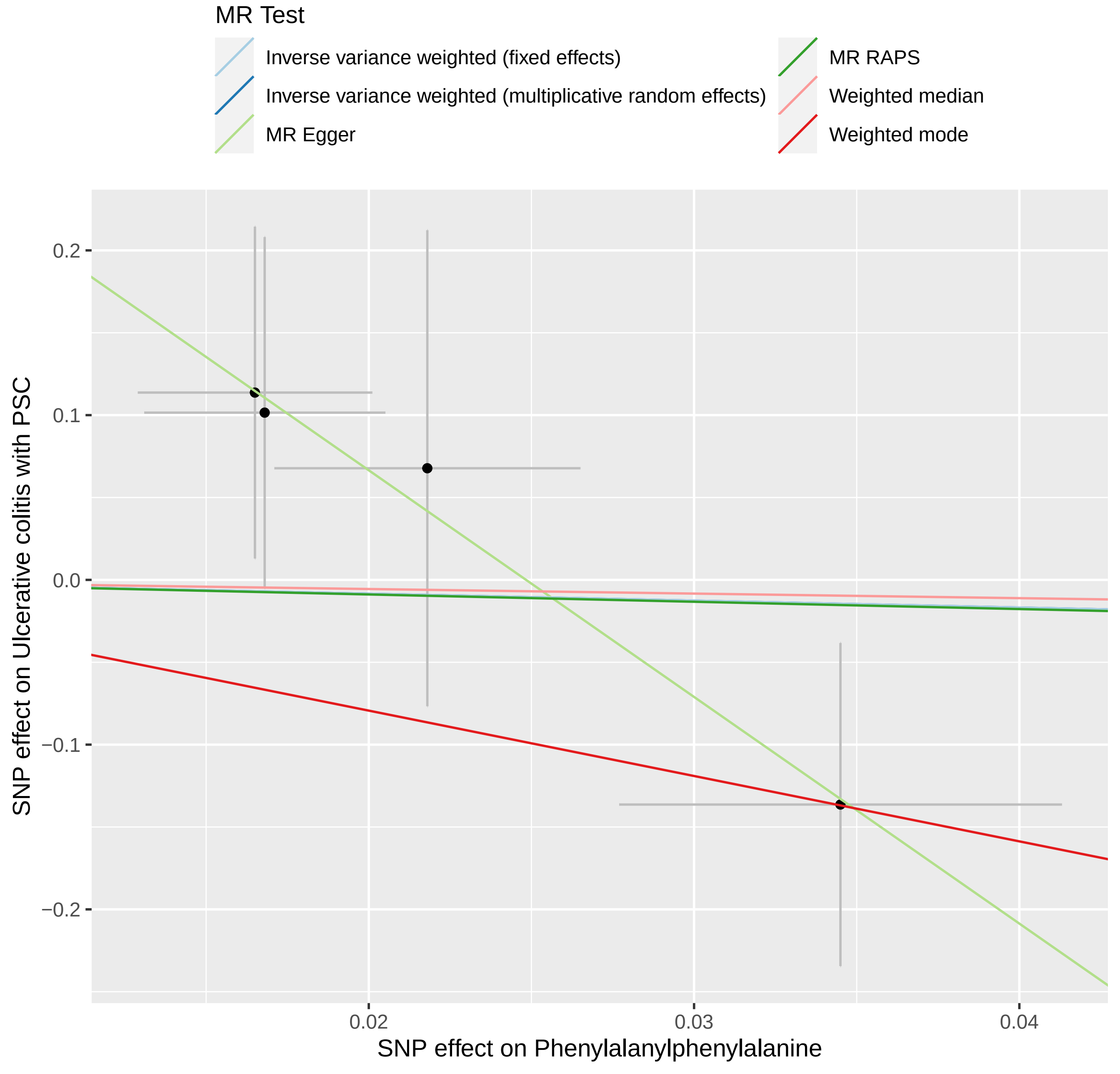

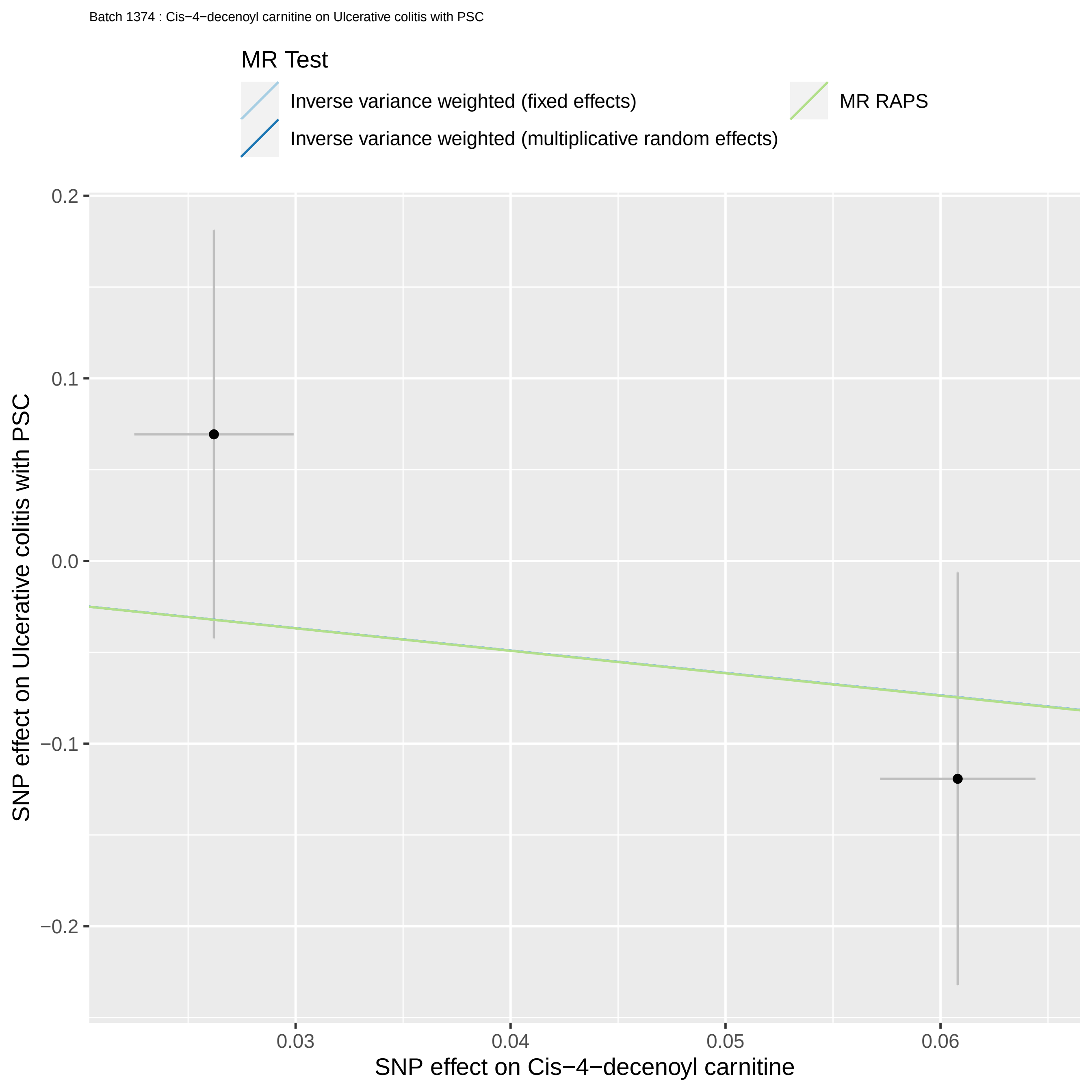

Batch 1374 : Cis−4−decenoyl carnitine on Ulcerative colitis with PSC
MR Test
Inverse variance weighted (fixed effects)
Inverse variance weighted (multiplicative random effects)
MR RAPS
0.2
0.1
0.0
−0.1
−0.2
SNP effect on Ulcerative colitis with PSC
0.03
0.04
0.05
0.06
SNP effect on Cis−4−decenoyl carnitine

Batch 1375 : 15−methylpalmitate (isobar with 2−methylpalmitate) on Ulcerative colitis with PSC

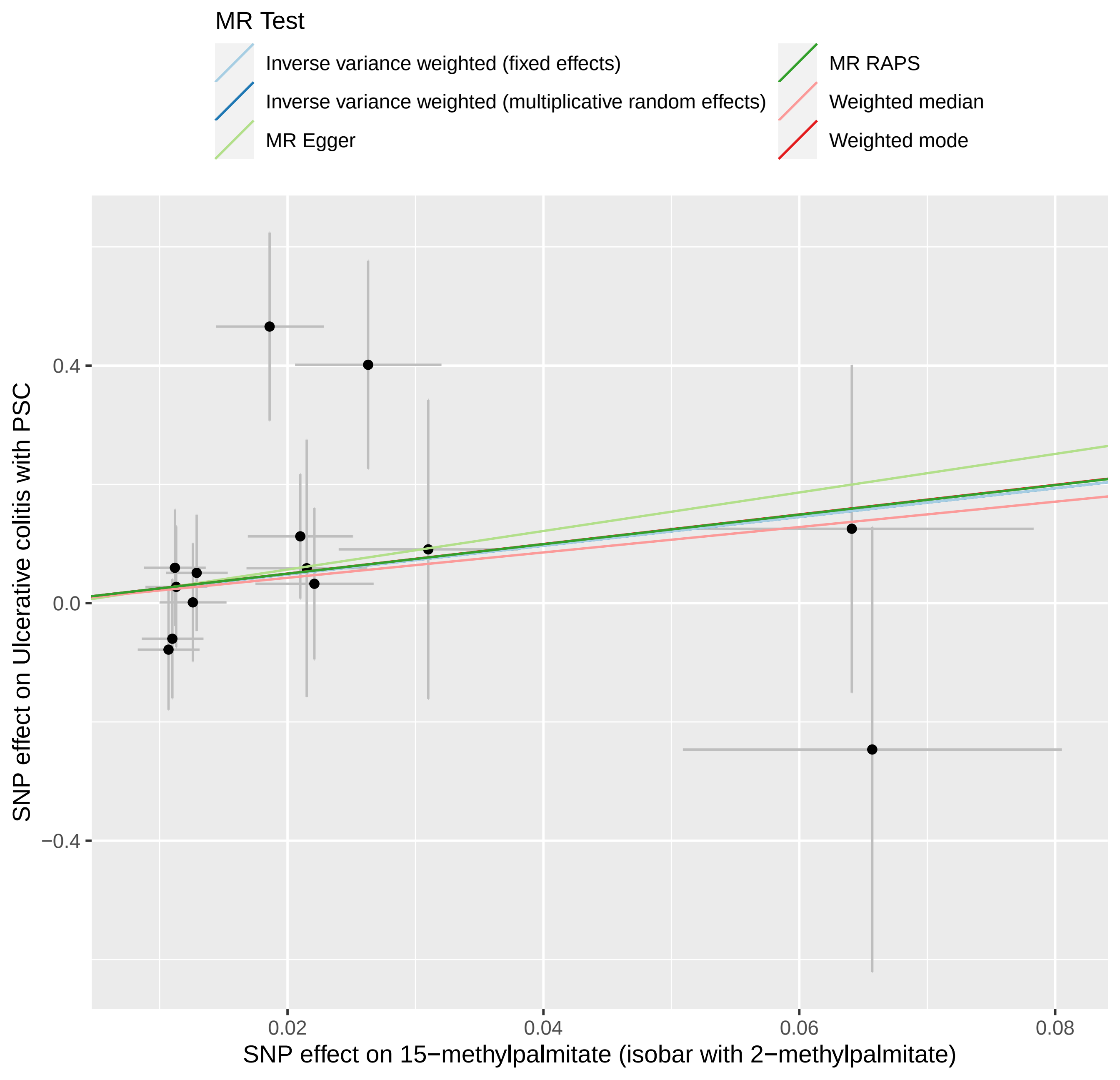

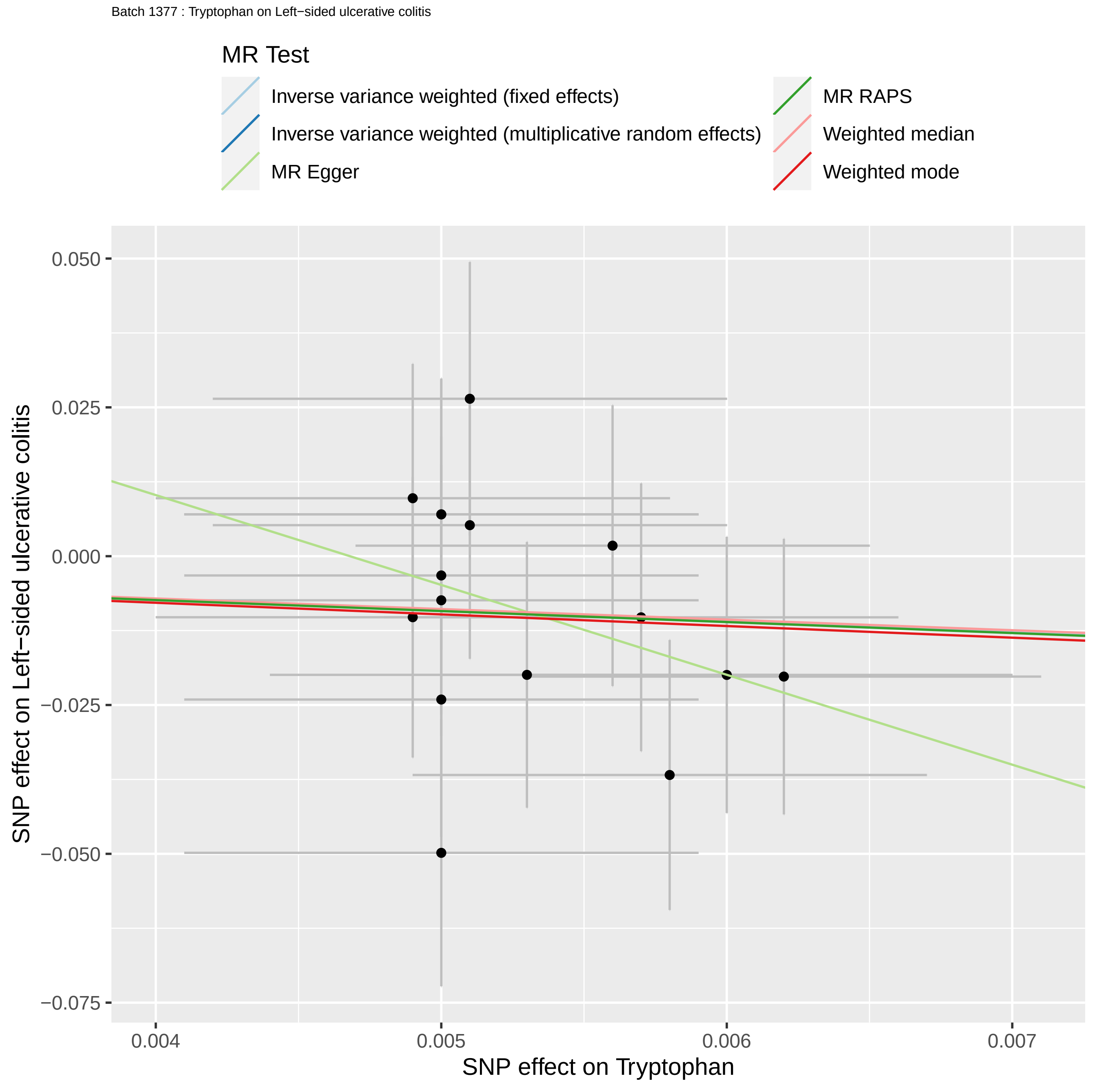
Batch 1377 : Tryptophan on Left−sided ulcerative colitis
MR Test
Inverse variance weighted (fixed effects)
Inverse variance weighted (multiplicative random effects)
MR Egger
MR RAPS
Weighted median
Weighted mode
0.050
0.025
0.000
−0.025
−0.050
−0.075
0.004
0.005
0.006
0.007
SNP effect on Tryptophan
SNP effect on Left−sided ulcerative colitis

Batch 1379 : Leucine on Left−sided ulcerative colitis

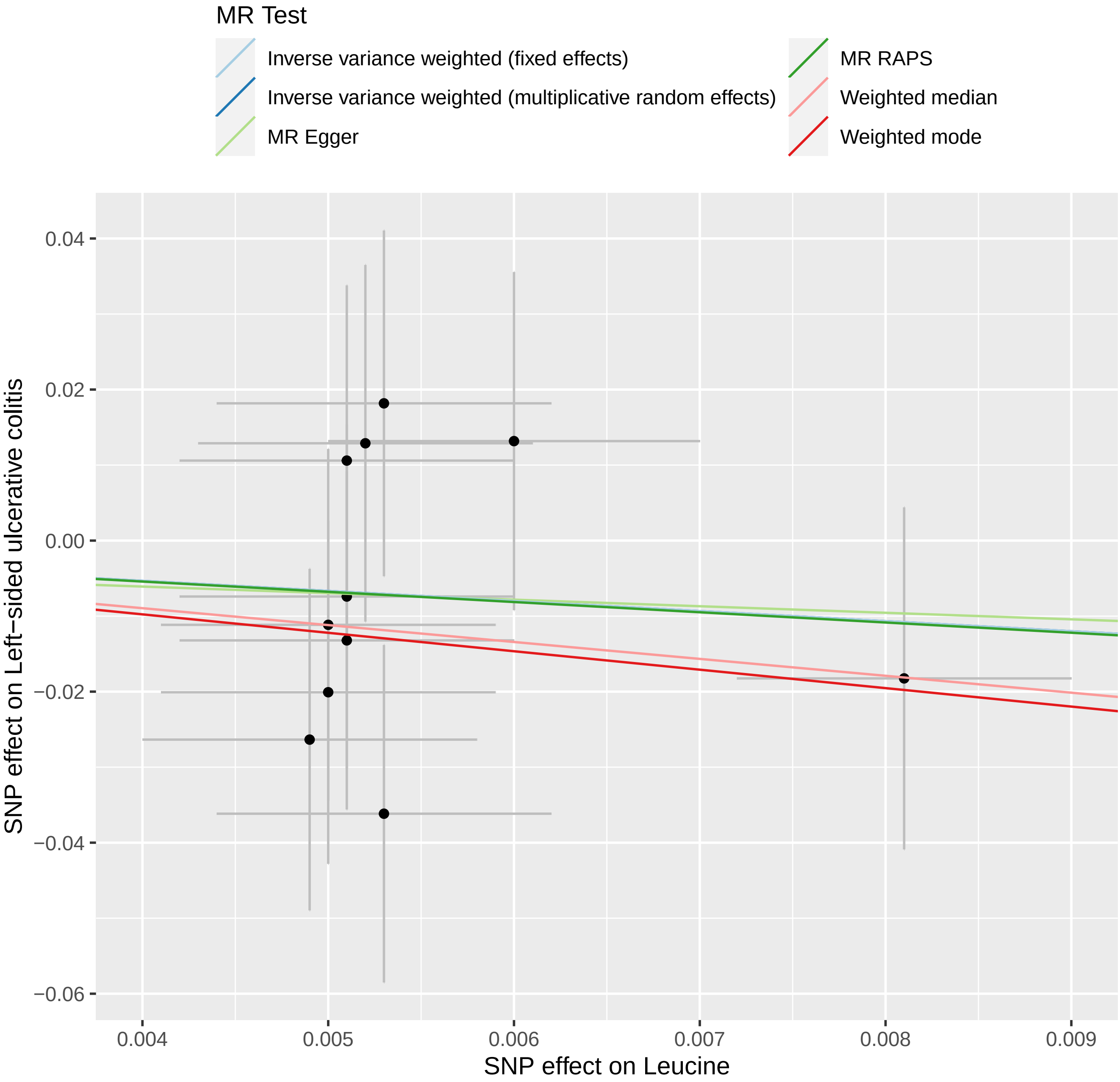

Batch 1383 : Lactate on Left−sided ulcerative colitis

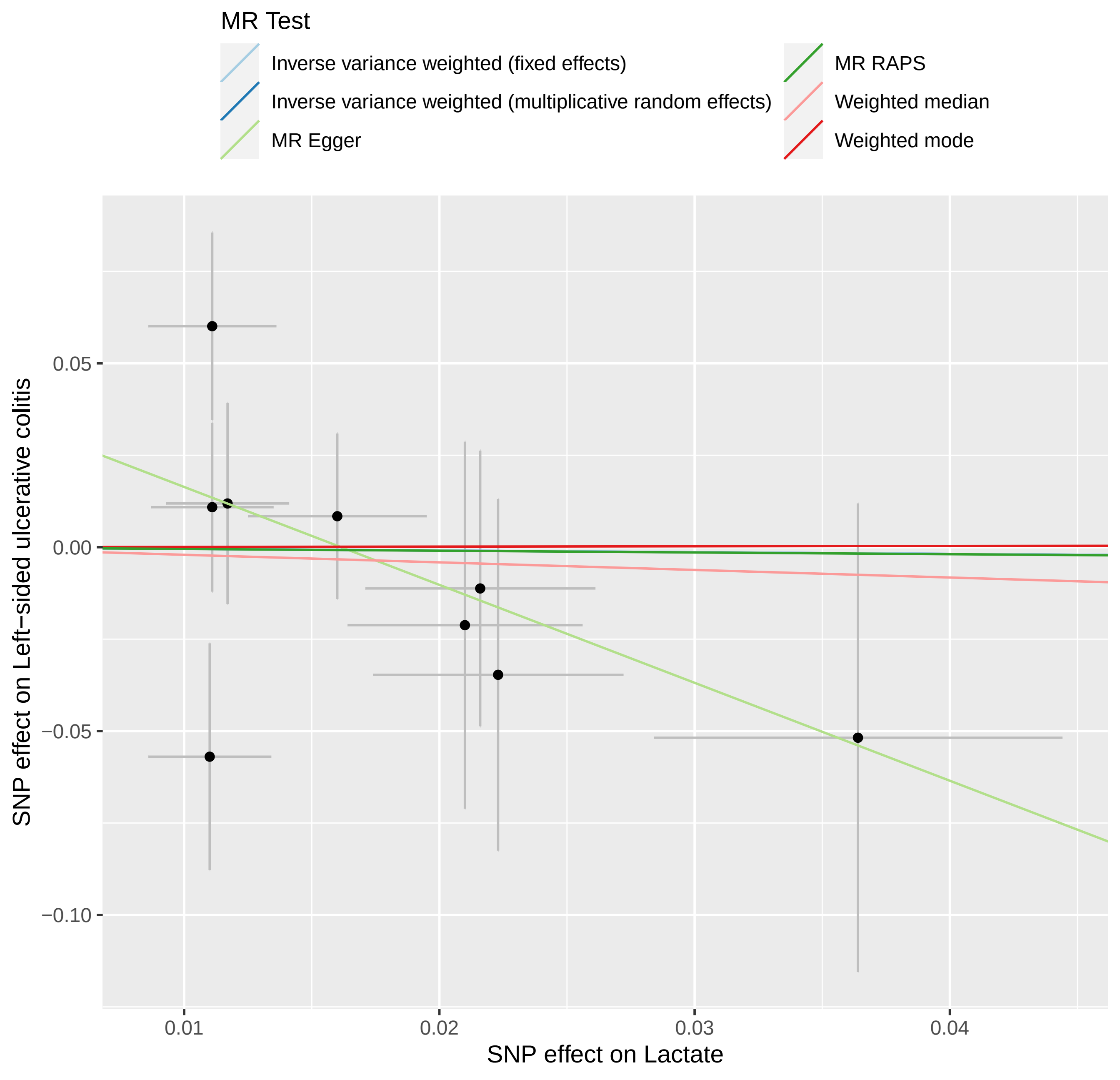

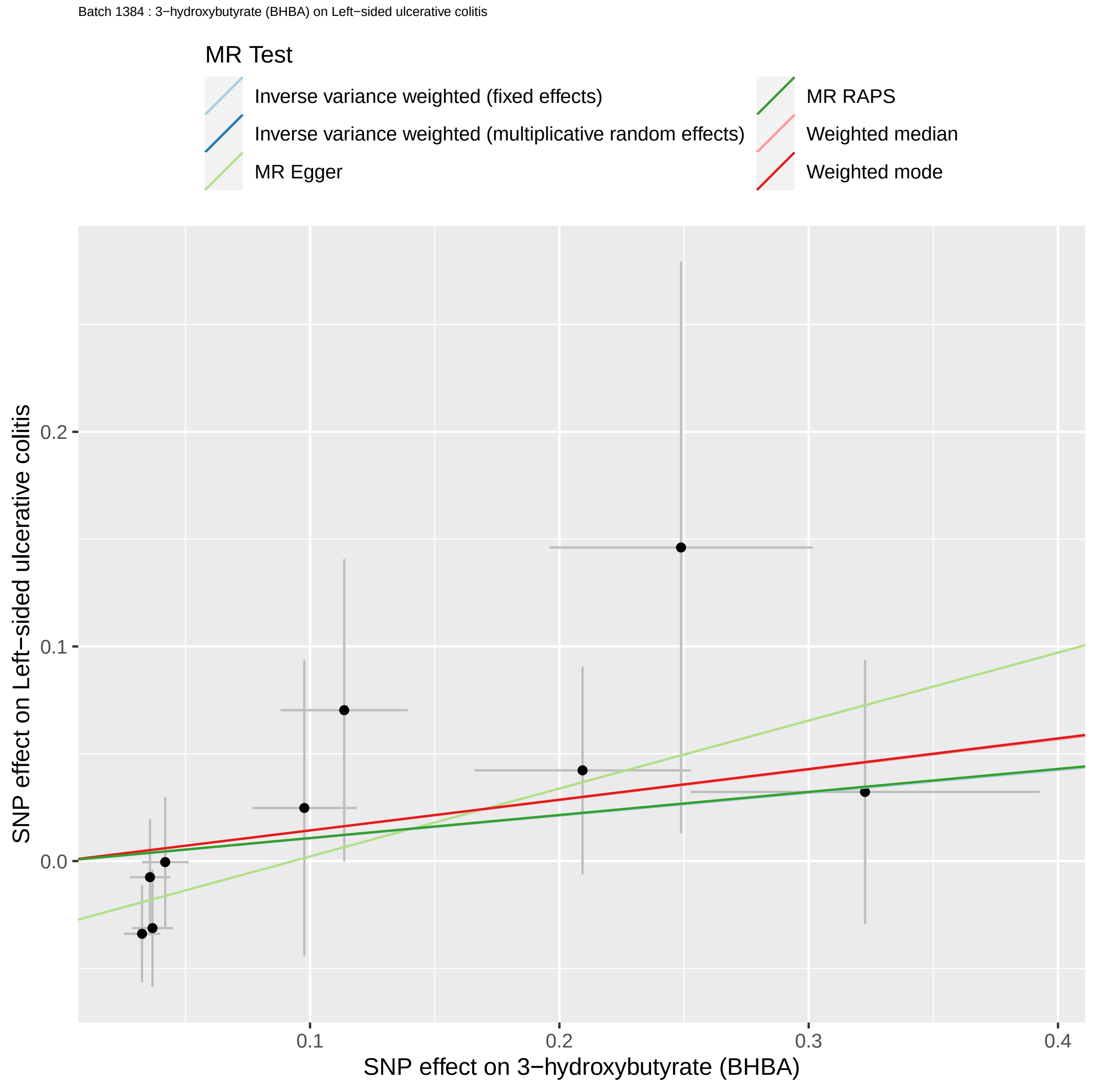
Batch 1384 : 3−hydroxybutyrate (BHBA) on Left−sided ulcerative colitis
MR Test
Inverse variance weighted (fixed effects)
Inverse variance weighted (multiplicative random effects)
MR Egger
MR RAPS
Weighted median
Weighted mode
0.2
0.1
0.0
0.1
0.2
0.3
0.4
SNP effect on Left−sided ulcerative colitis
SNP effect on 3−hydroxybutyrate (BHBA)

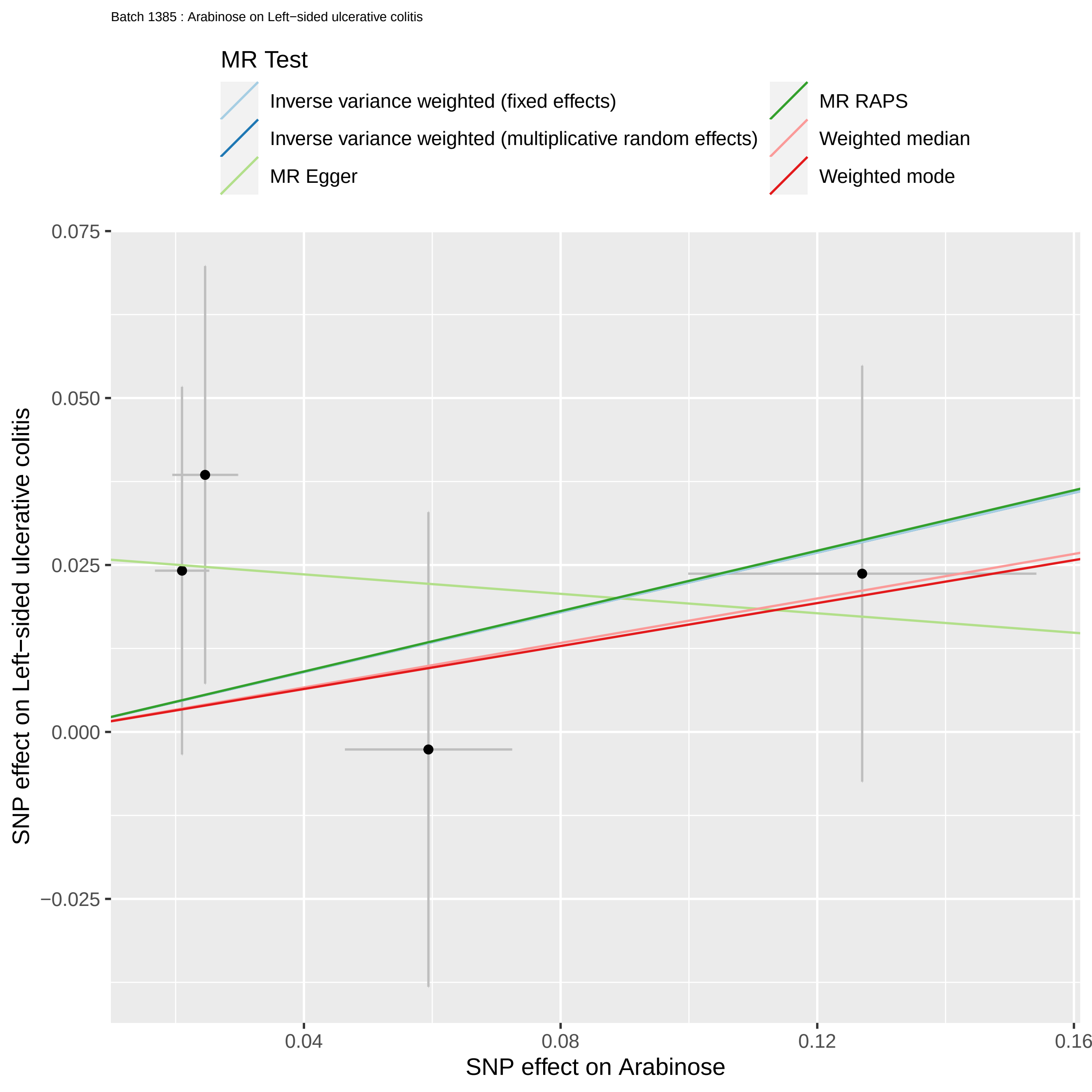
Batch 1385 : Arabinose on Left−sided ulcerative colitis
MR Test
Inverse variance weighted (fixed effects)
Inverse variance weighted (multiplicative random effects)
MR Egger
MR RAPS
Weighted median
Weighted mode
0.075
0.050
0.025
0.000
−0.025
0.04
0.08
0.12
0.16
SNP effect on Arabinose
SNP effect on Left−sided ulcerative colitis

Batch 1386 : Fructose on Left−sided ulcerative colitis

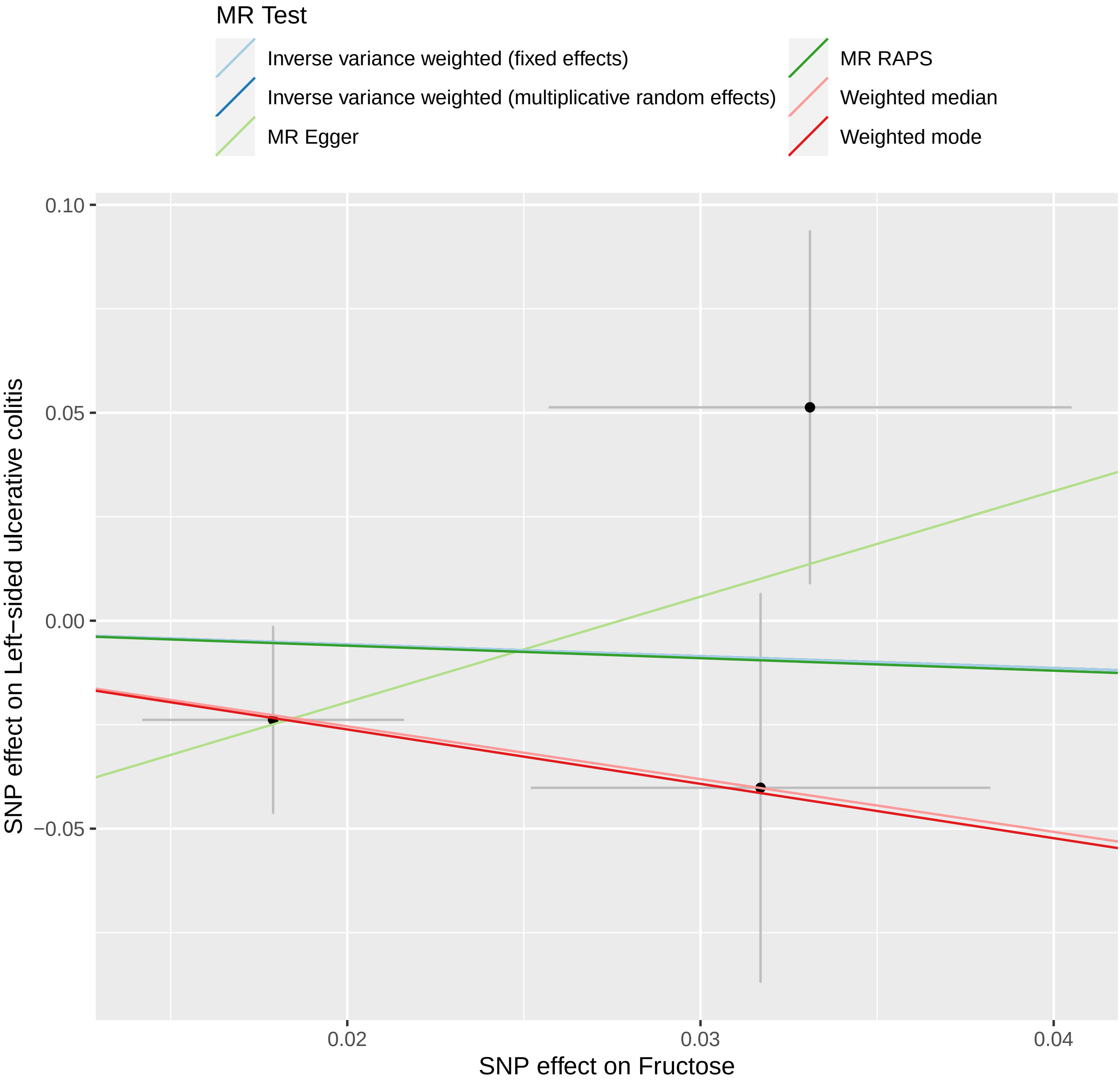

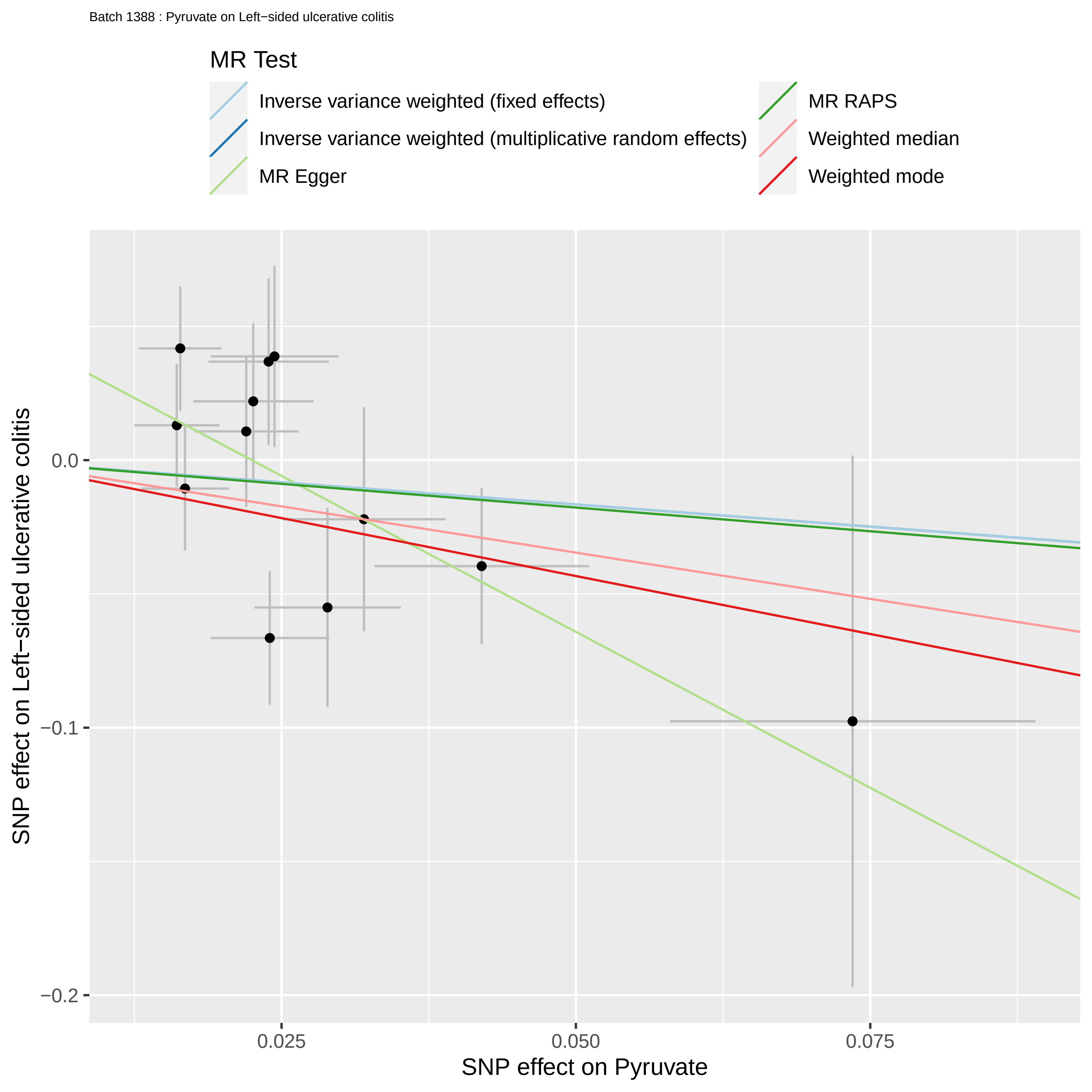
Batch 1388 : Pyruvate on Left−sided ulcerative colitis
MR Test
Inverse variance weighted (fixed effects)
Inverse variance weighted (multiplicative random effects)
MR Egger
MR RAPS
Weighted median
Weighted mode
SNP effect on Left−sided ulcerative colitis
0.0
−0.1
−0.2
0.025
0.050
0.075
SNP effect on Pyruvate

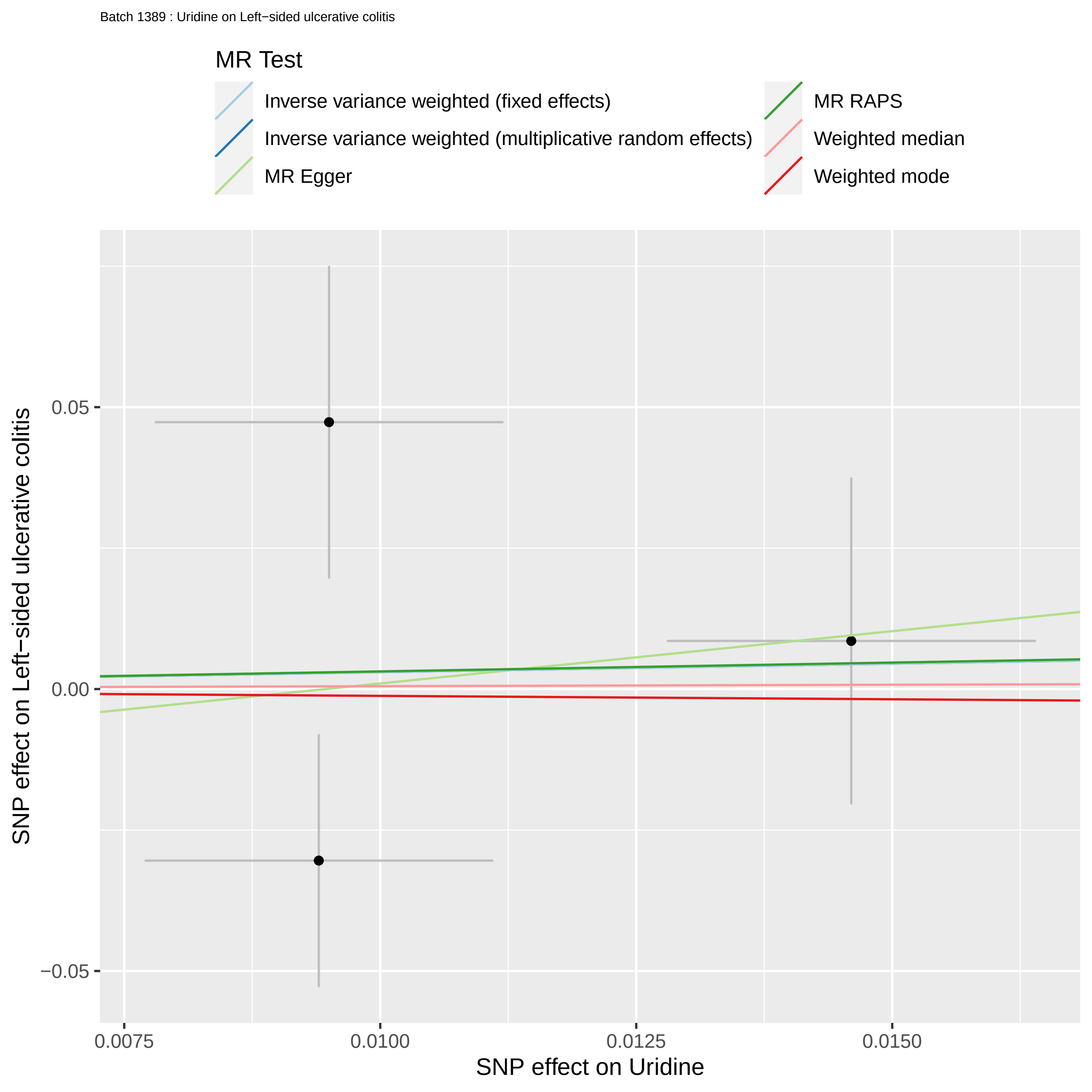
Batch 1389 : Uridine on Left−sided ulcerative colitis
MR Test
Inverse variance weighted (fixed effects)
Inverse variance weighted (multiplicative random effects)
MR Egger
MR RAPS
Weighted median
Weighted mode
0.05
0.00
−0.05
0.0075
0.0100
0.0125
0.0150
SNP effect on Uridine
SNP effect on Left−sided ulcerative colitis

Batch 1390 : Linoleate (18:2n6) on Left−sided ulcerative colitis

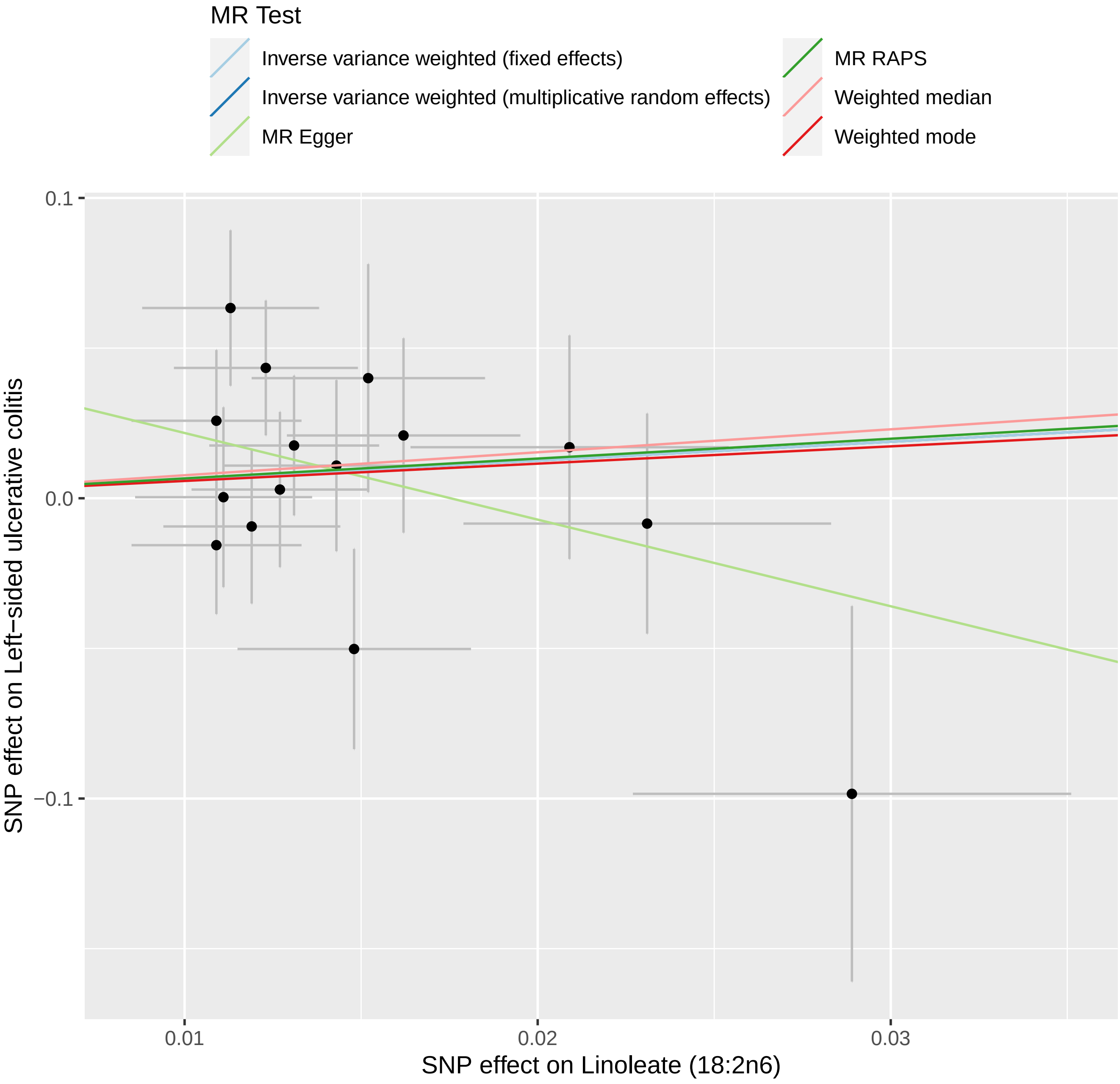

Batch 1391 : Allantoin on Left−sided ulcerative colitis

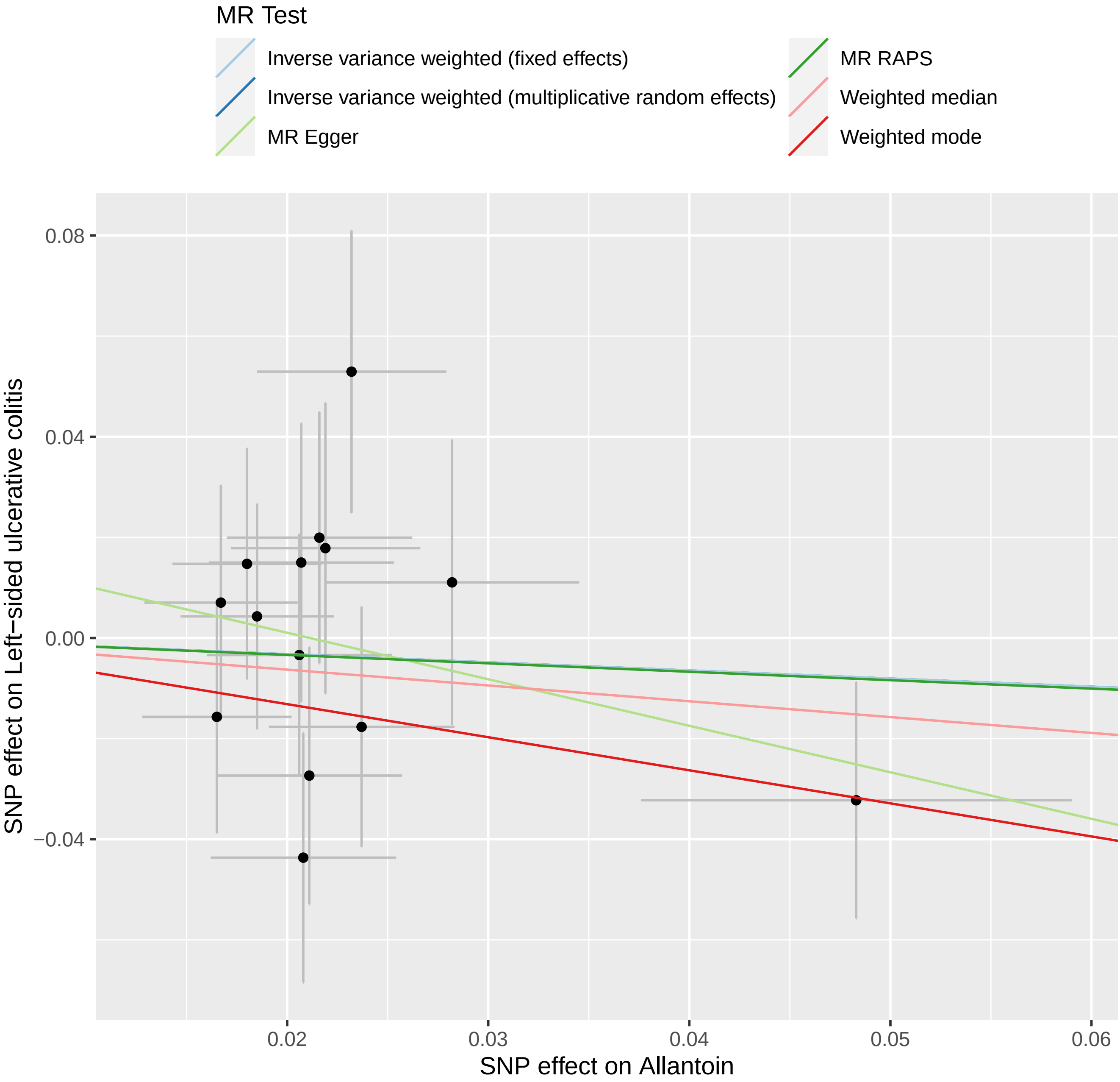

Batch 1396 : Isoleucine on Left−sided ulcerative colitis

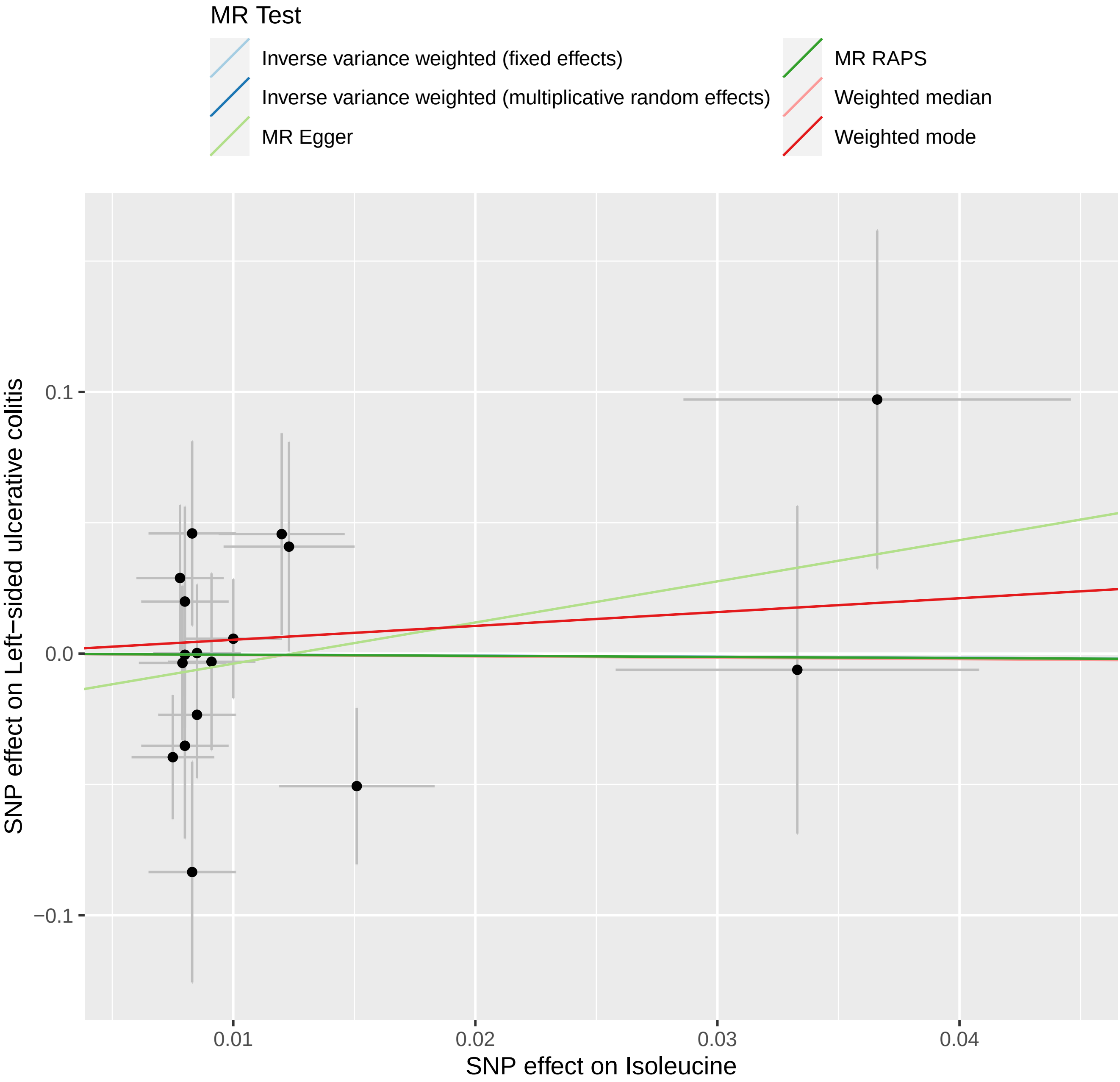

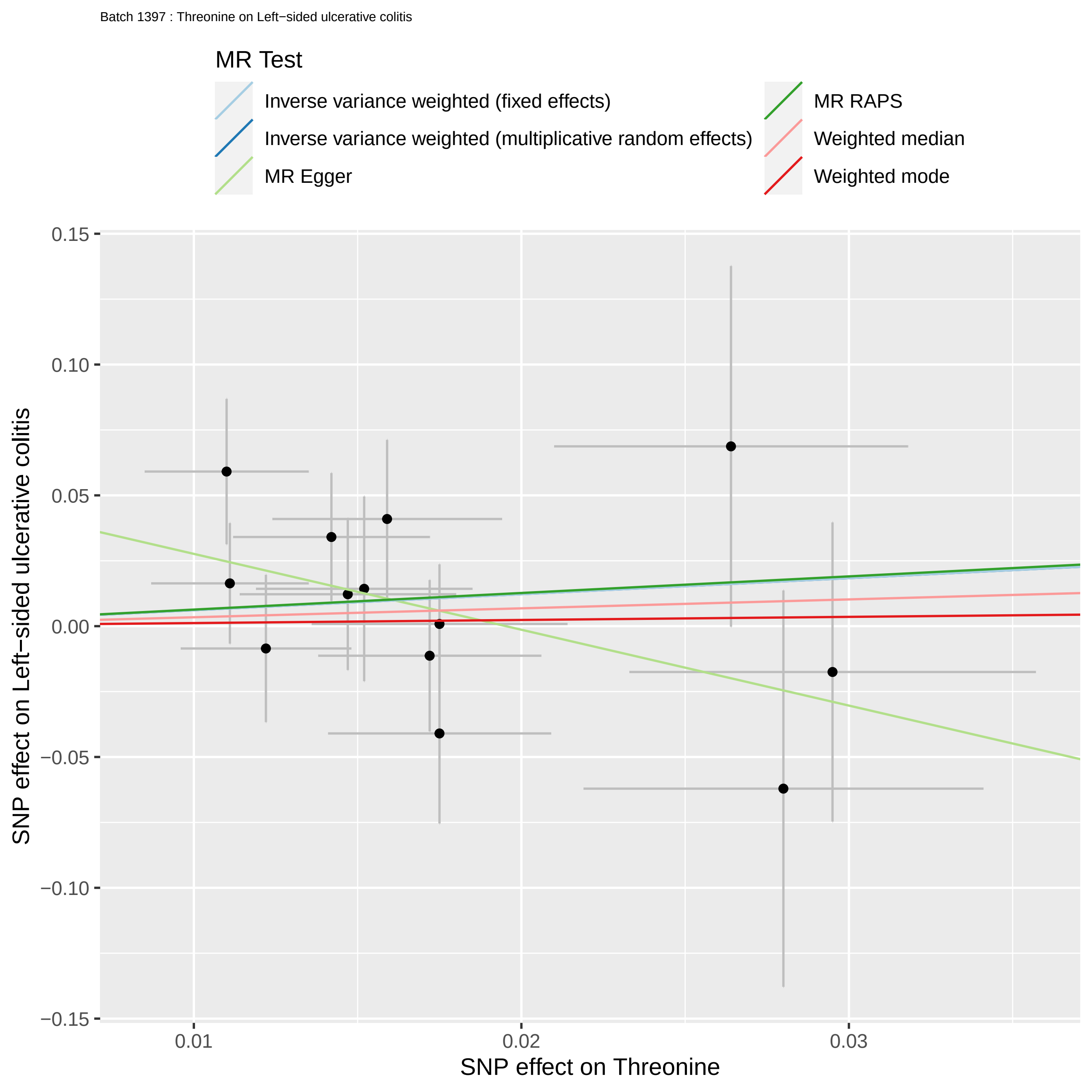
Batch 1397 : Threonine on Left−sided ulcerative colitis
MR Test
Inverse variance weighted (fixed effects)
Inverse variance weighted (multiplicative random effects)
MR Egger
MR RAPS
Weighted median
Weighted mode
0.15
0.10
0.05
0.00
−0.05
−0.10
−0.15
0.01
0.02
0.03
SNP effect on Threonine
SNP effect on Left−sided ulcerative colitis

Batch 1398 : Tyrosine on Left−sided ulcerative colitis

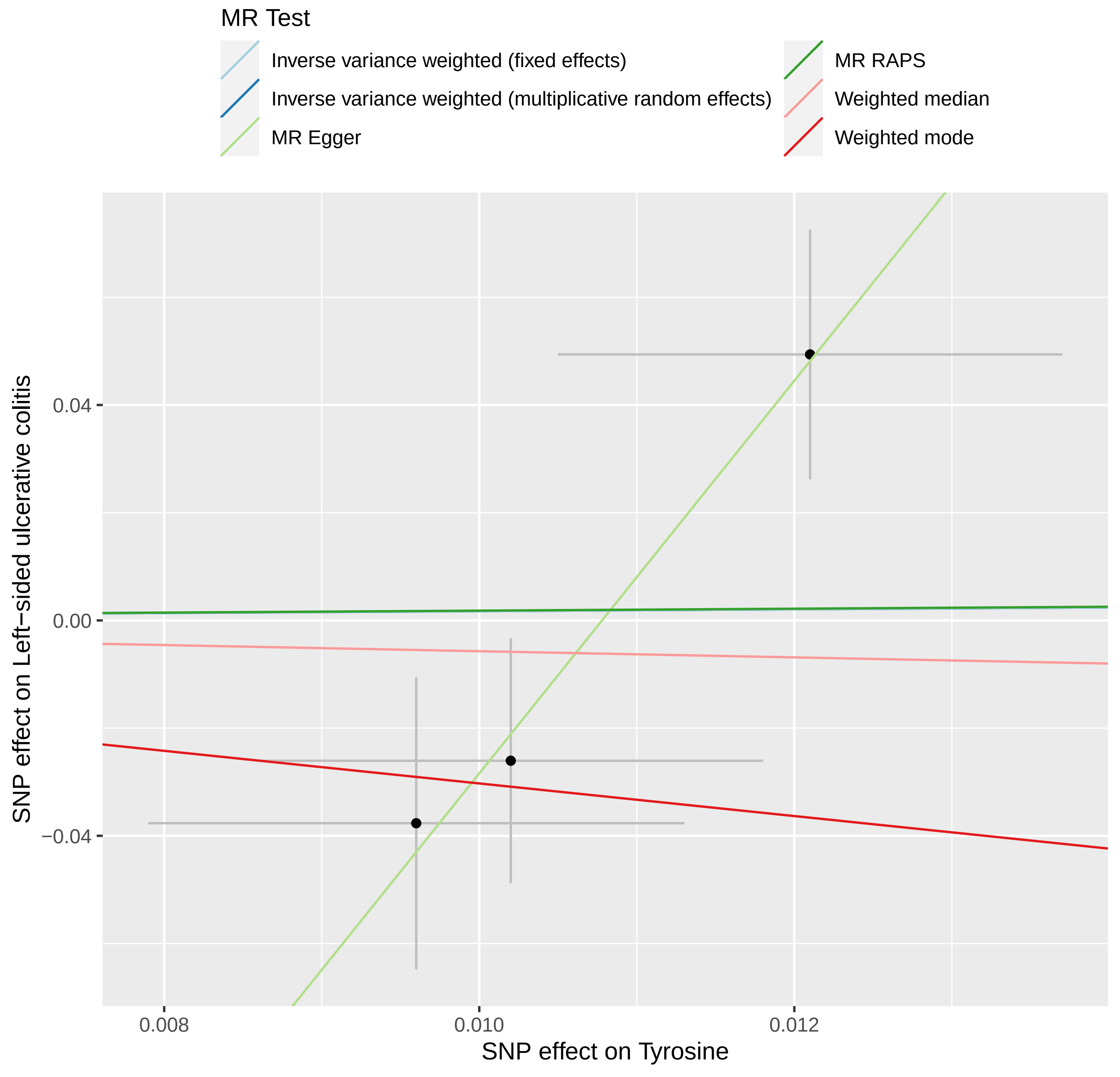

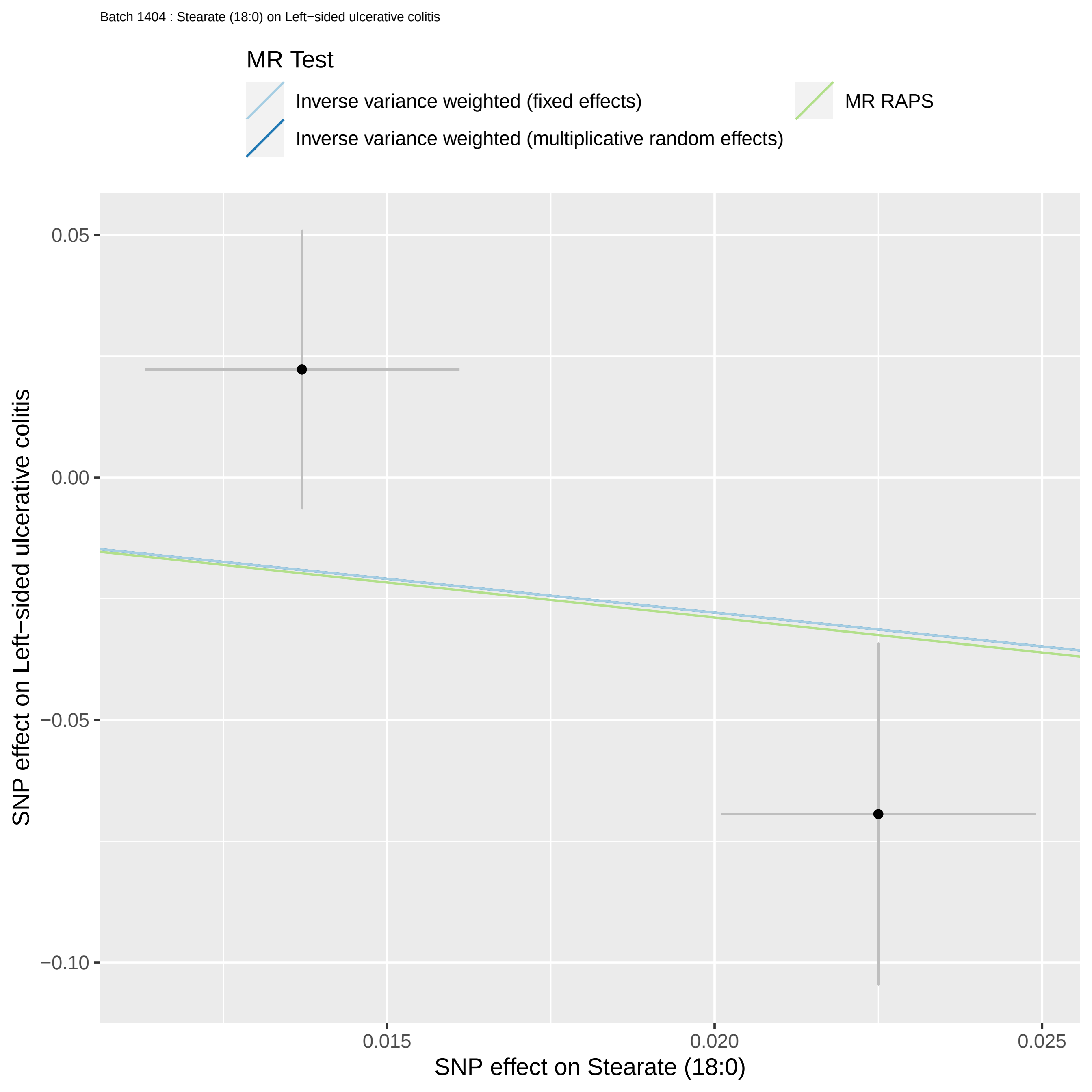
Batch 1404 : Stearate (18:0) on Left−sided ulcerative colitis
MR Test
Inverse variance weighted (fixed effects)
Inverse variance weighted (multiplicative random effects)
MR RAPS
0.05
0.00
−0.05
−0.10
0.015
0.020
0.025
SNP effect on Stearate (18:0)
SNP effect on Left−sided ulcerative colitis

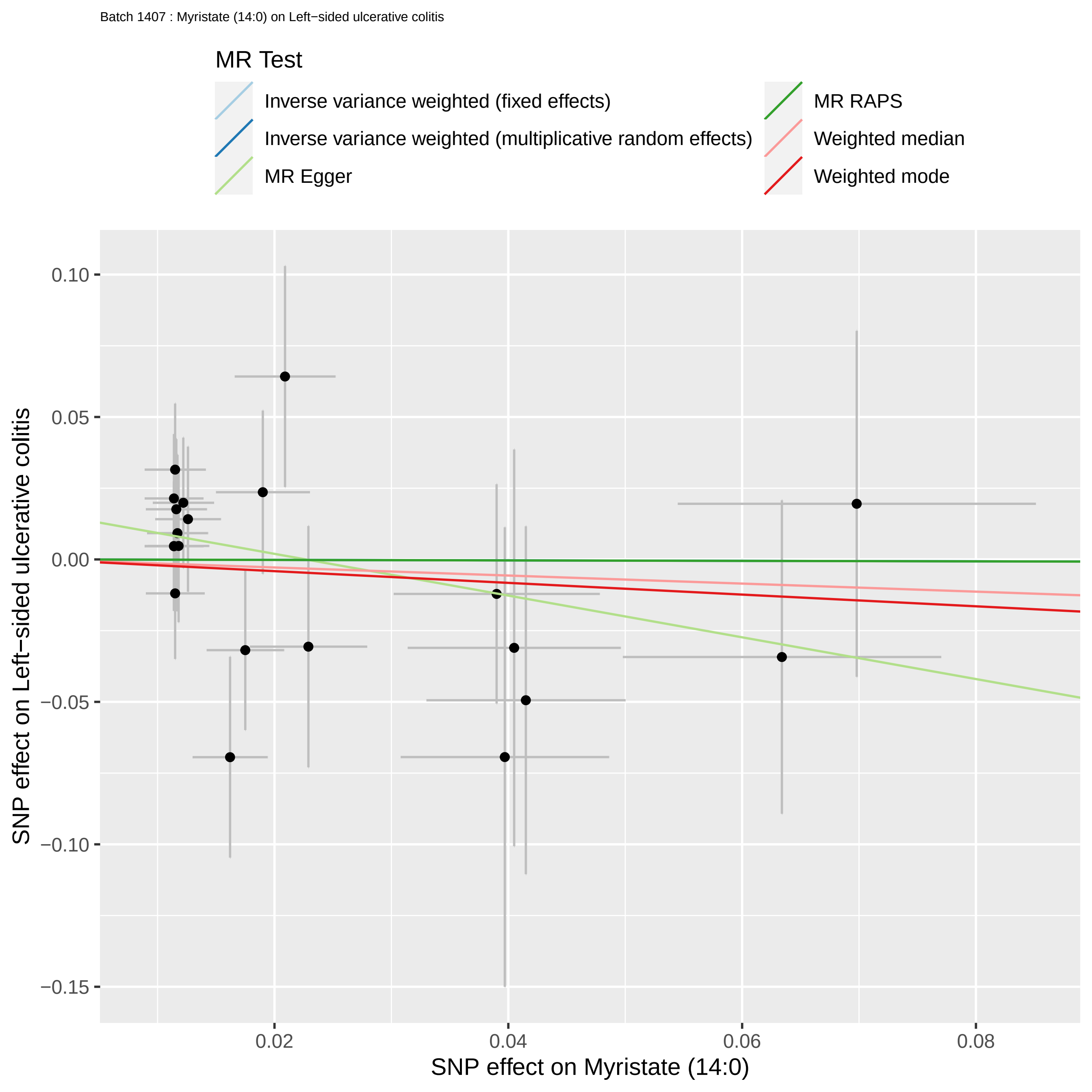

Batch 1407 : Myristate (14:0) on Left−sided ulcerative colitis
MR Test
Inverse variance weighted (fixed effects)
Inverse variance weighted (multiplicative random effects)
MR Egger
MR RAPS
Weighted median
Weighted mode
0.10
0.05
0.00
−0.05
−0.10
−0.15
0.02
0.04
0.06
0.08
SNP effect on Left−sided ulcerative colitis
SNP effect on Myristate (14:0)

Batch 1409 : Ornithine on Left−sided ulcerative colitis

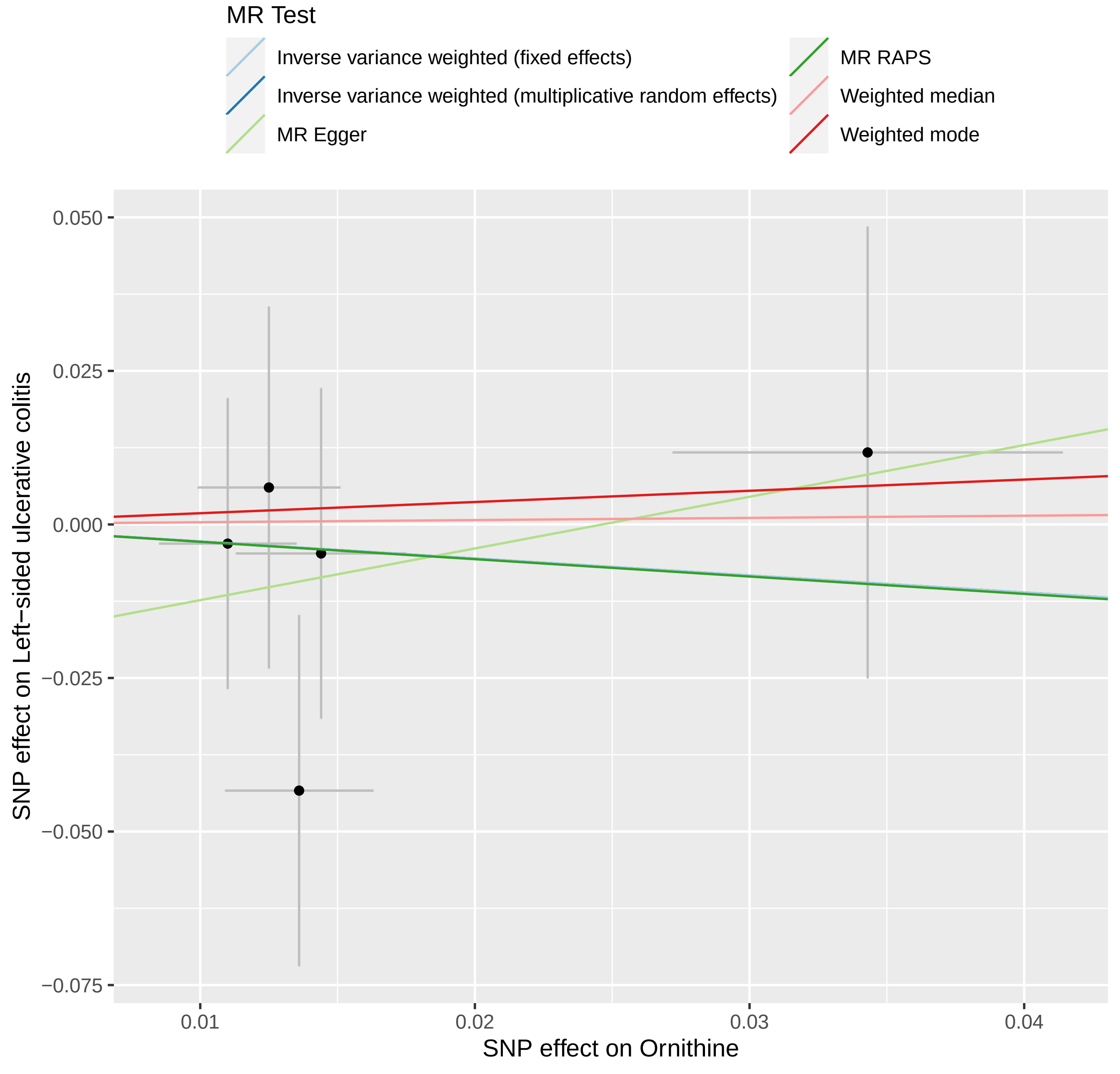

Batch 1412 : 4−acetamidobutanoate on Left−sided ulcerative colitis

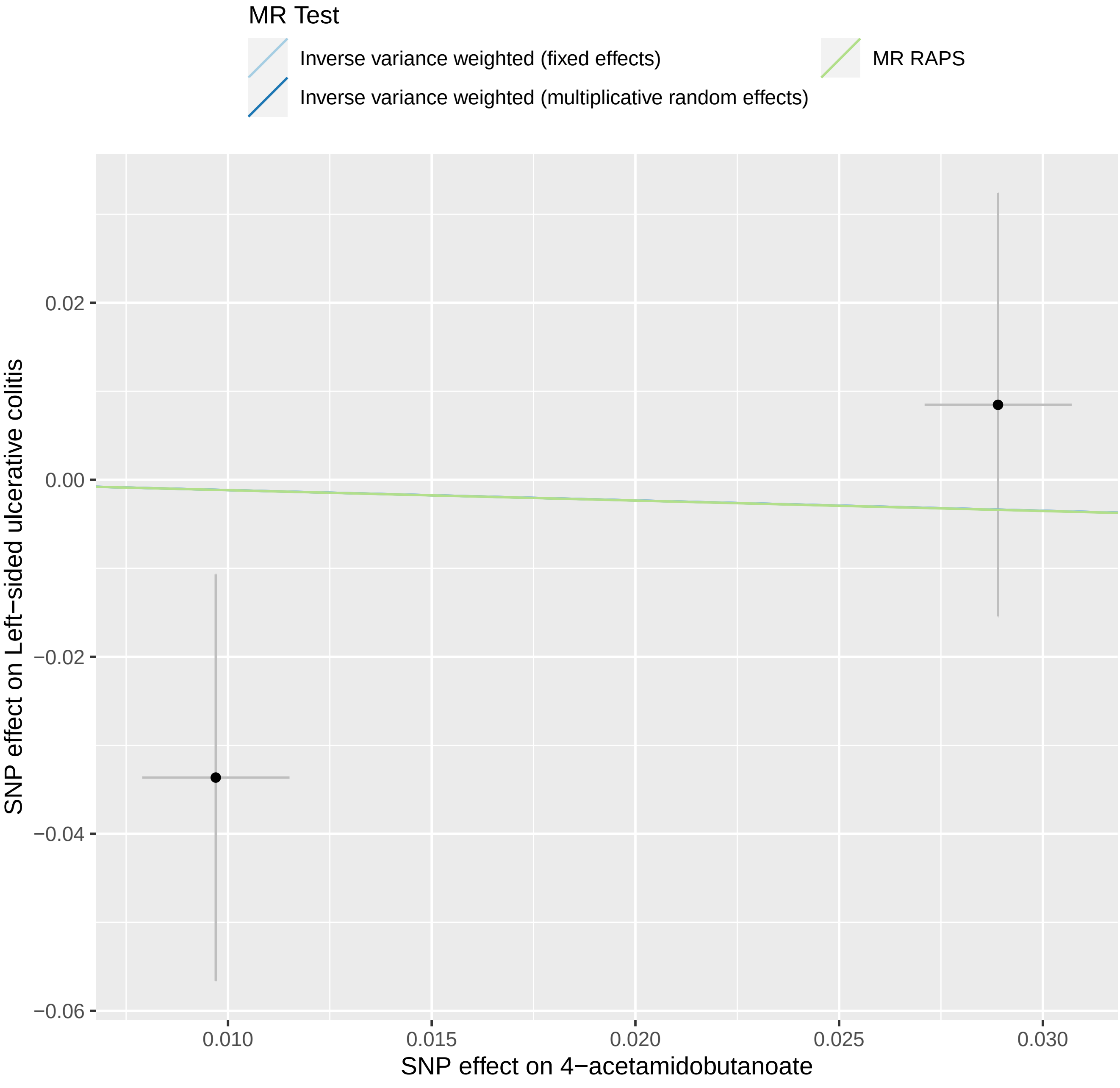

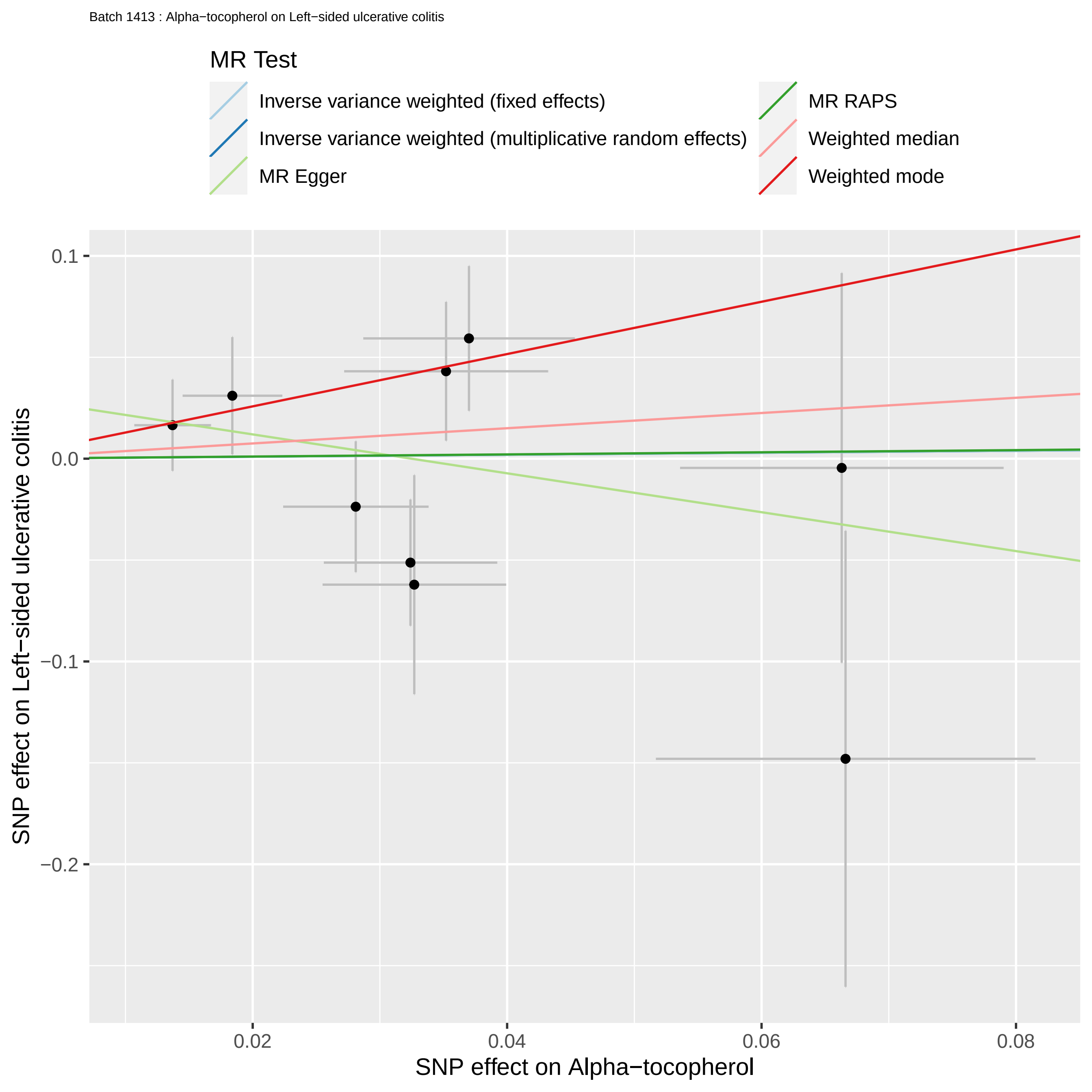
Batch 1413 : Alpha−tocopherol on Left−sided ulcerative colitis
MR Test
Inverse variance weighted (fixed effects)
Inverse variance weighted (multiplicative random effects)
MR Egger
MR RAPS
Weighted median
Weighted mode
0.1
0.0
−0.1
−0.2
0.02
0.04
0.06
0.08
SNP effect on Left−sided ulcerative colitis
SNP effect on Alpha−tocopherol

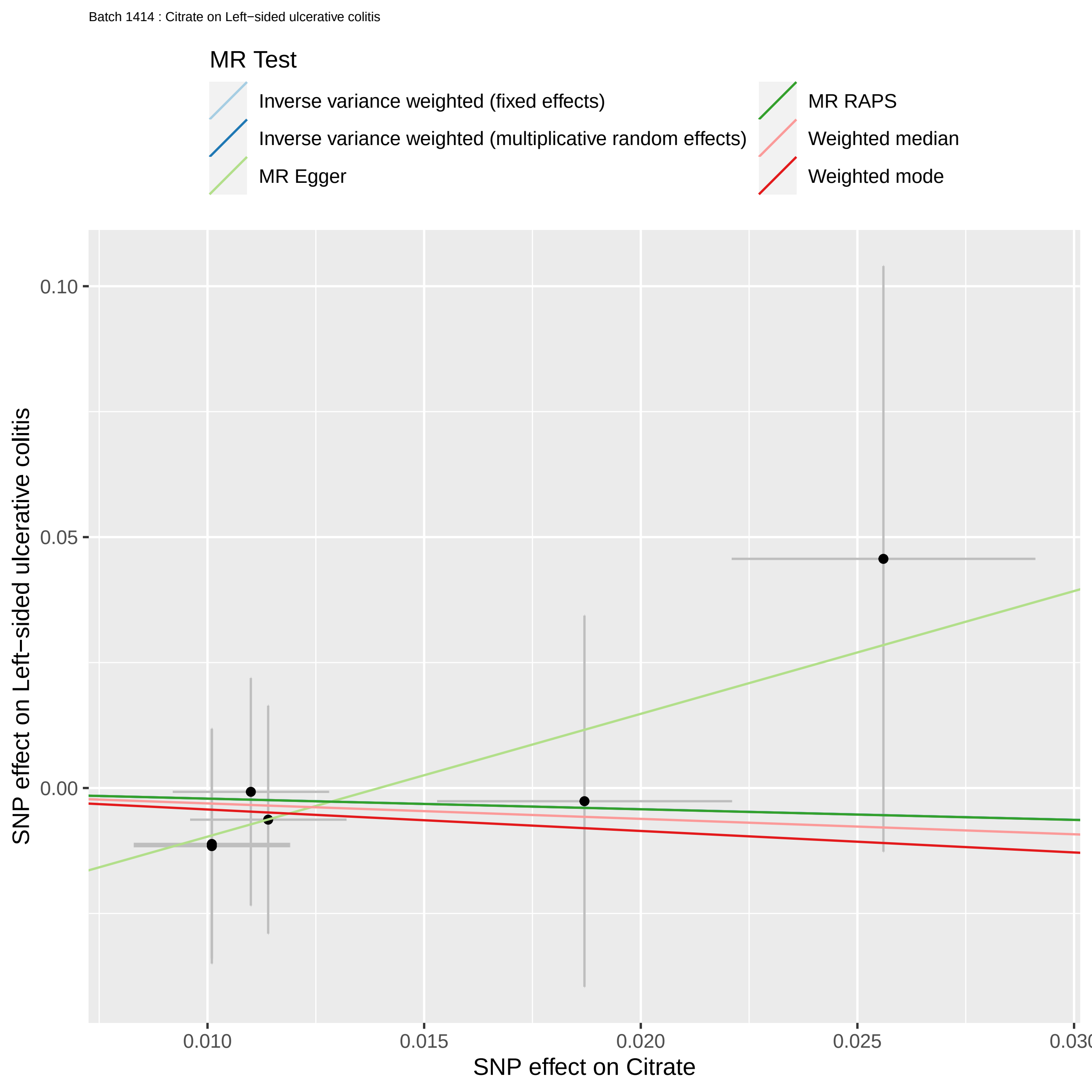

Batch 1414 : Citrate on Left−sided ulcerative colitis
MR Test
Inverse variance weighted (fixed effects)
Inverse variance weighted (multiplicative random effects)
MR Egger
MR RAPS
Weighted median
Weighted mode
0.10
0.05
0.00
0.010
0.015
0.020
0.025
0.030
SNP effect on Citrate
SNP effect on Left−sided ulcerative colitis

Batch 1415 : Glycerate on Left−sided ulcerative colitis

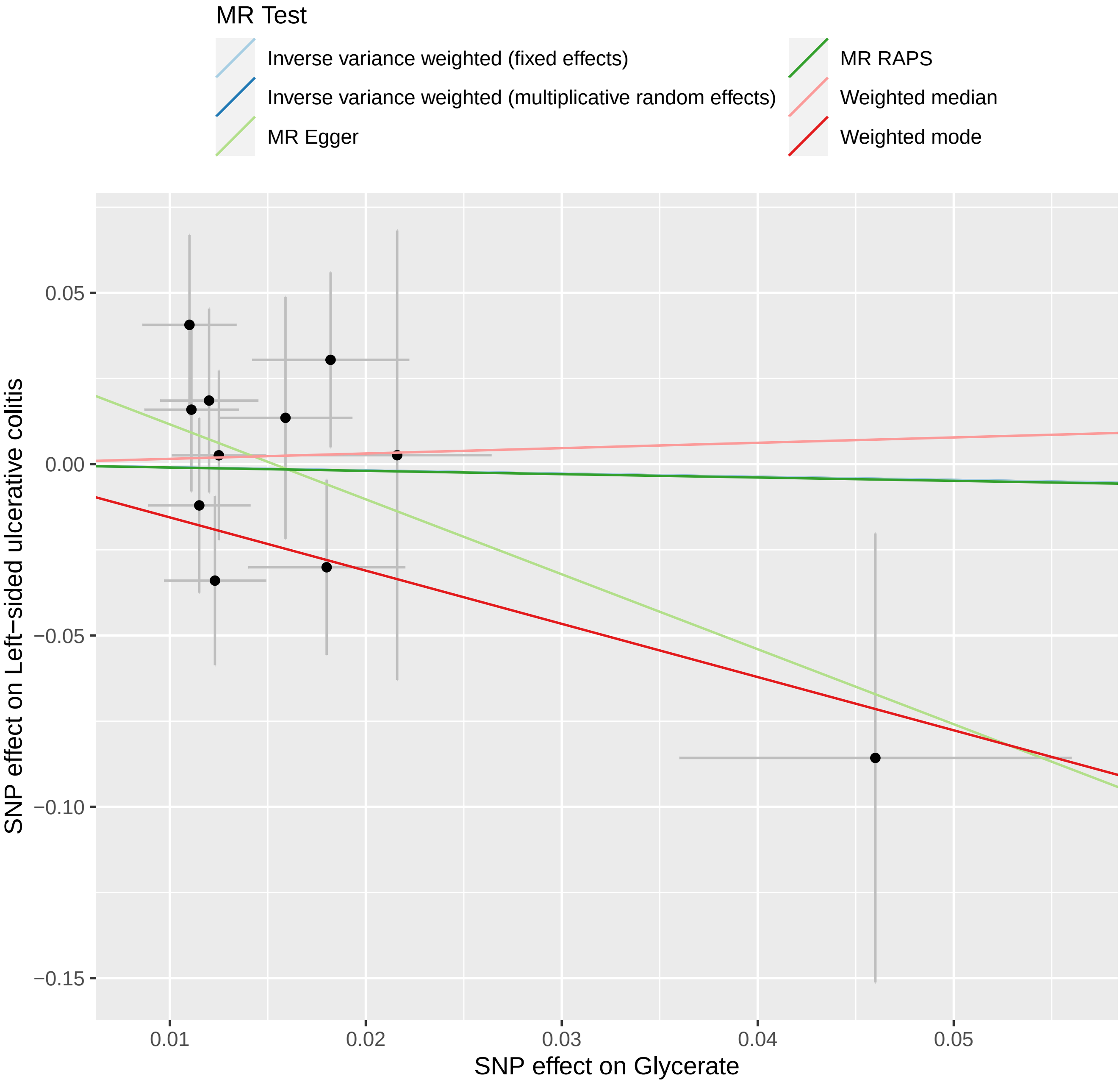

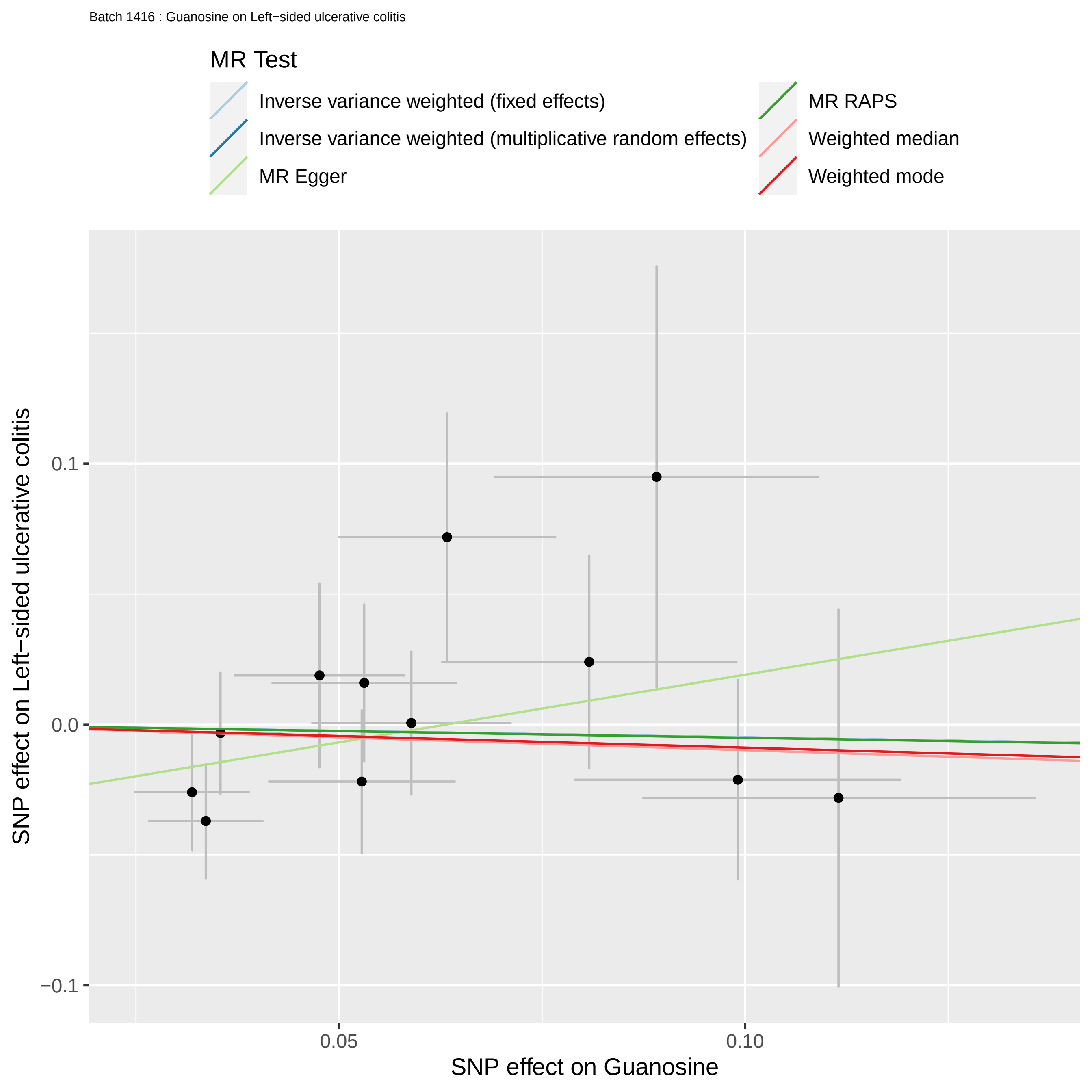

Batch 1416 : Guanosine on Left−sided ulcerative colitis
MR Test
Inverse variance weighted (fixed effects)
Inverse variance weighted (multiplicative random effects)
MR Egger
MR RAPS
Weighted median
Weighted mode
0.1
0.0
−0.1
0.05
0.10
SNP effect on Left−sided ulcerative colitis
SNP effect on Guanosine

Batch 1419 : Ursodeoxycholate on Left−sided ulcerative colitis

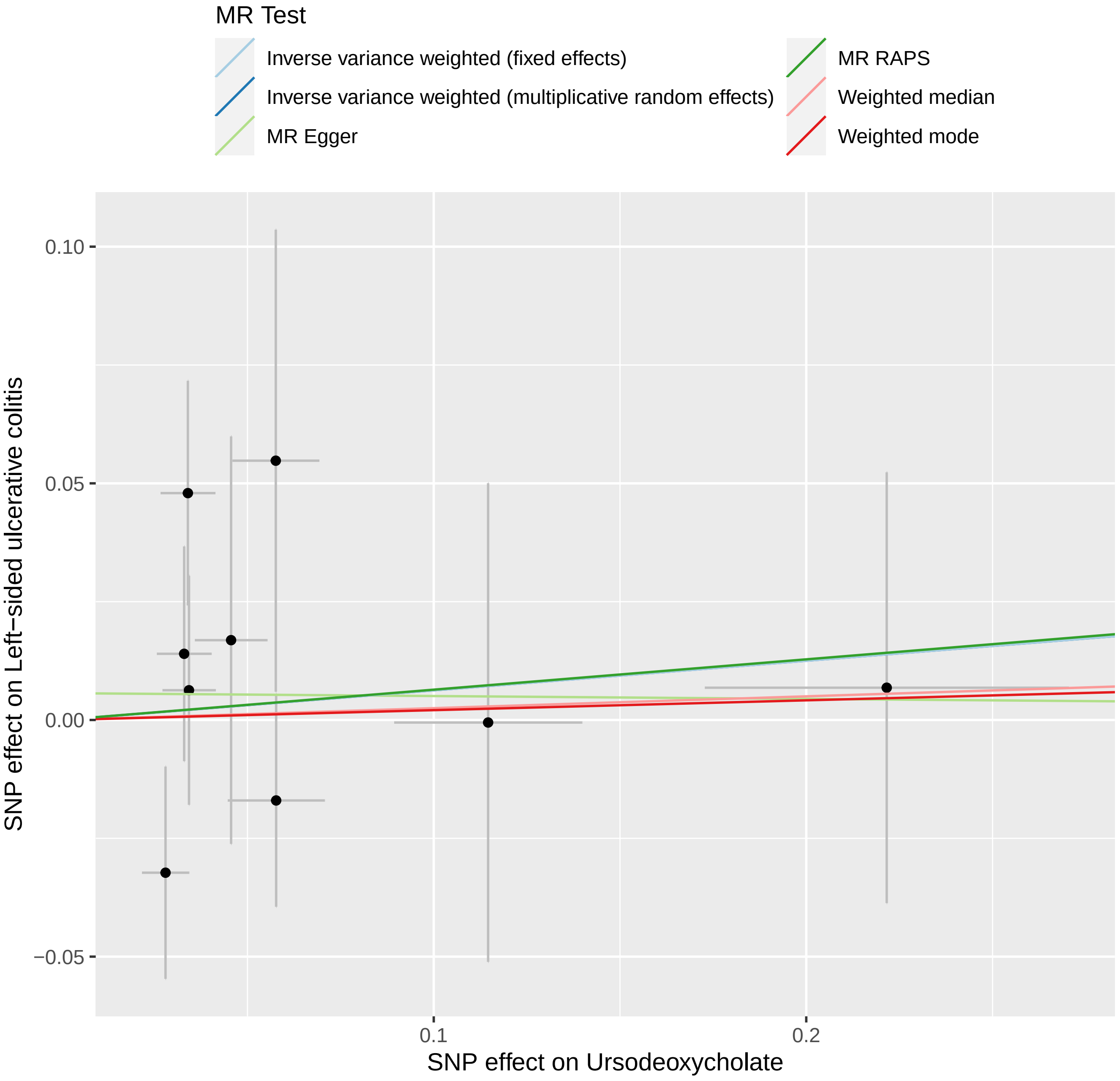

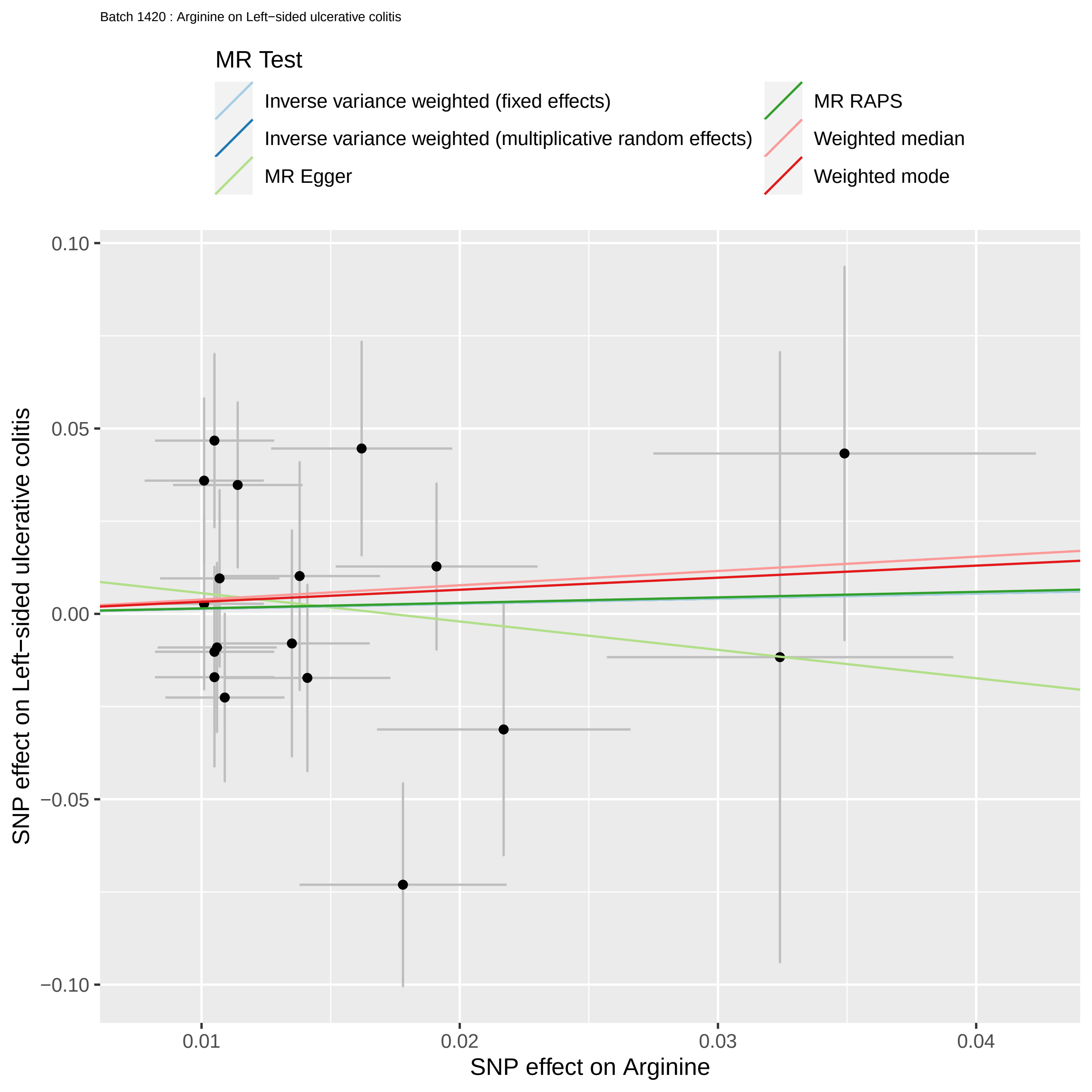

Batch 1420 : Arginine on Left−sided ulcerative colitis
MR Test
Inverse variance weighted (fixed effects)
Inverse variance weighted (multiplicative random effects)
MR Egger
MR RAPS
Weighted median
Weighted mode
0.10
0.05
0.00
−0.05
−0.10
0.01
0.02
0.03
0.04
SNP effect on Left−sided ulcerative colitis
SNP effect on Arginine

Batch 1421 : Ascorbate (Vitamin C) on Left−sided ulcerative colitis

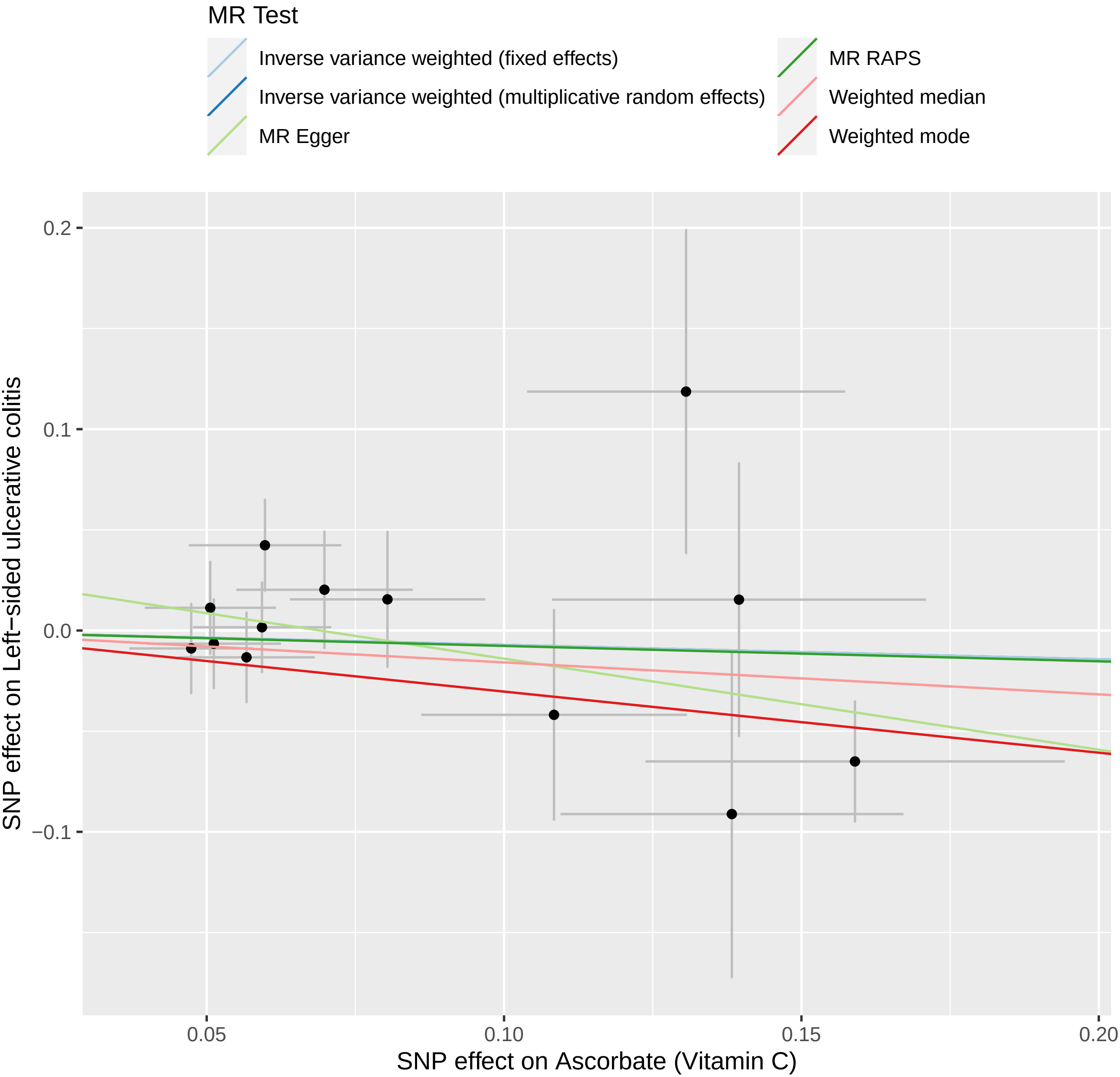

Batch 1422 : Heptanoate (7:0) on Left−sided ulcerative colitis

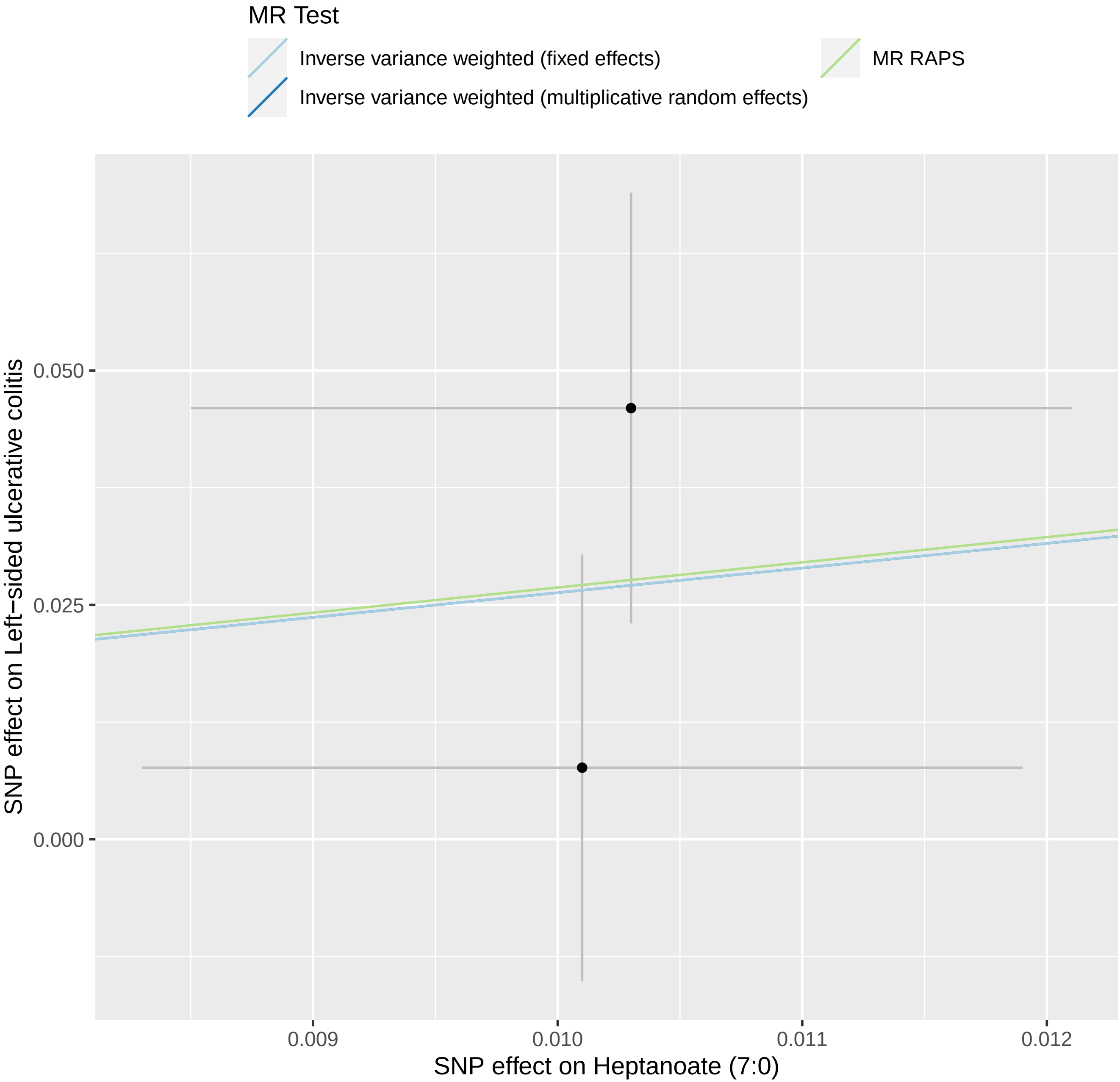

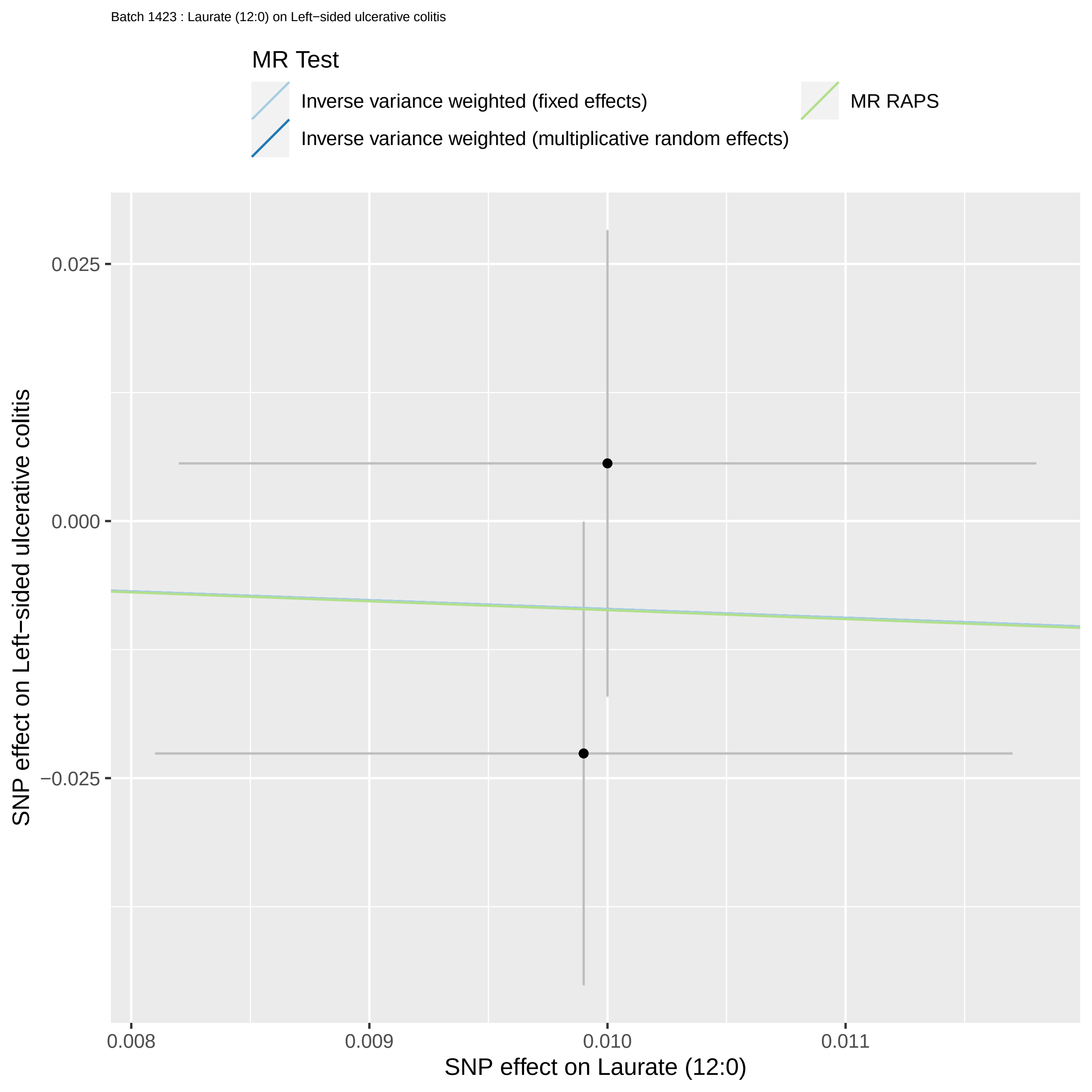

Batch 1423 : Laurate (12:0) on Left−sided ulcerative colitis
MR Test
Inverse variance weighted (fixed effects)
Inverse variance weighted (multiplicative random effects)
MR RAPS
0.025
0.000
−0.025
0.008
0.009
0.010
0.011
SNP effect on Laurate (12:0)
SNP effect on Left−sided ulcerative colitis

Batch 1425 : Urea on Left−sided ulcerative colitis

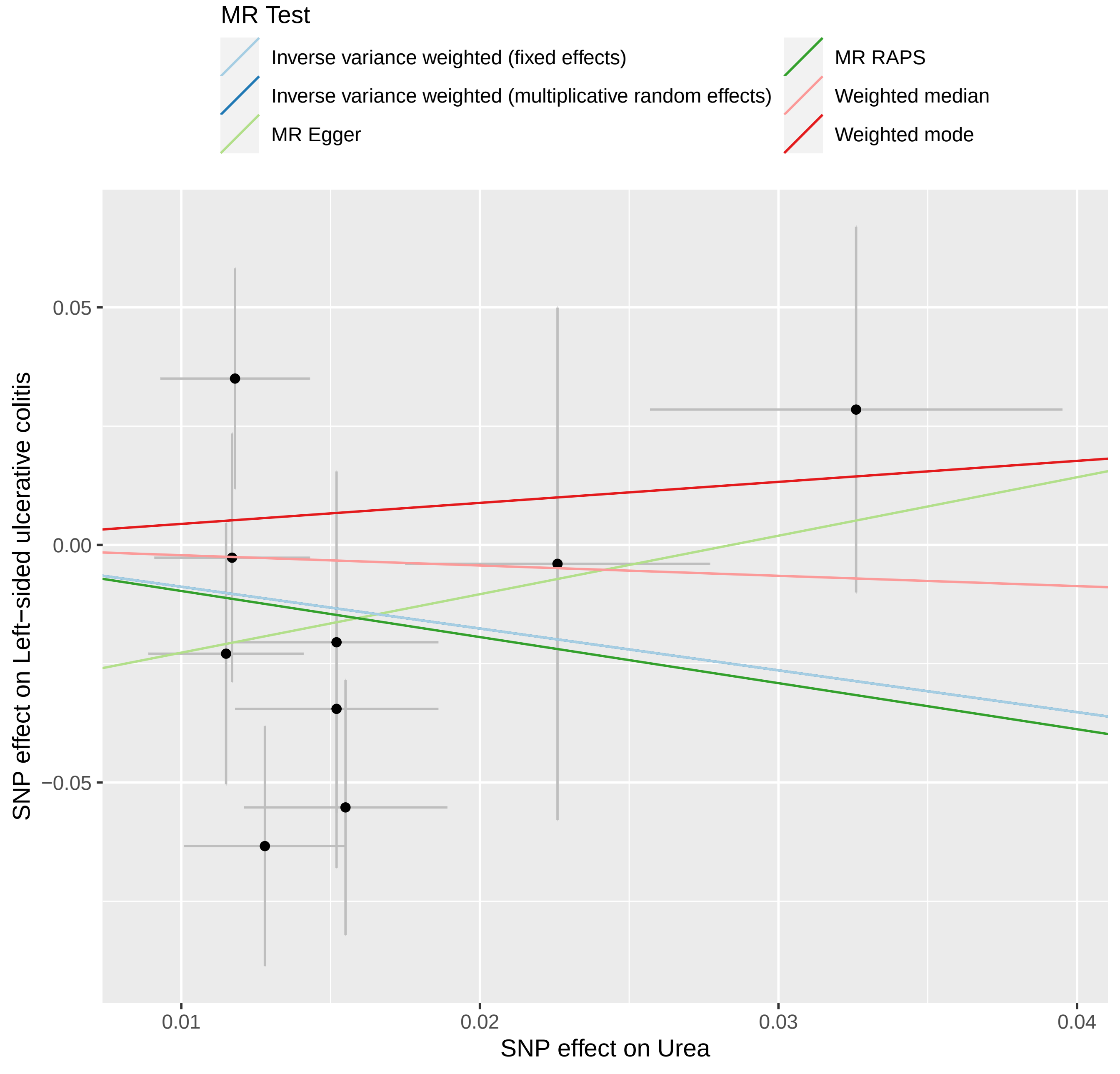

Batch 1426 : Cortisol on Left−sided ulcerative colitis

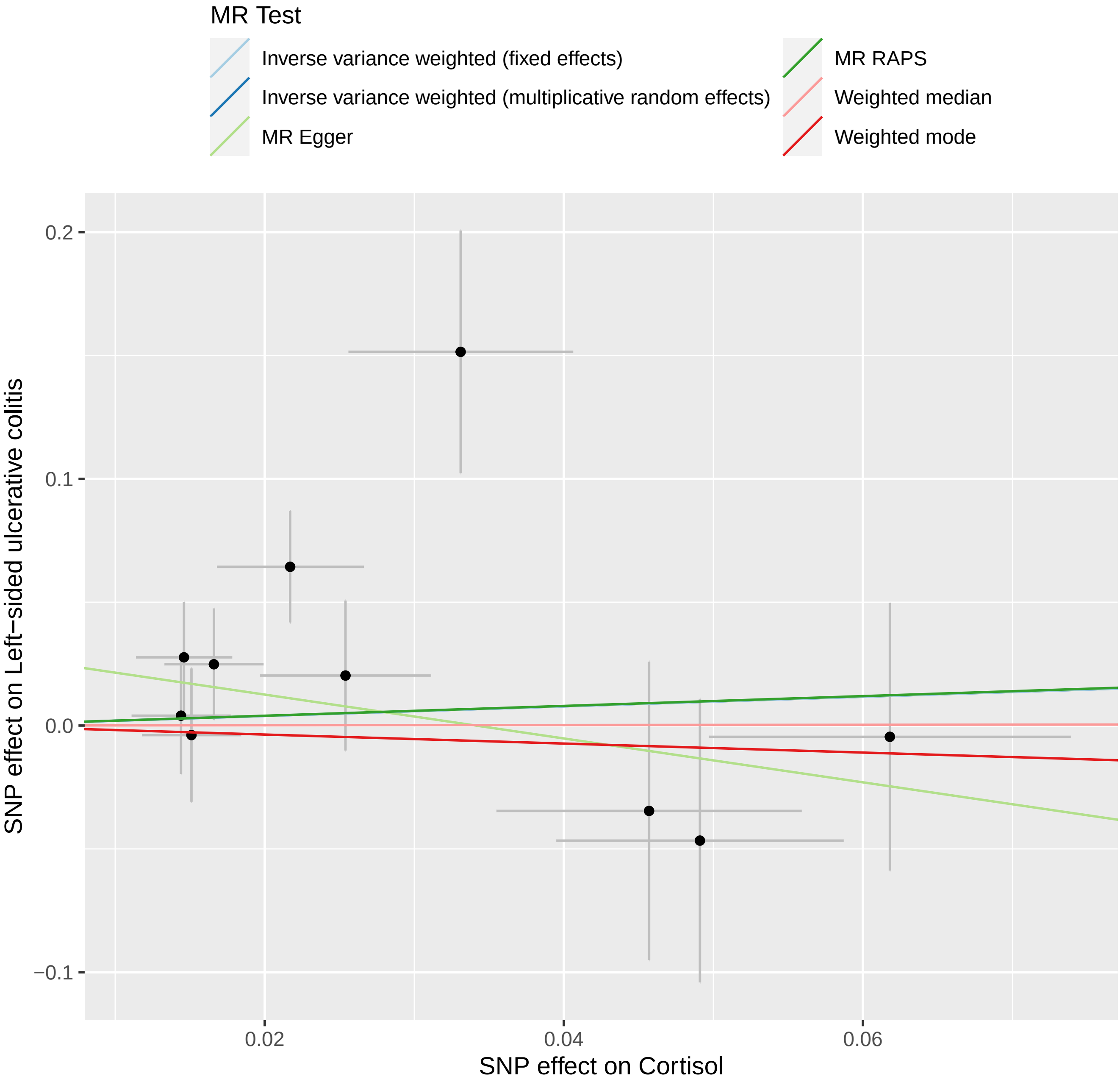

Batch 1429 : Citrulline on Left−sided ulcerative colitis

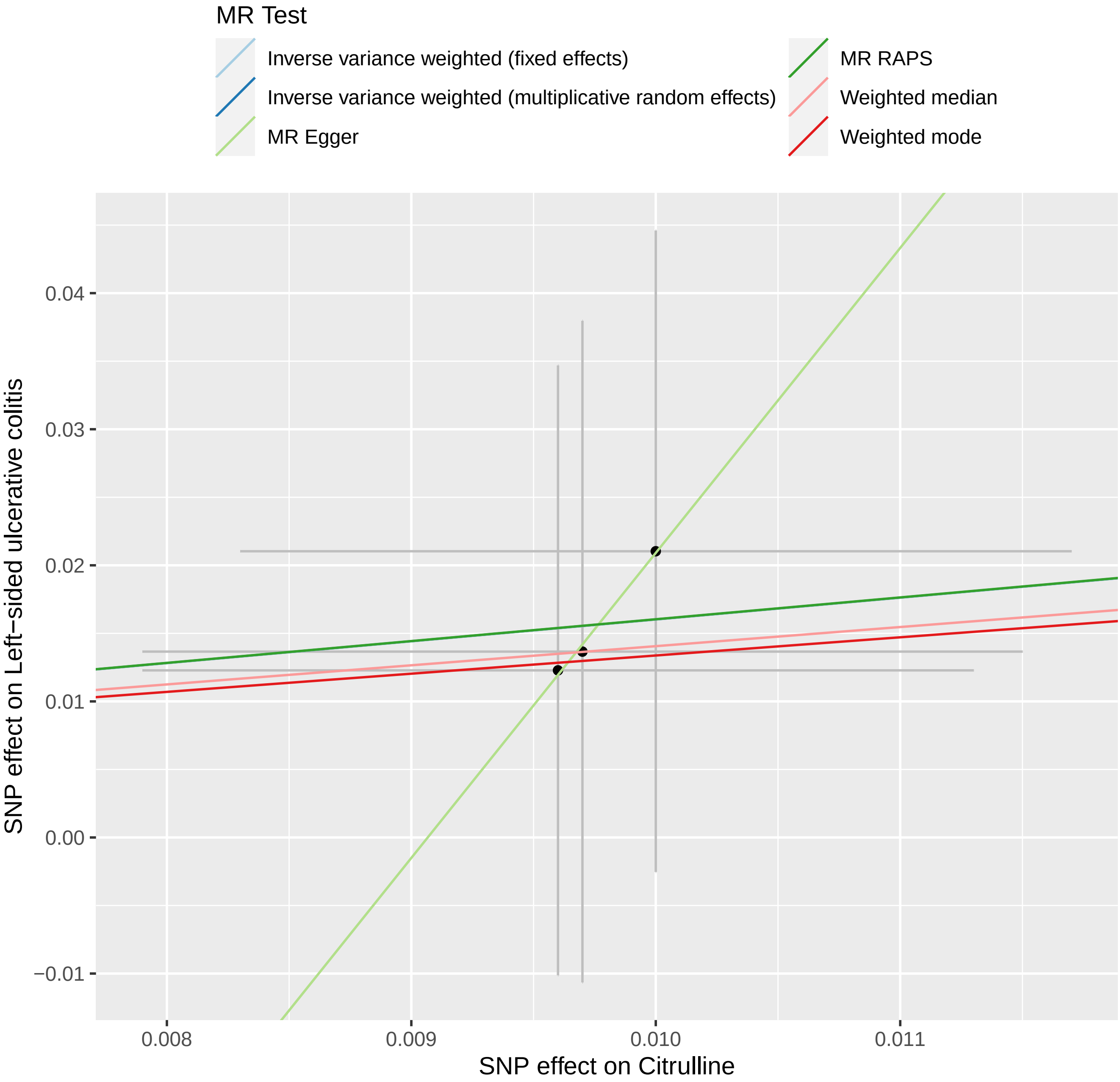

Batch 1431 : Serotonin (5HT) on Left−sided ulcerative colitis

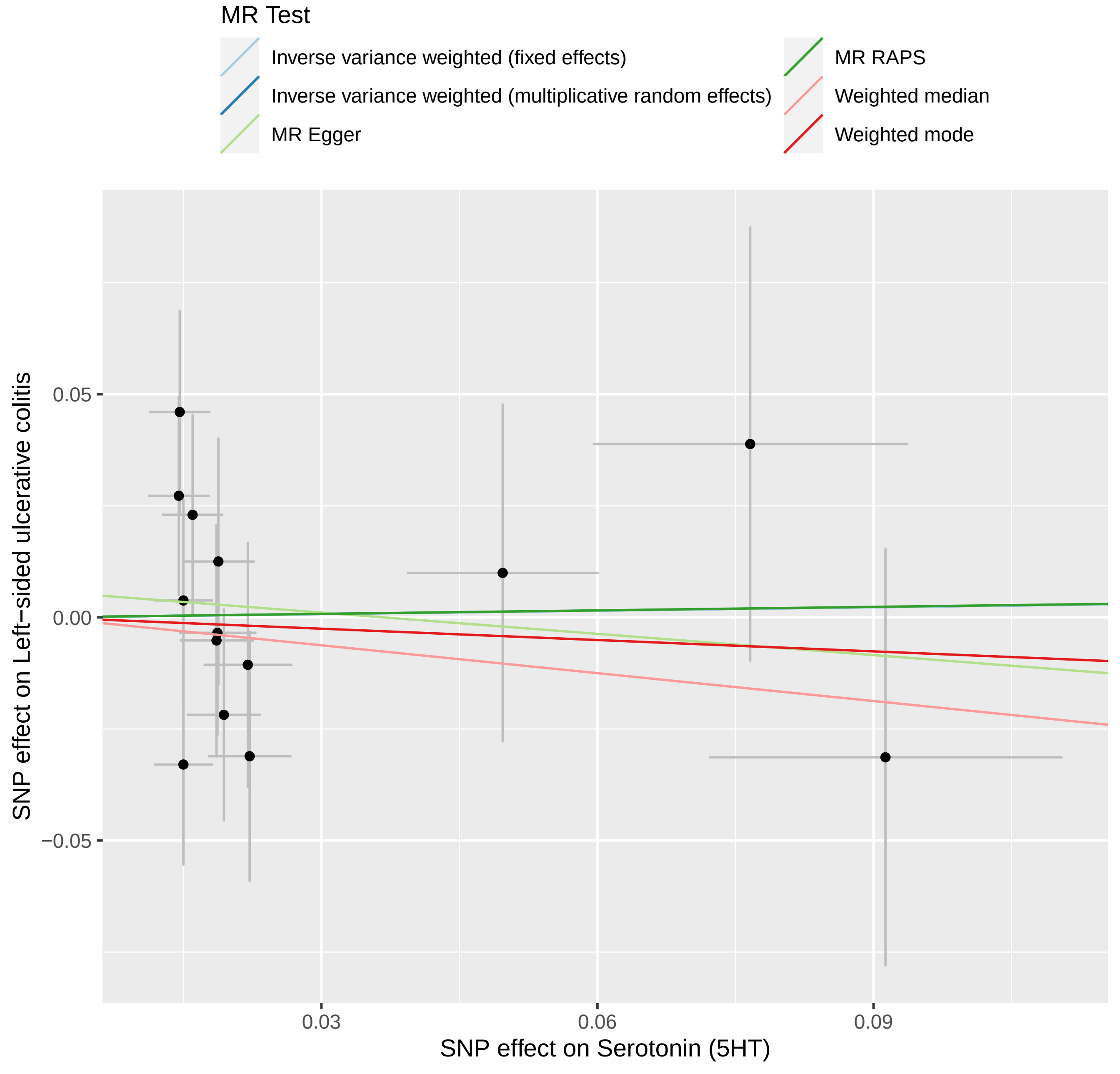

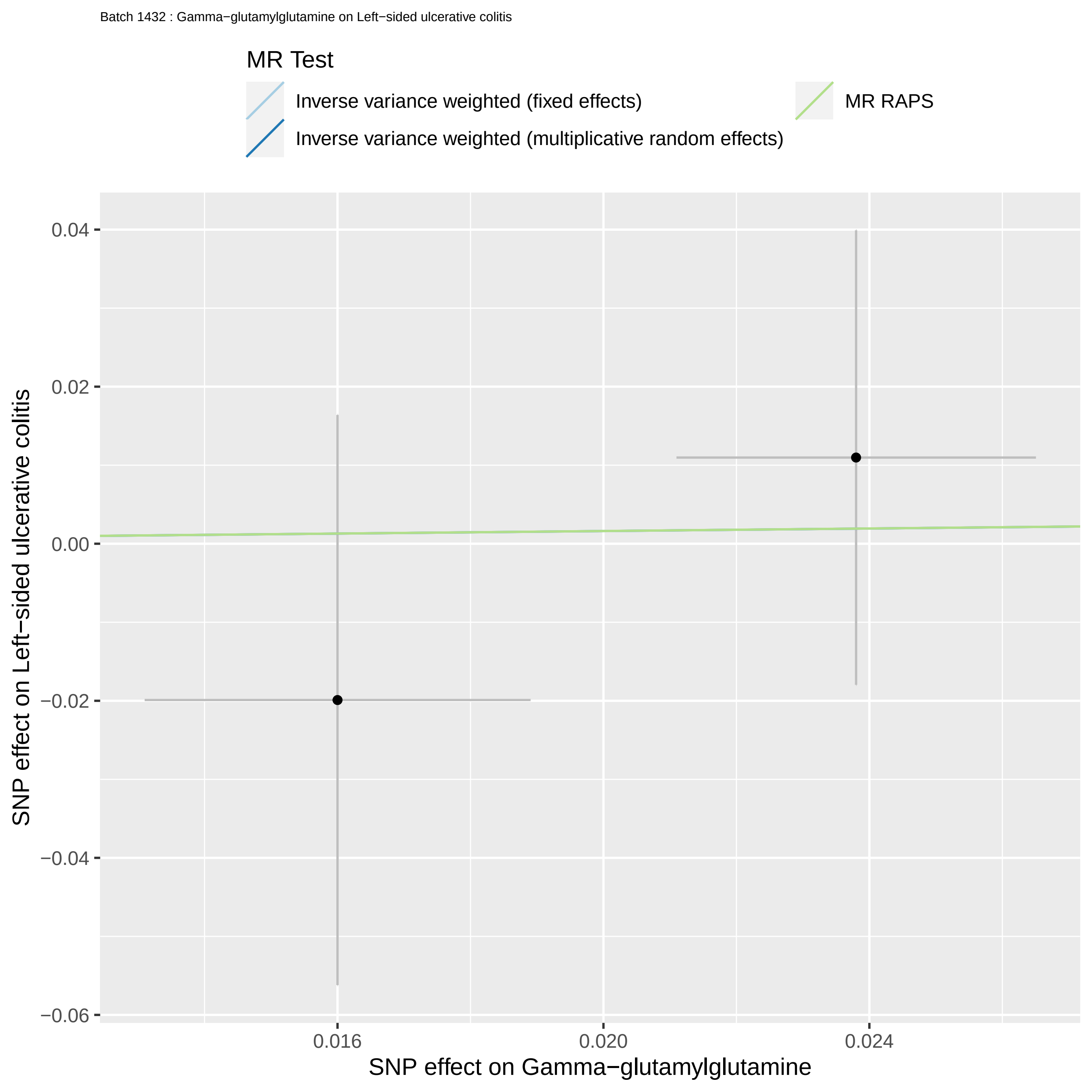

Batch 1432 : Gamma−glutamylglutamine on Left−sided ulcerative colitis
MR Test
Inverse variance weighted (fixed effects)
Inverse variance weighted (multiplicative random effects)
MR RAPS
0.04
0.02
0.00
−0.02
−0.04
−0.06
0.016
0.020
0.024
SNP effect on Left−sided ulcerative colitis
SNP effect on Gamma−glutamylglutamine

Batch 1433 : Gamma−glutamyltyrosine on Left−sided ulcerative colitis

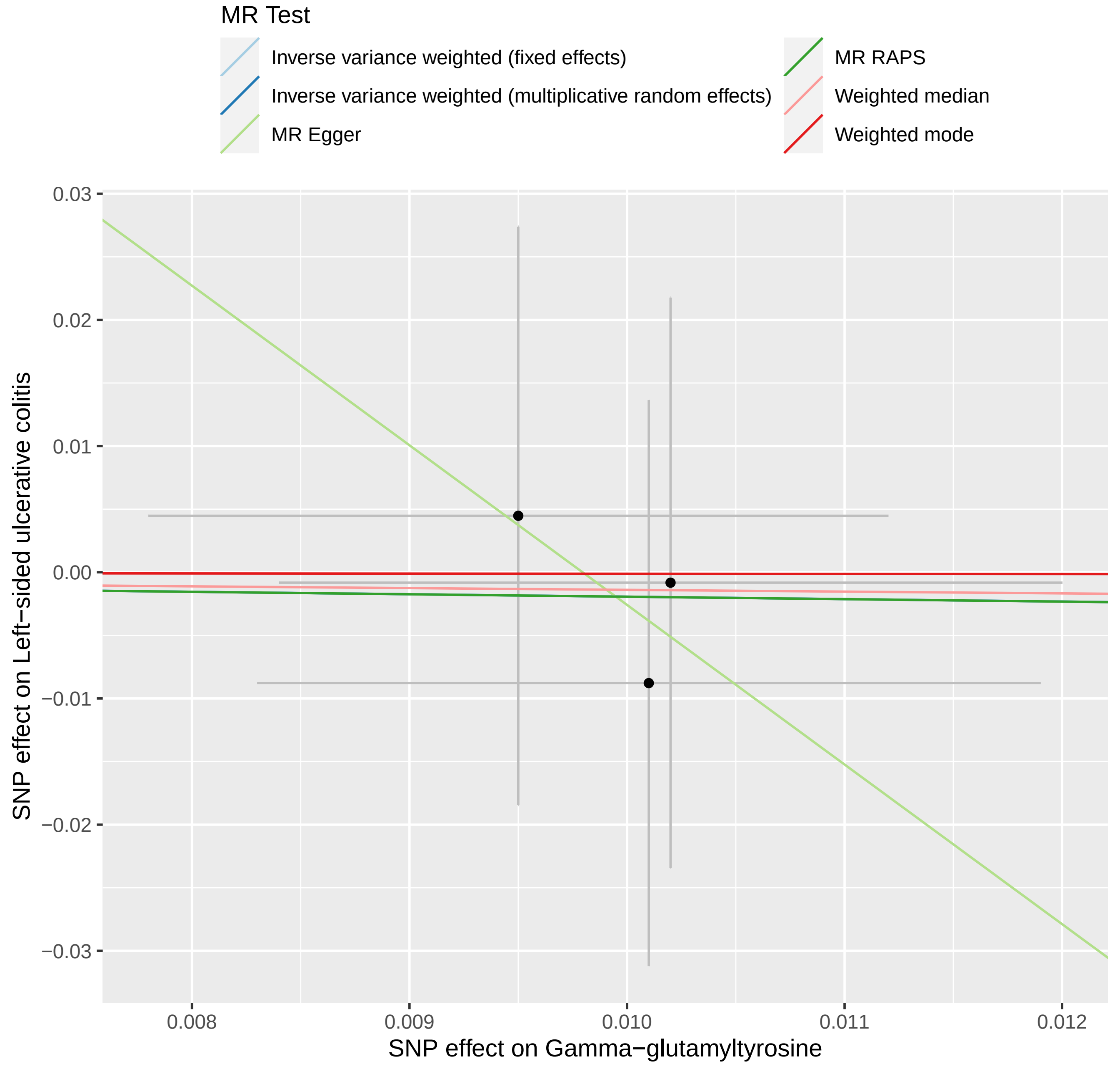

Batch 1435 : Betaine on Left−sided ulcerative colitis

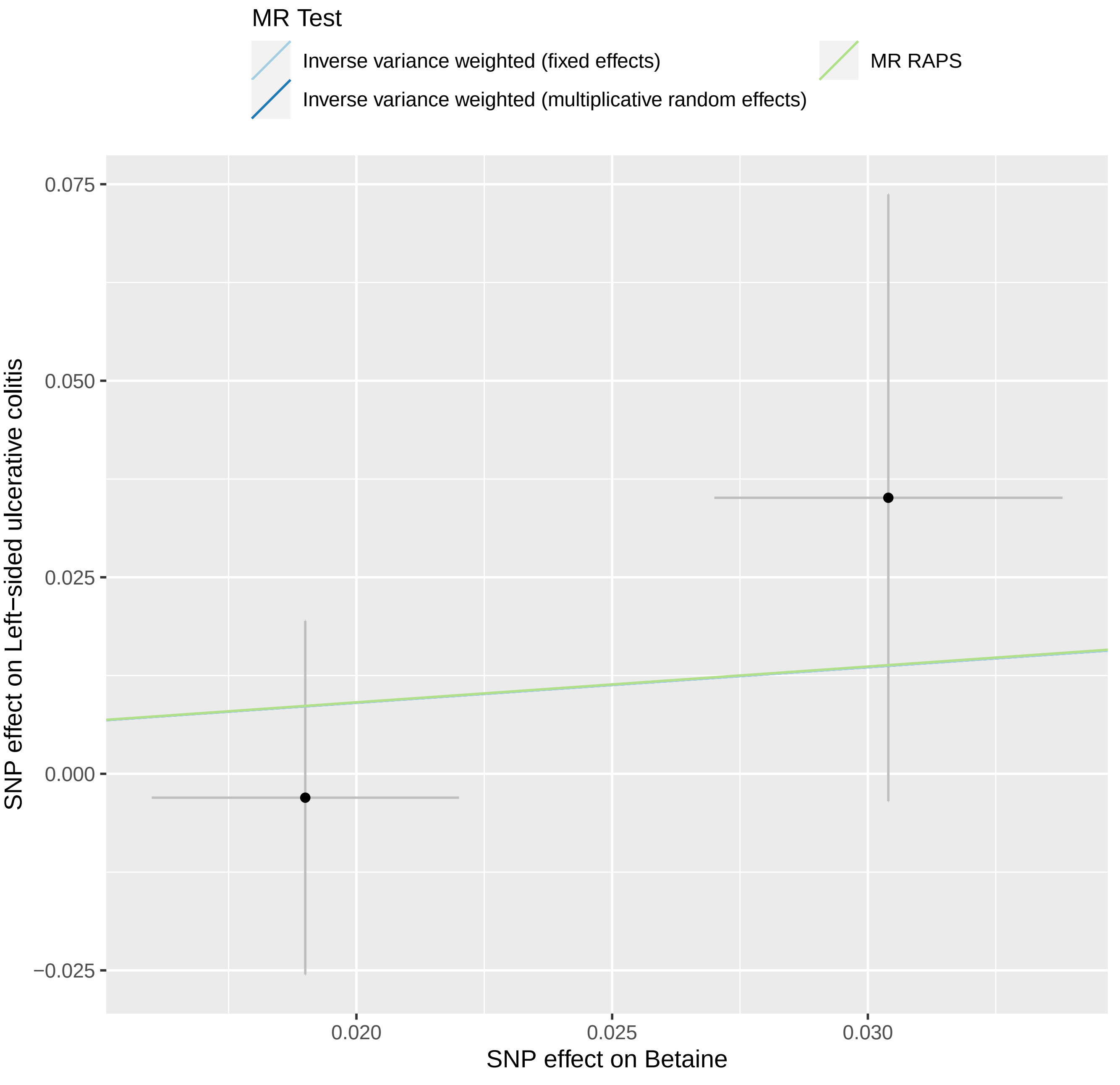

Batch 1436 : Xanthine on Left−sided ulcerative colitis

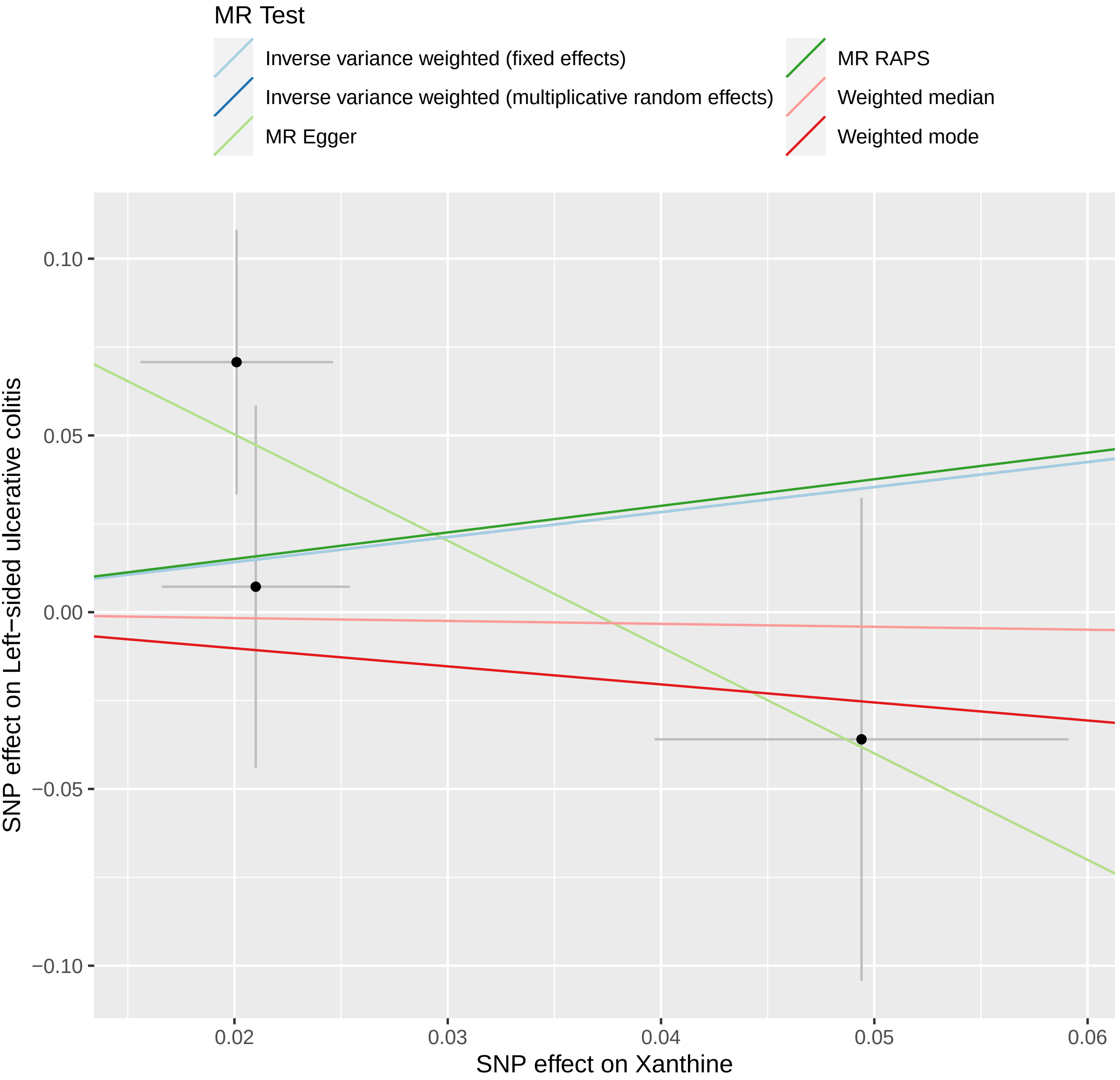

Batch 1437 : Phosphate on Left−sided ulcerative colitis

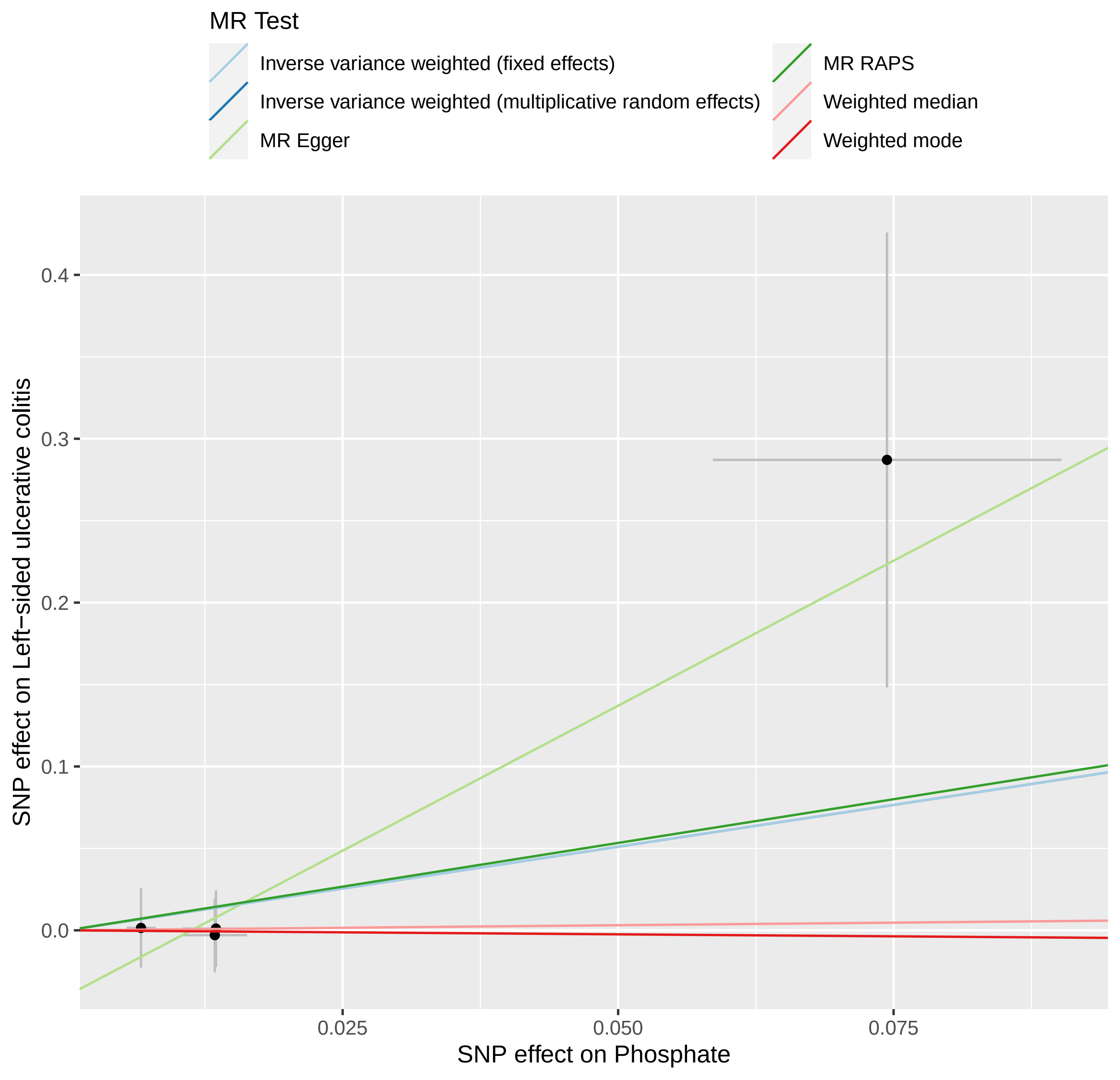

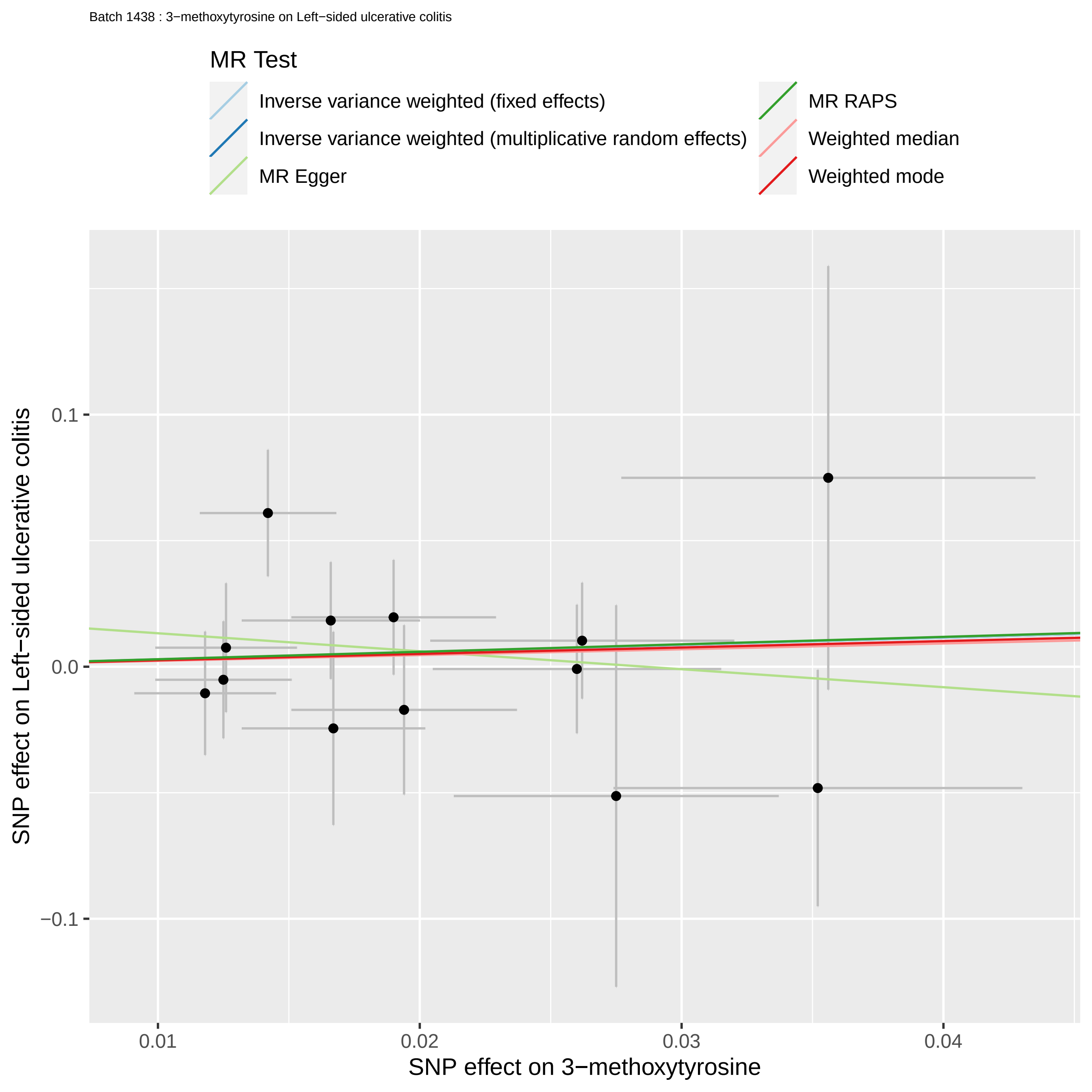

Batch 1438 : 3−methoxytyrosine on Left−sided ulcerative colitis
MR Test
Inverse variance weighted (fixed effects)
Inverse variance weighted (multiplicative random effects)
MR Egger
MR RAPS
Weighted median
Weighted mode
0.1
0.0
−0.1
0.01
0.02
0.03
0.04
SNP effect on 3−methoxytyrosine
SNP effect on Left−sided ulcerative colitis

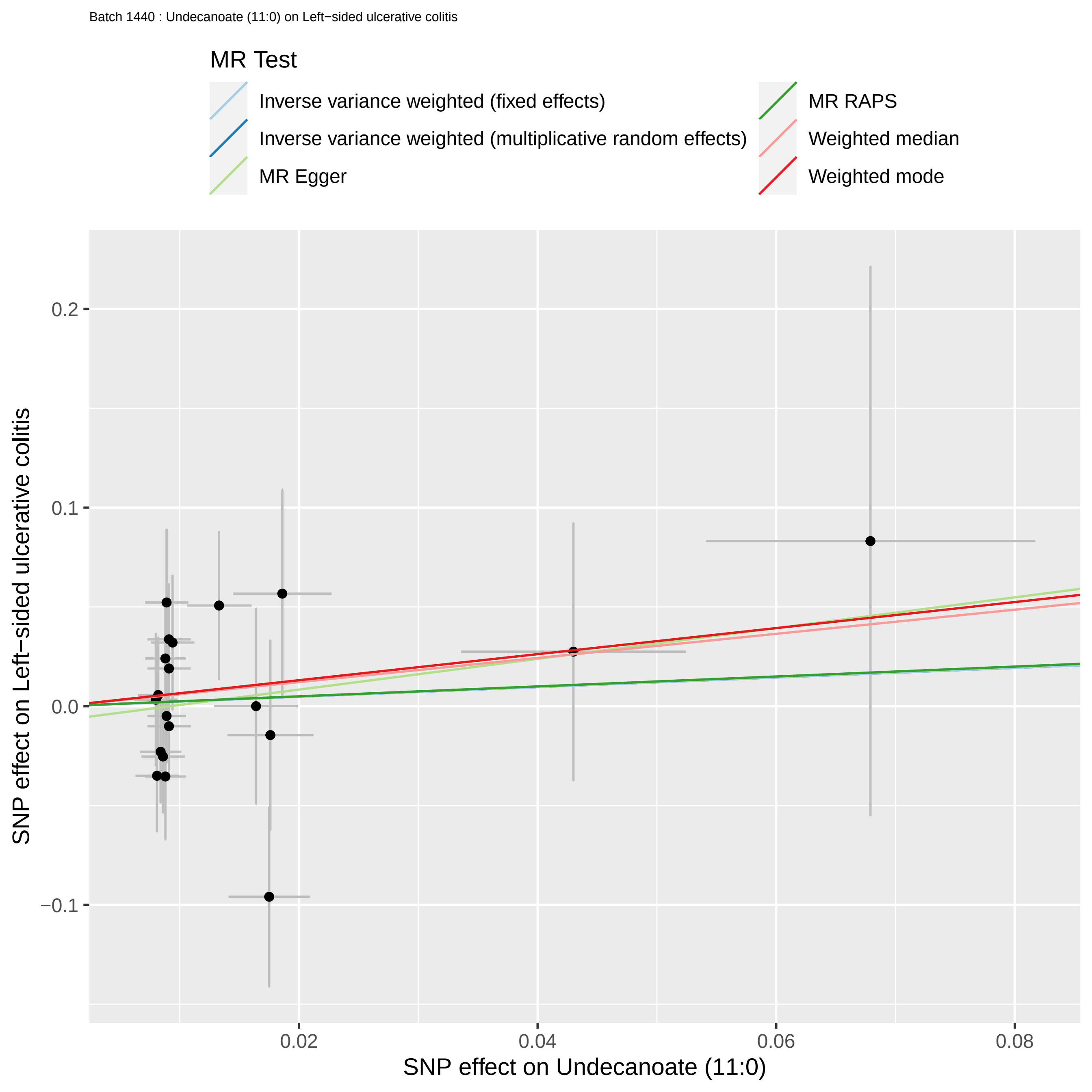
Batch 1440 : Undecanoate (11:0) on Left−sided ulcerative colitis
MR Test
Inverse variance weighted (fixed effects)
Inverse variance weighted (multiplicative random effects)
MR Egger
MR RAPS
Weighted median
Weighted mode
0.2
0.1
0.0
−0.1
0.02
0.04
0.06
0.08
SNP effect on Undecanoate (11:0)
SNP effect on Left−sided ulcerative colitis

Batch 1442 : Taurodeoxycholate on Left−sided ulcerative colitis

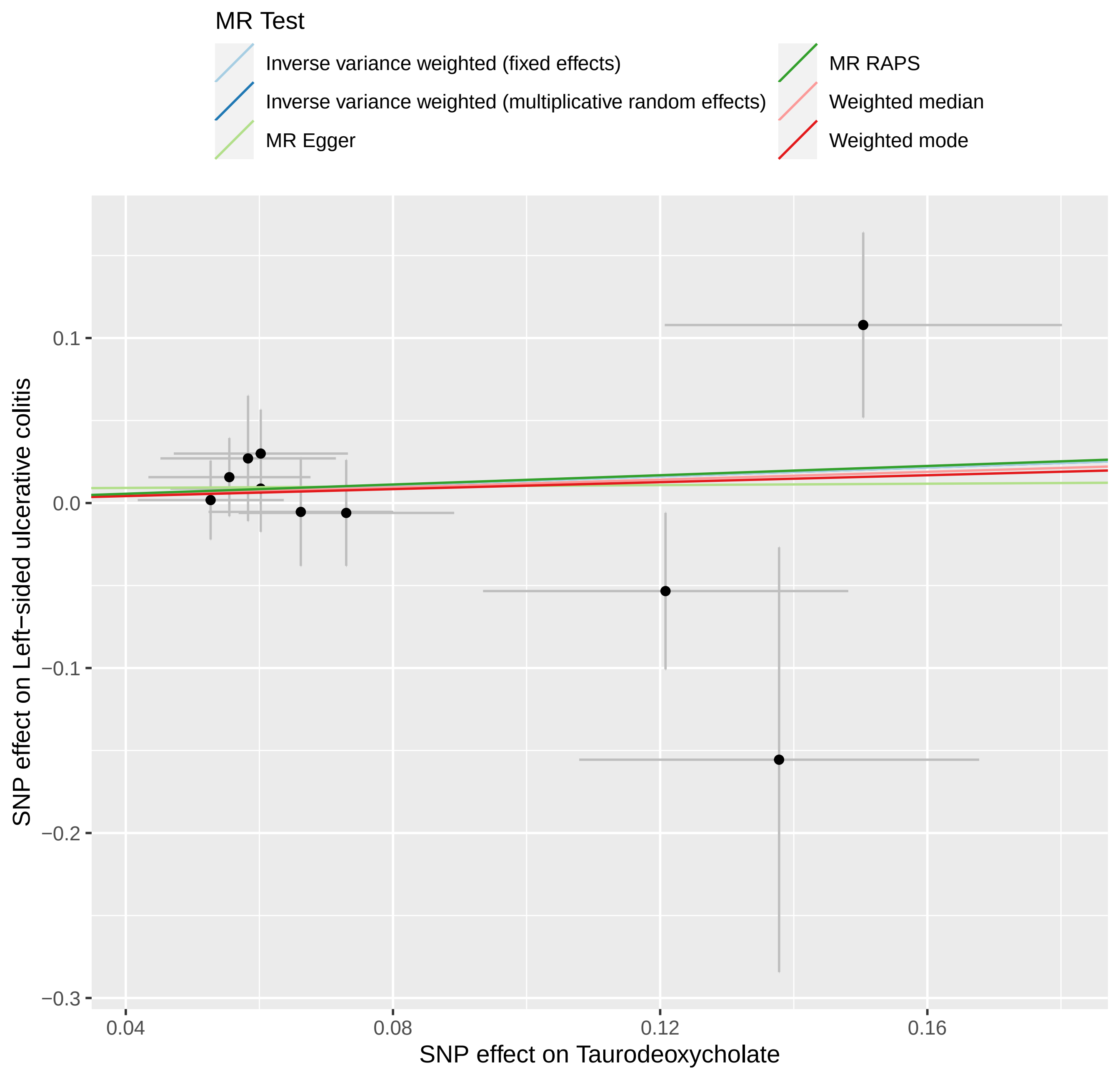

Batch 1443 : Glycerol on Left−sided ulcerative colitis

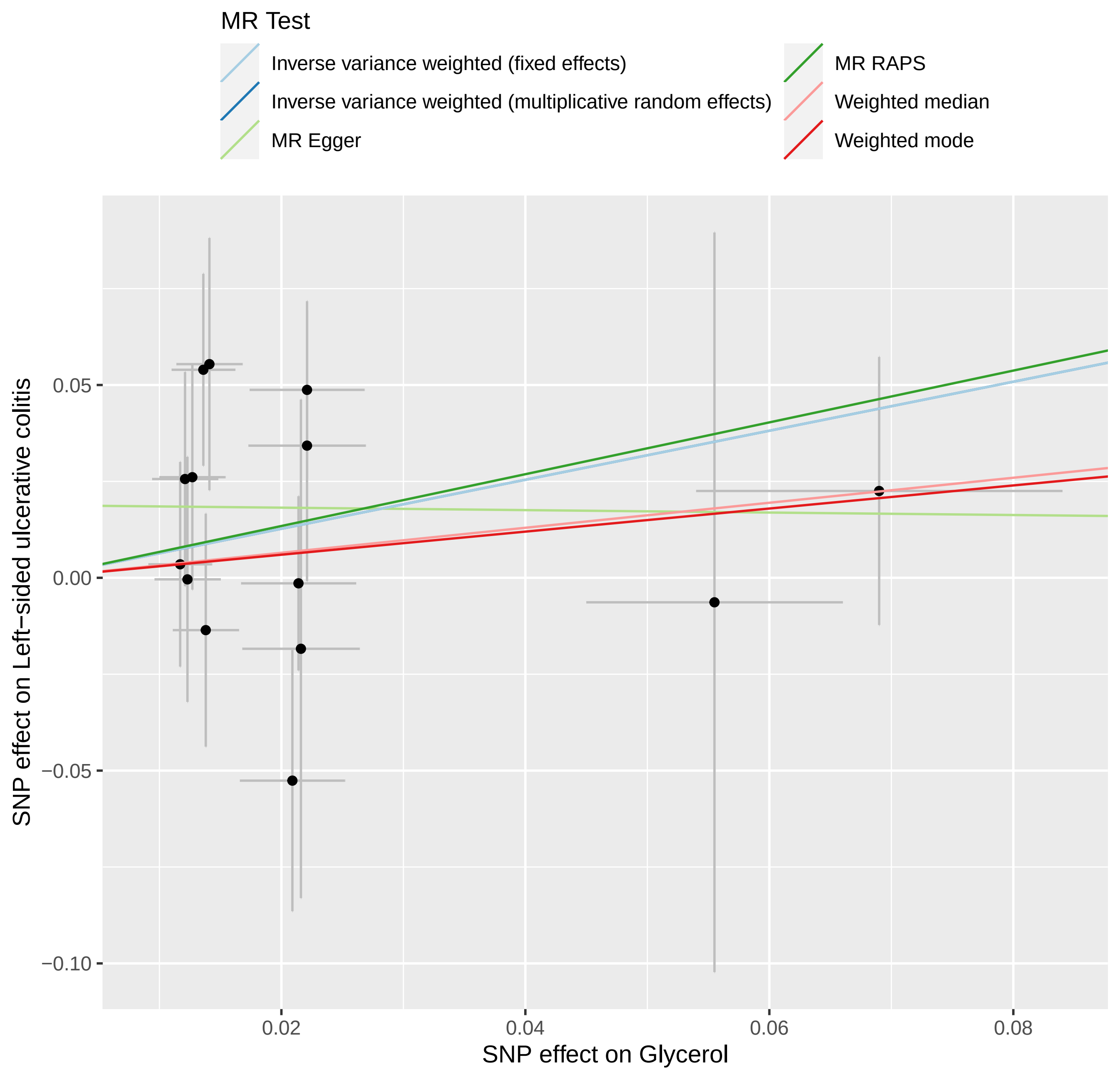

Batch 1444 : Kynurenine on Left−sided ulcerative colitis

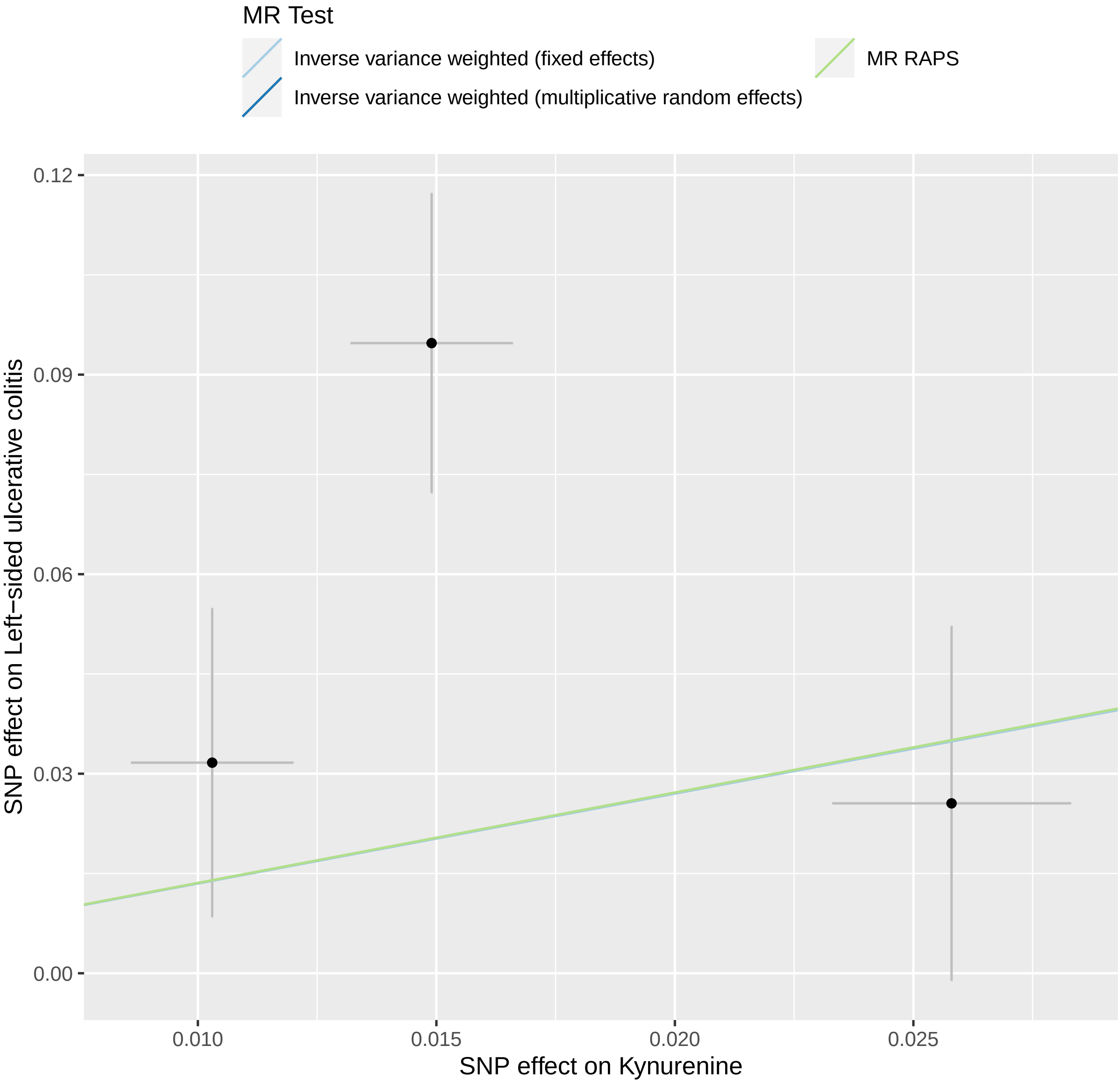

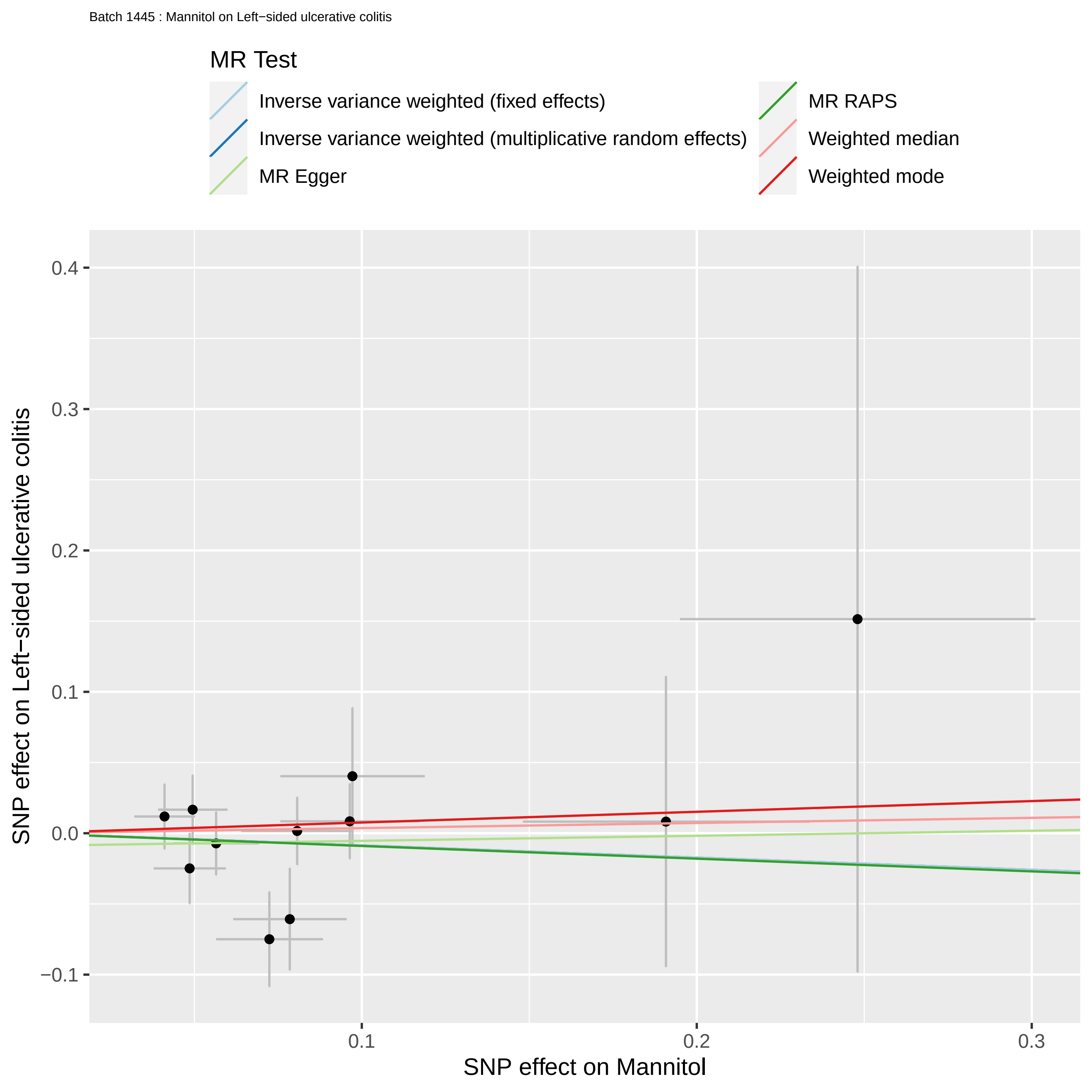
Batch 1445 : Mannitol on Left−sided ulcerative colitis
MR Test
Inverse variance weighted (fixed effects)
Inverse variance weighted (multiplicative random effects)
MR Egger
MR RAPS
Weighted median
Weighted mode
SNP effect on Left−sided ulcerative colitis
0.4
0.3
0.2
0.1
0.0
−0.1
0.1
0.2
0.3
SNP effect on Mannitol

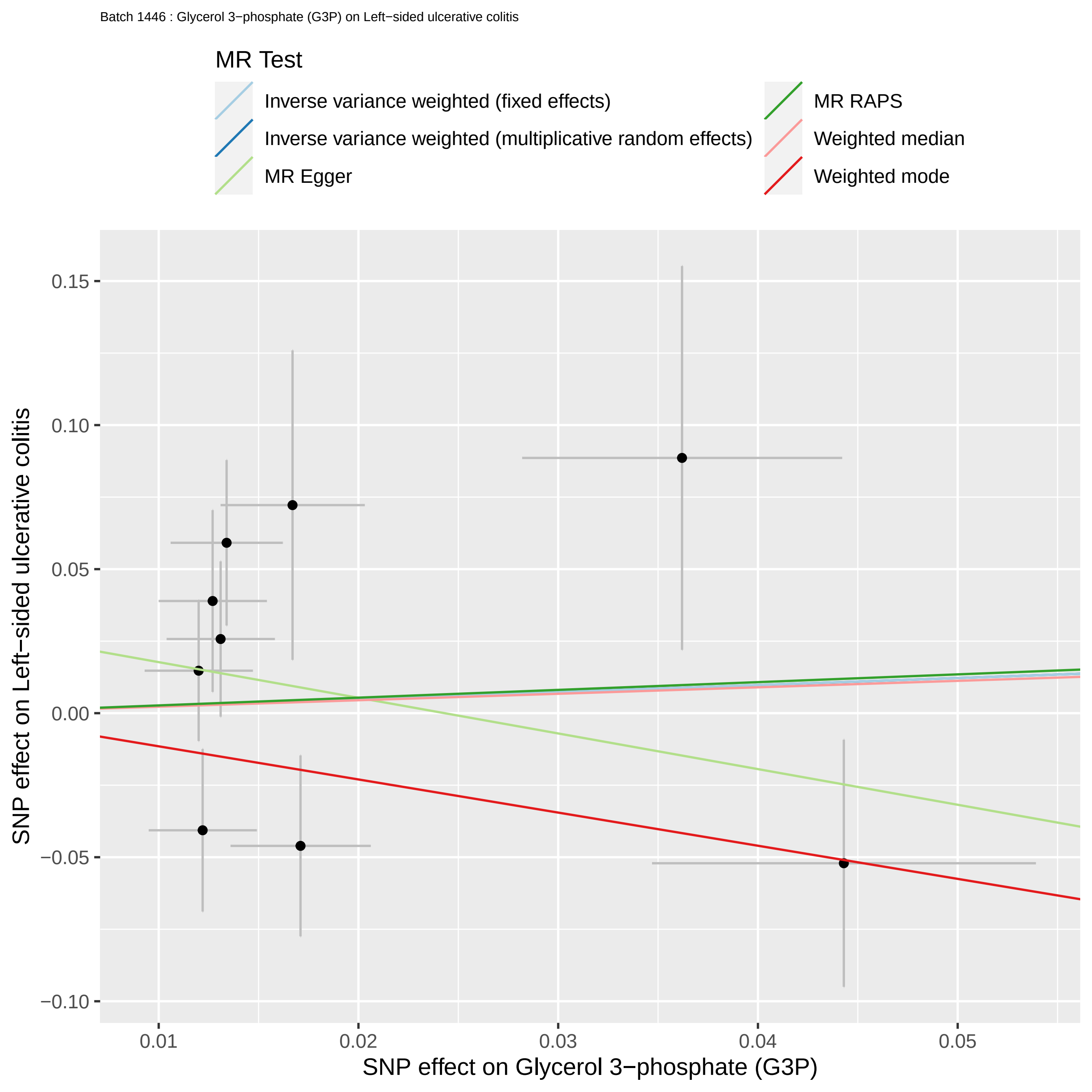
Batch 1446 : Glycerol 3−phosphate (G3P) on Left−sided ulcerative colitis
MR Test
Inverse variance weighted (fixed effects)
Inverse variance weighted (multiplicative random effects)
MR Egger
MR RAPS
Weighted median
Weighted mode
SNP effect on Left−sided ulcerative colitis
0.15
0.10
0.05
0.00
−0.05
−0.10
0.01
0.02
0.03
0.04
0.05
SNP effect on Glycerol 3−phosphate (G3P)

Batch 1448 : Carnitine on Left−sided ulcerative colitis

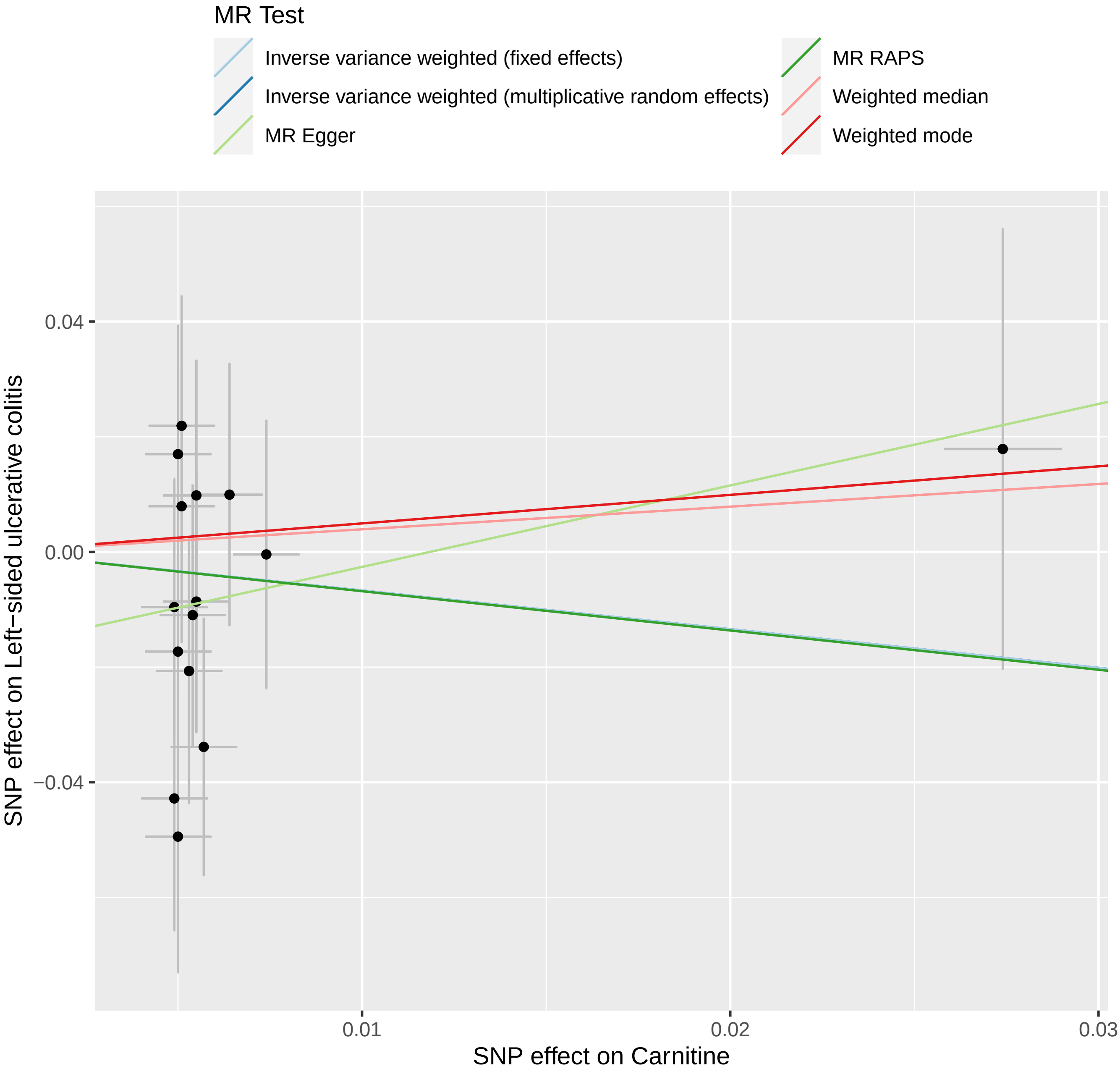

Batch 1449 : Choline on Left−sided ulcerative colitis

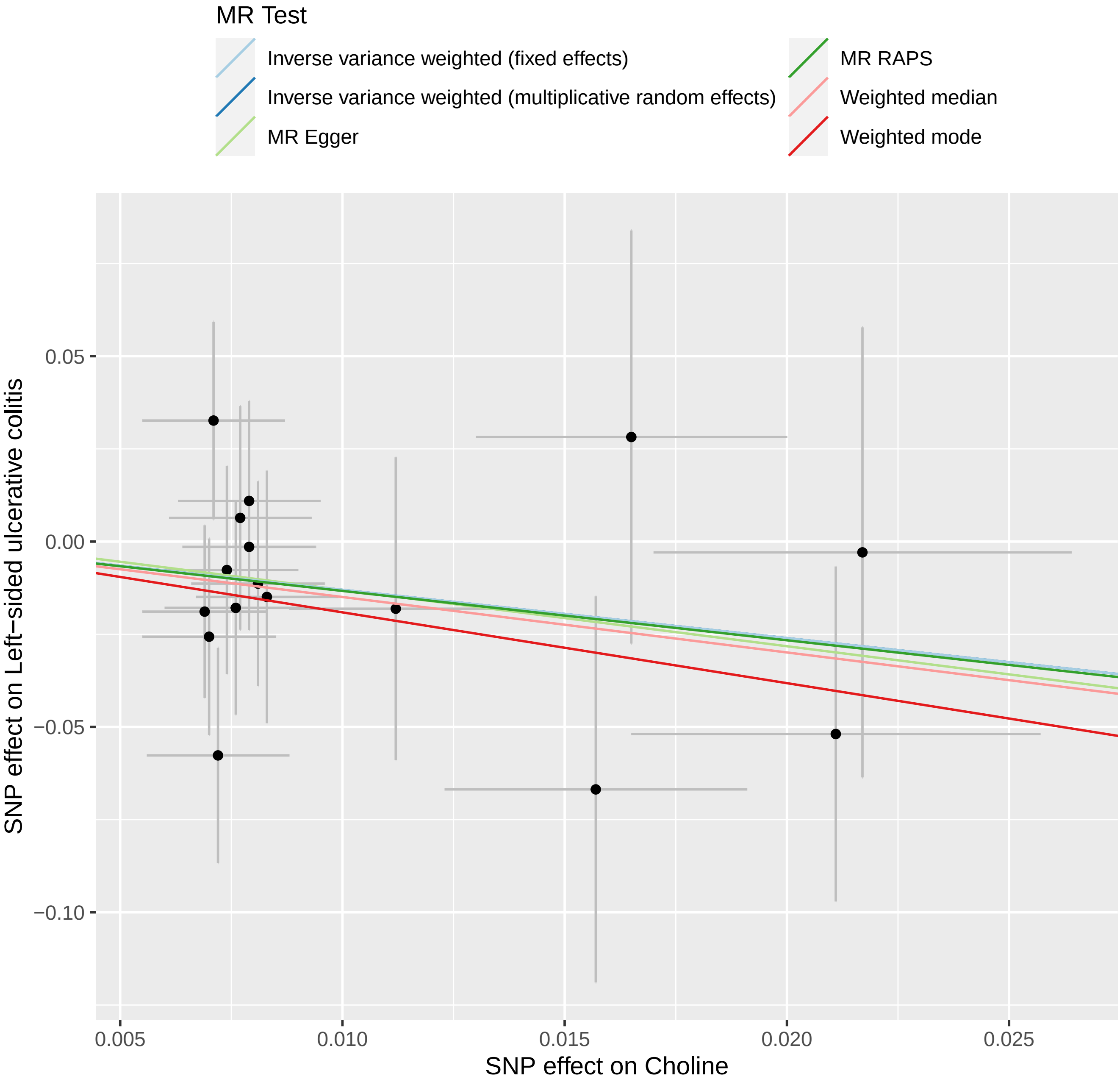

Batch 1452 : 3−methyl−2−oxovalerate on Left−sided ulcerative colitis

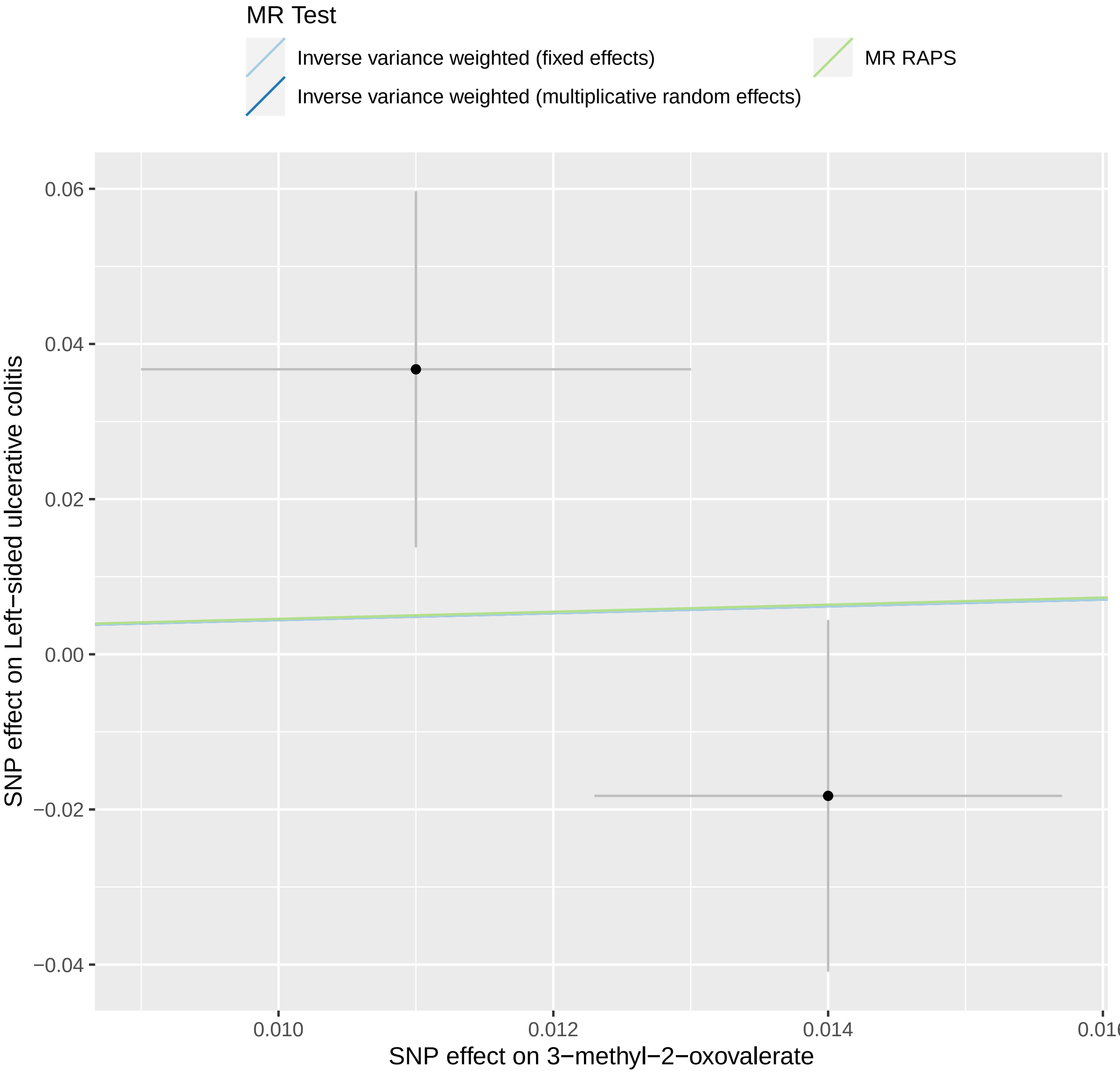

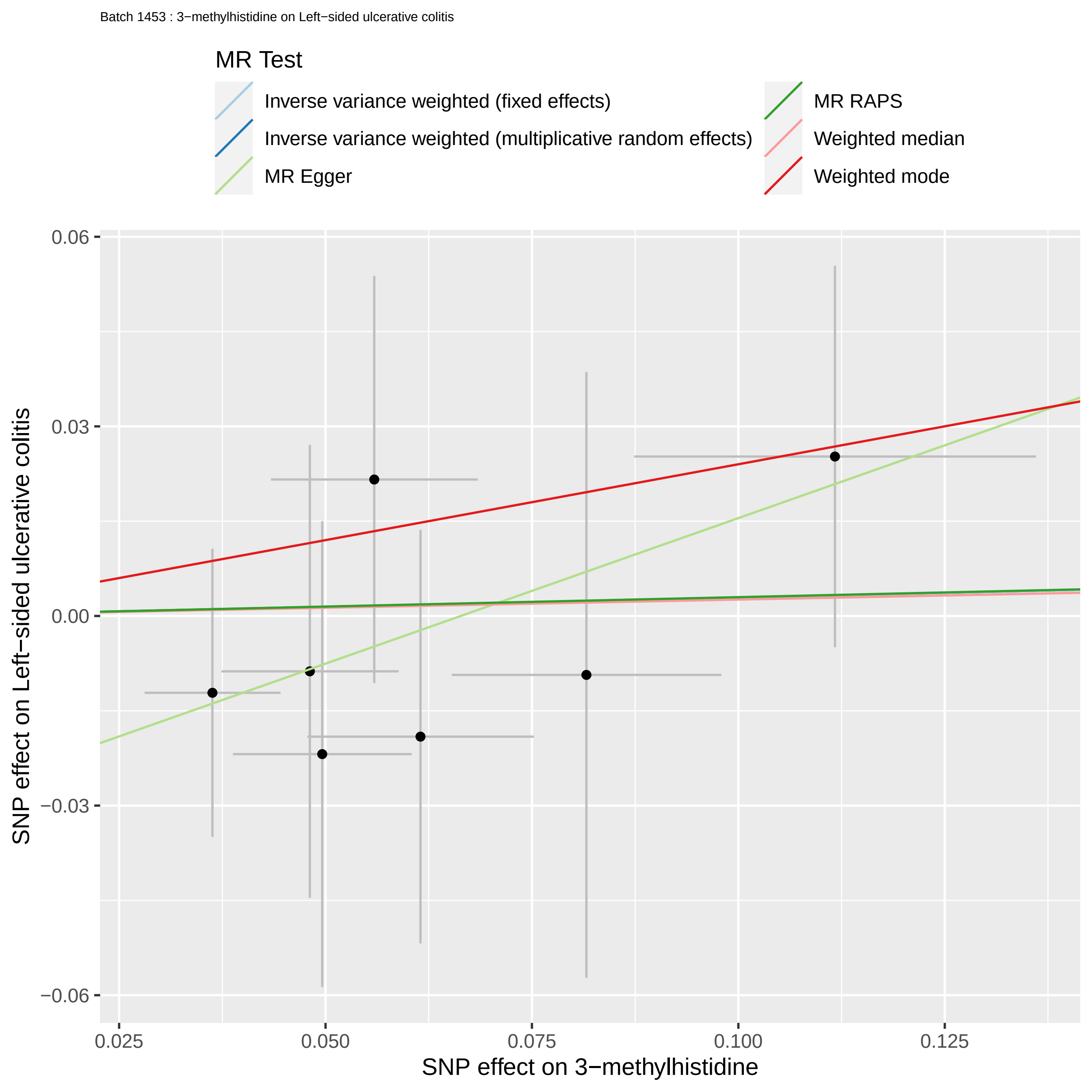
Batch 1453 : 3−methylhistidine on Left−sided ulcerative colitis
MR Test
Inverse variance weighted (fixed effects)
Inverse variance weighted (multiplicative random effects)
MR Egger
MR RAPS
Weighted median
Weighted mode
0.06
0.03
0.00
−0.03
−0.06
0.025
0.050
0.075
0.100
0.125
SNP effect on Left−sided ulcerative colitis
SNP effect on 3−methylhistidine

Batch 1455 : Phenylacetate on Left−sided ulcerative colitis

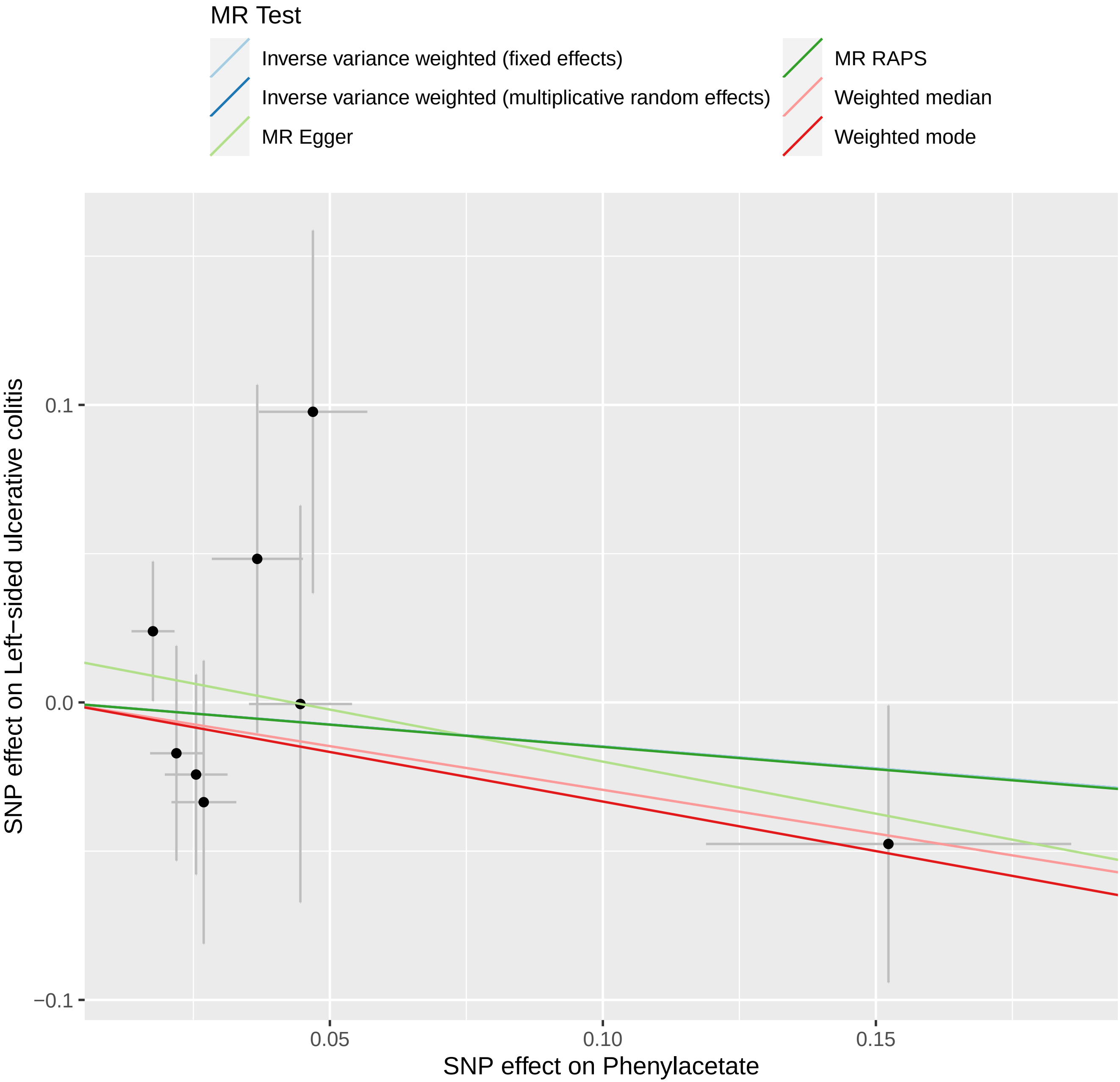

Batch 1456 : Glycerophosphorylcholine (GPC) on Left−sided ulcerative colitis

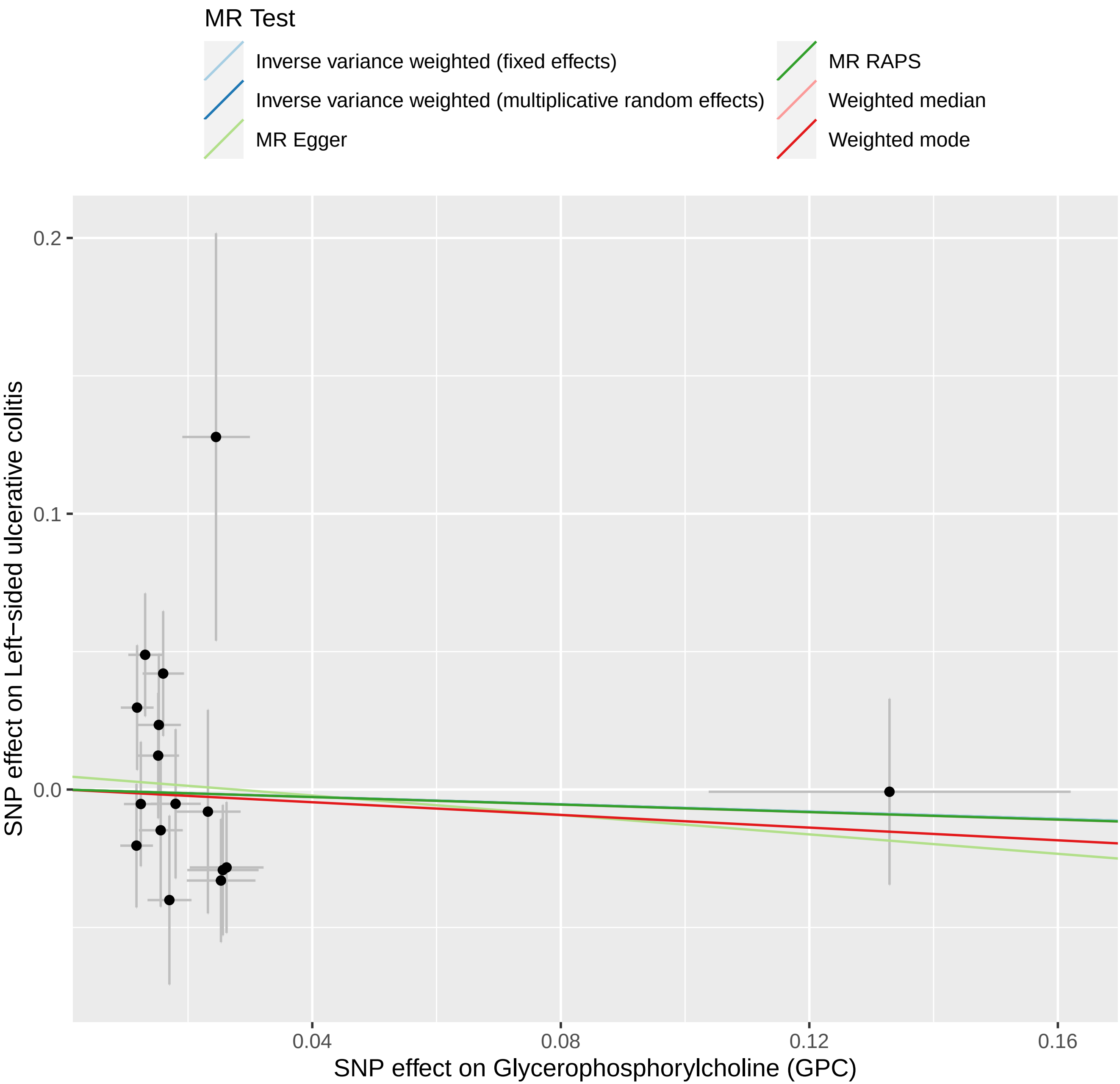

Batch 1457 : Aspartate on Left−sided ulcerative colitis

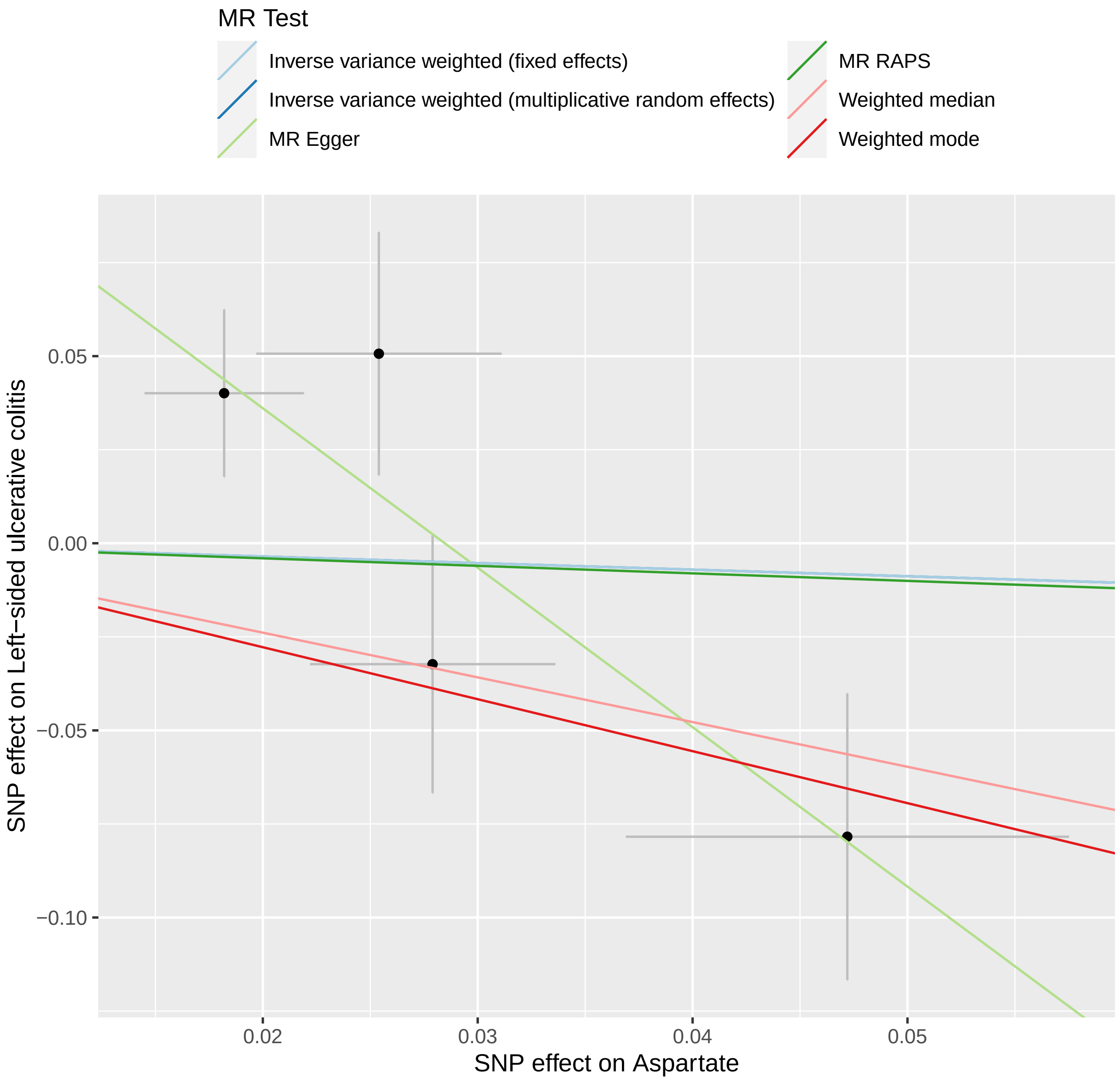

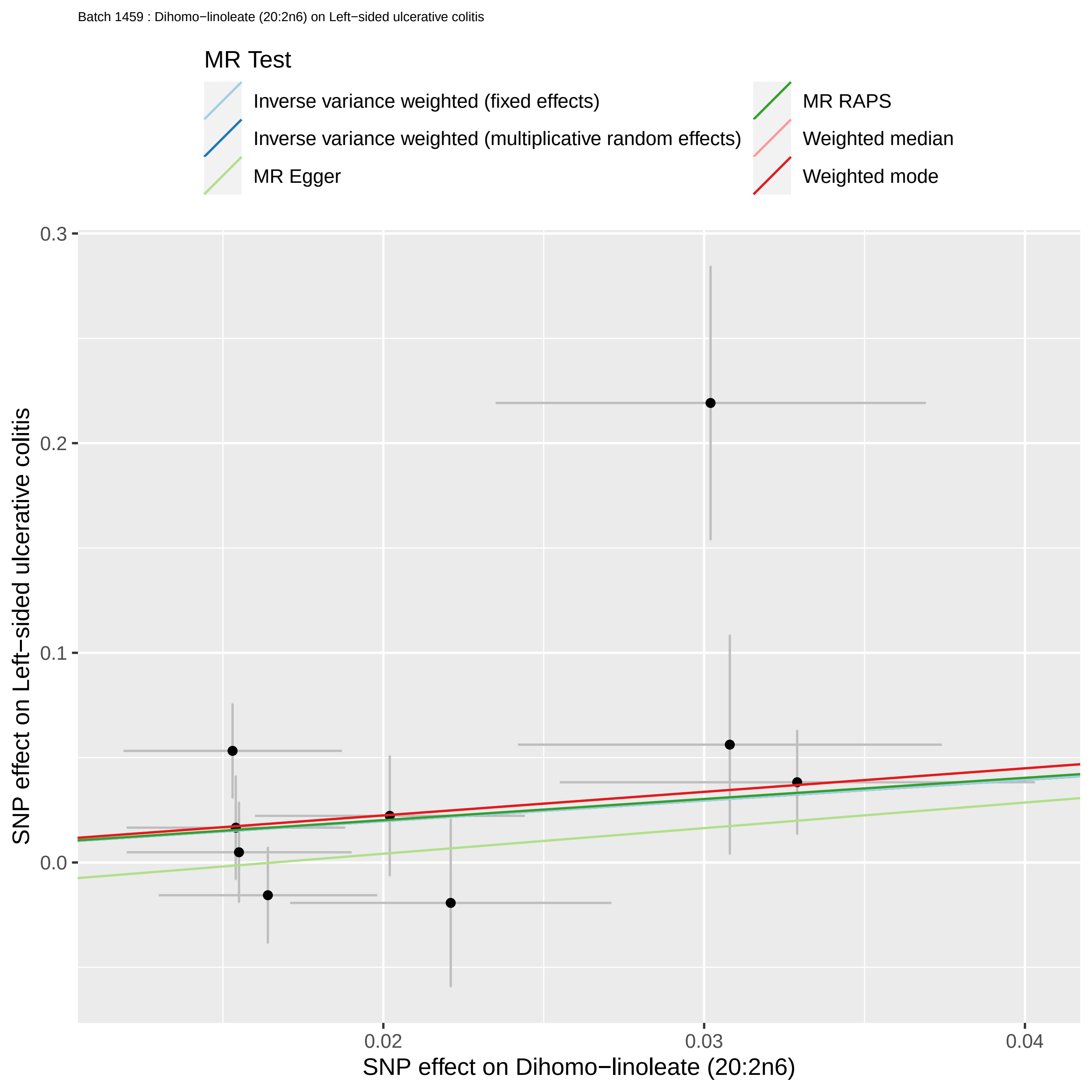
Batch 1459 : Dihomo−linoleate (20:2n6) on Left−sided ulcerative colitis
MR Test
Inverse variance weighted (fixed effects)
Inverse variance weighted (multiplicative random effects)
MR Egger
MR RAPS
Weighted median
Weighted mode
0.3
0.2
0.1
0.0
0.02
0.03
0.04
SNP effect on Left−sided ulcerative colitis
SNP effect on Dihomo−linoleate (20:2n6)

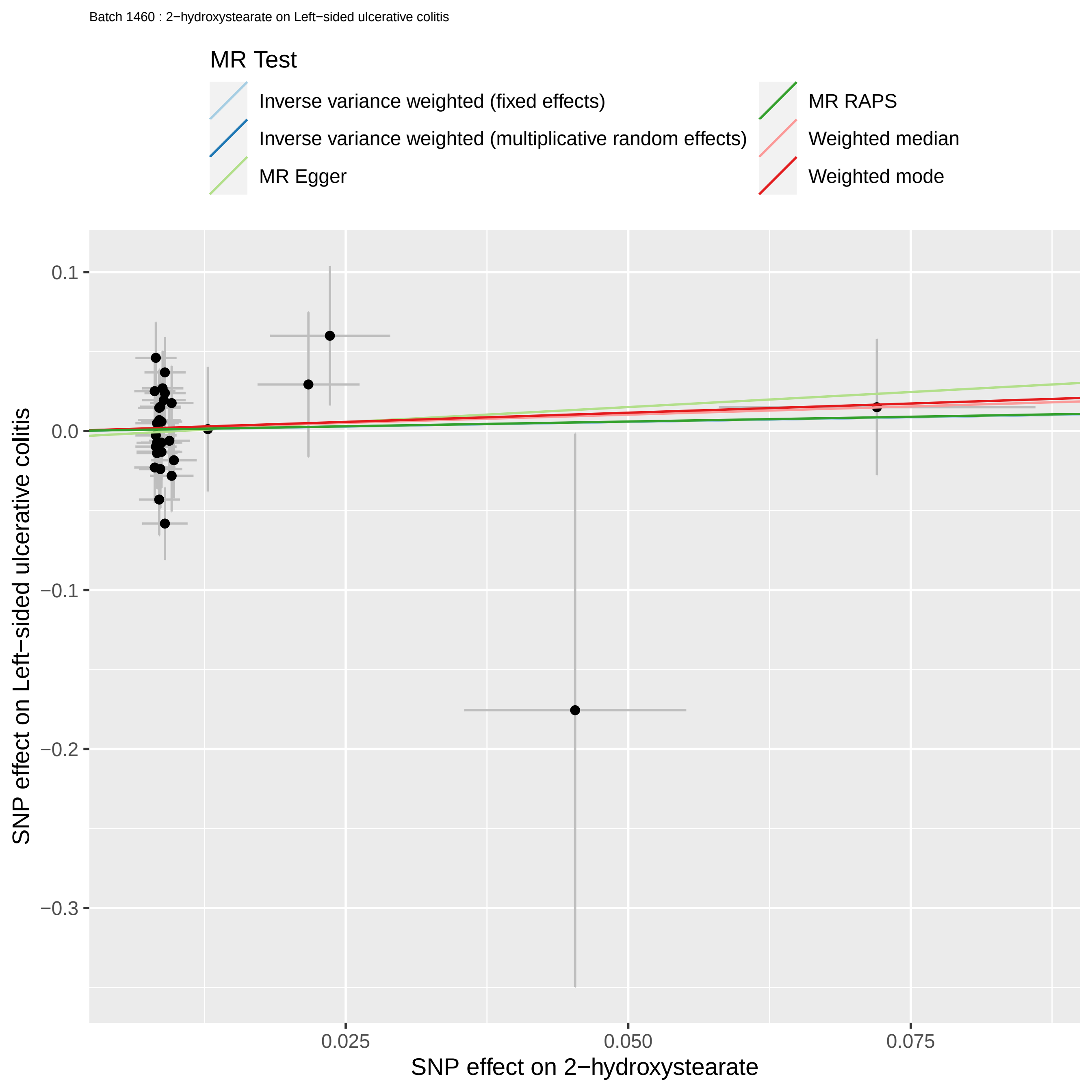
Batch 1460 : 2−hydroxystearate on Left−sided ulcerative colitis
MR Test
Inverse variance weighted (fixed effects)
Inverse variance weighted (multiplicative random effects)
MR Egger
MR RAPS
Weighted median
Weighted mode
0.1
0.0
−0.1
−0.2
−0.3
0.025
0.050
0.075
SNP effect on Left−sided ulcerative colitis
SNP effect on 2−hydroxystearate

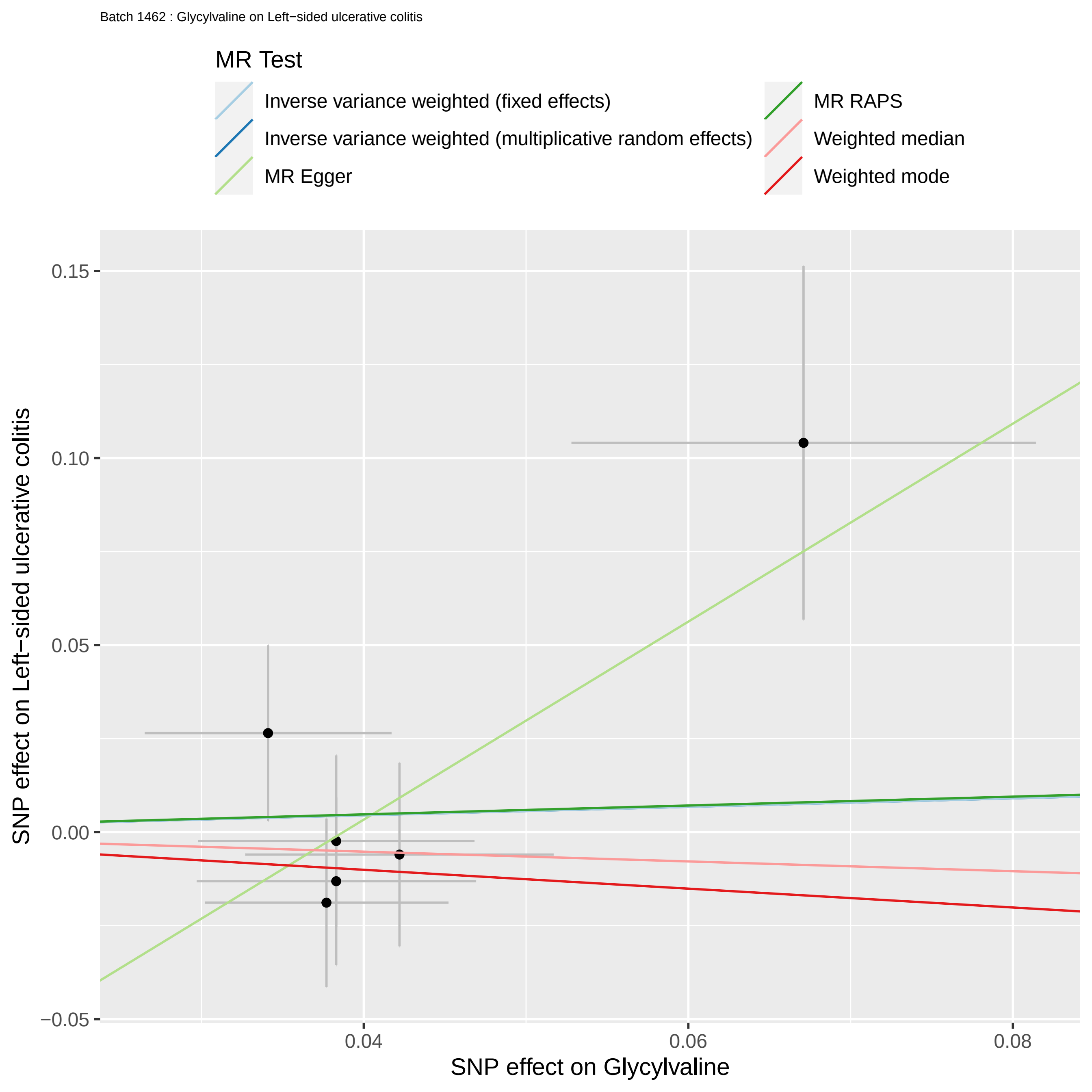
Batch 1462 : Glycylvaline on Left−sided ulcerative colitis
MR Test
Inverse variance weighted (fixed effects)
Inverse variance weighted (multiplicative random effects)
MR Egger
MR RAPS
Weighted median
Weighted mode
SNP effect on Left−sided ulcerative colitis
0.15
0.10
0.05
0.00
−0.05
SNP effect on Glycylvaline
0.04
0.06
0.08

Batch 1463 : Gamma−glutamylleucine on Left−sided ulcerative colitis

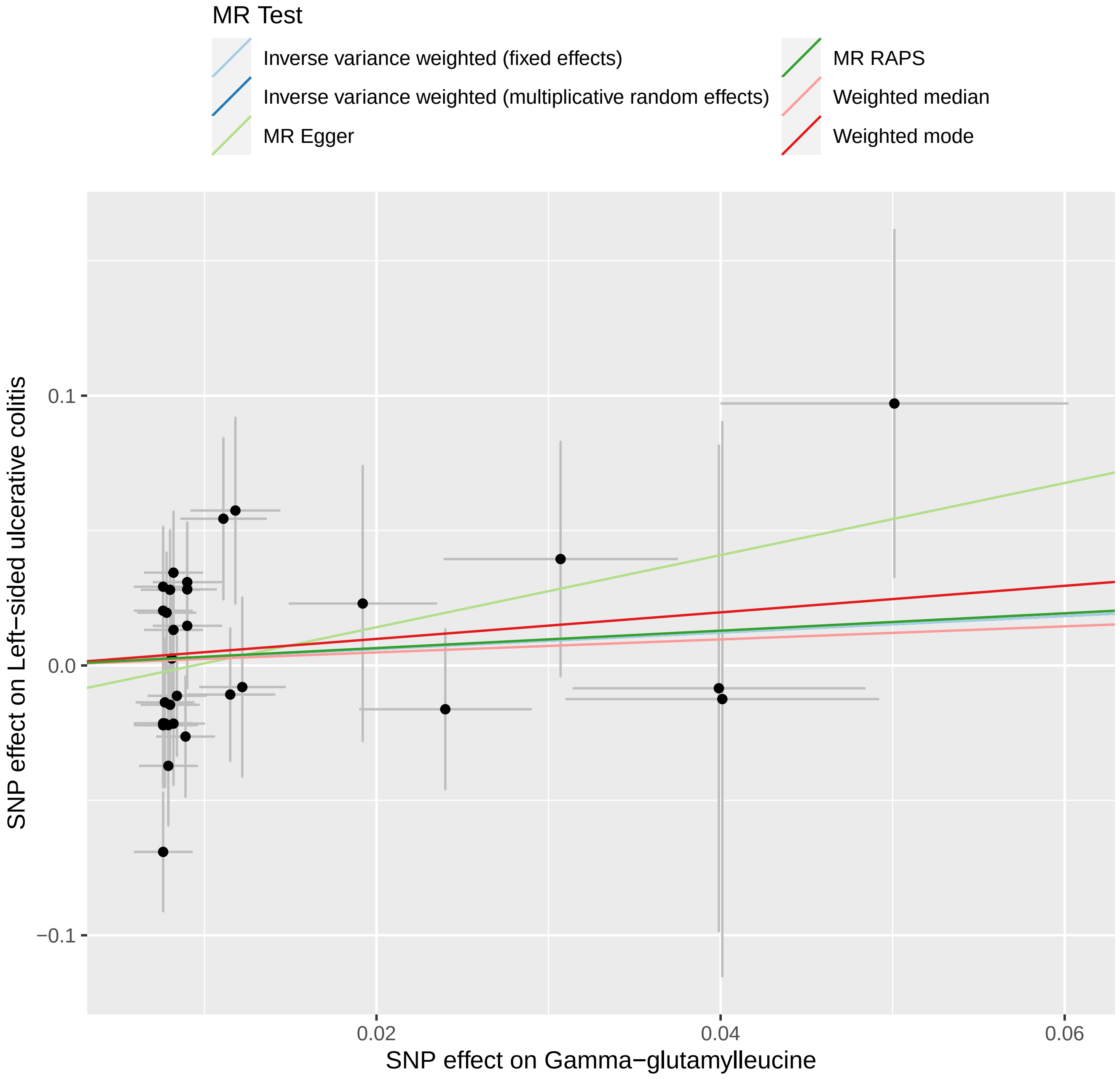

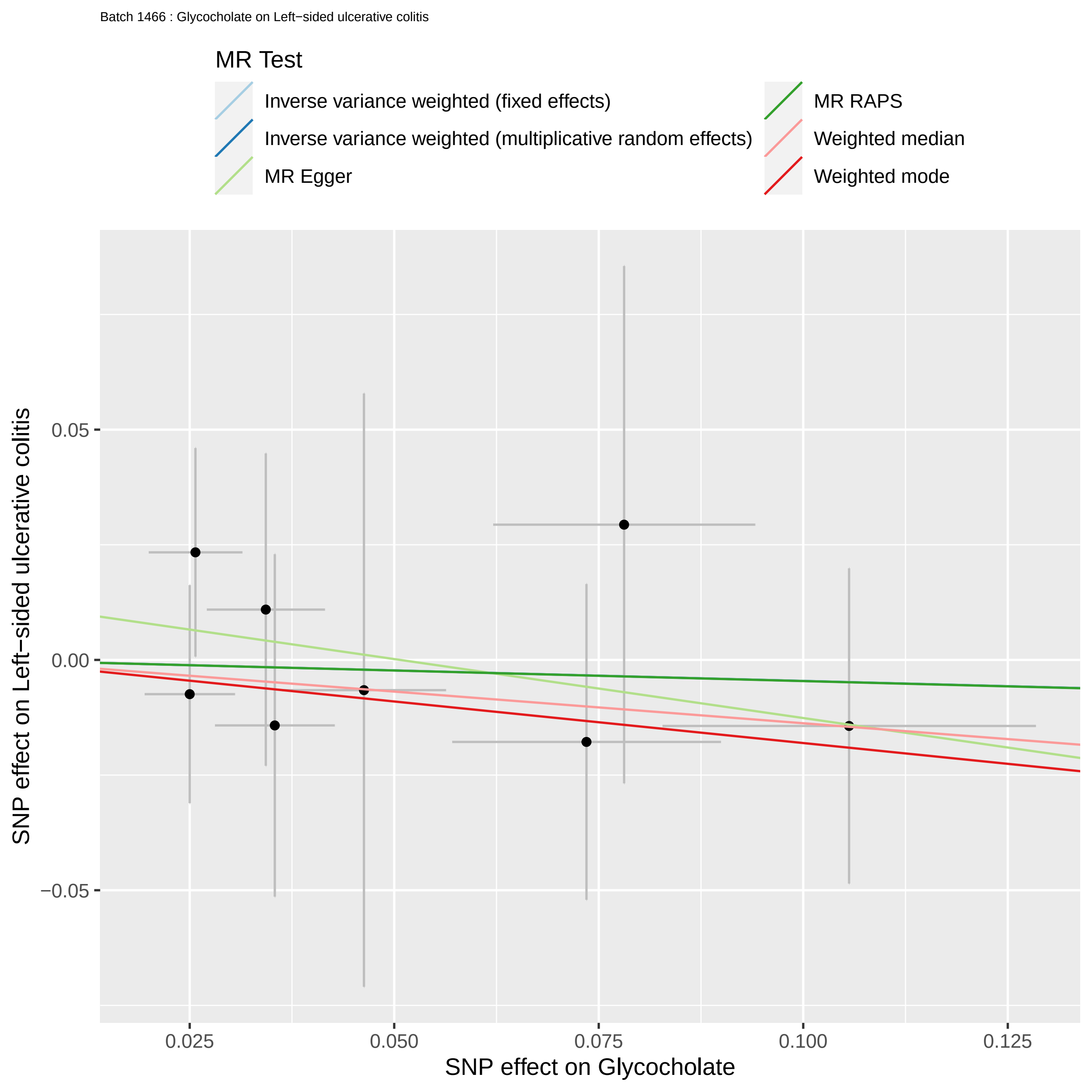

Batch 1466 : Glycocholate on Left−sided ulcerative colitis
MR Test
Inverse variance weighted (fixed effects)
Inverse variance weighted (multiplicative random effects)
MR Egger
MR RAPS
Weighted median
Weighted mode
0.05
0.00
−0.05
0.025
0.050
0.075
0.100
0.125
SNP effect on Glycocholate
SNP effect on Left−sided ulcerative colitis

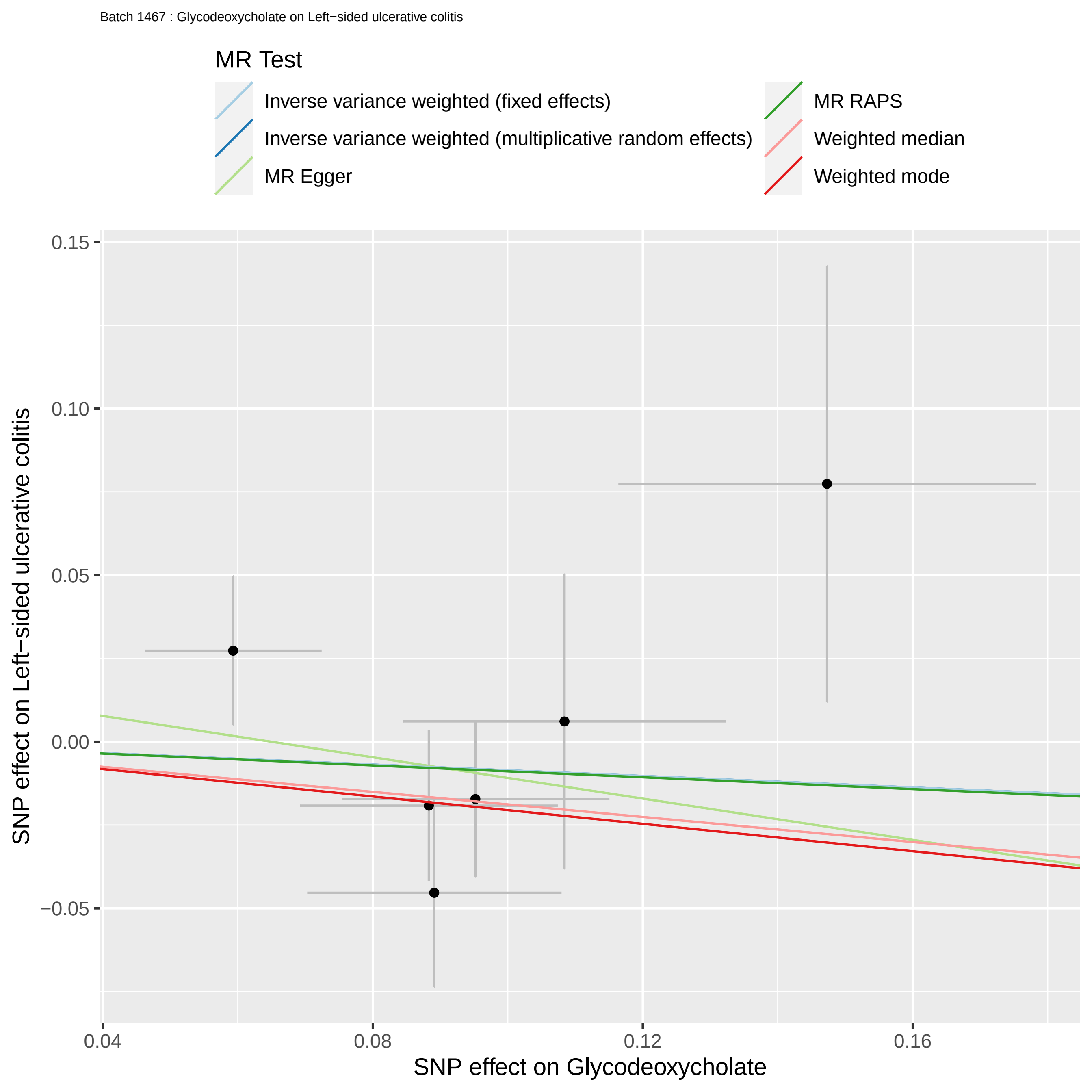

Batch 1467 : Glycodeoxycholate on Left−sided ulcerative colitis
MR Test
Inverse variance weighted (fixed effects)
Inverse variance weighted (multiplicative random effects)
MR Egger
MR RAPS
Weighted median
Weighted mode
0.15
0.10
0.05
0.00
−0.05
0.04
0.08
0.12
0.16
SNP effect on Glycodeoxycholate
SNP effect on Left−sided ulcerative colitis

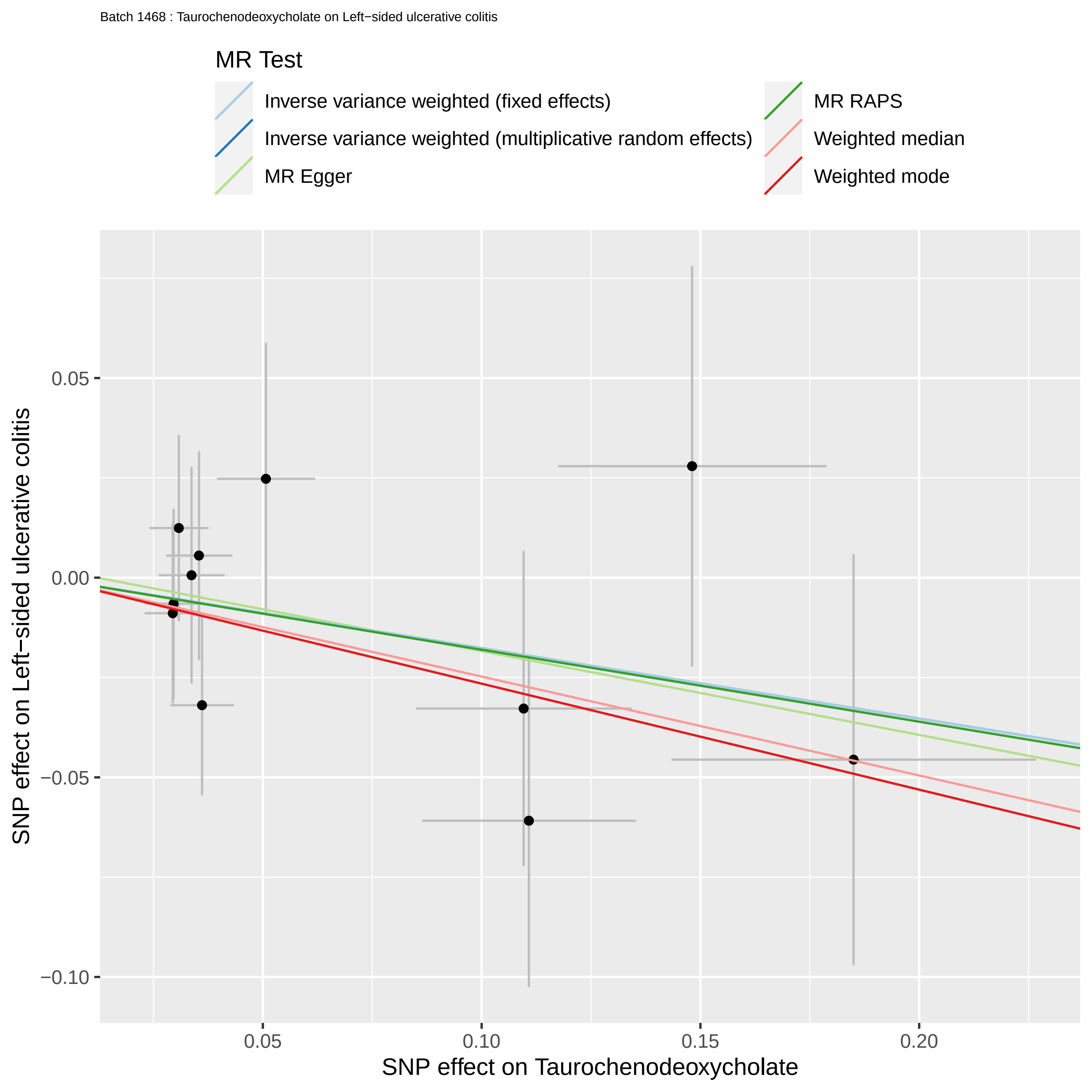

Batch 1468 : Taurochenodeoxycholate on Left−sided ulcerative colitis
MR Test
Inverse variance weighted (fixed effects)
Inverse variance weighted (multiplicative random effects)
MR Egger
MR RAPS
Weighted median
Weighted mode
0.05
0.00
−0.05
−0.10
0.05
0.10
0.15
0.20
SNP effect on Taurochenodeoxycholate
SNP effect on Left−sided ulcerative colitis

Batch 1469 : Taurocholate on Left−sided ulcerative colitis

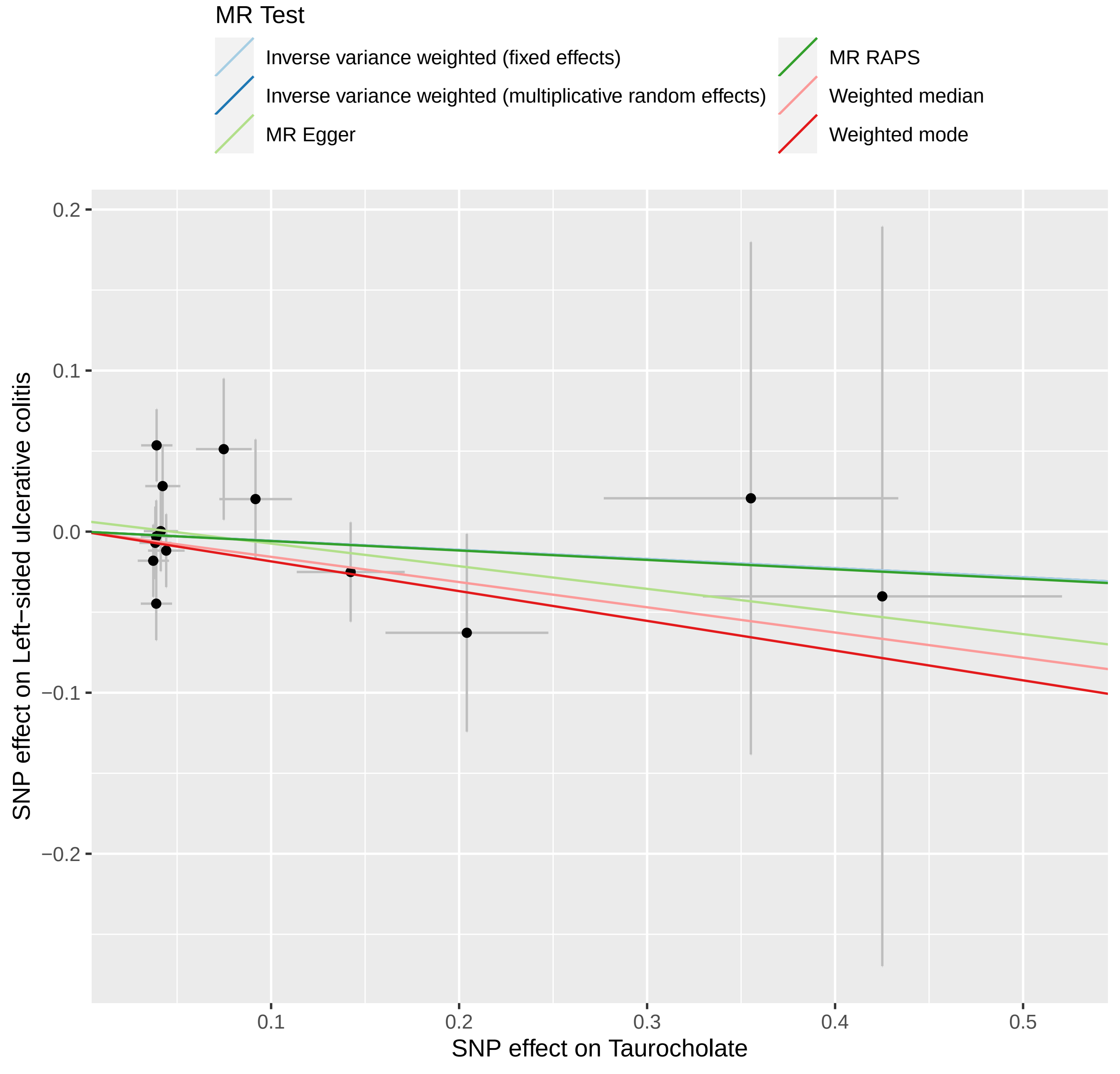

Batch 1471 : 1−stearoylglycerophosphoinositol on Left−sided ulcerative colitis

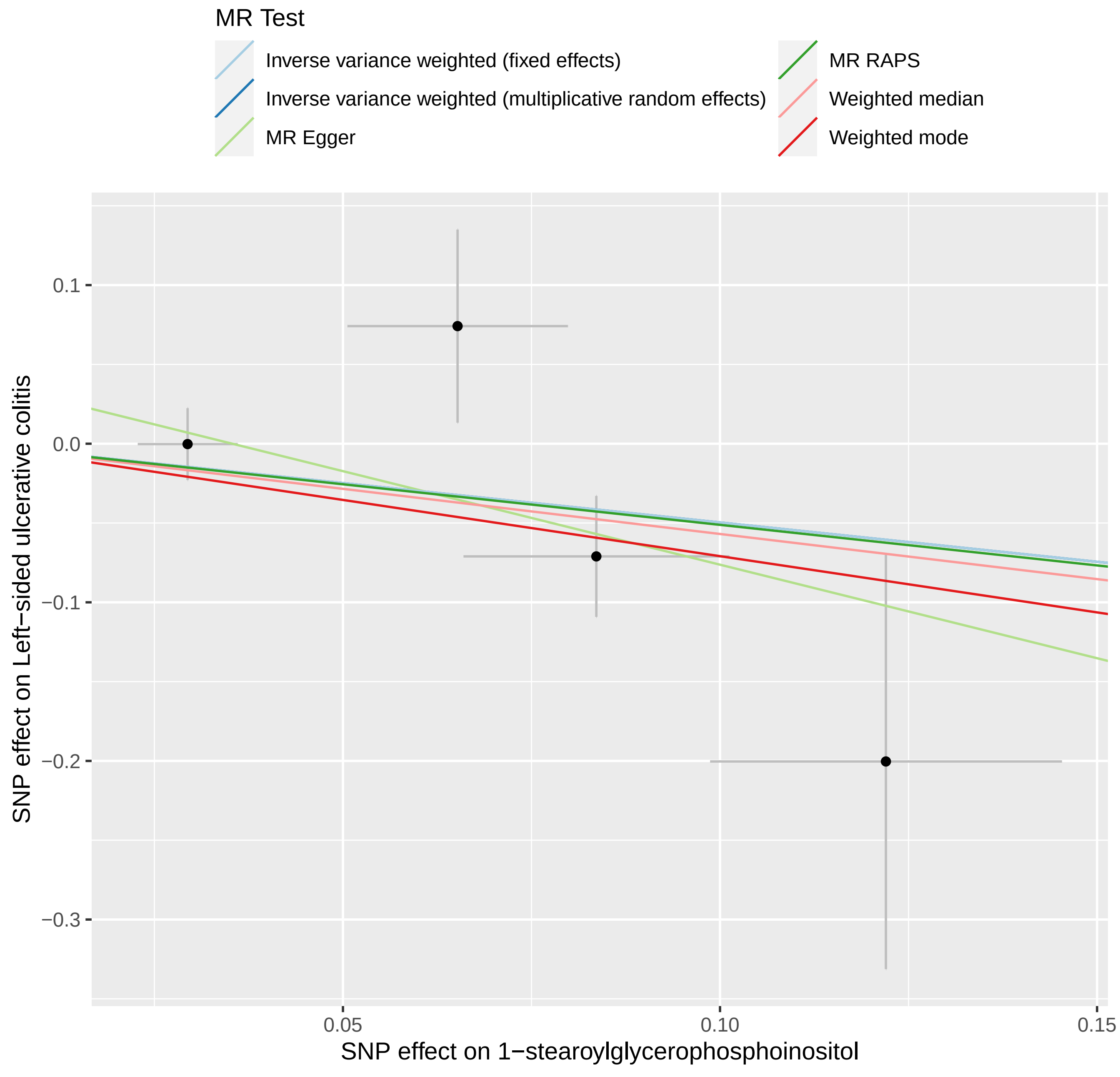

Batch 1472 : Myo−inositol on Left−sided ulcerative colitis

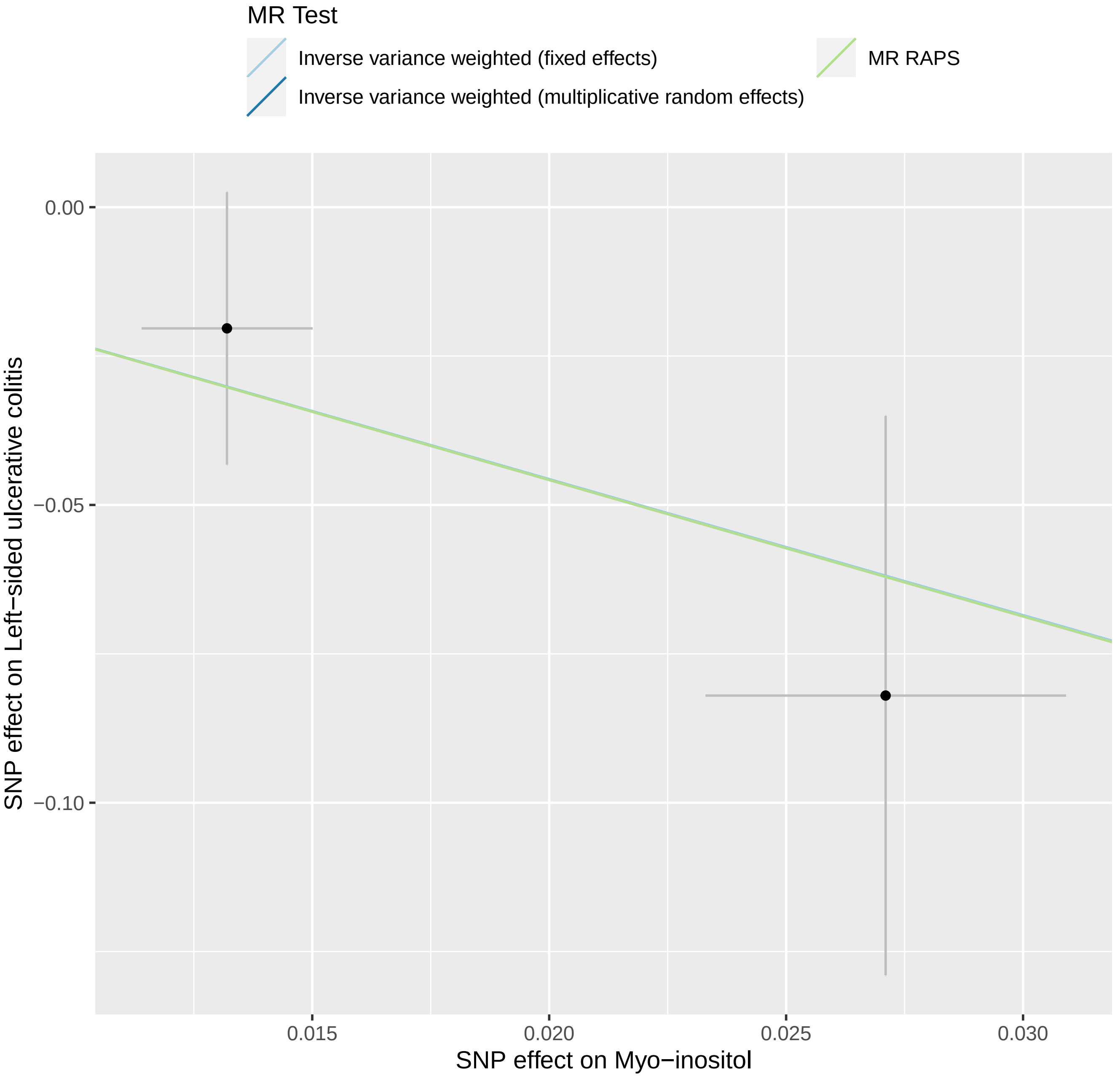

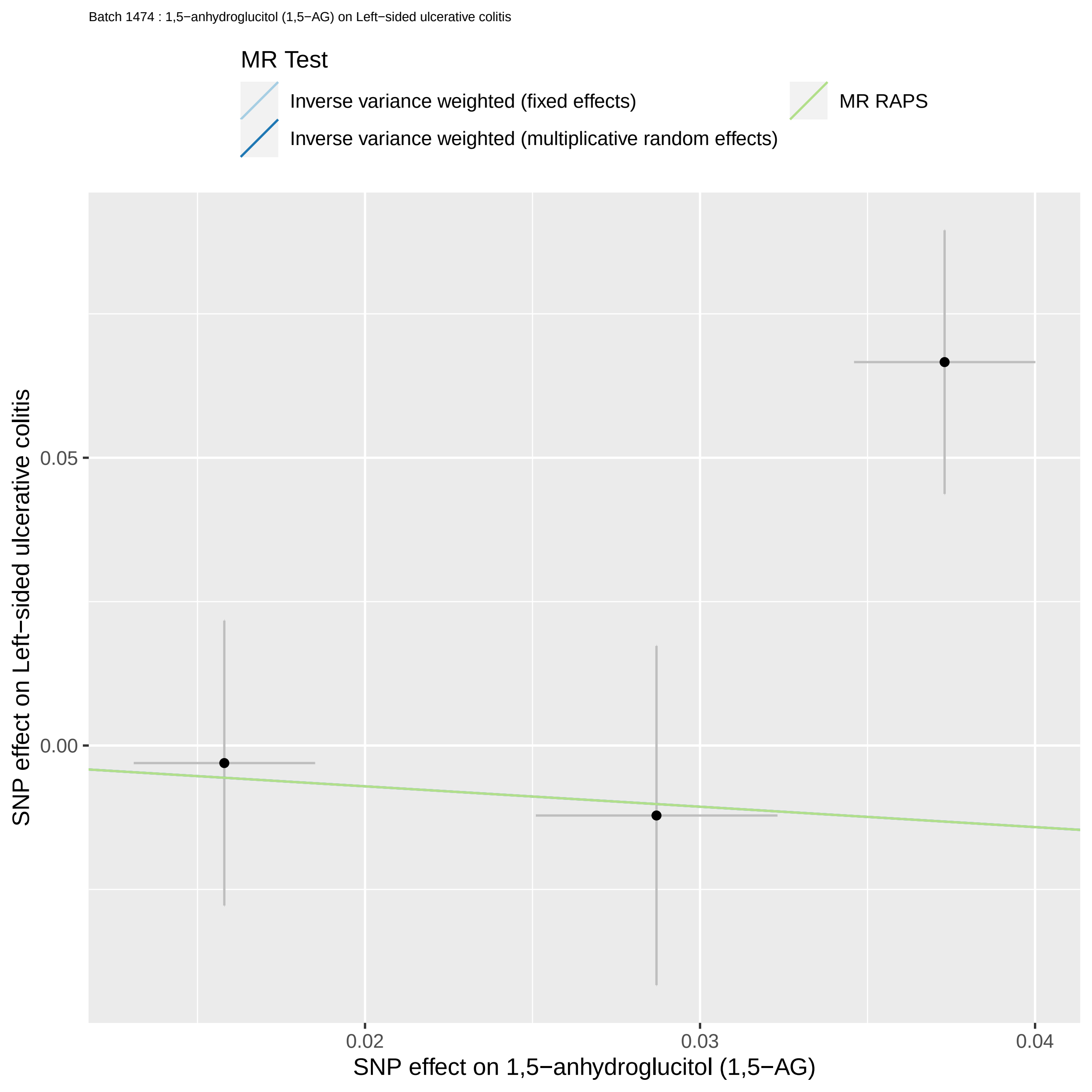
Batch 1474 : 1,5−anhydroglucitol (1,5−AG) on Left−sided ulcerative colitis
MR Test
Inverse variance weighted (fixed effects)
Inverse variance weighted (multiplicative random effects)
MR RAPS
SNP effect on Left−sided ulcerative colitis
0.05
0.00
0.02
0.03
0.04
SNP effect on 1,5−anhydroglucitol (1,5−AG)

Batch 1475 : 2−hydroxybutyrate (AHB) on Left−sided ulcerative colitis

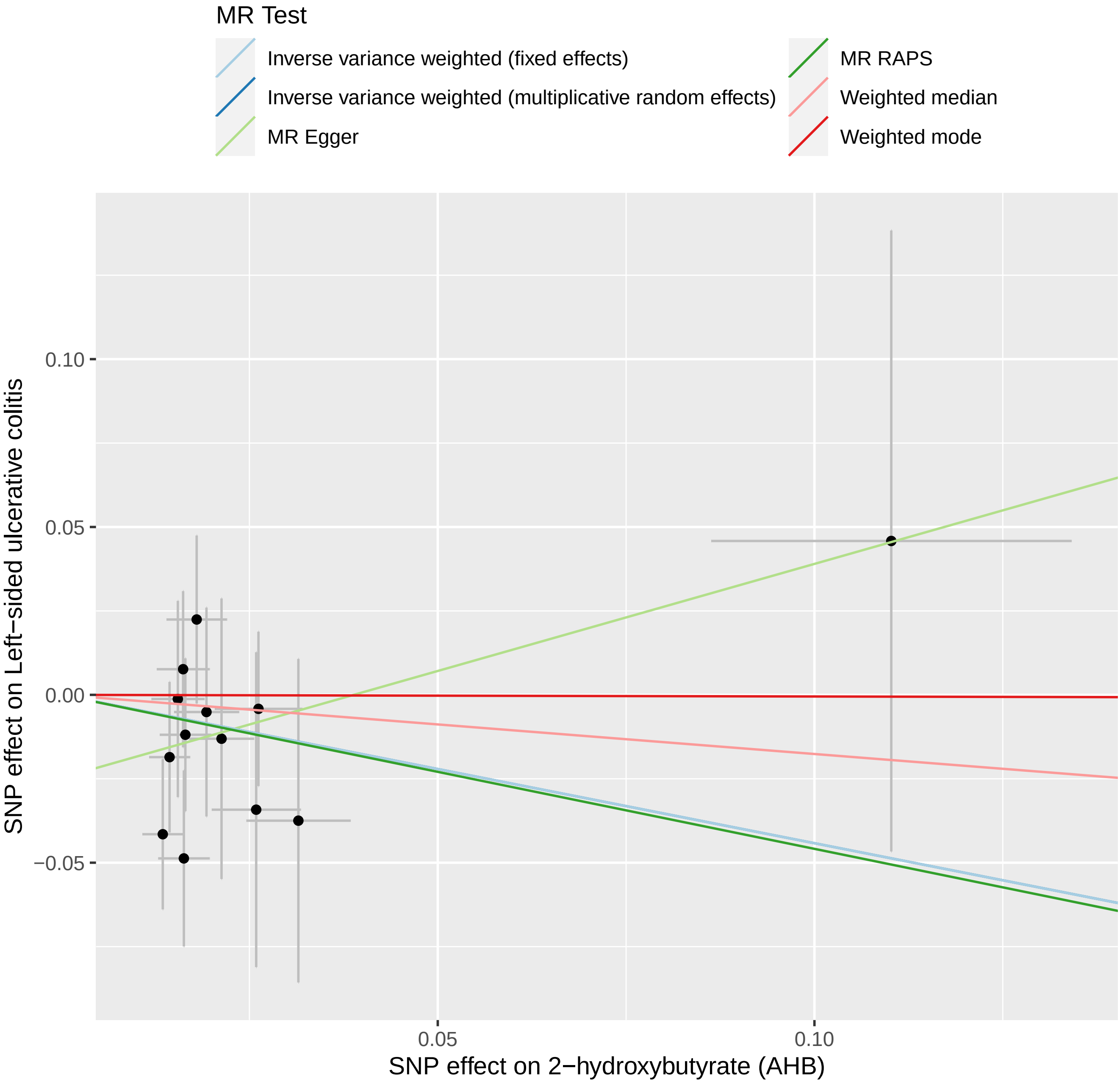

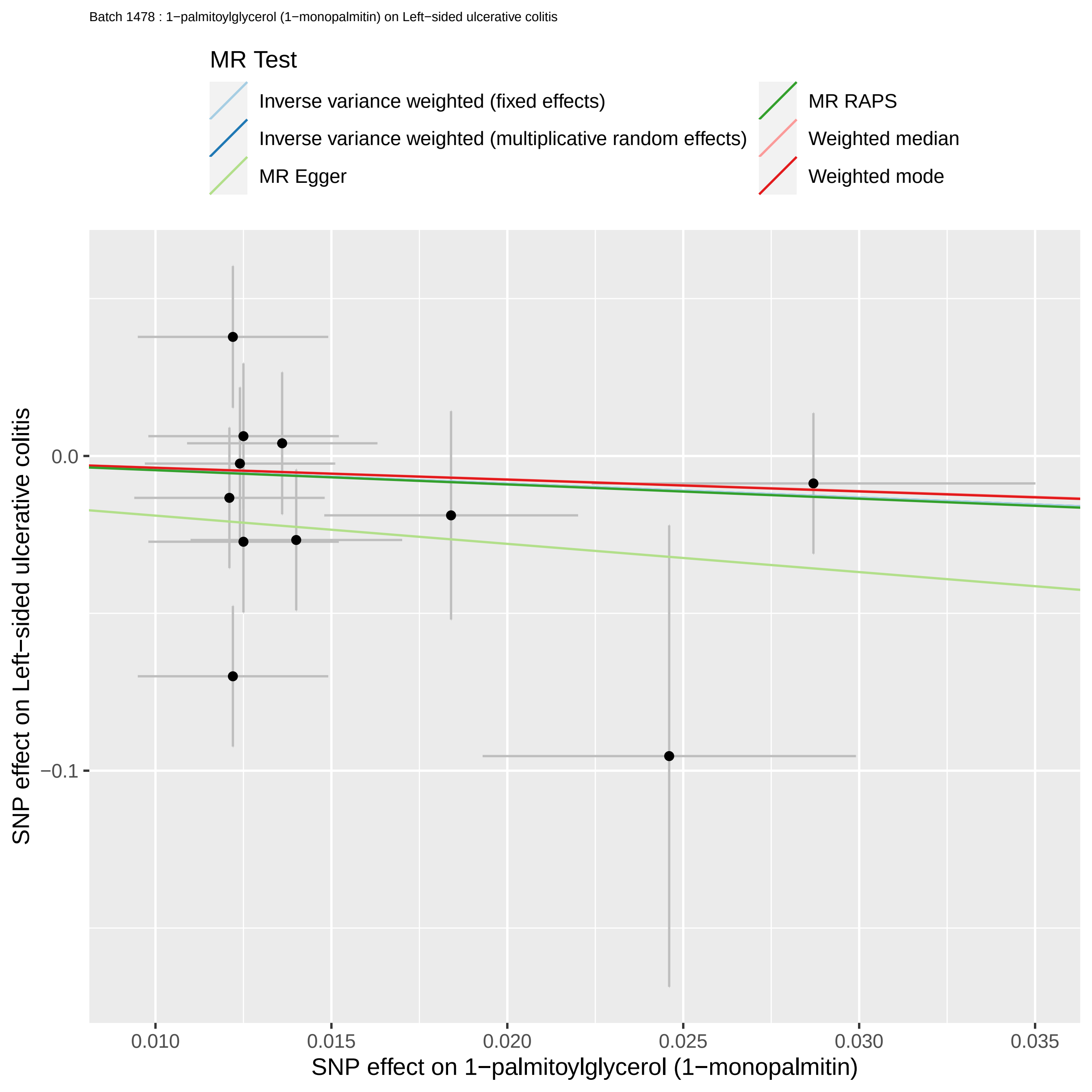
Batch 1478 : 1−palmitoylglycerol (1−monopalmitin) on Left−sided ulcerative colitis
MR Test
Inverse variance weighted (fixed effects)
Inverse variance weighted (multiplicative random effects)
MR Egger
MR RAPS
Weighted median
Weighted mode
SNP effect on Left−sided ulcerative colitis
0.0
−0.1
0.010
0.015
0.020
0.025
0.030
0.035
SNP effect on 1−palmitoylglycerol (1−monopalmitin)

Batch 1481 : 2−hydroxyisobutyrate on Left−sided ulcerative colitis

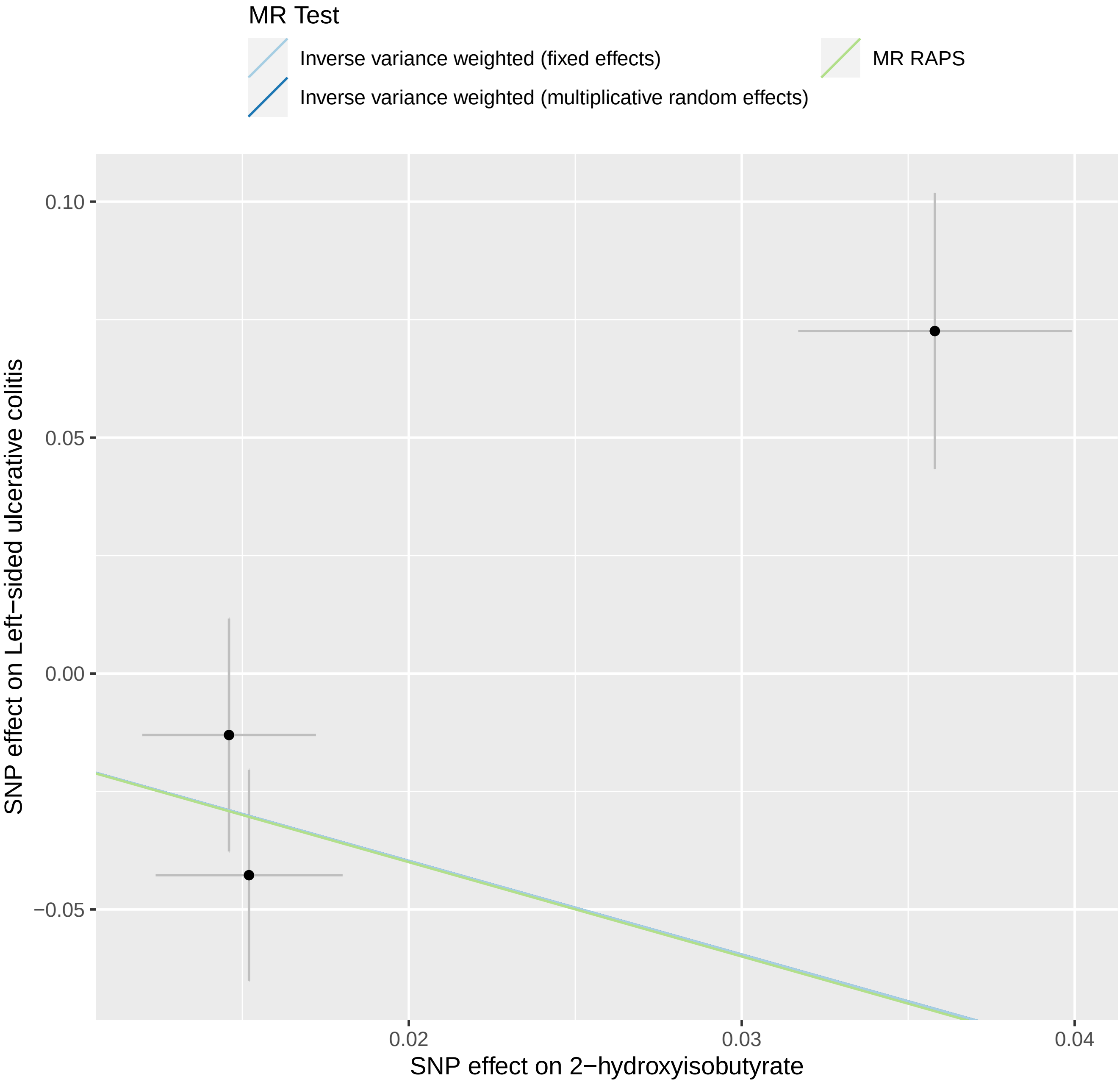

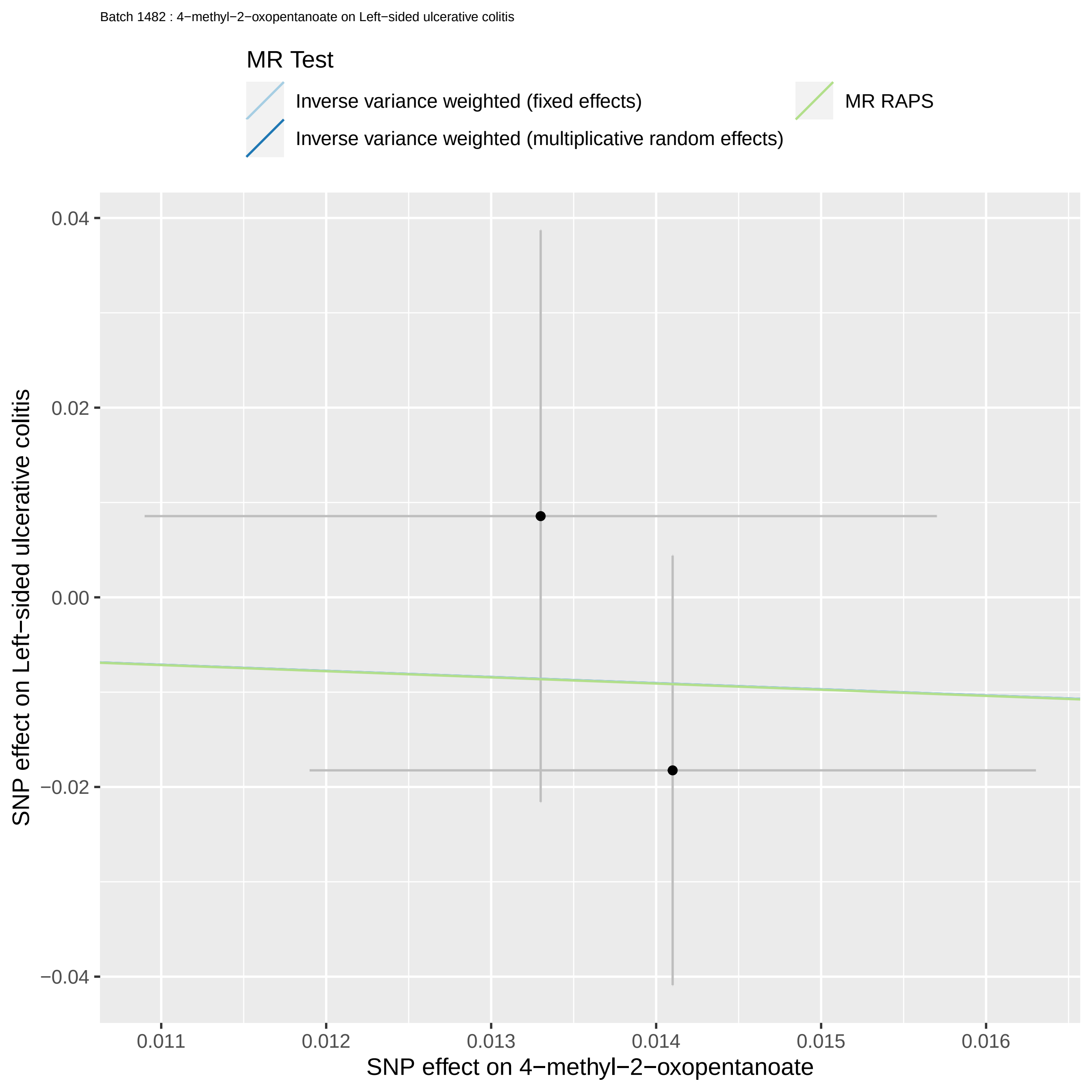

Batch 1482 : 4−methyl−2−oxopentanoate on Left−sided ulcerative colitis
MR Test
Inverse variance weighted (fixed effects)
Inverse variance weighted (multiplicative random effects)
MR RAPS
0.04
0.02
0.00
−0.02
−0.04
SNP effect on Left−sided ulcerative colitis
0.011
0.012
0.013
0.014
0.015
0.016
SNP effect on 4−methyl−2−oxopentanoate

Batch 1486 : Levulinate (4−oxovalerate) on Left−sided ulcerative colitis

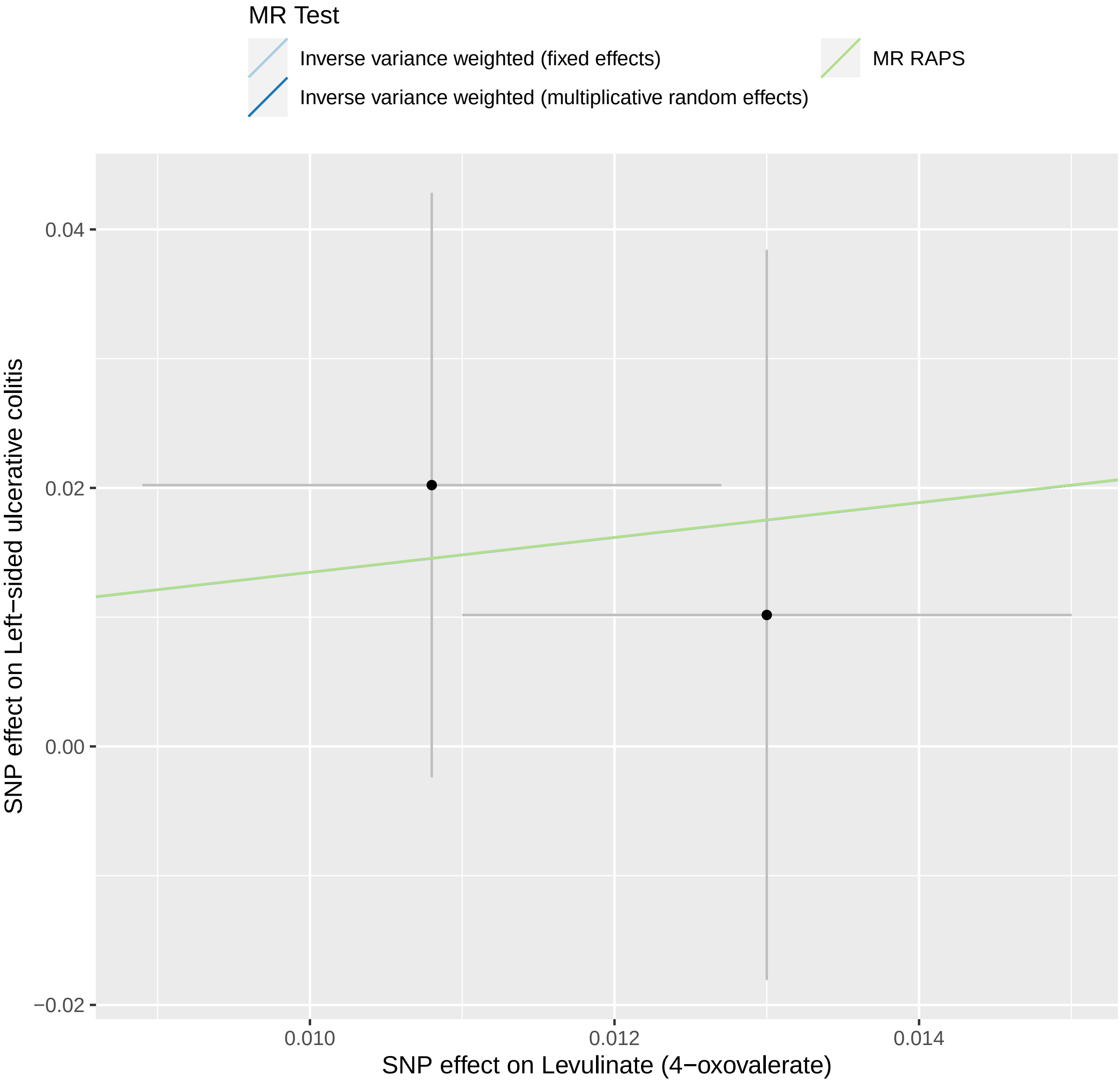

Batch 1488 : Cholate on Left−sided ulcerative colitis

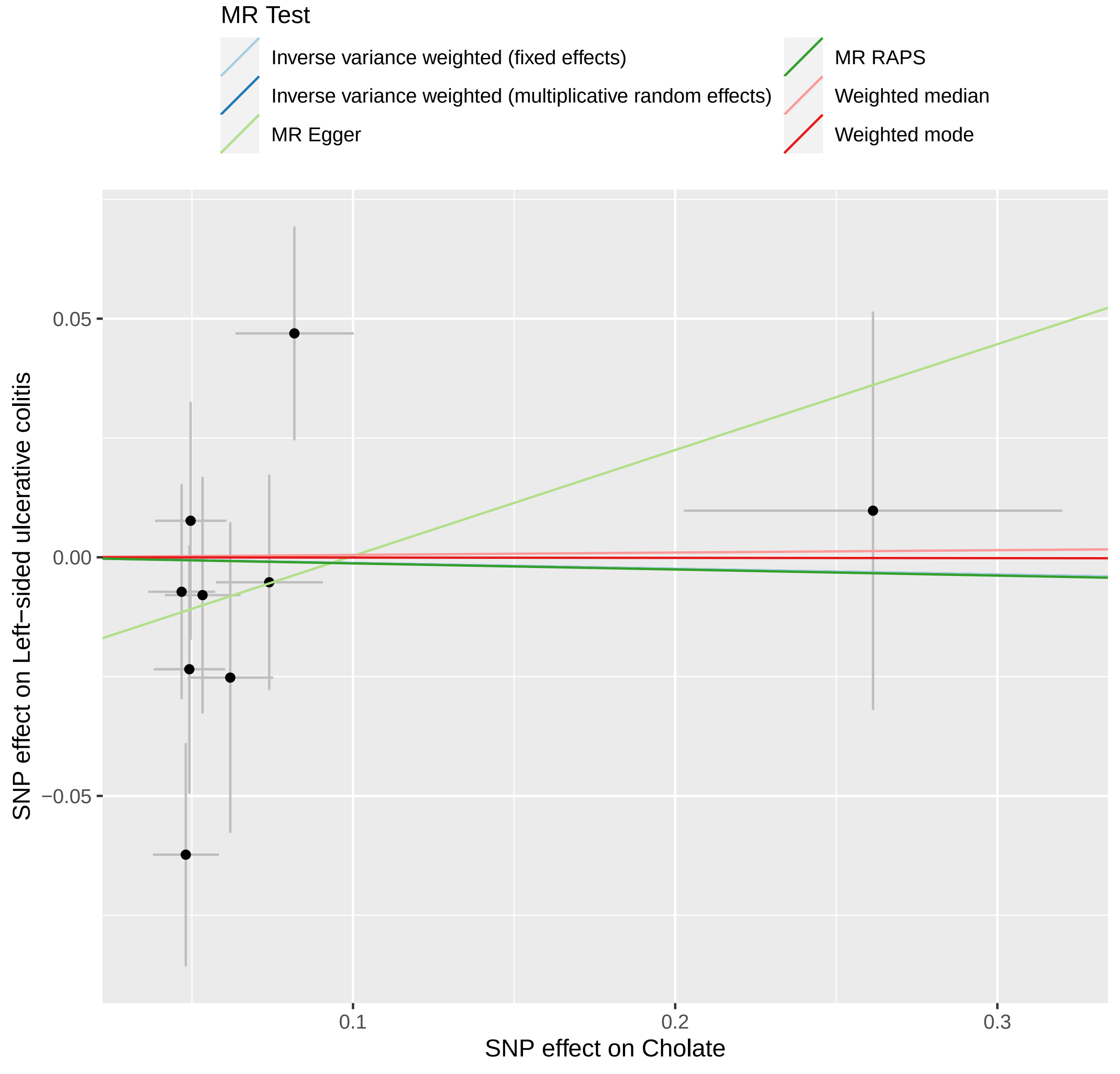

Batch 1491 : Hyodeoxycholate on Left−sided ulcerative colitis

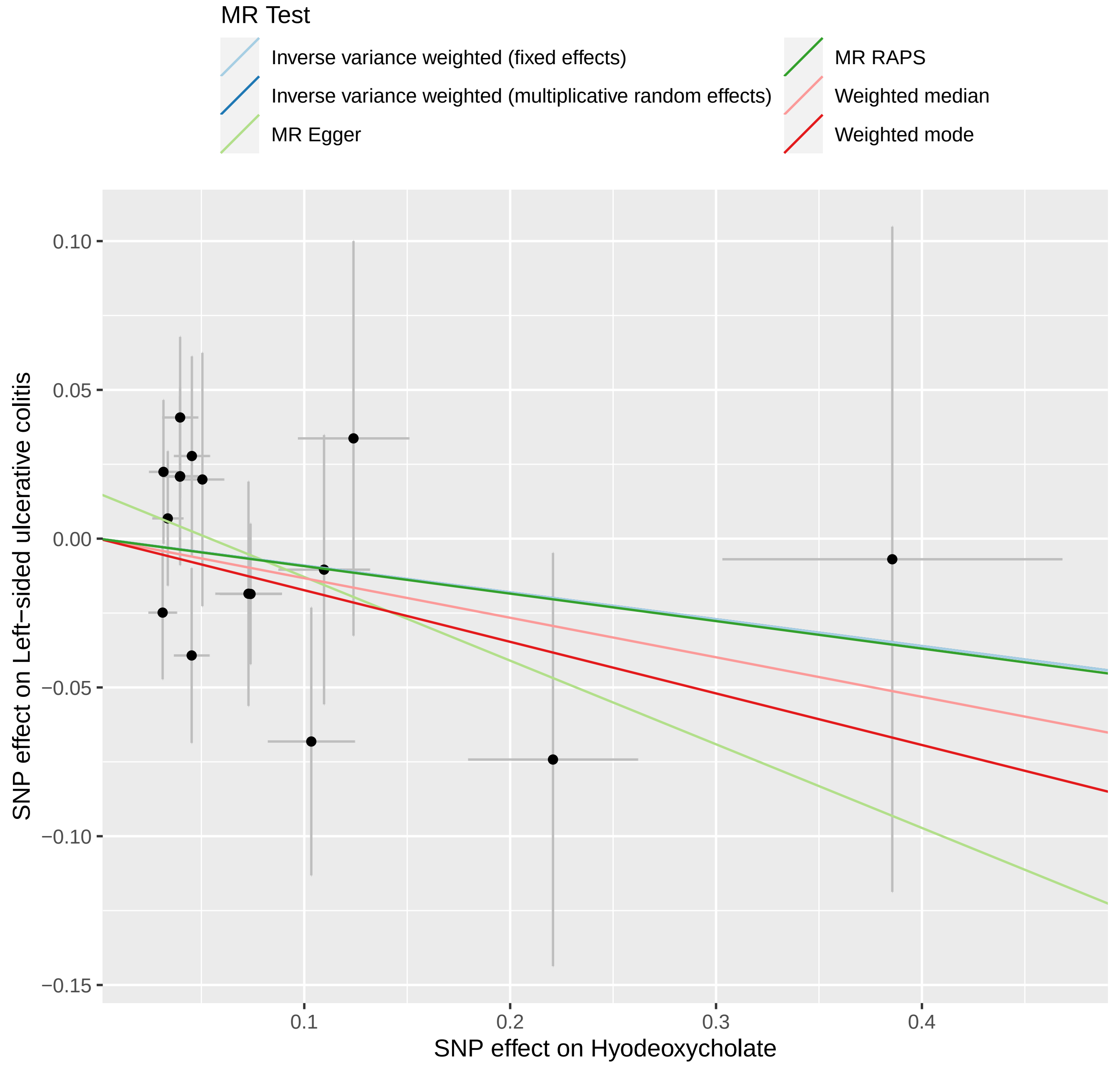

Batch 1492 : 3−indoxyl sulfate on Left−sided ulcerative colitis

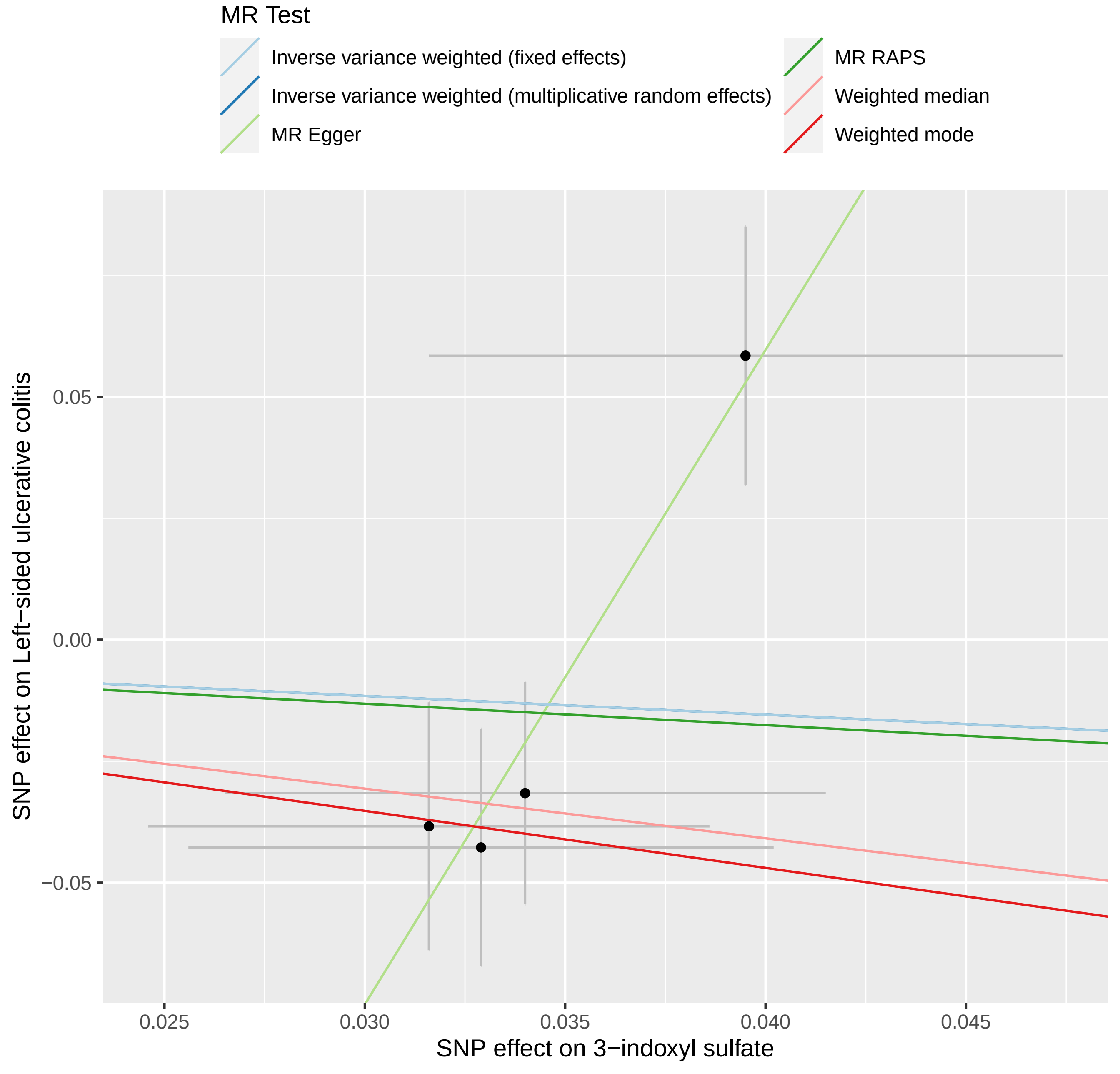

Batch 1496 : Erythrose on Left−sided ulcerative colitis

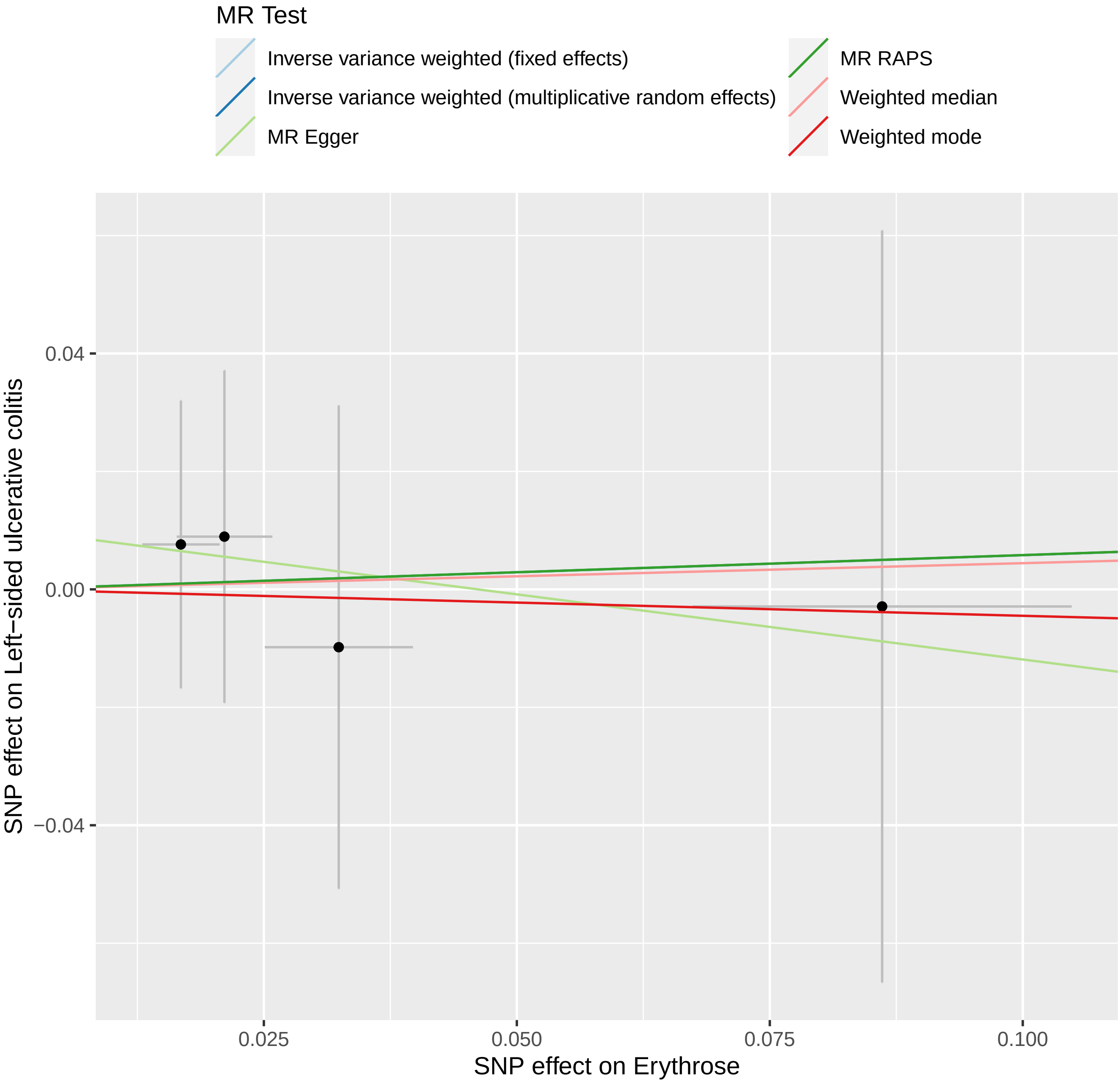

Batch 1497 : Threonate on Left−sided ulcerative colitis

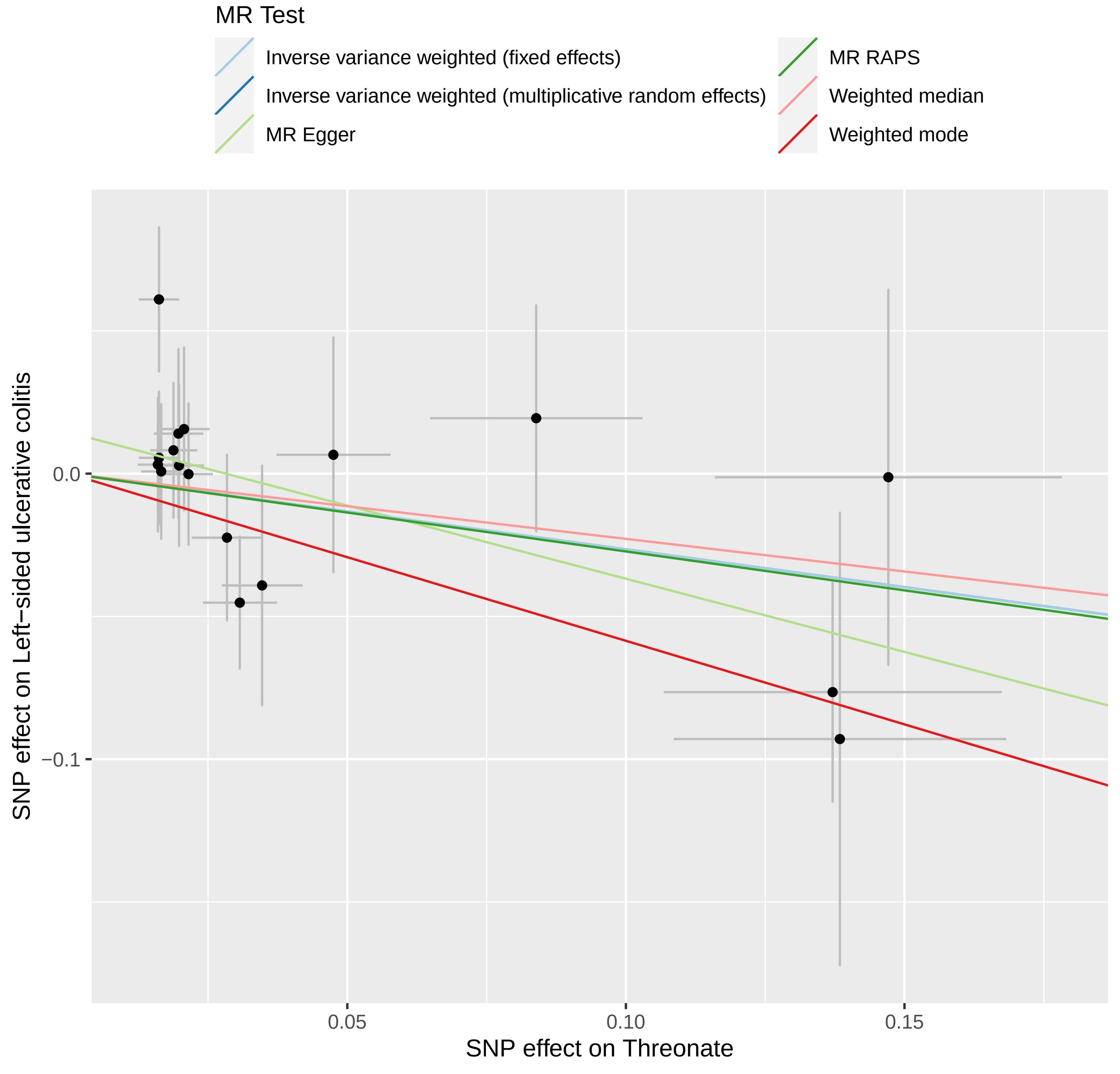

Batch 1498 : Cysteine on Left−sided ulcerative colitis

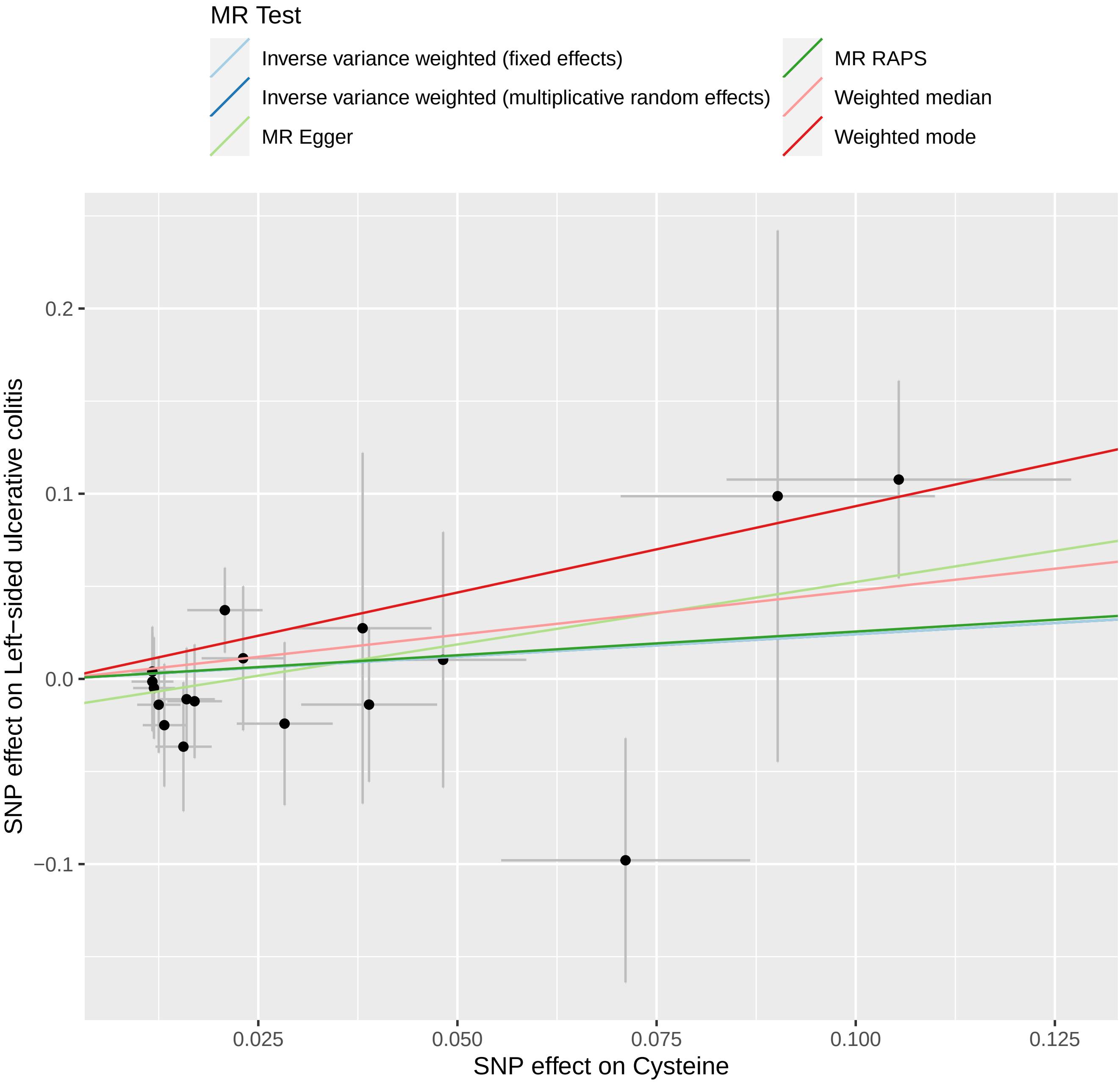

Batch 1499 : Pyroglutamylglycine on Left−sided ulcerative colitis

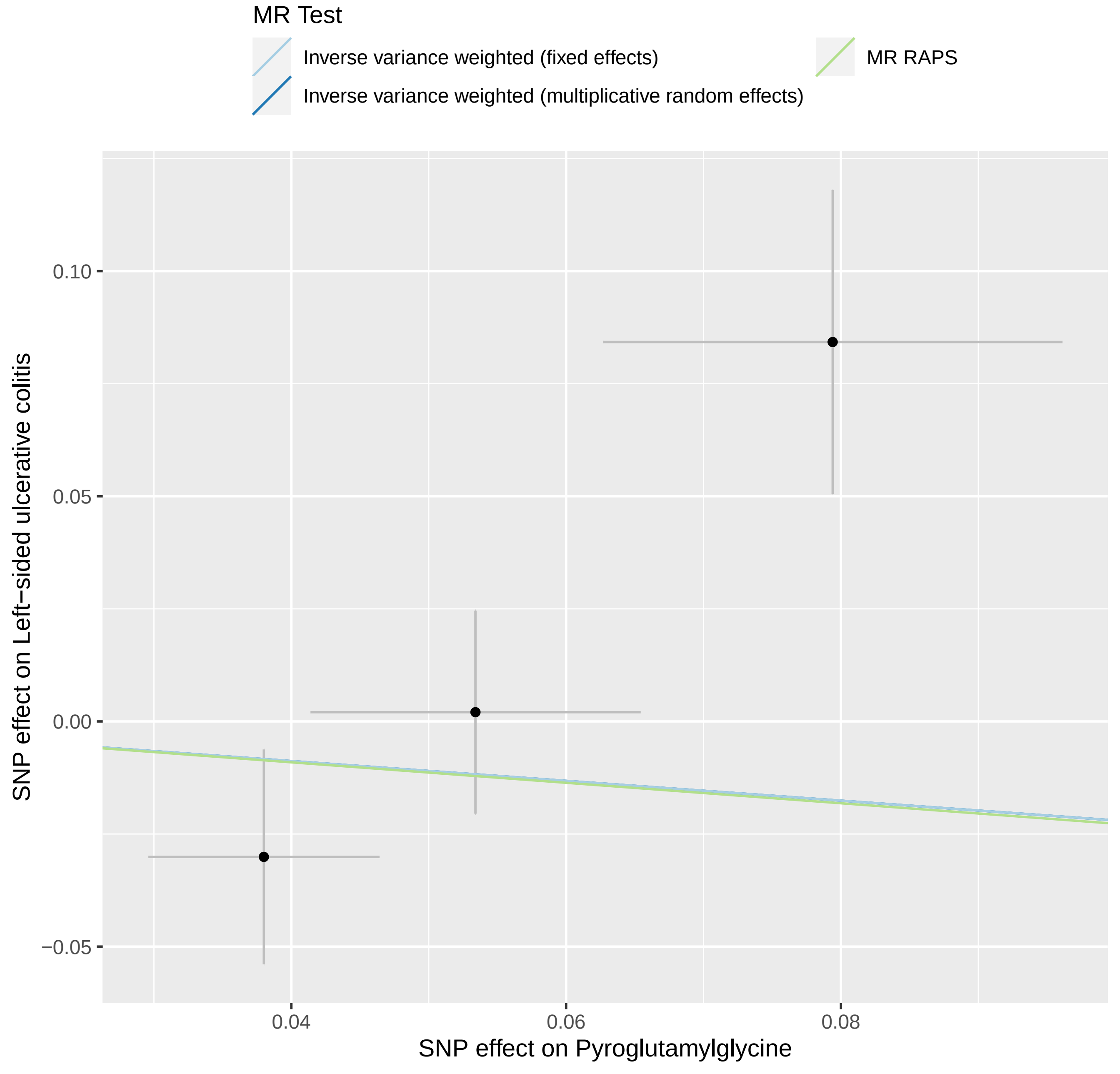

Batch 1501 : DSGEGDFXAEGGGVR* on Left−sided ulcerative colitis

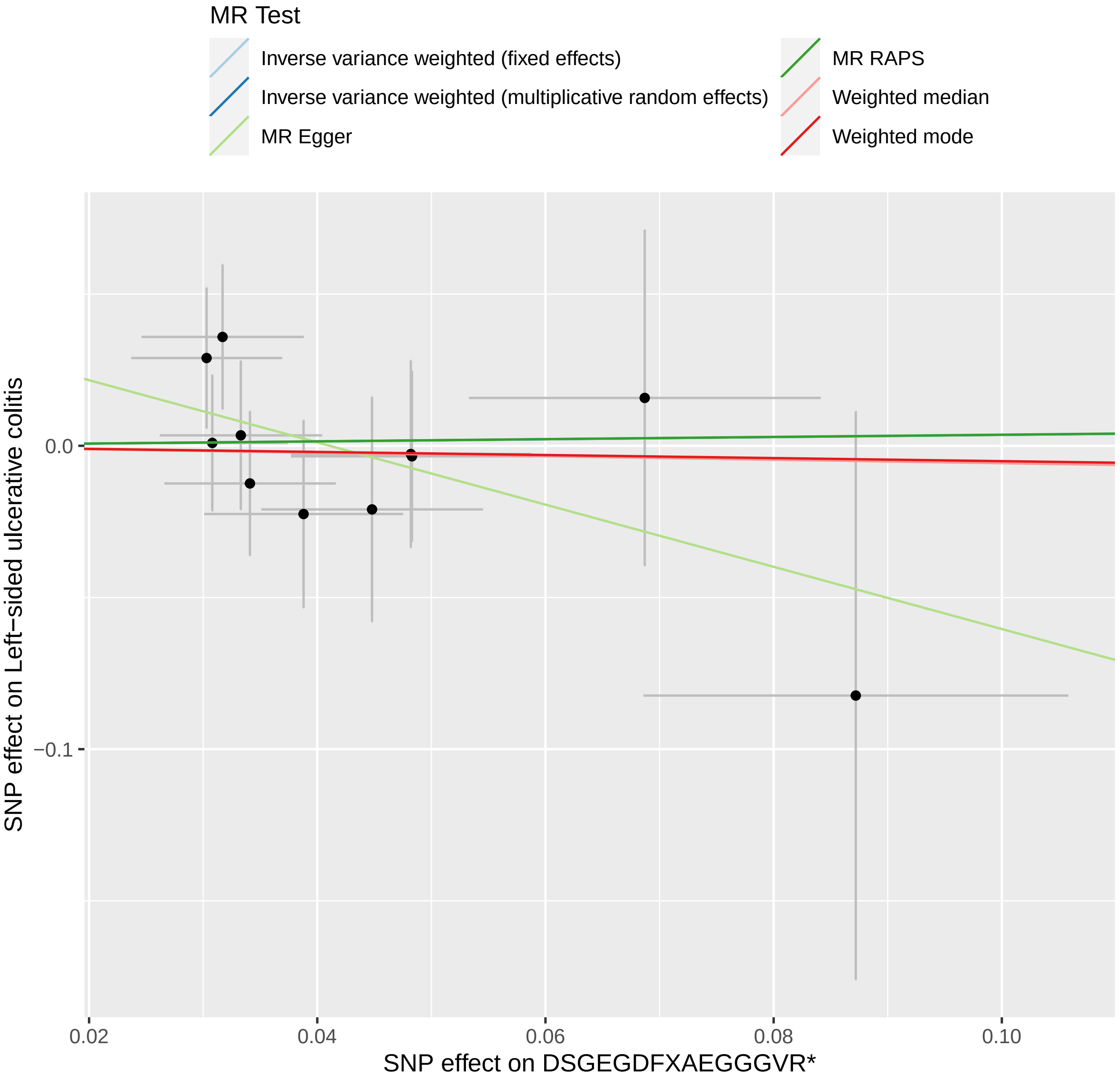

Batch 1502 : Pyridoxate on Left−sided ulcerative colitis

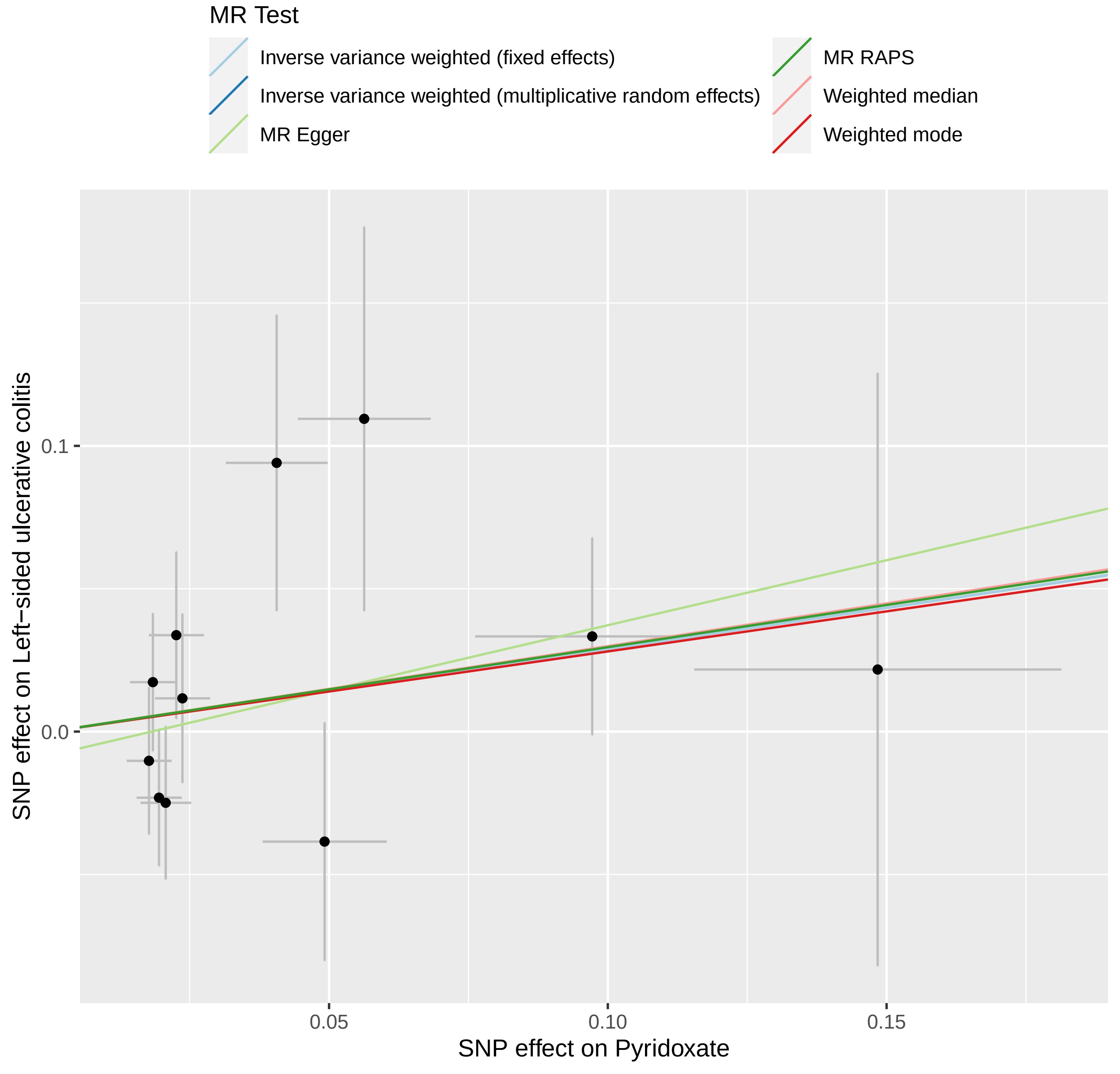

Batch 1503 : Androsterone sulfate on Left−sided ulcerative colitis

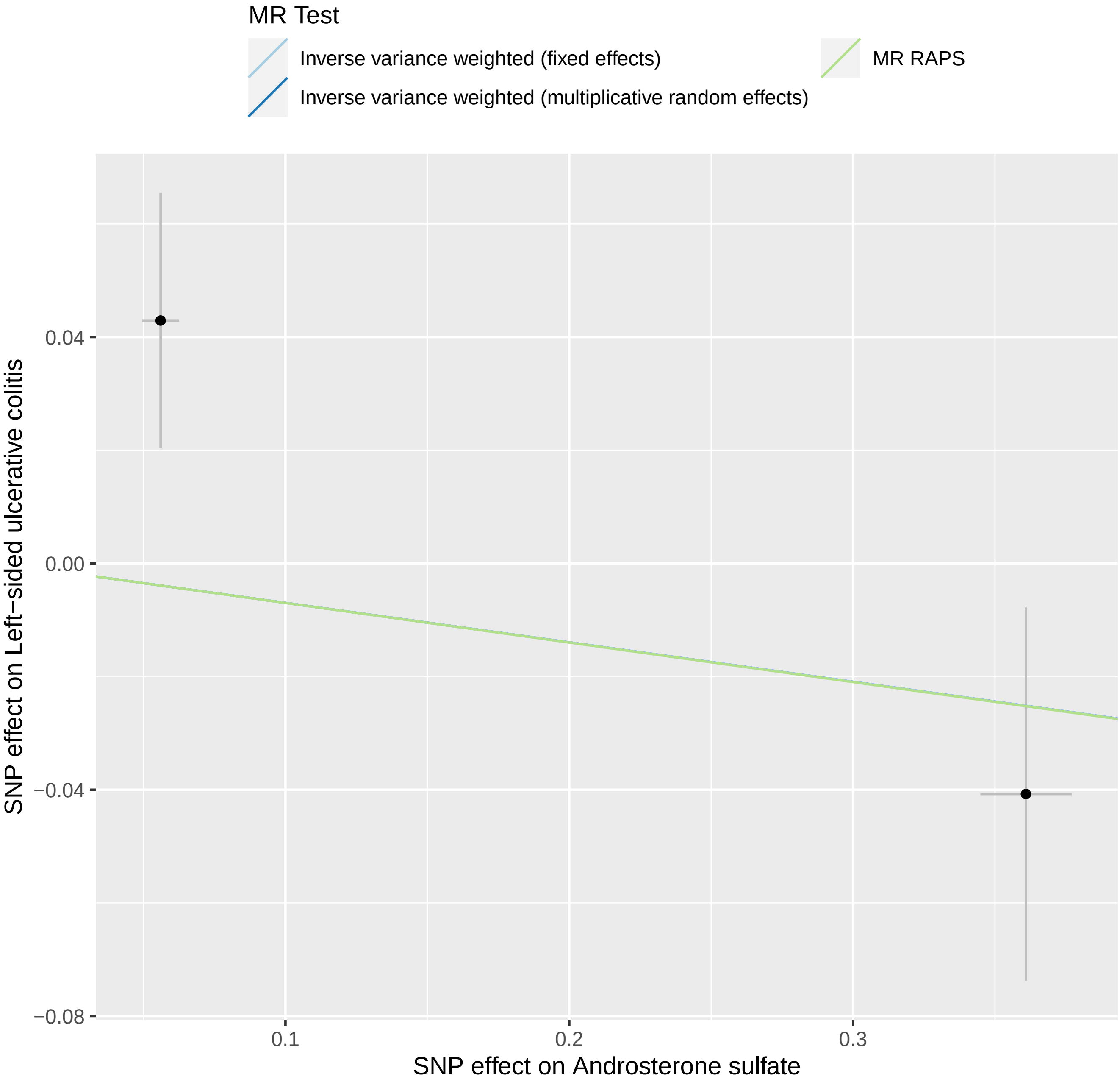

Batch 1507 : Serine on Left−sided ulcerative colitis

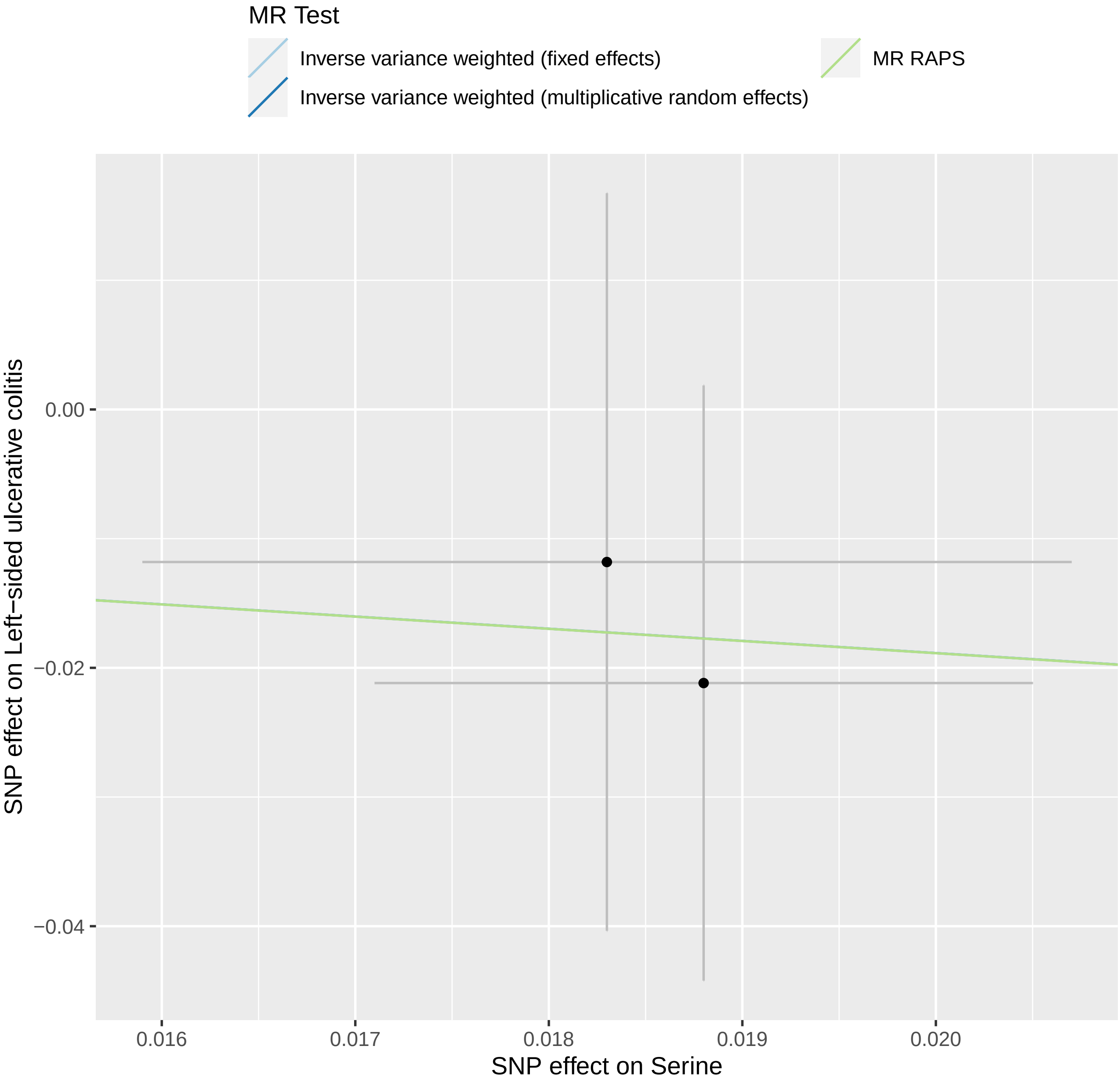

Batch 1508 : Trans−4−hydroxyproline on Left−sided ulcerative colitis

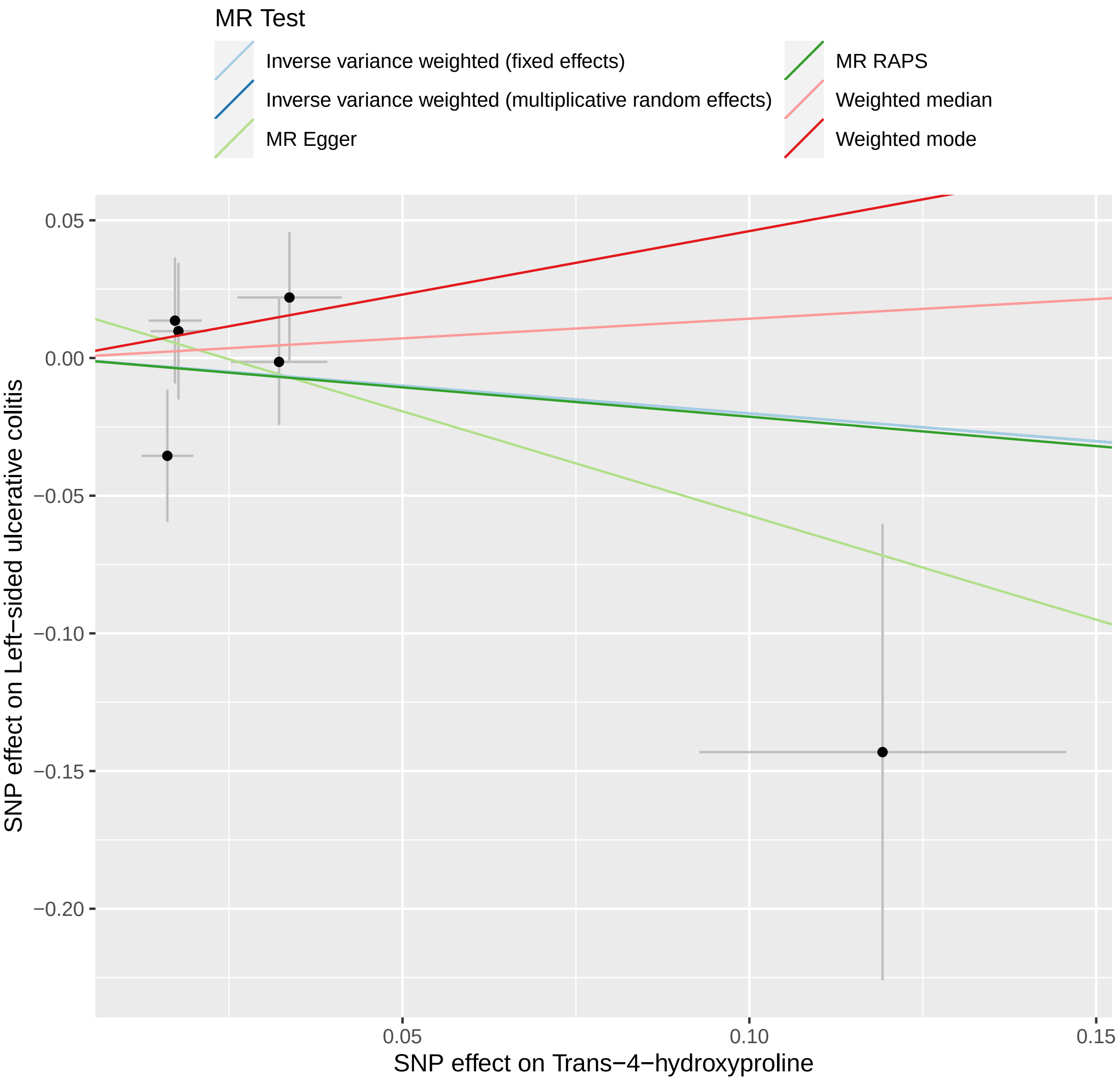

Batch 1510 : Hexanoylcarnitine on Left−sided ulcerative colitis

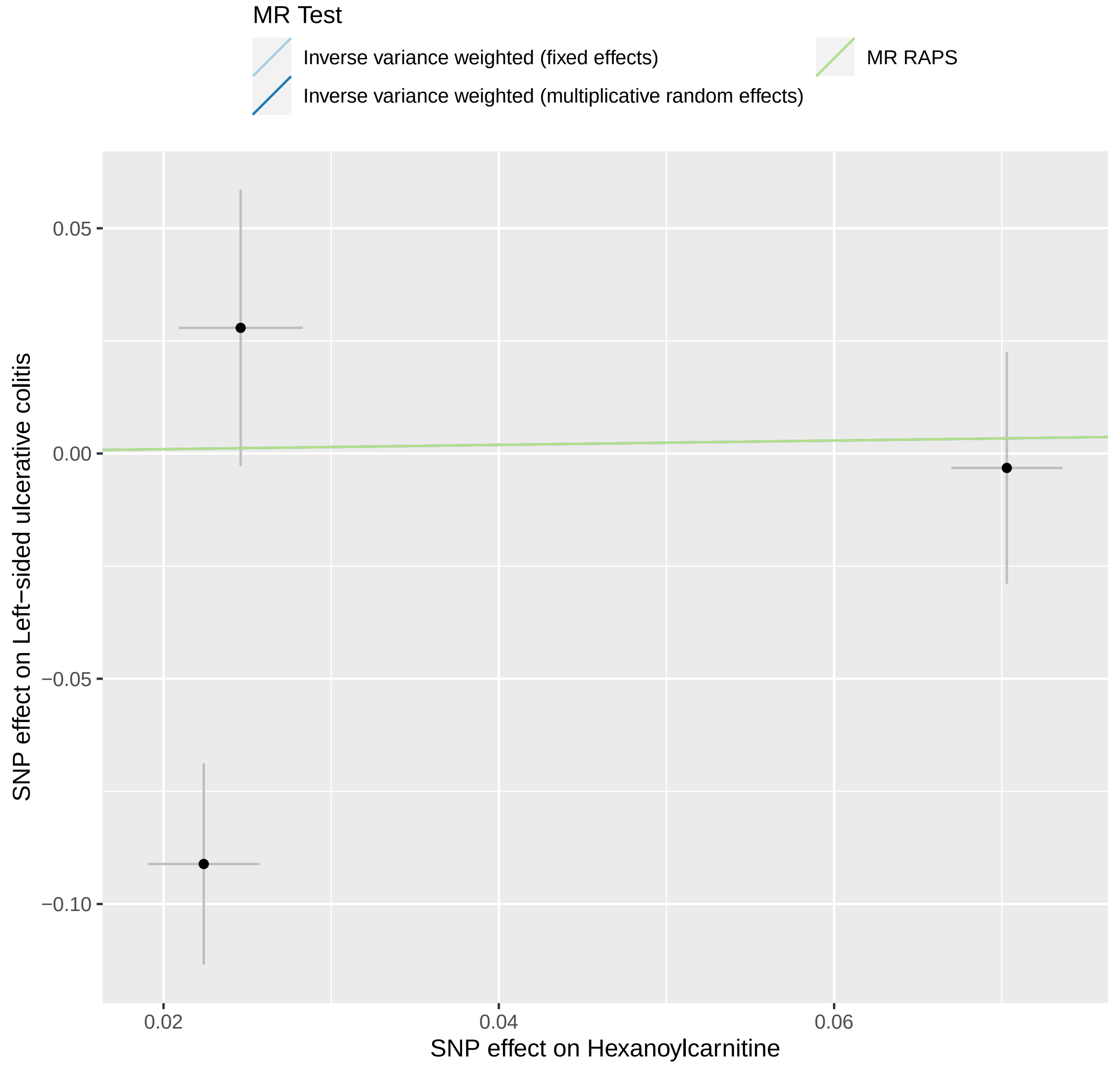

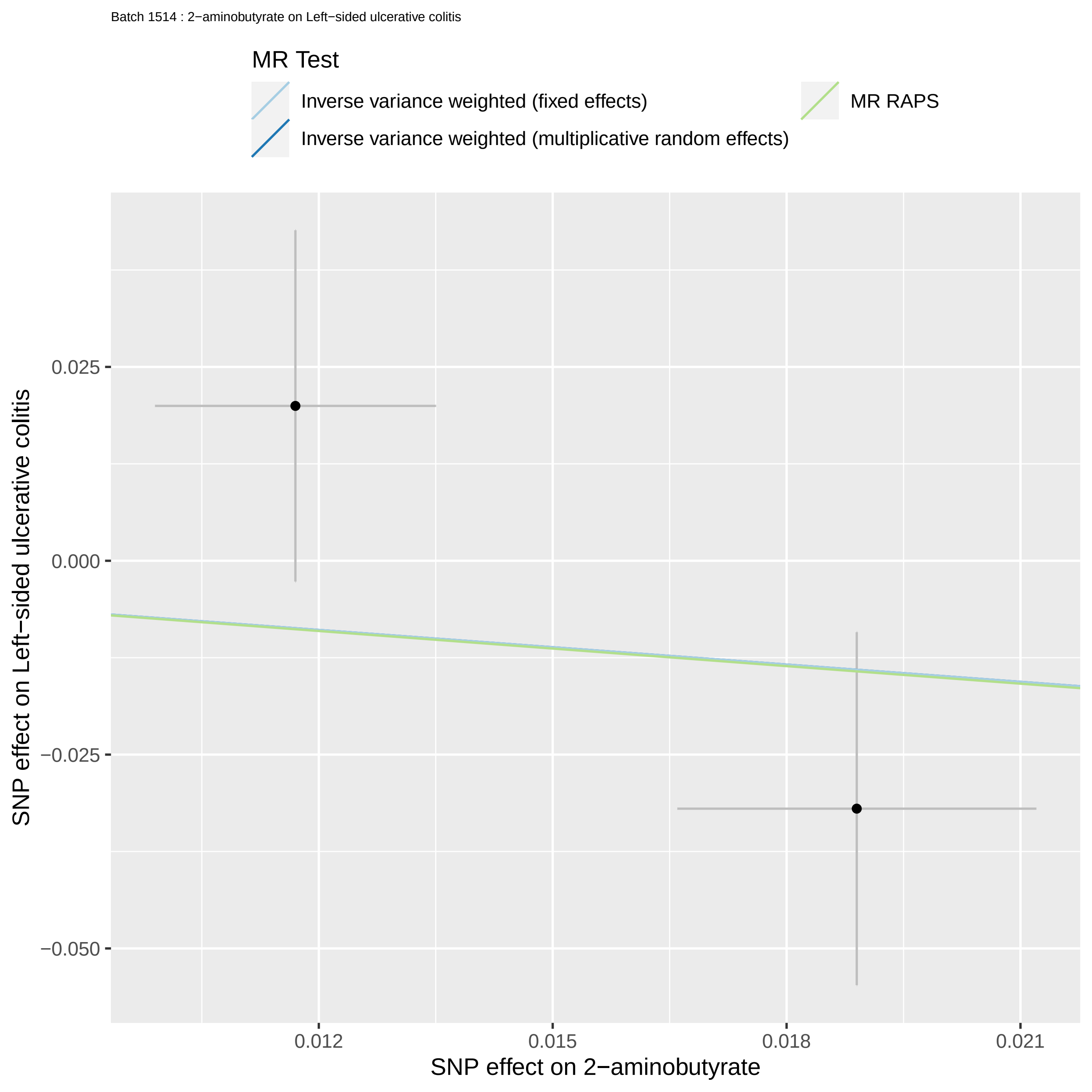

Batch 1514 : 2−aminobutyrate on Left−sided ulcerative colitis
MR Test
Inverse variance weighted (fixed effects)
Inverse variance weighted (multiplicative random effects)
MR RAPS
0.025
0.000
−0.025
−0.050
0.012
0.015
0.018
0.021
SNP effect on Left−sided ulcerative colitis
SNP effect on 2−aminobutyrate

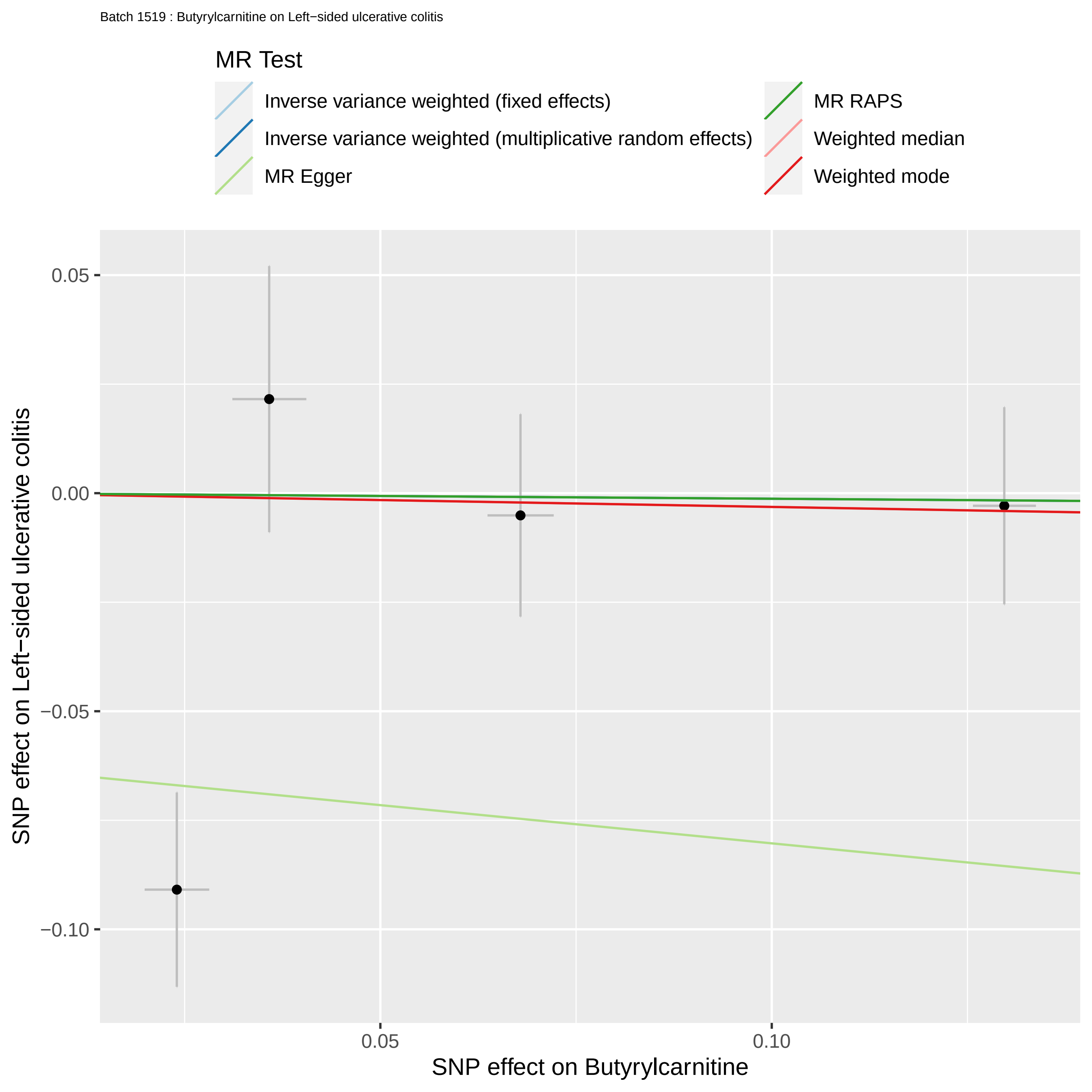

Batch 1519 : Butyrylcarnitine on Left−sided ulcerative colitis
MR Test
Inverse variance weighted (fixed effects)
Inverse variance weighted (multiplicative random effects)
MR Egger
MR RAPS
Weighted median
Weighted mode
0.05
0.00
−0.05
−0.10
0.05
0.10
SNP effect on Butyrylcarnitine
SNP effect on Left−sided ulcerative colitis

Batch 1522 : Propionylcarnitine on Left−sided ulcerative colitis

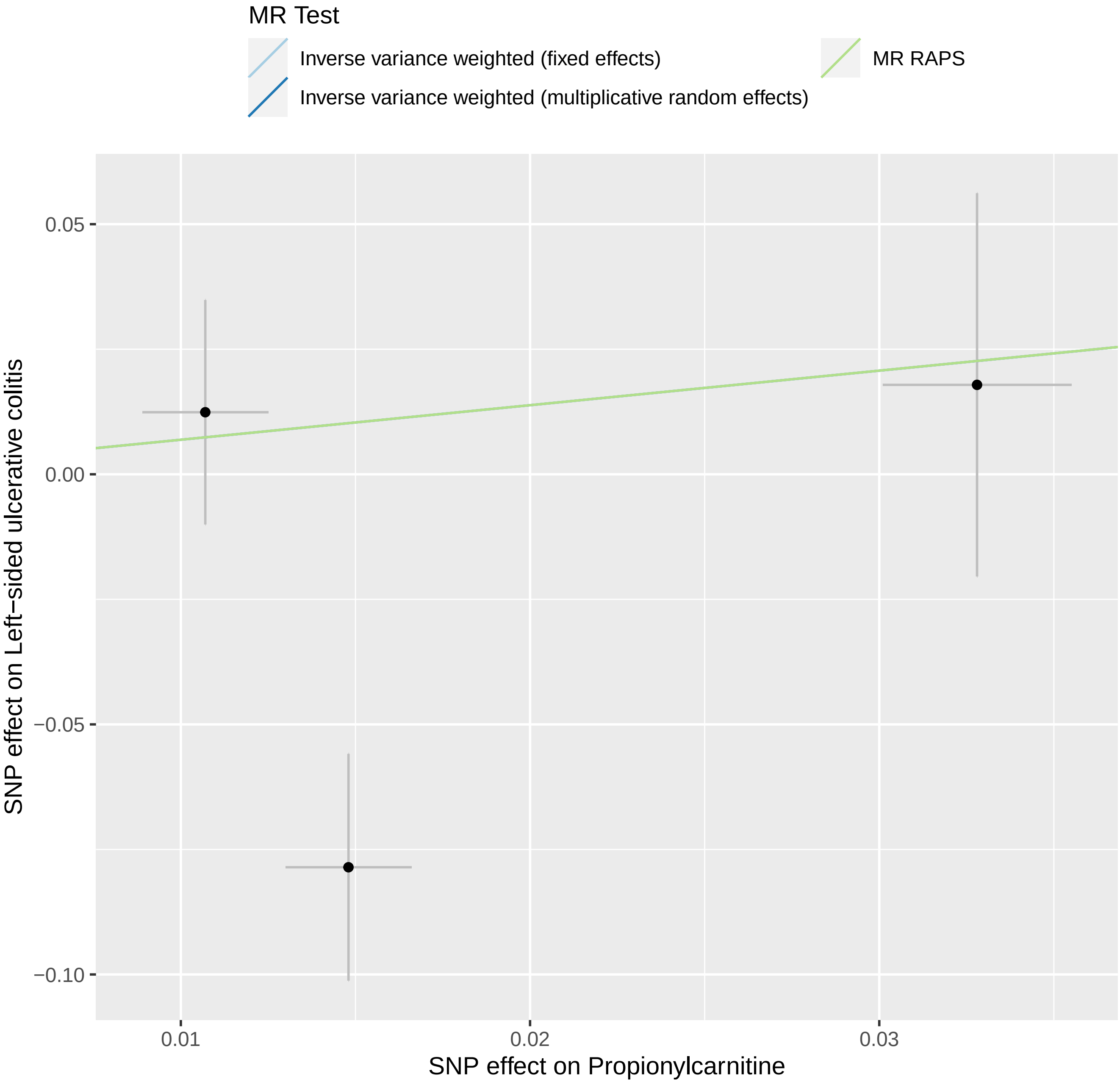

Batch 1523 : Caproate (6:0) on Left−sided ulcerative colitis

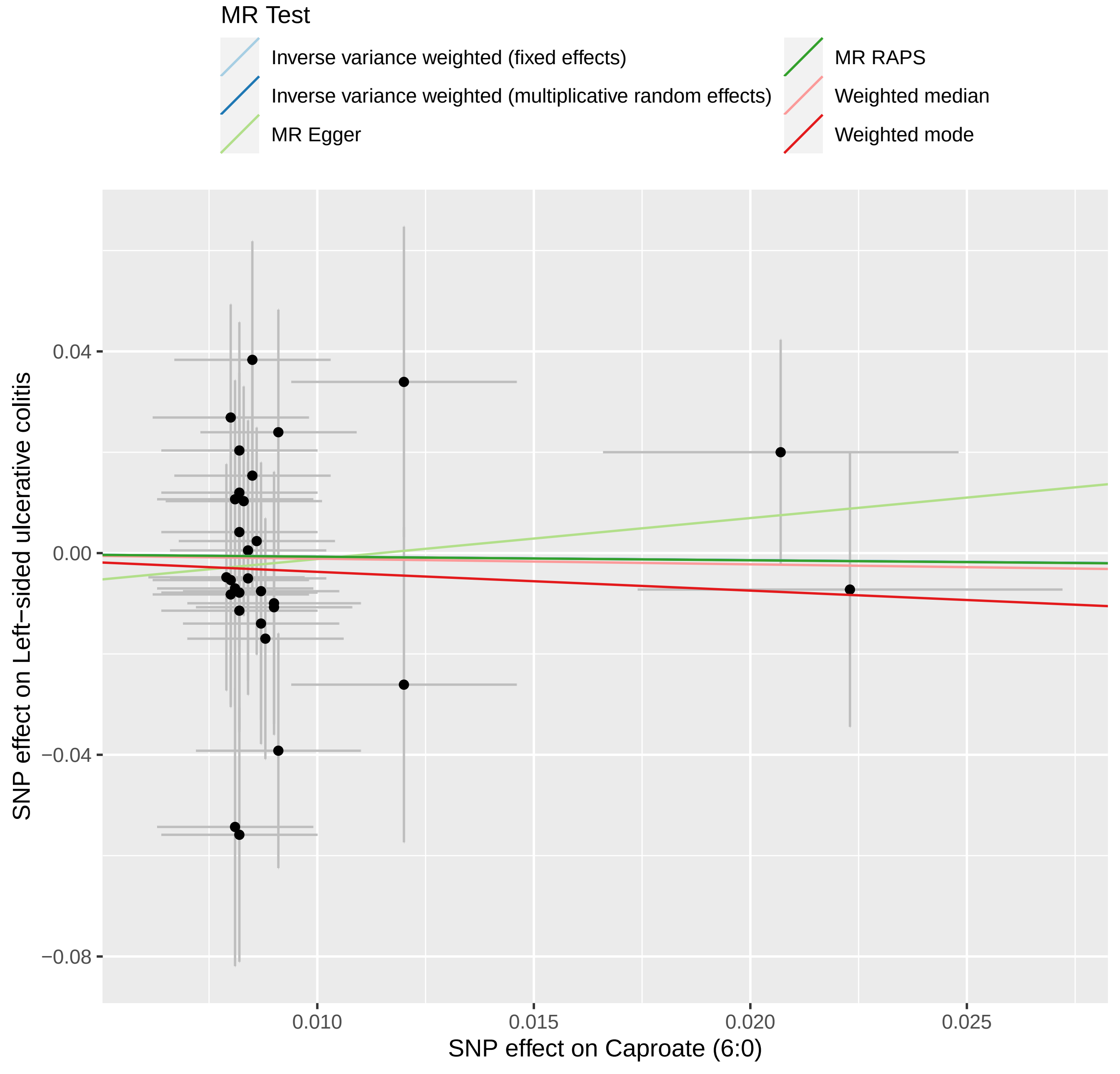

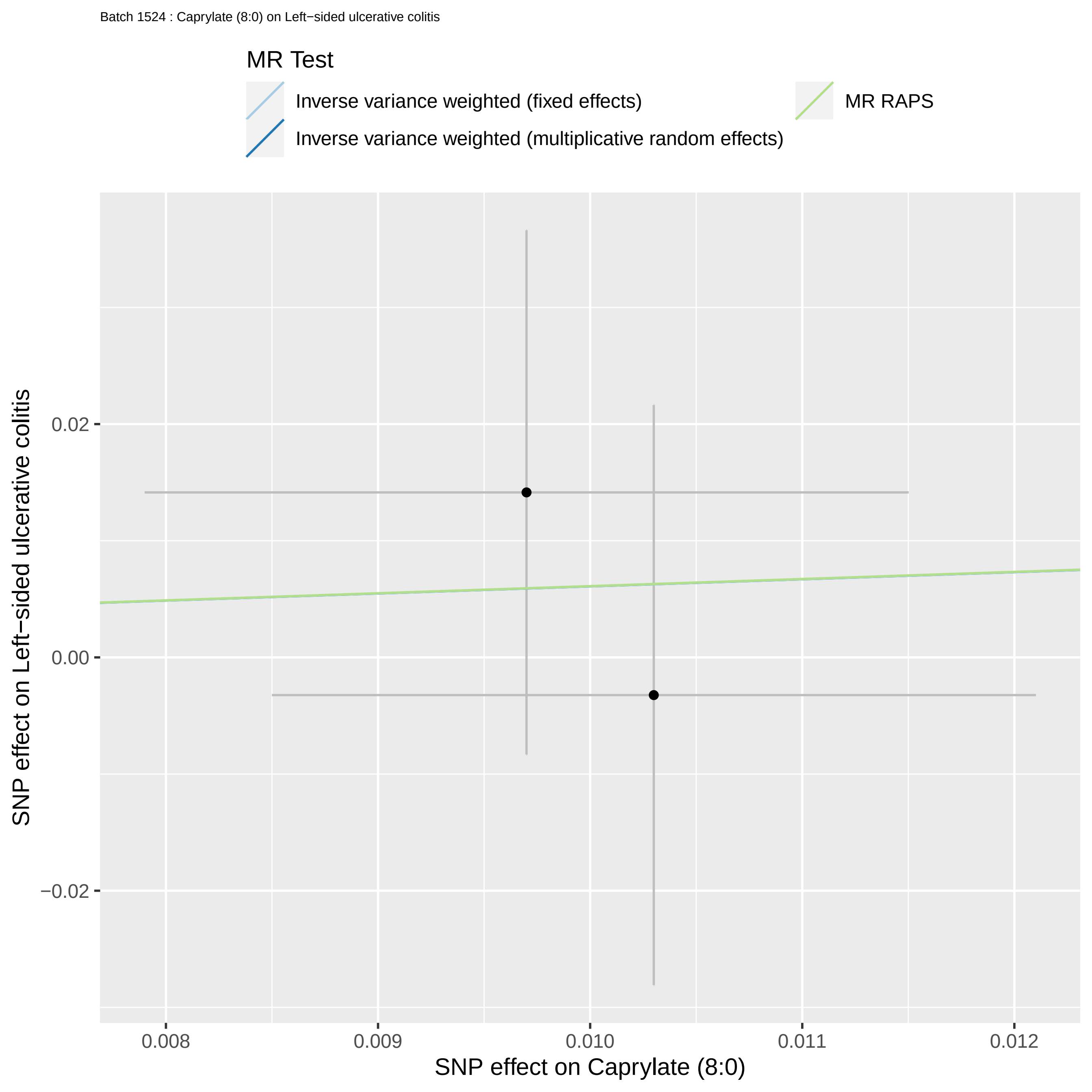
Batch 1524 : Caprylate (8:0) on Left−sided ulcerative colitis
MR Test
Inverse variance weighted (fixed effects)
Inverse variance weighted (multiplicative random effects)
MR RAPS
0.02
0.00
−0.02
0.008
0.009
0.010
0.011
0.012
SNP effect on Left−sided ulcerative colitis
SNP effect on Caprylate (8:0)

Batch 1525 : 10−undecenoate (11:1n1) on Left−sided ulcerative colitis

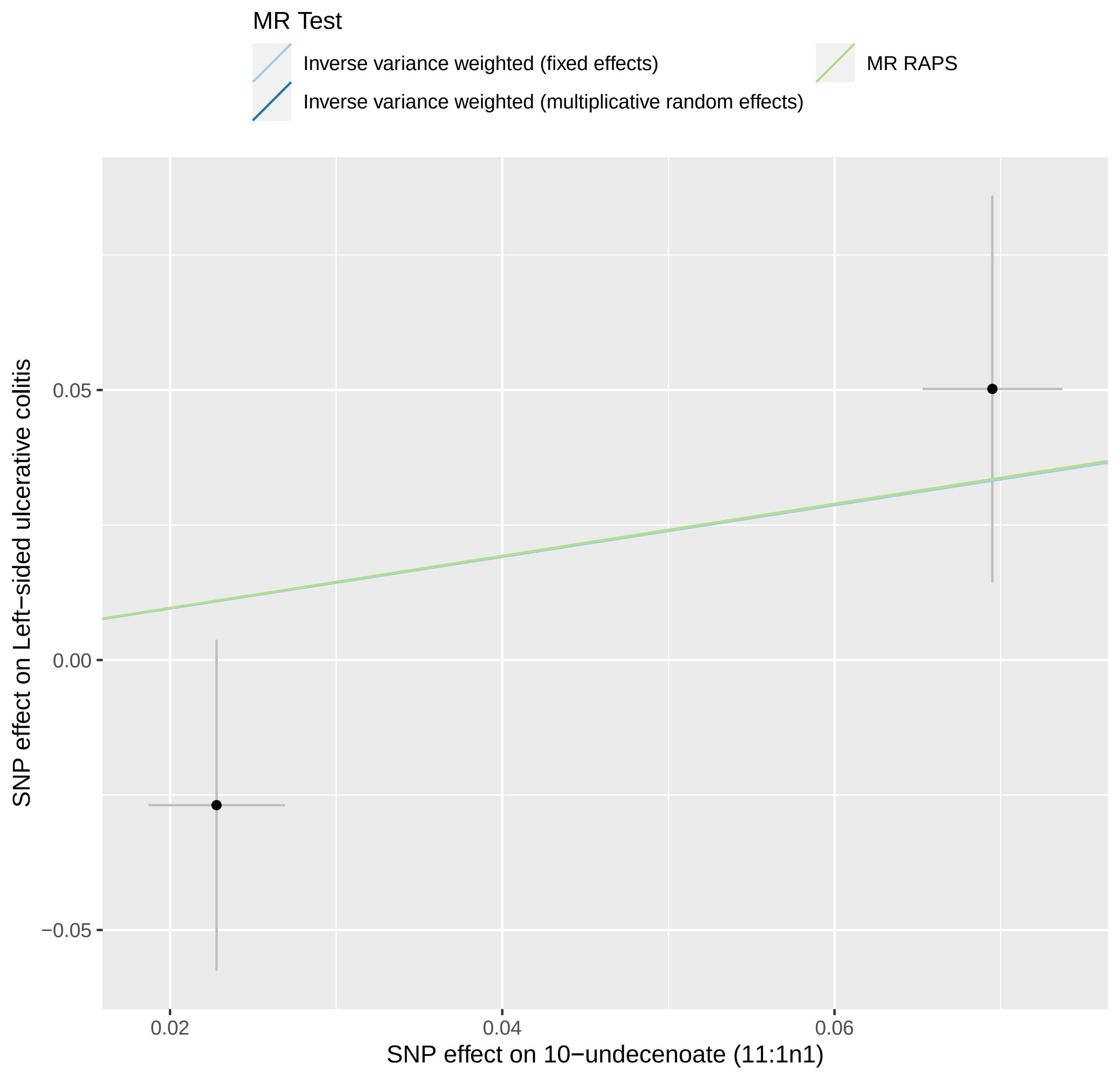

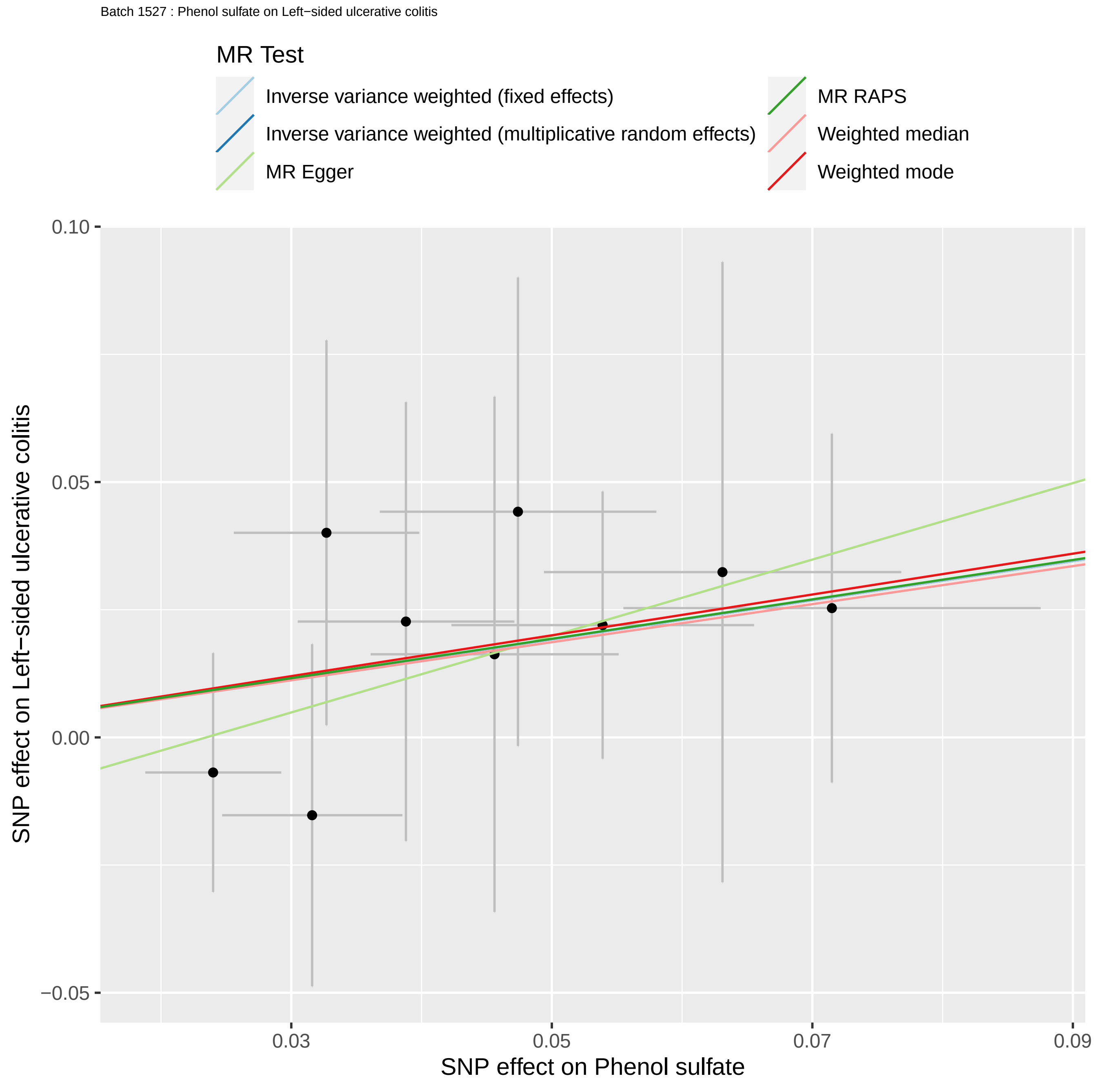
Batch 1527 : Phenol sulfate on Left−sided ulcerative colitis
MR Test
Inverse variance weighted (fixed effects)
Inverse variance weighted (multiplicative random effects)
MR Egger
MR RAPS
Weighted median
Weighted mode
0.10
0.05
0.00
−0.05
0.03
0.05
0.07
0.09
SNP effect on Left−sided ulcerative colitis
SNP effect on Phenol sulfate

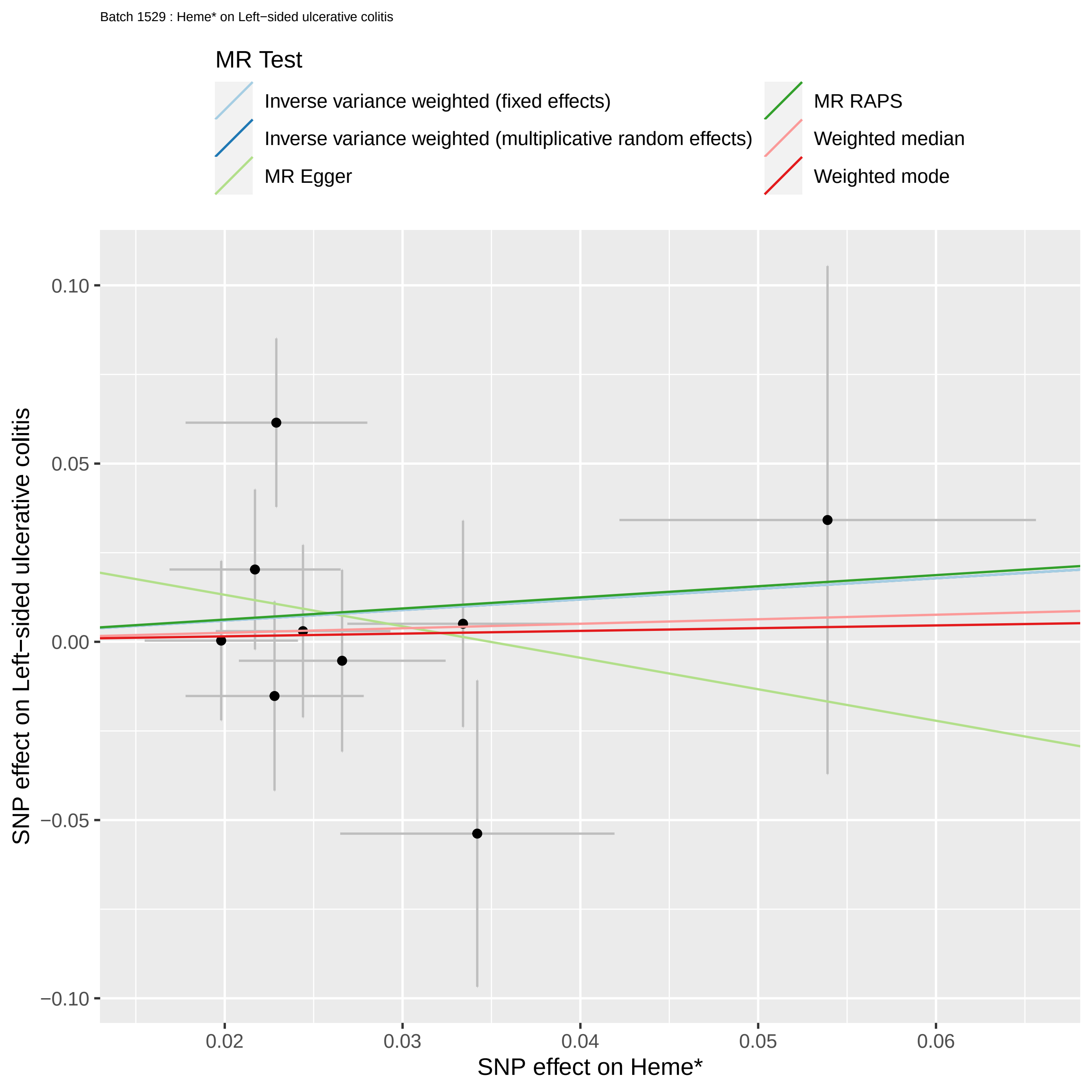
Batch 1529 : Heme* on Left−sided ulcerative colitis
MR Test
Inverse variance weighted (fixed effects)
Inverse variance weighted (multiplicative random effects)
MR Egger
MR RAPS
Weighted median
Weighted mode
0.10
0.05
0.00
−0.05
−0.10
0.02
0.03
0.04
0.05
0.06
SNP effect on Heme*
SNP effect on Left−sided ulcerative colitis

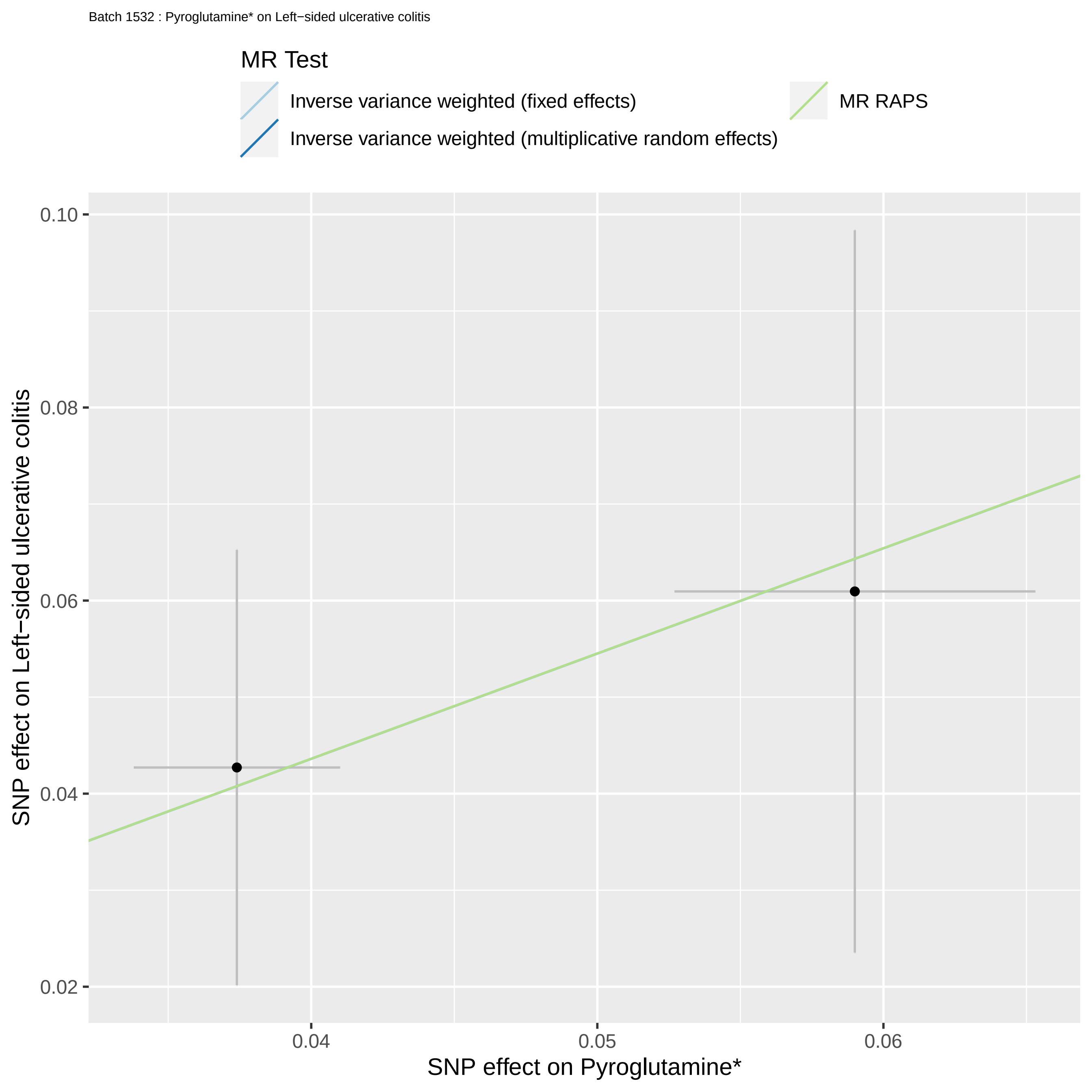
Batch 1532 : Pyroglutamine* on Left−sided ulcerative colitis
MR Test
Inverse variance weighted (fixed effects)
Inverse variance weighted (multiplicative random effects)
MR RAPS
0.10
0.08
0.06
0.04
0.02
0.04
0.05
0.06
SNP effect on Left−sided ulcerative colitis
SNP effect on Pyroglutamine*

Batch 1535 : X−11422−−xanthine on Left−sided ulcerative colitis

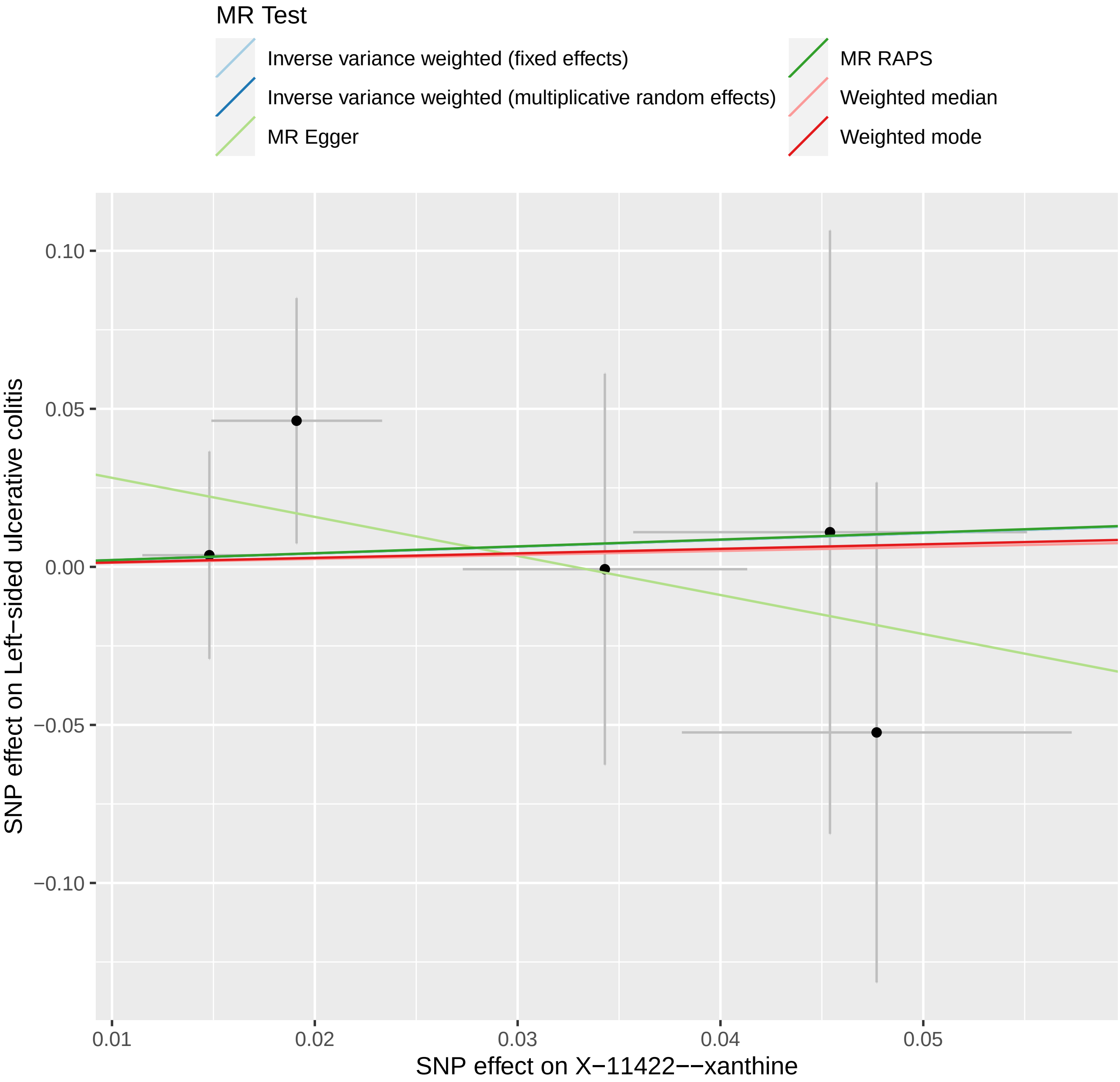

Batch 1538 : HWESASXX* on Left−sided ulcerative colitis

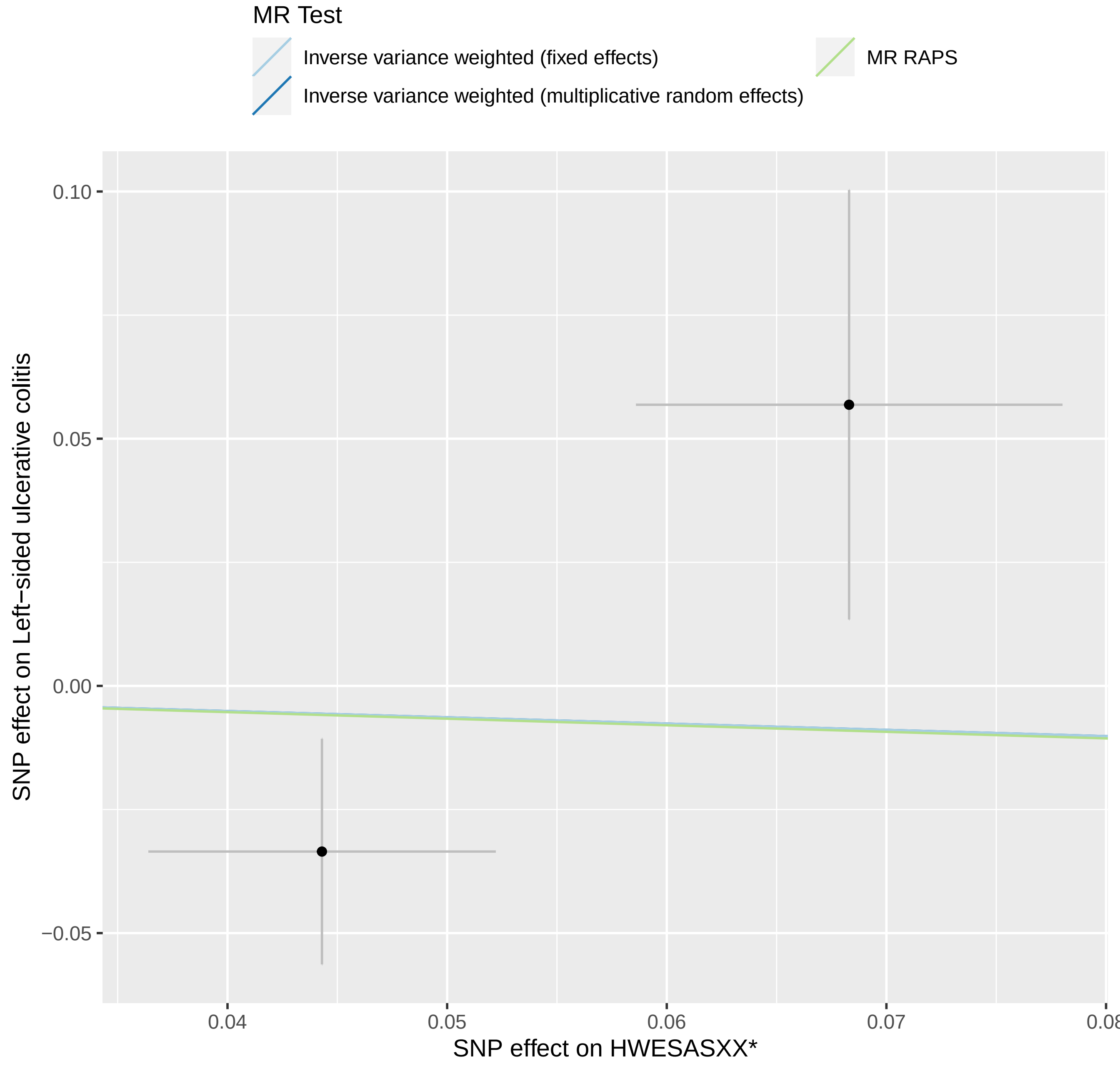

Batch 1539 : X−11593−−O−methylascorbate* on Left−sided ulcerative colitis

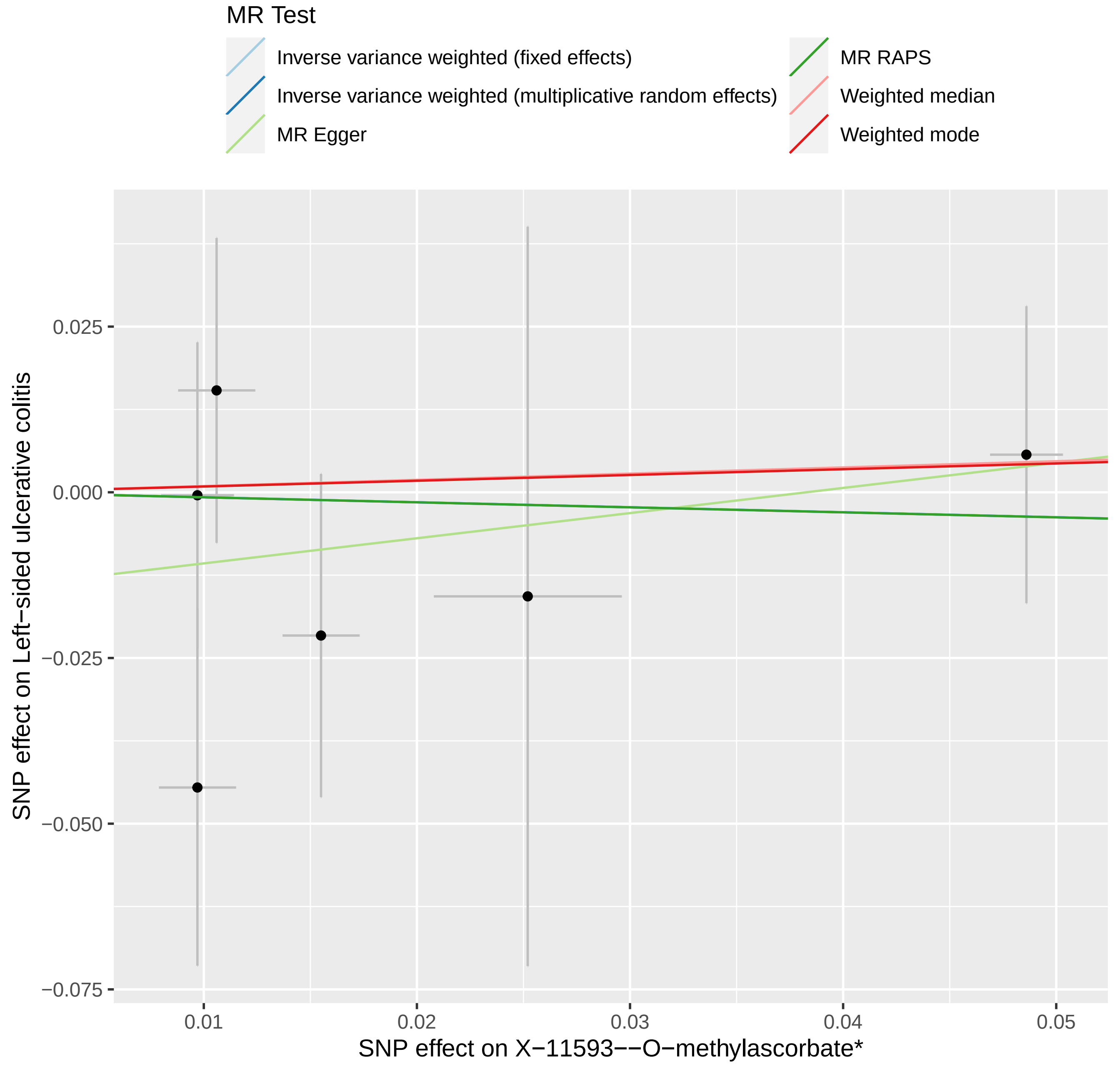

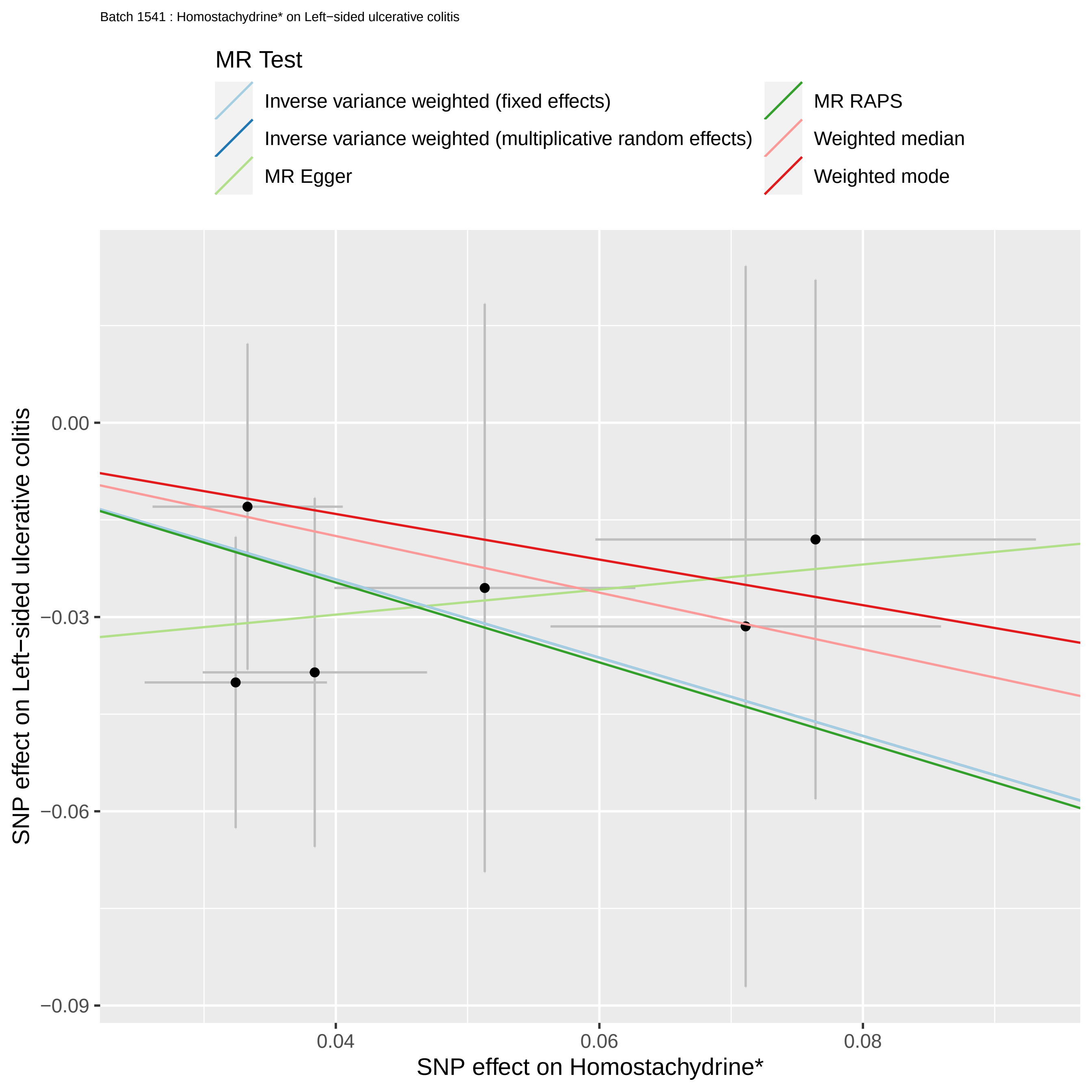

Batch 1541 : Homostachydrine* on Left−sided ulcerative colitis
MR Test
Inverse variance weighted (fixed effects)
Inverse variance weighted (multiplicative random effects)
MR Egger
MR RAPS
Weighted median
Weighted mode
0.00
−0.03
−0.06
−0.09
0.04
0.06
0.08
SNP effect on Left−sided ulcerative colitis
SNP effect on Homostachydrine*

Batch 1542 : ADSGEGDFXAEGGGVR* on Left−sided ulcerative colitis

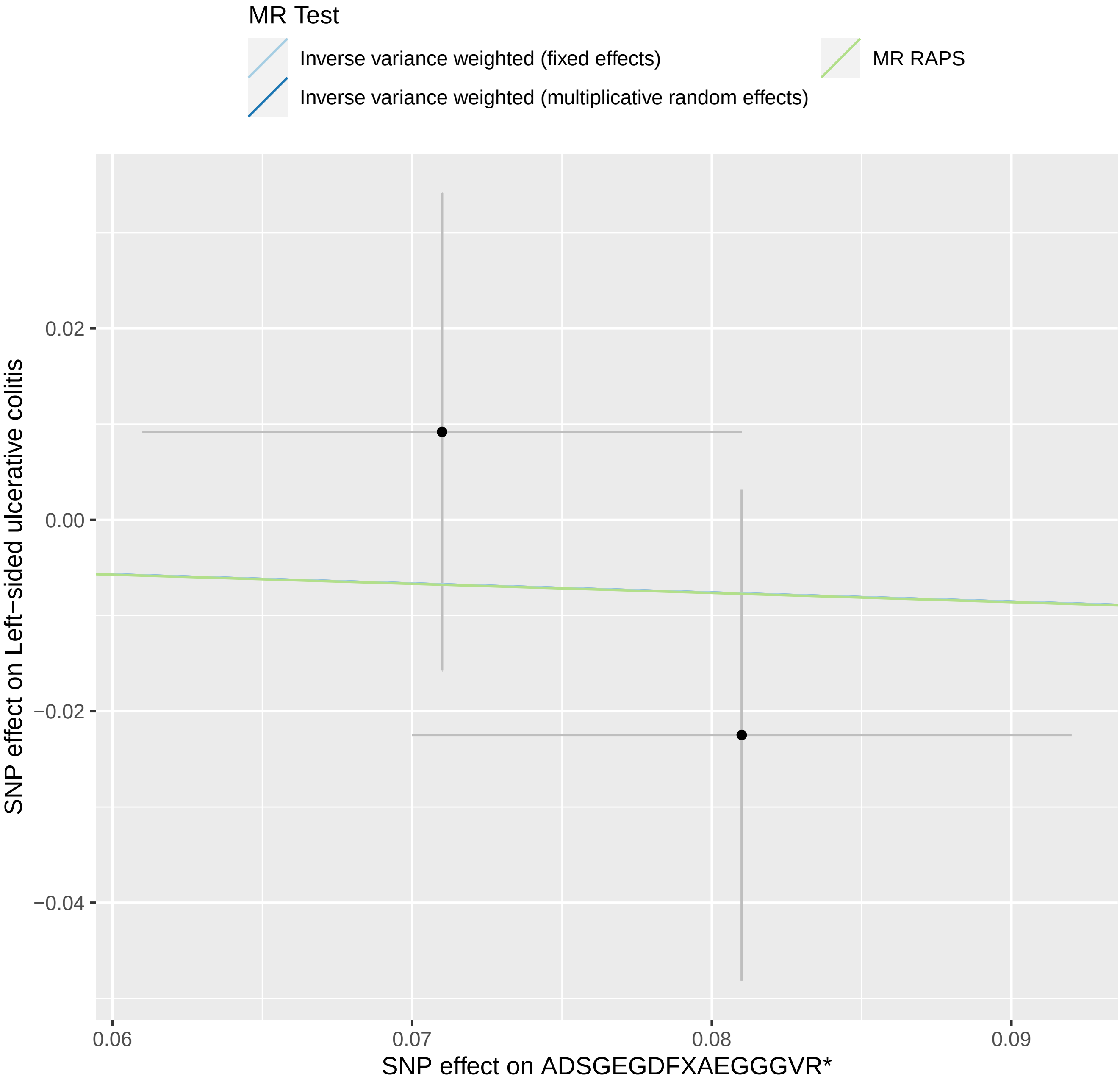

Batch 1544 : X−11793−−oxidized bilirubin* on Left−sided ulcerative colitis

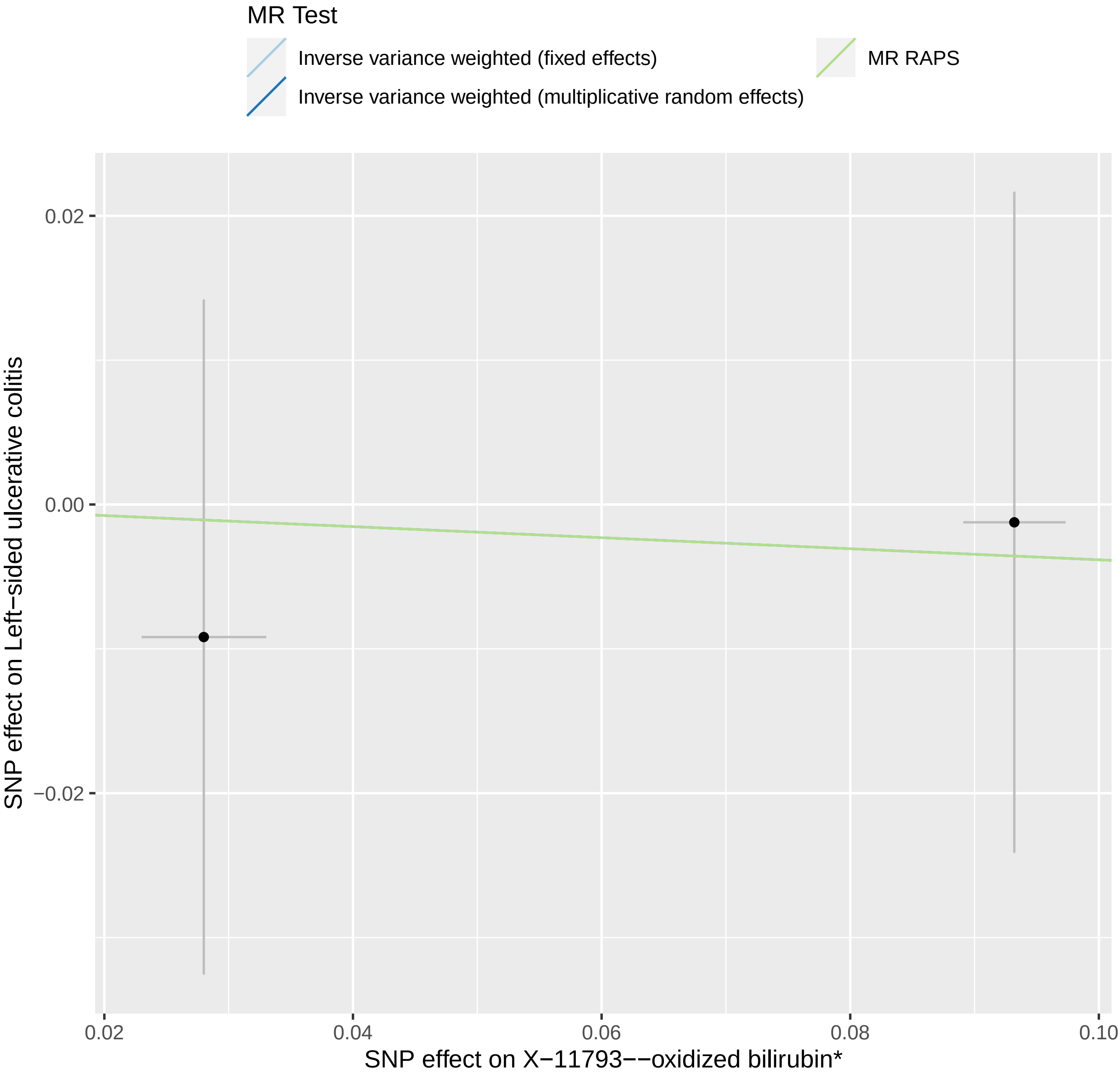

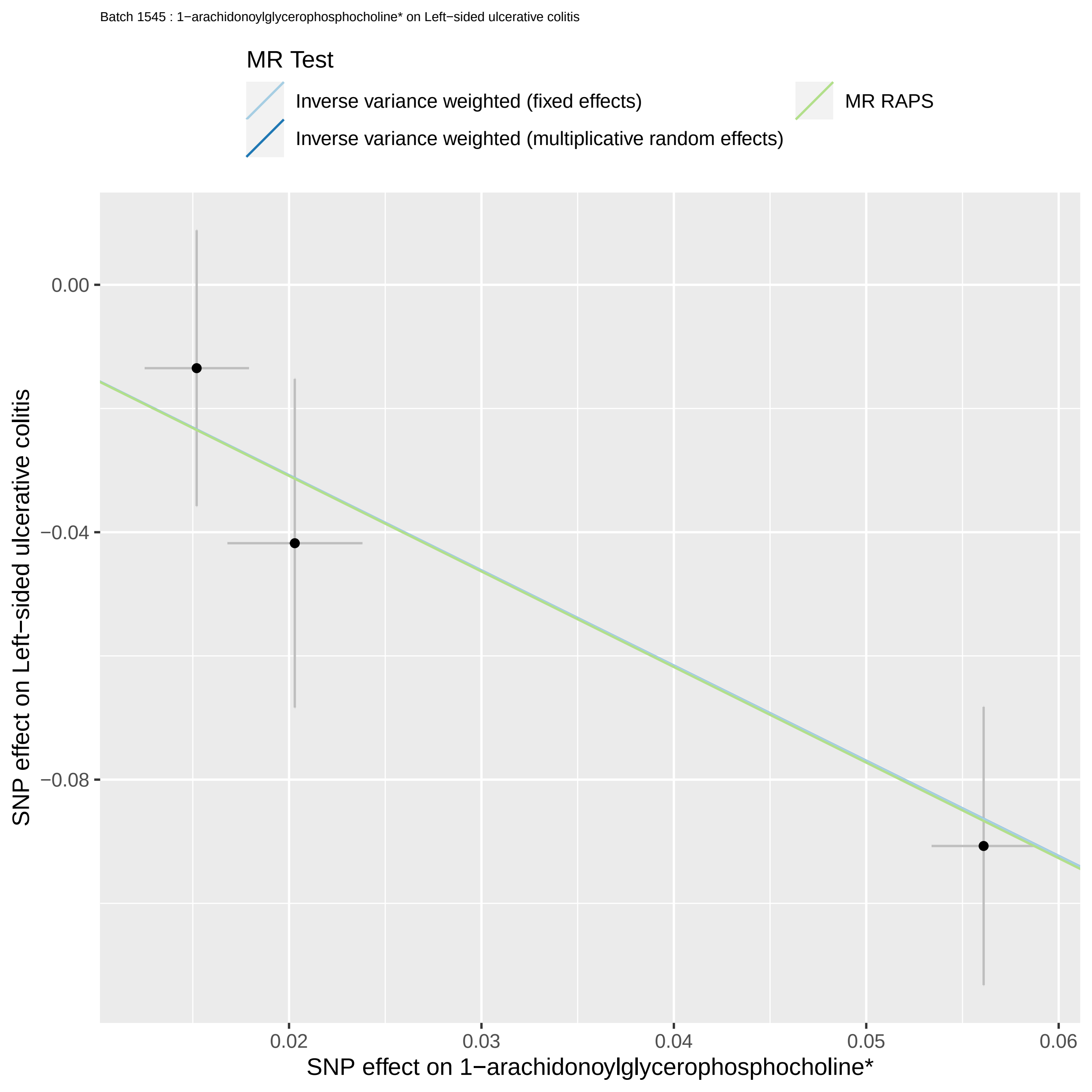
Batch 1545 : 1−arachidonoylglycerophosphocholine* on Left−sided ulcerative colitis
MR Test
Inverse variance weighted (fixed effects)
Inverse variance weighted (multiplicative random effects)
MR RAPS
0.00
−0.04
−0.08
0.02
0.03
0.04
0.05
0.06
SNP effect on Left−sided ulcerative colitis
SNP effect on 1−arachidonoylglycerophosphocholine*

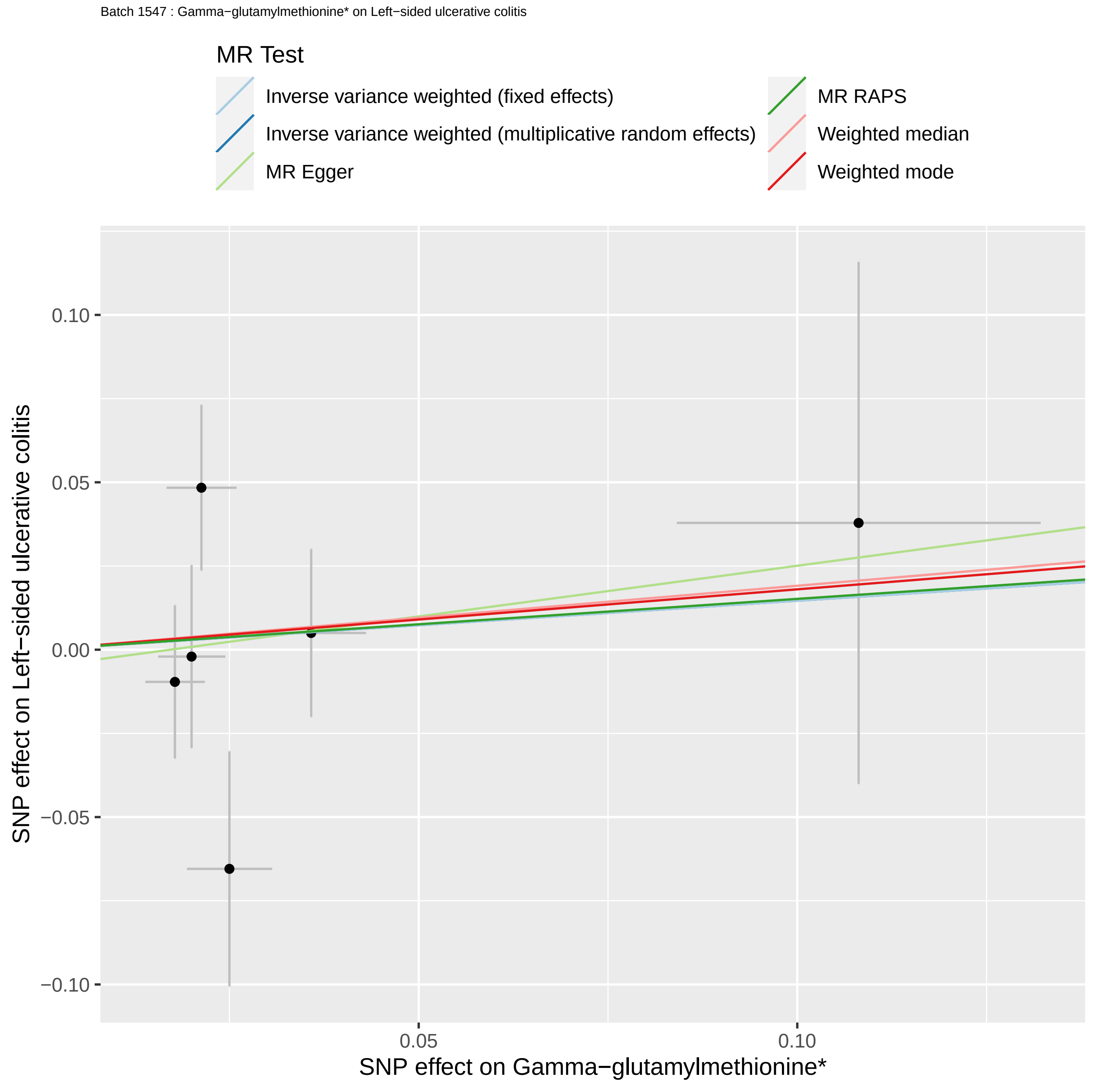
Batch 1547 : Gamma−glutamylmethionine* on Left−sided ulcerative colitis
MR Test
Inverse variance weighted (fixed effects)
Inverse variance weighted (multiplicative random effects)
MR Egger
MR RAPS
Weighted median
Weighted mode
0.10
0.05
0.00
−0.05
−0.10
0.05
0.10
SNP effect on Left−sided ulcerative colitis
SNP effect on Gamma−glutamylmethionine*

Batch 1548 : Gamma−glutamylthreonine* on Left−sided ulcerative colitis

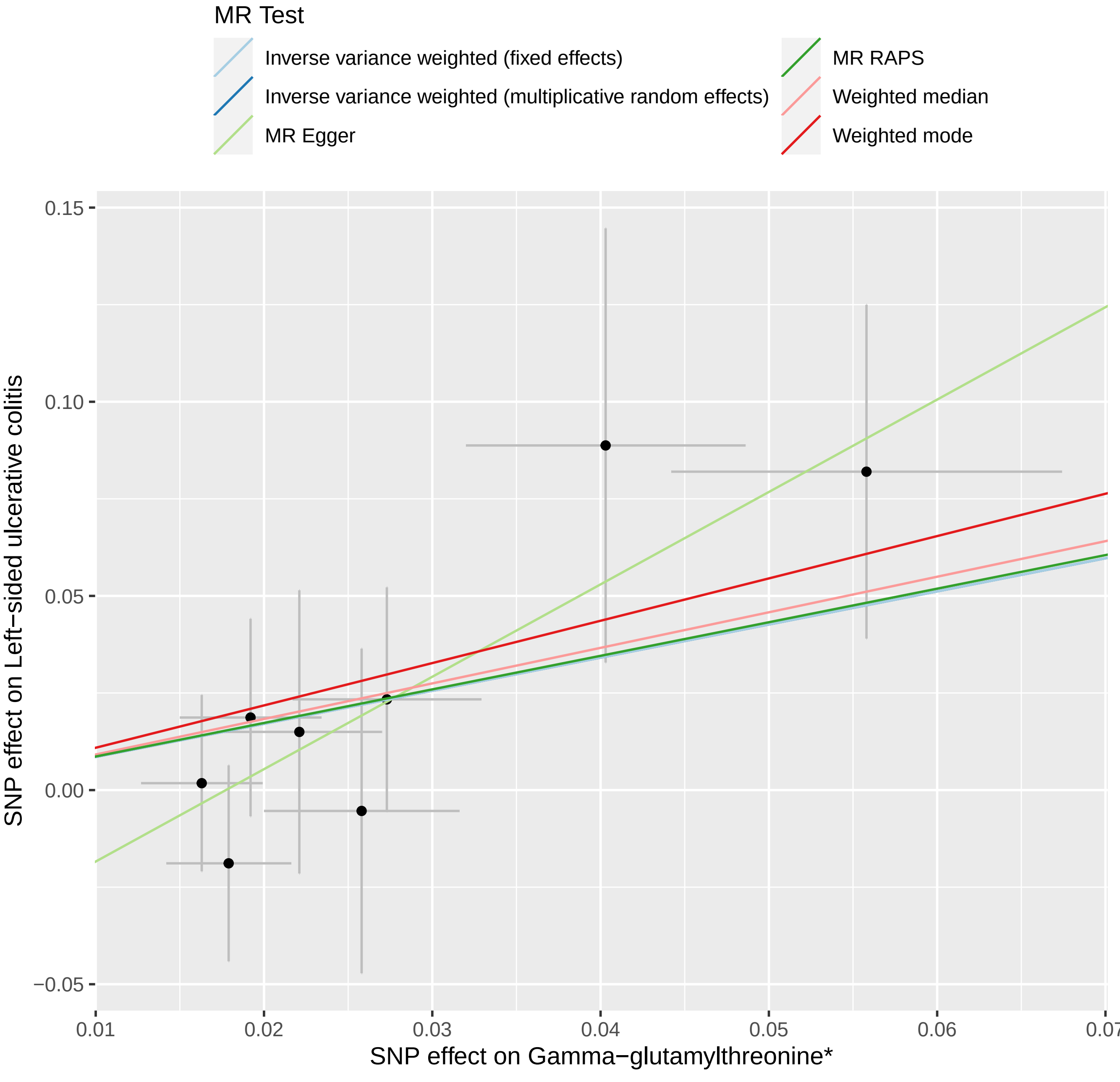

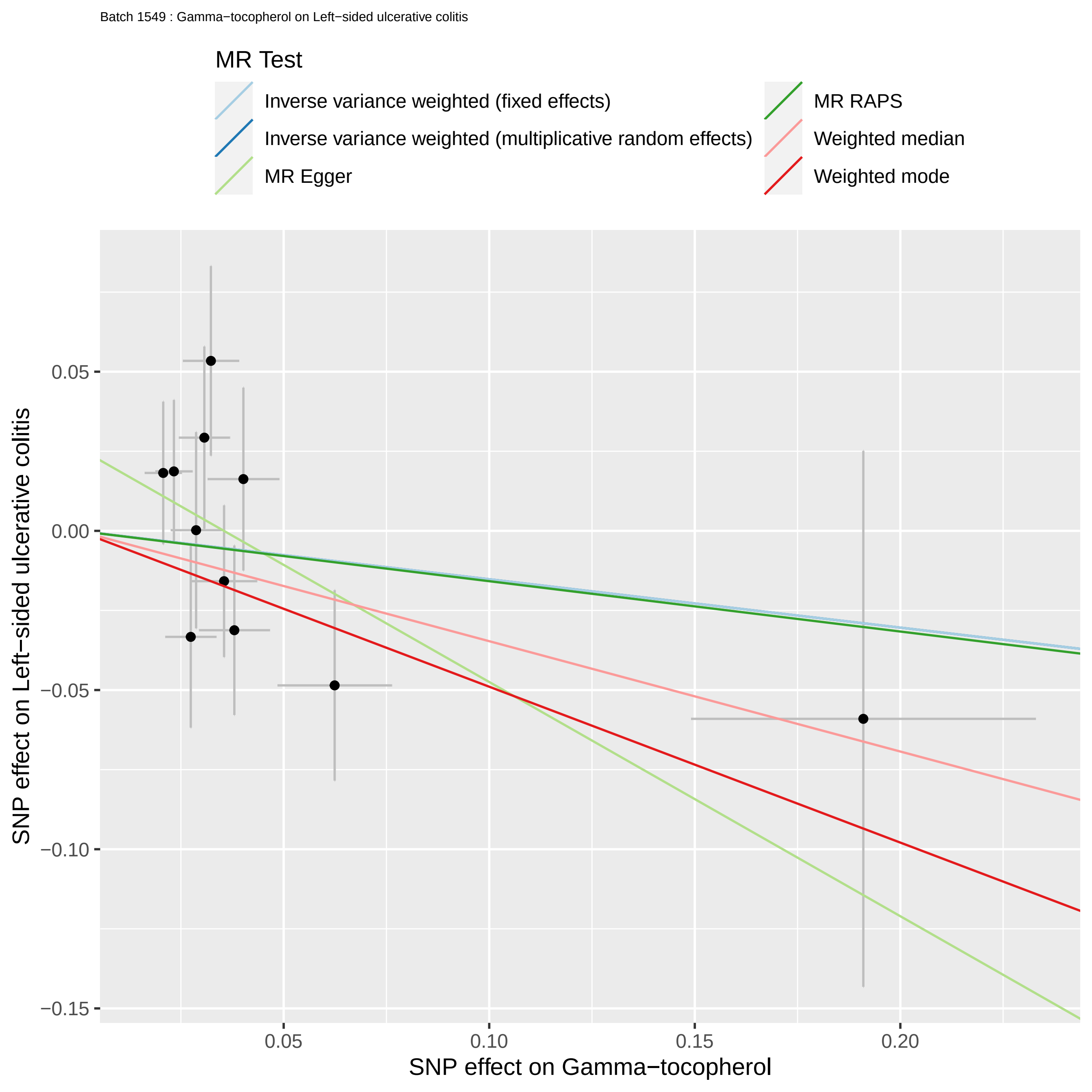
Batch 1549 : Gamma−tocopherol on Left−sided ulcerative colitis
MR Test
Inverse variance weighted (fixed effects)
Inverse variance weighted (multiplicative random effects)
MR Egger
MR RAPS
Weighted median
Weighted mode
0.05
0.00
−0.05
−0.10
−0.15
SNP effect on Left−sided ulcerative colitis
0.05
0.10
0.15
0.20
SNP effect on Gamma−tocopherol

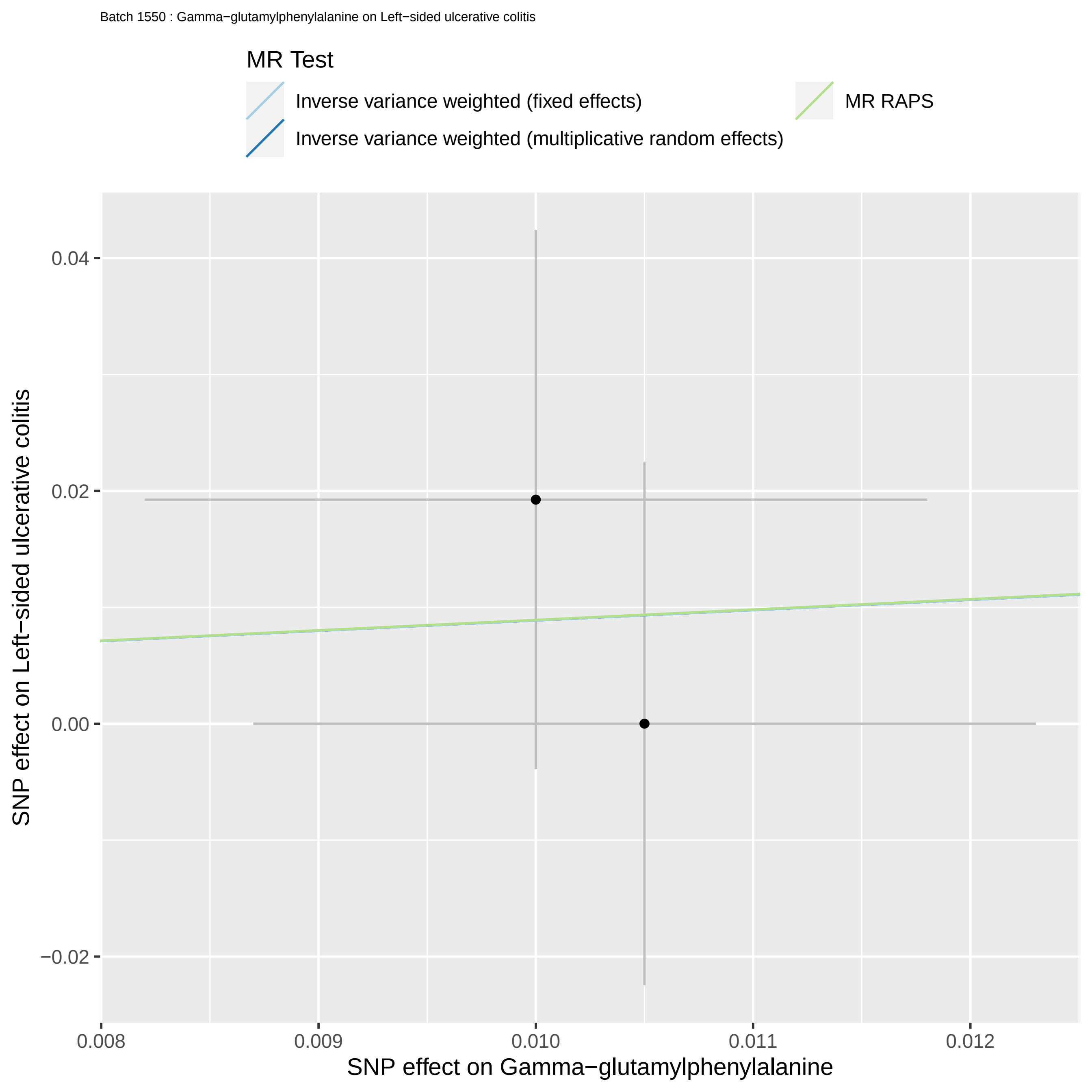
Batch 1550 : Gamma−glutamylphenylalanine on Left−sided ulcerative colitis
MR Test
Inverse variance weighted (fixed effects)
Inverse variance weighted (multiplicative random effects)
MR RAPS
0.04
0.02
0.00
−0.02
SNP effect on Left−sided ulcerative colitis
0.008
0.009
0.010
0.011
0.012
SNP effect on Gamma−glutamylphenylalanine

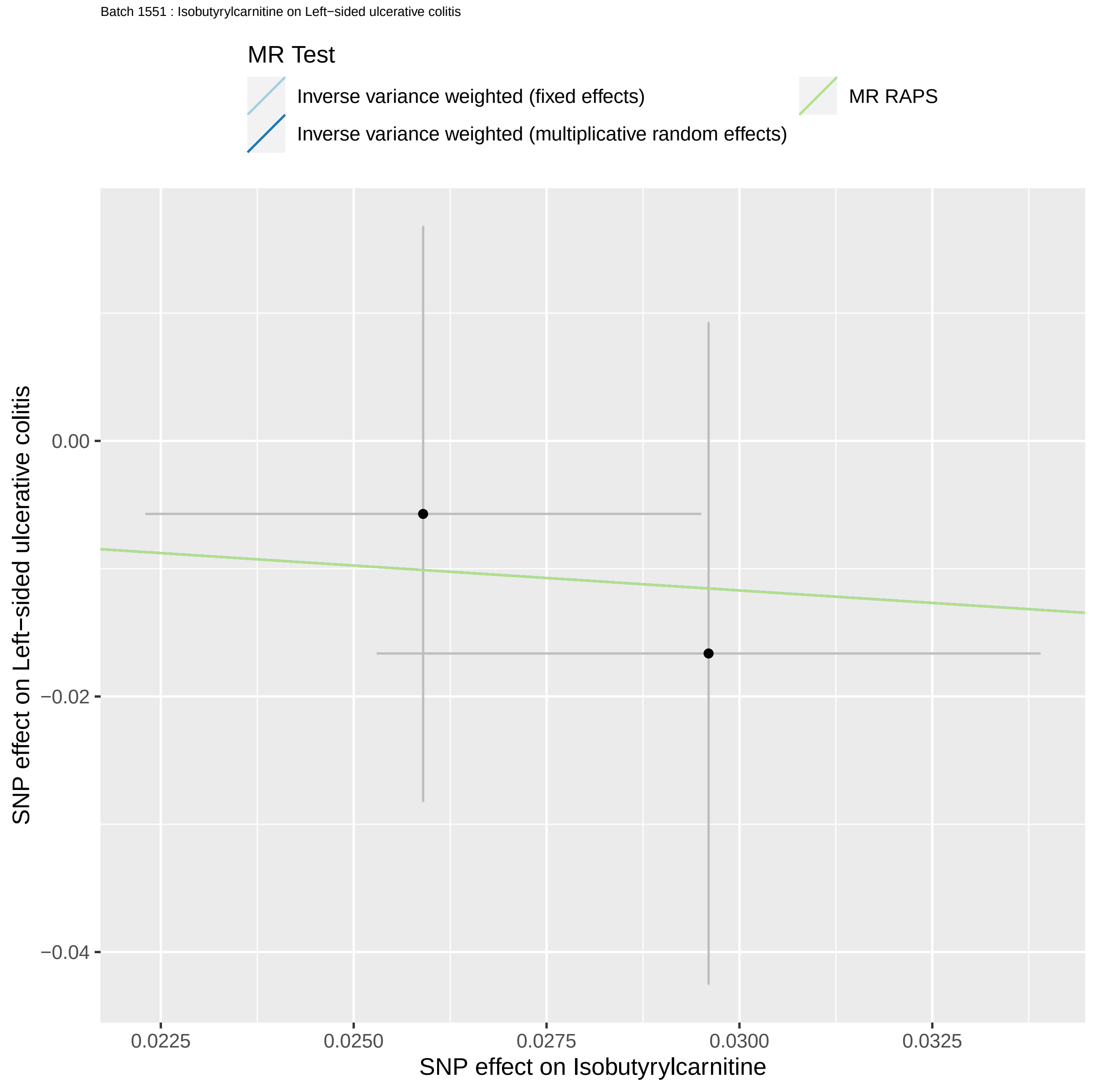

Batch 1551 : Isobutyrylcarnitine on Left−sided ulcerative colitis
MR Test
Inverse variance weighted (fixed effects)
Inverse variance weighted (multiplicative random effects)
MR RAPS
SNP effect on Left−sided ulcerative colitis
0.00
−0.02
−0.04
0.0225
0.0250
0.0275
0.0300
0.0325
SNP effect on Isobutyrylcarnitine

Batch 1552 : Pseudouridine on Left−sided ulcerative colitis

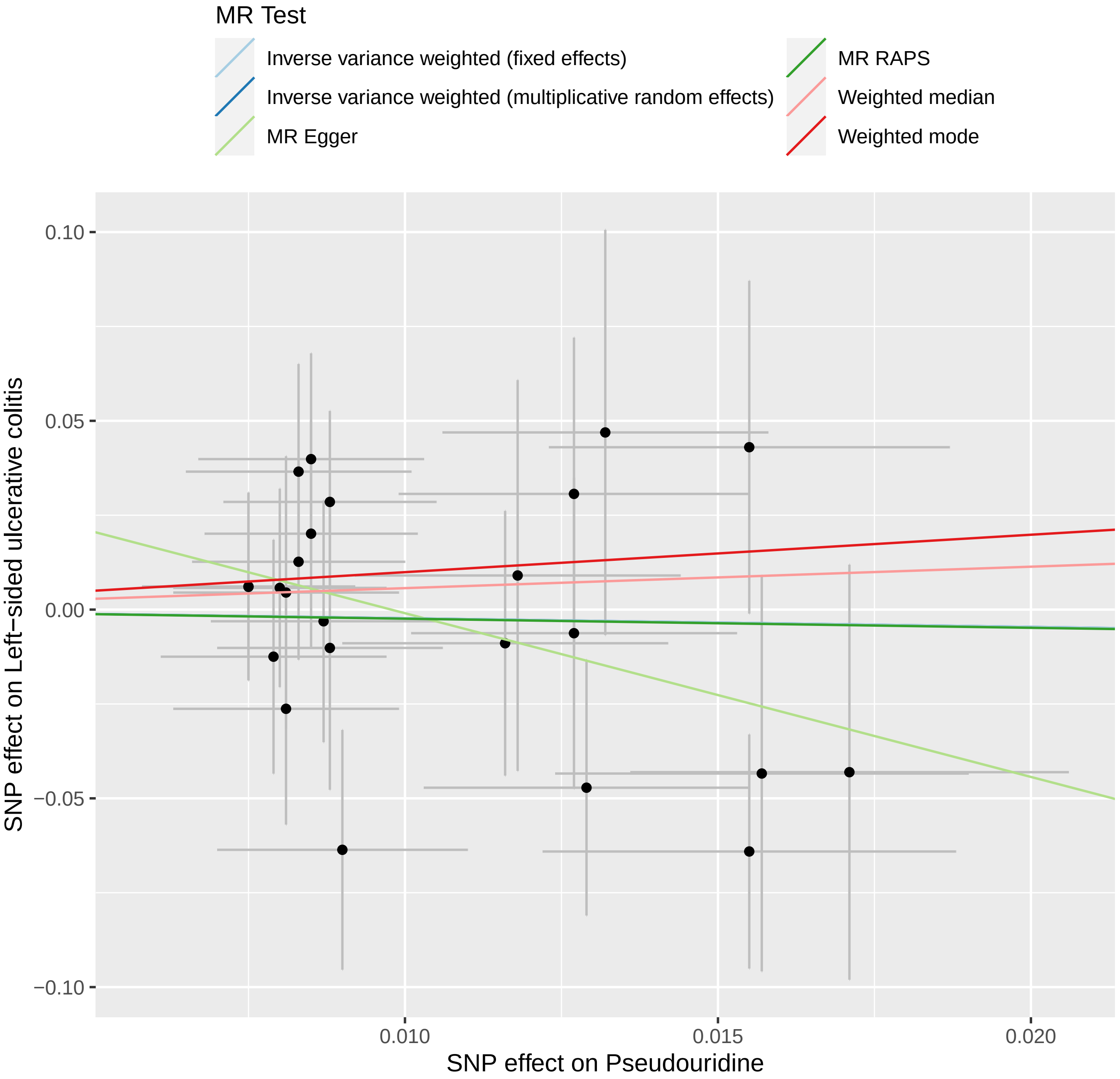

Batch 1553 : Valerate on Left−sided ulcerative colitis

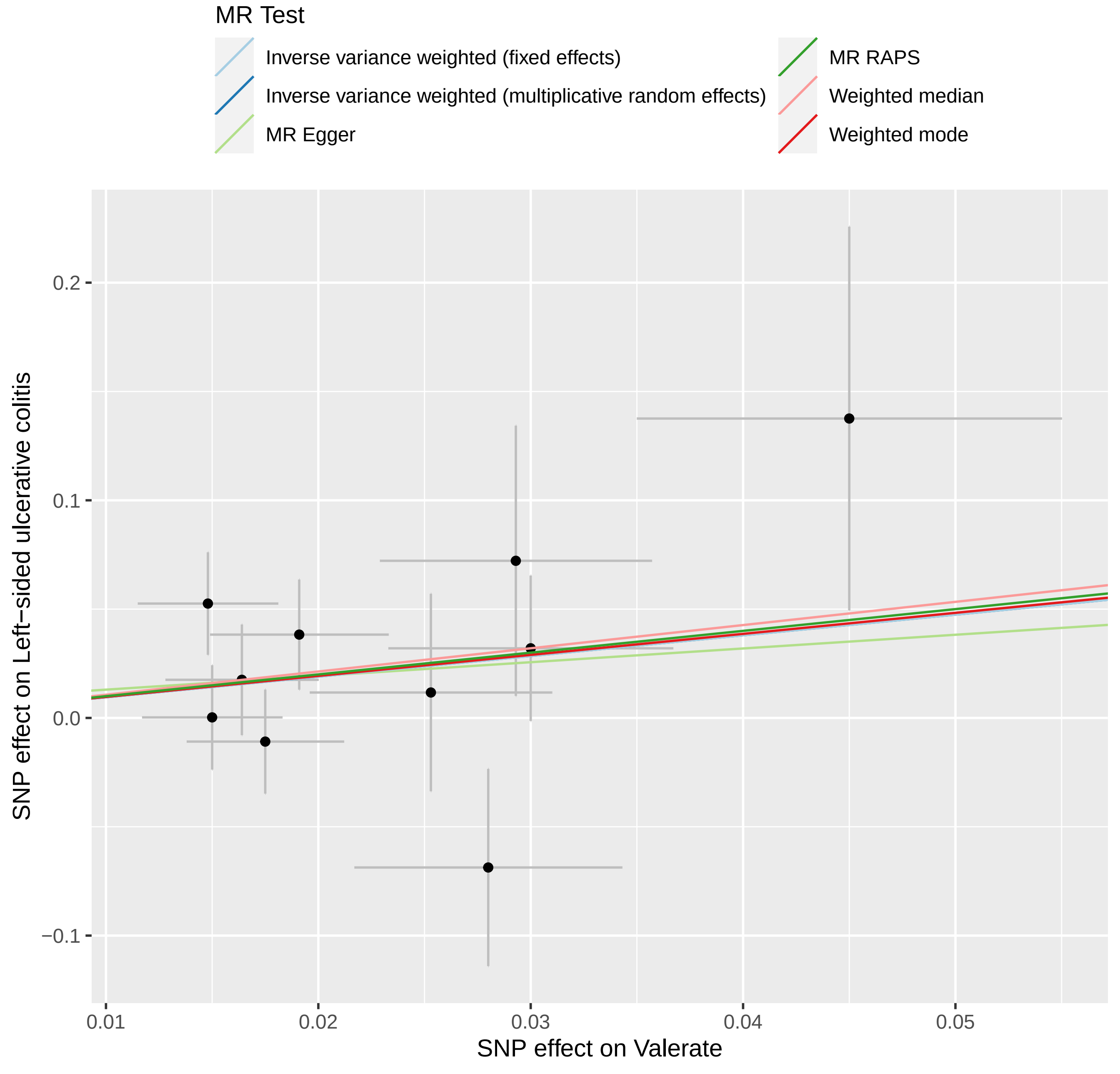

Batch 1556 : Erythronate* on Left−sided ulcerative colitis

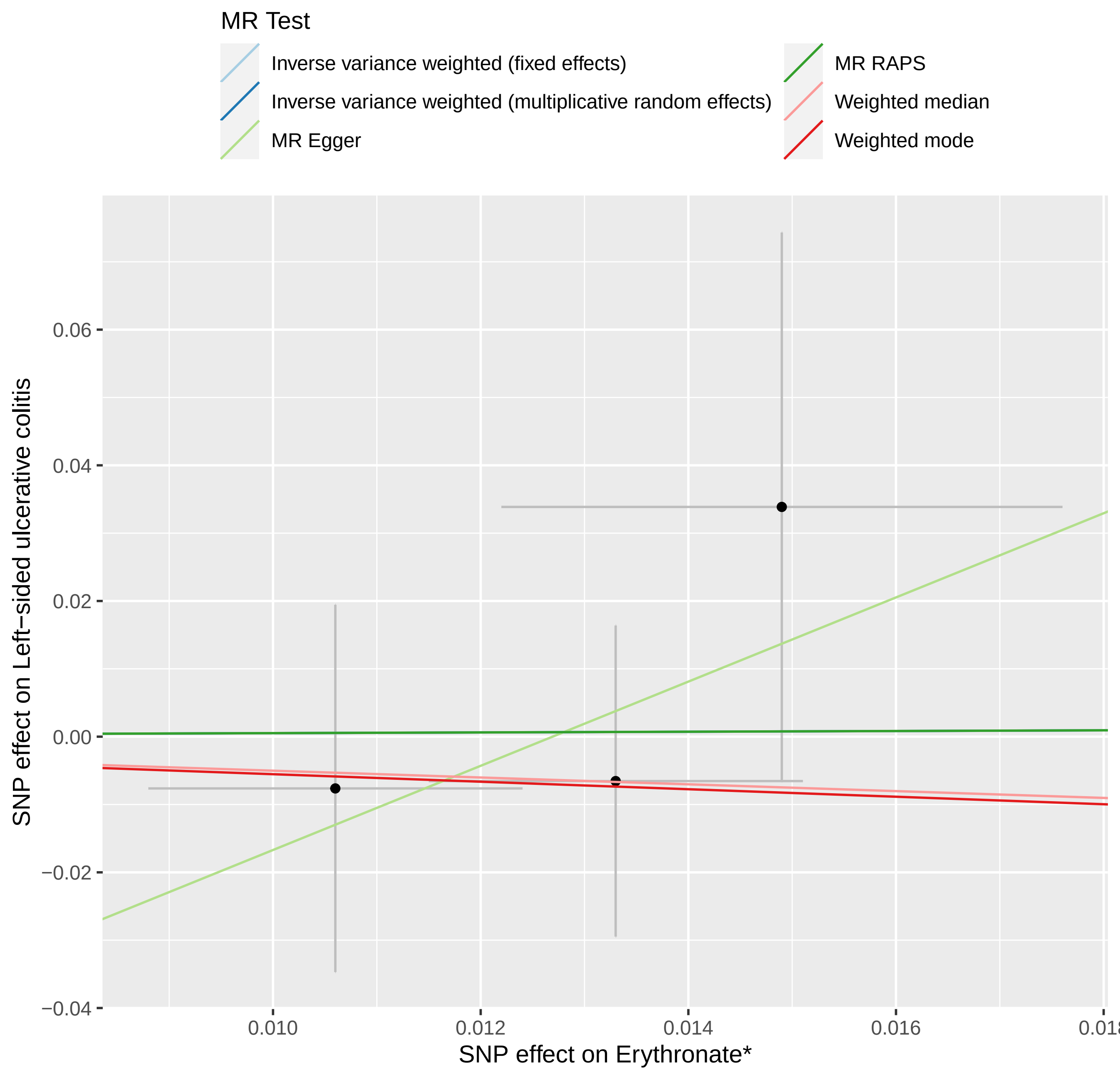

Batch 1557 : Lathosterol on Left−sided ulcerative colitis

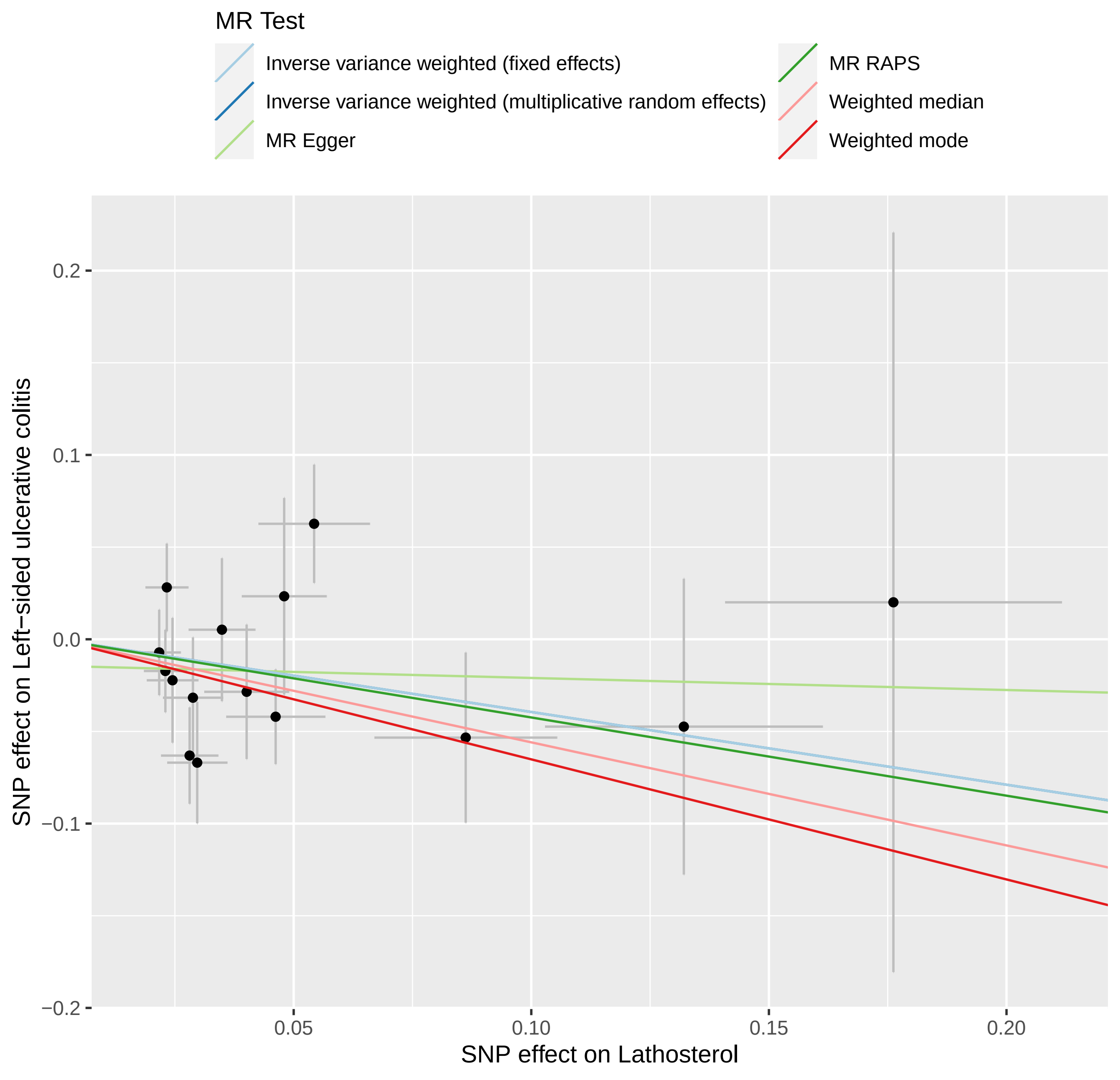

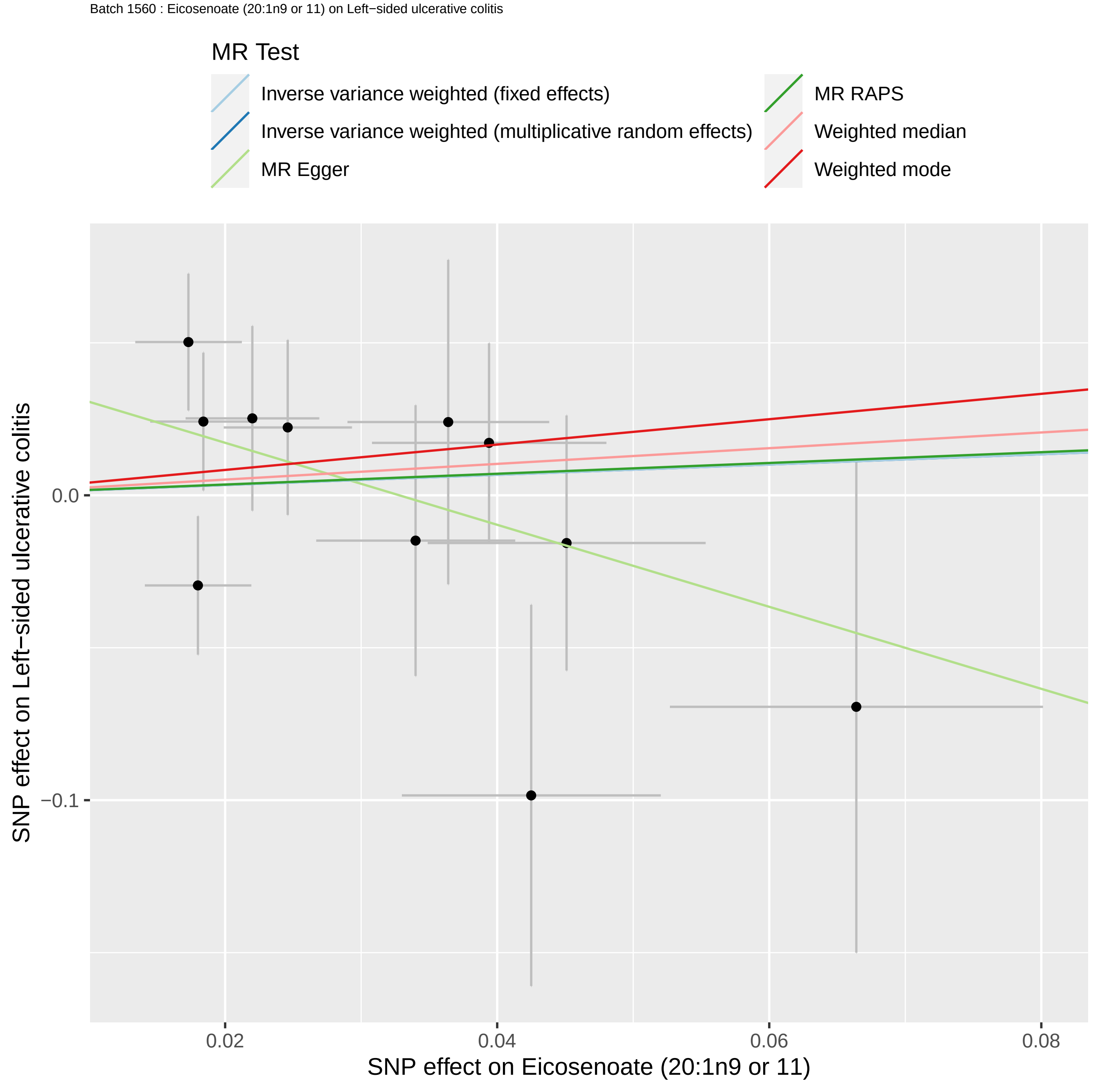

Batch 1560 : Eicosenoate (20:1n9 or 11) on Left−sided ulcerative colitis
MR Test
Inverse variance weighted (fixed effects)
Inverse variance weighted (multiplicative random effects)
MR Egger
MR RAPS
Weighted median
Weighted mode
SNP effect on Left−sided ulcerative colitis
0.0
−0.1
0.02
0.04
0.06
0.08
SNP effect on Eicosenoate (20:1n9 or 11)

Batch 1561 : X−12244−−N−acetylcarnosine on Left−sided ulcerative colitis

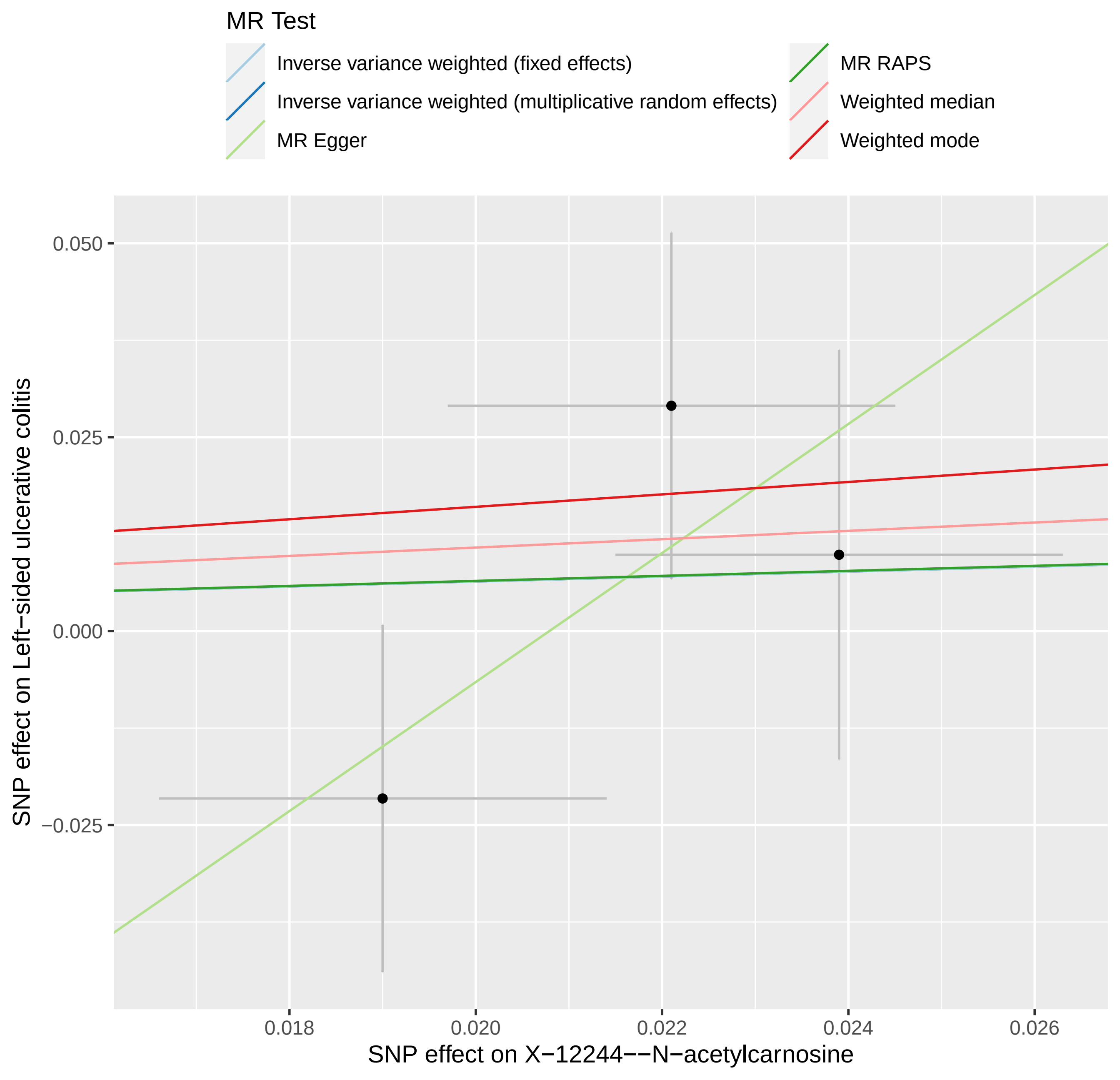

Batch 1563 : 1−eicosatrienoylglycerophosphocholine* on Left−sided ulcerative colitis

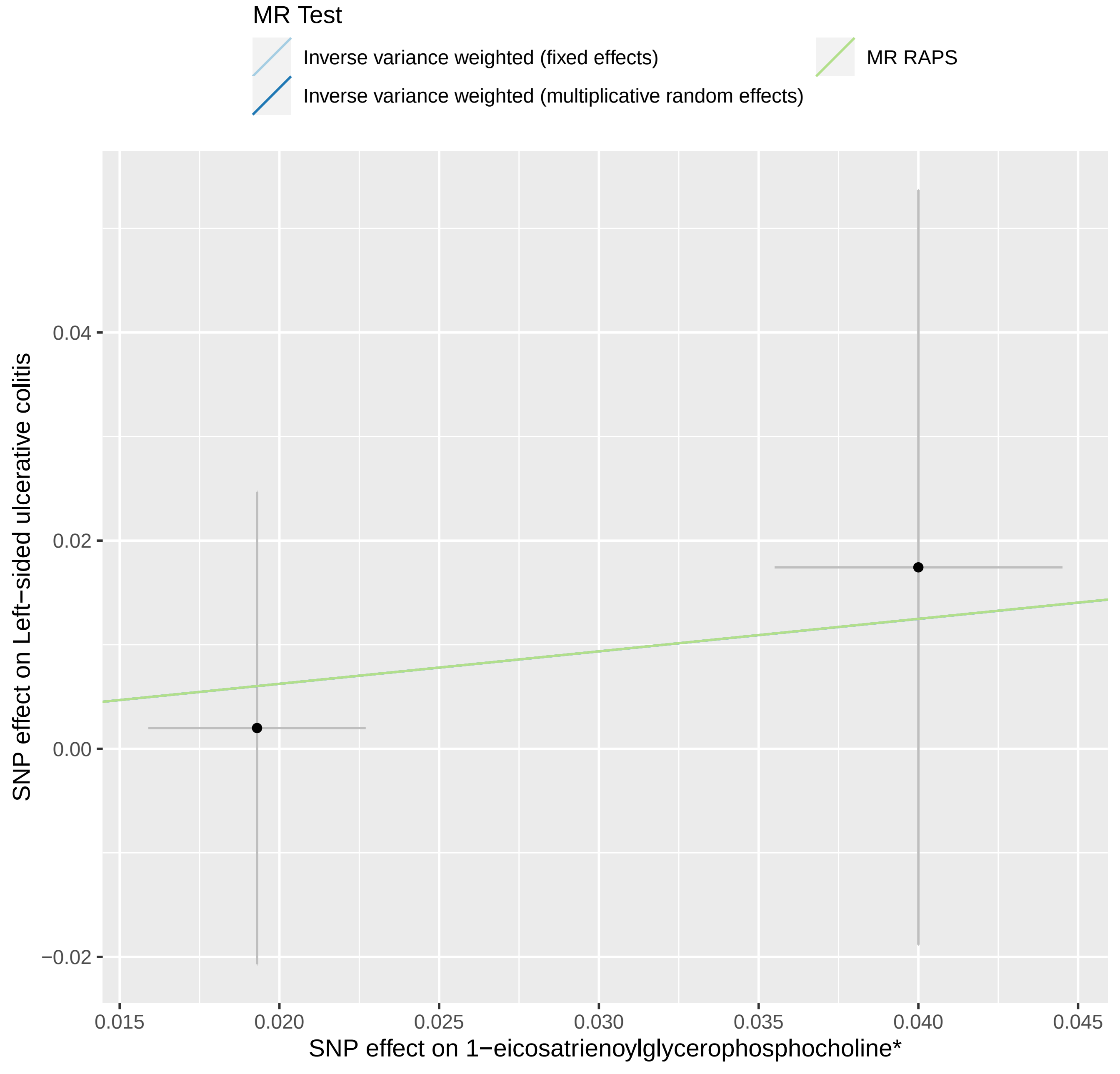

Batch 1564 : 1−docosahexaenoylglycerophosphocholine* on Left−sided ulcerative colitis

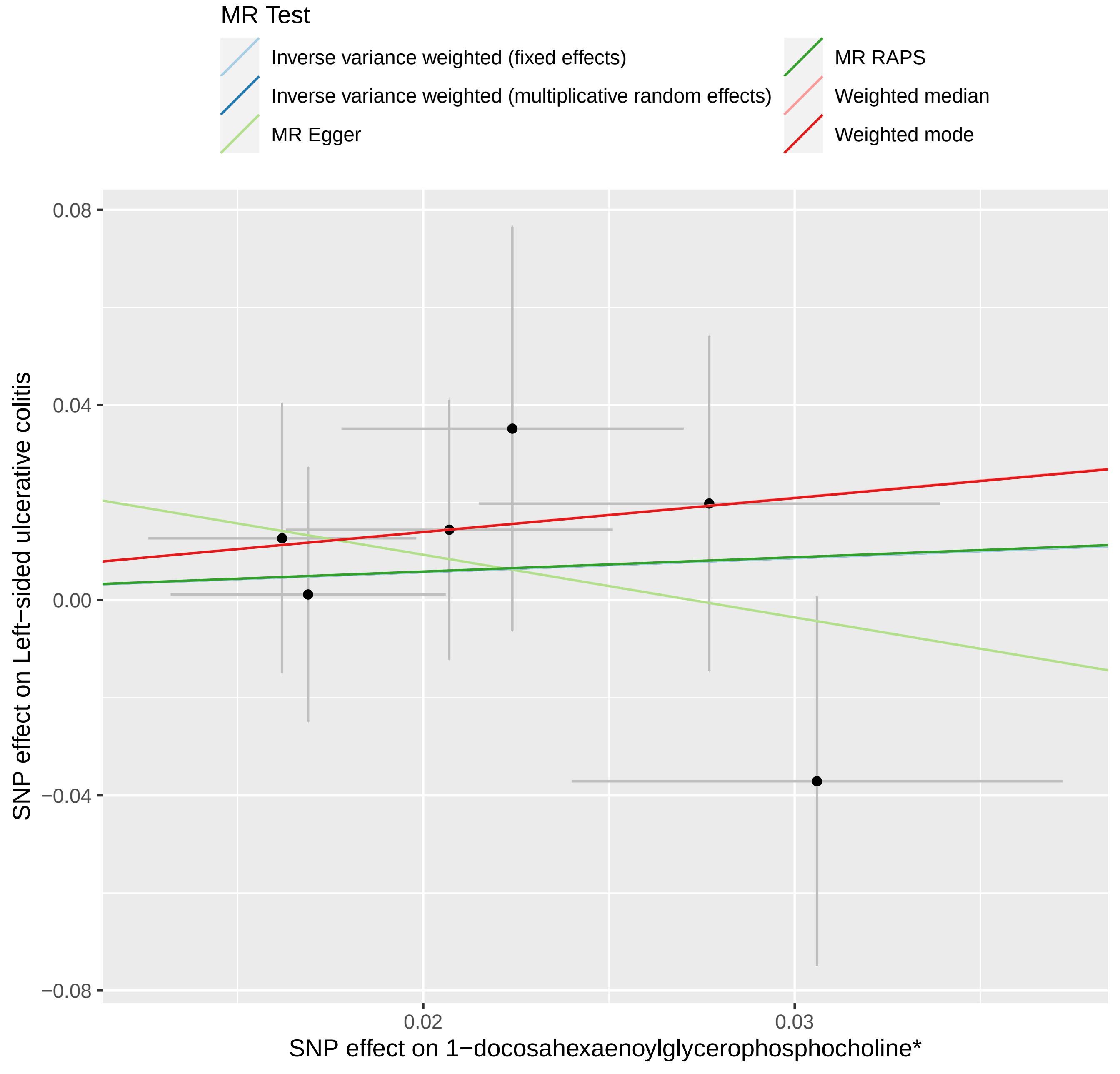

Batch 1568 : Octanoylcarnitine on Left−sided ulcerative colitis

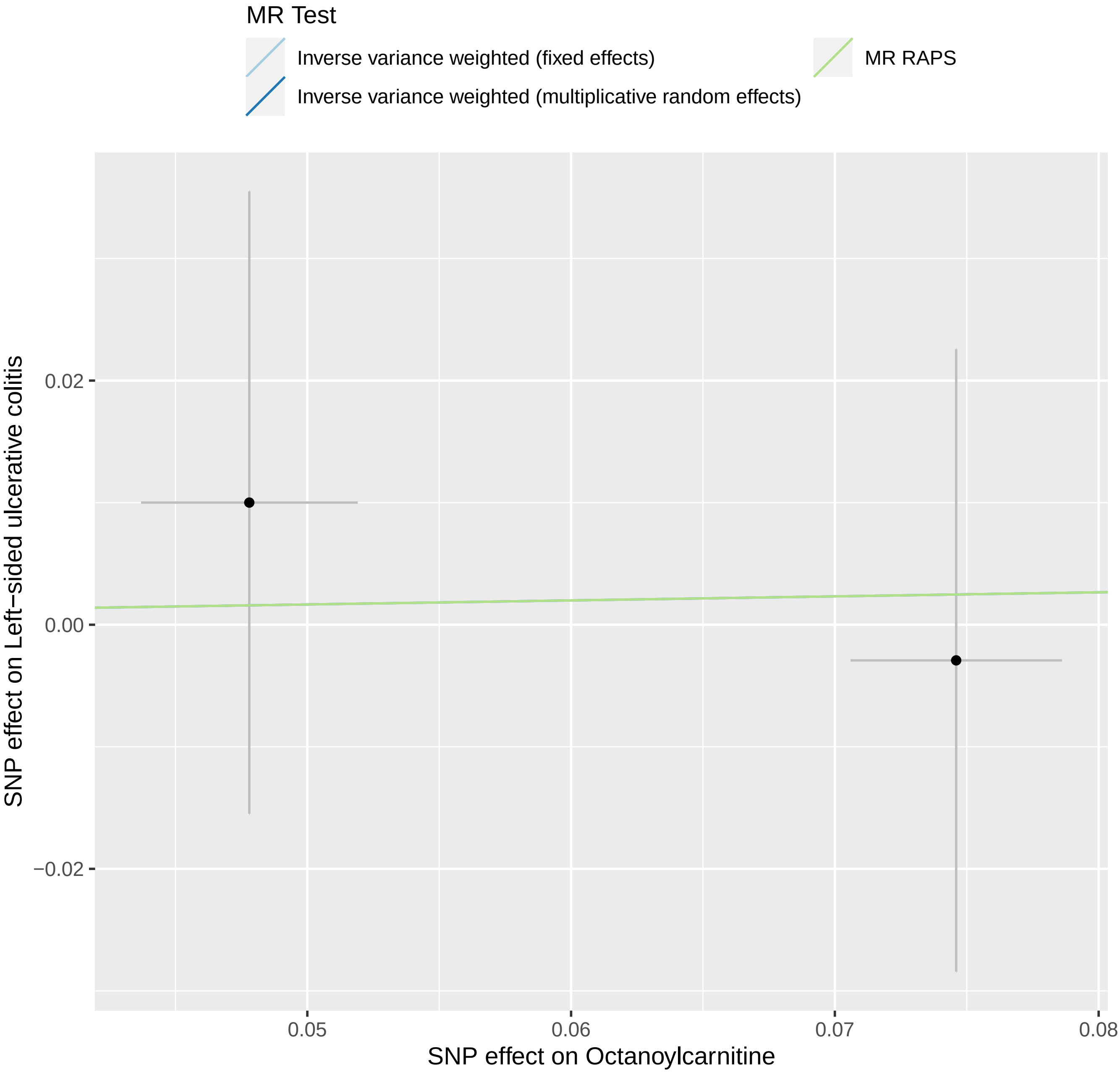

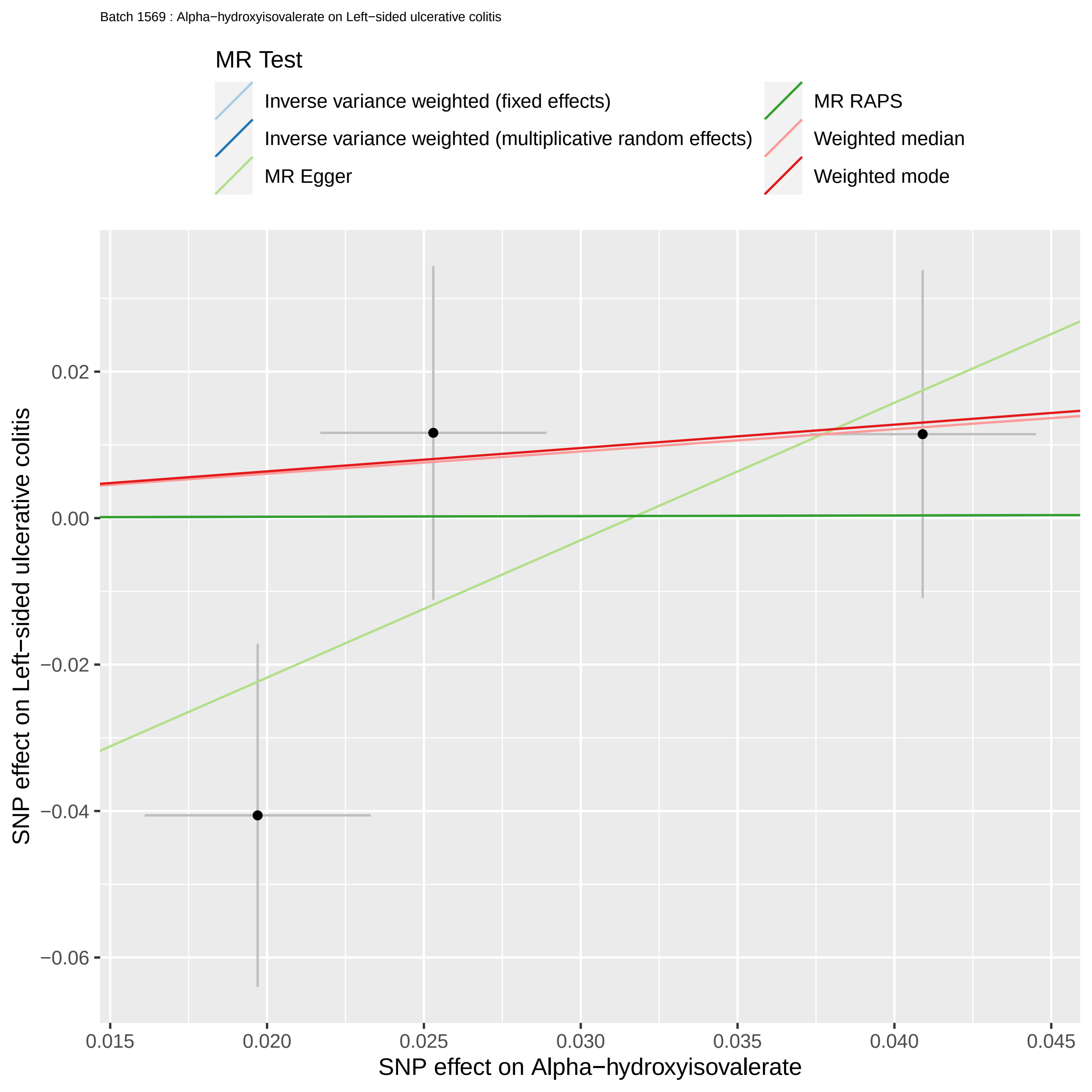

Batch 1569 : Alpha−hydroxyisovalerate on Left−sided ulcerative colitis
MR Test
Inverse variance weighted (fixed effects)
Inverse variance weighted (multiplicative random effects)
MR Egger
MR RAPS
Weighted median
Weighted mode
0.02
0.00
−0.02
−0.04
−0.06
0.015
0.020
0.025
0.030
0.035
0.040
0.045
SNP effect on Alpha−hydroxyisovalerate
SNP effect on Left−sided ulcerative colitis

Batch 1570 : N−acetylthreonine on Left−sided ulcerative colitis

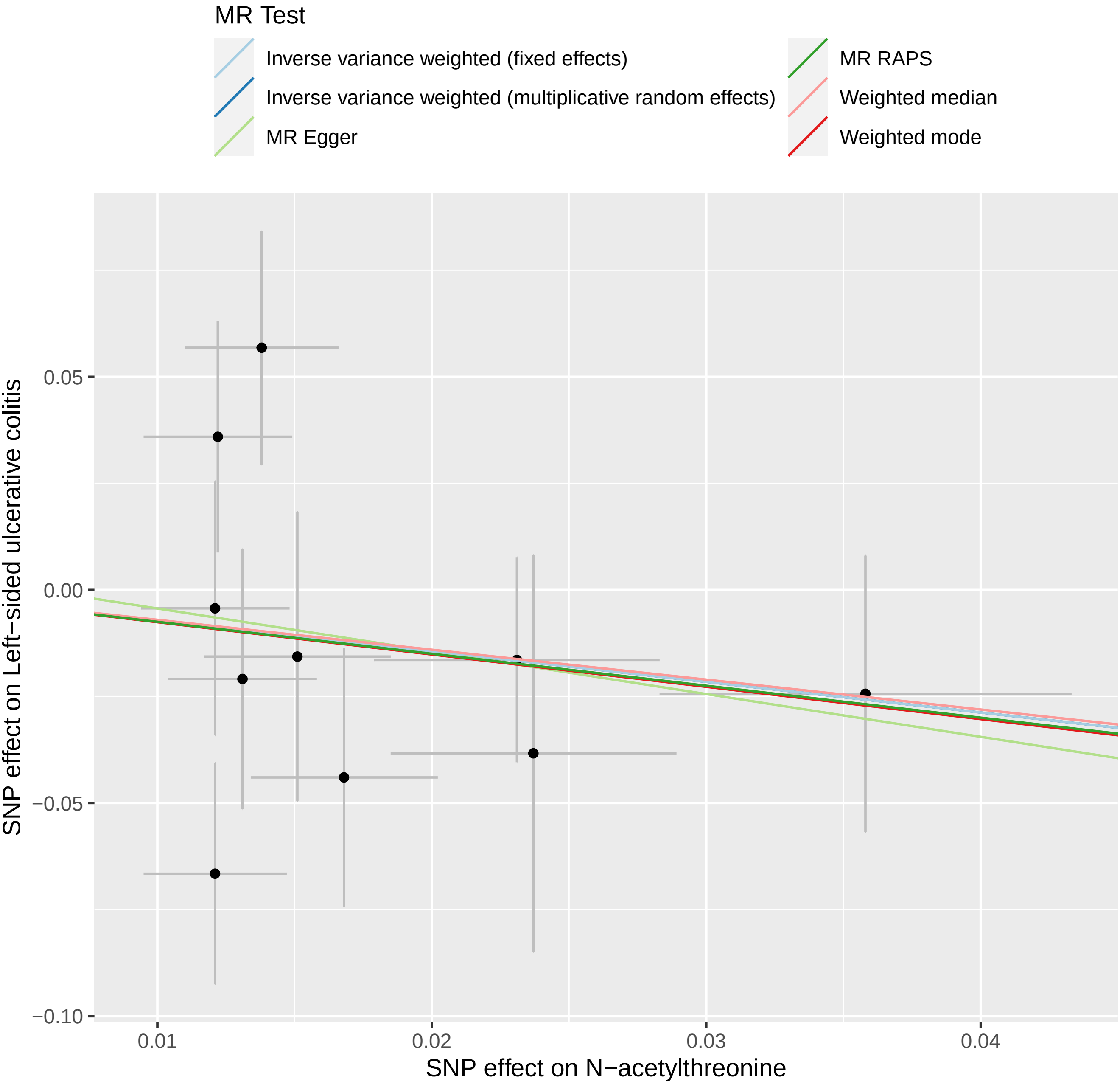

Batch 1571 : Decanoylcarnitine on Left−sided ulcerative colitis

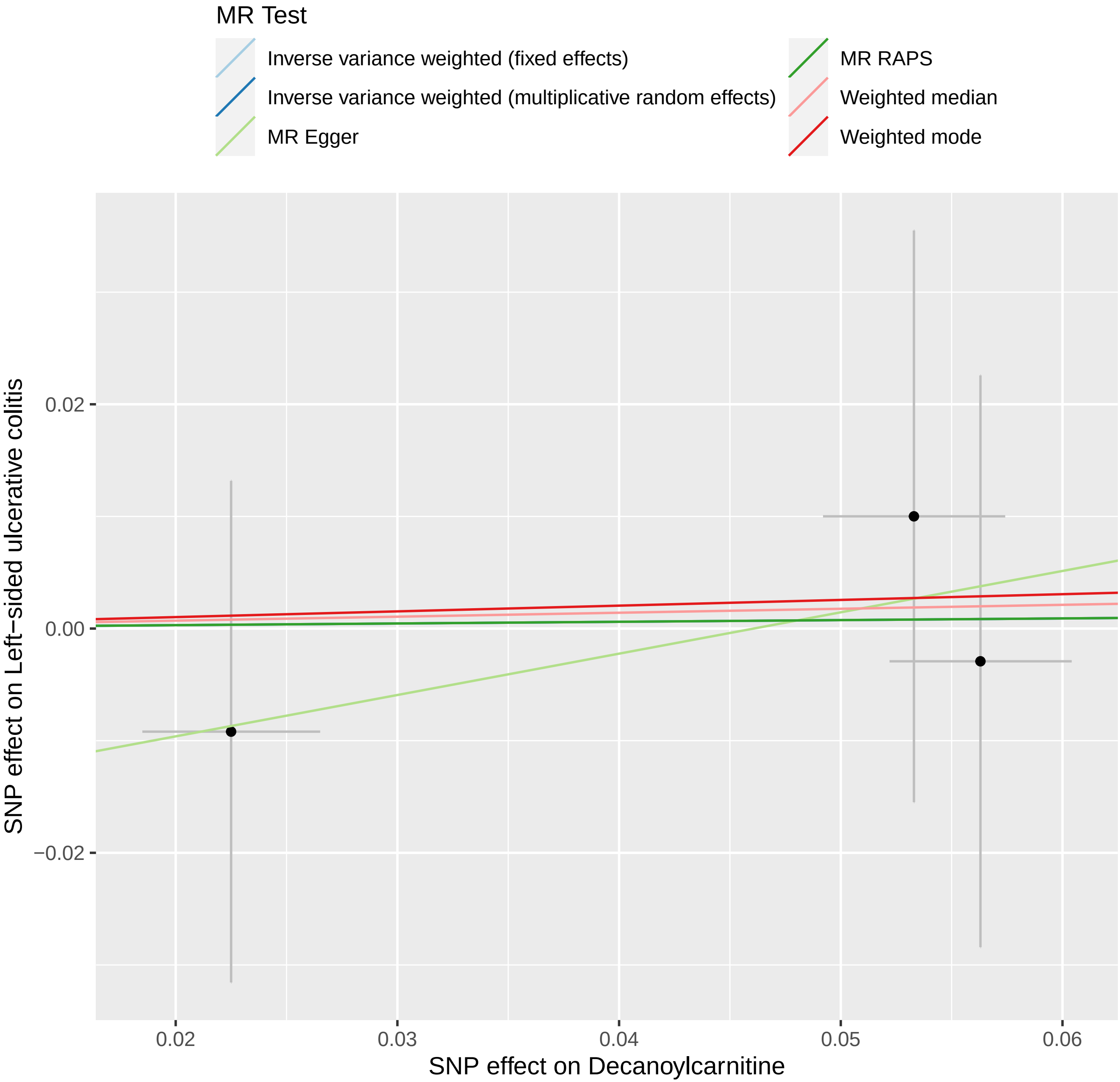

Batch 1572 : 1−palmitoylglycerophosphocholine on Left−sided ulcerative colitis

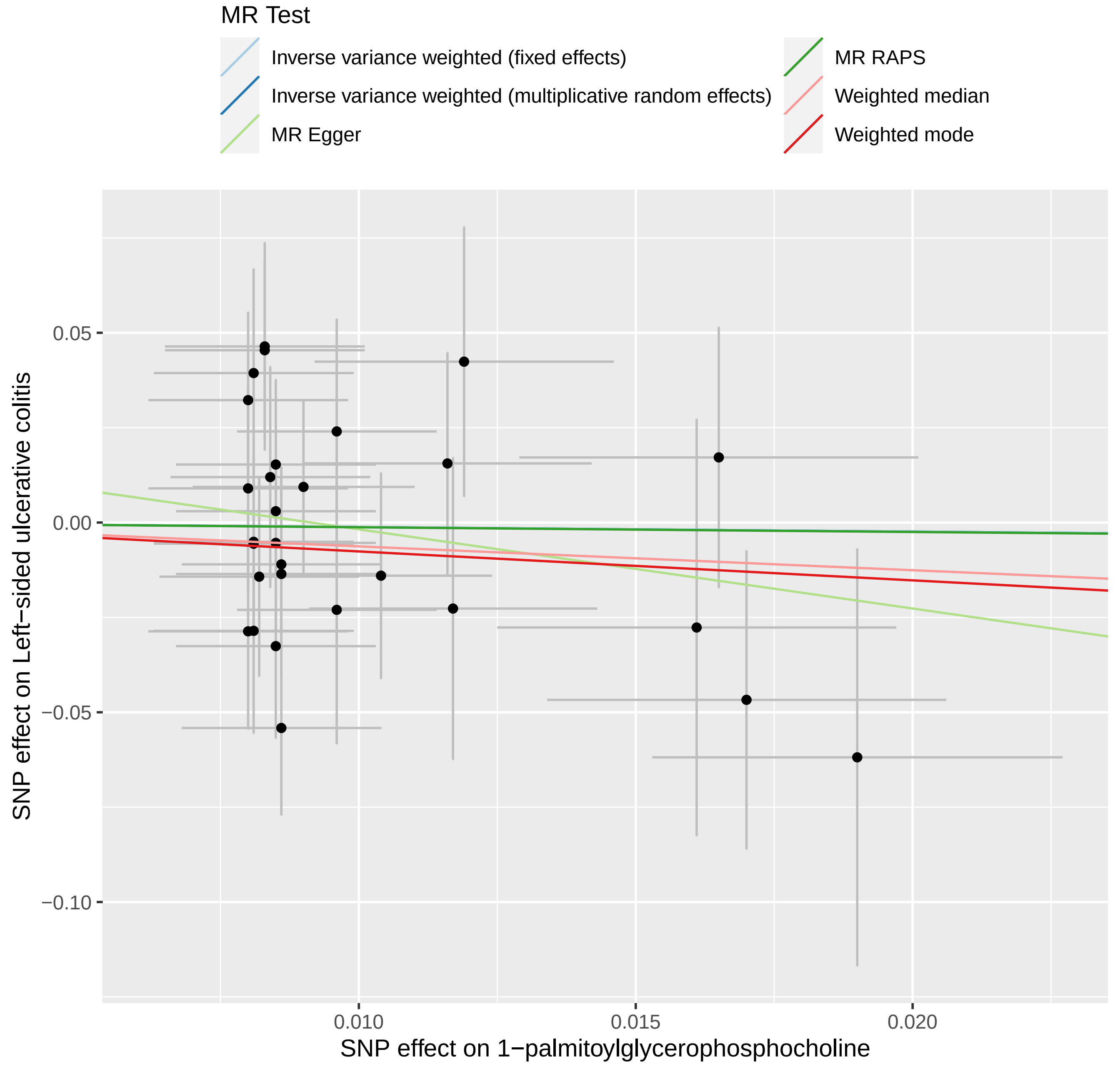

Batch 1573 : 1−heptadecanoylglycerophosphocholine on Left−sided ulcerative colitis

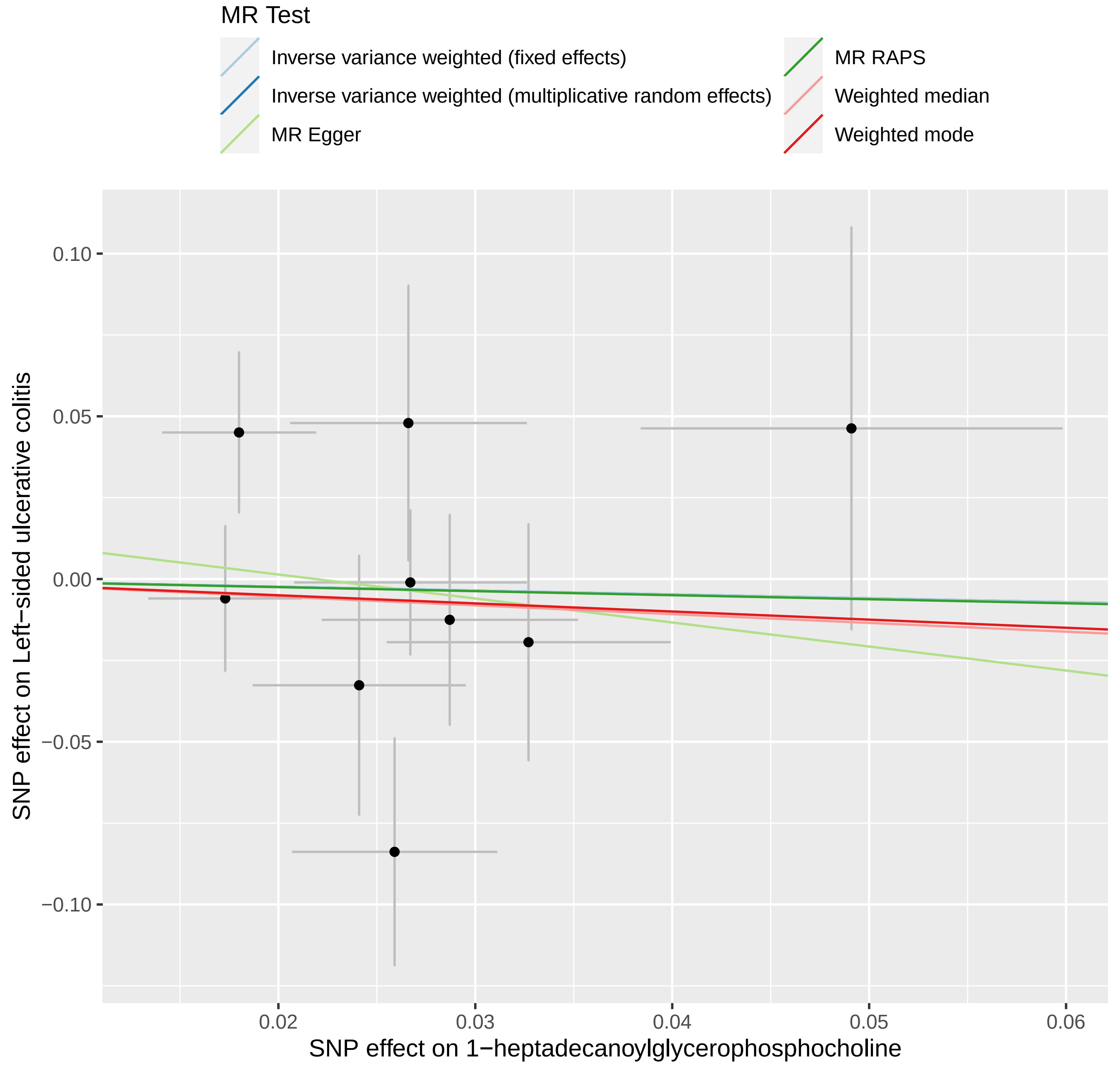

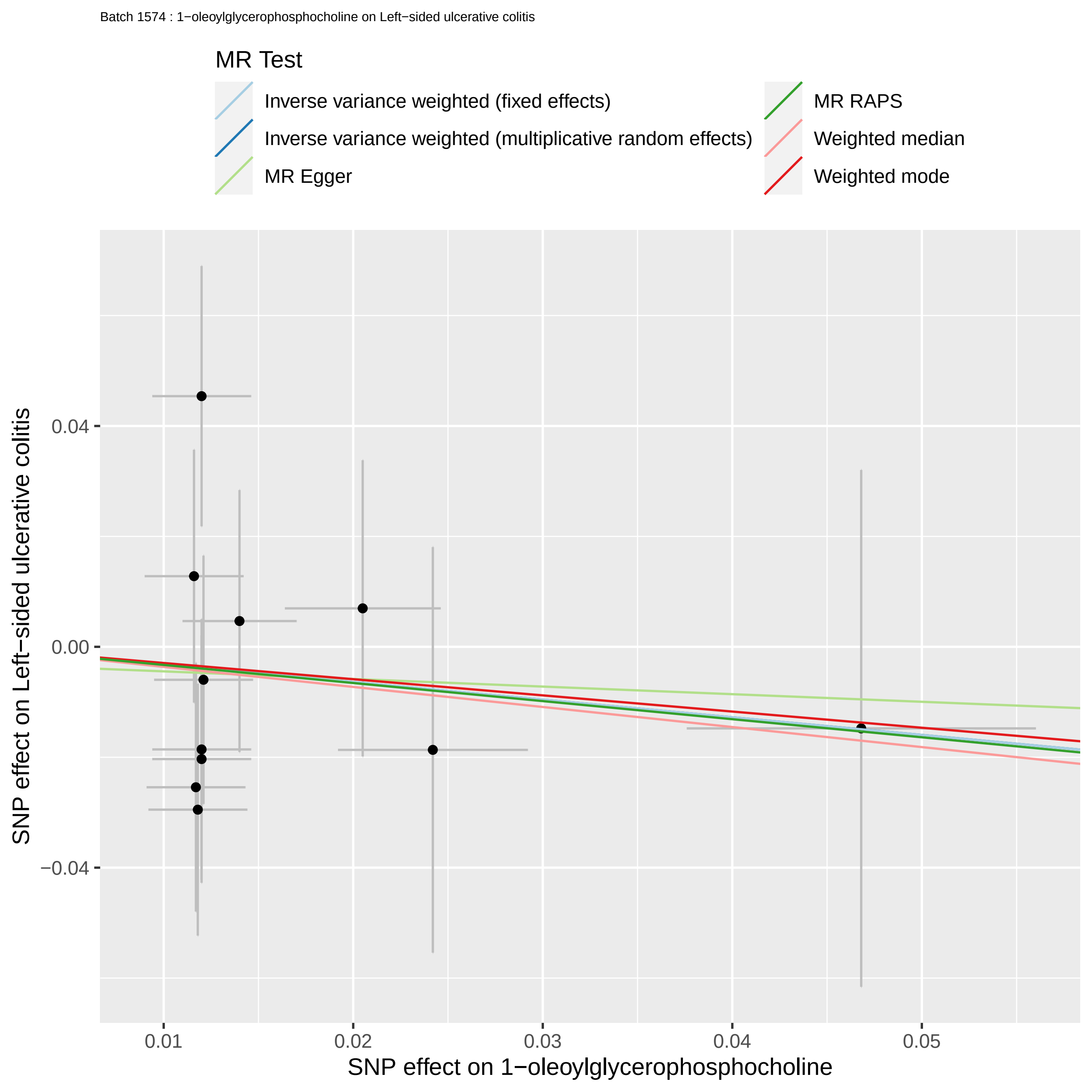
Batch 1574 : 1−oleoylglycerophosphocholine on Left−sided ulcerative colitis
MR Test
Inverse variance weighted (fixed effects)
Inverse variance weighted (multiplicative random effects)
MR Egger
MR RAPS
Weighted median
Weighted mode
0.04
0.00
−0.04
0.01
0.02
0.03
0.04
0.05
SNP effect on Left−sided ulcerative colitis
SNP effect on 1−oleoylglycerophosphocholine

Batch 1575 : 1−stearoylglycerophosphocholine on Left−sided ulcerative colitis

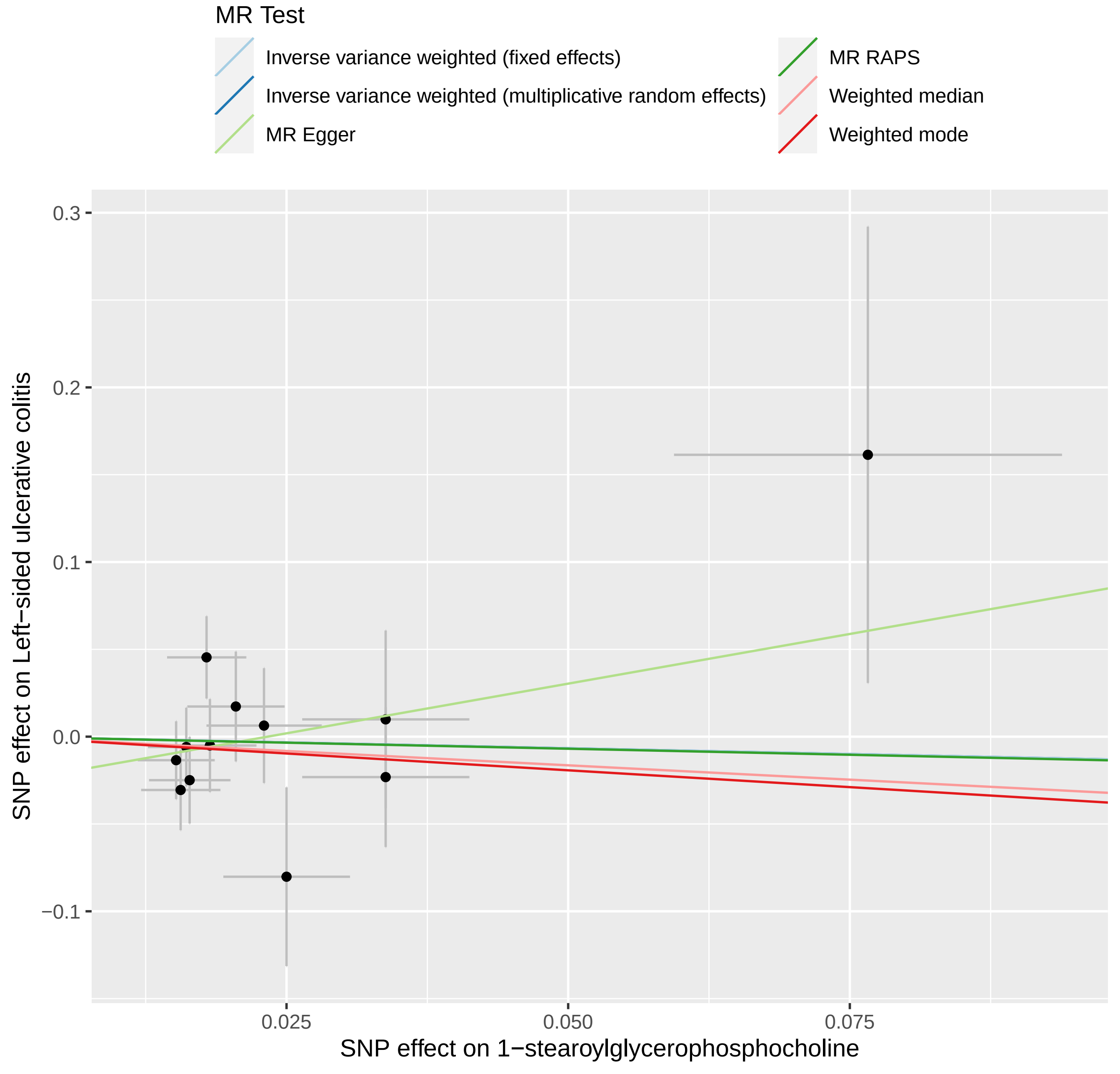

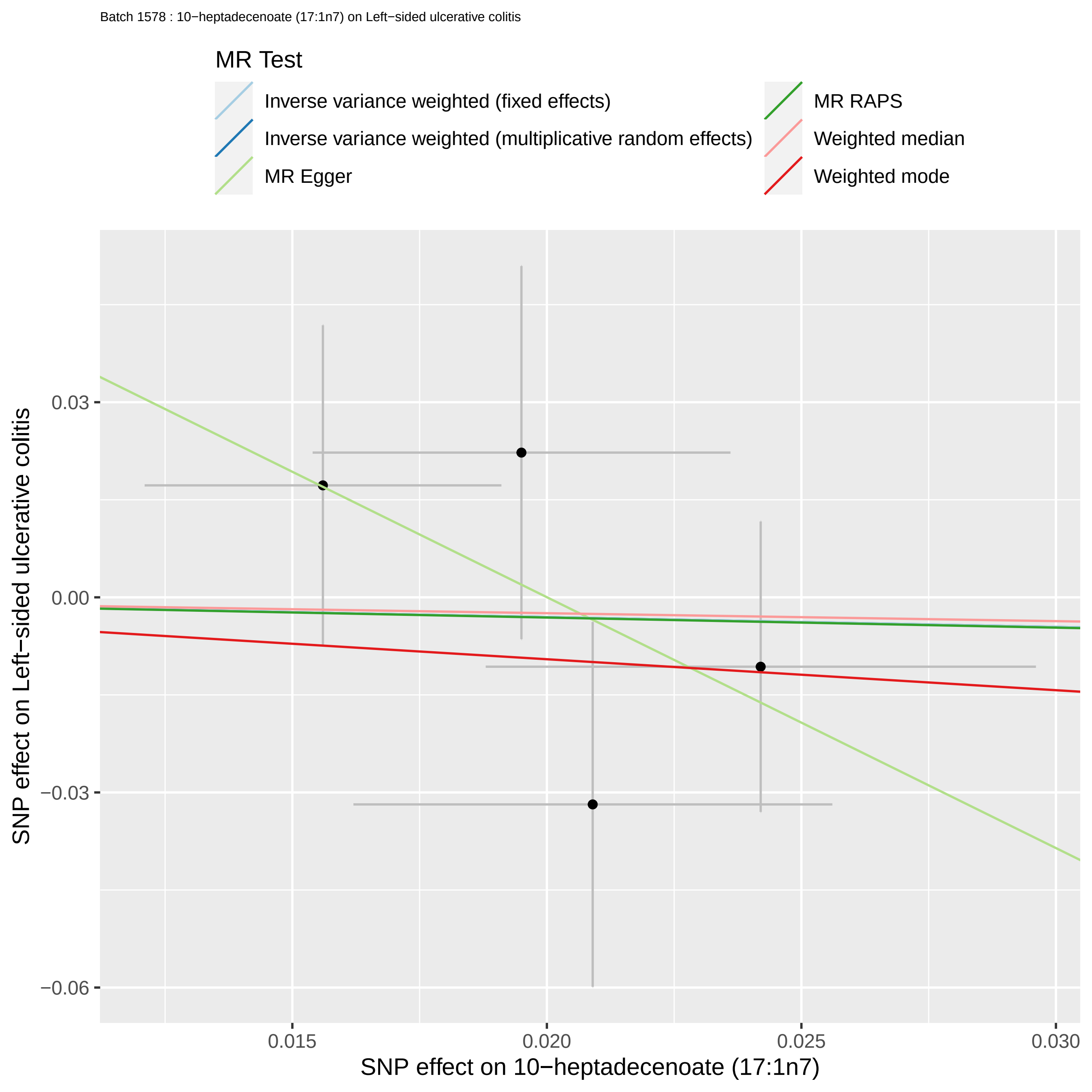
Batch 1578 : 10−heptadecenoate (17:1n7) on Left−sided ulcerative colitis
MR Test
Inverse variance weighted (fixed effects)
Inverse variance weighted (multiplicative random effects)
MR Egger
MR RAPS
Weighted median
Weighted mode
0.03
0.00
−0.03
−0.06
0.015
0.020
0.025
0.030
SNP effect on 10−heptadecenoate (17:1n7)
SNP effect on Left−sided ulcerative colitis

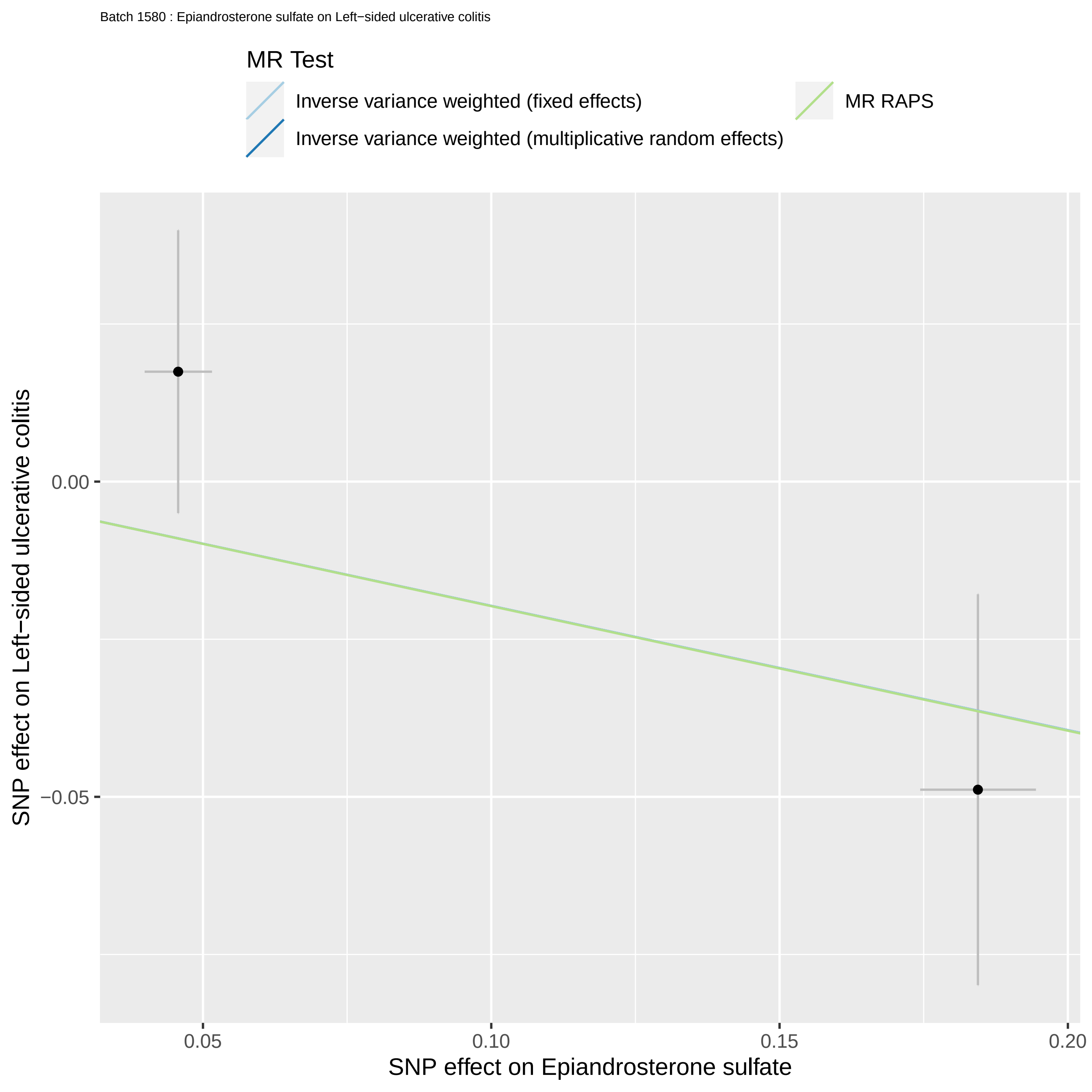

Batch 1580 : Epiandrosterone sulfate on Left−sided ulcerative colitis
MR Test
Inverse variance weighted (fixed effects)
Inverse variance weighted (multiplicative random effects)
MR RAPS
0.00
−0.05
0.05
0.10
0.15
0.20
SNP effect on Left−sided ulcerative colitis
SNP effect on Epiandrosterone sulfate

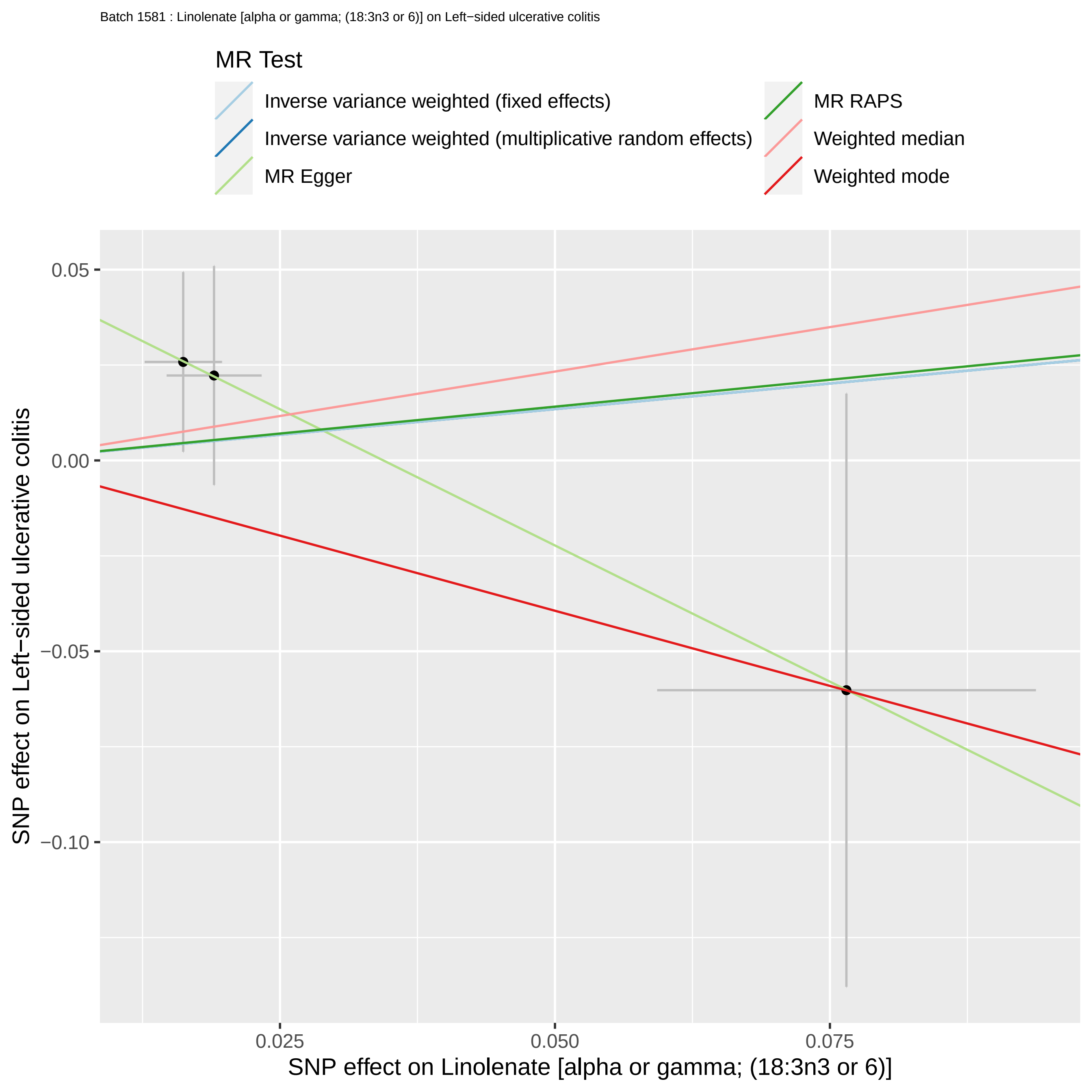
Batch 1581 : Linolenate [alpha or gamma; (18:3n3 or 6)] on Left−sided ulcerative colitis
MR Test
Inverse variance weighted (fixed effects)
Inverse variance weighted (multiplicative random effects)
MR Egger
MR RAPS
Weighted median
Weighted mode
0.05
0.00
−0.05
−0.10
0.025
0.050
0.075
SNP effect on Left−sided ulcerative colitis
SNP effect on Linolenate [alpha or gamma; (18:3n3 or 6)]

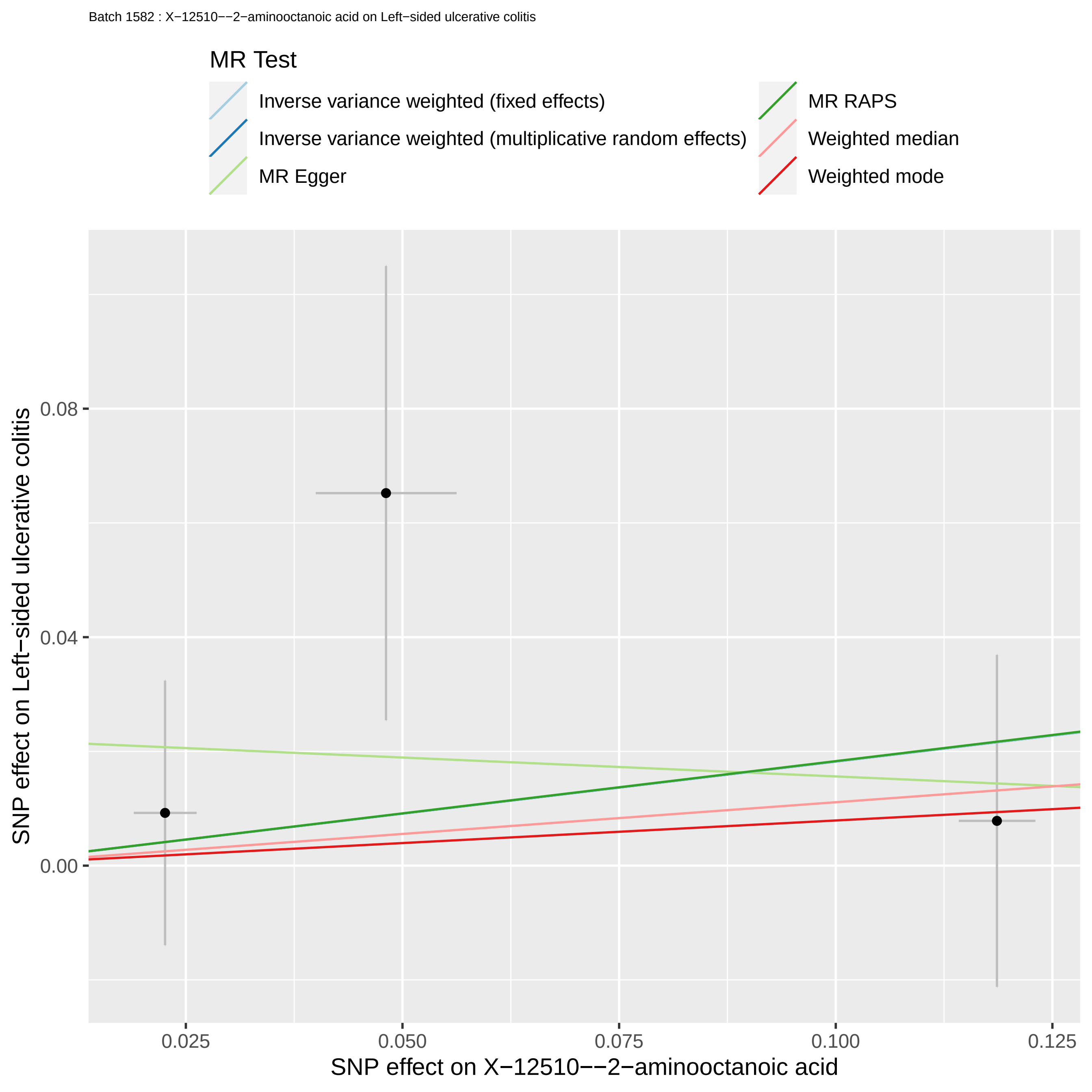
Batch 1582 : X−12510−−2−aminooctanoic acid on Left−sided ulcerative colitis
MR Test
Inverse variance weighted (fixed effects)
Inverse variance weighted (multiplicative random effects)
MR Egger
MR RAPS
Weighted median
Weighted mode
SNP effect on Left−sided ulcerative colitis
0.08
0.04
0.00
0.025
0.050
0.075
0.100
0.125
SNP effect on X−12510−−2−aminooctanoic acid

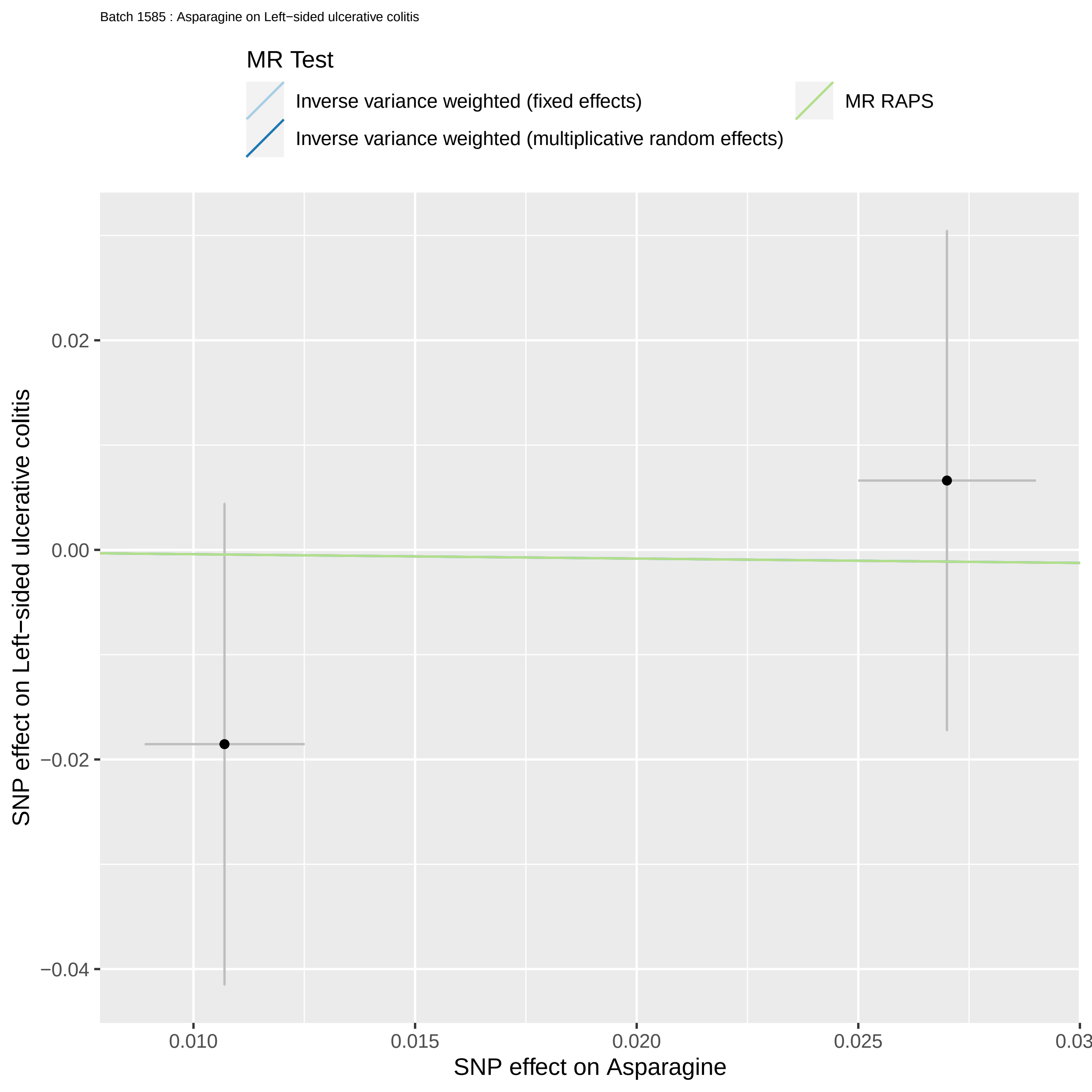

Batch 1585 : Asparagine on Left−sided ulcerative colitis
MR Test
Inverse variance weighted (fixed effects)
Inverse variance weighted (multiplicative random effects)
MR RAPS
0.02
0.00
−0.02
−0.04
SNP effect on Left−sided ulcerative colitis
0.010
0.015
0.020
0.025
0.03
SNP effect on Asparagine

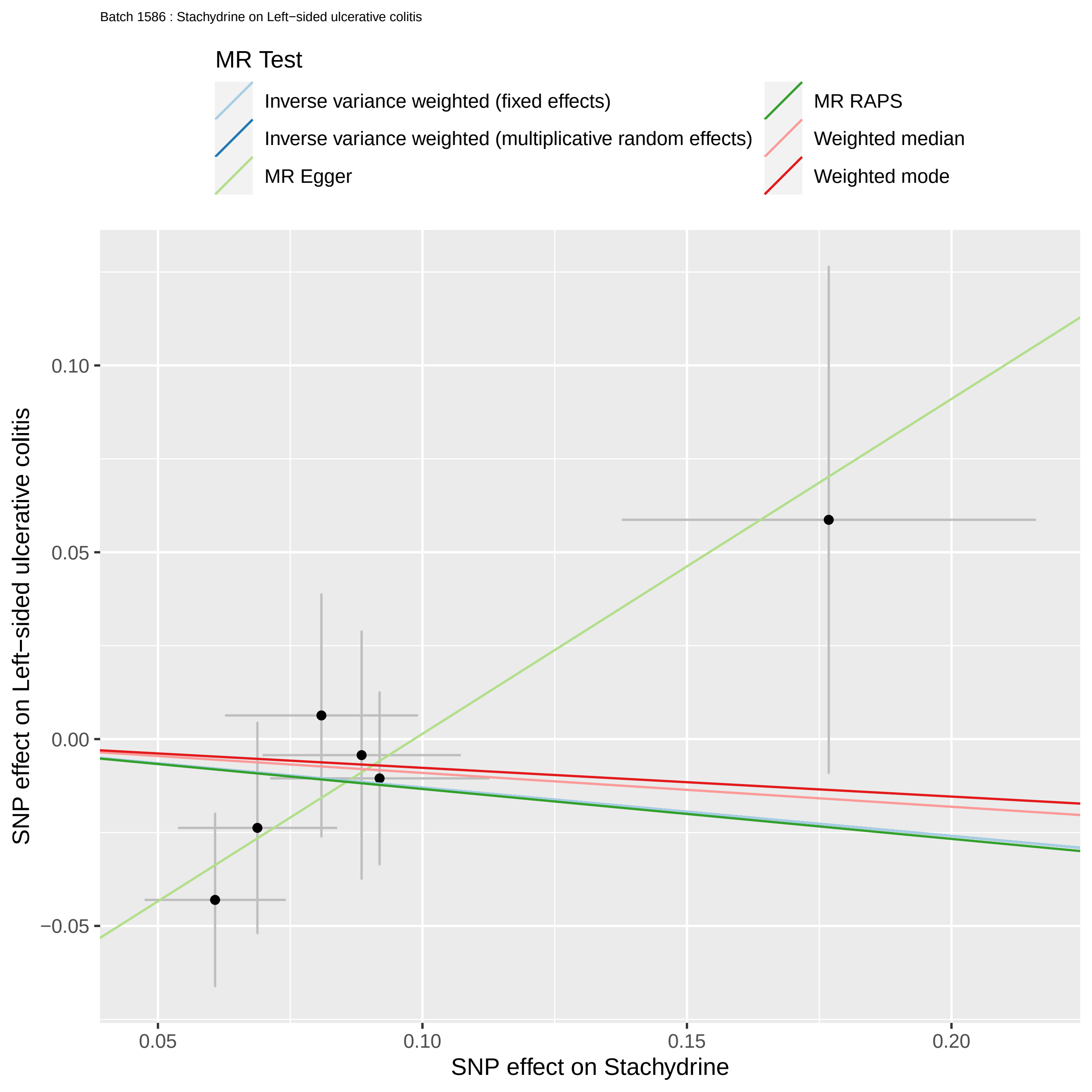

Batch 1586 : Stachydrine on Left−sided ulcerative colitis
MR Test
Inverse variance weighted (fixed effects)
Inverse variance weighted (multiplicative random effects)
MR Egger
MR RAPS
Weighted median
Weighted mode
0.10
0.05
0.00
−0.05
0.05
0.10
0.15
0.20
SNP effect on Stachydrine
SNP effect on Left−sided ulcerative colitis

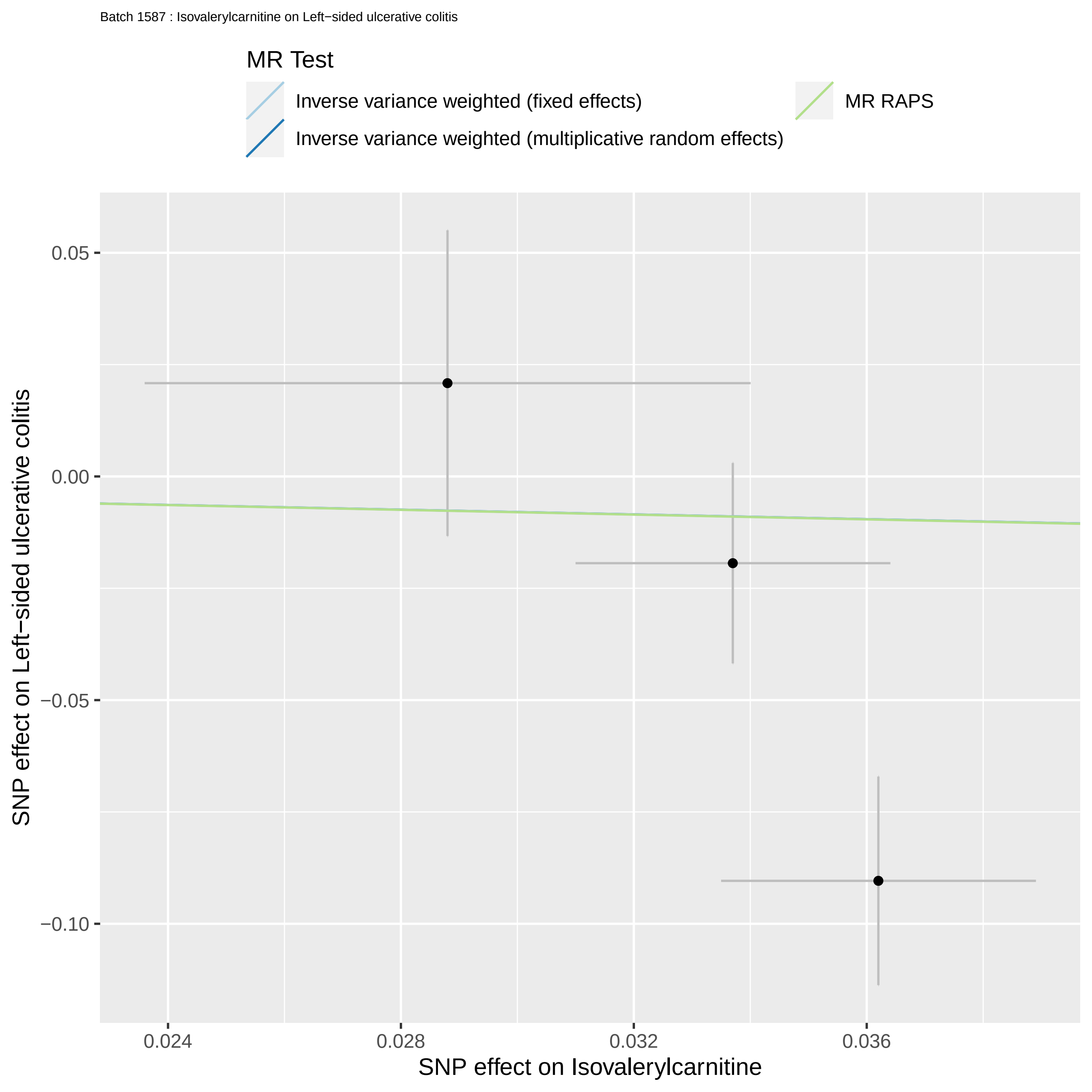
Batch 1587 : Isovalerylcarnitine on Left−sided ulcerative colitis
MR Test
Inverse variance weighted (fixed effects)
Inverse variance weighted (multiplicative random effects)
MR RAPS
0.05
0.00
−0.05
−0.10
0.024
0.028
0.032
0.036
SNP effect on Isovalerylcarnitine
SNP effect on Left−sided ulcerative colitis

Batch 1590 : 1−linoleoylglycerophosphocholine on Left−sided ulcerative colitis

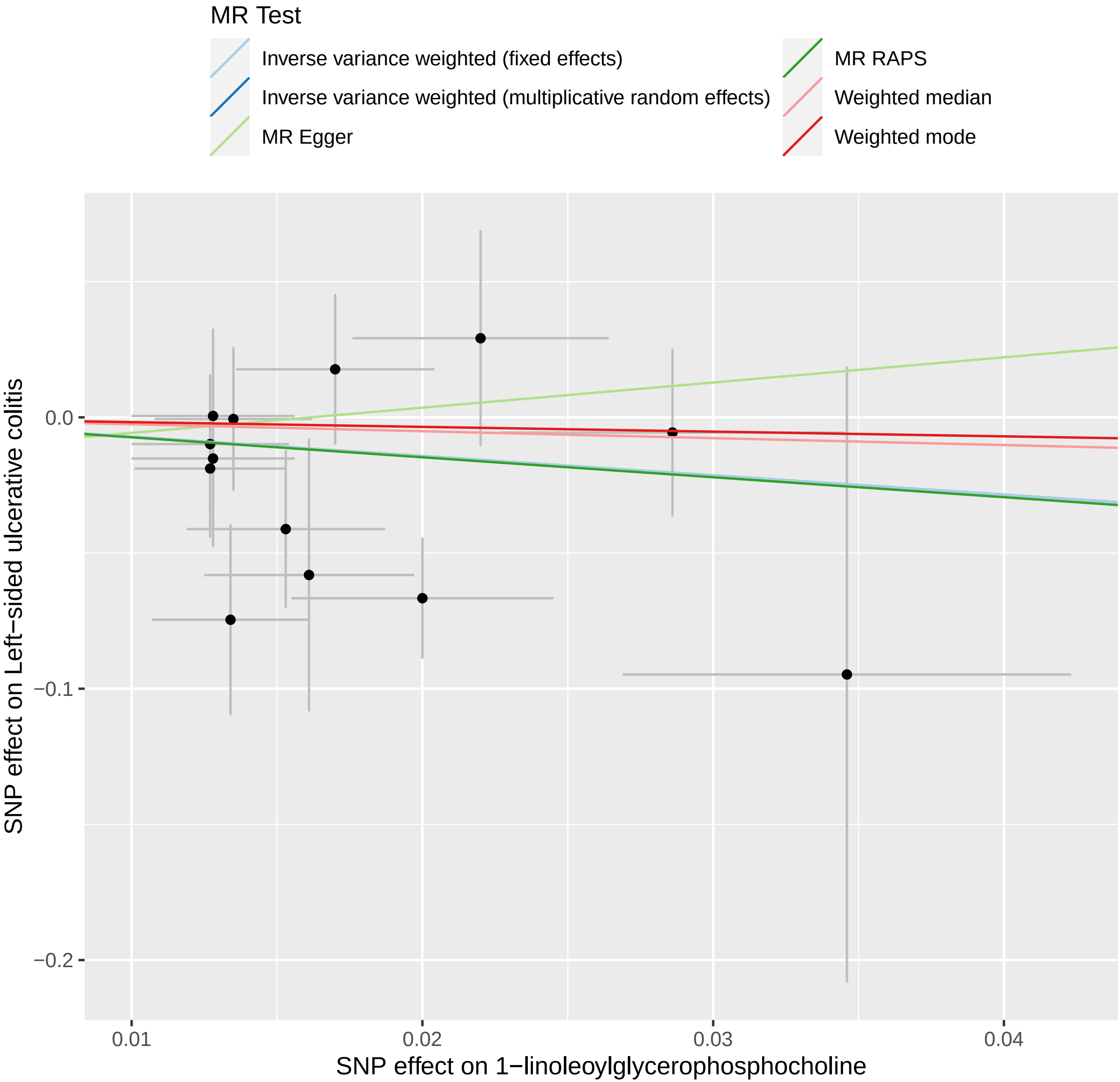

Batch 1591 : Bradykinin, des−arg(9) on Left−sided ulcerative colitis

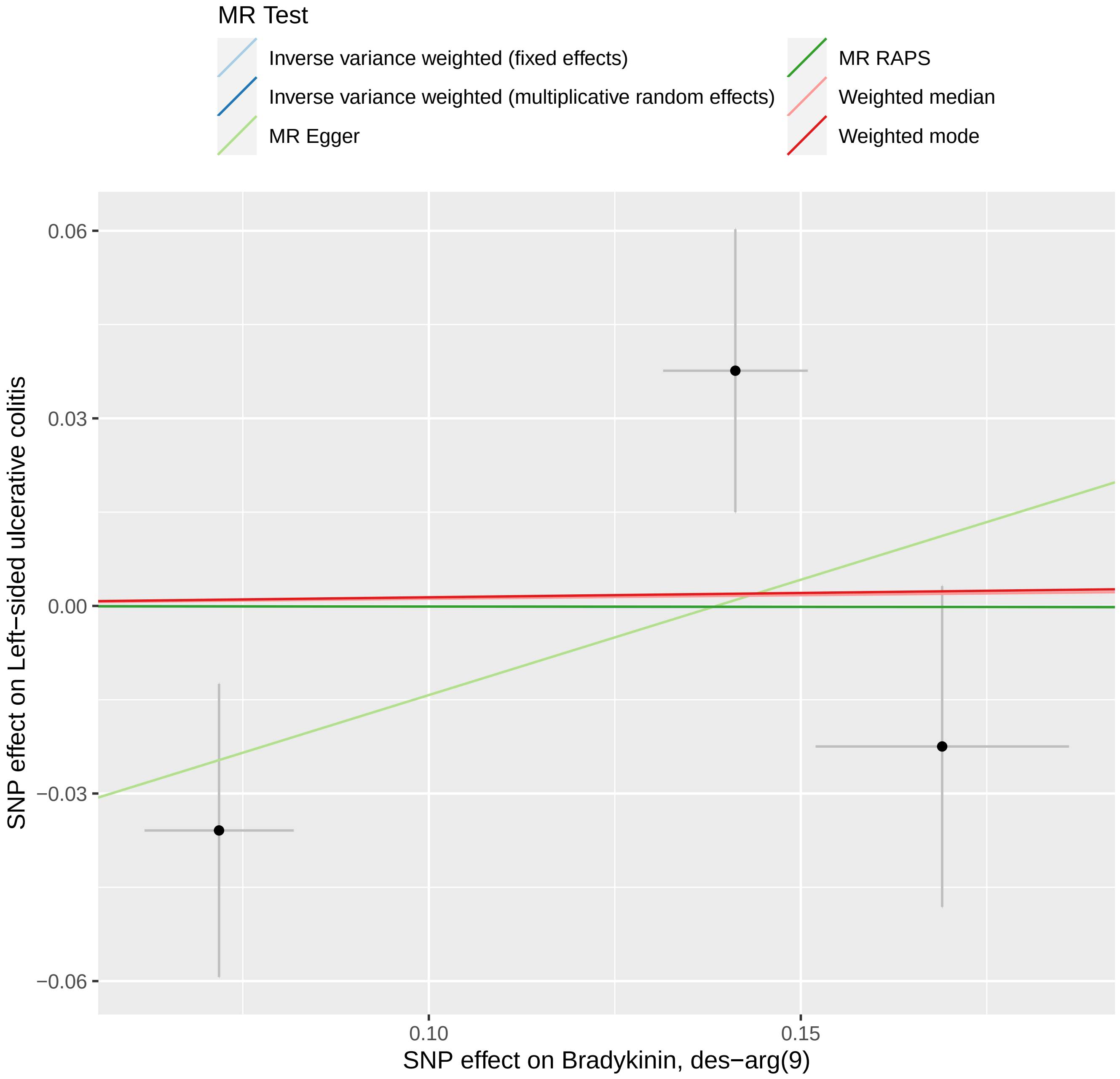

Batch 1592 : Gamma−glutamylisoleucine* on Left−sided ulcerative colitis

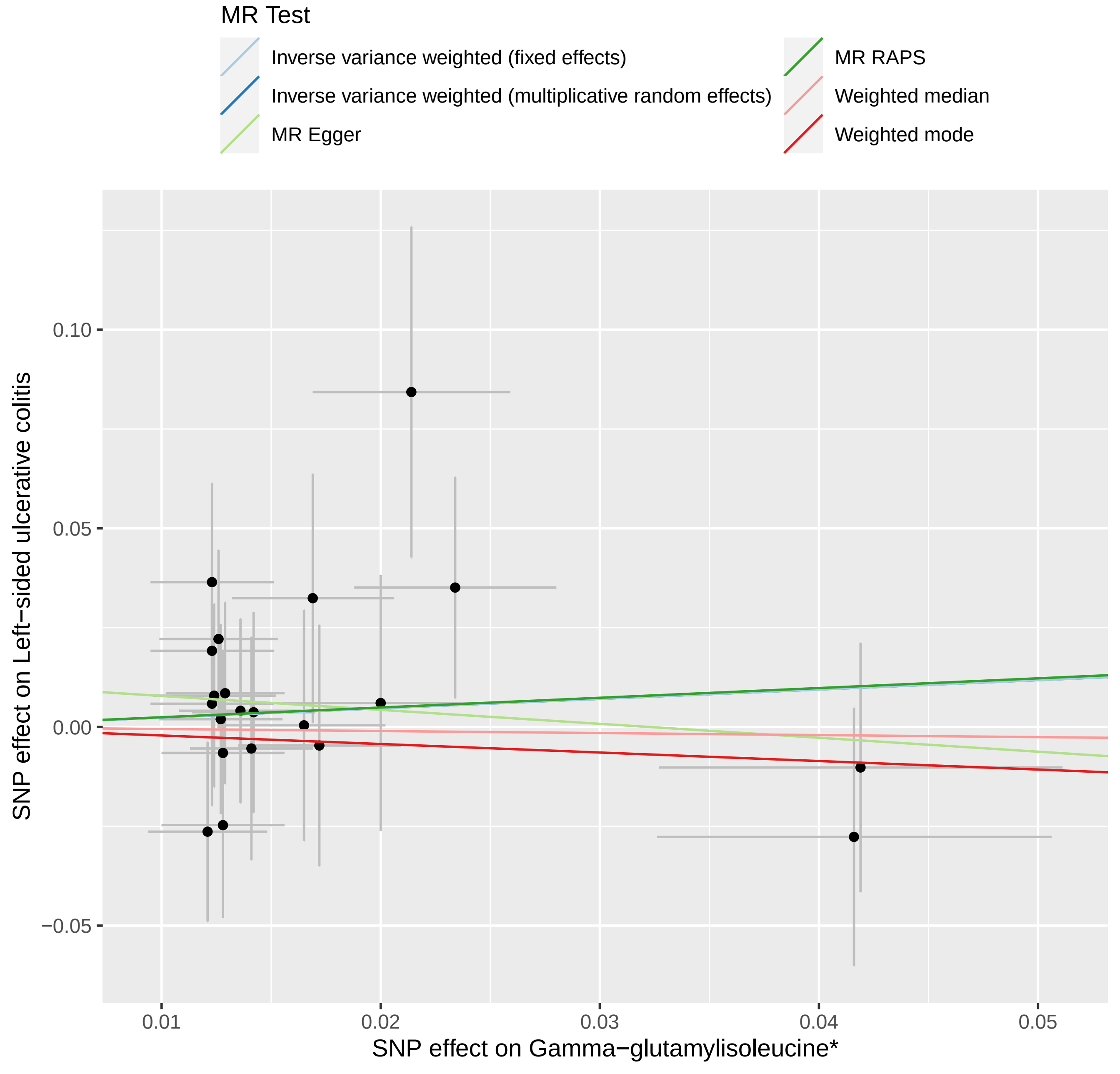

Batch 1595 : Isovalerate on Left−sided ulcerative colitis

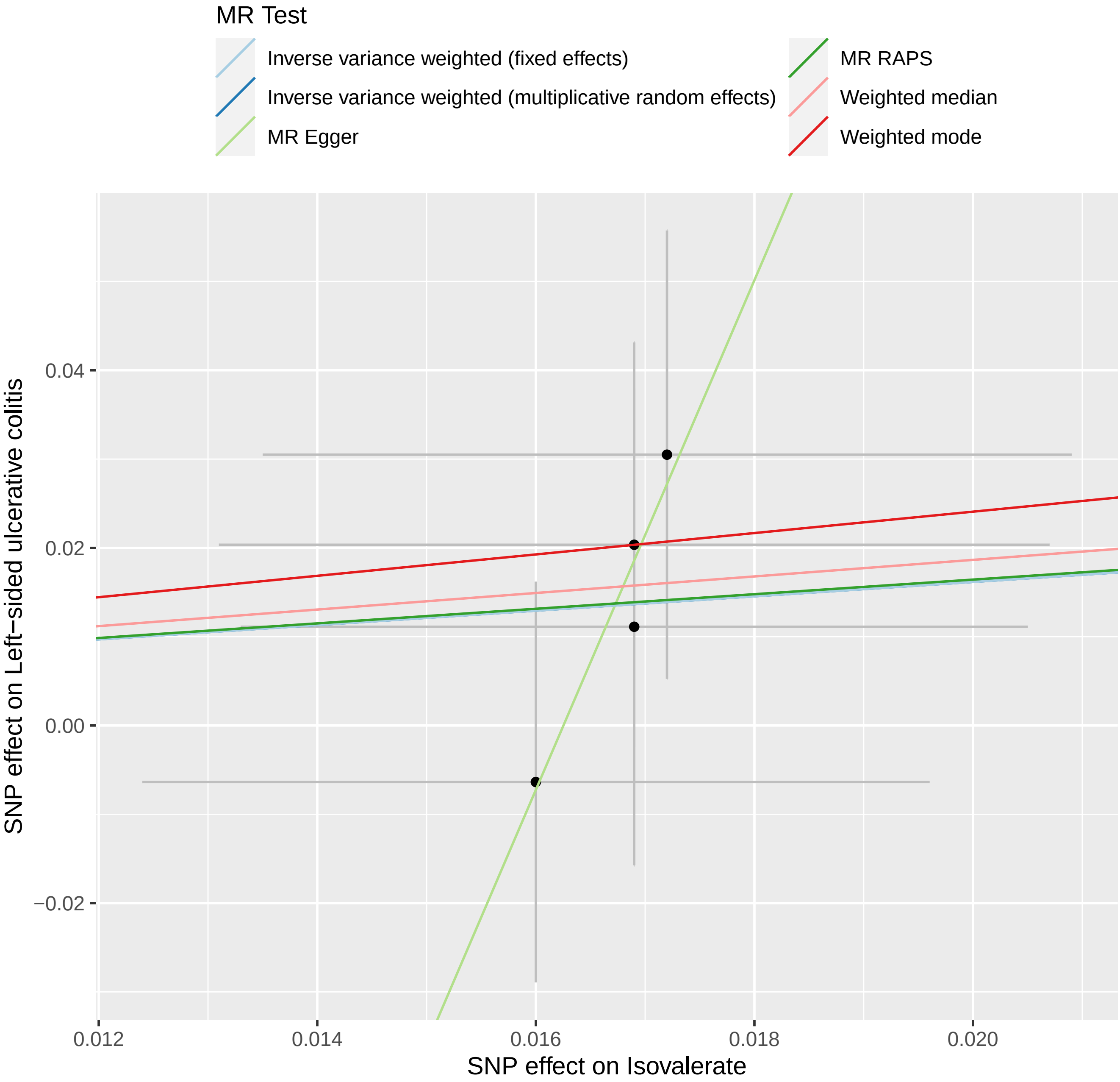

Batch 1596 : X−13183−−stearamide on Left−sided ulcerative colitis

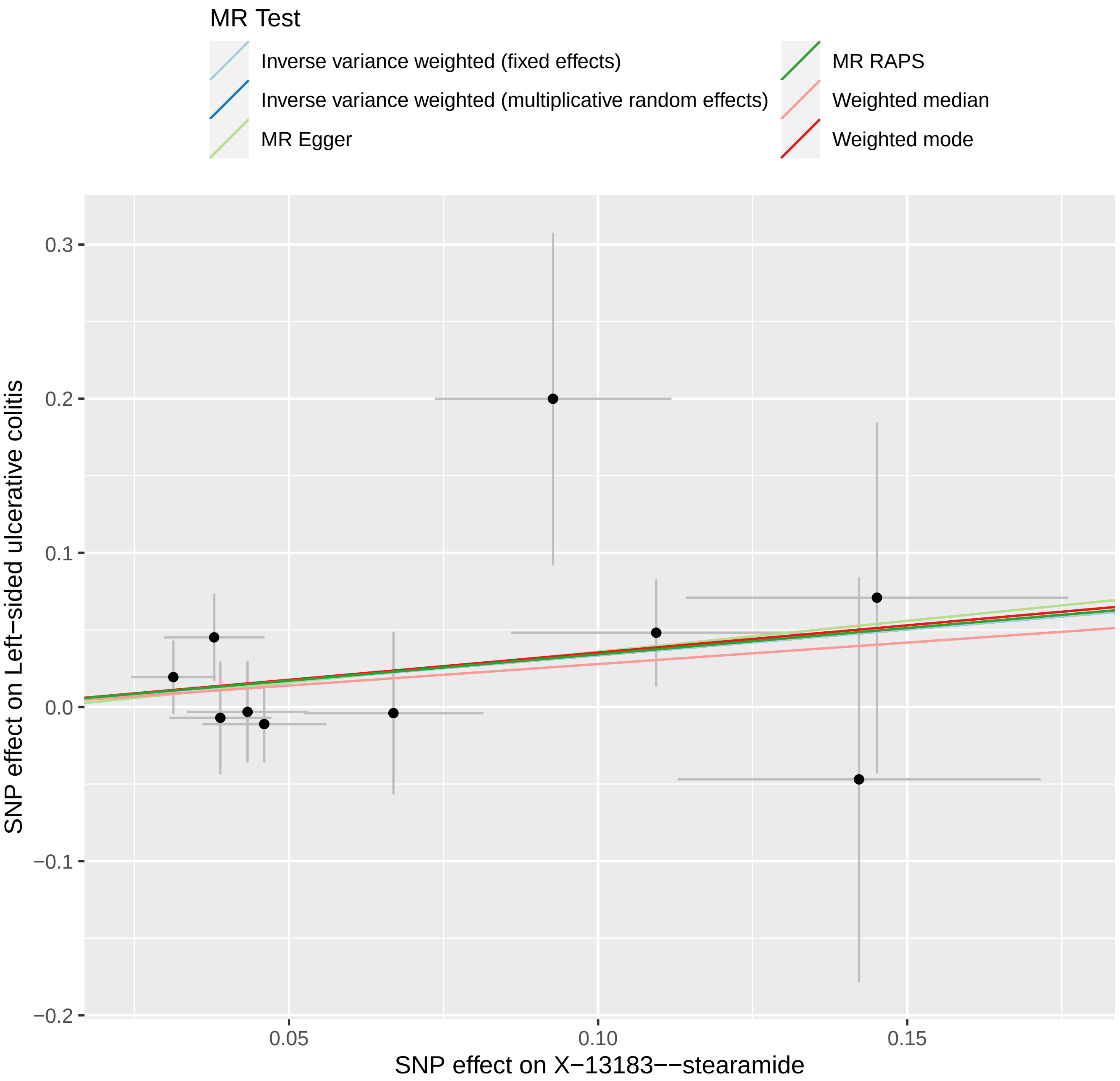

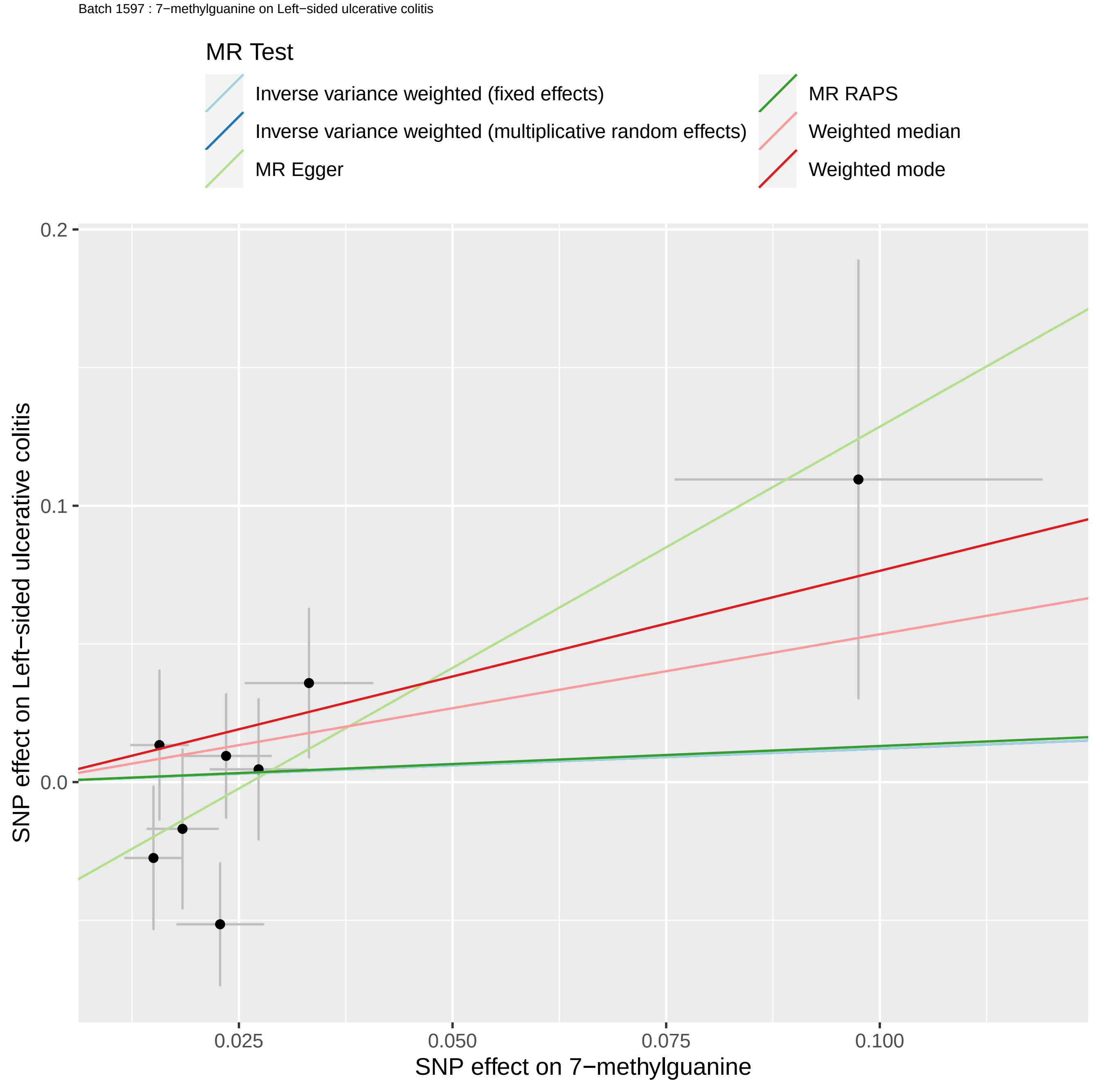
Batch 1597 : 7−methylguanine on Left−sided ulcerative colitis
MR Test
Inverse variance weighted (fixed effects)
Inverse variance weighted (multiplicative random effects)
MR Egger
MR RAPS
Weighted median
Weighted mode
0.2
0.1
0.0
0.025
0.050
0.075
0.100
SNP effect on Left−sided ulcerative colitis
SNP effect on 7−methylguanine

Batch 1604 : X−13431−−nonanoylcarnitine* on Left−sided ulcerative colitis

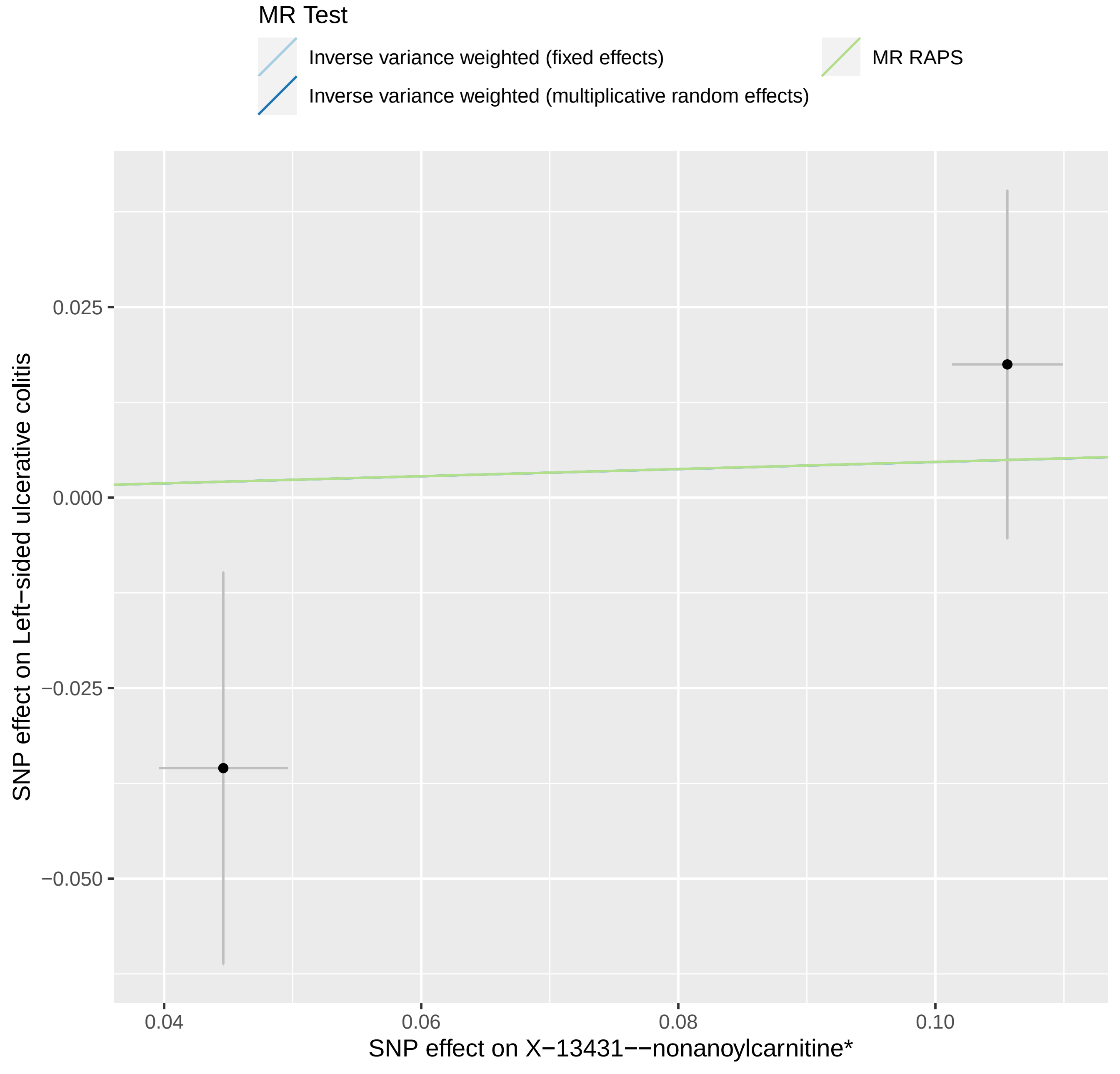

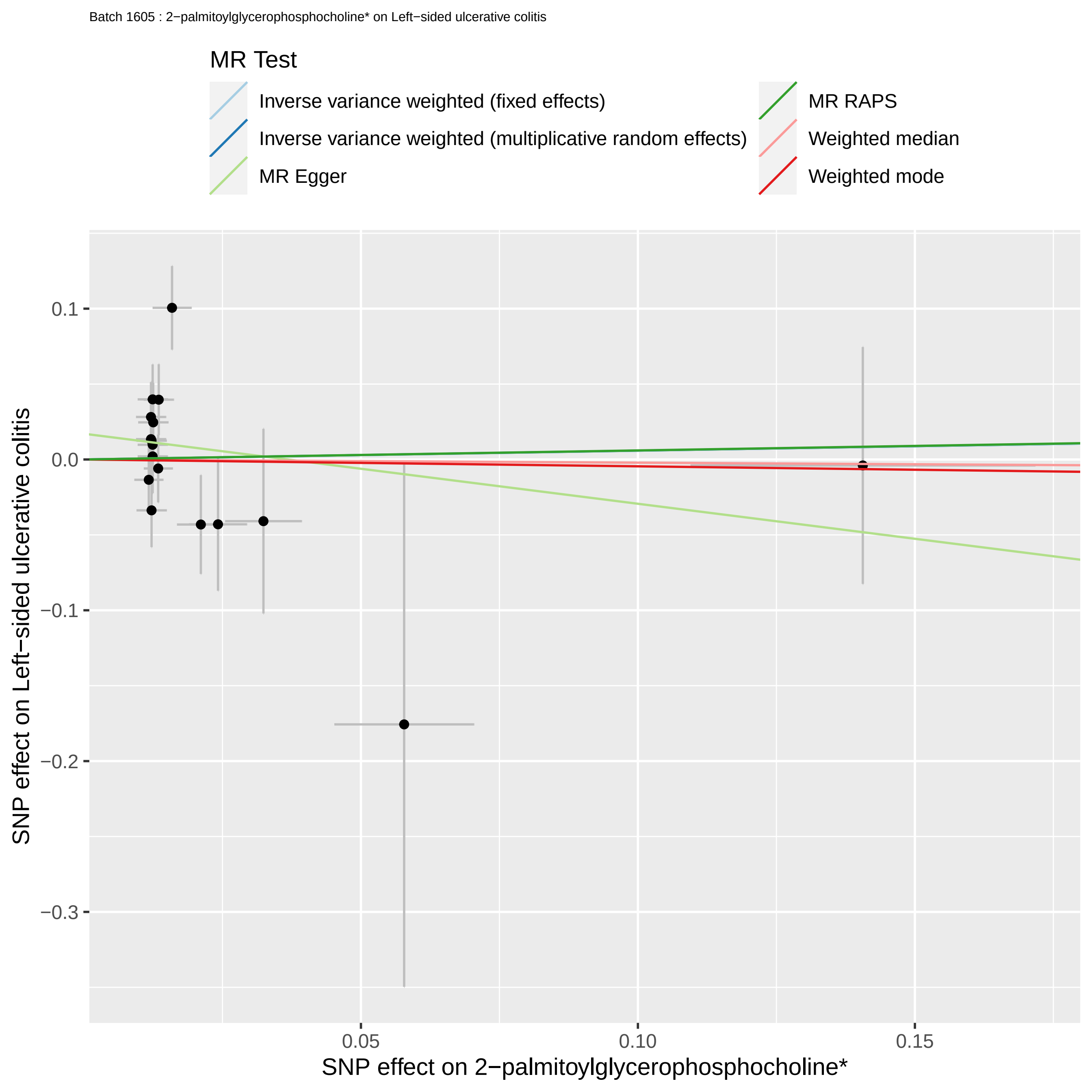
Batch 1605 : 2−palmitoylglycerophosphocholine* on Left−sided ulcerative colitis
MR Test
Inverse variance weighted (fixed effects)
Inverse variance weighted (multiplicative random effects)
MR Egger
MR RAPS
Weighted median
Weighted mode
0.1
0.0
−0.1
−0.2
−0.3
0.05
0.10
0.15
SNP effect on Left−sided ulcerative colitis
SNP effect on 2−palmitoylglycerophosphocholine*

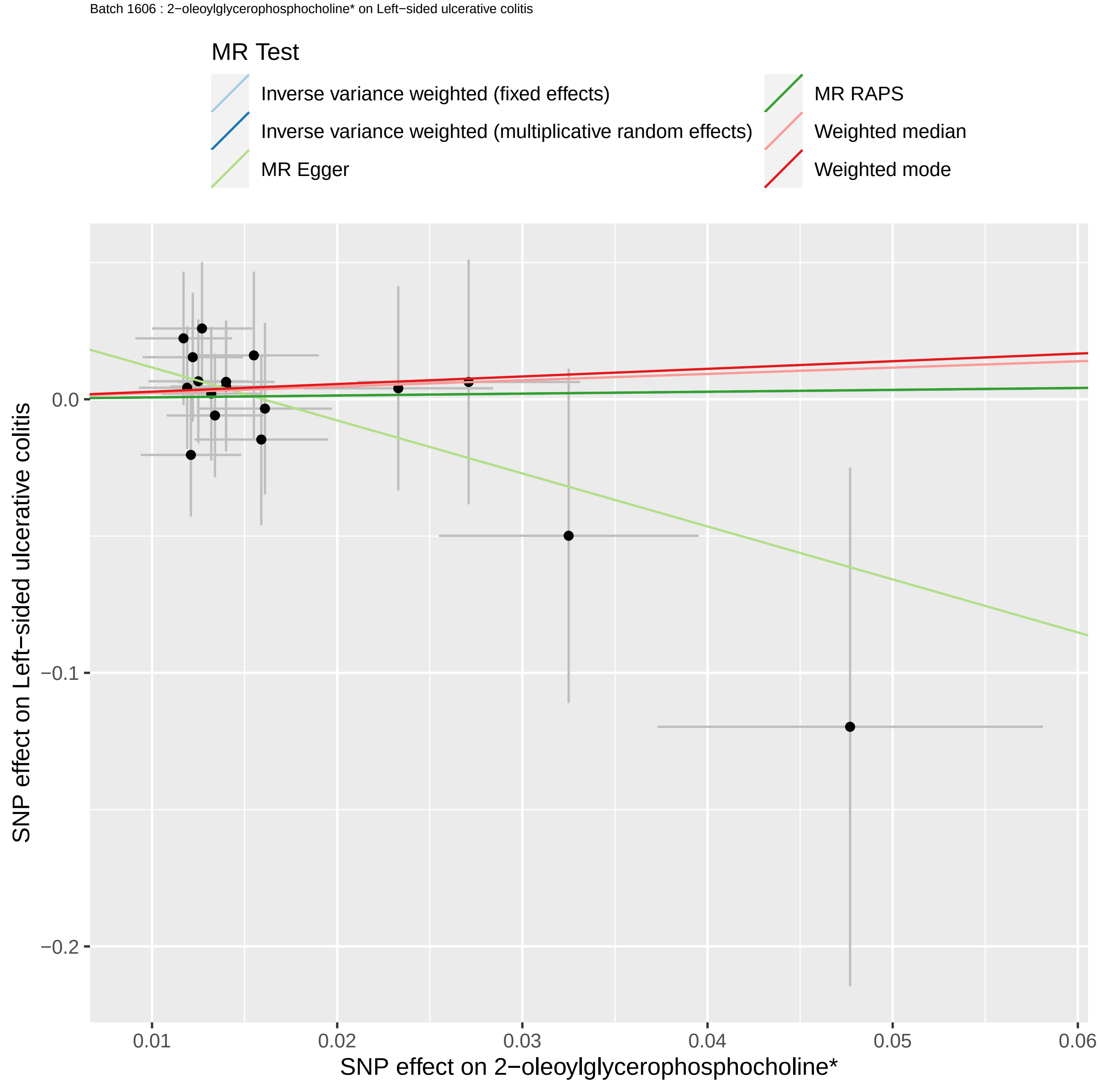

Batch 1606 : 2−oleoylglycerophosphocholine* on Left−sided ulcerative colitis
MR Test
Inverse variance weighted (fixed effects)
Inverse variance weighted (multiplicative random effects)
MR Egger
MR RAPS
Weighted median
Weighted mode
0.0
−0.1
−0.2
0.01
0.02
0.03
0.04
0.05
0.06
SNP effect on Left−sided ulcerative colitis
SNP effect on 2−oleoylglycerophosphocholine*

Batch 1609 : 1−palmitoylglycerophosphoinositol* on Left−sided ulcerative colitis

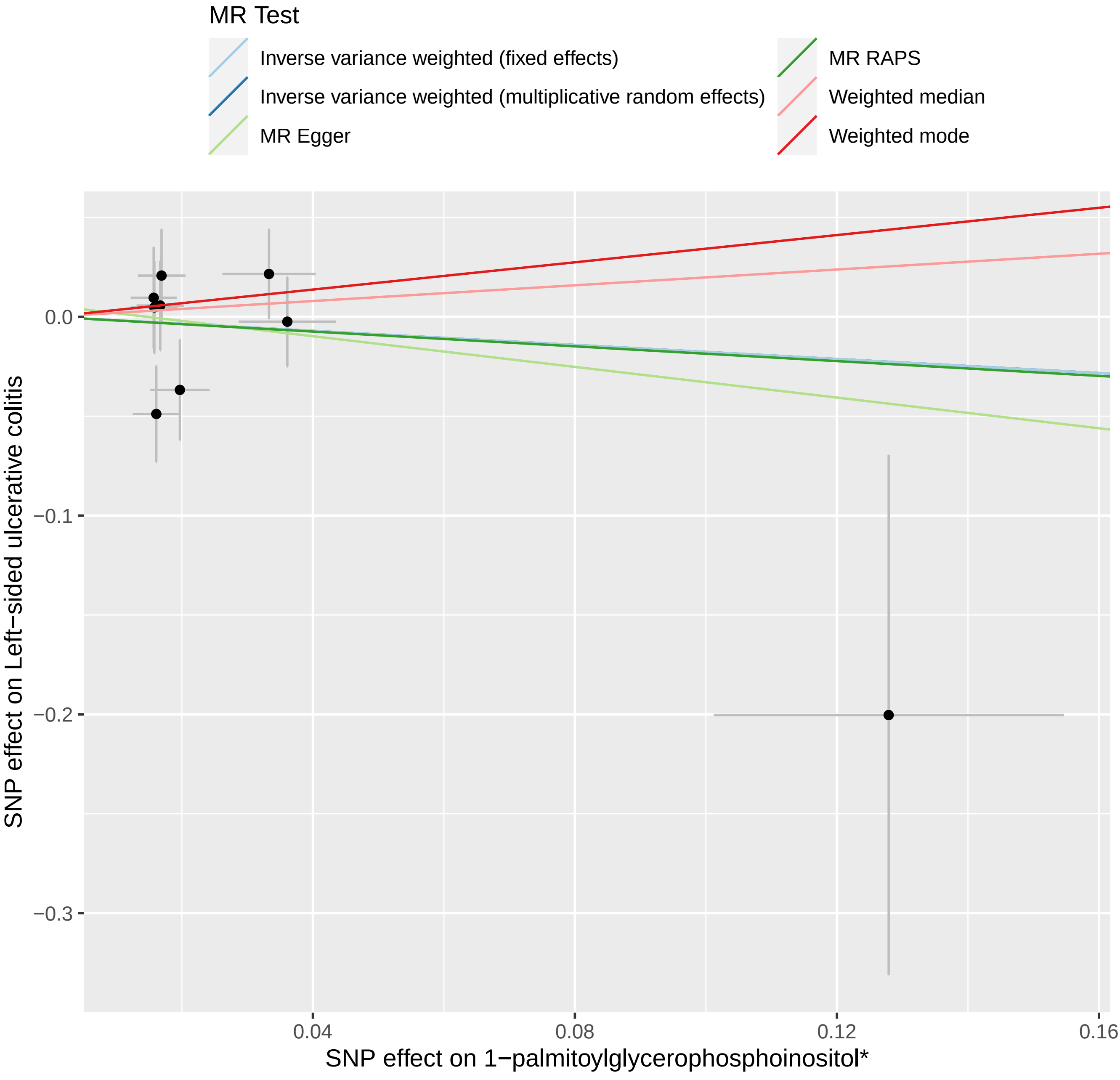

Batch 1612 : Glutaroyl carnitine on Left−sided ulcerative colitis

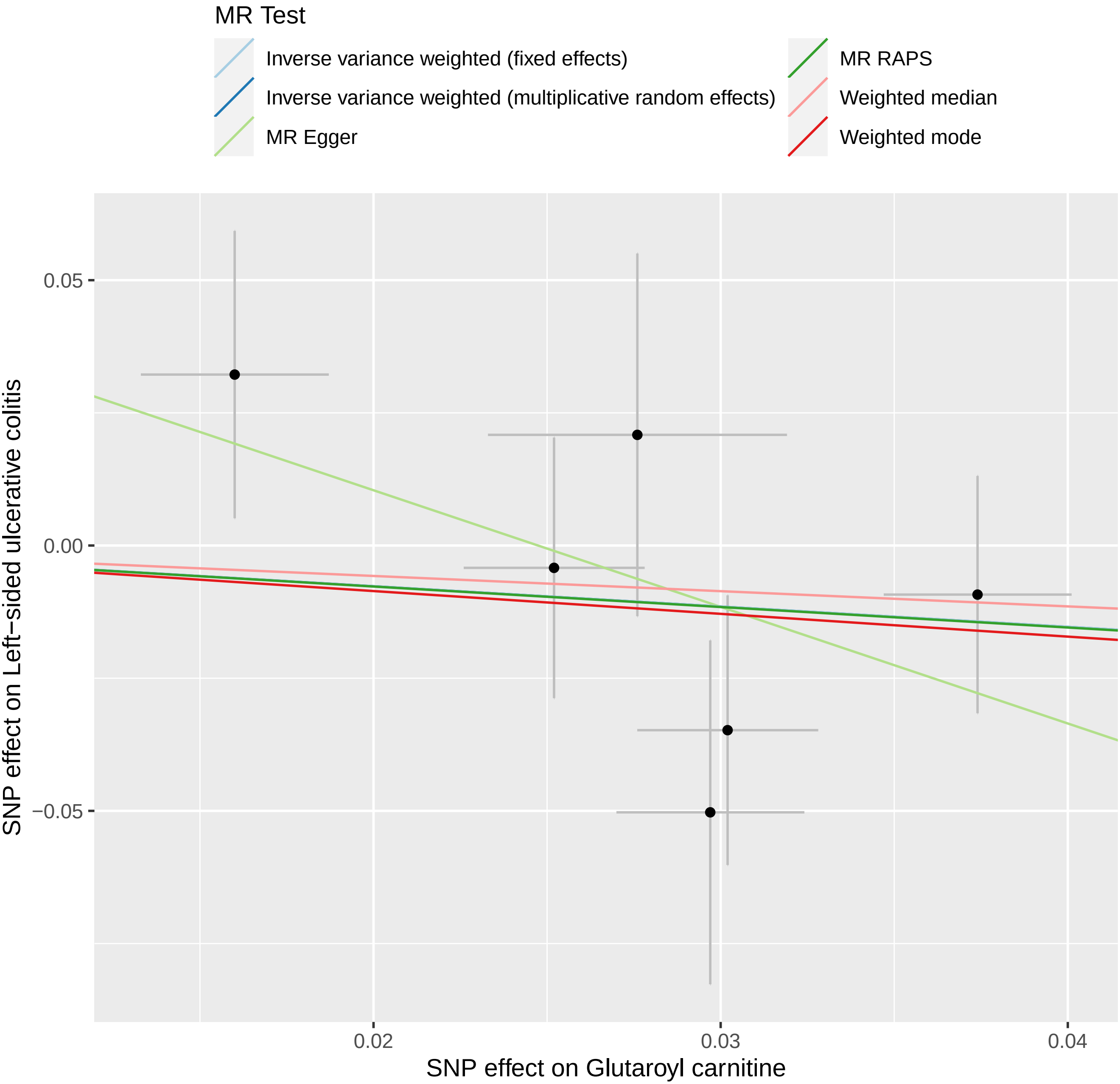

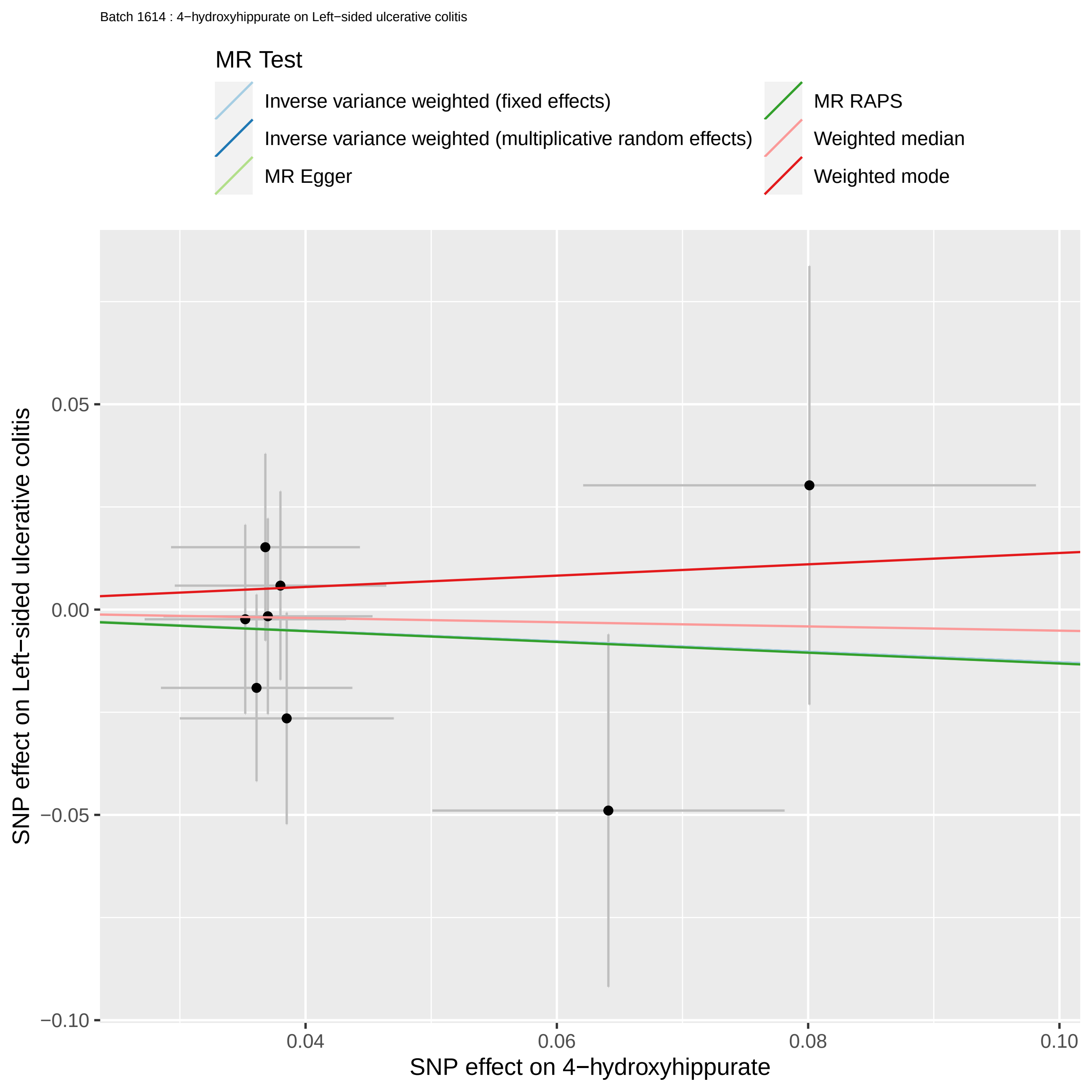

Batch 1614 : 4−hydroxyhippurate on Left−sided ulcerative colitis
MR Test
Inverse variance weighted (fixed effects)
Inverse variance weighted (multiplicative random effects)
MR Egger
MR RAPS
Weighted median
Weighted mode
SNP effect on Left−sided ulcerative colitis
0.05
0.00
−0.05
−0.10
0.04
0.06
0.08
0.10
SNP effect on 4−hydroxyhippurate

Batch 1615 : 1−myristoylglycerophosphocholine on Left−sided ulcerative colitis

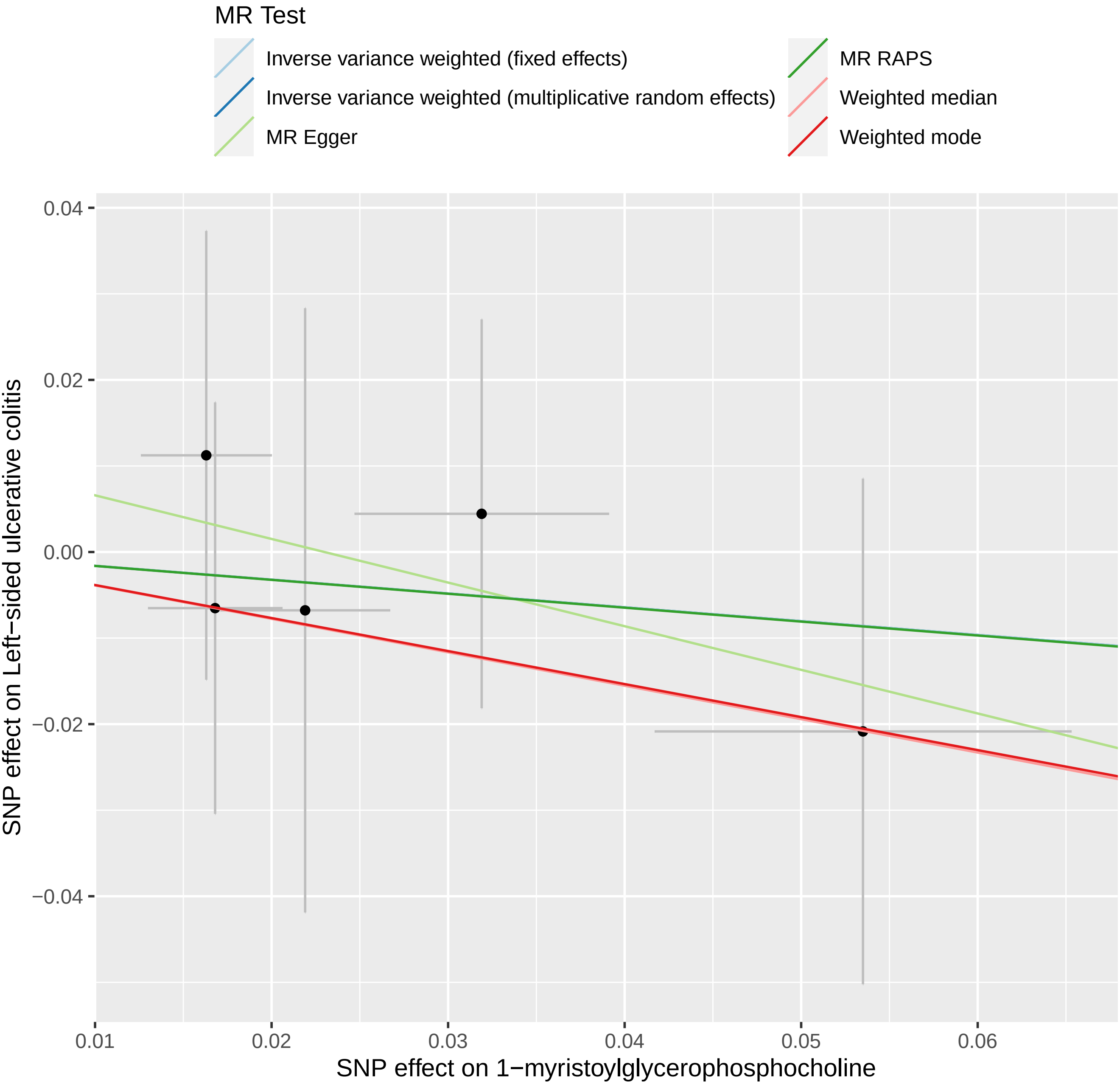

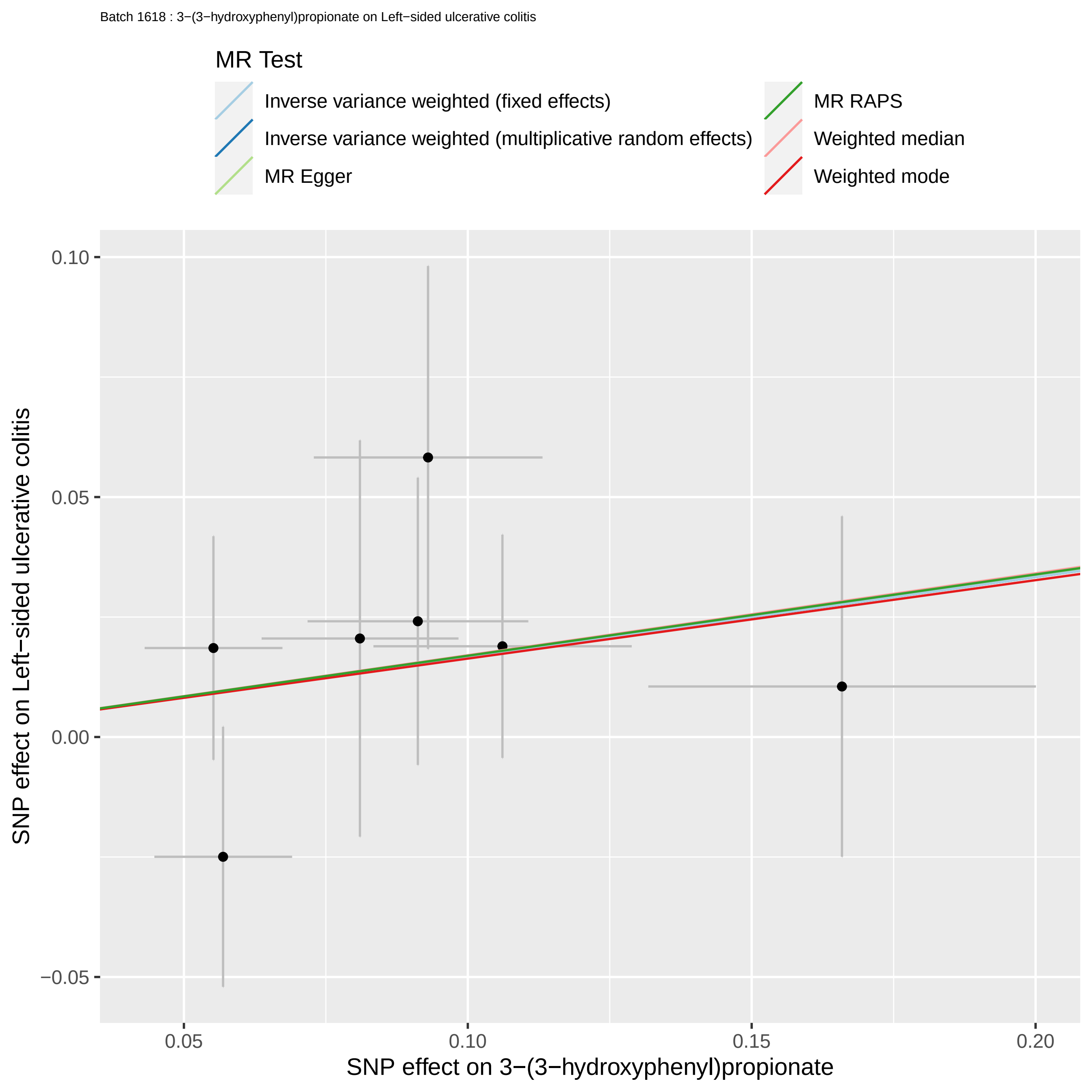
Batch 1618 : 3−(3−hydroxyphenyl)propionate on Left−sided ulcerative colitis
MR Test
Inverse variance weighted (fixed effects)
Inverse variance weighted (multiplicative random effects)
MR Egger
MR RAPS
Weighted median
Weighted mode
0.10
0.05
0.00
−0.05
0.05
0.10
0.15
0.20
SNP effect on Left−sided ulcerative colitis
SNP effect on 3−(3−hydroxyphenyl)propionate

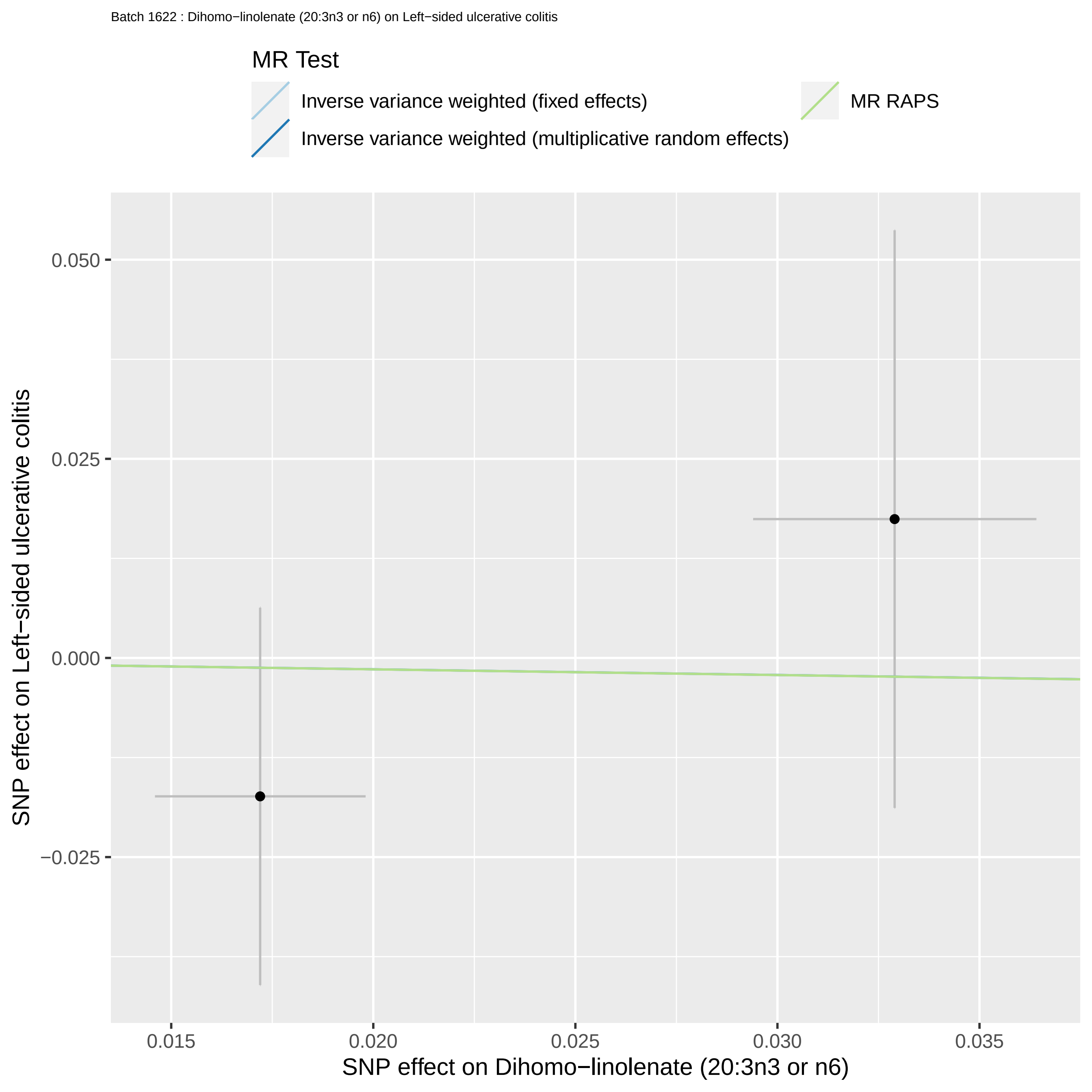
Batch 1622 : Dihomo−linolenate (20:3n3 or n6) on Left−sided ulcerative colitis
MR Test
Inverse variance weighted (fixed effects)
Inverse variance weighted (multiplicative random effects)
MR RAPS
0.050
0.025
0.000
−0.025
0.015
0.020
0.025
0.030
0.035
SNP effect on Left−sided ulcerative colitis
SNP effect on Dihomo−linolenate (20:3n3 or n6)

Batch 1623 : Threitol on Left−sided ulcerative colitis

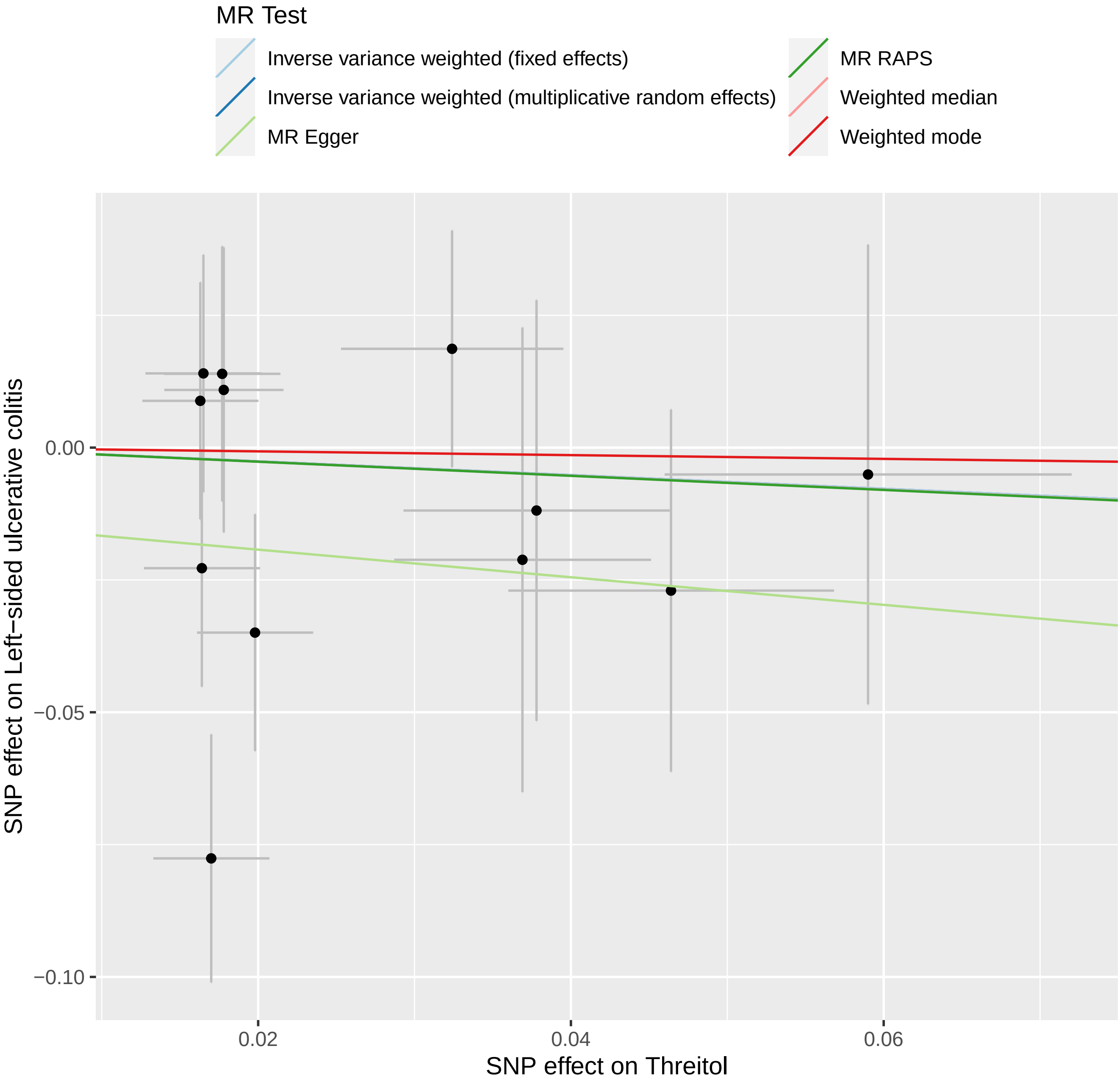

Batch 1624 : P−cresol sulfate on Left−sided ulcerative colitis

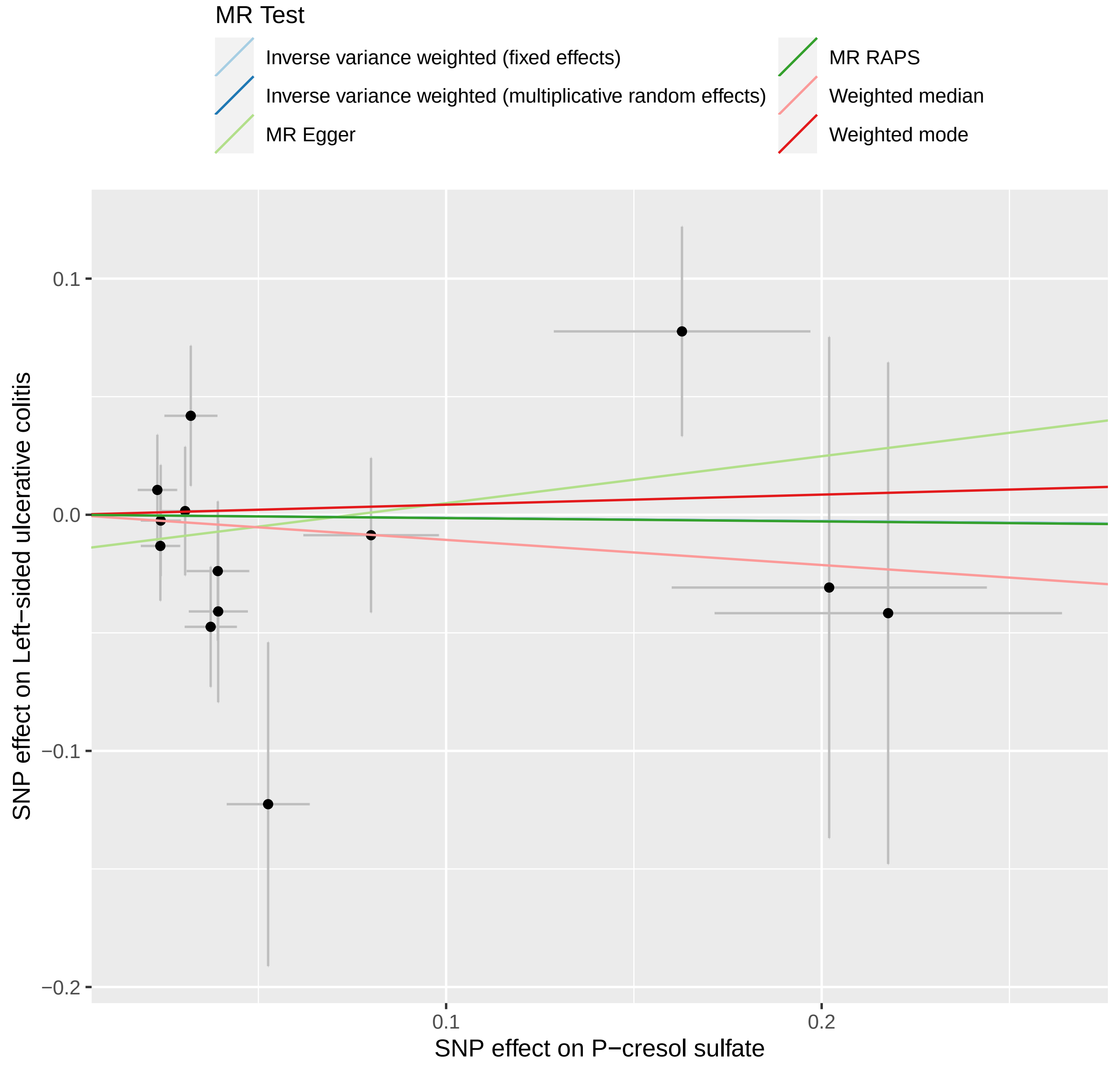

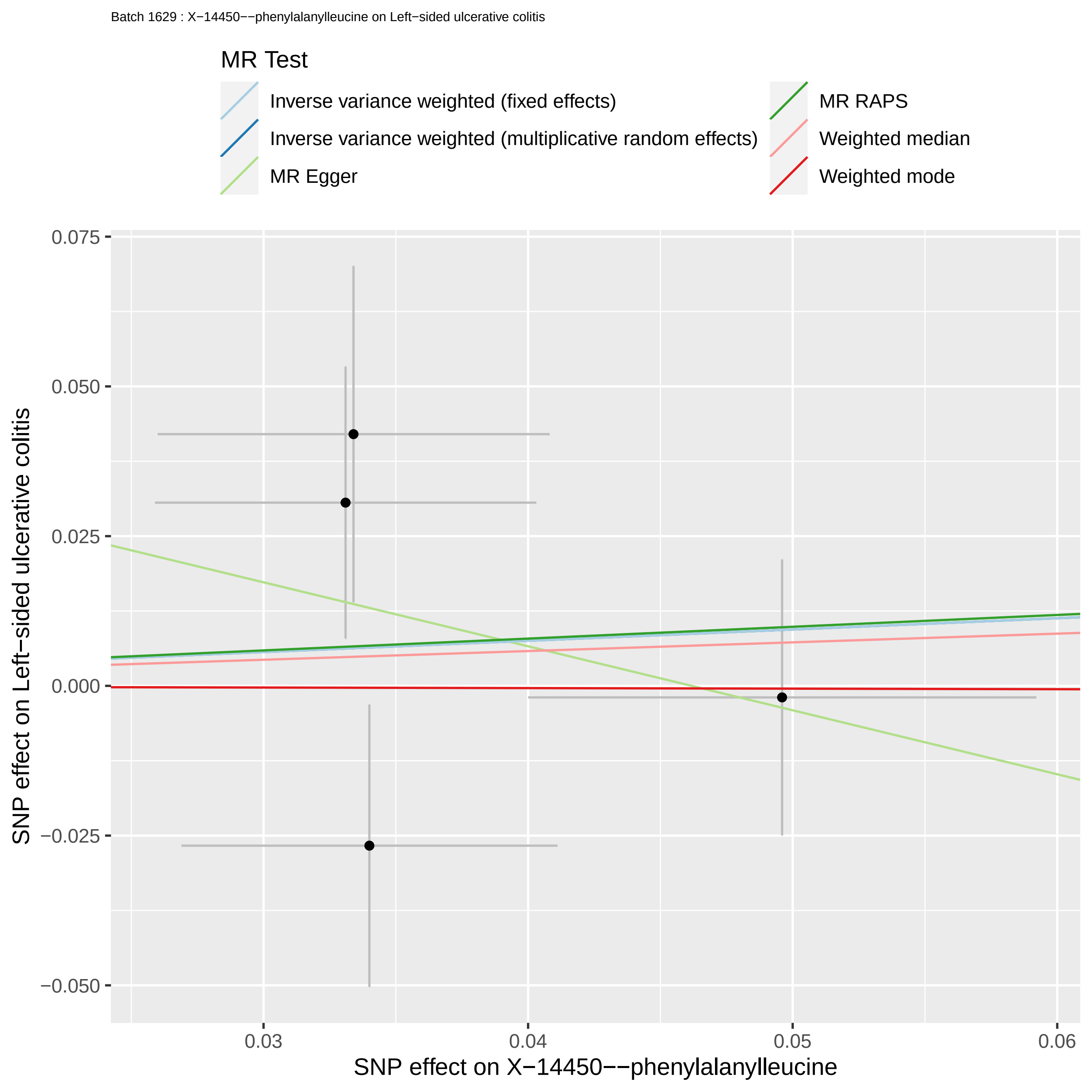

Batch 1629 : X−14450−−phenylalanylleucine on Left−sided ulcerative colitis
MR Test
Inverse variance weighted (fixed effects)
Inverse variance weighted (multiplicative random effects)
MR Egger
MR RAPS
Weighted median
Weighted mode
0.075
0.050
0.025
0.000
−0.025
−0.050
0.03
0.04
0.05
0.06
SNP effect on Left−sided ulcerative colitis
SNP effect on X−14450−−phenylalanylleucine

Batch 1630 : Gamma−glutamylglutamate on Left−sided ulcerative colitis

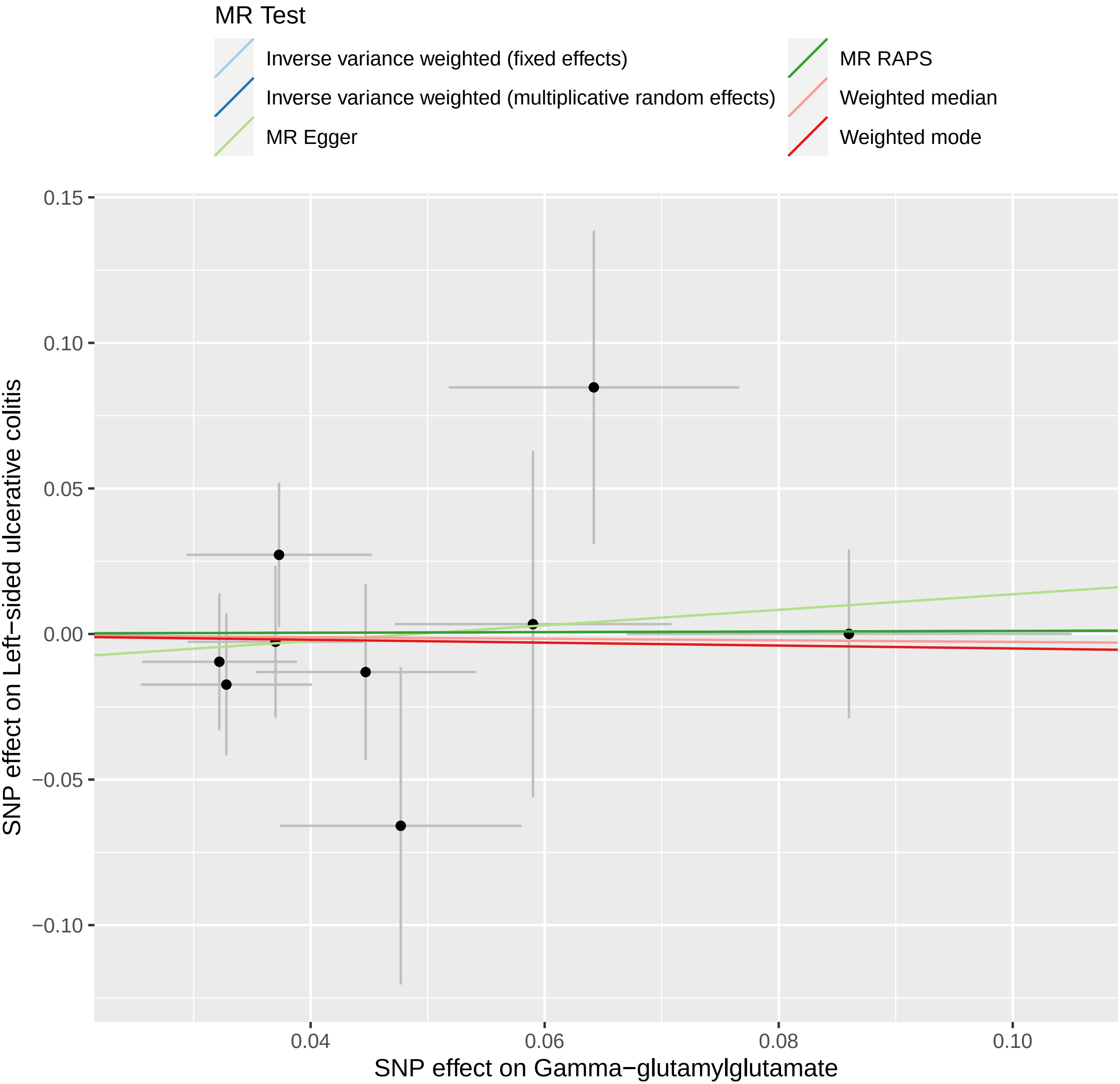

Batch 1631 : Octadecanedioate on Left−sided ulcerative colitis

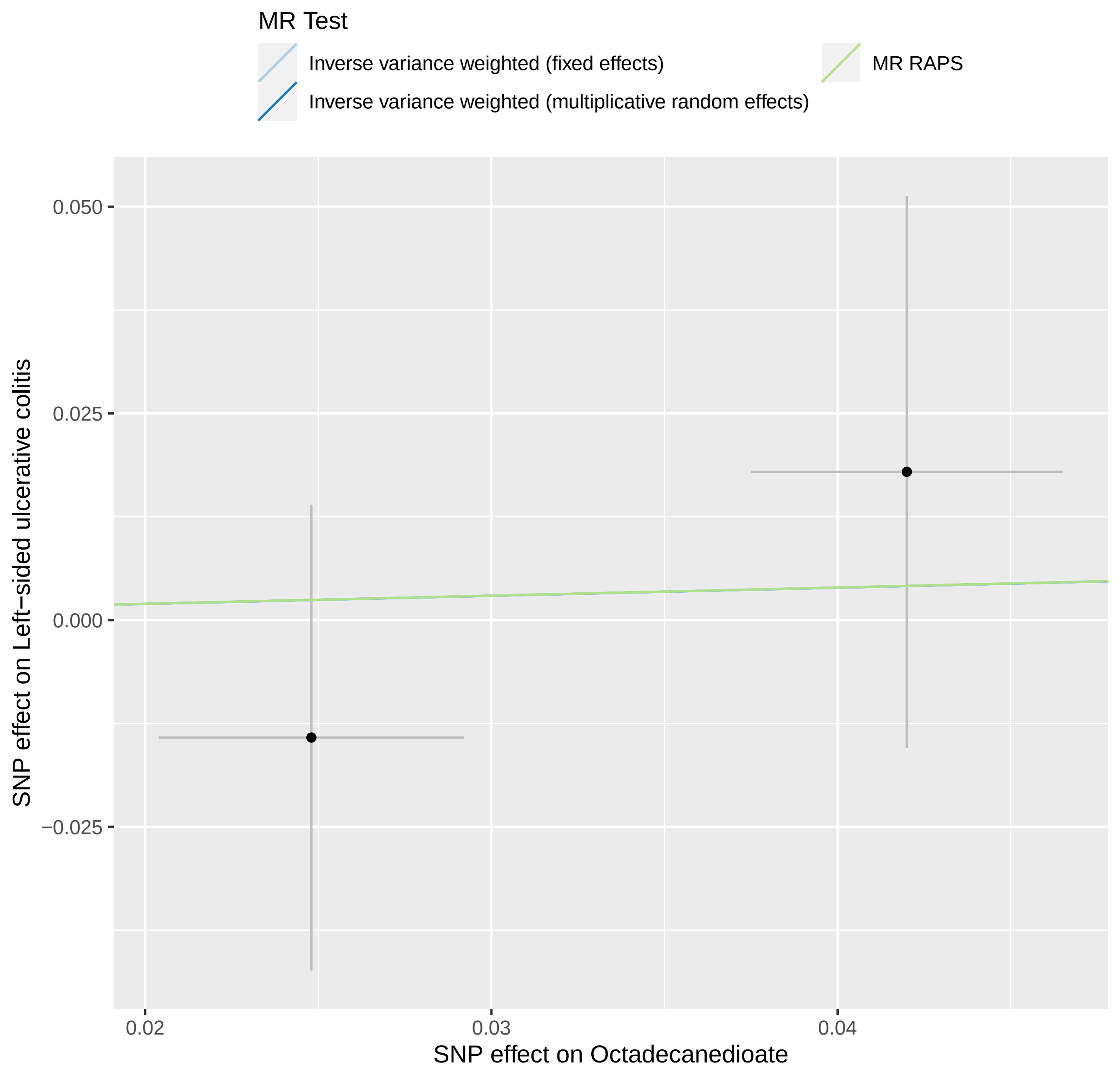

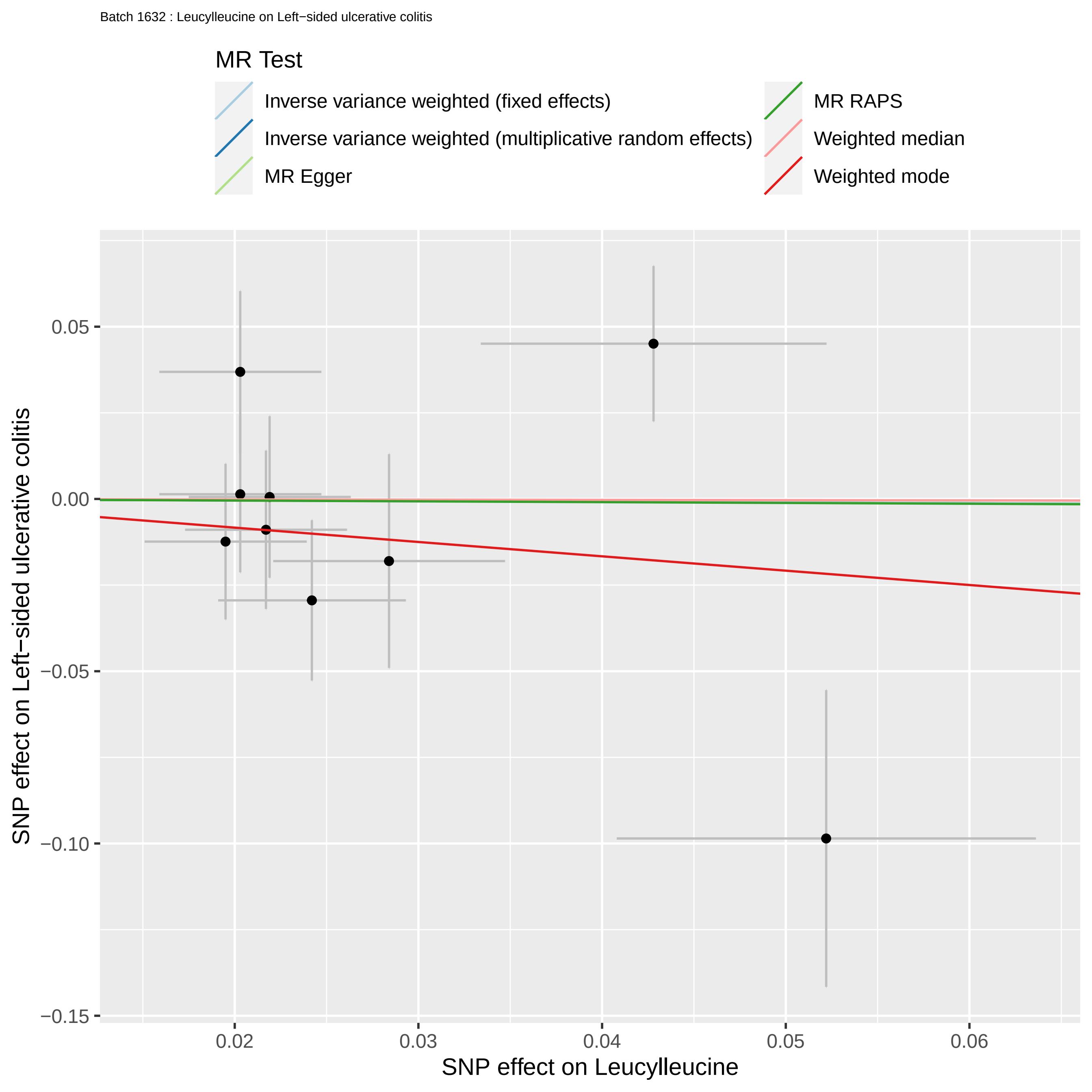

Batch 1632 : Leucylleucine on Left−sided ulcerative colitis
MR Test
Inverse variance weighted (fixed effects)
Inverse variance weighted (multiplicative random effects)
MR Egger
MR RAPS
Weighted median
Weighted mode
0.05
0.00
−0.05
−0.10
−0.15
0.02
0.03
0.04
0.05
0.06
SNP effect on Left−sided ulcerative colitis
SNP effect on Leucylleucine

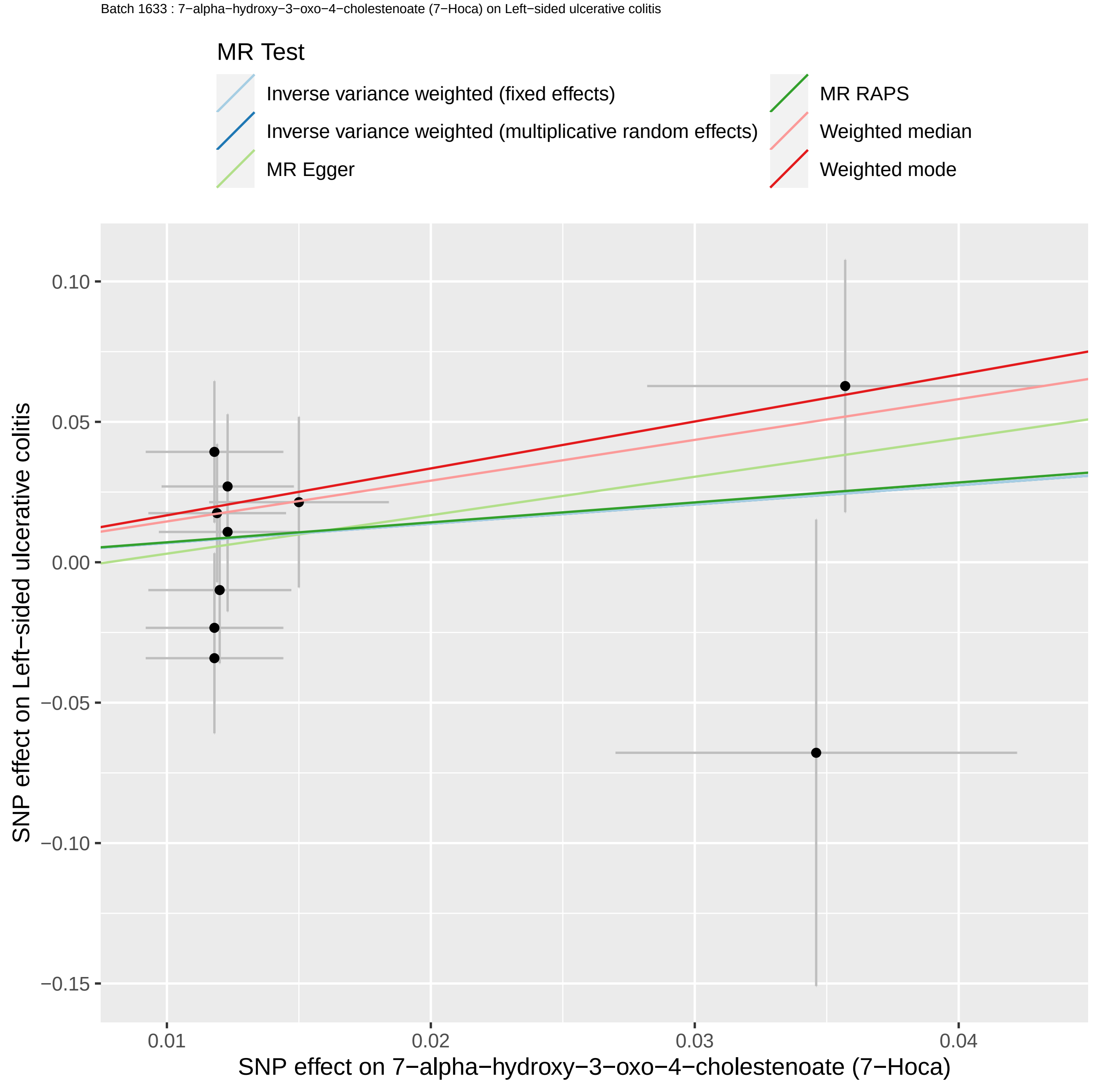
Batch 1633 : 7−alpha−hydroxy−3−oxo−4−cholestenoate (7−Hoca) on Left−sided ulcerative colitis
MR Test
Inverse variance weighted (fixed effects)
Inverse variance weighted (multiplicative random effects)
MR Egger
MR RAPS
Weighted median
Weighted mode
0.10
0.05
0.00
−0.05
−0.10
−0.15
0.01
0.02
0.03
0.04
SNP effect on Left−sided ulcerative colitis
SNP effect on 7−alpha−hydroxy−3−oxo−4−cholestenoate (7−Hoca)

Batch 1634 : N−Butyl Oleate on Left−sided ulcerative colitis

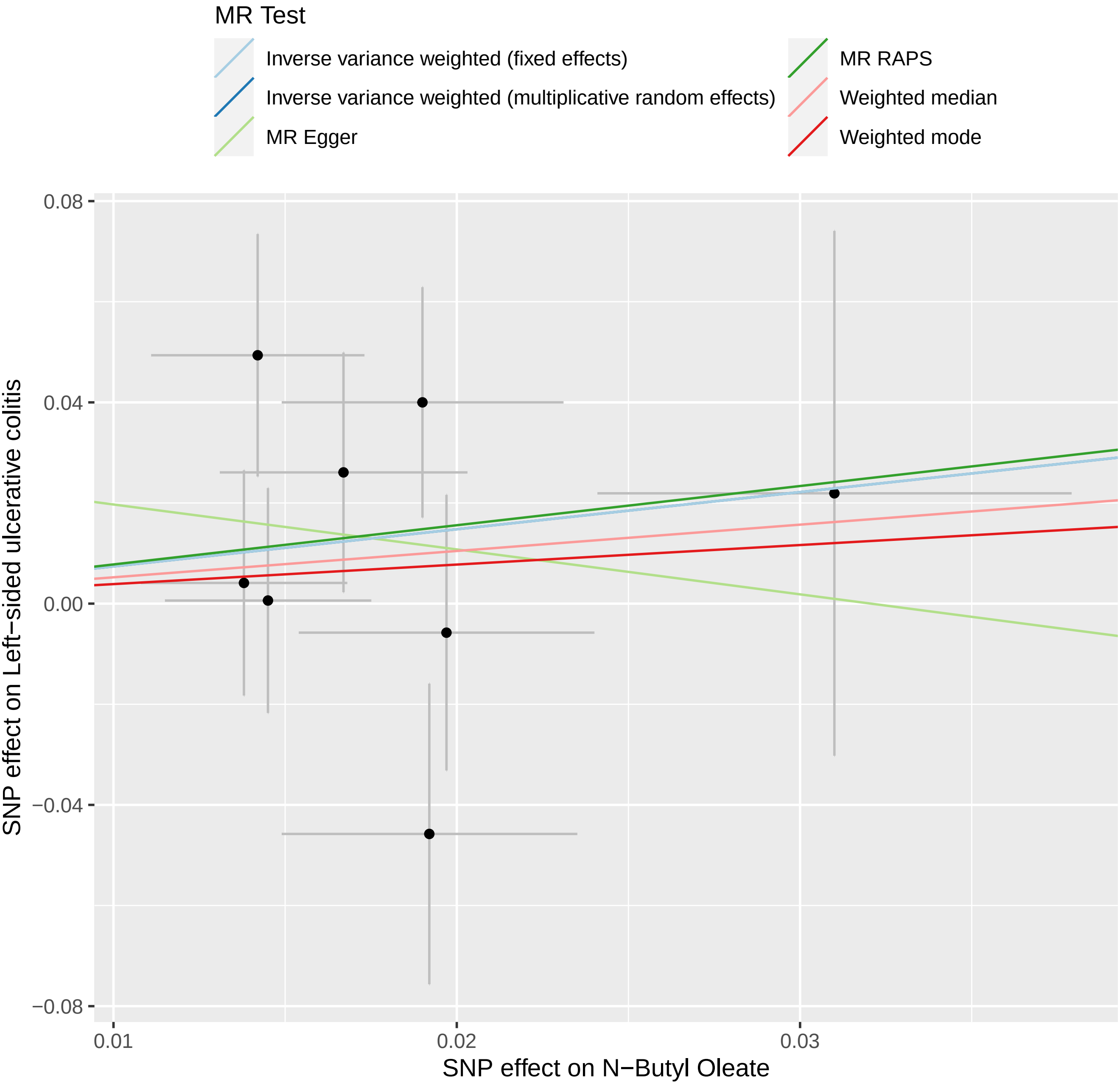

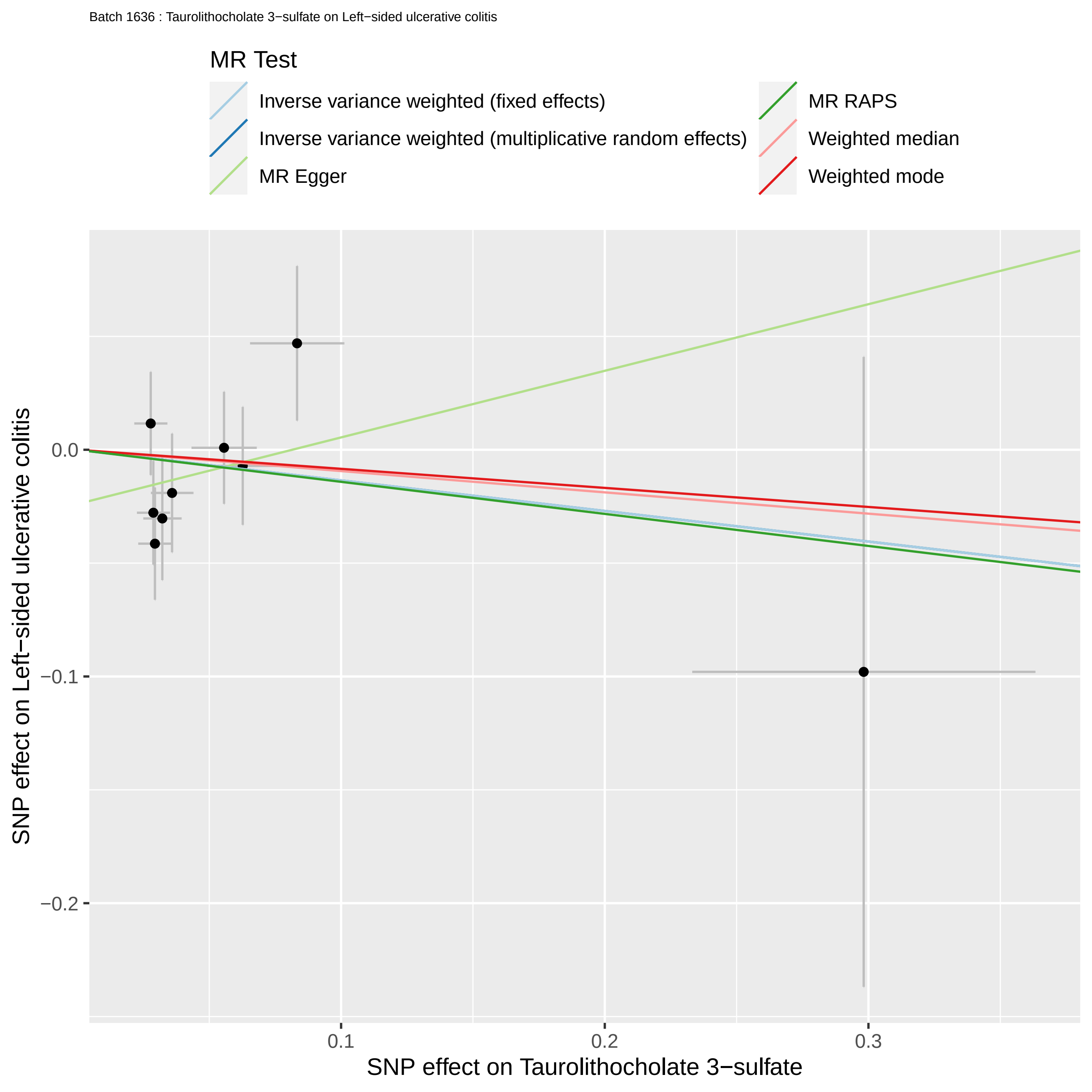

Batch 1636 : Taurolithocholate 3−sulfate on Left−sided ulcerative colitis
MR Test
Inverse variance weighted (fixed effects)
Inverse variance weighted (multiplicative random effects)
MR Egger
MR RAPS
Weighted median
Weighted mode
0.0
−0.1
−0.2
0.1
0.2
0.3
SNP effect on Left−sided ulcerative colitis
SNP effect on Taurolithocholate 3−sulfate

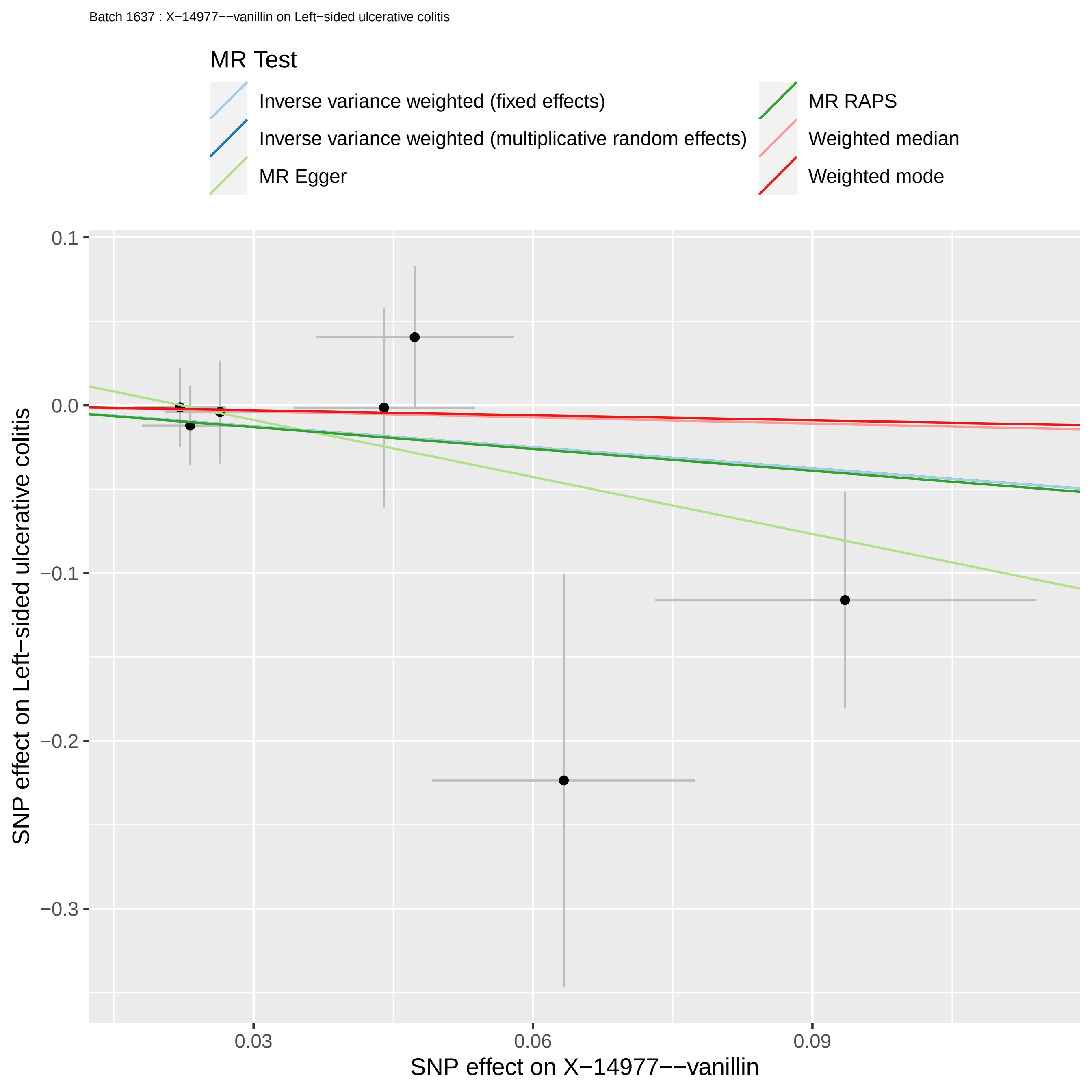
Batch 1637 : X−14977−−vanillin on Left−sided ulcerative colitis
MR Test
Inverse variance weighted (fixed effects)
Inverse variance weighted (multiplicative random effects)
MR Egger
MR RAPS
Weighted median
Weighted mode
0.1
0.0
−0.1
−0.2
−0.3
0.03
0.06
0.09
SNP effect on Left−sided ulcerative colitis
SNP effect on X−14977−−vanillin

Batch 1638 : Succinylcarnitine on Left−sided ulcerative colitis

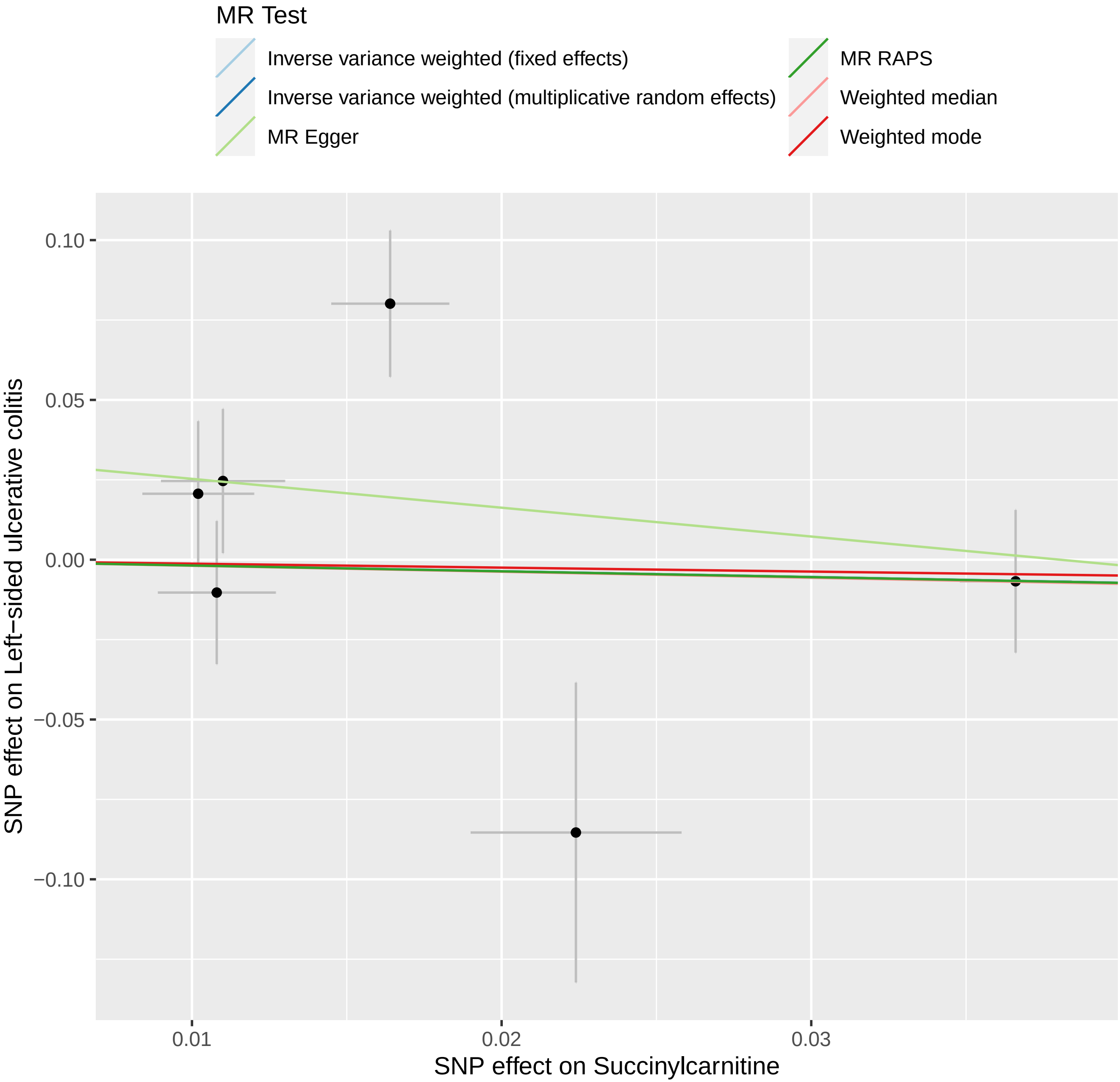

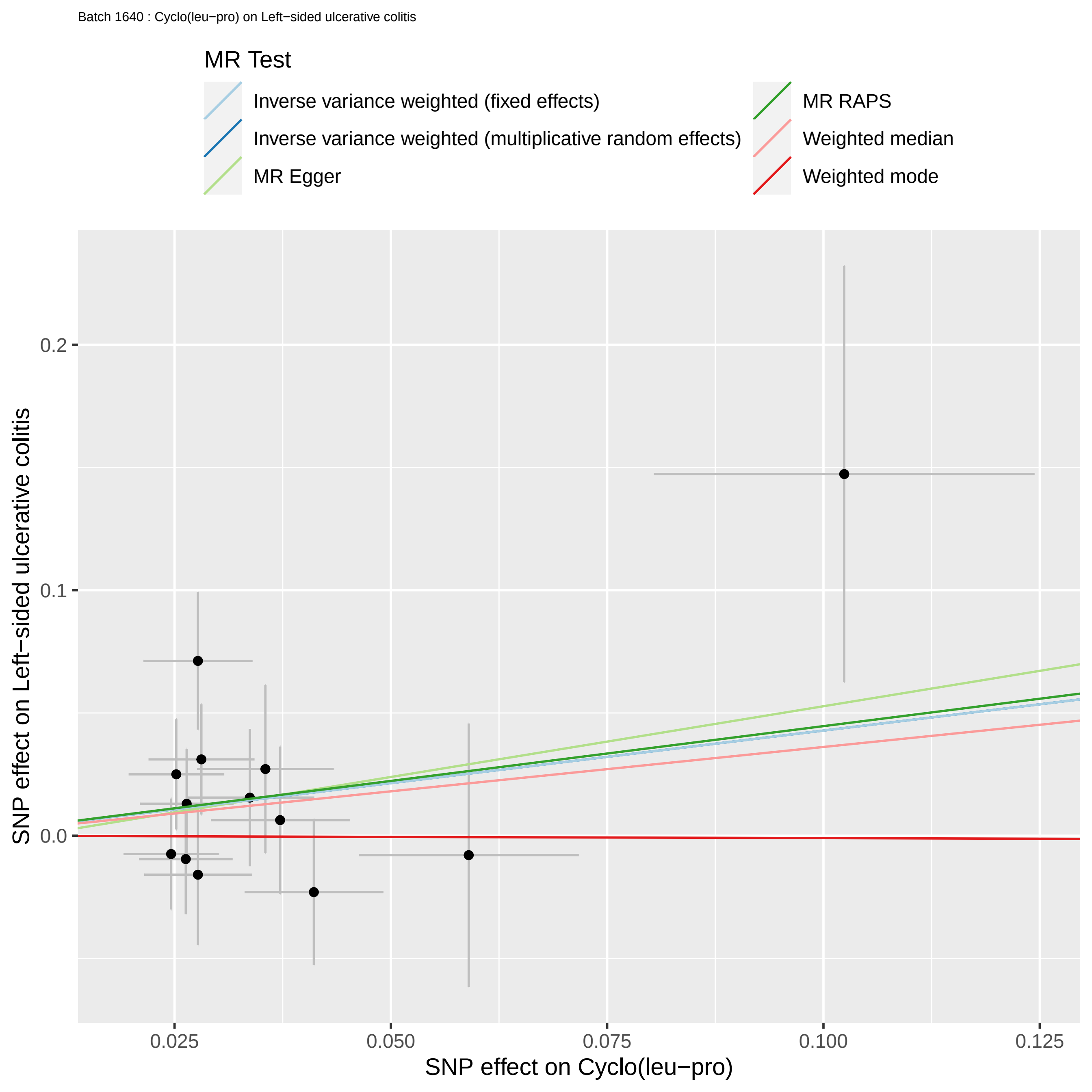

Batch 1640 : Cyclo(leu−pro) on Left−sided ulcerative colitis
MR Test
Inverse variance weighted (fixed effects)
Inverse variance weighted (multiplicative random effects)
MR Egger
MR RAPS
Weighted median
Weighted mode
SNP effect on Left−sided ulcerative colitis
0.2
0.1
0.0
0.025
0.050
0.075
0.100
0.125
SNP effect on Cyclo(leu−pro)

Batch 1641 : Chiro−inositol on Left−sided ulcerative colitis

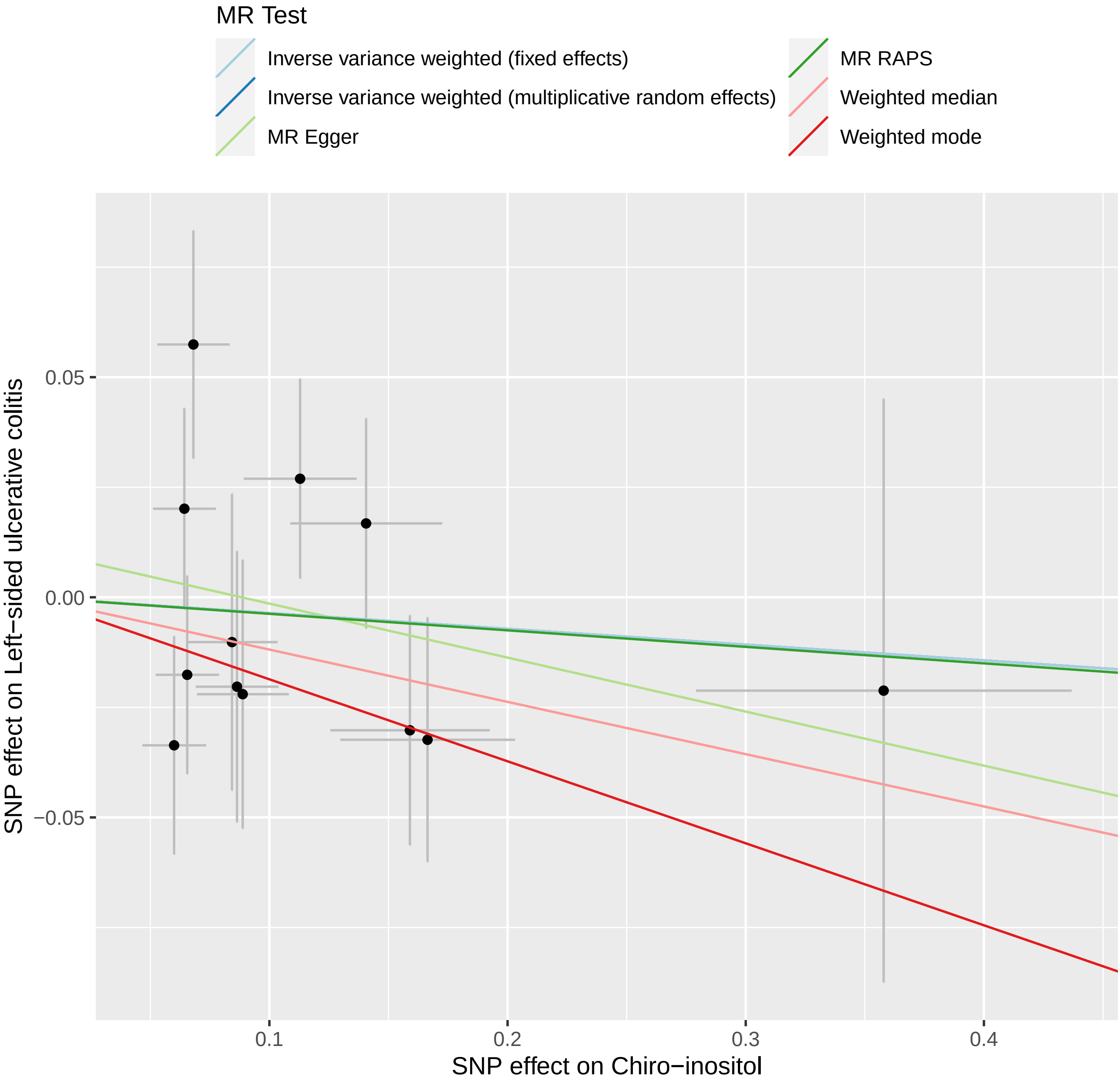

Batch 1645 : 2−hydroxyglutarate on Left−sided ulcerative colitis

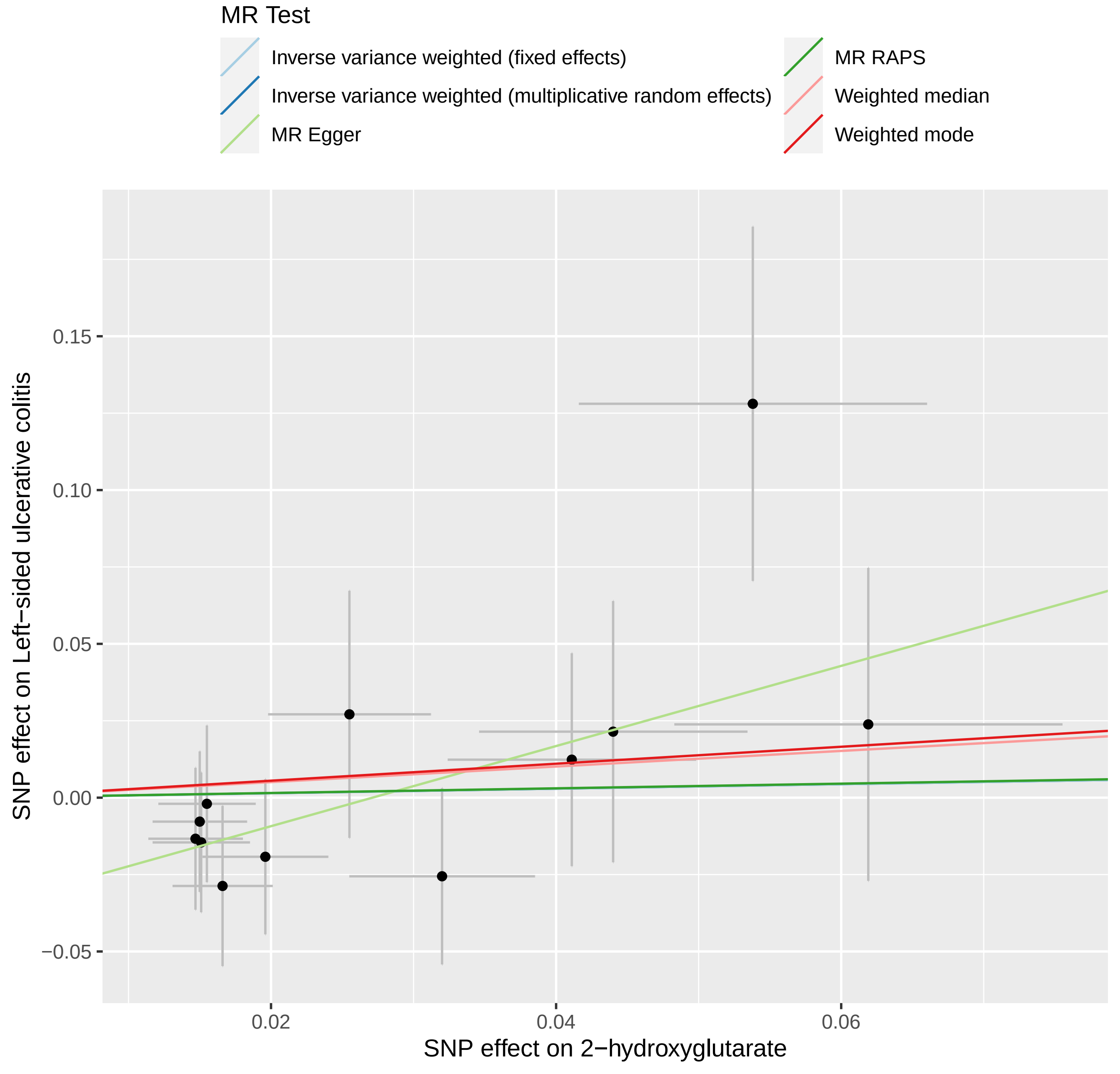

Batch 1646 : Ergothioneine on Left−sided ulcerative colitis

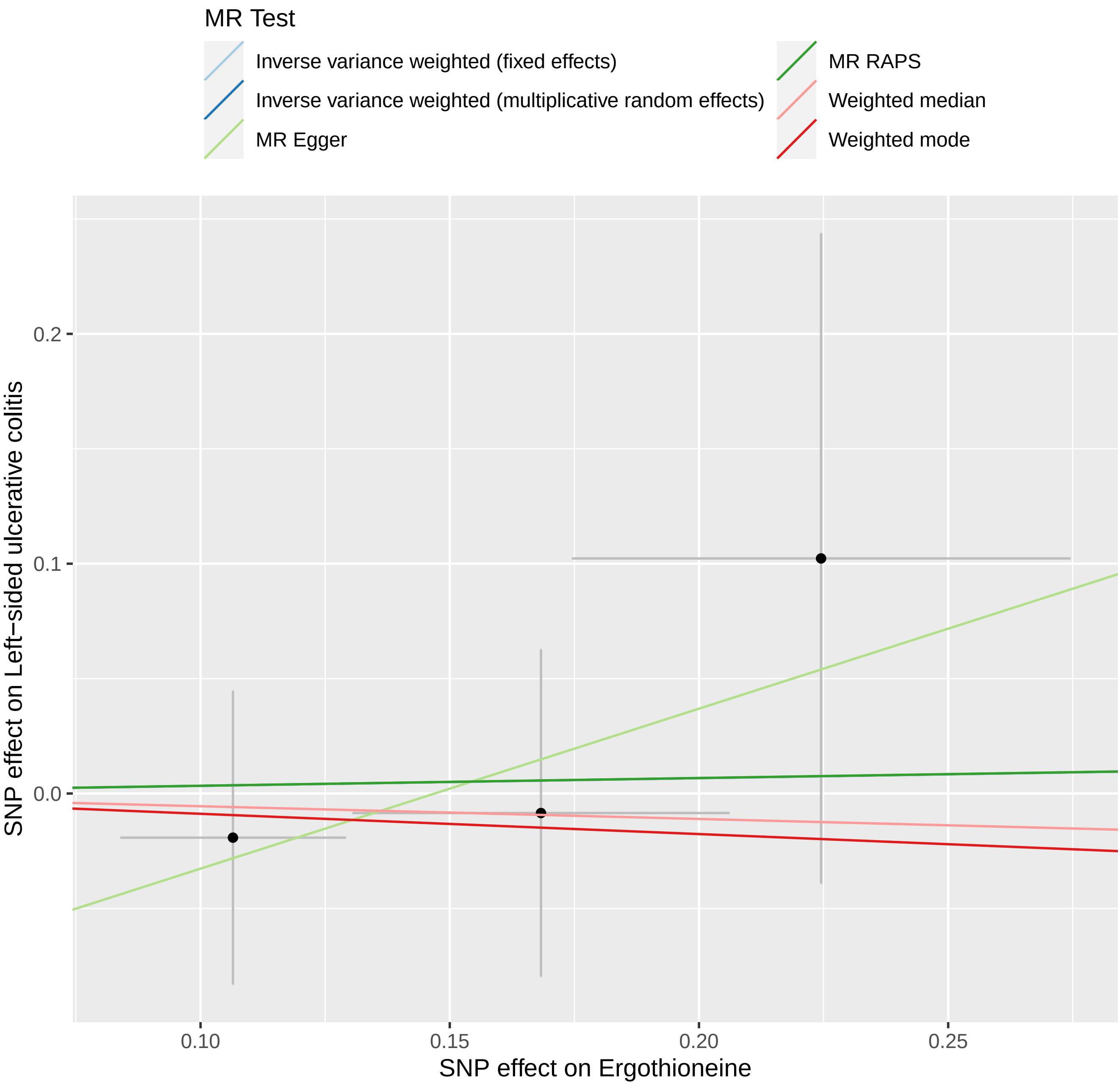

Batch 1648 : Phenylalanylphenylalanine on Left−sided ulcerative colitis

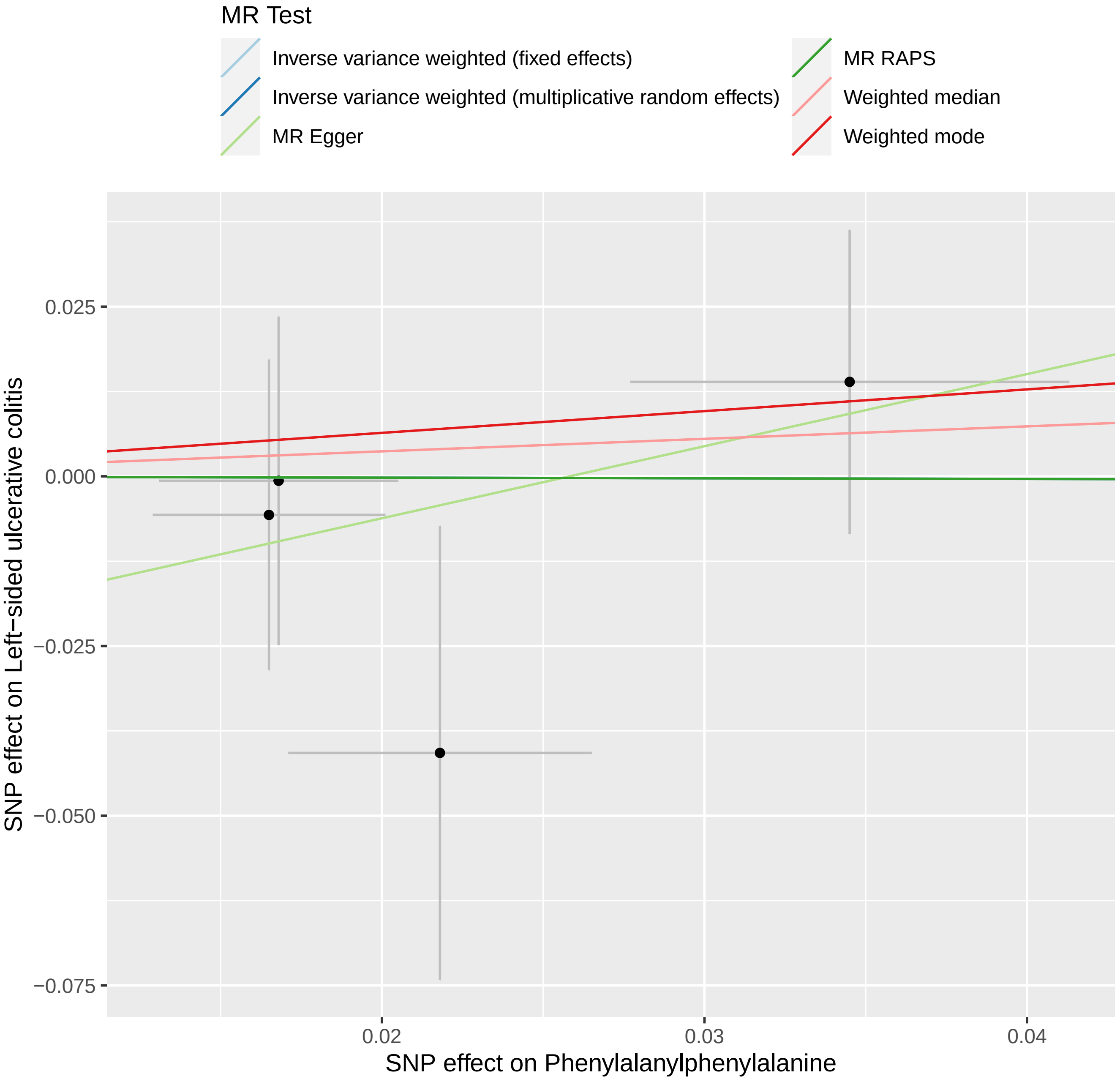

Batch 1649 : Cis−4−decenoyl carnitine on Left−sided ulcerative colitis

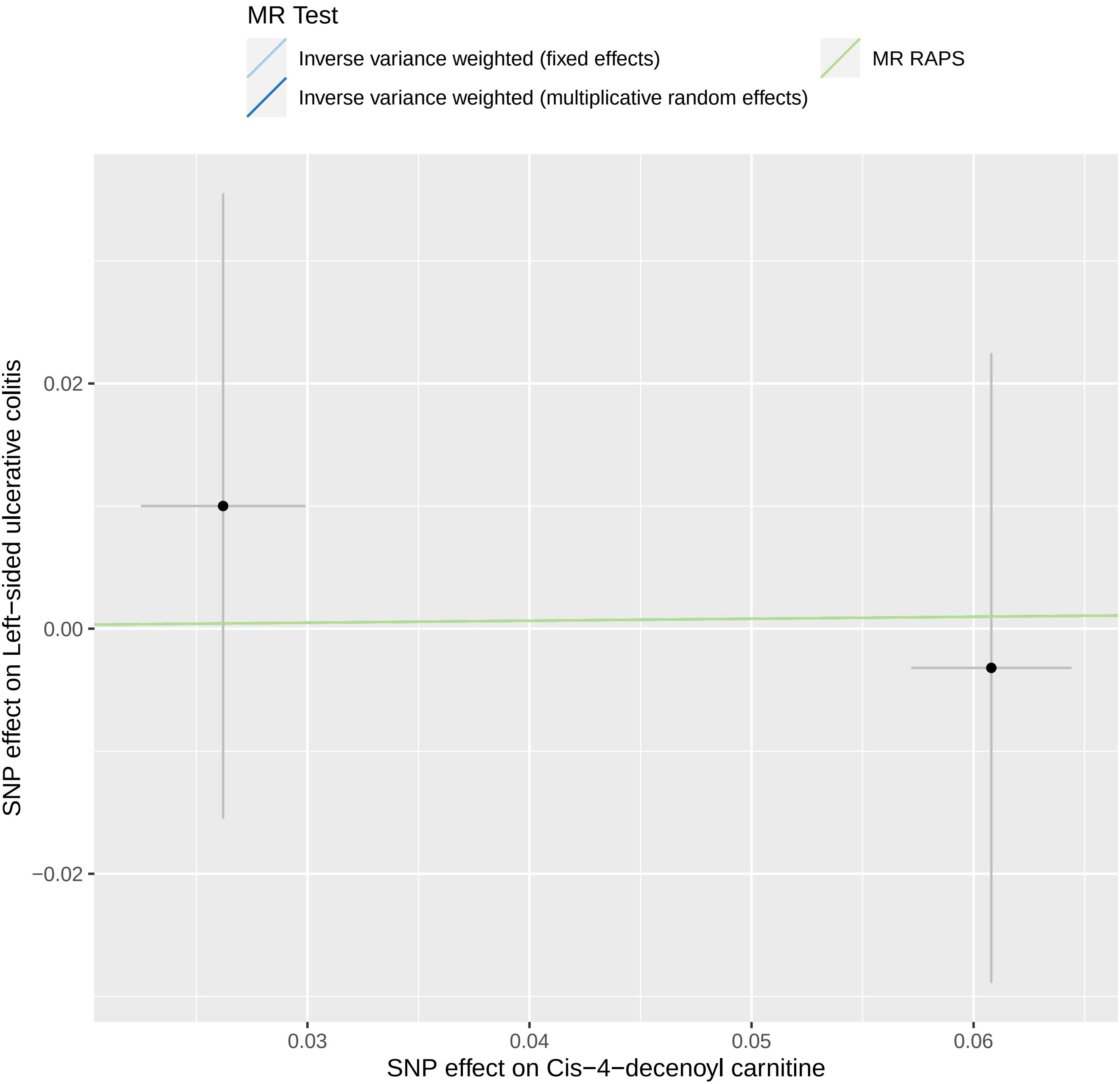

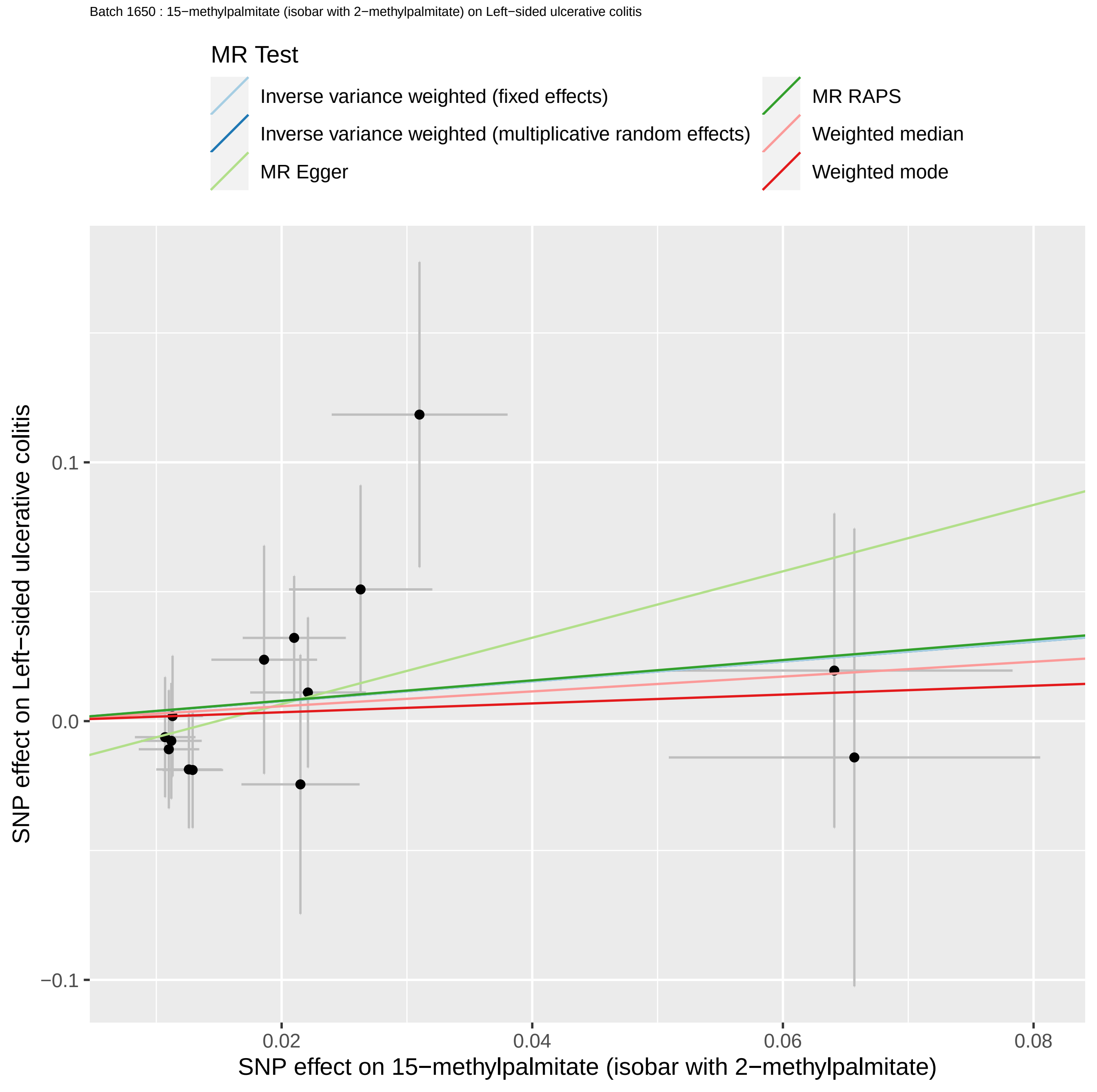

Batch 1650 : 15−methylpalmitate (isobar with 2−methylpalmitate) on Left−sided ulcerative colitis
MR Test
Inverse variance weighted (fixed effects)
Inverse variance weighted (multiplicative random effects)
MR Egger
MR RAPS
Weighted median
Weighted mode
SNP effect on Left−sided ulcerative colitis
0.1
0.0
−0.1
0.02
0.04
0.06
0.08
SNP effect on 15−methylpalmitate (isobar with 2−methylpalmitate)

Batch 1652 : Tryptophan on Arthropathy in ulcerative colitis

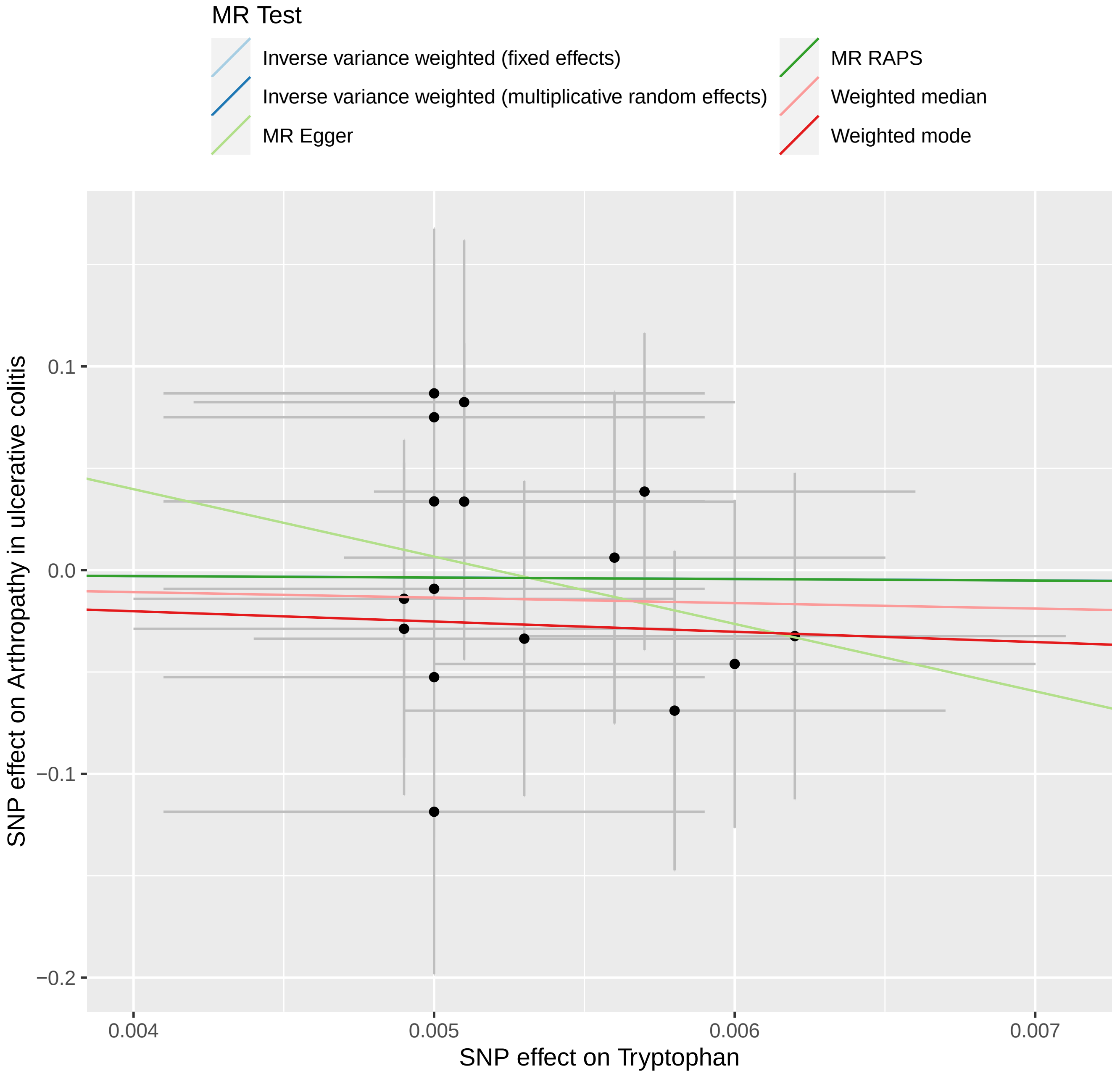

Batch 1654 : Leucine on Arthropathy in ulcerative colitis

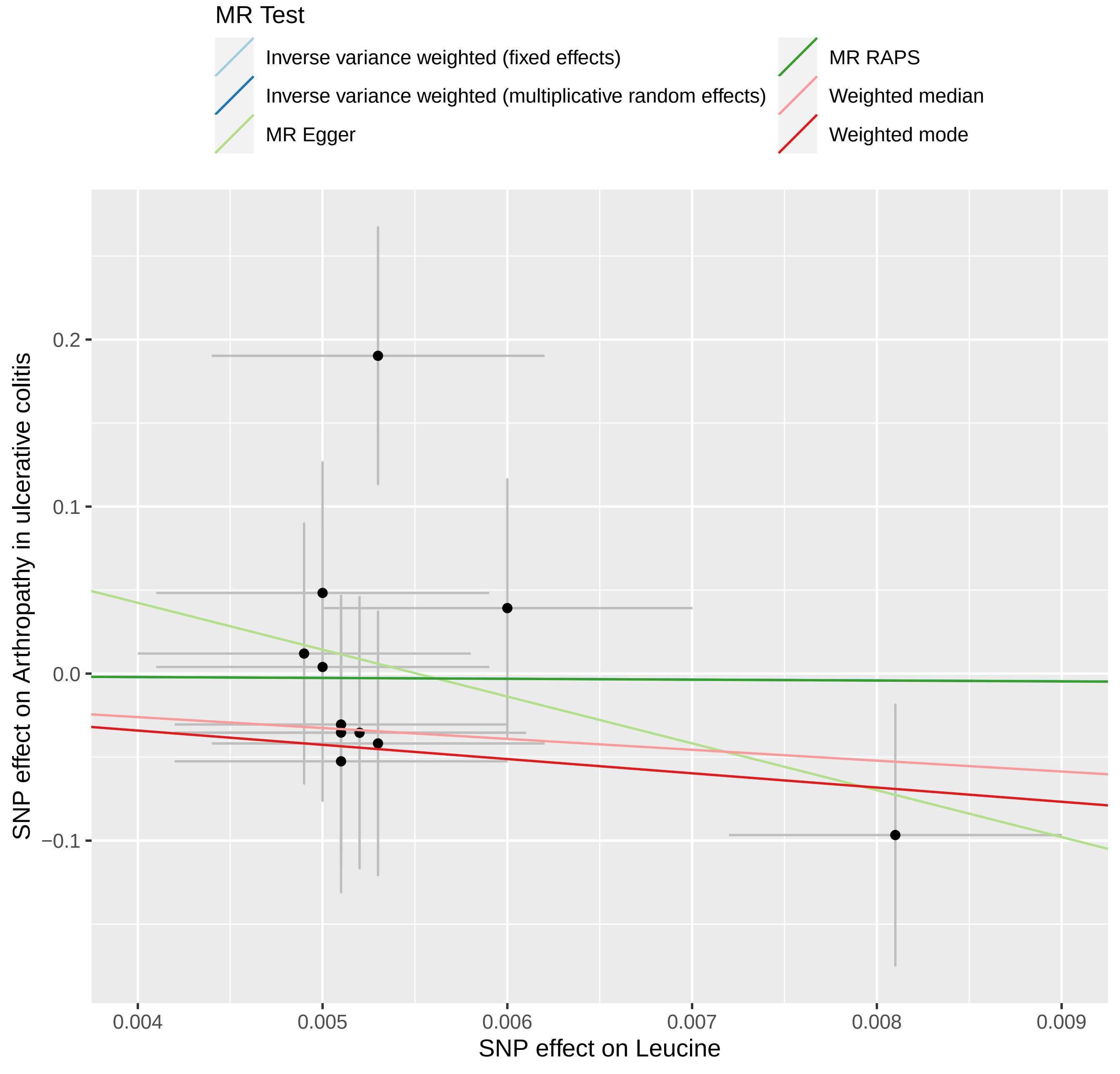

Batch 1658 : Lactate on Arthropathy in ulcerative colitis

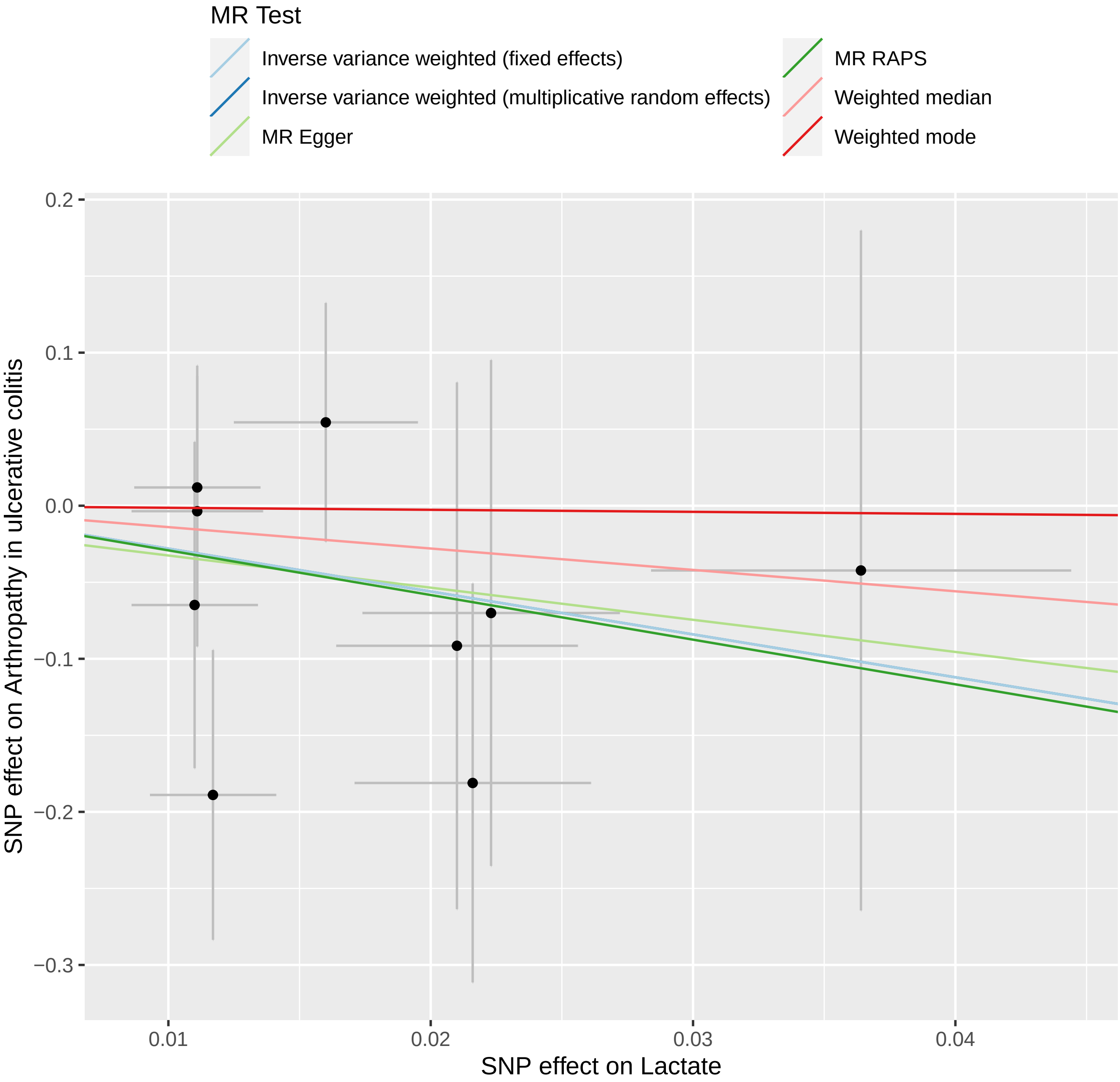

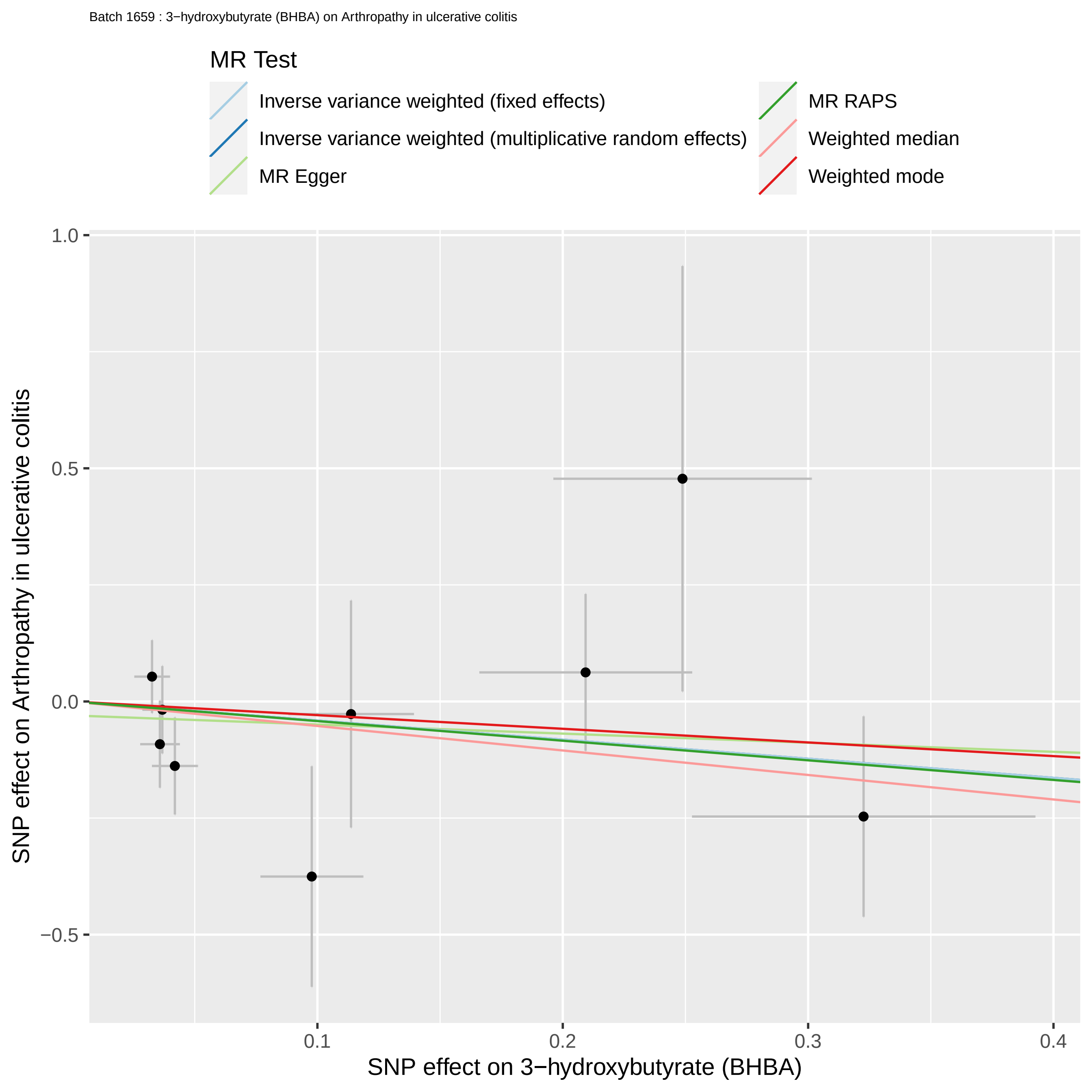
Batch 1659 : 3−hydroxybutyrate (BHBA) on Arthropathy in ulcerative colitis
MR Test
Inverse variance weighted (fixed effects)
Inverse variance weighted (multiplicative random effects)
MR Egger
MR RAPS
Weighted median
Weighted mode
1.0
0.5
0.0
−0.5
0.1
0.2
0.3
0.4
SNP effect on Arthropathy in ulcerative colitis
SNP effect on 3−hydroxybutyrate (BHBA)

Batch 1660 : Arabinose on Arthropathy in ulcerative colitis

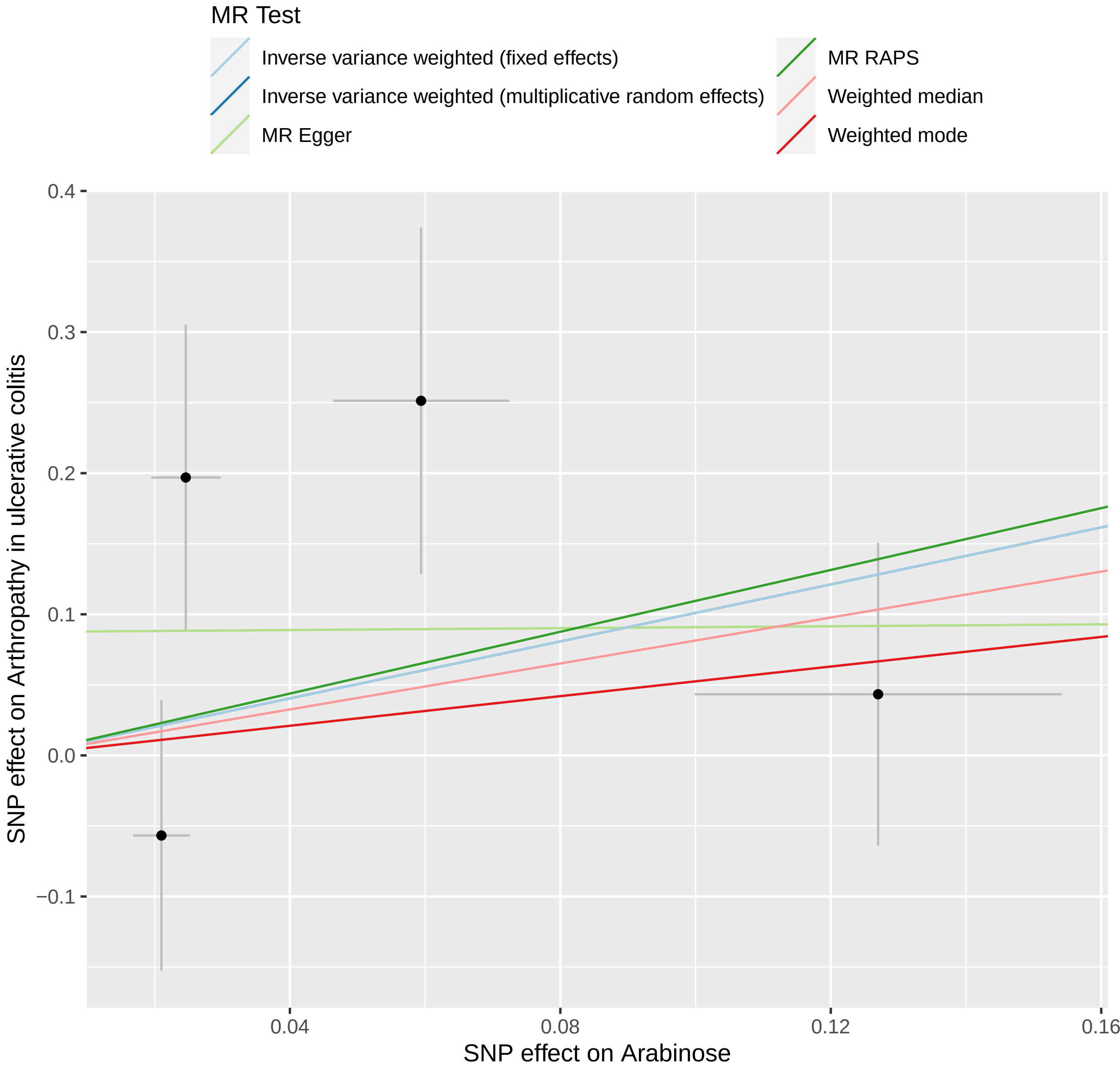

Batch 1661 : Fructose on Arthropathy in ulcerative colitis

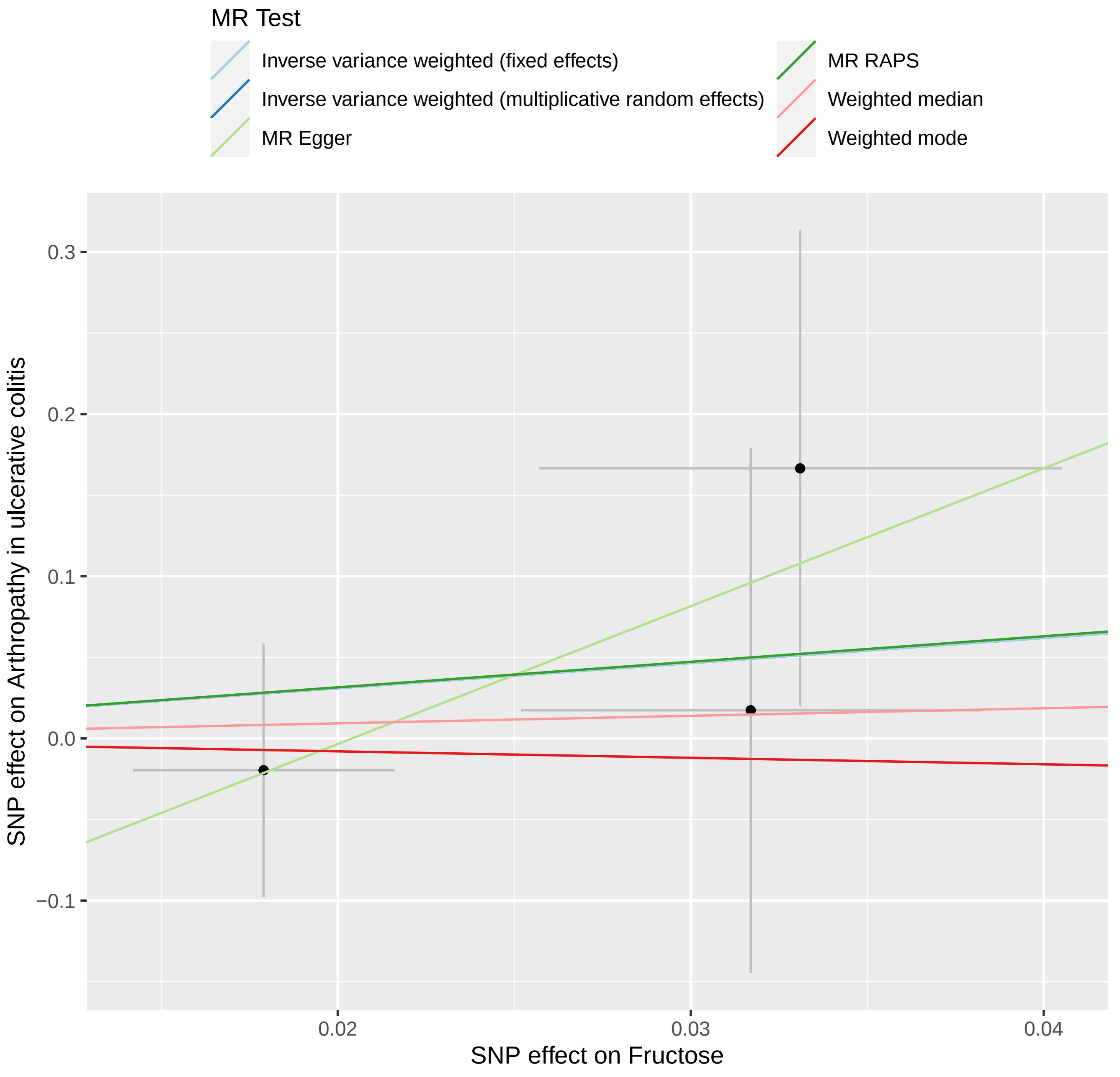

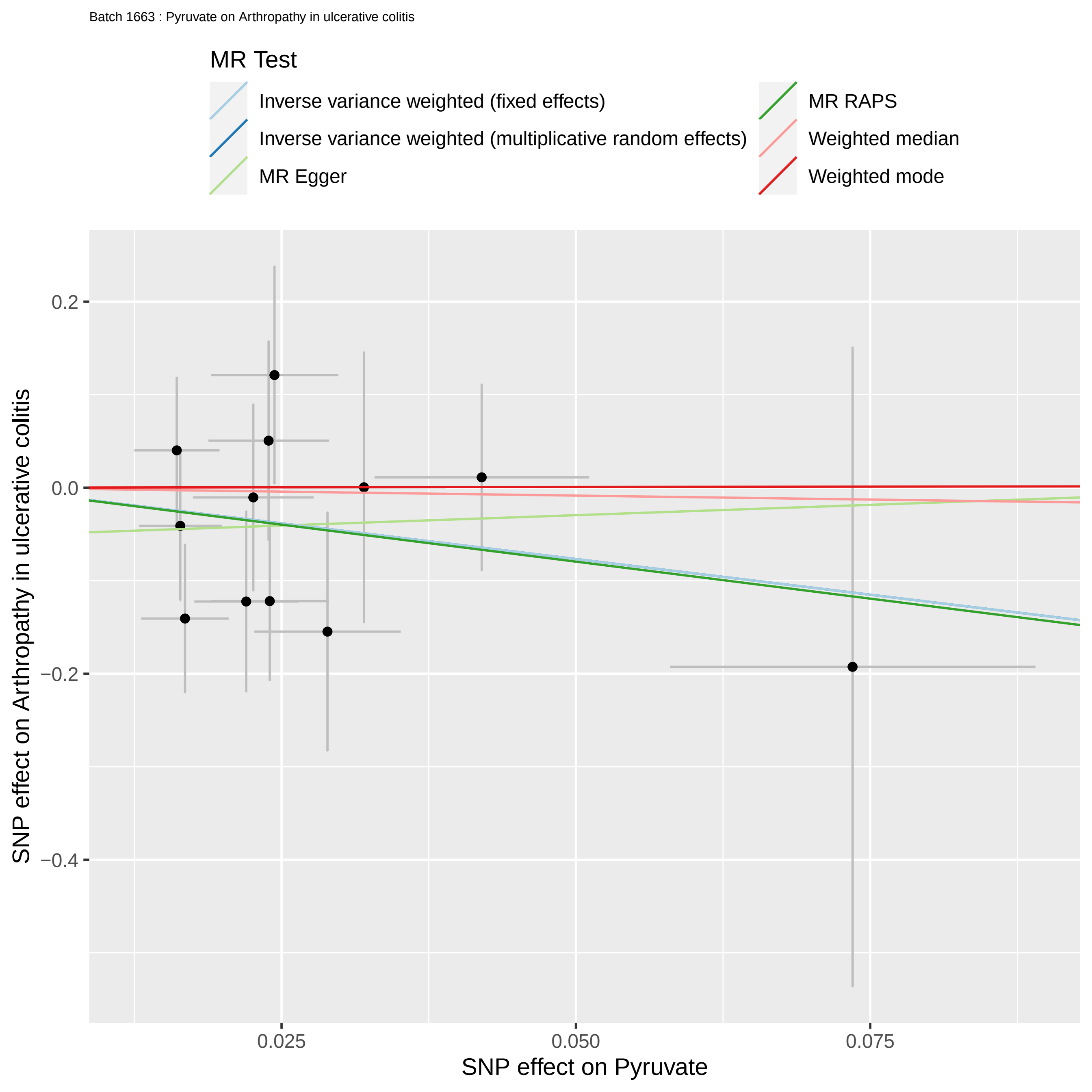

Batch 1663 : Pyruvate on Arthropathy in ulcerative colitis
MR Test
Inverse variance weighted (fixed effects)
Inverse variance weighted (multiplicative random effects)
MR Egger
MR RAPS
Weighted median
Weighted mode
0.2
0.0
−0.2
−0.4
0.025
0.050
0.075
SNP effect on Arthropathy in ulcerative colitis
SNP effect on Pyruvate

Batch 1664 : Uridine on Arthropathy in ulcerative colitis

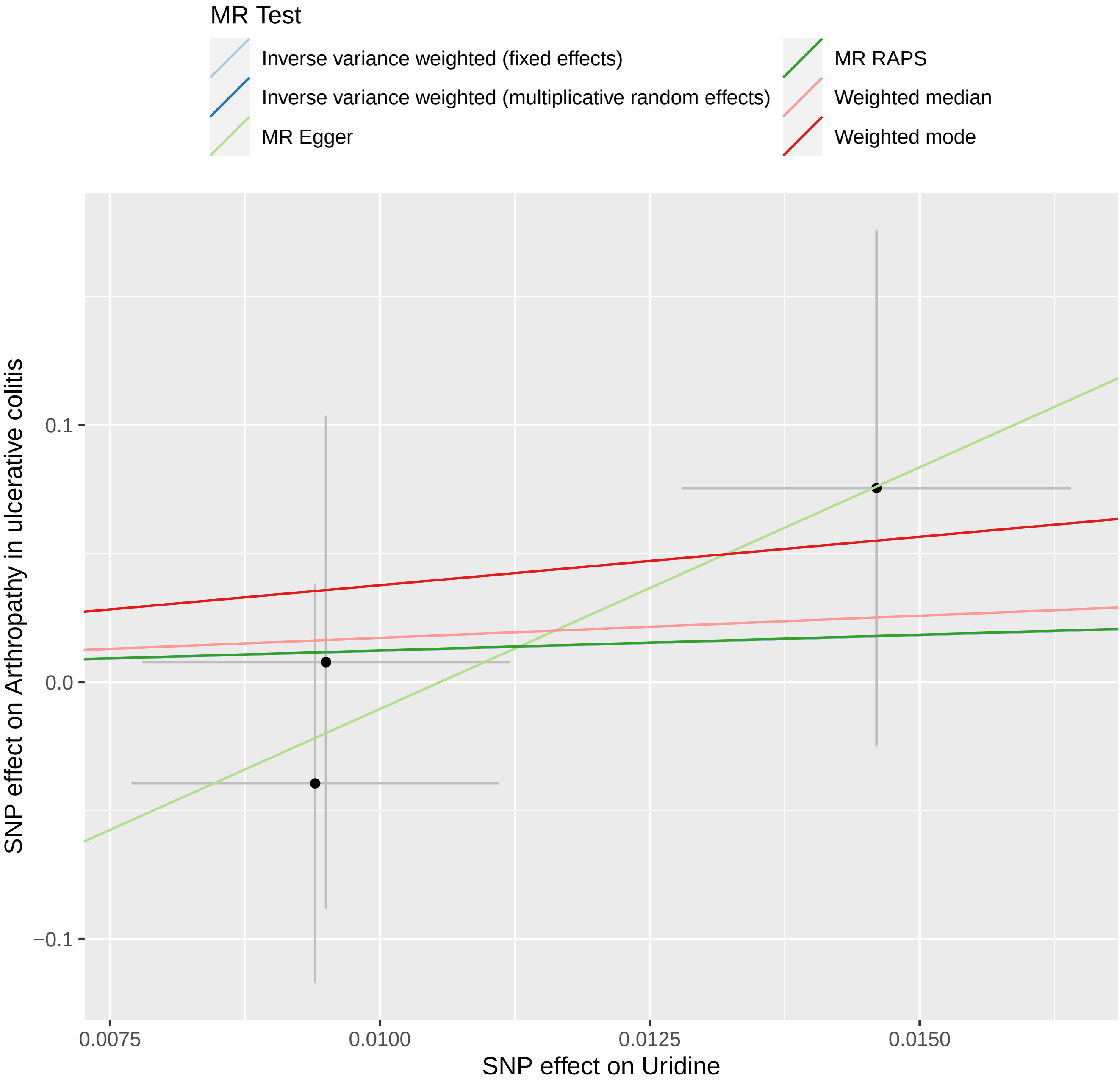

Batch 1665 : Linoleate (18:2n6) on Arthropathy in ulcerative colitis

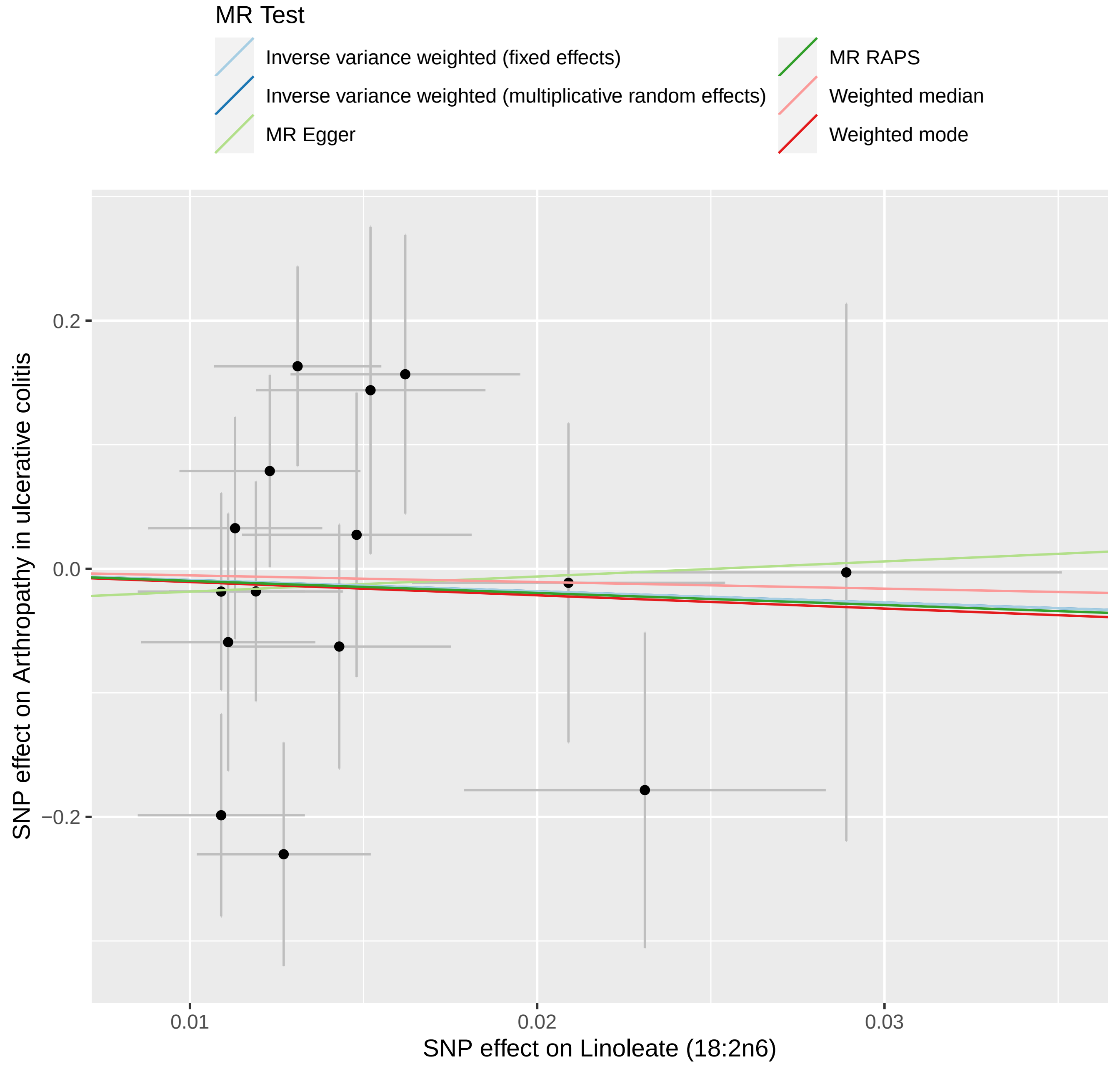

Batch 1666 : Allantoin on Arthropathy in ulcerative colitis

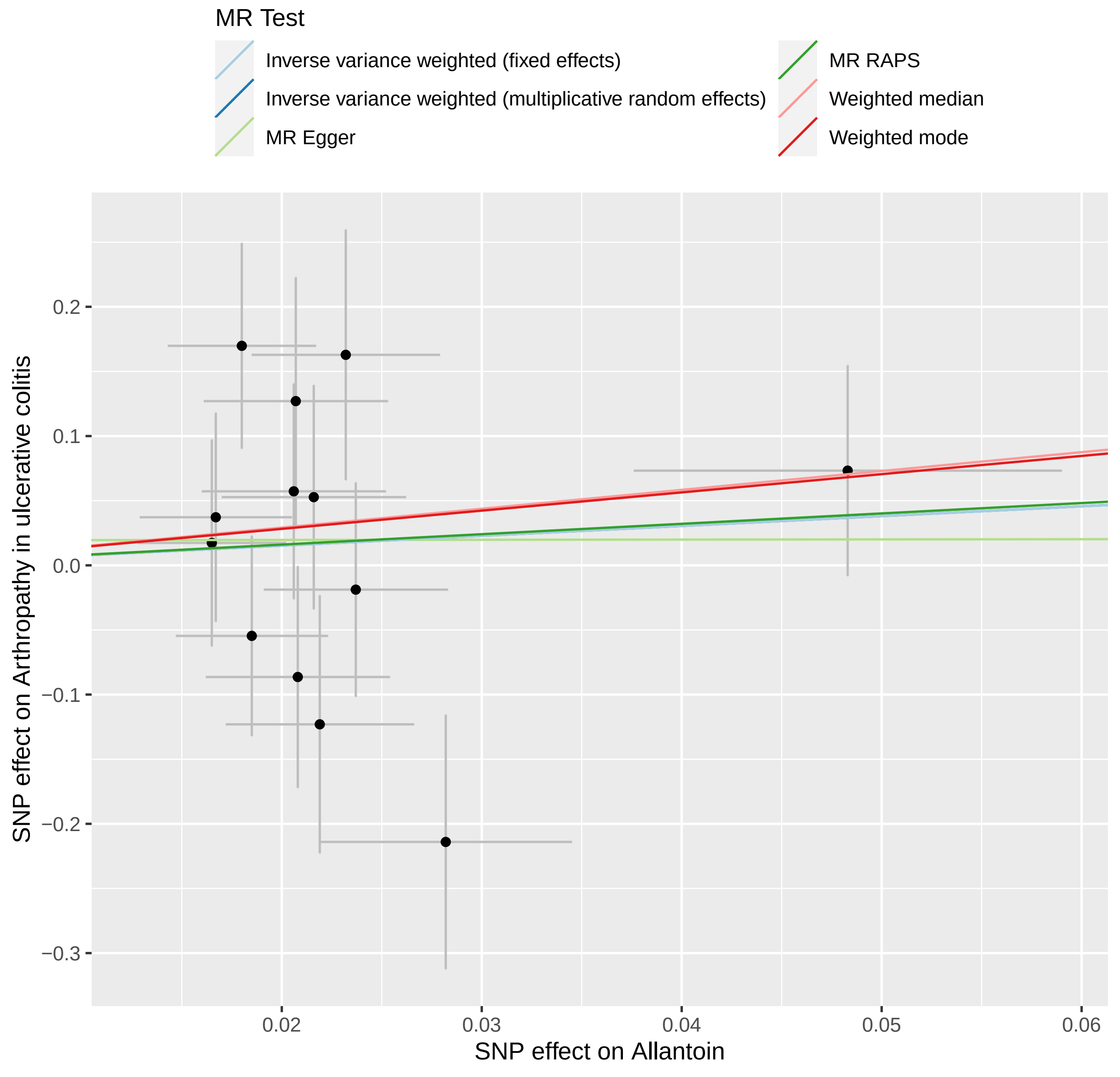

Batch 1671 : Isoleucine on Arthropathy in ulcerative colitis

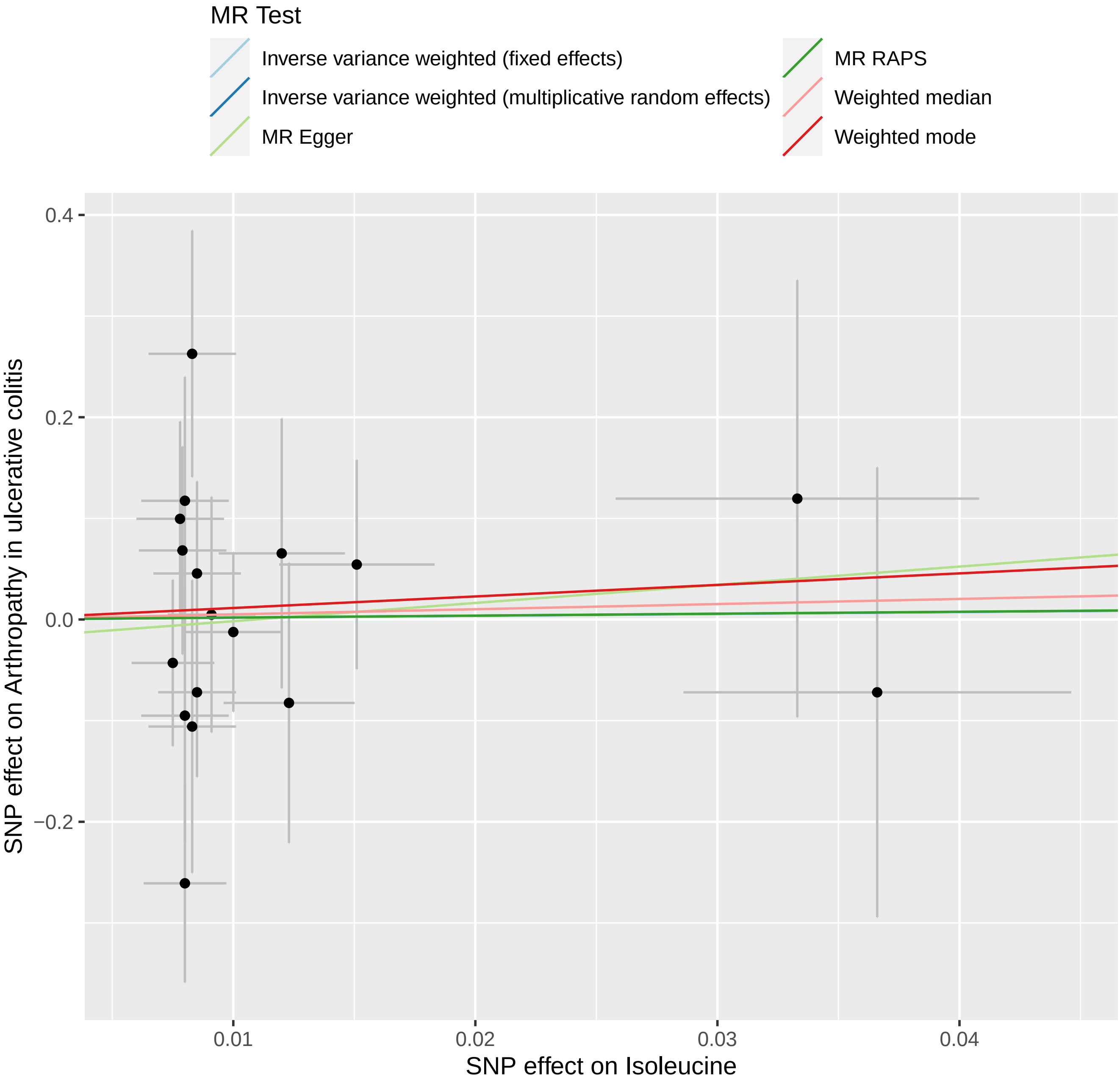

Batch 1672 : Threonine on Arthropathy in ulcerative colitis

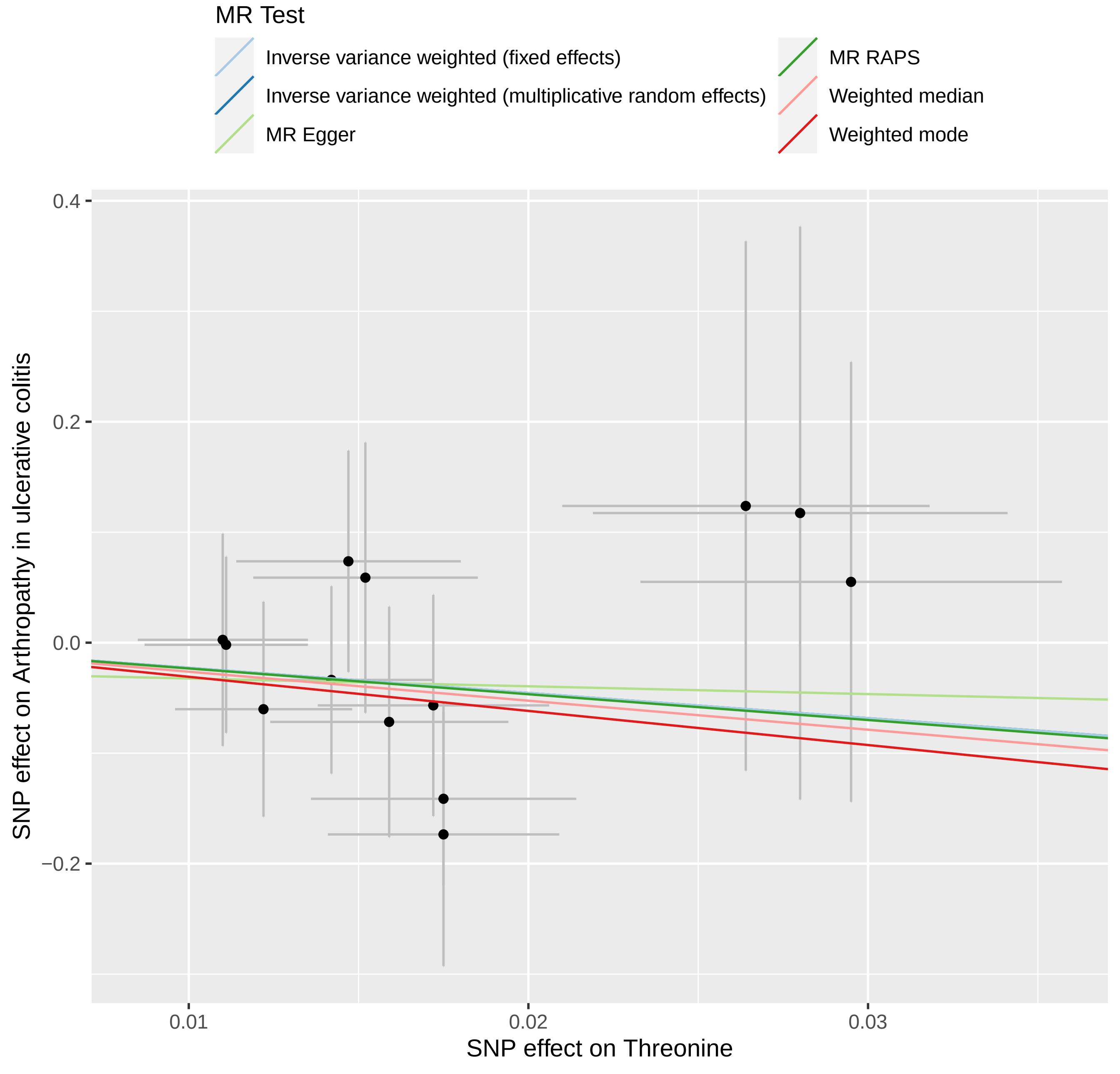

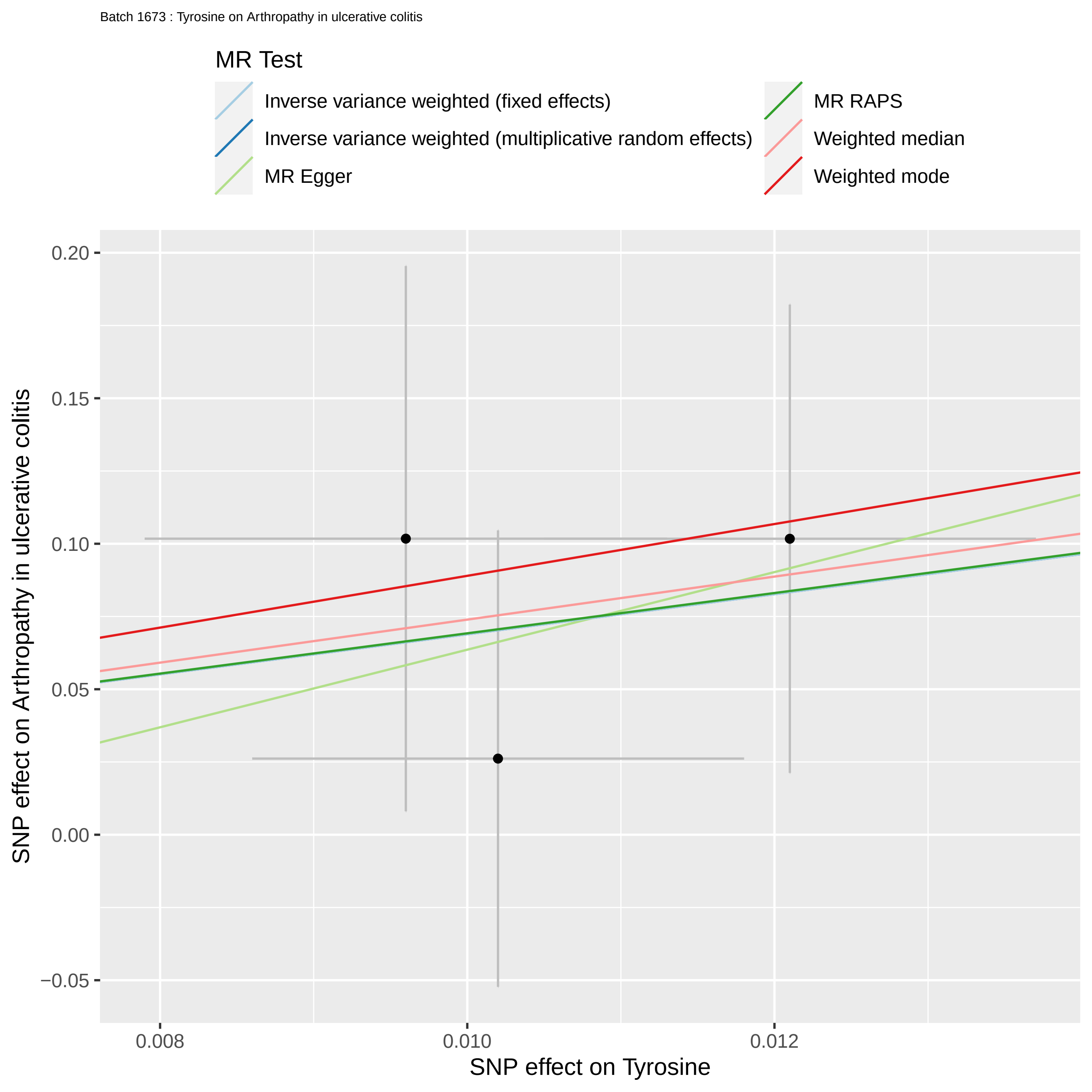

Batch 1673 : Tyrosine on Arthropathy in ulcerative colitis
MR Test
Inverse variance weighted (fixed effects)
Inverse variance weighted (multiplicative random effects)
MR Egger
MR RAPS
Weighted median
Weighted mode
0.20
0.15
0.10
0.05
0.00
−0.05
0.008
0.010
0.012
SNP effect on Tyrosine
SNP effect on Arthropathy in ulcerative colitis

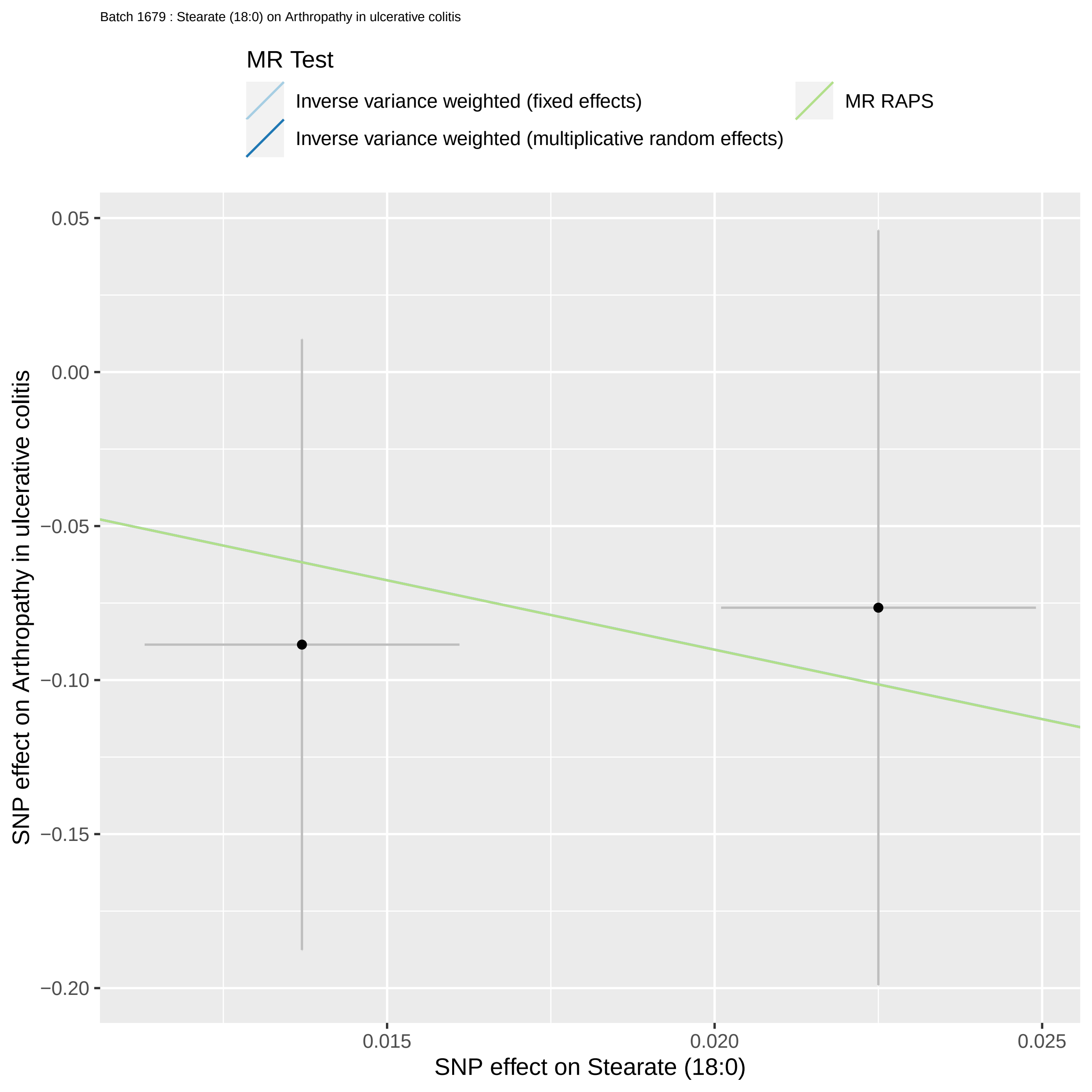

Batch 1679 : Stearate (18:0) on Arthropathy in ulcerative colitis
MR Test
Inverse variance weighted (fixed effects)
Inverse variance weighted (multiplicative random effects)
MR RAPS
0.05
0.00
−0.05
−0.10
−0.15
−0.20
0.015
0.020
0.025
SNP effect on Arthropathy in ulcerative colitis
SNP effect on Stearate (18:0)

Batch 1682 : Myristate (14:0) on Arthropathy in ulcerative colitis

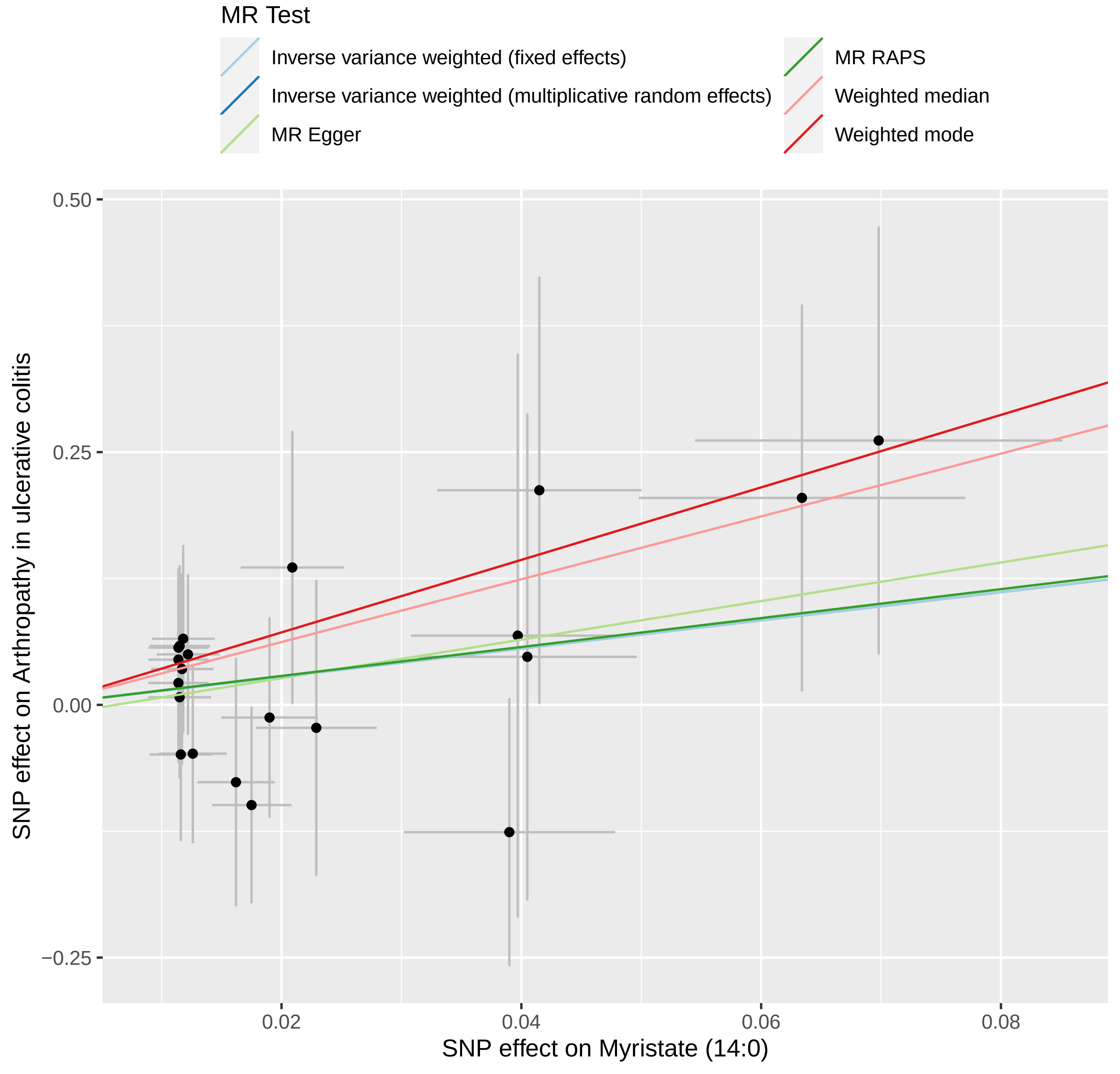

Batch 1684 : Ornithine on Arthropathy in ulcerative colitis

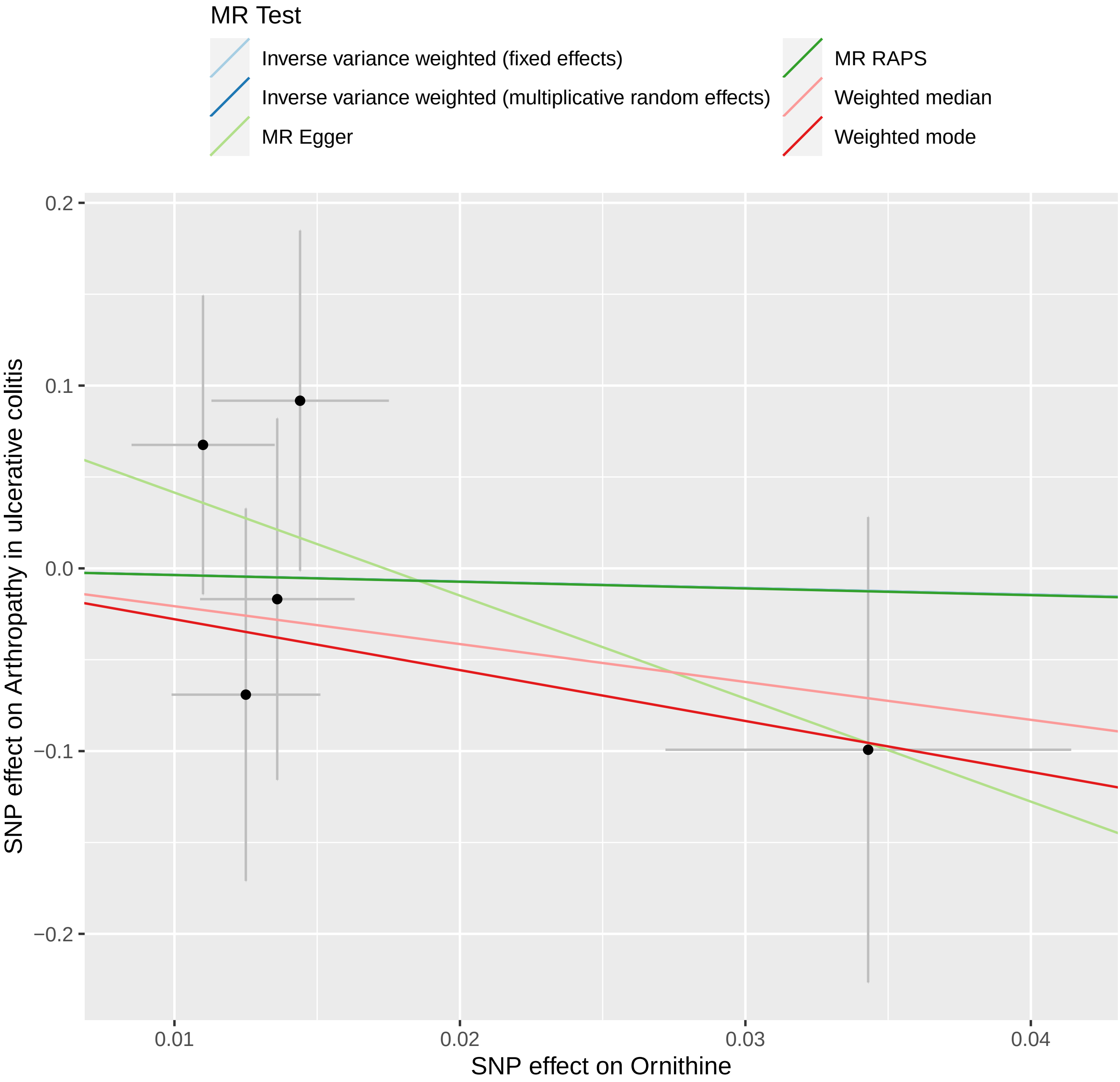

Batch 1688 : Alpha−tocopherol on Arthropathy in ulcerative colitis

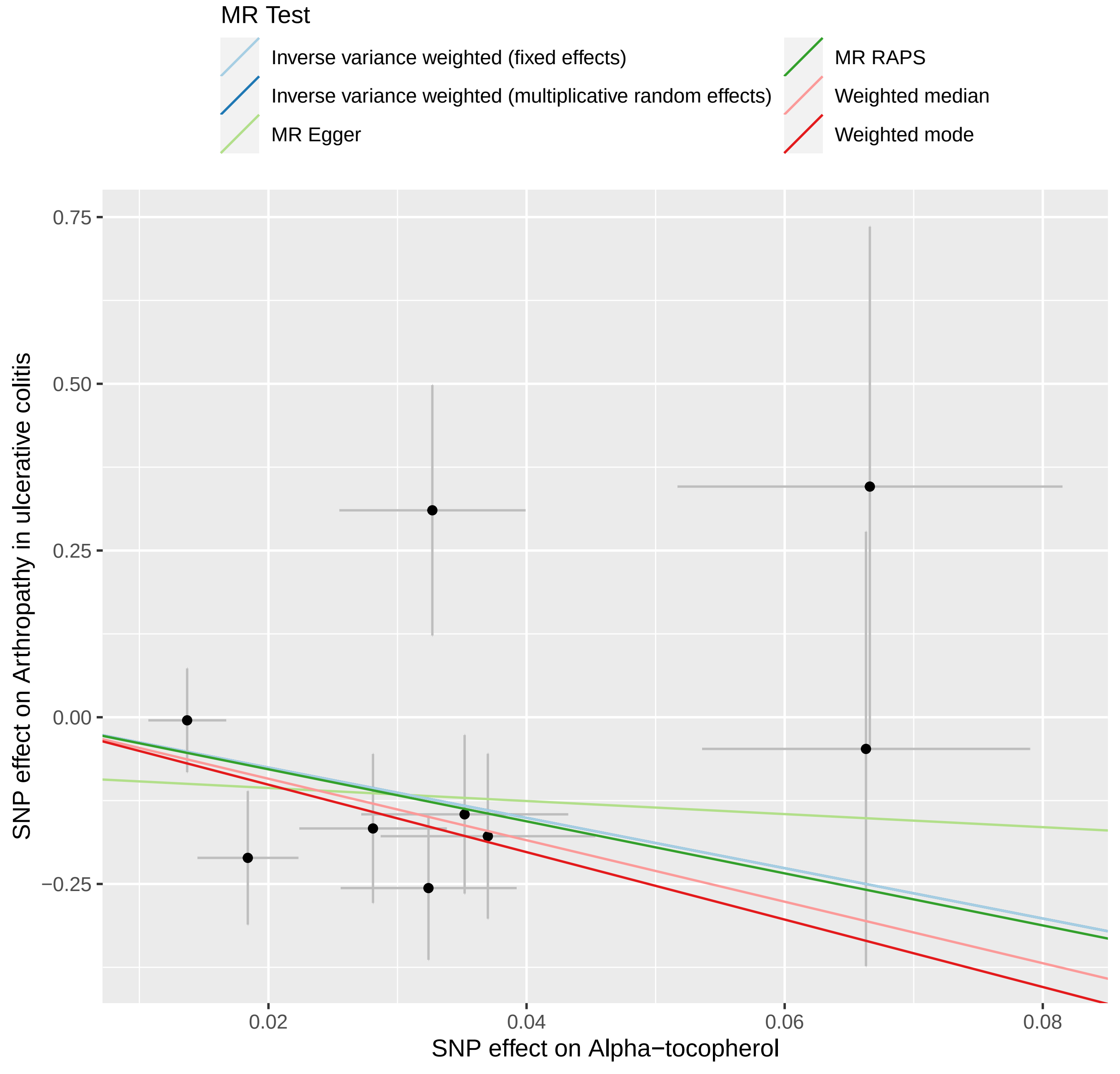

Batch 1689 : Citrate on Arthropathy in ulcerative colitis

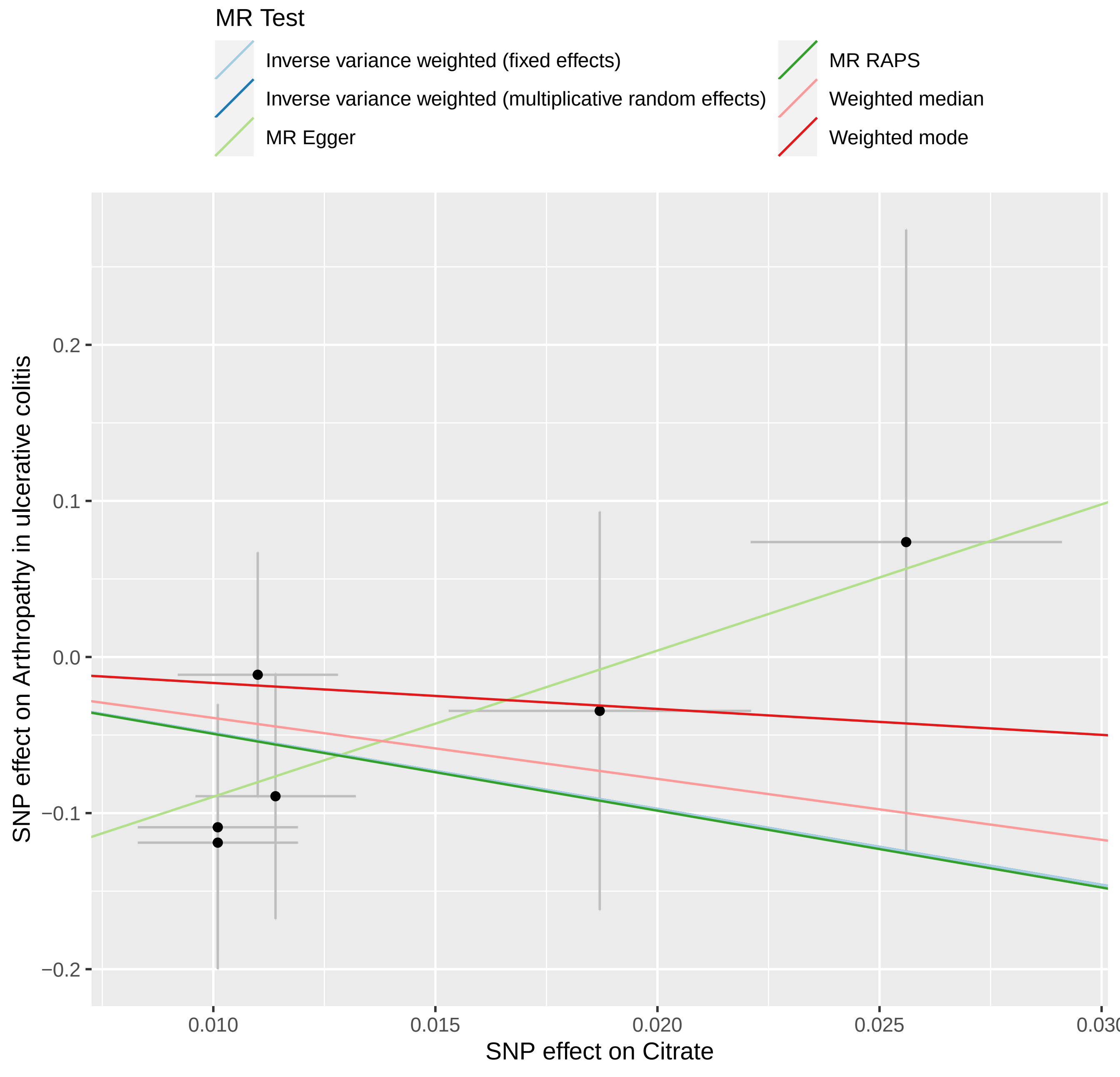

Batch 1690 : Glycerate on Arthropathy in ulcerative colitis

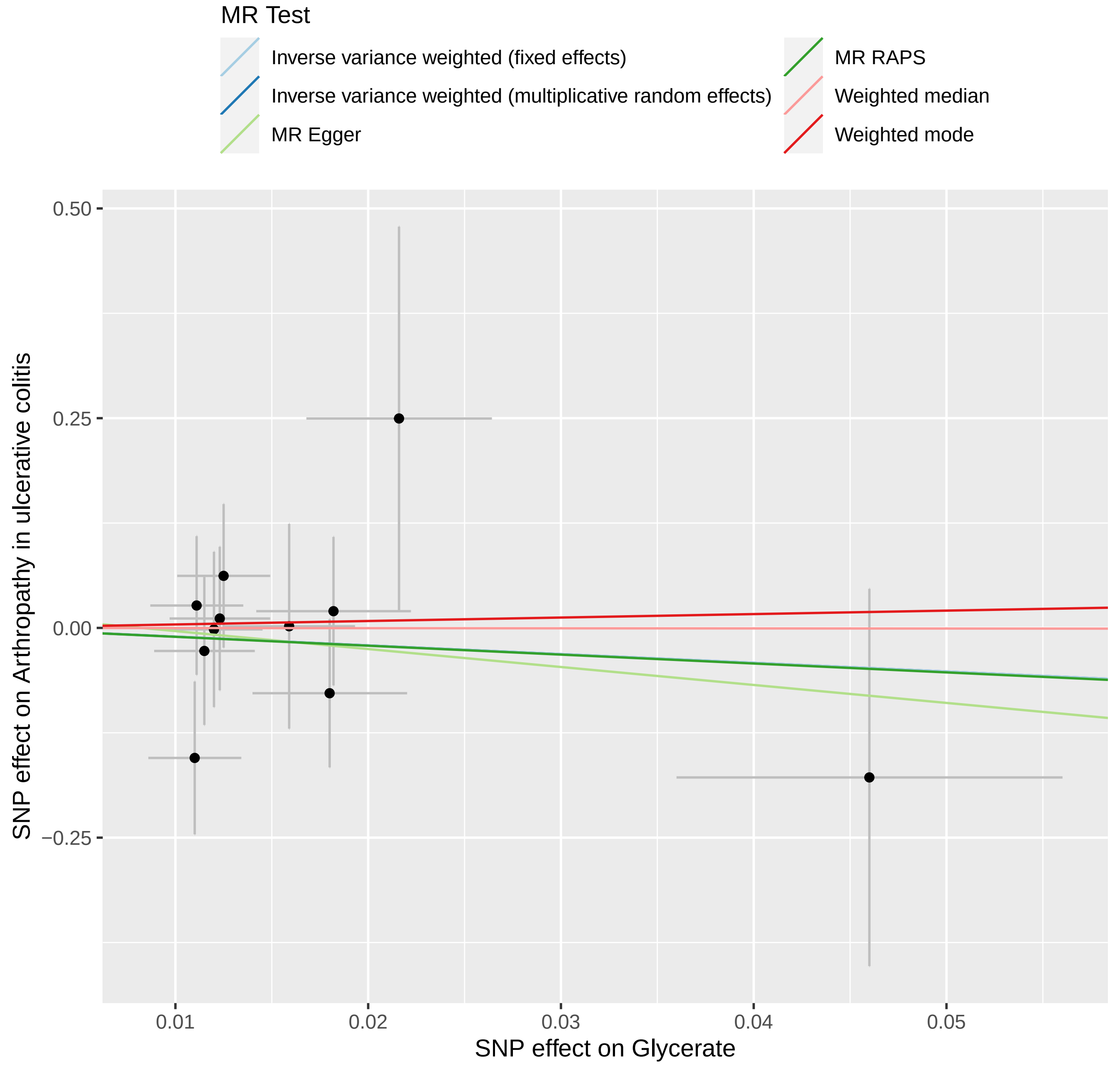

Batch 1691 : Guanosine on Arthropathy in ulcerative colitis

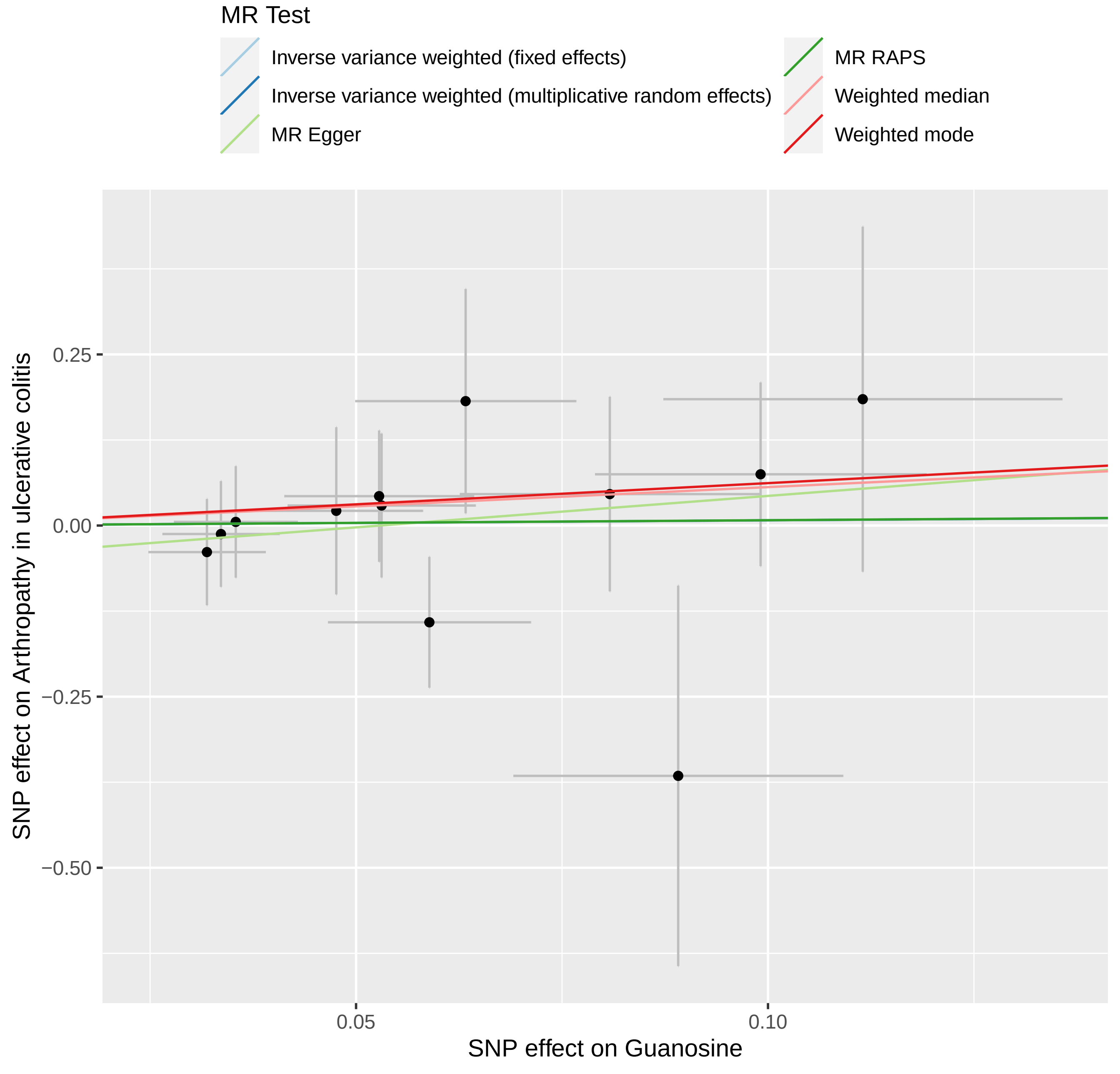

Batch 1694 : Ursodeoxycholate on Arthropathy in ulcerative colitis

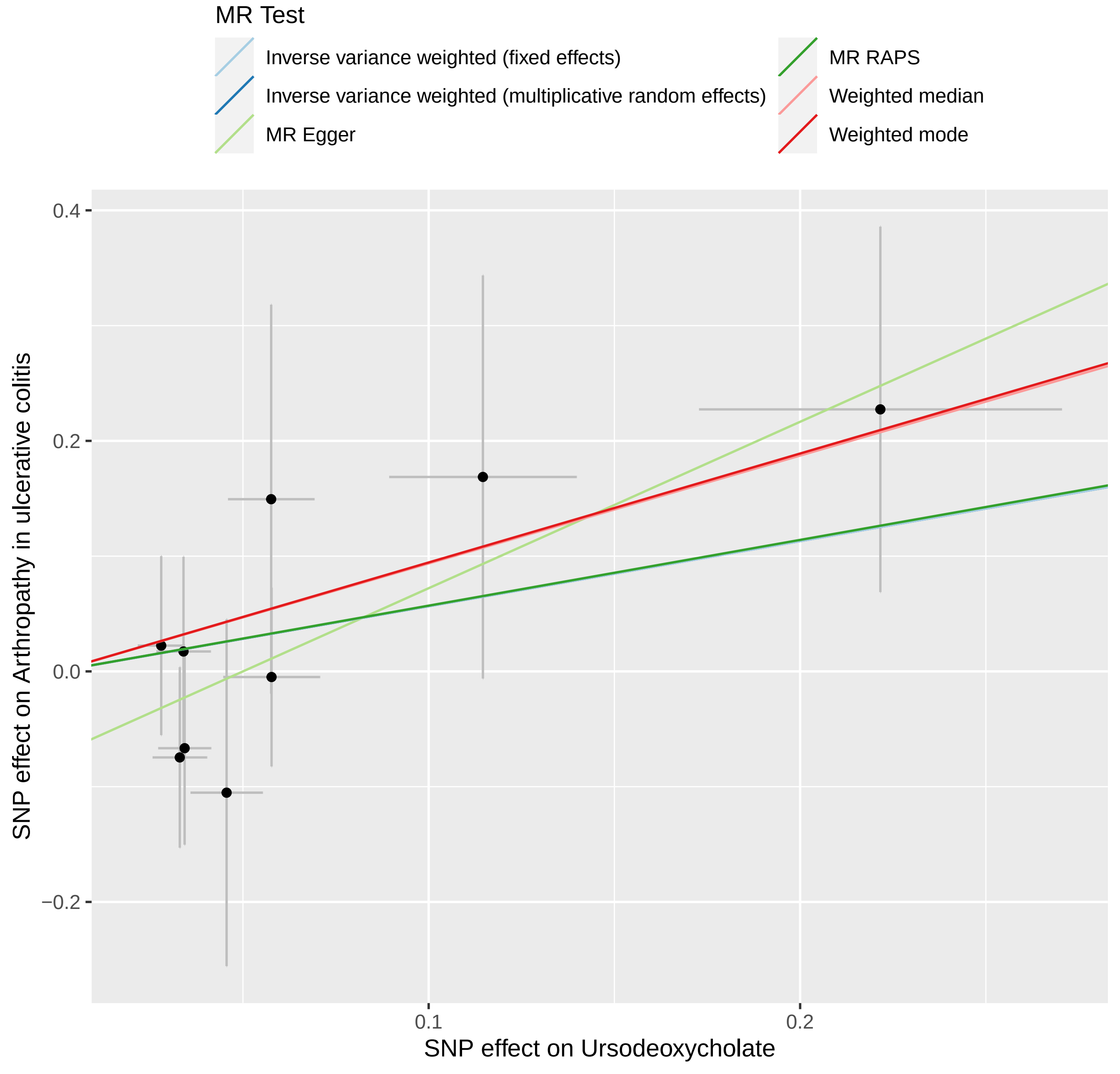

Batch 1695 : Arginine on Arthropathy in ulcerative colitis

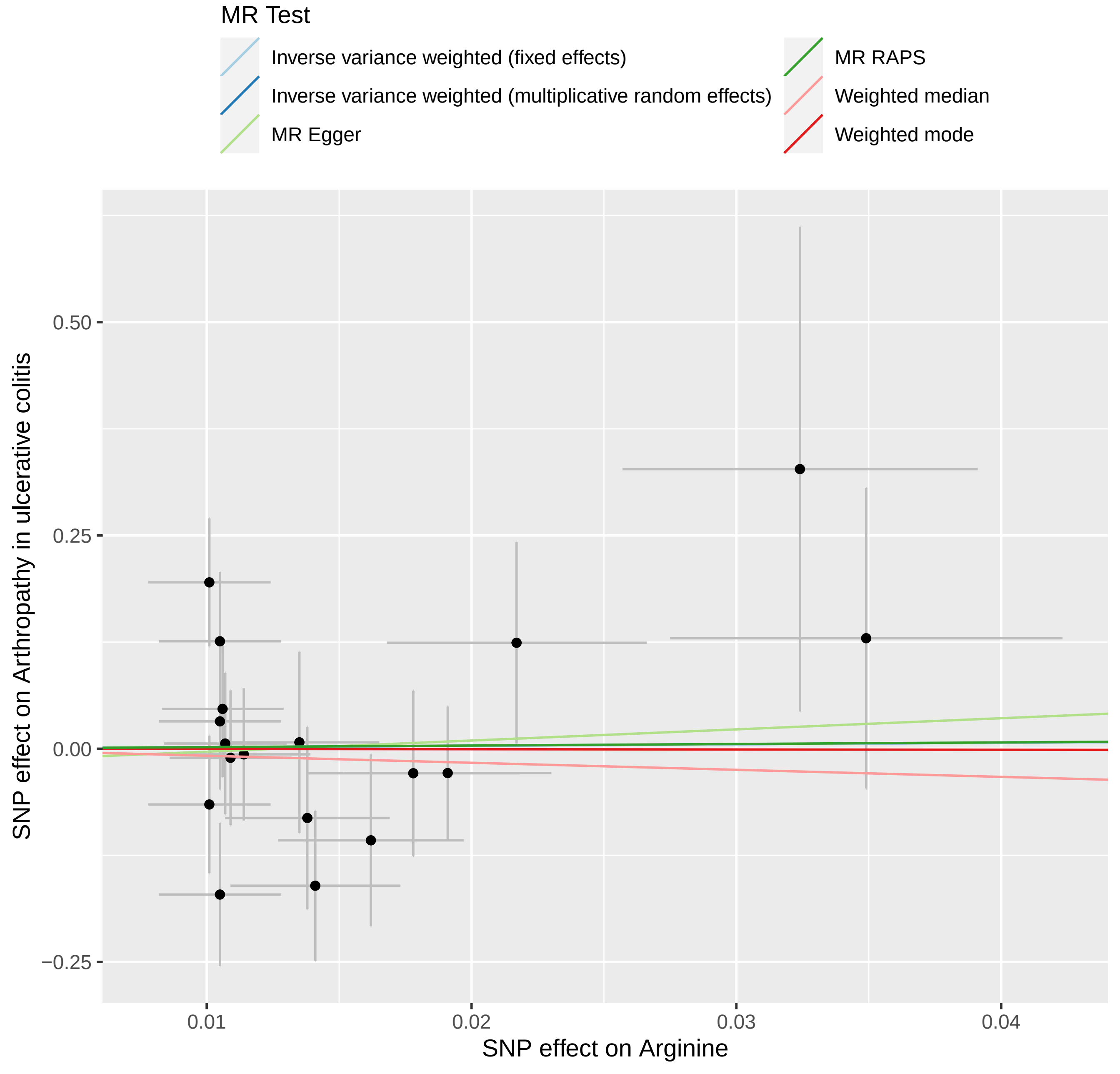

Batch 1696 : Ascorbate (Vitamin C) on Arthropathy in ulcerative colitis

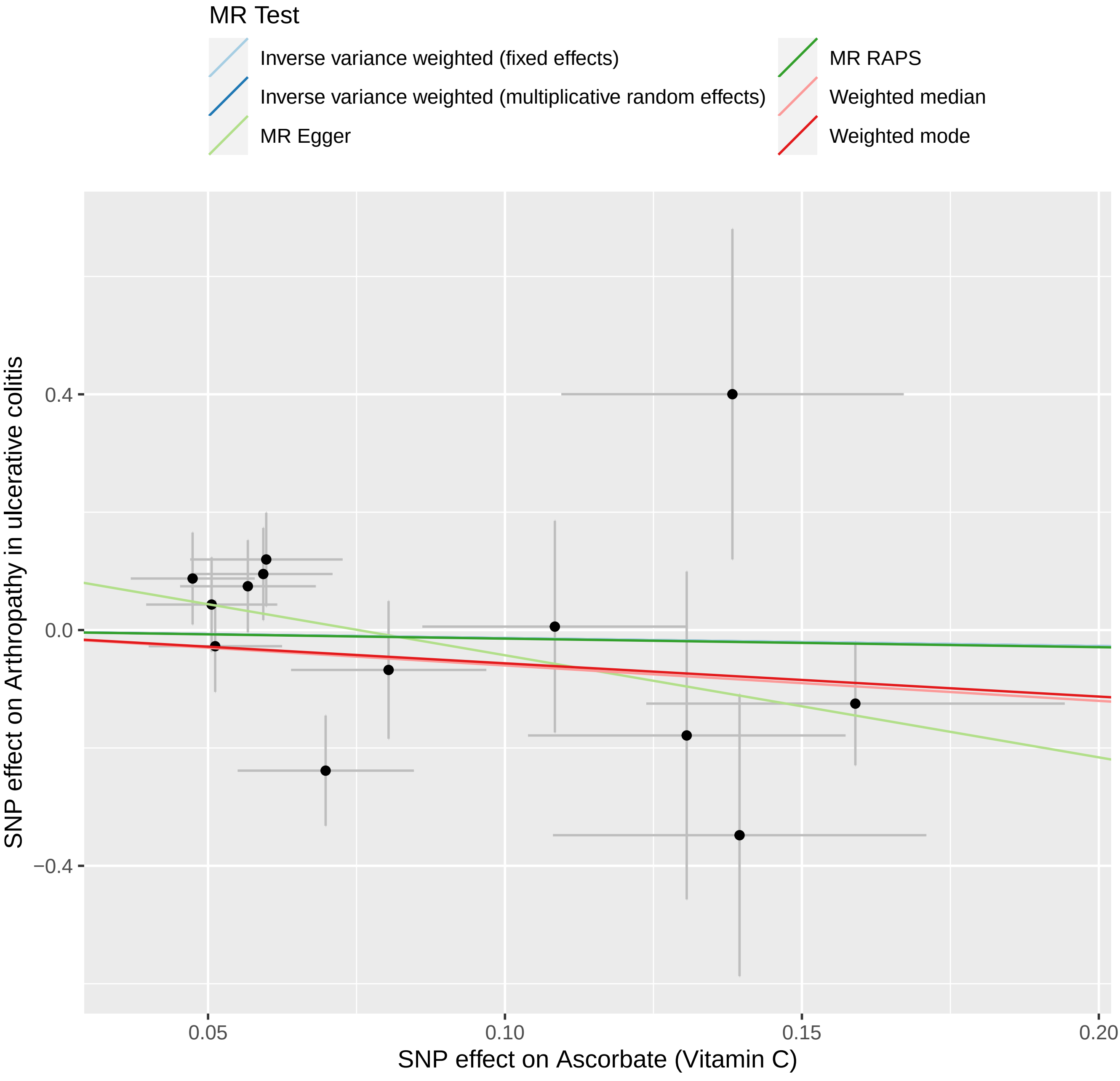

Batch 1697 : Heptanoate (7:0) on Arthropathy in ulcerative colitis

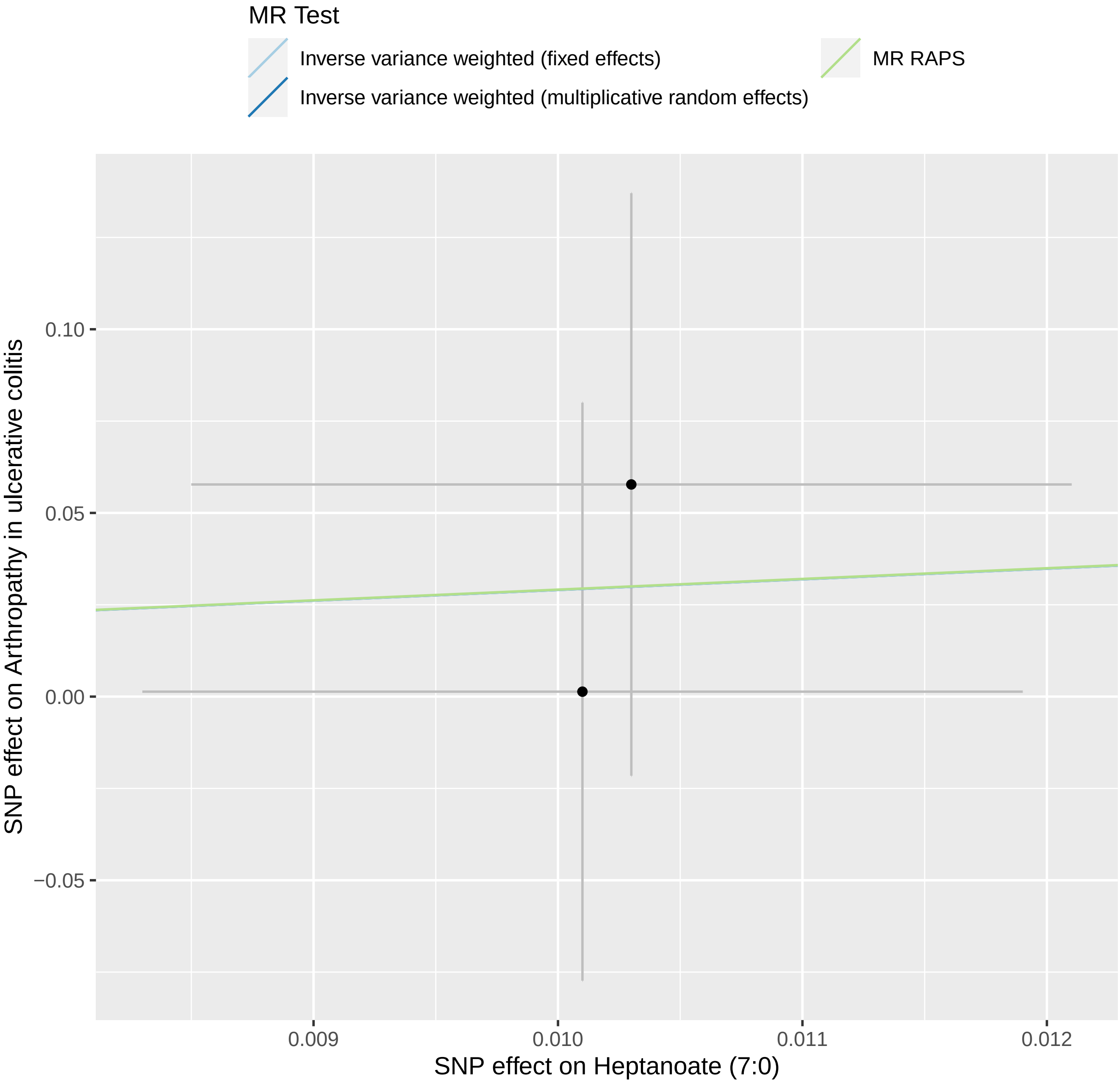

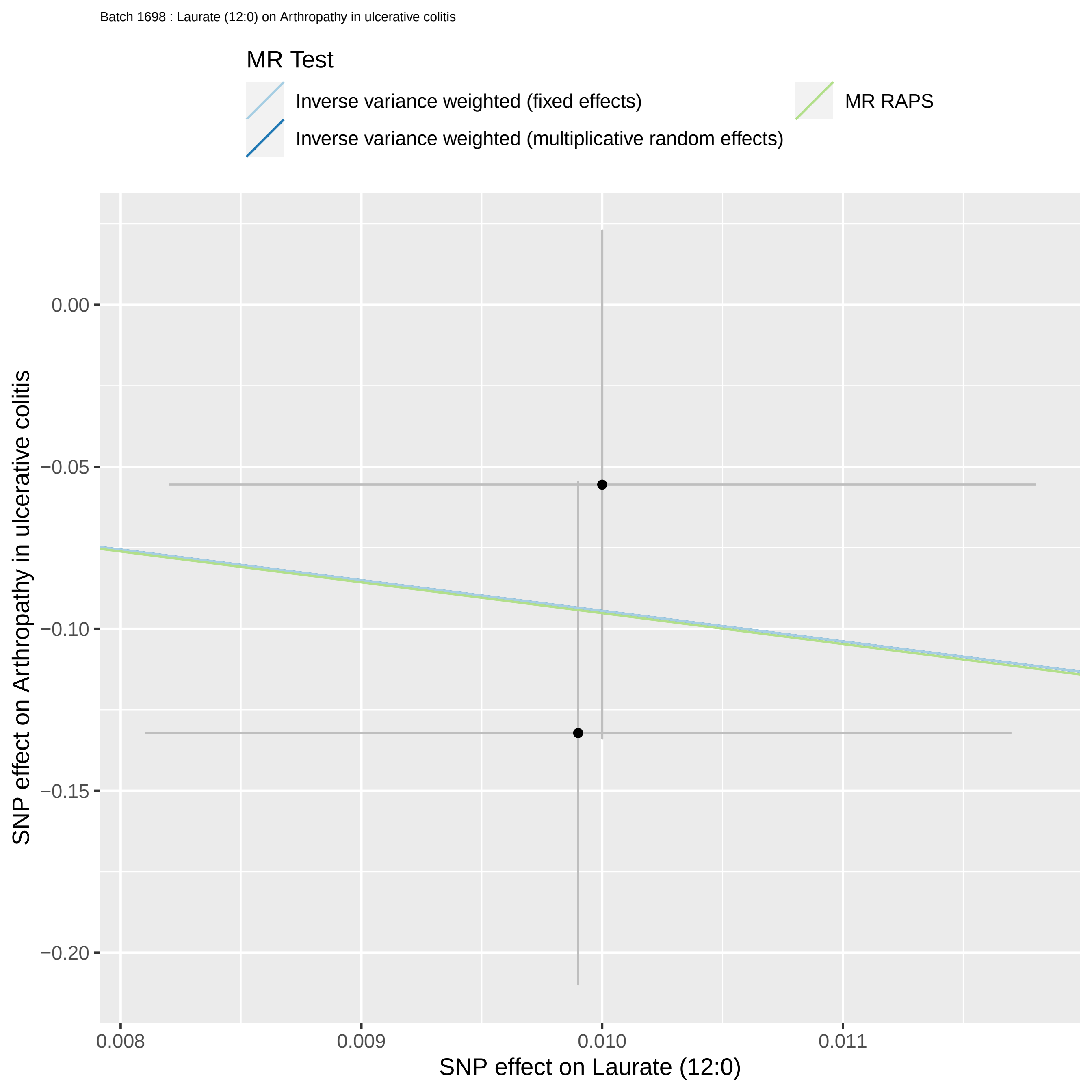

Batch 1698 : Laurate (12:0) on Arthropathy in ulcerative colitis
MR Test
Inverse variance weighted (fixed effects)
Inverse variance weighted (multiplicative random effects)
MR RAPS
0.00
−0.05
−0.10
−0.15
−0.20
SNP effect on Arthropathy in ulcerative colitis
0.008
0.009
0.010
0.011
SNP effect on Laurate (12:0)

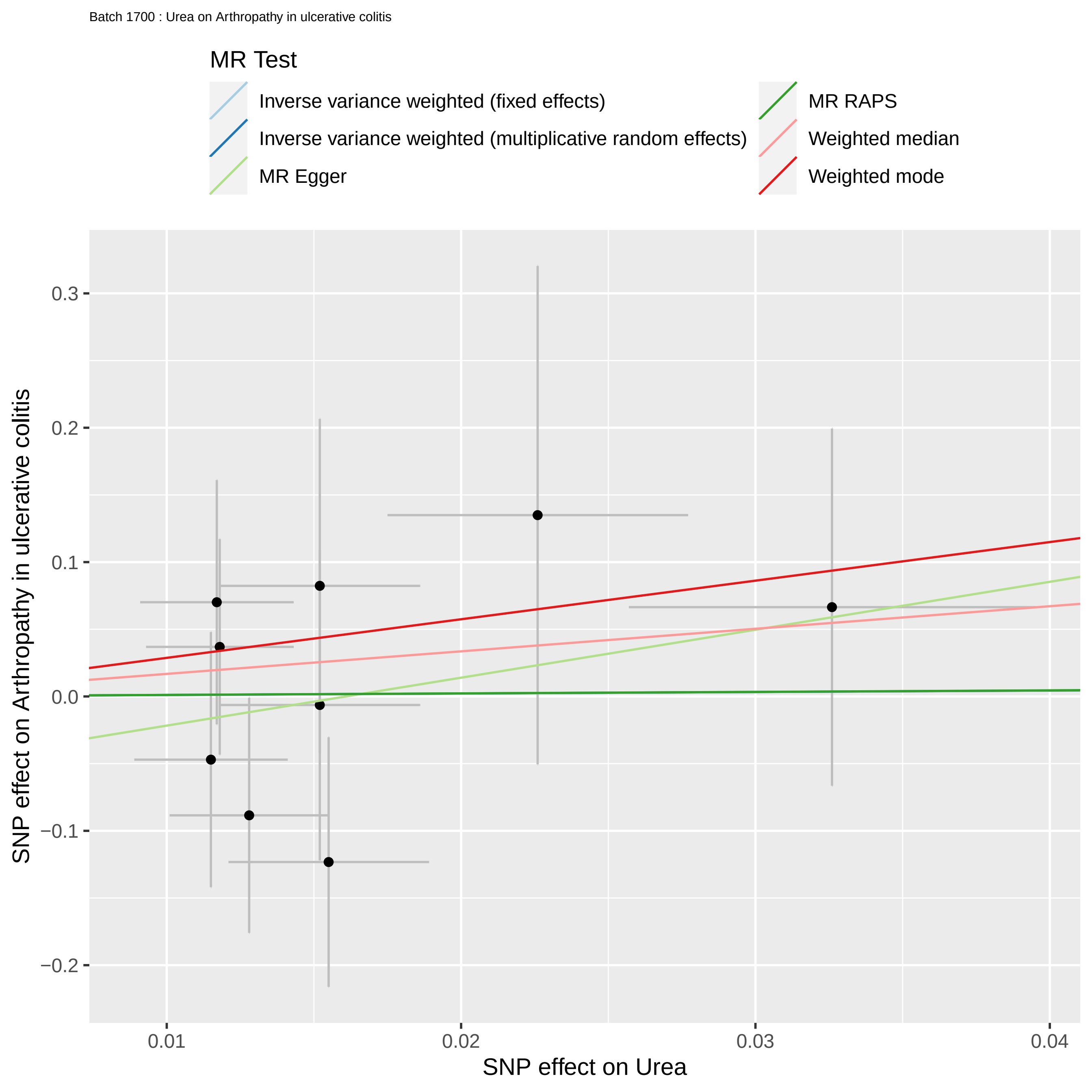
Batch 1700 : Urea on Arthropathy in ulcerative colitis
MR Test
Inverse variance weighted (fixed effects)
Inverse variance weighted (multiplicative random effects)
MR Egger
MR RAPS
Weighted median
Weighted mode
0.3
0.2
0.1
0.0
−0.1
−0.2
0.01
0.02
0.03
0.04
SNP effect on Arthropathy in ulcerative colitis
SNP effect on Urea

Batch 1701 : Cortisol on Arthropathy in ulcerative colitis

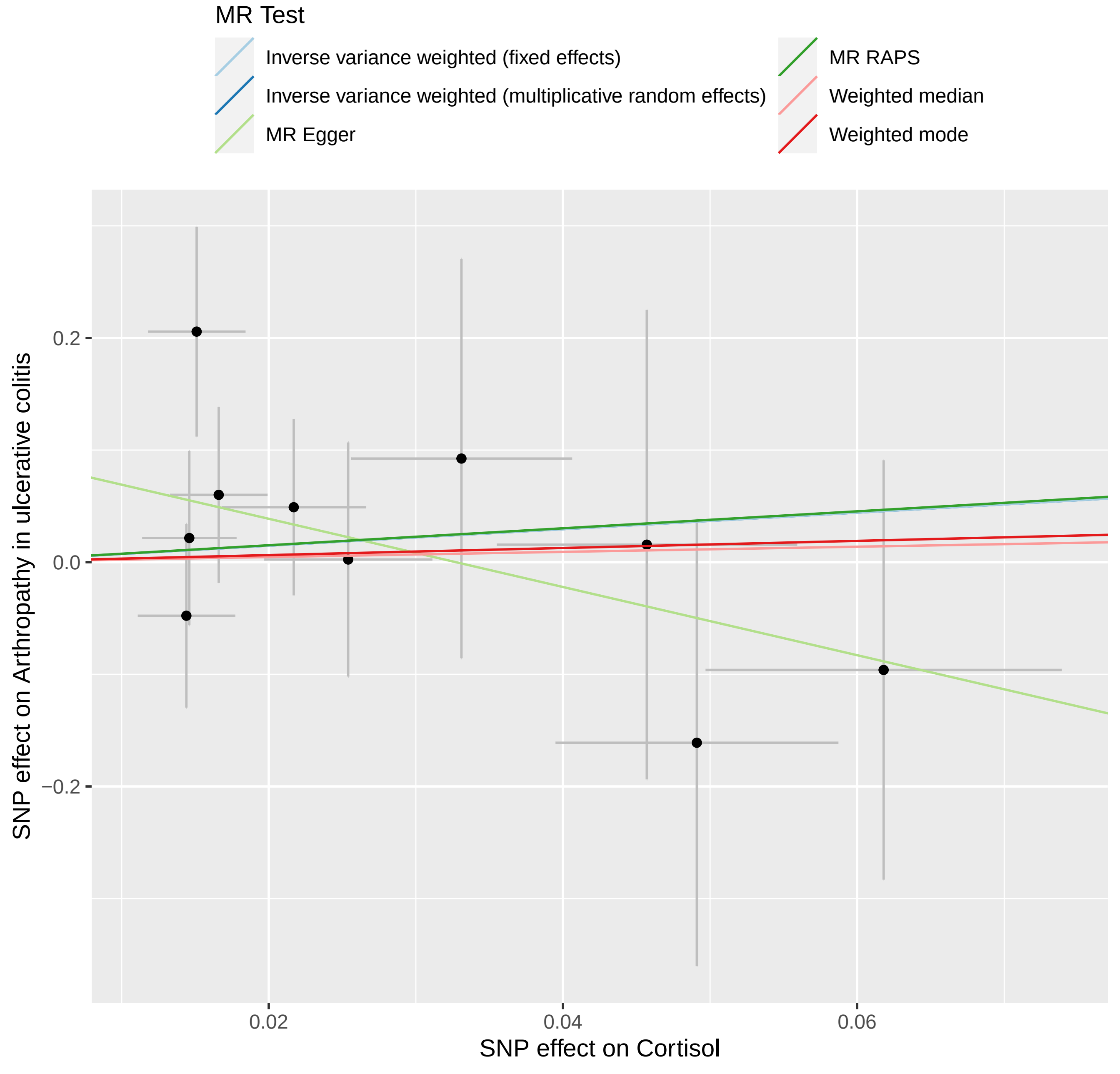

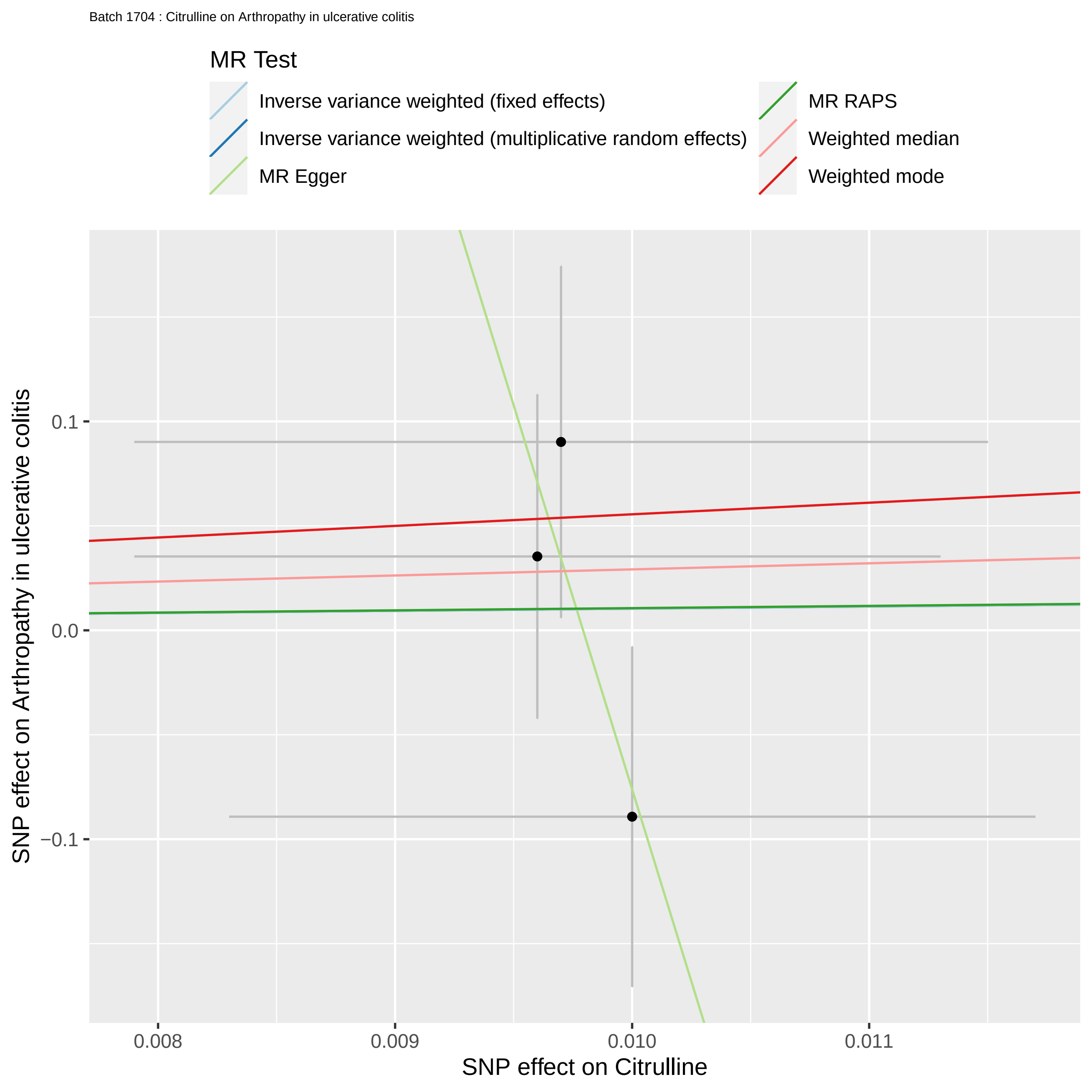
Batch 1704 : Citrulline on Arthropathy in ulcerative colitis
MR Test
Inverse variance weighted (fixed effects)
Inverse variance weighted (multiplicative random effects)
MR Egger
MR RAPS
Weighted median
Weighted mode
0.1
0.0
−0.1
0.008
0.009
0.010
0.011
SNP effect on Citrulline
SNP effect on Arthropathy in ulcerative colitis

Batch 1706 : Serotonin (5HT) on Arthropathy in ulcerative colitis

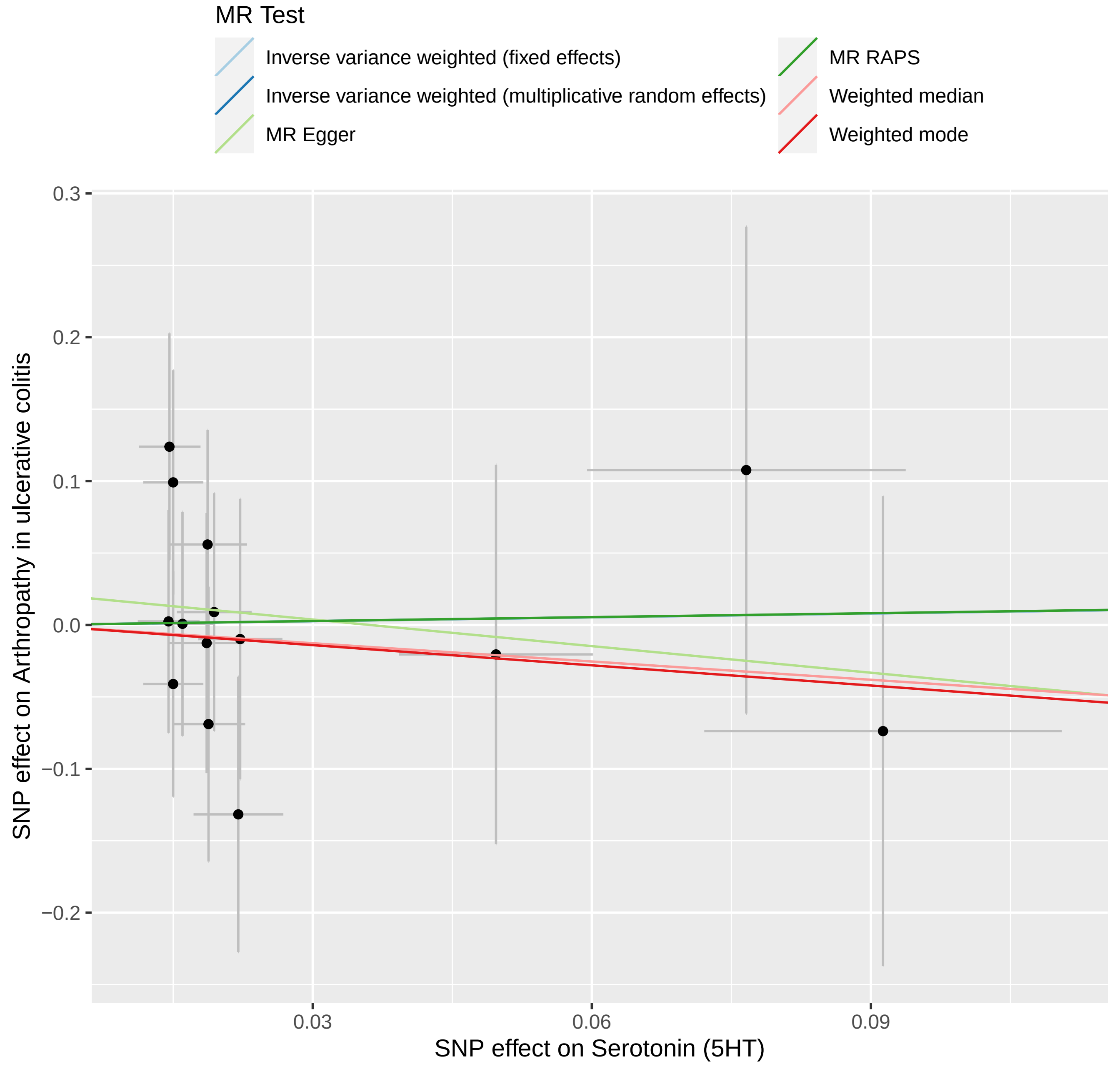

Batch 1707 : Gamma−glutamylglutamine on Arthropathy in ulcerative colitis

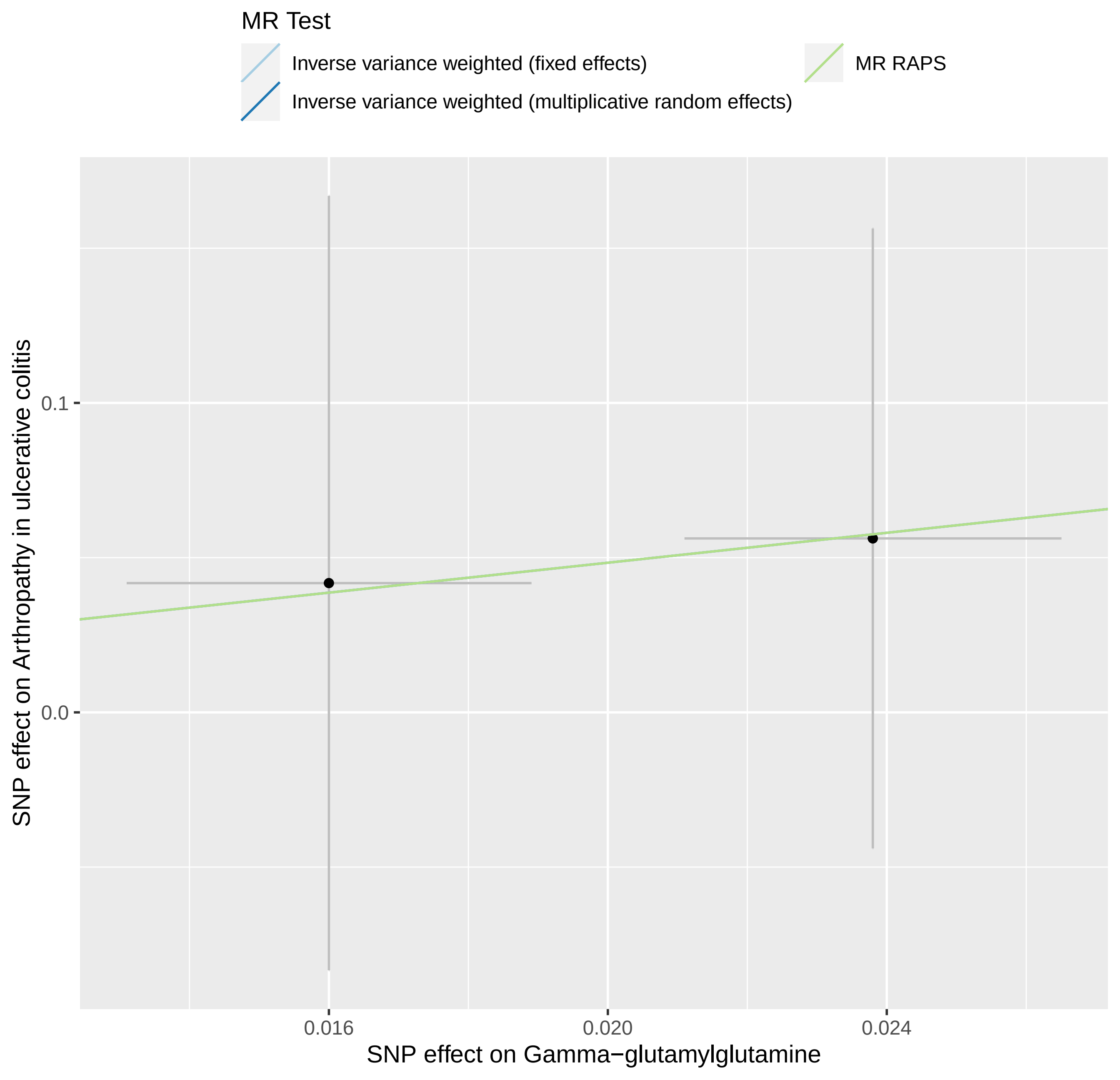

Batch 1708 : Gamma−glutamyltyrosine on Arthropathy in ulcerative colitis

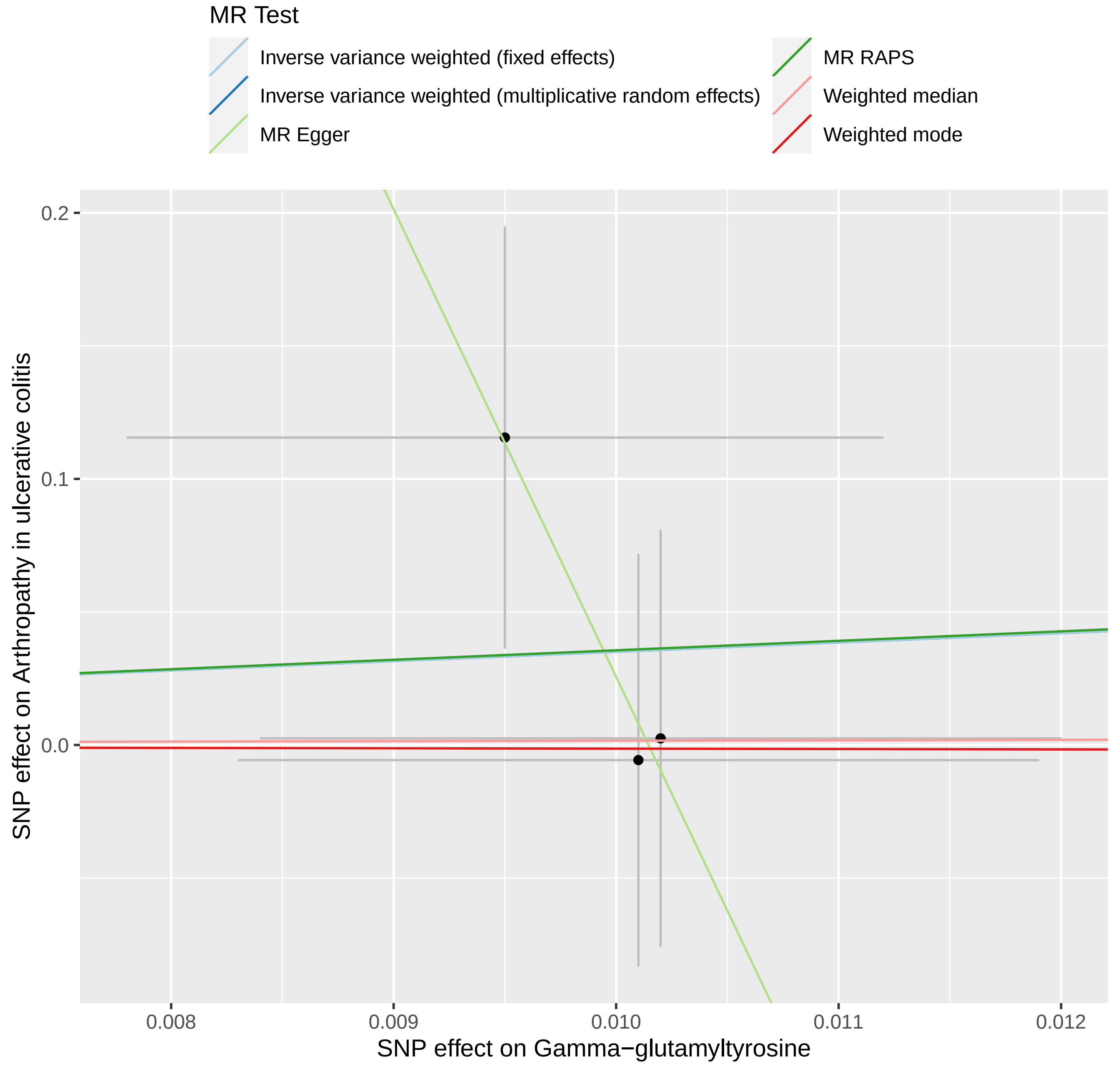

Batch 1710 : Betaine on Arthropathy in ulcerative colitis

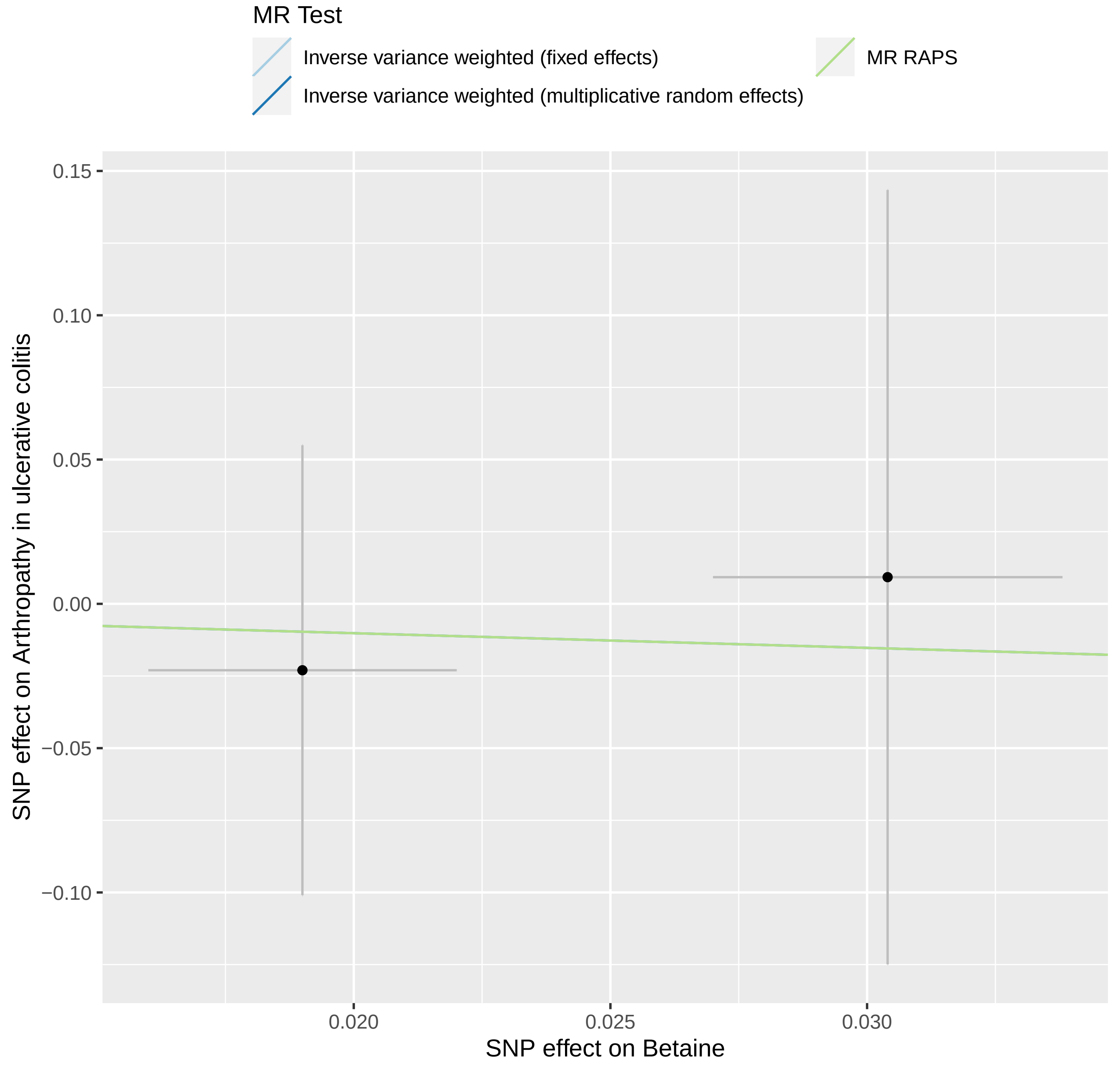

Batch 1711 : Xanthine on Arthropathy in ulcerative colitis

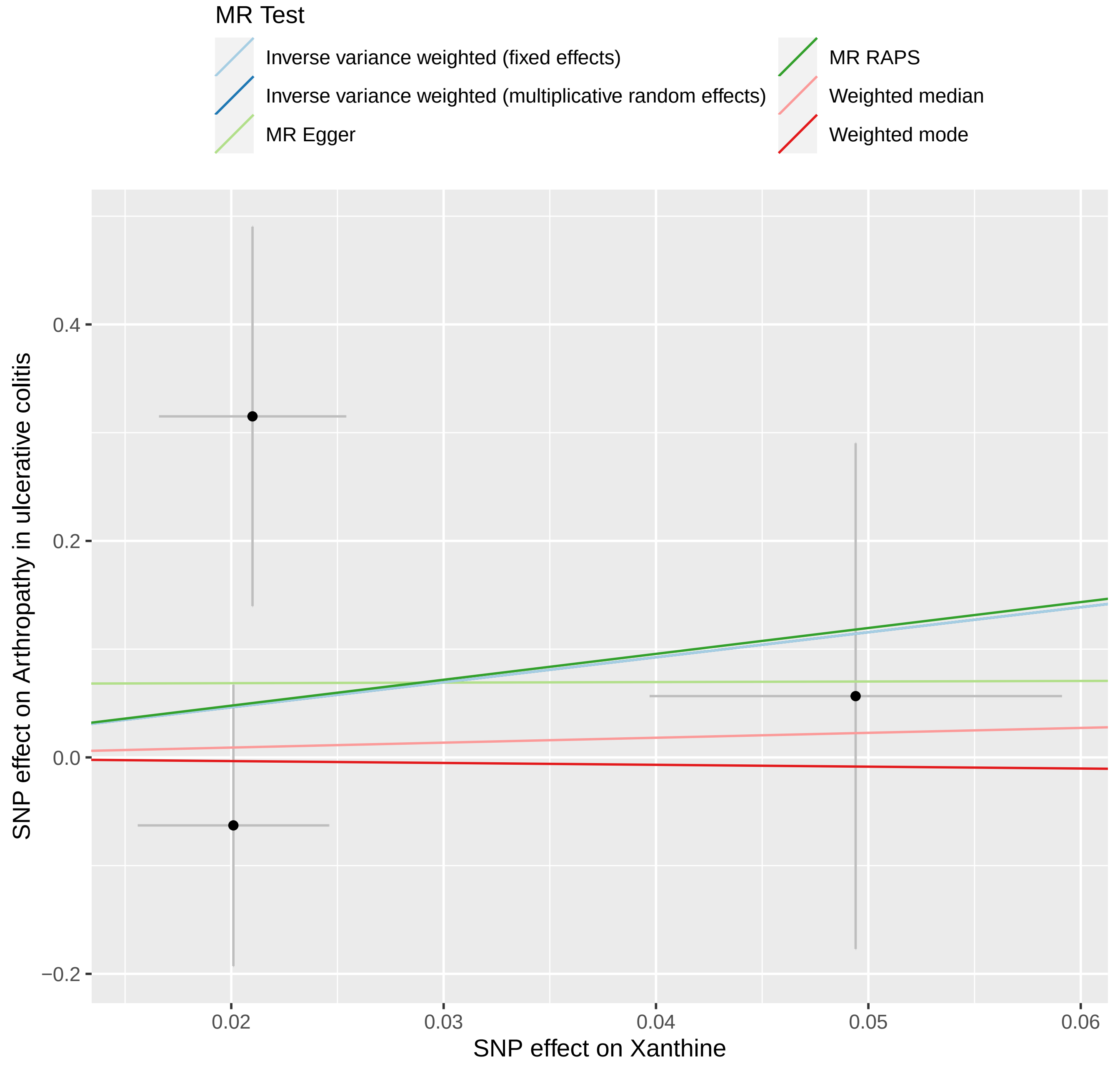

Batch 1712 : Phosphate on Arthropathy in ulcerative colitis

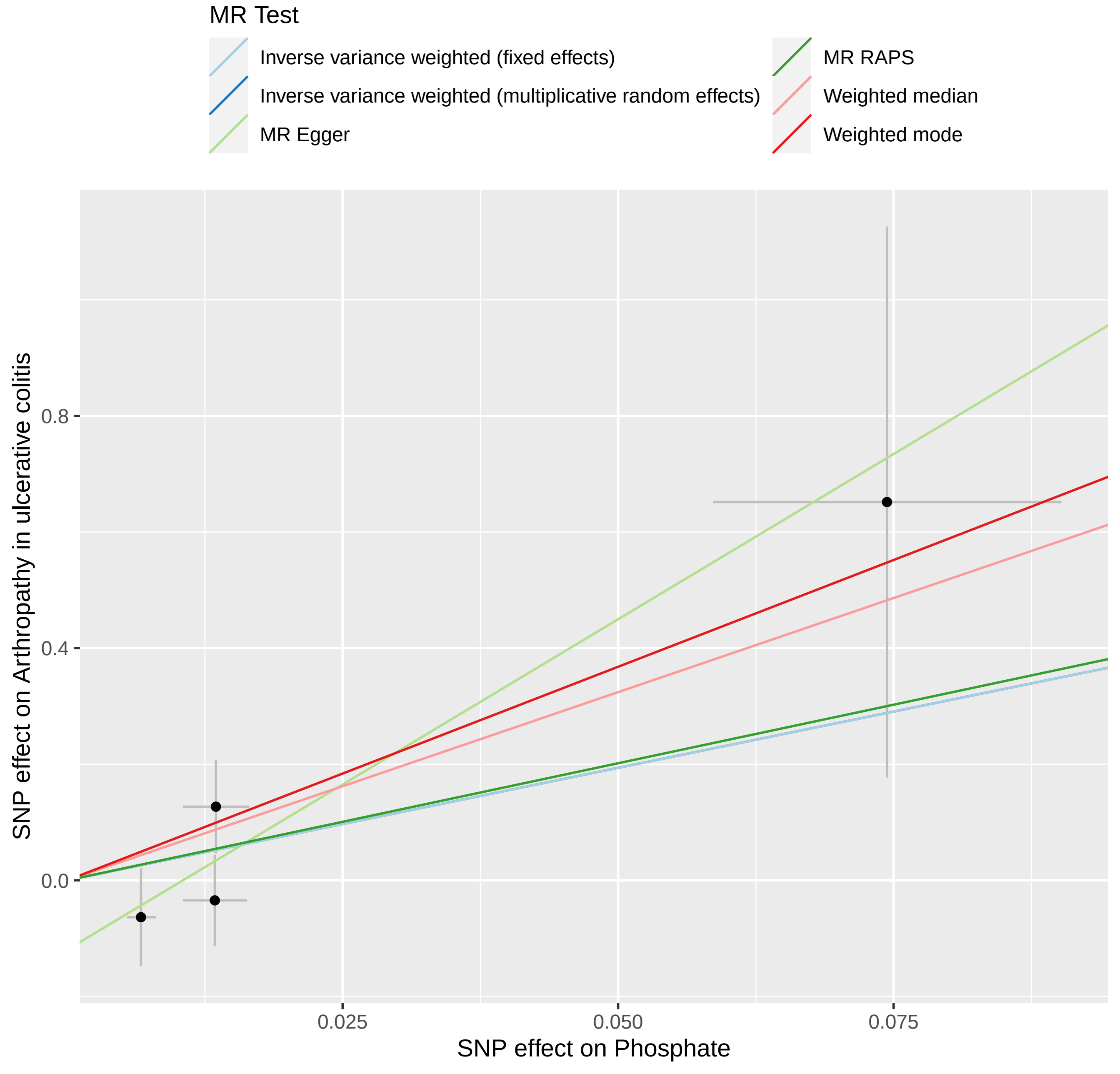

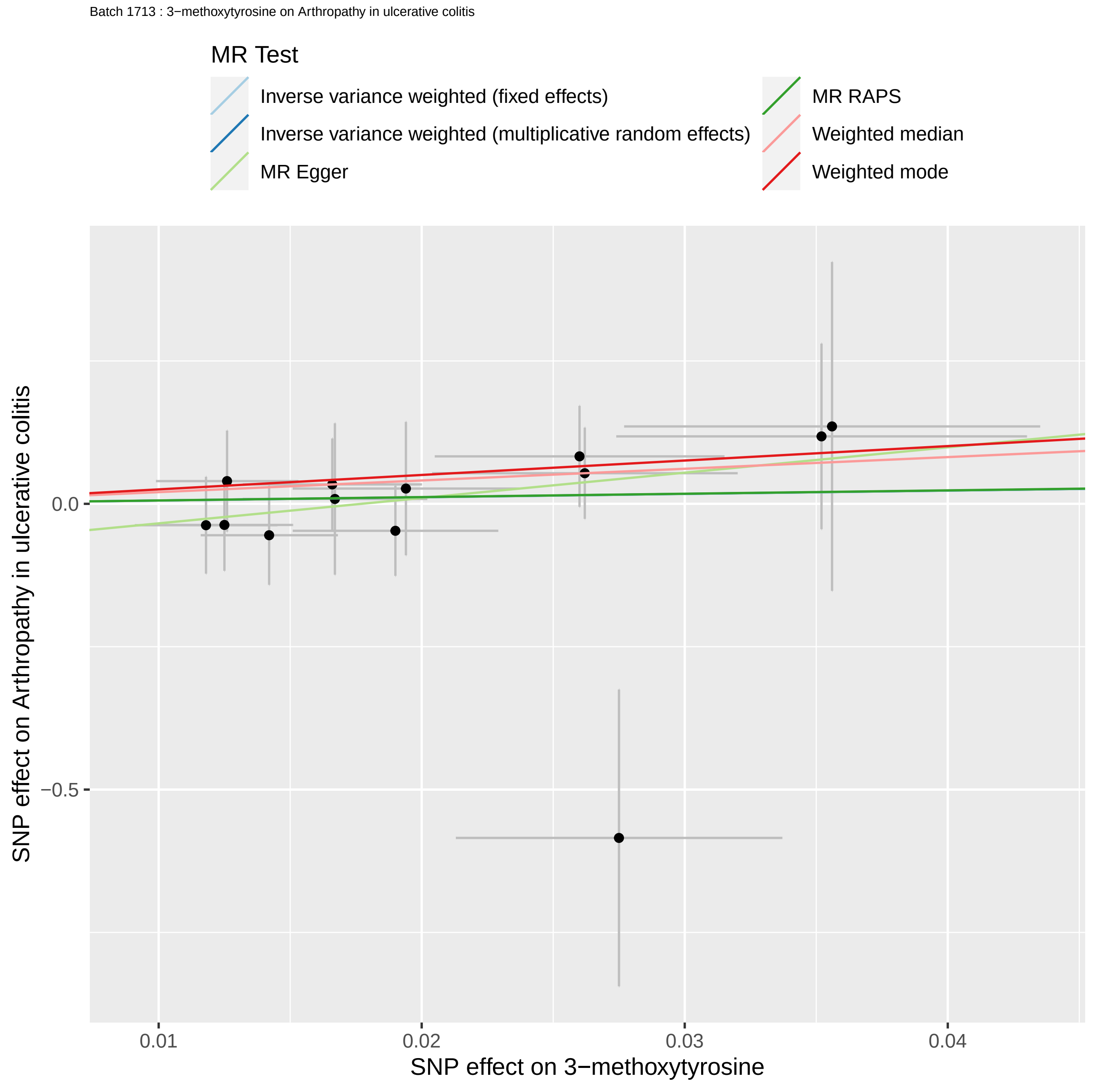
Batch 1713 : 3−methoxytyrosine on Arthropathy in ulcerative colitis
MR Test
Inverse variance weighted (fixed effects)
Inverse variance weighted (multiplicative random effects)
MR Egger
MR RAPS
Weighted median
Weighted mode
SNP effect on Arthropathy in ulcerative colitis
0.0
−0.5
0.01
0.02
0.03
0.04
SNP effect on 3−methoxytyrosine

Batch 1715 : Undecanoate (11:0) on Arthropathy in ulcerative colitis

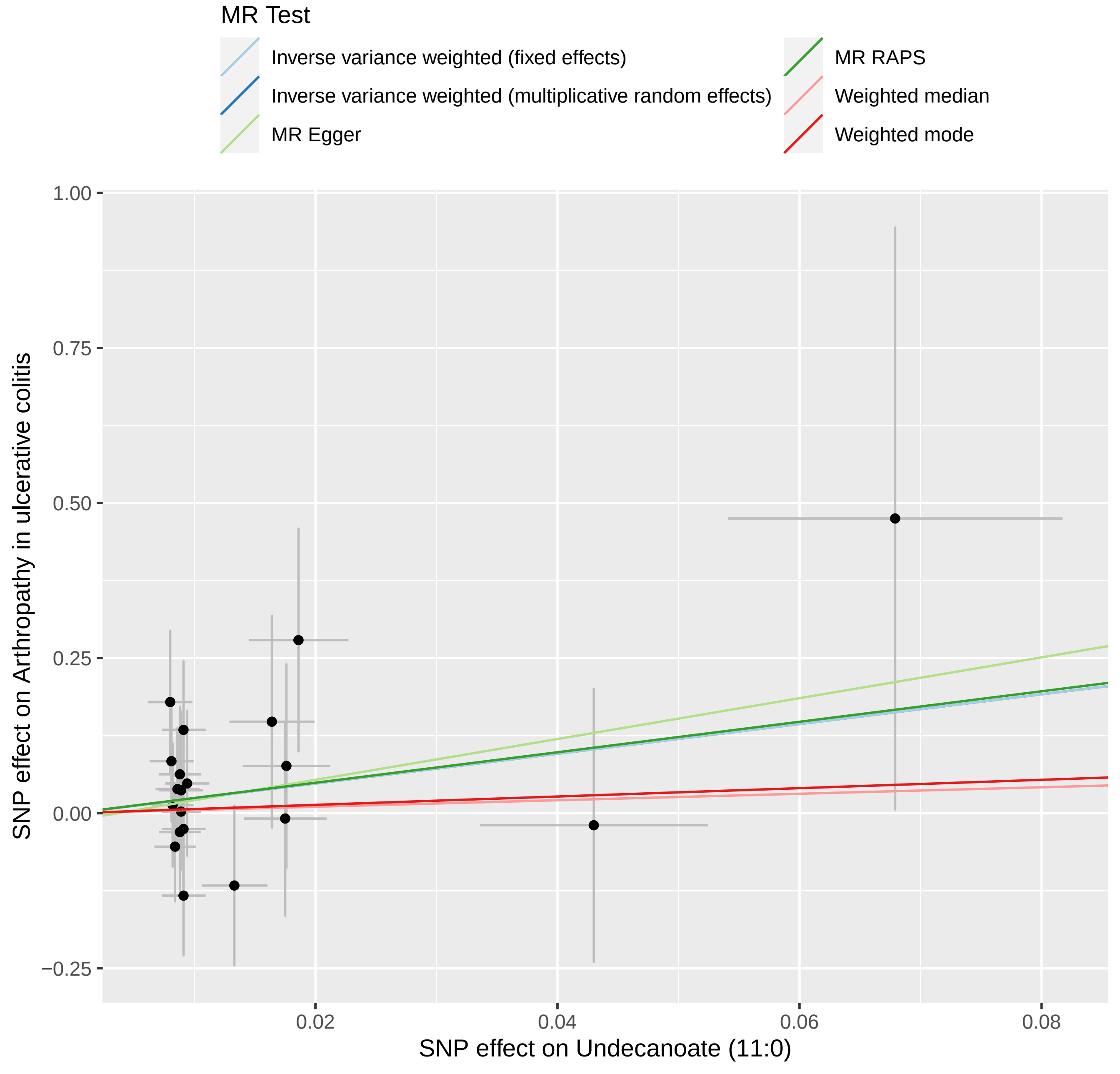

Batch 1717 : Taurodeoxycholate on Arthropathy in ulcerative colitis

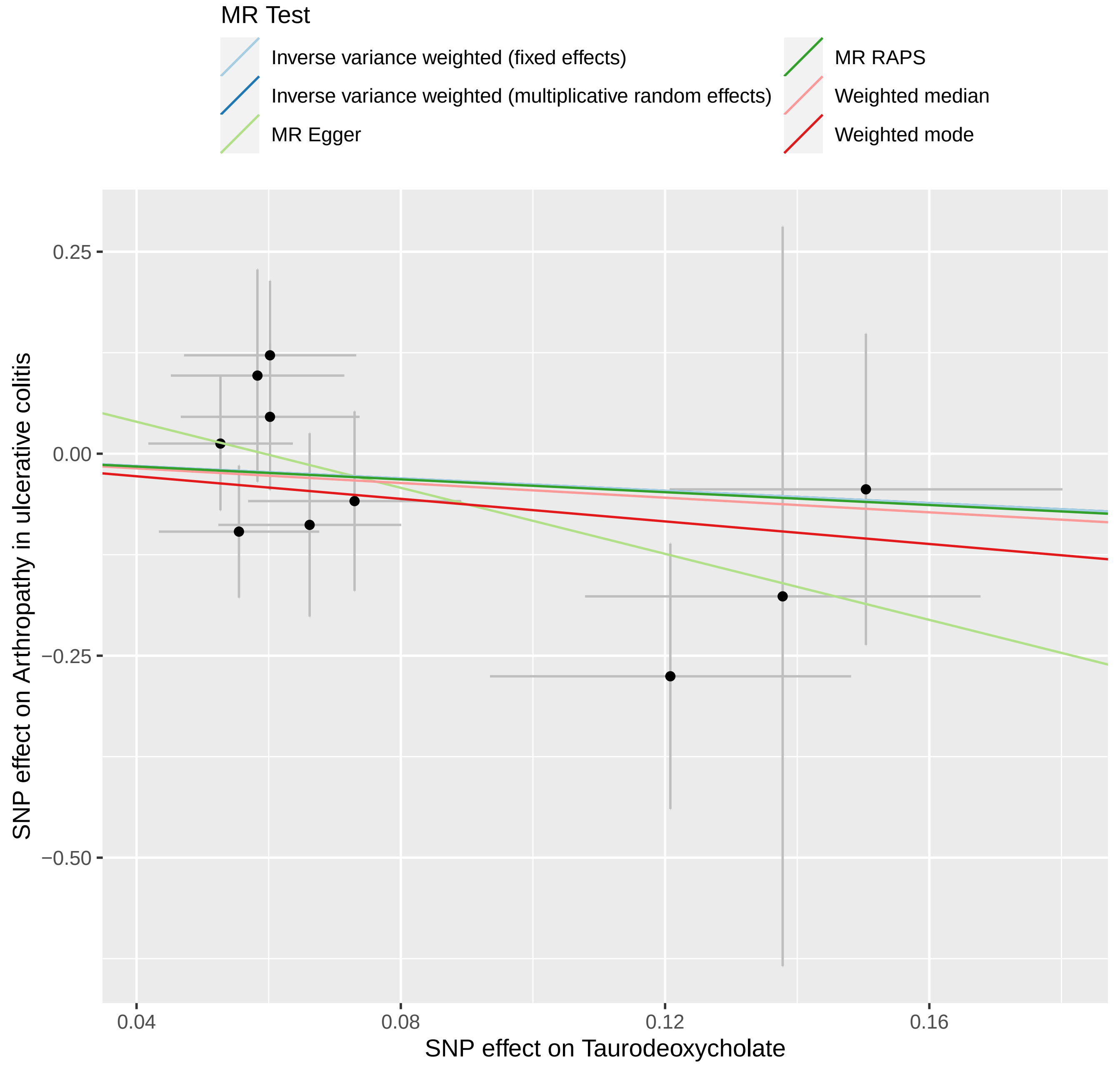

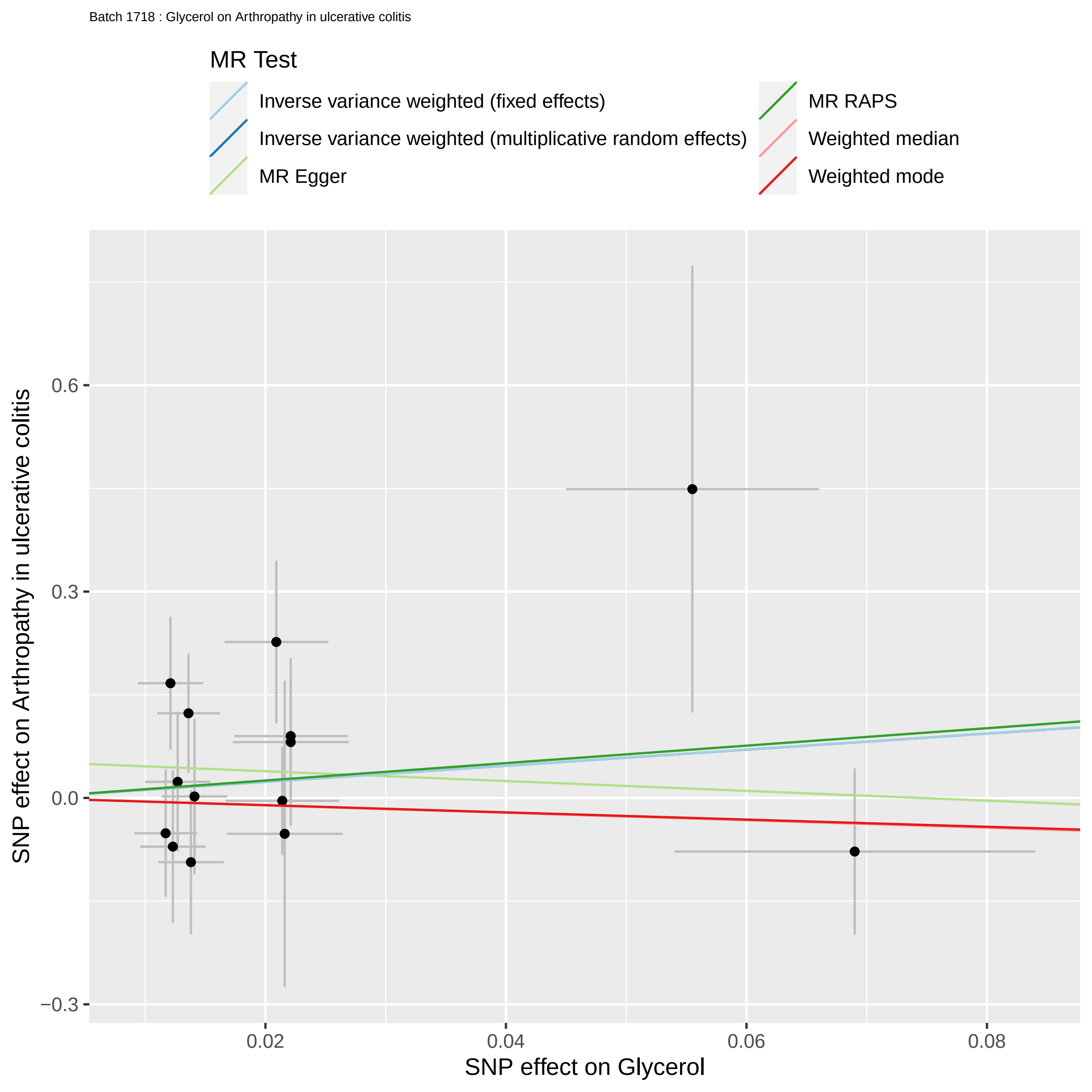
Batch 1718 : Glycerol on Arthropathy in ulcerative colitis
MR Test
Inverse variance weighted (fixed effects)
Inverse variance weighted (multiplicative random effects)
MR Egger
MR RAPS
Weighted median
Weighted mode
SNP effect on Arthropathy in ulcerative colitis
0.6
0.3
0.0
−0.3
0.02
0.04
0.06
0.08
SNP effect on Glycerol

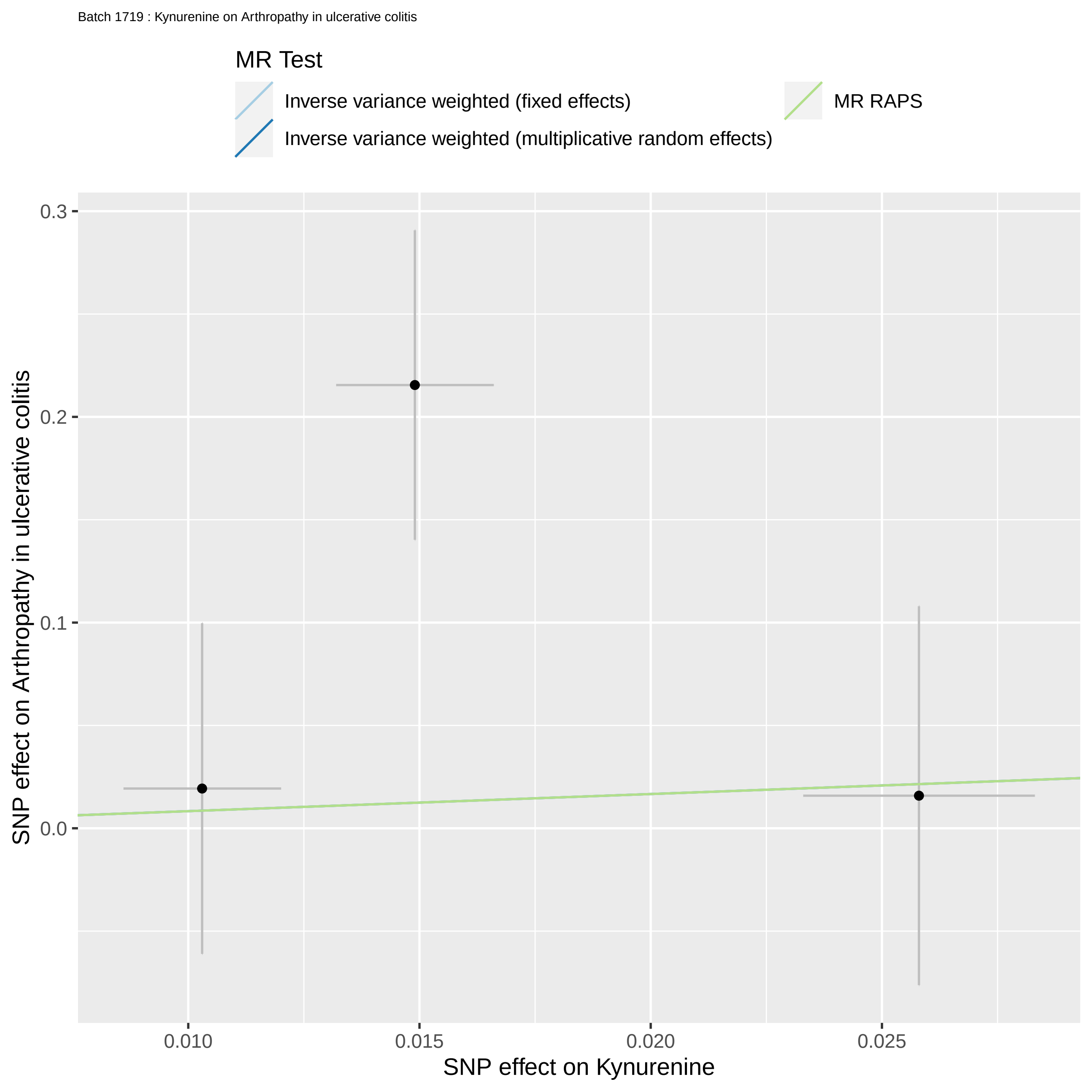

Batch 1719 : Kynurenine on Arthropathy in ulcerative colitis
MR Test
Inverse variance weighted (fixed effects)
Inverse variance weighted (multiplicative random effects)
MR RAPS
0.3
0.2
0.1
0.0
0.010
0.015
0.020
0.025
SNP effect on Arthropathy in ulcerative colitis
SNP effect on Kynurenine

Batch 1720 : Mannitol on Arthropathy in ulcerative colitis

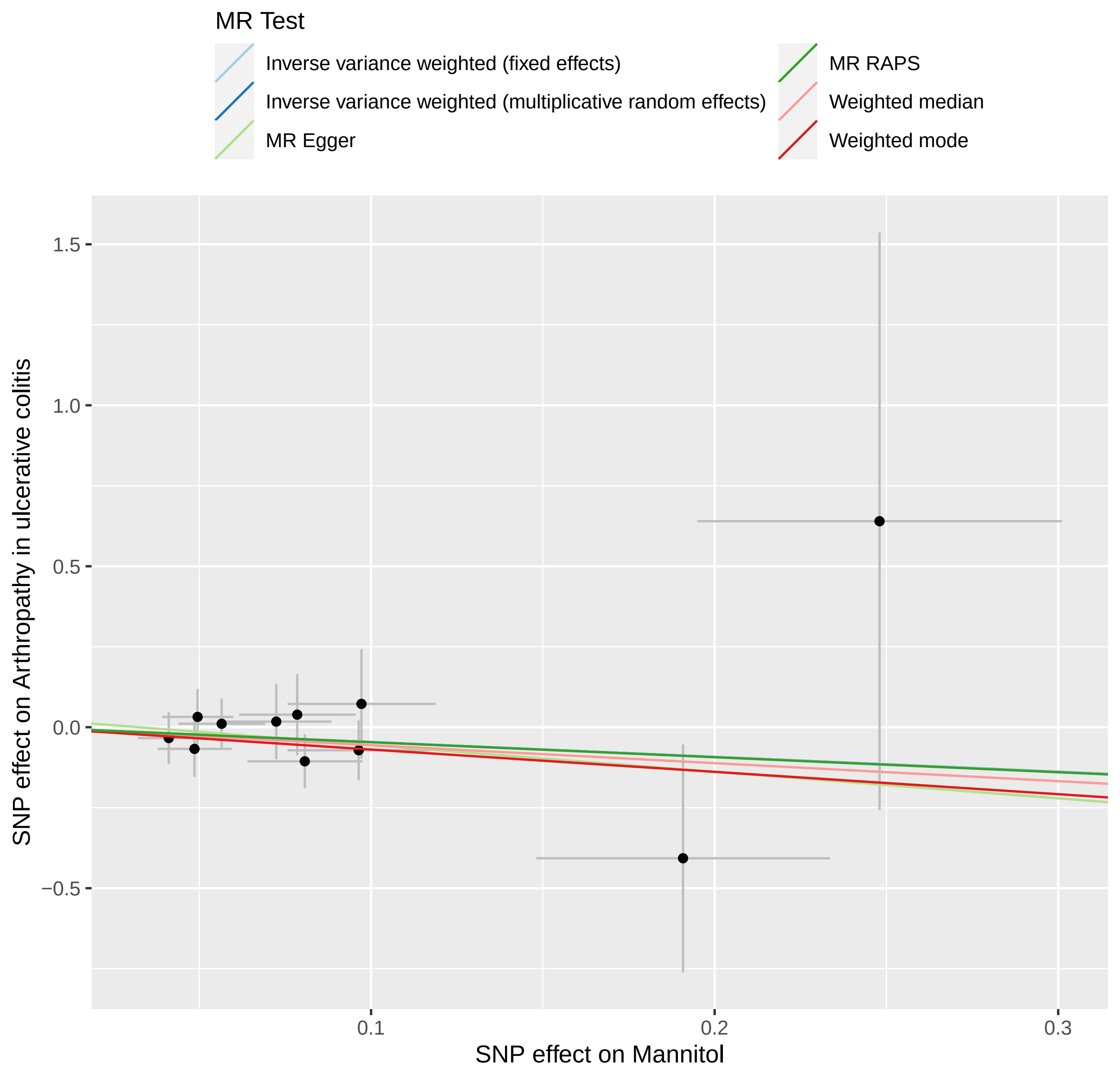

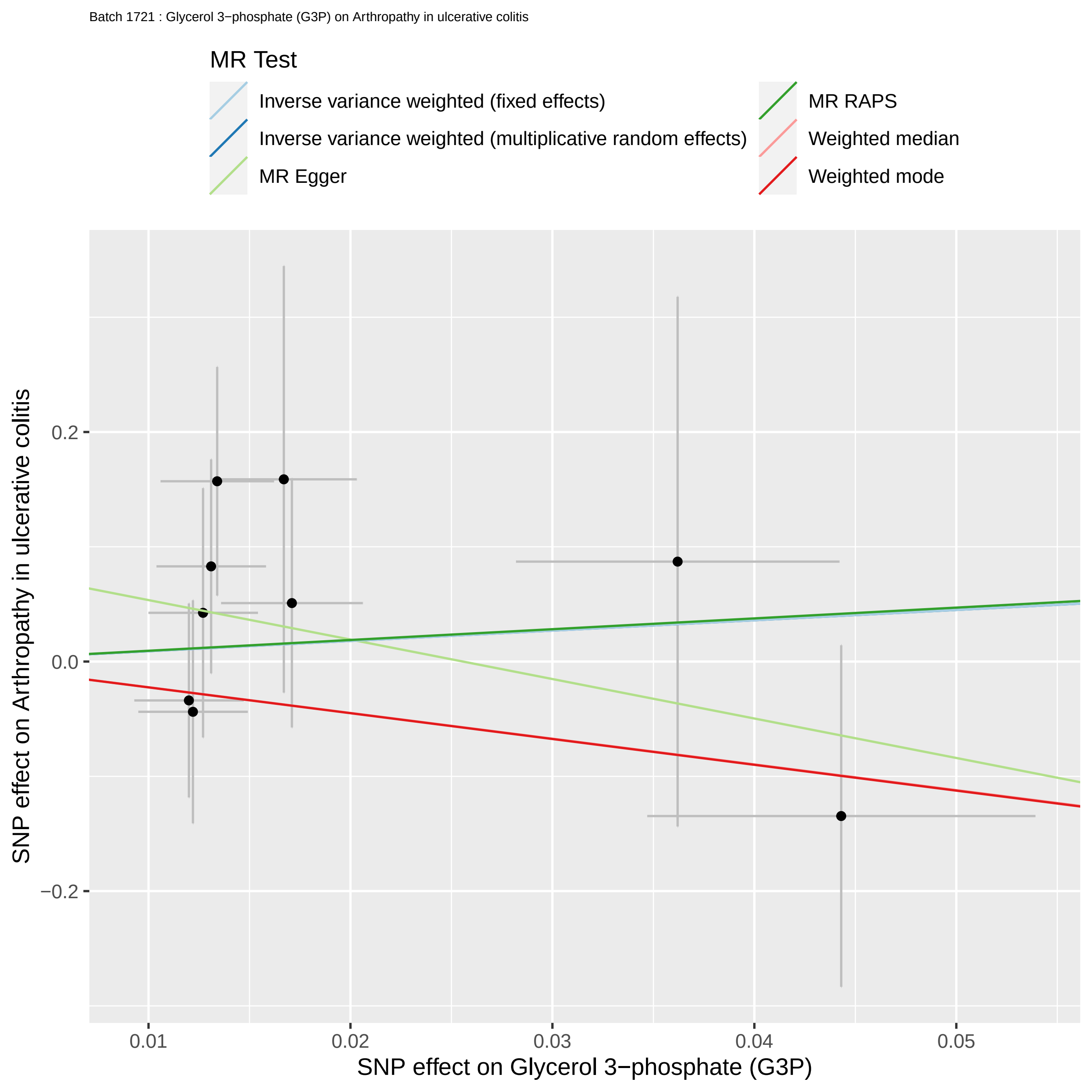

Batch 1721 : Glycerol 3−phosphate (G3P) on Arthropathy in ulcerative colitis
MR Test
Inverse variance weighted (fixed effects)
Inverse variance weighted (multiplicative random effects)
MR Egger
MR RAPS
Weighted median
Weighted mode
0.2
0.0
−0.2
0.01
0.02
0.03
0.04
0.05
SNP effect on Arthropathy in ulcerative colitis
SNP effect on Glycerol 3−phosphate (G3P)

Batch 1723 : Carnitine on Arthropathy in ulcerative colitis

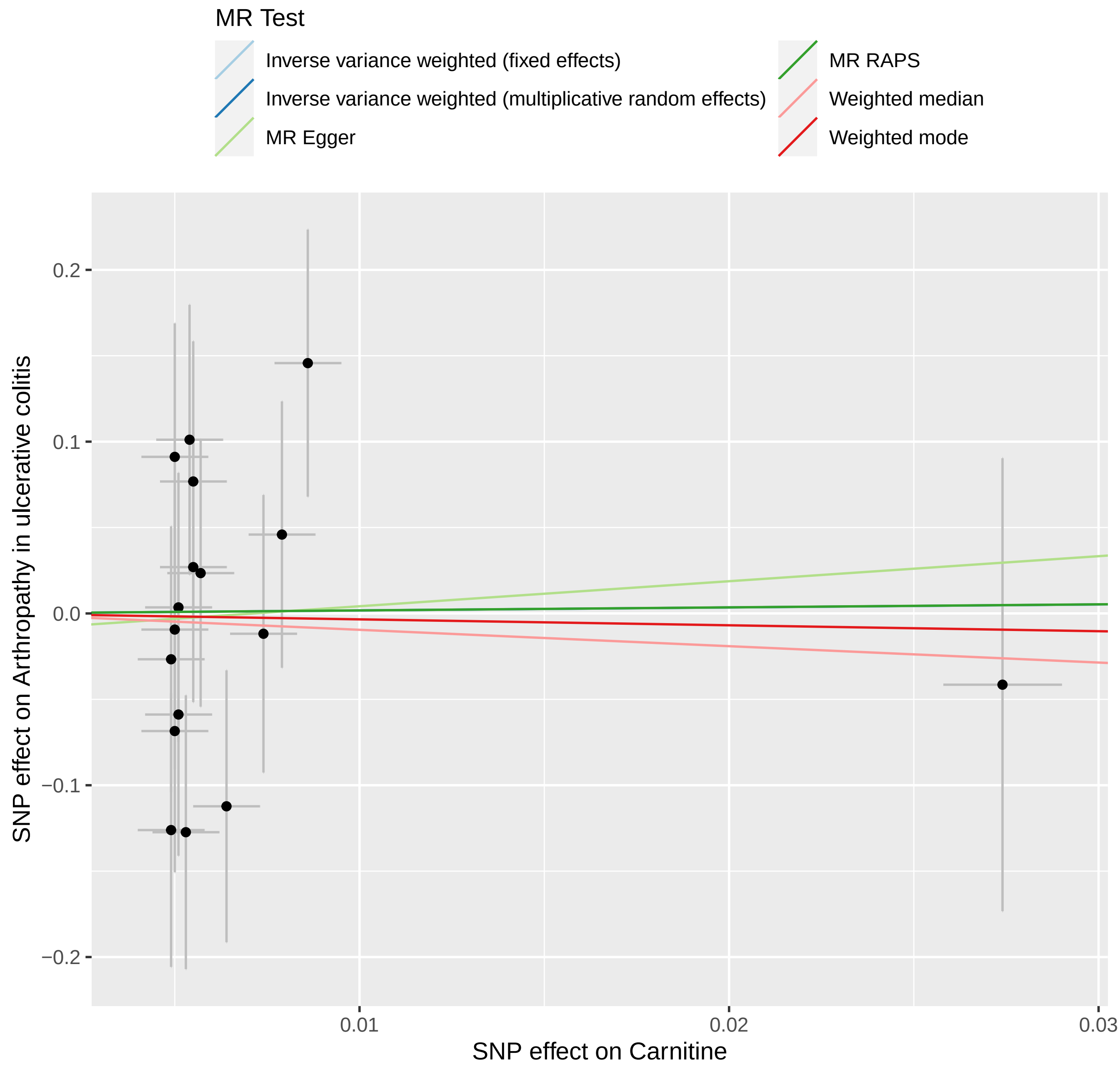

Batch 1724 : Choline on Arthropathy in ulcerative colitis

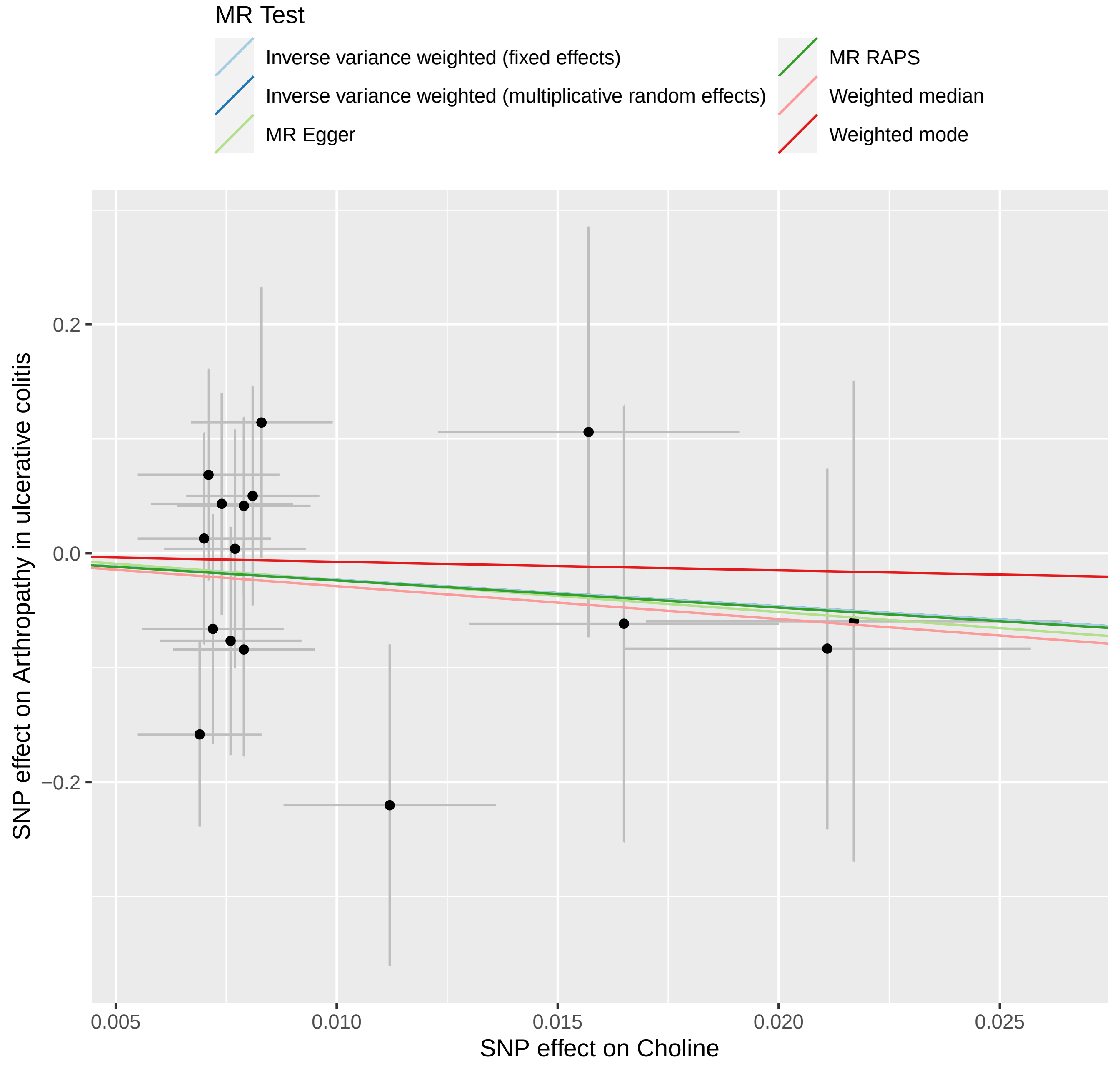

Batch 1727 : 3−methyl−2−oxovalerate on Arthropathy in ulcerative colitis

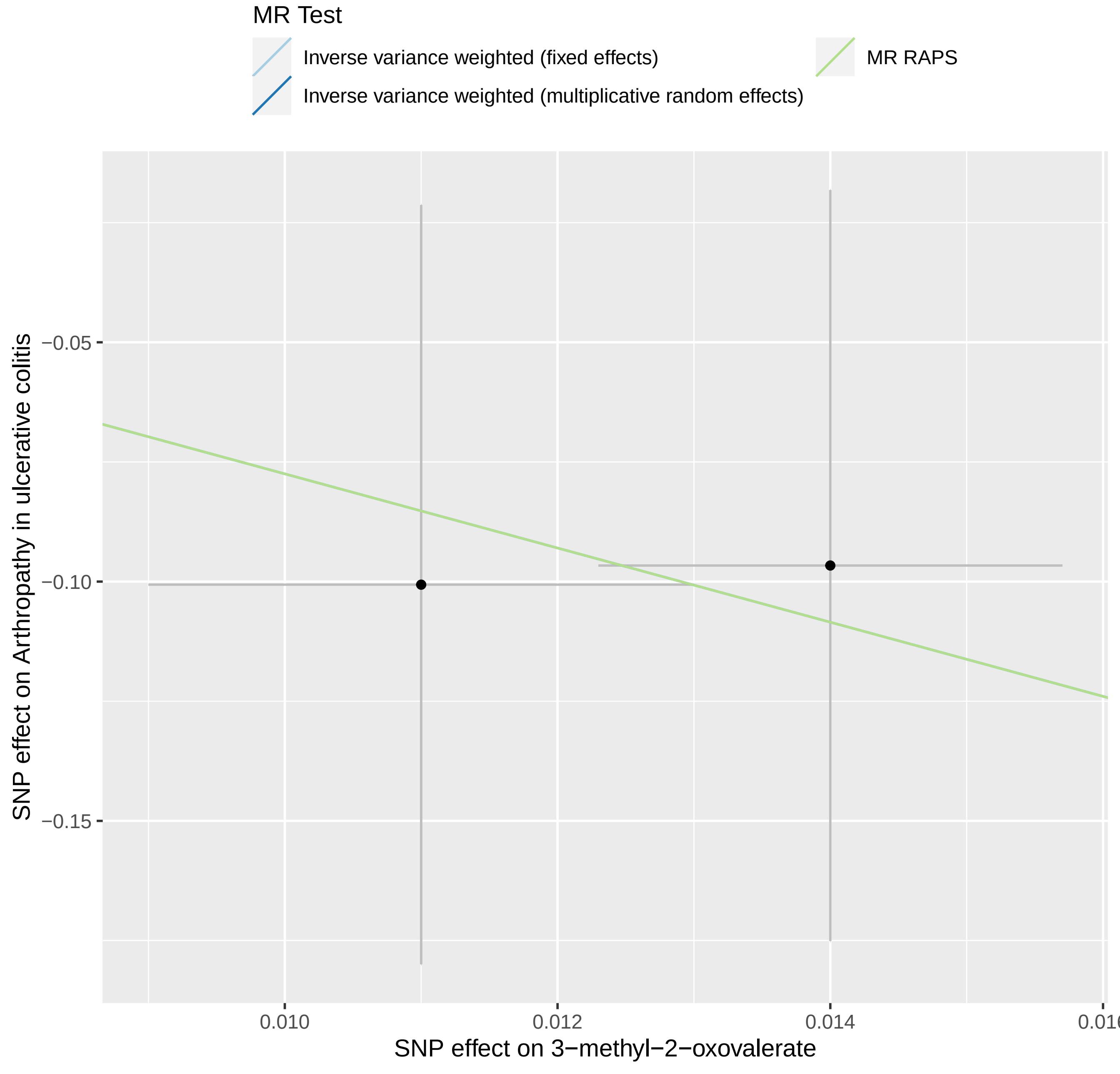

Batch 1728 : 3−methylhistidine on Arthropathy in ulcerative colitis

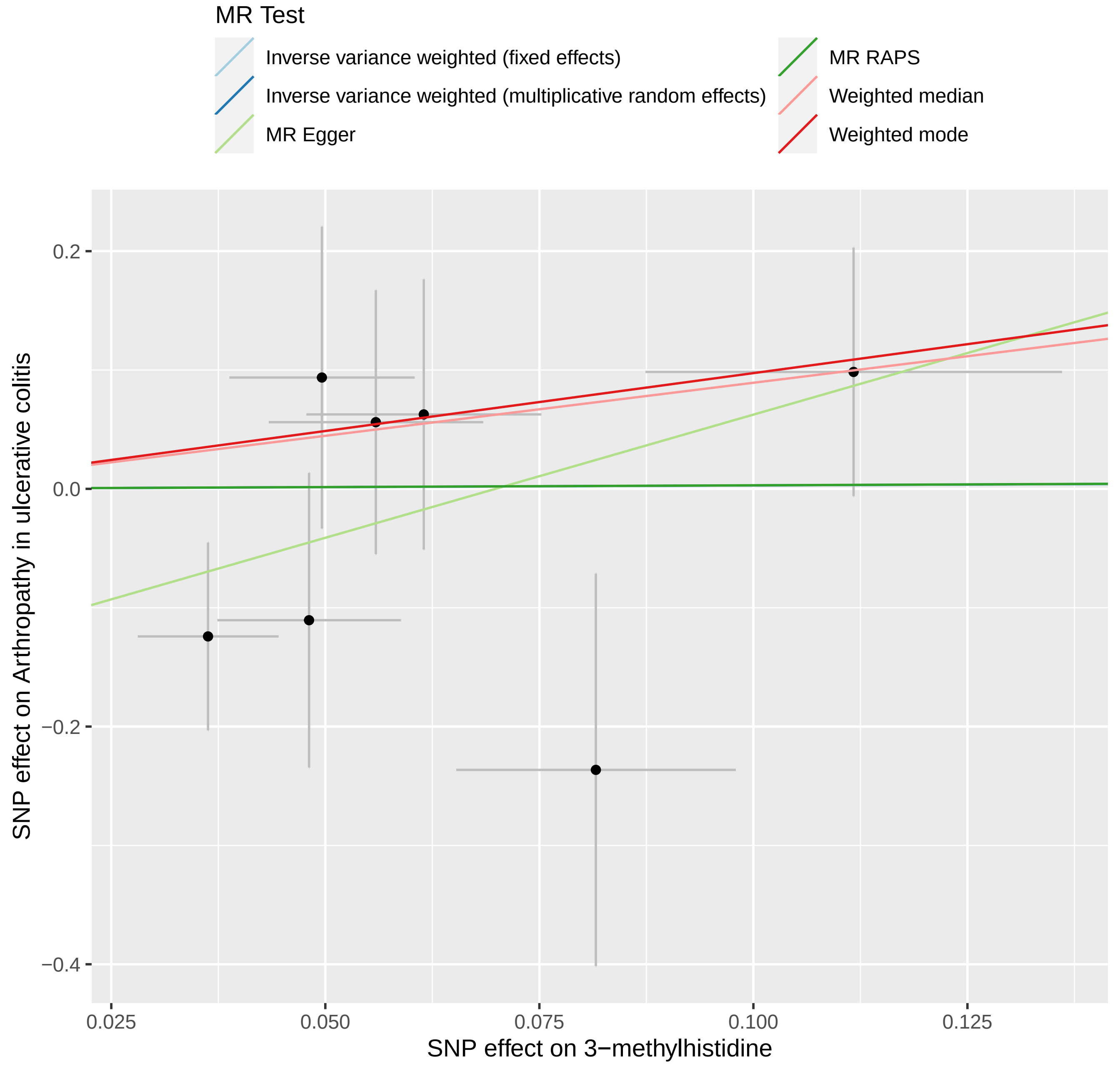

Batch 1730 : Phenylacetate on Arthropathy in ulcerative colitis

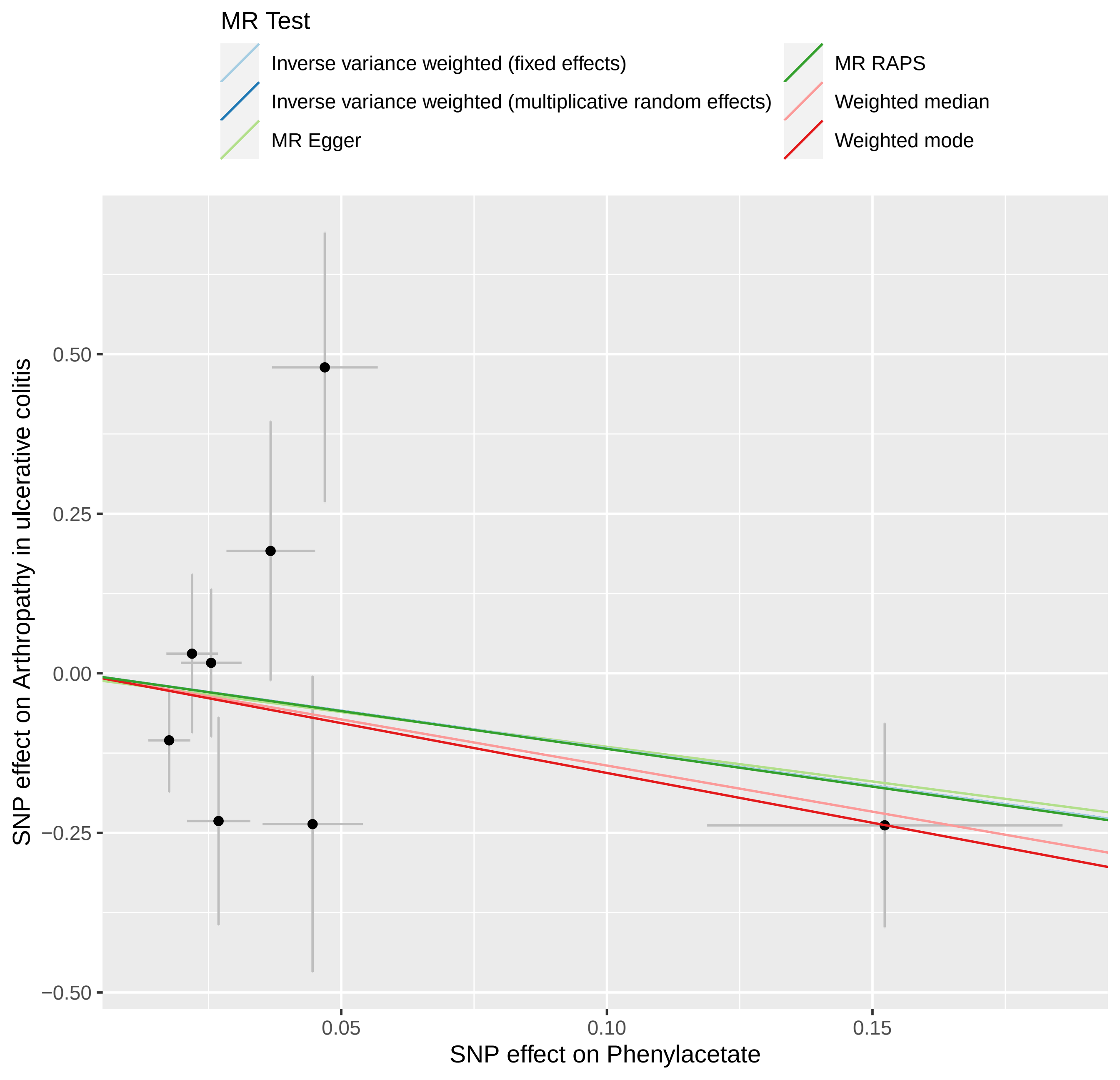

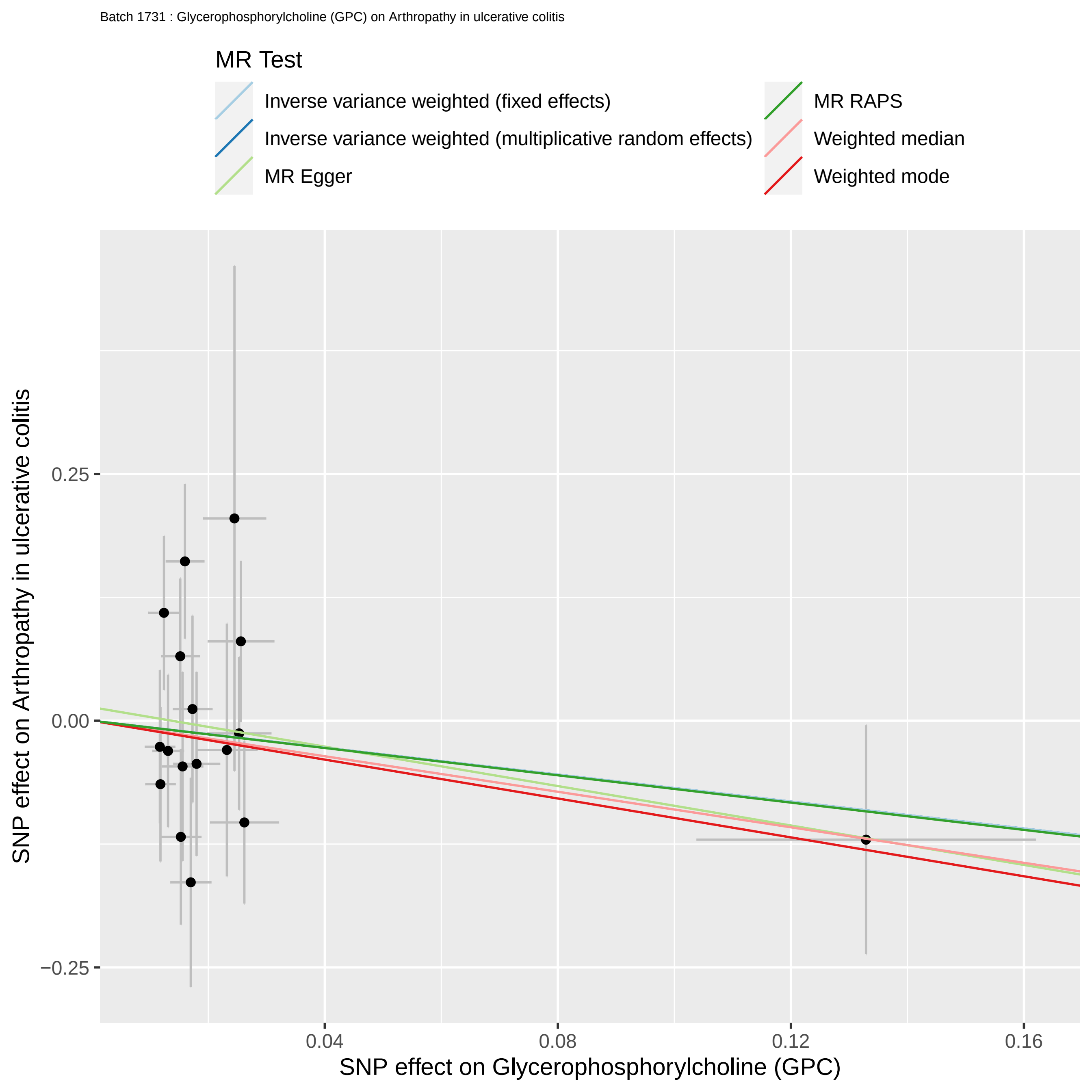
Batch 1731 : Glycerophosphorylcholine (GPC) on Arthropathy in ulcerative colitis
MR Test
Inverse variance weighted (fixed effects)
Inverse variance weighted (multiplicative random effects)
MR Egger
MR RAPS
Weighted median
Weighted mode
SNP effect on Arthropathy in ulcerative colitis
0.25
0.00
−0.25
0.04
0.08
0.12
0.16
SNP effect on Glycerophosphorylcholine (GPC)

Batch 1732 : Aspartate on Arthropathy in ulcerative colitis

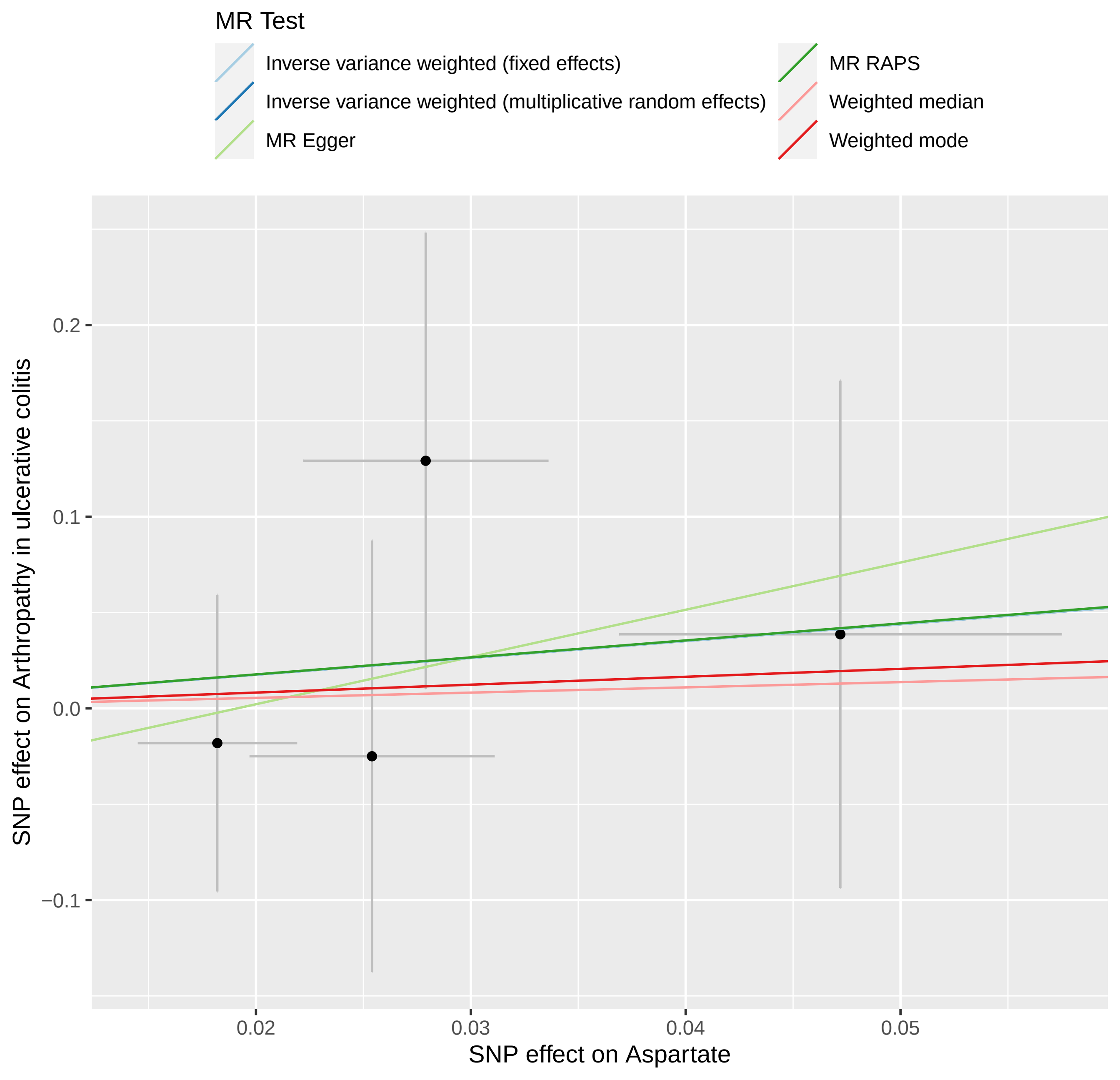

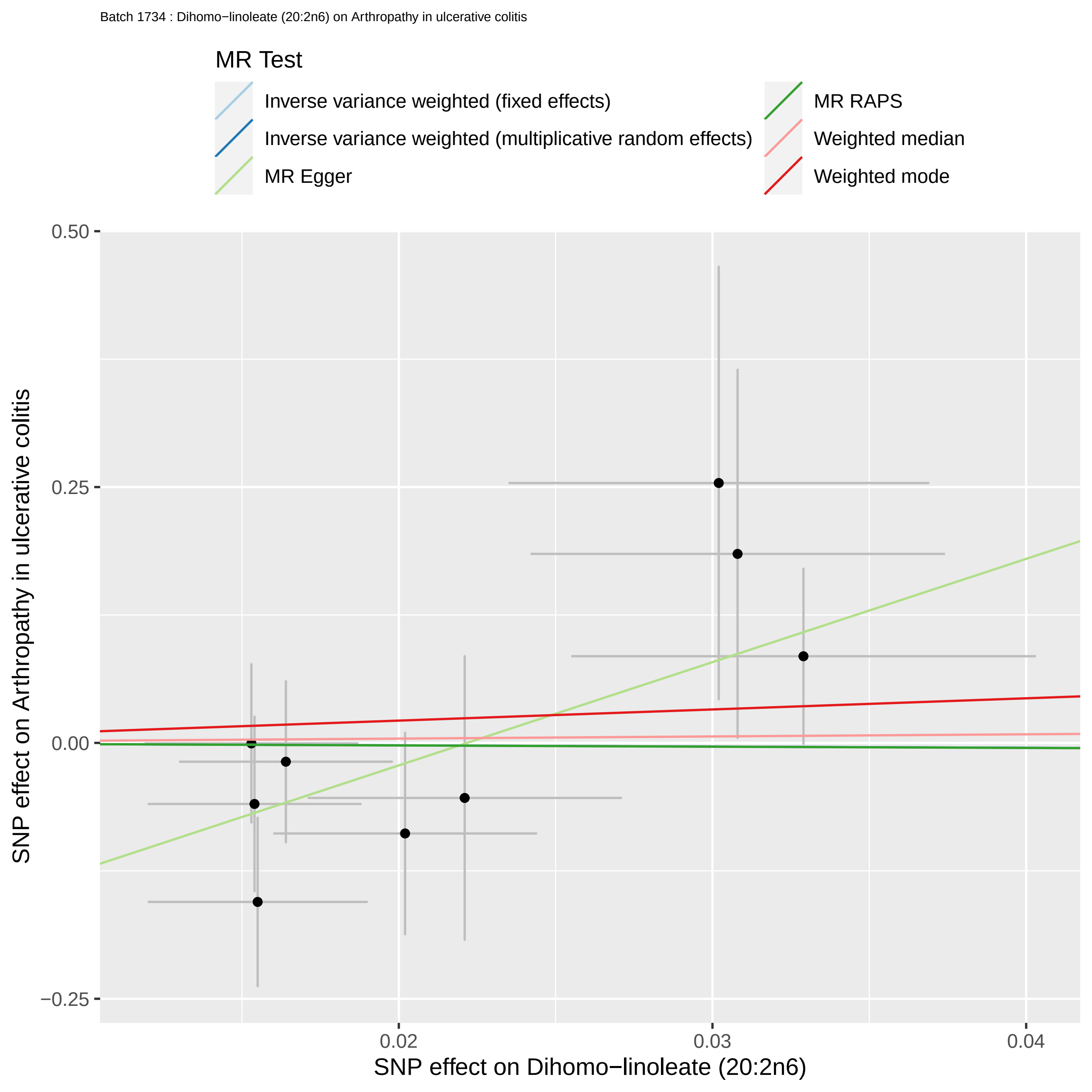

Batch 1734 : Dihomo−linoleate (20:2n6) on Arthropathy in ulcerative colitis
MR Test
Inverse variance weighted (fixed effects)
Inverse variance weighted (multiplicative random effects)
MR Egger
MR RAPS
Weighted median
Weighted mode
0.50
0.25
0.00
−0.25
0.02
0.03
0.04
SNP effect on Arthropathy in ulcerative colitis
SNP effect on Dihomo−linoleate (20:2n6)

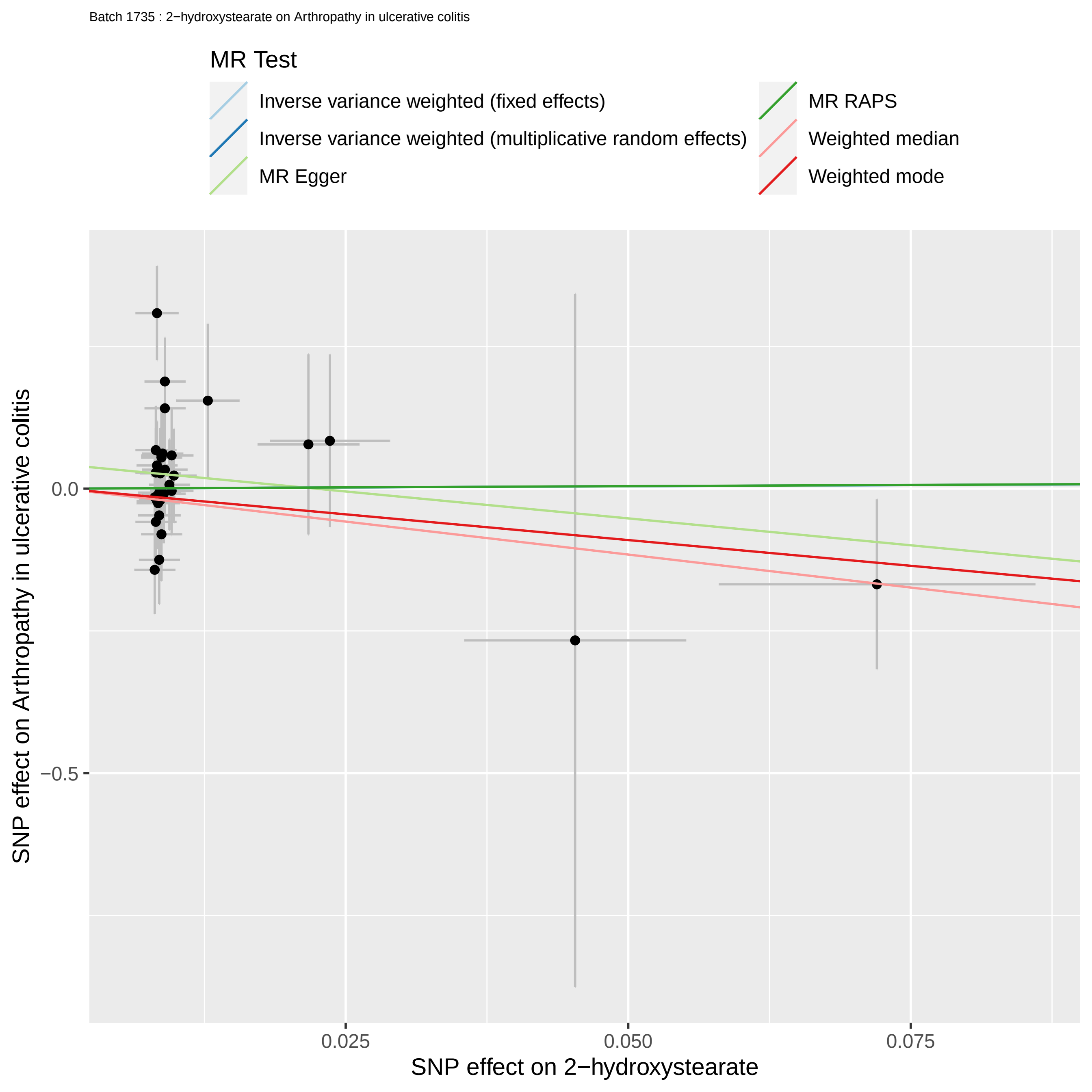

Batch 1735 : 2−hydroxystearate on Arthropathy in ulcerative colitis
MR Test
Inverse variance weighted (fixed effects)
Inverse variance weighted (multiplicative random effects)
MR Egger
MR RAPS
Weighted median
Weighted mode
0.0
−0.5
0.025
0.050
0.075
SNP effect on Arthropathy in ulcerative colitis
SNP effect on 2−hydroxystearate

Batch 1737 : Glycylvaline on Arthropathy in ulcerative colitis

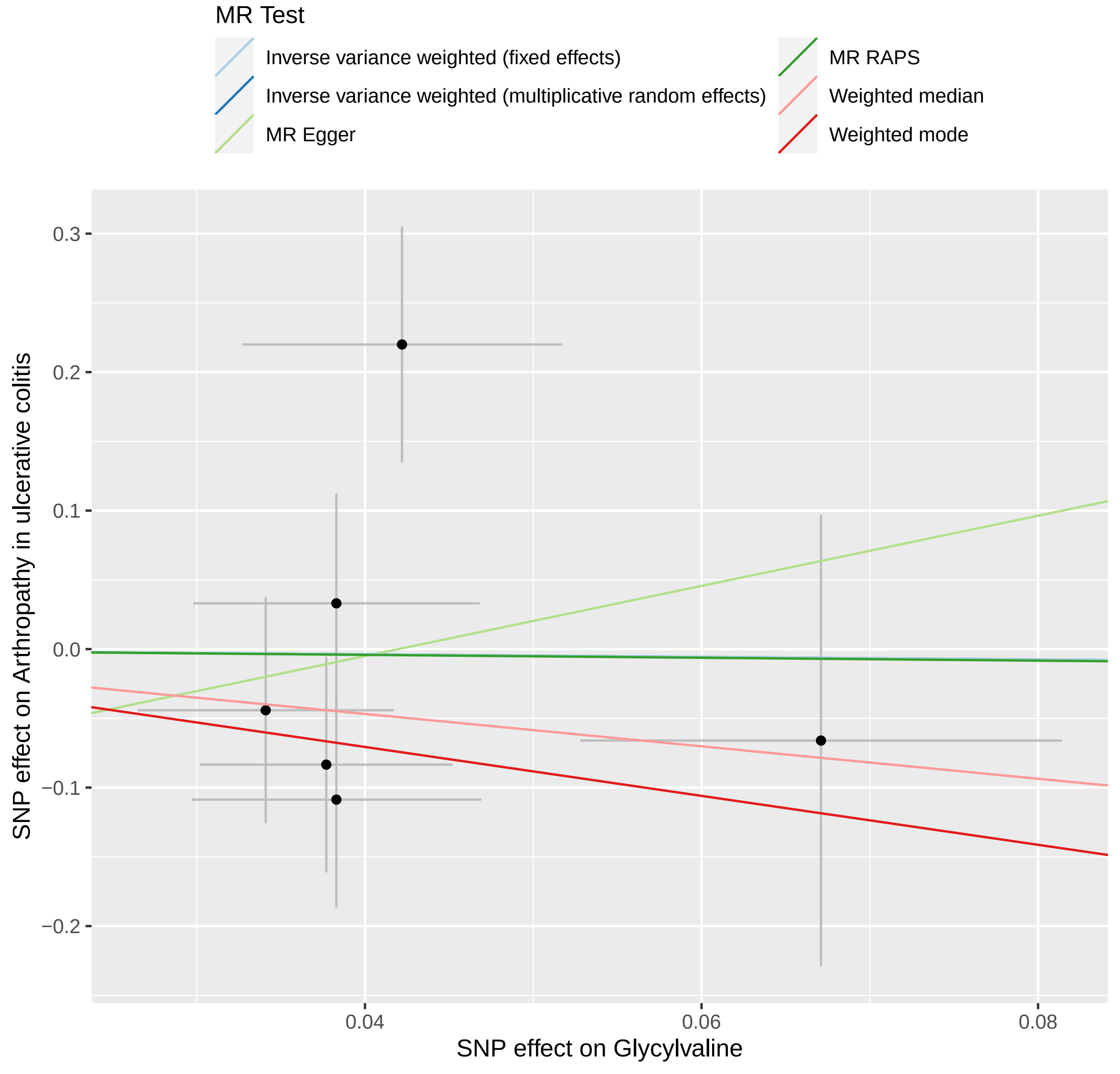

Batch 1738 : Gamma−glutamylleucine on Arthropathy in ulcerative colitis

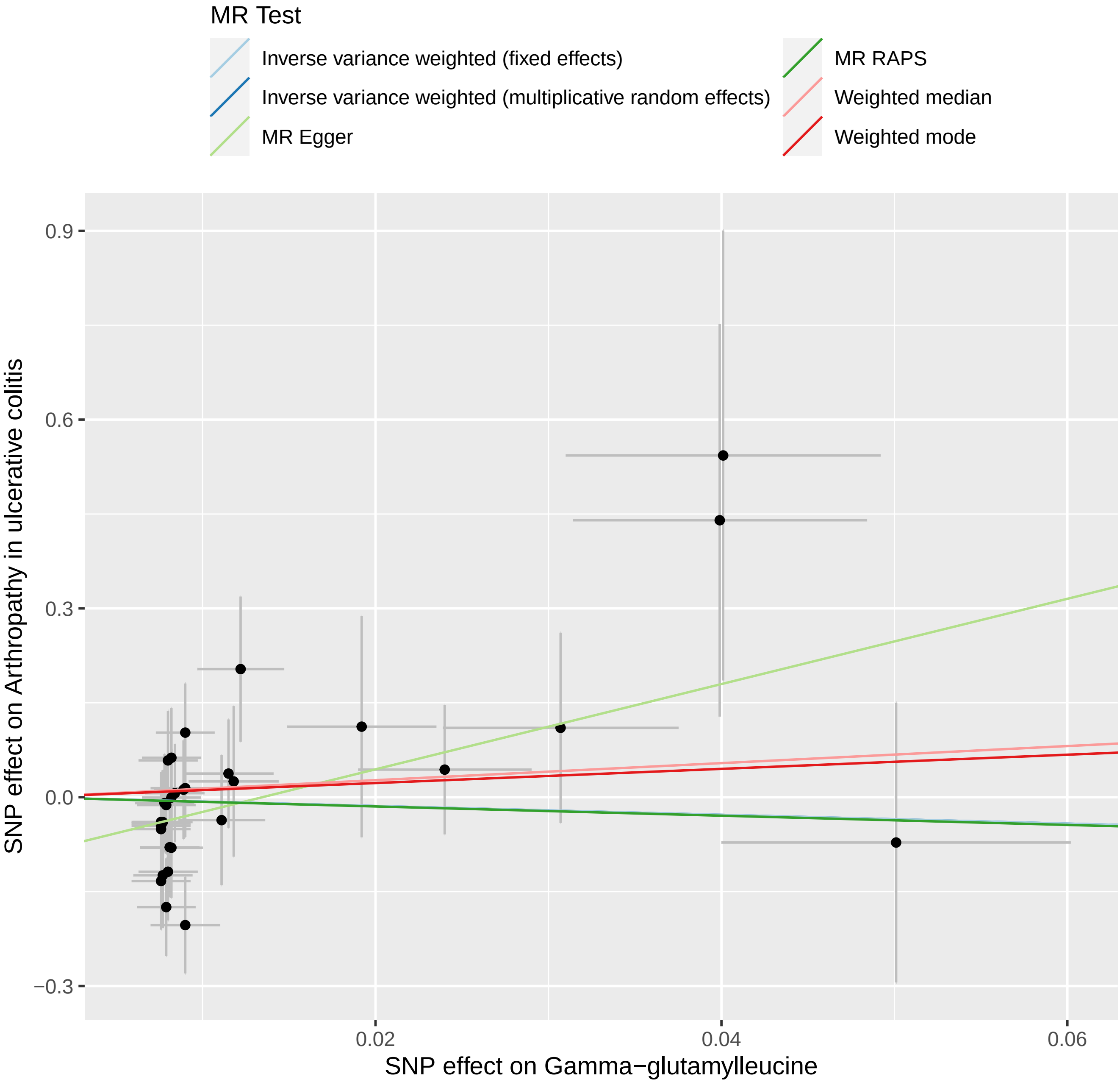

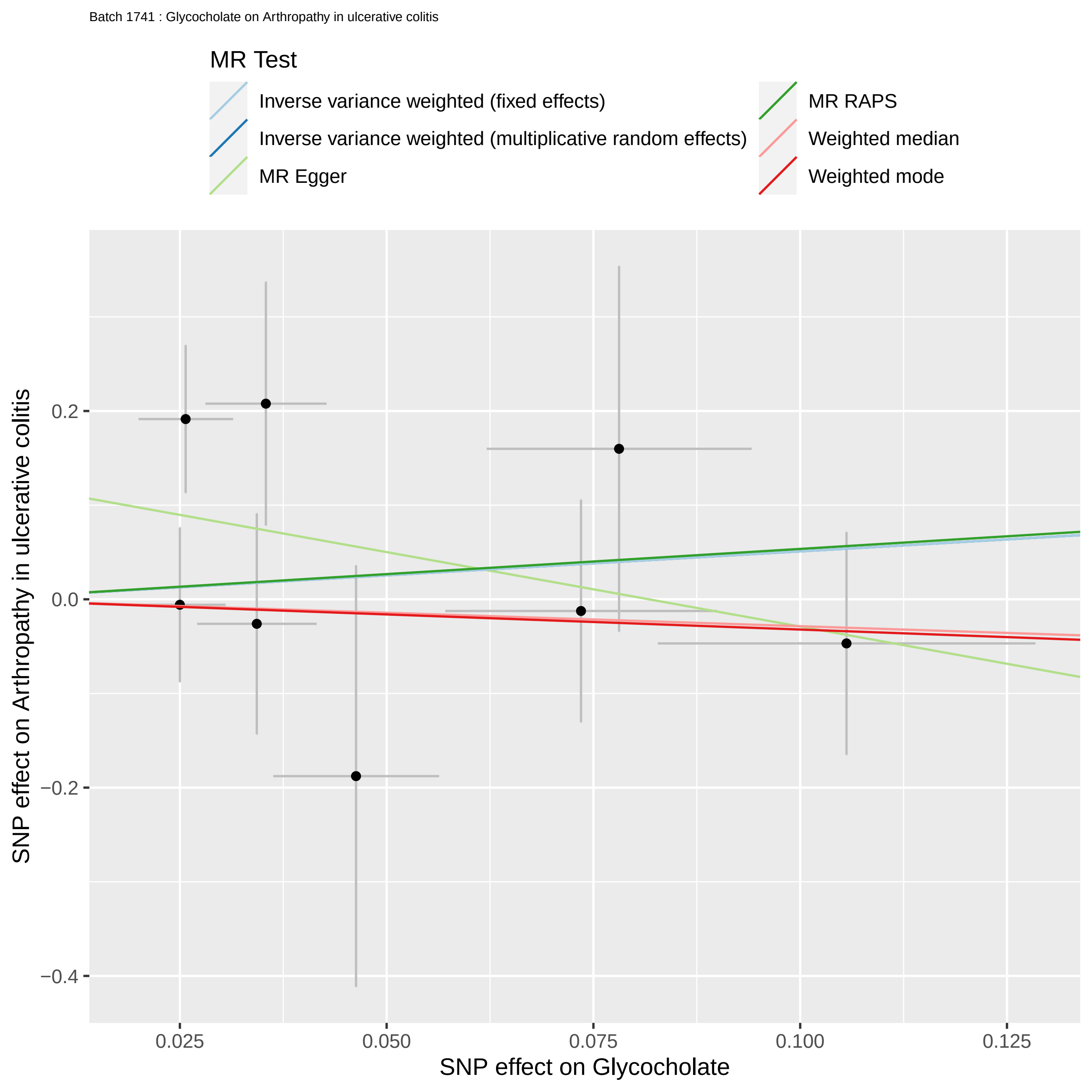
Batch 1741 : Glycocholate on Arthropathy in ulcerative colitis
MR Test
Inverse variance weighted (fixed effects)
Inverse variance weighted (multiplicative random effects)
MR Egger
MR RAPS
Weighted median
Weighted mode
0.2
0.0
−0.2
−0.4
SNP effect on Arthropathy in ulcerative colitis
0.025
0.050
0.075
0.100
0.125
SNP effect on Glycocholate

Batch 1742 : Glycodeoxycholate on Arthropathy in ulcerative colitis

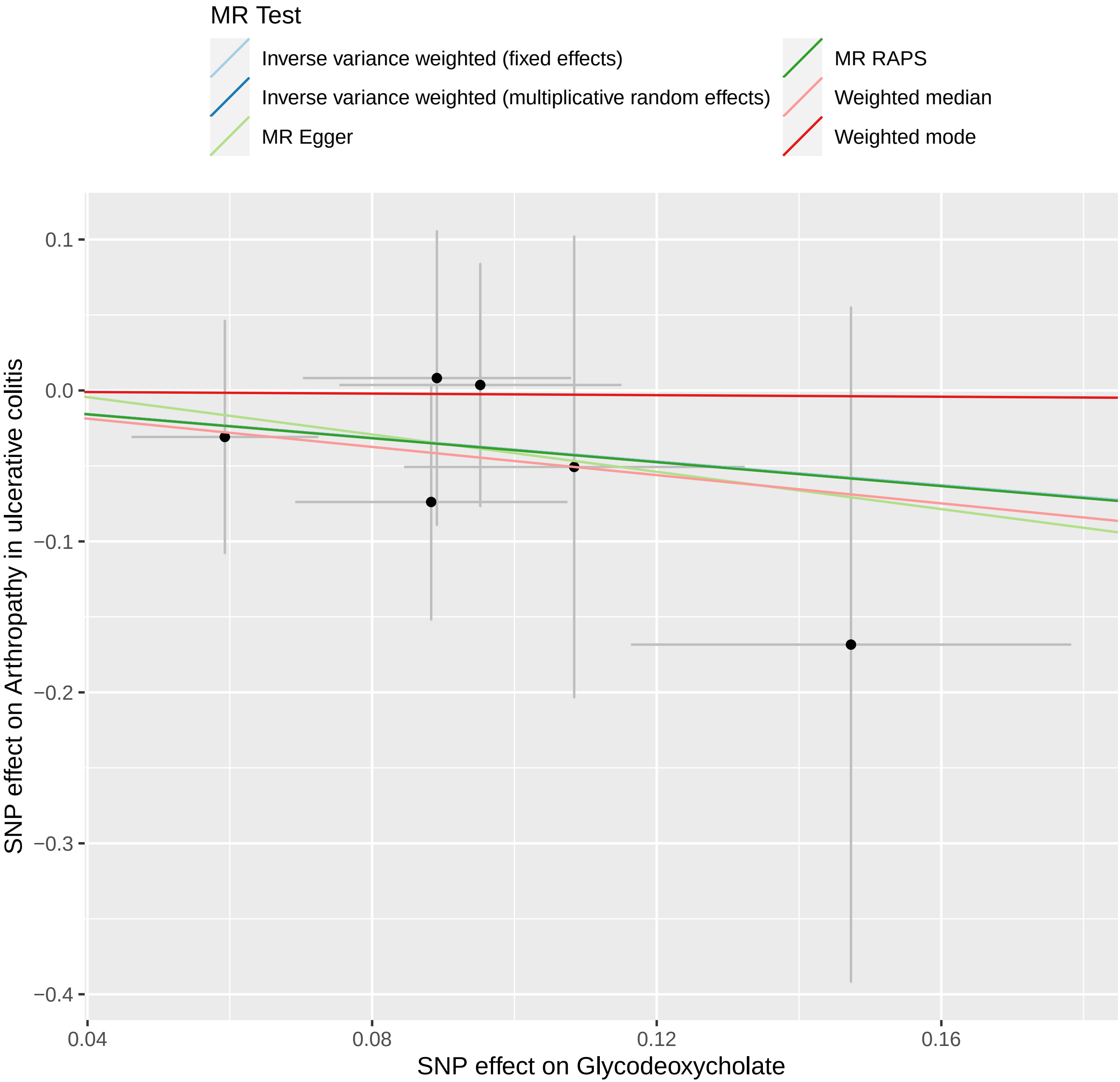

Batch 1743 : Taurochenodeoxycholate on Arthropathy in ulcerative colitis

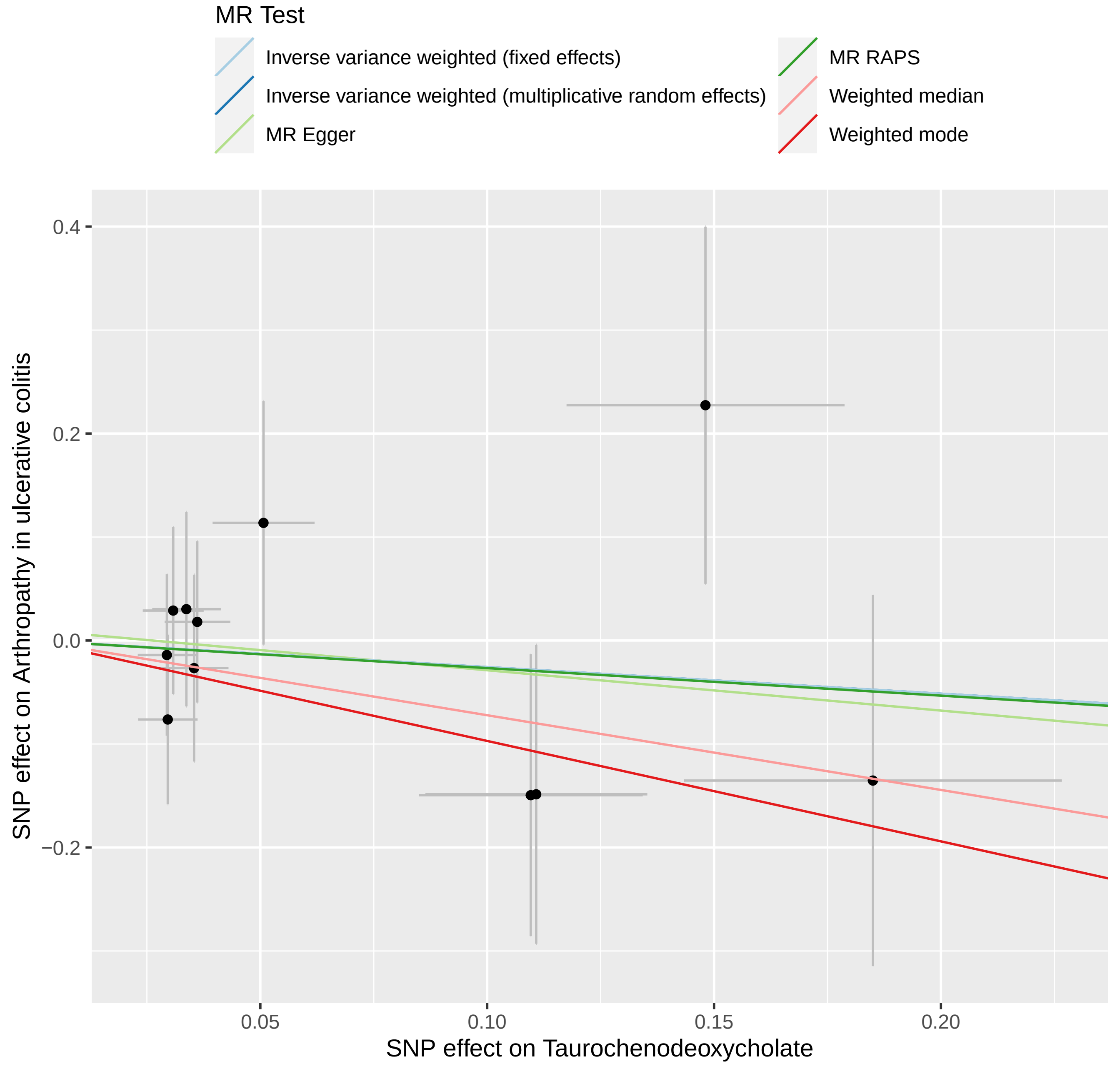

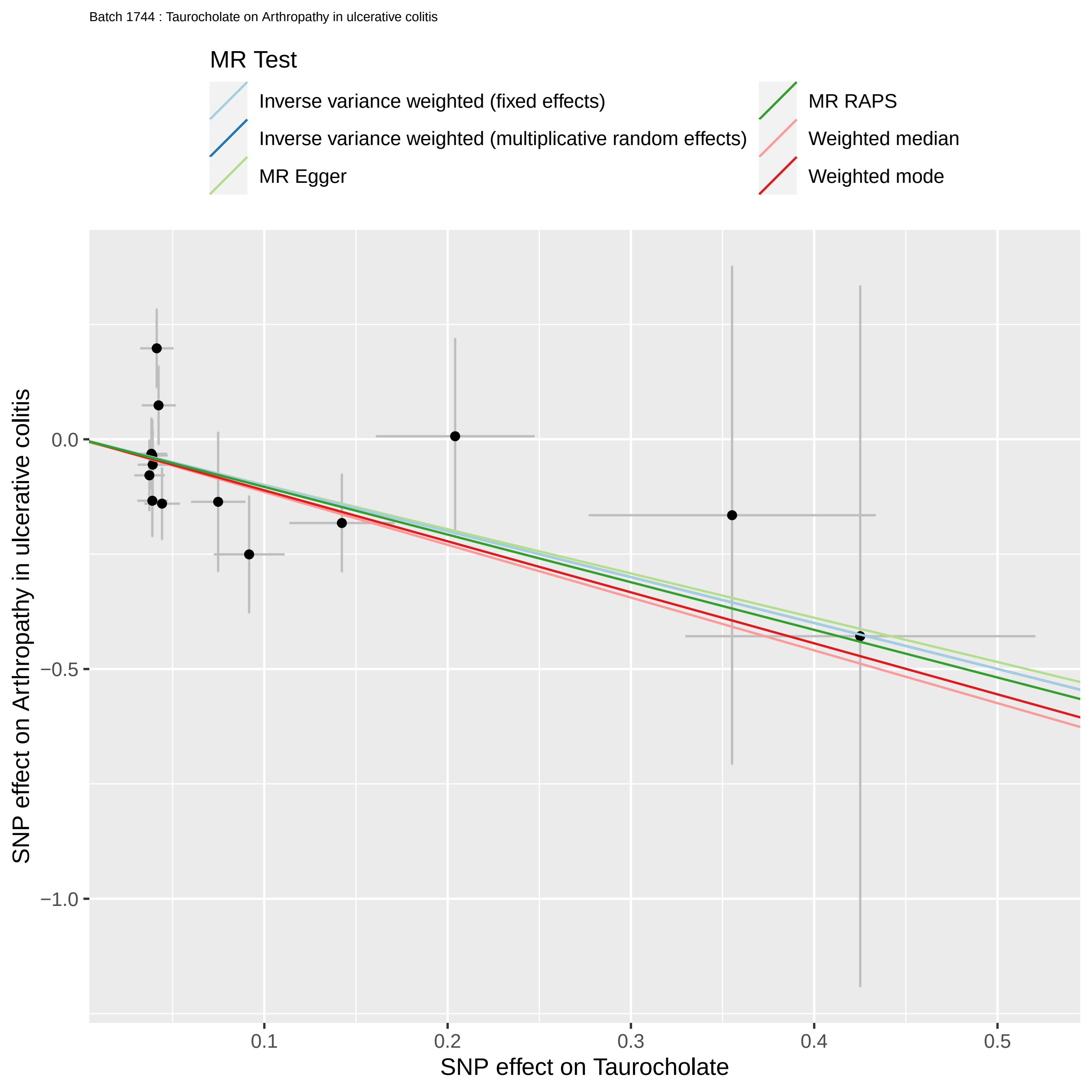
Batch 1744 : Taurocholate on Arthropathy in ulcerative colitis
MR Test
Inverse variance weighted (fixed effects)
Inverse variance weighted (multiplicative random effects)
MR Egger
MR RAPS
Weighted median
Weighted mode
0.0
−0.5
−1.0
0.1
0.2
0.3
0.4
0.5
SNP effect on Arthropathy in ulcerative colitis
SNP effect on Taurocholate

Batch 1746 : 1−stearoylglycerophosphoinositol on Arthropathy in ulcerative colitis

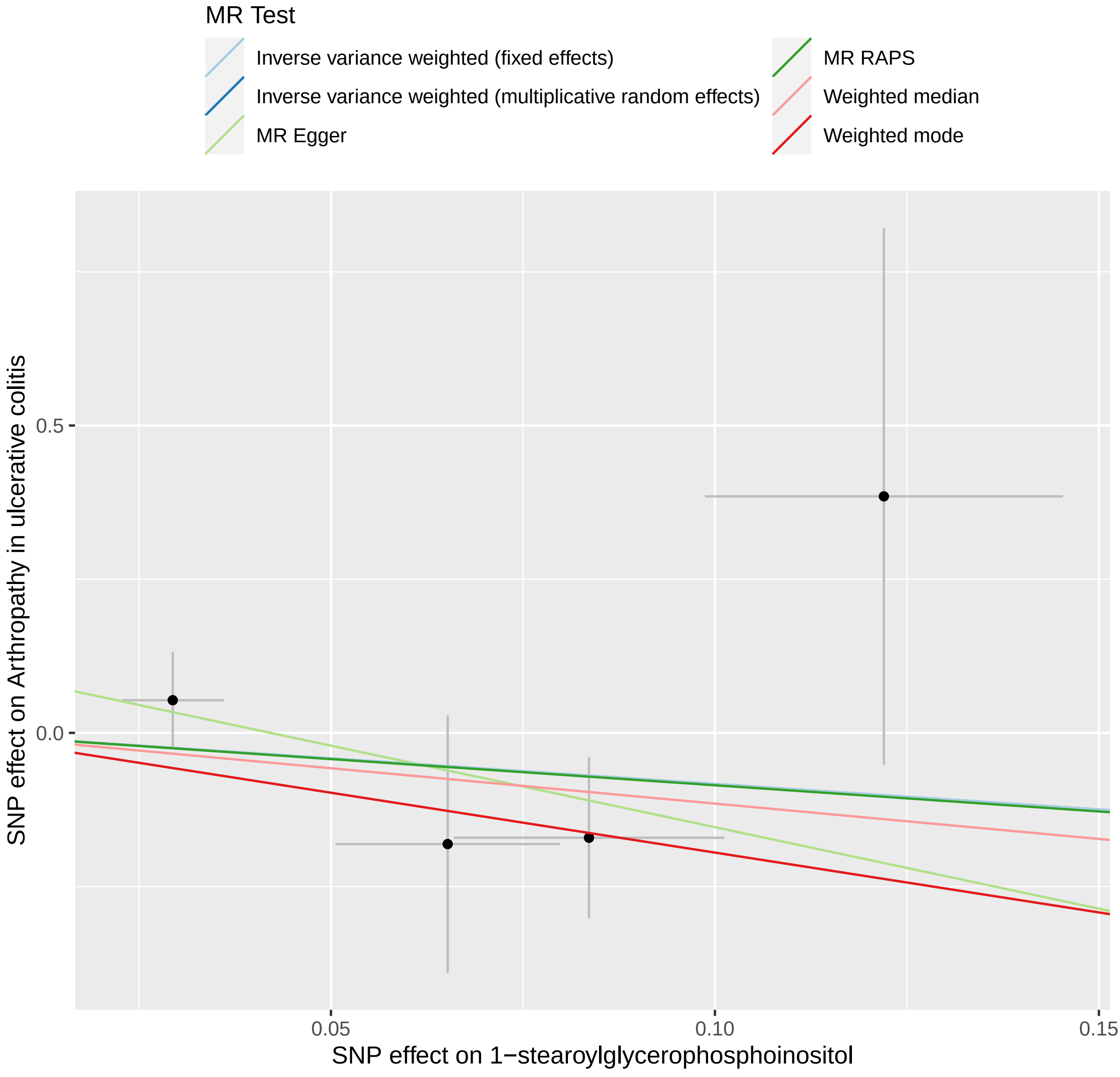

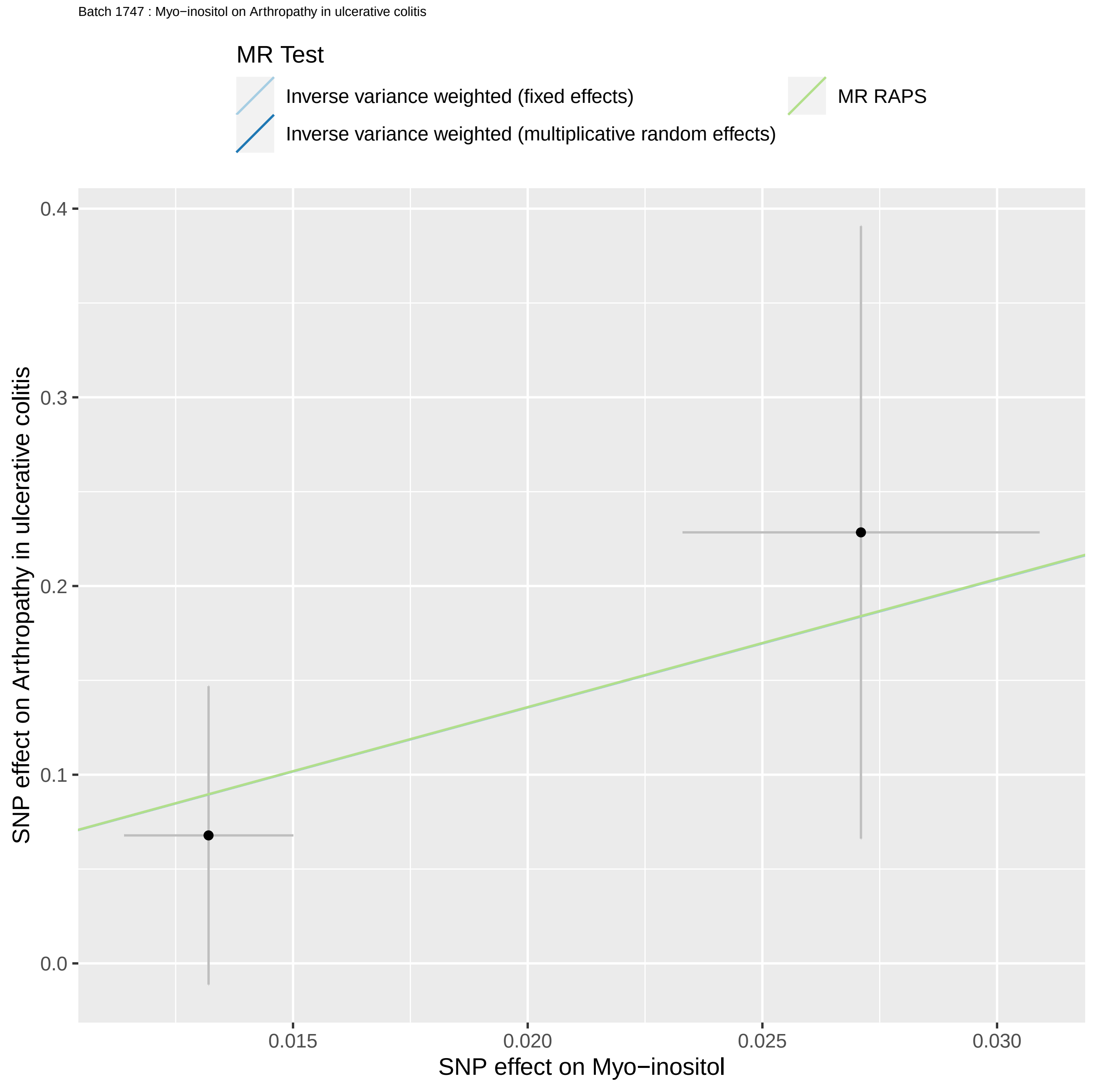
Batch 1747 : Myo−inositol on Arthropathy in ulcerative colitis
MR Test
Inverse variance weighted (fixed effects)
Inverse variance weighted (multiplicative random effects)
MR RAPS
SNP effect on Arthropathy in ulcerative colitis
0.0
0.1
0.2
0.3
0.4
0.015
0.020
0.025
0.030
SNP effect on Myo−inositol

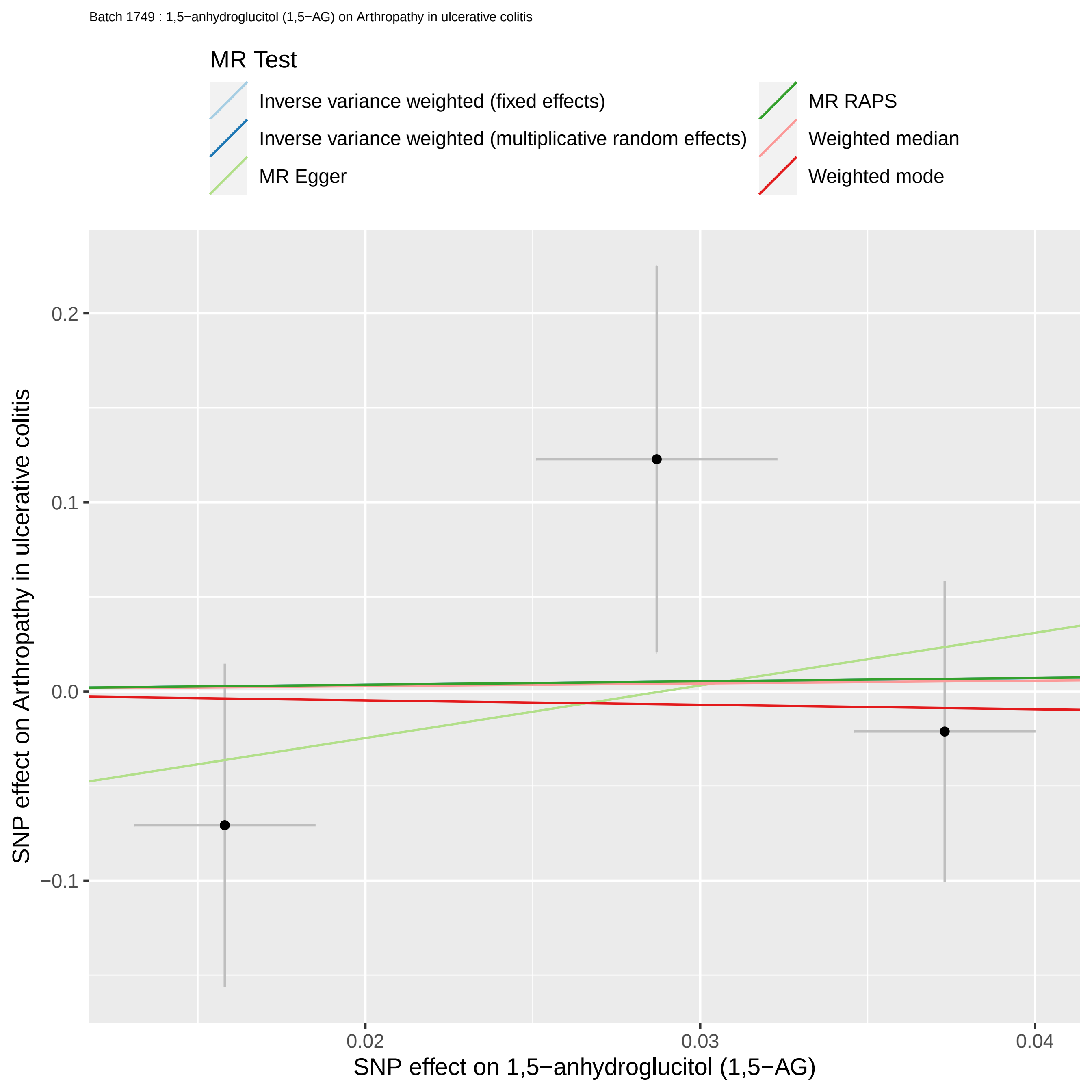
Batch 1749 : 1,5−anhydroglucitol (1,5−AG) on Arthropathy in ulcerative colitis
MR Test
Inverse variance weighted (fixed effects)
Inverse variance weighted (multiplicative random effects)
MR Egger
MR RAPS
Weighted median
Weighted mode
0.2
0.1
0.0
−0.1
0.02
0.03
0.04
SNP effect on Arthropathy in ulcerative colitis
SNP effect on 1,5−anhydroglucitol (1,5−AG)

Batch 1750 : 2−hydroxybutyrate (AHB) on Arthropathy in ulcerative colitis

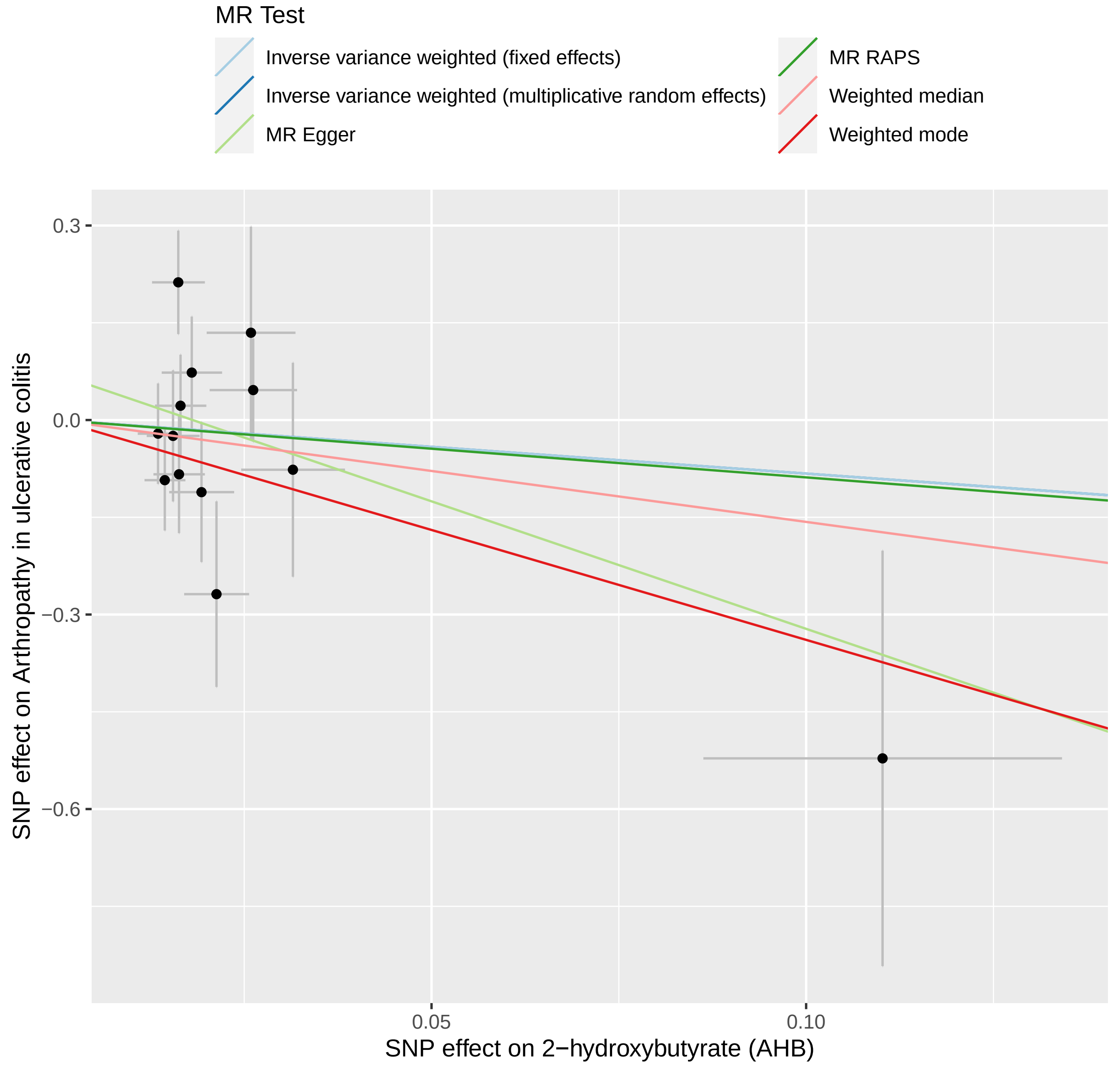

Batch 1753 : 1−palmitoylglycerol (1−monopalmitin) on Arthropathy in ulcerative colitis

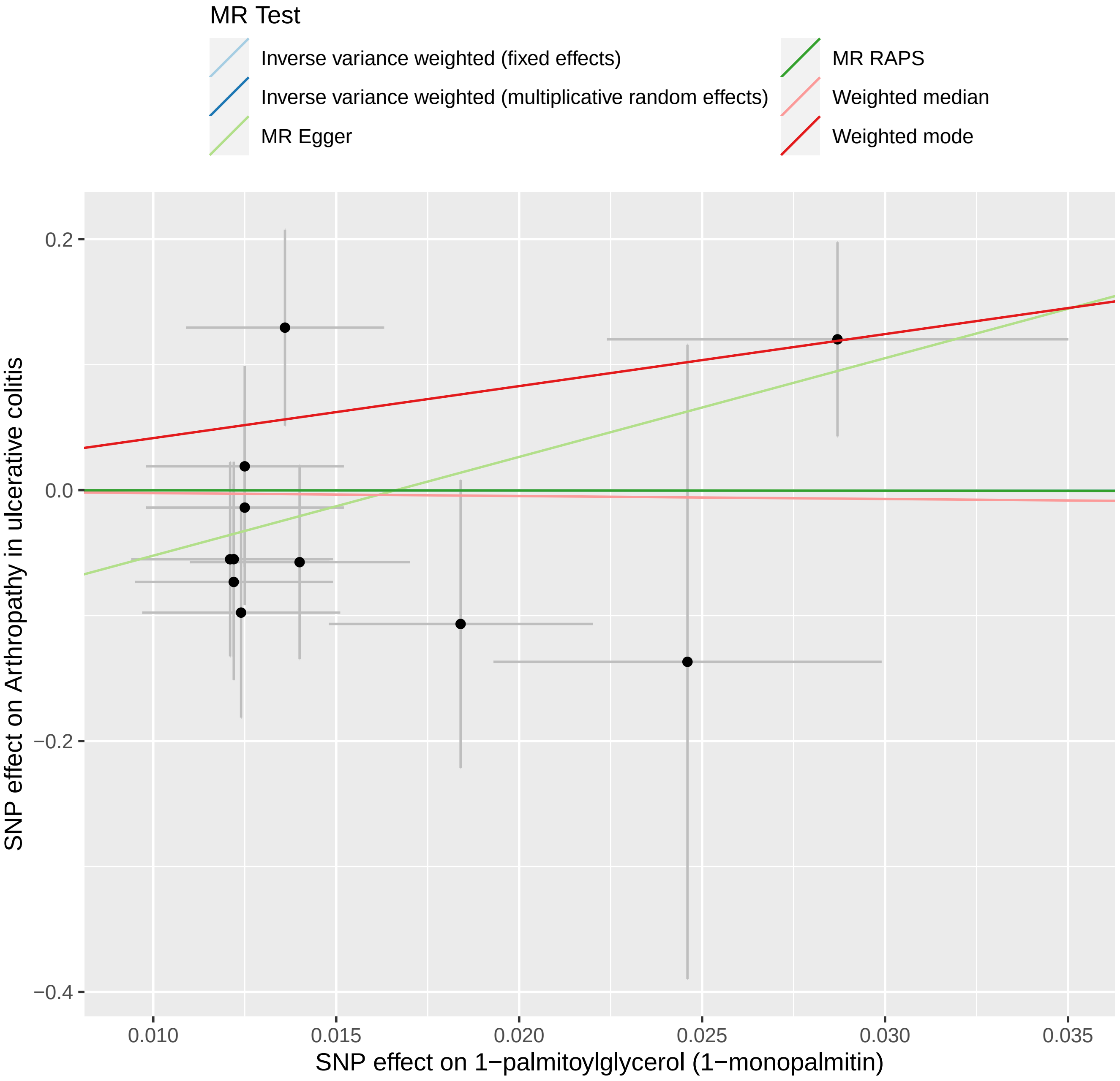

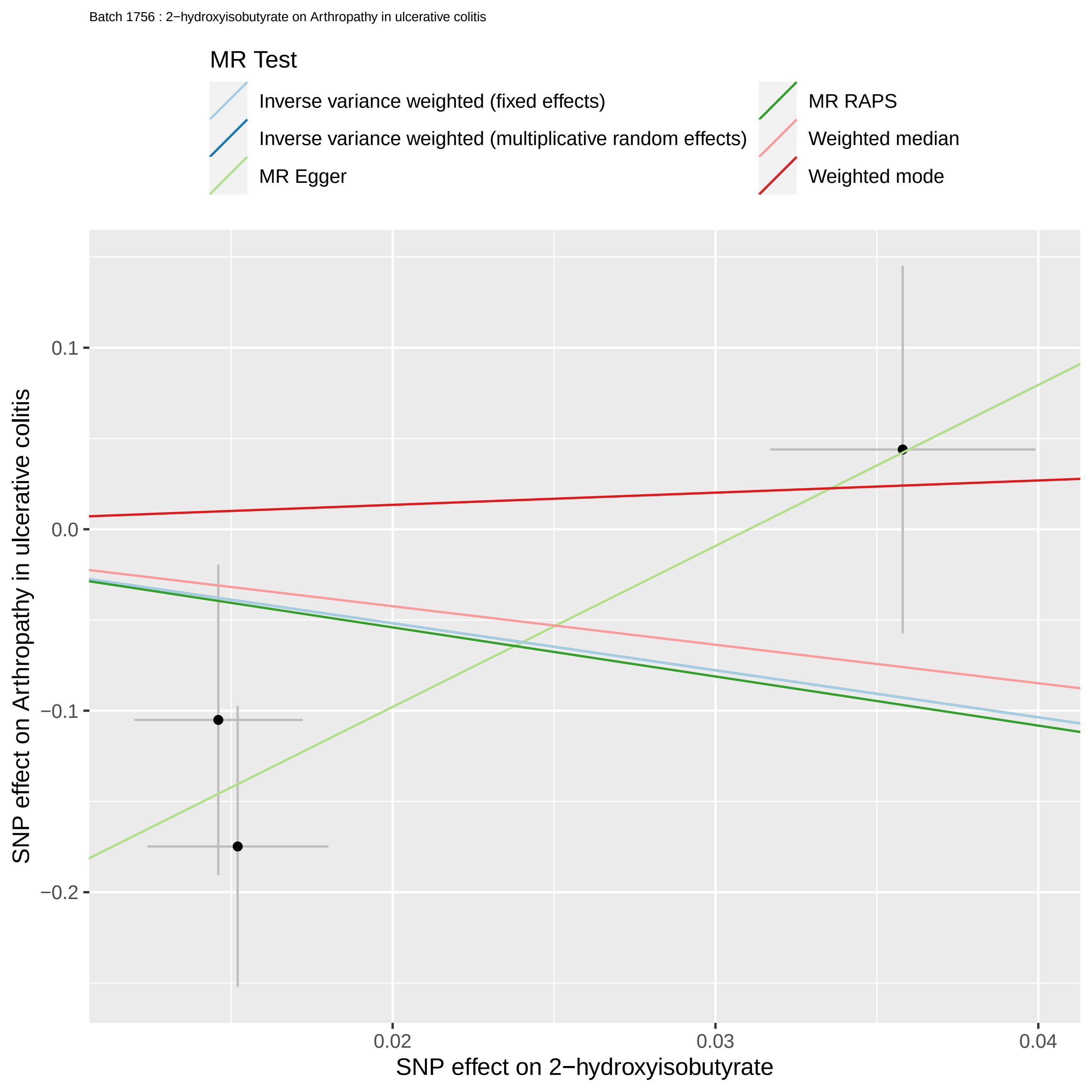
Batch 1756 : 2−hydroxyisobutyrate on Arthropathy in ulcerative colitis
MR Test
Inverse variance weighted (fixed effects)
Inverse variance weighted (multiplicative random effects)
MR Egger
MR RAPS
Weighted median
Weighted mode
0.1
0.0
−0.1
−0.2
0.02
0.03
0.04
SNP effect on Arthropathy in ulcerative colitis
SNP effect on 2−hydroxyisobutyrate

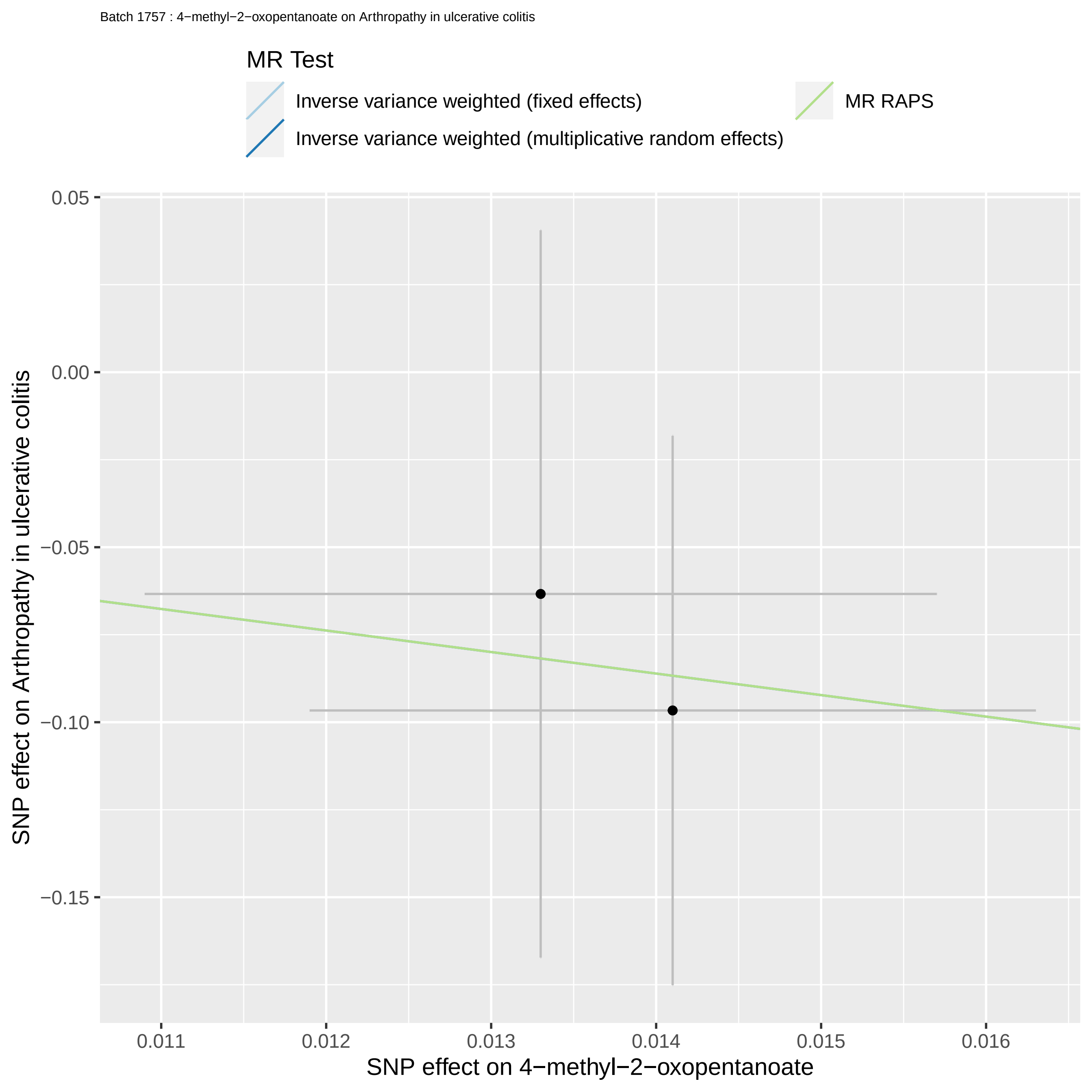

Batch 1757 : 4−methyl−2−oxopentanoate on Arthropathy in ulcerative colitis
MR Test
Inverse variance weighted (fixed effects)
Inverse variance weighted (multiplicative random effects)
MR RAPS
0.05
0.00
−0.05
−0.10
−0.15
0.011
0.012
0.013
0.014
0.015
0.016
SNP effect on Arthropathy in ulcerative colitis
SNP effect on 4−methyl−2−oxopentanoate

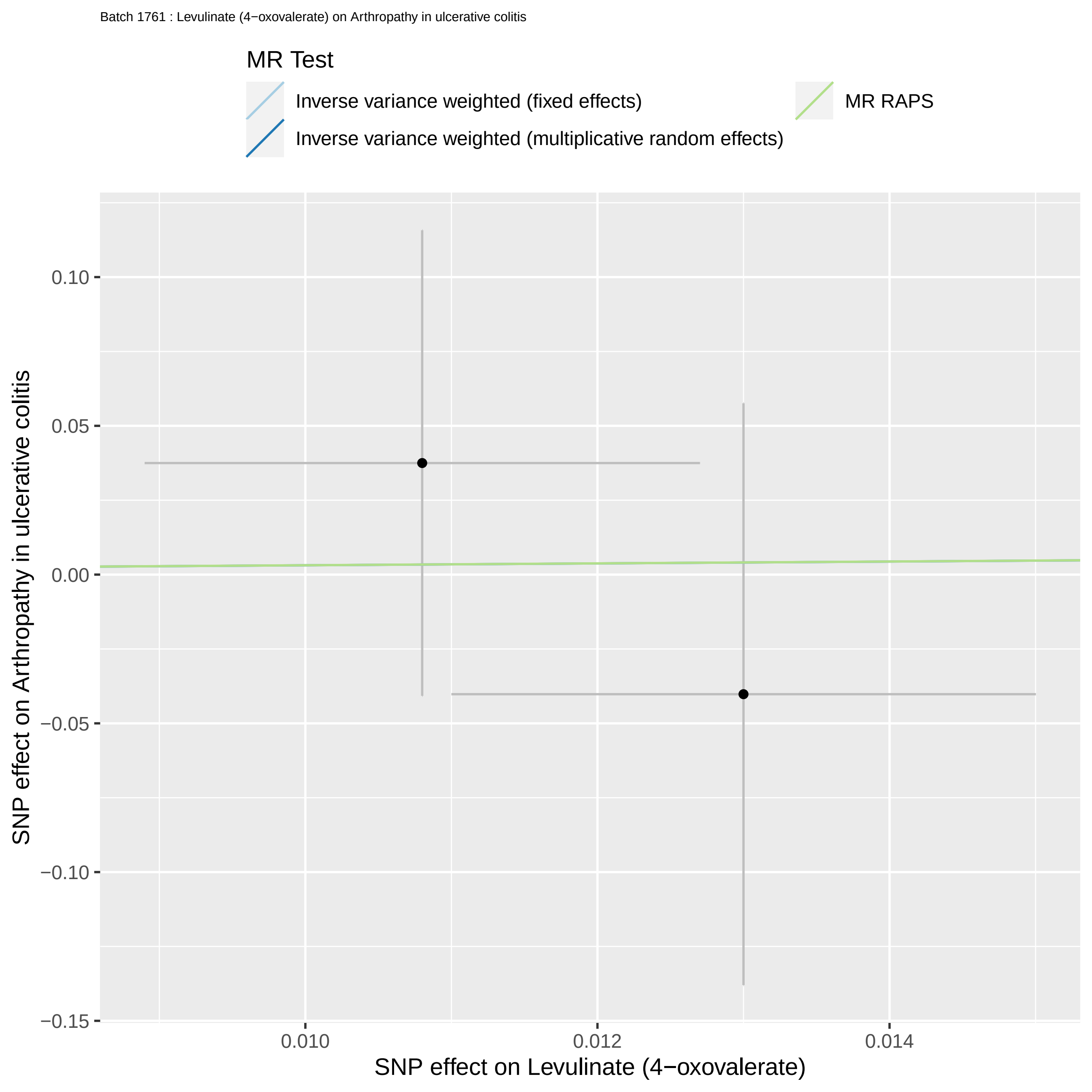
Batch 1761 : Levulinate (4−oxovalerate) on Arthropathy in ulcerative colitis
MR Test
Inverse variance weighted (fixed effects)
Inverse variance weighted (multiplicative random effects)
MR RAPS
0.10
0.05
0.00
−0.05
−0.10
−0.15
0.010
0.012
0.014
SNP effect on Arthropathy in ulcerative colitis
SNP effect on Levulinate (4−oxovalerate)

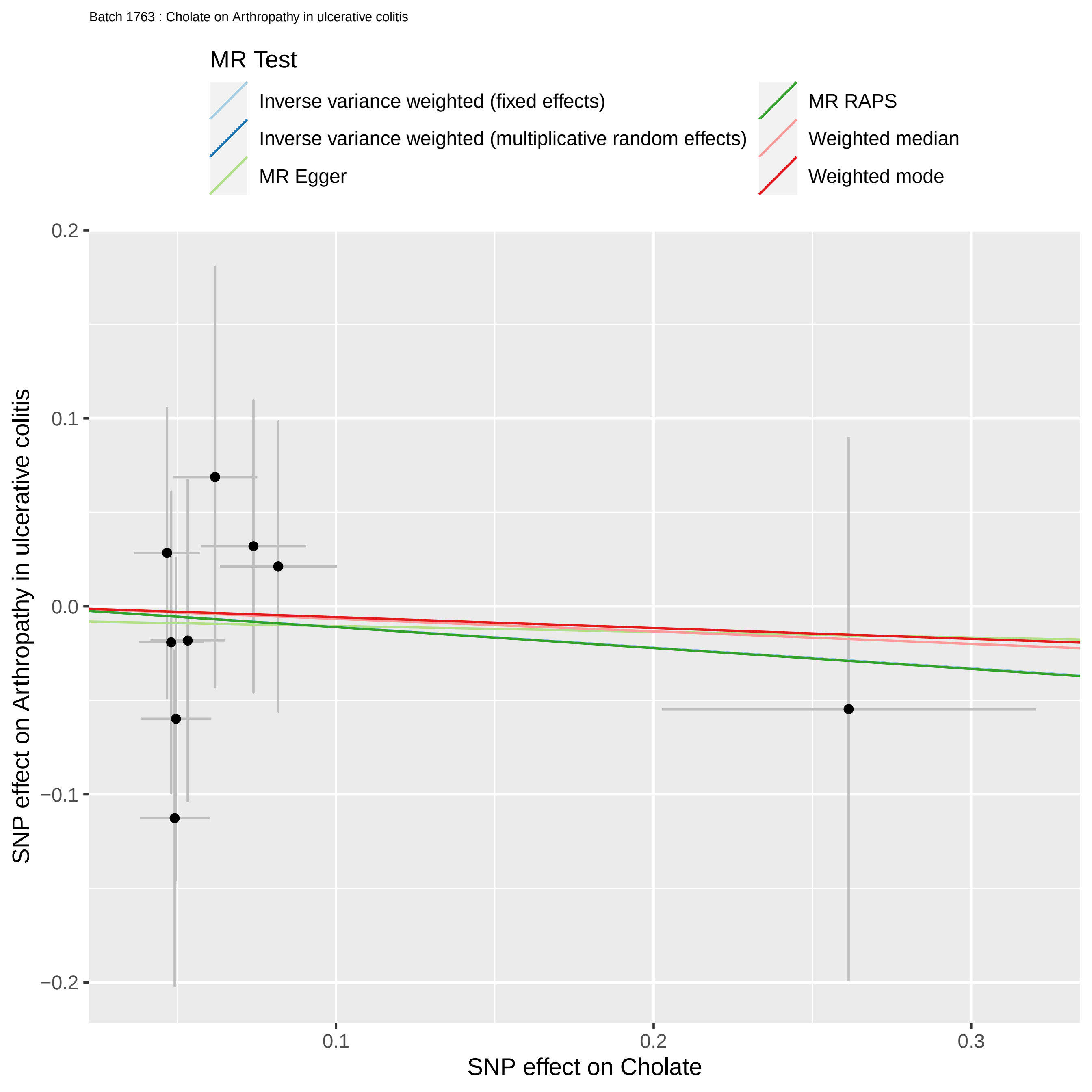

Batch 1763 : Cholate on Arthropathy in ulcerative colitis
MR Test
Inverse variance weighted (fixed effects)
Inverse variance weighted (multiplicative random effects)
MR Egger
MR RAPS
Weighted median
Weighted mode
0.2
0.1
0.0
−0.1
−0.2
0.1
0.2
0.3
SNP effect on Arthropathy in ulcerative colitis
SNP effect on Cholate

Batch 1766 : Hyodeoxycholate on Arthropathy in ulcerative colitis

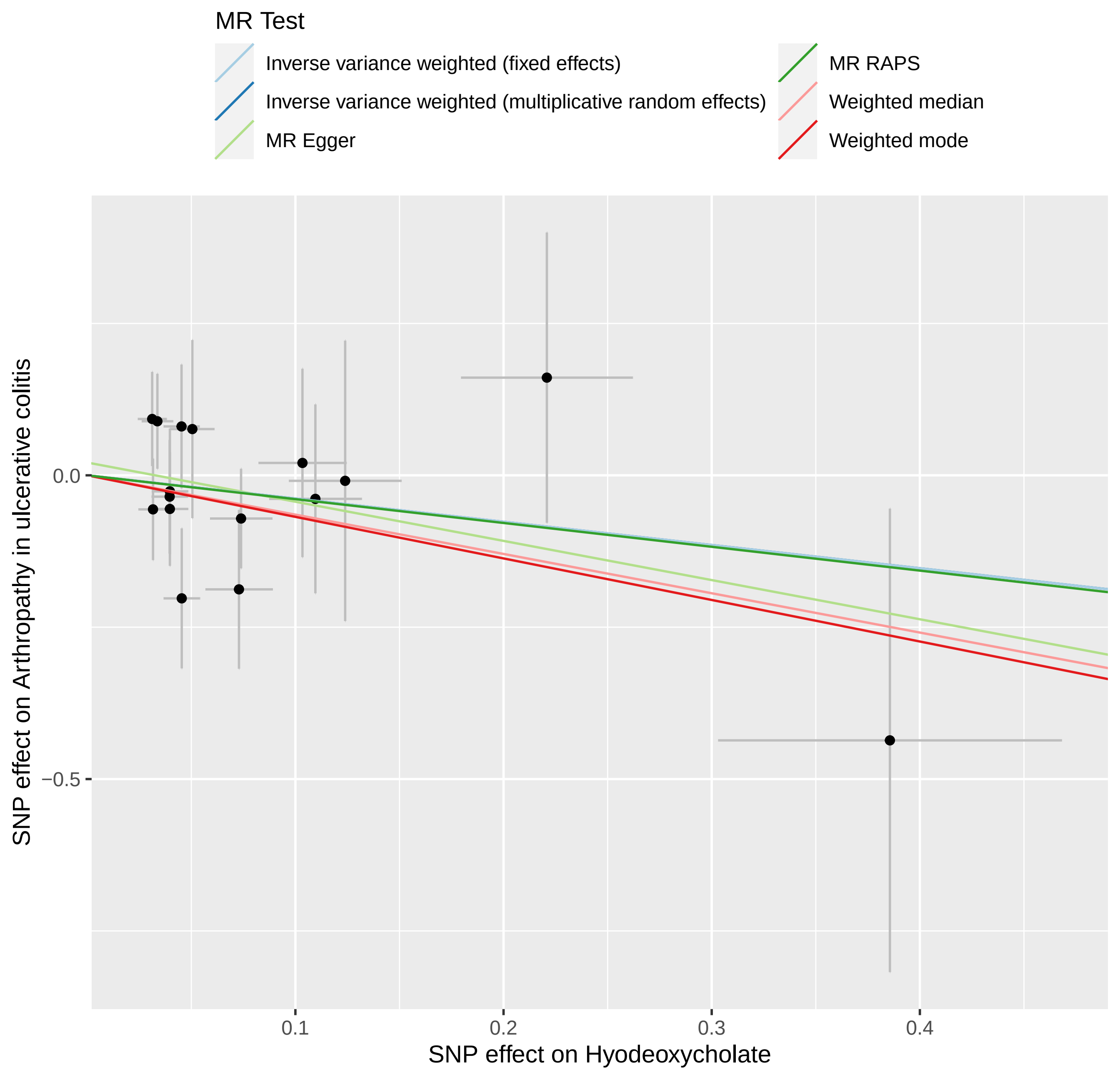

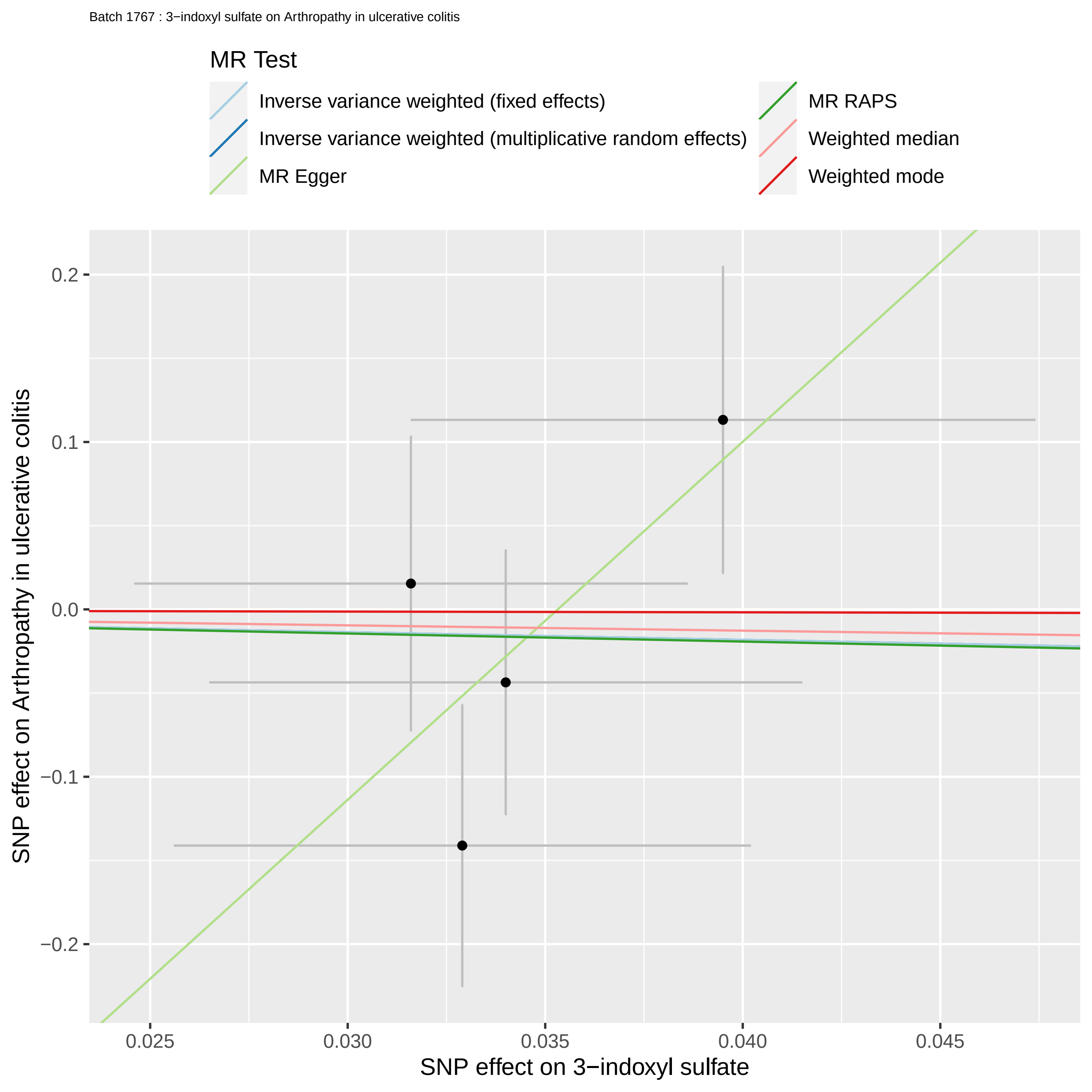

Batch 1767 : 3−indoxyl sulfate on Arthropathy in ulcerative colitis
MR Test
Inverse variance weighted (fixed effects)
Inverse variance weighted (multiplicative random effects)
MR Egger
MR RAPS
Weighted median
Weighted mode
0.2
0.1
0.0
−0.1
−0.2
0.025
0.030
0.035
0.040
0.045
SNP effect on Arthropathy in ulcerative colitis
SNP effect on 3−indoxyl sulfate

Batch 1771 : Erythrose on Arthropathy in ulcerative colitis

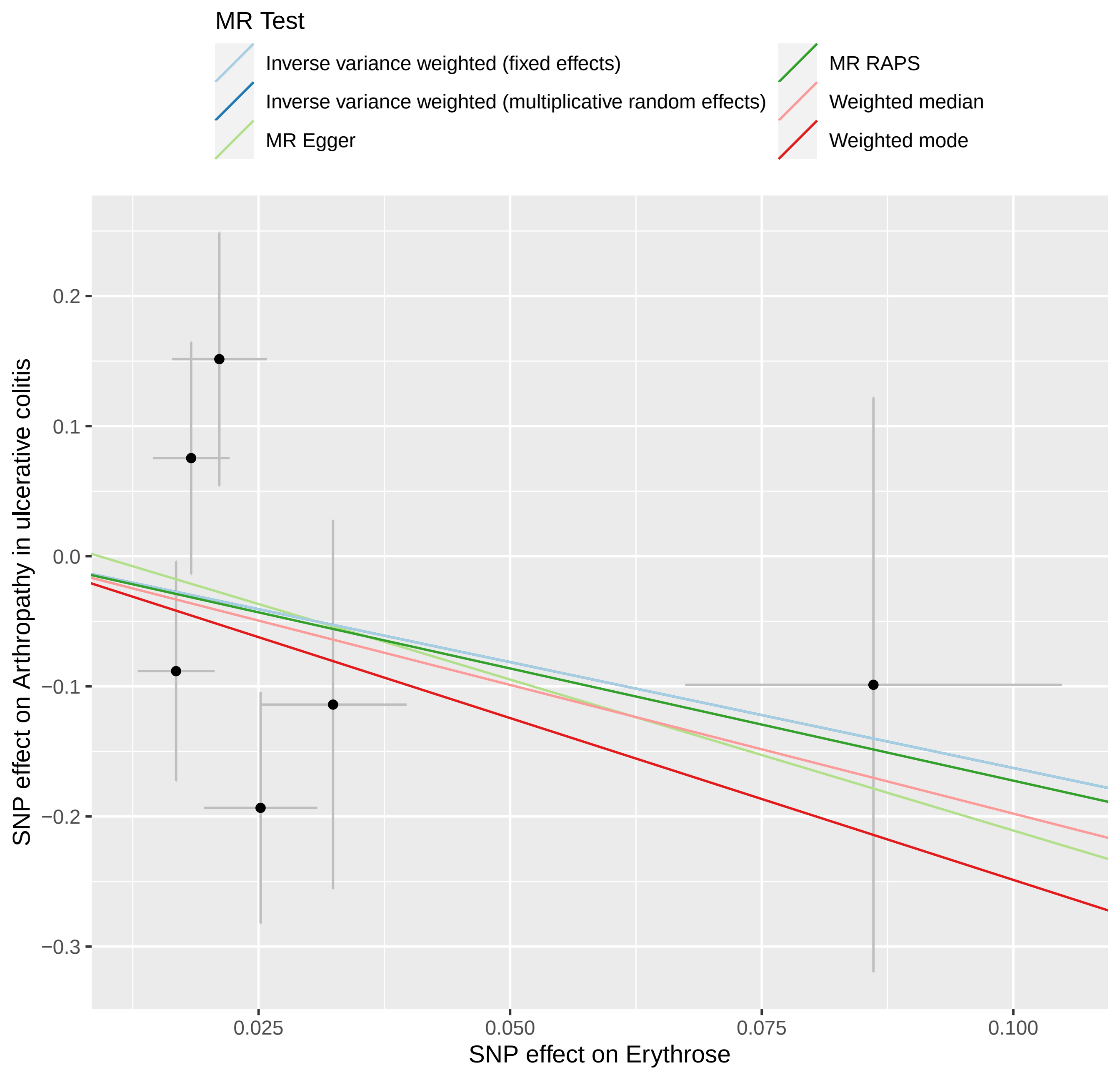

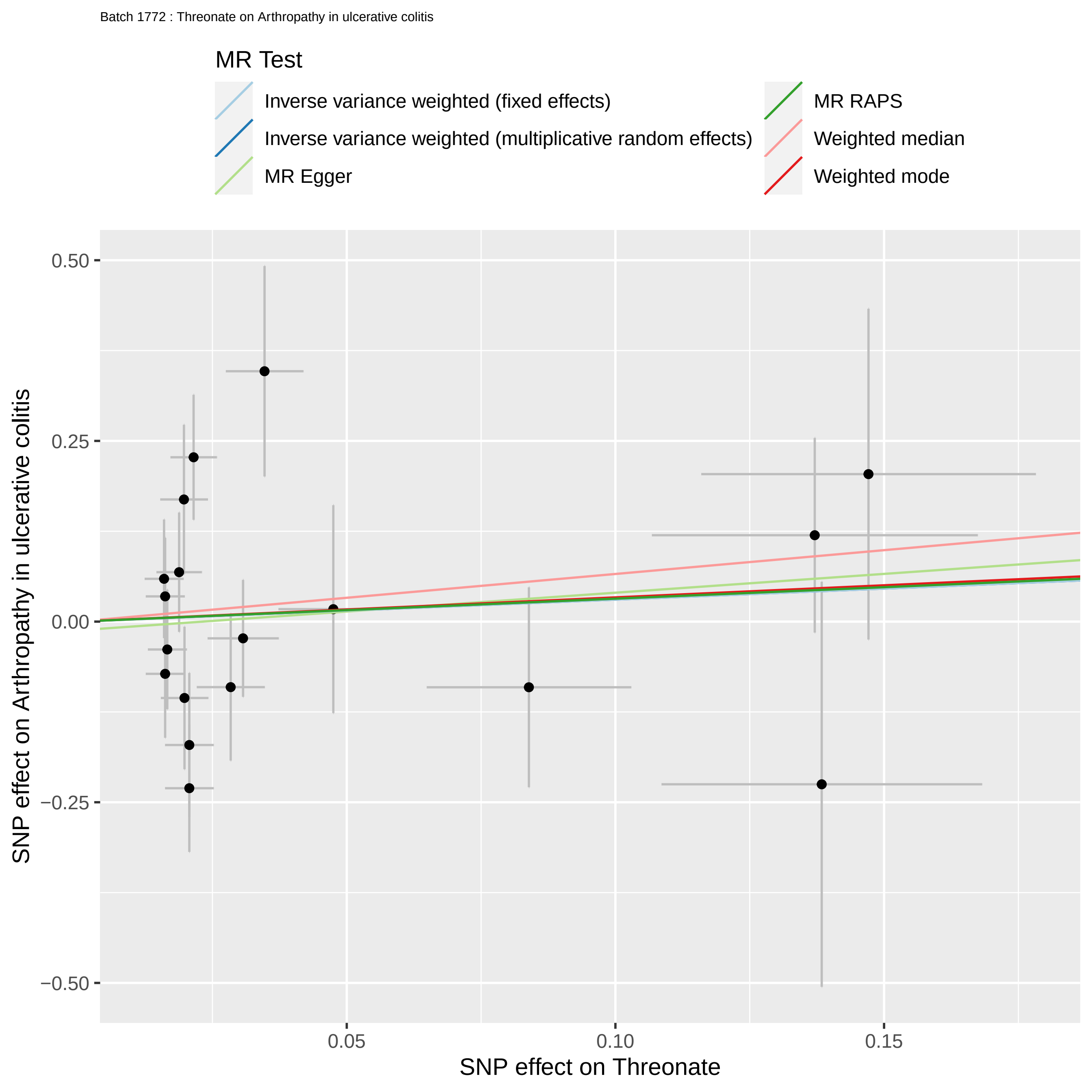
Batch 1772 : Threonate on Arthropathy in ulcerative colitis
MR Test
Inverse variance weighted (fixed effects)
Inverse variance weighted (multiplicative random effects)
MR Egger
MR RAPS
Weighted median
Weighted mode
0.50
0.25
0.00
−0.25
−0.50
SNP effect on Arthropathy in ulcerative colitis
0.05
0.10
0.15
SNP effect on Threonate

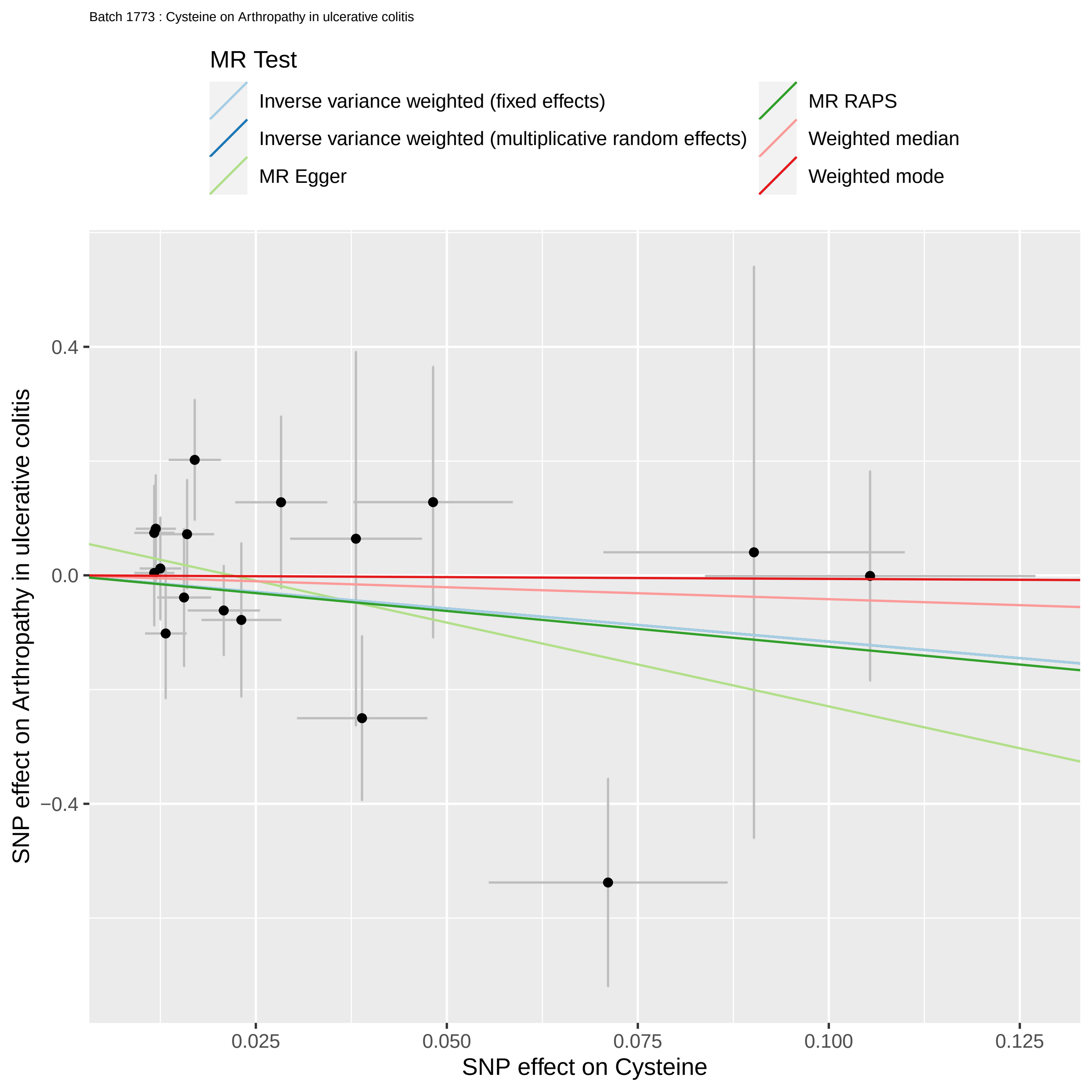

Batch 1773 : Cysteine on Arthropathy in ulcerative colitis
MR Test
Inverse variance weighted (fixed effects)
Inverse variance weighted (multiplicative random effects)
MR Egger
MR RAPS
Weighted median
Weighted mode
SNP effect on Arthropathy in ulcerative colitis
0.4
0.0
−0.4
SNP effect on Cysteine
0.025
0.050
0.075
0.100
0.125

Batch 1774 : Pyroglutamylglycine on Arthropathy in ulcerative colitis

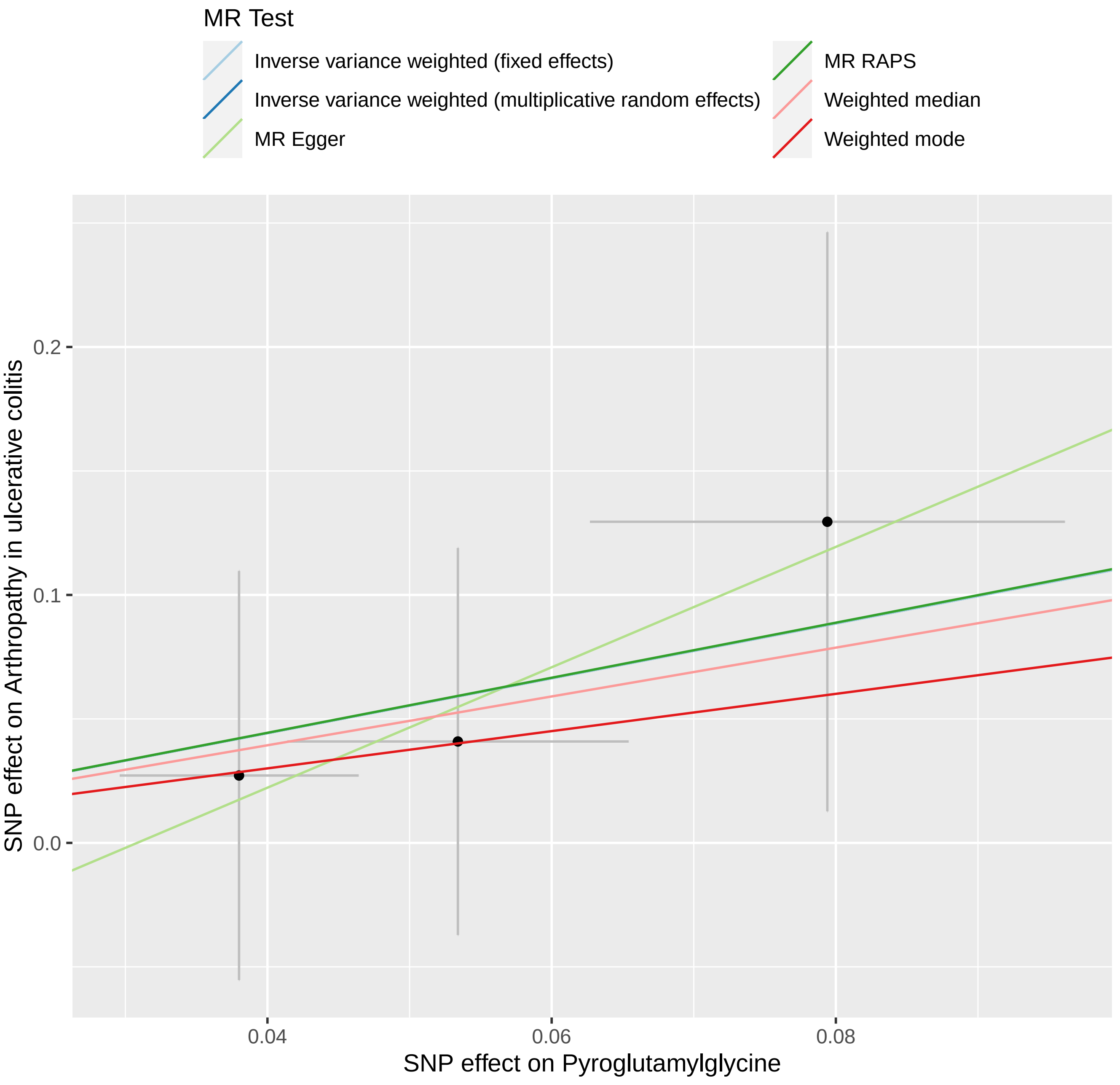

Batch 1776 : DSGEGDFXAEGGGVR* on Arthropathy in ulcerative colitis

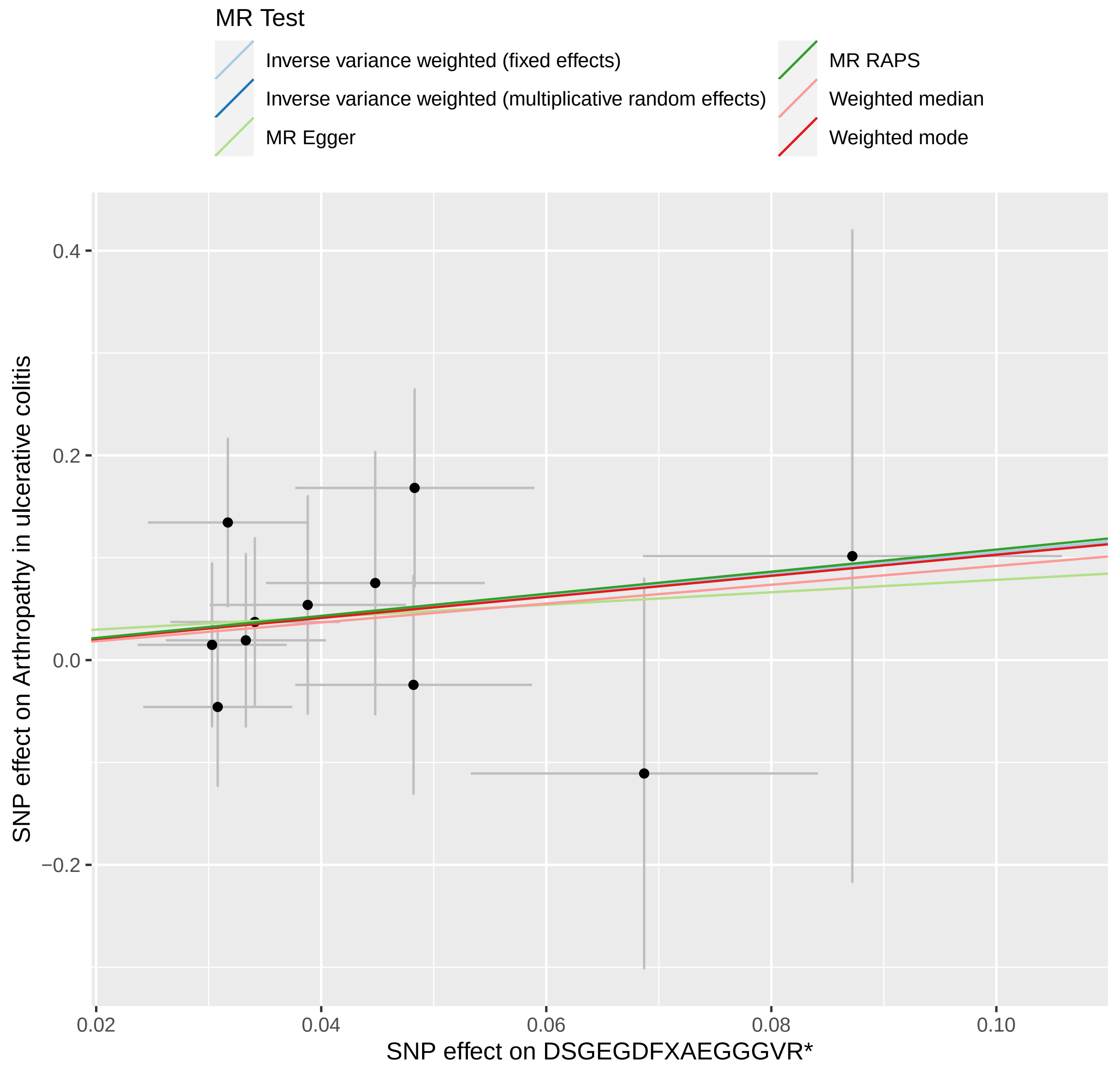

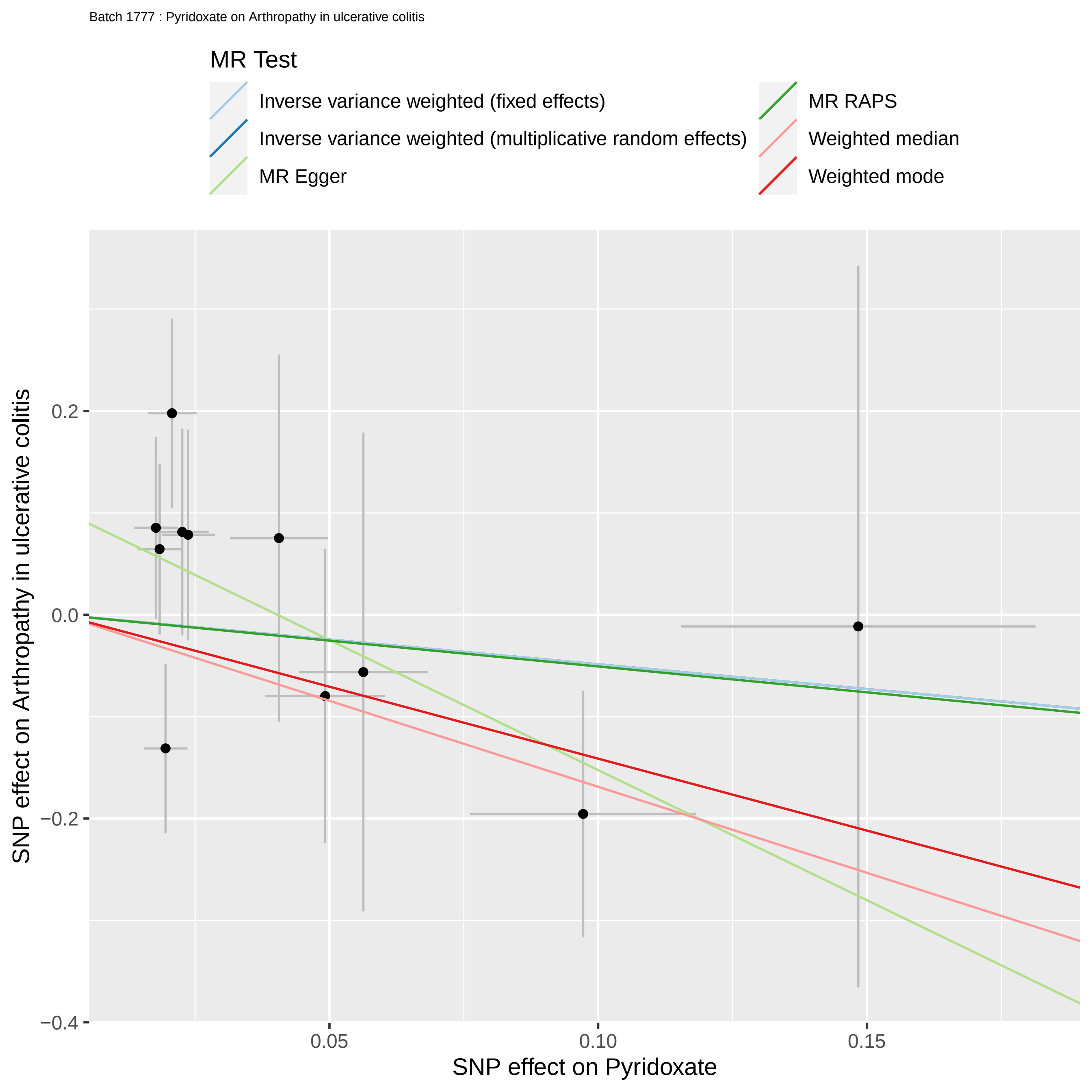

Batch 1777 : Pyridoxate on Arthropathy in ulcerative colitis
MR Test
Inverse variance weighted (fixed effects)
Inverse variance weighted (multiplicative random effects)
MR Egger
MR RAPS
Weighted median
Weighted mode
0.2
0.0
−0.2
−0.4
0.05
0.10
0.15
SNP effect on Arthropathy in ulcerative colitis
SNP effect on Pyridoxate

Batch 1778 : Androsterone sulfate on Arthropathy in ulcerative colitis

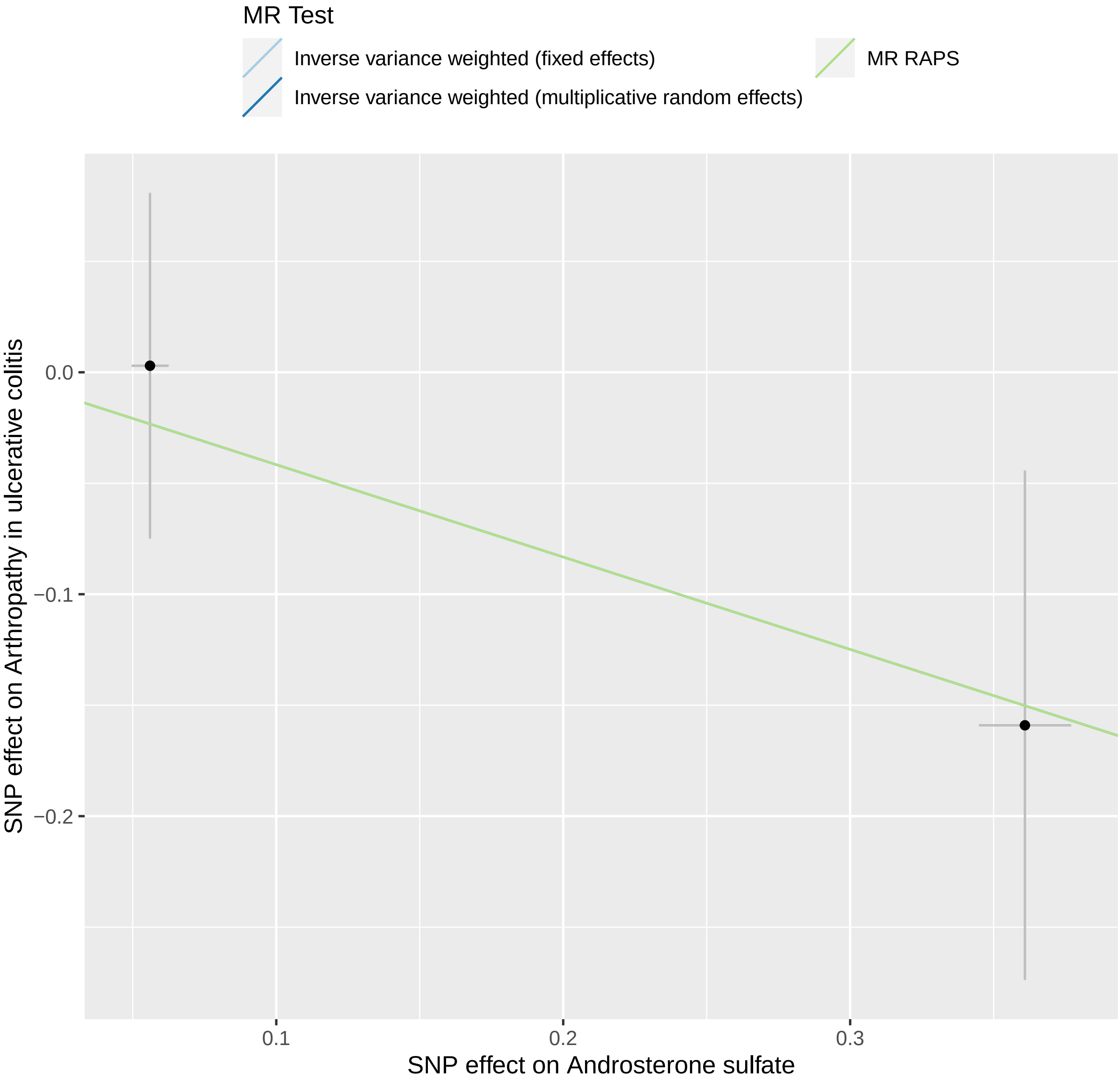

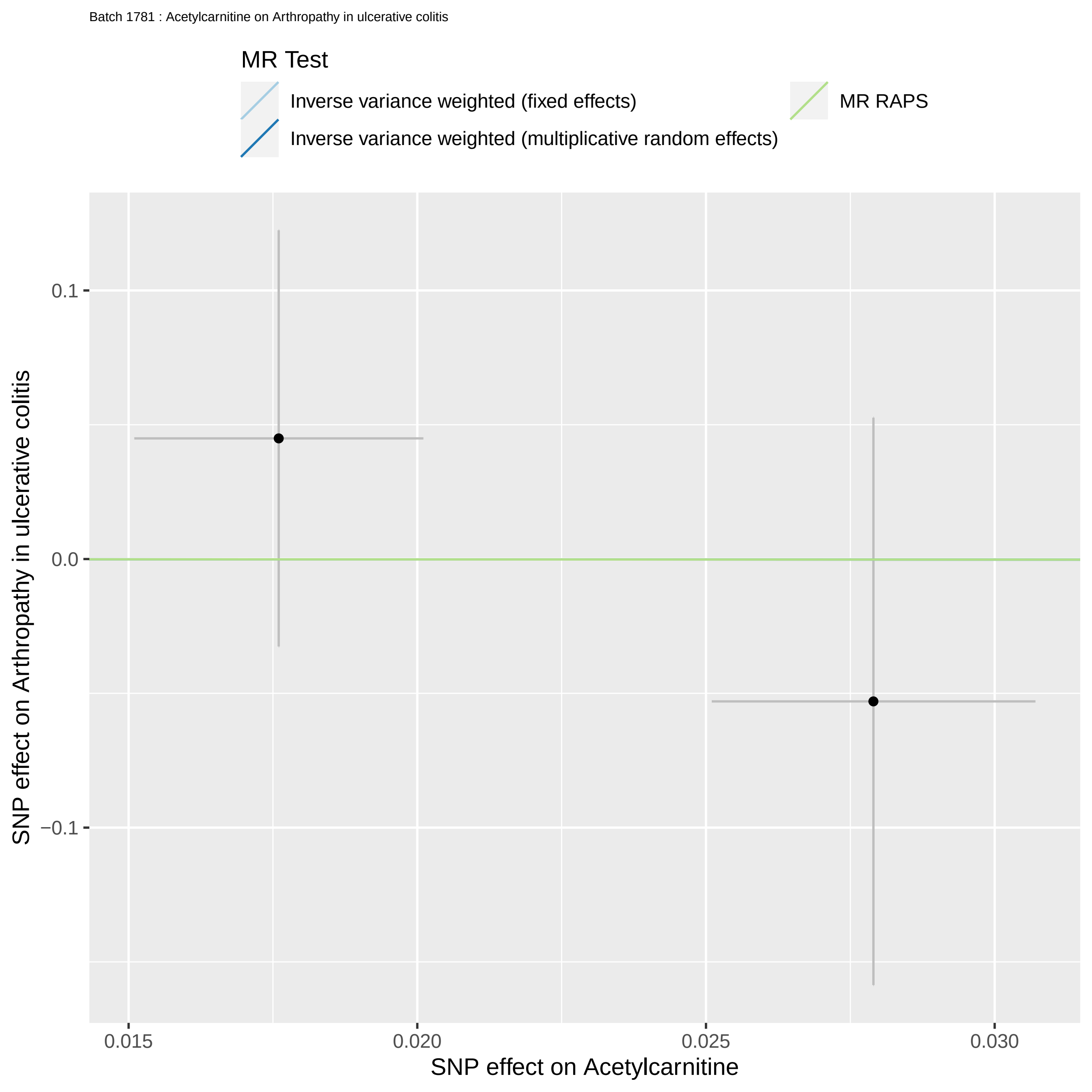

Batch 1781 : Acetylcarnitine on Arthropathy in ulcerative colitis
MR Test
Inverse variance weighted (fixed effects)
Inverse variance weighted (multiplicative random effects)
MR RAPS
0.1
0.0
−0.1
0.015
0.020
0.025
0.030
SNP effect on Acetylcarnitine
SNP effect on Arthropathy in ulcerative colitis

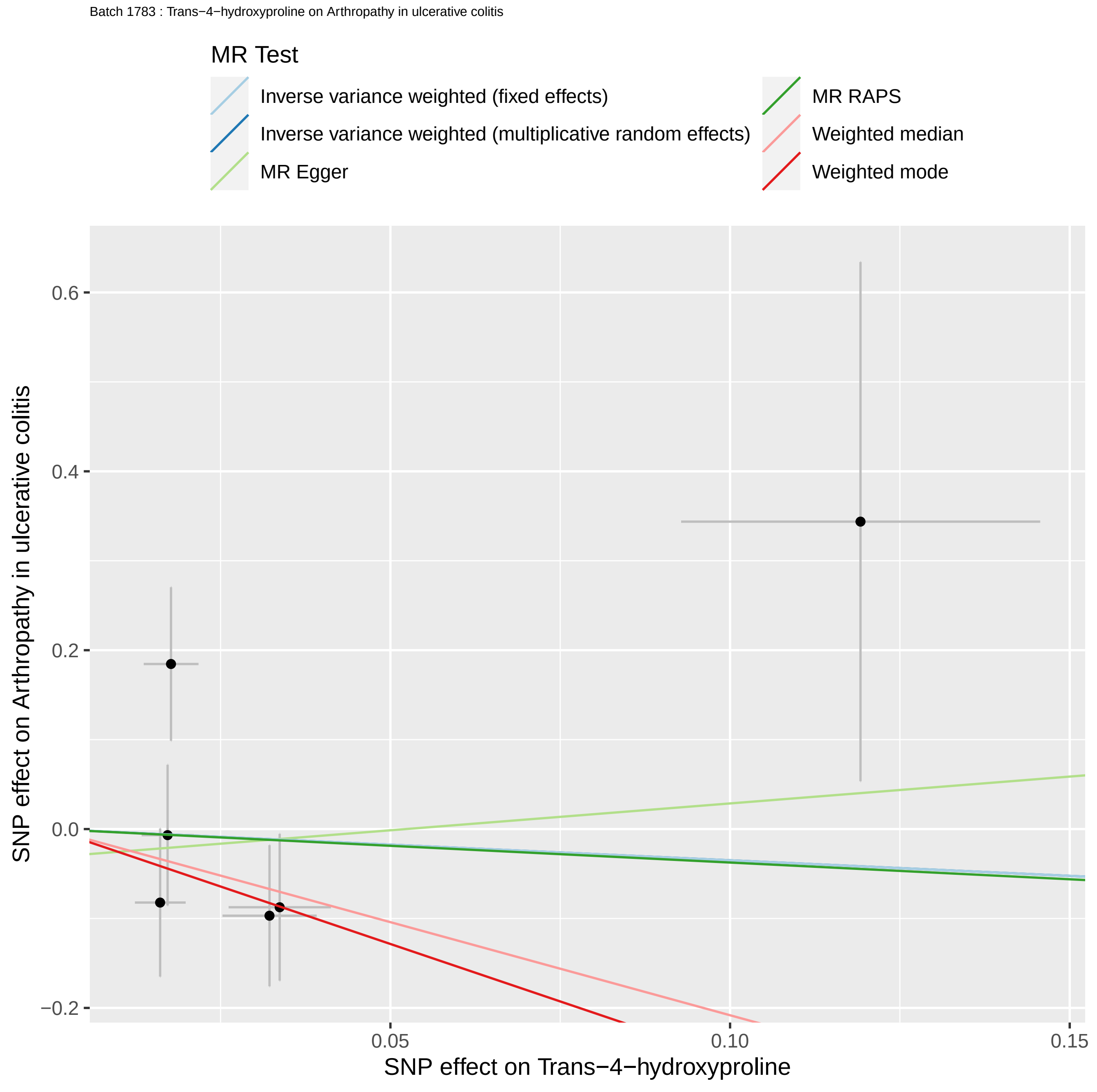
Batch 1783 : Trans−4−hydroxyproline on Arthropathy in ulcerative colitis
MR Test
Inverse variance weighted (fixed effects)
Inverse variance weighted (multiplicative random effects)
MR Egger
MR RAPS
Weighted median
Weighted mode
0.6
0.4
0.2
0.0
−0.2
0.05
0.10
0.15
SNP effect on Arthropathy in ulcerative colitis
SNP effect on Trans−4−hydroxyproline

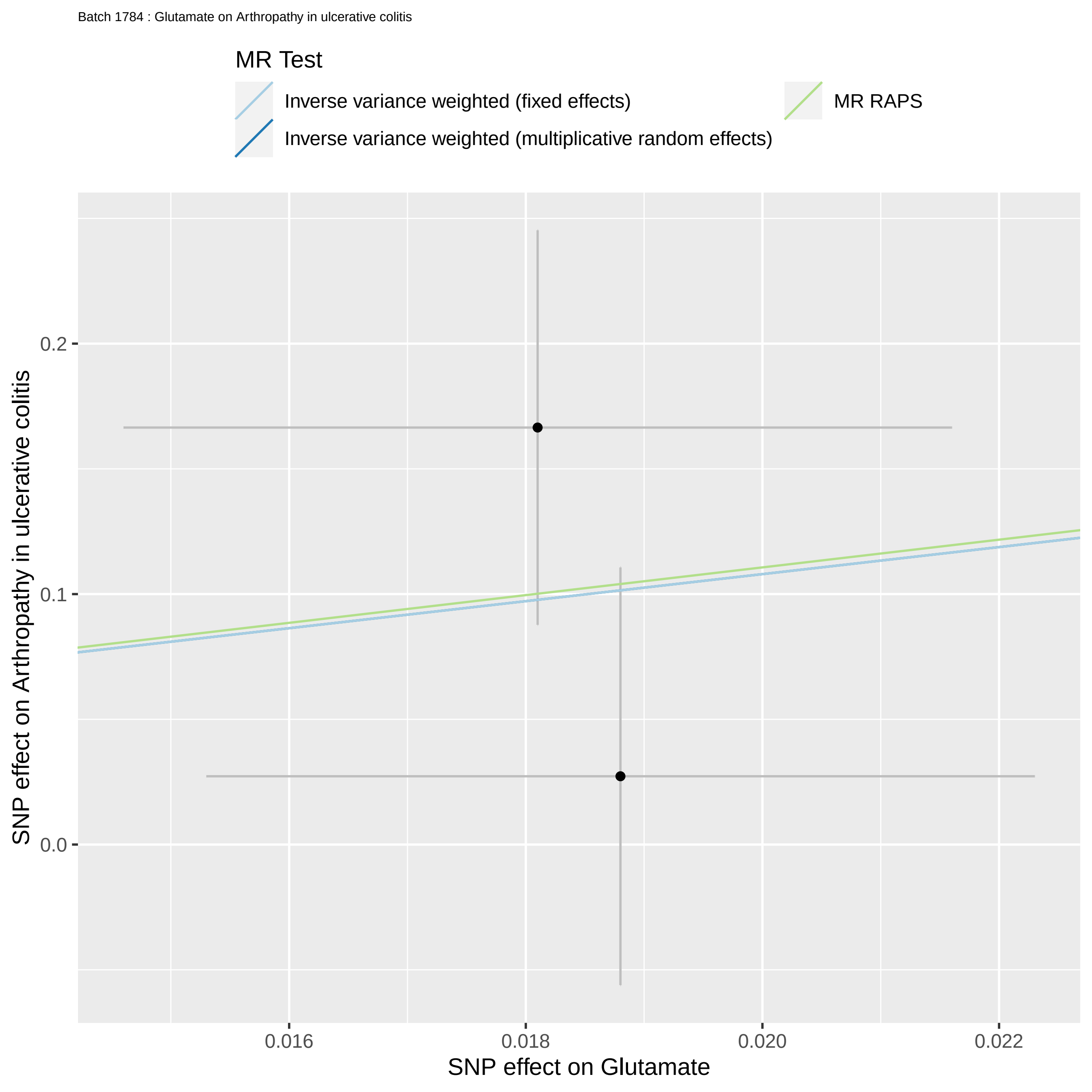

Batch 1784 : Glutamate on Arthropathy in ulcerative colitis
MR Test
Inverse variance weighted (fixed effects)
Inverse variance weighted (multiplicative random effects)
MR RAPS
SNP effect on Arthropathy in ulcerative colitis
0.2
0.1
0.0
0.016
0.018
0.020
0.022
SNP effect on Glutamate

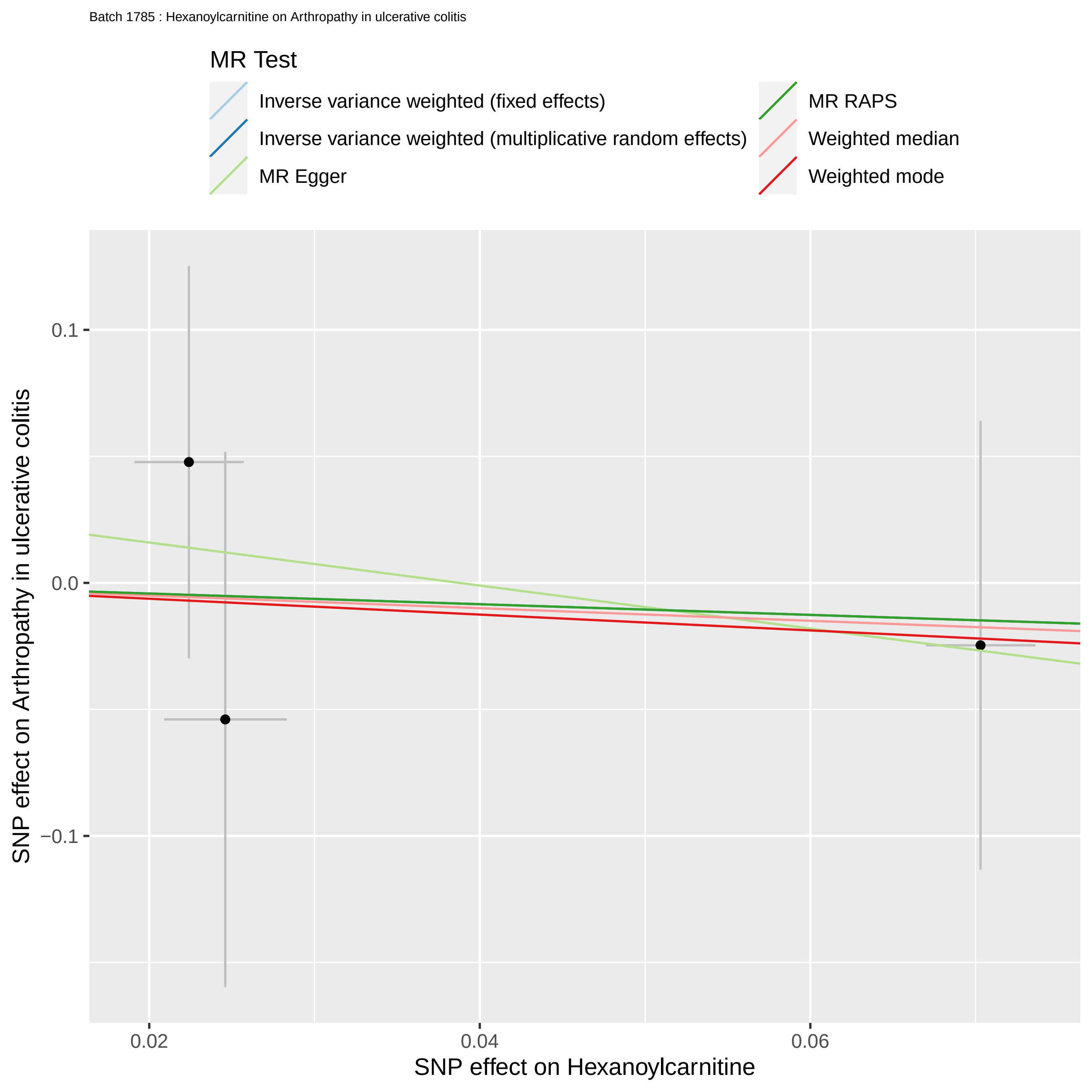

Batch 1785 : Hexanoylcarnitine on Arthropathy in ulcerative colitis
MR Test
Inverse variance weighted (fixed effects)
Inverse variance weighted (multiplicative random effects)
MR Egger
MR RAPS
Weighted median
Weighted mode
0.1
0.0
−0.1
0.02
0.04
0.06
SNP effect on Arthropathy in ulcerative colitis
SNP effect on Hexanoylcarnitine

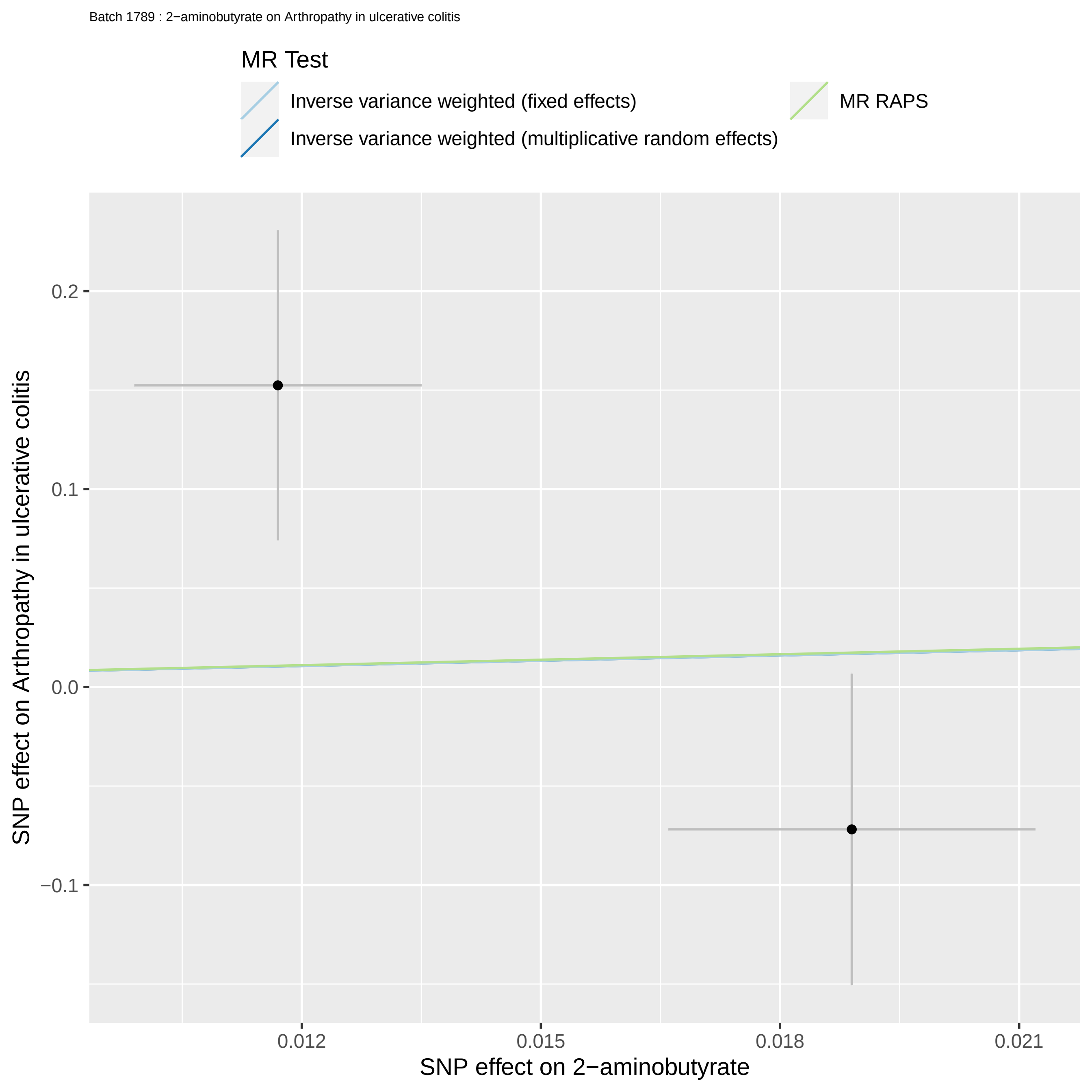
Batch 1789 : 2−aminobutyrate on Arthropathy in ulcerative colitis
MR Test
Inverse variance weighted (fixed effects)
Inverse variance weighted (multiplicative random effects)
MR RAPS
SNP effect on Arthropathy in ulcerative colitis
0.2
0.1
0.0
−0.1
0.012
0.015
0.018
0.021
SNP effect on 2−aminobutyrate

Batch 1794 : Butyrylcarnitine on Arthropathy in ulcerative colitis

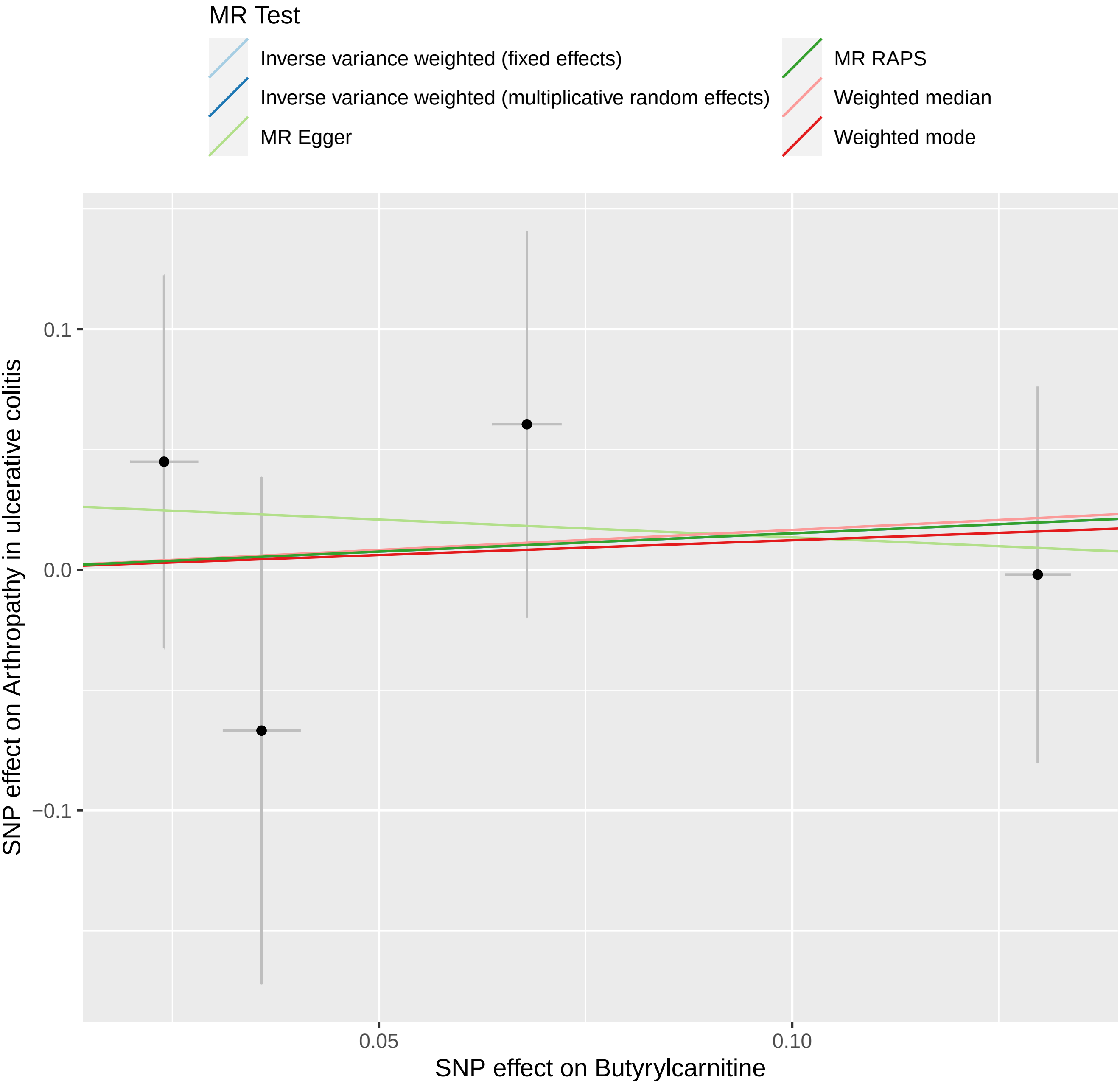

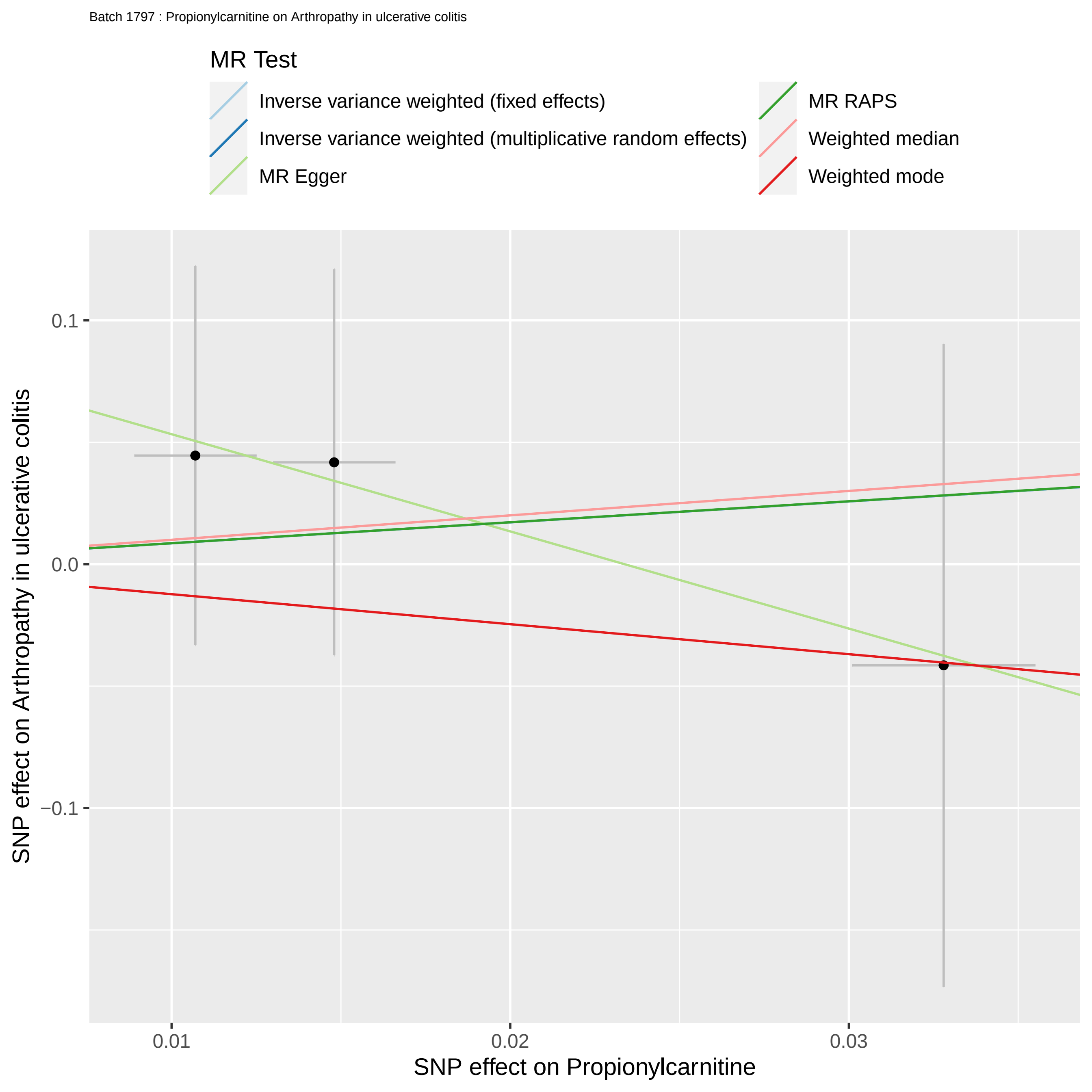

Batch 1797 : Propionylcarnitine on Arthropathy in ulcerative colitis
MR Test
Inverse variance weighted (fixed effects)
Inverse variance weighted (multiplicative random effects)
MR Egger
MR RAPS
Weighted median
Weighted mode
0.1
0.0
−0.1
0.01
0.02
0.03
SNP effect on Arthropathy in ulcerative colitis
SNP effect on Propionylcarnitine

Batch 1798 : Caproate (6:0) on Arthropathy in ulcerative colitis

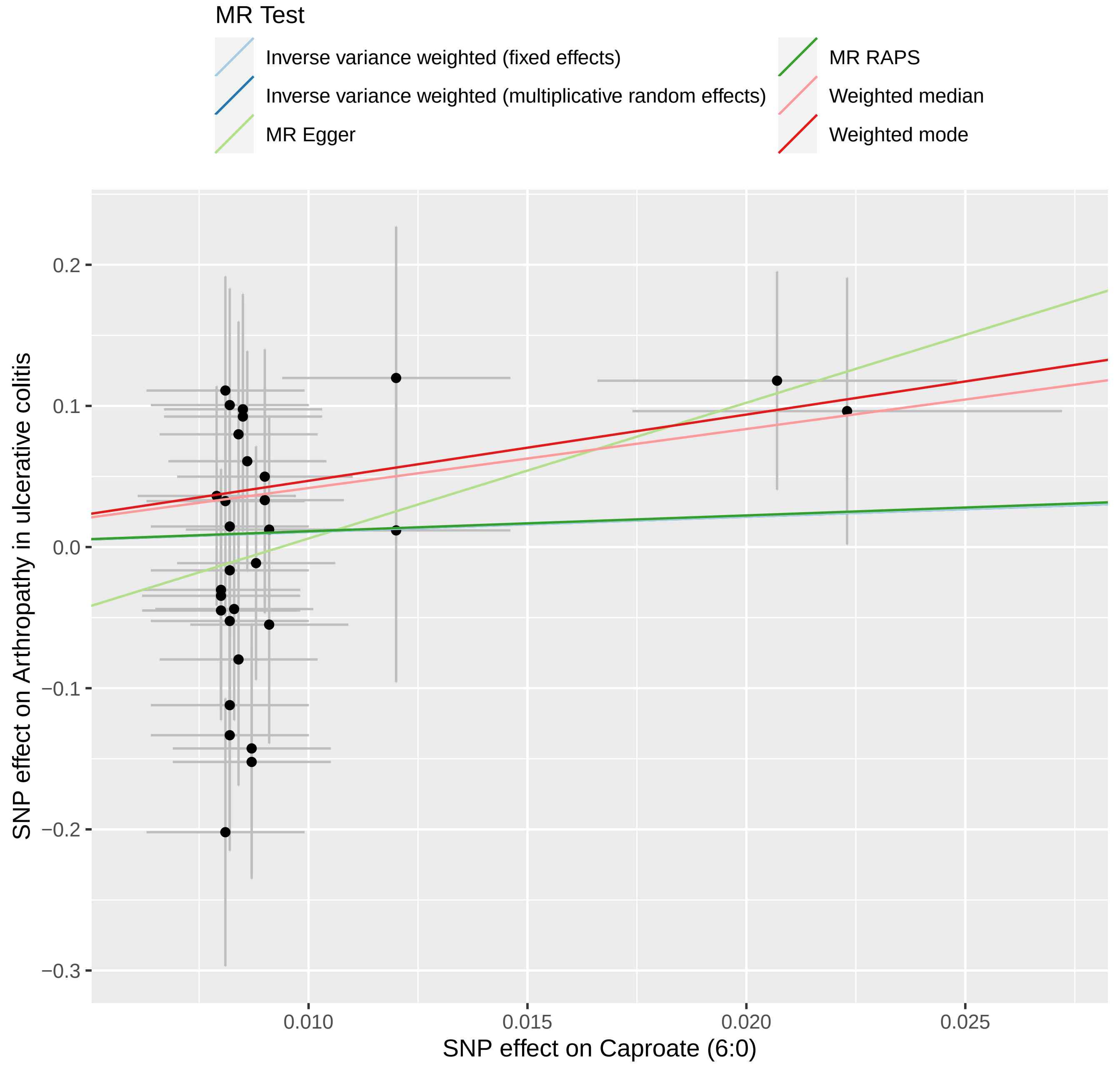

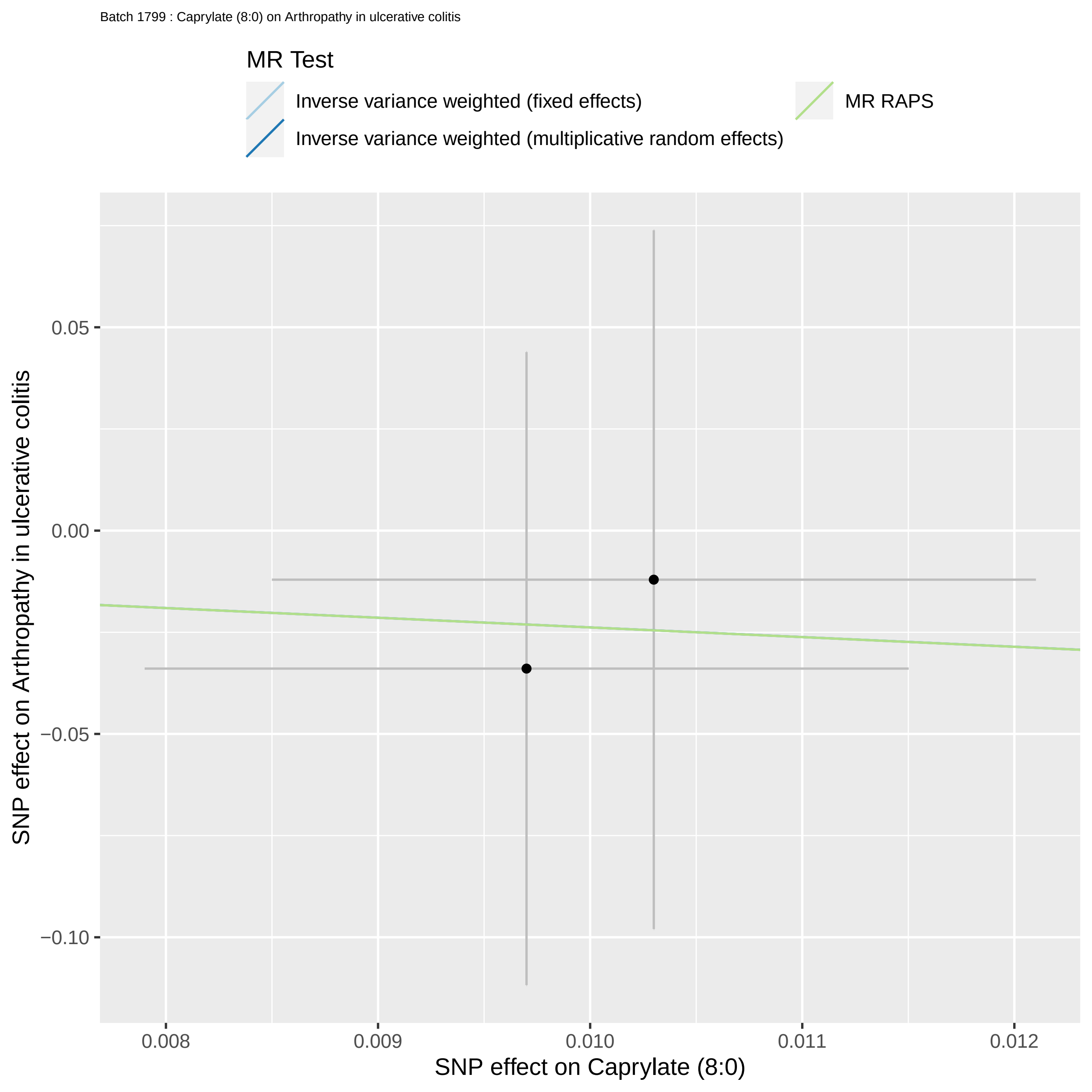
Batch 1799 : Caprylate (8:0) on Arthropathy in ulcerative colitis
MR Test
Inverse variance weighted (fixed effects)
Inverse variance weighted (multiplicative random effects)
MR RAPS
0.05
0.00
−0.05
−0.10
0.008
0.009
0.010
0.011
0.012
SNP effect on Caprylate (8:0)
SNP effect on Arthropathy in ulcerative colitis

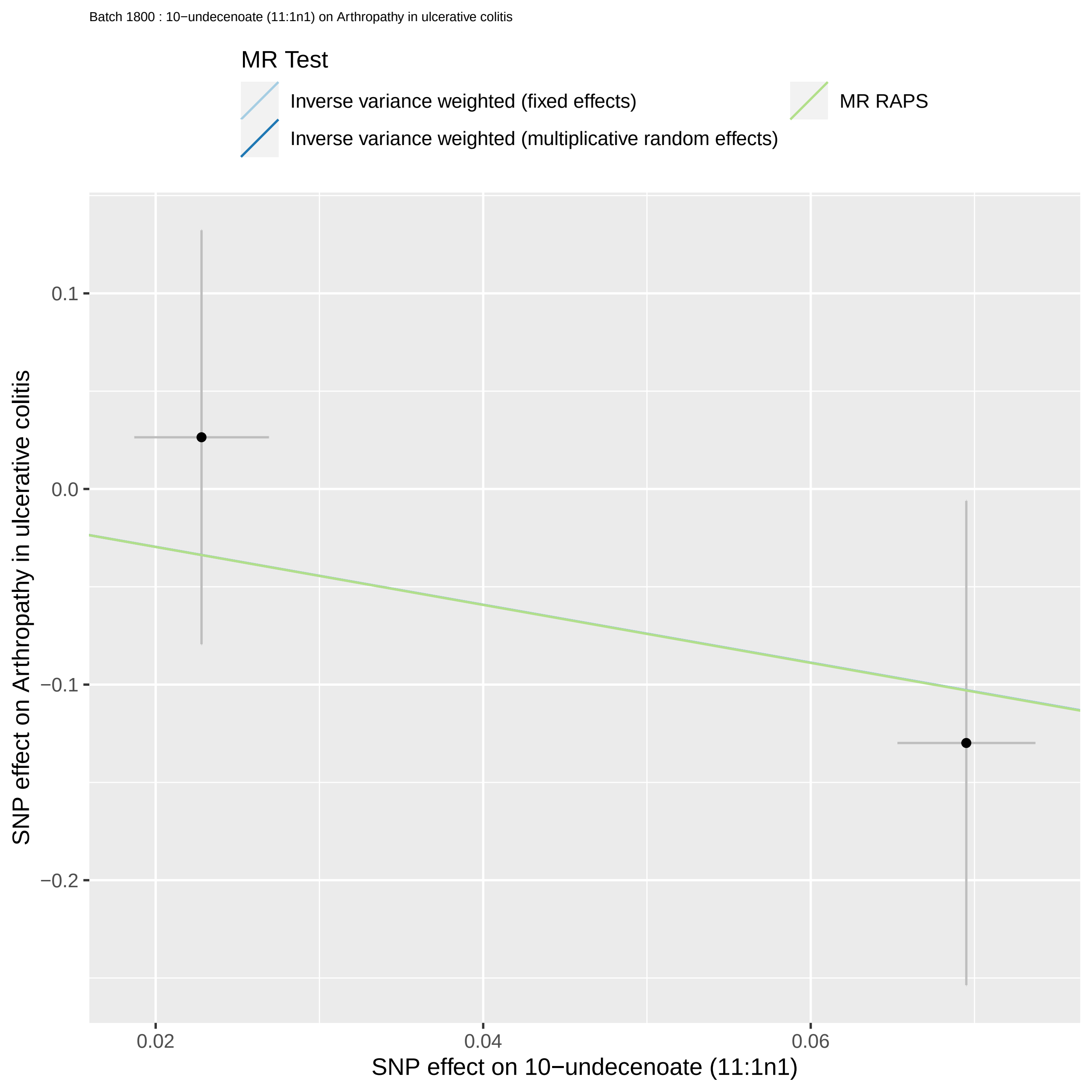

Batch 1800 : 10−undecenoate (11:1n1) on Arthropathy in ulcerative colitis
MR Test
Inverse variance weighted (fixed effects)
Inverse variance weighted (multiplicative random effects)
MR RAPS
0.1
0.0
−0.1
−0.2
SNP effect on Arthropathy in ulcerative colitis
0.02
0.04
0.06
SNP effect on 10−undecenoate (11:1n1)

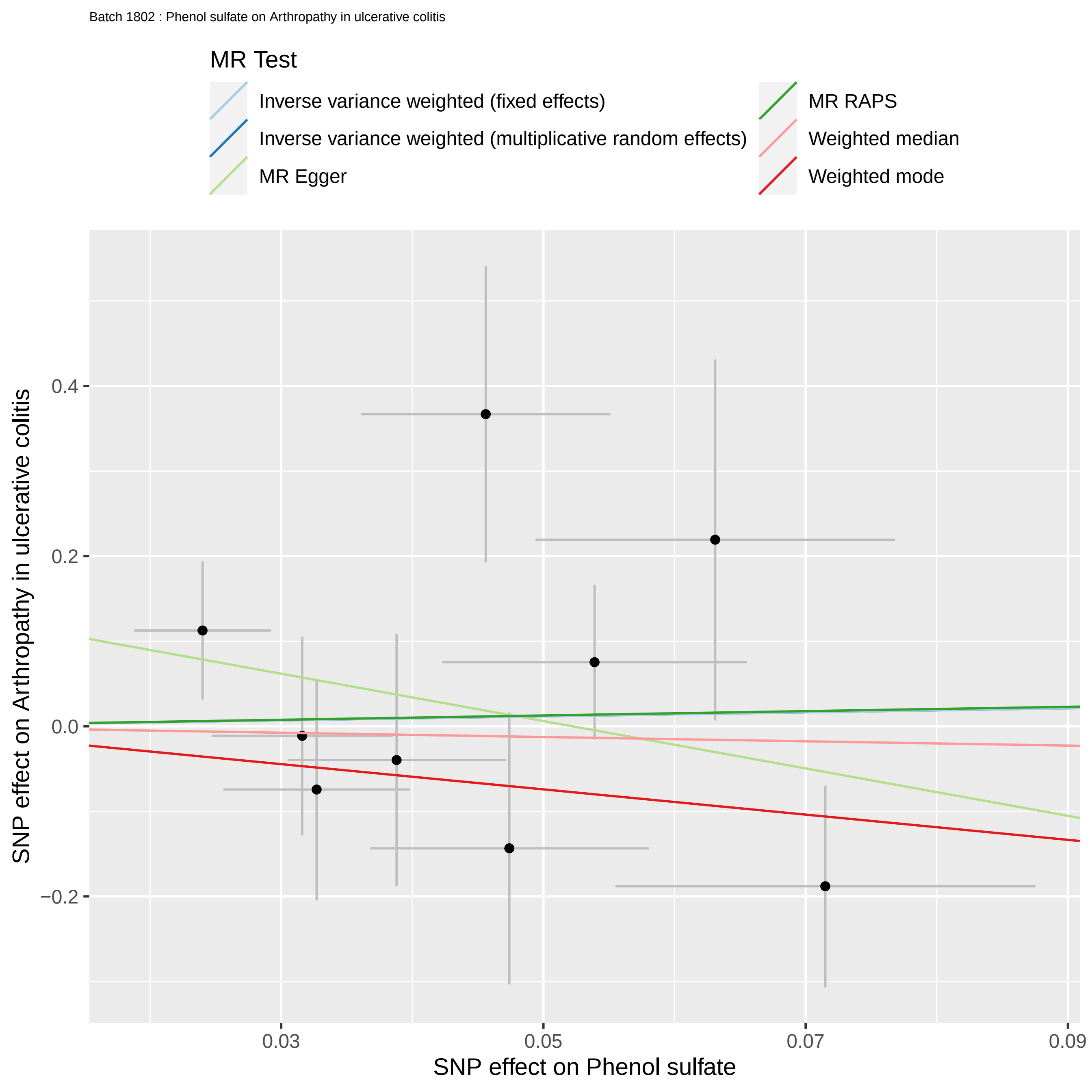

Batch 1802 : Phenol sulfate on Arthropathy in ulcerative colitis
MR Test
Inverse variance weighted (fixed effects)
Inverse variance weighted (multiplicative random effects)
MR Egger
MR RAPS
Weighted median
Weighted mode
0.4
0.2
0.0
−0.2
0.03
0.05
0.07
0.09
SNP effect on Arthropathy in ulcerative colitis
SNP effect on Phenol sulfate

Batch 1804 : Heme* on Arthropathy in ulcerative colitis

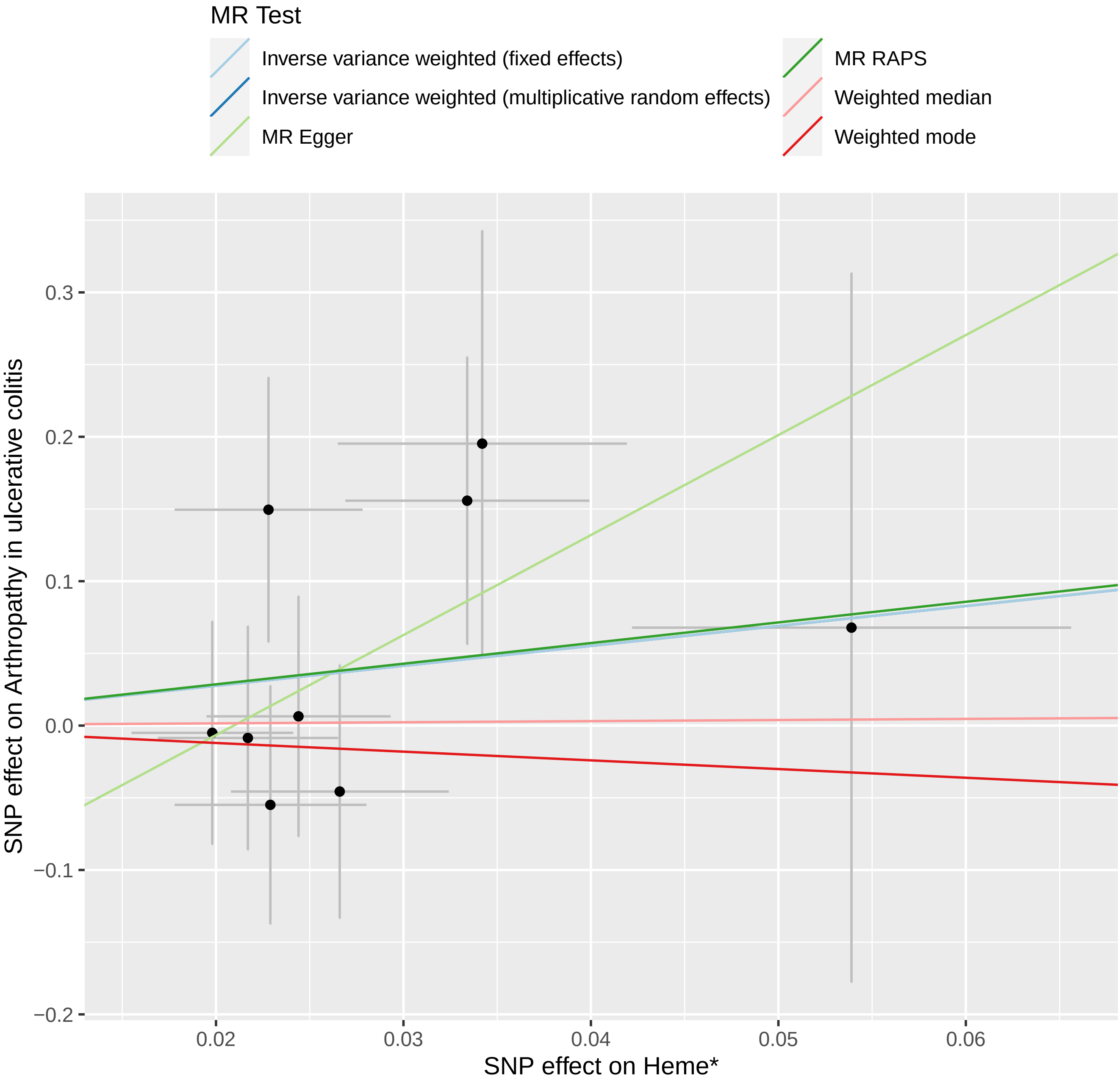

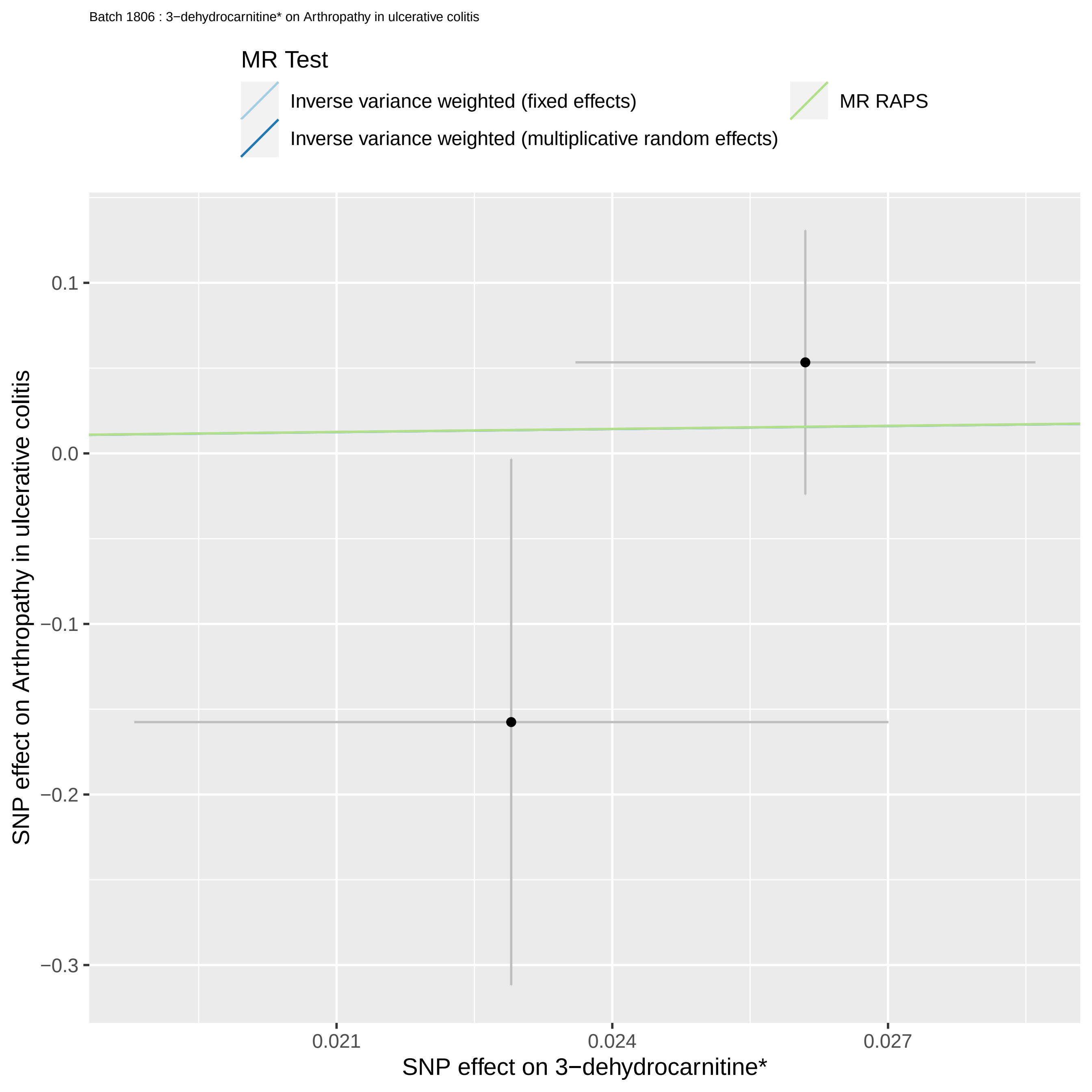

Batch 1806 : 3−dehydrocarnitine* on Arthropathy in ulcerative colitis
MR Test
Inverse variance weighted (fixed effects)
Inverse variance weighted (multiplicative random effects)
MR RAPS
0.1
0.0
−0.1
−0.2
−0.3
0.021
0.024
0.027
SNP effect on Arthropathy in ulcerative colitis
SNP effect on 3−dehydrocarnitine*

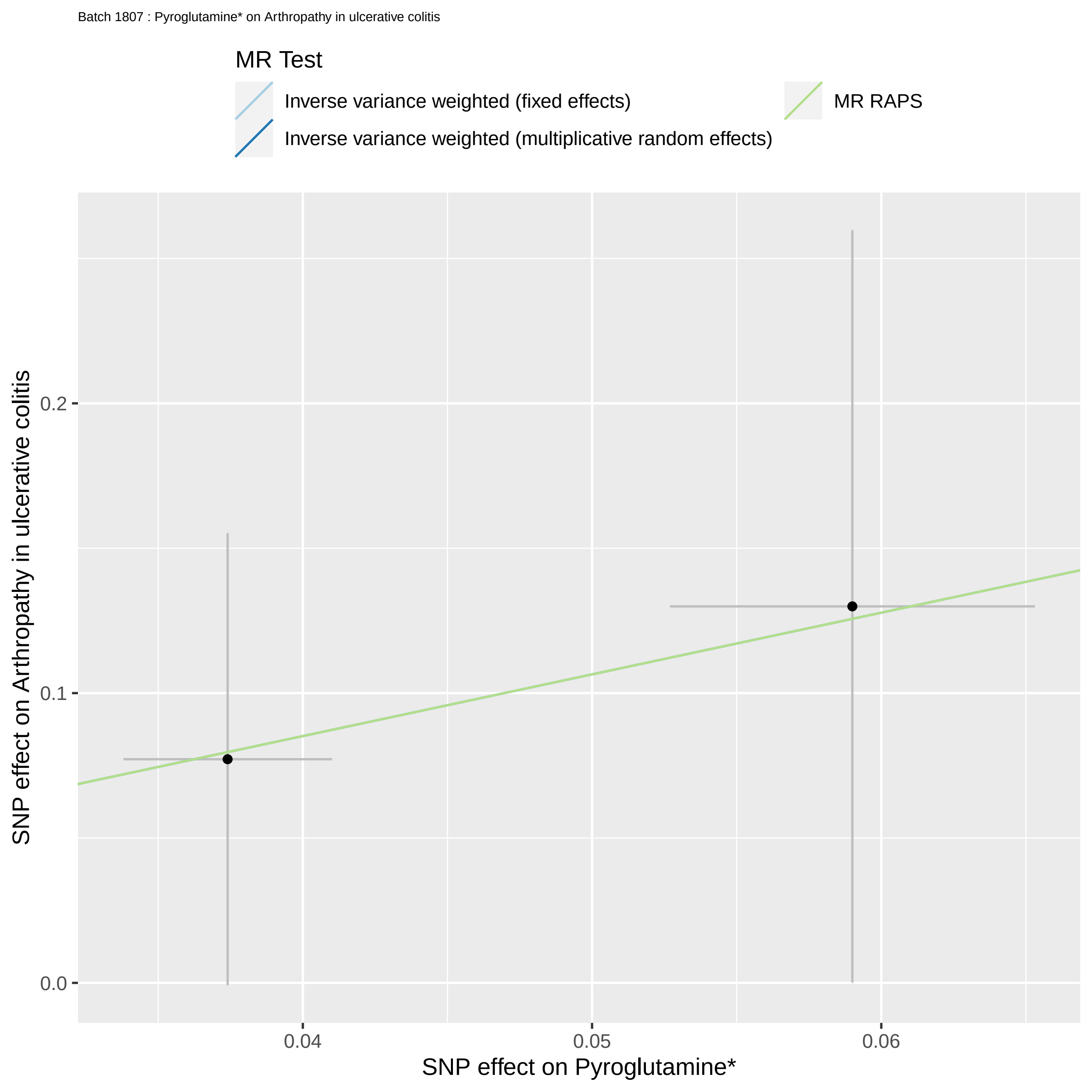

Batch 1807 : Pyroglutamine* on Arthropathy in ulcerative colitis
MR Test
Inverse variance weighted (fixed effects)
Inverse variance weighted (multiplicative random effects)
MR RAPS
0.2
0.1
0.0
0.04
0.05
0.06
SNP effect on Arthropathy in ulcerative colitis
SNP effect on Pyroglutamine*

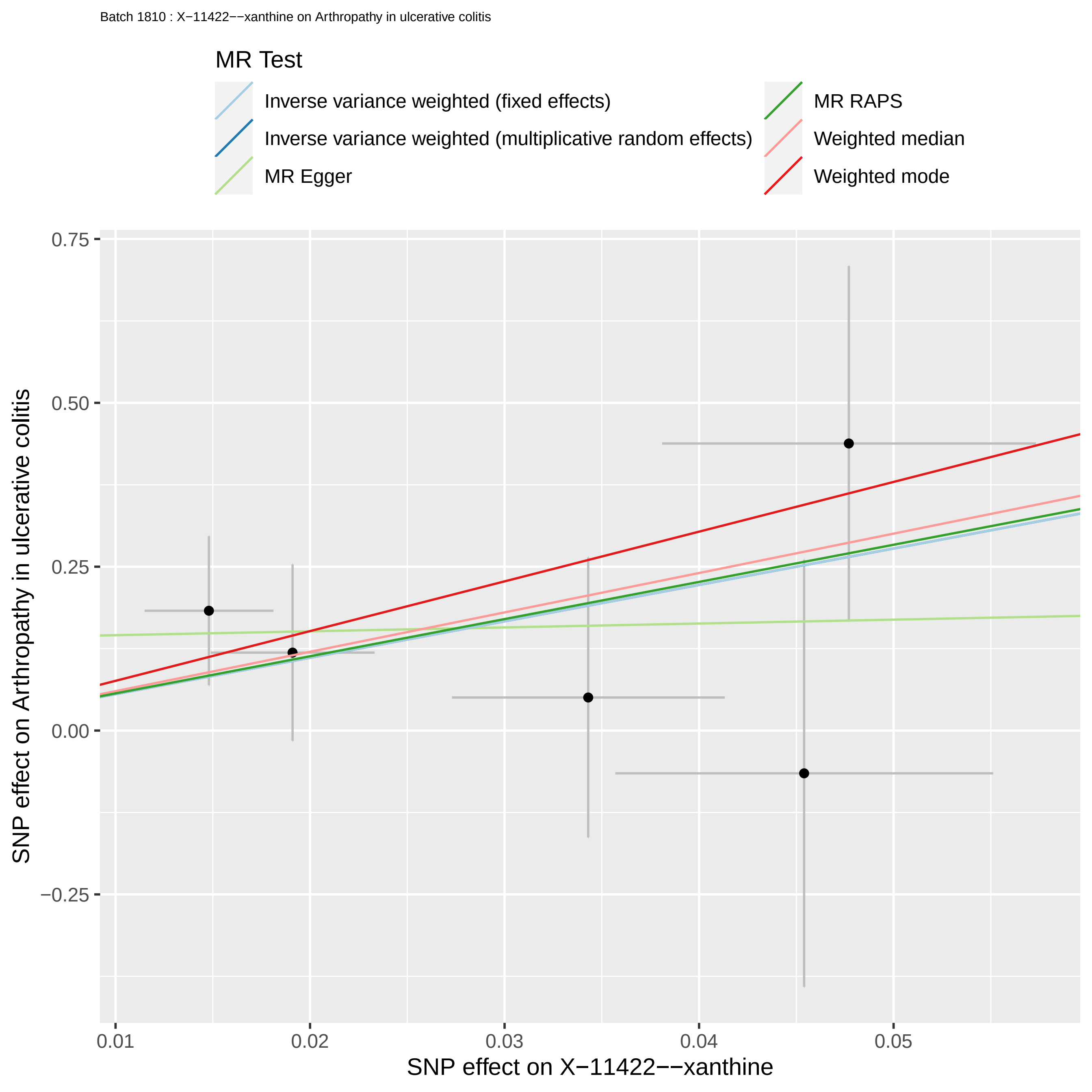
Batch 1810 : X−11422−−xanthine on Arthropathy in ulcerative colitis
MR Test
Inverse variance weighted (fixed effects)
Inverse variance weighted (multiplicative random effects)
MR Egger
MR RAPS
Weighted median
Weighted mode
SNP effect on Arthropathy in ulcerative colitis
0.75
0.50
0.25
0.00
−0.25
0.01
0.02
0.03
0.04
0.05
SNP effect on X−11422−−xanthine

Batch 1813 : HWESASXX* on Arthropathy in ulcerative colitis

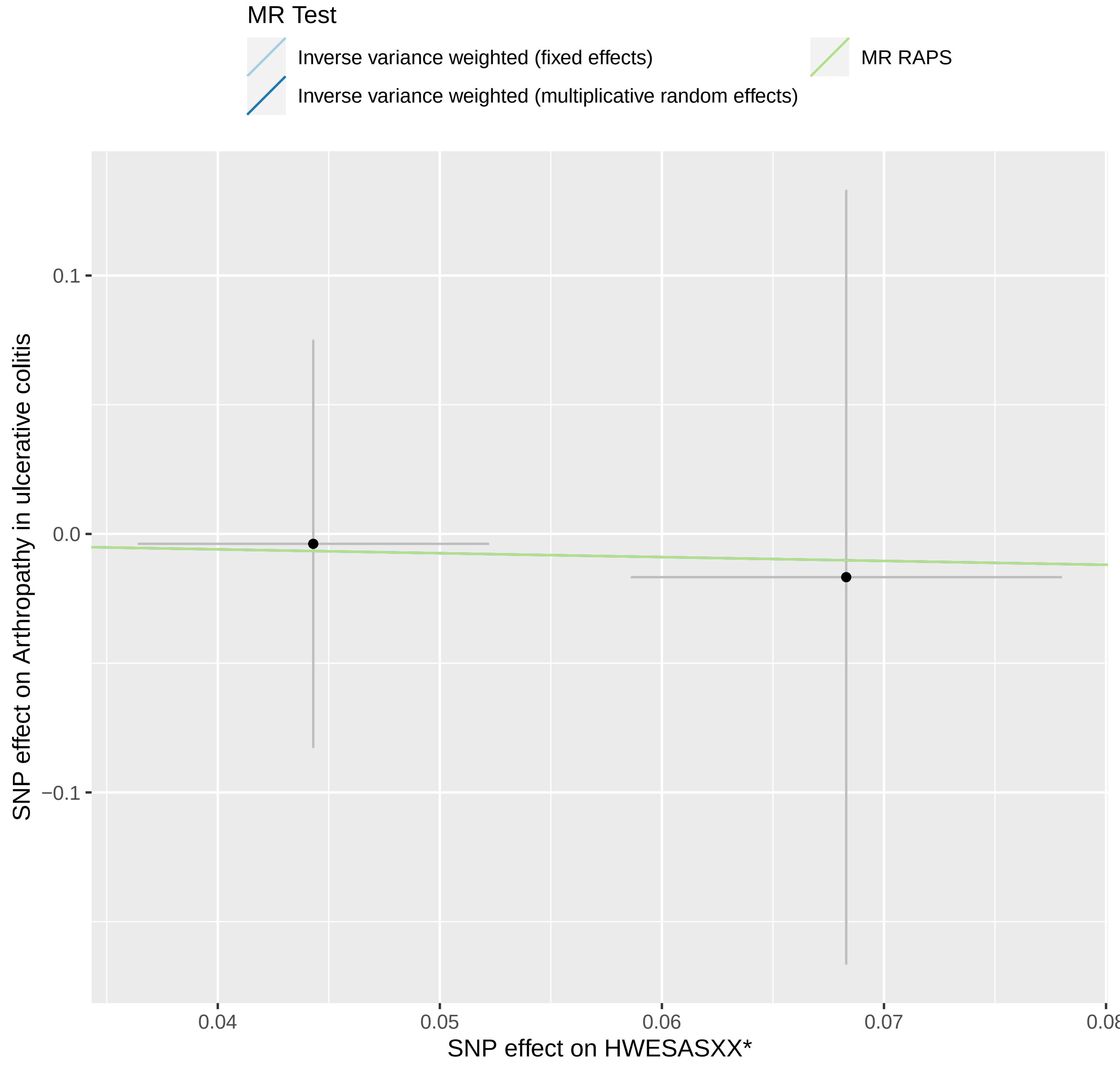

Batch 1814 : X−11593−−O−methylascorbate* on Arthropathy in ulcerative colitis

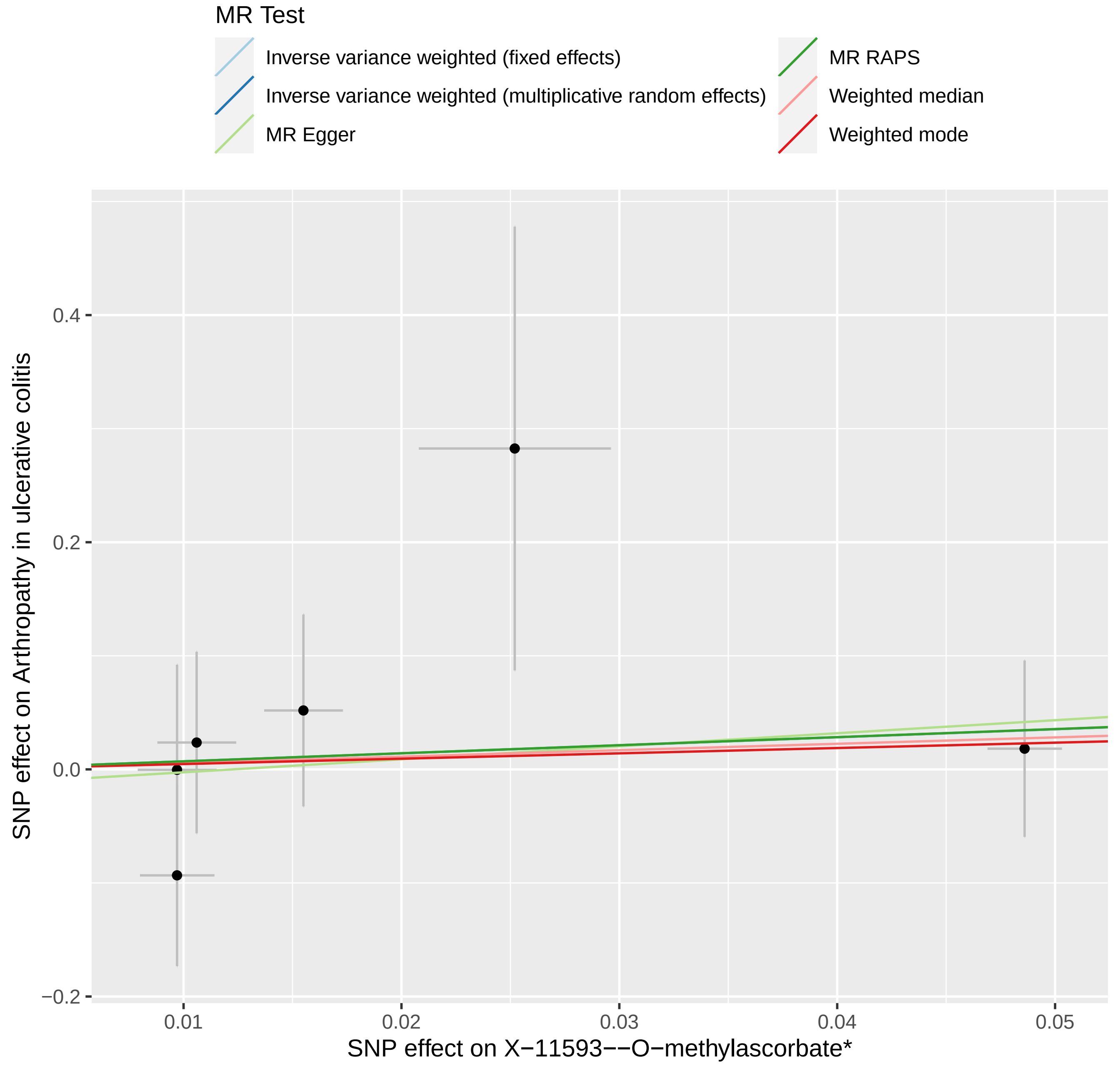

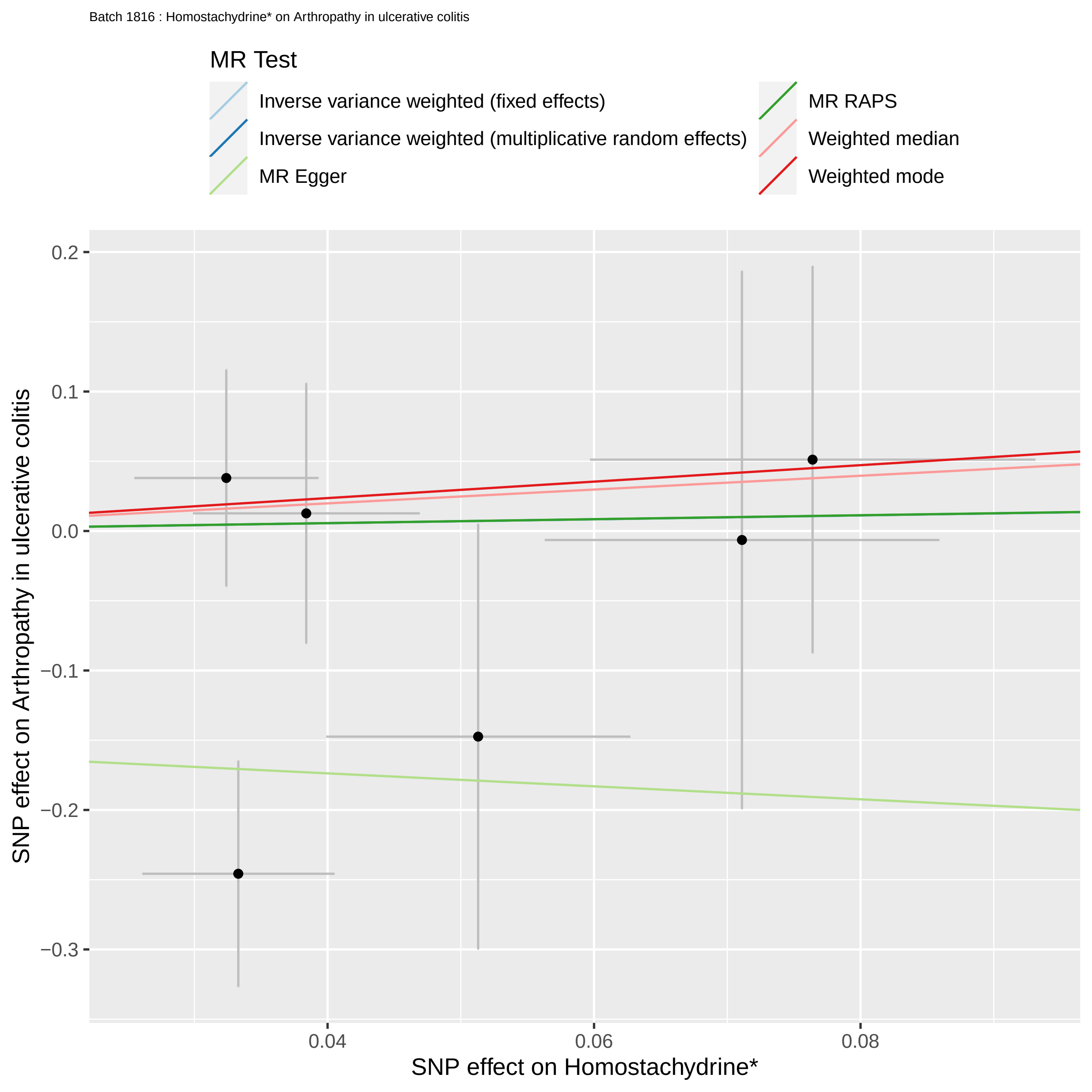
Batch 1816 : Homostachydrine* on Arthropathy in ulcerative colitis
MR Test
Inverse variance weighted (fixed effects)
Inverse variance weighted (multiplicative random effects)
MR Egger
MR RAPS
Weighted median
Weighted mode
0.2
0.1
0.0
−0.1
−0.2
−0.3
0.04
0.06
0.08
SNP effect on Arthropathy in ulcerative colitis
SNP effect on Homostachydrine*

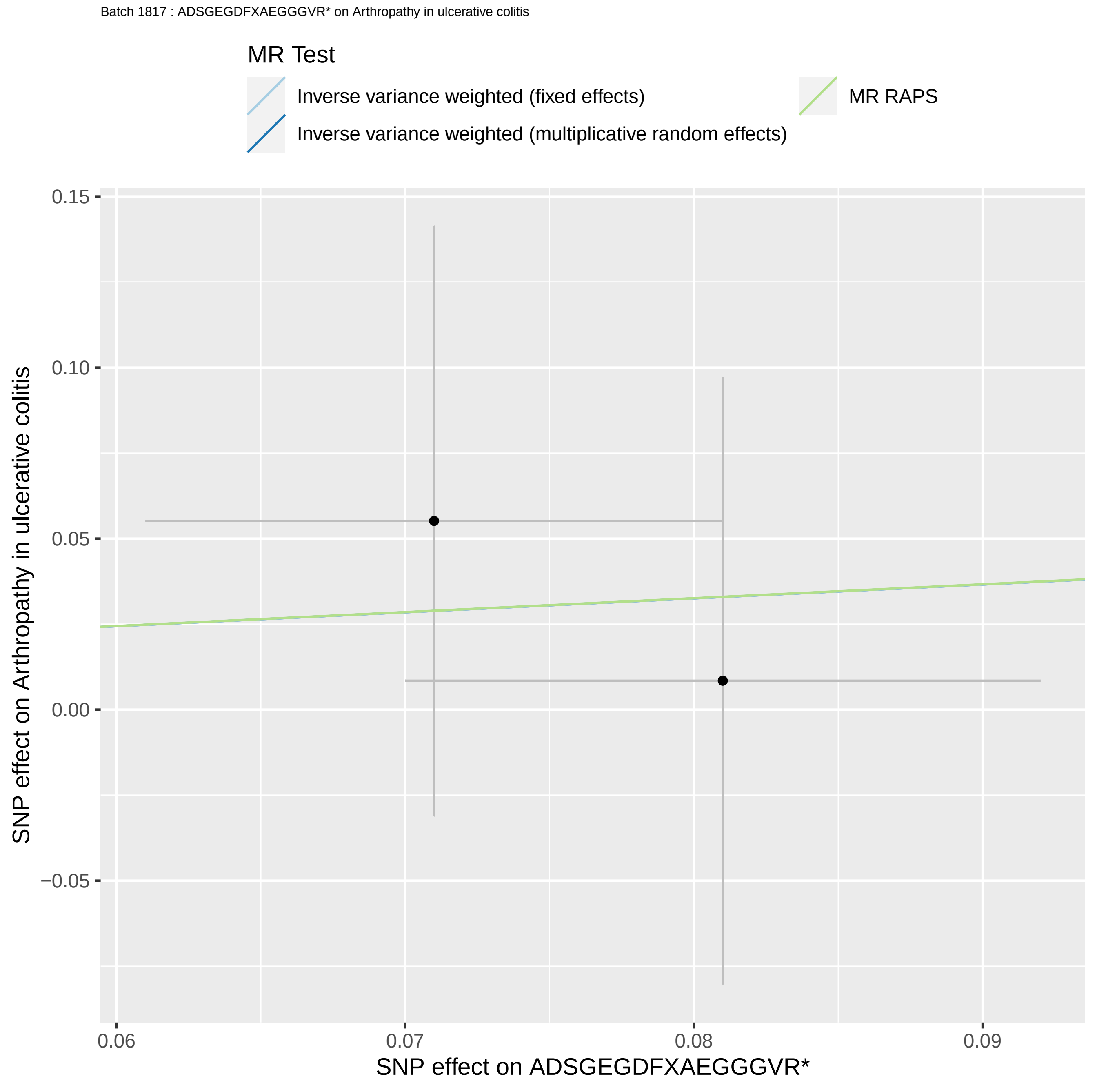

Batch 1817 : ADSGEGDFXAEGGGVR* on Arthropathy in ulcerative colitis
MR Test
Inverse variance weighted (fixed effects)
Inverse variance weighted (multiplicative random effects)
MR RAPS
0.15
0.10
0.05
0.00
−0.05
SNP effect on Arthropathy in ulcerative colitis
0.06
0.07
0.08
0.09
SNP effect on ADSGEGDFXAEGGGVR*

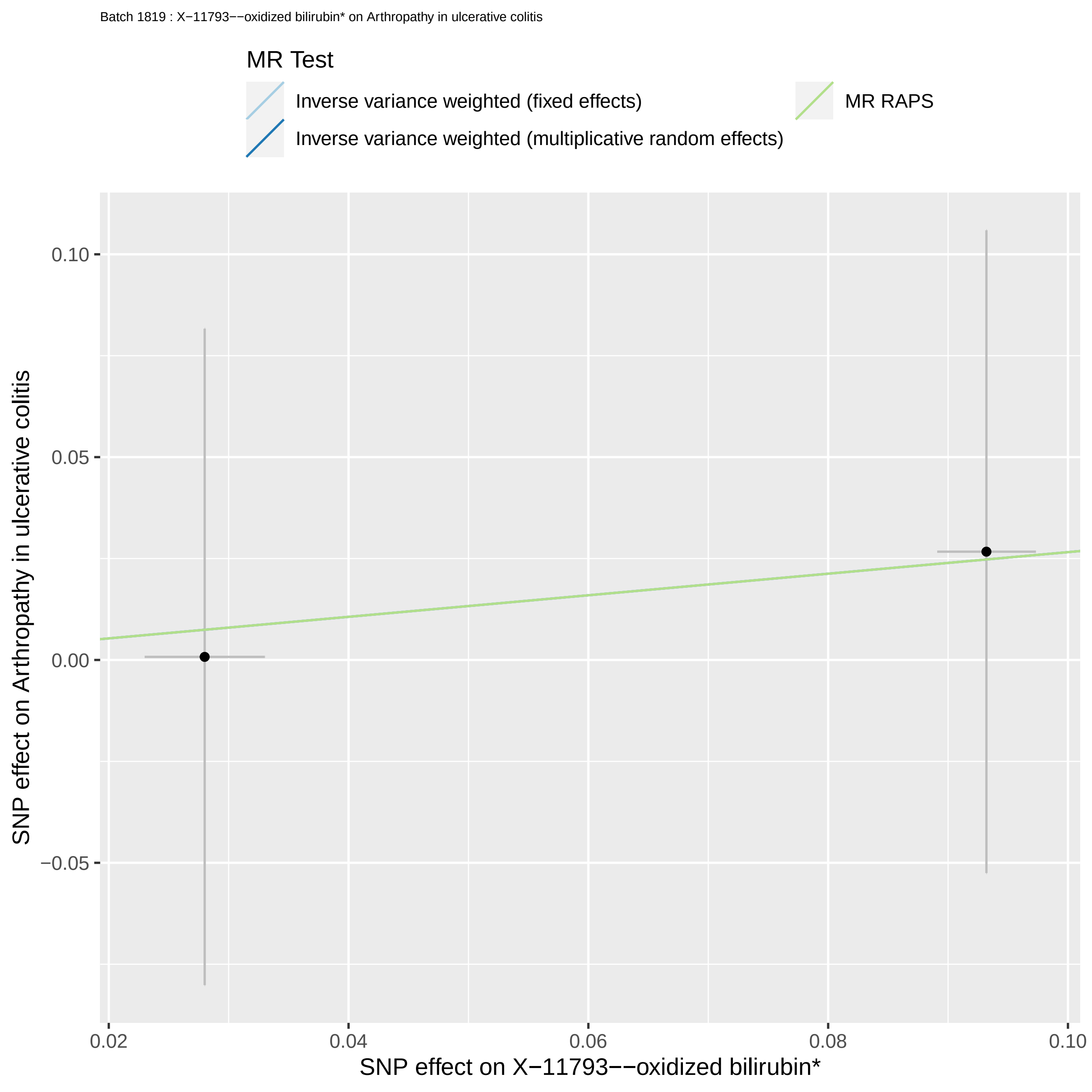

Batch 1819 : X−11793−−oxidized bilirubin* on Arthropathy in ulcerative colitis
MR Test
Inverse variance weighted (fixed effects)
Inverse variance weighted (multiplicative random effects)
MR RAPS
0.10
0.05
0.00
−0.05
0.02
0.04
0.06
0.08
0.10
SNP effect on Arthropathy in ulcerative colitis
SNP effect on X−11793−−oxidized bilirubin*

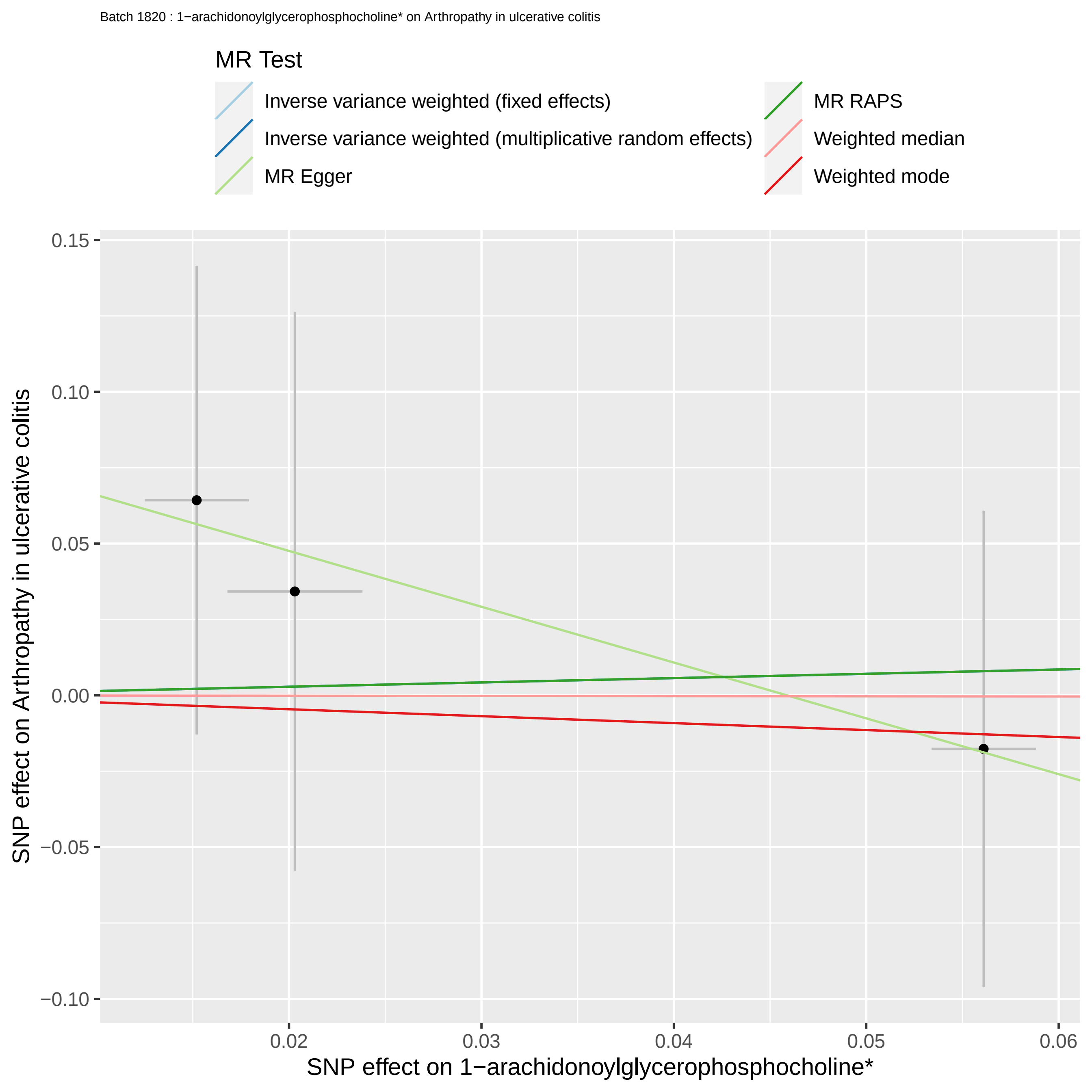

Batch 1820 : 1−arachidonoylglycerophosphocholine* on Arthropathy in ulcerative colitis
MR Test
Inverse variance weighted (fixed effects)
Inverse variance weighted (multiplicative random effects)
MR Egger
MR RAPS
Weighted median
Weighted mode
0.15
0.10
0.05
0.00
−0.05
−0.10
0.02
0.03
0.04
0.05
0.06
SNP effect on Arthropathy in ulcerative colitis
SNP effect on 1−arachidonoylglycerophosphocholine*

Batch 1822 : Gamma−glutamylmethionine* on Arthropathy in ulcerative colitis

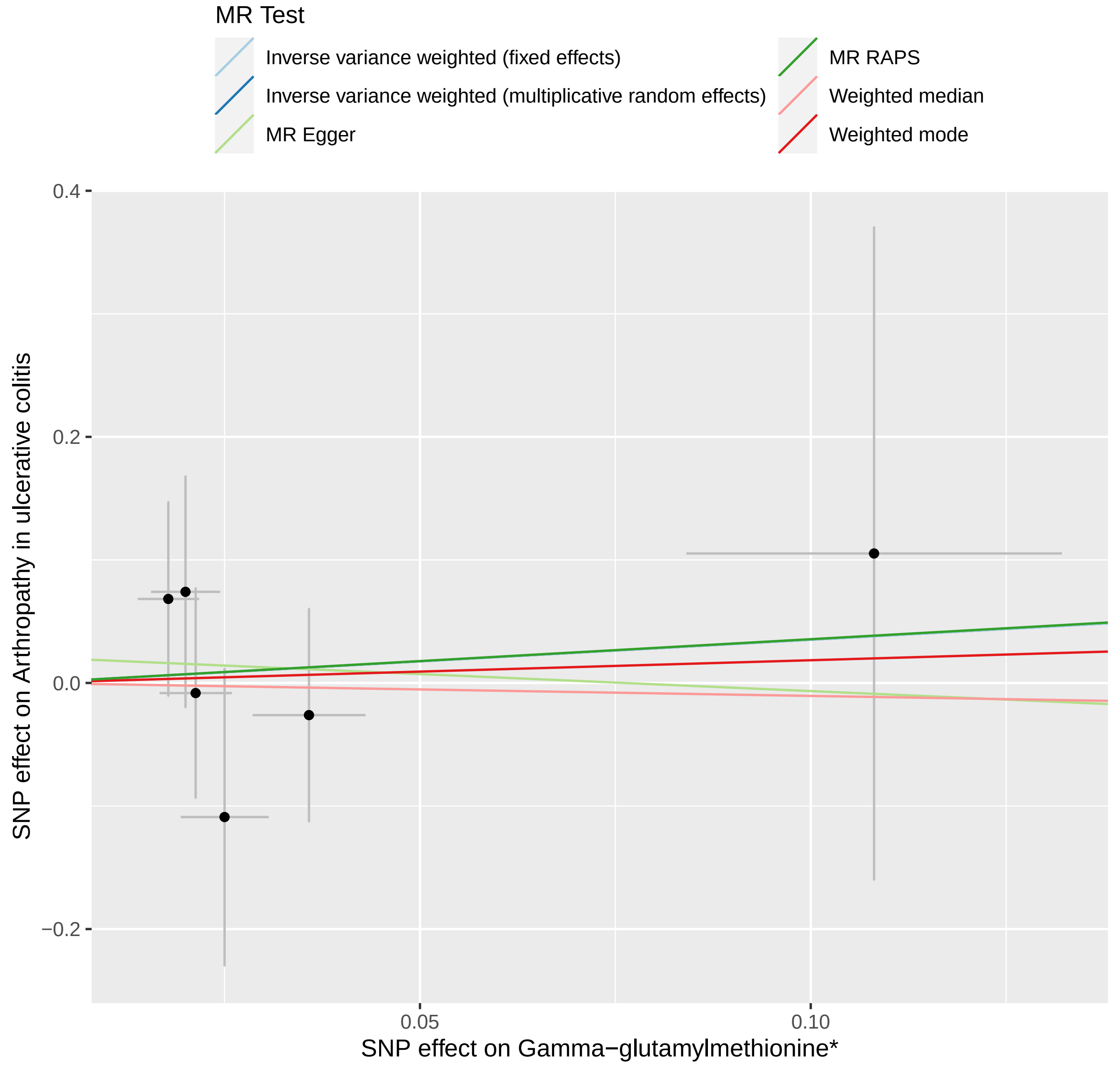

Batch 1823 : Gamma−glutamylthreonine* on Arthropathy in ulcerative colitis

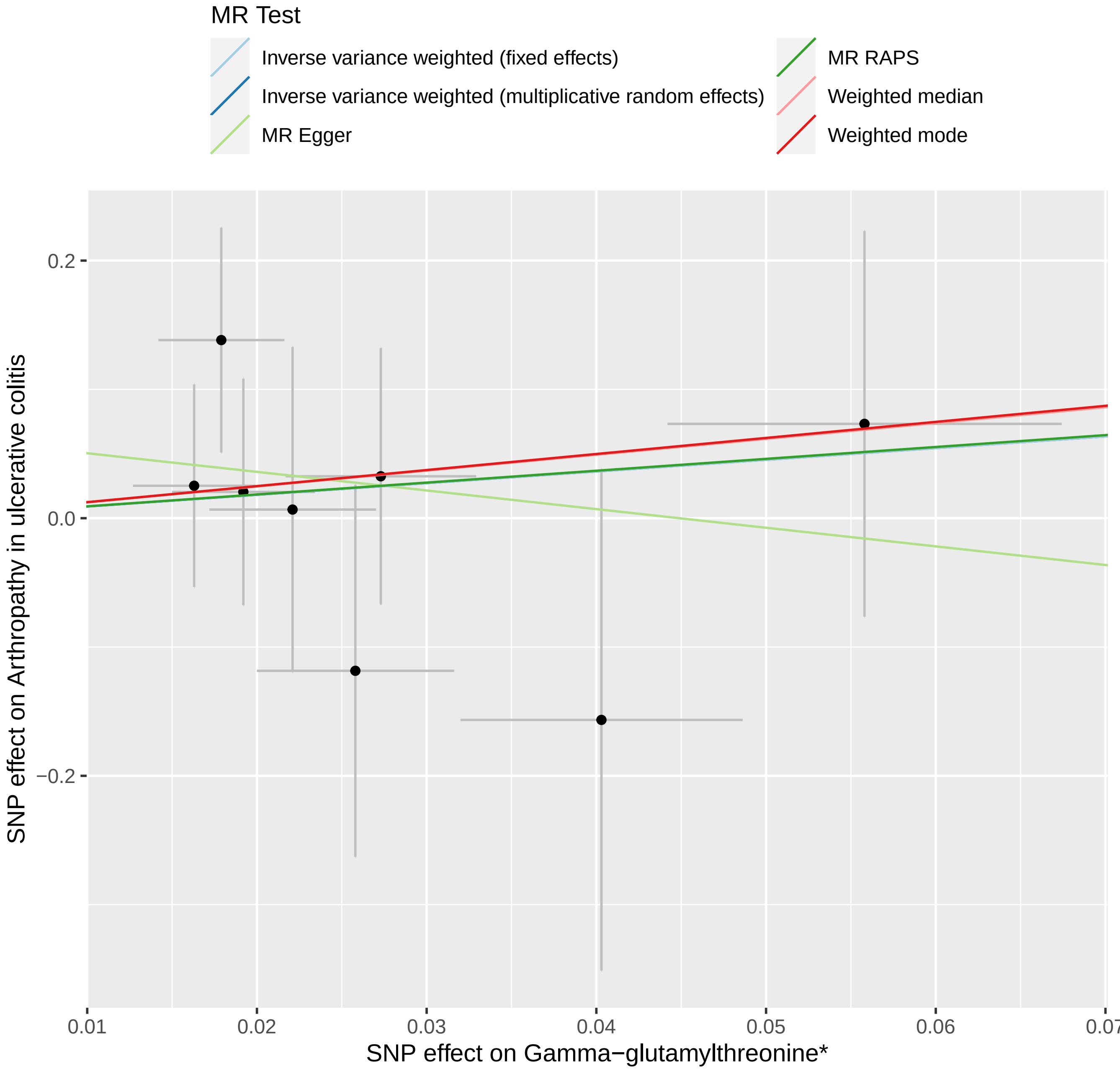

Batch 1824 : Gamma−tocopherol on Arthropathy in ulcerative colitis

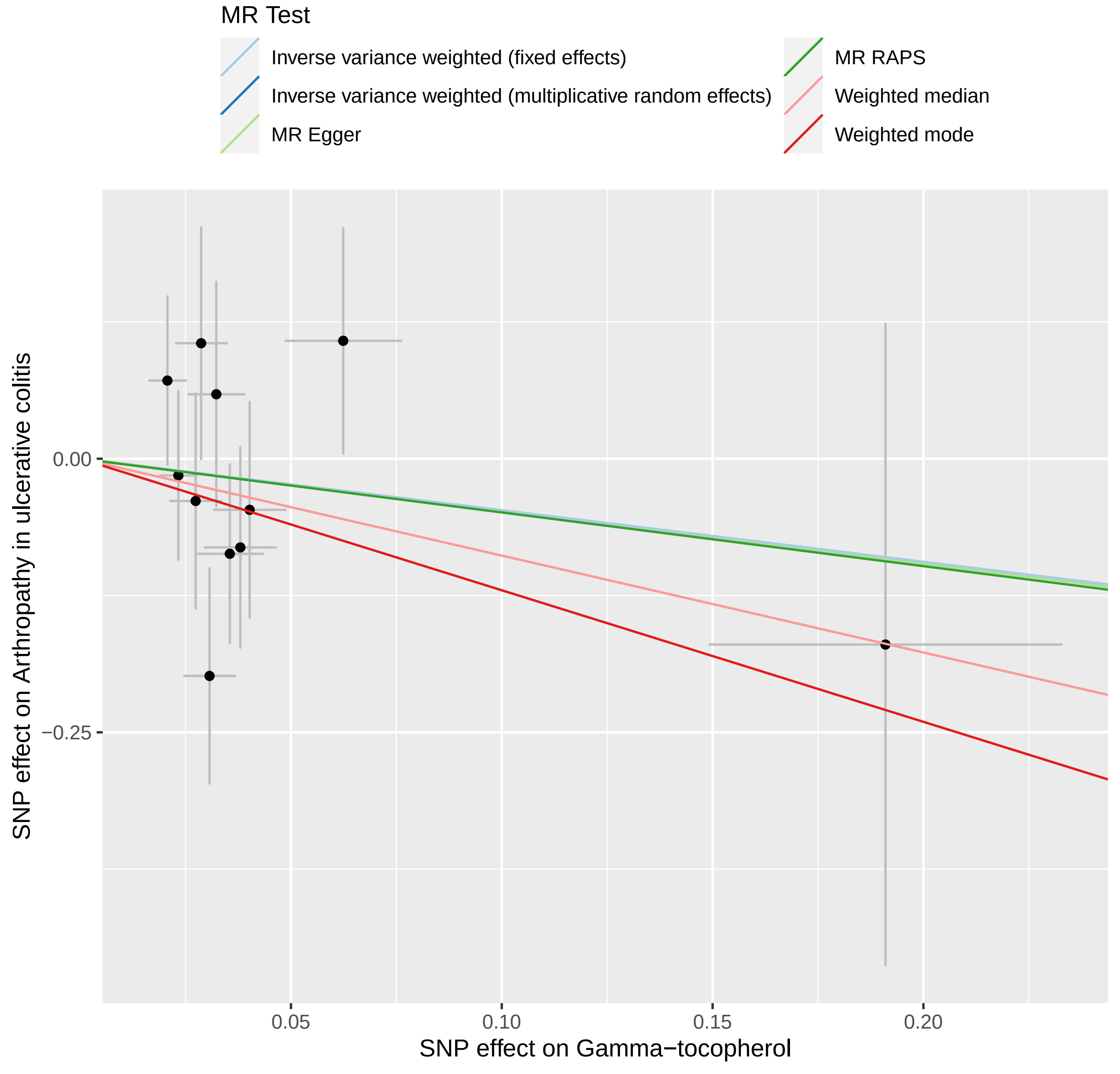

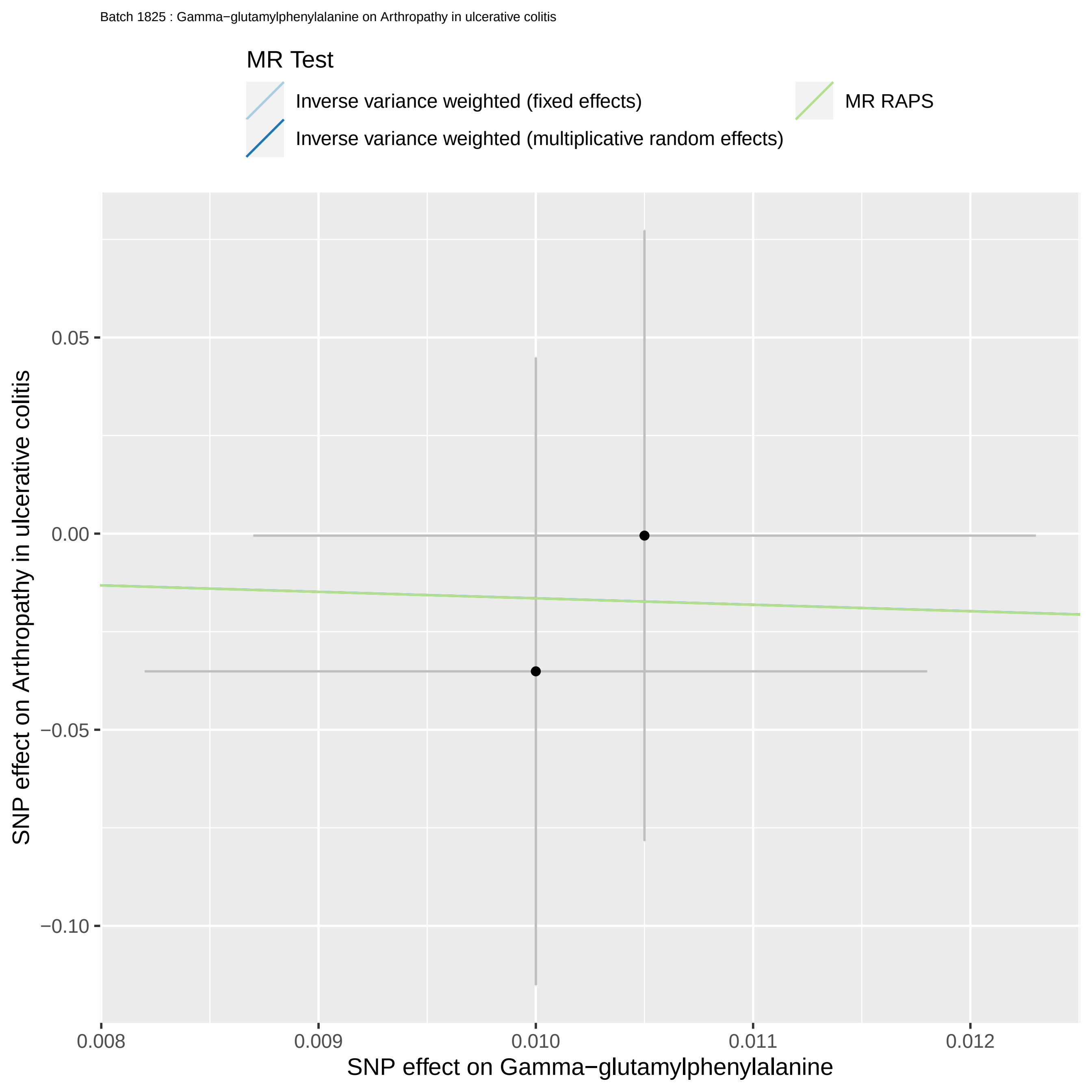

Batch 1825 : Gamma−glutamylphenylalanine on Arthropathy in ulcerative colitis
MR Test
Inverse variance weighted (fixed effects)
Inverse variance weighted (multiplicative random effects)
MR RAPS
0.05
0.00
−0.05
−0.10
0.008
0.009
0.010
0.011
0.012
SNP effect on Gamma−glutamylphenylalanine
SNP effect on Arthropathy in ulcerative colitis

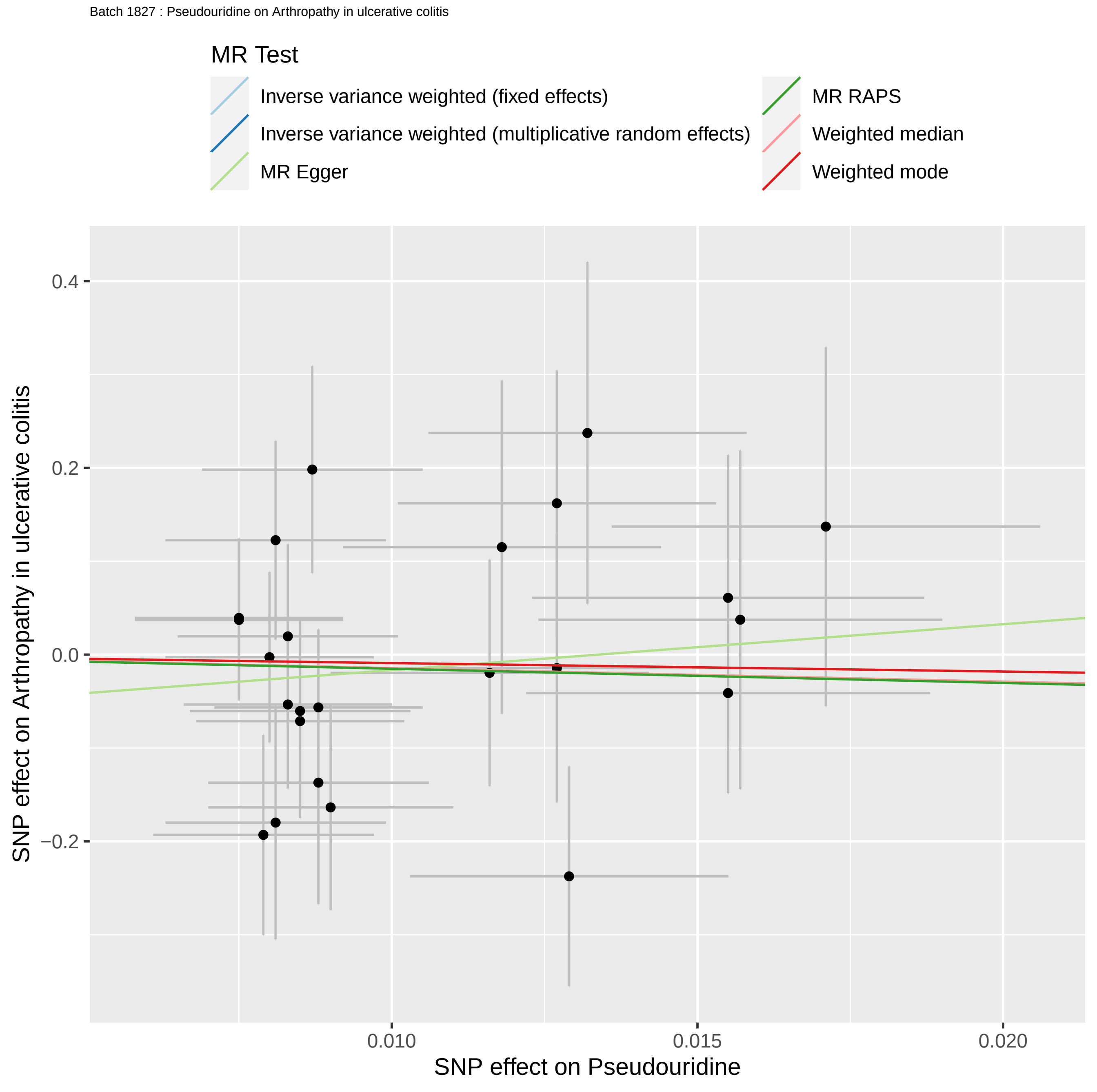

Batch 1827 : Pseudouridine on Arthropathy in ulcerative colitis
MR Test
Inverse variance weighted (fixed effects)
Inverse variance weighted (multiplicative random effects)
MR Egger
MR RAPS
Weighted median
Weighted mode
0.4
0.2
0.0
−0.2
0.010
0.015
0.020
SNP effect on Pseudouridine
SNP effect on Arthropathy in ulcerative colitis

Batch 1828 : Valerate on Arthropathy in ulcerative colitis

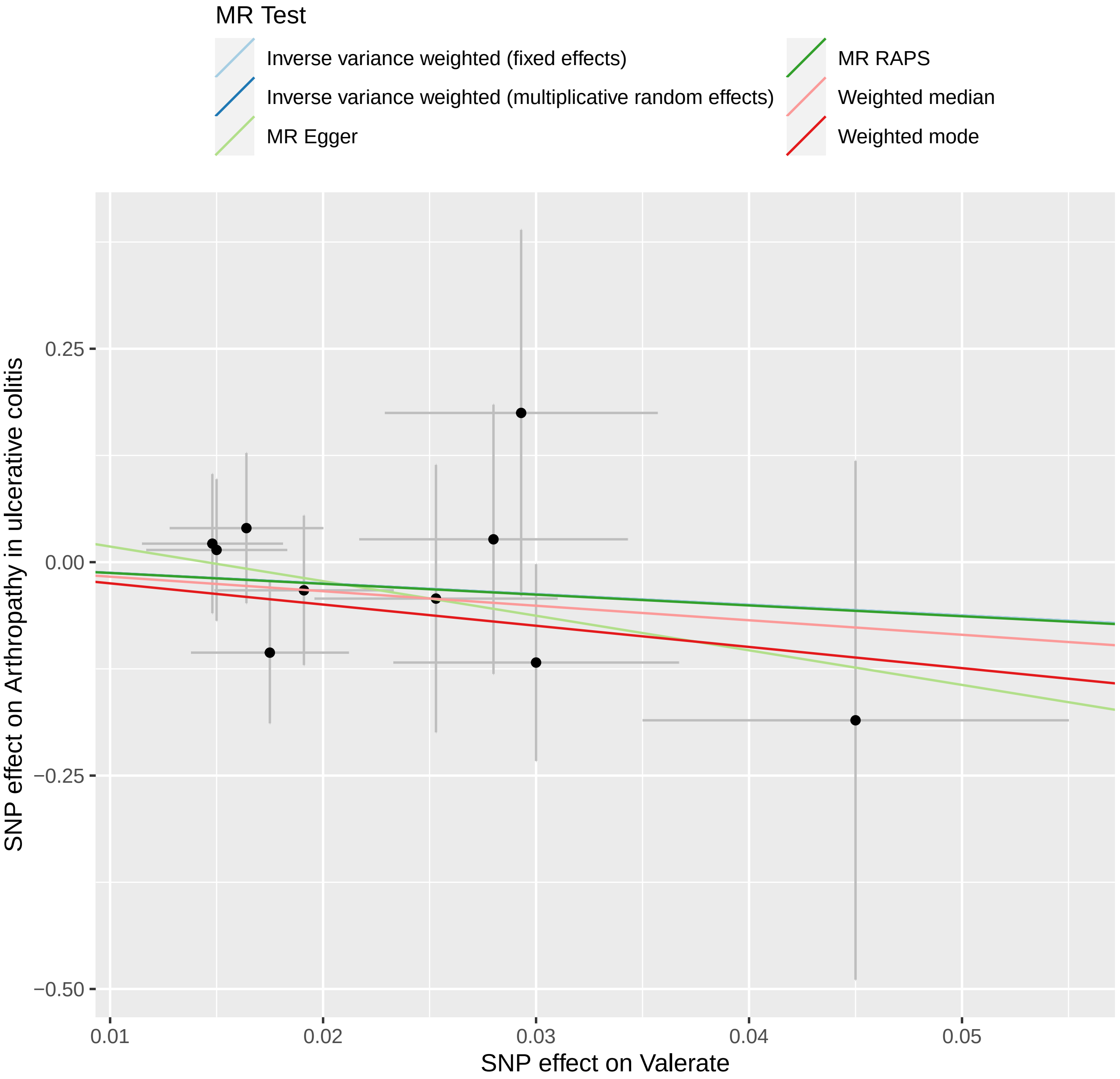

Batch 1831 : Erythronate* on Arthropathy in ulcerative colitis

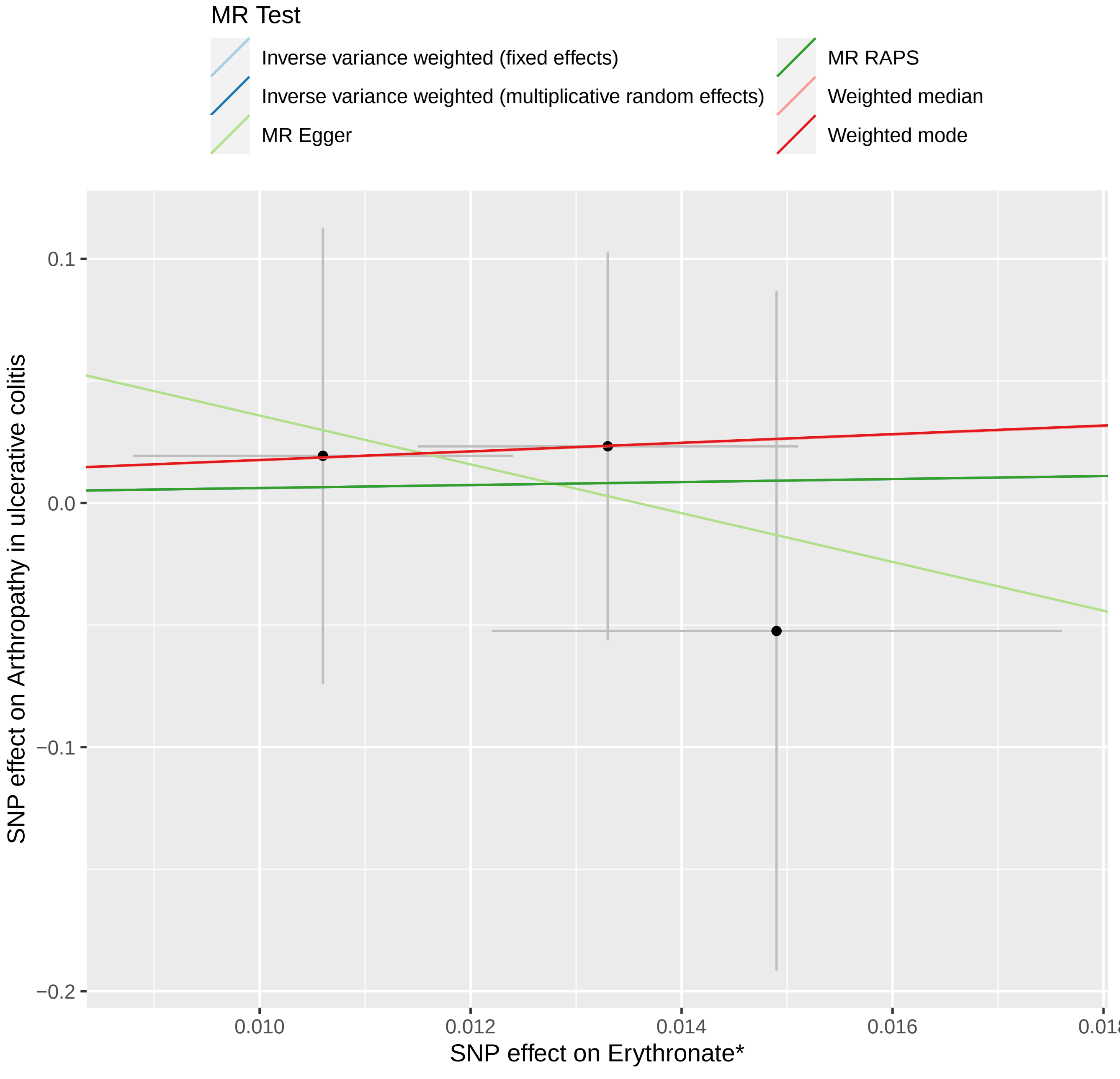

Batch 1832 : Lathosterol on Arthropathy in ulcerative colitis

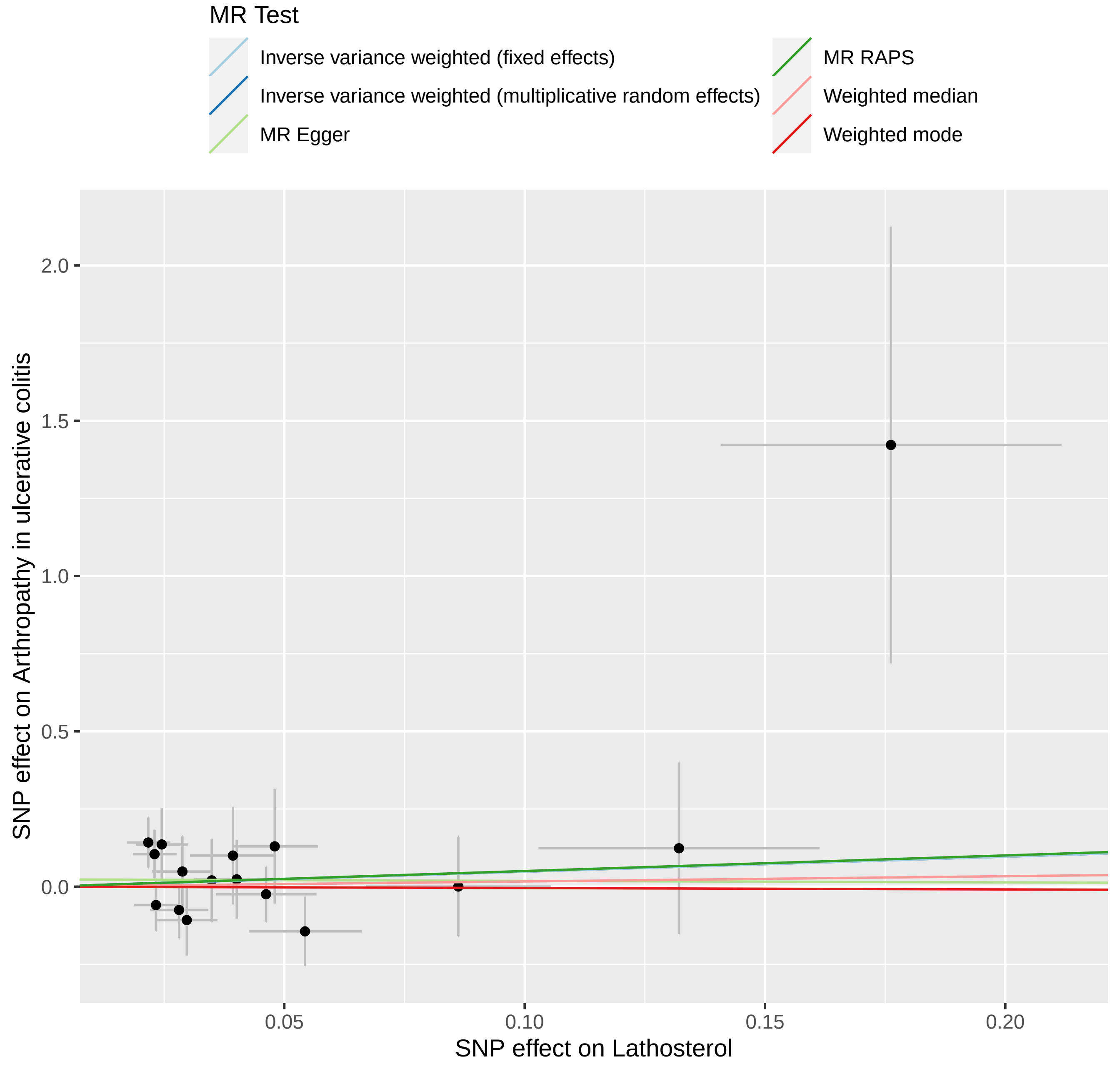

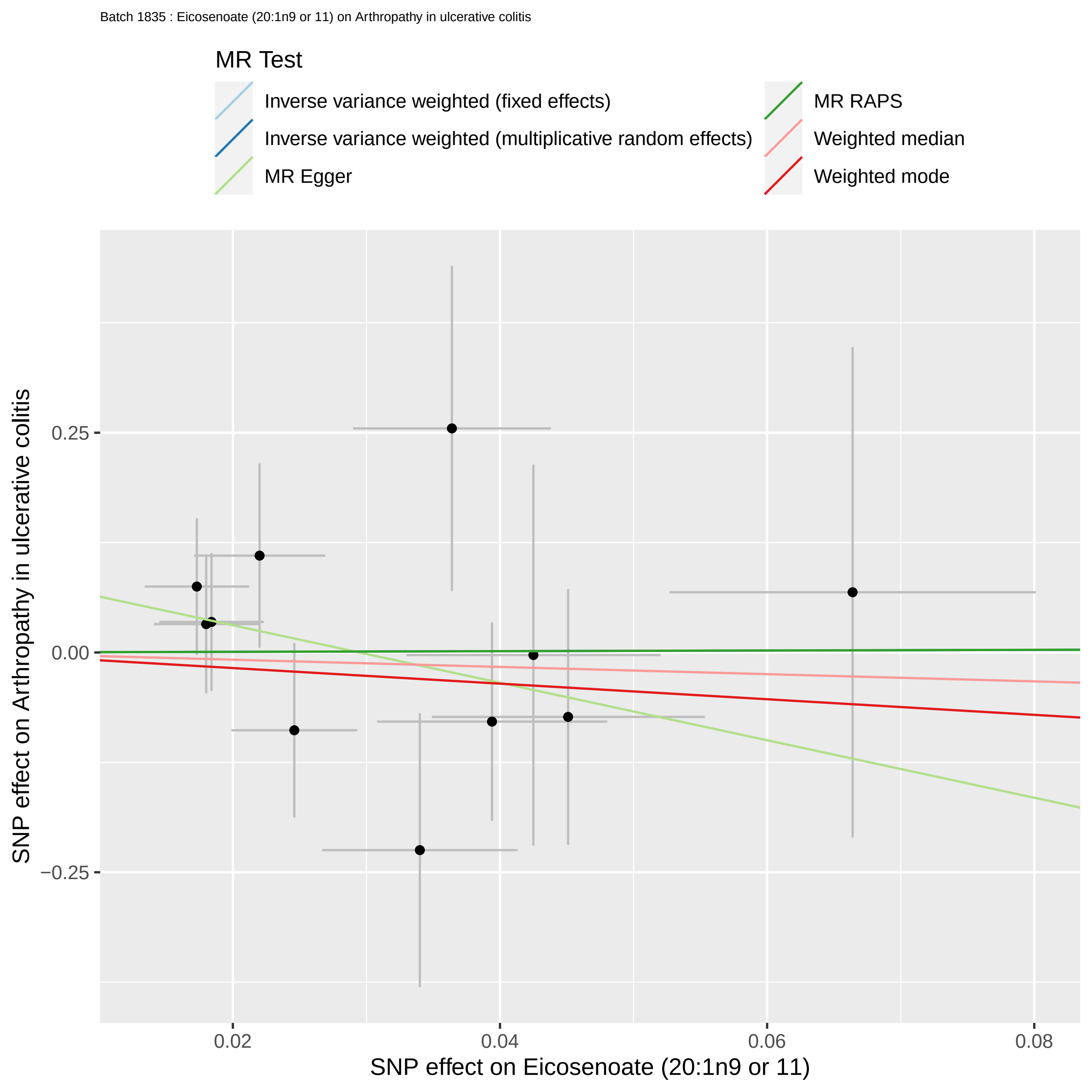
Batch 1835 : Eicosenoate (20:1n9 or 11) on Arthropathy in ulcerative colitis
MR Test
Inverse variance weighted (fixed effects)
Inverse variance weighted (multiplicative random effects)
MR Egger
MR RAPS
Weighted median
Weighted mode
0.25
0.00
−0.25
0.02
0.04
0.06
0.08
SNP effect on Arthropathy in ulcerative colitis
SNP effect on Eicosenoate (20:1n9 or 11)

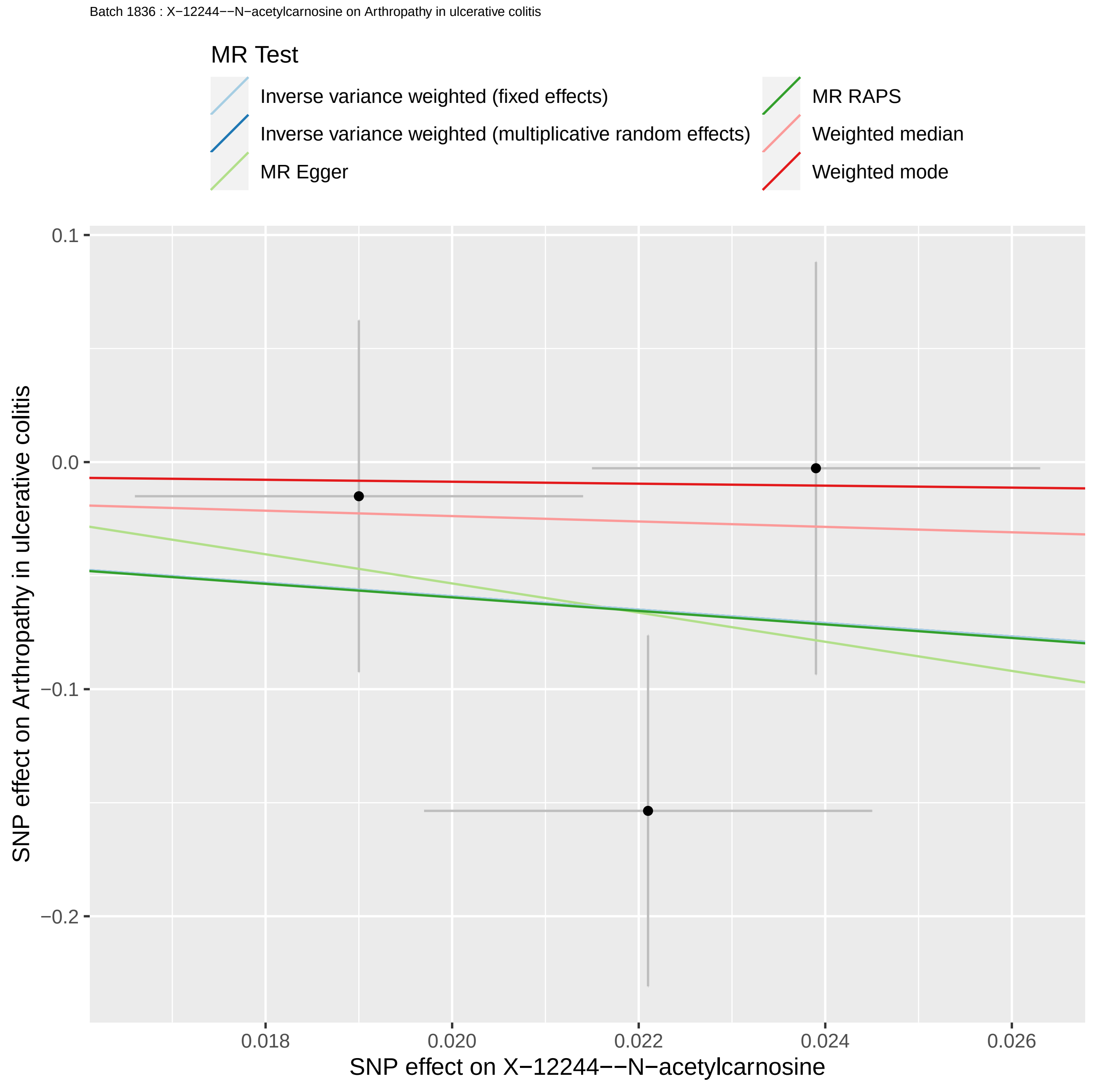

Batch 1836 : X−12244−−N−acetylcarnosine on Arthropathy in ulcerative colitis
MR Test
Inverse variance weighted (fixed effects)
Inverse variance weighted (multiplicative random effects)
MR Egger
MR RAPS
Weighted median
Weighted mode
0.1
0.0
−0.1
−0.2
0.018
0.020
0.022
0.024
0.026
SNP effect on Arthropathy in ulcerative colitis
SNP effect on X−12244−−N−acetylcarnosine

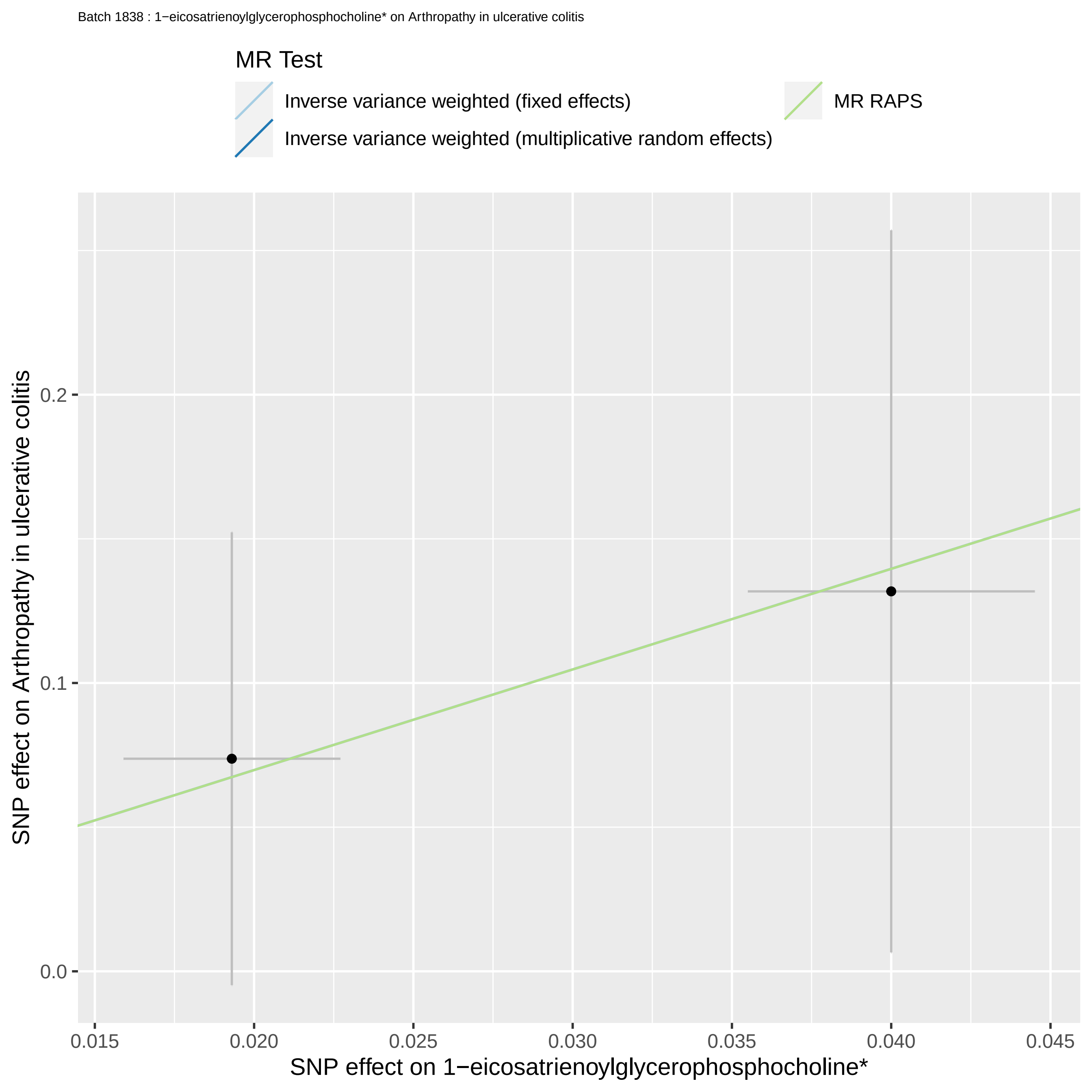
Batch 1838 : 1−eicosatrienoylglycerophosphocholine* on Arthropathy in ulcerative colitis
MR Test
Inverse variance weighted (fixed effects)
Inverse variance weighted (multiplicative random effects)
MR RAPS
0.2
0.1
0.0
0.015
0.020
0.025
0.030
0.035
0.040
0.045
SNP effect on Arthropathy in ulcerative colitis
SNP effect on 1−eicosatrienoylglycerophosphocholine*

Batch 1839 : 1−docosahexaenoylglycerophosphocholine* on Arthropathy in ulcerative colitis

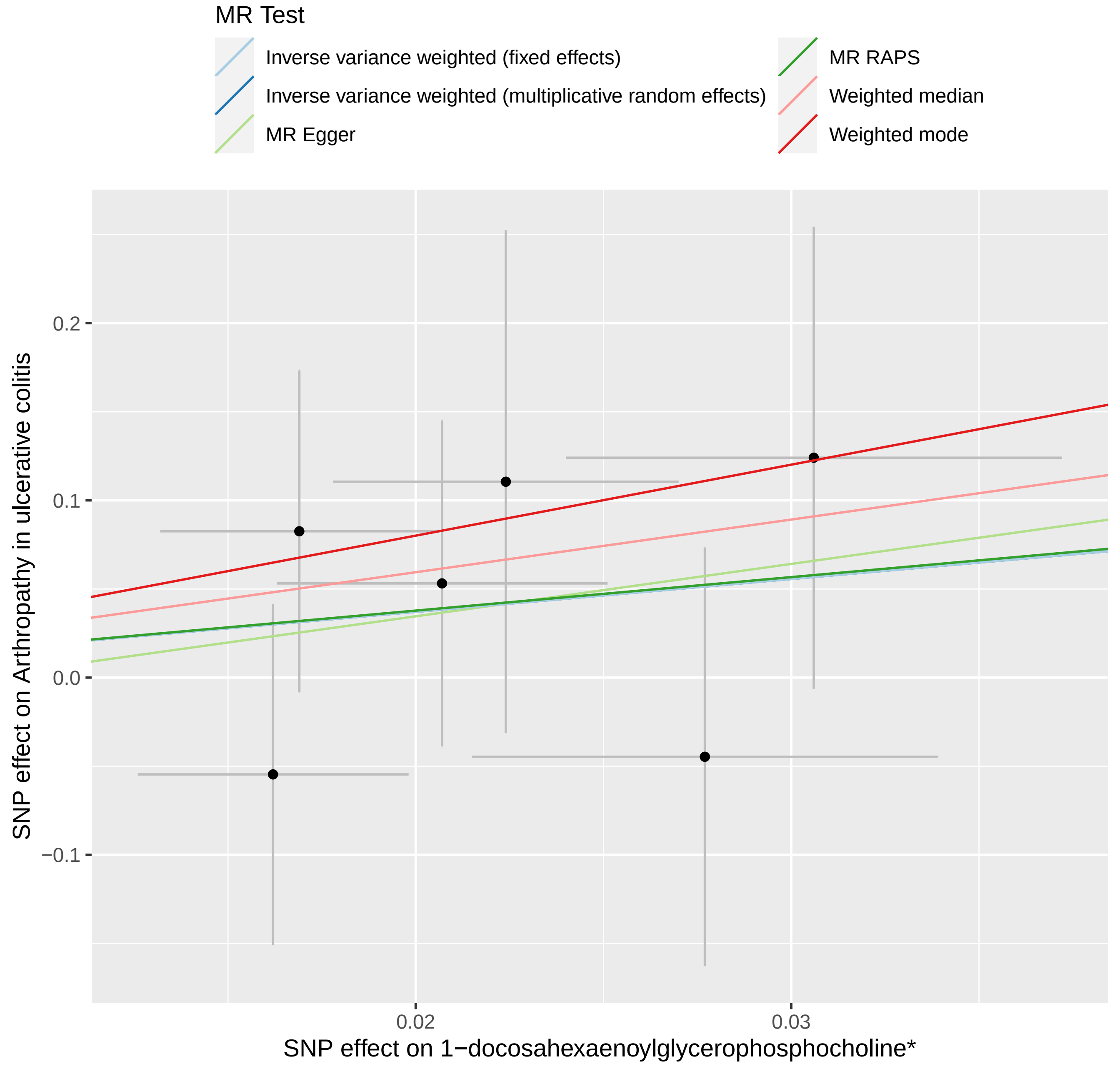

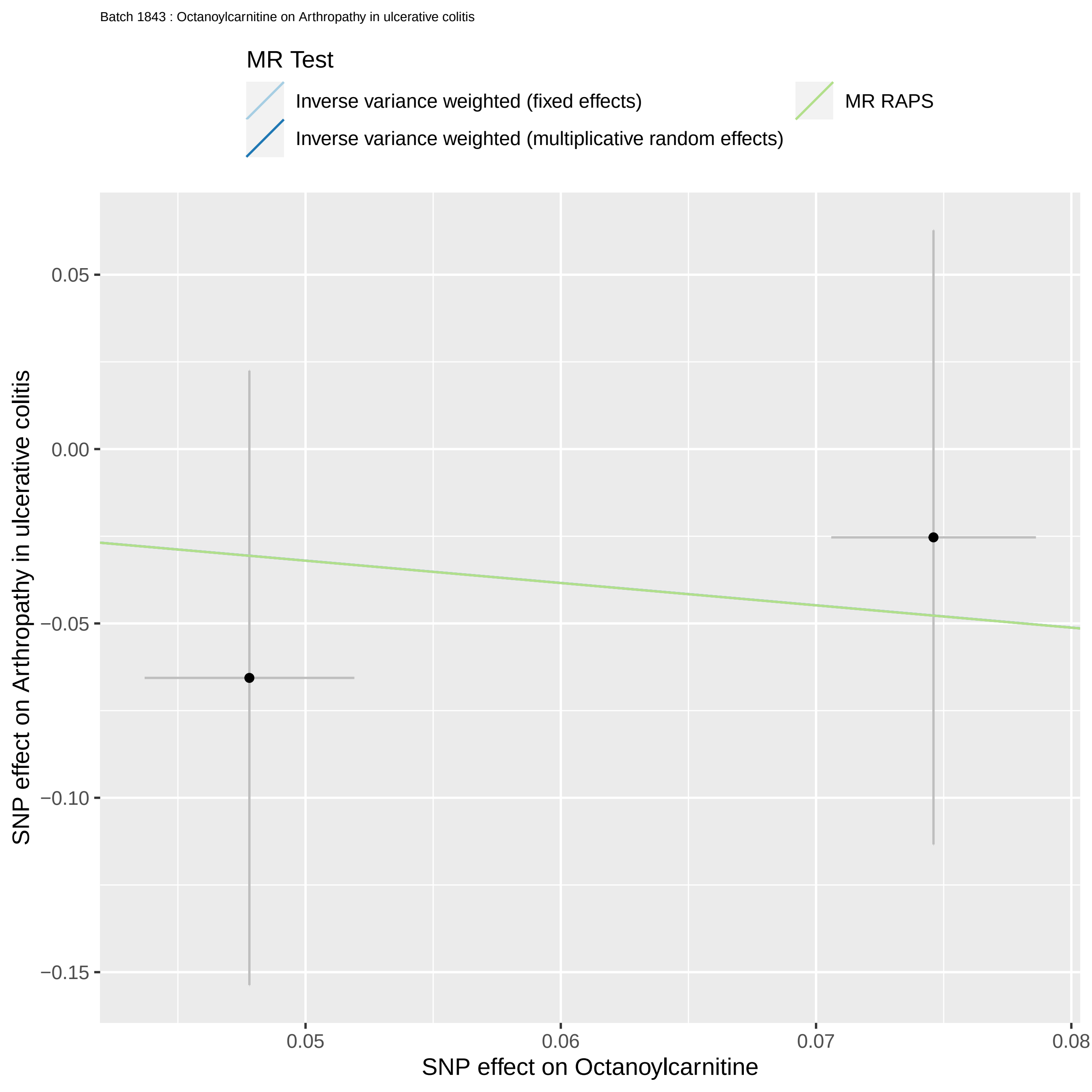

Batch 1843 : Octanoylcarnitine on Arthropathy in ulcerative colitis
MR Test
Inverse variance weighted (fixed effects)
Inverse variance weighted (multiplicative random effects)
MR RAPS
SNP effect on Arthropathy in ulcerative colitis
0.05
0.00
−0.05
−0.10
−0.15
0.05
0.06
0.07
0.08
SNP effect on Octanoylcarnitine

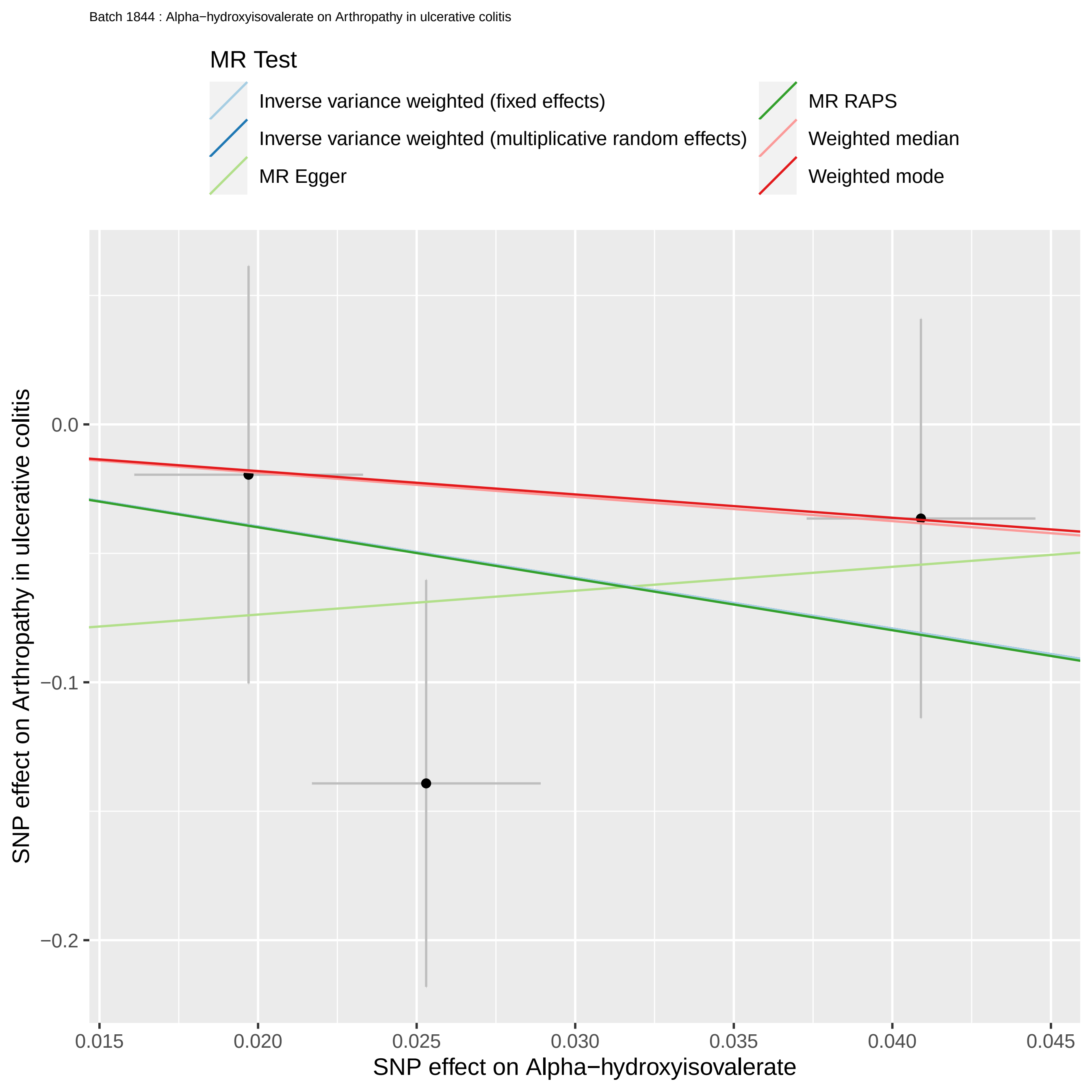
Batch 1844 : Alpha−hydroxyisovalerate on Arthropathy in ulcerative colitis
MR Test
Inverse variance weighted (fixed effects)
Inverse variance weighted (multiplicative random effects)
MR Egger
MR RAPS
Weighted median
Weighted mode
SNP effect on Arthropathy in ulcerative colitis
0.0
−0.1
−0.2
0.015
0.020
0.025
0.030
0.035
0.040
0.045
SNP effect on Alpha−hydroxyisovalerate

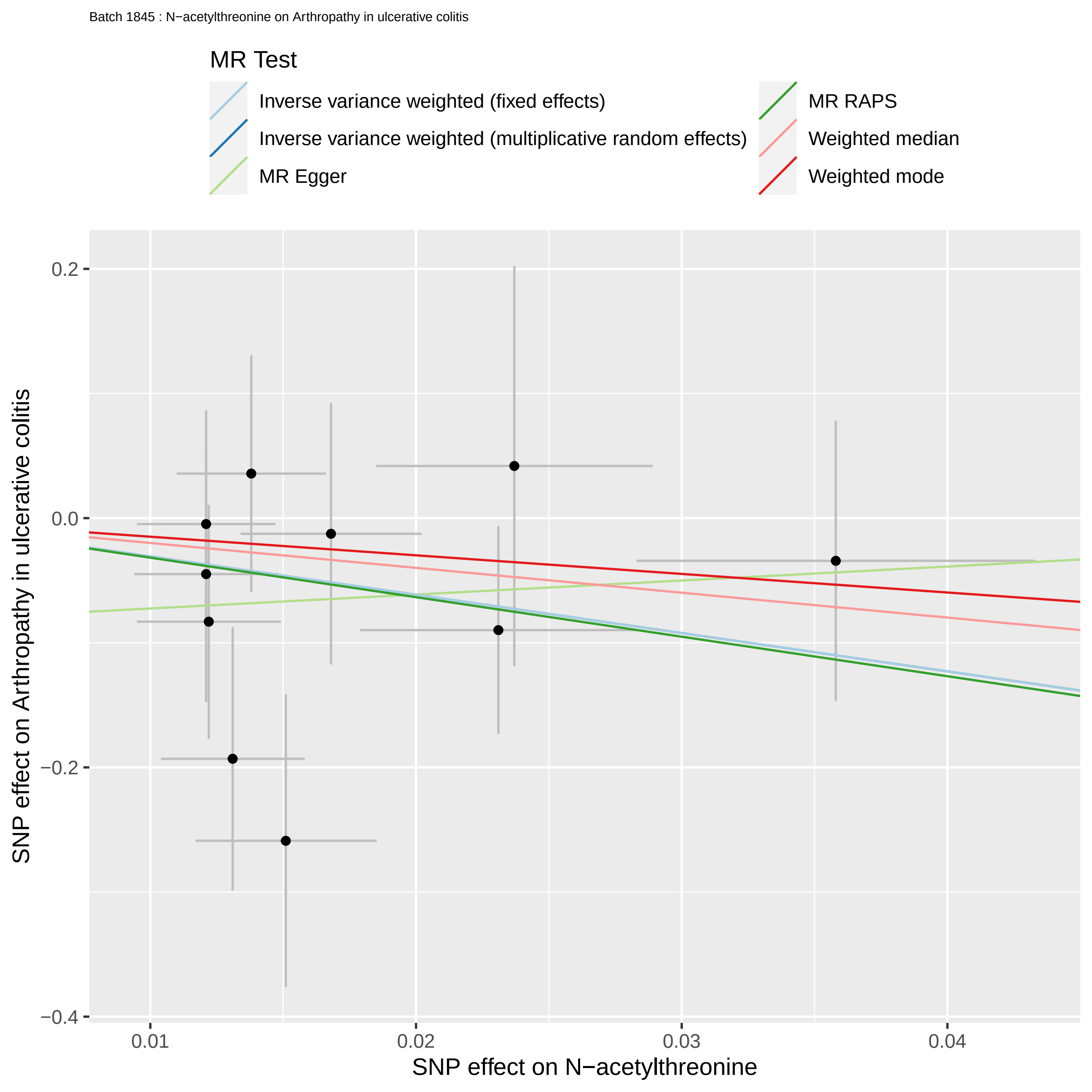
Batch 1845 : N−acetylthreonine on Arthropathy in ulcerative colitis
MR Test
Inverse variance weighted (fixed effects)
Inverse variance weighted (multiplicative random effects)
MR Egger
MR RAPS
Weighted median
Weighted mode
0.2
0.0
−0.2
−0.4
0.01
0.02
0.03
0.04
SNP effect on Arthropathy in ulcerative colitis
SNP effect on N−acetylthreonine

Batch 1846 : Decanoylcarnitine on Arthropathy in ulcerative colitis

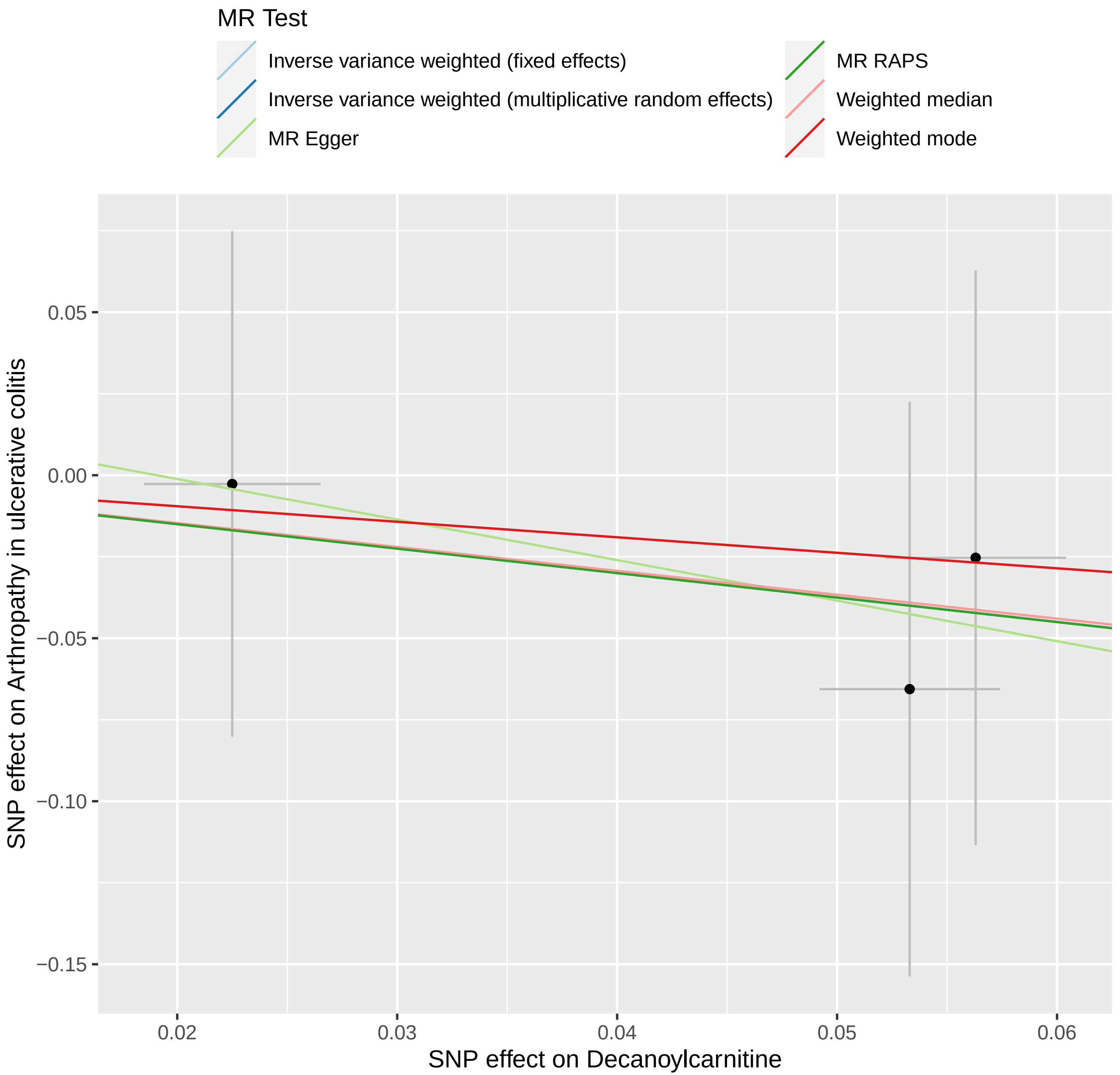

Batch 1847 : 1−palmitoylglycerophosphocholine on Arthropathy in ulcerative colitis

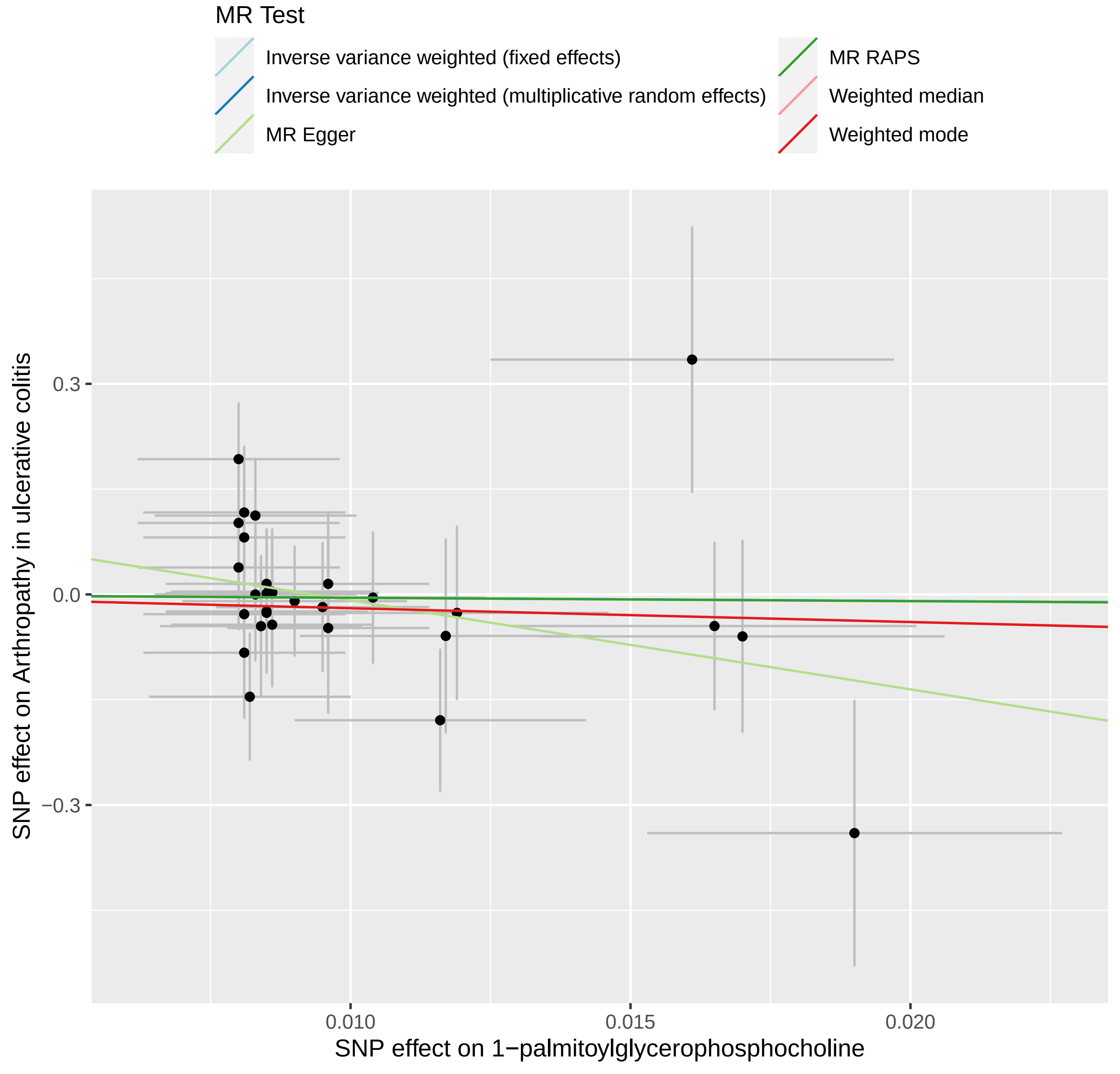

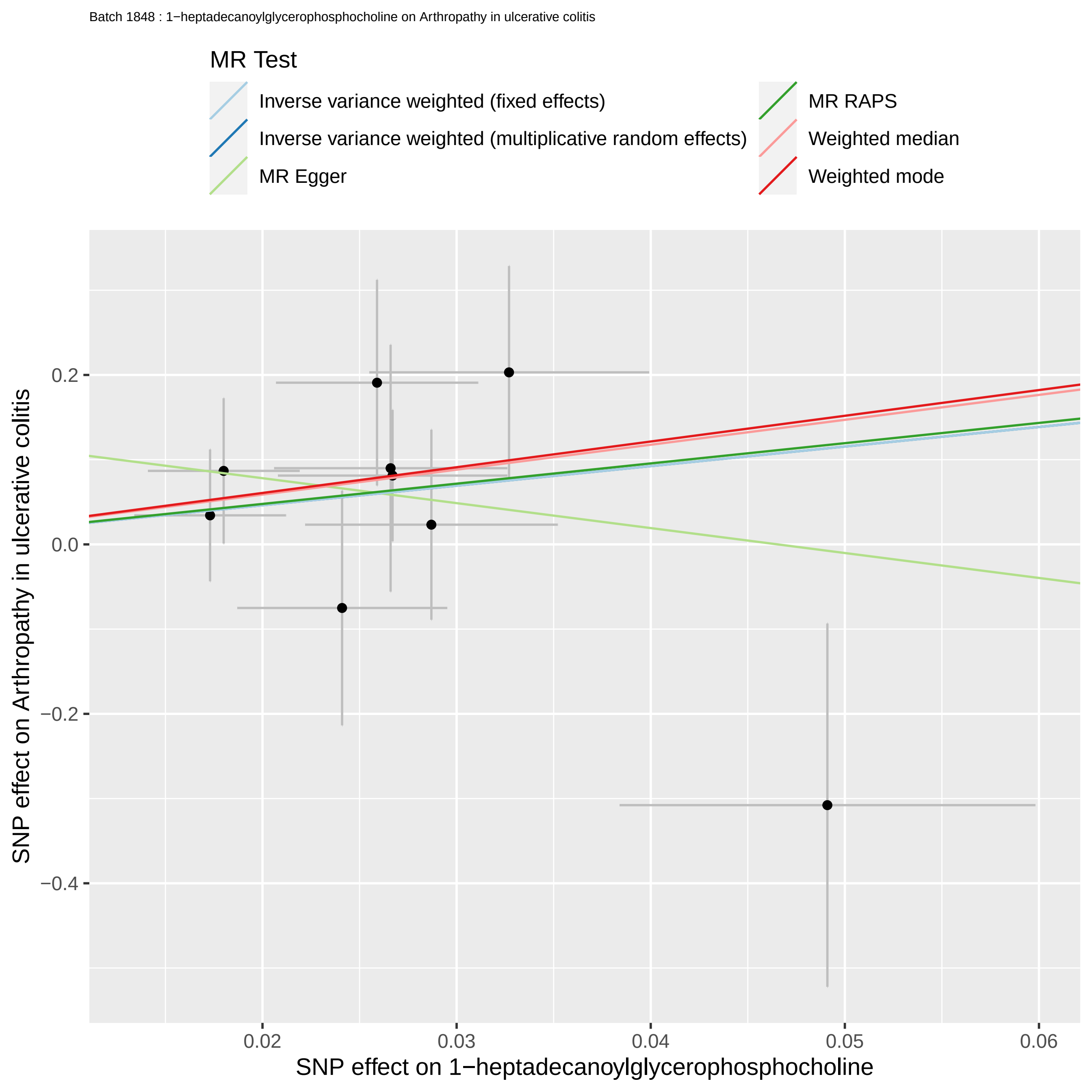

Batch 1848 : 1−heptadecanoylglycerophosphocholine on Arthropathy in ulcerative colitis
MR Test
Inverse variance weighted (fixed effects)
Inverse variance weighted (multiplicative random effects)
MR Egger
MR RAPS
Weighted median
Weighted mode
0.2
0.0
−0.2
−0.4
0.02
0.03
0.04
0.05
0.06
SNP effect on 1−heptadecanoylglycerophosphocholine
SNP effect on Arthropathy in ulcerative colitis

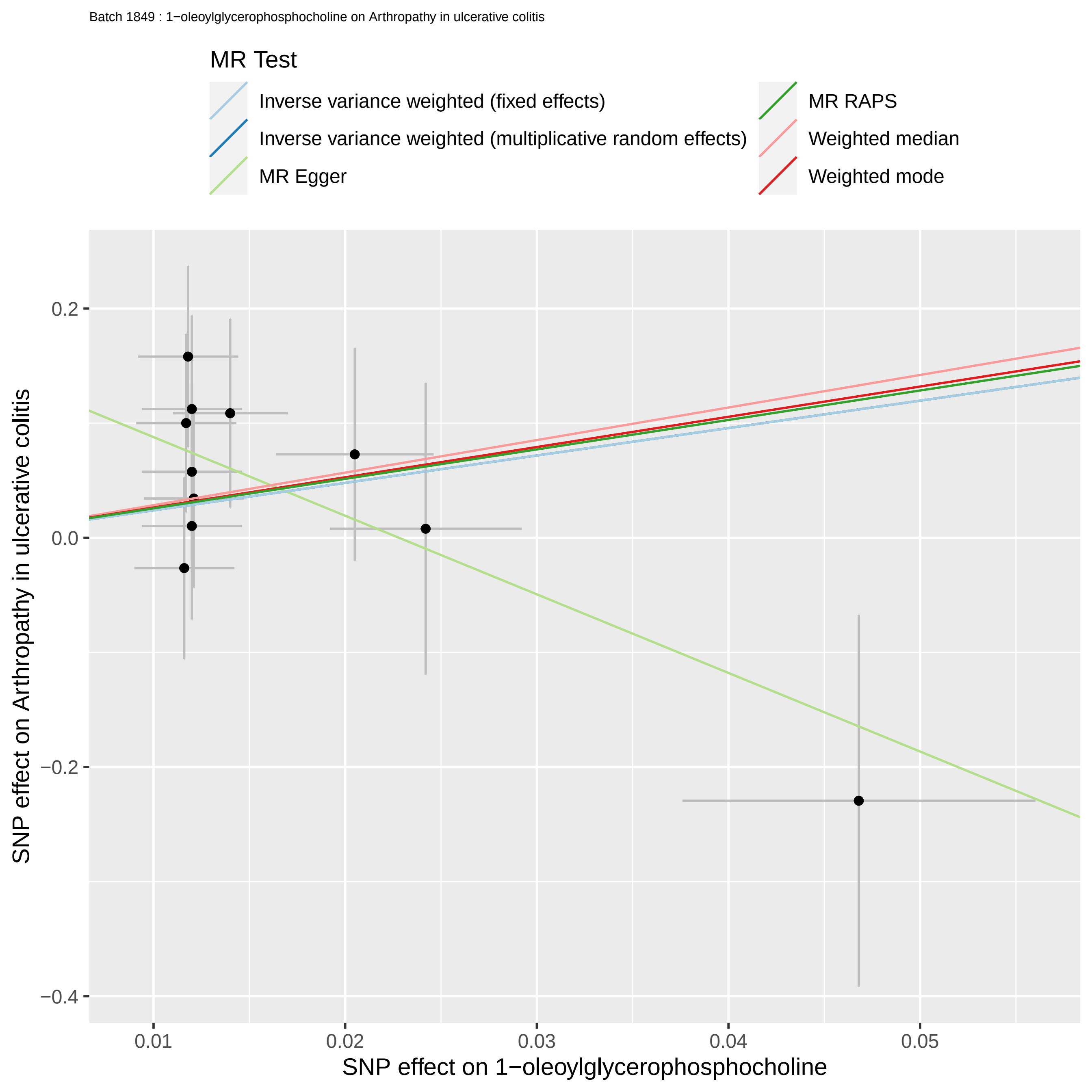

Batch 1849 : 1−oleoylglycerophosphocholine on Arthropathy in ulcerative colitis
MR Test
Inverse variance weighted (fixed effects)
Inverse variance weighted (multiplicative random effects)
MR Egger
MR RAPS
Weighted median
Weighted mode
0.2
0.0
−0.2
−0.4
0.01
0.02
0.03
0.04
0.05
SNP effect on Arthropathy in ulcerative colitis
SNP effect on 1−oleoylglycerophosphocholine

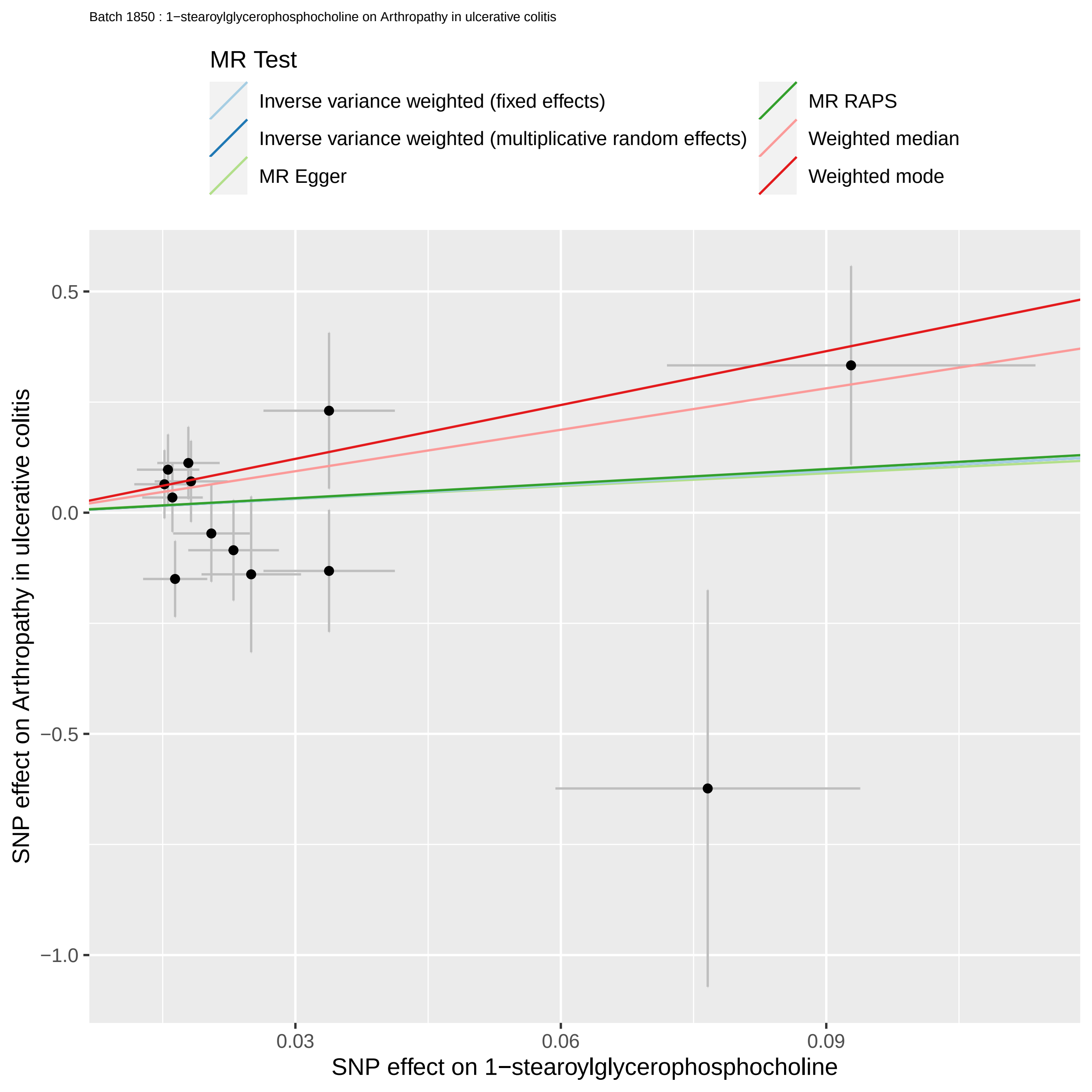
Batch 1850 : 1−stearoylglycerophosphocholine on Arthropathy in ulcerative colitis
MR Test
Inverse variance weighted (fixed effects)
Inverse variance weighted (multiplicative random effects)
MR Egger
MR RAPS
Weighted median
Weighted mode
0.5
0.0
−0.5
−1.0
0.03
0.06
0.09
SNP effect on Arthropathy in ulcerative colitis
SNP effect on 1−stearoylglycerophosphocholine

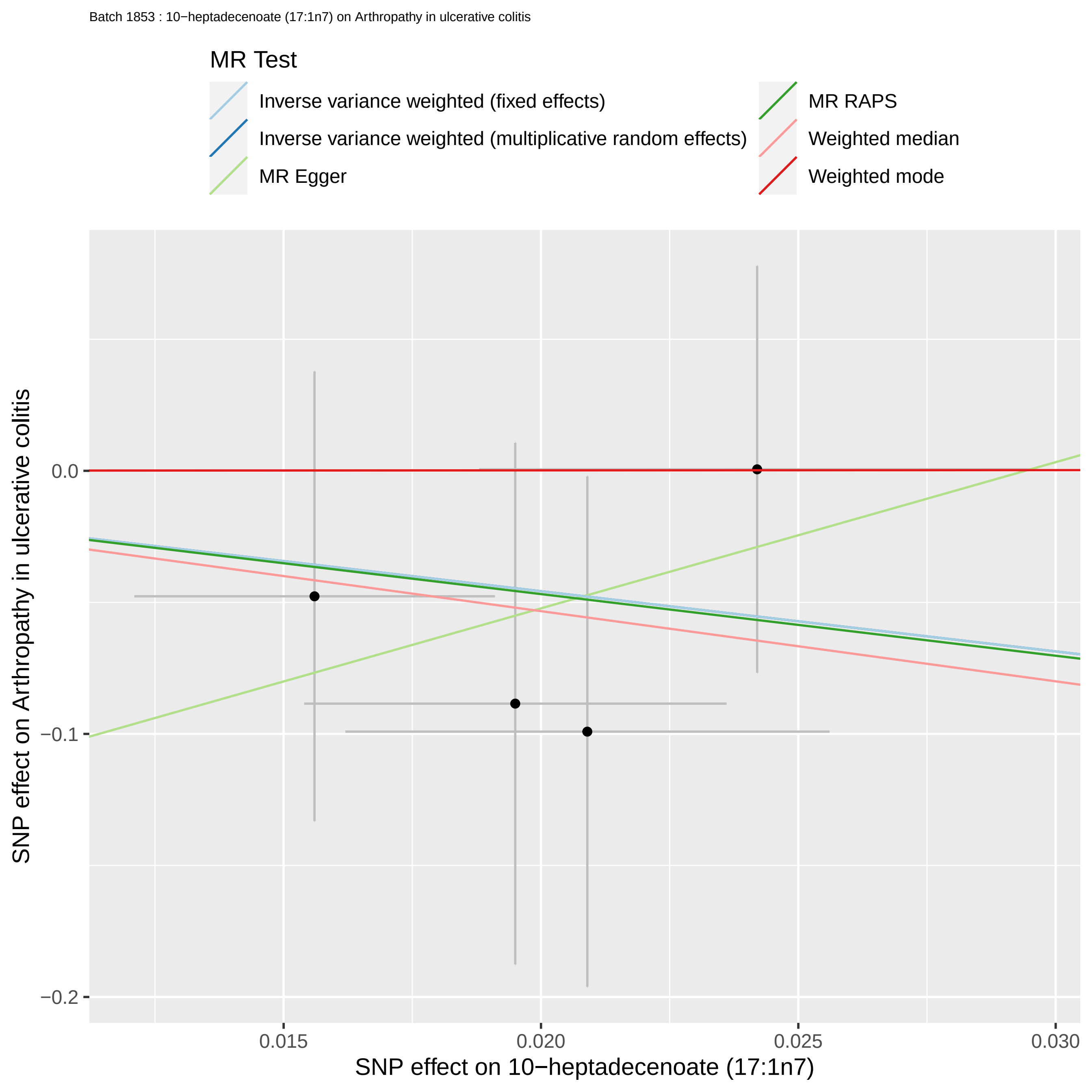

Batch 1853 : 10−heptadecenoate (17:1n7) on Arthropathy in ulcerative colitis
MR Test
Inverse variance weighted (fixed effects)
Inverse variance weighted (multiplicative random effects)
MR Egger
MR RAPS
Weighted median
Weighted mode
SNP effect on Arthropathy in ulcerative colitis
0.0
−0.1
−0.2
0.015
0.020
0.025
0.030
SNP effect on 10−heptadecenoate (17:1n7)

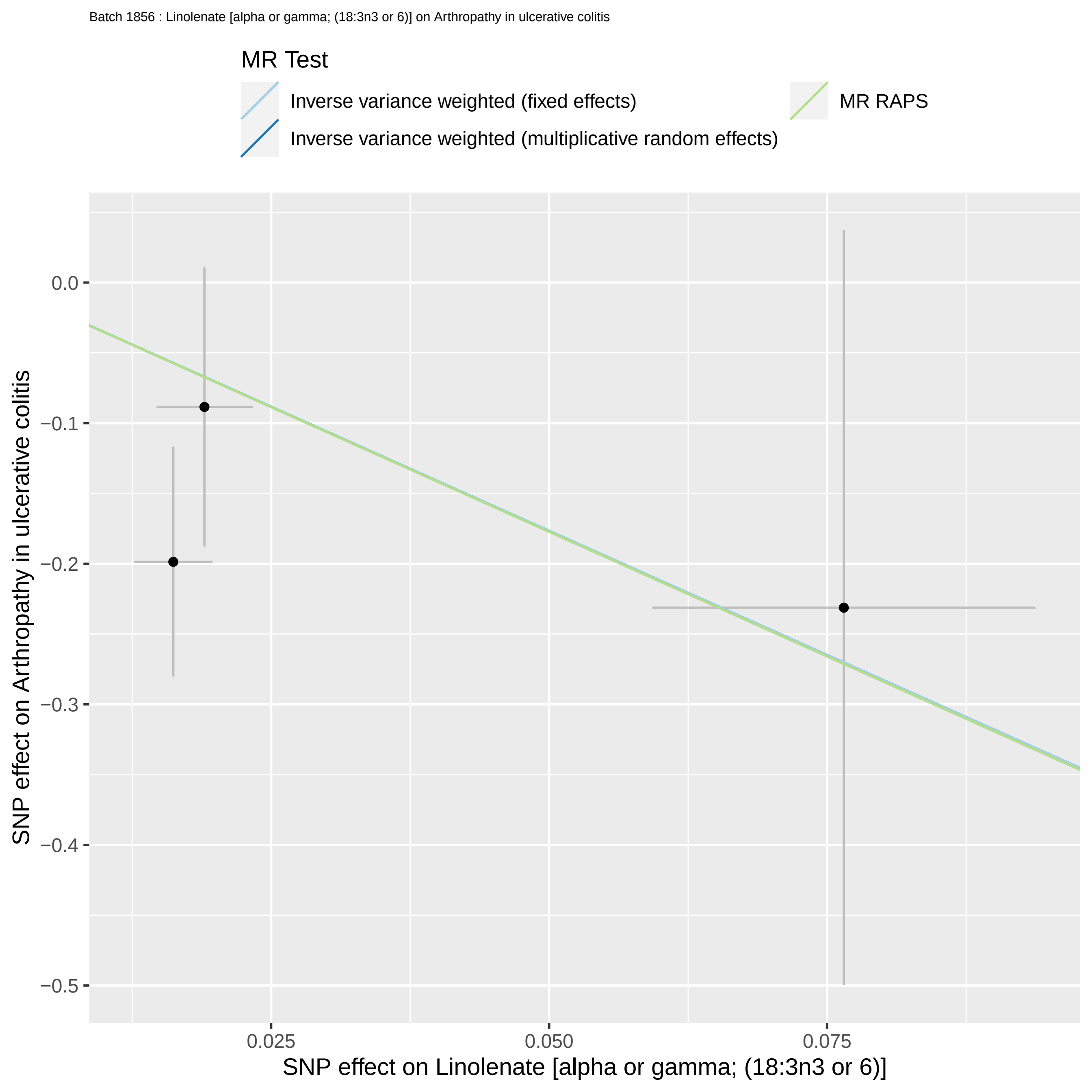

Batch 1856 : Linolenate [alpha or gamma; (18:3n3 or 6)] on Arthropathy in ulcerative colitis
MR Test
Inverse variance weighted (fixed effects)
Inverse variance weighted (multiplicative random effects)
MR RAPS
0.0
−0.1
−0.2
−0.3
−0.4
−0.5
SNP effect on Arthropathy in ulcerative colitis
0.025
0.050
0.075
SNP effect on Linolenate [alpha or gamma; (18:3n3 or 6)]

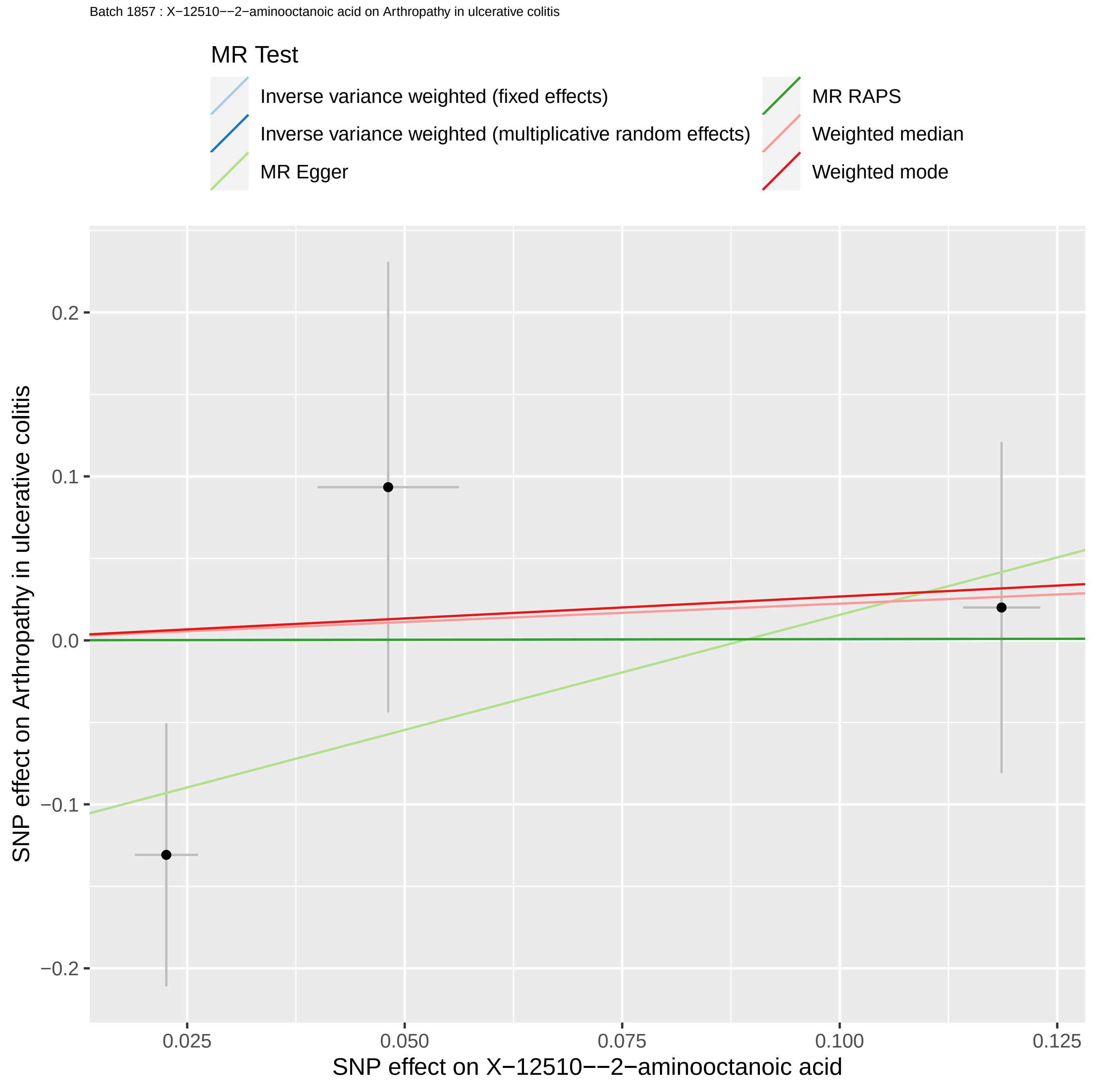
Batch 1857 : X−12510−−2−aminooctanoic acid on Arthropathy in ulcerative colitis
MR Test
Inverse variance weighted (fixed effects)
Inverse variance weighted (multiplicative random effects)
MR Egger
MR RAPS
Weighted median
Weighted mode
0.2
0.1
0.0
−0.1
−0.2
0.025
0.050
0.075
0.100
0.125
SNP effect on Arthropathy in ulcerative colitis
SNP effect on X−12510−−2−aminooctanoic acid

Batch 1860 : Asparagine on Arthropathy in ulcerative colitis

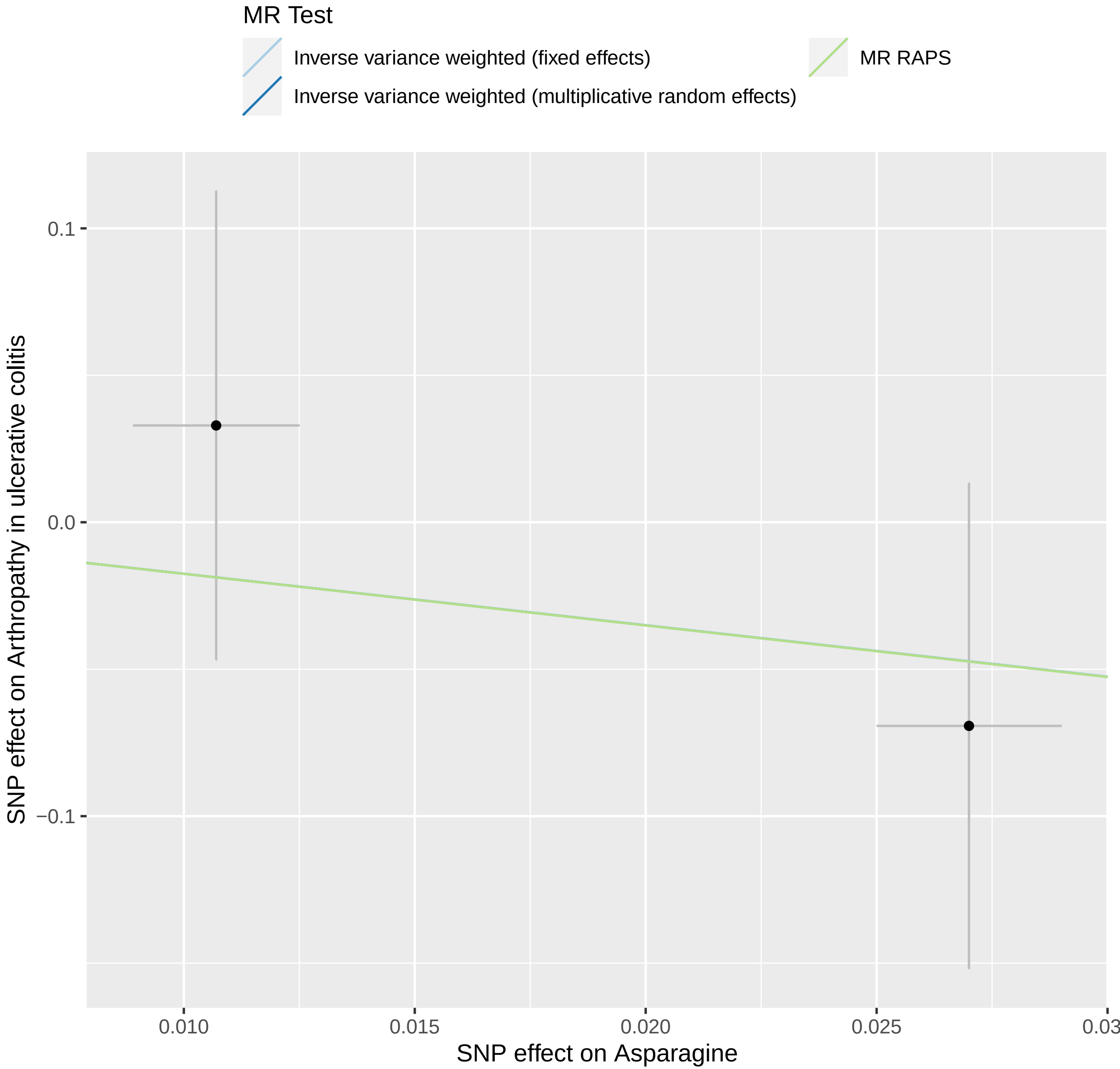

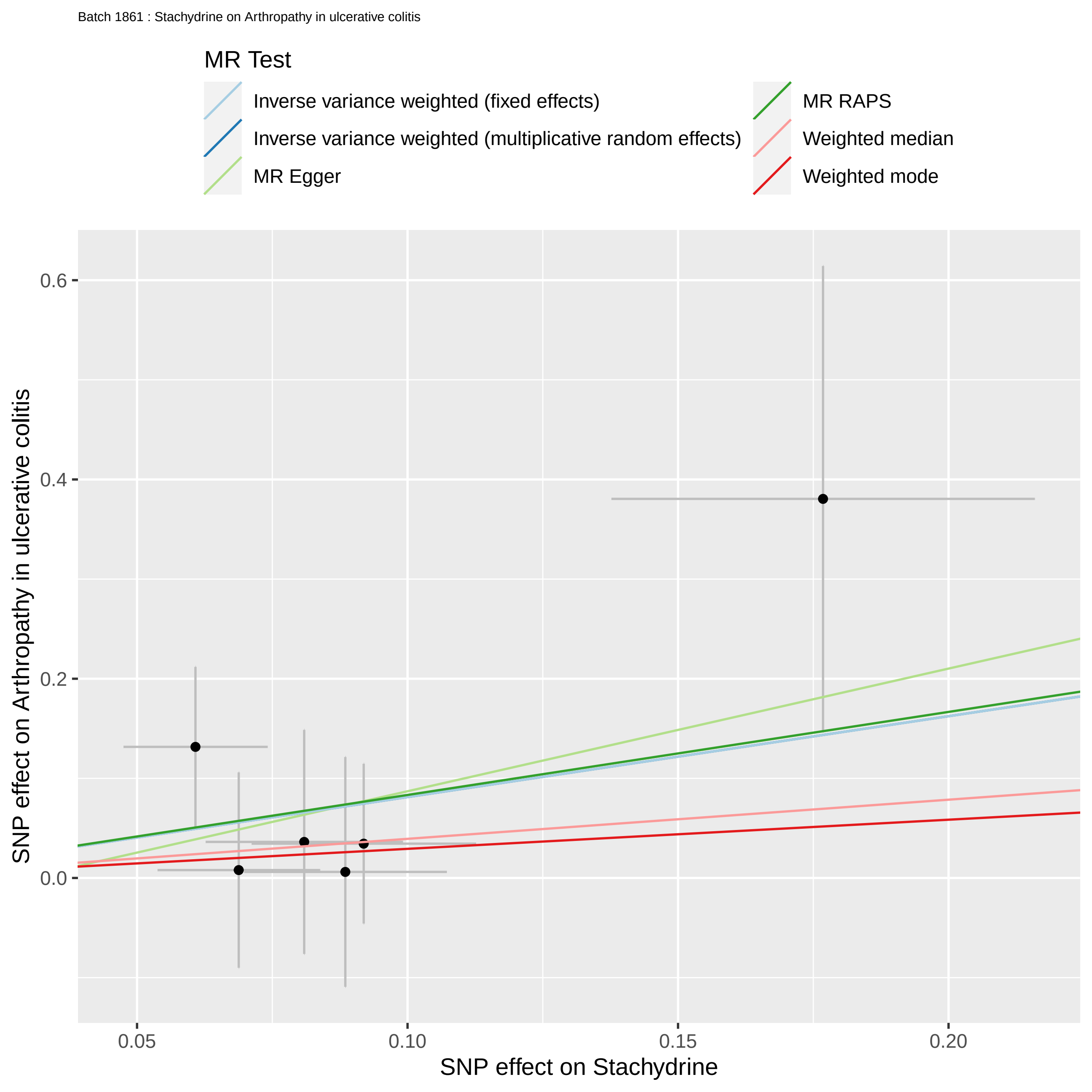
Batch 1861 : Stachydrine on Arthropathy in ulcerative colitis
MR Test
Inverse variance weighted (fixed effects)
Inverse variance weighted (multiplicative random effects)
MR Egger
MR RAPS
Weighted median
Weighted mode
SNP effect on Arthropathy in ulcerative colitis
SNP effect on Stachydrine
0.0
0.2
0.4
0.6
0.05
0.10
0.15
0.20

Batch 1862 : Isovalerylcarnitine on Arthropathy in ulcerative colitis

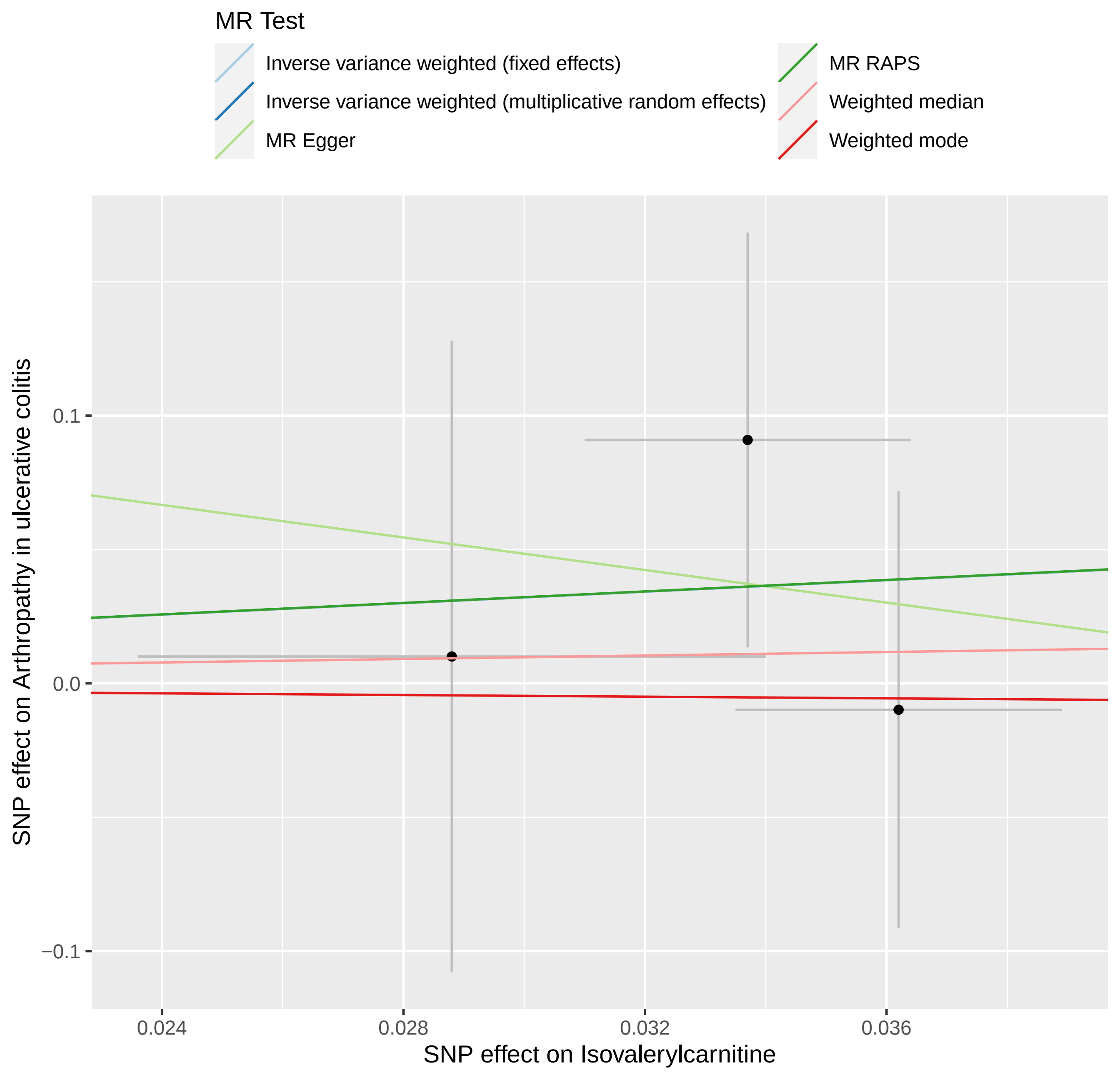

Batch 1865 : 1−linoleoylglycerophosphocholine on Arthropathy in ulcerative colitis

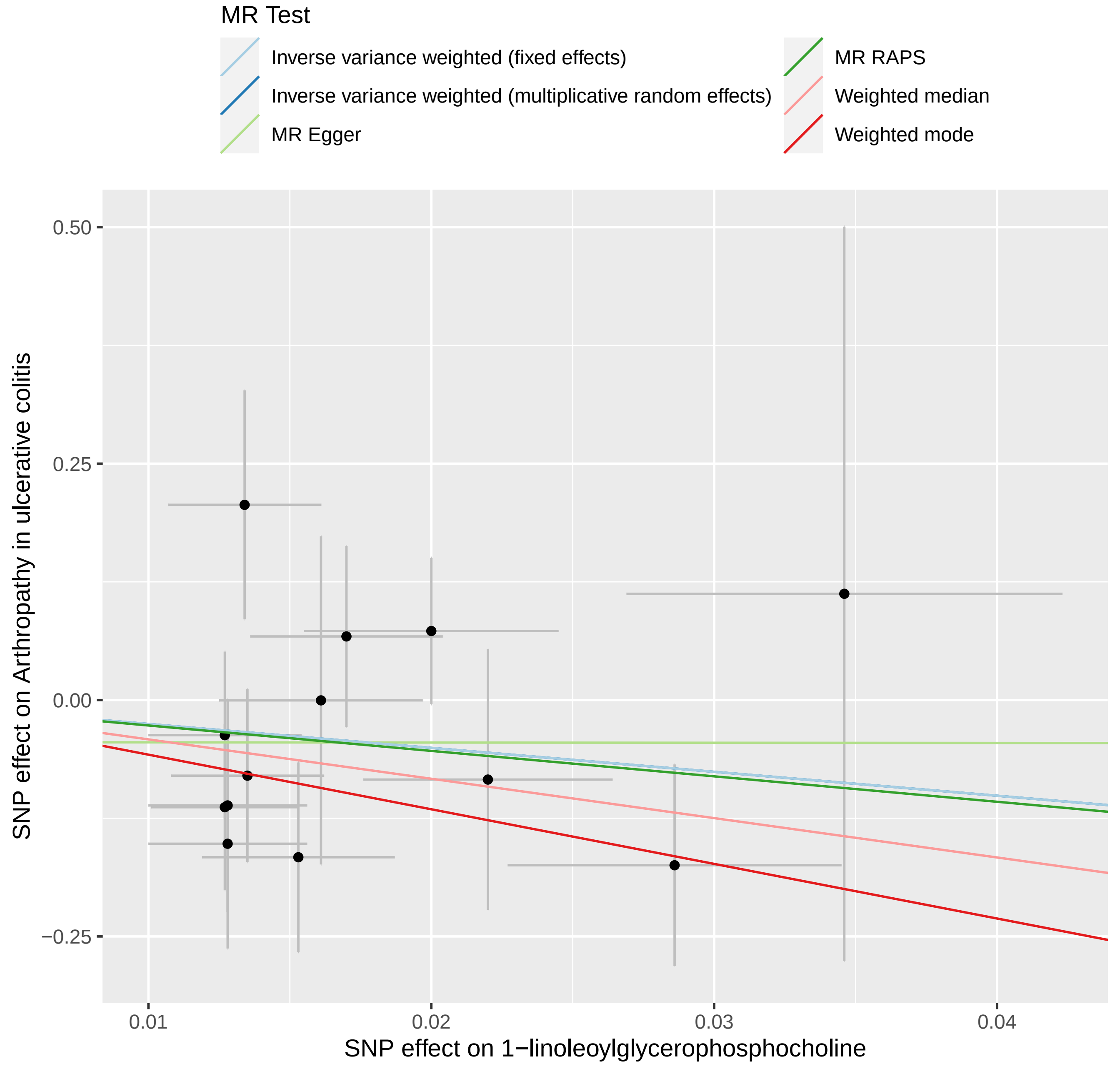

Batch 1866 : Bradykinin, des−arg(9) on Arthropathy in ulcerative colitis

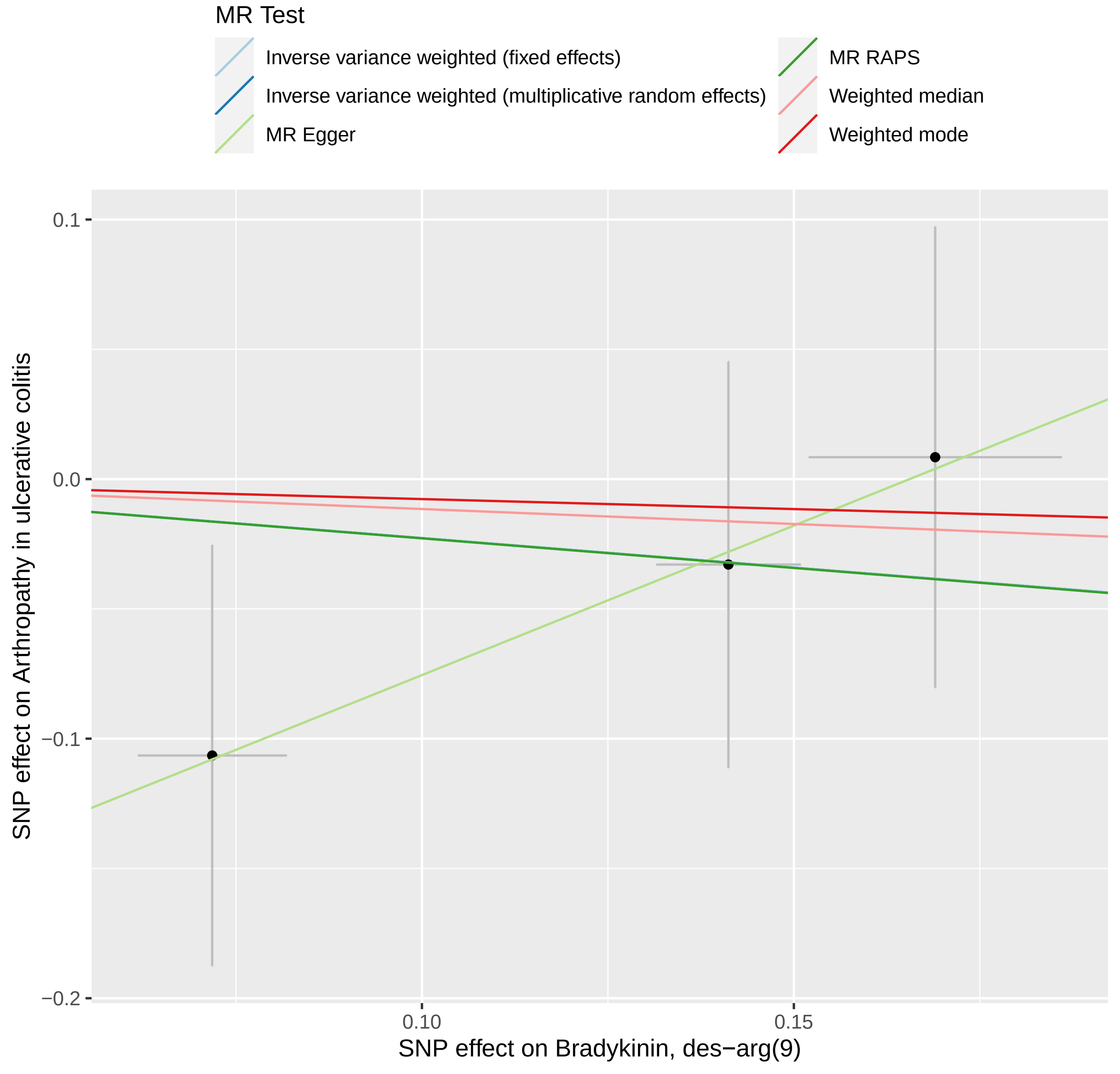

Batch 1867 : Gamma−glutamylisoleucine* on Arthropathy in ulcerative colitis

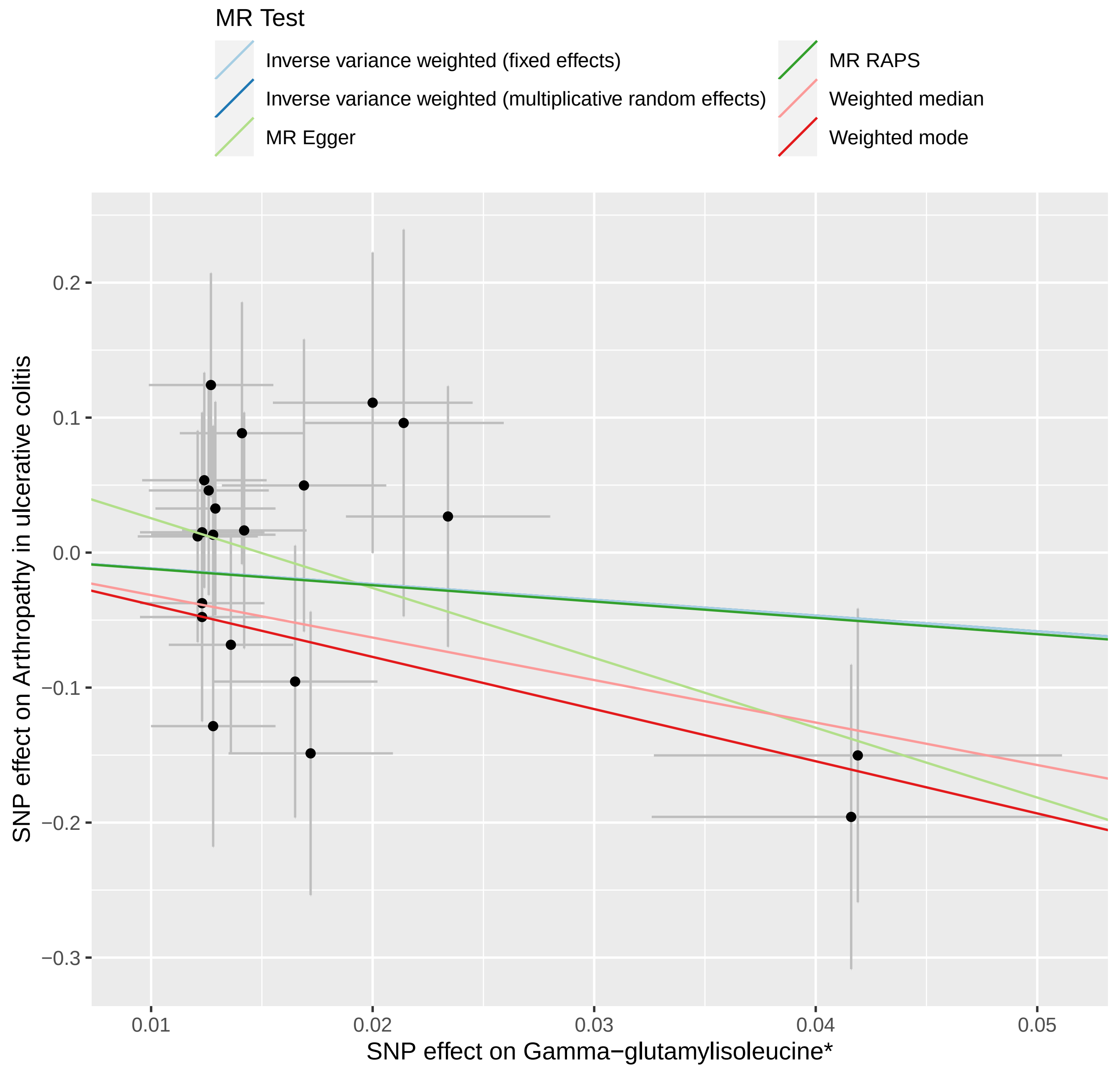

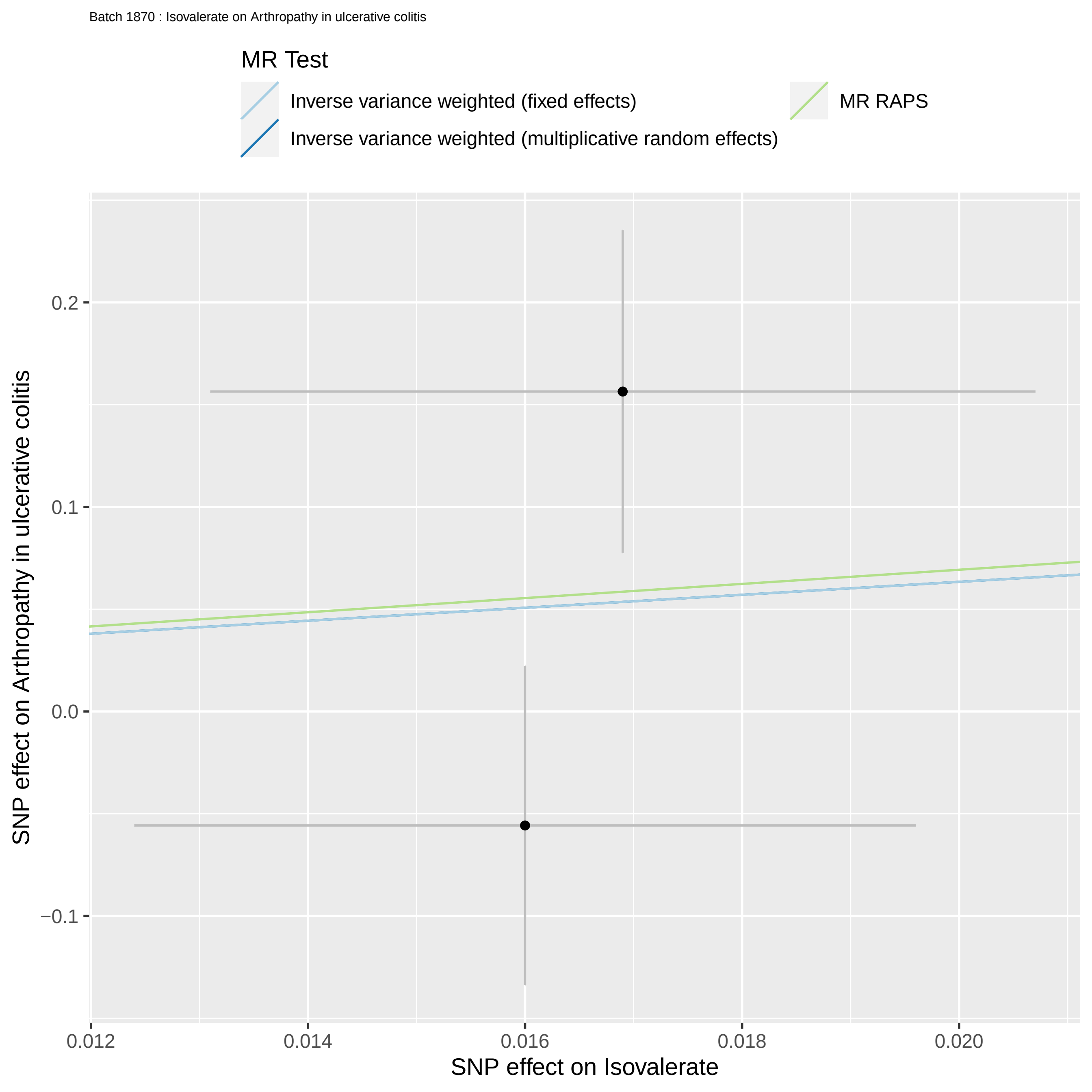
Batch 1870 : Isovalerate on Arthropathy in ulcerative colitis
MR Test
Inverse variance weighted (fixed effects)
Inverse variance weighted (multiplicative random effects)
MR RAPS
0.2
0.1
0.0
−0.1
0.012
0.014
0.016
0.018
0.020
SNP effect on Arthropathy in ulcerative colitis
SNP effect on Isovalerate

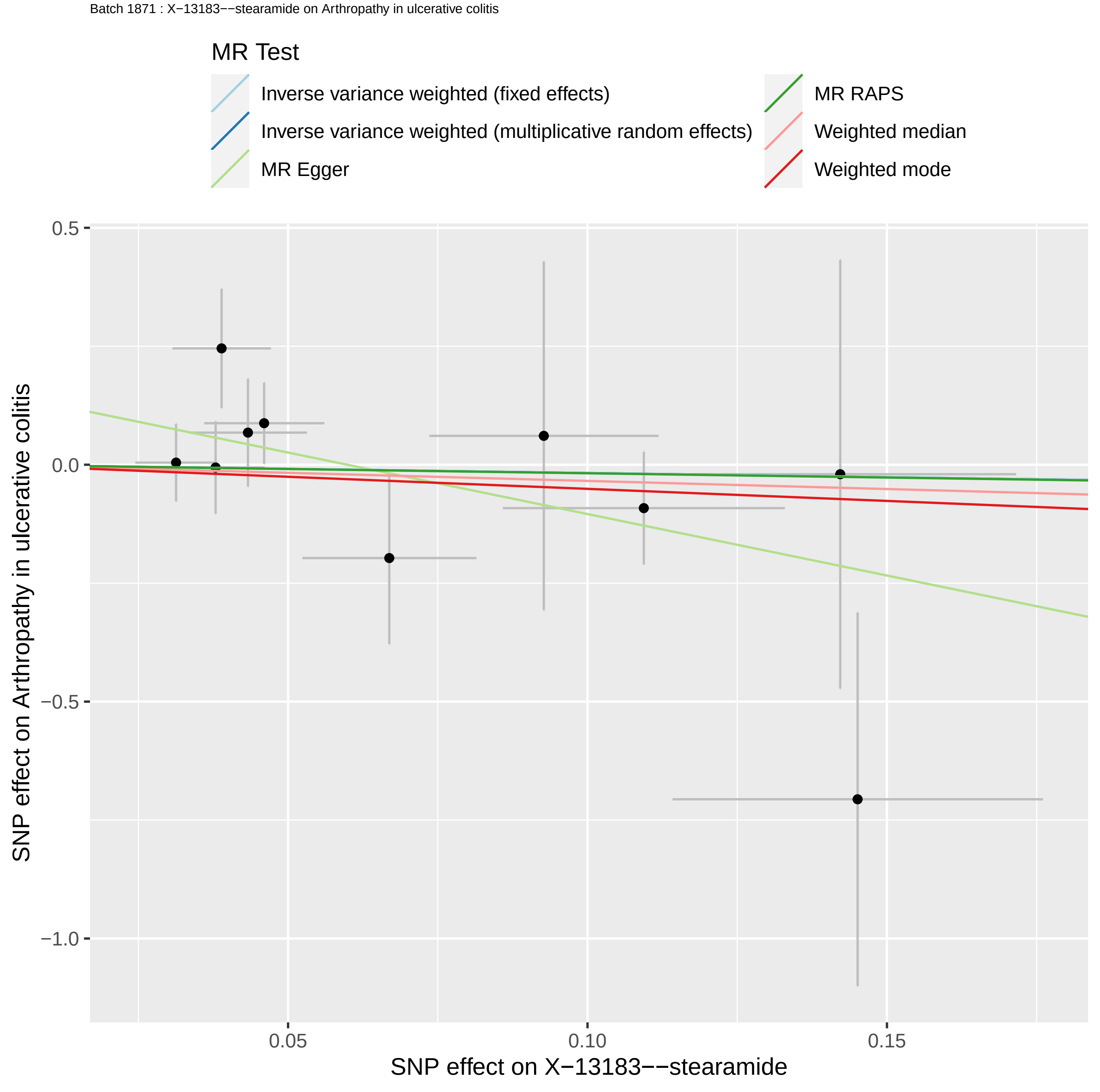

Batch 1871 : X−13183−−stearamide on Arthropathy in ulcerative colitis
MR Test
Inverse variance weighted (fixed effects)
Inverse variance weighted (multiplicative random effects)
MR Egger
MR RAPS
Weighted median
Weighted mode
0.5
0.0
−0.5
−1.0
0.05
0.10
0.15
SNP effect on Arthropathy in ulcerative colitis
SNP effect on X−13183−−stearamide

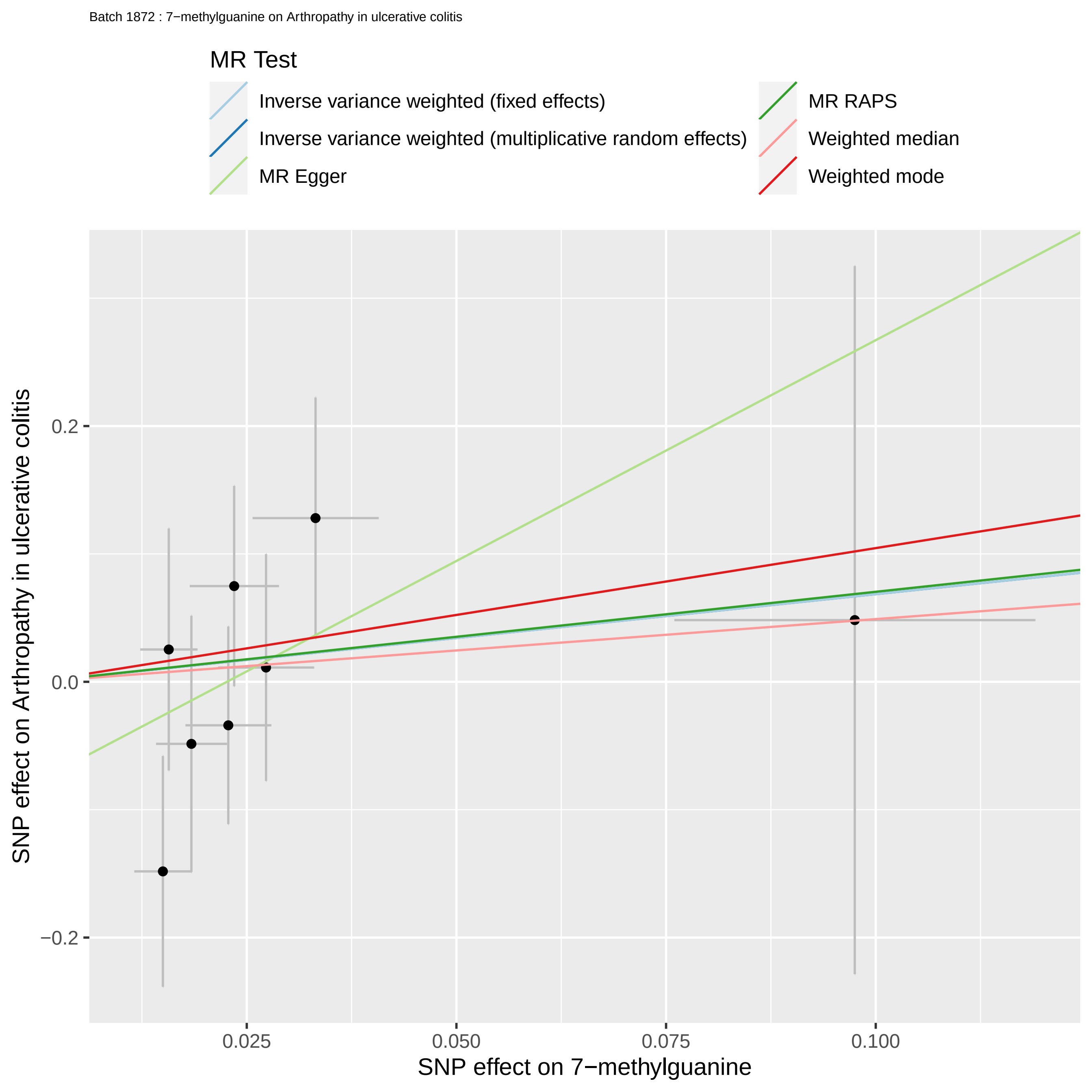

Batch 1872 : 7−methylguanine on Arthropathy in ulcerative colitis
MR Test
Inverse variance weighted (fixed effects)
Inverse variance weighted (multiplicative random effects)
MR Egger
MR RAPS
Weighted median
Weighted mode
0.2
0.0
−0.2
0.025
0.050
0.075
0.100
SNP effect on Arthropathy in ulcerative colitis
SNP effect on 7−methylguanine

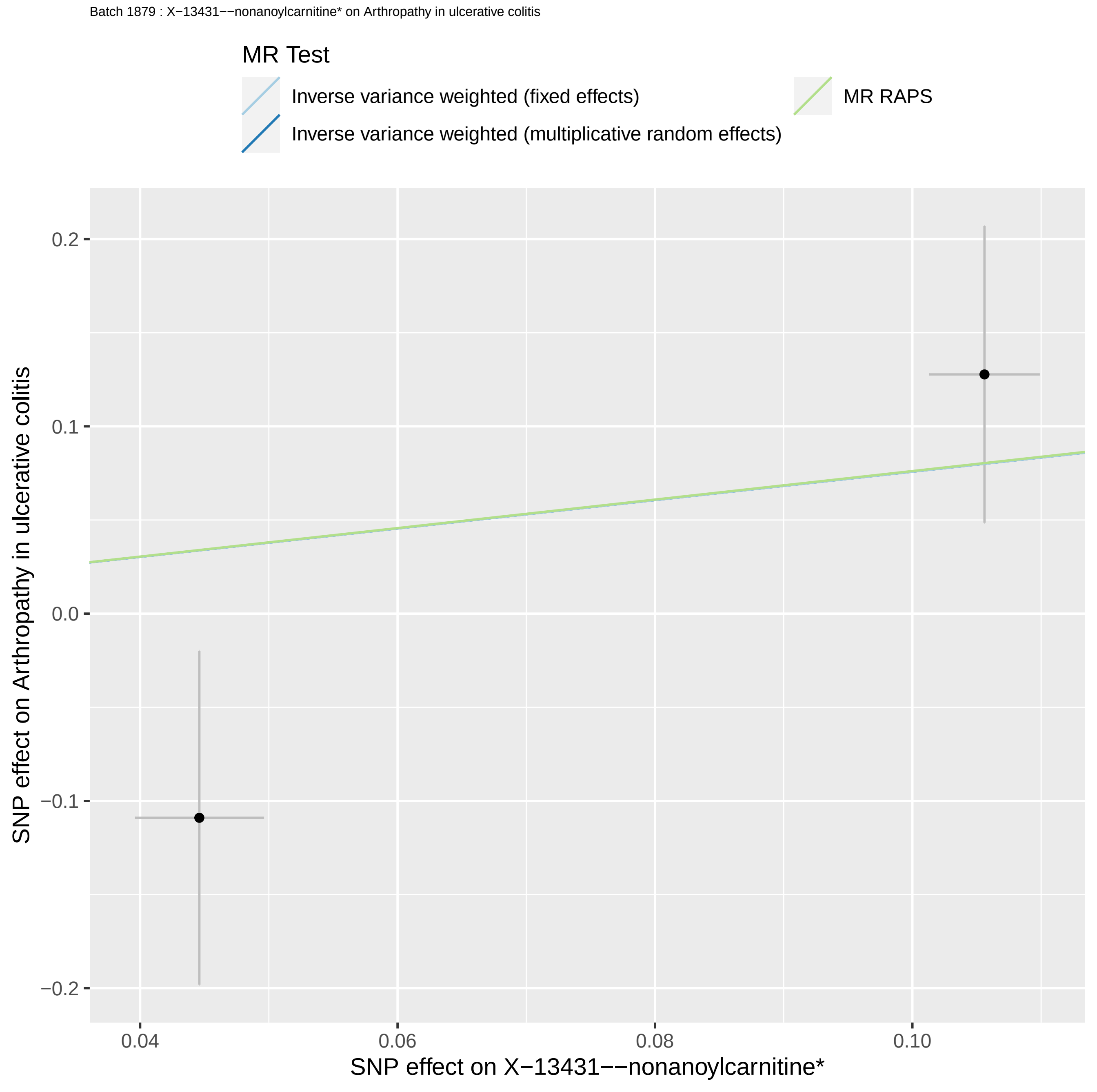
Batch 1879 : X−13431−−nonanoylcarnitine* on Arthropathy in ulcerative colitis
MR Test
Inverse variance weighted (fixed effects)
Inverse variance weighted (multiplicative random effects)
MR RAPS
SNP effect on Arthropathy in ulcerative colitis
0.2
0.1
0.0
−0.1
−0.2
0.04
0.06
0.08
0.10
SNP effect on X−13431−−nonanoylcarnitine*

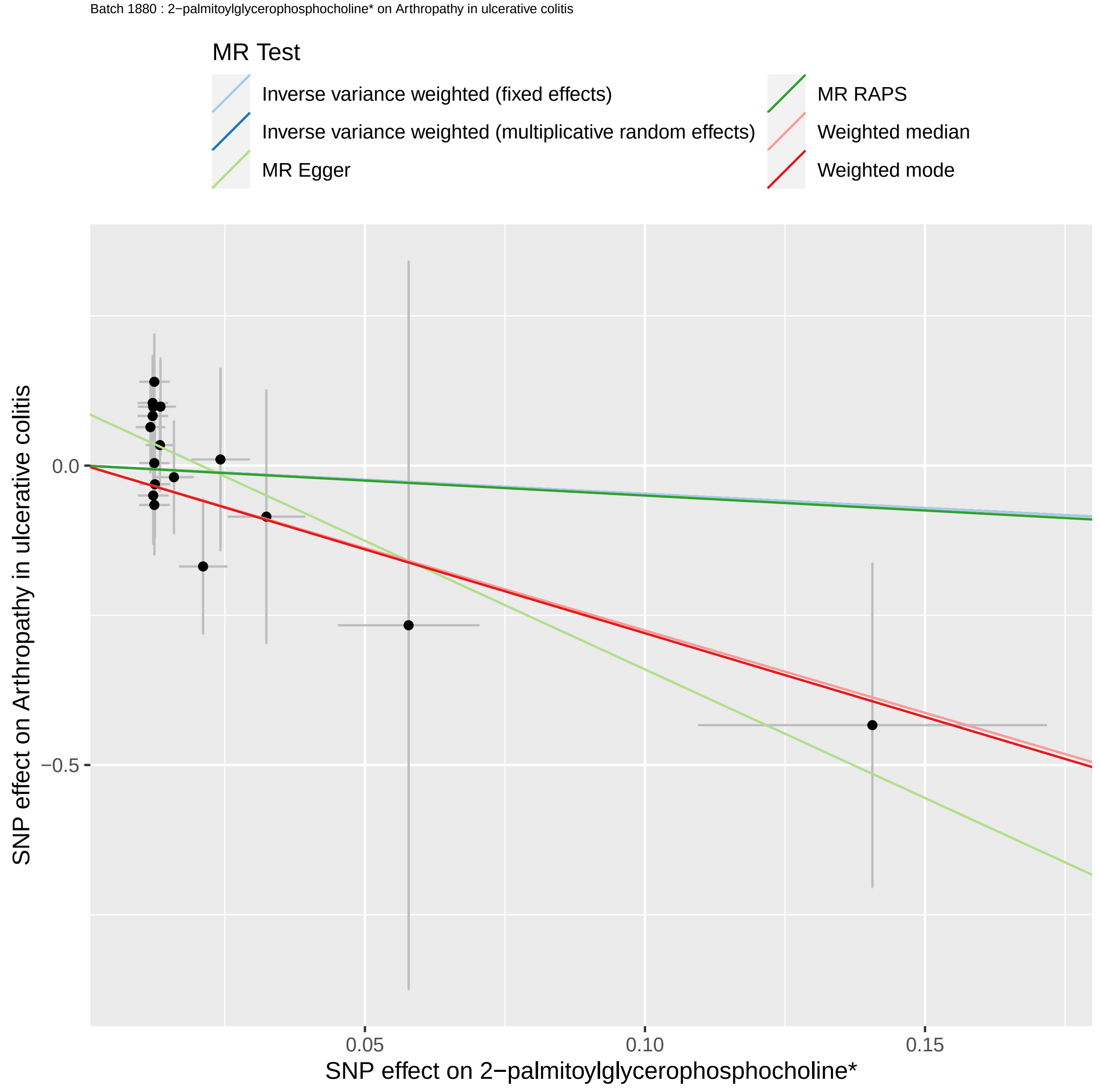

Batch 1880 : 2−palmitoylglycerophosphocholine* on Arthropathy in ulcerative colitis
MR Test
Inverse variance weighted (fixed effects)
Inverse variance weighted (multiplicative random effects)
MR Egger
MR RAPS
Weighted median
Weighted mode
SNP effect on Arthropathy in ulcerative colitis
0.0
−0.5
0.05
0.10
0.15
SNP effect on 2−palmitoylglycerophosphocholine*

Batch 1881 : 2−oleoylglycerophosphocholine* on Arthropathy in ulcerative colitis

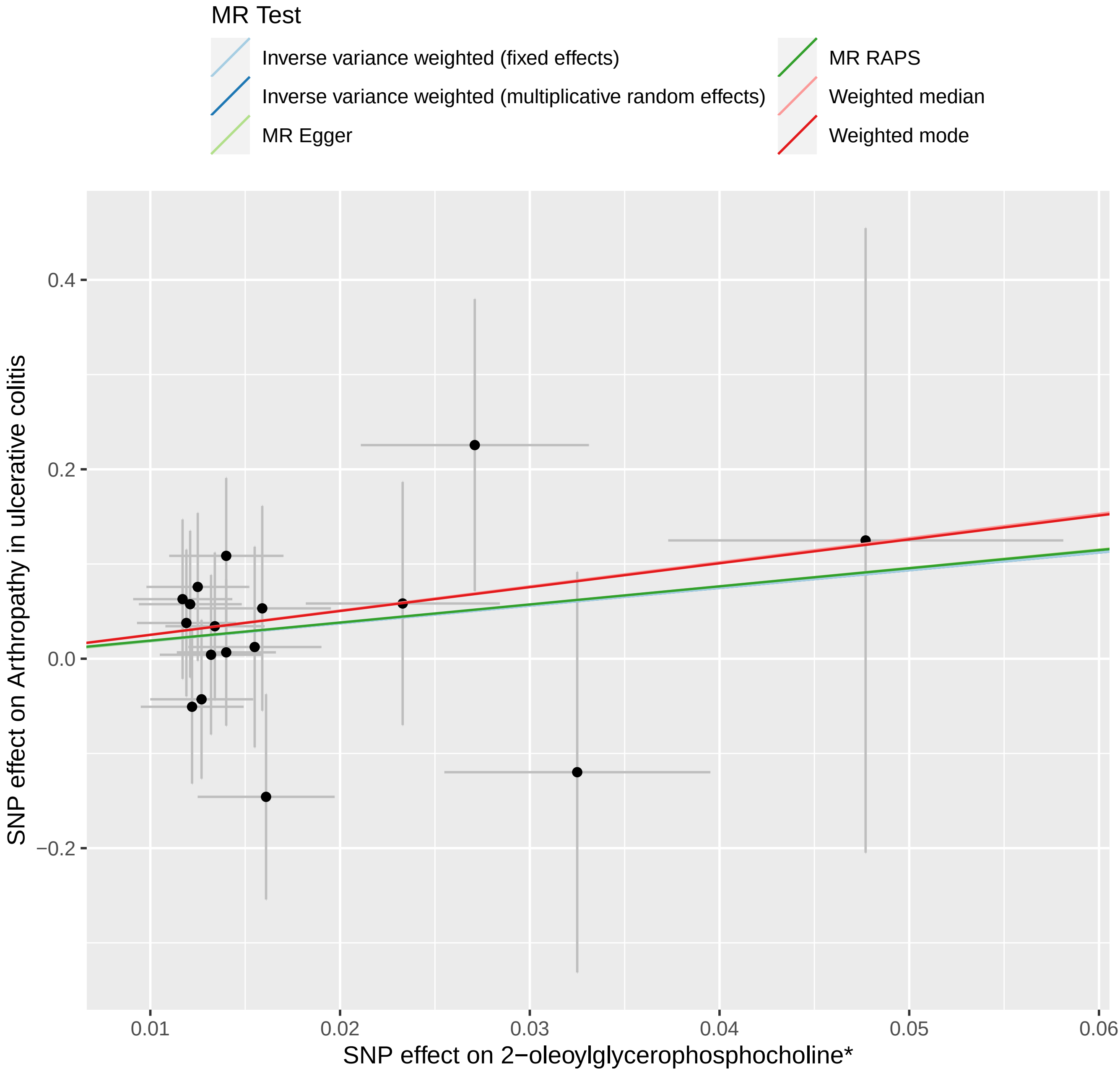

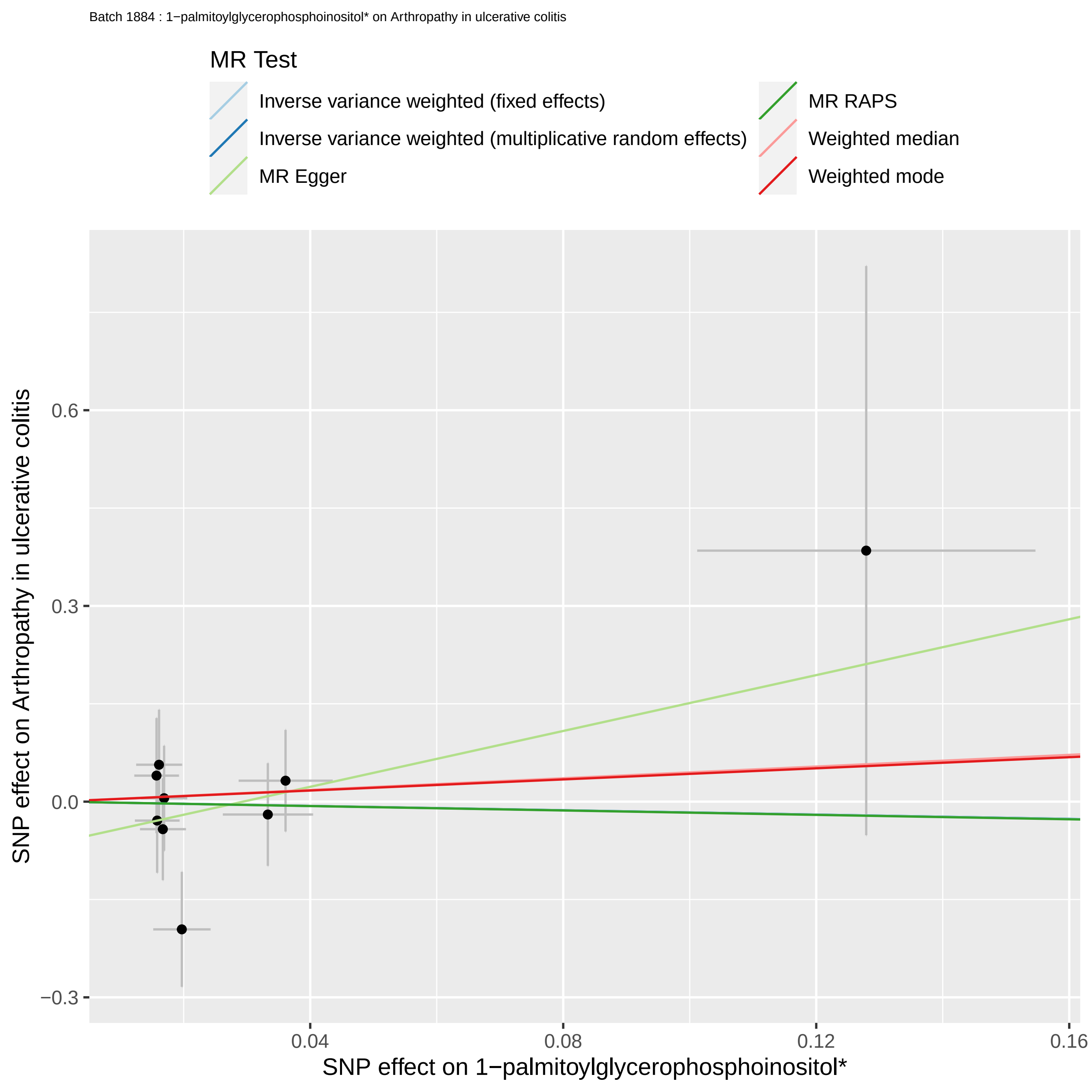

Batch 1884 : 1−palmitoylglycerophosphoinositol* on Arthropathy in ulcerative colitis
MR Test
Inverse variance weighted (fixed effects)
Inverse variance weighted (multiplicative random effects)
MR Egger
MR RAPS
Weighted median
Weighted mode
SNP effect on Arthropathy in ulcerative colitis
0.6
0.3
0.0
−0.3
0.04
0.08
0.12
0.16
SNP effect on 1−palmitoylglycerophosphoinositol*

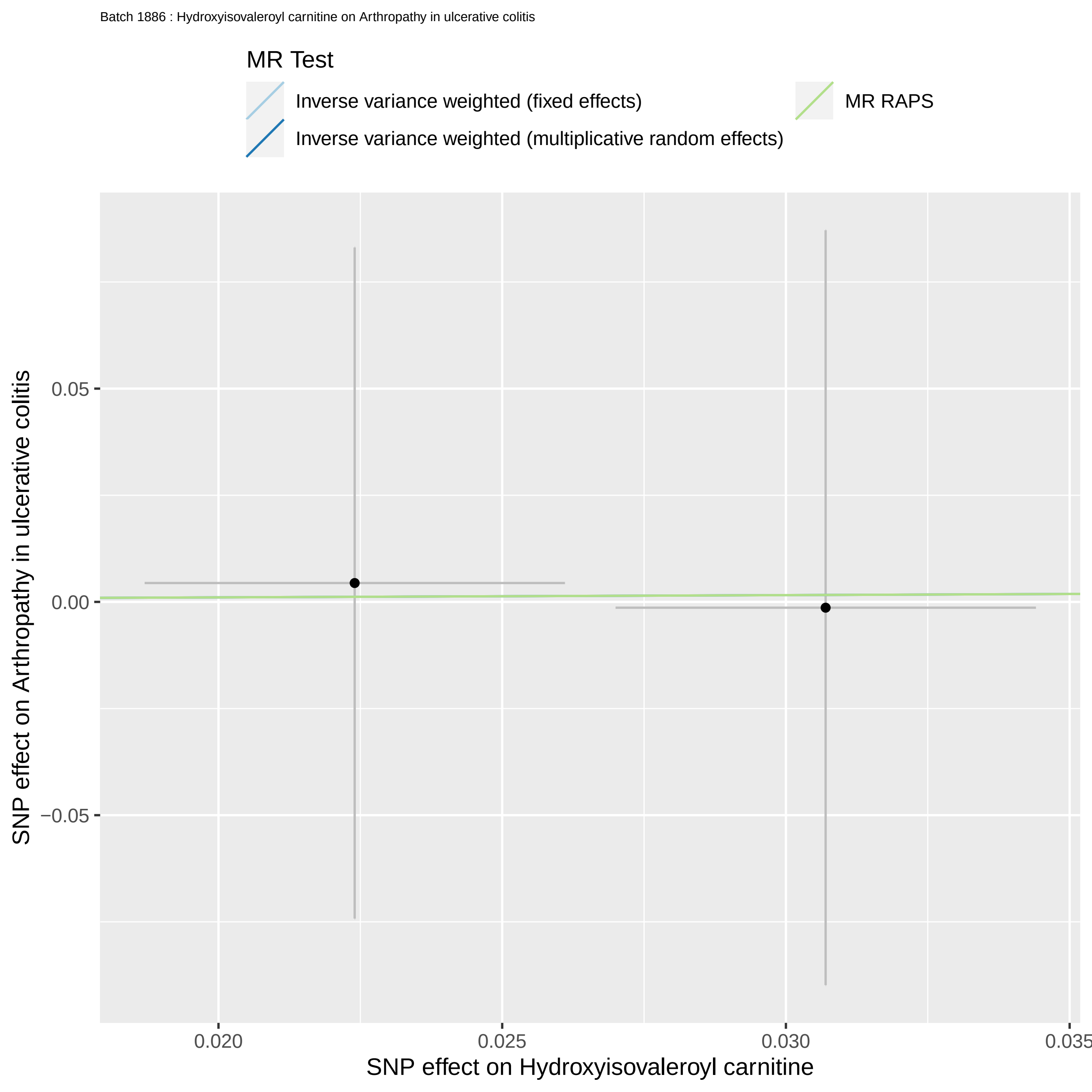

Batch 1886 : Hydroxyisovaleroyl carnitine on Arthropathy in ulcerative colitis
MR Test
Inverse variance weighted (fixed effects)
Inverse variance weighted (multiplicative random effects)
MR RAPS
0.05
0.00
−0.05
0.020
0.025
0.030
0.035
SNP effect on Arthropathy in ulcerative colitis
SNP effect on Hydroxyisovaleroyl carnitine

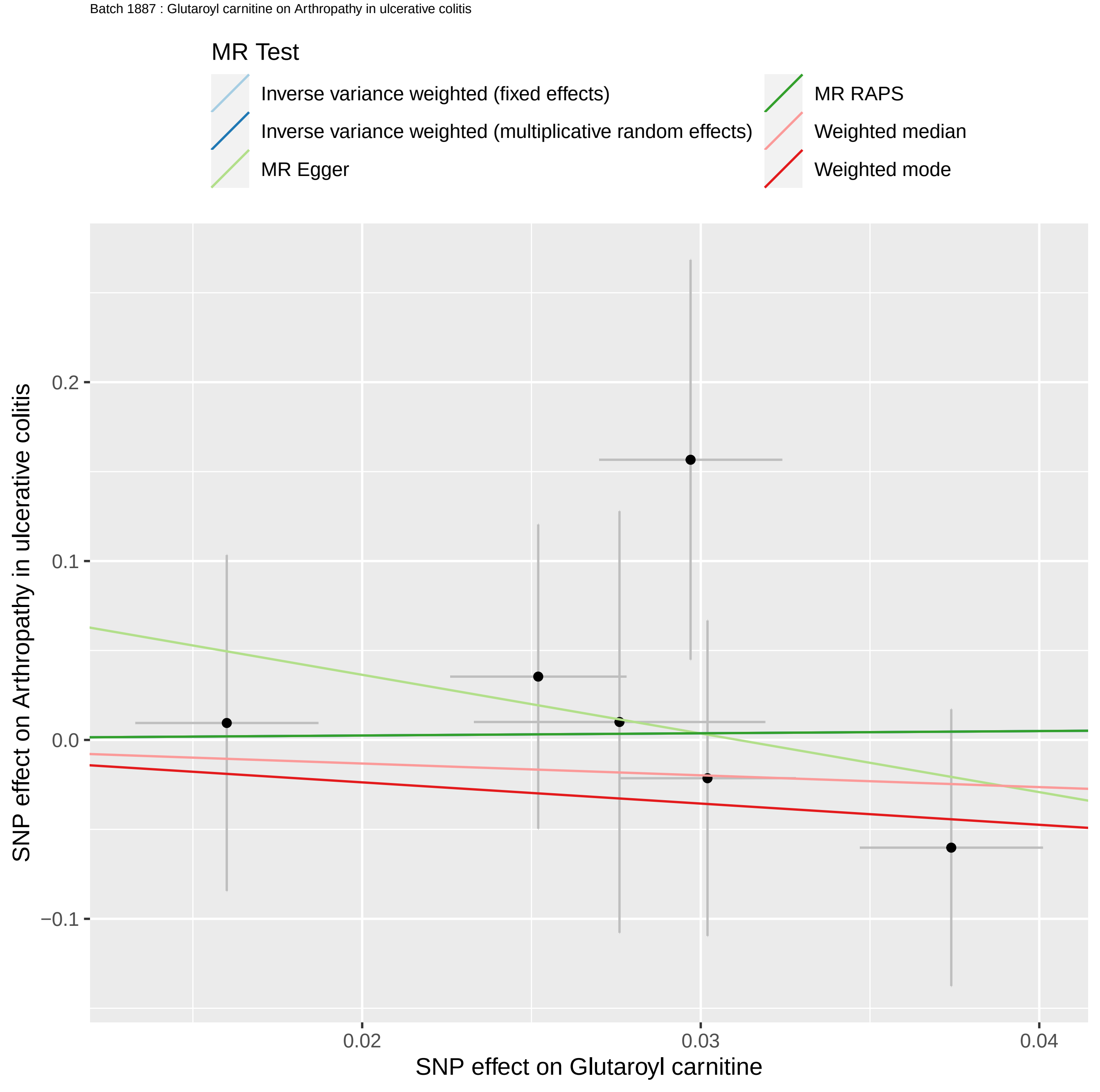
Batch 1887 : Glutaroyl carnitine on Arthropathy in ulcerative colitis
MR Test
Inverse variance weighted (fixed effects)
Inverse variance weighted (multiplicative random effects)
MR Egger
MR RAPS
Weighted median
Weighted mode
SNP effect on Arthropathy in ulcerative colitis
0.2
0.1
0.0
−0.1
0.02
0.03
0.04
SNP effect on Glutaroyl carnitine

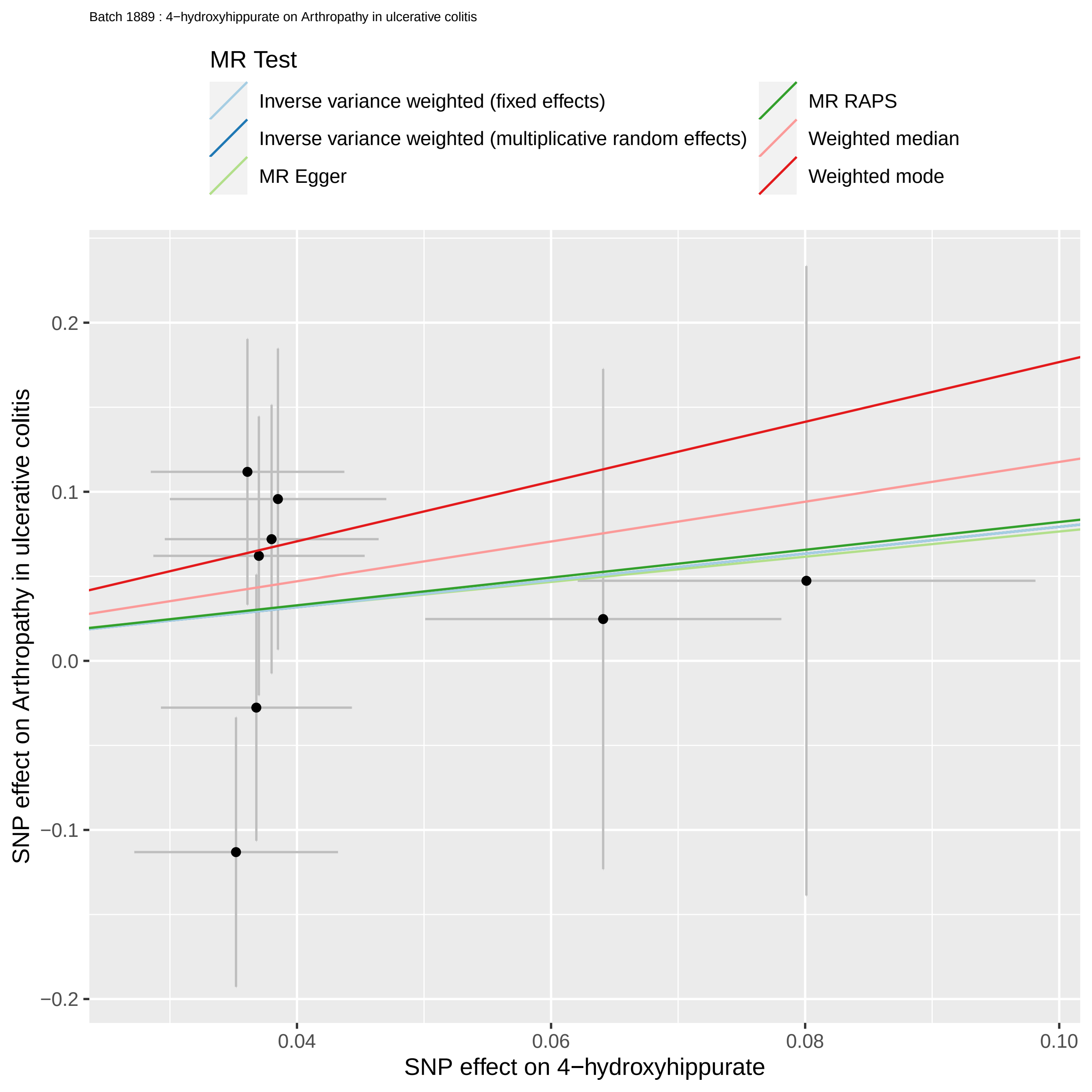

Batch 1889 : 4−hydroxyhippurate on Arthropathy in ulcerative colitis
MR Test
Inverse variance weighted (fixed effects)
Inverse variance weighted (multiplicative random effects)
MR Egger
MR RAPS
Weighted median
Weighted mode
0.2
0.1
0.0
−0.1
−0.2
0.04
0.06
0.08
0.10
SNP effect on Arthropathy in ulcerative colitis
SNP effect on 4−hydroxyhippurate

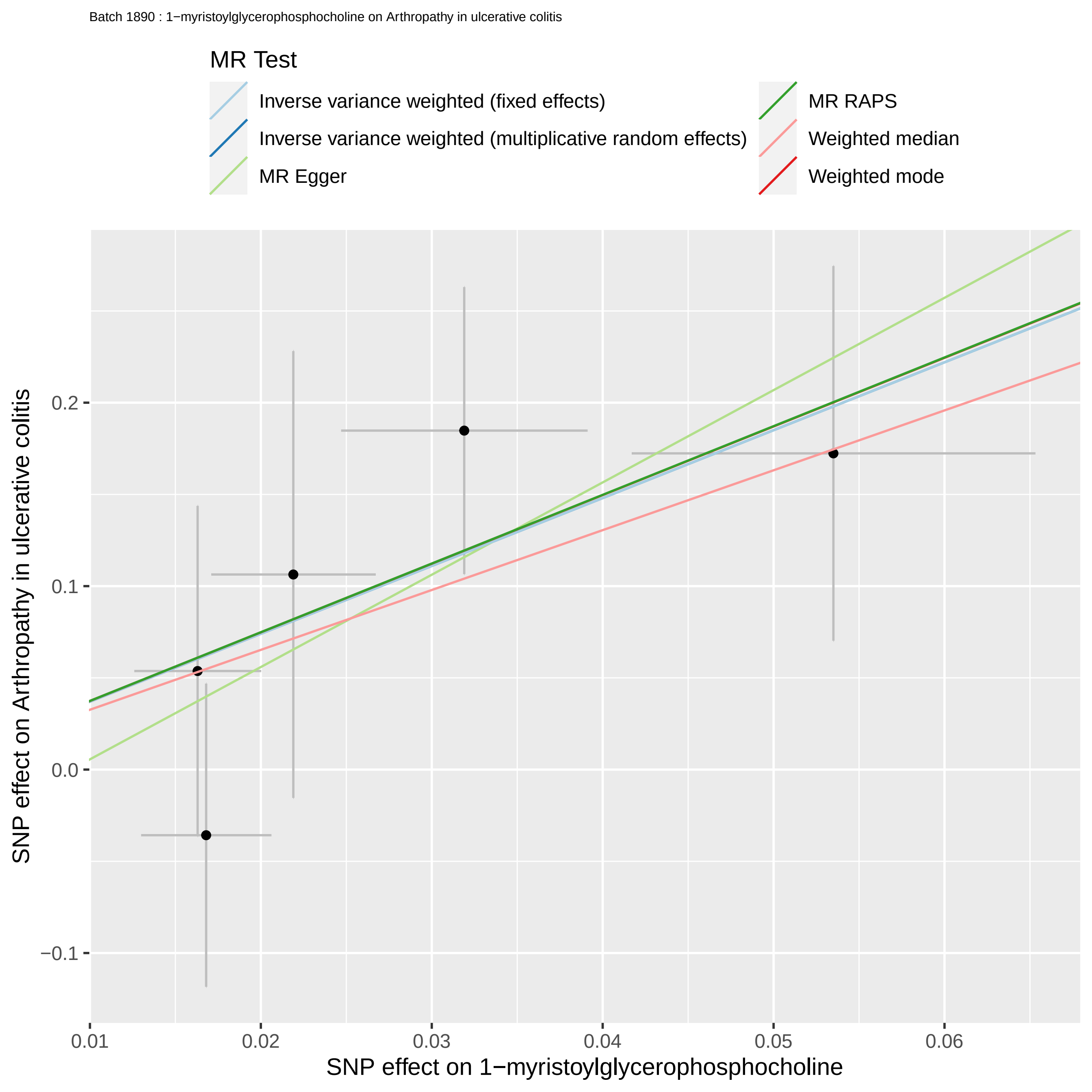

Batch 1890 : 1−myristoylglycerophosphocholine on Arthropathy in ulcerative colitis
MR Test
Inverse variance weighted (fixed effects)
Inverse variance weighted (multiplicative random effects)
MR Egger
MR RAPS
Weighted median
Weighted mode
SNP effect on Arthropathy in ulcerative colitis
0.2
0.1
0.0
−0.1
0.01
0.02
0.03
0.04
0.05
0.06
SNP effect on 1−myristoylglycerophosphocholine

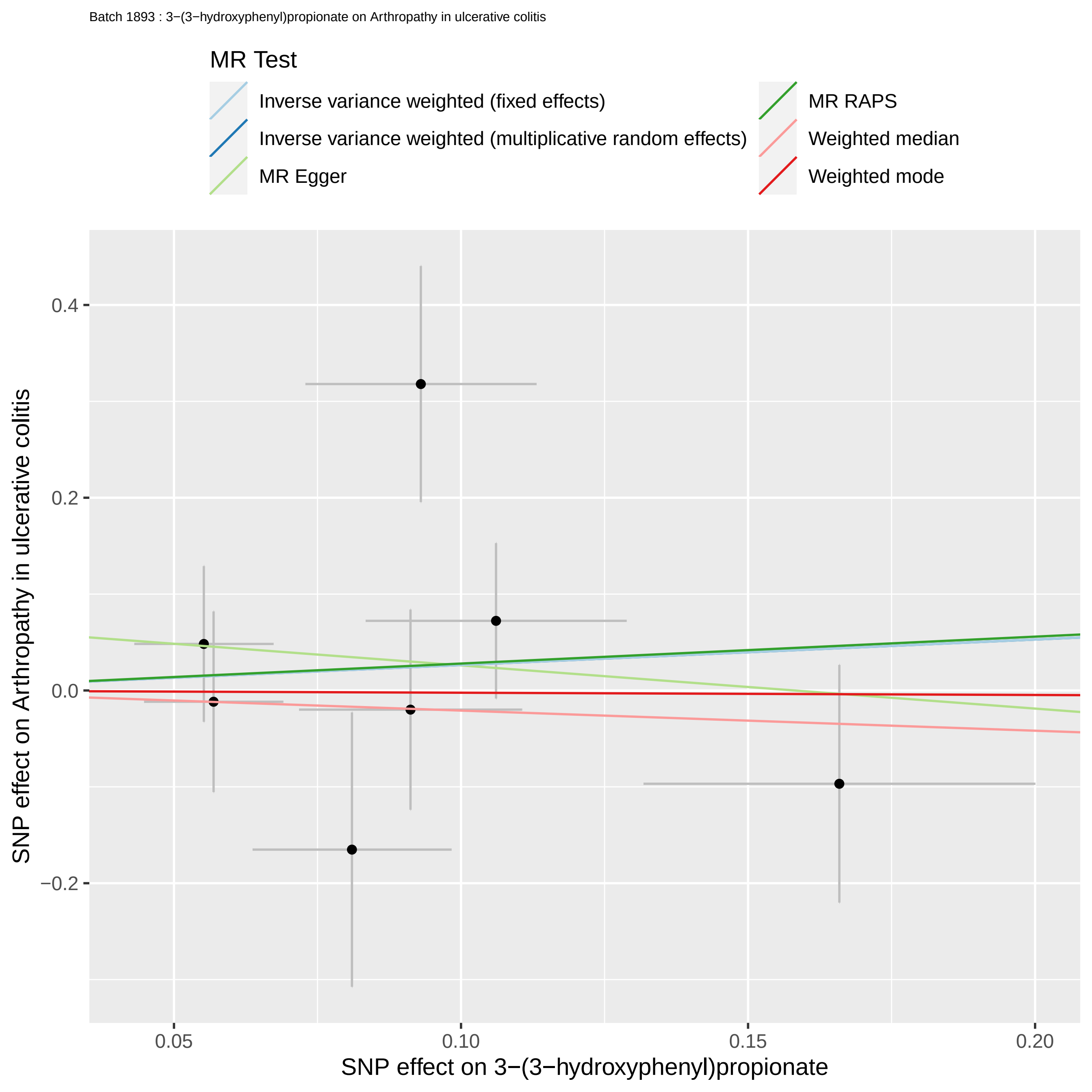

Batch 1893 : 3−(3−hydroxyphenyl)propionate on Arthropathy in ulcerative colitis
MR Test
Inverse variance weighted (fixed effects)
Inverse variance weighted (multiplicative random effects)
MR Egger
MR RAPS
Weighted median
Weighted mode
0.4
0.2
0.0
−0.2
0.05
0.10
0.15
0.20
SNP effect on Arthropathy in ulcerative colitis
SNP effect on 3−(3−hydroxyphenyl)propionate

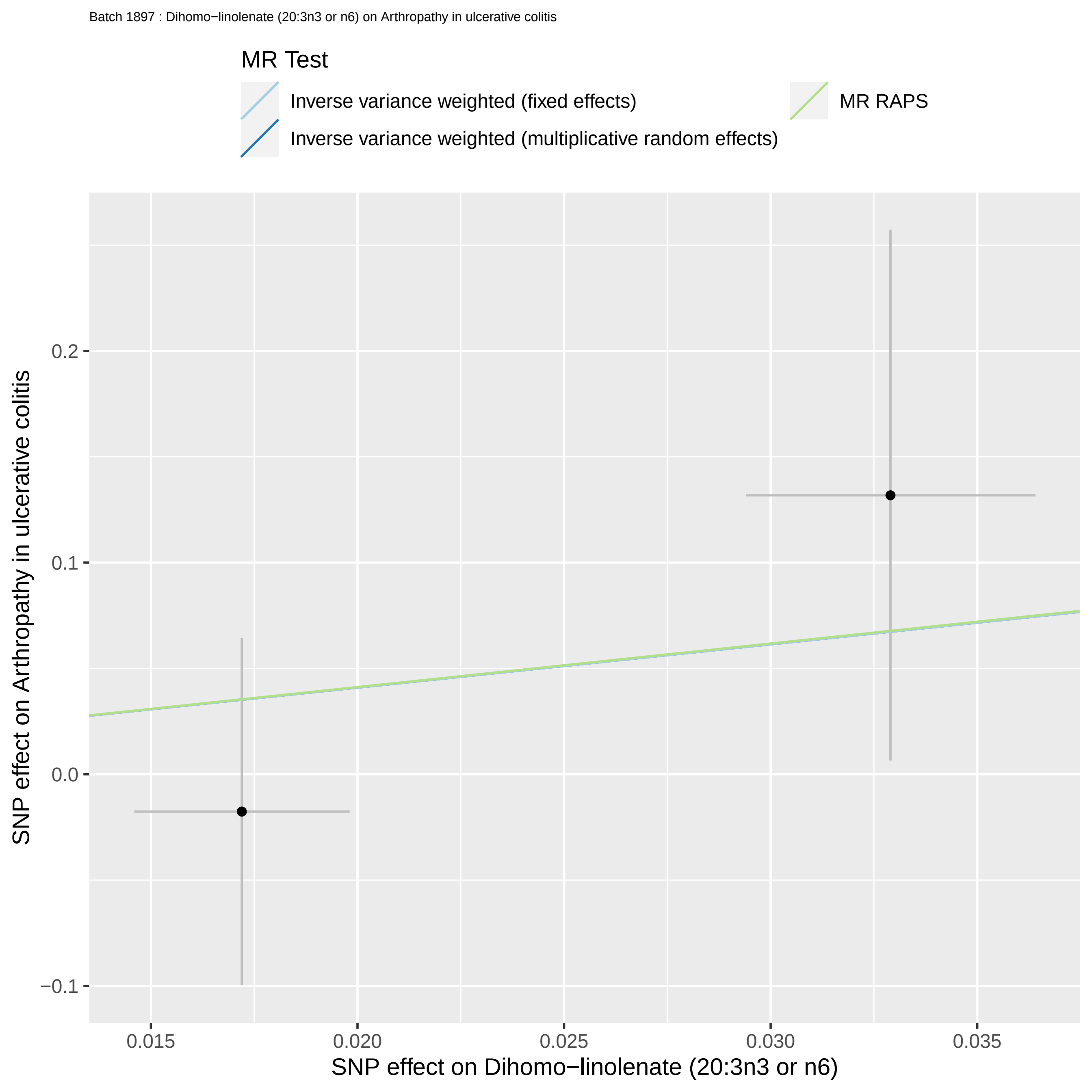

Batch 1897 : Dihomo−linolenate (20:3n3 or n6) on Arthropathy in ulcerative colitis
MR Test
Inverse variance weighted (fixed effects)
Inverse variance weighted (multiplicative random effects)
MR RAPS
SNP effect on Arthropathy in ulcerative colitis
0.2
0.1
0.0
−0.1
0.015
0.020
0.025
0.030
0.035
SNP effect on Dihomo−linolenate (20:3n3 or n6)

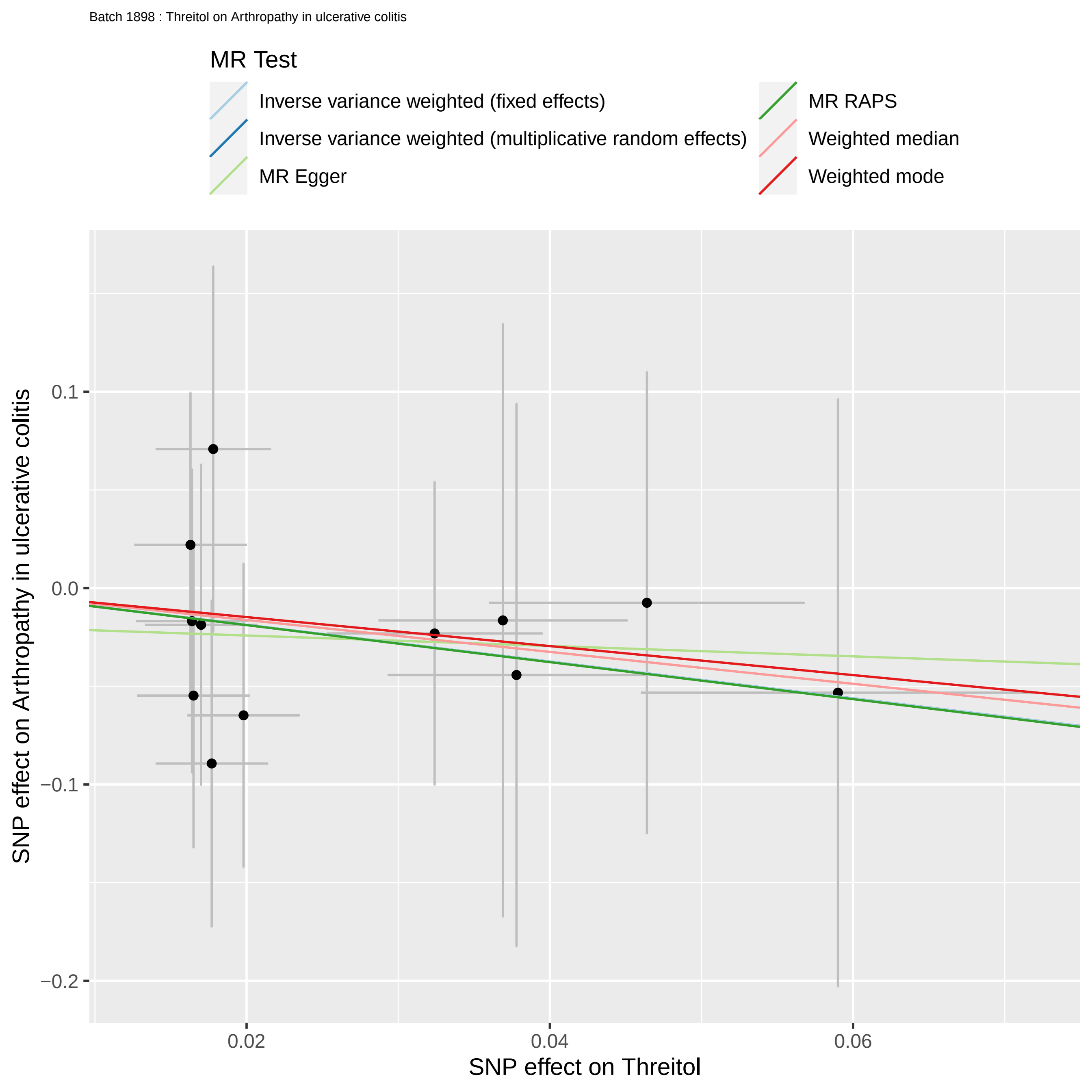

Batch 1898 : Threitol on Arthropathy in ulcerative colitis
MR Test
Inverse variance weighted (fixed effects)
Inverse variance weighted (multiplicative random effects)
MR Egger
MR RAPS
Weighted median
Weighted mode
0.1
0.0
−0.1
−0.2
0.02
0.04
0.06
SNP effect on Arthropathy in ulcerative colitis
SNP effect on Threitol

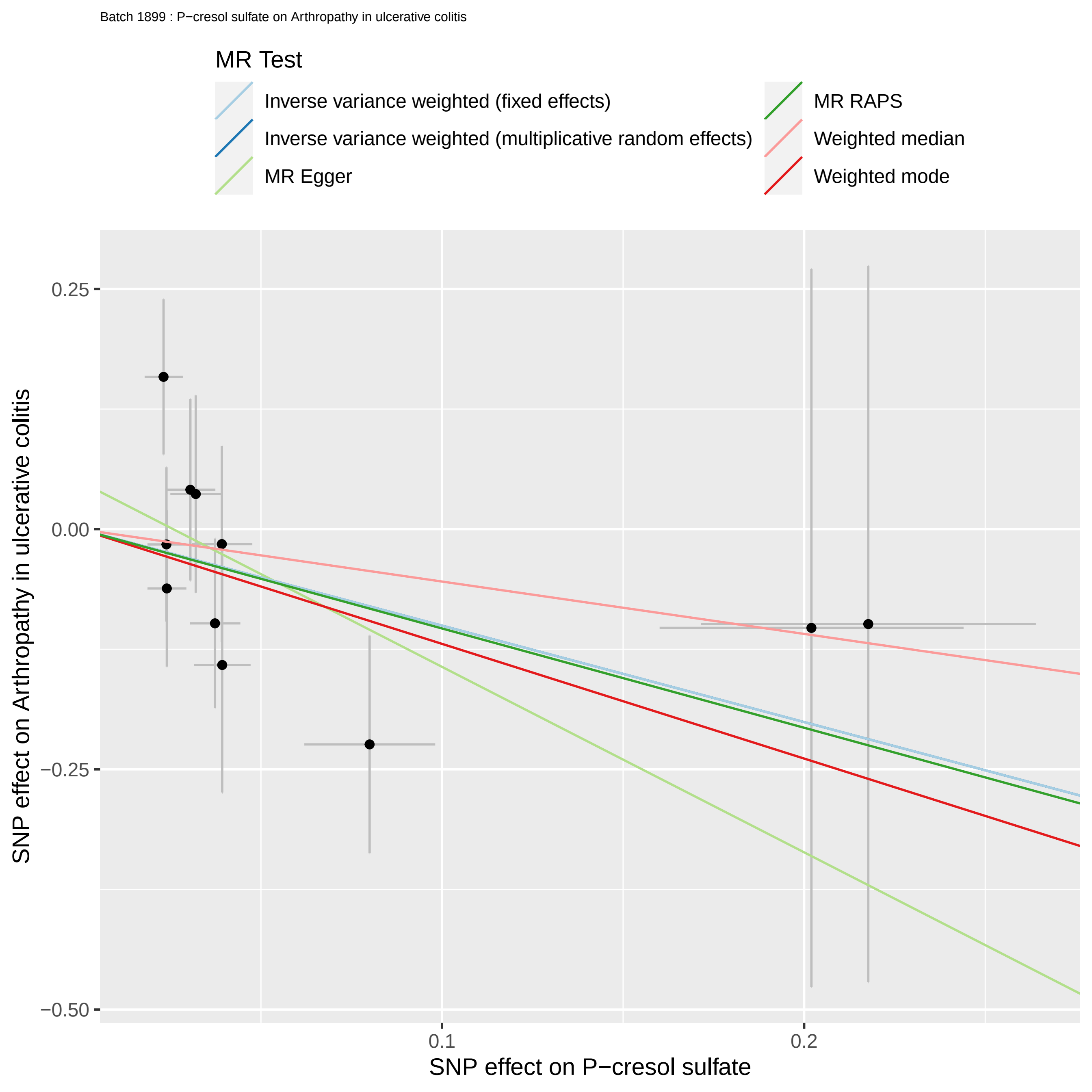
Batch 1899 : P−cresol sulfate on Arthropathy in ulcerative colitis
MR Test
Inverse variance weighted (fixed effects)
Inverse variance weighted (multiplicative random effects)
MR Egger
MR RAPS
Weighted median
Weighted mode
0.25
0.00
−0.25
−0.50
0.1
0.2
SNP effect on Arthropathy in ulcerative colitis
SNP effect on P−cresol sulfate

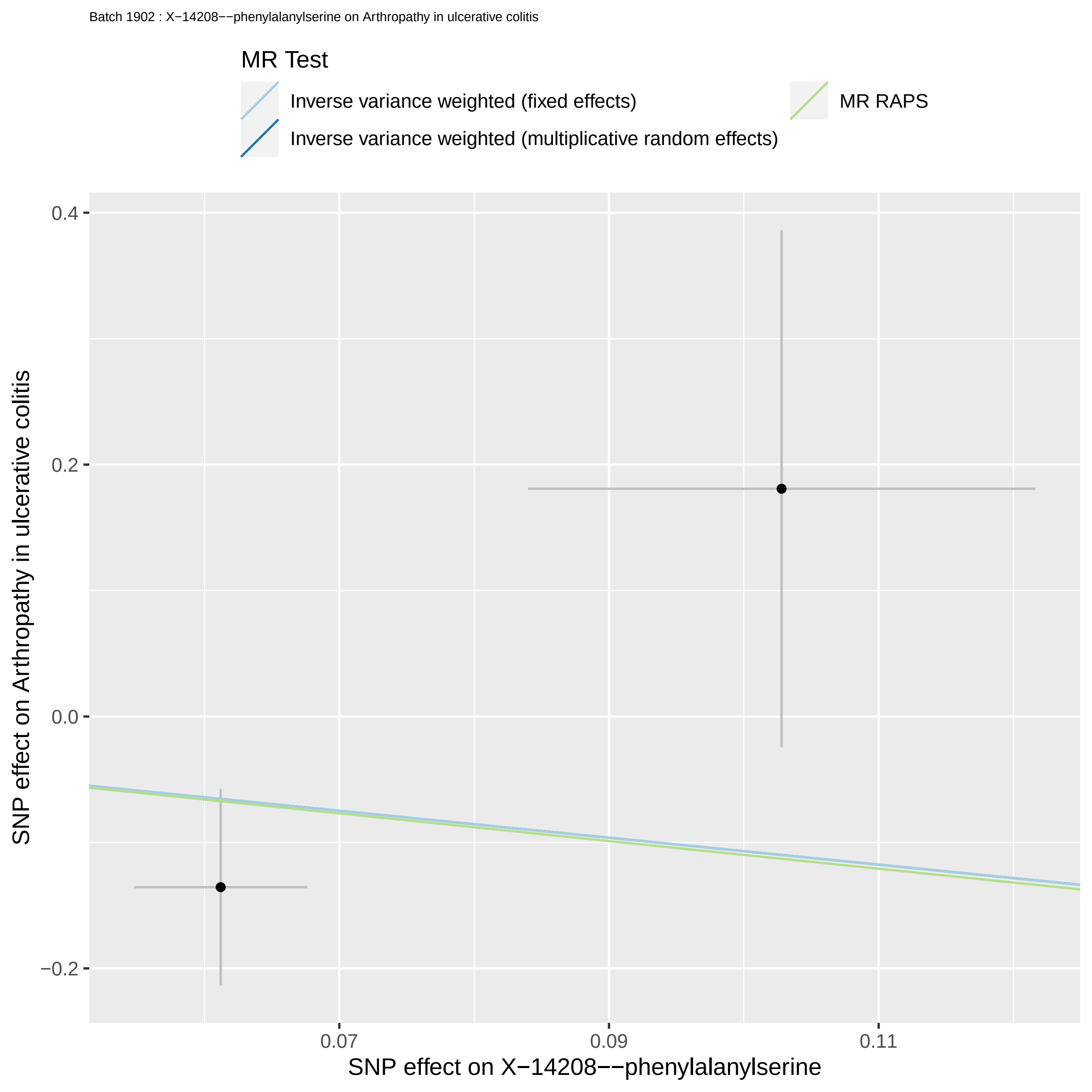

Batch 1902 : X−14208−−phenylalanylserine on Arthropathy in ulcerative colitis
MR Test
Inverse variance weighted (fixed effects)
Inverse variance weighted (multiplicative random effects)
MR RAPS
0.4
0.2
0.0
−0.2
0.07
0.09
0.11
SNP effect on Arthropathy in ulcerative colitis
SNP effect on X−14208−−phenylalanylserine

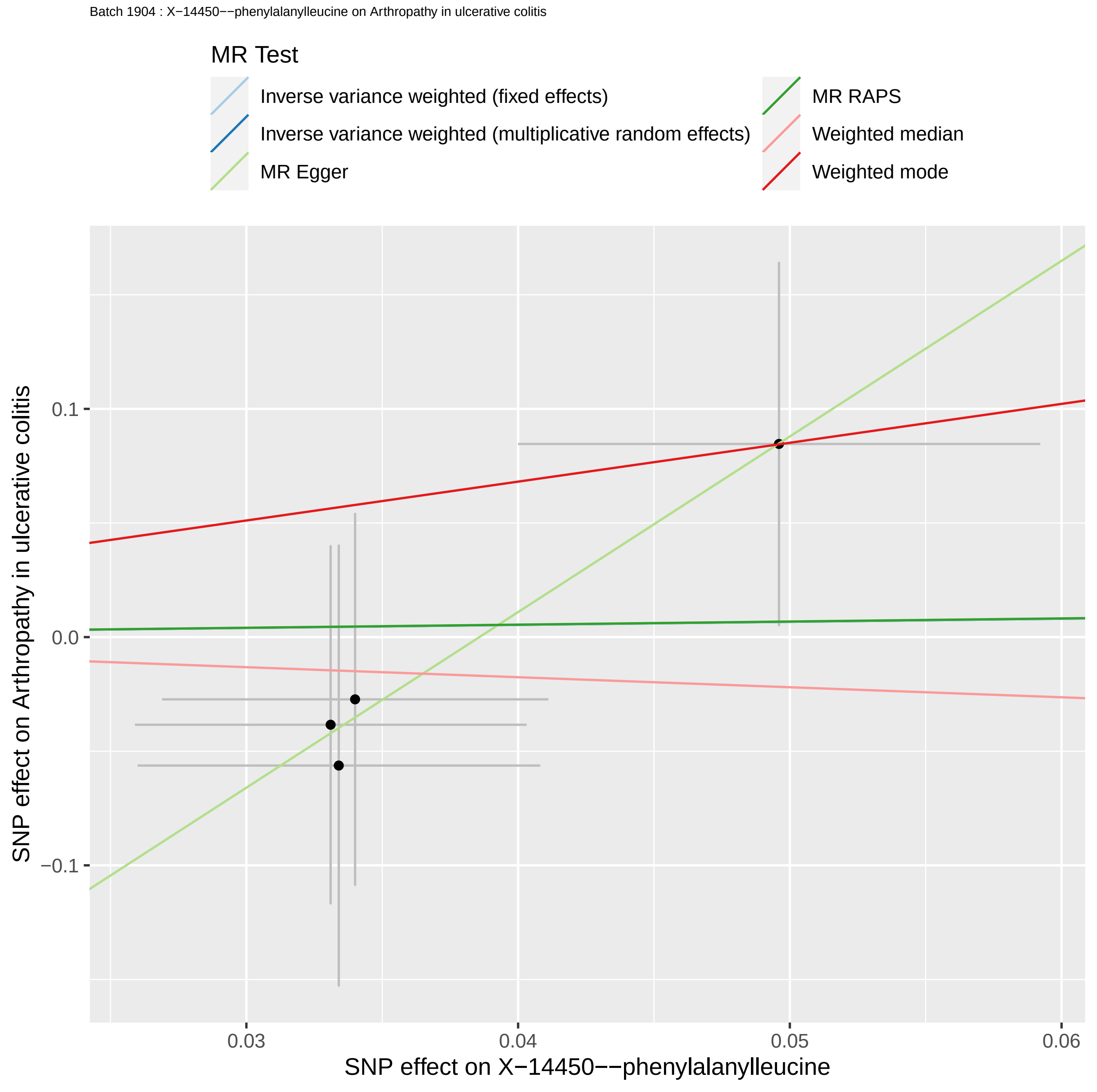

Batch 1904 : X−14450−−phenylalanylleucine on Arthropathy in ulcerative colitis
MR Test
Inverse variance weighted (fixed effects)
Inverse variance weighted (multiplicative random effects)
MR Egger
MR RAPS
Weighted median
Weighted mode
0.1
0.0
−0.1
0.03
0.04
0.05
0.06
SNP effect on Arthropathy in ulcerative colitis
SNP effect on X−14450−−phenylalanylleucine

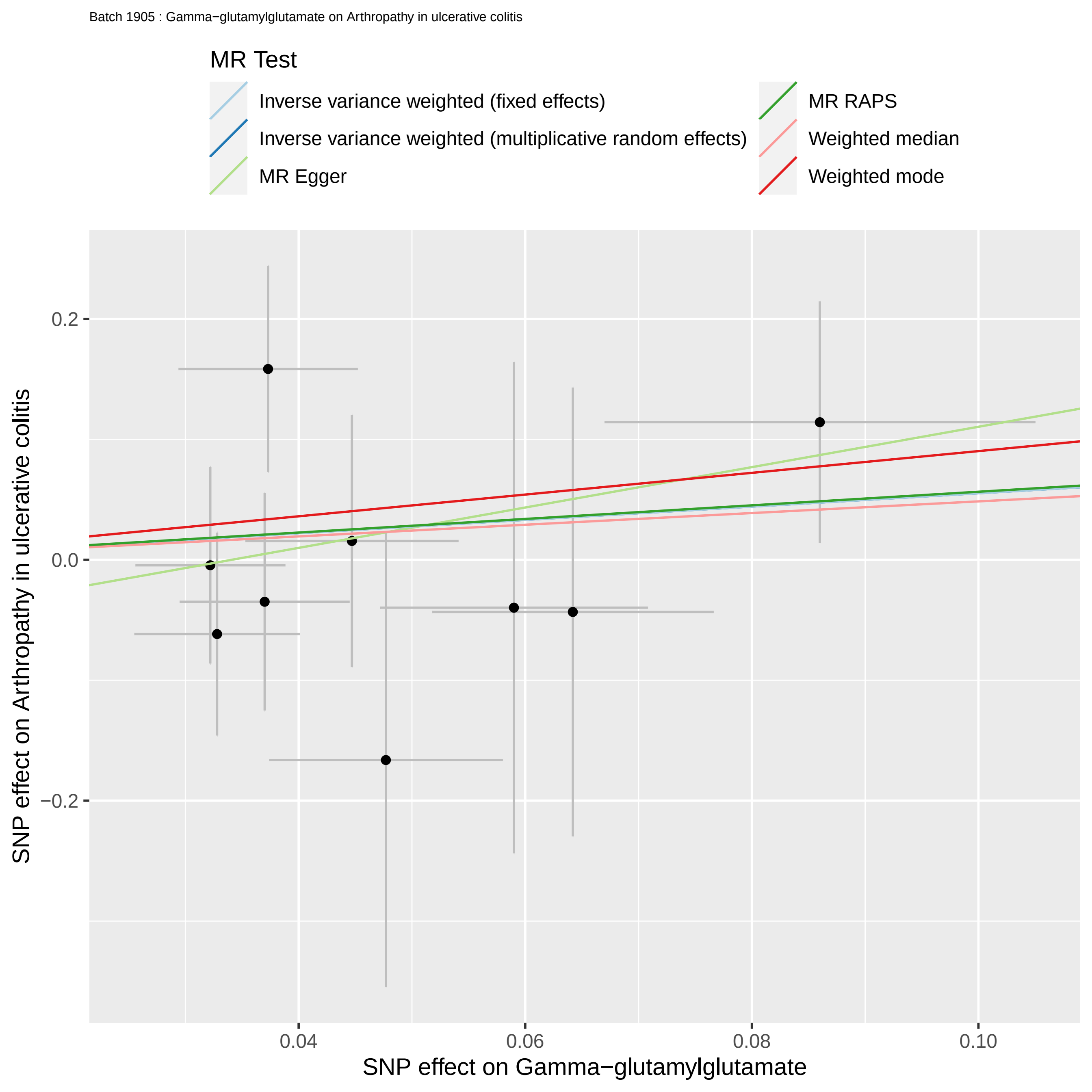

Batch 1905 : Gamma−glutamylglutamate on Arthropathy in ulcerative colitis
MR Test
Inverse variance weighted (fixed effects)
Inverse variance weighted (multiplicative random effects)
MR Egger
MR RAPS
Weighted median
Weighted mode
0.2
0.0
−0.2
0.04
0.06
0.08
0.10
SNP effect on Arthropathy in ulcerative colitis
SNP effect on Gamma−glutamylglutamate

Batch 1906 : Octadecanedioate on Arthropathy in ulcerative colitis

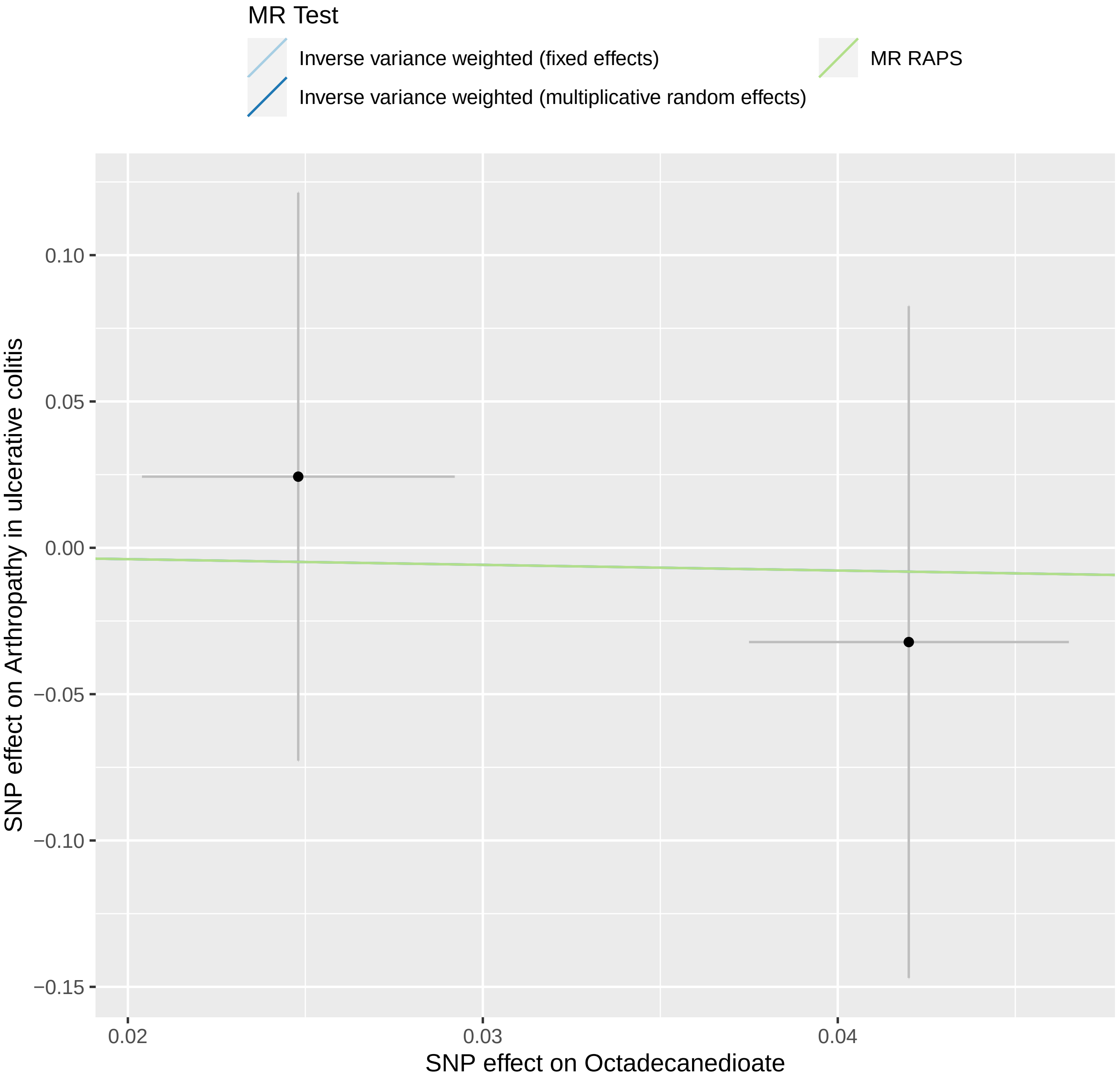

Batch 1907 : Leucylleucine on Arthropathy in ulcerative colitis

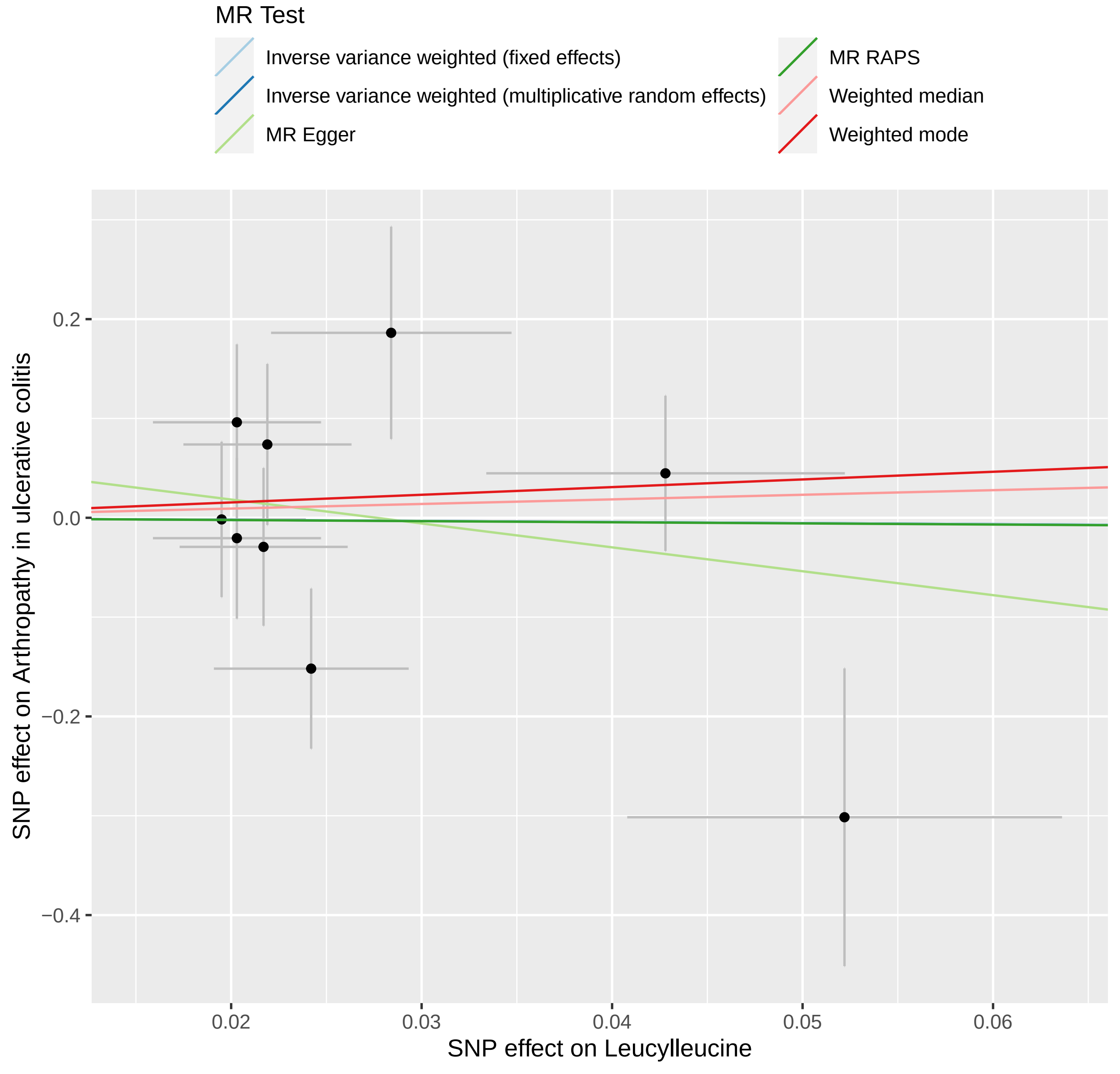

Batch 1908 : 7−alpha−hydroxy−3−oxo−4−cholestenoate (7−Hoca) on Arthropathy in ulcerative colitis

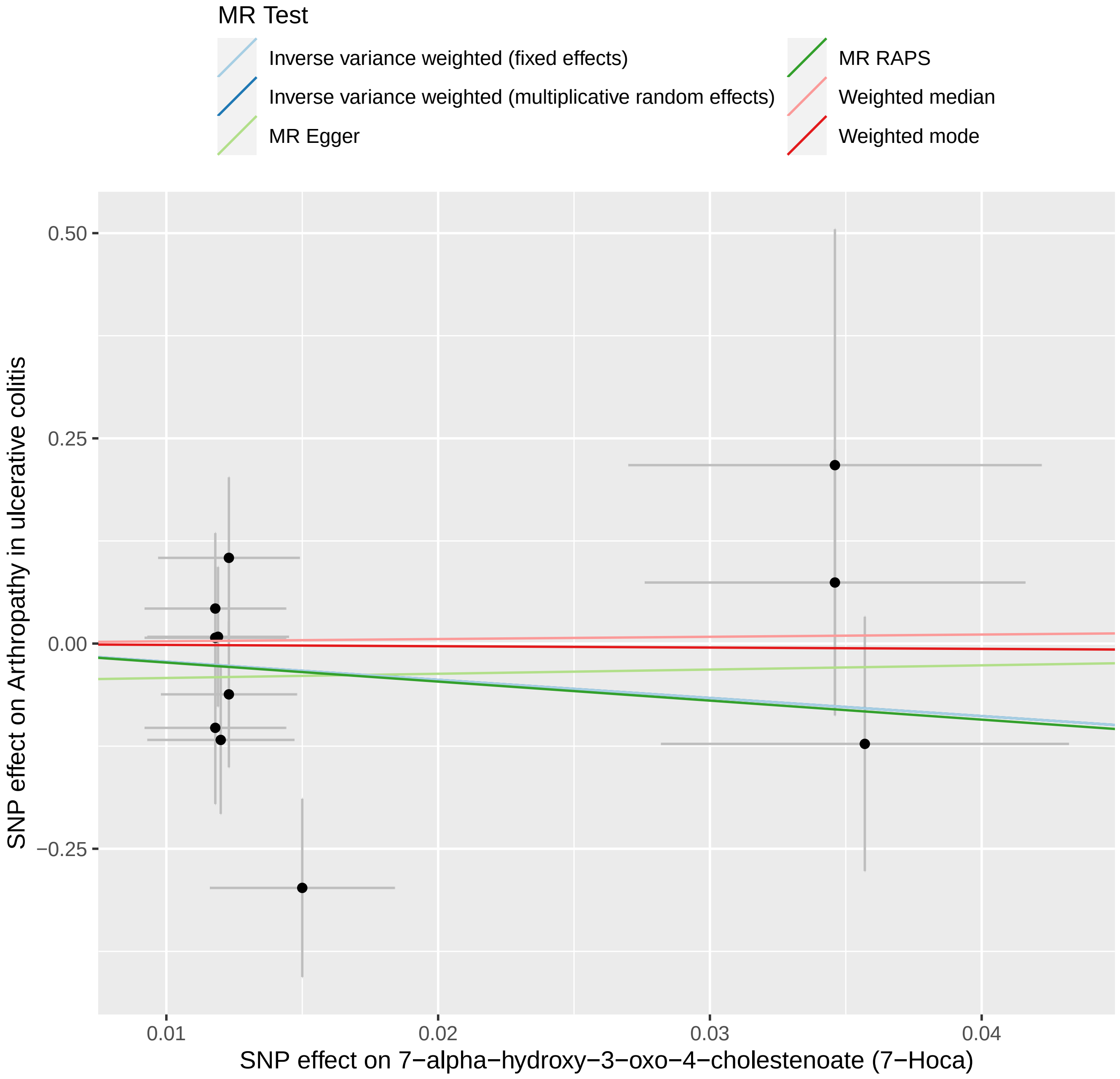

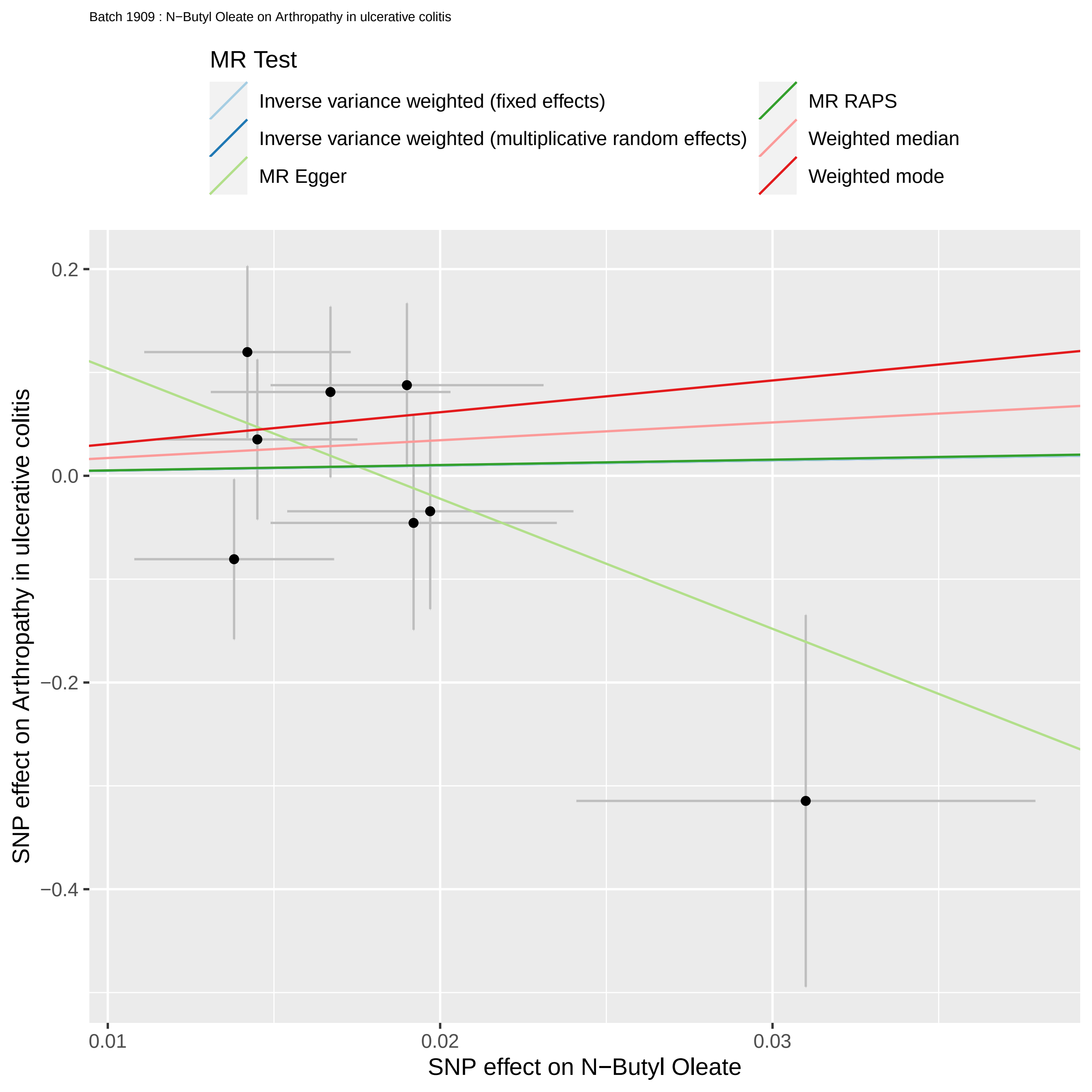
Batch 1909 : N−Butyl Oleate on Arthropathy in ulcerative colitis
MR Test
Inverse variance weighted (fixed effects)
Inverse variance weighted (multiplicative random effects)
MR Egger
MR RAPS
Weighted median
Weighted mode
0.2
0.0
−0.2
−0.4
0.01
0.02
0.03
SNP effect on Arthropathy in ulcerative colitis
SNP effect on N−Butyl Oleate

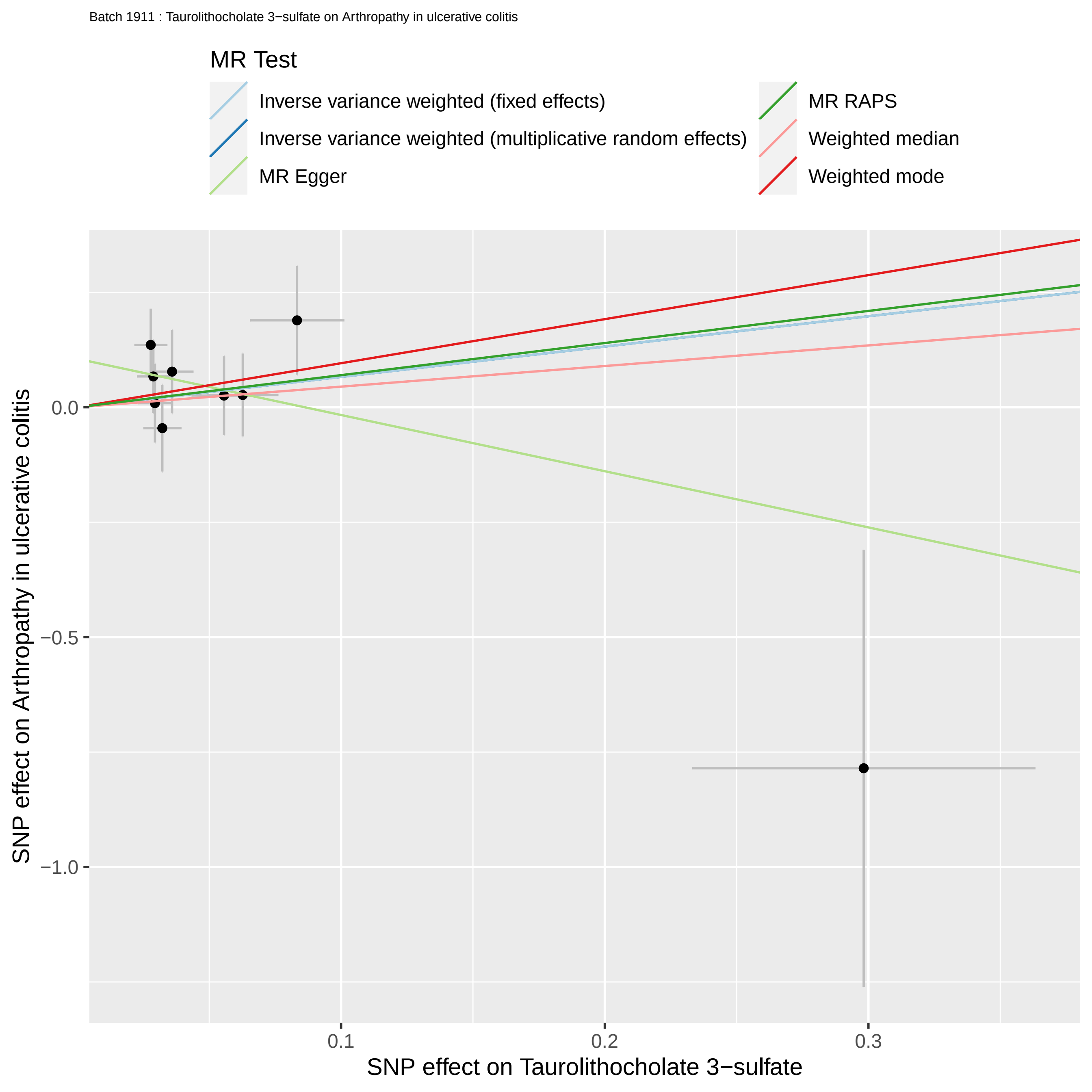
Batch 1911 : Taurolithocholate 3−sulfate on Arthropathy in ulcerative colitis
MR Test
Inverse variance weighted (fixed effects)
Inverse variance weighted (multiplicative random effects)
MR Egger
MR RAPS
Weighted median
Weighted mode
0.0
−0.5
−1.0
0.1
0.2
0.3
SNP effect on Arthropathy in ulcerative colitis
SNP effect on Taurolithocholate 3−sulfate

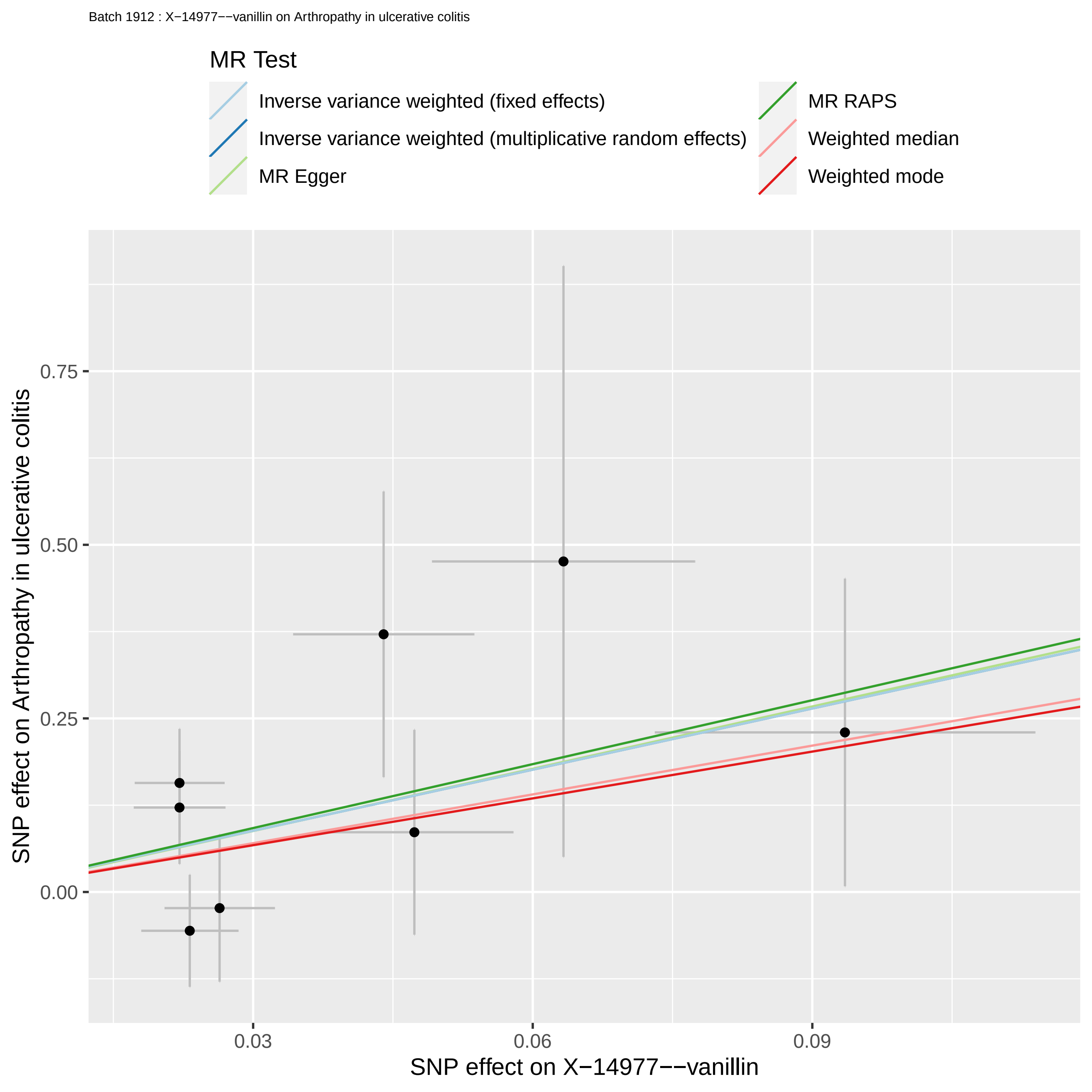

Batch 1912 : X−14977−−vanillin on Arthropathy in ulcerative colitis
MR Test
Inverse variance weighted (fixed effects)
Inverse variance weighted (multiplicative random effects)
MR Egger
MR RAPS
Weighted median
Weighted mode
0.75
0.50
0.25
0.00
0.03
0.06
0.09
SNP effect on Arthropathy in ulcerative colitis
SNP effect on X−14977−−vanillin

Batch 1913 : Succinylcarnitine on Arthropathy in ulcerative colitis

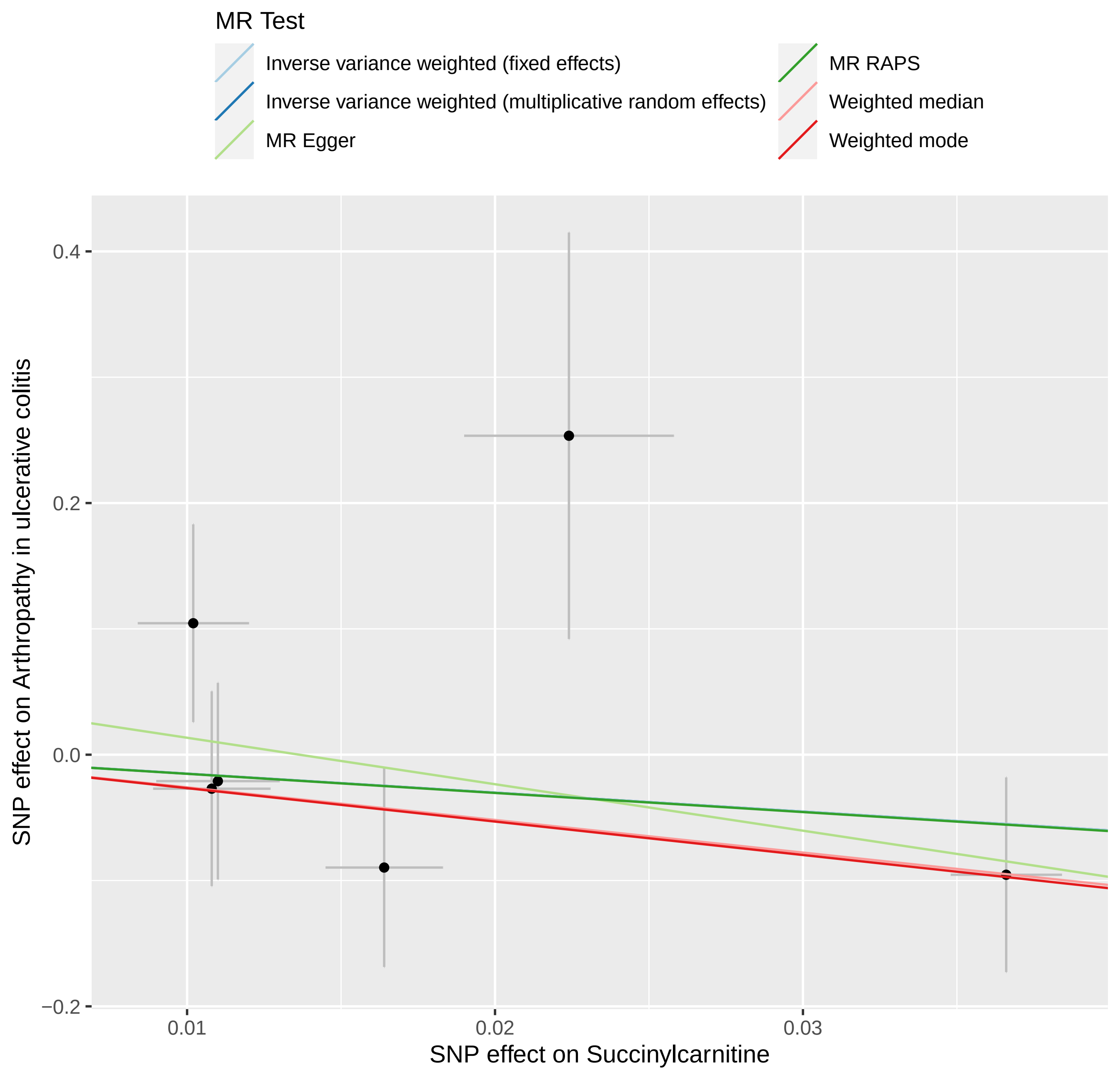

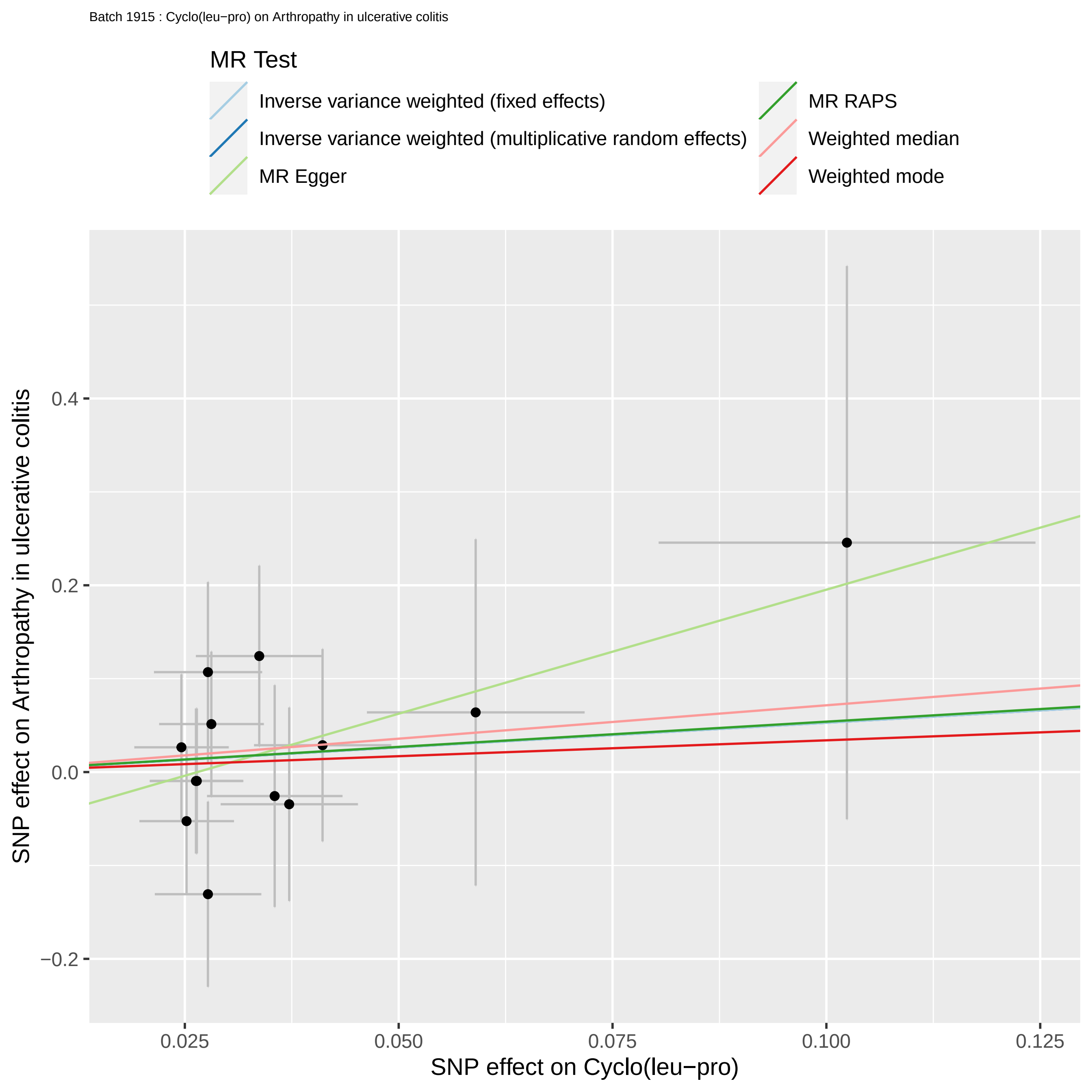

Batch 1915 : Cyclo(leu−pro) on Arthropathy in ulcerative colitis
MR Test
Inverse variance weighted (fixed effects)
Inverse variance weighted (multiplicative random effects)
MR Egger
MR RAPS
Weighted median
Weighted mode
0.4
0.2
0.0
−0.2
0.025
0.050
0.075
0.100
0.125
SNP effect on Arthropathy in ulcerative colitis
SNP effect on Cyclo(leu−pro)

Batch 1916 : Chiro−inositol on Arthropathy in ulcerative colitis

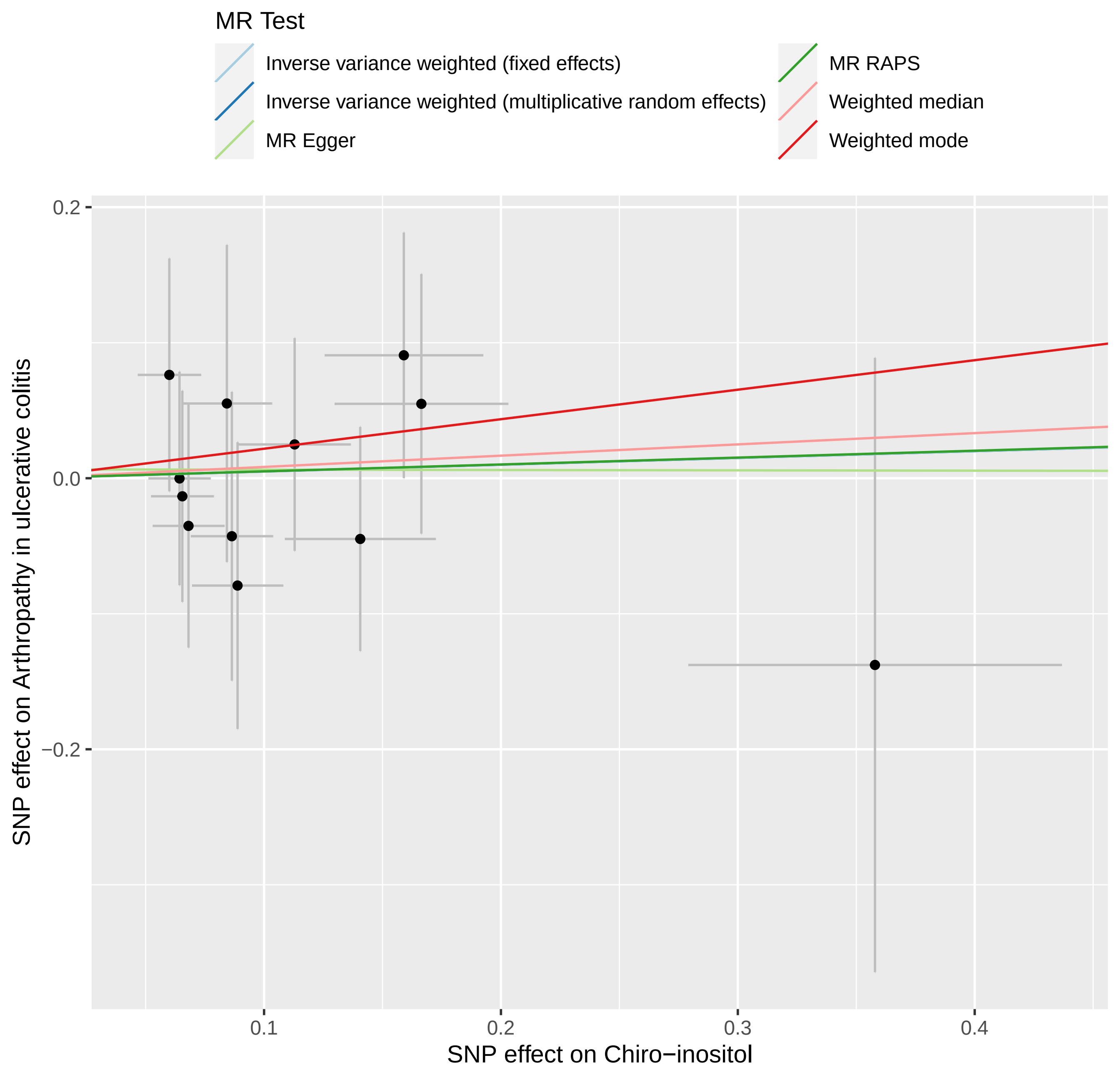

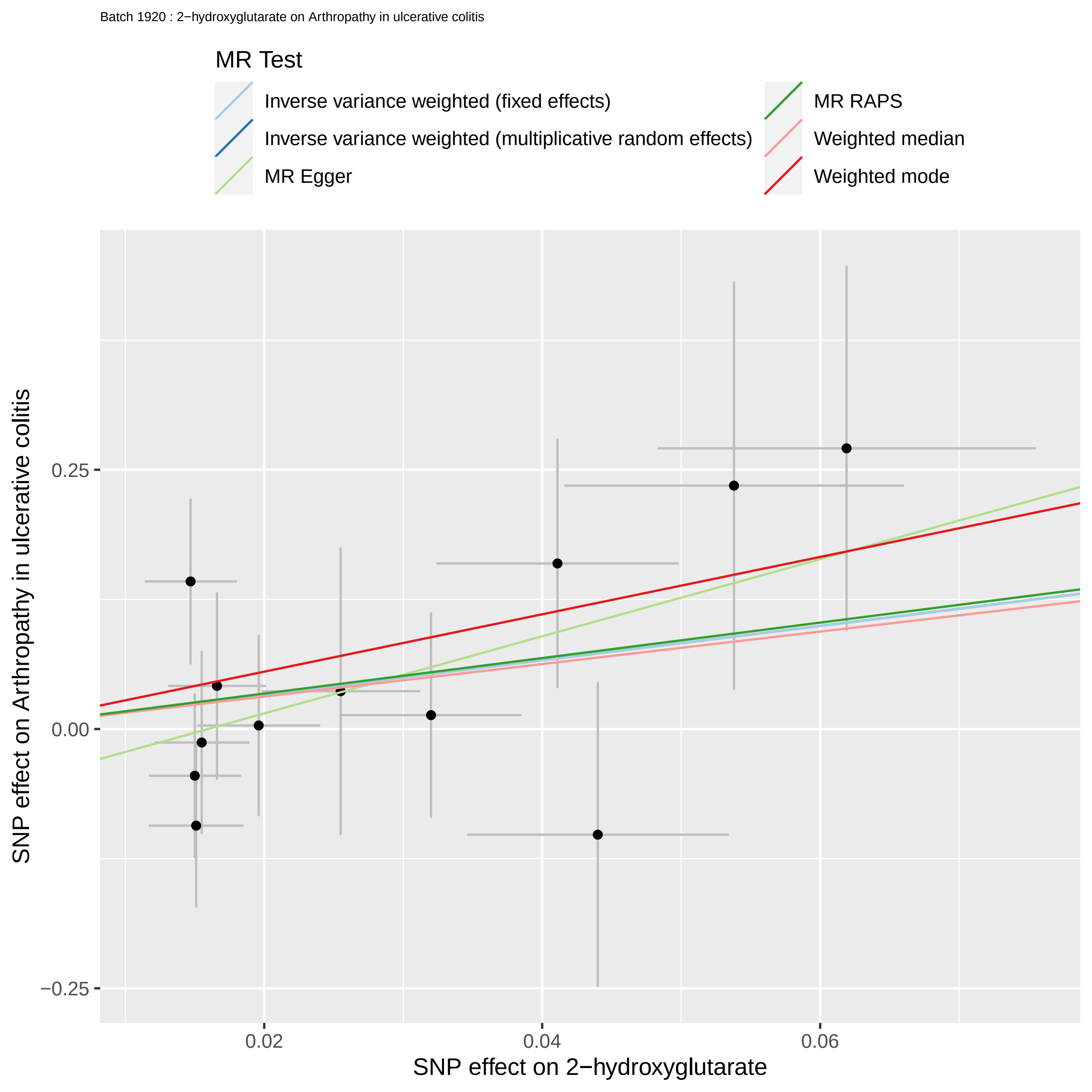
Batch 1920 : 2−hydroxyglutarate on Arthropathy in ulcerative colitis
MR Test
Inverse variance weighted (fixed effects)
Inverse variance weighted (multiplicative random effects)
MR Egger
MR RAPS
Weighted median
Weighted mode
SNP effect on Arthropathy in ulcerative colitis
0.25
0.00
−0.25
0.02
0.04
0.06
SNP effect on 2−hydroxyglutarate

Batch 1921 : Ergothioneine on Arthropathy in ulcerative colitis

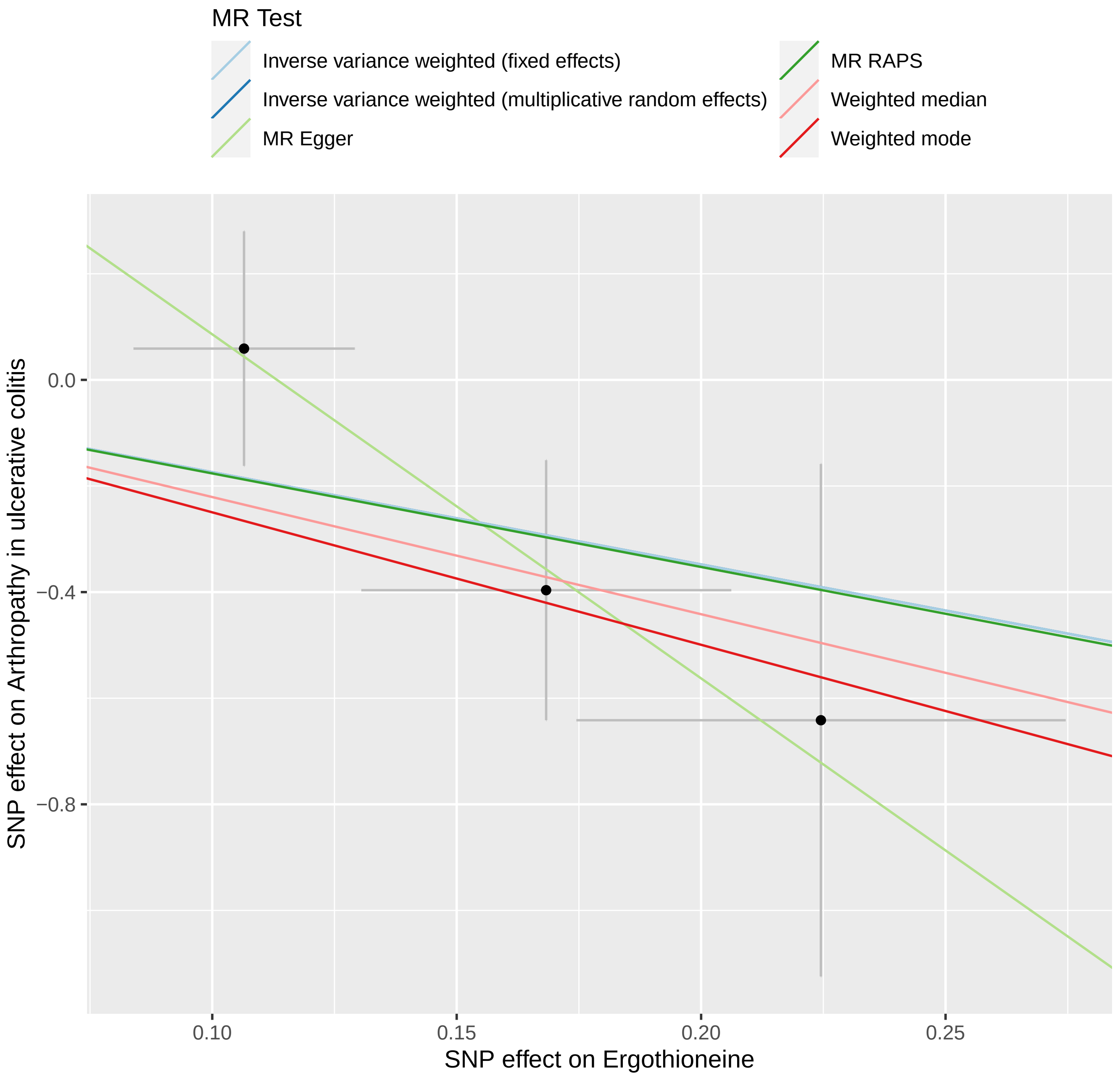

Batch 1923 : Phenylalanylphenylalanine on Arthropathy in ulcerative colitis

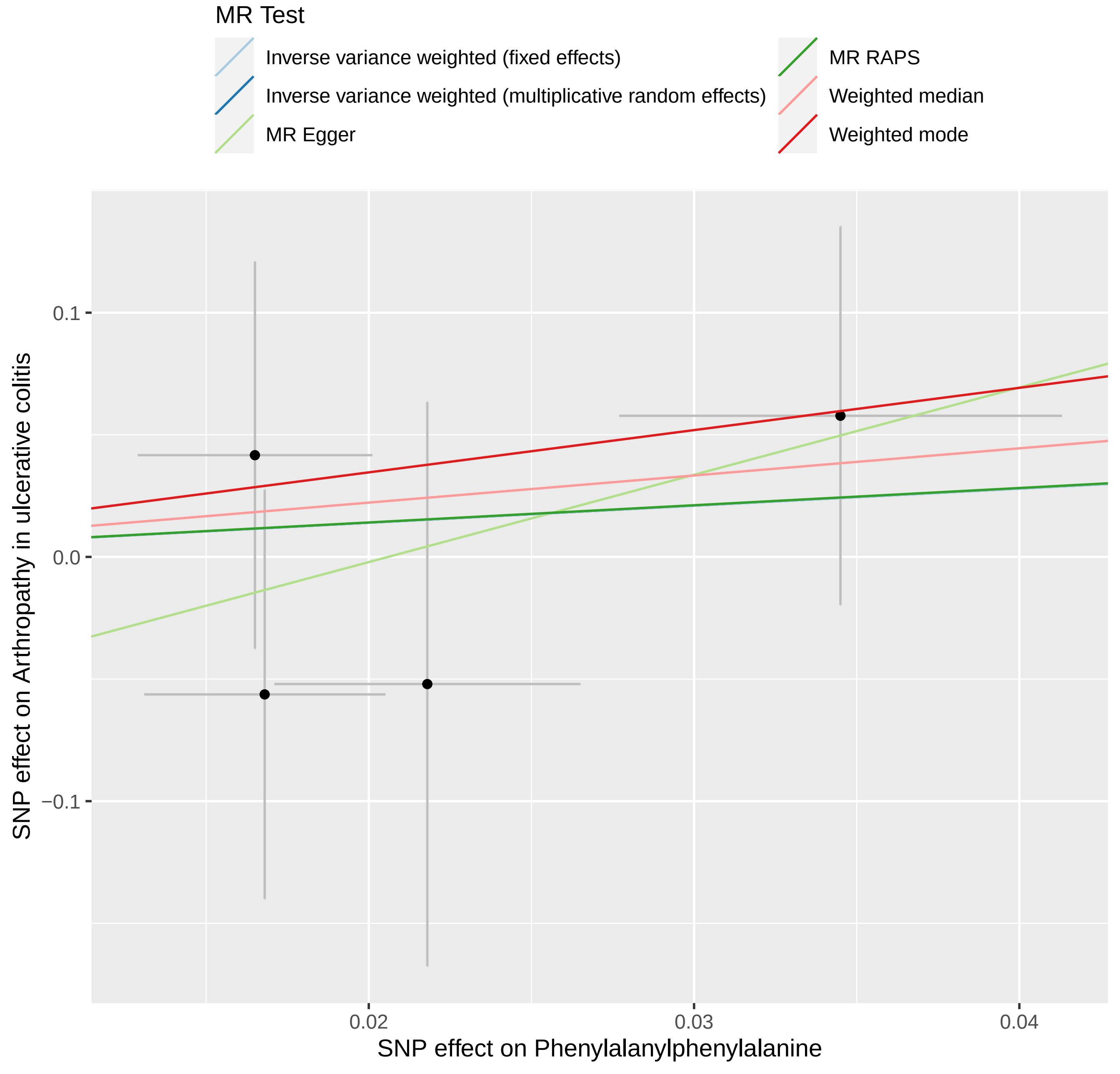

Batch 1924 : Cis−4−decenoyl carnitine on Arthropathy in ulcerative colitis

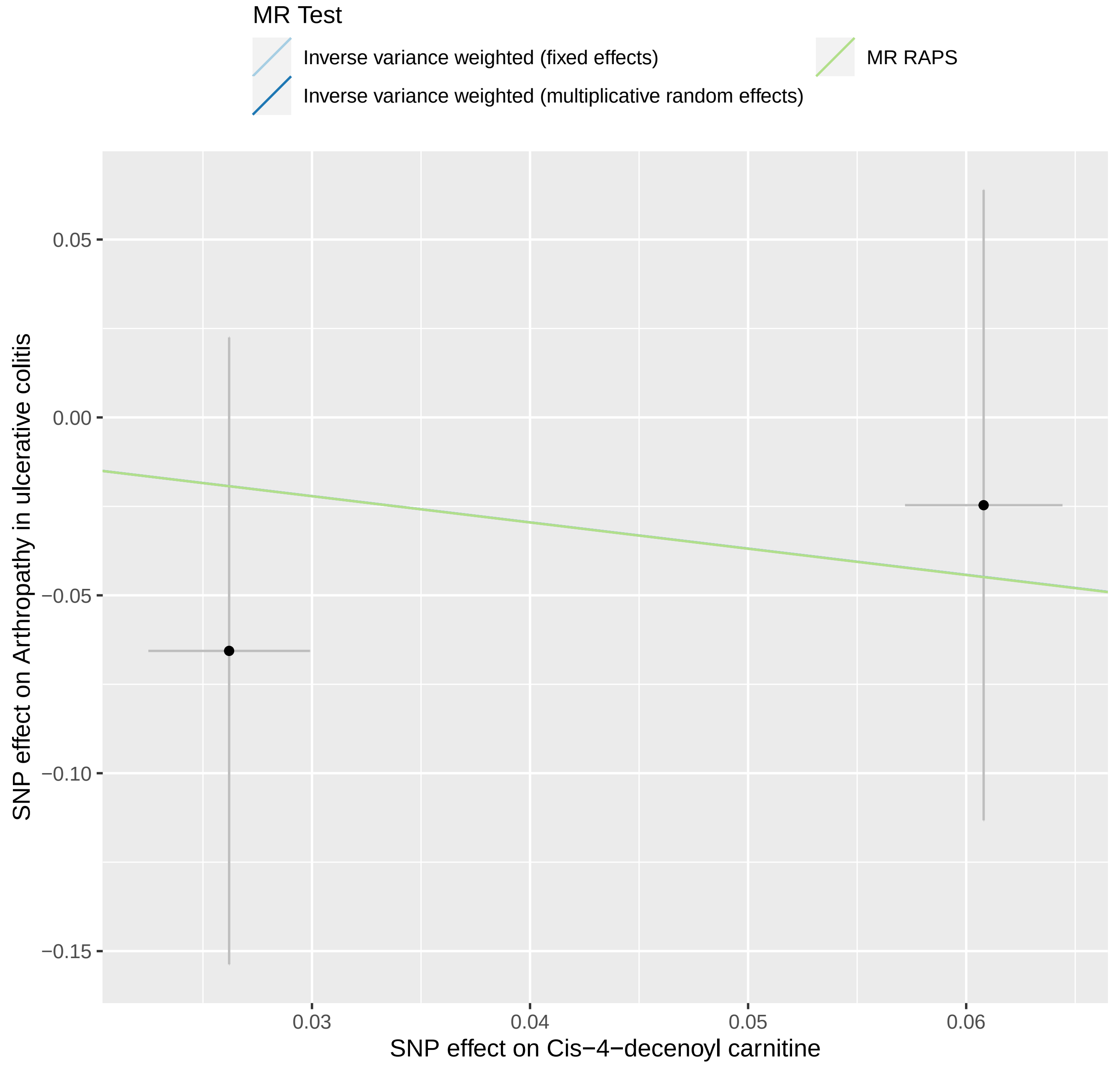

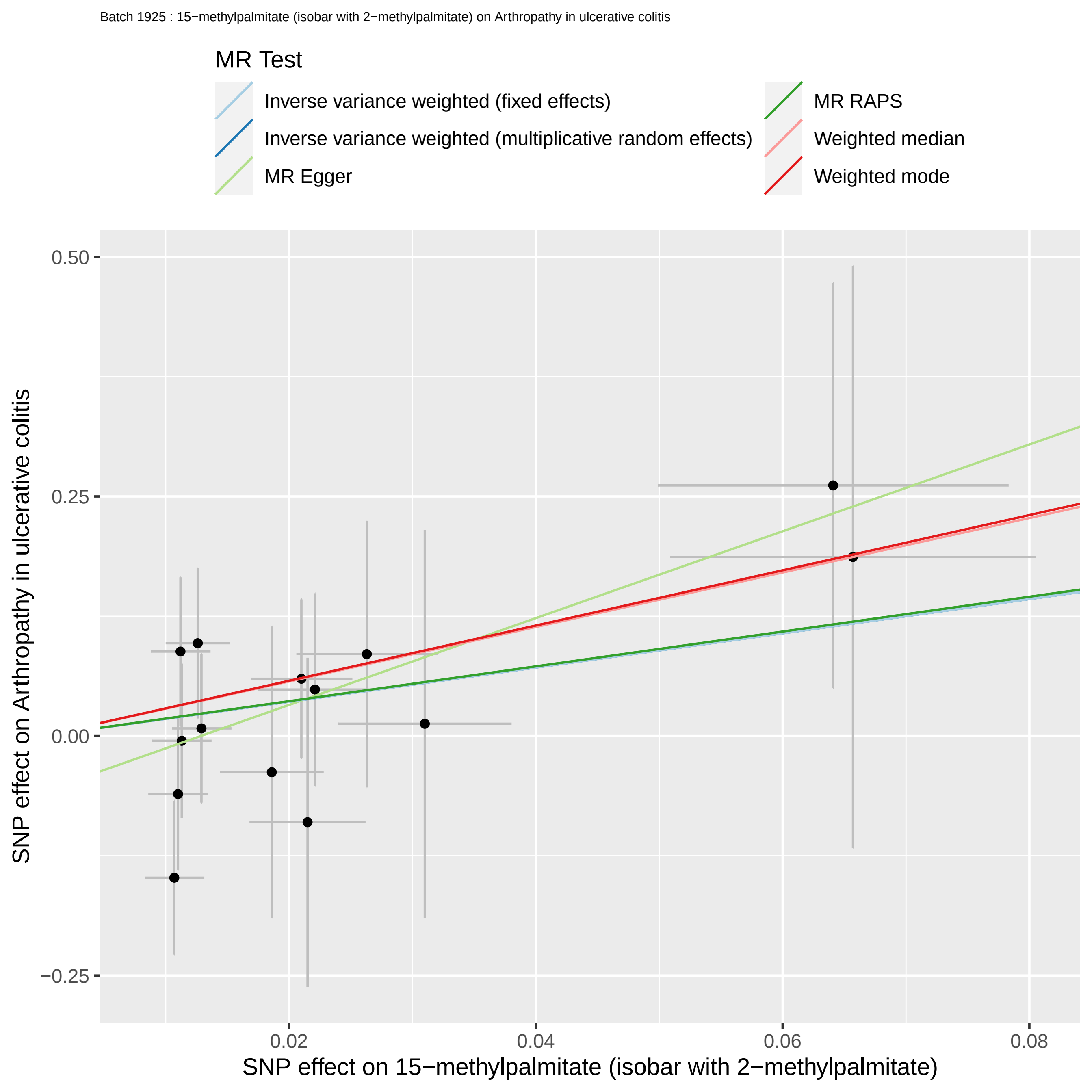

Batch 1925 : 15−methylpalmitate (isobar with 2−methylpalmitate) on Arthropathy in ulcerative colitis
MR Test
Inverse variance weighted (fixed effects)
Inverse variance weighted (multiplicative random effects)
MR Egger
MR RAPS
Weighted median
Weighted mode
0.50
0.25
0.00
−0.25
0.02
0.04
0.06
0.08
SNP effect on Arthropathy in ulcerative colitis
SNP effect on 15−methylpalmitate (isobar with 2−methylpalmitate)

Batch 1927 : Tryptophan on Ulcerative pancolitis

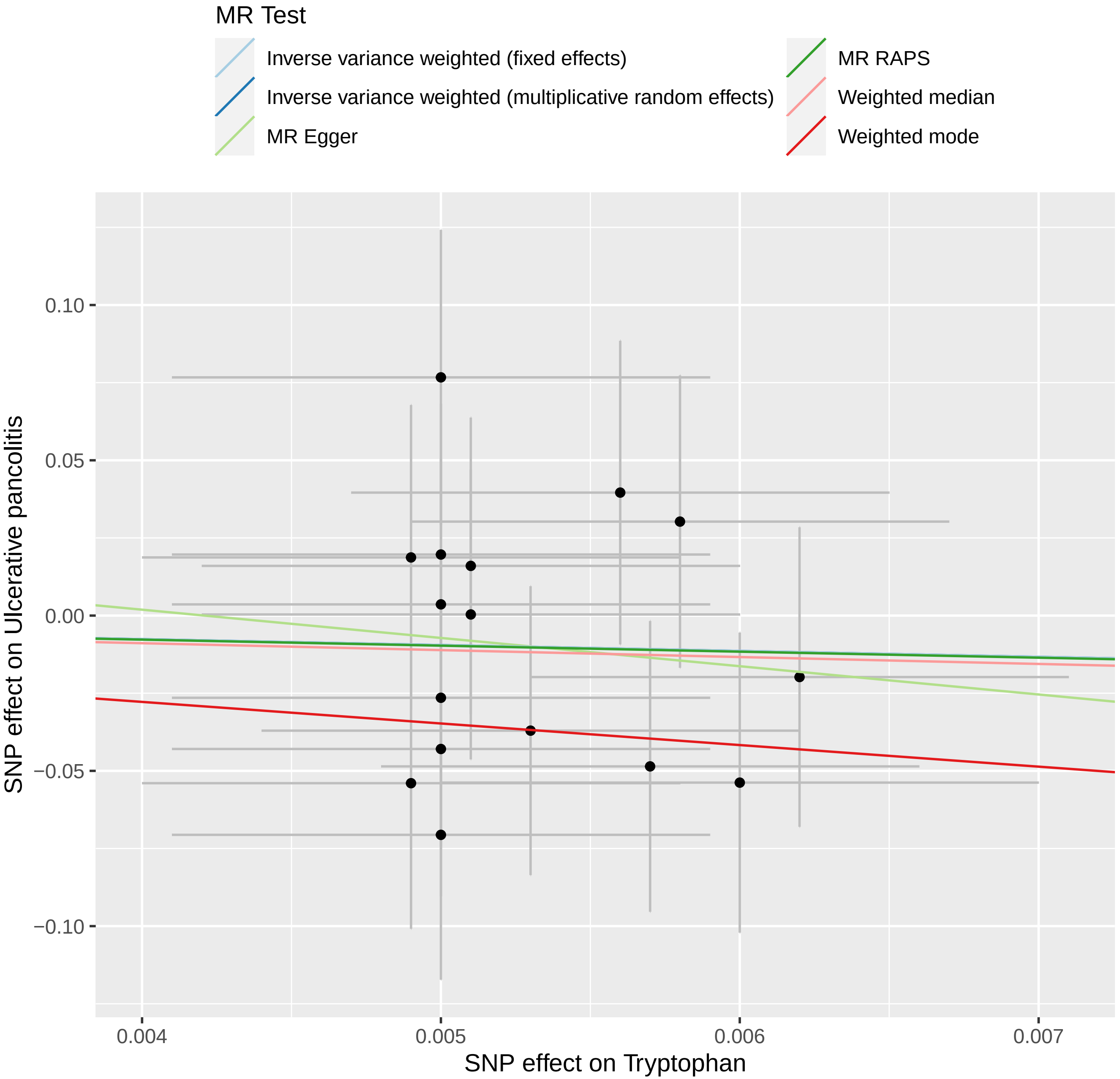

Batch 1929 : Leucine on Ulcerative pancolitis

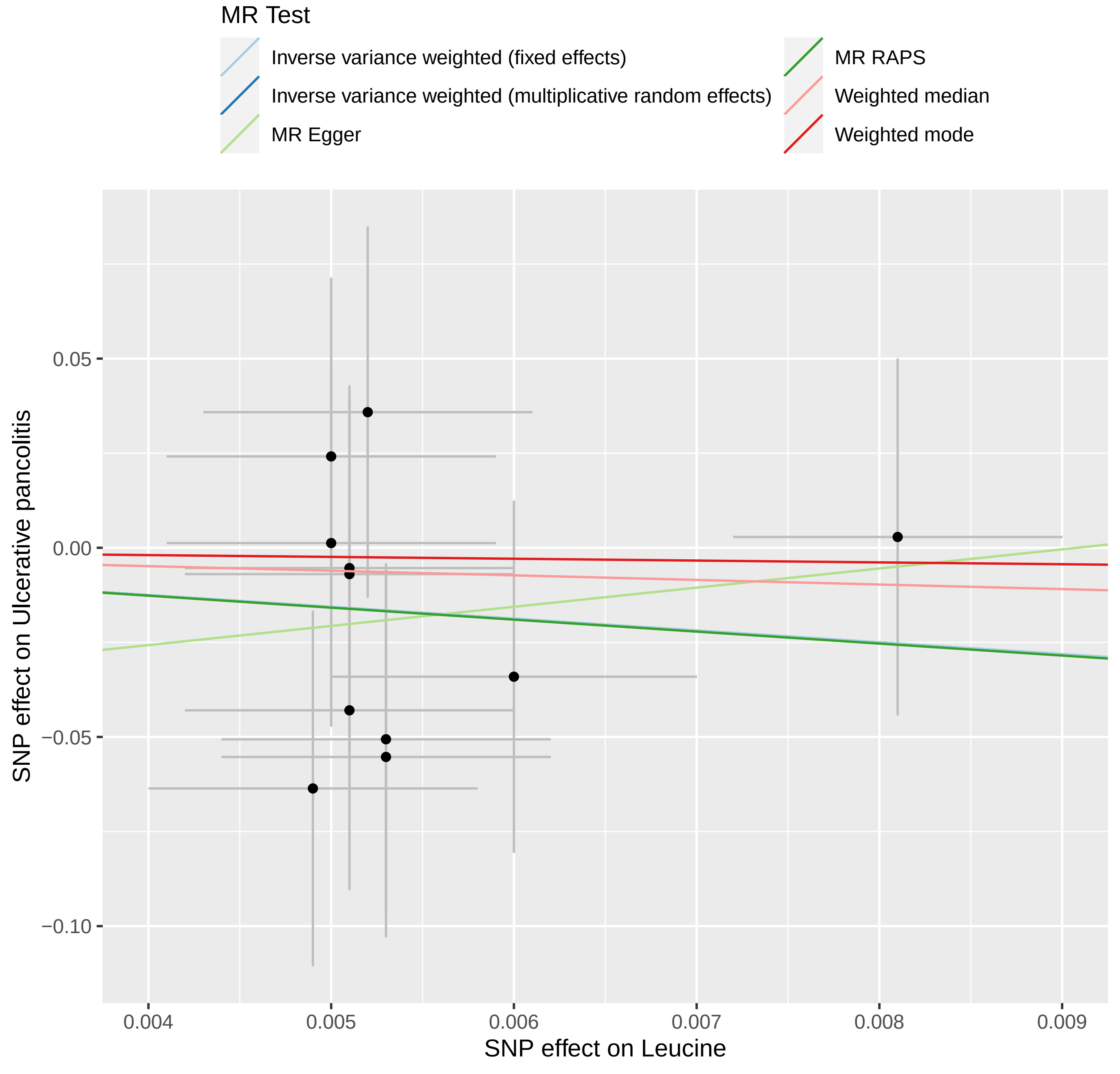

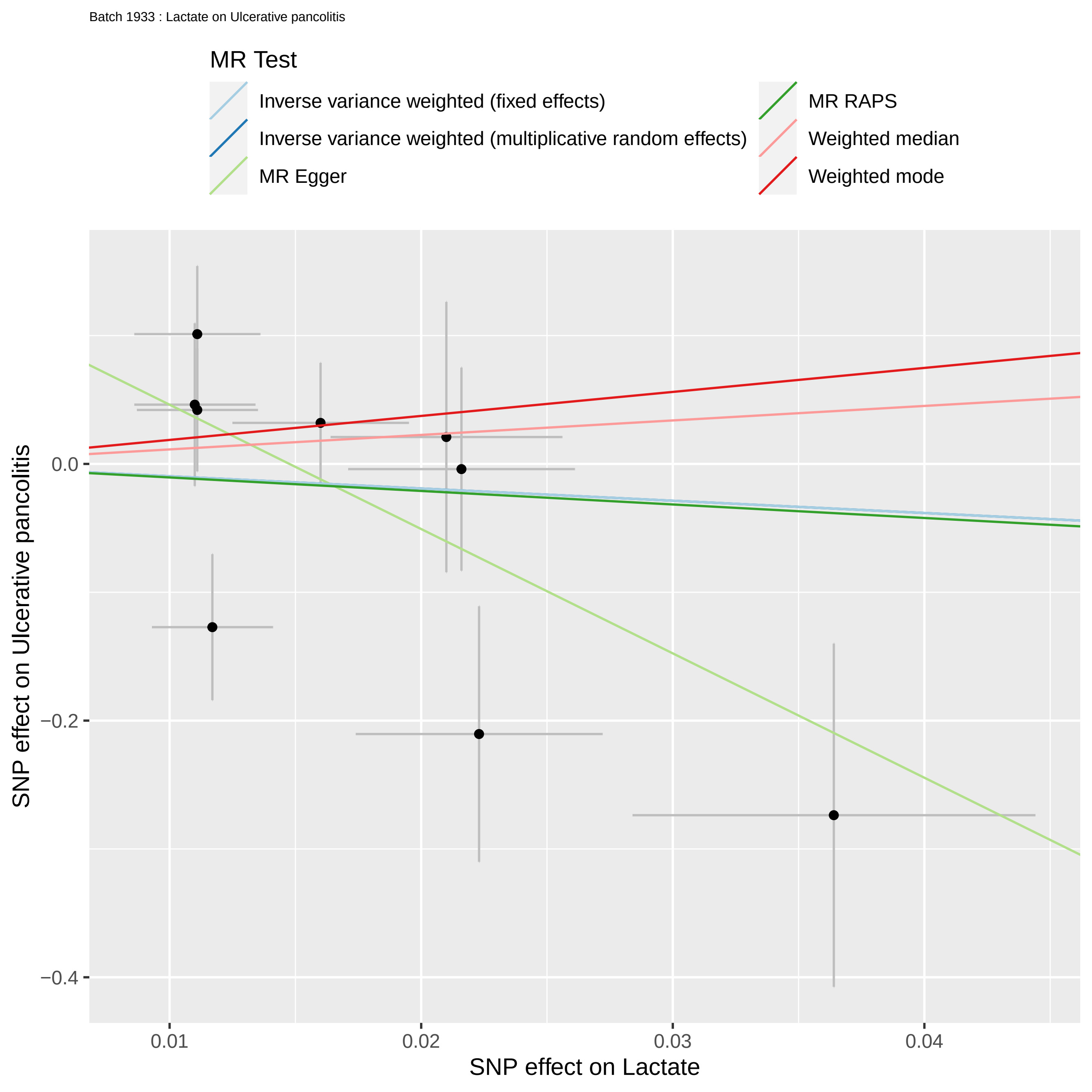
Batch 1933 : Lactate on Ulcerative pancolitis
MR Test
Inverse variance weighted (fixed effects)
Inverse variance weighted (multiplicative random effects)
MR Egger
MR RAPS
Weighted median
Weighted mode
0.0
−0.2
−0.4
0.01
0.02
0.03
0.04
SNP effect on Lactate
SNP effect on Ulcerative pancolitis

Batch 1934 : 3−hydroxybutyrate (BHBA) on Ulcerative pancolitis

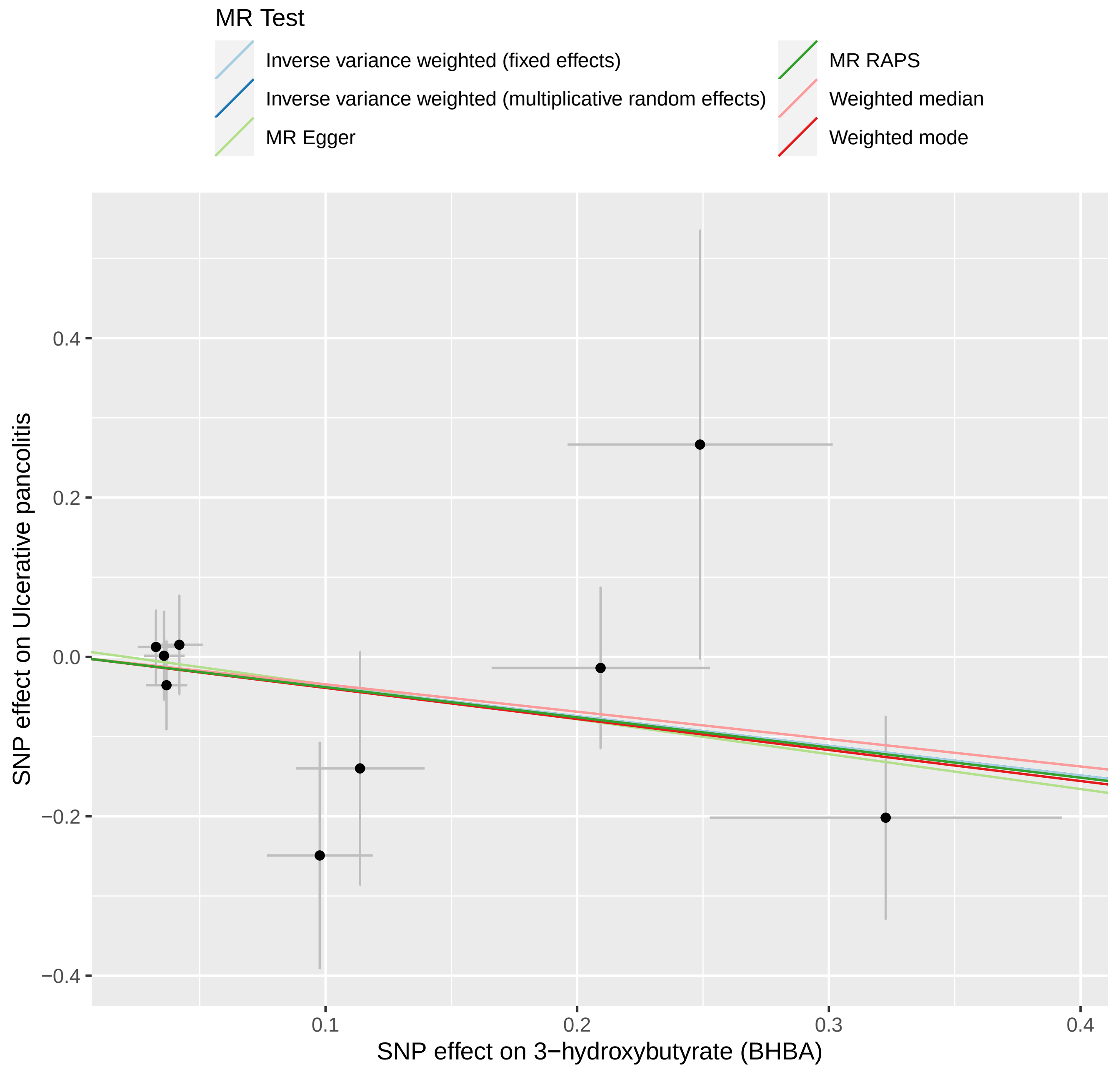

Batch 1935 : Arabinose on Ulcerative pancolitis

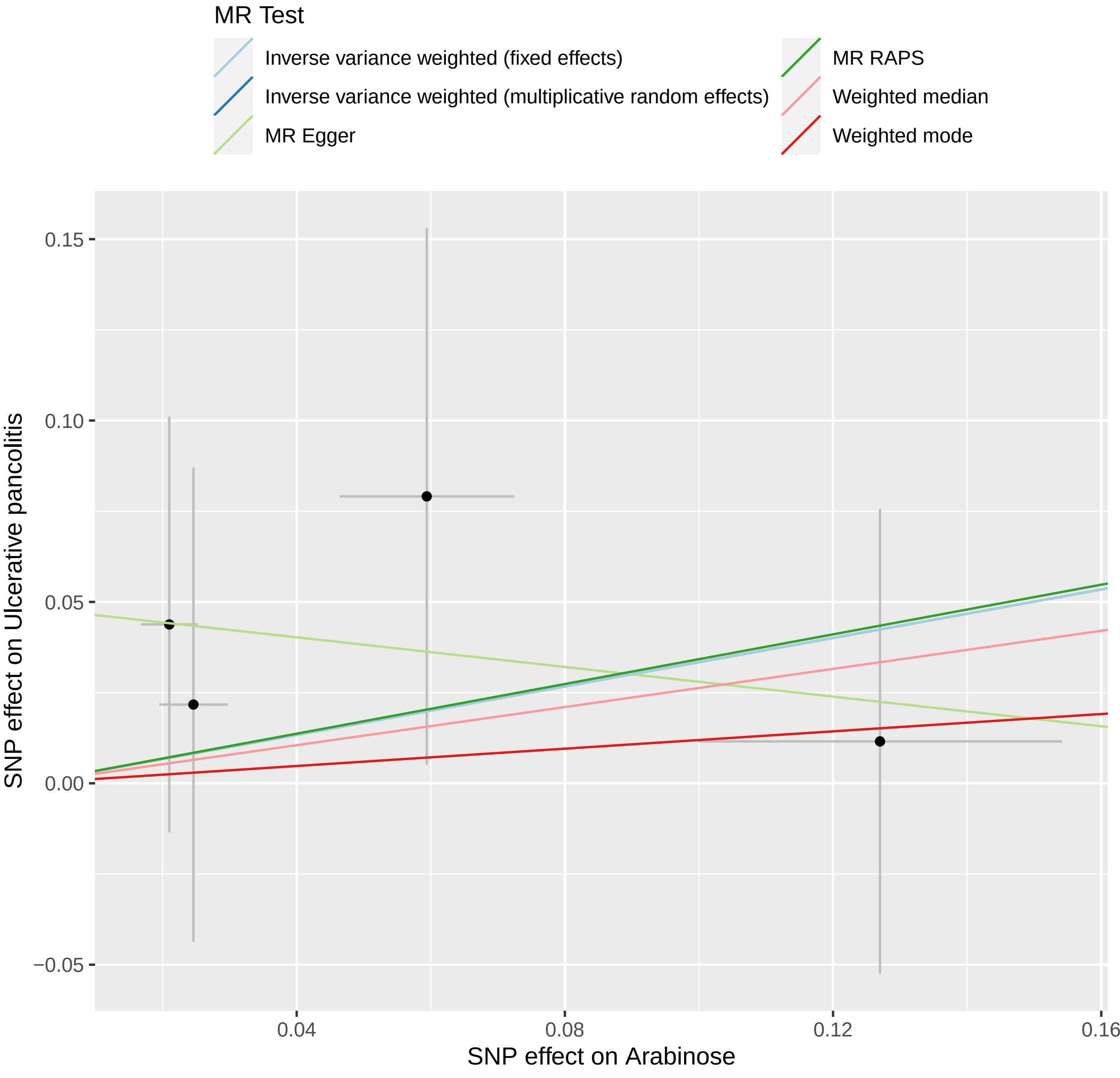

Batch 1936 : Fructose on Ulcerative pancolitis

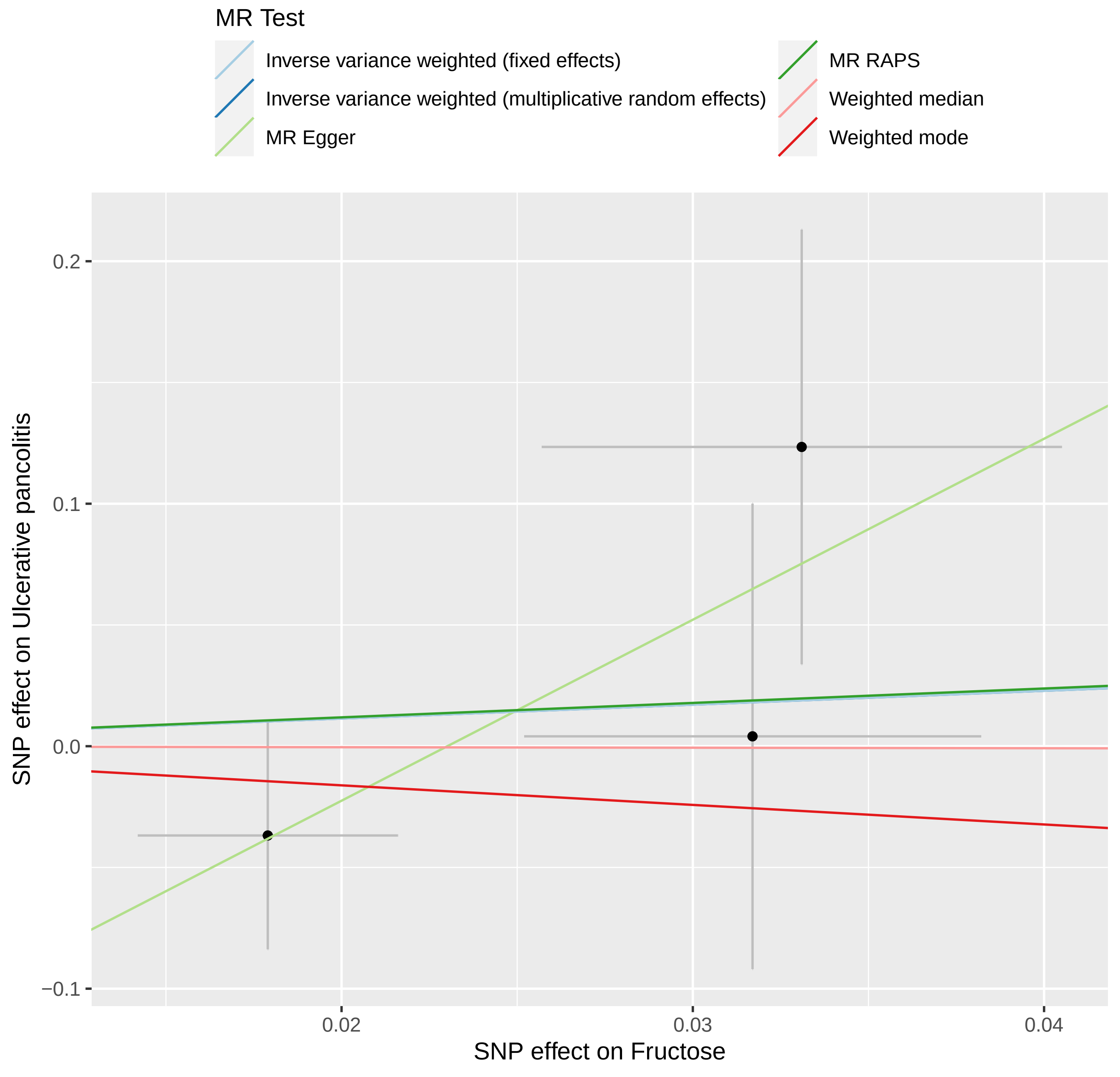

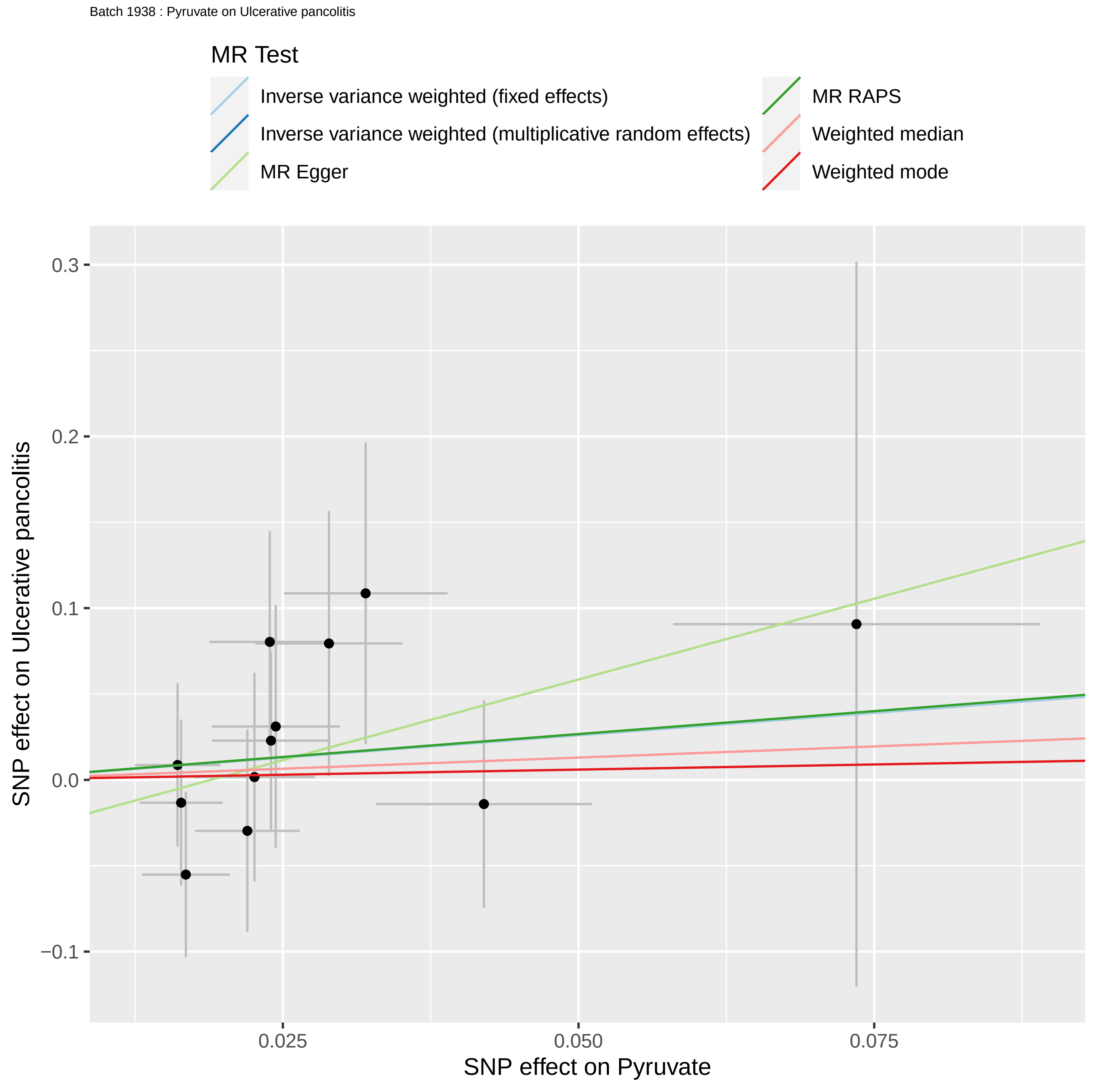

Batch 1938 : Pyruvate on Ulcerative pancolitis
MR Test
Inverse variance weighted (fixed effects)
Inverse variance weighted (multiplicative random effects)
MR Egger
MR RAPS
Weighted median
Weighted mode
0.3
0.2
0.1
0.0
−0.1
0.025
0.050
0.075
SNP effect on Pyruvate
SNP effect on Ulcerative pancolitis

Batch 1939 : Uridine on Ulcerative pancolitis

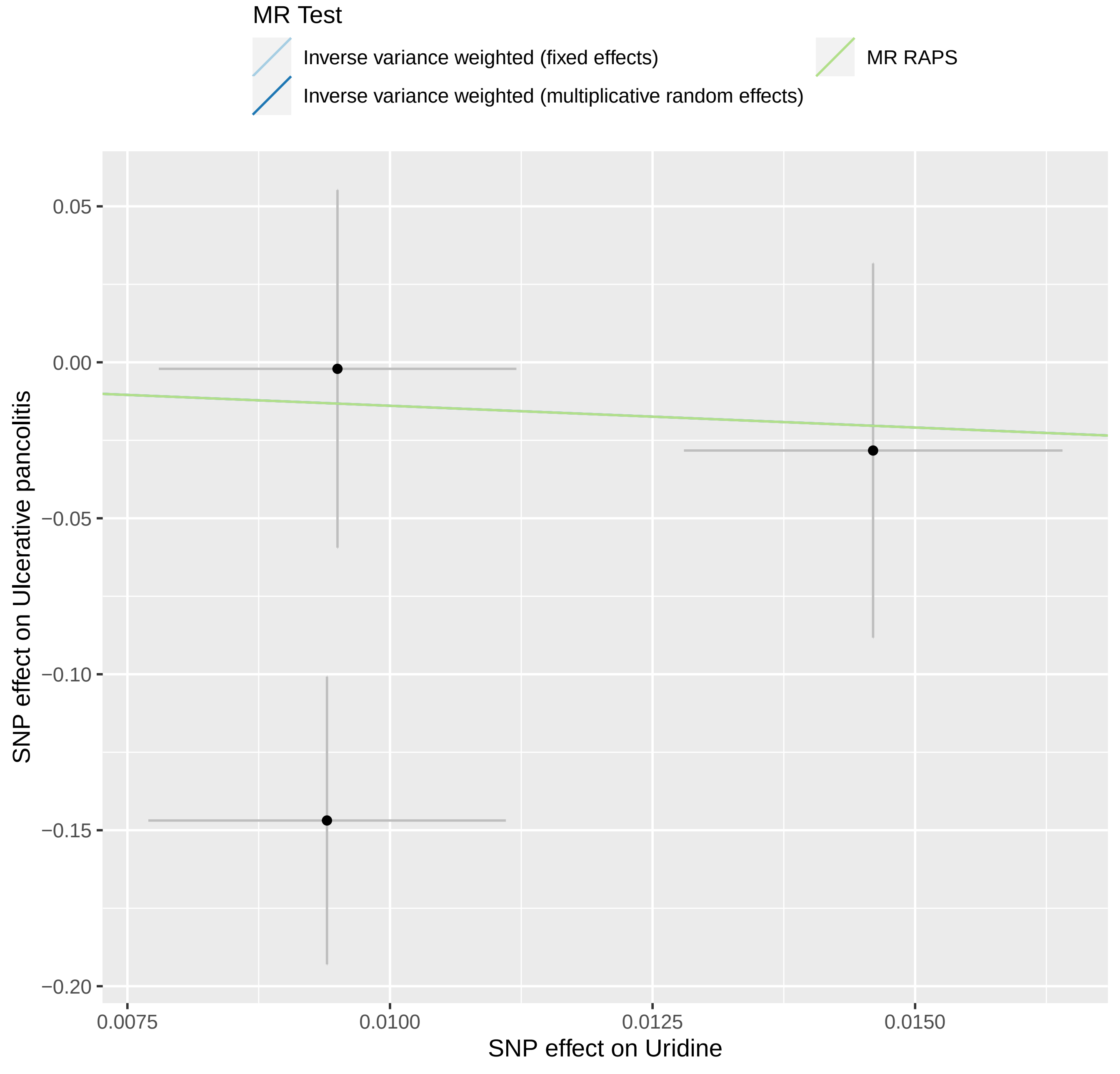

Batch 1940 : Linoleate (18:2n6) on Ulcerative pancolitis

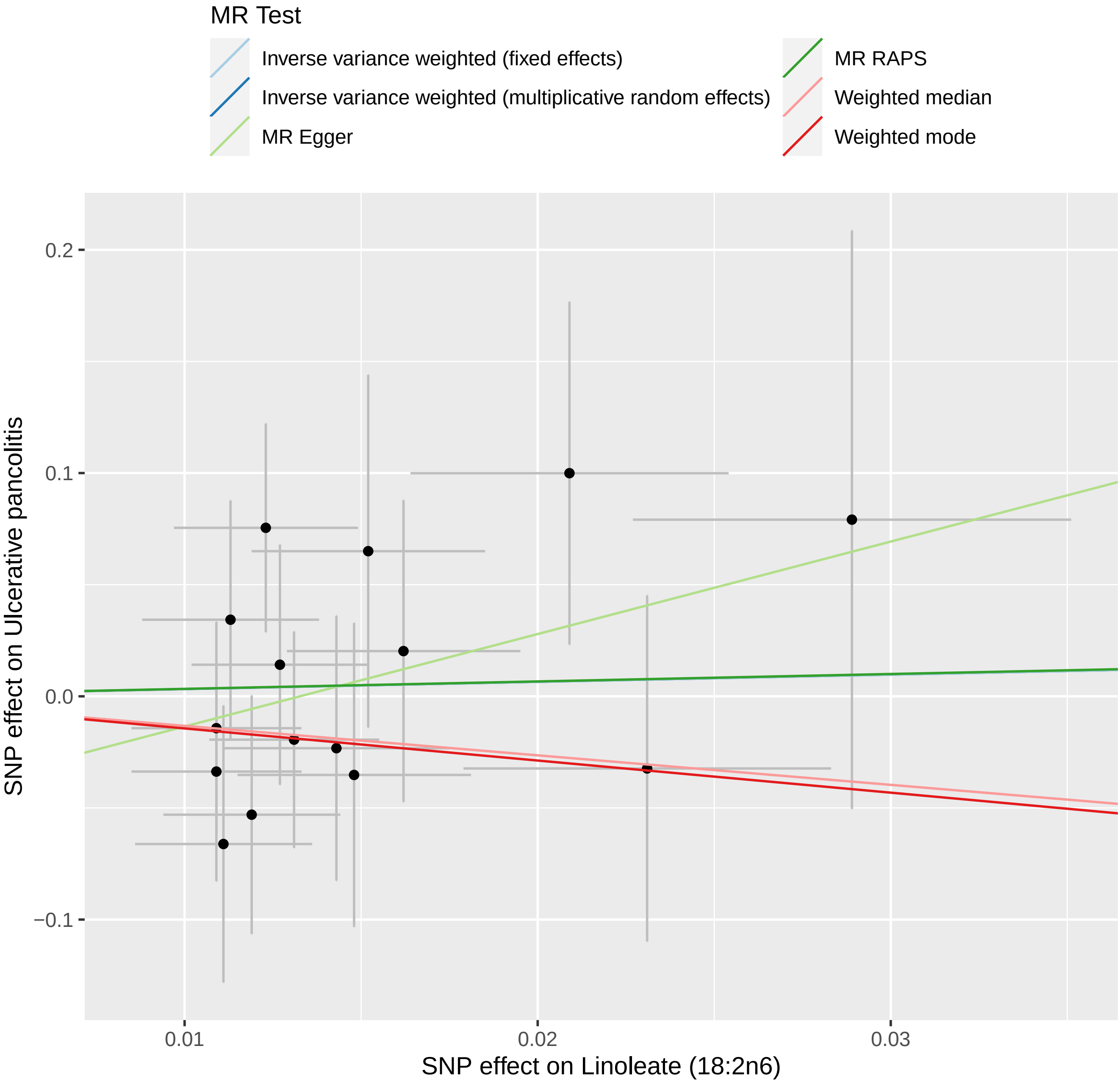

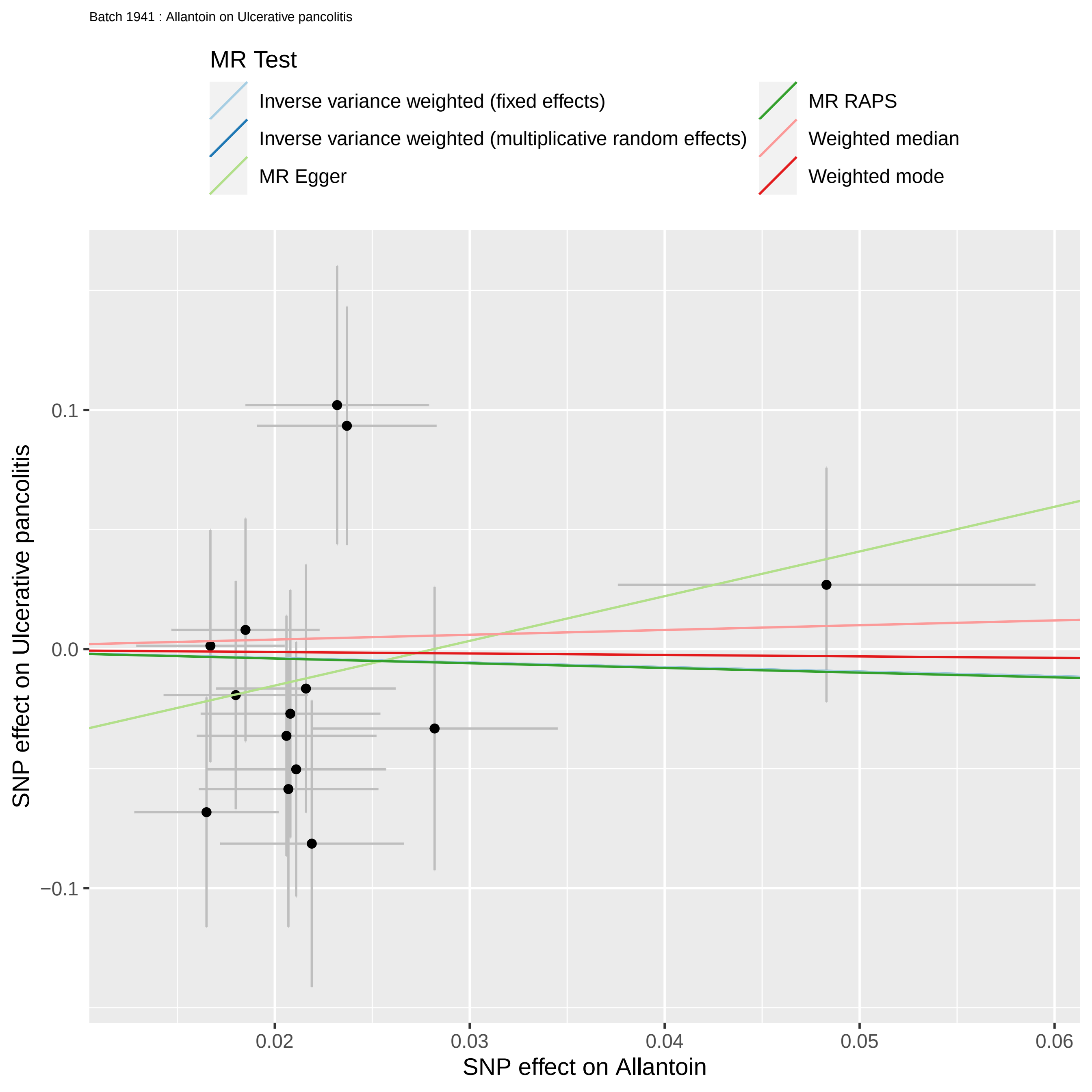

Batch 1941 : Allantoin on Ulcerative pancolitis
MR Test
Inverse variance weighted (fixed effects)
Inverse variance weighted (multiplicative random effects)
MR Egger
MR RAPS
Weighted median
Weighted mode
0.1
0.0
−0.1
0.02
0.03
0.04
0.05
0.06
SNP effect on Allantoin
SNP effect on Ulcerative pancolitis

Batch 1946 : Isoleucine on Ulcerative pancolitis

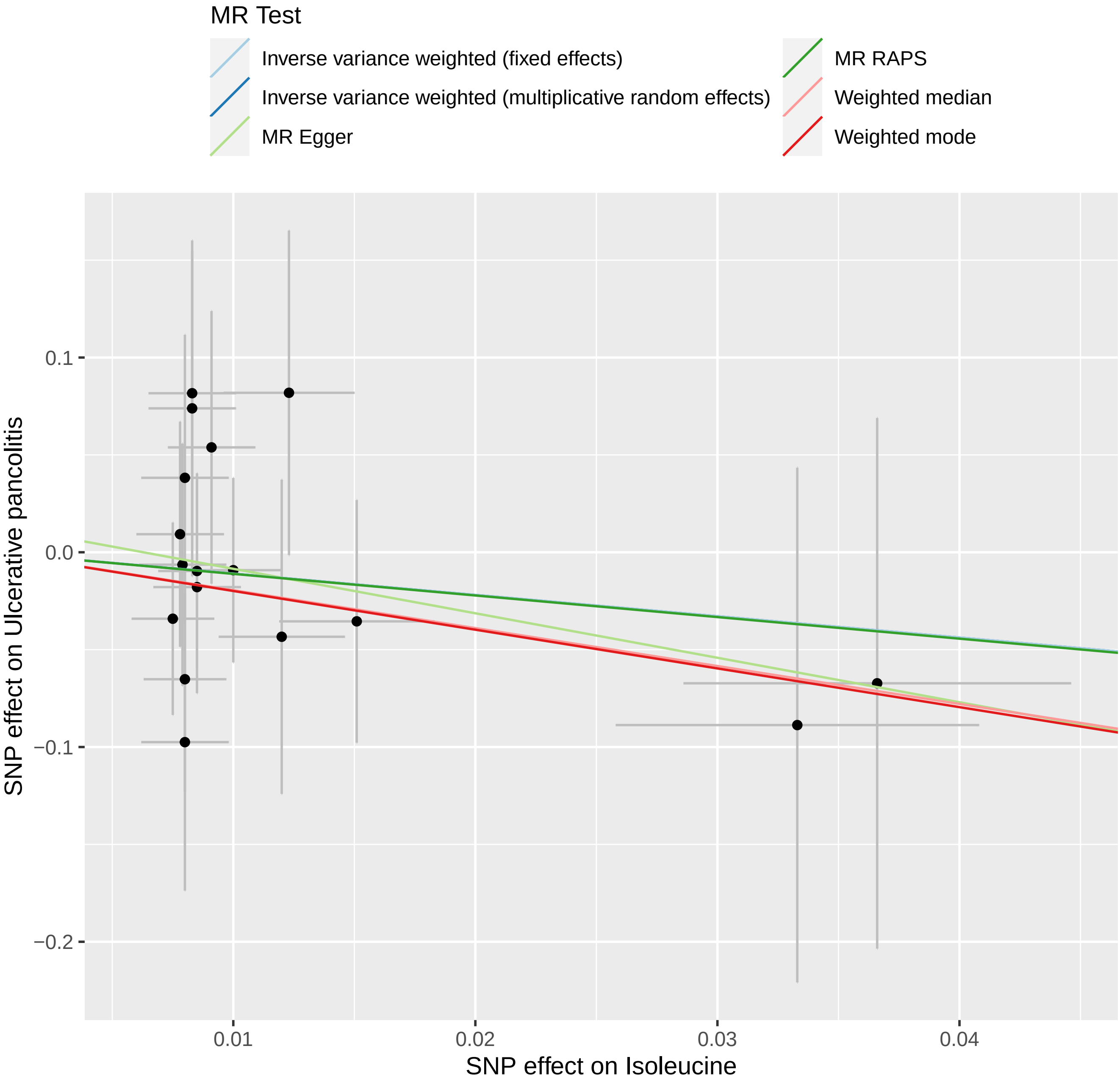

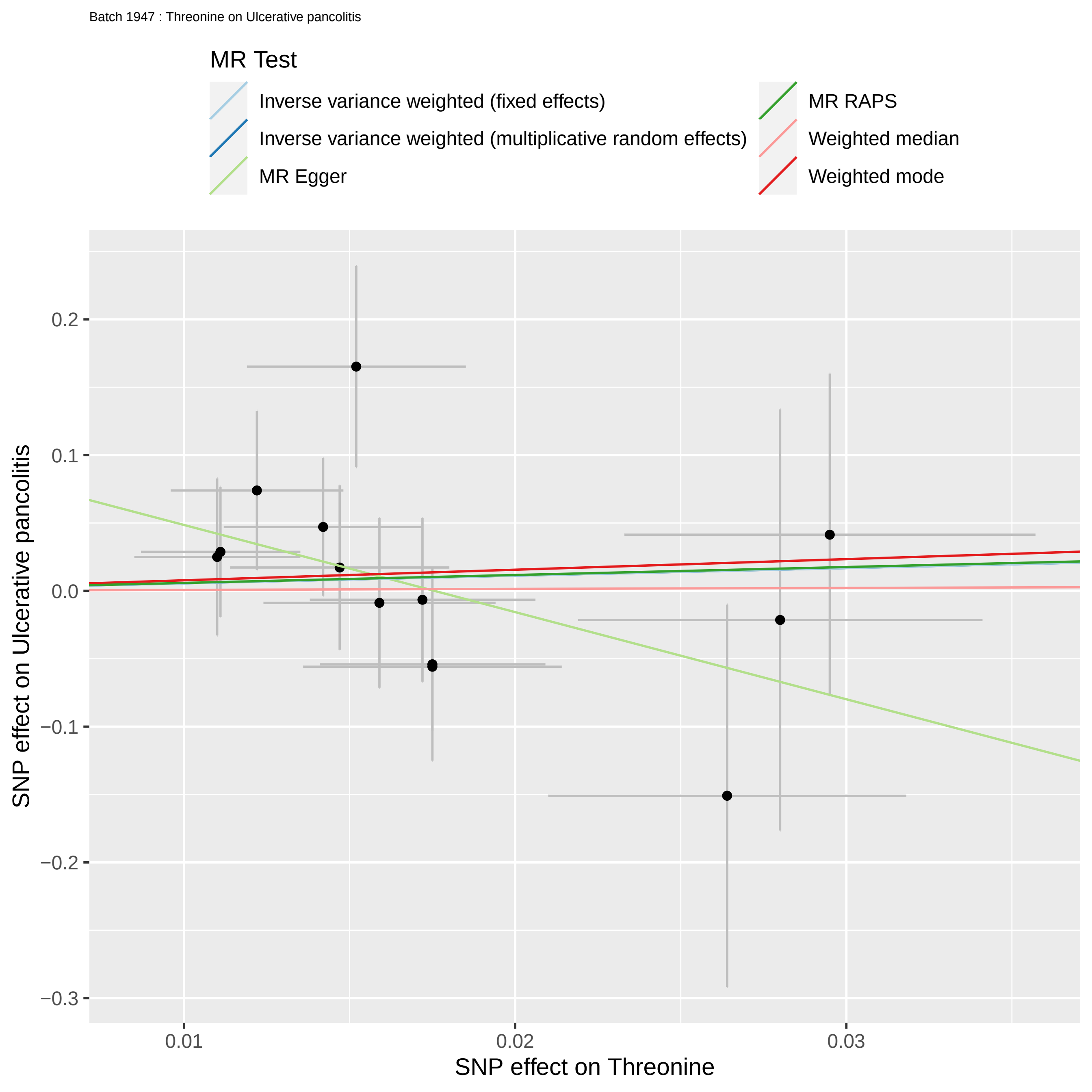

Batch 1947 : Threonine on Ulcerative pancolitis
MR Test
Inverse variance weighted (fixed effects)
Inverse variance weighted (multiplicative random effects)
MR Egger
MR RAPS
Weighted median
Weighted mode
0.2
0.1
0.0
−0.1
−0.2
−0.3
0.01
0.02
0.03
SNP effect on Ulcerative pancolitis
SNP effect on Threonine

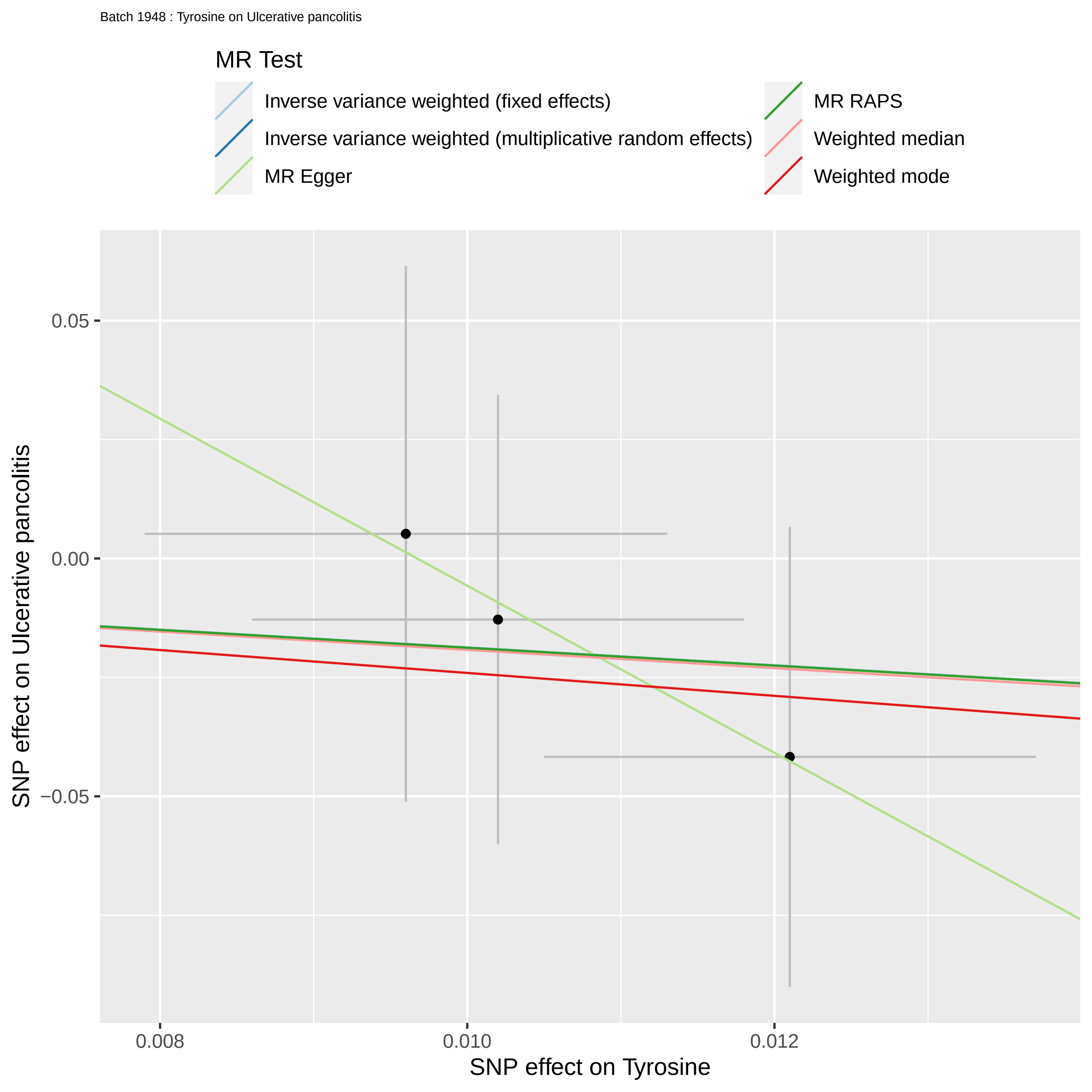
Batch 1948 : Tyrosine on Ulcerative pancolitis
MR Test
Inverse variance weighted (fixed effects)
Inverse variance weighted (multiplicative random effects)
MR Egger
MR RAPS
Weighted median
Weighted mode
0.05
0.00
−0.05
0.008
0.010
0.012
SNP effect on Tyrosine
SNP effect on Ulcerative pancolitis

Batch 1954 : Stearate (18:0) on Ulcerative pancolitis

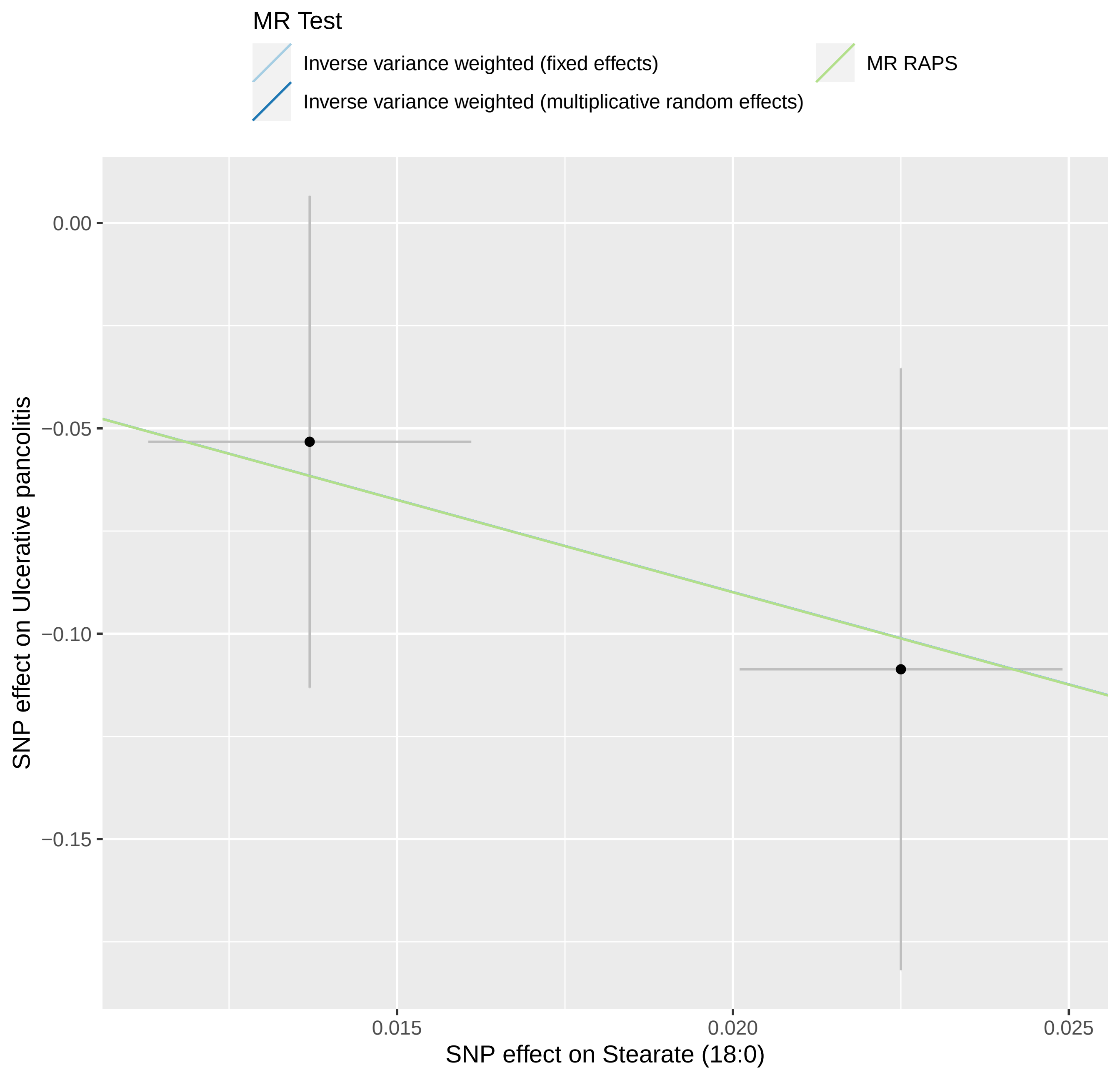

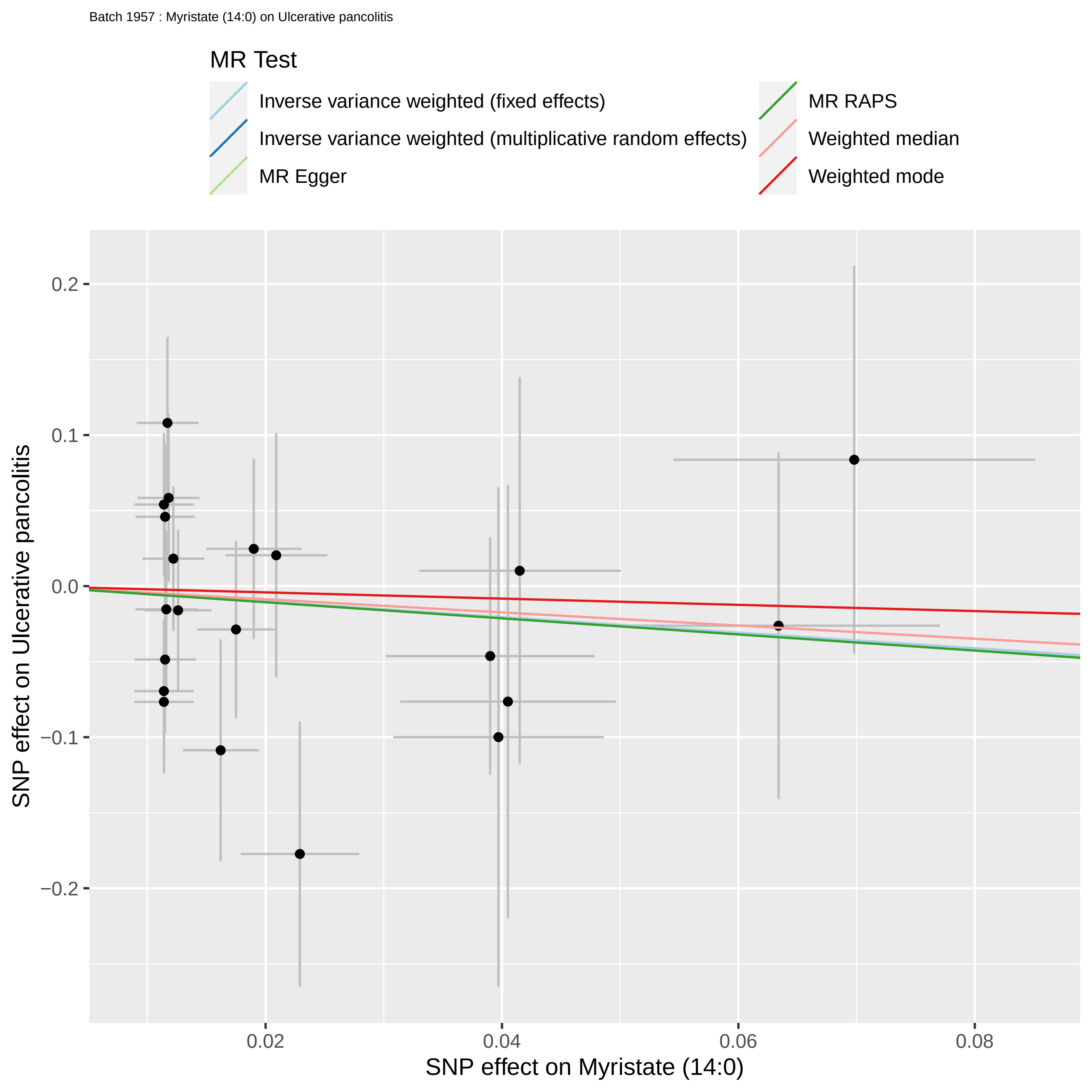
Batch 1957 : Myristate (14:0) on Ulcerative pancolitis
MR Test
Inverse variance weighted (fixed effects)
Inverse variance weighted (multiplicative random effects)
MR Egger
MR RAPS
Weighted median
Weighted mode
0.2
0.1
0.0
−0.1
−0.2
0.02
0.04
0.06
0.08
SNP effect on Ulcerative pancolitis
SNP effect on Myristate (14:0)

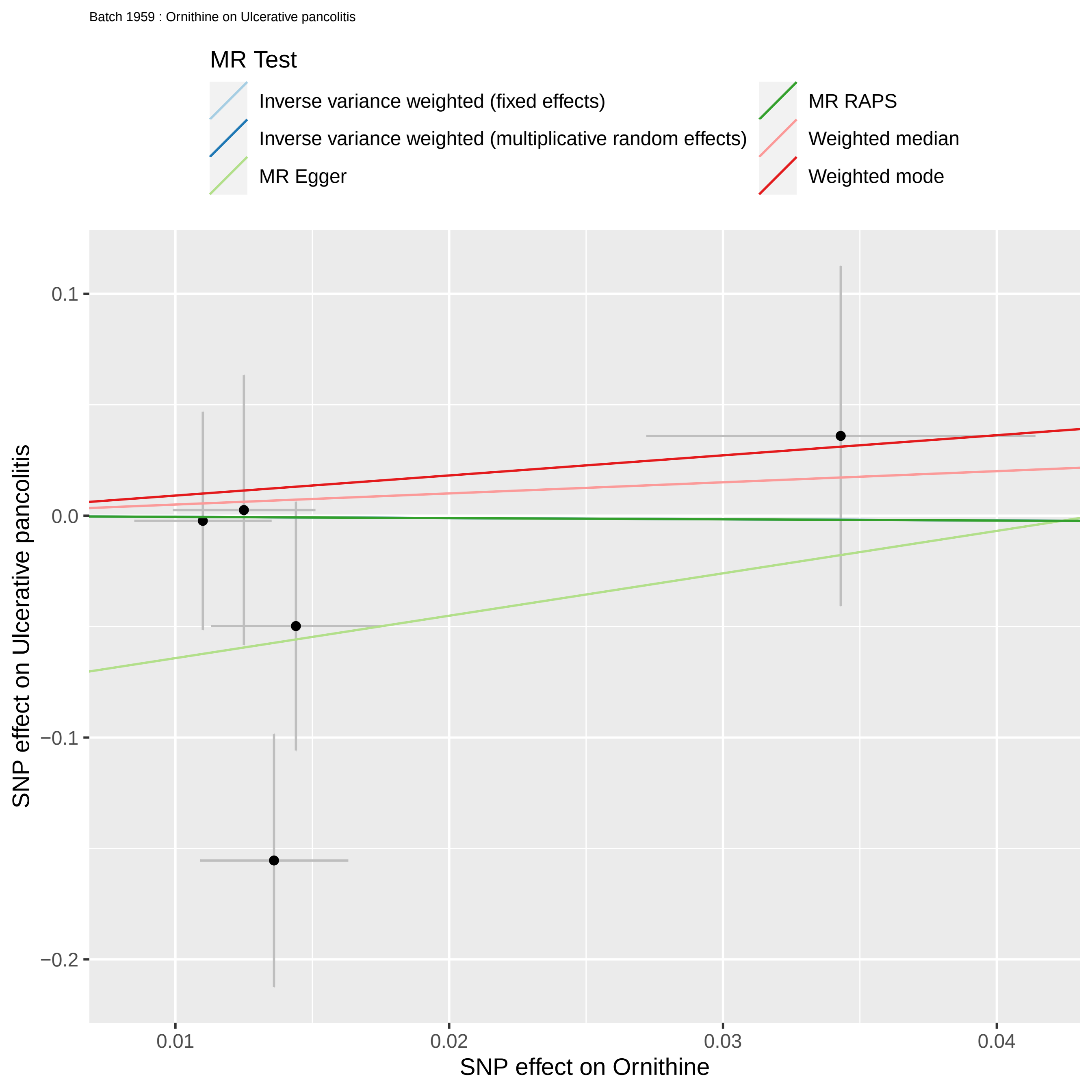
Batch 1959 : Ornithine on Ulcerative pancolitis
MR Test
Inverse variance weighted (fixed effects)
Inverse variance weighted (multiplicative random effects)
MR Egger
MR RAPS
Weighted median
Weighted mode
0.1
0.0
−0.1
−0.2
0.01
0.02
0.03
0.04
SNP effect on Ornithine
SNP effect on Ulcerative pancolitis

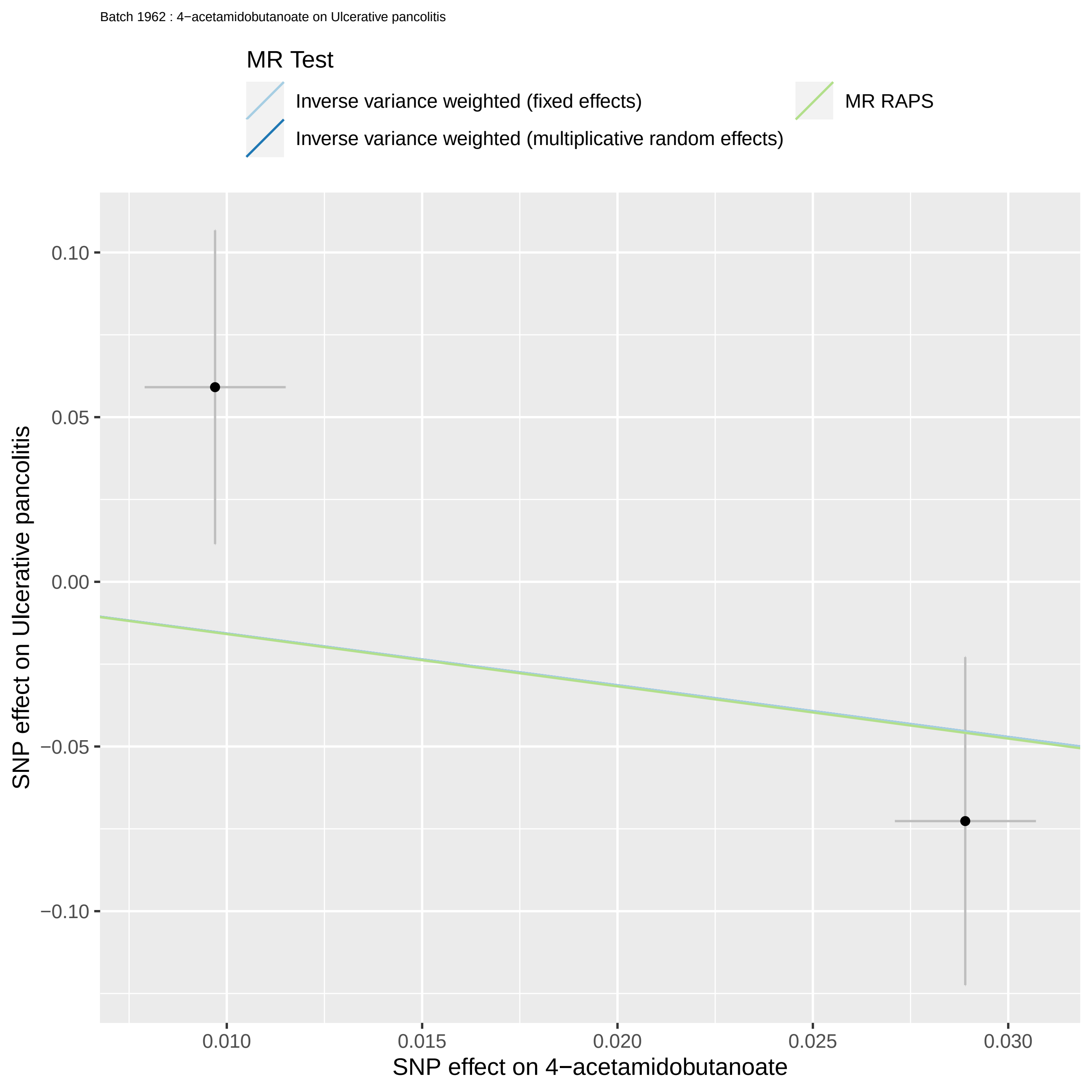

Batch 1962 : 4−acetamidobutanoate on Ulcerative pancolitis
MR Test
Inverse variance weighted (fixed effects)
Inverse variance weighted (multiplicative random effects)
MR RAPS
0.10
0.05
0.00
−0.05
−0.10
0.010
0.015
0.020
0.025
0.030
SNP effect on Ulcerative pancolitis
SNP effect on 4−acetamidobutanoate

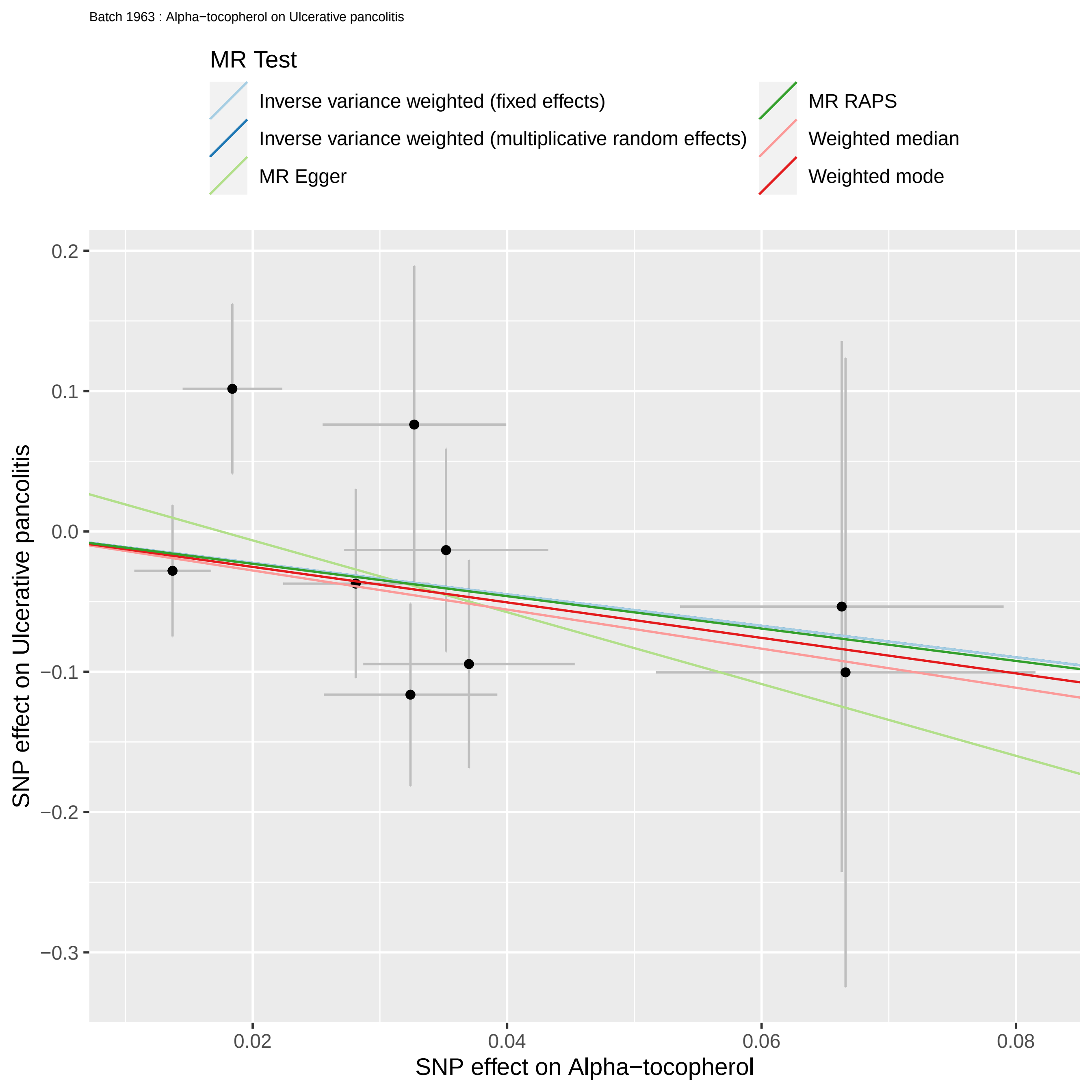
Batch 1963 : Alpha−tocopherol on Ulcerative pancolitis
MR Test
Inverse variance weighted (fixed effects)
Inverse variance weighted (multiplicative random effects)
MR Egger
MR RAPS
Weighted median
Weighted mode
0.2
0.1
0.0
−0.1
−0.2
−0.3
0.02
0.04
0.06
0.08
SNP effect on Ulcerative pancolitis
SNP effect on Alpha−tocopherol

Batch 1964 : Citrate on Ulcerative pancolitis

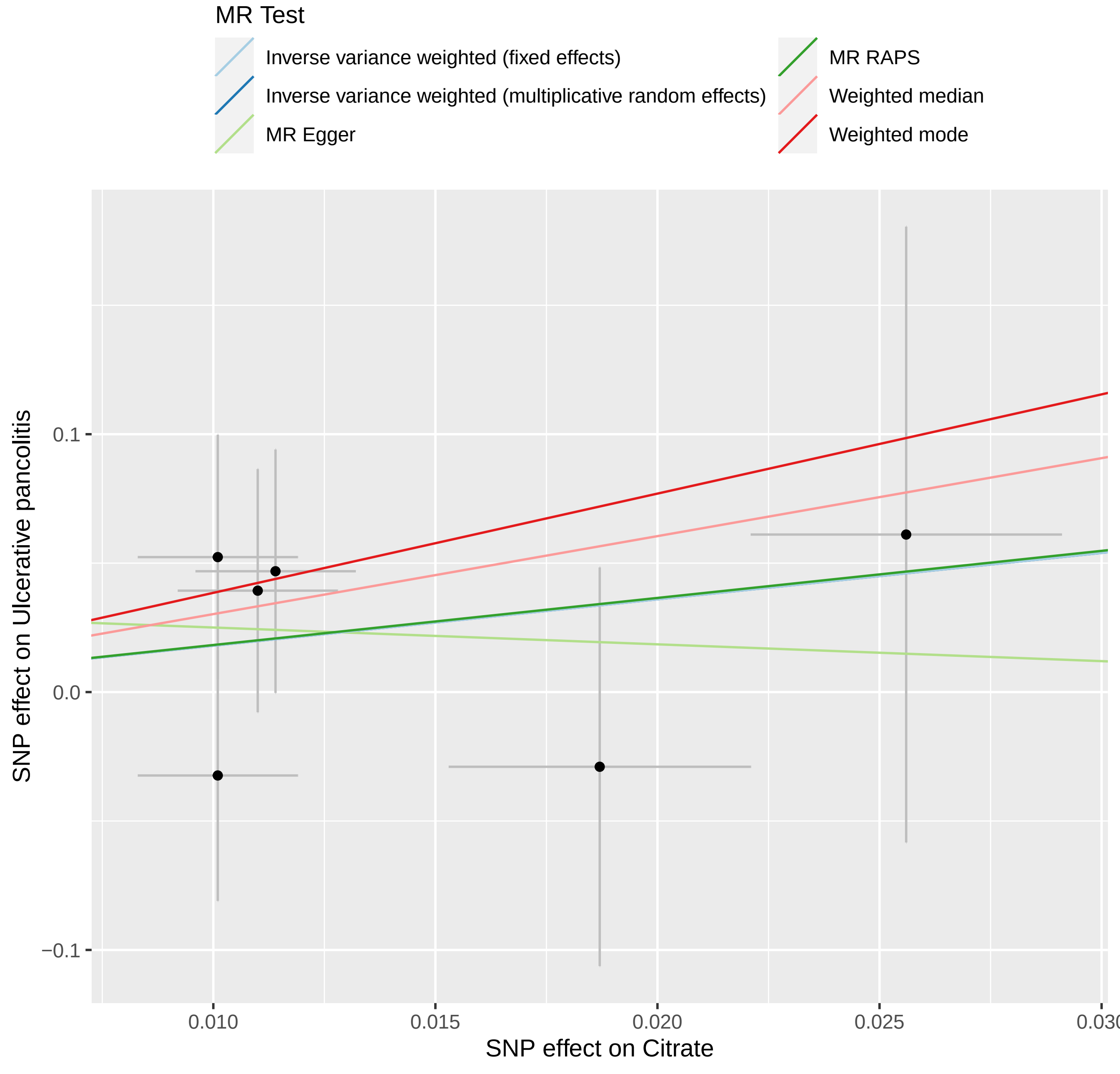

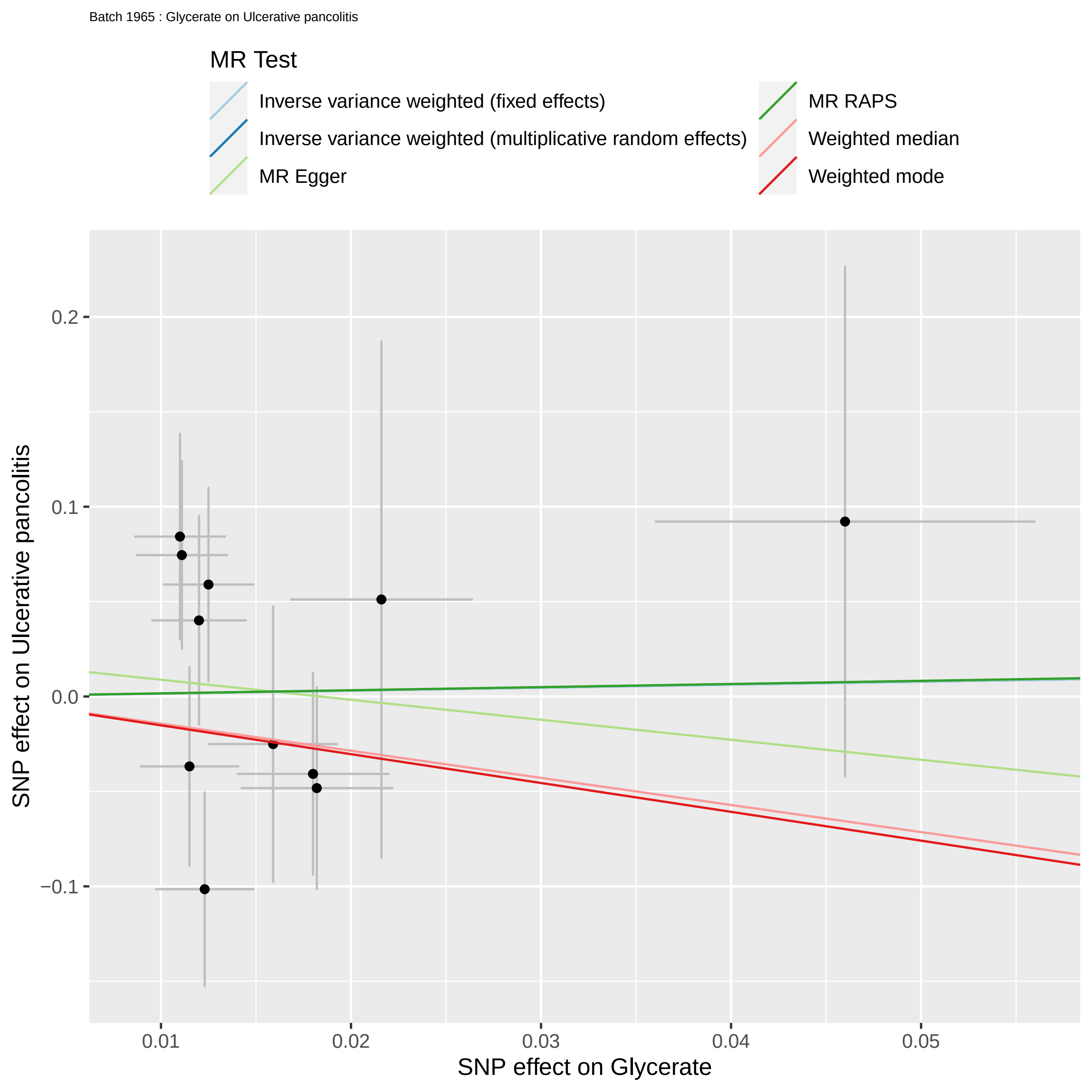

Batch 1965 : Glycerate on Ulcerative pancolitis
MR Test
Inverse variance weighted (fixed effects)
Inverse variance weighted (multiplicative random effects)
MR Egger
MR RAPS
Weighted median
Weighted mode
0.2
0.1
0.0
−0.1
SNP effect on Ulcerative pancolitis
0.01
0.02
0.03
0.04
0.05
SNP effect on Glycerate

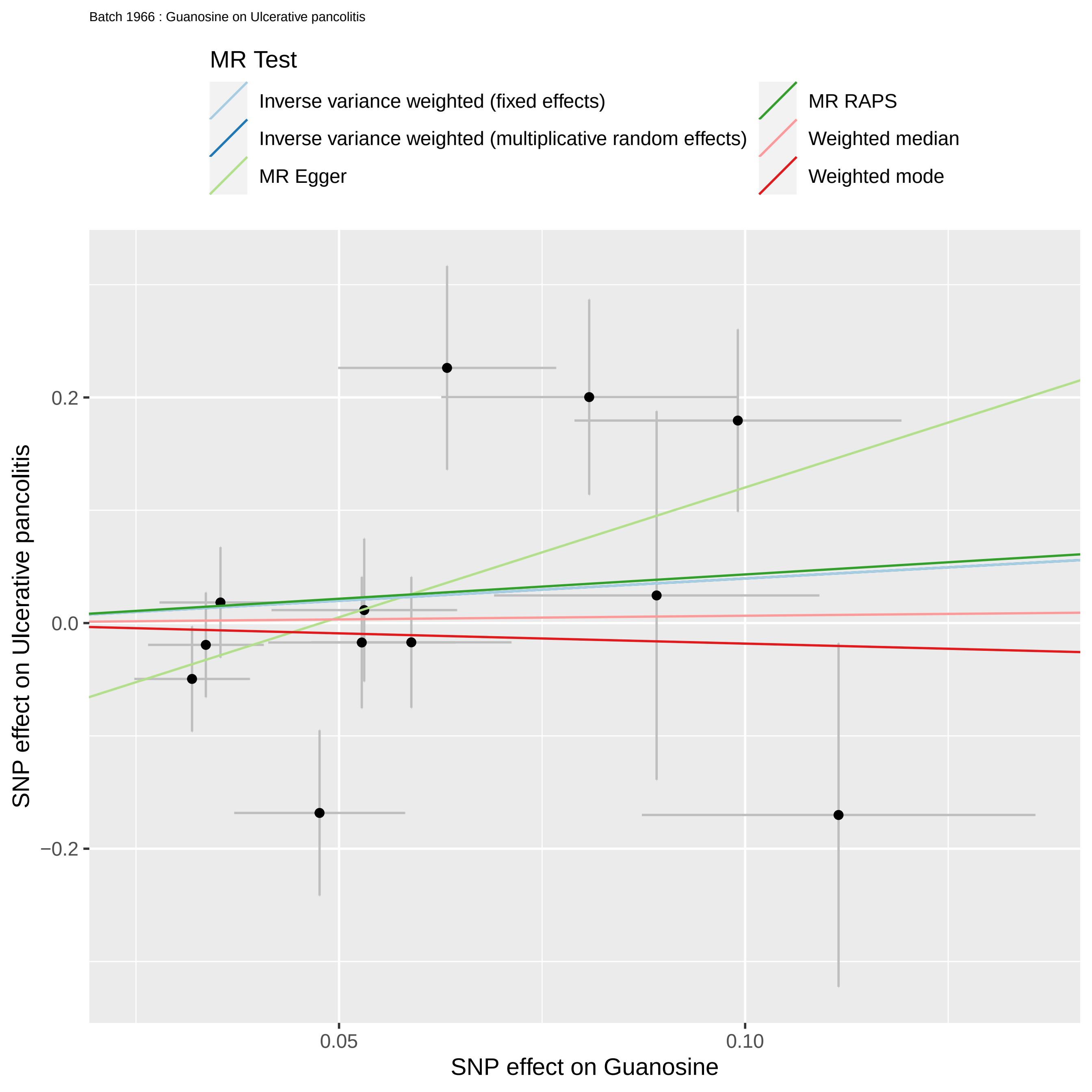

Batch 1966 : Guanosine on Ulcerative pancolitis
MR Test
Inverse variance weighted (fixed effects)
Inverse variance weighted (multiplicative random effects)
MR Egger
MR RAPS
Weighted median
Weighted mode
SNP effect on Ulcerative pancolitis
0.2
0.0
−0.2
0.05
0.10
SNP effect on Guanosine

Batch 1969 : Ursodeoxycholate on Ulcerative pancolitis

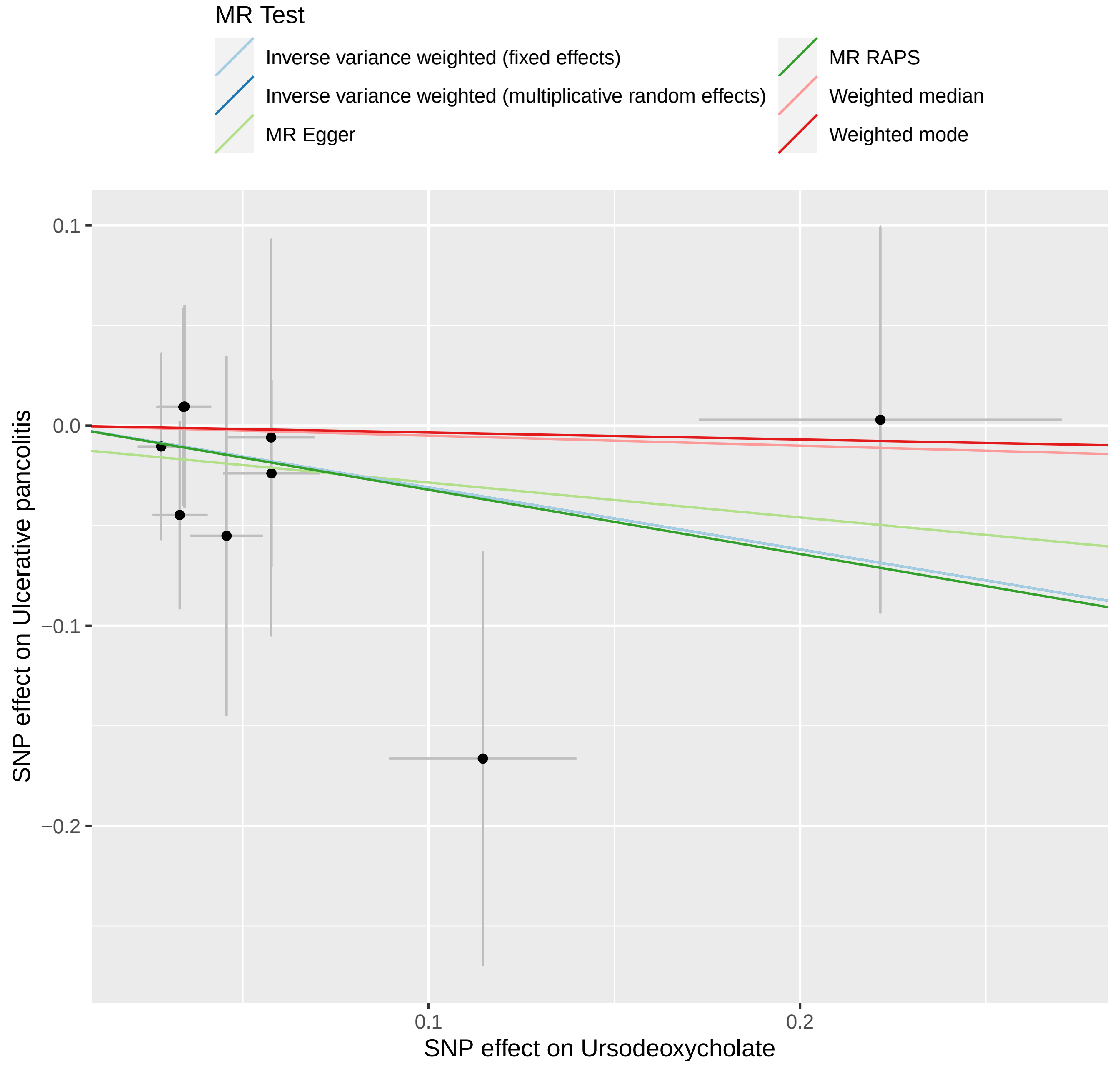

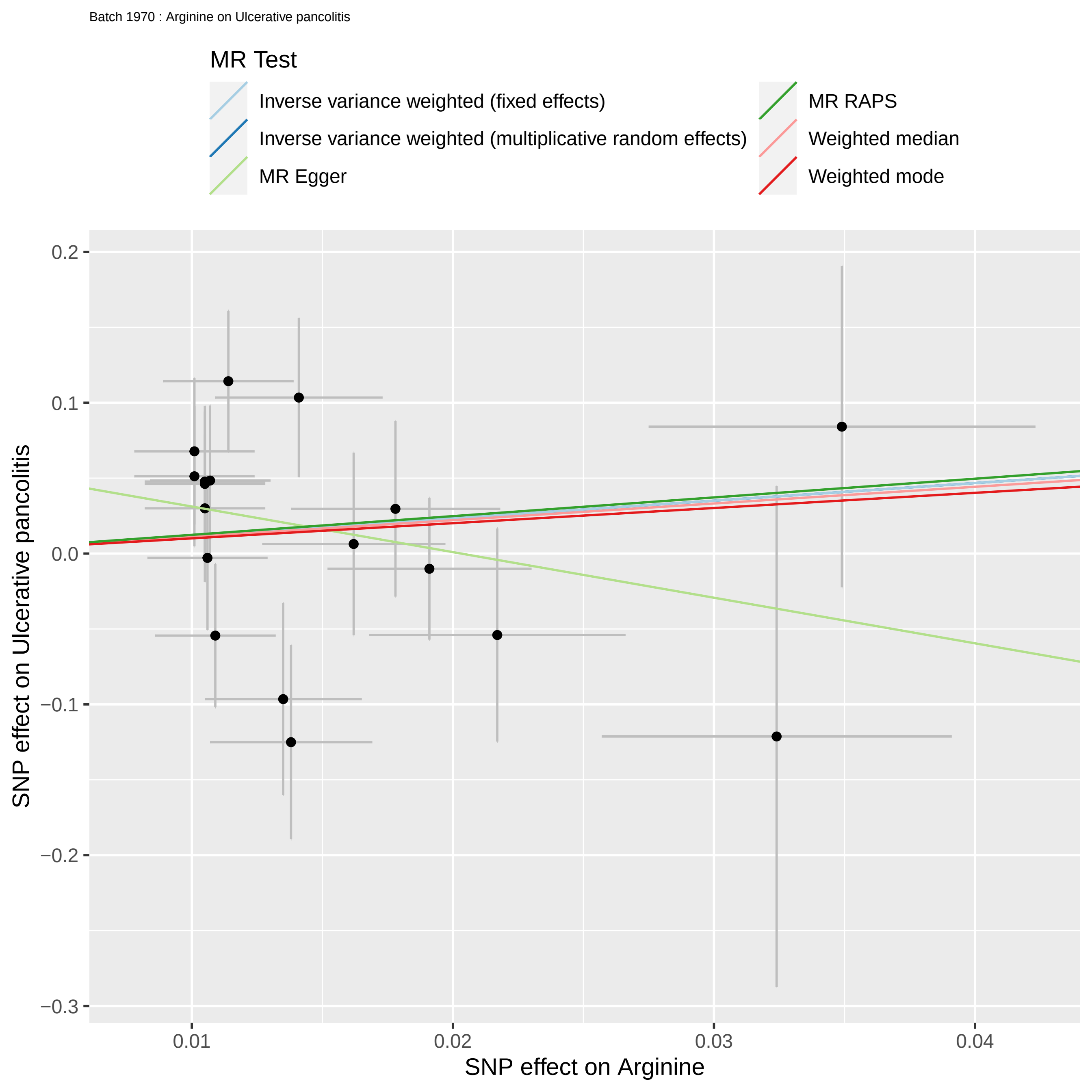
Batch 1970 : Arginine on Ulcerative pancolitis
MR Test
Inverse variance weighted (fixed effects)
Inverse variance weighted (multiplicative random effects)
MR Egger
MR RAPS
Weighted median
Weighted mode
SNP effect on Ulcerative pancolitis
SNP effect on Arginine
0.2
0.1
0.0
−0.1
−0.2
−0.3
0.01
0.02
0.03
0.04

Batch 1971 : Ascorbate (Vitamin C) on Ulcerative pancolitis

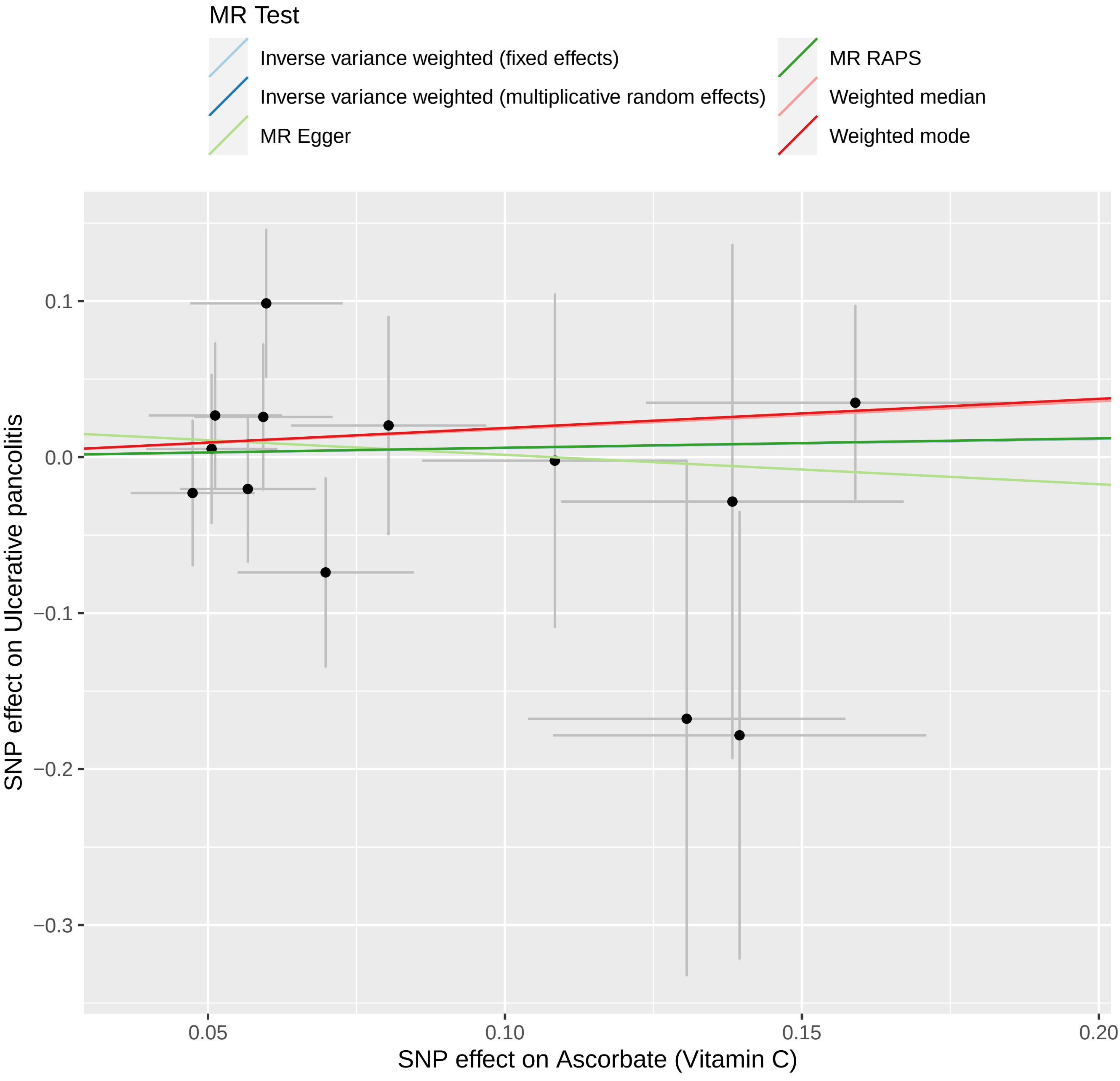

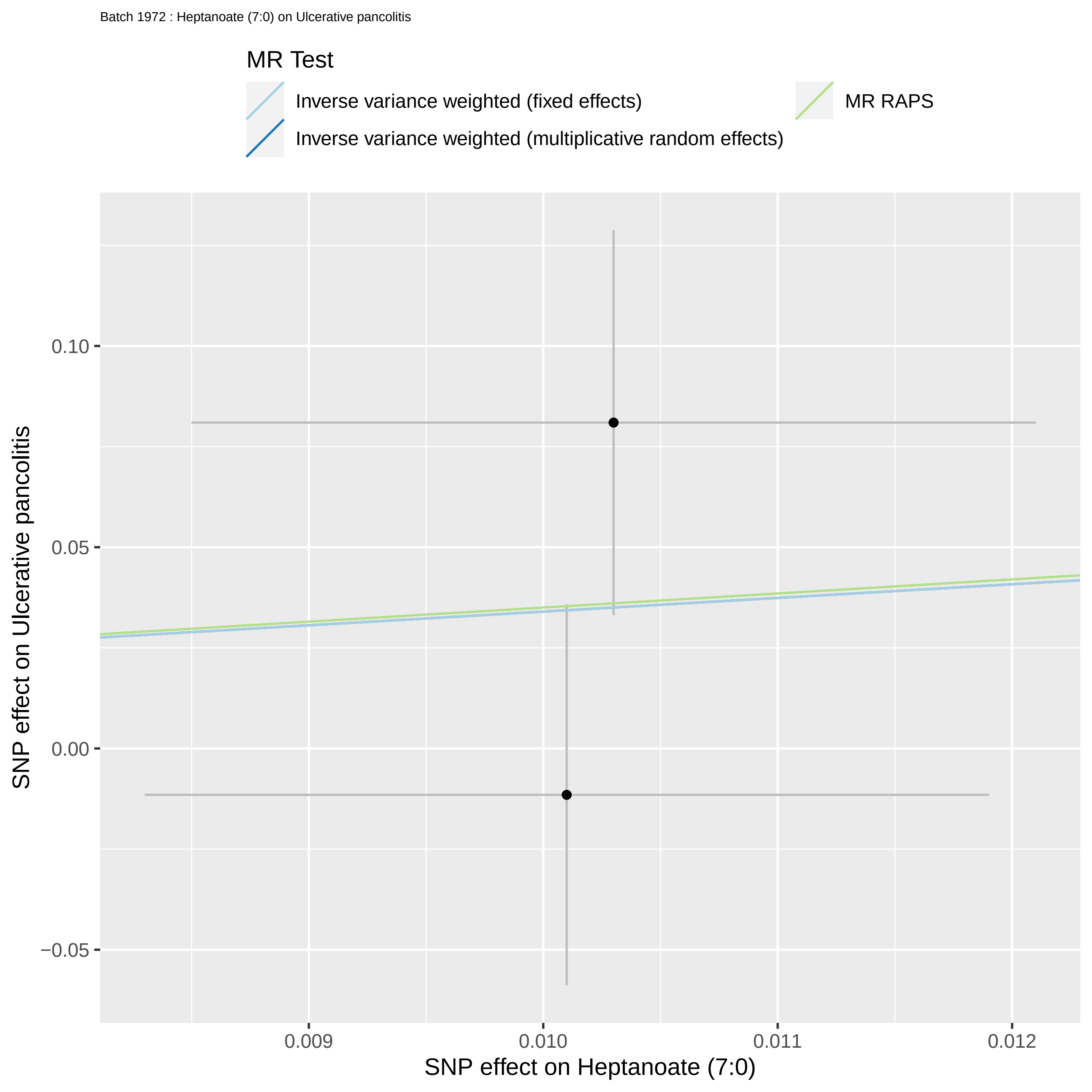

Batch 1972 : Heptanoate (7:0) on Ulcerative pancolitis
MR Test
Inverse variance weighted (fixed effects)
Inverse variance weighted (multiplicative random effects)
MR RAPS
0.10
0.05
0.00
−0.05
SNP effect on Ulcerative pancolitis
0.009
0.010
0.011
0.012
SNP effect on Heptanoate (7:0)

Batch 1973 : Laurate (12:0) on Ulcerative pancolitis

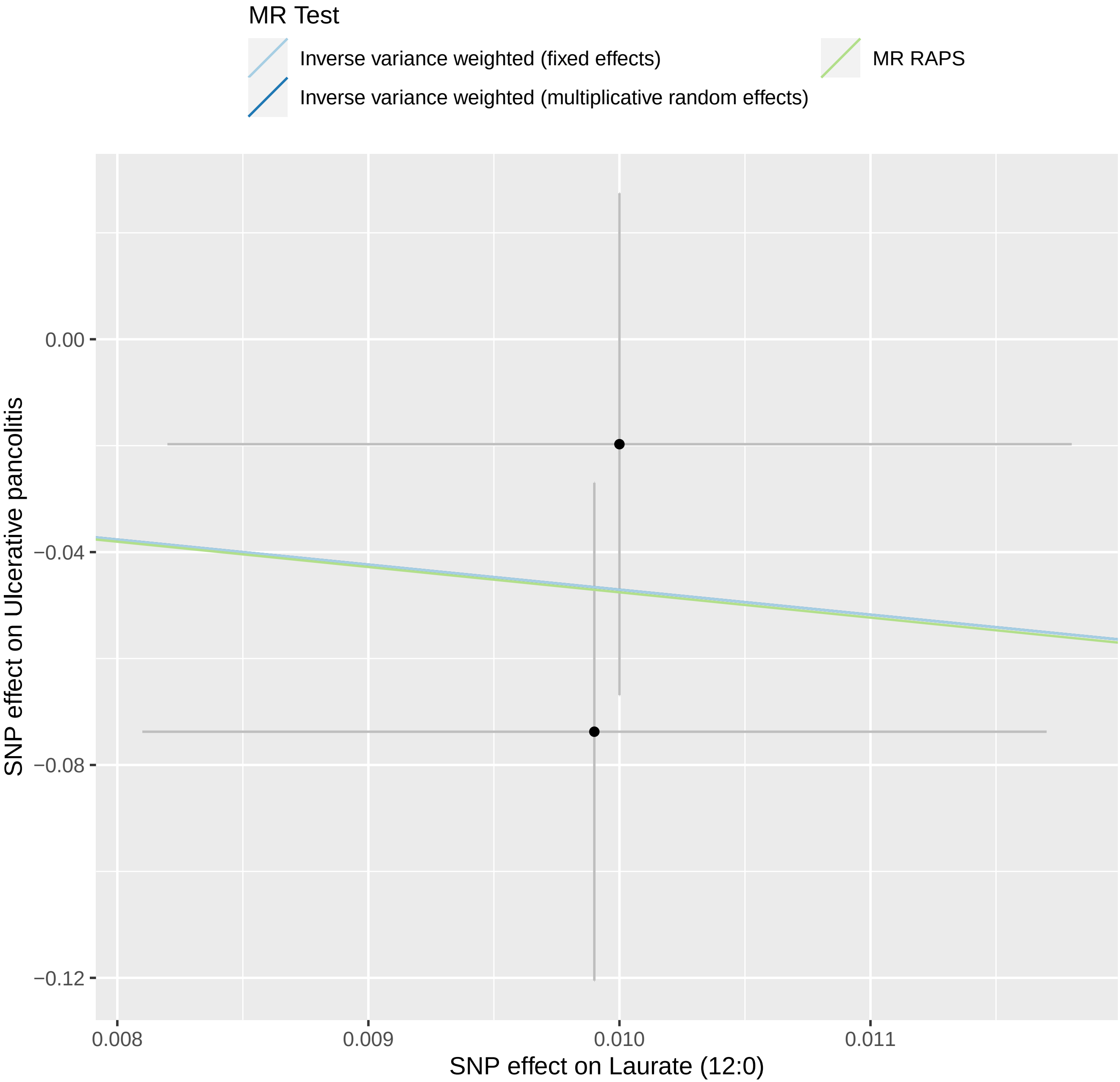

Batch 1975 : Urea on Ulcerative pancolitis

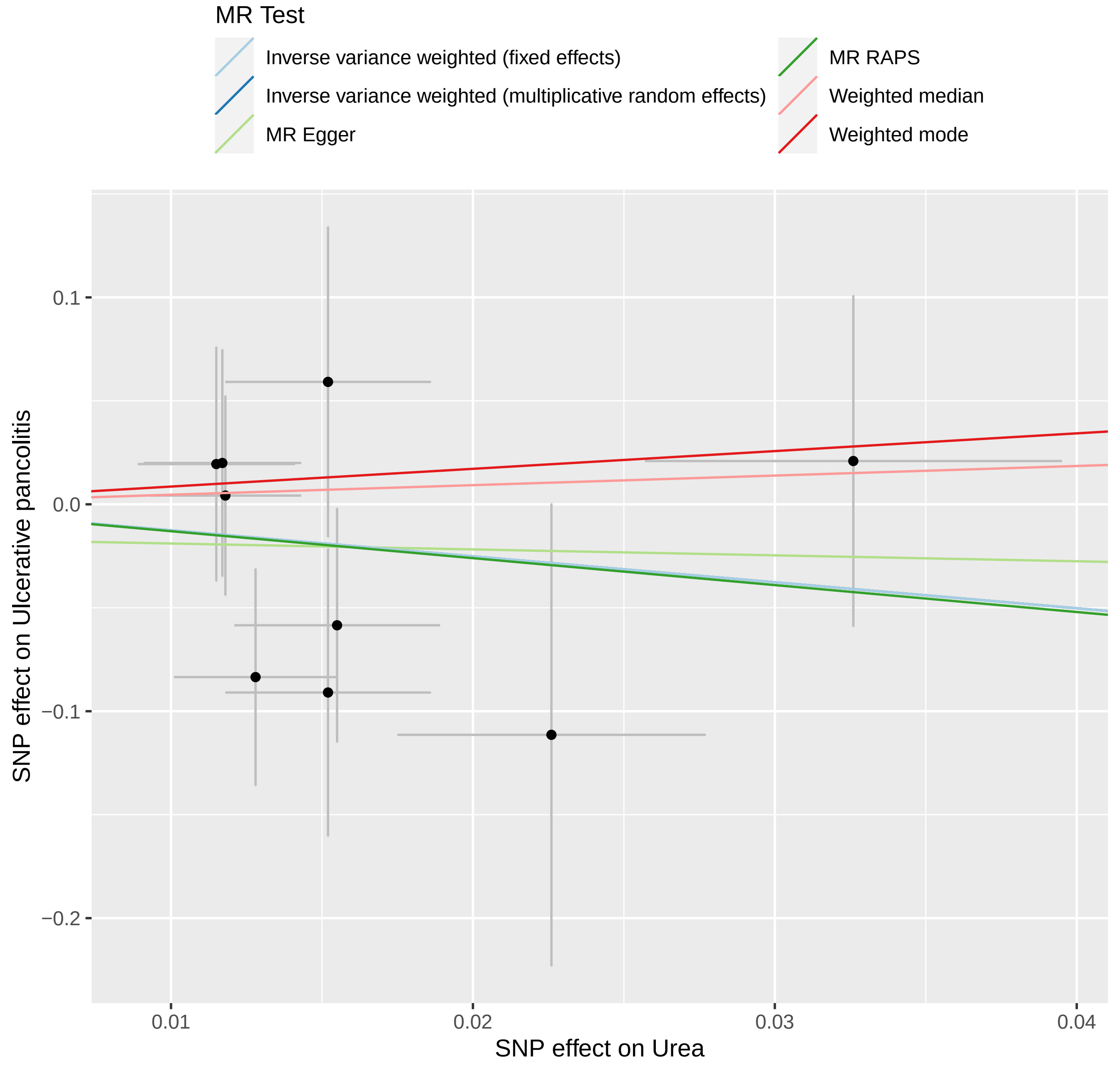

Batch 1976 : Cortisol on Ulcerative pancolitis

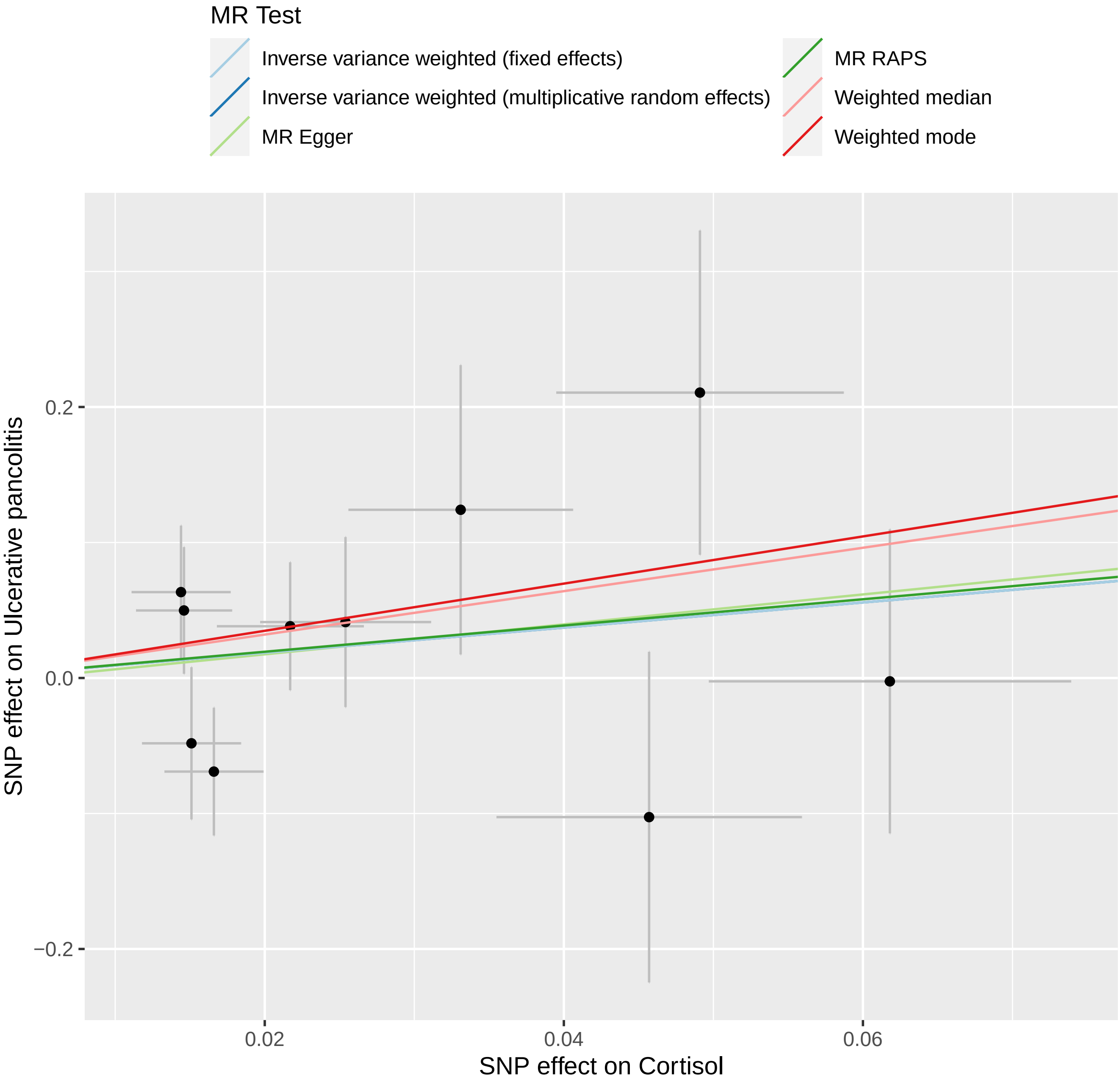

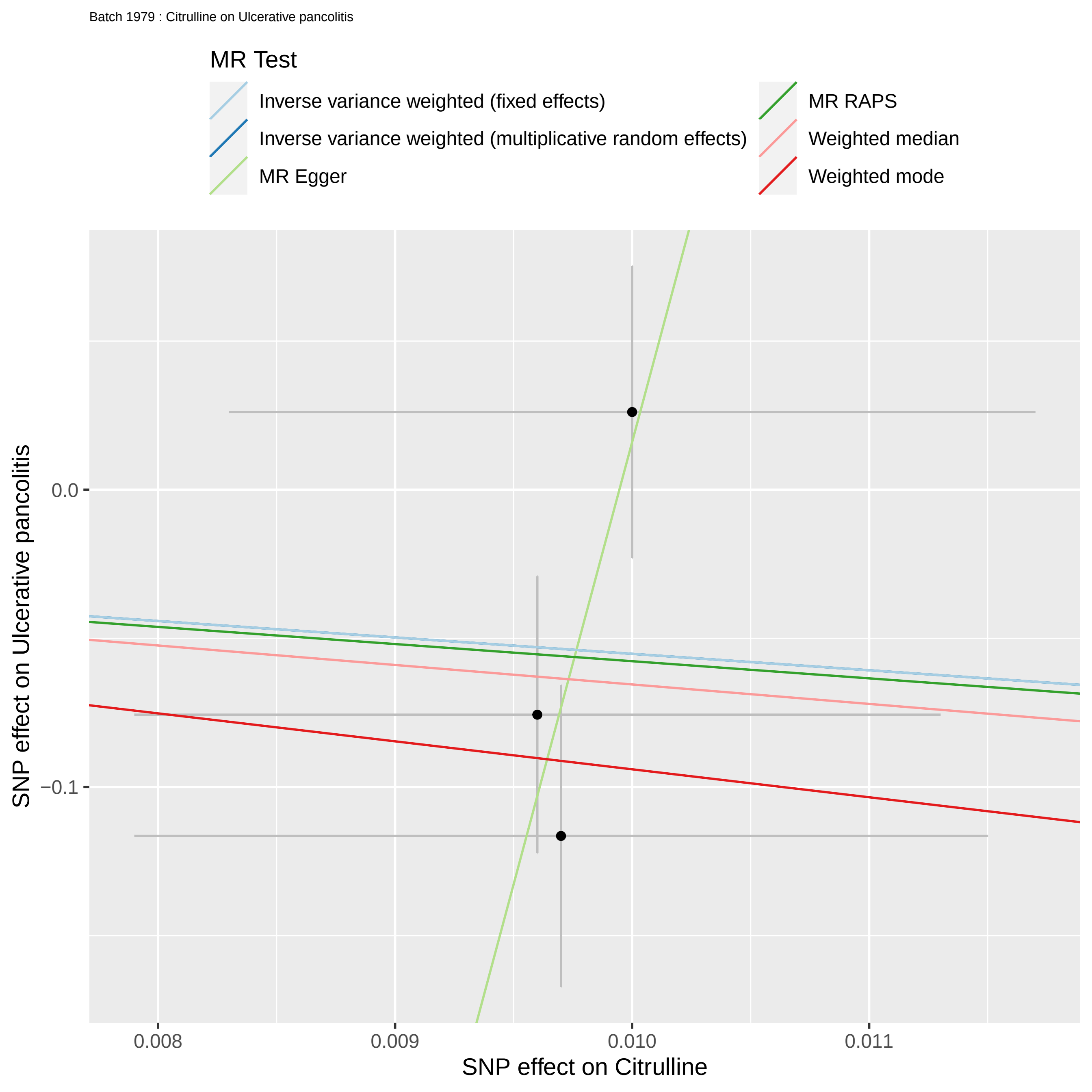
Batch 1979 : Citrulline on Ulcerative pancolitis
MR Test
Inverse variance weighted (fixed effects)
Inverse variance weighted (multiplicative random effects)
MR Egger
MR RAPS
Weighted median
Weighted mode
0.0
−0.1
0.008
0.009
0.010
0.011
SNP effect on Citrulline
SNP effect on Ulcerative pancolitis

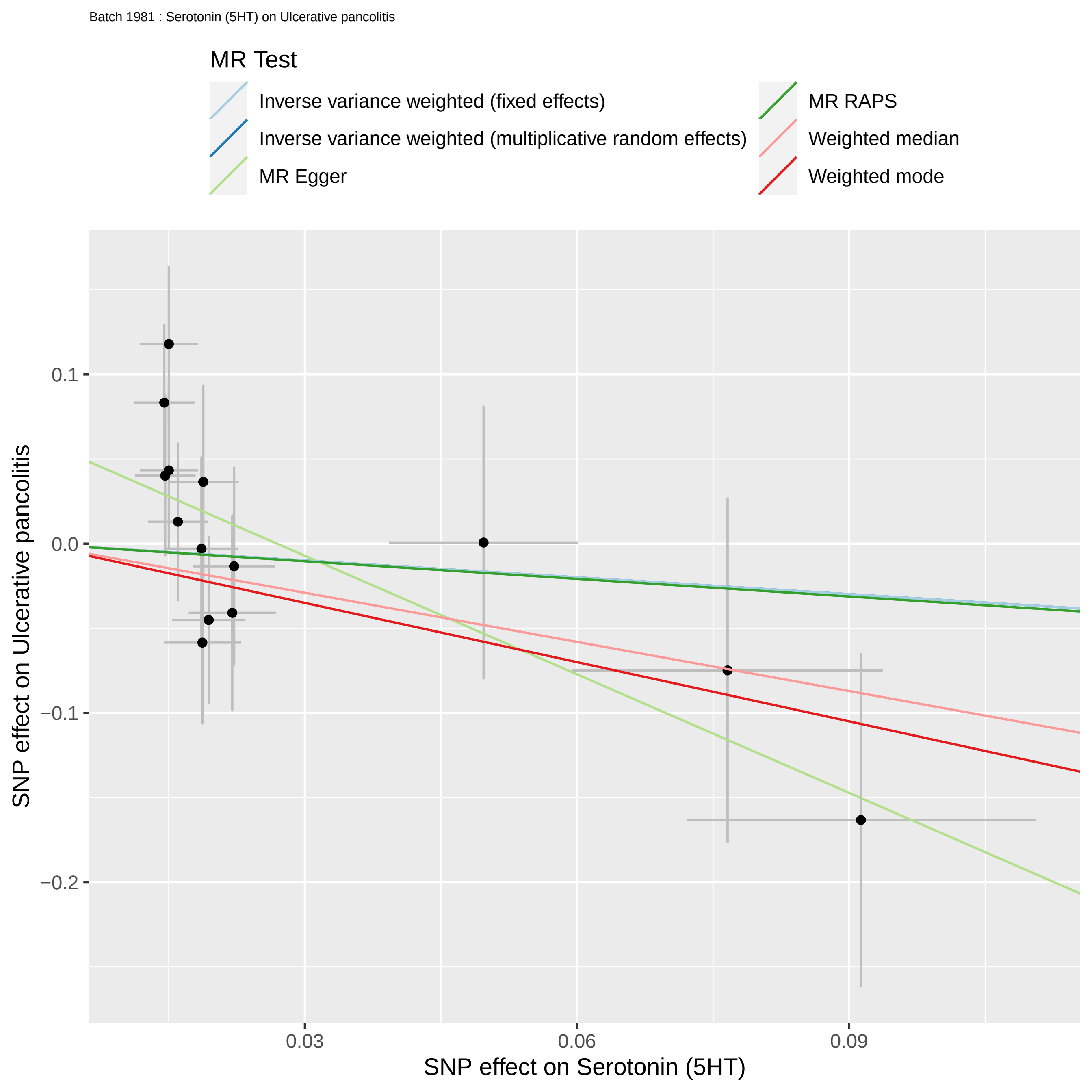
Batch 1981 : Serotonin (5HT) on Ulcerative pancolitis
MR Test
Inverse variance weighted (fixed effects)
Inverse variance weighted (multiplicative random effects)
MR Egger
MR RAPS
Weighted median
Weighted mode
SNP effect on Ulcerative pancolitis
0.1
0.0
−0.1
−0.2
0.03
0.06
0.09
SNP effect on Serotonin (5HT)

Batch 1982 : Gamma−glutamylglutamine on Ulcerative pancolitis

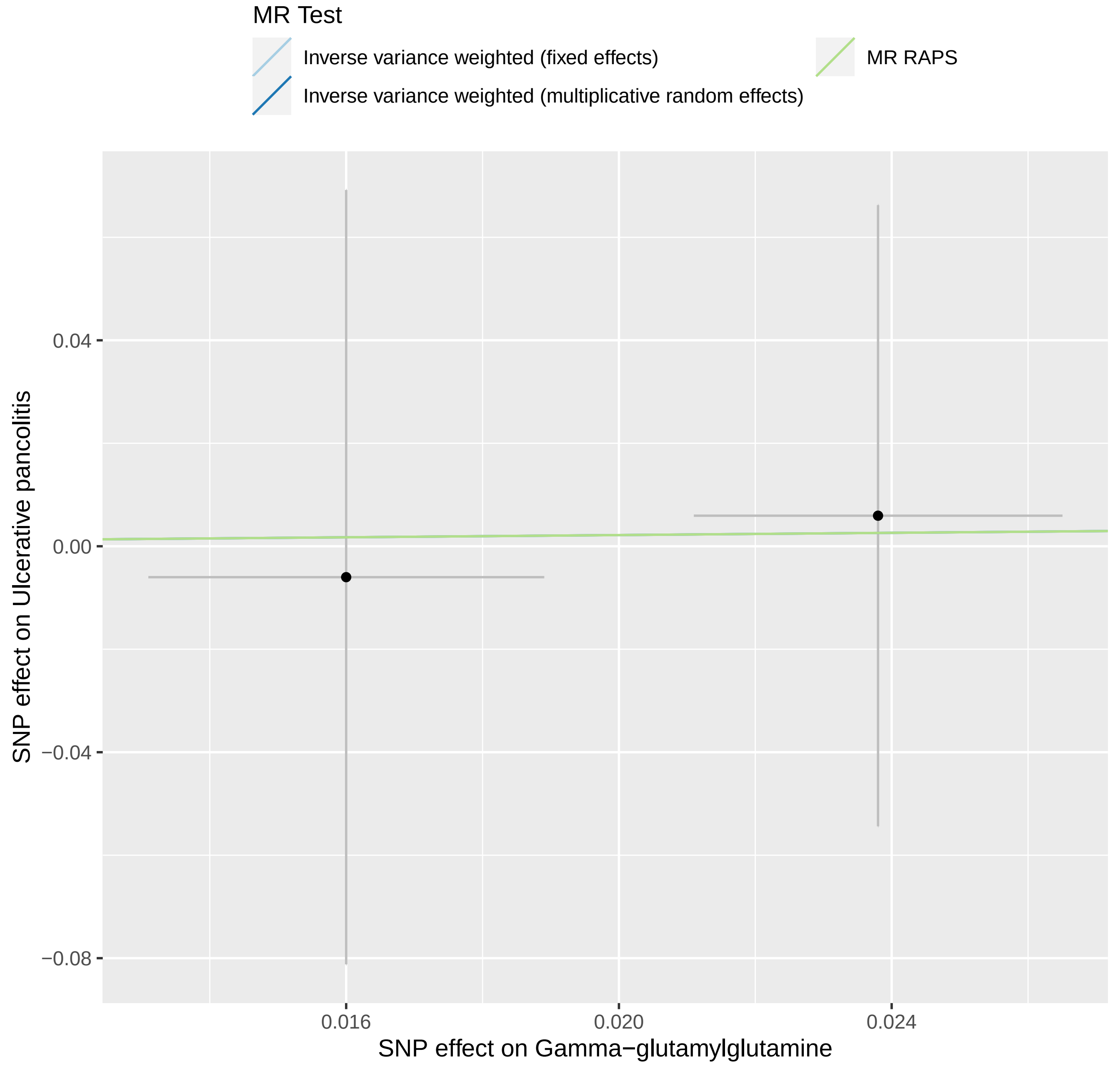

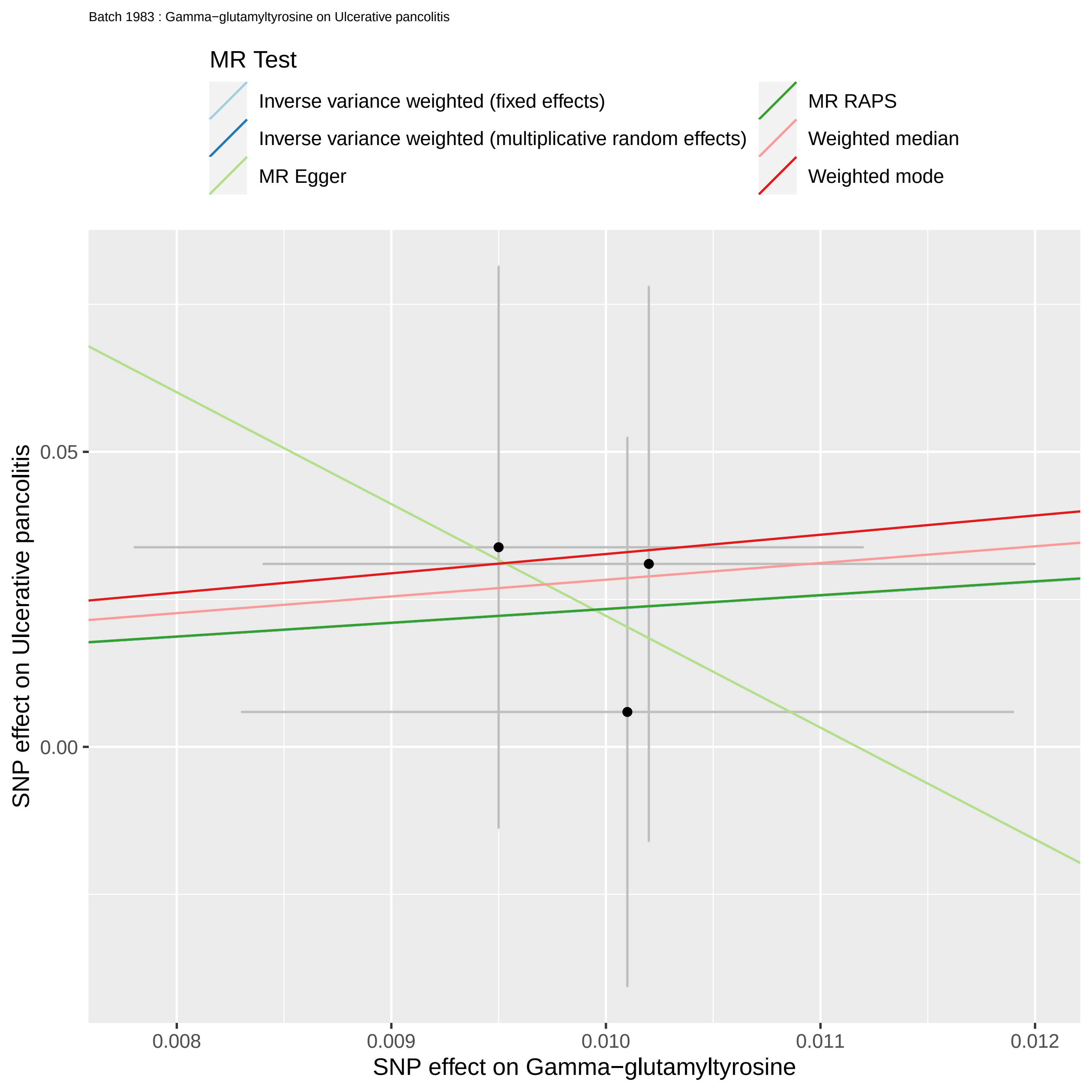
Batch 1983 : Gamma−glutamyltyrosine on Ulcerative pancolitis
MR Test
Inverse variance weighted (fixed effects)
Inverse variance weighted (multiplicative random effects)
MR Egger
MR RAPS
Weighted median
Weighted mode
0.05
0.00
SNP effect on Ulcerative pancolitis
0.008
0.009
0.010
0.011
0.012
SNP effect on Gamma−glutamyltyrosine

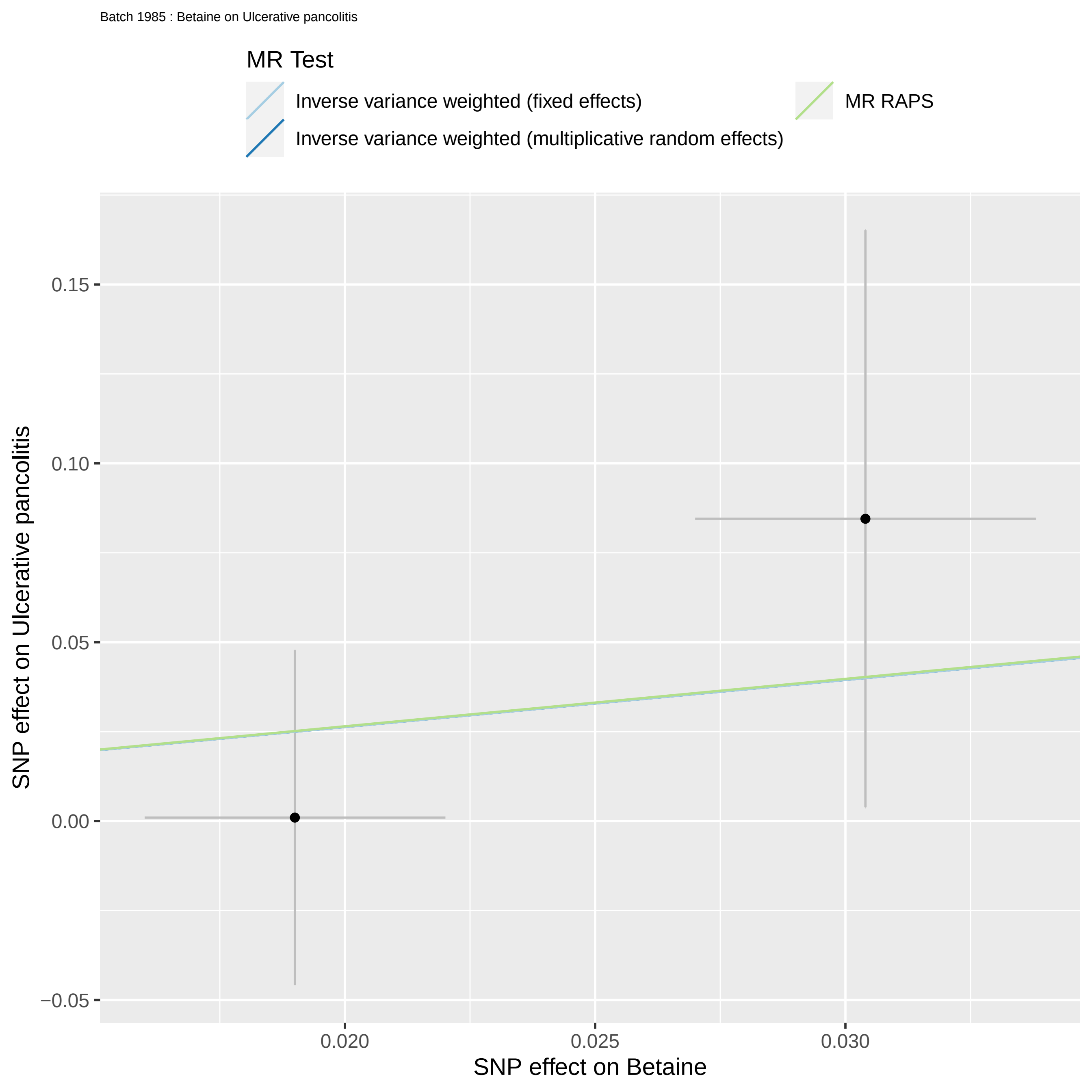

Batch 1985 : Betaine on Ulcerative pancolitis
MR Test
Inverse variance weighted (fixed effects)
Inverse variance weighted (multiplicative random effects)
MR RAPS
0.15
0.10
0.05
0.00
−0.05
SNP effect on Ulcerative pancolitis
0.020
0.025
0.030
SNP effect on Betaine

Batch 1986 : Xanthine on Ulcerative pancolitis

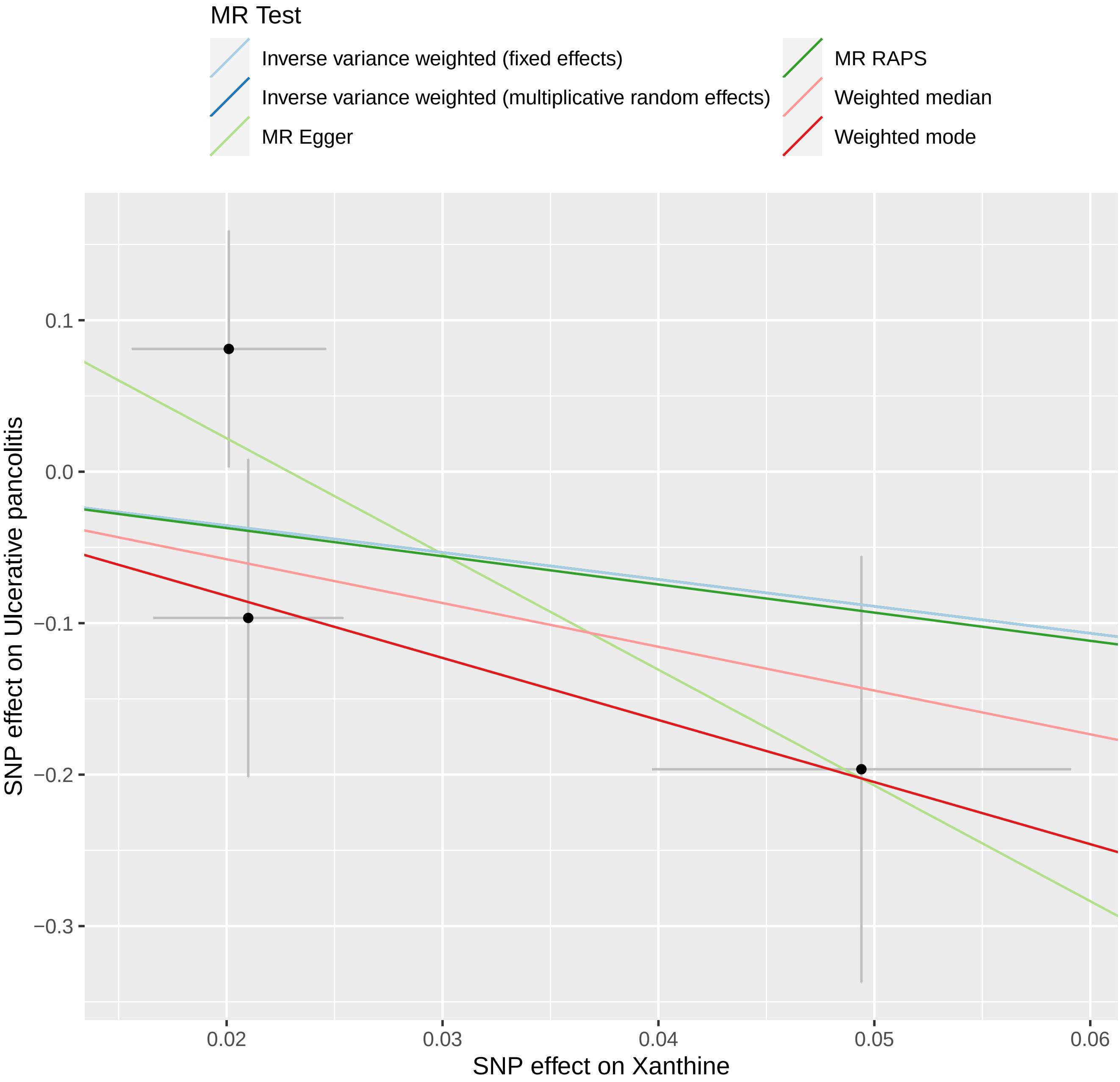

Batch 1987 : Phosphate on Ulcerative pancolitis

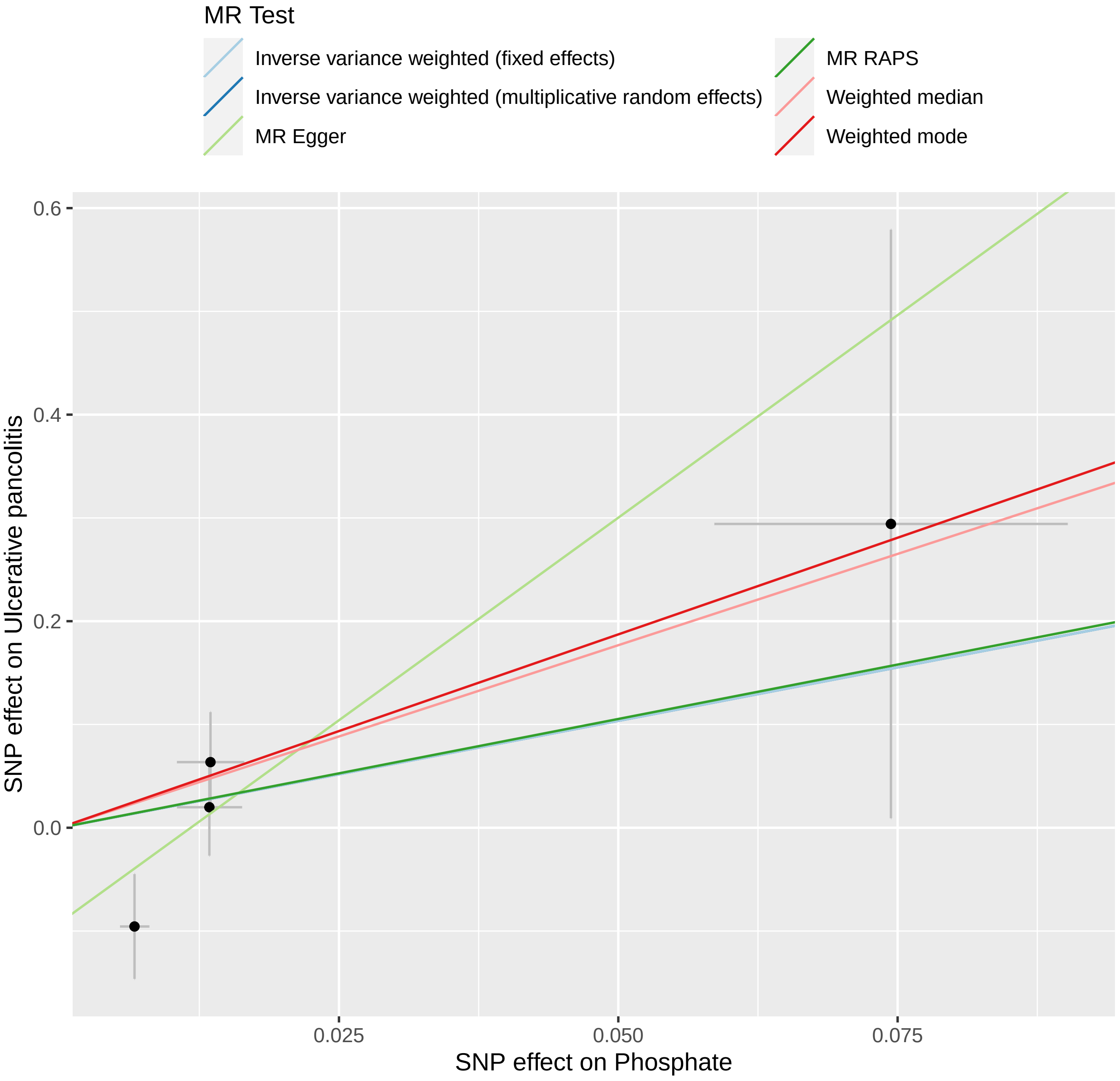

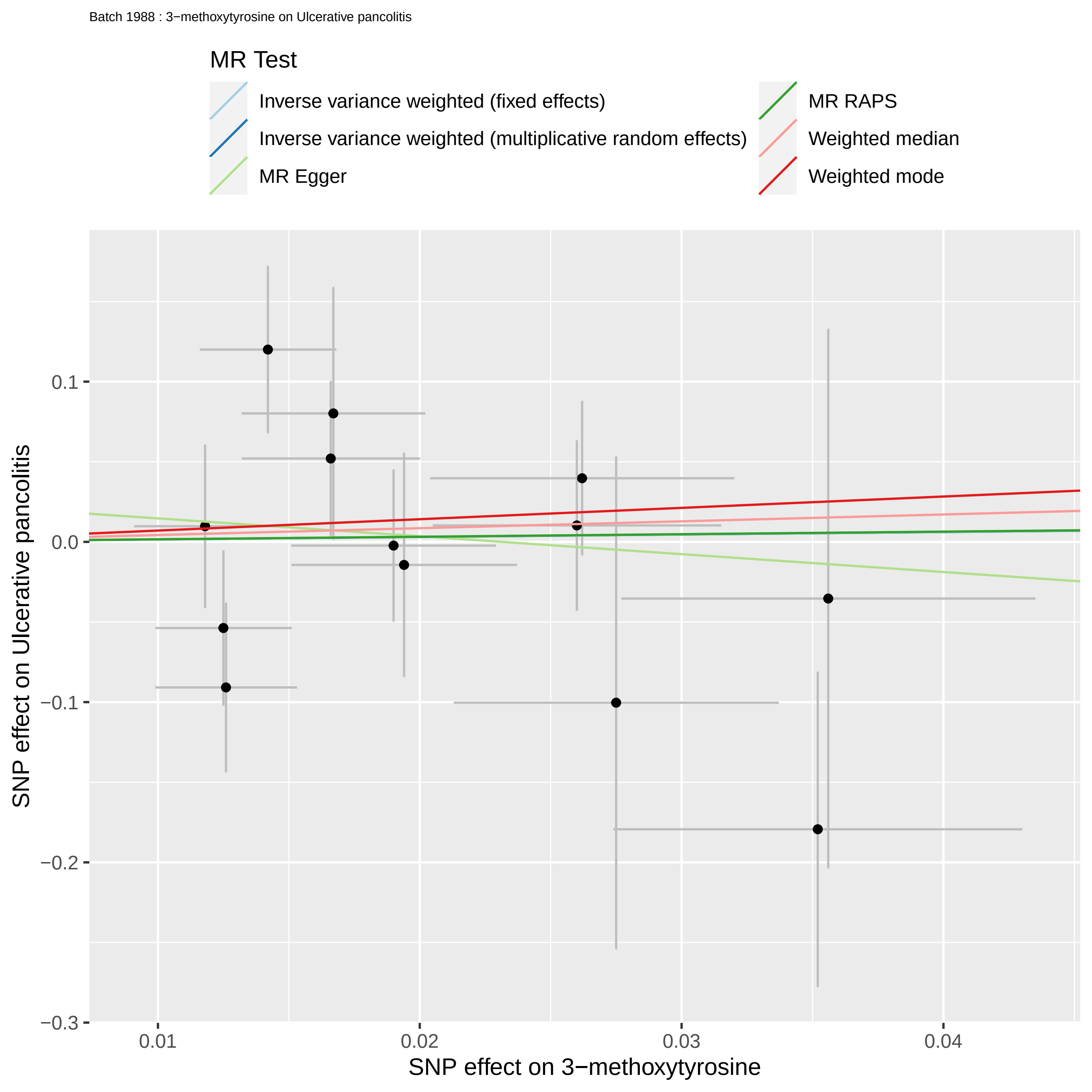

Batch 1988 : 3−methoxytyrosine on Ulcerative pancolitis
MR Test
Inverse variance weighted (fixed effects)
Inverse variance weighted (multiplicative random effects)
MR Egger
MR RAPS
Weighted median
Weighted mode
SNP effect on Ulcerative pancolitis
0.1
0.0
−0.1
−0.2
−0.3
0.01
0.02
0.03
0.04
SNP effect on 3−methoxytyrosine

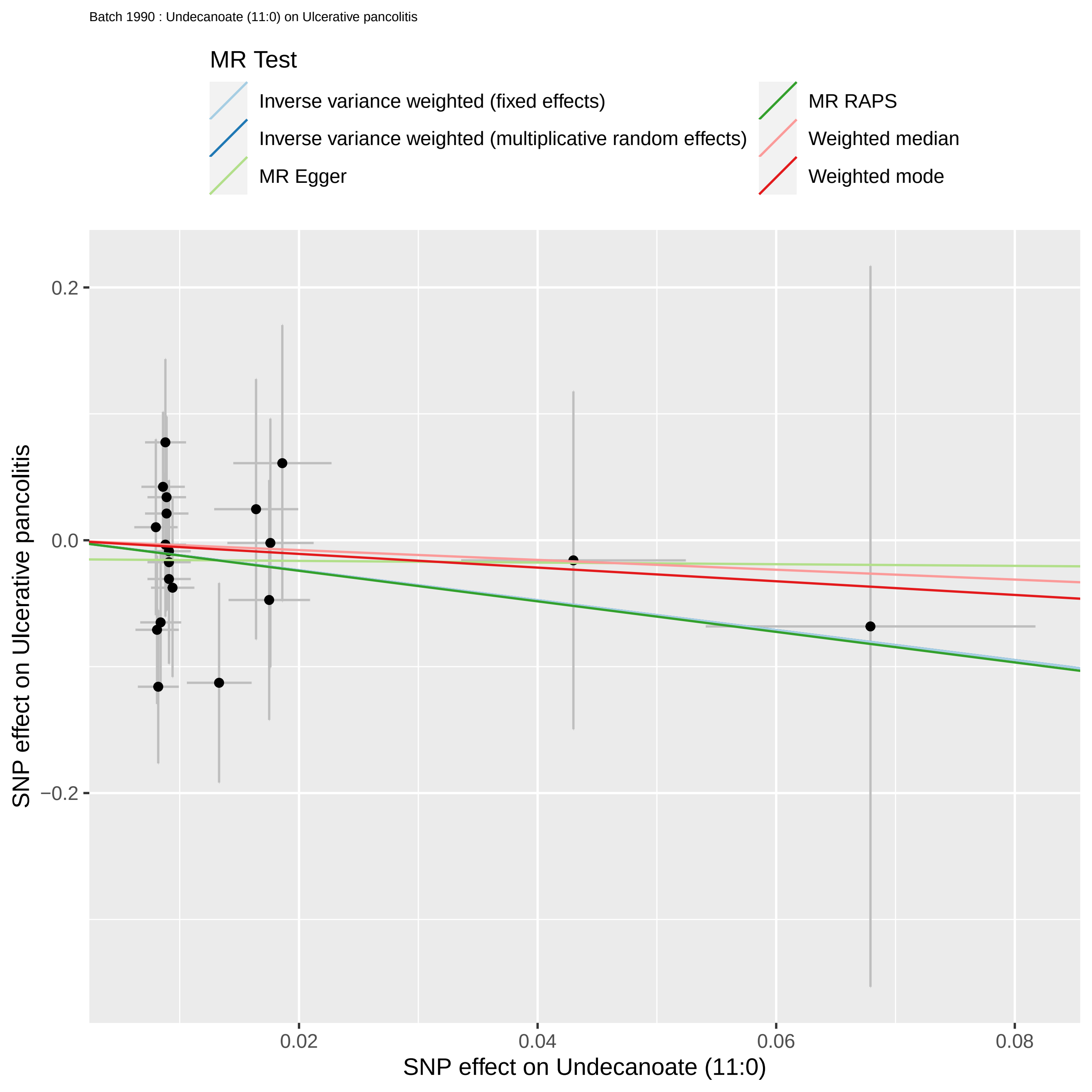

Batch 1990 : Undecanoate (11:0) on Ulcerative pancolitis
MR Test
Inverse variance weighted (fixed effects)
Inverse variance weighted (multiplicative random effects)
MR Egger
MR RAPS
Weighted median
Weighted mode
0.2
0.0
−0.2
0.02
0.04
0.06
0.08
SNP effect on Ulcerative pancolitis
SNP effect on Undecanoate (11:0)

Batch 1992 : Taurodeoxycholate on Ulcerative pancolitis

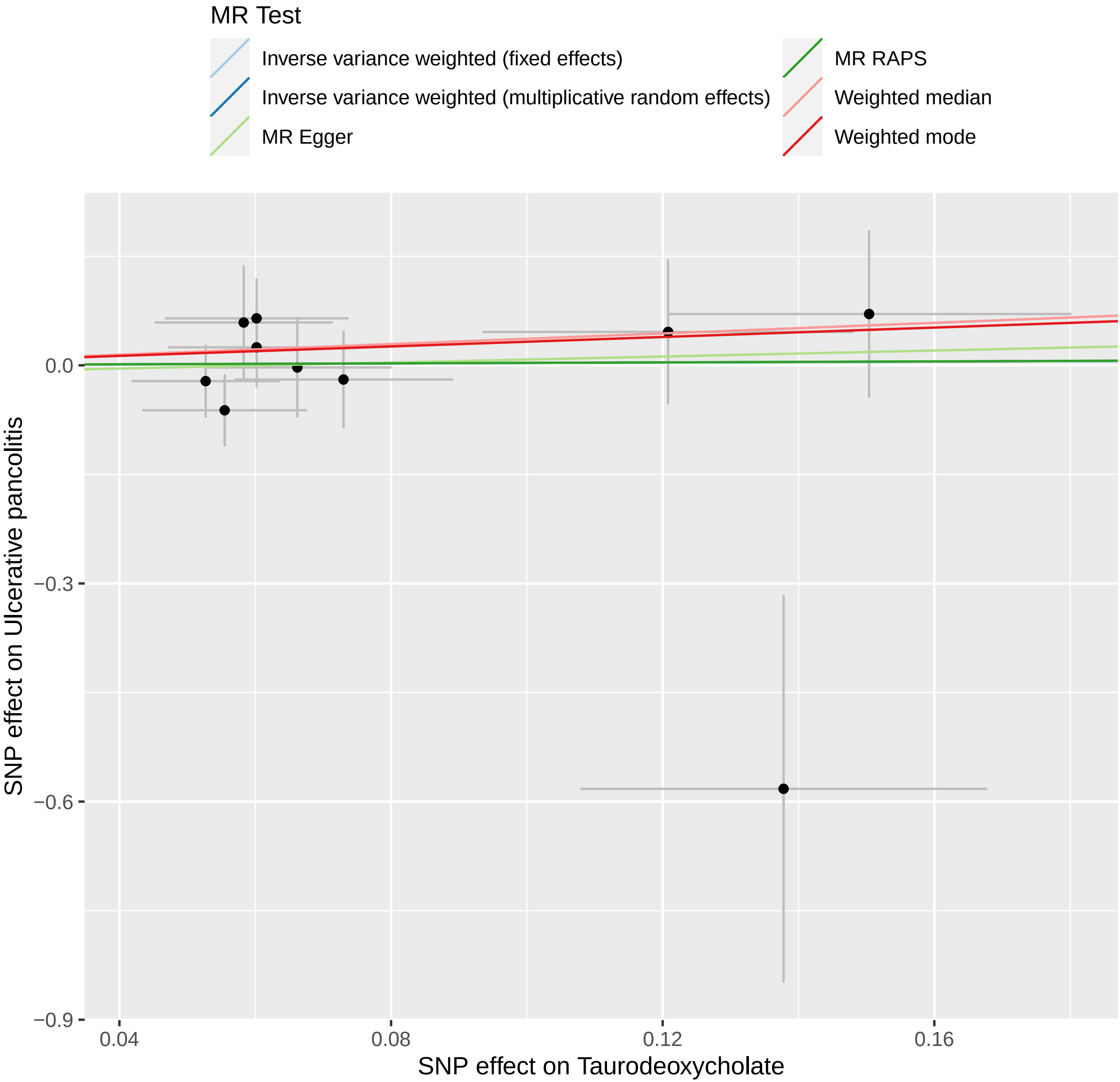

Batch 1993 : Glycerol on Ulcerative pancolitis

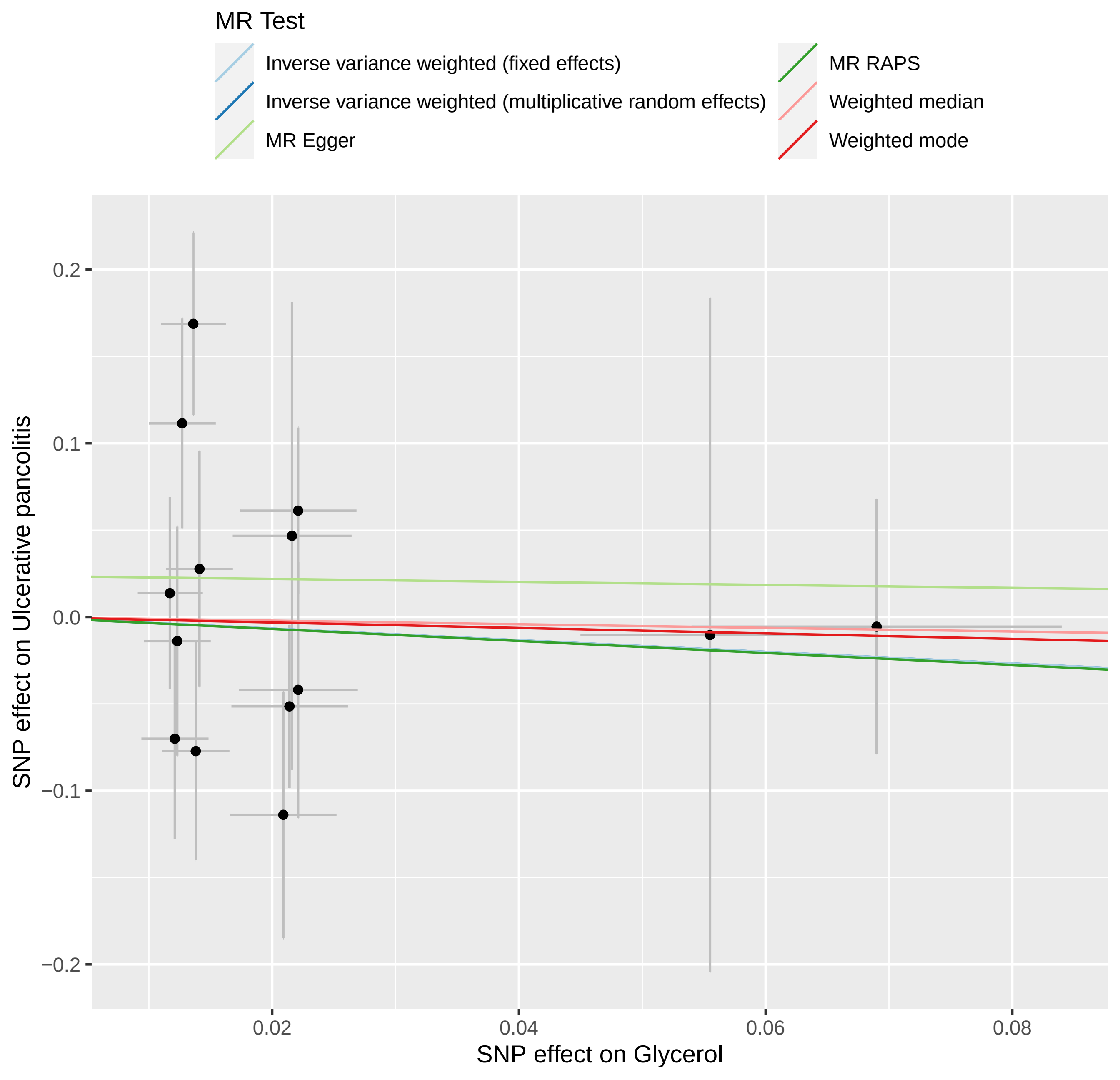

Batch 1994 : Kynurenine on Ulcerative pancolitis

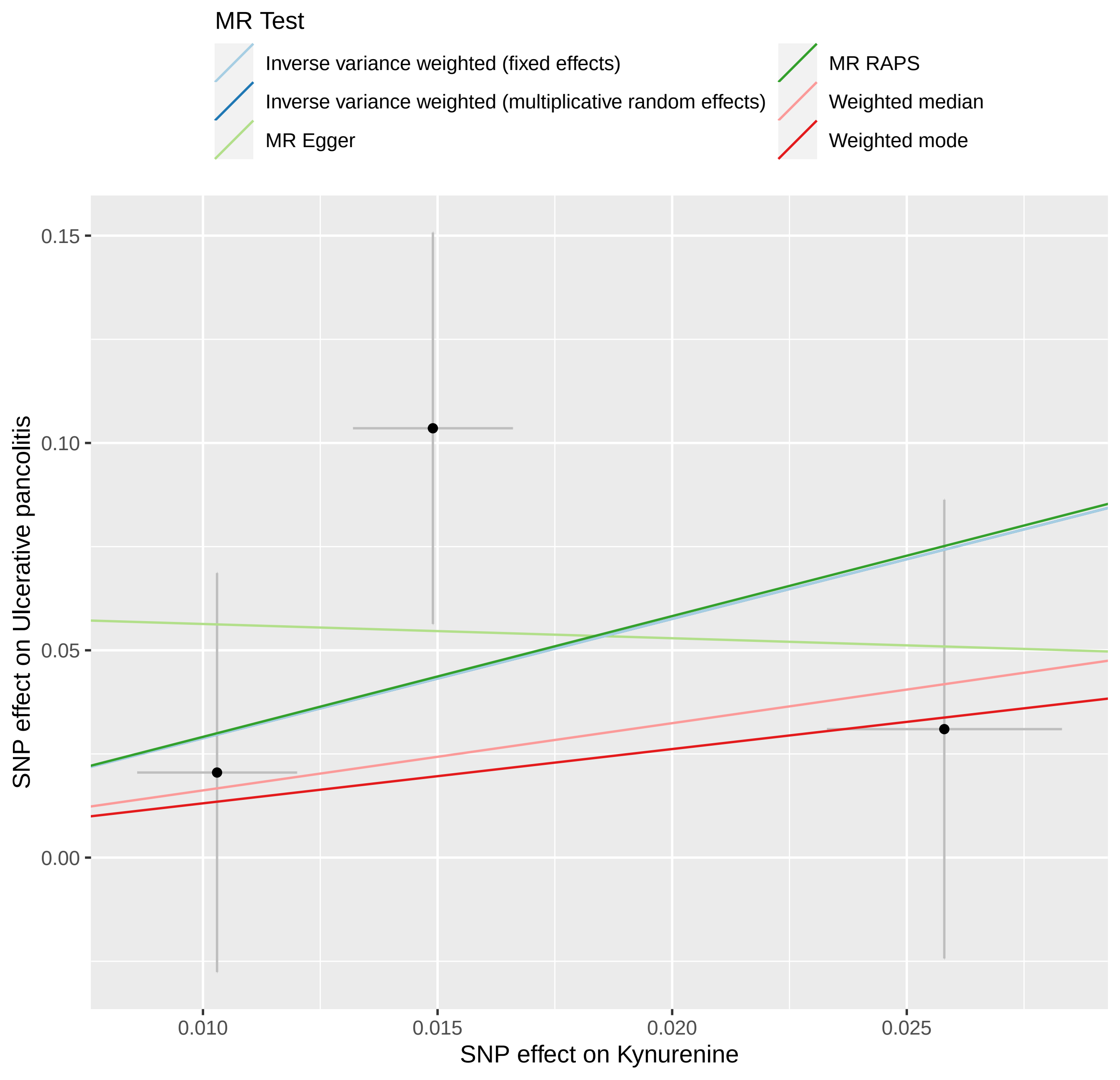

Batch 1995 : Mannitol on Ulcerative pancolitis

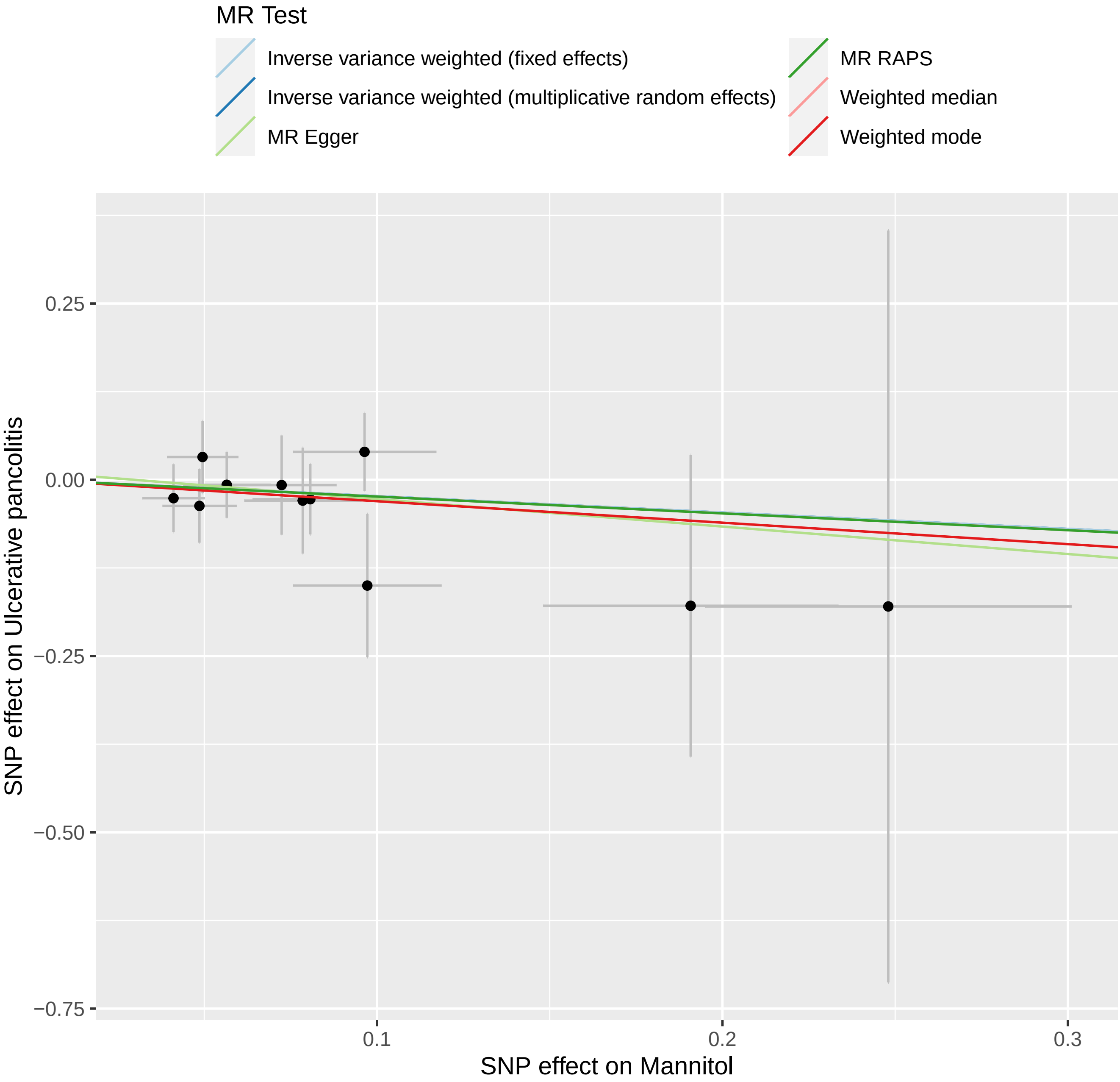

Batch 1996 : Glycerol 3−phosphate (G3P) on Ulcerative pancolitis

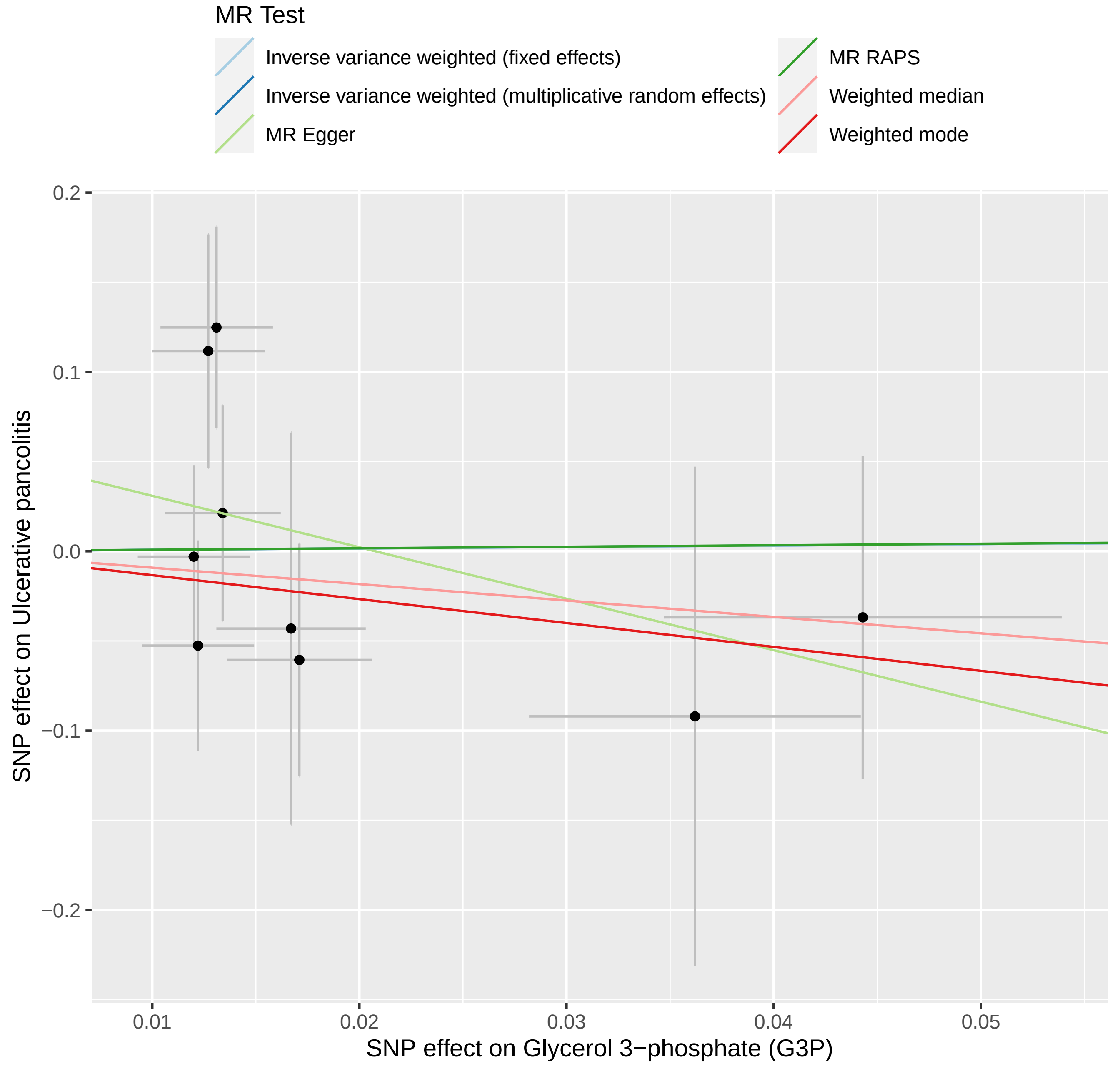

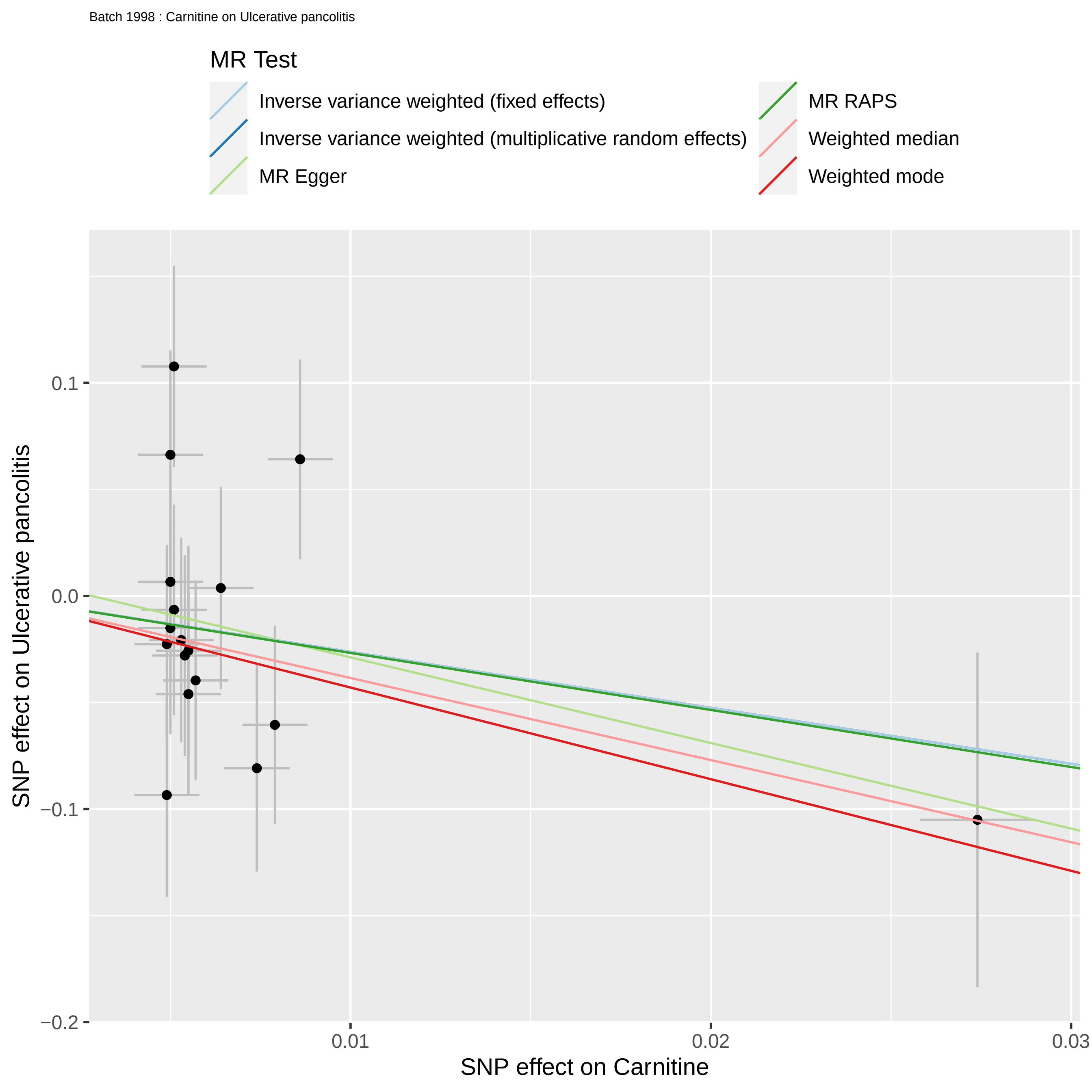

Batch 1998 : Carnitine on Ulcerative pancolitis
MR Test
Inverse variance weighted (fixed effects)
Inverse variance weighted (multiplicative random effects)
MR Egger
MR RAPS
Weighted median
Weighted mode
0.1
0.0
−0.1
−0.2
0.01
0.02
0.03
SNP effect on Carnitine
SNP effect on Ulcerative pancolitis

Batch 1999 : Choline on Ulcerative pancolitis

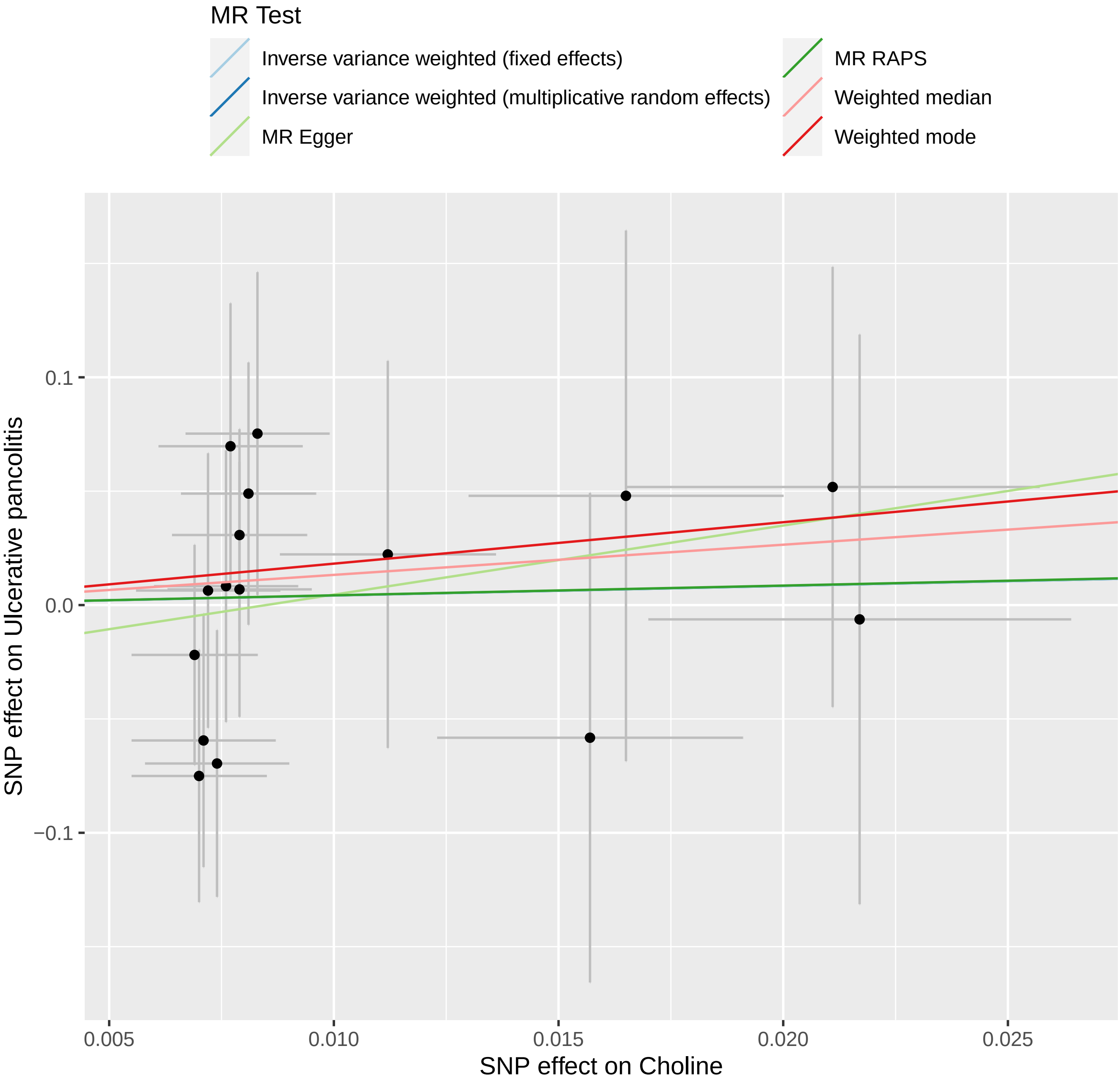

Batch 2002 : 3−methyl−2−oxovalerate on Ulcerative pancolitis

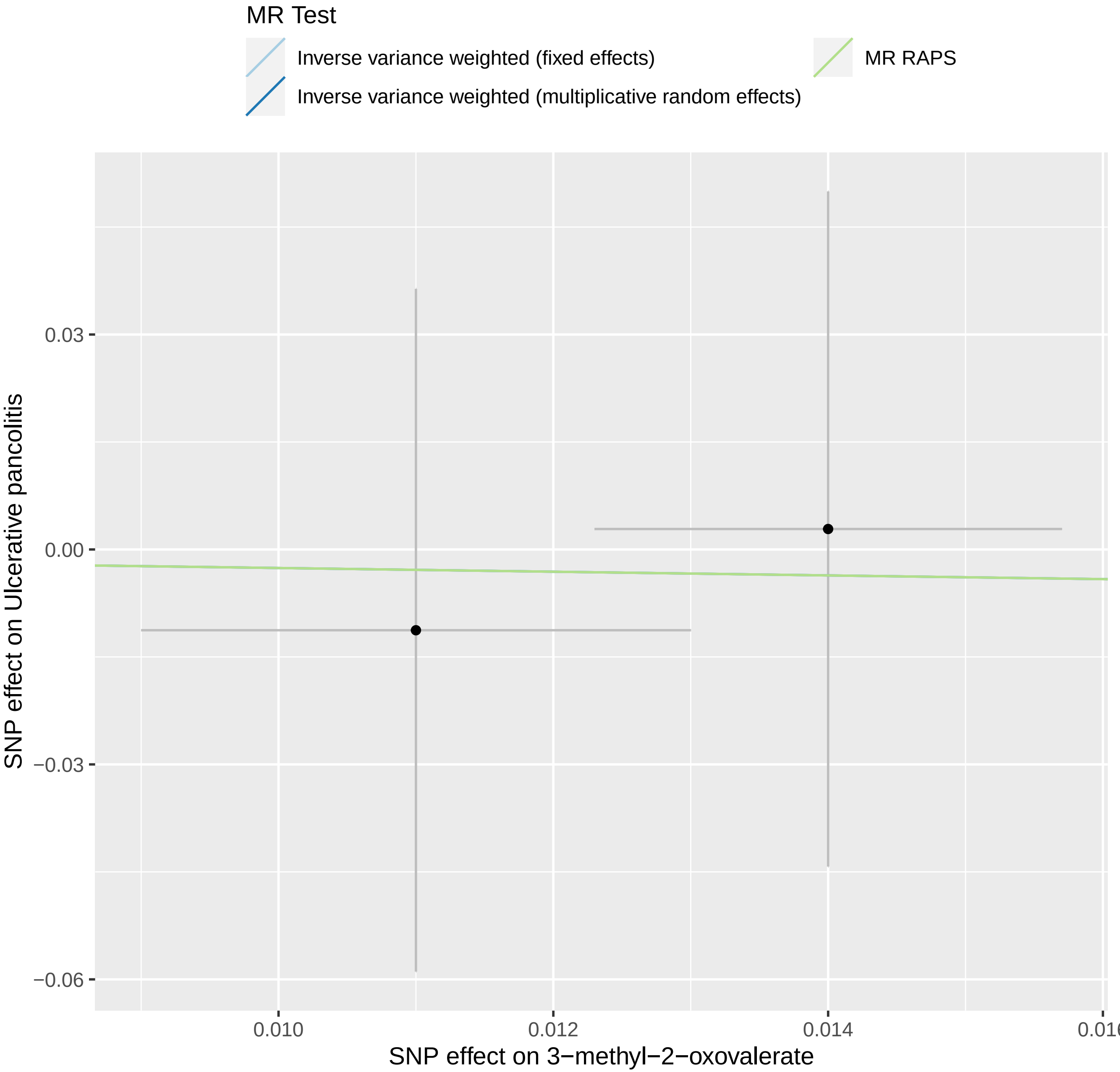

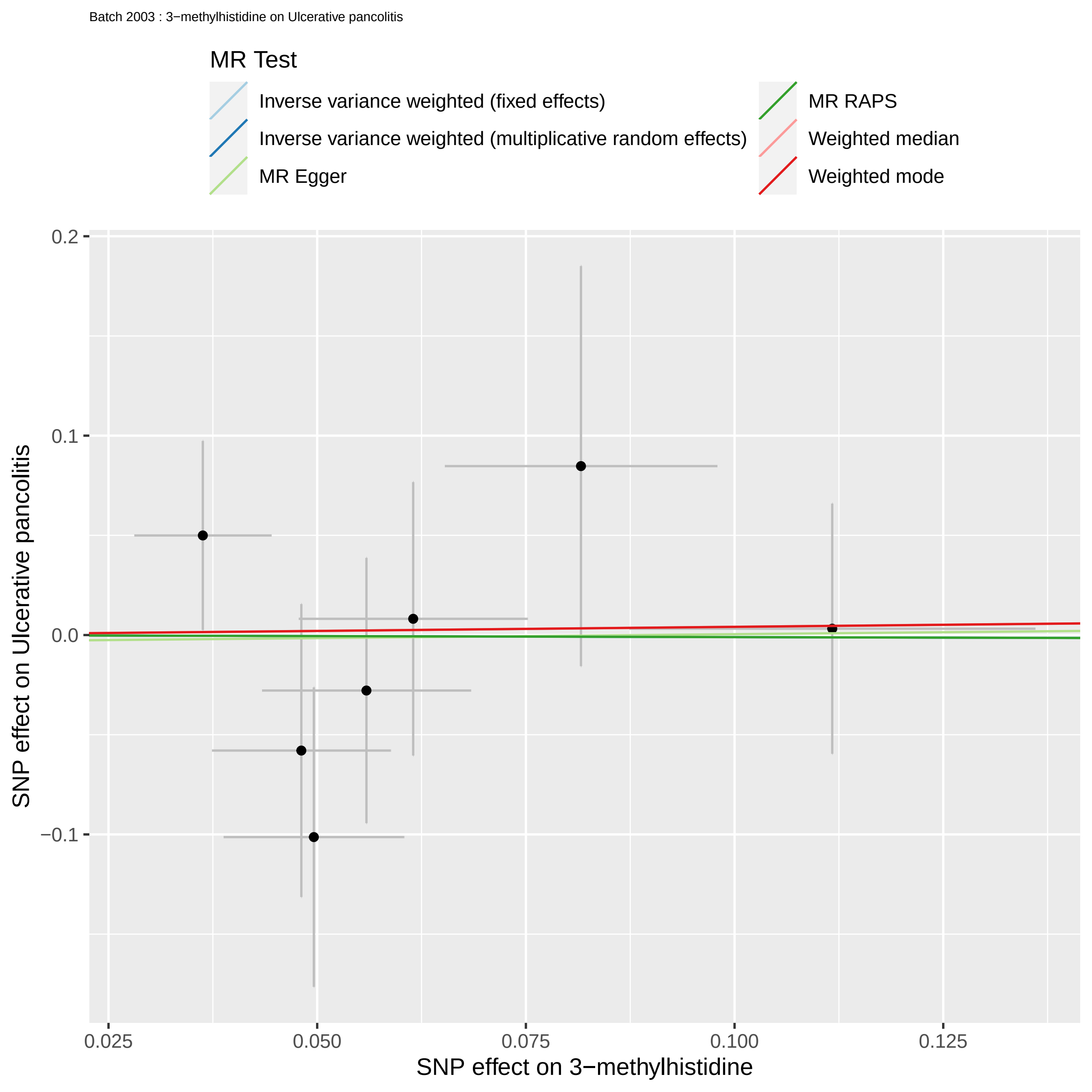
Batch 2003 : 3−methylhistidine on Ulcerative pancolitis
MR Test
Inverse variance weighted (fixed effects)
Inverse variance weighted (multiplicative random effects)
MR Egger
MR RAPS
Weighted median
Weighted mode
0.2
0.1
0.0
−0.1
0.025
0.050
0.075
0.100
0.125
SNP effect on Ulcerative pancolitis
SNP effect on 3−methylhistidine

Batch 2005 : Phenylacetate on Ulcerative pancolitis

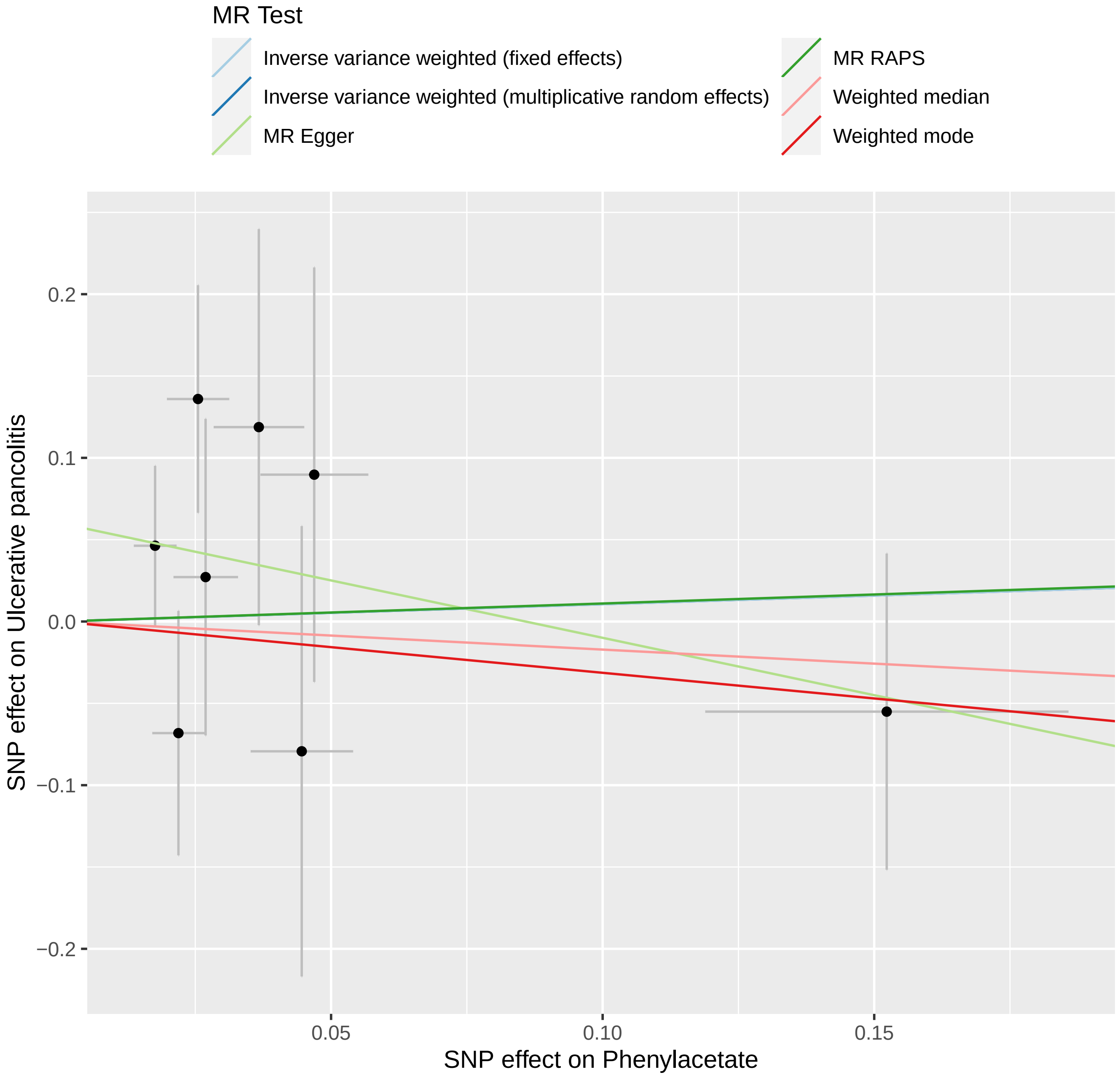

Batch 2006 : Glycerophosphorylcholine (GPC) on Ulcerative pancolitis

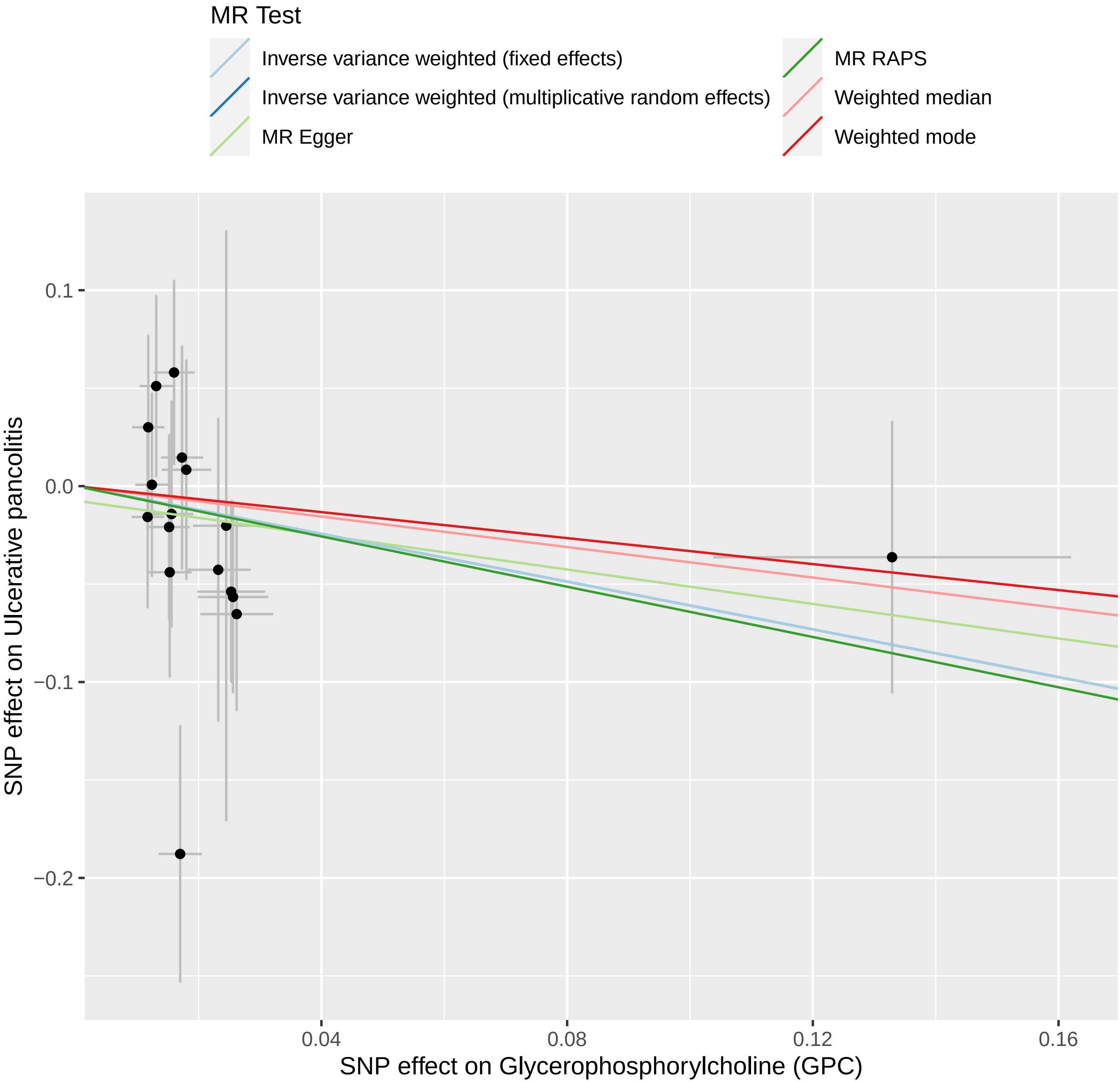

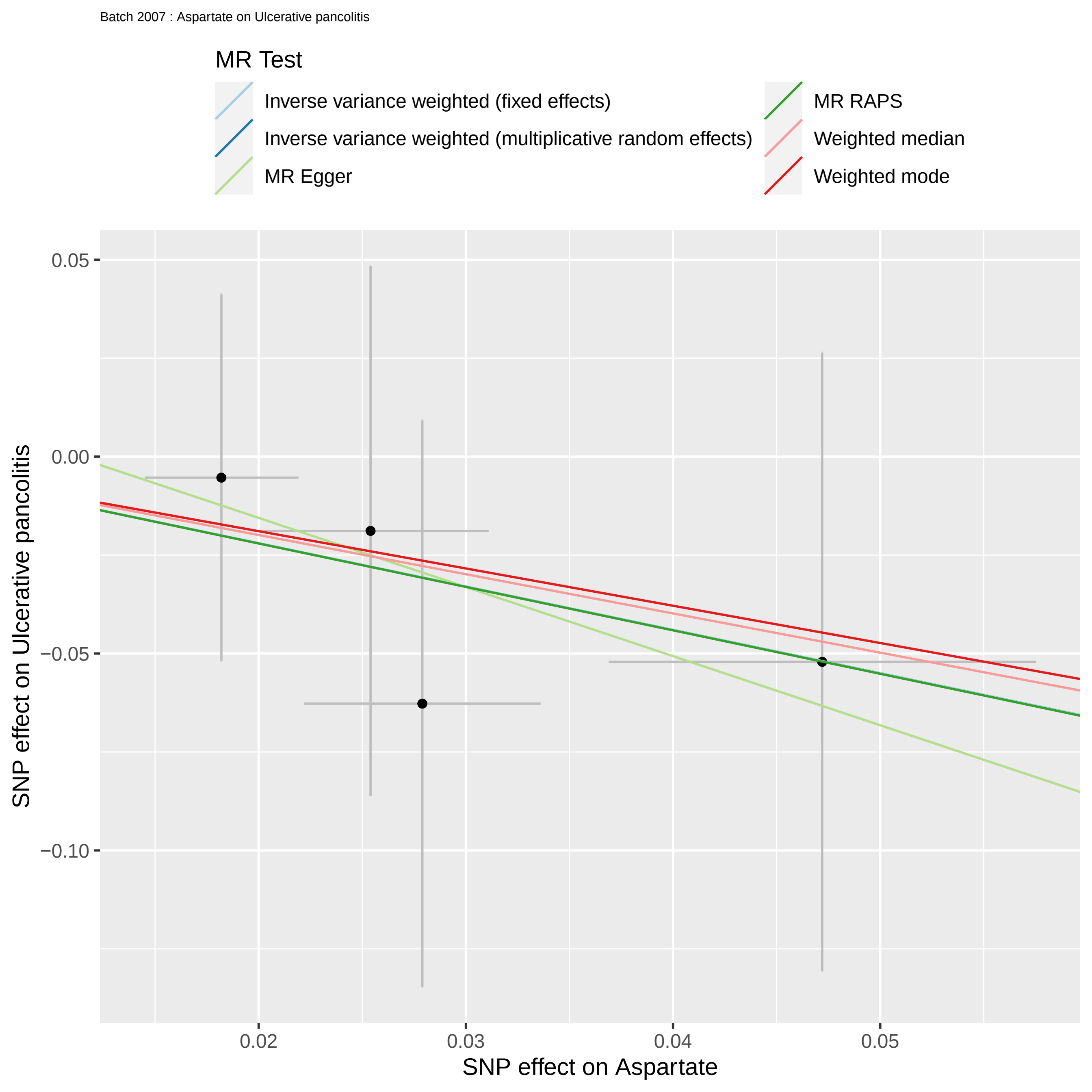

Batch 2007 : Aspartate on Ulcerative pancolitis
MR Test
Inverse variance weighted (fixed effects)
Inverse variance weighted (multiplicative random effects)
MR Egger
MR RAPS
Weighted median
Weighted mode
0.05
0.00
−0.05
−0.10
0.02
0.03
0.04
0.05
SNP effect on Ulcerative pancolitis
SNP effect on Aspartate

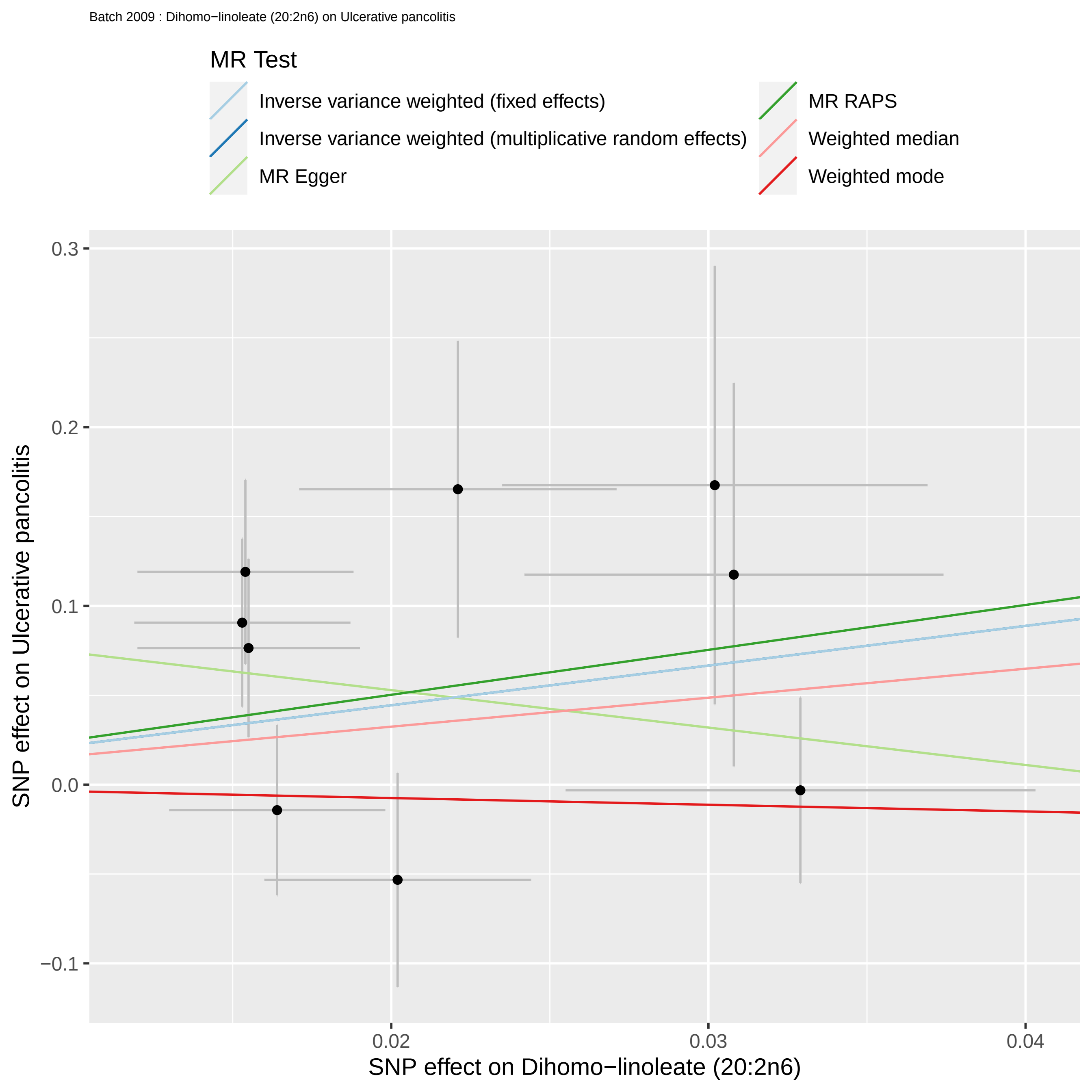

Batch 2009 : Dihomo−linoleate (20:2n6) on Ulcerative pancolitis
MR Test
Inverse variance weighted (fixed effects)
Inverse variance weighted (multiplicative random effects)
MR Egger
MR RAPS
Weighted median
Weighted mode
0.3
0.2
0.1
0.0
−0.1
0.02
0.03
0.04
SNP effect on Ulcerative pancolitis
SNP effect on Dihomo−linoleate (20:2n6)

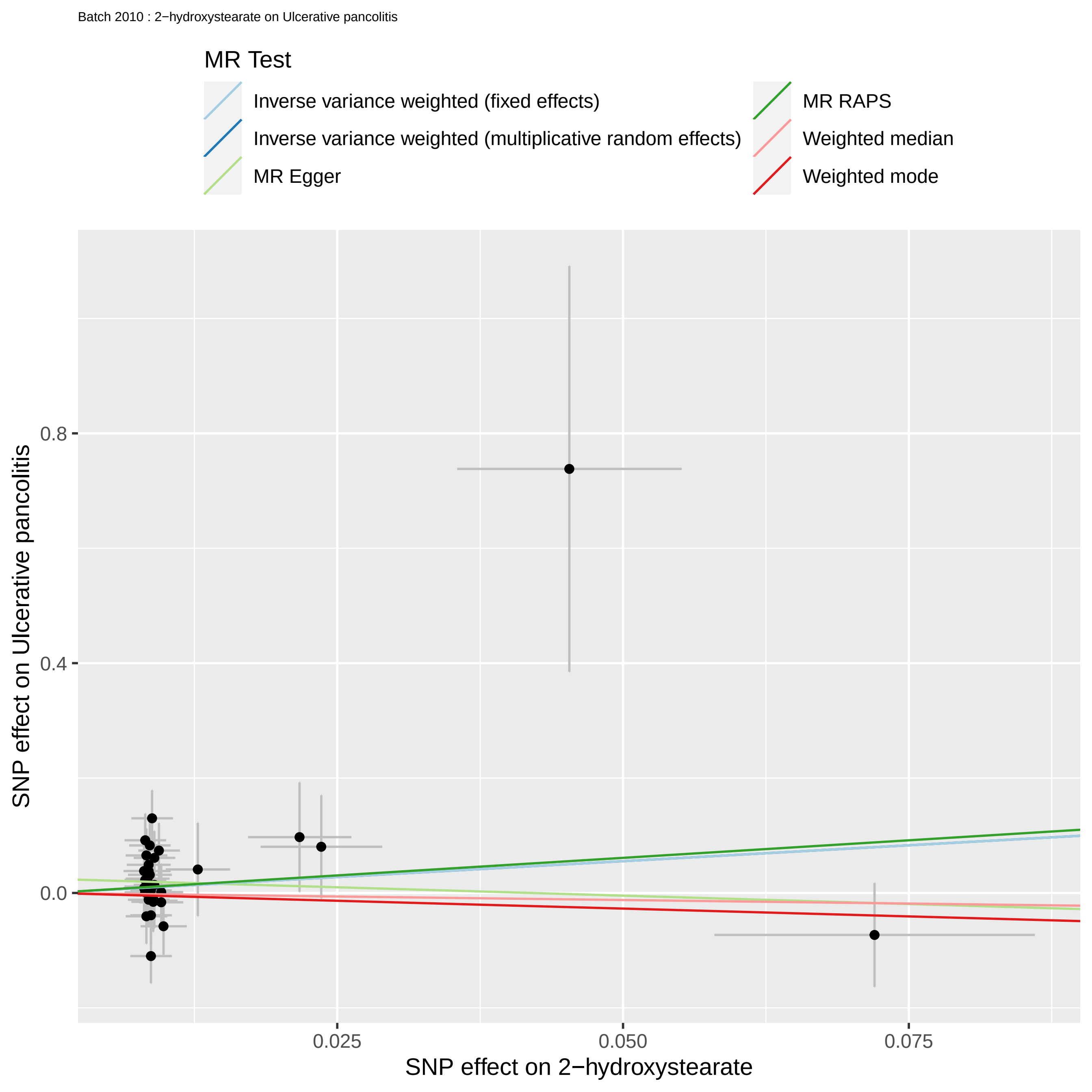

Batch 2010 : 2−hydroxystearate on Ulcerative pancolitis
MR Test
Inverse variance weighted (fixed effects)
Inverse variance weighted (multiplicative random effects)
MR Egger
MR RAPS
Weighted median
Weighted mode
SNP effect on Ulcerative pancolitis
0.0
0.4
0.8
0.025
0.050
0.075
SNP effect on 2−hydroxystearate

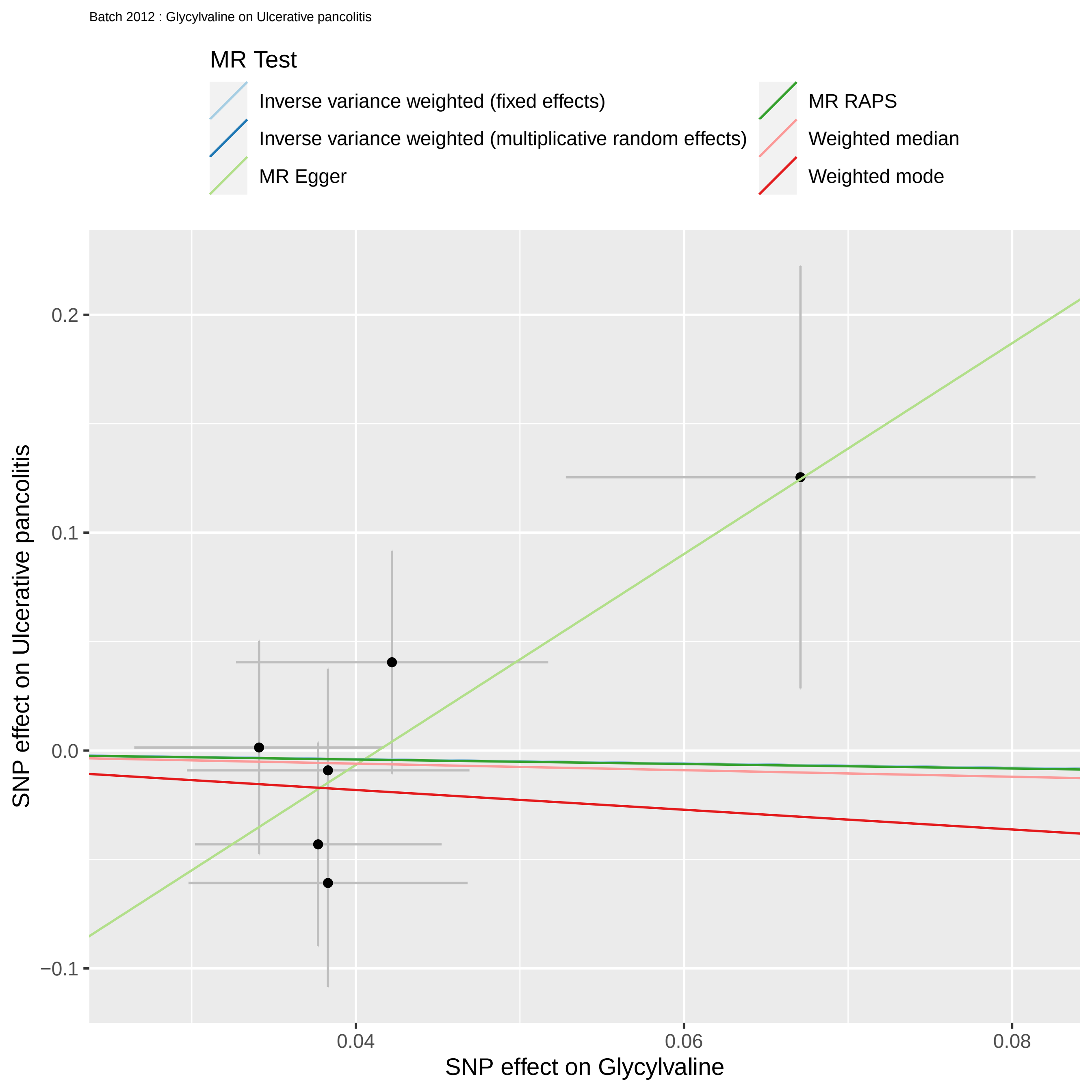

Batch 2012 : Glycylvaline on Ulcerative pancolitis
MR Test
Inverse variance weighted (fixed effects)
Inverse variance weighted (multiplicative random effects)
MR Egger
MR RAPS
Weighted median
Weighted mode
0.2
0.1
0.0
−0.1
0.04
0.06
0.08
SNP effect on Ulcerative pancolitis
SNP effect on Glycylvaline

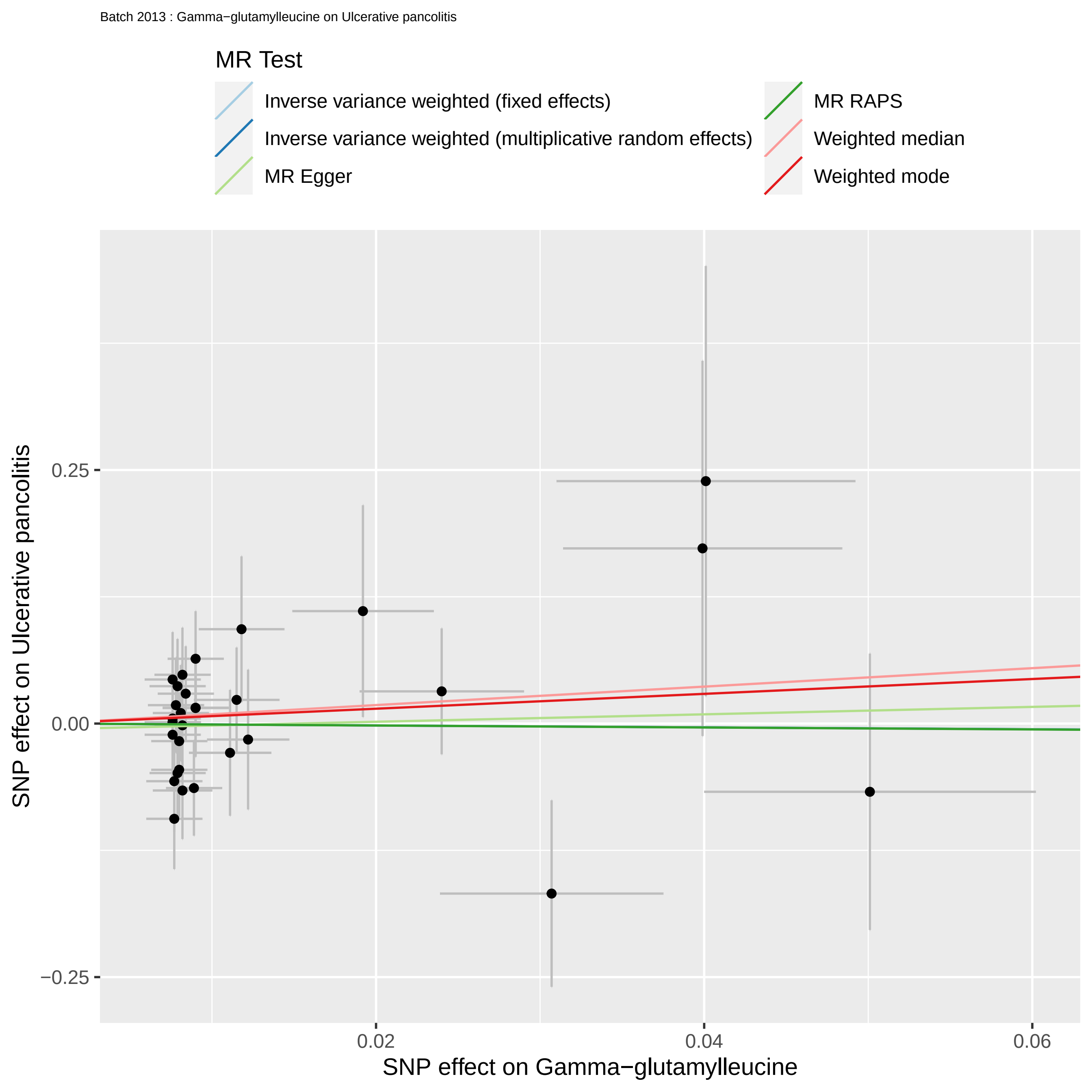
Batch 2013 : Gamma−glutamylleucine on Ulcerative pancolitis
MR Test
Inverse variance weighted (fixed effects)
Inverse variance weighted (multiplicative random effects)
MR Egger
MR RAPS
Weighted median
Weighted mode
SNP effect on Ulcerative pancolitis
0.25
0.00
−0.25
0.02
0.04
0.06
SNP effect on Gamma−glutamylleucine

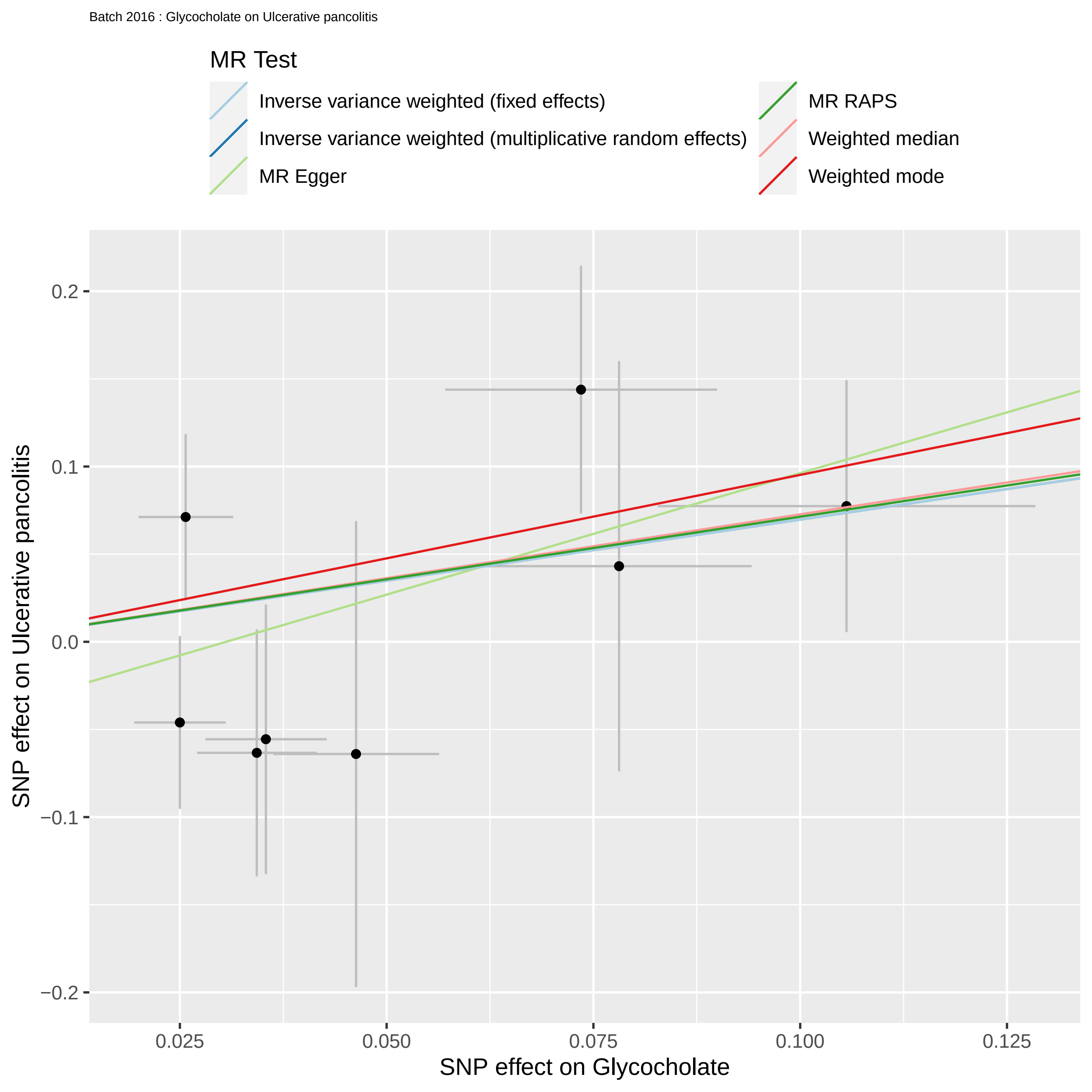

Batch 2016 : Glycocholate on Ulcerative pancolitis
MR Test
Inverse variance weighted (fixed effects)
Inverse variance weighted (multiplicative random effects)
MR Egger
MR RAPS
Weighted median
Weighted mode
0.2
0.1
0.0
−0.1
−0.2
SNP effect on Ulcerative pancolitis
0.025
0.050
0.075
0.100
0.125
SNP effect on Glycocholate

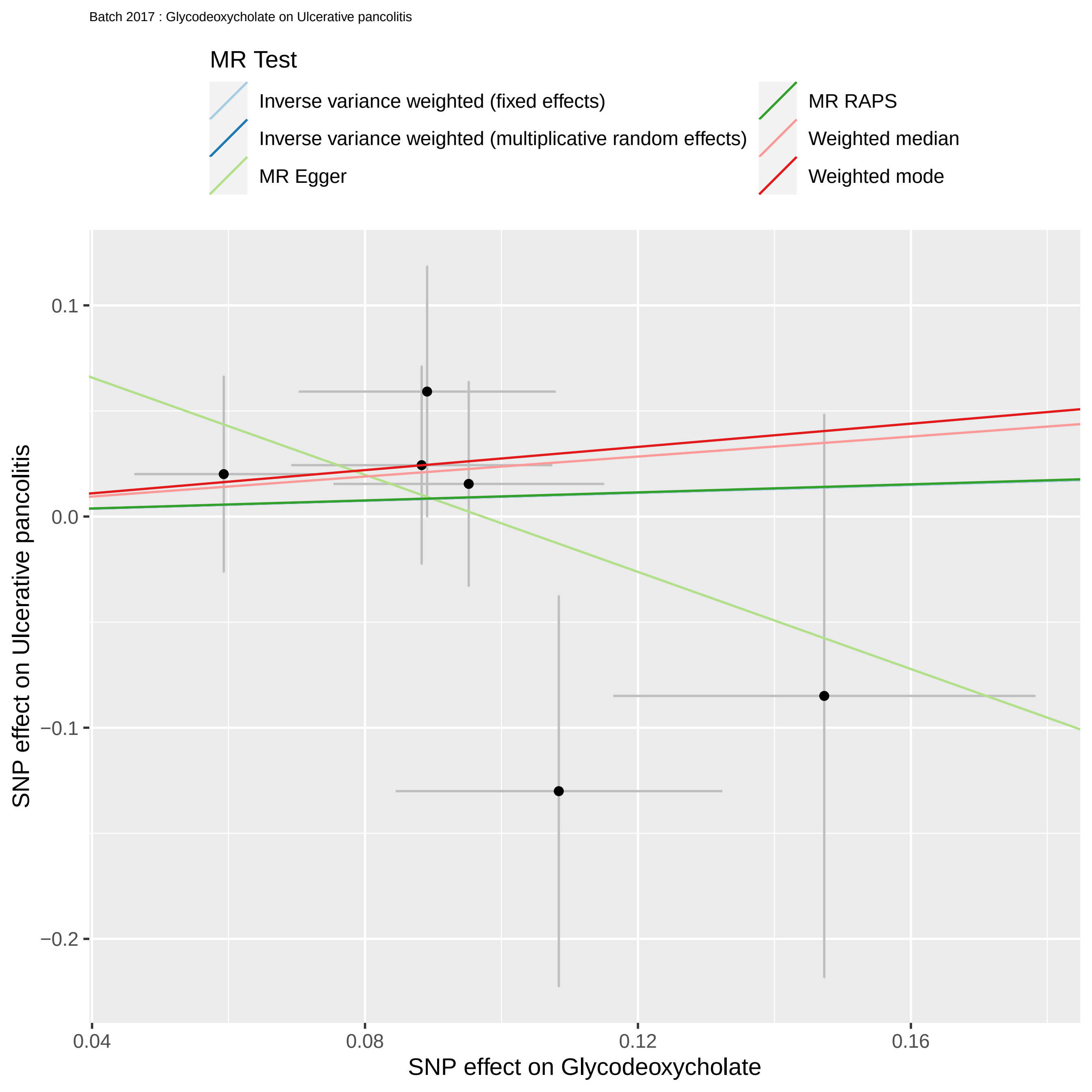

Batch 2017 : Glycodeoxycholate on Ulcerative pancolitis
MR Test
Inverse variance weighted (fixed effects)
Inverse variance weighted (multiplicative random effects)
MR Egger
MR RAPS
Weighted median
Weighted mode
SNP effect on Ulcerative pancolitis
0.1
0.0
−0.1
−0.2
0.04
0.08
0.12
0.16
SNP effect on Glycodeoxycholate

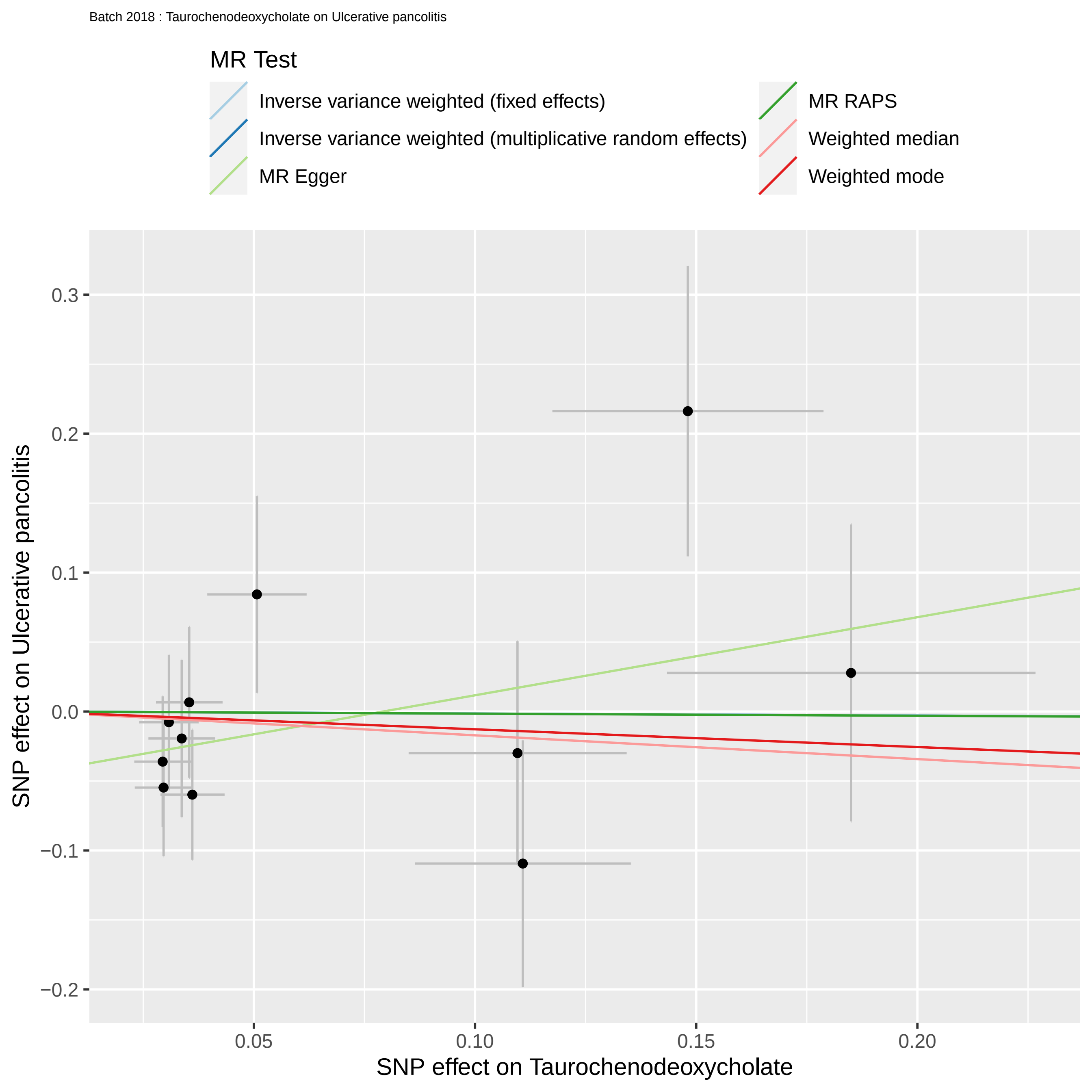
Batch 2018 : Taurochenodeoxycholate on Ulcerative pancolitis
MR Test
Inverse variance weighted (fixed effects)
Inverse variance weighted (multiplicative random effects)
MR Egger
MR RAPS
Weighted median
Weighted mode
SNP effect on Ulcerative pancolitis
0.3
0.2
0.1
0.0
−0.1
−0.2
0.05
0.10
0.15
0.20
SNP effect on Taurochenodeoxycholate

Batch 2019 : Taurocholate on Ulcerative pancolitis

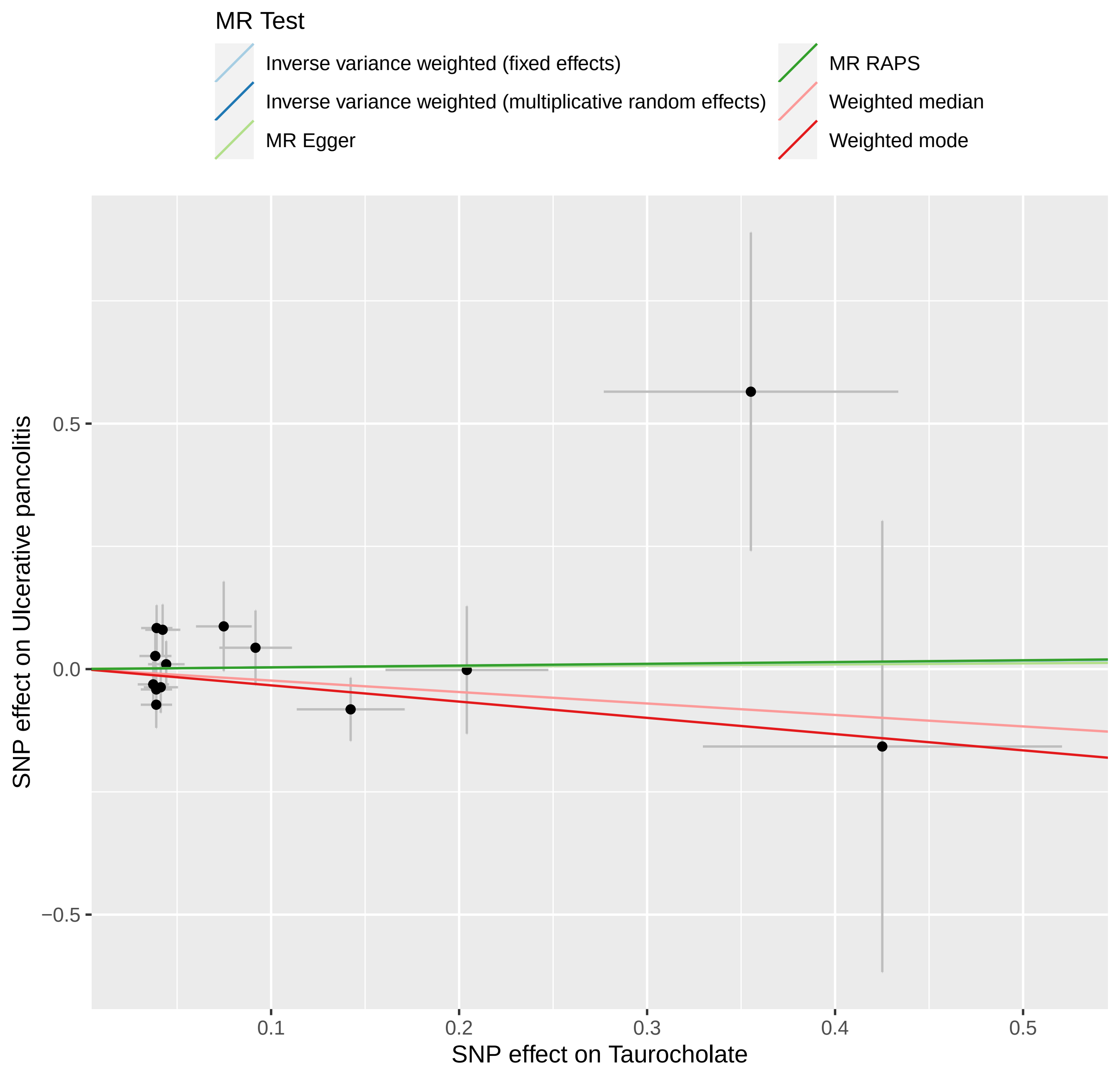

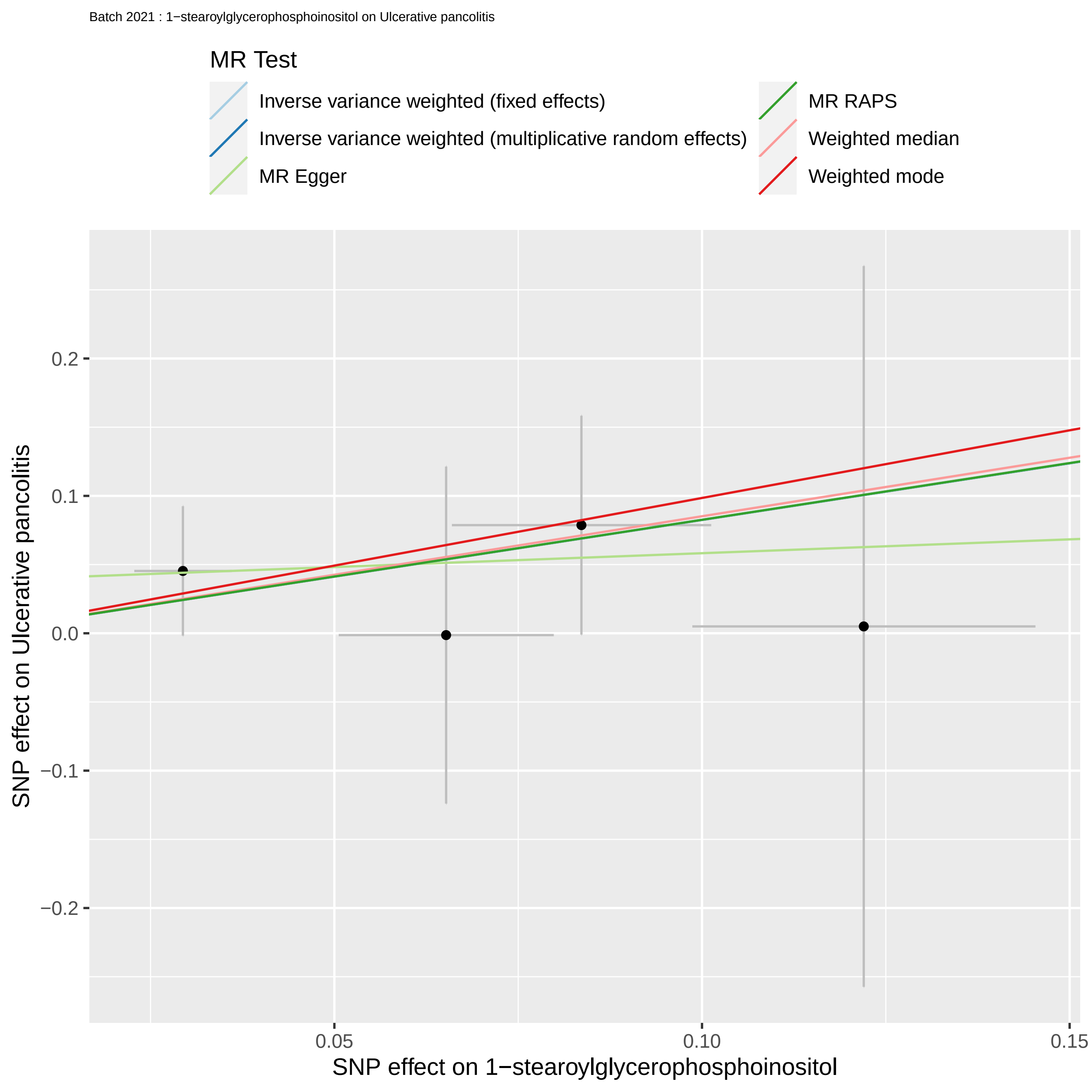

Batch 2021 : 1−stearoylglycerophosphoinositol on Ulcerative pancolitis
MR Test
Inverse variance weighted (fixed effects)
Inverse variance weighted (multiplicative random effects)
MR Egger
MR RAPS
Weighted median
Weighted mode
0.2
0.1
0.0
−0.1
−0.2
0.05
0.10
0.15
SNP effect on Ulcerative pancolitis
SNP effect on 1−stearoylglycerophosphoinositol

Batch 2022 : Myo−inositol on Ulcerative pancolitis

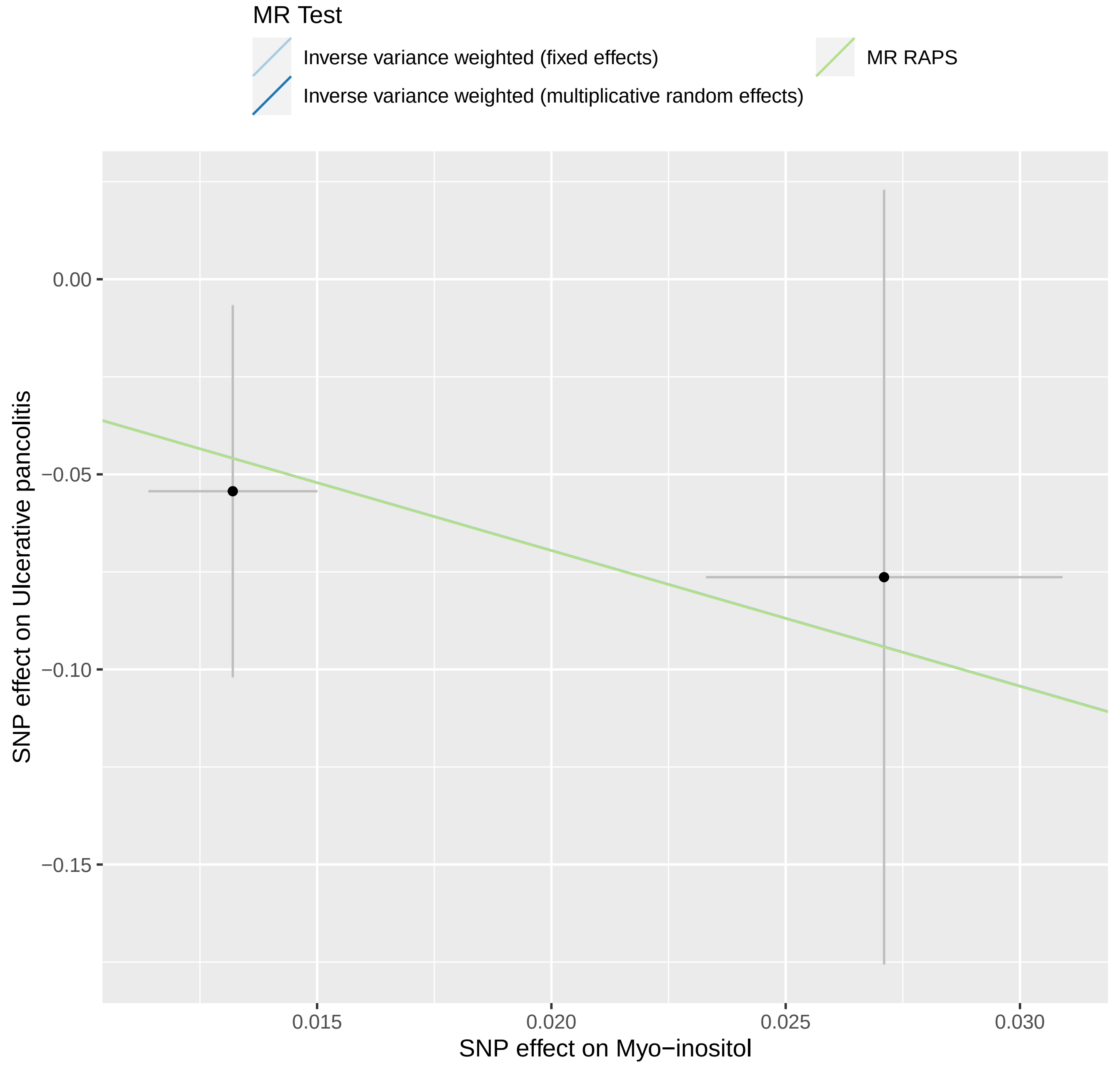

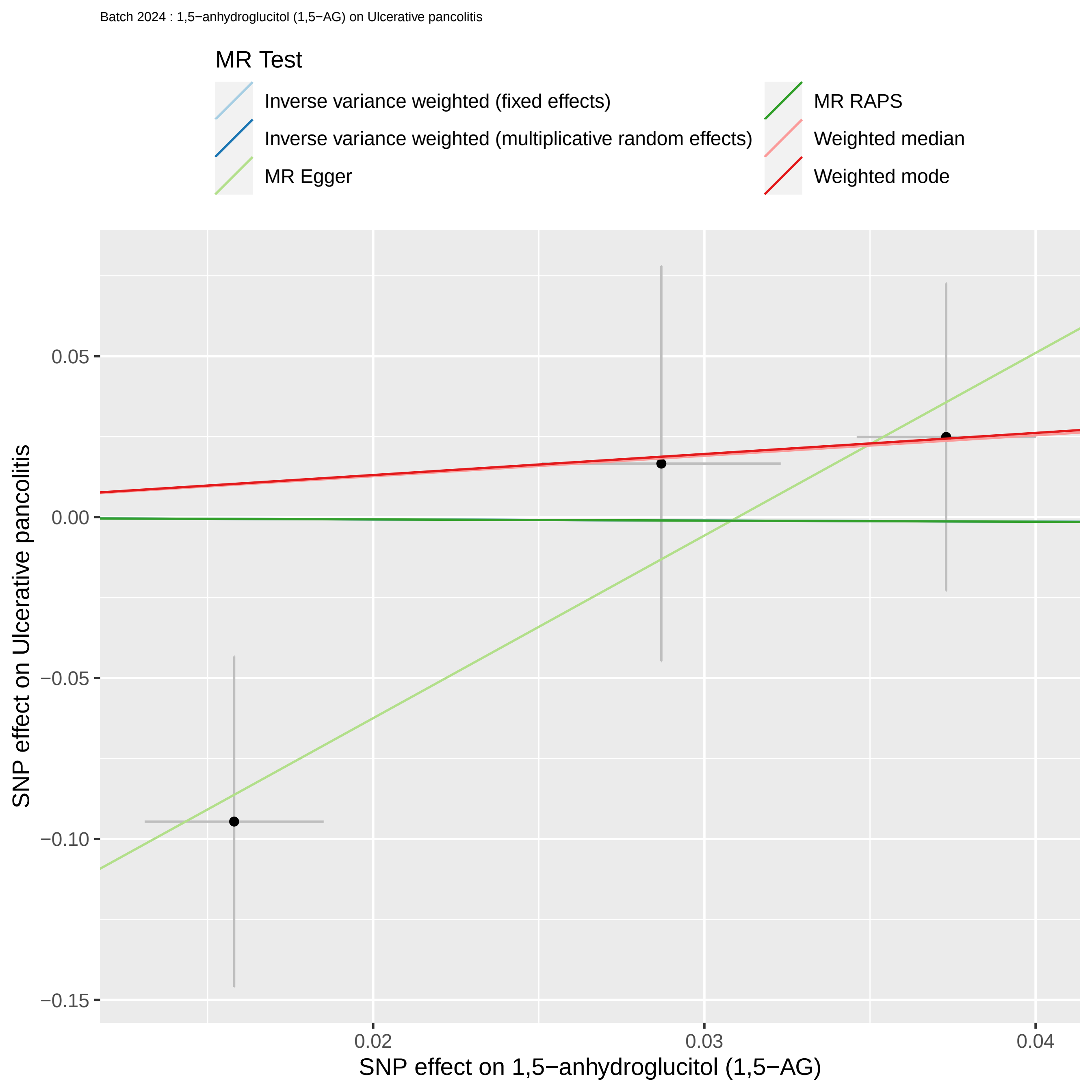
Batch 2024 : 1,5−anhydroglucitol (1,5−AG) on Ulcerative pancolitis
MR Test
Inverse variance weighted (fixed effects)
Inverse variance weighted (multiplicative random effects)
MR Egger
MR RAPS
Weighted median
Weighted mode
SNP effect on Ulcerative pancolitis
0.05
0.00
−0.05
−0.10
−0.15
0.02
0.03
0.04
SNP effect on 1,5−anhydroglucitol (1,5−AG)

Batch 2025 : 2−hydroxybutyrate (AHB) on Ulcerative pancolitis

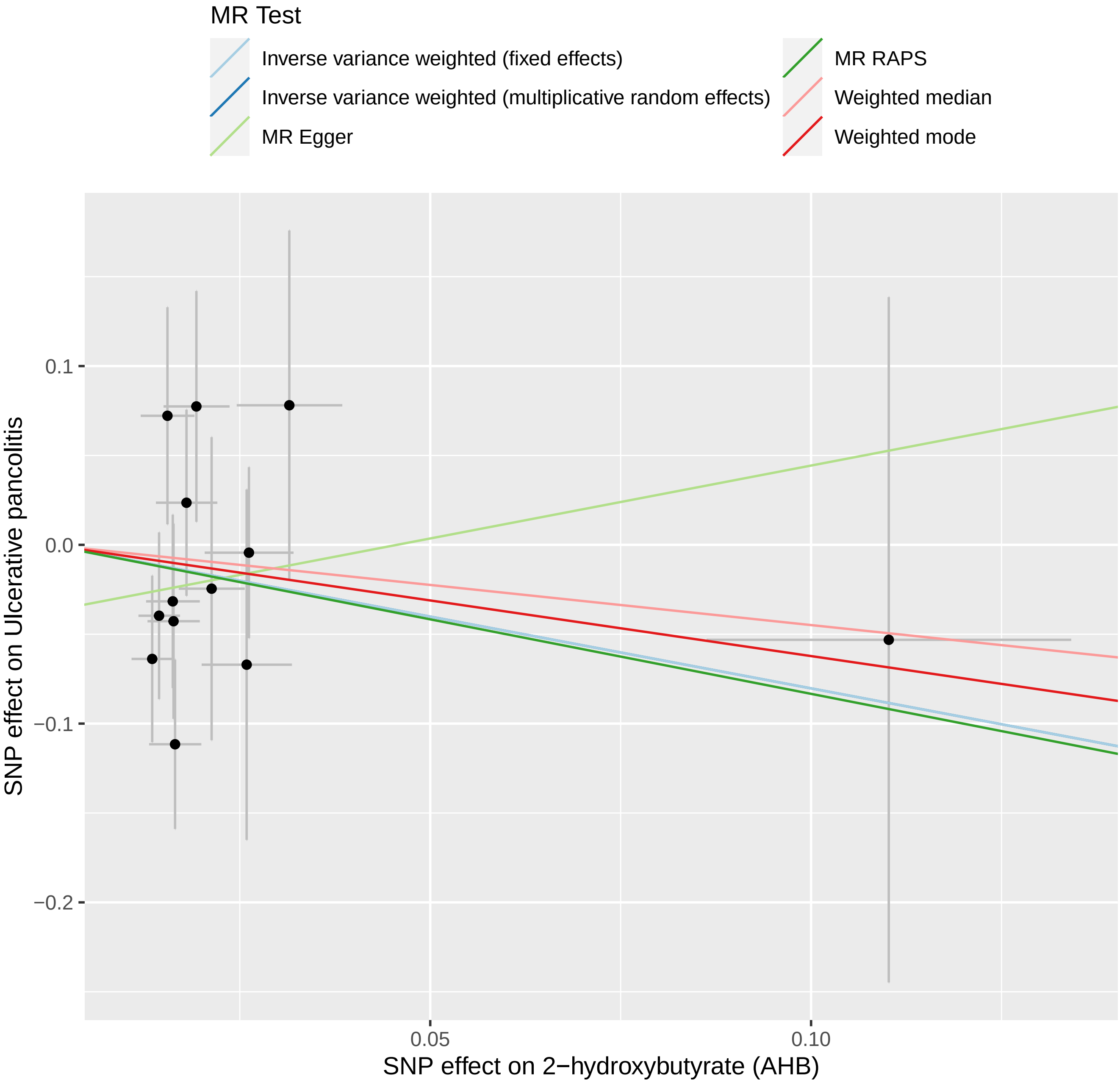

Batch 2028 : 1−palmitoylglycerol (1−monopalmitin) on Ulcerative pancolitis

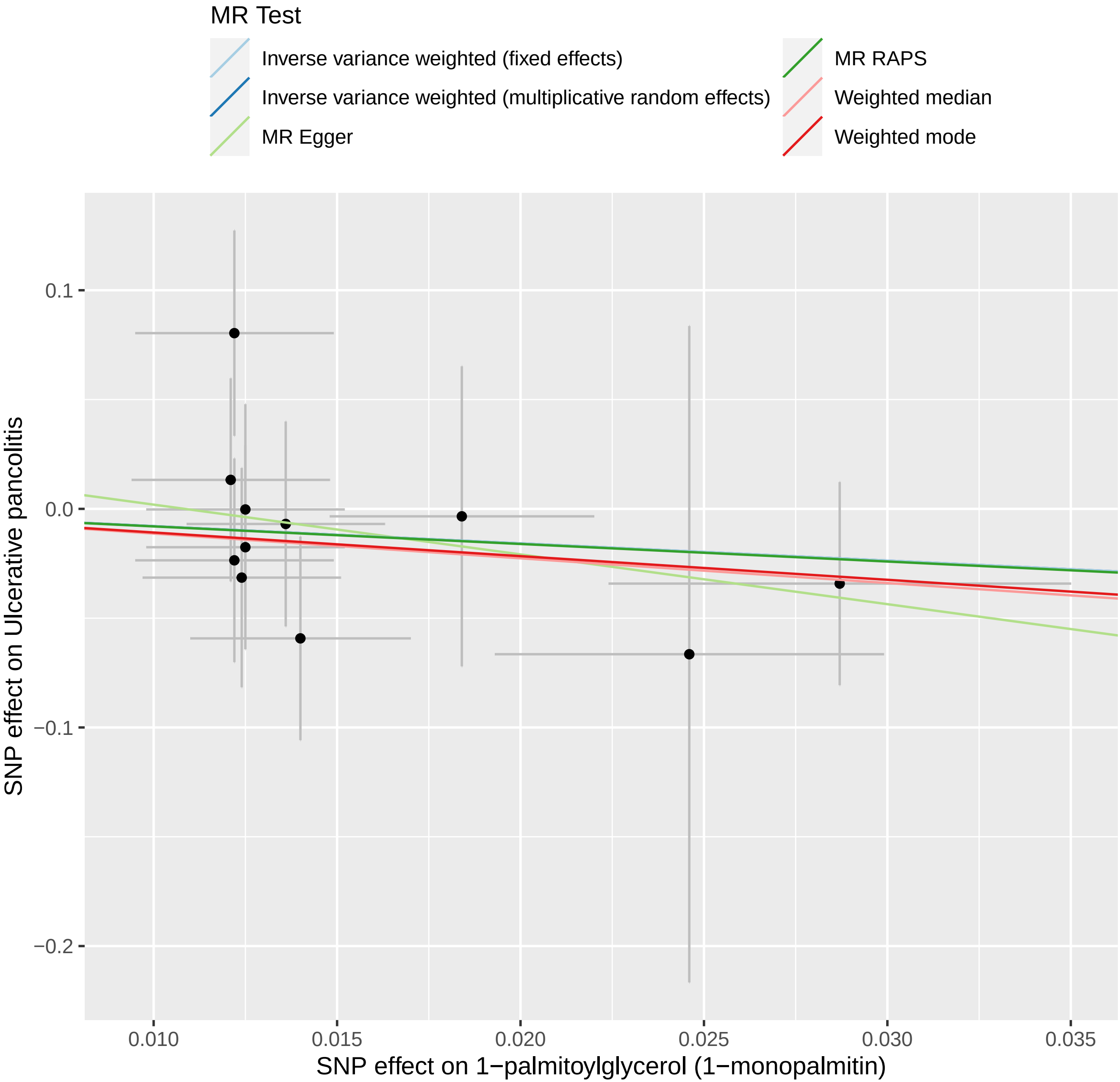

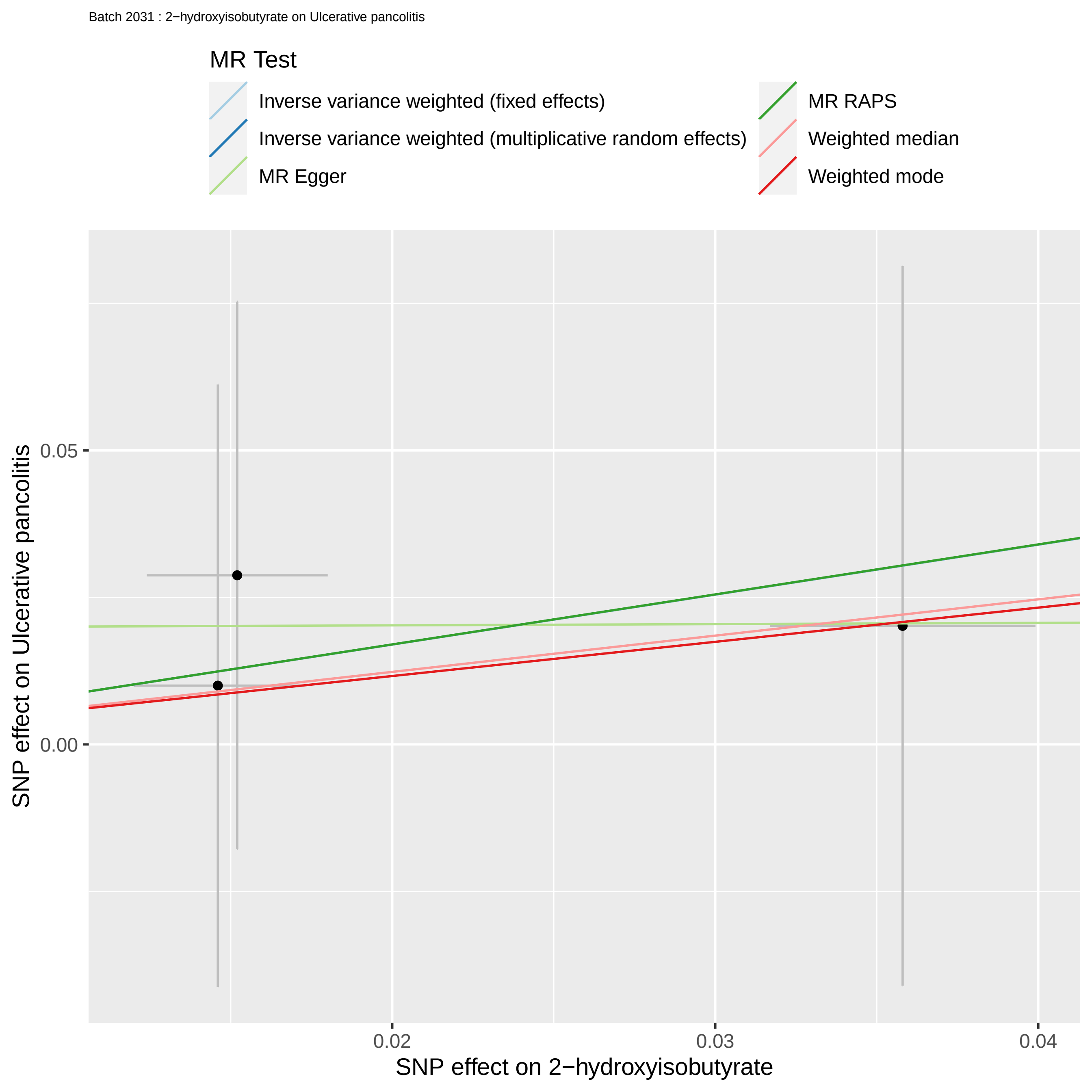
Batch 2031 : 2−hydroxyisobutyrate on Ulcerative pancolitis
MR Test
Inverse variance weighted (fixed effects)
Inverse variance weighted (multiplicative random effects)
MR Egger
MR RAPS
Weighted median
Weighted mode
SNP effect on Ulcerative pancolitis
0.05
0.00
0.02
0.03
0.04
SNP effect on 2−hydroxyisobutyrate

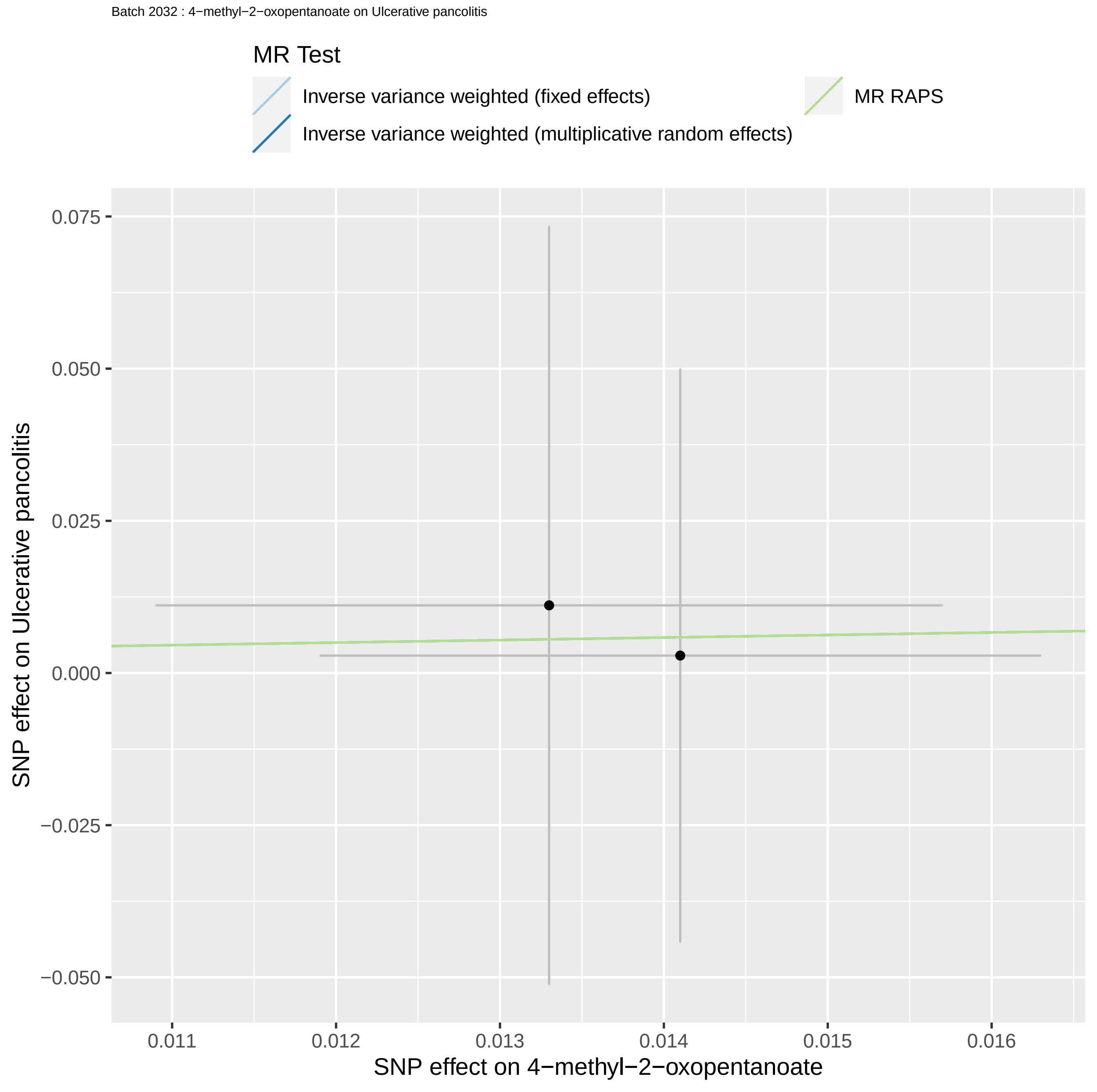
Batch 2032 : 4−methyl−2−oxopentanoate on Ulcerative pancolitis
MR Test
Inverse variance weighted (fixed effects)
Inverse variance weighted (multiplicative random effects)
MR RAPS
0.075
0.050
0.025
0.000
−0.025
−0.050
SNP effect on Ulcerative pancolitis
0.011
0.012
0.013
0.014
0.015
0.016
SNP effect on 4−methyl−2−oxopentanoate

Batch 2036 : Levulinate (4−oxovalerate) on Ulcerative pancolitis

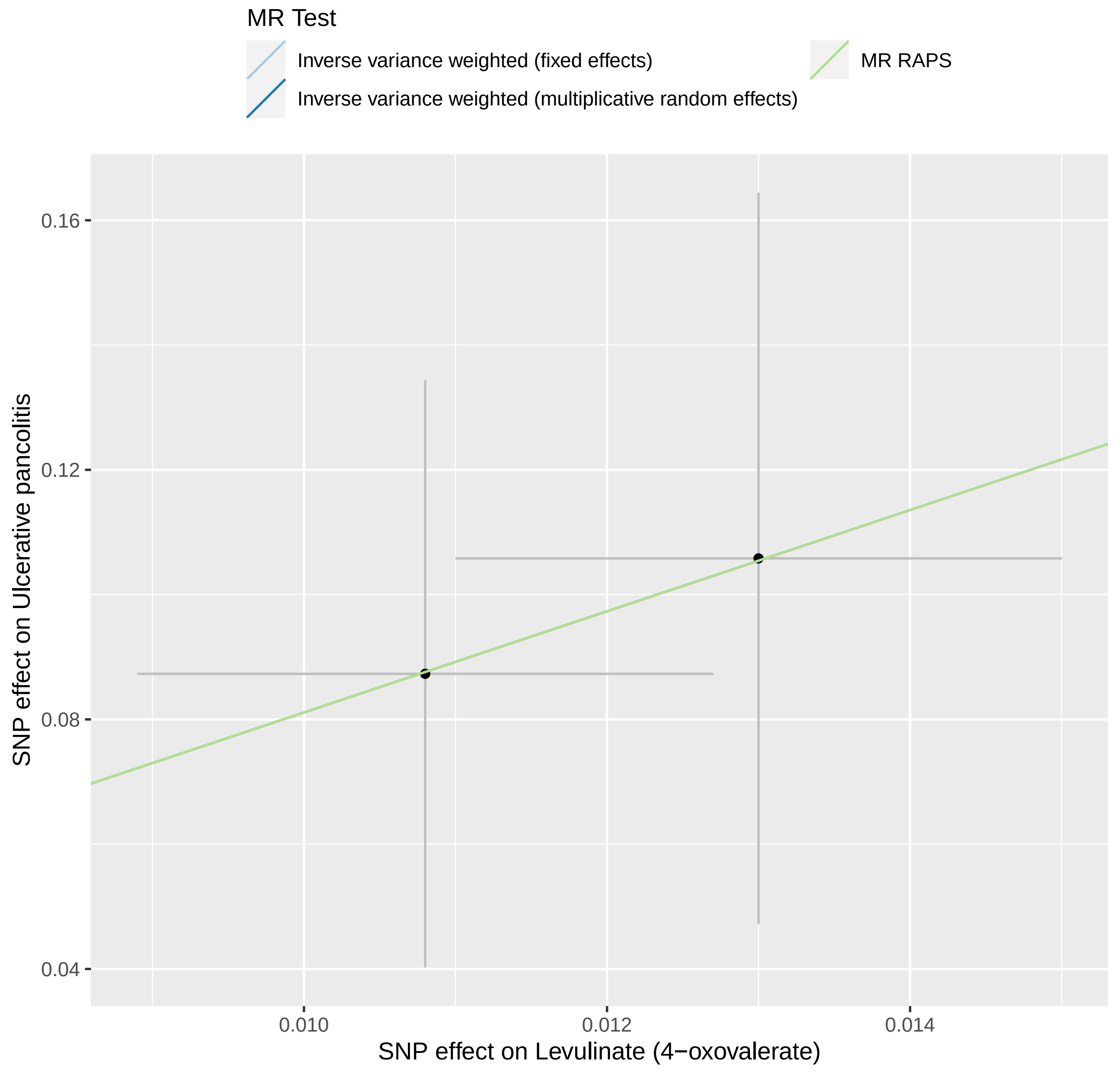

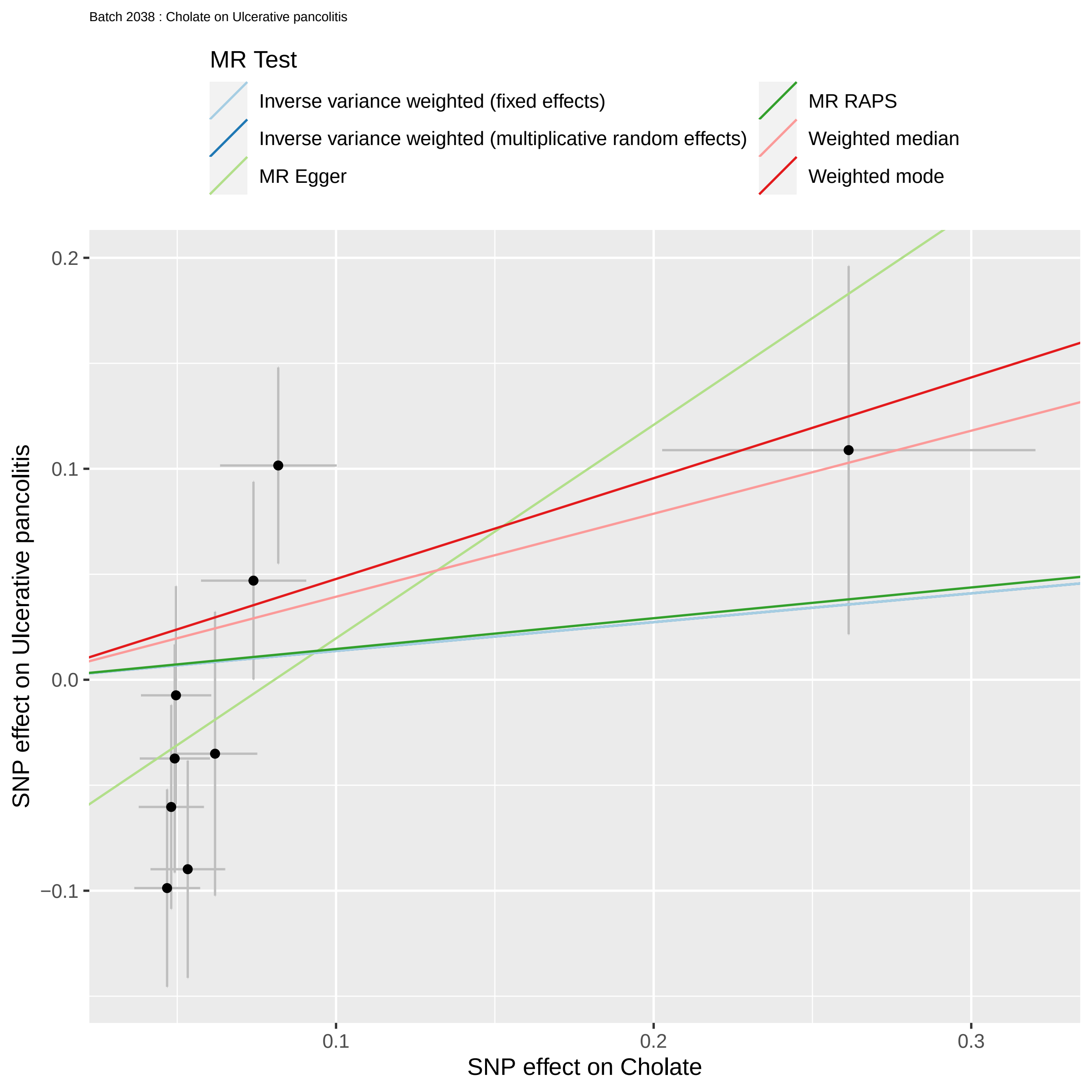

Batch 2038 : Cholate on Ulcerative pancolitis
MR Test
Inverse variance weighted (fixed effects)
Inverse variance weighted (multiplicative random effects)
MR Egger
MR RAPS
Weighted median
Weighted mode
0.2
0.1
0.0
−0.1
SNP effect on Ulcerative pancolitis
0.1
0.2
0.3
SNP effect on Cholate

Batch 2041 : Hyodeoxycholate on Ulcerative pancolitis

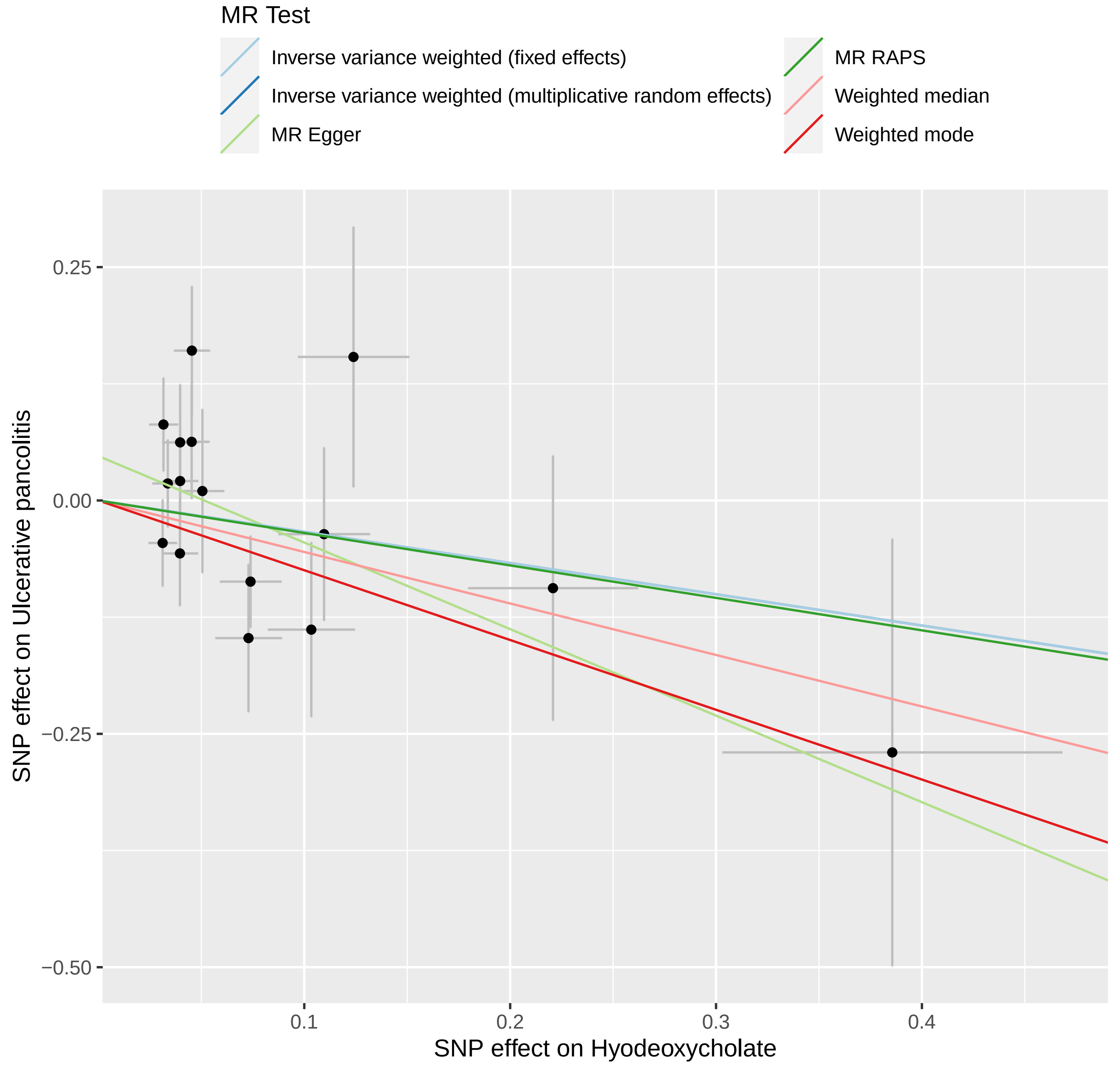

Batch 2042 : 3−indoxyl sulfate on Ulcerative pancolitis

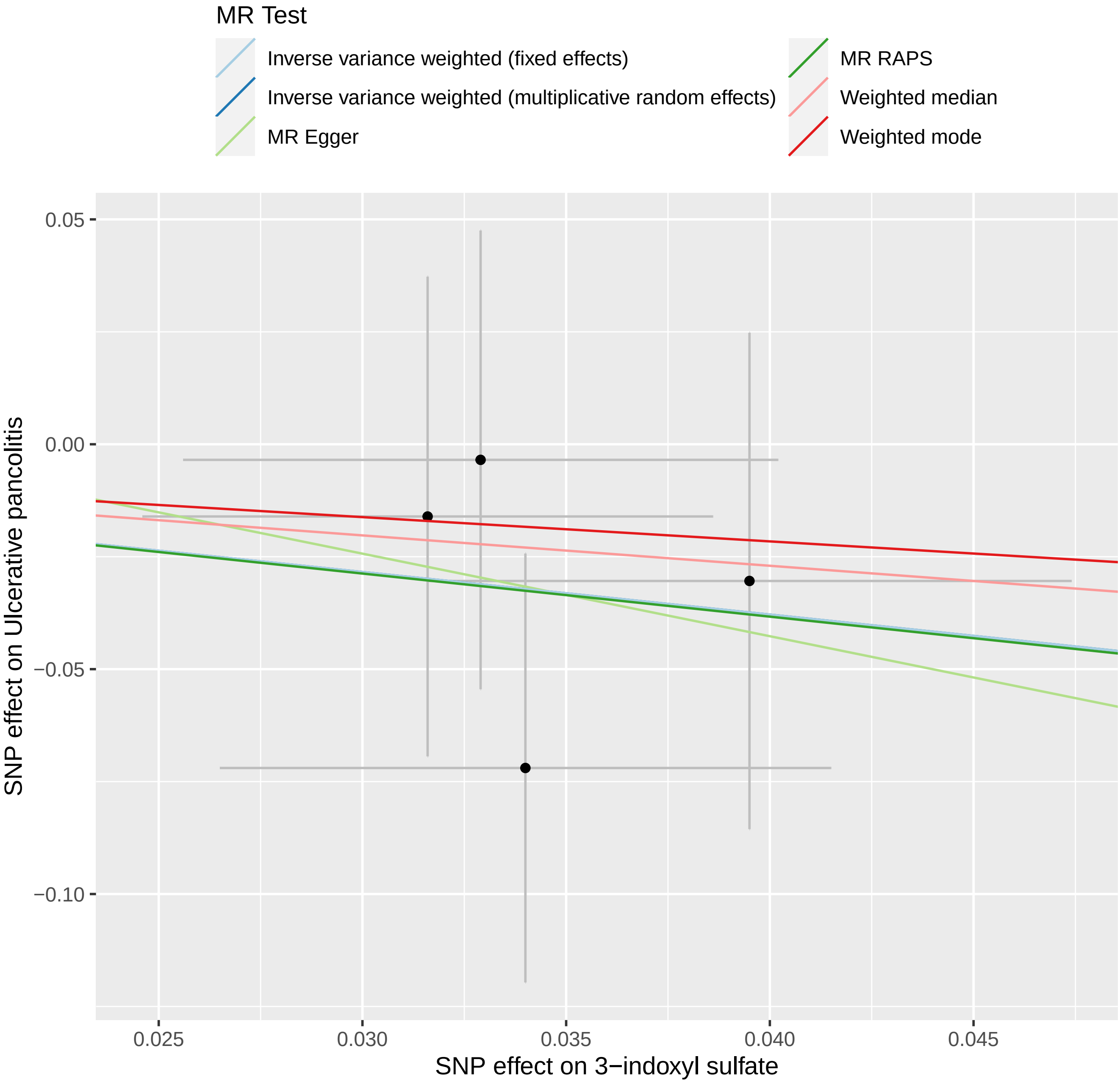

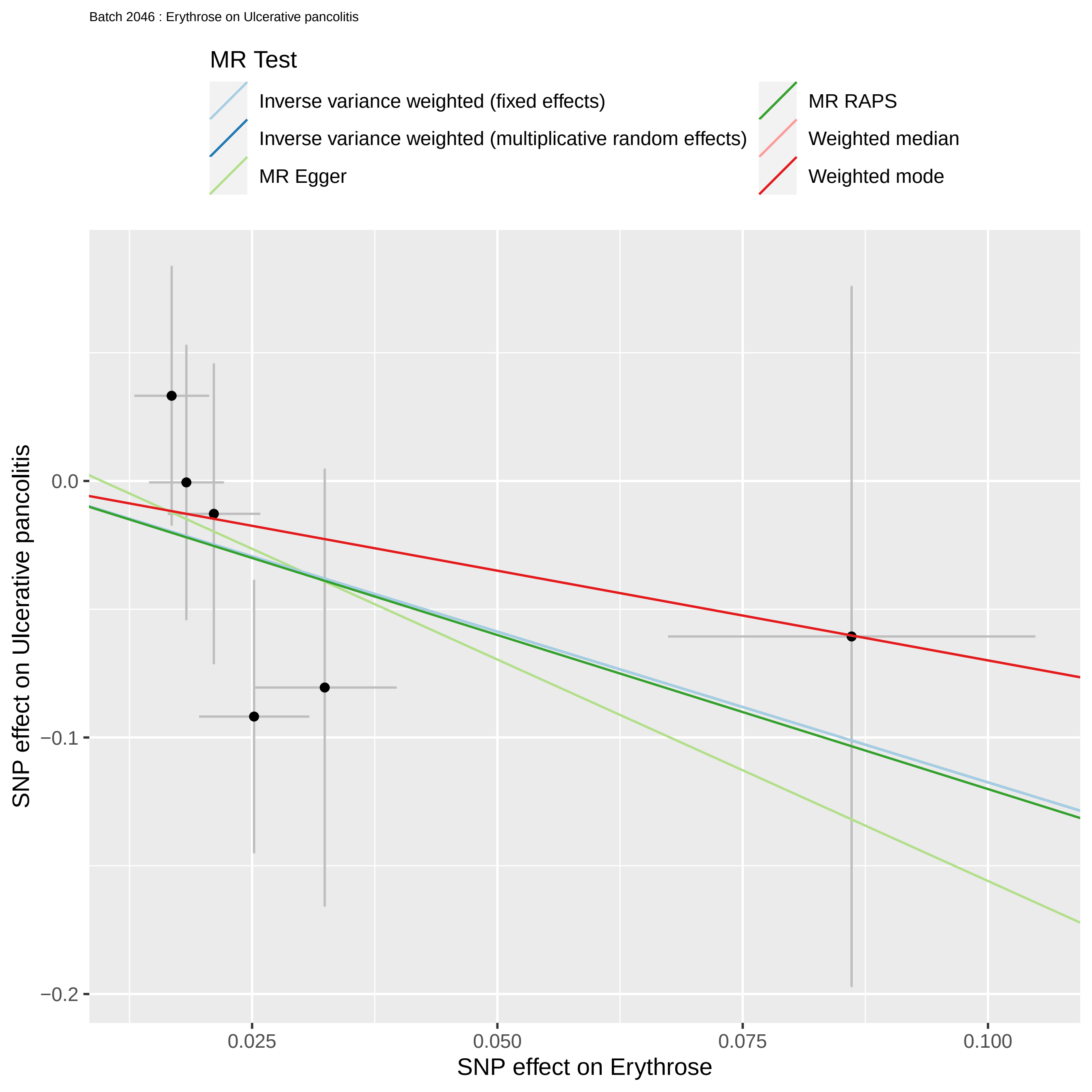

Batch 2046 : Erythrose on Ulcerative pancolitis
MR Test
Inverse variance weighted (fixed effects)
Inverse variance weighted (multiplicative random effects)
MR Egger
MR RAPS
Weighted median
Weighted mode
0.0
−0.1
−0.2
0.025
0.050
0.075
0.100
SNP effect on Erythrose
SNP effect on Ulcerative pancolitis

Batch 2047 : Threonate on Ulcerative pancolitis

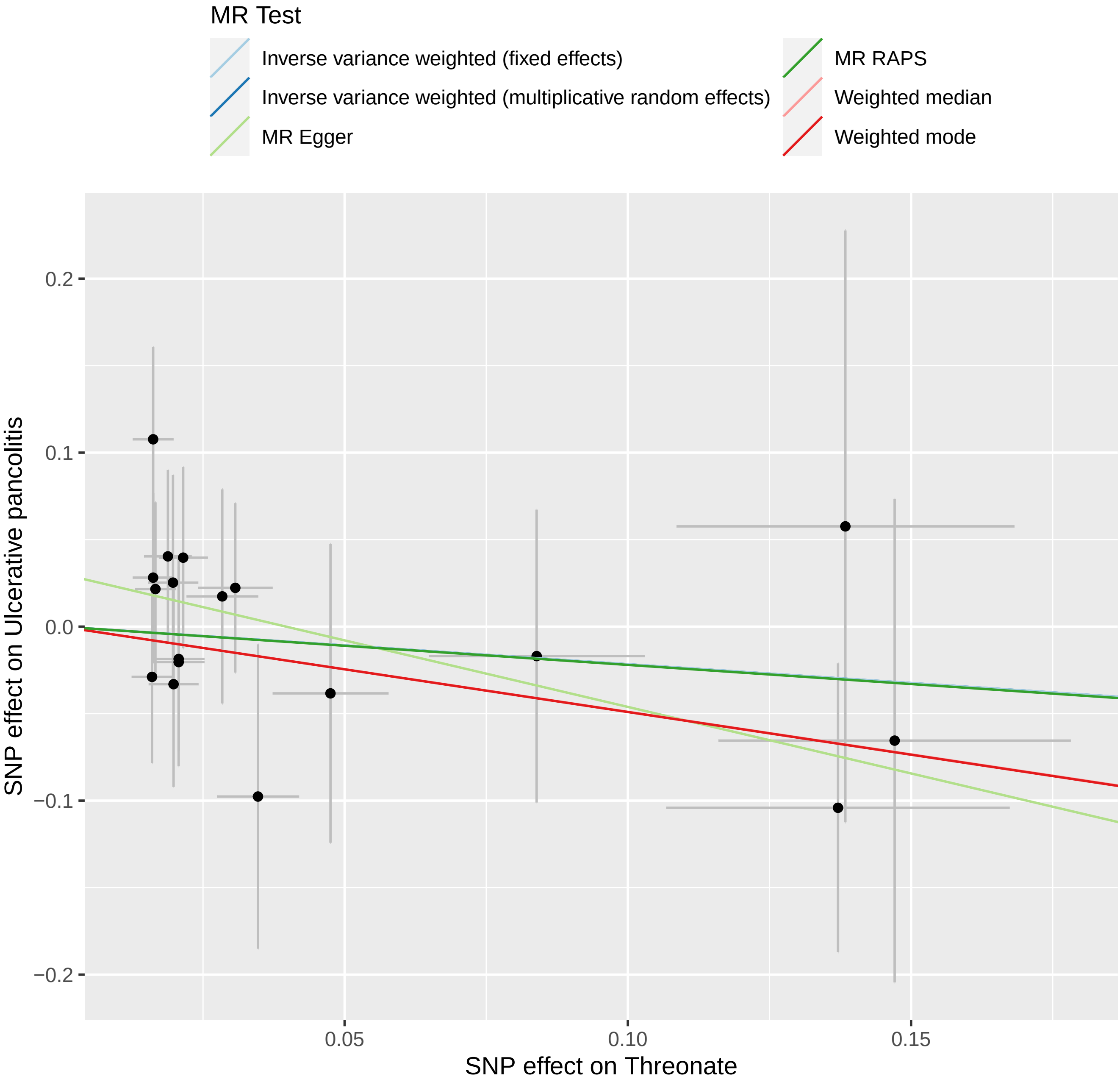

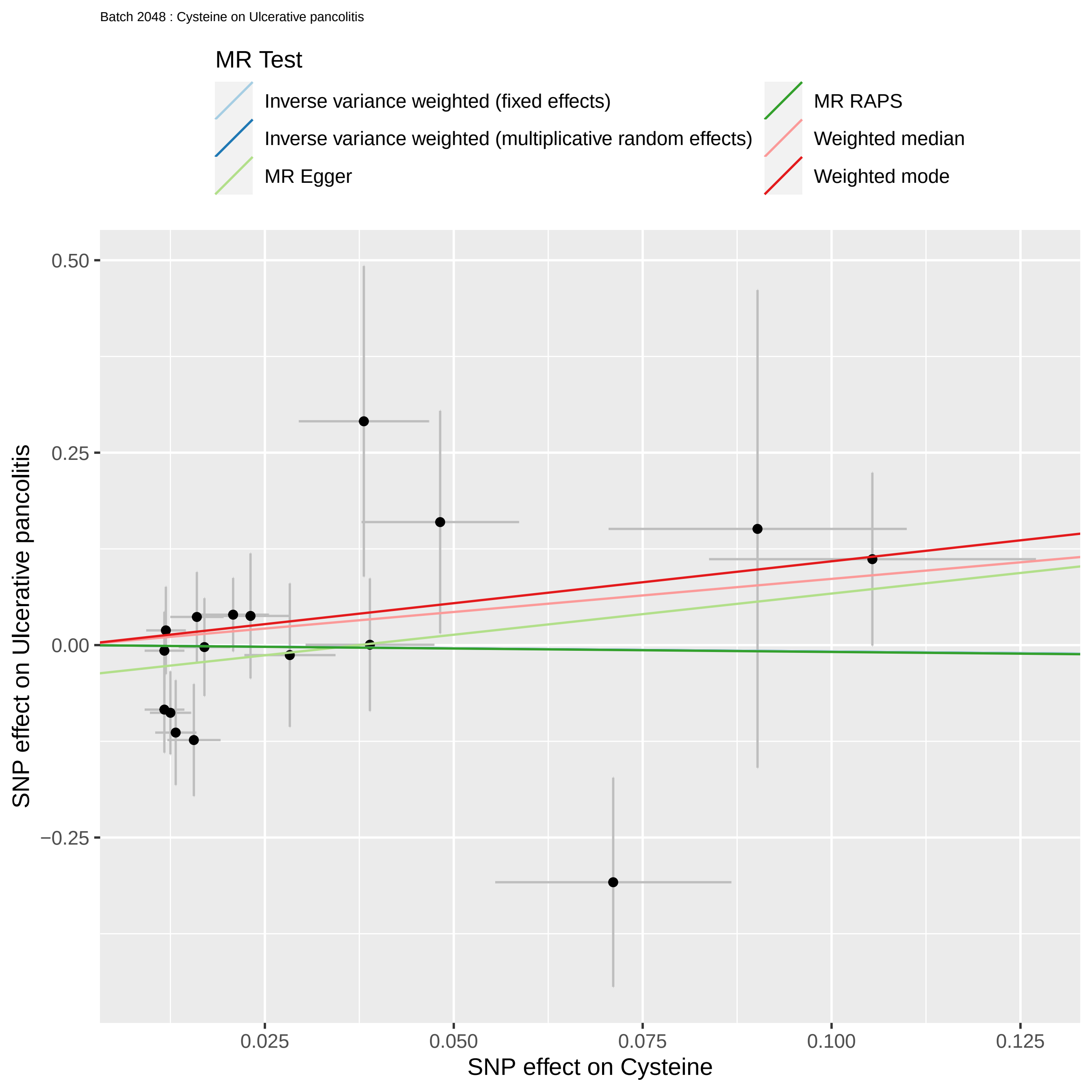
Batch 2048 : Cysteine on Ulcerative pancolitis
MR Test
Inverse variance weighted (fixed effects)
Inverse variance weighted (multiplicative random effects)
MR Egger
MR RAPS
Weighted median
Weighted mode
SNP effect on Ulcerative pancolitis
0.50
0.25
0.00
−0.25
0.025
0.050
0.075
0.100
0.125
SNP effect on Cysteine

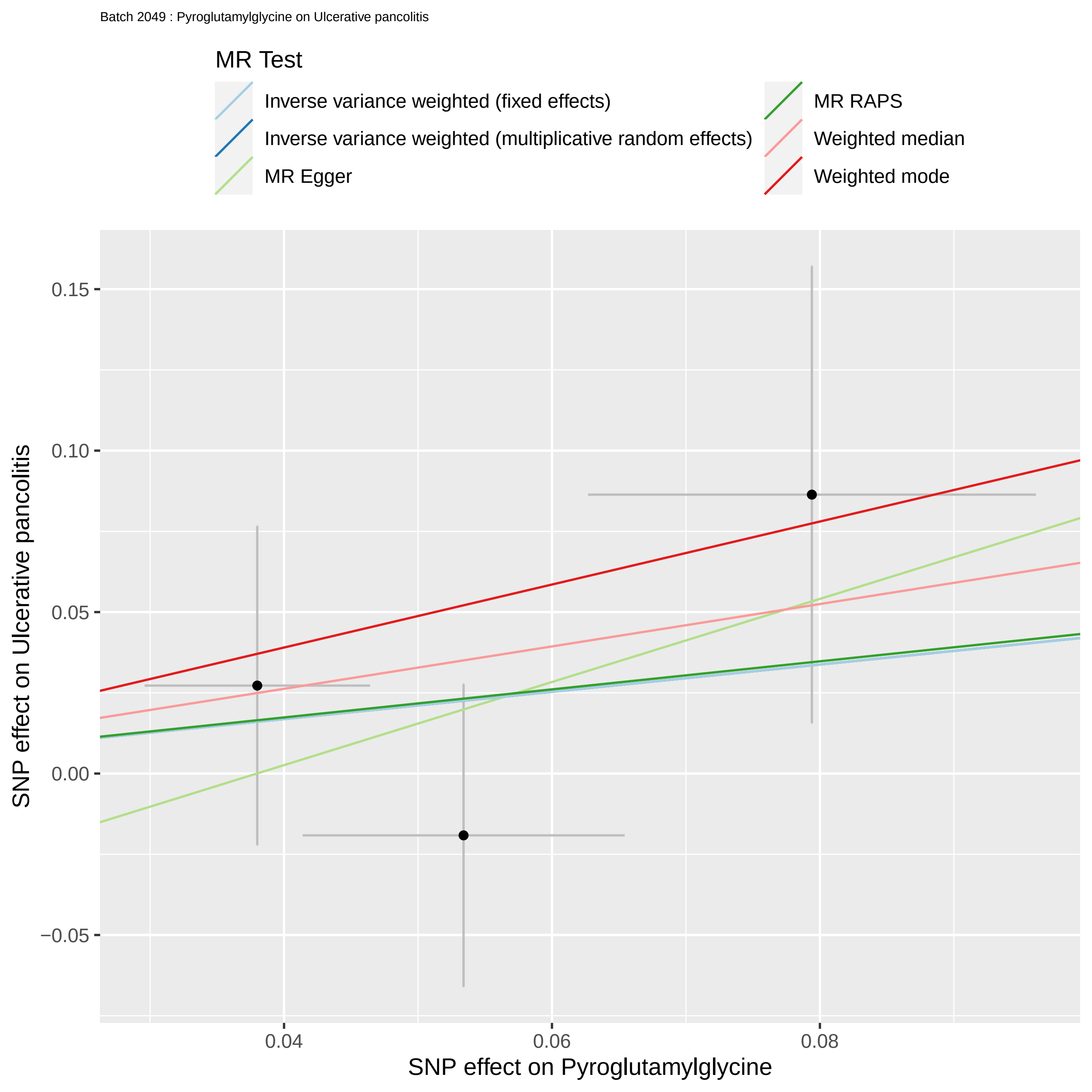

Batch 2049 : Pyroglutamylglycine on Ulcerative pancolitis
MR Test
Inverse variance weighted (fixed effects)
Inverse variance weighted (multiplicative random effects)
MR Egger
MR RAPS
Weighted median
Weighted mode
0.15
0.10
0.05
0.00
−0.05
0.04
0.06
0.08
SNP effect on Ulcerative pancolitis
SNP effect on Pyroglutamylglycine

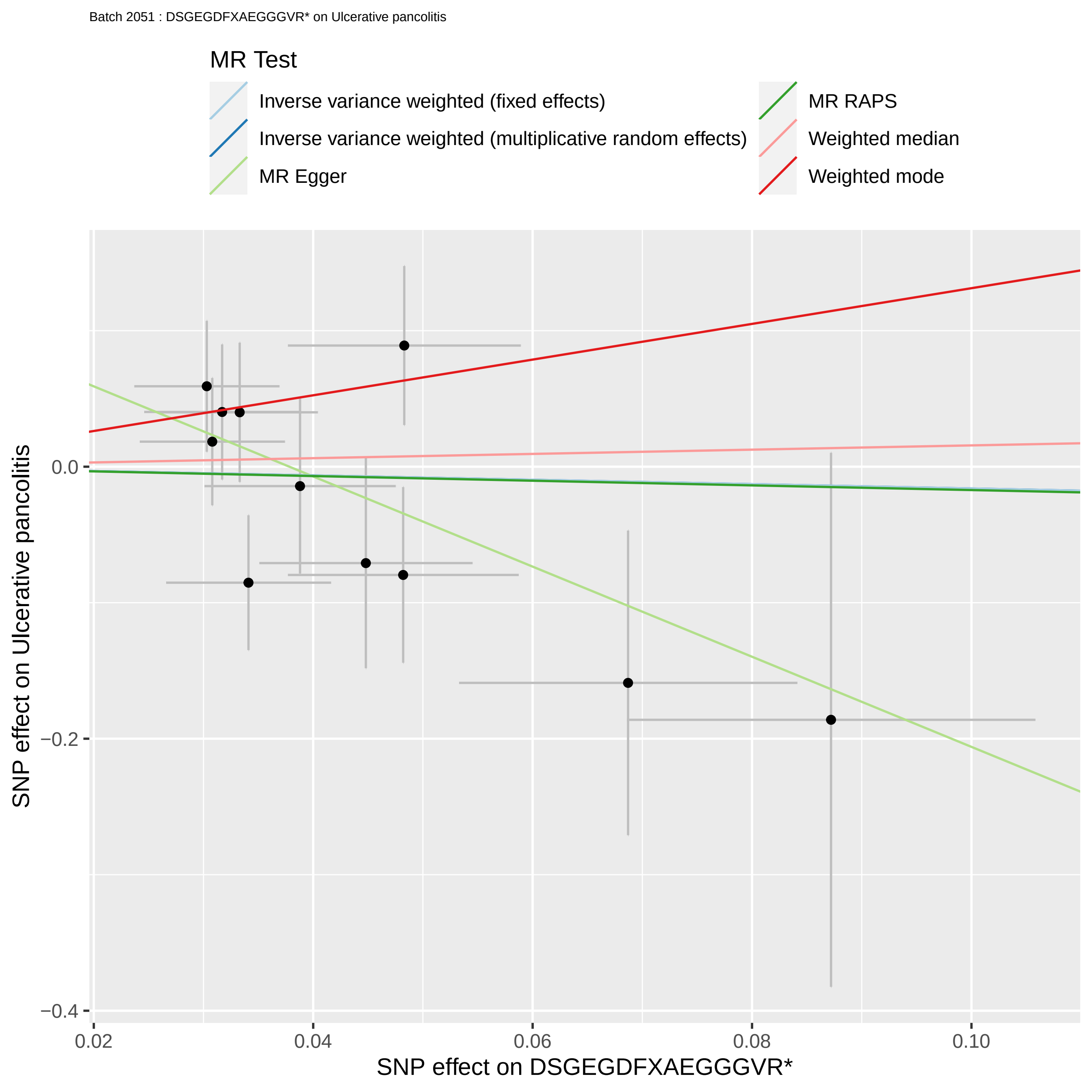

Batch 2051 : DSGEGDFXAEGGGVR* on Ulcerative pancolitis
MR Test
Inverse variance weighted (fixed effects)
Inverse variance weighted (multiplicative random effects)
MR Egger
MR RAPS
Weighted median
Weighted mode
SNP effect on Ulcerative pancolitis
0.0
−0.2
−0.4
0.02
0.04
0.06
0.08
0.10
SNP effect on DSGEGDFXAEGGGVR*

Batch 2052 : Pyridoxate on Ulcerative pancolitis

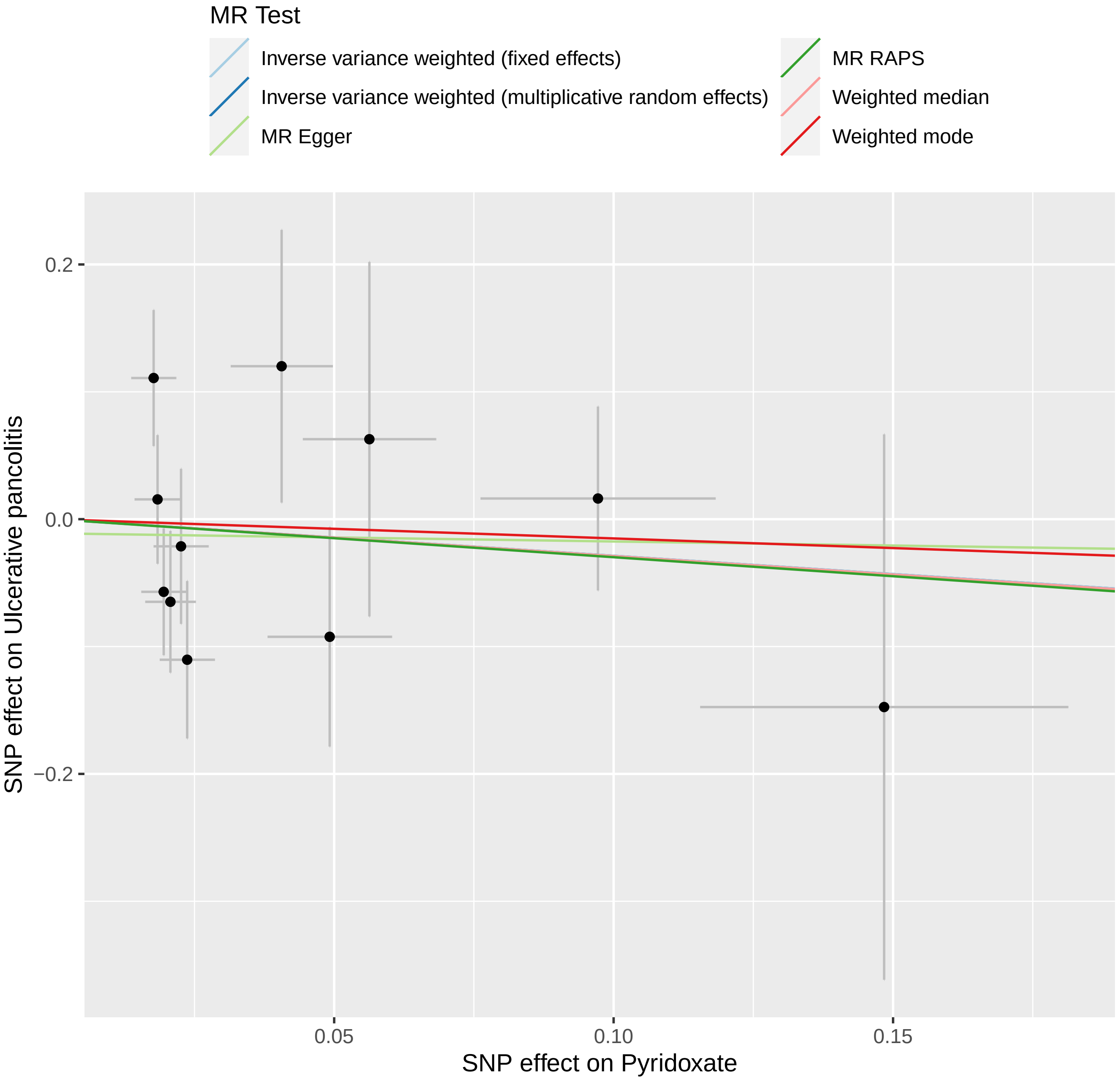

Batch 2053 : Androsterone sulfate on Ulcerative pancolitis

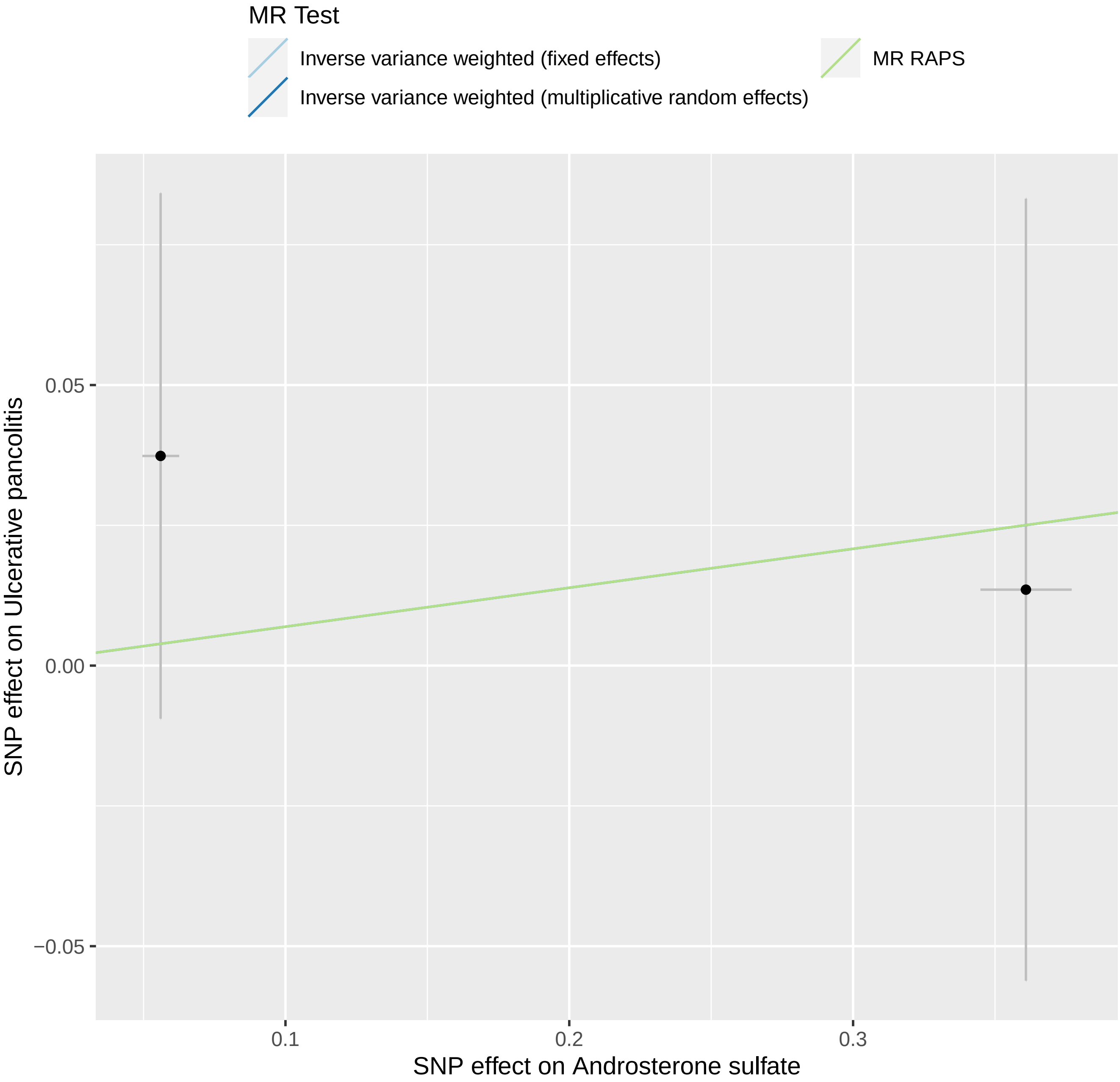

Batch 2056 : Acetylcarnitine on Ulcerative pancolitis

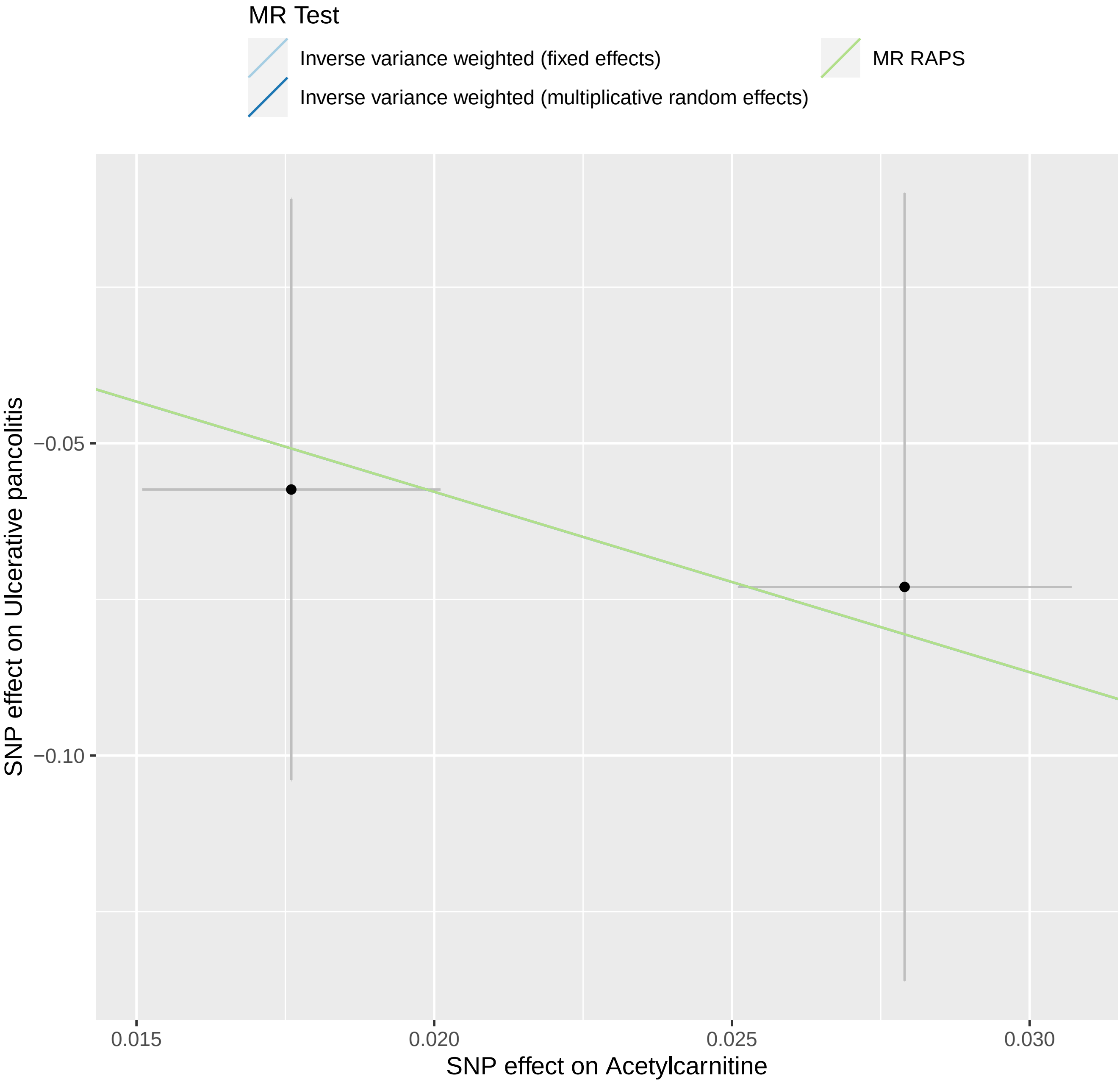

Batch 2057 : Serine on Ulcerative pancolitis

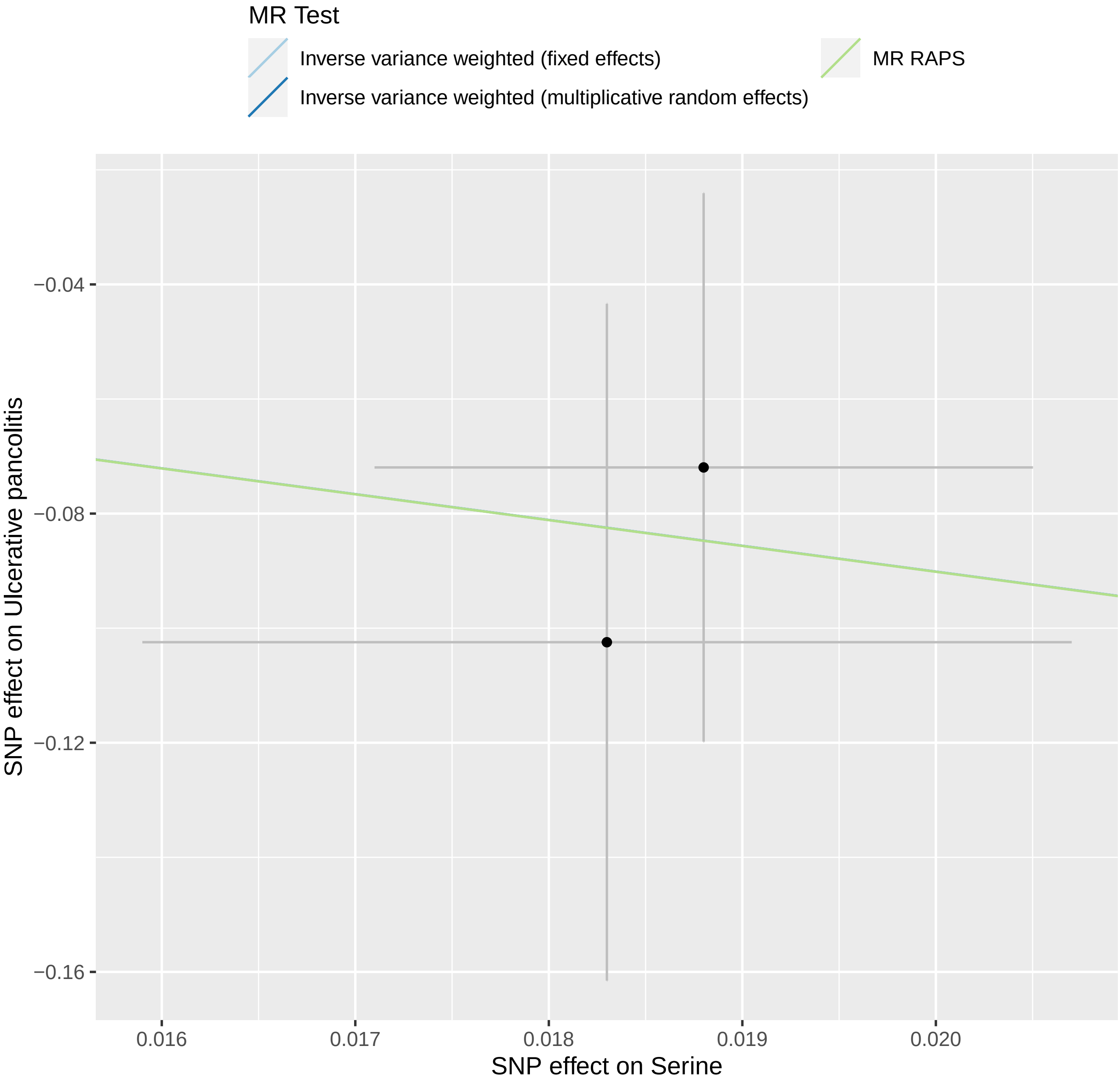

Batch 2058 : Trans−4−hydroxyproline on Ulcerative pancolitis

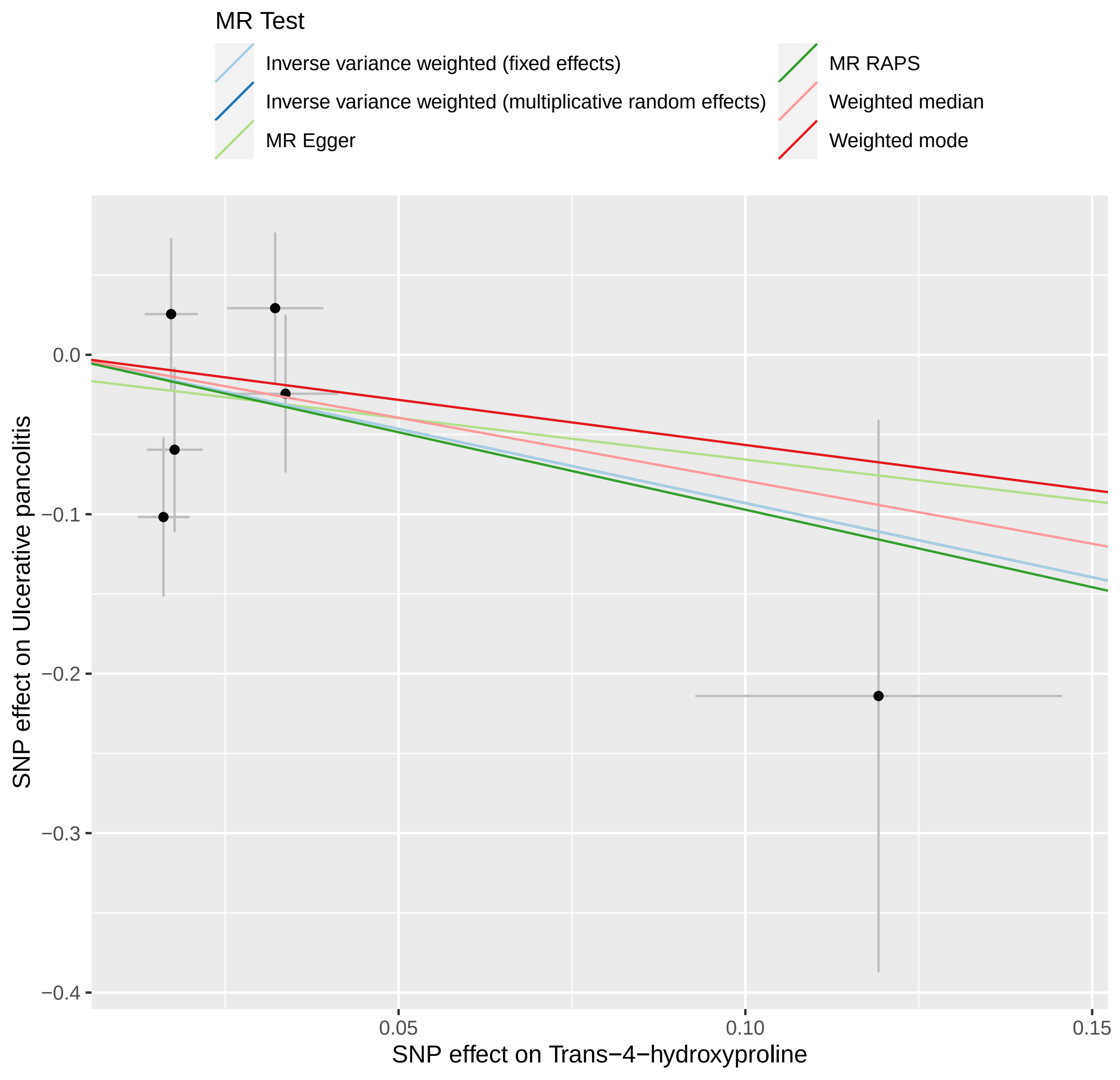

Batch 2059 : Glutamate on Ulcerative pancolitis

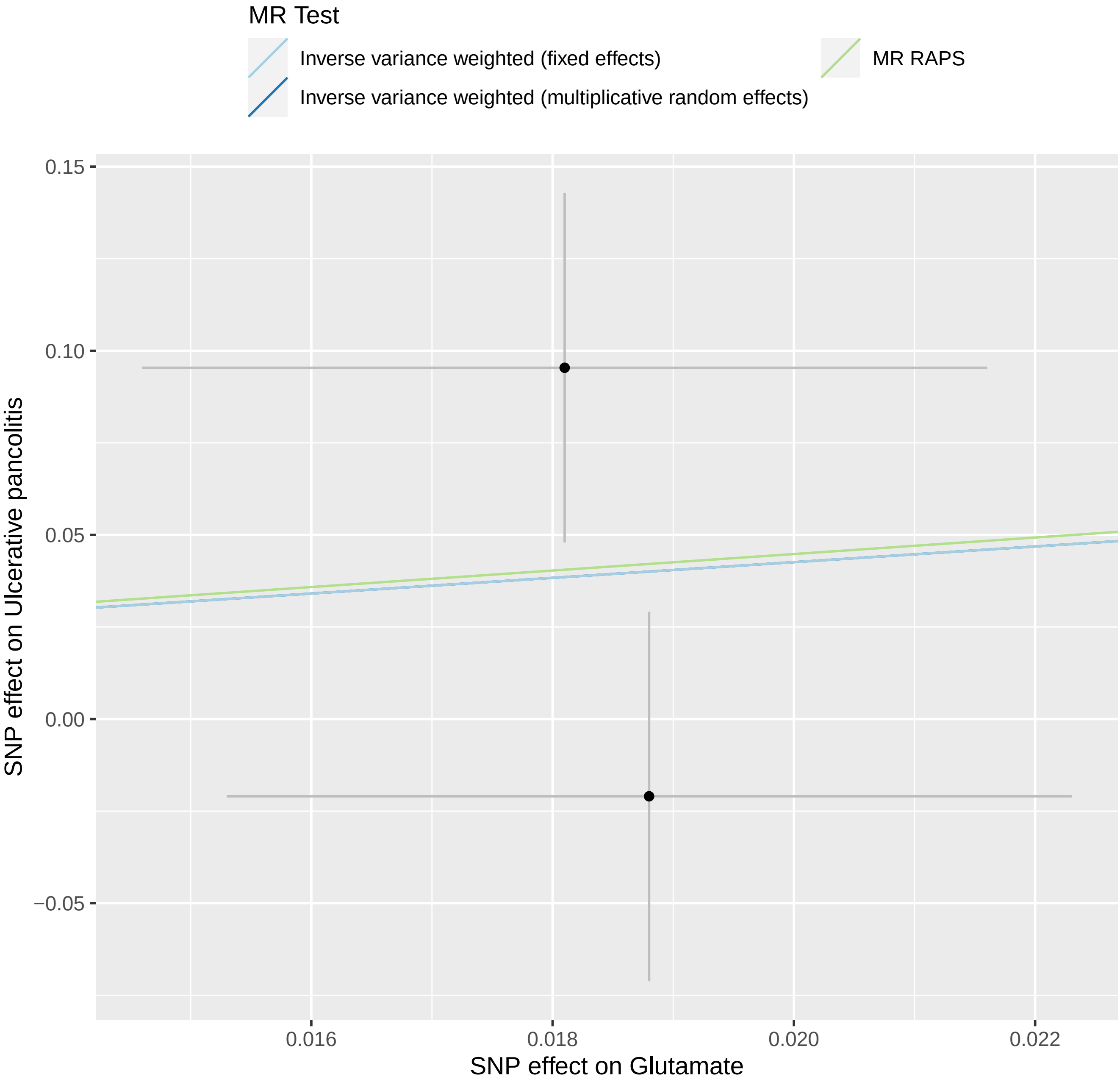

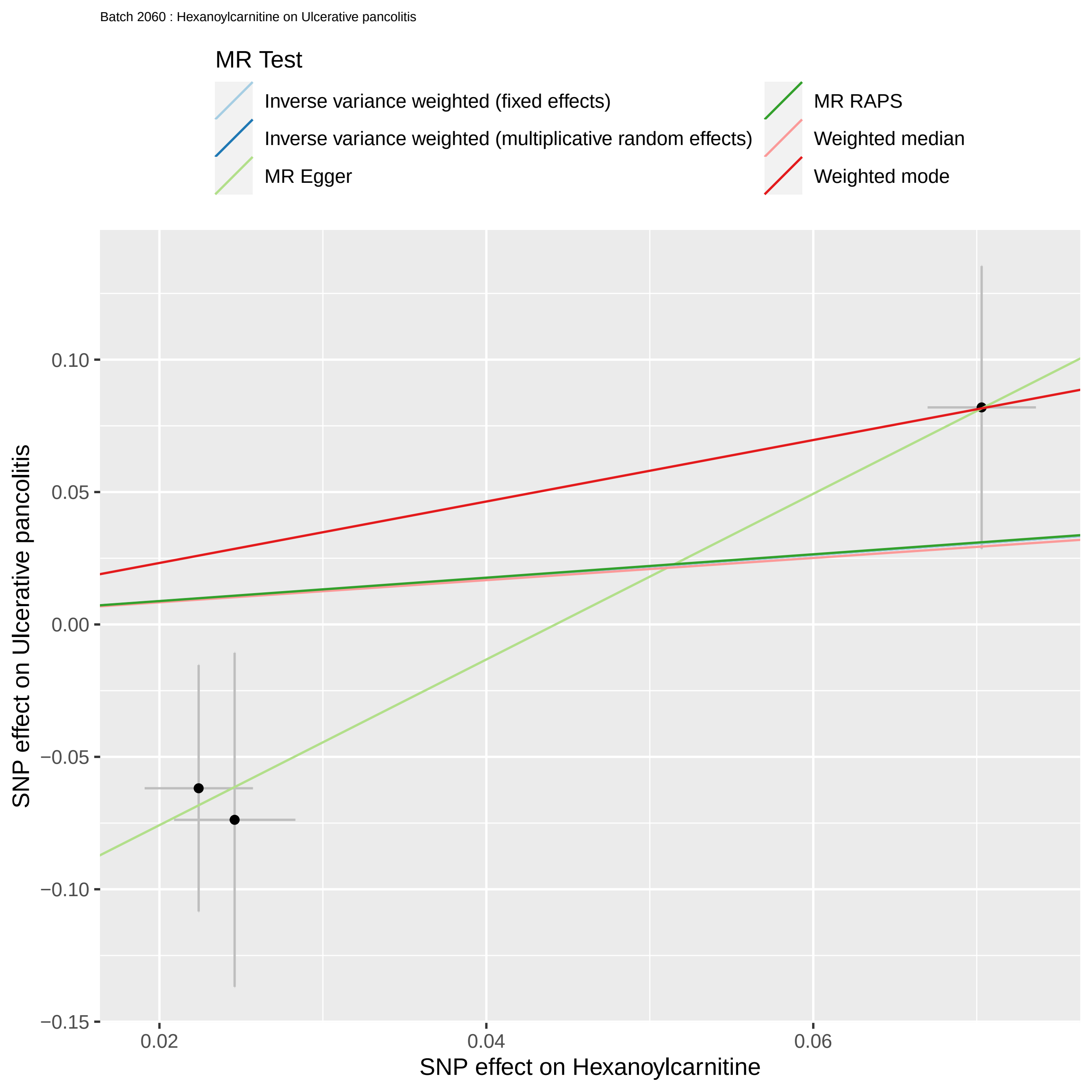
Batch 2060 : Hexanoylcarnitine on Ulcerative pancolitis
MR Test
Inverse variance weighted (fixed effects)
Inverse variance weighted (multiplicative random effects)
MR Egger
MR RAPS
Weighted median
Weighted mode
SNP effect on Ulcerative pancolitis
0.10
0.05
0.00
−0.05
−0.10
−0.15
0.02
0.04
0.06
SNP effect on Hexanoylcarnitine

Batch 2064 : 2−aminobutyrate on Ulcerative pancolitis

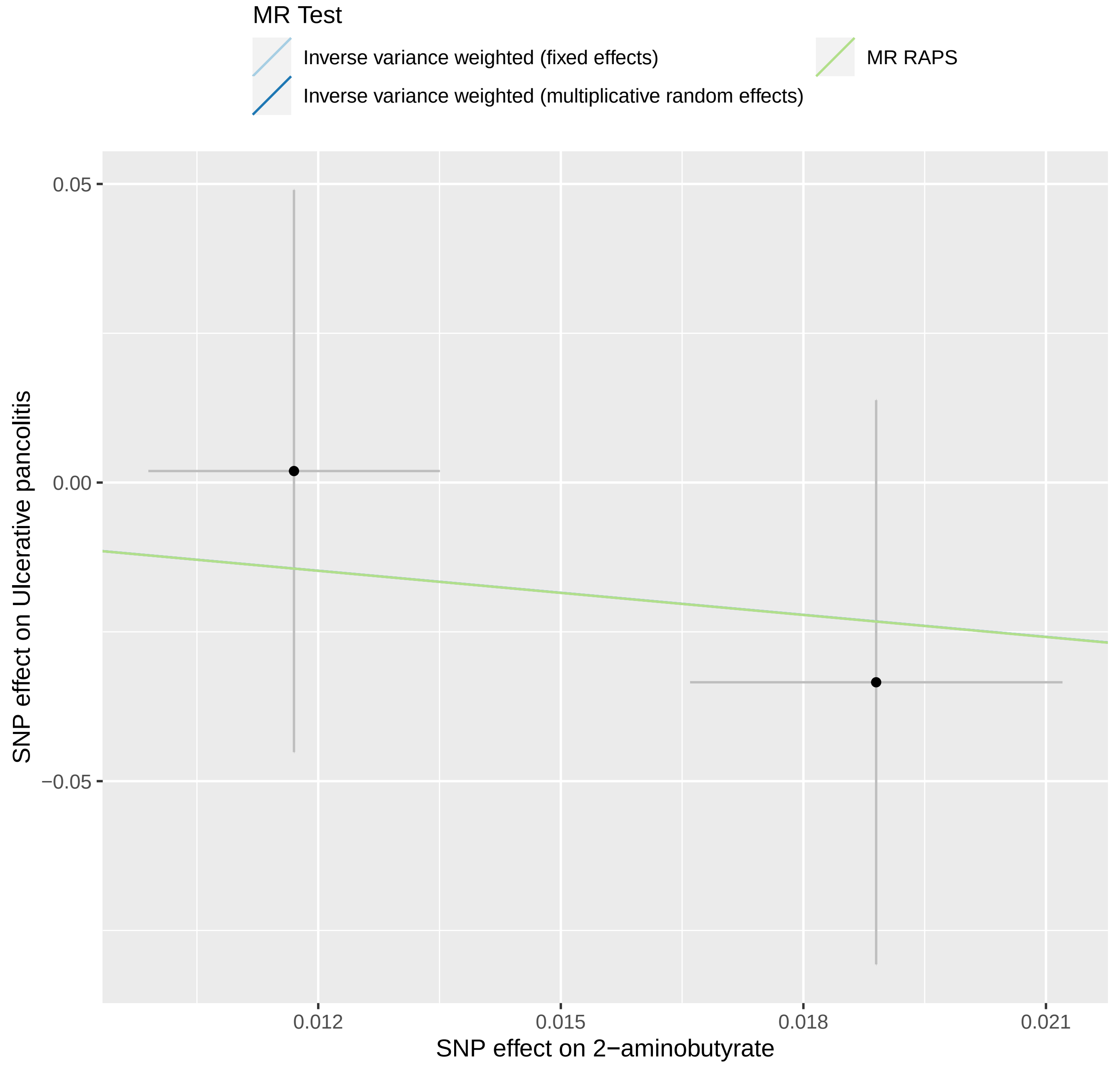

Batch 2069 : Butyrylcarnitine on Ulcerative pancolitis

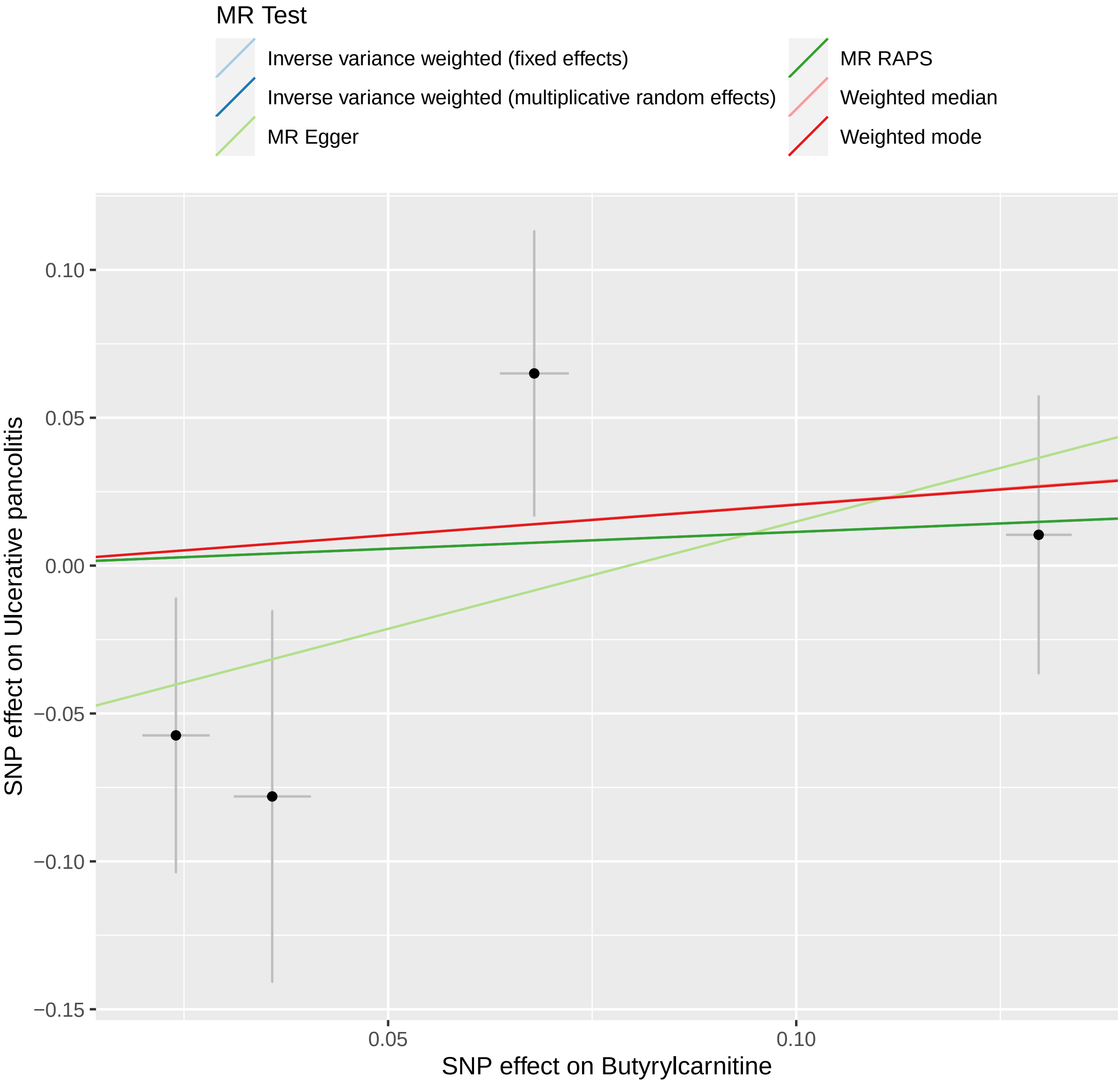

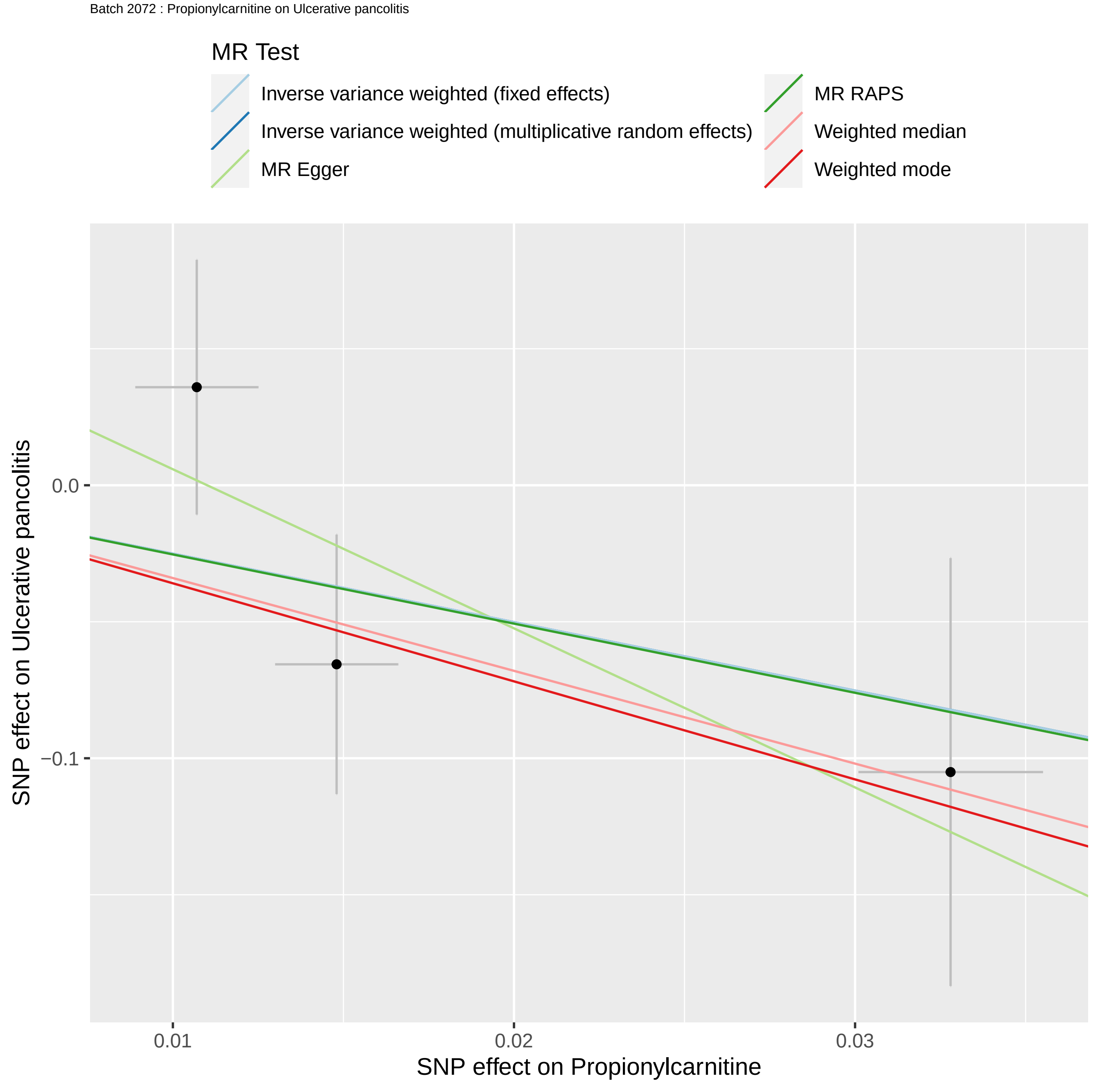

Batch 2072 : Propionylcarnitine on Ulcerative pancolitis
MR Test
Inverse variance weighted (fixed effects)
Inverse variance weighted (multiplicative random effects)
MR Egger
MR RAPS
Weighted median
Weighted mode
SNP effect on Ulcerative pancolitis
0.0
−0.1
0.01
0.02
0.03
SNP effect on Propionylcarnitine

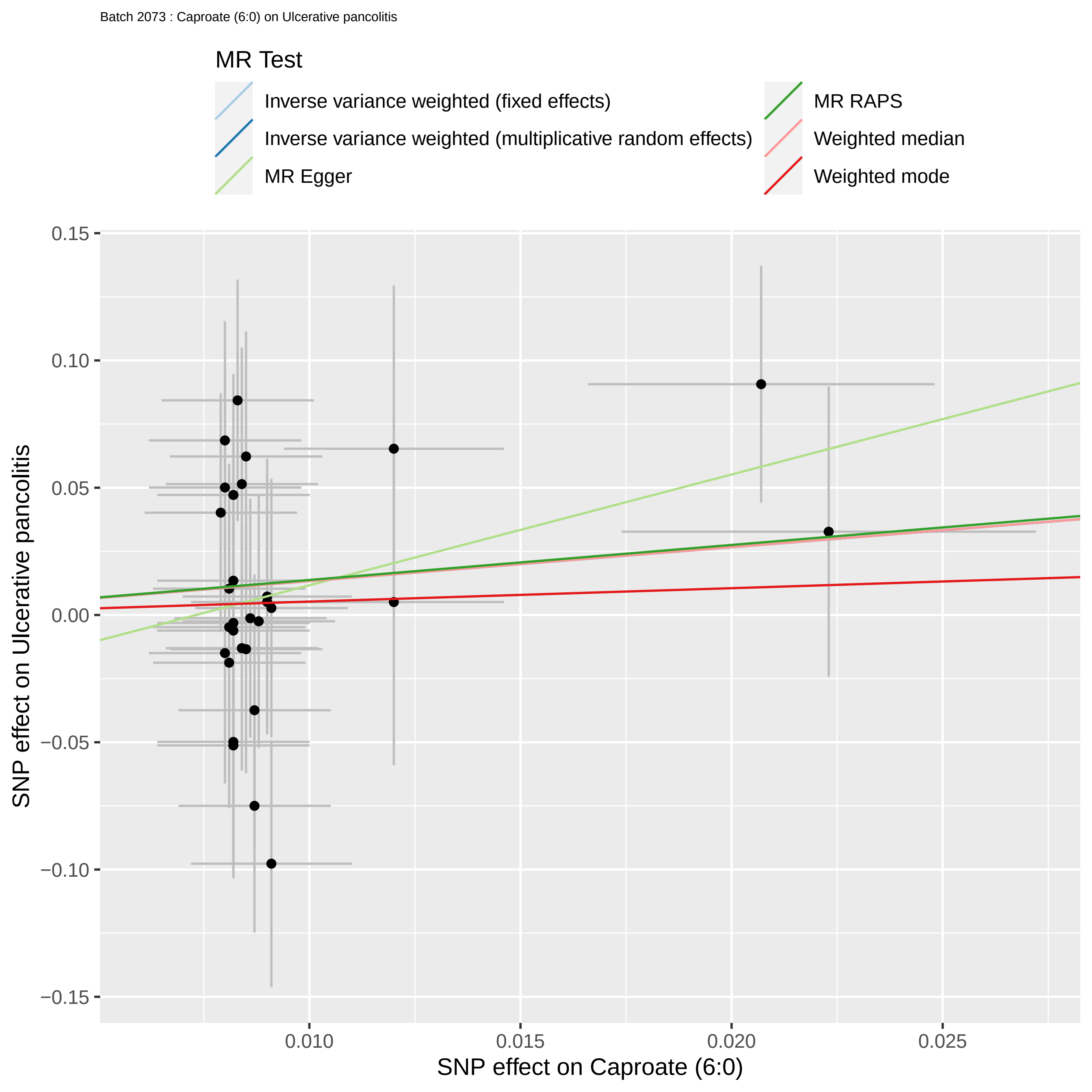
Batch 2073 : Caproate (6:0) on Ulcerative pancolitis
MR Test
Inverse variance weighted (fixed effects)
Inverse variance weighted (multiplicative random effects)
MR Egger
MR RAPS
Weighted median
Weighted mode
SNP effect on Ulcerative pancolitis
SNP effect on Caproate (6:0)
0.15
0.10
0.05
0.00
−0.05
−0.10
−0.15
0.010
0.015
0.020
0.025

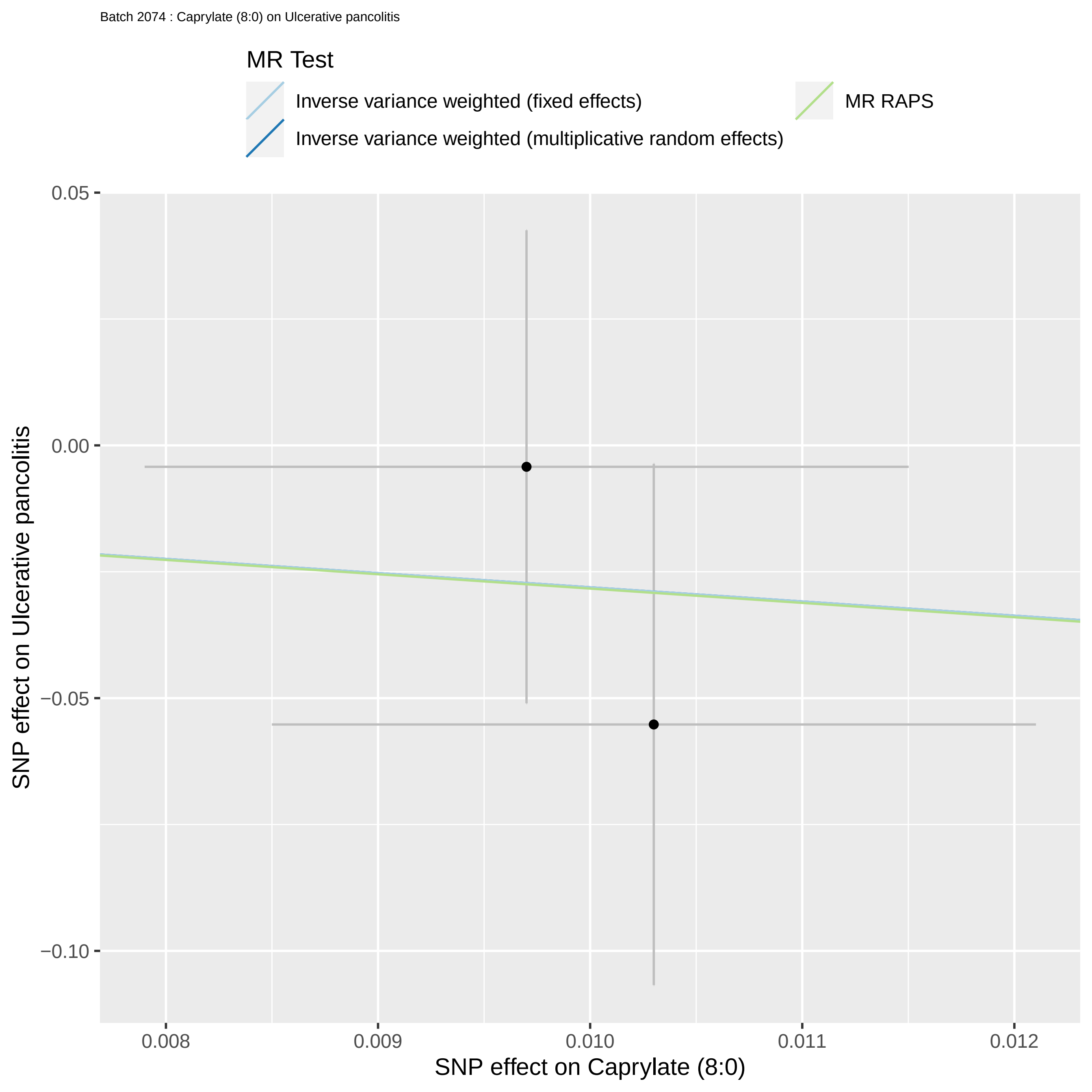

Batch 2074 : Caprylate (8:0) on Ulcerative pancolitis
MR Test
Inverse variance weighted (fixed effects)
Inverse variance weighted (multiplicative random effects)
MR RAPS
0.05
0.00
−0.05
−0.10
0.008
0.009
0.010
0.011
0.012
SNP effect on Caprylate (8:0)
SNP effect on Ulcerative pancolitis

Batch 2075 : 10−undecenoate (11:1n1) on Ulcerative pancolitis

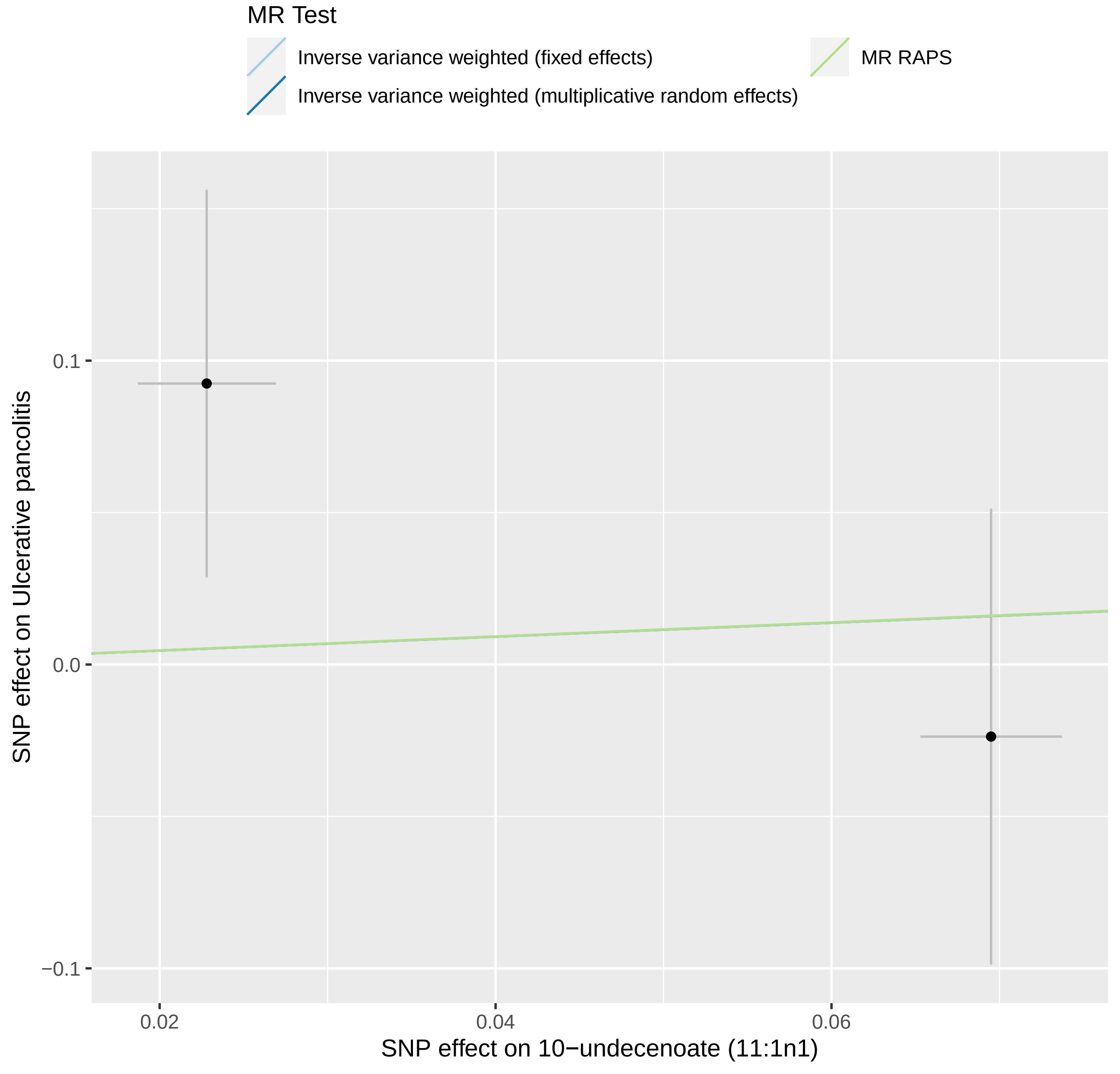

Batch 2077 : Phenol sulfate on Ulcerative pancolitis

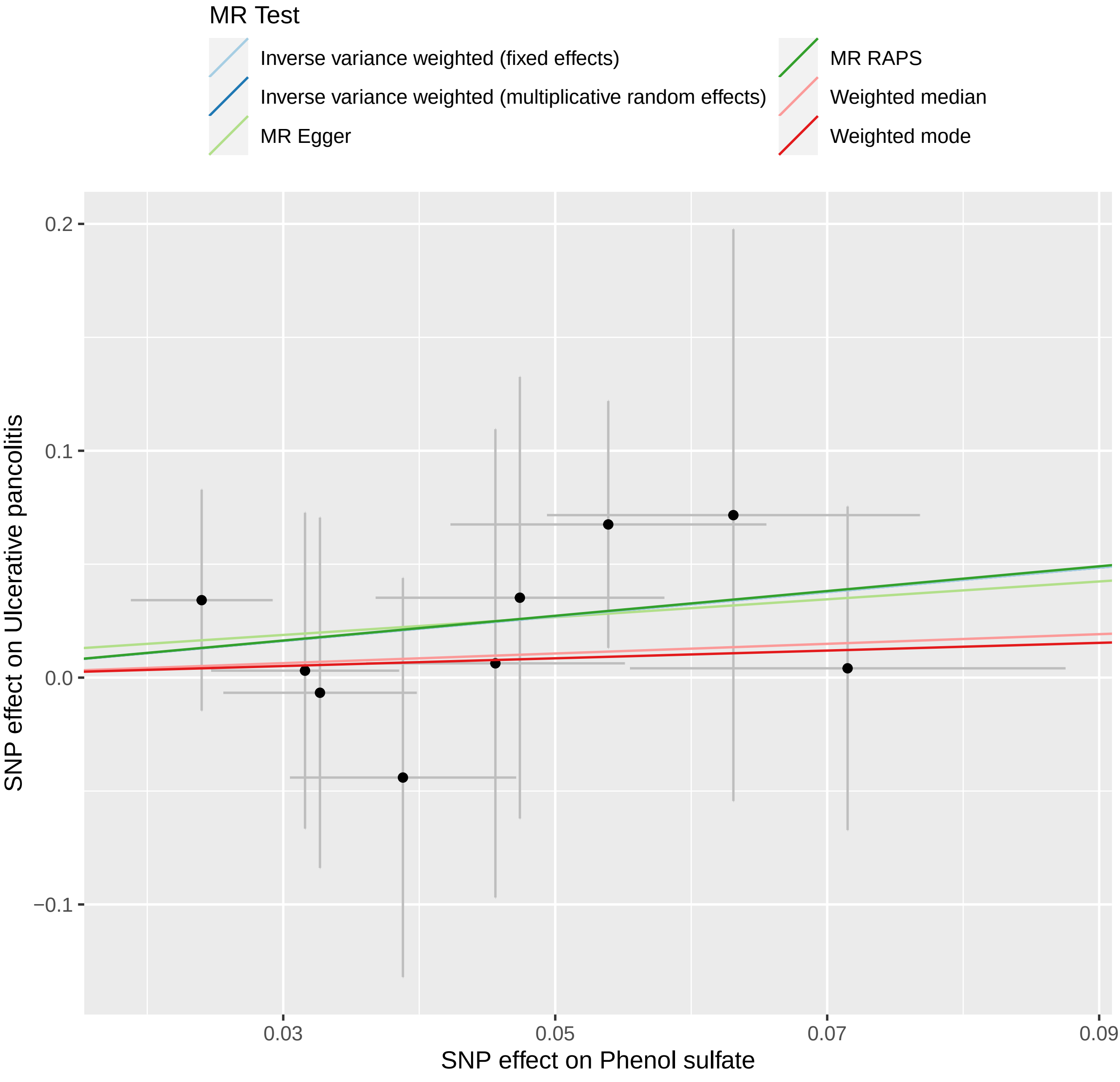

Batch 2079 : Heme* on Ulcerative pancolitis

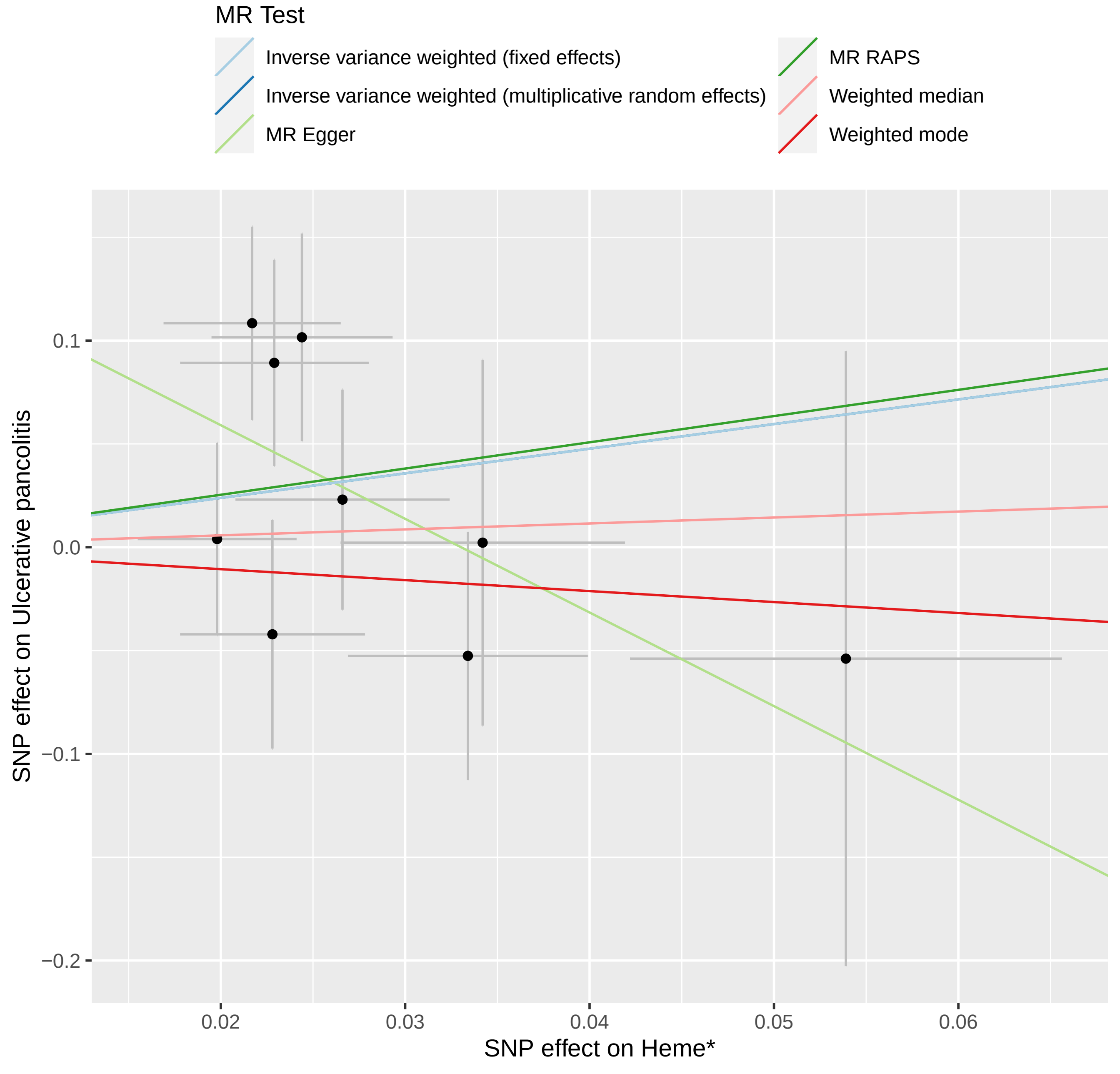

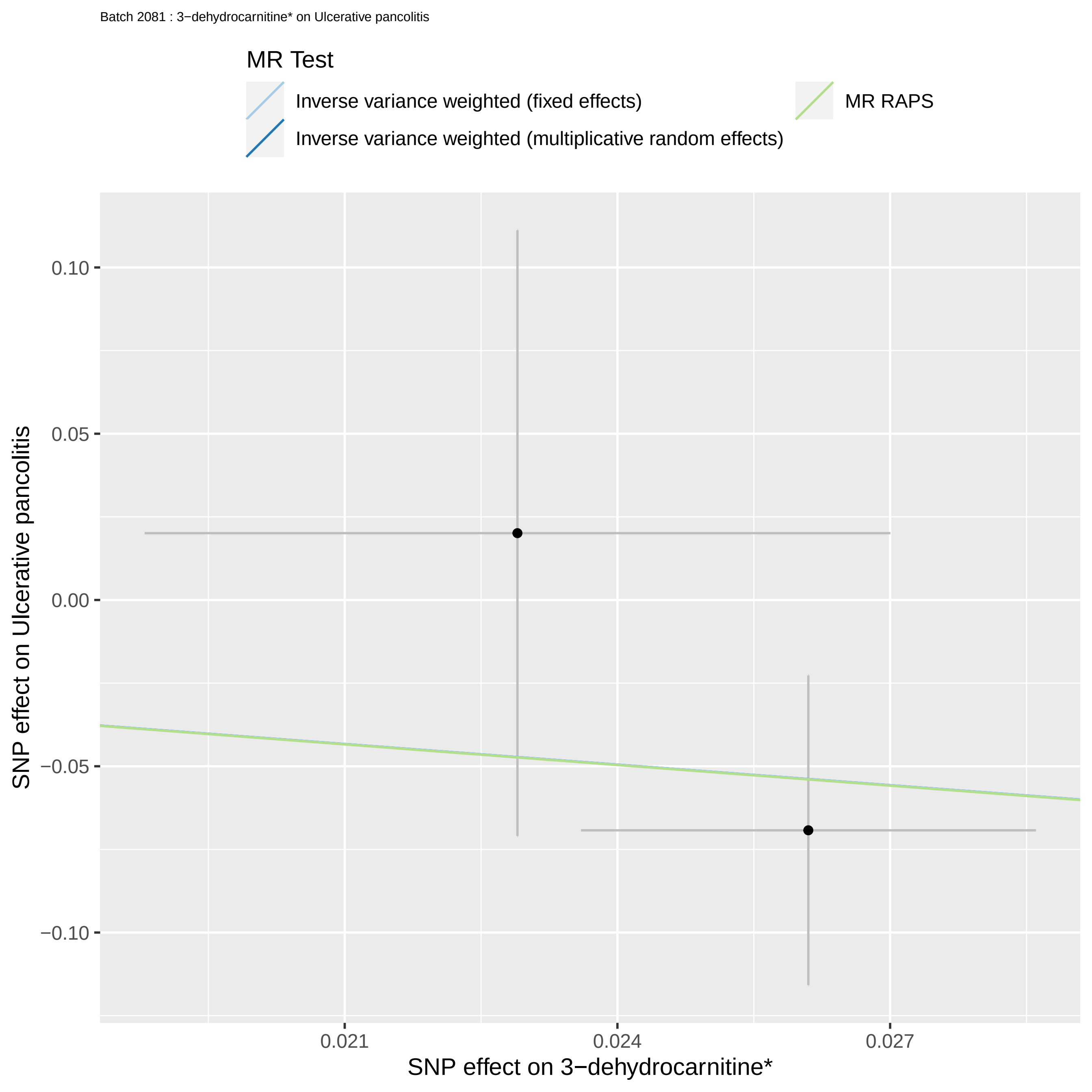

Batch 2081 : 3−dehydrocarnitine* on Ulcerative pancolitis
MR Test
Inverse variance weighted (fixed effects)
Inverse variance weighted (multiplicative random effects)
MR RAPS
0.10
0.05
0.00
−0.05
−0.10
SNP effect on Ulcerative pancolitis
0.021
0.024
0.027
SNP effect on 3−dehydrocarnitine*

Batch 2082 : Pyroglutamine* on Ulcerative pancolitis

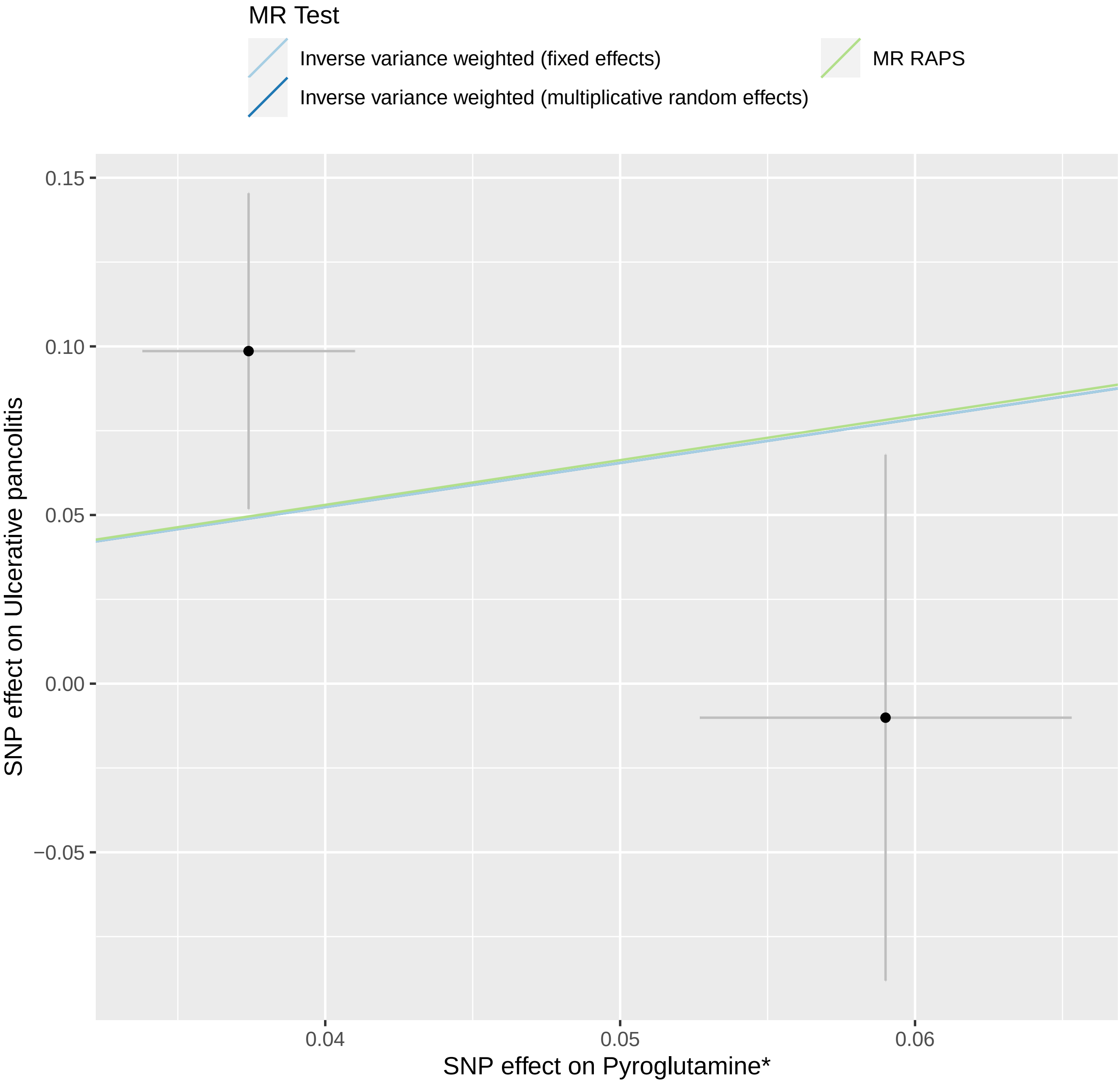

Batch 2085 : X−11422−−xanthine on Ulcerative pancolitis

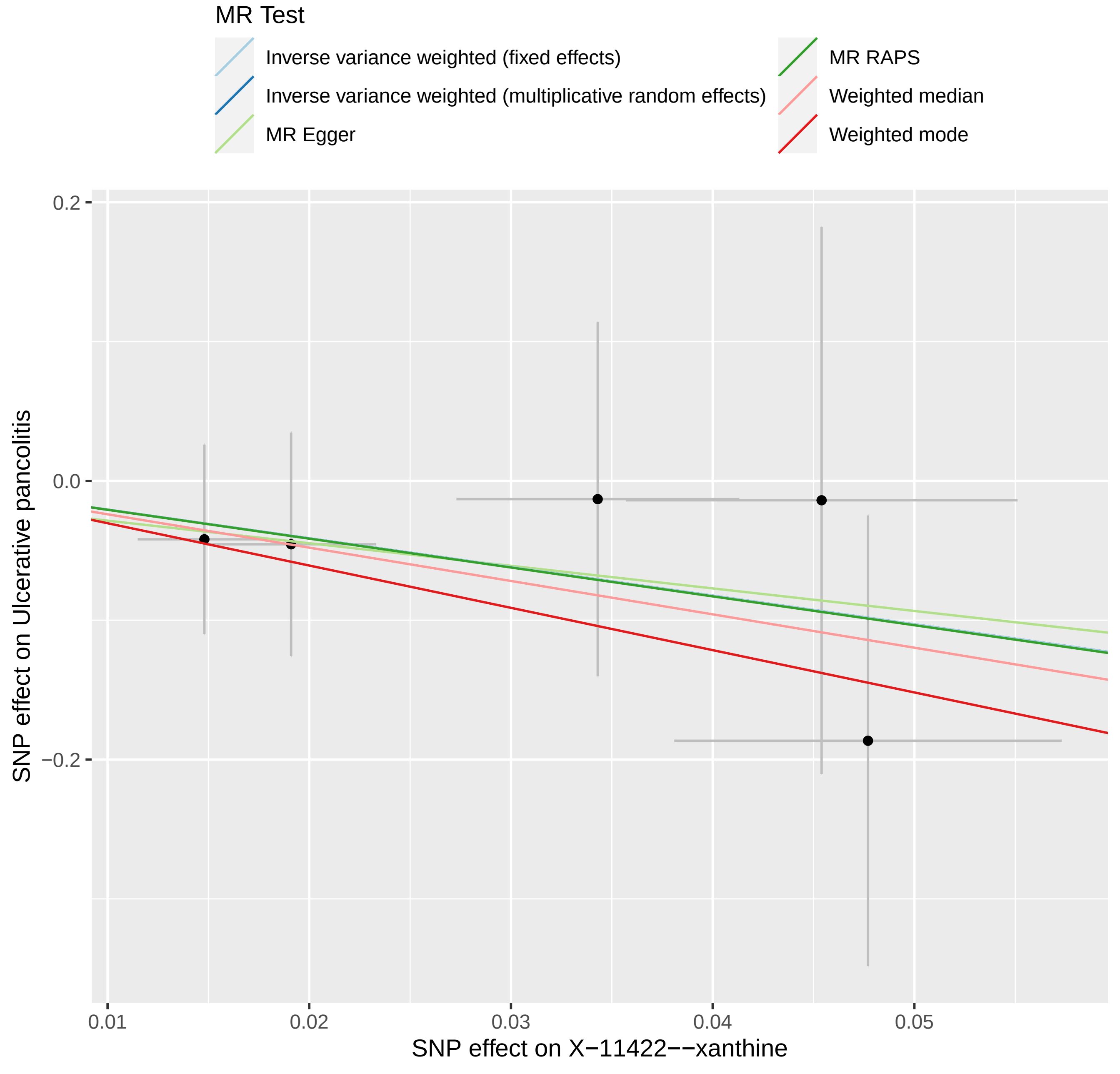

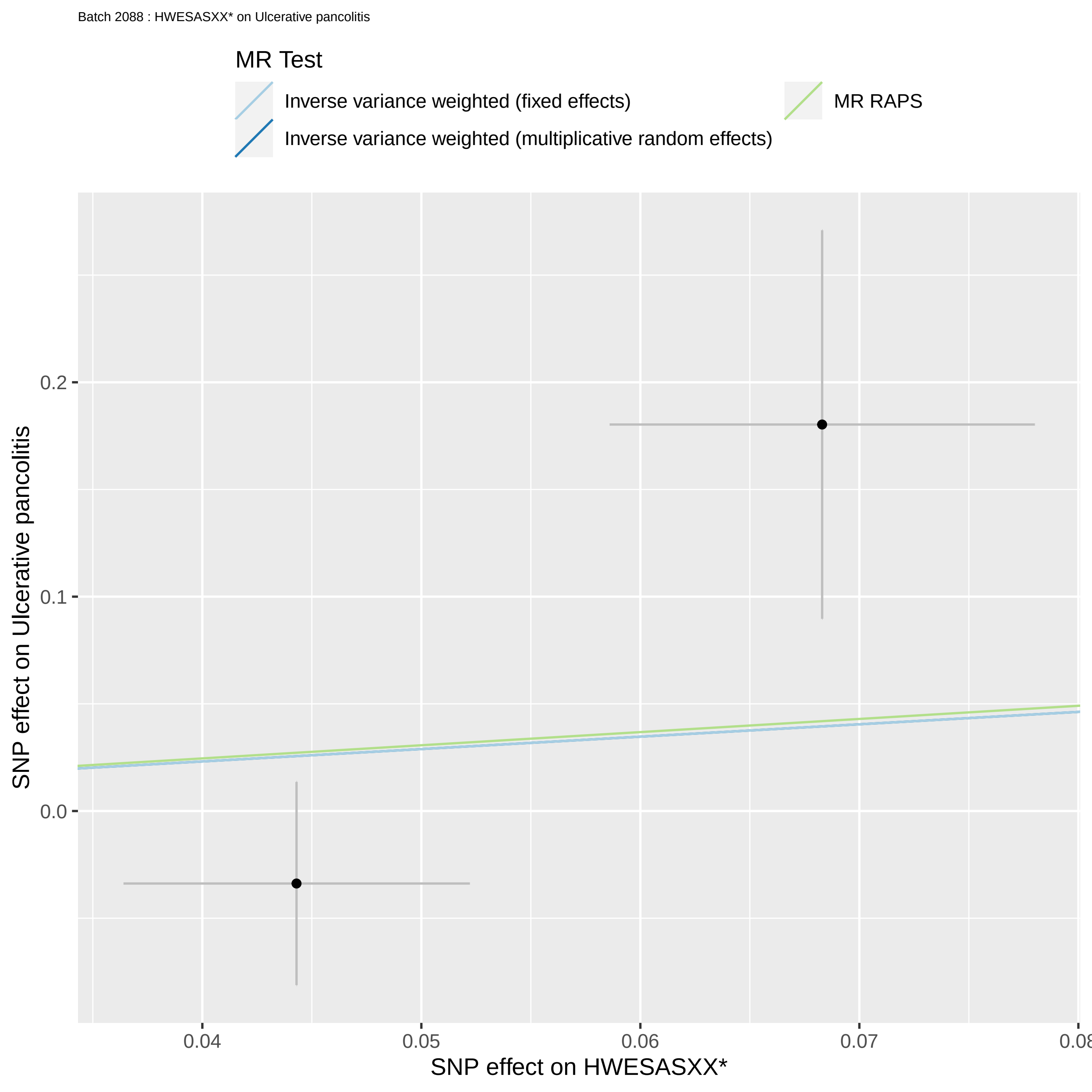

Batch 2088 : HWESASXX* on Ulcerative pancolitis
MR Test
Inverse variance weighted (fixed effects)
Inverse variance weighted (multiplicative random effects)
MR RAPS
0.2
0.1
0.0
0.04
0.05
0.06
0.07
SNP effect on HWESASXX*
SNP effect on Ulcerative pancolitis

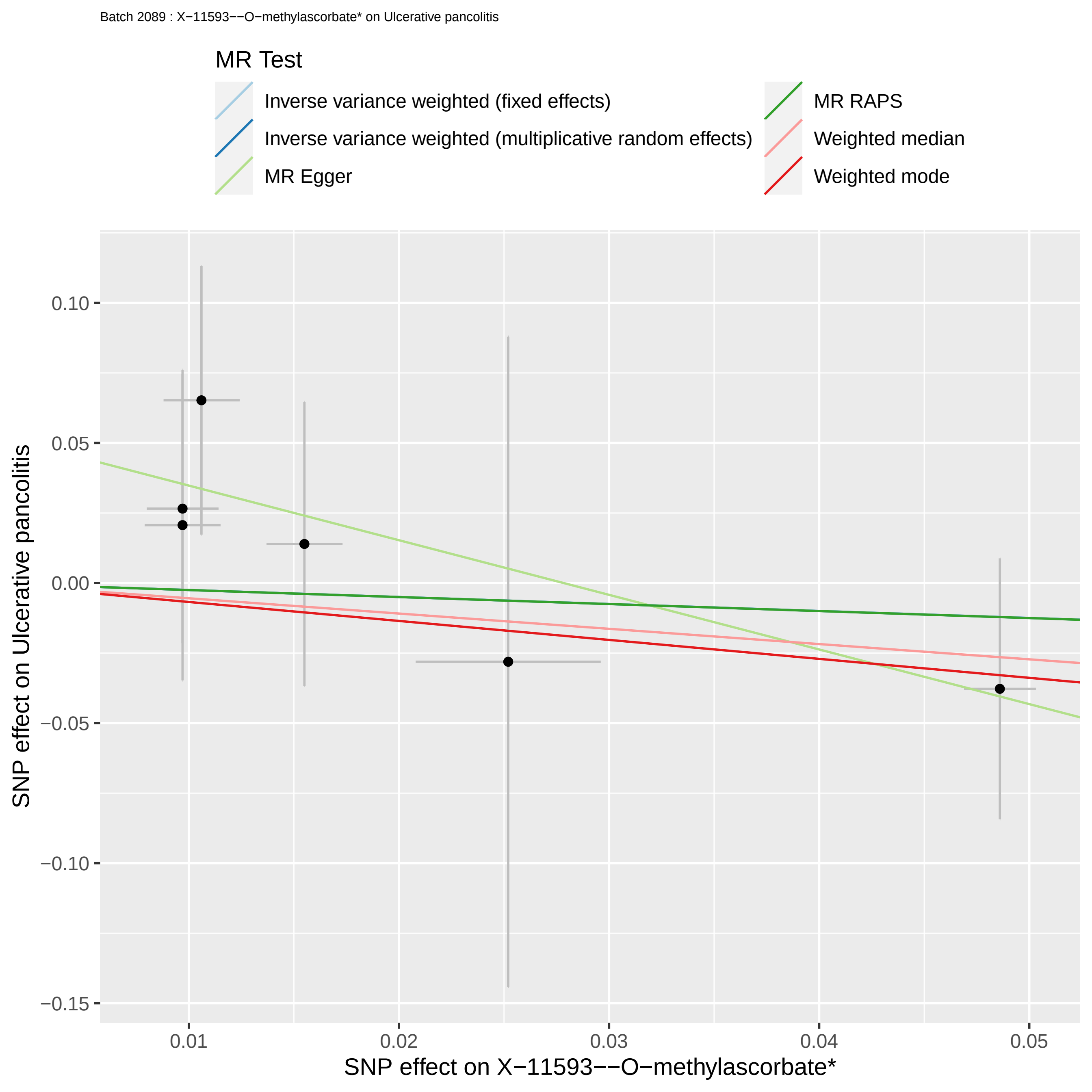
Batch 2089 : X−11593−−O−methylascorbate* on Ulcerative pancolitis
MR Test
Inverse variance weighted (fixed effects)
Inverse variance weighted (multiplicative random effects)
MR Egger
MR RAPS
Weighted median
Weighted mode
SNP effect on Ulcerative pancolitis
0.10
0.05
0.00
−0.05
−0.10
−0.15
0.01
0.02
0.03
0.04
0.05
SNP effect on X−11593−−O−methylascorbate*

Batch 2091 : Homostachydrine* on Ulcerative pancolitis

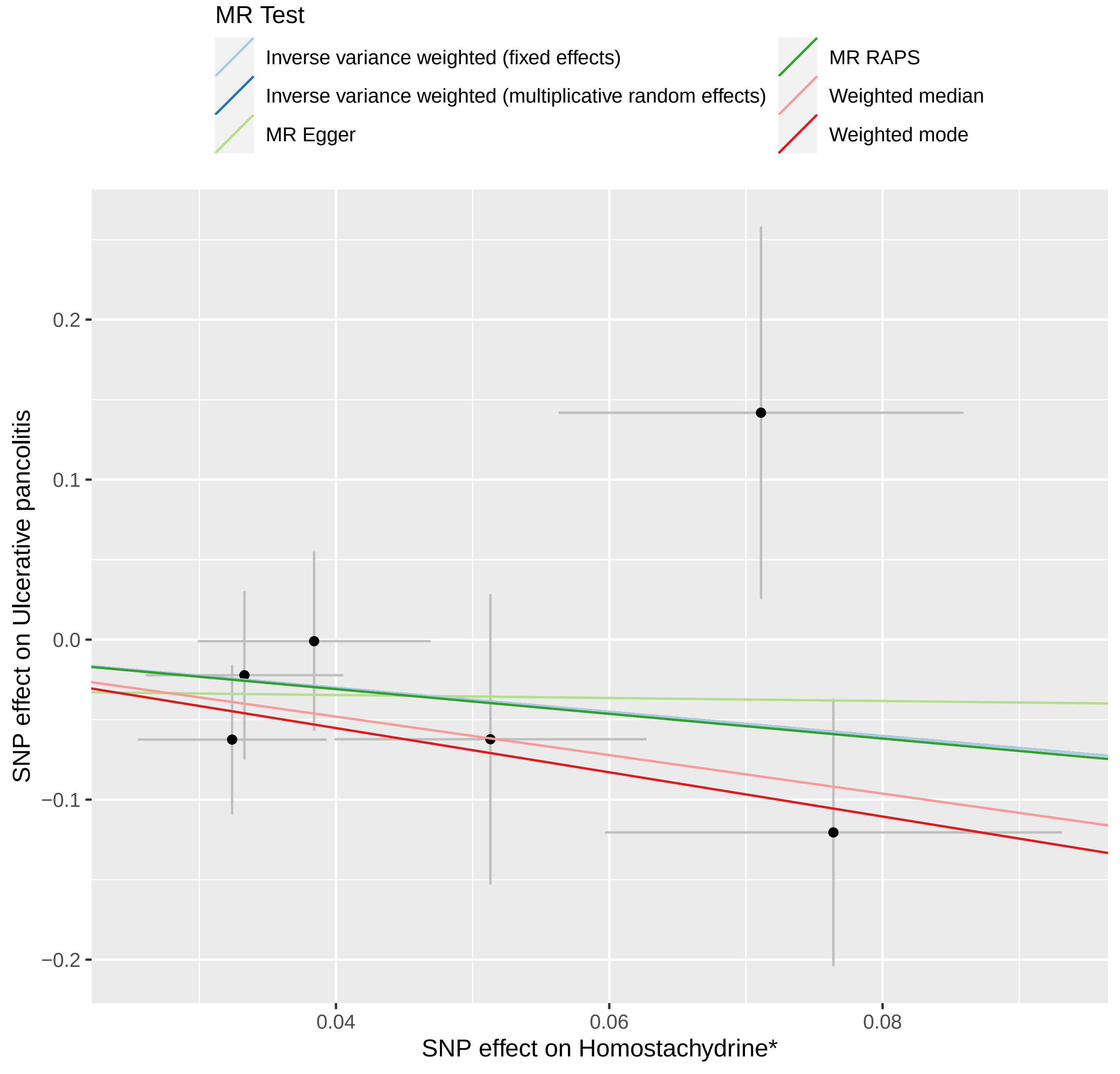

Batch 2092 : ADSGEGDFXAEGGGVR* on Ulcerative pancolitis

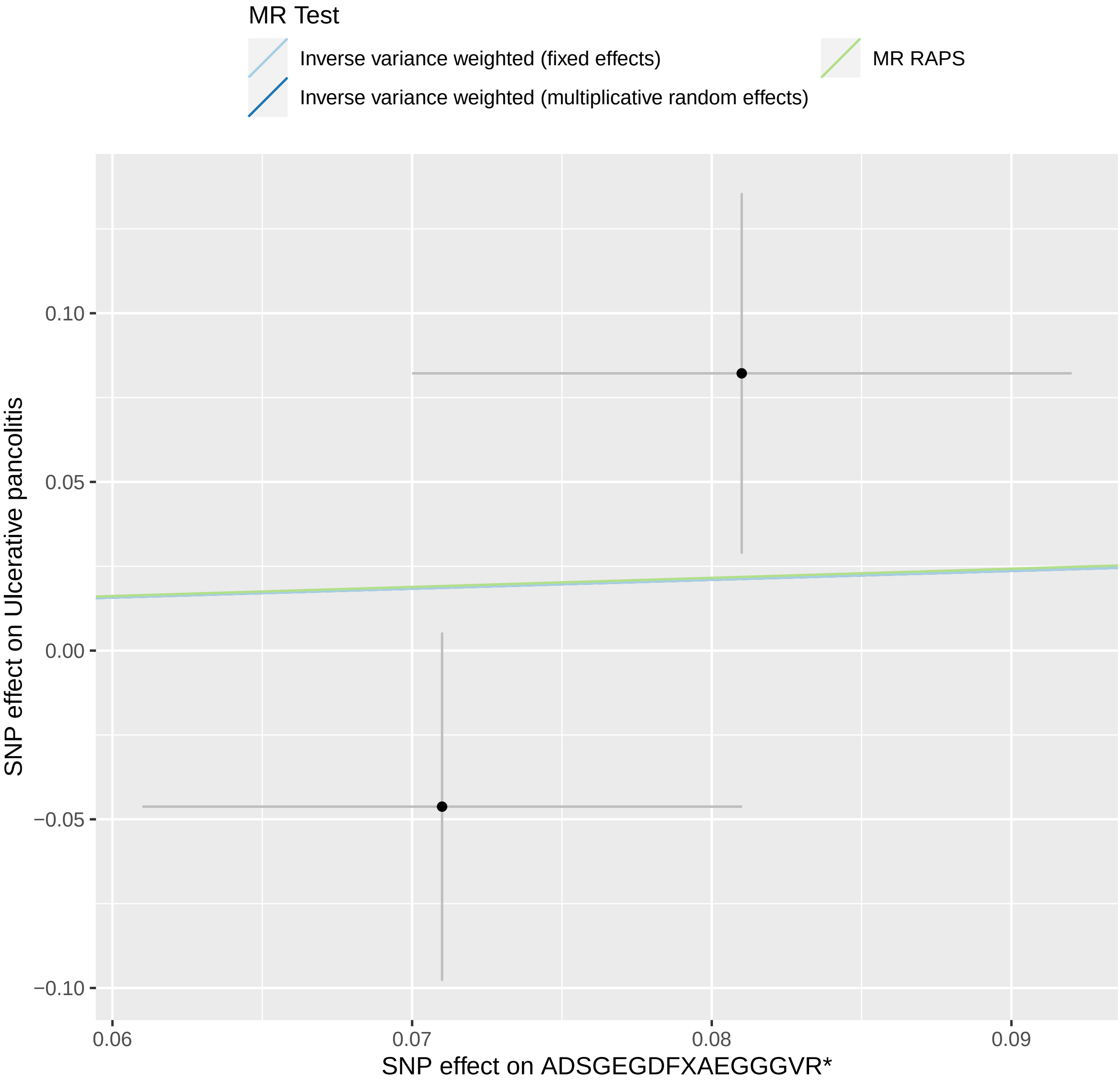

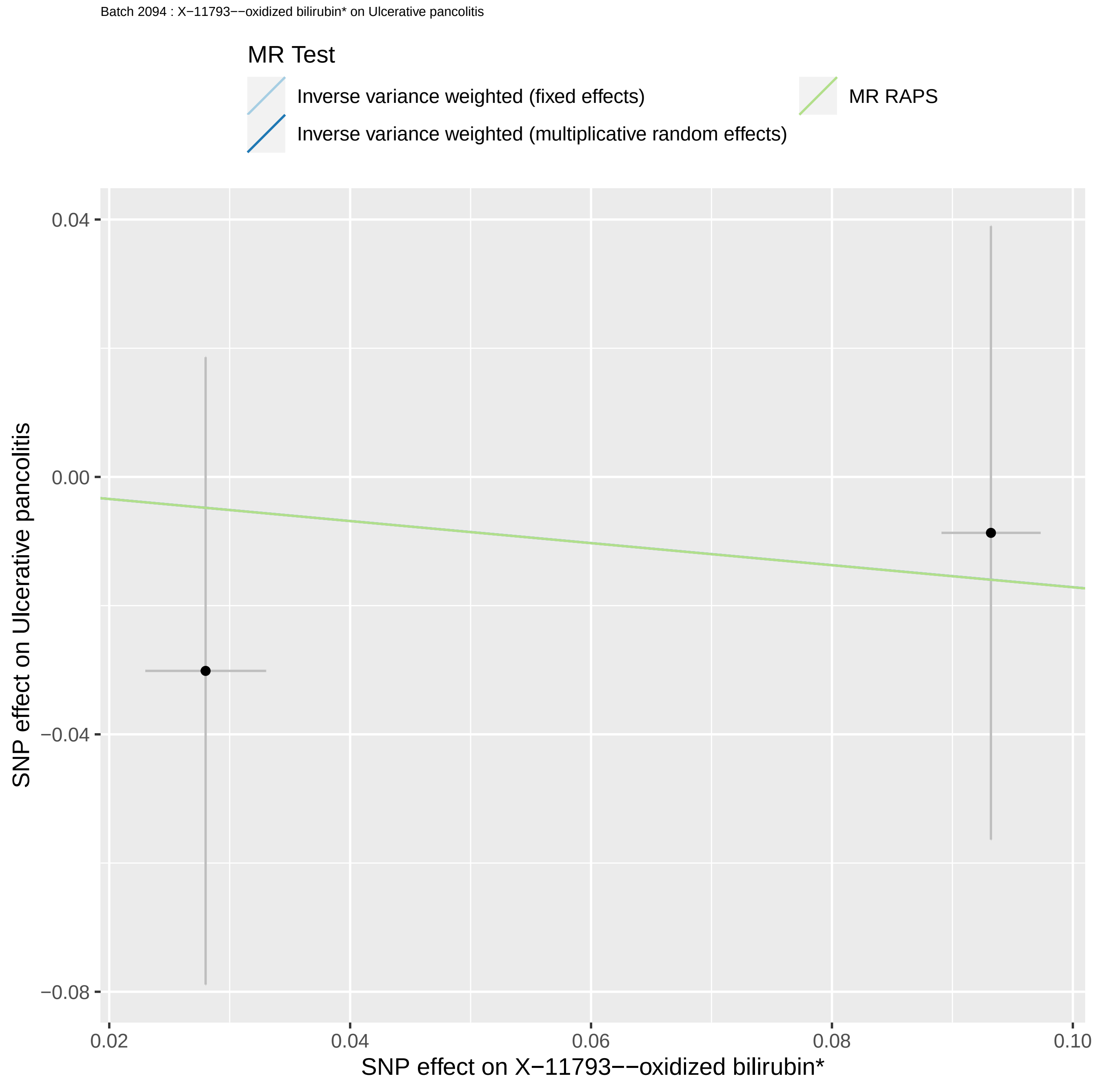
Batch 2094 : X−11793−−oxidized bilirubin* on Ulcerative pancolitis
MR Test
Inverse variance weighted (fixed effects)
Inverse variance weighted (multiplicative random effects)
MR RAPS
0.04
0.00
−0.04
−0.08
0.02
0.04
0.06
0.08
0.10
SNP effect on Ulcerative pancolitis
SNP effect on X−11793−−oxidized bilirubin*

Batch 2095 : 1−arachidonoylglycerophosphocholine* on Ulcerative pancolitis

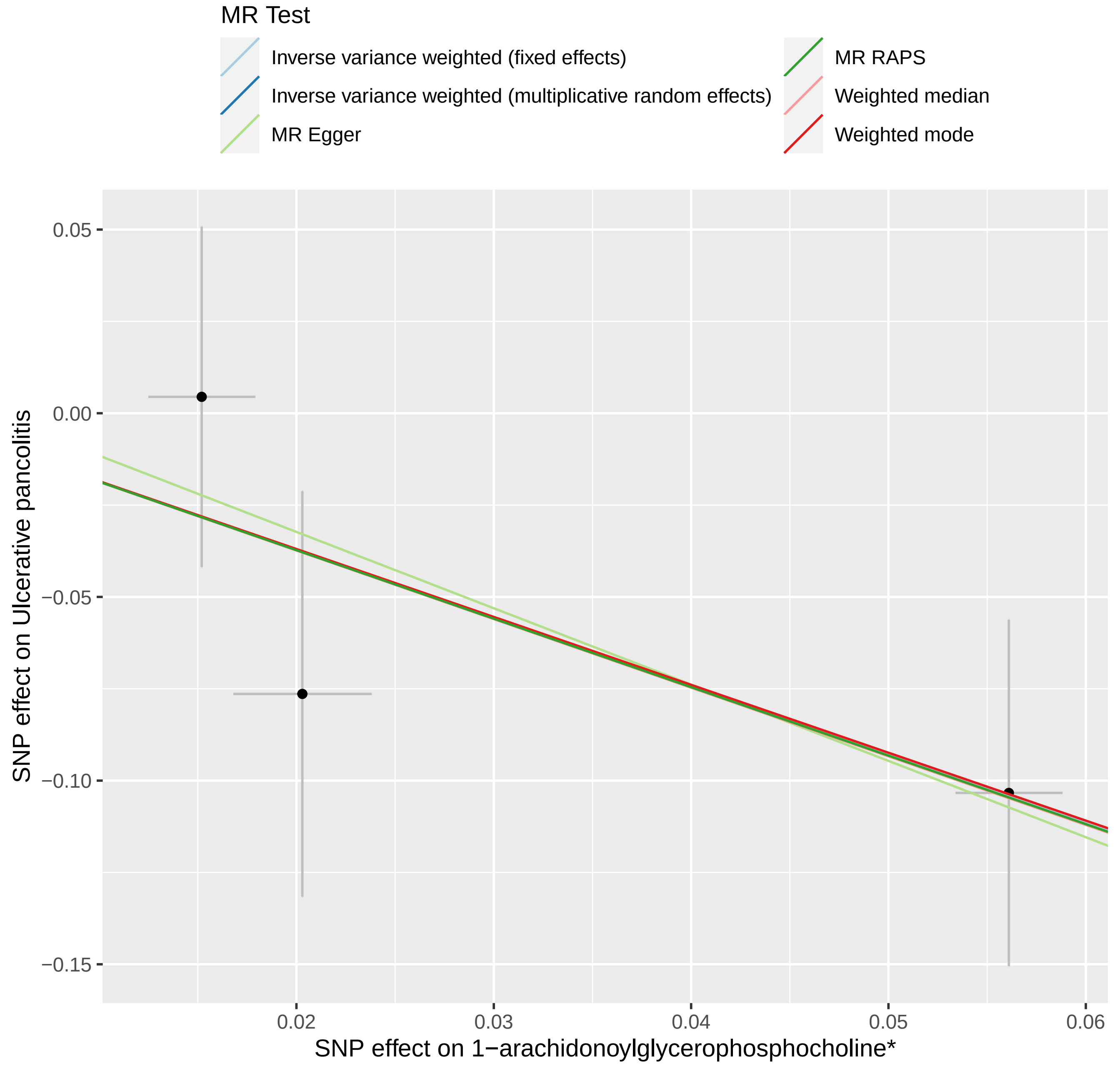

Batch 2097 : Gamma−glutamylmethionine* on Ulcerative pancolitis

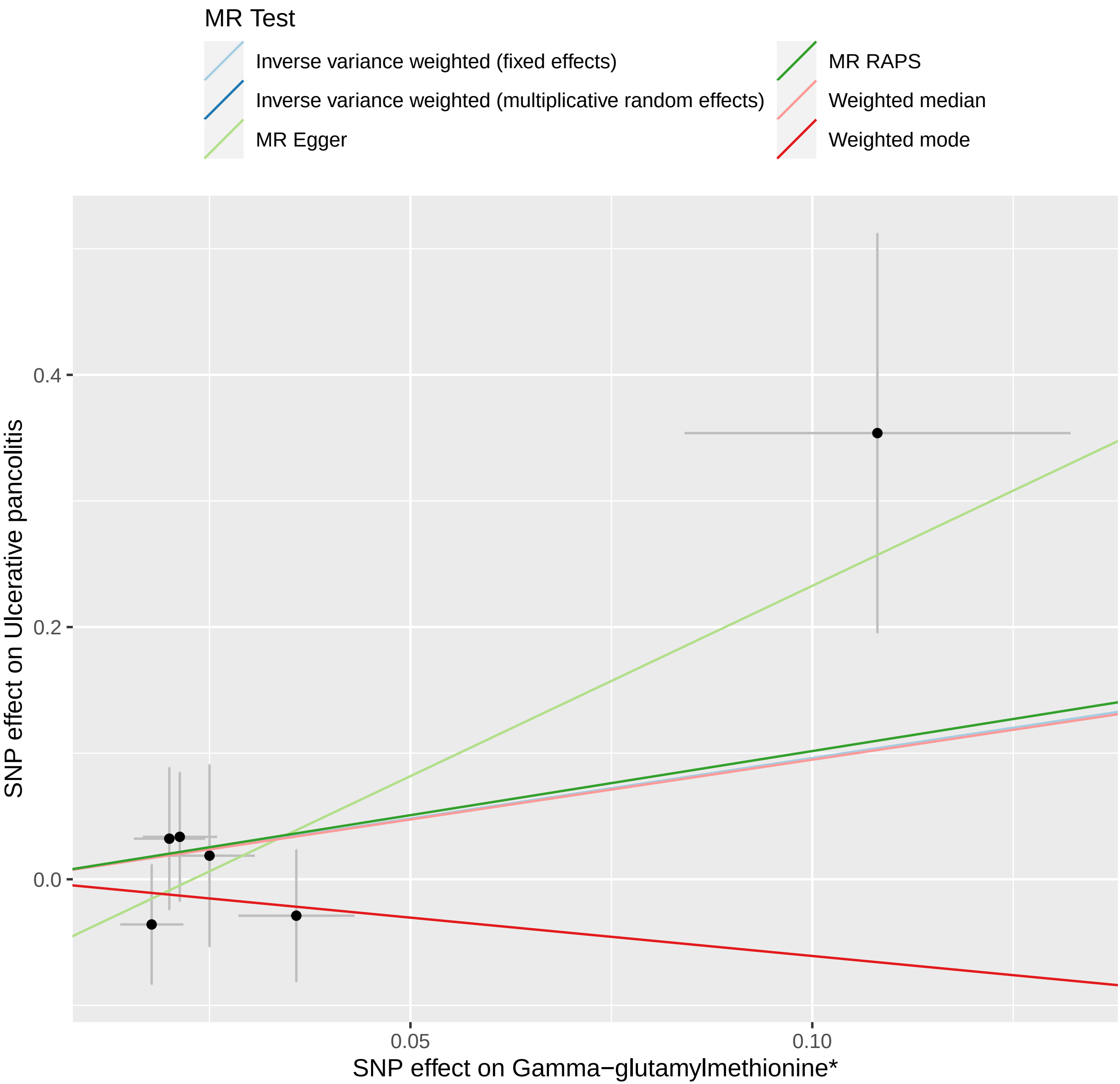

Batch 2098 : Gamma−glutamylthreonine* on Ulcerative pancolitis

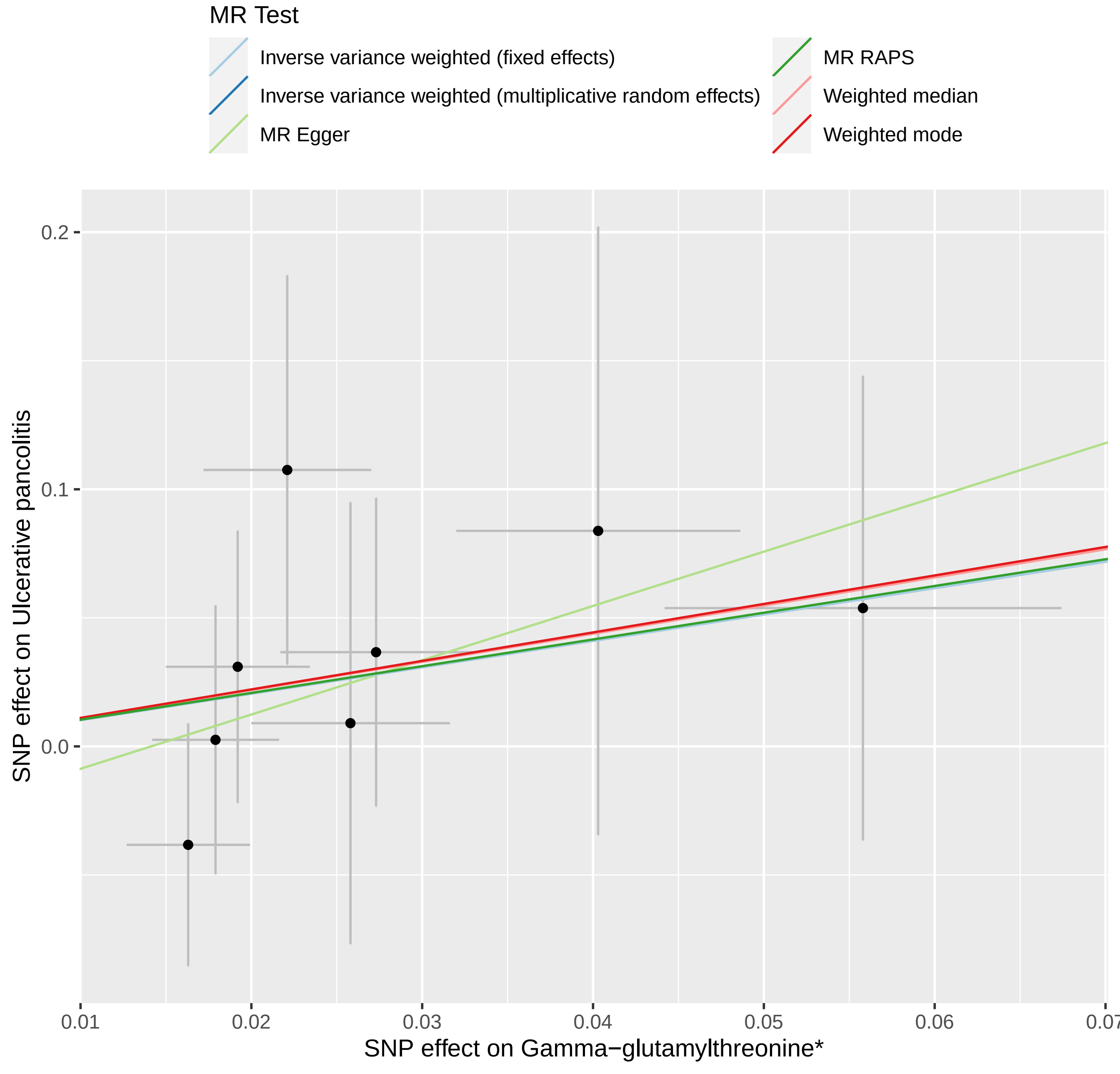

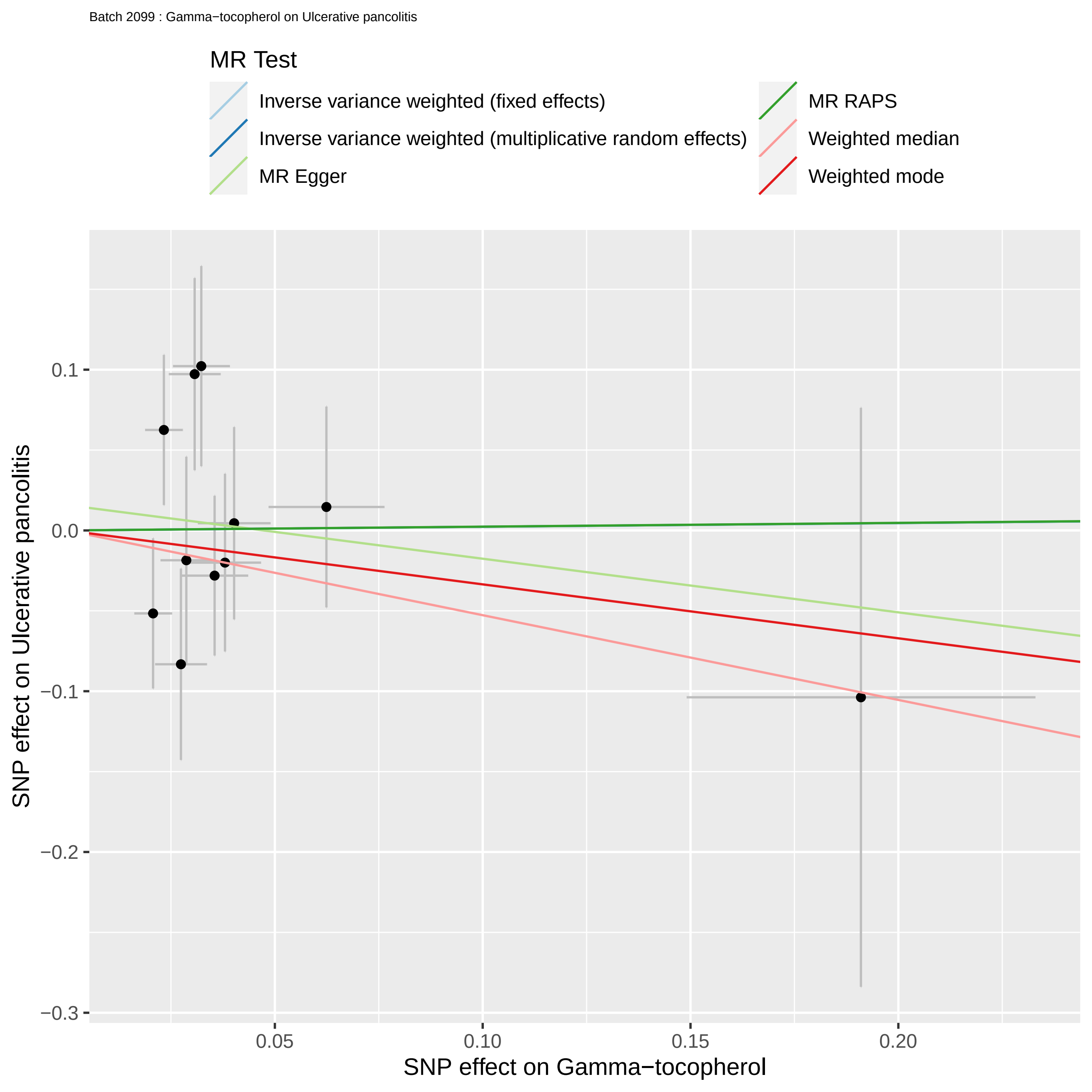

Batch 2099 : Gamma−tocopherol on Ulcerative pancolitis
MR Test
Inverse variance weighted (fixed effects)
Inverse variance weighted (multiplicative random effects)
MR Egger
MR RAPS
Weighted median
Weighted mode
0.1
0.0
−0.1
−0.2
−0.3
0.05
0.10
0.15
0.20
SNP effect on Ulcerative pancolitis
SNP effect on Gamma−tocopherol

Batch 2100 : Gamma−glutamylphenylalanine on Ulcerative pancolitis

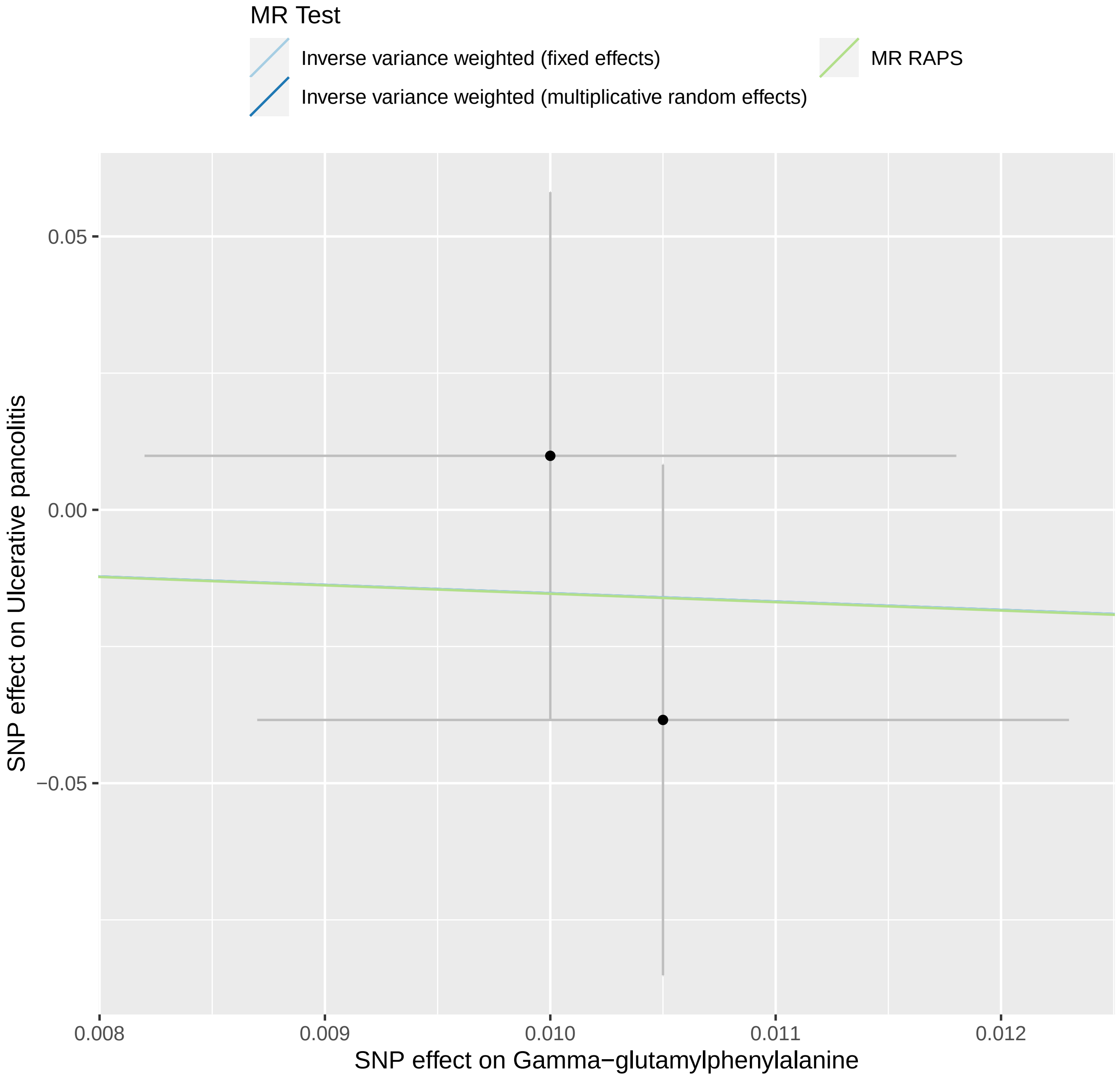

Batch 2101 : Isobutyrylcarnitine on Ulcerative pancolitis

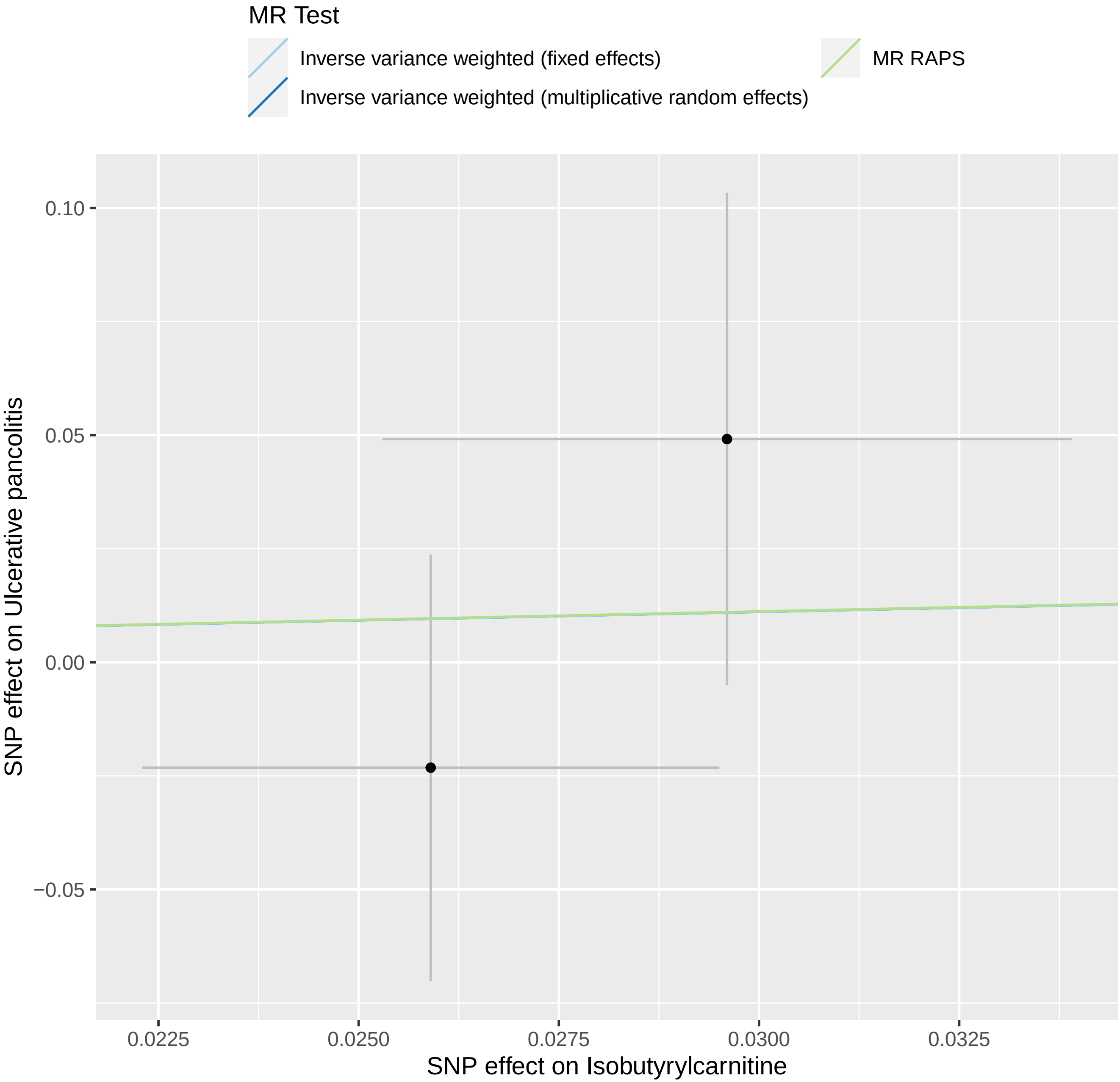

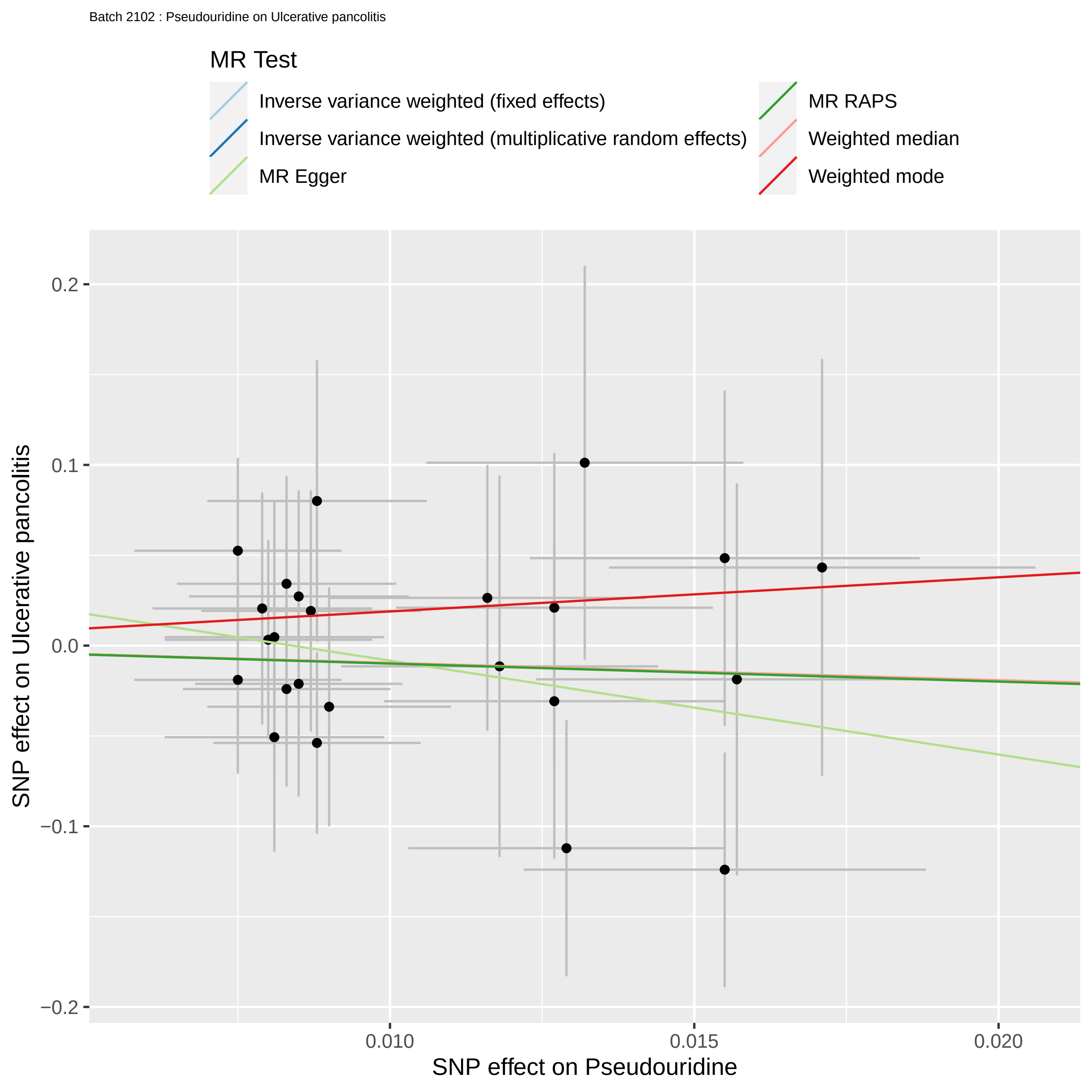

Batch 2102 : Pseudouridine on Ulcerative pancolitis
MR Test
Inverse variance weighted (fixed effects)
Inverse variance weighted (multiplicative random effects)
MR Egger
MR RAPS
Weighted median
Weighted mode
SNP effect on Ulcerative pancolitis
0.2
0.1
0.0
−0.1
−0.2
0.010
0.015
0.020
SNP effect on Pseudouridine

Batch 2103 : Valerate on Ulcerative pancolitis

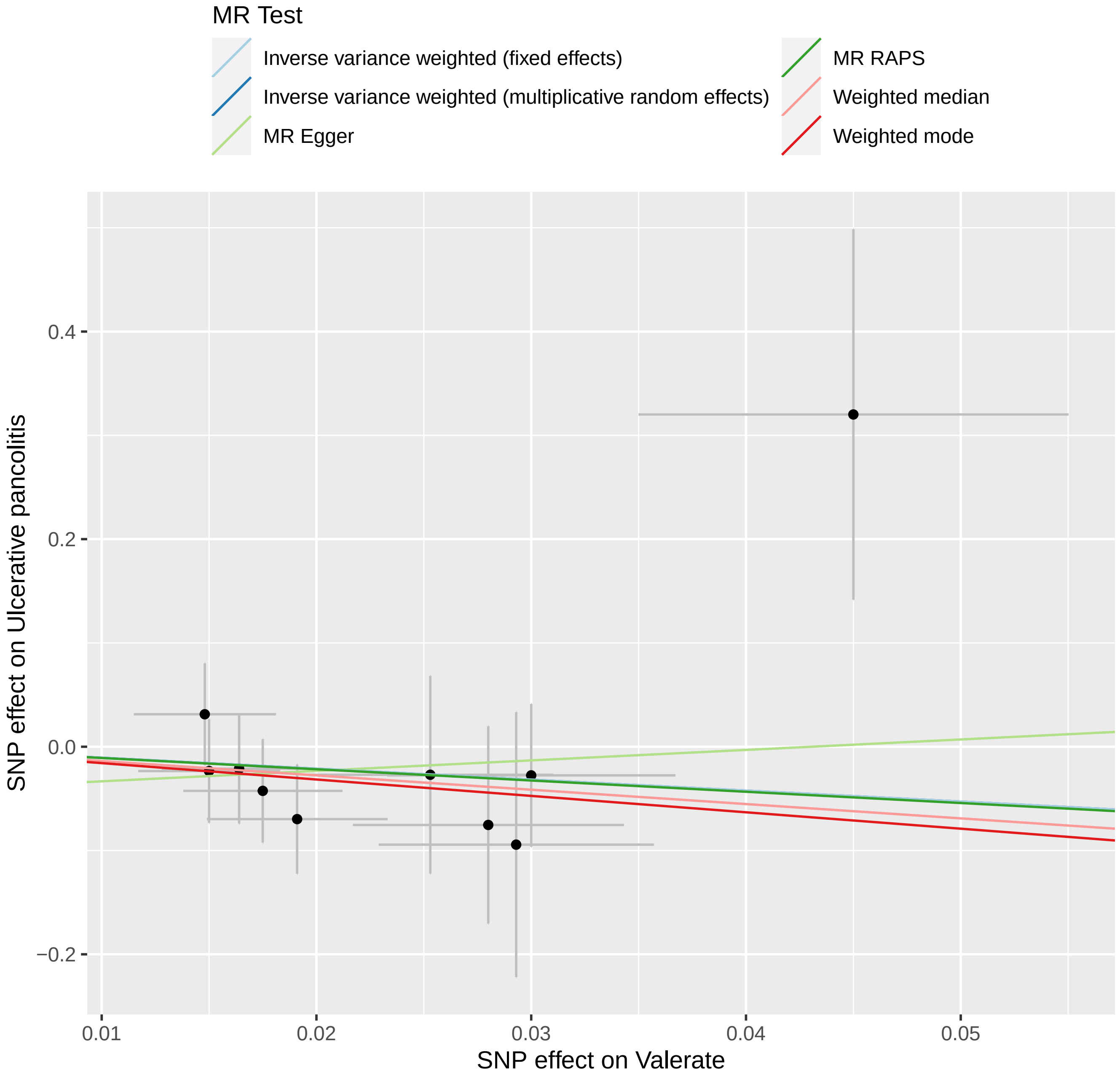

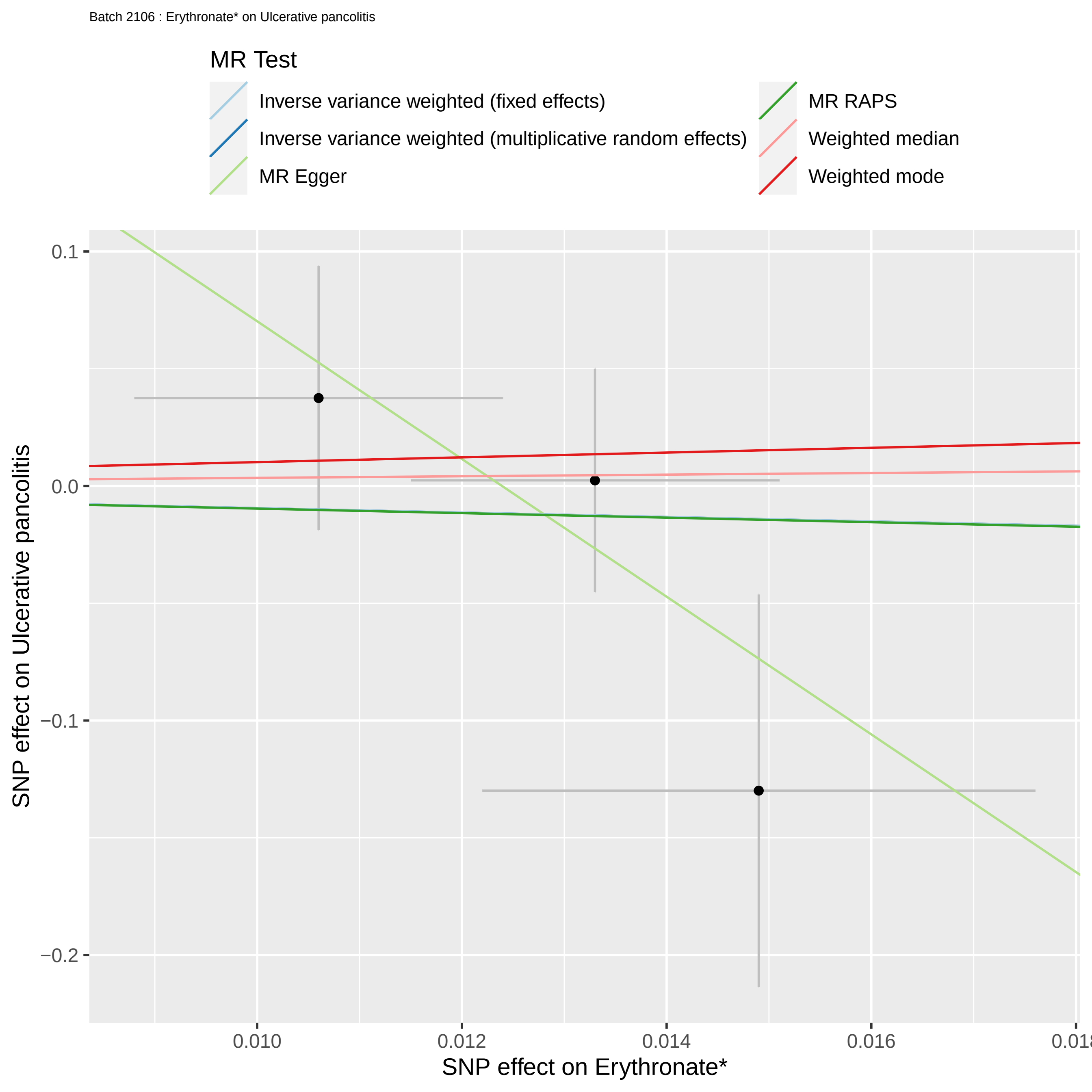

Batch 2106 : Erythronate* on Ulcerative pancolitis
MR Test
Inverse variance weighted (fixed effects)
Inverse variance weighted (multiplicative random effects)
MR Egger
MR RAPS
Weighted median
Weighted mode
0.1
0.0
−0.1
−0.2
SNP effect on Ulcerative pancolitis
0.010
0.012
0.014
0.016
SNP effect on Erythronate*

Batch 2107 : Lathosterol on Ulcerative pancolitis

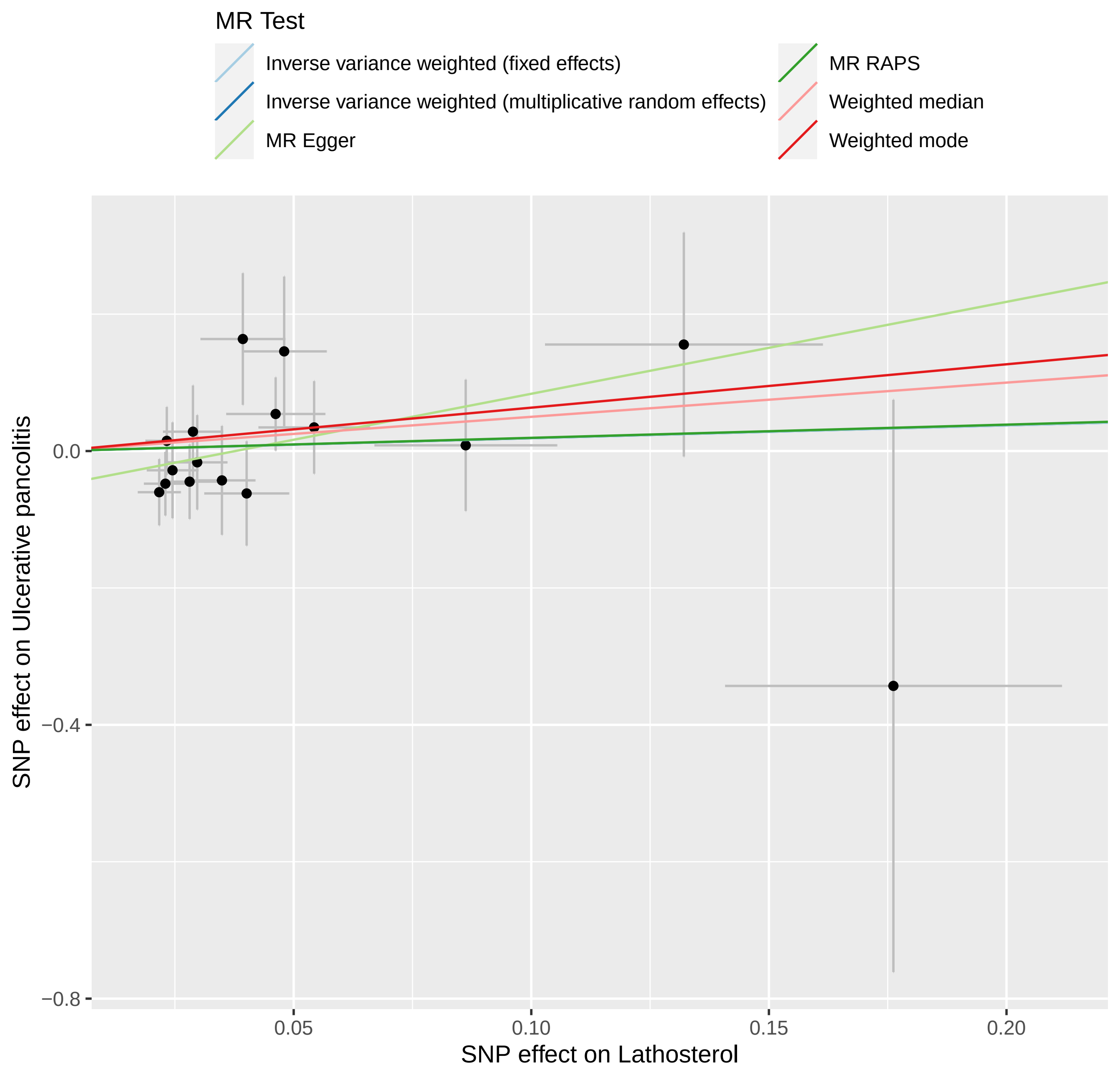

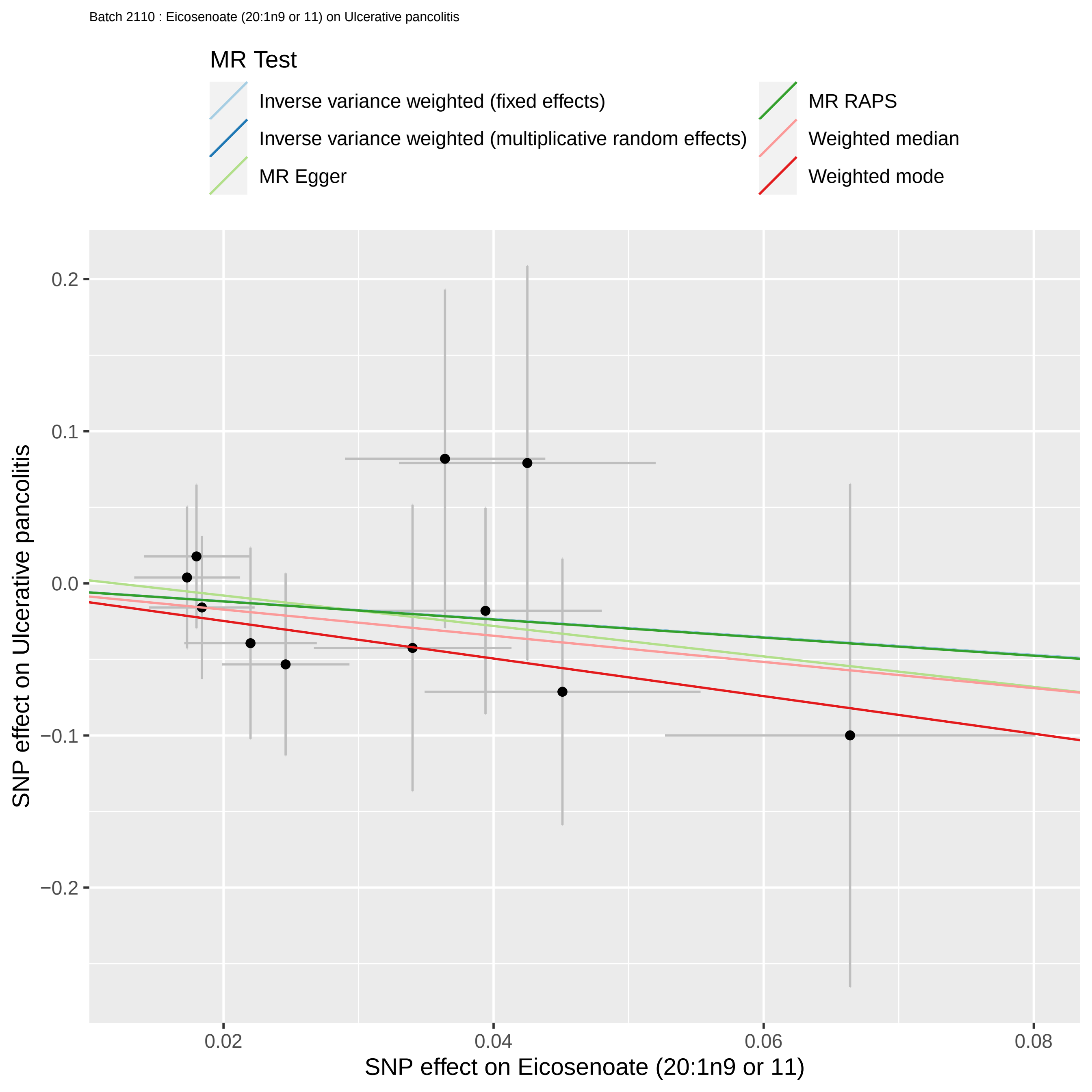

Batch 2110 : Eicosenoate (20:1n9 or 11) on Ulcerative pancolitis
MR Test
Inverse variance weighted (fixed effects)
Inverse variance weighted (multiplicative random effects)
MR Egger
MR RAPS
Weighted median
Weighted mode
0.2
0.1
0.0
−0.1
−0.2
0.02
0.04
0.06
0.08
SNP effect on Ulcerative pancolitis
SNP effect on Eicosenoate (20:1n9 or 11)

Batch 2111 : X−12244−−N−acetylcarnosine on Ulcerative pancolitis

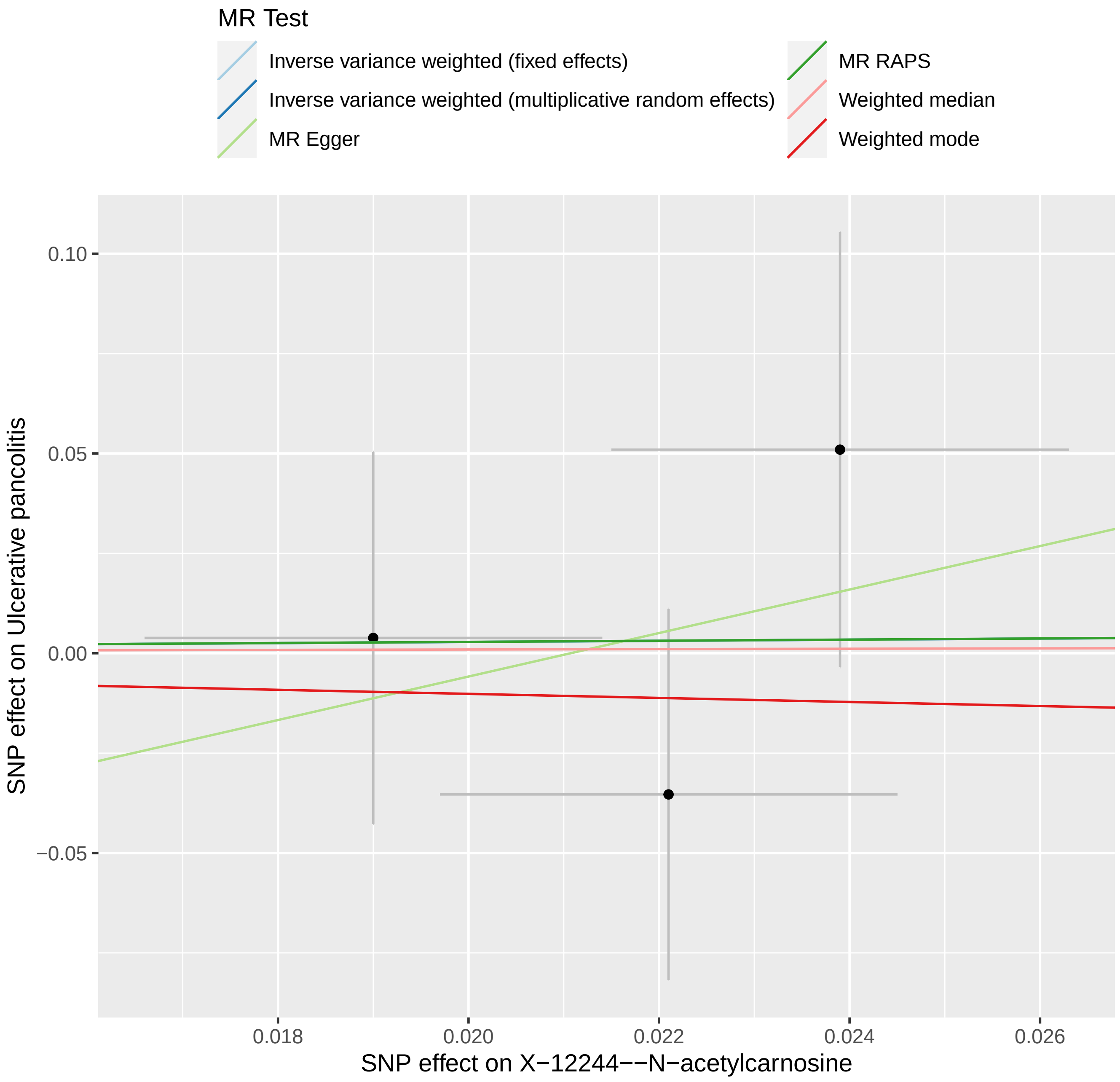

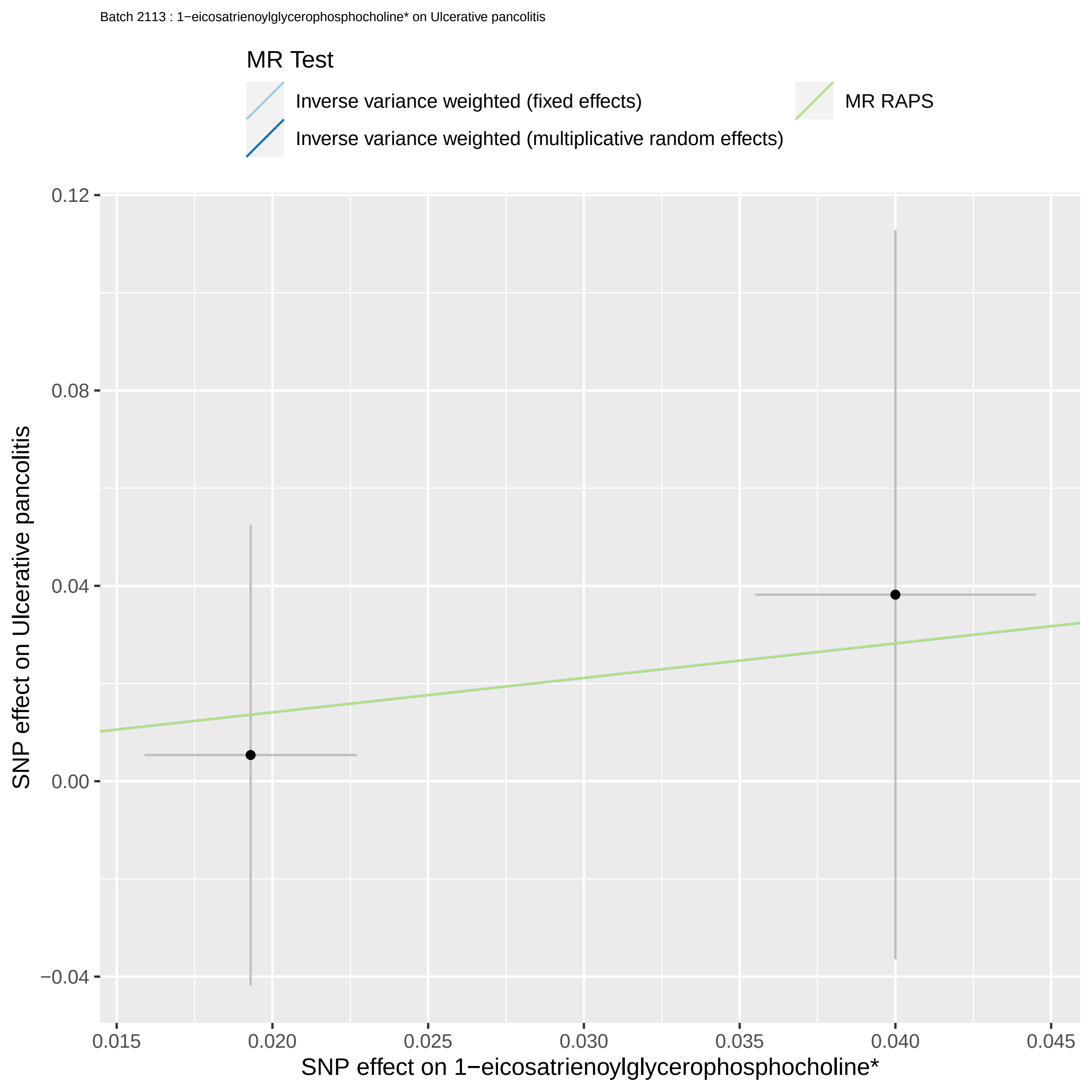

Batch 2113 : 1−eicosatrienoylglycerophosphocholine* on Ulcerative pancolitis
MR Test
Inverse variance weighted (fixed effects)
Inverse variance weighted (multiplicative random effects)
MR RAPS
0.12
0.08
0.04
0.00
−0.04
0.015
0.020
0.025
0.030
0.035
0.040
0.045
SNP effect on Ulcerative pancolitis
SNP effect on 1−eicosatrienoylglycerophosphocholine*

Batch 2114 : 1−docosahexaenoylglycerophosphocholine* on Ulcerative pancolitis

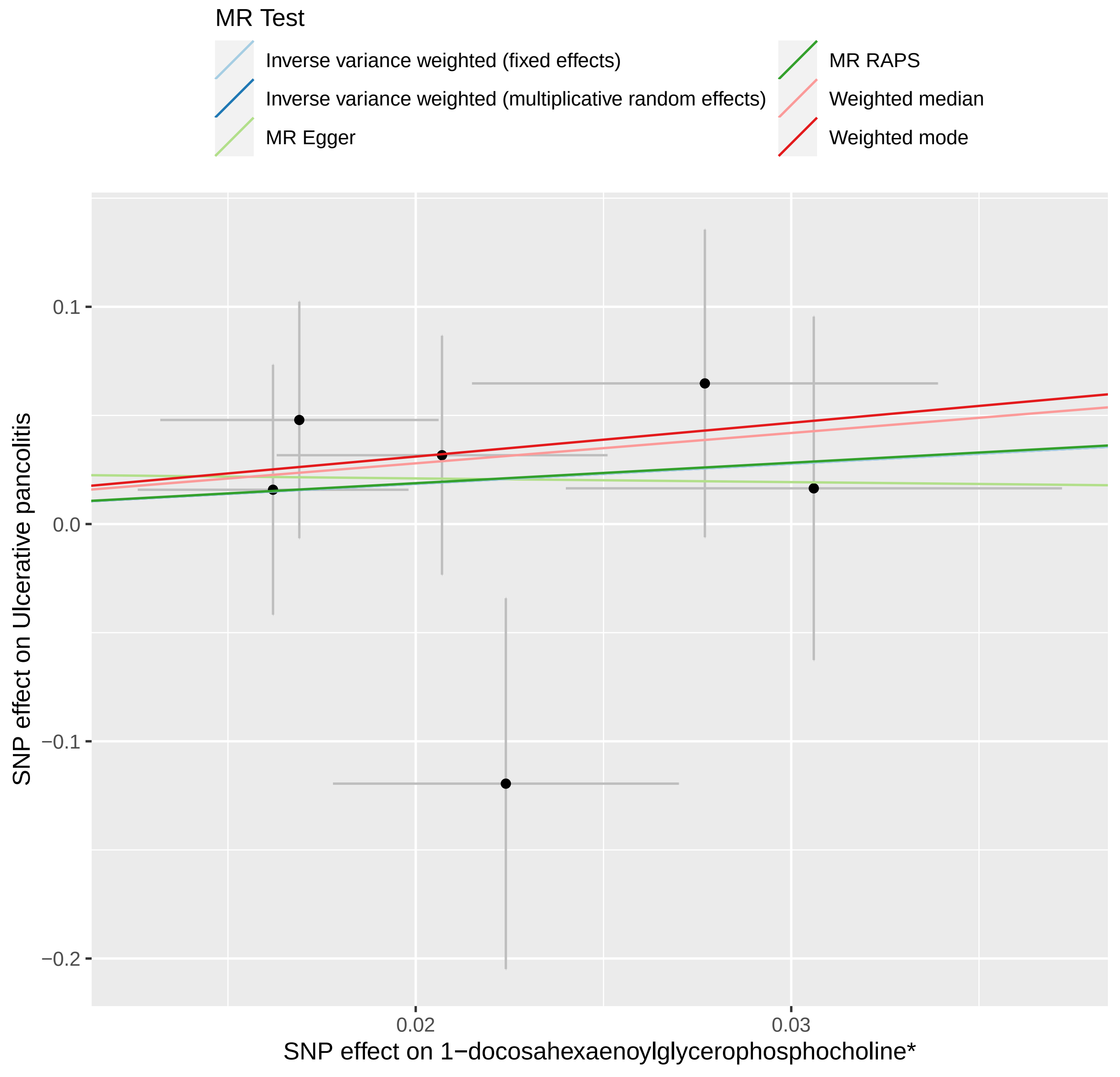

Batch 2118 : Octanoylcarnitine on Ulcerative pancolitis

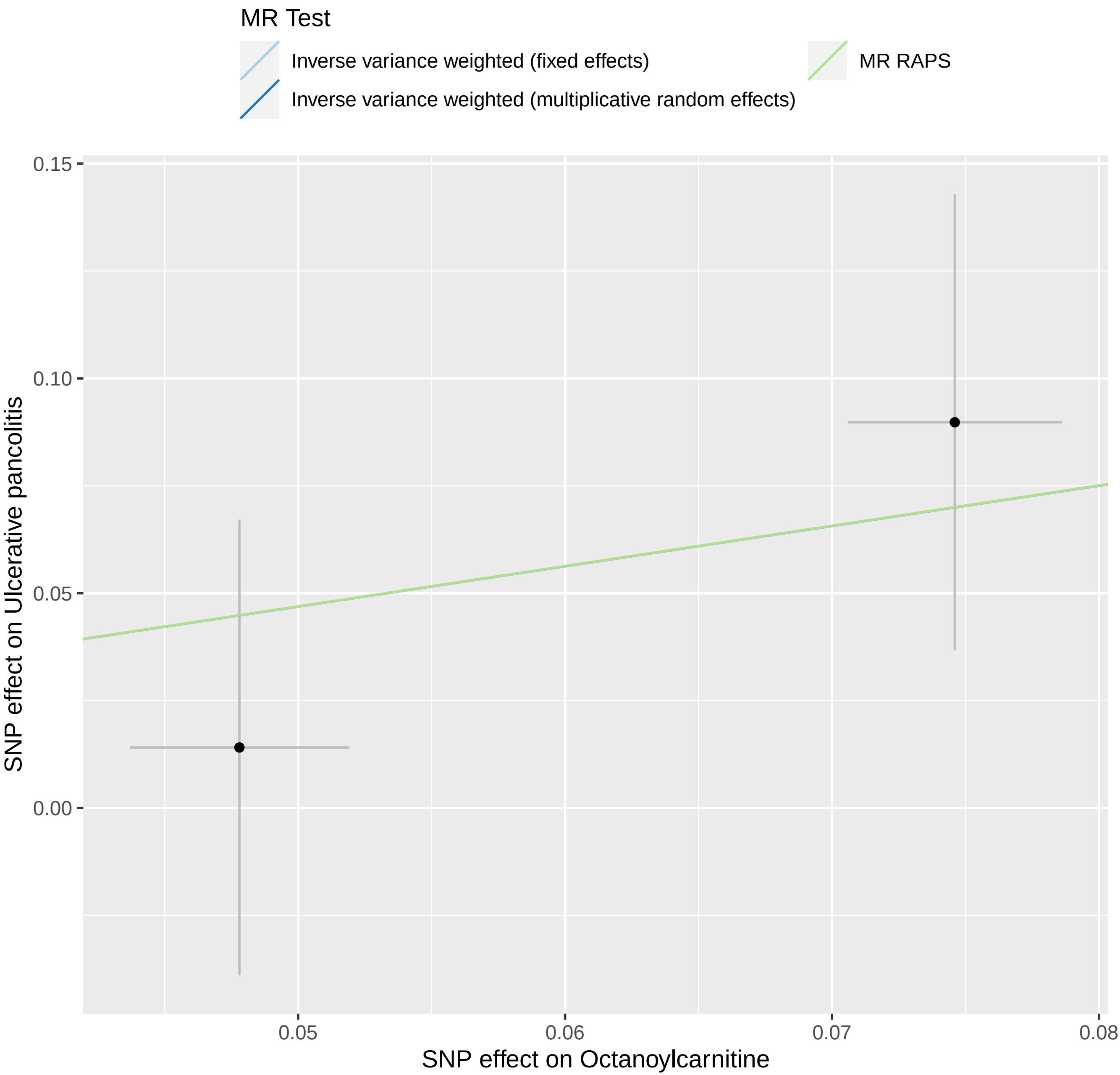

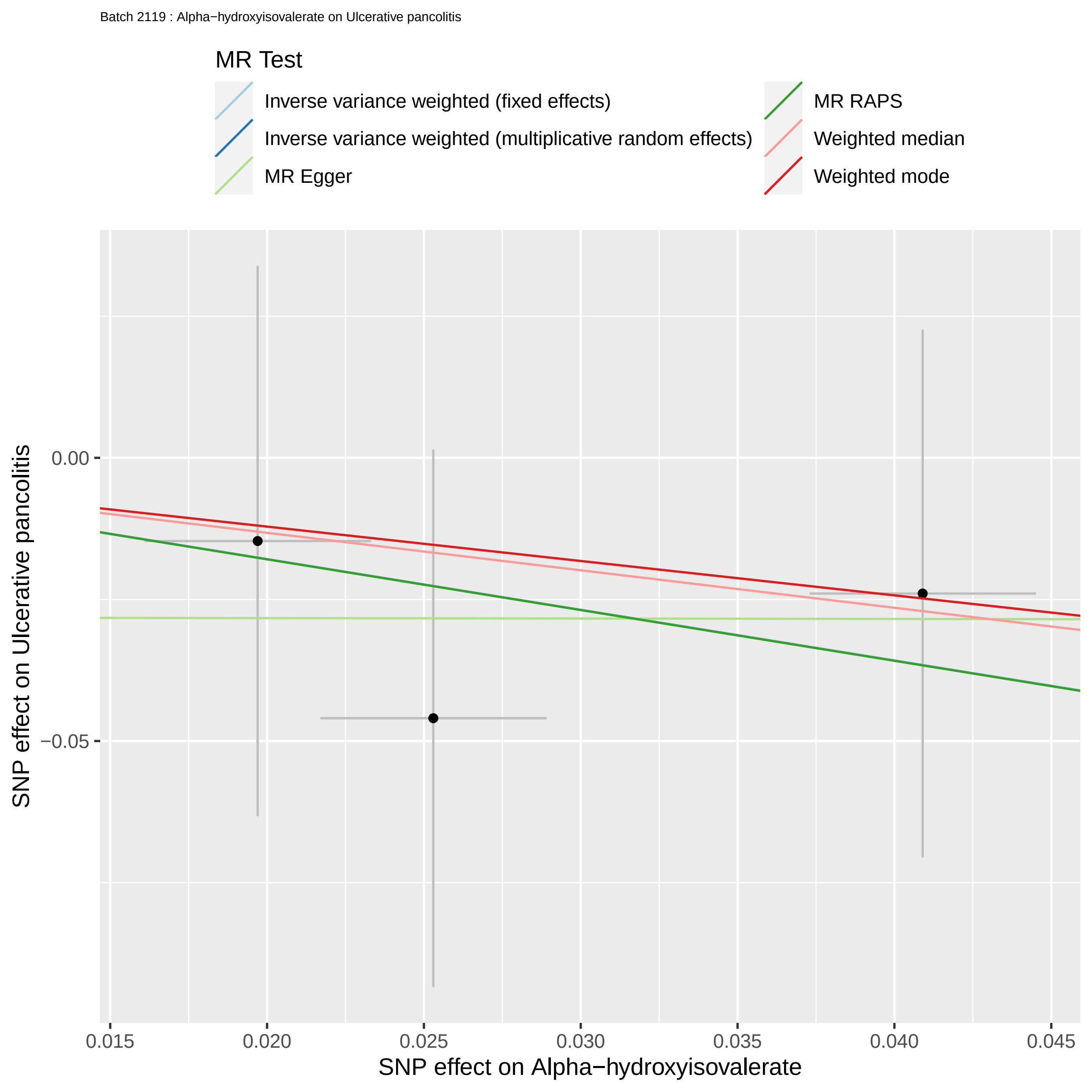

Batch 2119 : Alpha−hydroxyisovalerate on Ulcerative pancolitis
MR Test
Inverse variance weighted (fixed effects)
Inverse variance weighted (multiplicative random effects)
MR Egger
MR RAPS
Weighted median
Weighted mode
0.00
−0.05
SNP effect on Ulcerative pancolitis
0.015
0.020
0.025
0.030
0.035
0.040
0.045
SNP effect on Alpha−hydroxyisovalerate

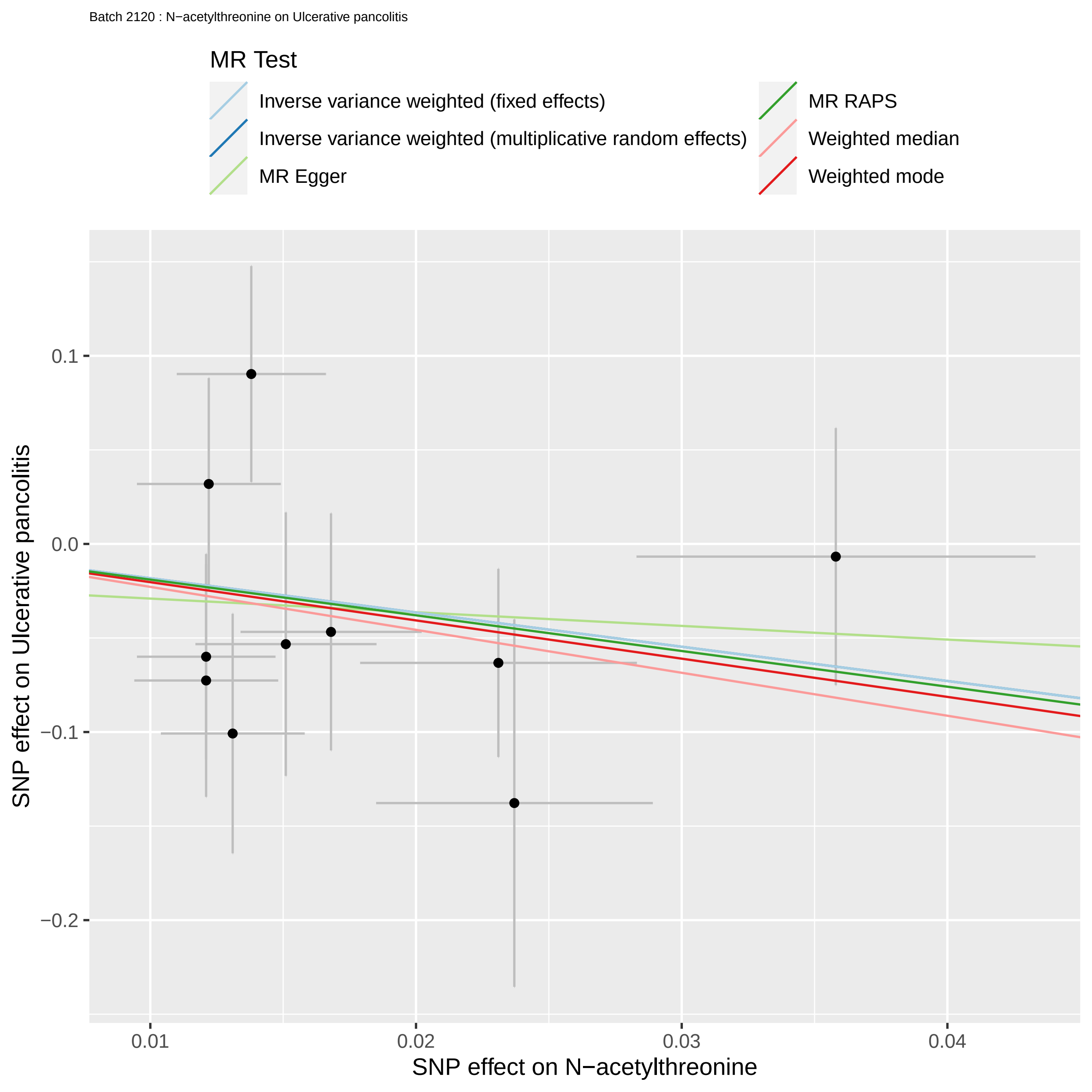

Batch 2120 : N−acetylthreonine on Ulcerative pancolitis
MR Test
Inverse variance weighted (fixed effects)
Inverse variance weighted (multiplicative random effects)
MR Egger
MR RAPS
Weighted median
Weighted mode
SNP effect on Ulcerative pancolitis
0.1
0.0
−0.1
−0.2
0.01
0.02
0.03
0.04
SNP effect on N−acetylthreonine

Batch 2121 : Decanoylcarnitine on Ulcerative pancolitis

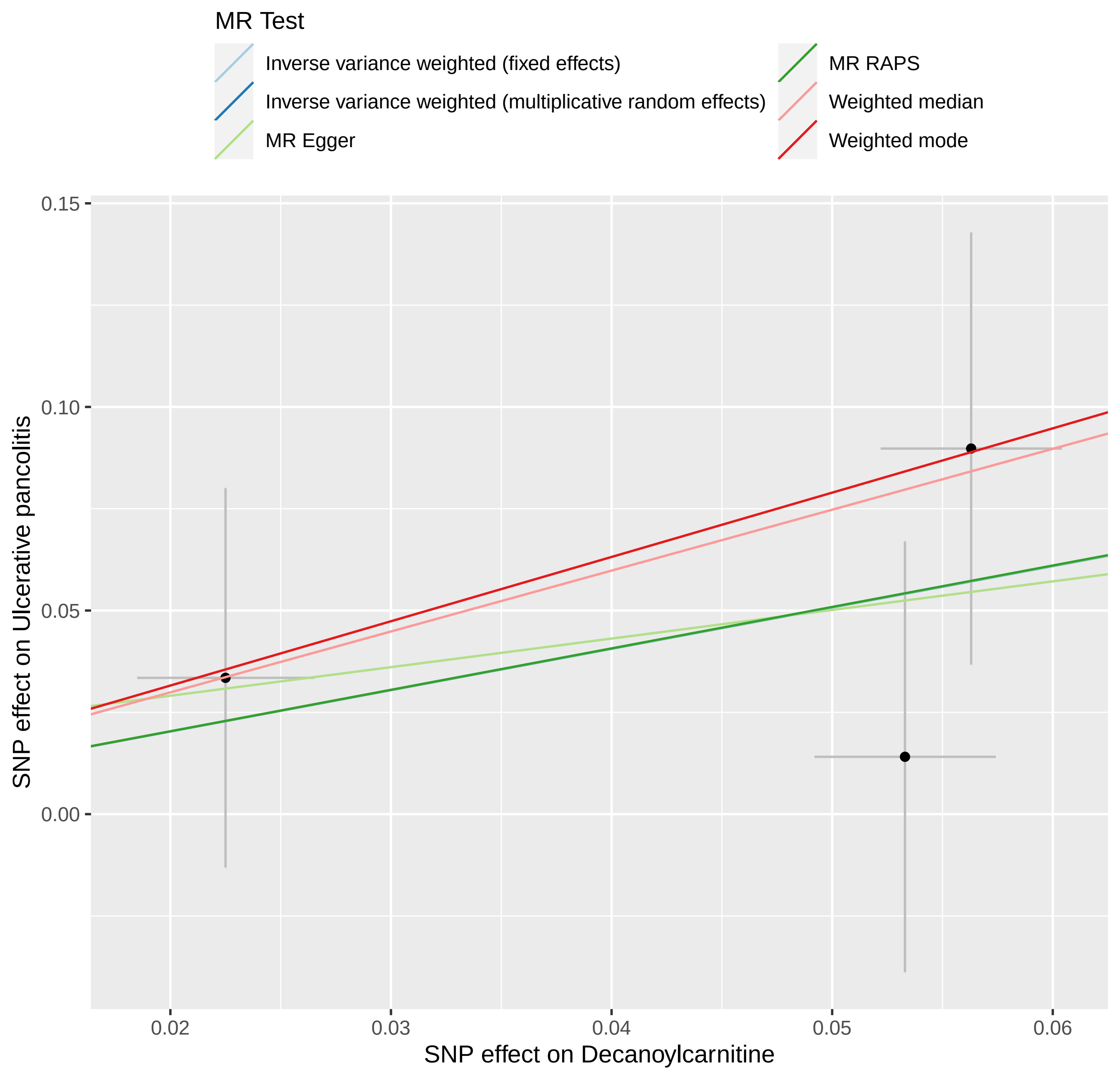

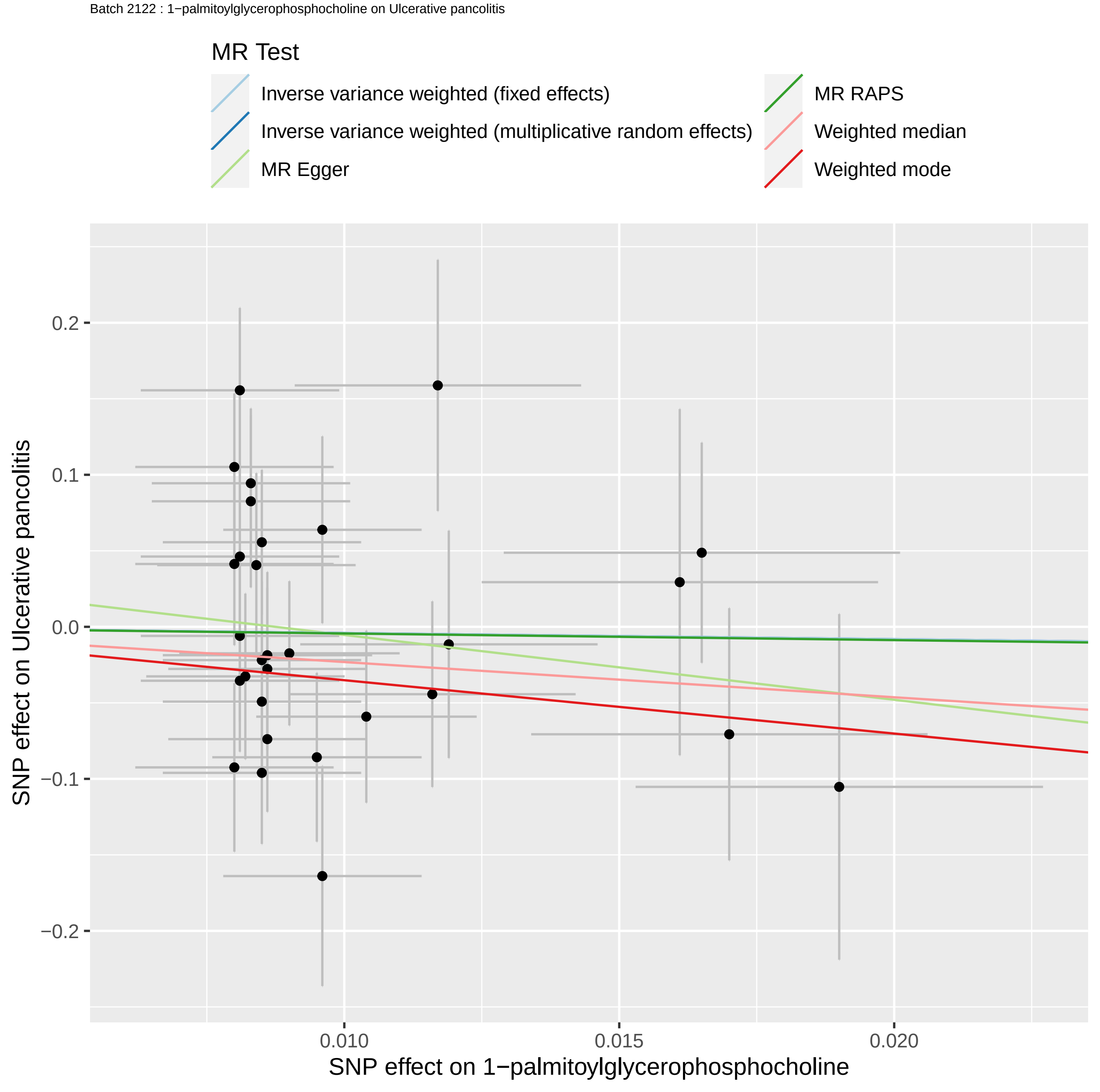
Batch 2122 : 1−palmitoylglycerophosphocholine on Ulcerative pancolitis
MR Test
Inverse variance weighted (fixed effects)
Inverse variance weighted (multiplicative random effects)
MR Egger
MR RAPS
Weighted median
Weighted mode
0.2
0.1
0.0
−0.1
−0.2
SNP effect on Ulcerative pancolitis
0.010
0.015
0.020
SNP effect on 1−palmitoylglycerophosphocholine

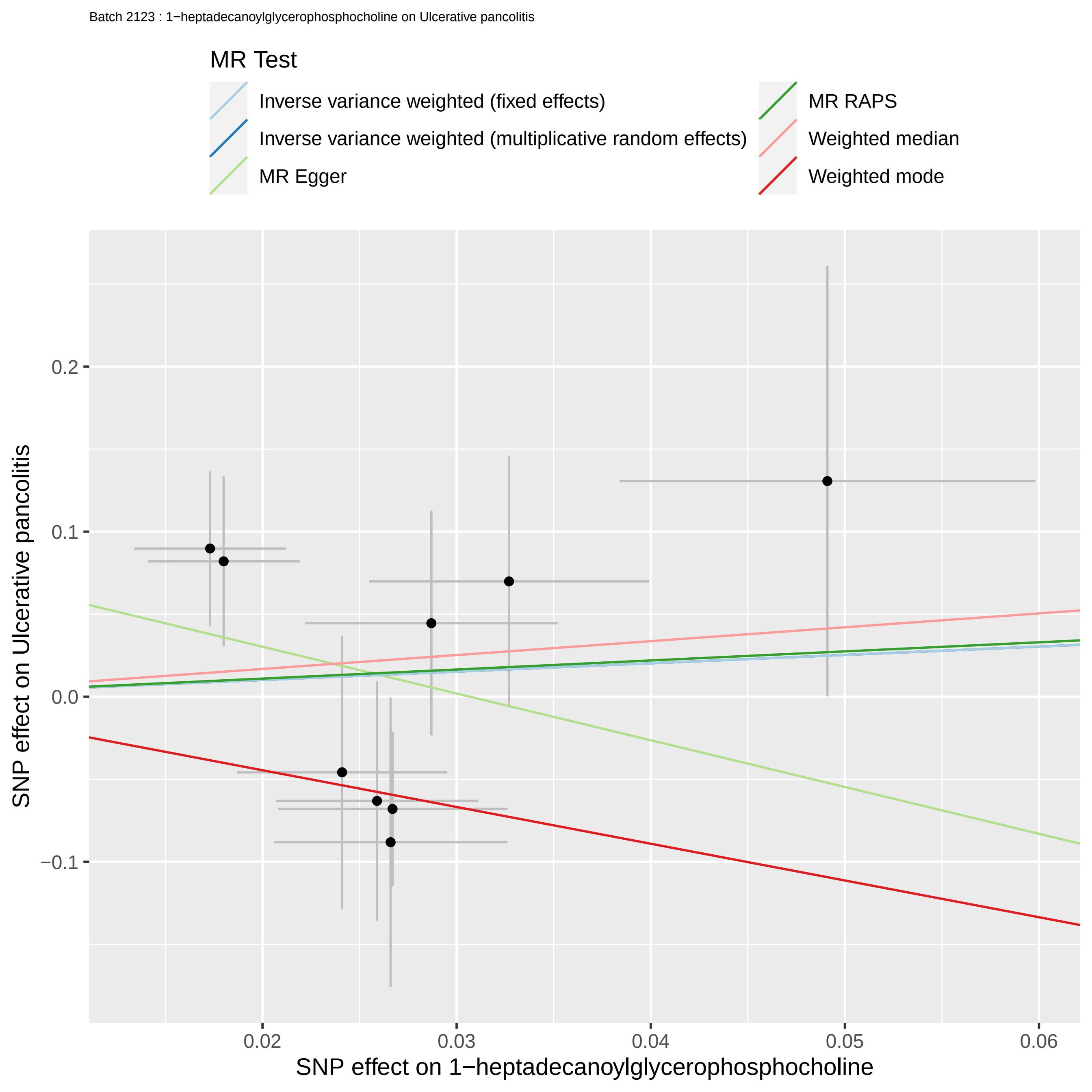
Batch 2123 : 1−heptadecanoylglycerophosphocholine on Ulcerative pancolitis
MR Test
Inverse variance weighted (fixed effects)
Inverse variance weighted (multiplicative random effects)
MR Egger
MR RAPS
Weighted median
Weighted mode
0.2
0.1
0.0
−0.1
0.02
0.03
0.04
0.05
0.06
SNP effect on Ulcerative pancolitis
SNP effect on 1−heptadecanoylglycerophosphocholine

Batch 2124 : 1−oleoylglycerophosphocholine on Ulcerative pancolitis

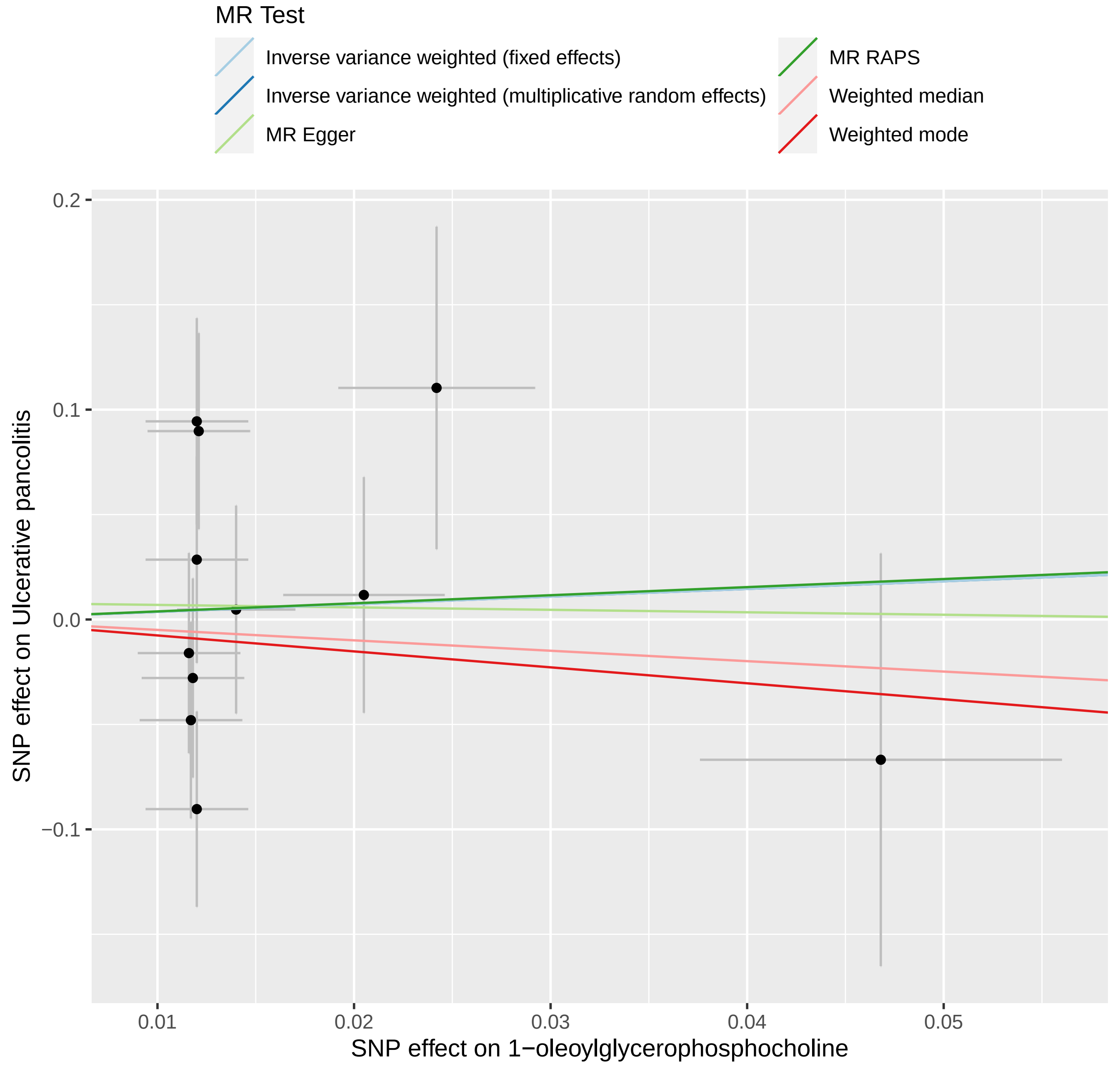

Batch 2125 : 1−stearoylglycerophosphocholine on Ulcerative pancolitis

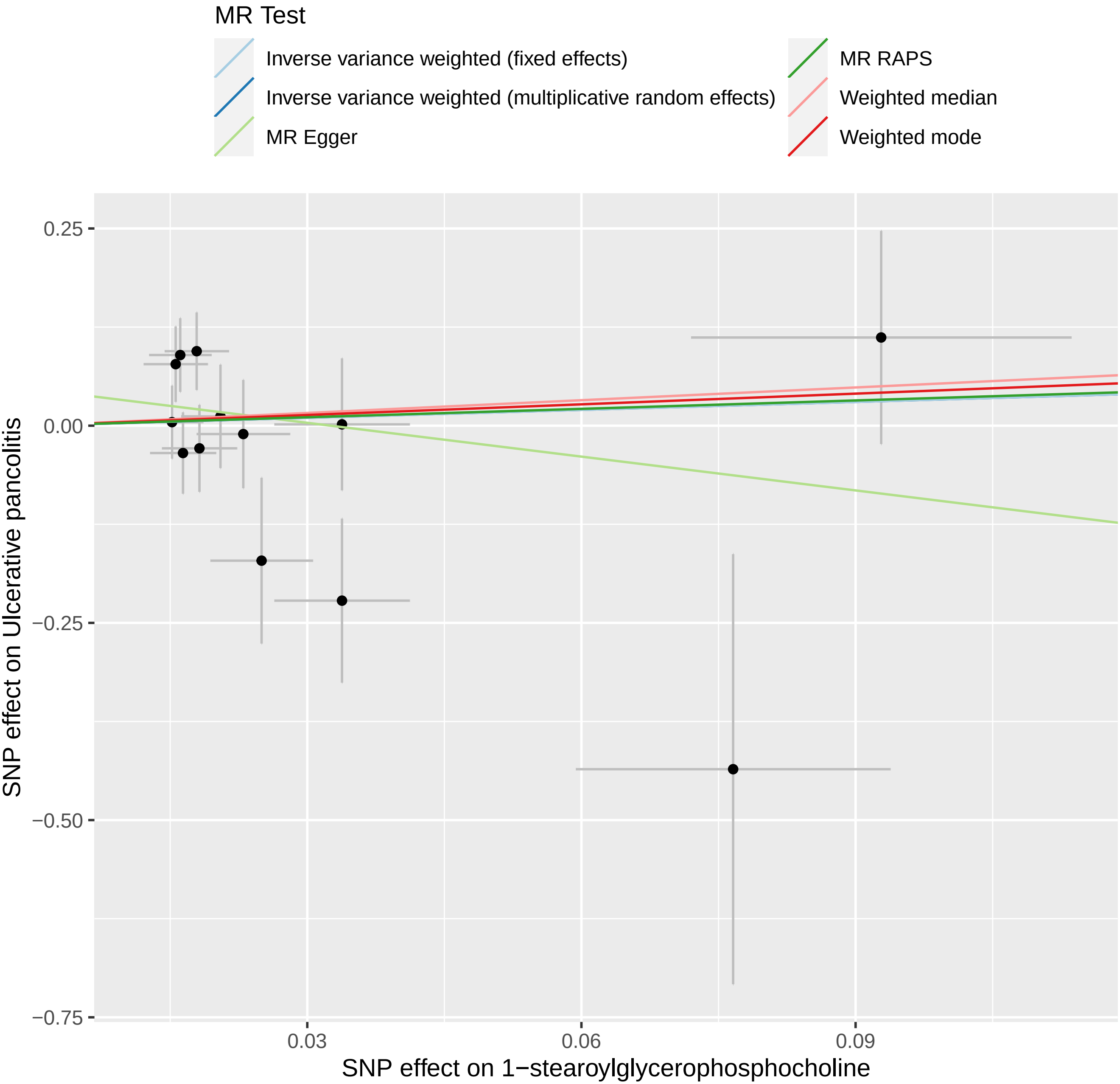

Batch 2128 : 10−heptadecenoate (17:1n7) on Ulcerative pancolitis

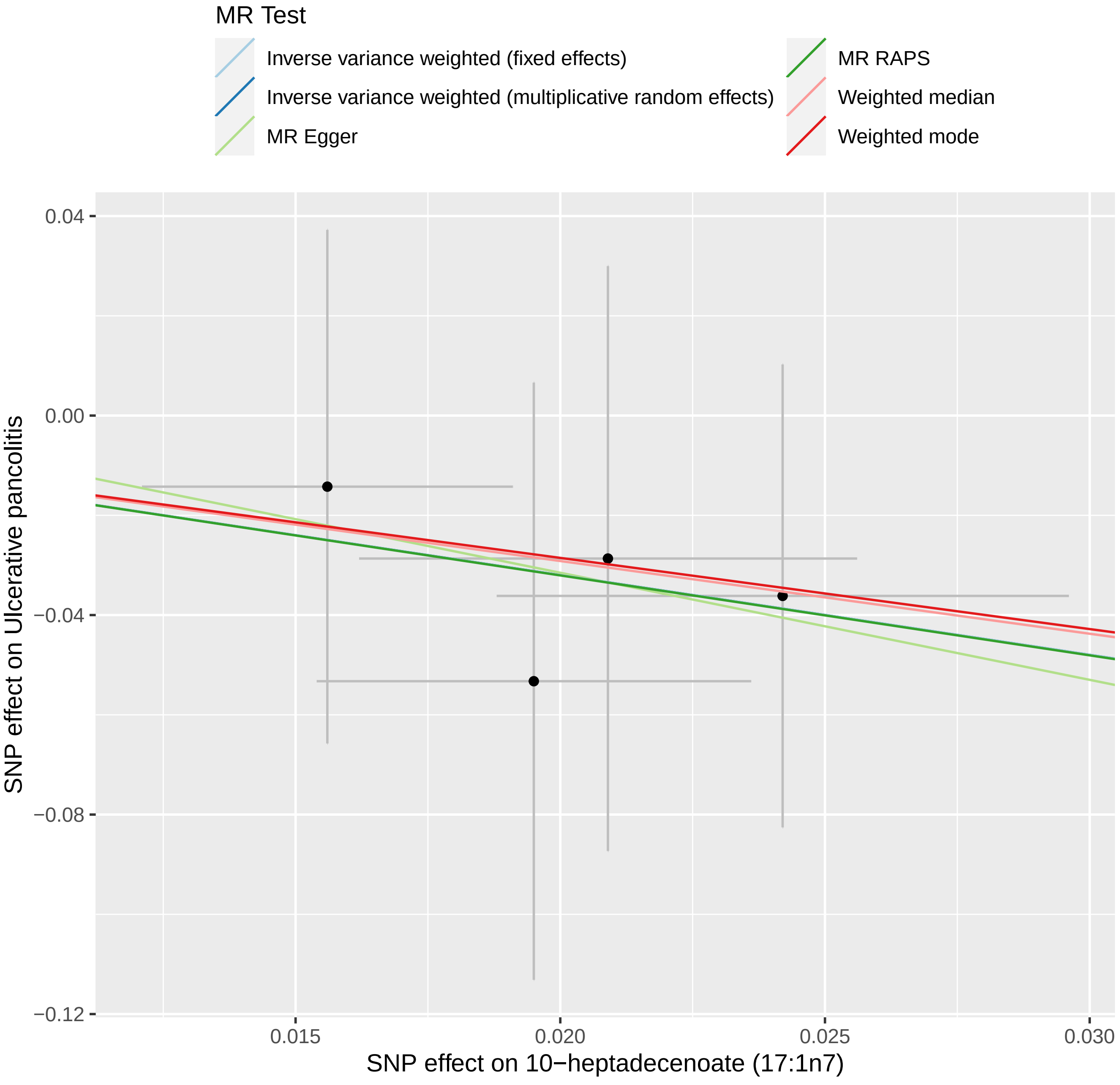

Batch 2130 : Epiandrosterone sulfate on Ulcerative pancolitis

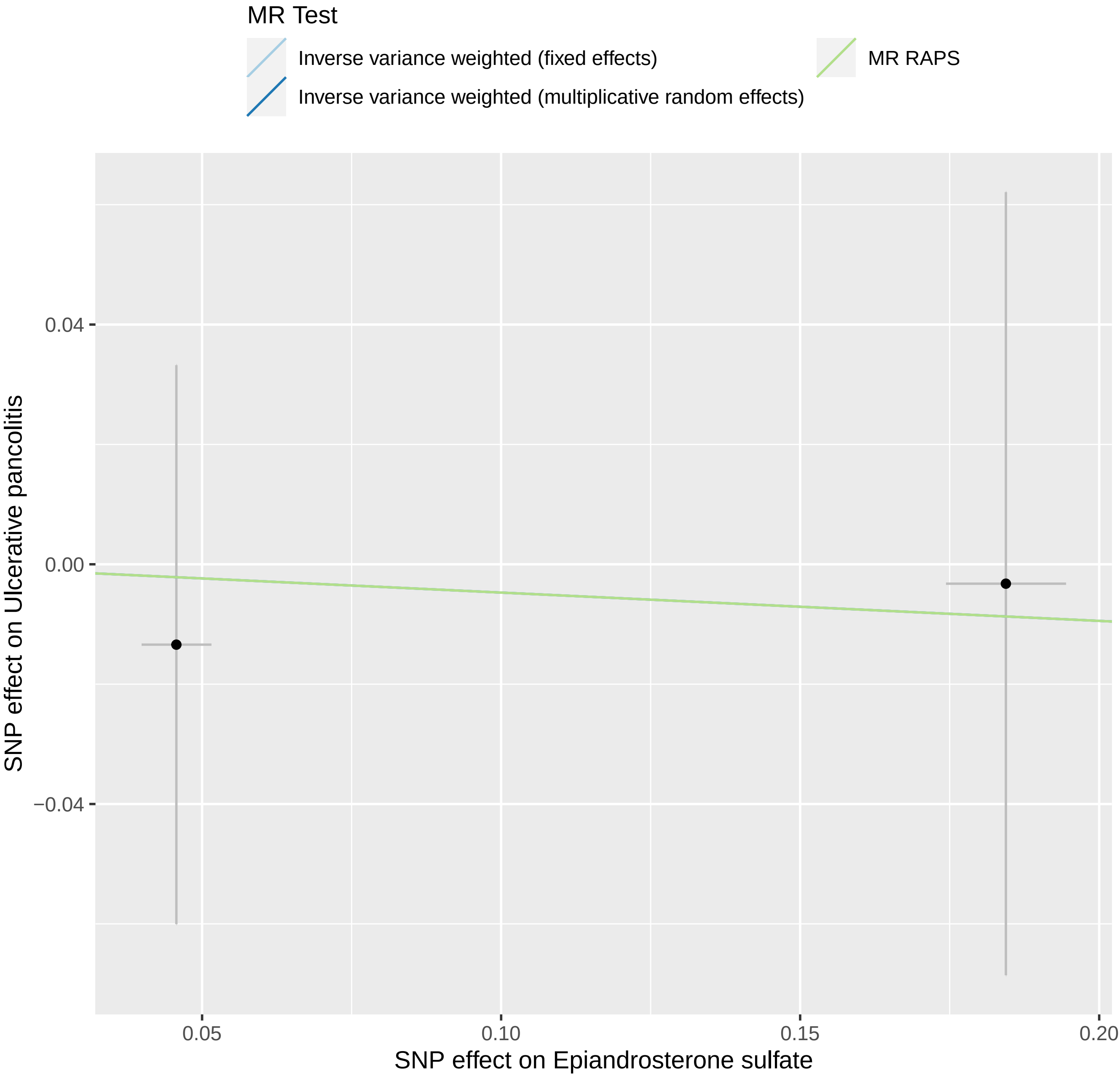

Batch 2131 : Linolenate [alpha or gamma; (18:3n3 or 6)] on Ulcerative pancolitis

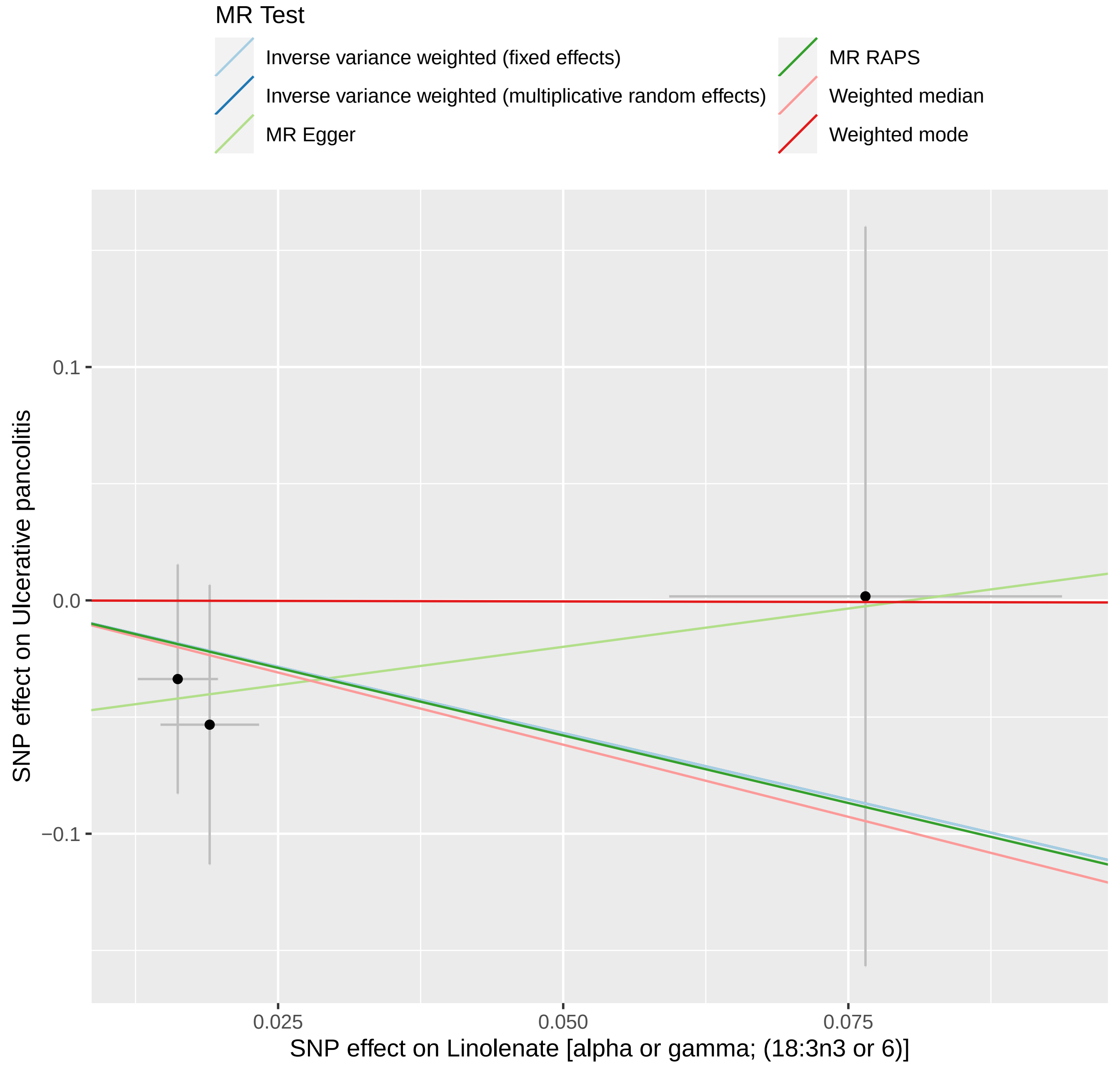

Batch 2132 : X−12510−−2−aminooctanoic acid on Ulcerative pancolitis

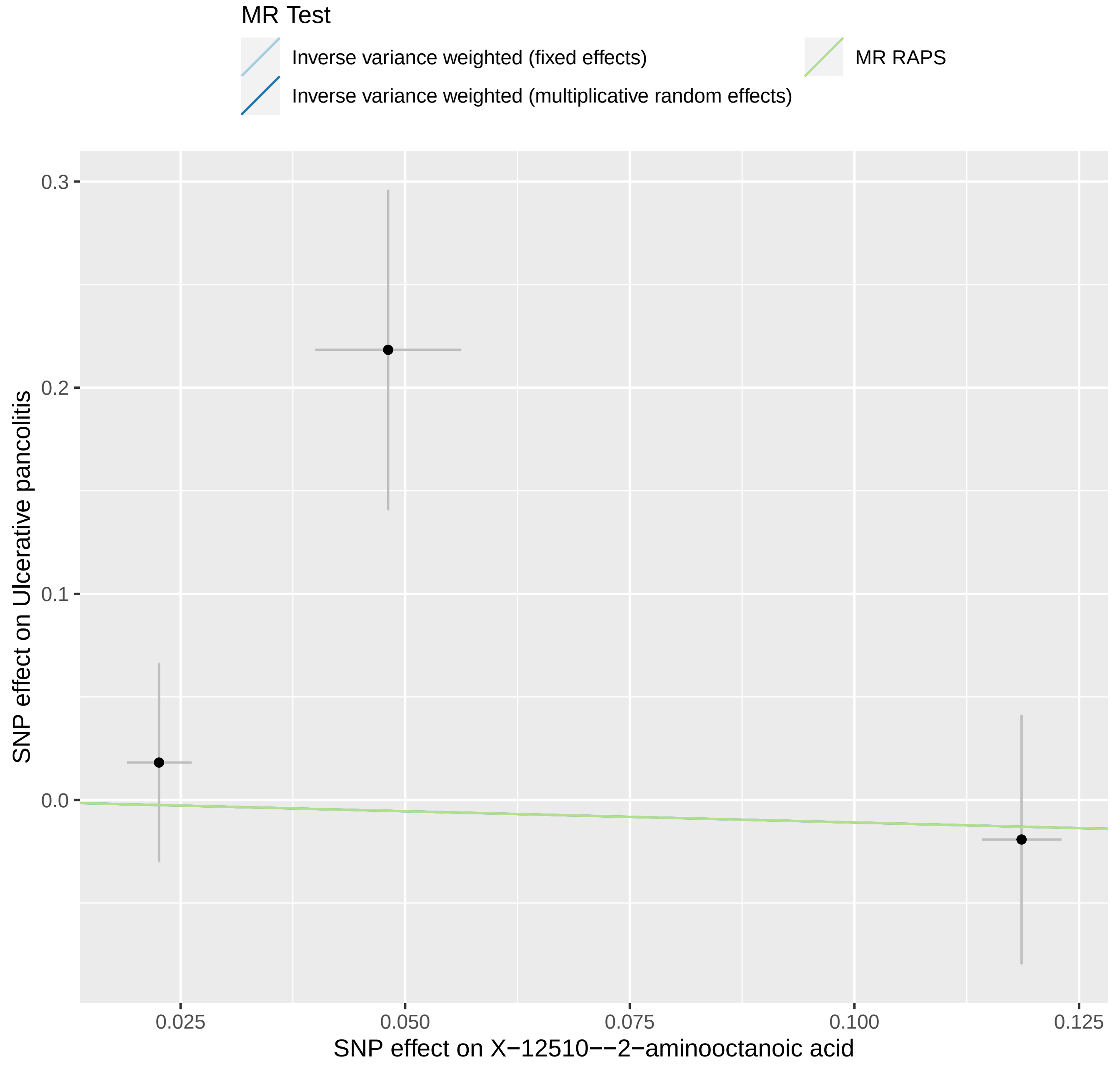

Batch 2135 : Asparagine on Ulcerative pancolitis

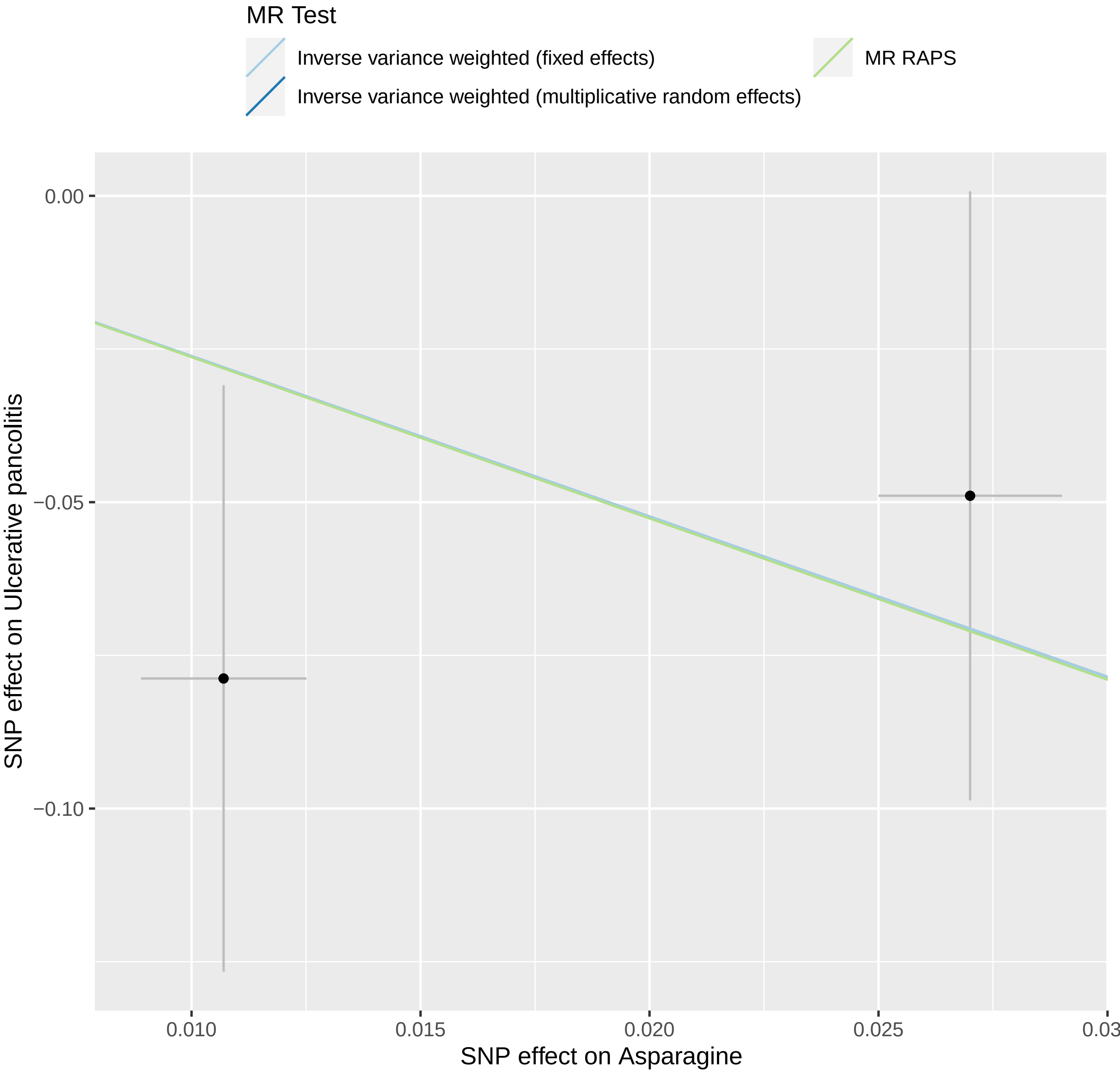

Batch 2136 : Stachydrine on Ulcerative pancolitis

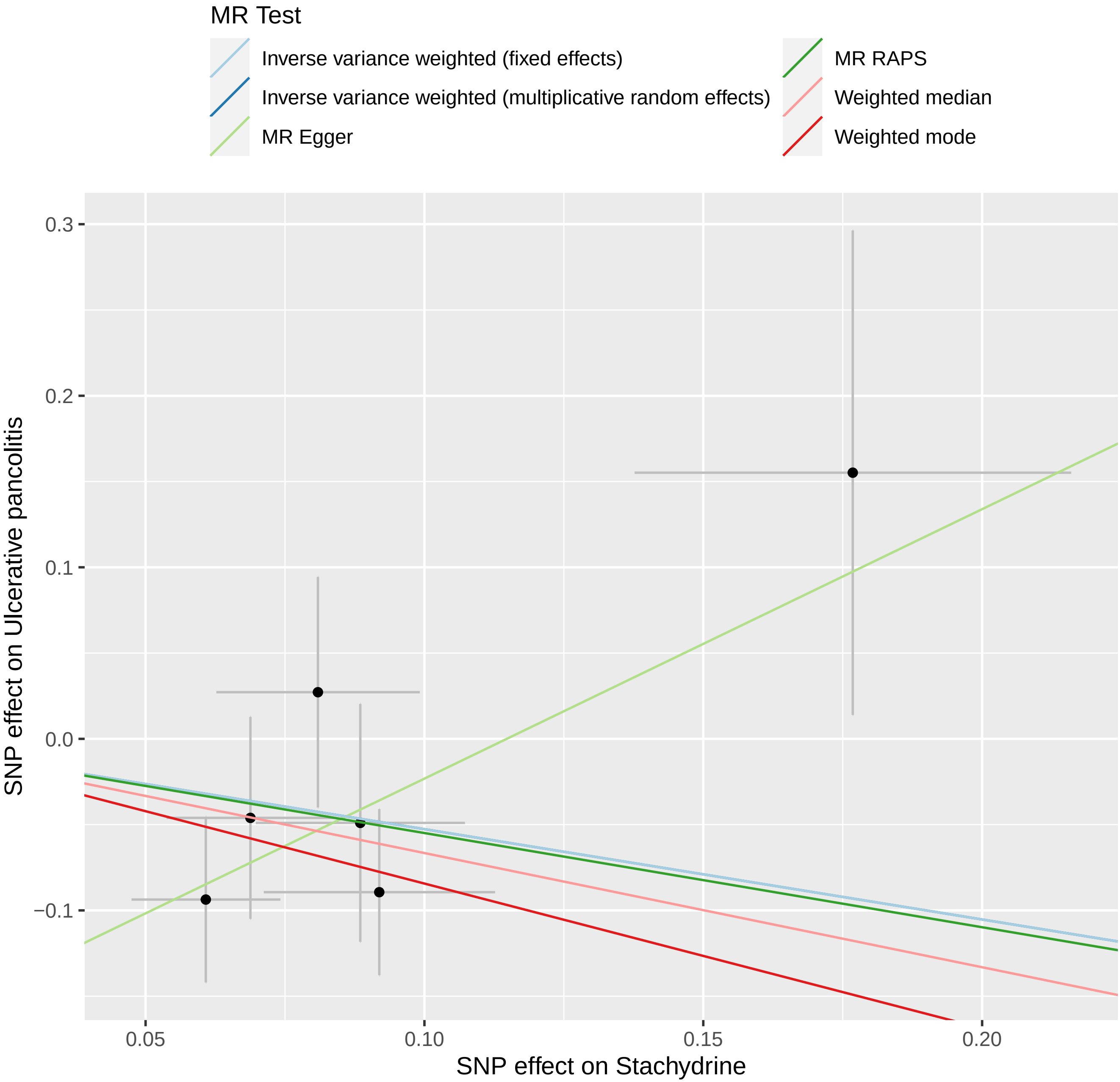

Batch 2137 : Isovalerylcarnitine on Ulcerative pancolitis

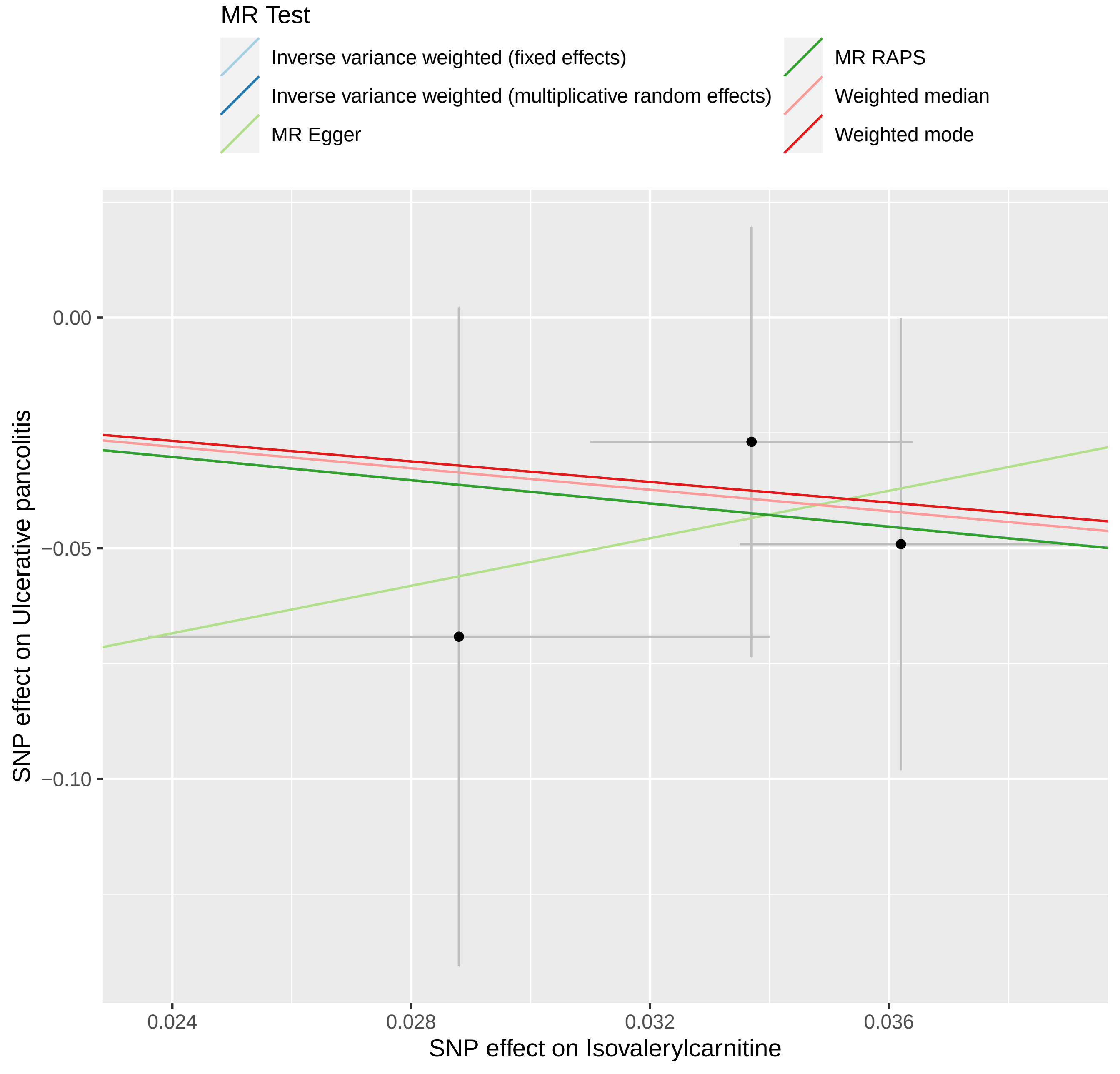

Batch 2140 : 1−linoleoylglycerophosphocholine on Ulcerative pancolitis

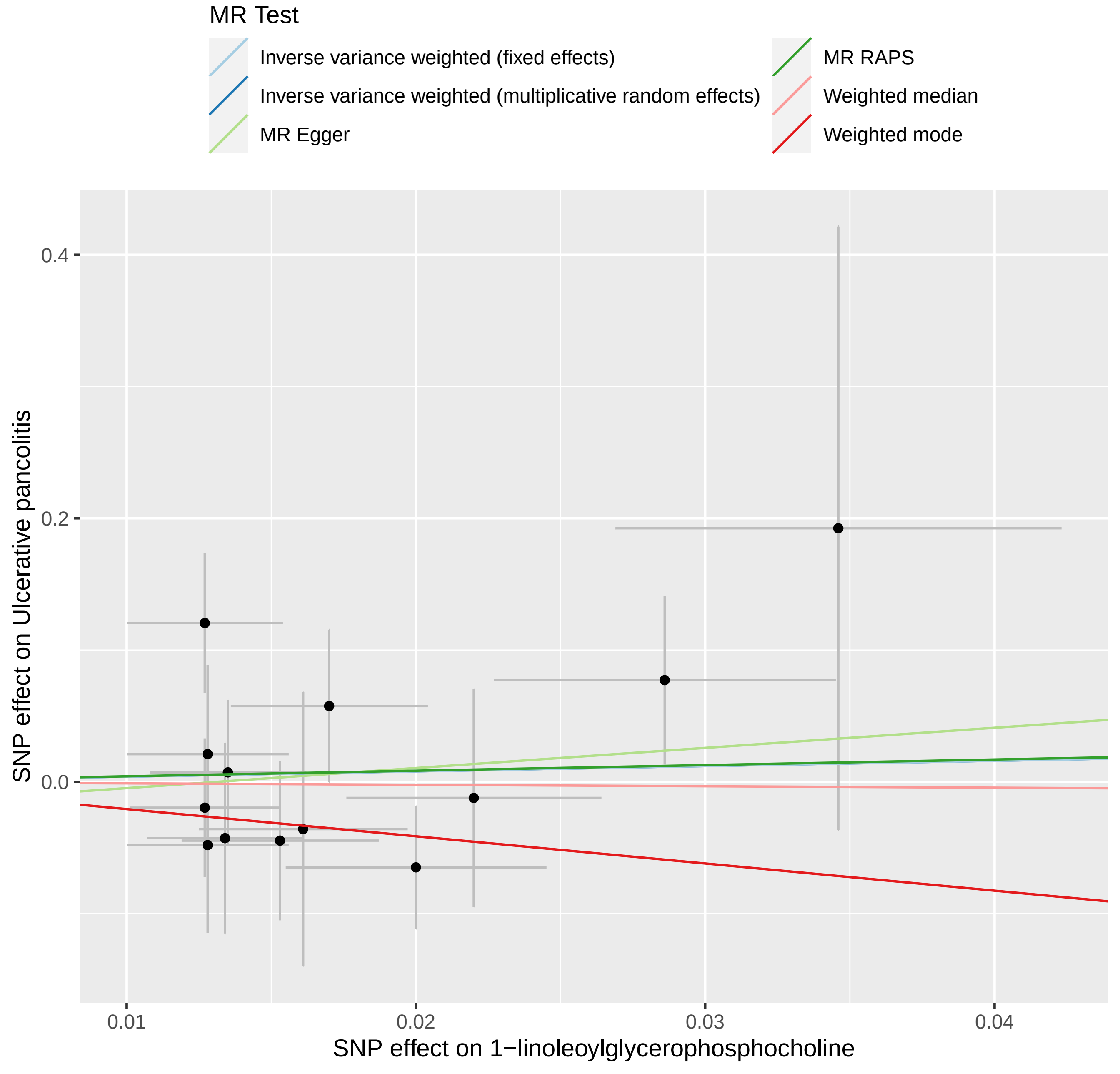

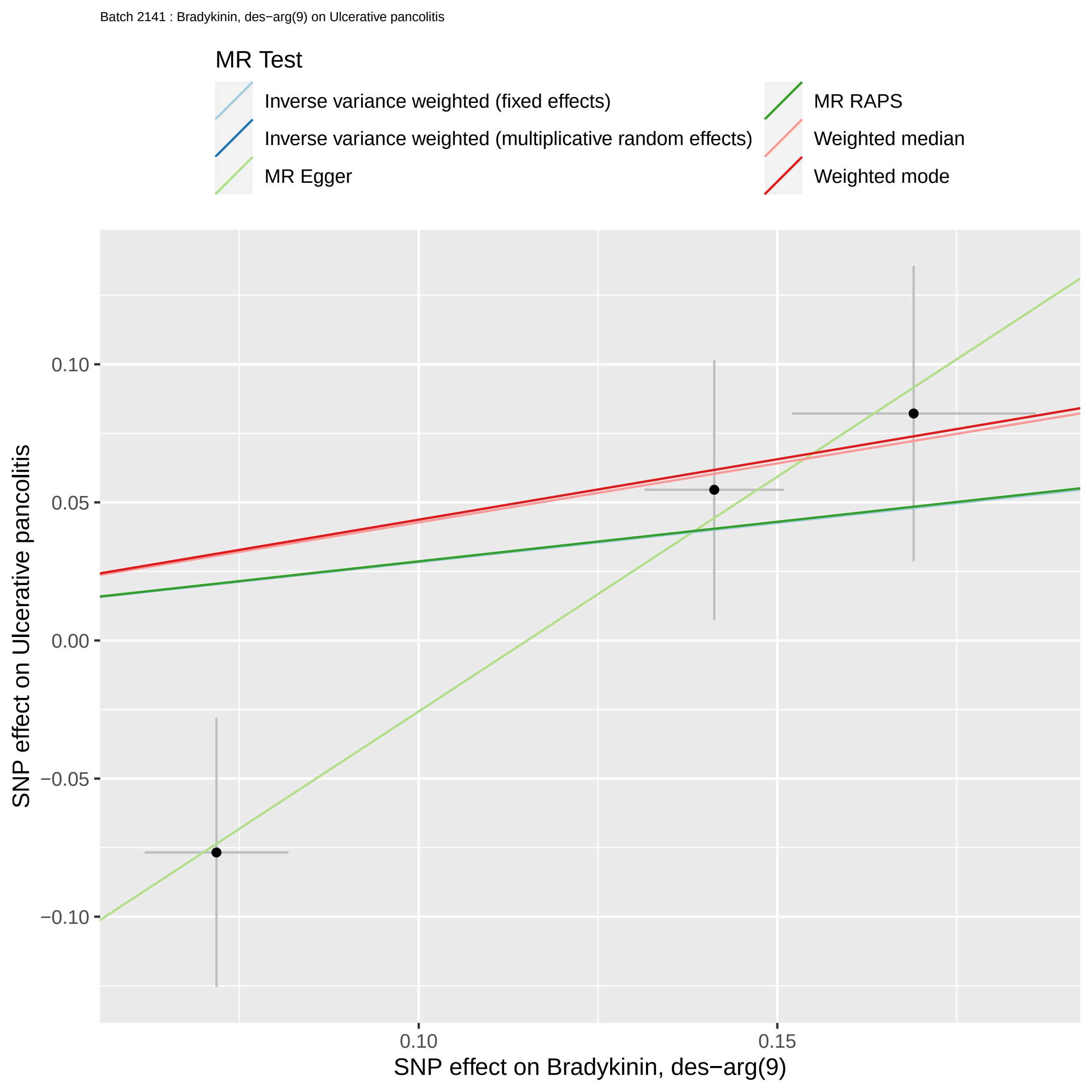

Batch 2141 : Bradykinin, des−arg(9) on Ulcerative pancolitis
MR Test
Inverse variance weighted (fixed effects)
Inverse variance weighted (multiplicative random effects)
MR Egger
MR RAPS
Weighted median
Weighted mode
0.10
0.05
0.00
−0.05
−0.10
0.10
0.15
SNP effect on Ulcerative pancolitis
SNP effect on Bradykinin, des−arg(9)

Batch 2142 : Gamma−glutamylisoleucine* on Ulcerative pancolitis

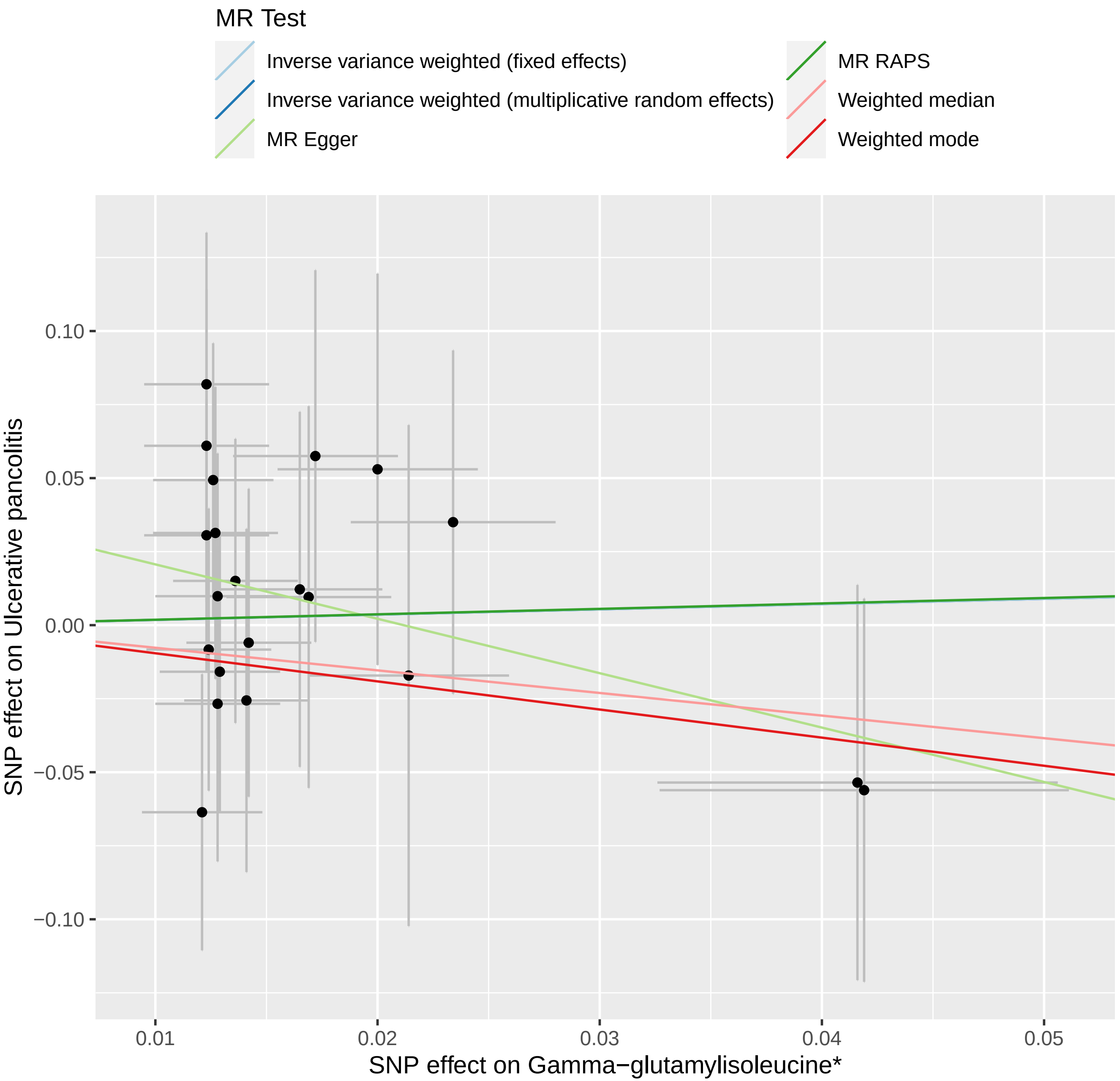

Batch 2145 : Isovalerate on Ulcerative pancolitis

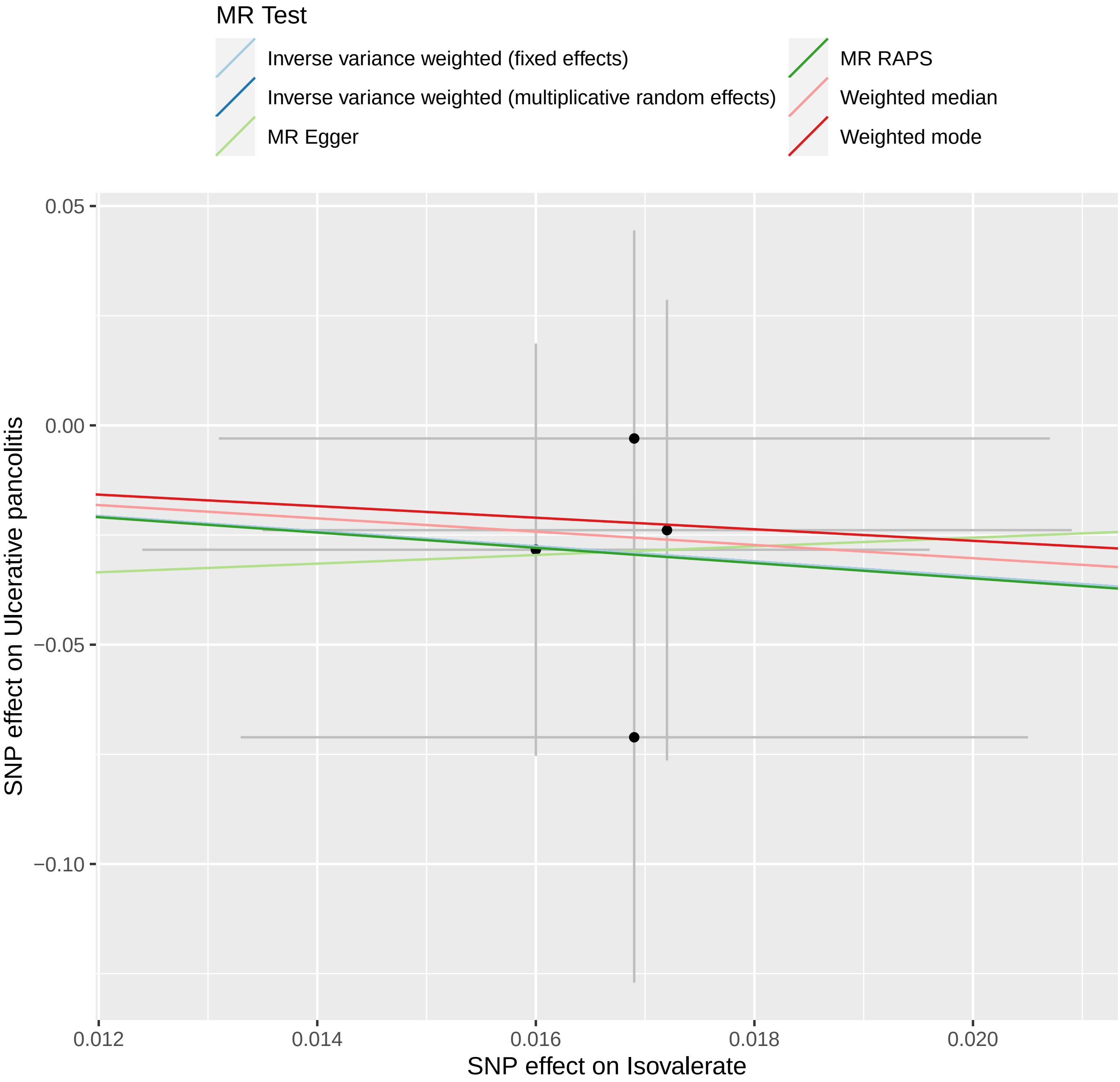

Batch 2146 : X−13183−−stearamide on Ulcerative pancolitis

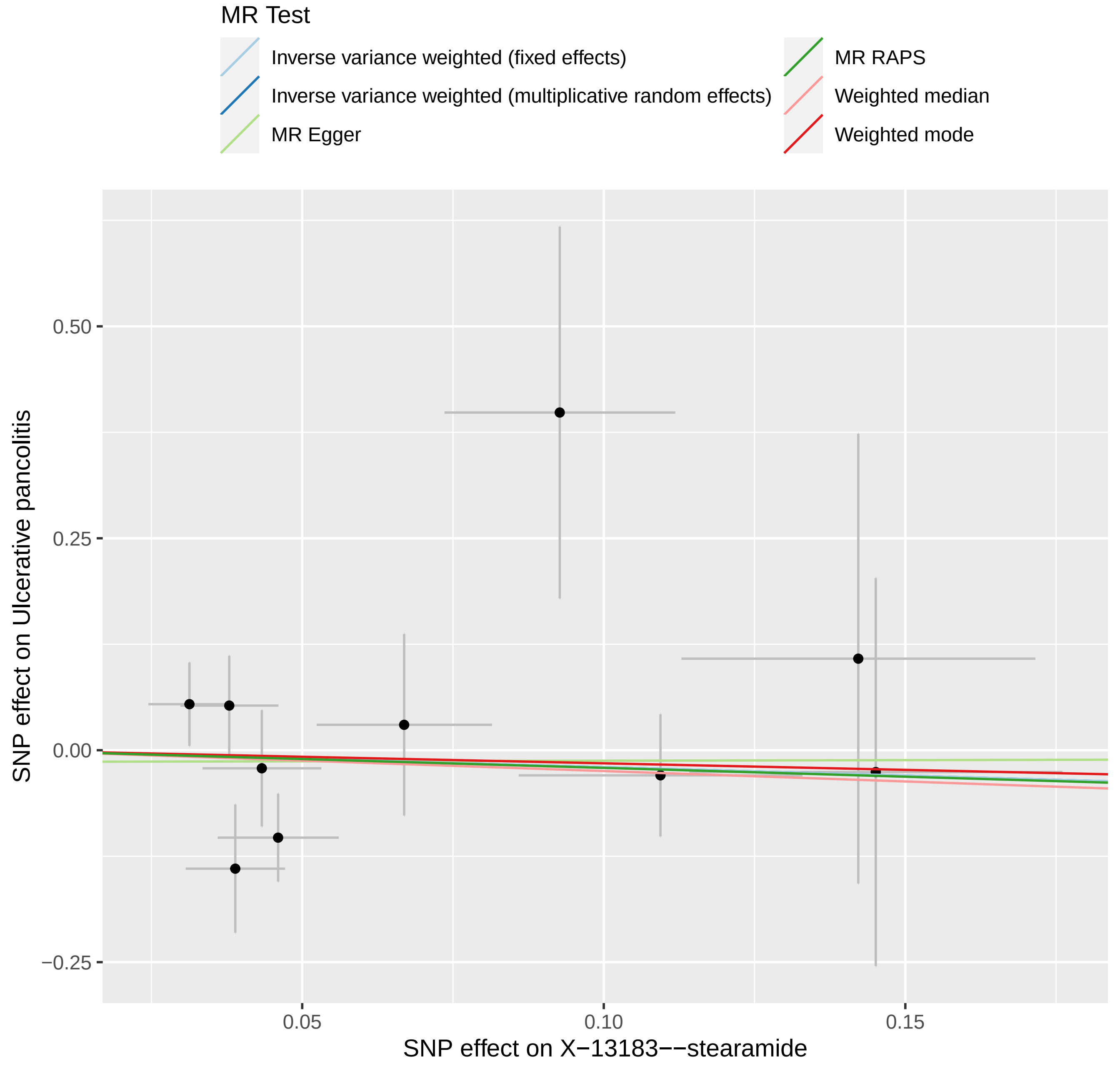

Batch 2147 : 7−methylguanine on Ulcerative pancolitis

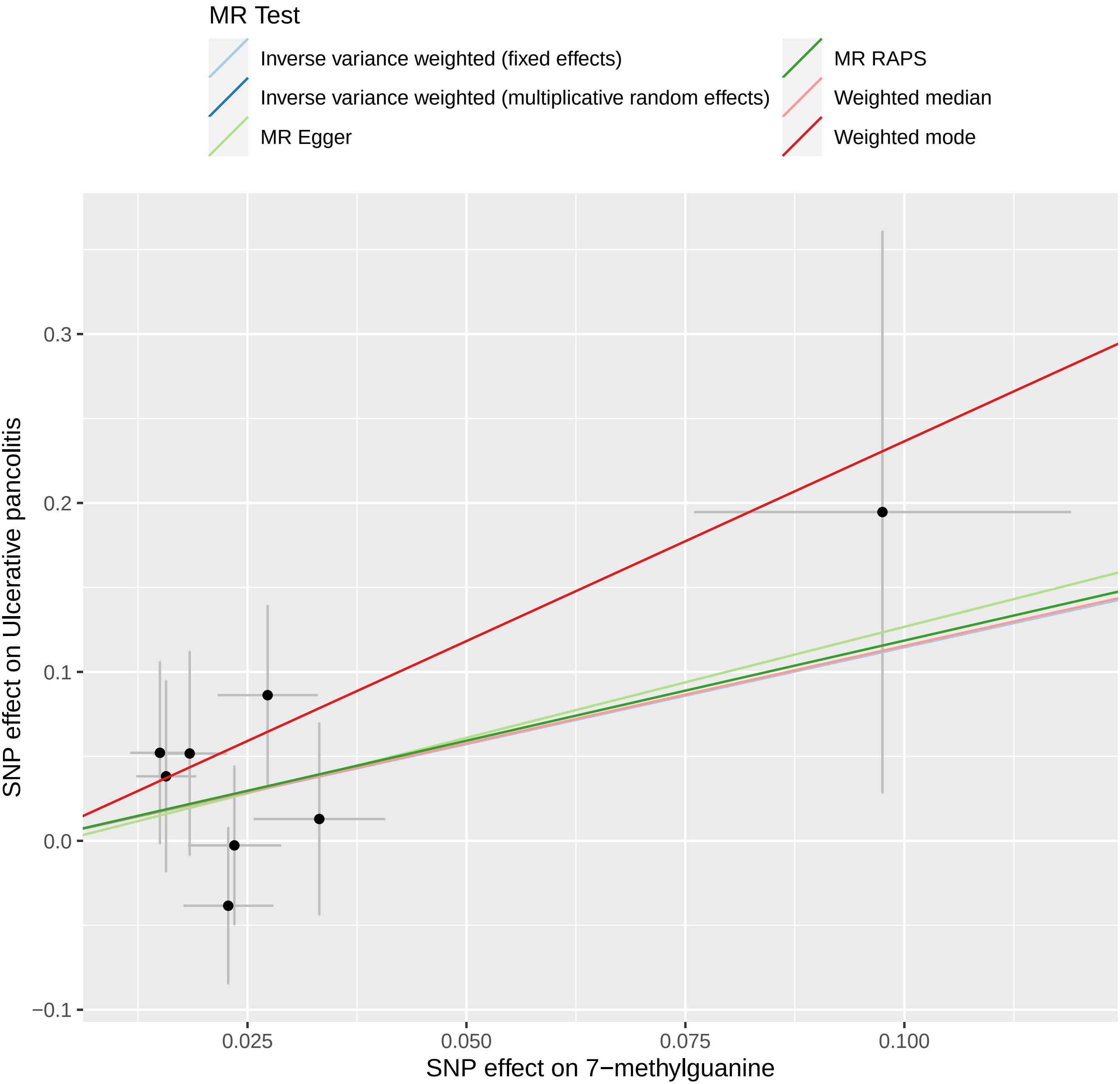

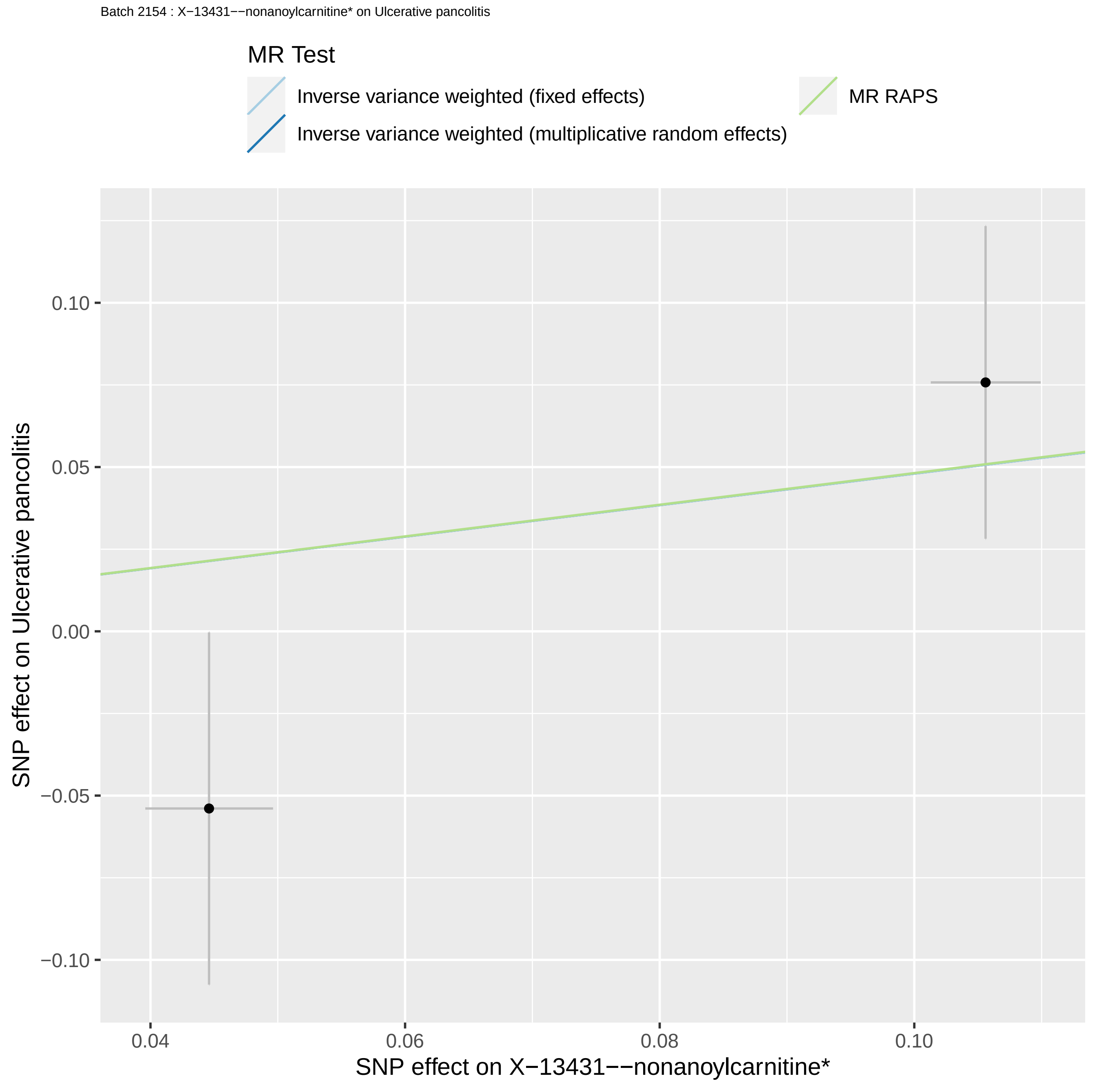
Batch 2154 : X−13431−−nonanoylcarnitine* on Ulcerative pancolitis
MR Test
Inverse variance weighted (fixed effects)
Inverse variance weighted (multiplicative random effects)
MR RAPS
0.10
0.05
0.00
−0.05
−0.10
0.04
0.06
0.08
0.10
SNP effect on Ulcerative pancolitis
SNP effect on X−13431−−nonanoylcarnitine*

Batch 2155 : 2−palmitoylglycerophosphocholine* on Ulcerative pancolitis

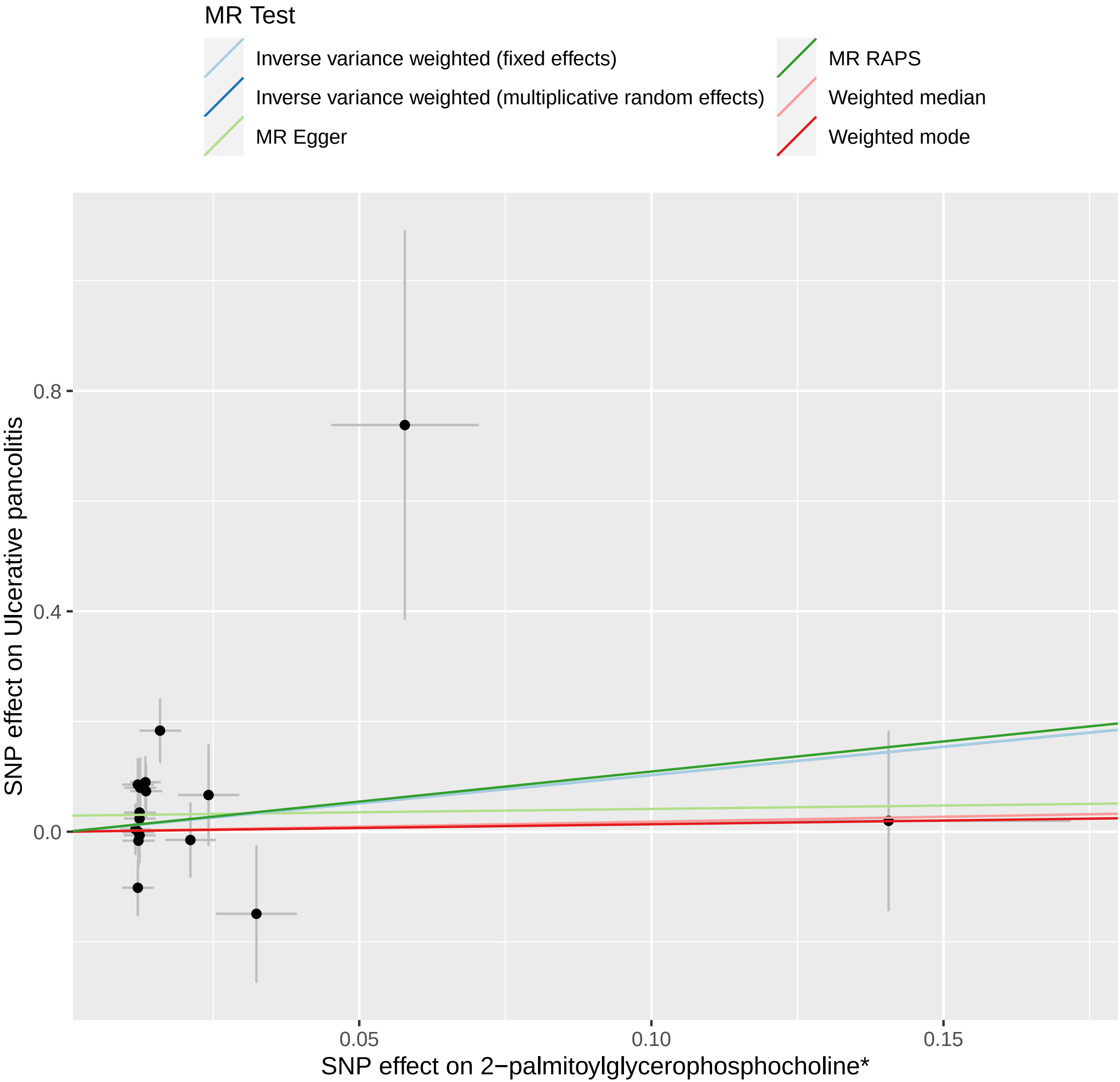

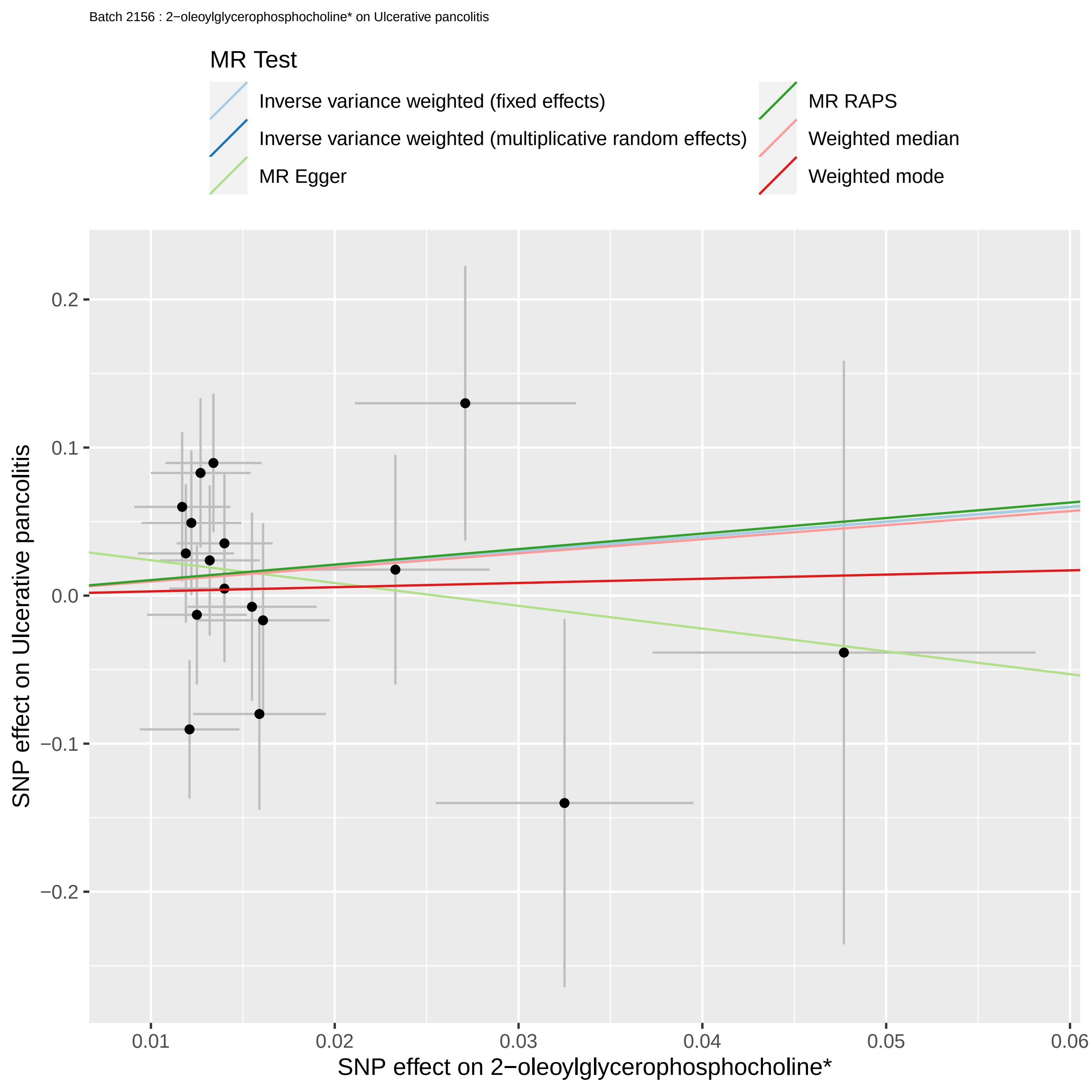

Batch 2156 : 2−oleoylglycerophosphocholine* on Ulcerative pancolitis
MR Test
Inverse variance weighted (fixed effects)
Inverse variance weighted (multiplicative random effects)
MR Egger
MR RAPS
Weighted median
Weighted mode
SNP effect on Ulcerative pancolitis
0.2
0.1
0.0
−0.1
−0.2
0.01
0.02
0.03
0.04
0.05
0.06
SNP effect on 2−oleoylglycerophosphocholine*

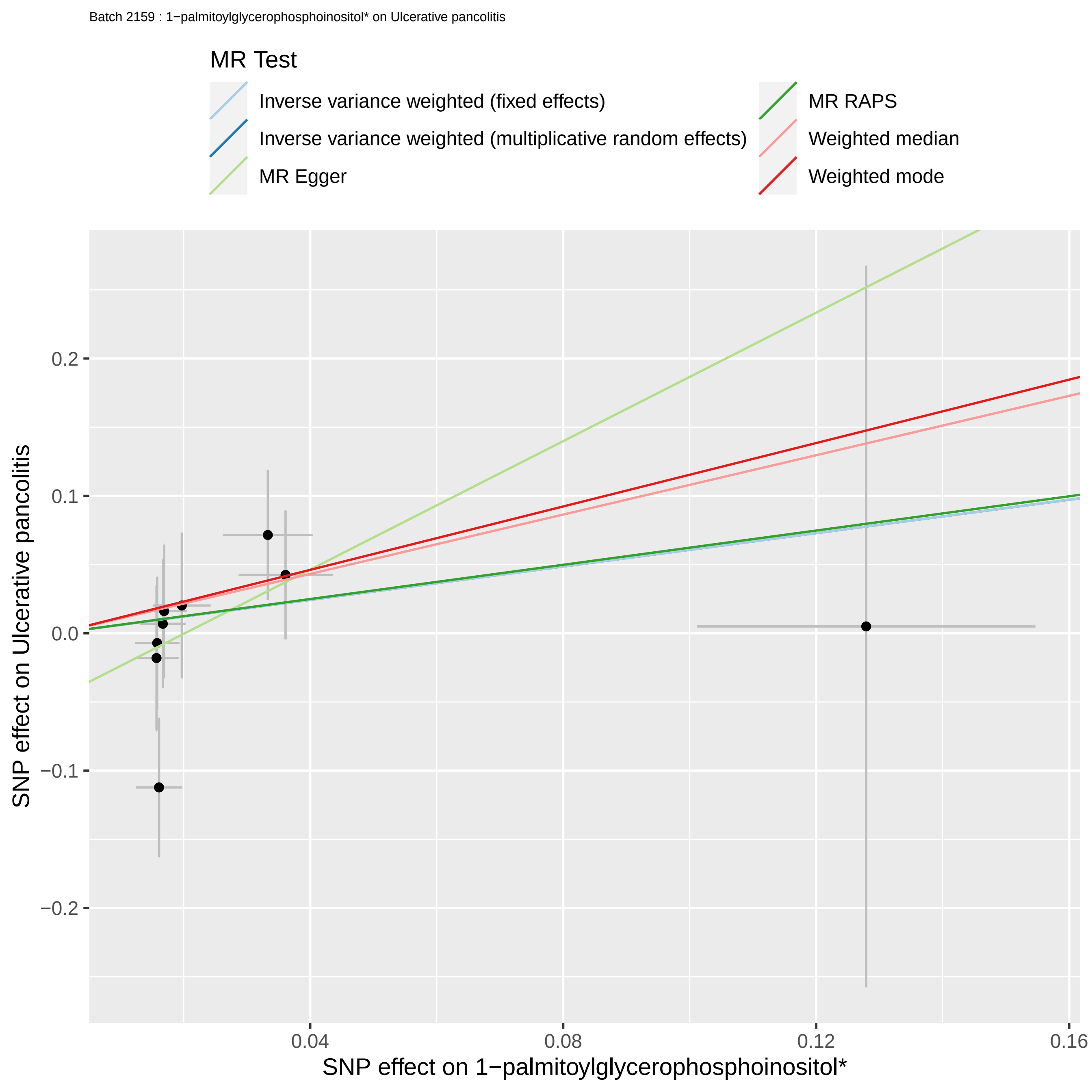

Batch 2159 : 1−palmitoylglycerophosphoinositol* on Ulcerative pancolitis
MR Test
Inverse variance weighted (fixed effects)
Inverse variance weighted (multiplicative random effects)
MR Egger
MR RAPS
Weighted median
Weighted mode
0.2
0.1
0.0
−0.1
−0.2
0.04
0.08
0.12
0.16
SNP effect on Ulcerative pancolitis
SNP effect on 1−palmitoylglycerophosphoinositol*

Batch 2161 : Hydroxyisovaleroyl carnitine on Ulcerative pancolitis

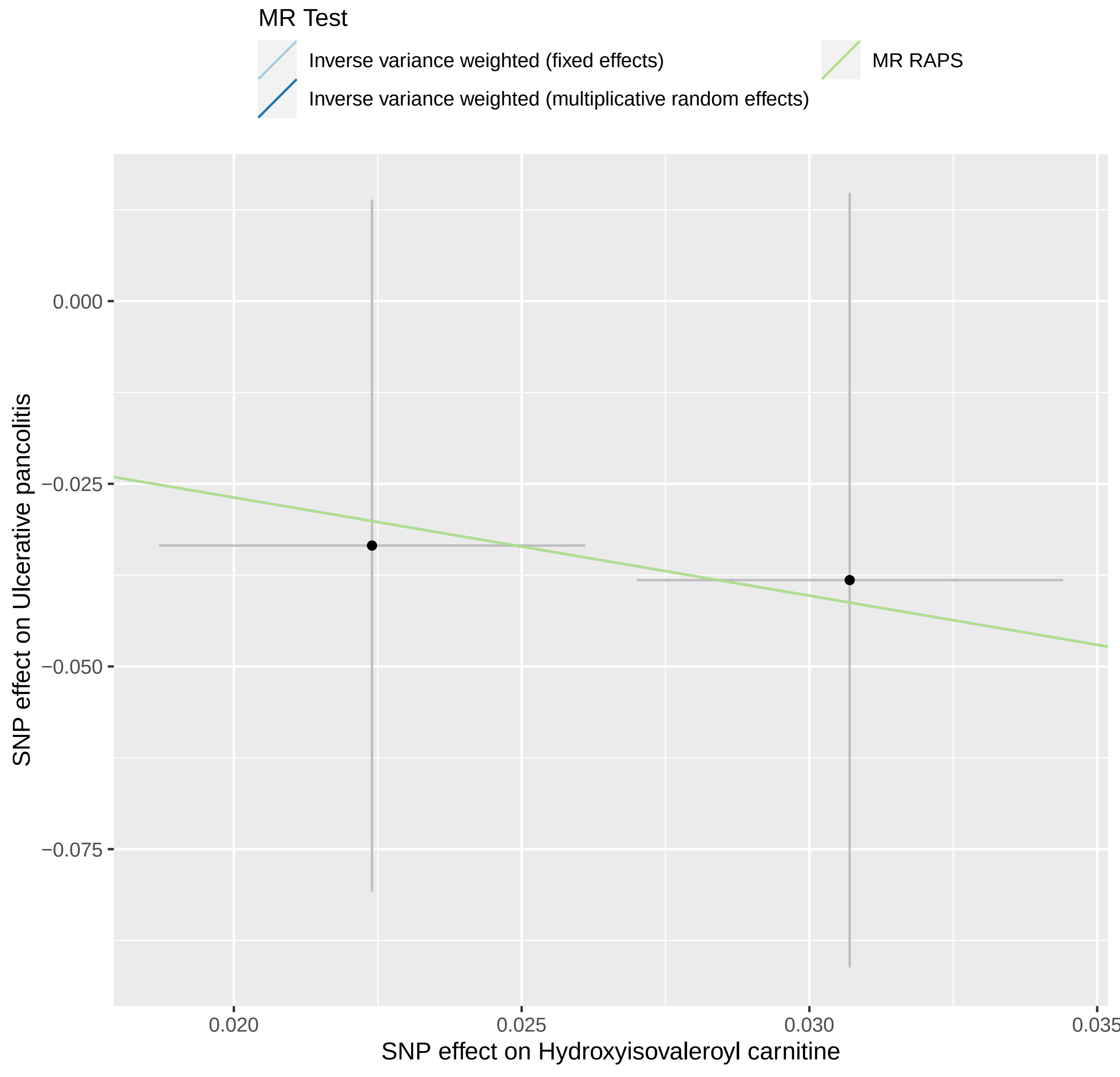

Batch 2162 : Glutaroyl carnitine on Ulcerative pancolitis

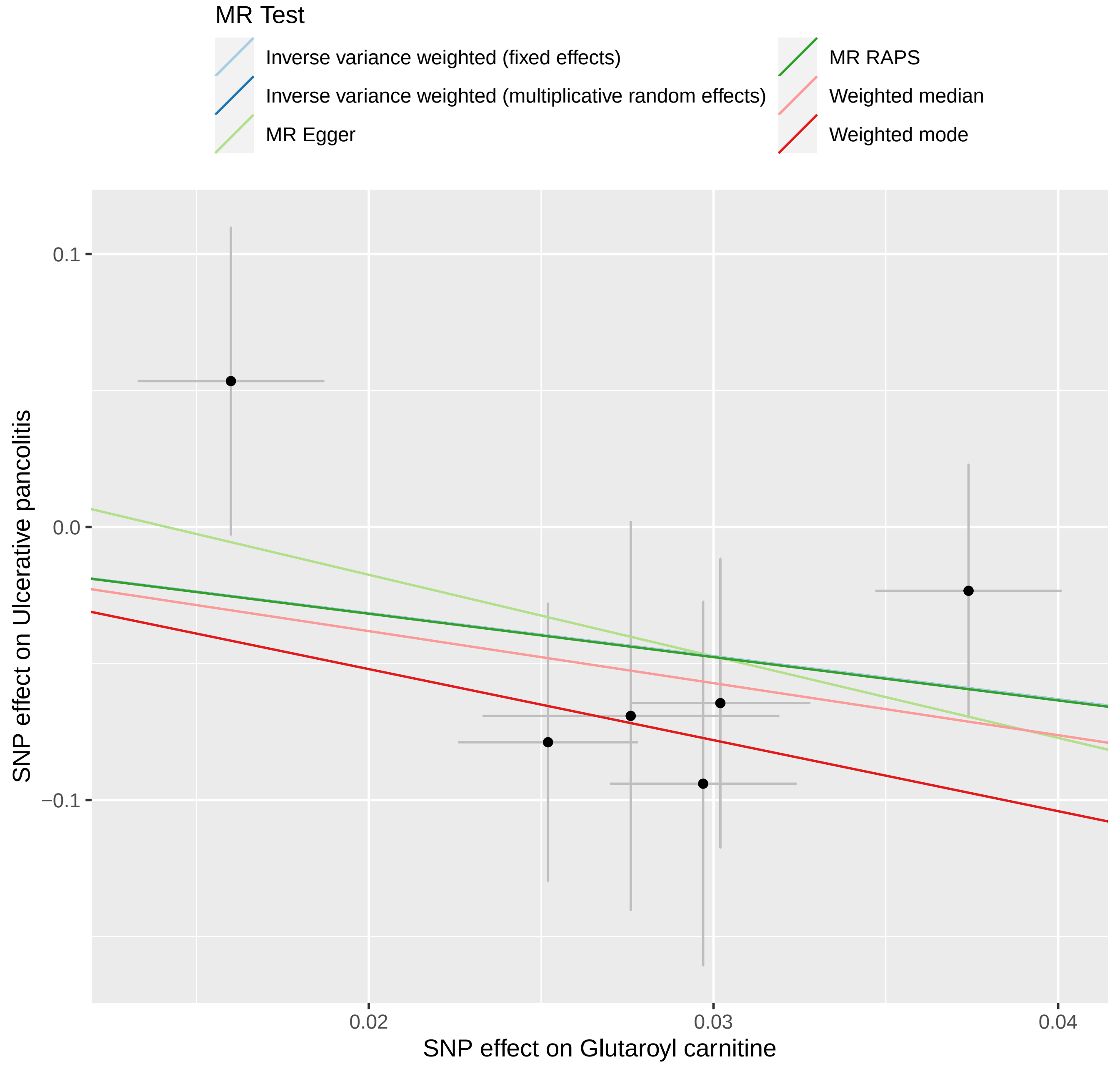

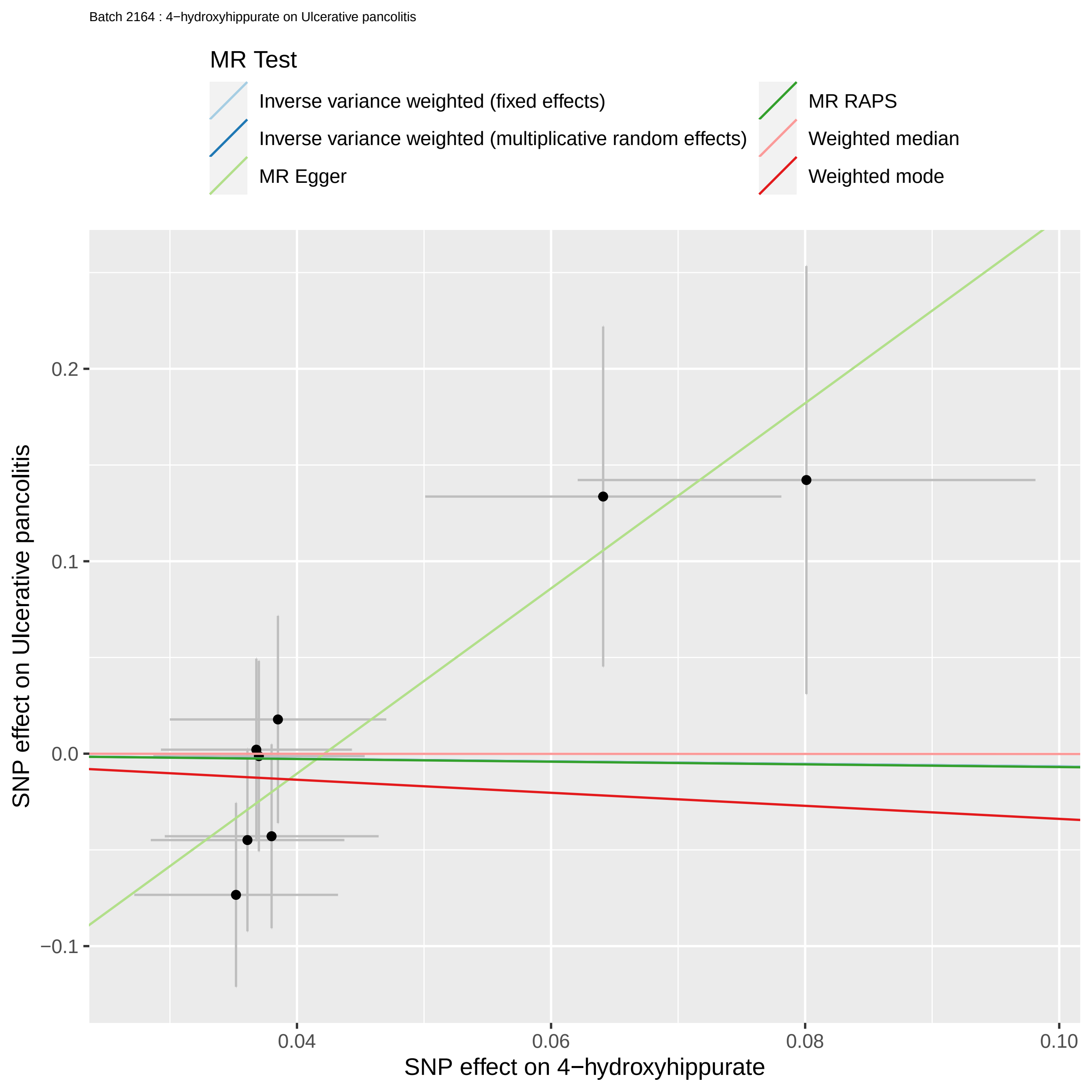

Batch 2164 : 4−hydroxyhippurate on Ulcerative pancolitis
MR Test
Inverse variance weighted (fixed effects)
Inverse variance weighted (multiplicative random effects)
MR Egger
MR RAPS
Weighted median
Weighted mode
SNP effect on Ulcerative pancolitis
0.2
0.1
0.0
−0.1
0.04
0.06
0.08
0.10
SNP effect on 4−hydroxyhippurate

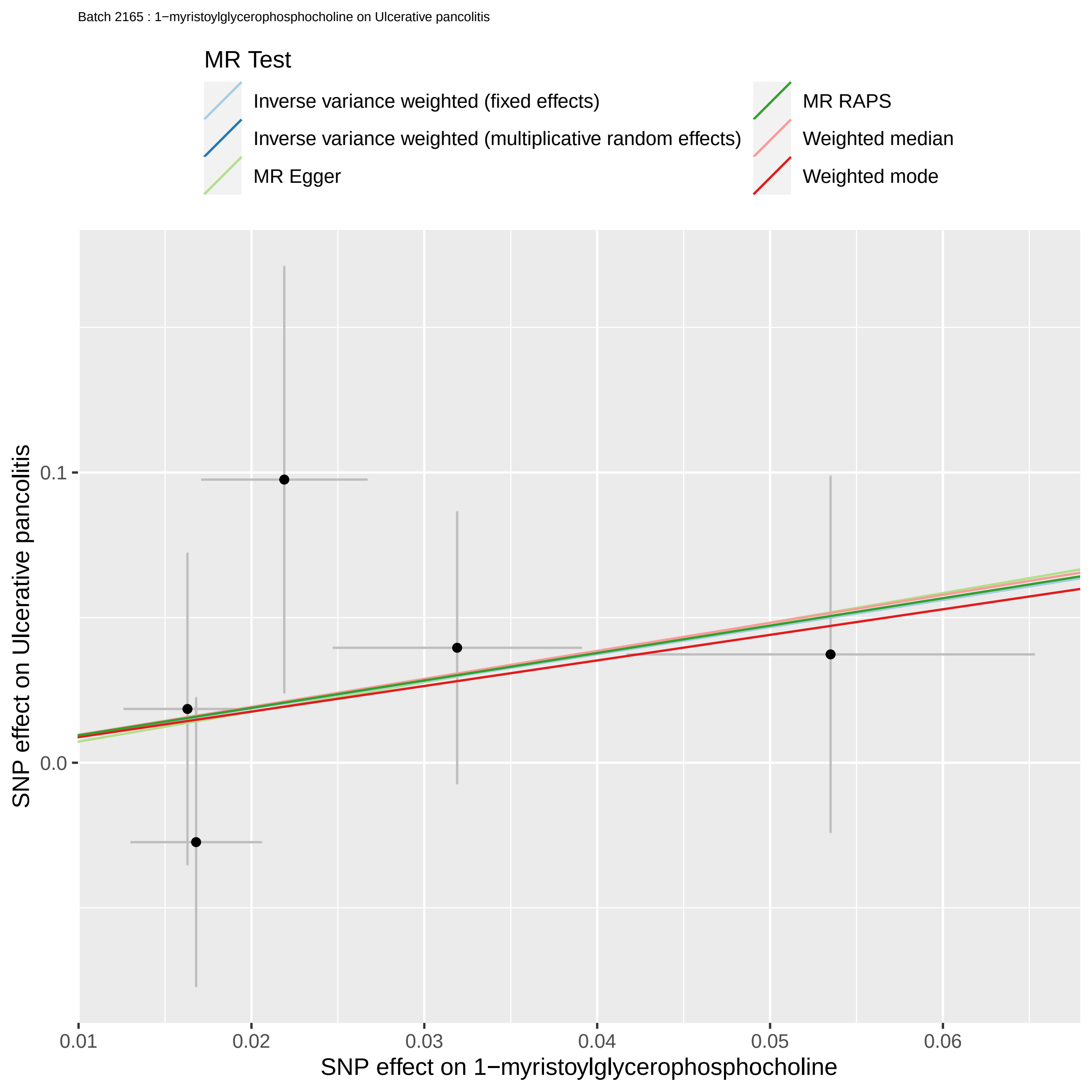

Batch 2165 : 1−myristoylglycerophosphocholine on Ulcerative pancolitis
MR Test
Inverse variance weighted (fixed effects)
Inverse variance weighted (multiplicative random effects)
MR Egger
MR RAPS
Weighted median
Weighted mode
0.1
0.0
0.01
0.02
0.03
0.04
0.05
0.06
SNP effect on Ulcerative pancolitis
SNP effect on 1−myristoylglycerophosphocholine

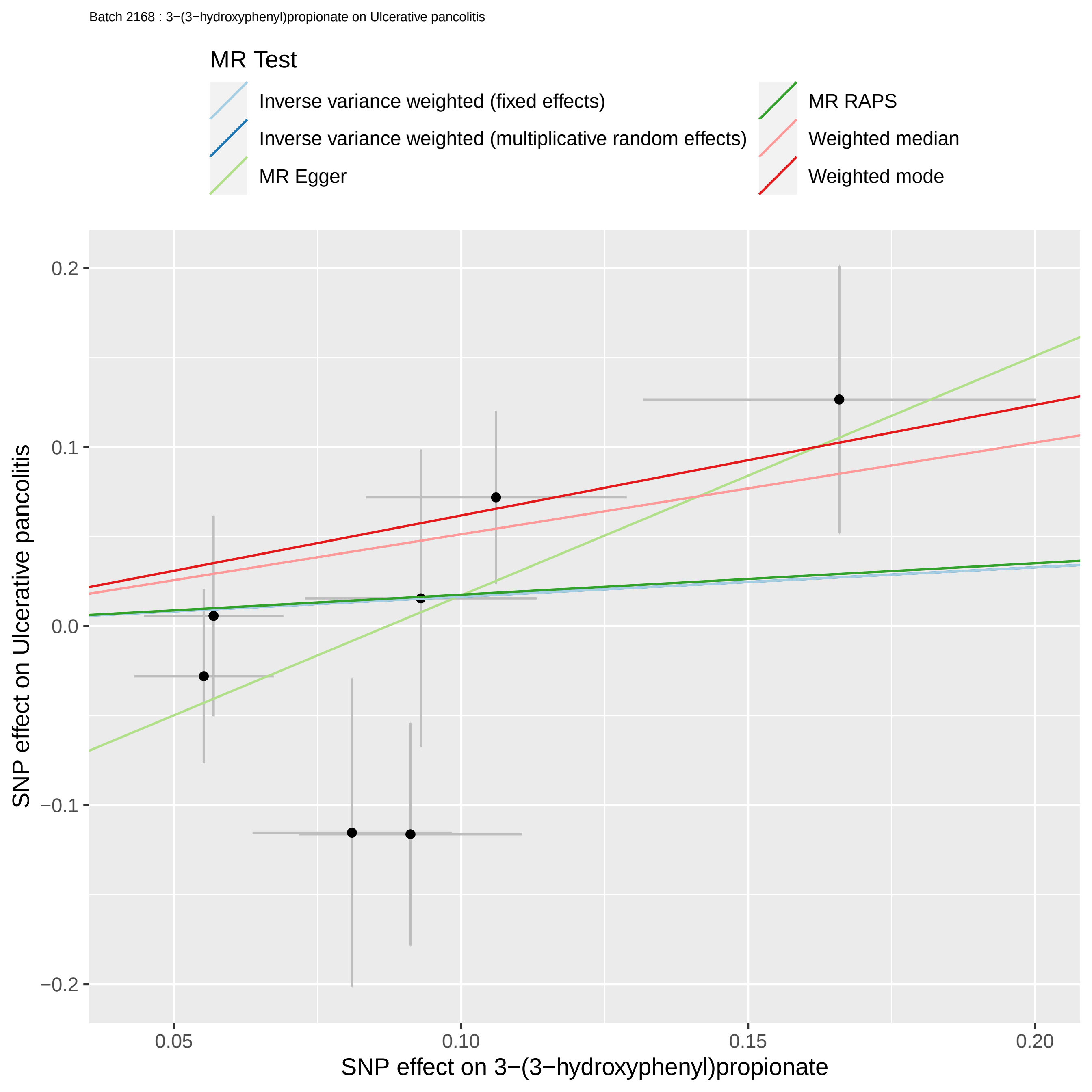

Batch 2168 : 3−(3−hydroxyphenyl)propionate on Ulcerative pancolitis
MR Test
Inverse variance weighted (fixed effects)
Inverse variance weighted (multiplicative random effects)
MR Egger
MR RAPS
Weighted median
Weighted mode
0.2
0.1
0.0
−0.1
−0.2
0.05
0.10
0.15
0.20
SNP effect on Ulcerative pancolitis
SNP effect on 3−(3−hydroxyphenyl)propionate

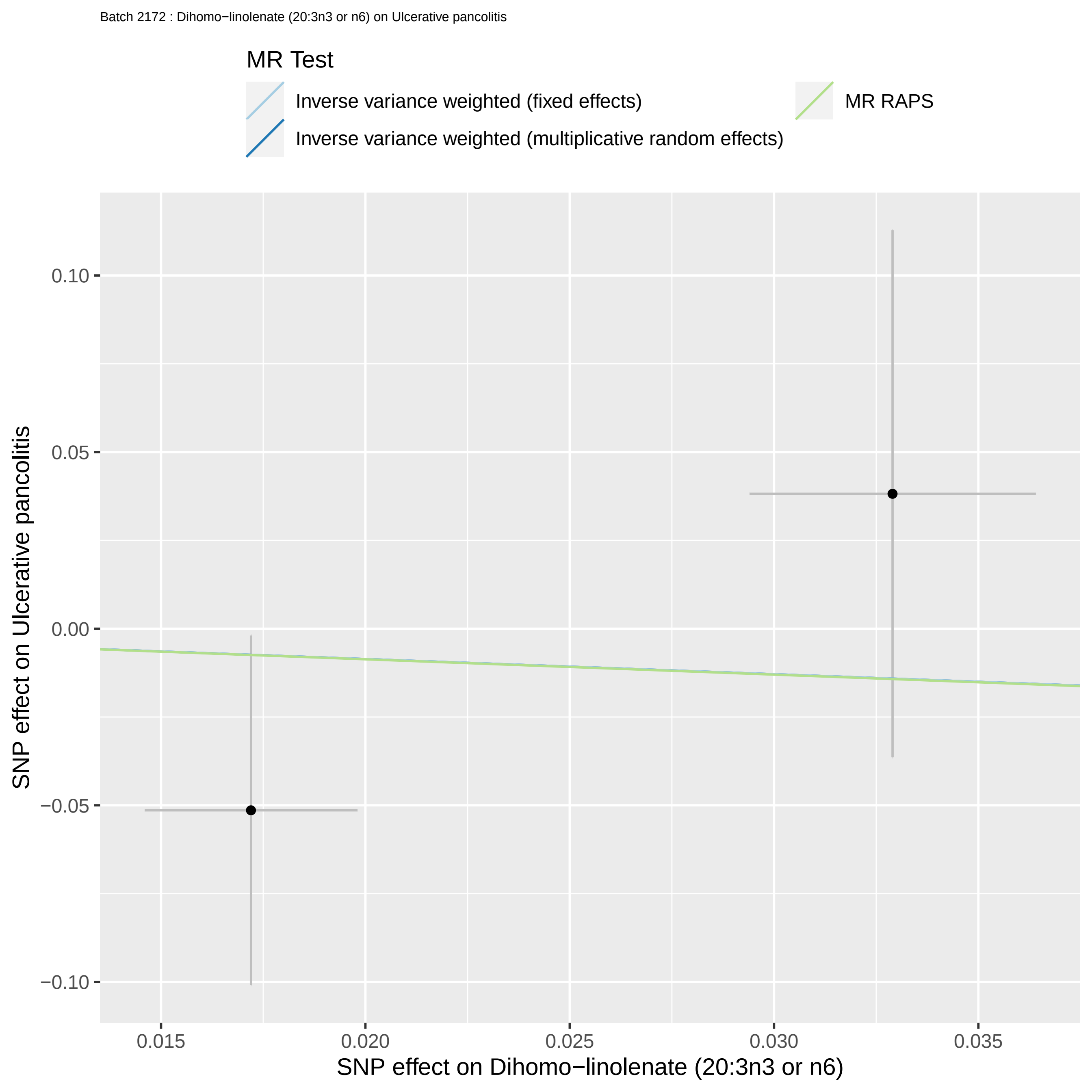

Batch 2172 : Dihomo−linolenate (20:3n3 or n6) on Ulcerative pancolitis
MR Test
Inverse variance weighted (fixed effects)
Inverse variance weighted (multiplicative random effects)
MR RAPS
0.10
0.05
0.00
−0.05
−0.10
0.015
0.020
0.025
0.030
0.035
SNP effect on Ulcerative pancolitis
SNP effect on Dihomo−linolenate (20:3n3 or n6)

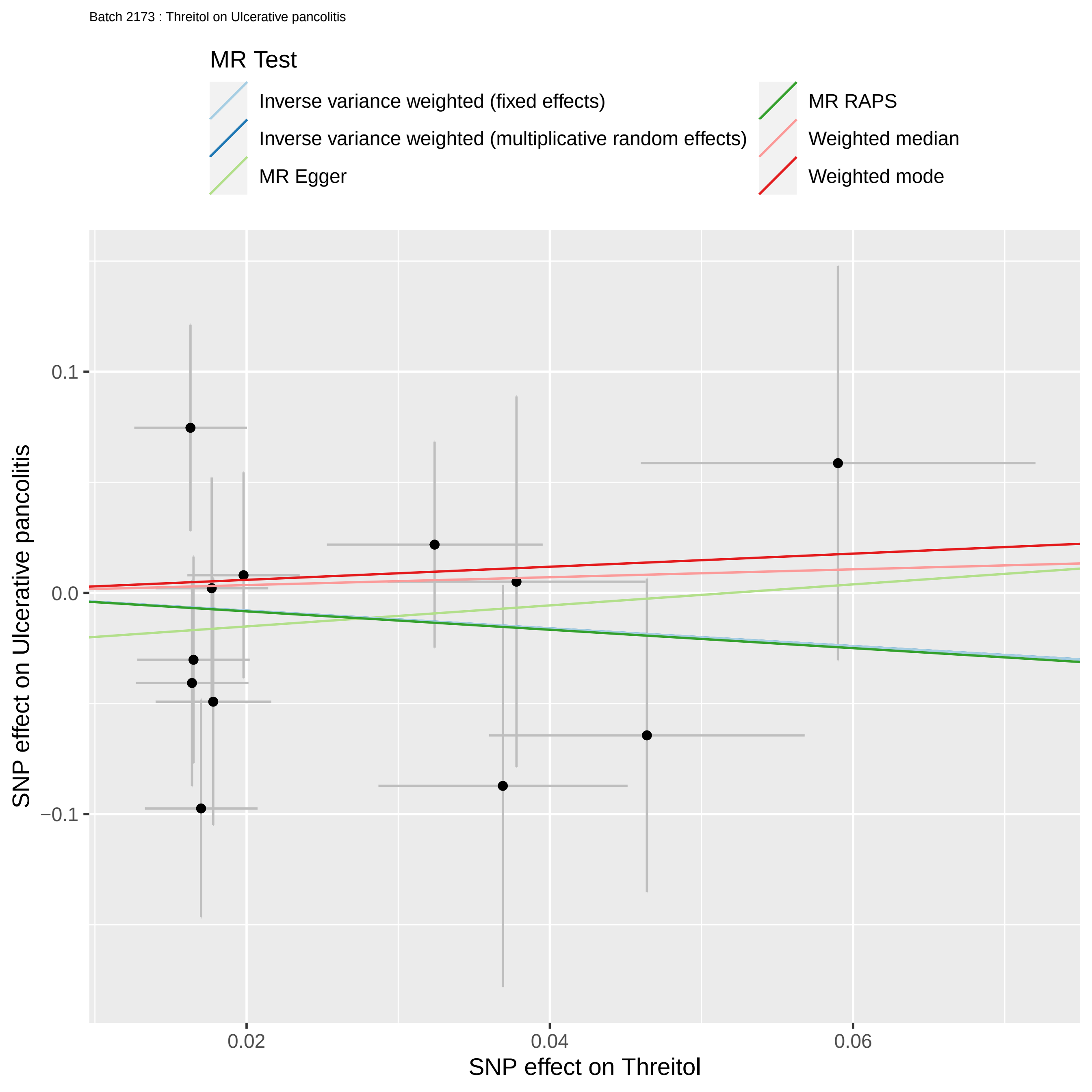

Batch 2173 : Threitol on Ulcerative pancolitis
MR Test
Inverse variance weighted (fixed effects)
Inverse variance weighted (multiplicative random effects)
MR Egger
MR RAPS
Weighted median
Weighted mode
0.1
0.0
−0.1
0.02
0.04
0.06
SNP effect on Ulcerative pancolitis
SNP effect on Threitol

Batch 2174 : P−cresol sulfate on Ulcerative pancolitis

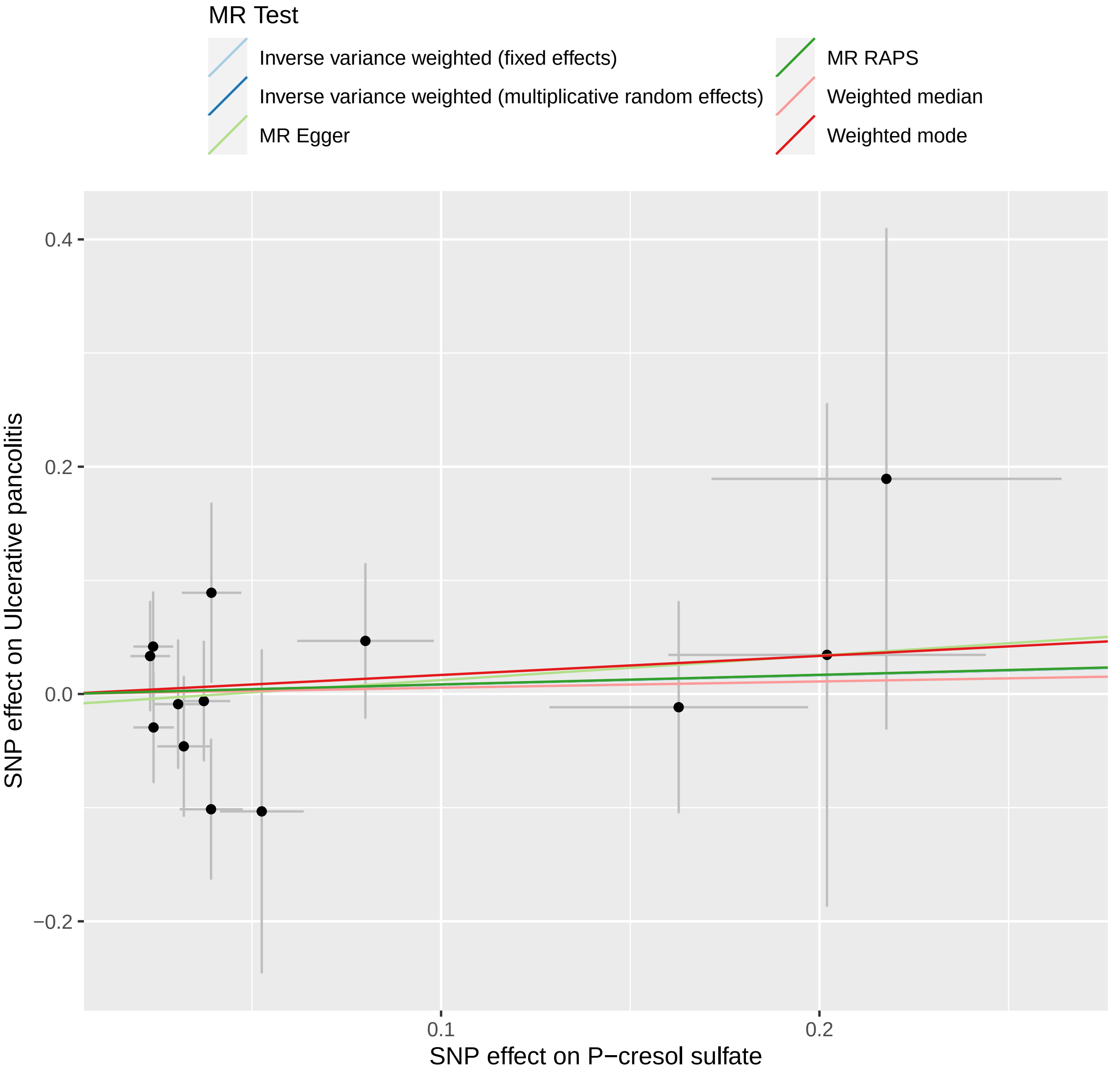

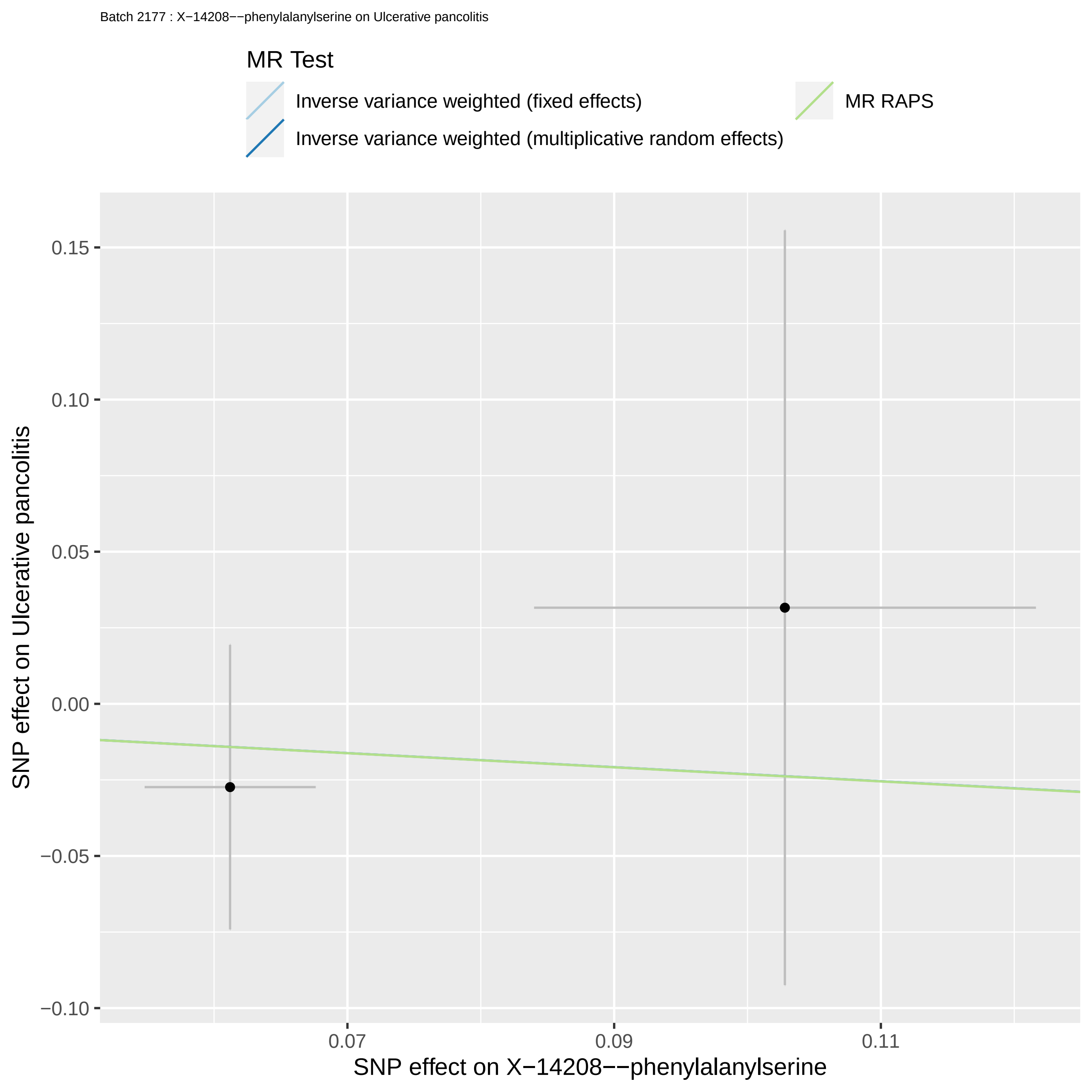

Batch 2177 : X−14208−−phenylalanylserine on Ulcerative pancolitis
MR Test
Inverse variance weighted (fixed effects)
Inverse variance weighted (multiplicative random effects)
MR RAPS
0.15
0.10
0.05
0.00
−0.05
−0.10
0.07
0.09
0.11
SNP effect on Ulcerative pancolitis
SNP effect on X−14208−−phenylalanylserine

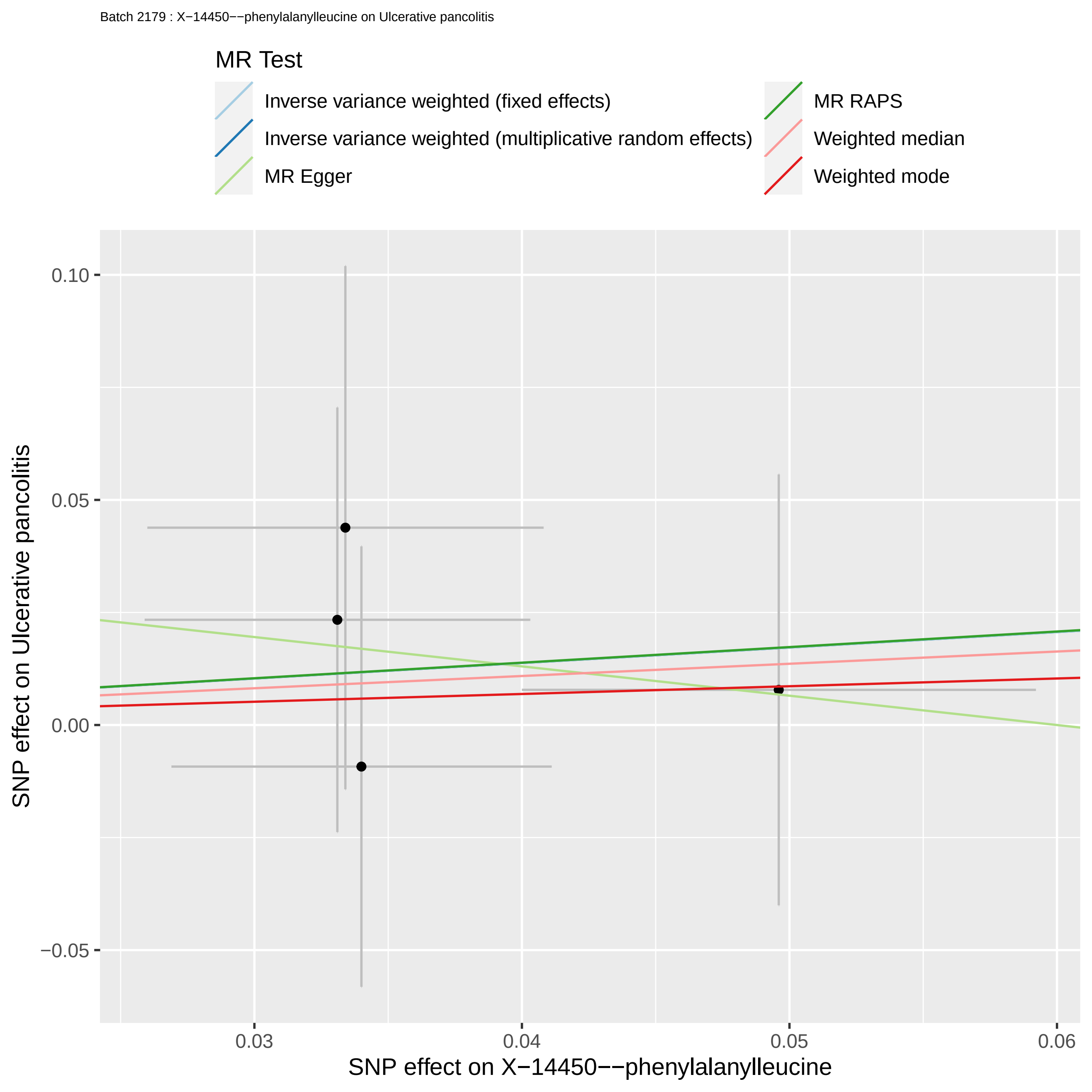
Batch 2179 : X−14450−−phenylalanylleucine on Ulcerative pancolitis
MR Test
Inverse variance weighted (fixed effects)
Inverse variance weighted (multiplicative random effects)
MR Egger
MR RAPS
Weighted median
Weighted mode
0.10
0.05
0.00
−0.05
0.03
0.04
0.05
0.06
SNP effect on Ulcerative pancolitis
SNP effect on X−14450−−phenylalanylleucine

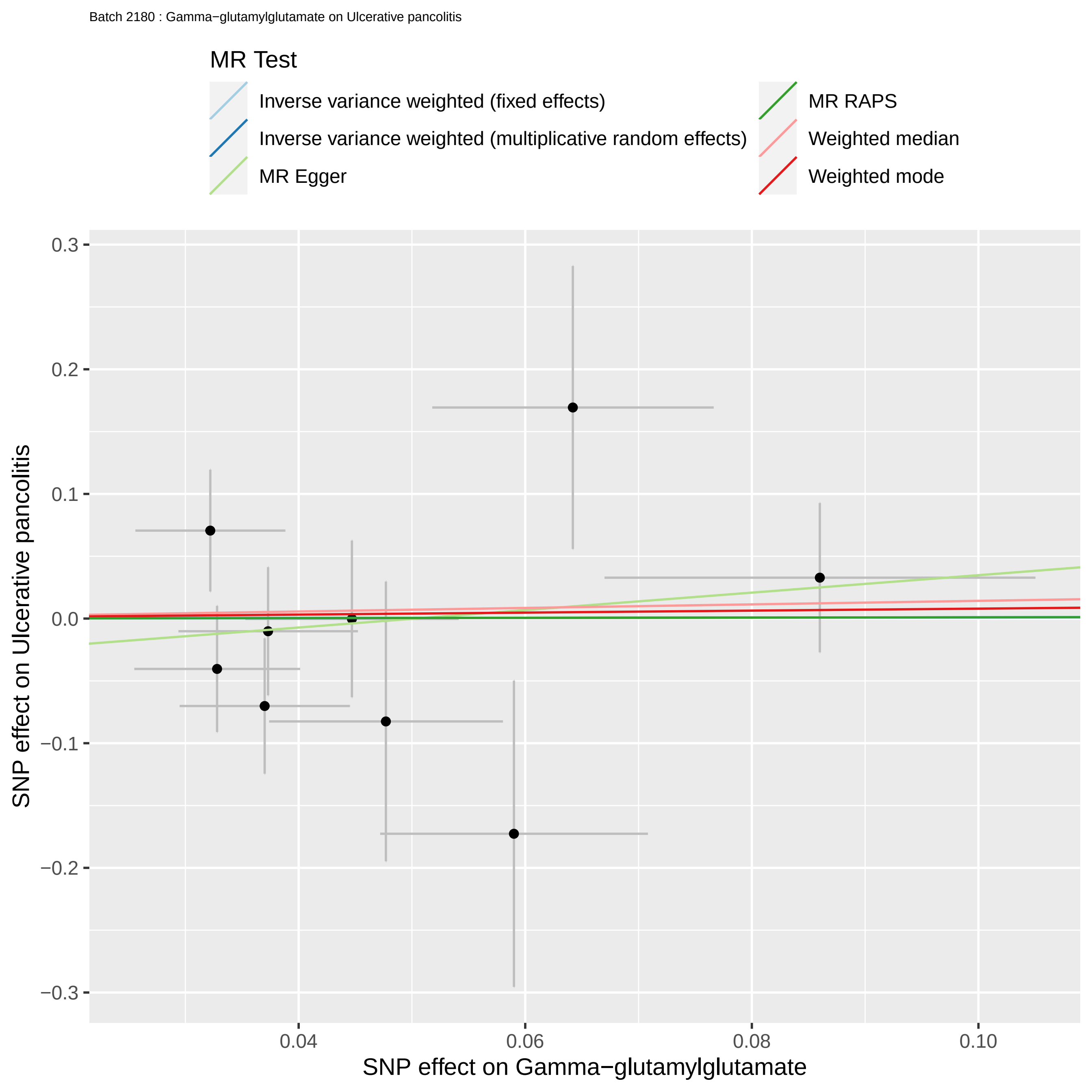

Batch 2180 : Gamma−glutamylglutamate on Ulcerative pancolitis
MR Test
Inverse variance weighted (fixed effects)
Inverse variance weighted (multiplicative random effects)
MR Egger
MR RAPS
Weighted median
Weighted mode
0.3
0.2
0.1
0.0
−0.1
−0.2
−0.3
0.04
0.06
0.08
0.10
SNP effect on Ulcerative pancolitis
SNP effect on Gamma−glutamylglutamate

Batch 2181 : Octadecanedioate on Ulcerative pancolitis

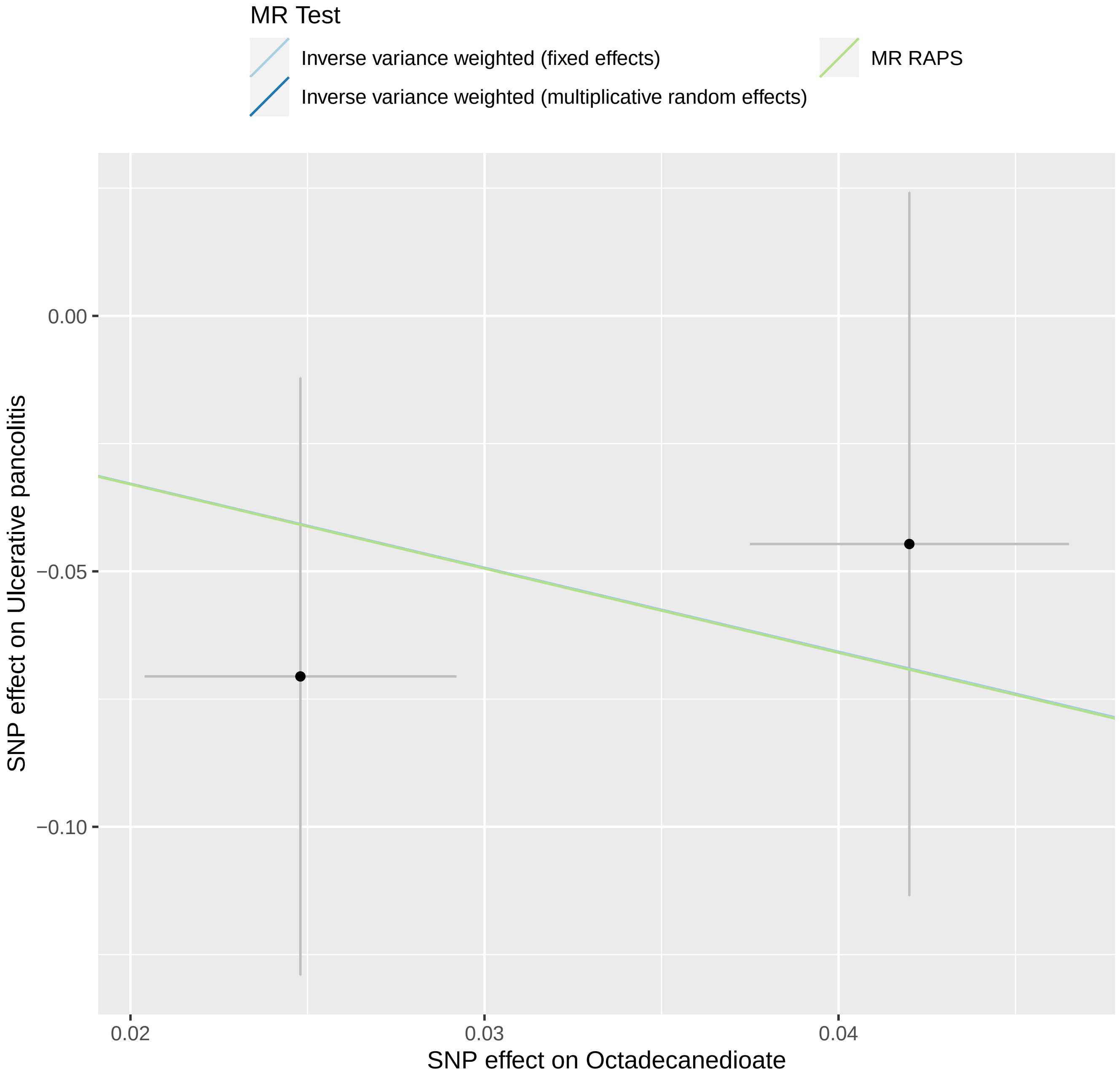

Batch 2182 : Leucylleucine on Ulcerative pancolitis

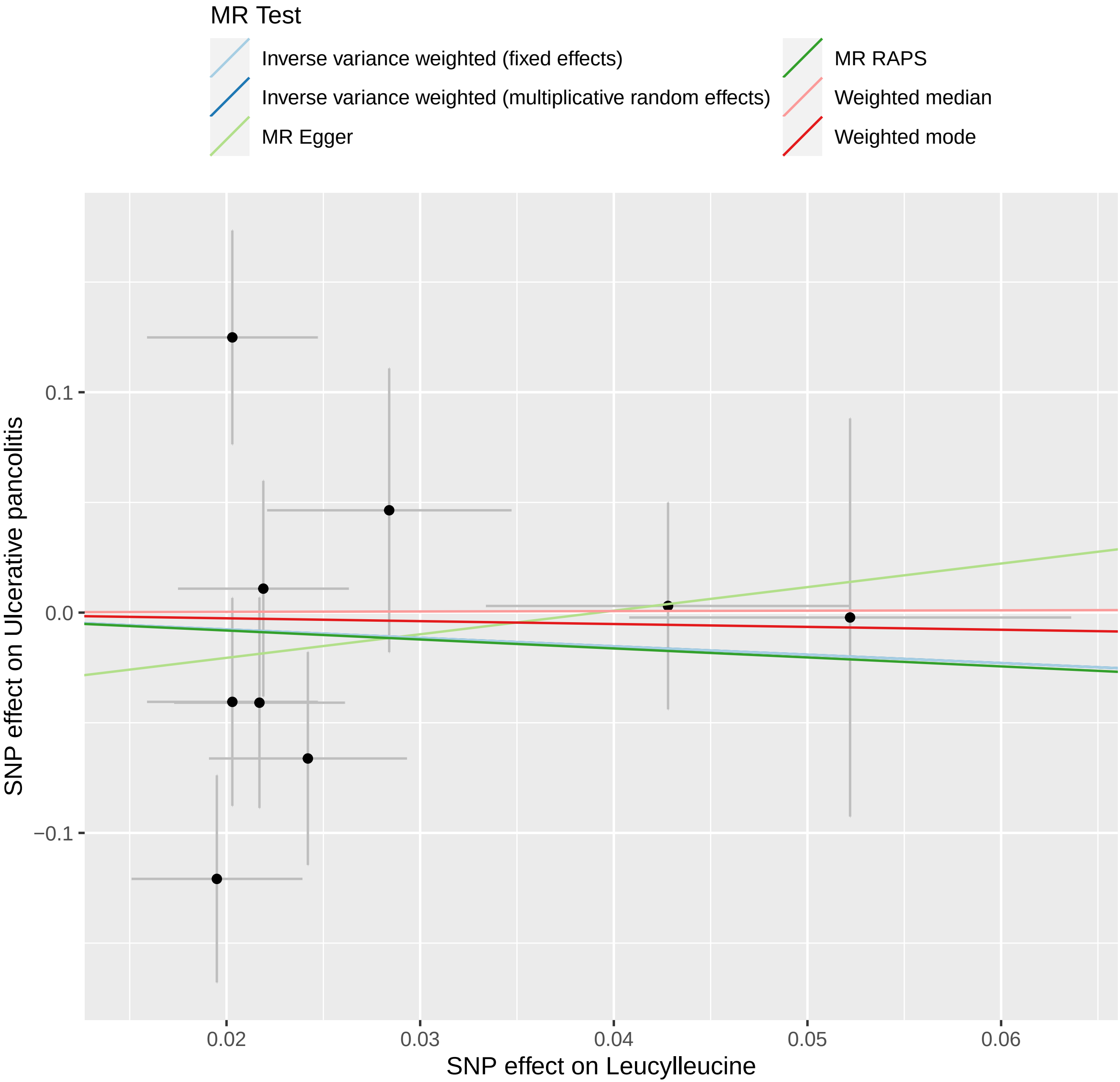

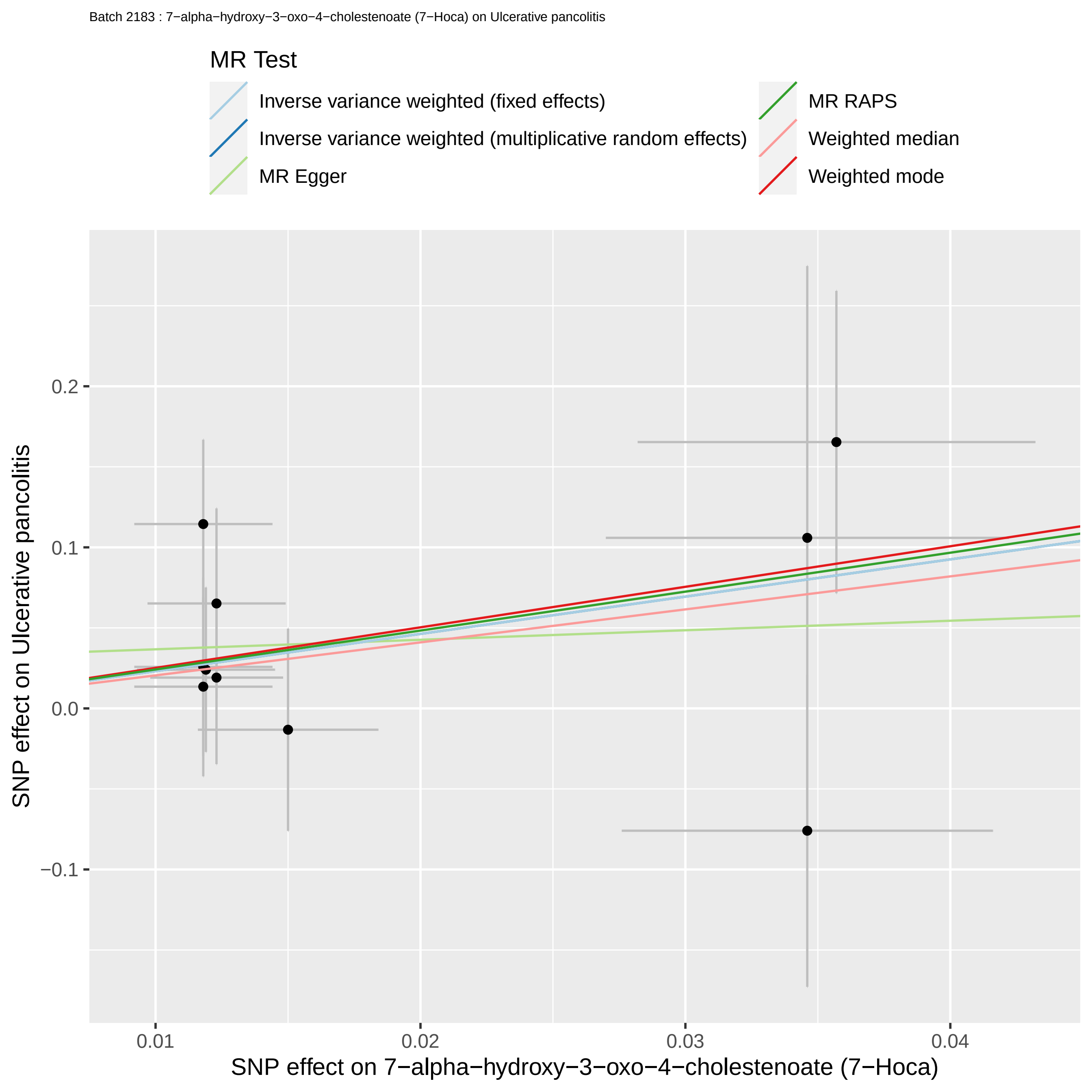

Batch 2183 : 7−alpha−hydroxy−3−oxo−4−cholestenoate (7−Hoca) on Ulcerative pancolitis
MR Test
Inverse variance weighted (fixed effects)
Inverse variance weighted (multiplicative random effects)
MR Egger
MR RAPS
Weighted median
Weighted mode
0.2
0.1
0.0
−0.1
0.01
0.02
0.03
0.04
SNP effect on Ulcerative pancolitis
SNP effect on 7−alpha−hydroxy−3−oxo−4−cholestenoate (7−Hoca)

Batch 2184 : N−Butyl Oleate on Ulcerative pancolitis

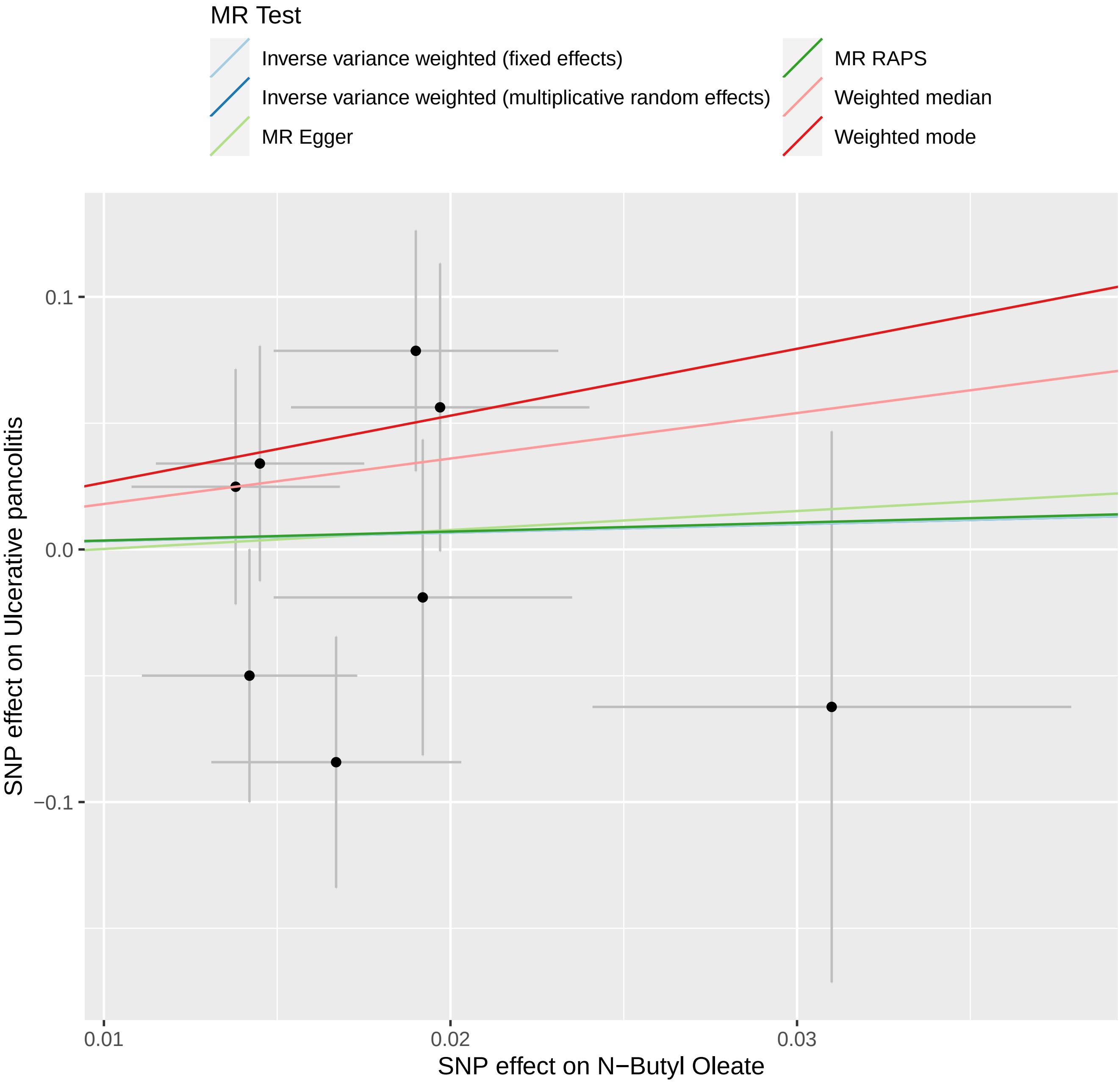

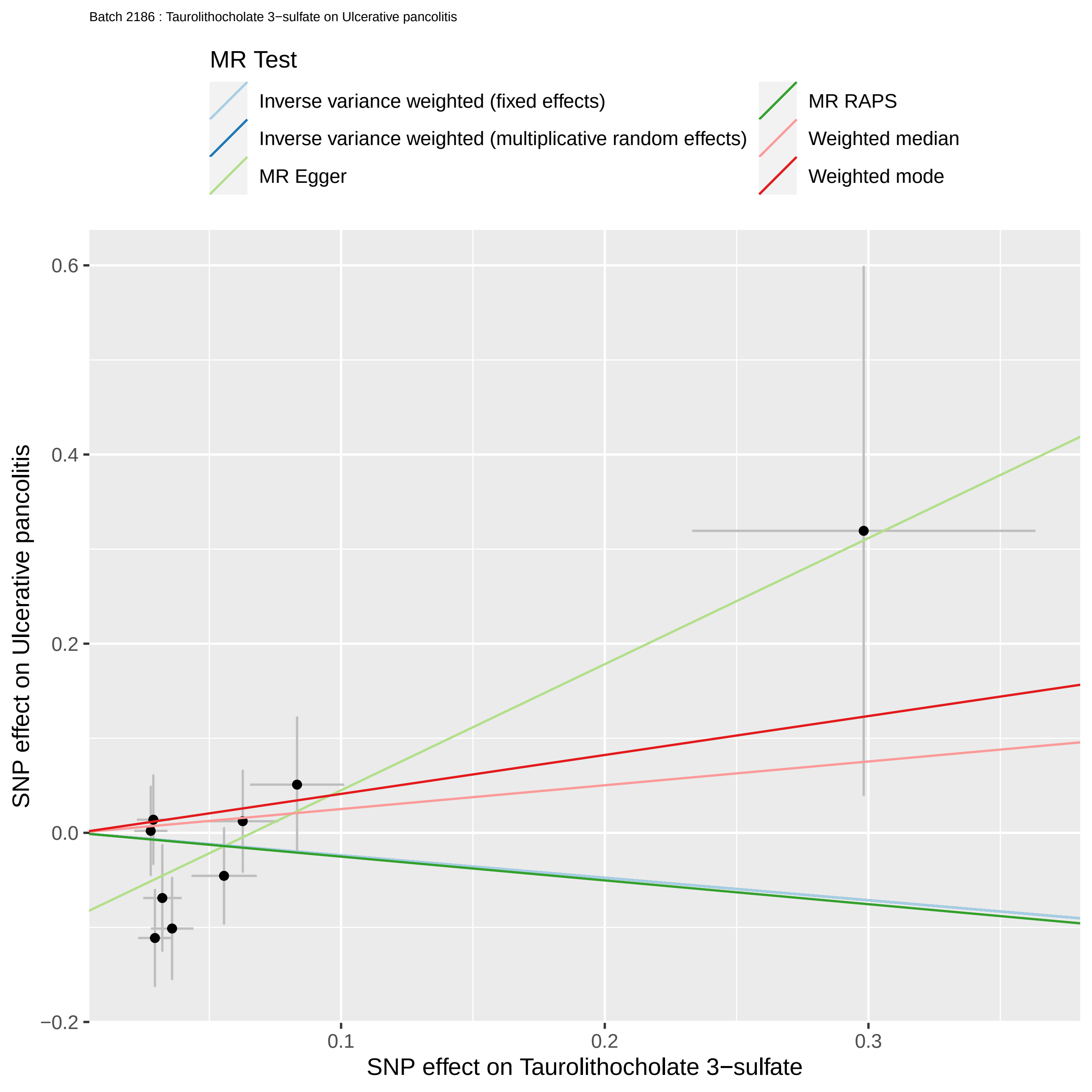
Batch 2186 : Taurolithocholate 3−sulfate on Ulcerative pancolitis
MR Test
Inverse variance weighted (fixed effects)
Inverse variance weighted (multiplicative random effects)
MR Egger
MR RAPS
Weighted median
Weighted mode
0.6
0.4
0.2
0.0
−0.2
0.1
0.2
0.3
SNP effect on Ulcerative pancolitis
SNP effect on Taurolithocholate 3−sulfate

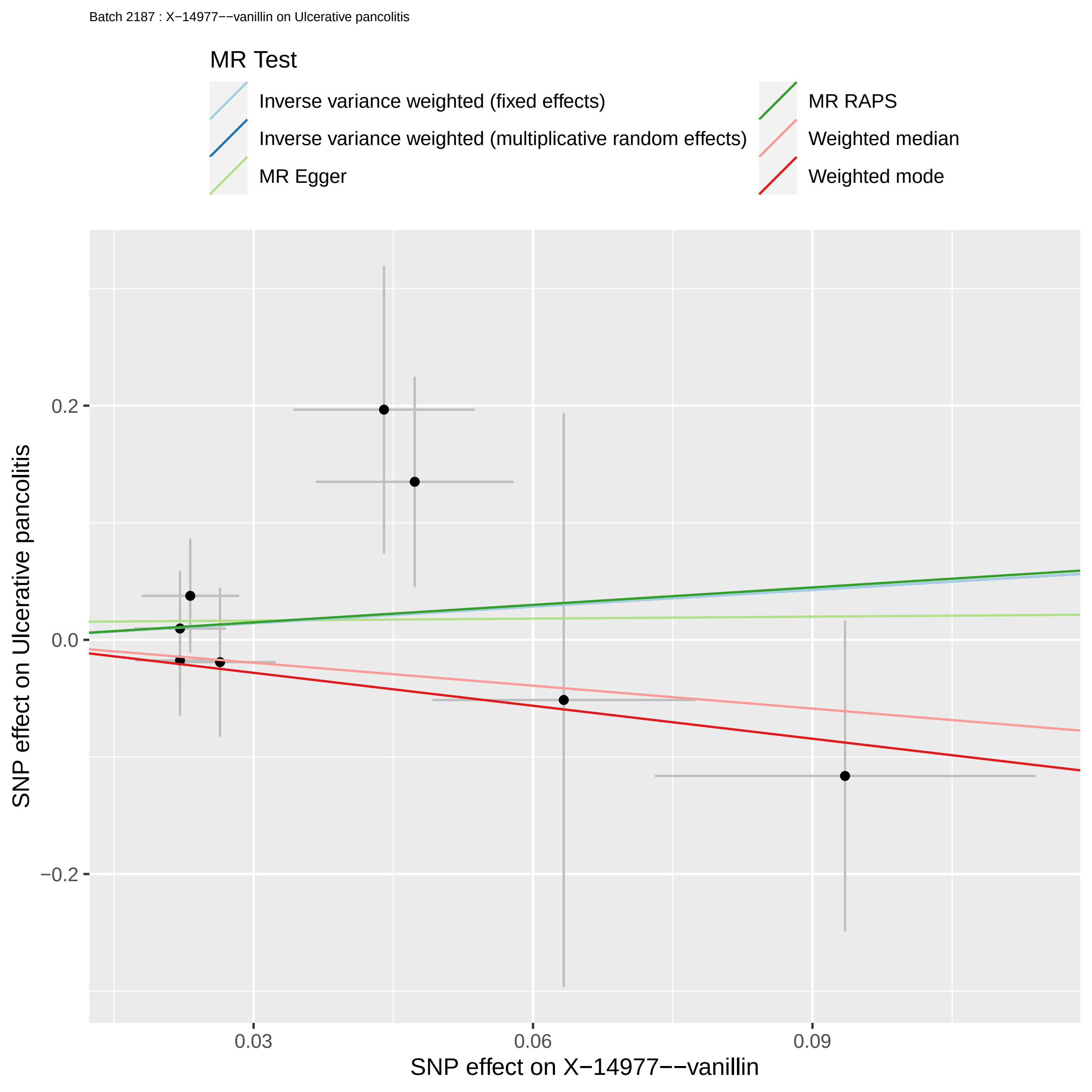

Batch 2187 : X−14977−−vanillin on Ulcerative pancolitis
MR Test
Inverse variance weighted (fixed effects)
Inverse variance weighted (multiplicative random effects)
MR Egger
MR RAPS
Weighted median
Weighted mode
0.2
0.0
−0.2
0.03
0.06
0.09
SNP effect on Ulcerative pancolitis
SNP effect on X−14977−−vanillin

Batch 2188 : Succinylcarnitine on Ulcerative pancolitis

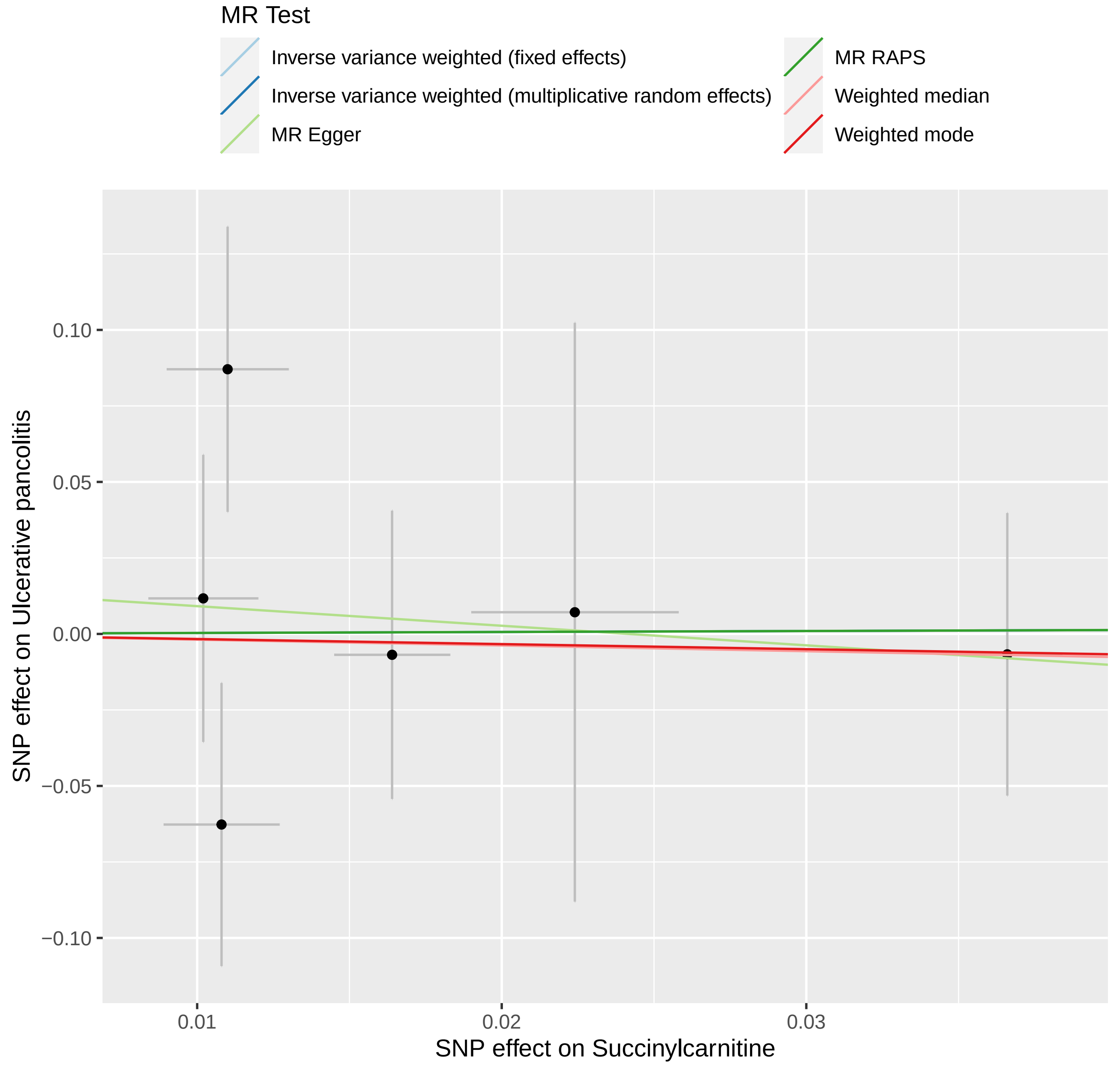

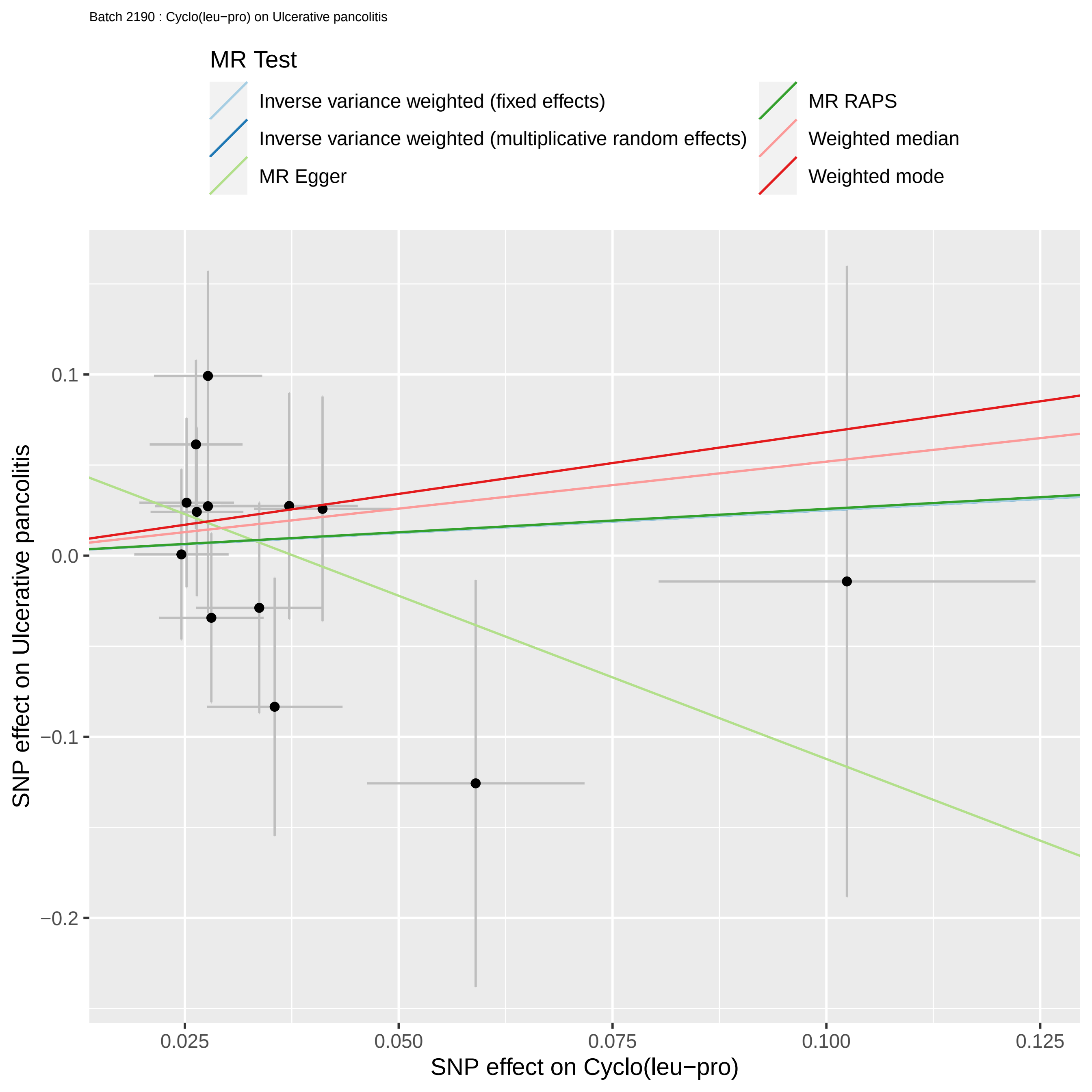

Batch 2190 : Cyclo(leu−pro) on Ulcerative pancolitis
MR Test
Inverse variance weighted (fixed effects)
Inverse variance weighted (multiplicative random effects)
MR Egger
MR RAPS
Weighted median
Weighted mode
0.1
0.0
−0.1
−0.2
0.025
0.050
0.075
0.100
0.125
SNP effect on Ulcerative pancolitis
SNP effect on Cyclo(leu−pro)

Batch 2191 : Chiro−inositol on Ulcerative pancolitis

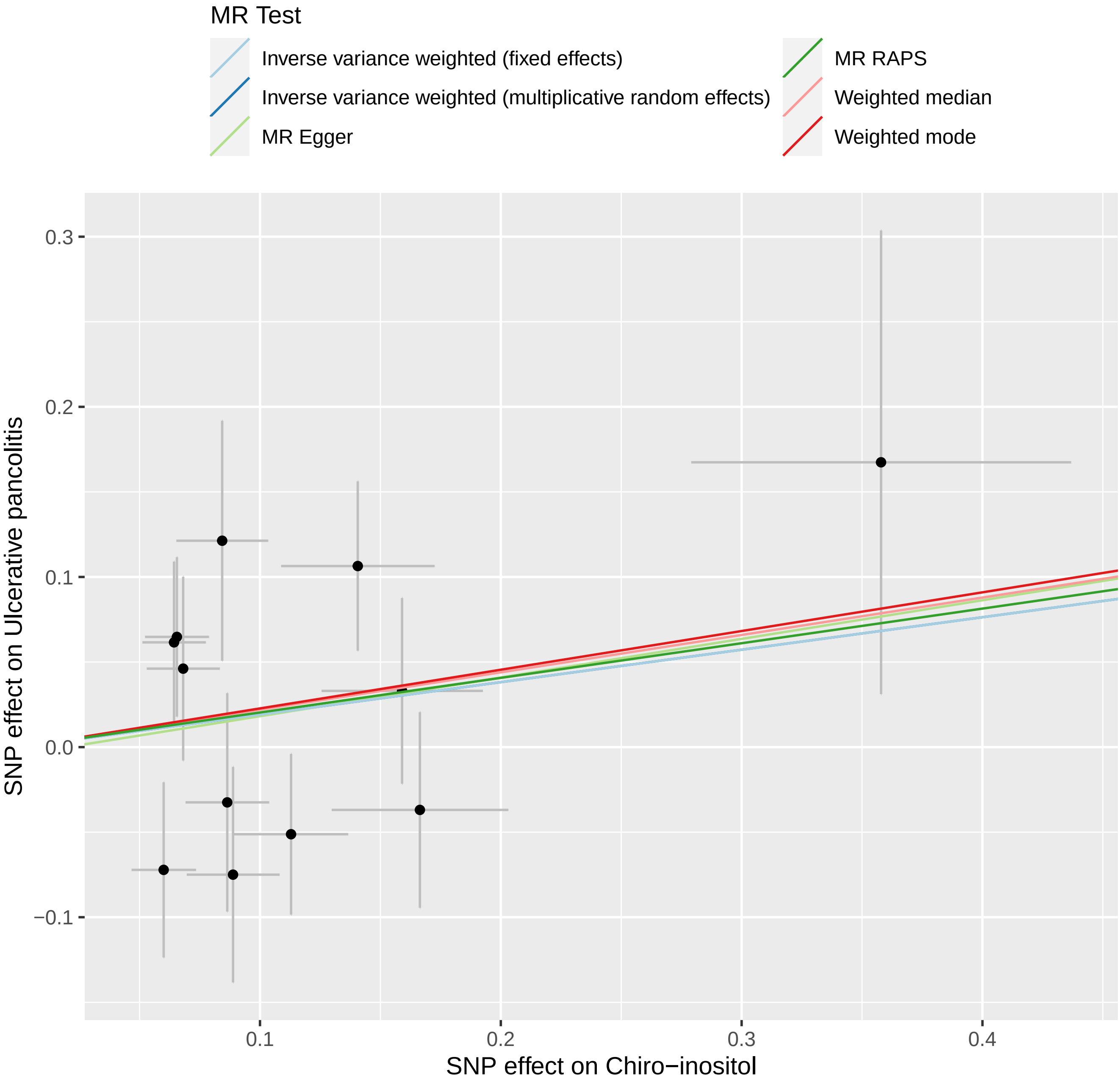

Batch 2195 : 2−hydroxyglutarate on Ulcerative pancolitis

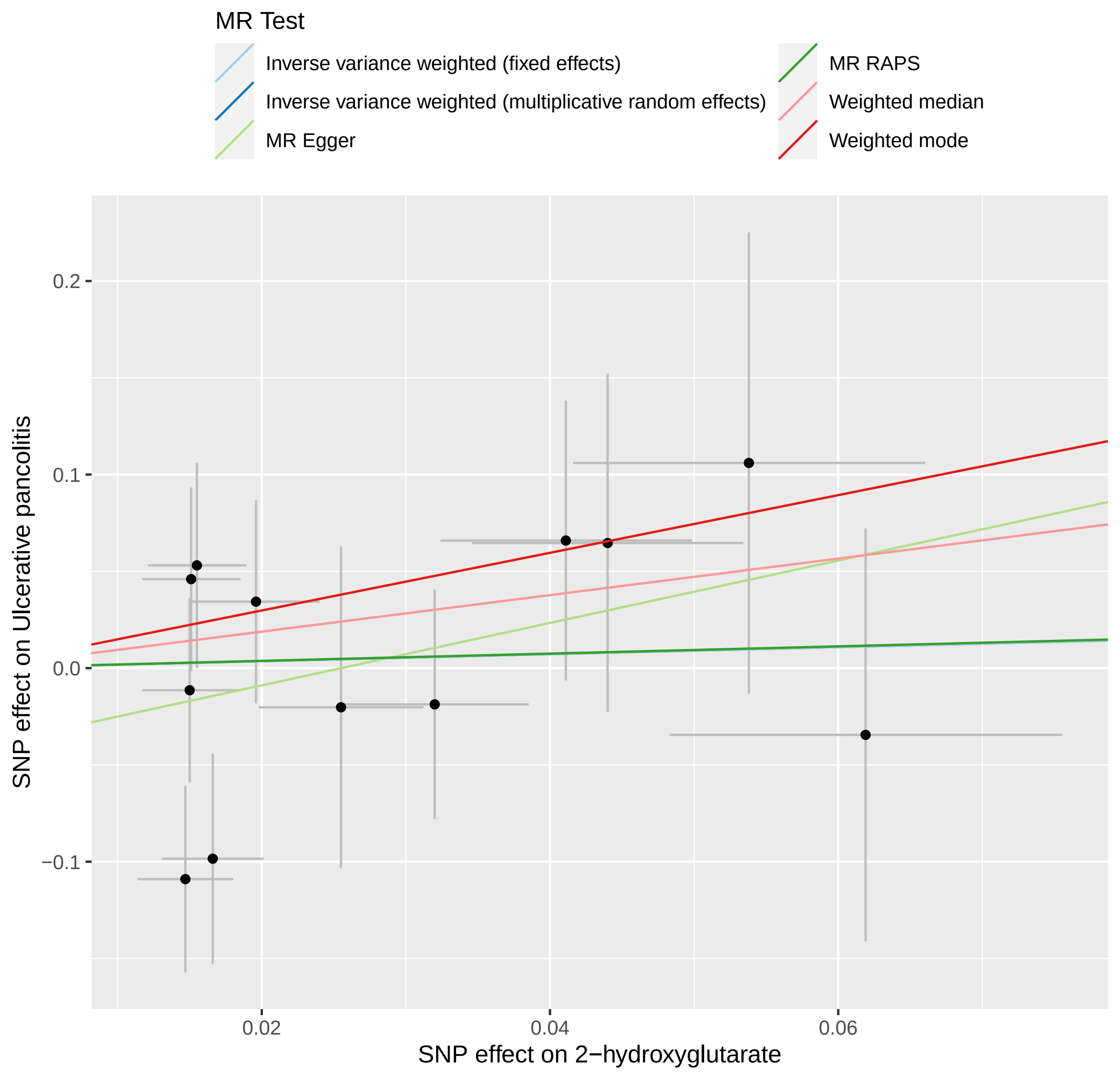

Batch 2196 : Ergothioneine on Ulcerative pancolitis

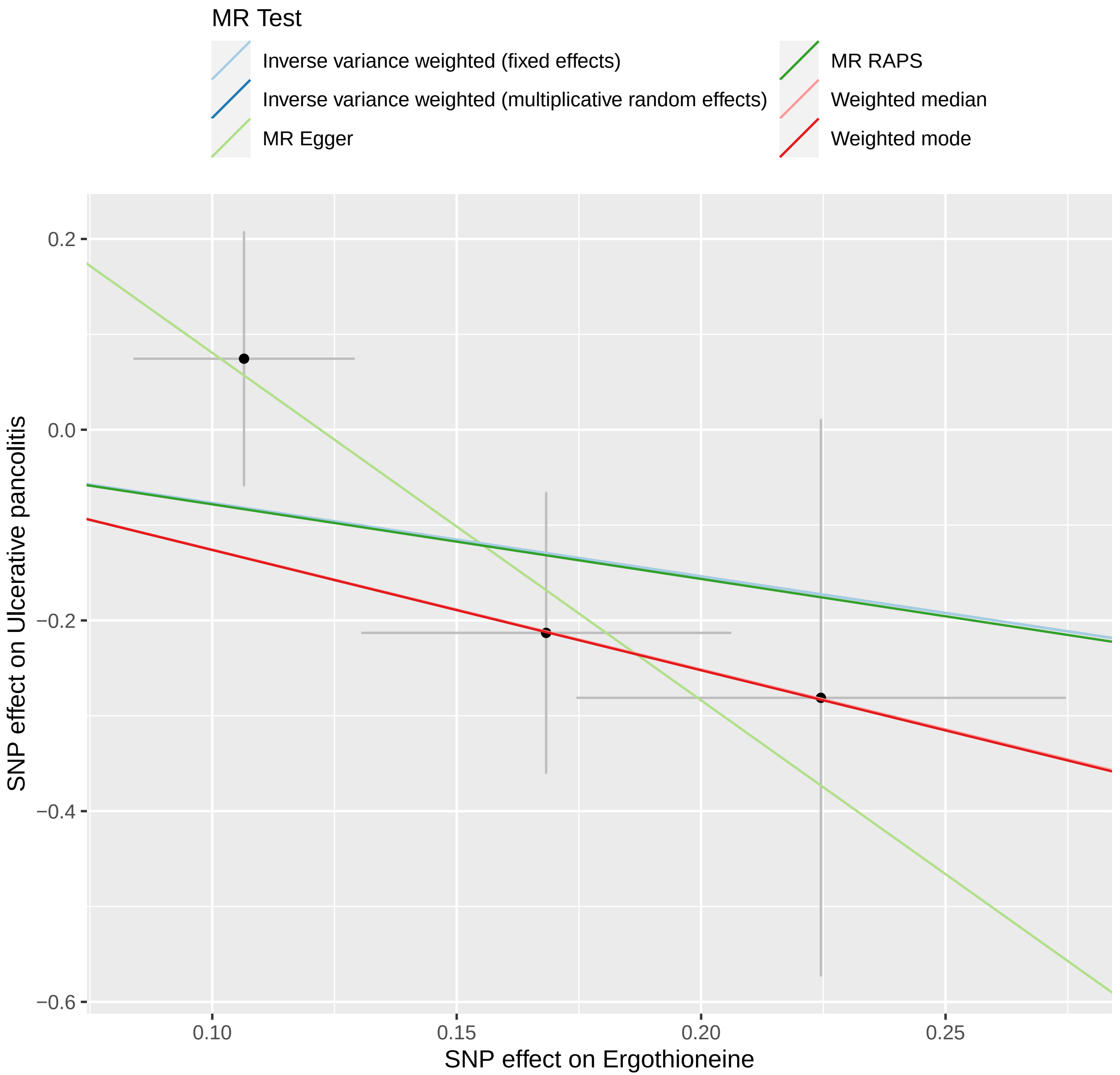

Batch 2198 : Phenylalanylphenylalanine on Ulcerative pancolitis

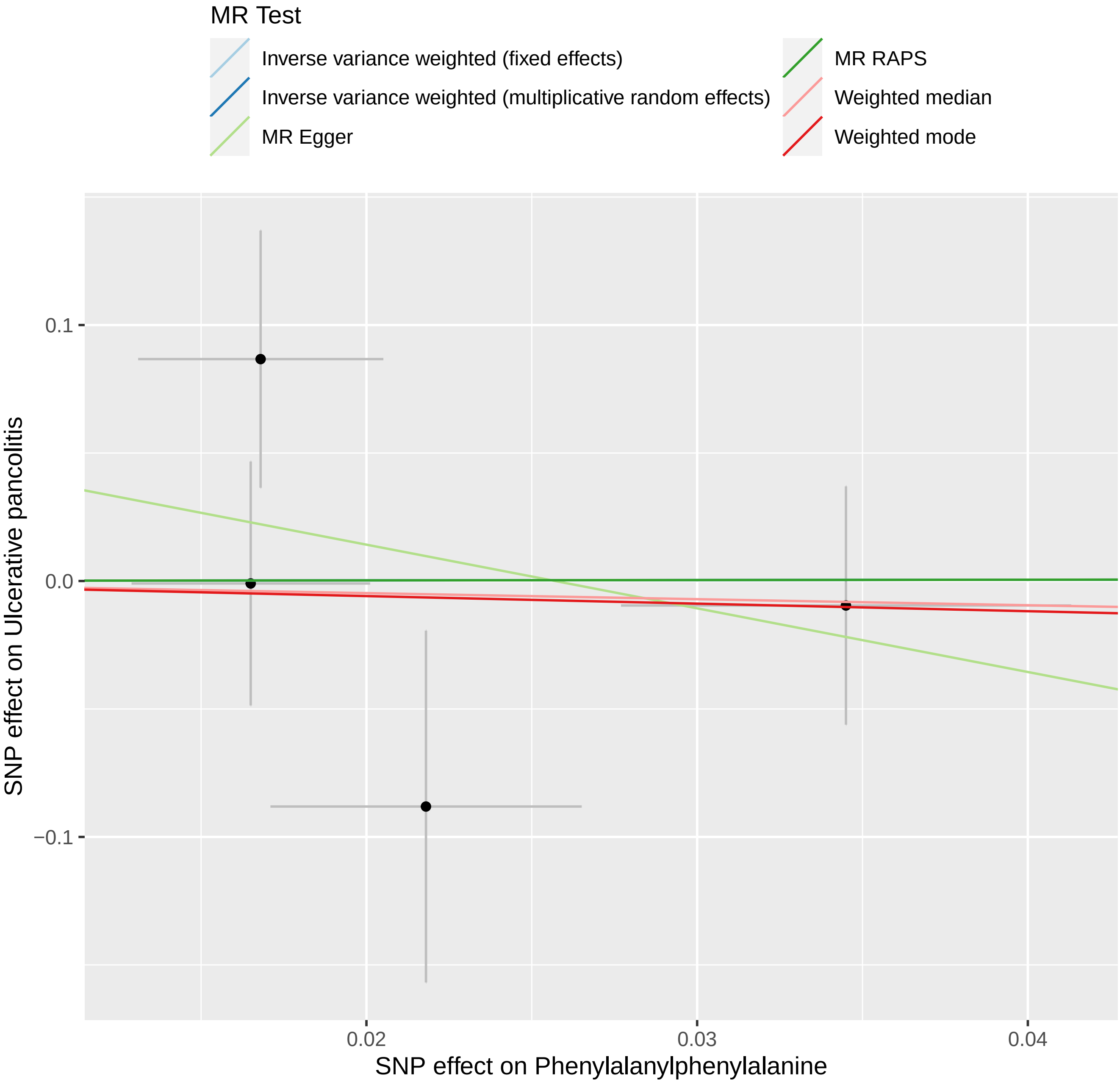

Batch 2199 : Cis−4−decenoyl carnitine on Ulcerative pancolitis

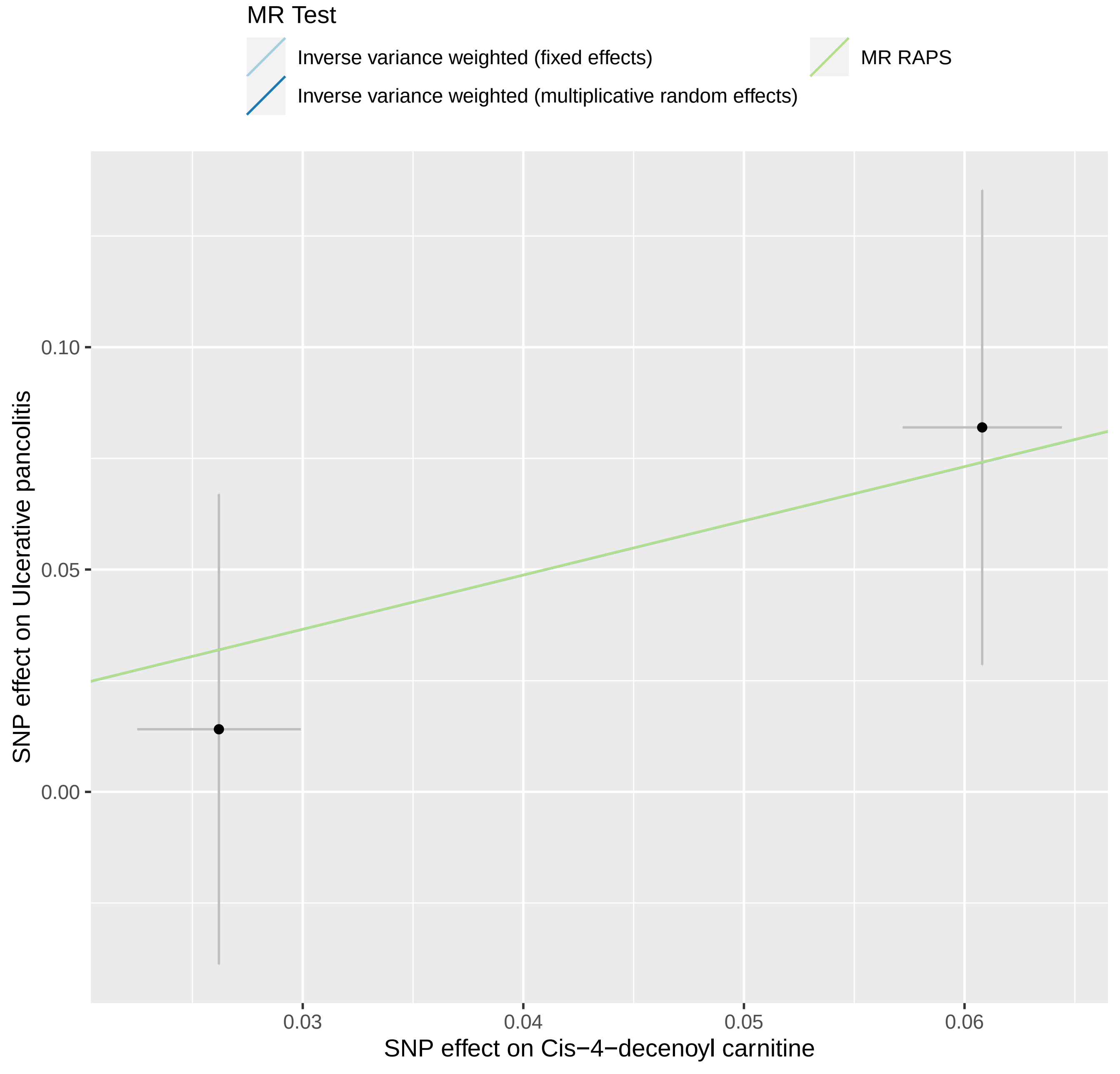

Batch 2200 : 15−methylpalmitate (isobar with 2−methylpalmitate) on Ulcerative pancolitis

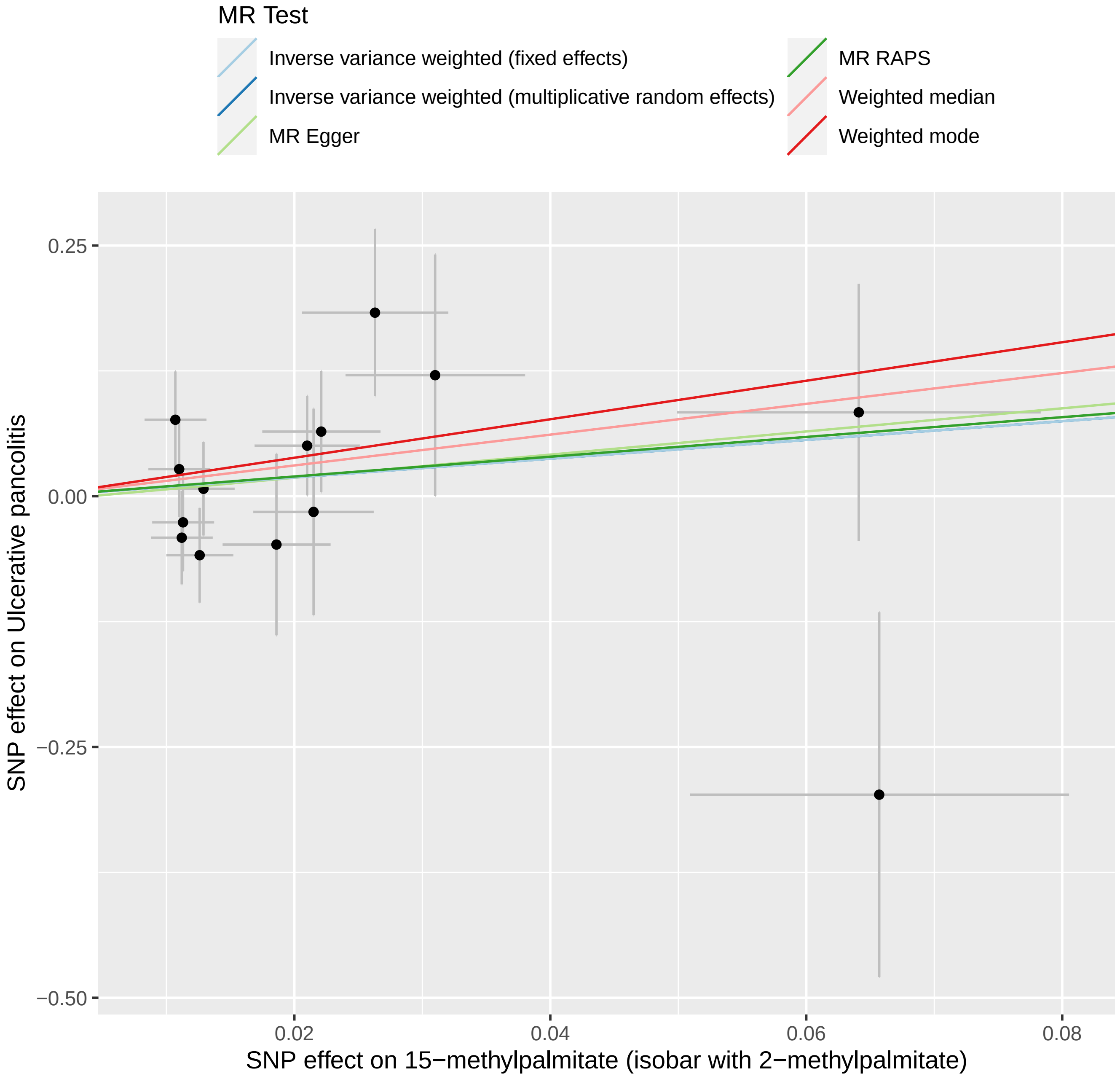

Batch 2202 : Tryptophan on Ulcerative proctitis

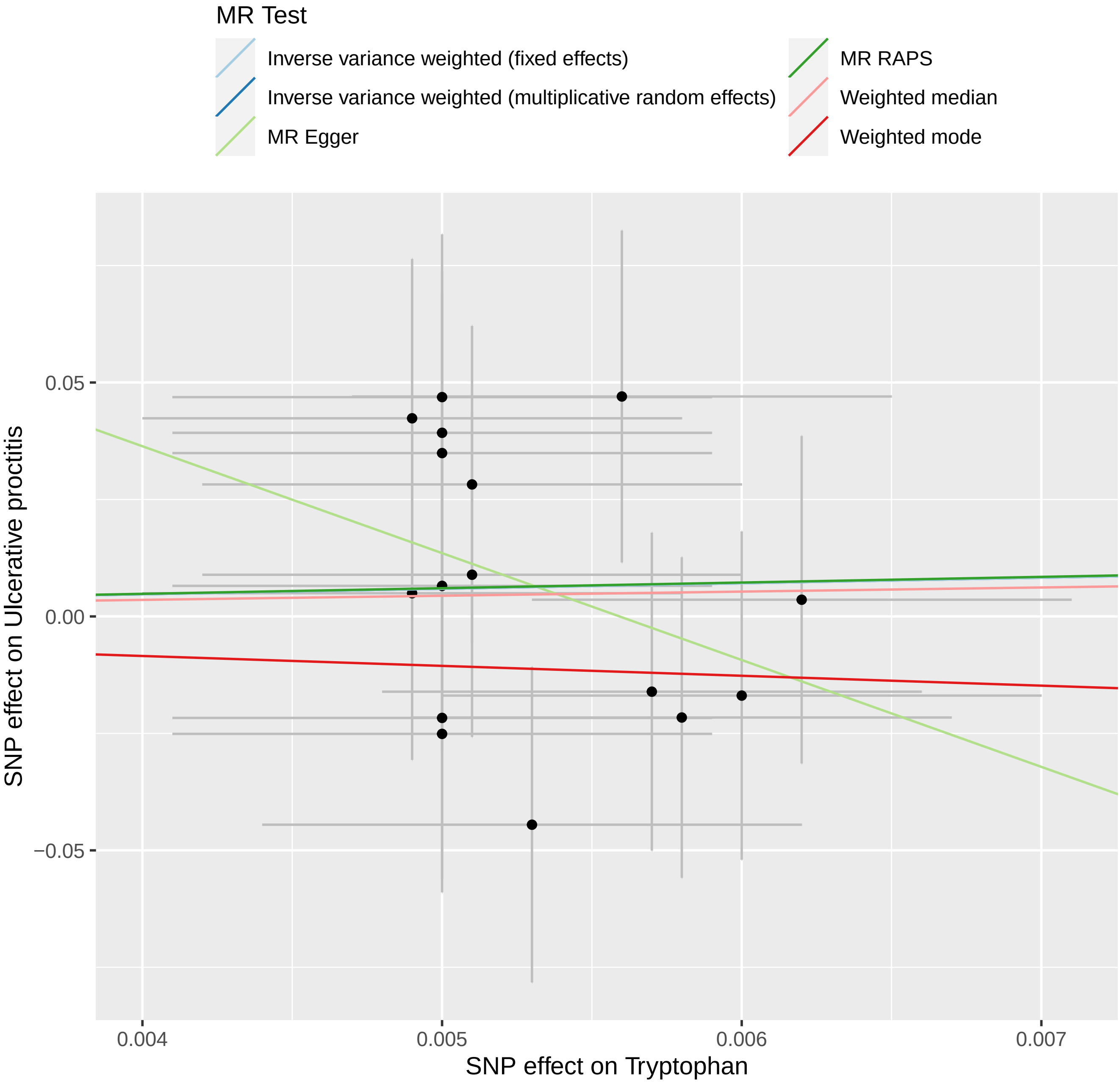

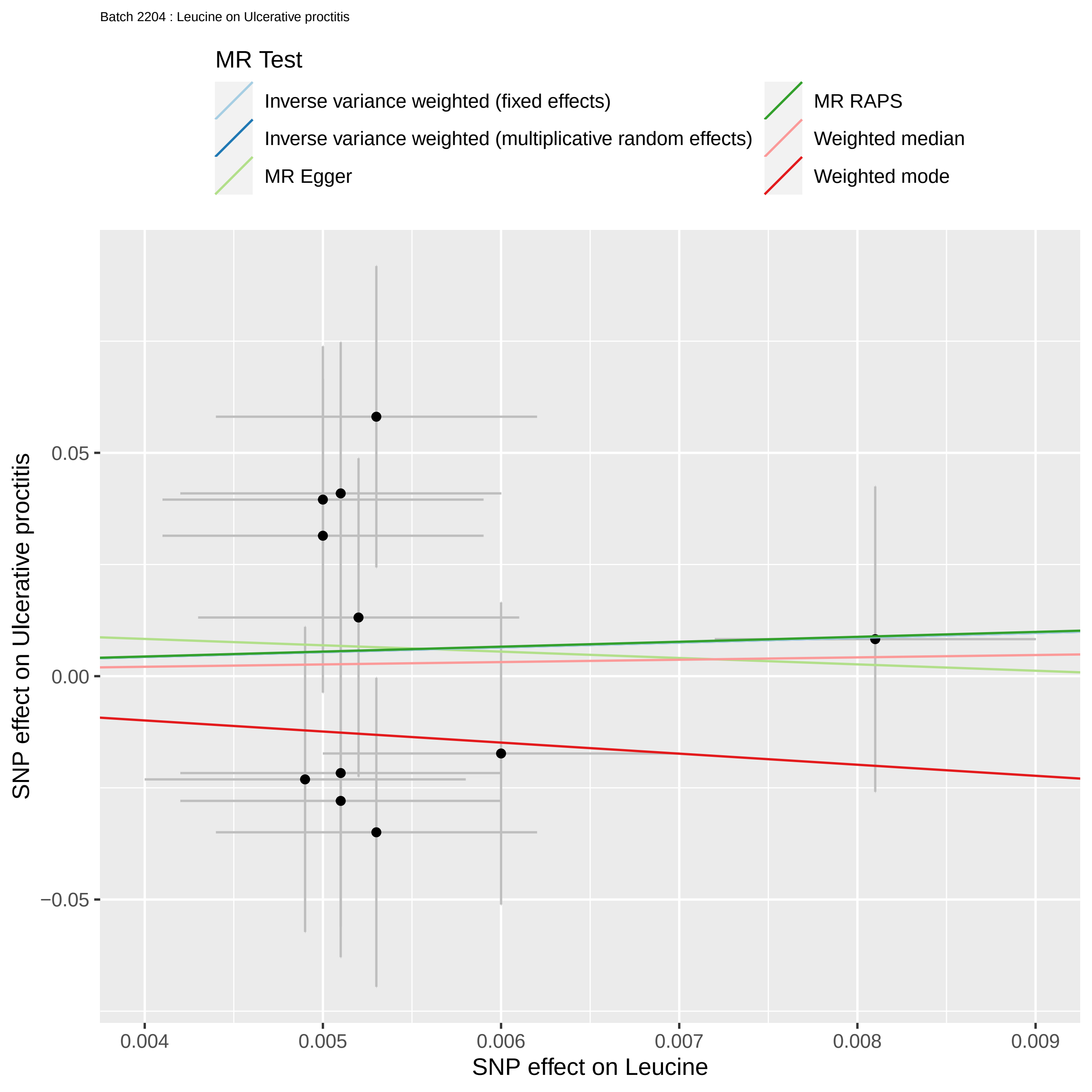

Batch 2204 : Leucine on Ulcerative proctitis
MR Test
Inverse variance weighted (fixed effects)
Inverse variance weighted (multiplicative random effects)
MR Egger
MR RAPS
Weighted median
Weighted mode
0.05
0.00
−0.05
0.004
0.005
0.006
0.007
0.008
0.009
SNP effect on Ulcerative proctitis
SNP effect on Leucine

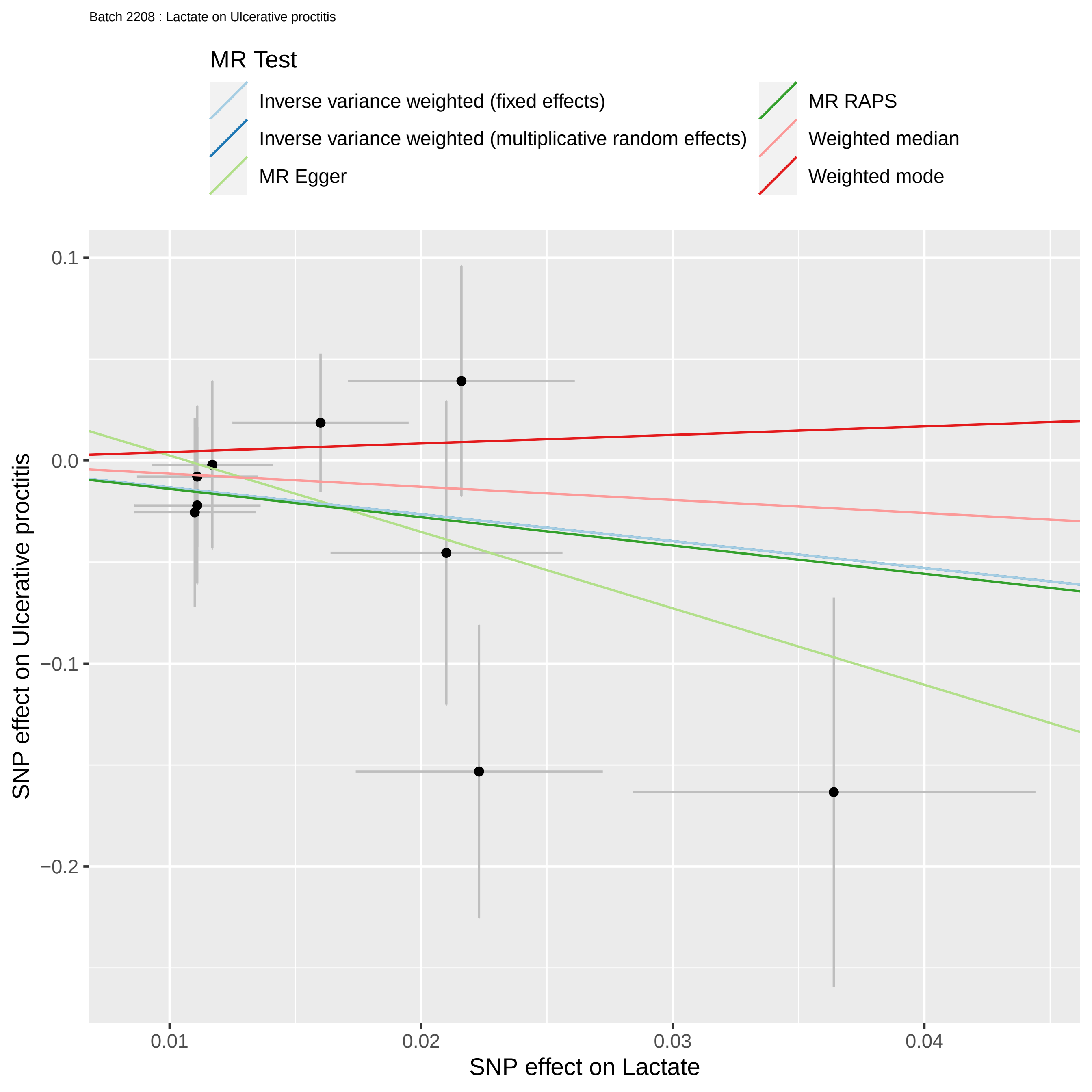
Batch 2208 : Lactate on Ulcerative proctitis
MR Test
Inverse variance weighted (fixed effects)
Inverse variance weighted (multiplicative random effects)
MR Egger
MR RAPS
Weighted median
Weighted mode
SNP effect on Ulcerative proctitis
0.1
0.0
−0.1
−0.2
0.01
0.02
0.03
0.04
SNP effect on Lactate

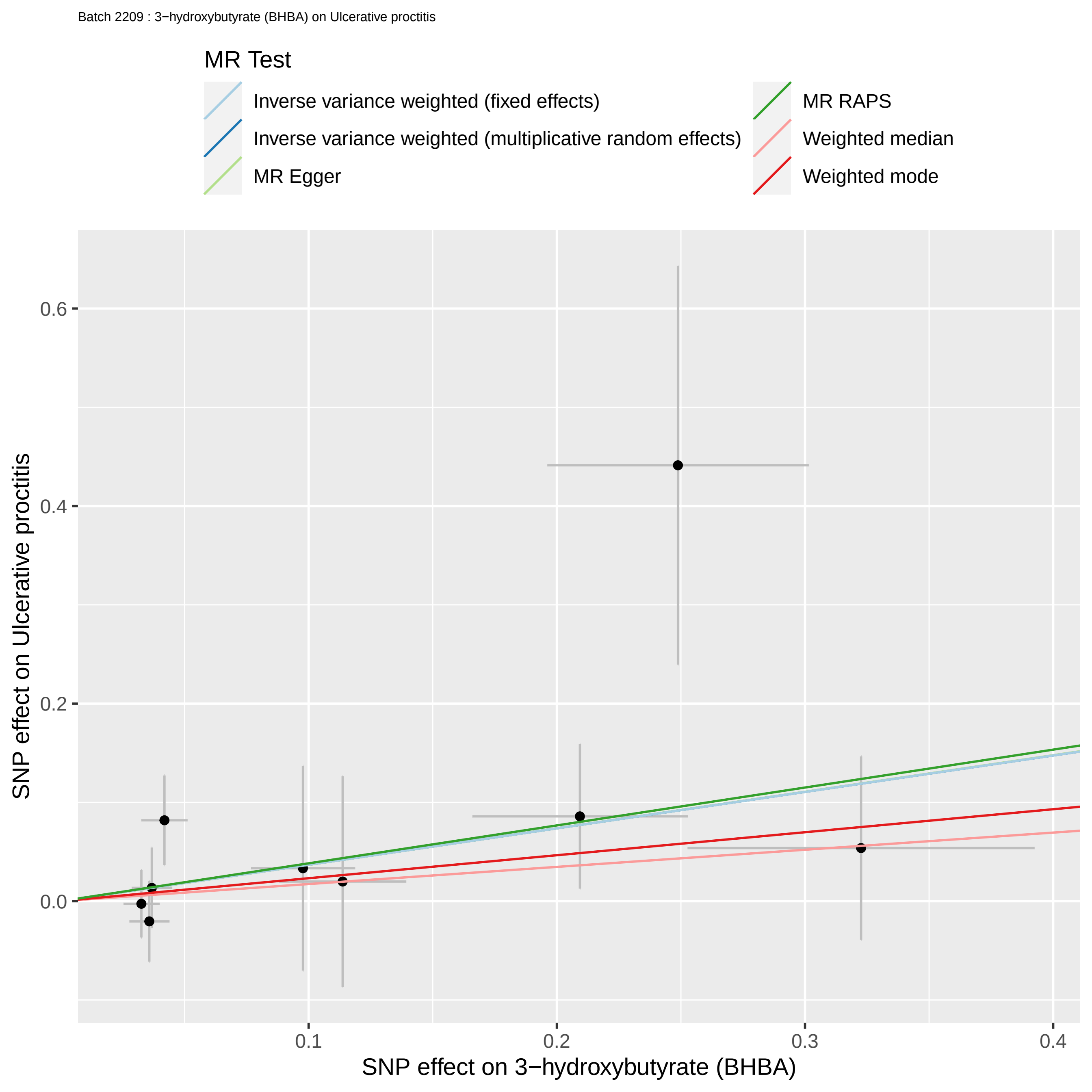

Batch 2209 : 3−hydroxybutyrate (BHBA) on Ulcerative proctitis
MR Test
Inverse variance weighted (fixed effects)
Inverse variance weighted (multiplicative random effects)
MR Egger
MR RAPS
Weighted median
Weighted mode
0.6
0.4
0.2
0.0
0.1
0.2
0.3
0.4
SNP effect on Ulcerative proctitis
SNP effect on 3−hydroxybutyrate (BHBA)

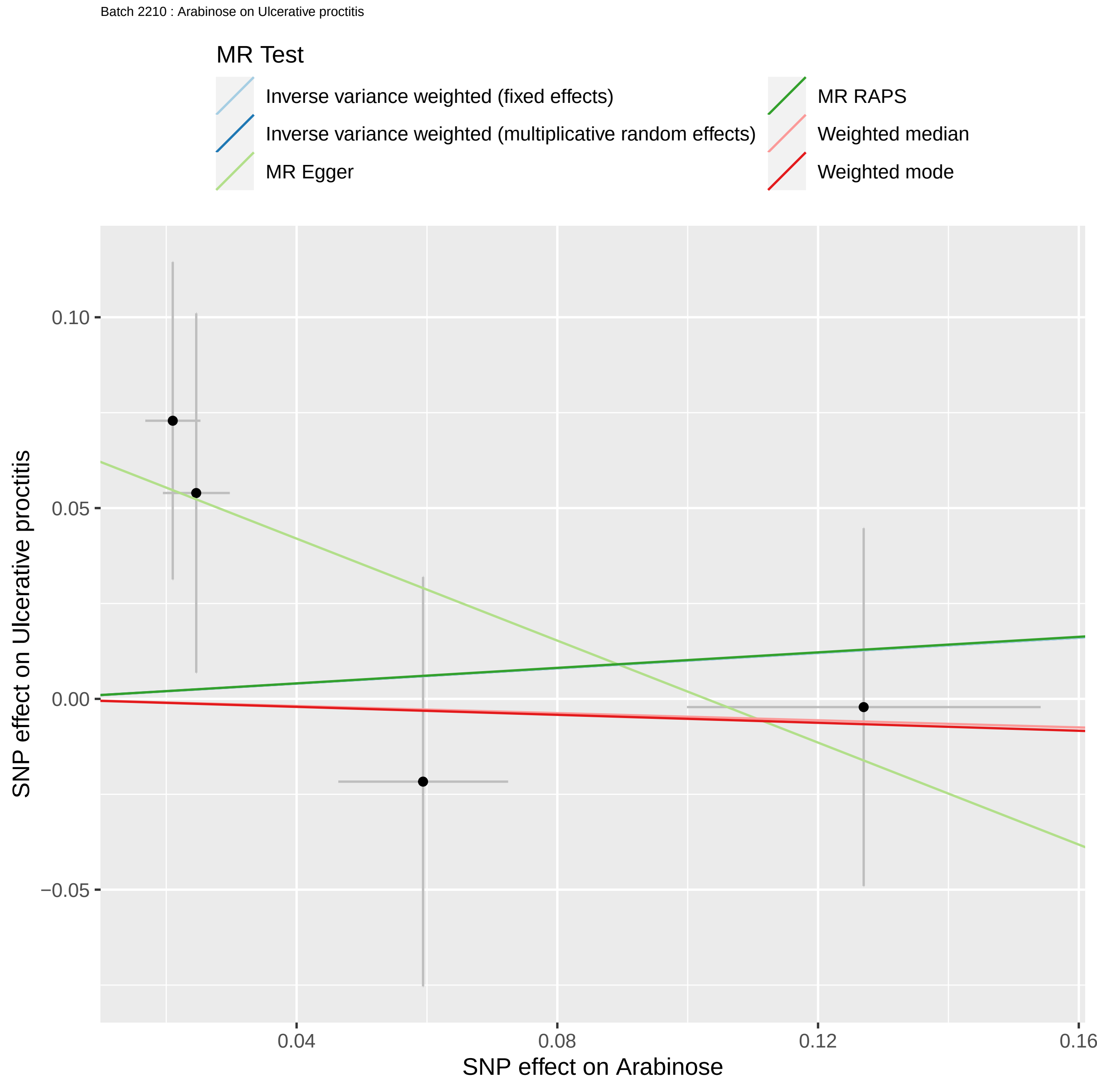
Batch 2210 : Arabinose on Ulcerative proctitis
MR Test
Inverse variance weighted (fixed effects)
Inverse variance weighted (multiplicative random effects)
MR Egger
MR RAPS
Weighted median
Weighted mode
0.10
0.05
0.00
−0.05
0.04
0.08
0.12
0.16
SNP effect on Ulcerative proctitis
SNP effect on Arabinose

Batch 2211 : Fructose on Ulcerative proctitis

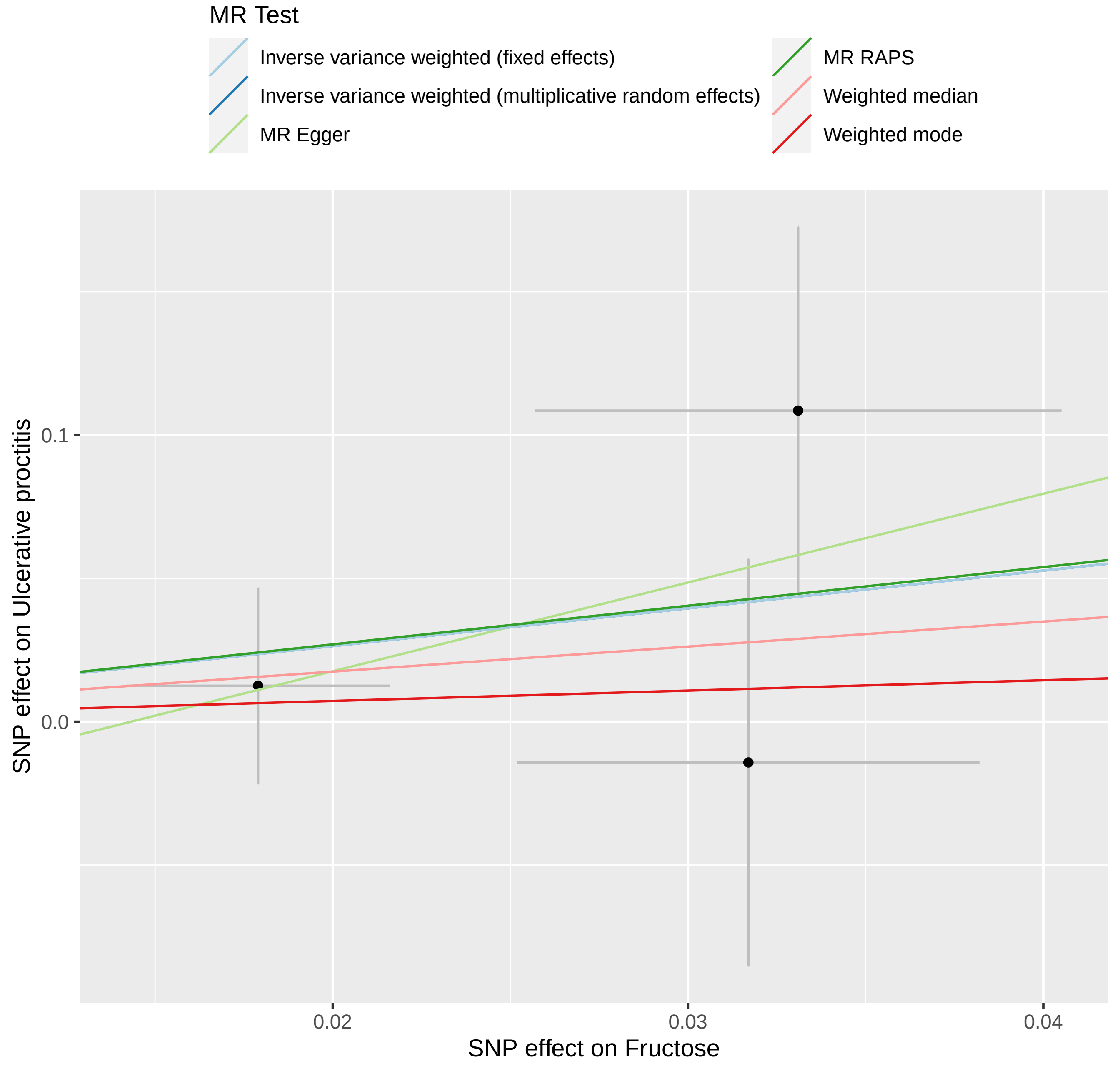

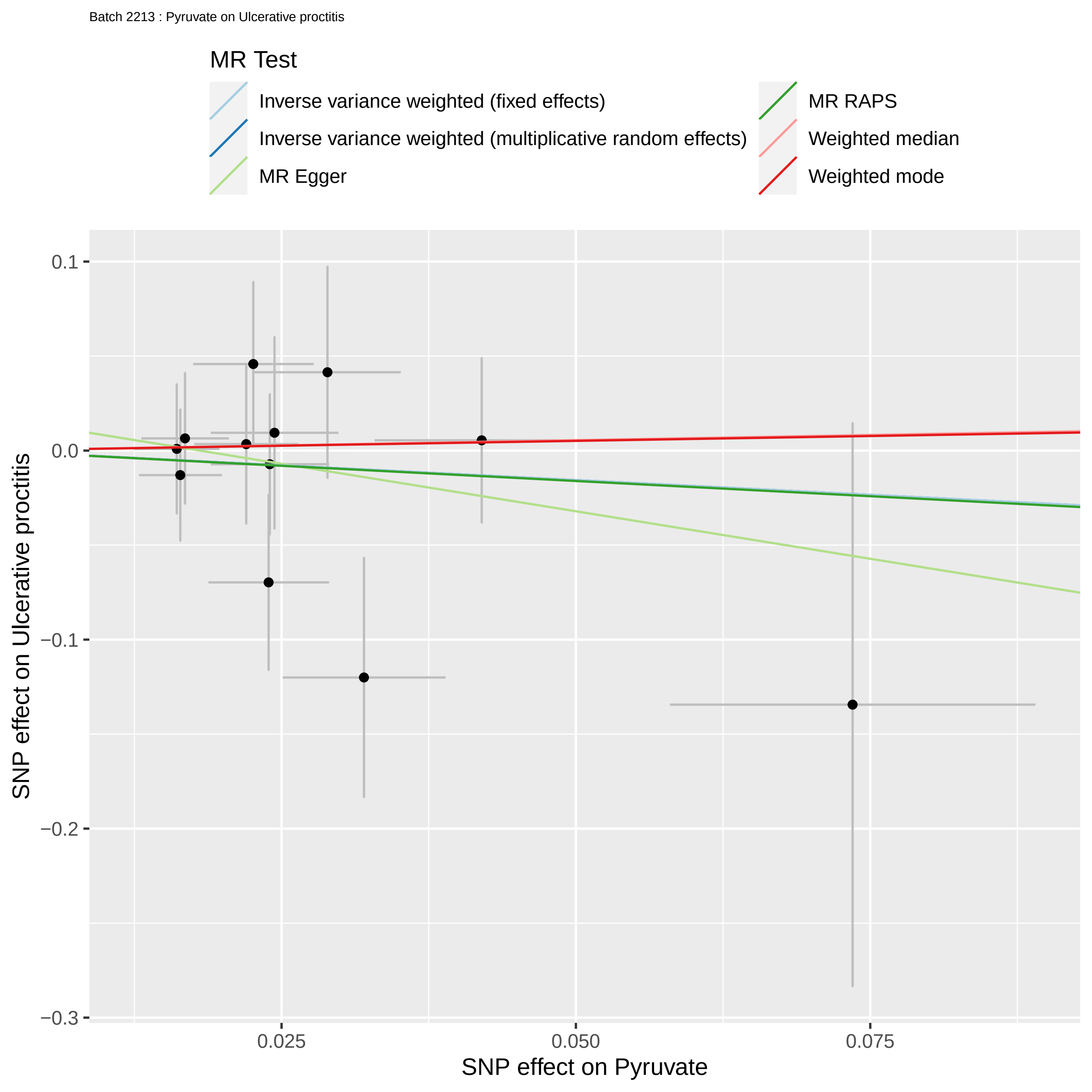
Batch 2213 : Pyruvate on Ulcerative proctitis
MR Test
Inverse variance weighted (fixed effects)
Inverse variance weighted (multiplicative random effects)
MR Egger
MR RAPS
Weighted median
Weighted mode
0.1
0.0
−0.1
−0.2
−0.3
0.025
0.050
0.075
SNP effect on Ulcerative proctitis
SNP effect on Pyruvate

Batch 2214 : Uridine on Ulcerative proctitis

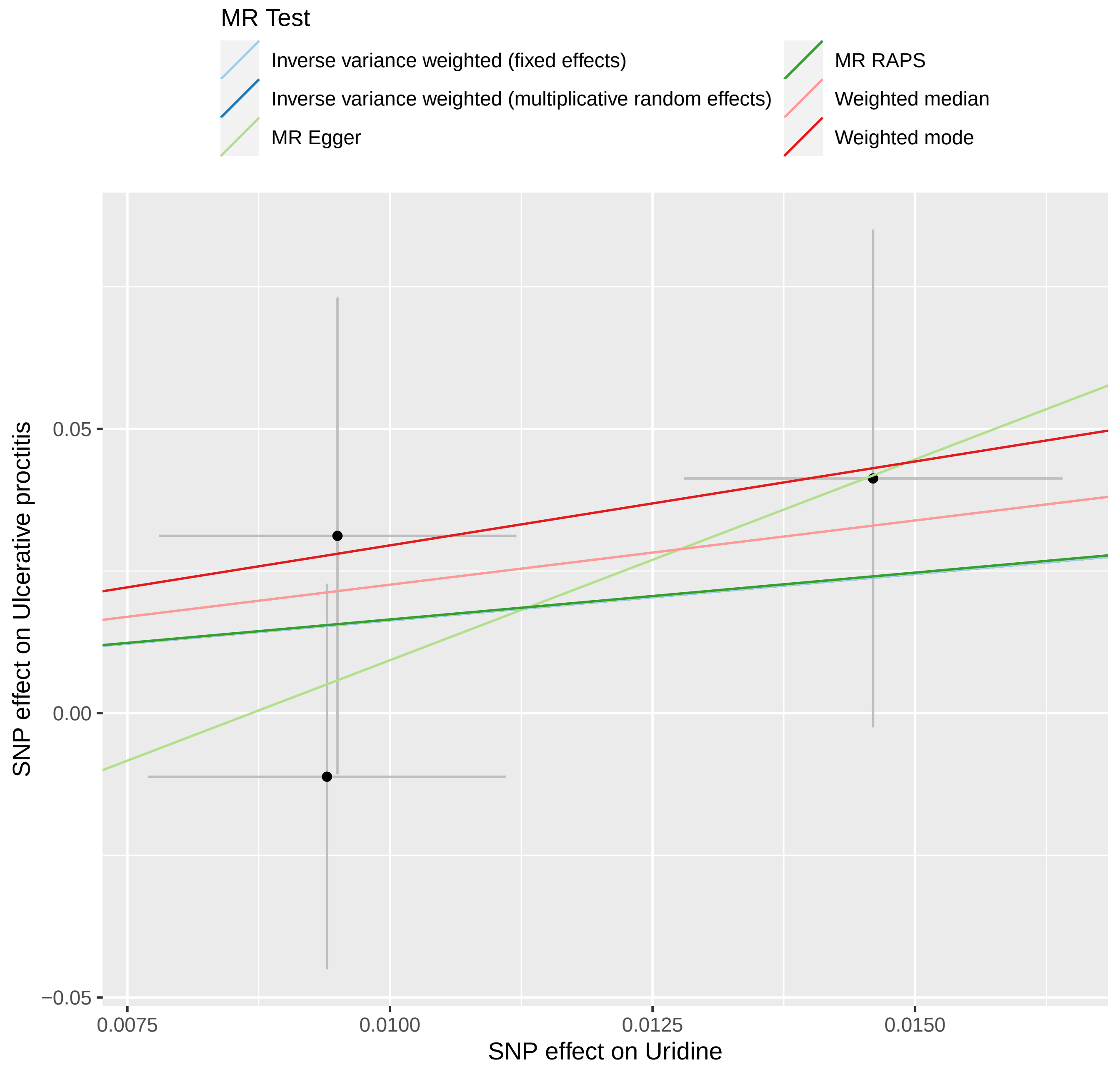

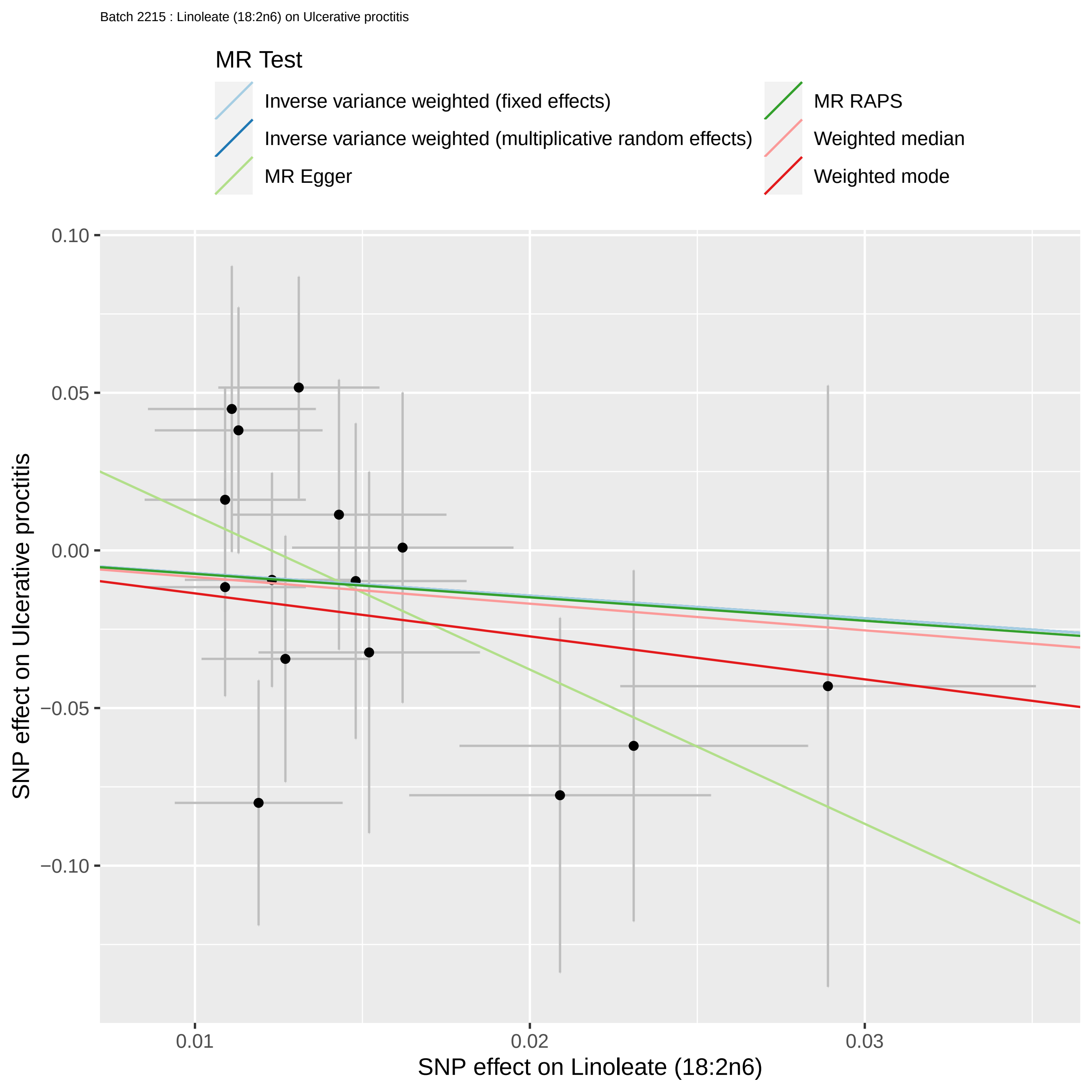
Batch 2215 : Linoleate (18:2n6) on Ulcerative proctitis
MR Test
Inverse variance weighted (fixed effects)
Inverse variance weighted (multiplicative random effects)
MR Egger
MR RAPS
Weighted median
Weighted mode
0.10
0.05
0.00
−0.05
−0.10
0.01
0.02
0.03
SNP effect on Ulcerative proctitis
SNP effect on Linoleate (18:2n6)

Batch 2216 : Allantoin on Ulcerative proctitis

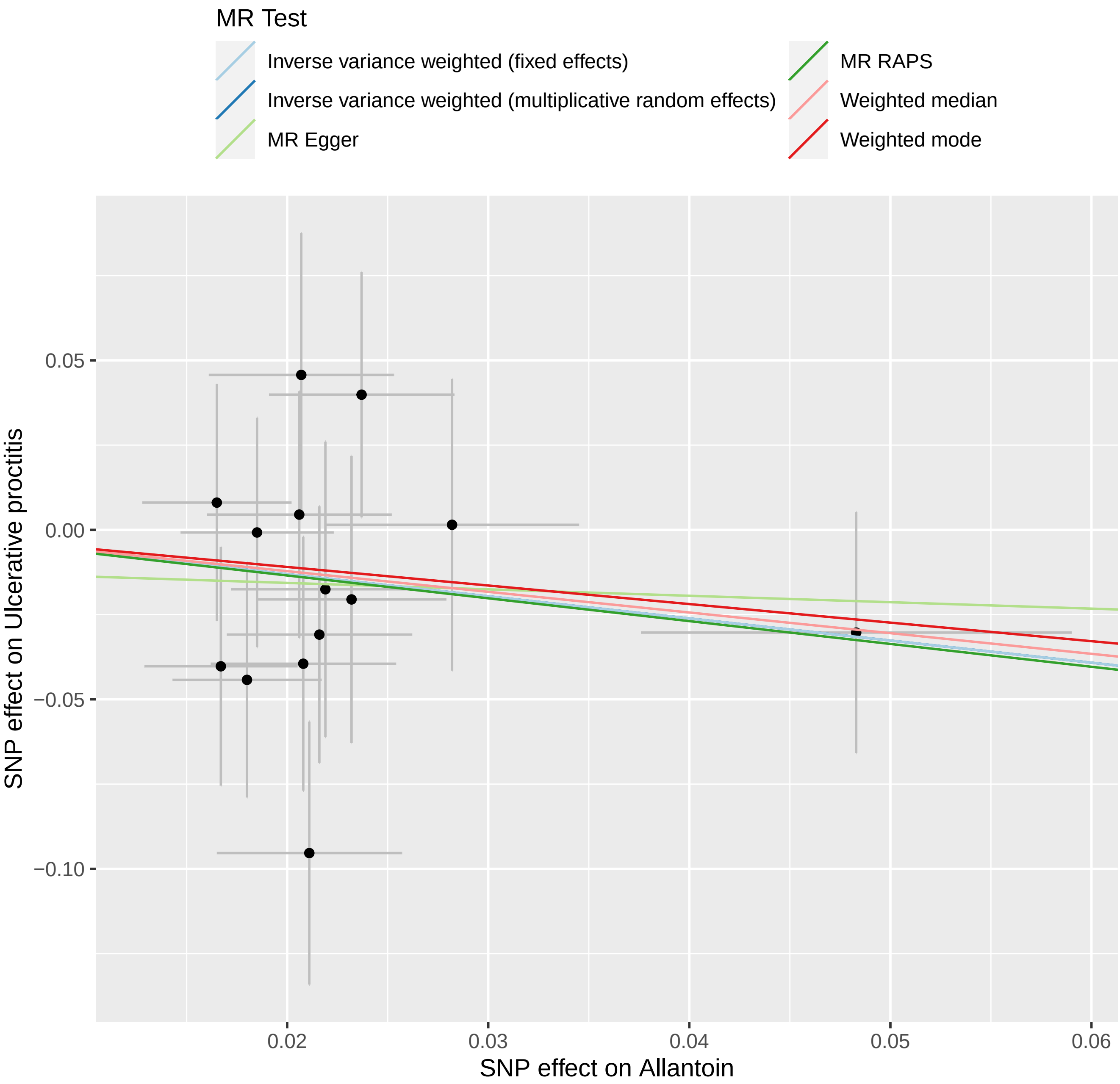

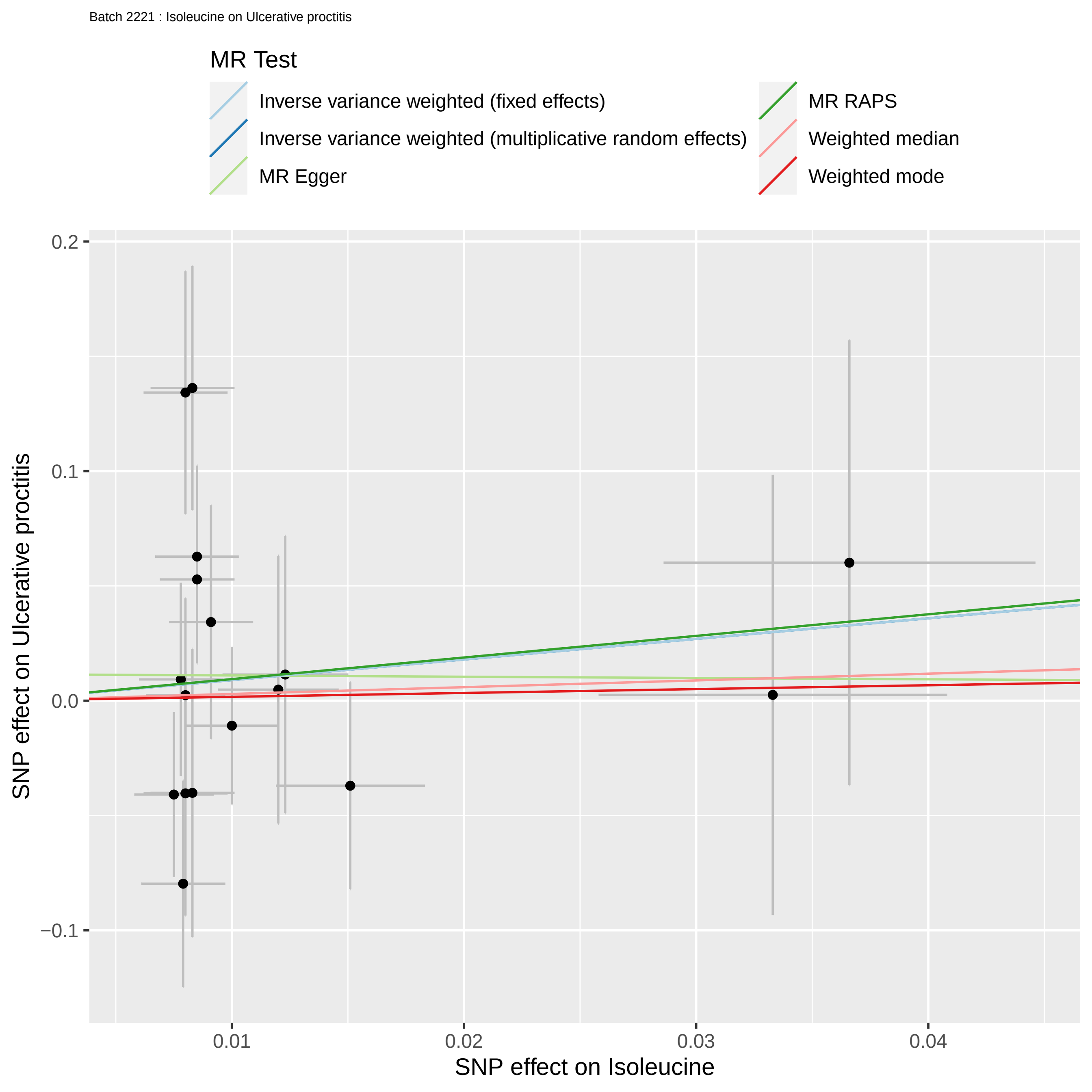

Batch 2221 : Isoleucine on Ulcerative proctitis
MR Test
Inverse variance weighted (fixed effects)
Inverse variance weighted (multiplicative random effects)
MR Egger
MR RAPS
Weighted median
Weighted mode
0.2
0.1
0.0
−0.1
0.01
0.02
0.03
0.04
SNP effect on Ulcerative proctitis
SNP effect on Isoleucine

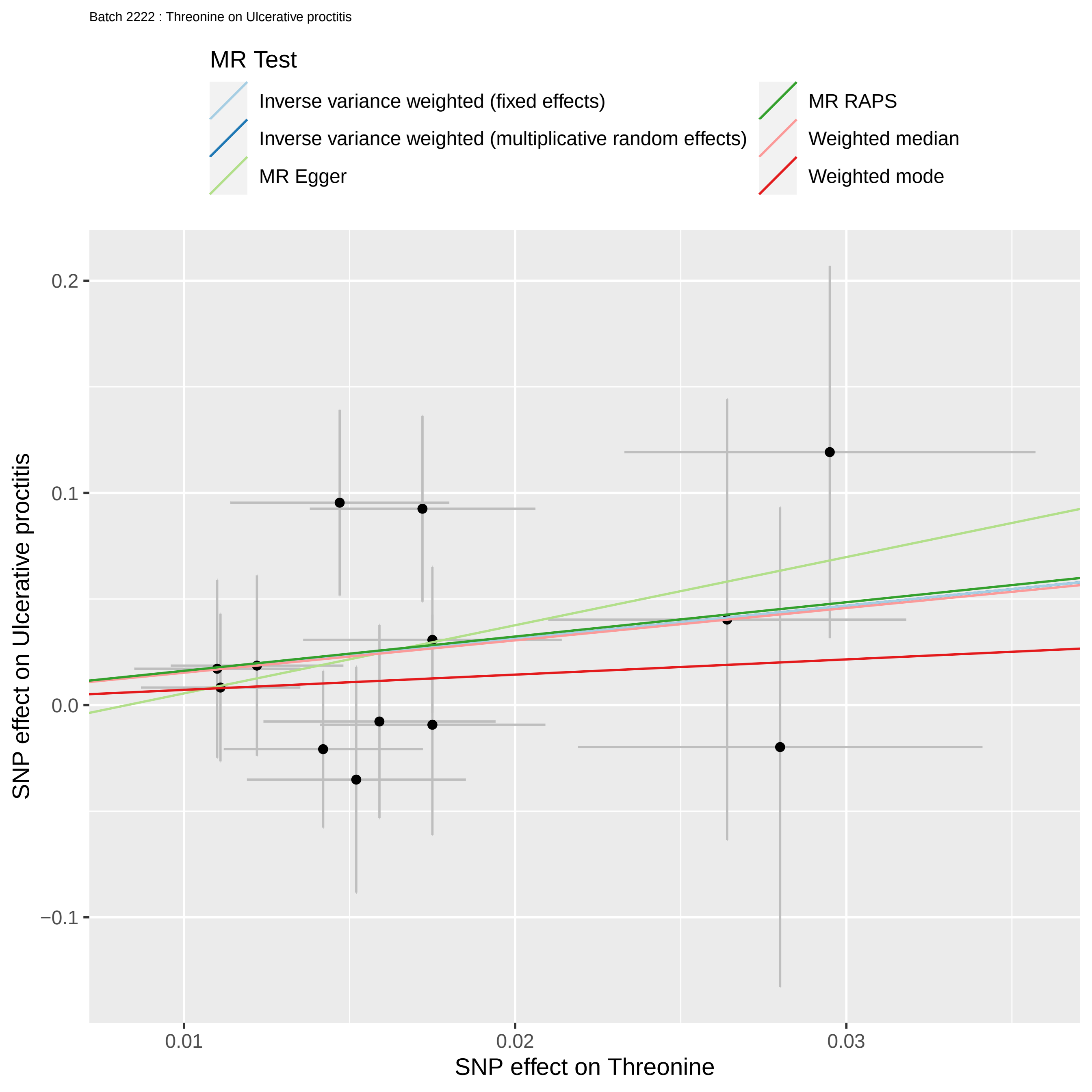
Batch 2222 : Threonine on Ulcerative proctitis
MR Test
Inverse variance weighted (fixed effects)
Inverse variance weighted (multiplicative random effects)
MR Egger
MR RAPS
Weighted median
Weighted mode
SNP effect on Ulcerative proctitis
0.2
0.1
0.0
−0.1
0.01
0.02
0.03
SNP effect on Threonine

Batch 2223 : Tyrosine on Ulcerative proctitis

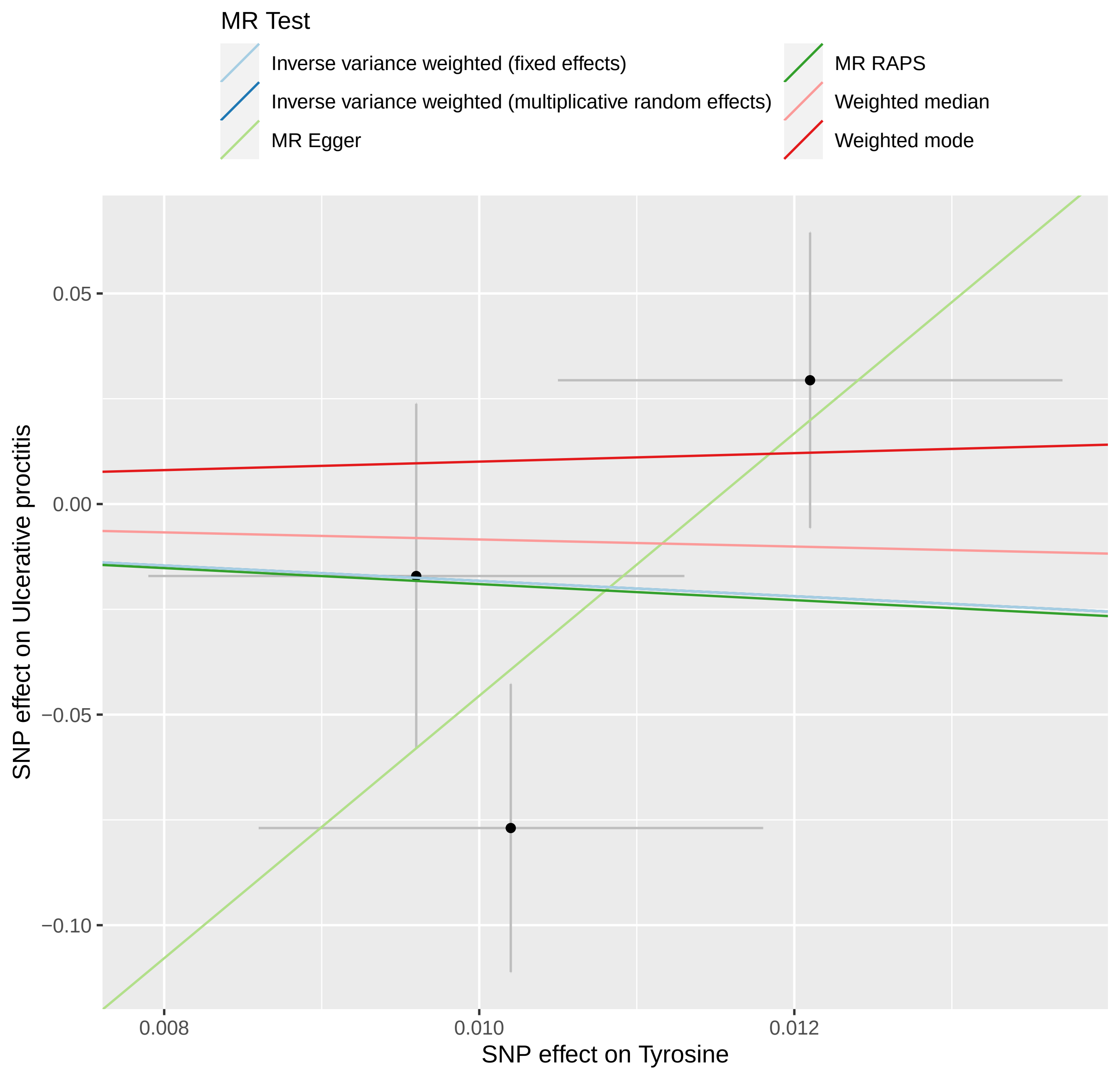

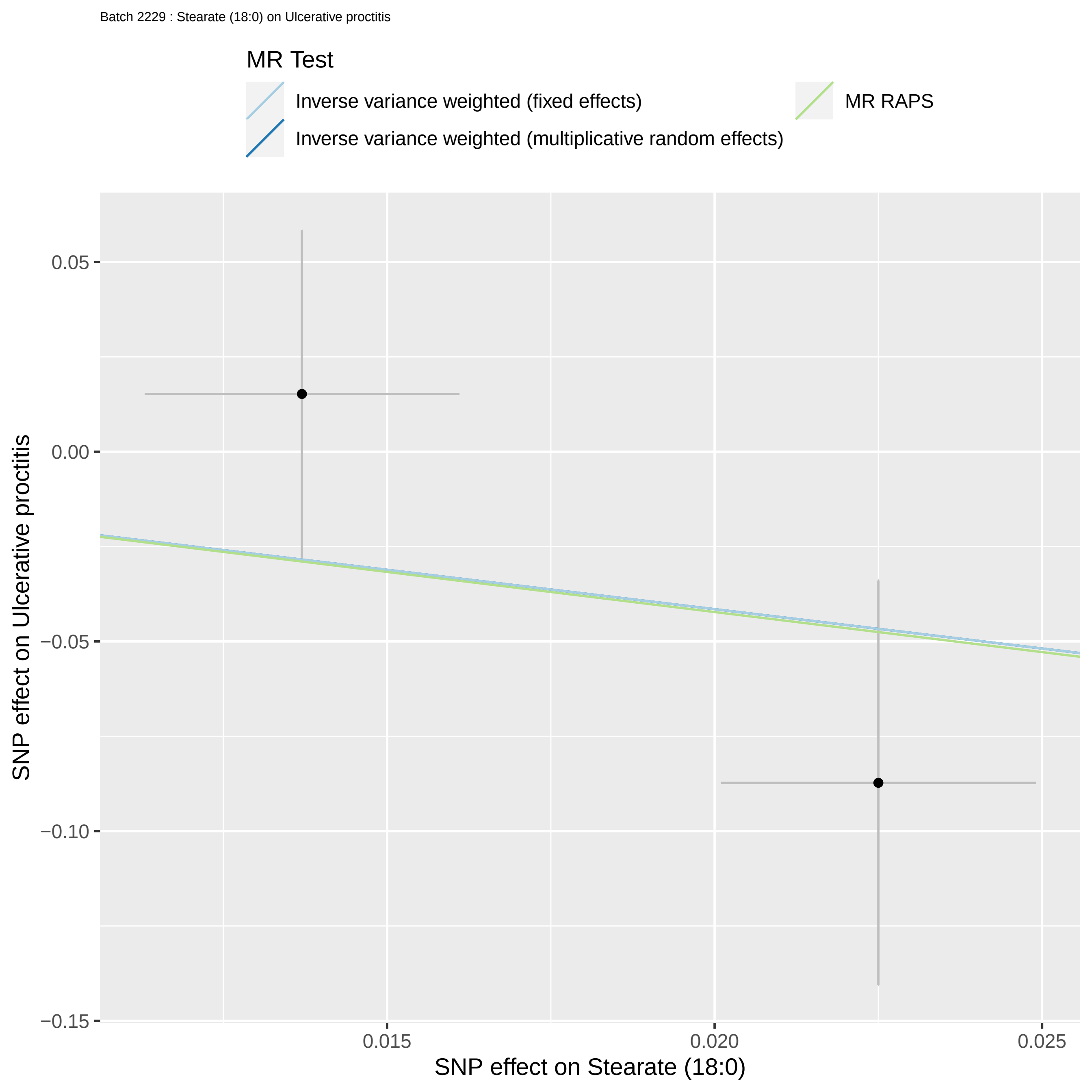

Batch 2229 : Stearate (18:0) on Ulcerative proctitis
MR Test
Inverse variance weighted (fixed effects)
Inverse variance weighted (multiplicative random effects)
MR RAPS
0.05
0.00
−0.05
−0.10
−0.15
0.015
0.020
0.025
SNP effect on Ulcerative proctitis
SNP effect on Stearate (18:0)

Batch 2232 : Myristate (14:0) on Ulcerative proctitis

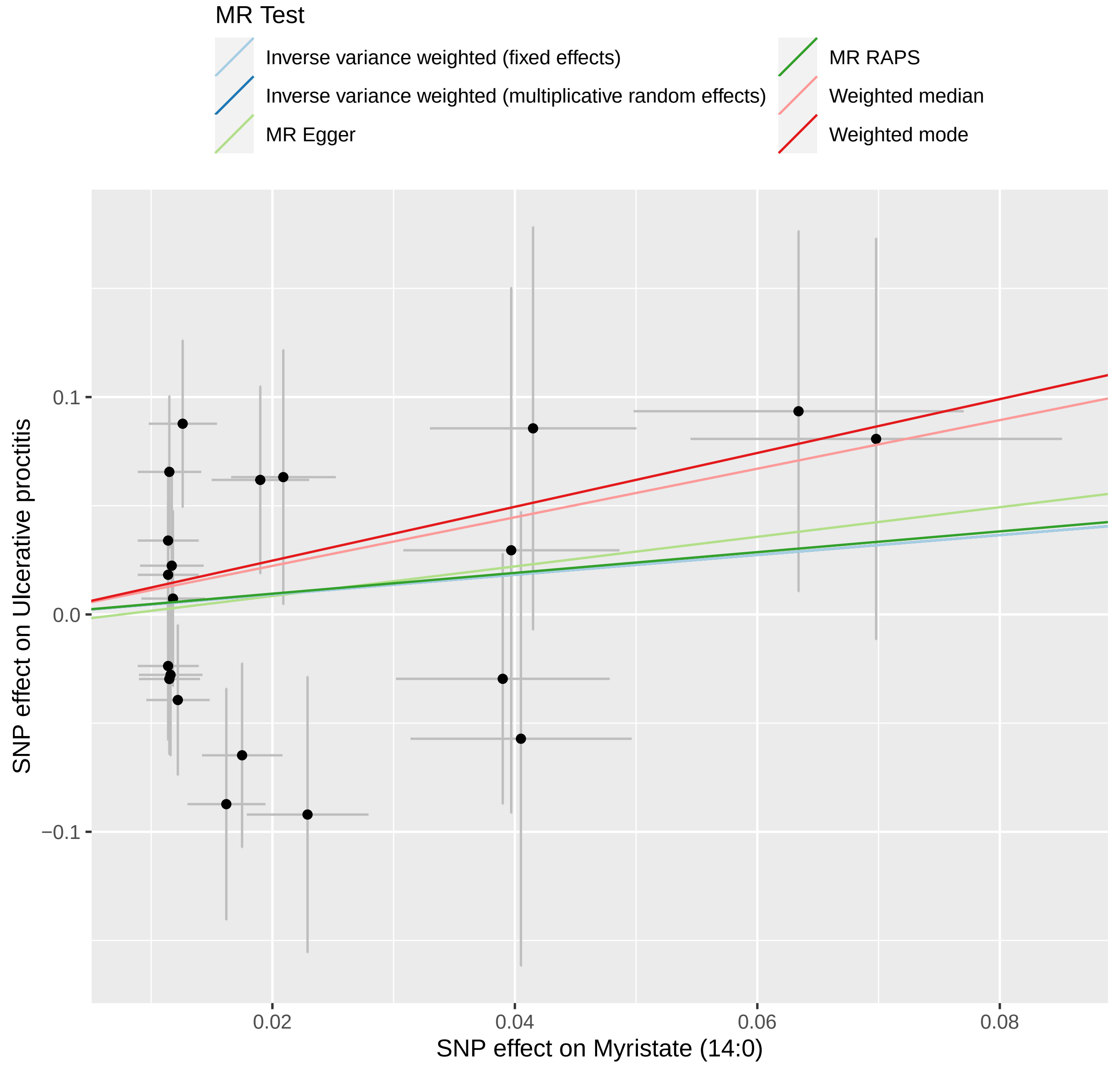

Batch 2234 : Ornithine on Ulcerative proctitis

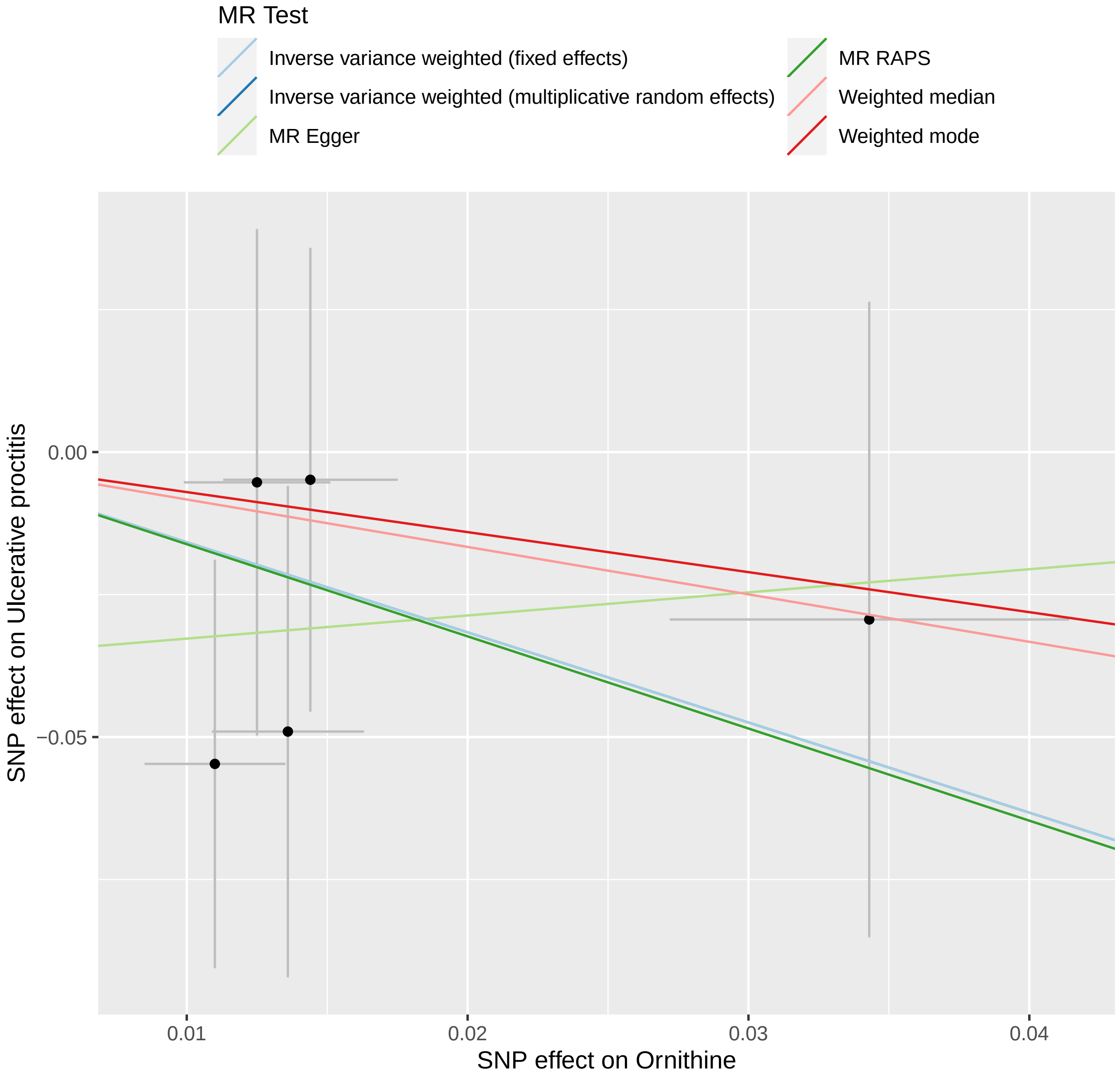

Batch 2237 : 4−acetamidobutanoate on Ulcerative proctitis

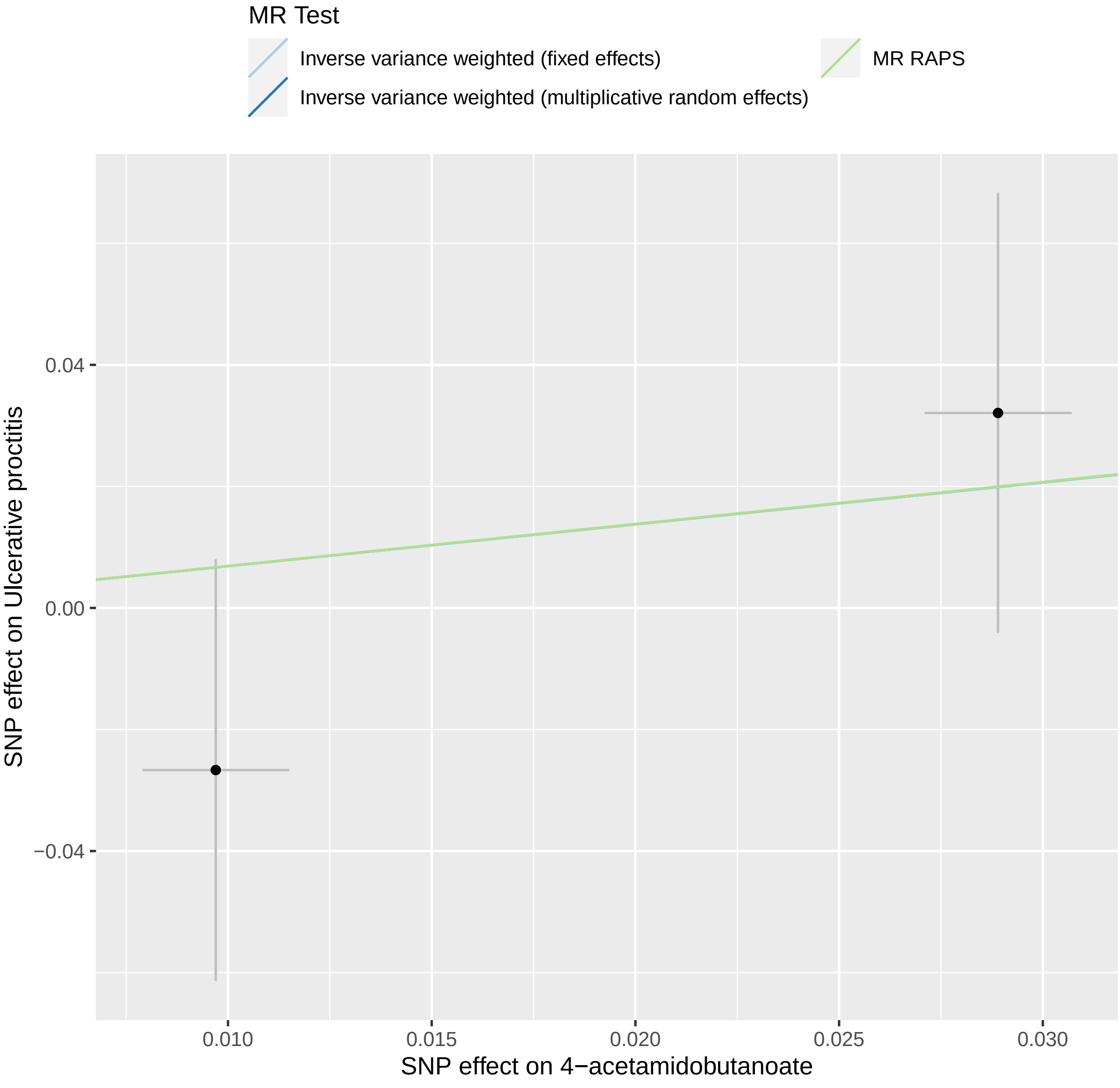

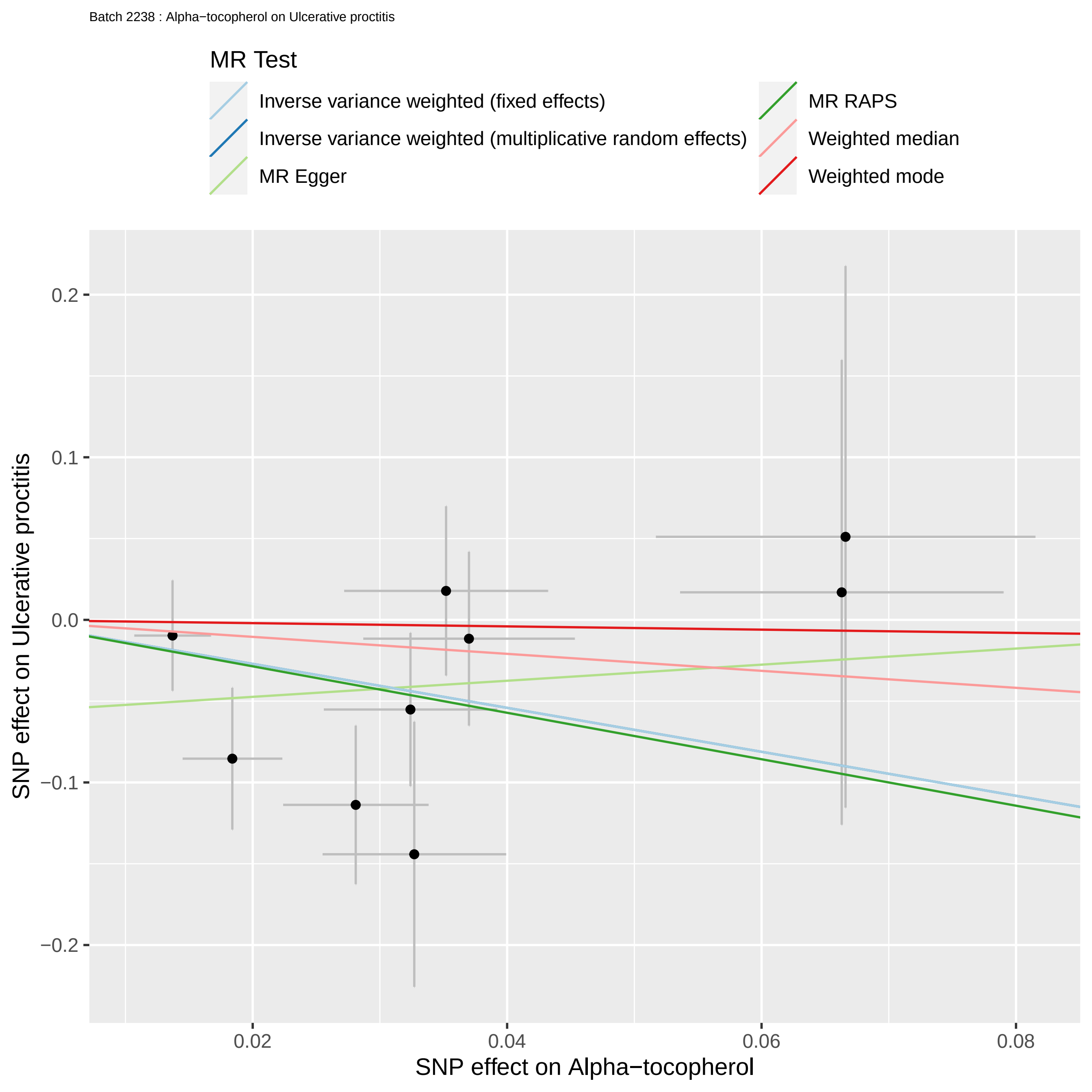
Batch 2238 : Alpha−tocopherol on Ulcerative proctitis
MR Test
Inverse variance weighted (fixed effects)
Inverse variance weighted (multiplicative random effects)
MR Egger
MR RAPS
Weighted median
Weighted mode
0.2
0.1
0.0
−0.1
−0.2
0.02
0.04
0.06
0.08
SNP effect on Ulcerative proctitis
SNP effect on Alpha−tocopherol

Batch 2239 : Citrate on Ulcerative proctitis

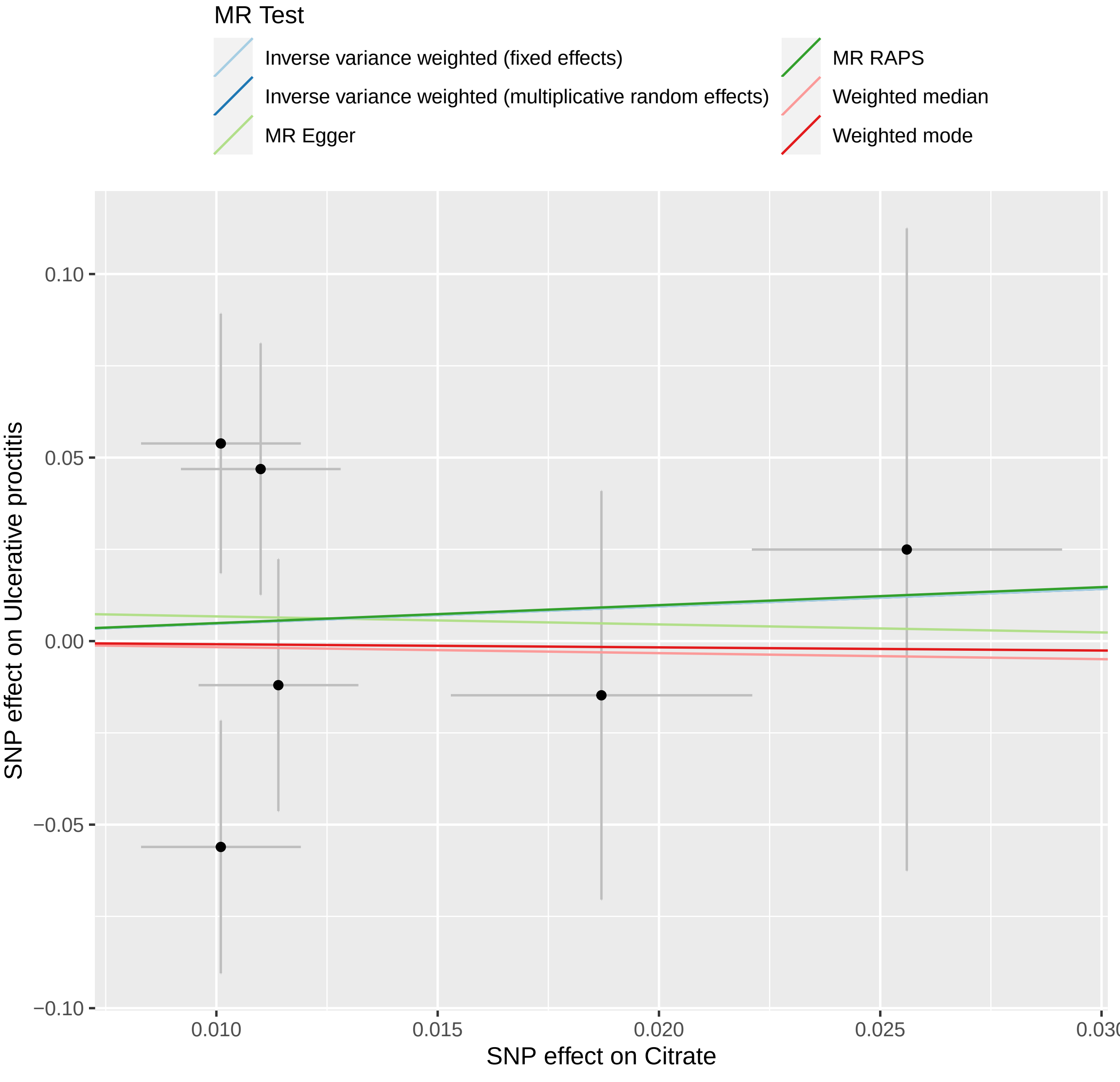

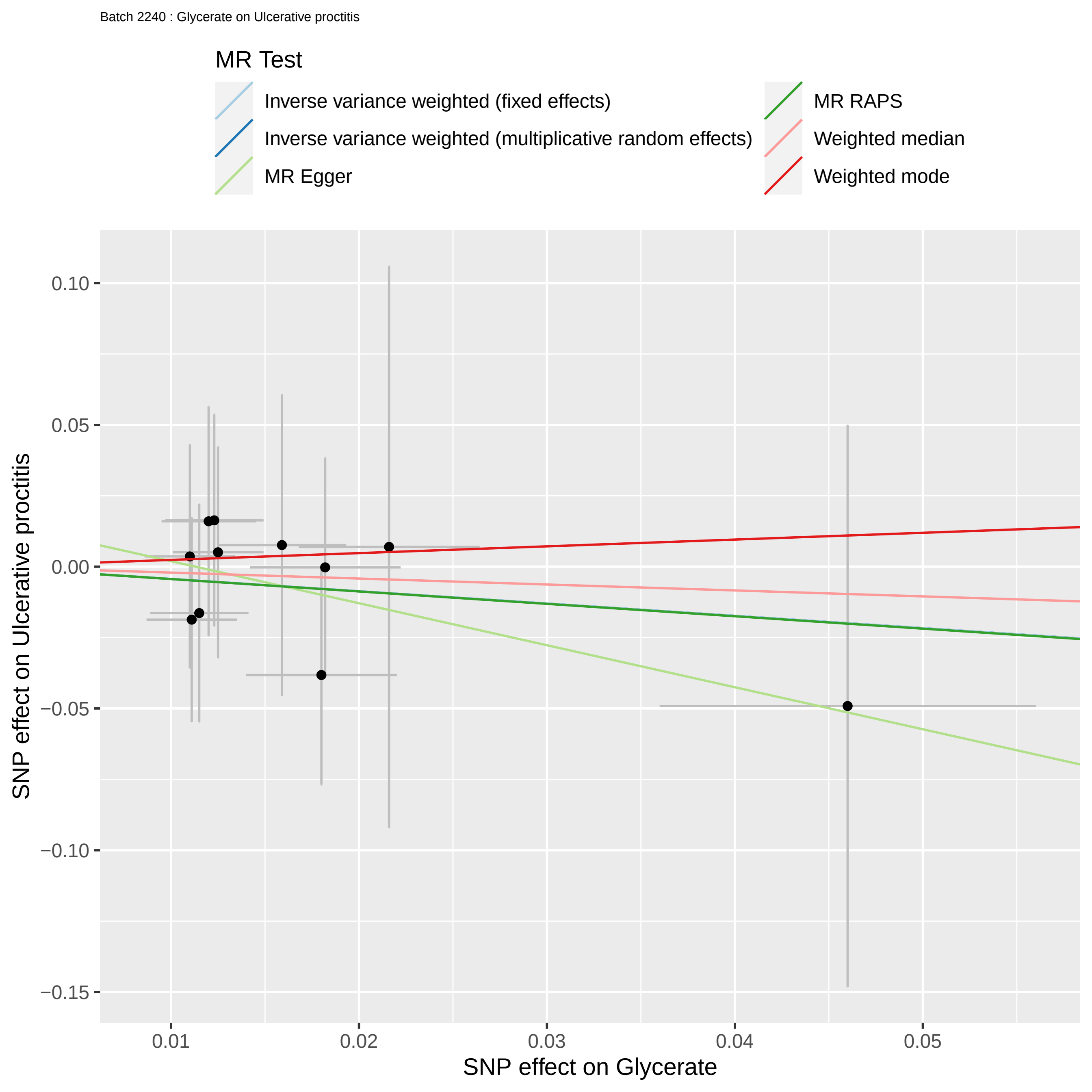

Batch 2240 : Glycerate on Ulcerative proctitis
MR Test
Inverse variance weighted (fixed effects)
Inverse variance weighted (multiplicative random effects)
MR Egger
MR RAPS
Weighted median
Weighted mode
0.10
0.05
0.00
−0.05
−0.10
−0.15
SNP effect on Ulcerative proctitis
0.01
0.02
0.03
0.04
0.05
SNP effect on Glycerate

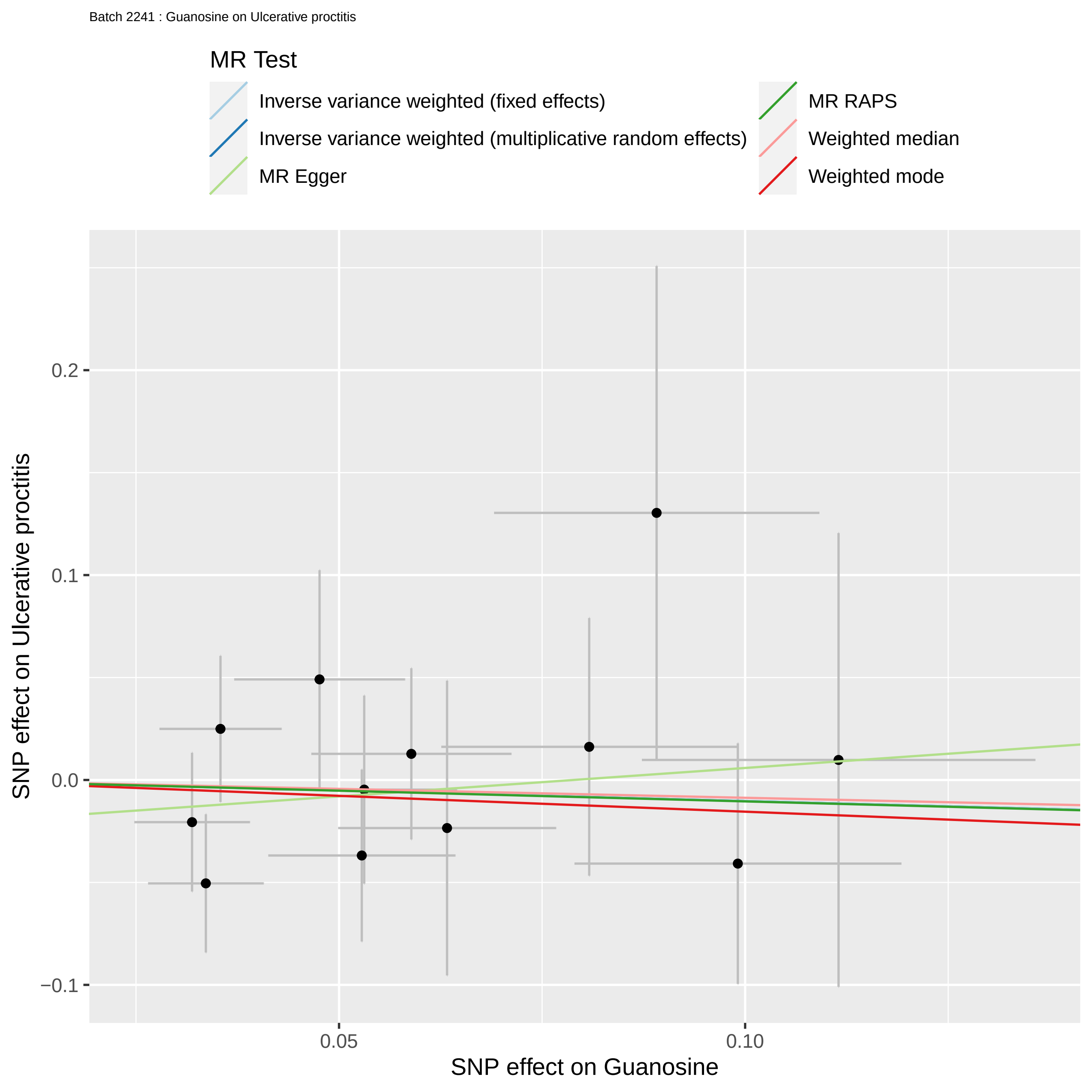

Batch 2241 : Guanosine on Ulcerative proctitis
MR Test
Inverse variance weighted (fixed effects)
Inverse variance weighted (multiplicative random effects)
MR Egger
MR RAPS
Weighted median
Weighted mode
0.2
0.1
0.0
−0.1
0.05
0.10
SNP effect on Guanosine
SNP effect on Ulcerative proctitis

Batch 2244 : Ursodeoxycholate on Ulcerative proctitis

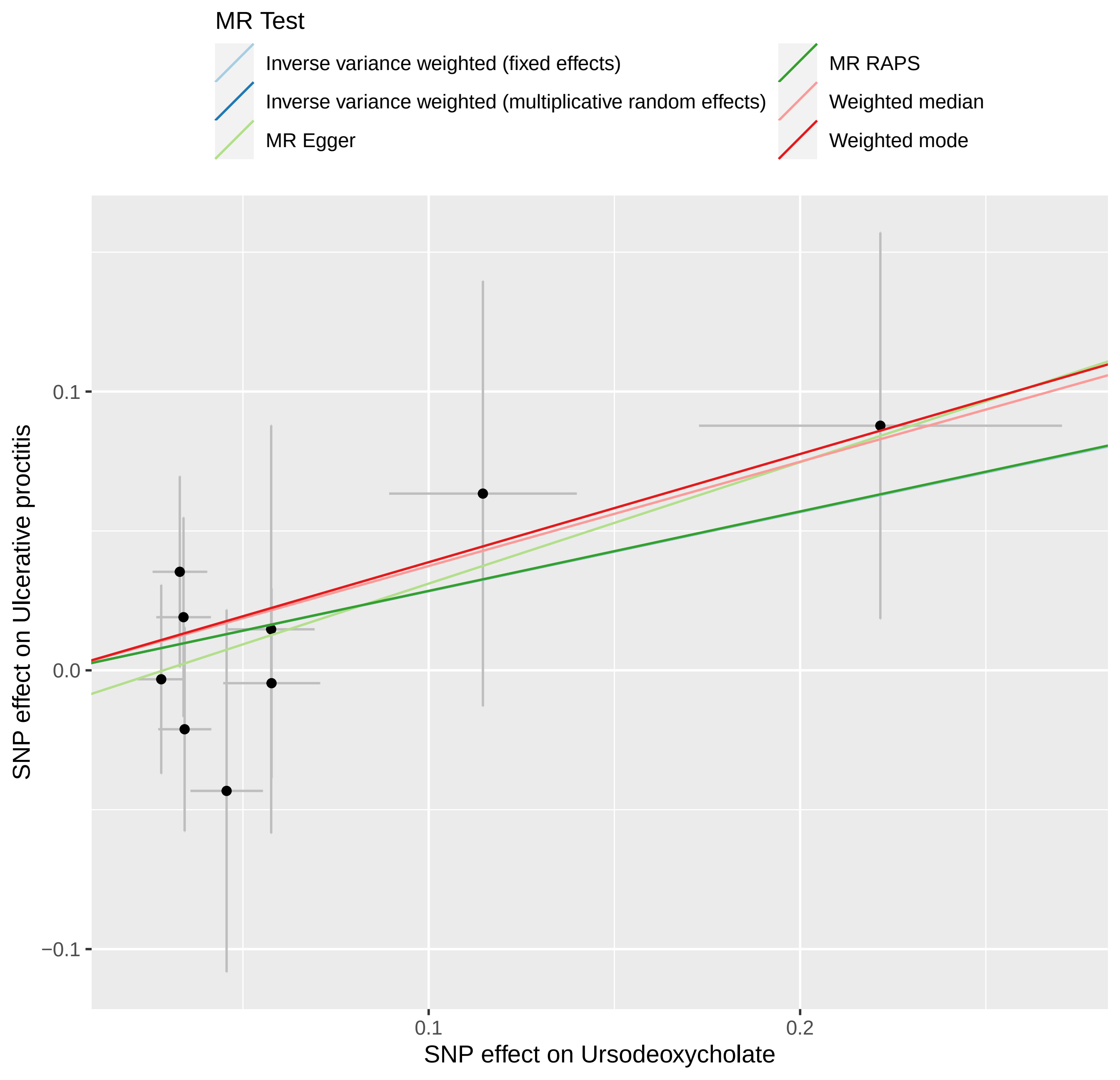

Batch 2245 : Arginine on Ulcerative proctitis

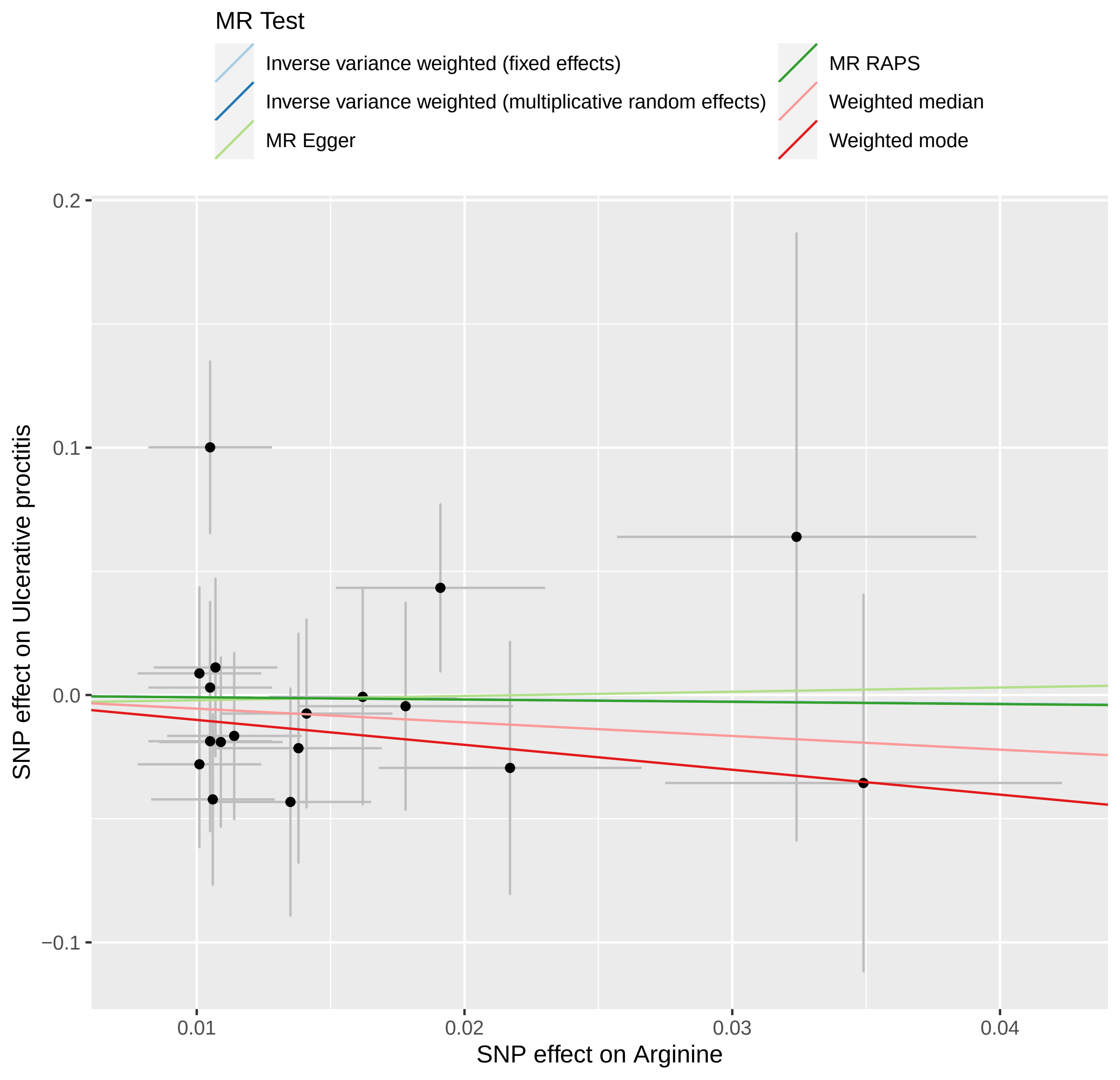

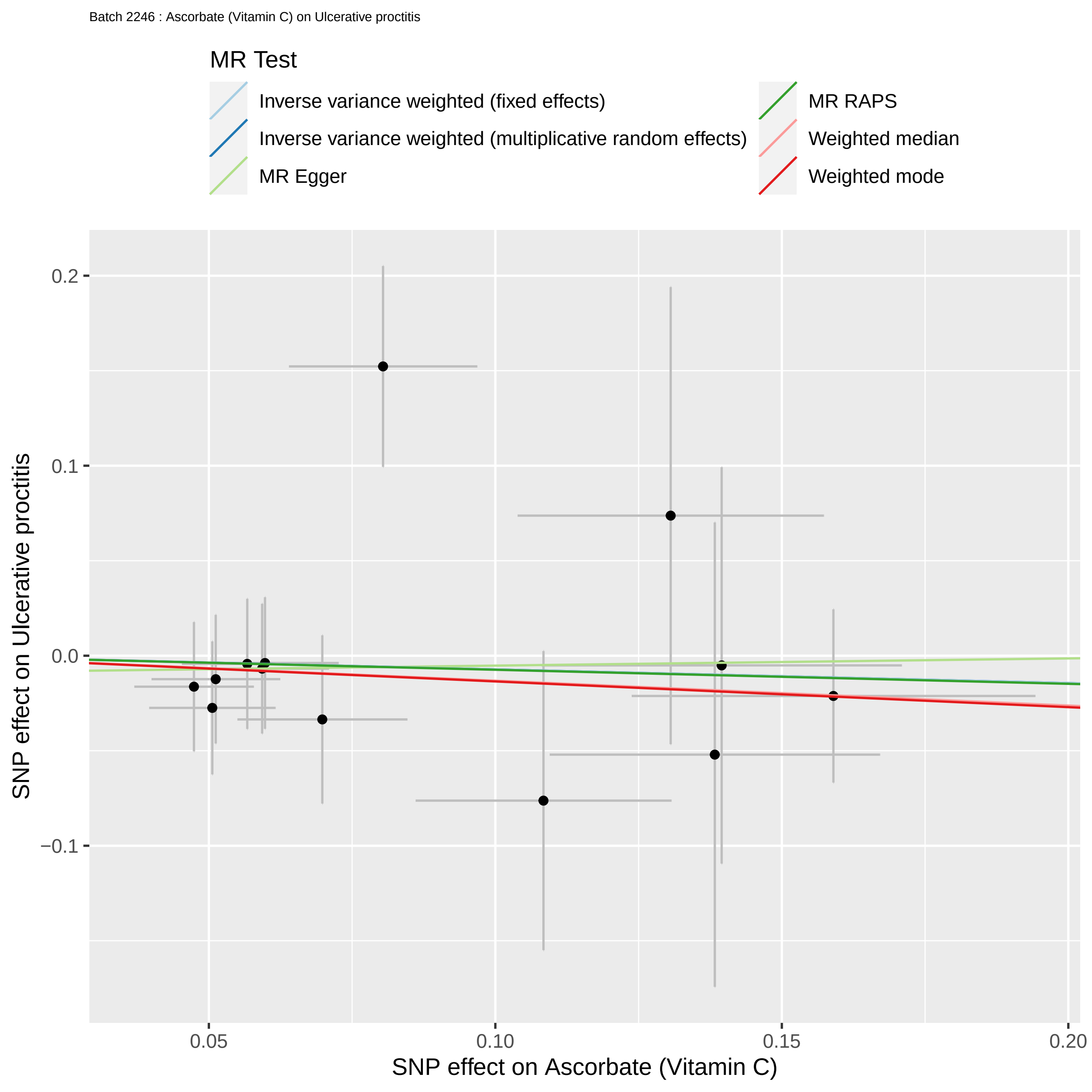
Batch 2246 : Ascorbate (Vitamin C) on Ulcerative proctitis
MR Test
Inverse variance weighted (fixed effects)
Inverse variance weighted (multiplicative random effects)
MR Egger
MR RAPS
Weighted median
Weighted mode
0.2
0.1
0.0
−0.1
0.05
0.10
0.15
0.20
SNP effect on Ulcerative proctitis
SNP effect on Ascorbate (Vitamin C)

Batch 2247 : Heptanoate (7:0) on Ulcerative proctitis

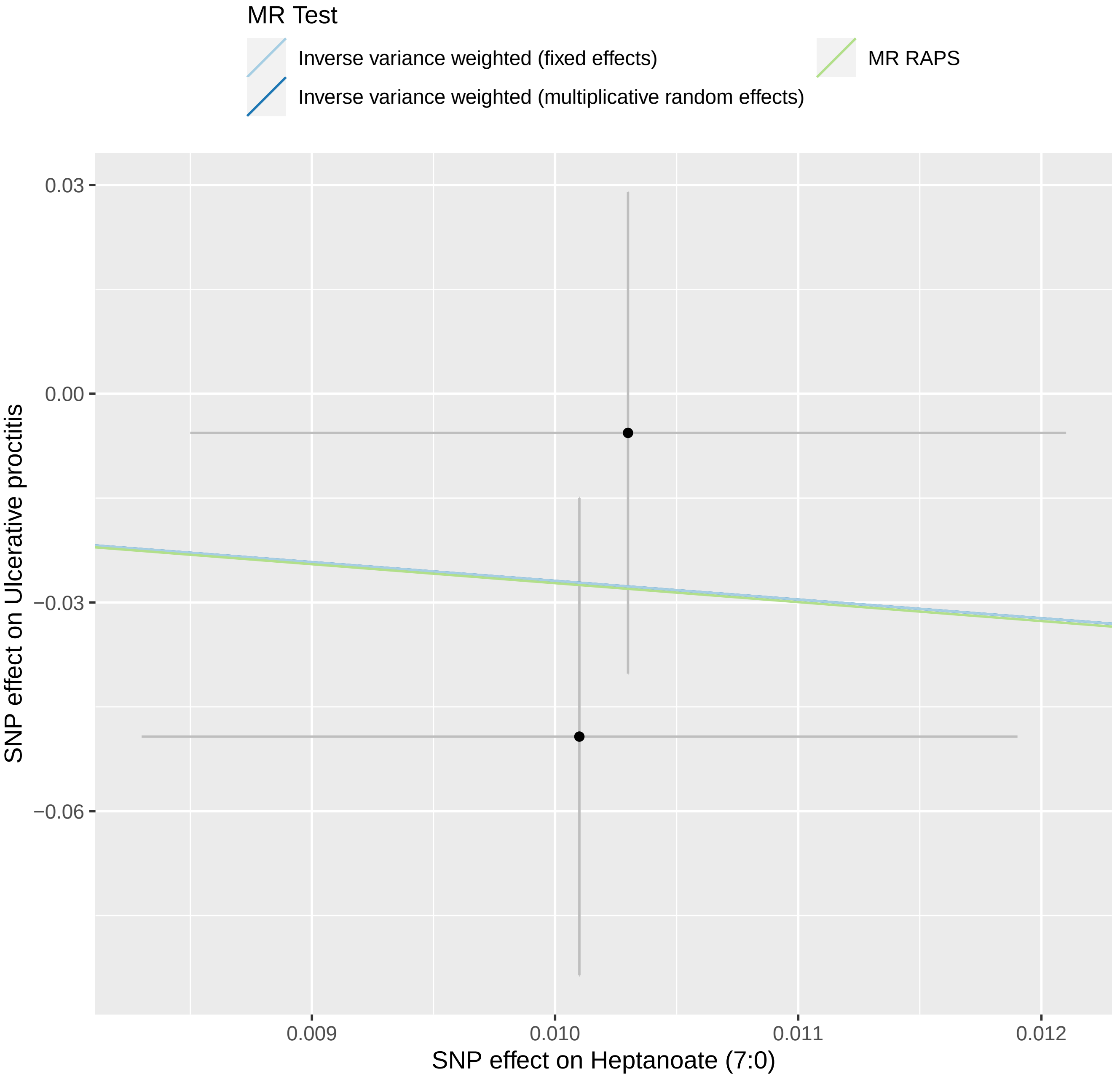

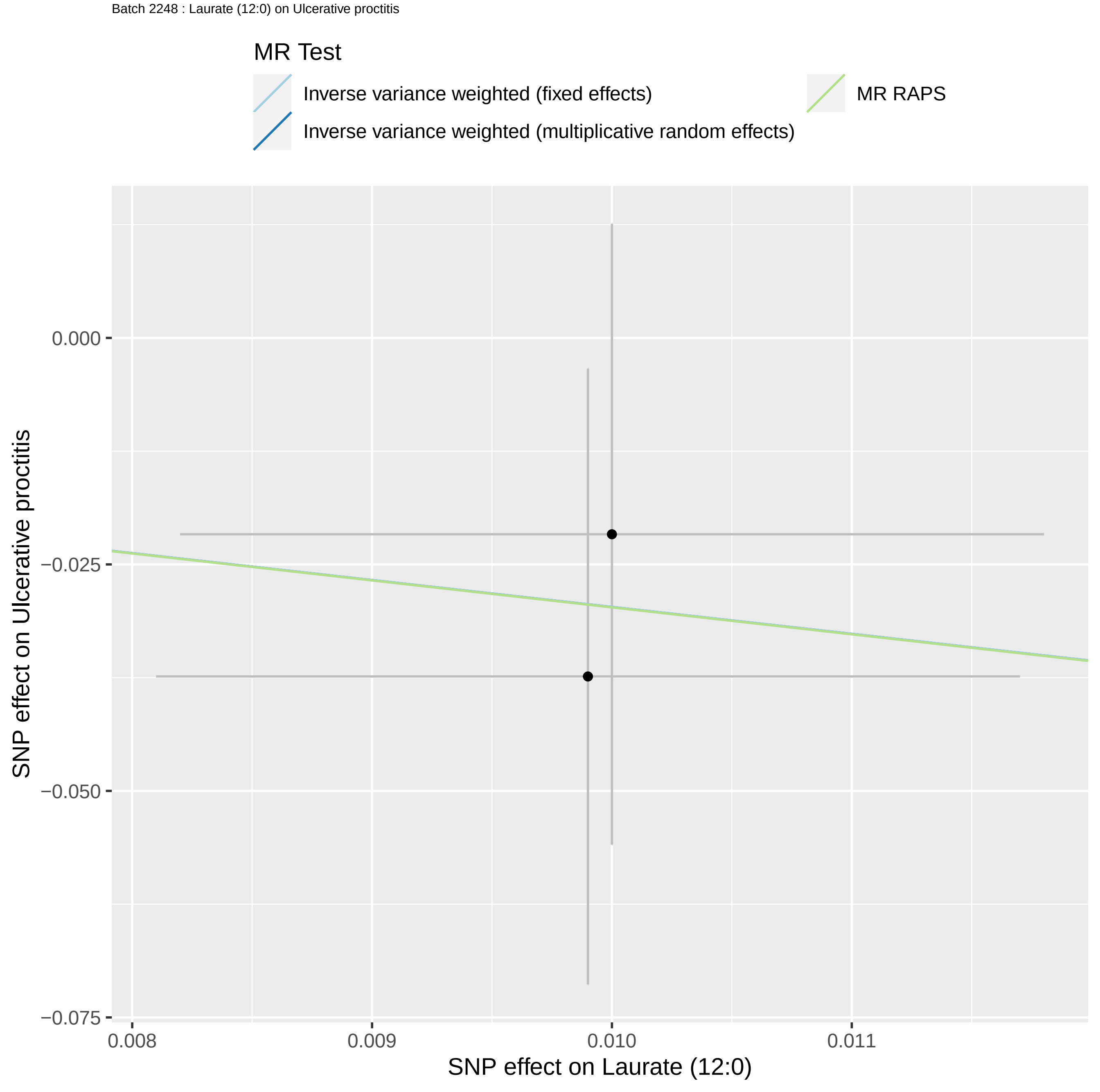

Batch 2248 : Laurate (12:0) on Ulcerative proctitis
MR Test
Inverse variance weighted (fixed effects)
Inverse variance weighted (multiplicative random effects)
MR RAPS
0.000
−0.025
−0.050
−0.075
SNP effect on Ulcerative proctitis
0.008
0.009
0.010
0.011
SNP effect on Laurate (12:0)

Batch 2250 : Urea on Ulcerative proctitis

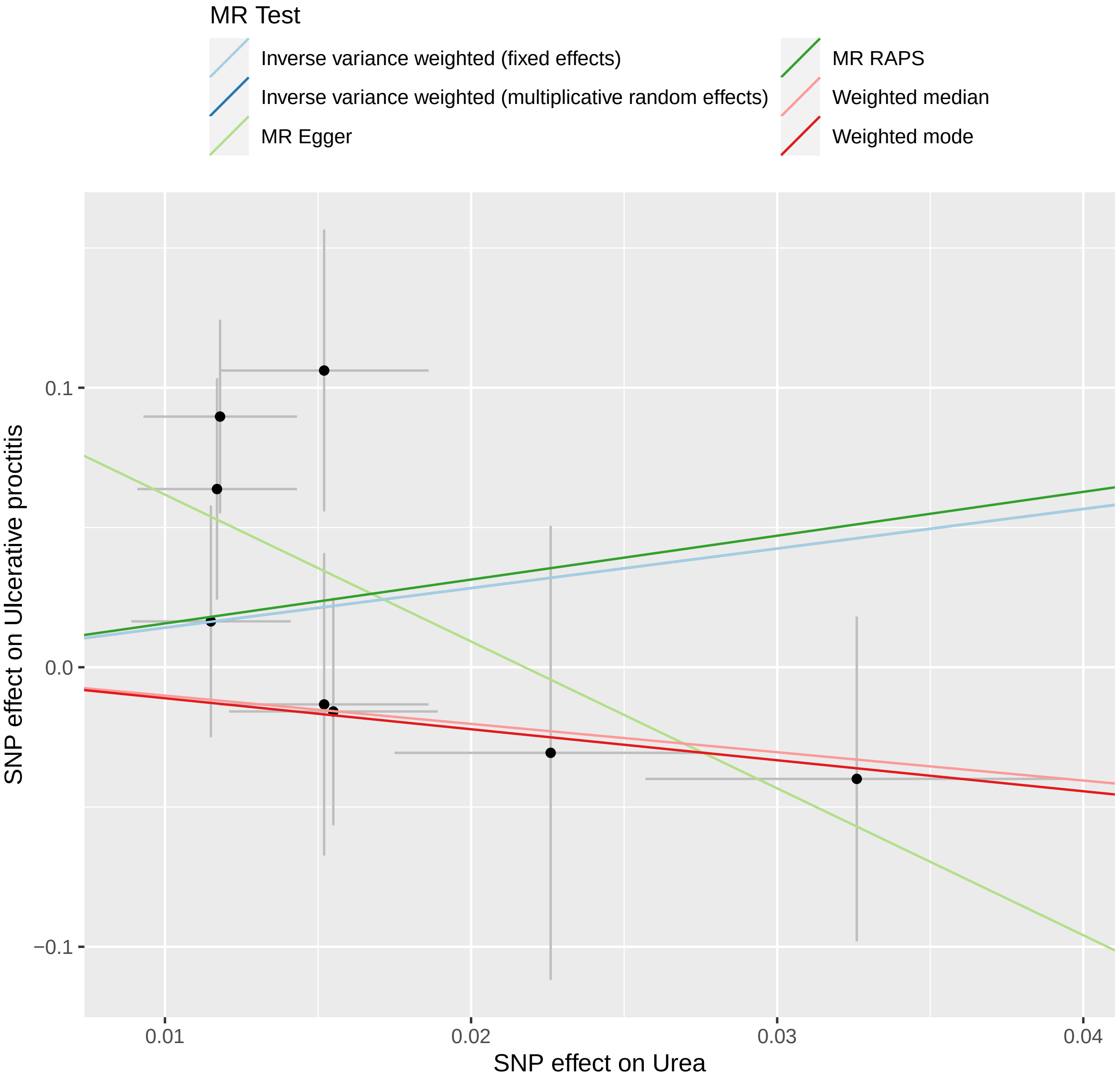

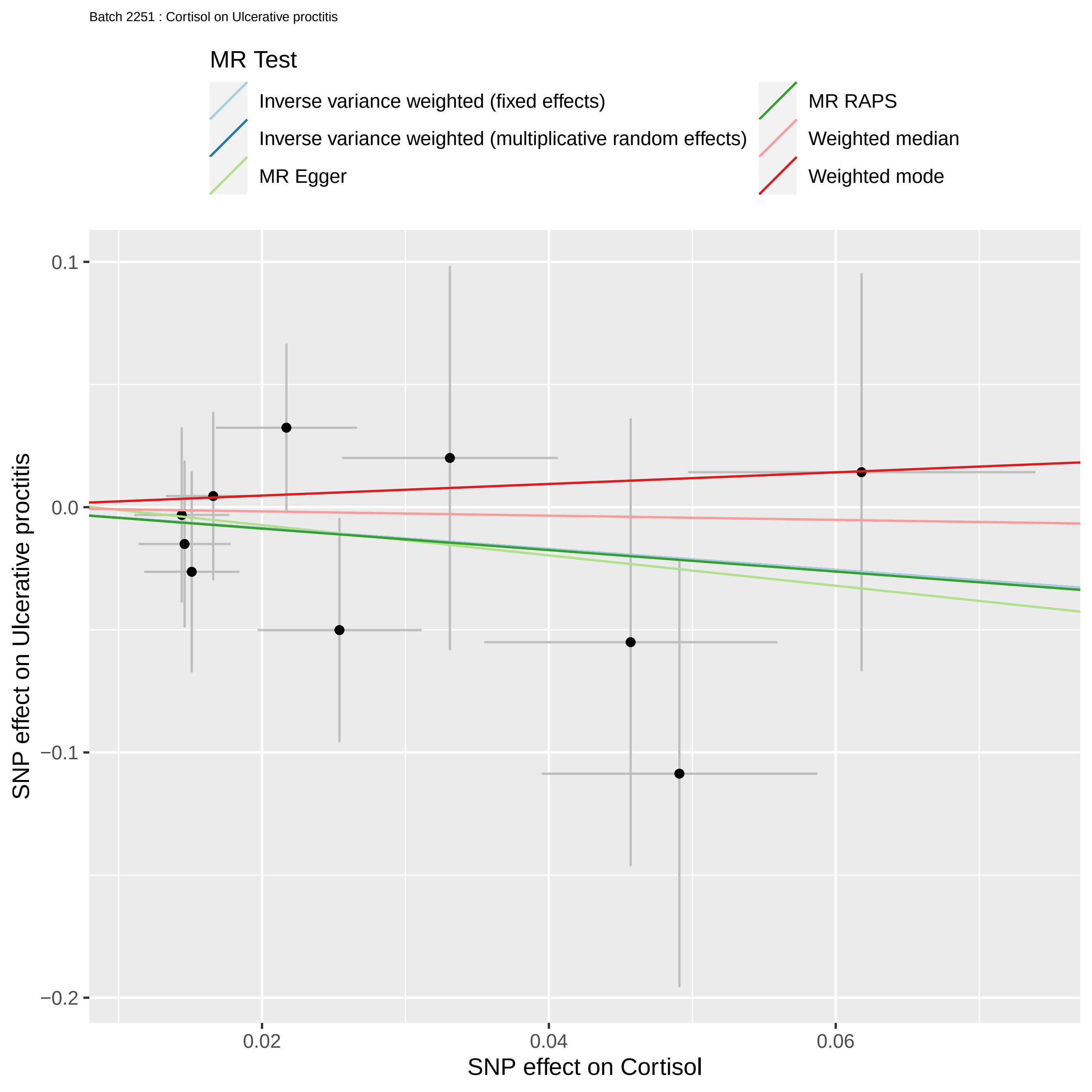

Batch 2251 : Cortisol on Ulcerative proctitis
MR Test
Inverse variance weighted (fixed effects)
Inverse variance weighted (multiplicative random effects)
MR Egger
MR RAPS
Weighted median
Weighted mode
0.1
0.0
−0.1
−0.2
0.02
0.04
0.06
SNP effect on Ulcerative proctitis
SNP effect on Cortisol

Batch 2254 : Citrulline on Ulcerative proctitis

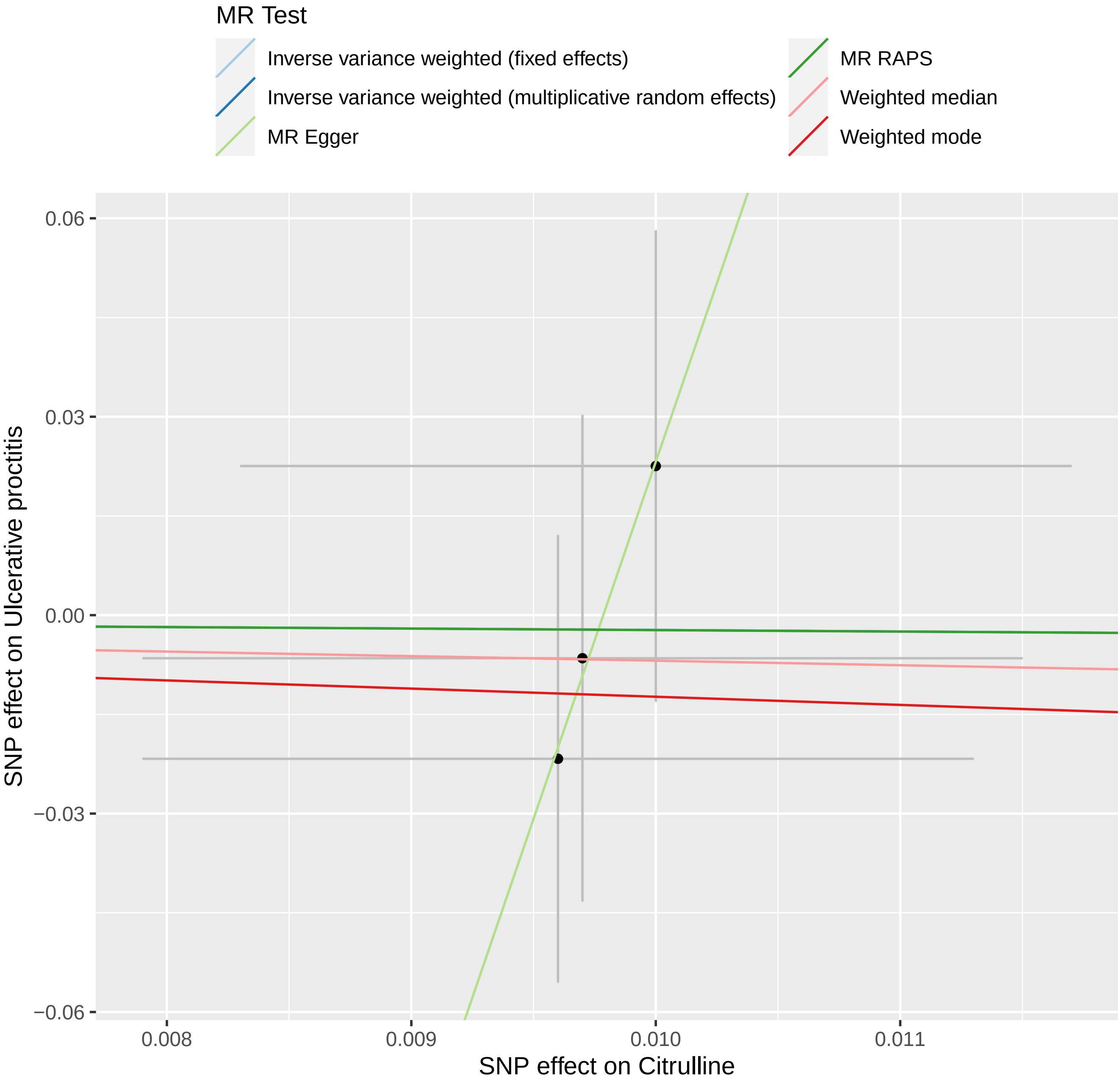

Batch 2256 : Serotonin (5HT) on Ulcerative proctitis

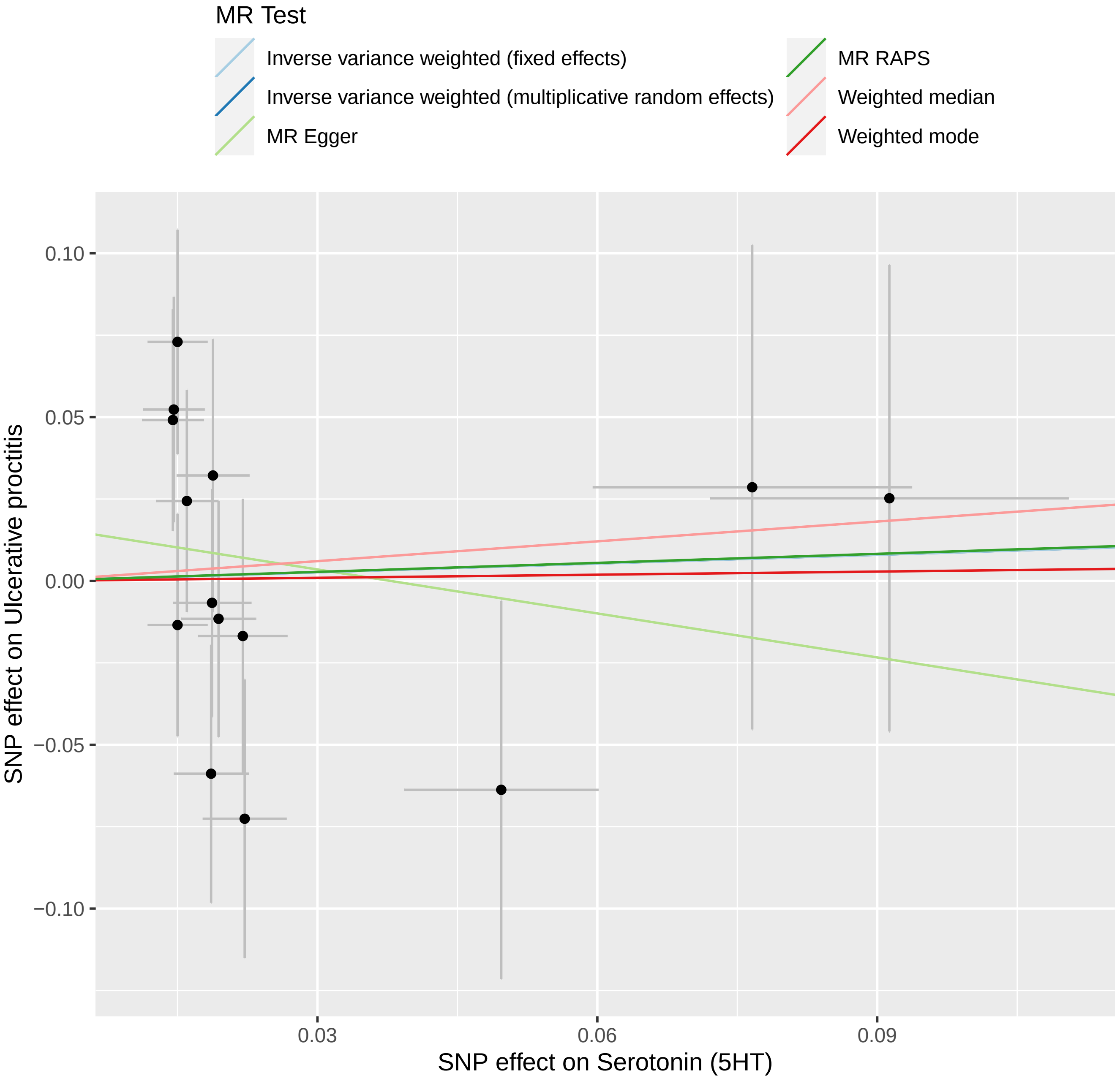

Batch 2257 : Gamma−glutamylglutamine on Ulcerative proctitis

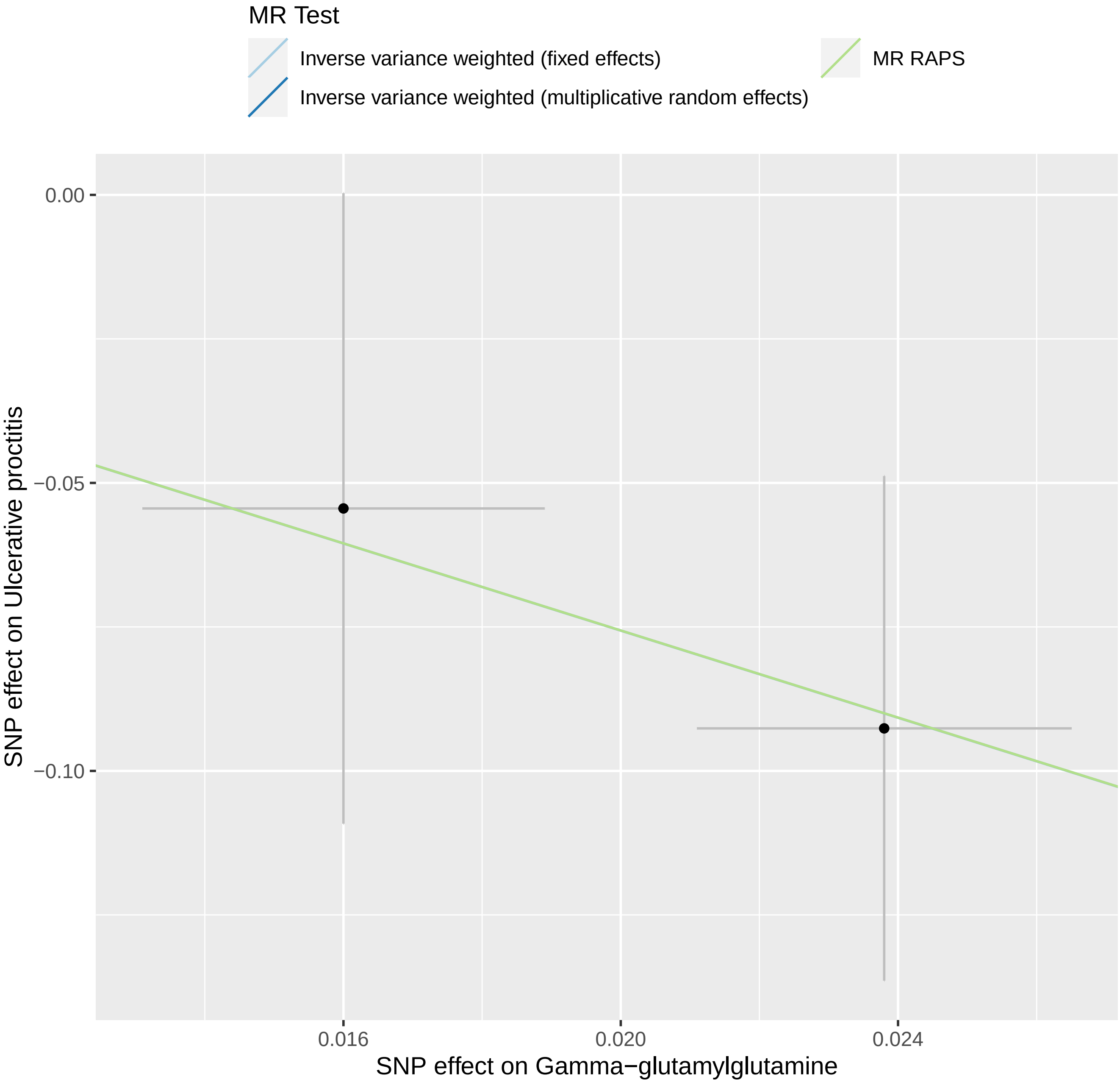

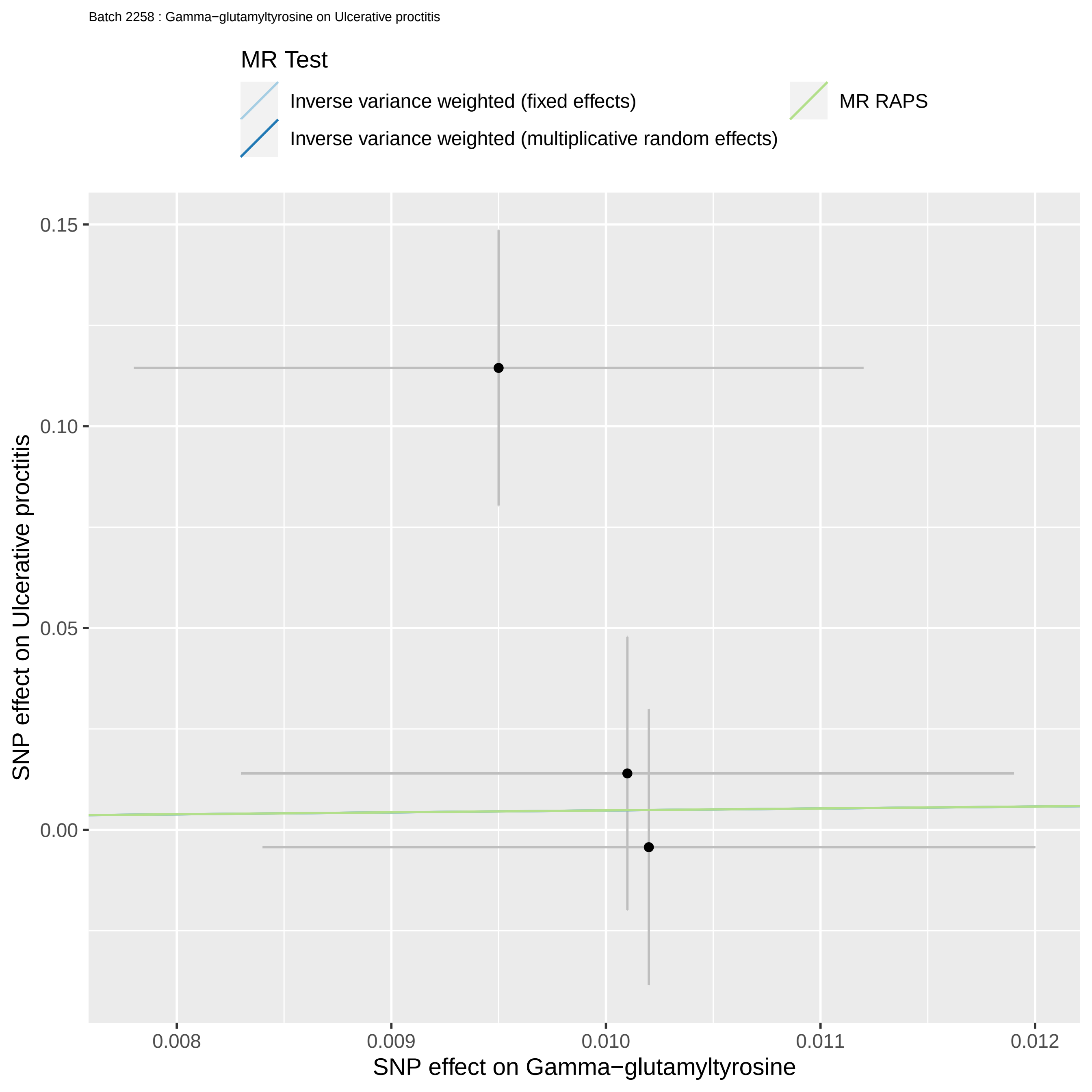

Batch 2258 : Gamma−glutamyltyrosine on Ulcerative proctitis
MR Test
Inverse variance weighted (fixed effects)
Inverse variance weighted (multiplicative random effects)
MR RAPS
SNP effect on Ulcerative proctitis
0.15
0.10
0.05
0.00
0.008
0.009
0.010
0.011
0.012
SNP effect on Gamma−glutamyltyrosine

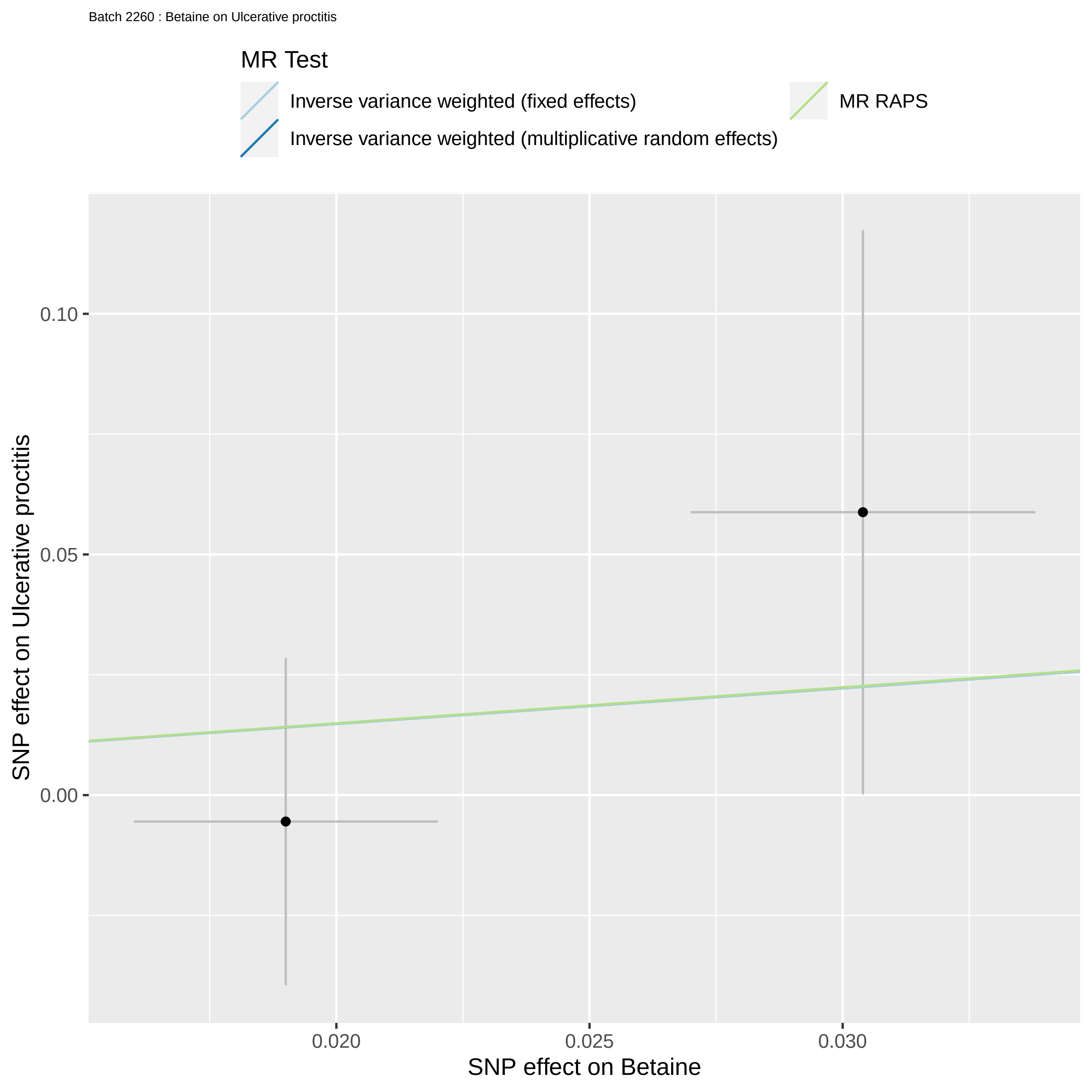
Batch 2260 : Betaine on Ulcerative proctitis
MR Test
Inverse variance weighted (fixed effects)
Inverse variance weighted (multiplicative random effects)
MR RAPS
0.10
0.05
0.00
0.020
0.025
0.030
SNP effect on Ulcerative proctitis
SNP effect on Betaine

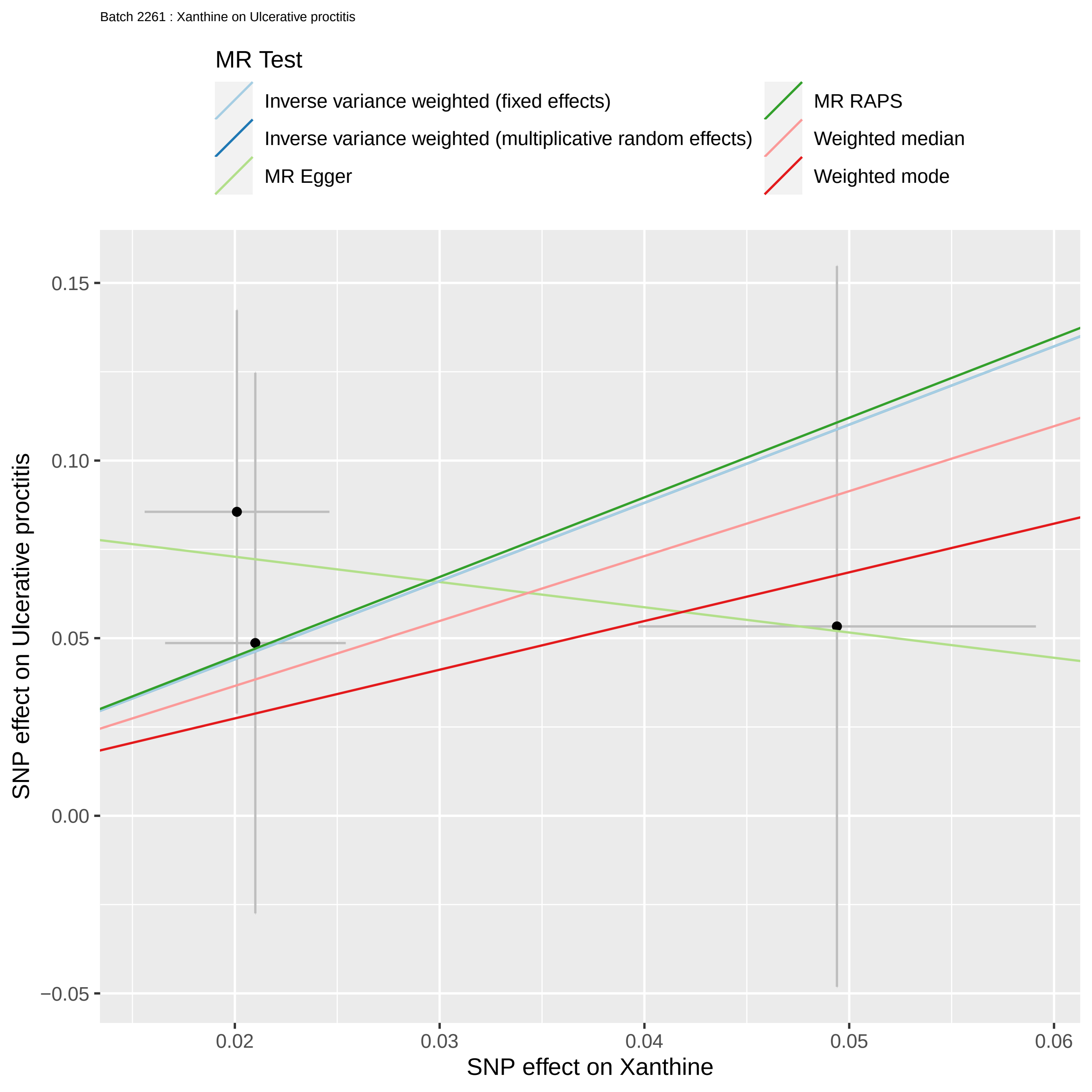

Batch 2261 : Xanthine on Ulcerative proctitis
MR Test
Inverse variance weighted (fixed effects)
Inverse variance weighted (multiplicative random effects)
MR Egger
MR RAPS
Weighted median
Weighted mode
0.15
0.10
0.05
0.00
−0.05
0.02
0.03
0.04
0.05
0.06
SNP effect on Ulcerative proctitis
SNP effect on Xanthine

Batch 2262 : Phosphate on Ulcerative proctitis

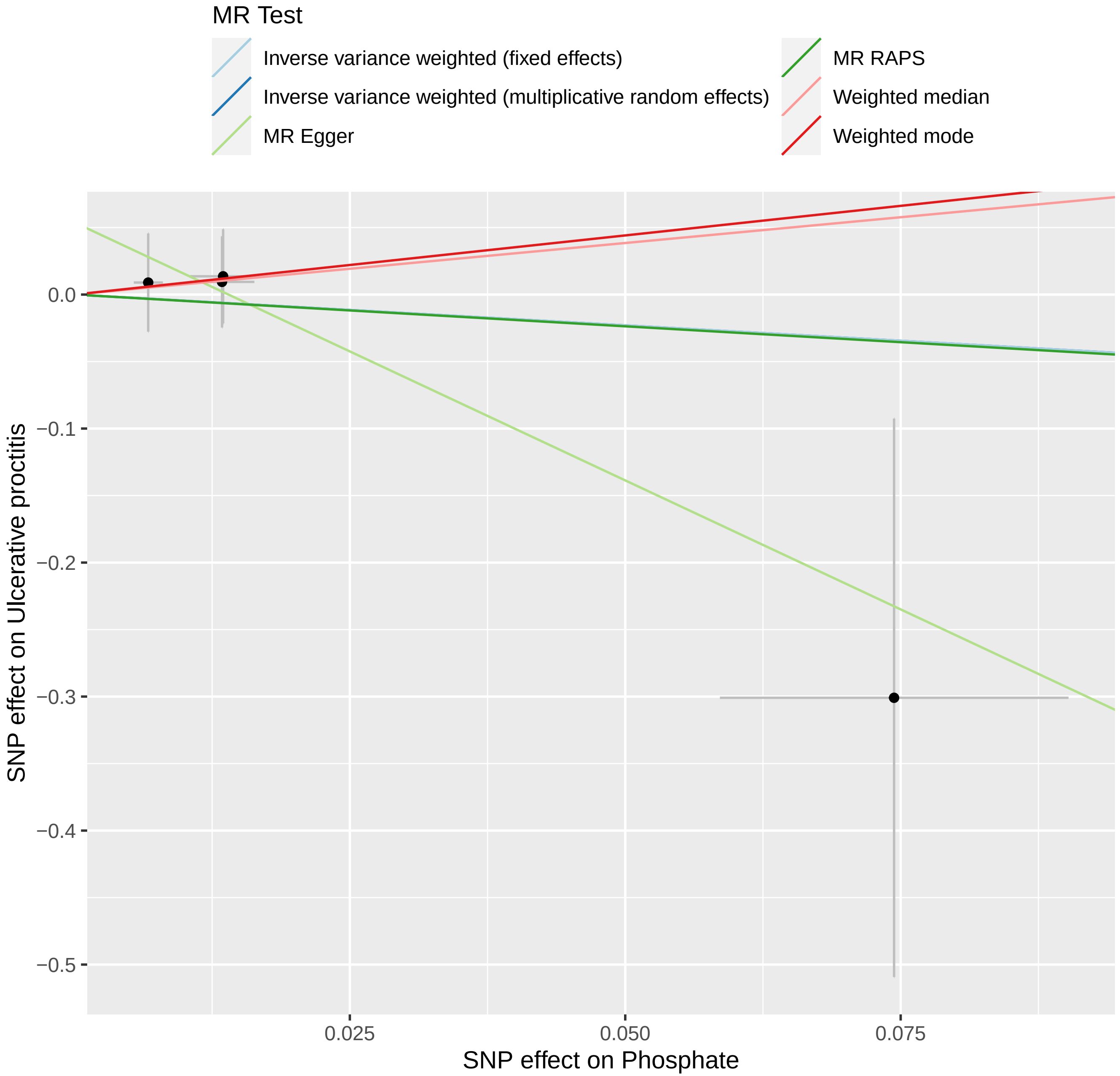

Batch 2263 : 3−methoxytyrosine on Ulcerative proctitis

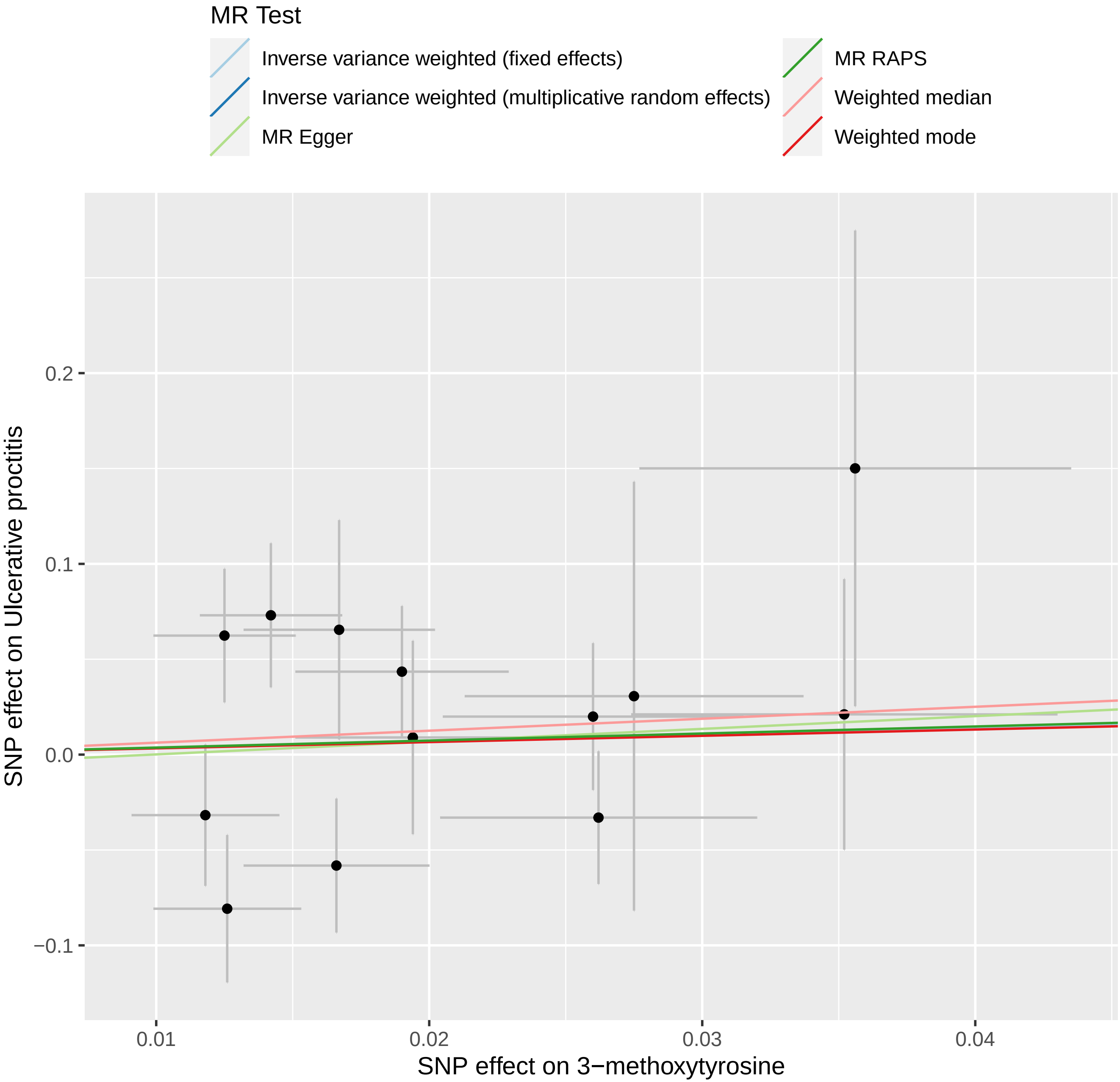

Batch 2265 : Undecanoate (11:0) on Ulcerative proctitis

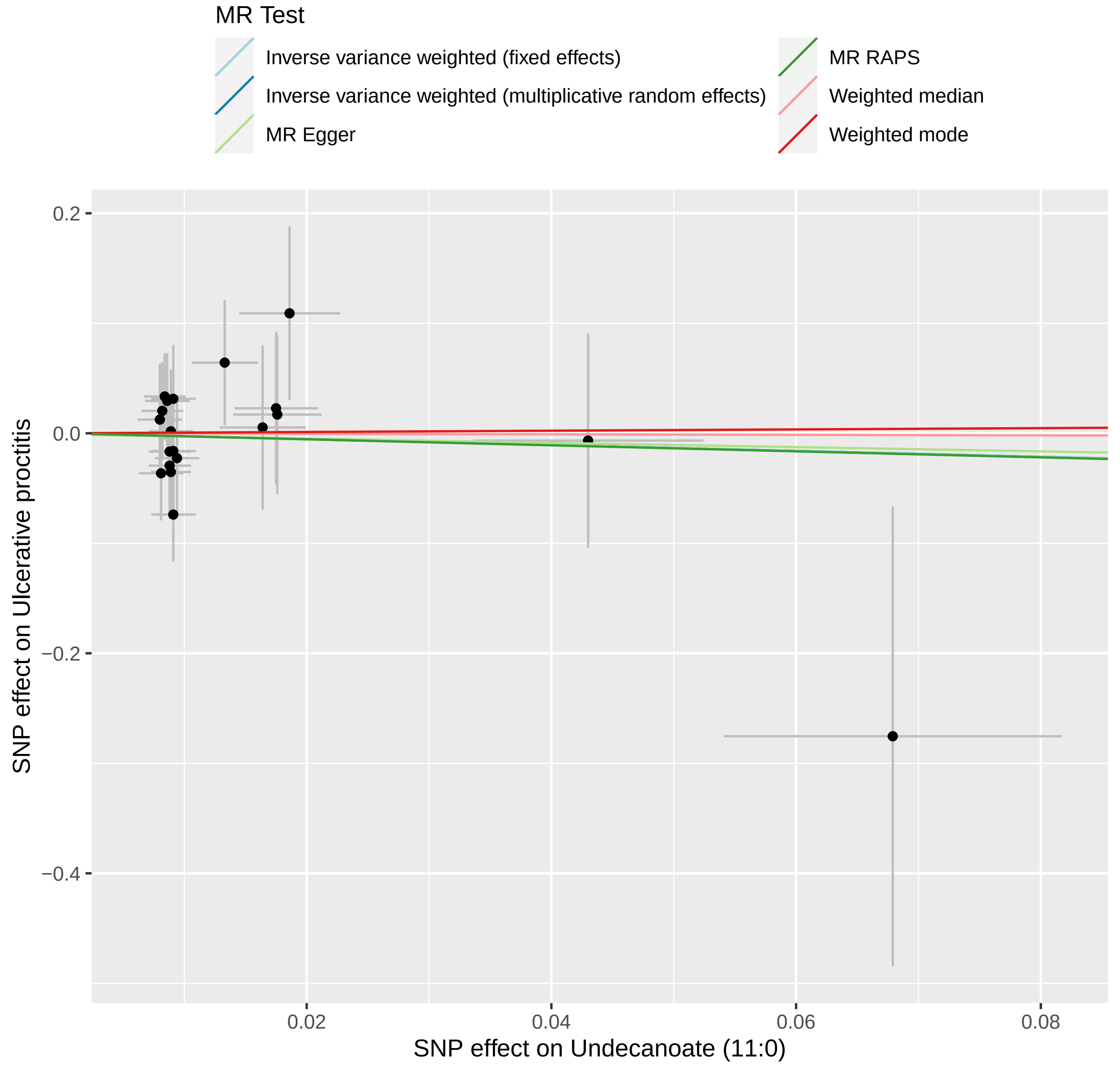

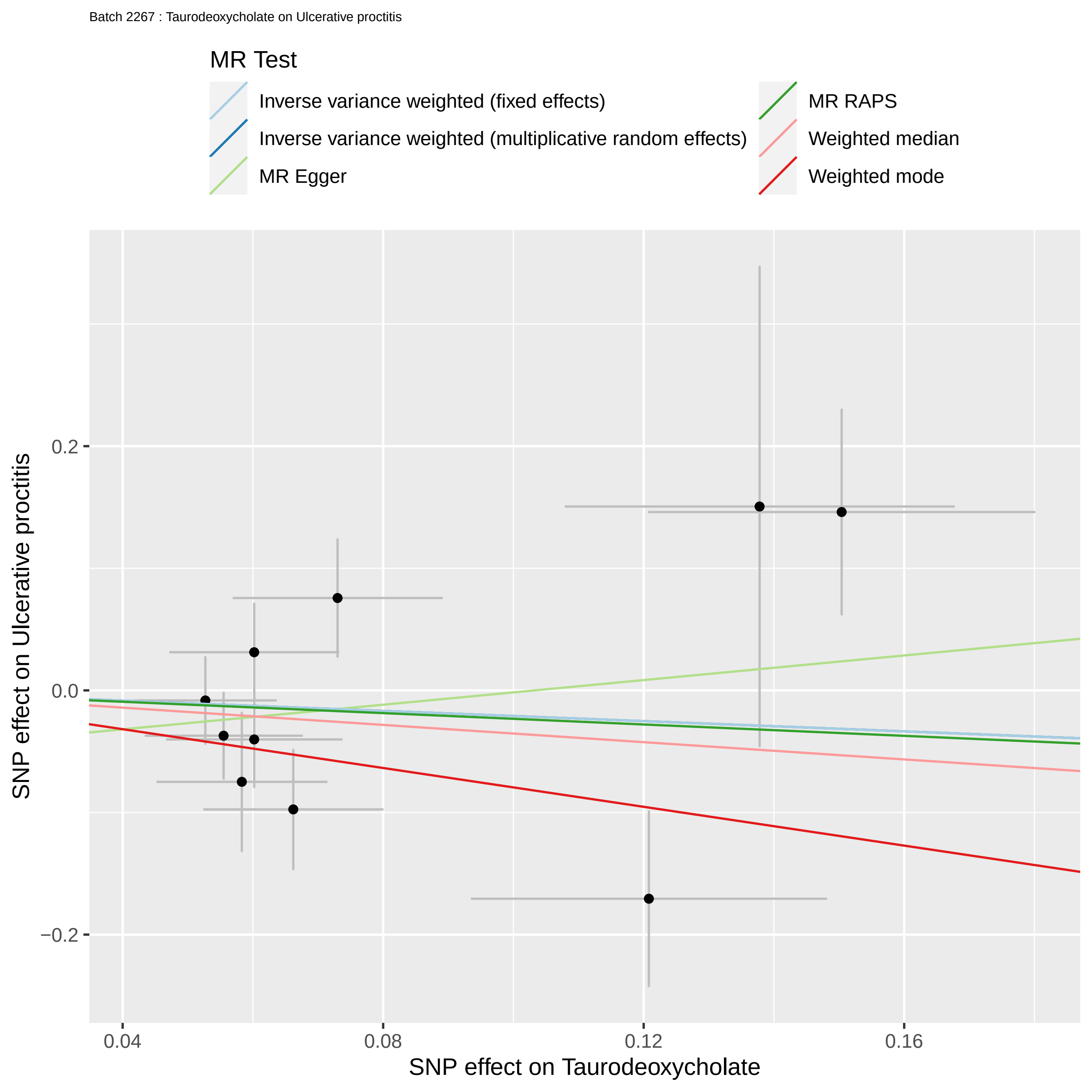

Batch 2267 : Taurodeoxycholate on Ulcerative proctitis
MR Test
Inverse variance weighted (fixed effects)
Inverse variance weighted (multiplicative random effects)
MR Egger
MR RAPS
Weighted median
Weighted mode
0.2
0.0
−0.2
0.04
0.08
0.12
0.16
SNP effect on Ulcerative proctitis
SNP effect on Taurodeoxycholate

Batch 2268 : Glycerol on Ulcerative proctitis

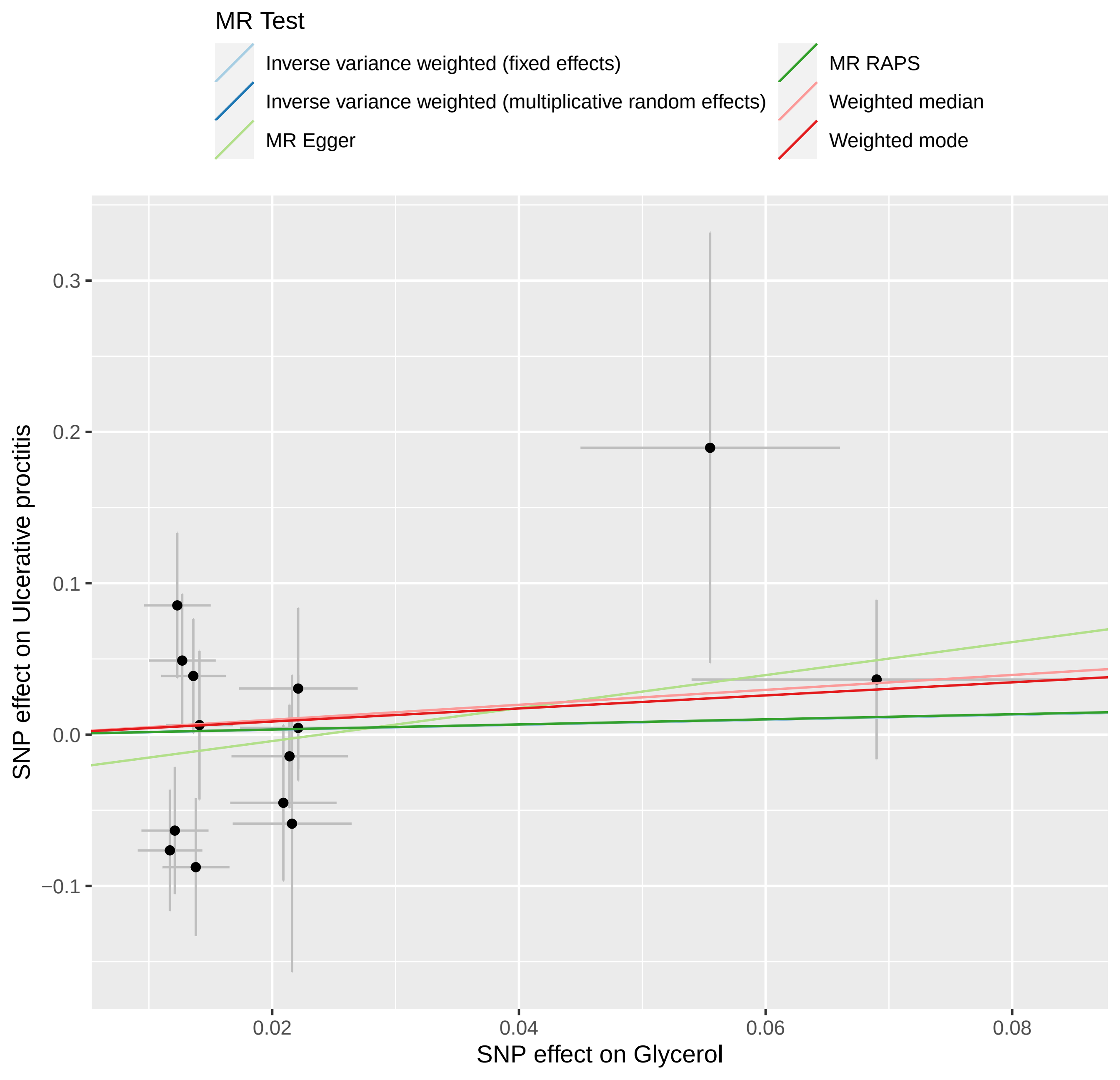

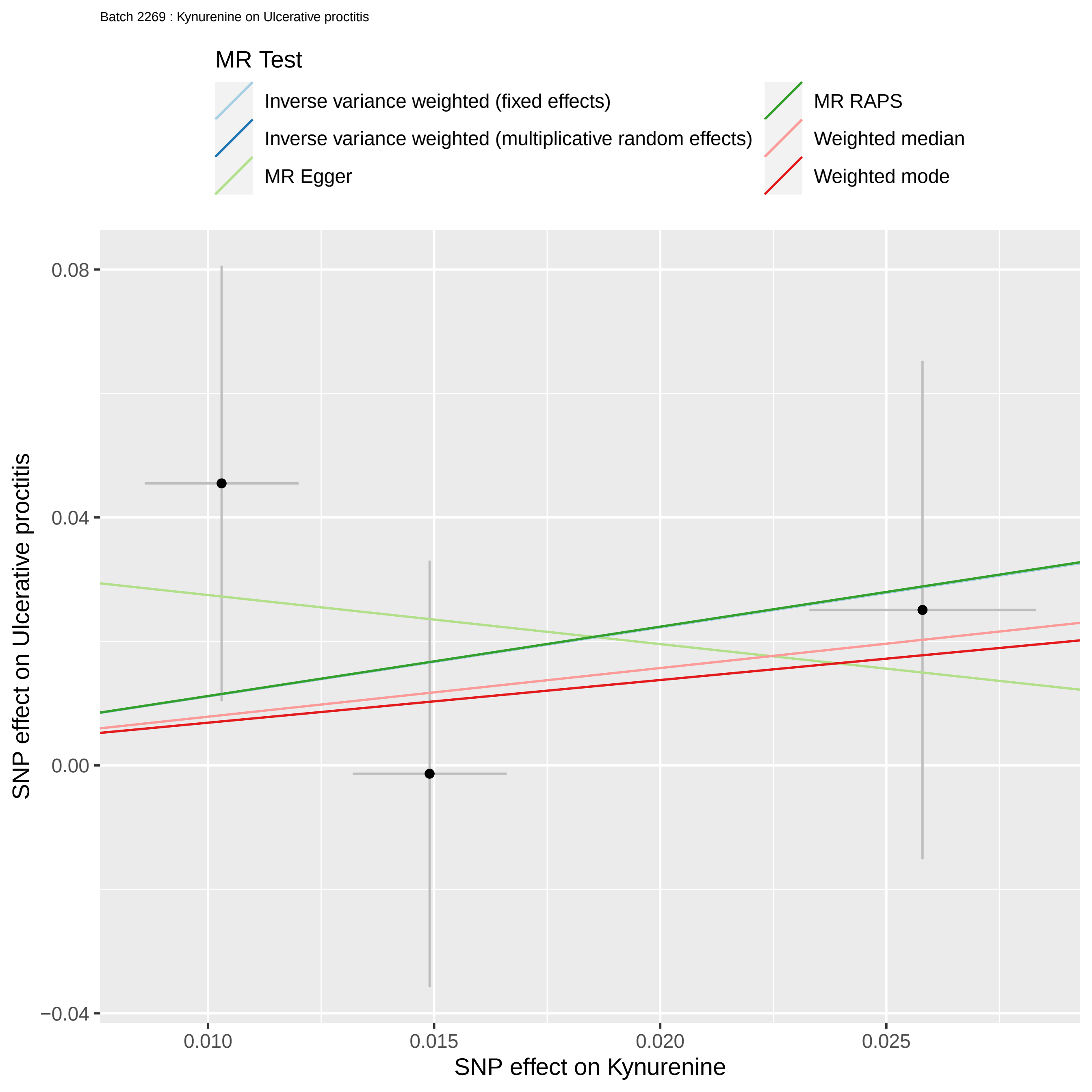
Batch 2269 : Kynurenine on Ulcerative proctitis
MR Test
Inverse variance weighted (fixed effects)
Inverse variance weighted (multiplicative random effects)
MR Egger
MR RAPS
Weighted median
Weighted mode
0.08
0.04
0.00
−0.04
0.010
0.015
0.020
0.025
SNP effect on Kynurenine
SNP effect on Ulcerative proctitis

Batch 2270 : Mannitol on Ulcerative proctitis

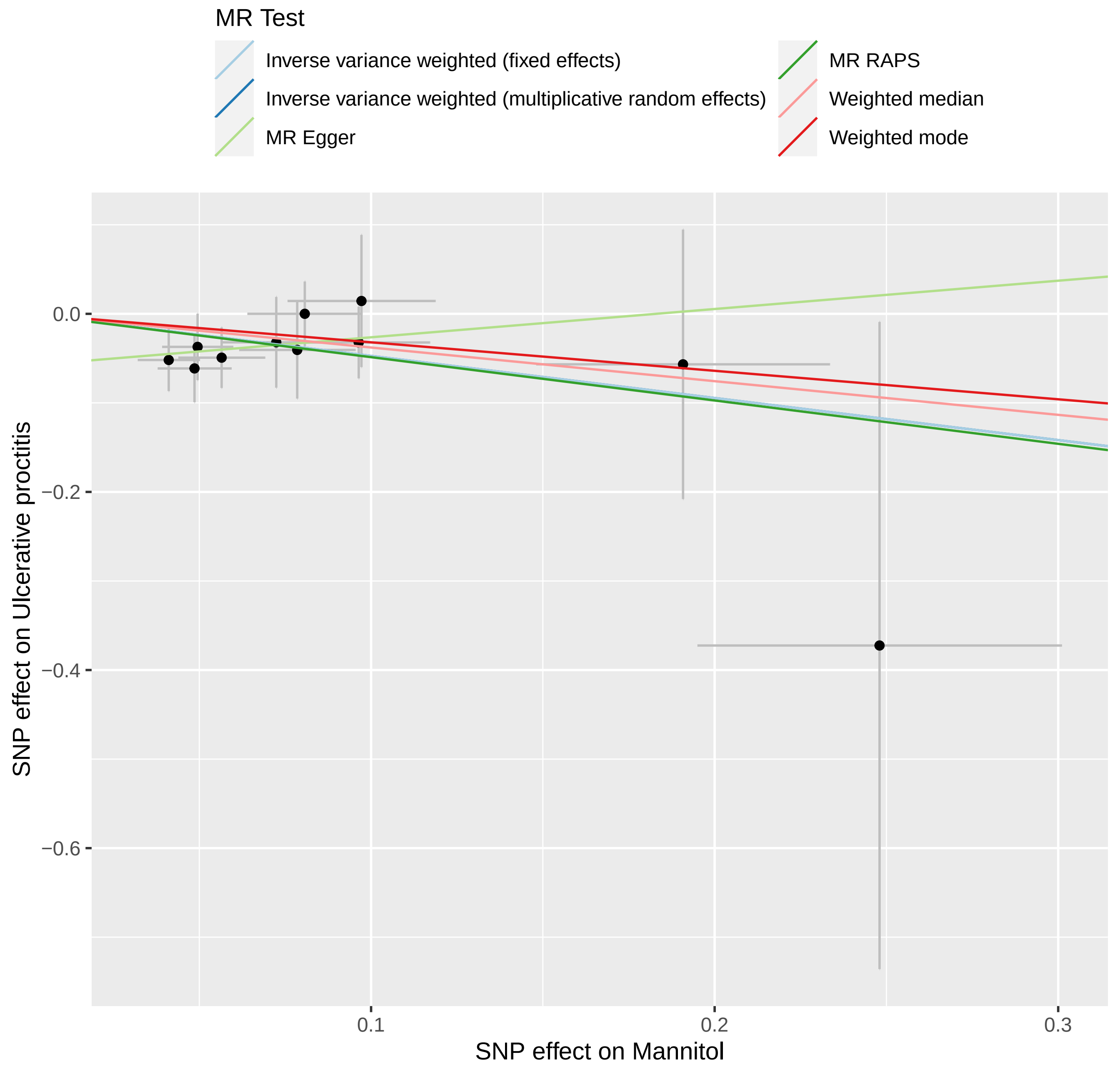

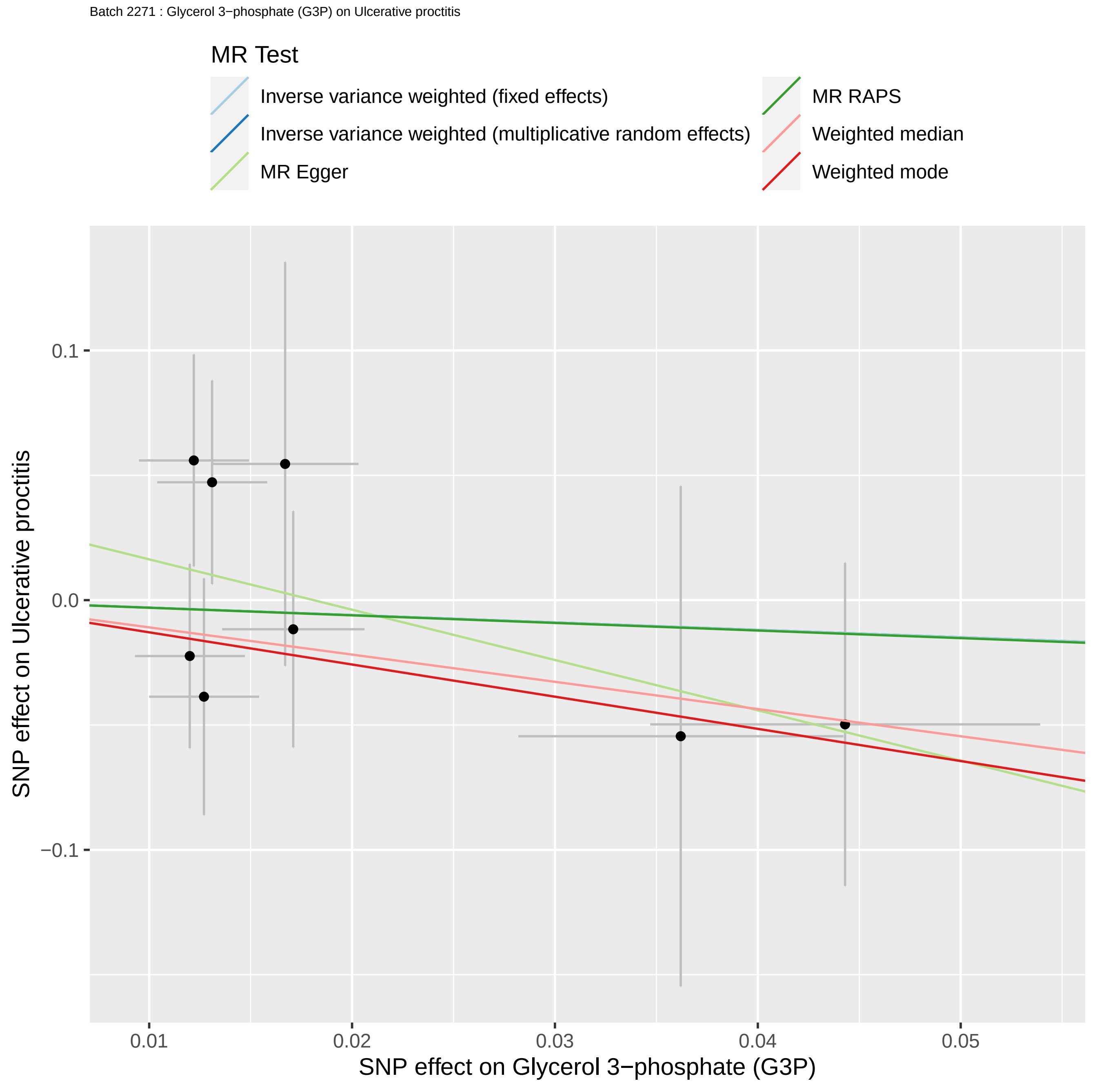

Batch 2271 : Glycerol 3−phosphate (G3P) on Ulcerative proctitis
MR Test
Inverse variance weighted (fixed effects)
Inverse variance weighted (multiplicative random effects)
MR Egger
MR RAPS
Weighted median
Weighted mode
0.1
0.0
−0.1
0.01
0.02
0.03
0.04
0.05
SNP effect on Ulcerative proctitis
SNP effect on Glycerol 3−phosphate (G3P)

Batch 2273 : Carnitine on Ulcerative proctitis

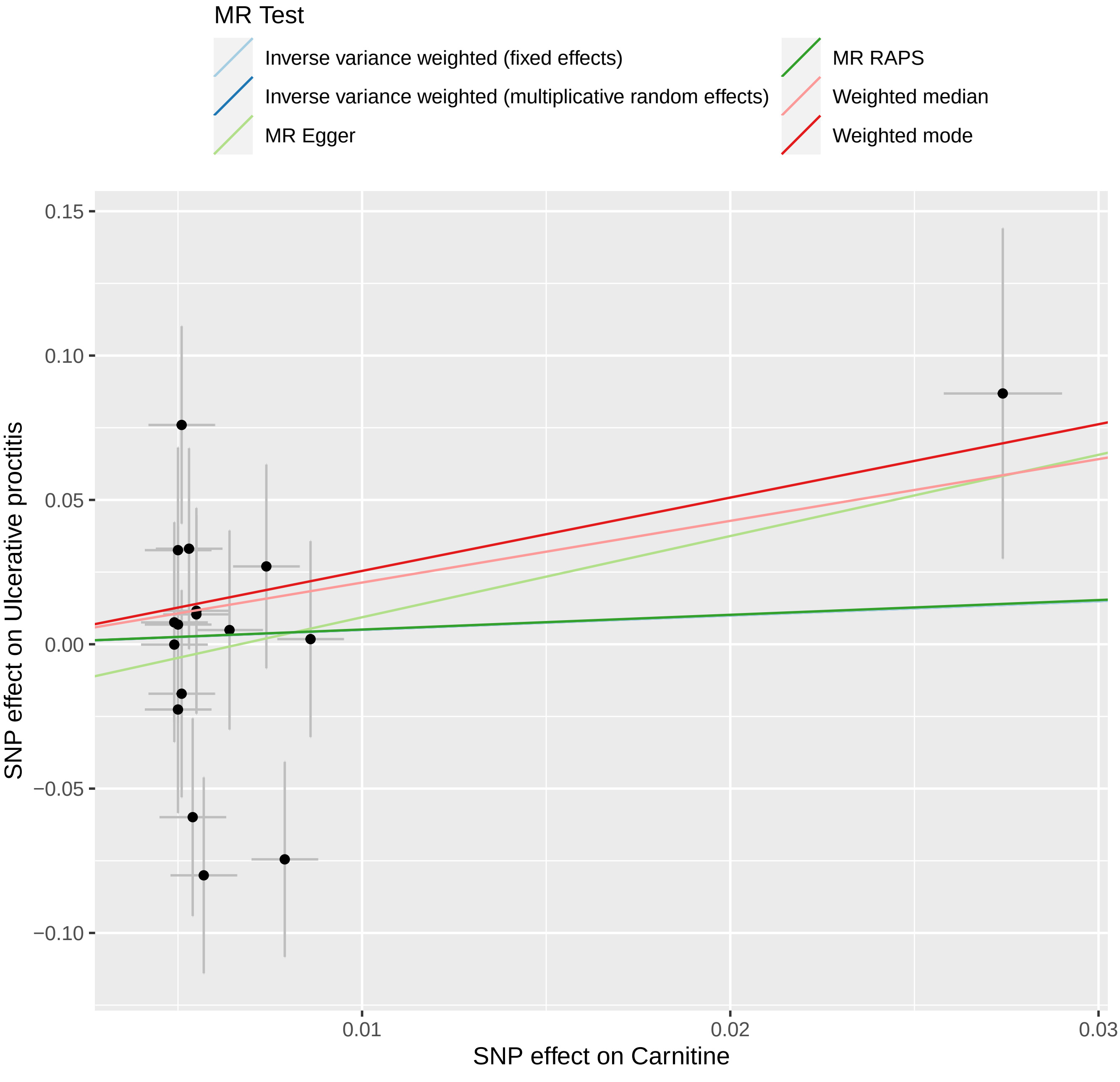

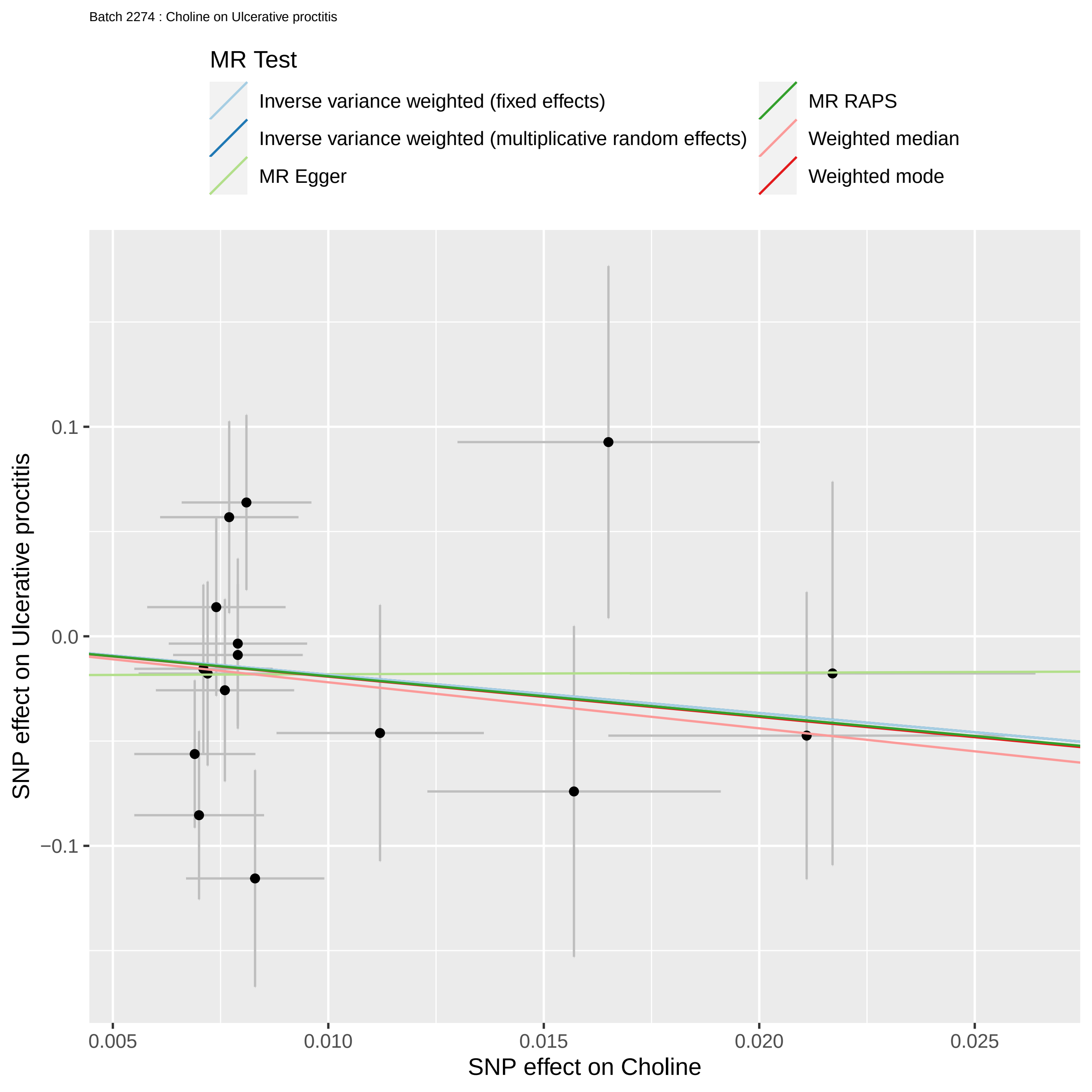
Batch 2274 : Choline on Ulcerative proctitis
MR Test
Inverse variance weighted (fixed effects)
Inverse variance weighted (multiplicative random effects)
MR Egger
MR RAPS
Weighted median
Weighted mode
SNP effect on Ulcerative proctitis
0.1
0.0
−0.1
0.005
0.010
0.015
0.020
0.025
SNP effect on Choline

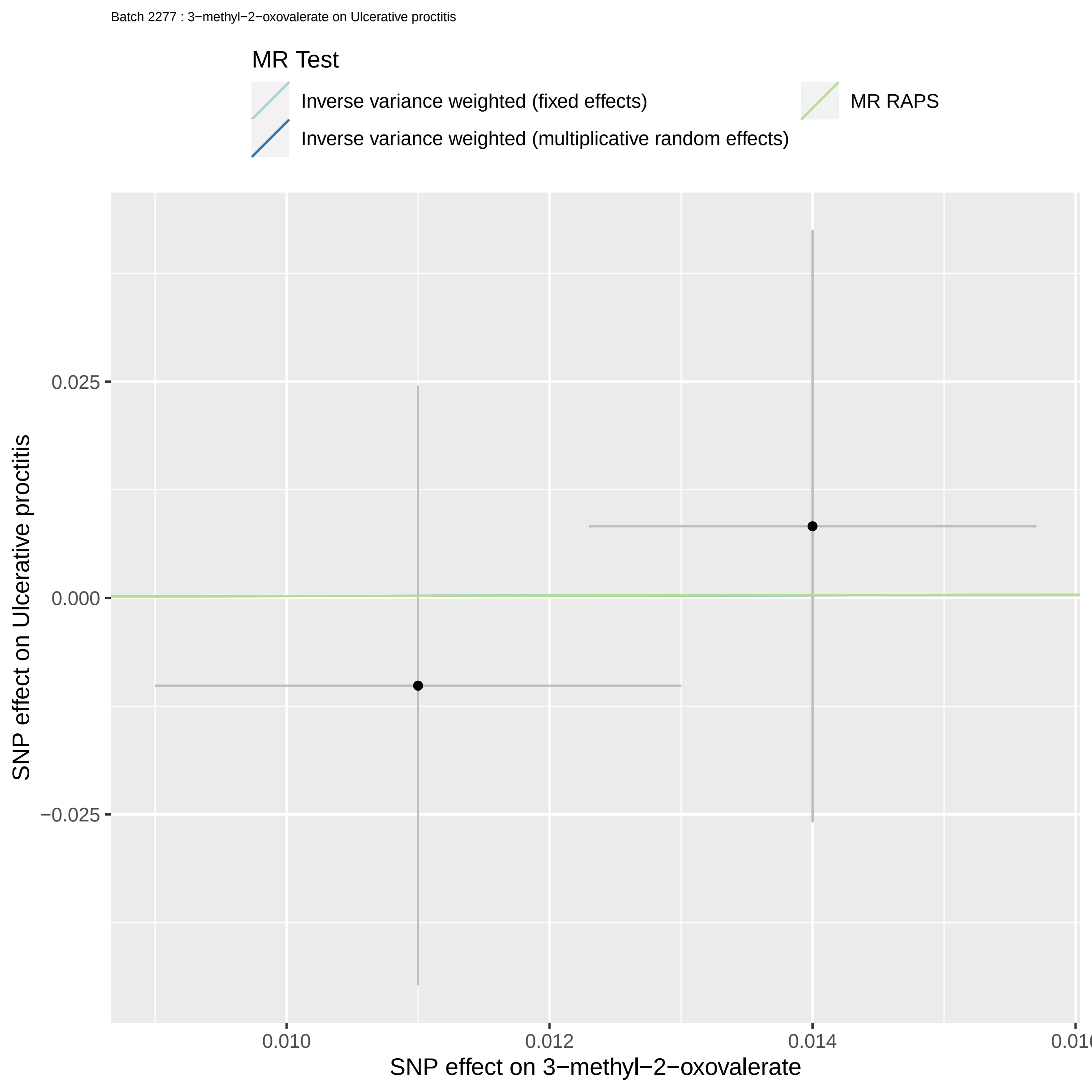

Batch 2277 : 3−methyl−2−oxovalerate on Ulcerative proctitis
MR Test
Inverse variance weighted (fixed effects)
Inverse variance weighted (multiplicative random effects)
MR RAPS
0.025
0.000
−0.025
0.010
0.012
0.014
SNP effect on Ulcerative proctitis
SNP effect on 3−methyl−2−oxovalerate

Batch 2278 : 3−methylhistidine on Ulcerative proctitis

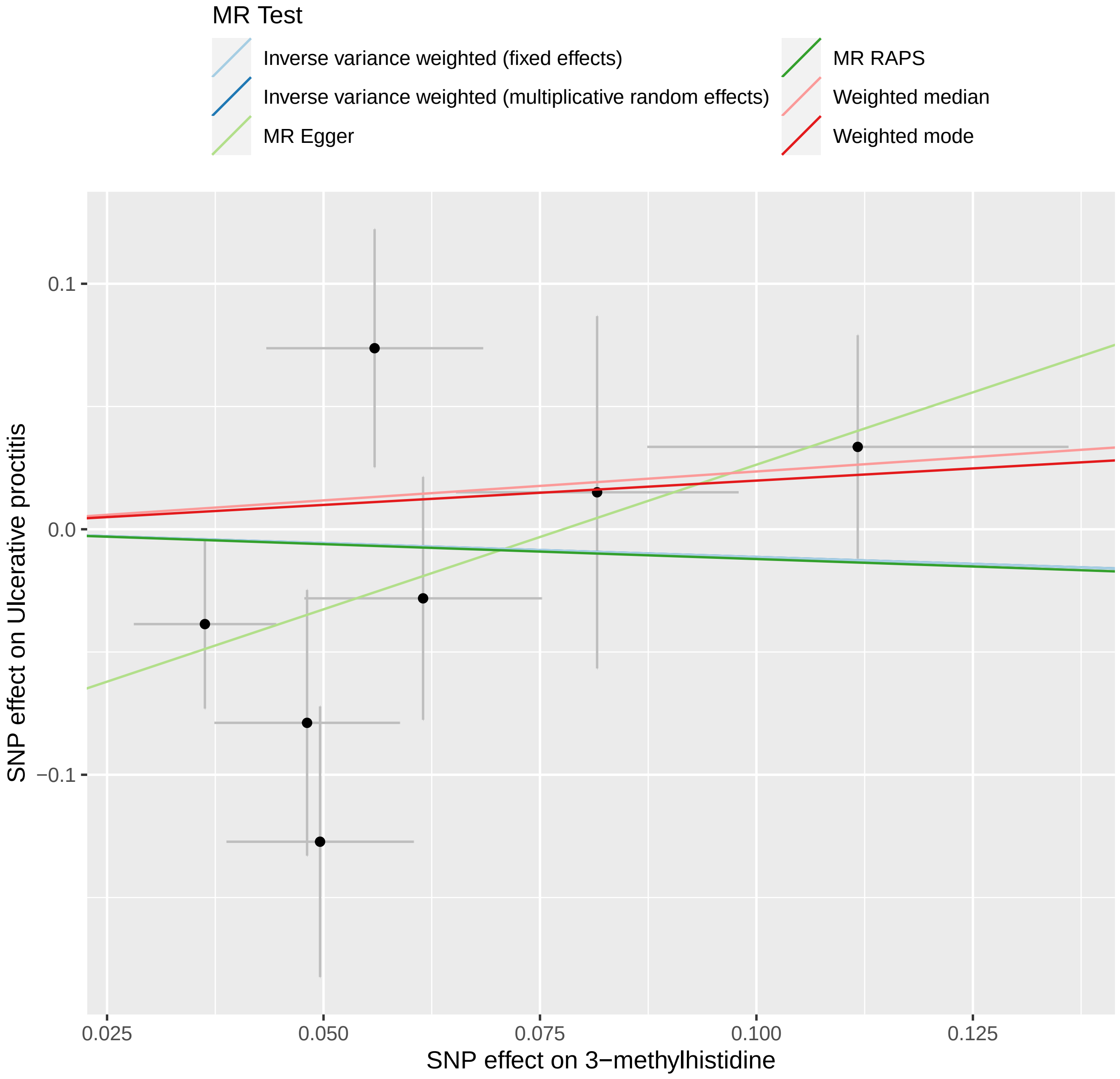

Batch 2280 : Phenylacetate on Ulcerative proctitis

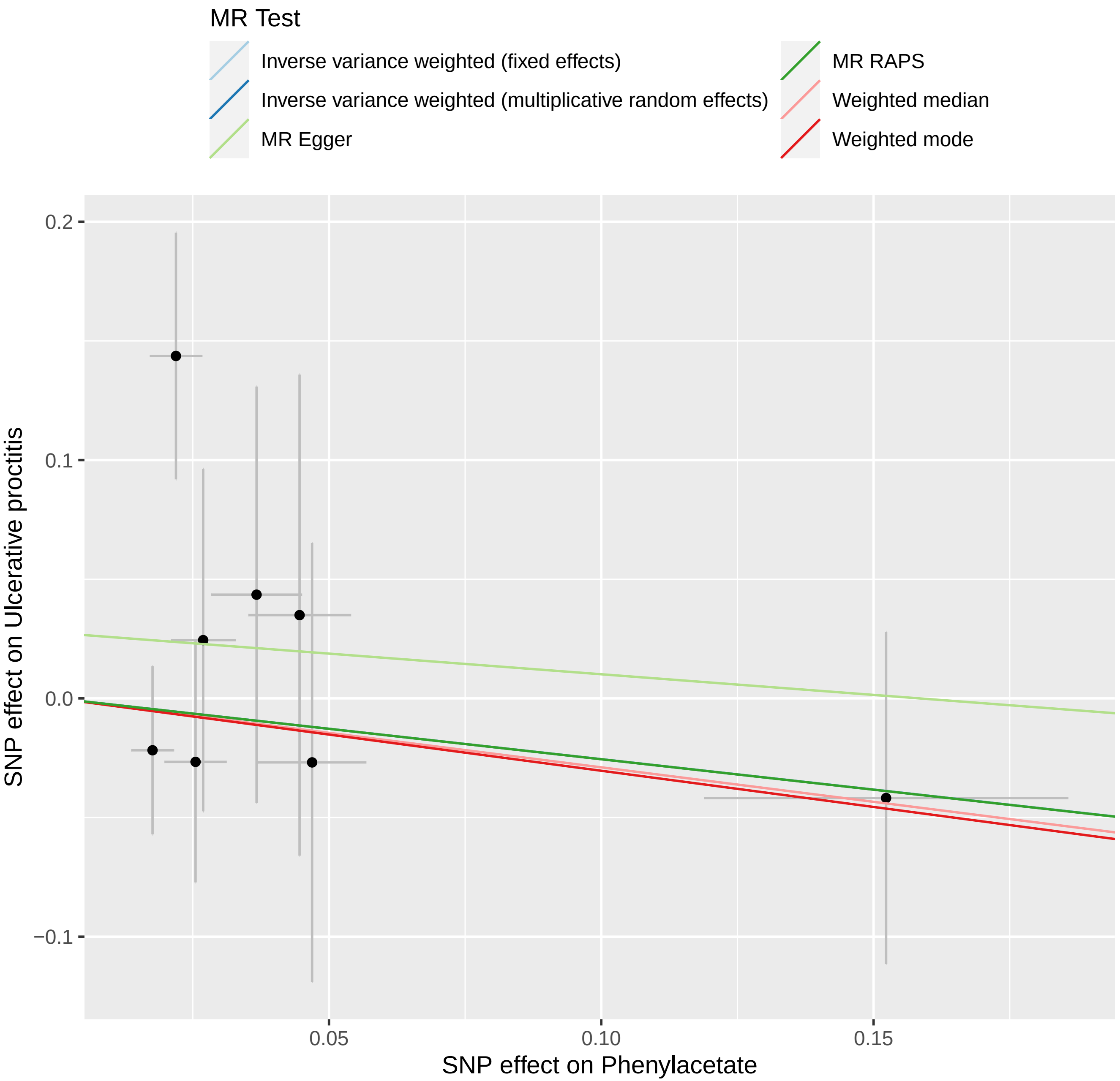

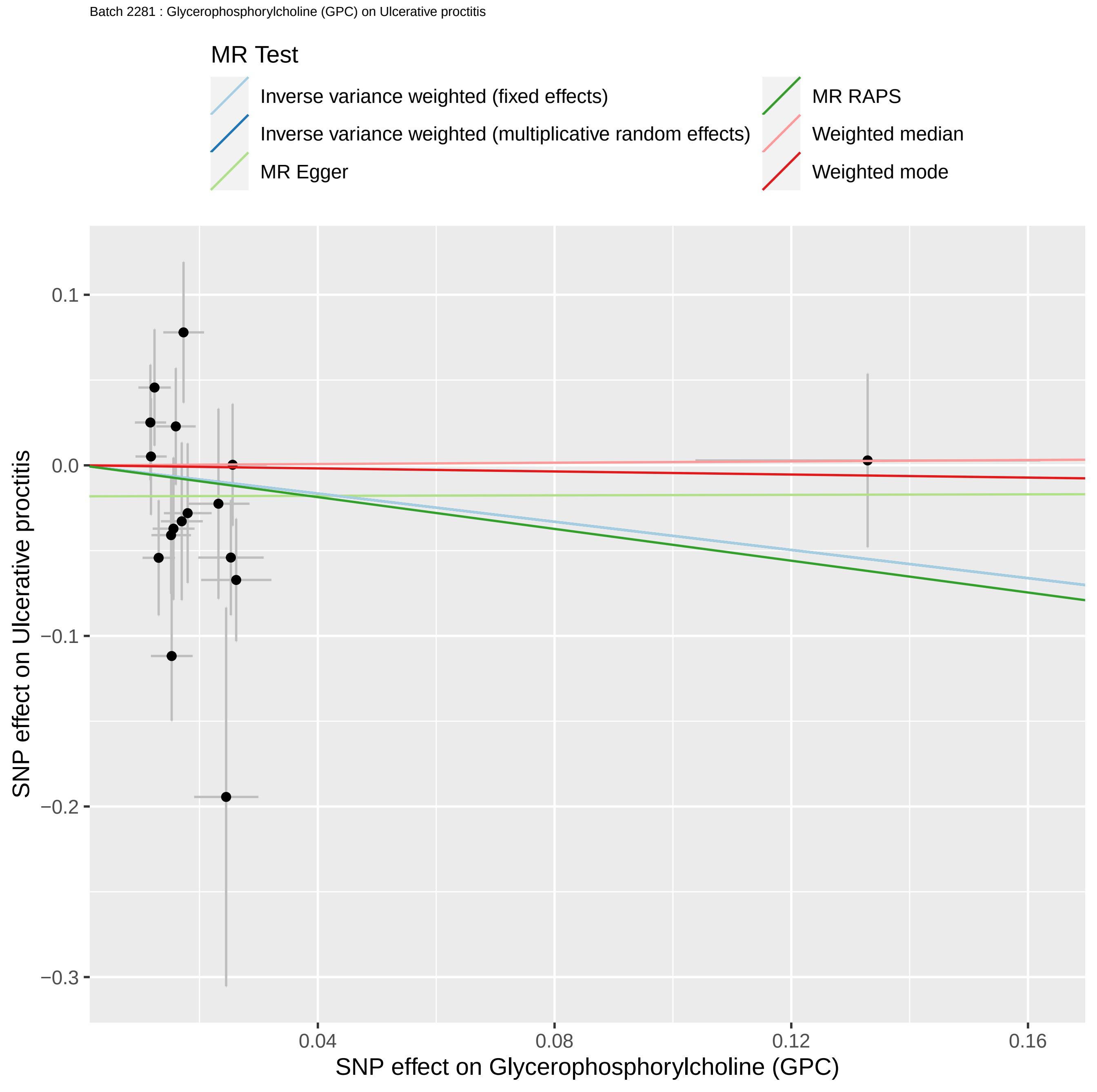
Batch 2281 : Glycerophosphorylcholine (GPC) on Ulcerative proctitis
MR Test
Inverse variance weighted (fixed effects)
Inverse variance weighted (multiplicative random effects)
MR Egger
MR RAPS
Weighted median
Weighted mode
0.1
0.0
−0.1
−0.2
−0.3
0.04
0.08
0.12
0.16
SNP effect on Ulcerative proctitis
SNP effect on Glycerophosphorylcholine (GPC)

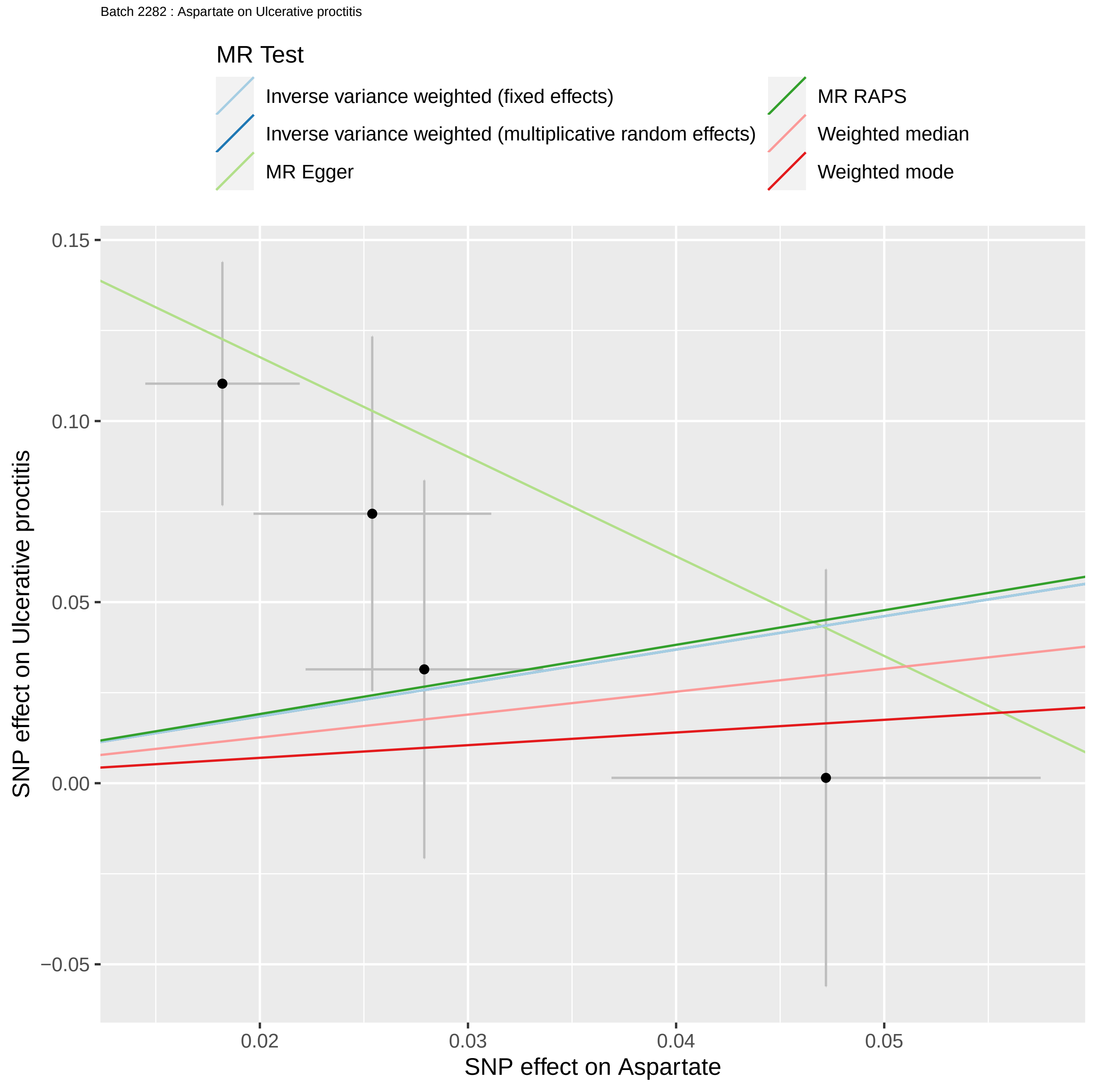
Batch 2282 : Aspartate on Ulcerative proctitis
MR Test
Inverse variance weighted (fixed effects)
Inverse variance weighted (multiplicative random effects)
MR Egger
MR RAPS
Weighted median
Weighted mode
0.15
0.10
0.05
0.00
−0.05
0.02
0.03
0.04
0.05
SNP effect on Ulcerative proctitis
SNP effect on Aspartate

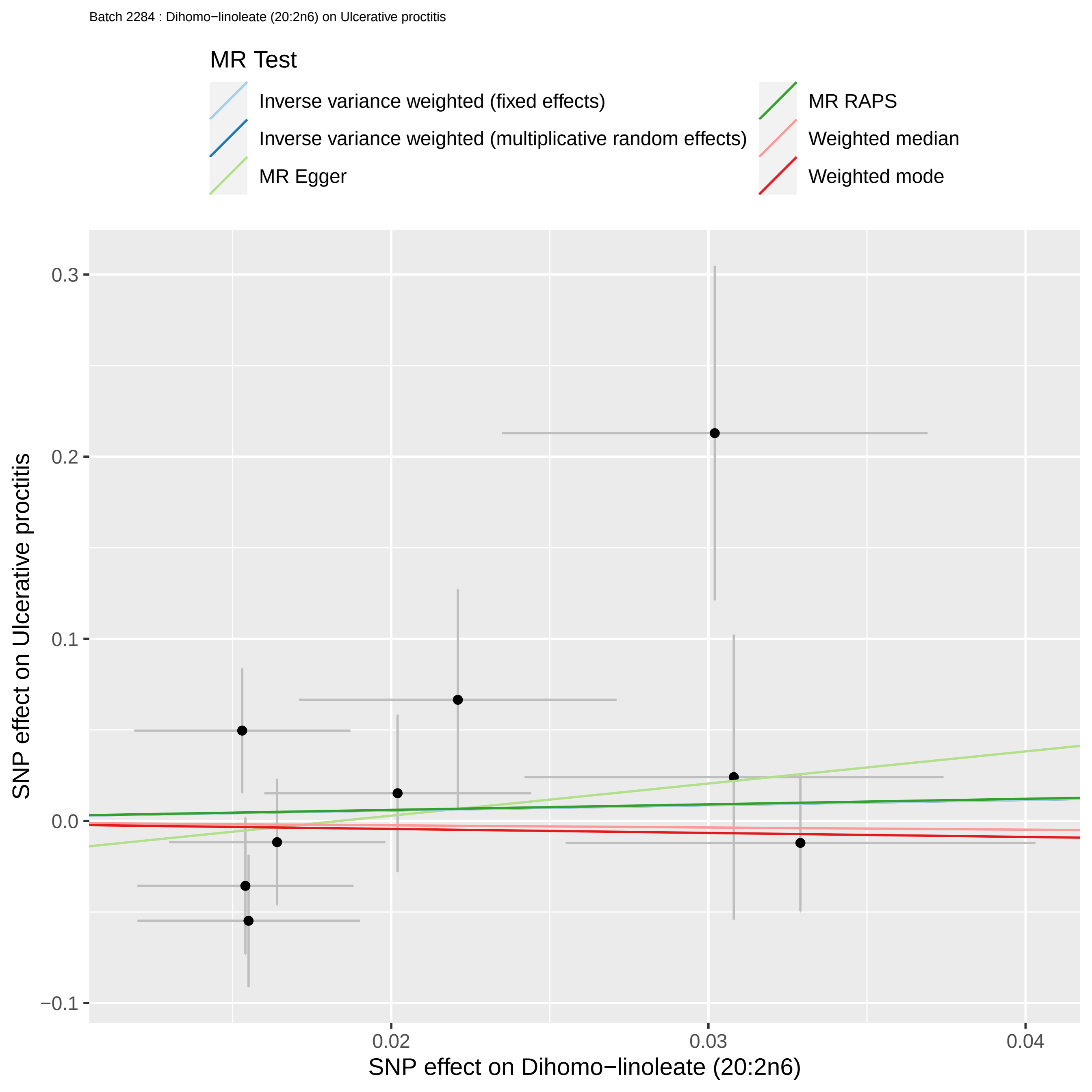

Batch 2284 : Dihomo−linoleate (20:2n6) on Ulcerative proctitis
MR Test
Inverse variance weighted (fixed effects)
Inverse variance weighted (multiplicative random effects)
MR Egger
MR RAPS
Weighted median
Weighted mode
SNP effect on Ulcerative proctitis
0.3
0.2
0.1
0.0
−0.1
0.02
0.03
0.04
SNP effect on Dihomo−linoleate (20:2n6)

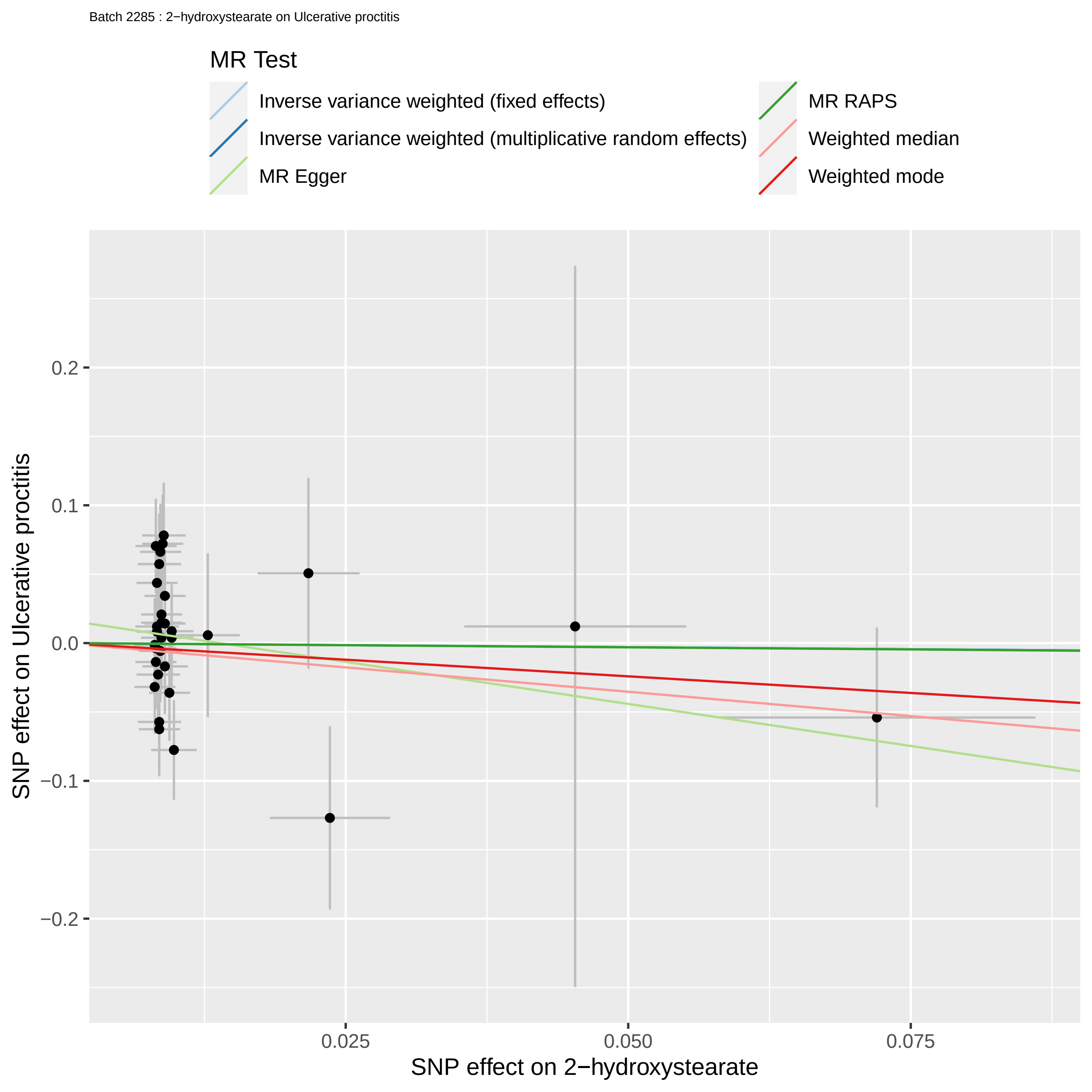

Batch 2285 : 2−hydroxystearate on Ulcerative proctitis
MR Test
Inverse variance weighted (fixed effects)
Inverse variance weighted (multiplicative random effects)
MR Egger
MR RAPS
Weighted median
Weighted mode
0.2
0.1
0.0
−0.1
−0.2
SNP effect on Ulcerative proctitis
0.025
0.050
0.075
SNP effect on 2−hydroxystearate

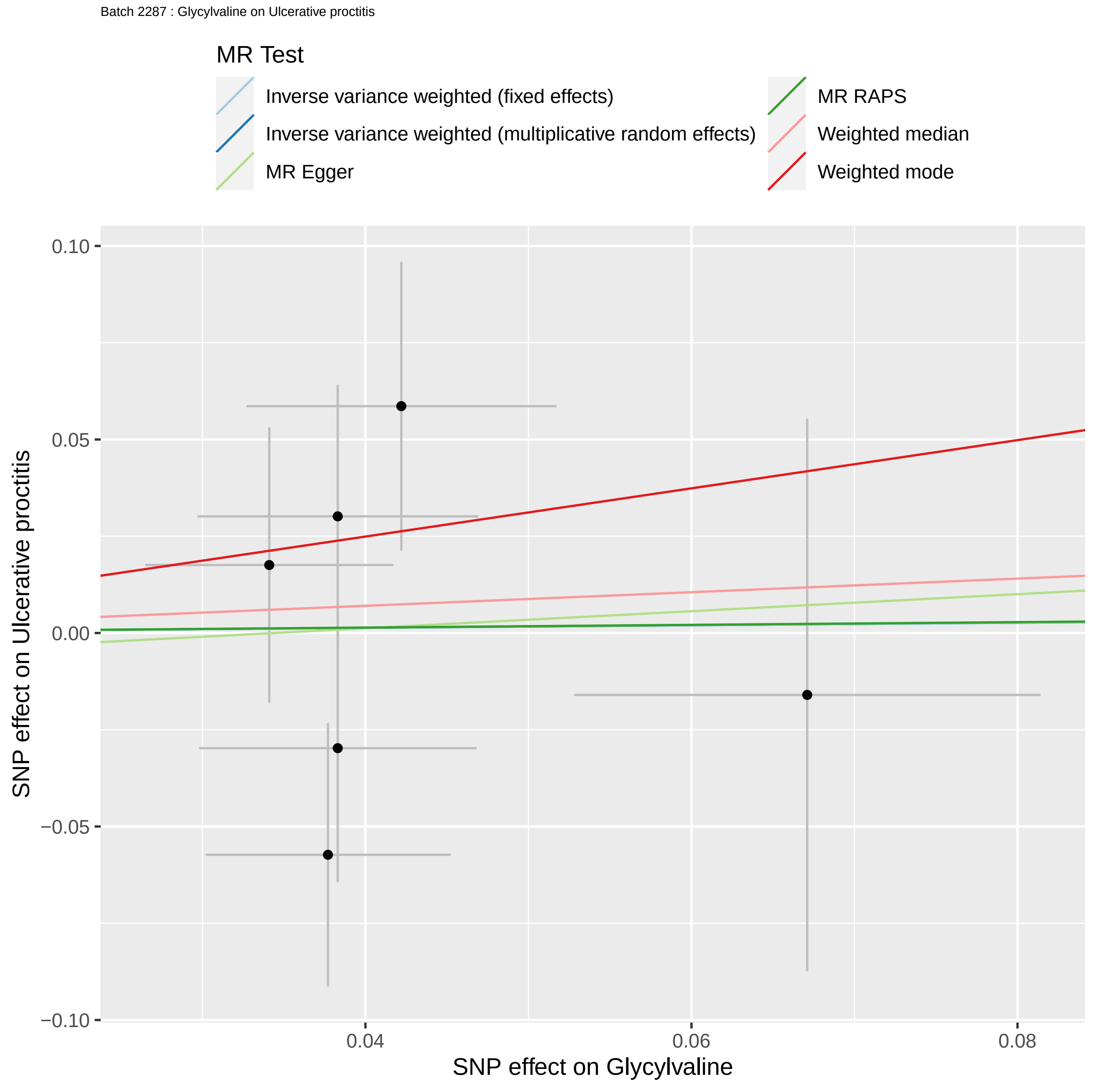

Batch 2287 : Glycylvaline on Ulcerative proctitis
MR Test
Inverse variance weighted (fixed effects)
Inverse variance weighted (multiplicative random effects)
MR Egger
MR RAPS
Weighted median
Weighted mode
0.10
0.05
0.00
−0.05
−0.10
0.04
0.06
0.08
SNP effect on Ulcerative proctitis
SNP effect on Glycylvaline

Batch 2288 : Gamma−glutamylleucine on Ulcerative proctitis

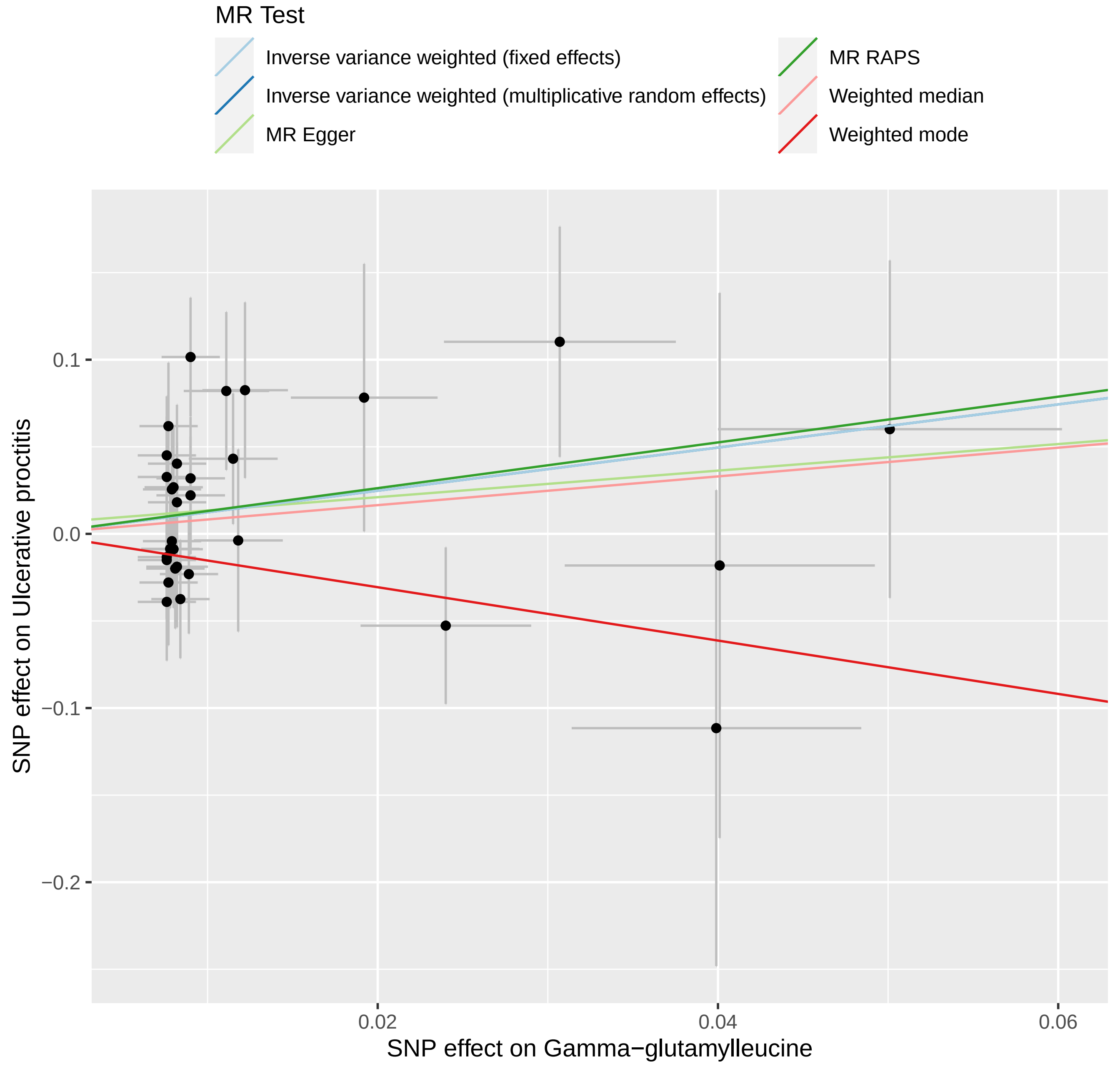

Batch 2291 : Glycocholate on Ulcerative proctitis

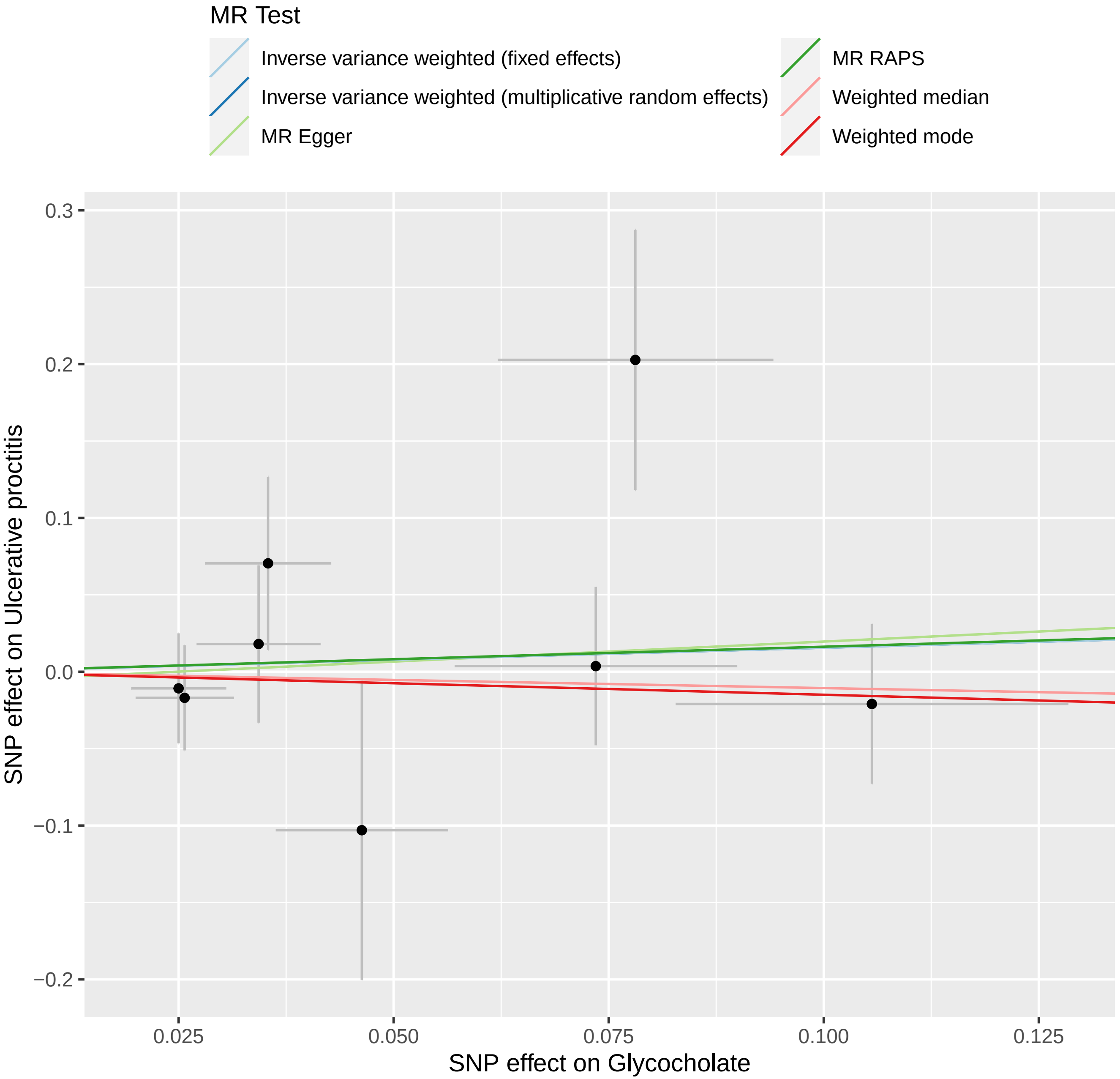

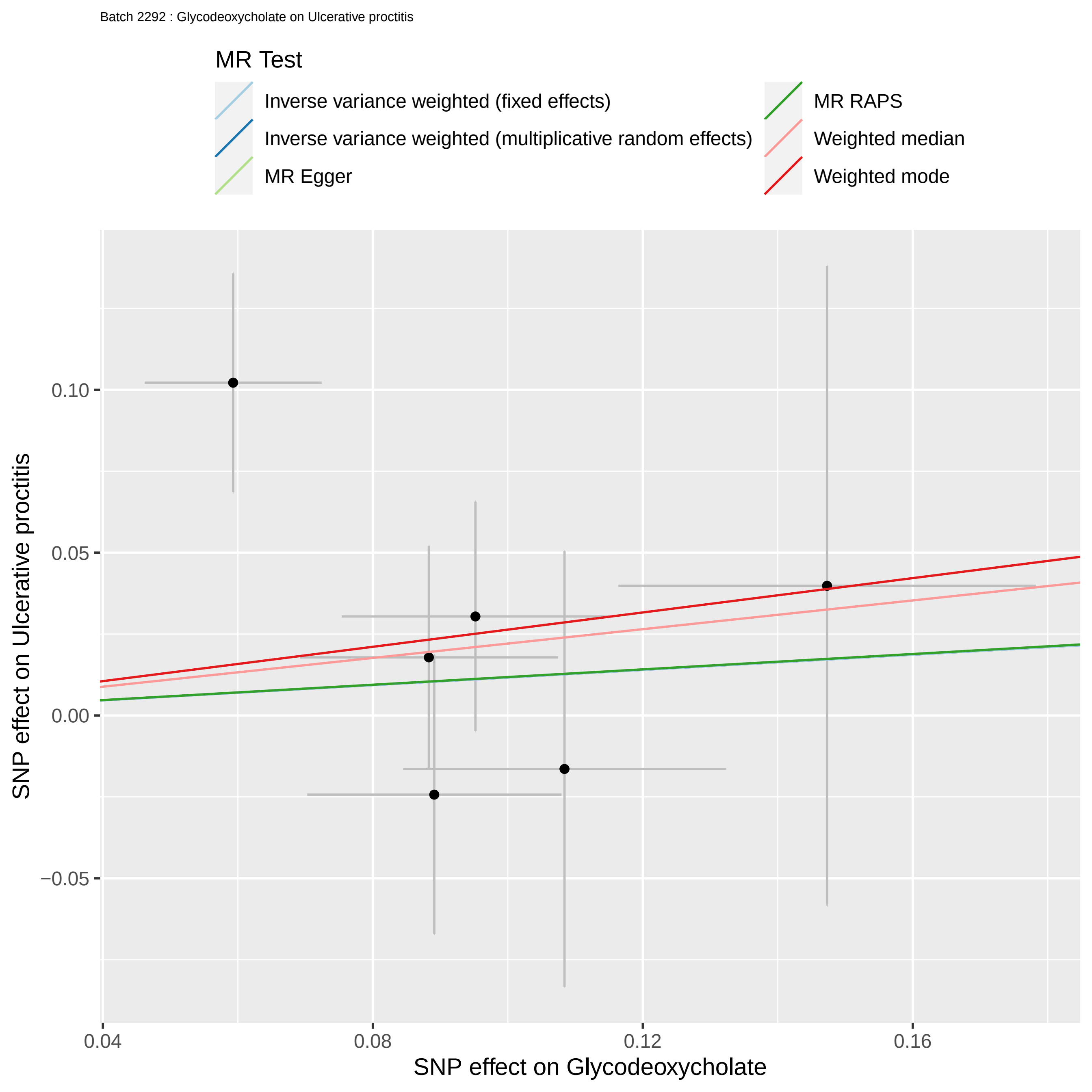

Batch 2292 : Glycodeoxycholate on Ulcerative proctitis
MR Test
Inverse variance weighted (fixed effects)
Inverse variance weighted (multiplicative random effects)
MR Egger
MR RAPS
Weighted median
Weighted mode
0.10
0.05
0.00
−0.05
SNP effect on Ulcerative proctitis
0.04
0.08
0.12
0.16
SNP effect on Glycodeoxycholate

Batch 2293 : Taurochenodeoxycholate on Ulcerative proctitis

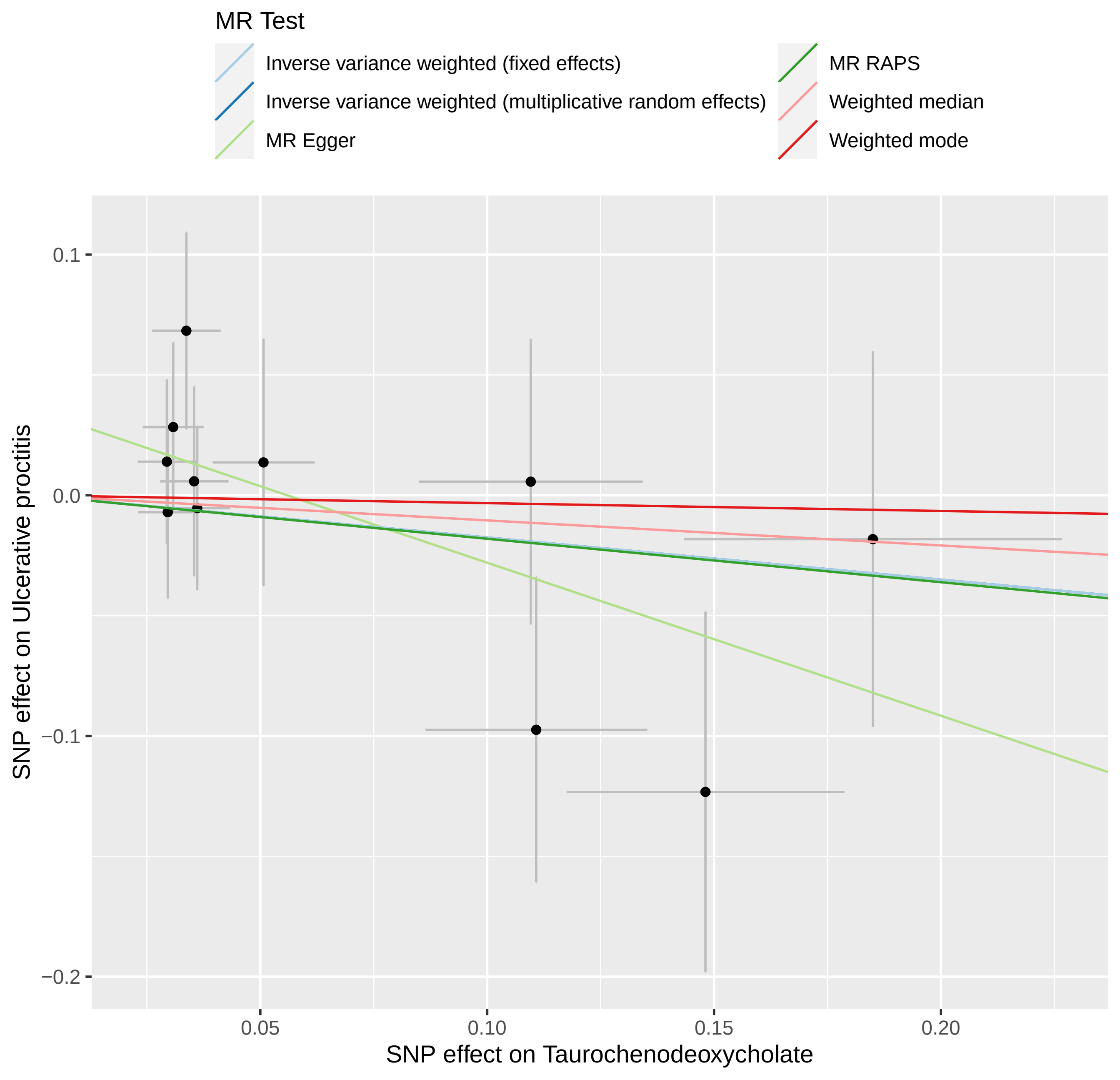

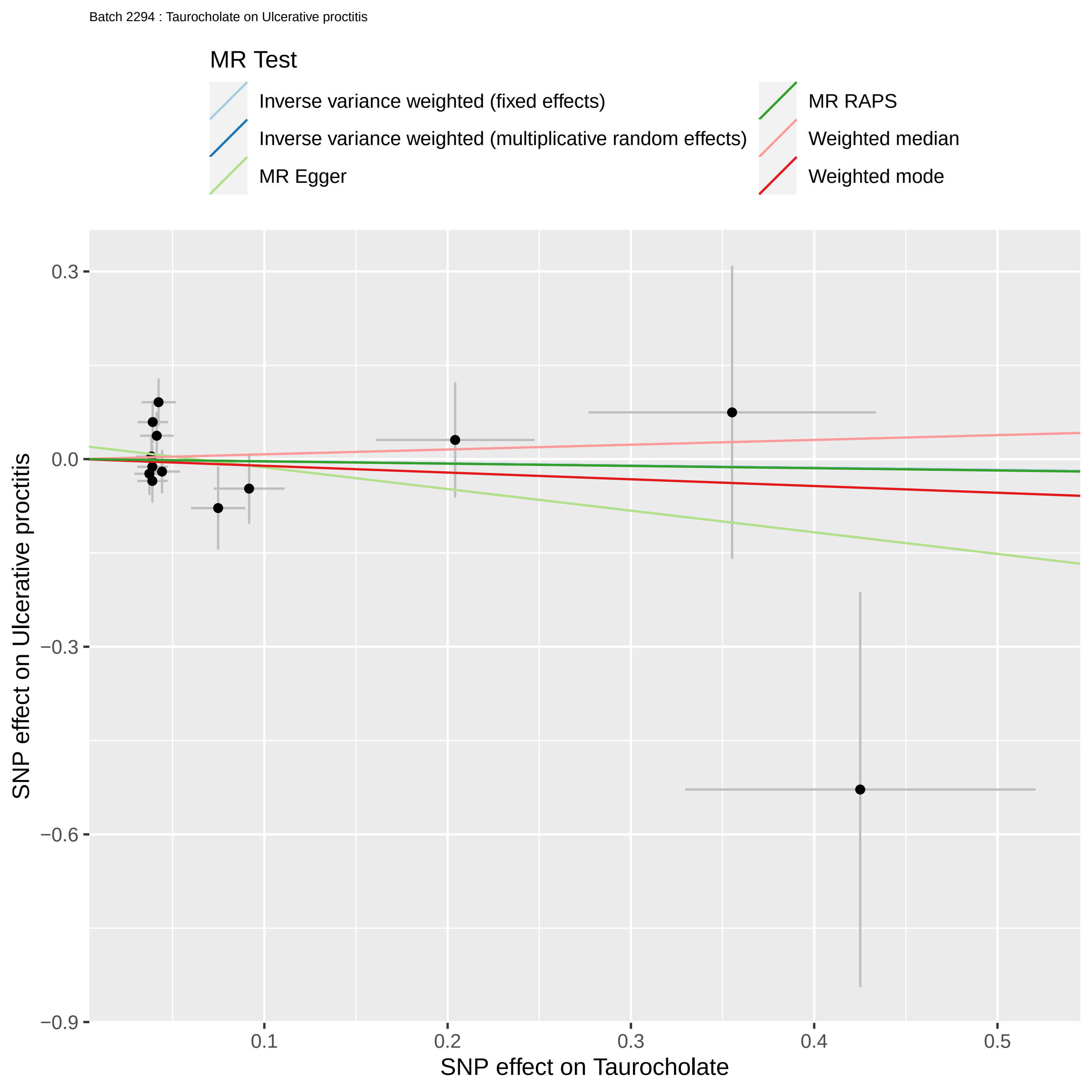

Batch 2294 : Taurocholate on Ulcerative proctitis
MR Test
Inverse variance weighted (fixed effects)
Inverse variance weighted (multiplicative random effects)
MR Egger
MR RAPS
Weighted median
Weighted mode
0.3
0.0
−0.3
−0.6
−0.9
0.1
0.2
0.3
0.4
0.5
SNP effect on Ulcerative proctitis
SNP effect on Taurocholate

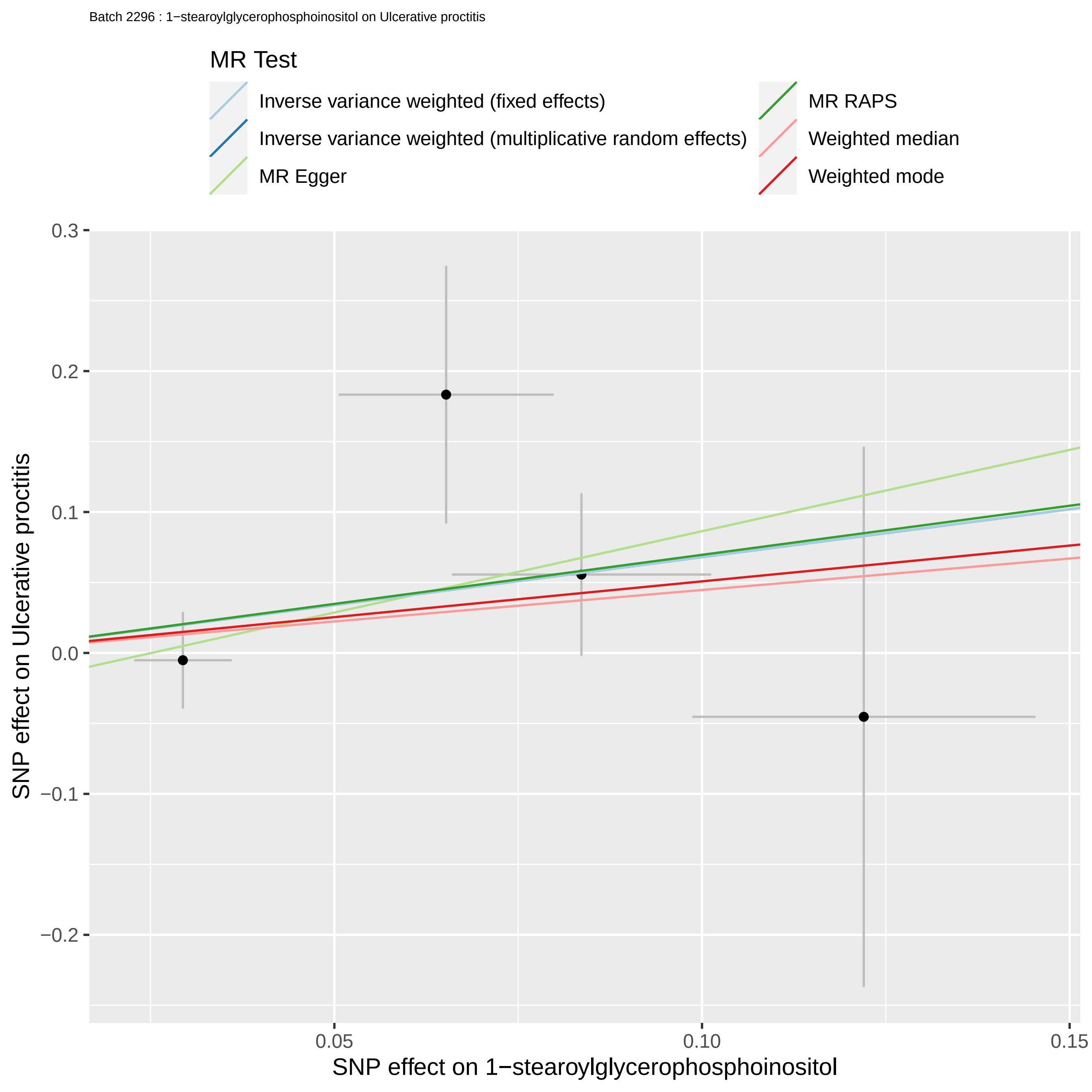

Batch 2296 : 1−stearoylglycerophosphoinositol on Ulcerative proctitis
MR Test
Inverse variance weighted (fixed effects)
Inverse variance weighted (multiplicative random effects)
MR Egger
MR RAPS
Weighted median
Weighted mode
0.3
0.2
0.1
0.0
−0.1
−0.2
0.05
0.10
0.15
SNP effect on Ulcerative proctitis
SNP effect on 1−stearoylglycerophosphoinositol

Batch 2297 : Myo−inositol on Ulcerative proctitis

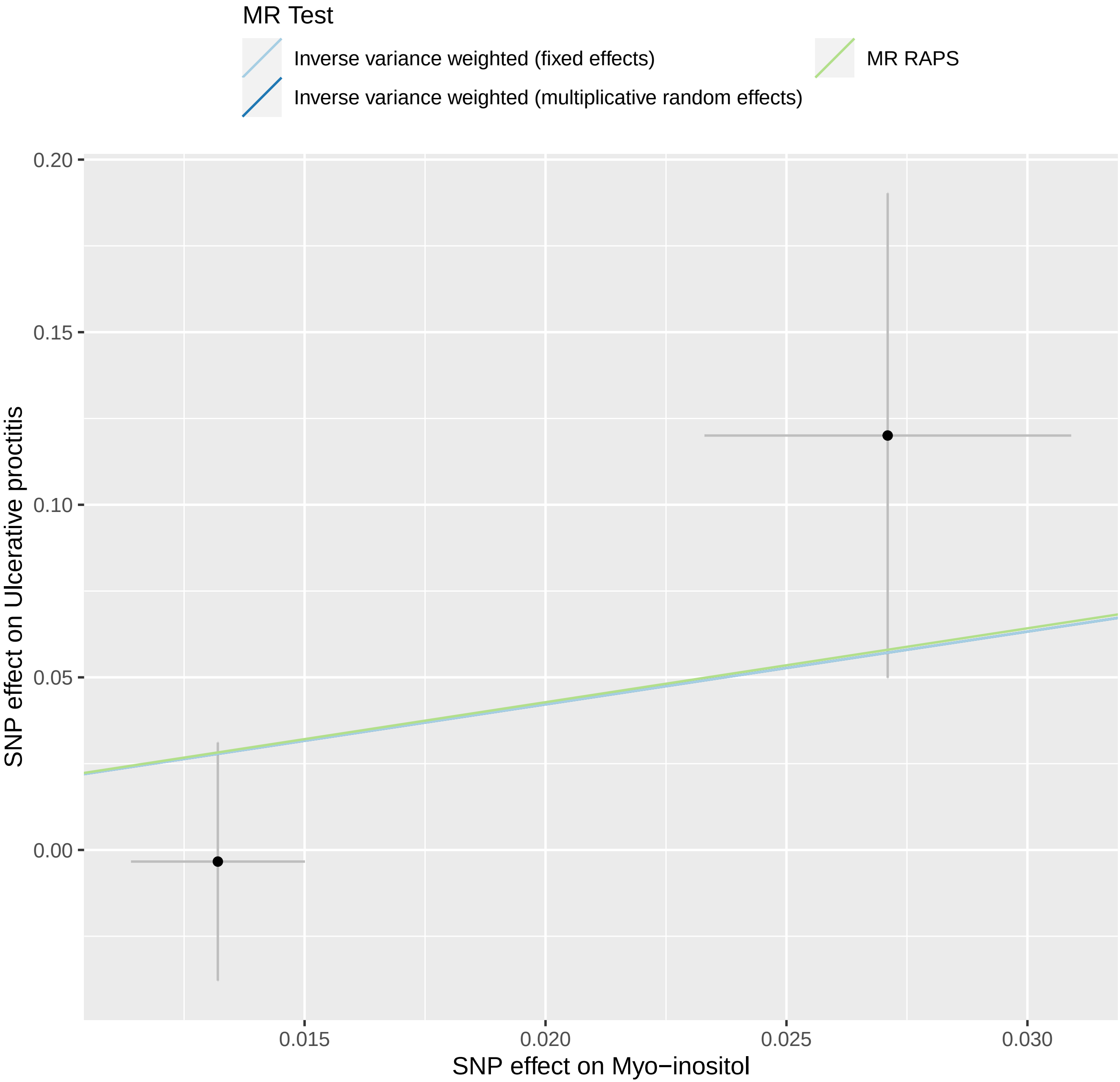

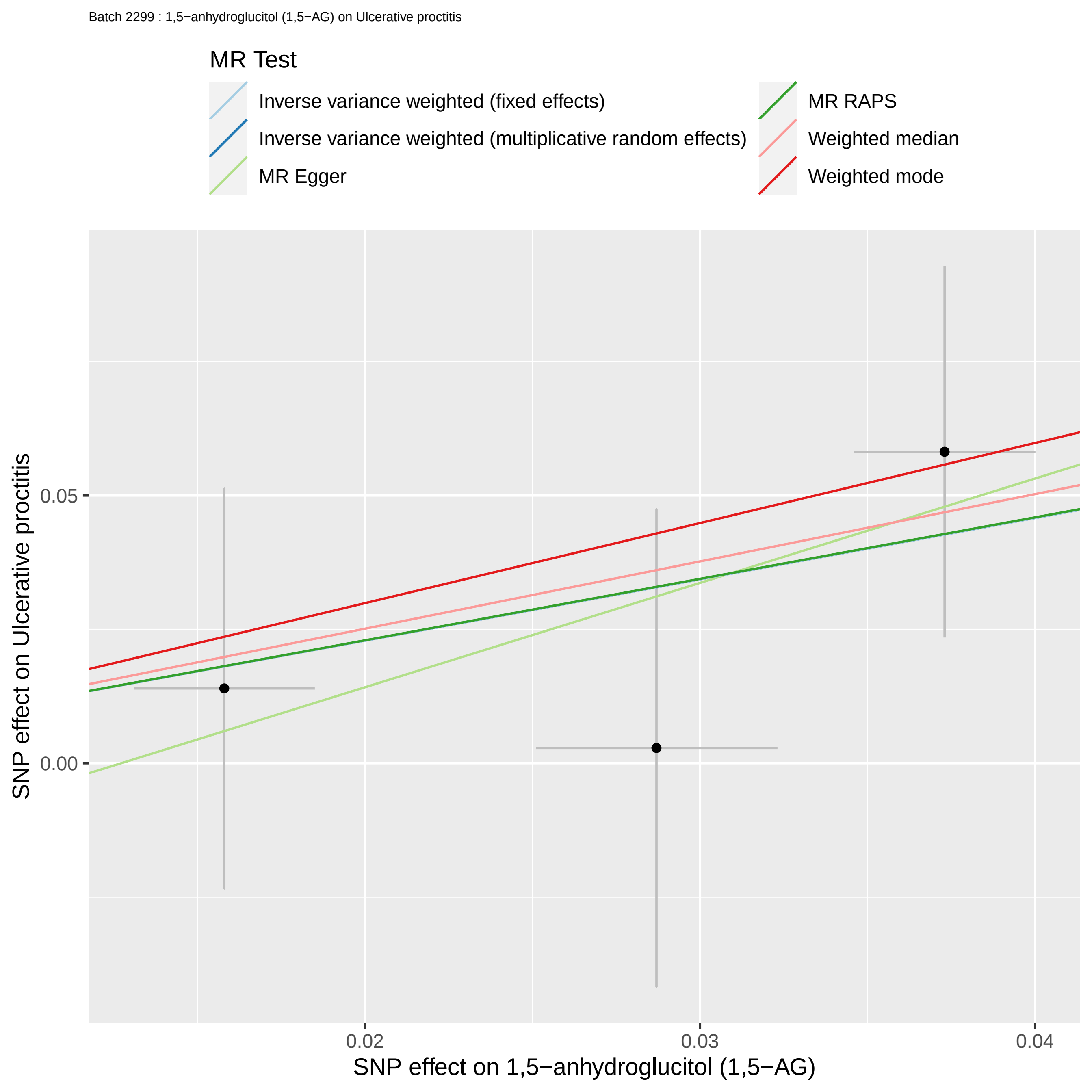

Batch 2299 : 1,5−anhydroglucitol (1,5−AG) on Ulcerative proctitis
MR Test
Inverse variance weighted (fixed effects)
Inverse variance weighted (multiplicative random effects)
MR Egger
MR RAPS
Weighted median
Weighted mode
0.05
0.00
0.02
0.03
0.04
SNP effect on Ulcerative proctitis
SNP effect on 1,5−anhydroglucitol (1,5−AG)

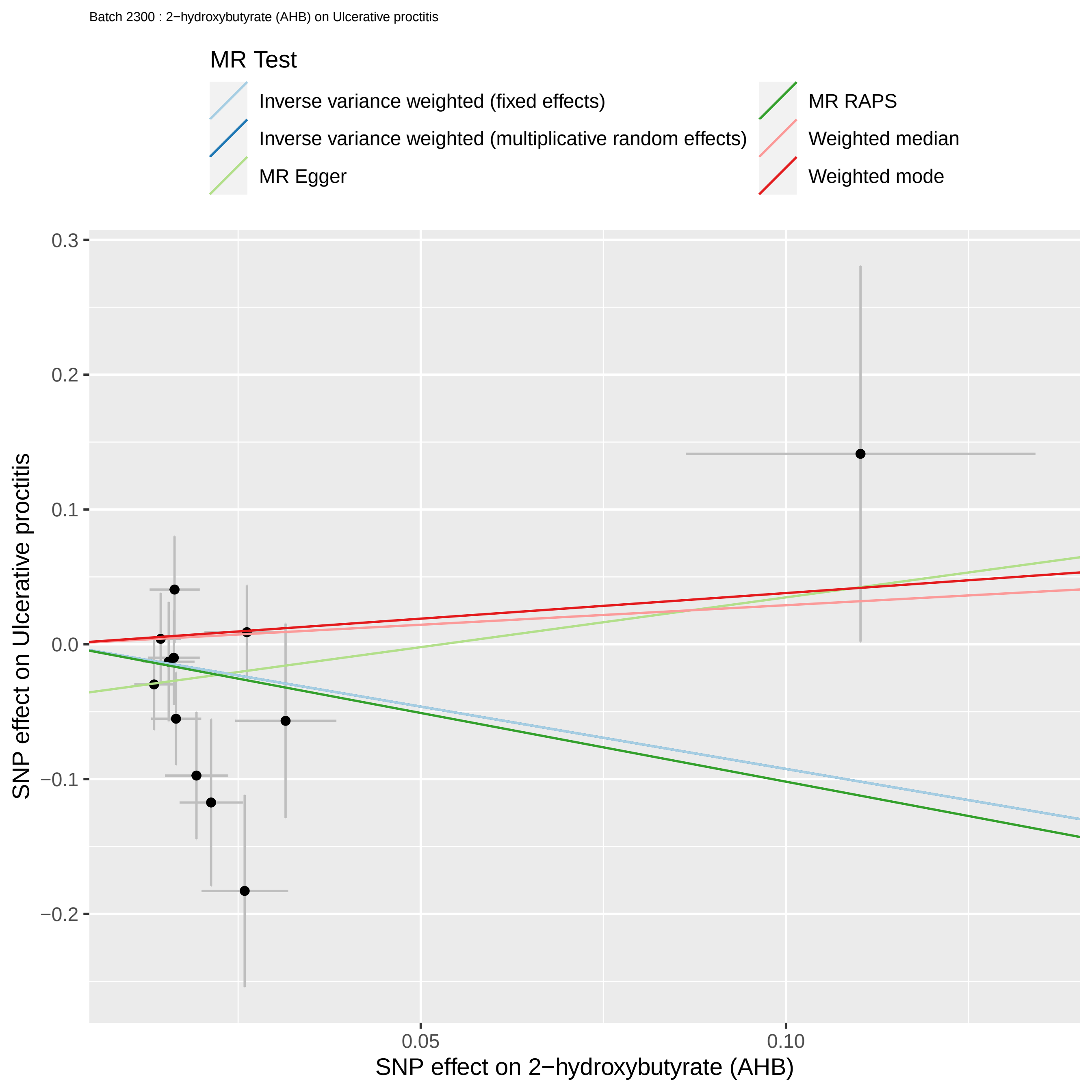

Batch 2300 : 2−hydroxybutyrate (AHB) on Ulcerative proctitis
MR Test
Inverse variance weighted (fixed effects)
Inverse variance weighted (multiplicative random effects)
MR Egger
MR RAPS
Weighted median
Weighted mode
0.3
0.2
0.1
0.0
−0.1
−0.2
0.05
0.10
SNP effect on Ulcerative proctitis
SNP effect on 2−hydroxybutyrate (AHB)

Batch 2303 : 1−palmitoylglycerol (1−monopalmitin) on Ulcerative proctitis

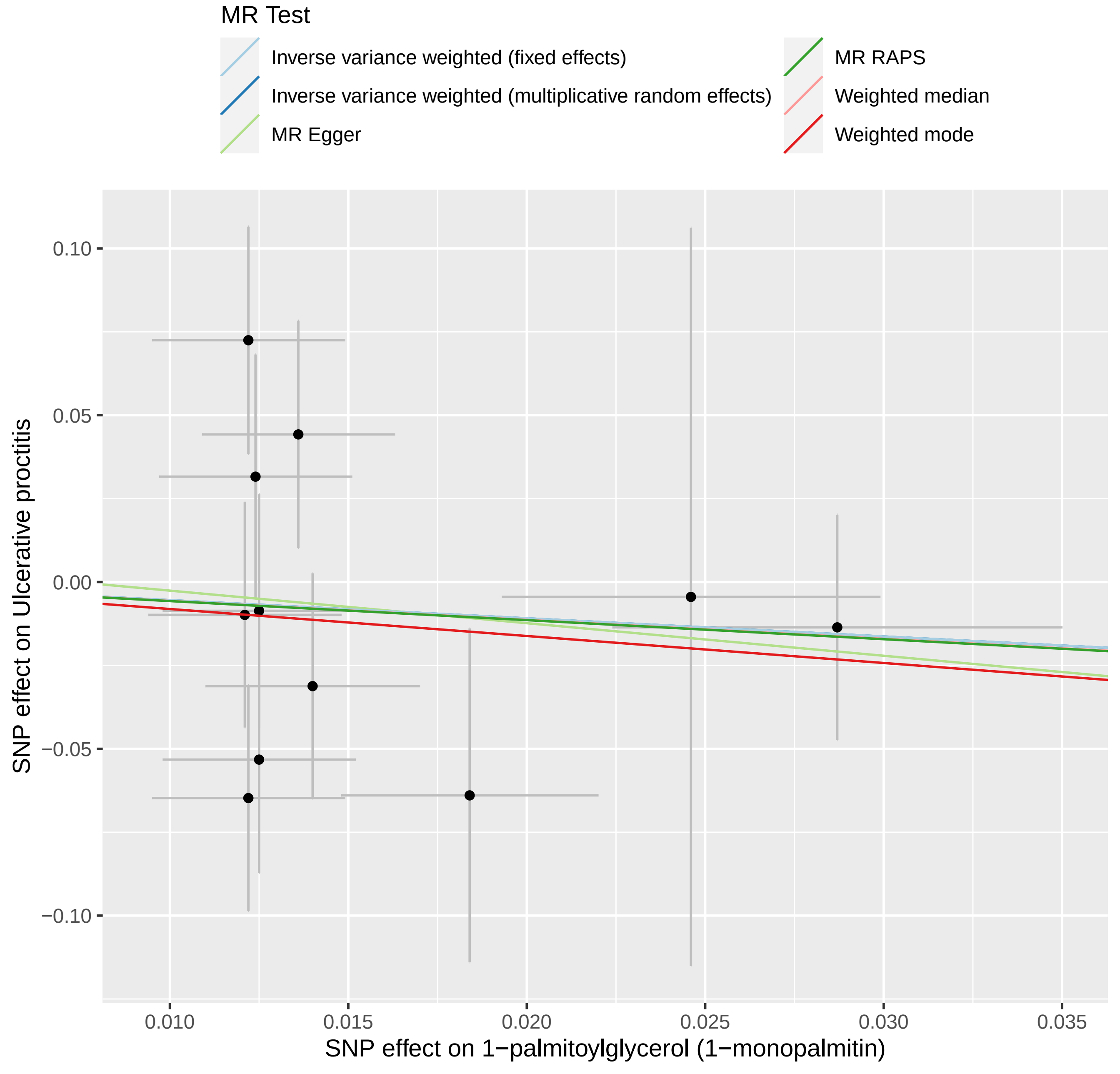

Batch 2306 : 2−hydroxyisobutyrate on Ulcerative proctitis

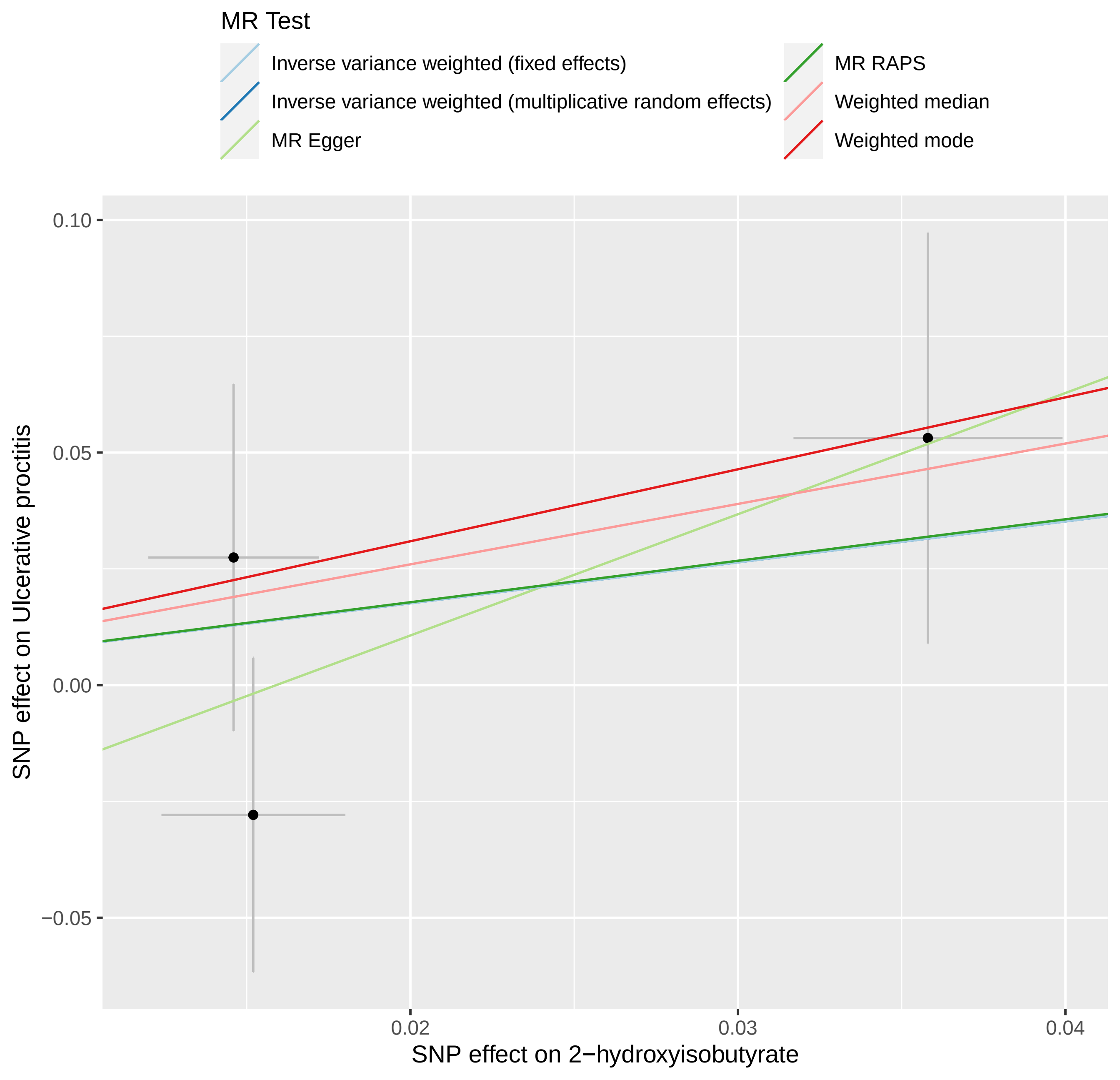

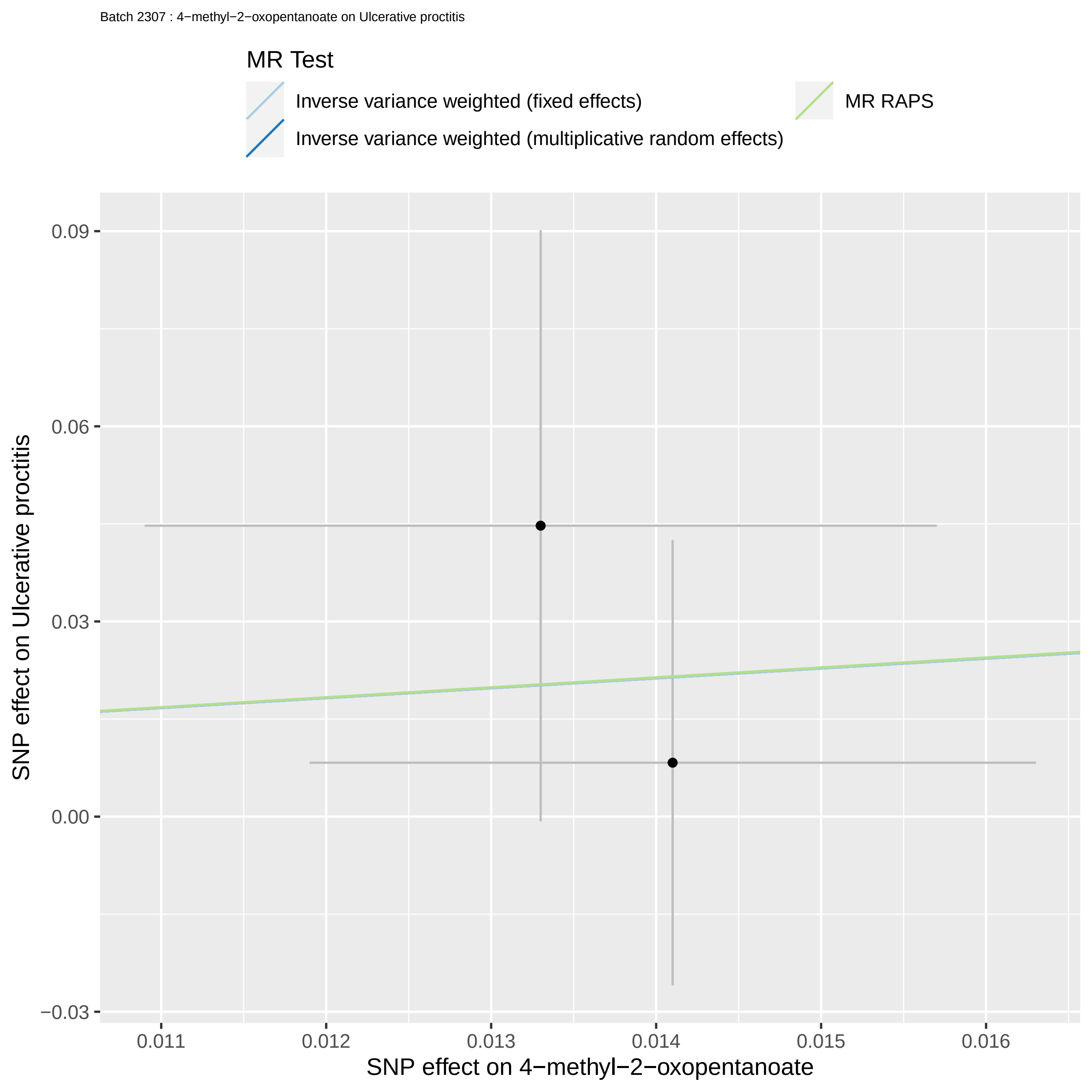
Batch 2307 : 4−methyl−2−oxopentanoate on Ulcerative proctitis
MR Test
Inverse variance weighted (fixed effects)
Inverse variance weighted (multiplicative random effects)
MR RAPS
0.09
0.06
0.03
0.00
−0.03
0.011
0.012
0.013
0.014
0.015
0.016
SNP effect on Ulcerative proctitis
SNP effect on 4−methyl−2−oxopentanoate

Batch 2311 : Levulinate (4−oxovalerate) on Ulcerative proctitis

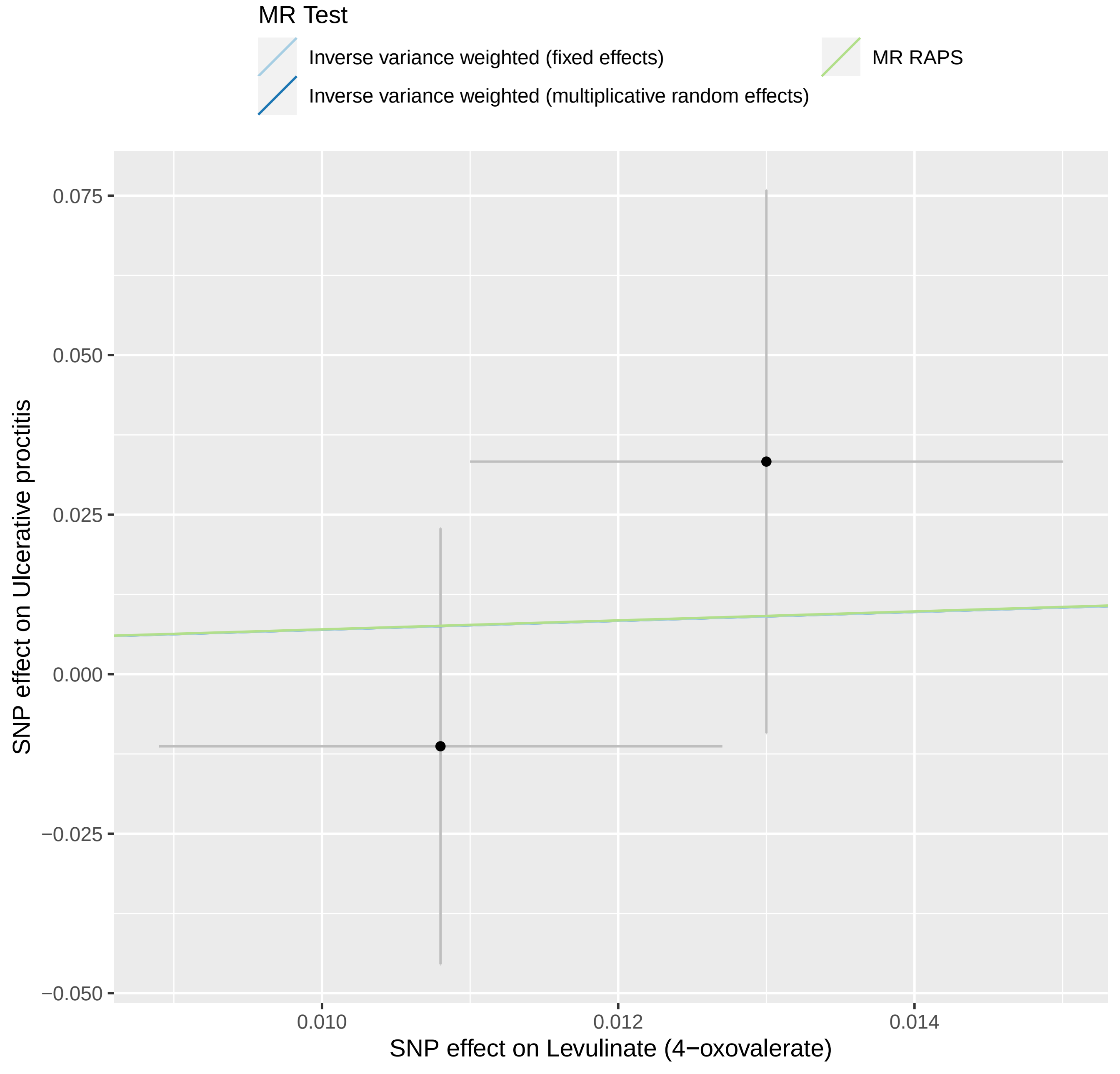

Batch 2313 : Cholate on Ulcerative proctitis

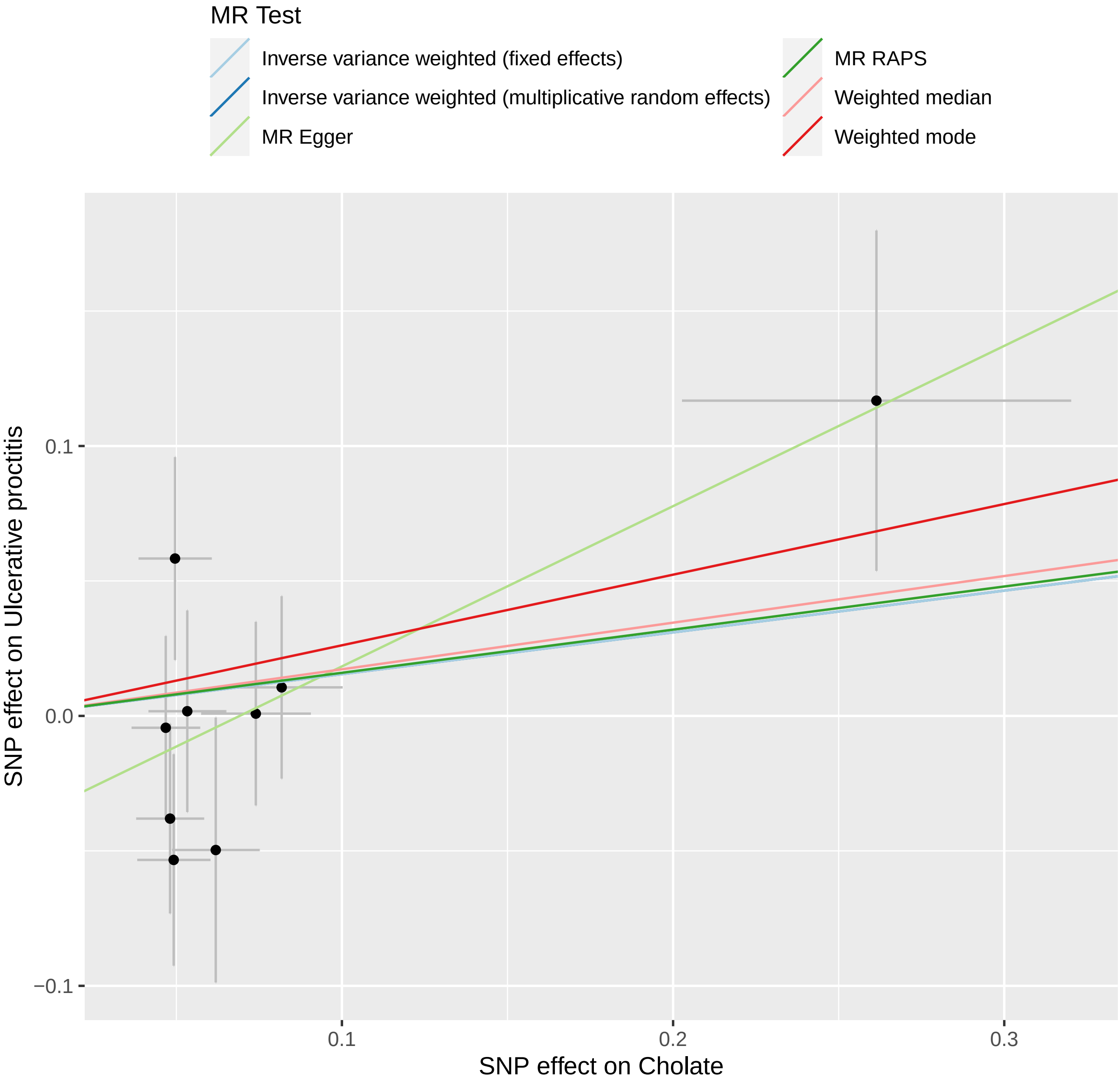

Batch 2316 : Hyodeoxycholate on Ulcerative proctitis

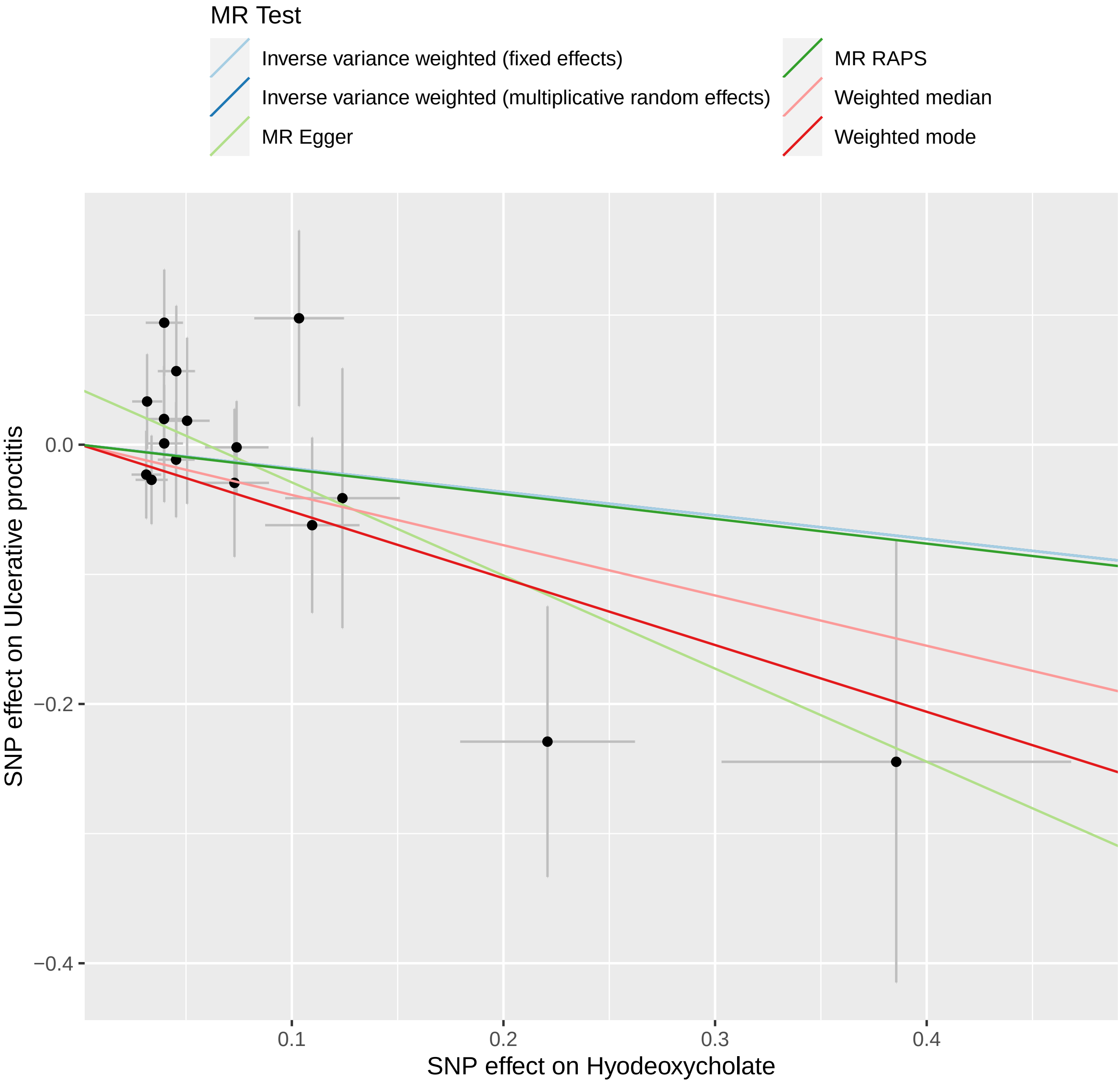

Batch 2317 : 3−indoxyl sulfate on Ulcerative proctitis

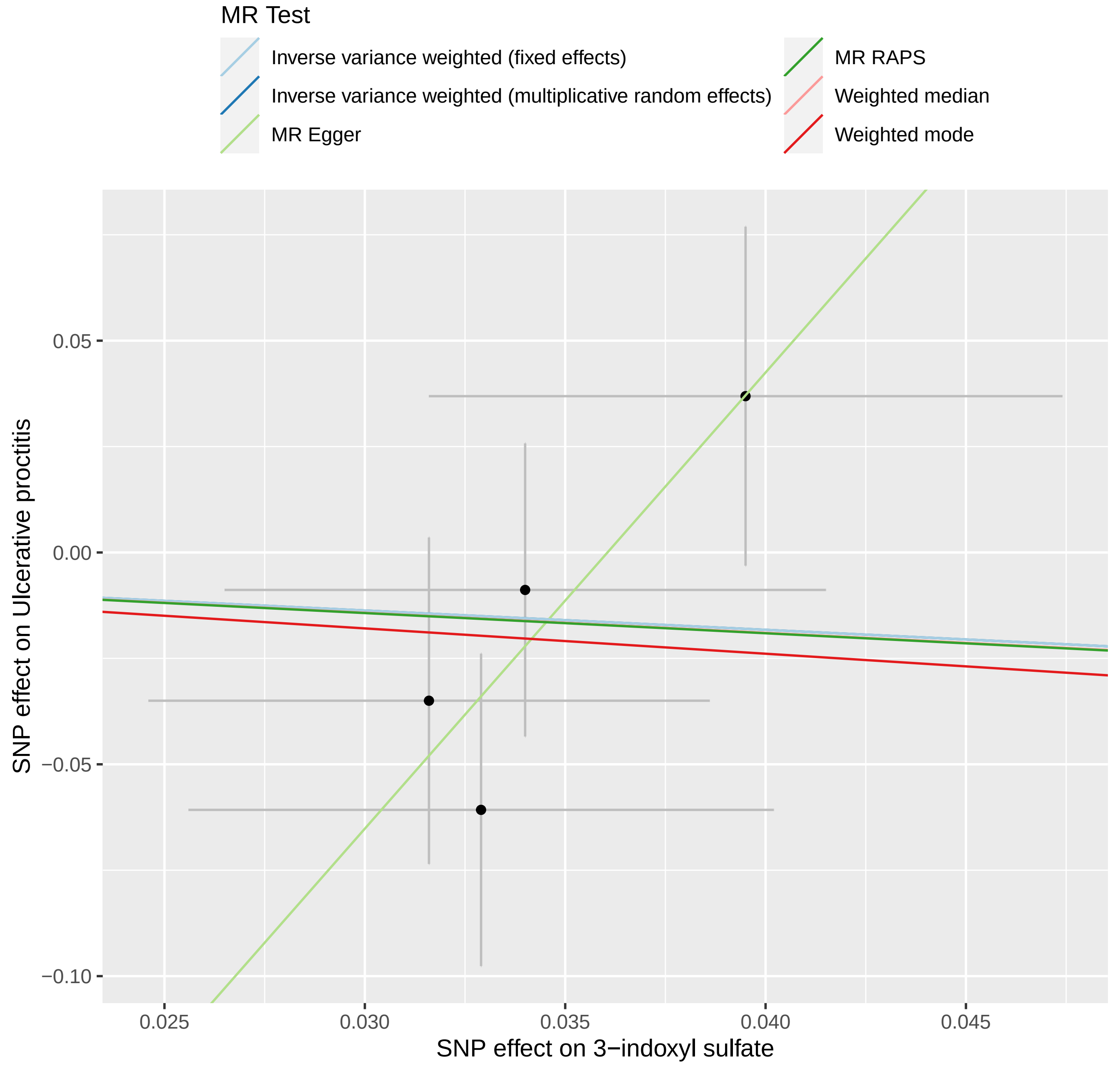

Batch 2321 : Erythrose on Ulcerative proctitis

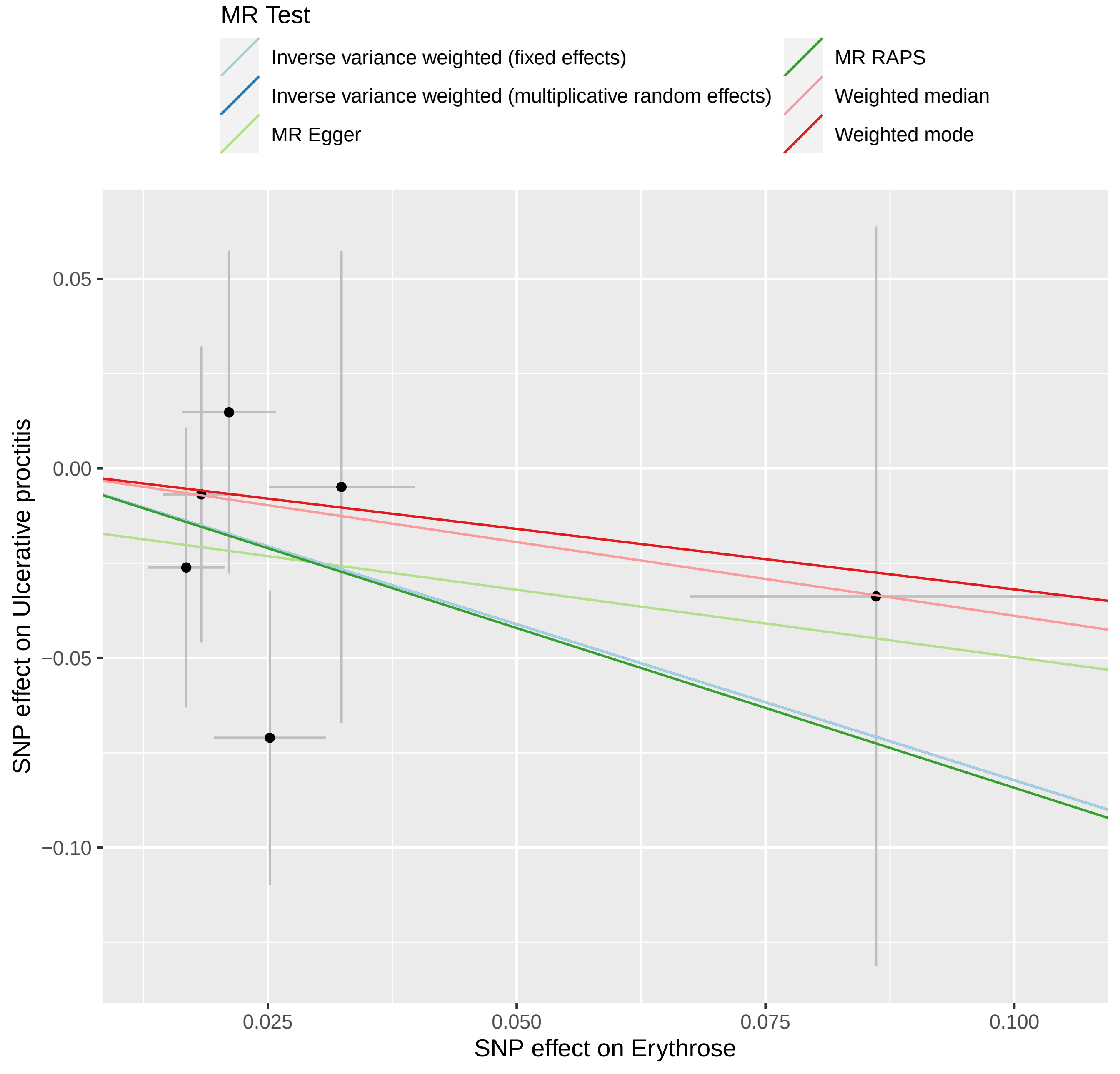

Batch 2322 : Threonate on Ulcerative proctitis

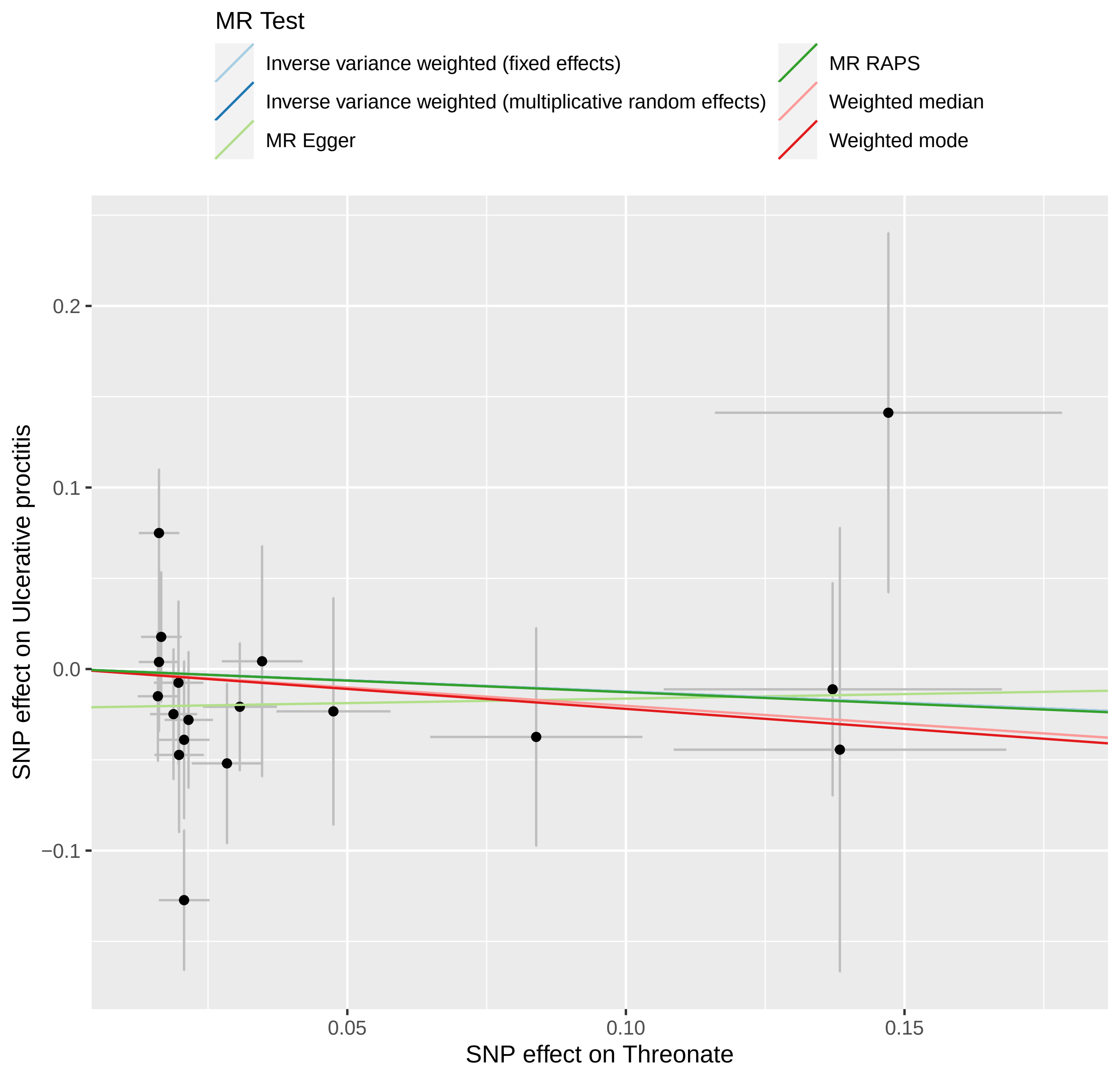

Batch 2323 : Cysteine on Ulcerative proctitis

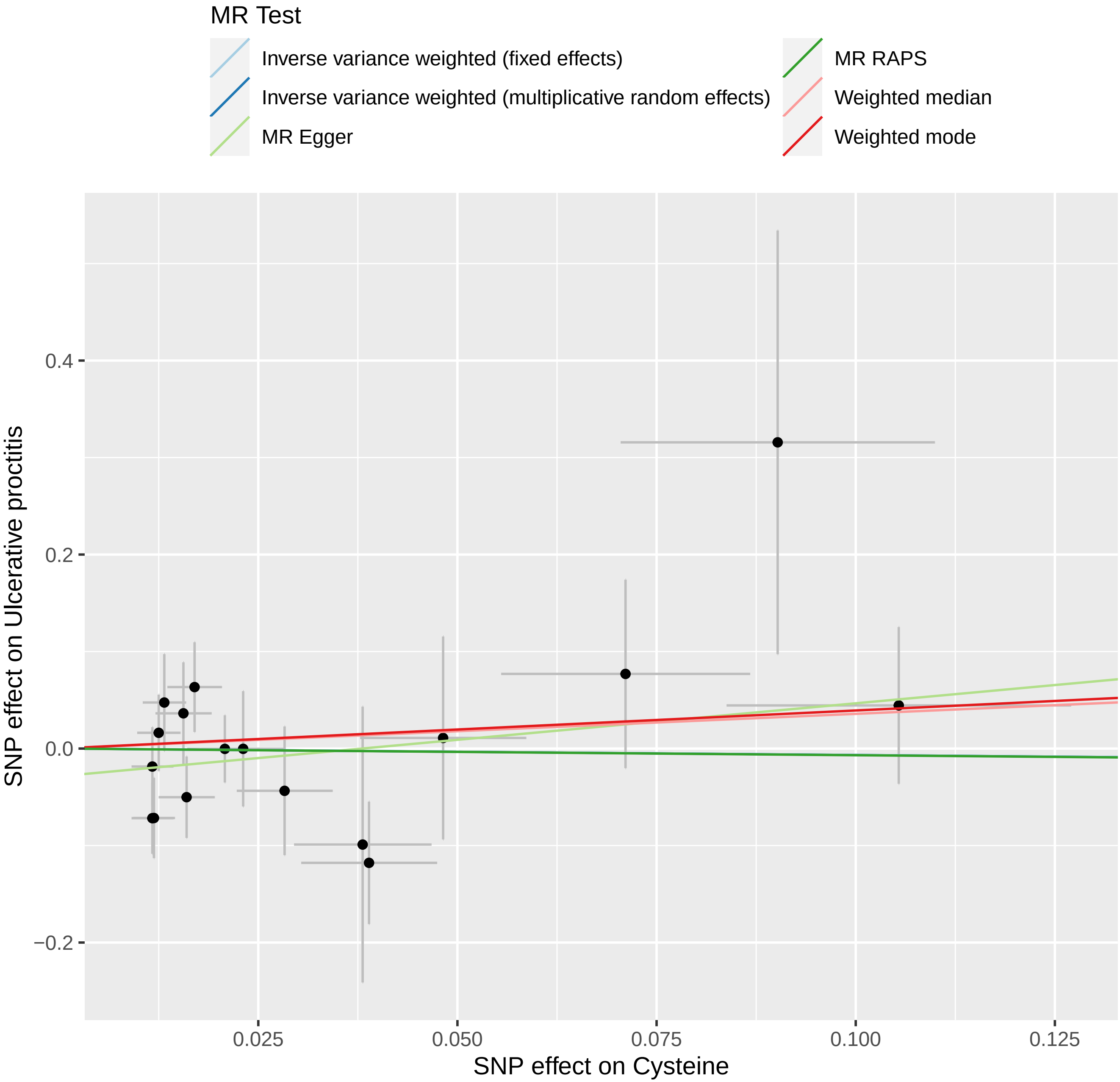

Batch 2324 : Pyroglutamylglycine on Ulcerative proctitis

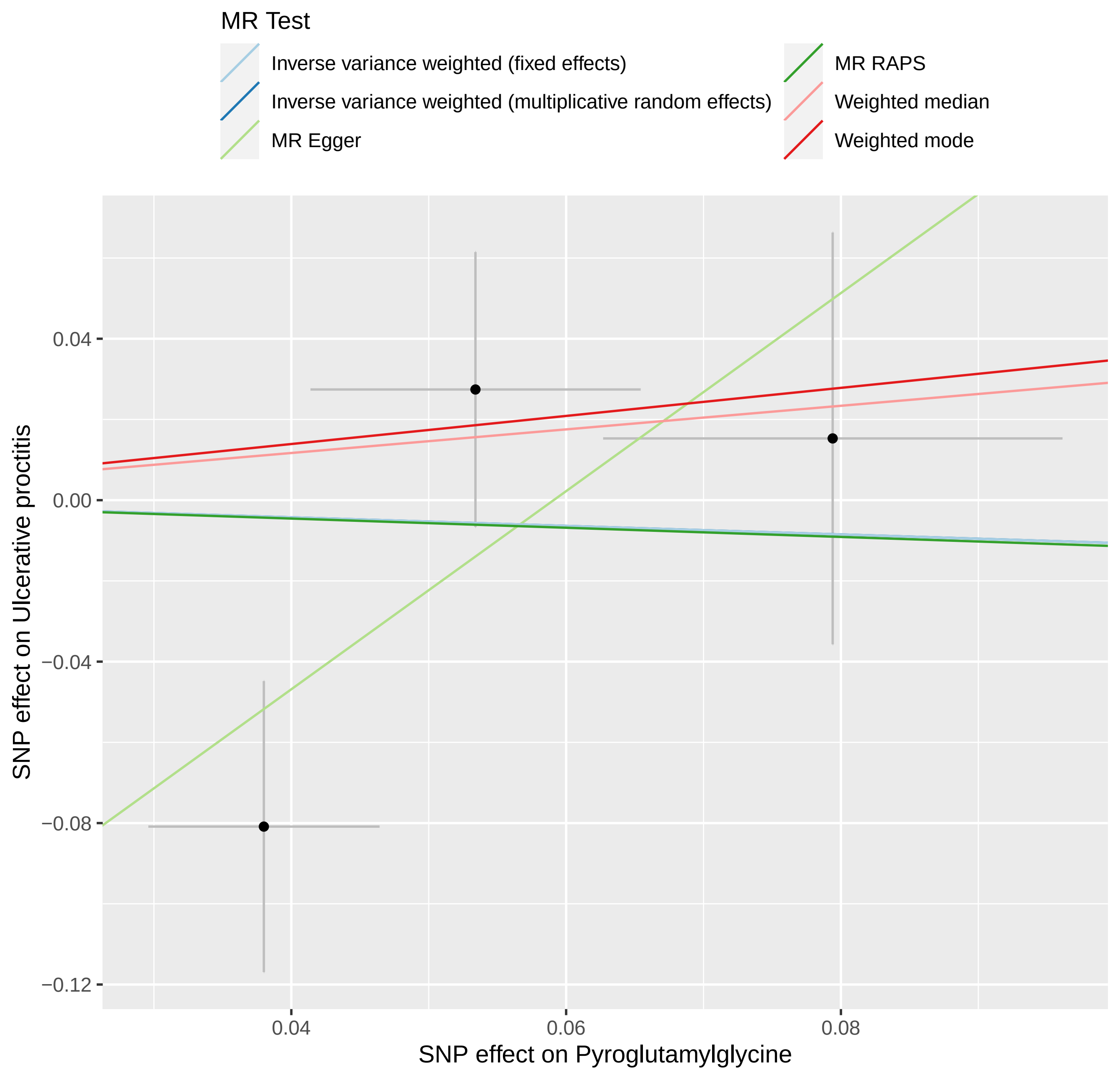

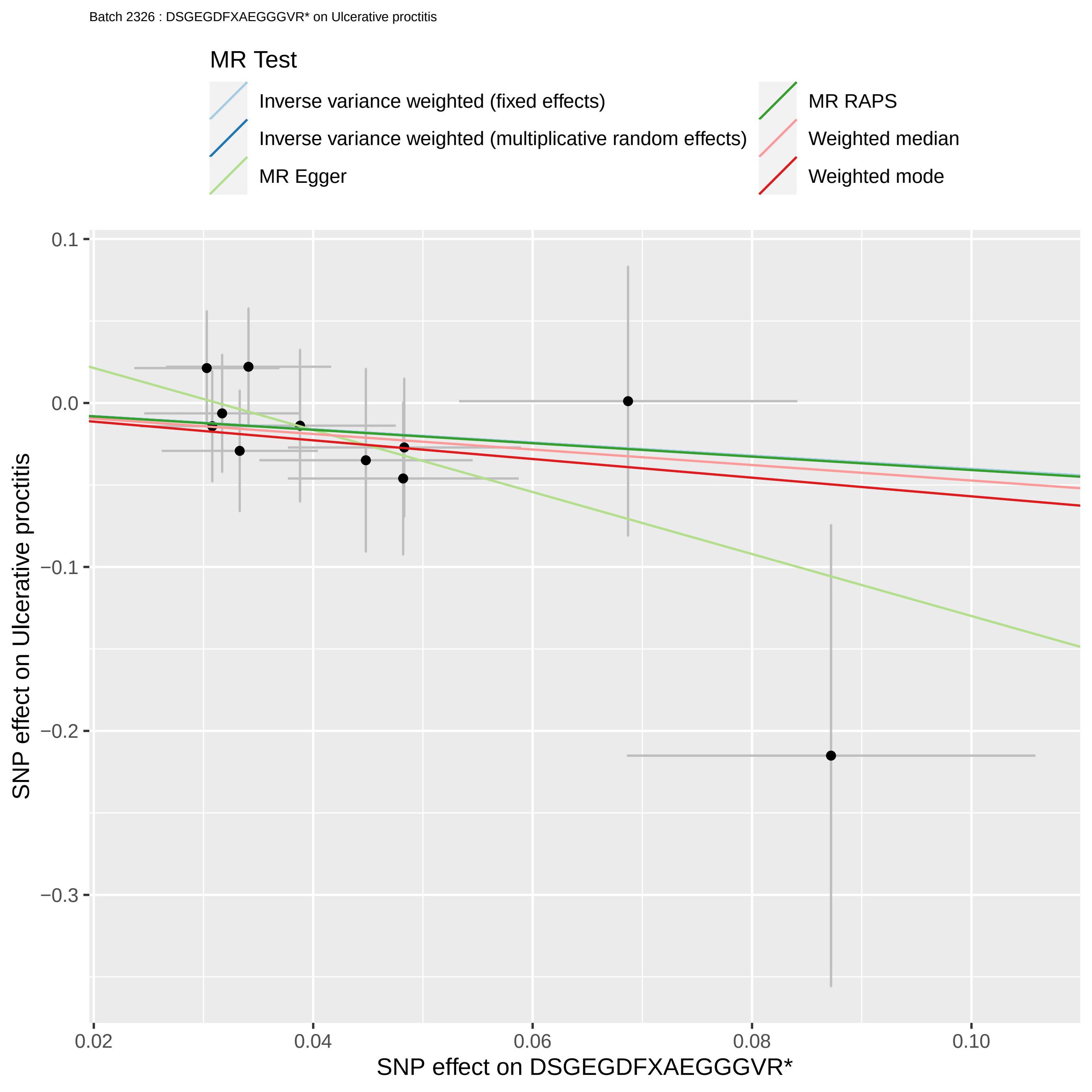
Batch 2326 : DSGEGDFXAEGGGVR* on Ulcerative proctitis
MR Test
Inverse variance weighted (fixed effects)
Inverse variance weighted (multiplicative random effects)
MR Egger
MR RAPS
Weighted median
Weighted mode
0.1
0.0
−0.1
−0.2
−0.3
0.02
0.04
0.06
0.08
0.10
SNP effect on DSGEGDFXAEGGGVR*
SNP effect on Ulcerative proctitis

Batch 2327 : Pyridoxate on Ulcerative proctitis

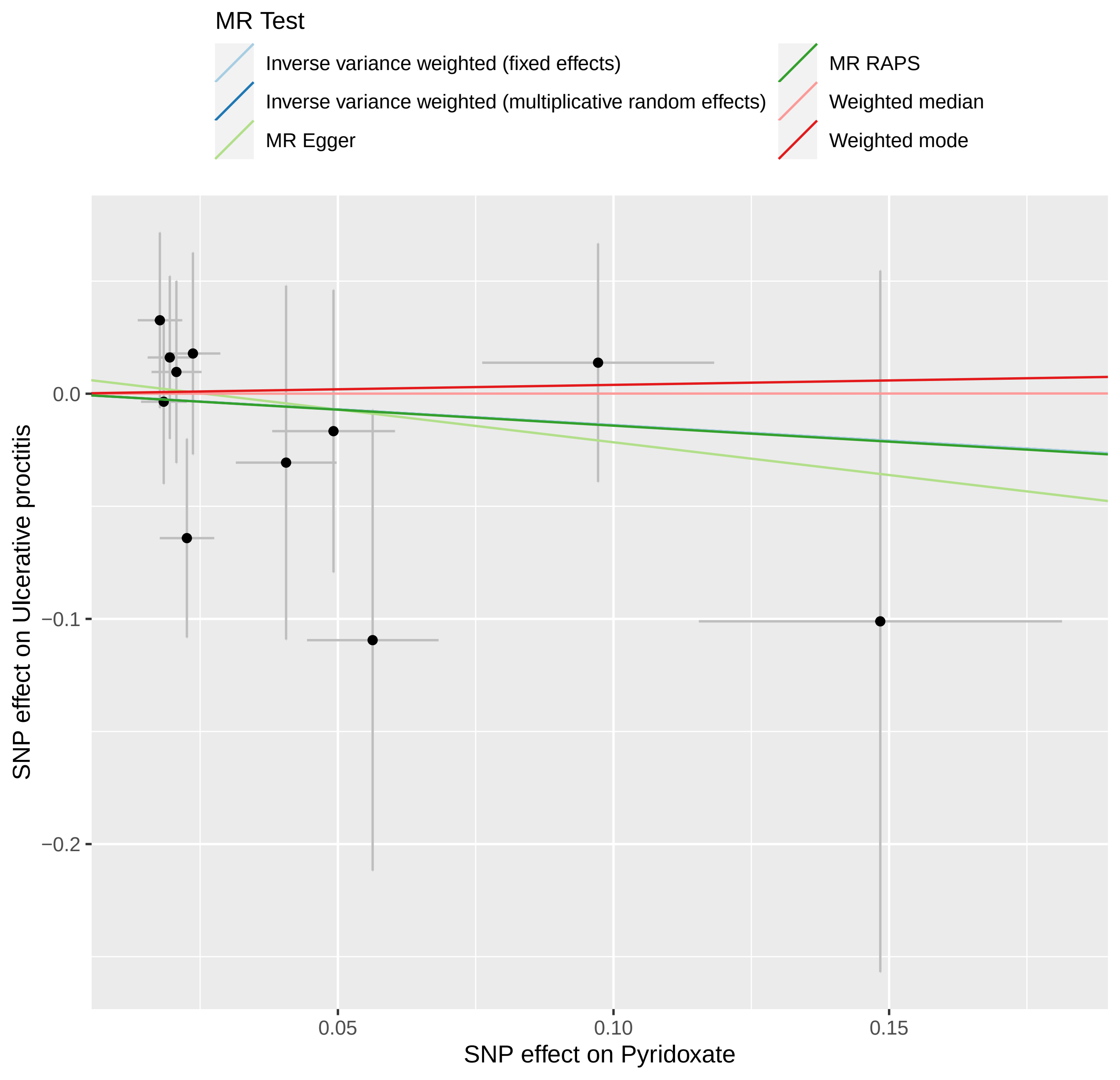

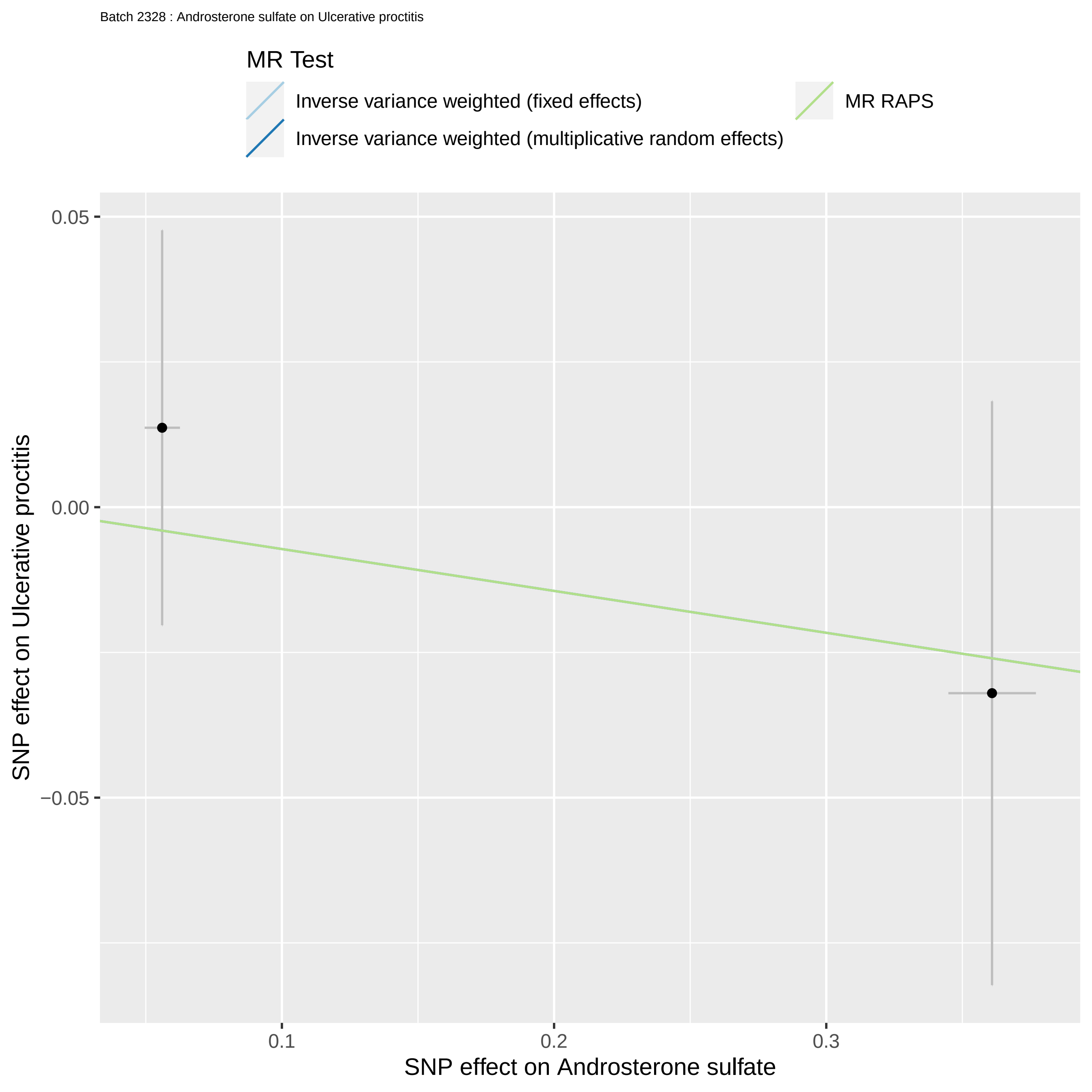

Batch 2328 : Androsterone sulfate on Ulcerative proctitis
MR Test
Inverse variance weighted (fixed effects)
Inverse variance weighted (multiplicative random effects)
MR RAPS
0.05
0.00
−0.05
0.1
0.2
0.3
SNP effect on Ulcerative proctitis
SNP effect on Androsterone sulfate

Batch 2331 : Acetylcarnitine on Ulcerative proctitis

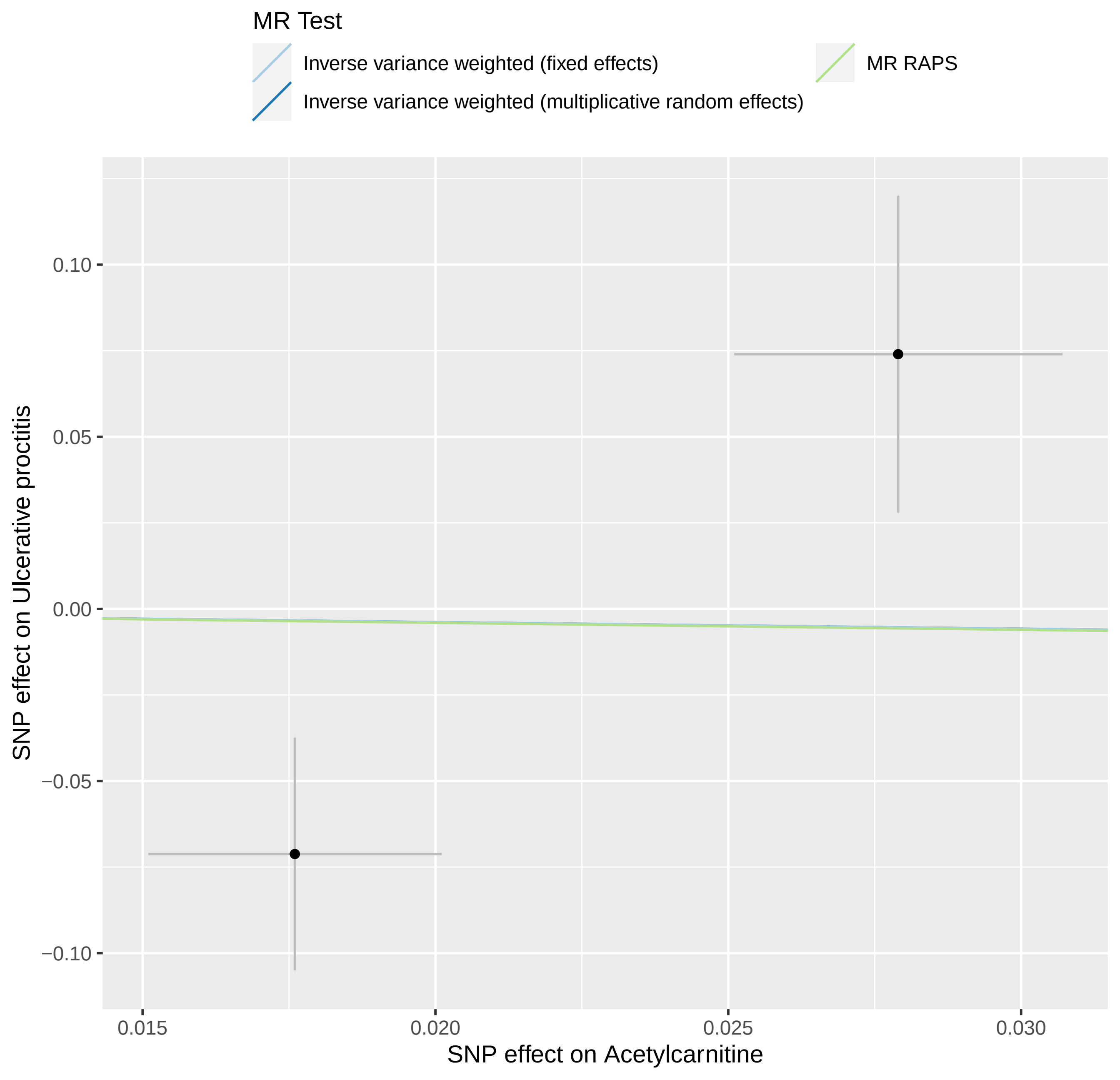

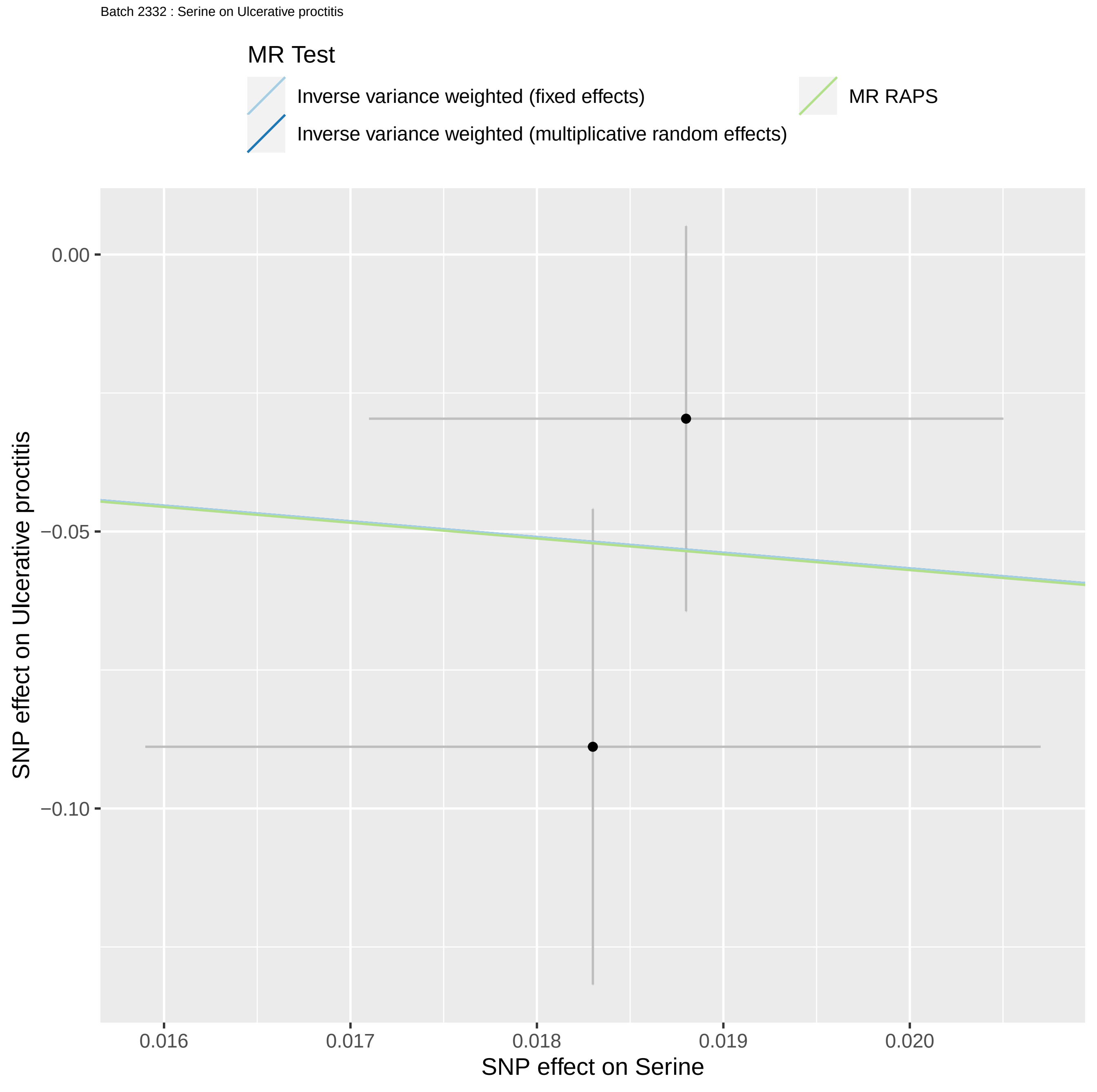
Batch 2332 : Serine on Ulcerative proctitis
MR Test
Inverse variance weighted (fixed effects)
Inverse variance weighted (multiplicative random effects)
MR RAPS
0.00
−0.05
−0.10
SNP effect on Ulcerative proctitis
0.016
0.017
0.018
0.019
0.020
SNP effect on Serine

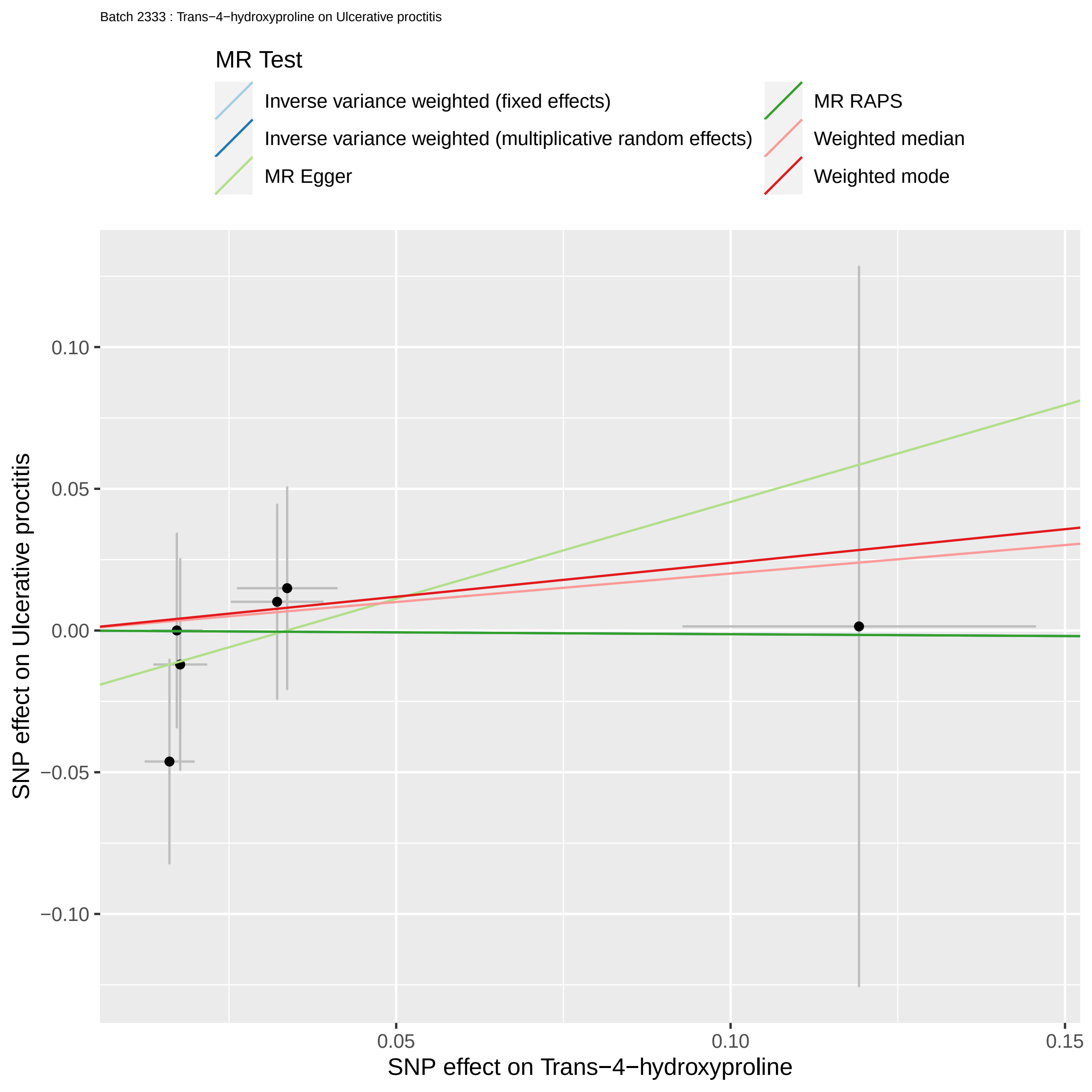

Batch 2333 : Trans−4−hydroxyproline on Ulcerative proctitis
MR Test
Inverse variance weighted (fixed effects)
Inverse variance weighted (multiplicative random effects)
MR Egger
MR RAPS
Weighted median
Weighted mode
0.10
0.05
0.00
−0.05
−0.10
0.05
0.10
0.15
SNP effect on Ulcerative proctitis
SNP effect on Trans−4−hydroxyproline

Batch 2334 : Glutamate on Ulcerative proctitis

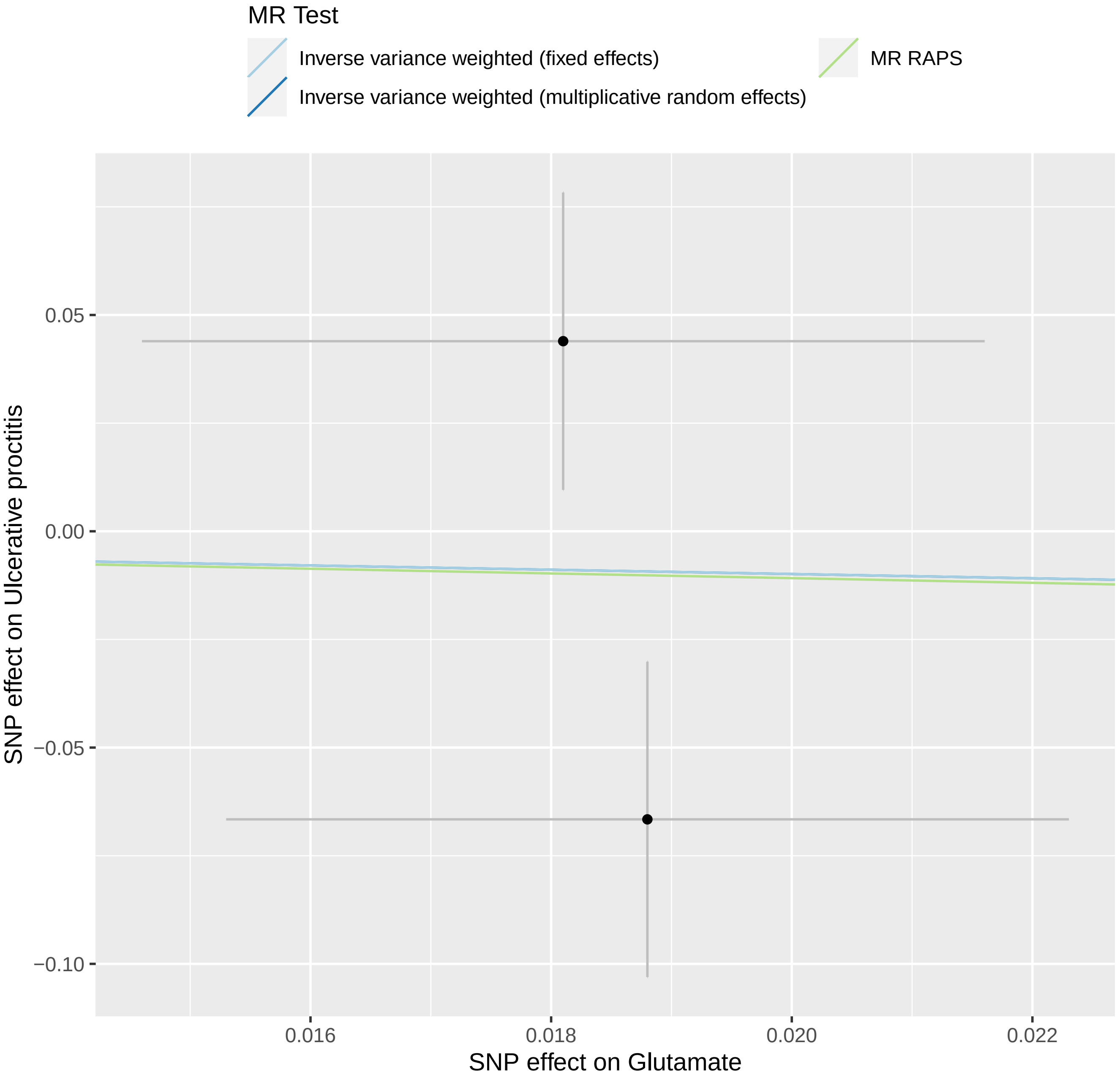

Batch 2335 : Hexanoylcarnitine on Ulcerative proctitis

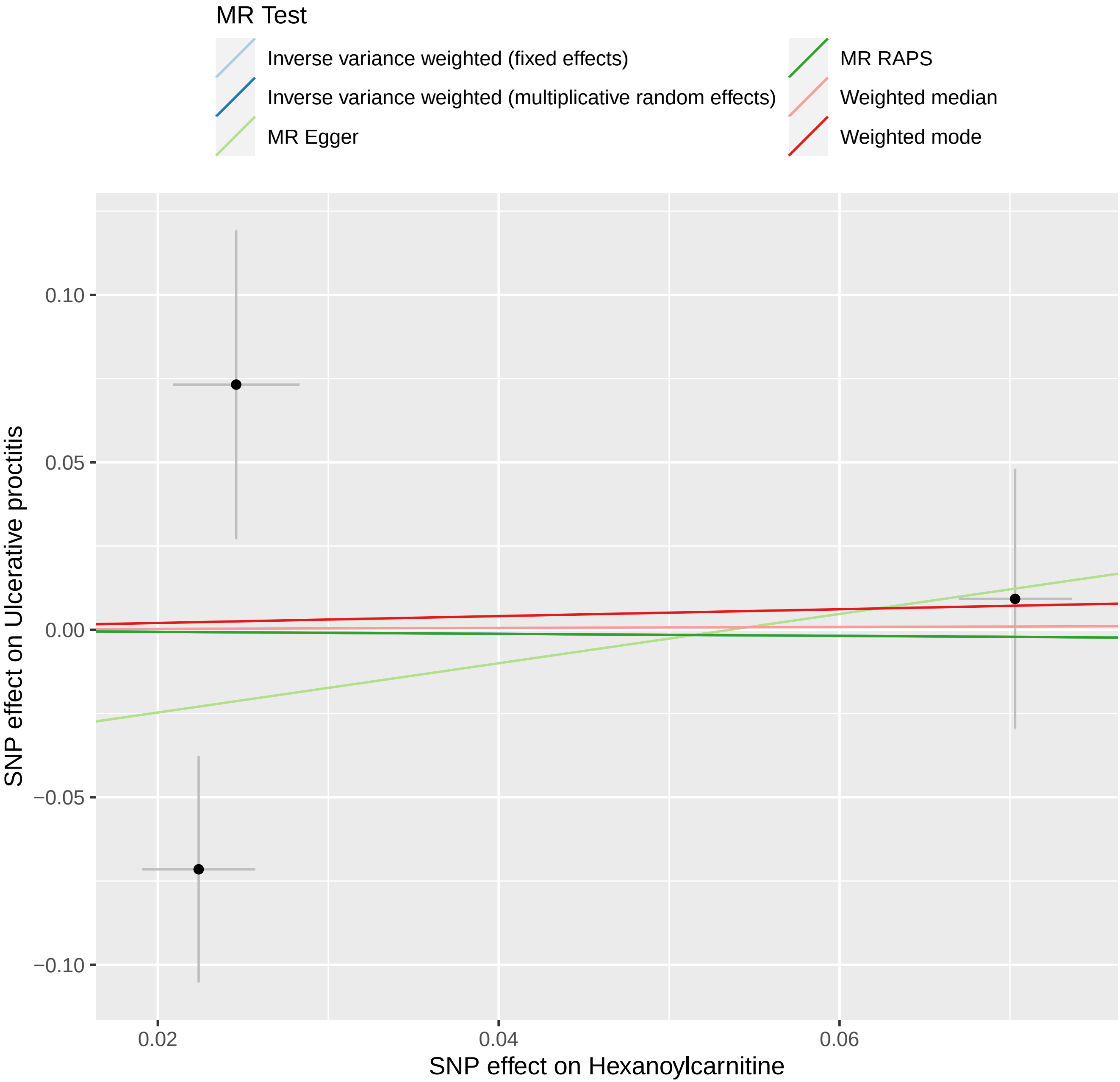

Batch 2339 : 2−aminobutyrate on Ulcerative proctitis

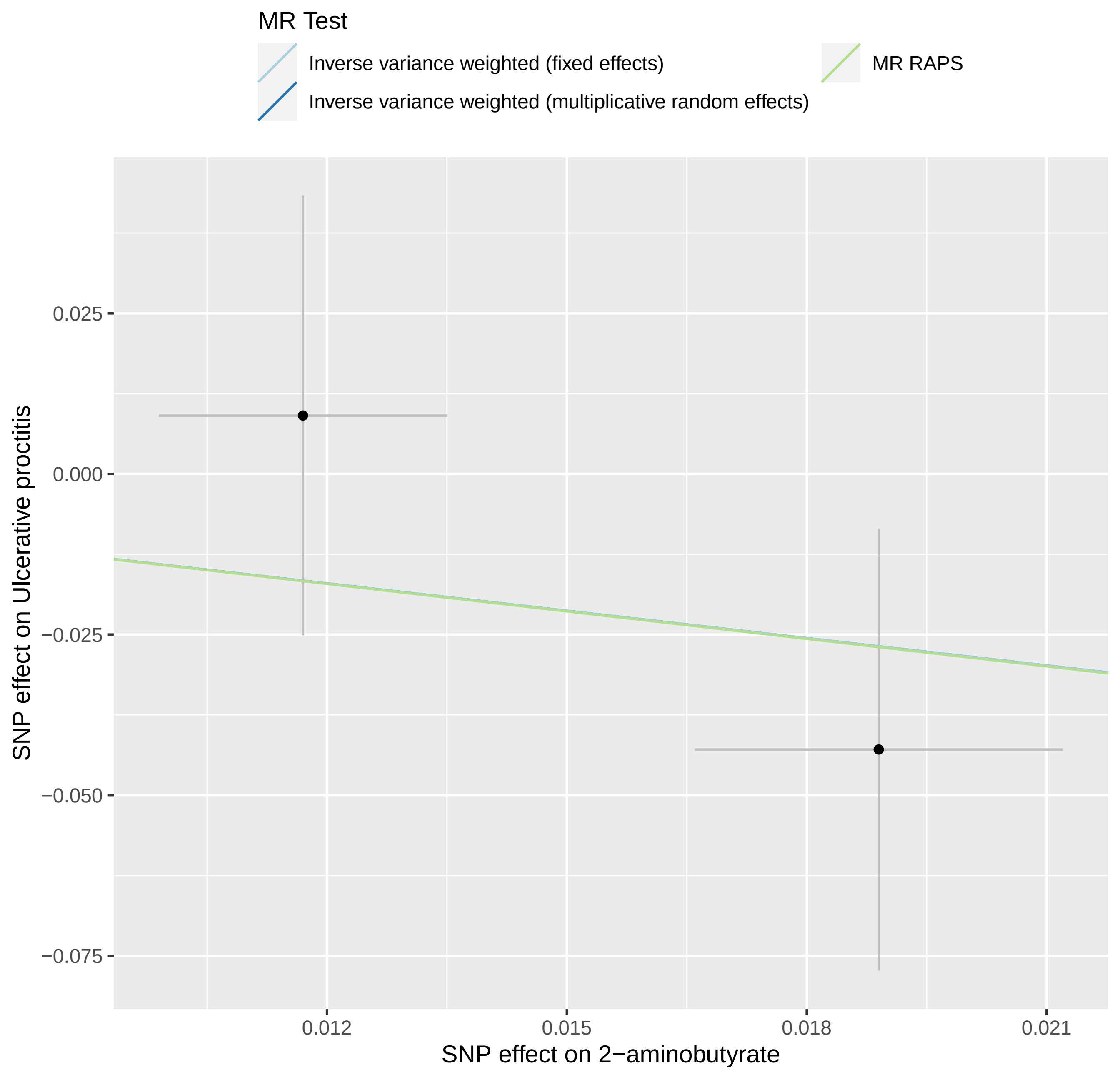

Batch 2344 : Butyrylcarnitine on Ulcerative proctitis

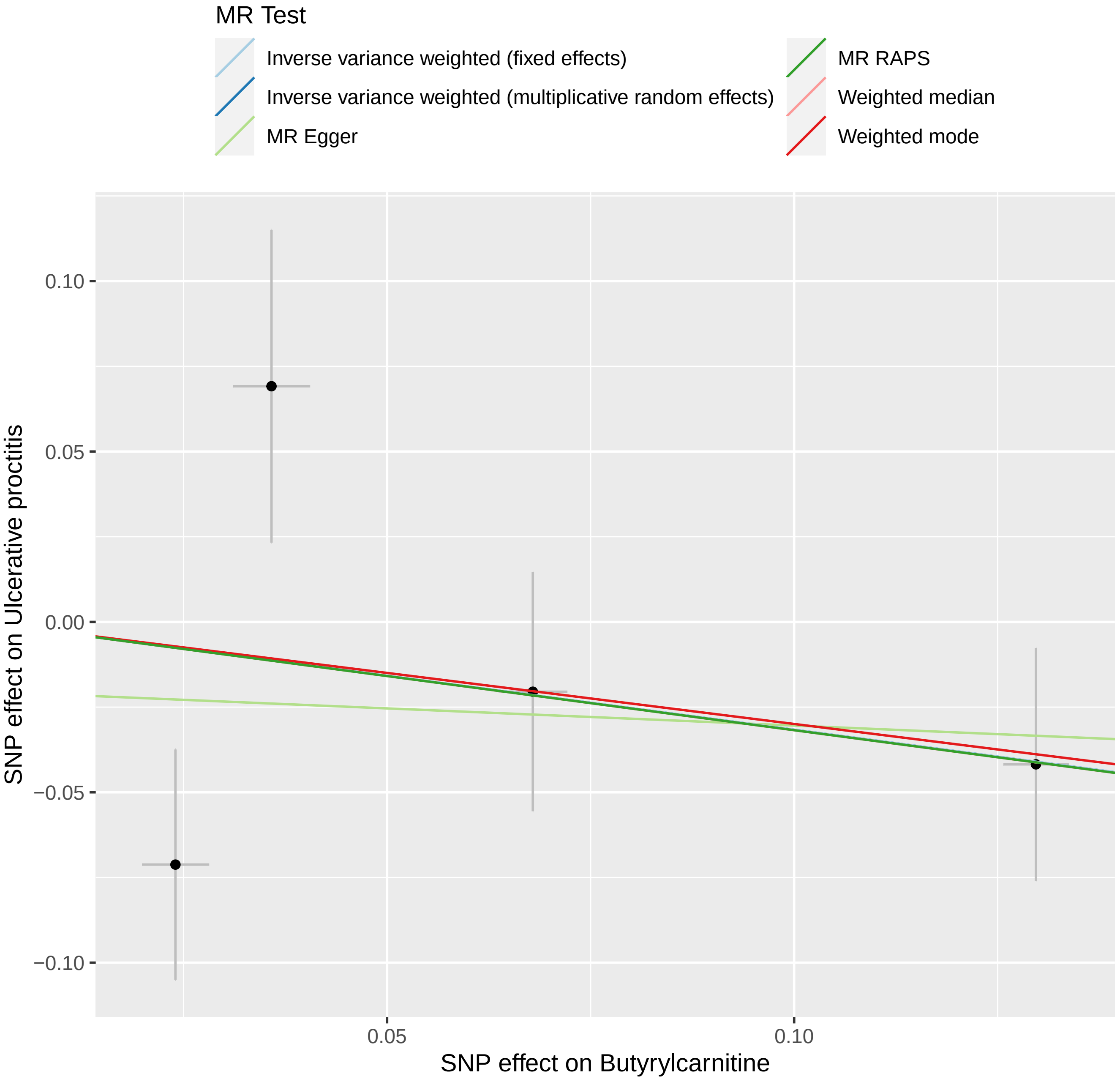

Batch 2347 : Propionylcarnitine on Ulcerative proctitis

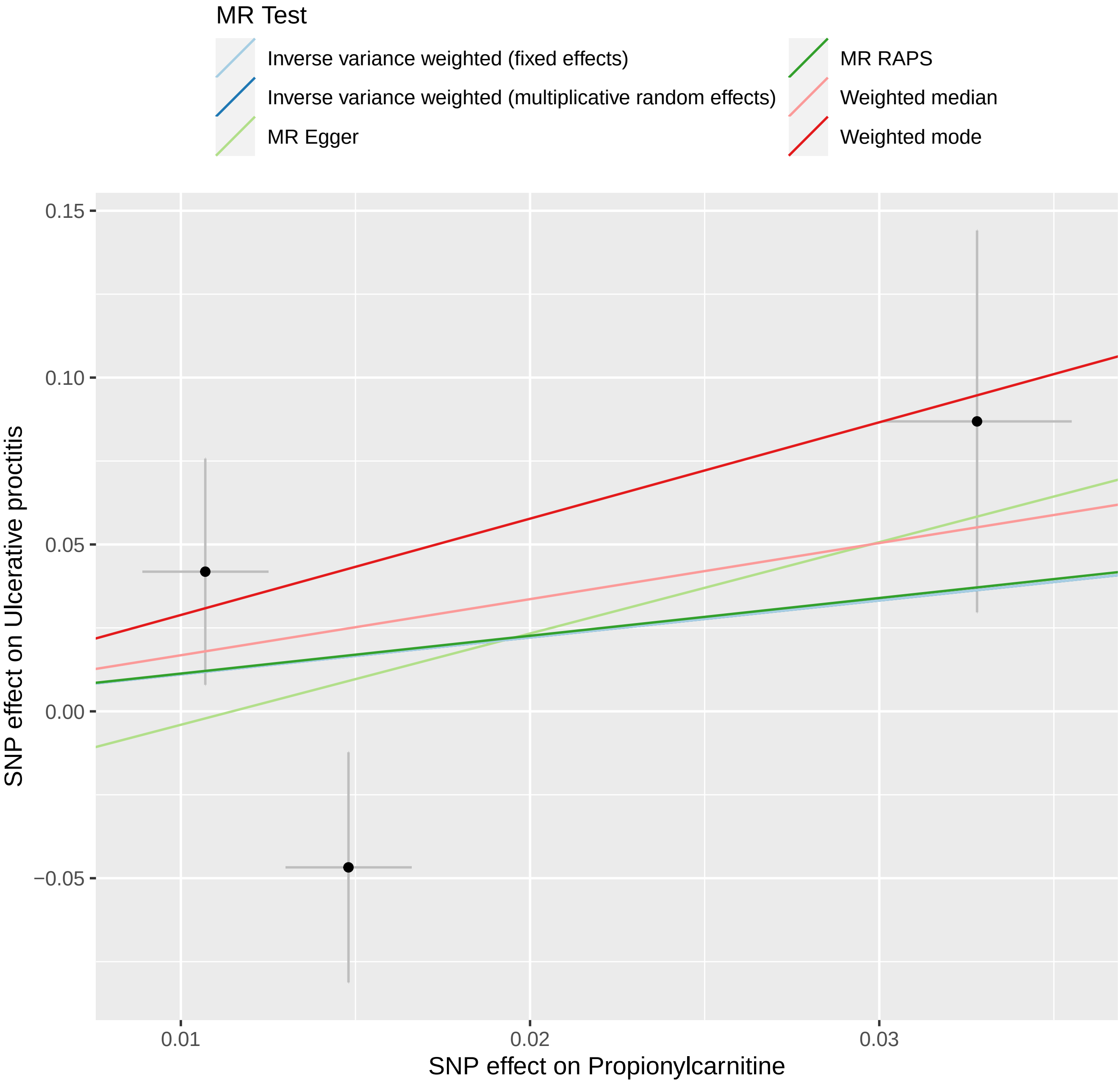

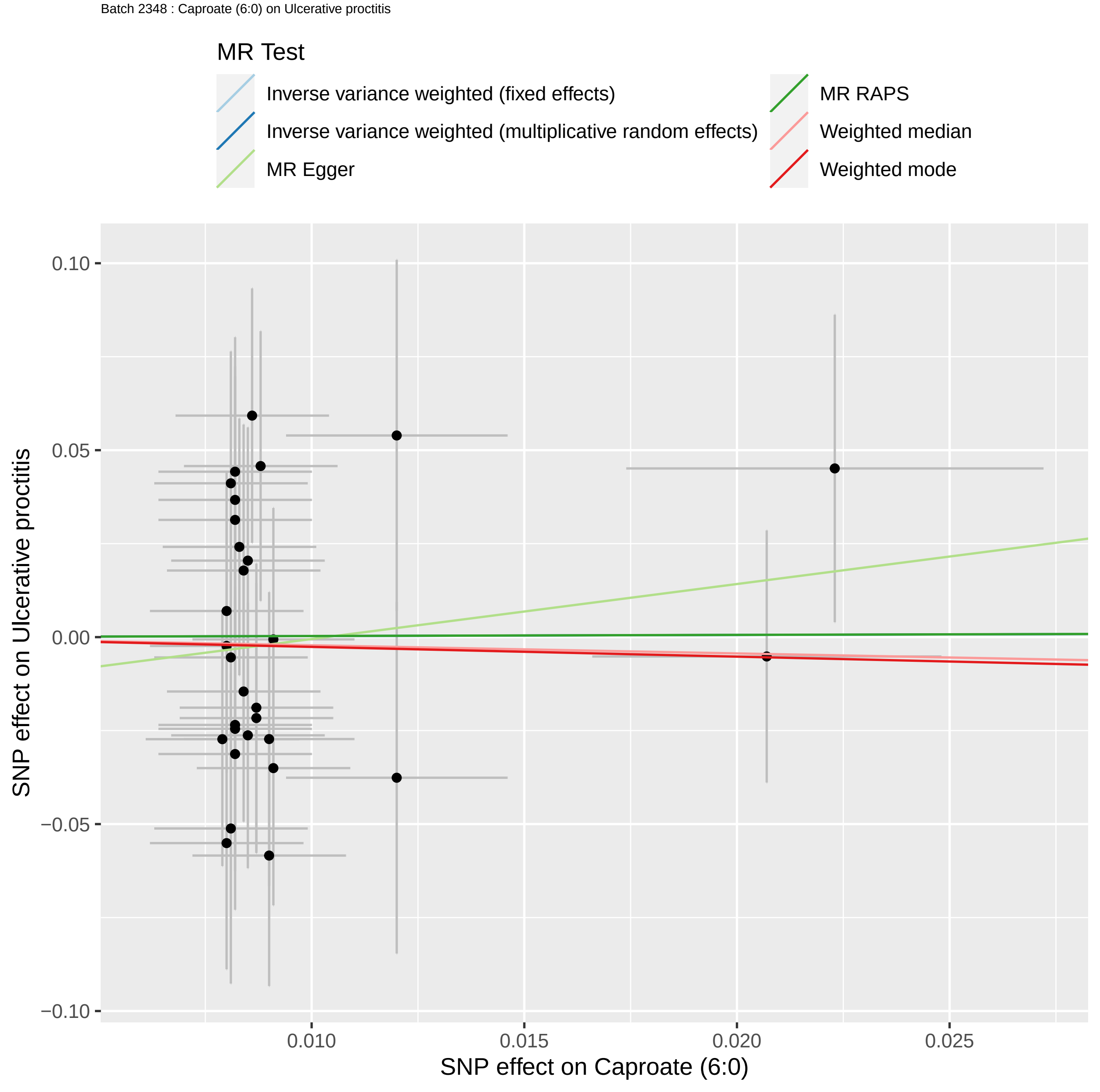
Batch 2348 : Caproate (6:0) on Ulcerative proctitis
MR Test
Inverse variance weighted (fixed effects)
Inverse variance weighted (multiplicative random effects)
MR Egger
MR RAPS
Weighted median
Weighted mode
0.10
0.05
0.00
−0.05
−0.10
0.010
0.015
0.020
0.025
SNP effect on Ulcerative proctitis
SNP effect on Caproate (6:0)

Batch 2349 : Caprylate (8:0) on Ulcerative proctitis

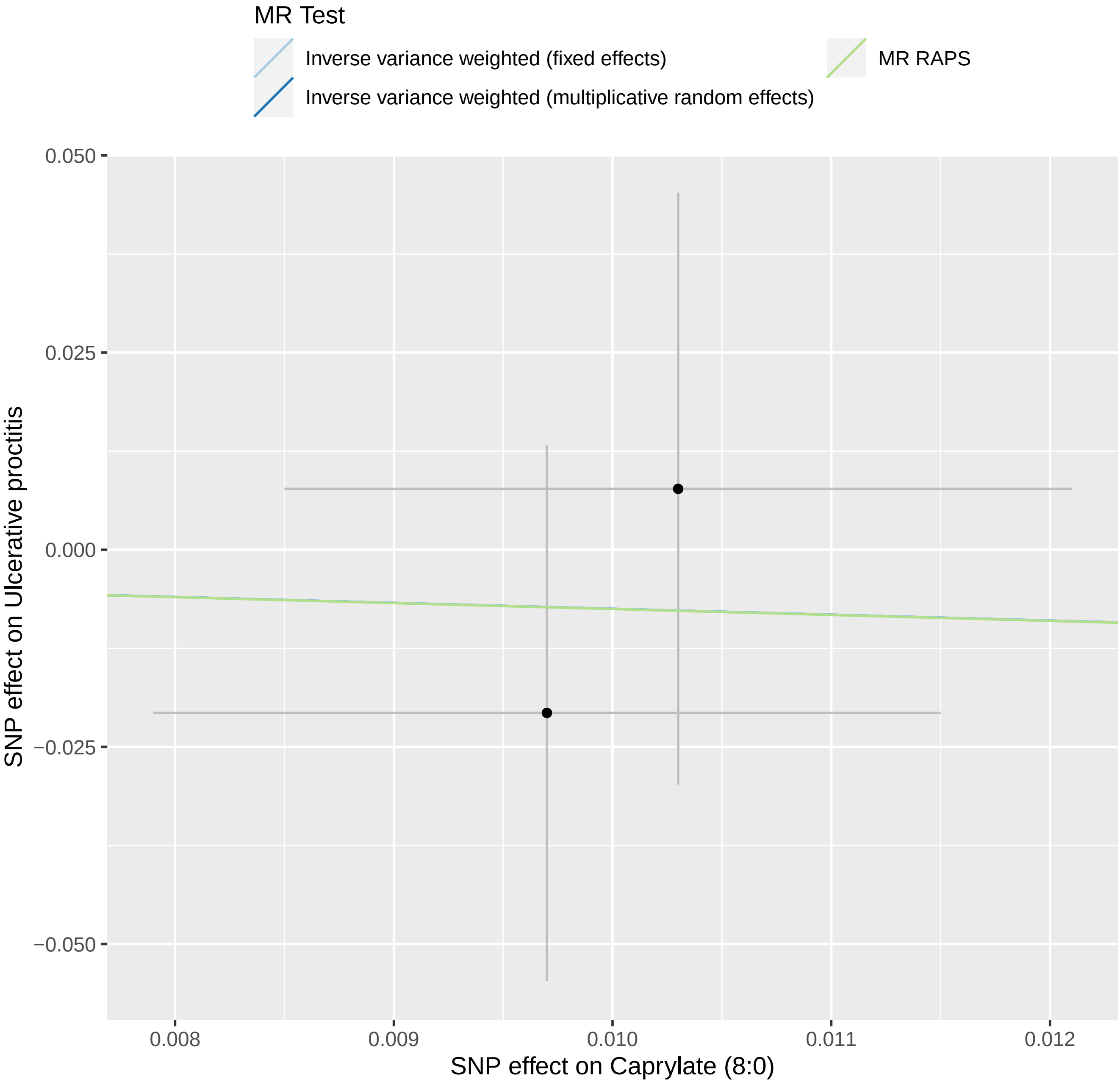

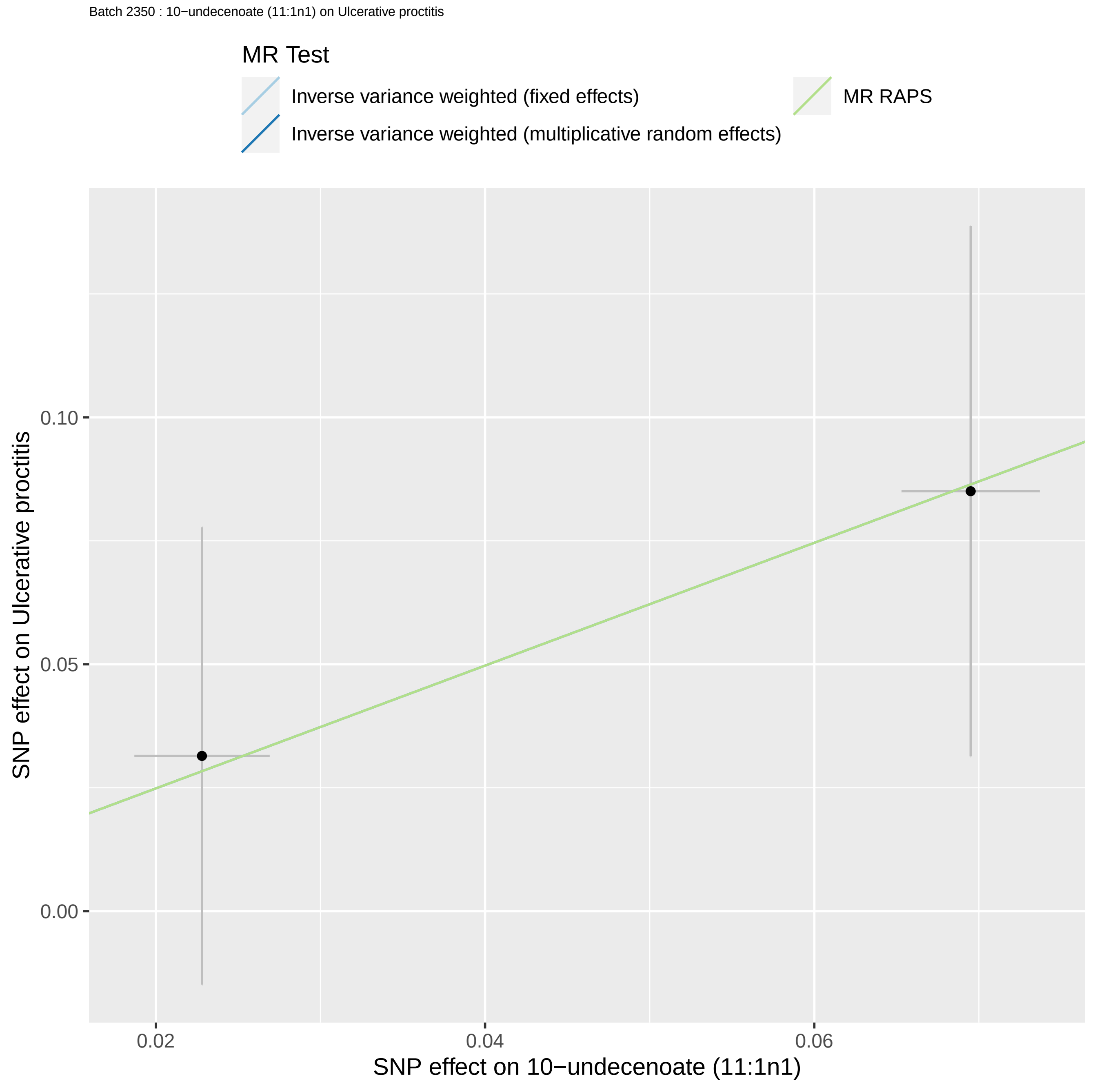
Batch 2350 : 10−undecenoate (11:1n1) on Ulcerative proctitis
MR Test
Inverse variance weighted (fixed effects)
Inverse variance weighted (multiplicative random effects)
MR RAPS
SNP effect on Ulcerative proctitis
0.10
0.05
0.00
0.02
0.04
0.06
SNP effect on 10−undecenoate (11:1n1)

Batch 2352 : Phenol sulfate on Ulcerative proctitis

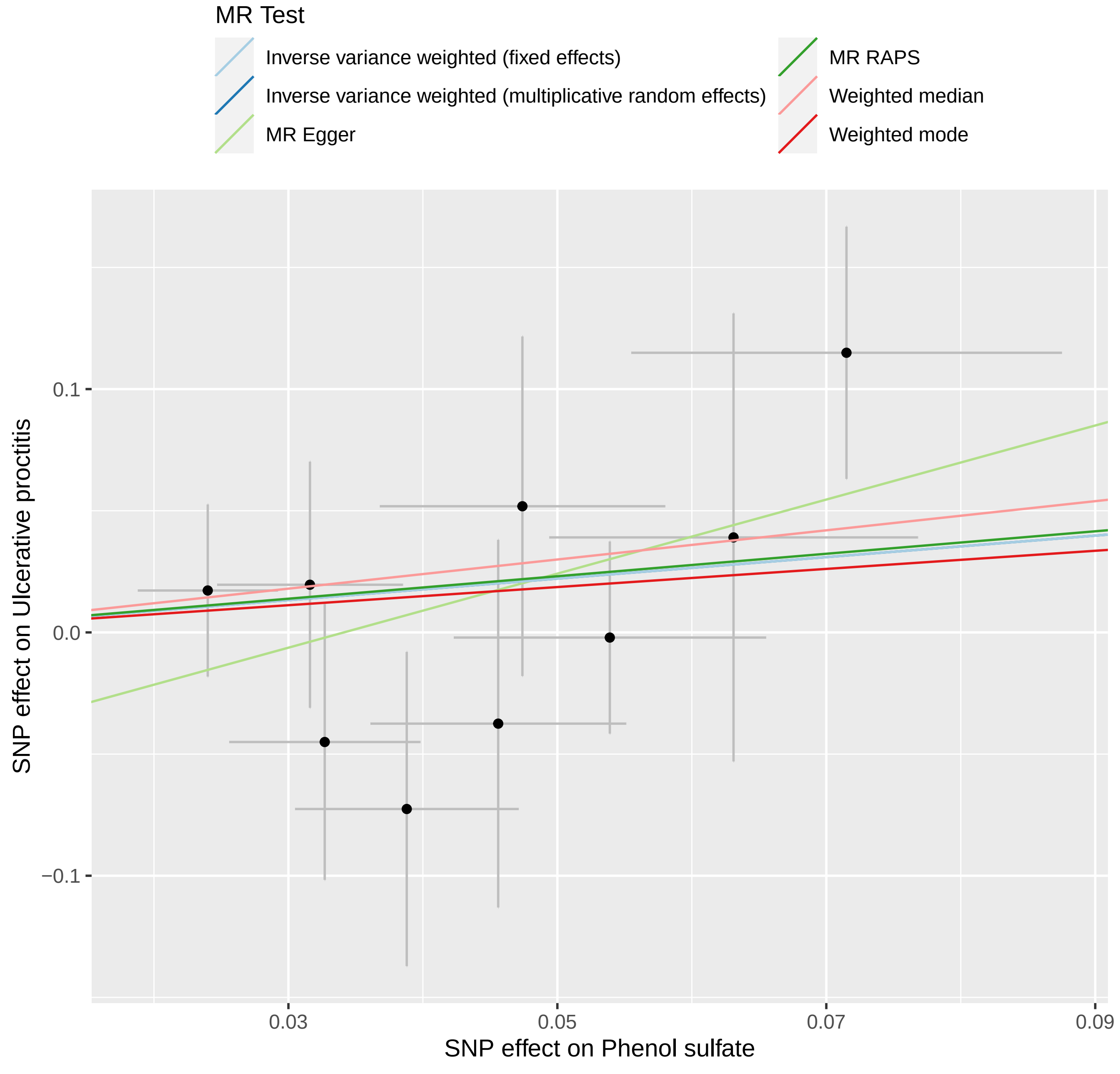

Batch 2354 : Heme* on Ulcerative proctitis

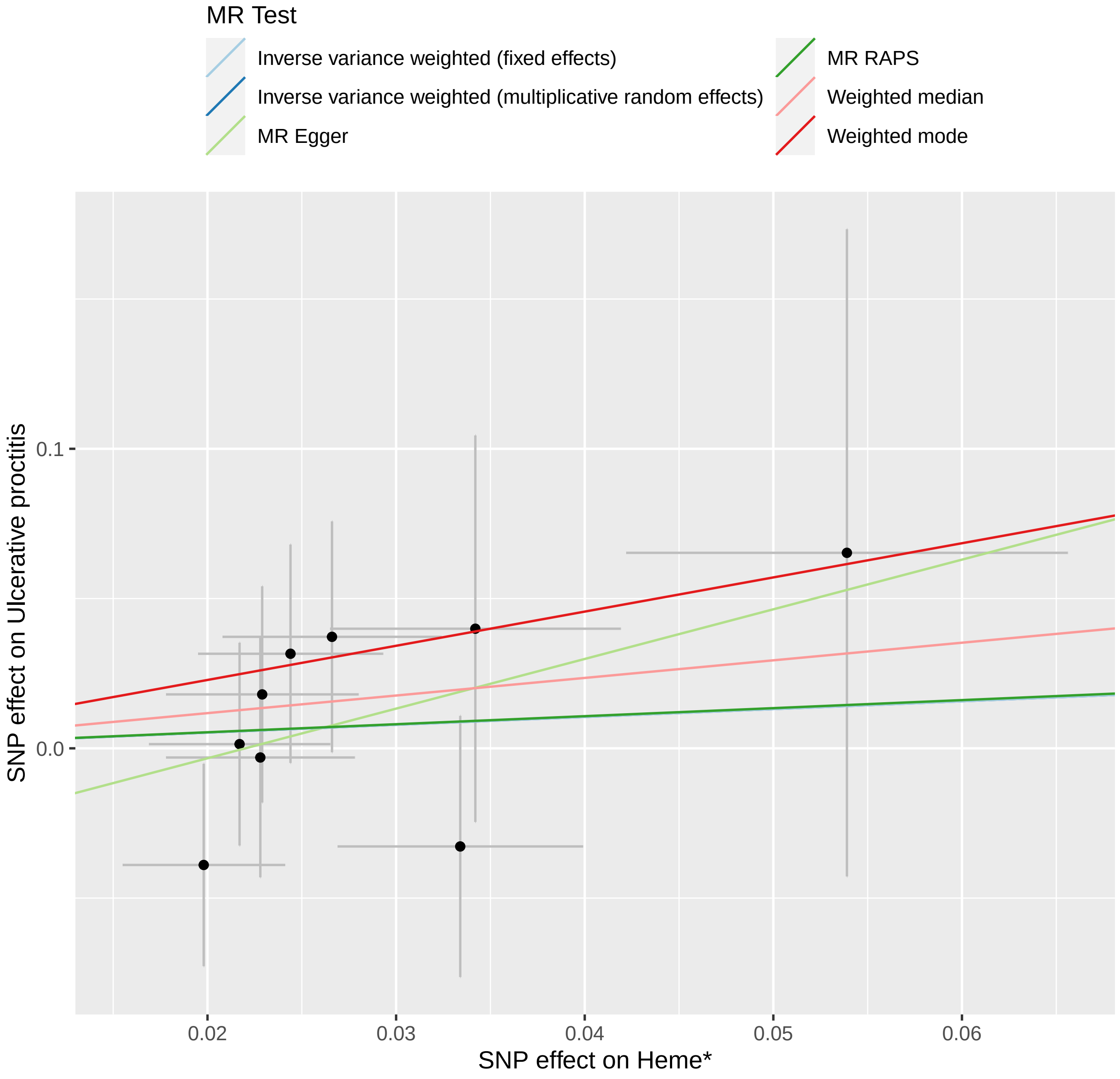

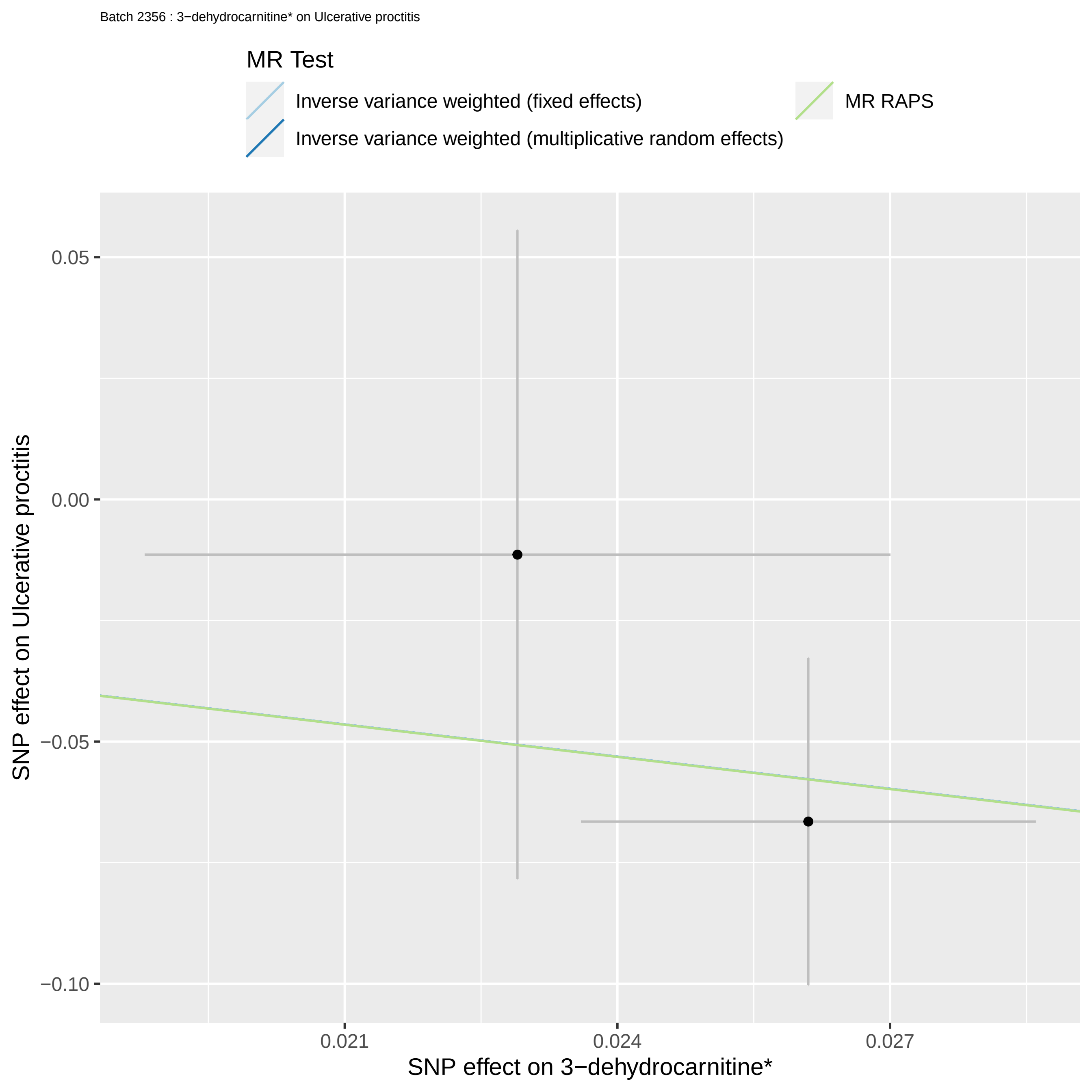

Batch 2356 : 3−dehydrocarnitine* on Ulcerative proctitis
MR Test
Inverse variance weighted (fixed effects)
Inverse variance weighted (multiplicative random effects)
MR RAPS
0.05
0.00
−0.05
−0.10
0.021
0.024
0.027
SNP effect on Ulcerative proctitis
SNP effect on 3−dehydrocarnitine*

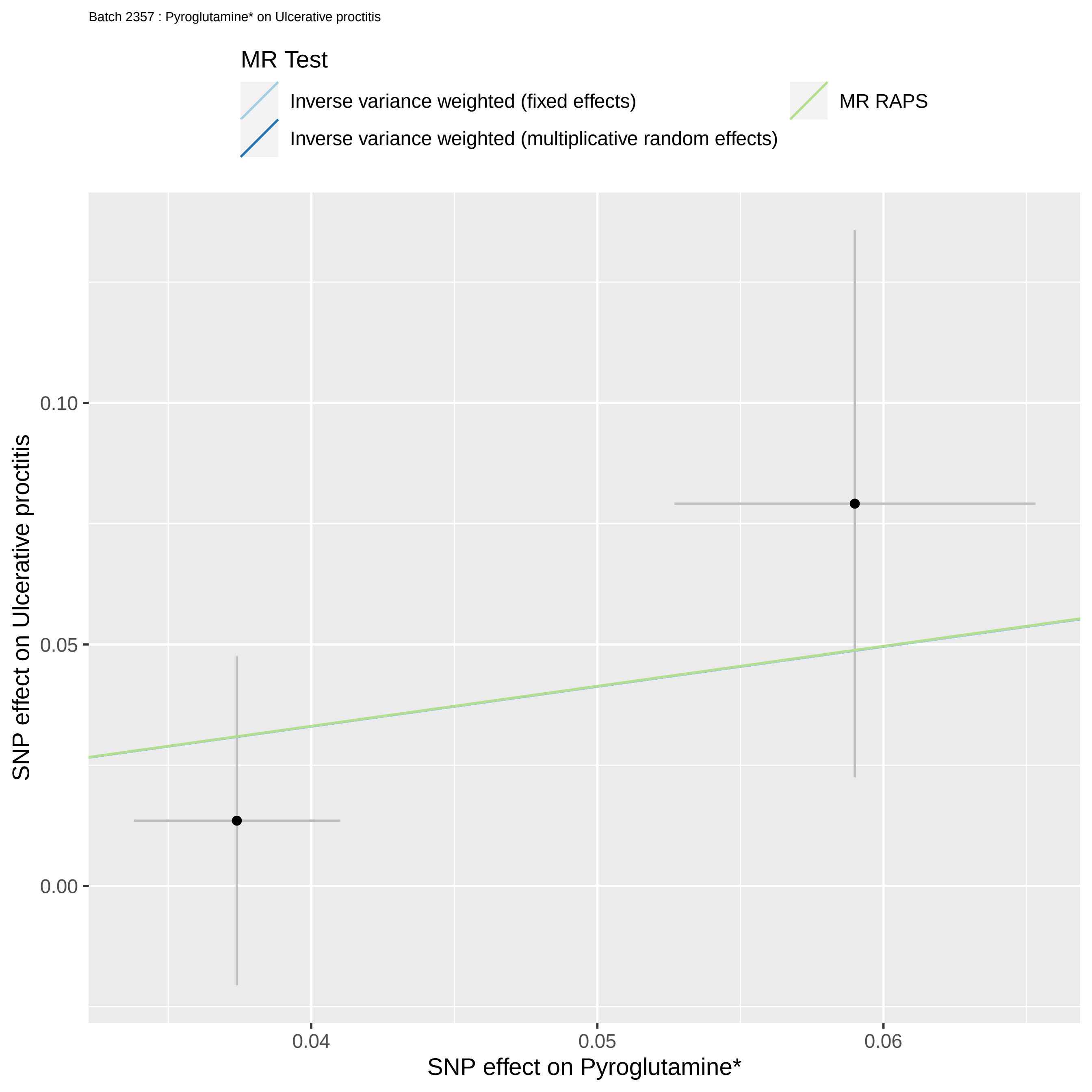
Batch 2357 : Pyroglutamine* on Ulcerative proctitis
MR Test
Inverse variance weighted (fixed effects)
Inverse variance weighted (multiplicative random effects)
MR RAPS
0.10
0.05
0.00
0.04
0.05
0.06
SNP effect on Ulcerative proctitis
SNP effect on Pyroglutamine*

Batch 2360 : X−11422−−xanthine on Ulcerative proctitis

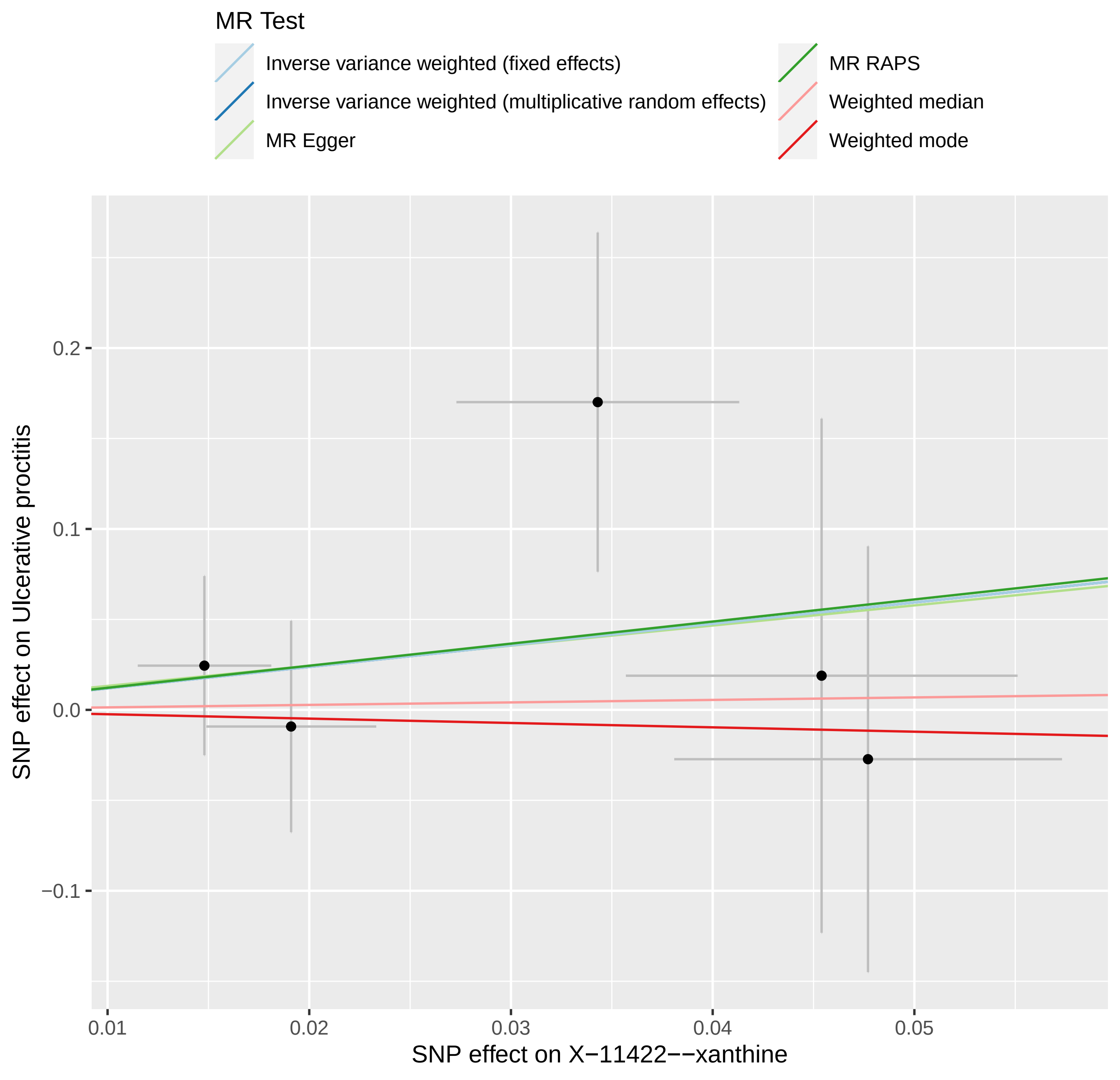

Batch 2363 : HWESASXX* on Ulcerative proctitis

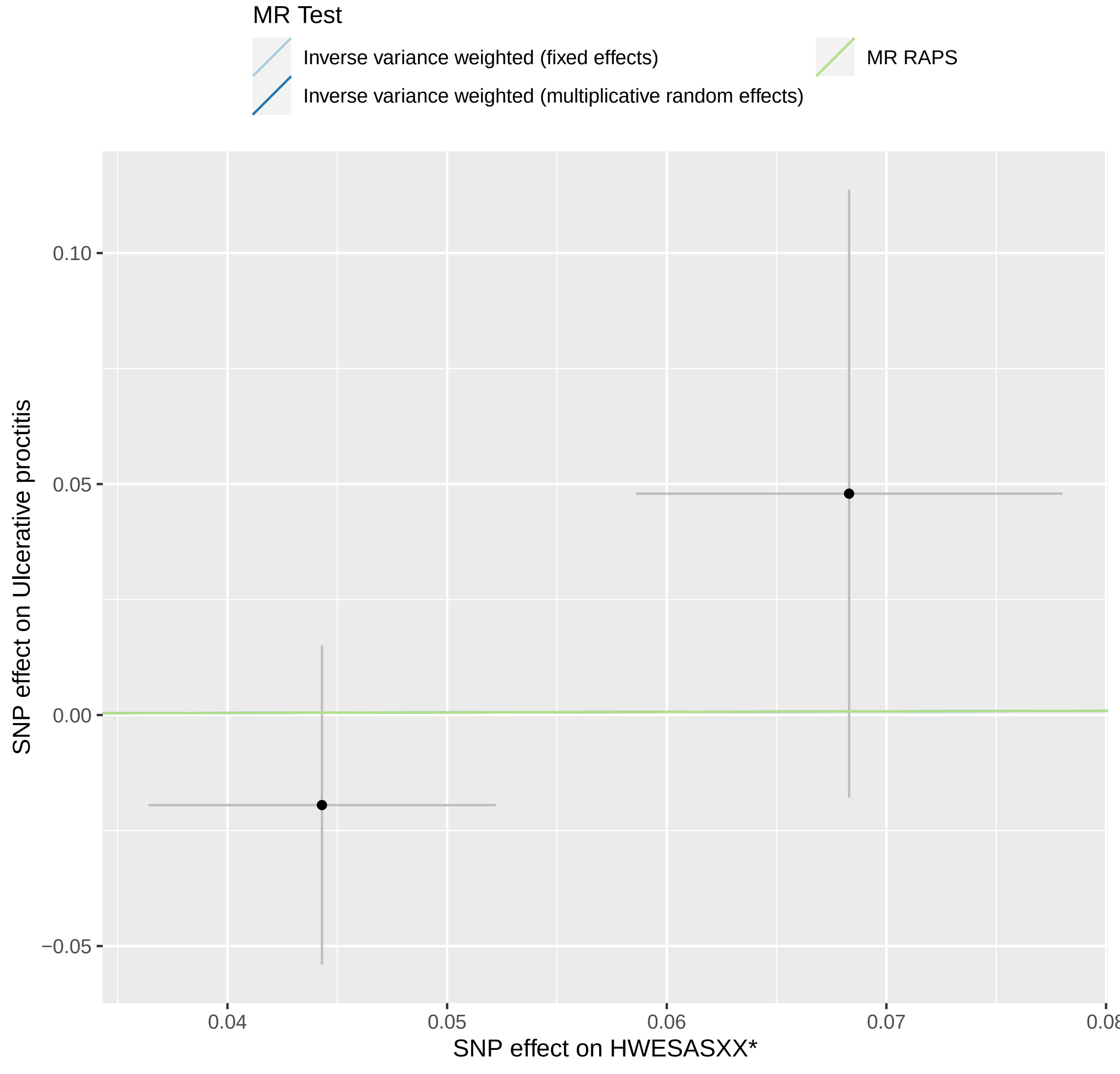

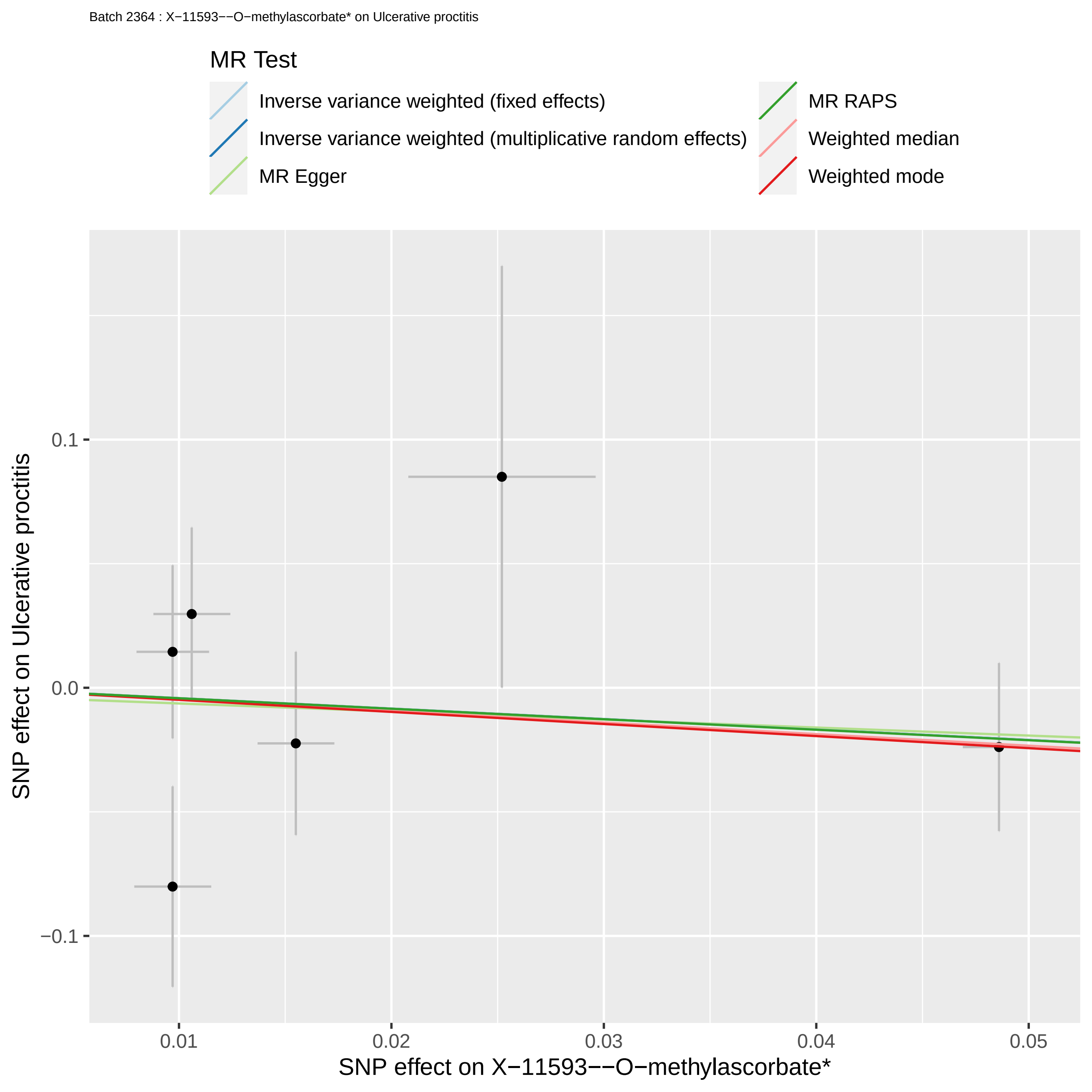

Batch 2364 : X−11593−−O−methylascorbate* on Ulcerative proctitis
MR Test
Inverse variance weighted (fixed effects)
Inverse variance weighted (multiplicative random effects)
MR Egger
MR RAPS
Weighted median
Weighted mode
SNP effect on Ulcerative proctitis
0.1
0.0
−0.1
0.01
0.02
0.03
0.04
0.05
SNP effect on X−11593−−O−methylascorbate*

Batch 2366 : Homostachydrine* on Ulcerative proctitis

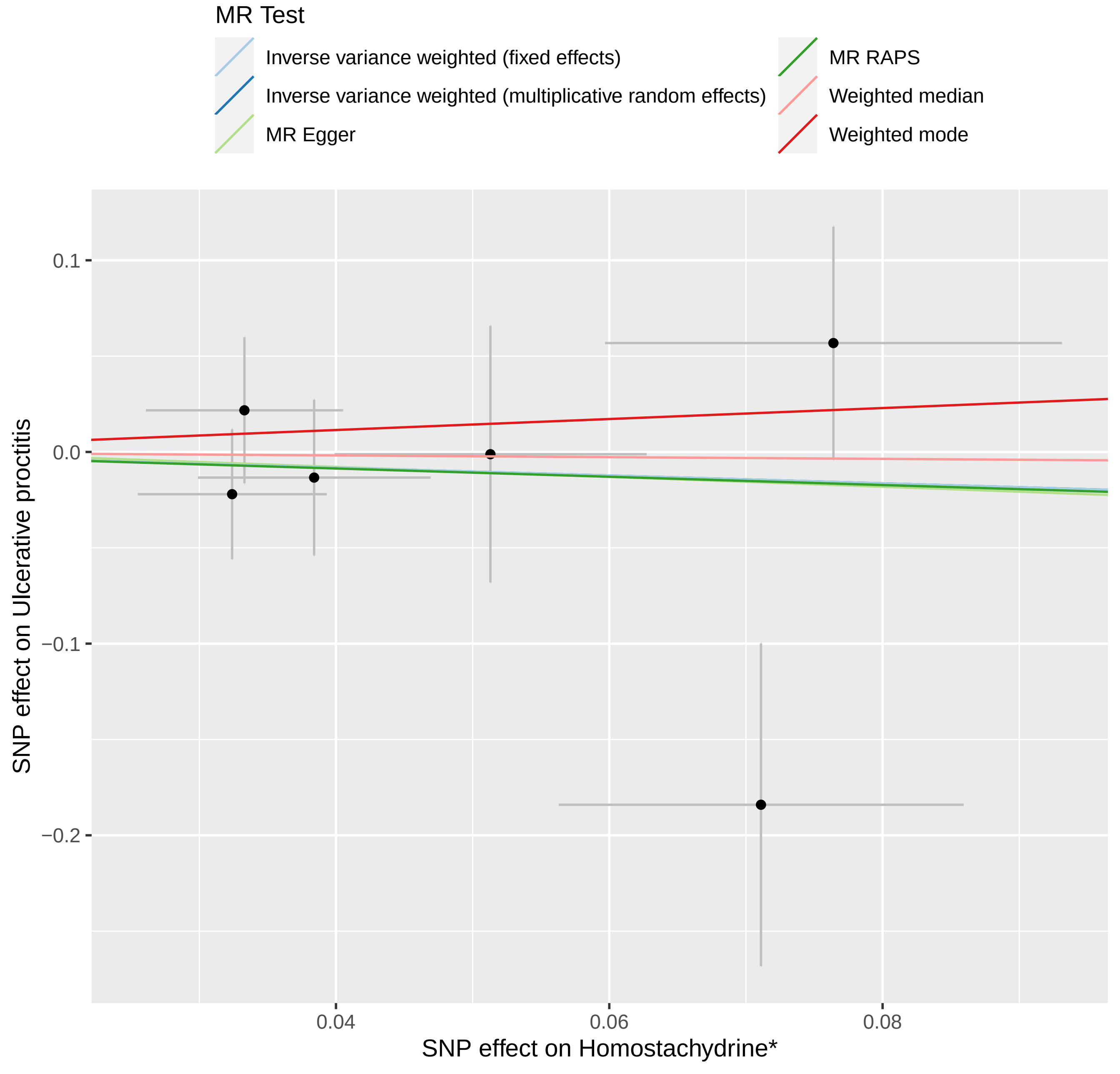

Batch 2367 : ADSGEGDFXAEGGGVR* on Ulcerative proctitis

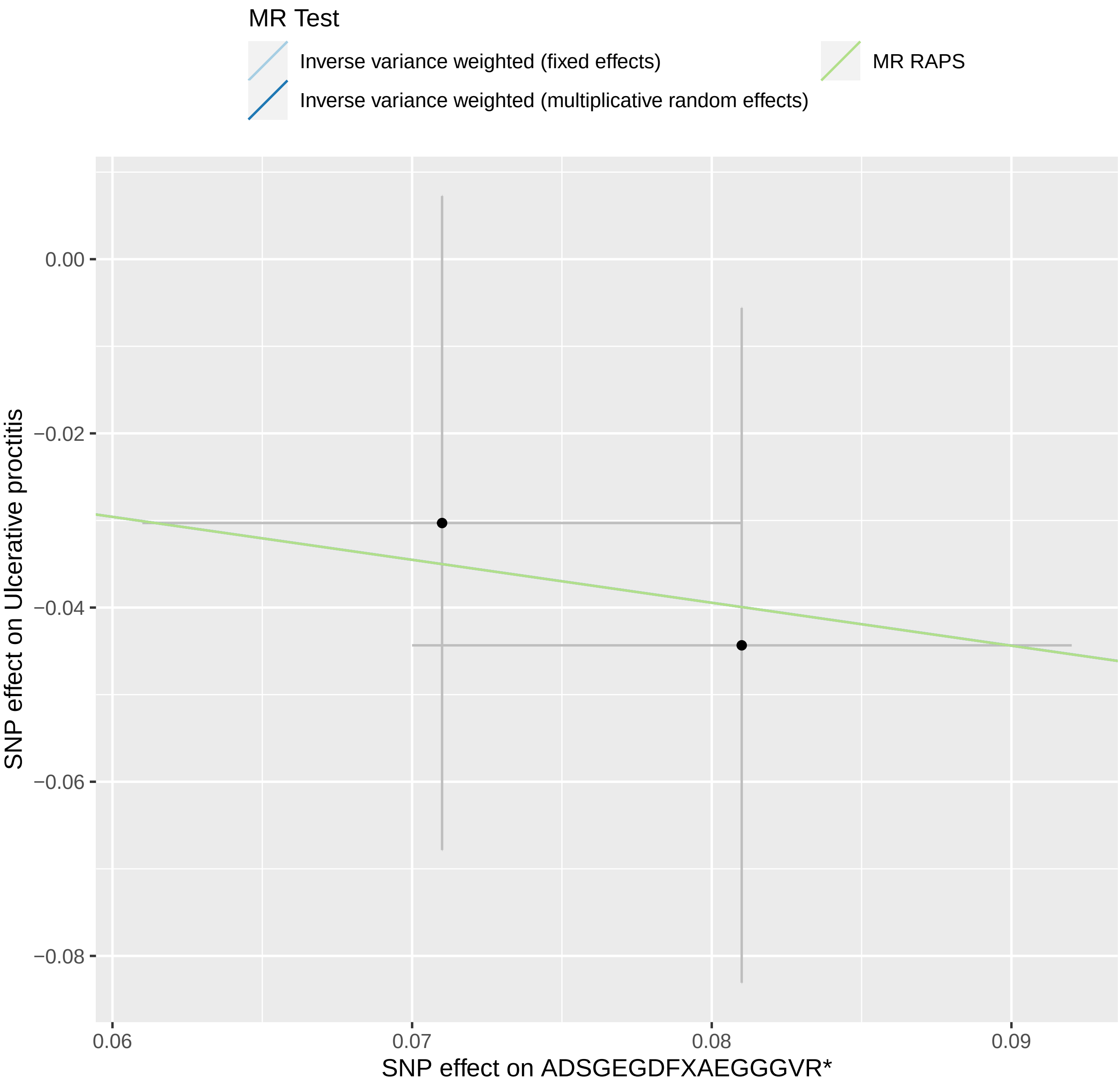

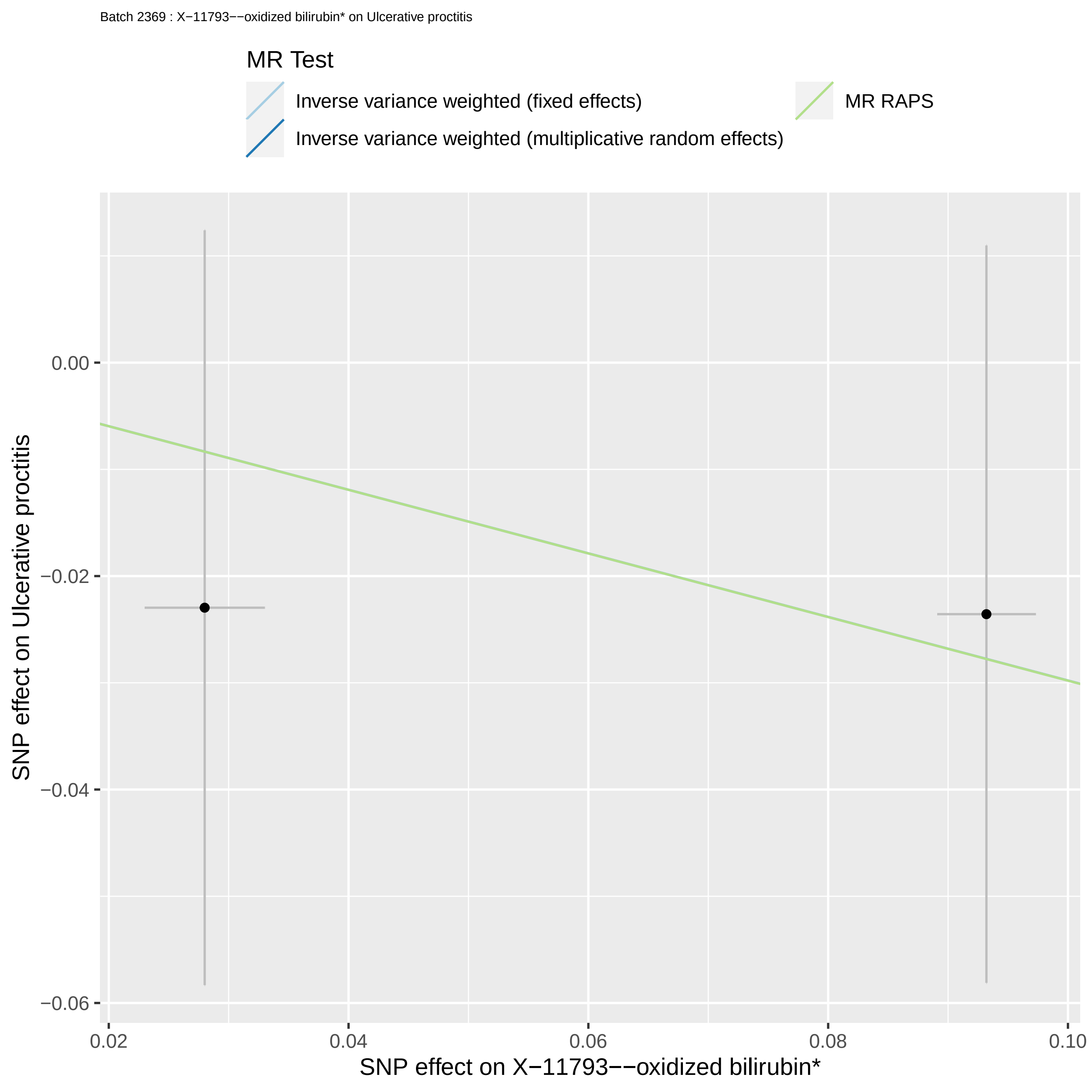

Batch 2369 : X−11793−−oxidized bilirubin* on Ulcerative proctitis
MR Test
Inverse variance weighted (fixed effects)
Inverse variance weighted (multiplicative random effects)
MR RAPS
0.00
−0.02
−0.04
−0.06
0.02
0.04
0.06
0.08
0.10
SNP effect on Ulcerative proctitis
SNP effect on X−11793−−oxidized bilirubin*

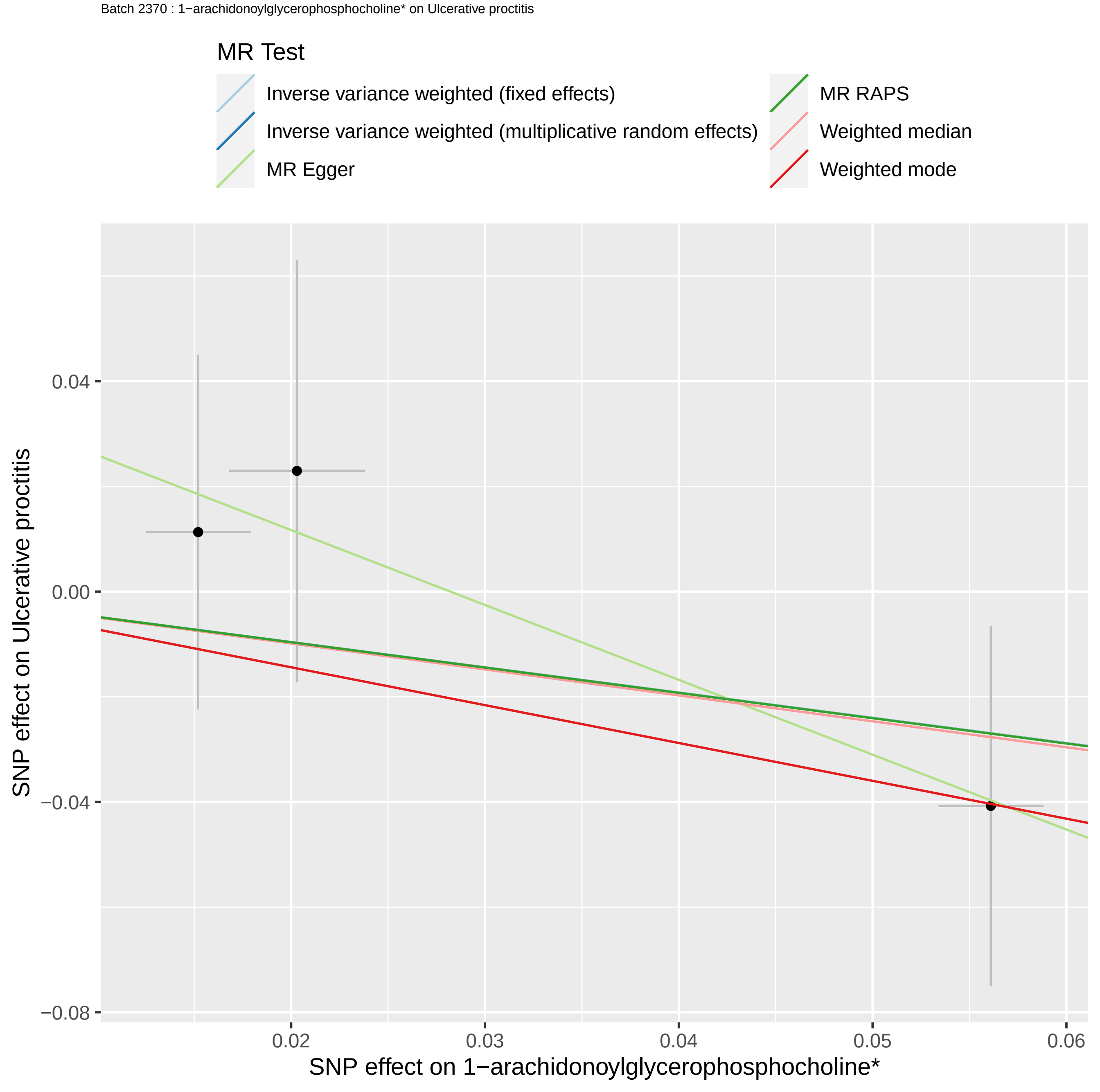

Batch 2370 : 1−arachidonoylglycerophosphocholine* on Ulcerative proctitis
MR Test
Inverse variance weighted (fixed effects)
Inverse variance weighted (multiplicative random effects)
MR Egger
MR RAPS
Weighted median
Weighted mode
0.04
0.00
−0.04
−0.08
0.02
0.03
0.04
0.05
0.06
SNP effect on Ulcerative proctitis
SNP effect on 1−arachidonoylglycerophosphocholine*

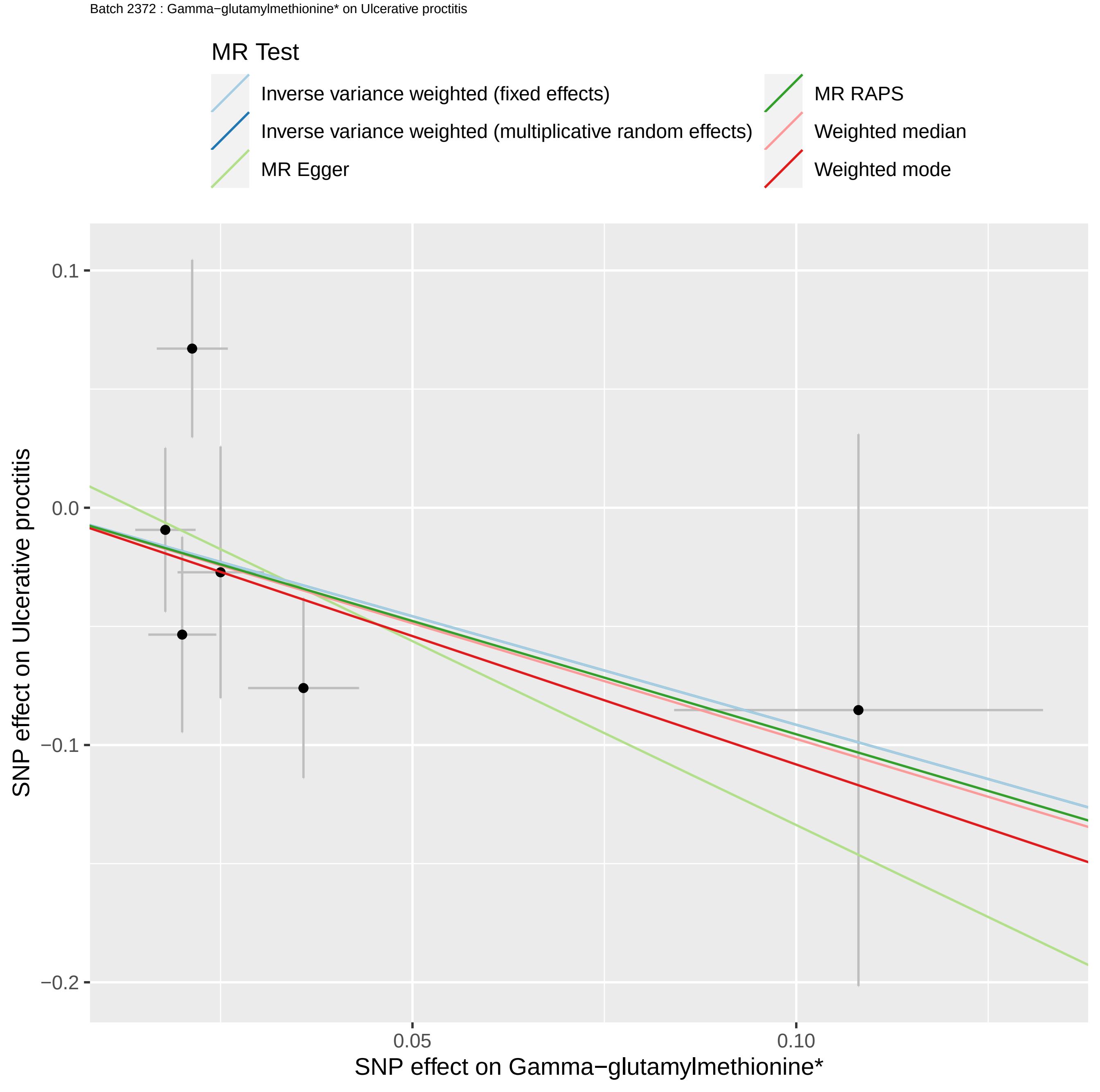

Batch 2372 : Gamma−glutamylmethionine* on Ulcerative proctitis
MR Test
Inverse variance weighted (fixed effects)
Inverse variance weighted (multiplicative random effects)
MR Egger
MR RAPS
Weighted median
Weighted mode
0.1
0.0
−0.1
−0.2
0.05
0.10
SNP effect on Ulcerative proctitis
SNP effect on Gamma−glutamylmethionine*

Batch 2373 : Gamma−glutamylthreonine* on Ulcerative proctitis

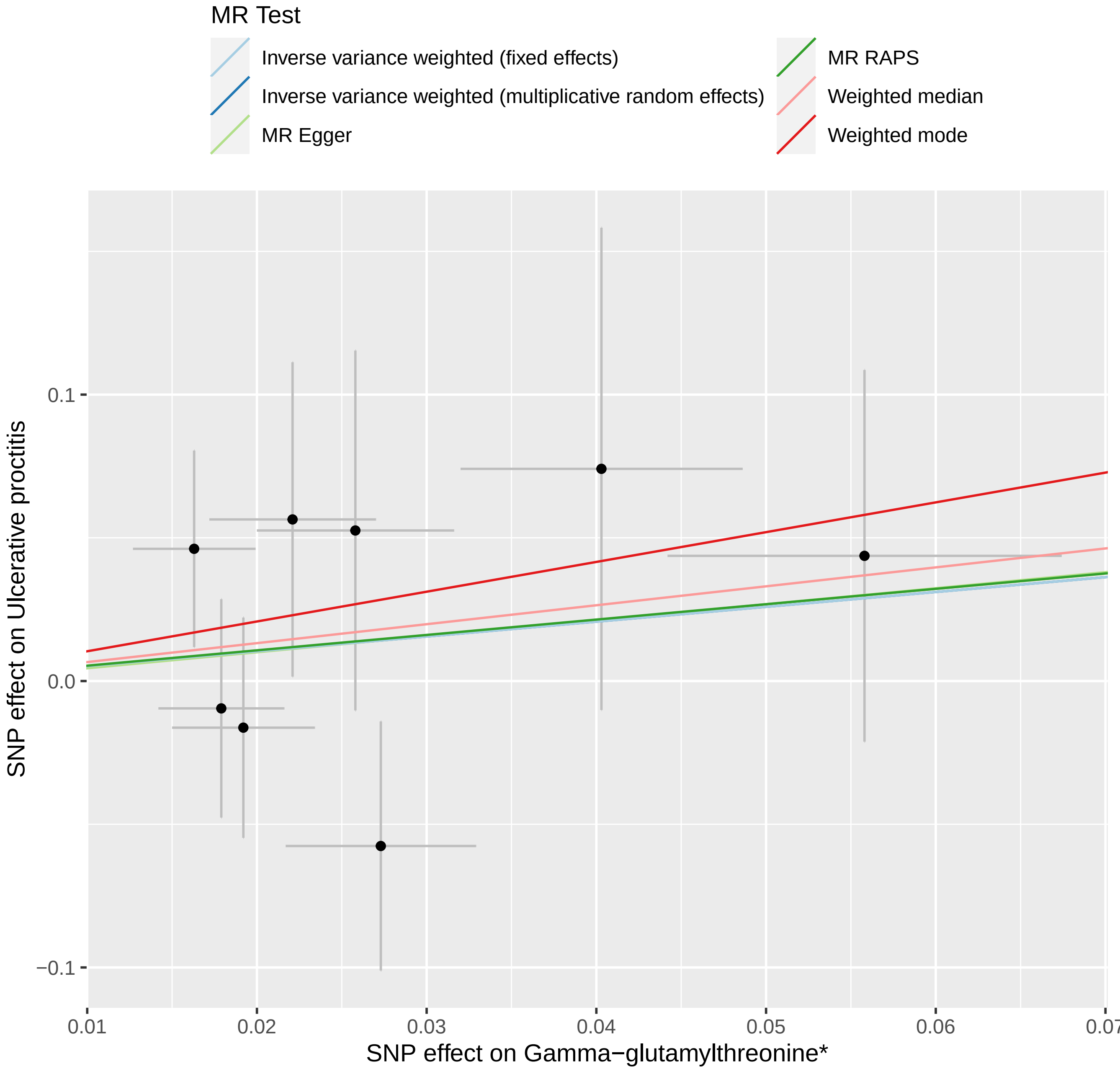

Batch 2374 : Gamma−tocopherol on Ulcerative proctitis

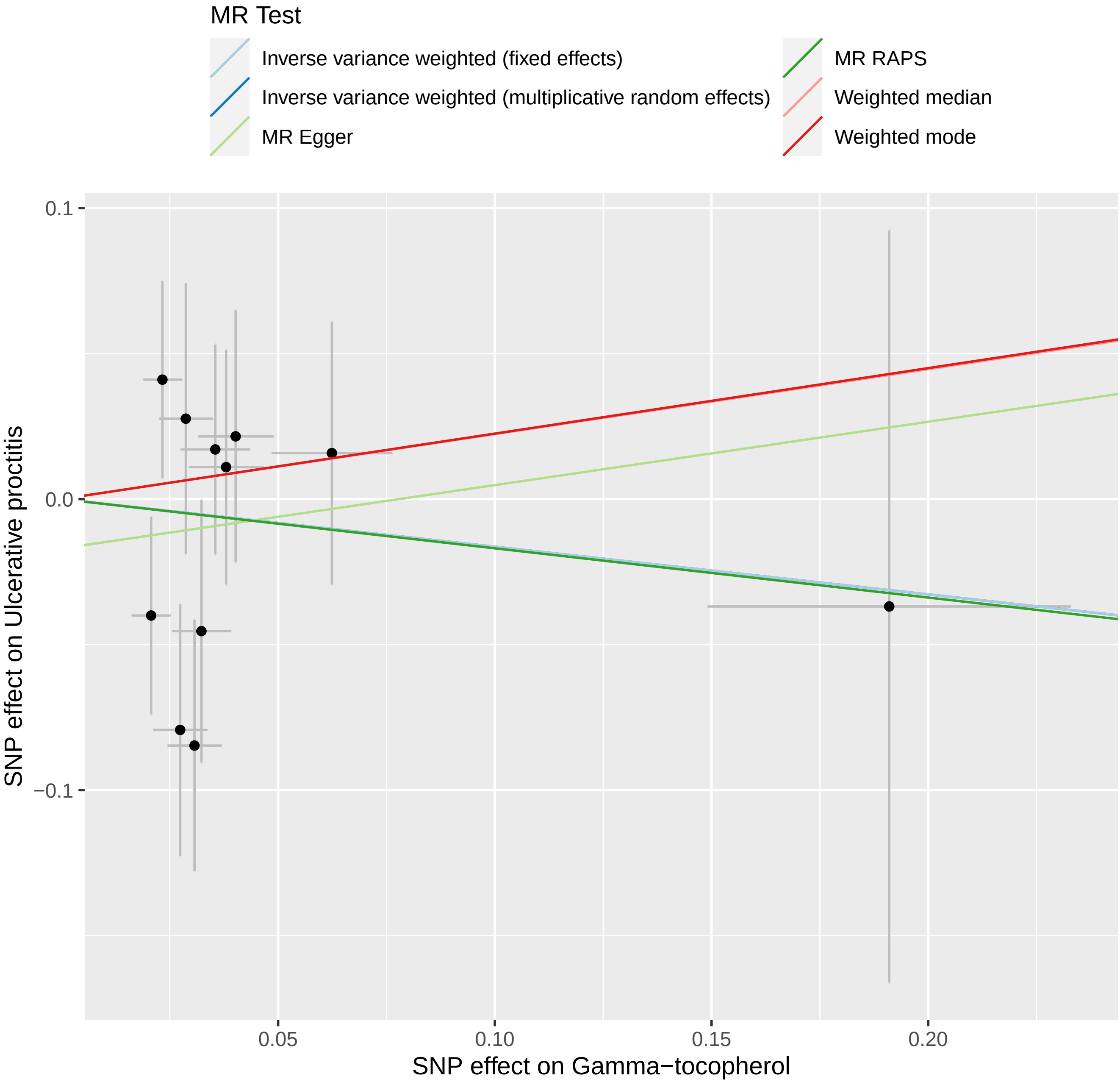

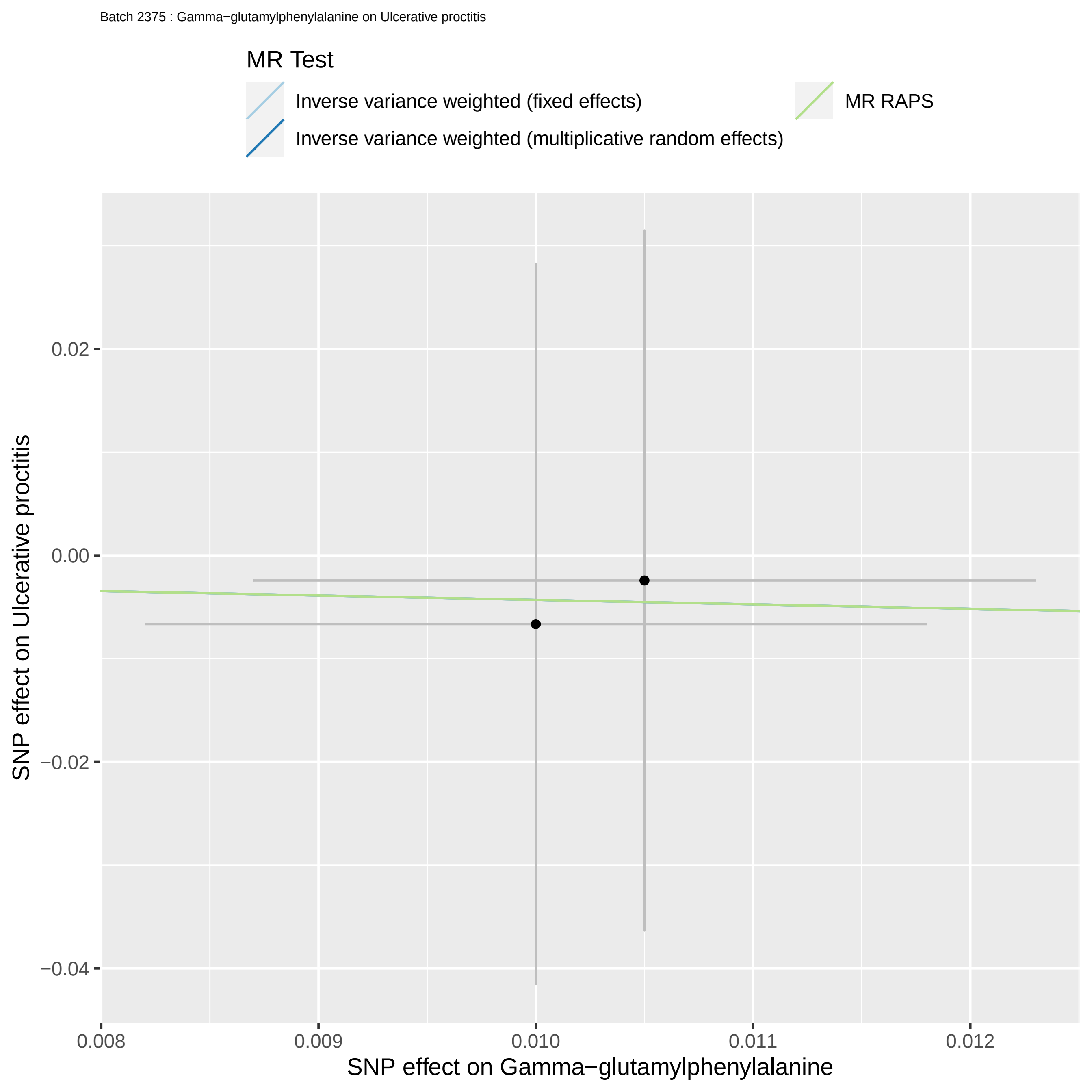
Batch 2375 : Gamma−glutamylphenylalanine on Ulcerative proctitis
MR Test
Inverse variance weighted (fixed effects)
Inverse variance weighted (multiplicative random effects)
MR RAPS
0.02
0.00
−0.02
−0.04
SNP effect on Ulcerative proctitis
0.008
0.009
0.010
0.011
0.012
SNP effect on Gamma−glutamylphenylalanine

Batch 2376 : Isobutyrylcarnitine on Ulcerative proctitis

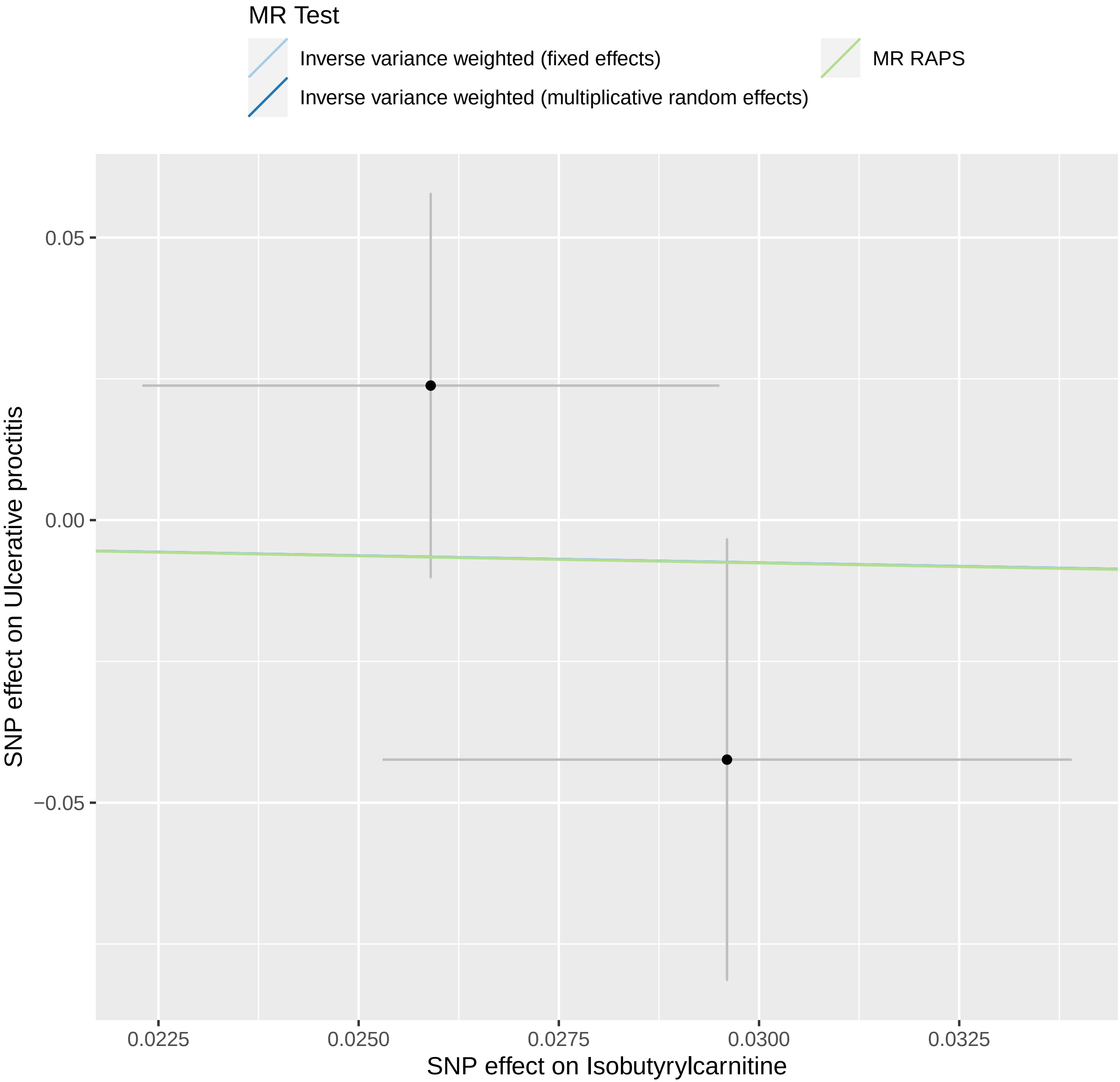

Batch 2377 : Pseudouridine on Ulcerative proctitis

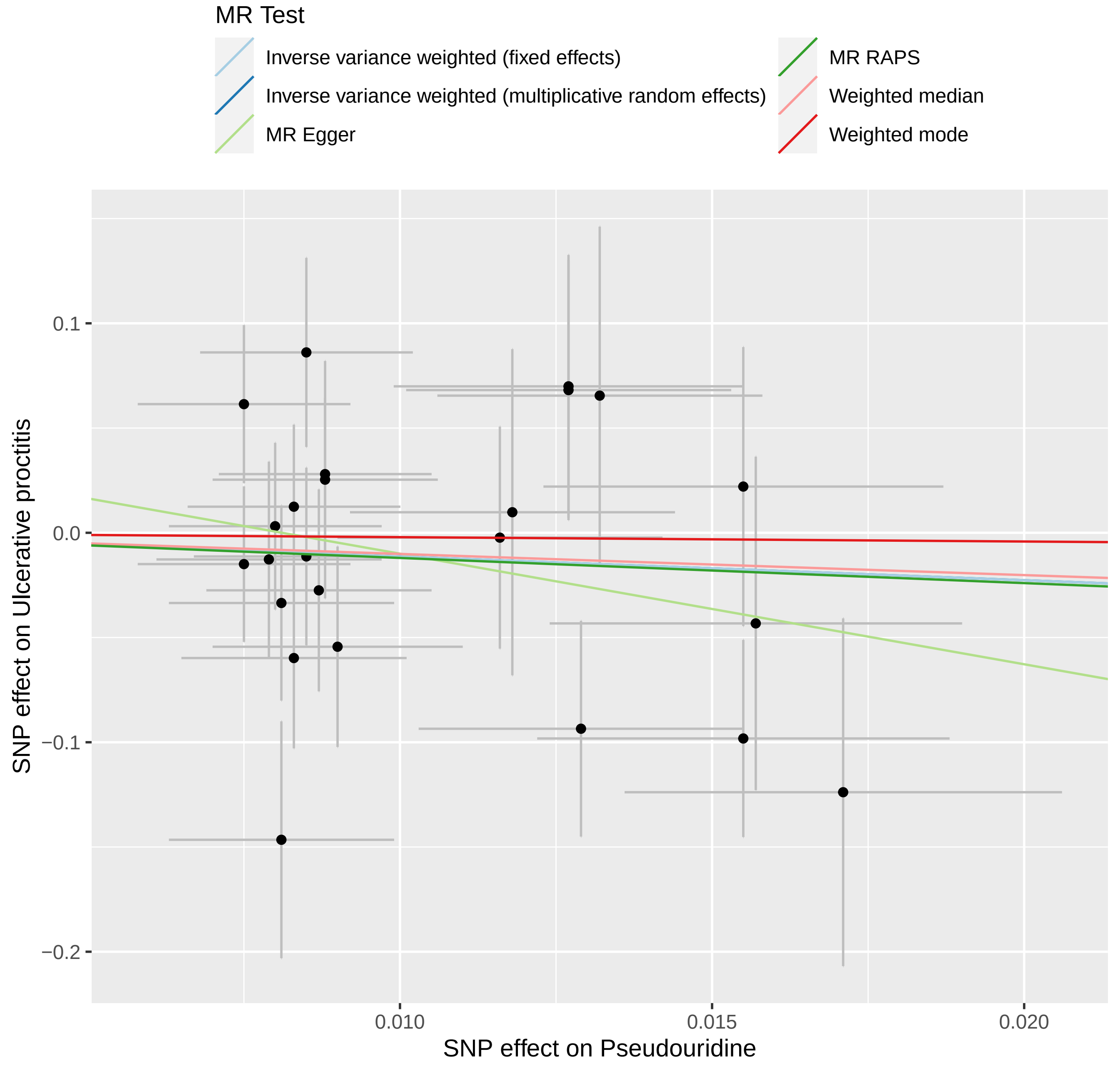

Batch 2378 : Valerate on Ulcerative proctitis

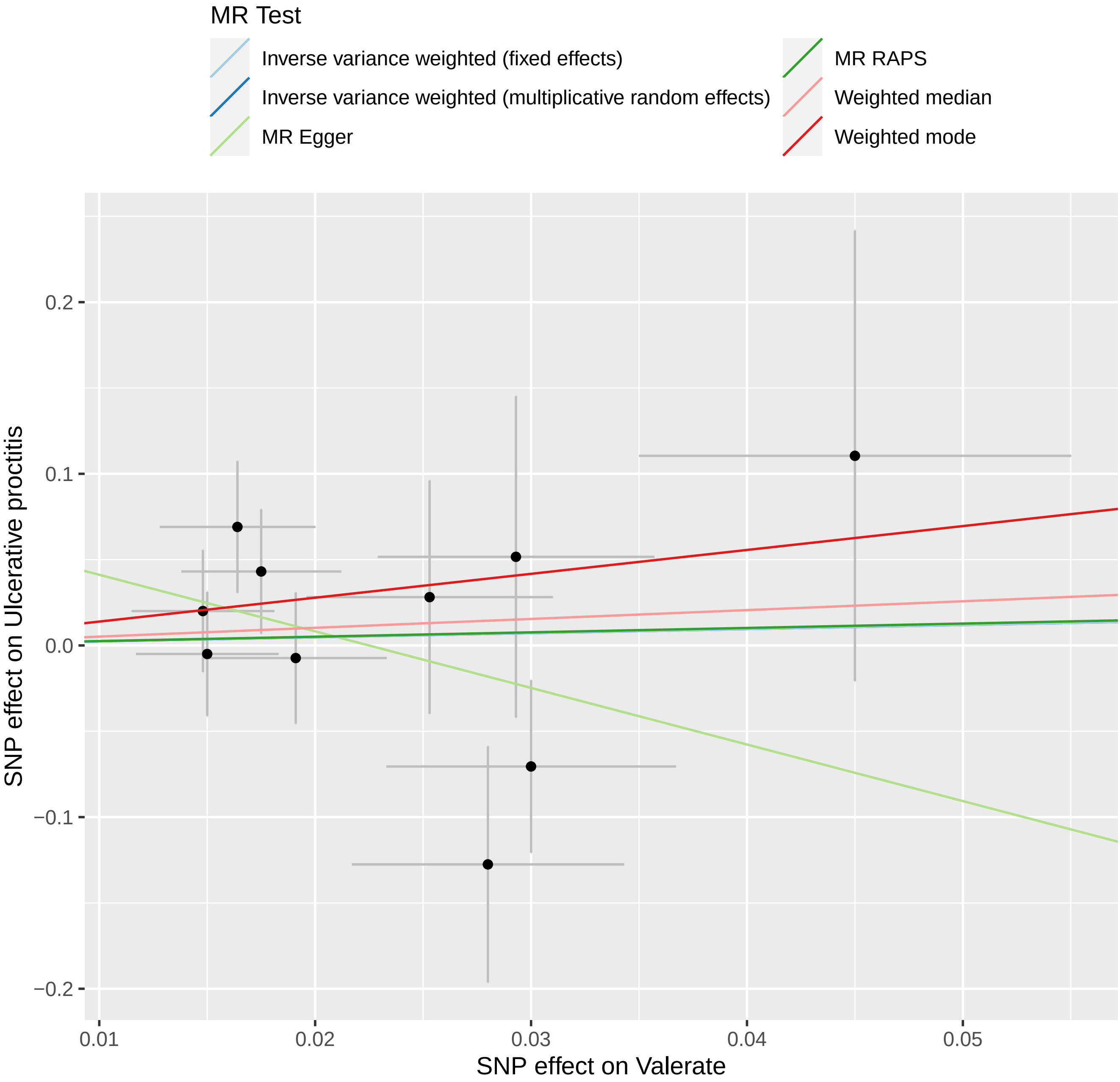

Batch 2381 : Erythronate* on Ulcerative proctitis

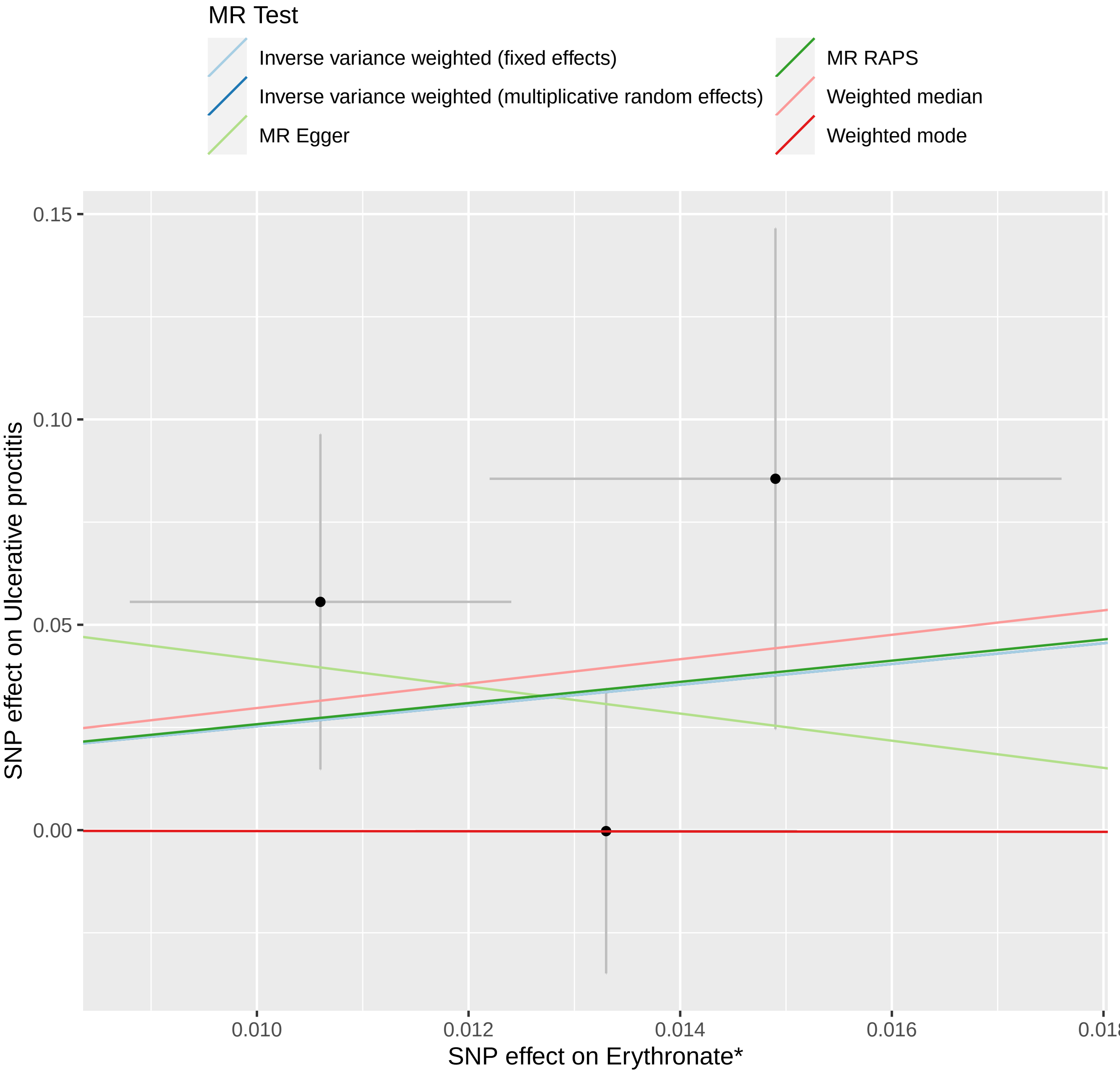

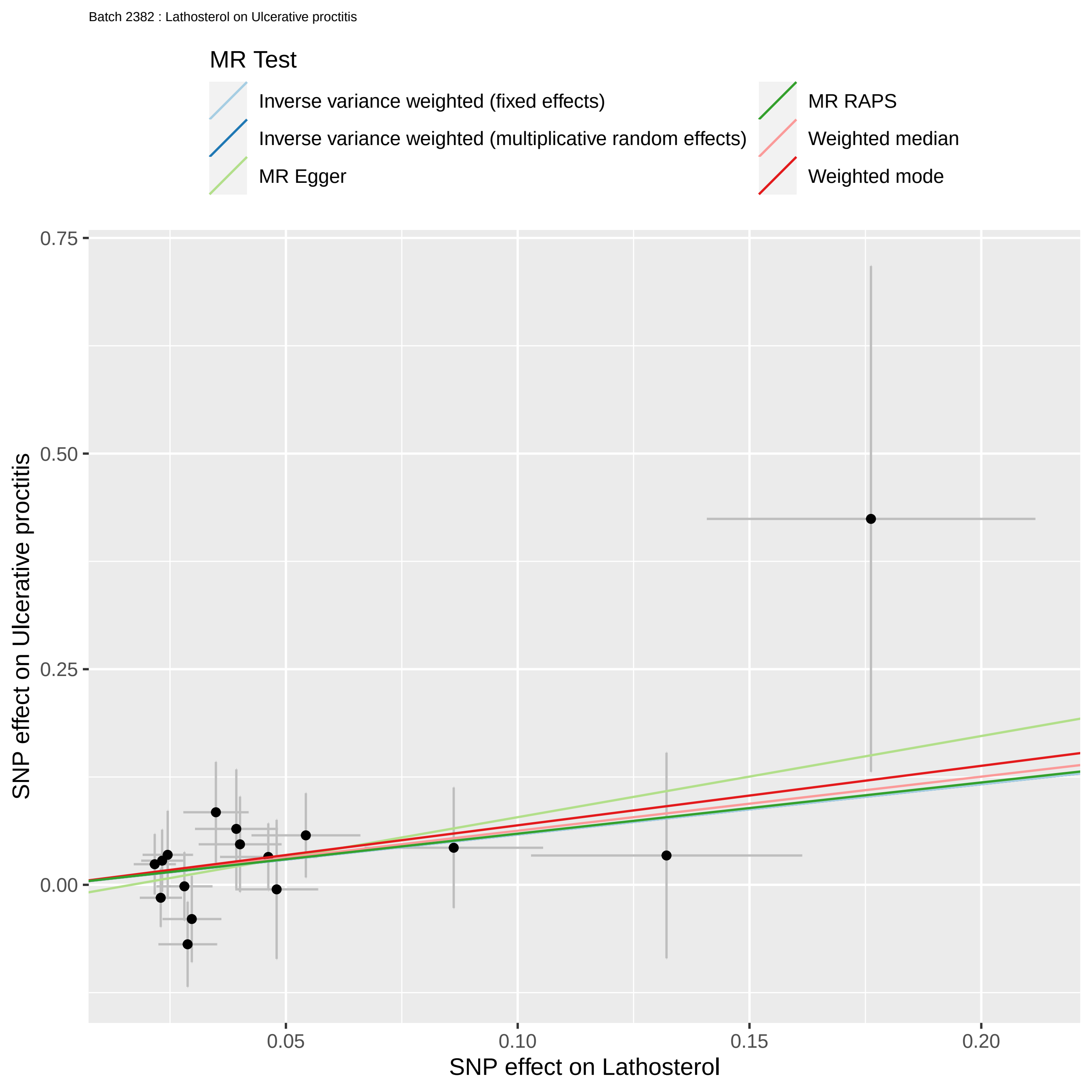

Batch 2382 : Lathosterol on Ulcerative proctitis
MR Test
Inverse variance weighted (fixed effects)
Inverse variance weighted (multiplicative random effects)
MR Egger
MR RAPS
Weighted median
Weighted mode
0.75
0.50
0.25
0.00
0.05
0.10
0.15
0.20
SNP effect on Ulcerative proctitis
SNP effect on Lathosterol

Batch 2385 : Eicosenoate (20:1n9 or 11) on Ulcerative proctitis

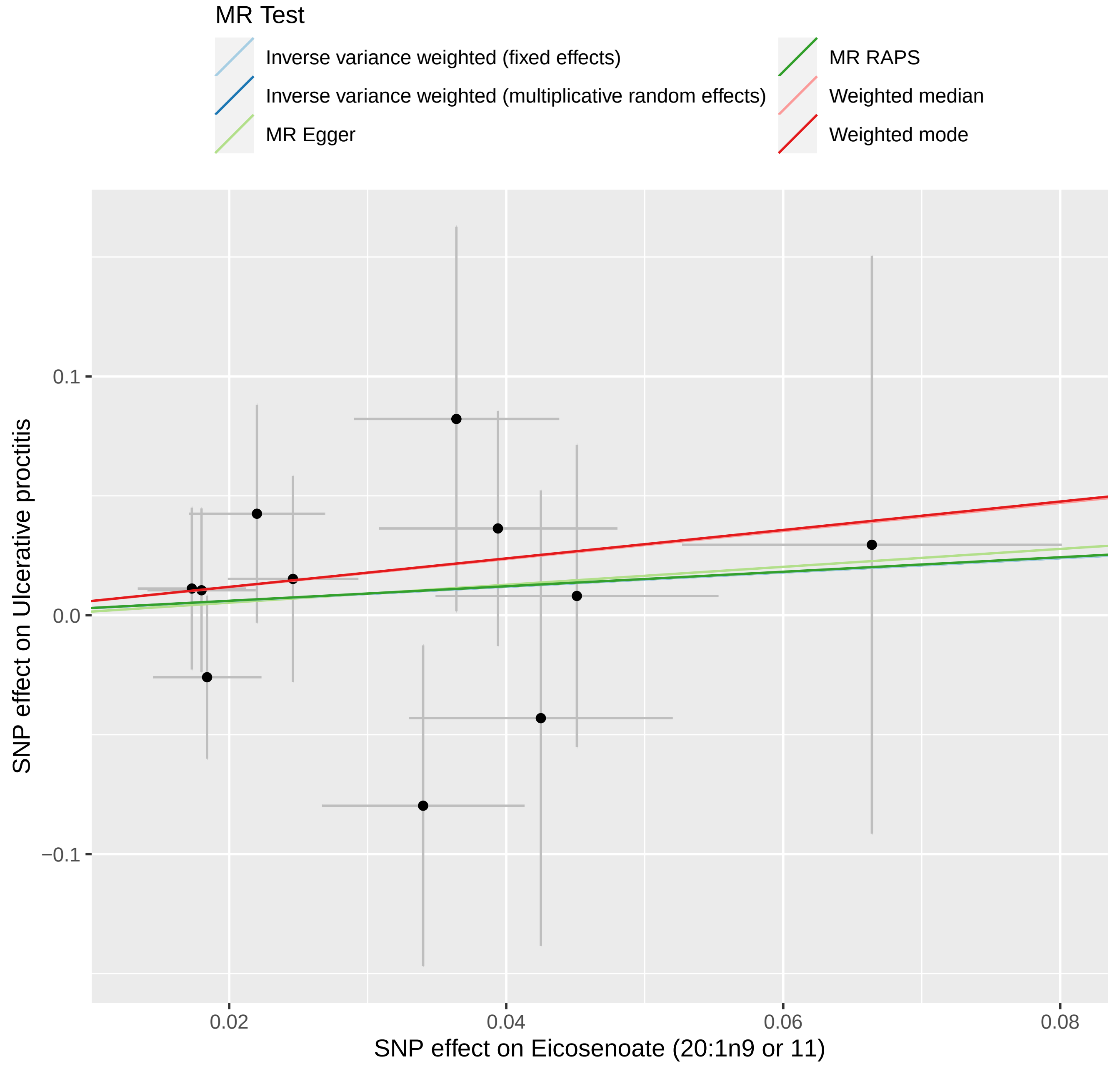

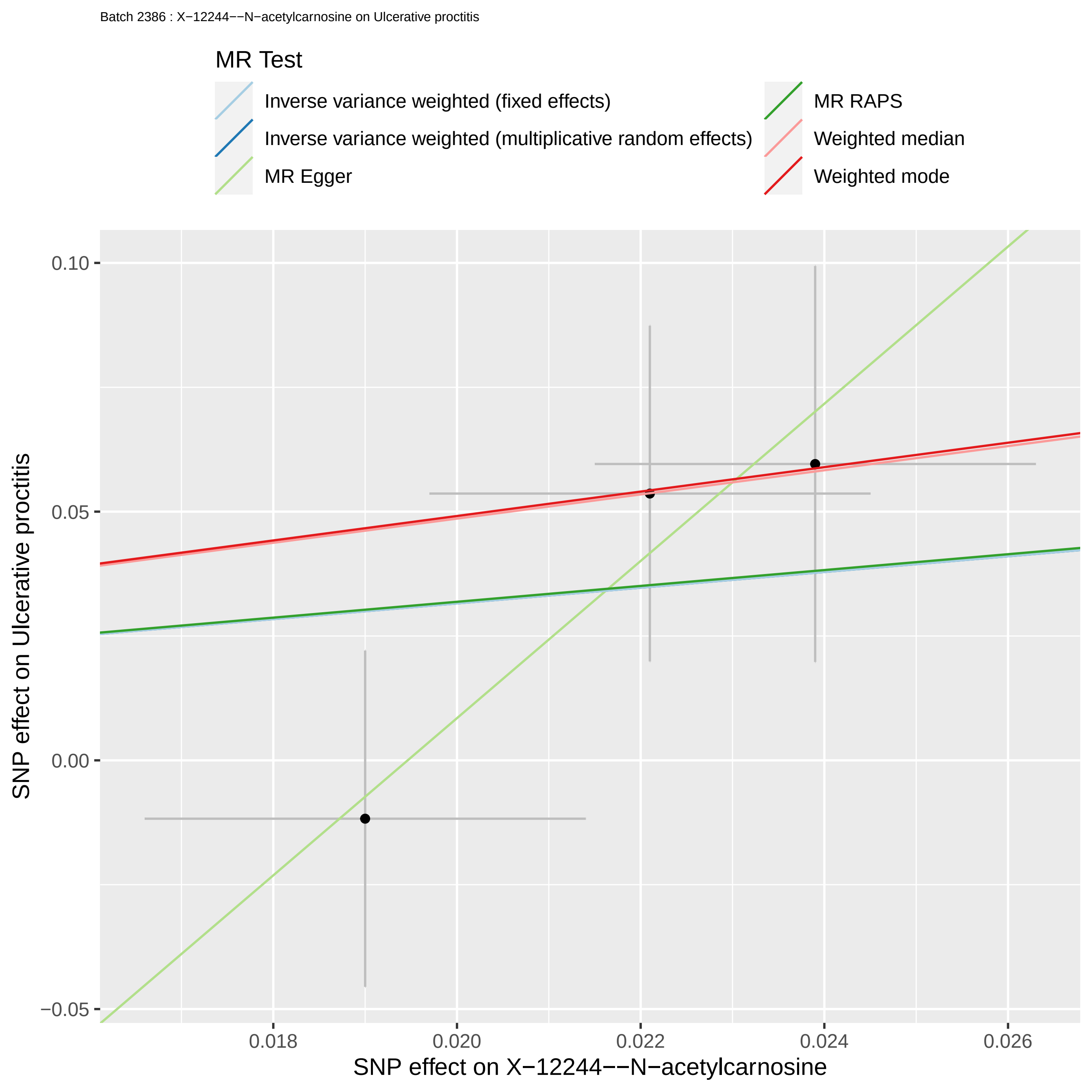

Batch 2386 : X−12244−−N−acetylcarnosine on Ulcerative proctitis
MR Test
Inverse variance weighted (fixed effects)
Inverse variance weighted (multiplicative random effects)
MR Egger
MR RAPS
Weighted median
Weighted mode
0.10
0.05
0.00
−0.05
0.018
0.020
0.022
0.024
0.026
SNP effect on Ulcerative proctitis
SNP effect on X−12244−−N−acetylcarnosine

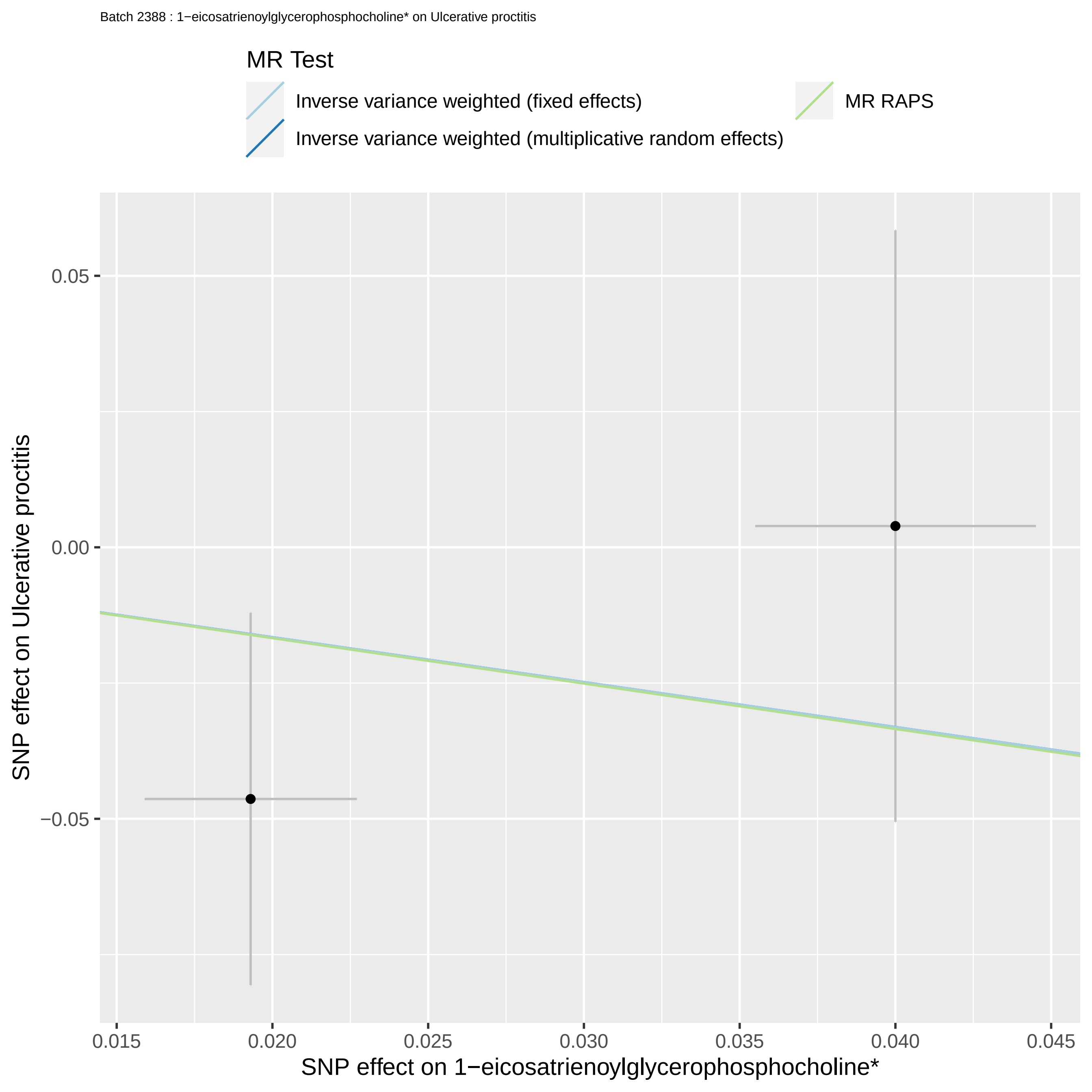

Batch 2388 : 1−eicosatrienoylglycerophosphocholine* on Ulcerative proctitis
MR Test
Inverse variance weighted (fixed effects)
Inverse variance weighted (multiplicative random effects)
MR RAPS
0.05
0.00
−0.05
0.015
0.020
0.025
0.030
0.035
0.040
0.045
SNP effect on Ulcerative proctitis
SNP effect on 1−eicosatrienoylglycerophosphocholine*

Batch 2389 : 1−docosahexaenoylglycerophosphocholine* on Ulcerative proctitis

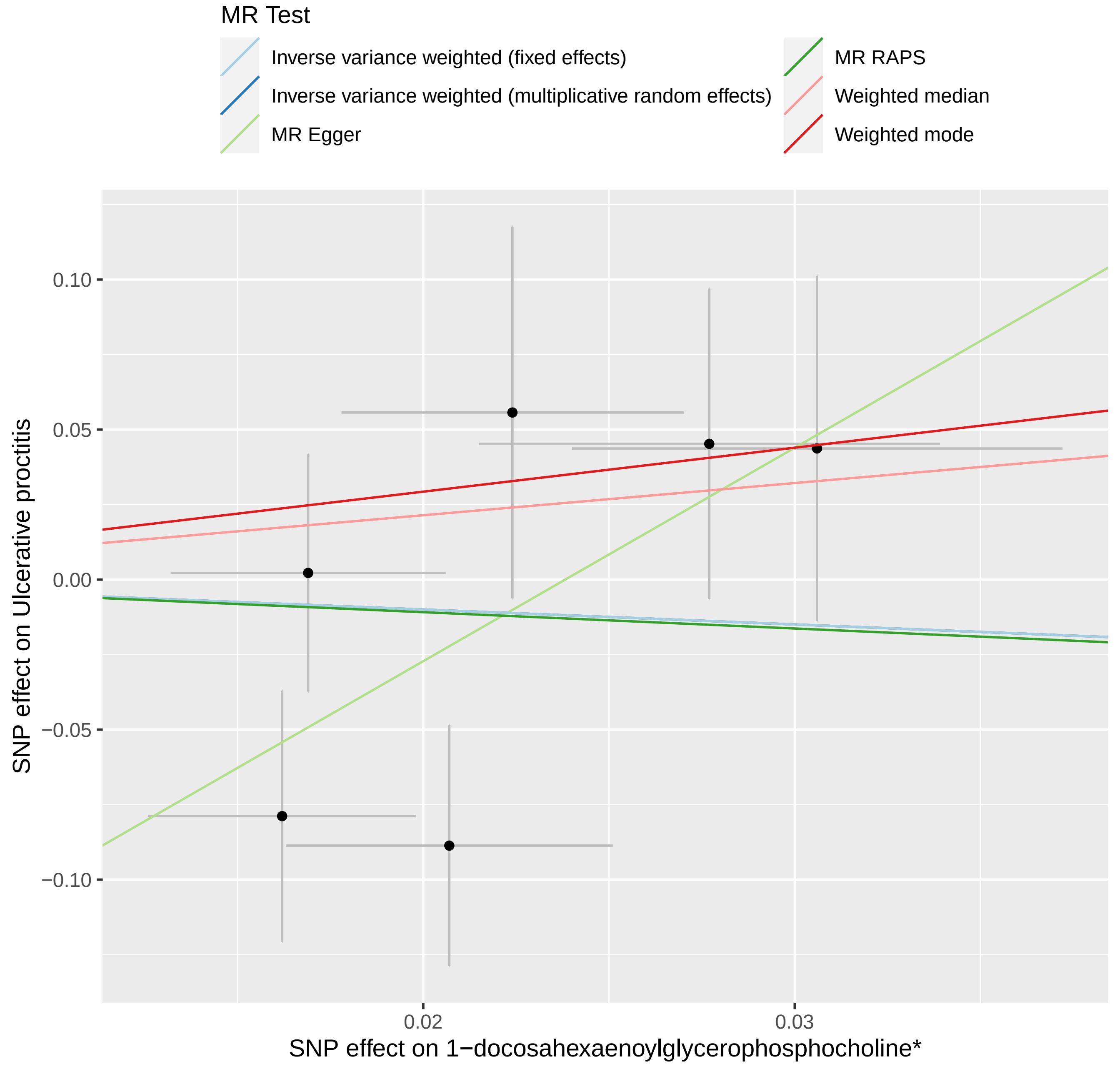

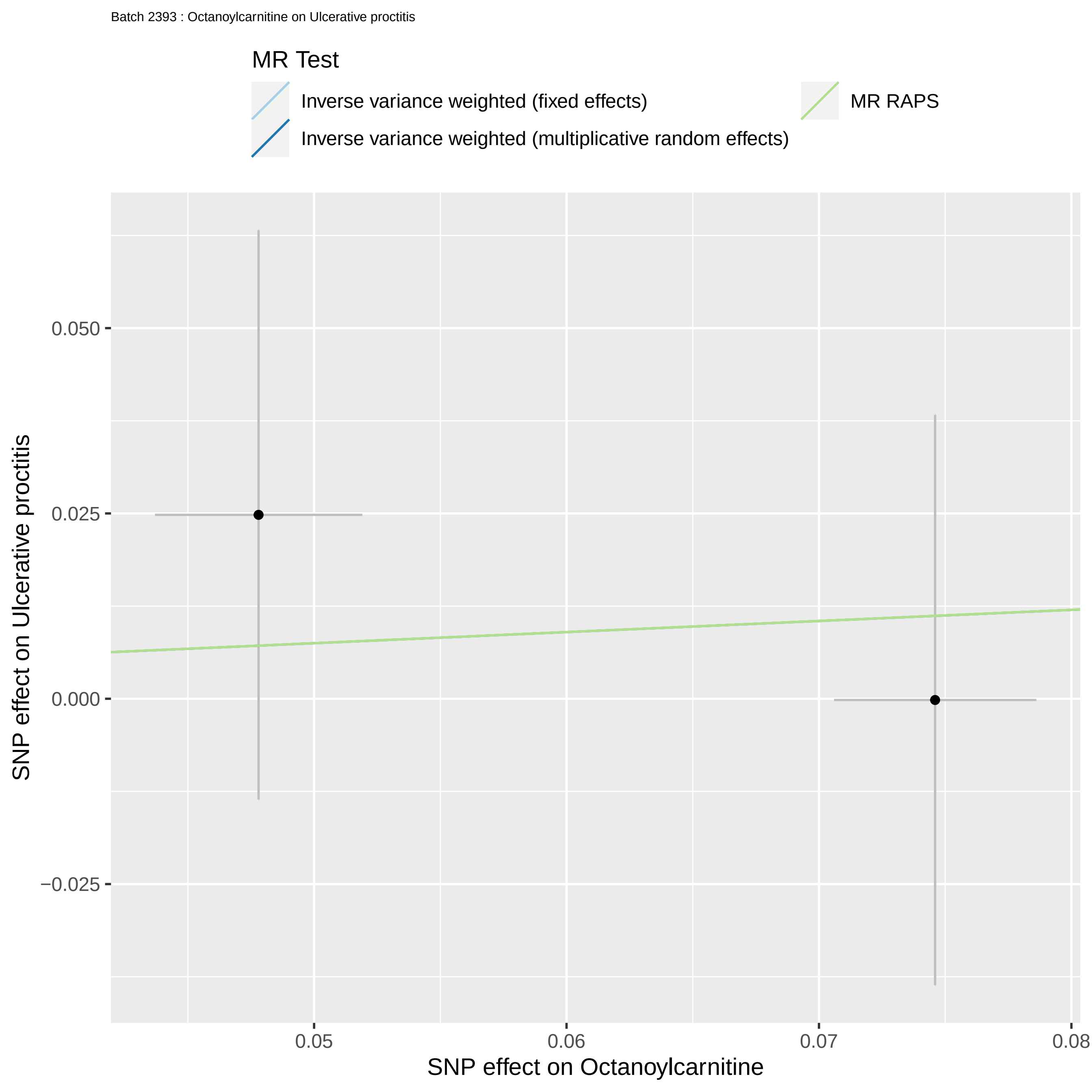

Batch 2393 : Octanoylcarnitine on Ulcerative proctitis
MR Test
Inverse variance weighted (fixed effects)
Inverse variance weighted (multiplicative random effects)
MR RAPS
0.050
0.025
0.000
−0.025
0.05
0.06
0.07
0.08
SNP effect on Octanoylcarnitine
SNP effect on Ulcerative proctitis

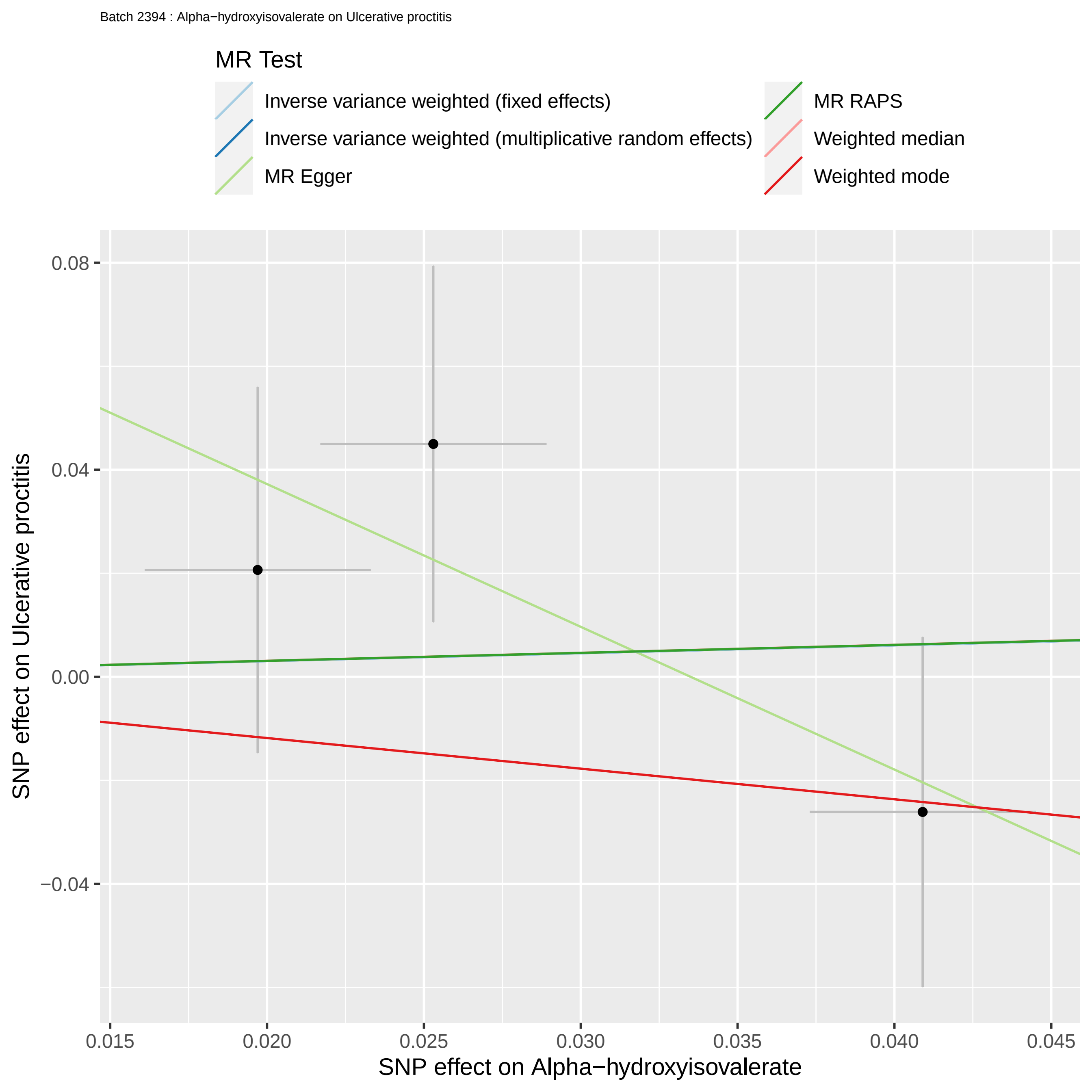
Batch 2394 : Alpha−hydroxyisovalerate on Ulcerative proctitis
MR Test
Inverse variance weighted (fixed effects)
Inverse variance weighted (multiplicative random effects)
MR Egger
MR RAPS
Weighted median
Weighted mode
0.08
0.04
0.00
−0.04
0.015
0.020
0.025
0.030
0.035
0.040
0.045
SNP effect on Ulcerative proctitis
SNP effect on Alpha−hydroxyisovalerate

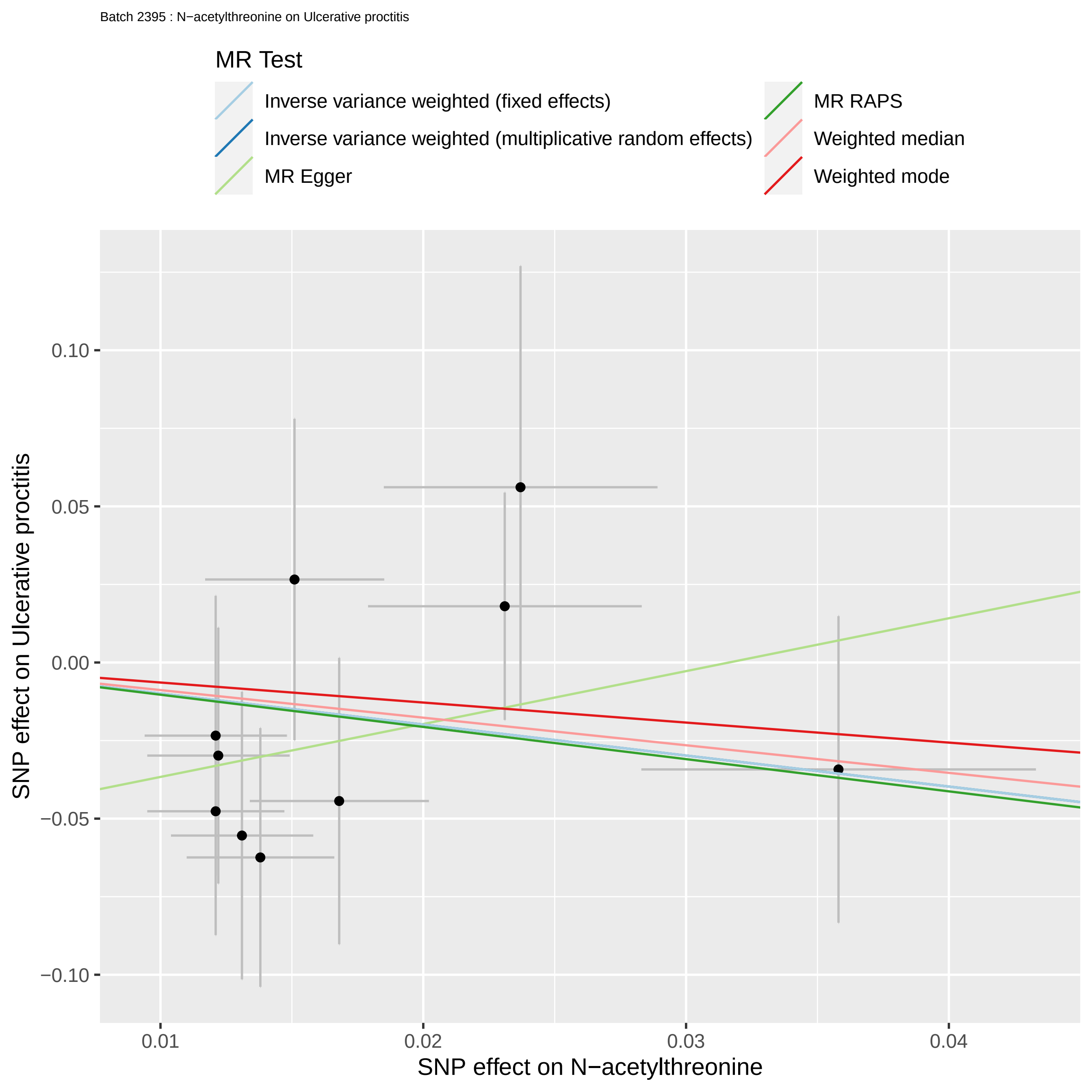

Batch 2395 : N−acetylthreonine on Ulcerative proctitis
MR Test
Inverse variance weighted (fixed effects)
Inverse variance weighted (multiplicative random effects)
MR Egger
MR RAPS
Weighted median
Weighted mode
0.10
0.05
0.00
−0.05
−0.10
SNP effect on Ulcerative proctitis
0.01
0.02
0.03
0.04
SNP effect on N−acetylthreonine

Batch 2396 : Decanoylcarnitine on Ulcerative proctitis

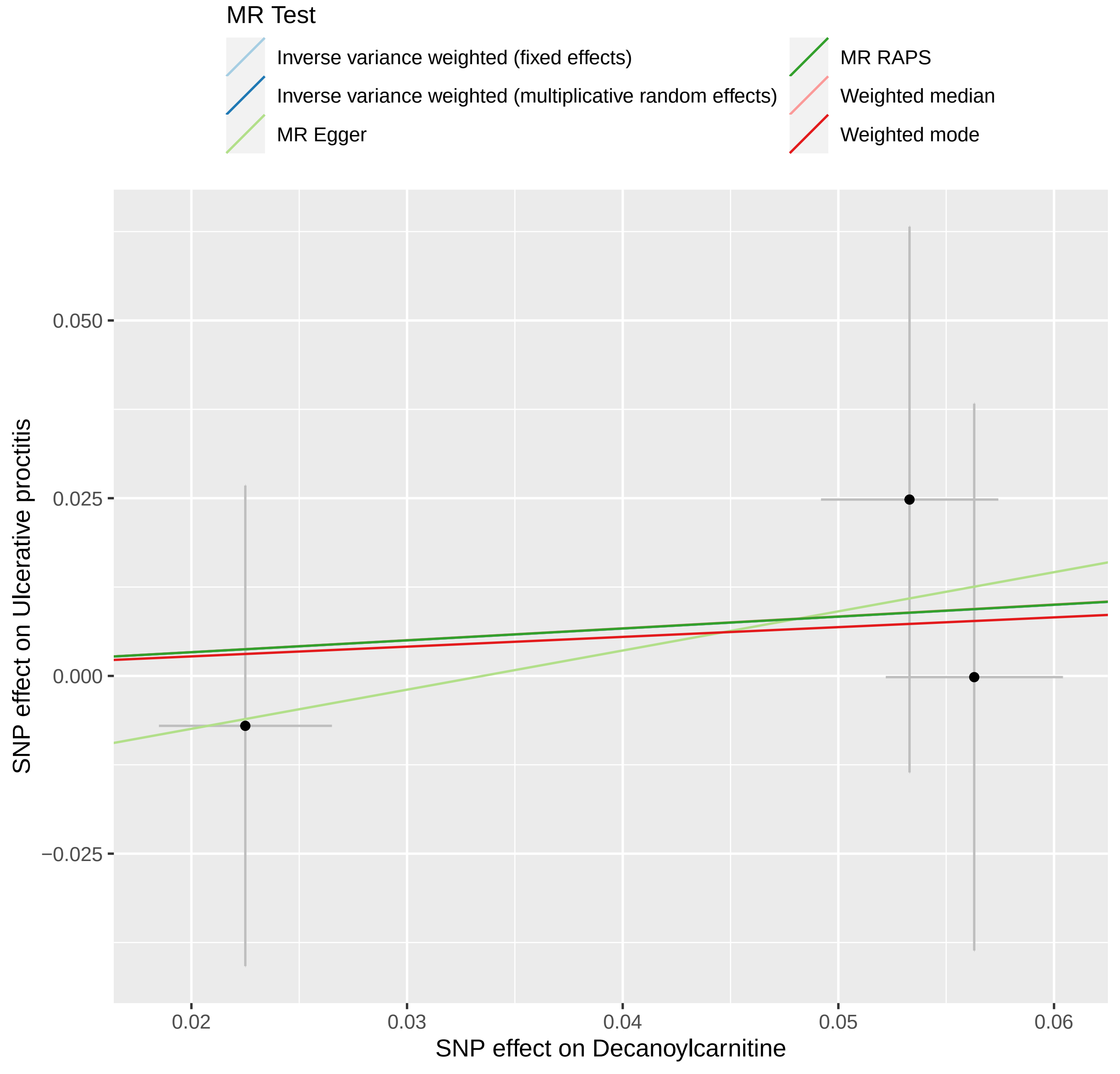

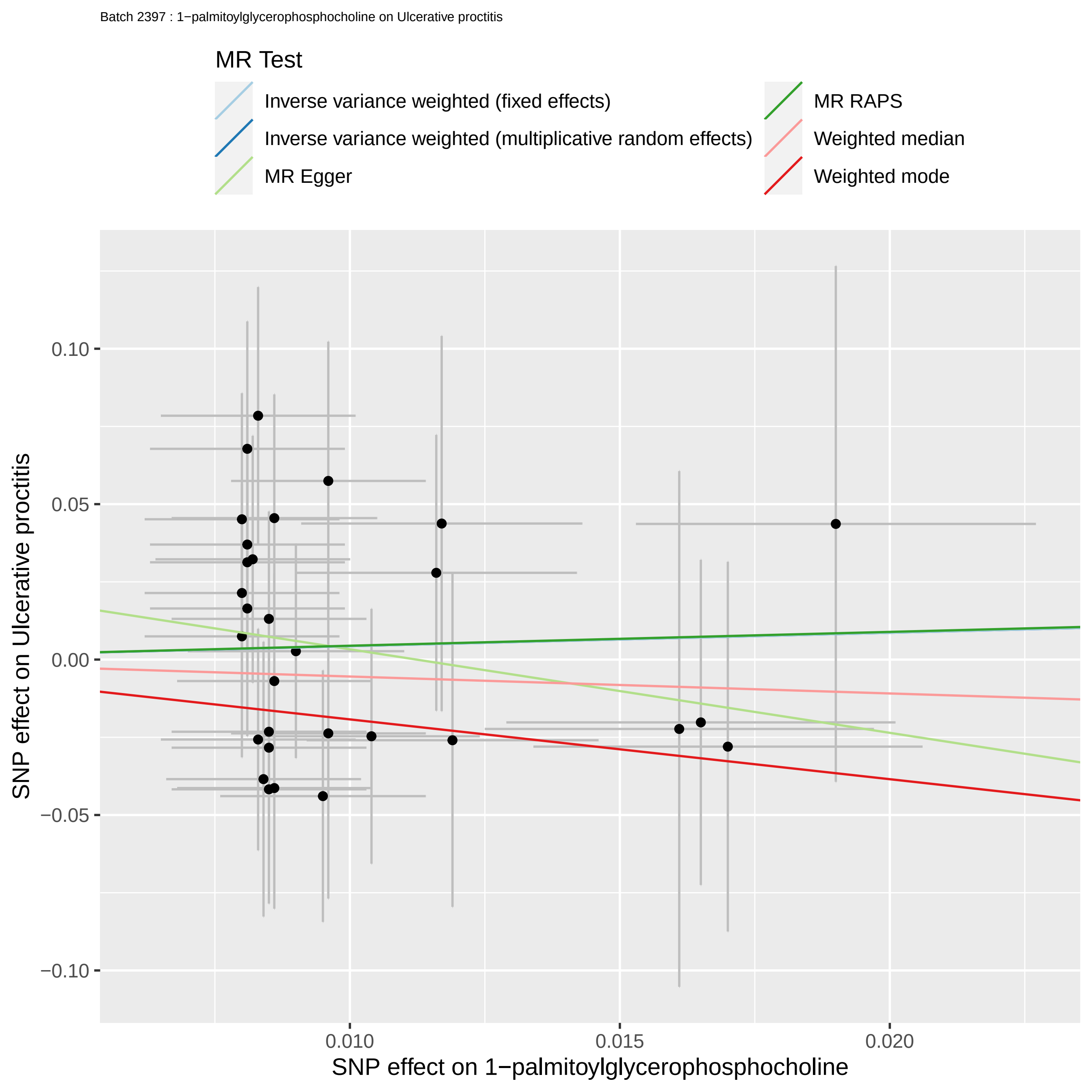

Batch 2397 : 1−palmitoylglycerophosphocholine on Ulcerative proctitis
MR Test
Inverse variance weighted (fixed effects)
Inverse variance weighted (multiplicative random effects)
MR Egger
MR RAPS
Weighted median
Weighted mode
0.10
0.05
0.00
−0.05
−0.10
0.010
0.015
0.020
SNP effect on Ulcerative proctitis
SNP effect on 1−palmitoylglycerophosphocholine

Batch 2398 : 1−heptadecanoylglycerophosphocholine on Ulcerative proctitis

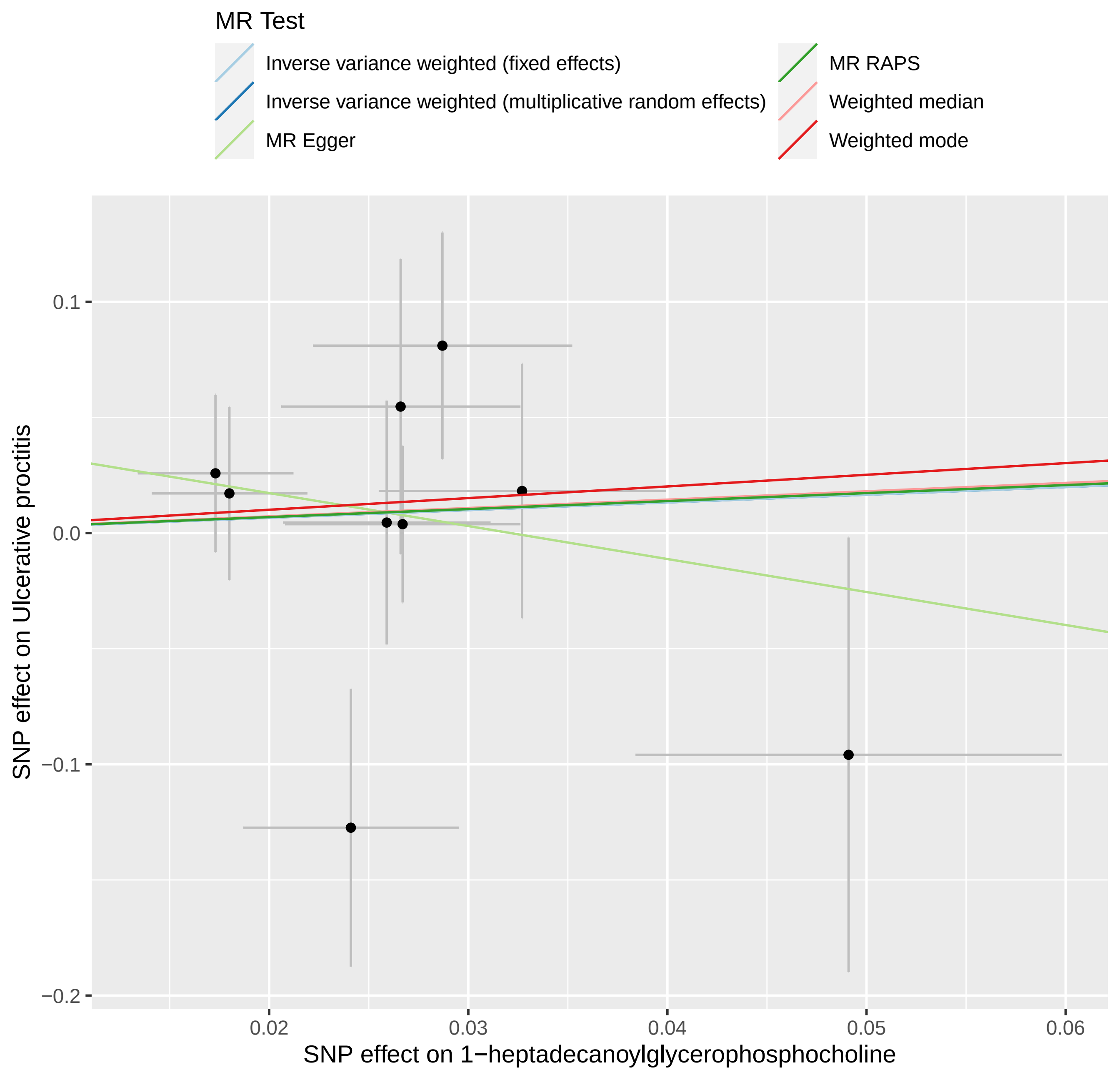

Batch 2399 : 1−oleoylglycerophosphocholine on Ulcerative proctitis

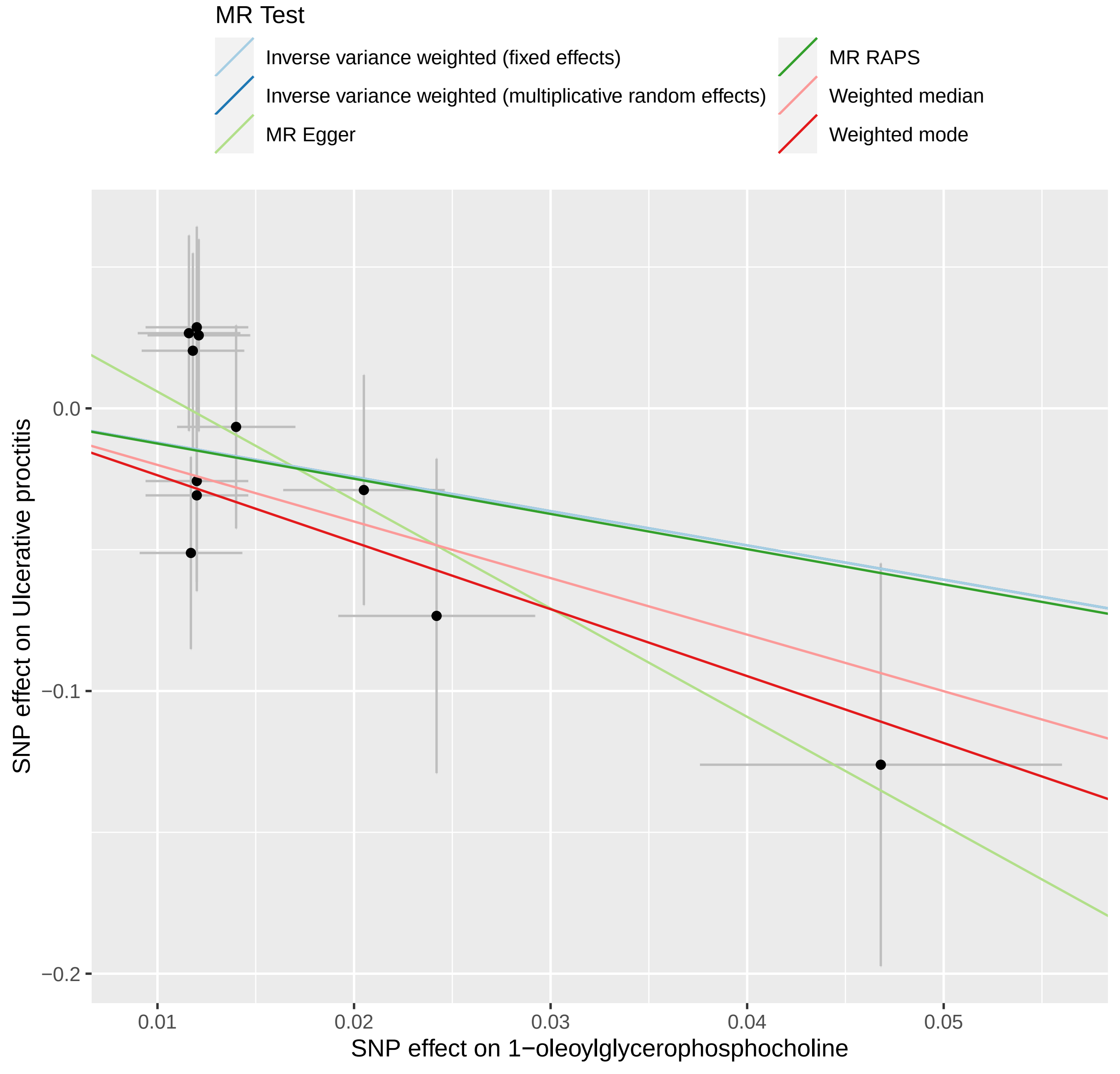

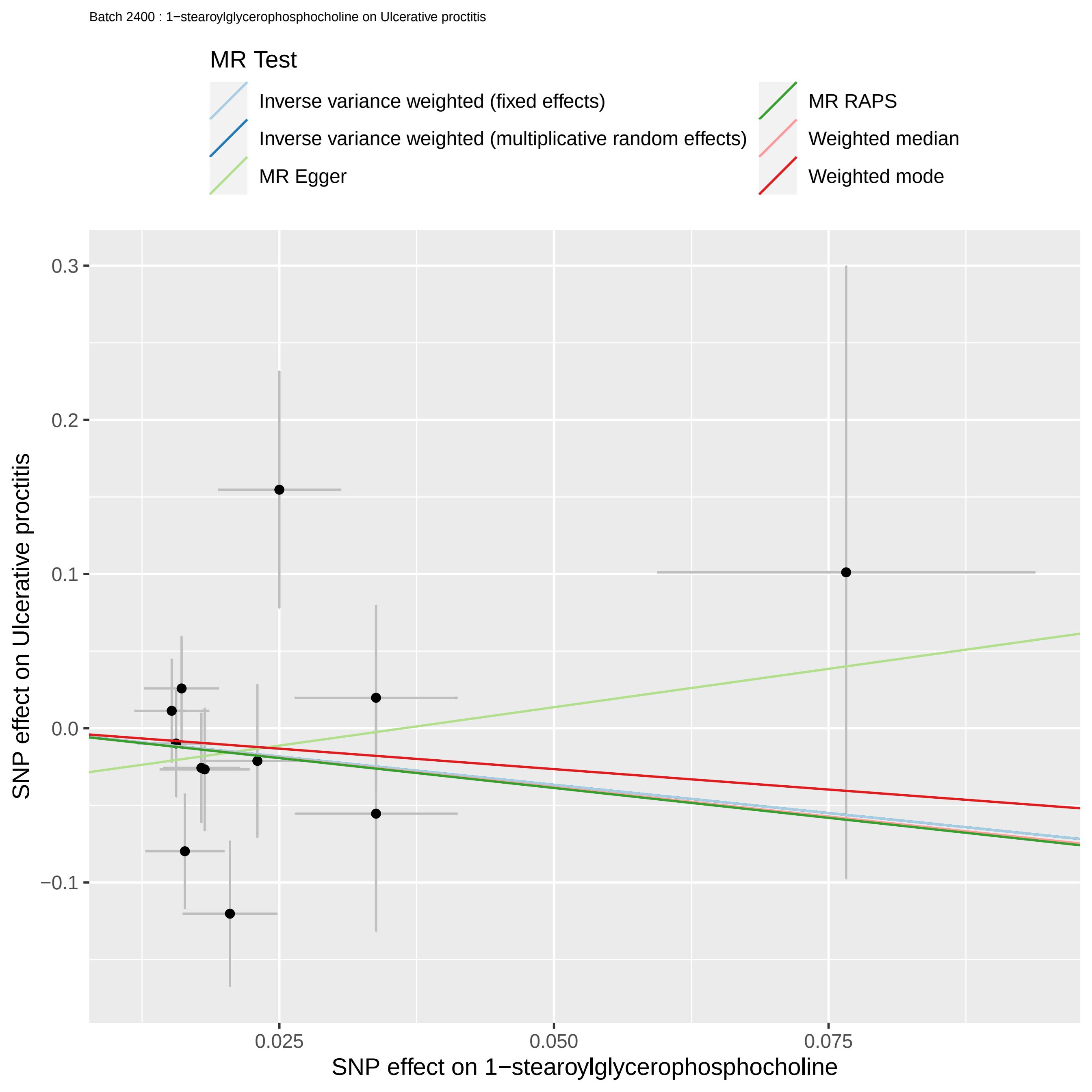
Batch 2400 : 1−stearoylglycerophosphocholine on Ulcerative proctitis
MR Test
Inverse variance weighted (fixed effects)
Inverse variance weighted (multiplicative random effects)
MR Egger
MR RAPS
Weighted median
Weighted mode
0.3
0.2
0.1
0.0
−0.1
0.025
0.050
0.075
SNP effect on Ulcerative proctitis
SNP effect on 1−stearoylglycerophosphocholine

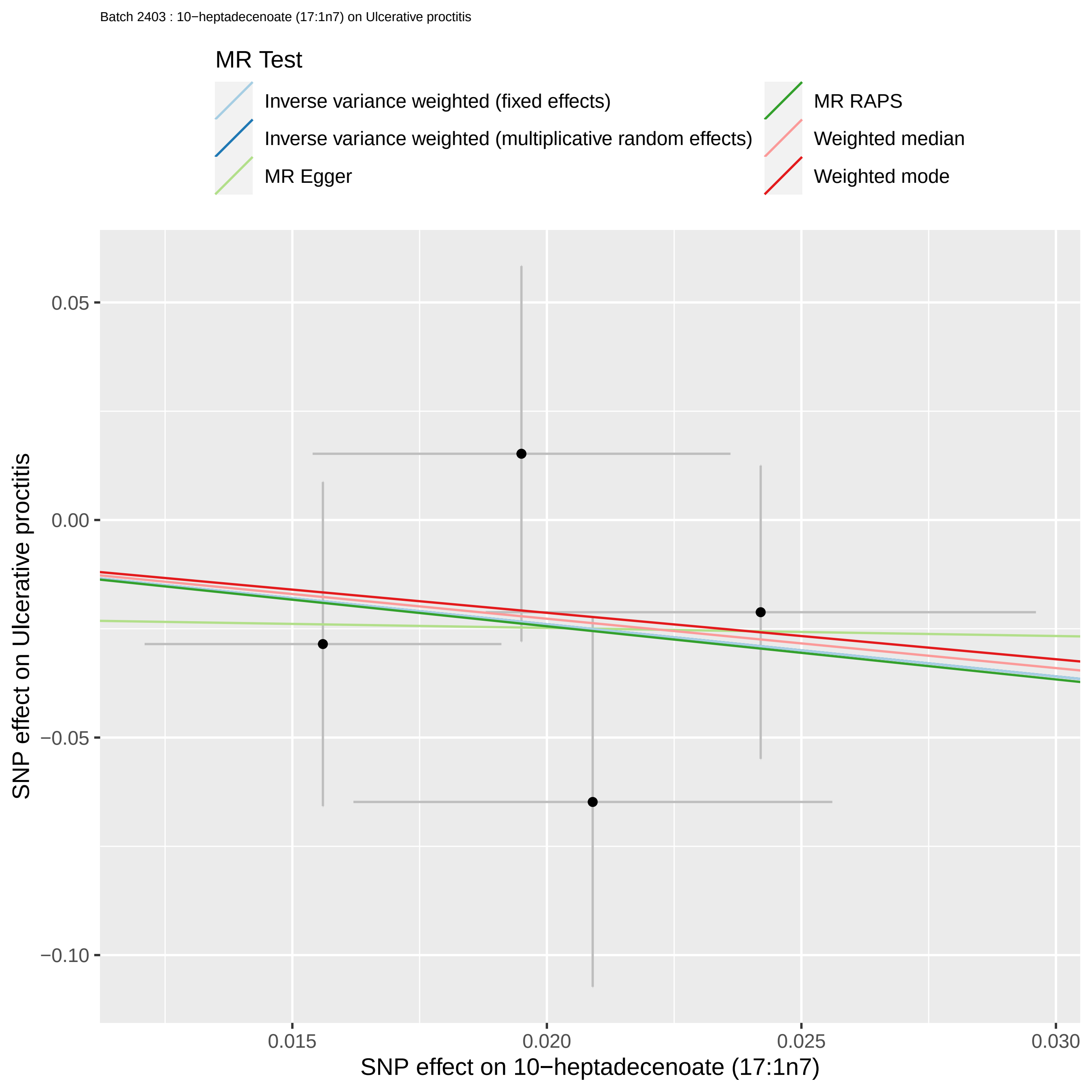

Batch 2403 : 10−heptadecenoate (17:1n7) on Ulcerative proctitis
MR Test
Inverse variance weighted (fixed effects)
Inverse variance weighted (multiplicative random effects)
MR Egger
MR RAPS
Weighted median
Weighted mode
0.05
0.00
−0.05
−0.10
0.015
0.020
0.025
0.030
SNP effect on Ulcerative proctitis
SNP effect on 10−heptadecenoate (17:1n7)

Batch 2405 : Epiandrosterone sulfate on Ulcerative proctitis

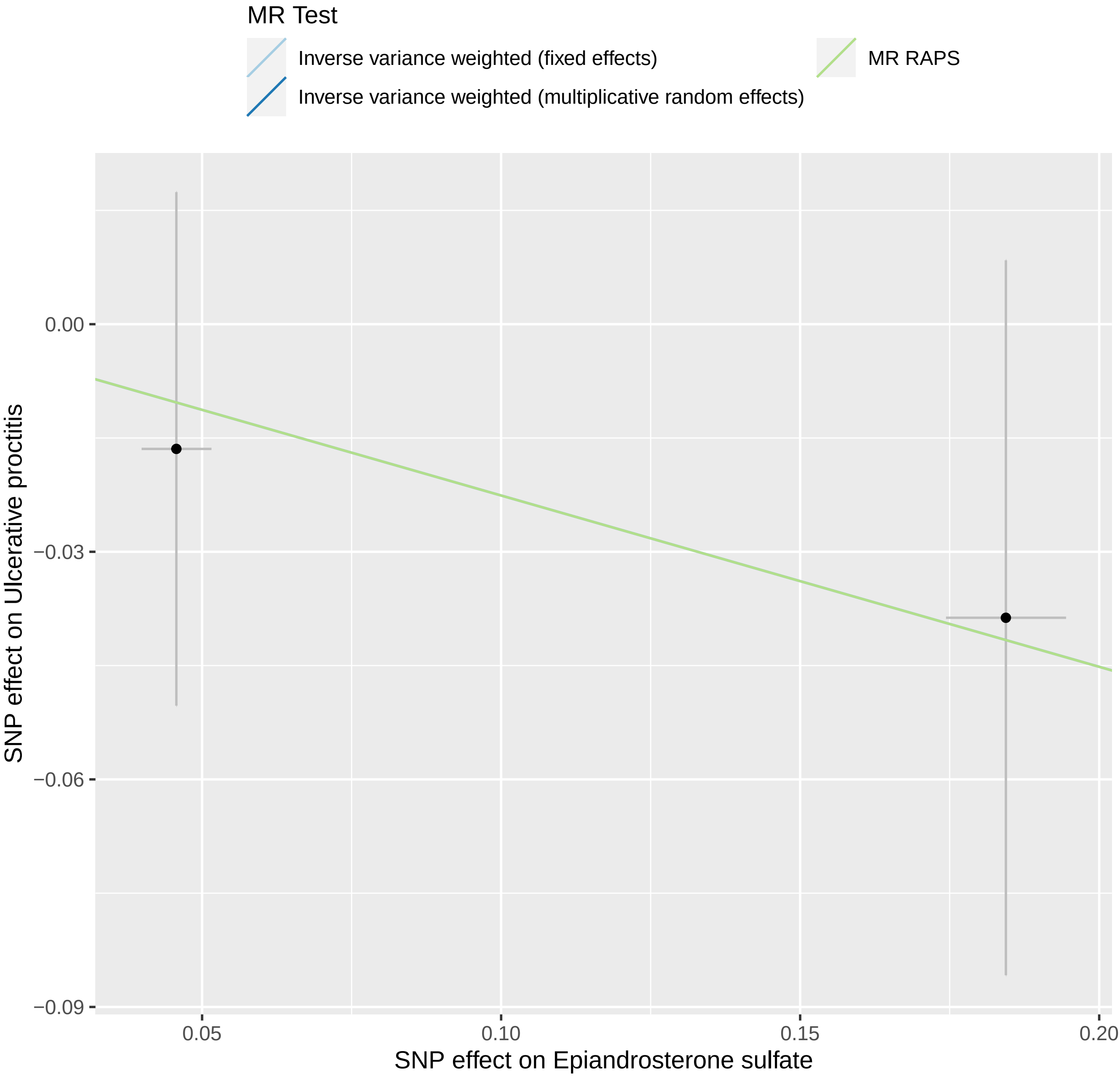

Batch 2406 : Linolenate [alpha or gamma; (18:3n3 or 6)] on Ulcerative proctitis

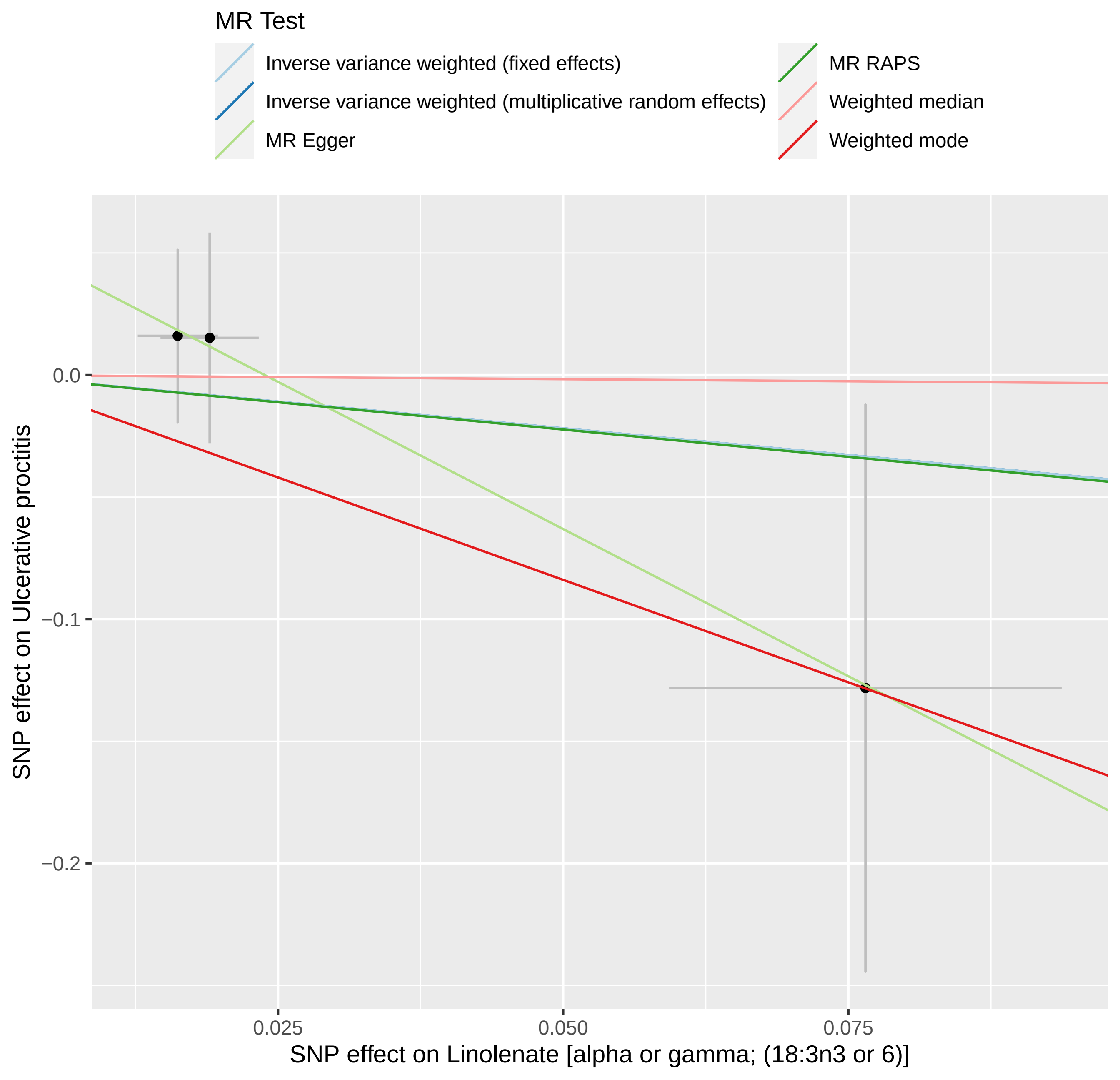

Batch 2407 : X−12510−−2−aminooctanoic acid on Ulcerative proctitis

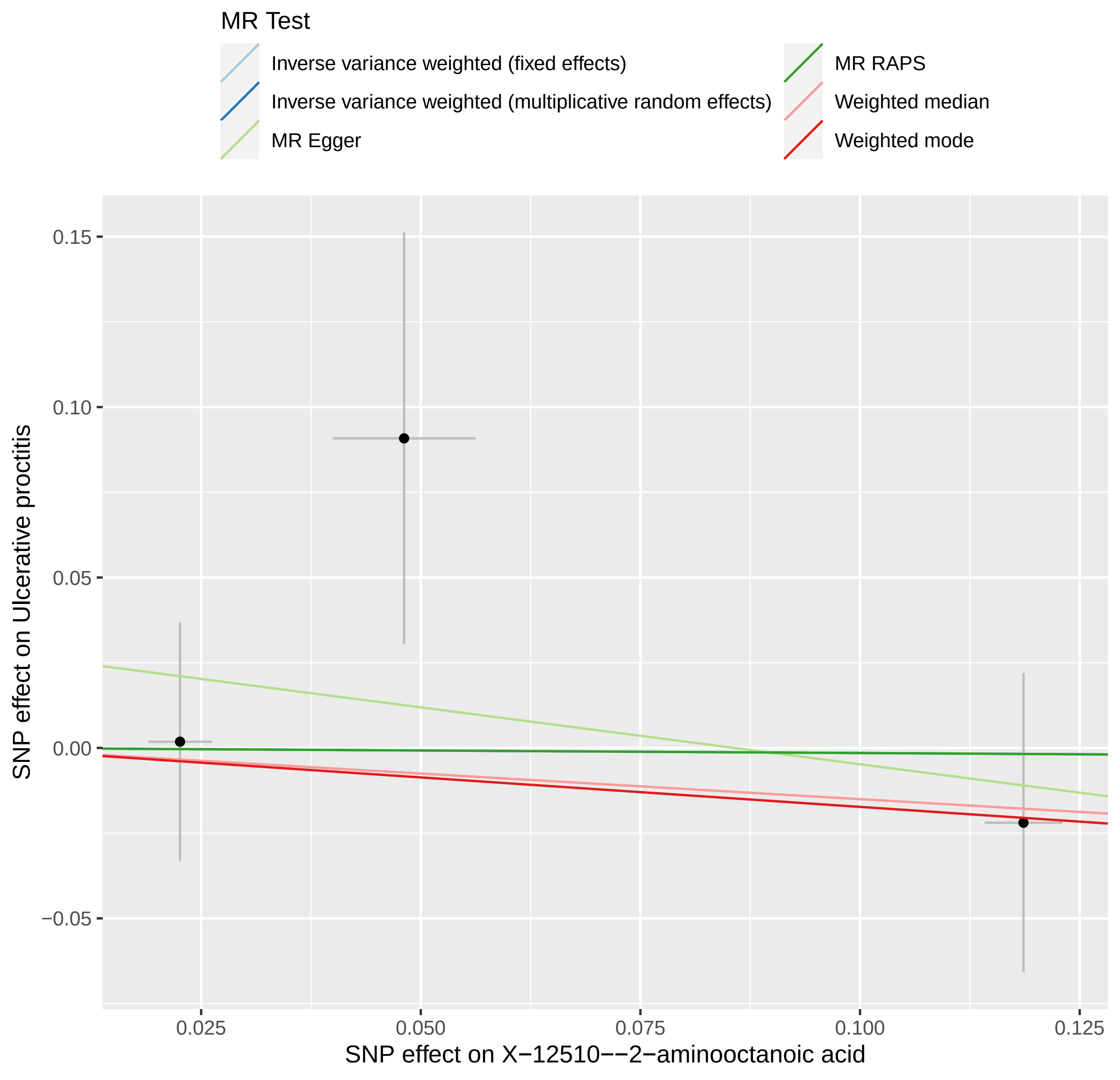

Batch 2410 : Asparagine on Ulcerative proctitis

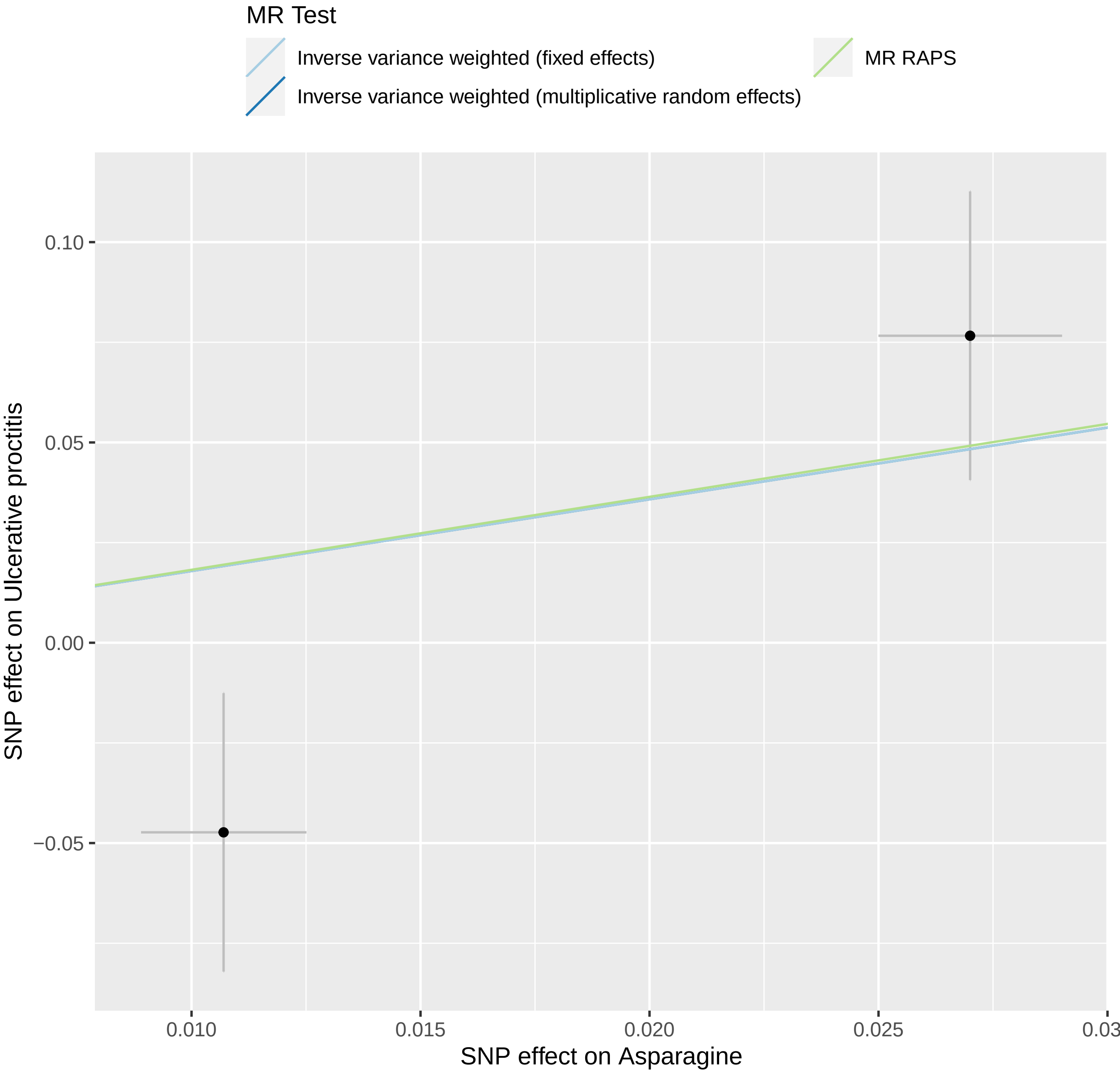

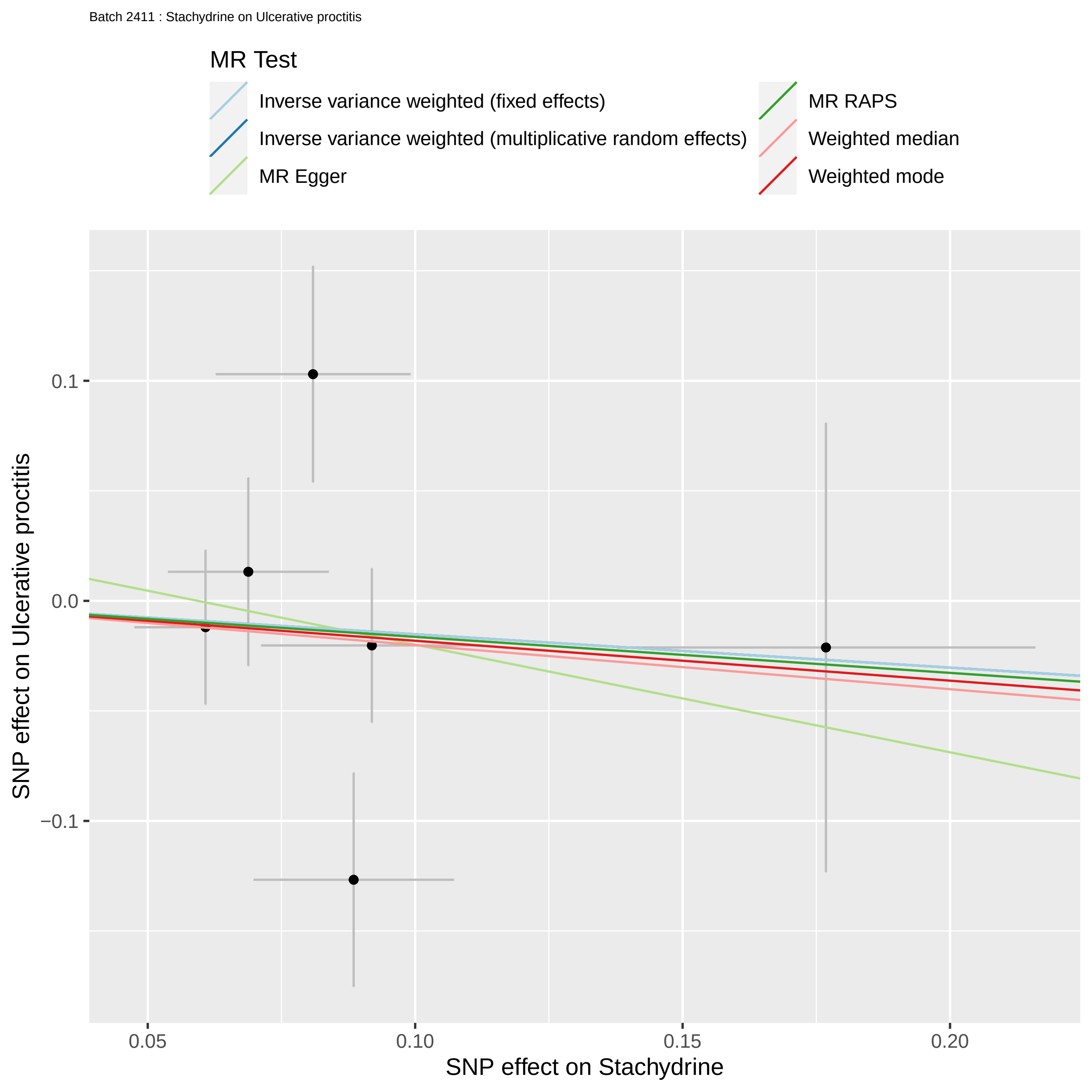

Batch 2411 : Stachydrine on Ulcerative proctitis
MR Test
Inverse variance weighted (fixed effects)
Inverse variance weighted (multiplicative random effects)
MR Egger
MR RAPS
Weighted median
Weighted mode
0.1
0.0
−0.1
0.05
0.10
0.15
0.20
SNP effect on Stachydrine
SNP effect on Ulcerative proctitis

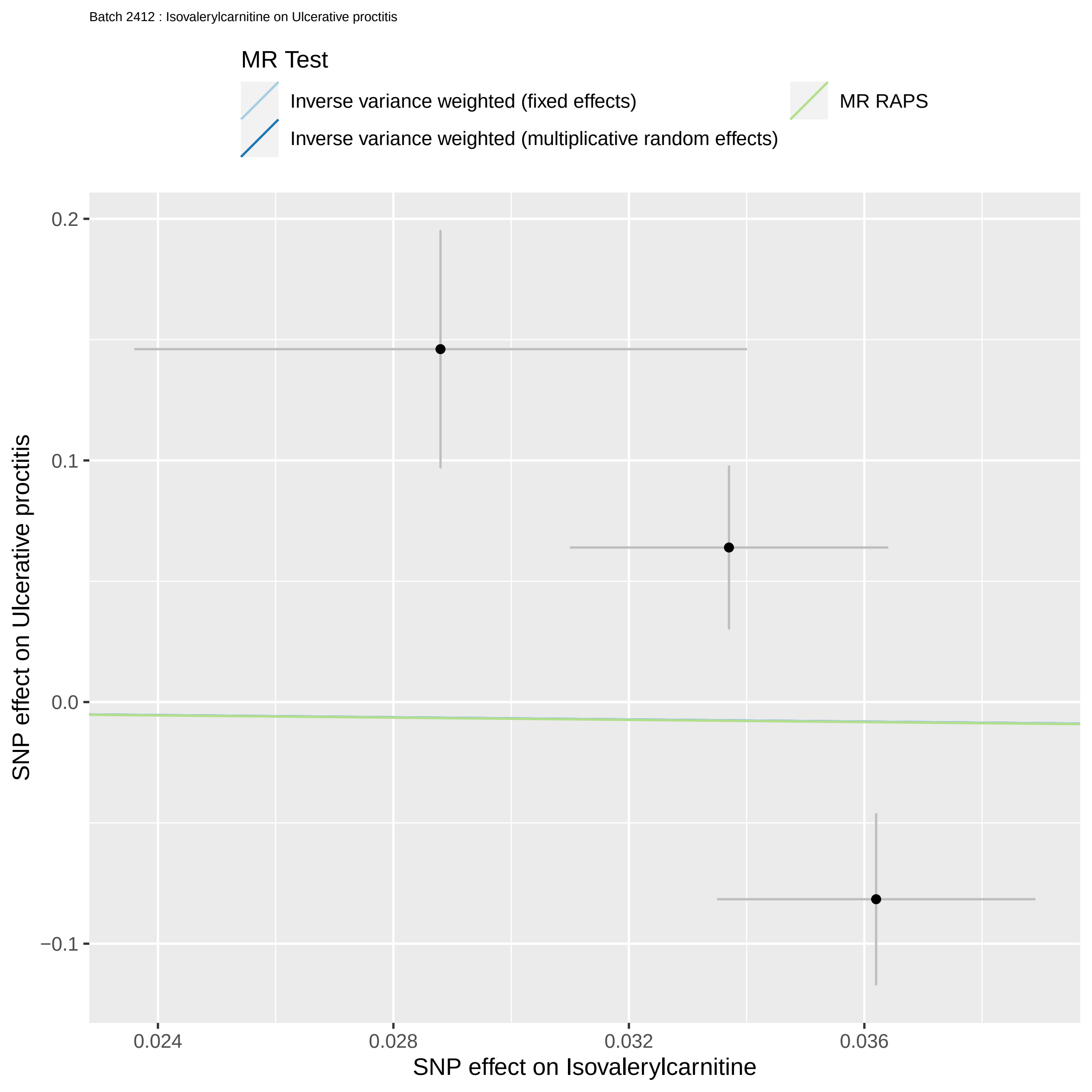

Batch 2412 : Isovalerylcarnitine on Ulcerative proctitis
MR Test
Inverse variance weighted (fixed effects)
Inverse variance weighted (multiplicative random effects)
MR RAPS
0.2
0.1
0.0
−0.1
0.024
0.028
0.032
0.036
SNP effect on Isovalerylcarnitine
SNP effect on Ulcerative proctitis

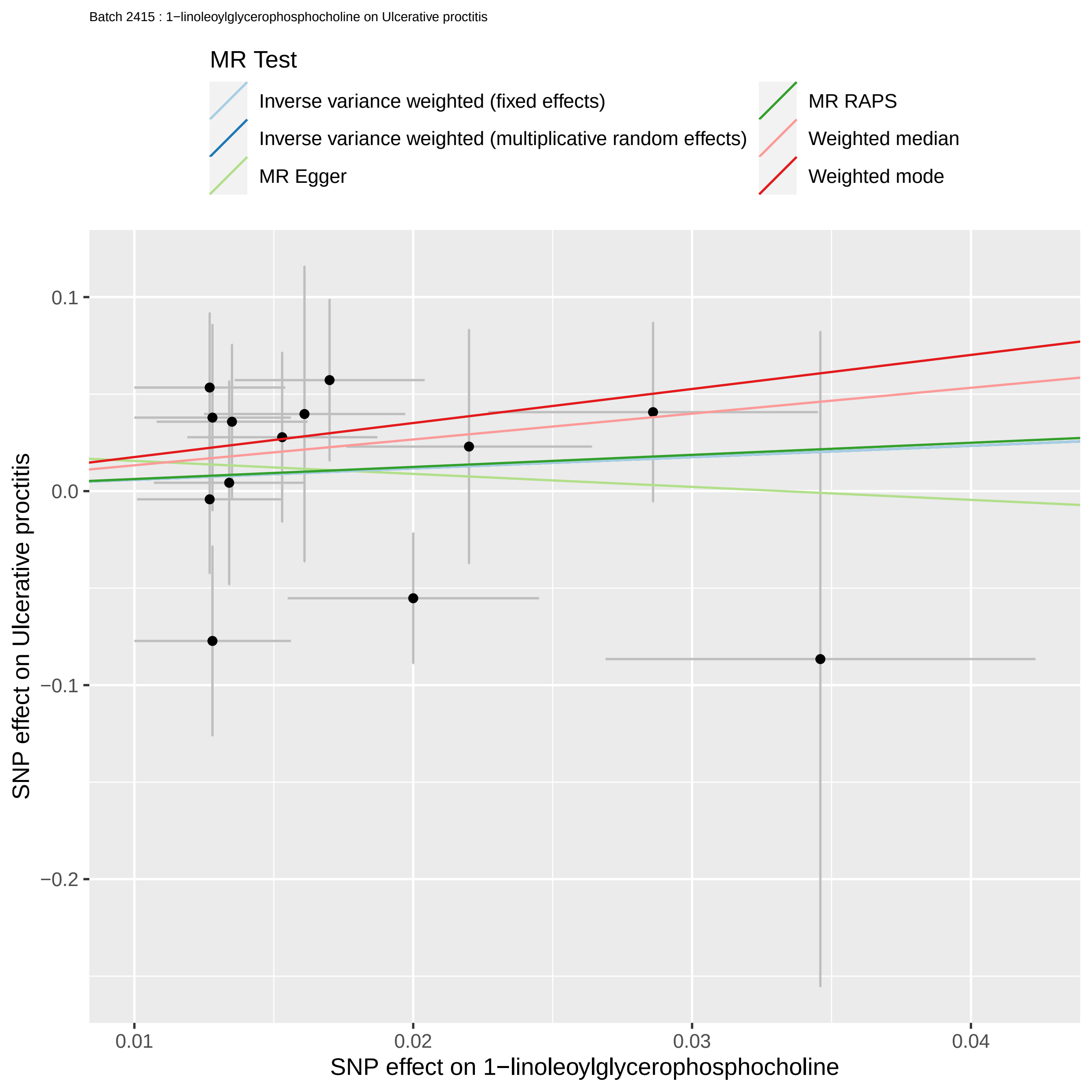

Batch 2415 : 1−linoleoylglycerophosphocholine on Ulcerative proctitis
MR Test
Inverse variance weighted (fixed effects)
Inverse variance weighted (multiplicative random effects)
MR Egger
MR RAPS
Weighted median
Weighted mode
0.1
0.0
−0.1
−0.2
0.01
0.02
0.03
0.04
SNP effect on Ulcerative proctitis
SNP effect on 1−linoleoylglycerophosphocholine

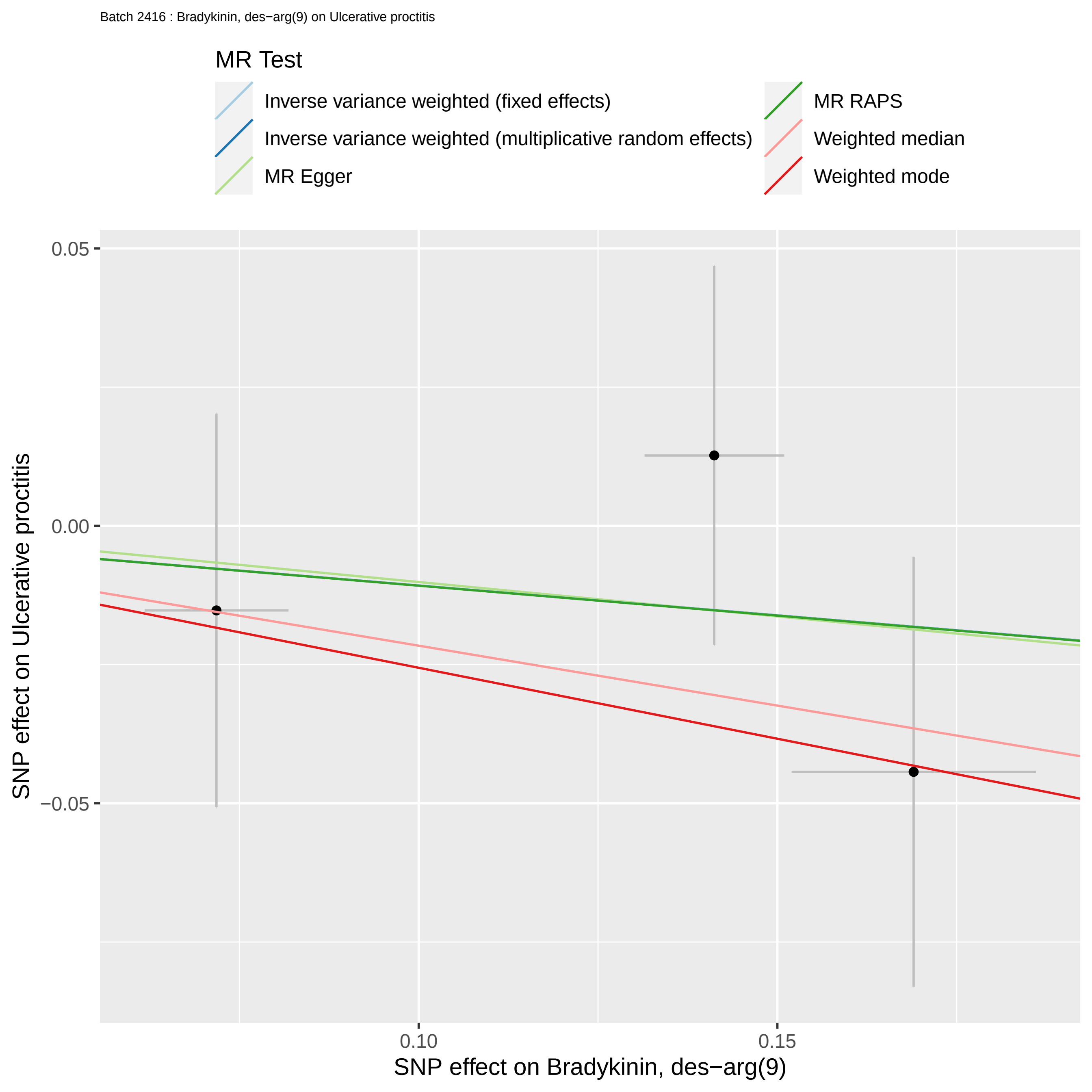

Batch 2416 : Bradykinin, des−arg(9) on Ulcerative proctitis
MR Test
Inverse variance weighted (fixed effects)
Inverse variance weighted (multiplicative random effects)
MR Egger
MR RAPS
Weighted median
Weighted mode
0.05
0.00
−0.05
0.10
0.15
SNP effect on Ulcerative proctitis
SNP effect on Bradykinin, des−arg(9)

Batch 2417 : Gamma−glutamylisoleucine* on Ulcerative proctitis

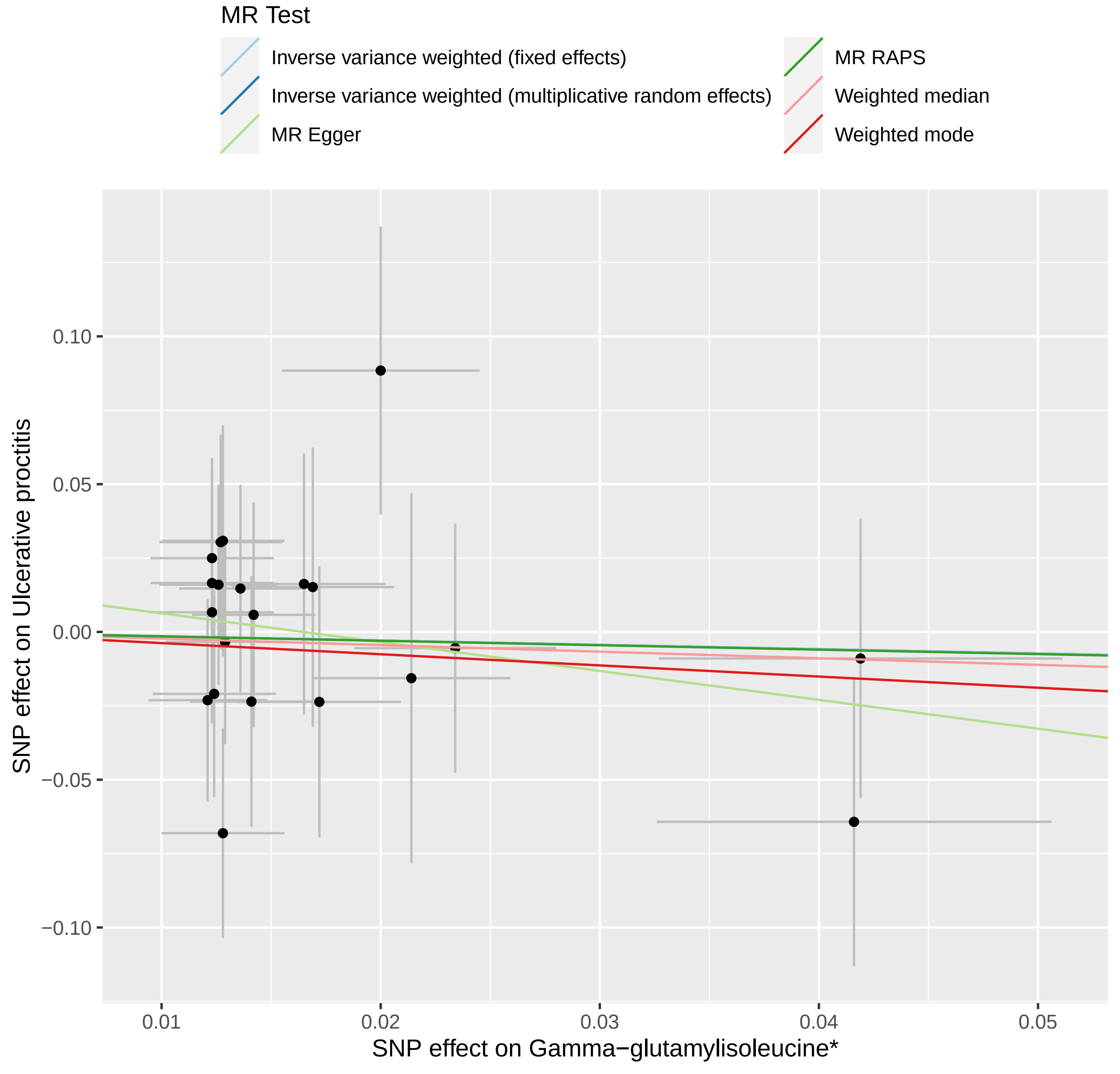

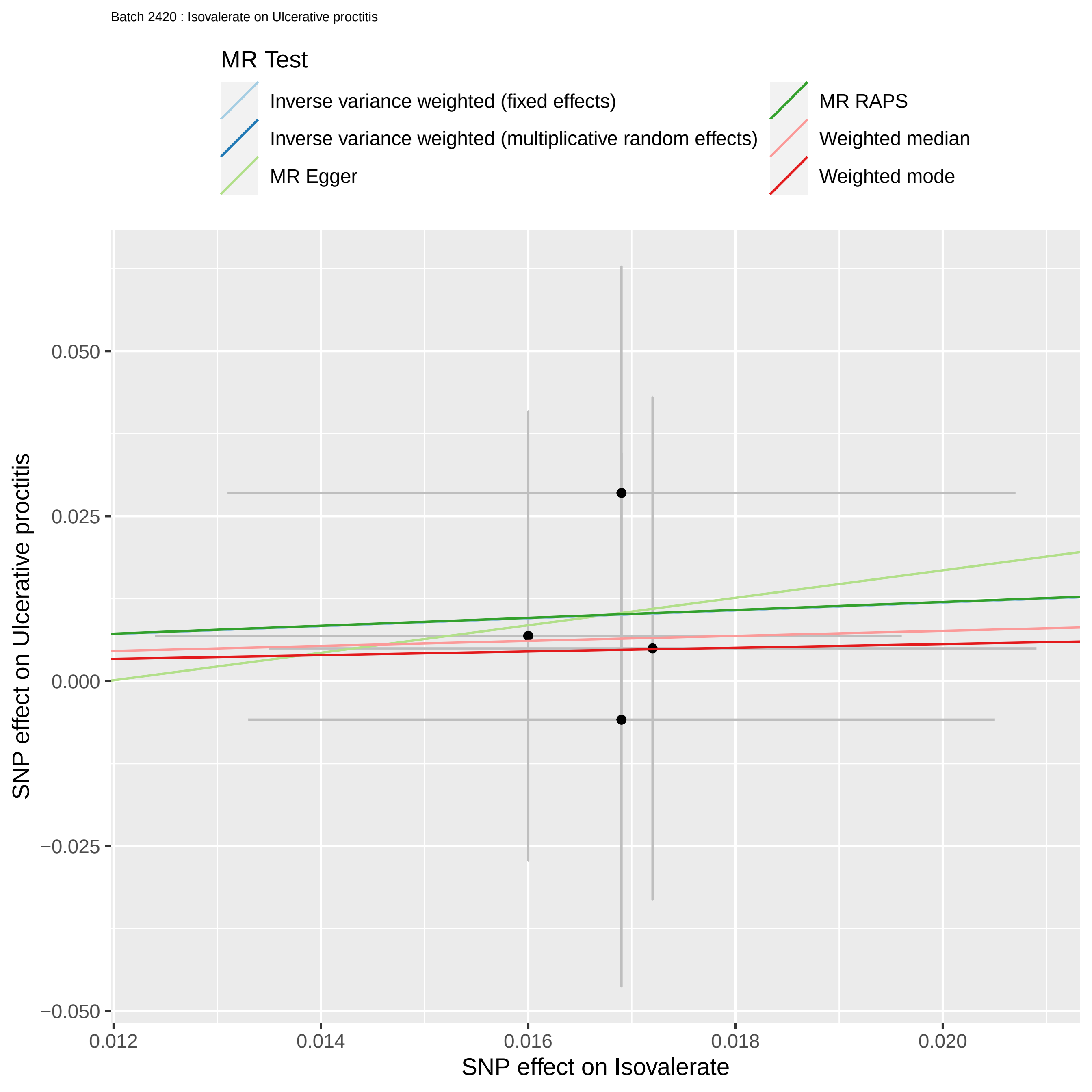
Batch 2420 : Isovalerate on Ulcerative proctitis
MR Test
Inverse variance weighted (fixed effects)
Inverse variance weighted (multiplicative random effects)
MR Egger
MR RAPS
Weighted median
Weighted mode
0.050
0.025
0.000
−0.025
−0.050
SNP effect on Ulcerative proctitis
0.012
0.014
0.016
0.018
0.020
SNP effect on Isovalerate

Batch 2421 : X−13183−−stearamide on Ulcerative proctitis

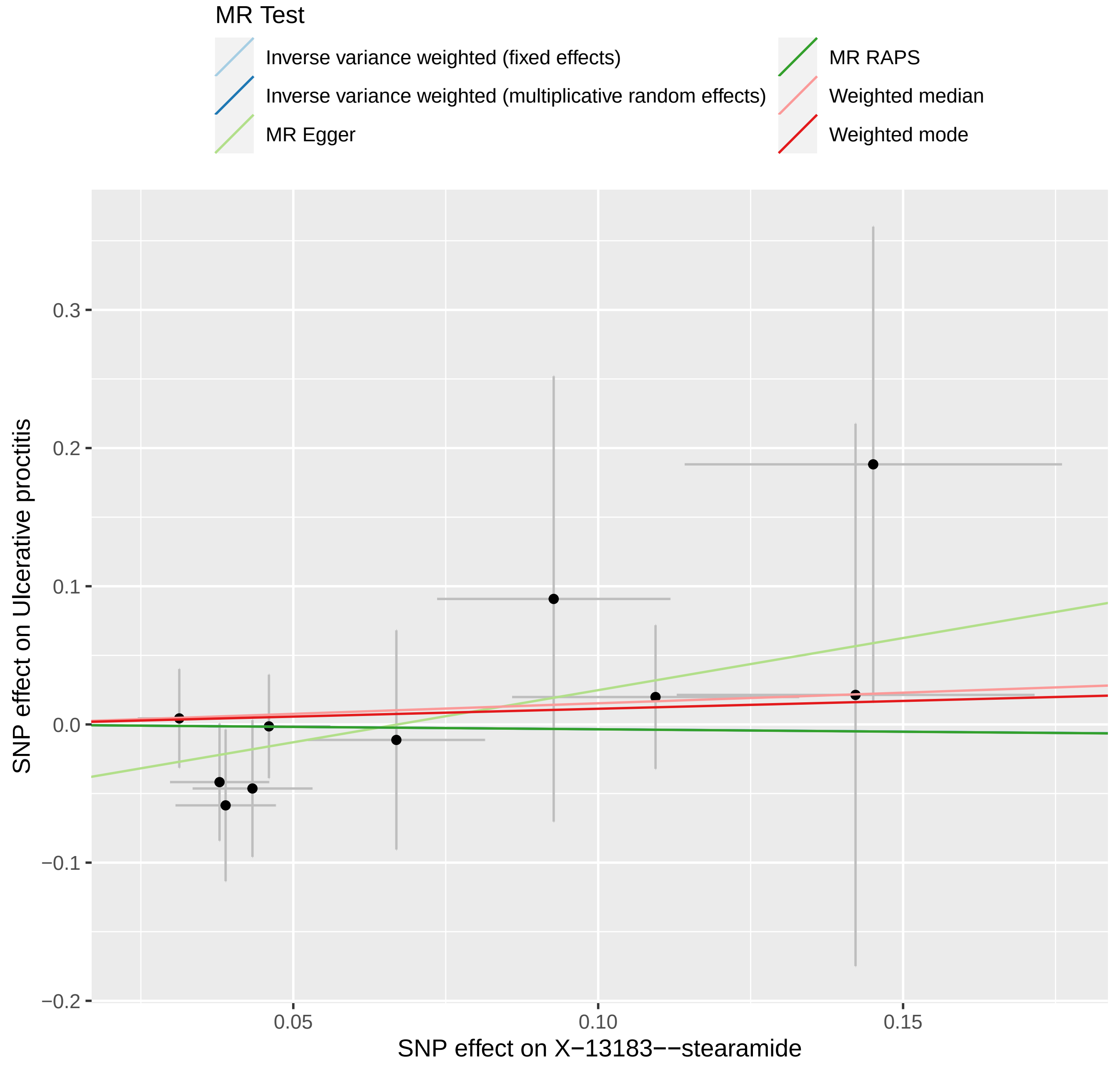

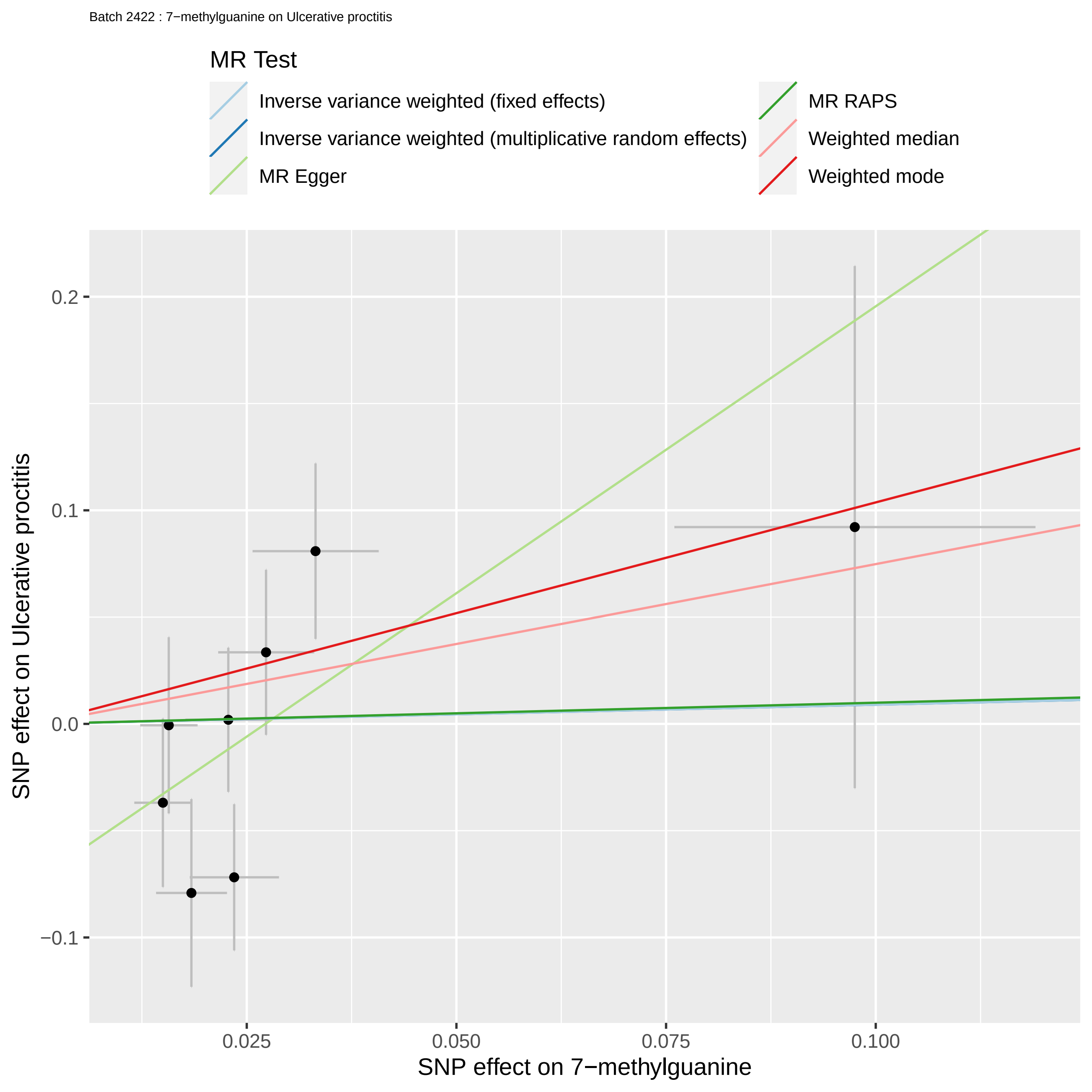

Batch 2422 : 7−methylguanine on Ulcerative proctitis
MR Test
Inverse variance weighted (fixed effects)
Inverse variance weighted (multiplicative random effects)
MR Egger
MR RAPS
Weighted median
Weighted mode
0.2
0.1
0.0
−0.1
0.025
0.050
0.075
0.100
SNP effect on Ulcerative proctitis
SNP effect on 7−methylguanine

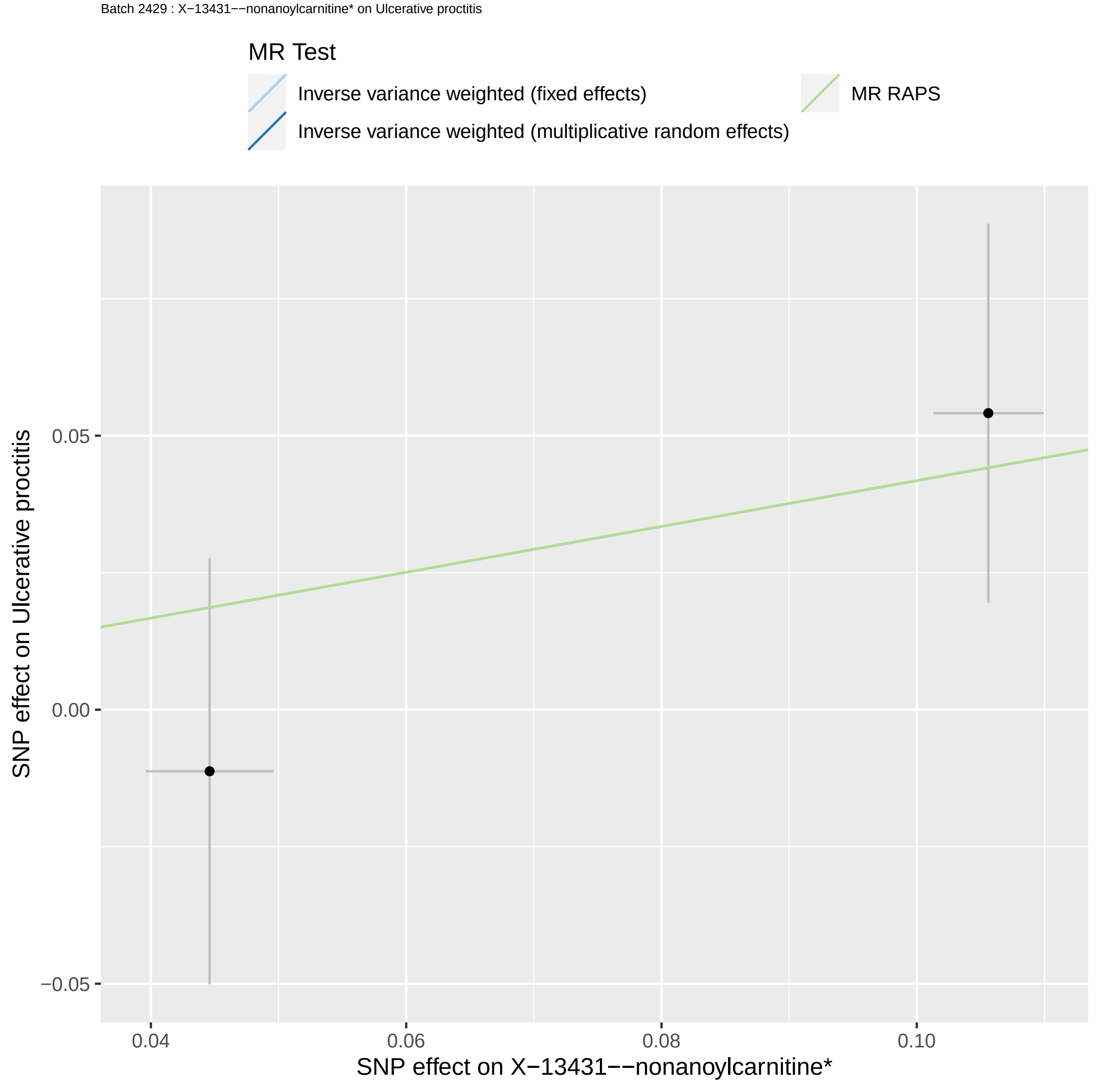
Batch 2429 : X−13431−−nonanoylcarnitine* on Ulcerative proctitis
MR Test
Inverse variance weighted (fixed effects)
Inverse variance weighted (multiplicative random effects)
MR RAPS
SNP effect on Ulcerative proctitis
0.05
0.00
−0.05
0.04
0.06
0.08
0.10
SNP effect on X−13431−−nonanoylcarnitine*

Batch 2430 : 2−palmitoylglycerophosphocholine* on Ulcerative proctitis

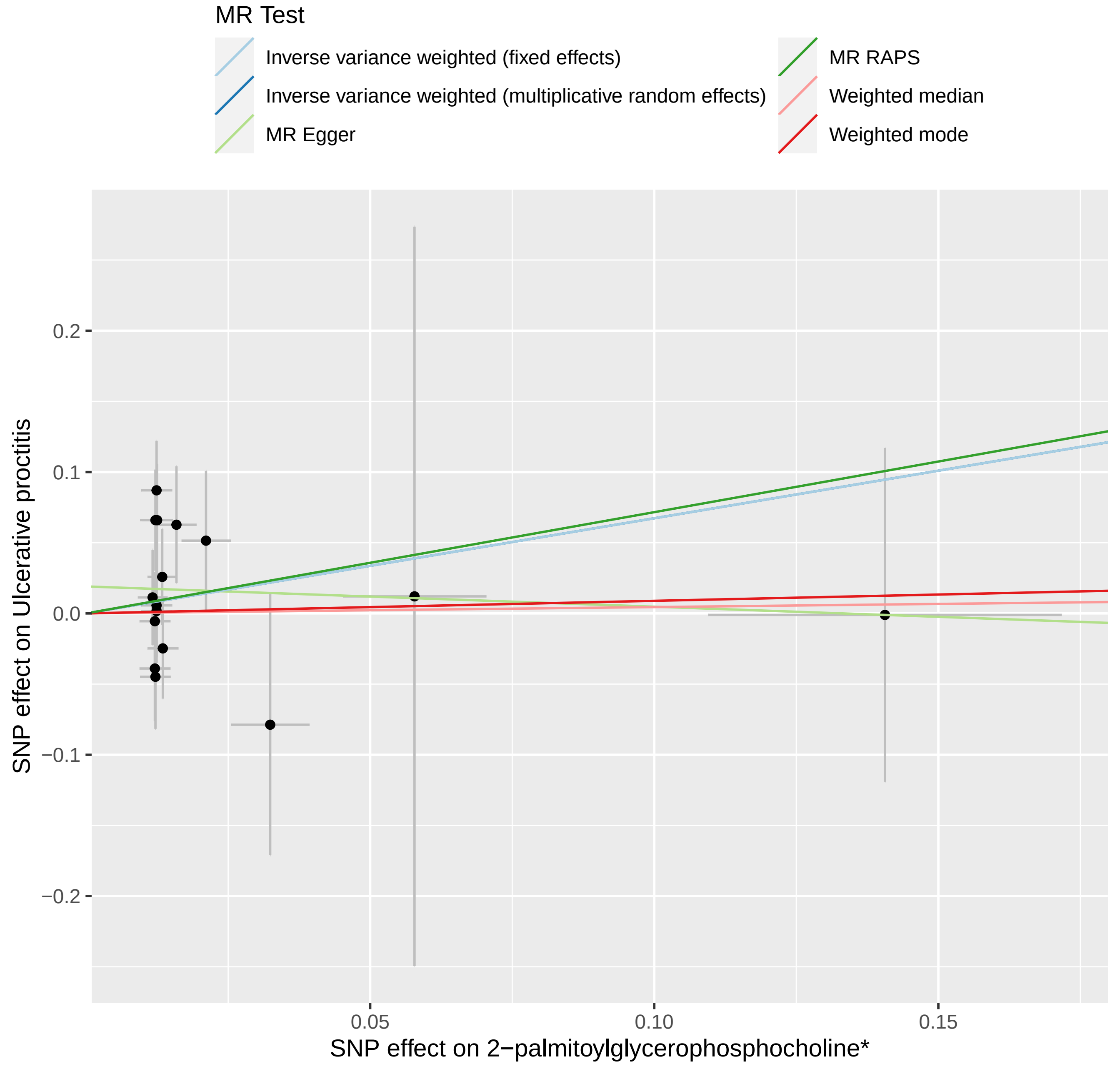

Batch 2431 : 2−oleoylglycerophosphocholine* on Ulcerative proctitis

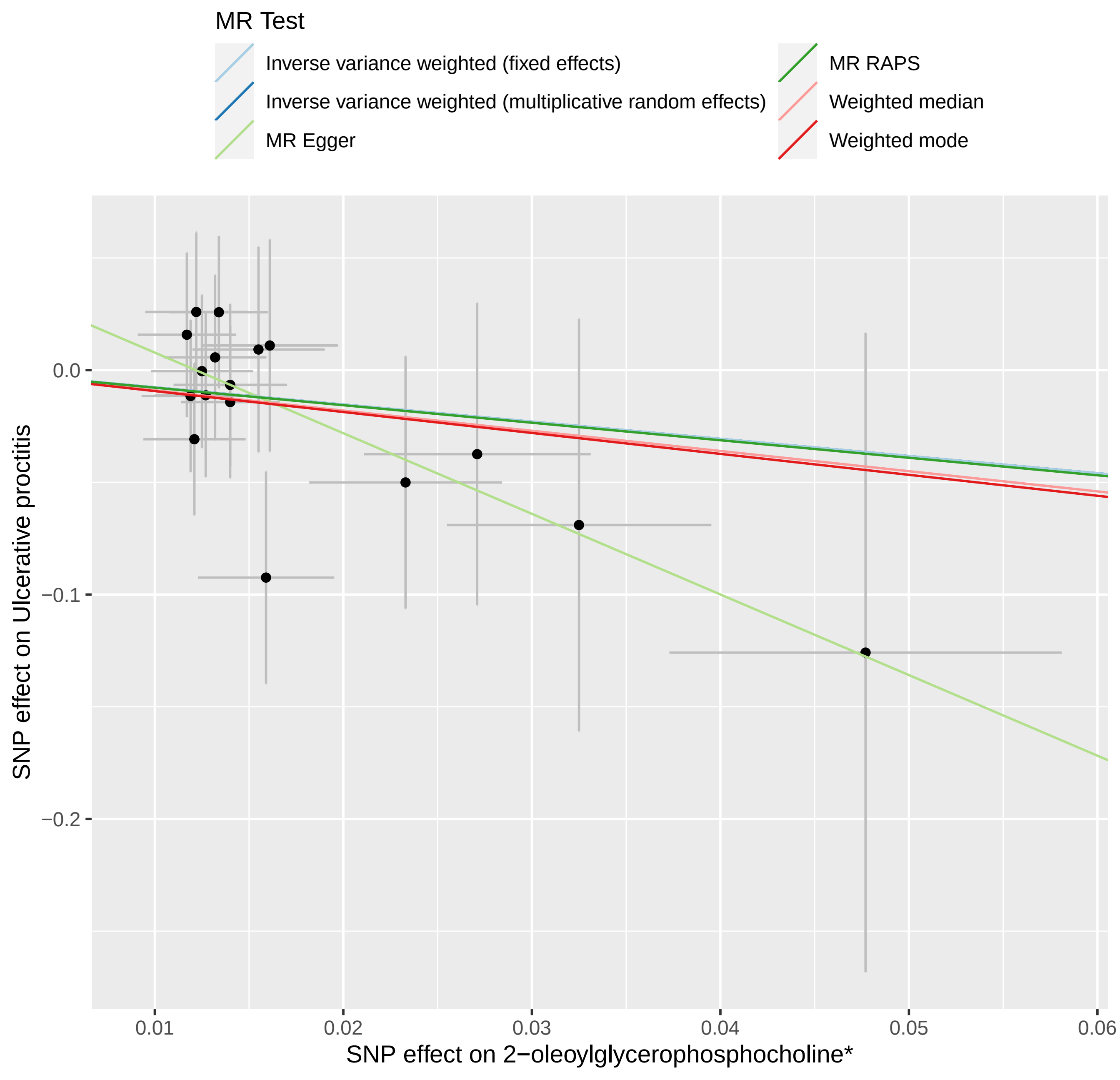

Batch 2434 : 1−palmitoylglycerophosphoinositol* on Ulcerative proctitis

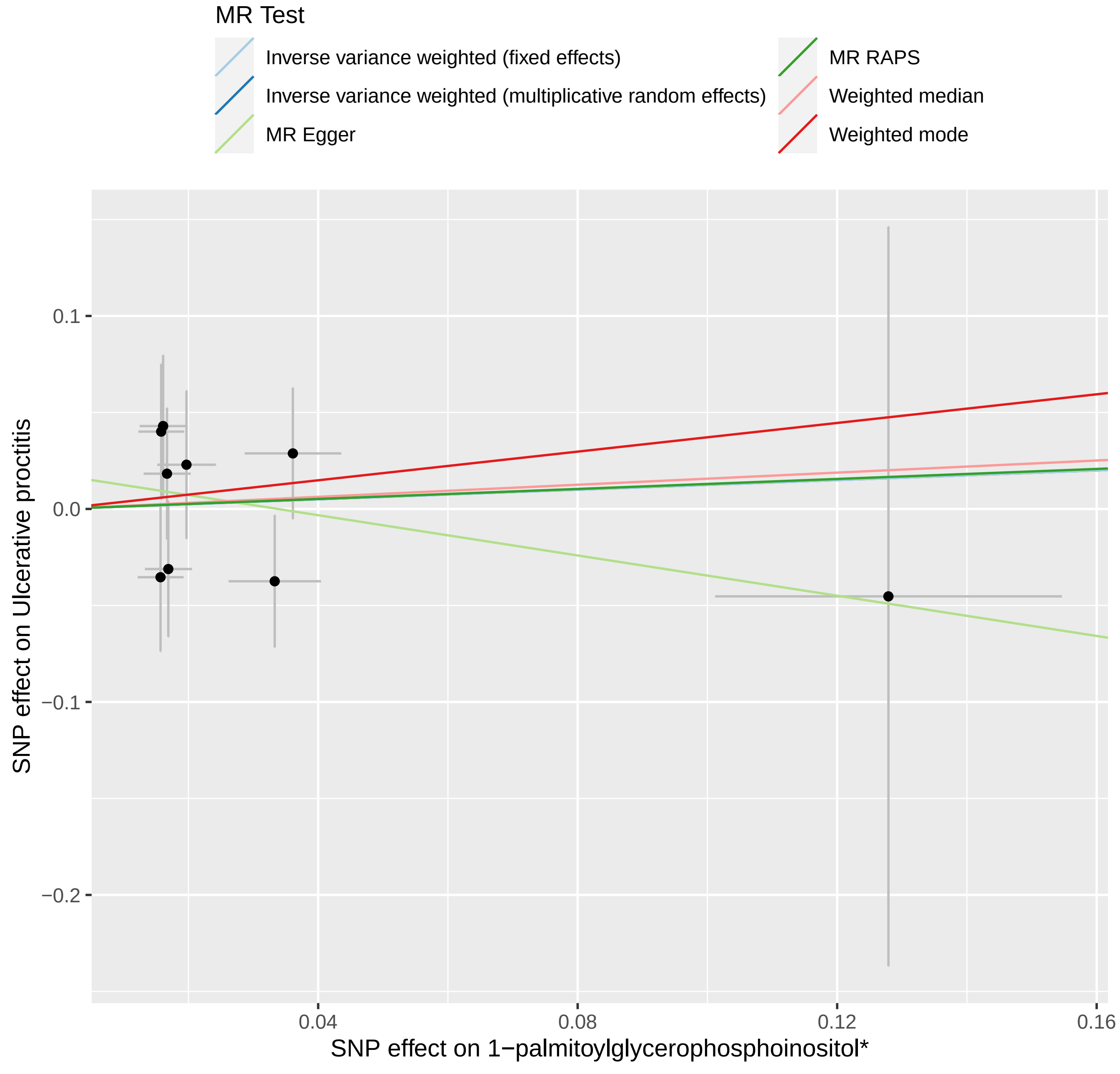

Batch 2437 : Glutaroyl carnitine on Ulcerative proctitis

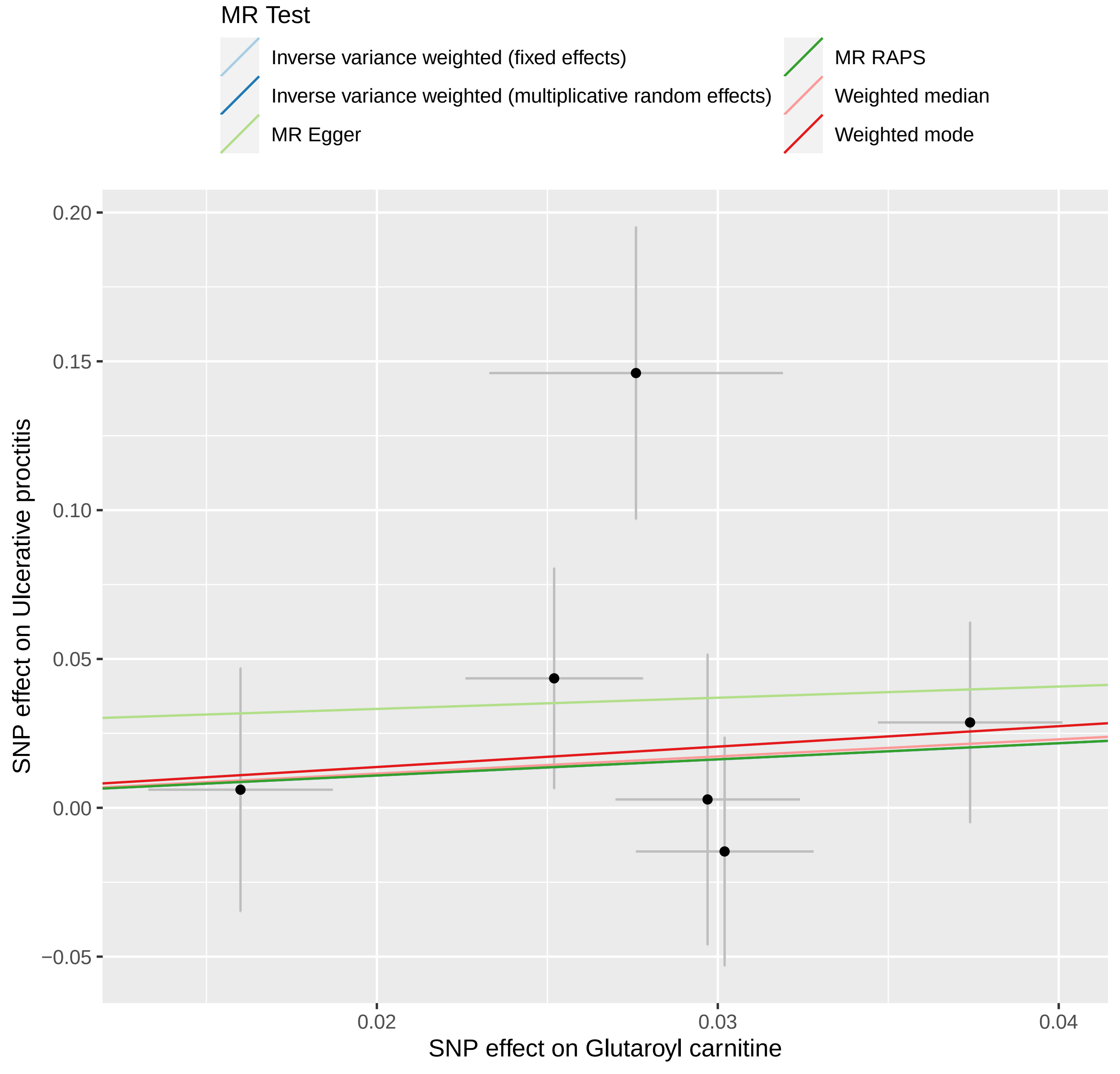

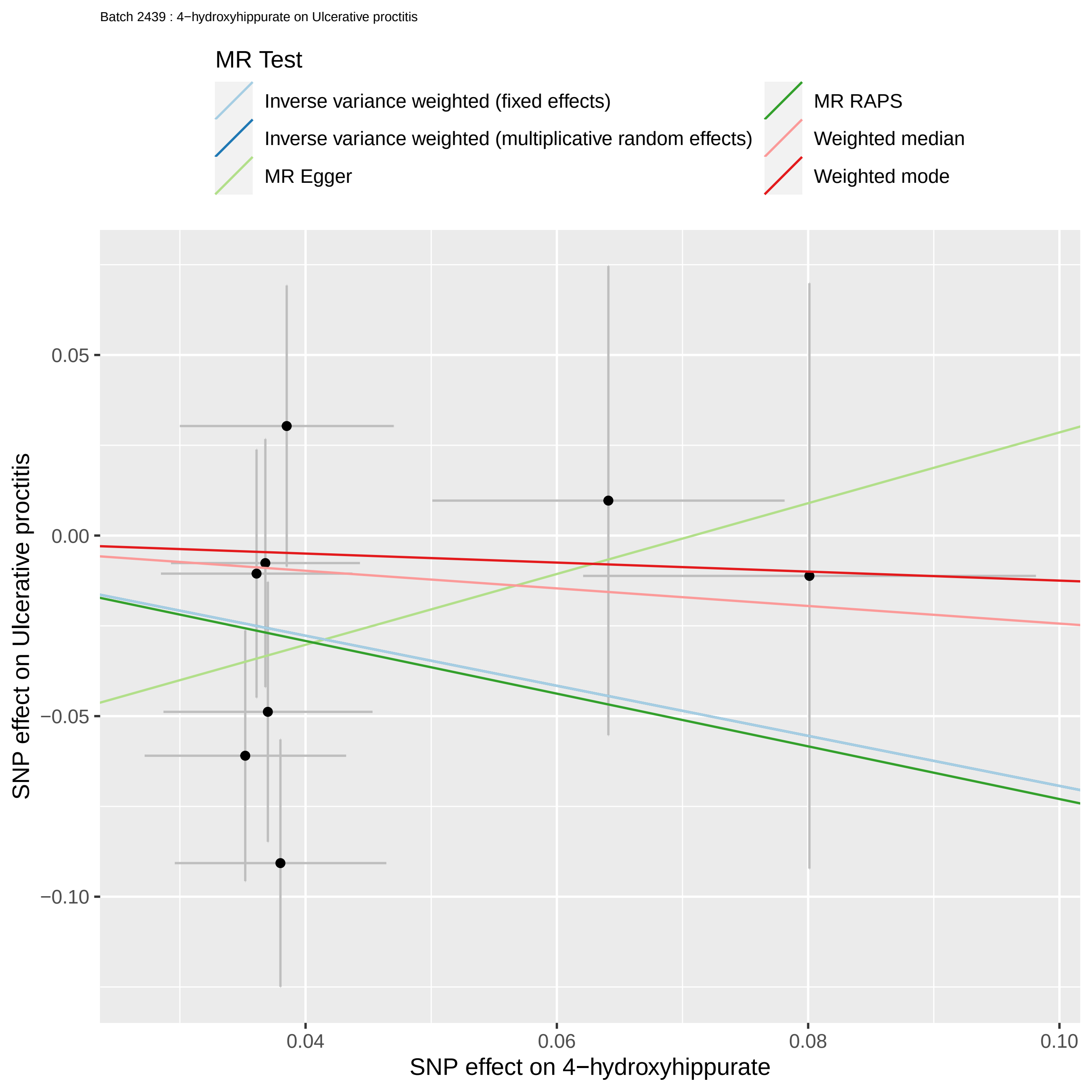

Batch 2439 : 4−hydroxyhippurate on Ulcerative proctitis
MR Test
Inverse variance weighted (fixed effects)
Inverse variance weighted (multiplicative random effects)
MR Egger
MR RAPS
Weighted median
Weighted mode
SNP effect on Ulcerative proctitis
0.05
0.00
−0.05
−0.10
0.04
0.06
0.08
0.10
SNP effect on 4−hydroxyhippurate

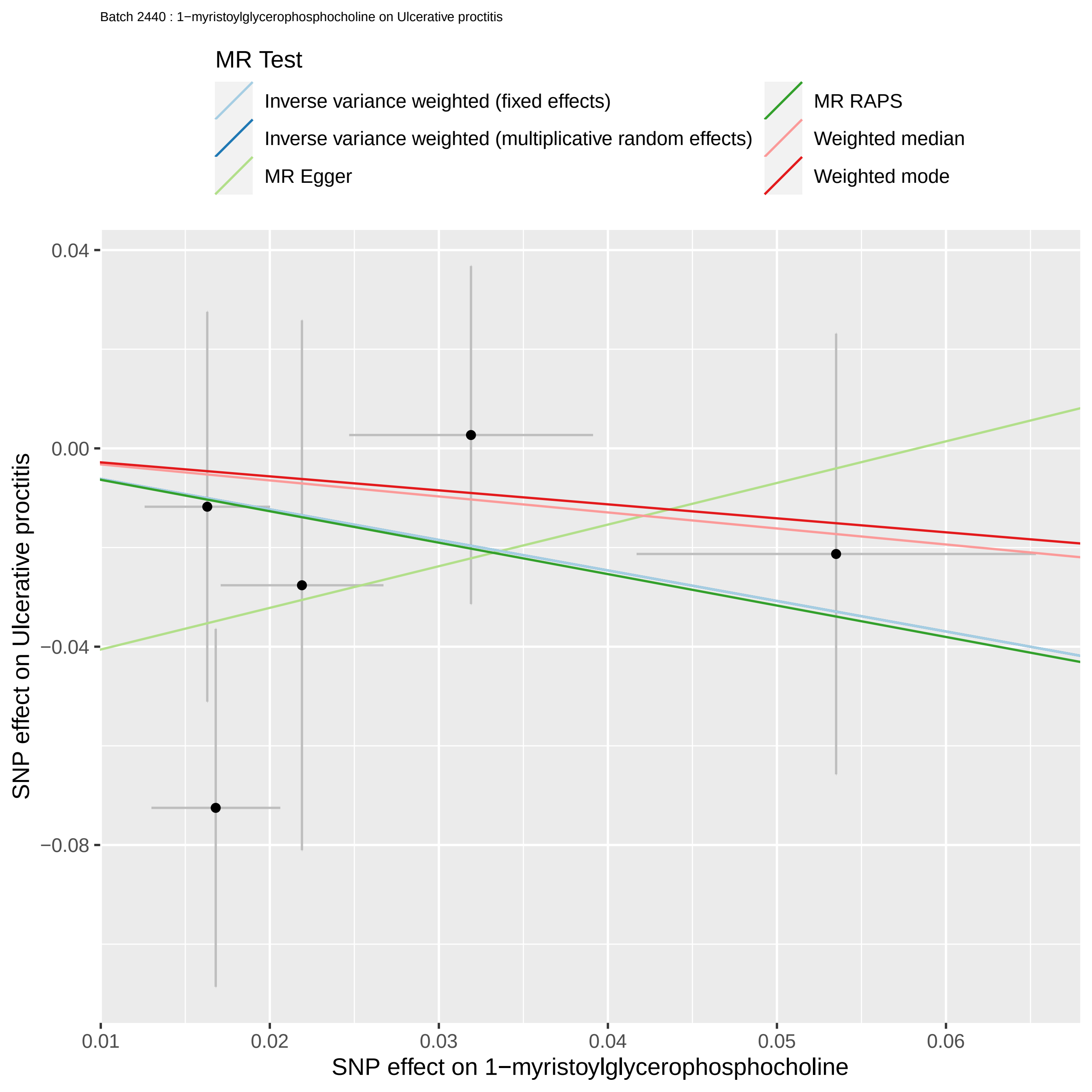

Batch 2440 : 1−myristoylglycerophosphocholine on Ulcerative proctitis
MR Test
Inverse variance weighted (fixed effects)
Inverse variance weighted (multiplicative random effects)
MR Egger
MR RAPS
Weighted median
Weighted mode
0.04
0.00
−0.04
−0.08
SNP effect on Ulcerative proctitis
0.01
0.02
0.03
0.04
0.05
0.06
SNP effect on 1−myristoylglycerophosphocholine

Batch 2443 : 3−(3−hydroxyphenyl)propionate on Ulcerative proctitis

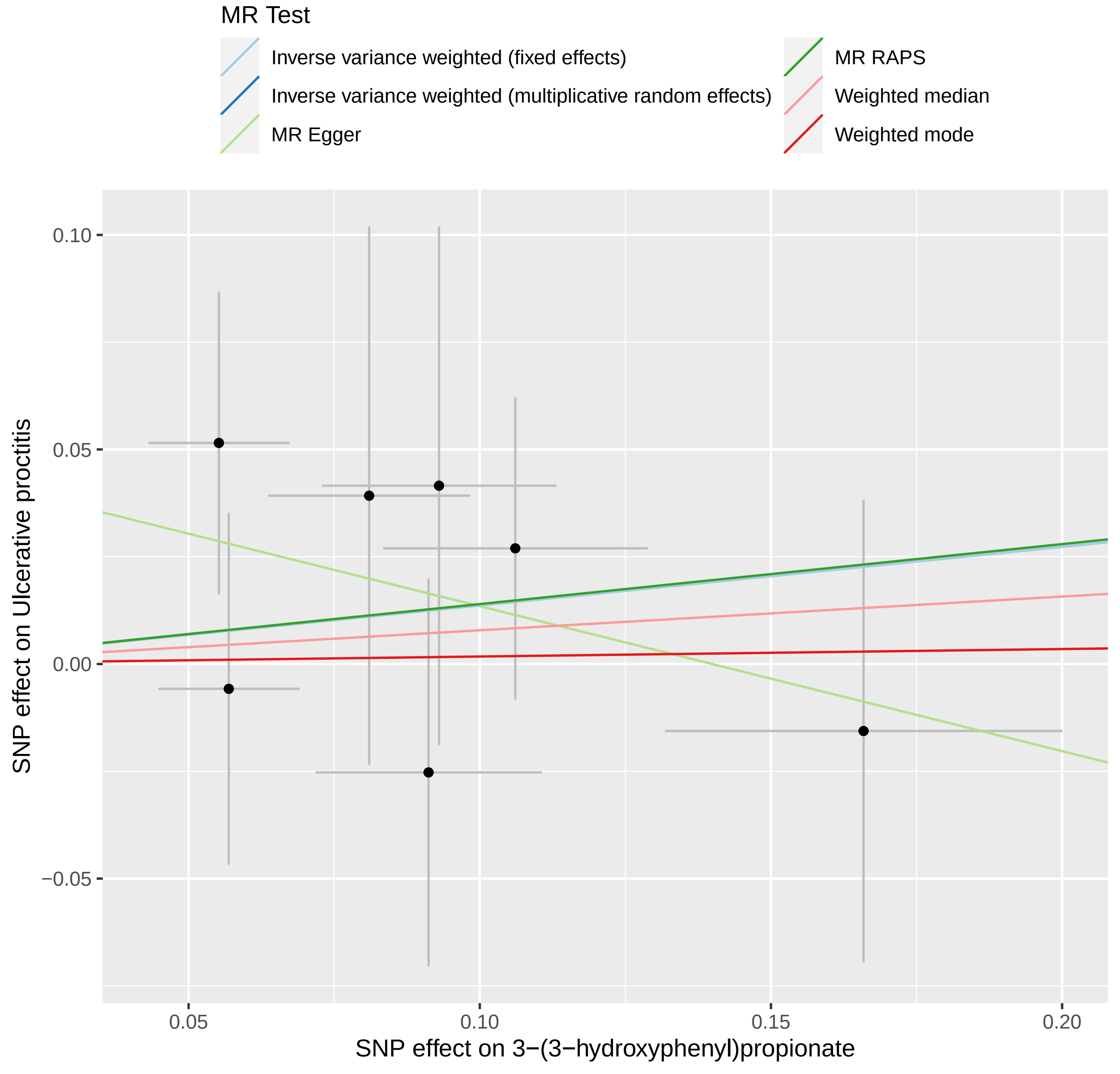

Batch 2447 : Dihomo−linolenate (20:3n3 or n6) on Ulcerative proctitis

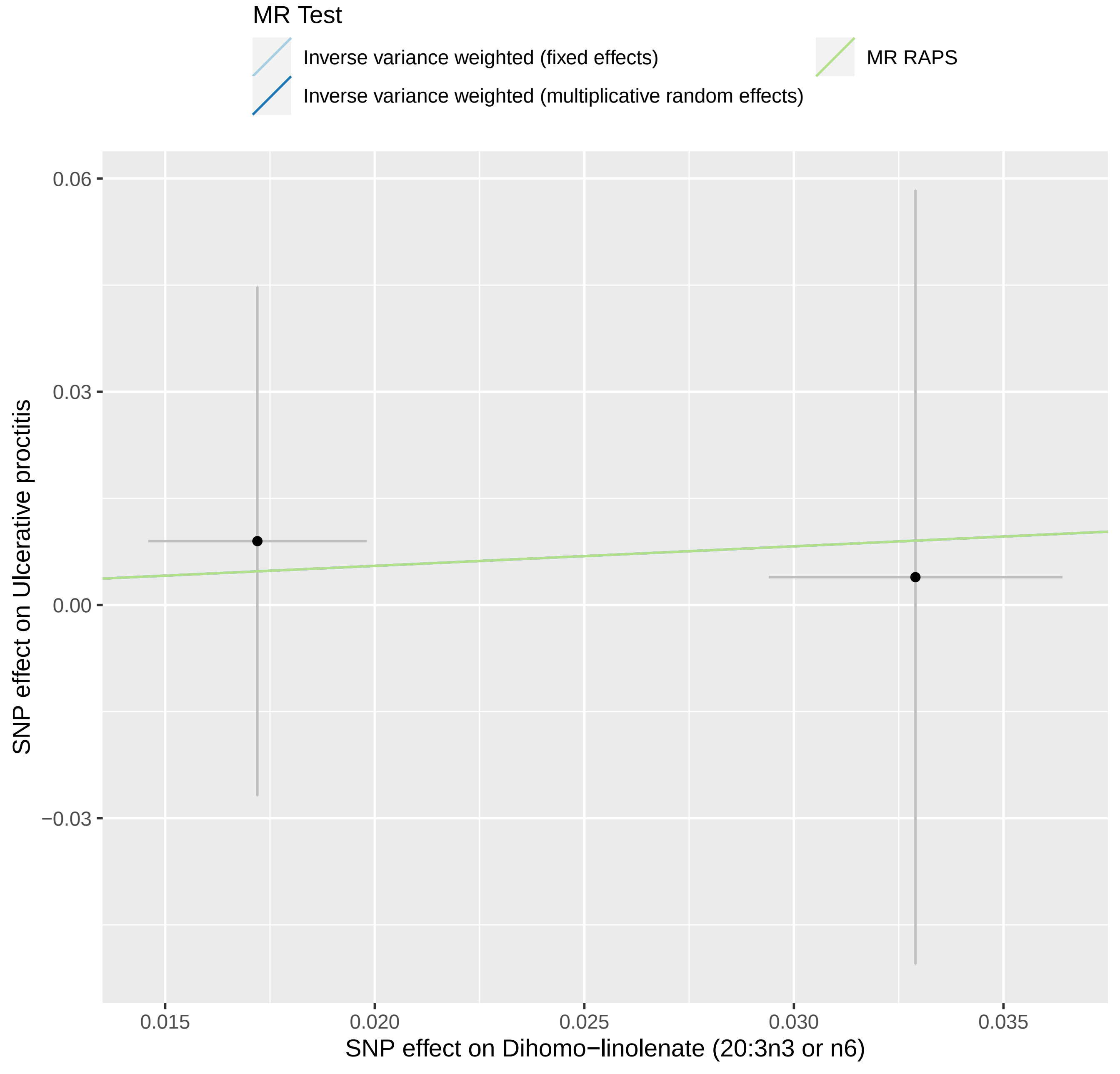

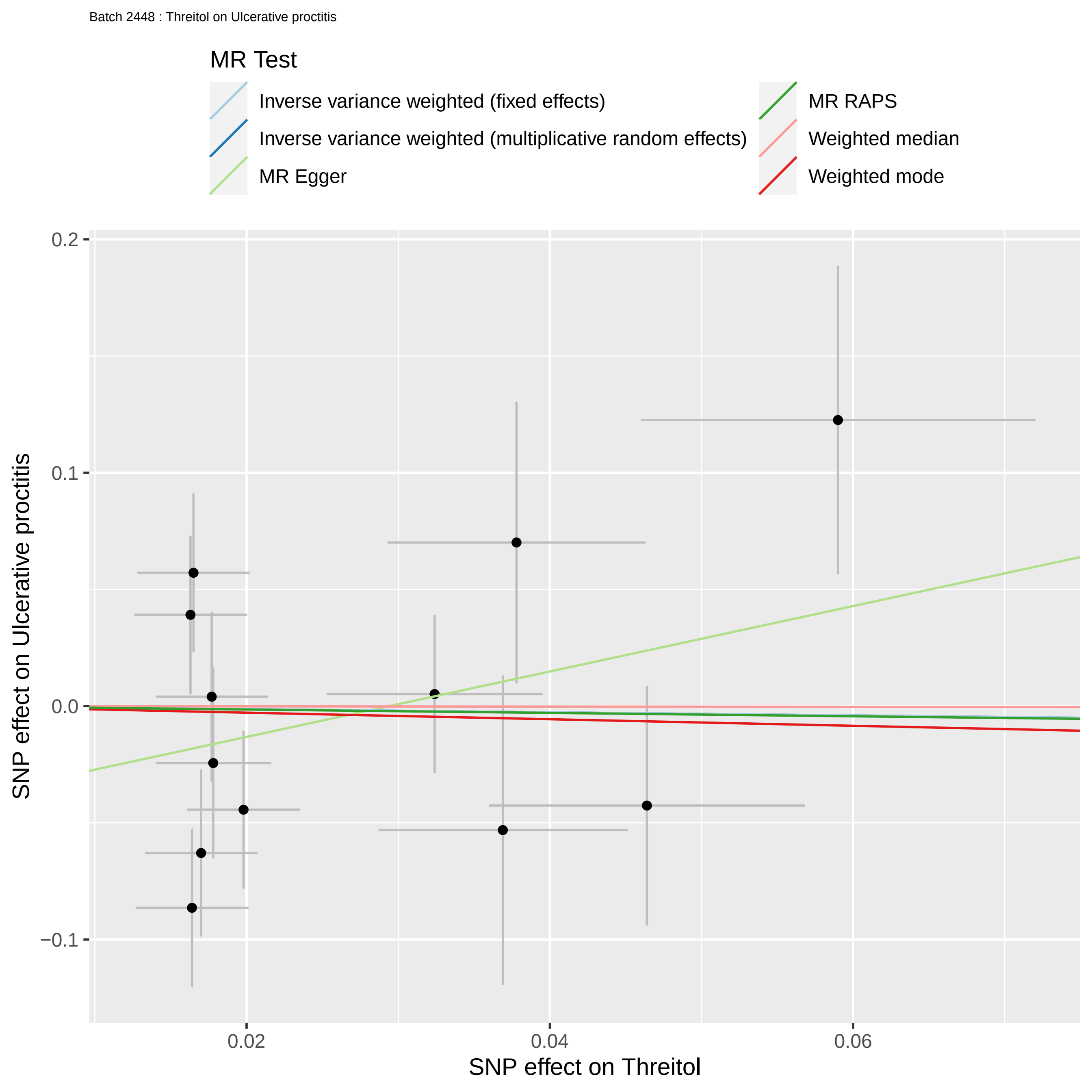
Batch 2448 : Threitol on Ulcerative proctitis
MR Test
Inverse variance weighted (fixed effects)
Inverse variance weighted (multiplicative random effects)
MR Egger
MR RAPS
Weighted median
Weighted mode
0.2
0.1
0.0
−0.1
0.02
0.04
0.06
SNP effect on Ulcerative proctitis
SNP effect on Threitol

Batch 2449 : P−cresol sulfate on Ulcerative proctitis

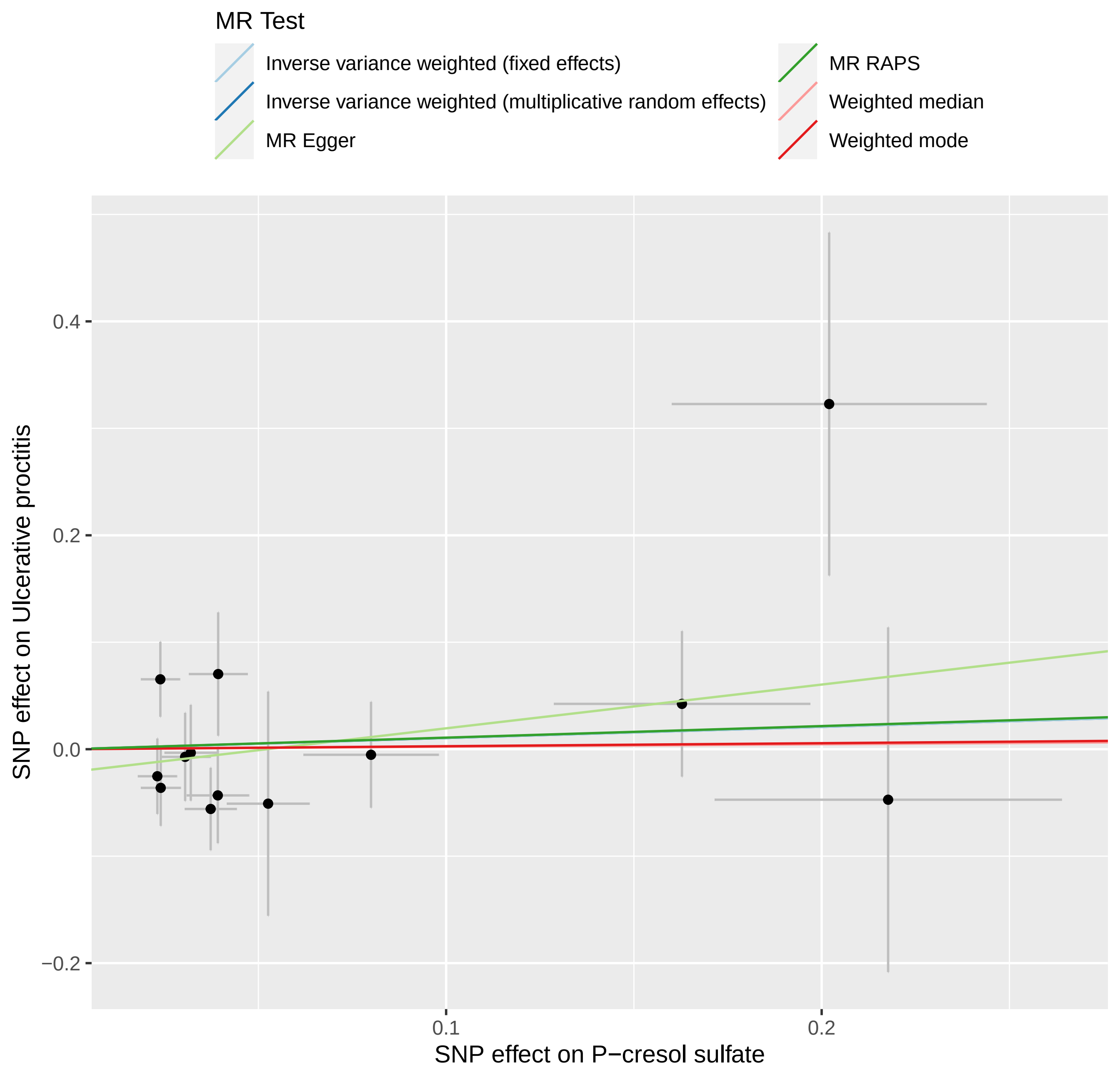

Batch 2452 : X−14208−−phenylalanylserine on Ulcerative proctitis

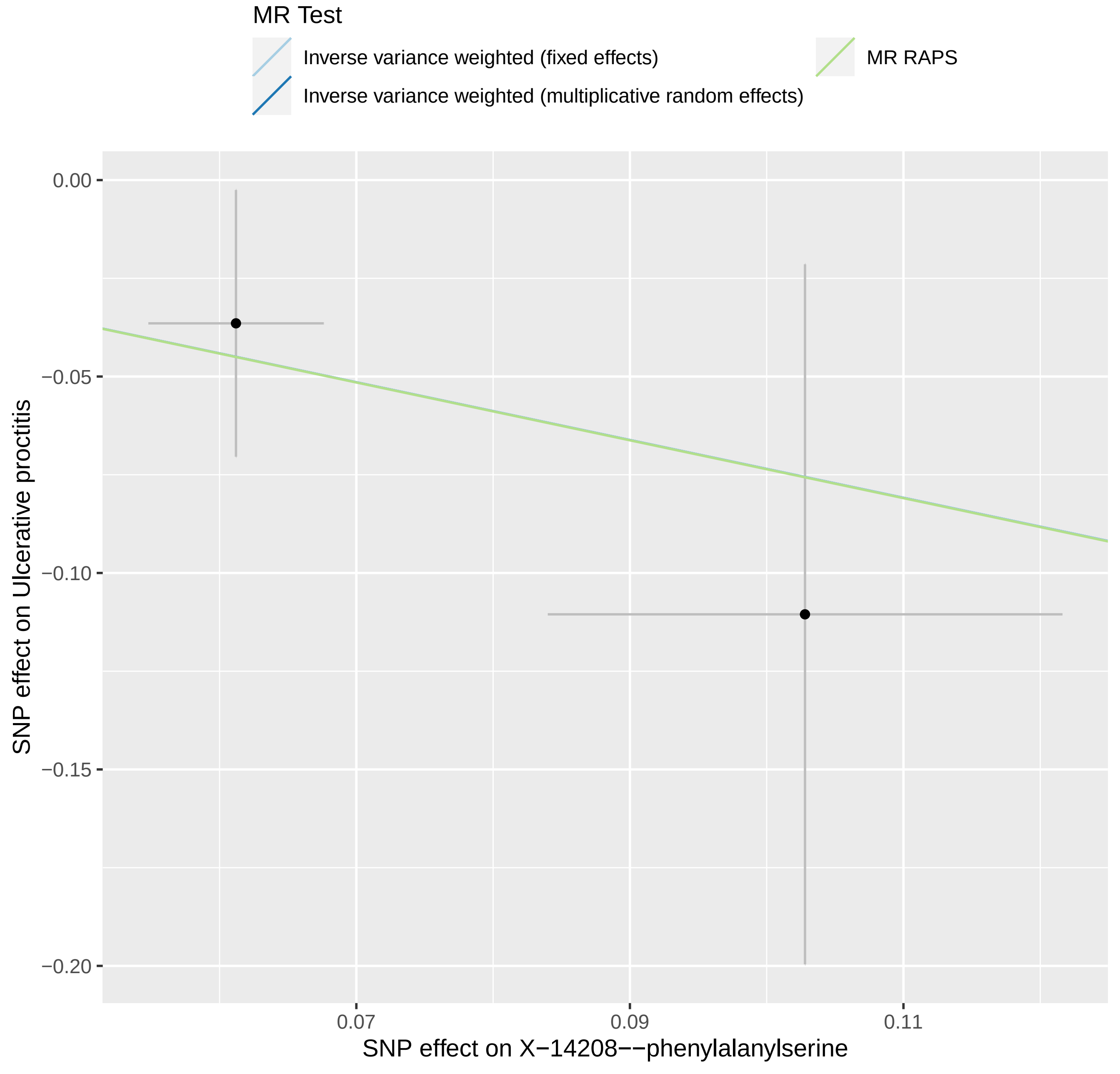

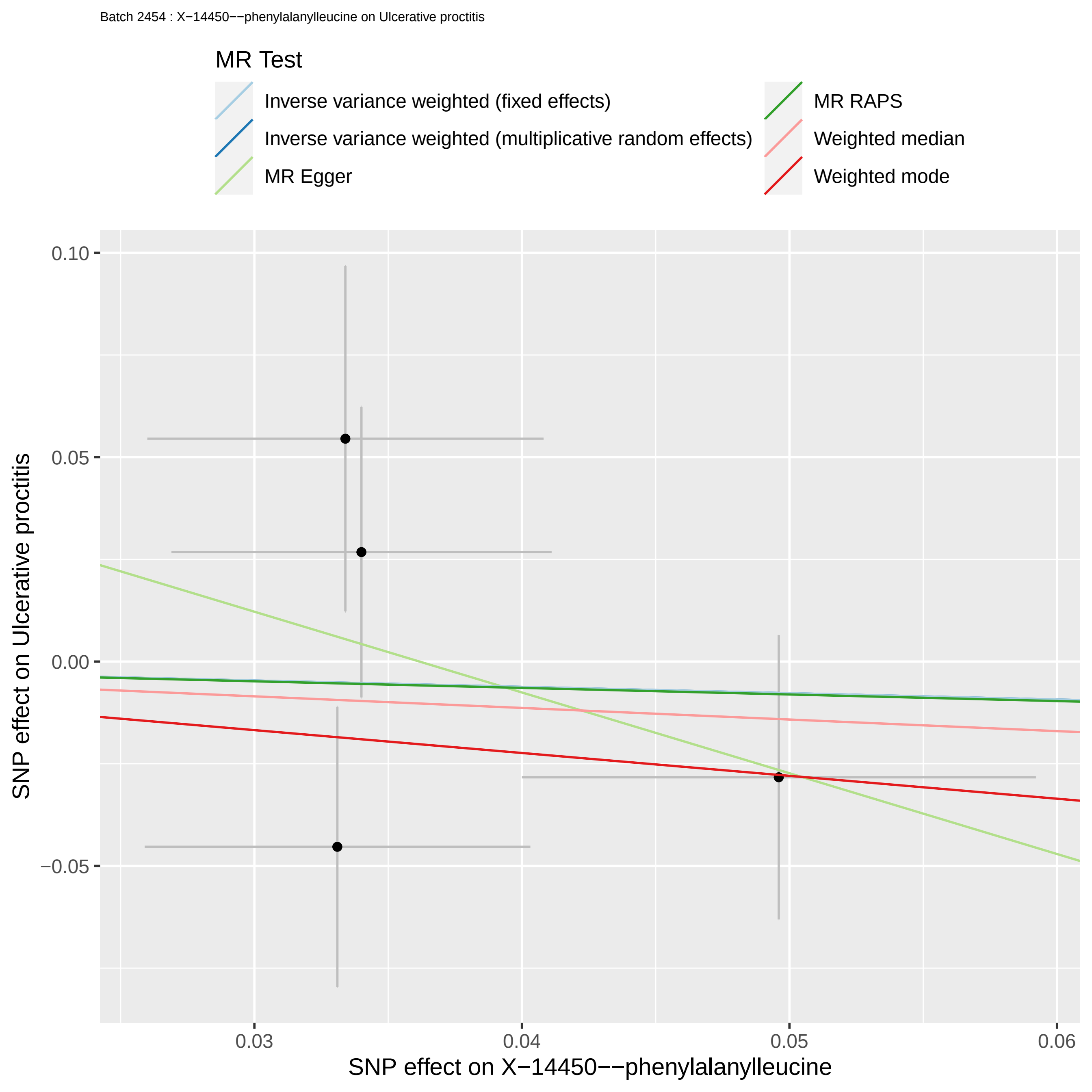

Batch 2454 : X−14450−−phenylalanylleucine on Ulcerative proctitis
MR Test
Inverse variance weighted (fixed effects)
Inverse variance weighted (multiplicative random effects)
MR Egger
MR RAPS
Weighted median
Weighted mode
0.10
0.05
0.00
−0.05
0.03
0.04
0.05
0.06
SNP effect on Ulcerative proctitis
SNP effect on X−14450−−phenylalanylleucine

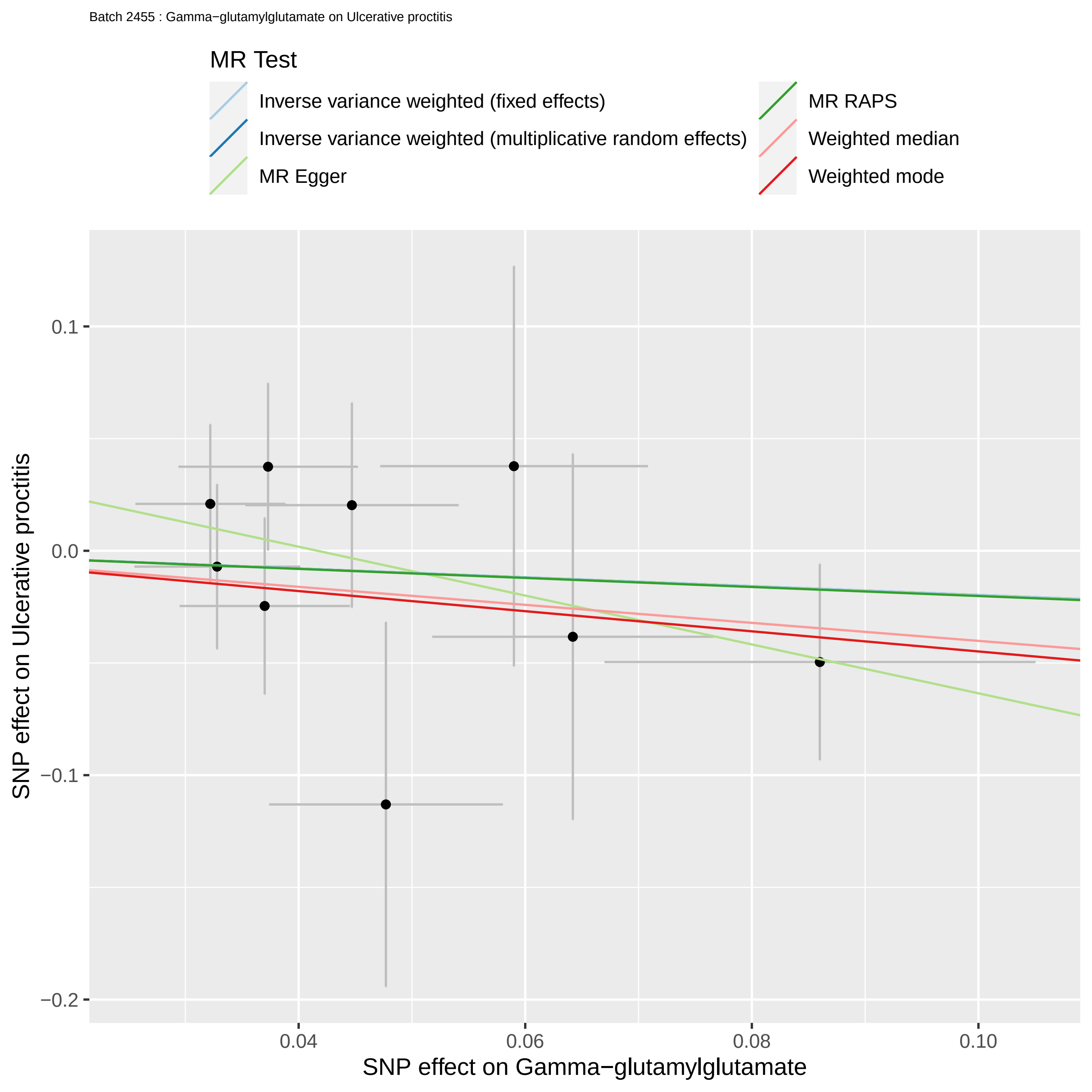

Batch 2455 : Gamma−glutamylglutamate on Ulcerative proctitis
MR Test
Inverse variance weighted (fixed effects)
Inverse variance weighted (multiplicative random effects)
MR Egger
MR RAPS
Weighted median
Weighted mode
0.1
0.0
−0.1
−0.2
0.04
0.06
0.08
0.10
SNP effect on Ulcerative proctitis
SNP effect on Gamma−glutamylglutamate

Batch 2456 : Octadecanedioate on Ulcerative proctitis

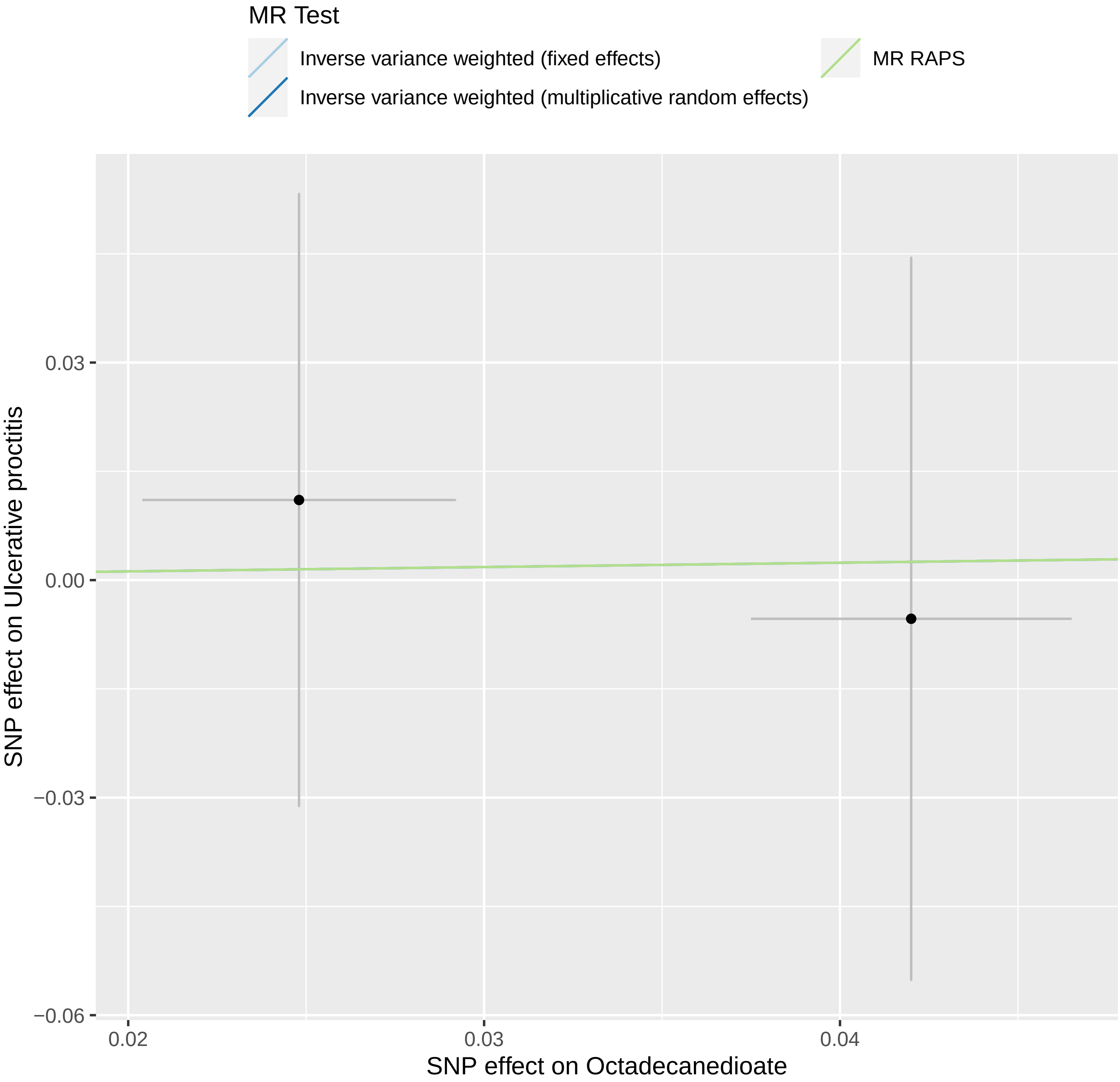

Batch 2457 : Leucylleucine on Ulcerative proctitis

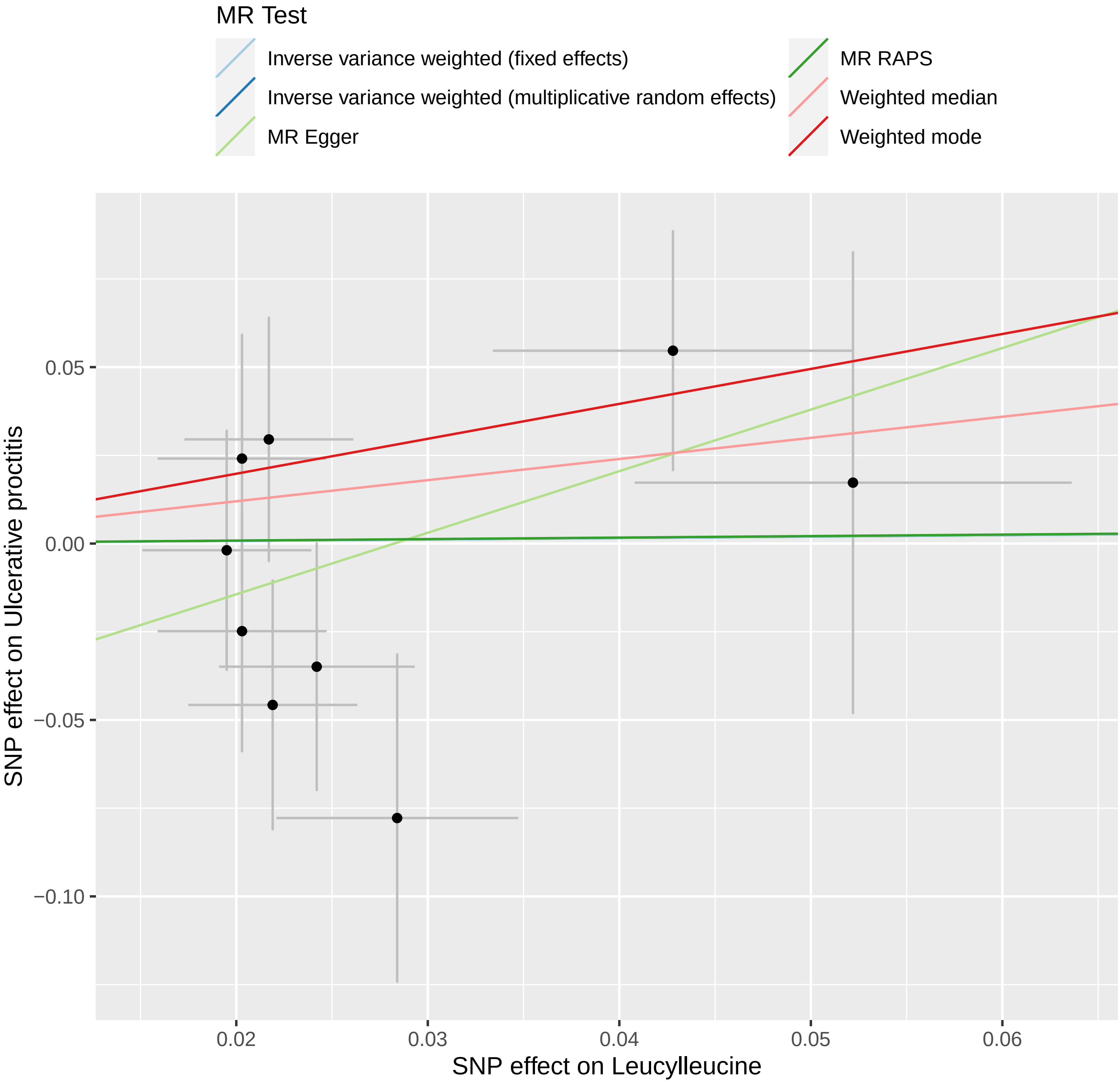

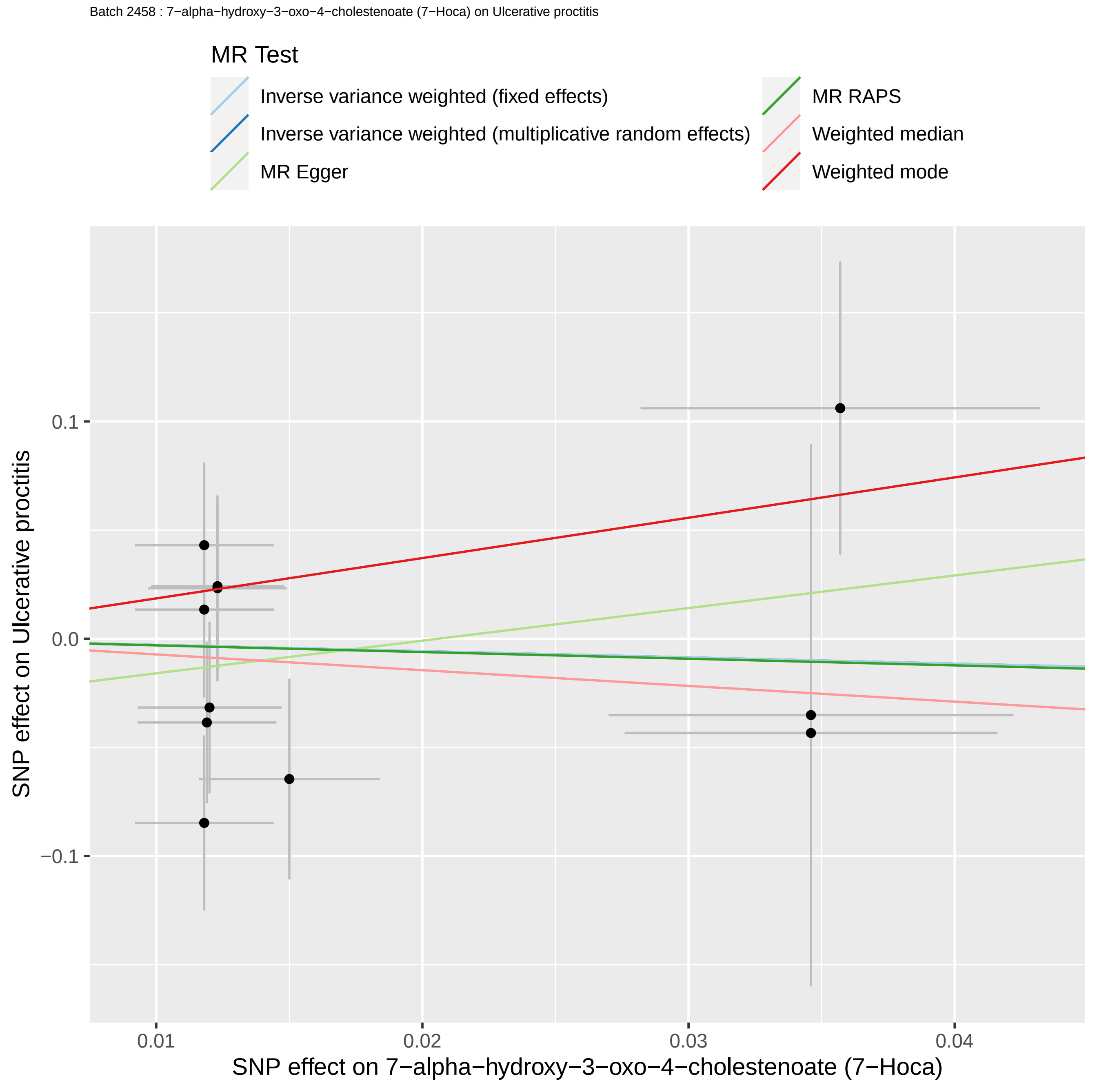

Batch 2458 : 7−alpha−hydroxy−3−oxo−4−cholestenoate (7−Hoca) on Ulcerative proctitis
MR Test
Inverse variance weighted (fixed effects)
Inverse variance weighted (multiplicative random effects)
MR Egger
MR RAPS
Weighted median
Weighted mode
0.1
0.0
−0.1
0.01
0.02
0.03
0.04
SNP effect on Ulcerative proctitis
SNP effect on 7−alpha−hydroxy−3−oxo−4−cholestenoate (7−Hoca)

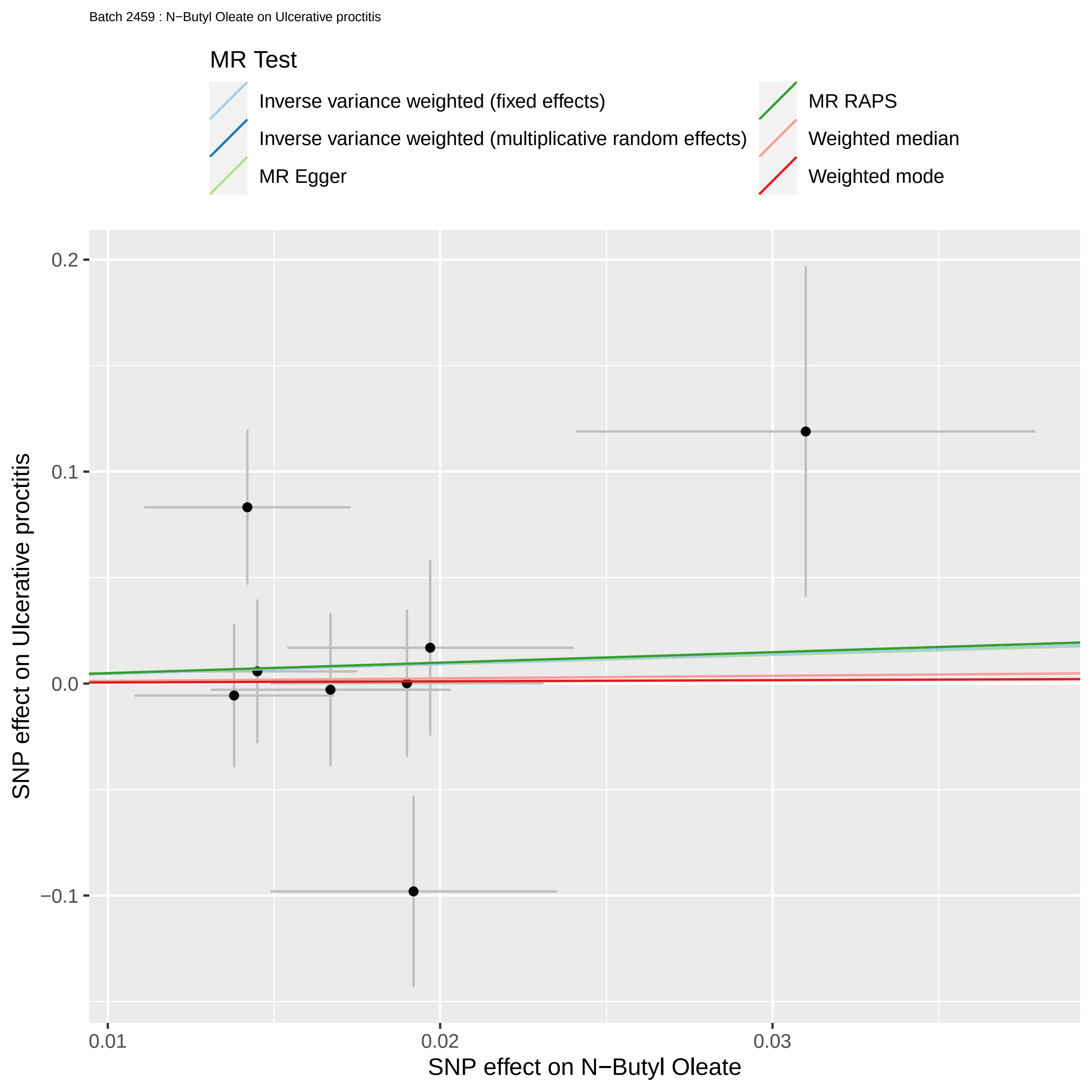

Batch 2459 : N−Butyl Oleate on Ulcerative proctitis
MR Test
Inverse variance weighted (fixed effects)
Inverse variance weighted (multiplicative random effects)
MR Egger
MR RAPS
Weighted median
Weighted mode
0.2
0.1
0.0
−0.1
0.01
0.02
0.03
SNP effect on Ulcerative proctitis
SNP effect on N−Butyl Oleate

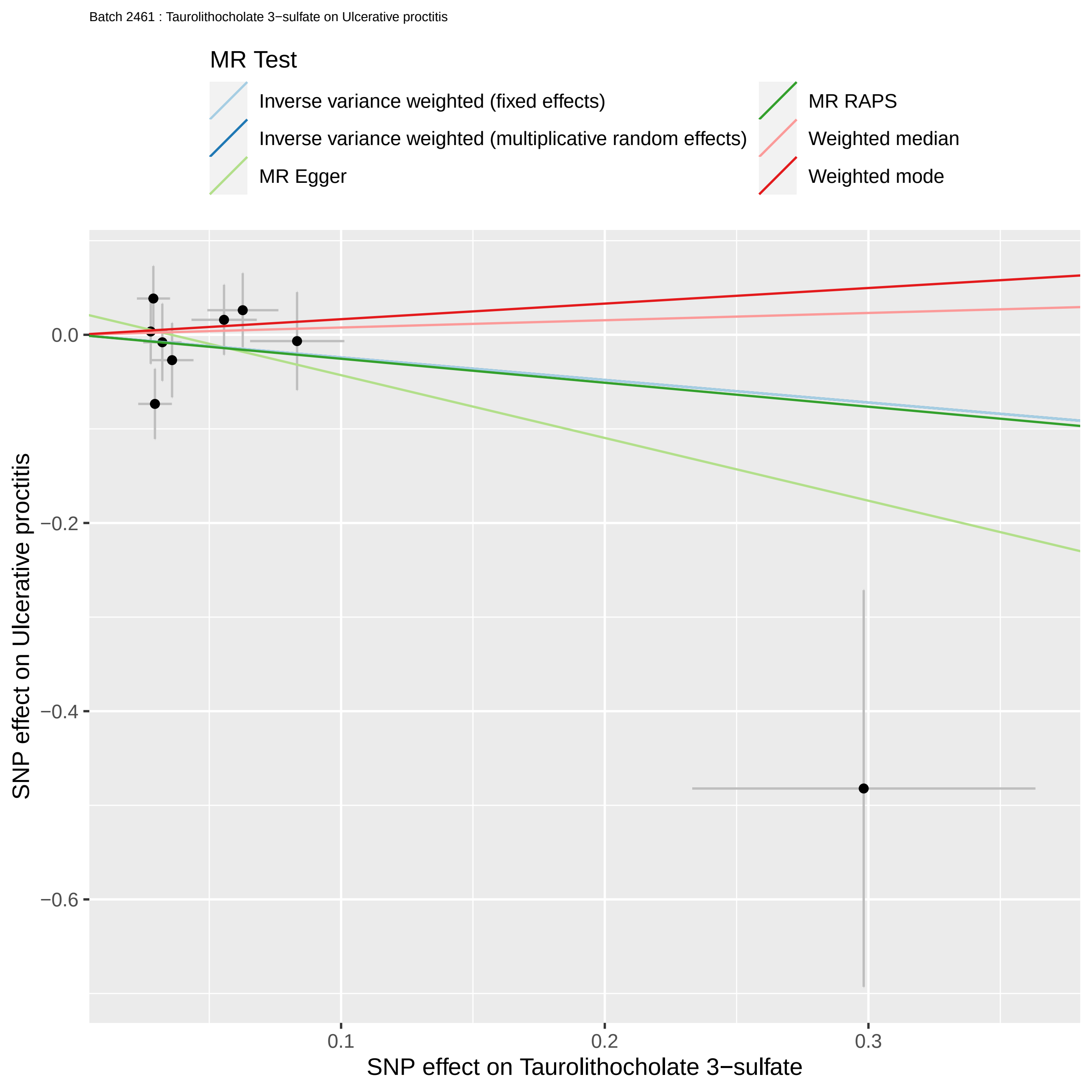

Batch 2461 : Taurolithocholate 3−sulfate on Ulcerative proctitis
MR Test
Inverse variance weighted (fixed effects)
Inverse variance weighted (multiplicative random effects)
MR Egger
MR RAPS
Weighted median
Weighted mode
0.0
−0.2
−0.4
−0.6
0.1
0.2
0.3
SNP effect on Ulcerative proctitis
SNP effect on Taurolithocholate 3−sulfate

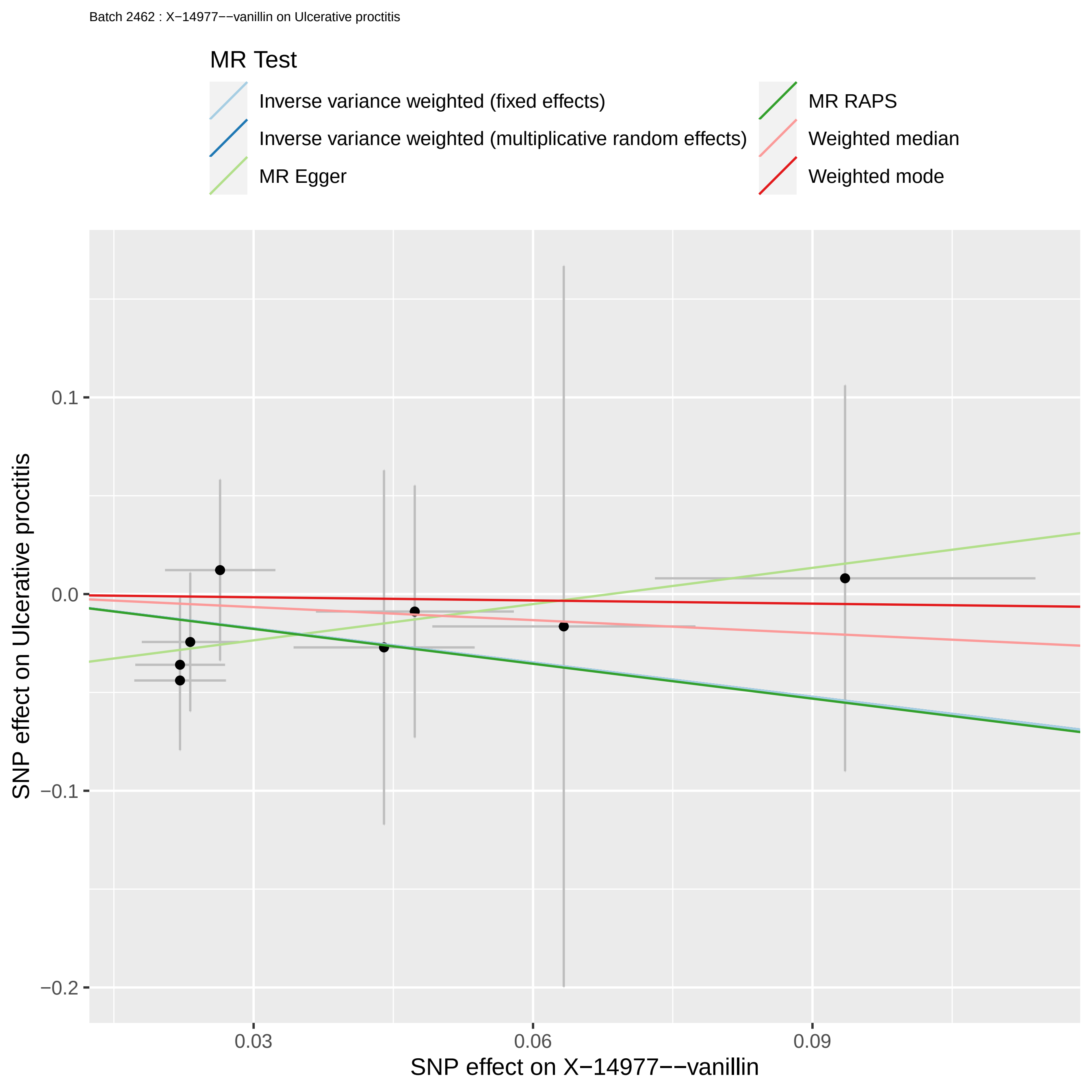

Batch 2462 : X−14977−−vanillin on Ulcerative proctitis
MR Test
Inverse variance weighted (fixed effects)
Inverse variance weighted (multiplicative random effects)
MR Egger
MR RAPS
Weighted median
Weighted mode
0.1
0.0
−0.1
−0.2
0.03
0.06
0.09
SNP effect on Ulcerative proctitis
SNP effect on X−14977−−vanillin

Batch 2463 : Succinylcarnitine on Ulcerative proctitis

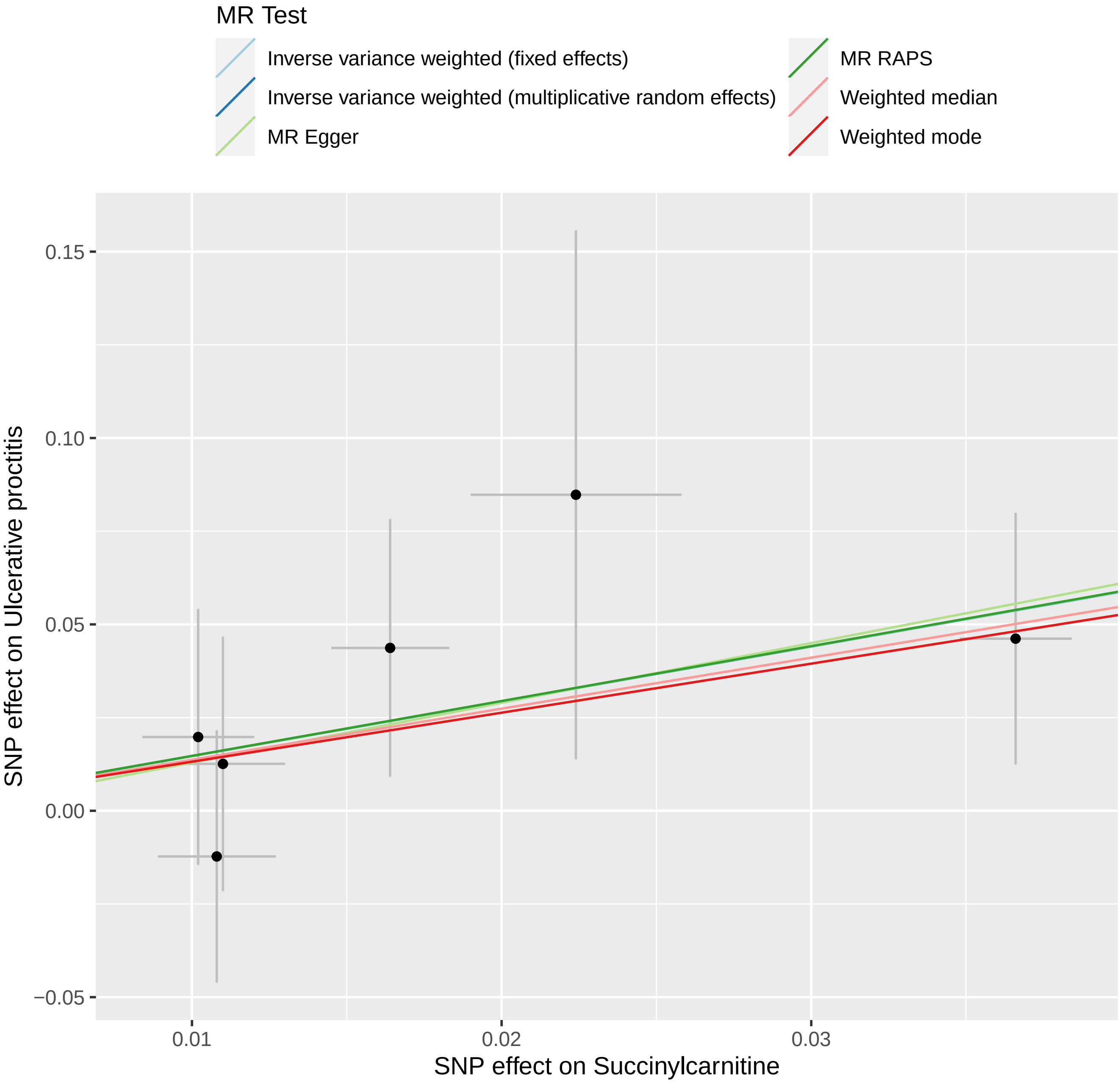

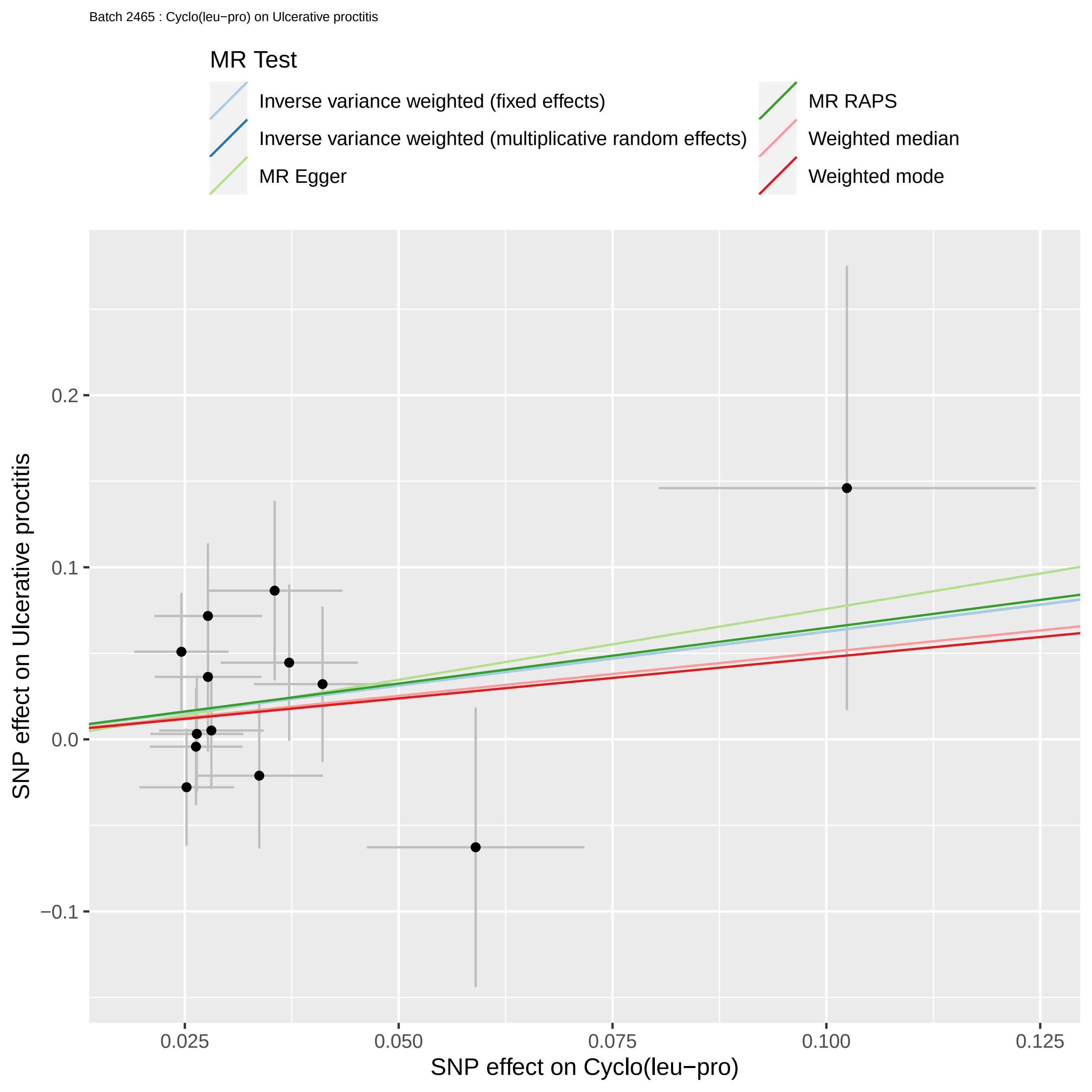

Batch 2465 : Cyclo(leu−pro) on Ulcerative proctitis
MR Test
Inverse variance weighted (fixed effects)
Inverse variance weighted (multiplicative random effects)
MR Egger
MR RAPS
Weighted median
Weighted mode
0.2
0.1
0.0
−0.1
0.025
0.050
0.075
0.100
0.125
SNP effect on Cyclo(leu−pro)
SNP effect on Ulcerative proctitis

Batch 2466 : Chiro−inositol on Ulcerative proctitis

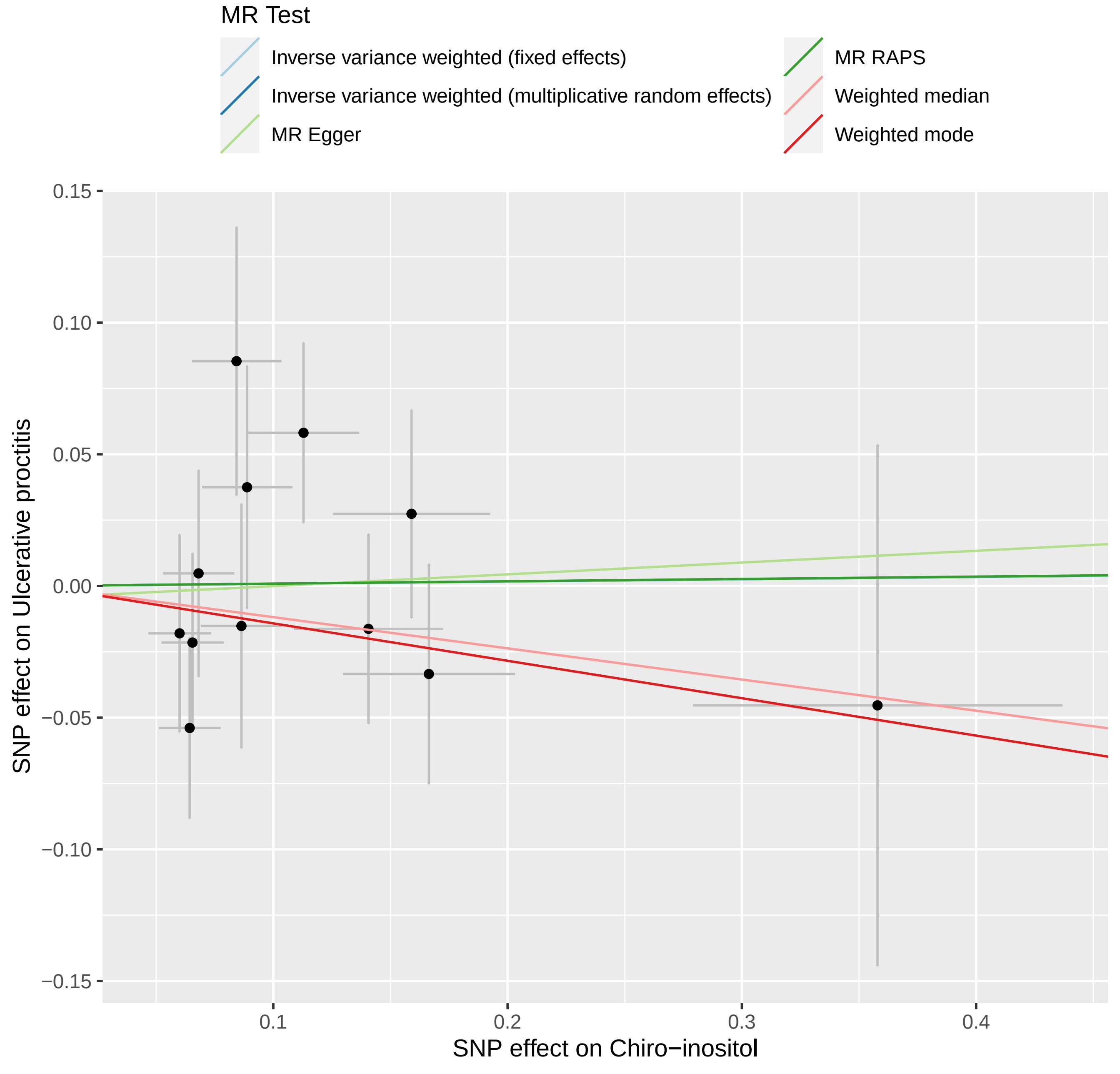

Batch 2470 : 2−hydroxyglutarate on Ulcerative proctitis

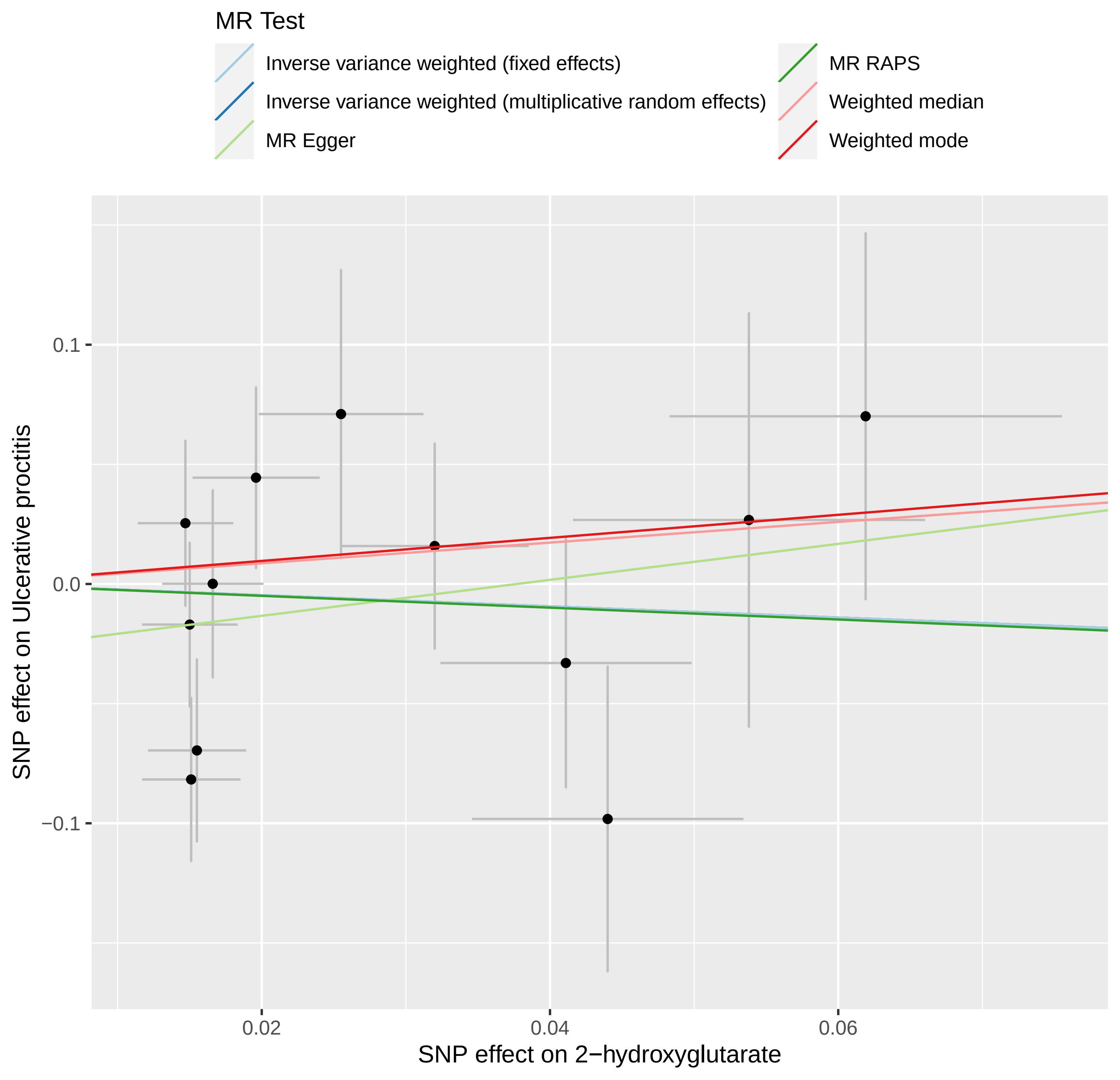

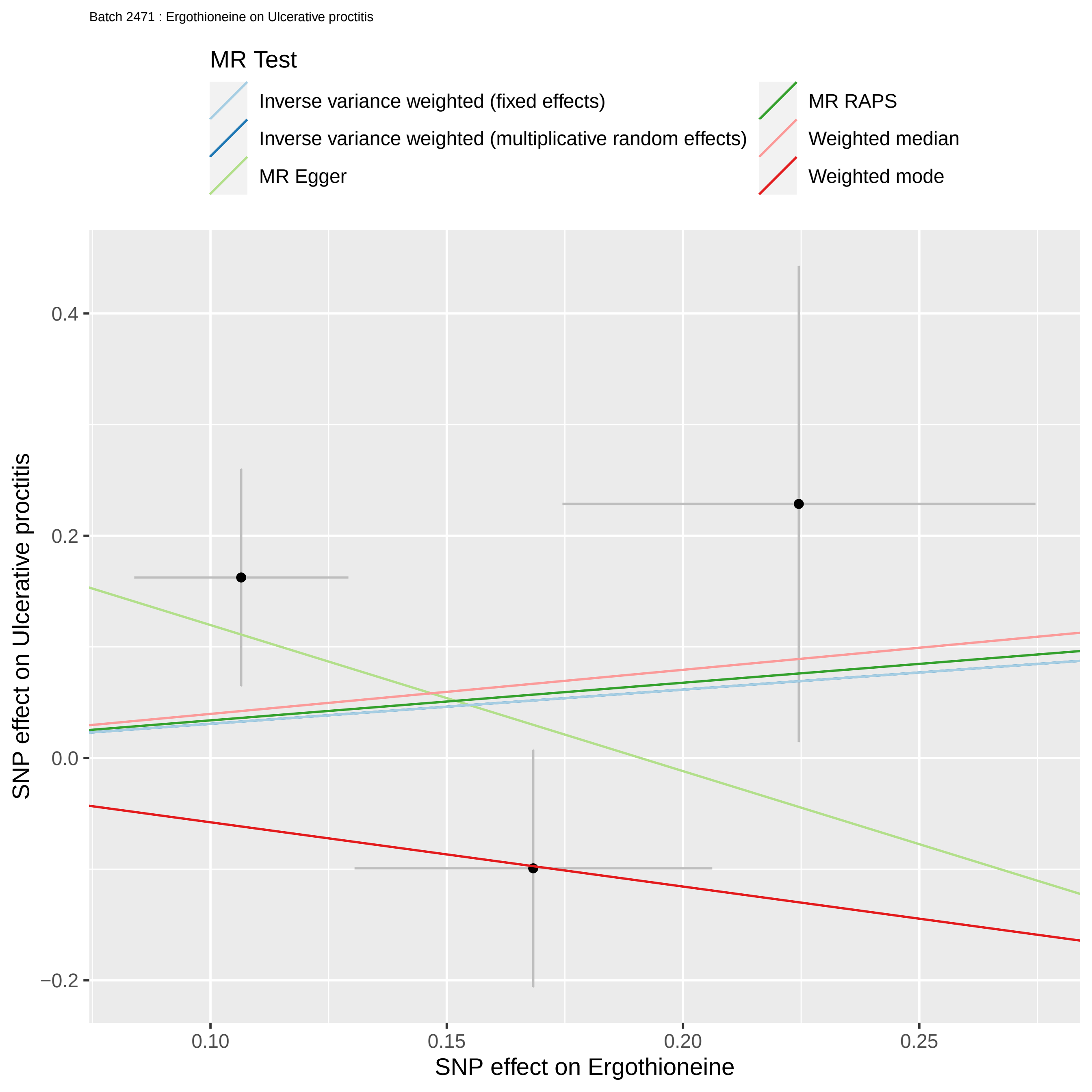
Batch 2471 : Ergothioneine on Ulcerative proctitis
MR Test
Inverse variance weighted (fixed effects)
Inverse variance weighted (multiplicative random effects)
MR Egger
MR RAPS
Weighted median
Weighted mode
0.4
0.2
0.0
−0.2
0.10
0.15
0.20
0.25
SNP effect on Ulcerative proctitis
SNP effect on Ergothioneine

Batch 2473 : Phenylalanylphenylalanine on Ulcerative proctitis

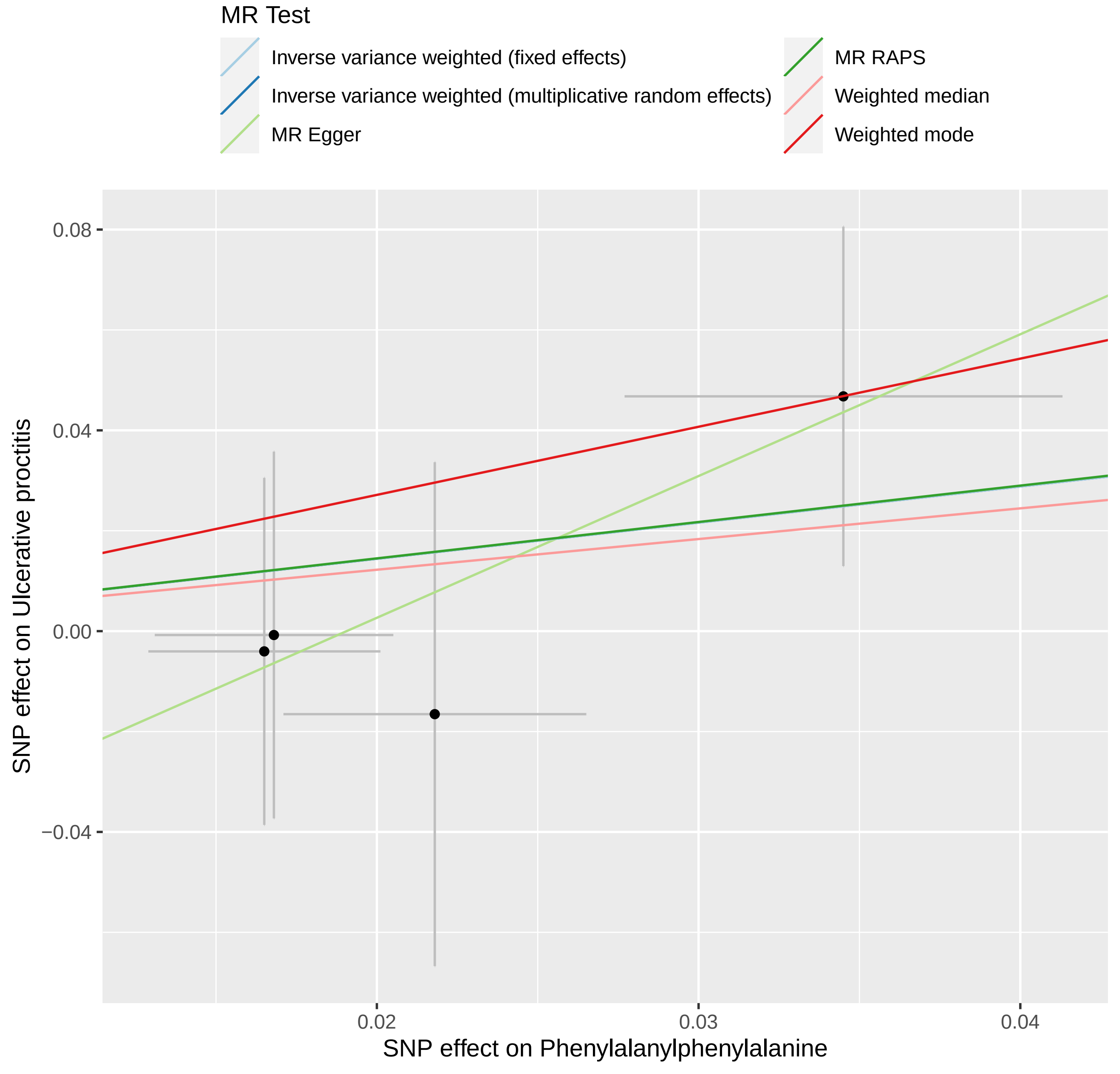

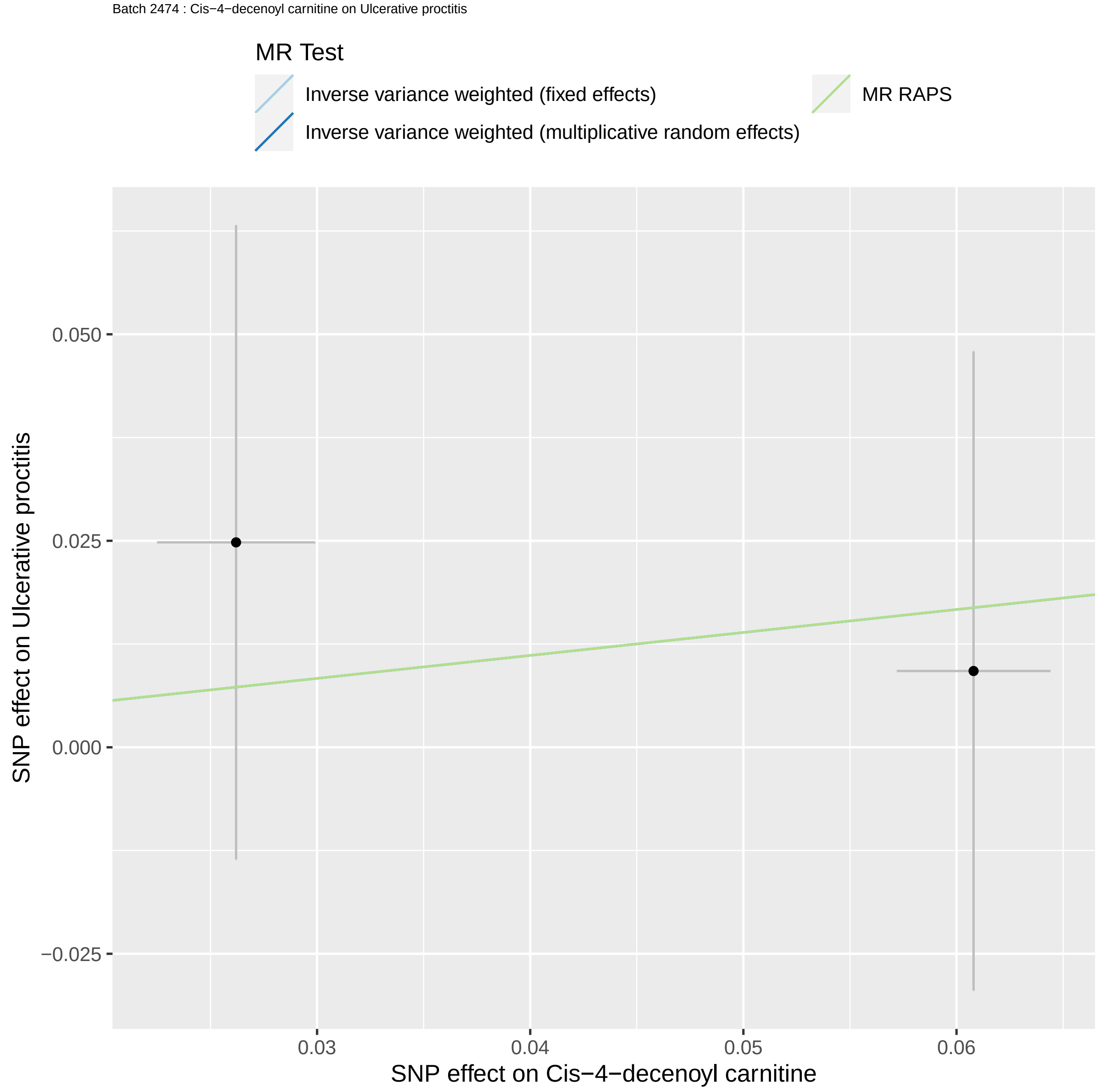

Batch 2474 : Cis−4−decenoyl carnitine on Ulcerative proctitis
MR Test
Inverse variance weighted (fixed effects)
Inverse variance weighted (multiplicative random effects)
MR RAPS
0.050
0.025
0.000
−0.025
0.03
0.04
0.05
0.06
SNP effect on Ulcerative proctitis
SNP effect on Cis−4−decenoyl carnitine

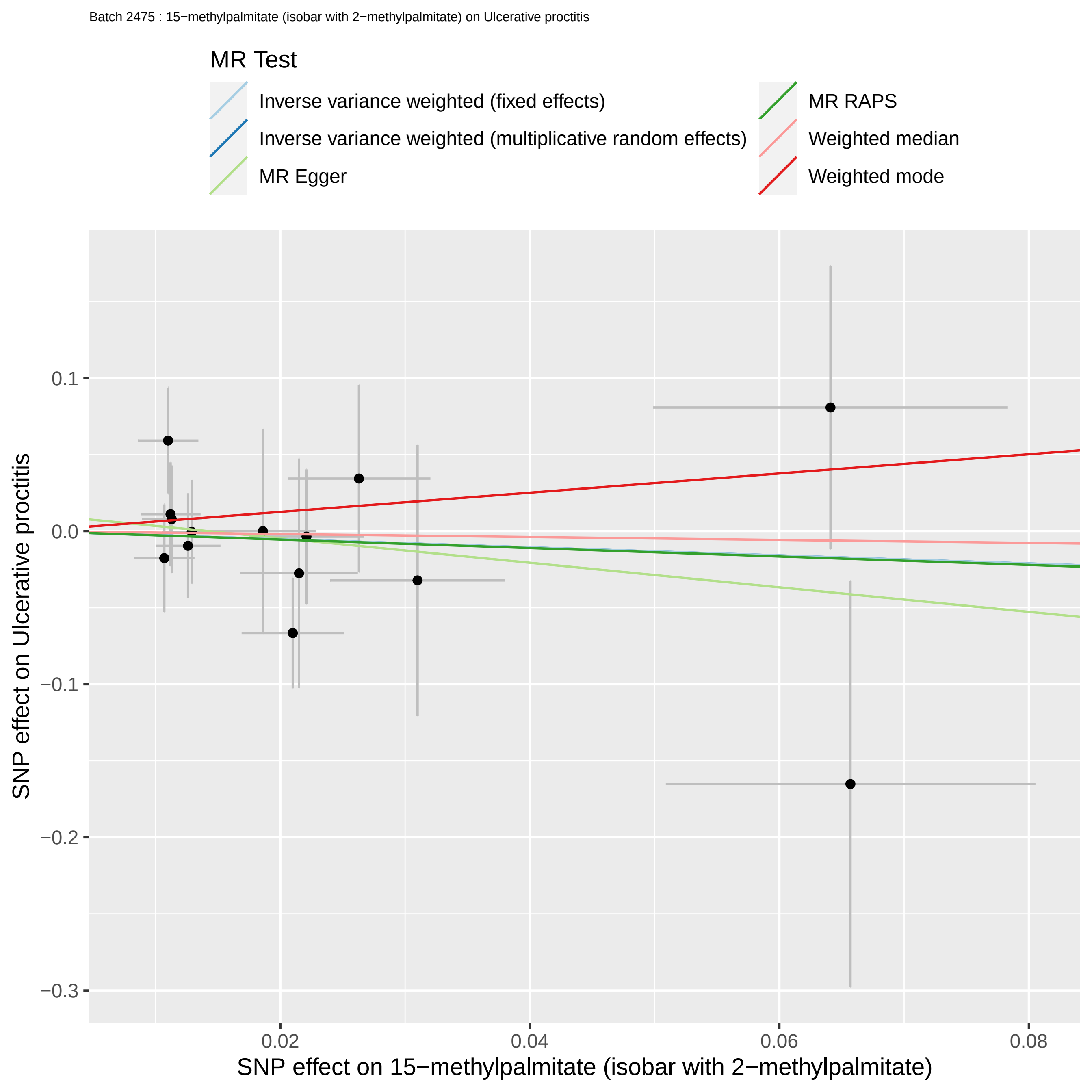
Batch 2475 : 15−methylpalmitate (isobar with 2−methylpalmitate) on Ulcerative proctitis
MR Test
Inverse variance weighted (fixed effects)
Inverse variance weighted (multiplicative random effects)
MR Egger
MR RAPS
Weighted median
Weighted mode
0.1
0.0
−0.1
−0.2
−0.3
0.02
0.04
0.06
0.08
SNP effect on Ulcerative proctitis
SNP effect on 15−methylpalmitate (isobar with 2−methylpalmitate)

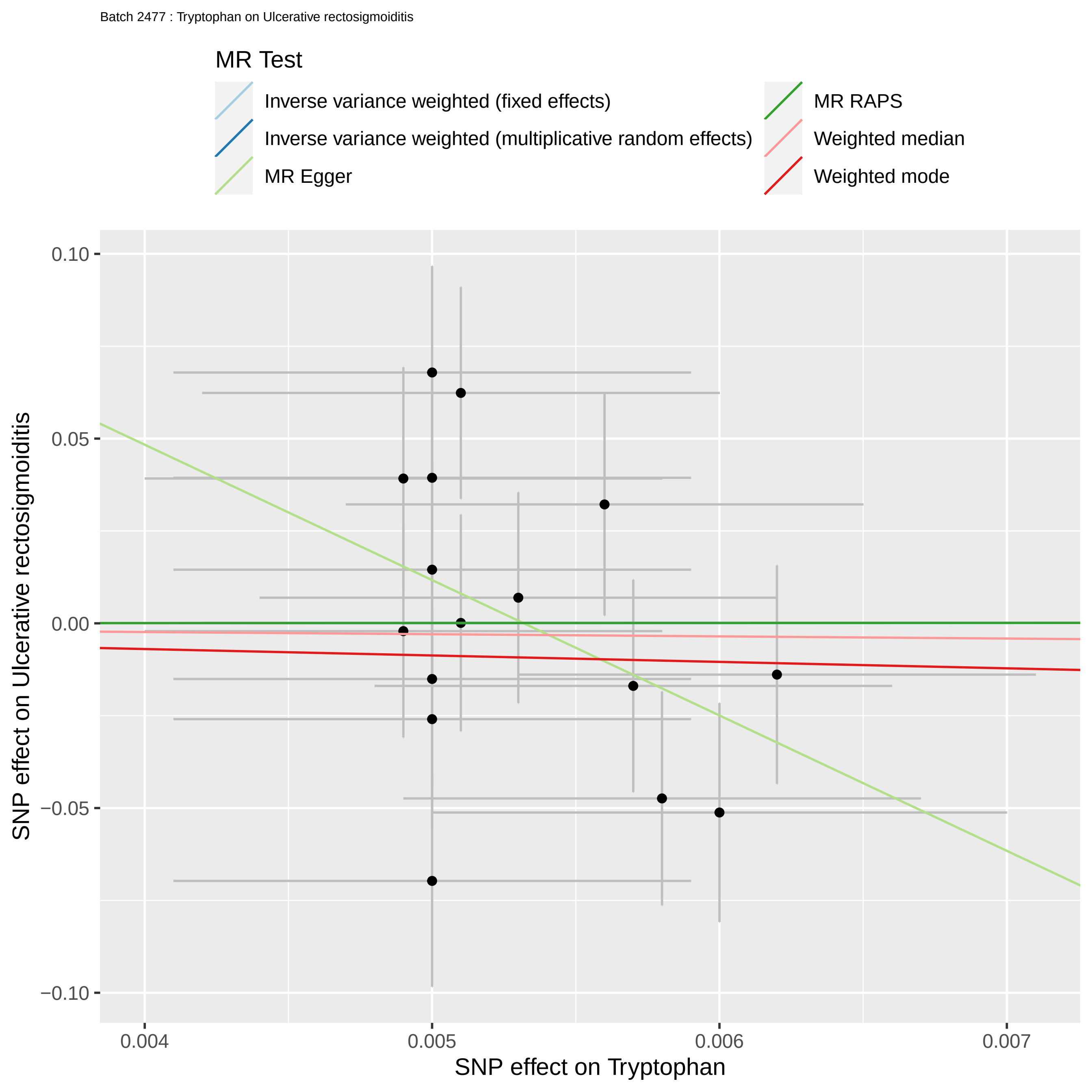

Batch 2477 : Tryptophan on Ulcerative rectosigmoiditis
MR Test
Inverse variance weighted (fixed effects)
Inverse variance weighted (multiplicative random effects)
MR Egger
MR RAPS
Weighted median
Weighted mode
0.10
0.05
0.00
−0.05
−0.10
0.004
0.005
0.006
0.007
SNP effect on Tryptophan
SNP effect on Ulcerative rectosigmoiditis

Batch 2479 : Leucine on Ulcerative rectosigmoiditis

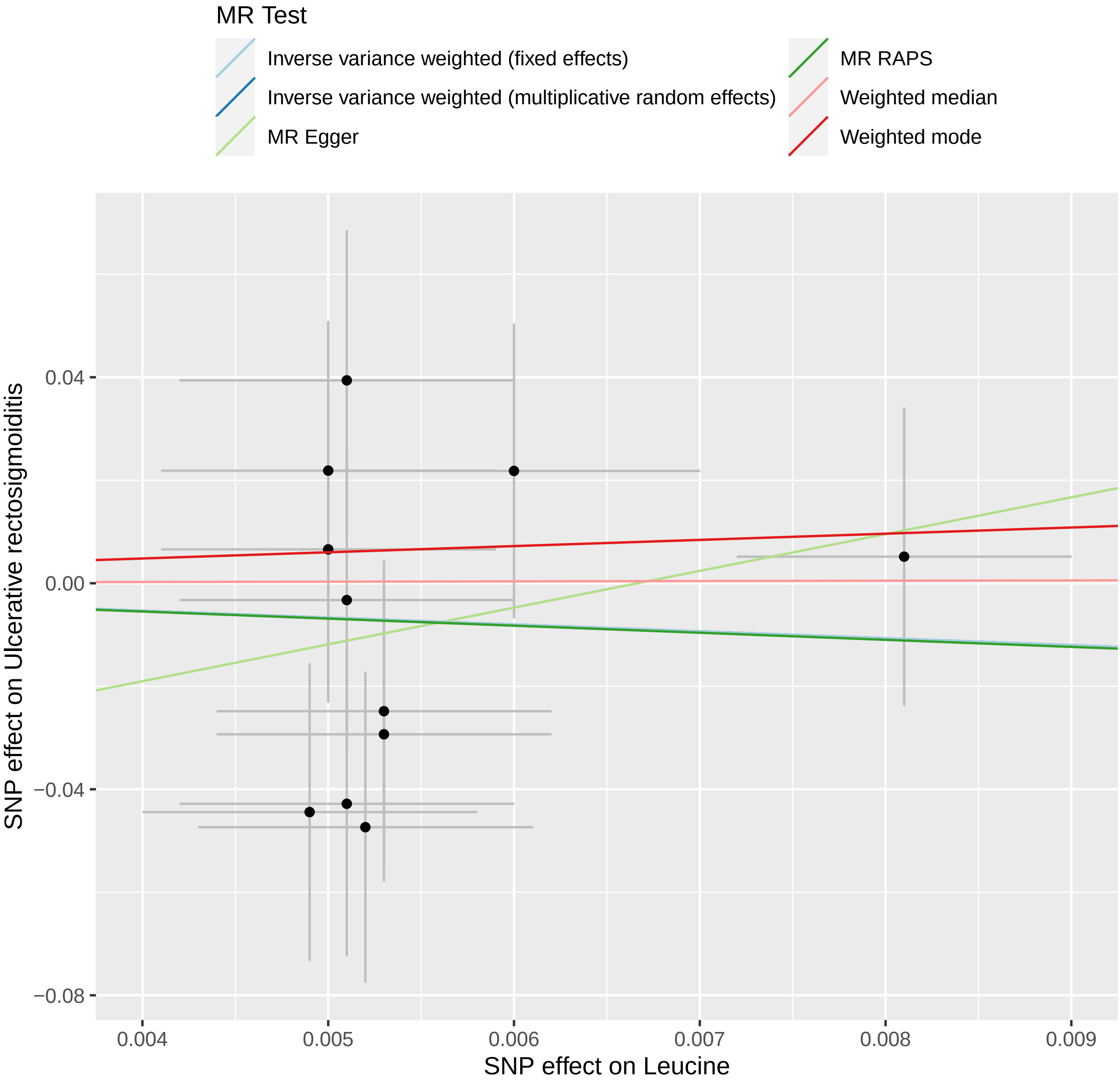

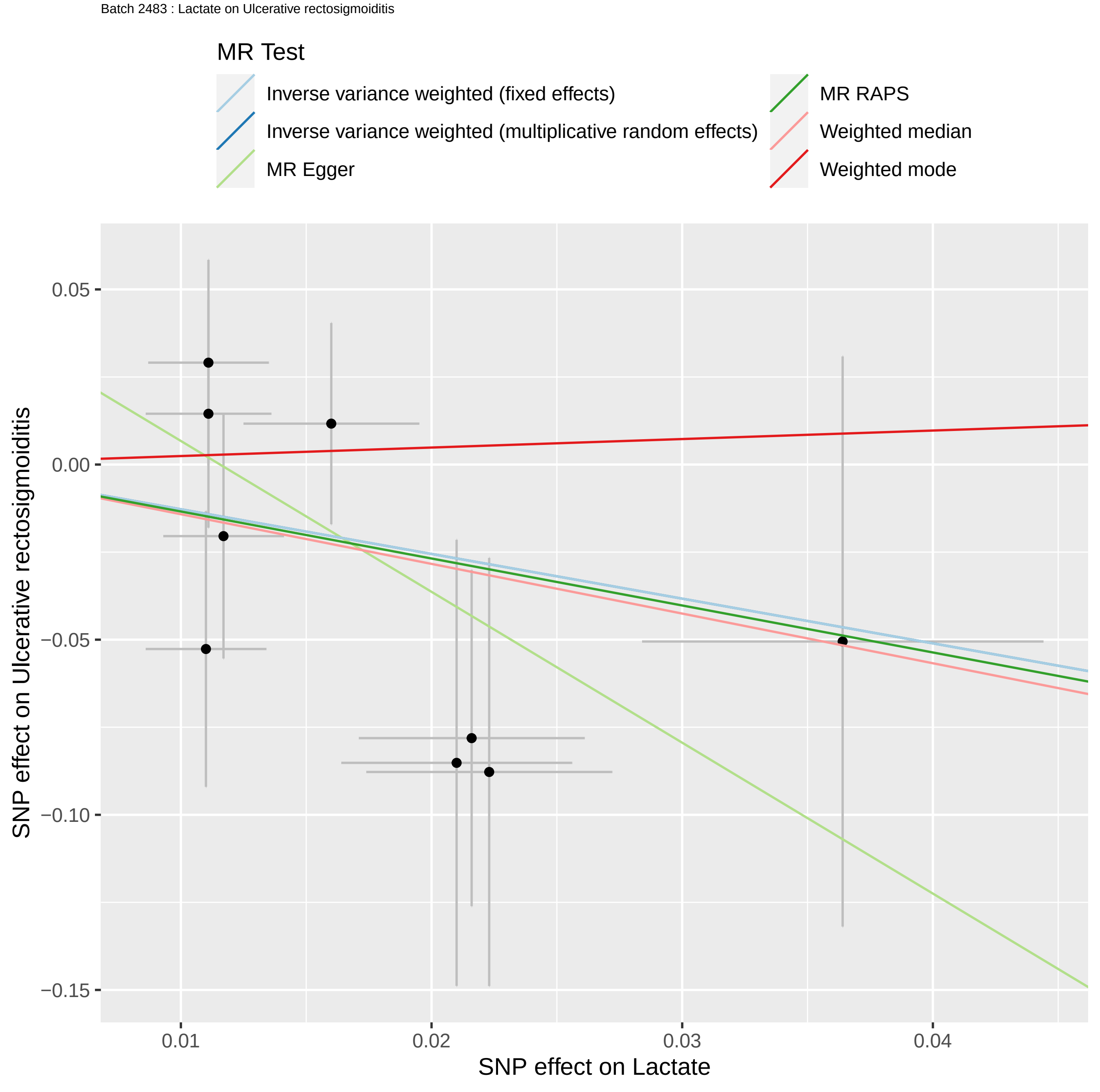

Batch 2483 : Lactate on Ulcerative rectosigmoiditis
MR Test
Inverse variance weighted (fixed effects)
Inverse variance weighted (multiplicative random effects)
MR Egger
MR RAPS
Weighted median
Weighted mode
0.05
0.00
−0.05
−0.10
−0.15
0.01
0.02
0.03
0.04
SNP effect on Ulcerative rectosigmoiditis
SNP effect on Lactate

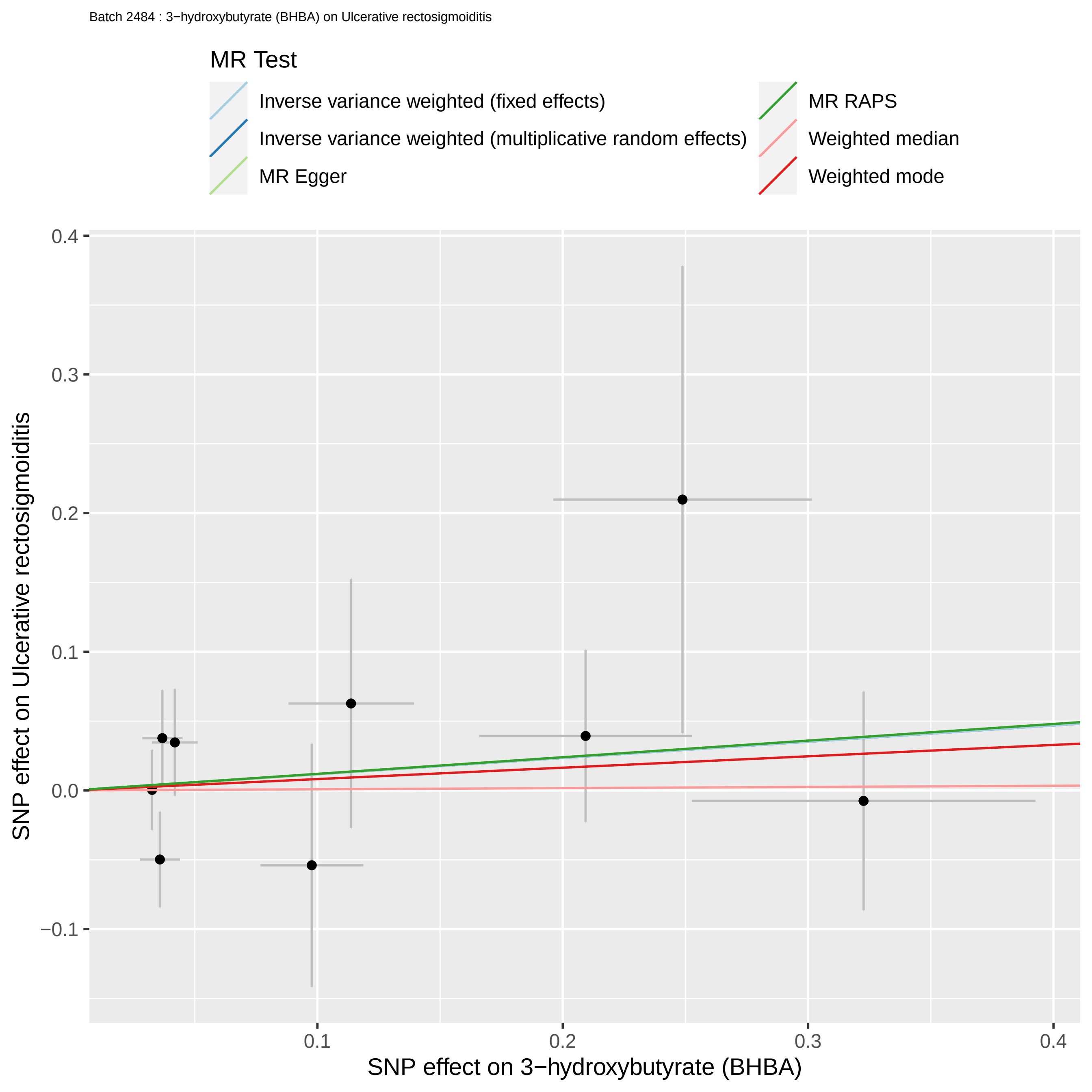
Batch 2484 : 3−hydroxybutyrate (BHBA) on Ulcerative rectosigmoiditis
MR Test
Inverse variance weighted (fixed effects)
Inverse variance weighted (multiplicative random effects)
MR Egger
MR RAPS
Weighted median
Weighted mode
0.4
0.3
0.2
0.1
0.0
−0.1
0.1
0.2
0.3
0.4
SNP effect on Ulcerative rectosigmoiditis
SNP effect on 3−hydroxybutyrate (BHBA)

Batch 2485 : Arabinose on Ulcerative rectosigmoiditis

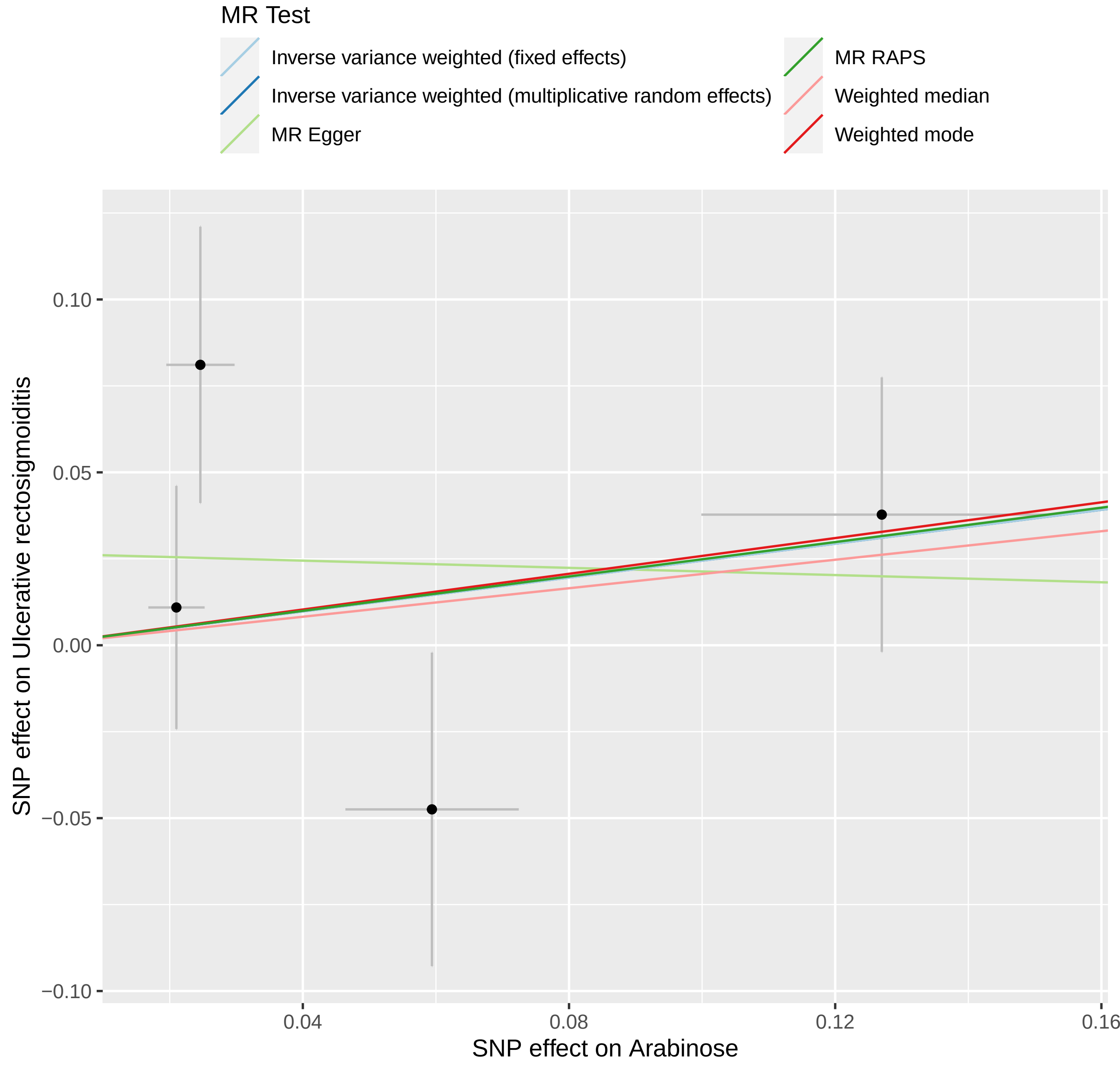

Batch 2486 : Fructose on Ulcerative rectosigmoiditis

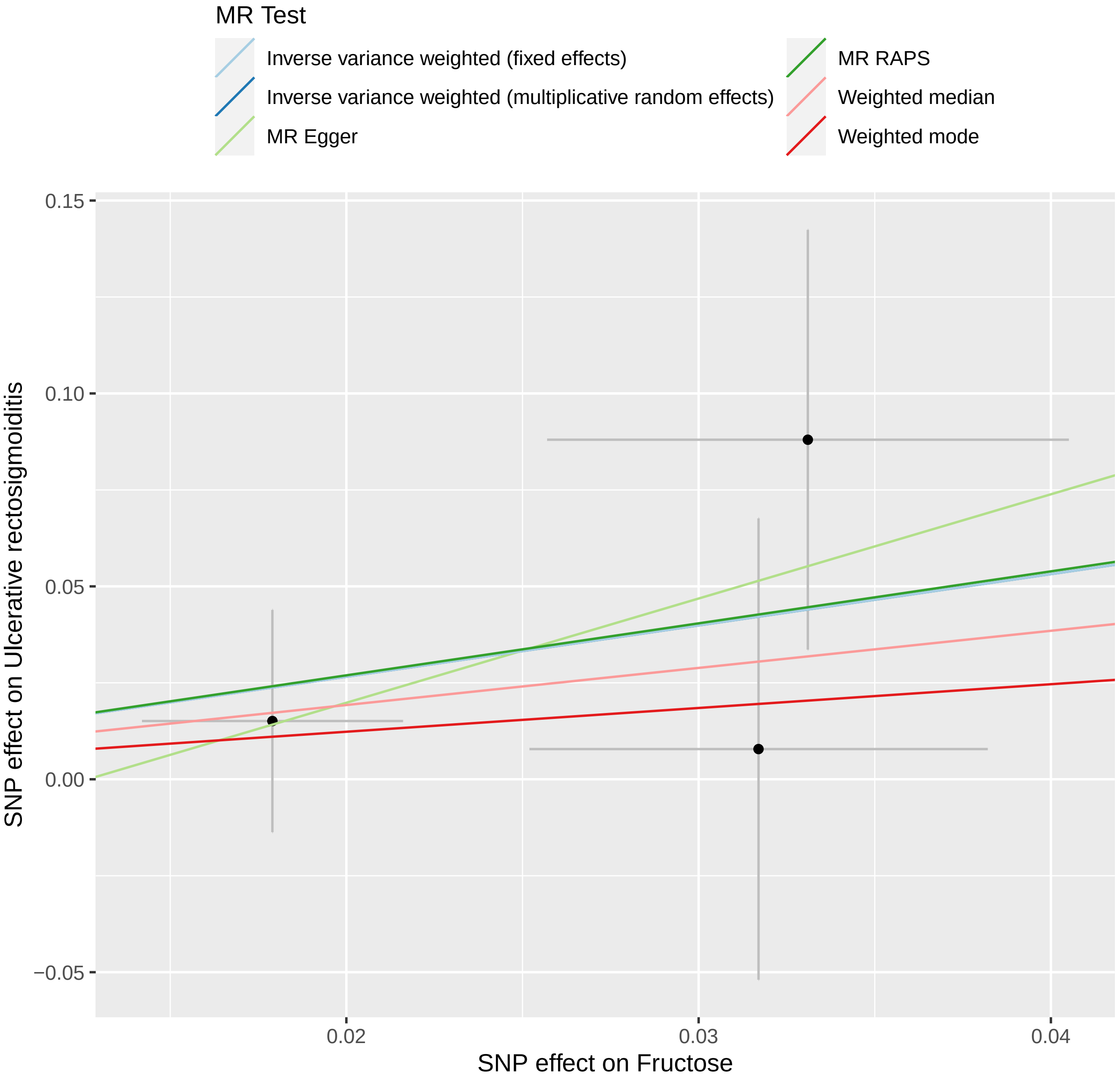

Batch 2488 : Pyruvate on Ulcerative rectosigmoiditis

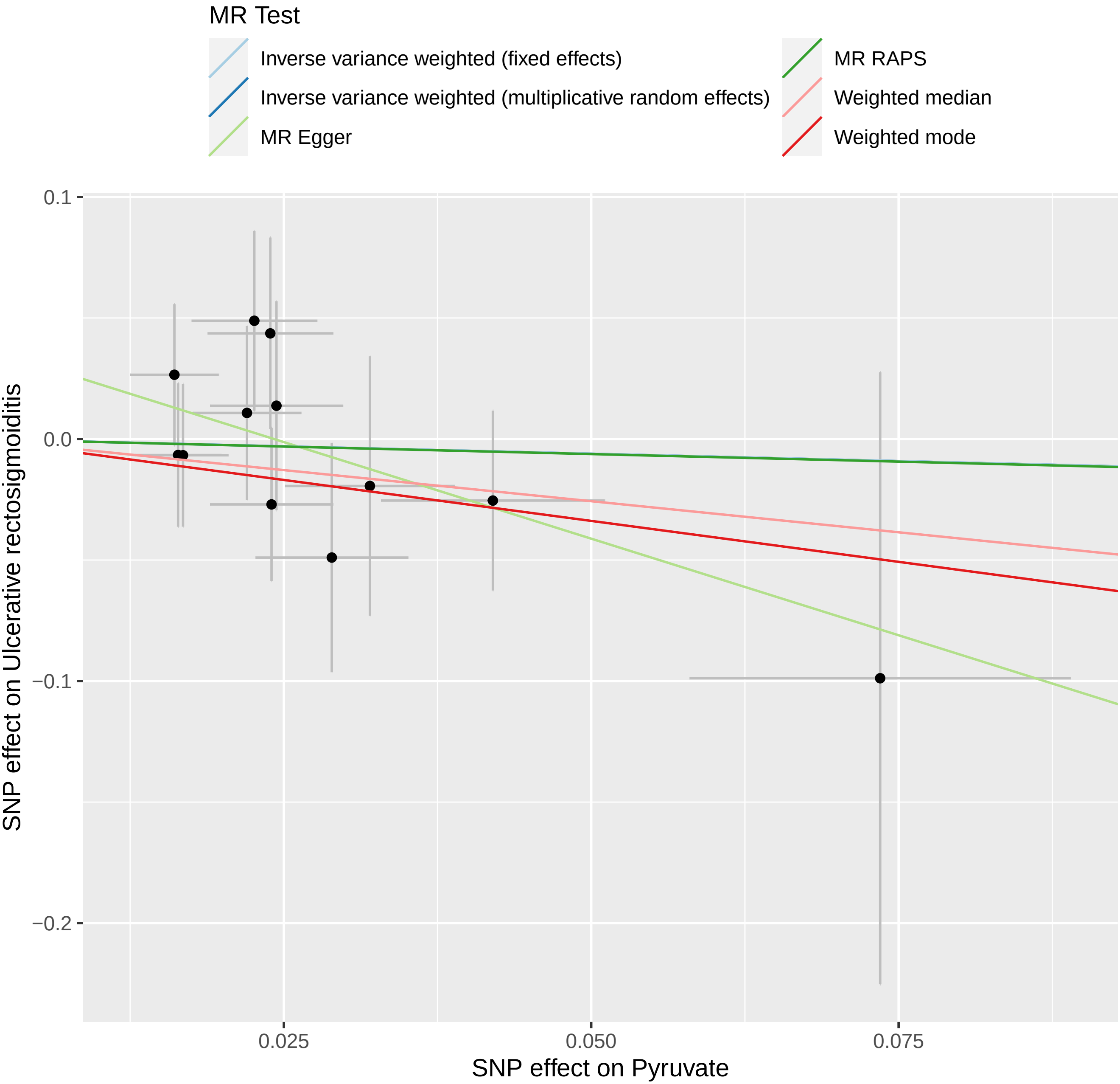

Batch 2489 : Uridine on Ulcerative rectosigmoiditis

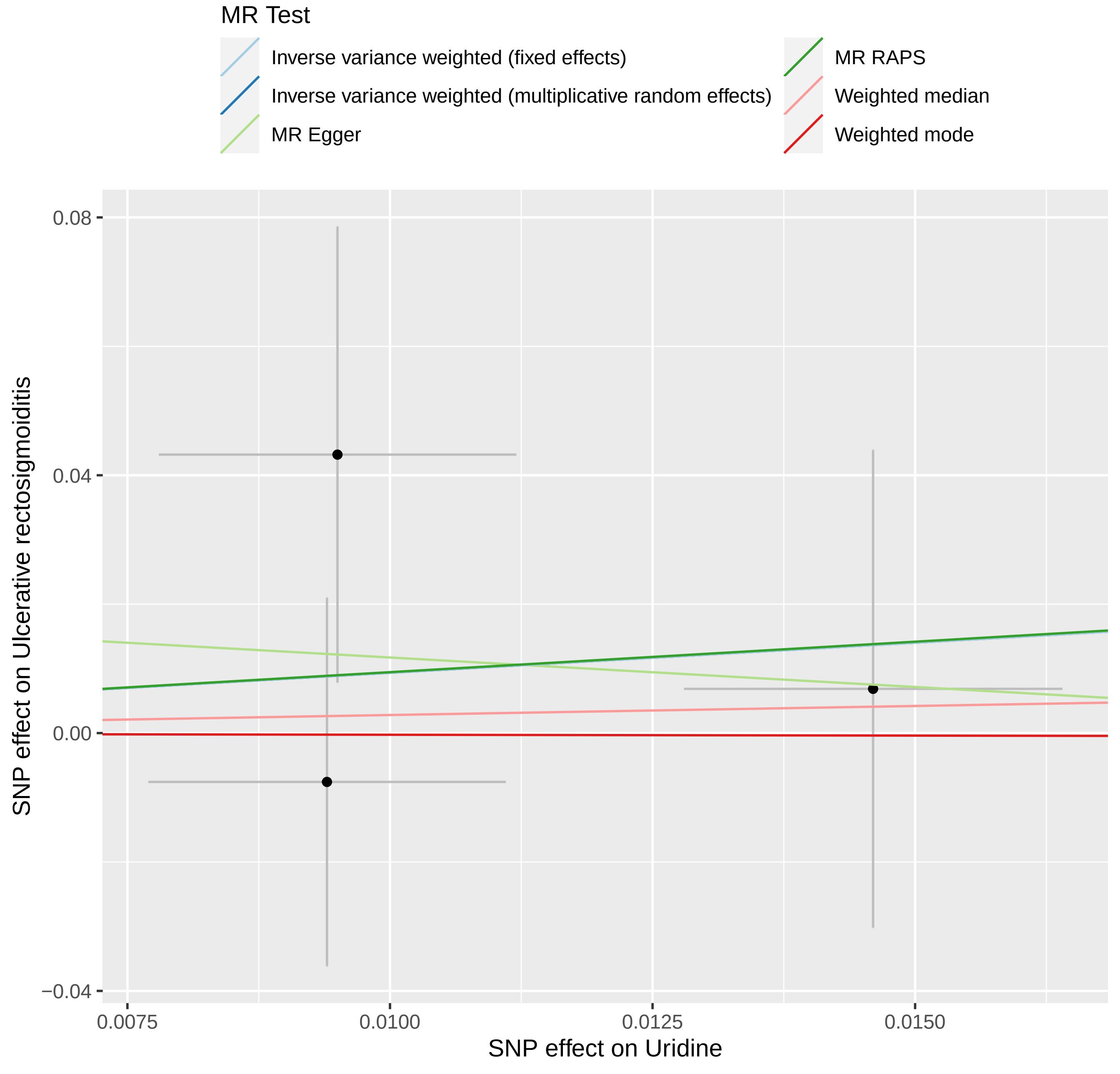

Batch 2490 : Linoleate (18:2n6) on Ulcerative rectosigmoiditis

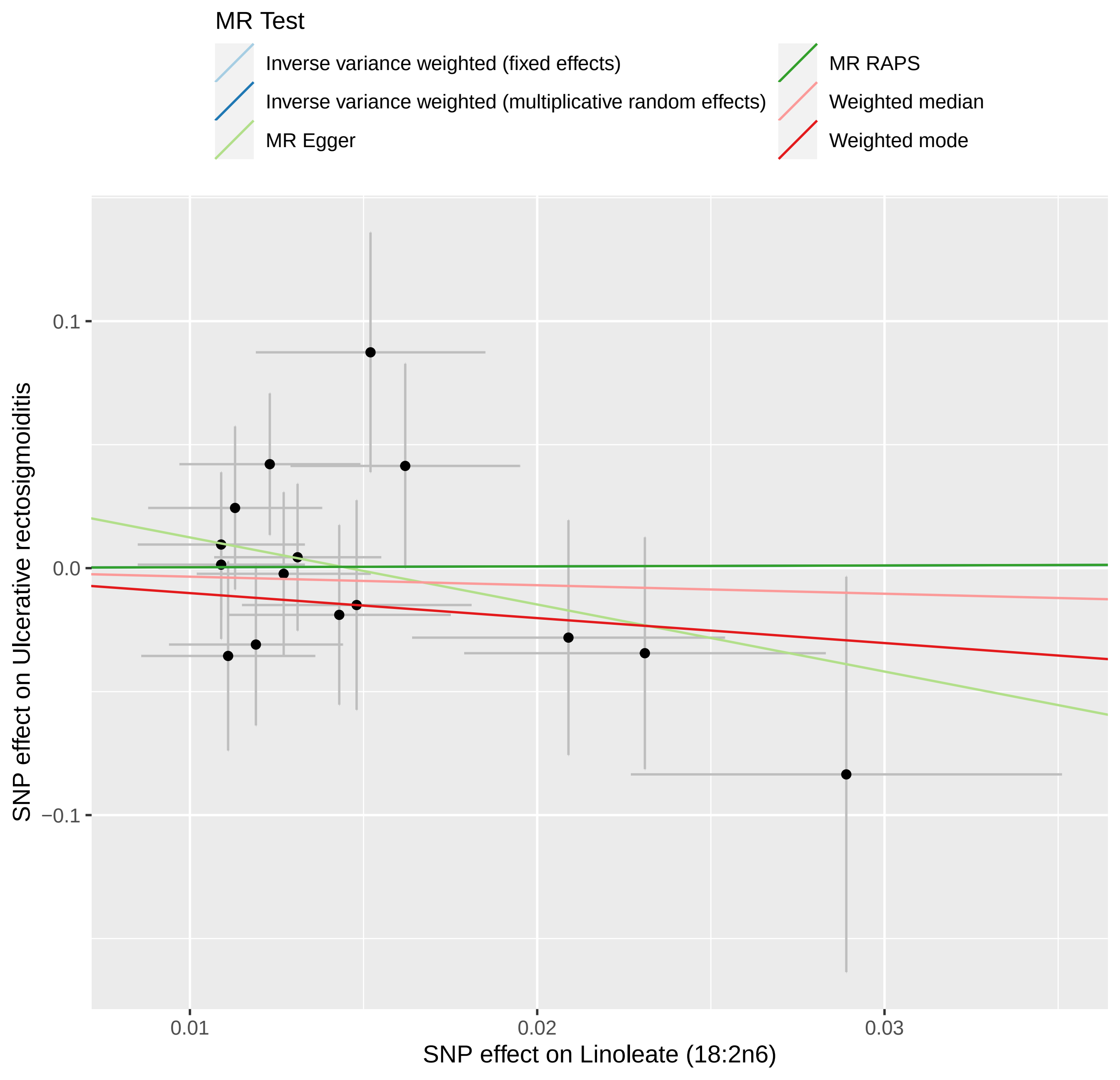

Batch 2491 : Allantoin on Ulcerative rectosigmoiditis

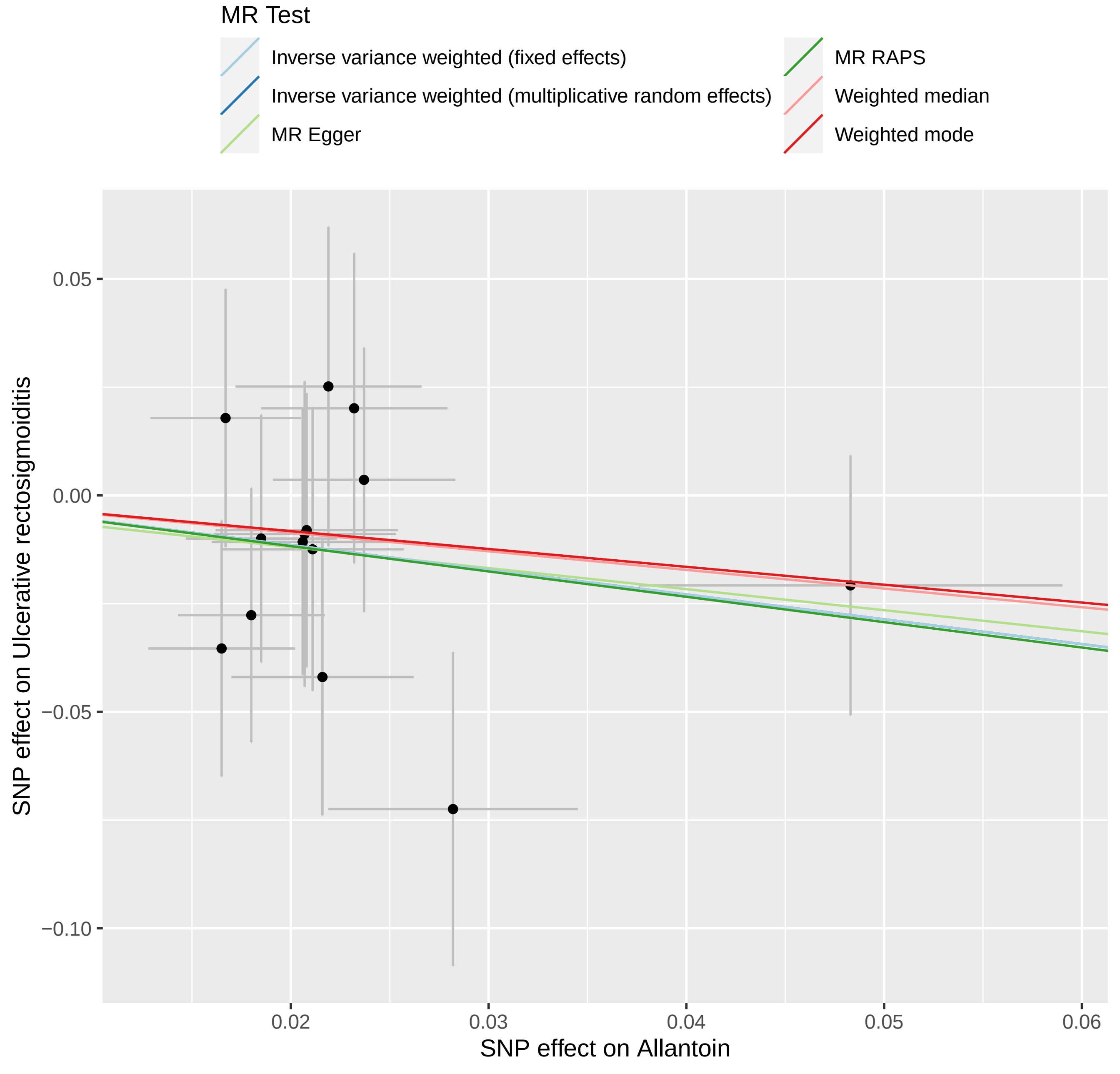

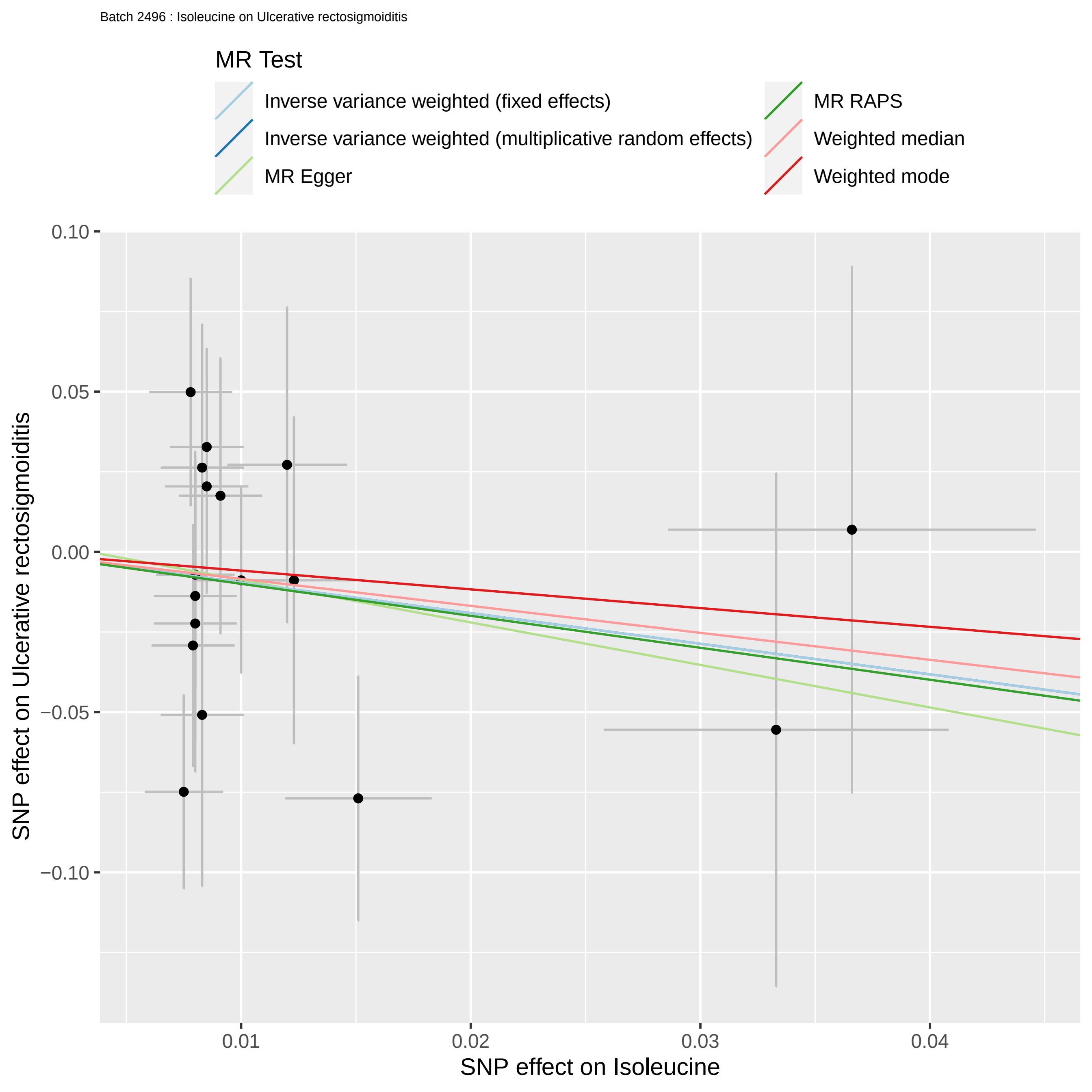

Batch 2496 : Isoleucine on Ulcerative rectosigmoiditis
MR Test
Inverse variance weighted (fixed effects)
Inverse variance weighted (multiplicative random effects)
MR Egger
MR RAPS
Weighted median
Weighted mode
0.10
0.05
0.00
−0.05
−0.10
0.01
0.02
0.03
0.04
SNP effect on Ulcerative rectosigmoiditis
SNP effect on Isoleucine

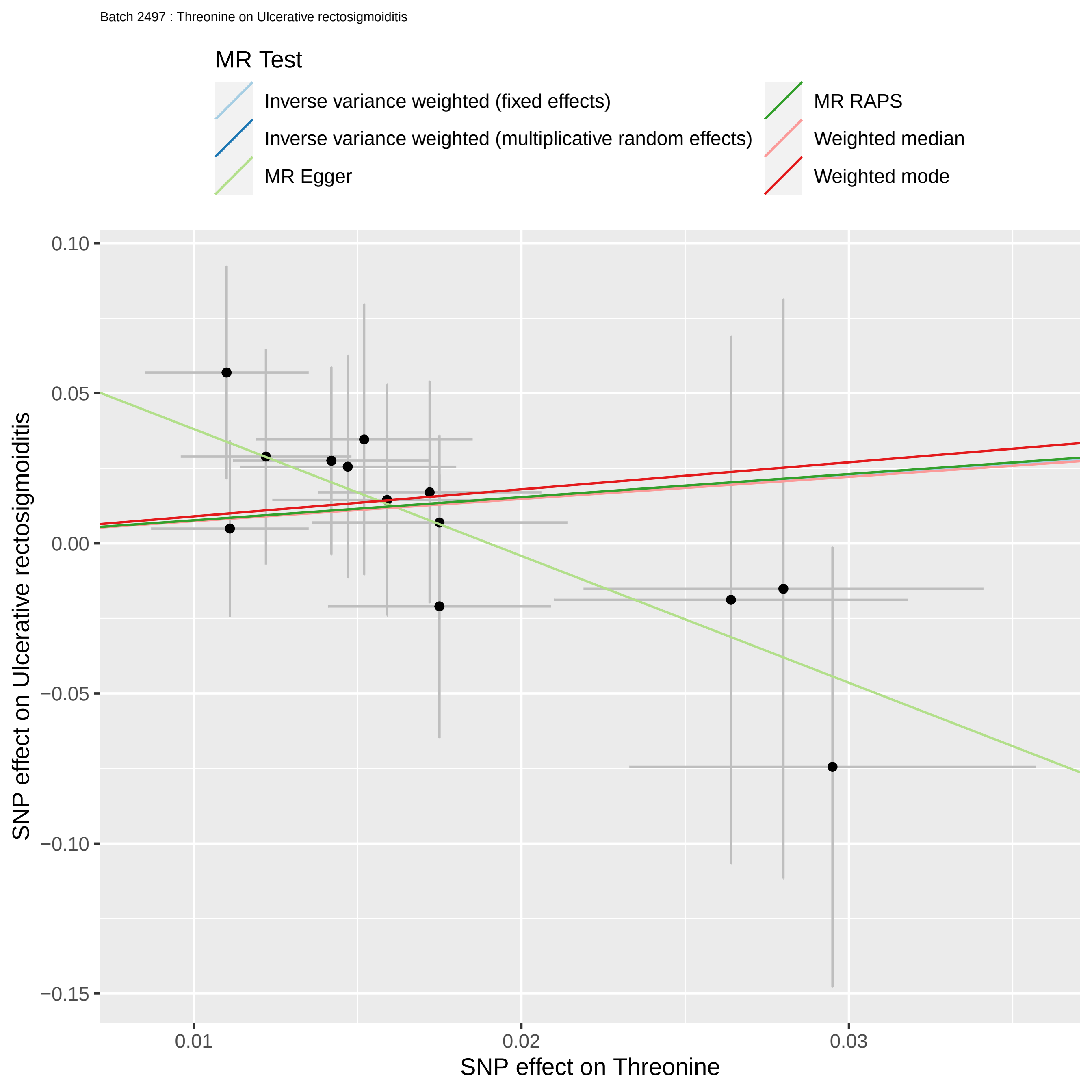

Batch 2497 : Threonine on Ulcerative rectosigmoiditis
MR Test
Inverse variance weighted (fixed effects)
Inverse variance weighted (multiplicative random effects)
MR Egger
MR RAPS
Weighted median
Weighted mode
0.10
0.05
0.00
−0.05
−0.10
−0.15
0.01
0.02
0.03
SNP effect on Ulcerative rectosigmoiditis
SNP effect on Threonine

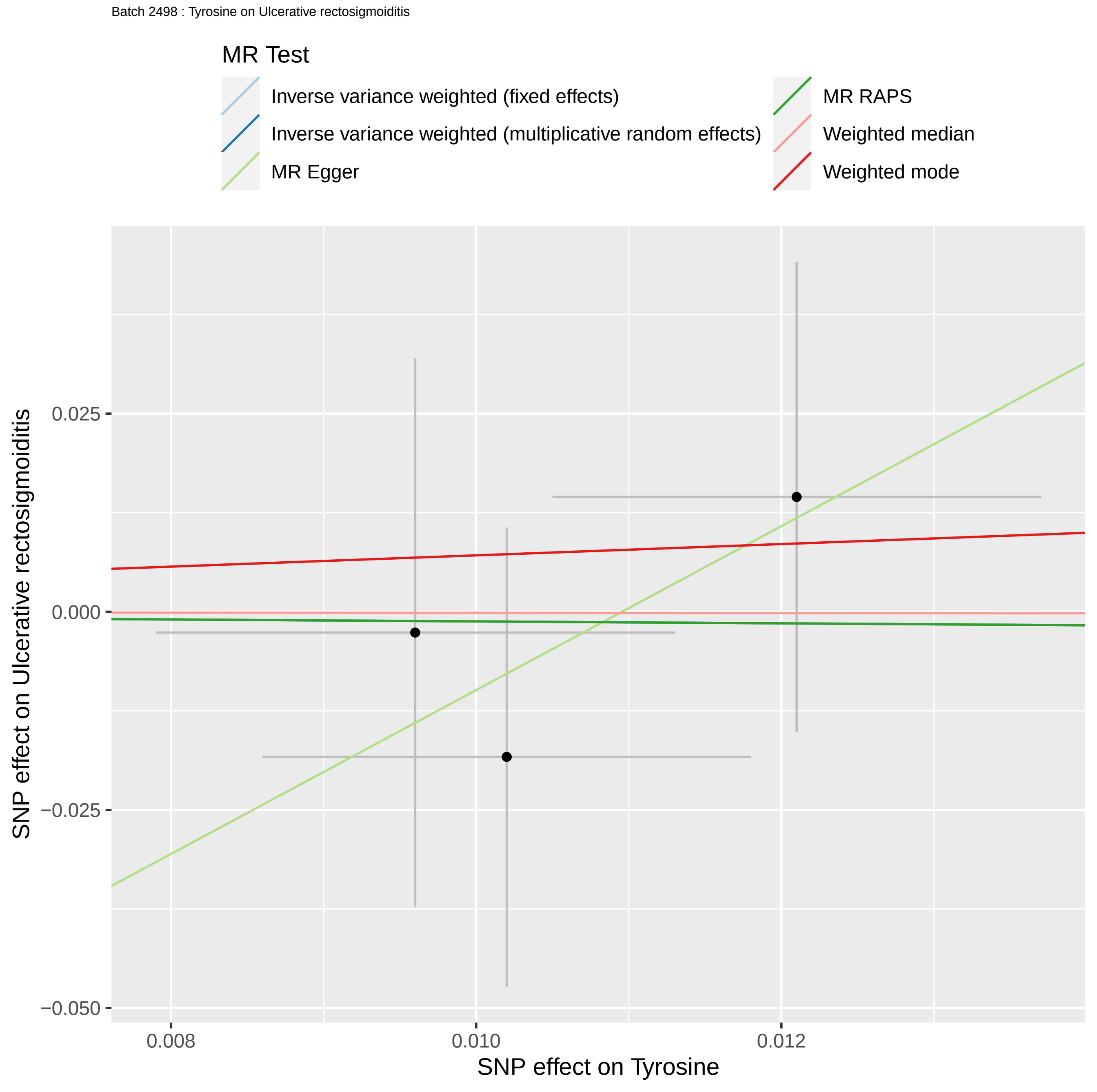
Batch 2498 : Tyrosine on Ulcerative rectosigmoiditis
MR Test
Inverse variance weighted (fixed effects)
Inverse variance weighted (multiplicative random effects)
MR Egger
MR RAPS
Weighted median
Weighted mode
SNP effect on Ulcerative rectosigmoiditis
0.025
0.000
−0.025
−0.050
0.008
0.010
0.012
SNP effect on Tyrosine

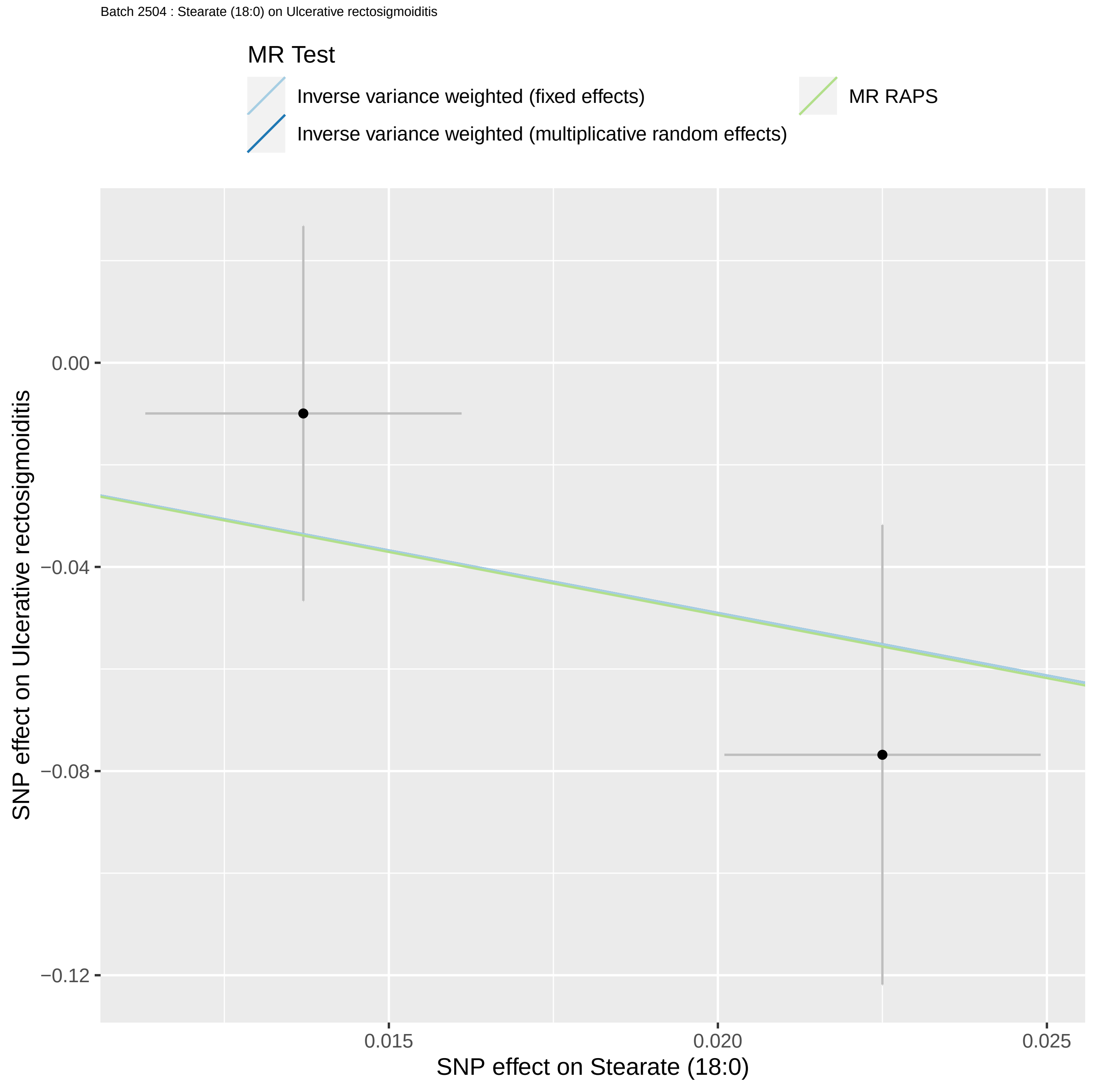

Batch 2504 : Stearate (18:0) on Ulcerative rectosigmoiditis
MR Test
Inverse variance weighted (fixed effects)
Inverse variance weighted (multiplicative random effects)
MR RAPS
0.00
−0.04
−0.08
−0.12
0.015
0.020
0.025
SNP effect on Stearate (18:0)
SNP effect on Ulcerative rectosigmoiditis

Batch 2507 : Myristate (14:0) on Ulcerative rectosigmoiditis

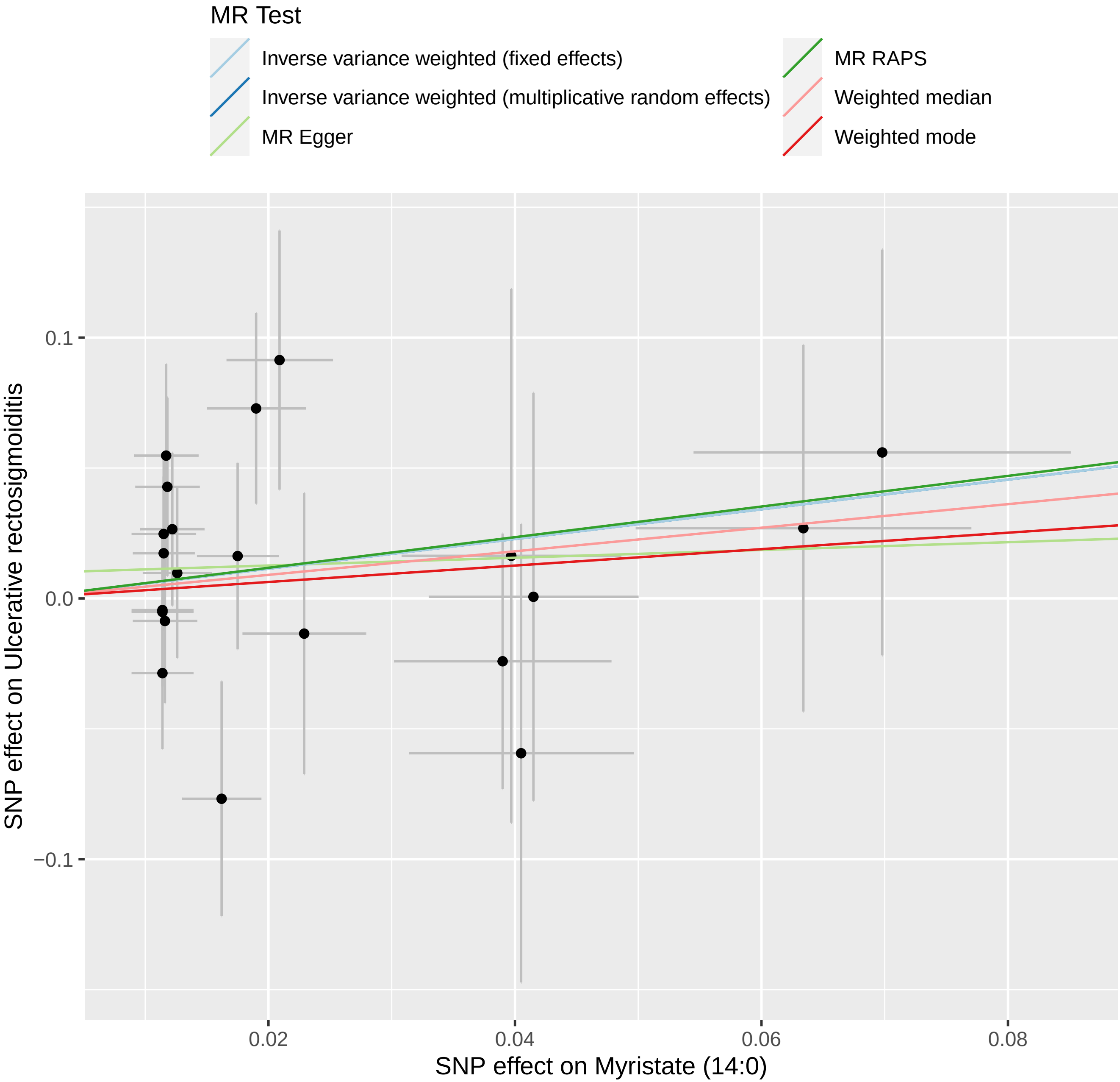

Batch 2509 : Ornithine on Ulcerative rectosigmoiditis

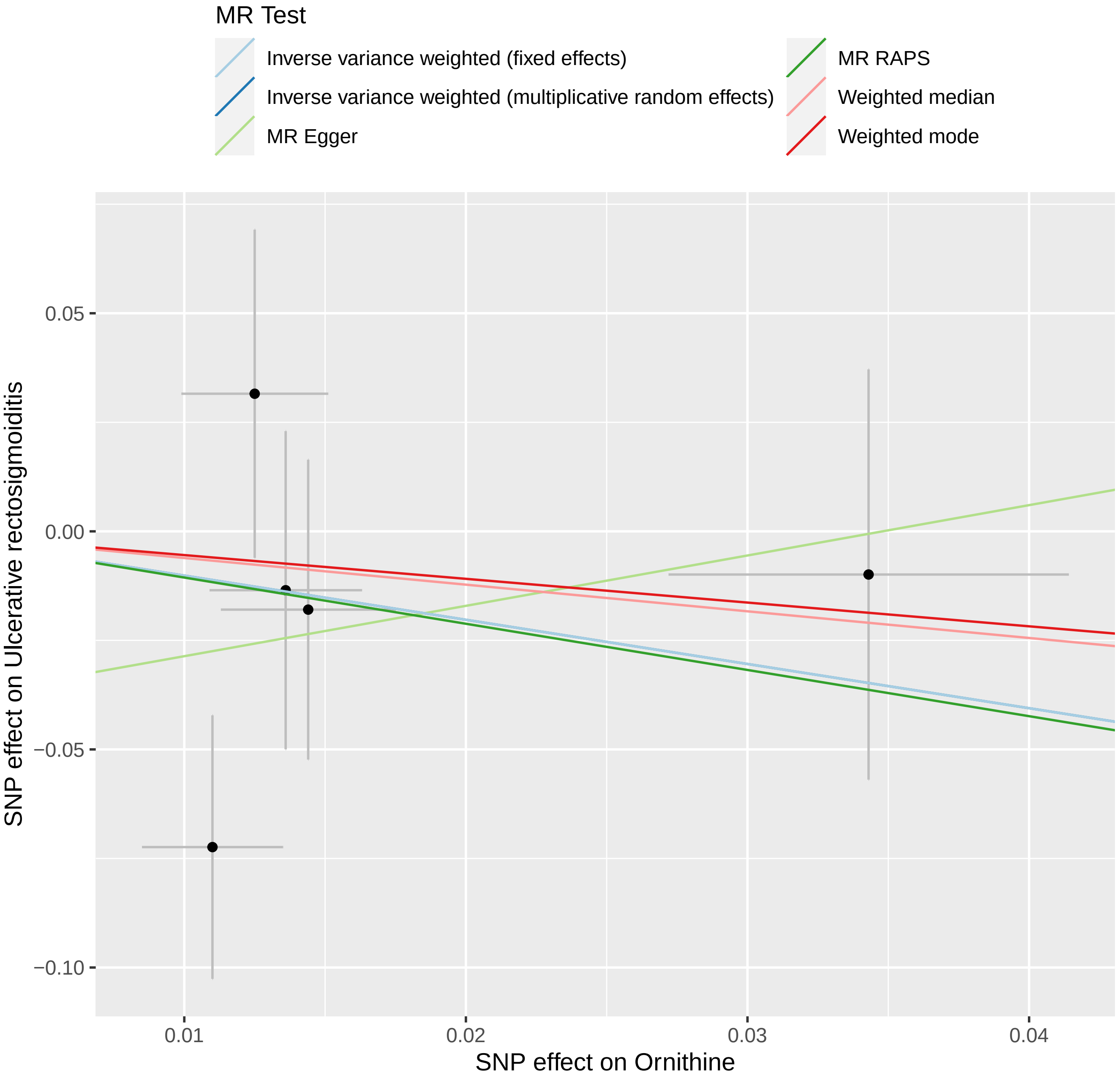

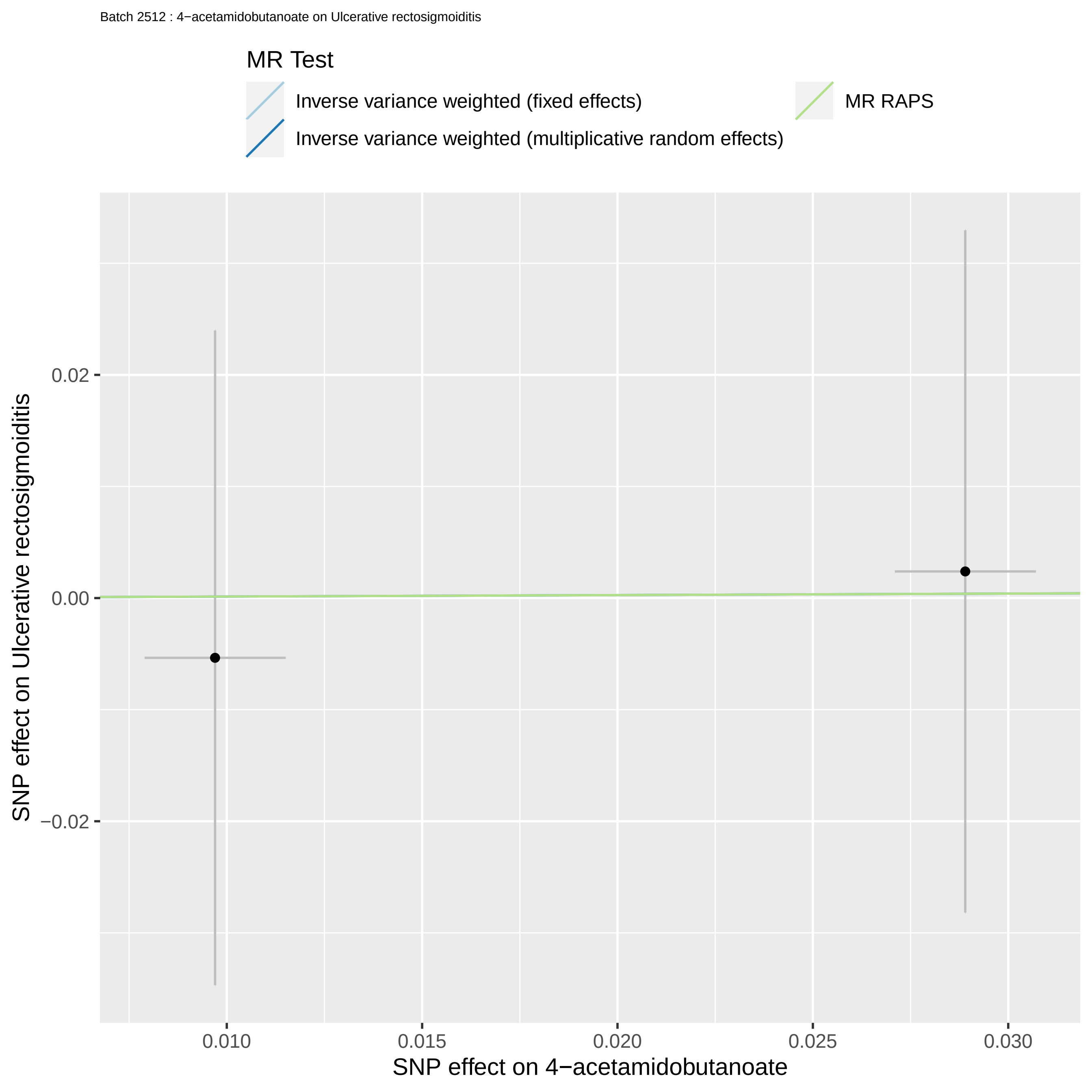

Batch 2512 : 4−acetamidobutanoate on Ulcerative rectosigmoiditis
MR Test
Inverse variance weighted (fixed effects)
Inverse variance weighted (multiplicative random effects)
MR RAPS
0.02
0.00
−0.02
0.010
0.015
0.020
0.025
0.030
SNP effect on Ulcerative rectosigmoiditis
SNP effect on 4−acetamidobutanoate

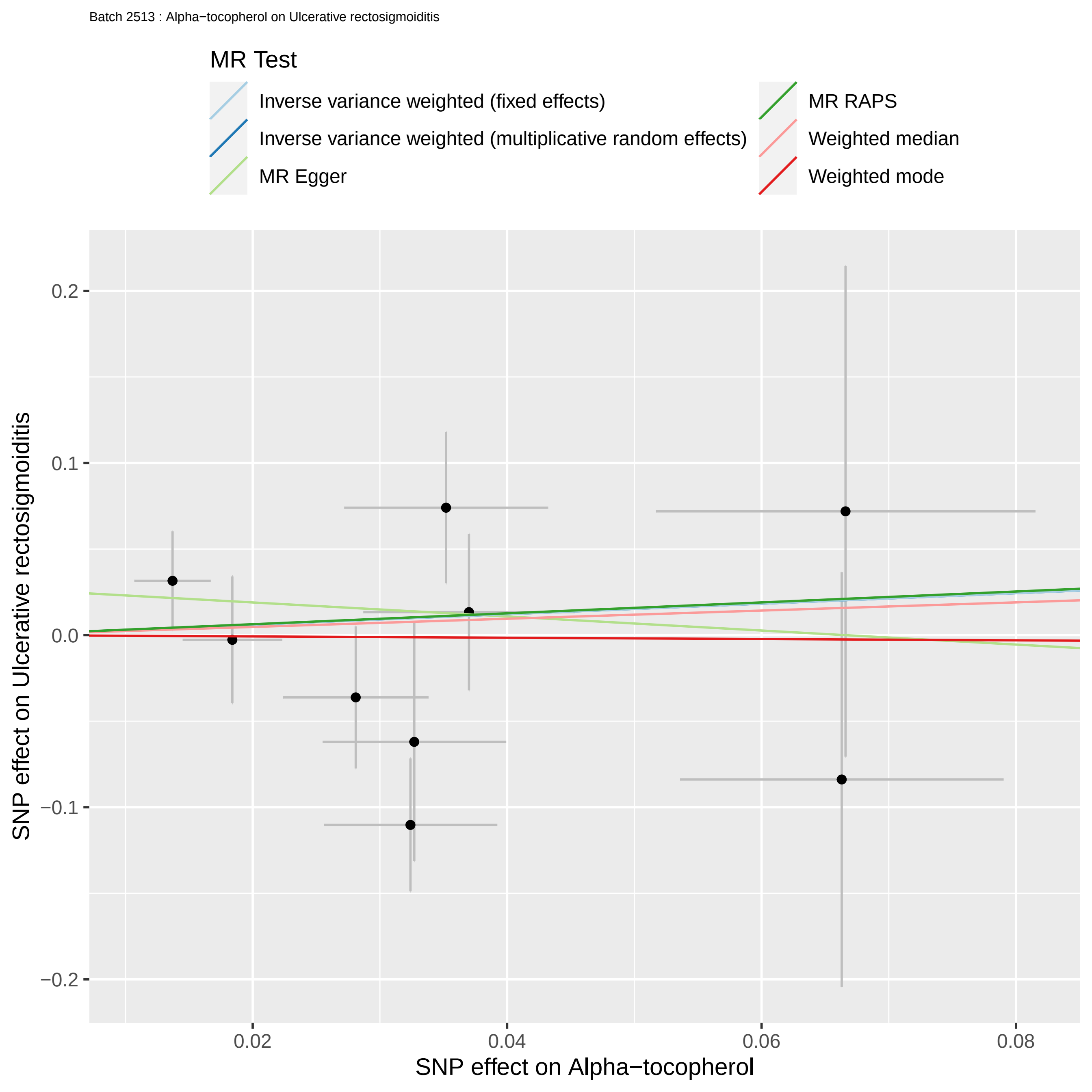

Batch 2513 : Alpha−tocopherol on Ulcerative rectosigmoiditis
MR Test
Inverse variance weighted (fixed effects)
Inverse variance weighted (multiplicative random effects)
MR Egger
MR RAPS
Weighted median
Weighted mode
0.2
0.1
0.0
−0.1
−0.2
0.02
0.04
0.06
0.08
SNP effect on Ulcerative rectosigmoiditis
SNP effect on Alpha−tocopherol

Batch 2514 : Citrate on Ulcerative rectosigmoiditis

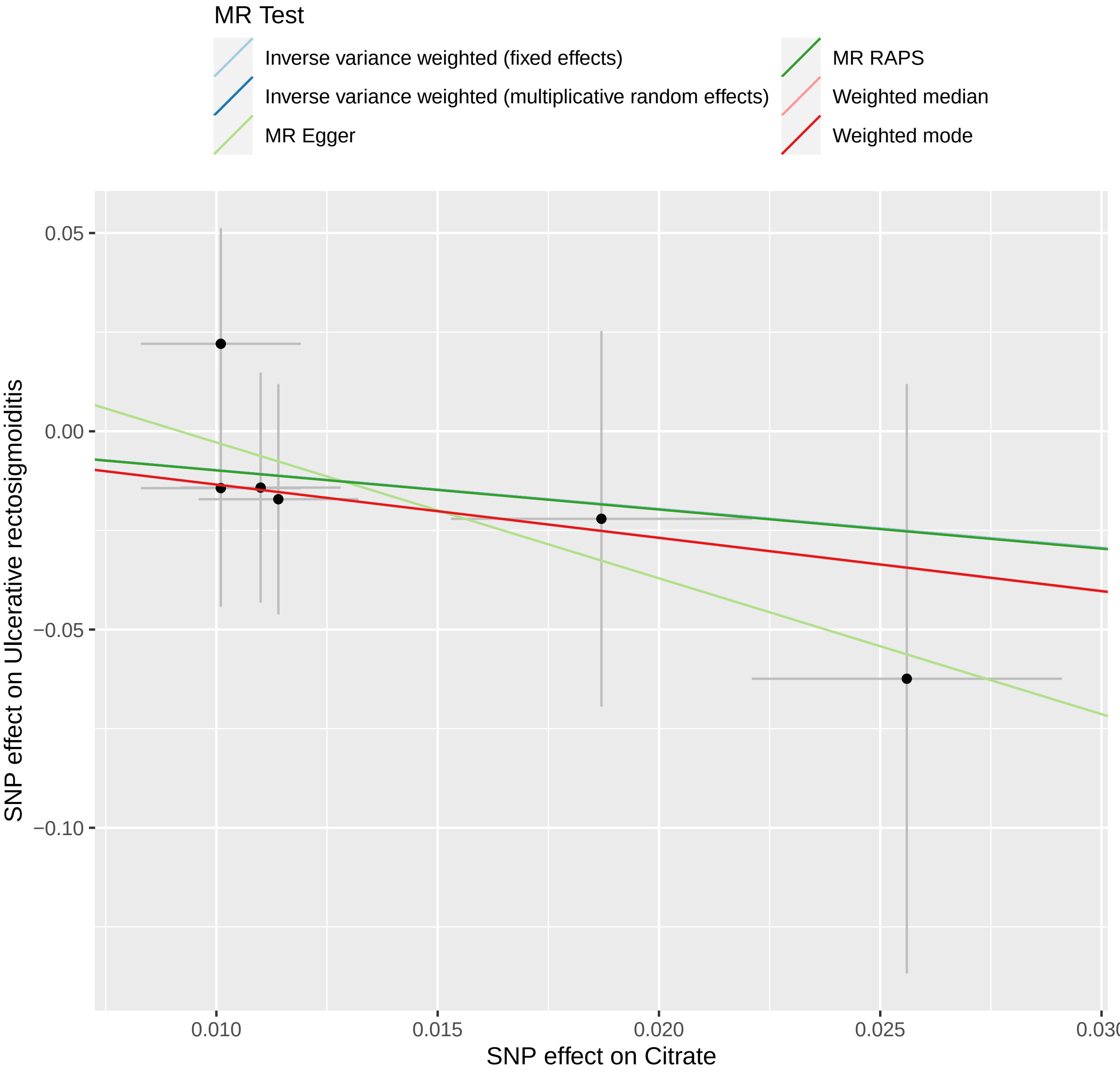

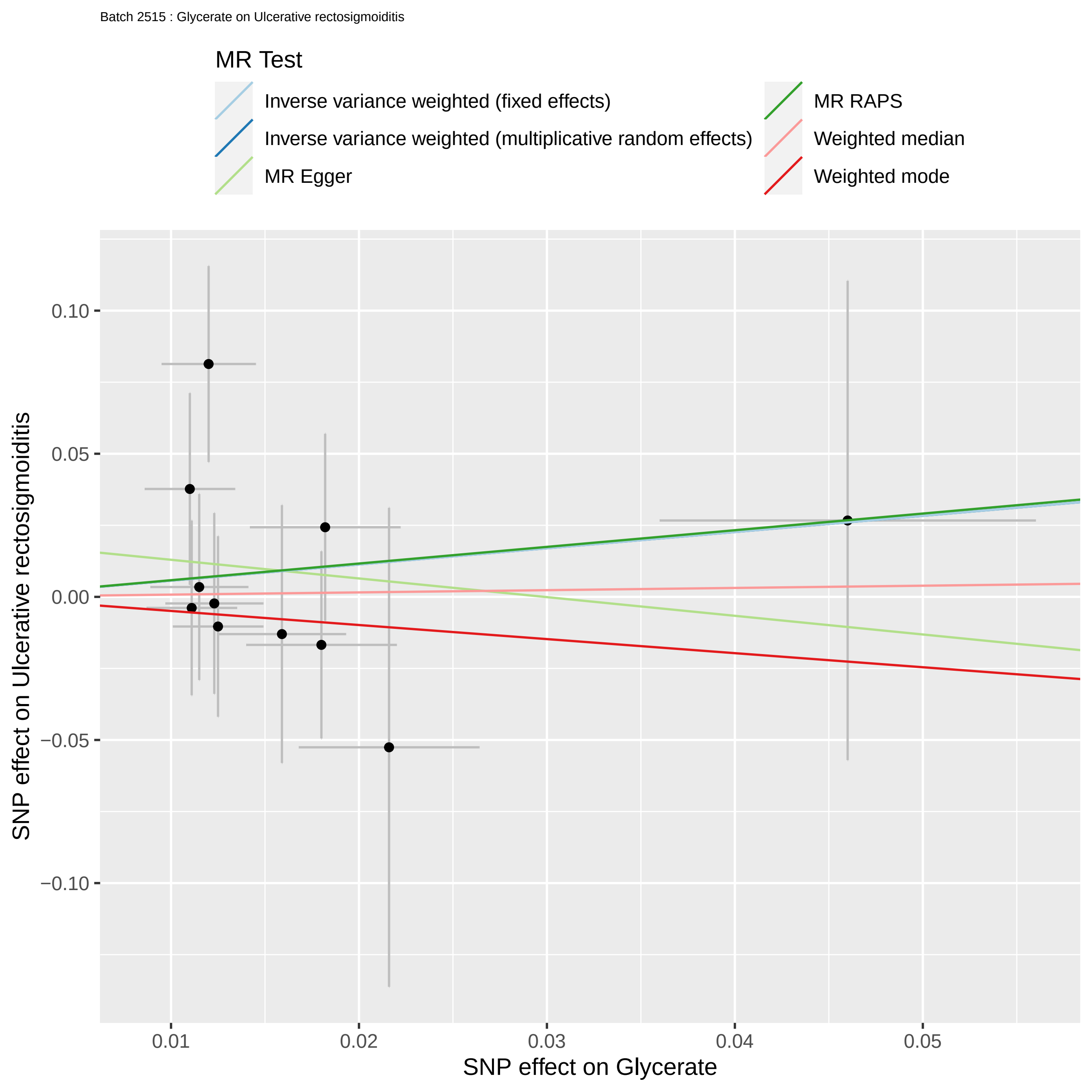

Batch 2515 : Glycerate on Ulcerative rectosigmoiditis
MR Test
Inverse variance weighted (fixed effects)
Inverse variance weighted (multiplicative random effects)
MR Egger
MR RAPS
Weighted median
Weighted mode
0.10
0.05
0.00
−0.05
−0.10
0.01
0.02
0.03
0.04
0.05
SNP effect on Ulcerative rectosigmoiditis
SNP effect on Glycerate

Batch 2516 : Guanosine on Ulcerative rectosigmoiditis

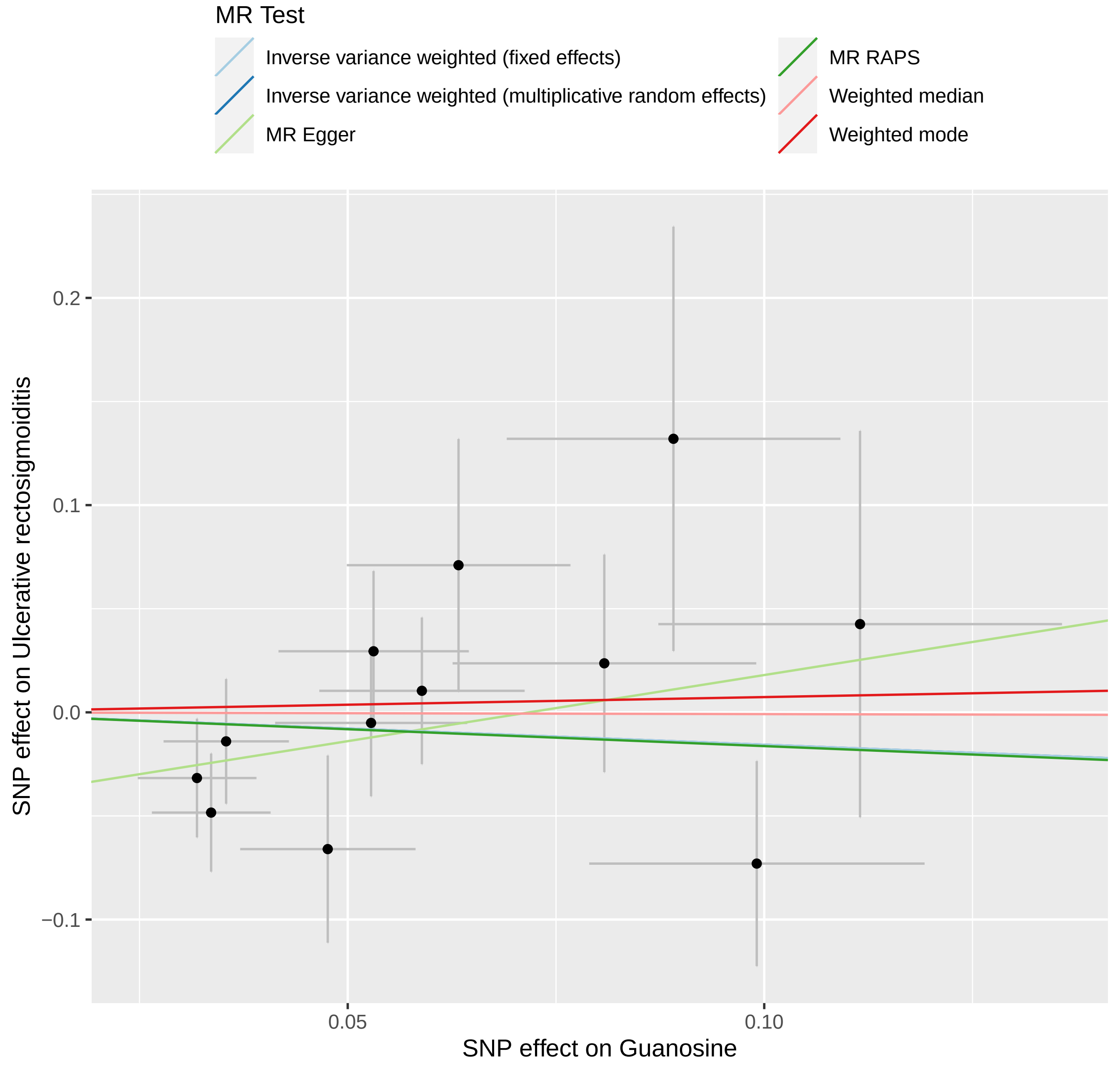

Batch 2519 : Ursodeoxycholate on Ulcerative rectosigmoiditis

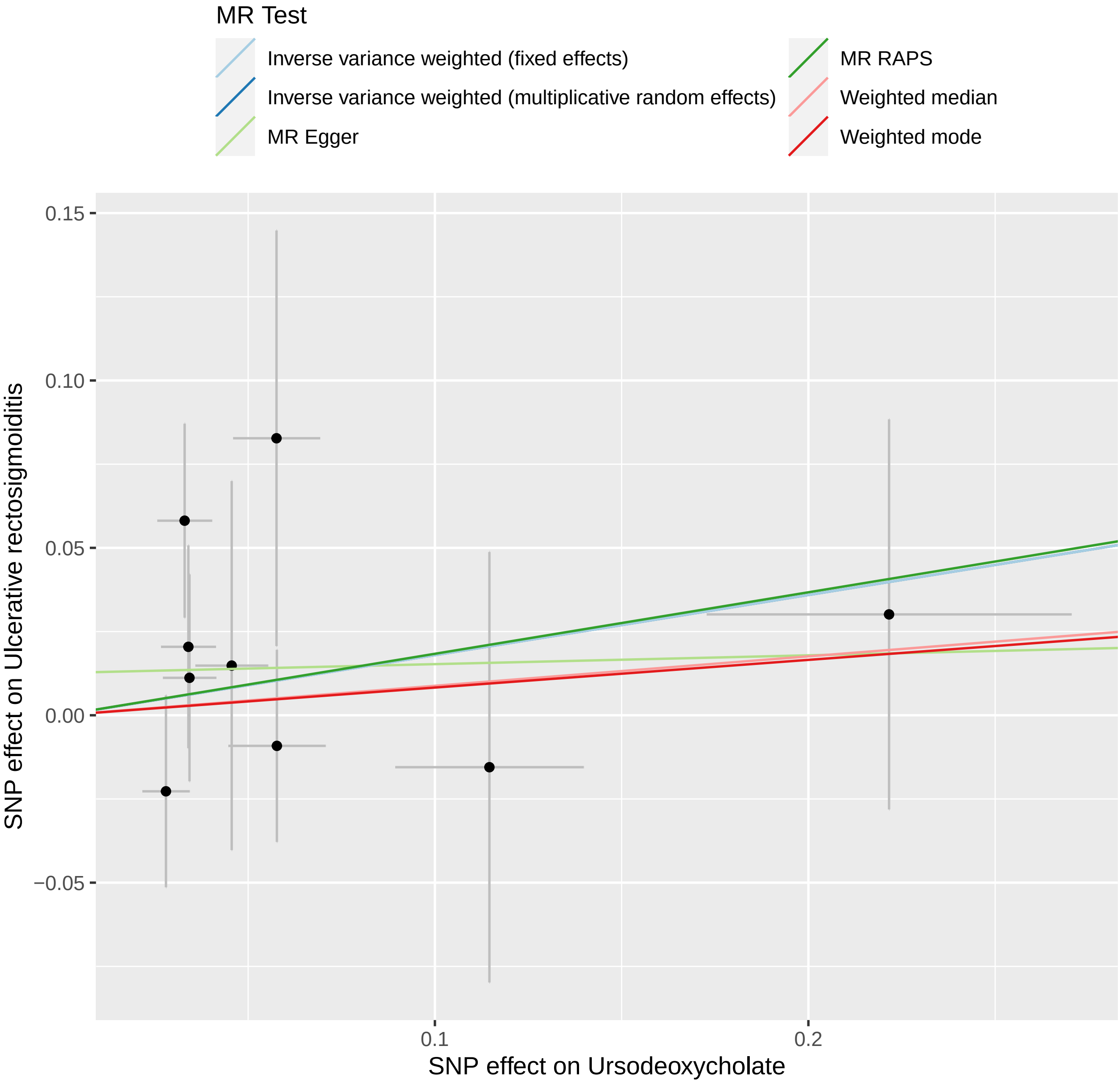

Batch 2520 : Arginine on Ulcerative rectosigmoiditis

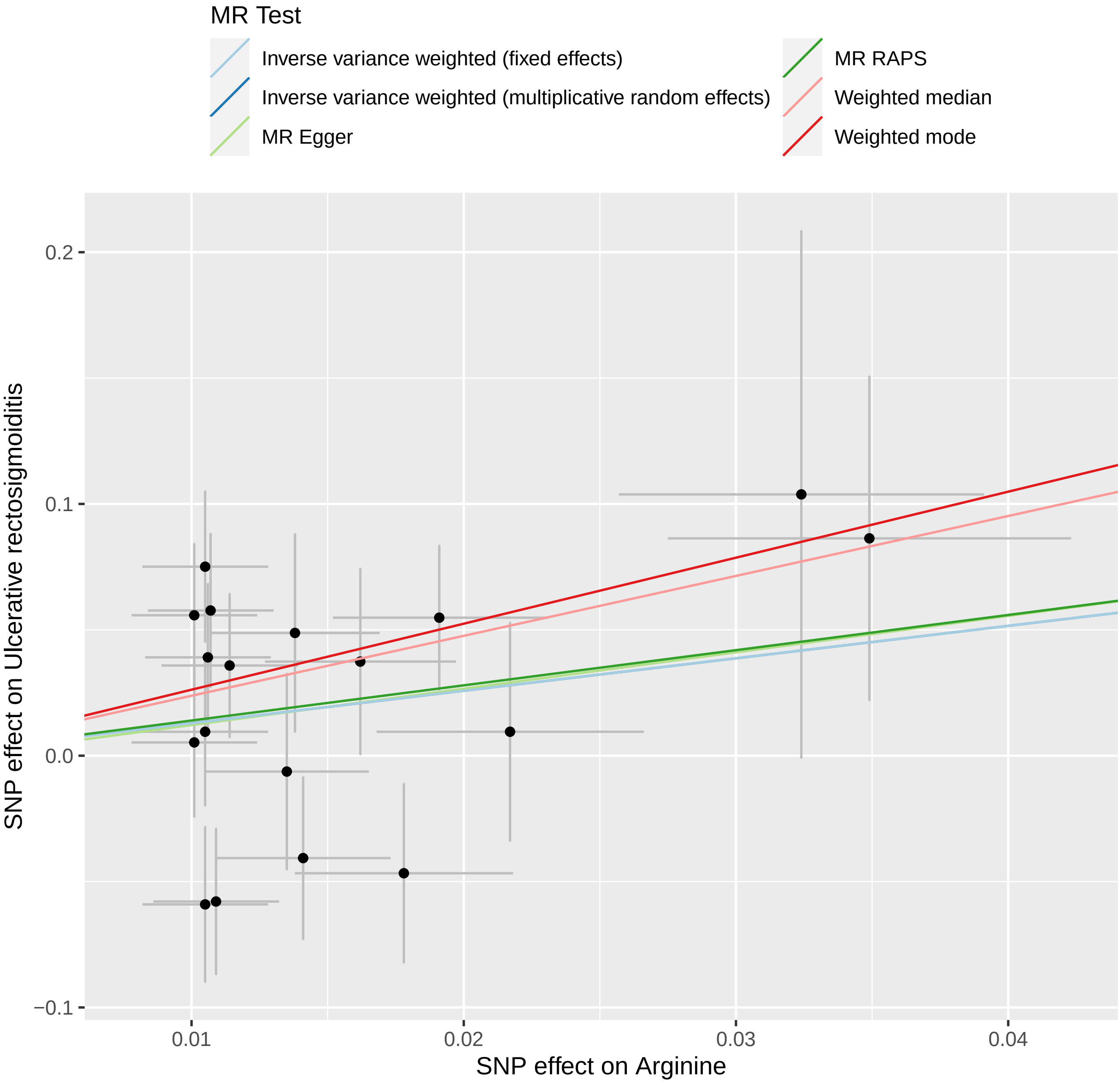

Batch 2521 : Ascorbate (Vitamin C) on Ulcerative rectosigmoiditis

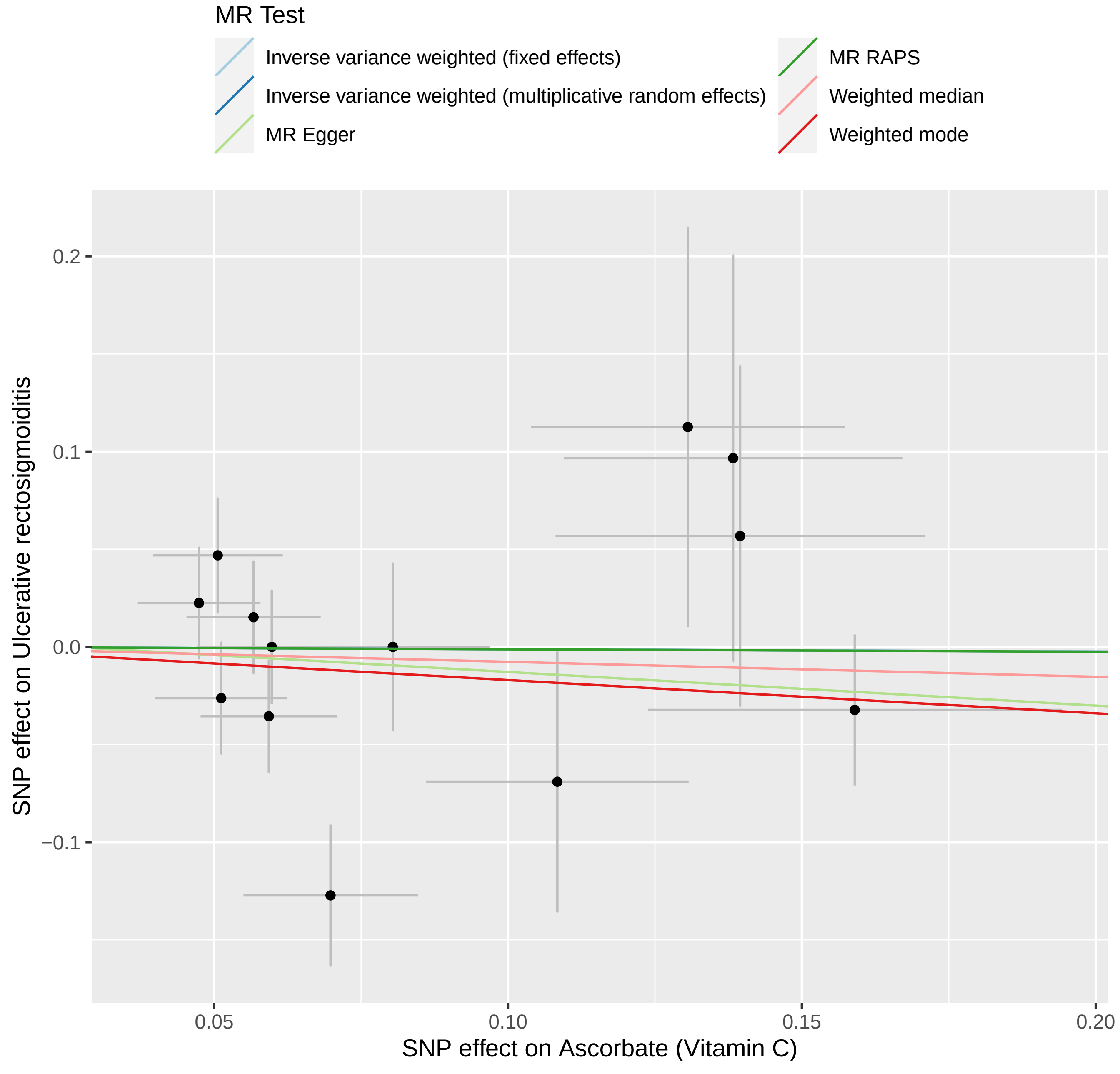

Batch 2522 : Heptanoate (7:0) on Ulcerative rectosigmoiditis

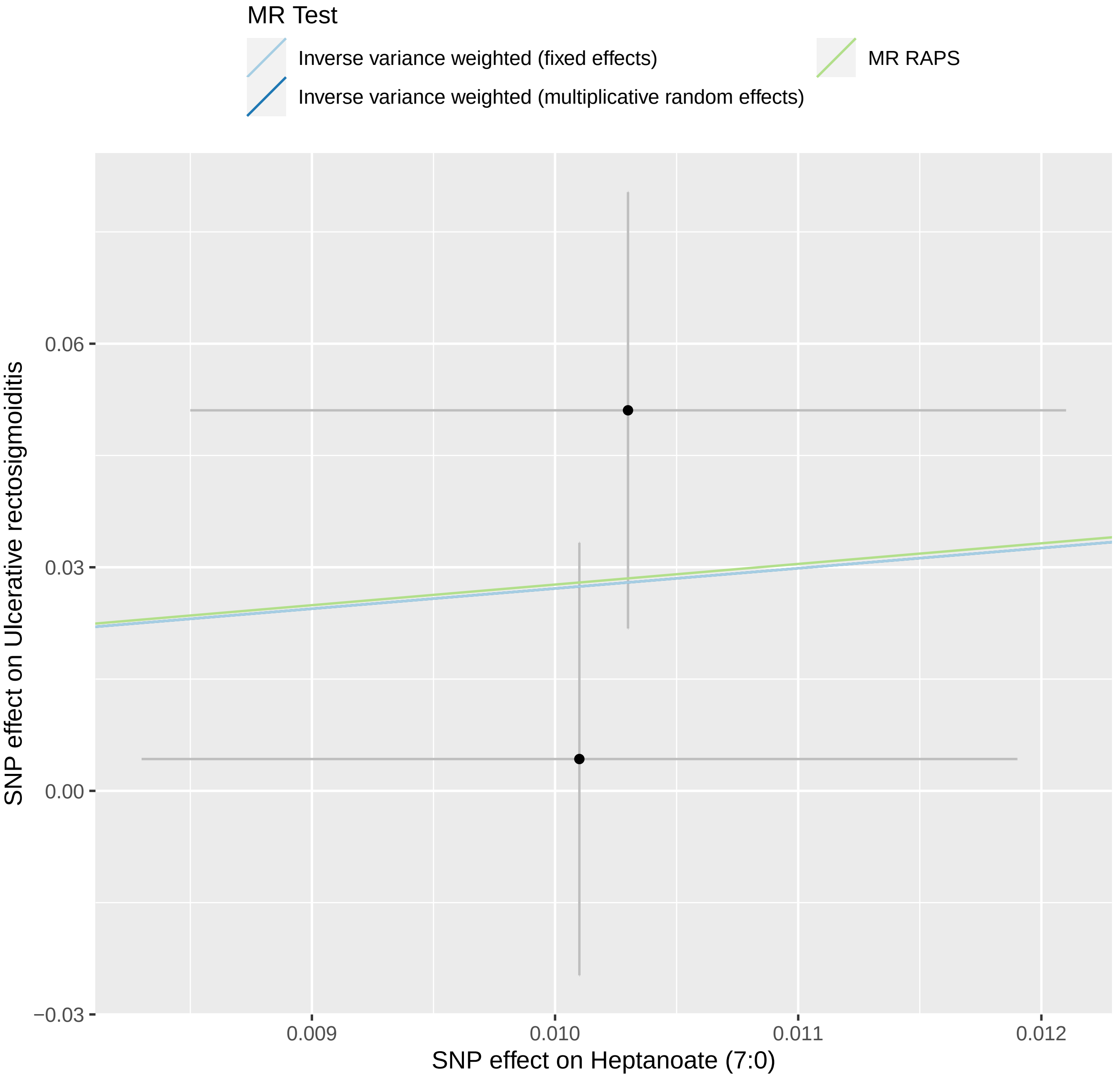

Batch 2523 : Laurate (12:0) on Ulcerative rectosigmoiditis

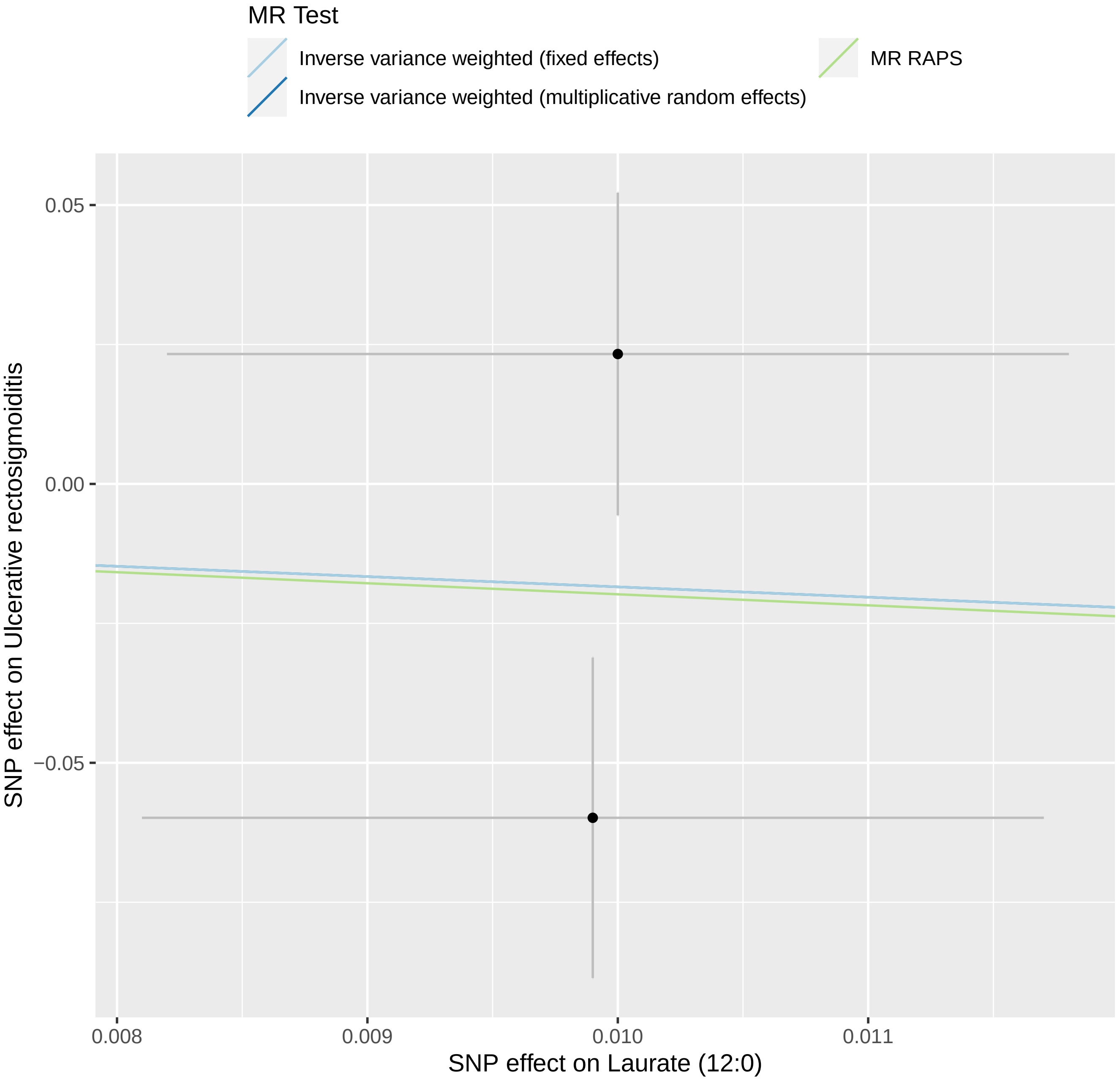

Batch 2525 : Urea on Ulcerative rectosigmoiditis

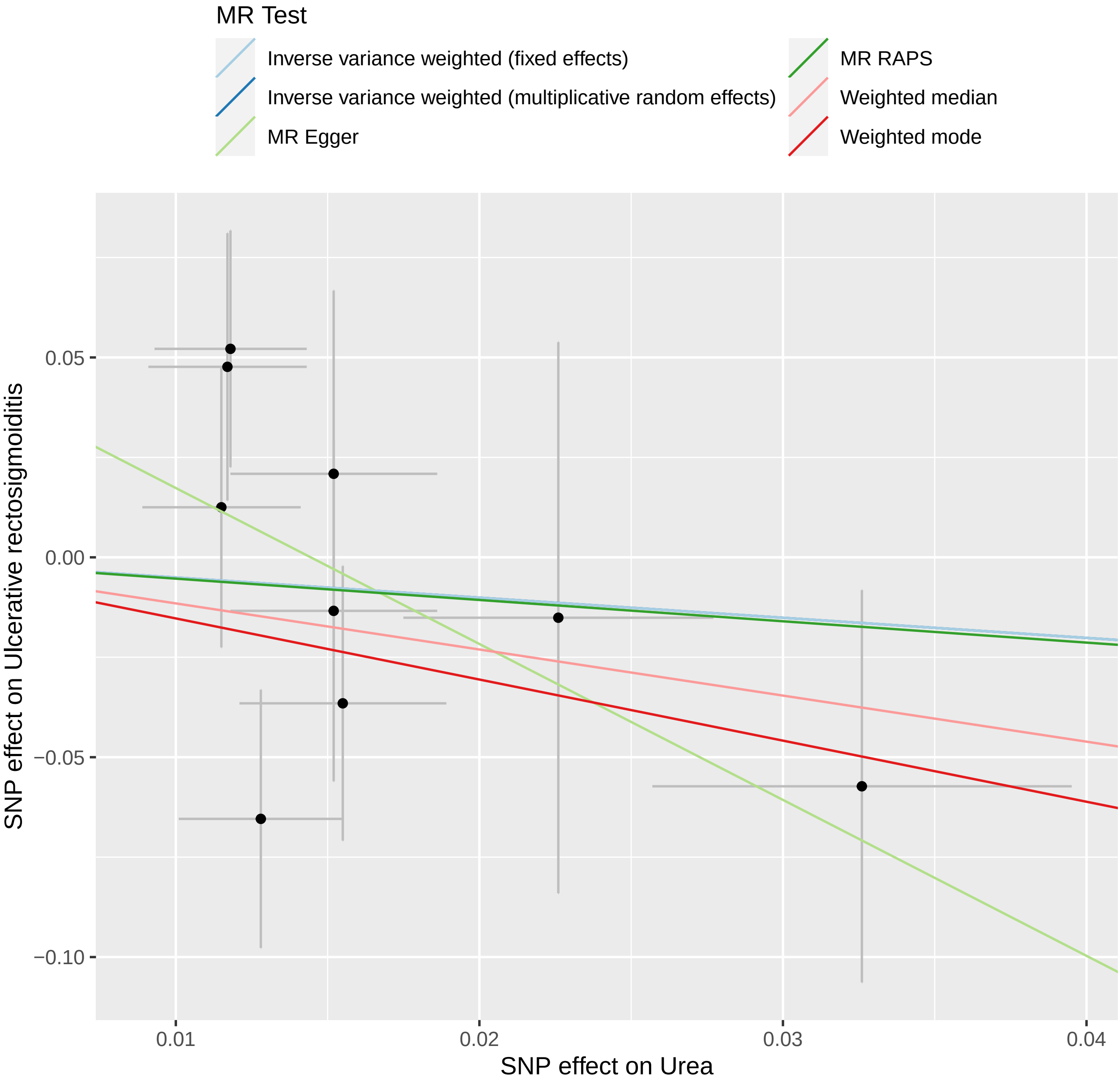

Batch 2526 : Cortisol on Ulcerative rectosigmoiditis

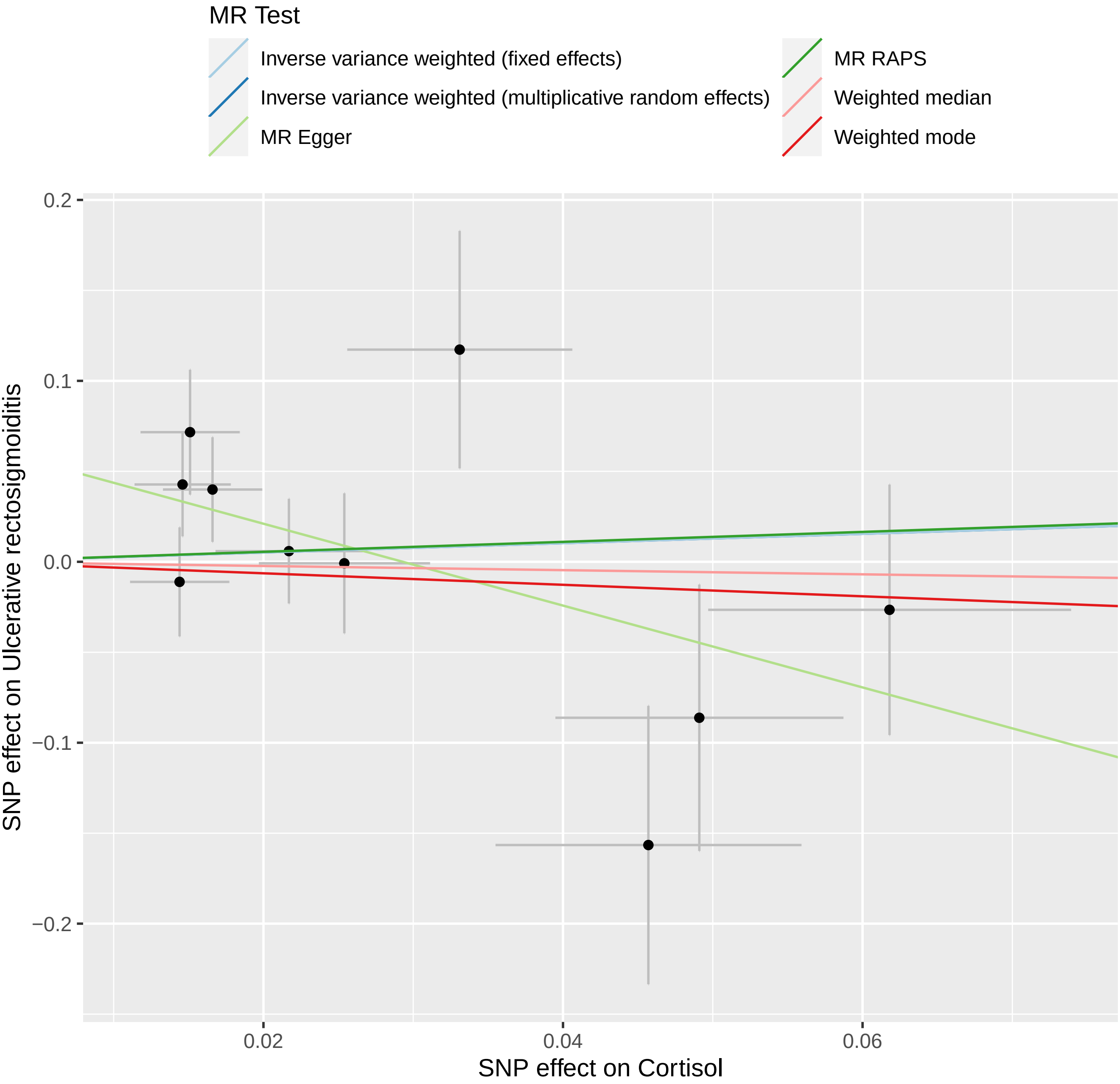

Batch 2529 : Citrulline on Ulcerative rectosigmoiditis

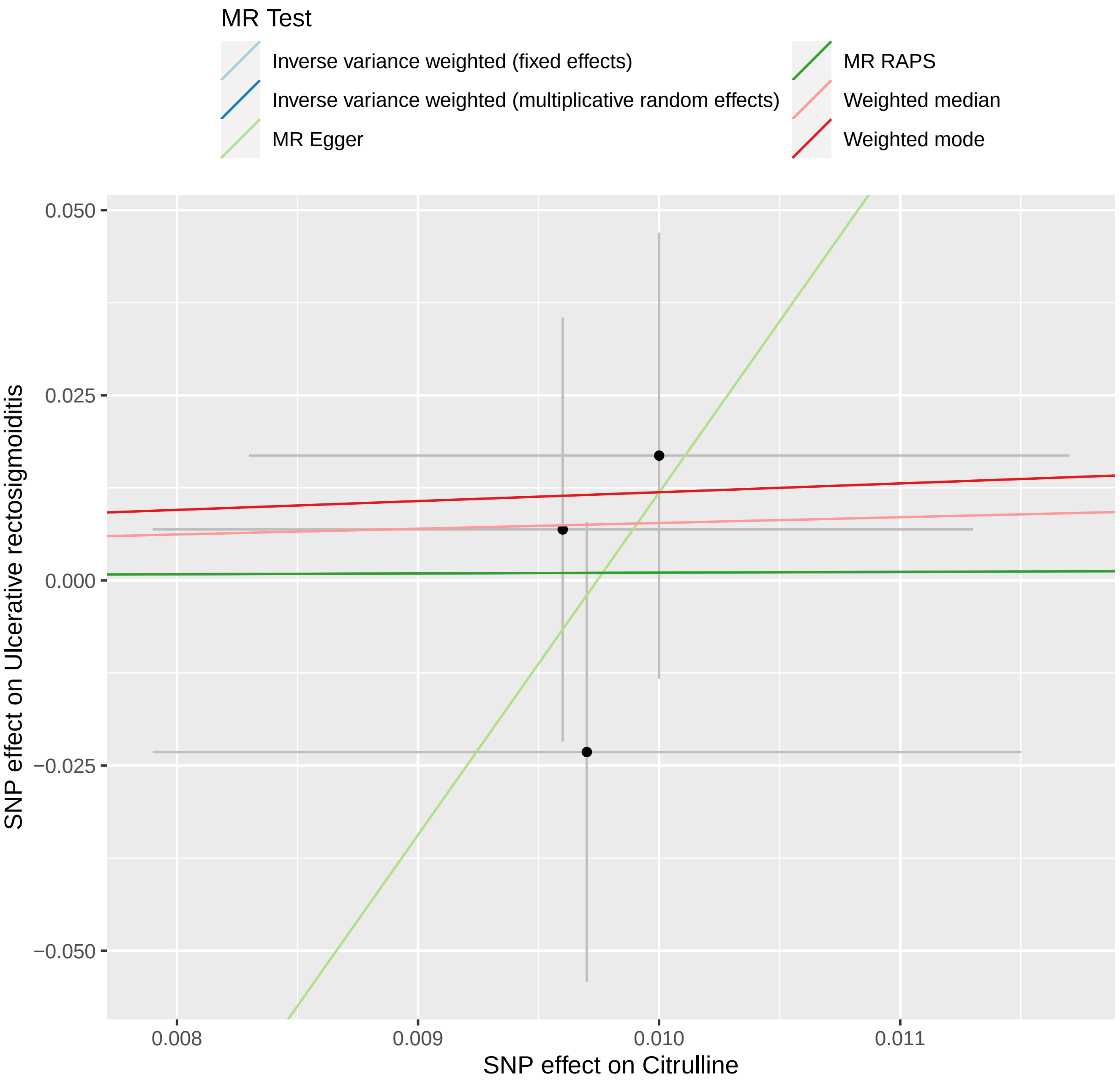

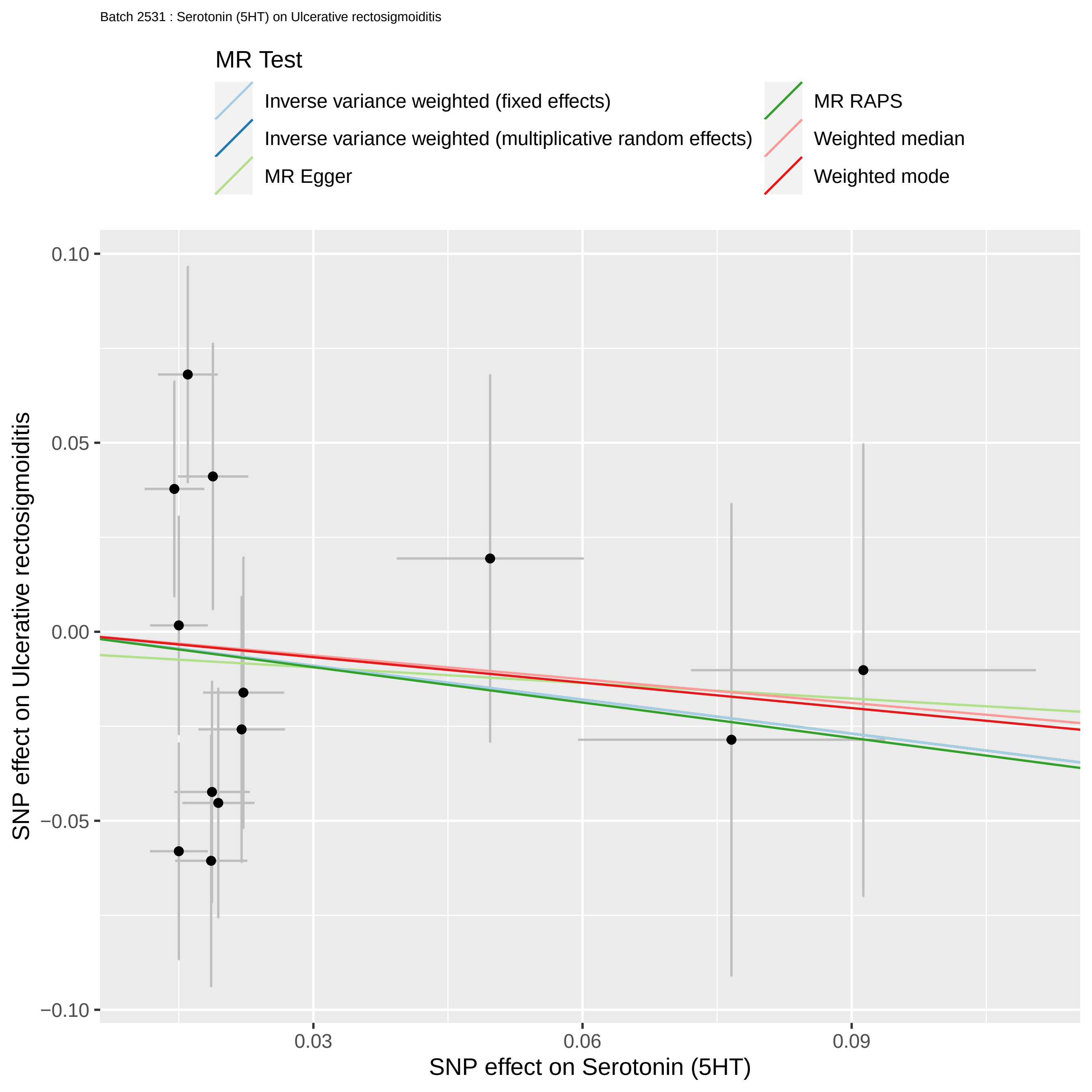
Batch 2531 : Serotonin (5HT) on Ulcerative rectosigmoiditis
MR Test
Inverse variance weighted (fixed effects)
Inverse variance weighted (multiplicative random effects)
MR Egger
MR RAPS
Weighted median
Weighted mode
0.10
0.05
0.00
−0.05
−0.10
0.03
0.06
0.09
SNP effect on Ulcerative rectosigmoiditis
SNP effect on Serotonin (5HT)

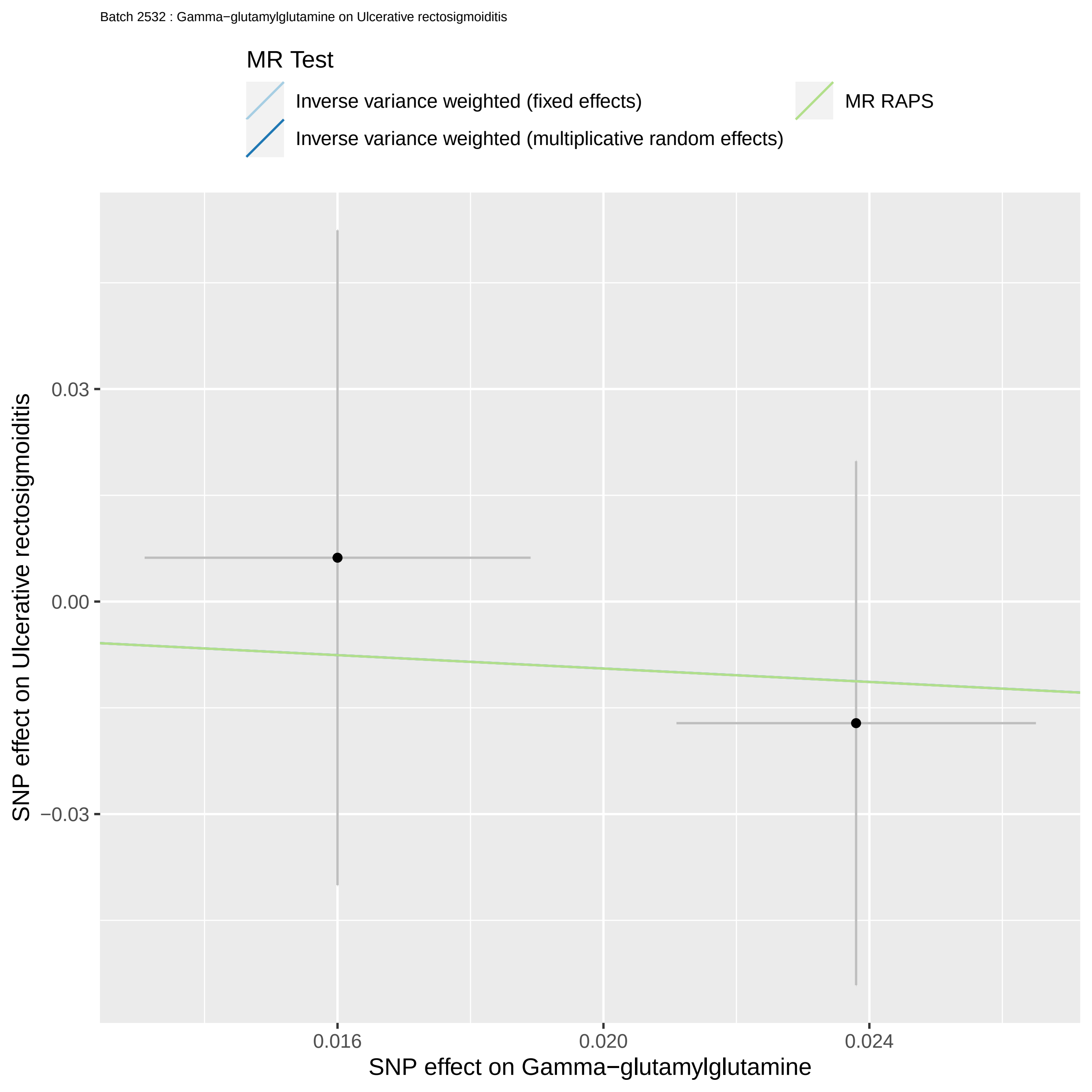

Batch 2532 : Gamma−glutamylglutamine on Ulcerative rectosigmoiditis
MR Test
Inverse variance weighted (fixed effects)
Inverse variance weighted (multiplicative random effects)
MR RAPS
0.03
0.00
−0.03
0.016
0.020
0.024
SNP effect on Gamma−glutamylglutamine
SNP effect on Ulcerative rectosigmoiditis

Batch 2533 : Gamma−glutamyltyrosine on Ulcerative rectosigmoiditis

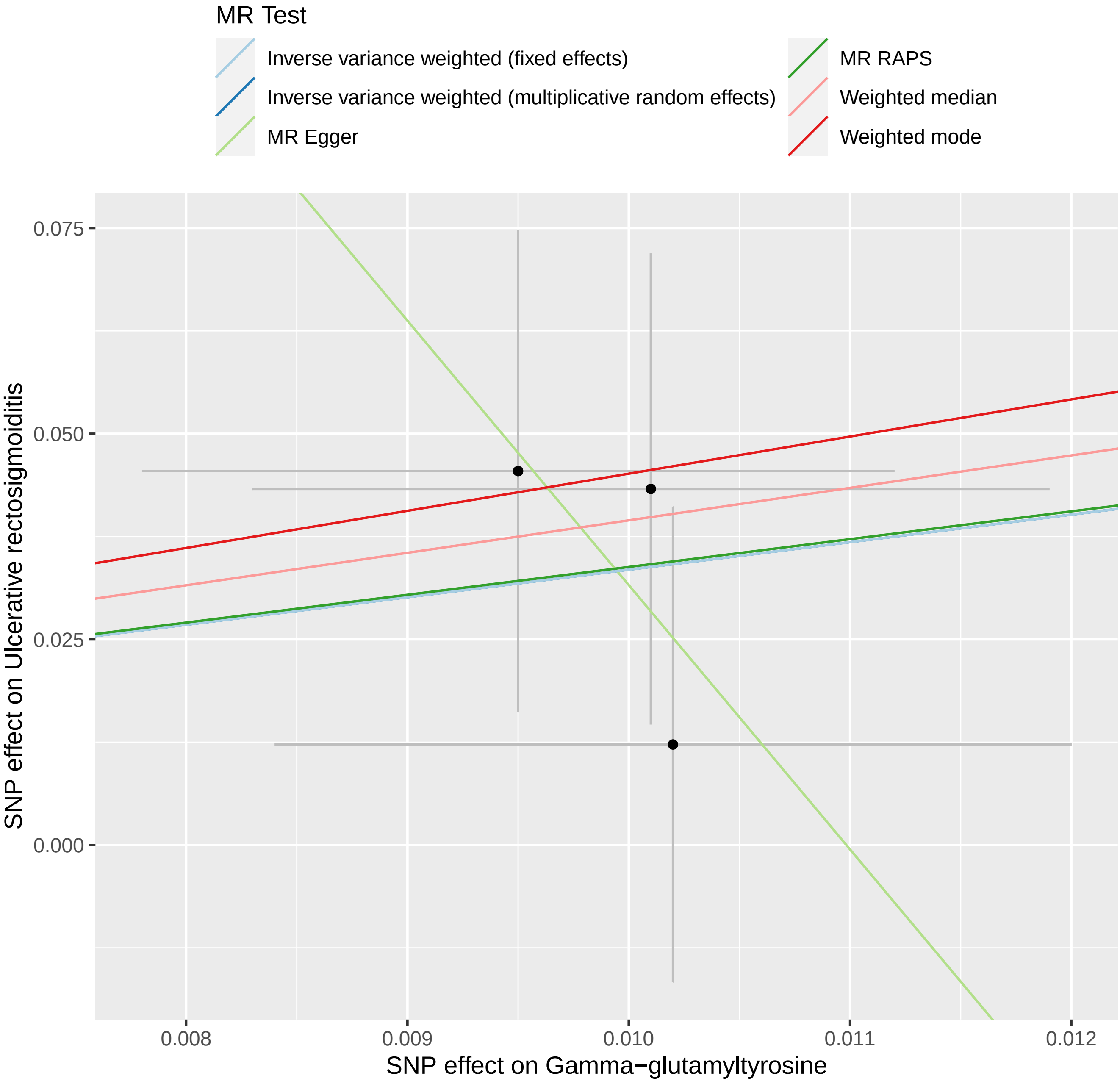

Batch 2535 : Betaine on Ulcerative rectosigmoiditis

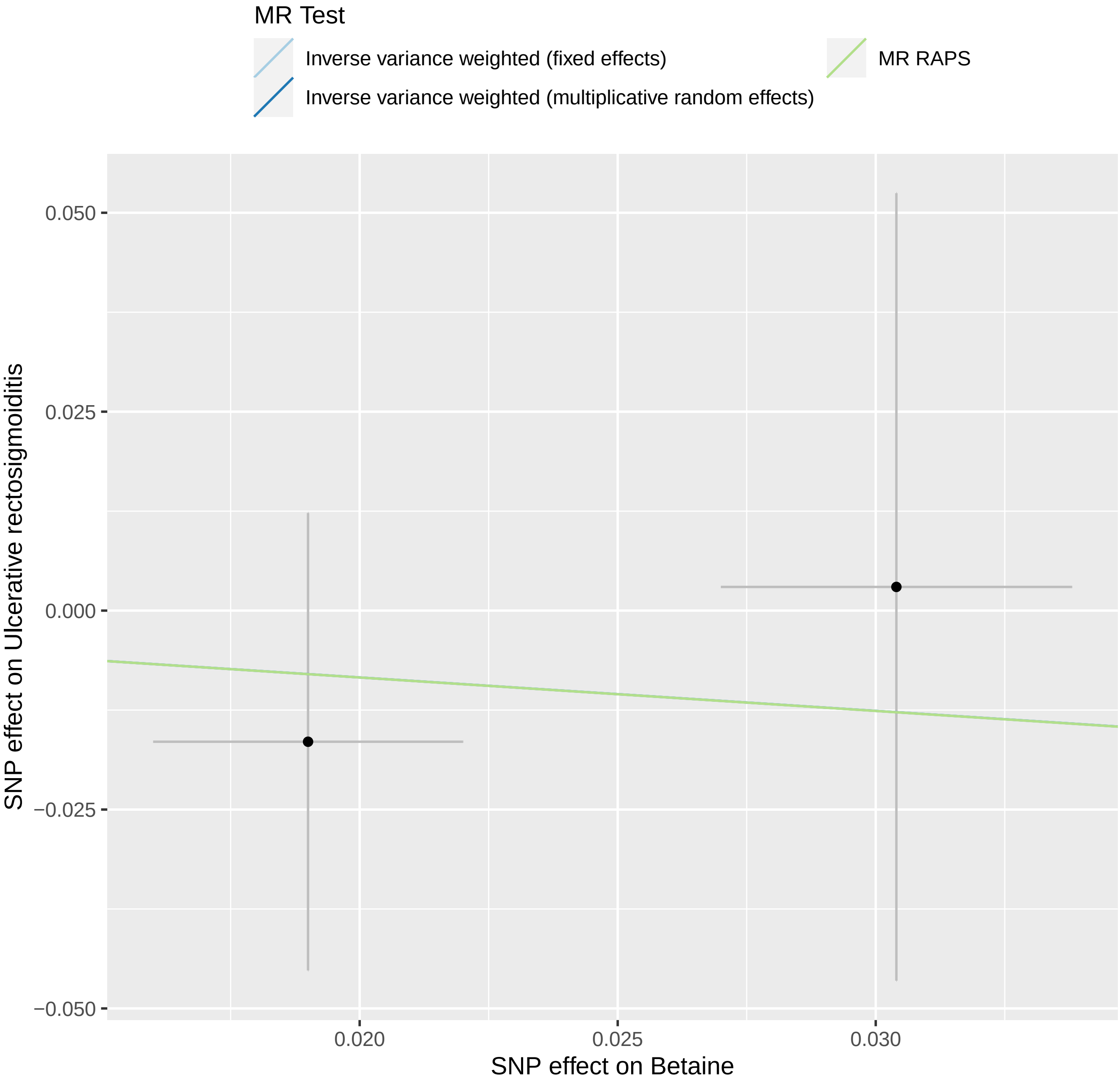

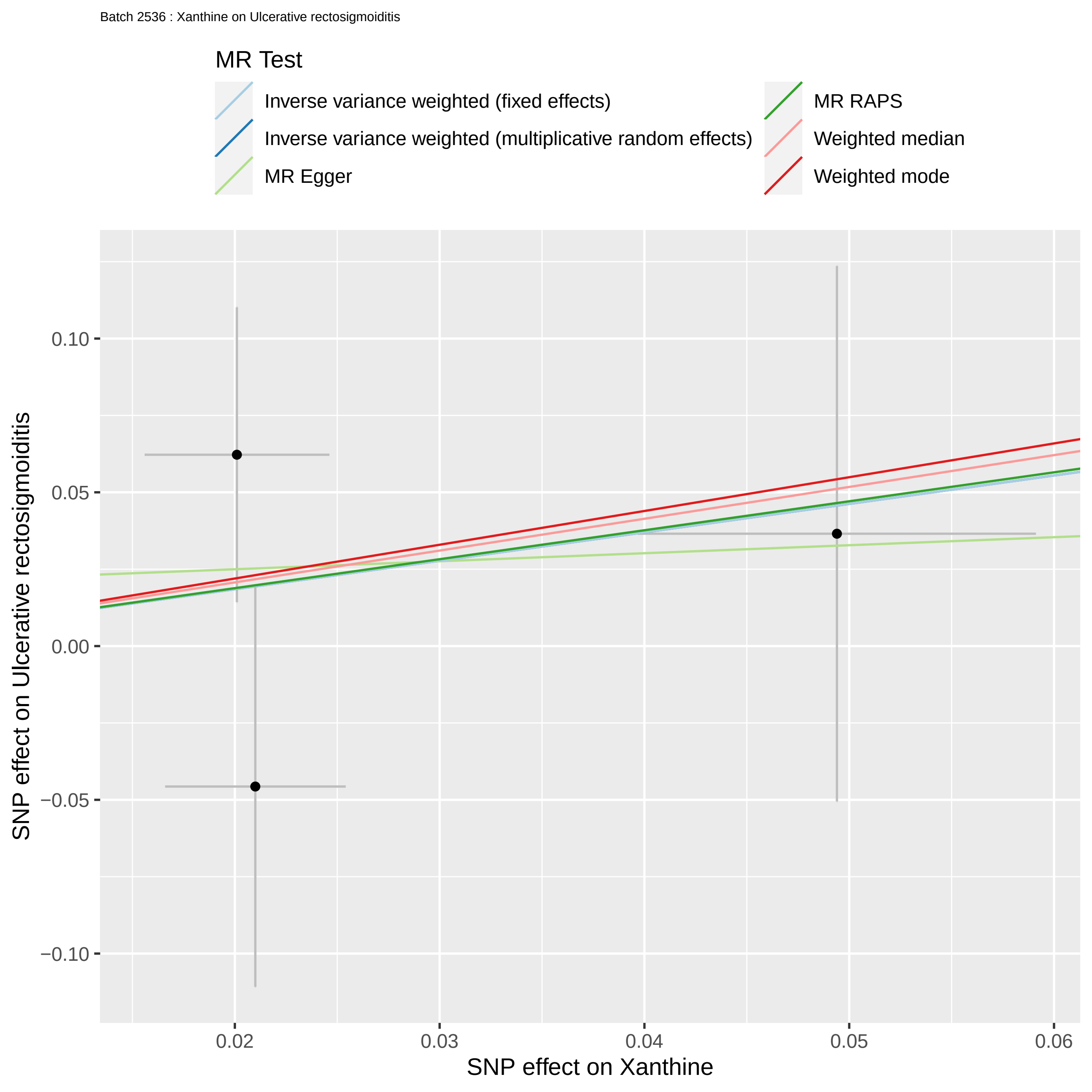
Batch 2536 : Xanthine on Ulcerative rectosigmoiditis
MR Test
Inverse variance weighted (fixed effects)
Inverse variance weighted (multiplicative random effects)
MR Egger
MR RAPS
Weighted median
Weighted mode
0.10
0.05
0.00
−0.05
−0.10
SNP effect on Ulcerative rectosigmoiditis
0.02
0.03
0.04
0.05
0.06
SNP effect on Xanthine

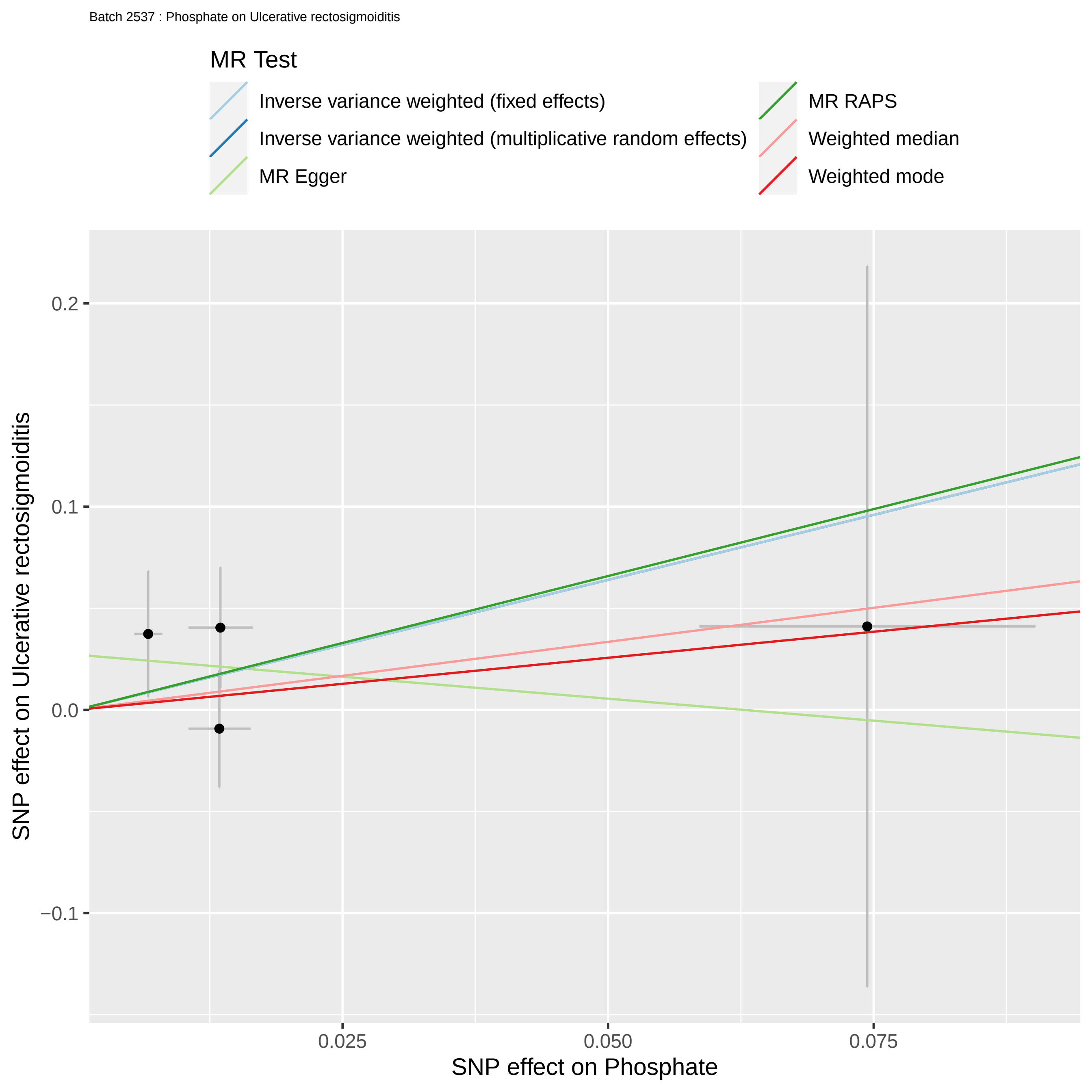

Batch 2537 : Phosphate on Ulcerative rectosigmoiditis
MR Test
Inverse variance weighted (fixed effects)
Inverse variance weighted (multiplicative random effects)
MR Egger
MR RAPS
Weighted median
Weighted mode
0.2
0.1
0.0
−0.1
0.025
0.050
0.075
SNP effect on Ulcerative rectosigmoiditis
SNP effect on Phosphate

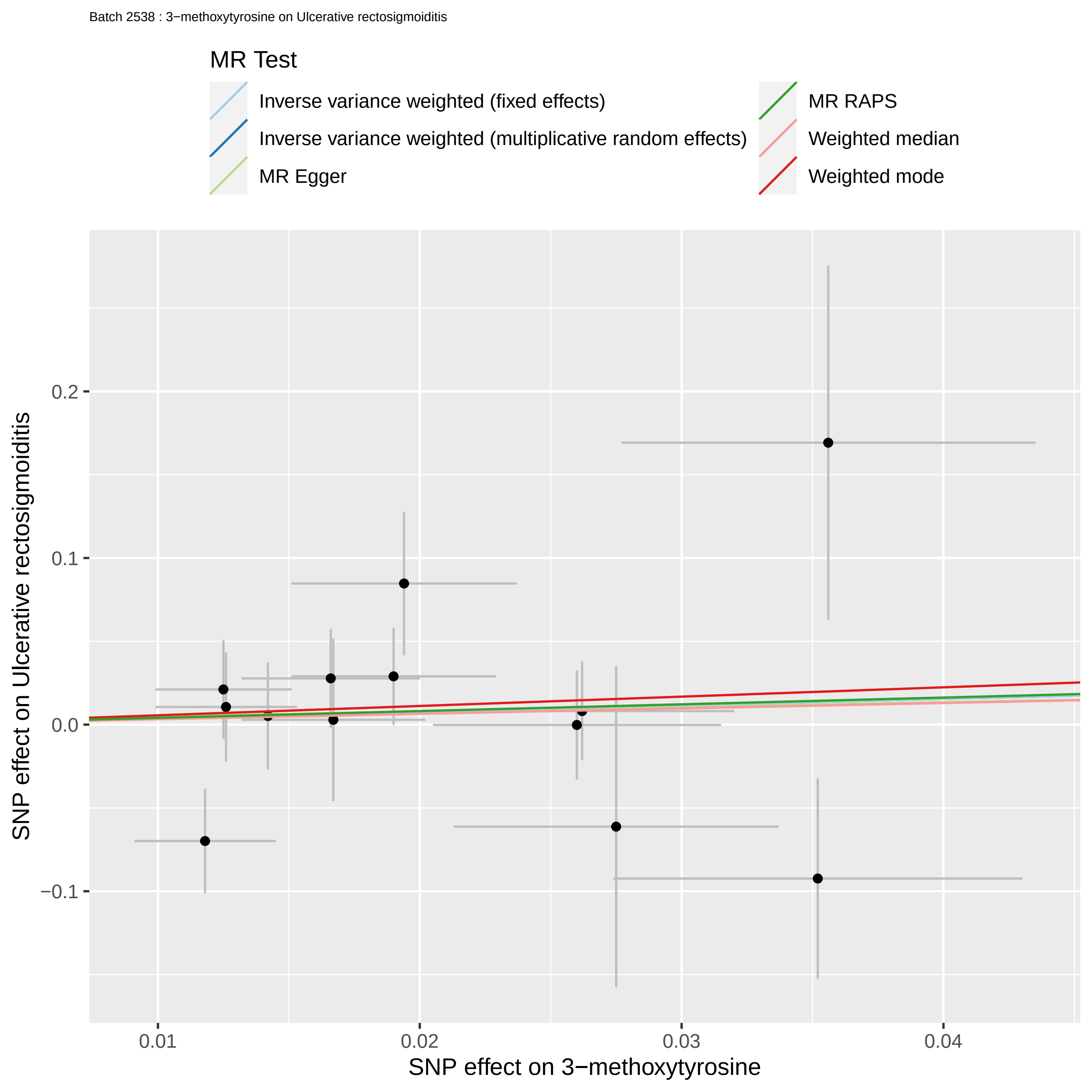

Batch 2538 : 3−methoxytyrosine on Ulcerative rectosigmoiditis
MR Test
Inverse variance weighted (fixed effects)
Inverse variance weighted (multiplicative random effects)
MR Egger
MR RAPS
Weighted median
Weighted mode
0.2
0.1
0.0
−0.1
0.01
0.02
0.03
0.04
SNP effect on Ulcerative rectosigmoiditis
SNP effect on 3−methoxytyrosine

Batch 2540 : Undecanoate (11:0) on Ulcerative rectosigmoiditis

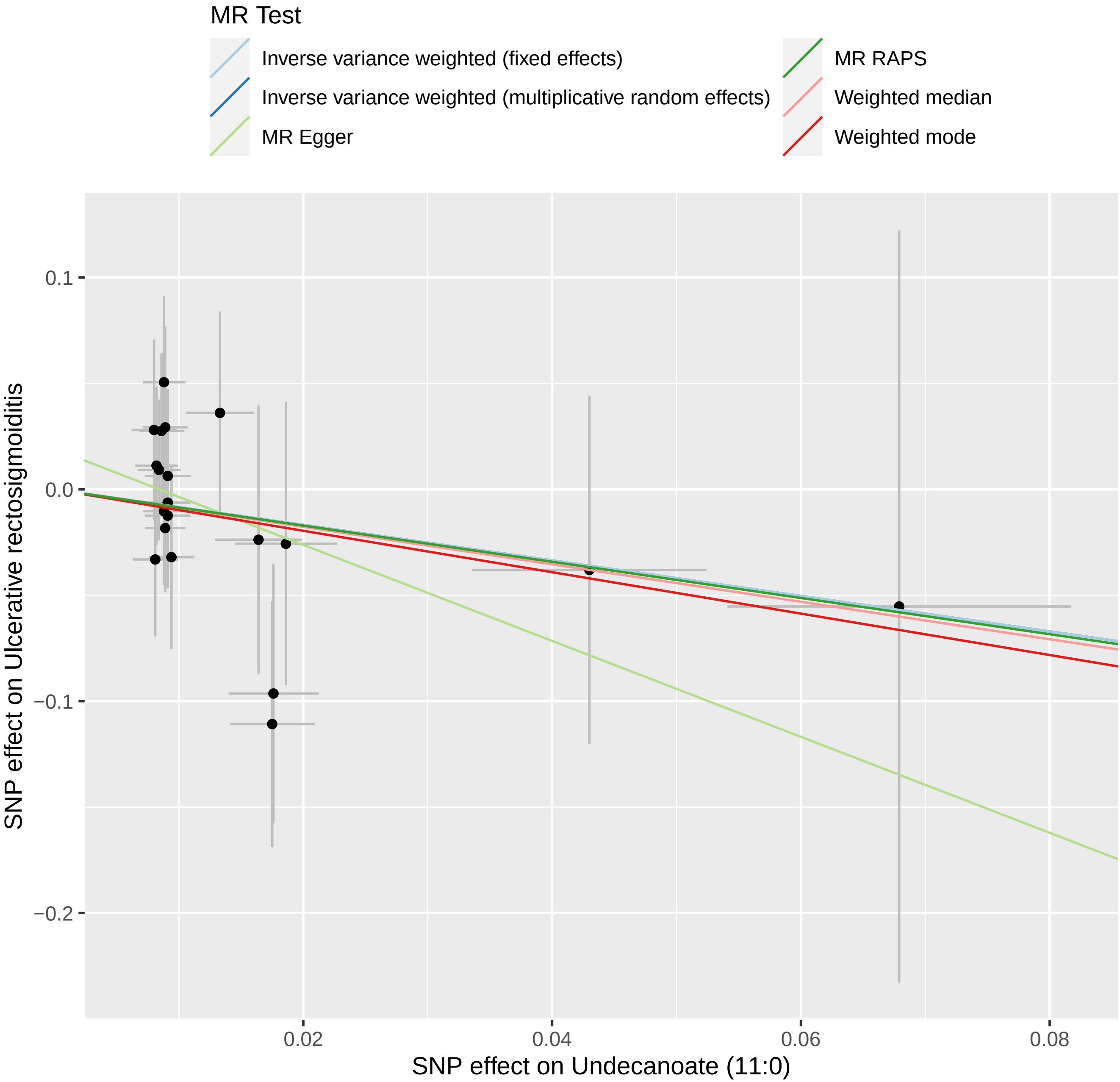

Batch 2542 : Taurodeoxycholate on Ulcerative rectosigmoiditis

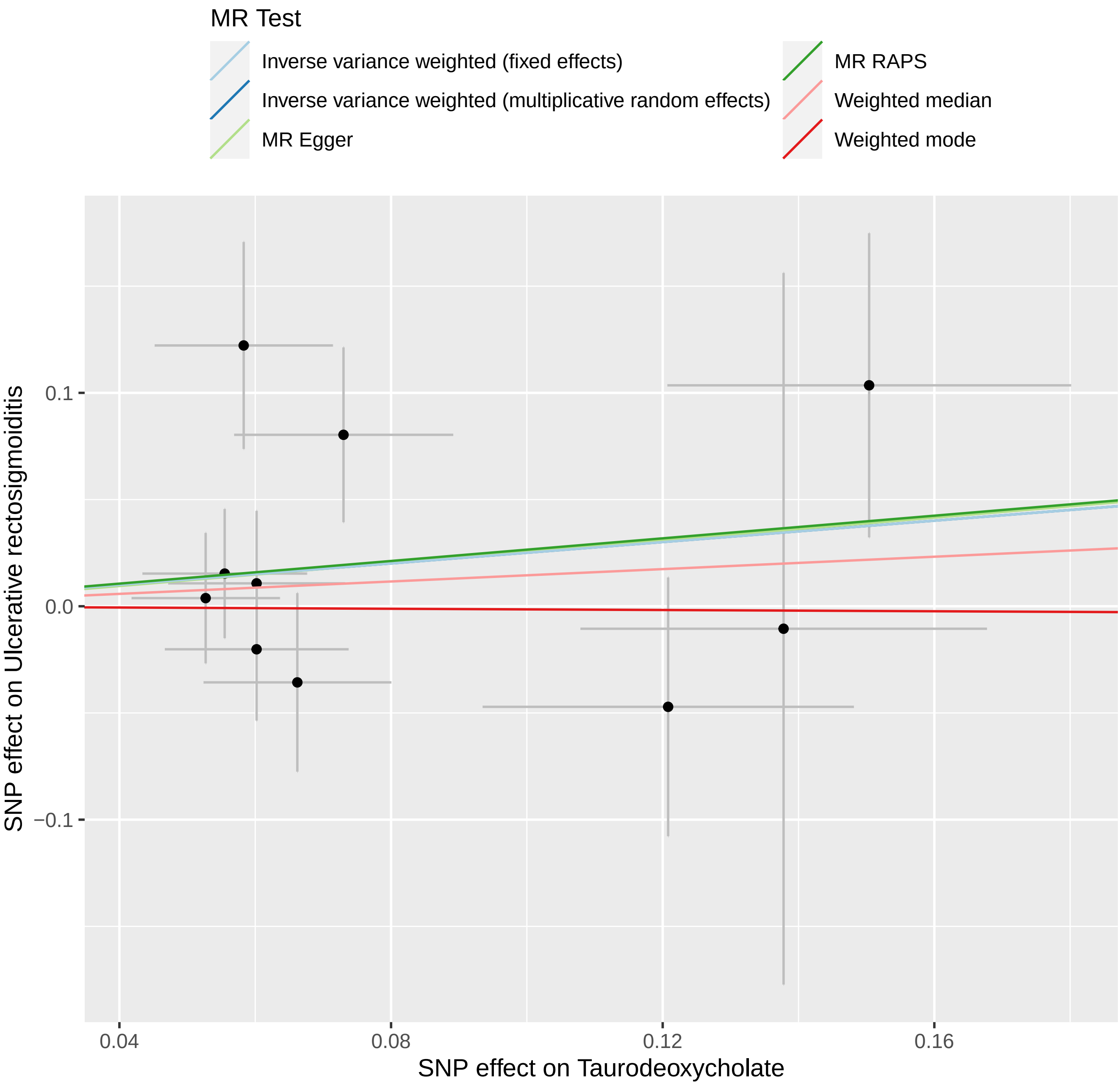

Batch 2543 : Glycerol on Ulcerative rectosigmoiditis

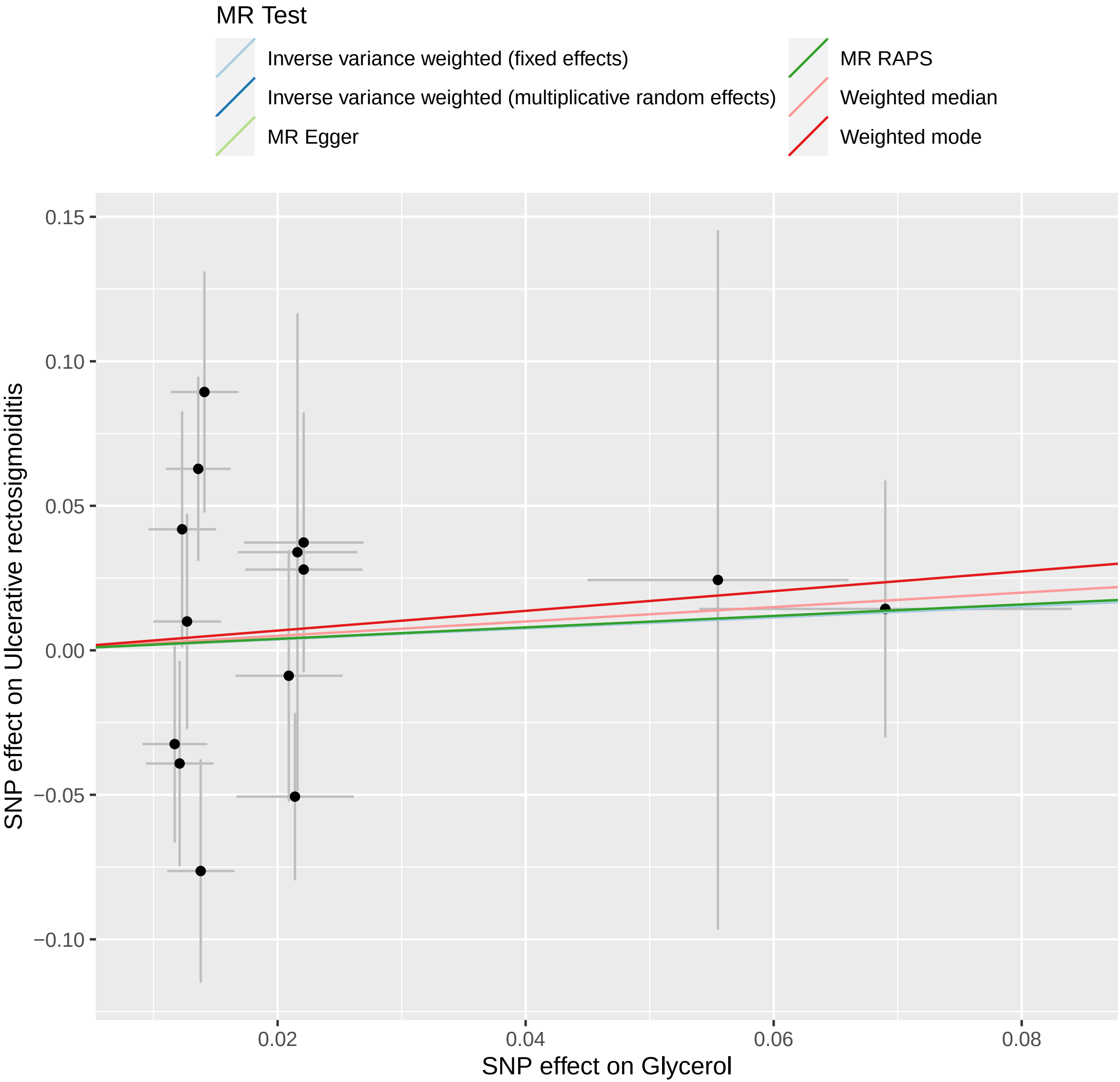

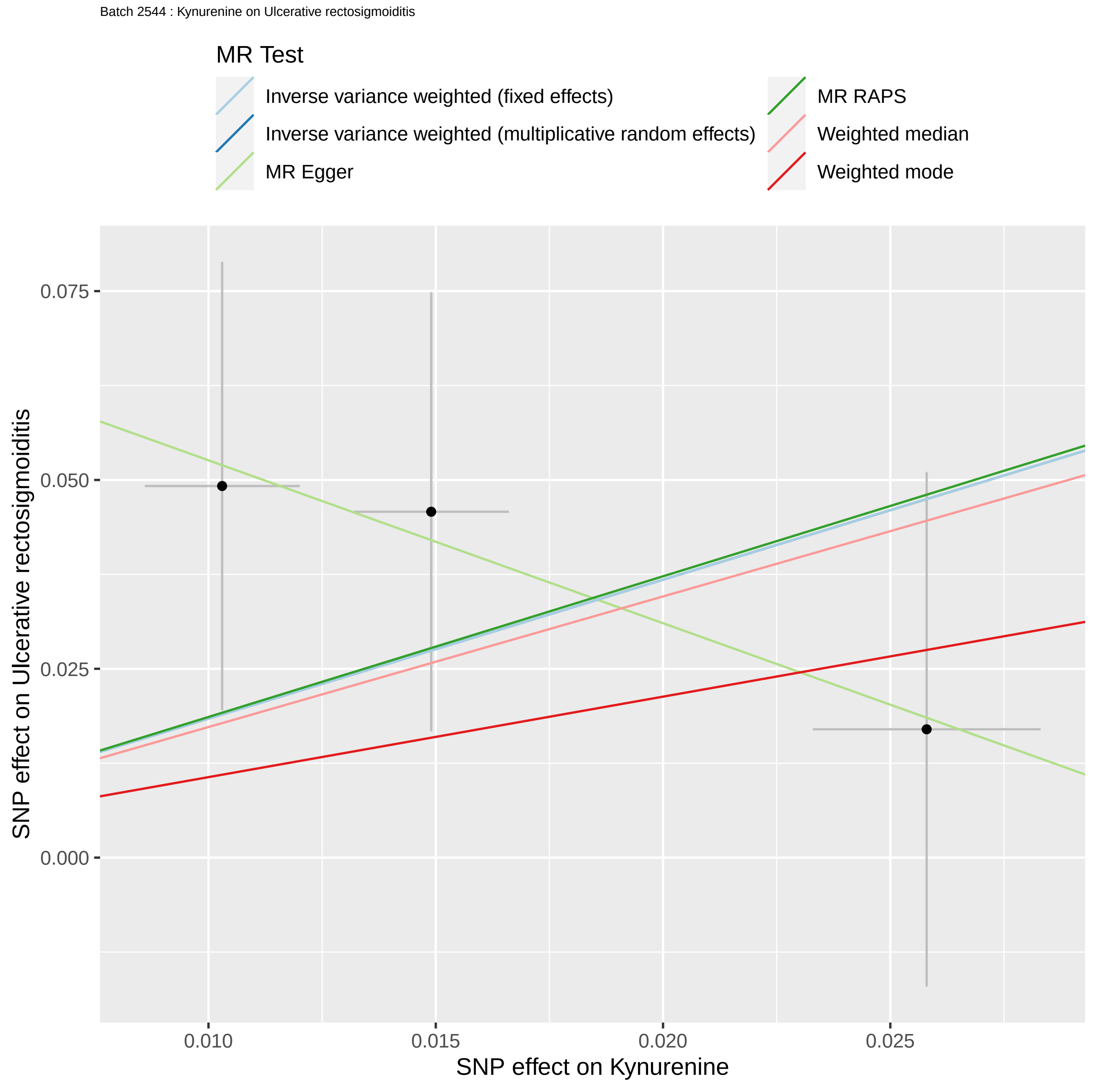
Batch 2544 : Kynurenine on Ulcerative rectosigmoiditis
MR Test
Inverse variance weighted (fixed effects)
Inverse variance weighted (multiplicative random effects)
MR Egger
MR RAPS
Weighted median
Weighted mode
0.075
0.050
0.025
0.000
0.010
0.015
0.020
0.025
SNP effect on Ulcerative rectosigmoiditis
SNP effect on Kynurenine

Batch 2545 : Mannitol on Ulcerative rectosigmoiditis

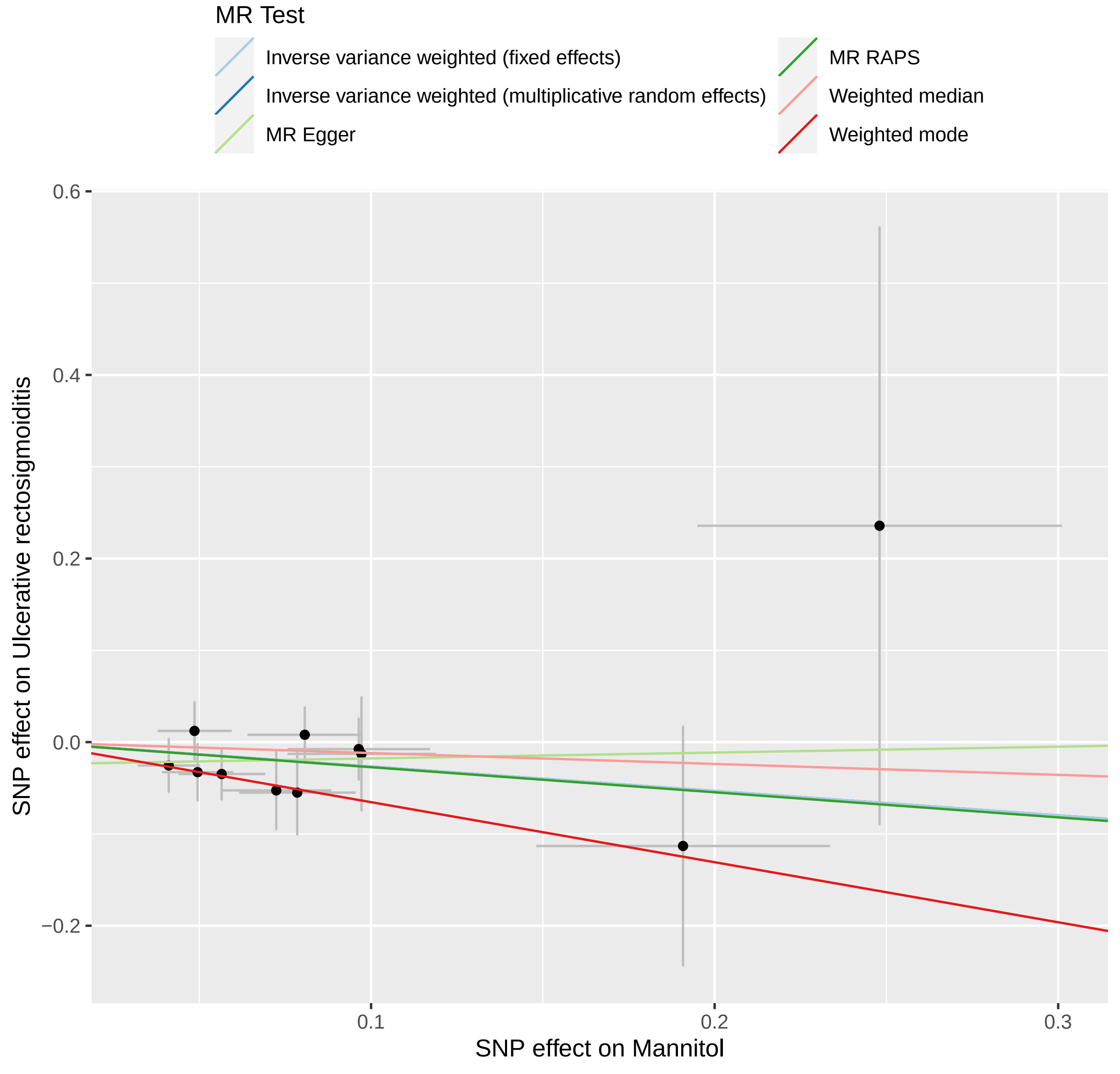

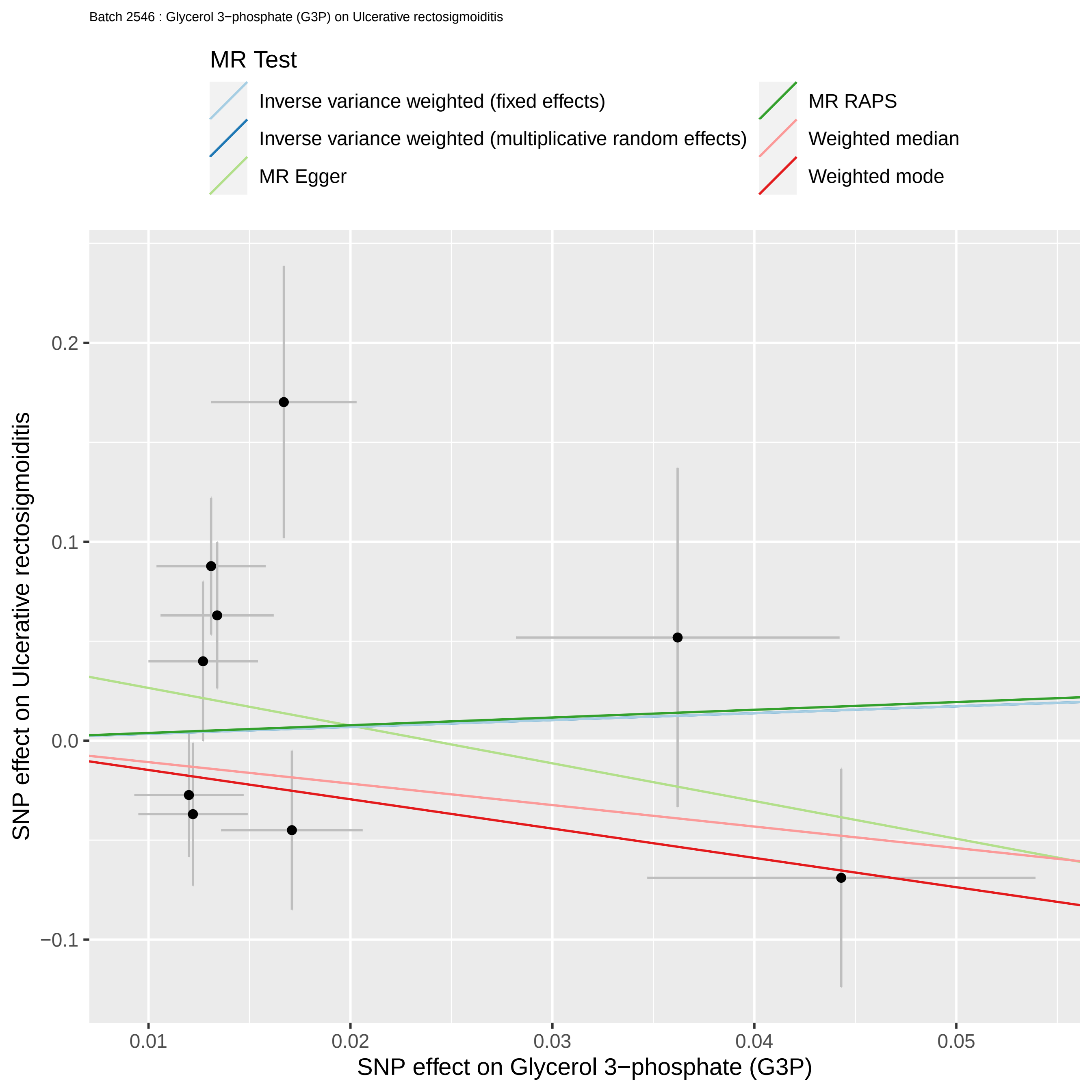

Batch 2546 : Glycerol 3−phosphate (G3P) on Ulcerative rectosigmoiditis
MR Test
Inverse variance weighted (fixed effects)
Inverse variance weighted (multiplicative random effects)
MR Egger
MR RAPS
Weighted median
Weighted mode
0.2
0.1
0.0
−0.1
0.01
0.02
0.03
0.04
0.05
SNP effect on Ulcerative rectosigmoiditis
SNP effect on Glycerol 3−phosphate (G3P)

Batch 2548 : Carnitine on Ulcerative rectosigmoiditis

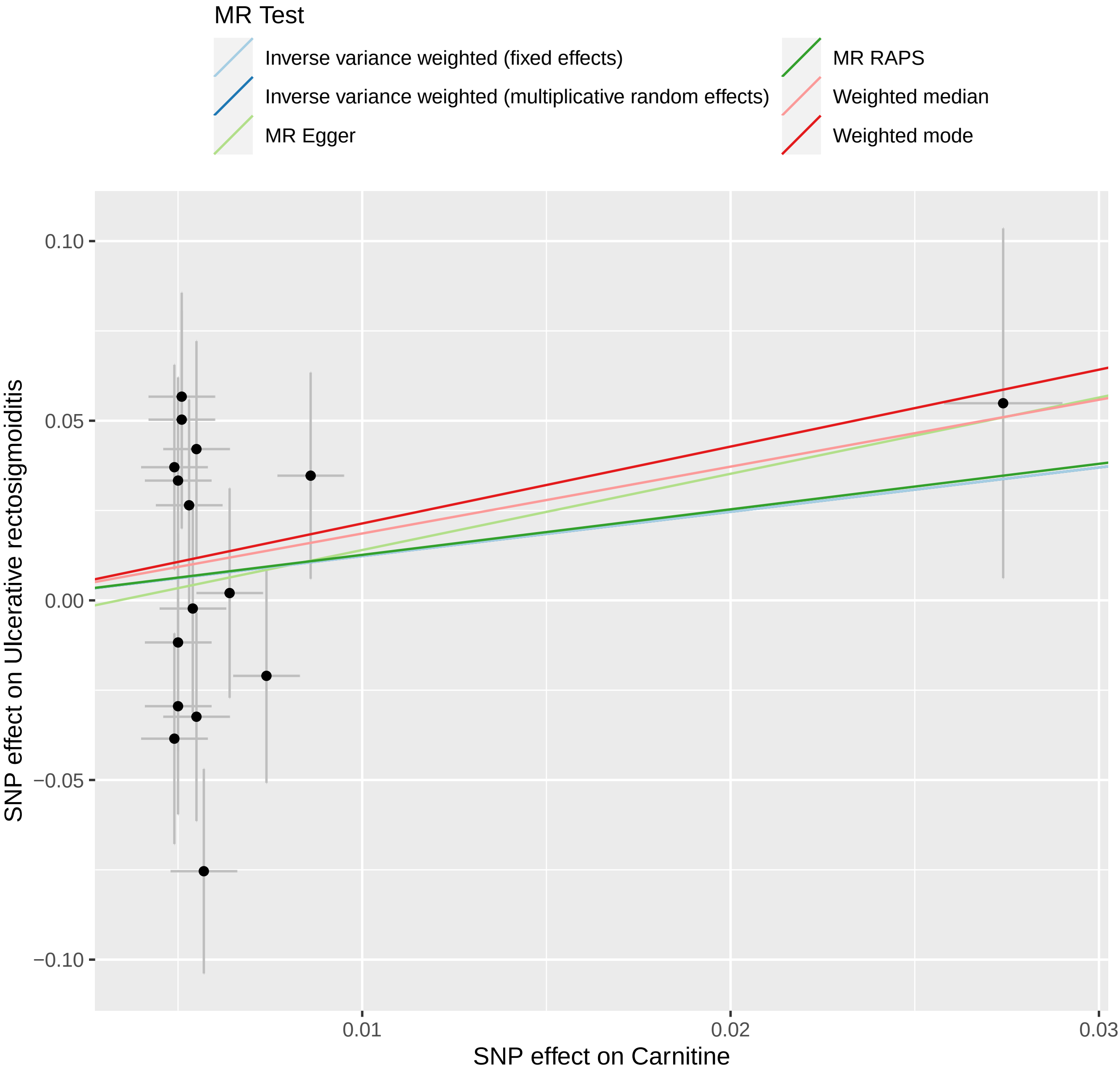

Batch 2549 : Choline on Ulcerative rectosigmoiditis

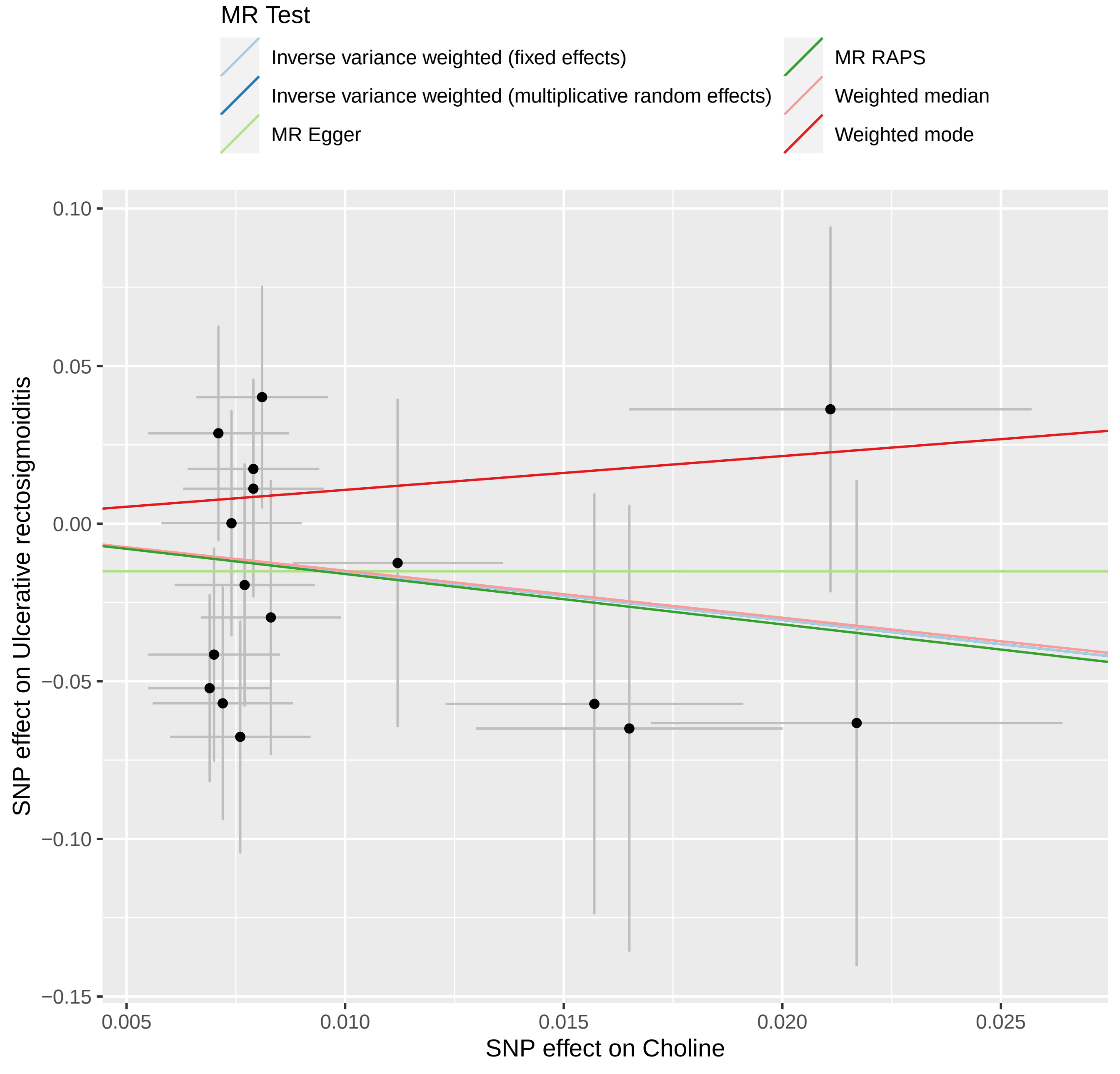

Batch 2552 : 3−methyl−2−oxovalerate on Ulcerative rectosigmoiditis

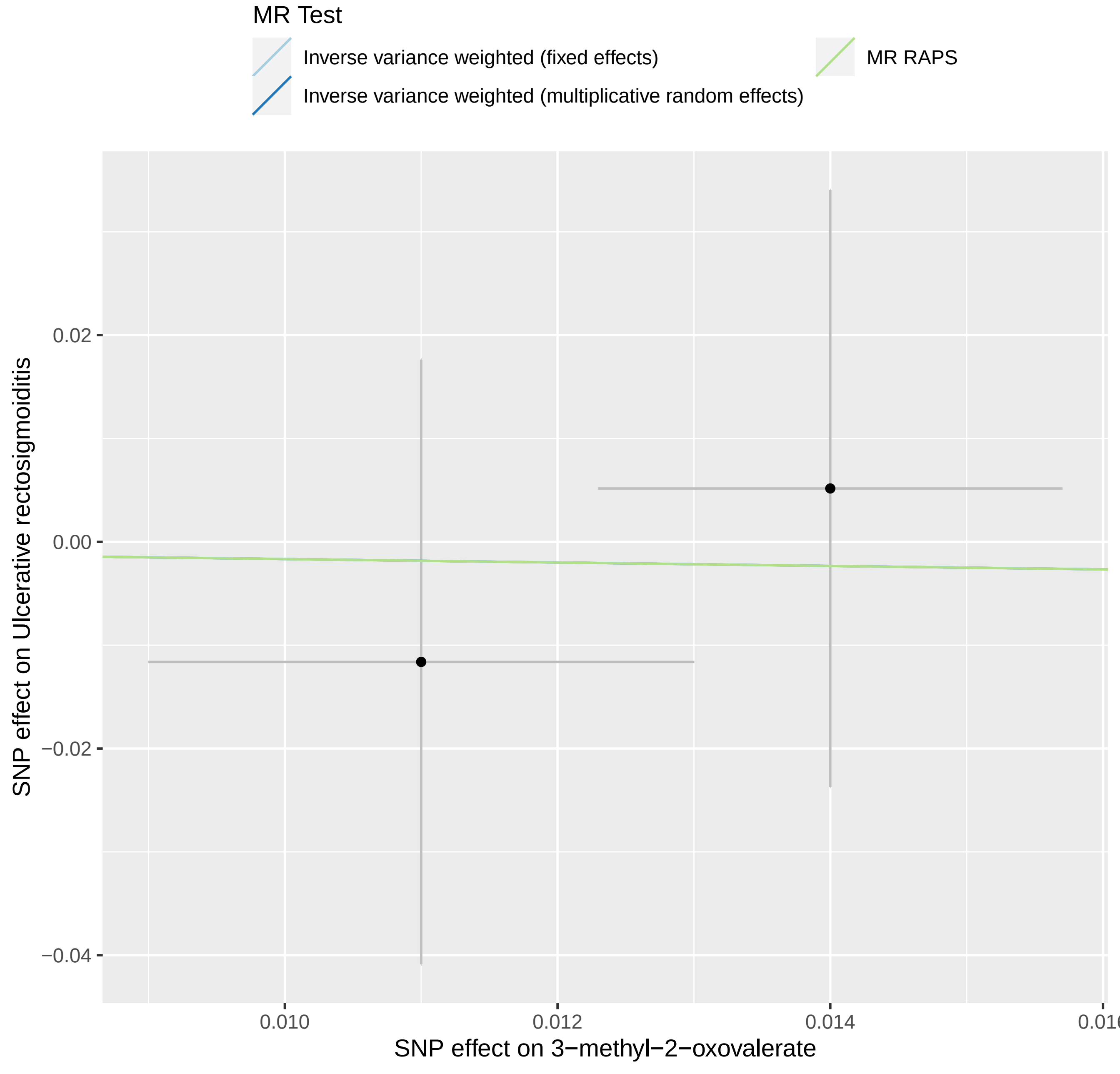

Batch 2553 : 3−methylhistidine on Ulcerative rectosigmoiditis

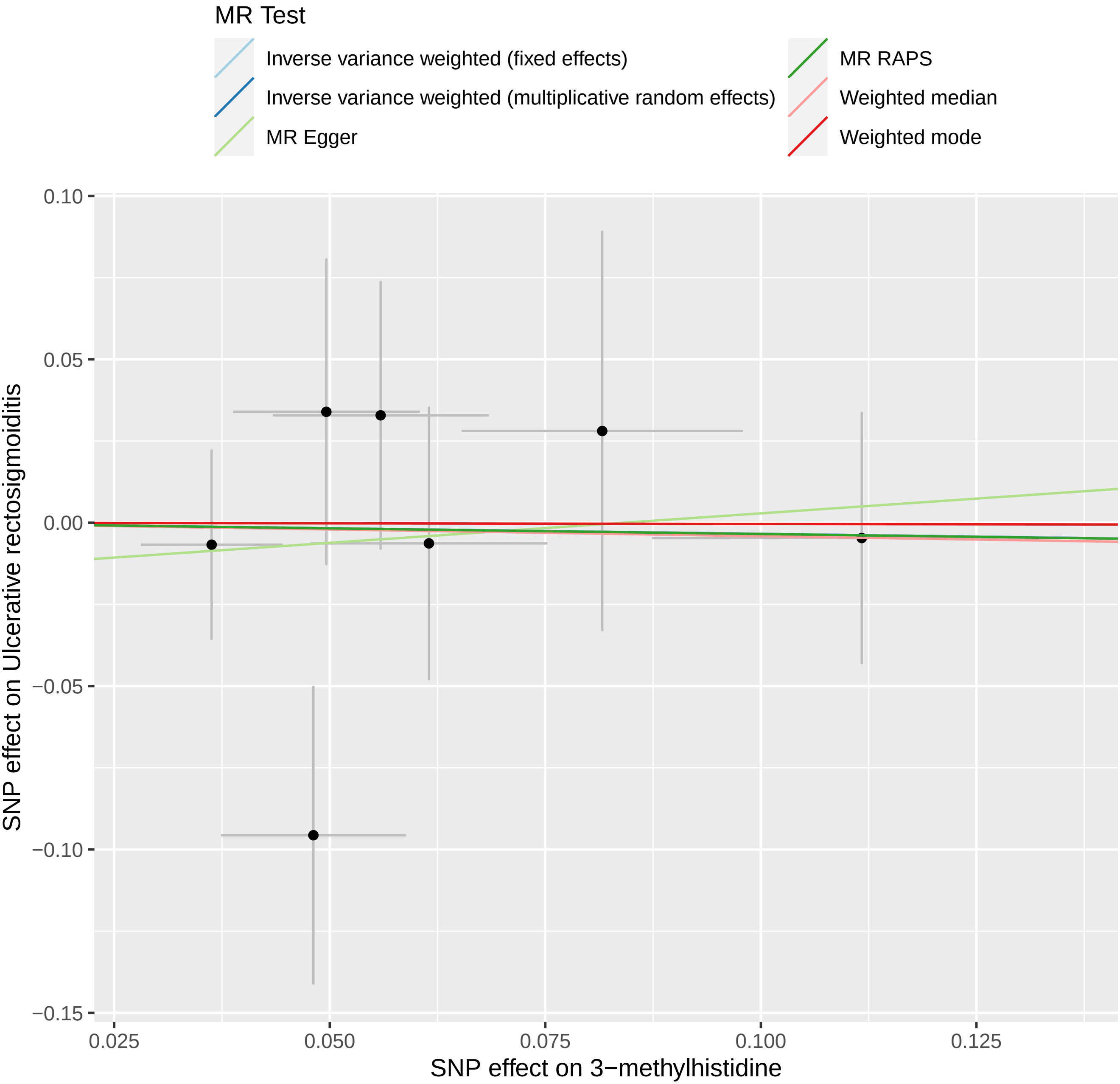

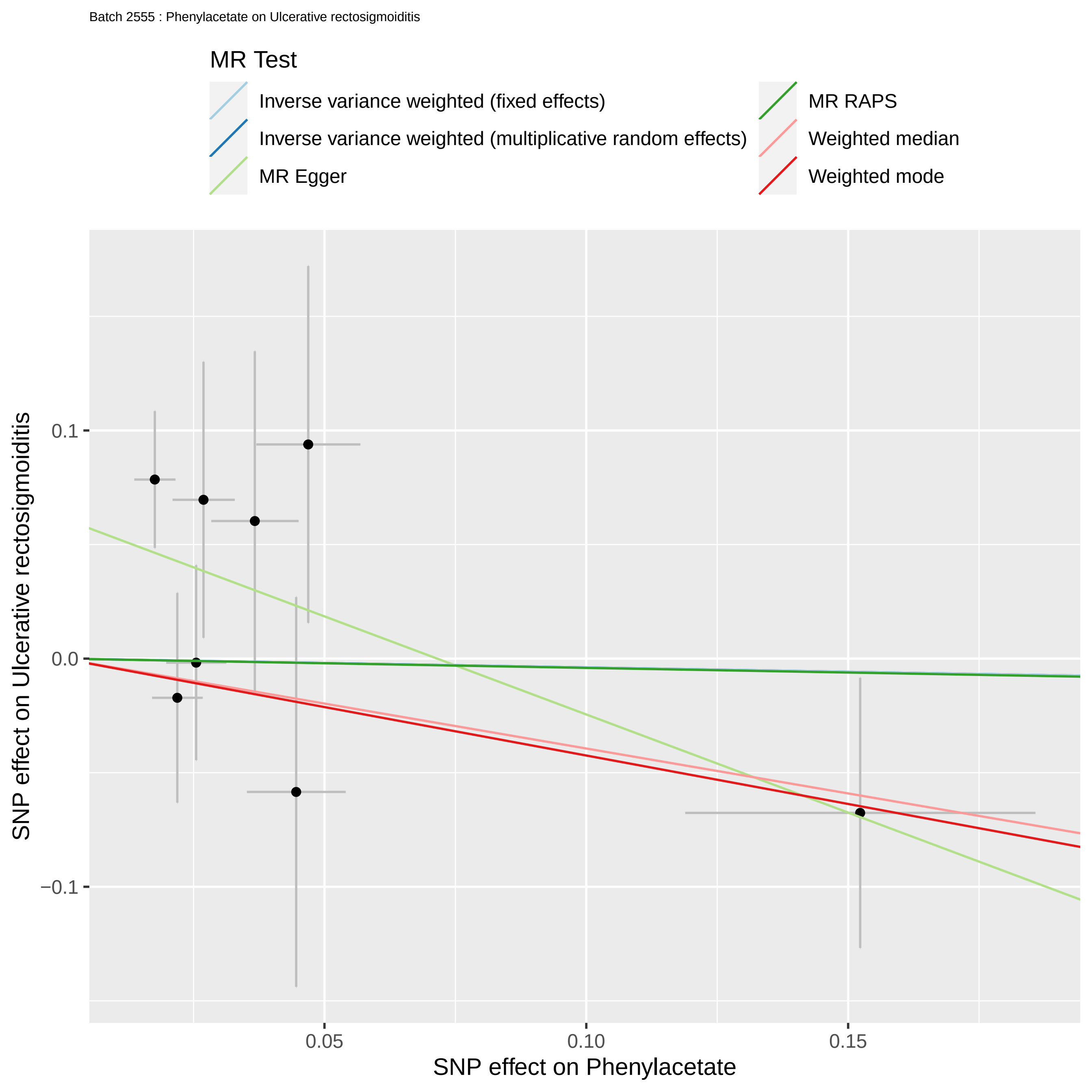
Batch 2555 : Phenylacetate on Ulcerative rectosigmoiditis
MR Test
Inverse variance weighted (fixed effects)
Inverse variance weighted (multiplicative random effects)
MR Egger
MR RAPS
Weighted median
Weighted mode
0.1
0.0
−0.1
0.05
0.10
0.15
SNP effect on Ulcerative rectosigmoiditis
SNP effect on Phenylacetate

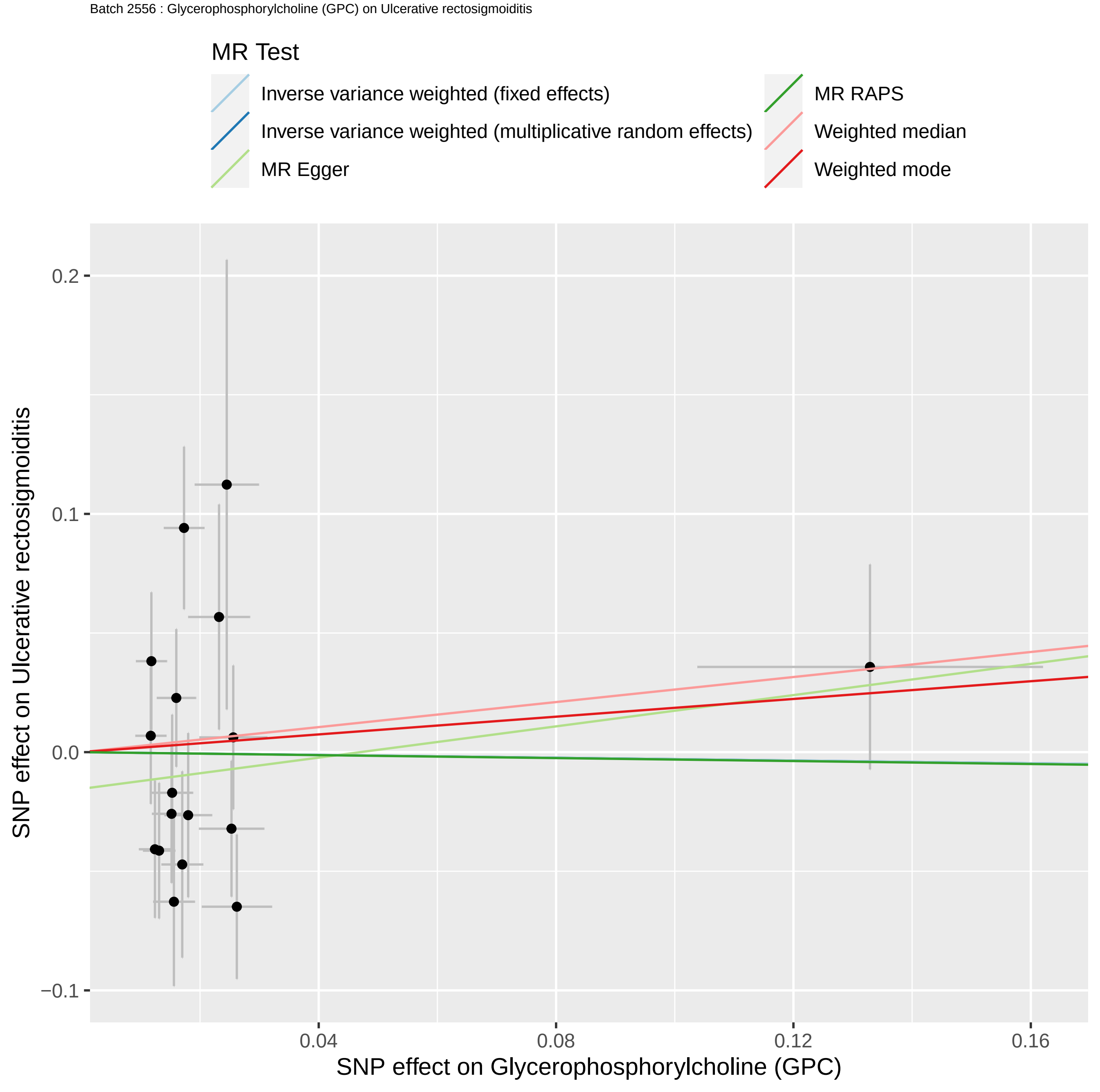

Batch 2556 : Glycerophosphorylcholine (GPC) on Ulcerative rectosigmoiditis
MR Test
Inverse variance weighted (fixed effects)
Inverse variance weighted (multiplicative random effects)
MR Egger
MR RAPS
Weighted median
Weighted mode
0.2
0.1
0.0
−0.1
0.04
0.08
0.12
0.16
SNP effect on Ulcerative rectosigmoiditis
SNP effect on Glycerophosphorylcholine (GPC)

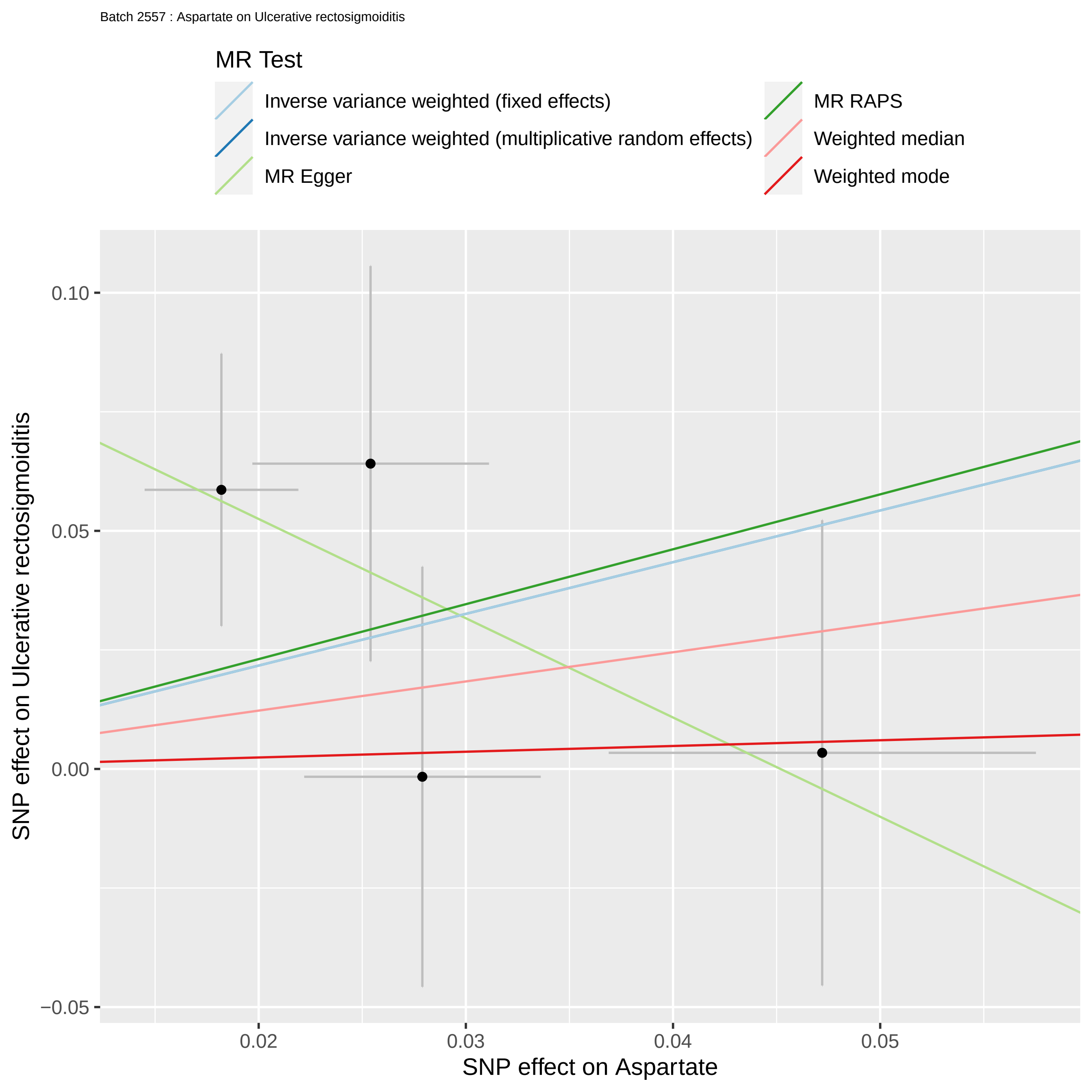

Batch 2557 : Aspartate on Ulcerative rectosigmoiditis
MR Test
Inverse variance weighted (fixed effects)
Inverse variance weighted (multiplicative random effects)
MR Egger
MR RAPS
Weighted median
Weighted mode
0.10
0.05
0.00
−0.05
0.02
0.03
0.04
0.05
SNP effect on Ulcerative rectosigmoiditis
SNP effect on Aspartate

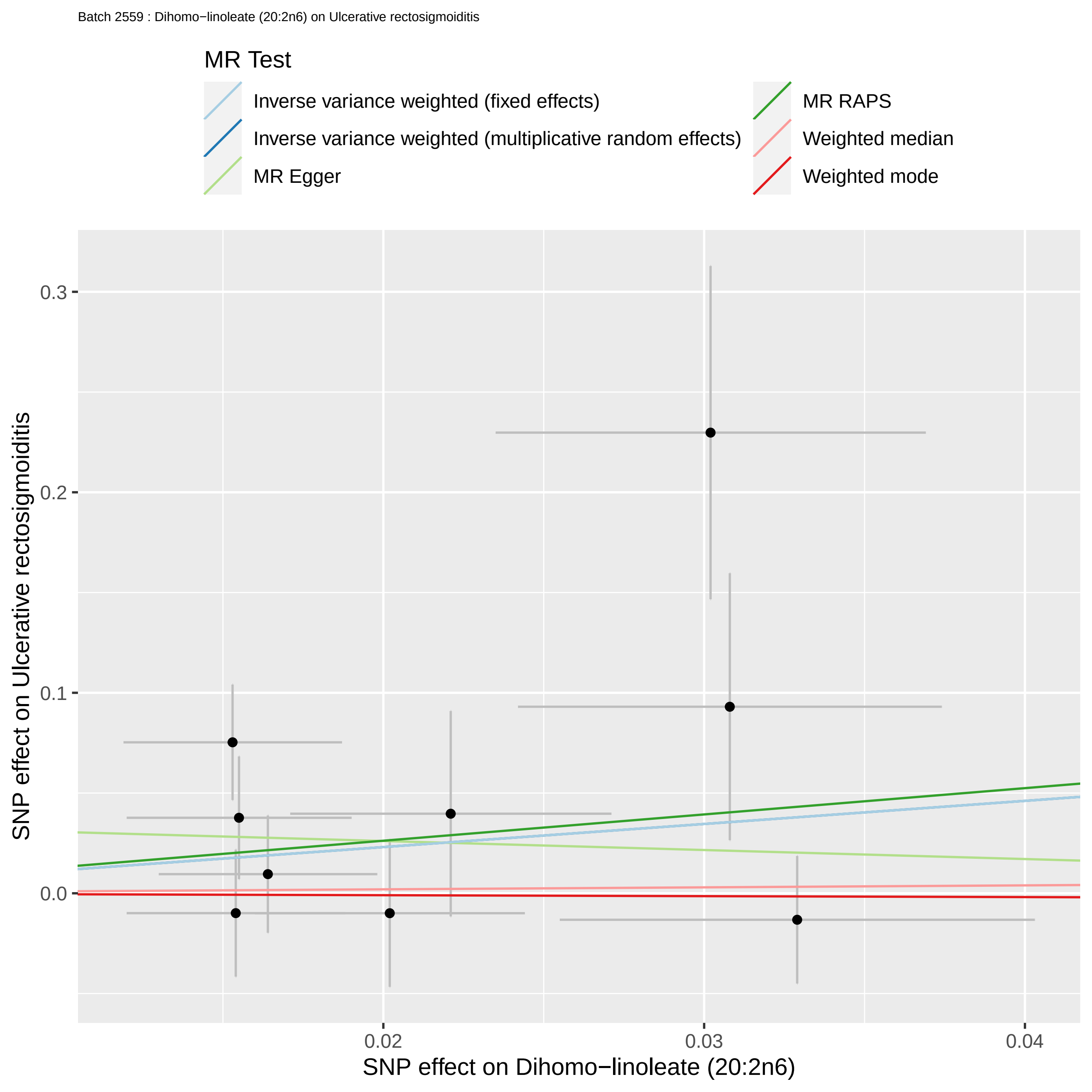

Batch 2559 : Dihomo−linoleate (20:2n6) on Ulcerative rectosigmoiditis
MR Test
Inverse variance weighted (fixed effects)
Inverse variance weighted (multiplicative random effects)
MR Egger
MR RAPS
Weighted median
Weighted mode
0.3
0.2
0.1
0.0
0.02
0.03
0.04
SNP effect on Ulcerative rectosigmoiditis
SNP effect on Dihomo−linoleate (20:2n6)

Batch 2560 : 2−hydroxystearate on Ulcerative rectosigmoiditis

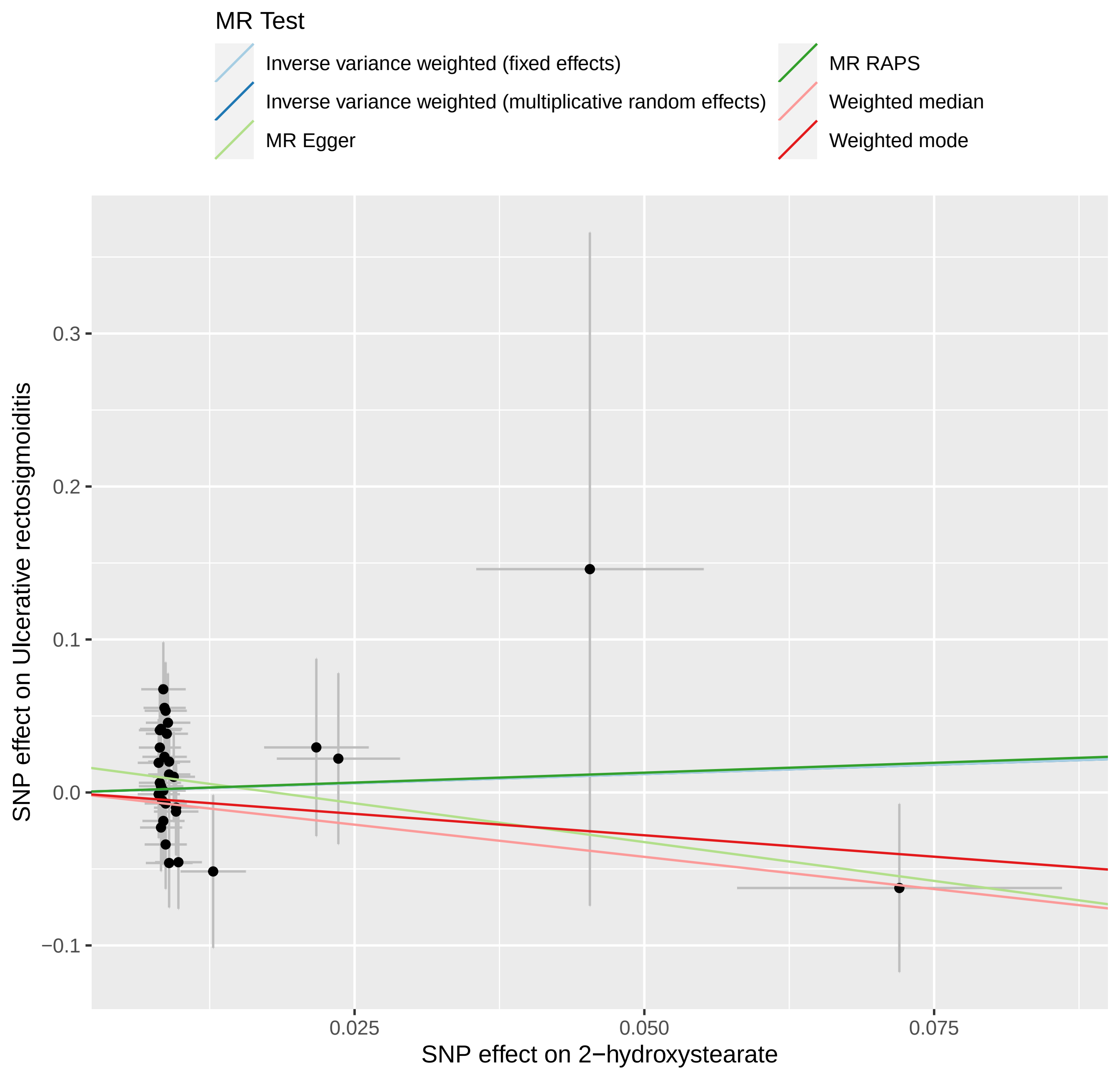

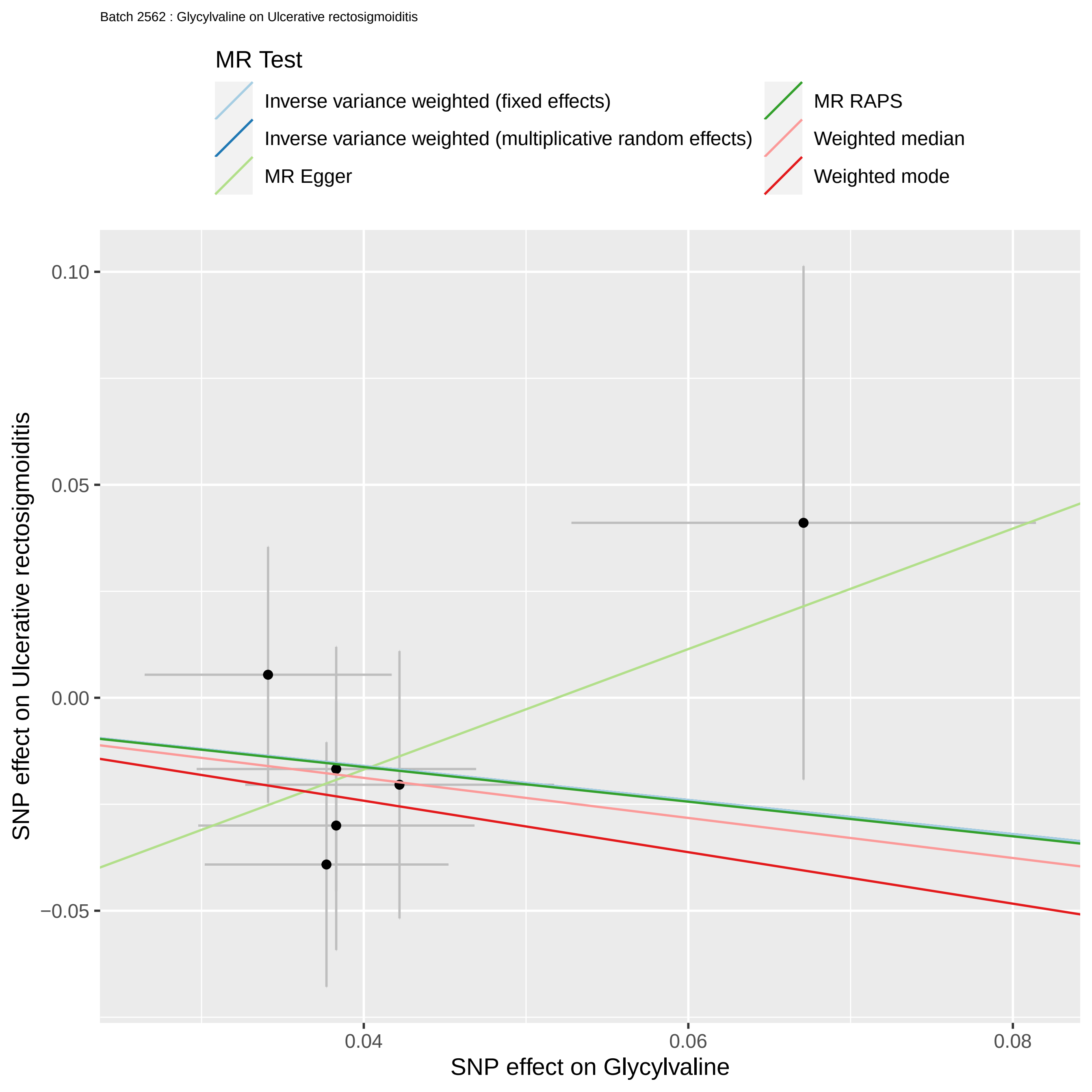

Batch 2562 : Glycylvaline on Ulcerative rectosigmoiditis
MR Test
Inverse variance weighted (fixed effects)
Inverse variance weighted (multiplicative random effects)
MR Egger
MR RAPS
Weighted median
Weighted mode
0.10
0.05
0.00
−0.05
0.04
0.06
0.08
SNP effect on Ulcerative rectosigmoiditis
SNP effect on Glycylvaline

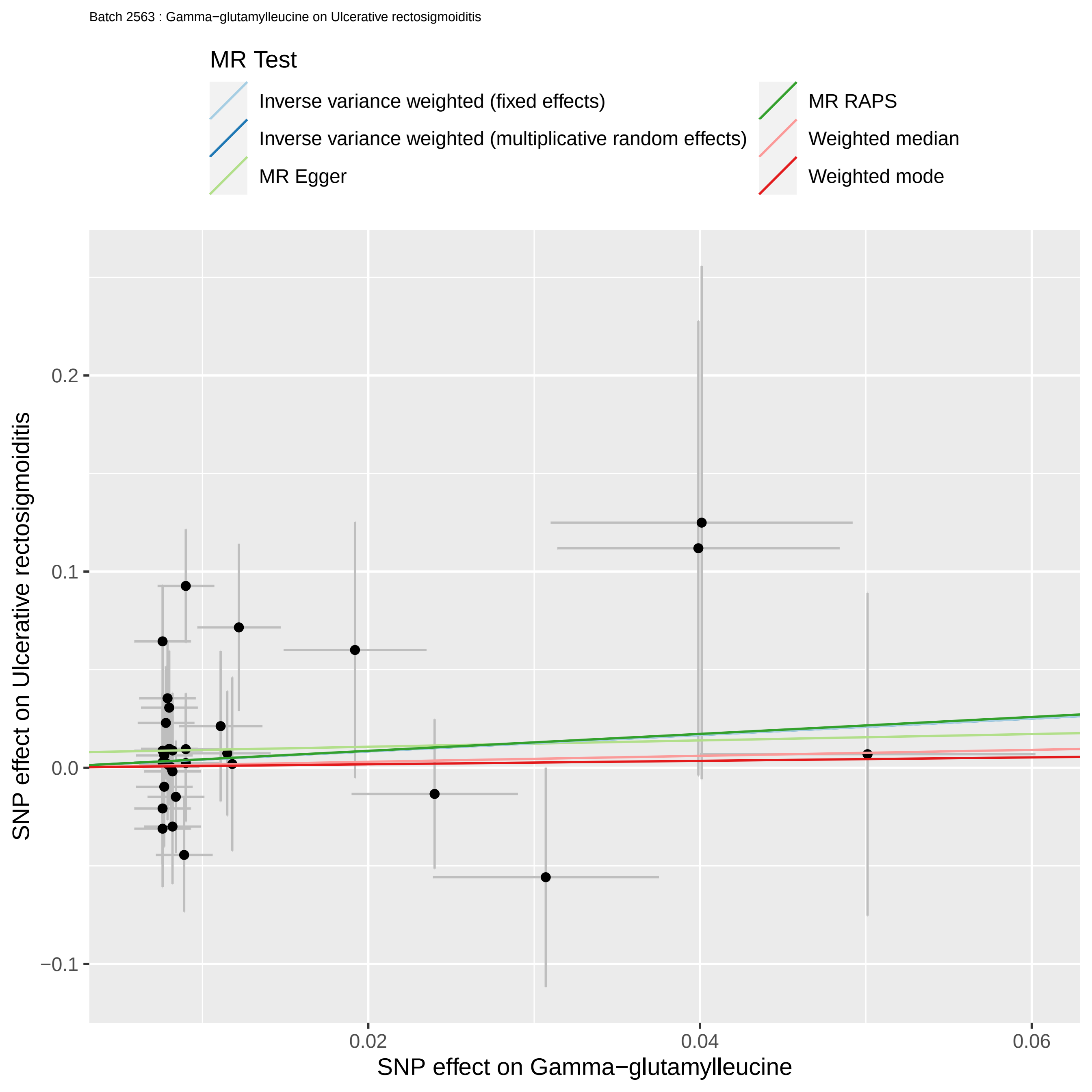

Batch 2563 : Gamma−glutamylleucine on Ulcerative rectosigmoiditis
MR Test
Inverse variance weighted (fixed effects)
Inverse variance weighted (multiplicative random effects)
MR Egger
MR RAPS
Weighted median
Weighted mode
0.2
0.1
0.0
−0.1
SNP effect on Ulcerative rectosigmoiditis
0.02
0.04
0.06
SNP effect on Gamma−glutamylleucine

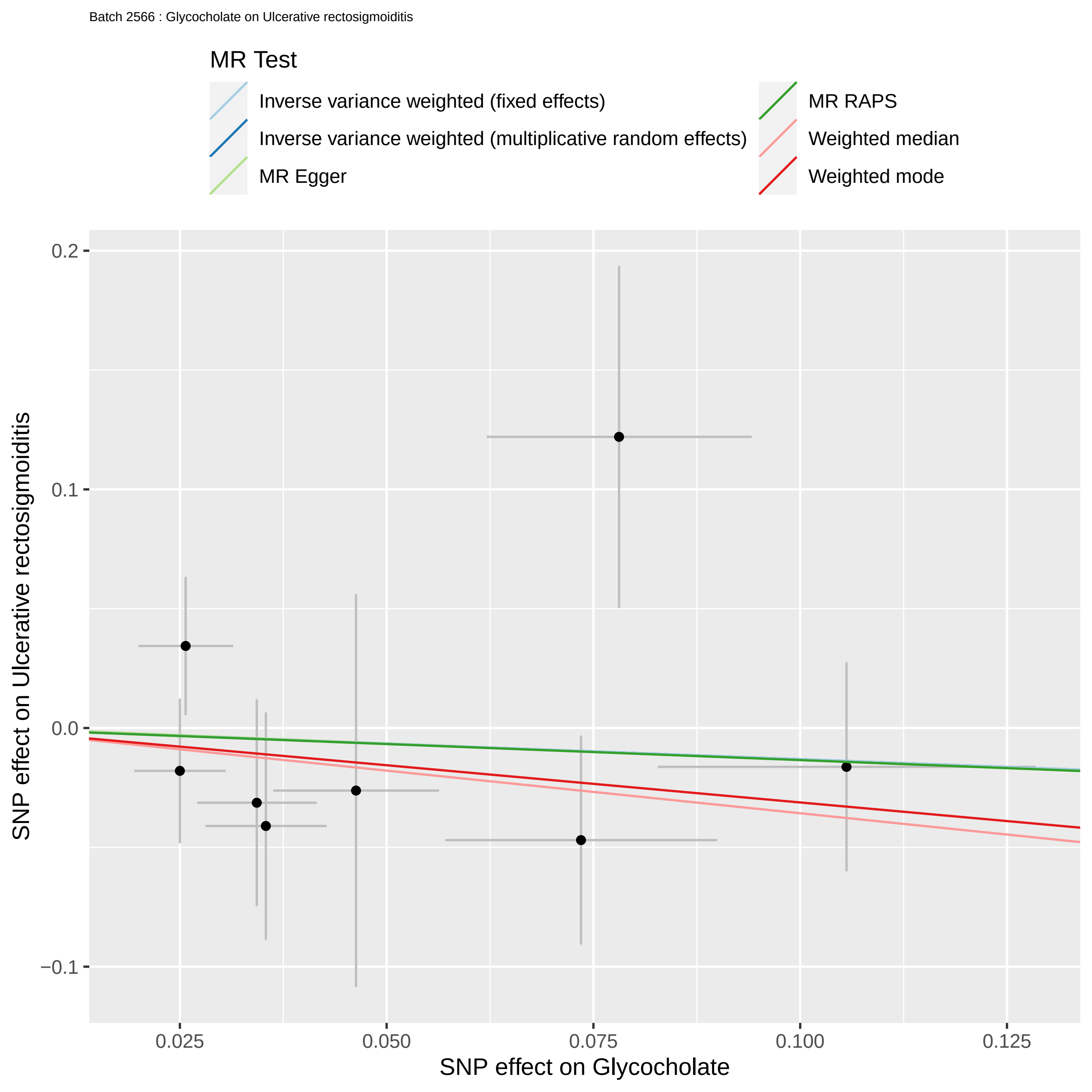

Batch 2566 : Glycocholate on Ulcerative rectosigmoiditis
MR Test
Inverse variance weighted (fixed effects)
Inverse variance weighted (multiplicative random effects)
MR Egger
MR RAPS
Weighted median
Weighted mode
0.2
0.1
0.0
−0.1
0.025
0.050
0.075
0.100
0.125
SNP effect on Ulcerative rectosigmoiditis
SNP effect on Glycocholate

Batch 2567 : Glycodeoxycholate on Ulcerative rectosigmoiditis

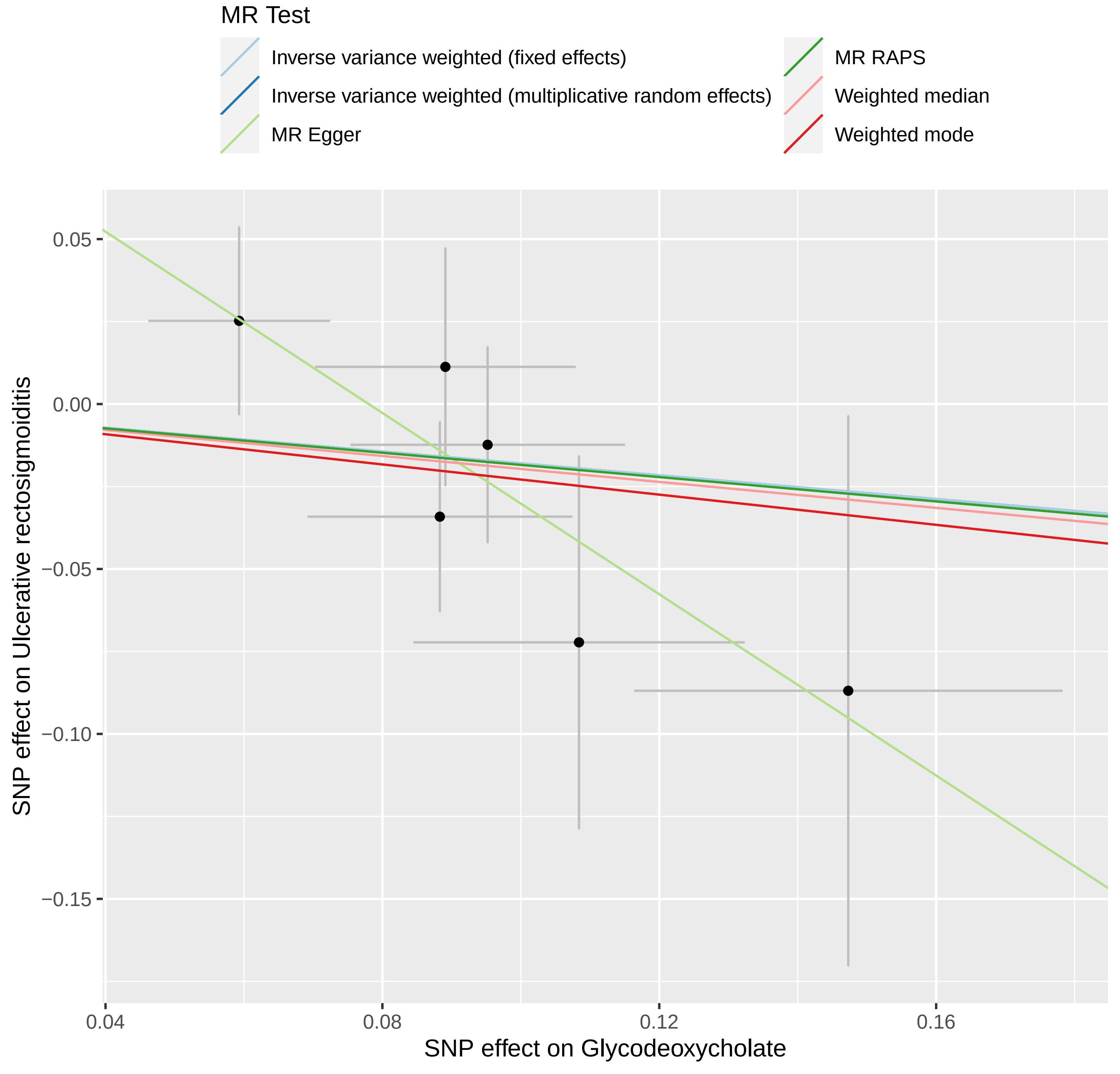

Batch 2568 : Taurochenodeoxycholate on Ulcerative rectosigmoiditis

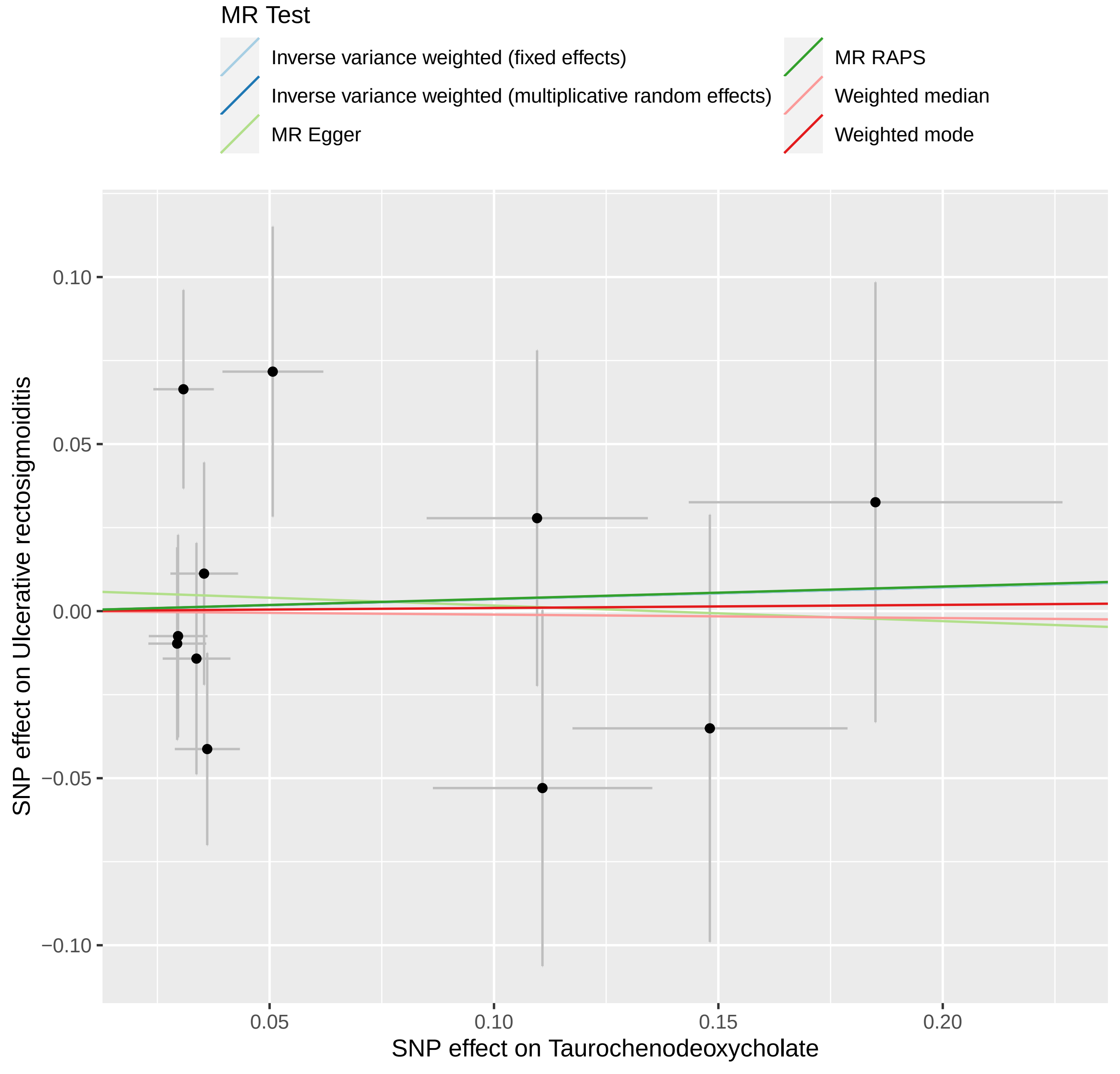

Batch 2569 : Taurocholate on Ulcerative rectosigmoiditis

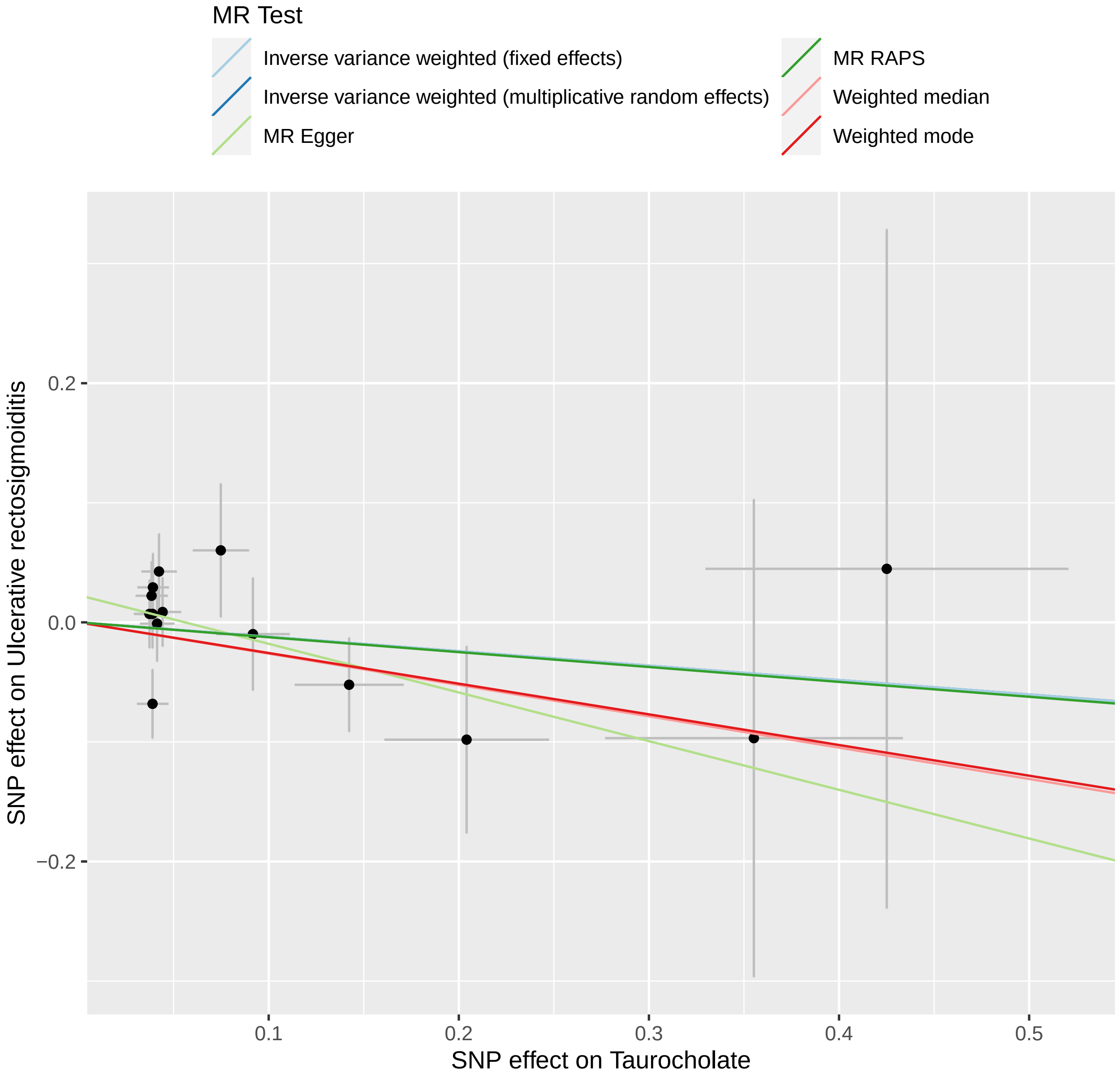

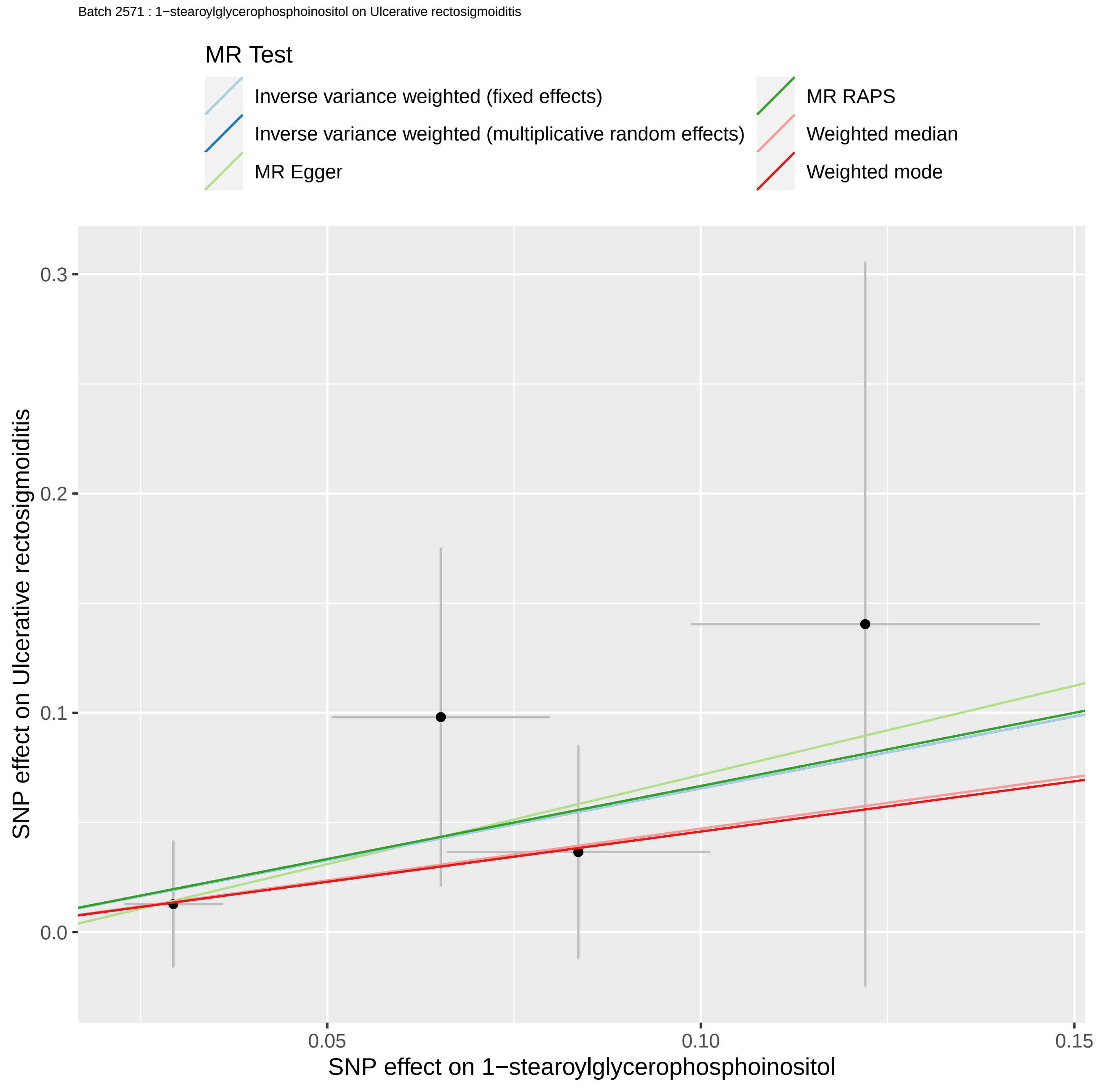
Batch 2571 : 1−stearoylglycerophosphoinositol on Ulcerative rectosigmoiditis
MR Test
Inverse variance weighted (fixed effects)
Inverse variance weighted (multiplicative random effects)
MR Egger
MR RAPS
Weighted median
Weighted mode
0.3
0.2
0.1
0.0
0.05
0.10
0.15
SNP effect on Ulcerative rectosigmoiditis
SNP effect on 1−stearoylglycerophosphoinositol

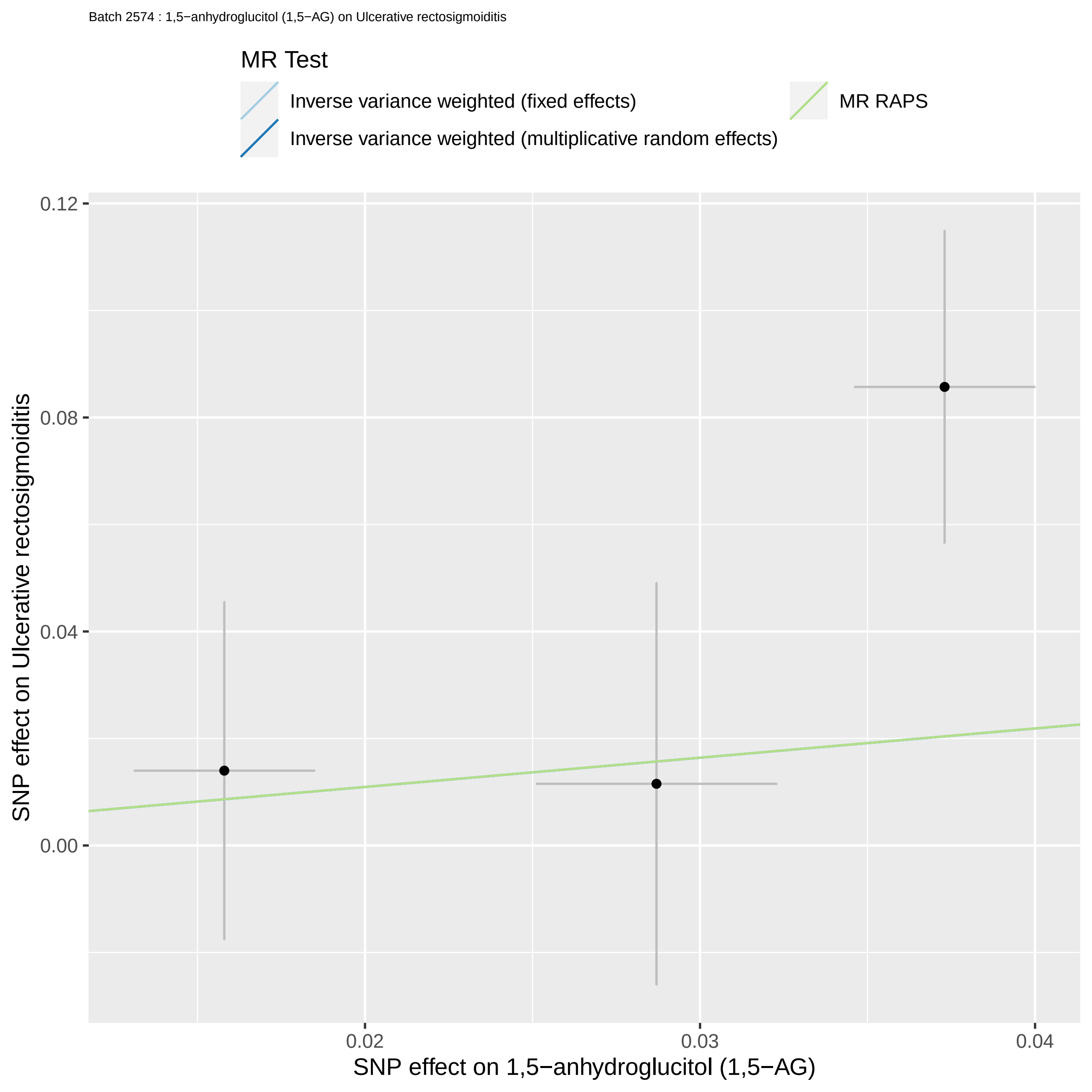

Batch 2574 : 1,5−anhydroglucitol (1,5−AG) on Ulcerative rectosigmoiditis
MR Test
Inverse variance weighted (fixed effects)
Inverse variance weighted (multiplicative random effects)
MR RAPS
0.12
0.08
0.04
0.00
0.02
0.03
0.04
SNP effect on Ulcerative rectosigmoiditis
SNP effect on 1,5−anhydroglucitol (1,5−AG)

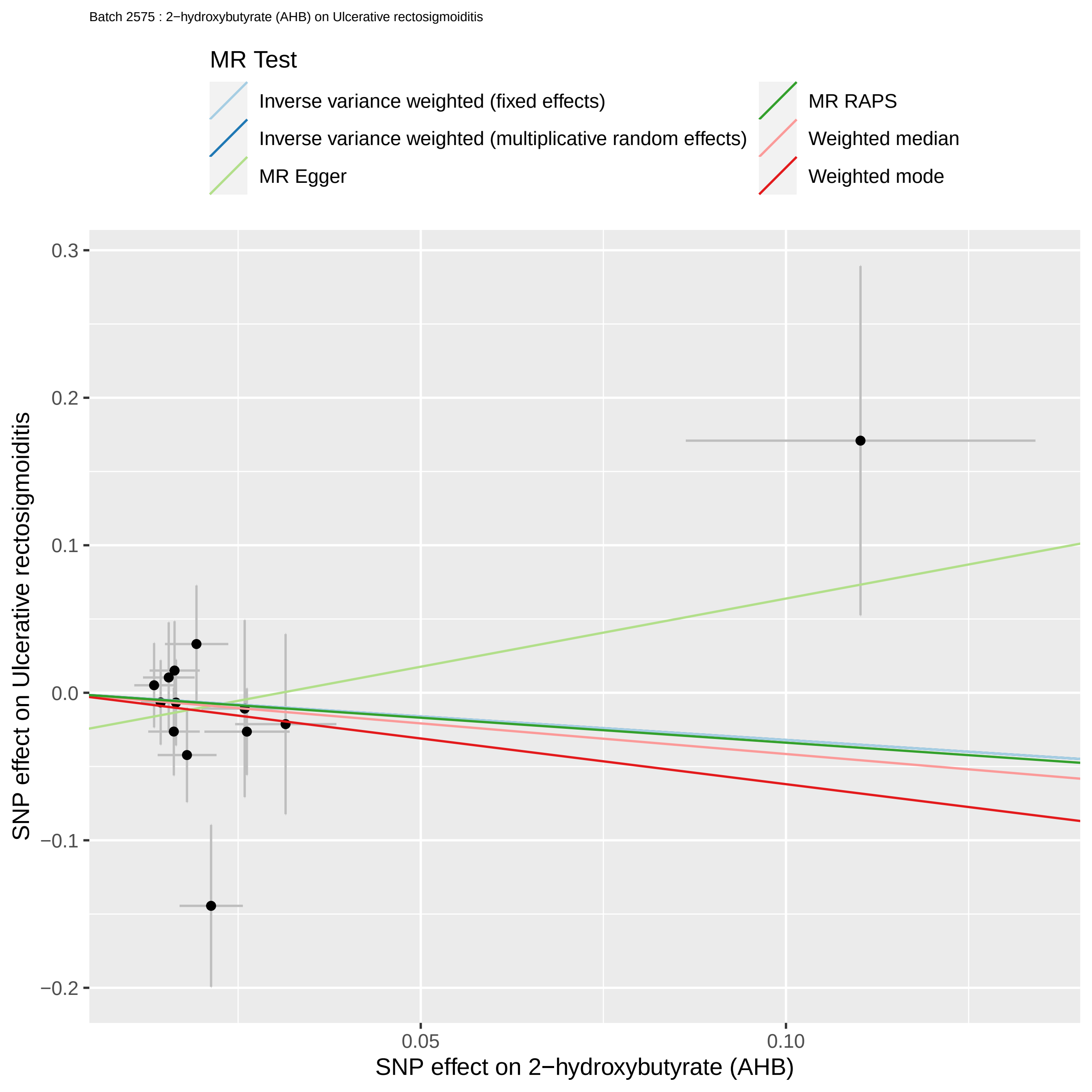
Batch 2575 : 2−hydroxybutyrate (AHB) on Ulcerative rectosigmoiditis
MR Test
Inverse variance weighted (fixed effects)
Inverse variance weighted (multiplicative random effects)
MR Egger
MR RAPS
Weighted median
Weighted mode
0.3
0.2
0.1
0.0
−0.1
−0.2
0.05
0.10
SNP effect on Ulcerative rectosigmoiditis
SNP effect on 2−hydroxybutyrate (AHB)

Batch 2578 : 1−palmitoylglycerol (1−monopalmitin) on Ulcerative rectosigmoiditis

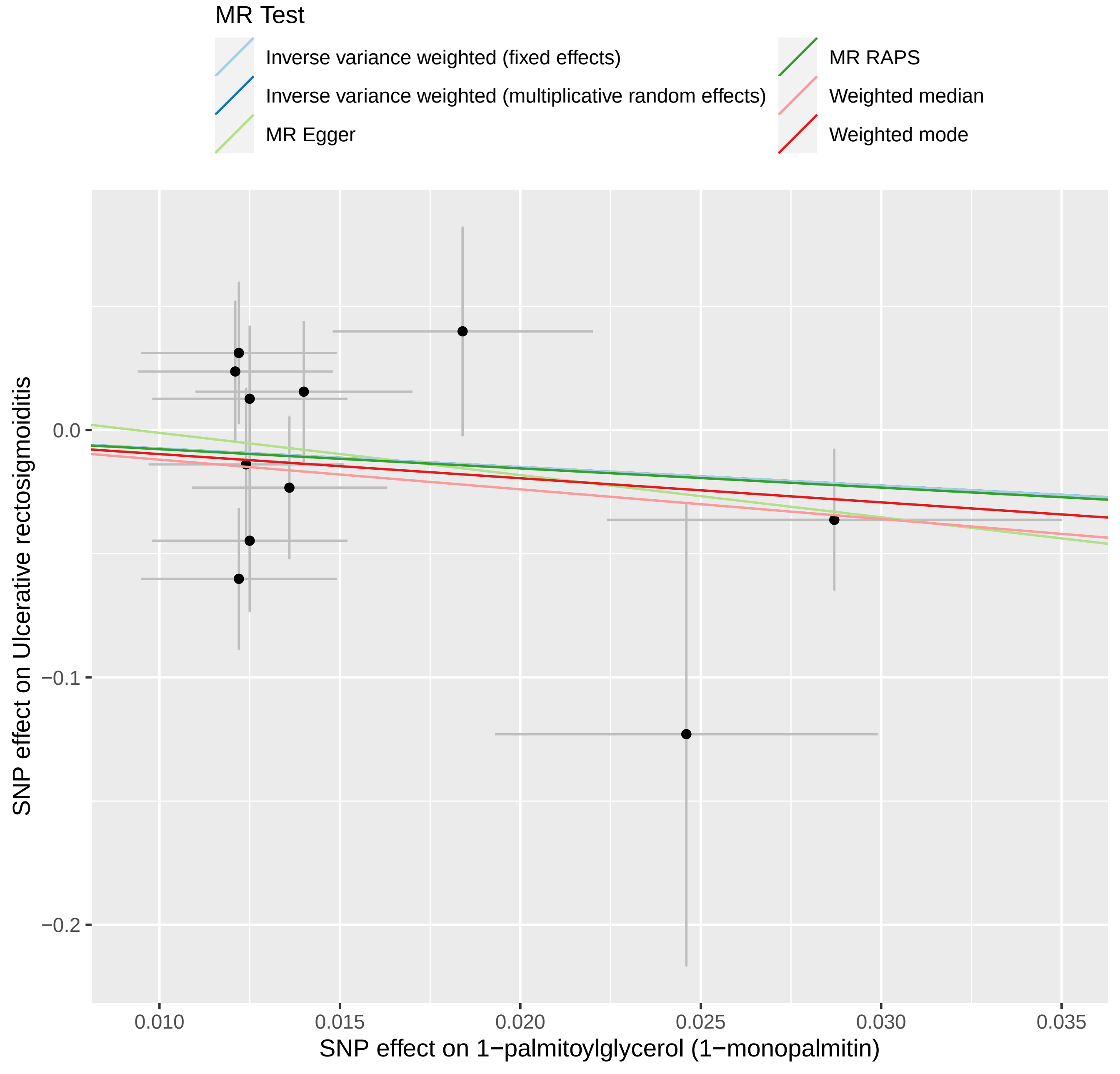

Batch 2581 : 2−hydroxyisobutyrate on Ulcerative rectosigmoiditis

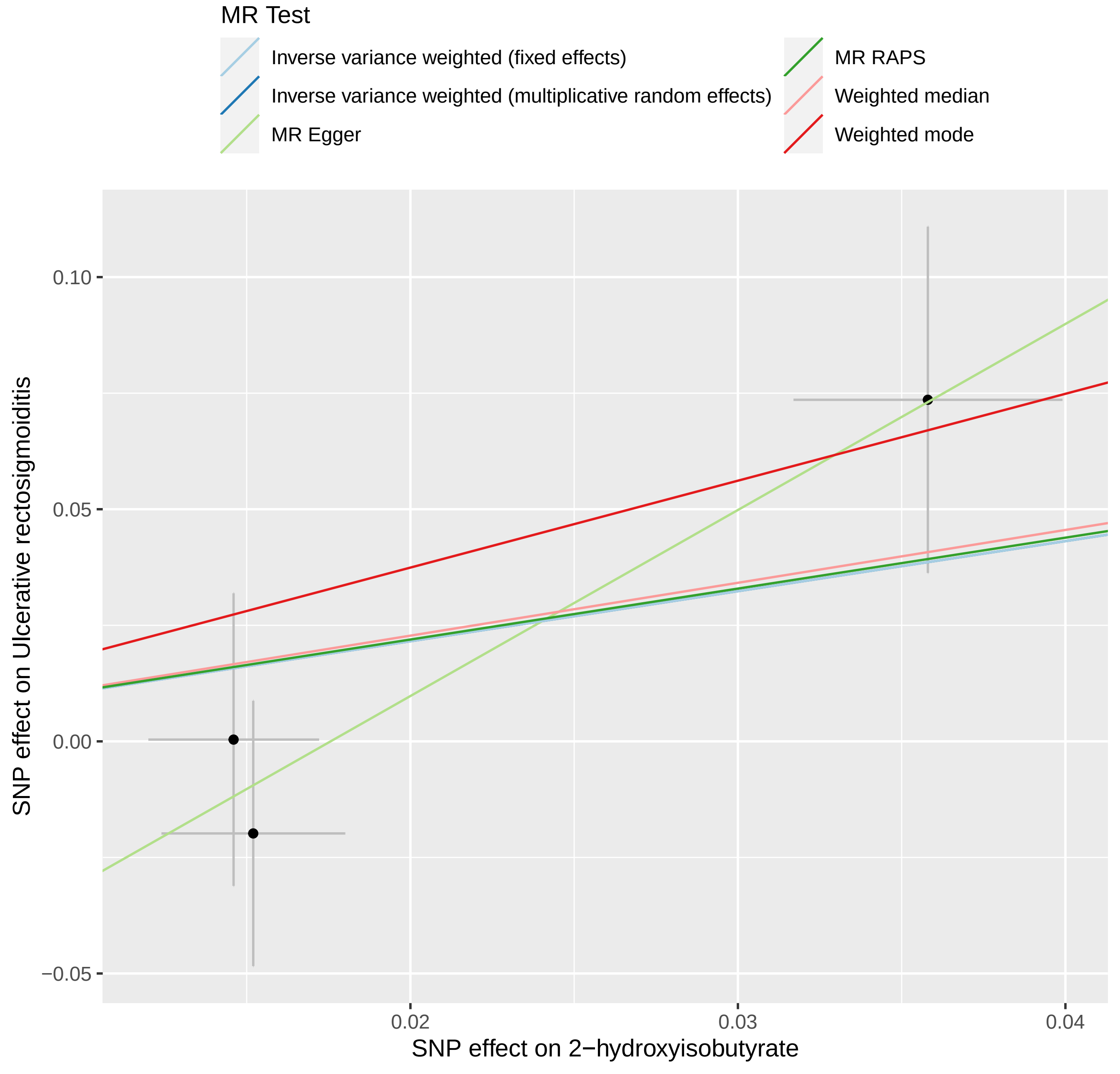

Batch 2582 : 4−methyl−2−oxopentanoate on Ulcerative rectosigmoiditis

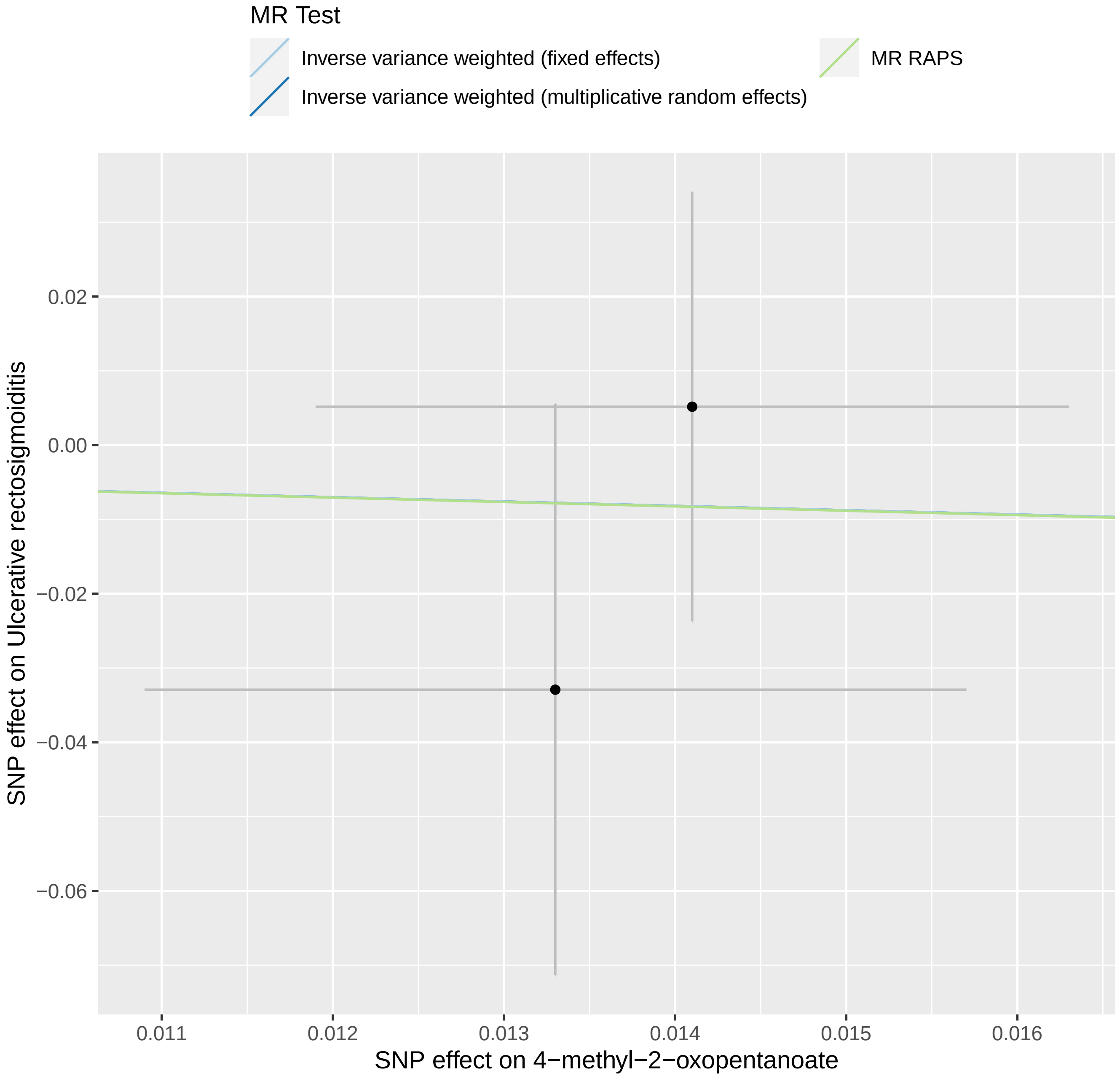

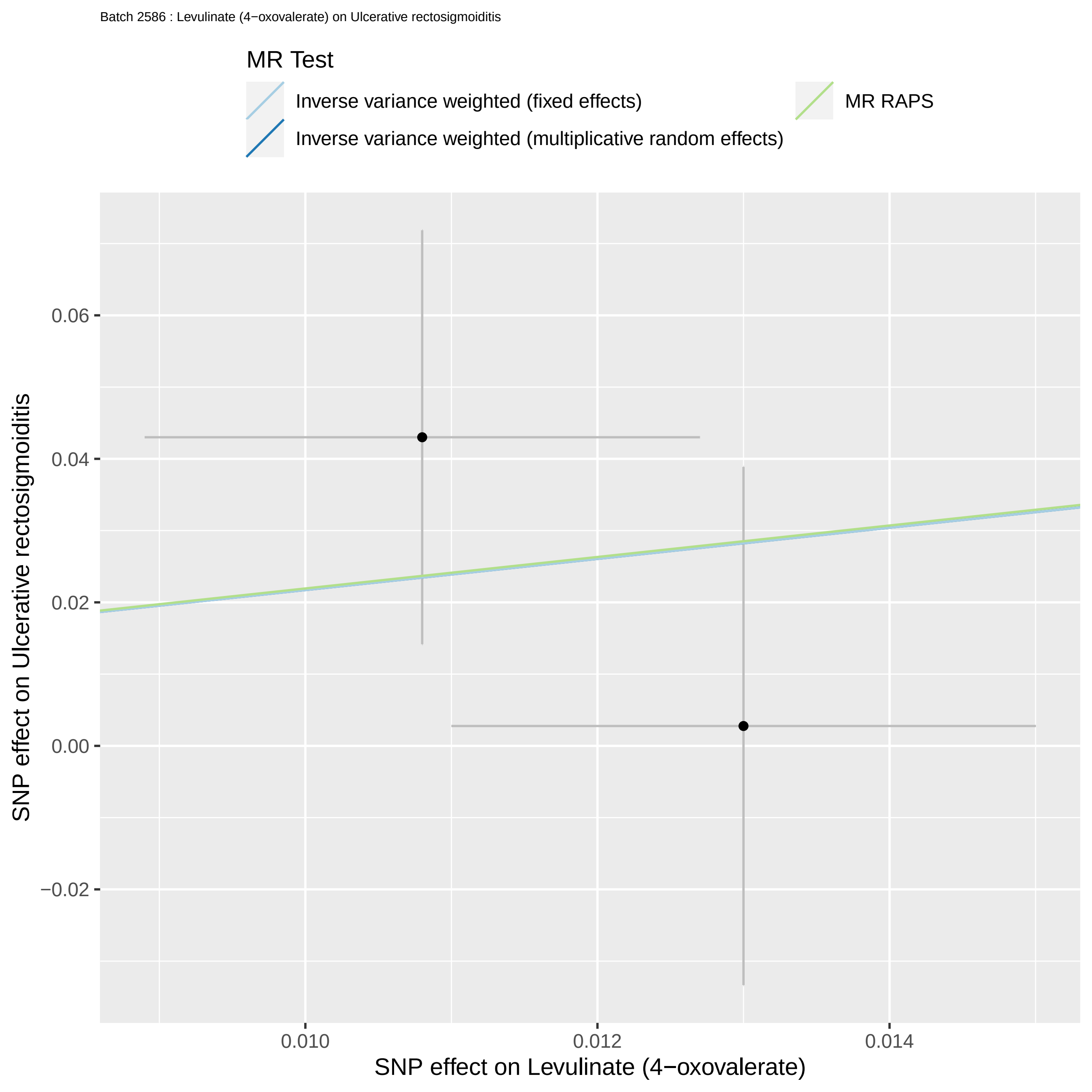

Batch 2586 : Levulinate (4−oxovalerate) on Ulcerative rectosigmoiditis
MR Test
Inverse variance weighted (fixed effects)
Inverse variance weighted (multiplicative random effects)
MR RAPS
SNP effect on Ulcerative rectosigmoiditis
0.06
0.04
0.02
0.00
−0.02
0.010
0.012
0.014
SNP effect on Levulinate (4−oxovalerate)

Batch 2588 : Cholate on Ulcerative rectosigmoiditis

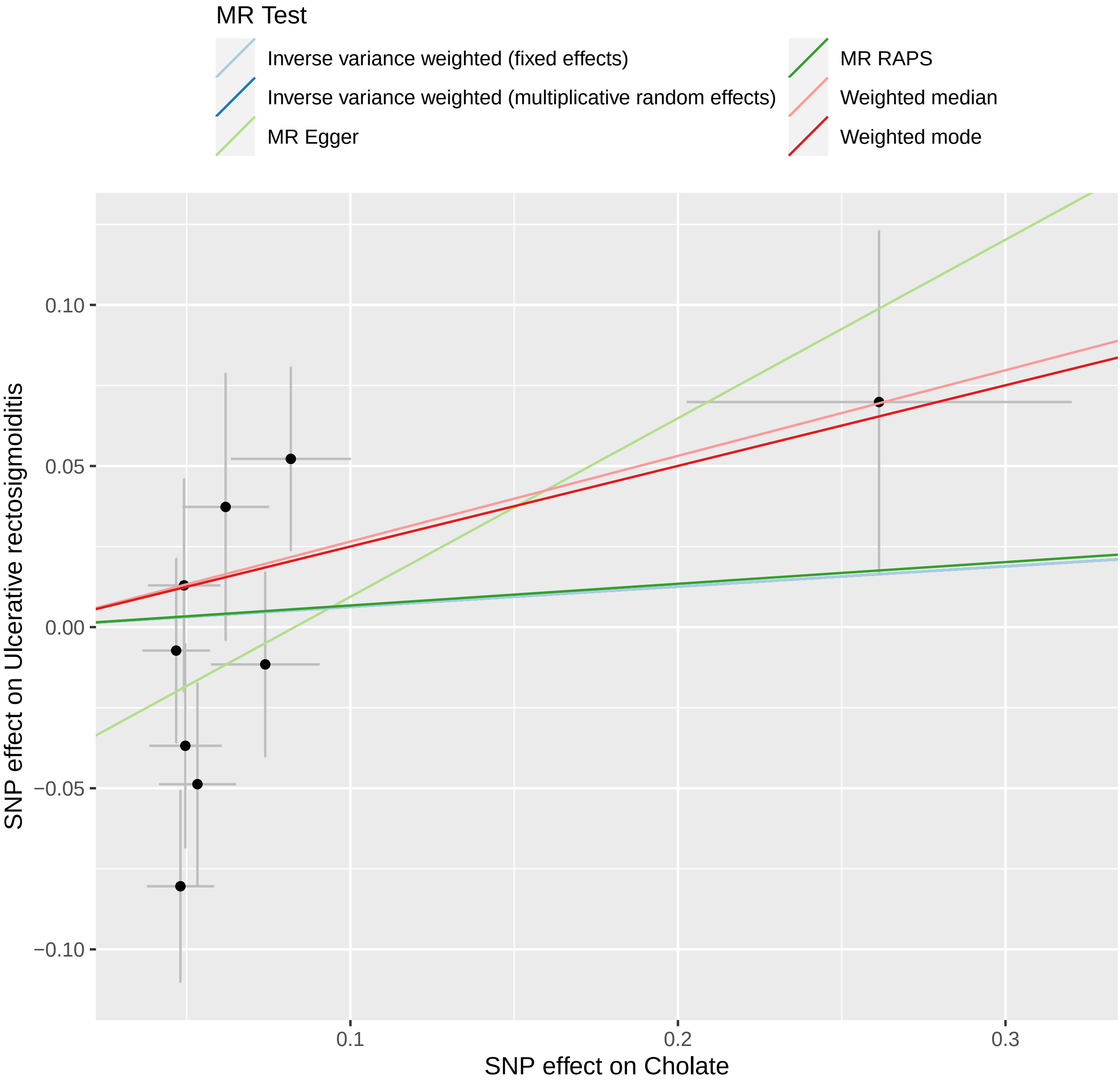

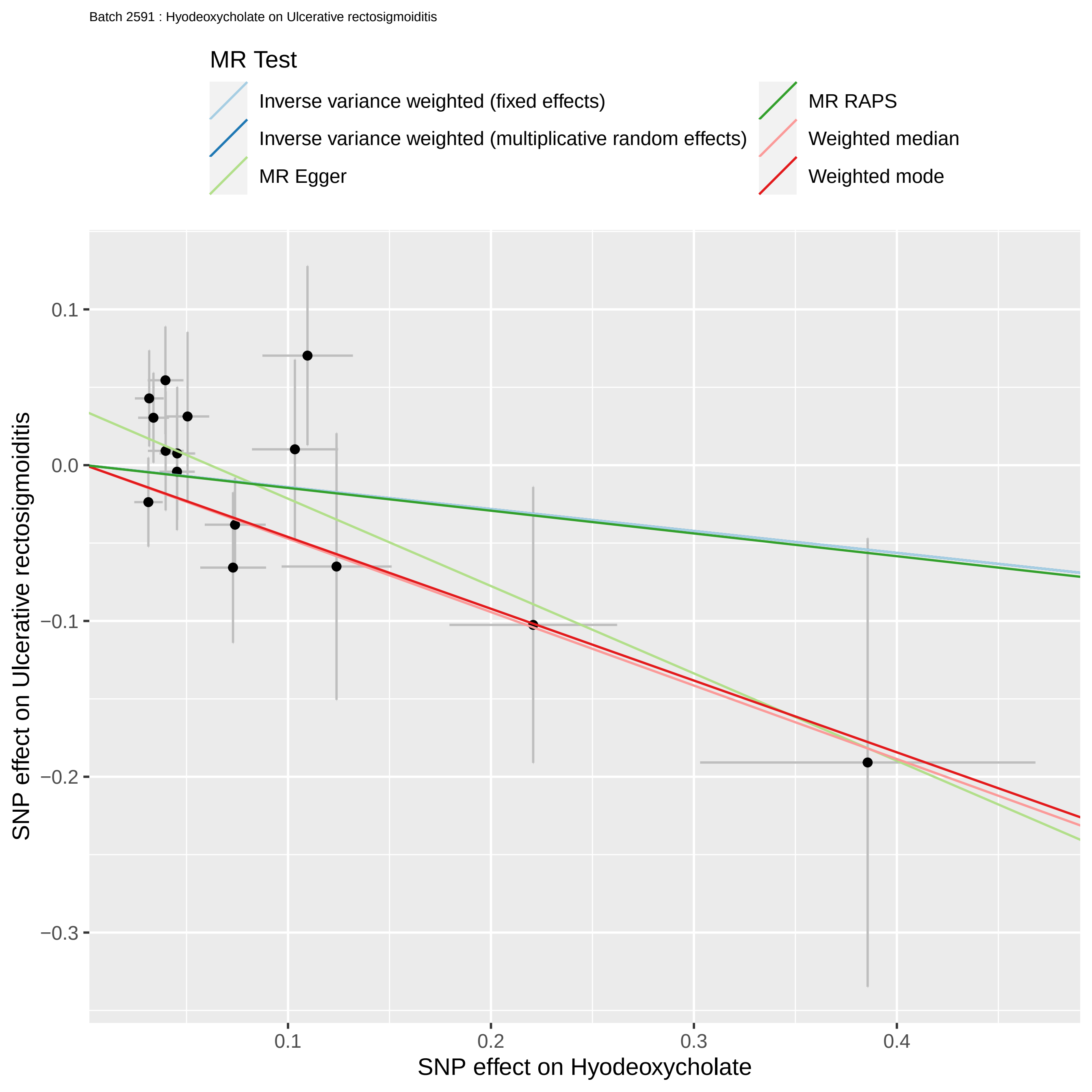
Batch 2591 : Hyodeoxycholate on Ulcerative rectosigmoiditis
MR Test
Inverse variance weighted (fixed effects)
Inverse variance weighted (multiplicative random effects)
MR Egger
MR RAPS
Weighted median
Weighted mode
0.1
0.0
−0.1
−0.2
−0.3
SNP effect on Ulcerative rectosigmoiditis
0.1
0.2
0.3
0.4
SNP effect on Hyodeoxycholate

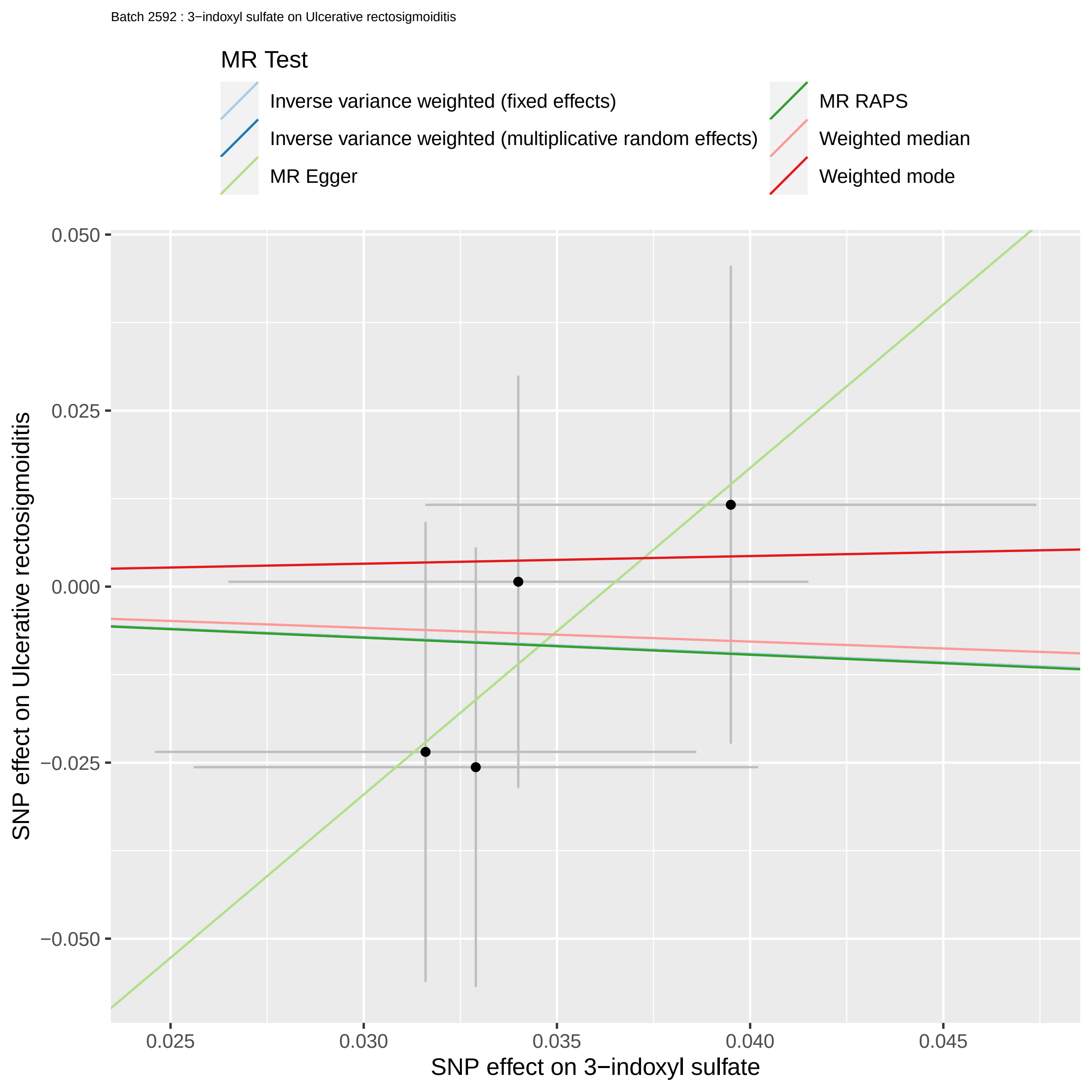
Batch 2592 : 3−indoxyl sulfate on Ulcerative rectosigmoiditis
MR Test
Inverse variance weighted (fixed effects)
Inverse variance weighted (multiplicative random effects)
MR Egger
MR RAPS
Weighted median
Weighted mode
0.050
0.025
0.000
−0.025
−0.050
0.025
0.030
0.035
0.040
0.045
SNP effect on Ulcerative rectosigmoiditis
SNP effect on 3−indoxyl sulfate

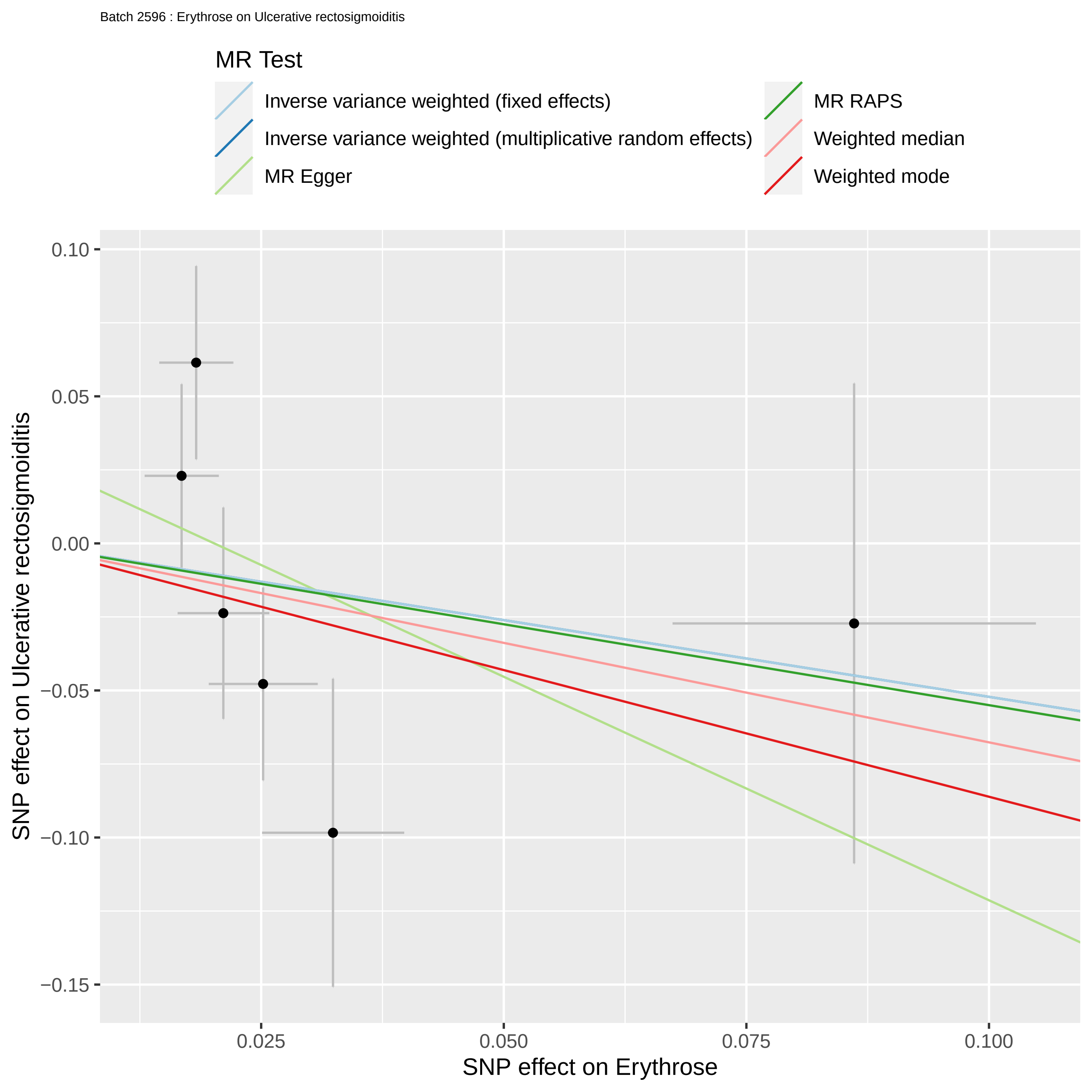

Batch 2596 : Erythrose on Ulcerative rectosigmoiditis
MR Test
Inverse variance weighted (fixed effects)
Inverse variance weighted (multiplicative random effects)
MR Egger
MR RAPS
Weighted median
Weighted mode
0.10
0.05
0.00
−0.05
−0.10
−0.15
0.025
0.050
0.075
0.100
SNP effect on Ulcerative rectosigmoiditis
SNP effect on Erythrose

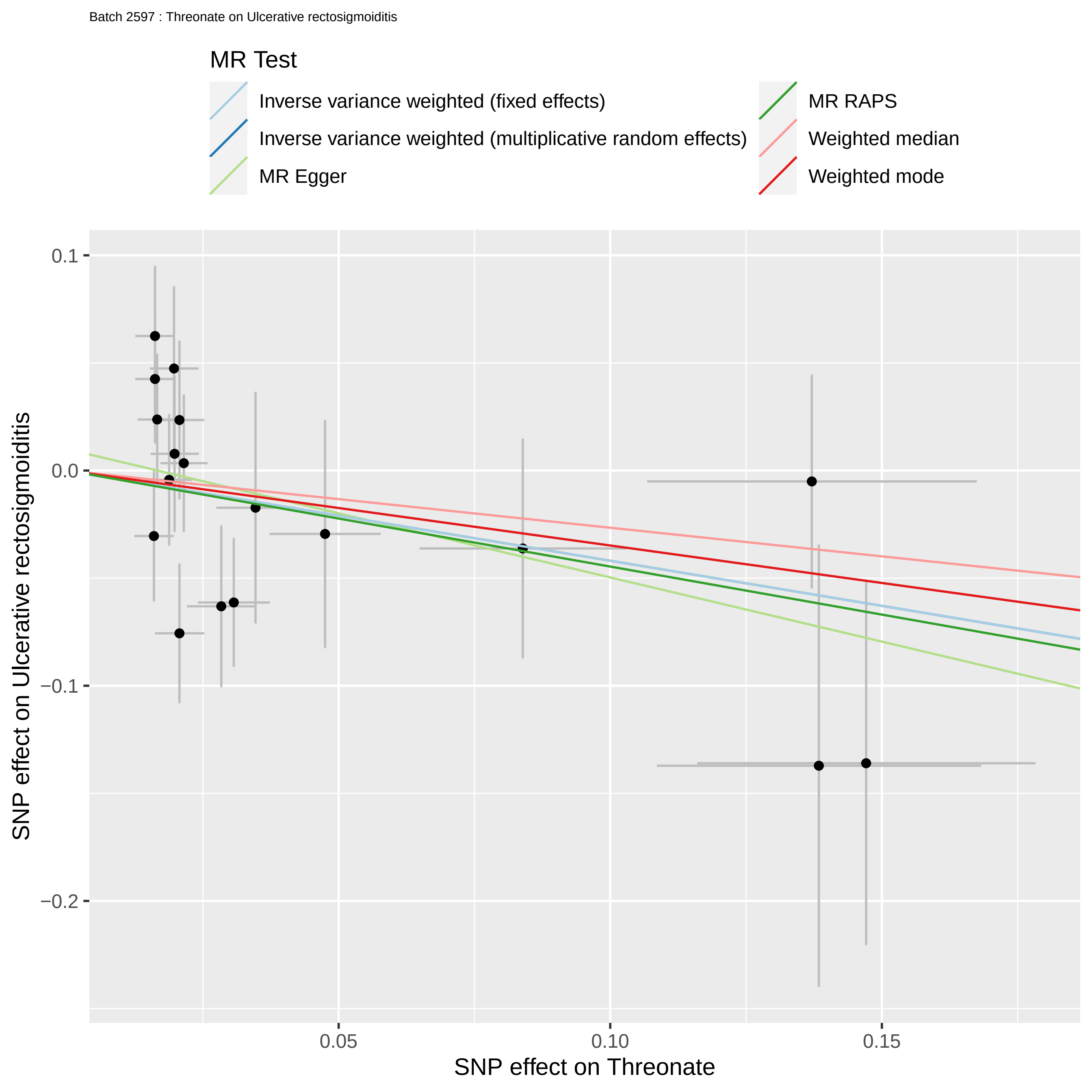
Batch 2597 : Threonate on Ulcerative rectosigmoiditis
MR Test
Inverse variance weighted (fixed effects)
Inverse variance weighted (multiplicative random effects)
MR Egger
MR RAPS
Weighted median
Weighted mode
0.1
0.0
−0.1
−0.2
0.05
0.10
0.15
SNP effect on Ulcerative rectosigmoiditis
SNP effect on Threonate

Batch 2598 : Cysteine on Ulcerative rectosigmoiditis

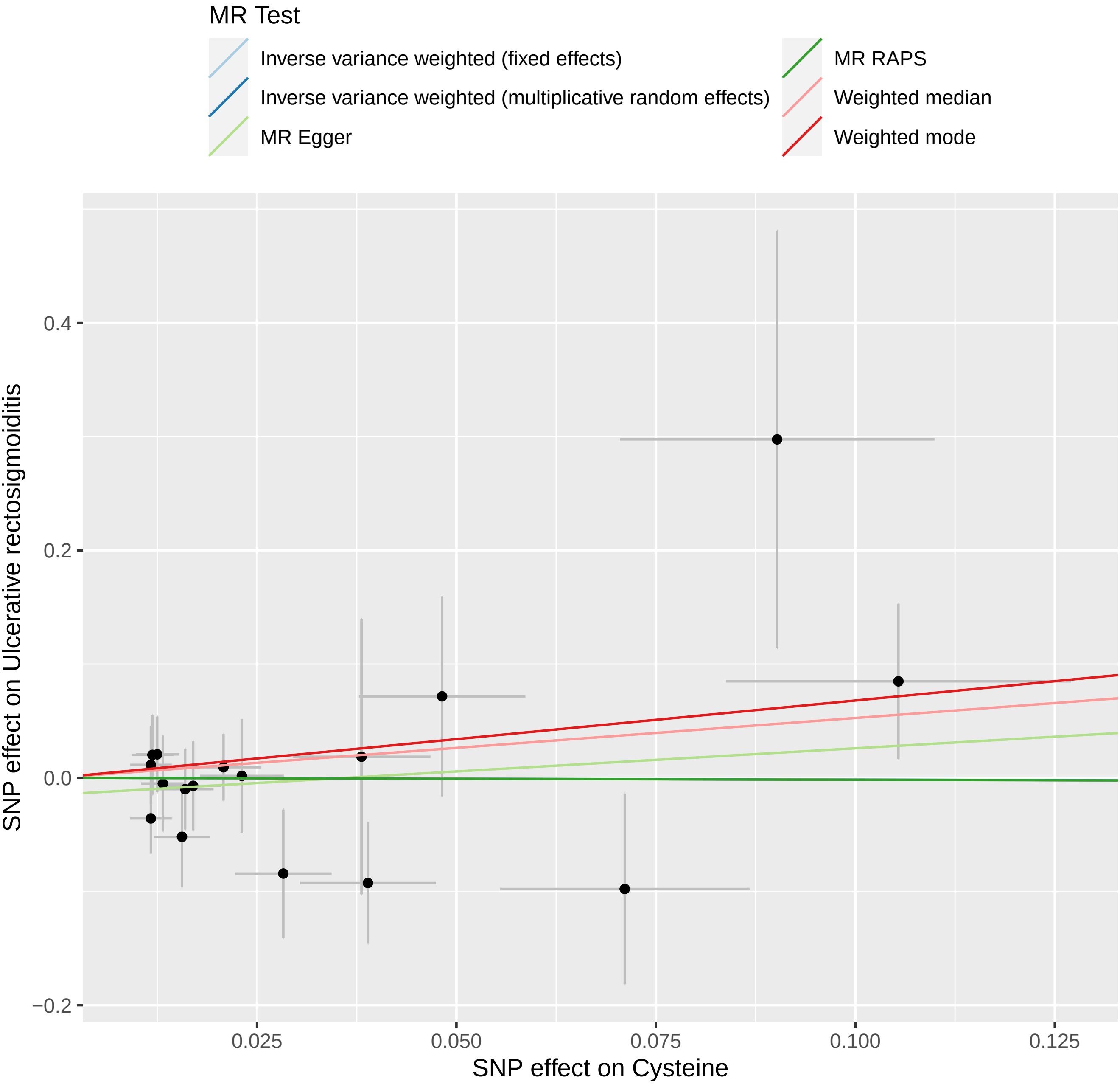

Batch 2599 : Pyroglutamylglycine on Ulcerative rectosigmoiditis

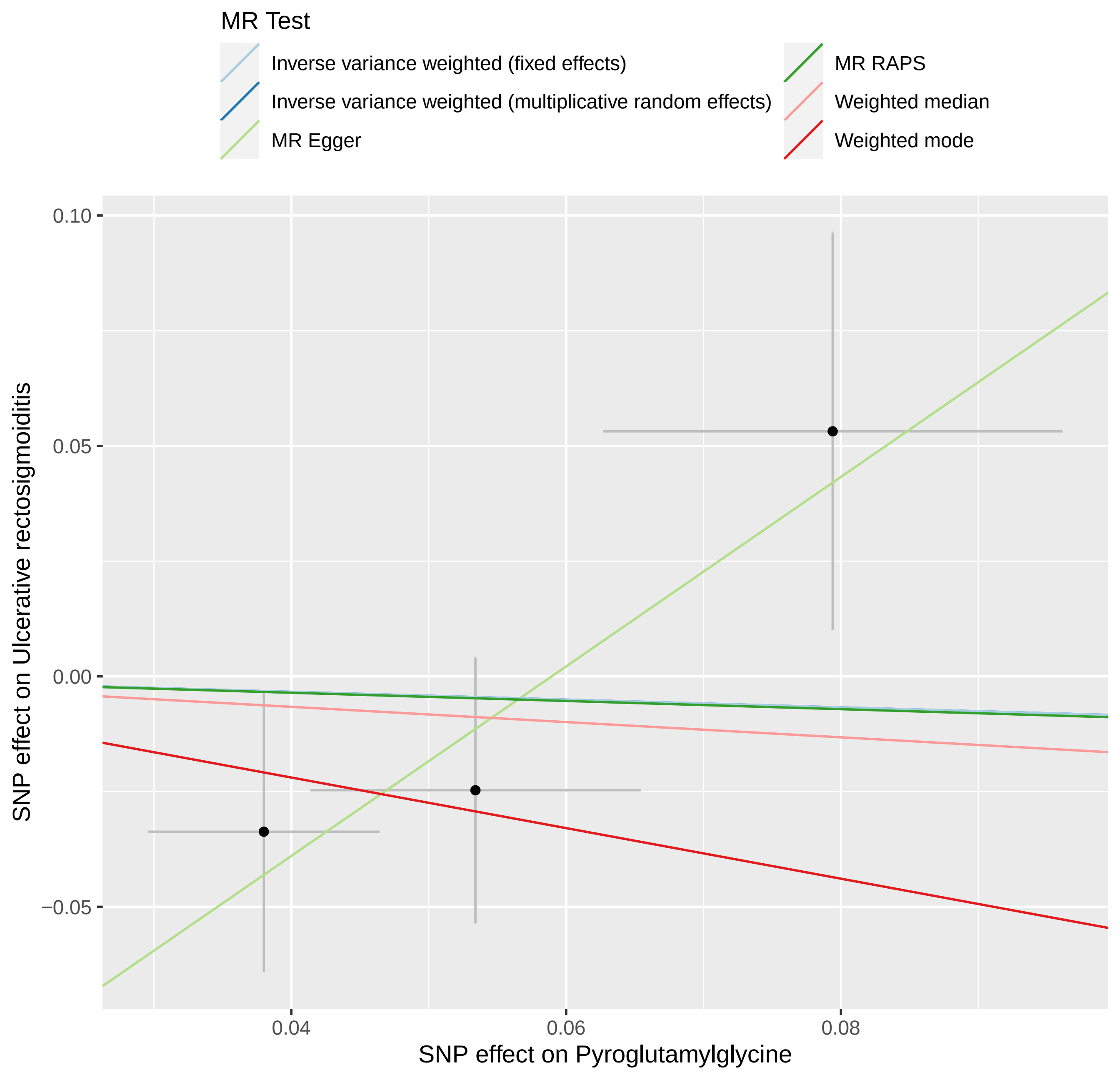

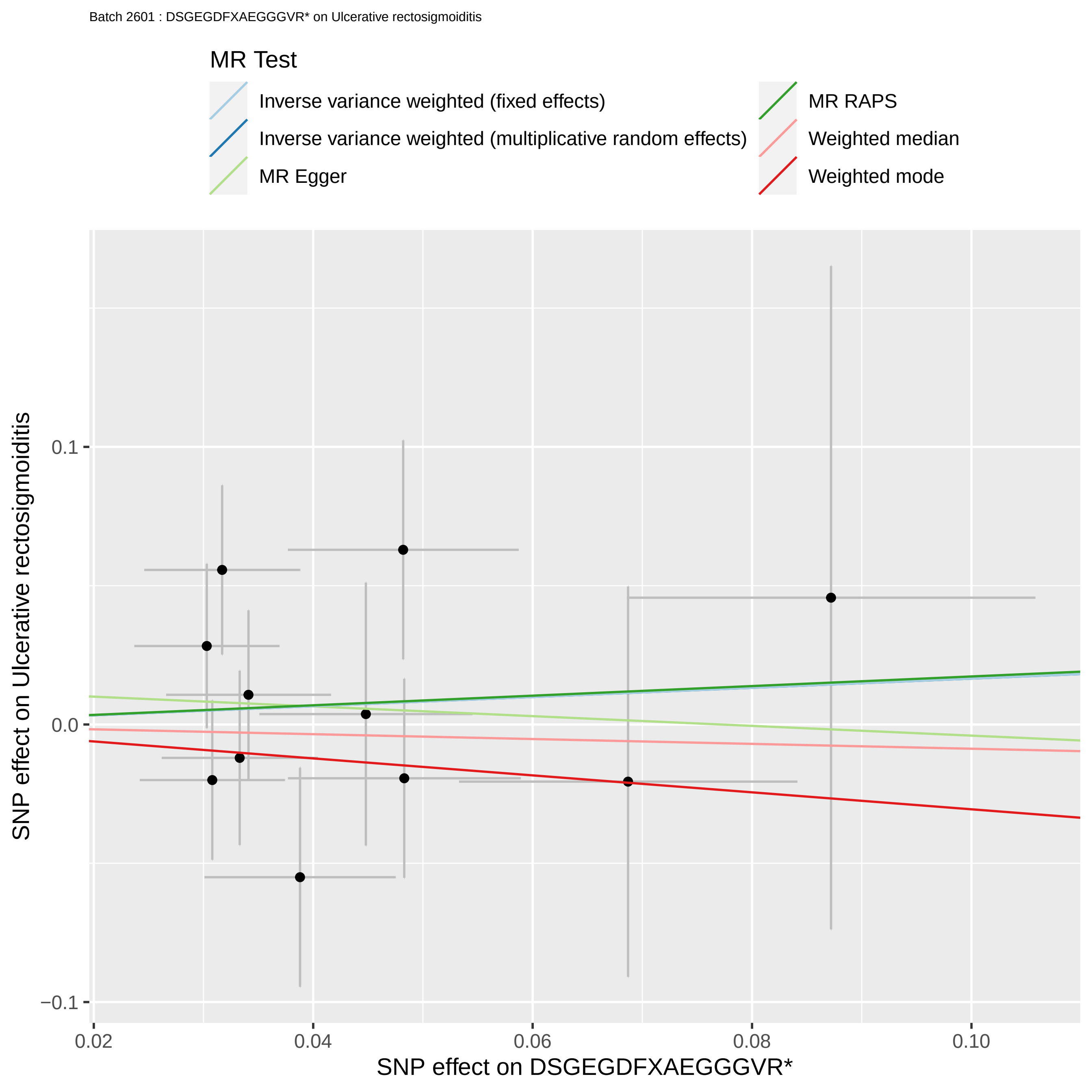

Batch 2601 : DSGEGDFXAEGGGVR* on Ulcerative rectosigmoiditis
MR Test
Inverse variance weighted (fixed effects)
Inverse variance weighted (multiplicative random effects)
MR Egger
MR RAPS
Weighted median
Weighted mode
0.1
0.0
−0.1
0.02
0.04
0.06
0.08
0.10
SNP effect on Ulcerative rectosigmoiditis
SNP effect on DSGEGDFXAEGGGVR*

Batch 2602 : Pyridoxate on Ulcerative rectosigmoiditis

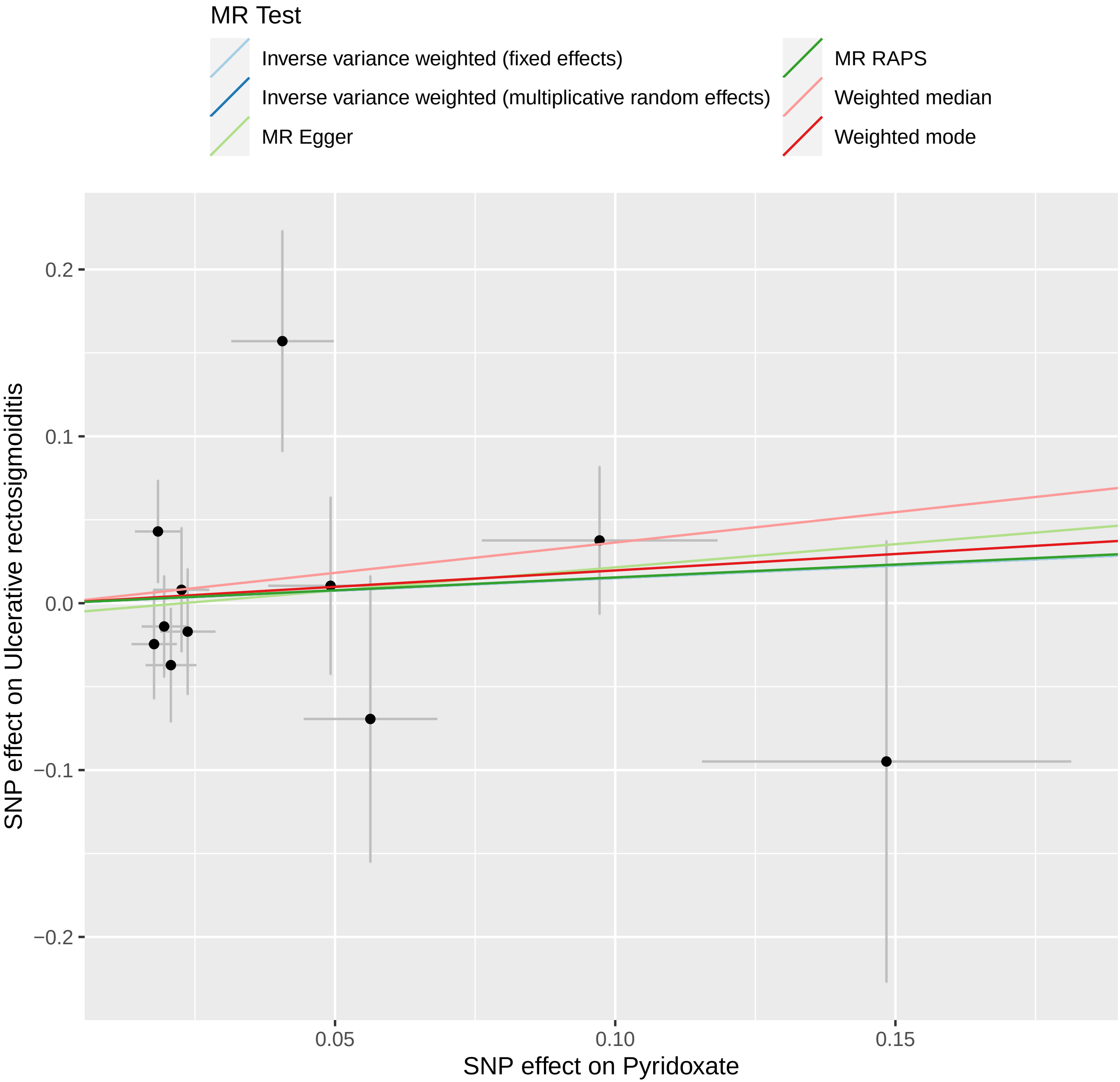

Batch 2603 : Androsterone sulfate on Ulcerative rectosigmoiditis

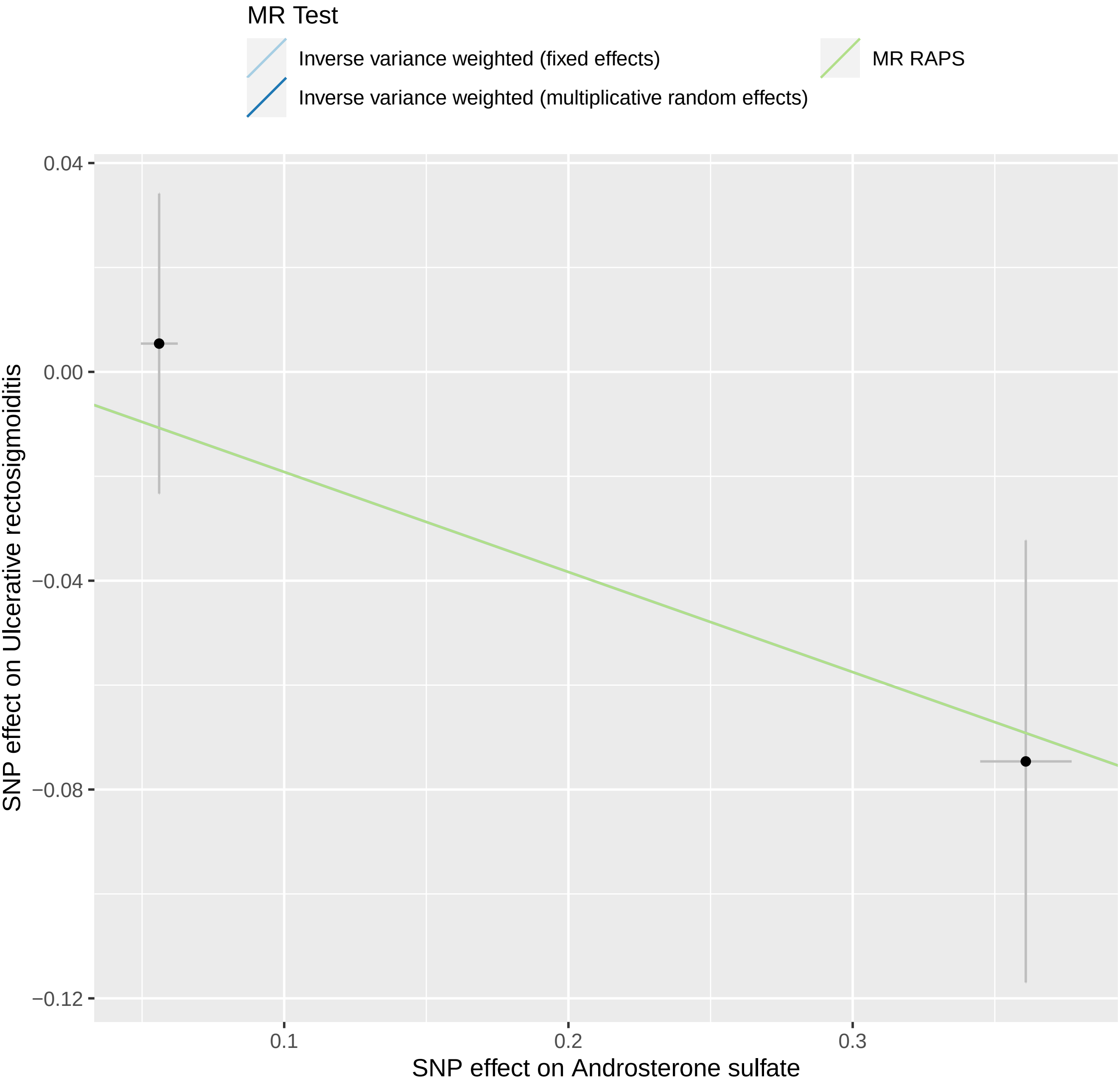

Batch 2607 : Serine on Ulcerative rectosigmoiditis

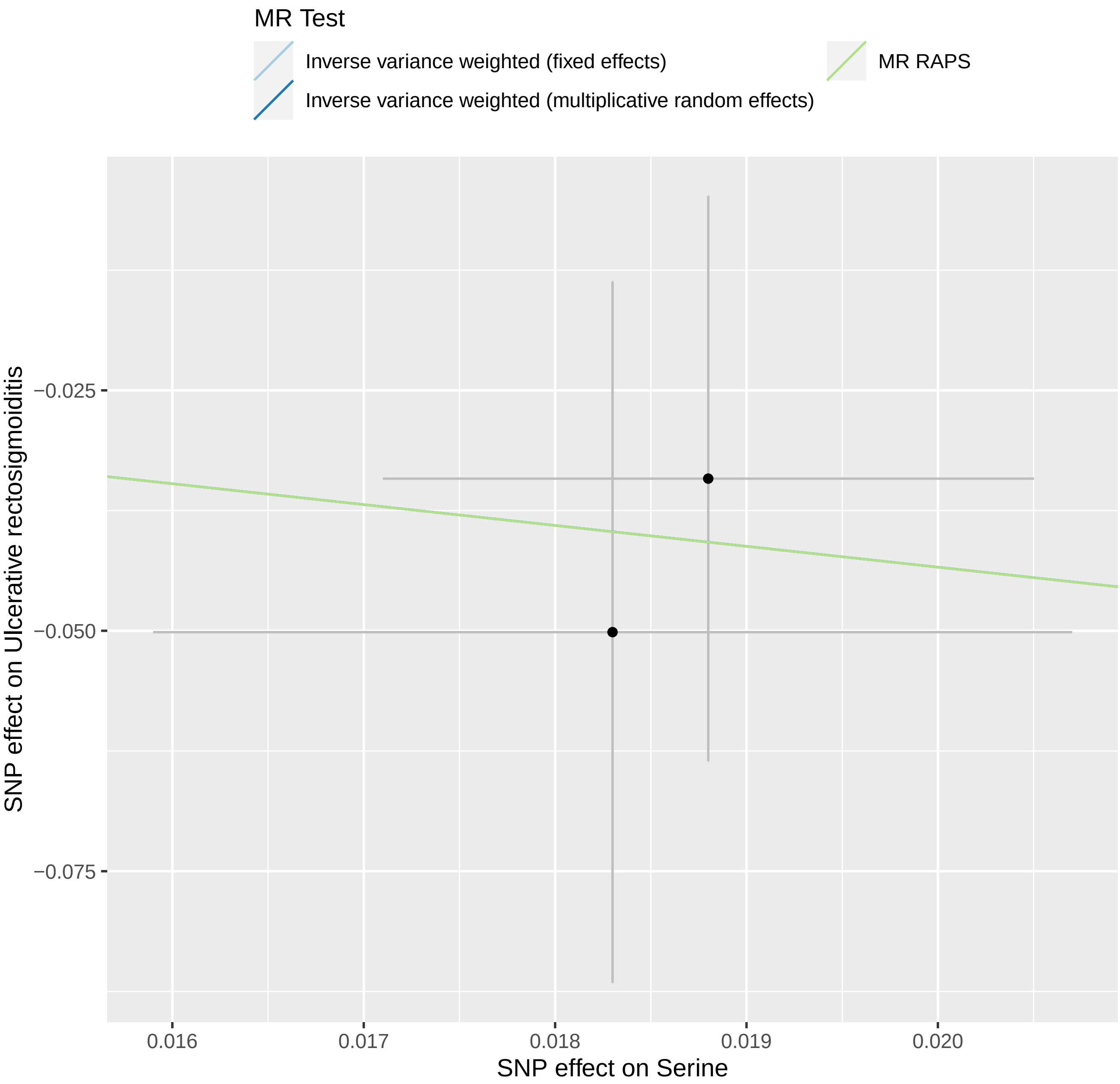

Batch 2608 : Trans−4−hydroxyproline on Ulcerative rectosigmoiditis

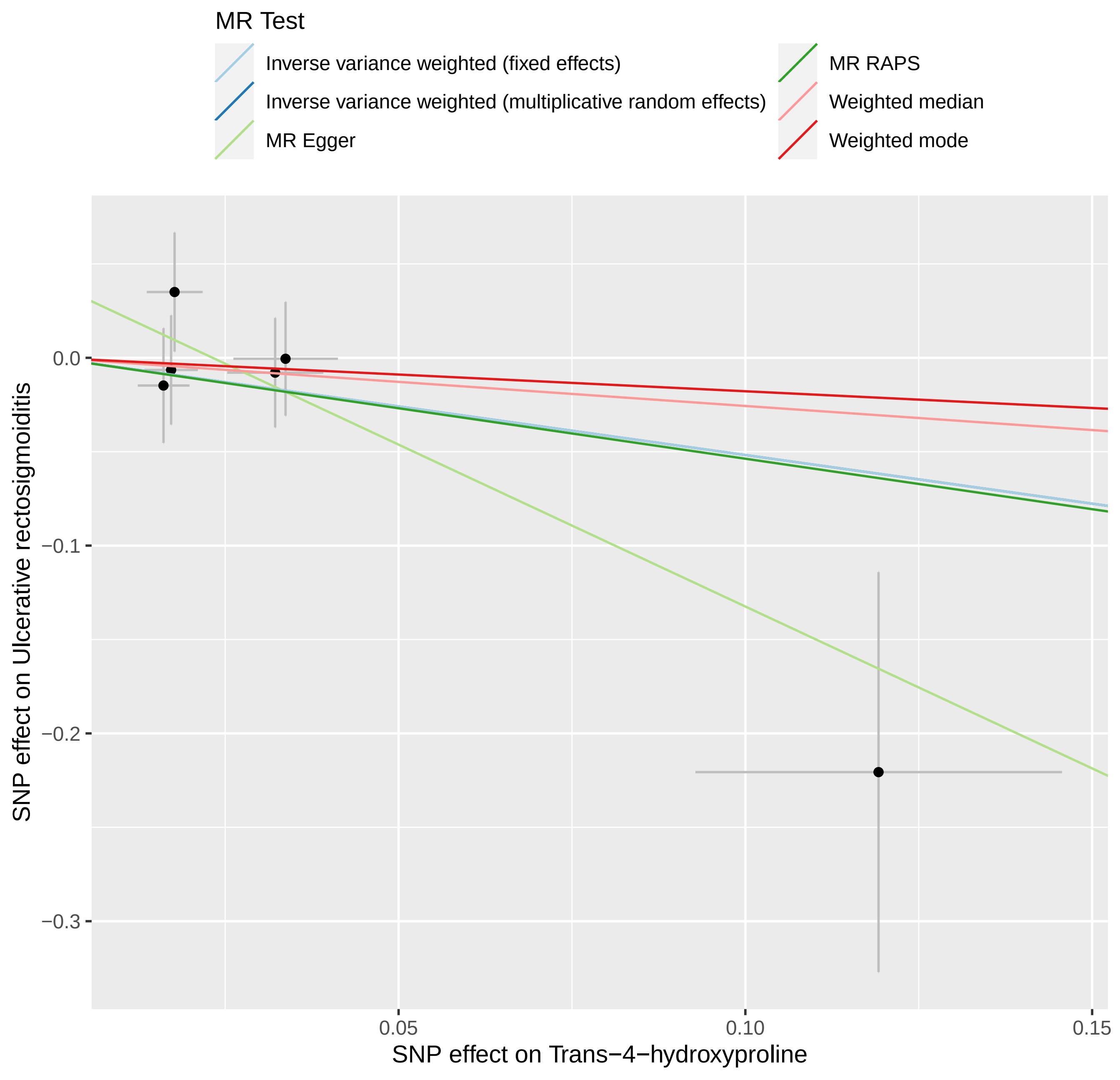

Batch 2610 : Hexanoylcarnitine on Ulcerative rectosigmoiditis

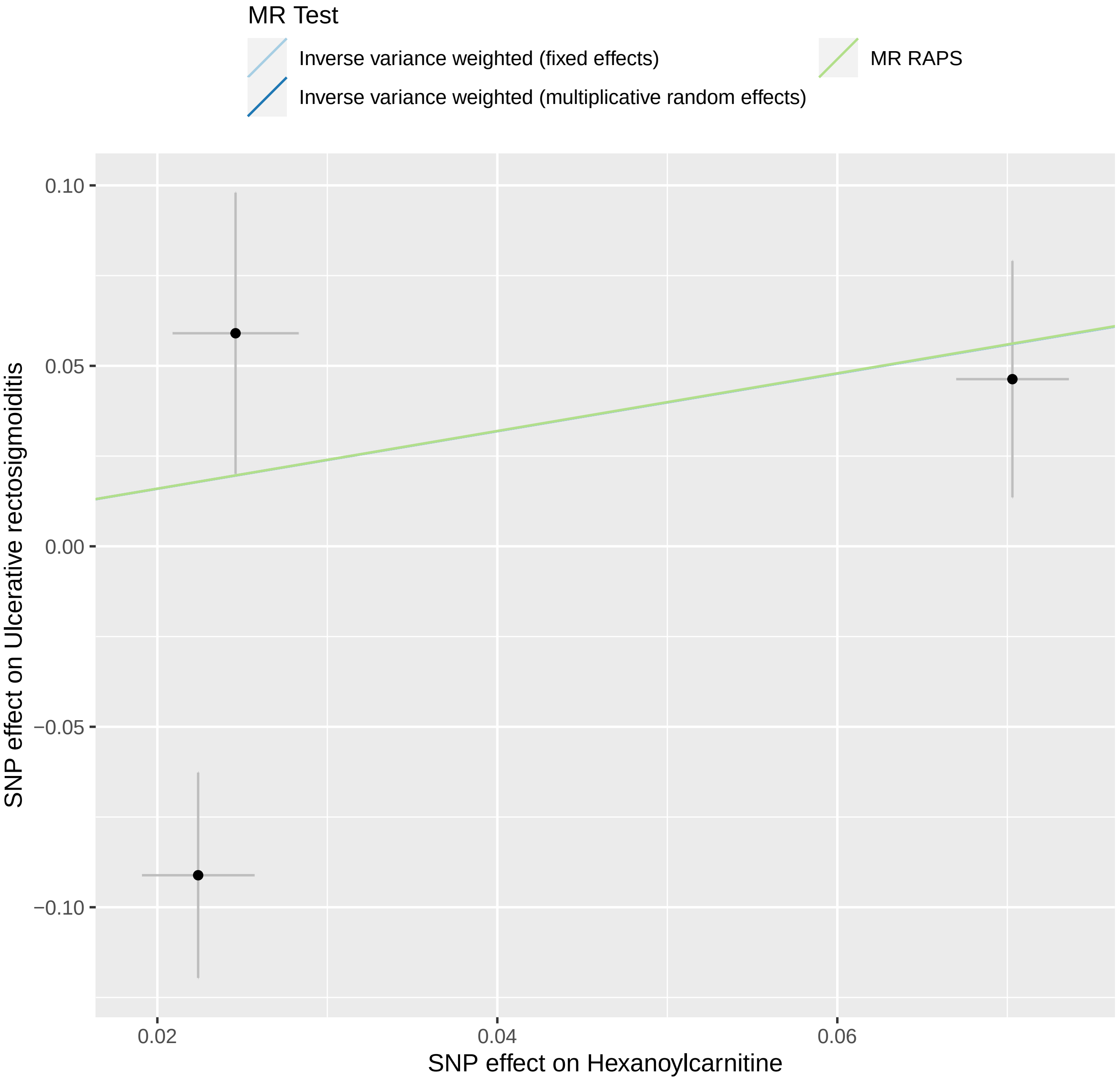

Batch 2619 : Butyrylcarnitine on Ulcerative rectosigmoiditis

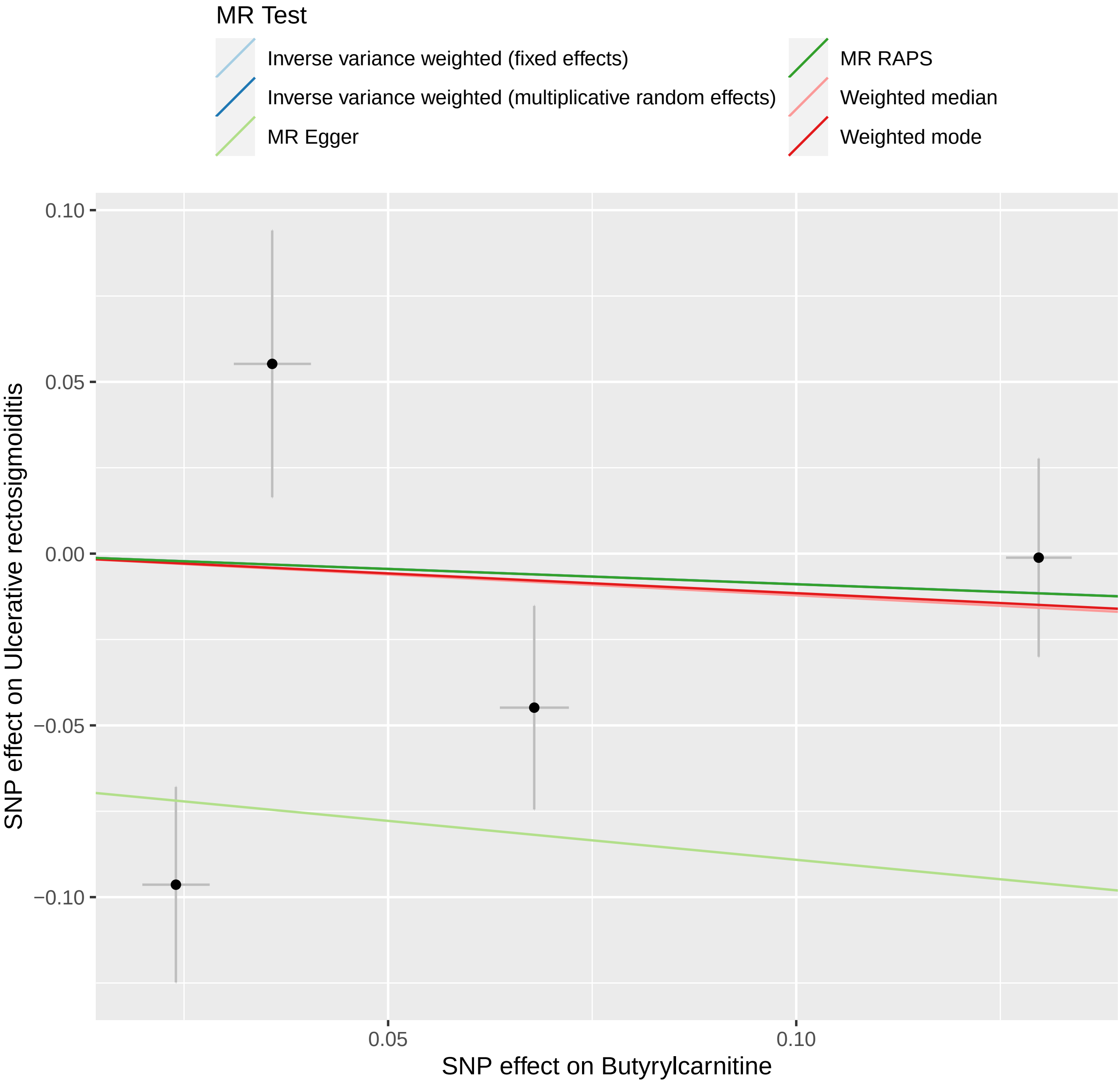

Batch 2622 : Propionylcarnitine on Ulcerative rectosigmoiditis

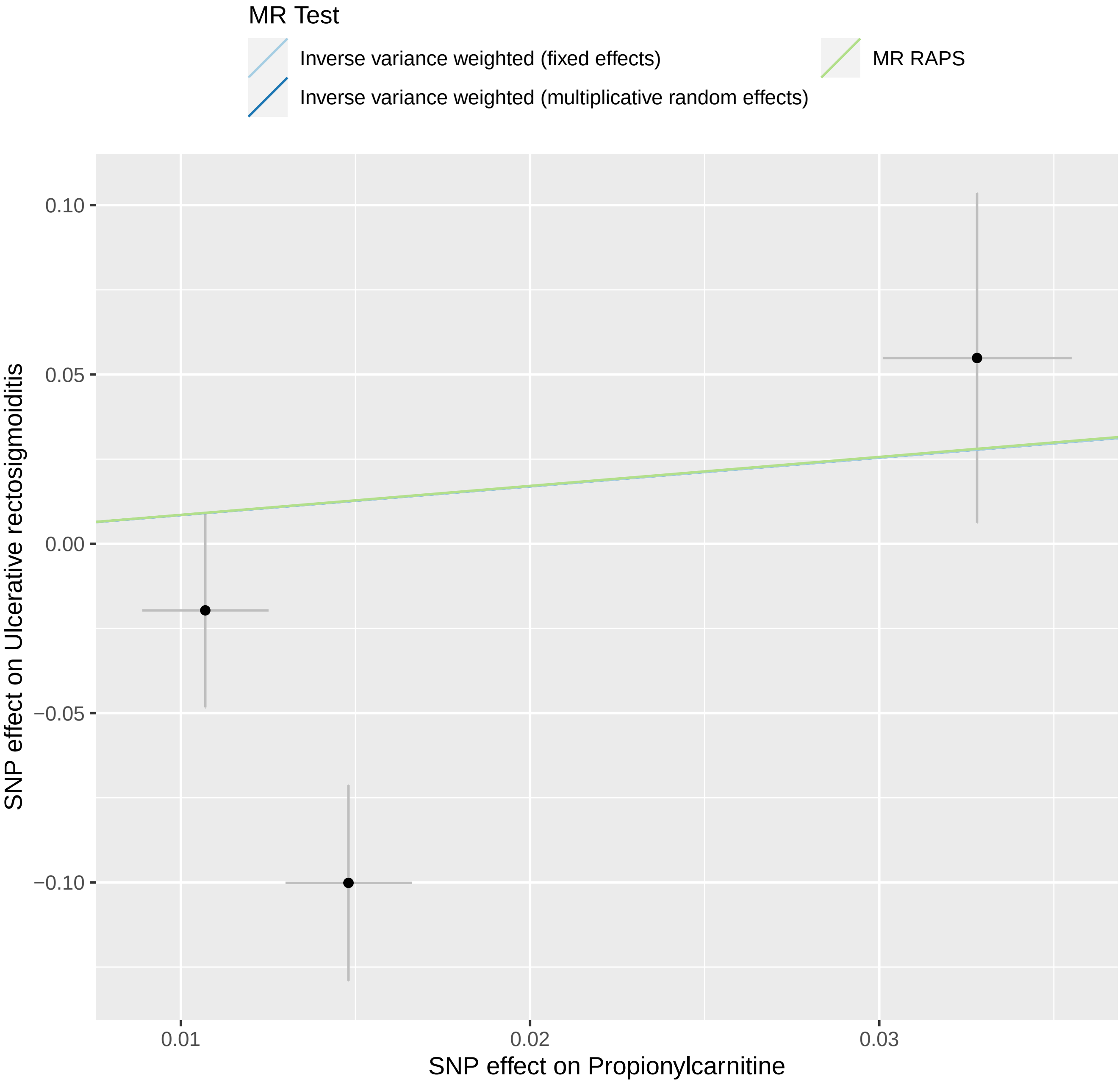

Batch 2623 : Caproate (6:0) on Ulcerative rectosigmoiditis

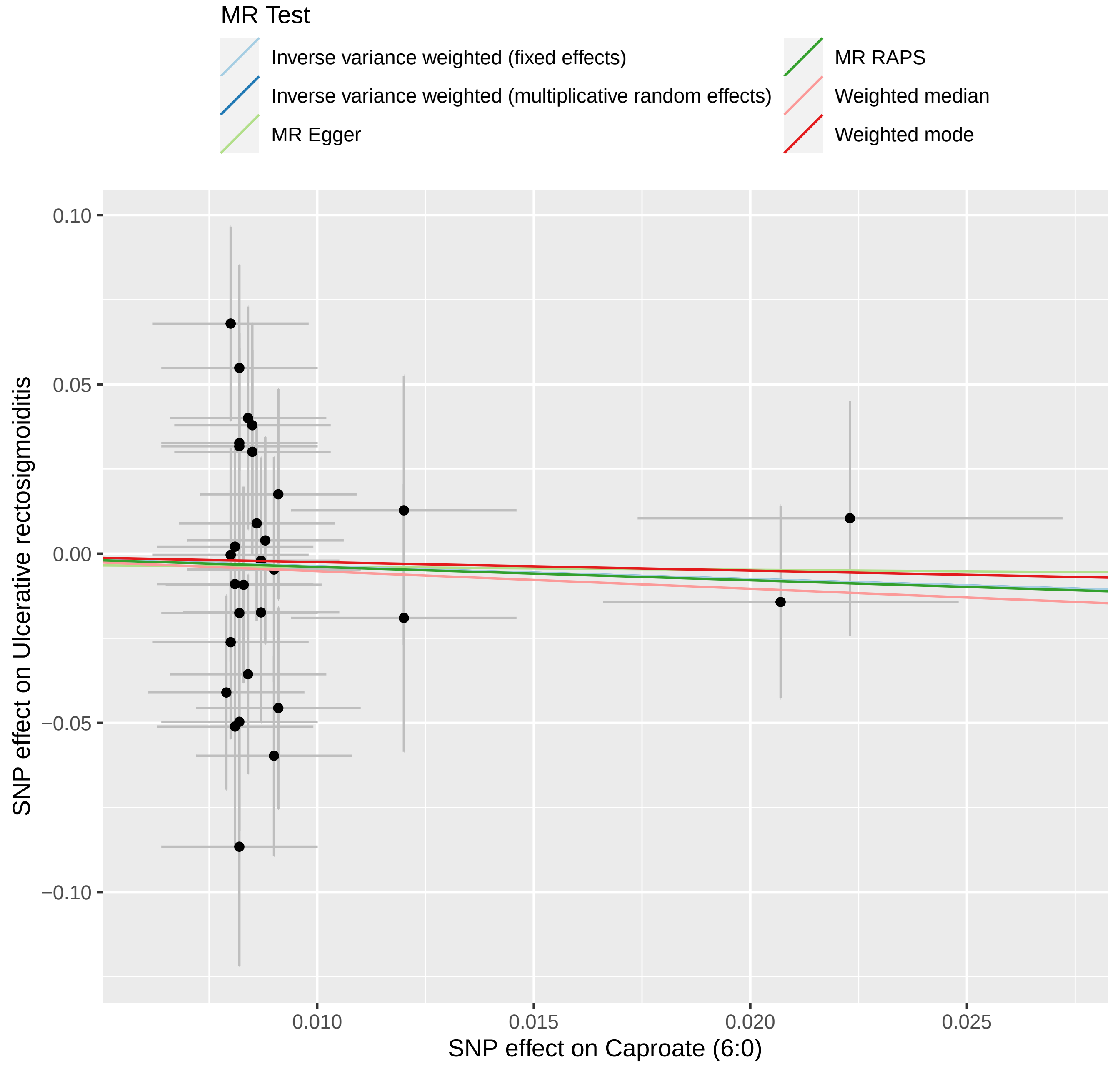

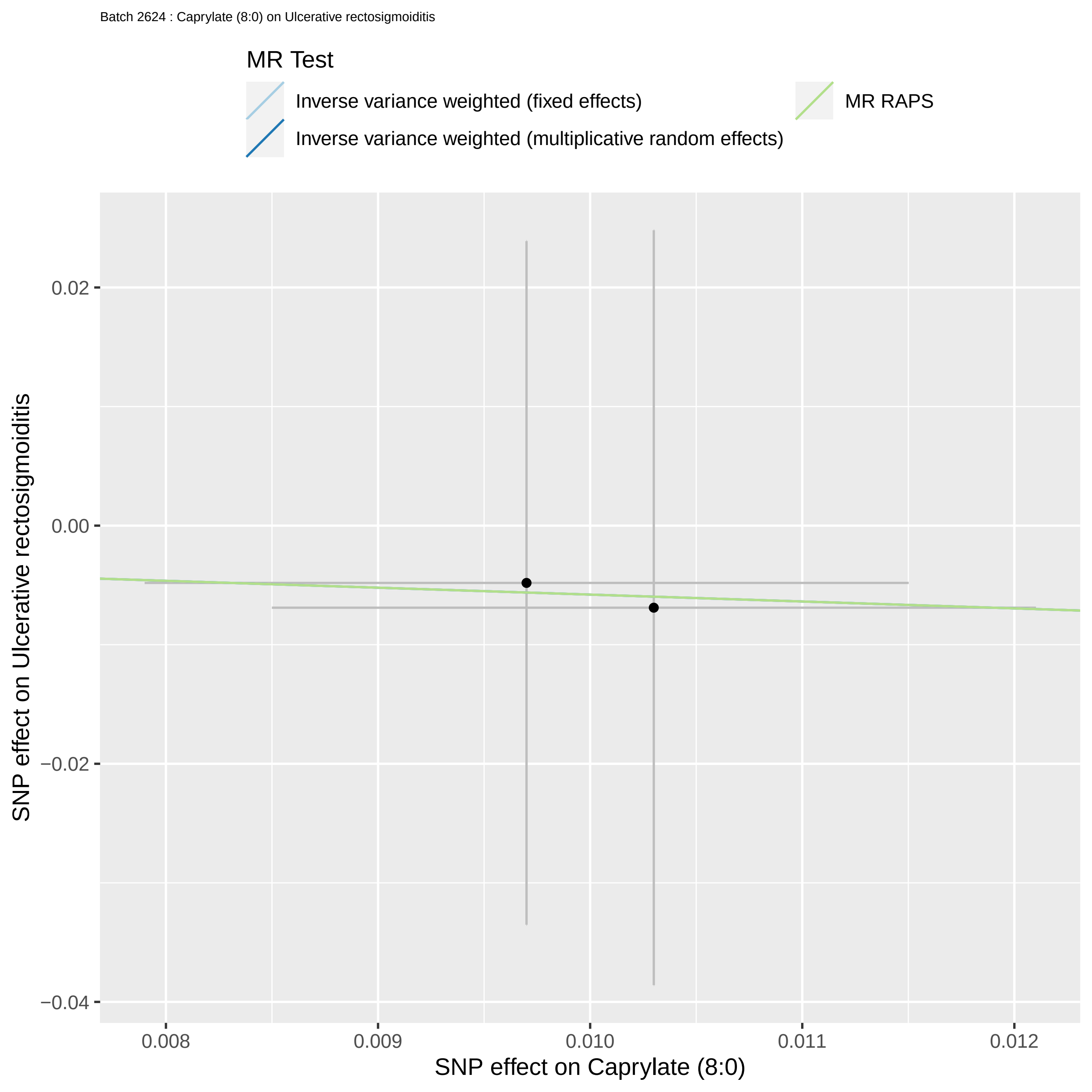

Batch 2624 : Caprylate (8:0) on Ulcerative rectosigmoiditis
MR Test
Inverse variance weighted (fixed effects)
Inverse variance weighted (multiplicative random effects)
MR RAPS
0.02
0.00
−0.02
−0.04
0.008
0.009
0.010
0.011
0.012
SNP effect on Caprylate (8:0)
SNP effect on Ulcerative rectosigmoiditis

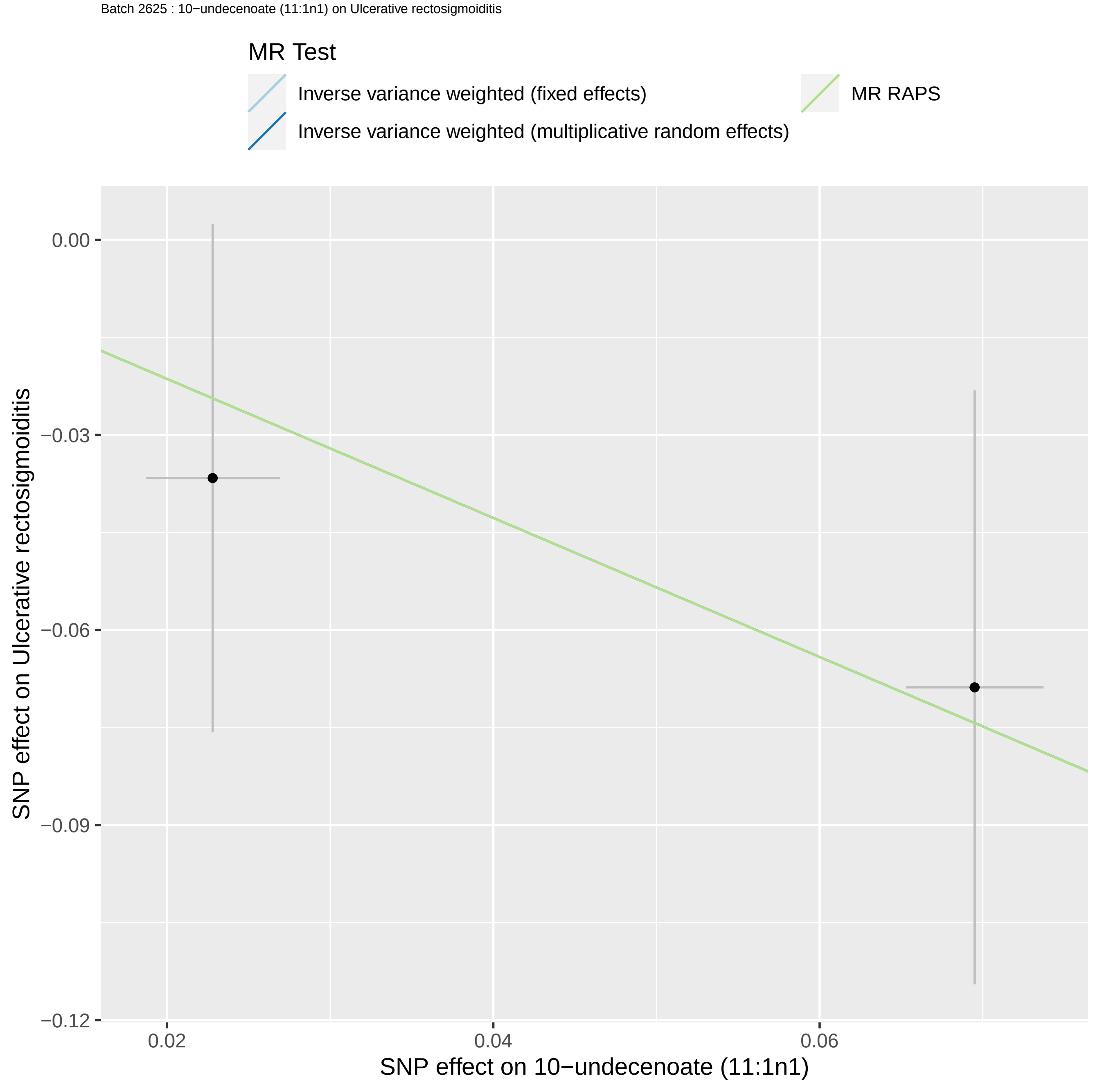

Batch 2625 : 10−undecenoate (11:1n1) on Ulcerative rectosigmoiditis
MR Test
Inverse variance weighted (fixed effects)
Inverse variance weighted (multiplicative random effects)
MR RAPS
0.00
−0.03
−0.06
−0.09
−0.12
0.02
0.04
0.06
SNP effect on Ulcerative rectosigmoiditis
SNP effect on 10−undecenoate (11:1n1)

Batch 2627 : Phenol sulfate on Ulcerative rectosigmoiditis

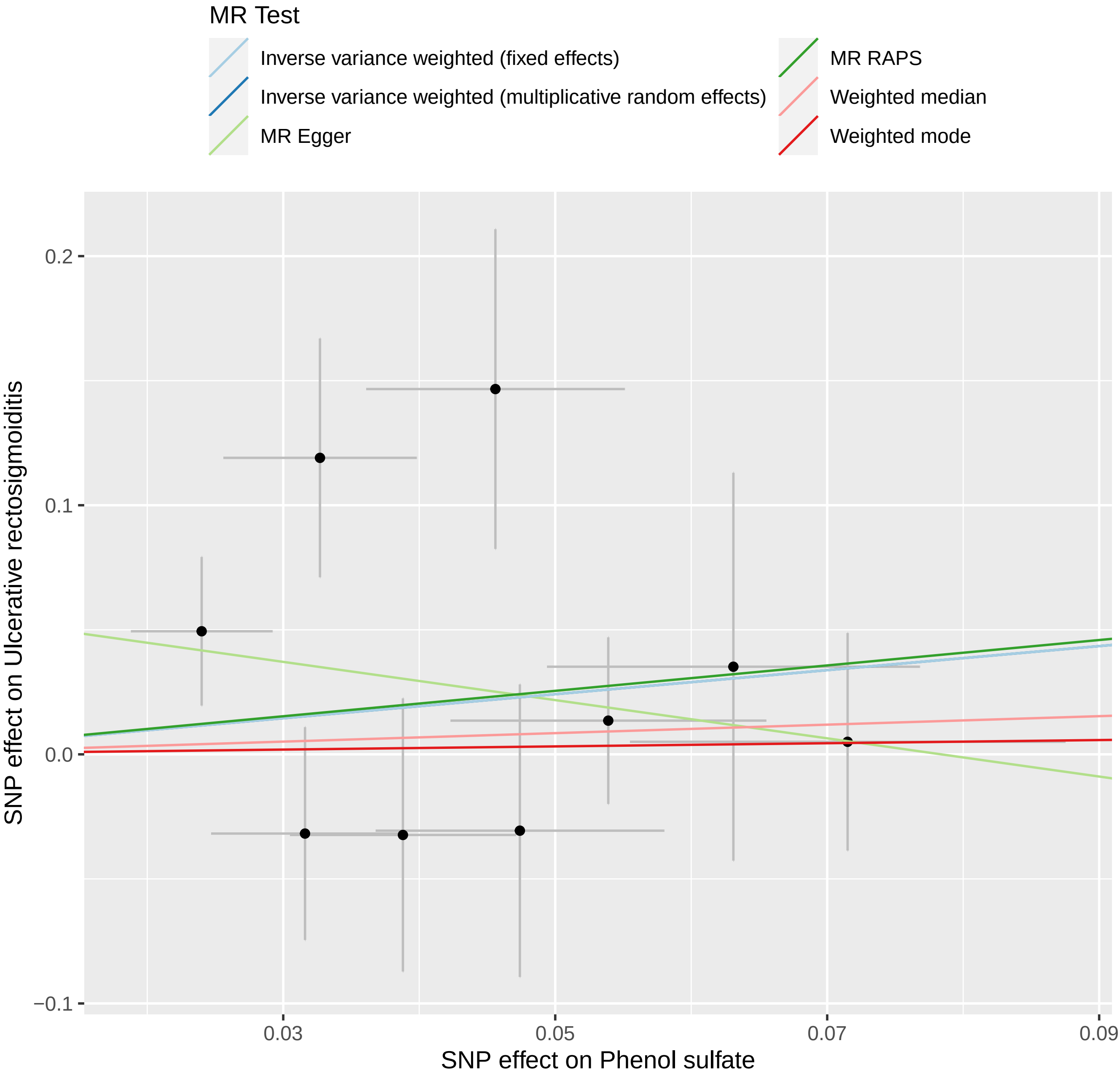

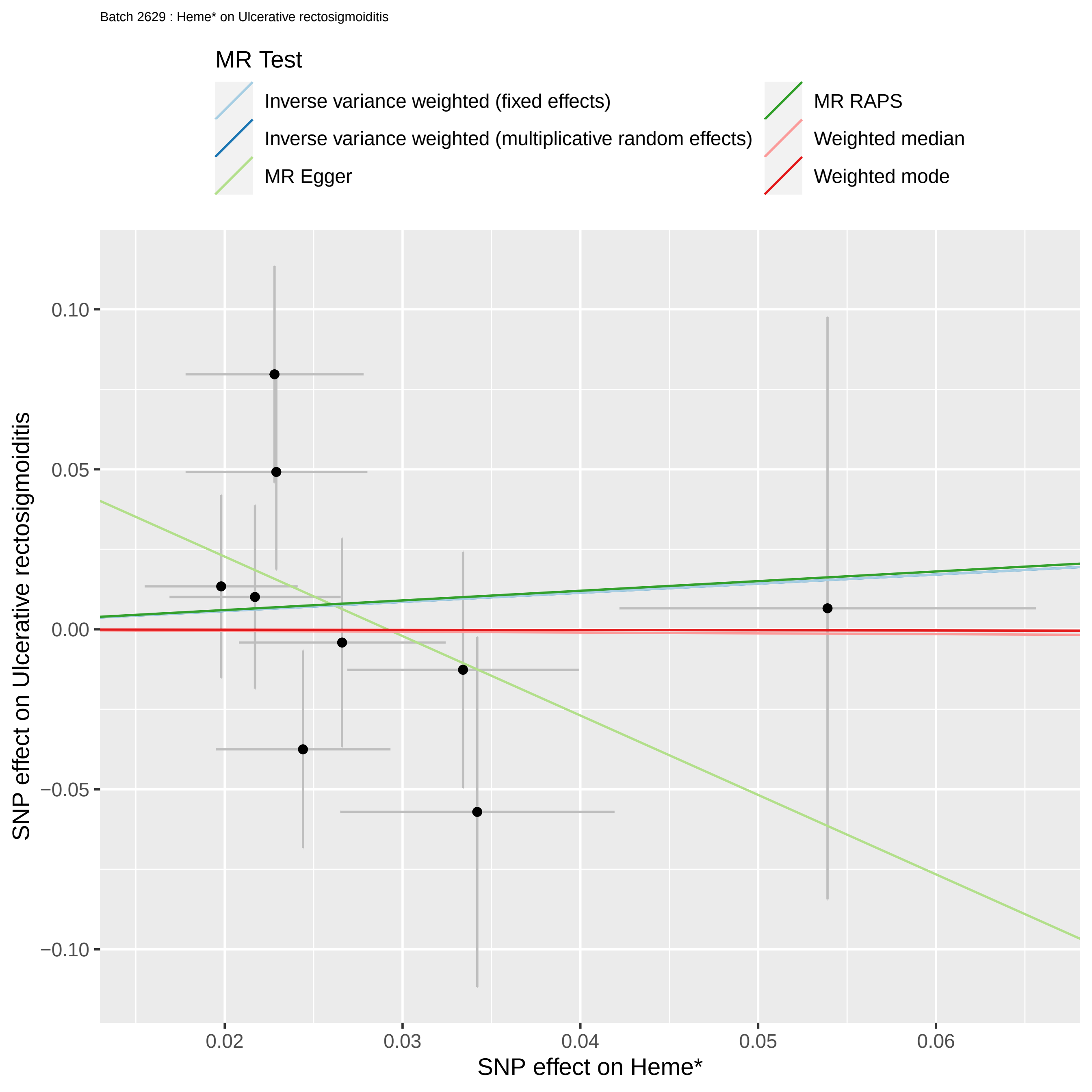

Batch 2629 : Heme* on Ulcerative rectosigmoiditis
MR Test
Inverse variance weighted (fixed effects)
Inverse variance weighted (multiplicative random effects)
MR Egger
MR RAPS
Weighted median
Weighted mode
SNP effect on Ulcerative rectosigmoiditis
0.10
0.05
0.00
−0.05
−0.10
0.02
0.03
0.04
0.05
0.06
SNP effect on Heme*

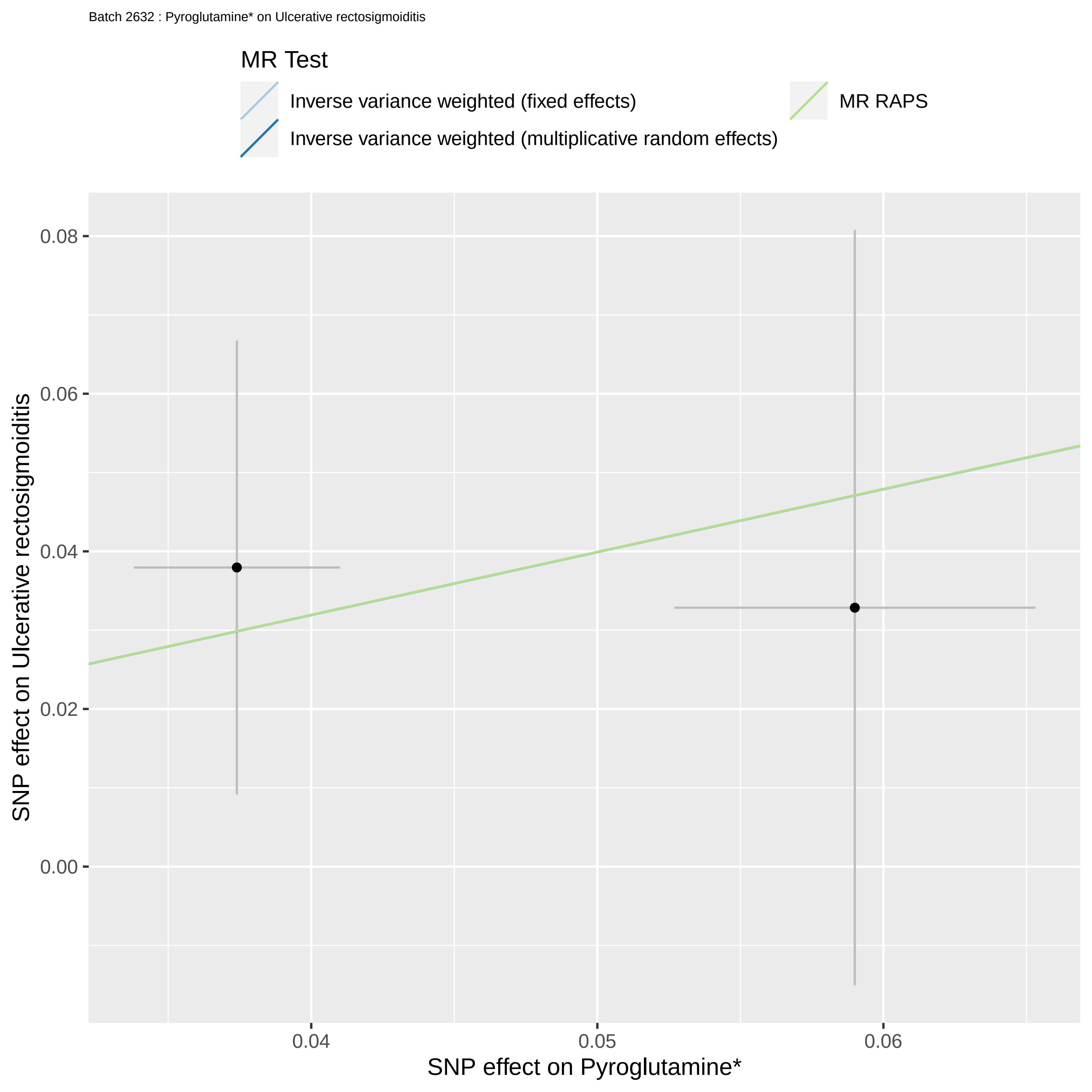
Batch 2632 : Pyroglutamine* on Ulcerative rectosigmoiditis
MR Test
Inverse variance weighted (fixed effects)
Inverse variance weighted (multiplicative random effects)
MR RAPS
0.08
0.06
0.04
0.02
0.00
0.04
0.05
0.06
SNP effect on Ulcerative rectosigmoiditis
SNP effect on Pyroglutamine*

Batch 2635 : X−11422−−xanthine on Ulcerative rectosigmoiditis

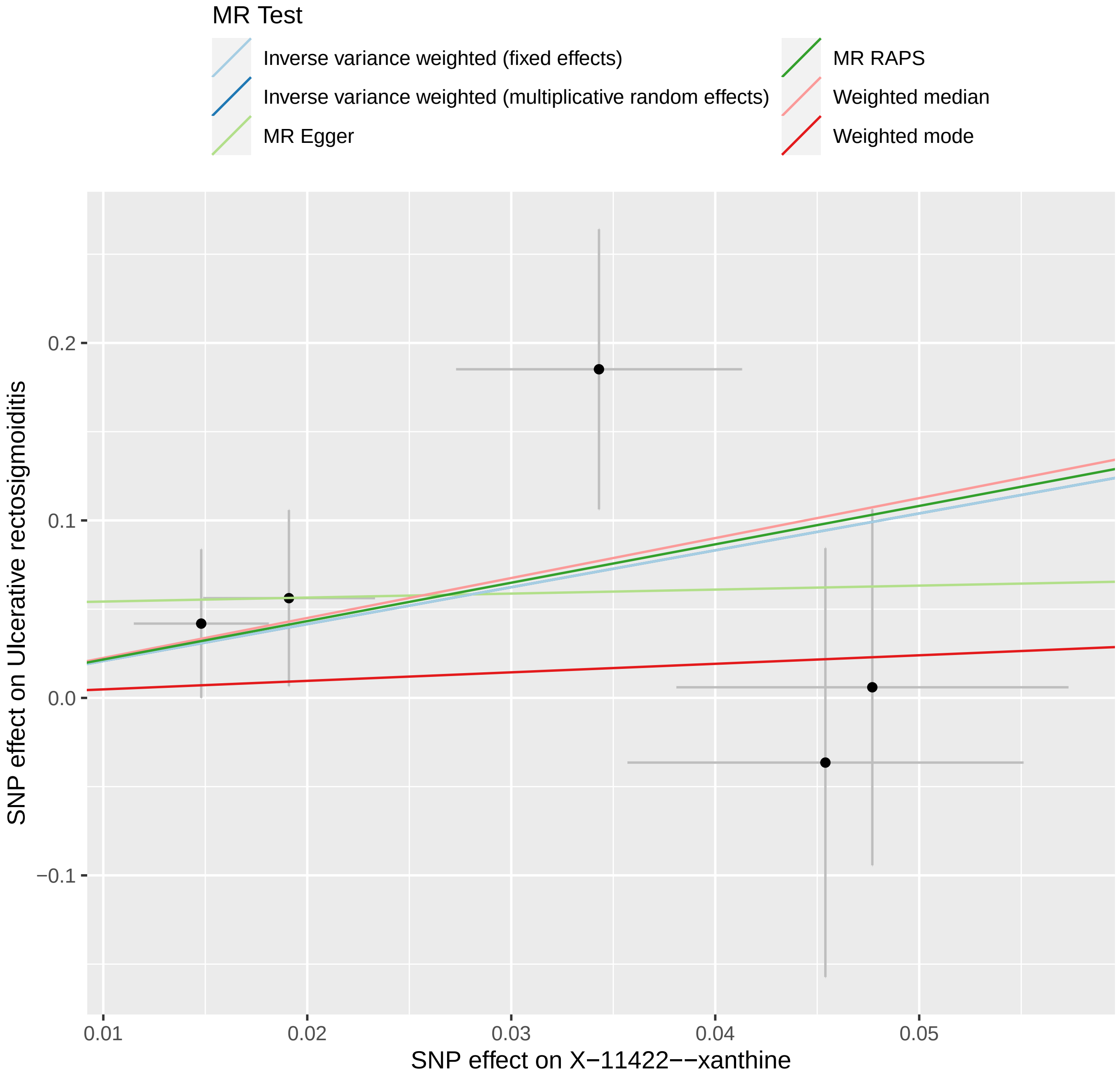

Batch 2638 : HWESASXX* on Ulcerative rectosigmoiditis

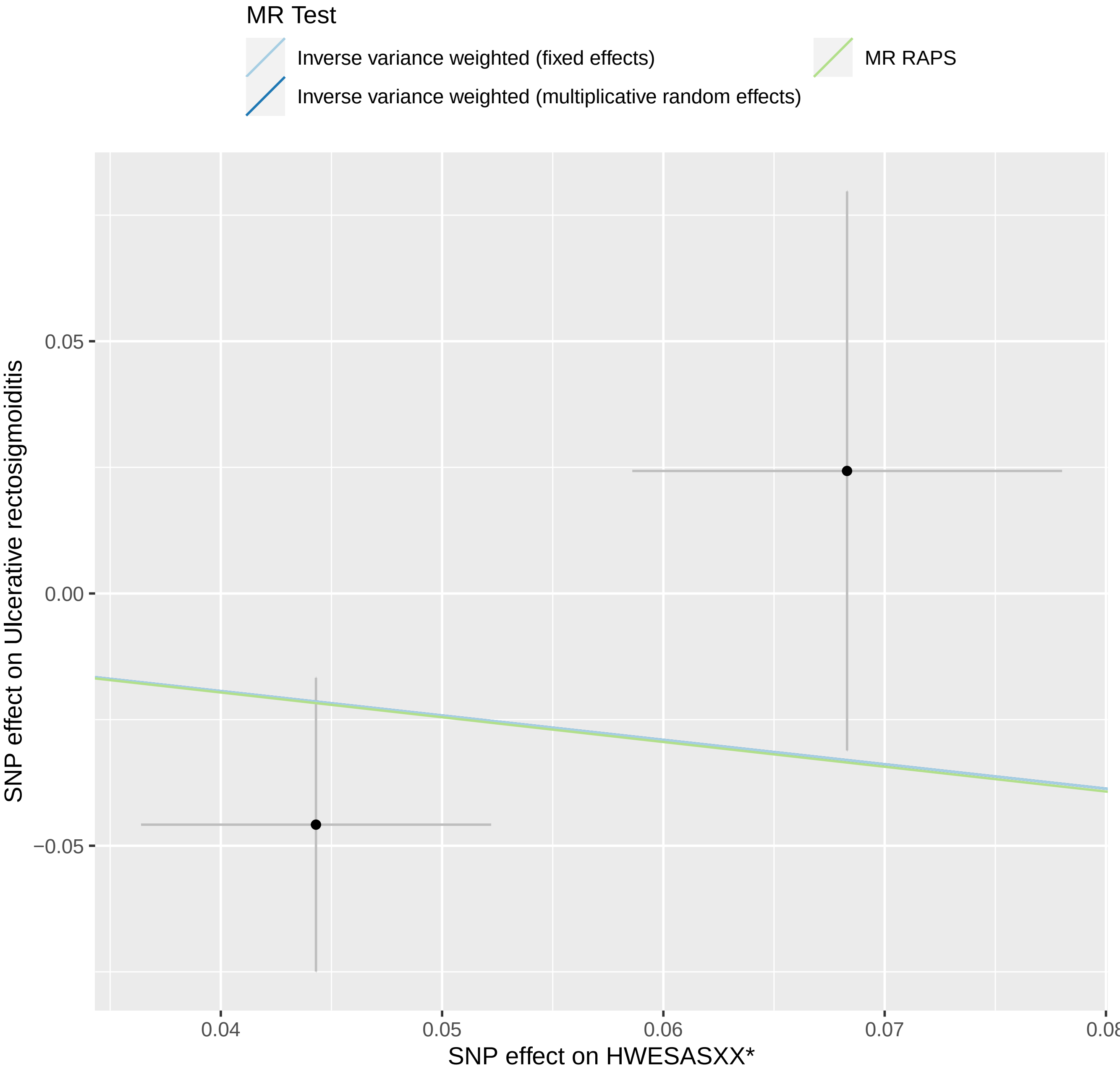

Batch 2639 : X−11593−−O−methylascorbate* on Ulcerative rectosigmoiditis

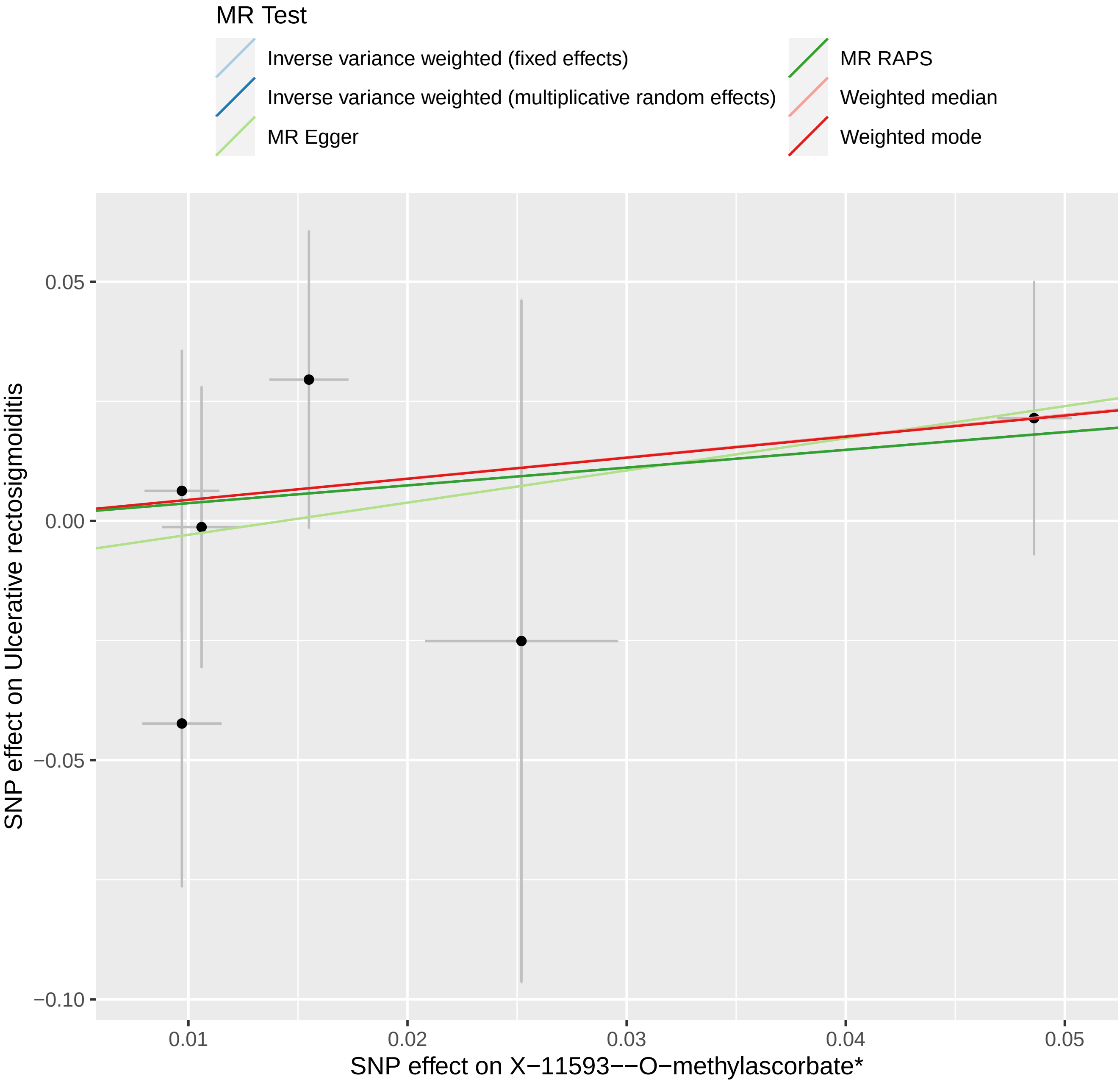

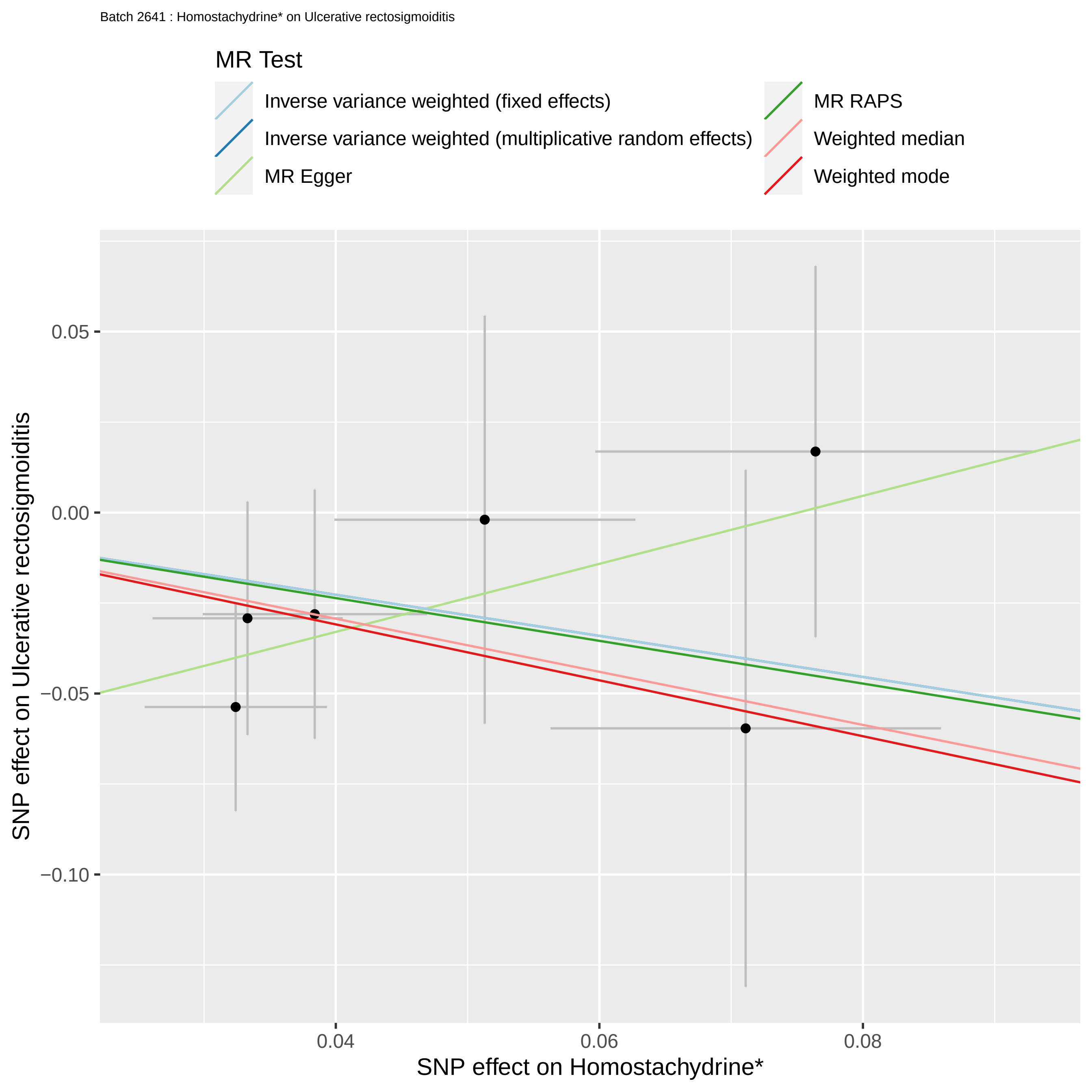

Batch 2641 : Homostachydrine* on Ulcerative rectosigmoiditis
MR Test
Inverse variance weighted (fixed effects)
Inverse variance weighted (multiplicative random effects)
MR Egger
MR RAPS
Weighted median
Weighted mode
0.05
0.00
−0.05
−0.10
0.04
0.06
0.08
SNP effect on Ulcerative rectosigmoiditis
SNP effect on Homostachydrine*

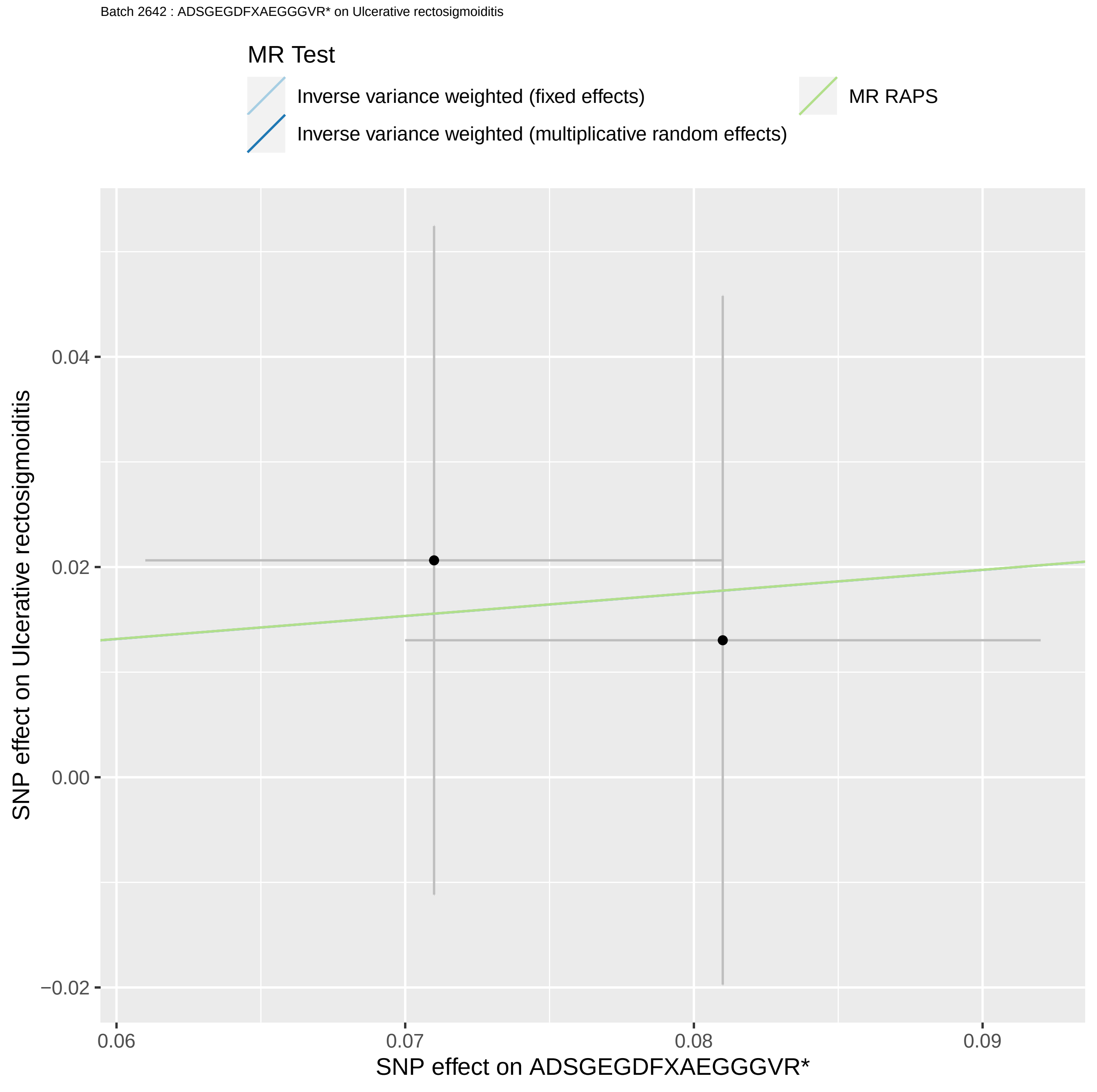
Batch 2642 : ADSGEGDFXAEGGGVR* on Ulcerative rectosigmoiditis
MR Test
Inverse variance weighted (fixed effects)
Inverse variance weighted (multiplicative random effects)
MR RAPS
0.04
0.02
0.00
−0.02
0.06
0.07
0.08
0.09
SNP effect on Ulcerative rectosigmoiditis
SNP effect on ADSGEGDFXAEGGGVR*

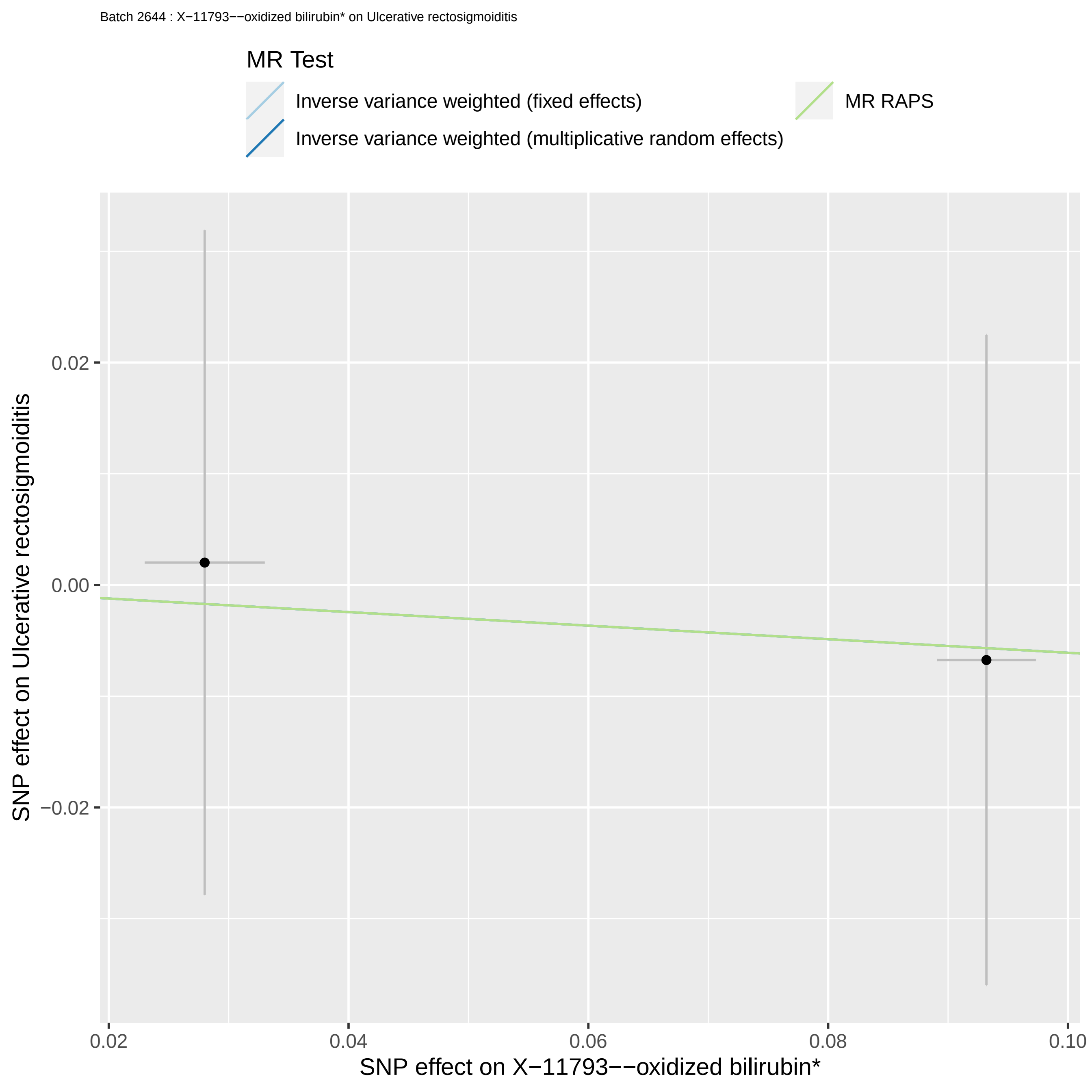

Batch 2644 : X−11793−−oxidized bilirubin* on Ulcerative rectosigmoiditis
MR Test
Inverse variance weighted (fixed effects)
Inverse variance weighted (multiplicative random effects)
MR RAPS
0.02
0.00
−0.02
SNP effect on Ulcerative rectosigmoiditis
0.02
0.04
0.06
0.08
0.10
SNP effect on X−11793−−oxidized bilirubin*

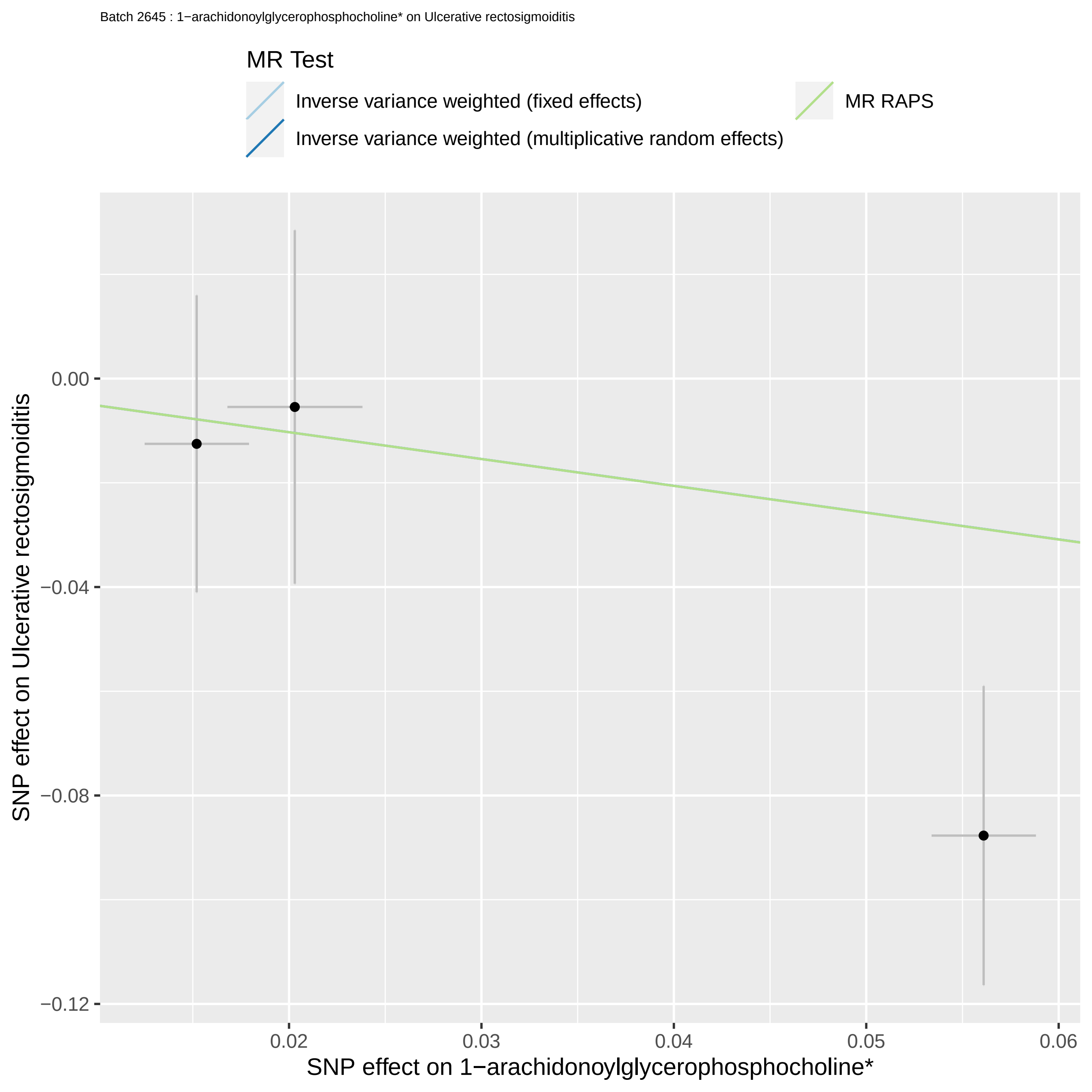
Batch 2645 : 1−arachidonoylglycerophosphocholine* on Ulcerative rectosigmoiditis
MR Test
Inverse variance weighted (fixed effects)
Inverse variance weighted (multiplicative random effects)
MR RAPS
0.00
−0.04
−0.08
−0.12
0.02
0.03
0.04
0.05
0.06
SNP effect on Ulcerative rectosigmoiditis
SNP effect on 1−arachidonoylglycerophosphocholine*

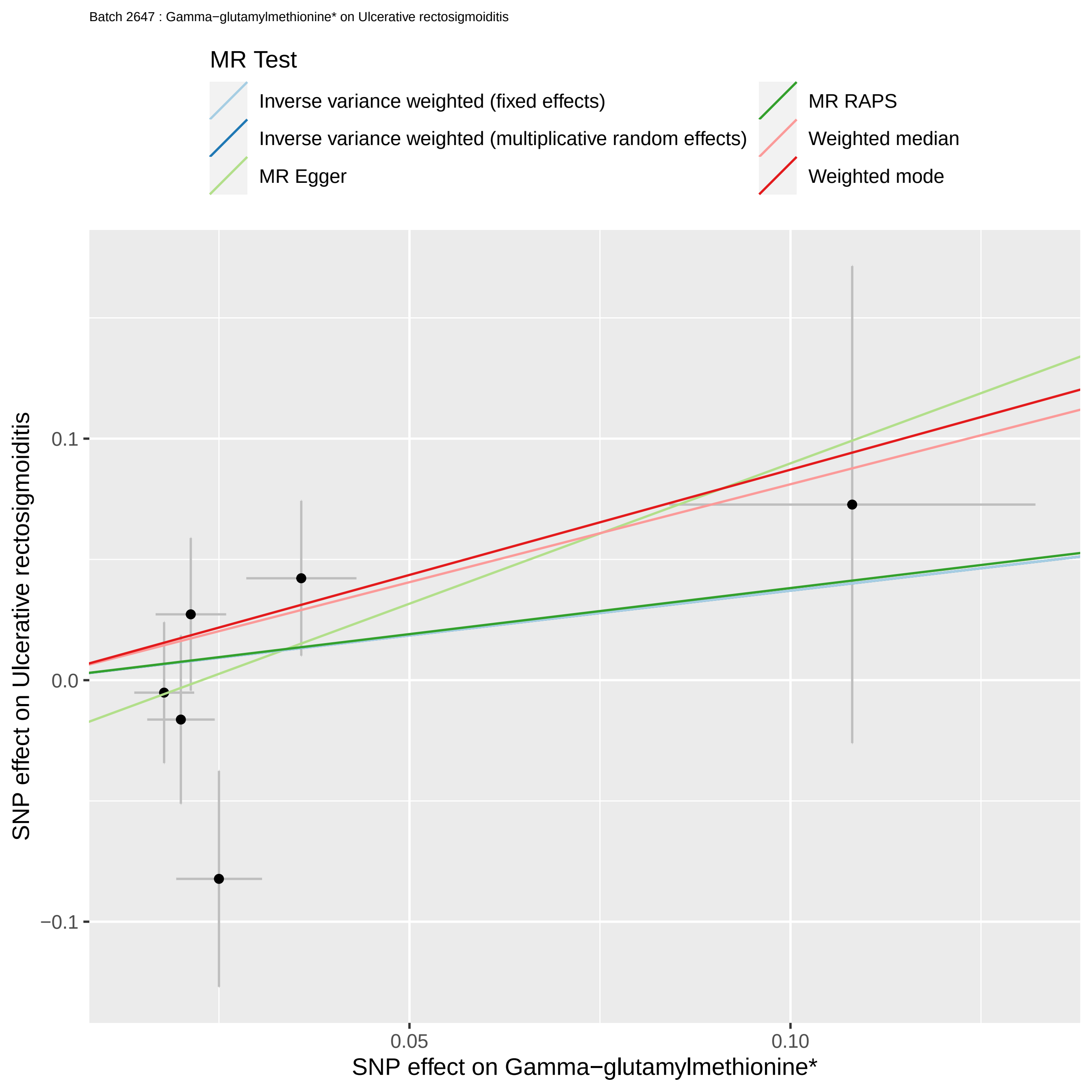

Batch 2647 : Gamma−glutamylmethionine* on Ulcerative rectosigmoiditis
MR Test
Inverse variance weighted (fixed effects)
Inverse variance weighted (multiplicative random effects)
MR Egger
MR RAPS
Weighted median
Weighted mode
0.1
0.0
−0.1
0.05
0.10
SNP effect on Gamma−glutamylmethionine*
SNP effect on Ulcerative rectosigmoiditis

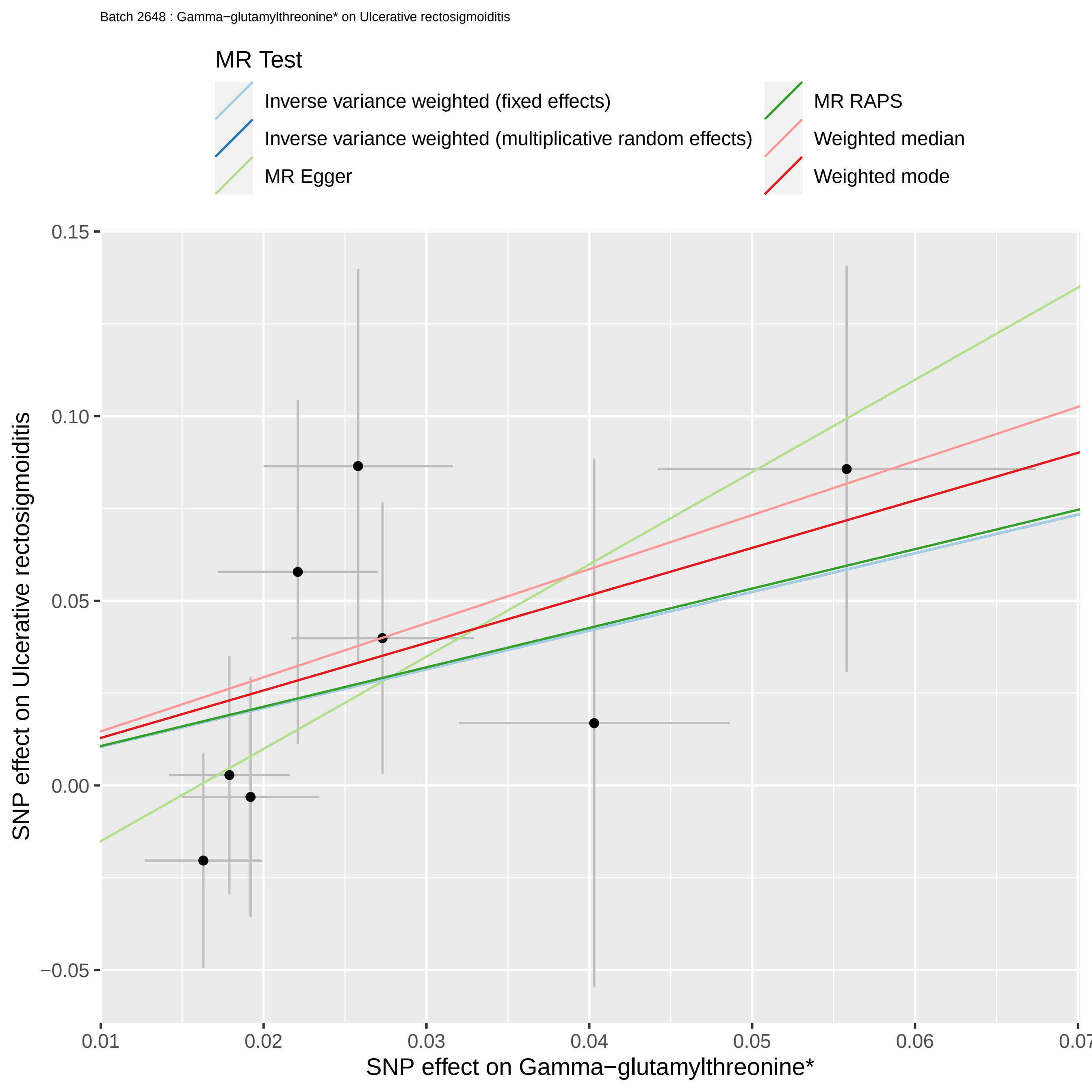

Batch 2648 : Gamma−glutamylthreonine* on Ulcerative rectosigmoiditis
MR Test
Inverse variance weighted (fixed effects)
Inverse variance weighted (multiplicative random effects)
MR Egger
MR RAPS
Weighted median
Weighted mode
0.15
0.10
0.05
0.00
−0.05
0.01
0.02
0.03
0.04
0.05
0.06
0.07
SNP effect on Ulcerative rectosigmoiditis
SNP effect on Gamma−glutamylthreonine*

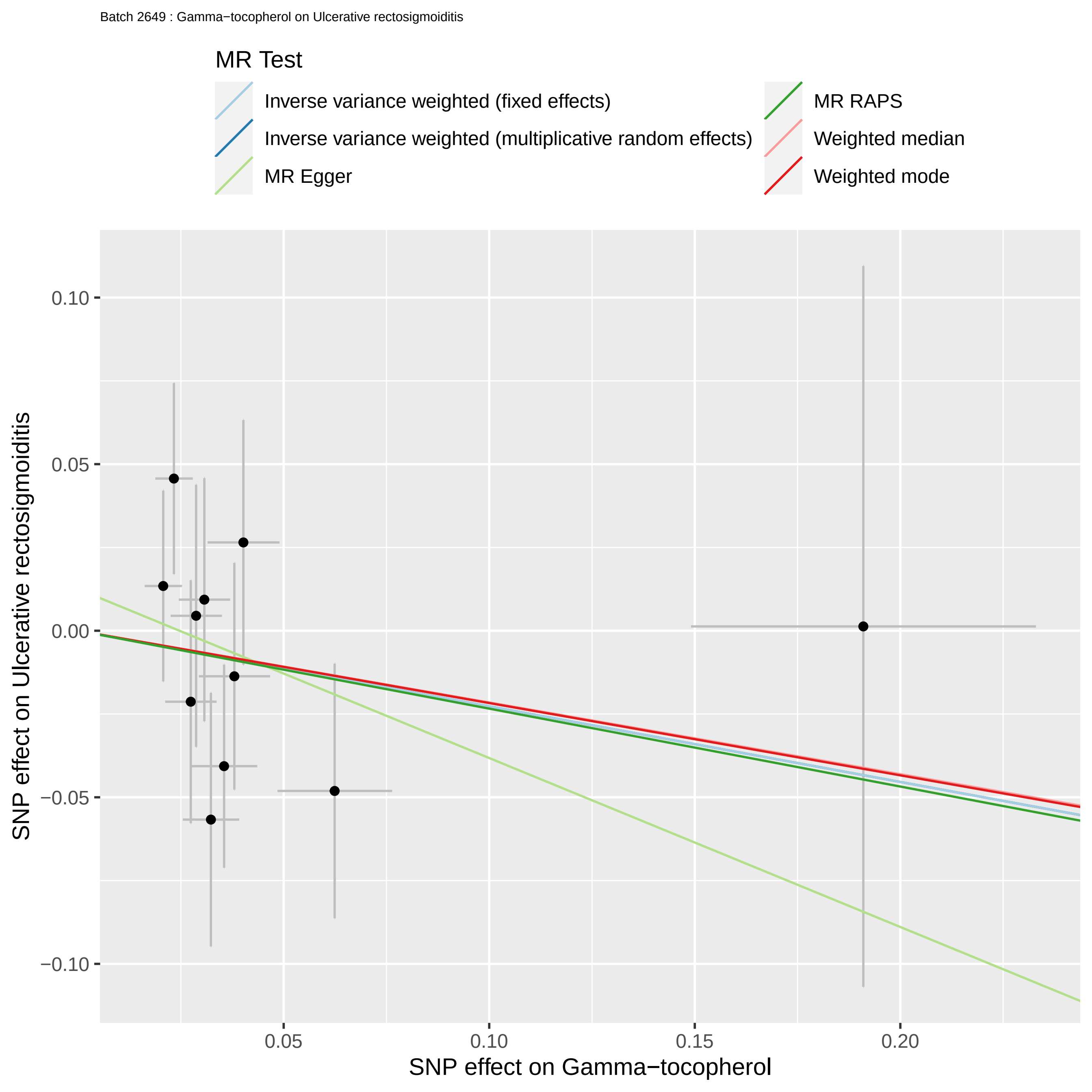

Batch 2649 : Gamma−tocopherol on Ulcerative rectosigmoiditis
MR Test
Inverse variance weighted (fixed effects)
Inverse variance weighted (multiplicative random effects)
MR Egger
MR RAPS
Weighted median
Weighted mode
0.10
0.05
0.00
−0.05
−0.10
SNP effect on Ulcerative rectosigmoiditis
0.05
0.10
0.15
0.20
SNP effect on Gamma−tocopherol

Batch 2650 : Gamma−glutamylphenylalanine on Ulcerative rectosigmoiditis

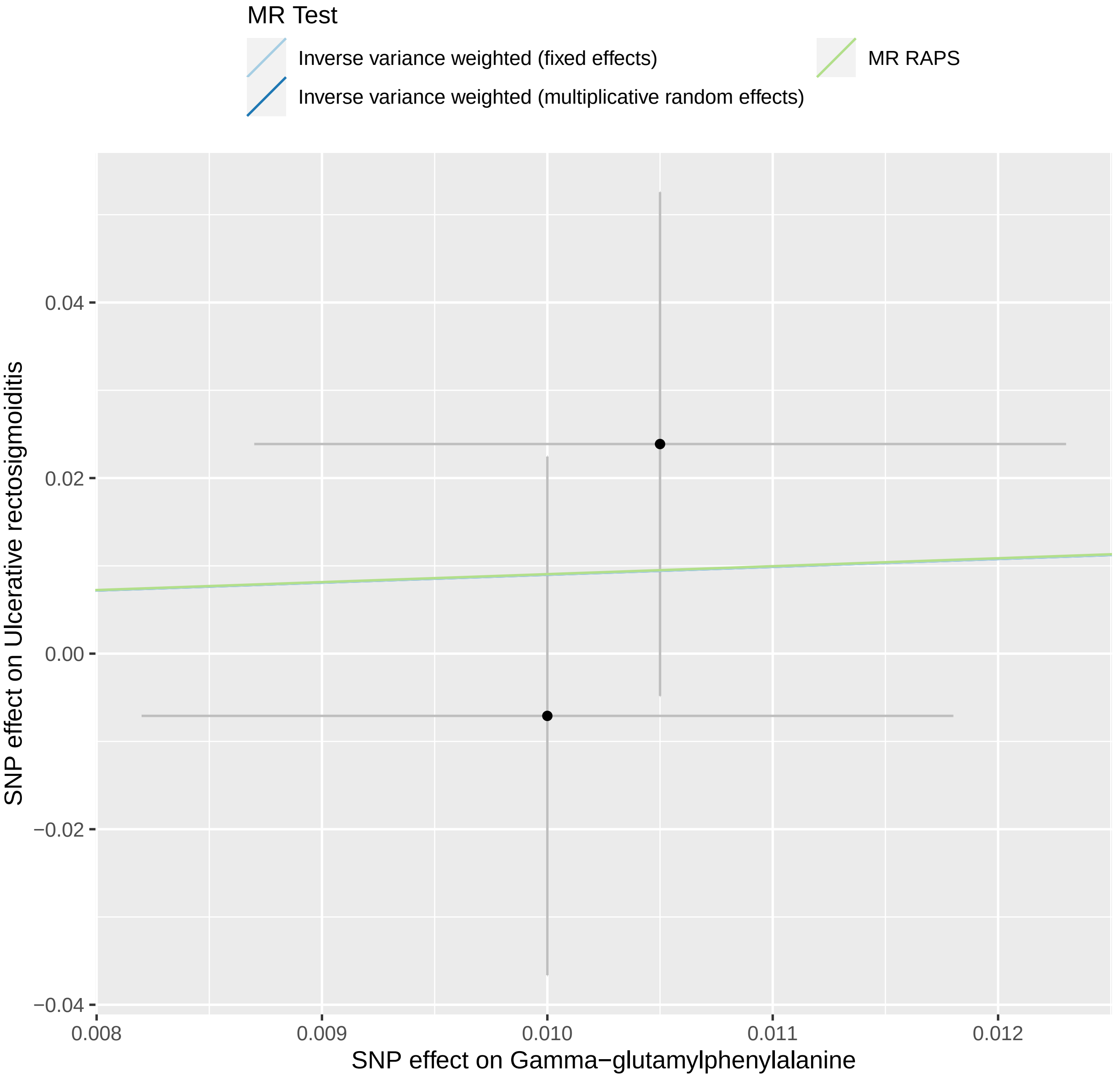

Batch 2651 : Isobutyrylcarnitine on Ulcerative rectosigmoiditis

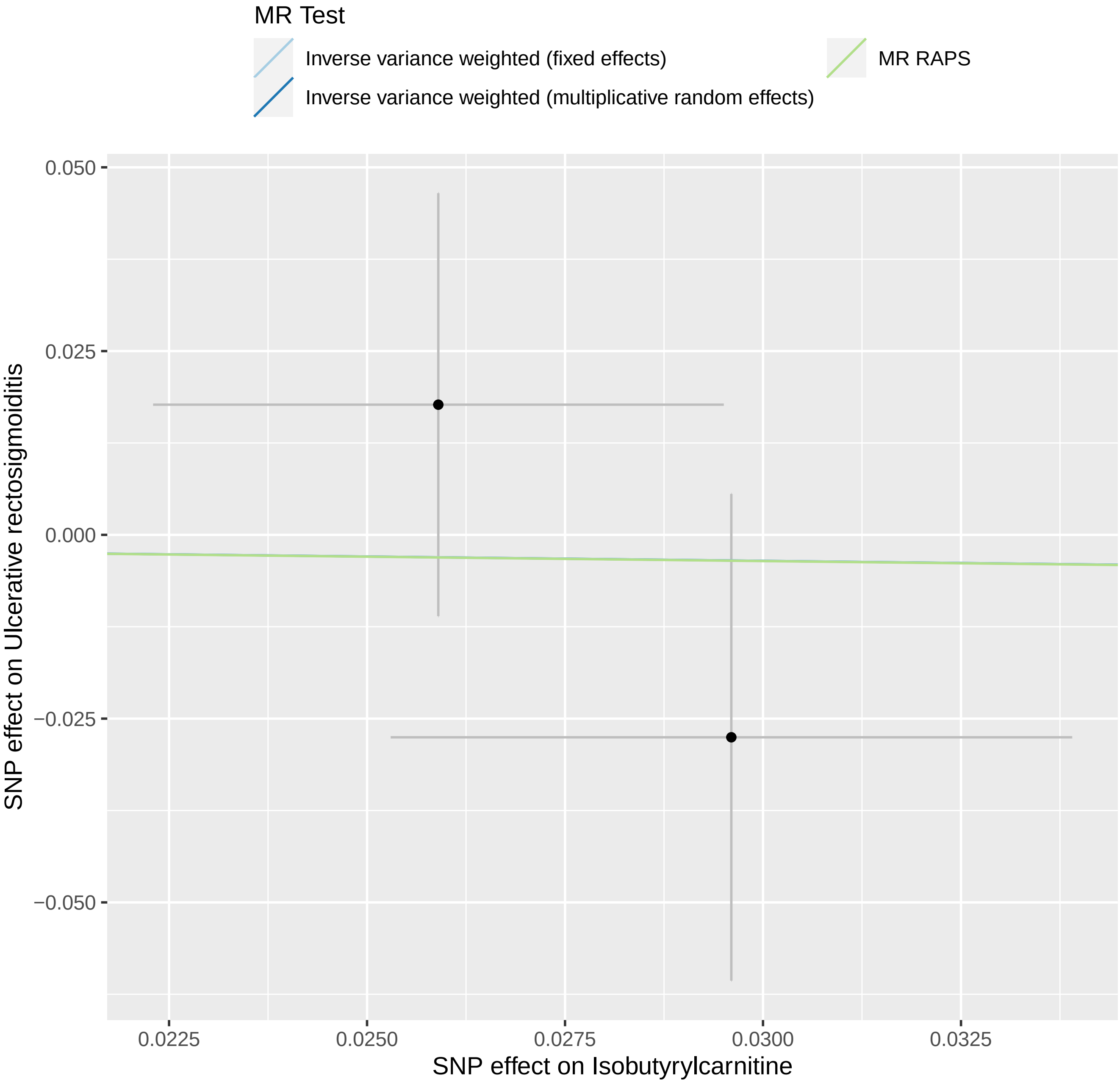

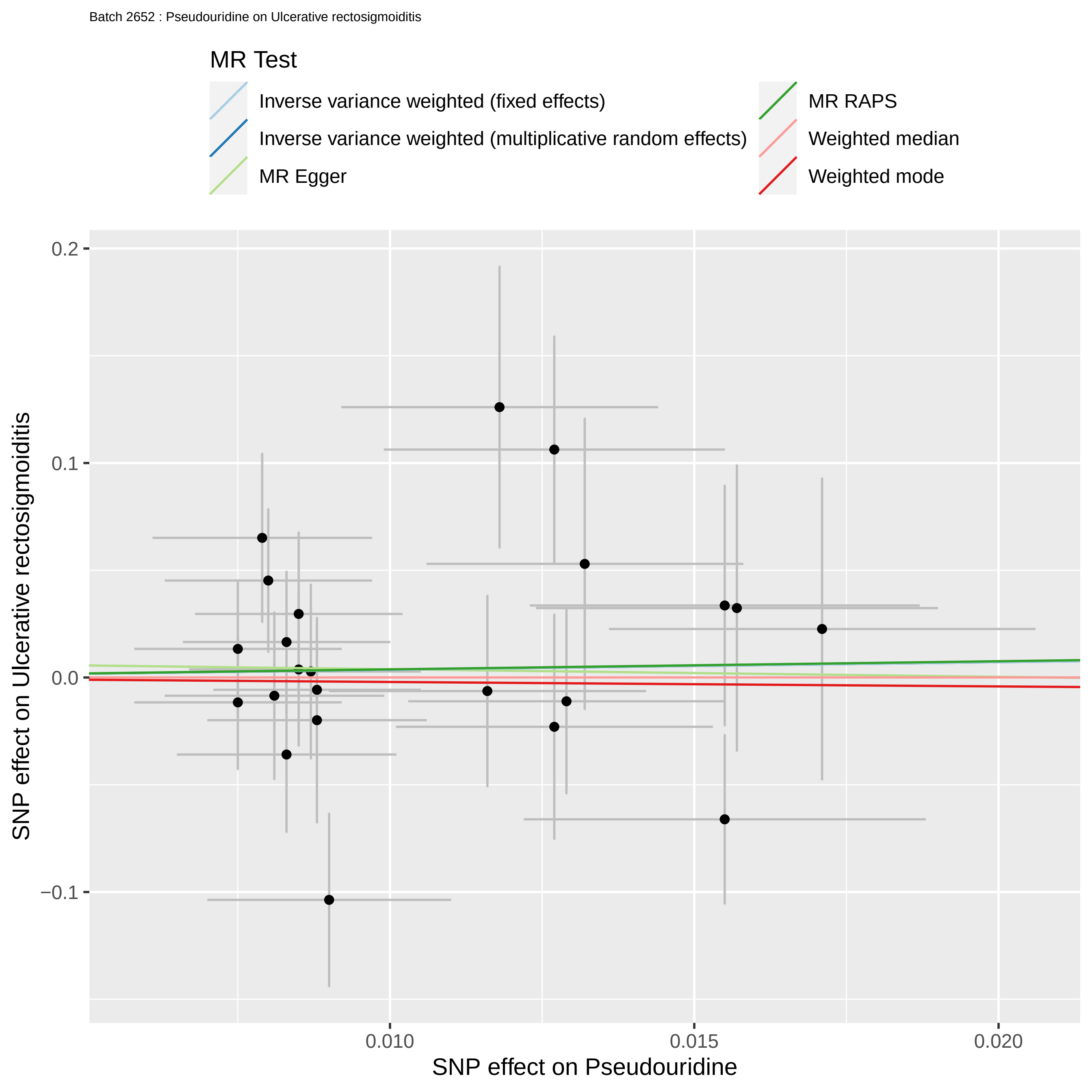
Batch 2652 : Pseudouridine on Ulcerative rectosigmoiditis
MR Test
Inverse variance weighted (fixed effects)
Inverse variance weighted (multiplicative random effects)
MR Egger
MR RAPS
Weighted median
Weighted mode
0.2
0.1
0.0
−0.1
0.010
0.015
0.020
SNP effect on Pseudouridine
SNP effect on Ulcerative rectosigmoiditis

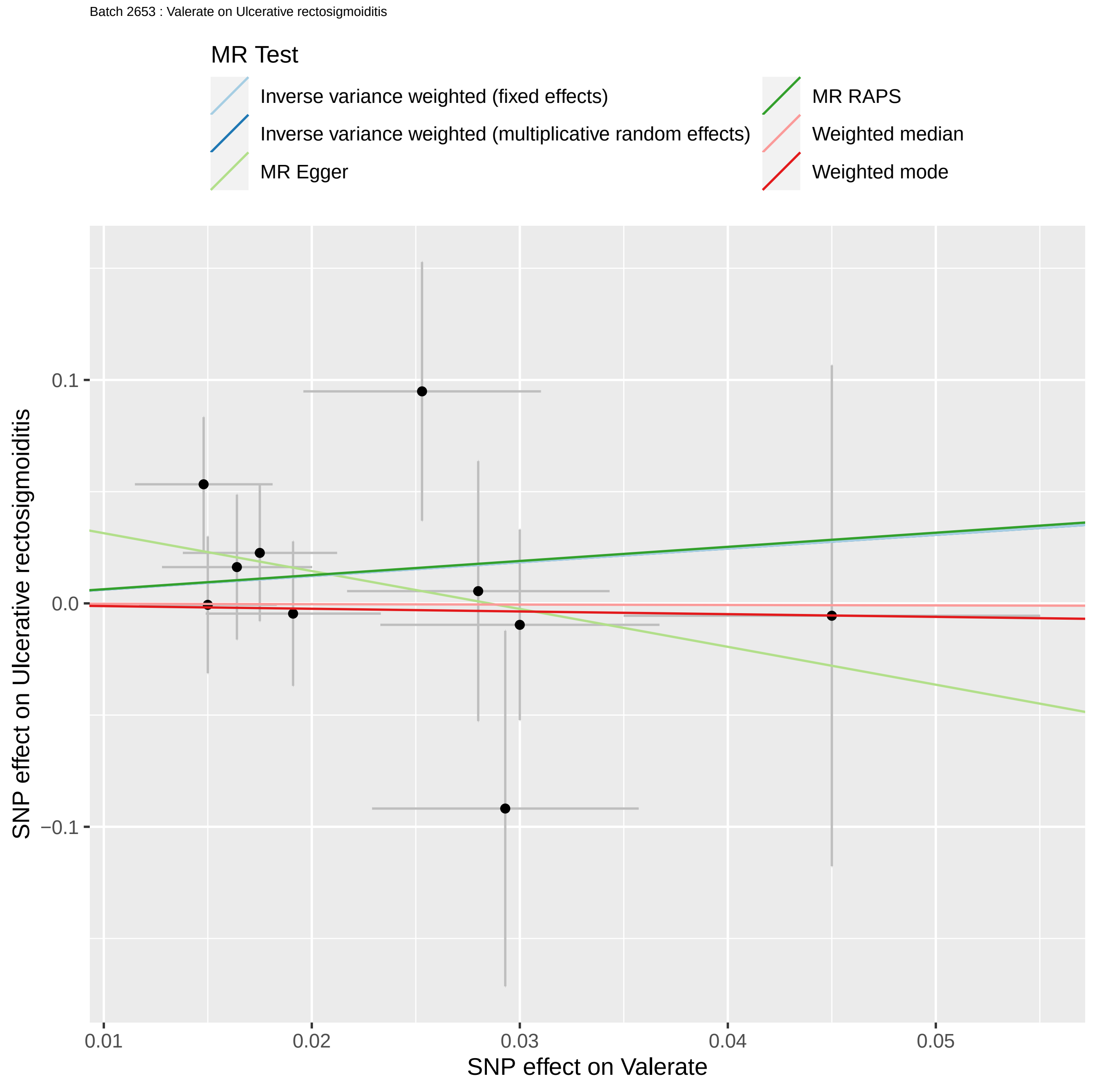
Batch 2653 : Valerate on Ulcerative rectosigmoiditis
MR Test
Inverse variance weighted (fixed effects)
Inverse variance weighted (multiplicative random effects)
MR Egger
MR RAPS
Weighted median
Weighted mode
0.1
0.0
−0.1
0.01
0.02
0.03
0.04
0.05
SNP effect on Ulcerative rectosigmoiditis
SNP effect on Valerate

Batch 2656 : Erythronate* on Ulcerative rectosigmoiditis

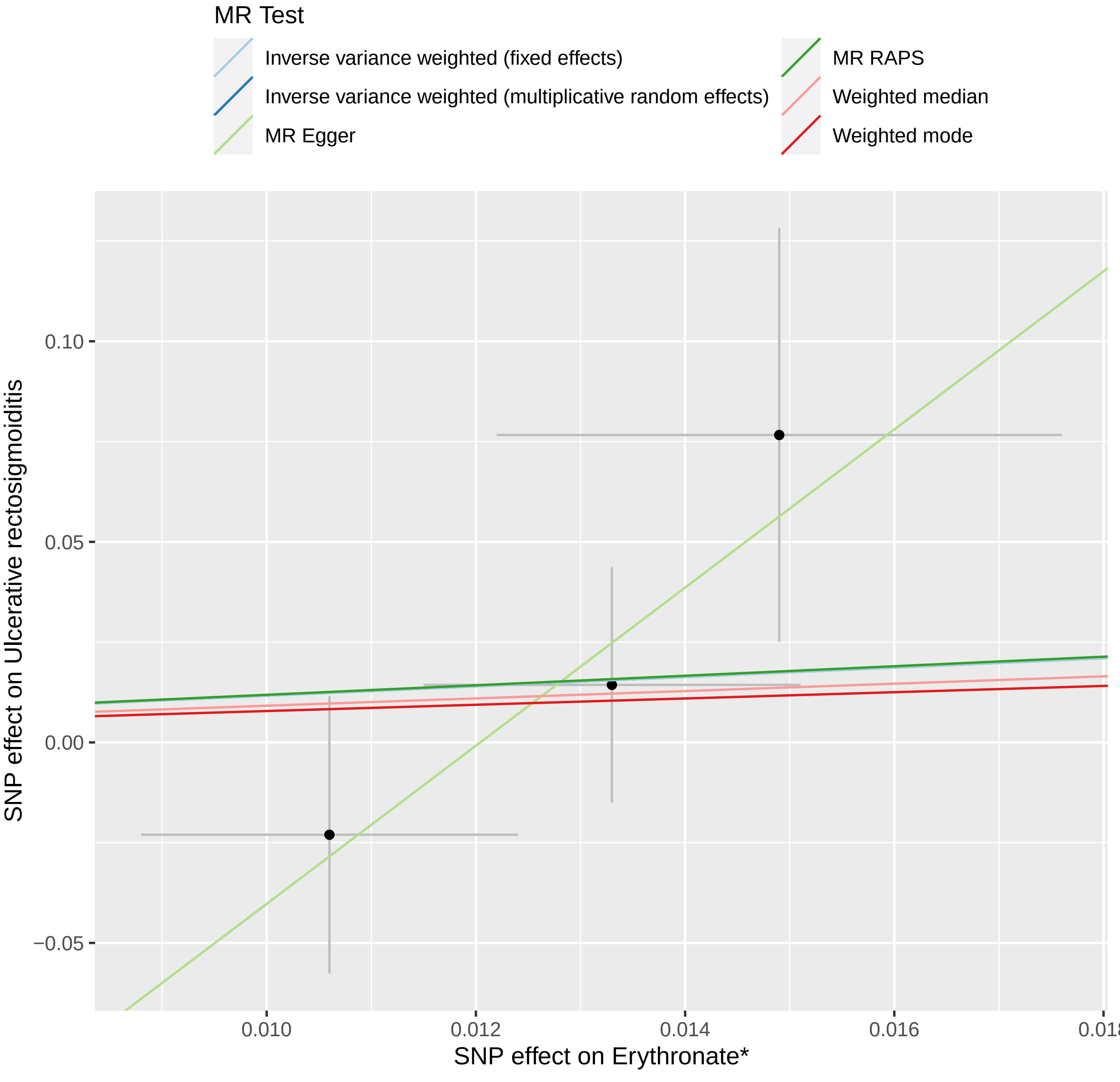

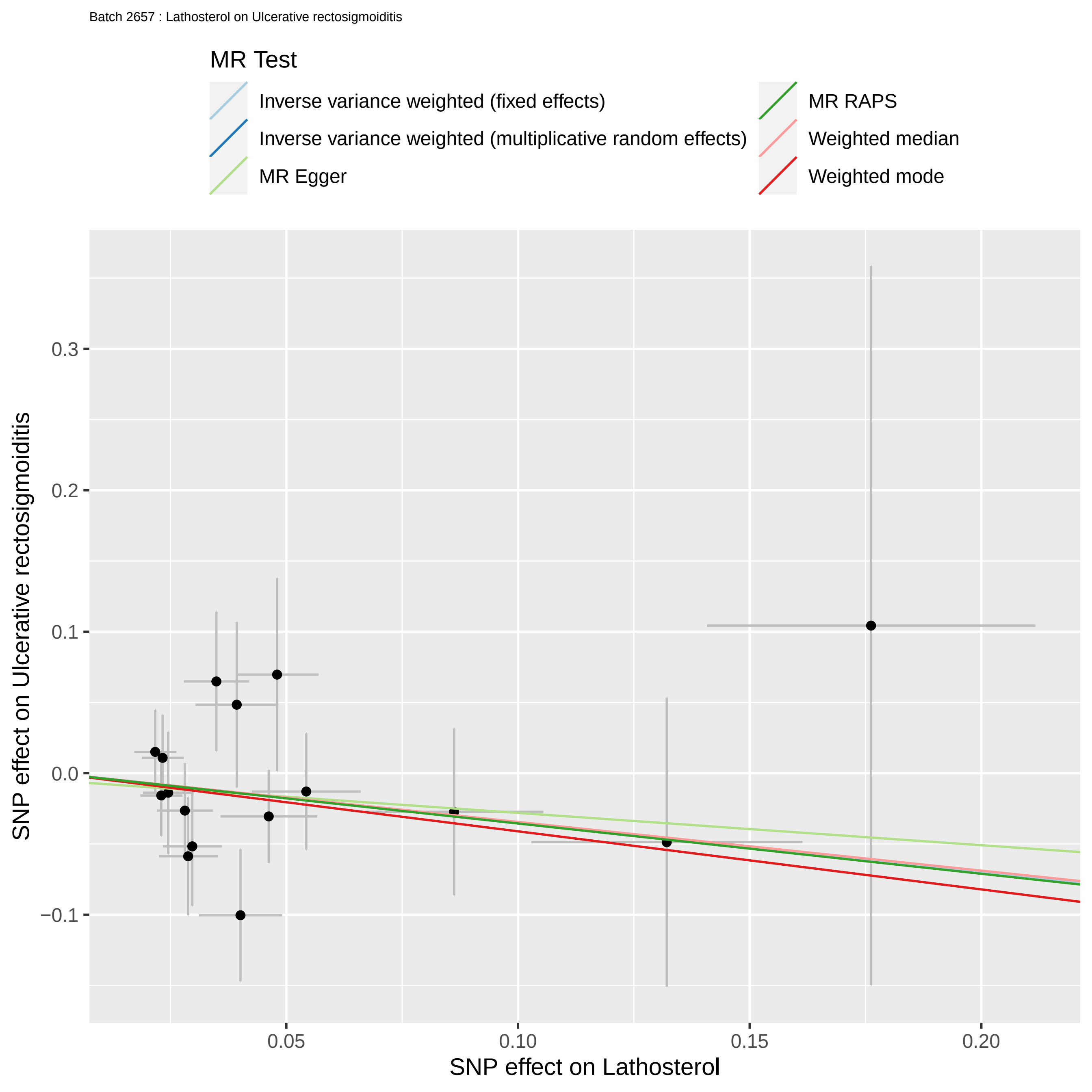

Batch 2657 : Lathosterol on Ulcerative rectosigmoiditis
MR Test
Inverse variance weighted (fixed effects)
Inverse variance weighted (multiplicative random effects)
MR Egger
MR RAPS
Weighted median
Weighted mode
SNP effect on Ulcerative rectosigmoiditis
0.3
0.2
0.1
0.0
−0.1
0.05
0.10
0.15
0.20
SNP effect on Lathosterol

Batch 2660 : Eicosenoate (20:1n9 or 11) on Ulcerative rectosigmoiditis

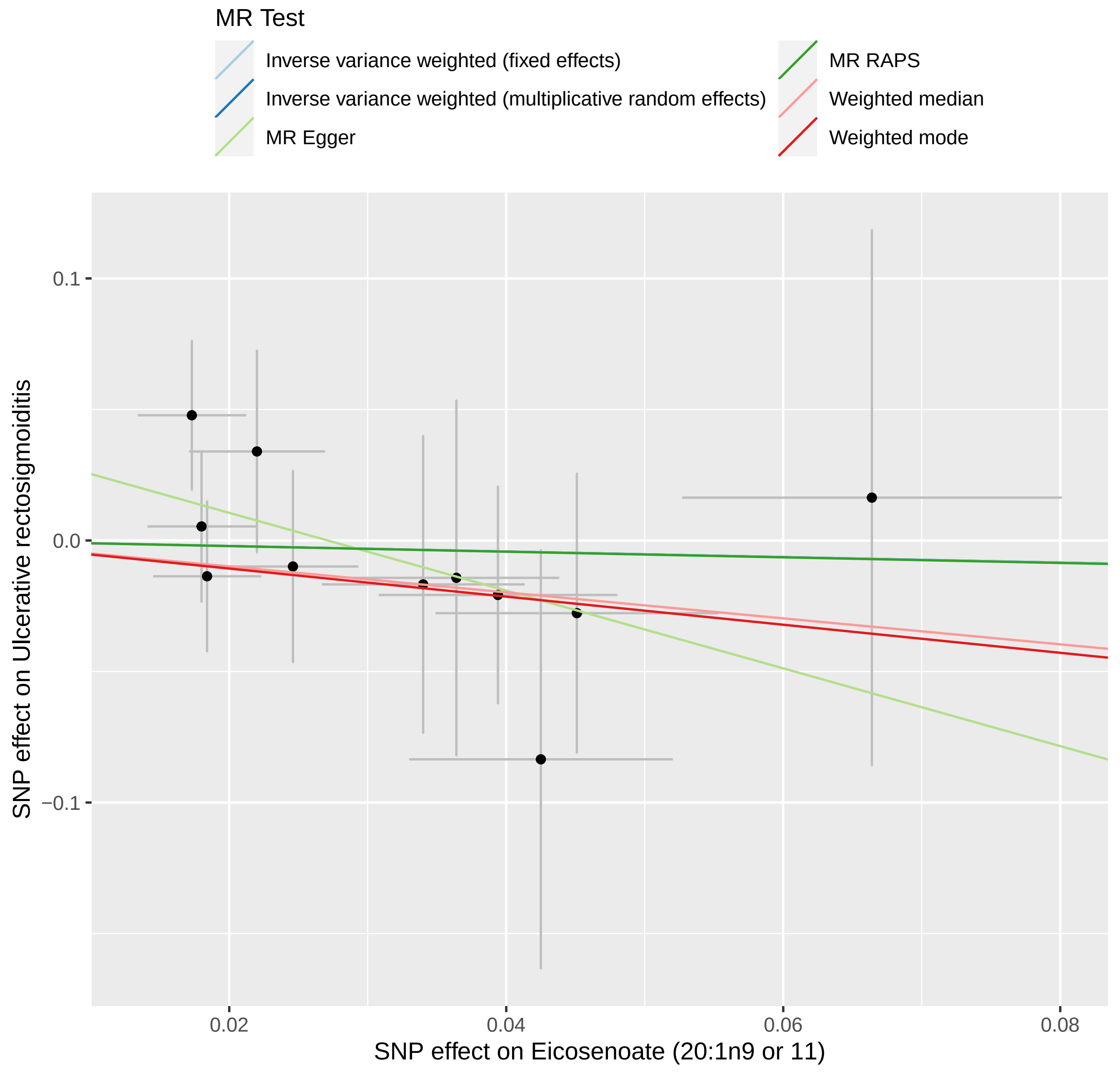

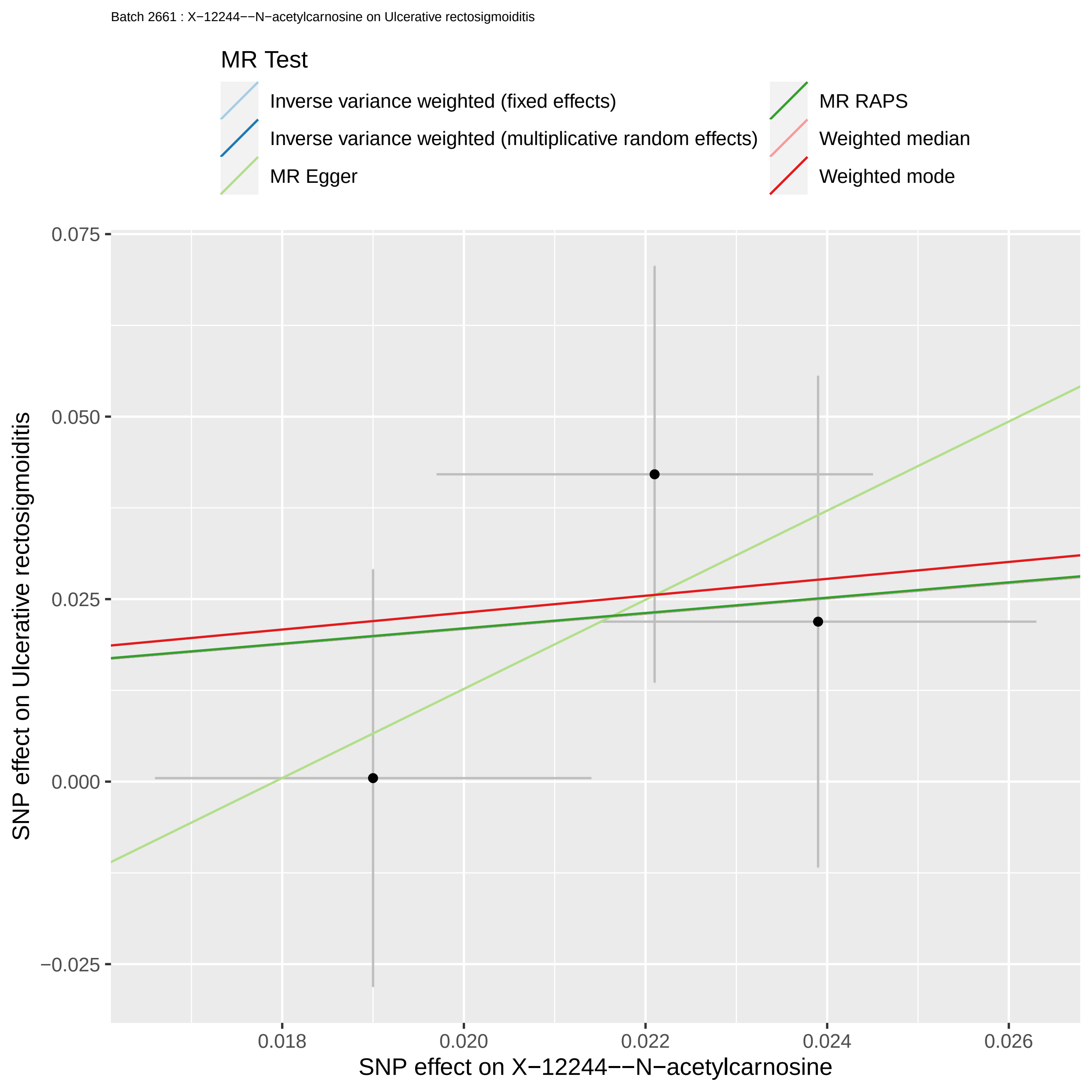

Batch 2661 : X−12244−−N−acetylcarnosine on Ulcerative rectosigmoiditis
MR Test
Inverse variance weighted (fixed effects)
Inverse variance weighted (multiplicative random effects)
MR Egger
MR RAPS
Weighted median
Weighted mode
0.075
0.050
0.025
0.000
−0.025
0.018
0.020
0.022
0.024
0.026
SNP effect on Ulcerative rectosigmoiditis
SNP effect on X−12244−−N−acetylcarnosine

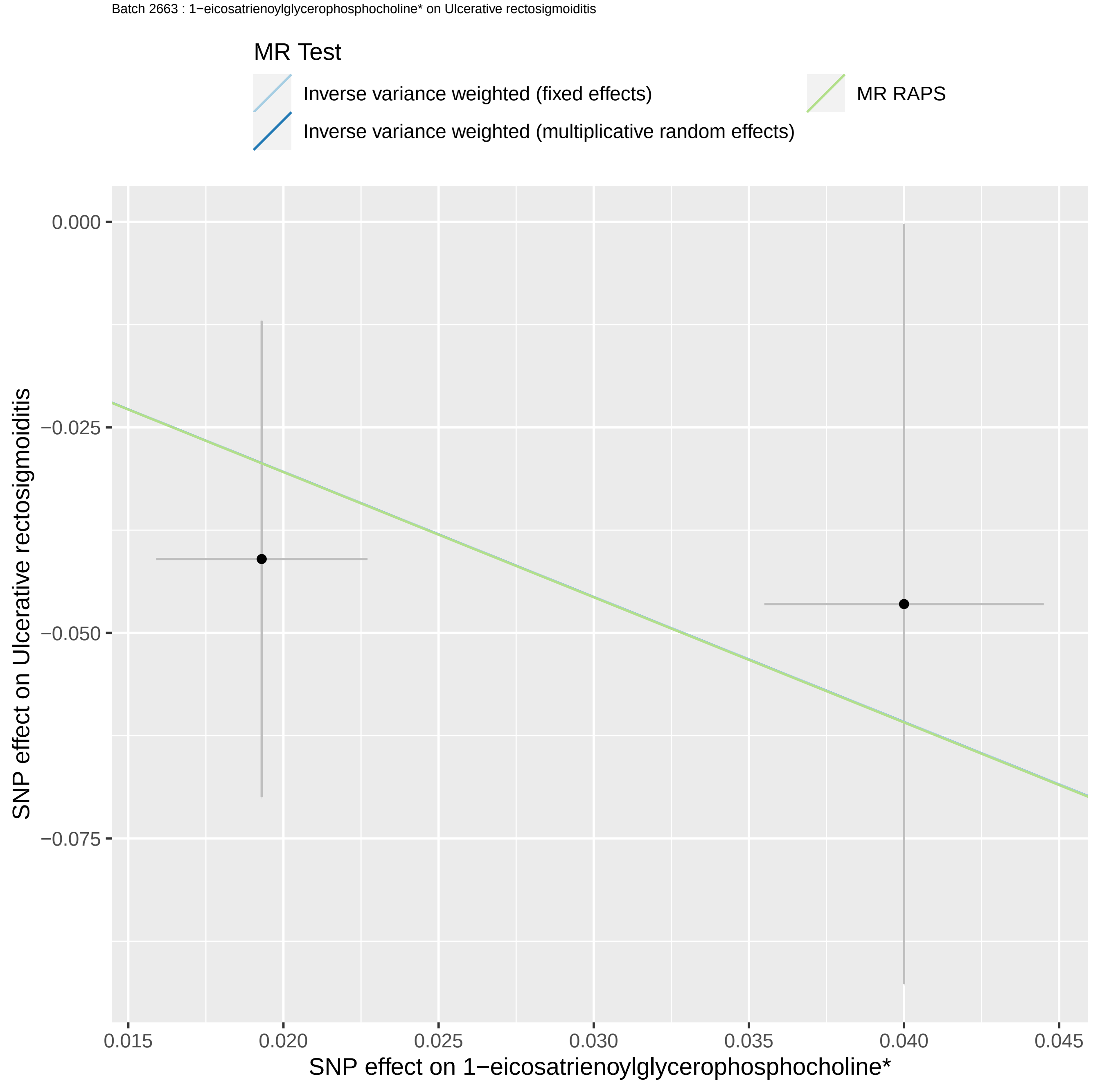

Batch 2663 : 1−eicosatrienoylglycerophosphocholine* on Ulcerative rectosigmoiditis
MR Test
Inverse variance weighted (fixed effects)
Inverse variance weighted (multiplicative random effects)
MR RAPS
0.000
−0.025
−0.050
−0.075
SNP effect on Ulcerative rectosigmoiditis
0.015
0.020
0.025
0.030
0.035
0.040
0.045
SNP effect on 1−eicosatrienoylglycerophosphocholine*

Batch 2664 : 1−docosahexaenoylglycerophosphocholine* on Ulcerative rectosigmoiditis

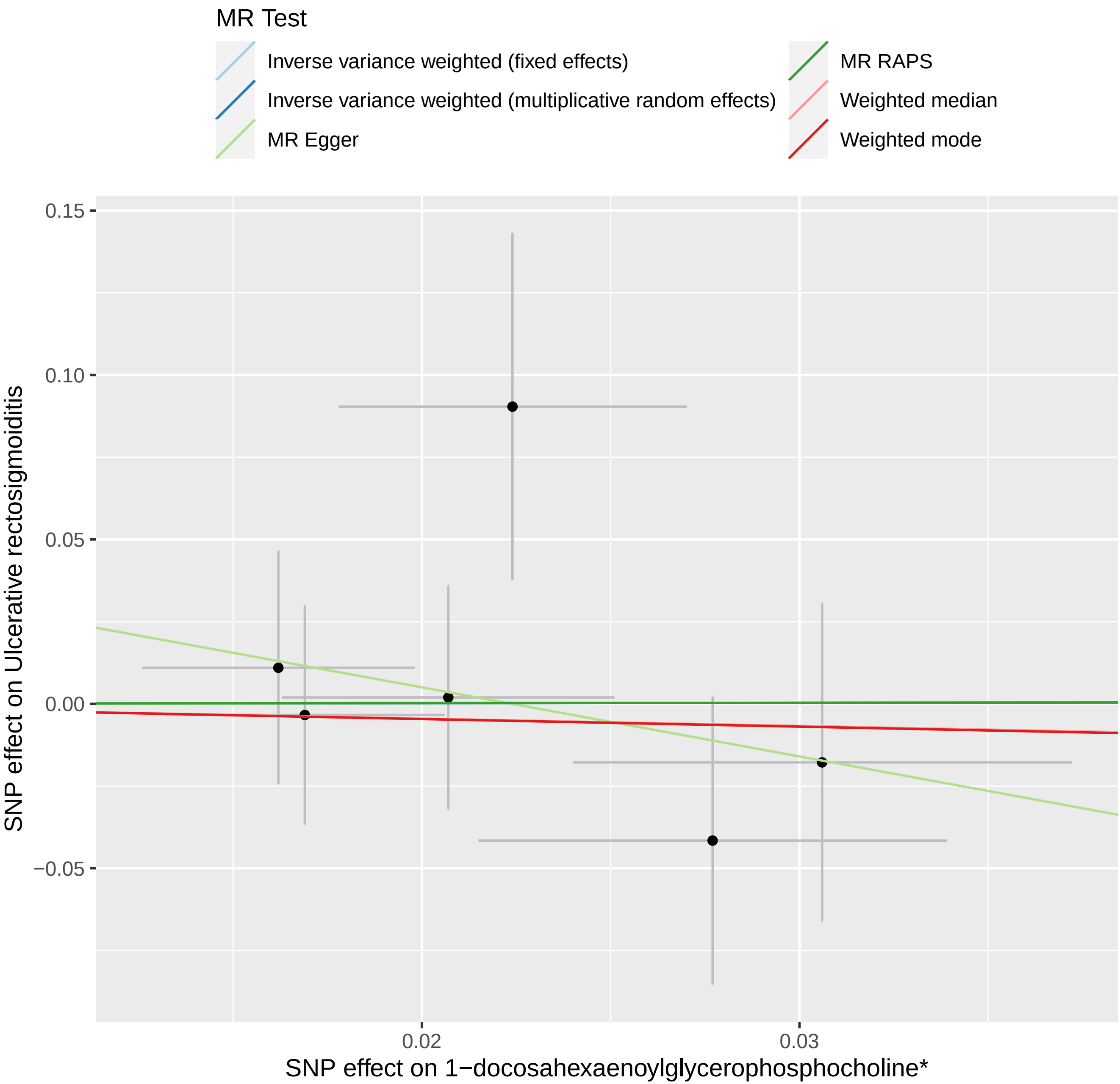

Batch 2668 : Octanoylcarnitine on Ulcerative rectosigmoiditis

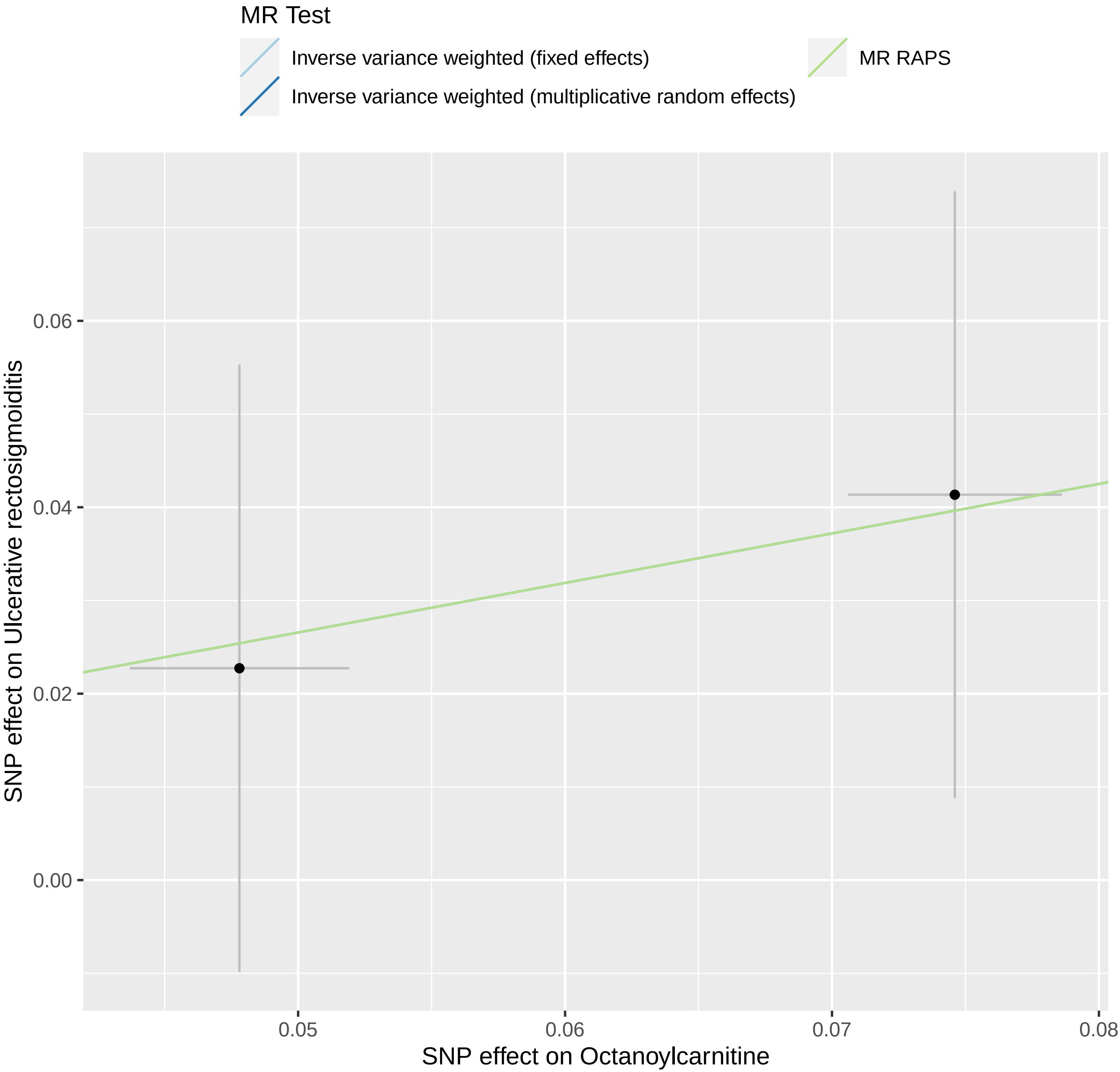

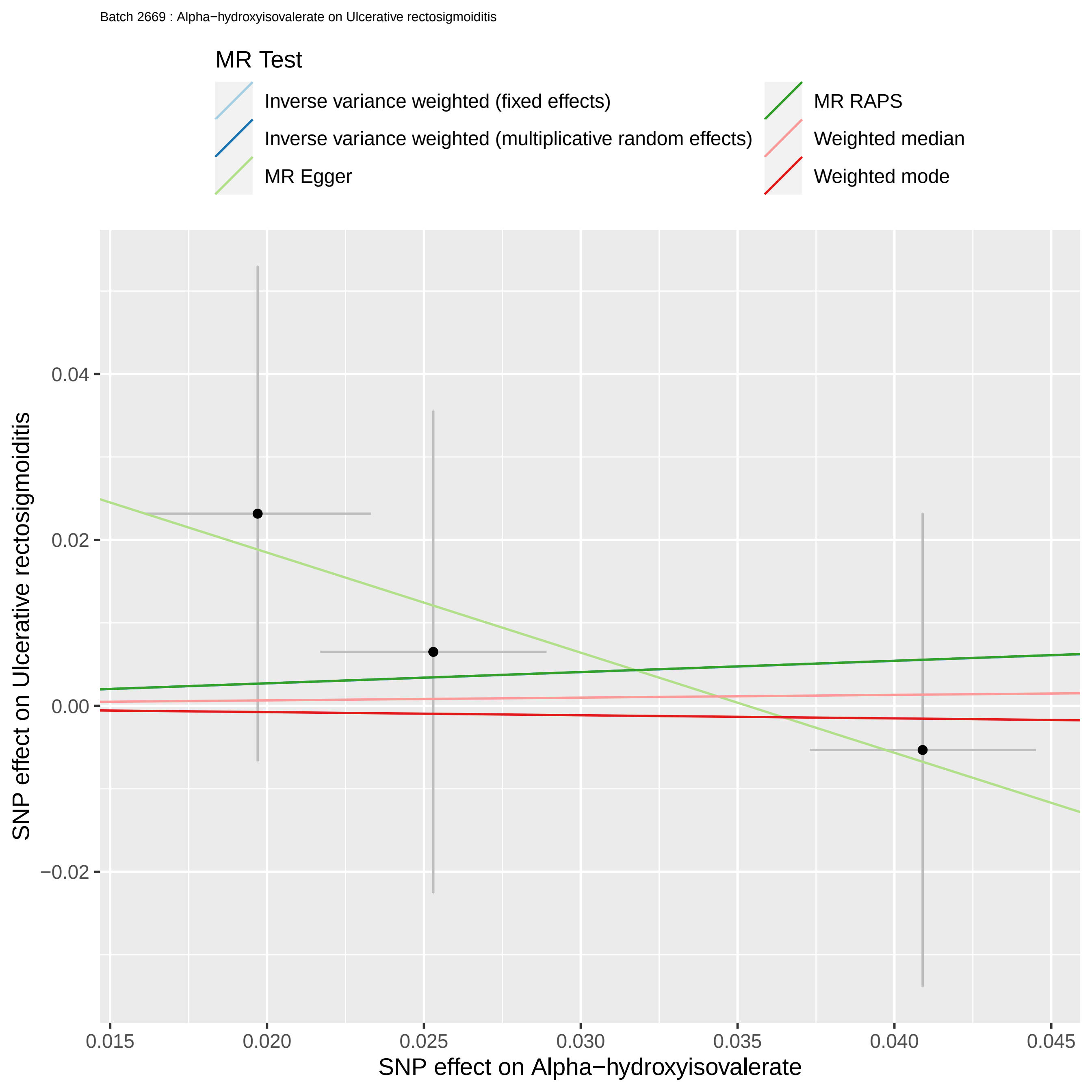
Batch 2669 : Alpha−hydroxyisovalerate on Ulcerative rectosigmoiditis
MR Test
Inverse variance weighted (fixed effects)
Inverse variance weighted (multiplicative random effects)
MR Egger
MR RAPS
Weighted median
Weighted mode
0.04
0.02
0.00
−0.02
0.015
0.020
0.025
0.030
0.035
0.040
0.045
SNP effect on Alpha−hydroxyisovalerate
SNP effect on Ulcerative rectosigmoiditis

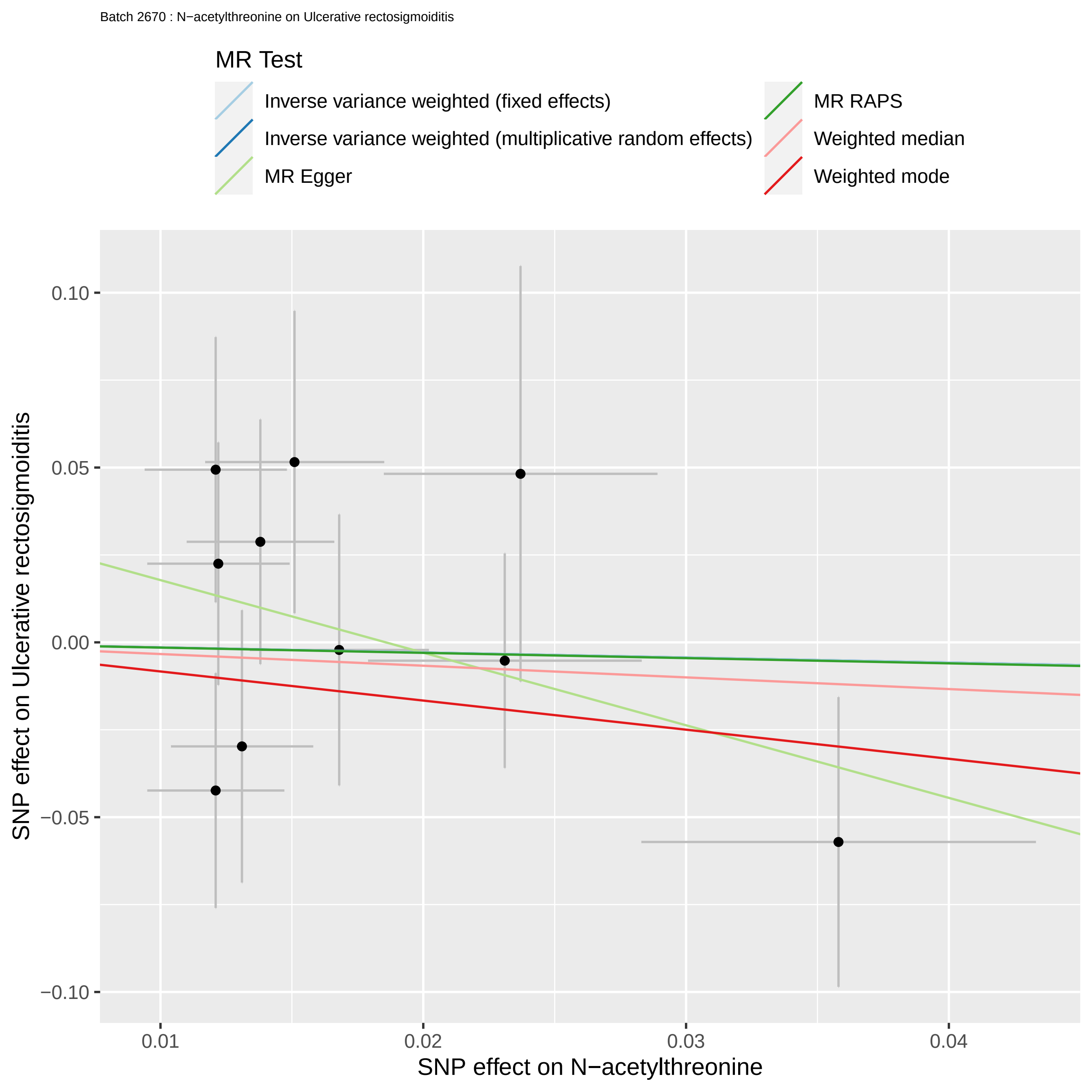

Batch 2670 : N−acetylthreonine on Ulcerative rectosigmoiditis
MR Test
Inverse variance weighted (fixed effects)
Inverse variance weighted (multiplicative random effects)
MR Egger
MR RAPS
Weighted median
Weighted mode
0.10
0.05
0.00
−0.05
−0.10
0.01
0.02
0.03
0.04
SNP effect on Ulcerative rectosigmoiditis
SNP effect on N−acetylthreonine

Batch 2671 : Decanoylcarnitine on Ulcerative rectosigmoiditis

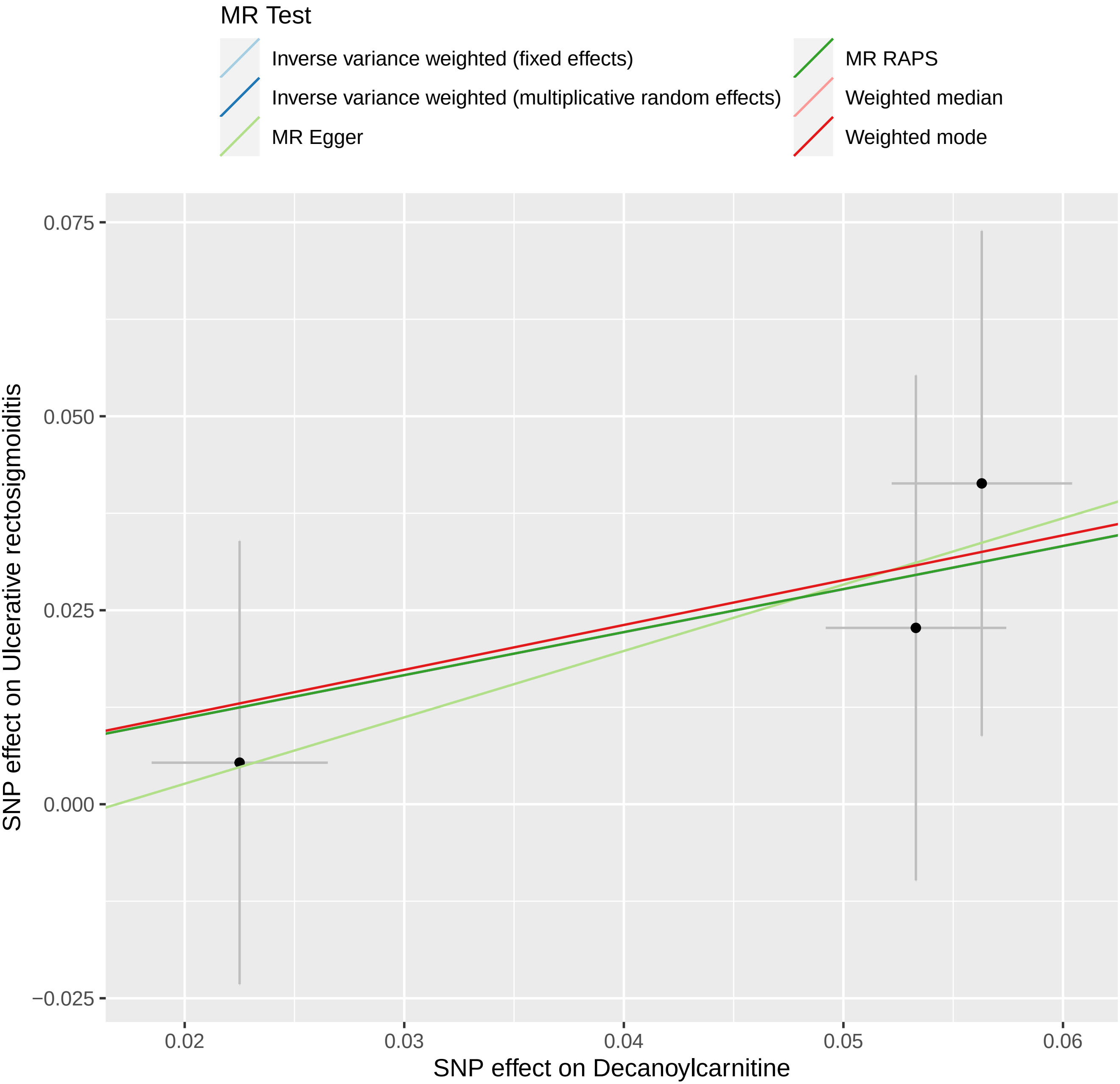

Batch 2672 : 1−palmitoylglycerophosphocholine on Ulcerative rectosigmoiditis

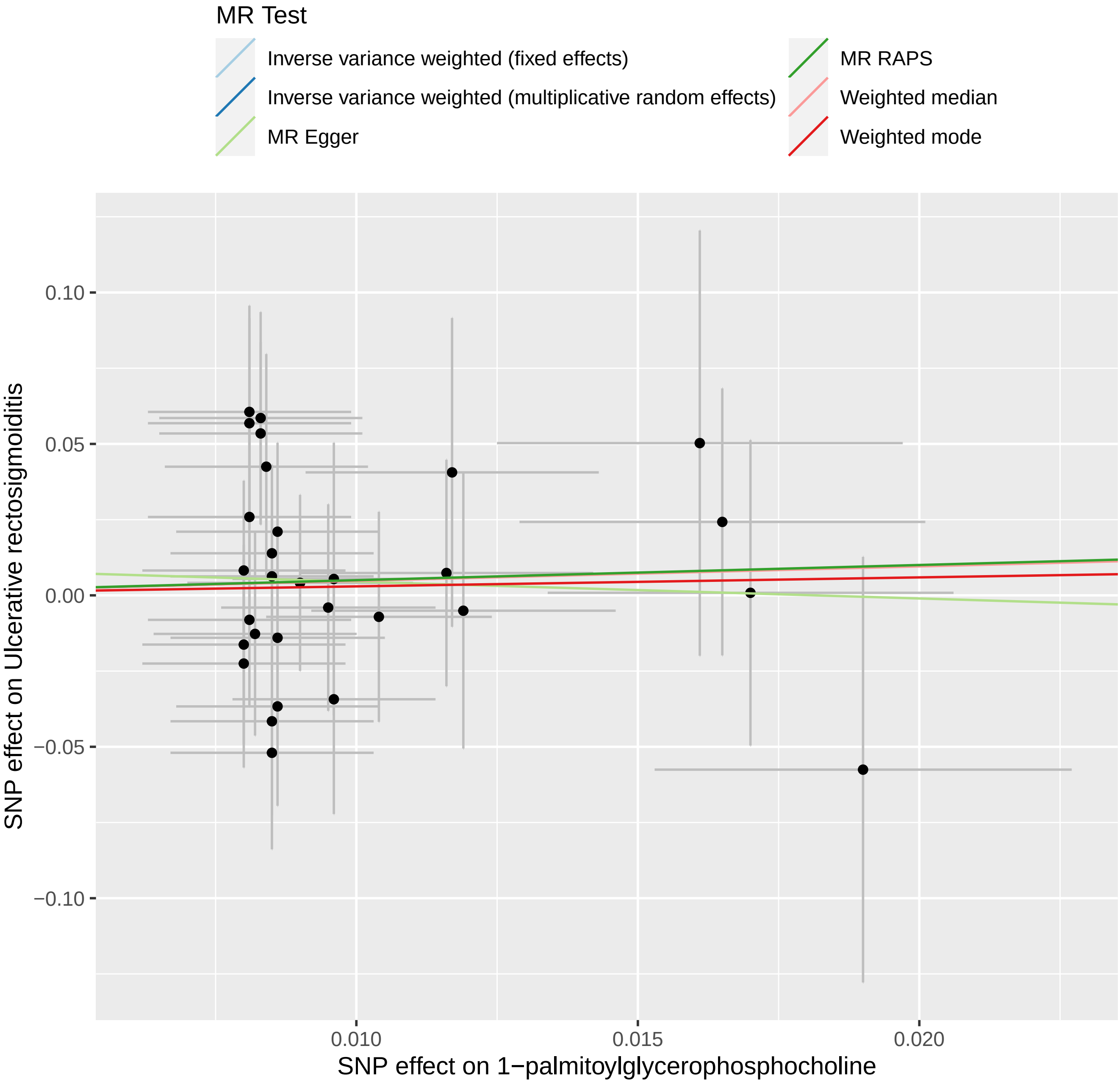

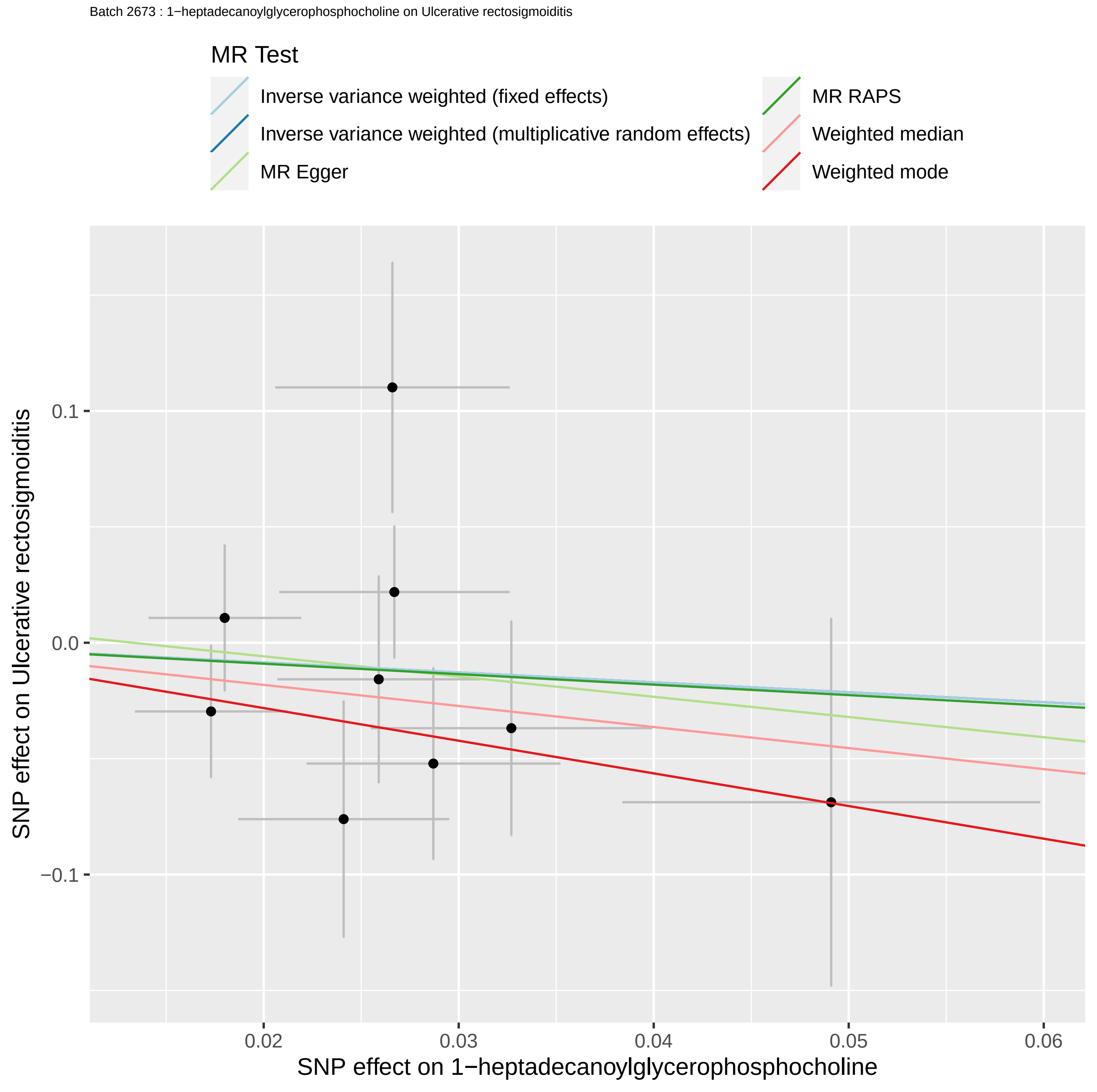

Batch 2673 : 1−heptadecanoylglycerophosphocholine on Ulcerative rectosigmoiditis
MR Test
Inverse variance weighted (fixed effects)
Inverse variance weighted (multiplicative random effects)
MR Egger
MR RAPS
Weighted median
Weighted mode
SNP effect on Ulcerative rectosigmoiditis
0.1
0.0
−0.1
0.02
0.03
0.04
0.05
0.06
SNP effect on 1−heptadecanoylglycerophosphocholine

Batch 2674 : 1−oleoylglycerophosphocholine on Ulcerative rectosigmoiditis

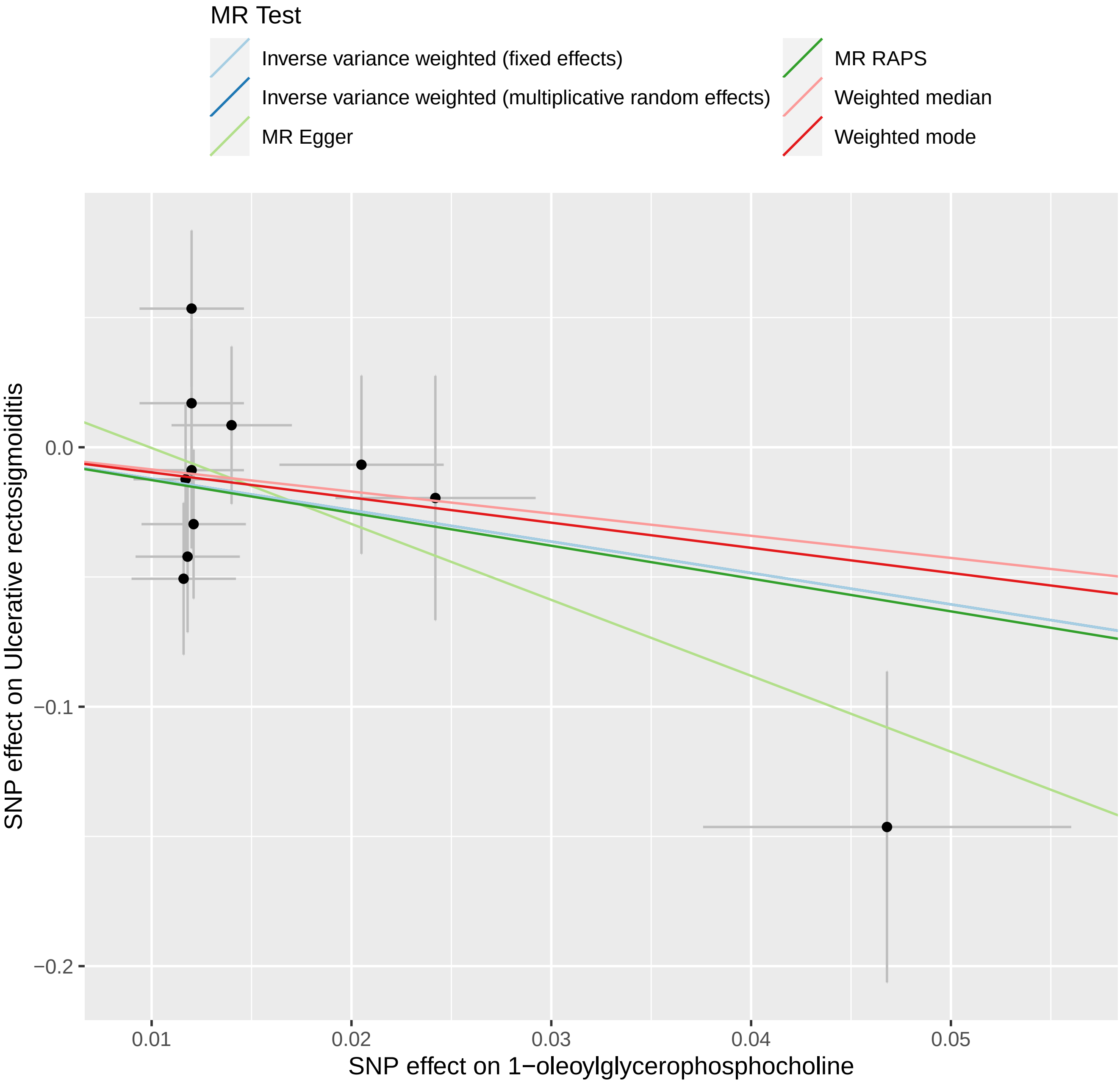

Batch 2675 : 1−stearoylglycerophosphocholine on Ulcerative rectosigmoiditis

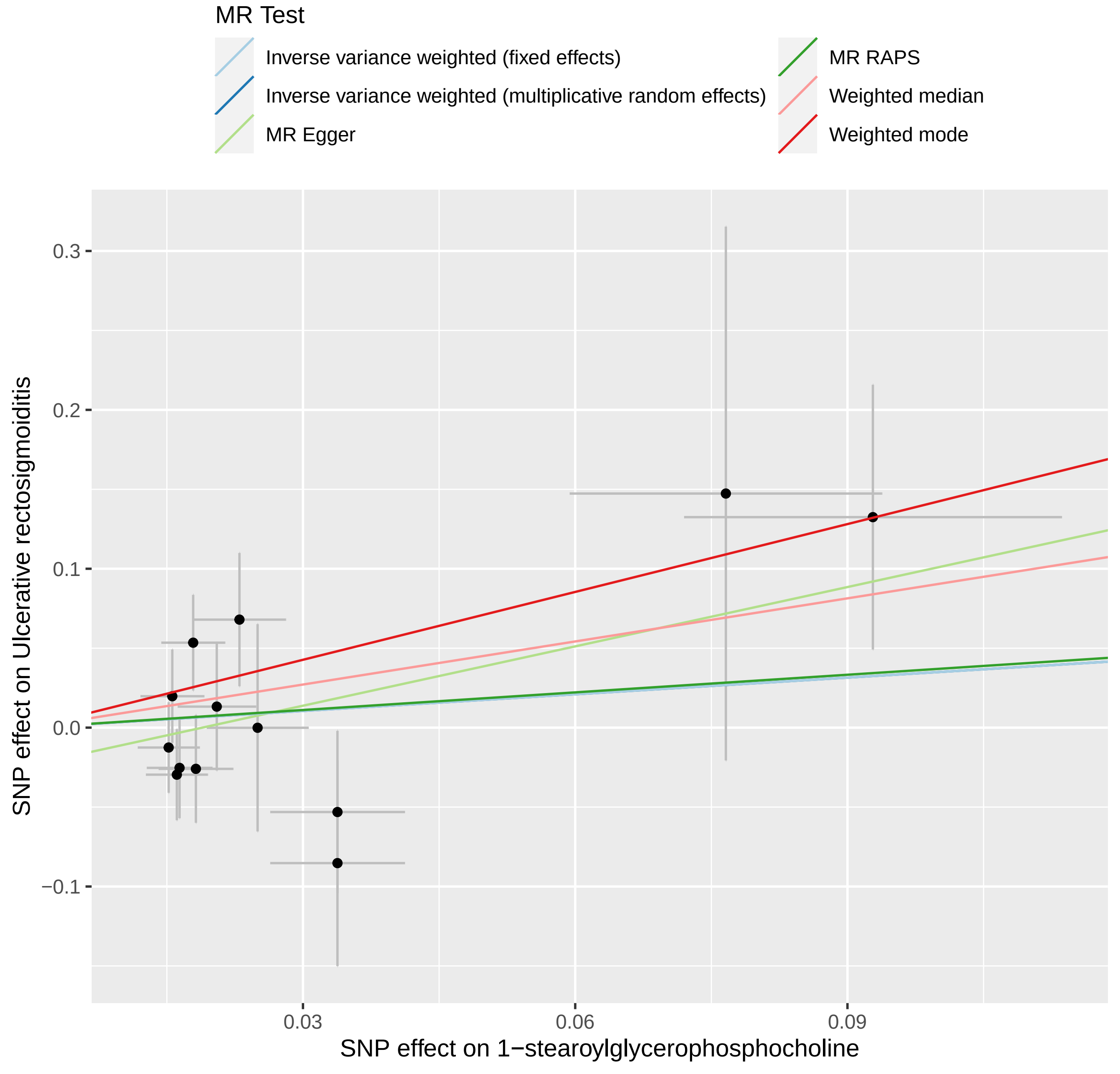

Batch 2678 : 10−heptadecenoate (17:1n7) on Ulcerative rectosigmoiditis

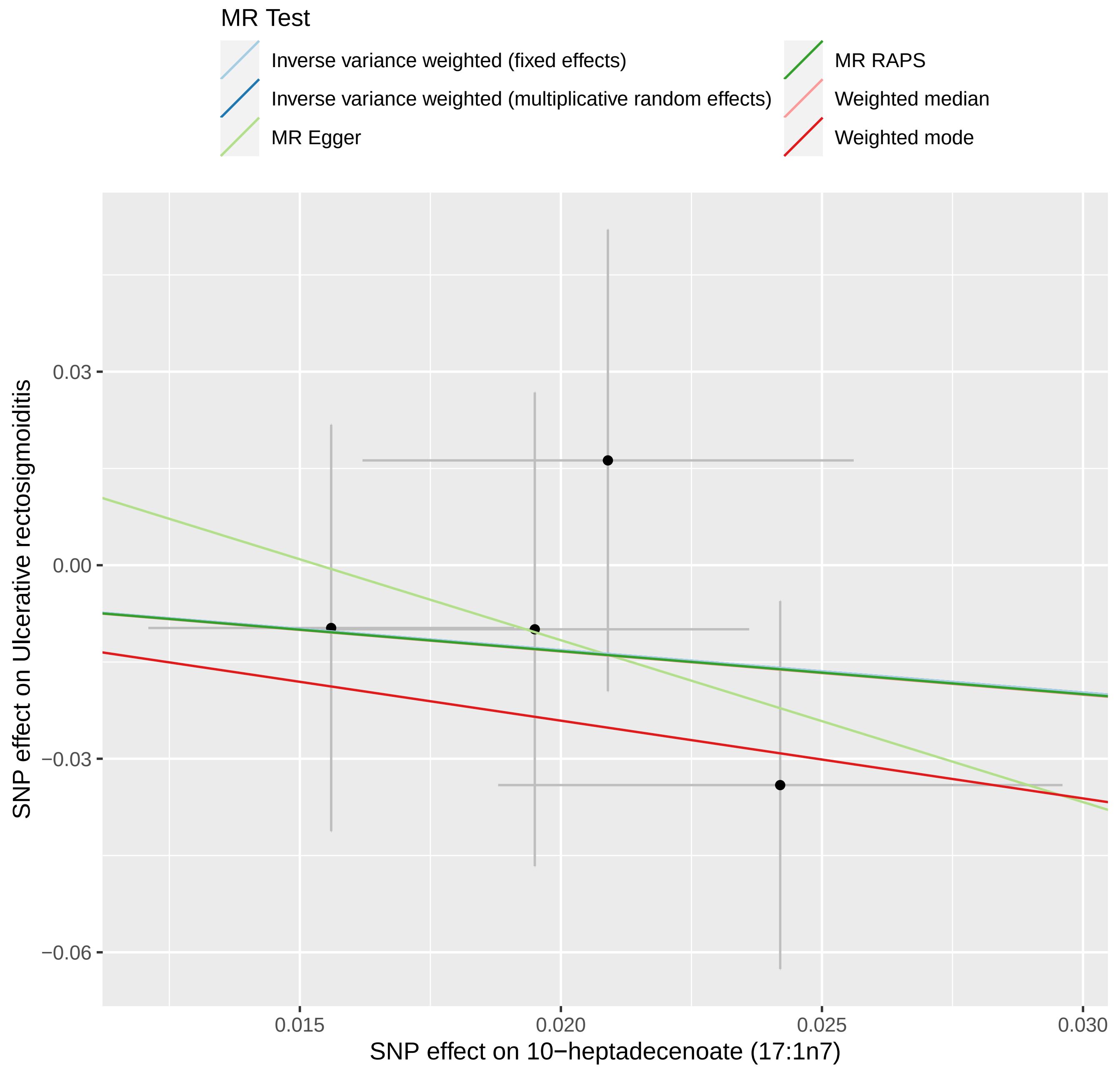

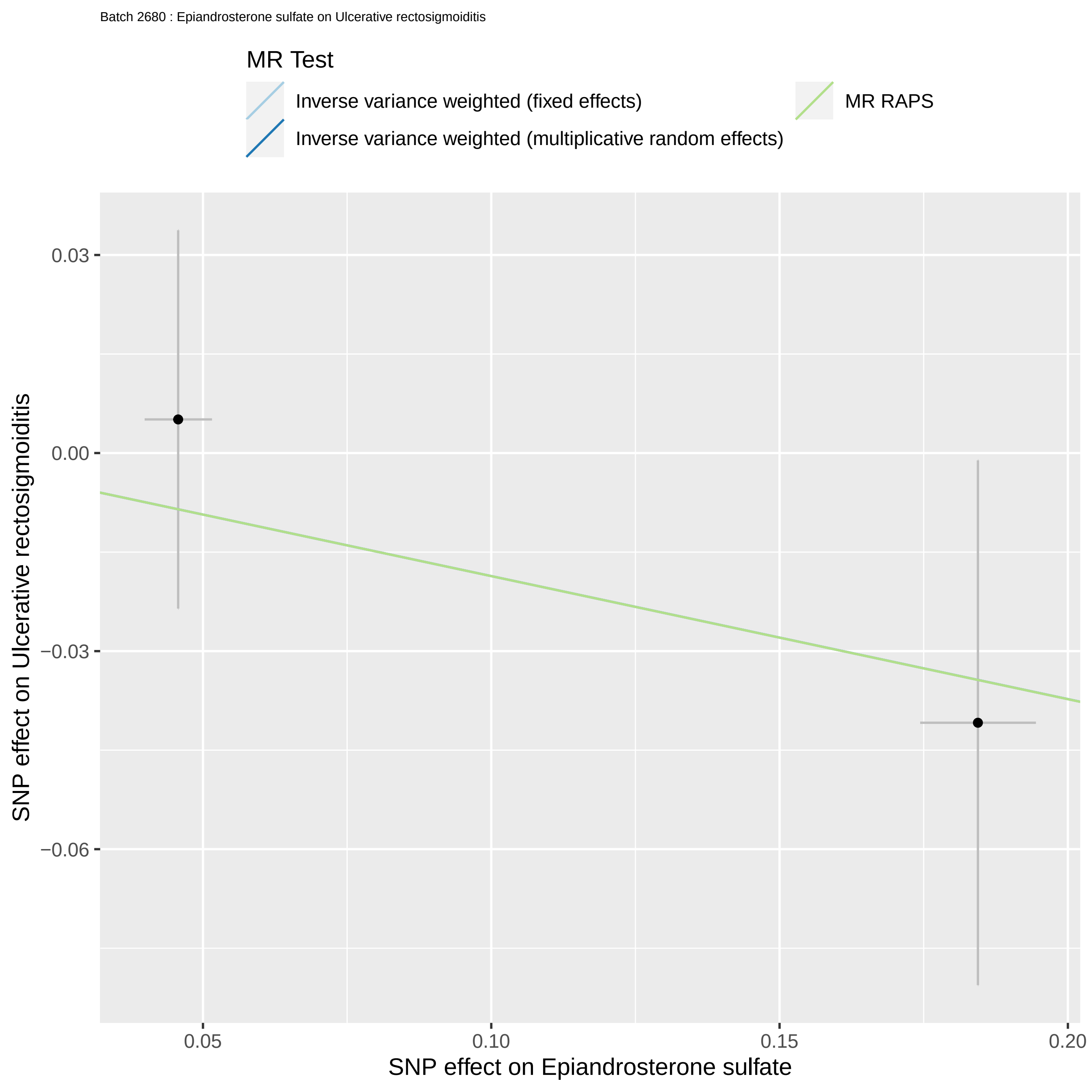

Batch 2680 : Epiandrosterone sulfate on Ulcerative rectosigmoiditis
MR Test
Inverse variance weighted (fixed effects)
Inverse variance weighted (multiplicative random effects)
MR RAPS
0.03
0.00
−0.03
−0.06
0.05
0.10
0.15
0.20
SNP effect on Ulcerative rectosigmoiditis
SNP effect on Epiandrosterone sulfate

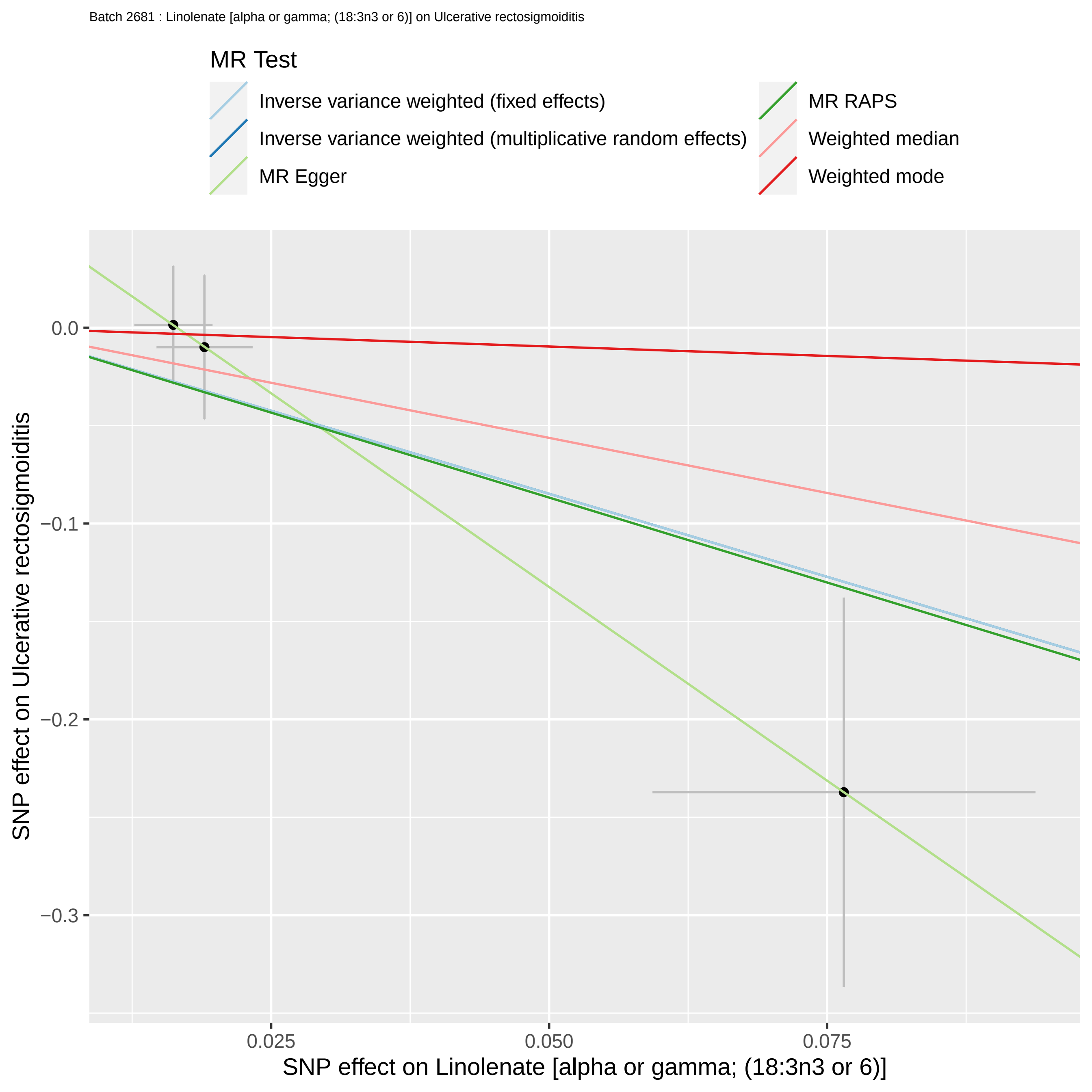

Batch 2681 : Linolenate [alpha or gamma; (18:3n3 or 6)] on Ulcerative rectosigmoiditis
MR Test
Inverse variance weighted (fixed effects)
Inverse variance weighted (multiplicative random effects)
MR Egger
MR RAPS
Weighted median
Weighted mode
0.0
−0.1
−0.2
−0.3
0.025
0.050
0.075
SNP effect on Linolenate [alpha or gamma; (18:3n3 or 6)]
SNP effect on Ulcerative rectosigmoiditis

Batch 2682 : X−12510−−2−aminooctanoic acid on Ulcerative rectosigmoiditis

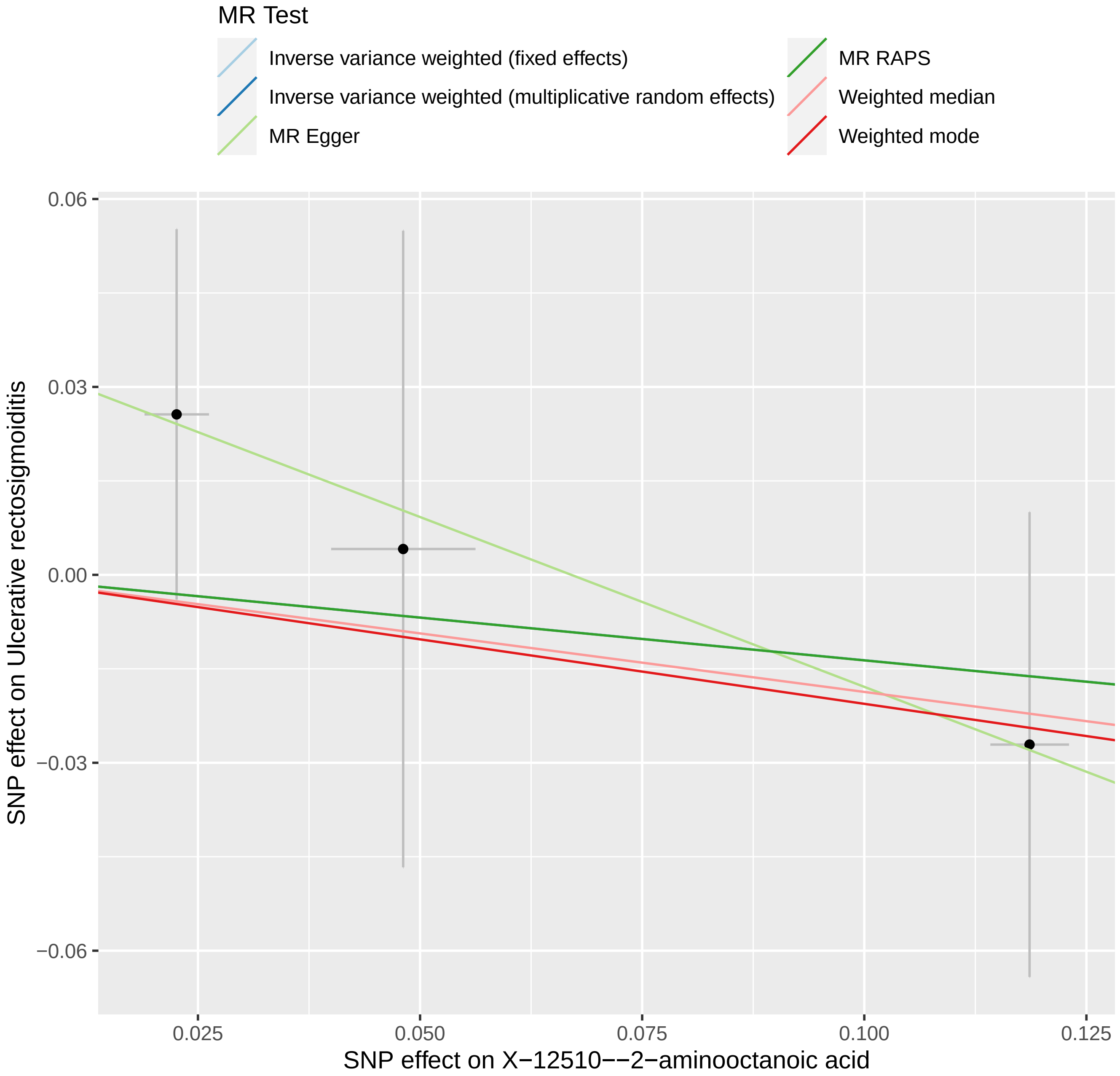

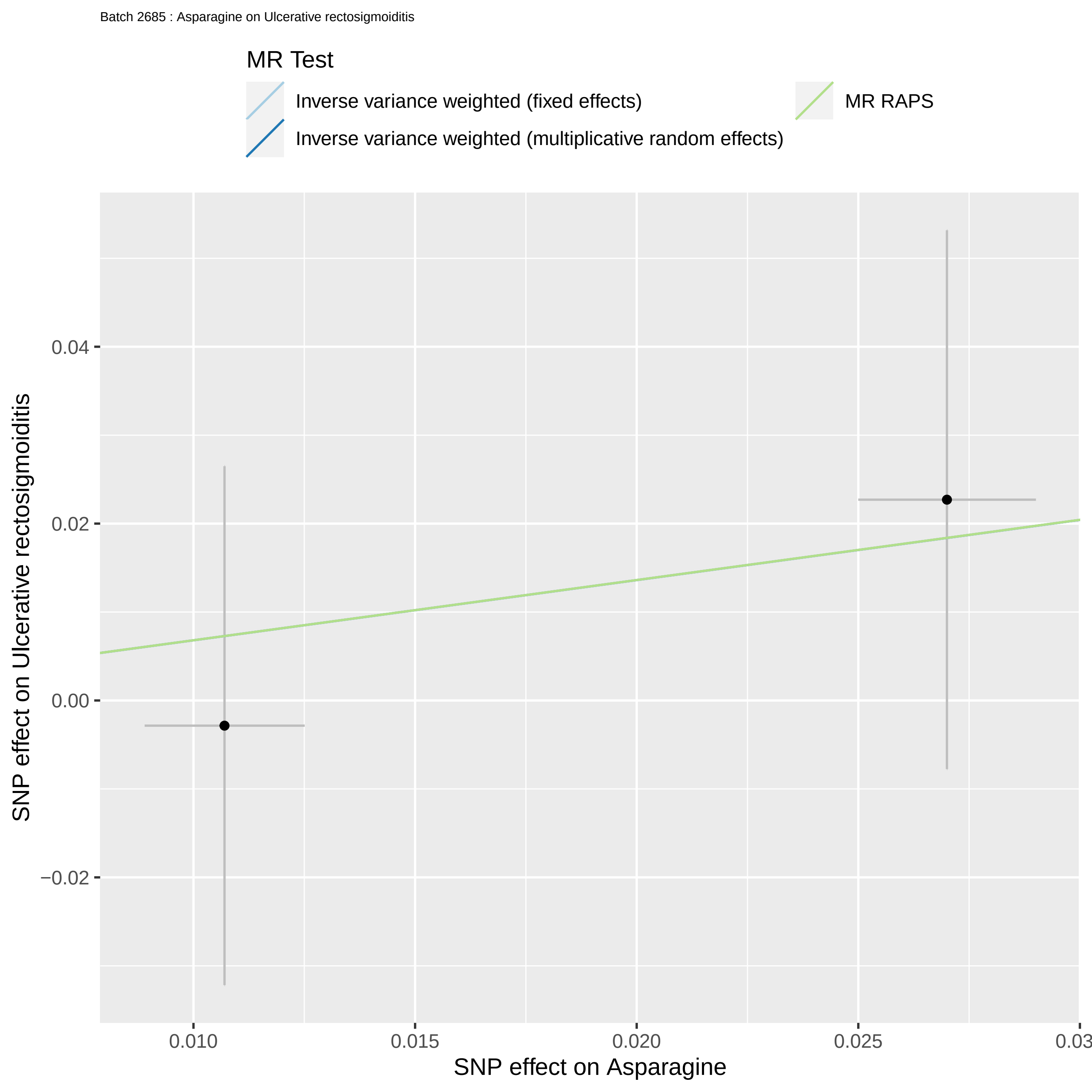

Batch 2685 : Asparagine on Ulcerative rectosigmoiditis
MR Test
Inverse variance weighted (fixed effects)
Inverse variance weighted (multiplicative random effects)
MR RAPS
0.04
0.02
0.00
−0.02
SNP effect on Ulcerative rectosigmoiditis
0.010
0.015
0.020
0.025
0.03
SNP effect on Asparagine

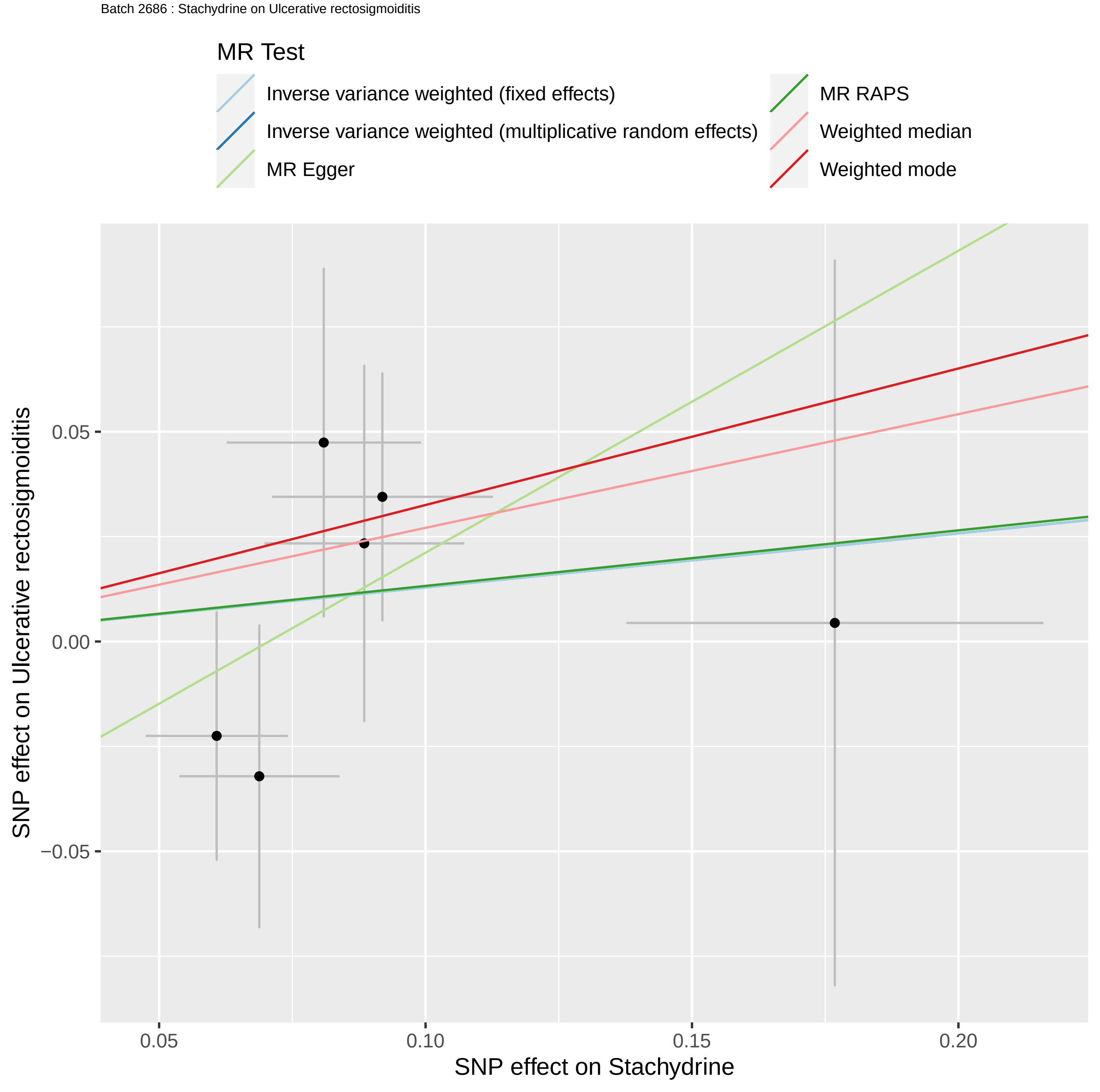

Batch 2686 : Stachydrine on Ulcerative rectosigmoiditis
MR Test
Inverse variance weighted (fixed effects)
Inverse variance weighted (multiplicative random effects)
MR Egger
MR RAPS
Weighted median
Weighted mode
0.05
0.00
−0.05
0.05
0.10
0.15
0.20
SNP effect on Stachydrine
SNP effect on Ulcerative rectosigmoiditis

Batch 2687 : Isovalerylcarnitine on Ulcerative rectosigmoiditis

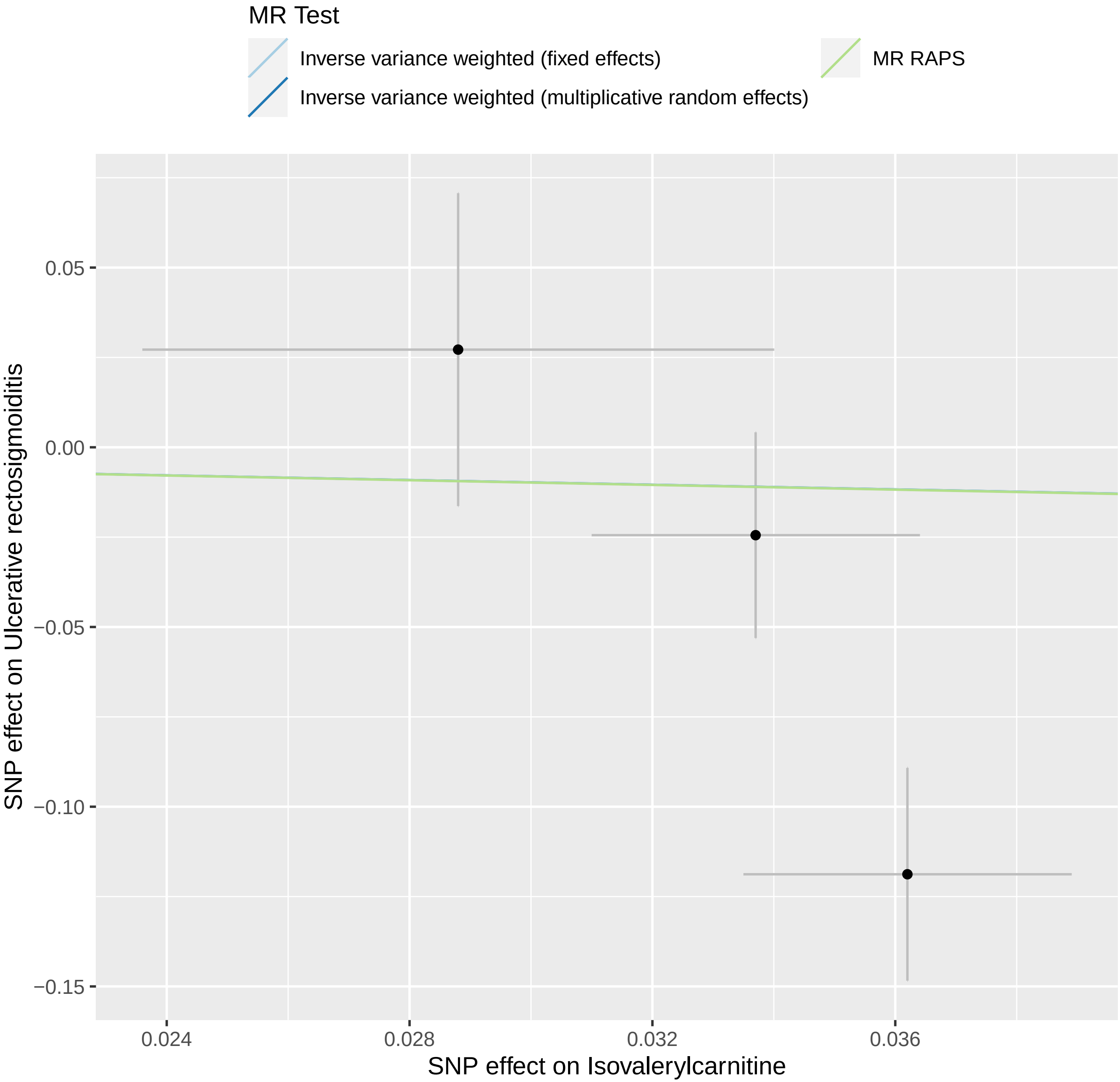

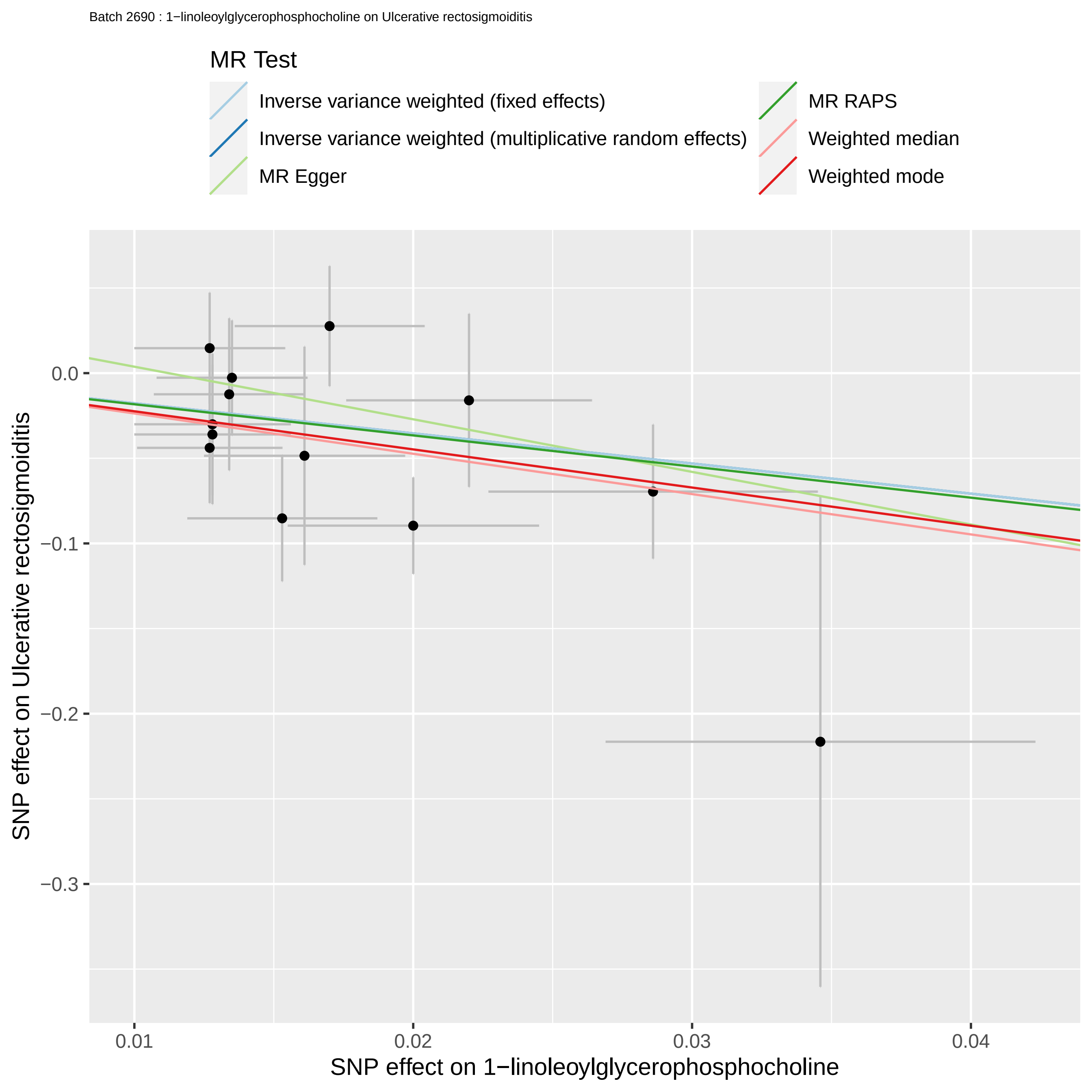

Batch 2690 : 1−linoleoylglycerophosphocholine on Ulcerative rectosigmoiditis
MR Test
Inverse variance weighted (fixed effects)
Inverse variance weighted (multiplicative random effects)
MR Egger
MR RAPS
Weighted median
Weighted mode
0.0
−0.1
−0.2
−0.3
0.01
0.02
0.03
0.04
SNP effect on Ulcerative rectosigmoiditis
SNP effect on 1−linoleoylglycerophosphocholine

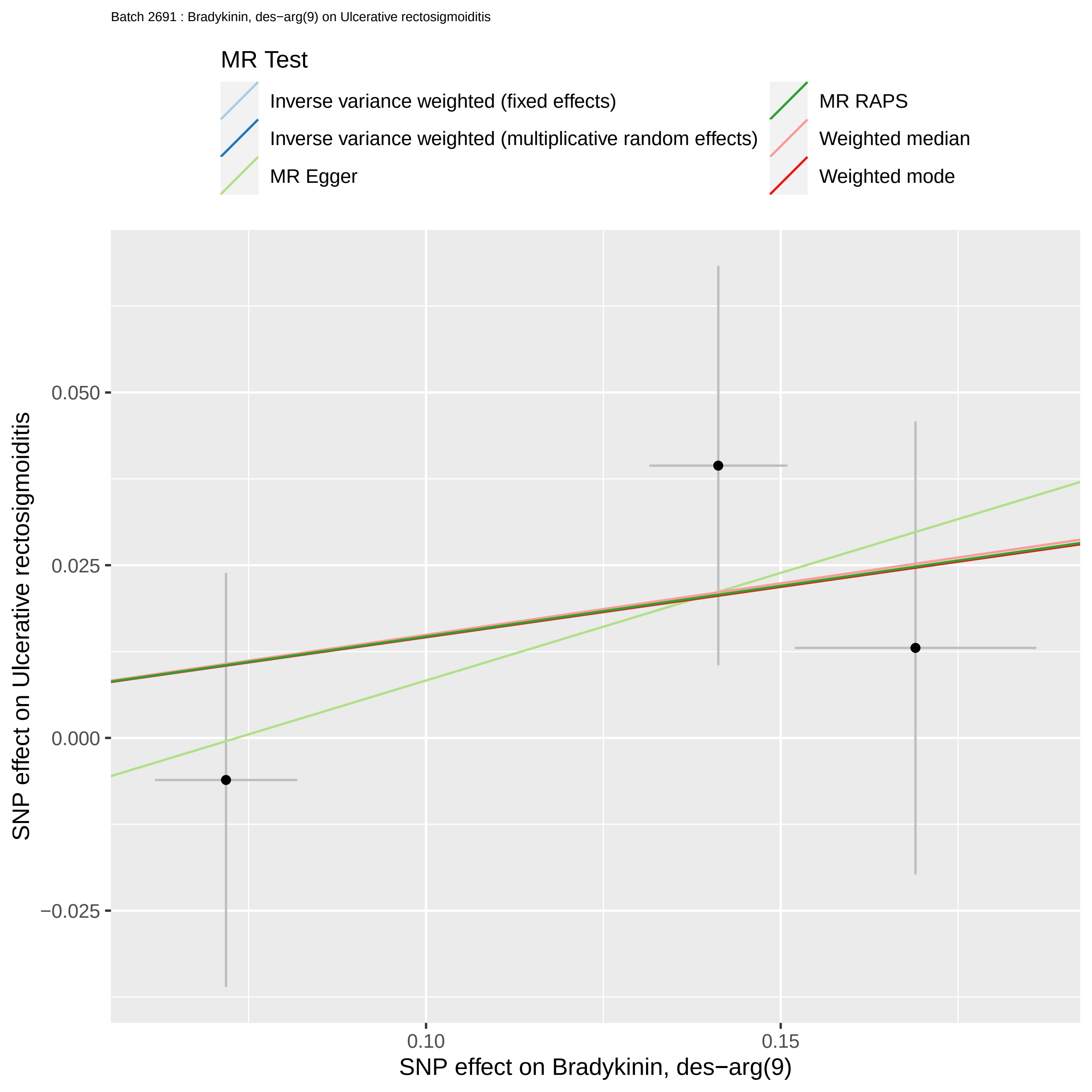

Batch 2691 : Bradykinin, des−arg(9) on Ulcerative rectosigmoiditis
MR Test
Inverse variance weighted (fixed effects)
Inverse variance weighted (multiplicative random effects)
MR Egger
MR RAPS
Weighted median
Weighted mode
0.050
0.025
0.000
−0.025
0.10
0.15
SNP effect on Ulcerative rectosigmoiditis
SNP effect on Bradykinin, des−arg(9)

Batch 2692 : Gamma−glutamylisoleucine* on Ulcerative rectosigmoiditis

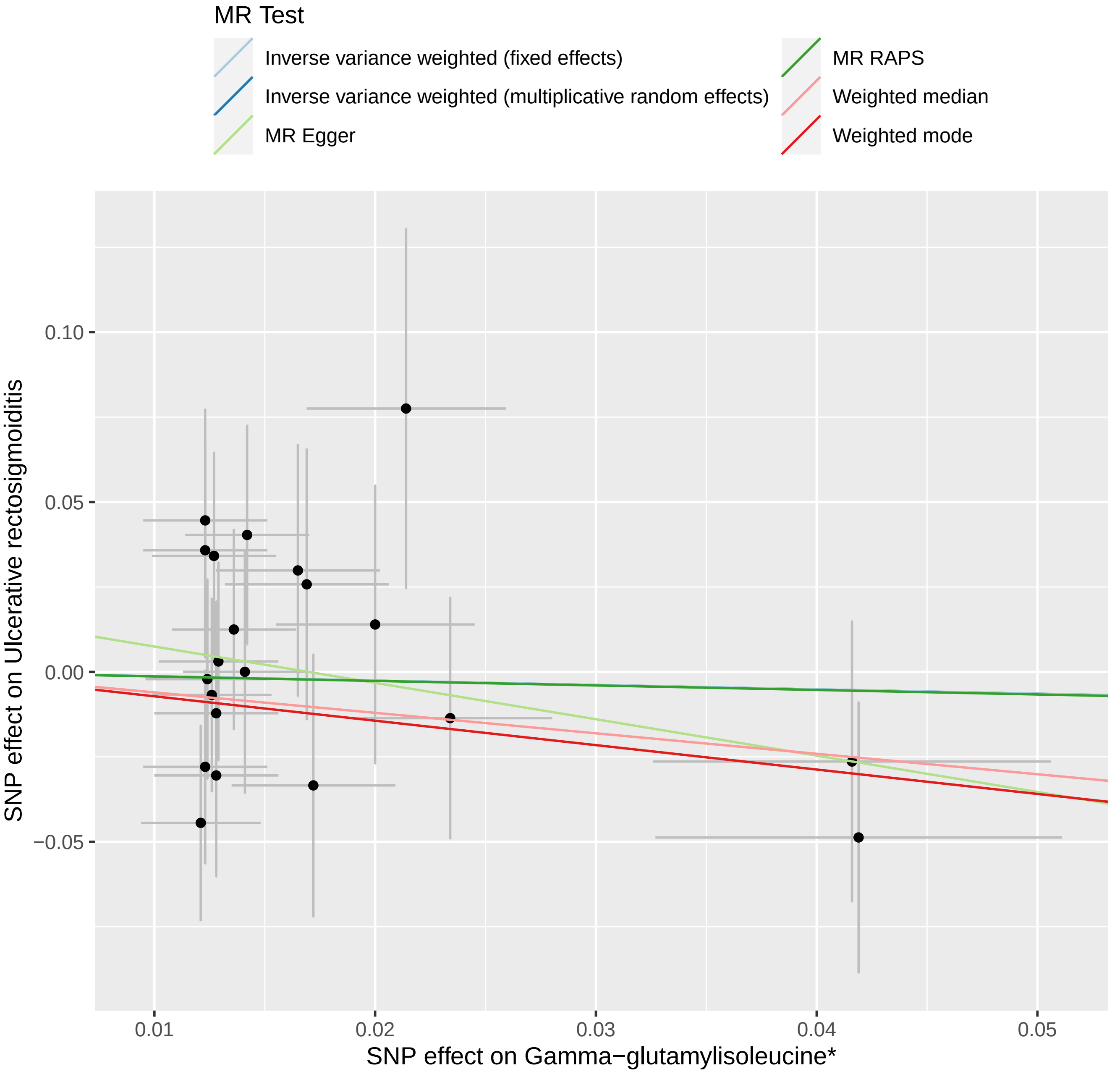

Batch 2695 : Isovalerate on Ulcerative rectosigmoiditis

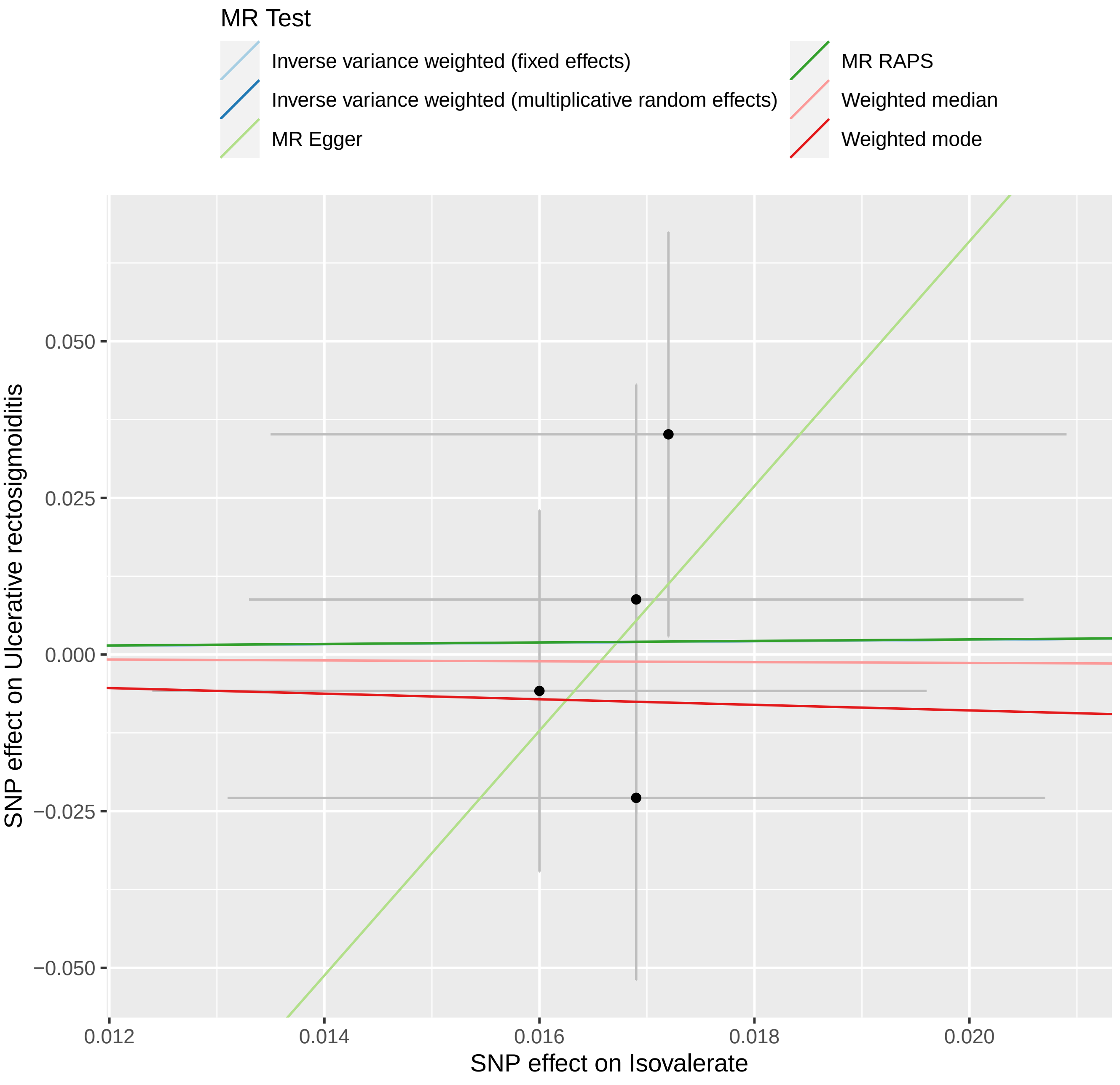

Batch 2696 : X−13183−−stearamide on Ulcerative rectosigmoiditis

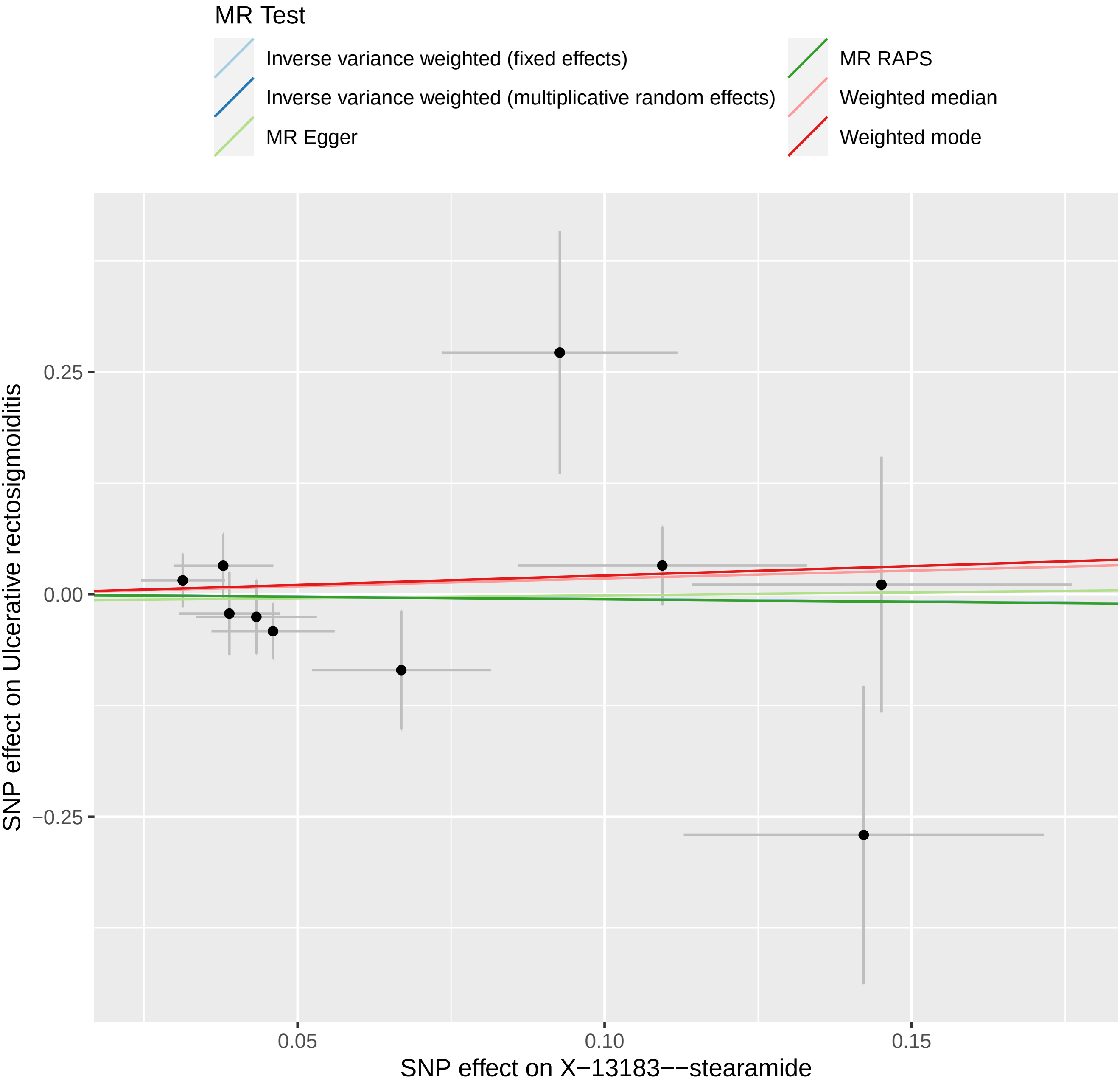

Batch 2697 : 7−methylguanine on Ulcerative rectosigmoiditis

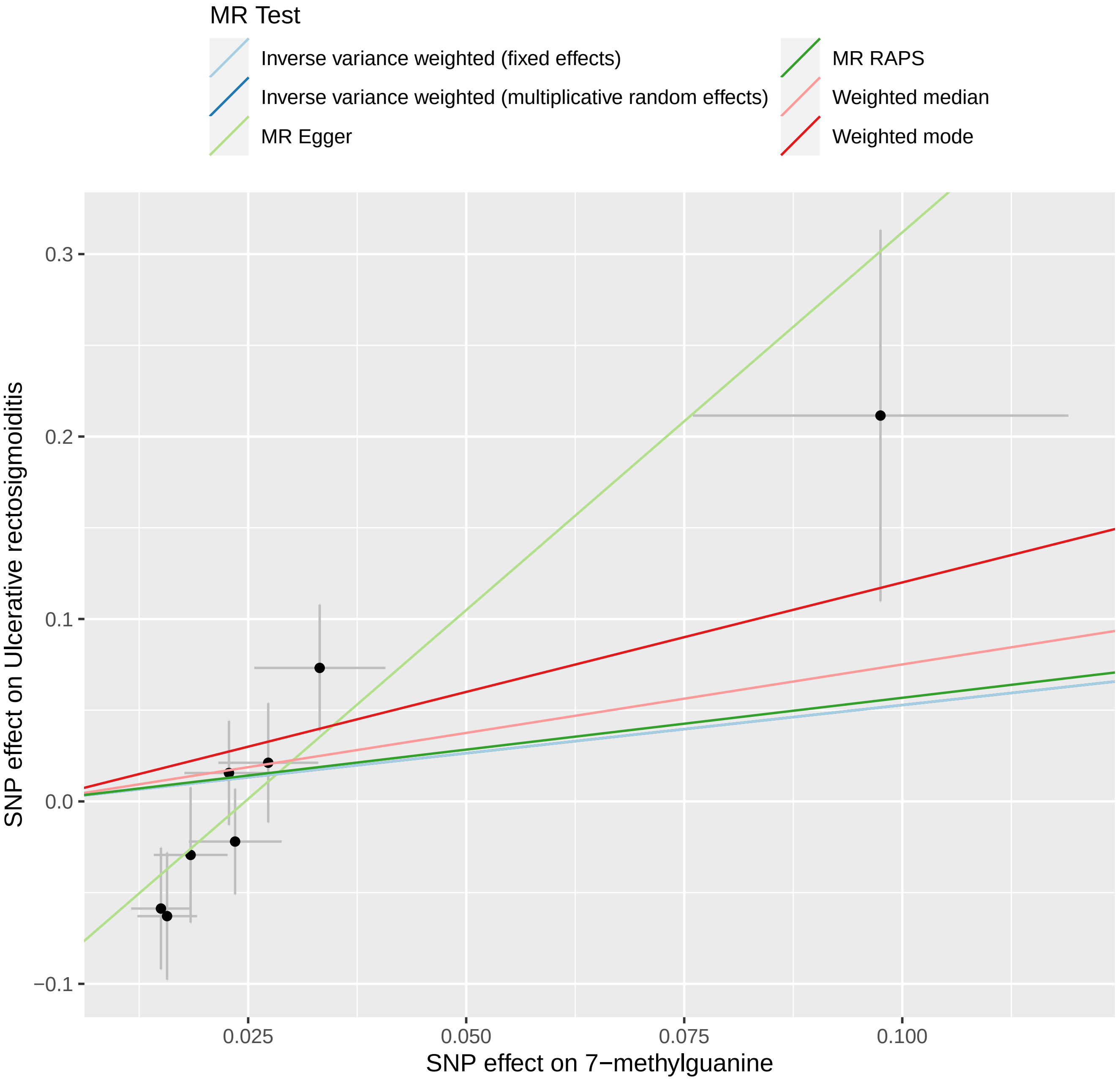

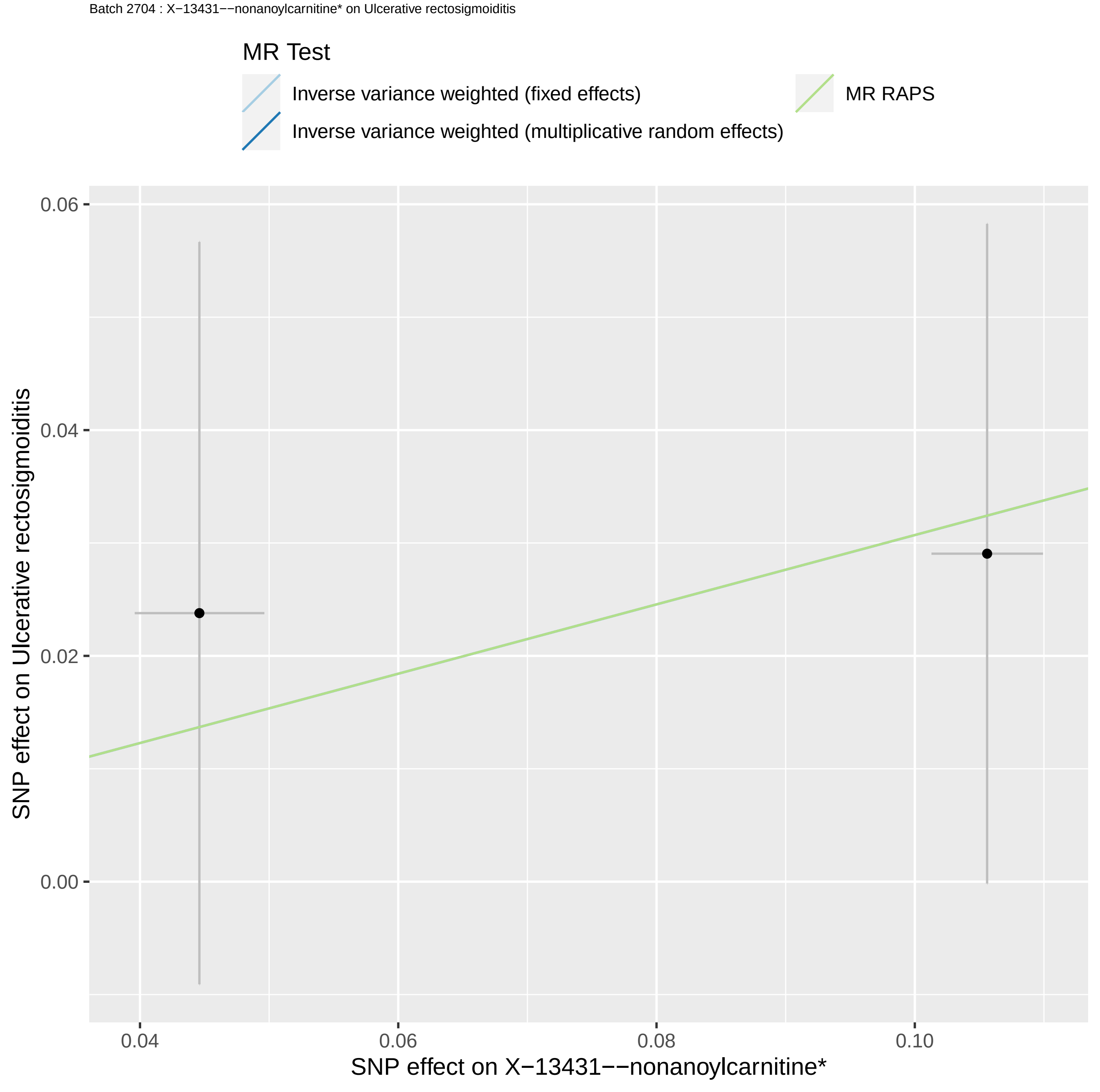

Batch 2704 : X−13431−−nonanoylcarnitine* on Ulcerative rectosigmoiditis
MR Test
Inverse variance weighted (fixed effects)
Inverse variance weighted (multiplicative random effects)
MR RAPS
0.06
0.04
0.02
0.00
0.04
0.06
0.08
0.10
SNP effect on Ulcerative rectosigmoiditis
SNP effect on X−13431−−nonanoylcarnitine*

Batch 2705 : 2−palmitoylglycerophosphocholine* on Ulcerative rectosigmoiditis

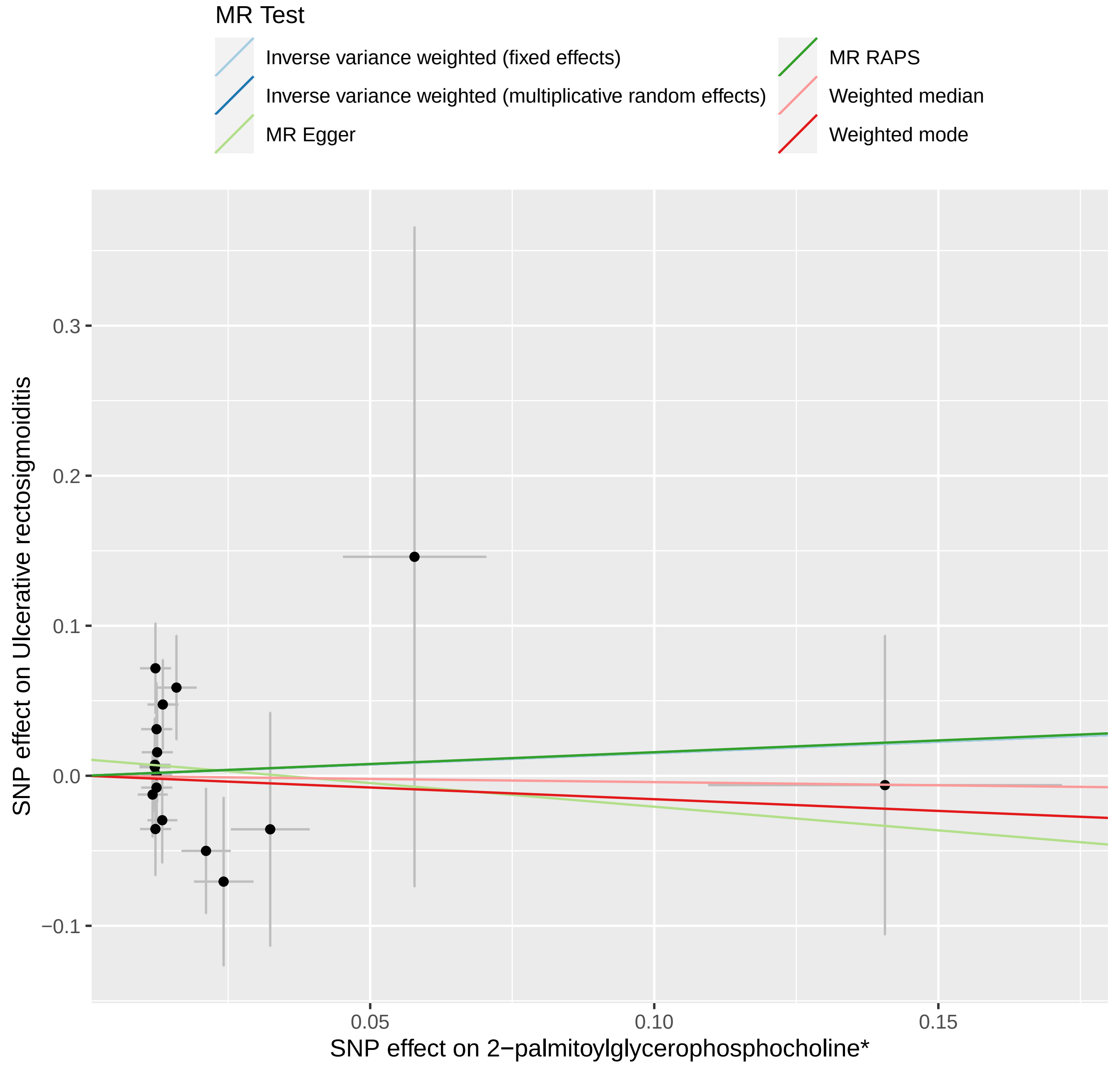

Batch 2706 : 2−oleoylglycerophosphocholine* on Ulcerative rectosigmoiditis

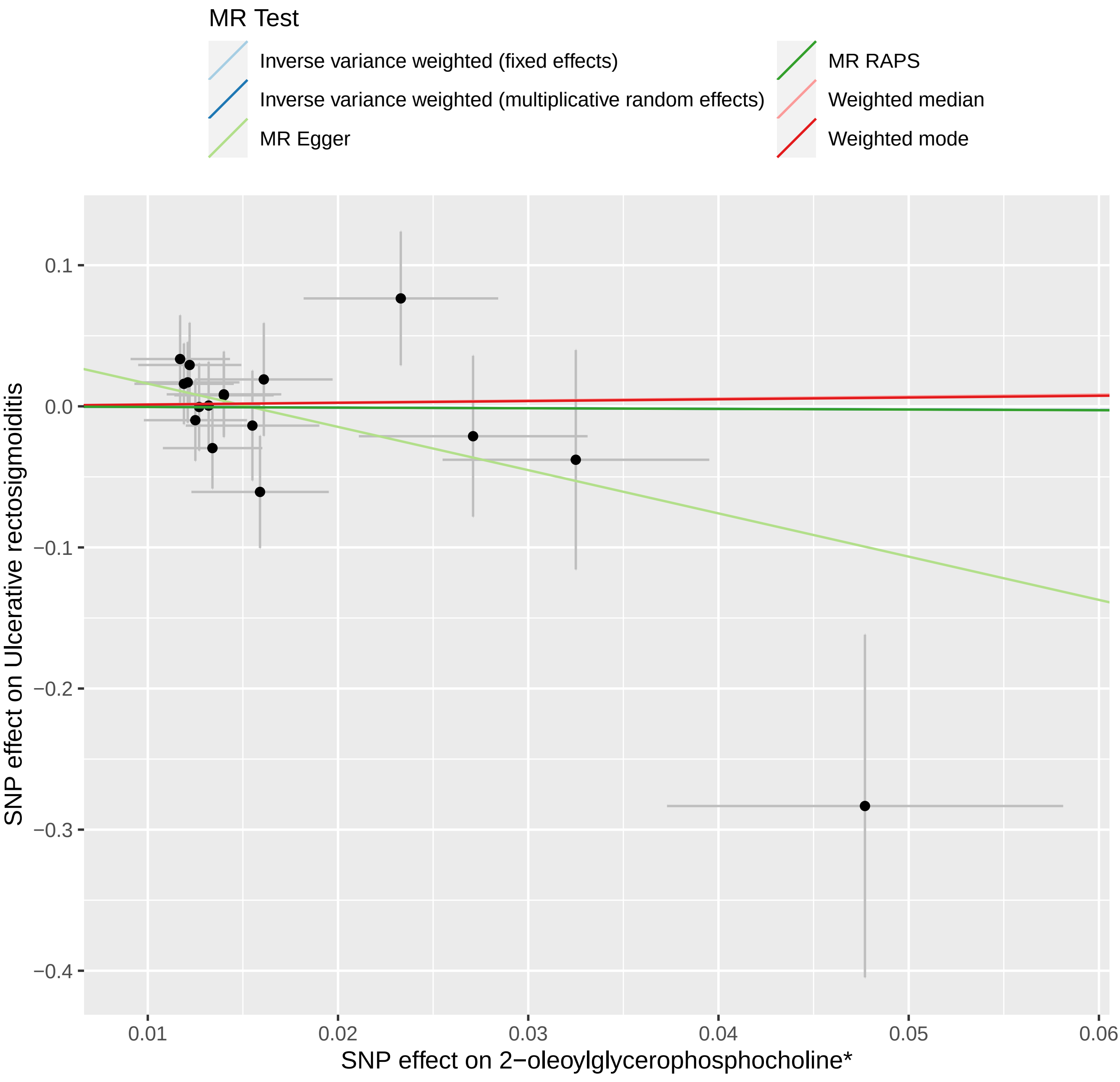

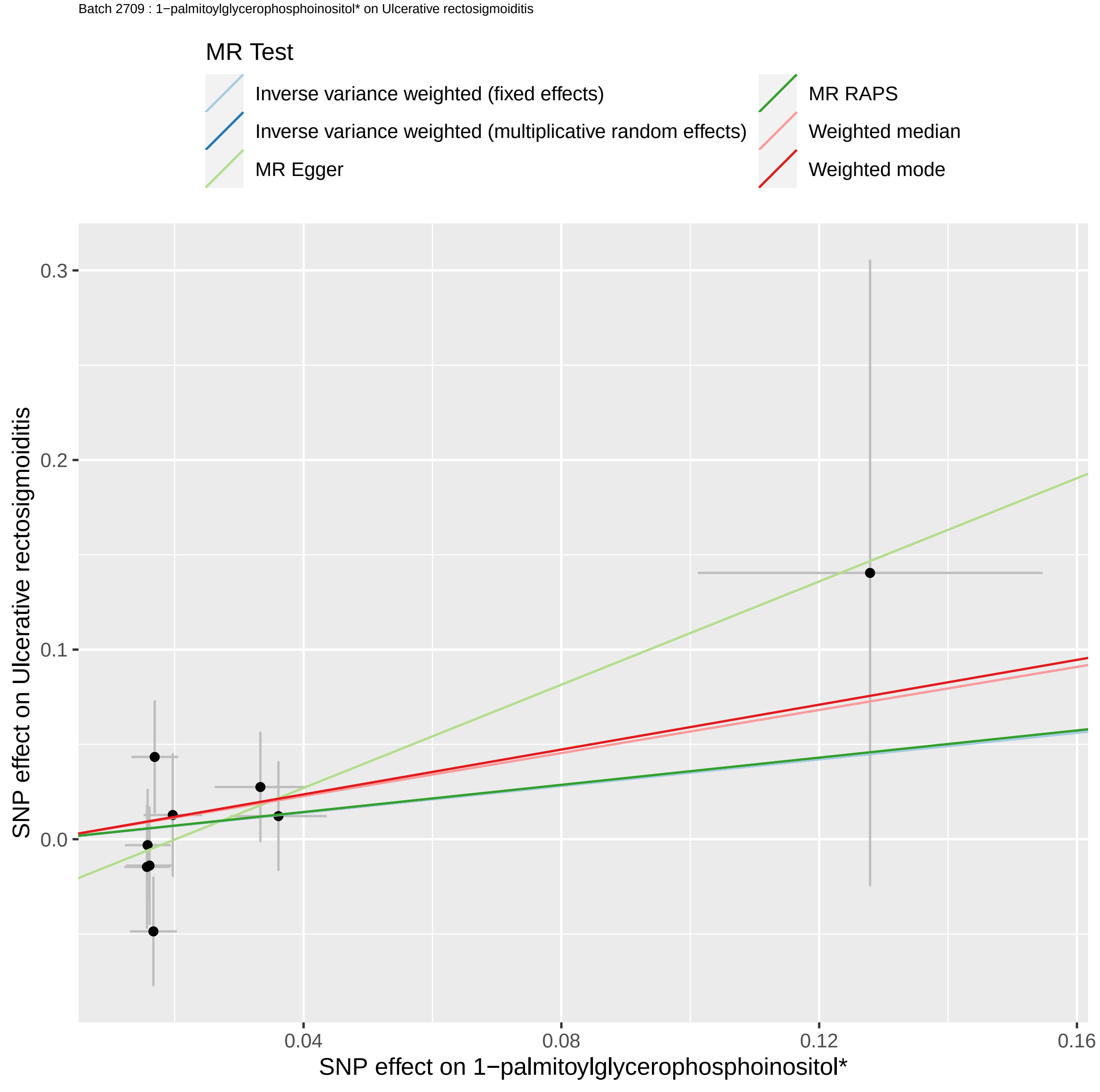

Batch 2709 : 1−palmitoylglycerophosphoinositol* on Ulcerative rectosigmoiditis
MR Test
Inverse variance weighted (fixed effects)
Inverse variance weighted (multiplicative random effects)
MR Egger
MR RAPS
Weighted median
Weighted mode
0.3
0.2
0.1
0.0
0.04
0.08
0.12
0.16
SNP effect on Ulcerative rectosigmoiditis
SNP effect on 1−palmitoylglycerophosphoinositol*

Batch 2712 : Glutaroyl carnitine on Ulcerative rectosigmoiditis

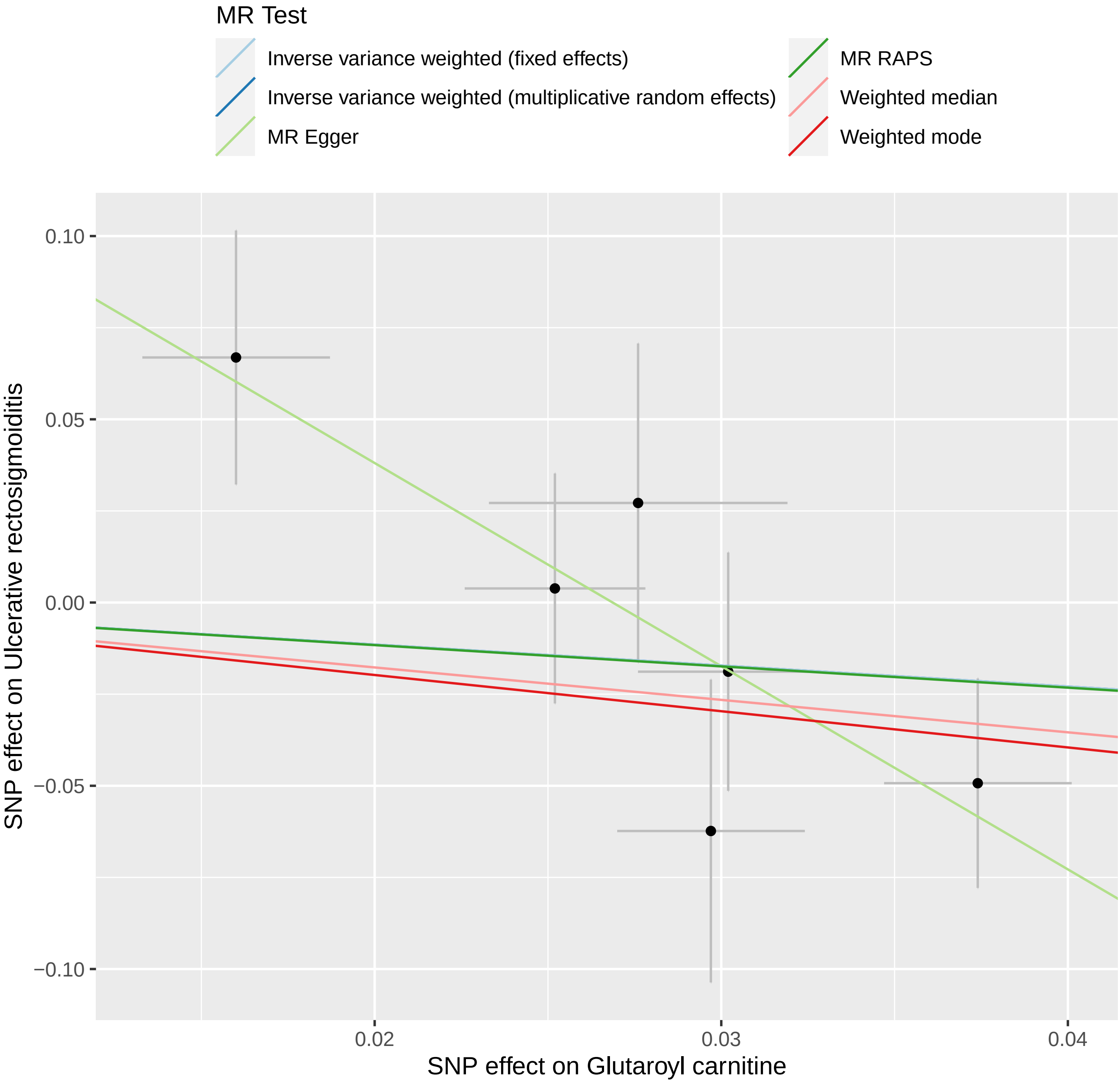

Batch 2714 : 4−hydroxyhippurate on Ulcerative rectosigmoiditis

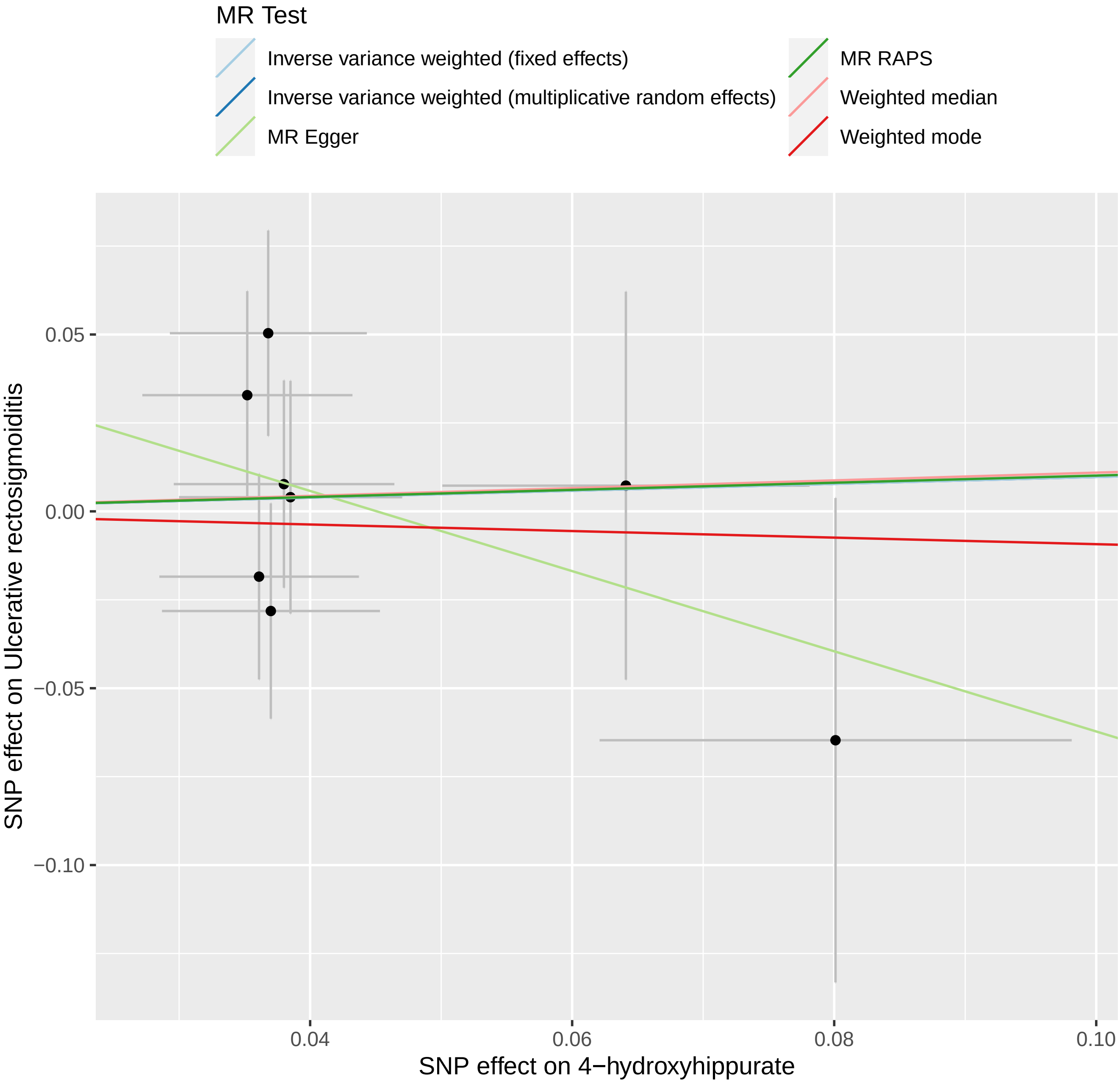

Batch 2715 : 1−myristoylglycerophosphocholine on Ulcerative rectosigmoiditis

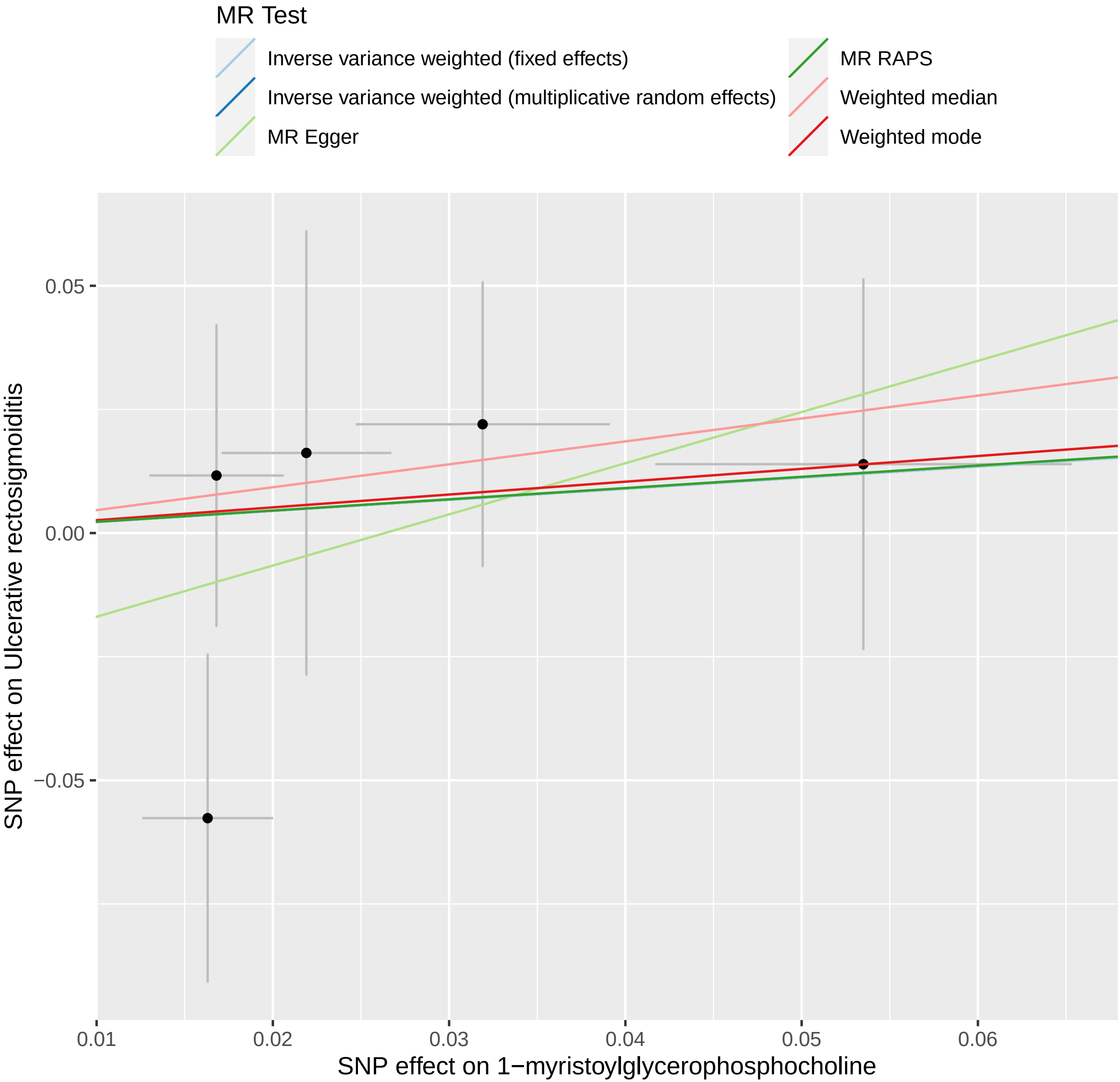

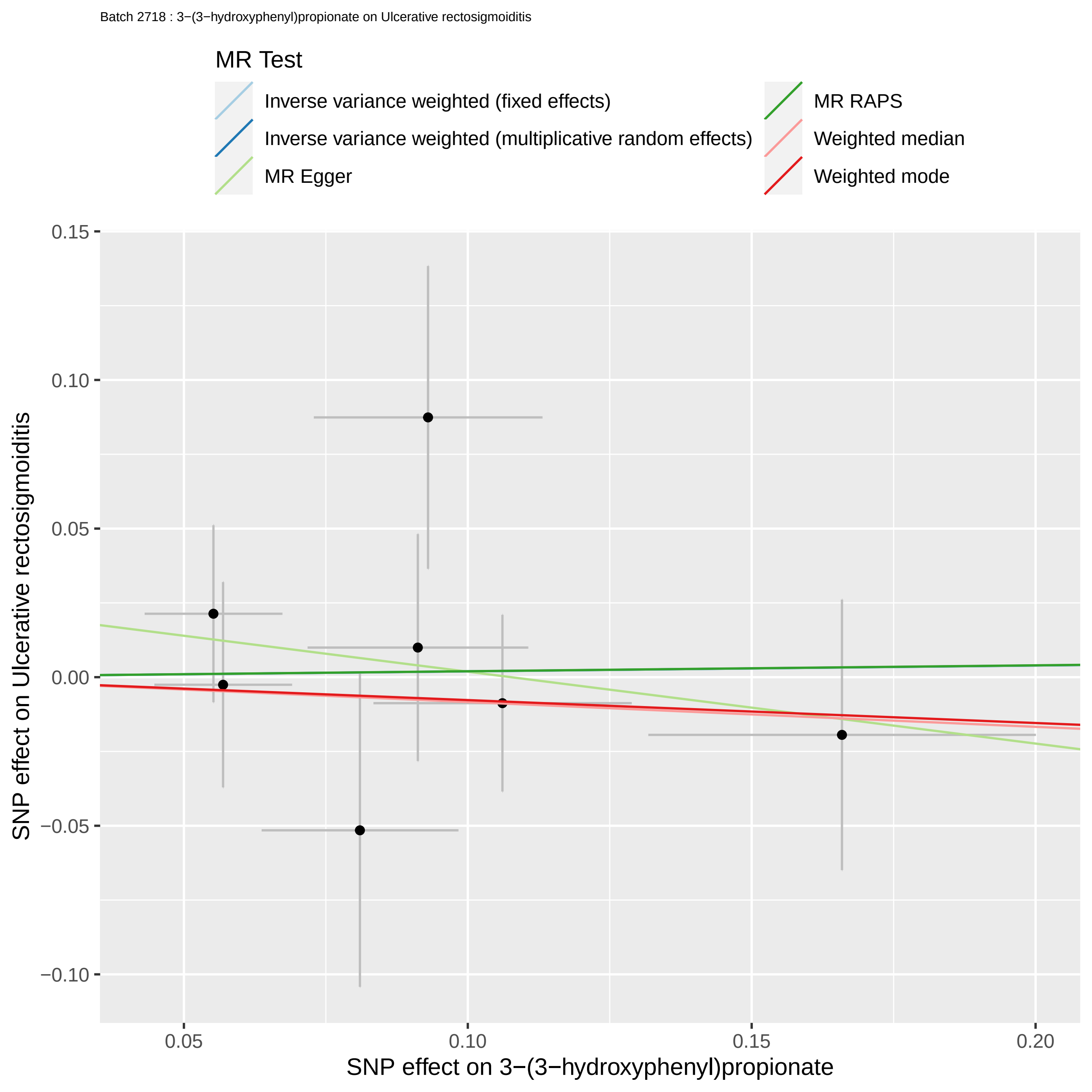
Batch 2718 : 3−(3−hydroxyphenyl)propionate on Ulcerative rectosigmoiditis
MR Test
Inverse variance weighted (fixed effects)
Inverse variance weighted (multiplicative random effects)
MR Egger
MR RAPS
Weighted median
Weighted mode
0.15
0.10
0.05
0.00
−0.05
−0.10
0.05
0.10
0.15
0.20
SNP effect on Ulcerative rectosigmoiditis
SNP effect on 3−(3−hydroxyphenyl)propionate

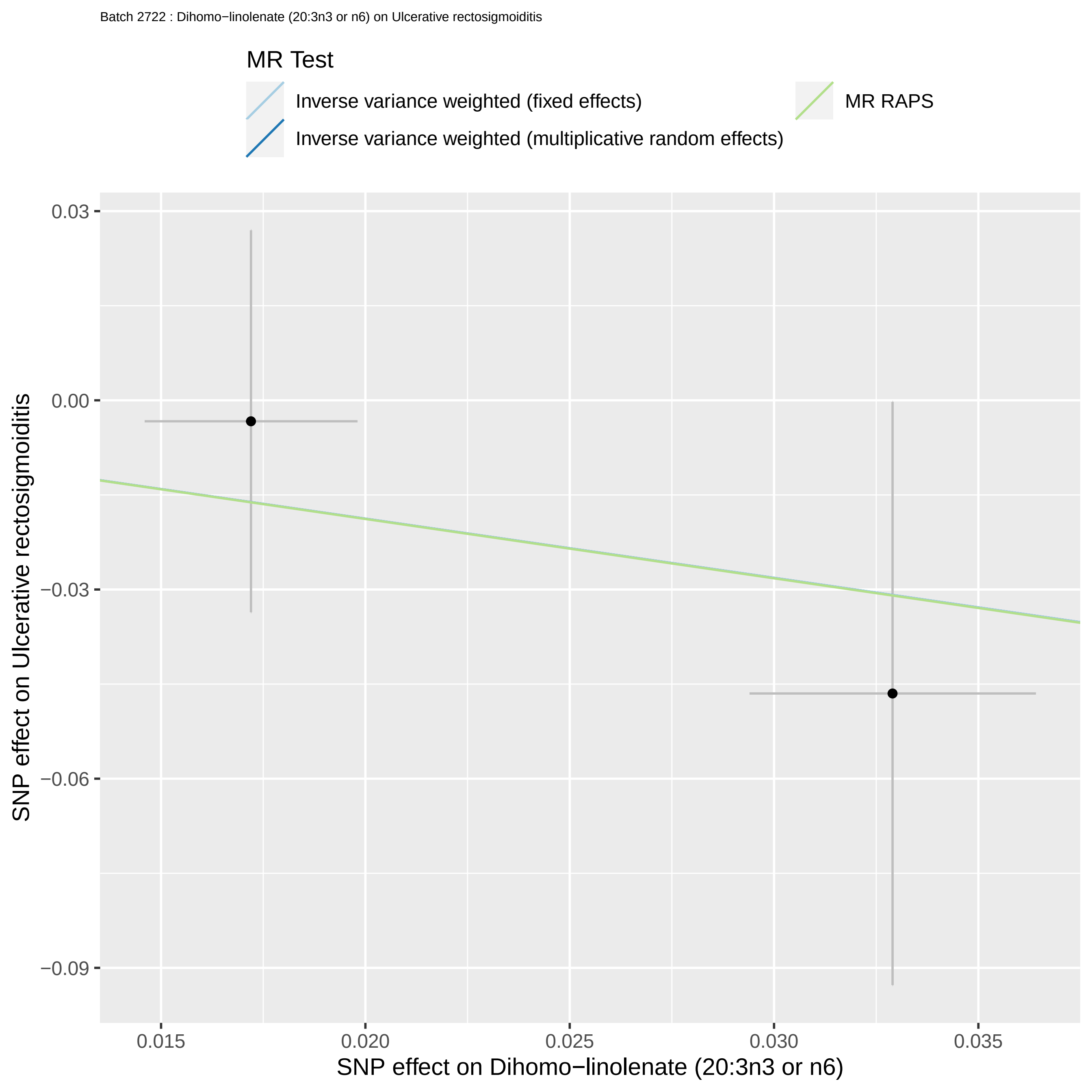

Batch 2722 : Dihomo−linolenate (20:3n3 or n6) on Ulcerative rectosigmoiditis
MR Test
Inverse variance weighted (fixed effects)
Inverse variance weighted (multiplicative random effects)
MR RAPS
0.03
0.00
−0.03
−0.06
−0.09
0.015
0.020
0.025
0.030
0.035
SNP effect on Ulcerative rectosigmoiditis
SNP effect on Dihomo−linolenate (20:3n3 or n6)

Batch 2723 : Threitol on Ulcerative rectosigmoiditis

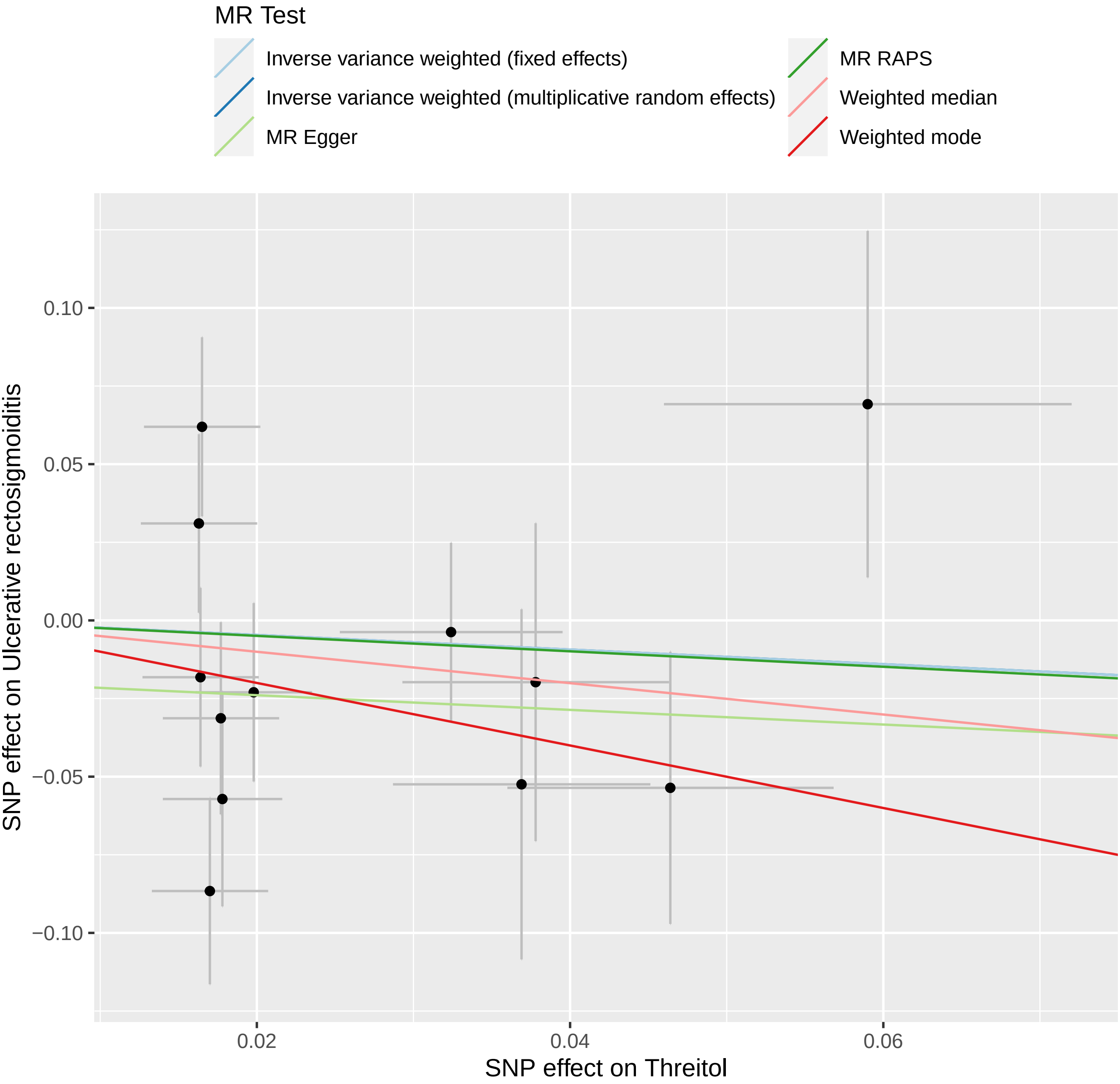

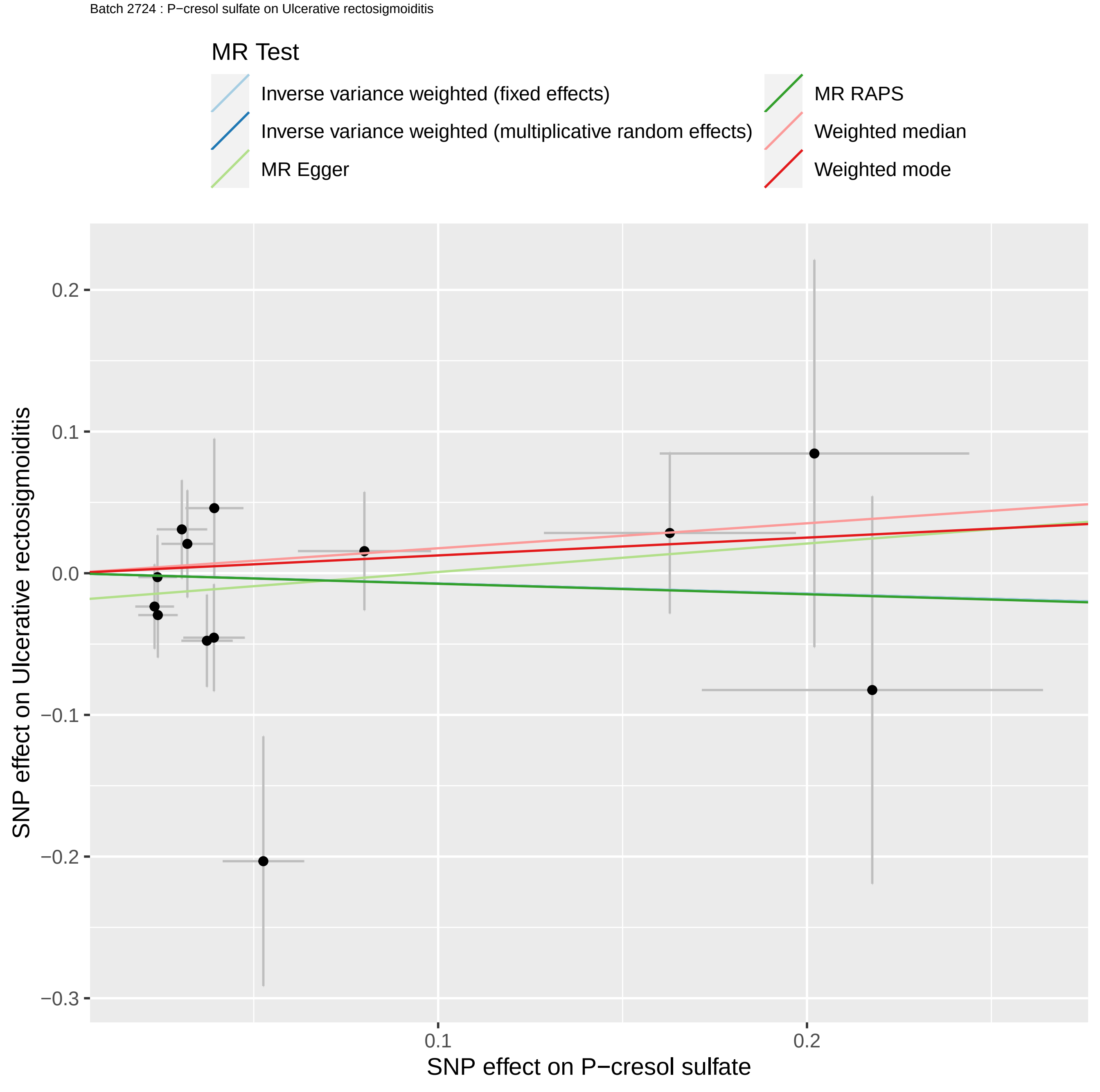
Batch 2724 : P−cresol sulfate on Ulcerative rectosigmoiditis
MR Test
Inverse variance weighted (fixed effects)
Inverse variance weighted (multiplicative random effects)
MR Egger
MR RAPS
Weighted median
Weighted mode
0.2
0.1
0.0
−0.1
−0.2
−0.3
0.1
0.2
SNP effect on Ulcerative rectosigmoiditis
SNP effect on P−cresol sulfate

Batch 2727 : X−14208−−phenylalanylserine on Ulcerative rectosigmoiditis

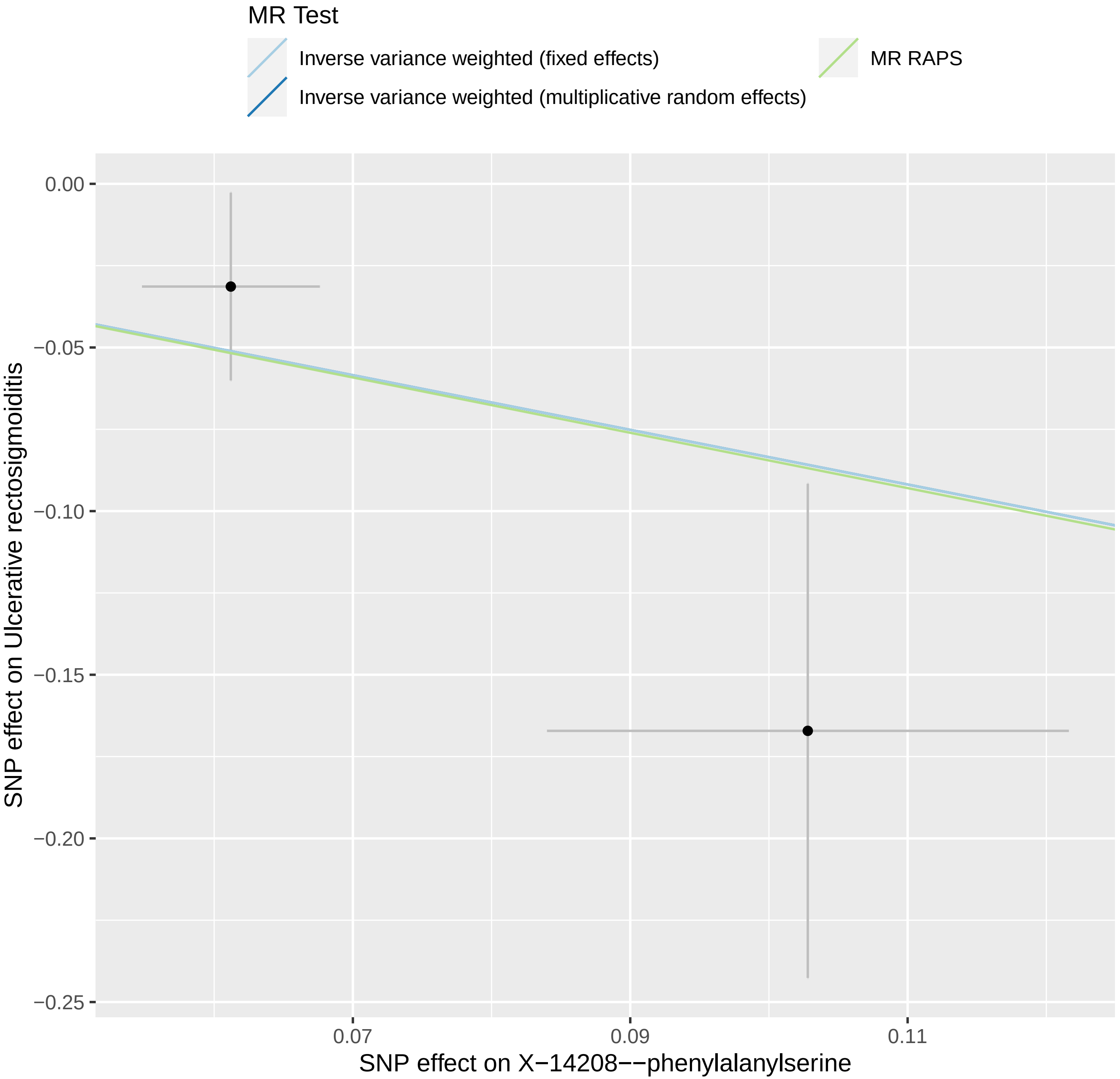

Batch 2729 : X−14450−−phenylalanylleucine on Ulcerative rectosigmoiditis

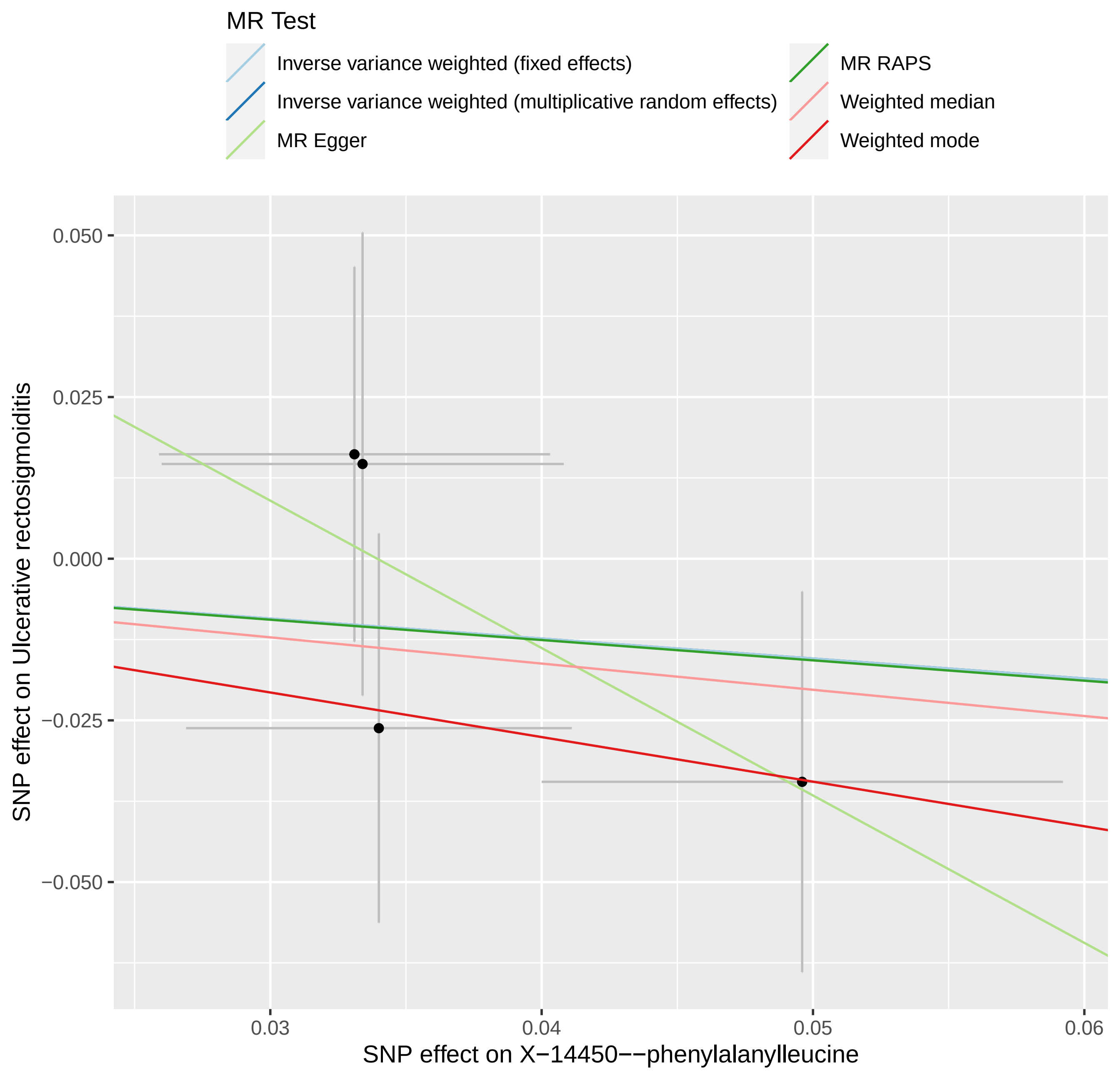

Batch 2730 : Gamma−glutamylglutamate on Ulcerative rectosigmoiditis

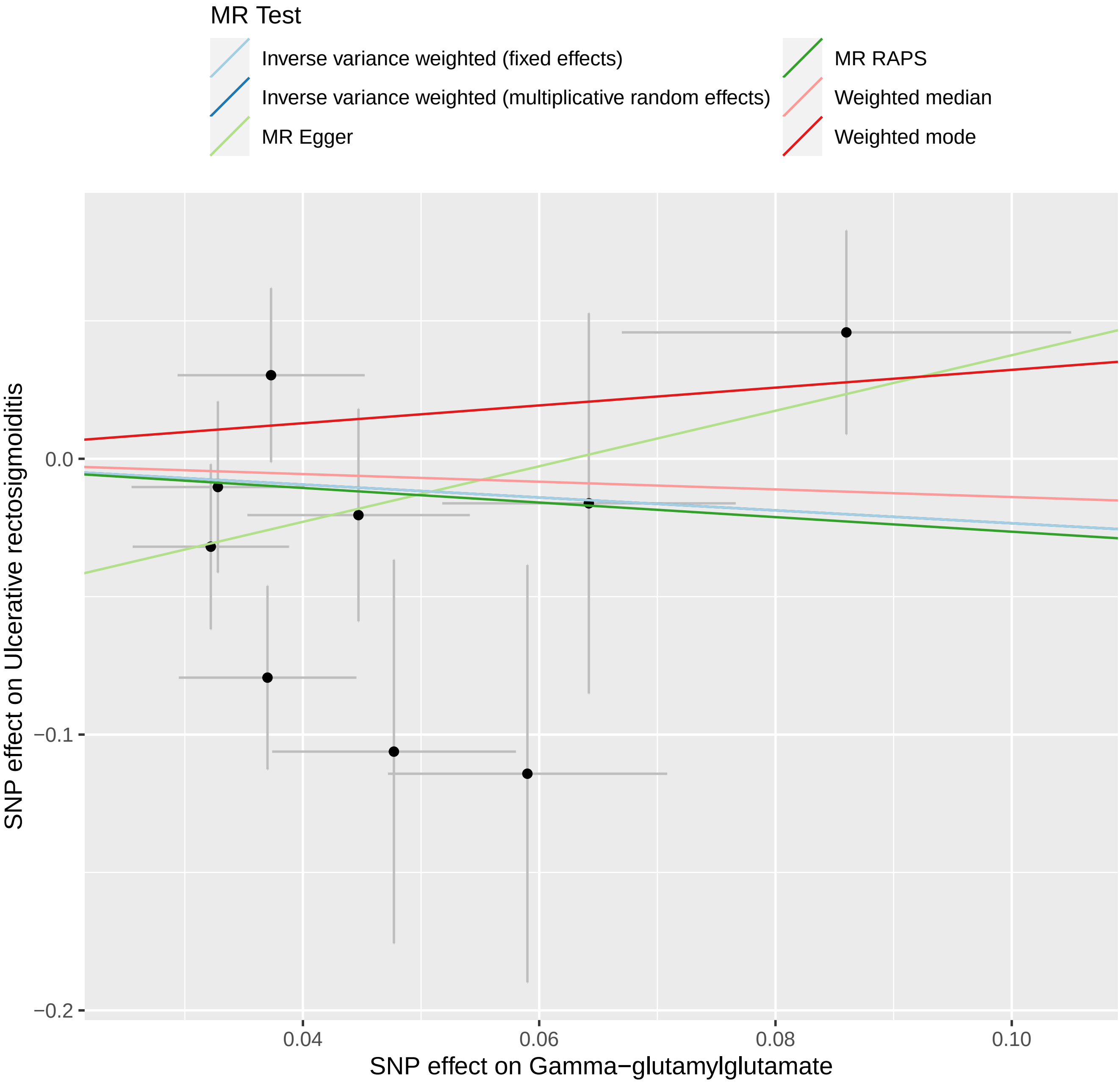

Batch 2731 : Octadecanedioate on Ulcerative rectosigmoiditis

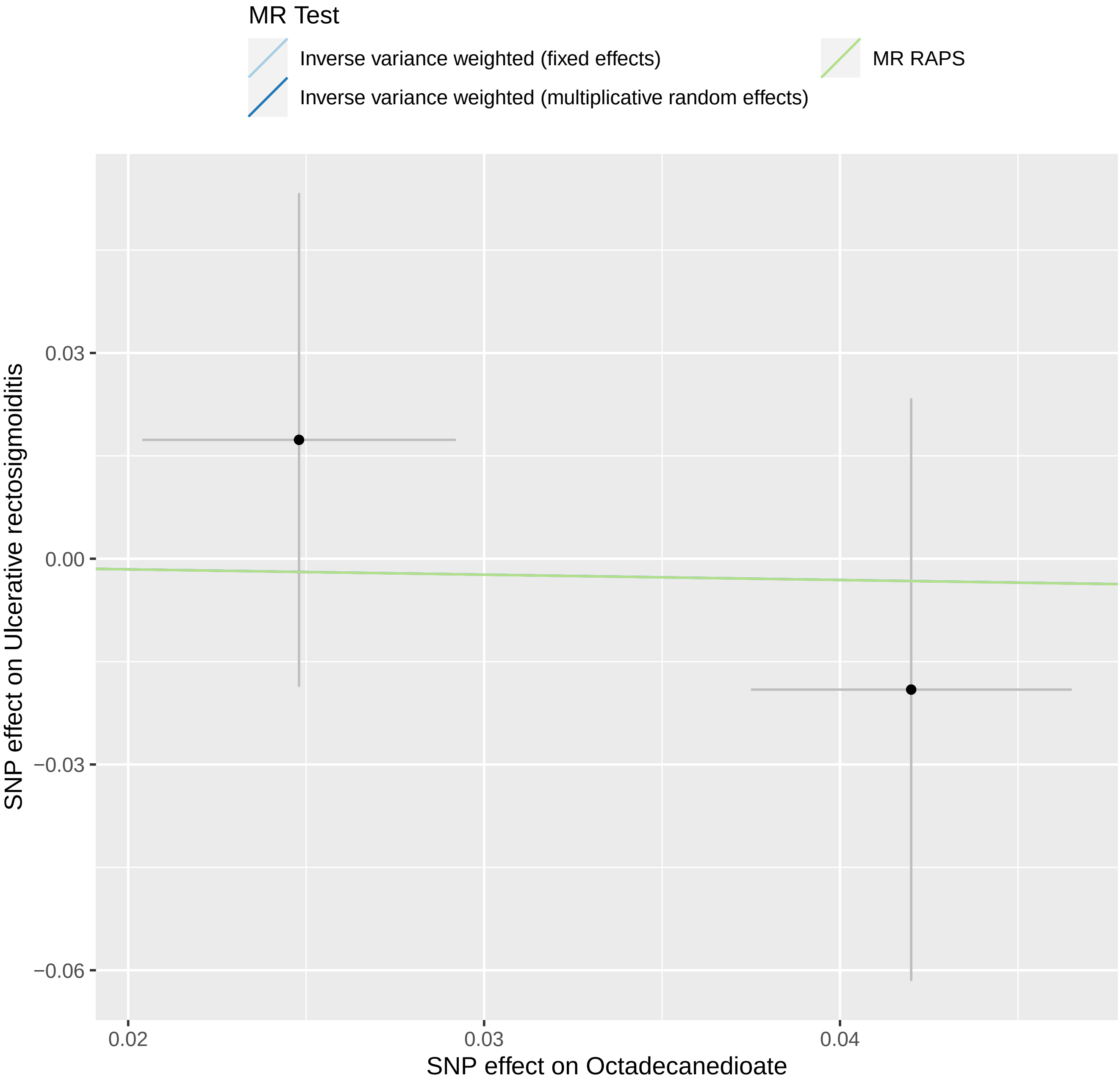

Batch 2732 : Leucylleucine on Ulcerative rectosigmoiditis

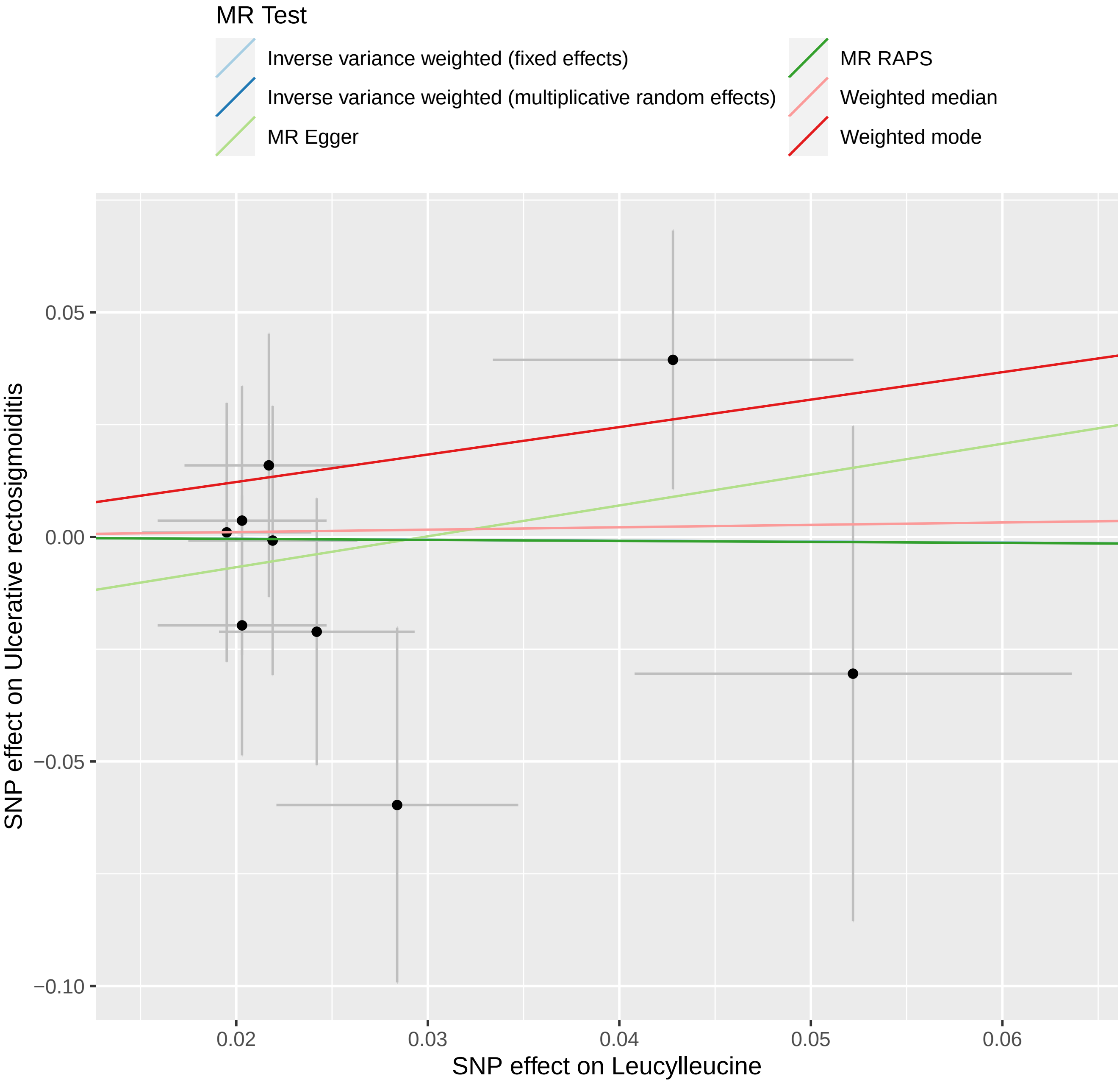

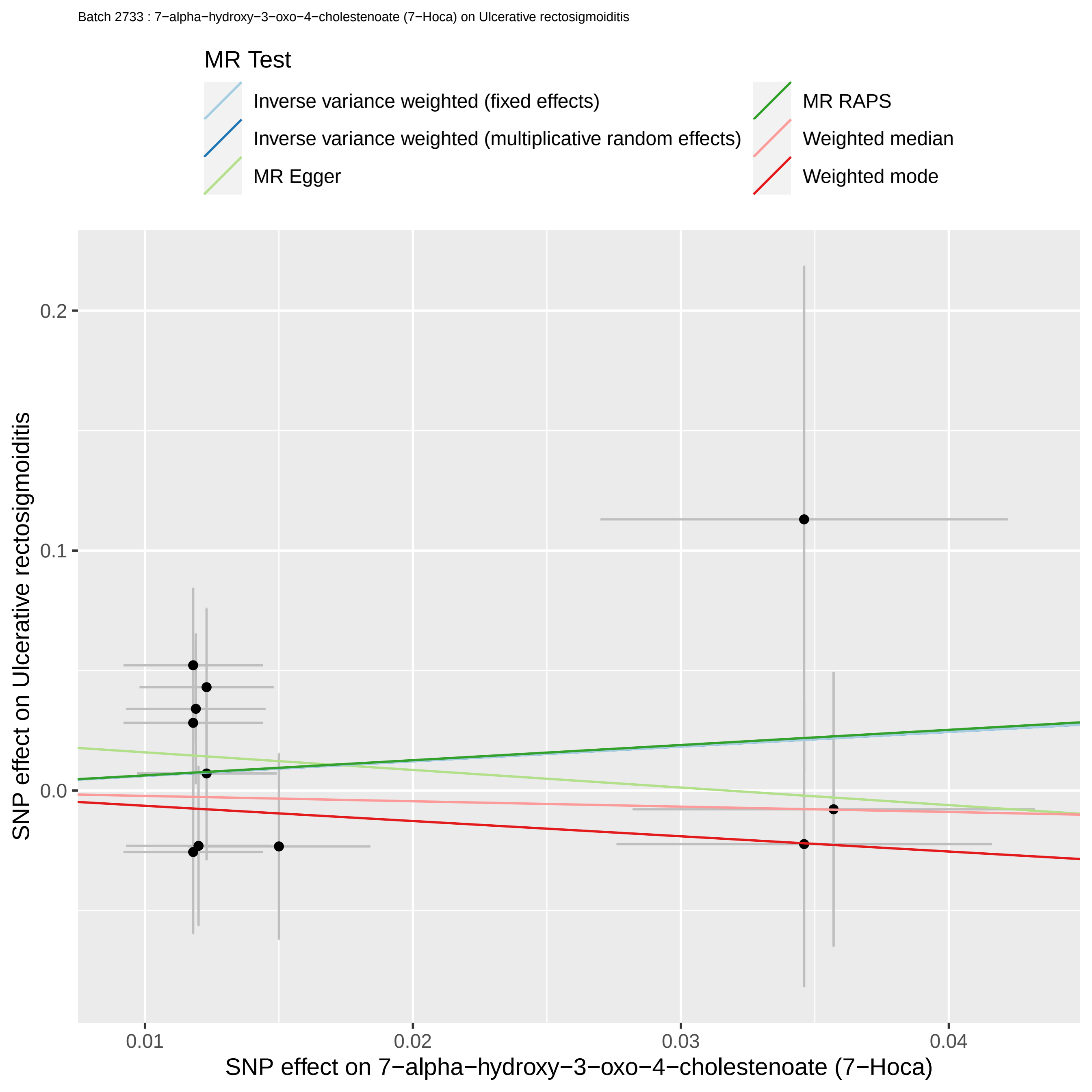

Batch 2733 : 7−alpha−hydroxy−3−oxo−4−cholestenoate (7−Hoca) on Ulcerative rectosigmoiditis
MR Test
Inverse variance weighted (fixed effects)
Inverse variance weighted (multiplicative random effects)
MR Egger
MR RAPS
Weighted median
Weighted mode
0.2
0.1
0.0
0.01
0.02
0.03
0.04
SNP effect on Ulcerative rectosigmoiditis
SNP effect on 7−alpha−hydroxy−3−oxo−4−cholestenoate (7−Hoca)

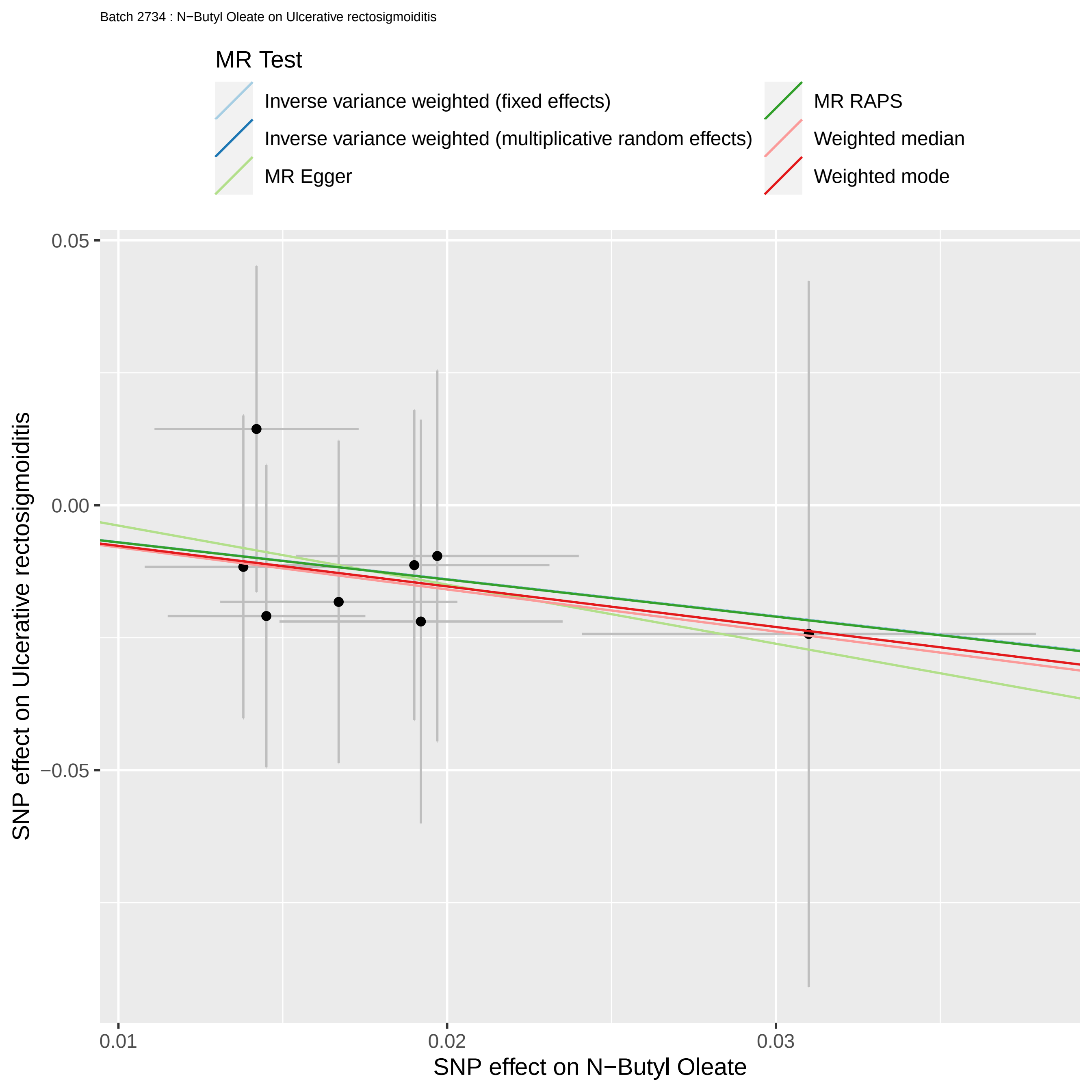
Batch 2734 : N−Butyl Oleate on Ulcerative rectosigmoiditis
MR Test
Inverse variance weighted (fixed effects)
Inverse variance weighted (multiplicative random effects)
MR Egger
MR RAPS
Weighted median
Weighted mode
0.05
0.00
−0.05
0.01
0.02
0.03
SNP effect on Ulcerative rectosigmoiditis
SNP effect on N−Butyl Oleate

Batch 2736 : Taurolithocholate 3−sulfate on Ulcerative rectosigmoiditis

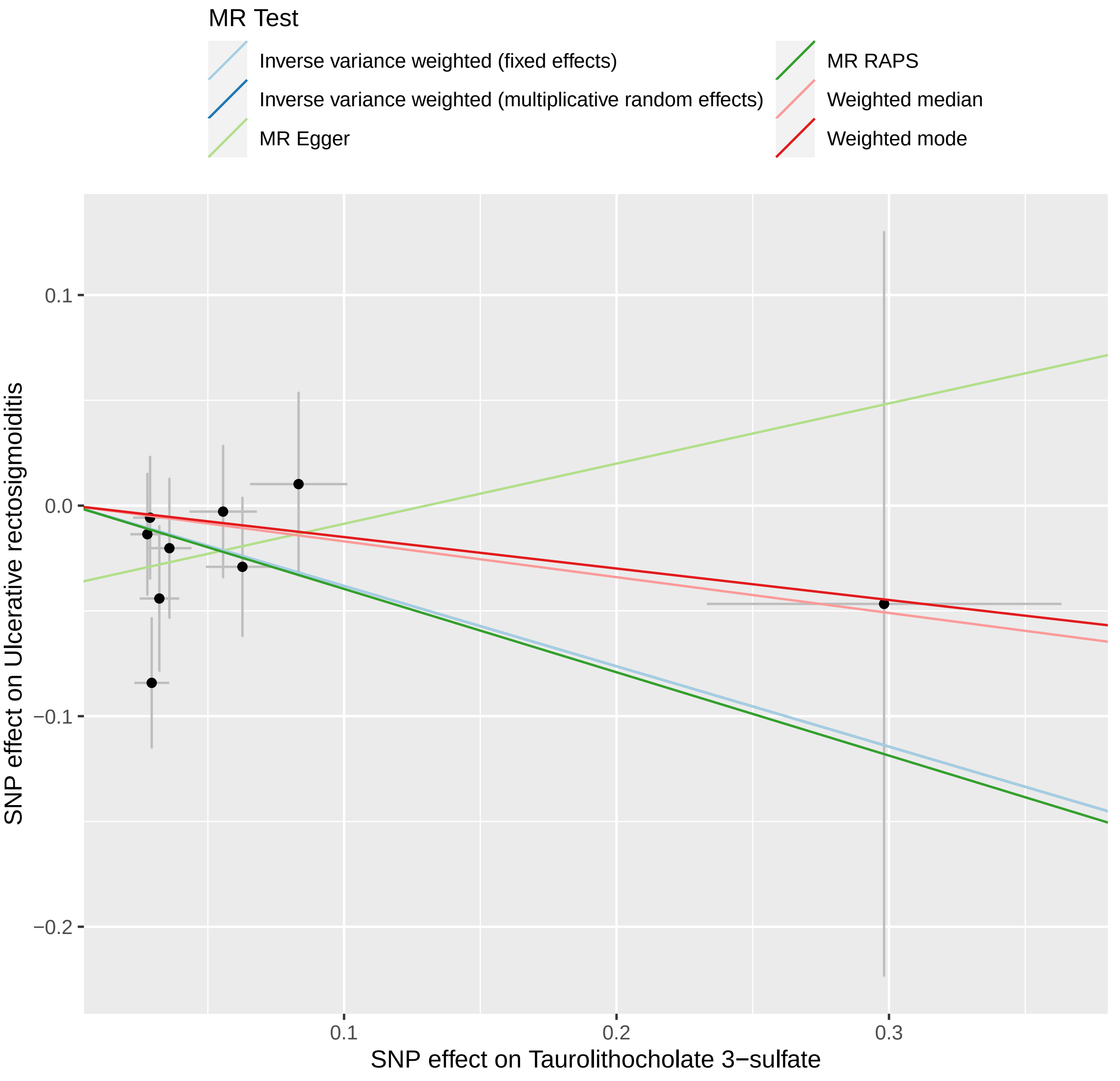

Batch 2737 : X−14977−−vanillin on Ulcerative rectosigmoiditis

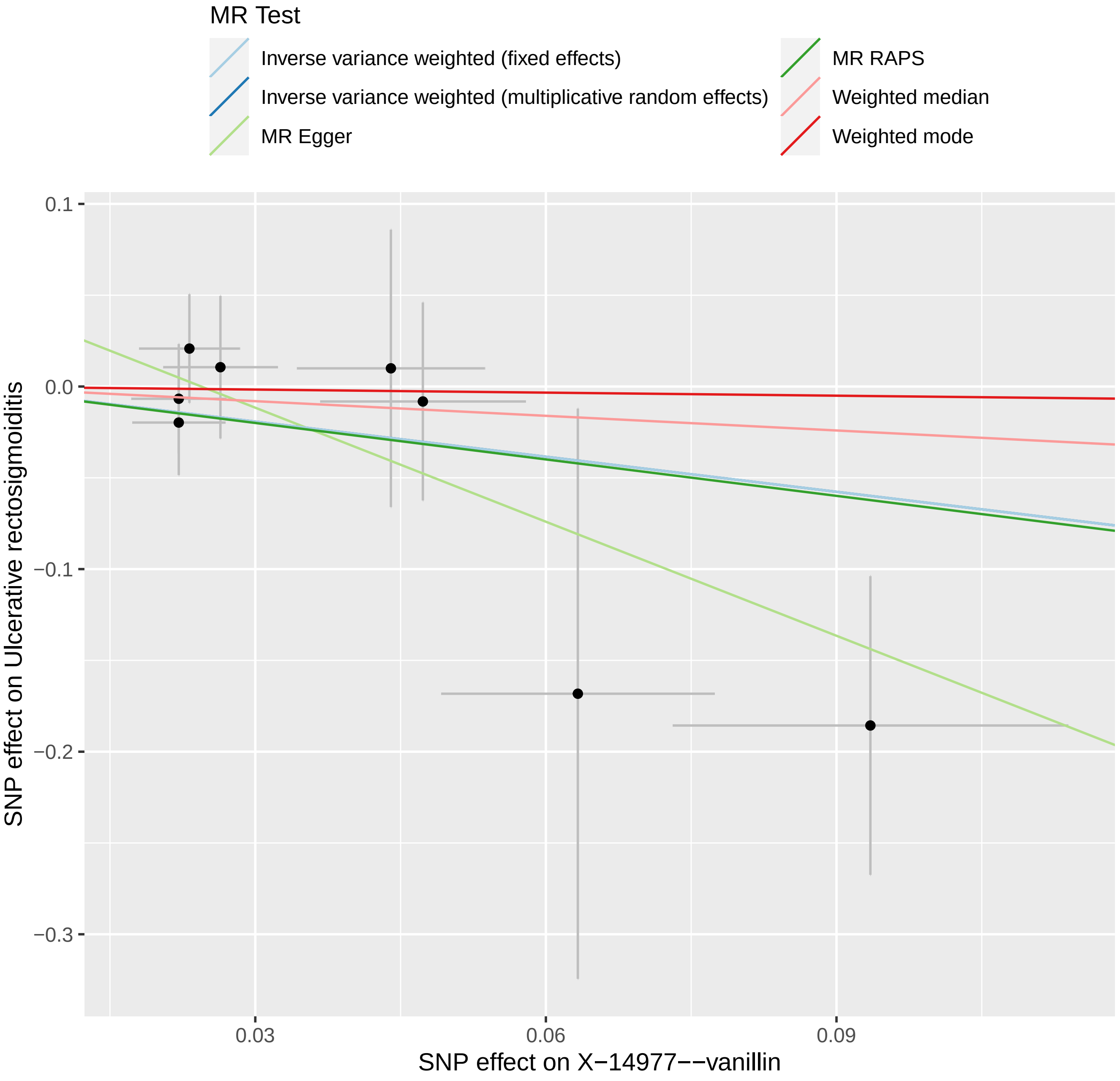

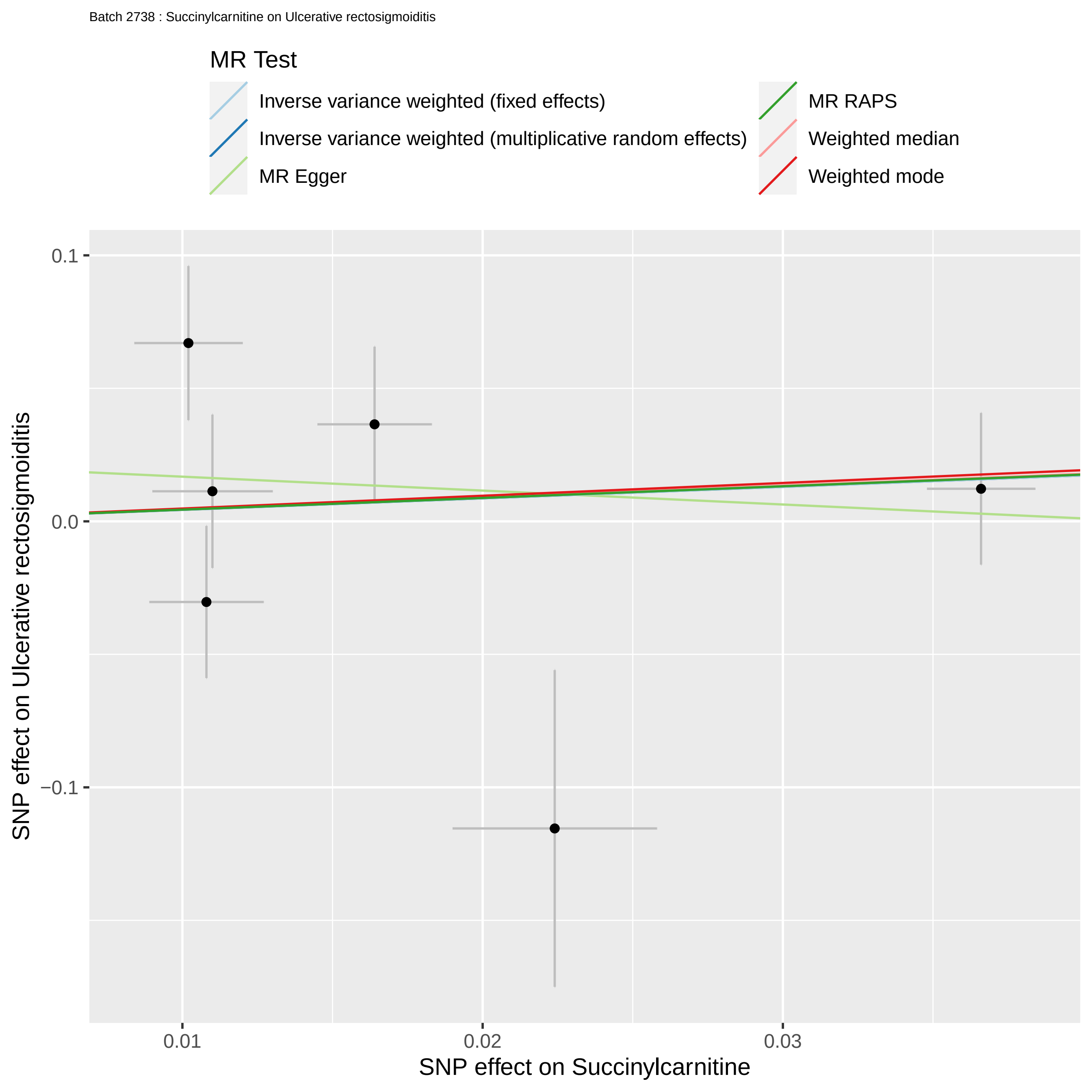
Batch 2738 : Succinylcarnitine on Ulcerative rectosigmoiditis
MR Test
Inverse variance weighted (fixed effects)
Inverse variance weighted (multiplicative random effects)
MR Egger
MR RAPS
Weighted median
Weighted mode
0.1
0.0
−0.1
0.01
0.02
0.03
SNP effect on Ulcerative rectosigmoiditis
SNP effect on Succinylcarnitine

Batch 2740 : Cyclo(leu−pro) on Ulcerative rectosigmoiditis

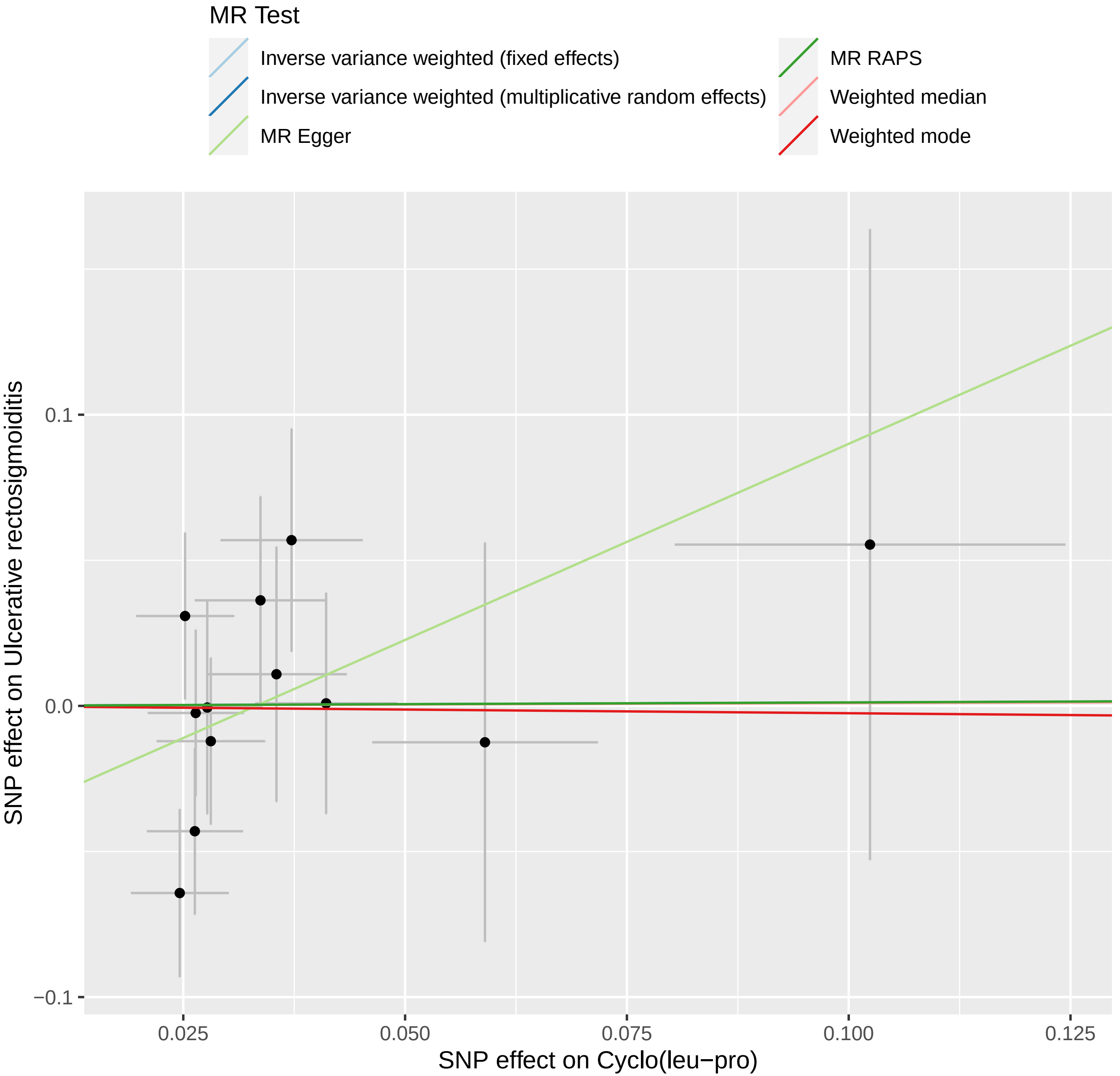

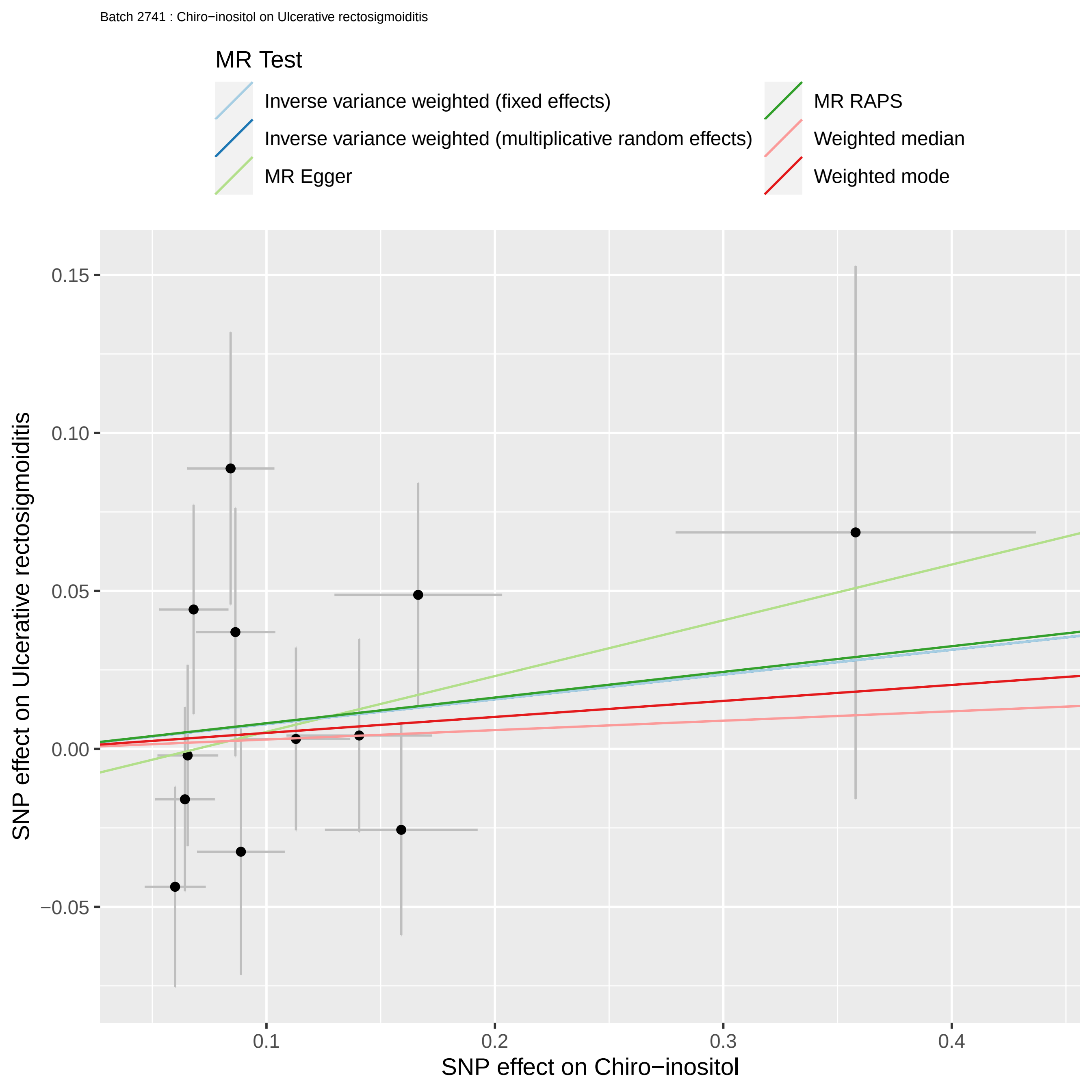

Batch 2741 : Chiro−inositol on Ulcerative rectosigmoiditis
MR Test
Inverse variance weighted (fixed effects)
Inverse variance weighted (multiplicative random effects)
MR Egger
MR RAPS
Weighted median
Weighted mode
SNP effect on Ulcerative rectosigmoiditis
0.15
0.10
0.05
0.00
−0.05
0.1
0.2
0.3
0.4
SNP effect on Chiro−inositol

Batch 2745 : 2−hydroxyglutarate on Ulcerative rectosigmoiditis

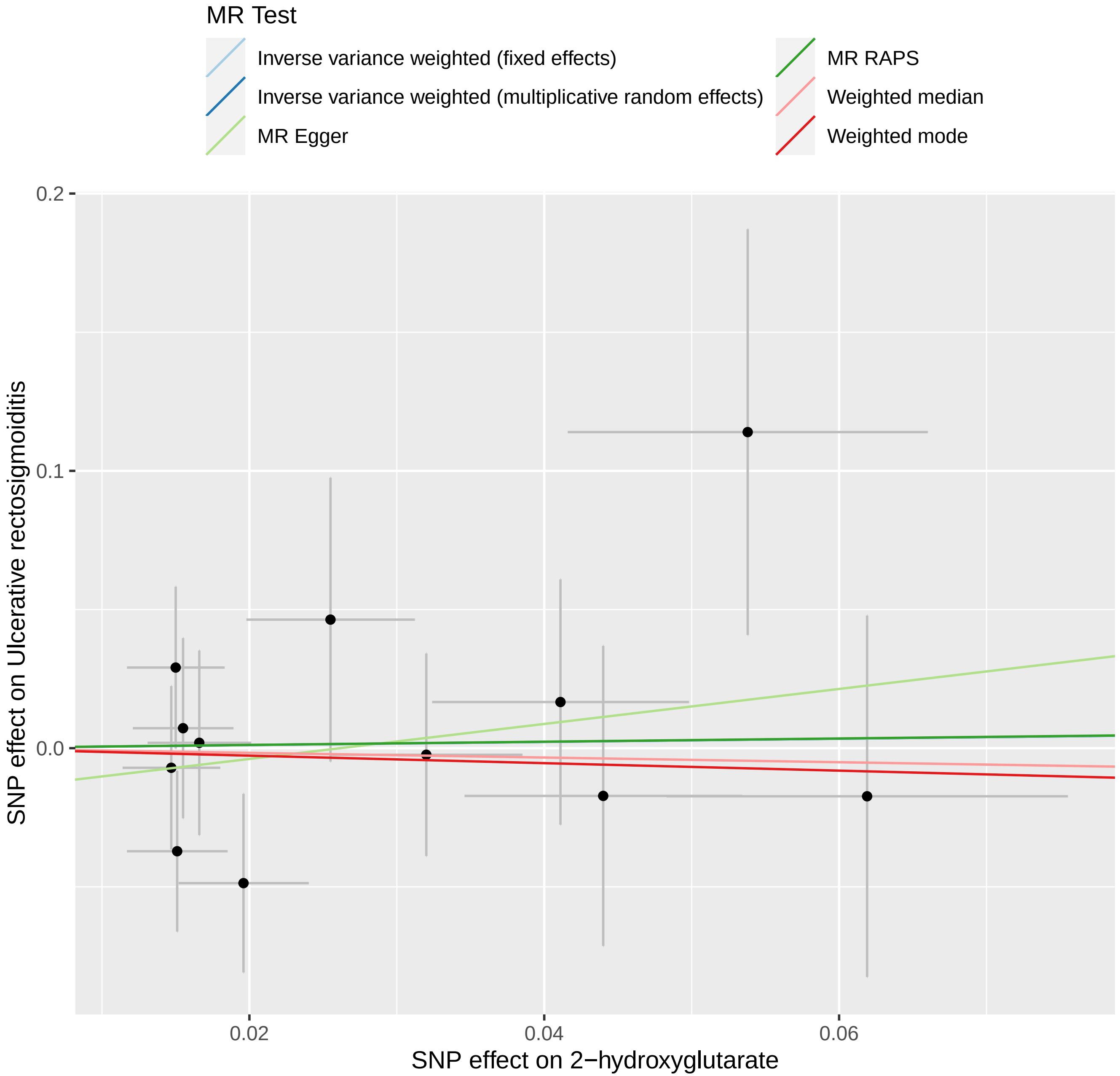

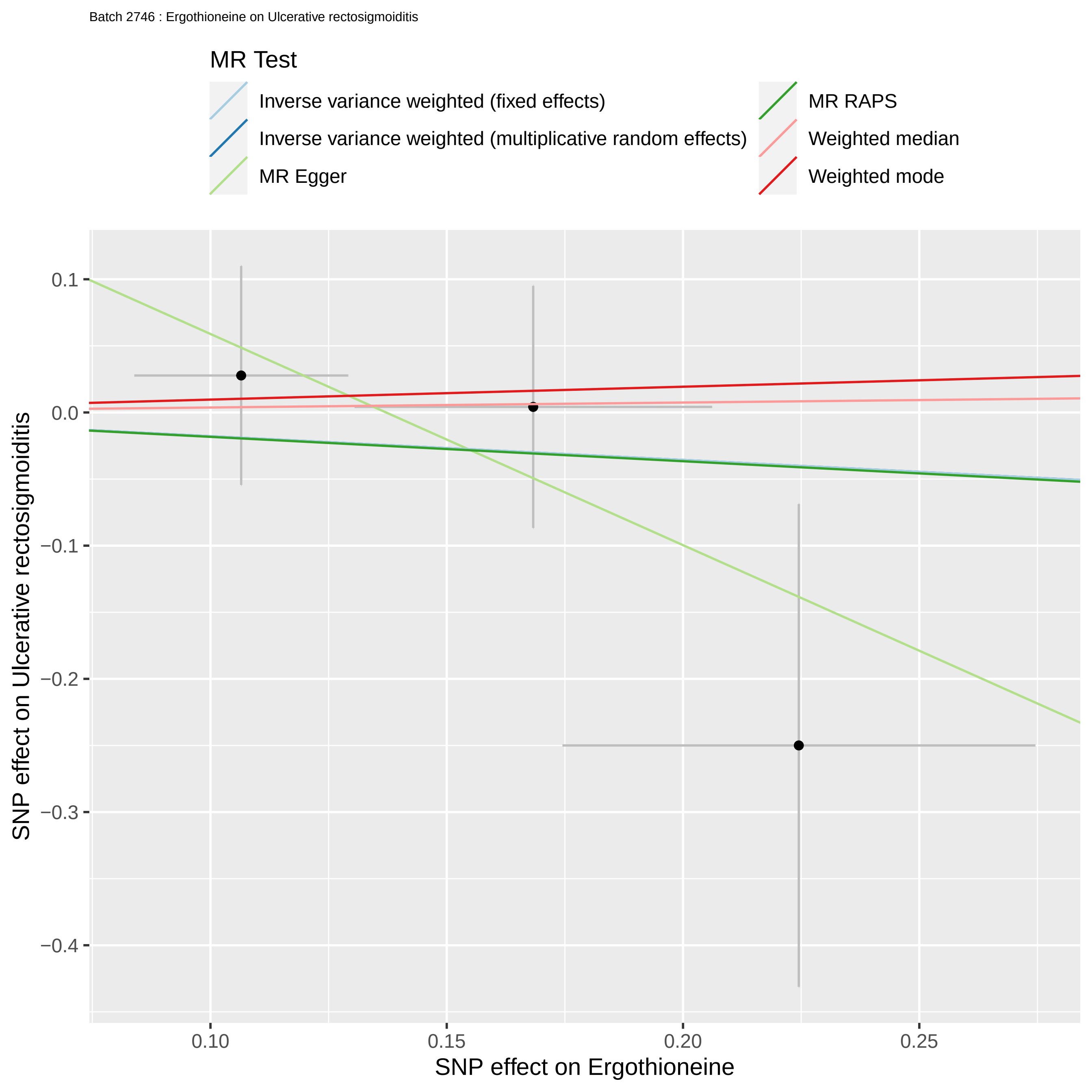

Batch 2746 : Ergothioneine on Ulcerative rectosigmoiditis
MR Test
Inverse variance weighted (fixed effects)
Inverse variance weighted (multiplicative random effects)
MR Egger
MR RAPS
Weighted median
Weighted mode
0.1
0.0
−0.1
−0.2
−0.3
−0.4
SNP effect on Ulcerative rectosigmoiditis
0.10
0.15
0.20
0.25
SNP effect on Ergothioneine

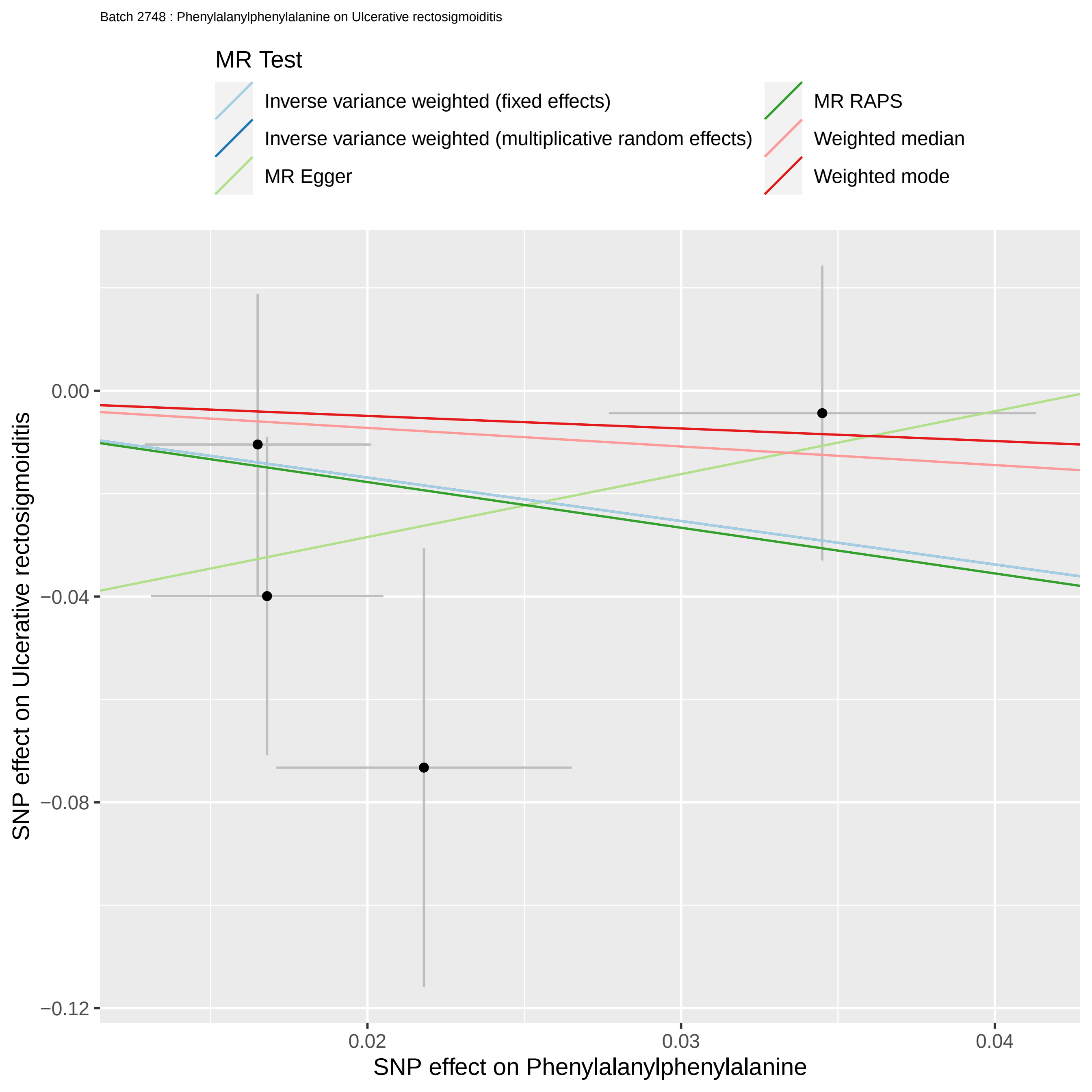

Batch 2748 : Phenylalanylphenylalanine on Ulcerative rectosigmoiditis
MR Test
Inverse variance weighted (fixed effects)
Inverse variance weighted (multiplicative random effects)
MR Egger
MR RAPS
Weighted median
Weighted mode
0.00
−0.04
−0.08
−0.12
0.02
0.03
0.04
SNP effect on Ulcerative rectosigmoiditis
SNP effect on Phenylalanylphenylalanine

Batch 2749 : Cis−4−decenoyl carnitine on Ulcerative rectosigmoiditis

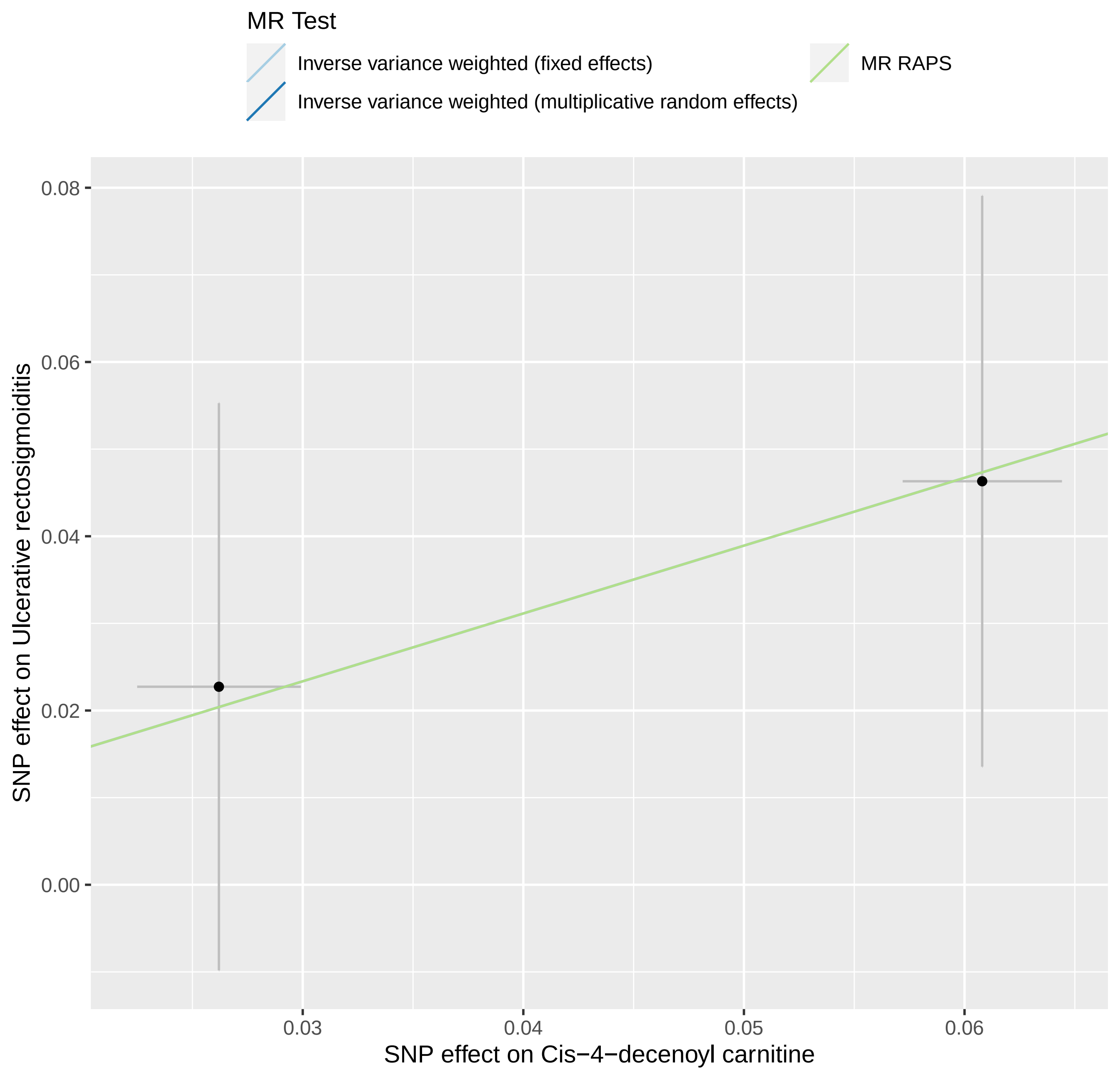

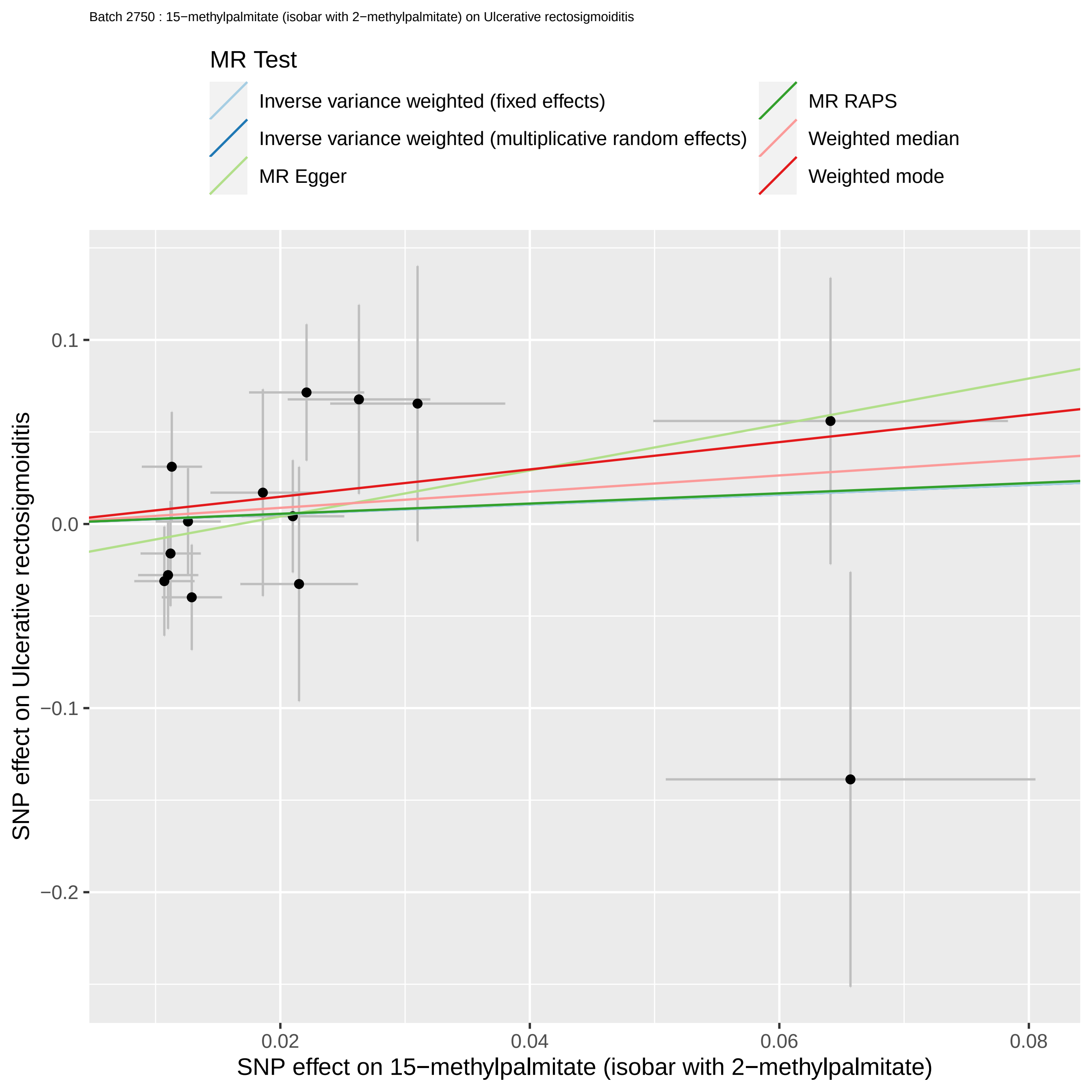
Batch 2750 : 15−methylpalmitate (isobar with 2−methylpalmitate) on Ulcerative rectosigmoiditis
MR Test
Inverse variance weighted (fixed effects)
Inverse variance weighted (multiplicative random effects)
MR Egger
MR RAPS
Weighted median
Weighted mode
0.1
0.0
−0.1
−0.2
0.02
0.04
0.06
0.08
SNP effect on 15−methylpalmitate (isobar with 2−methylpalmitate)
SNP effect on Ulcerative rectosigmoiditis
