# Supplementary material for: From serum metabolites to the gut: revealing metabolic clues to susceptibility to subtypes of Crohn’s disease and ulcerative colitis
Source: Front Endocrinol (Lausanne). 2024 Aug 8;15:1375896. doi: 10.3389/fendo.2024.1375896 (PMC11338916; doi:10.3389/fendo.2024.1375896)
Supplement: Supplementary Figure 3 — Leave-one-out Plots. [file DataSheet_3.pdf]

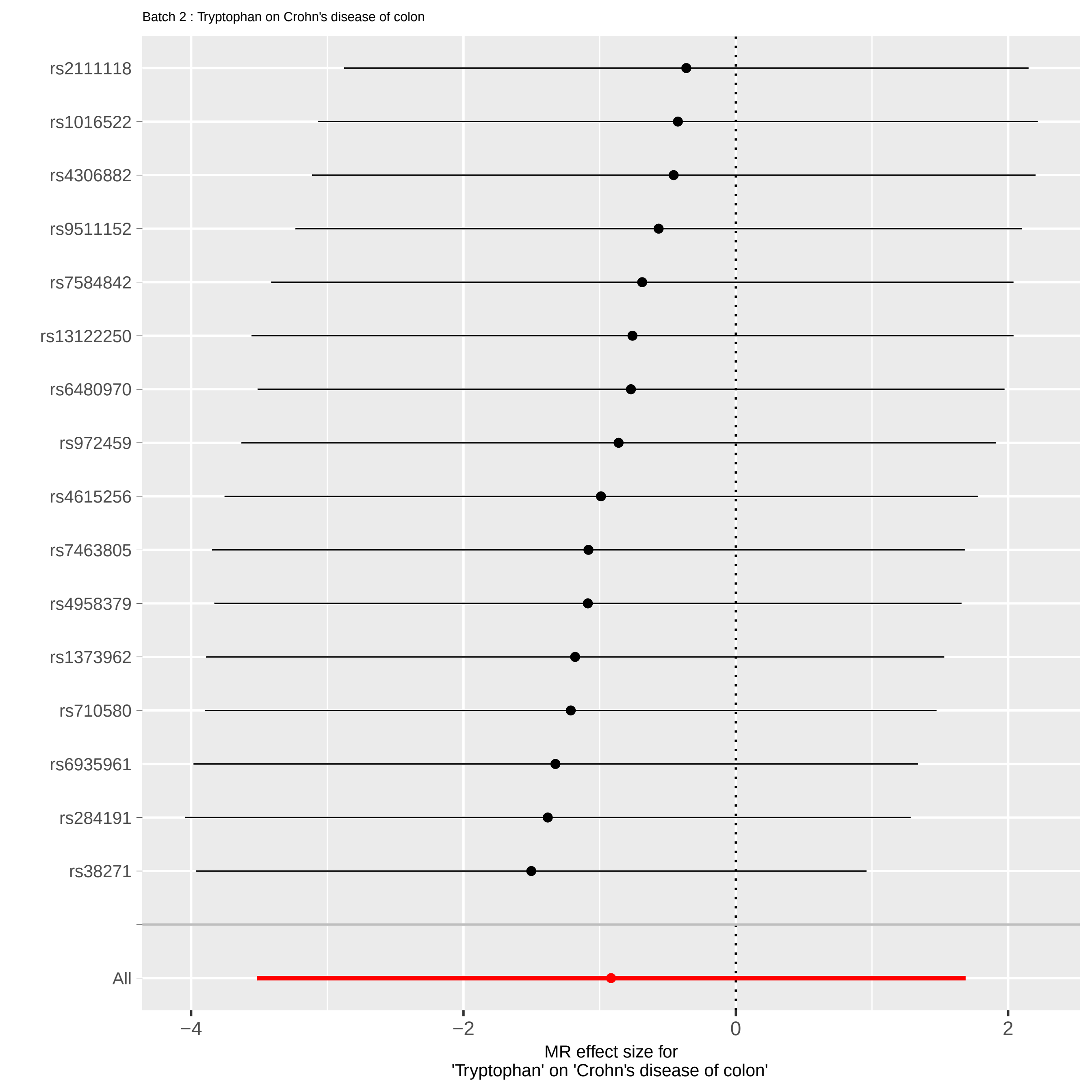
Batch 2 : Tryptophan on Crohn's disease of colon
rs2111118
rs1016522
rs4306882
rs9511152
rs7584842
rs13122250
rs6480970
rs972459
rs4615256
rs7463805
rs4958379
rs1373962
rs710580
rs6935961
rs284191
rs38271
All
−4
−2
0
2
MR effect size for
'Tryptophan' on 'Crohn's disease of colon'

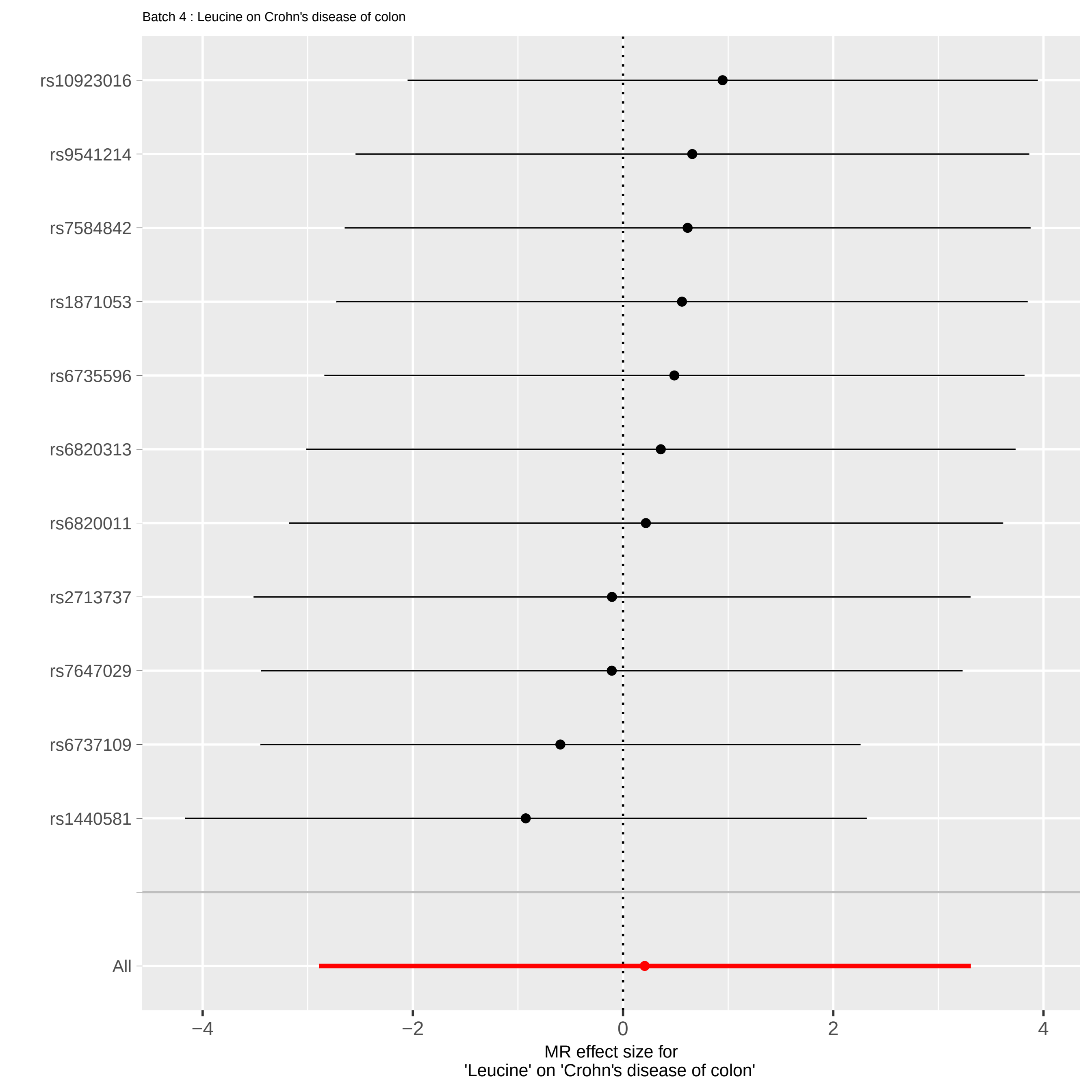
Batch 4 : Leucine on Crohn's disease of colon
rs10923016
rs9541214
rs7584842
rs1871053
rs6735596
rs6820313
rs6820011
rs2713737
rs7647029
rs6737109
rs1440581
All
−4
−2
0
2
4
MR effect size for
'Leucine' on 'Crohn's disease of colon'

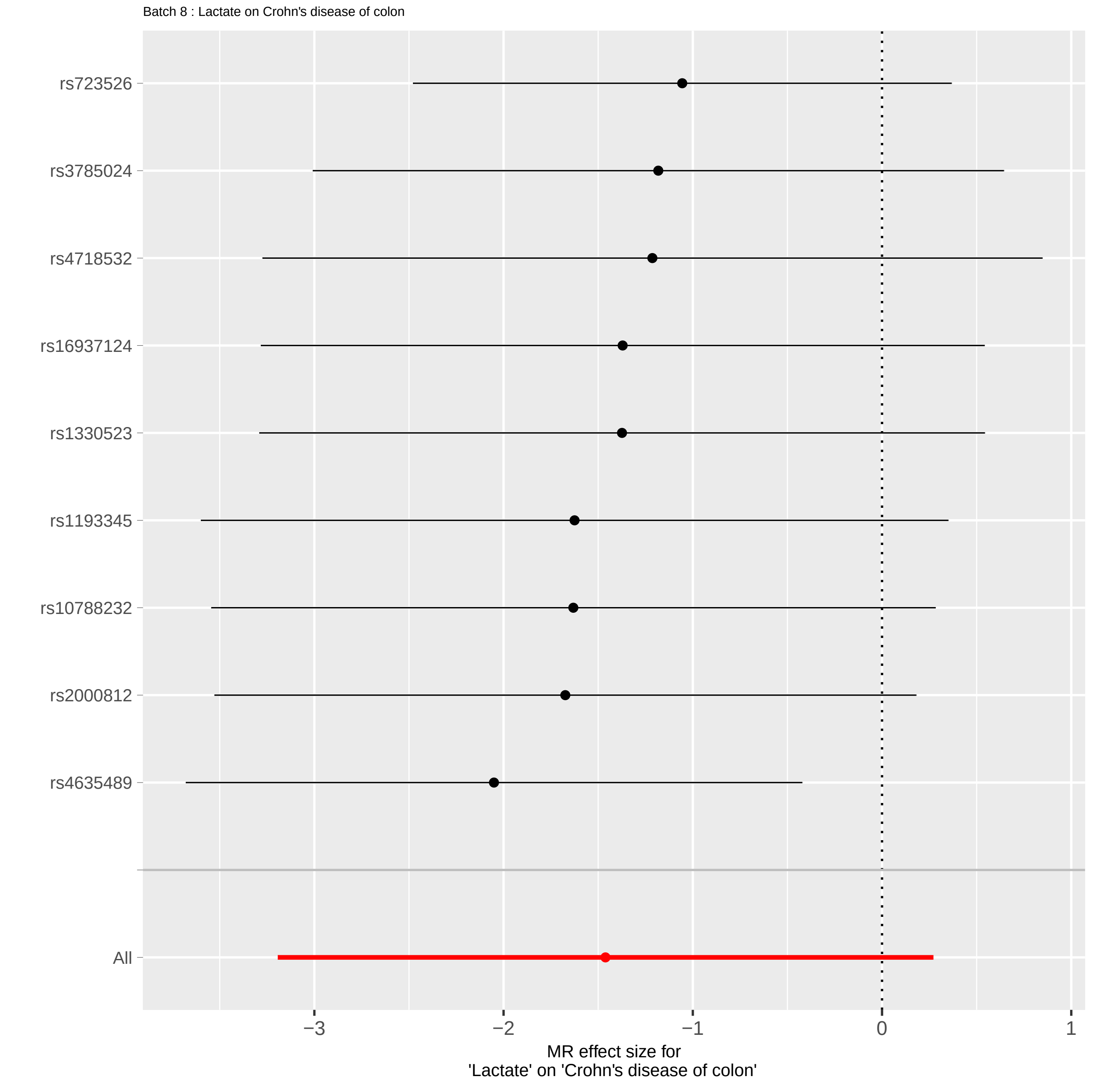

Batch 8 : Lactate on Crohn's disease of colon
rs723526
rs3785024
rs4718532
rs16937124
rs1330523
rs1193345
rs10788232
rs2000812
rs4635489
All
−3
−2
−1
0
1
MR effect size for
'Lactate' on 'Crohn's disease of colon'

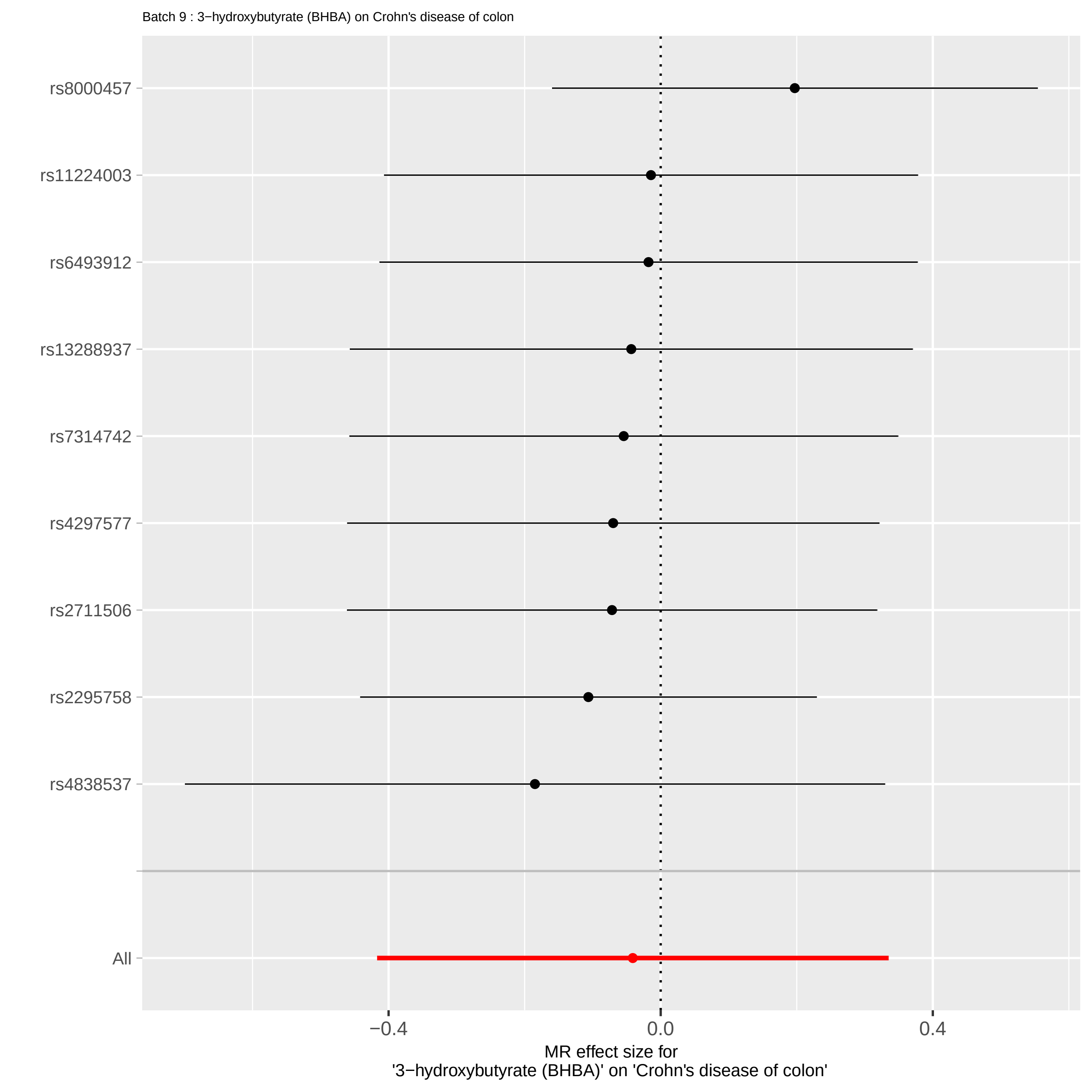
Batch 9 : 3−hydroxybutyrate (BHBA) on Crohn's disease of colon
rs8000457
rs11224003
rs6493912
rs13288937
rs7314742
rs4297577
rs2711506
rs2295758
rs4838537
All
−0.4
0.0
0.4
MR effect size for
'3−hydroxybutyrate (BHBA)' on 'Crohn's disease of colon'

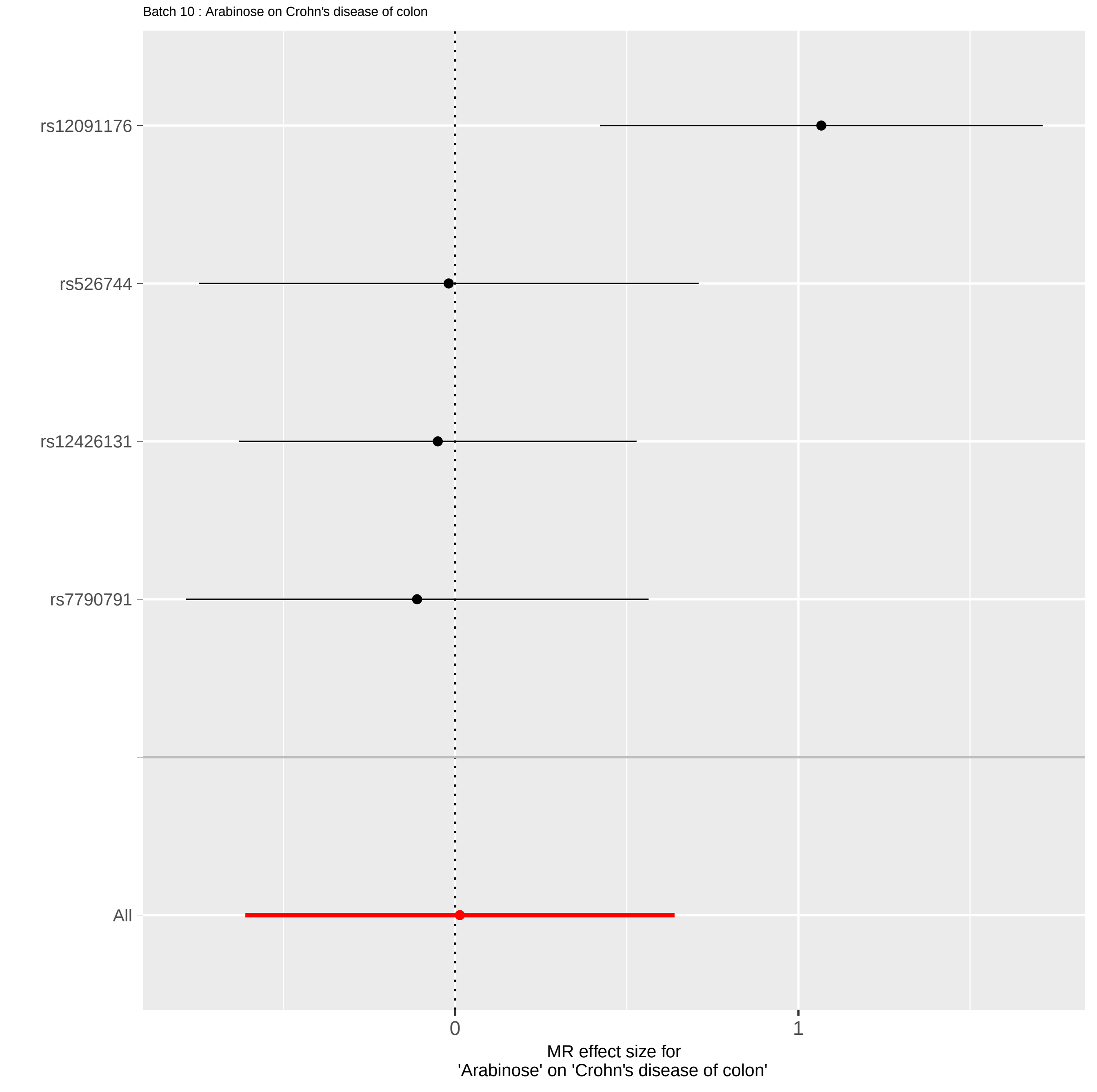
Batch 10 : Arabinose on Crohn's disease of colon
rs12091176
rs526744
rs12426131
rs7790791
All
0
1
MR effect size for
'Arabinose' on 'Crohn's disease of colon'

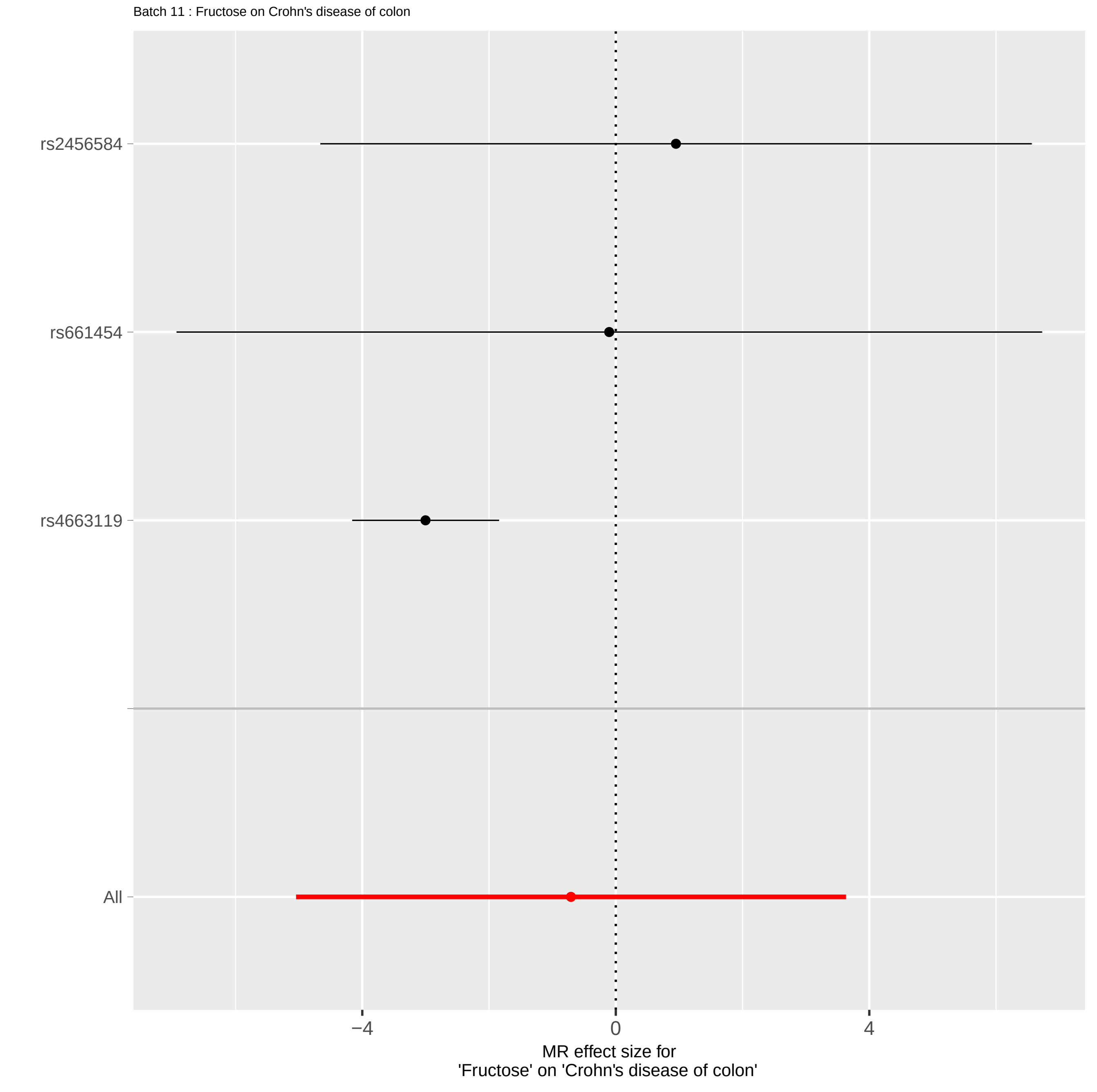
Batch 11 : Fructose on Crohn's disease of colon
rs2456584
rs661454
rs4663119
All
−4
0
4
MR effect size for
'Fructose' on 'Crohn's disease of colon'

Batch 13 : Pyruvate on Crohn's disease of colon

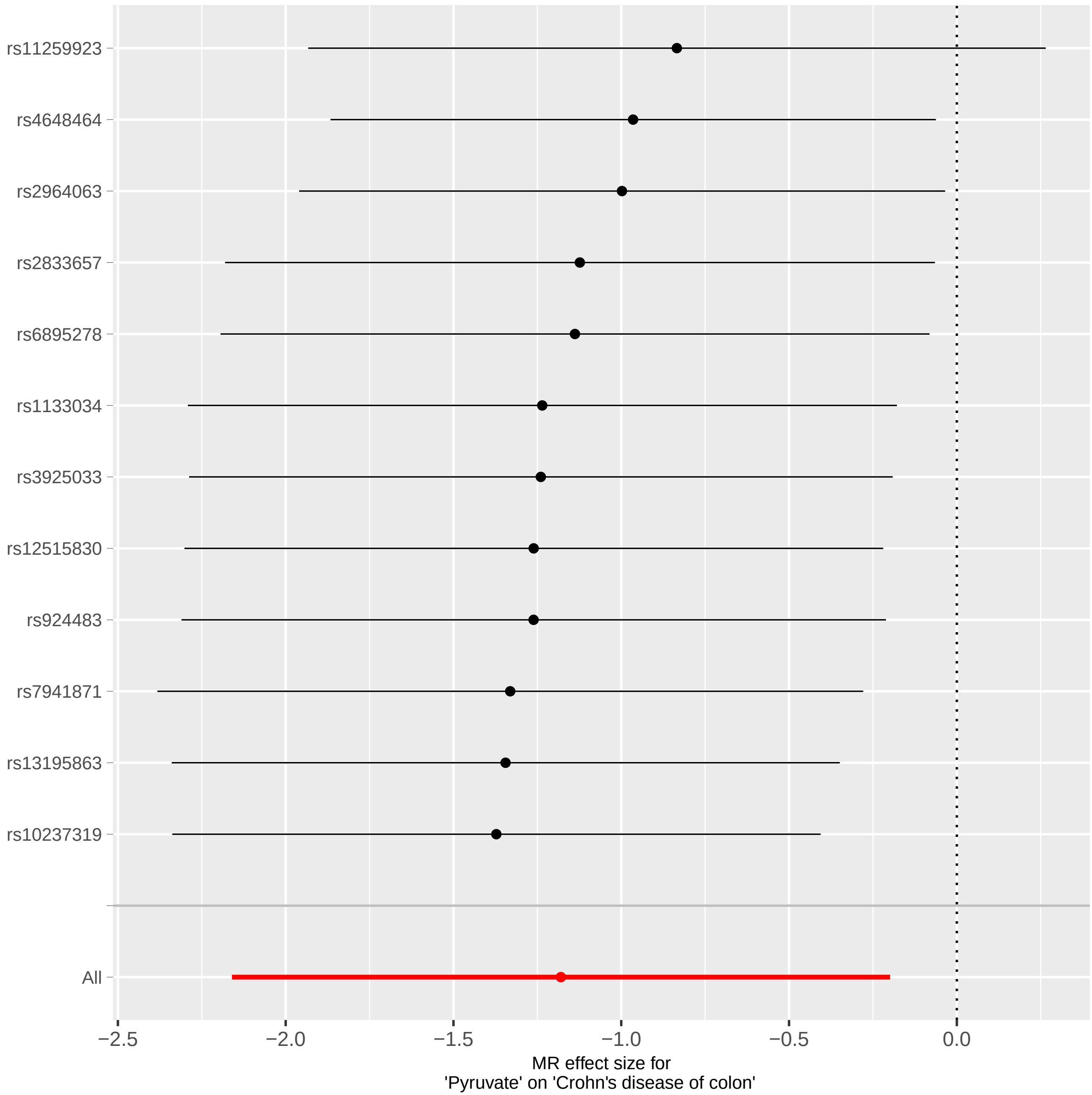

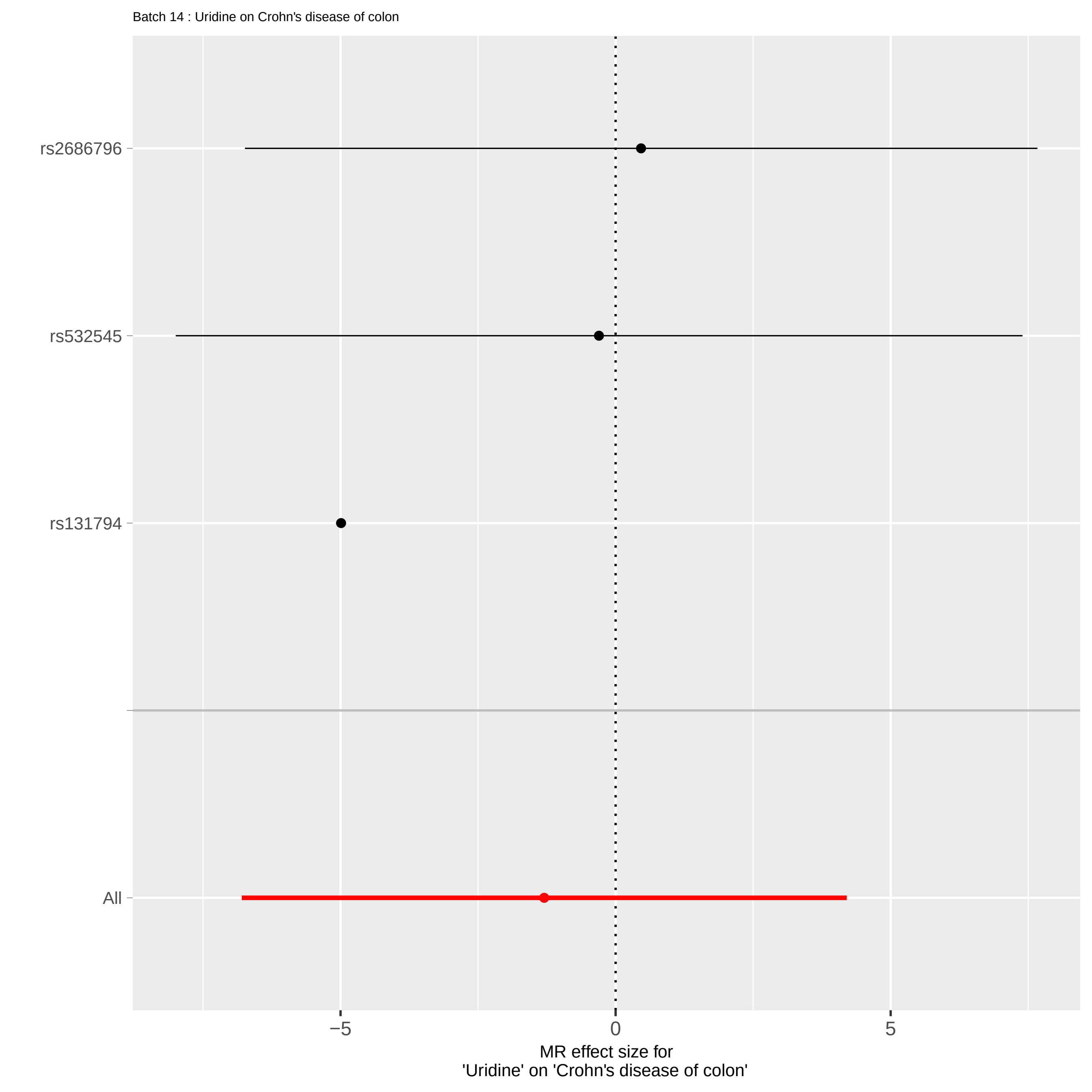
Batch 14 : Uridine on Crohn's disease of colon
rs2686796
rs532545
rs131794
All
−5
0
5
MR effect size for
'Uridine' on 'Crohn's disease of colon'

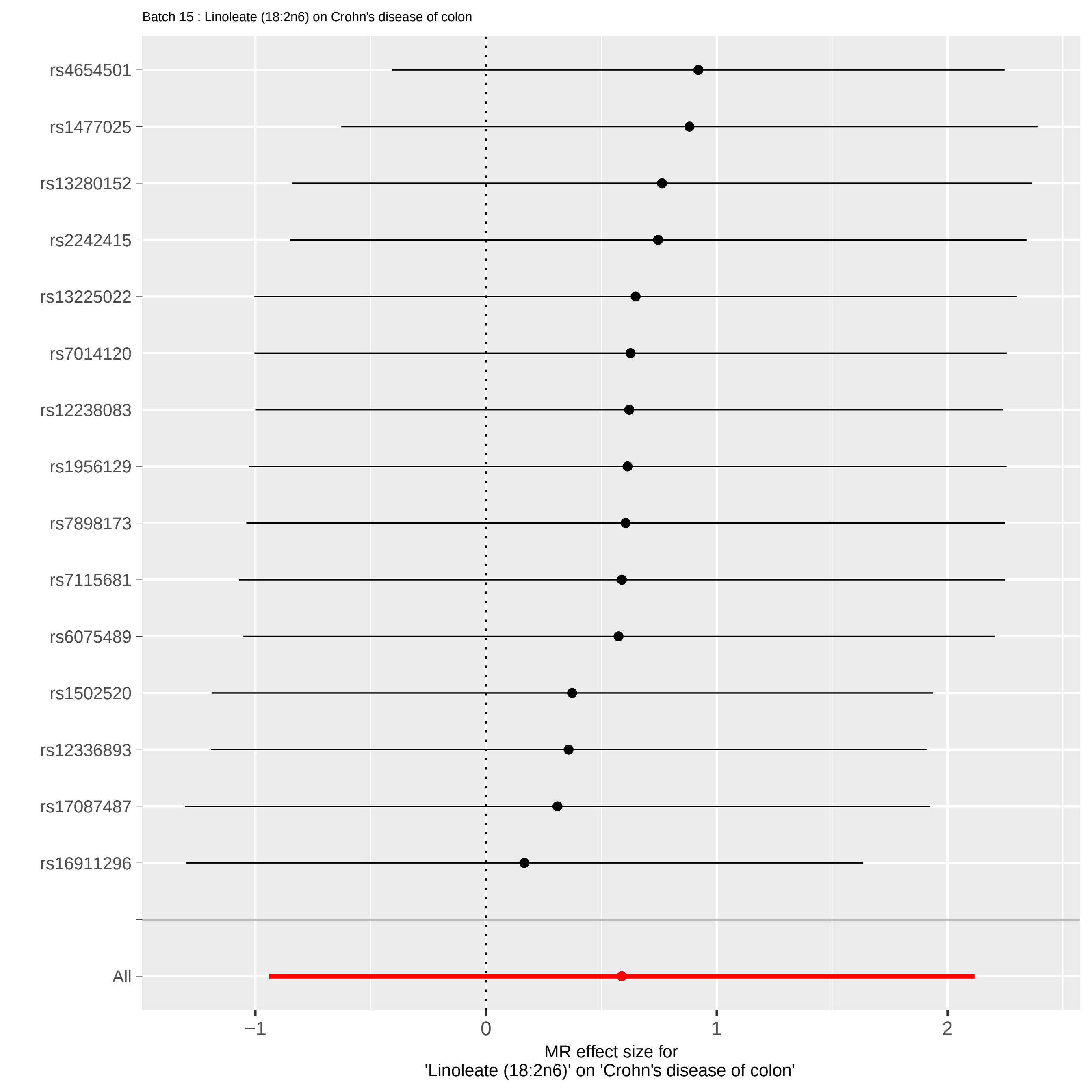

Batch 15 : Linoleate (18:2n6) on Crohn's disease of colon
rs4654501
rs1477025
rs13280152
rs2242415
rs13225022
rs7014120
rs12238083
rs1956129
rs7898173
rs7115681
rs6075489
rs1502520
rs12336893
rs17087487
rs16911296
All
−1
0
1
2
MR effect size for
'Linoleate (18:2n6)' on 'Crohn's disease of colon'

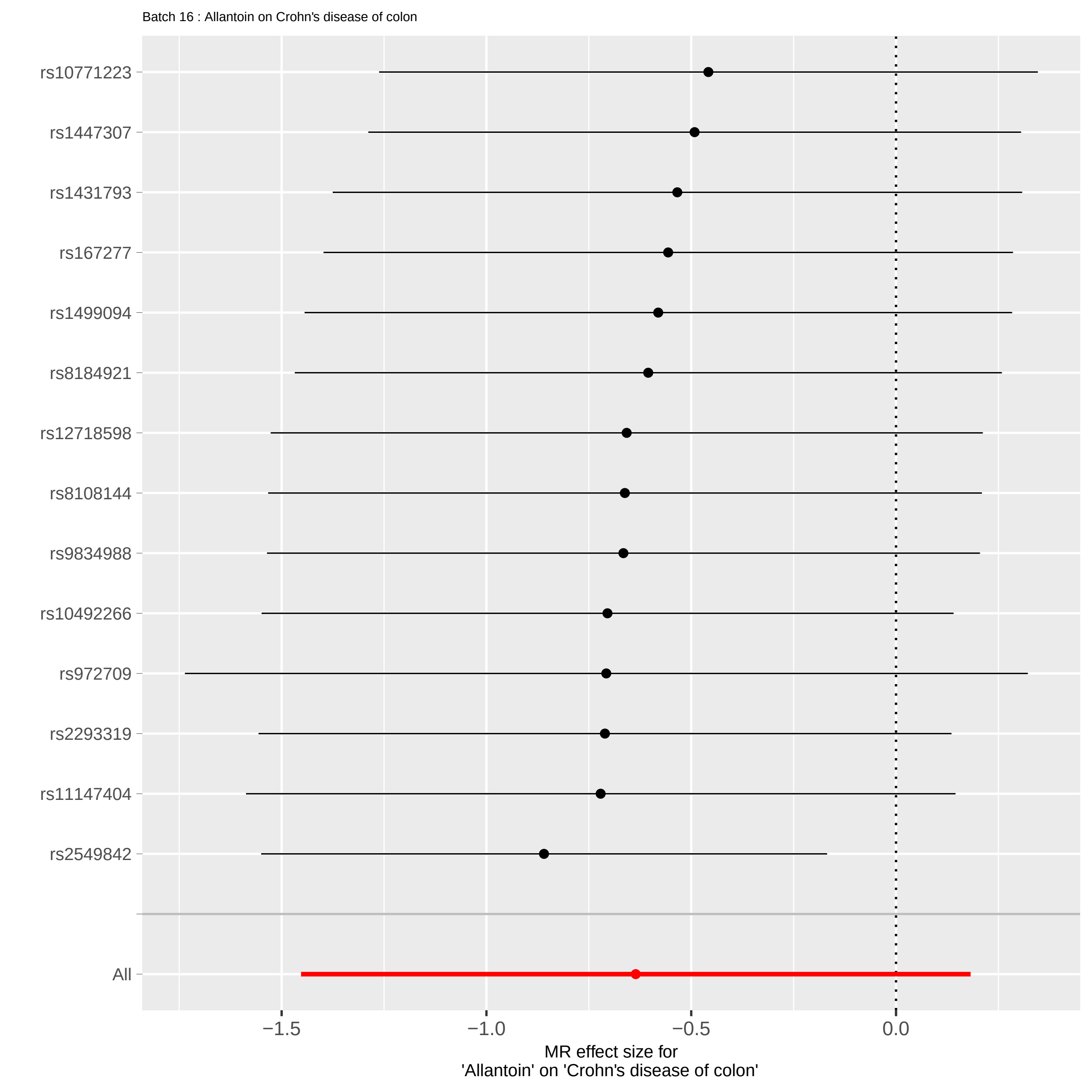

Batch 16 : Allantoin on Crohn's disease of colon
rs10771223
rs1447307
rs1431793
rs167277
rs1499094
rs8184921
rs12718598
rs8108144
rs9834988
rs10492266
rs972709
rs2293319
rs11147404
rs2549842
All
−1.5
−1.0
−0.5
0.0
MR effect size for
'Allantoin' on 'Crohn's disease of colon'

Batch 21 : Isoleucine on Crohn's disease of colon

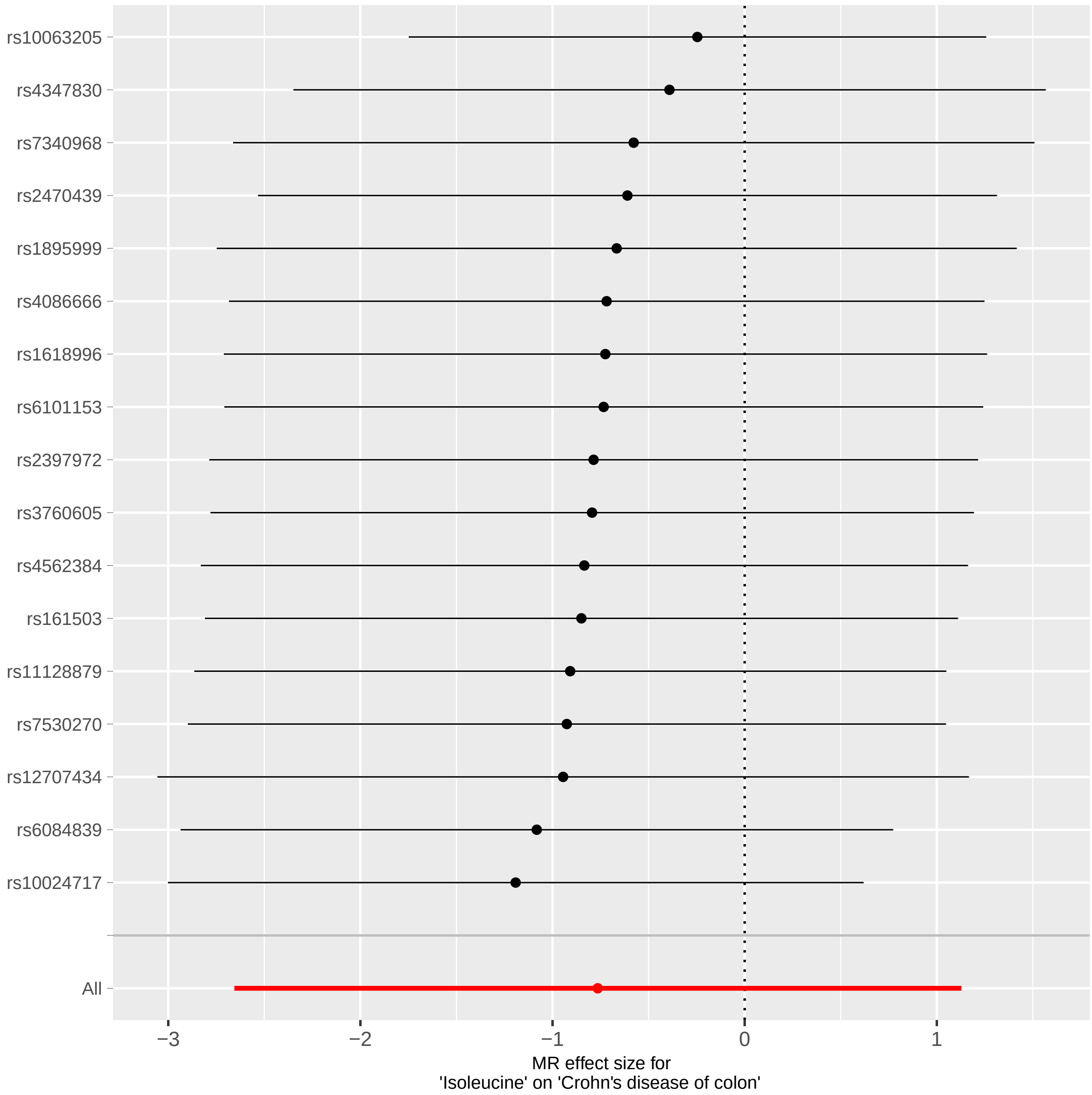

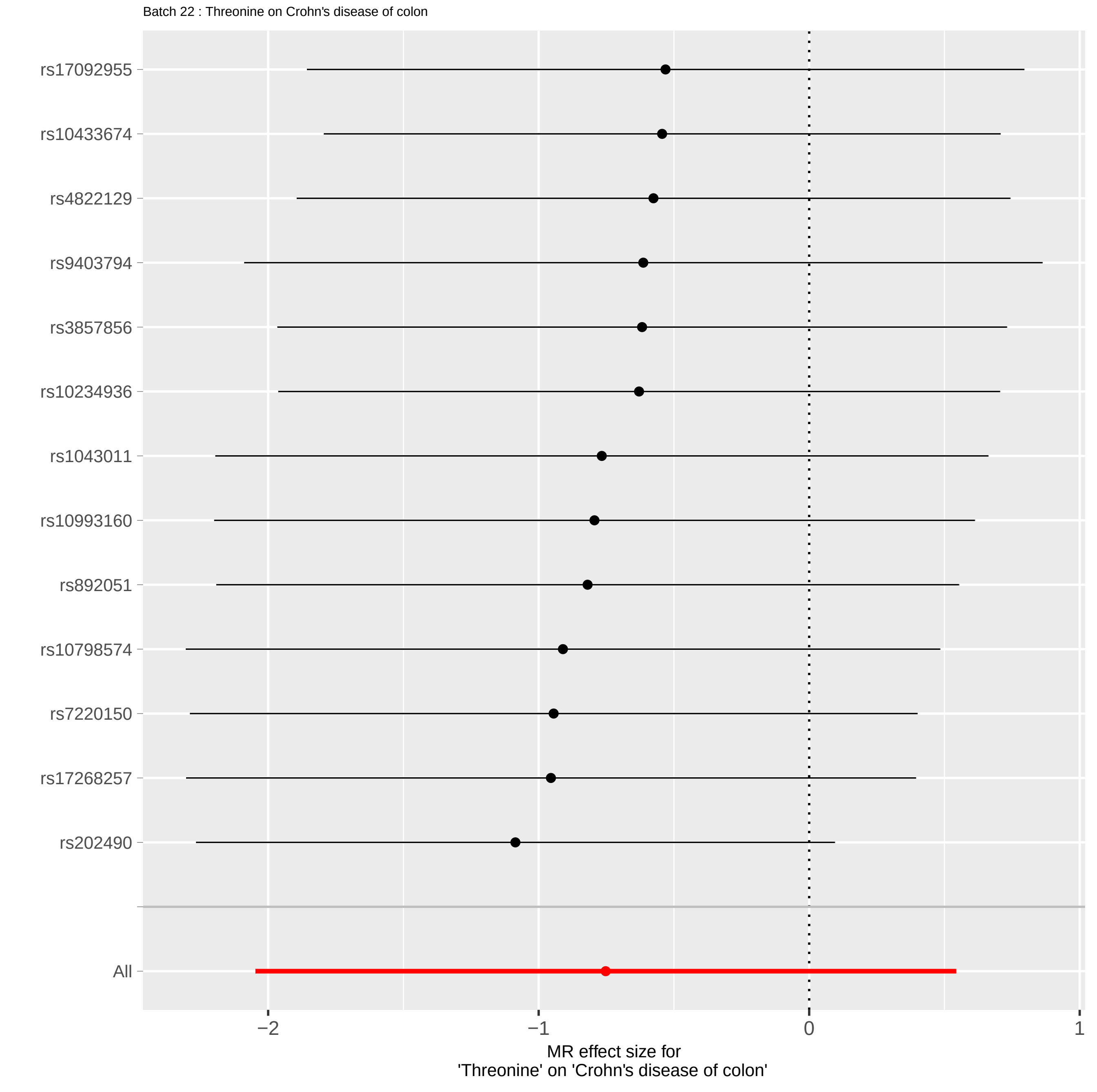
Batch 22 : Threonine on Crohn's disease of colon
rs17092955
rs10433674
rs4822129
rs9403794
rs3857856
rs10234936
rs1043011
rs10993160
rs892051
rs10798574
rs7220150
rs17268257
rs202490
All
−2
−1
0
1
MR effect size for
'Threonine' on 'Crohn's disease of colon'

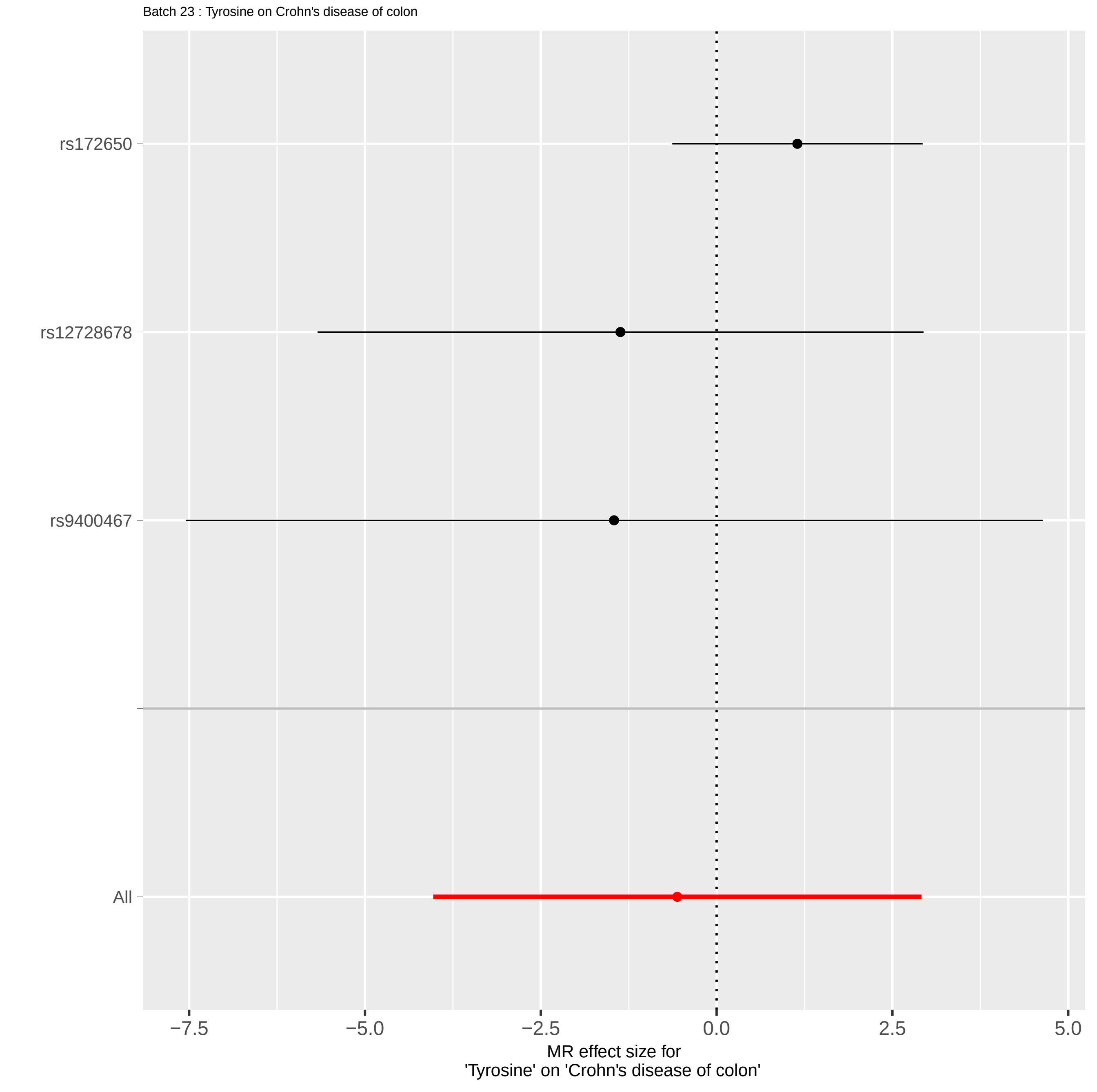

Batch 23 : Tyrosine on Crohn's disease of colon
rs172650
rs12728678
rs9400467
All
−7.5
−5.0
−2.5
0.0
2.5
5.0
MR effect size for
'Tyrosine' on 'Crohn's disease of colon'

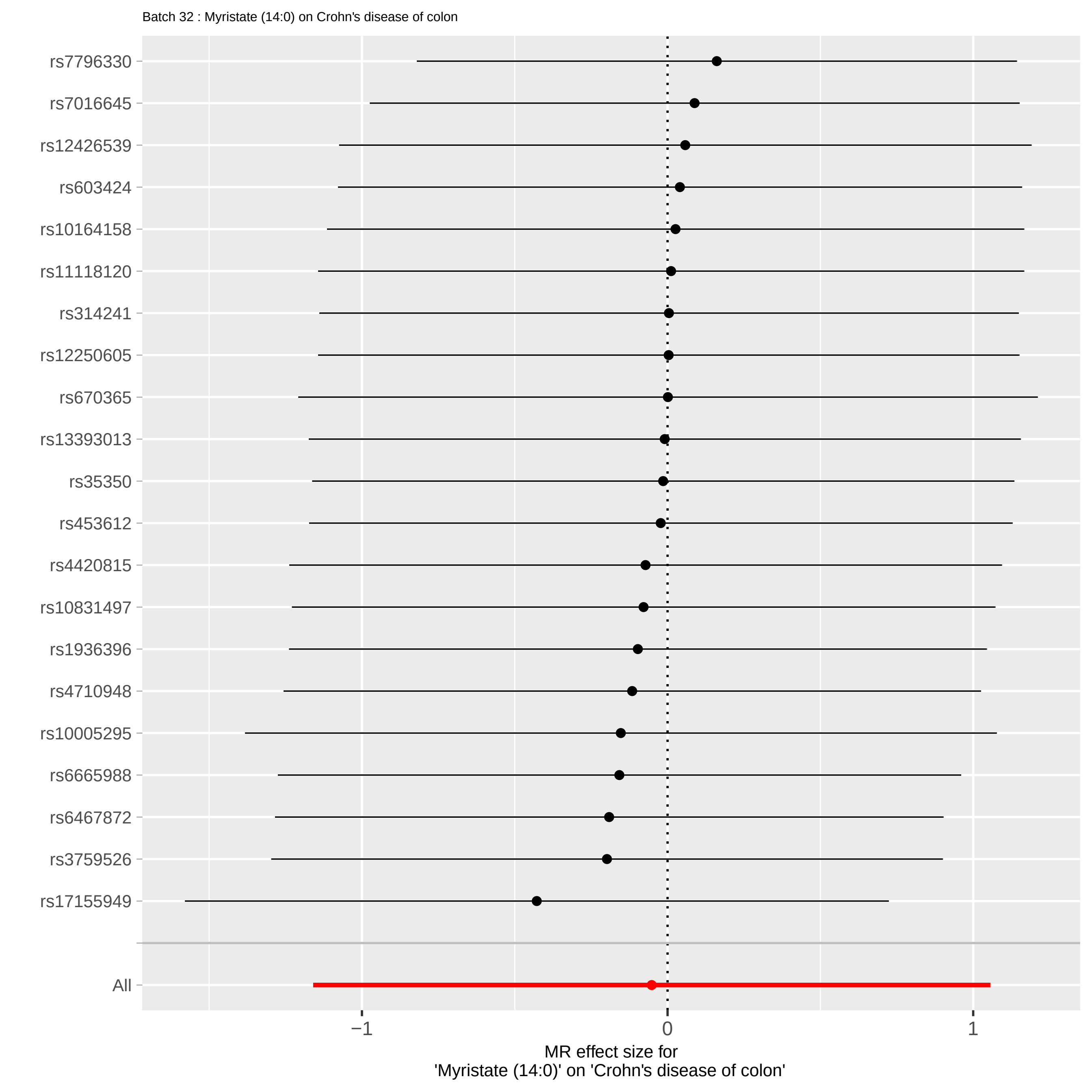

Batch 32 : Myristate (14:0) on Crohn's disease of colon
rs7796330
rs7016645
rs12426539
rs603424
rs10164158
rs11118120
rs314241
rs12250605
rs670365
rs13393013
rs35350
rs453612
rs4420815
rs10831497
rs1936396
rs4710948
rs10005295
rs6665988
rs6467872
rs3759526
rs17155949
All
−1
0
1
MR effect size for
'Myristate (14:0)' on 'Crohn's disease of colon'

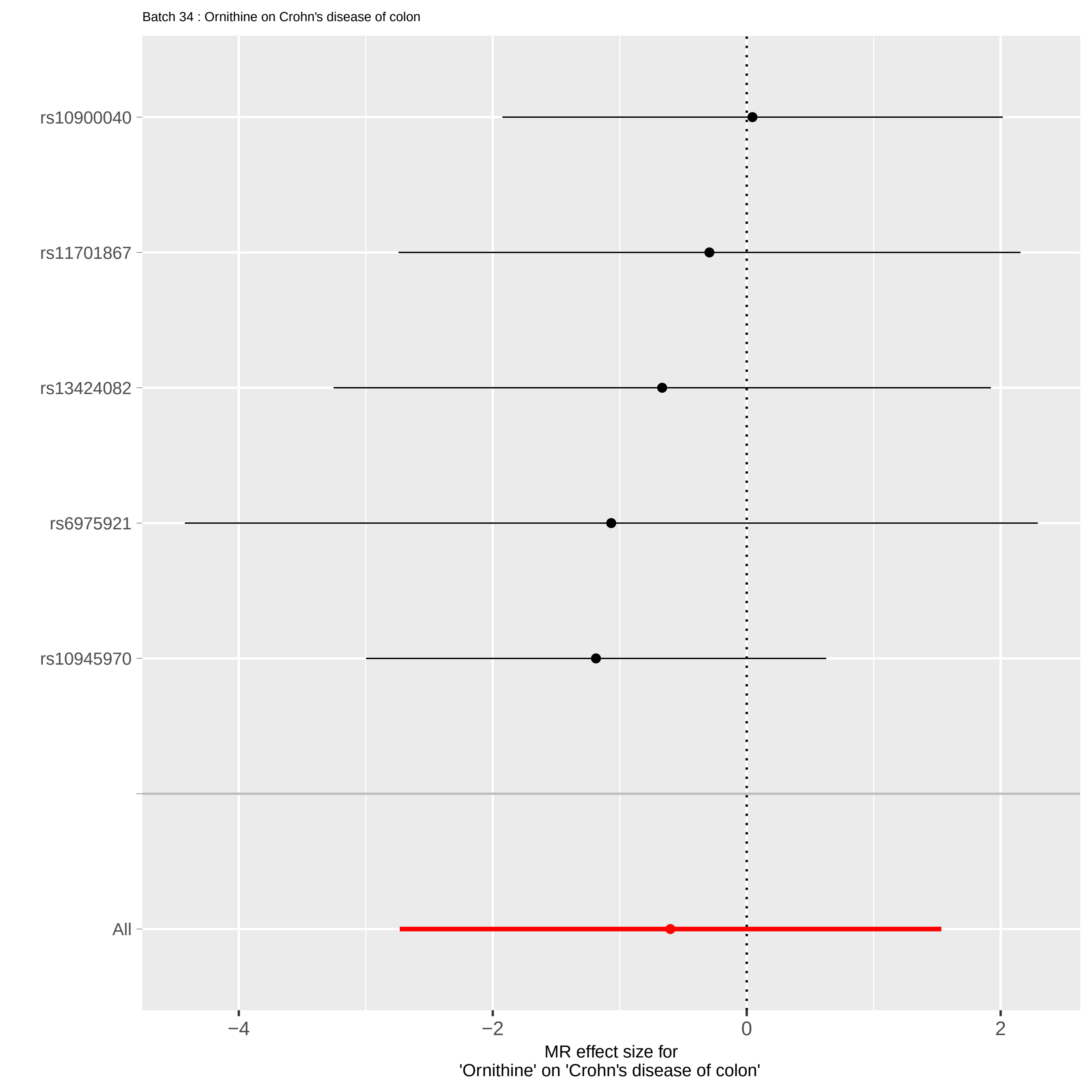

Batch 34 : Ornithine on Crohn's disease of colon
rs10900040
rs11701867
rs13424082
rs6975921
rs10945970
All
−4
−2
0
2
MR effect size for
'Ornithine' on 'Crohn's disease of colon'

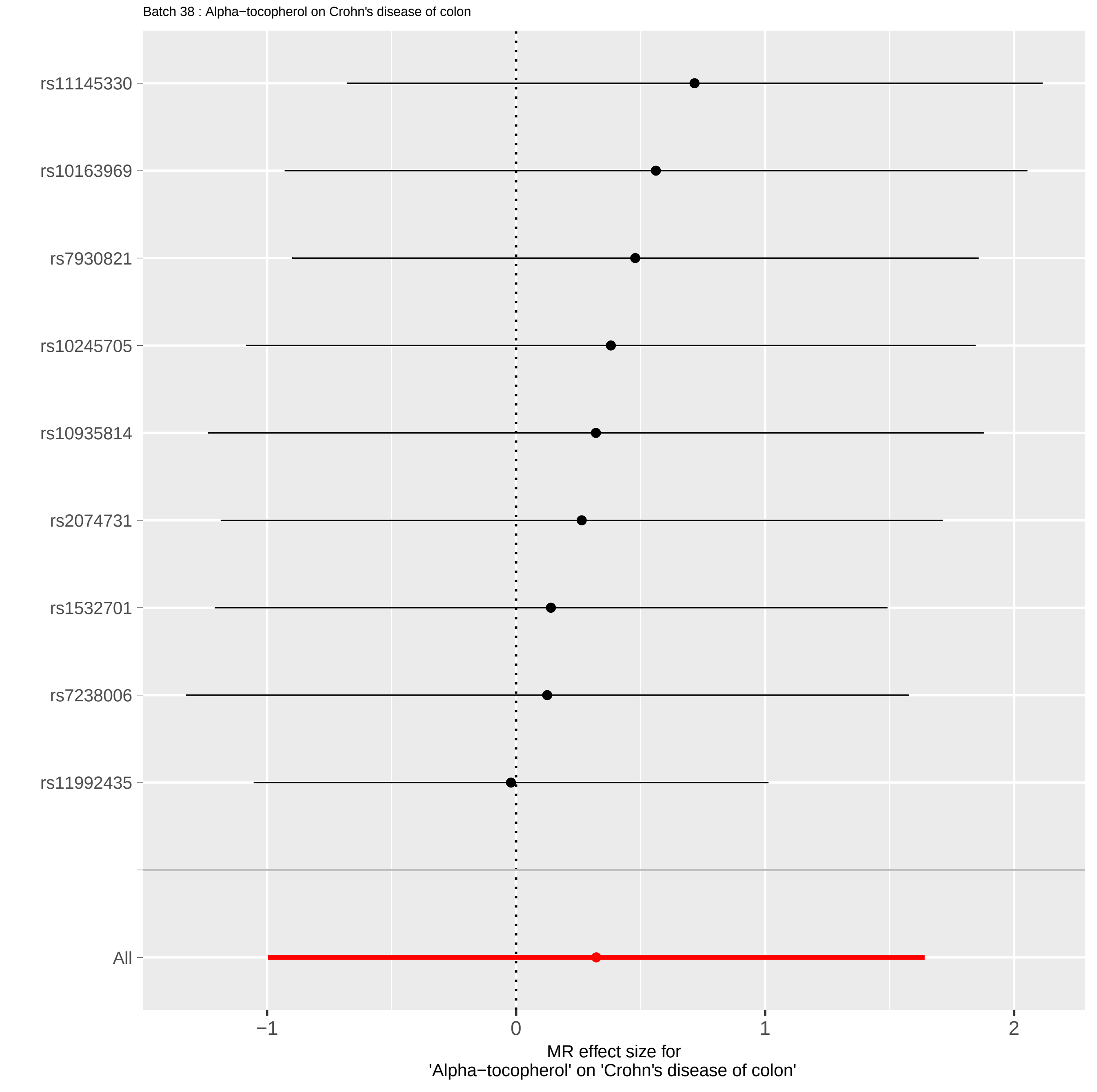

Batch 38 : Alpha−tocopherol on Crohn's disease of colon
rs11145330
rs10163969
rs7930821
rs10245705
rs10935814
rs2074731
rs1532701
rs7238006
rs11992435
All
−1
0
1
2
MR effect size for
'Alpha−tocopherol' on 'Crohn's disease of colon'

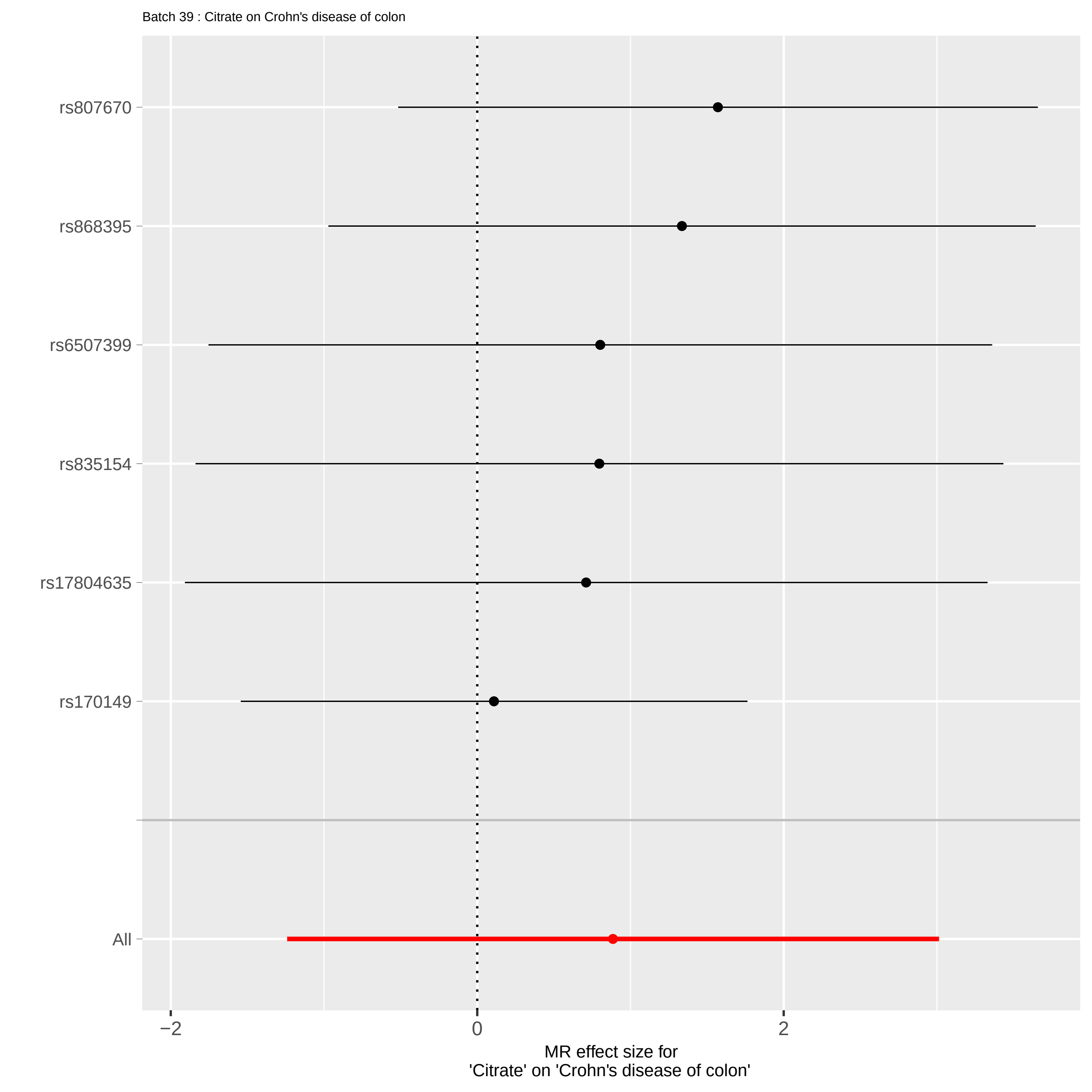
Batch 39 : Citrate on Crohn's disease of colon
rs807670
rs868395
rs6507399
rs835154
rs17804635
rs170149
All
−2
0
2
MR effect size for
'Citrate' on 'Crohn's disease of colon'

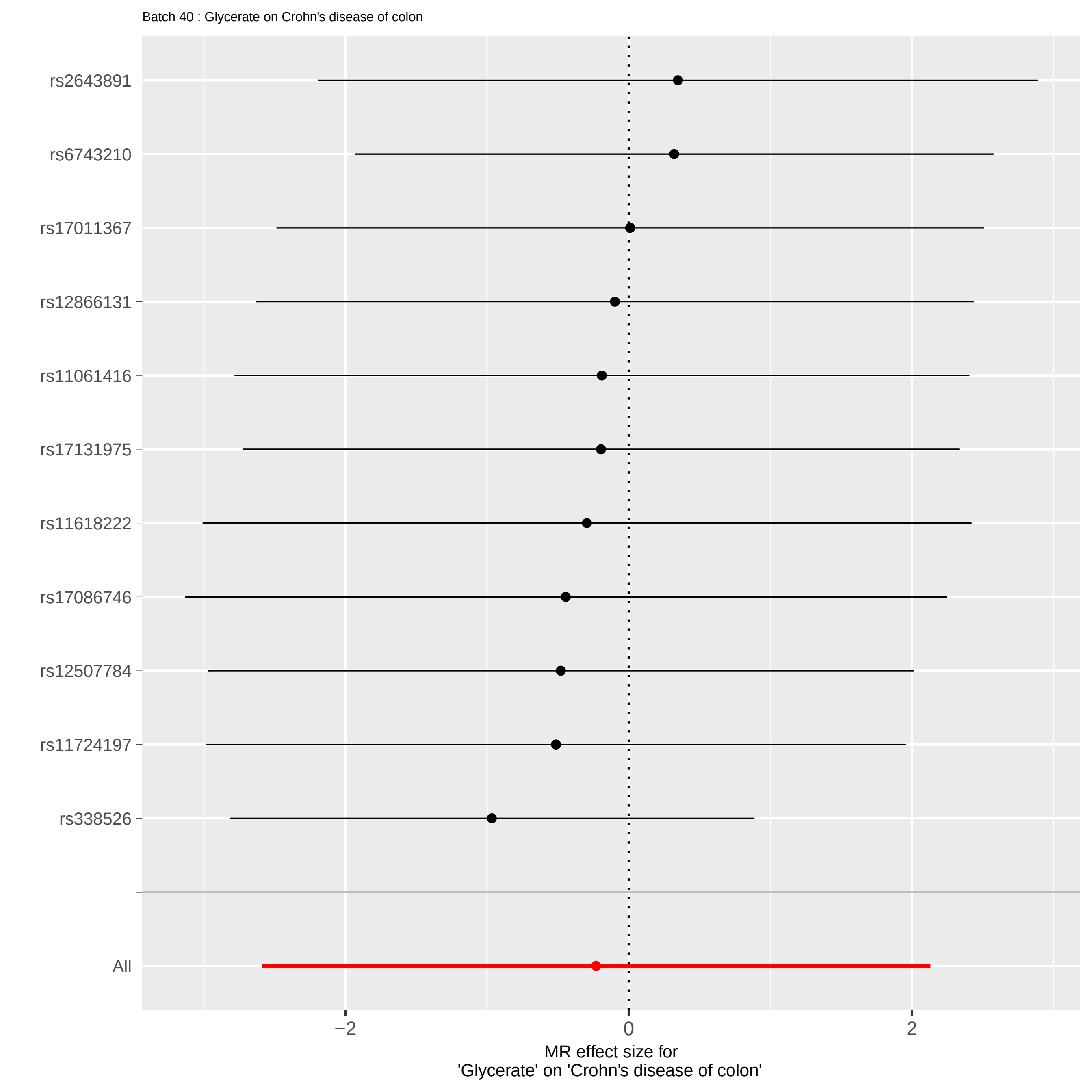
Batch 40 : Glycerate on Crohn's disease of colon
rs2643891
rs6743210
rs17011367
rs12866131
rs11061416
rs17131975
rs11618222
rs17086746
rs12507784
rs11724197
rs338526
All
−2
0
2
MR effect size for
'Glycerate' on 'Crohn's disease of colon'

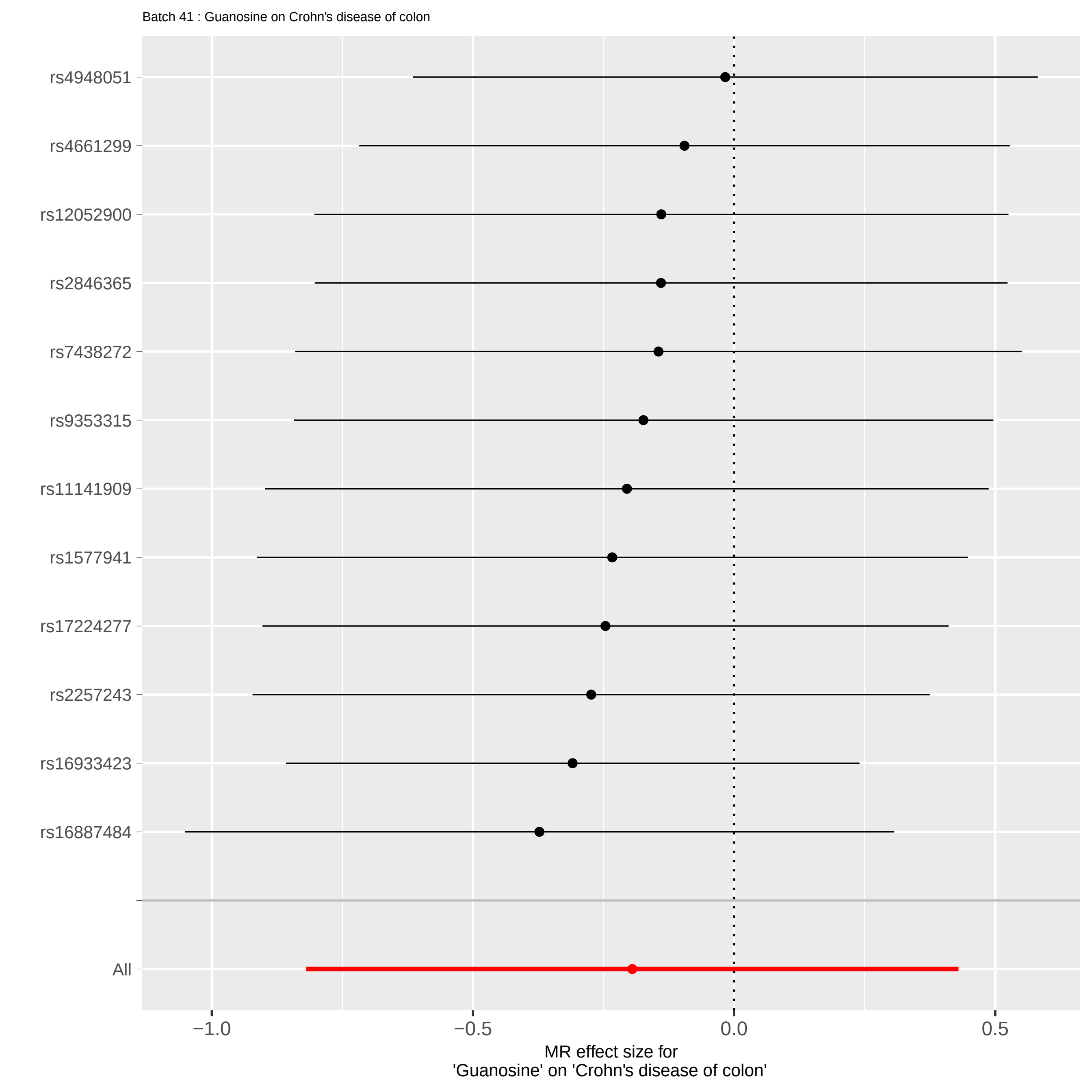

Batch 41 : Guanosine on Crohn's disease of colon
rs4948051
rs4661299
rs12052900
rs2846365
rs7438272
rs9353315
rs11141909
rs1577941
rs17224277
rs2257243
rs16933423
rs16887484
All
−1.0
−0.5
0.0
0.5
MR effect size for
'Guanosine' on 'Crohn's disease of colon'

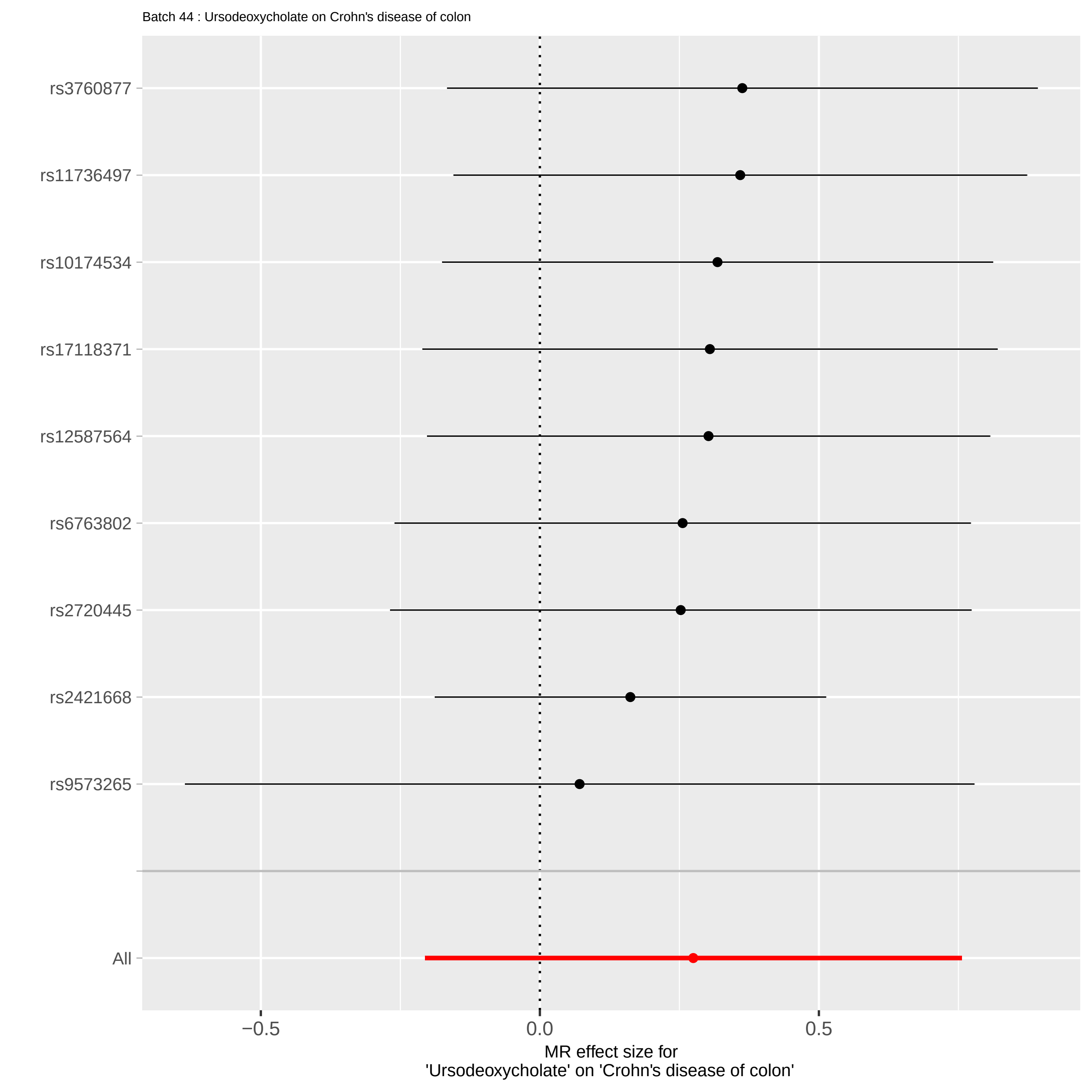

Batch 44 : Ursodeoxycholate on Crohn's disease of colon
rs3760877
rs11736497
rs10174534
rs17118371
rs12587564
rs6763802
rs2720445
rs2421668
rs9573265
All
−0.5
0.0
0.5
MR effect size for
'Ursodeoxycholate' on 'Crohn's disease of colon'

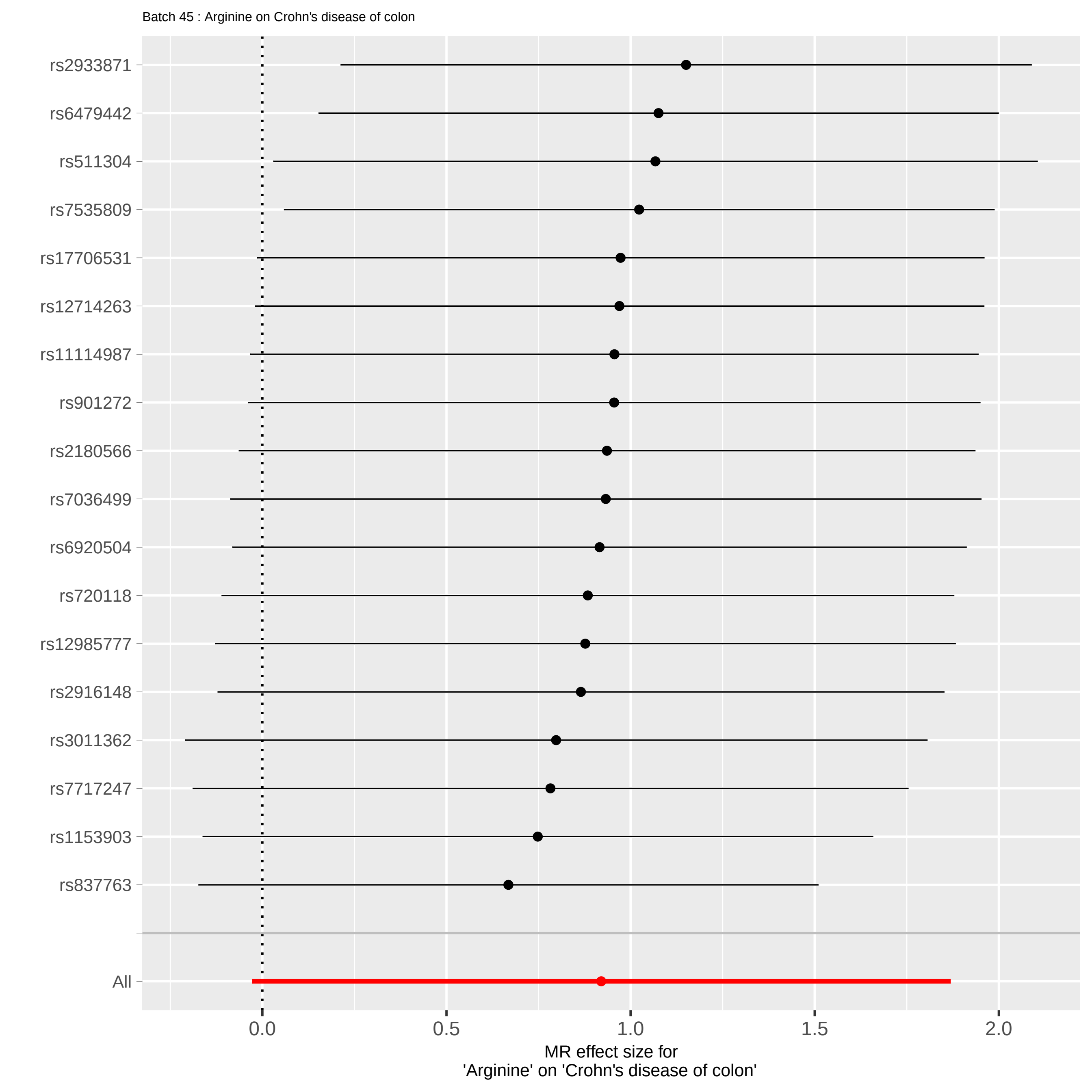

Batch 45 : Arginine on Crohn's disease of colon
rs2933871
rs6479442
rs511304
rs7535809
rs17706531
rs12714263
rs11114987
rs901272
rs2180566
rs7036499
rs6920504
rs720118
rs12985777
rs2916148
rs3011362
rs7717247
rs1153903
rs837763
All
0.0
0.5
1.0
1.5
2.0
MR effect size for
'Arginine' on 'Crohn's disease of colon'

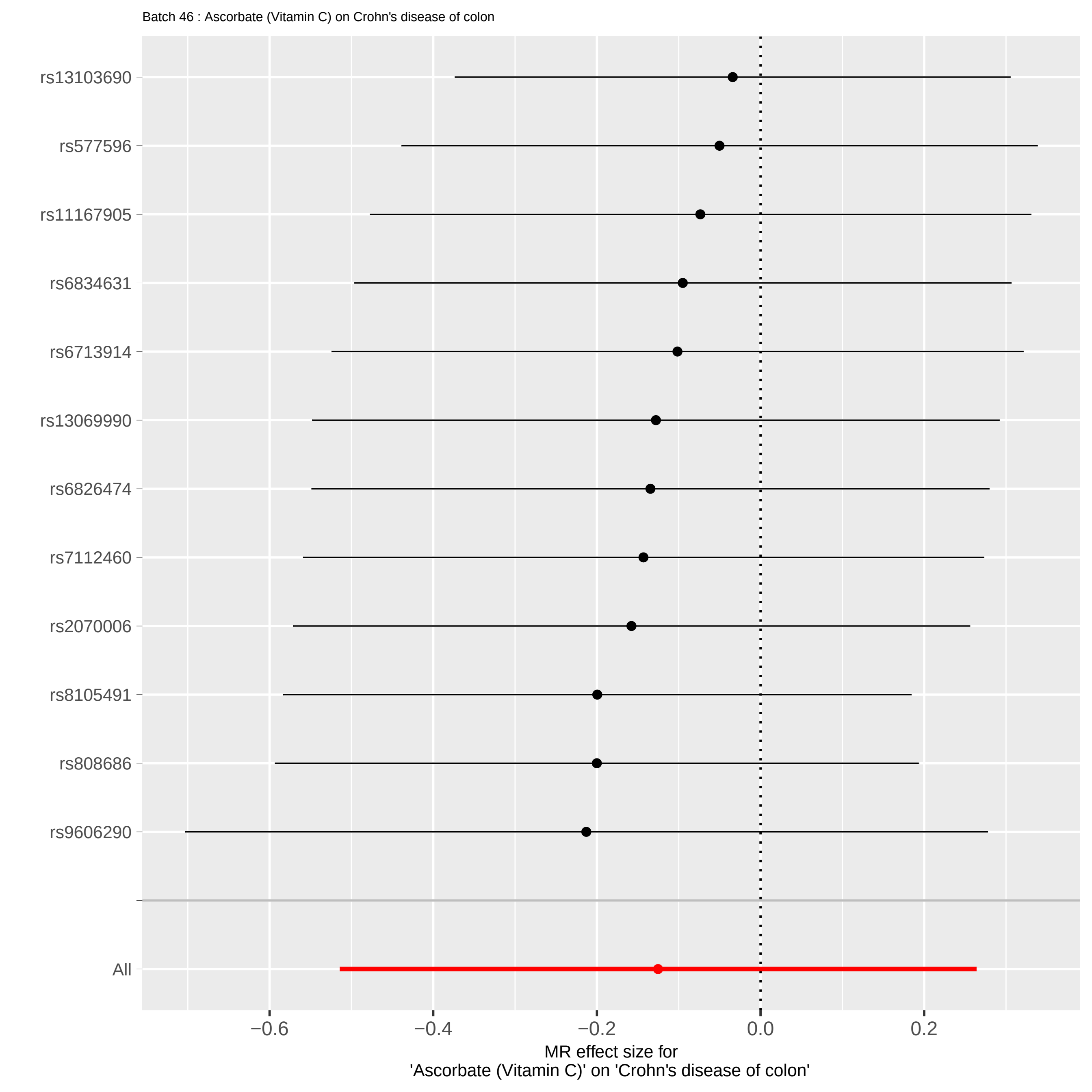
Batch 46 : Ascorbate (Vitamin C) on Crohn's disease of colon
rs13103690
rs577596
rs11167905
rs6834631
rs6713914
rs13069990
rs6826474
rs7112460
rs2070006
rs8105491
rs808686
rs9606290
All
−0.6
−0.4
−0.2
0.0
0.2
MR effect size for
'Ascorbate (Vitamin C)' on 'Crohn's disease of colon'

Batch 50 : Urea on Crohn's disease of colon

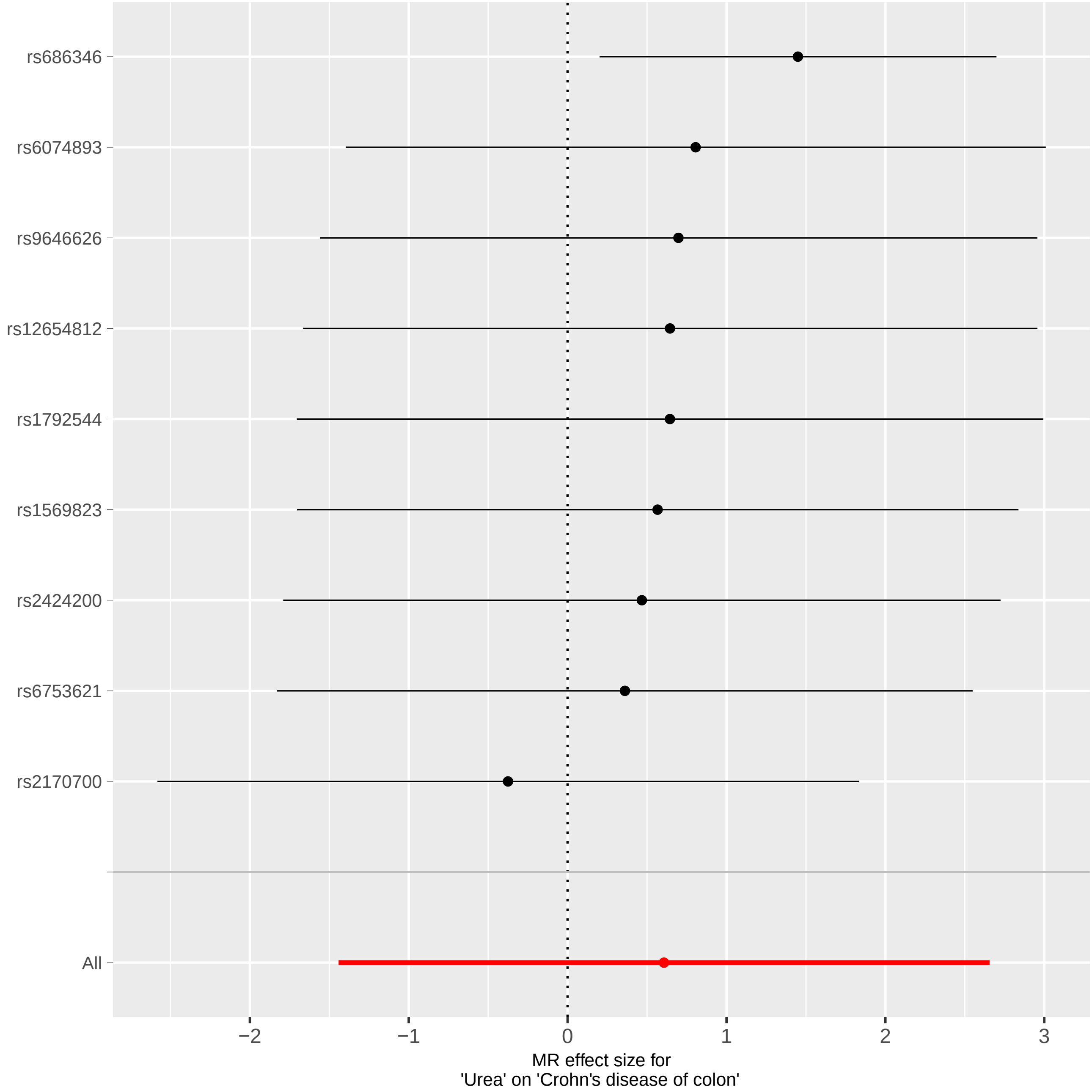

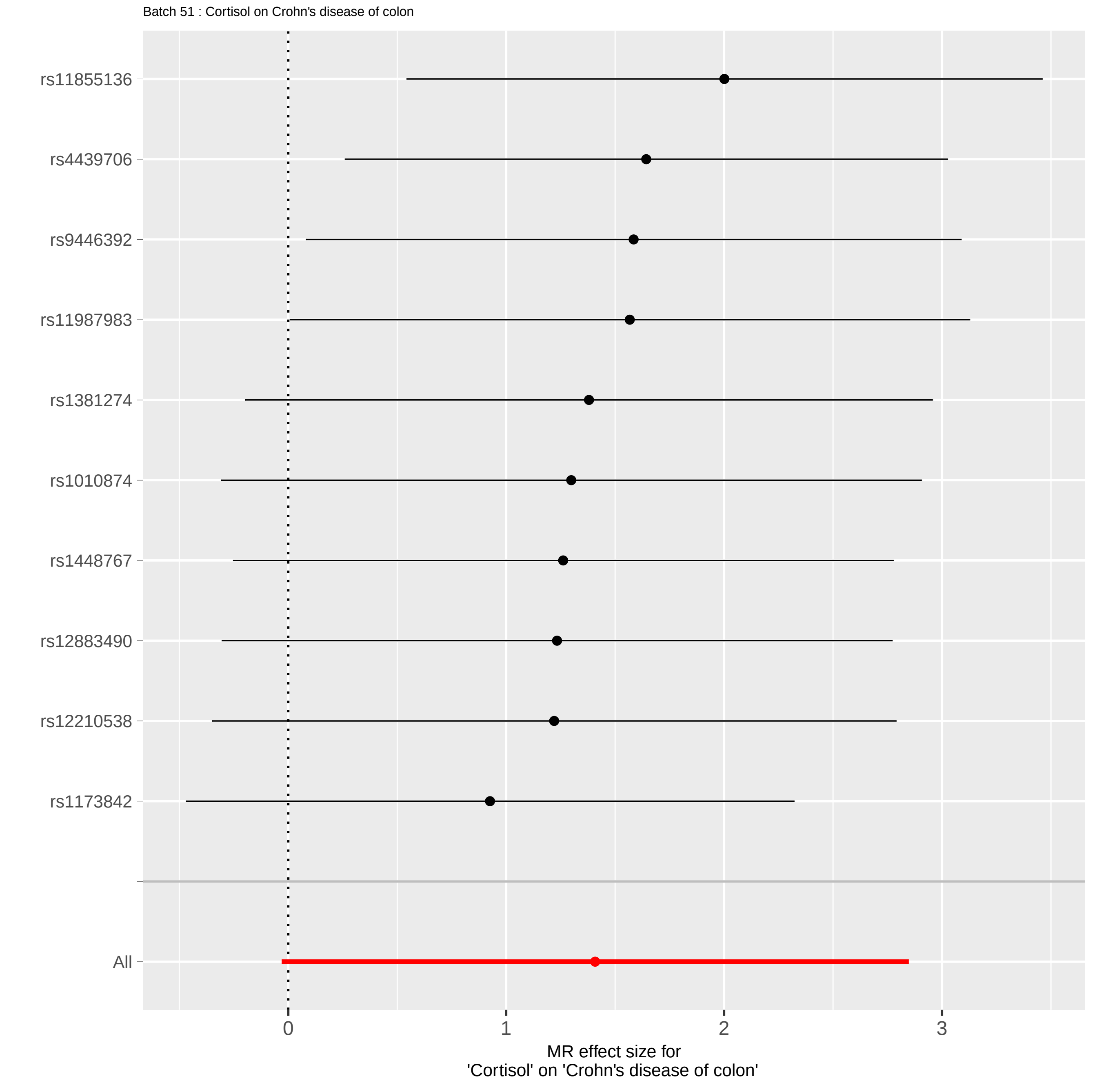

Batch 51 : Cortisol on Crohn's disease of colon
rs11855136
rs4439706
rs9446392
rs11987983
rs1381274
rs1010874
rs1448767
rs12883490
rs12210538
rs1173842
All
0
1
2
3
MR effect size for
'Cortisol' on 'Crohn's disease of colon'

Batch 54 : Citrulline on Crohn's disease of colon

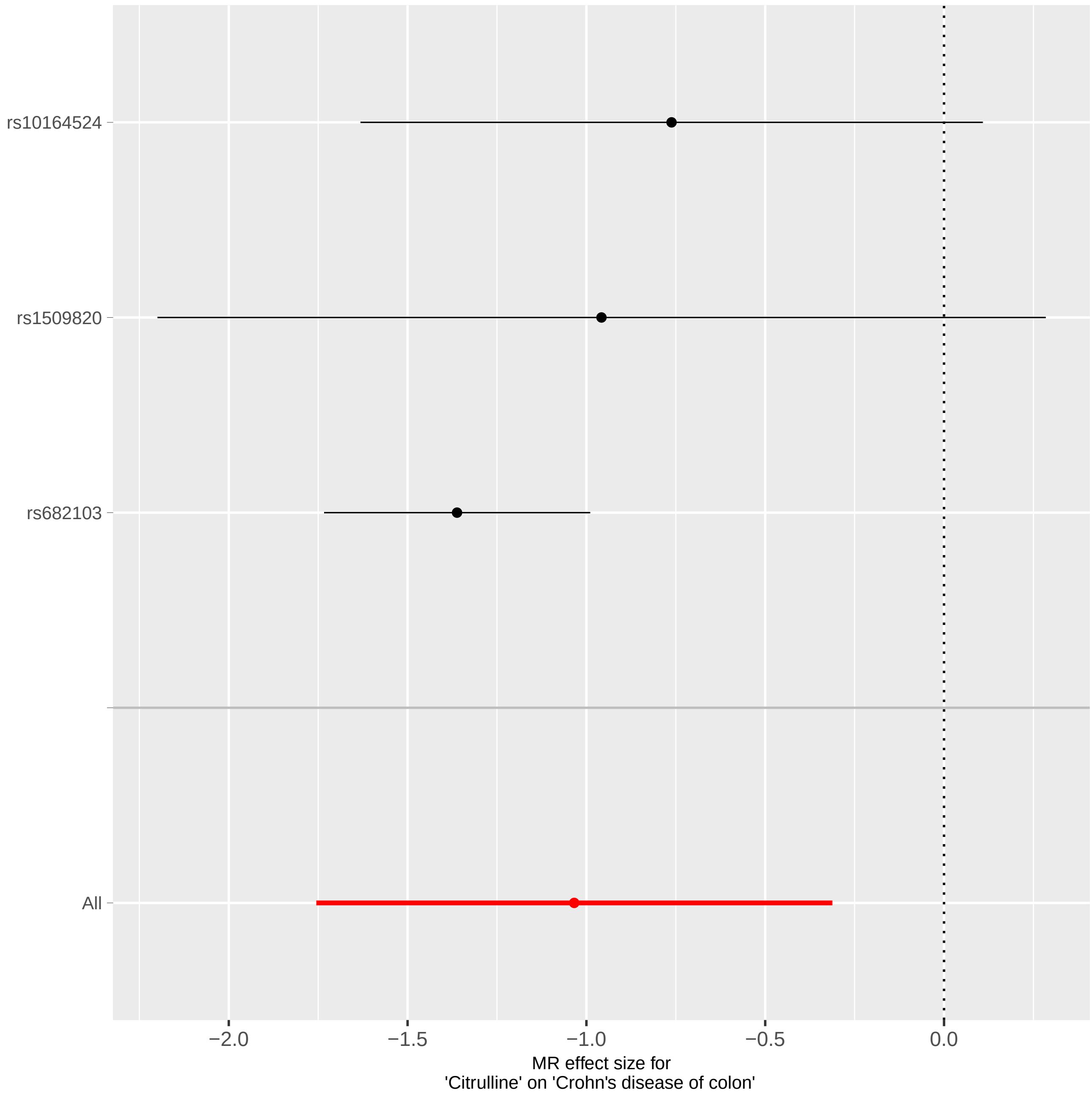

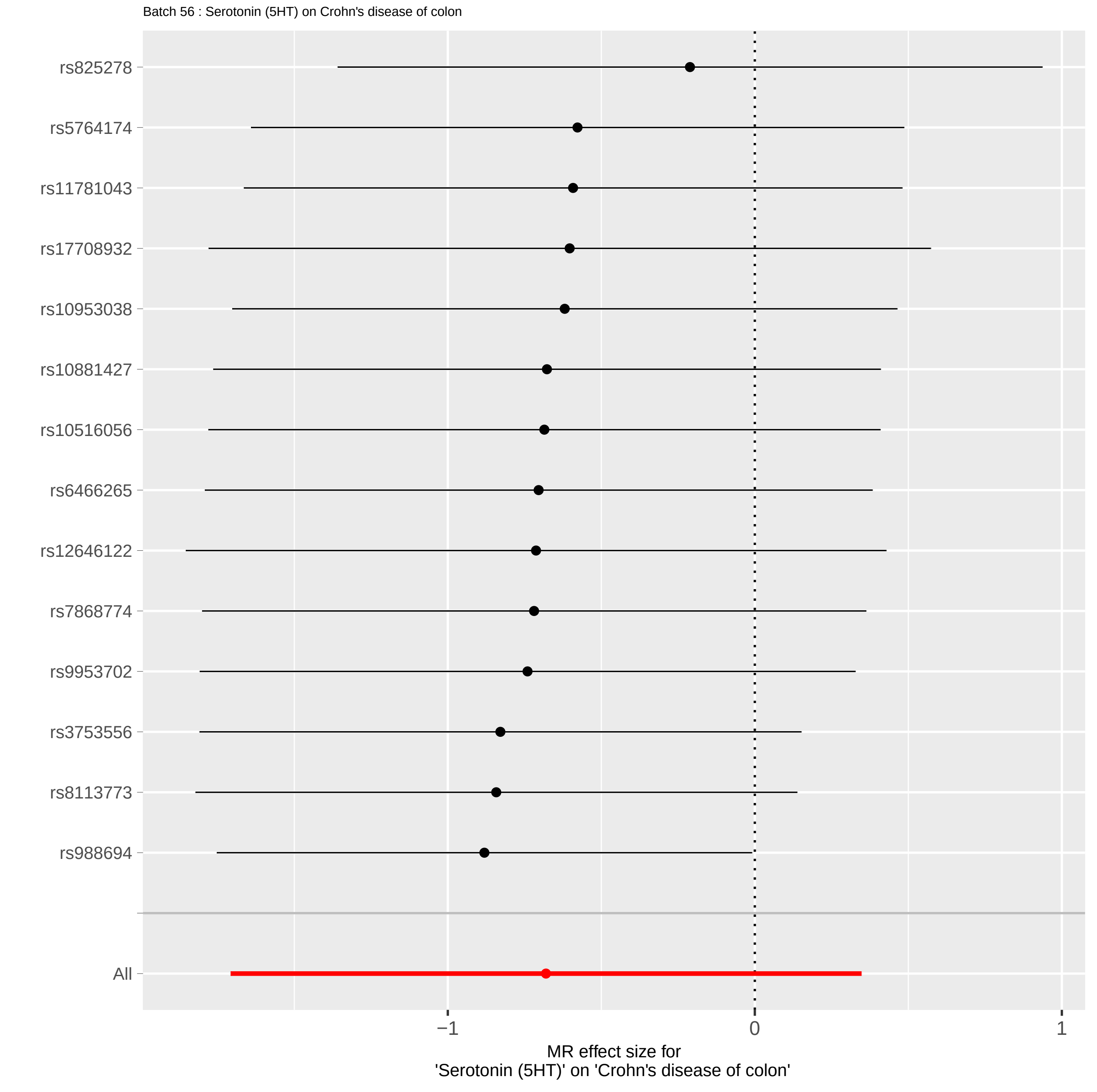
Batch 56 : Serotonin (5HT) on Crohn's disease of colon
rs825278
rs5764174
rs11781043
rs17708932
rs10953038
rs10881427
rs10516056
rs6466265
rs12646122
rs7868774
rs9953702
rs3753556
rs8113773
rs988694
All
−1
0
1
MR effect size for
'Serotonin (5HT)' on 'Crohn's disease of colon'

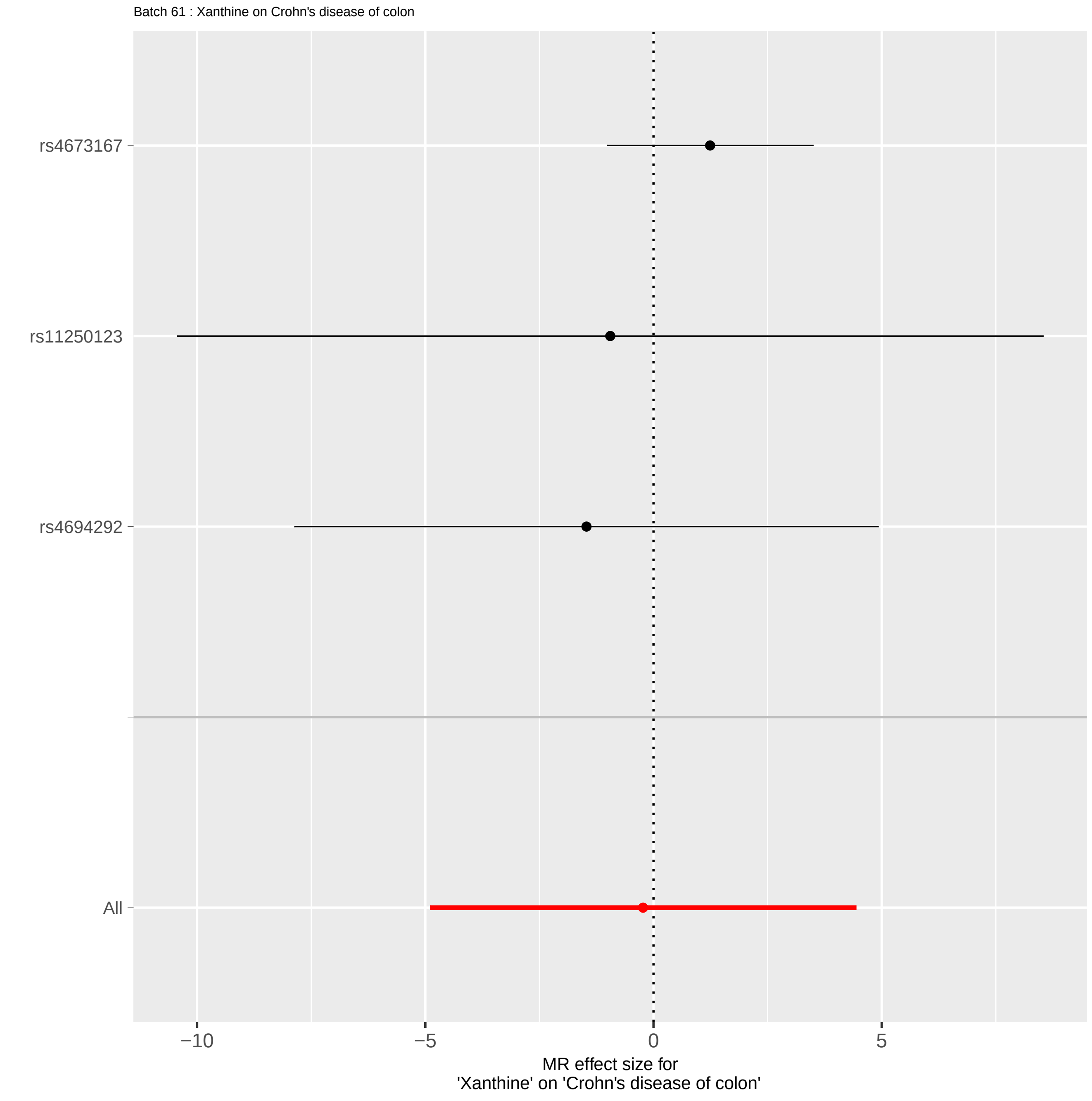

Batch 61 : Xanthine on Crohn's disease of colon
rs4673167
rs11250123
rs4694292
All
−10
−5
0
5
MR effect size for
'Xanthine' on 'Crohn's disease of colon'

Batch 62 : Phosphate on Crohn's disease of colon

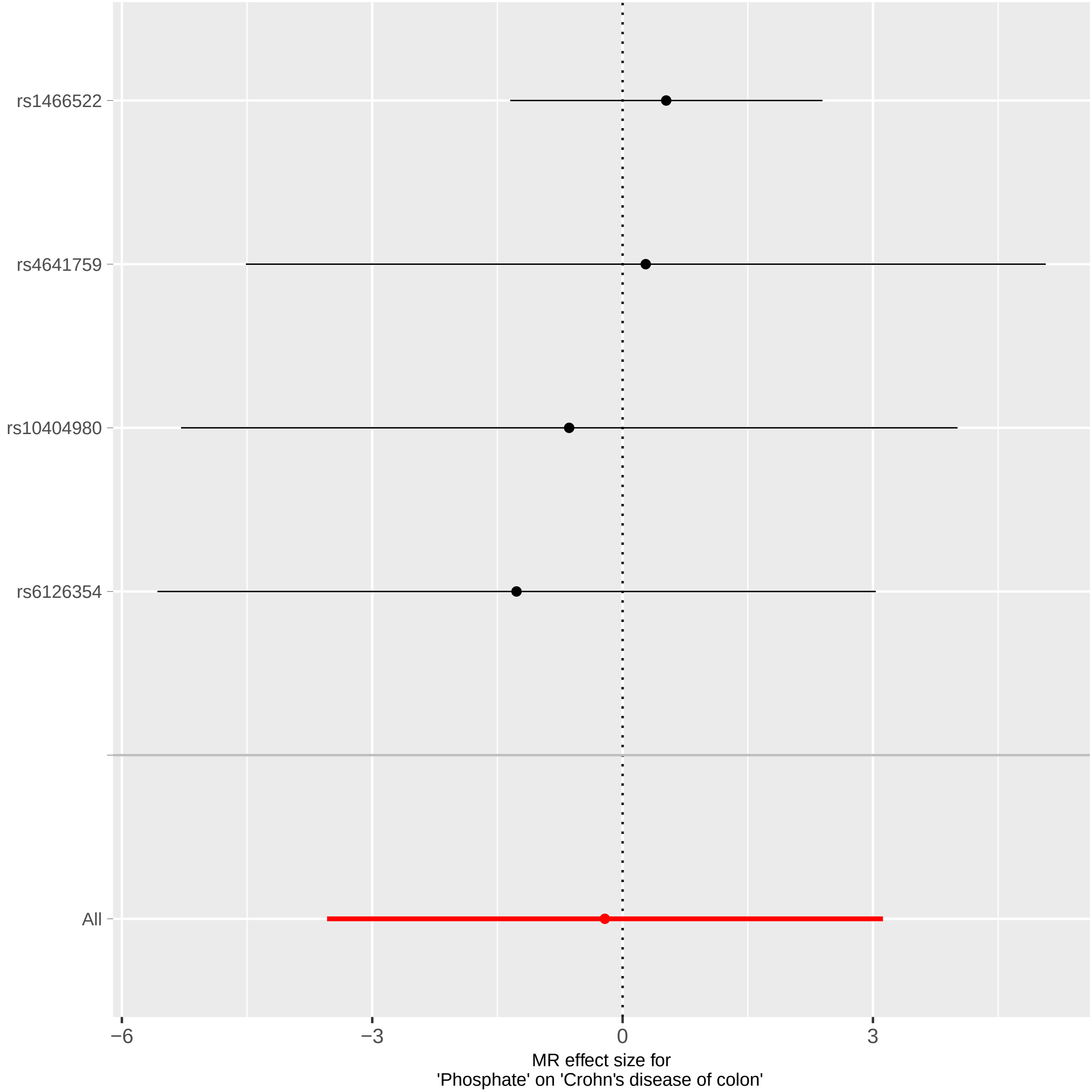

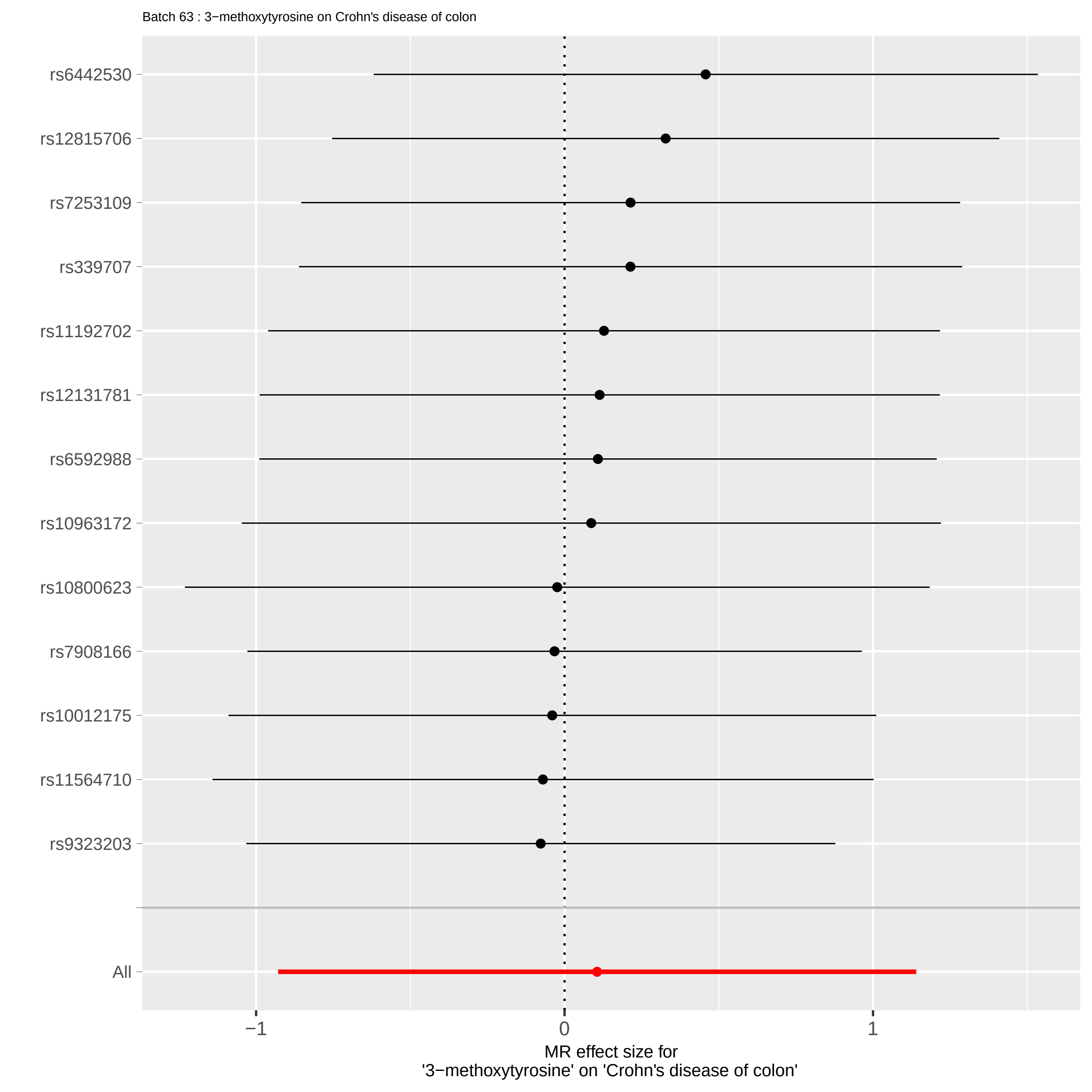
Batch 63 : 3−methoxytyrosine on Crohn's disease of colon
rs6442530
rs12815706
rs7253109
rs339707
rs11192702
rs12131781
rs6592988
rs10963172
rs10800623
rs7908166
rs10012175
rs11564710
rs9323203
All
−1
0
1
MR effect size for
'3−methoxytyrosine' on 'Crohn's disease of colon'

Batch 65 : Undecanoate (11:0) on Crohn's disease of colon

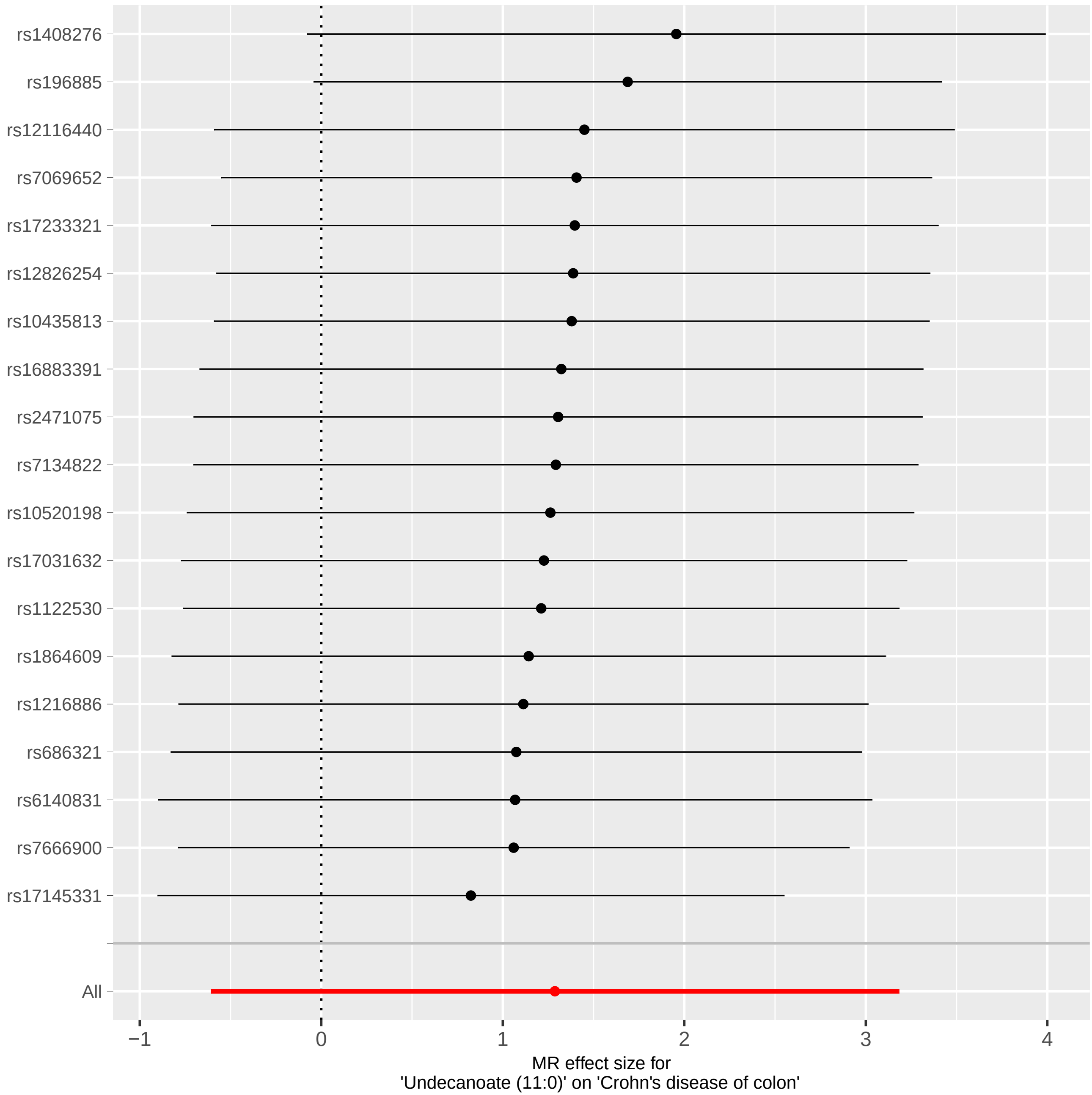

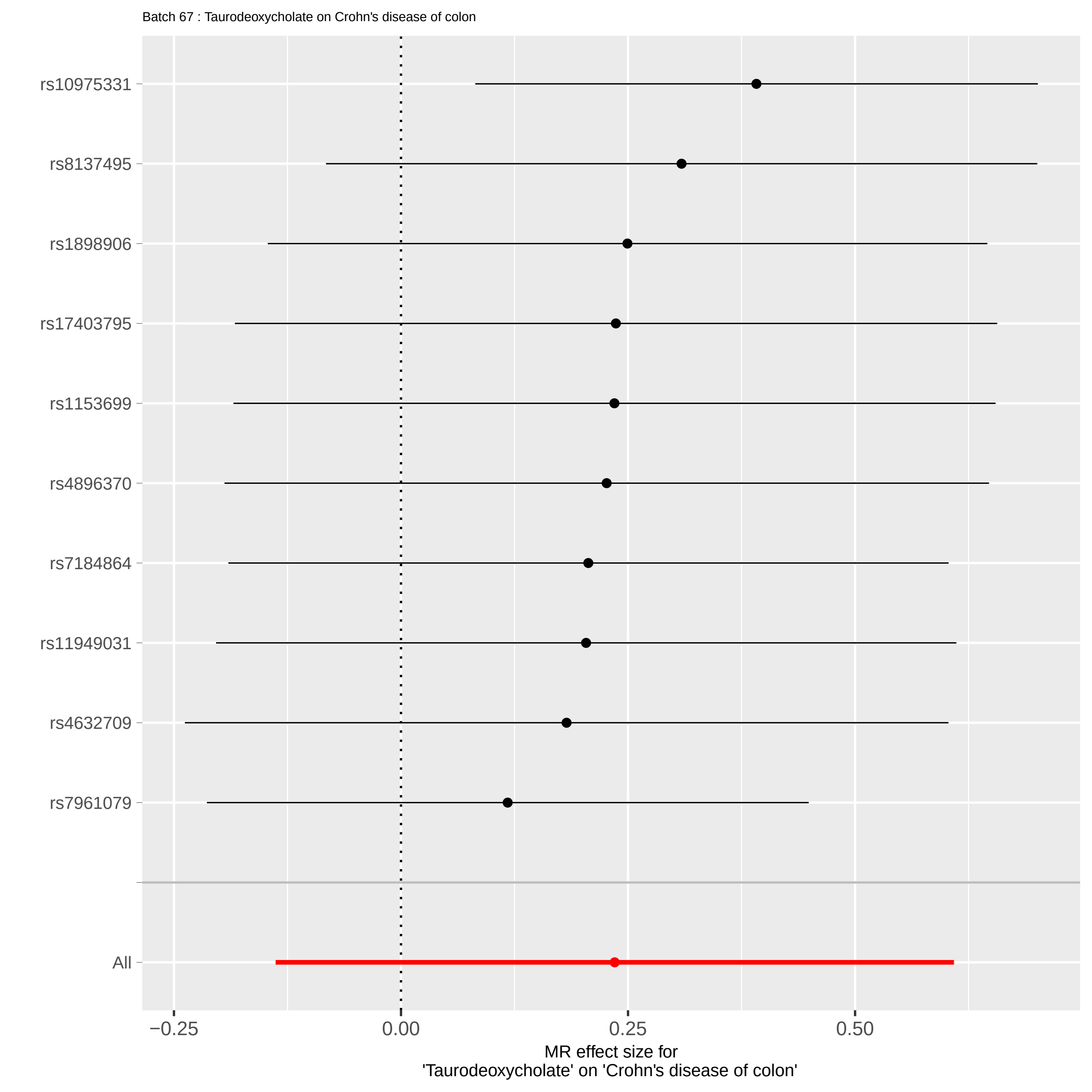

Batch 67 : Taurodeoxycholate on Crohn's disease of colon
rs10975331
rs8137495
rs1898906
rs17403795
rs1153699
rs4896370
rs7184864
rs11949031
rs4632709
rs7961079
All
−0.25
0.00
0.25
0.50
MR effect size for
'Taurodeoxycholate' on 'Crohn's disease of colon'

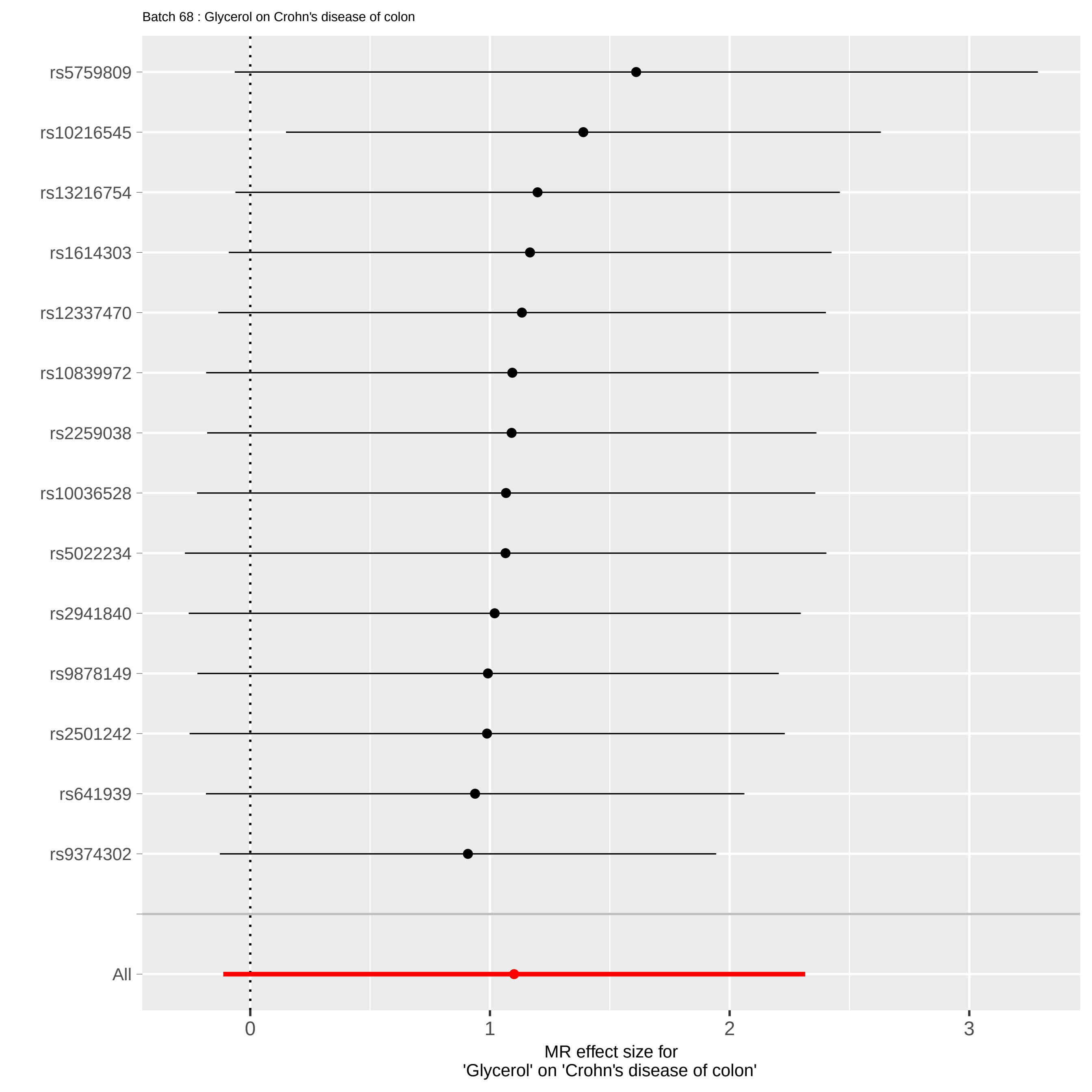

Batch 68 : Glycerol on Crohn's disease of colon
rs5759809
rs10216545
rs13216754
rs1614303
rs12337470
rs10839972
rs2259038
rs10036528
rs5022234
rs2941840
rs9878149
rs2501242
rs641939
rs9374302
All
0
1
2
3
MR effect size for
'Glycerol' on 'Crohn's disease of colon'

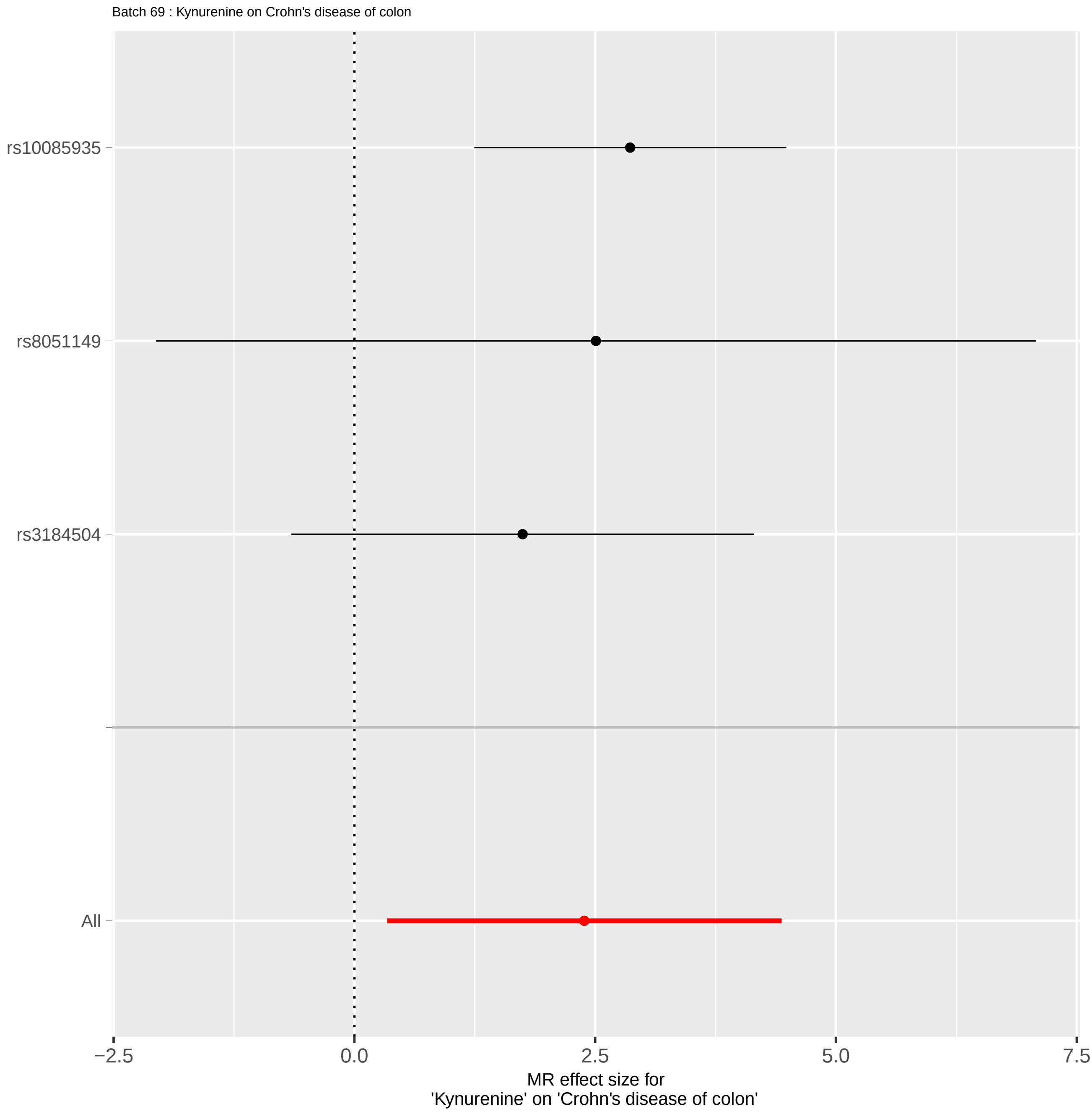

Batch 69 : Kynurenine on Crohn's disease of colon
rs10085935
rs8051149
rs3184504
All
−2.5
0.0
2.5
5.0
7.5
MR effect size for
'Kynurenine' on 'Crohn's disease of colon'

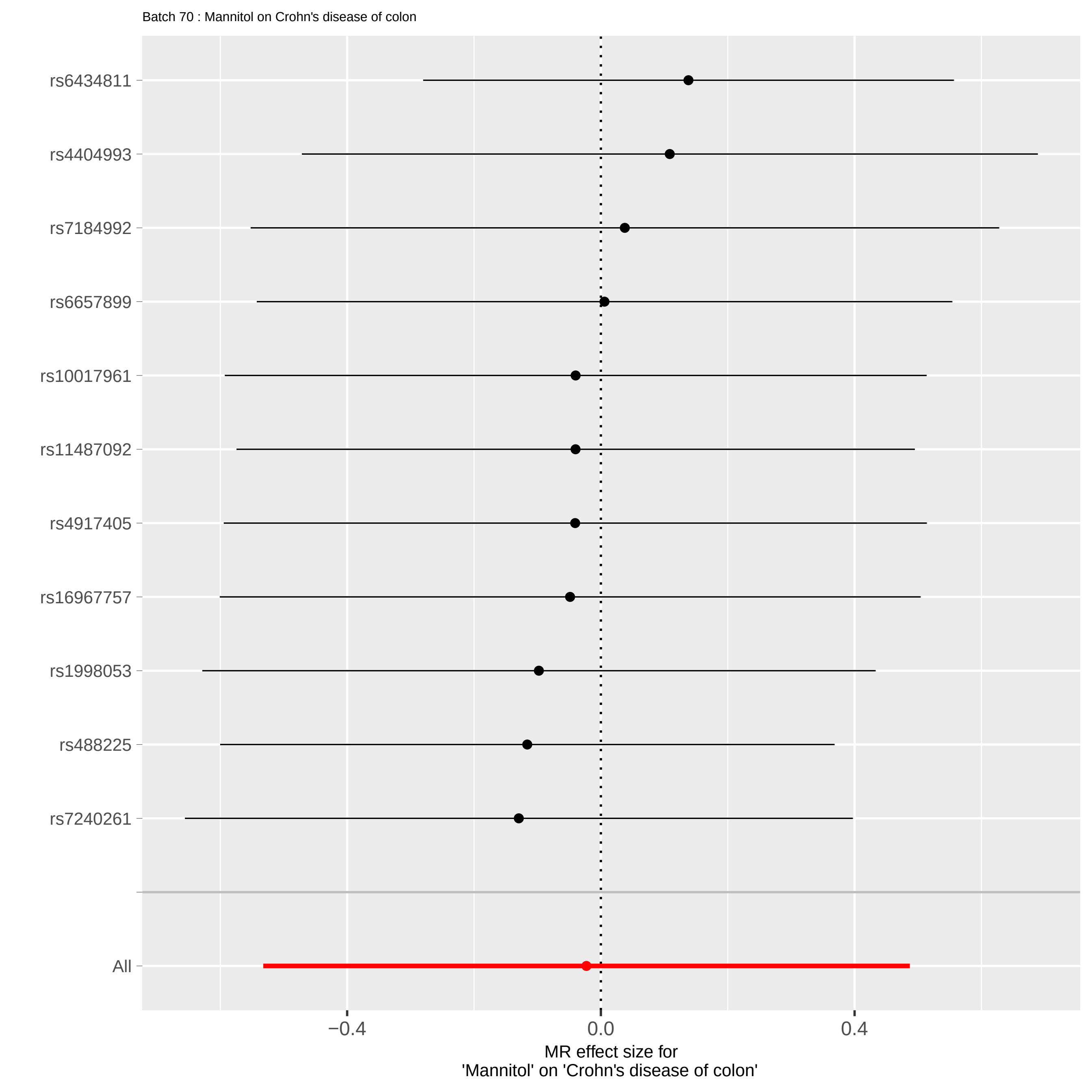

Batch 70 : Mannitol on Crohn's disease of colon
rs6434811
rs4404993
rs7184992
rs6657899
rs10017961
rs11487092
rs4917405
rs16967757
rs1998053
rs488225
rs7240261
All
−0.4
0.0
0.4
MR effect size for
'Mannitol' on 'Crohn's disease of colon'

Batch 71 : Glycerol 3−phosphate (G3P) on Crohn's disease of colon

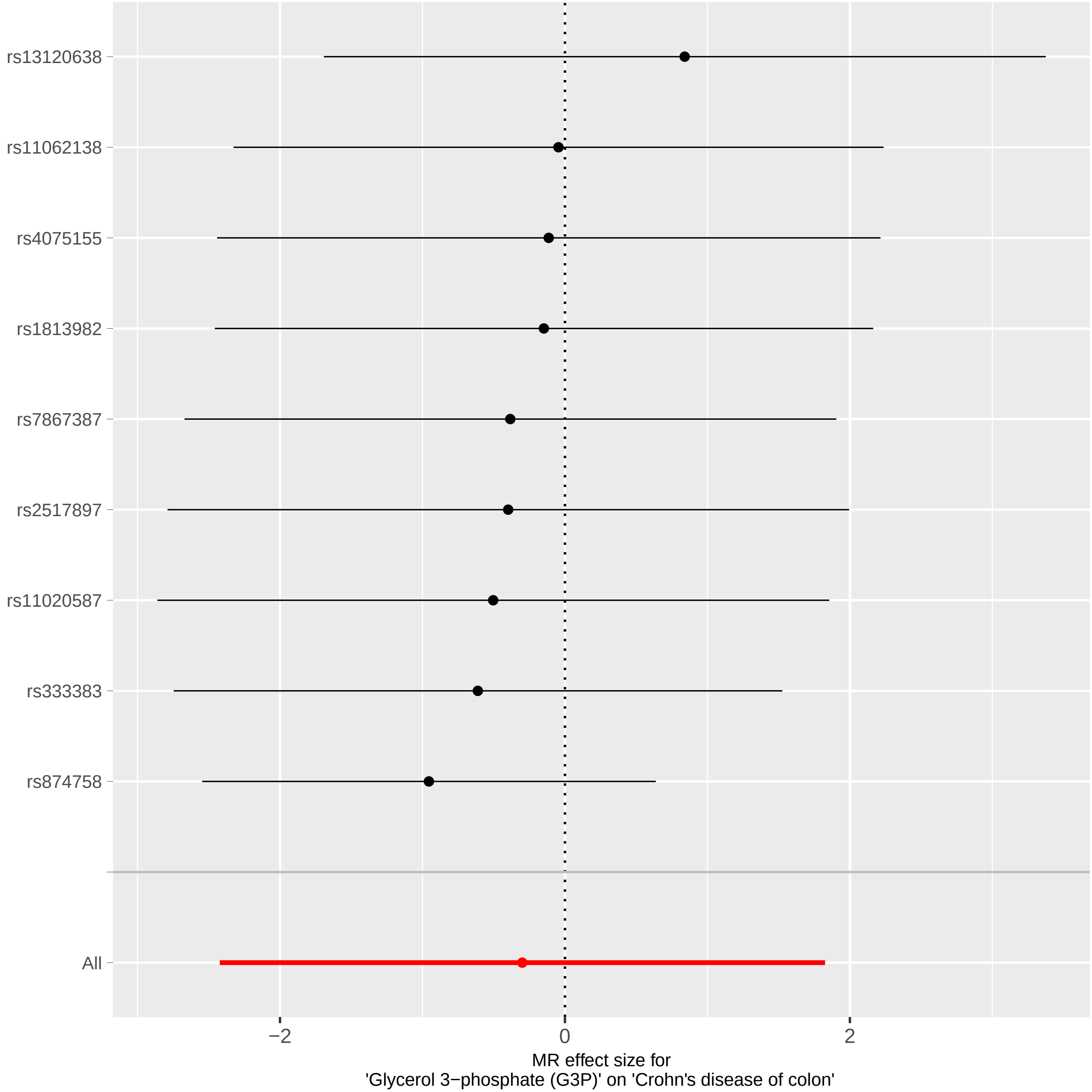

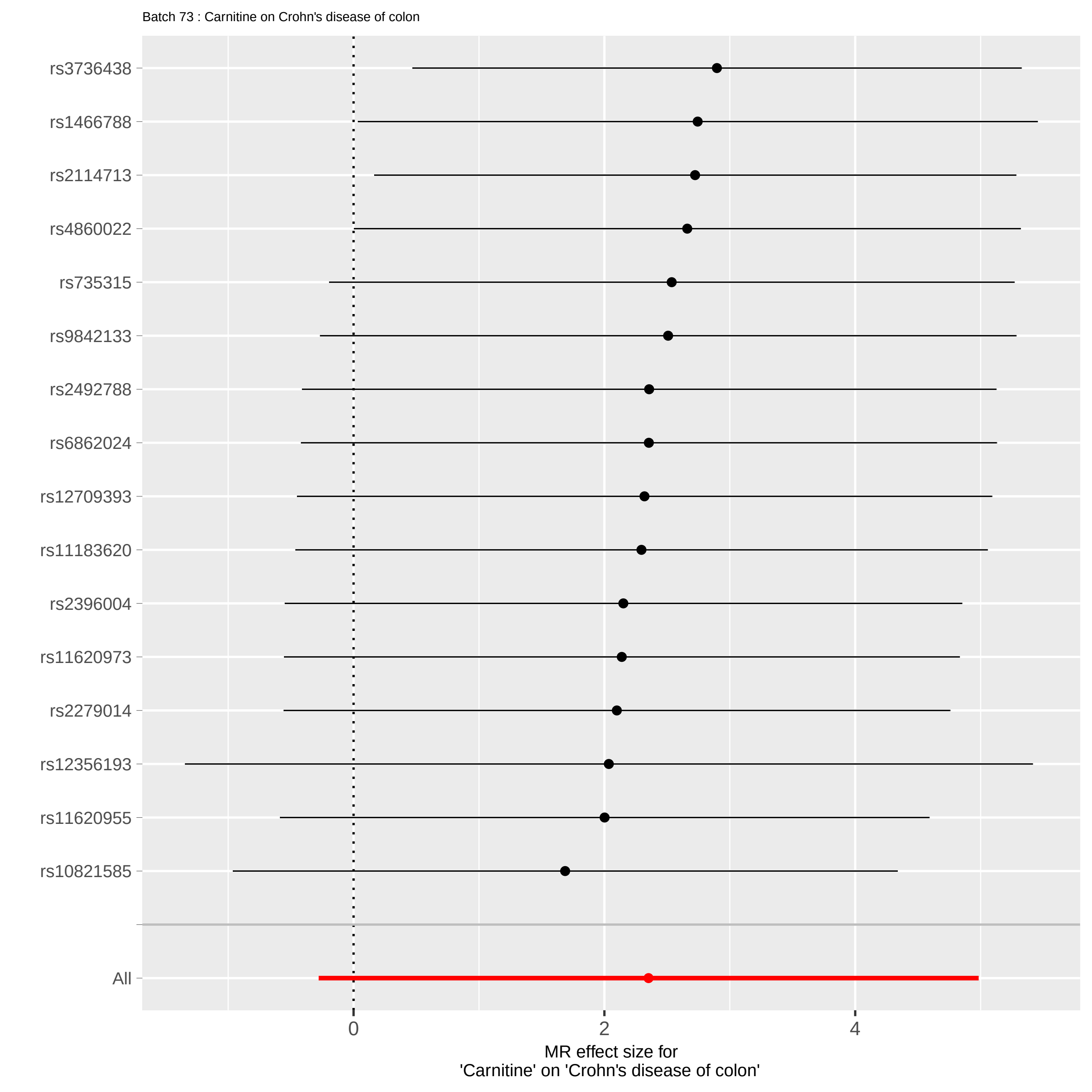
Batch 73 : Carnitine on Crohn's disease of colon
rs3736438
rs1466788
rs2114713
rs4860022
rs735315
rs9842133
rs2492788
rs6862024
rs12709393
rs11183620
rs2396004
rs11620973
rs2279014
rs12356193
rs11620955
rs10821585
All
0
2
4
MR effect size for
'Carnitine' on 'Crohn's disease of colon'

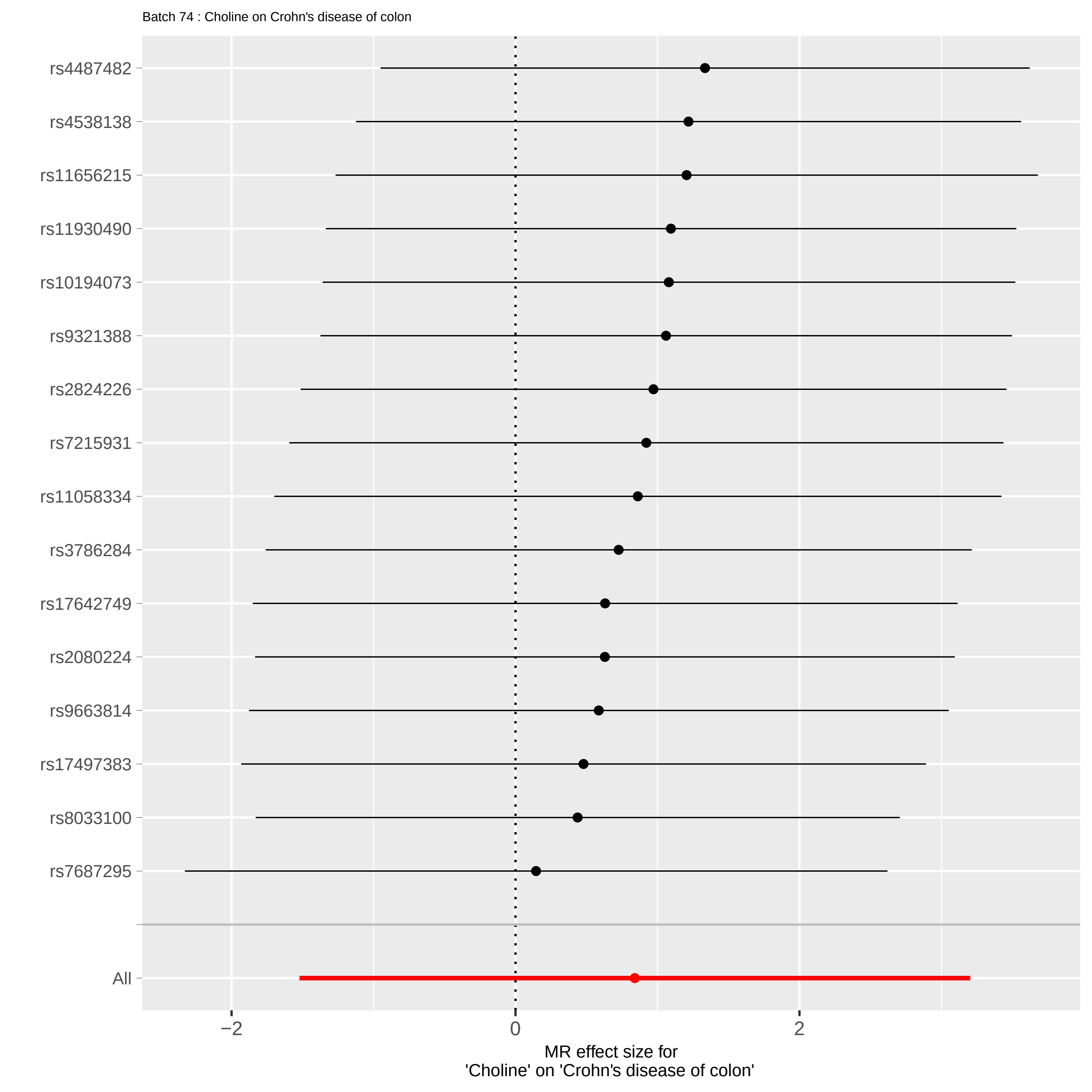

Batch 74 : Choline on Crohn's disease of colon
rs4487482
rs4538138
rs11656215
rs11930490
rs10194073
rs9321388
rs2824226
rs7215931
rs11058334
rs3786284
rs17642749
rs2080224
rs9663814
rs17497383
rs8033100
rs7687295
All
−2
0
2
MR effect size for
'Choline' on 'Crohn's disease of colon'

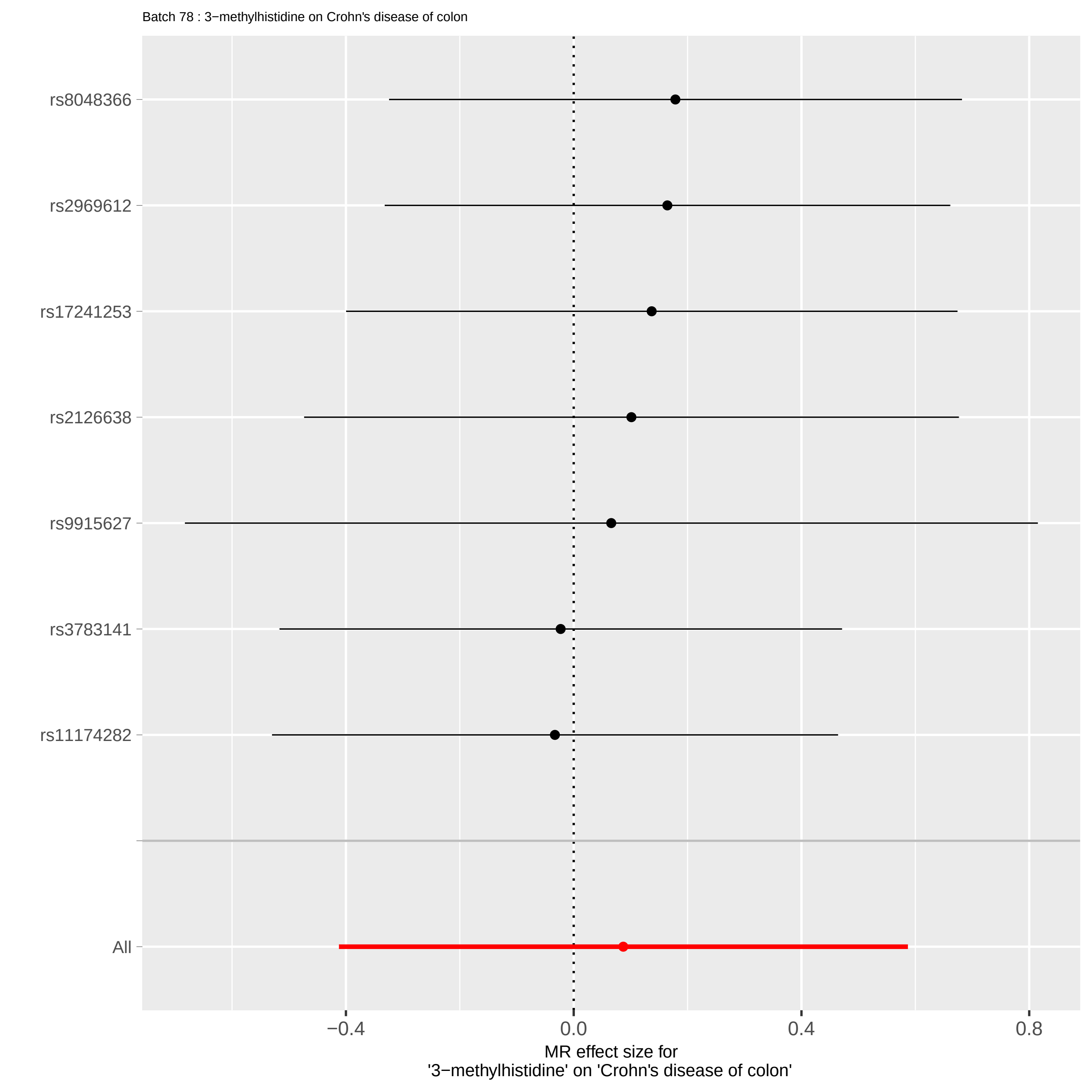
Batch 78 : 3−methylhistidine on Crohn's disease of colon
rs8048366
rs2969612
rs17241253
rs2126638
rs9915627
rs3783141
rs11174282
All
−0.4
0.0
0.4
0.8
MR effect size for
'3−methylhistidine' on 'Crohn's disease of colon'

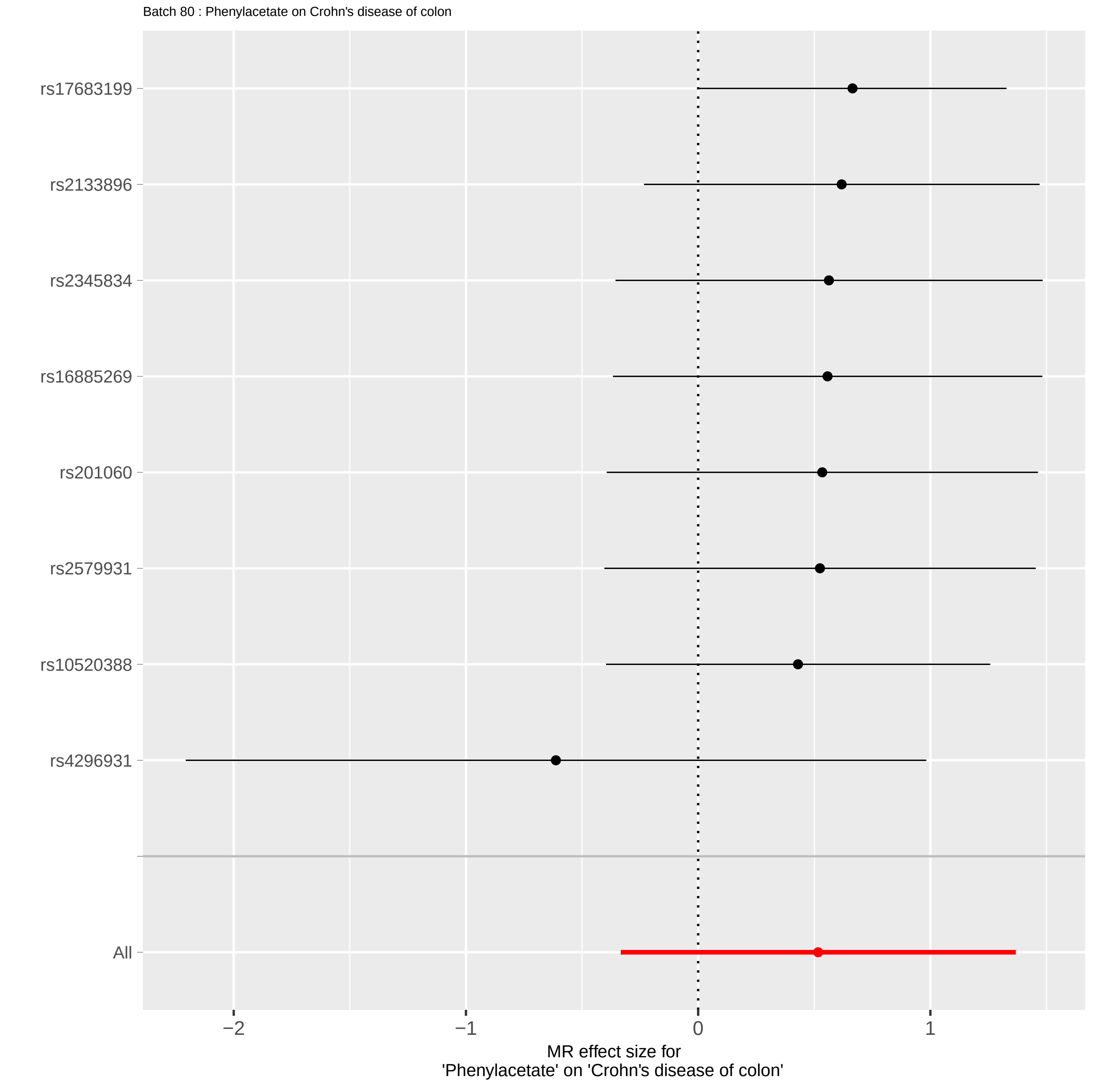

Batch 80 : Phenylacetate on Crohn's disease of colon
rs17683199
rs2133896
rs2345834
rs16885269
rs201060
rs2579931
rs10520388
rs4296931
All
−2
−1
0
1
MR effect size for
'Phenylacetate' on 'Crohn's disease of colon'

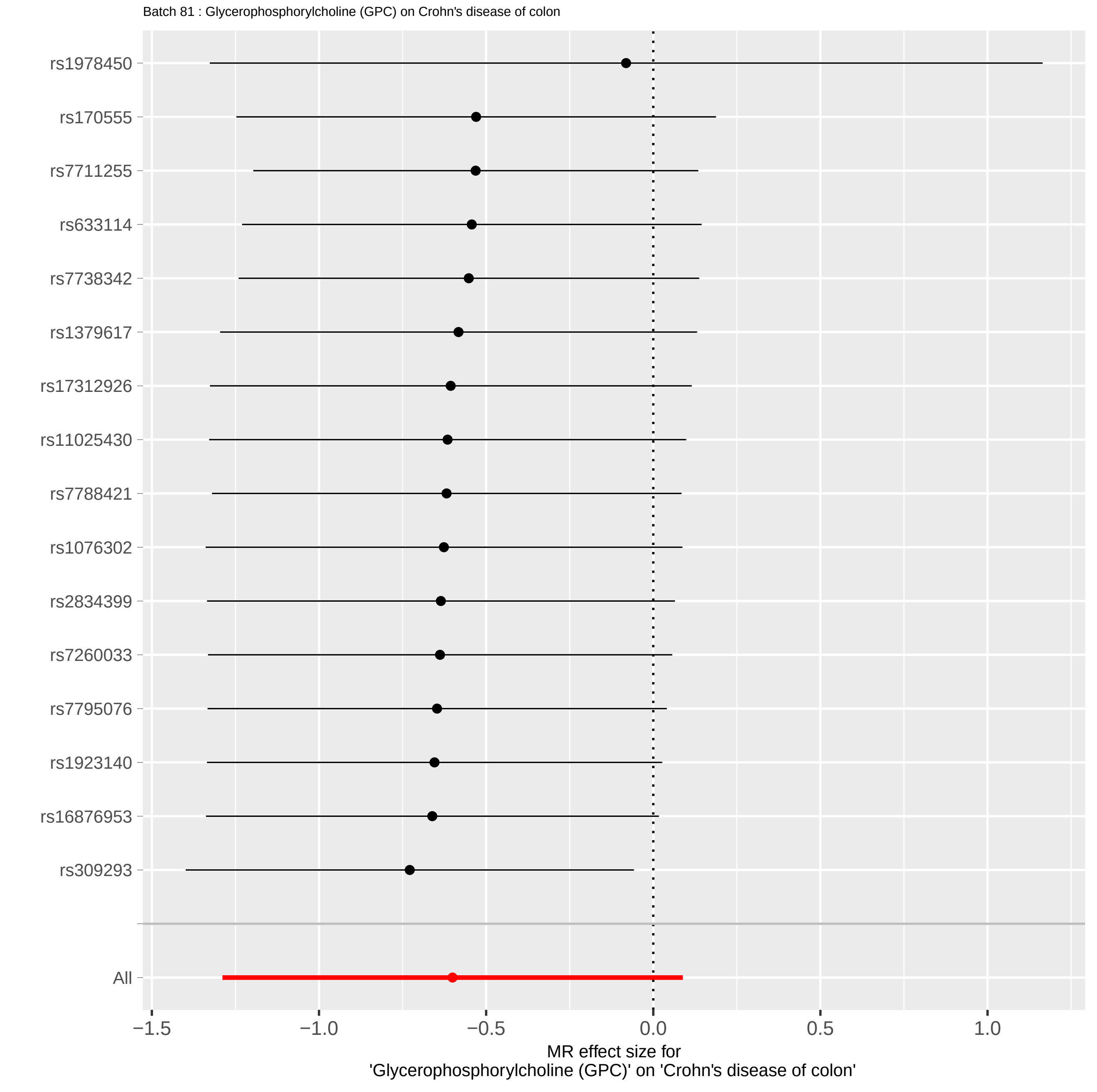

Batch 81 : Glycerophosphorylcholine (GPC) on Crohn's disease of colon
rs1978450
rs170555
rs7711255
rs633114
rs7738342
rs1379617
rs17312926
rs11025430
rs7788421
rs1076302
rs2834399
rs7260033
rs7795076
rs1923140
rs16876953
rs309293
All
−1.5
−1.0
−0.5
0.0
0.5
1.0
MR effect size for
'Glycerophosphorylcholine (GPC)' on 'Crohn's disease of colon'

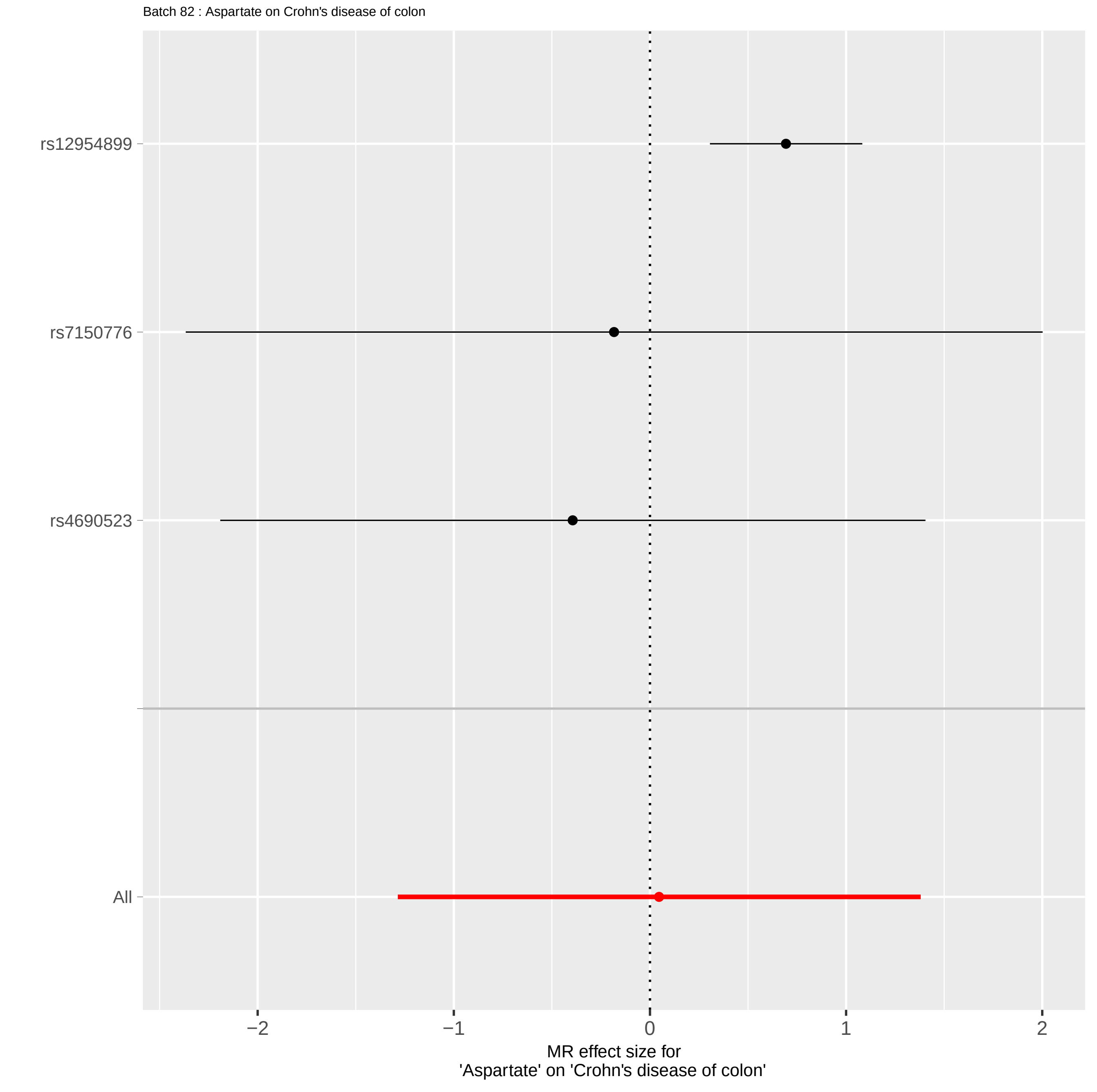

Batch 82 : Aspartate on Crohn's disease of colon
rs12954899
rs7150776
rs4690523
All
−2
−1
0
1
2
MR effect size for
'Aspartate' on 'Crohn's disease of colon'

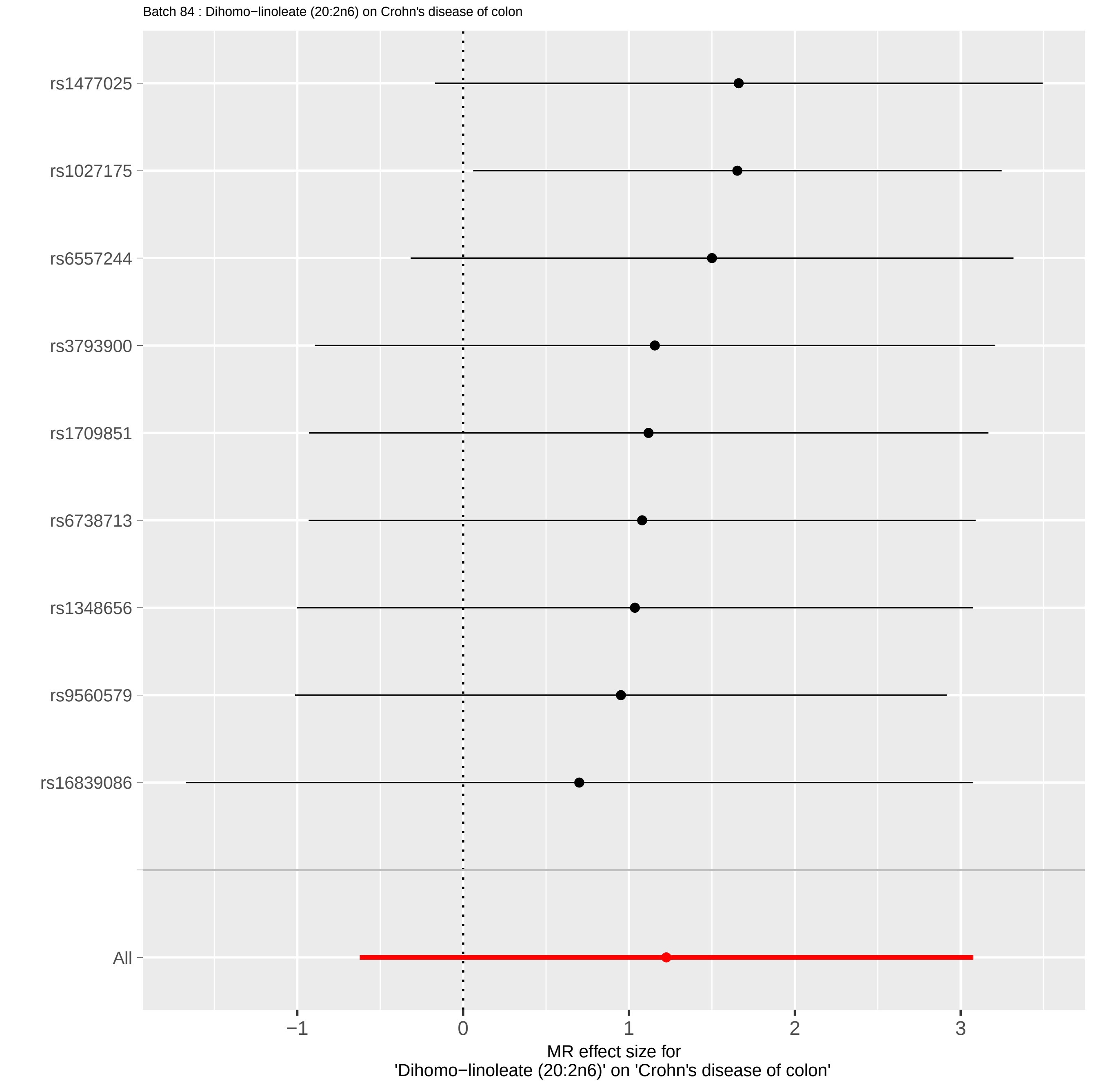

Batch 84 : Dihomo−linoleate (20:2n6) on Crohn's disease of colon
rs1477025
rs1027175
rs6557244
rs3793900
rs1709851
rs6738713
rs1348656
rs9560579
rs16839086
All
−1
0
1
2
3
MR effect size for
'Dihomo−linoleate (20:2n6)' on 'Crohn's disease of colon'

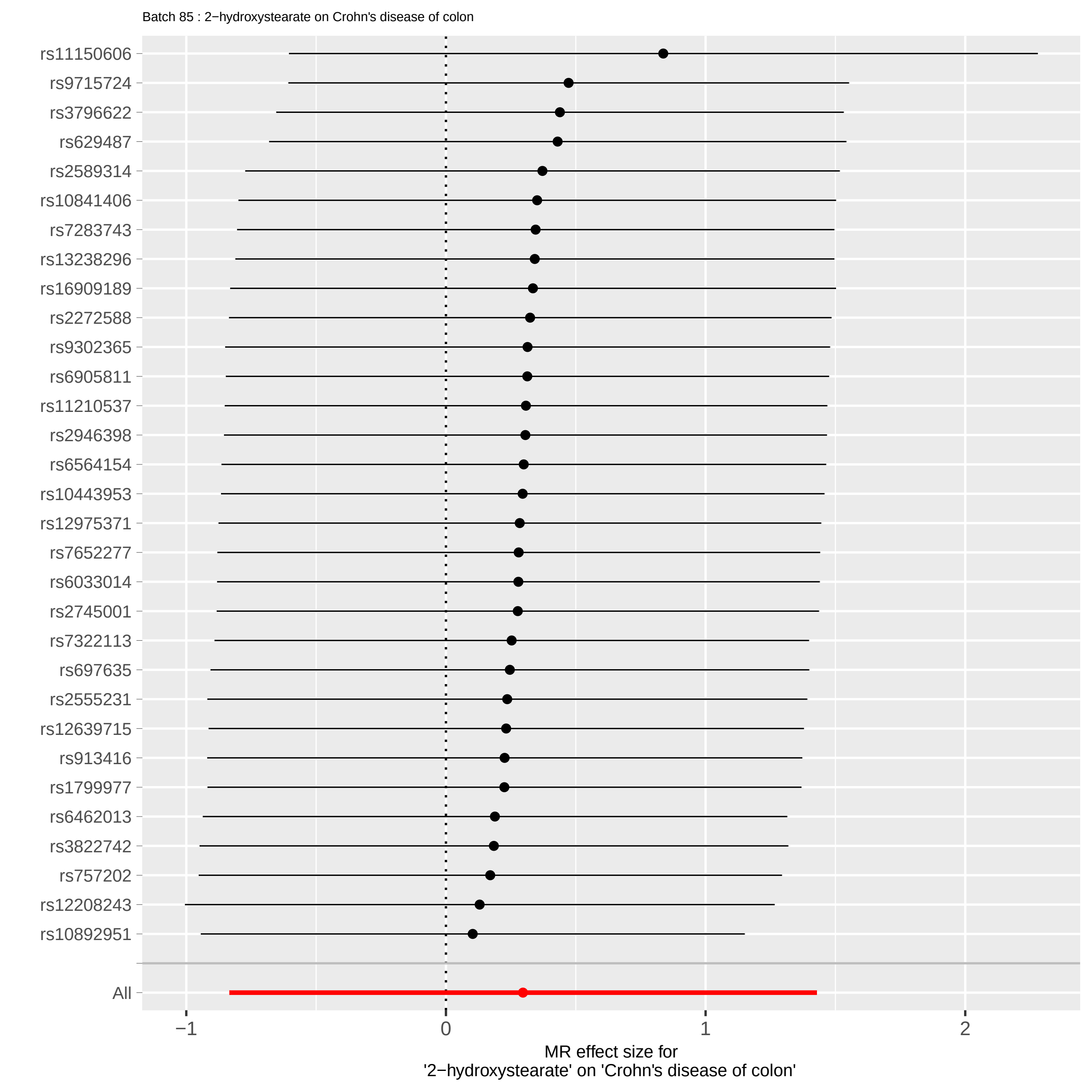

Batch 85 : 2−hydroxystearate on Crohn's disease of colon
rs11150606
rs9715724
rs3796622
rs629487
rs2589314
rs10841406
rs7283743
rs13238296
rs16909189
rs2272588
rs9302365
rs6905811
rs11210537
rs2946398
rs6564154
rs10443953
rs12975371
rs7652277
rs6033014
rs2745001
rs7322113
rs697635
rs2555231
rs12639715
rs913416
rs1799977
rs6462013
rs3822742
rs757202
rs12208243
rs10892951
All
−1
0
1
2
MR effect size for
'2−hydroxystearate' on 'Crohn's disease of colon'

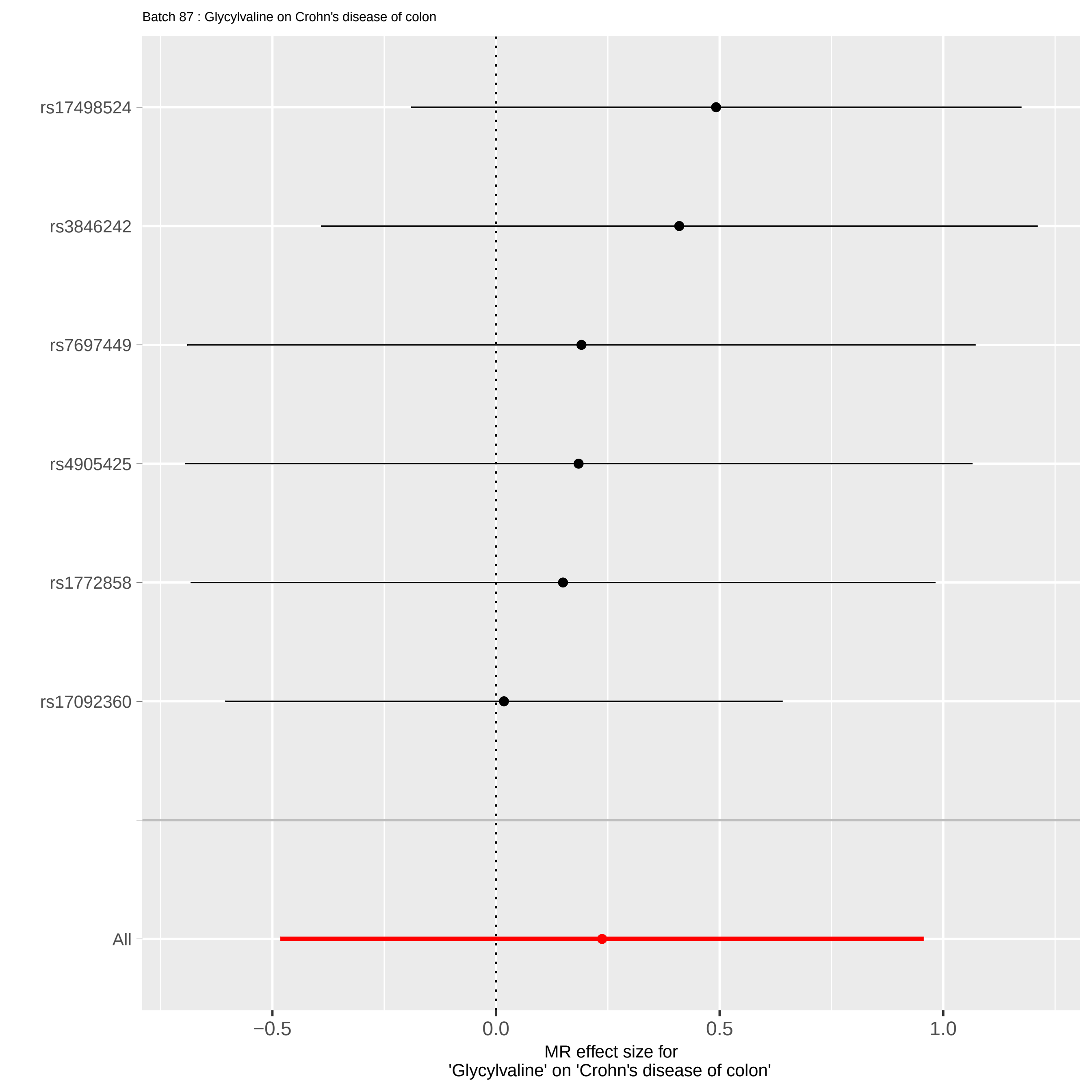

Batch 87 : Glycylvaline on Crohn's disease of colon
rs17498524
rs3846242
rs7697449
rs4905425
rs1772858
rs17092360
All
−0.5
0.0
0.5
1.0
MR effect size for
'Glycylvaline' on 'Crohn's disease of colon'

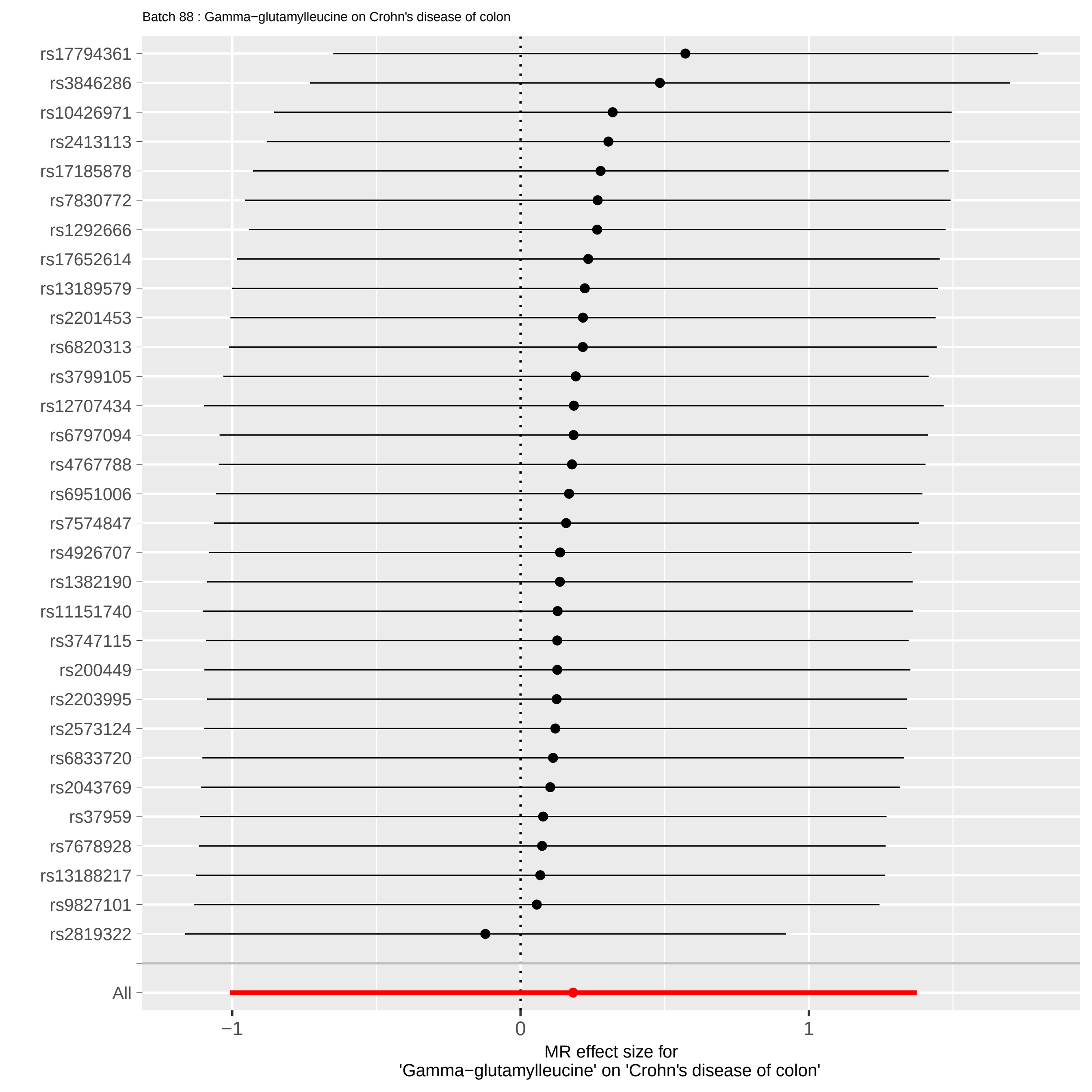

Batch 88 : Gamma−glutamylleucine on Crohn's disease of colon
rs17794361
rs3846286
rs10426971
rs2413113
rs17185878
rs7830772
rs1292666
rs17652614
rs13189579
rs2201453
rs6820313
rs3799105
rs12707434
rs6797094
rs4767788
rs6951006
rs7574847
rs4926707
rs1382190
rs11151740
rs3747115
rs200449
rs2203995
rs2573124
rs6833720
rs2043769
rs37959
rs7678928
rs13188217
rs9827101
rs2819322
All
−1
0
1
MR effect size for
'Gamma−glutamylleucine' on 'Crohn's disease of colon'

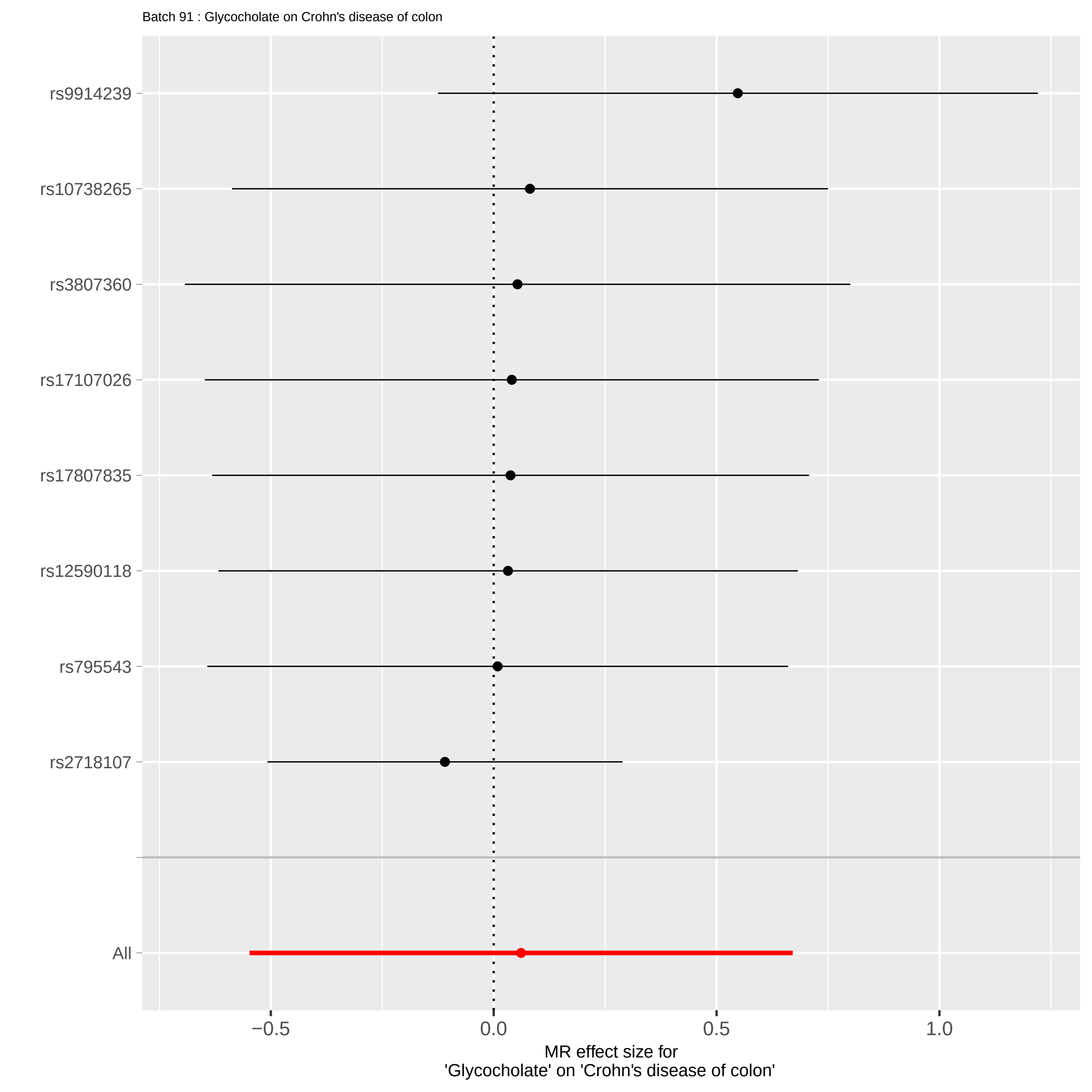

Batch 91 : Glycocholate on Crohn's disease of colon
rs9914239
rs10738265
rs3807360
rs17107026
rs17807835
rs12590118
rs795543
rs2718107
All
−0.5
0.0
0.5
1.0
MR effect size for
'Glycocholate' on 'Crohn's disease of colon'

Batch 92 : Glycodeoxycholate on Crohn's disease of colon

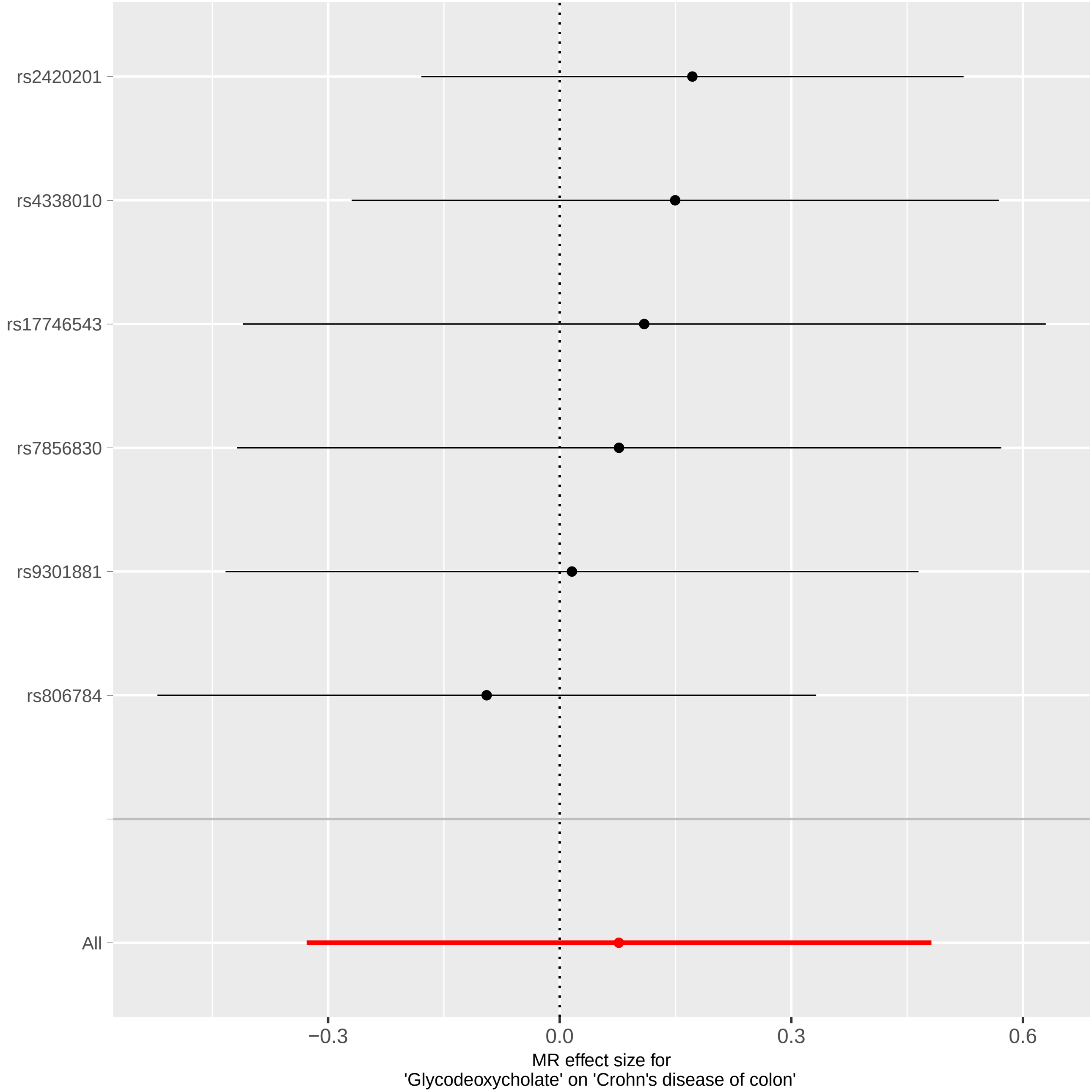

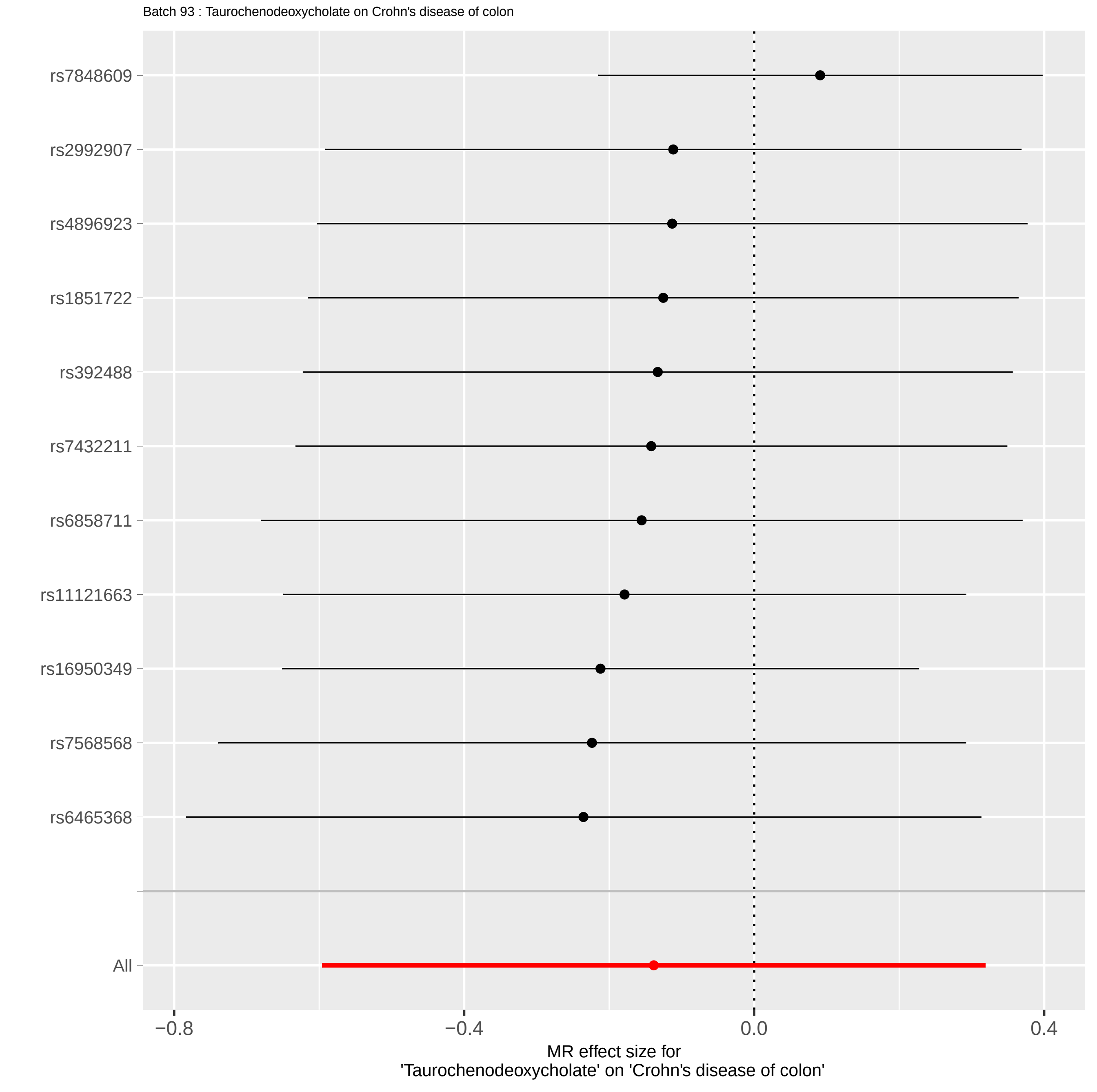
Batch 93 : Taurochenodeoxycholate on Crohn's disease of colon
rs7848609
rs2992907
rs4896923
rs1851722
rs392488
rs7432211
rs6858711
rs11121663
rs16950349
rs7568568
rs6465368
All
−0.8
−0.4
0.0
0.4
MR effect size for
'Taurochenodeoxycholate' on 'Crohn's disease of colon'

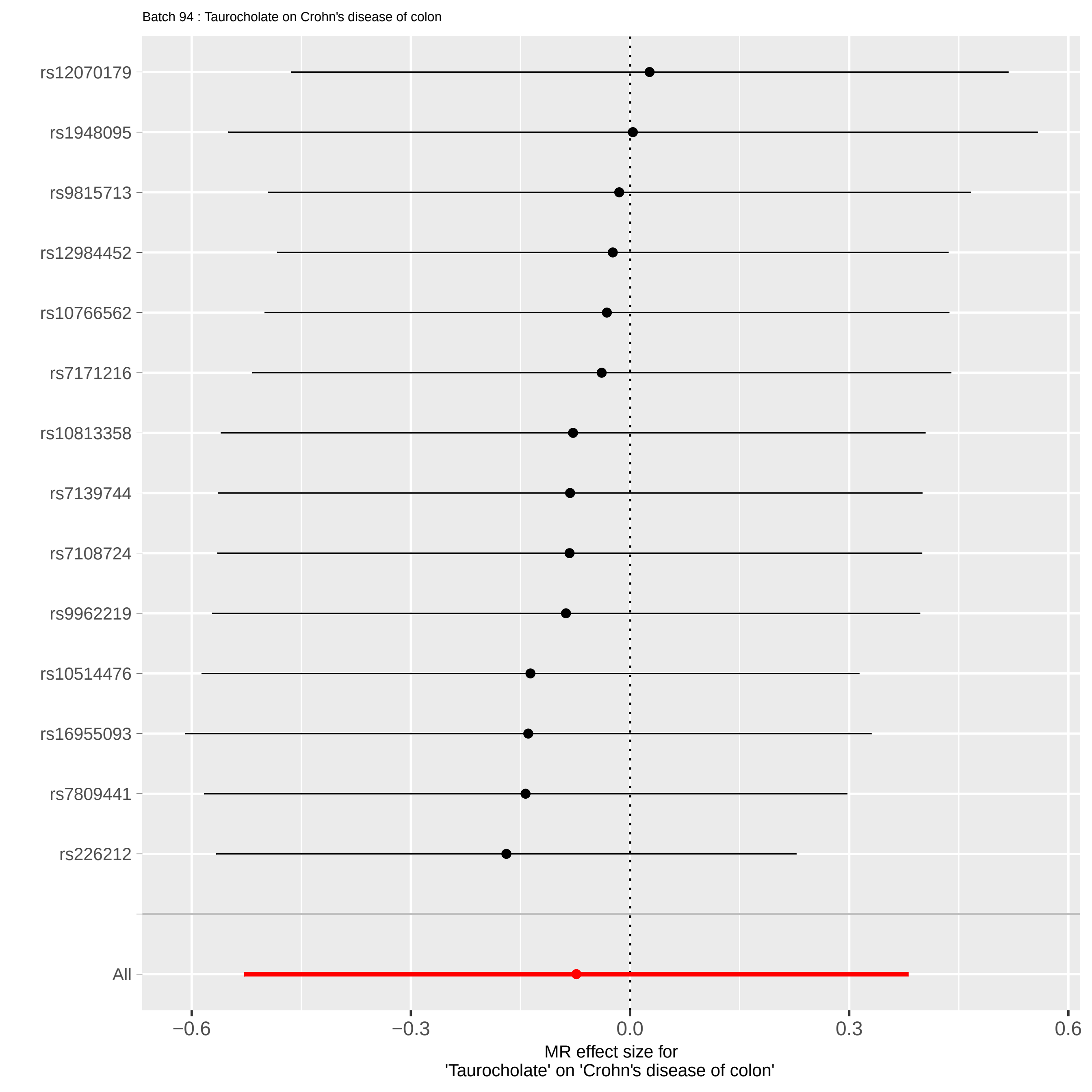

Batch 94 : Taurocholate on Crohn's disease of colon
rs12070179
rs1948095
rs9815713
rs12984452
rs10766562
rs7171216
rs10813358
rs7139744
rs7108724
rs9962219
rs10514476
rs16955093
rs7809441
rs226212
All
−0.6
−0.3
0.0
0.3
0.6
MR effect size for
'Taurocholate' on 'Crohn's disease of colon'

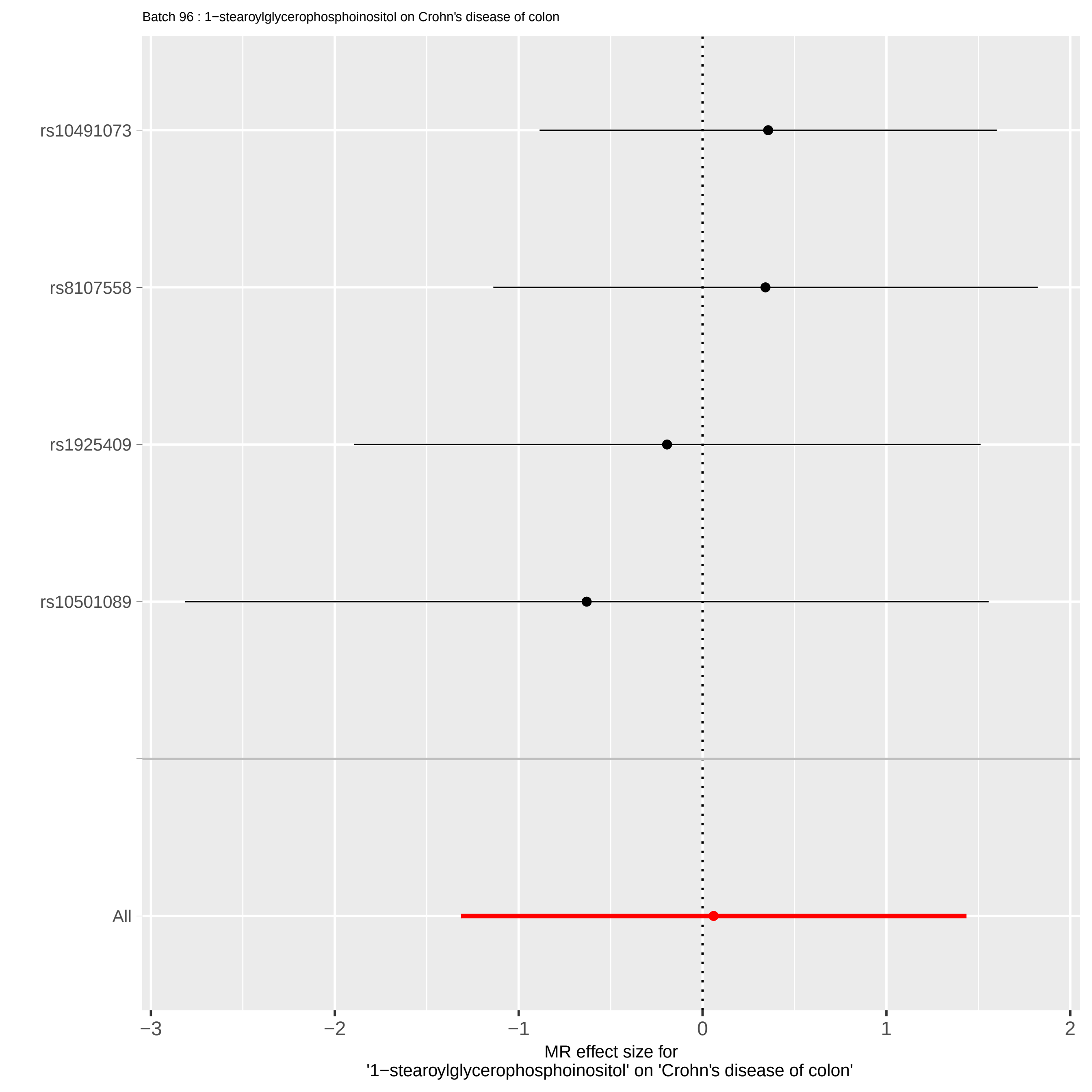

Batch 96 : 1−stearoylglycerophosphoinositol on Crohn's disease of colon
rs10491073
rs8107558
rs1925409
rs10501089
All
−3
−2
−1
0
1
2
MR effect size for
'1−stearoylglycerophosphoinositol' on 'Crohn's disease of colon'

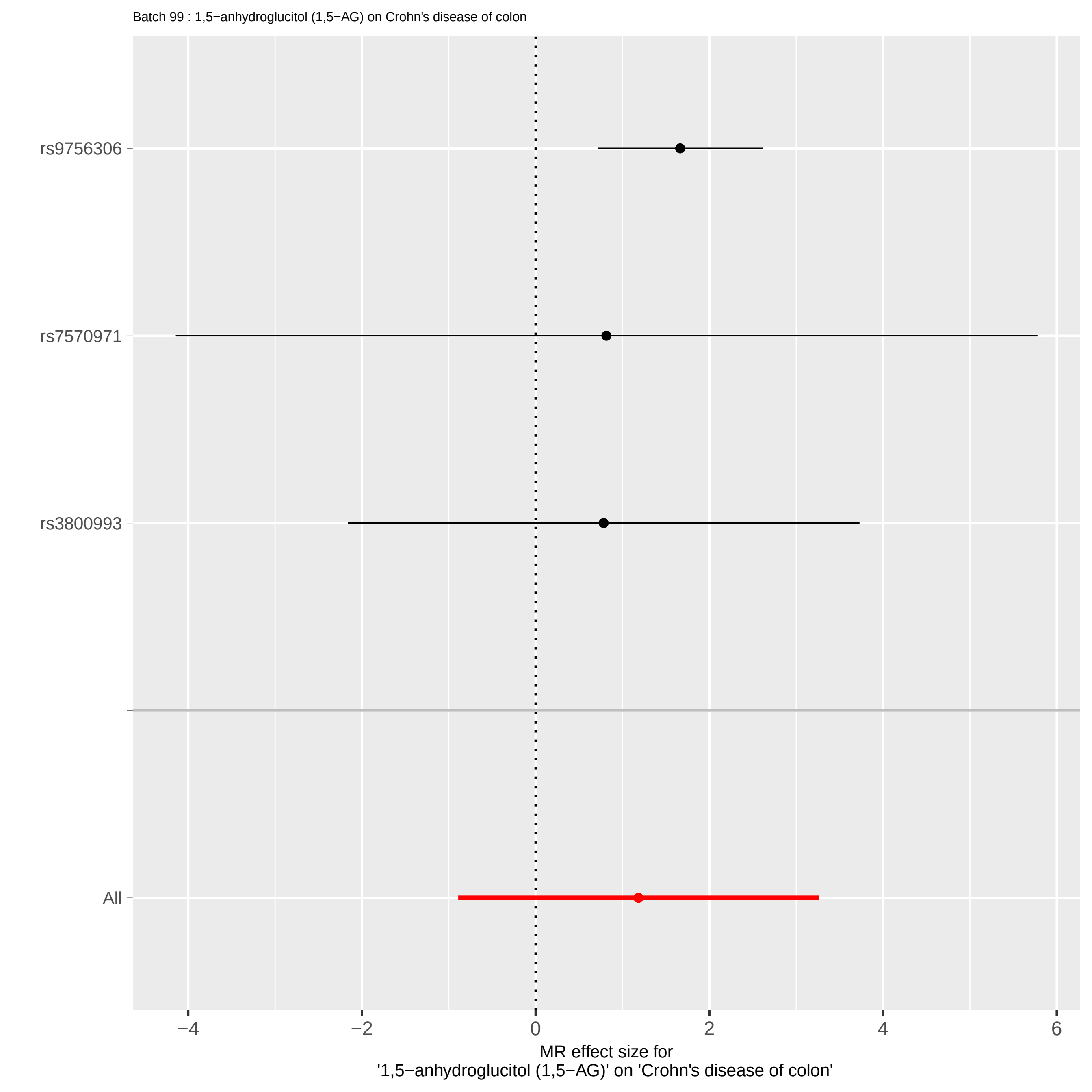

Batch 99 : 1,5−anhydroglucitol (1,5−AG) on Crohn's disease of colon
rs9756306
rs7570971
rs3800993
All
−4
−2
0
2
4
6
MR effect size for
'1,5−anhydroglucitol (1,5−AG)' on 'Crohn's disease of colon'

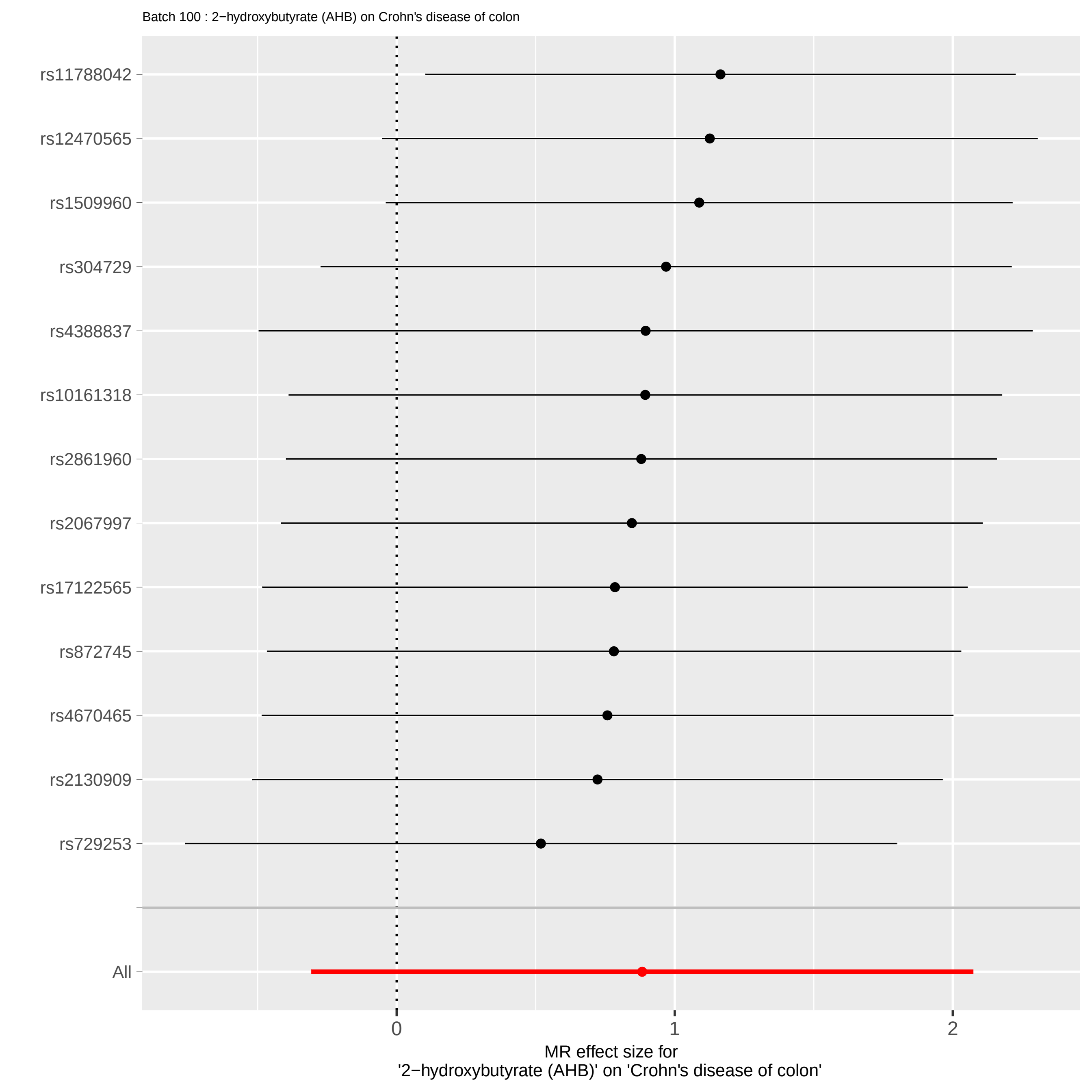
Batch 100 : 2−hydroxybutyrate (AHB) on Crohn's disease of colon
rs11788042
rs12470565
rs1509960
rs304729
rs4388837
rs10161318
rs2861960
rs2067997
rs17122565
rs872745
rs4670465
rs2130909
rs729253
All
0
1
2
MR effect size for
'2−hydroxybutyrate (AHB)' on 'Crohn's disease of colon'

Batch 103 : 1−palmitoylglycerol (1−monopalmitin) on Crohn's disease of colon

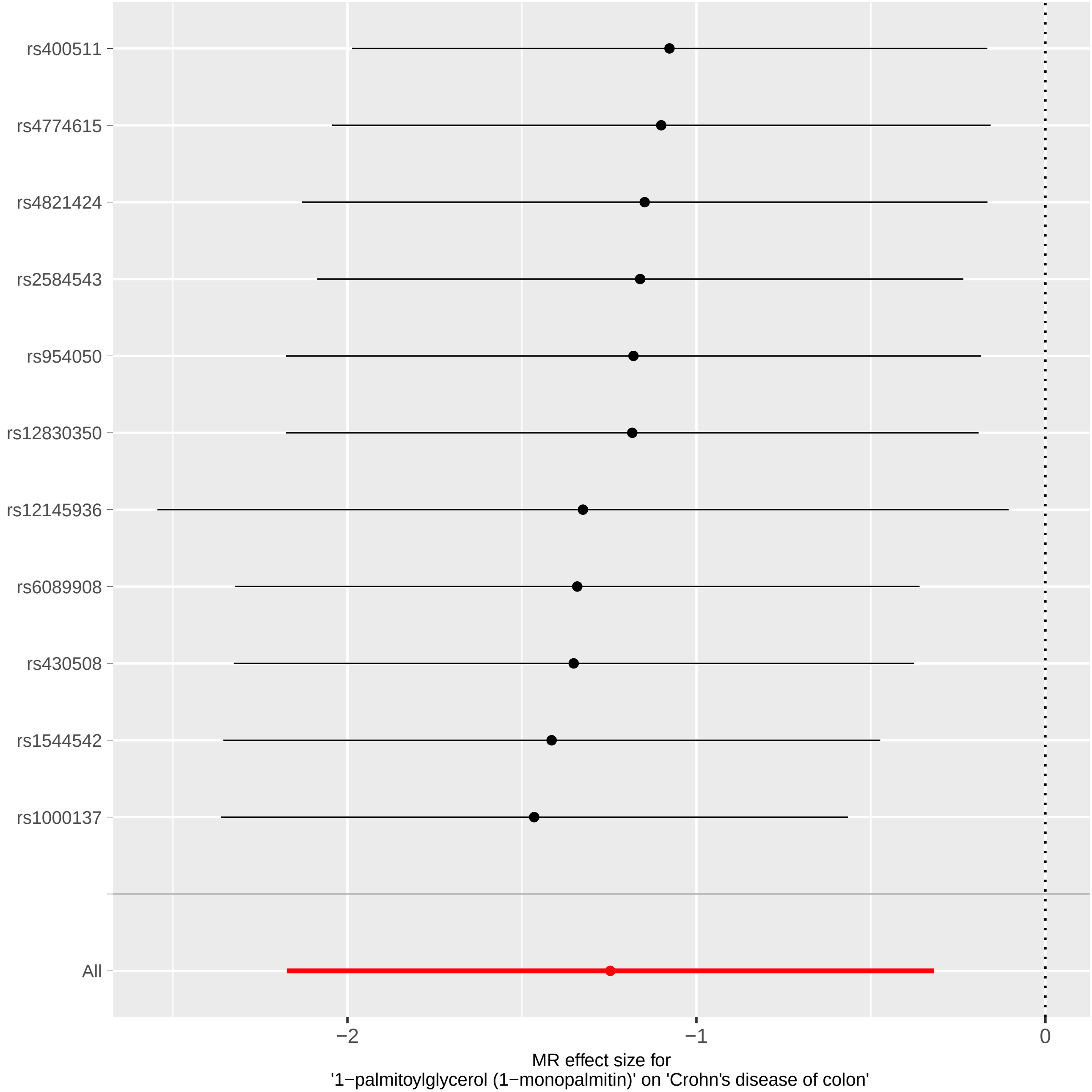

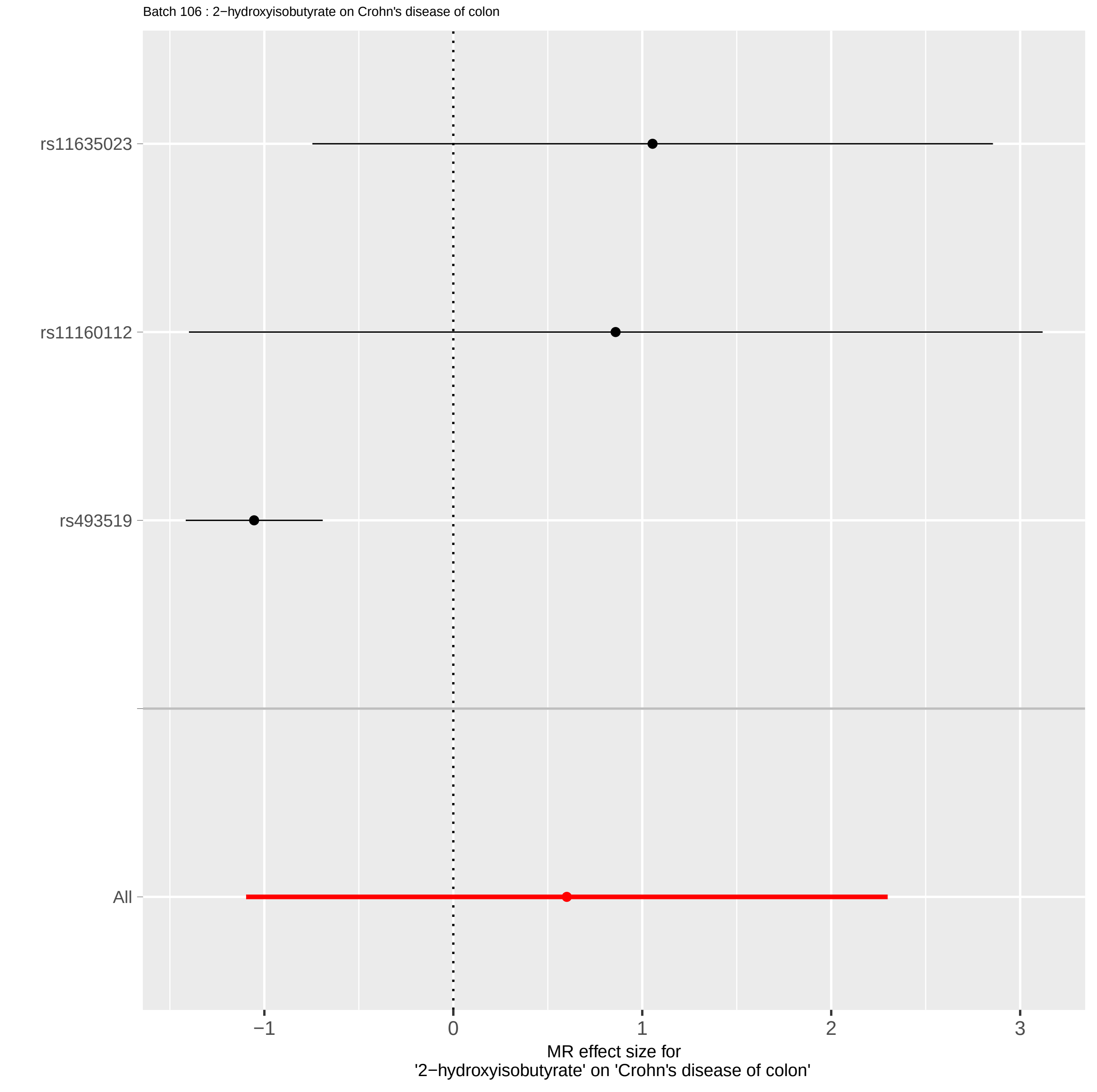

Batch 106 : 2−hydroxyisobutyrate on Crohn's disease of colon
rs11635023
rs11160112
rs493519
All
−1
0
1
2
3
MR effect size for
'2−hydroxyisobutyrate' on 'Crohn's disease of colon'

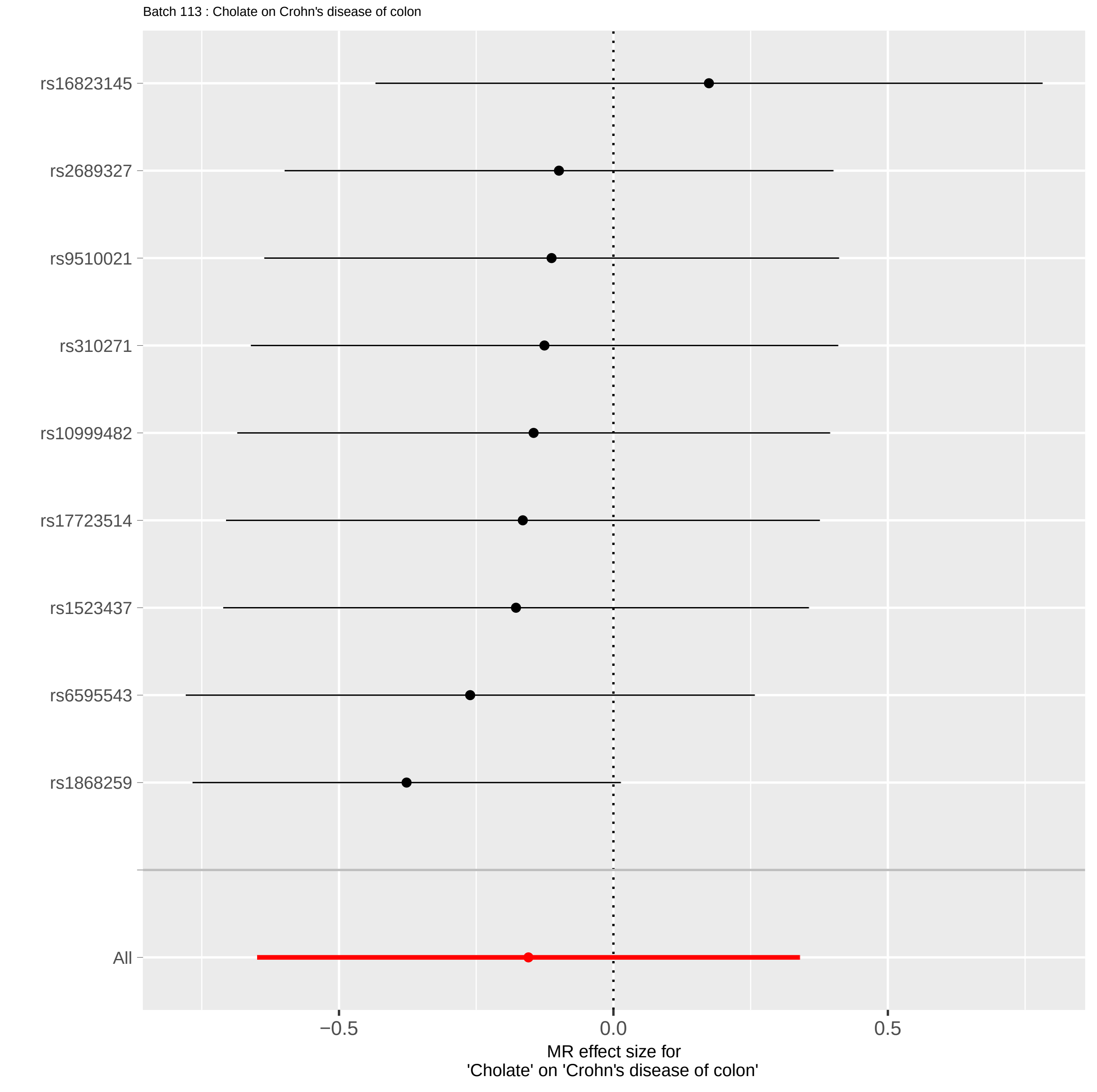
Batch 113 : Cholate on Crohn's disease of colon
rs16823145
rs2689327
rs9510021
rs310271
rs10999482
rs17723514
rs1523437
rs6595543
rs1868259
All
−0.5
0.0
0.5
MR effect size for
'Cholate' on 'Crohn's disease of colon'

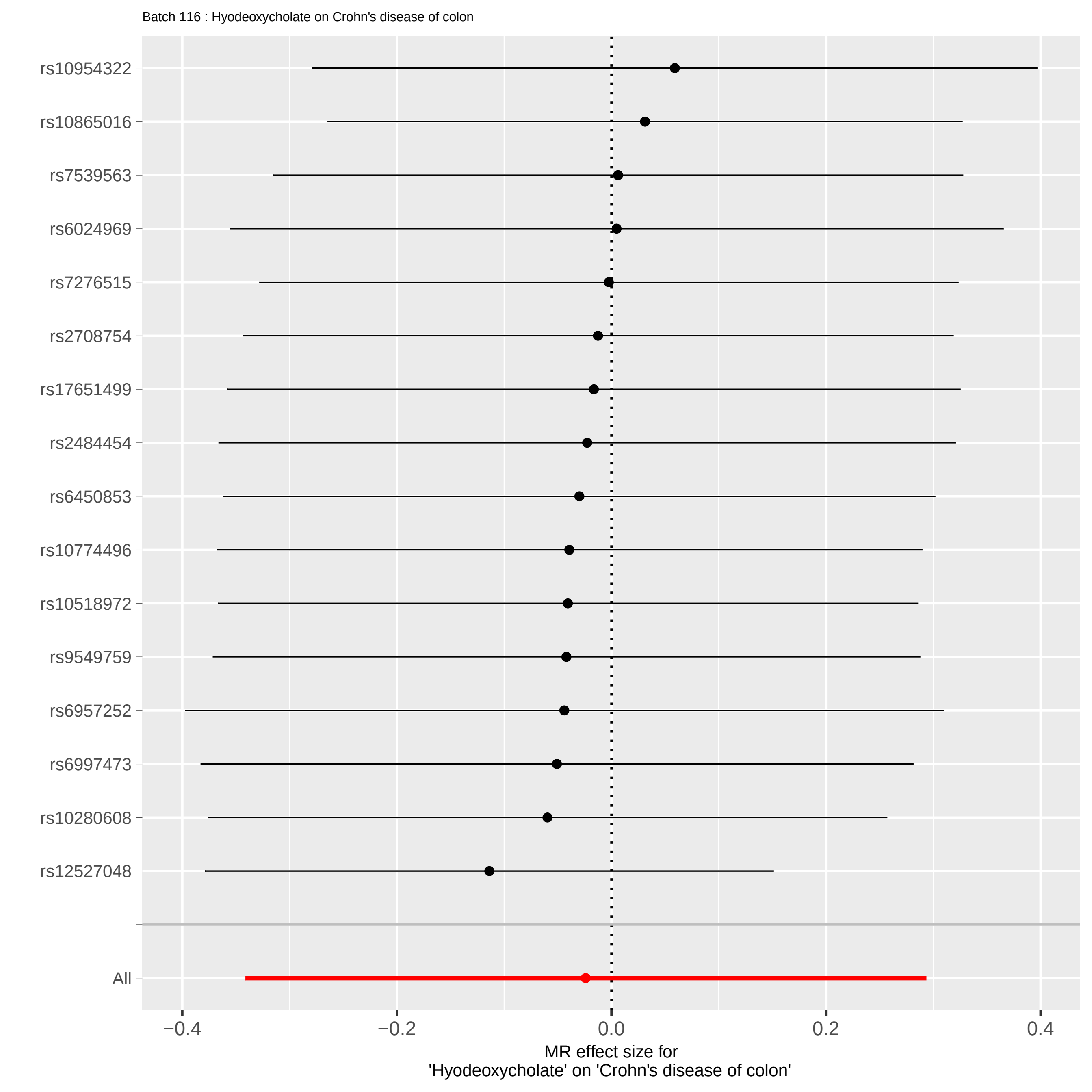
Batch 116 : Hyodeoxycholate on Crohn's disease of colon
rs10954322
rs10865016
rs7539563
rs6024969
rs7276515
rs2708754
rs17651499
rs2484454
rs6450853
rs10774496
rs10518972
rs9549759
rs6957252
rs6997473
rs10280608
rs12527048
All
−0.4
−0.2
0.0
0.2
0.4
MR effect size for
'Hyodeoxycholate' on 'Crohn's disease of colon'

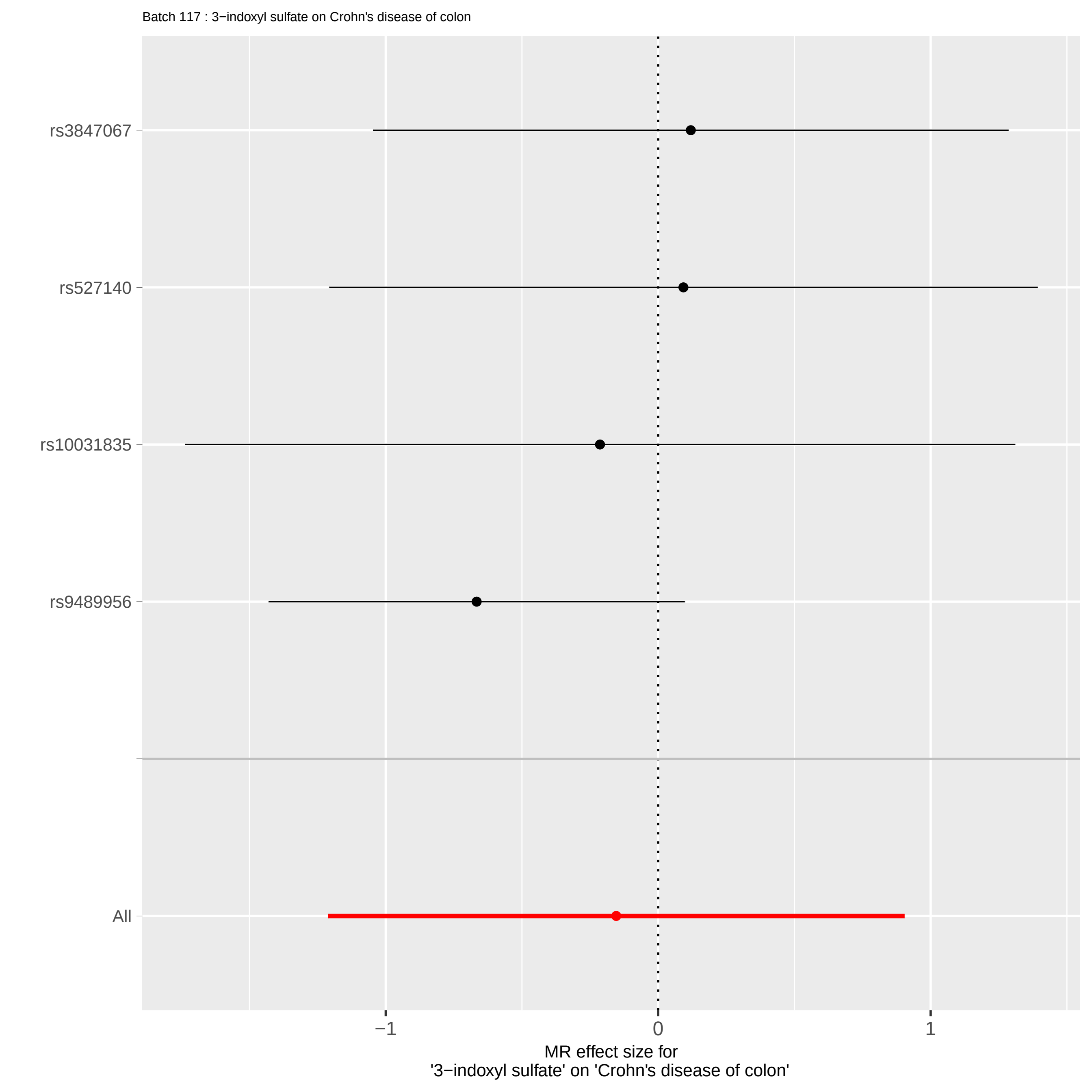
Batch 117 : 3−indoxyl sulfate on Crohn's disease of colon
rs3847067
rs527140
rs10031835
rs9489956
All
−1
0
1
MR effect size for
'3−indoxyl sulfate' on 'Crohn's disease of colon'

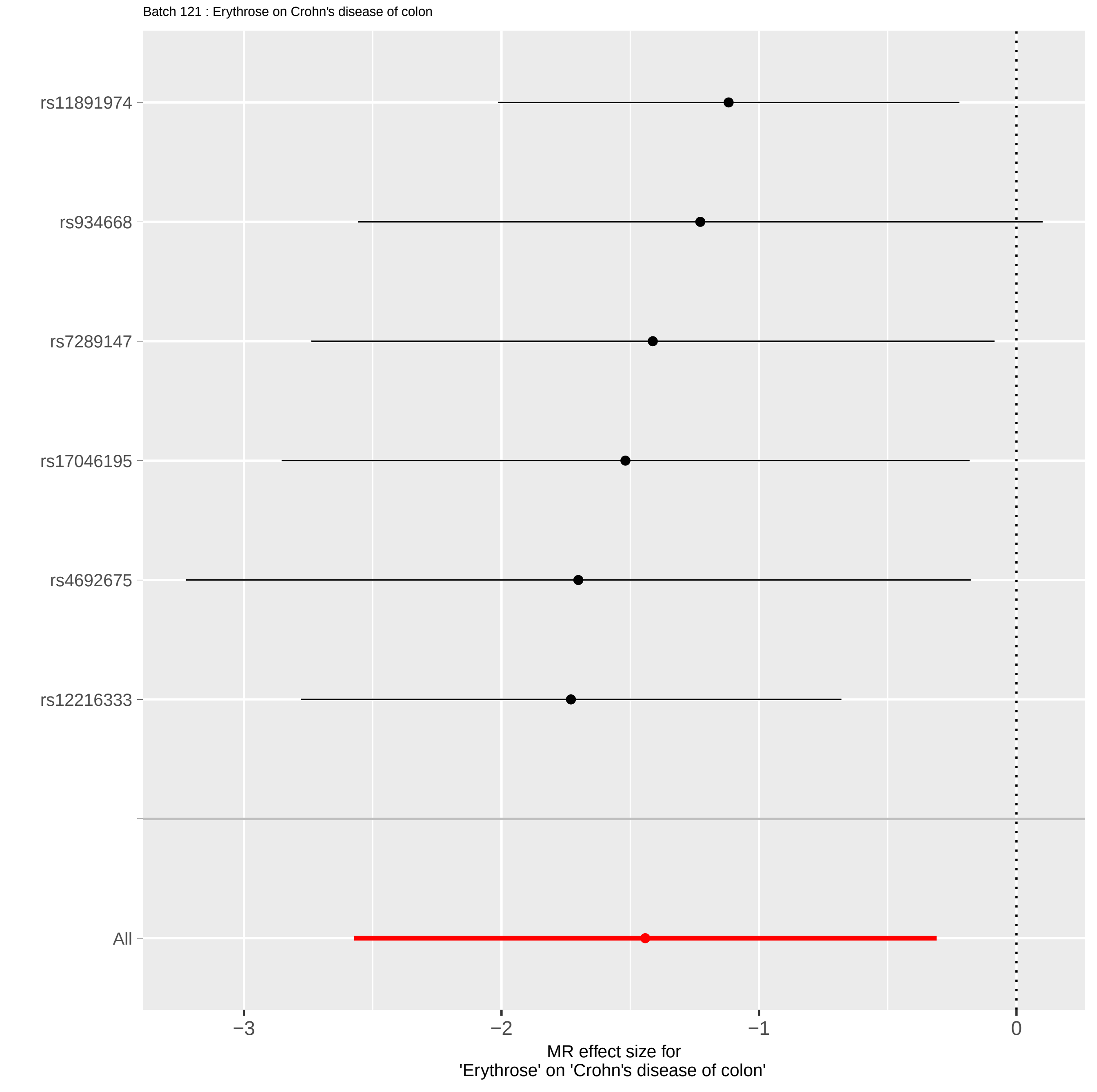

Batch 121 : Erythrose on Crohn's disease of colon
rs11891974
rs934668
rs7289147
rs17046195
rs4692675
rs12216333
All
−3
−2
−1
0
MR effect size for
'Erythrose' on 'Crohn's disease of colon'

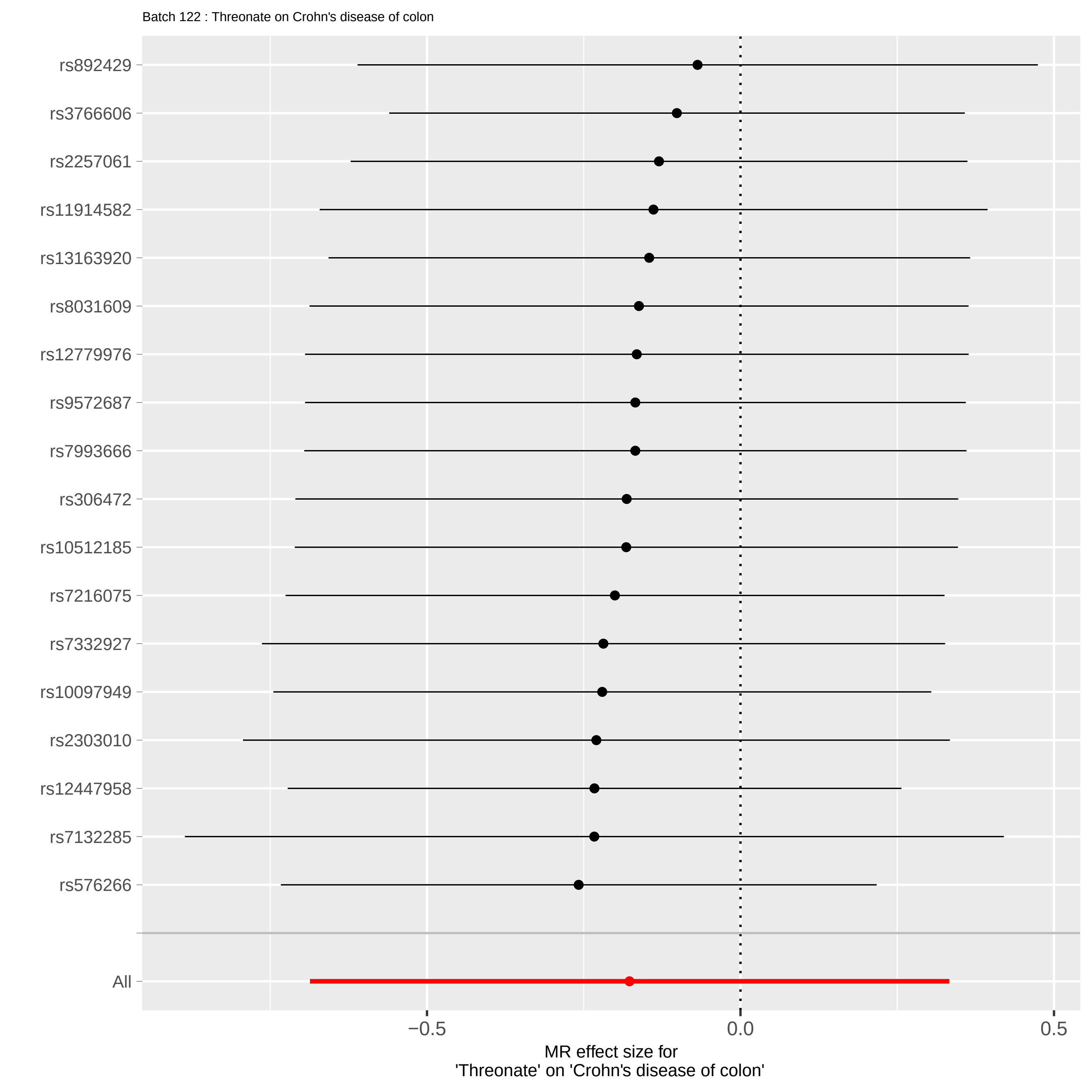

Batch 122 : Threonate on Crohn's disease of colon
rs892429
rs3766606
rs2257061
rs11914582
rs13163920
rs8031609
rs12779976
rs9572687
rs7993666
rs306472
rs10512185
rs7216075
rs7332927
rs10097949
rs2303010
rs12447958
rs7132285
rs576266
All
−0.5
0.0
0.5
MR effect size for
'Threonate' on 'Crohn's disease of colon'

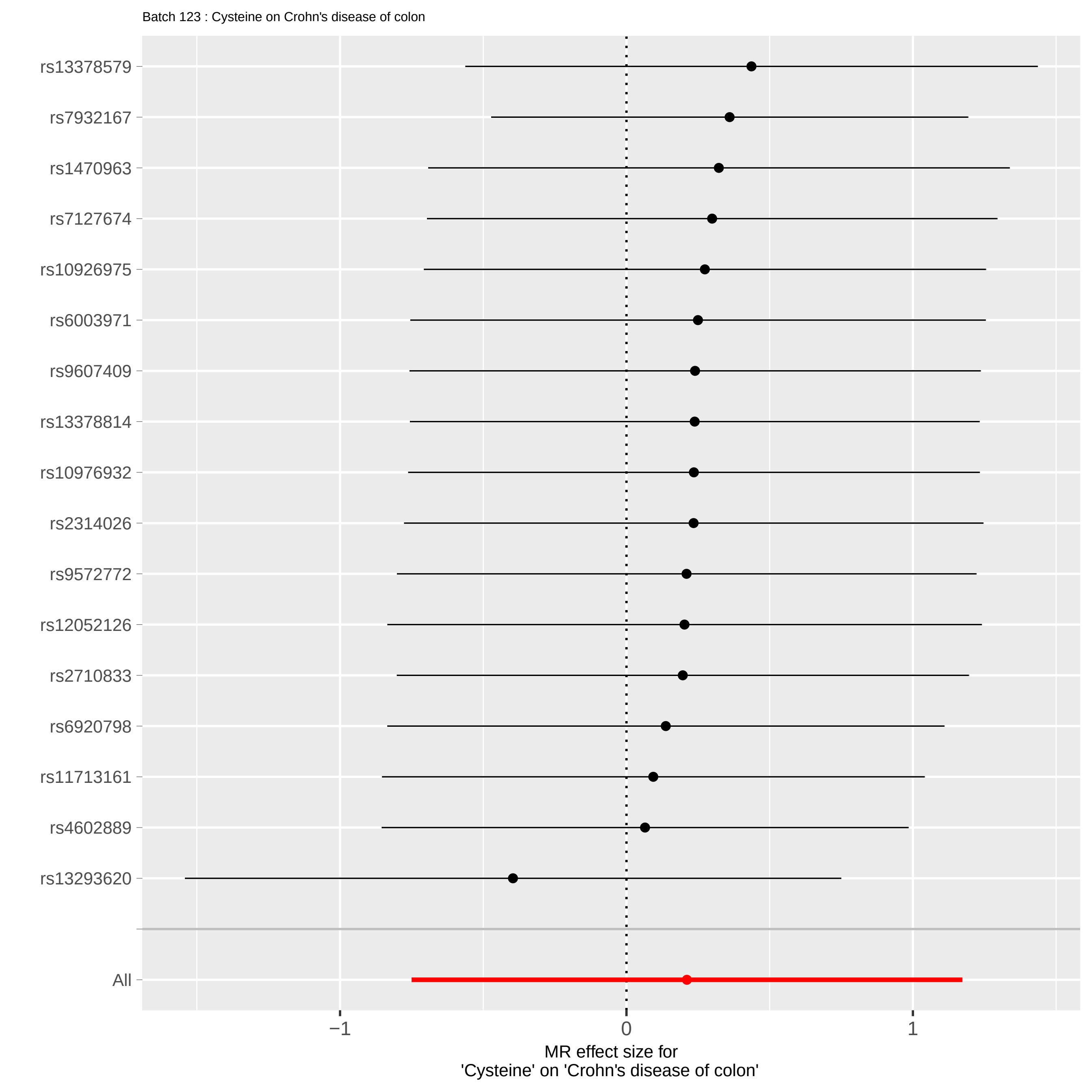
Batch 123 : Cysteine on Crohn's disease of colon
rs13378579
rs7932167
rs1470963
rs7127674
rs10926975
rs6003971
rs9607409
rs13378814
rs10976932
rs2314026
rs9572772
rs12052126
rs2710833
rs6920798
rs11713161
rs4602889
rs13293620
All
−1
0
1
MR effect size for
'Cysteine' on 'Crohn's disease of colon'

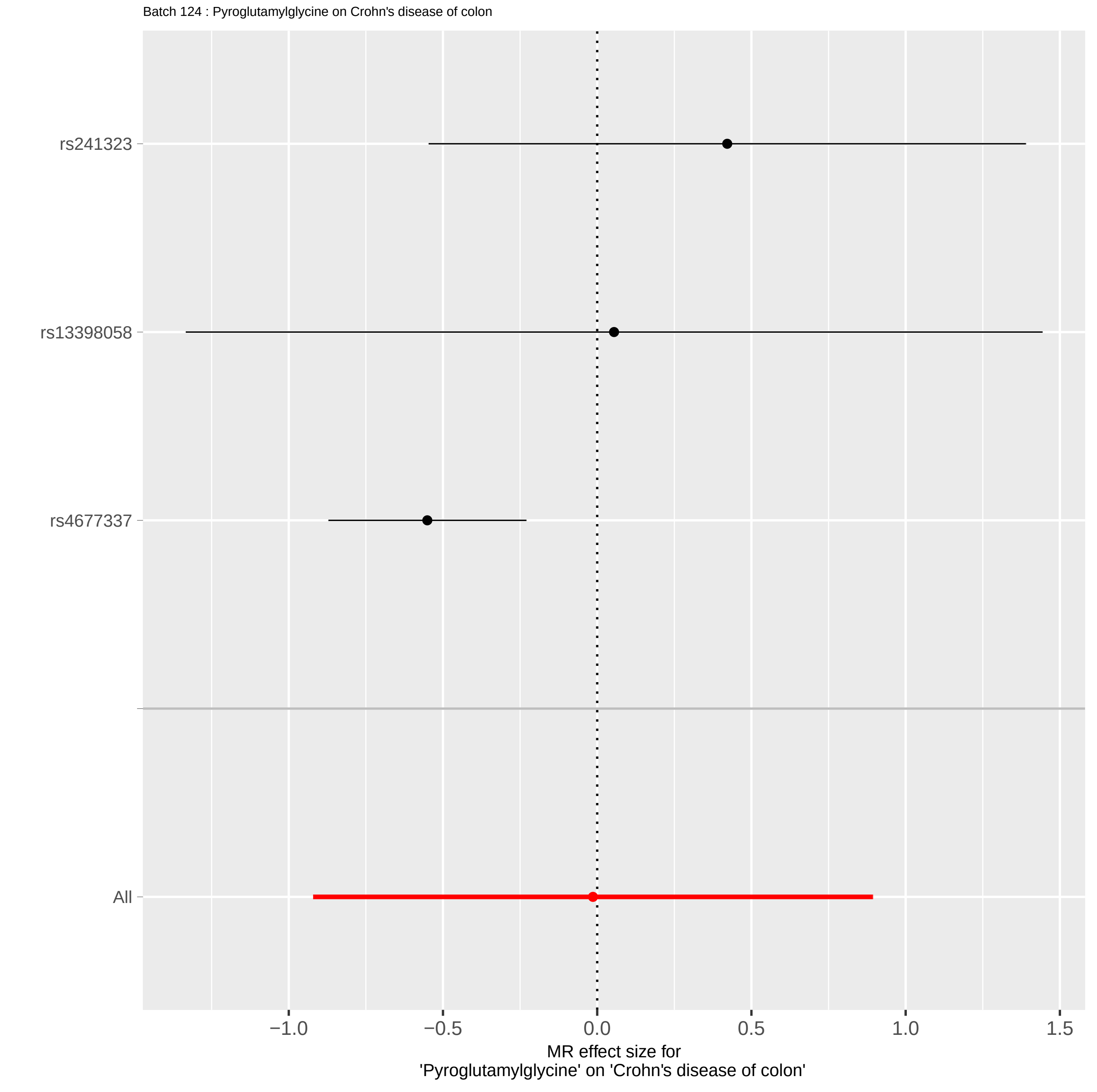

Batch 124 : Pyroglutamylglycine on Crohn's disease of colon
rs241323
rs13398058
rs4677337
All
−1.0
−0.5
0.0
0.5
1.0
1.5
MR effect size for
'Pyroglutamylglycine' on 'Crohn's disease of colon'

Batch 126 : DSGEGDFXAEGGGVR* on Crohn's disease of colon

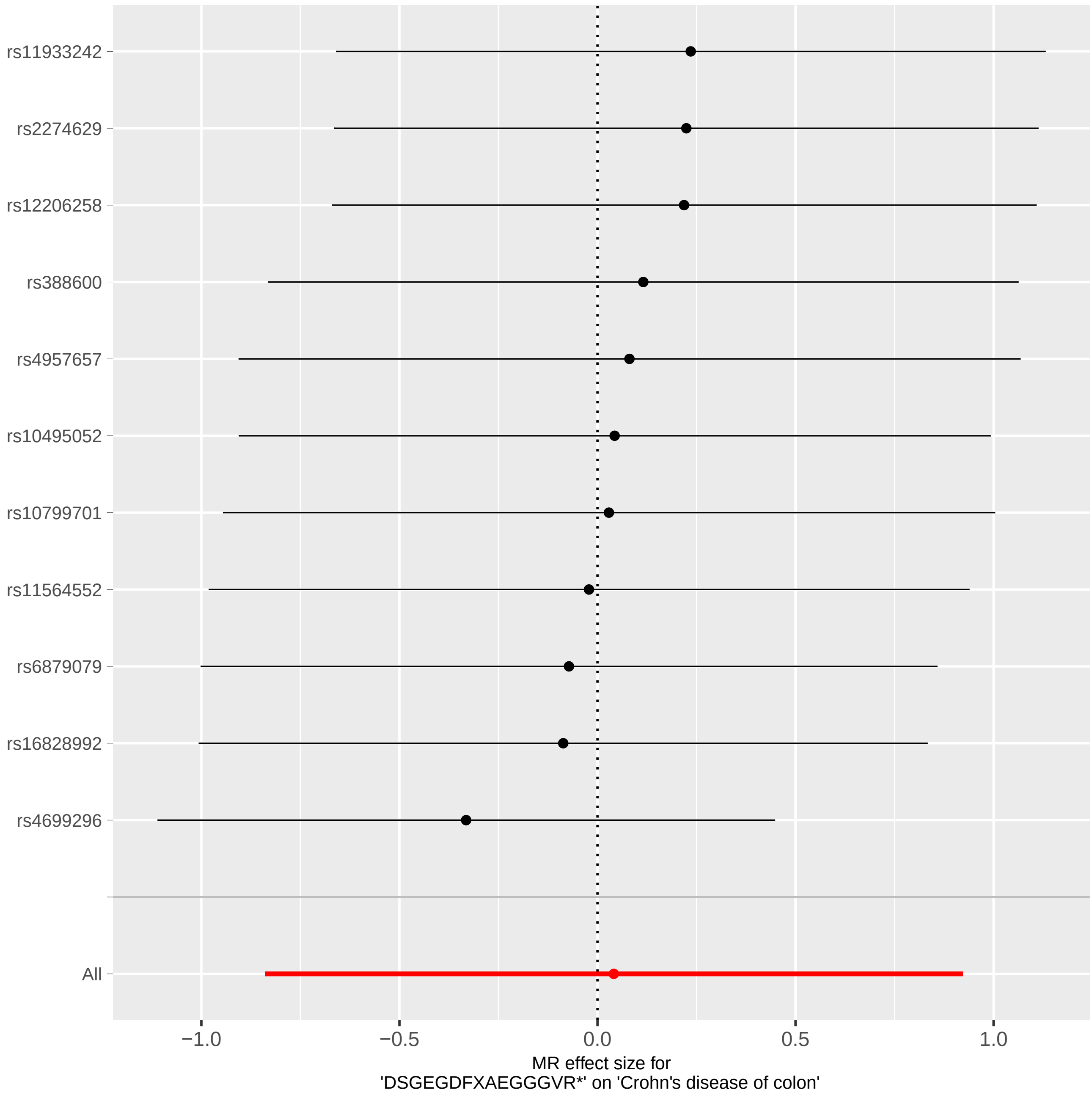

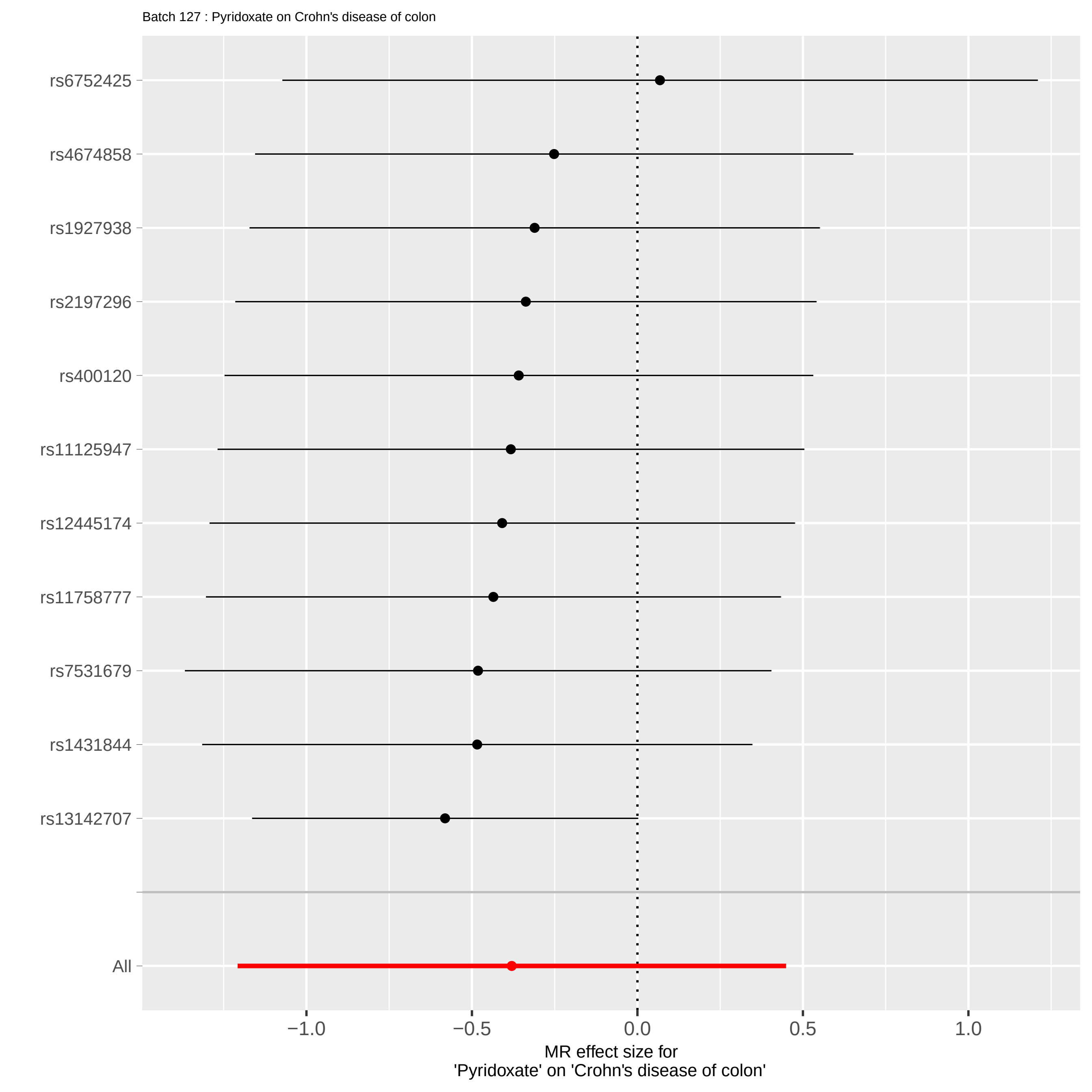
Batch 127 : Pyridoxate on Crohn's disease of colon
rs6752425
rs4674858
rs1927938
rs2197296
rs400120
rs11125947
rs12445174
rs11758777
rs7531679
rs1431844
rs13142707
All
−1.0
−0.5
0.0
0.5
1.0
MR effect size for
'Pyridoxate' on 'Crohn's disease of colon'

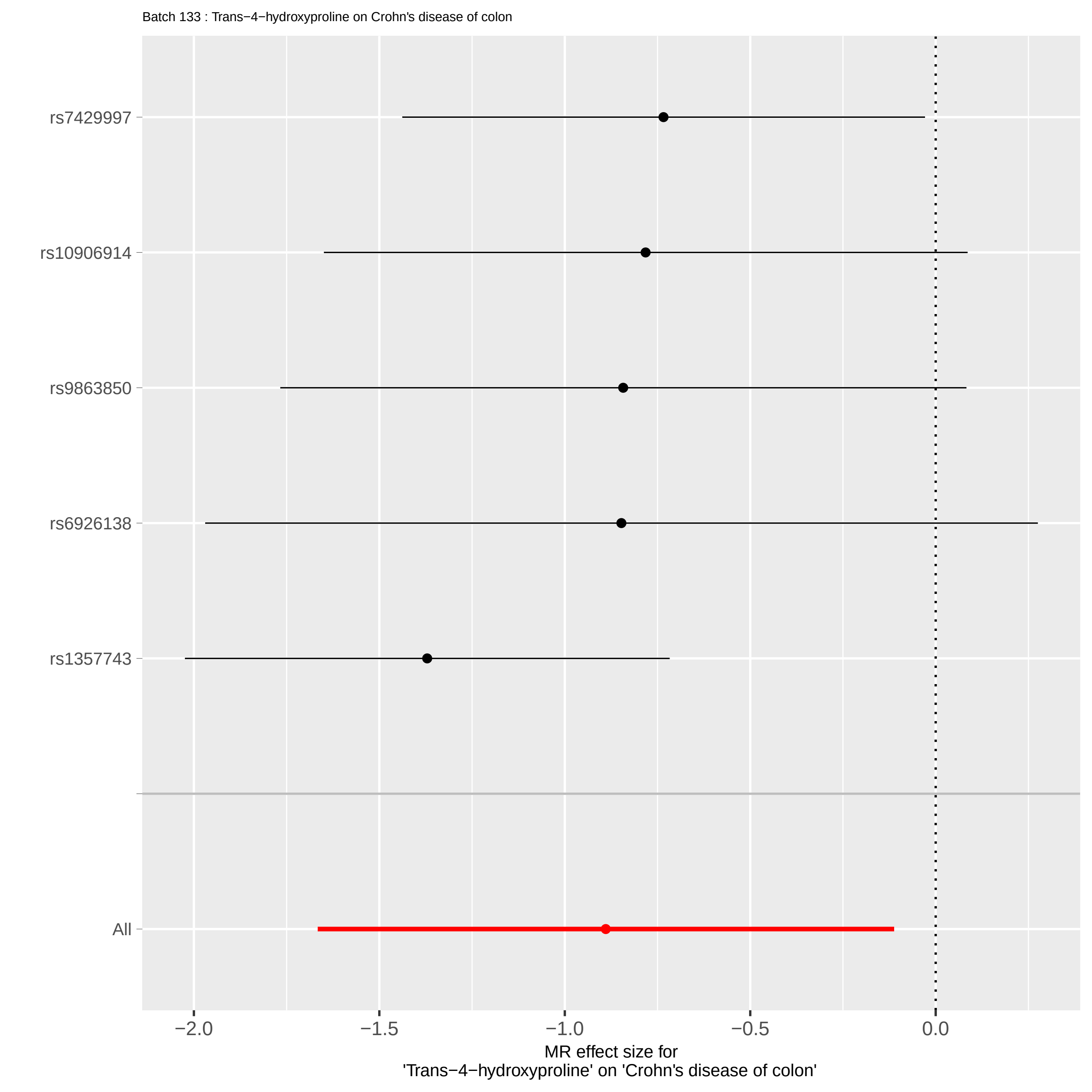

Batch 133 : Trans−4−hydroxyproline on Crohn's disease of colon
rs7429997
rs10906914
rs9863850
rs6926138
rs1357743
All
−2.0
−1.5
−1.0
−0.5
0.0
MR effect size for
'Trans−4−hydroxyproline' on 'Crohn's disease of colon'

Batch 144 : Butyrylcarnitine on Crohn's disease of colon

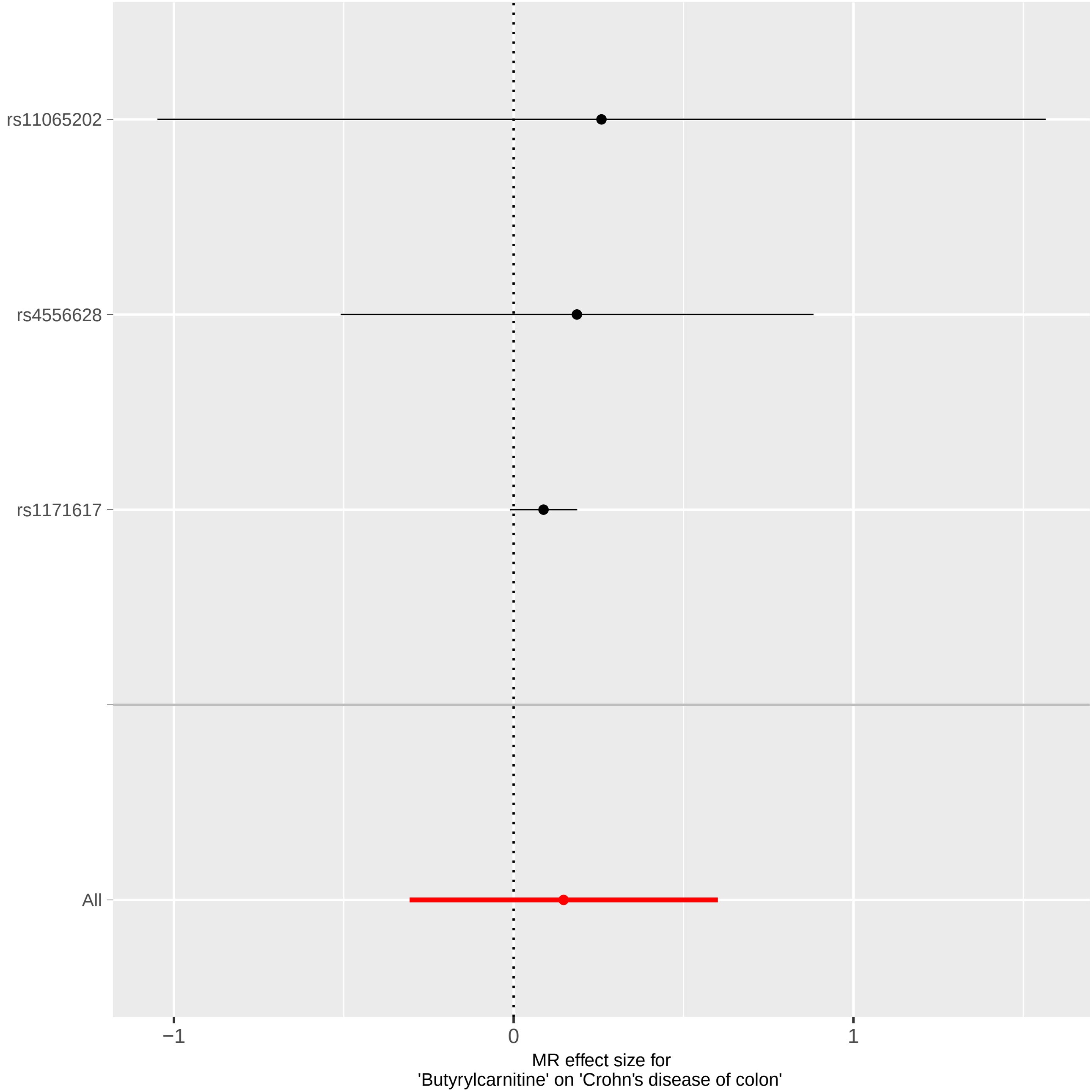

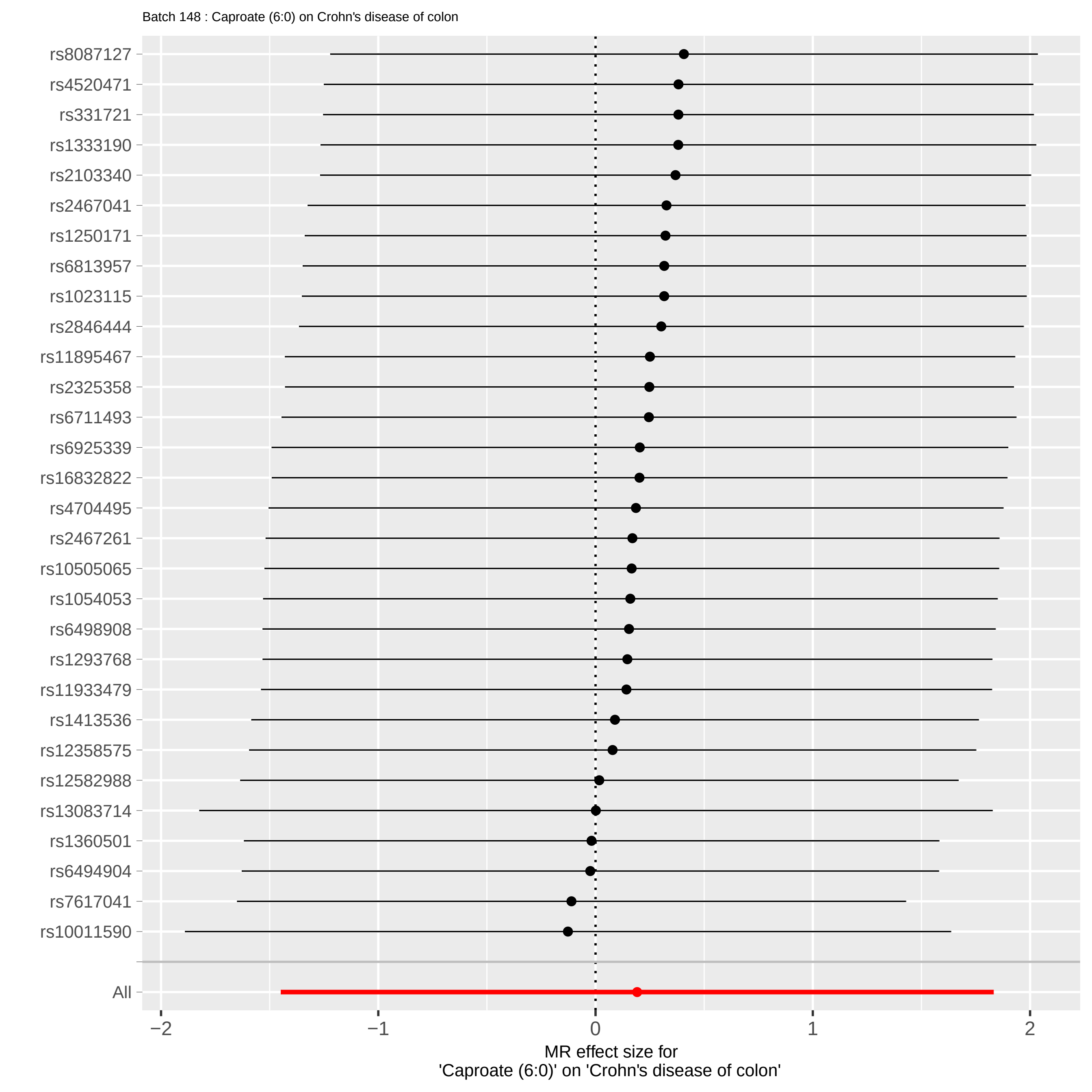

Batch 148 : Caproate (6:0) on Crohn's disease of colon
rs8087127
rs4520471
rs331721
rs1333190
rs2103340
rs2467041
rs1250171
rs6813957
rs1023115
rs2846444
rs11895467
rs2325358
rs6711493
rs6925339
rs16832822
rs4704495
rs2467261
rs10505065
rs1054053
rs6498908
rs1293768
rs11933479
rs1413536
rs12358575
rs12582988
rs13083714
rs1360501
rs6494904
rs7617041
rs10011590
All
−2
−1
0
1
2
MR effect size for
'Caproate (6:0)' on 'Crohn's disease of colon'

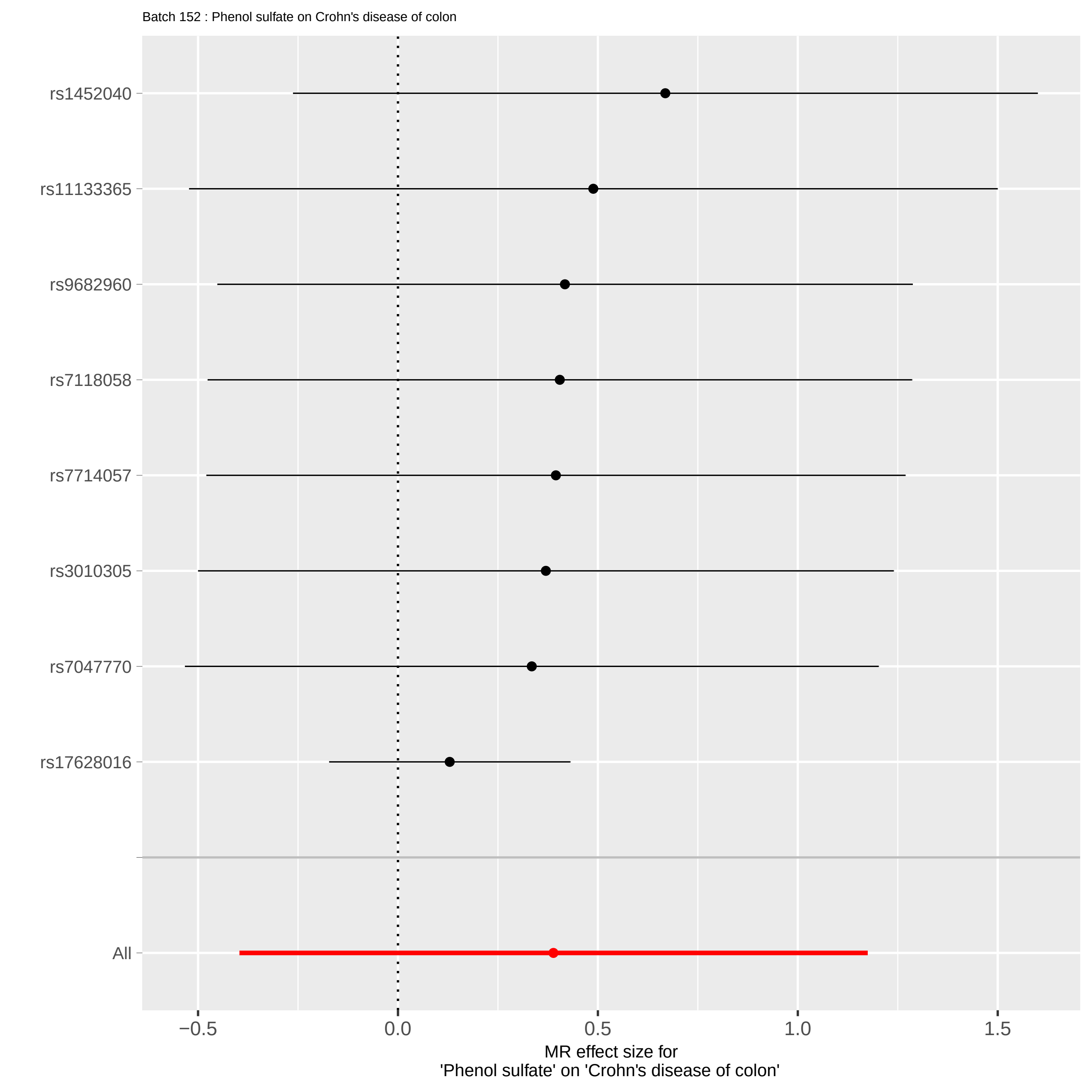
Batch 152 : Phenol sulfate on Crohn's disease of colon
rs1452040
rs11133365
rs9682960
rs7118058
rs7714057
rs3010305
rs7047770
rs17628016
All
−0.5
0.0
0.5
1.0
1.5
MR effect size for
'Phenol sulfate' on 'Crohn's disease of colon'

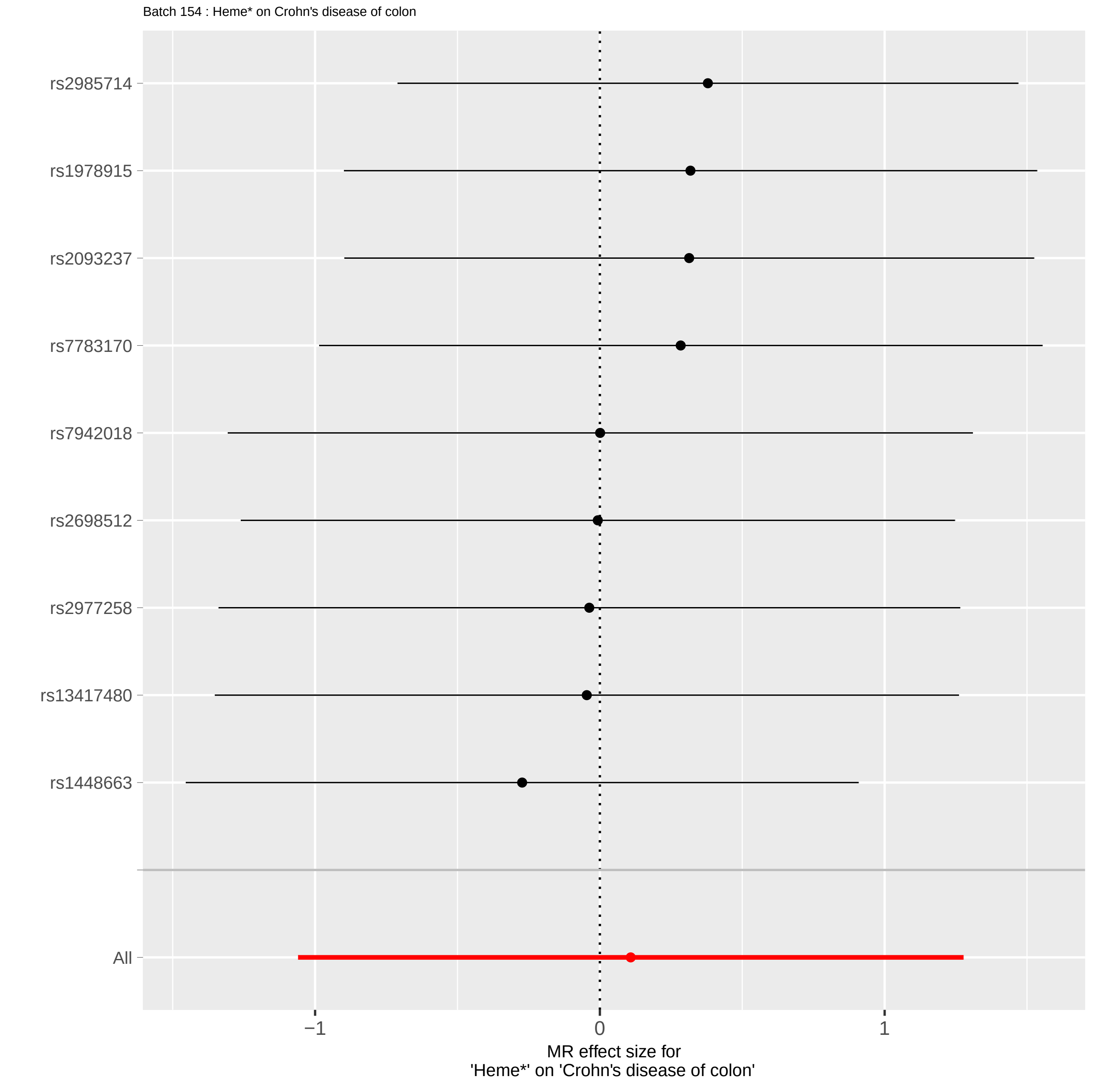
Batch 154 : Heme* on Crohn's disease of colon
rs2985714
rs1978915
rs2093237
rs7783170
rs7942018
rs2698512
rs2977258
rs13417480
rs1448663
All
−1
0
1
MR effect size for
'Heme*' on 'Crohn's disease of colon'

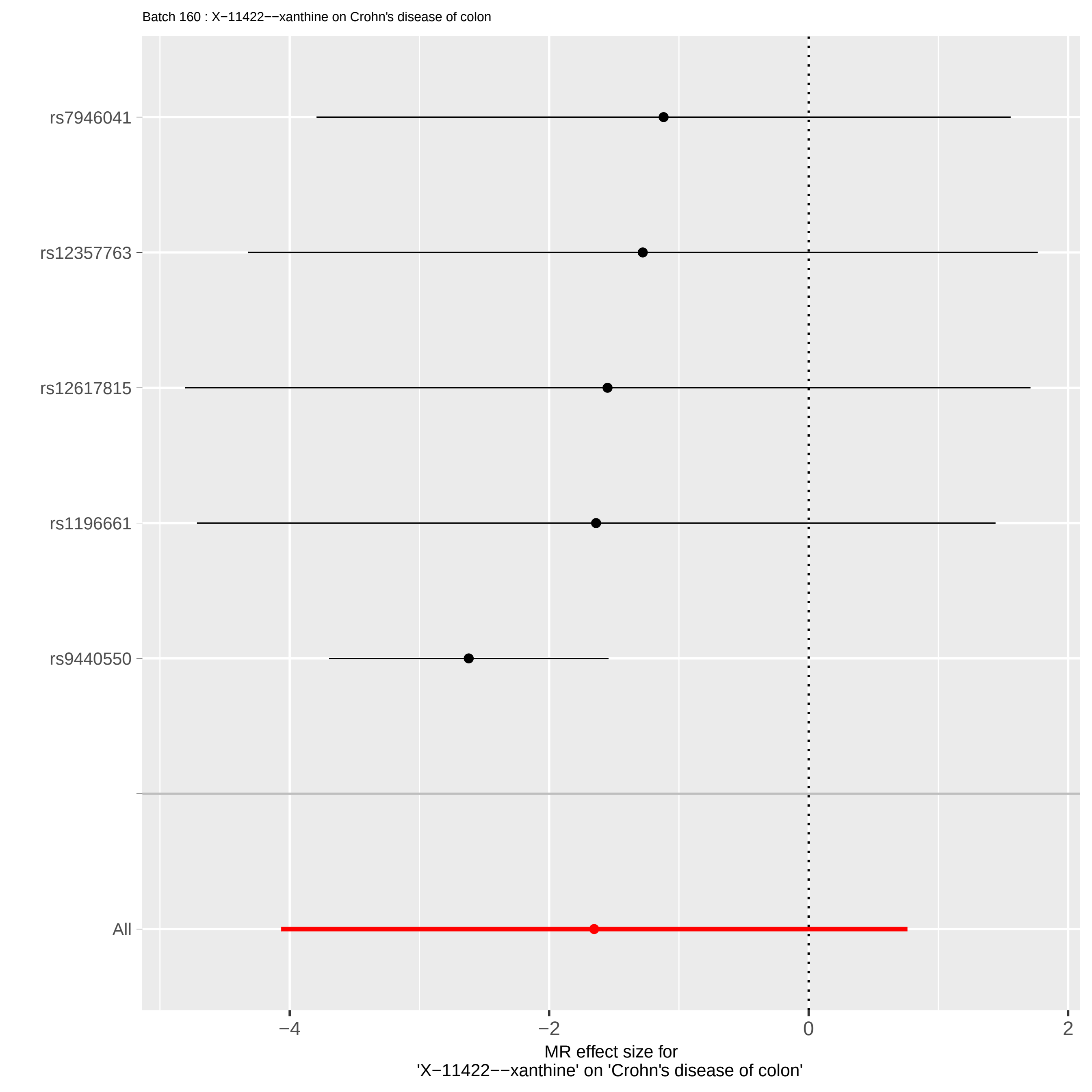
Batch 160 : X−11422−−xanthine on Crohn's disease of colon
rs7946041
rs12357763
rs12617815
rs1196661
rs9440550
All
−4
−2
0
2
MR effect size for
'X−11422−−xanthine' on 'Crohn's disease of colon'

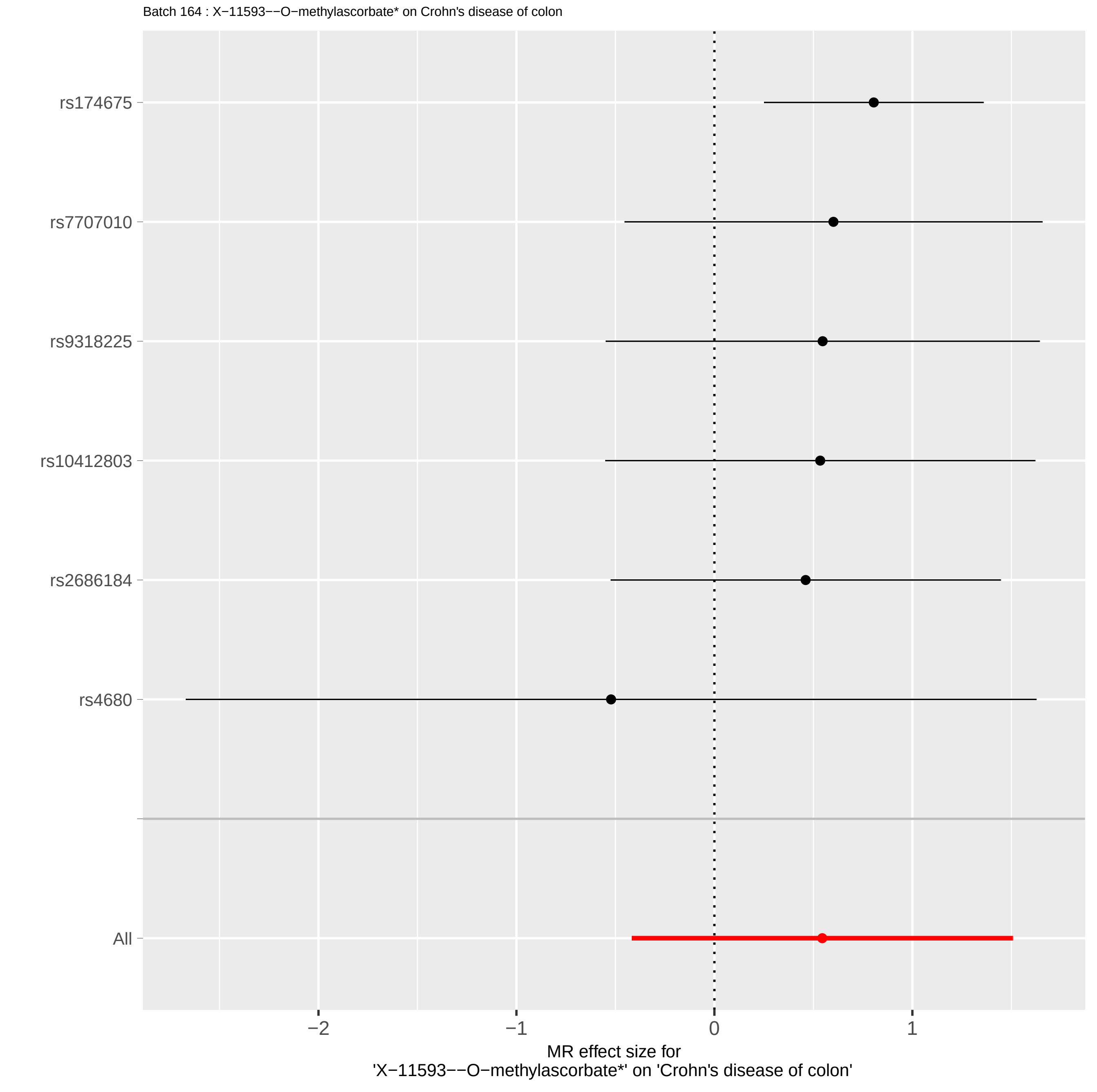
Batch 164 : X−11593−−O−methylascorbate* on Crohn's disease of colon
rs174675
rs7707010
rs9318225
rs10412803
rs2686184
rs4680
All
−2
−1
0
1
MR effect size for
'X−11593−−O−methylascorbate*' on 'Crohn's disease of colon'

Batch 166 : Homostachydrine* on Crohn's disease of colon

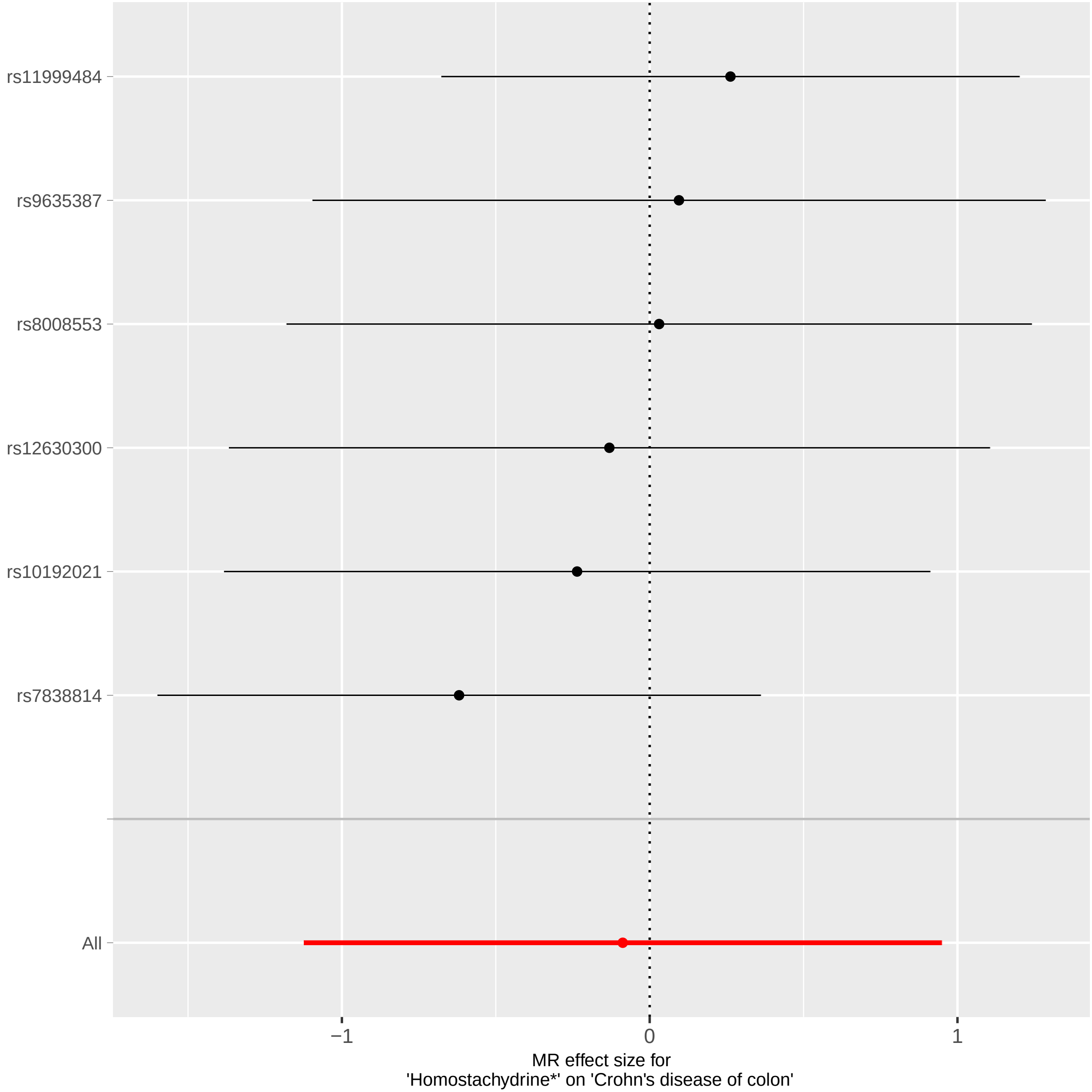

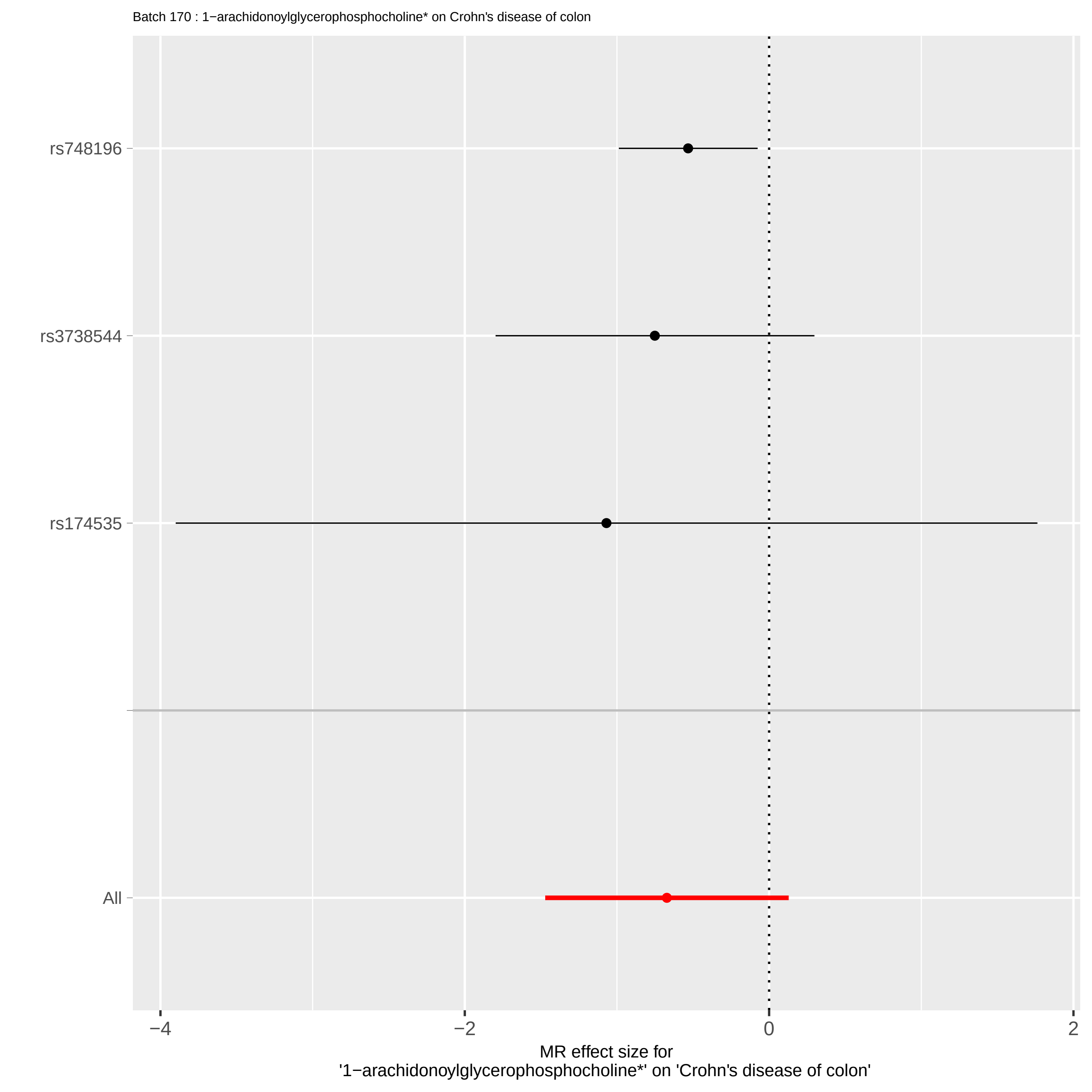

Batch 170 : 1−arachidonoylglycerophosphocholine* on Crohn's disease of colon
rs748196
rs3738544
rs174535
All
−4
−2
0
2
MR effect size for
'1−arachidonoylglycerophosphocholine*' on 'Crohn's disease of colon'

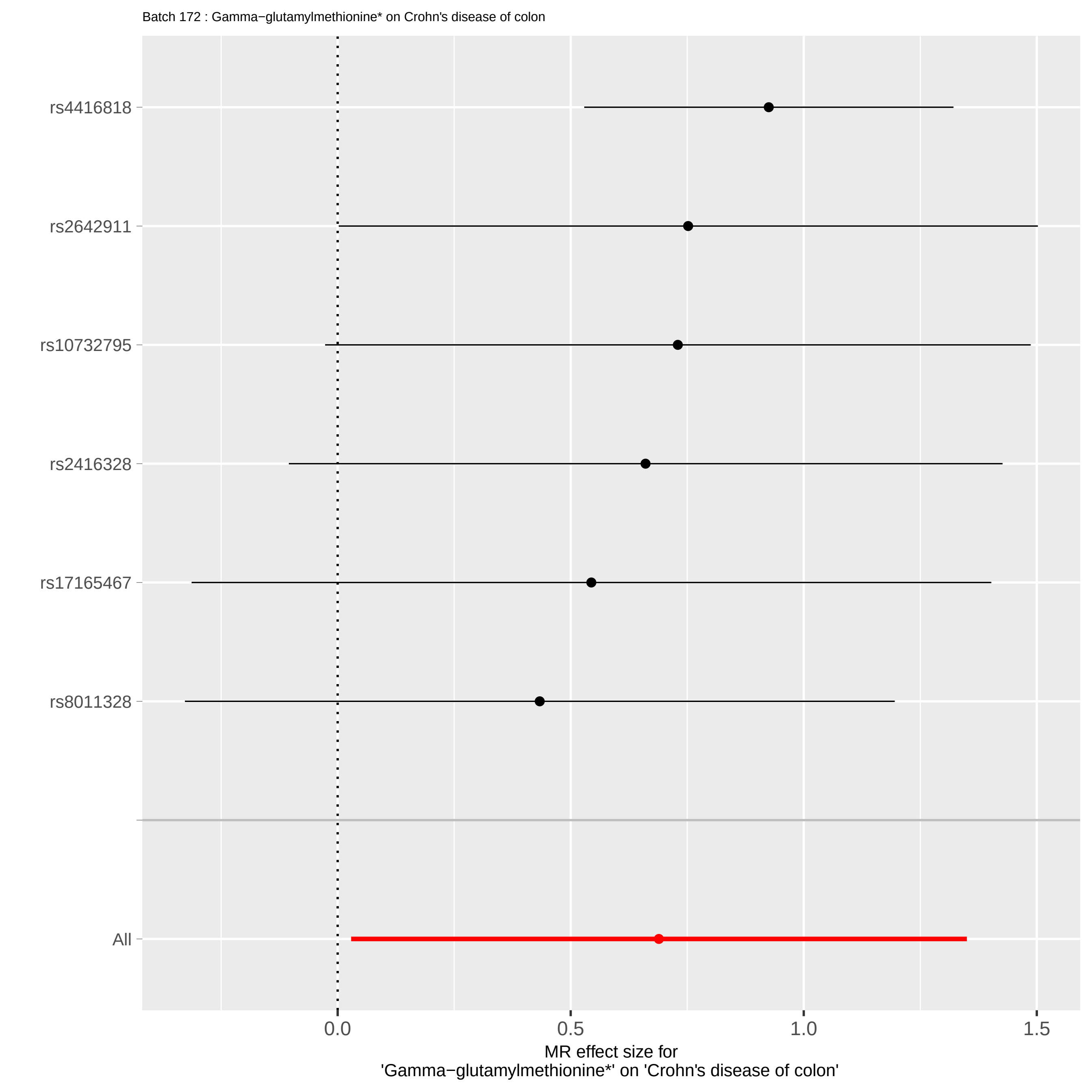

Batch 172 : Gamma−glutamylmethionine* on Crohn's disease of colon
rs4416818
rs2642911
rs10732795
rs2416328
rs17165467
rs8011328
All
0.0
0.5
1.0
1.5
MR effect size for
'Gamma−glutamylmethionine*' on 'Crohn's disease of colon'

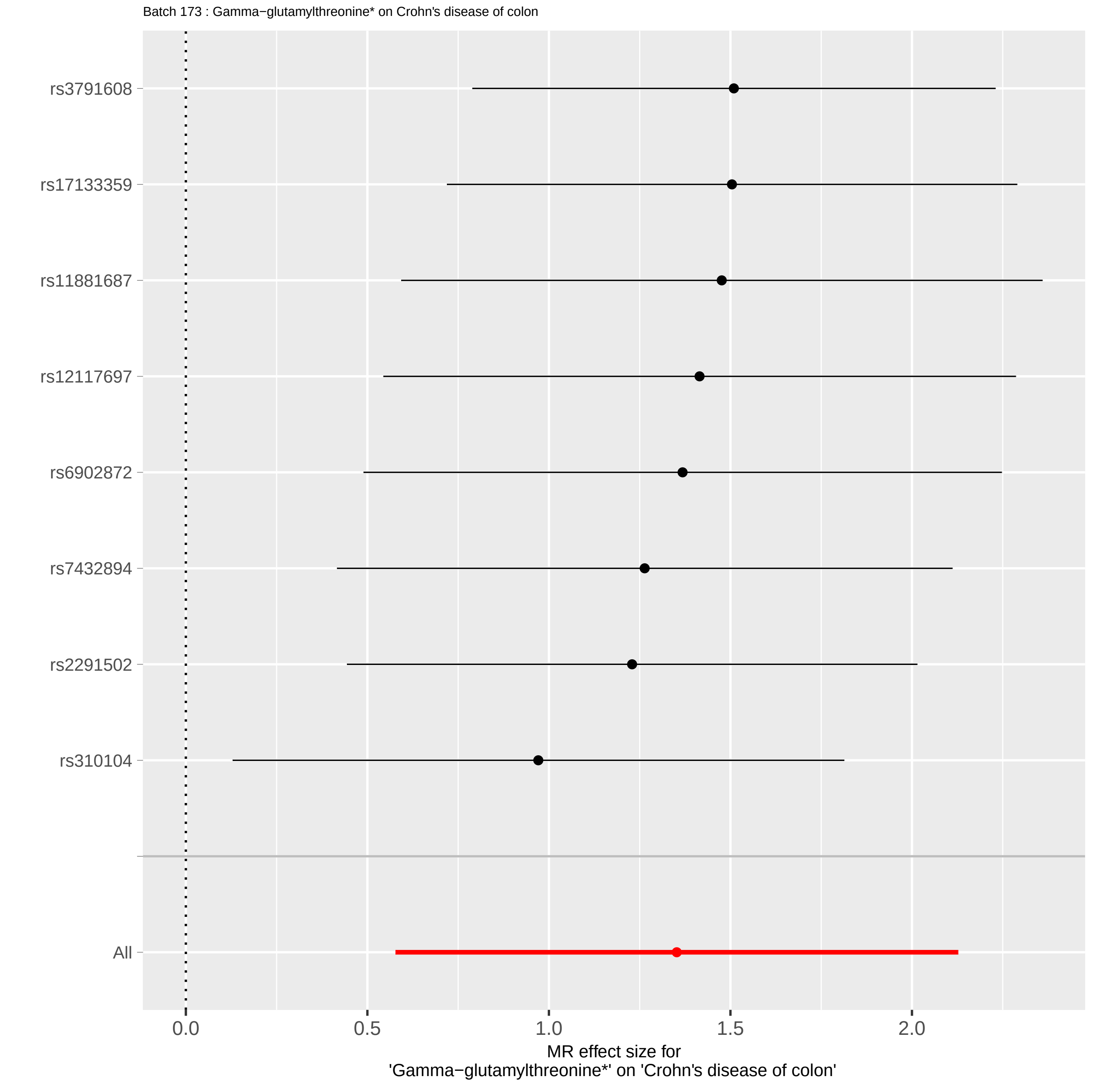
Batch 173 : Gamma−glutamylthreonine* on Crohn's disease of colon
rs3791608
rs17133359
rs11881687
rs12117697
rs6902872
rs7432894
rs2291502
rs310104
All
0.0
0.5
1.0
1.5
2.0
MR effect size for
'Gamma−glutamylthreonine*' on 'Crohn's disease of colon'

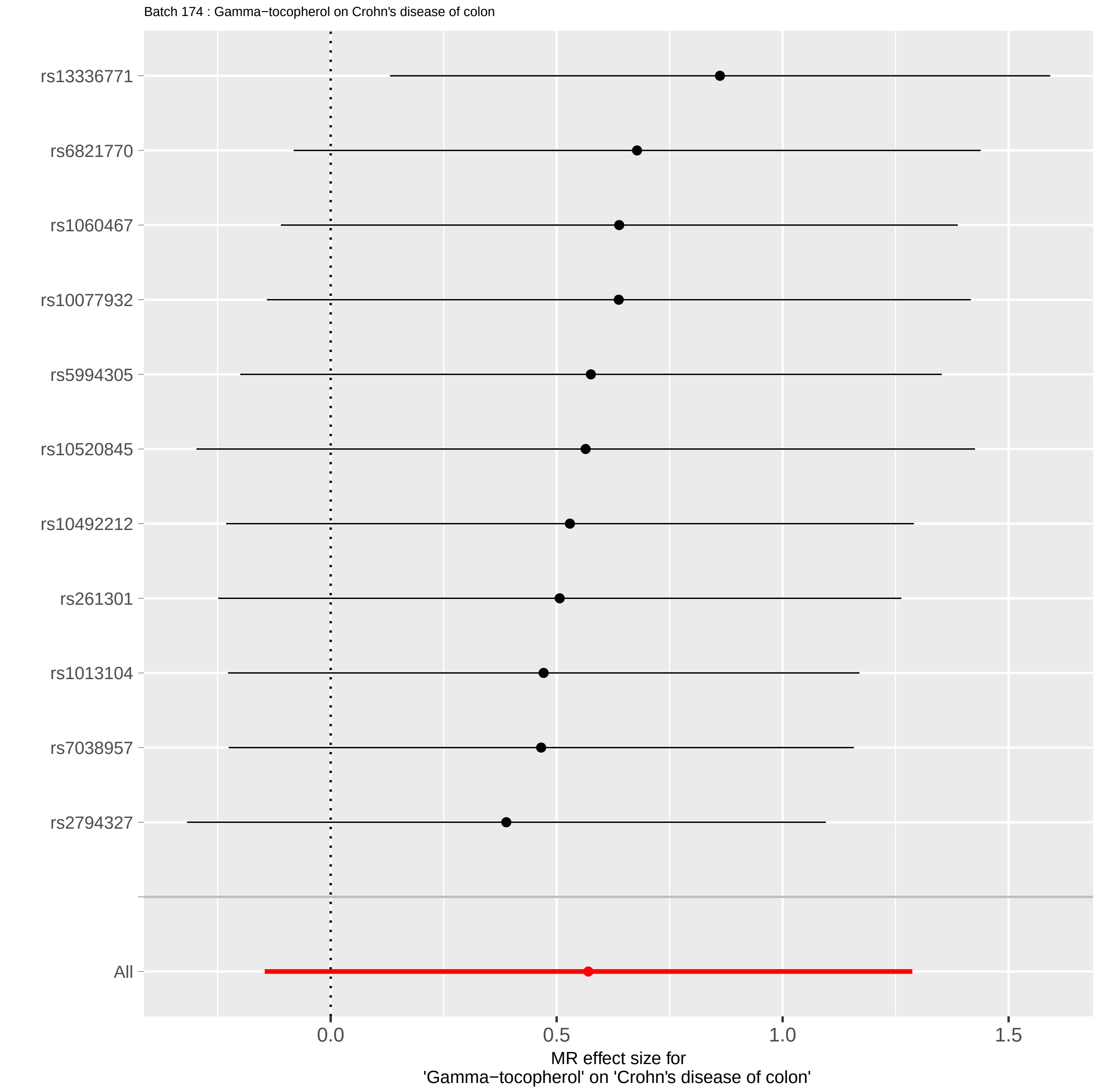
Batch 174 : Gamma−tocopherol on Crohn's disease of colon
rs13336771
rs6821770
rs1060467
rs10077932
rs5994305
rs10520845
rs10492212
rs261301
rs1013104
rs7038957
rs2794327
All
0.0
0.5
1.0
1.5
MR effect size for
'Gamma−tocopherol' on 'Crohn's disease of colon'

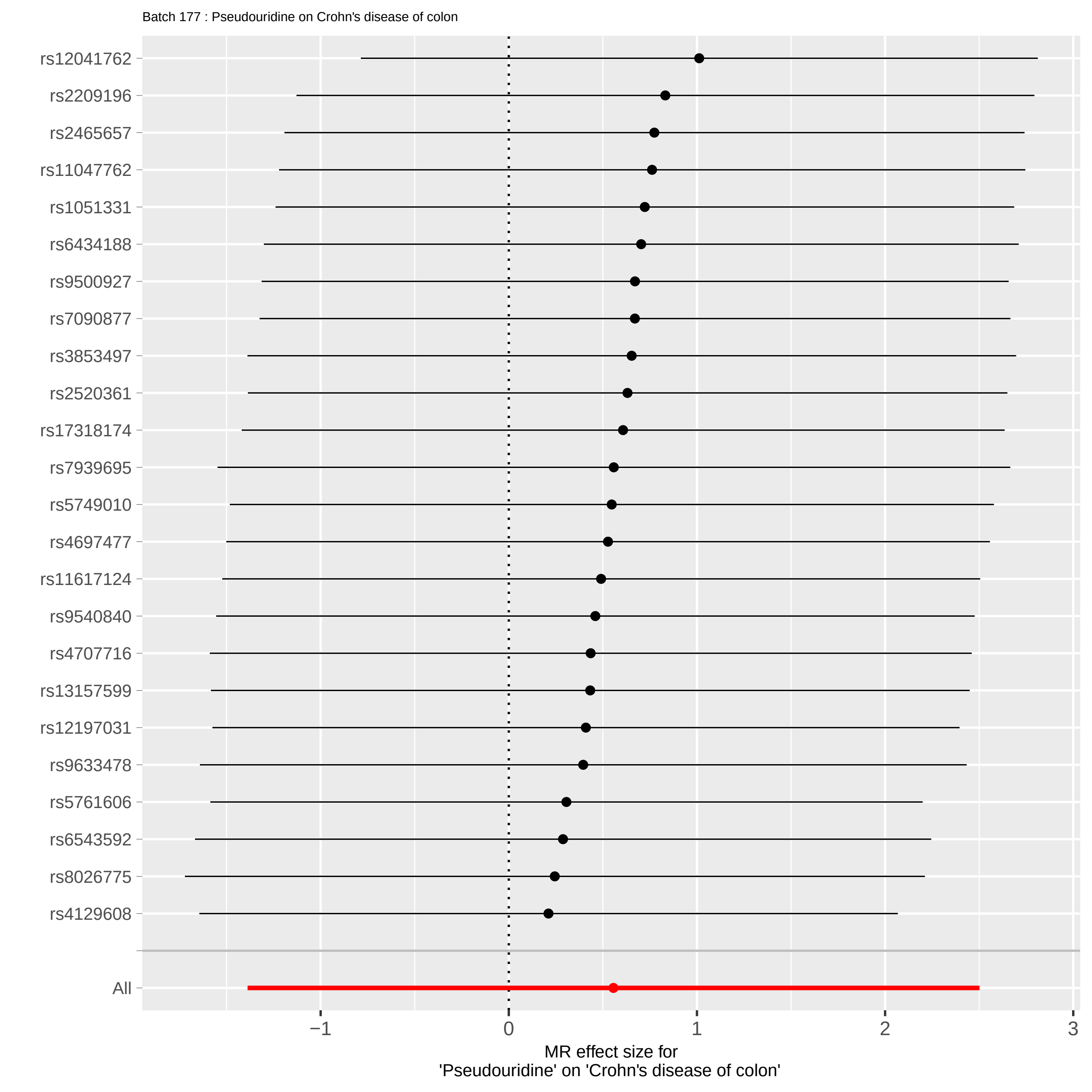

Batch 177 : Pseudouridine on Crohn's disease of colon
rs12041762
rs2209196
rs2465657
rs11047762
rs1051331
rs6434188
rs9500927
rs7090877
rs3853497
rs2520361
rs17318174
rs7939695
rs5749010
rs4697477
rs11617124
rs9540840
rs4707716
rs13157599
rs12197031
rs9633478
rs5761606
rs6543592
rs8026775
rs4129608
All
−1
0
1
2
3
MR effect size for
'Pseudouridine' on 'Crohn's disease of colon'

Batch 178 : Valerate on Crohn's disease of colon

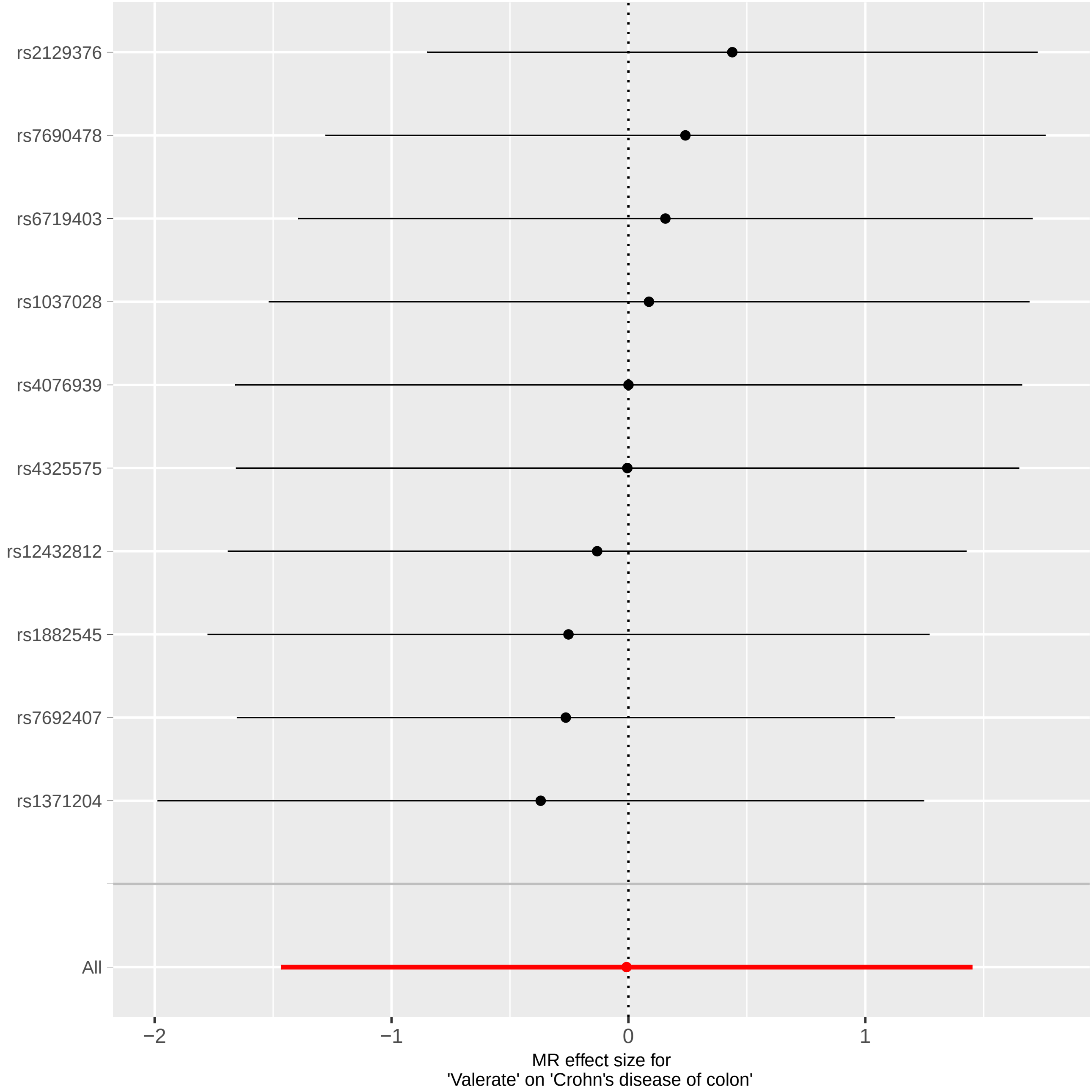

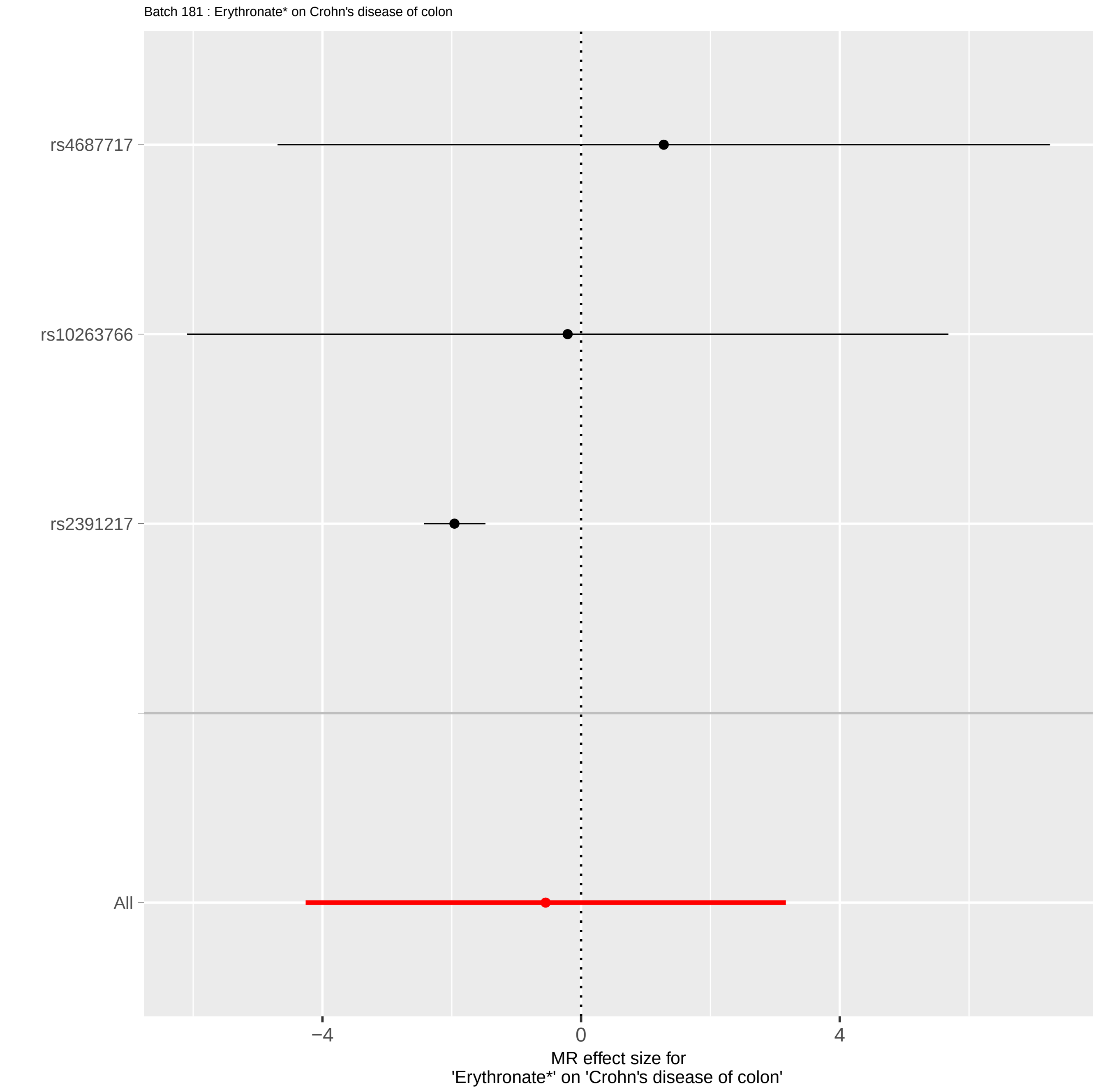
Batch 181 : Erythronate* on Crohn's disease of colon
rs4687717
rs10263766
rs2391217
All
−4
0
4
MR effect size for
'Erythronate*' on 'Crohn's disease of colon'

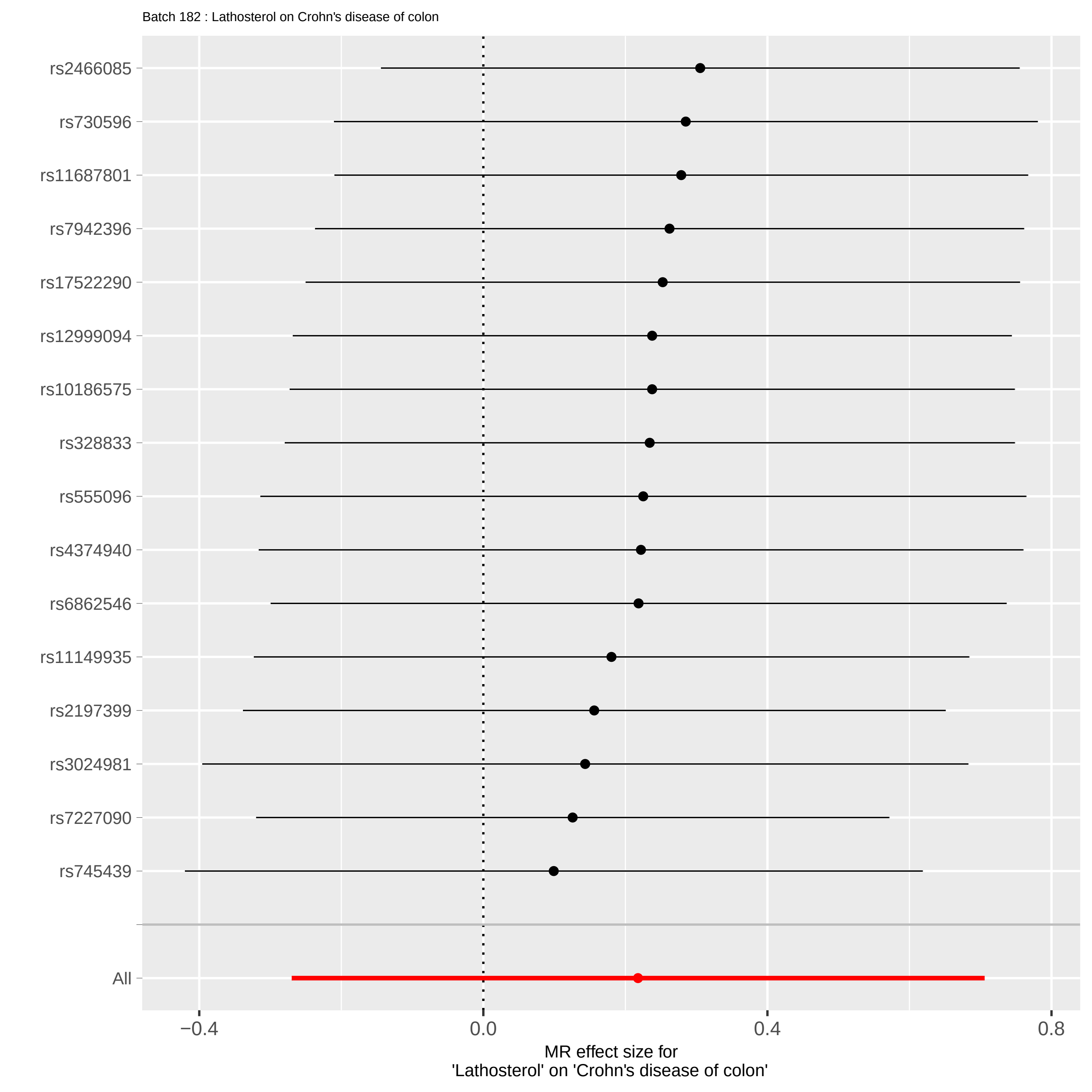
Batch 182 : Lathosterol on Crohn's disease of colon
rs2466085
rs730596
rs11687801
rs7942396
rs17522290
rs12999094
rs10186575
rs328833
rs555096
rs4374940
rs6862546
rs11149935
rs2197399
rs3024981
rs7227090
rs745439
All
−0.4
0.0
0.4
0.8
MR effect size for
'Lathosterol' on 'Crohn's disease of colon'

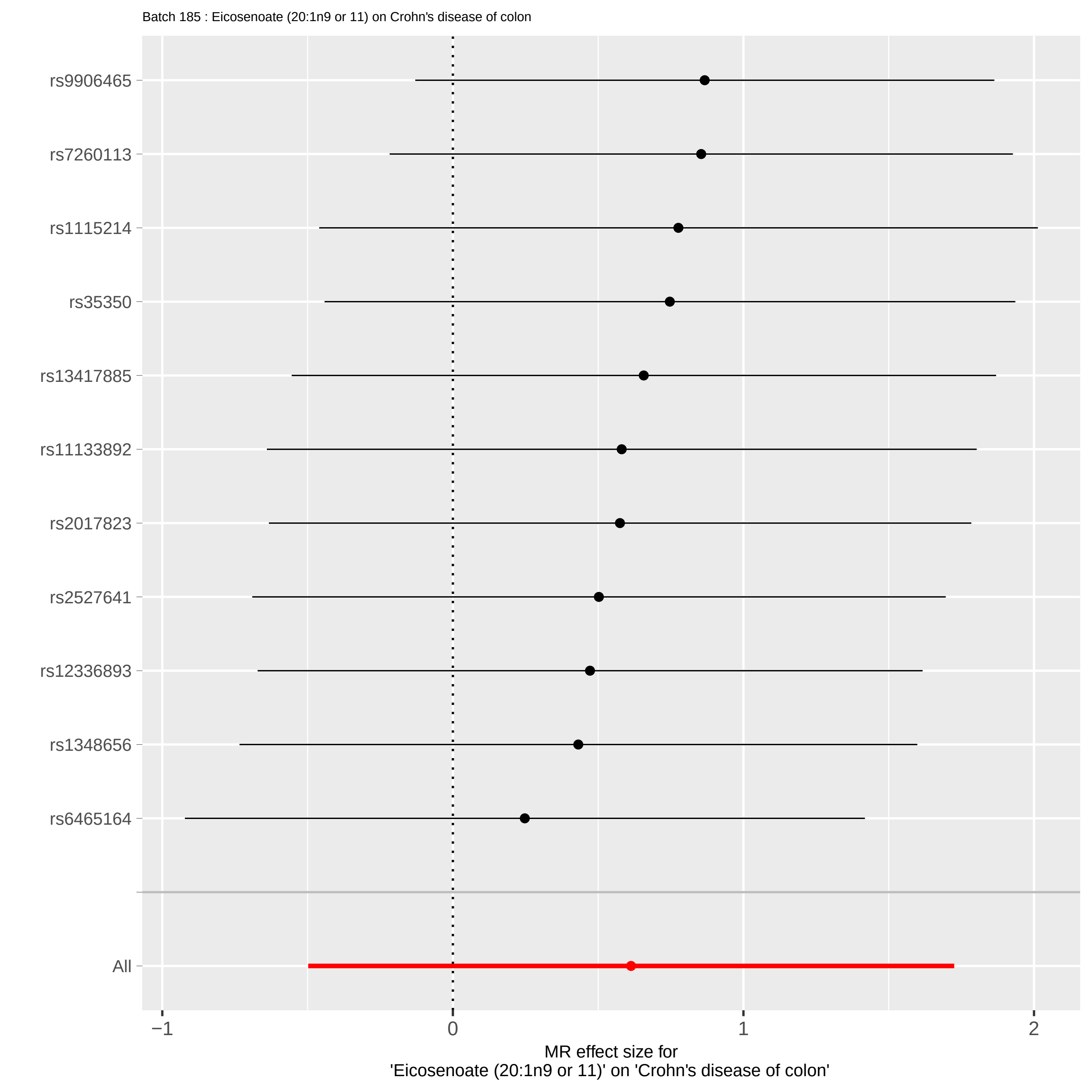

Batch 185 : Eicosenoate (20:1n9 or 11) on Crohn's disease of colon
rs9906465
rs7260113
rs1115214
rs35350
rs13417885
rs11133892
rs2017823
rs2527641
rs12336893
rs1348656
rs6465164
All
−1
0
1
2
MR effect size for
'Eicosenoate (20:1n9 or 11)' on 'Crohn's disease of colon'

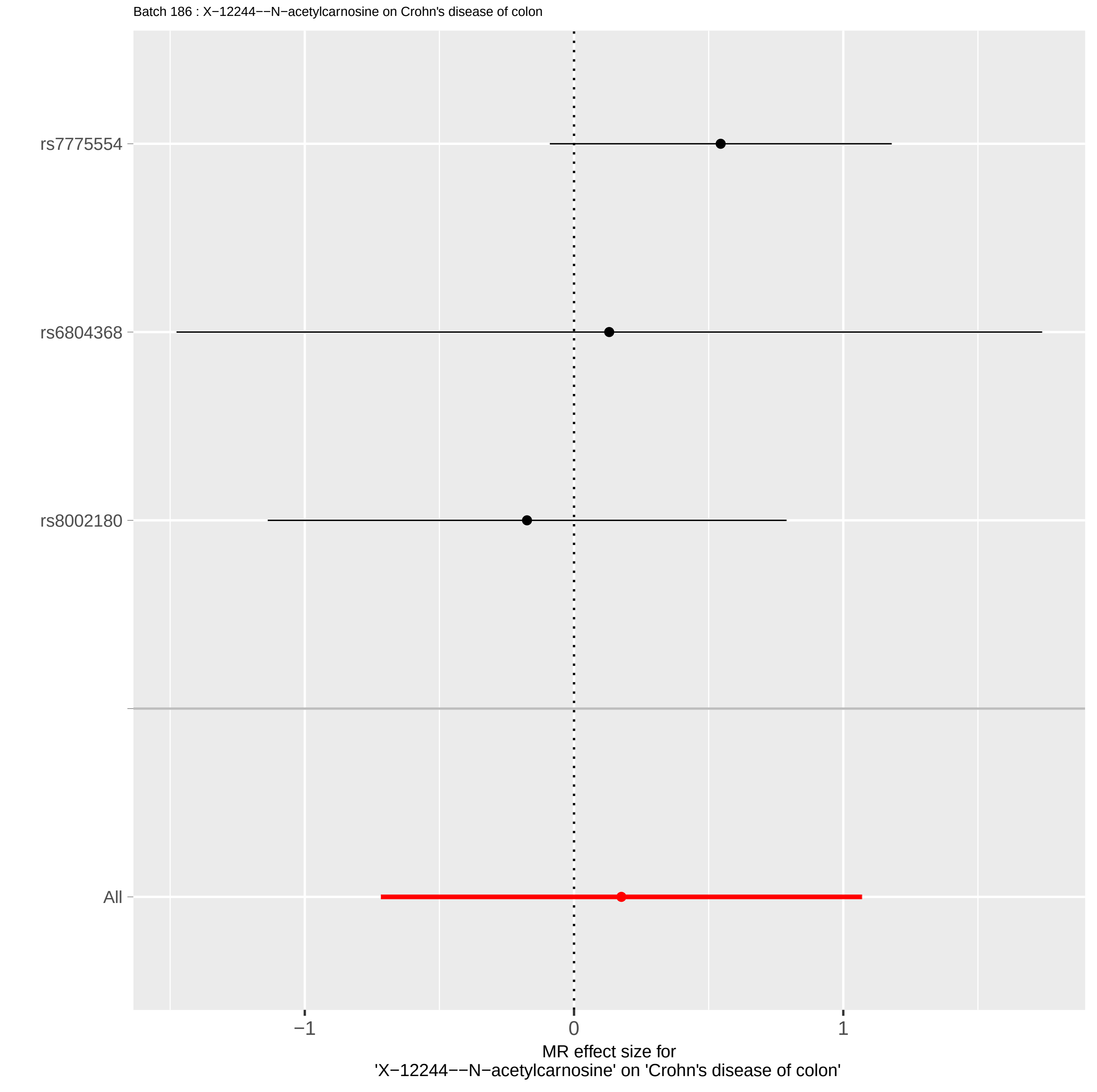

Batch 186 : X−12244−−N−acetylcarnosine on Crohn's disease of colon
rs7775554
rs6804368
rs8002180
All
−1
0
1
MR effect size for
'X−12244−−N−acetylcarnosine' on 'Crohn's disease of colon'

Batch 189 : 1−docosahexaenoylglycerophosphocholine* on Crohn's disease of colon

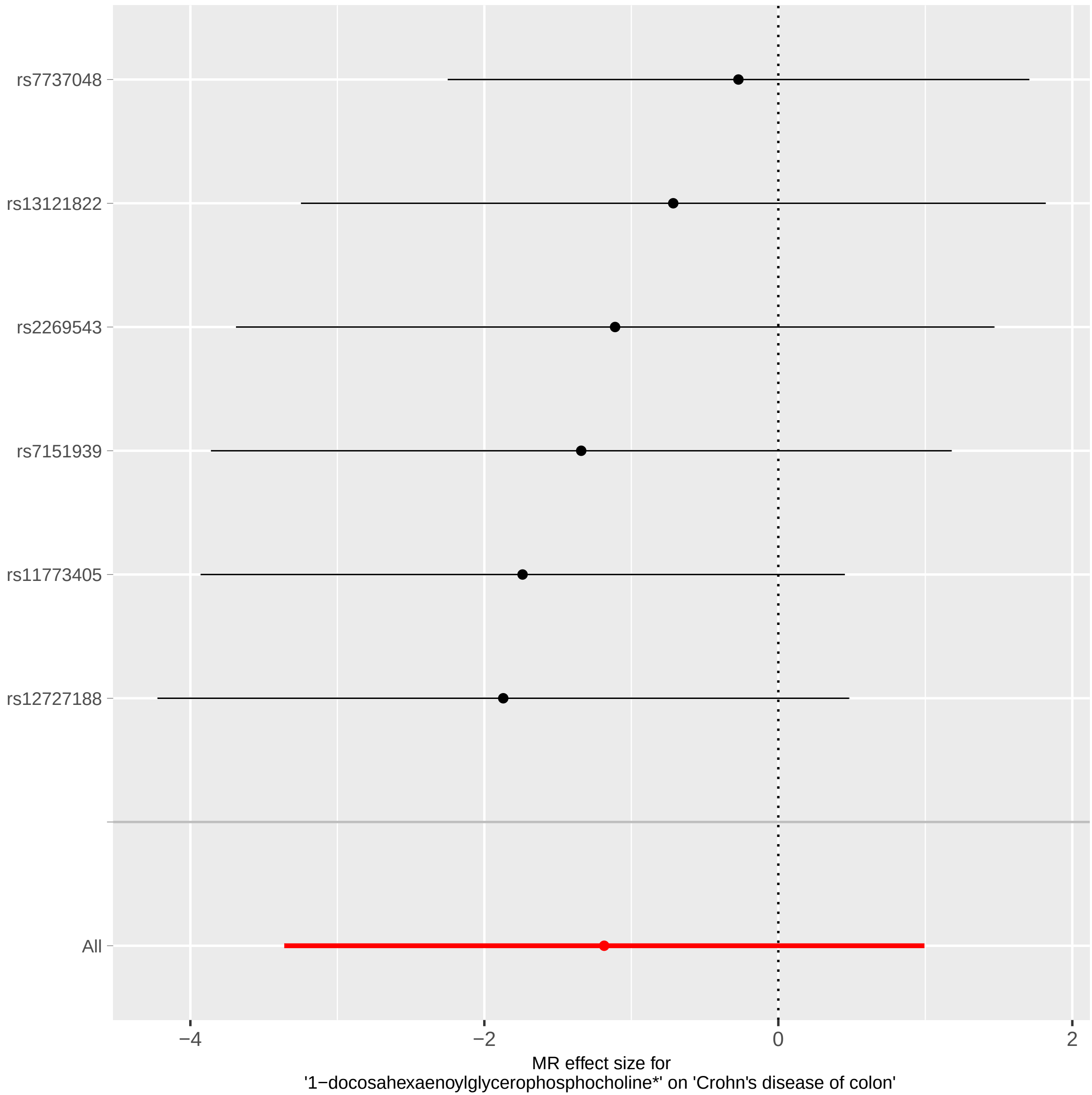

Batch 194 : Alpha−hydroxyisovalerate on Crohn's disease of colon

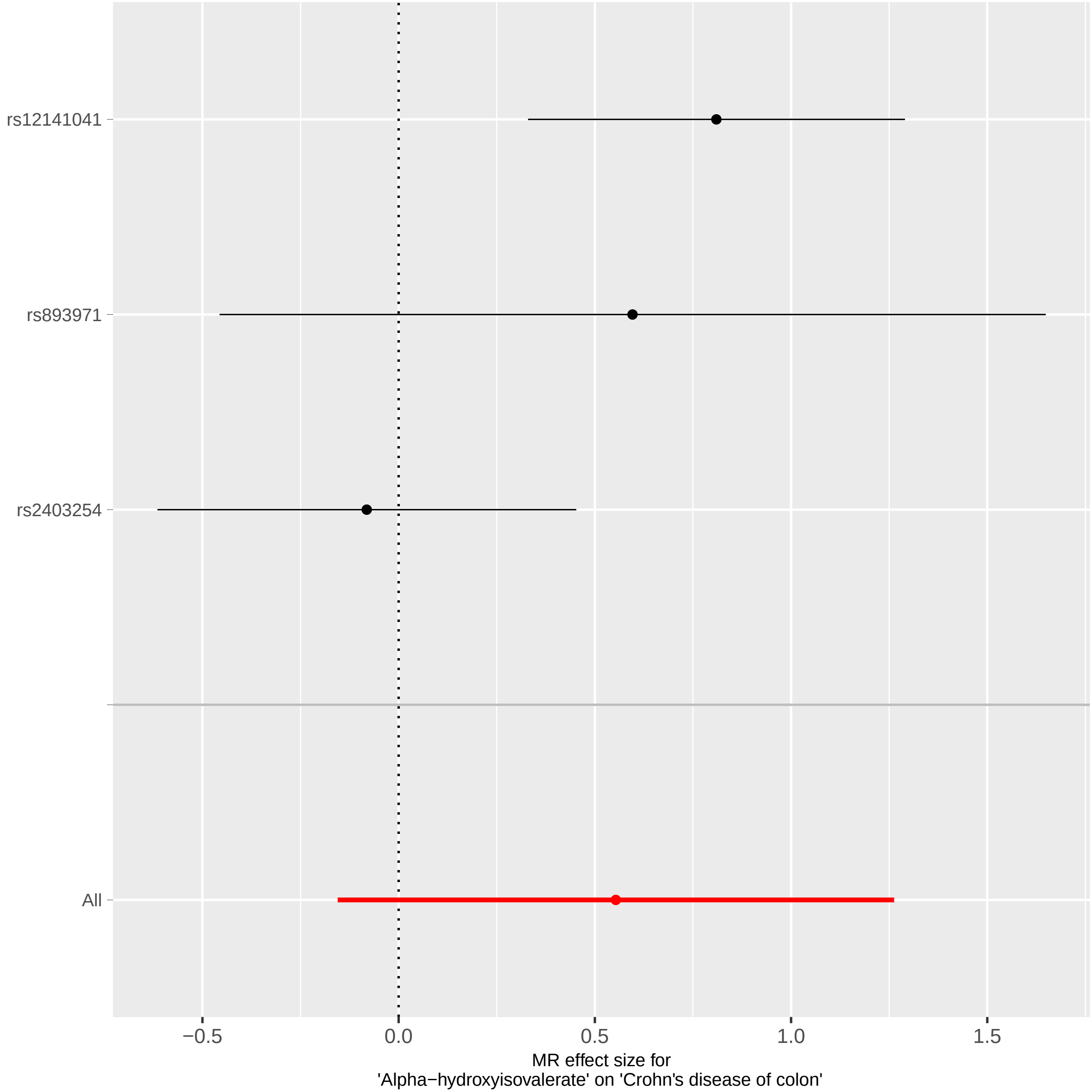

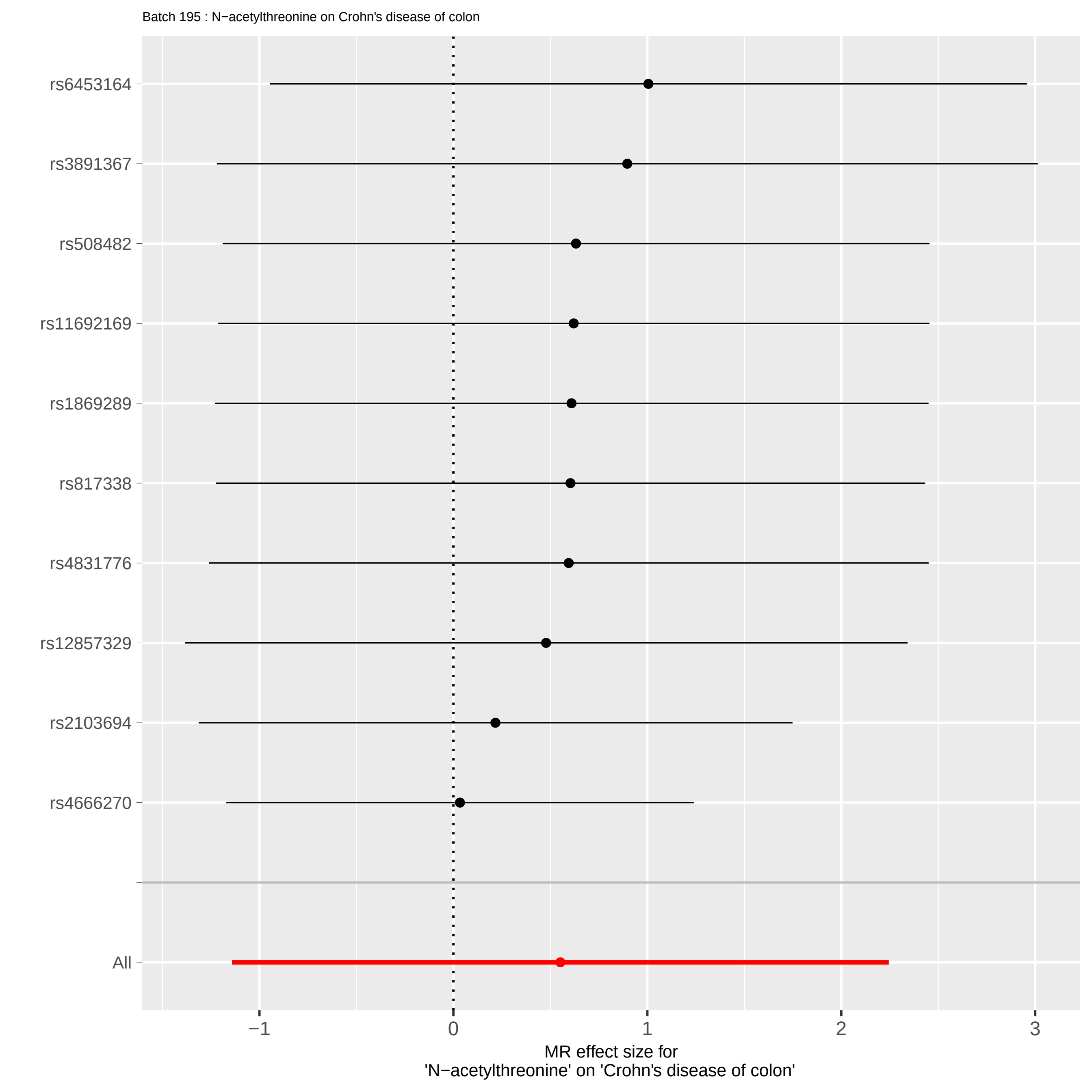

Batch 195 : N−acetylthreonine on Crohn's disease of colon
rs6453164
rs3891367
rs508482
rs11692169
rs1869289
rs817338
rs4831776
rs12857329
rs2103694
rs4666270
All
−1
0
1
2
3
MR effect size for
'N−acetylthreonine' on 'Crohn's disease of colon'

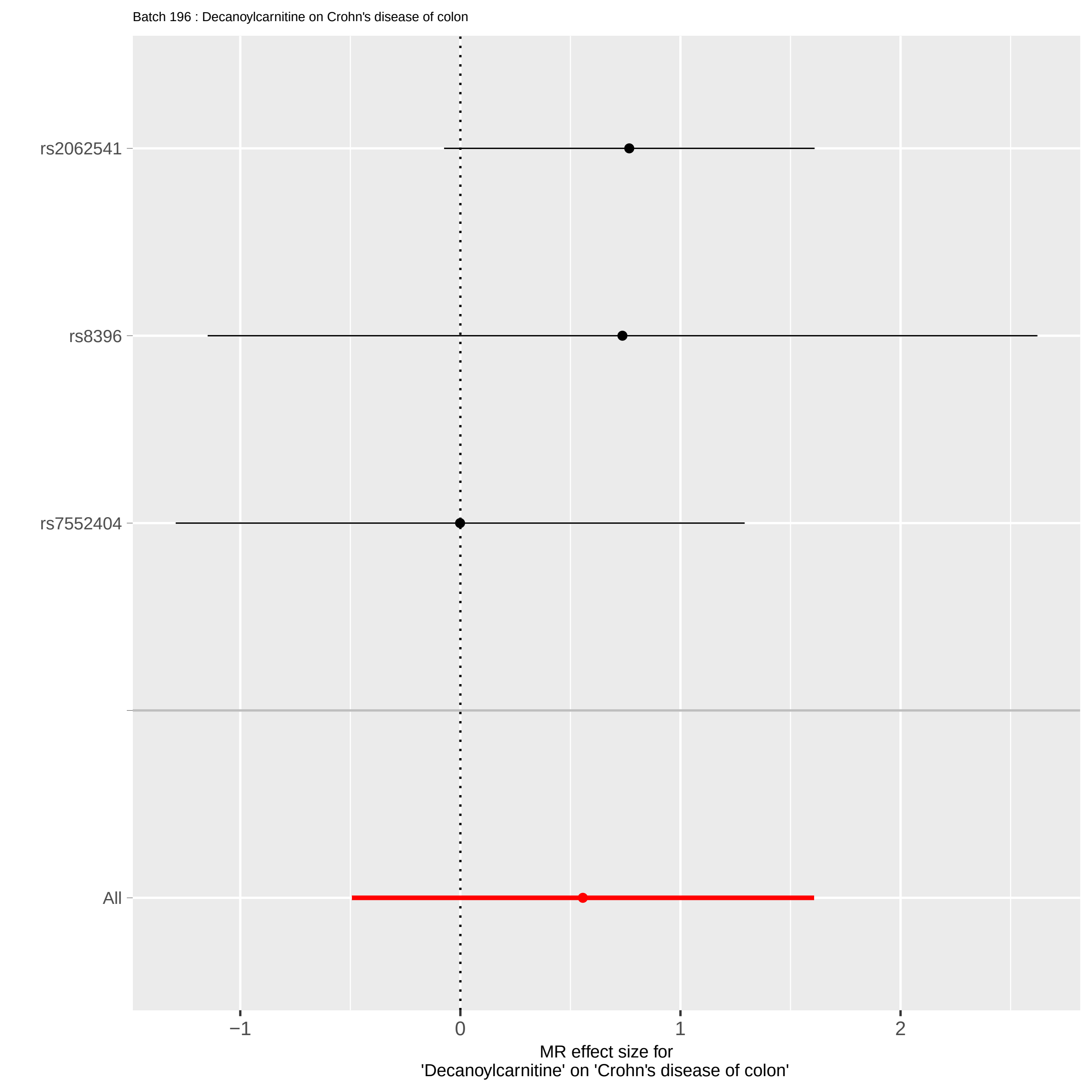
Batch 196 : Decanoylcarnitine on Crohn's disease of colon
rs2062541
rs8396
rs7552404
All
−1
0
1
2
MR effect size for
'Decanoylcarnitine' on 'Crohn's disease of colon'

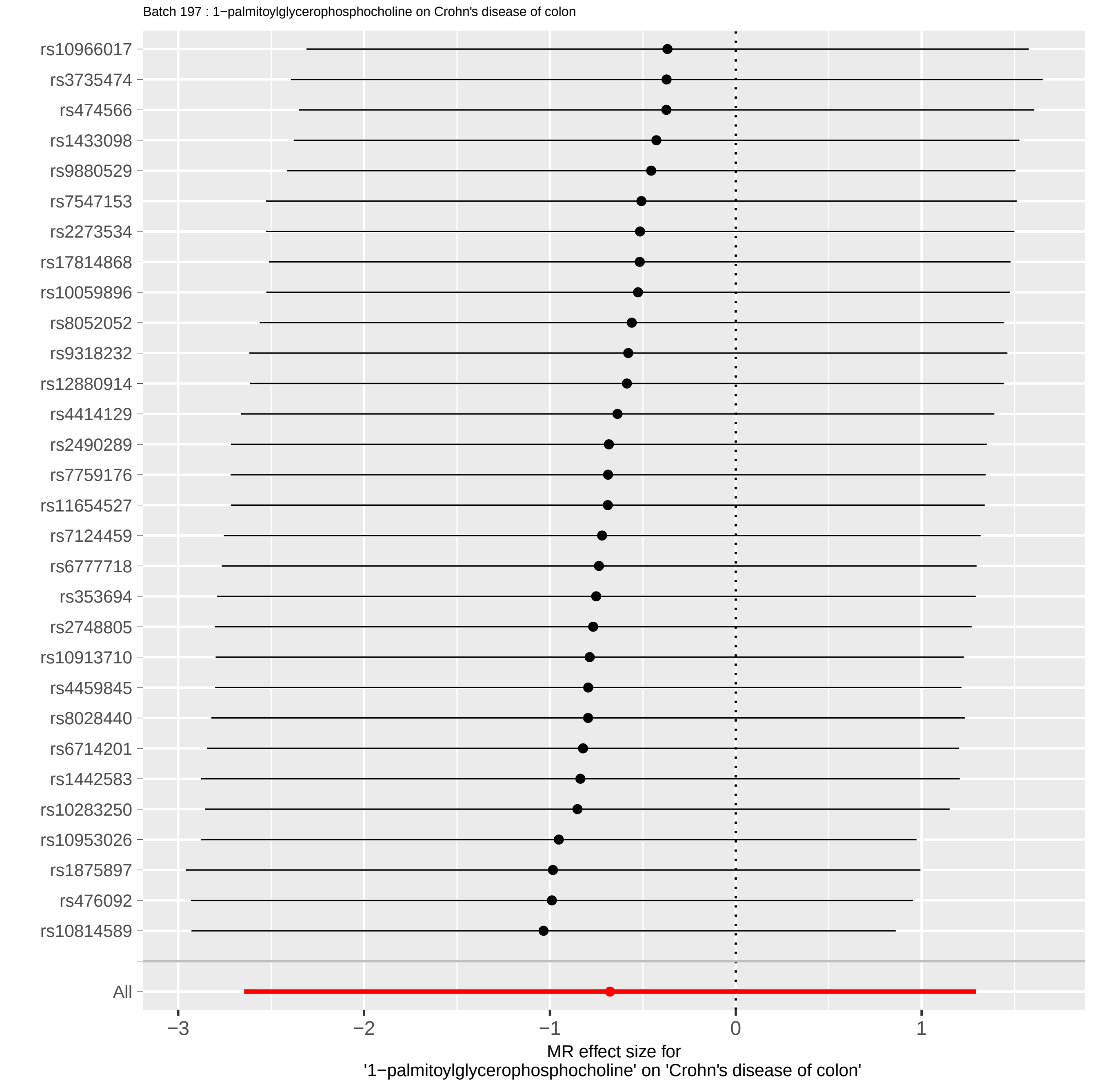

Batch 197 : 1−palmitoylglycerophosphocholine on Crohn's disease of colon
rs10966017
rs3735474
rs474566
rs1433098
rs9880529
rs7547153
rs2273534
rs17814868
rs10059896
rs8052052
rs9318232
rs12880914
rs4414129
rs2490289
rs7759176
rs11654527
rs7124459
rs6777718
rs353694
rs2748805
rs10913710
rs4459845
rs8028440
rs6714201
rs1442583
rs10283250
rs10953026
rs1875897
rs476092
rs10814589
All
−3
−2
−1
0
1
MR effect size for
'1−palmitoylglycerophosphocholine' on 'Crohn's disease of colon'

Batch 198 : 1−heptadecanoylglycerophosphocholine on Crohn's disease of colon

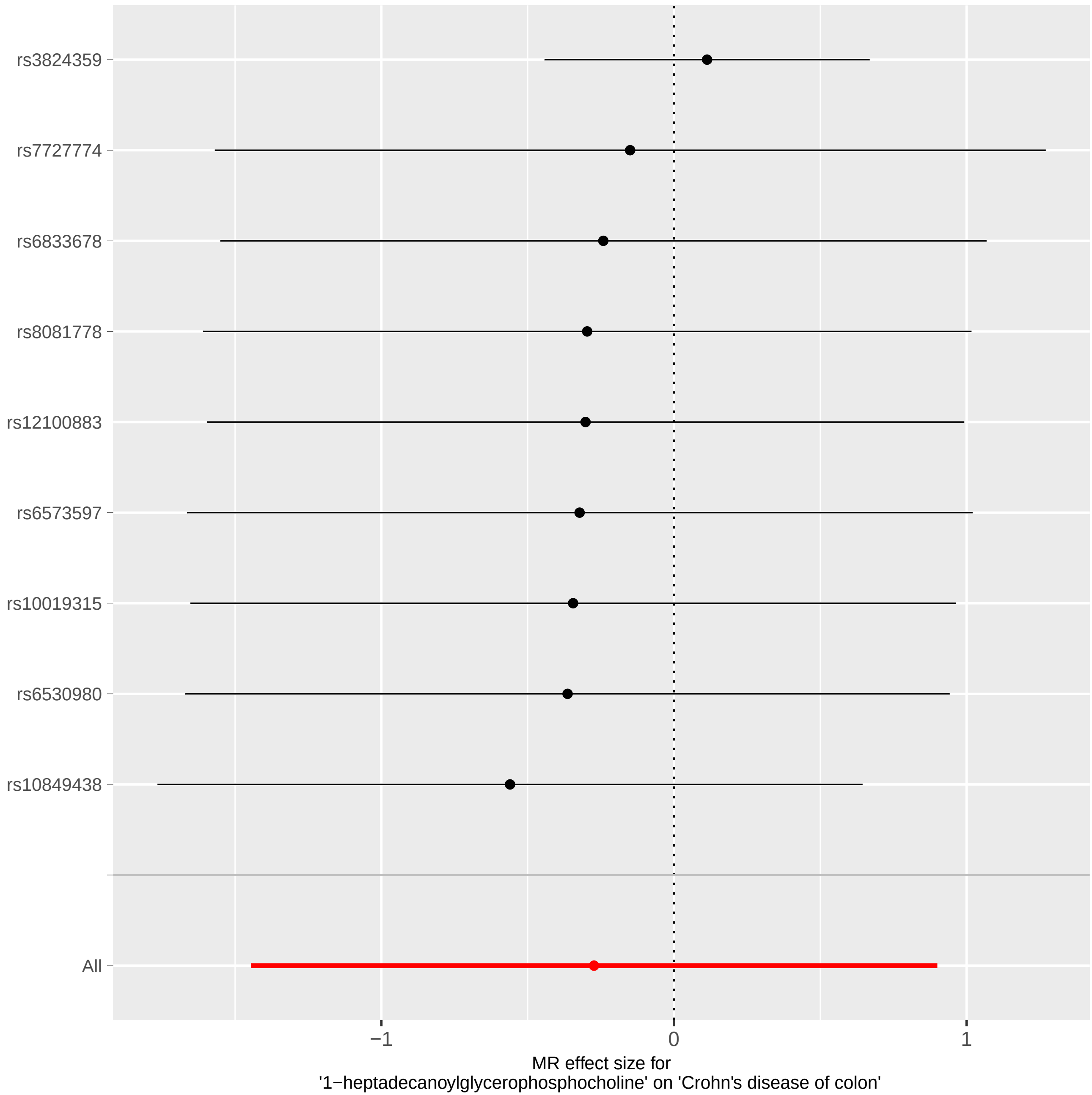

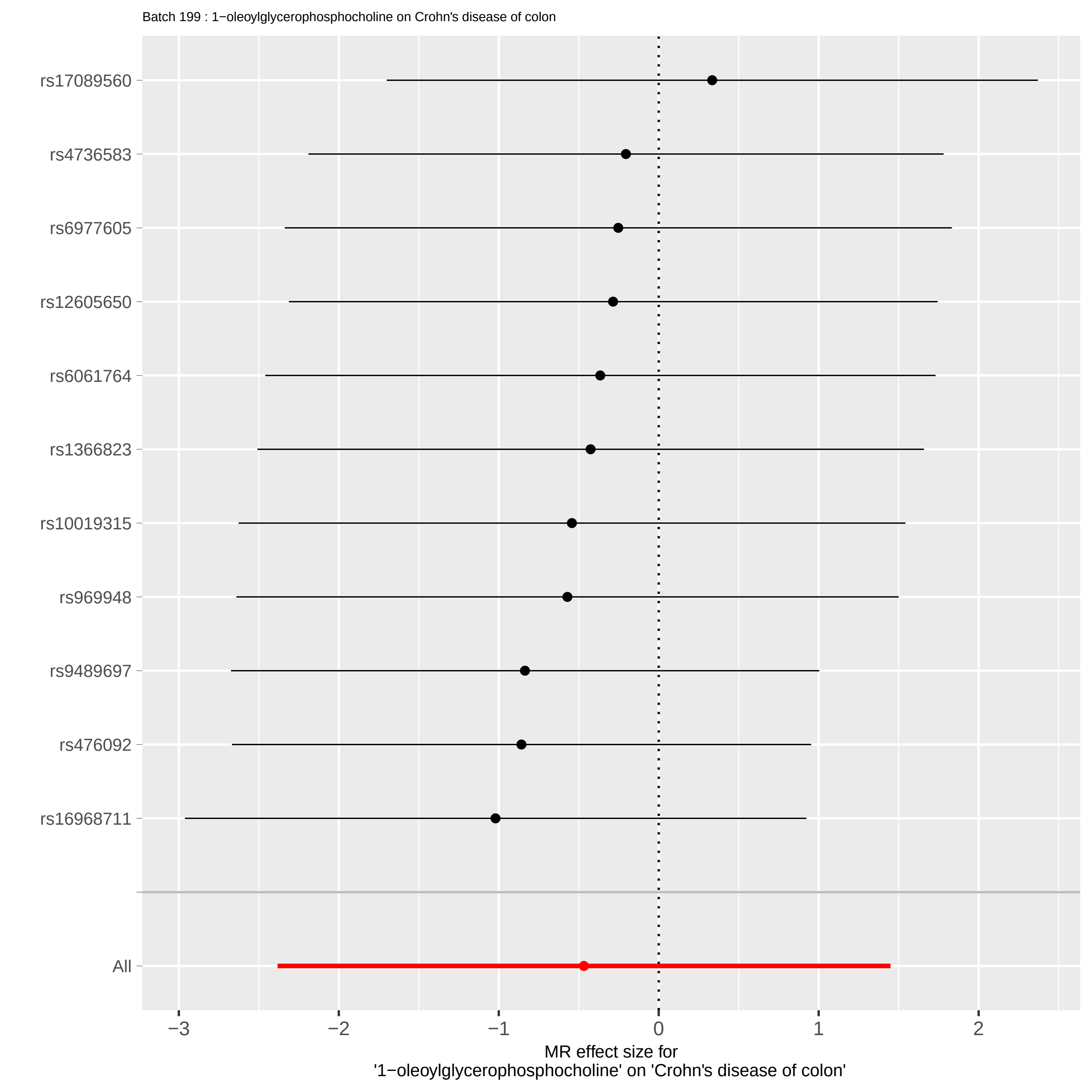

Batch 199 : 1−oleoylglycerophosphocholine on Crohn's disease of colon
rs17089560
rs4736583
rs6977605
rs12605650
rs6061764
rs1366823
rs10019315
rs969948
rs9489697
rs476092
rs16968711
All
−3
−2
−1
0
1
2
MR effect size for
'1−oleoylglycerophosphocholine' on 'Crohn's disease of colon'

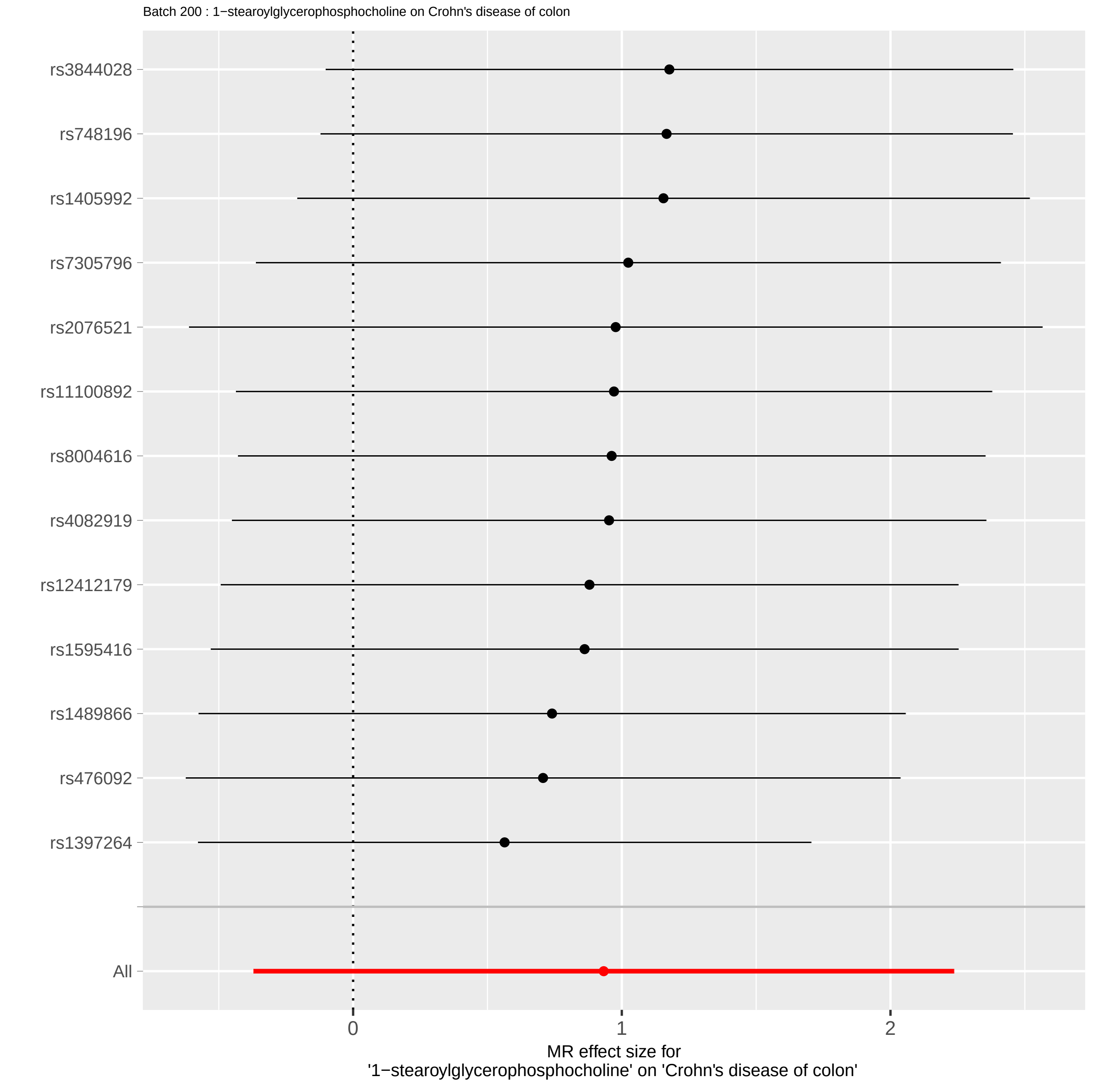

Batch 200 : 1−stearoylglycerophosphocholine on Crohn's disease of colon
rs3844028
rs748196
rs1405992
rs7305796
rs2076521
rs11100892
rs8004616
rs4082919
rs12412179
rs1595416
rs1489866
rs476092
rs1397264
All
0
1
2
MR effect size for
'1−stearoylglycerophosphocholine' on 'Crohn's disease of colon'

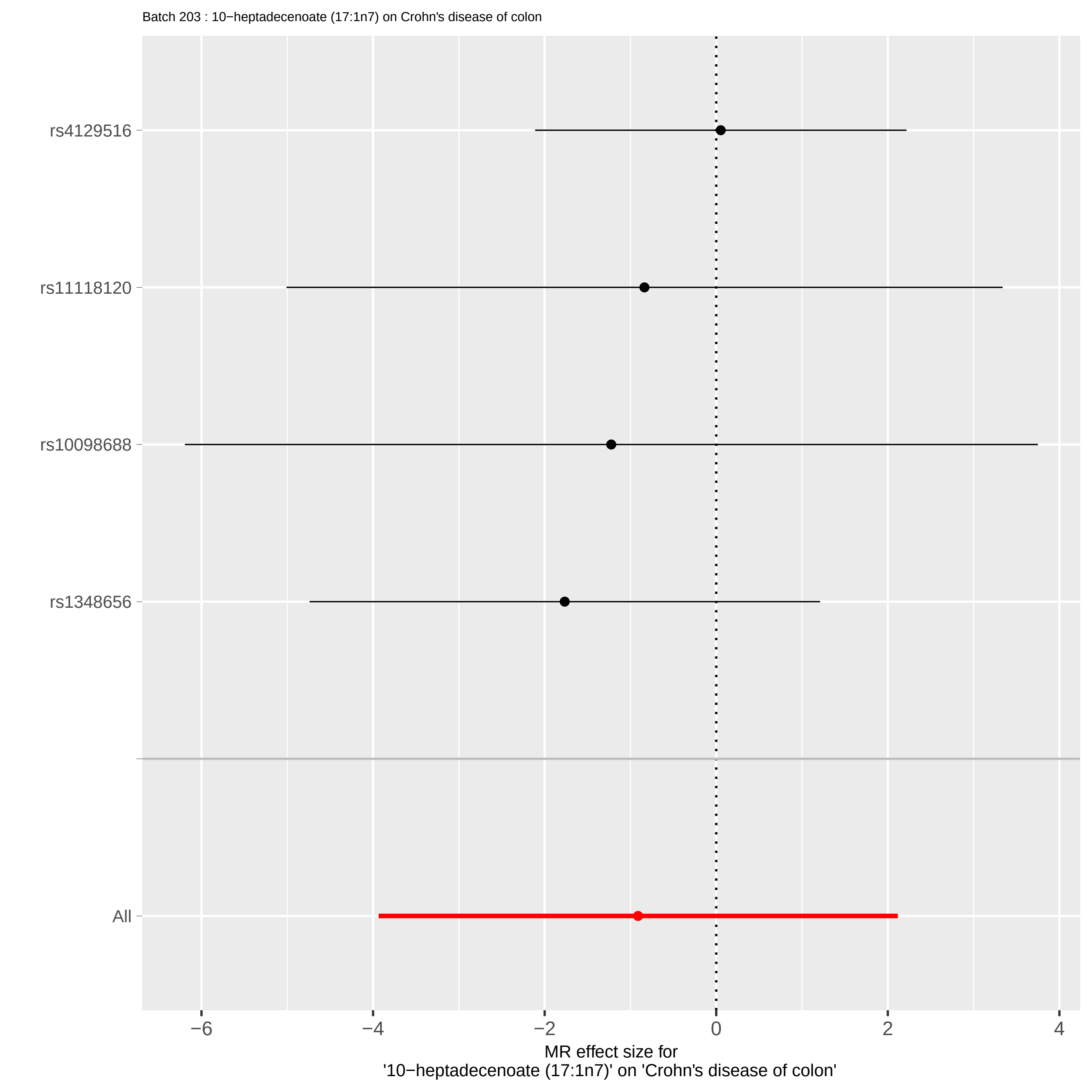

Batch 203 : 10−heptadecenoate (17:1n7) on Crohn's disease of colon
rs4129516
rs11118120
rs10098688
rs1348656
All
−6
−4
−2
0
2
4
MR effect size for
'10−heptadecenoate (17:1n7)' on 'Crohn's disease of colon'

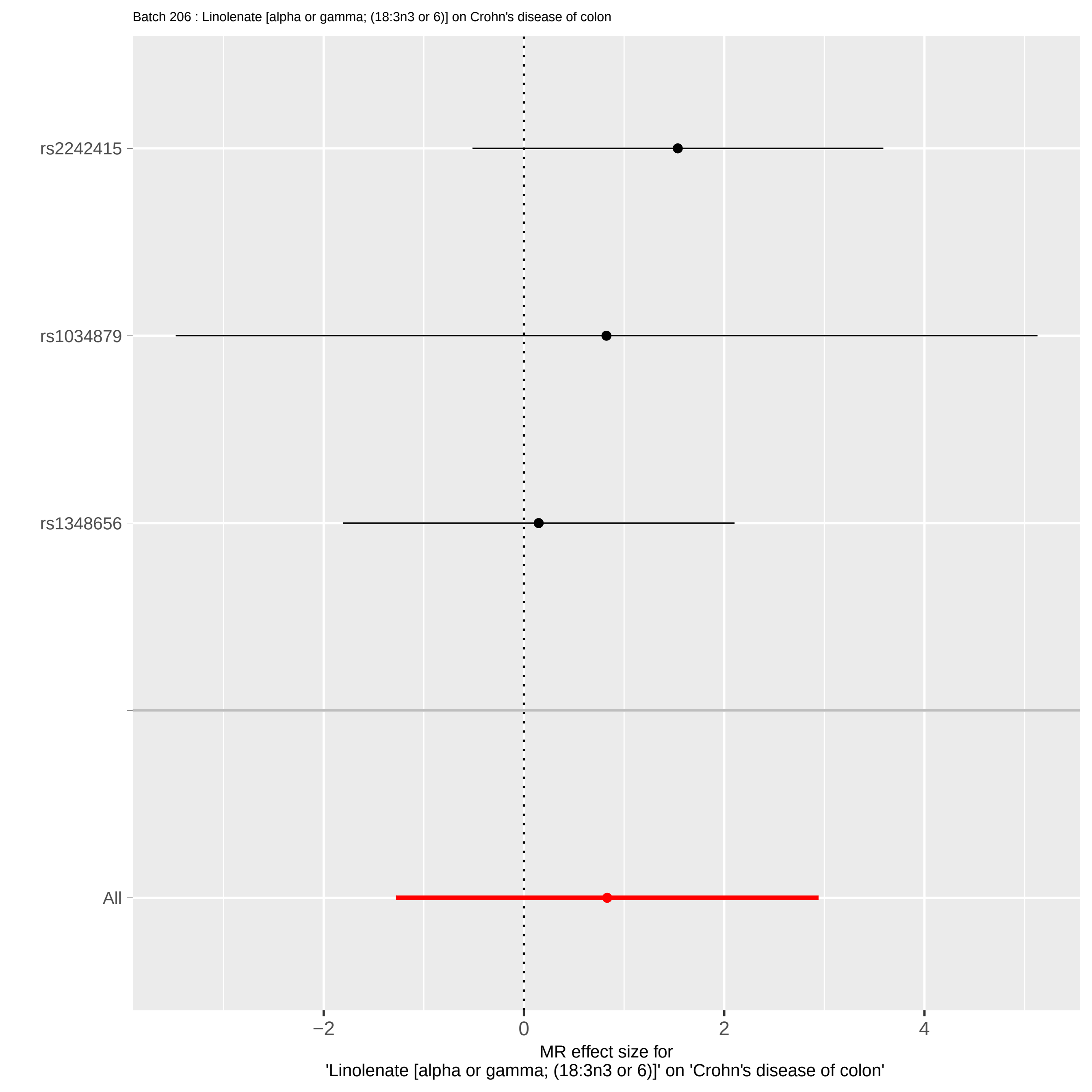

Batch 206 : Linolenate [alpha or gamma; (18:3n3 or 6)] on Crohn's disease of colon
rs2242415
rs1034879
rs1348656
All
−2
0
2
4
MR effect size for
'Linolenate [alpha or gamma; (18:3n3 or 6)]' on 'Crohn's disease of colon'

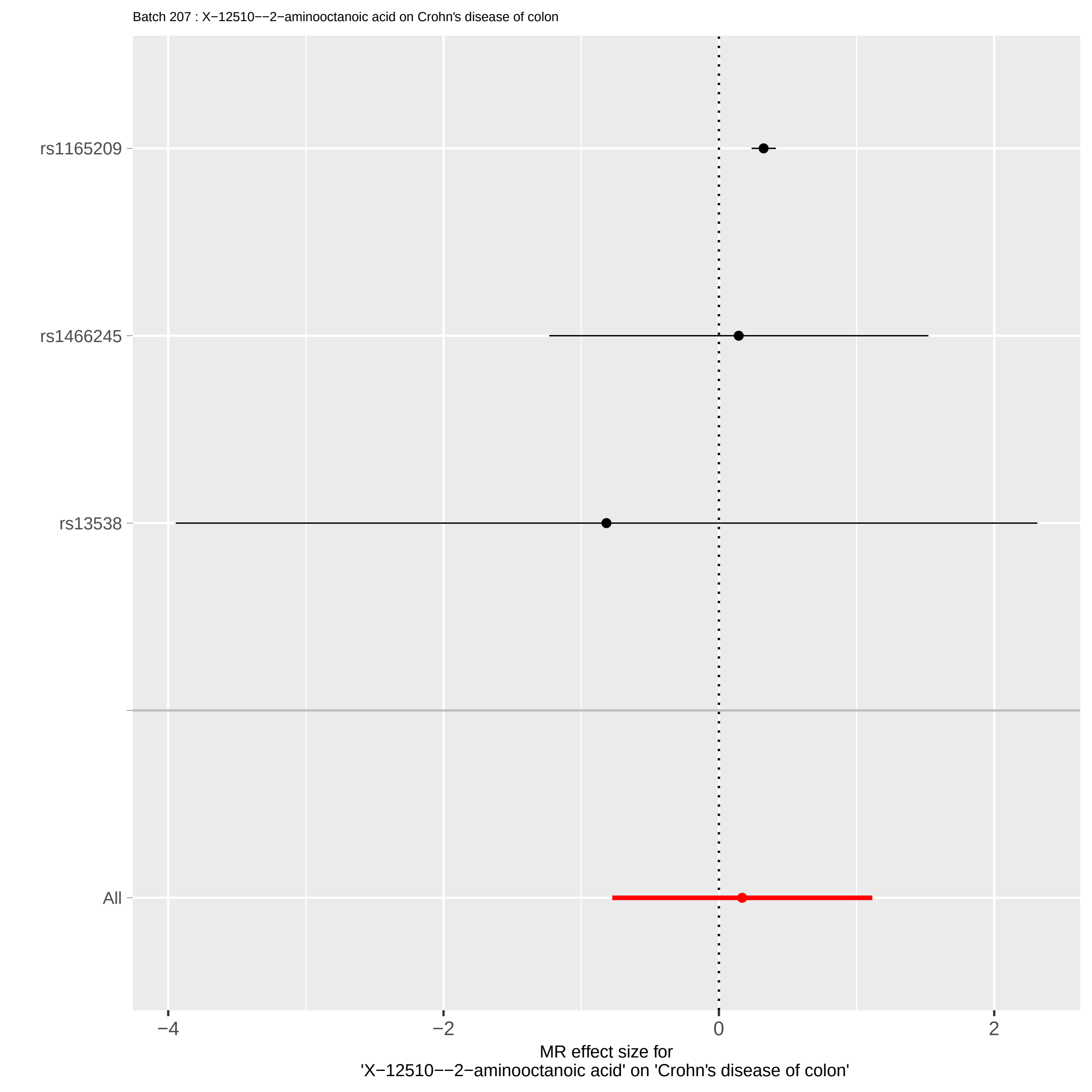
Batch 207 : X−12510−−2−aminooctanoic acid on Crohn's disease of colon
rs1165209
rs1466245
rs13538
All
−4
−2
0
2
MR effect size for
'X−12510−−2−aminooctanoic acid' on 'Crohn's disease of colon'

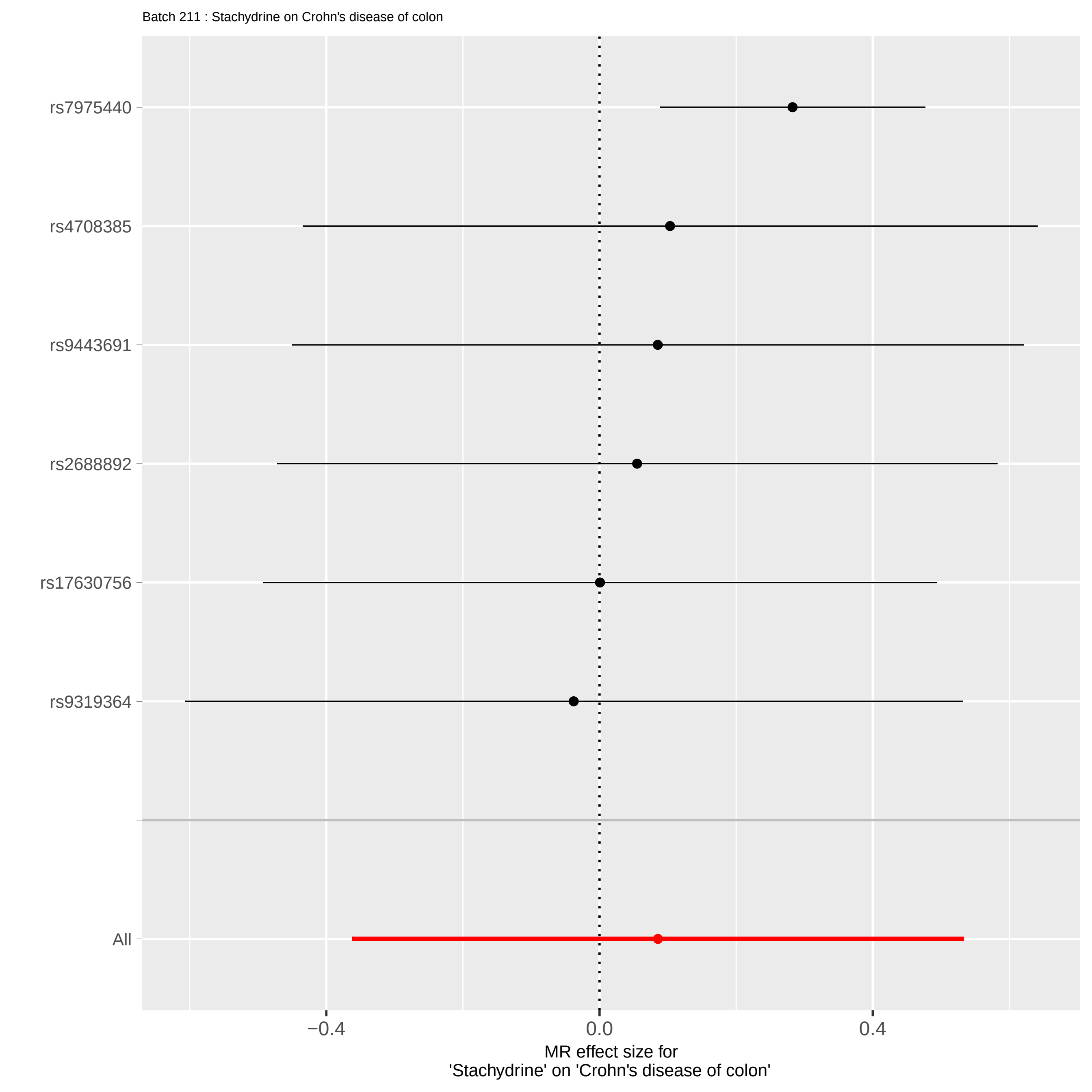

Batch 211 : Stachydrine on Crohn's disease of colon
rs7975440
rs4708385
rs9443691
rs2688892
rs17630756
rs9319364
All
−0.4
0.0
0.4
MR effect size for
'Stachydrine' on 'Crohn's disease of colon'

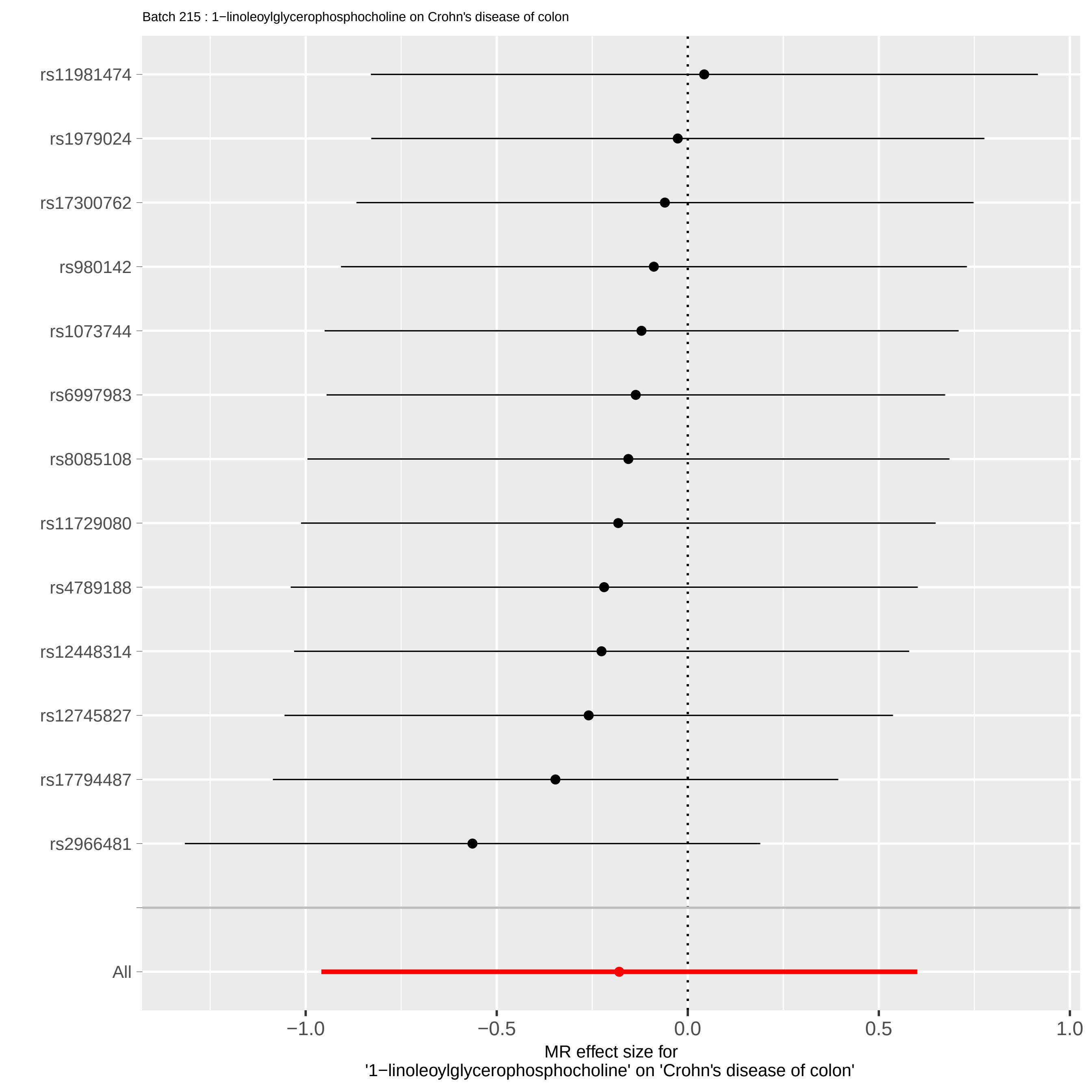

Batch 215 : 1−linoleoylglycerophosphocholine on Crohn's disease of colon
rs11981474
rs1979024
rs17300762
rs980142
rs1073744
rs6997983
rs8085108
rs11729080
rs4789188
rs12448314
rs12745827
rs17794487
rs2966481
All
−1.0
−0.5
0.0
0.5
1.0
MR effect size for
'1−linoleoylglycerophosphocholine' on 'Crohn's disease of colon'

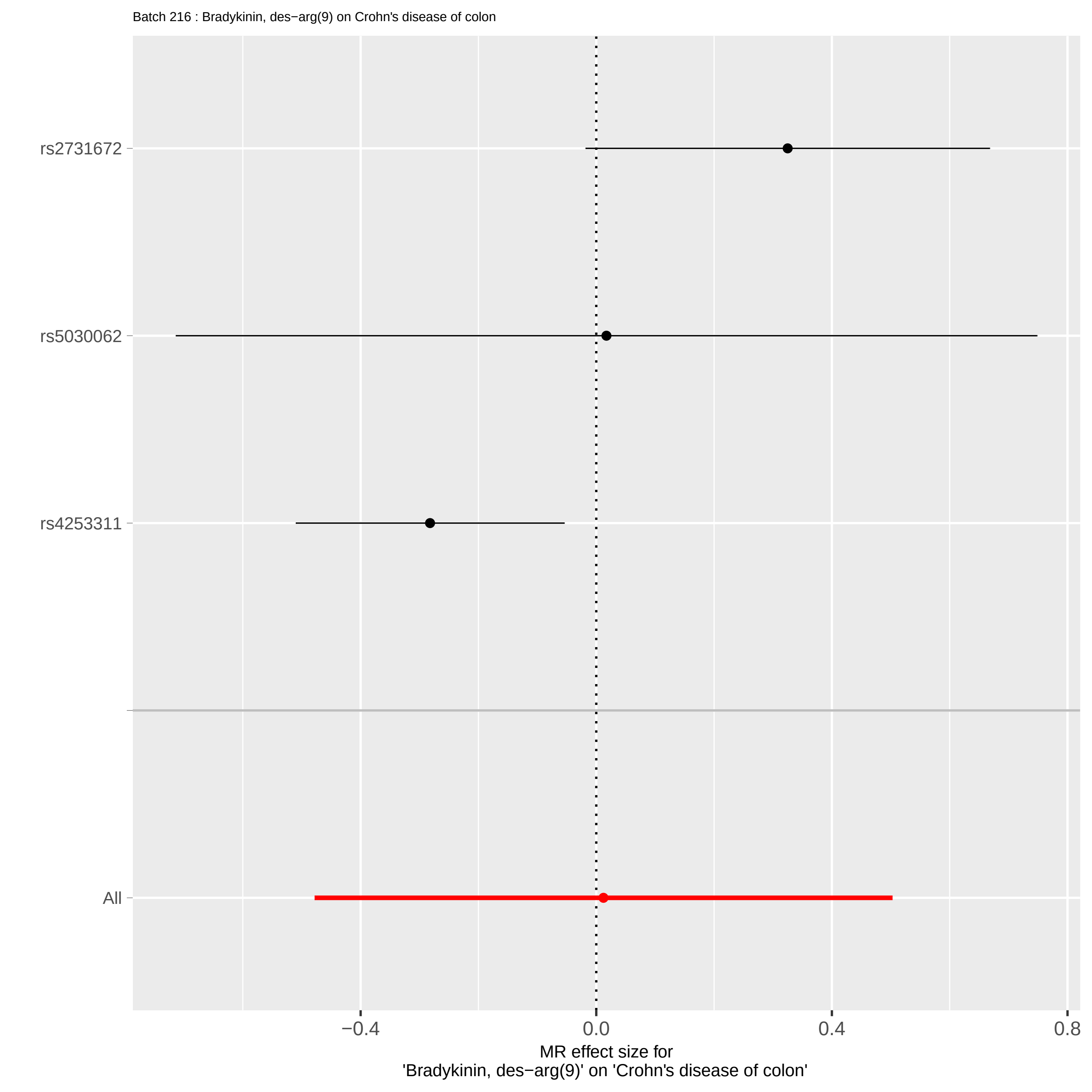
Batch 216 : Bradykinin, des−arg(9) on Crohn's disease of colon
rs2731672
rs5030062
rs4253311
All
−0.4
0.0
0.4
0.8
MR effect size for
'Bradykinin, des−arg(9)' on 'Crohn's disease of colon'

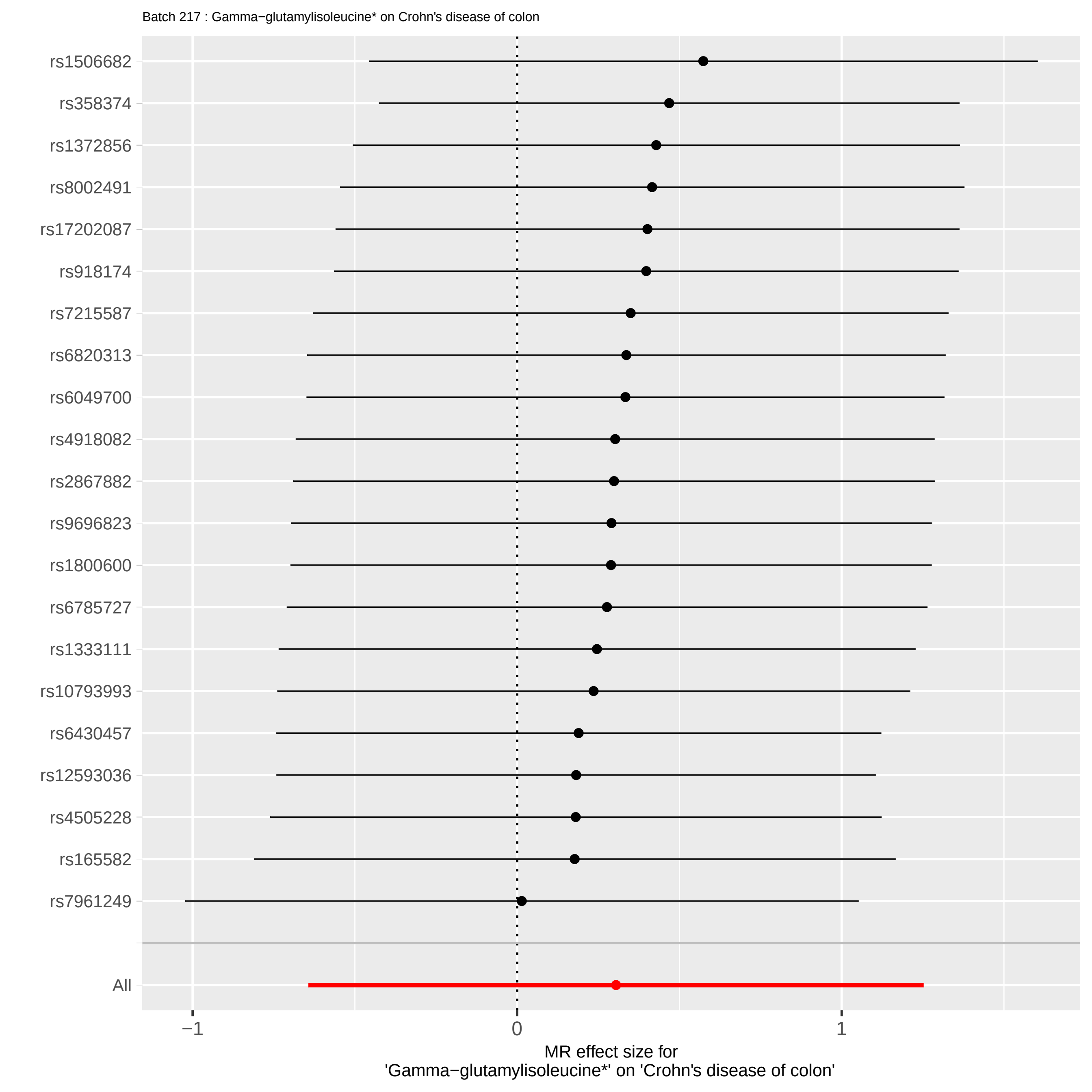
Batch 217 : Gamma−glutamylisoleucine* on Crohn's disease of colon
rs1506682
rs358374
rs1372856
rs8002491
rs17202087
rs918174
rs7215587
rs6820313
rs6049700
rs4918082
rs2867882
rs9696823
rs1800600
rs6785727
rs1333111
rs10793993
rs6430457
rs12593036
rs4505228
rs165582
rs7961249
All
−1
0
1
MR effect size for
'Gamma−glutamylisoleucine*' on 'Crohn's disease of colon'

Batch 220 : Isovalerate on Crohn's disease of colon

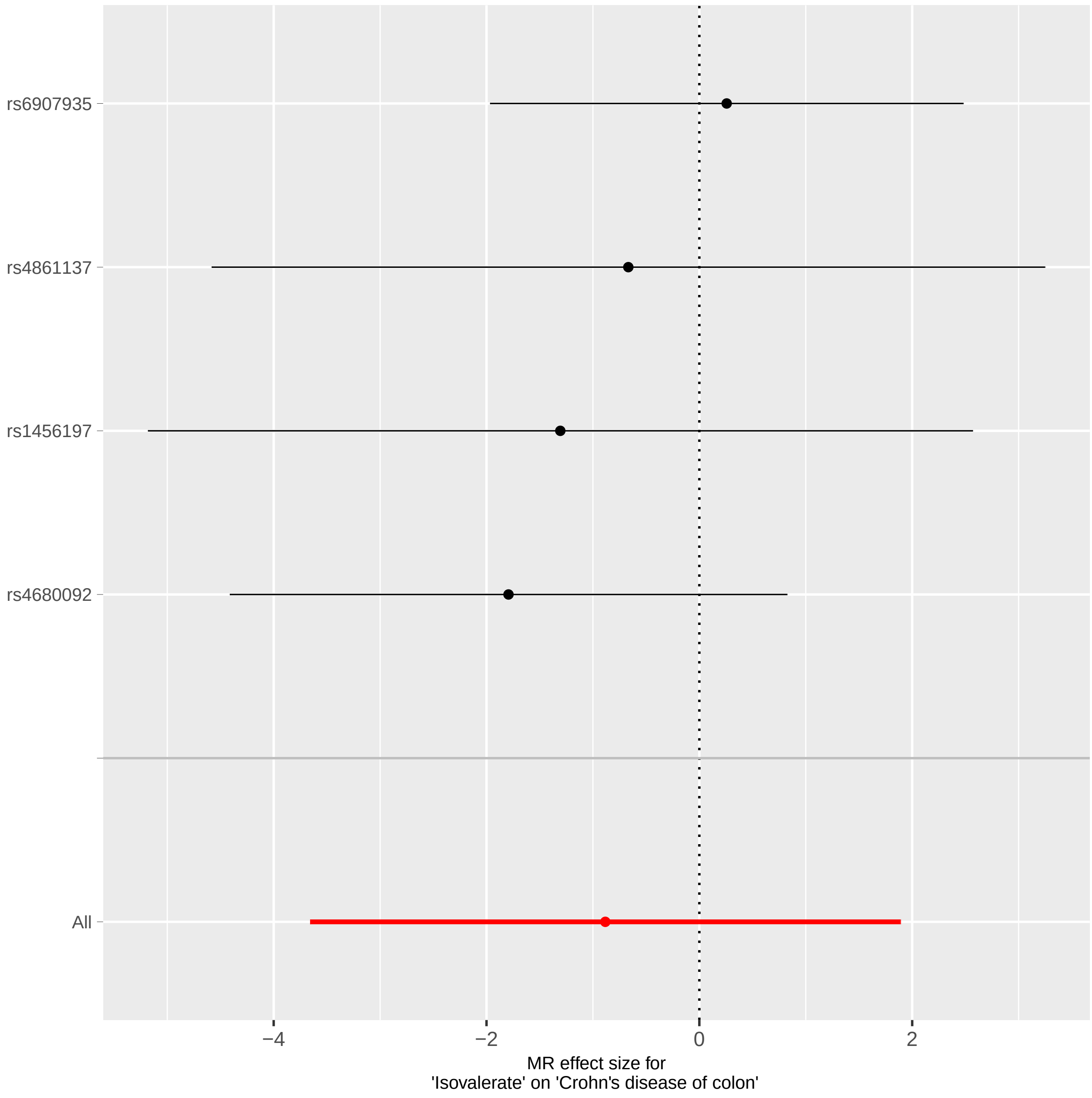

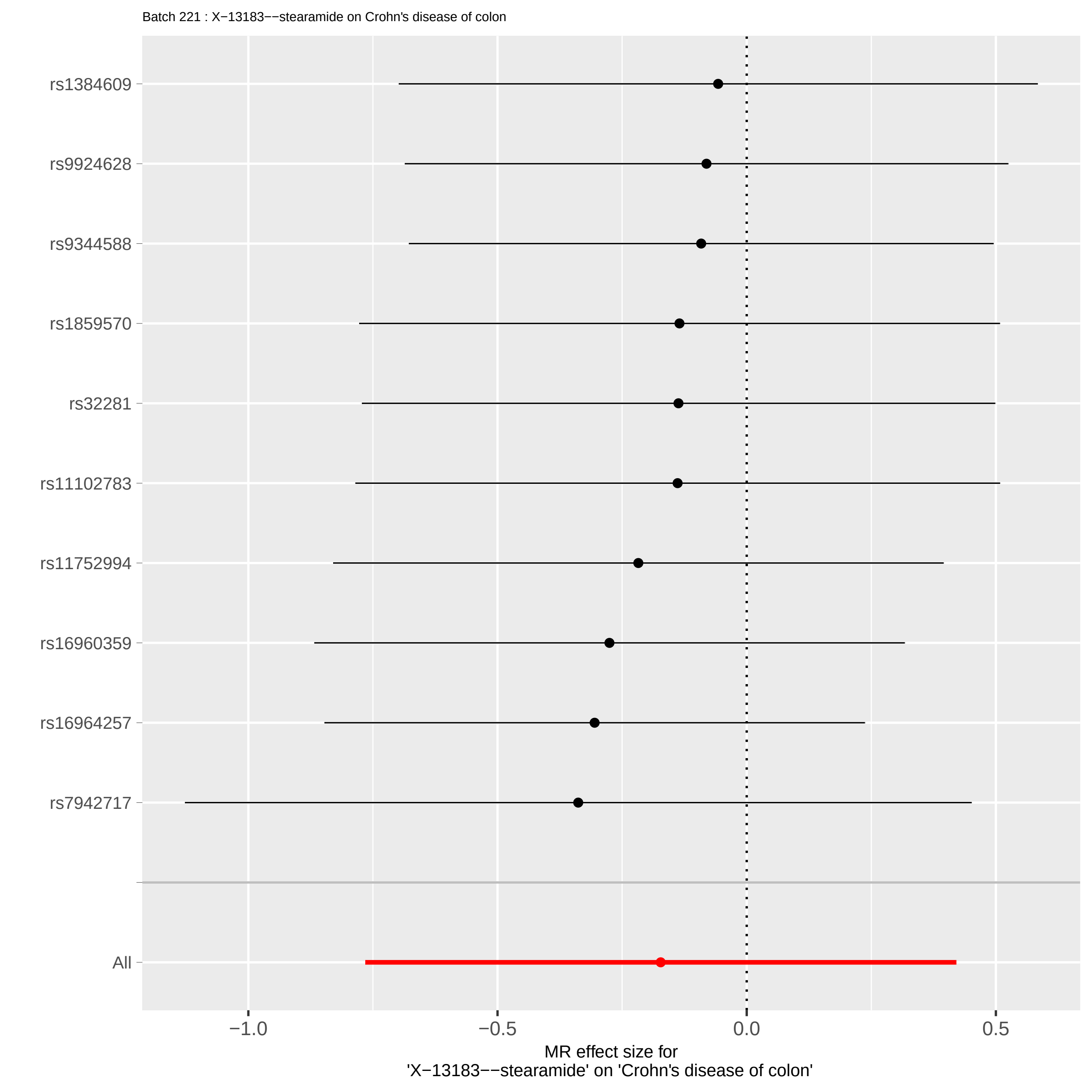
Batch 221 : X−13183−−stearamide on Crohn's disease of colon
rs1384609
rs9924628
rs9344588
rs1859570
rs32281
rs11102783
rs11752994
rs16960359
rs16964257
rs7942717
All
−1.0
−0.5
0.0
0.5
MR effect size for
'X−13183−−stearamide' on 'Crohn's disease of colon'

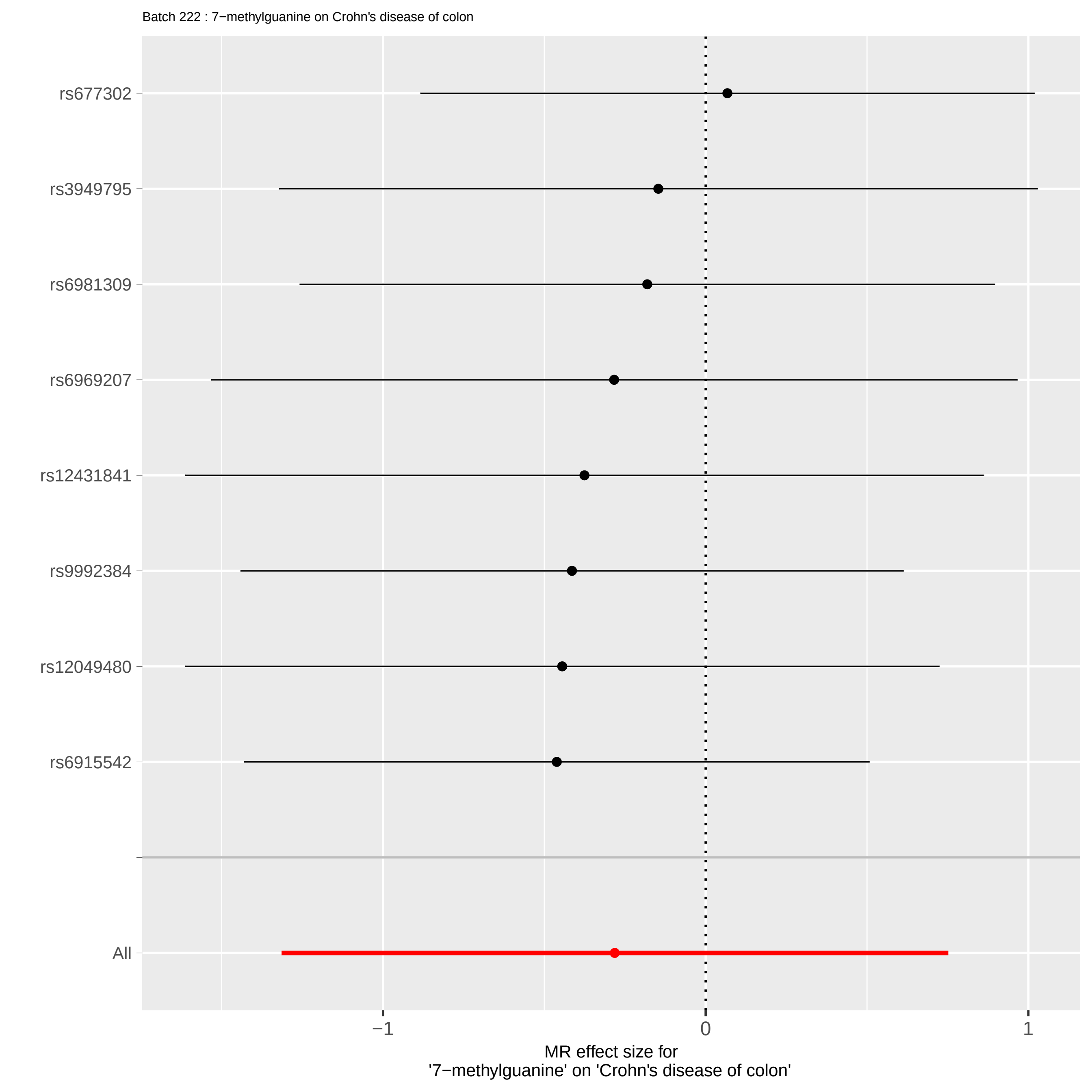

Batch 222 : 7−methylguanine on Crohn's disease of colon
rs677302
rs3949795
rs6981309
rs6969207
rs12431841
rs9992384
rs12049480
rs6915542
All
−1
0
1
MR effect size for
'7−methylguanine' on 'Crohn's disease of colon'

Batch 230 : 2−palmitoylglycerophosphocholine* on Crohn's disease of colon

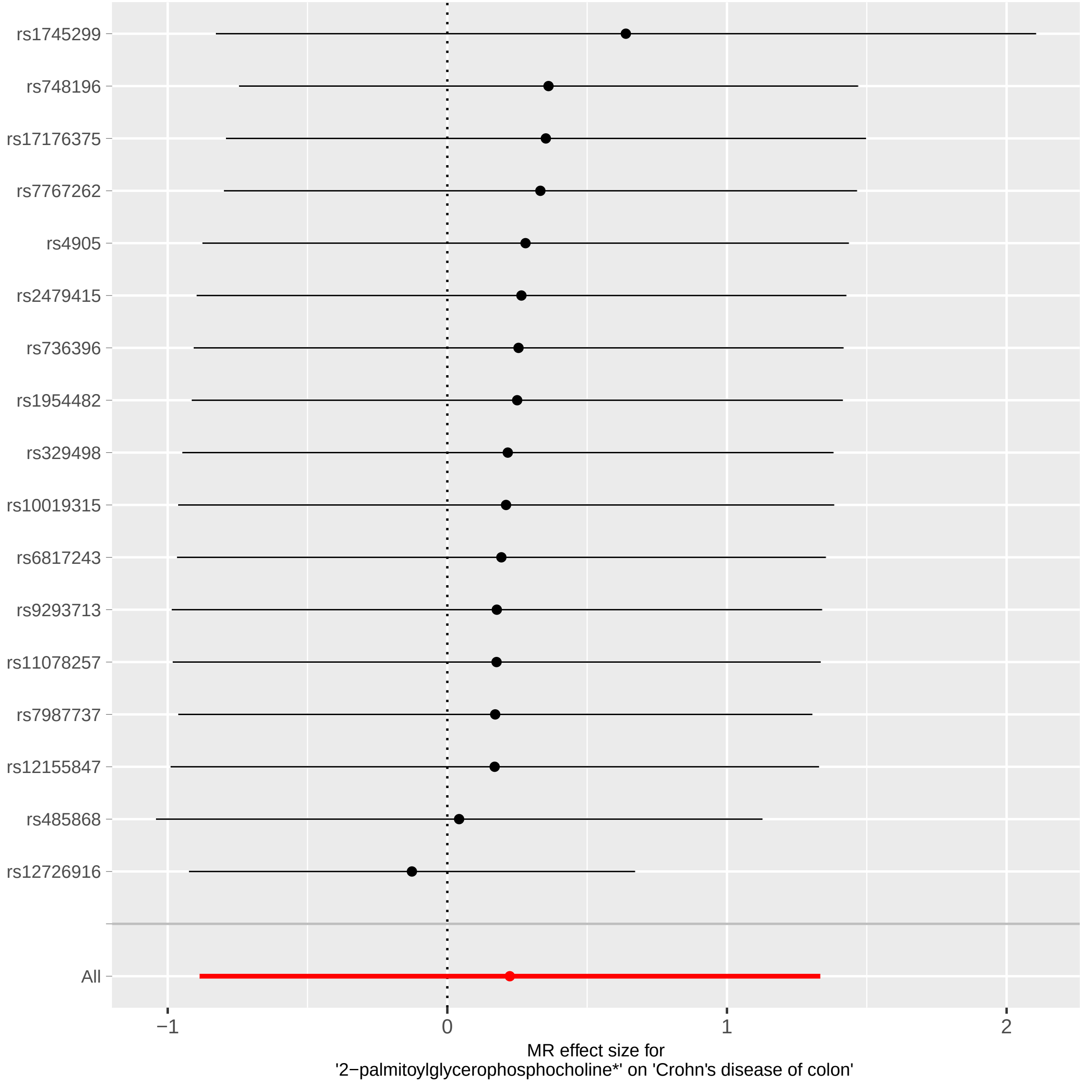

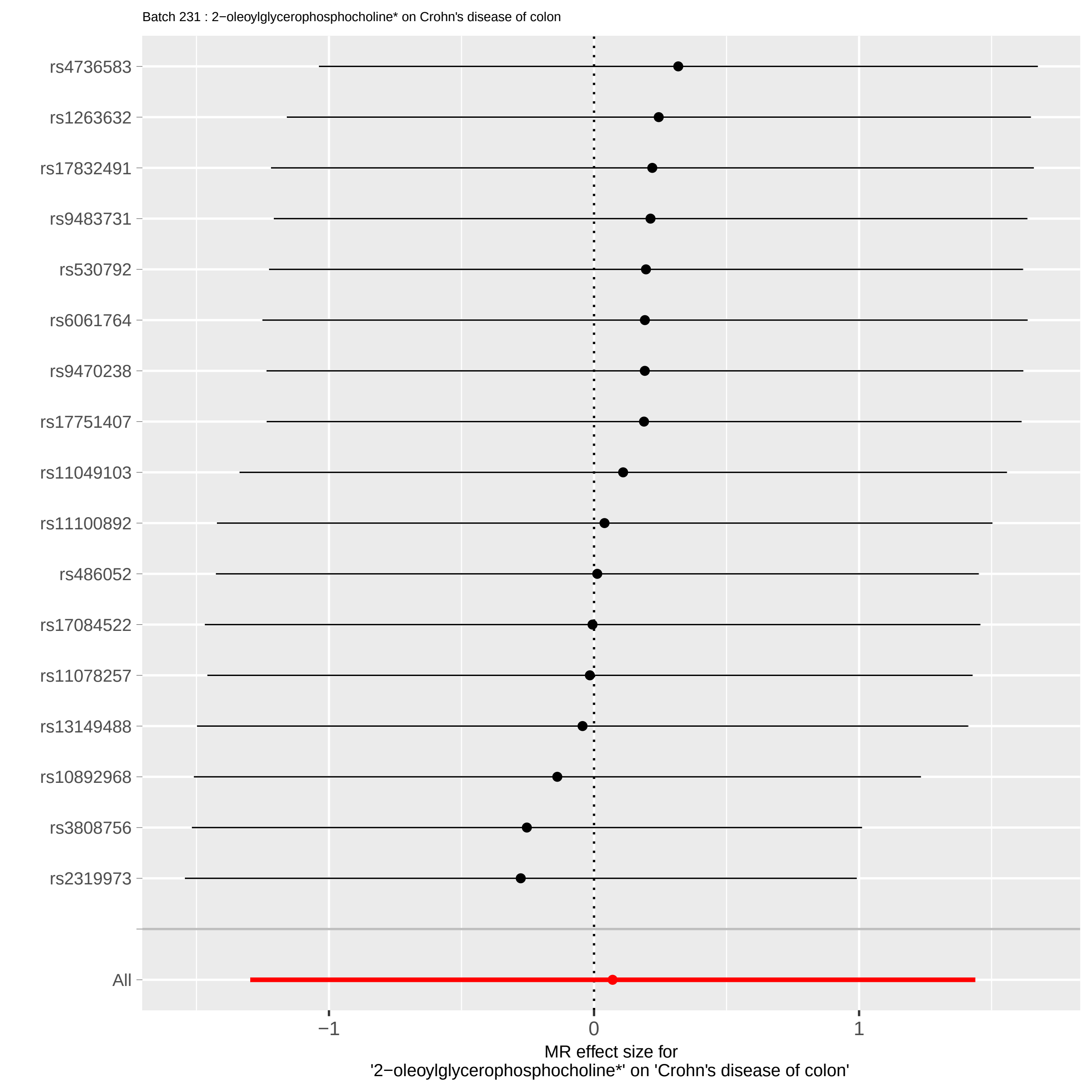

Batch 231 : 2−oleoylglycerophosphocholine* on Crohn's disease of colon
rs4736583
rs1263632
rs17832491
rs9483731
rs530792
rs6061764
rs9470238
rs17751407
rs11049103
rs11100892
rs486052
rs17084522
rs11078257
rs13149488
rs10892968
rs3808756
rs2319973
All
−1
0
1
MR effect size for
'2−oleoylglycerophosphocholine*' on 'Crohn's disease of colon'

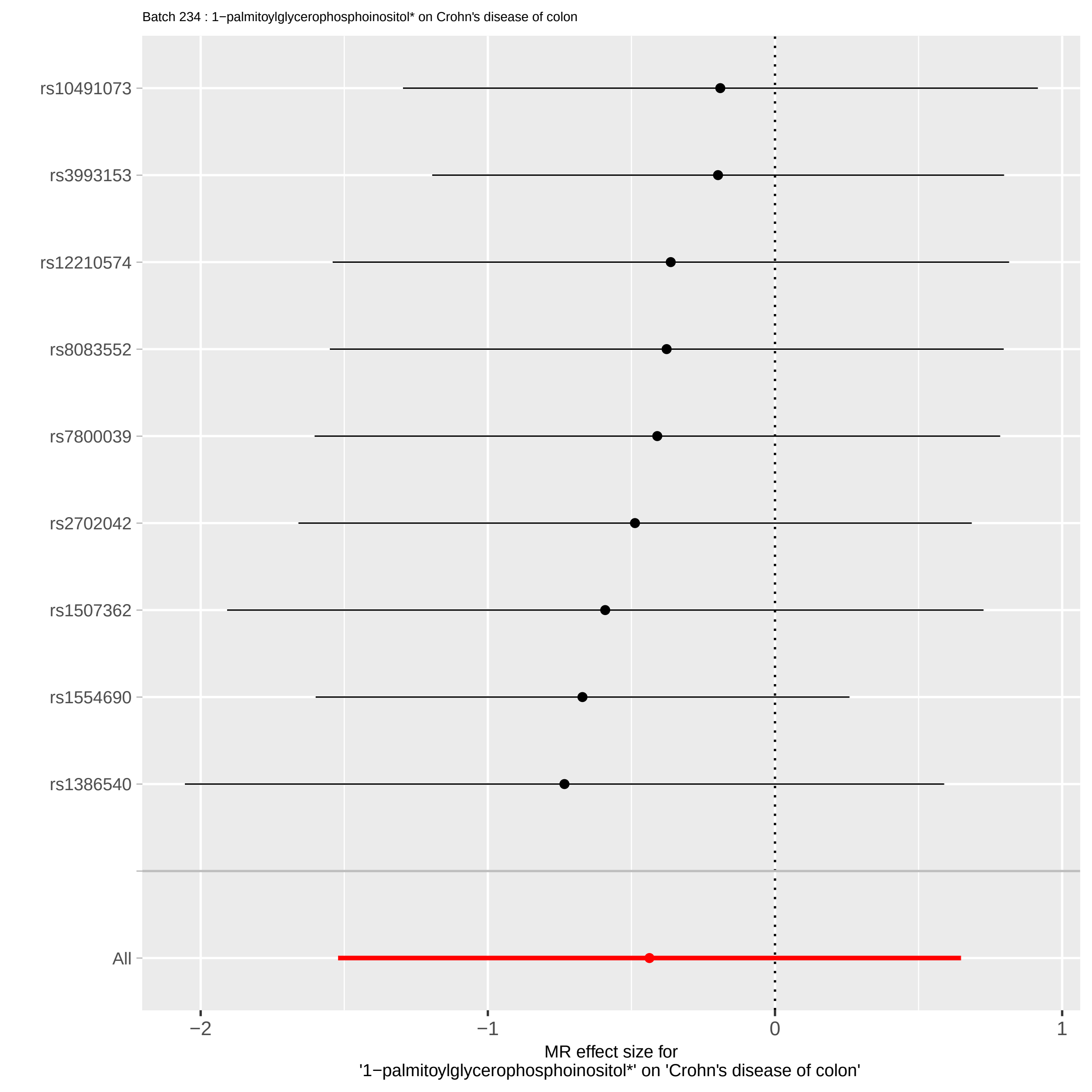
Batch 234 : 1−palmitoylglycerophosphoinositol* on Crohn's disease of colon
rs10491073
rs3993153
rs12210574
rs8083552
rs7800039
rs2702042
rs1507362
rs1554690
rs1386540
All
−2
−1
0
1
MR effect size for
'1−palmitoylglycerophosphoinositol*' on 'Crohn's disease of colon'

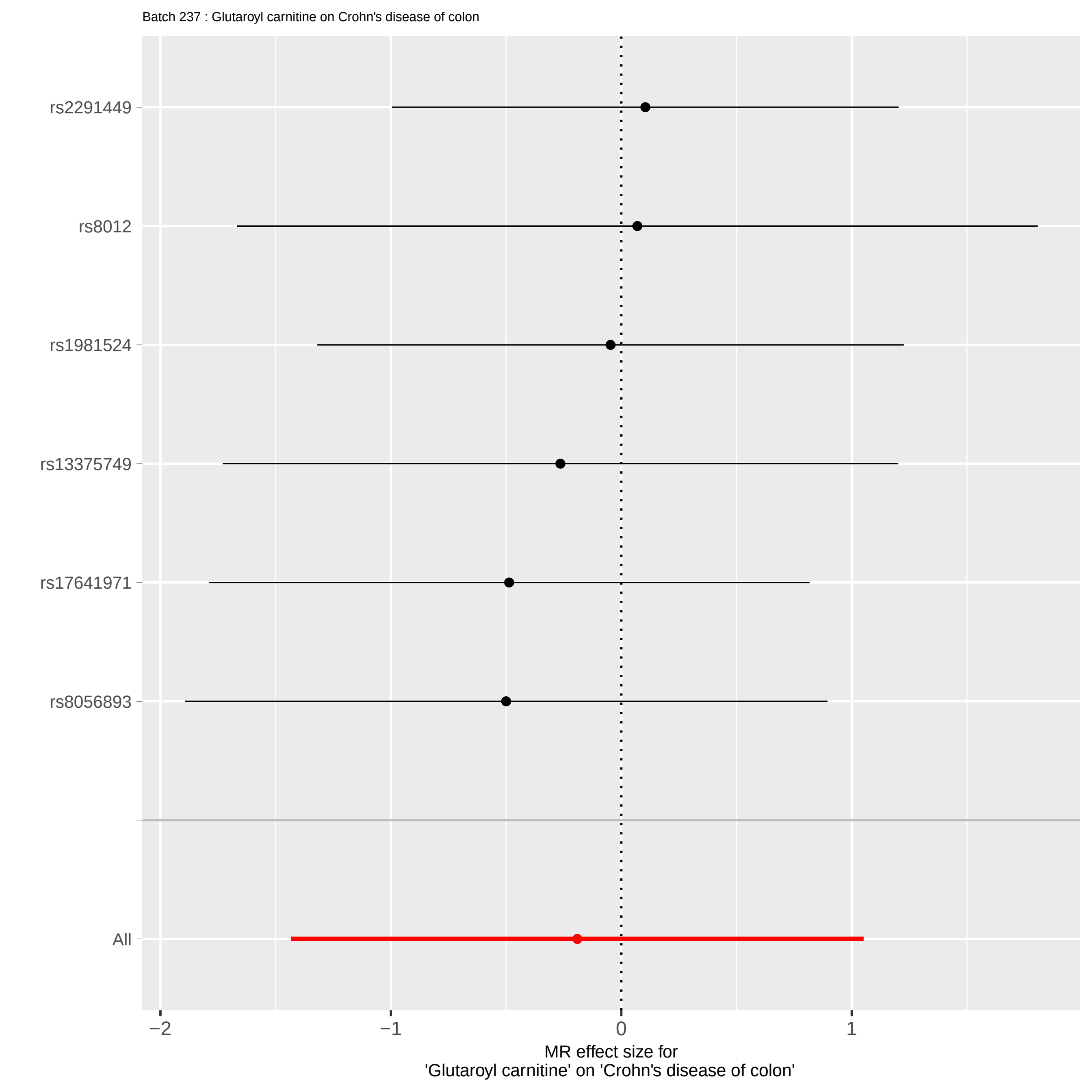
Batch 237 : Glutaroyl carnitine on Crohn's disease of colon
rs2291449
rs8012
rs1981524
rs13375749
rs17641971
rs8056893
All
−2
−1
0
1
MR effect size for
'Glutaroyl carnitine' on 'Crohn's disease of colon'

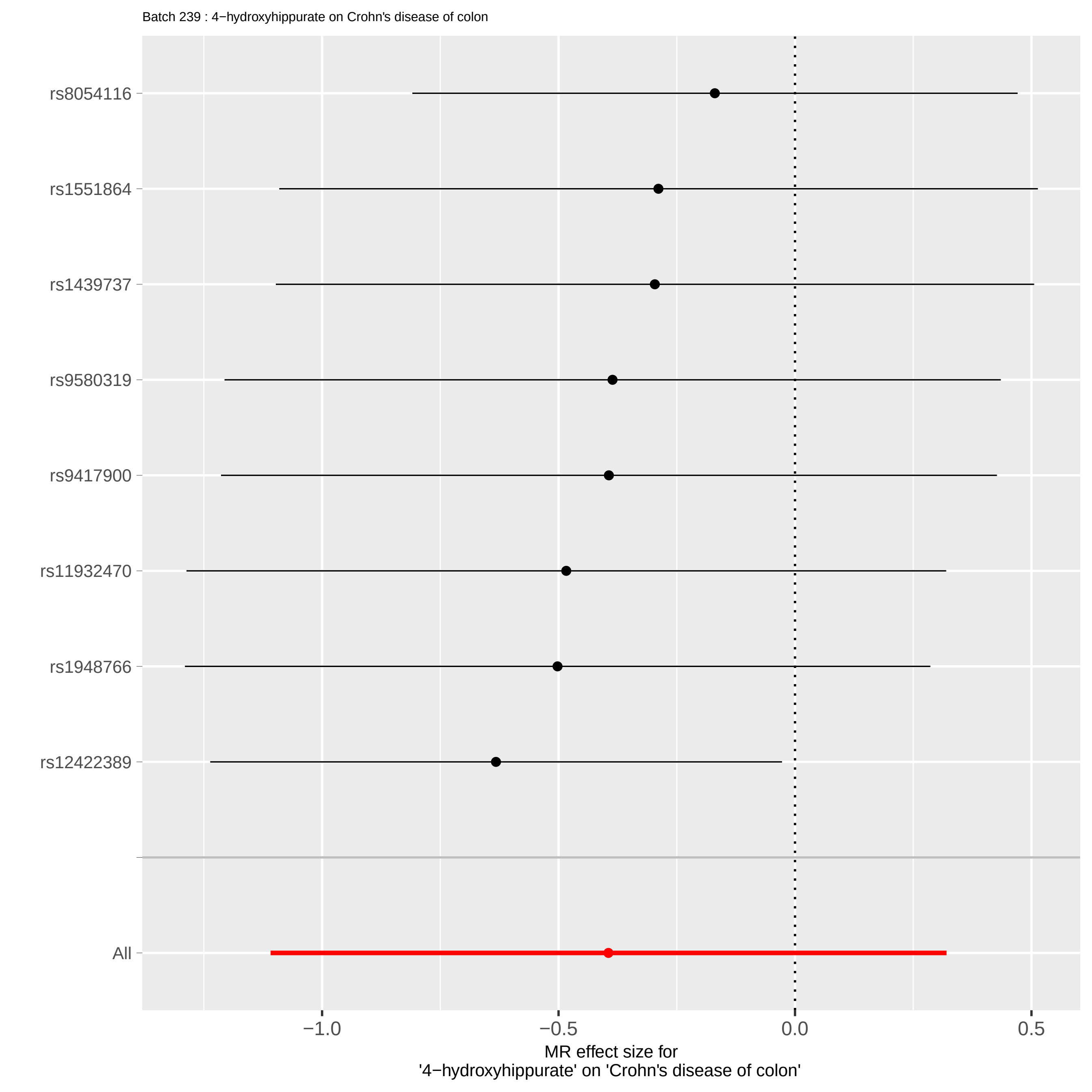

Batch 239 : 4−hydroxyhippurate on Crohn's disease of colon
rs8054116
rs1551864
rs1439737
rs9580319
rs9417900
rs11932470
rs1948766
rs12422389
All
−1.0
−0.5
0.0
0.5
MR effect size for
'4−hydroxyhippurate' on 'Crohn's disease of colon'

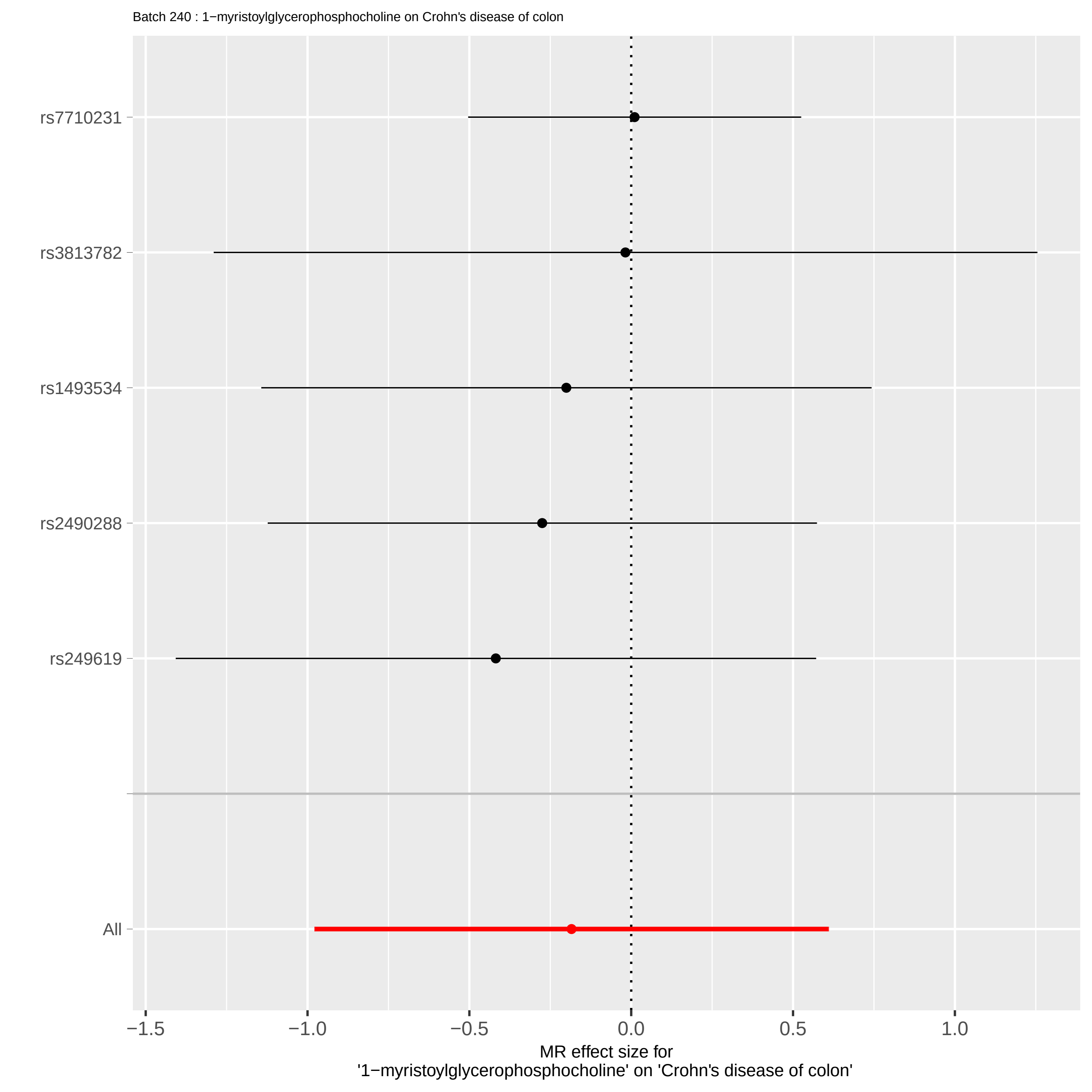

Batch 240 : 1−myristoylglycerophosphocholine on Crohn's disease of colon
rs7710231
rs3813782
rs1493534
rs2490288
rs249619
All
−1.5
−1.0
−0.5
0.0
0.5
1.0
MR effect size for
'1−myristoylglycerophosphocholine' on 'Crohn's disease of colon'

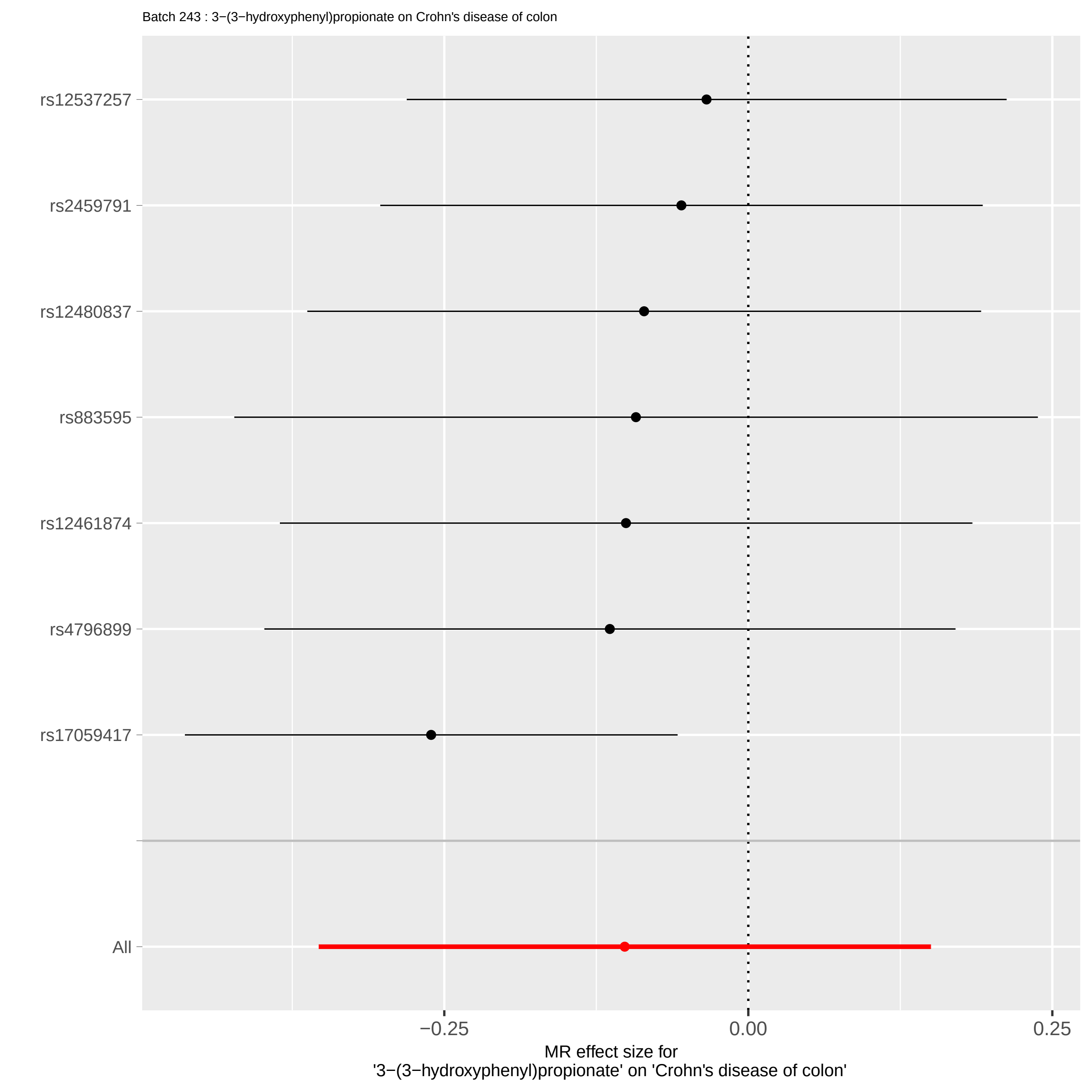

Batch 243 : 3−(3−hydroxyphenyl)propionate on Crohn's disease of colon
rs12537257
rs2459791
rs12480837
rs883595
rs12461874
rs4796899
rs17059417
All
−0.25
0.00
0.25
MR effect size for
'3−(3−hydroxyphenyl)propionate' on 'Crohn's disease of colon'

Batch 248 : Threitol on Crohn's disease of colon

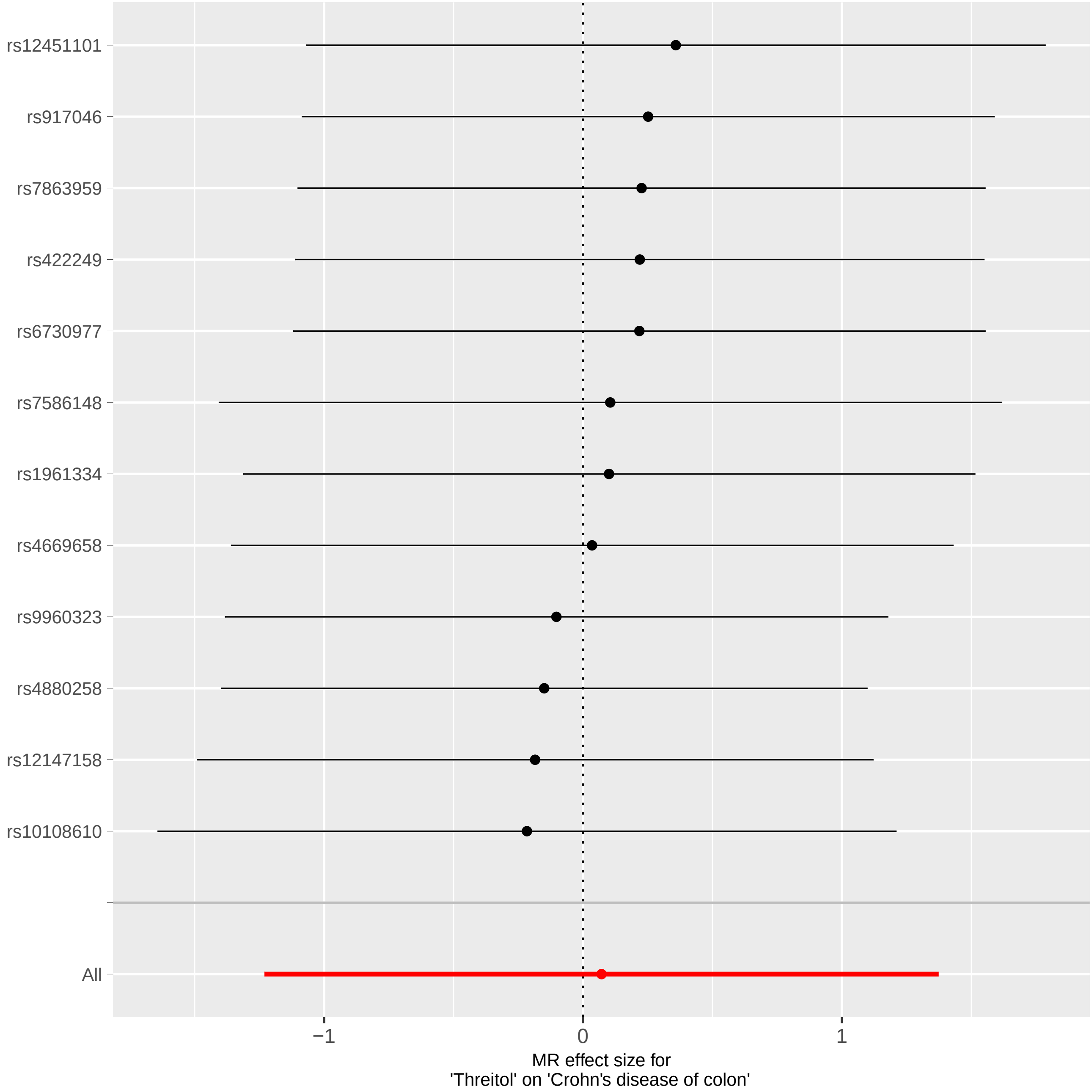

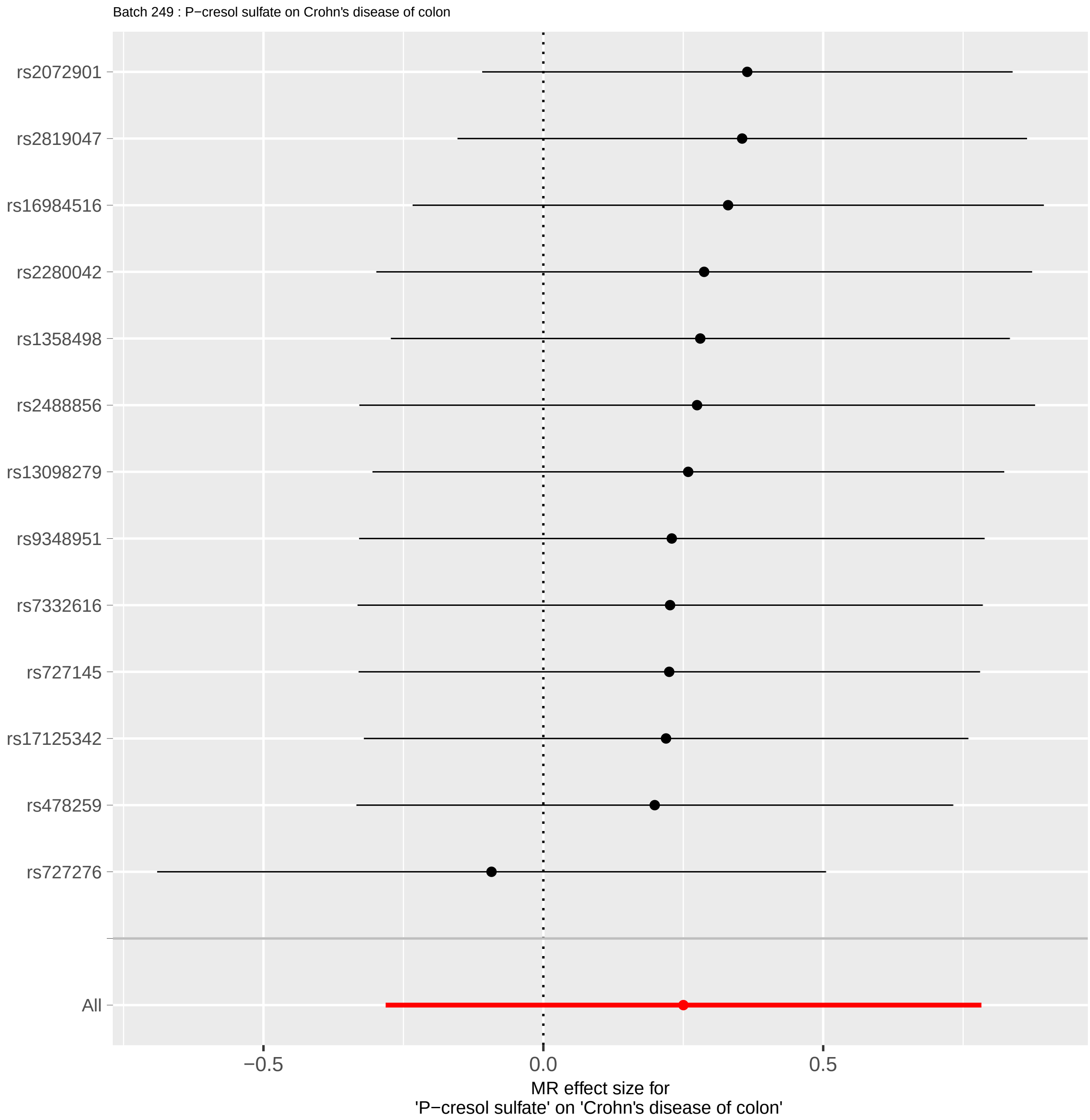

Batch 249 : P−cresol sulfate on Crohn's disease of colon
rs2072901
rs2819047
rs16984516
rs2280042
rs1358498
rs2488856
rs13098279
rs9348951
rs7332616
rs727145
rs17125342
rs478259
rs727276
All
−0.5
0.0
0.5
MR effect size for
'P−cresol sulfate' on 'Crohn's disease of colon'

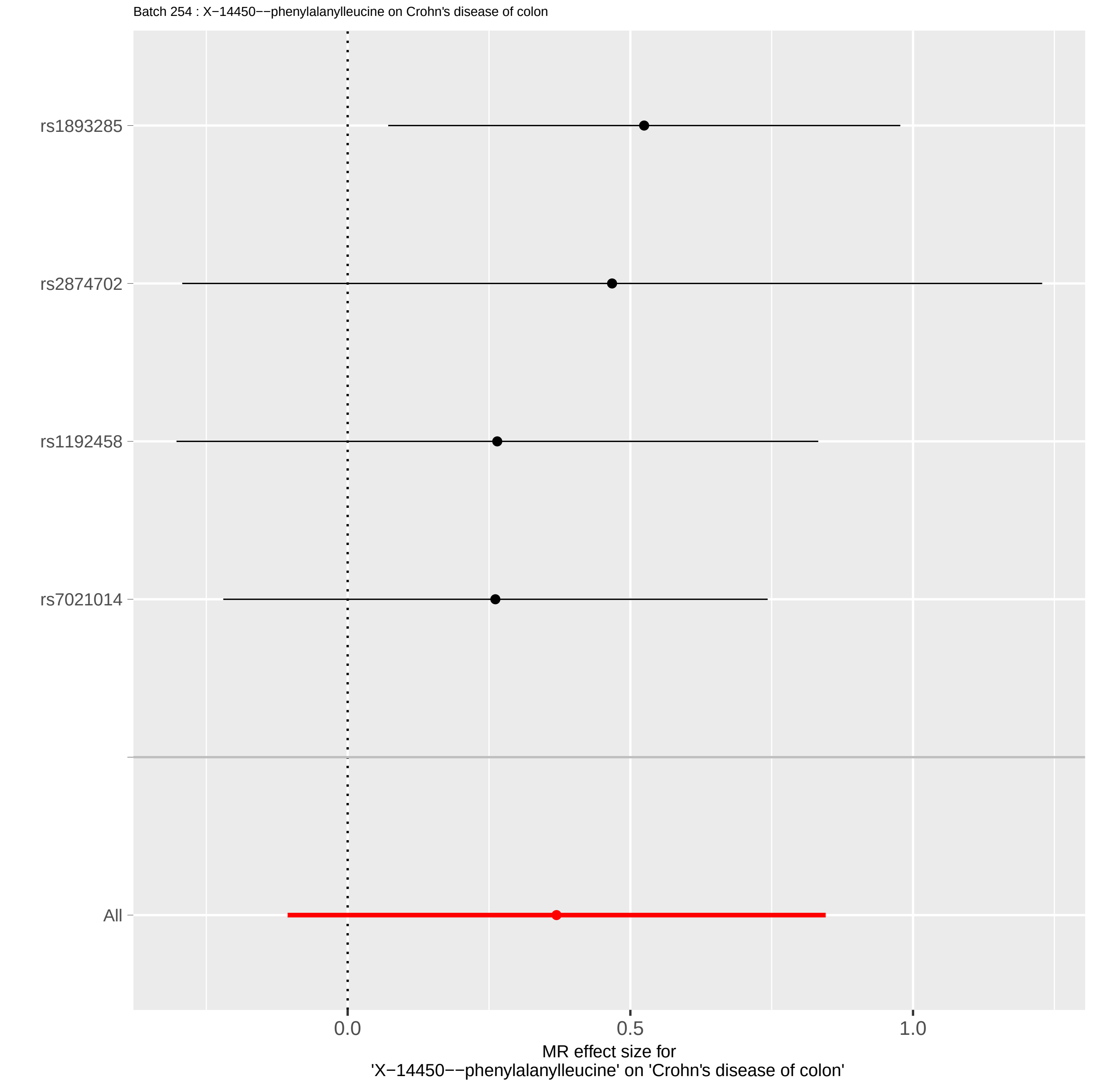

Batch 254 : X−14450−−phenylalanylleucine on Crohn's disease of colon
rs1893285
rs2874702
rs1192458
rs7021014
All
0.0
0.5
1.0
MR effect size for
'X−14450−−phenylalanylleucine' on 'Crohn's disease of colon'

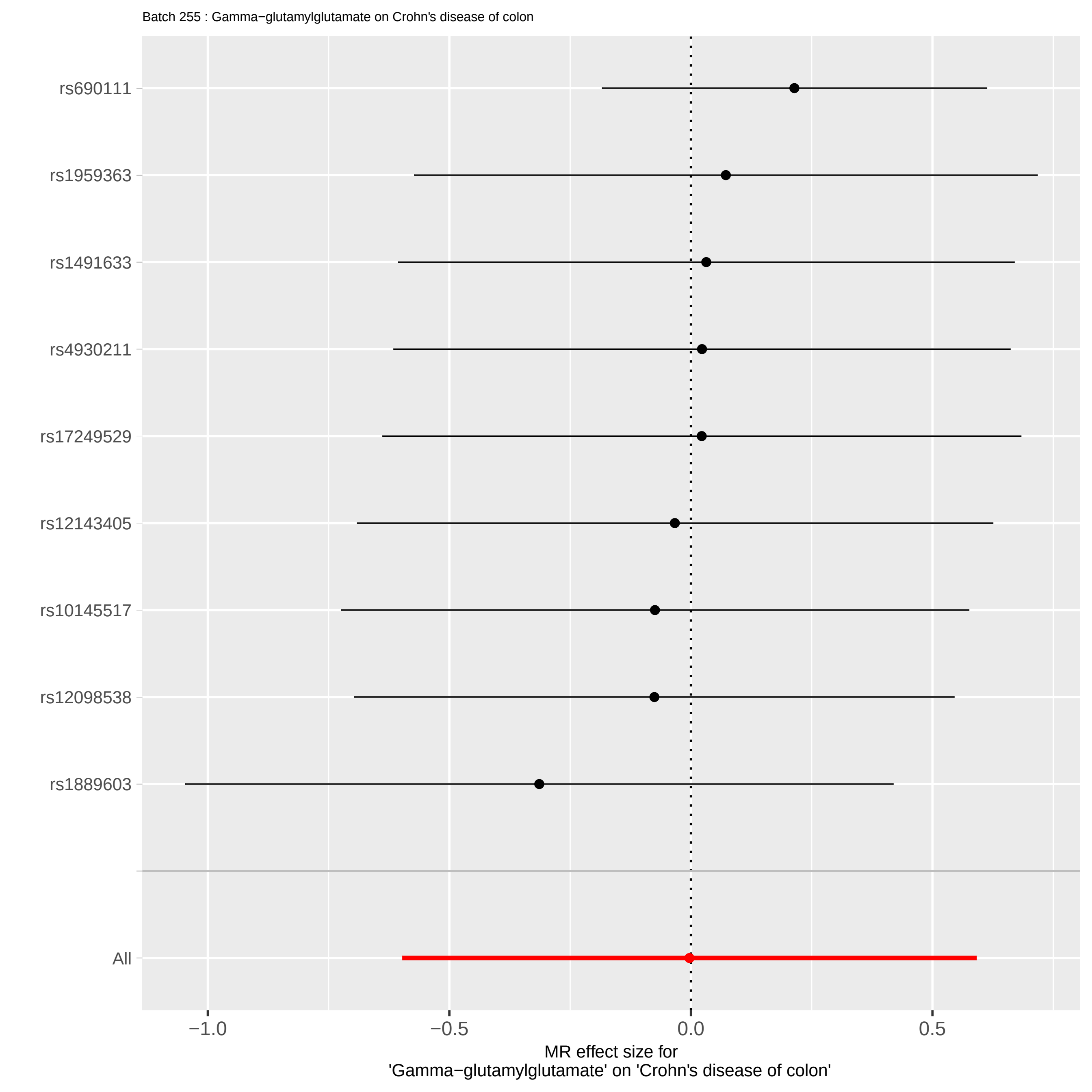
Batch 255 : Gamma−glutamylglutamate on Crohn's disease of colon
rs690111
rs1959363
rs1491633
rs4930211
rs17249529
rs12143405
rs10145517
rs12098538
rs1889603
All
−1.0
−0.5
0.0
0.5
MR effect size for
'Gamma−glutamylglutamate' on 'Crohn's disease of colon'

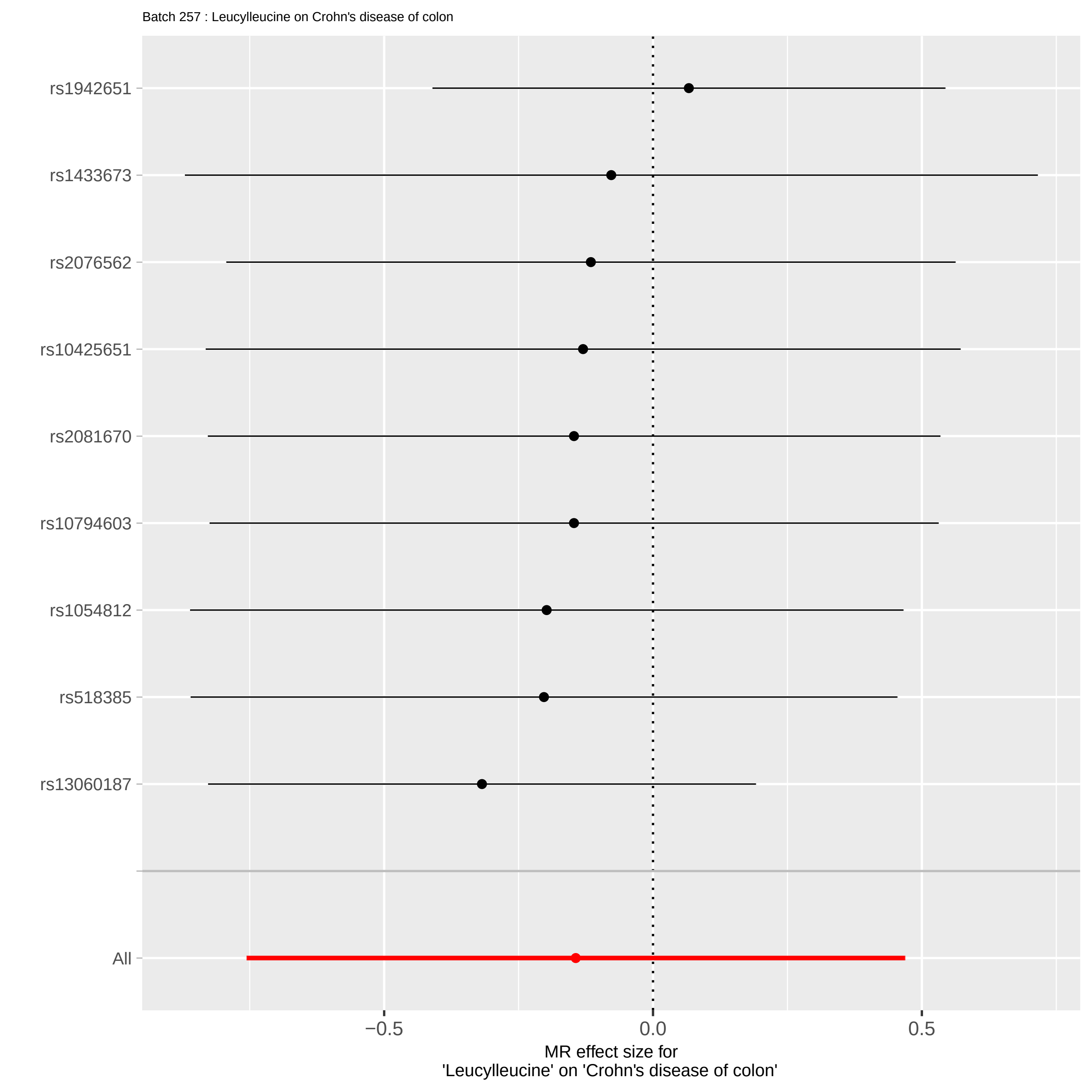

Batch 257 : Leucylleucine on Crohn's disease of colon
rs1942651
rs1433673
rs2076562
rs10425651
rs2081670
rs10794603
rs1054812
rs518385
rs13060187
All
−0.5
0.0
0.5
MR effect size for
'Leucylleucine' on 'Crohn's disease of colon'

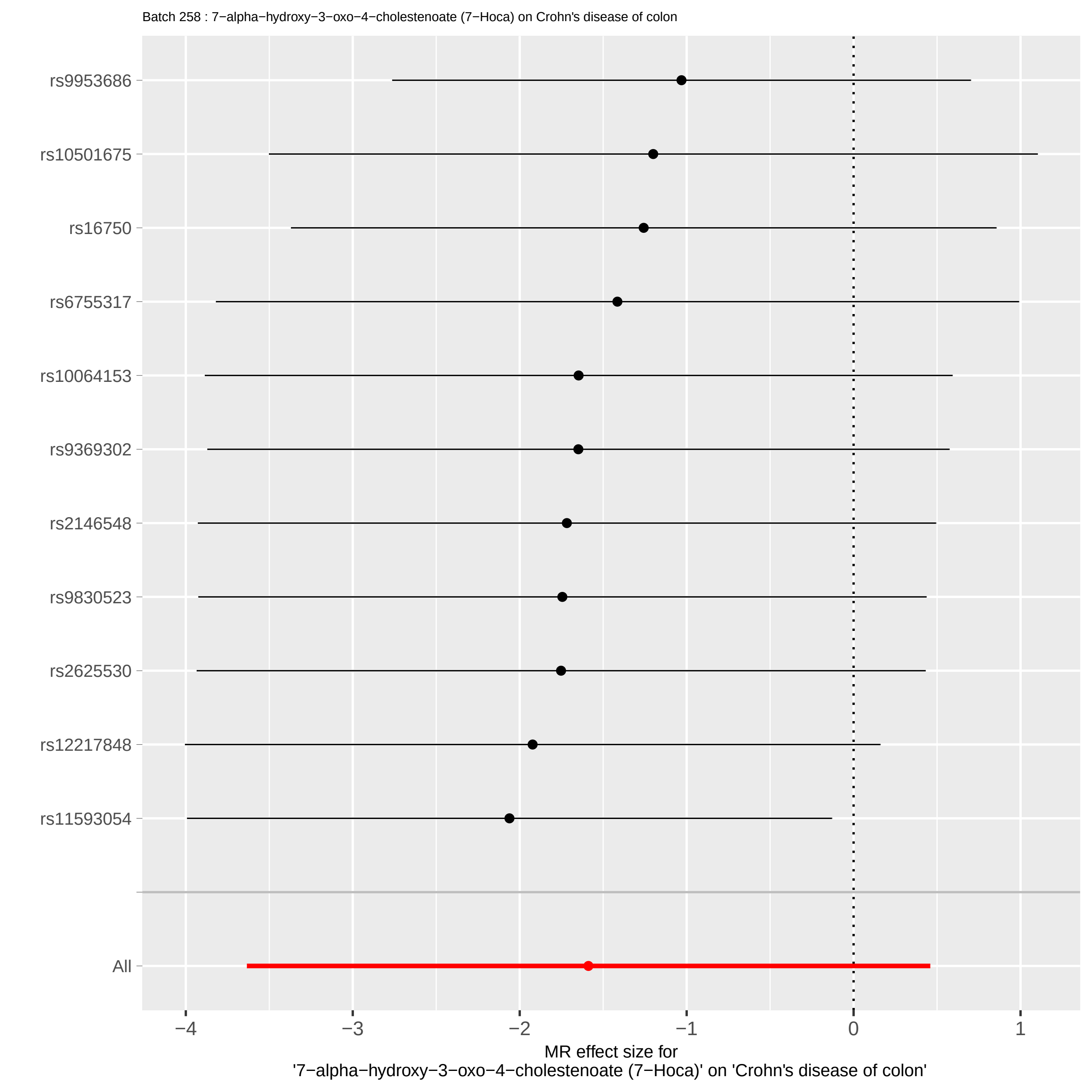

Batch 258 : 7−alpha−hydroxy−3−oxo−4−cholestenoate (7−Hoca) on Crohn's disease of colon
rs9953686
rs10501675
rs16750
rs6755317
rs10064153
rs9369302
rs2146548
rs9830523
rs2625530
rs12217848
rs11593054
All
−4
−3
−2
−1
0
1
MR effect size for
'7−alpha−hydroxy−3−oxo−4−cholestenoate (7−Hoca)' on 'Crohn's disease of colon'

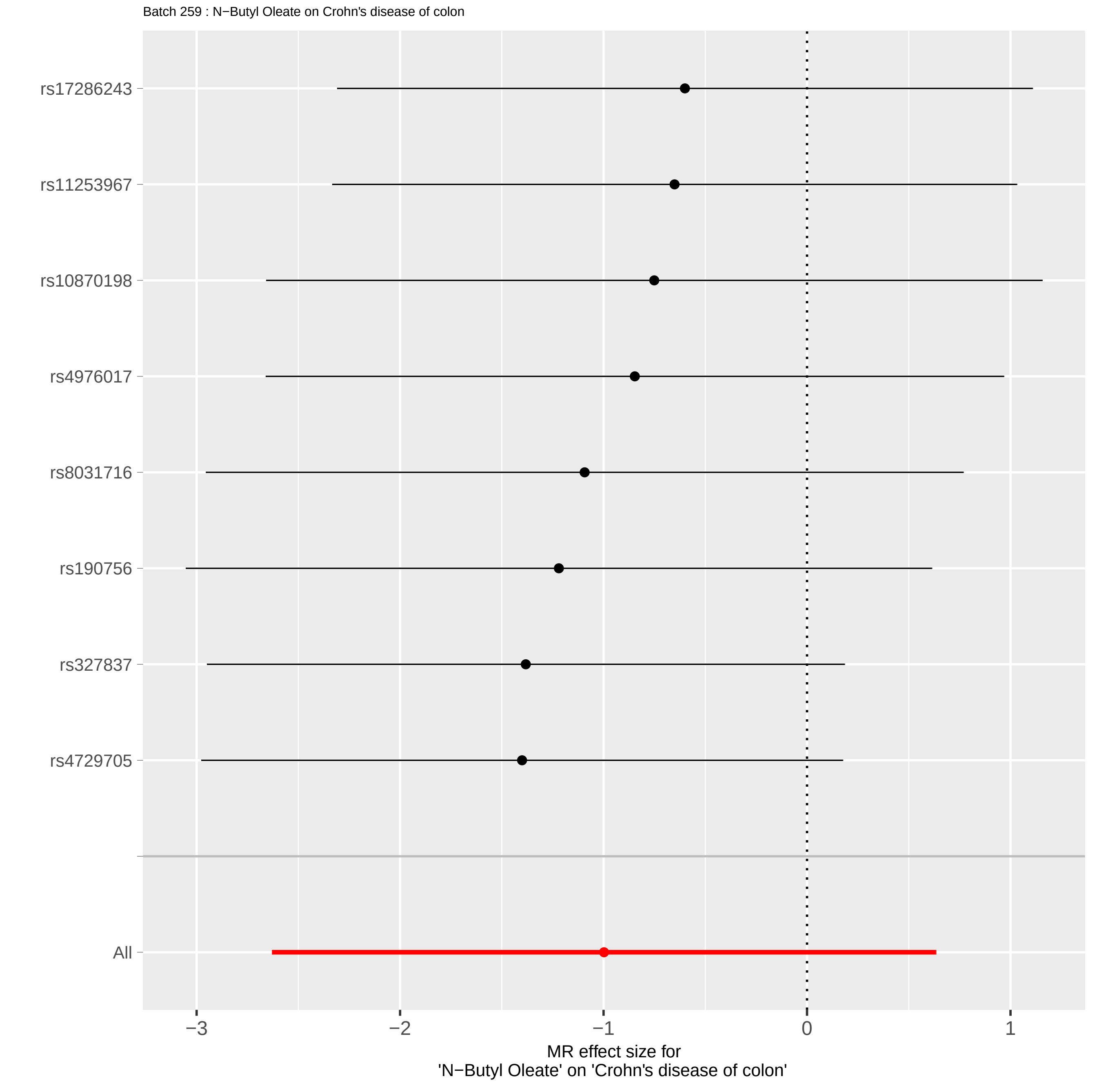

Batch 259 : N−Butyl Oleate on Crohn's disease of colon
rs17286243
rs11253967
rs10870198
rs4976017
rs8031716
rs190756
rs327837
rs4729705
All
−3
−2
−1
0
1
MR effect size for
'N−Butyl Oleate' on 'Crohn's disease of colon'

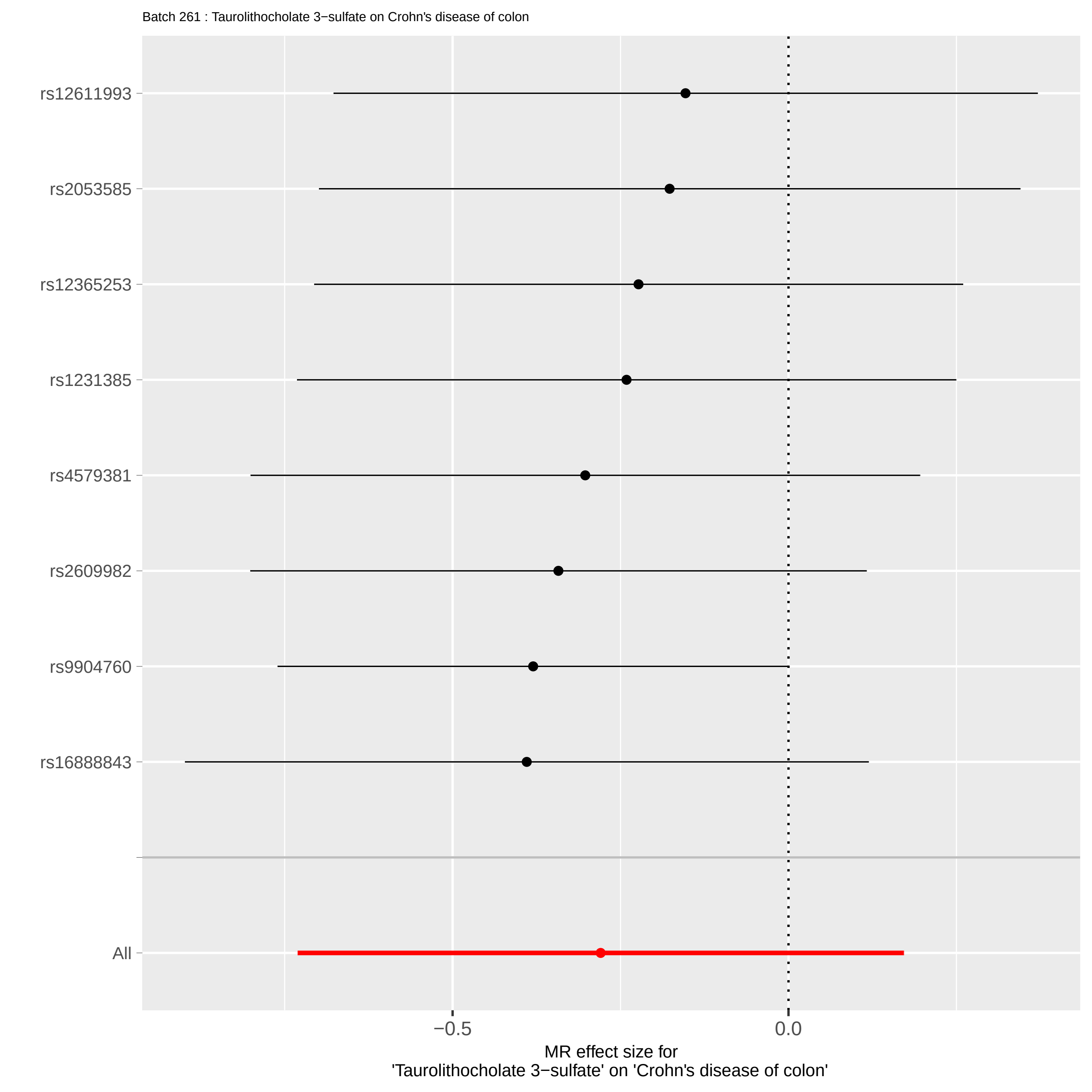

Batch 261 : Taurolithocholate 3−sulfate on Crohn's disease of colon
rs12611993
rs2053585
rs12365253
rs1231385
rs4579381
rs2609982
rs9904760
rs16888843
All
−0.5
0.0
MR effect size for
'Taurolithocholate 3−sulfate' on 'Crohn's disease of colon'

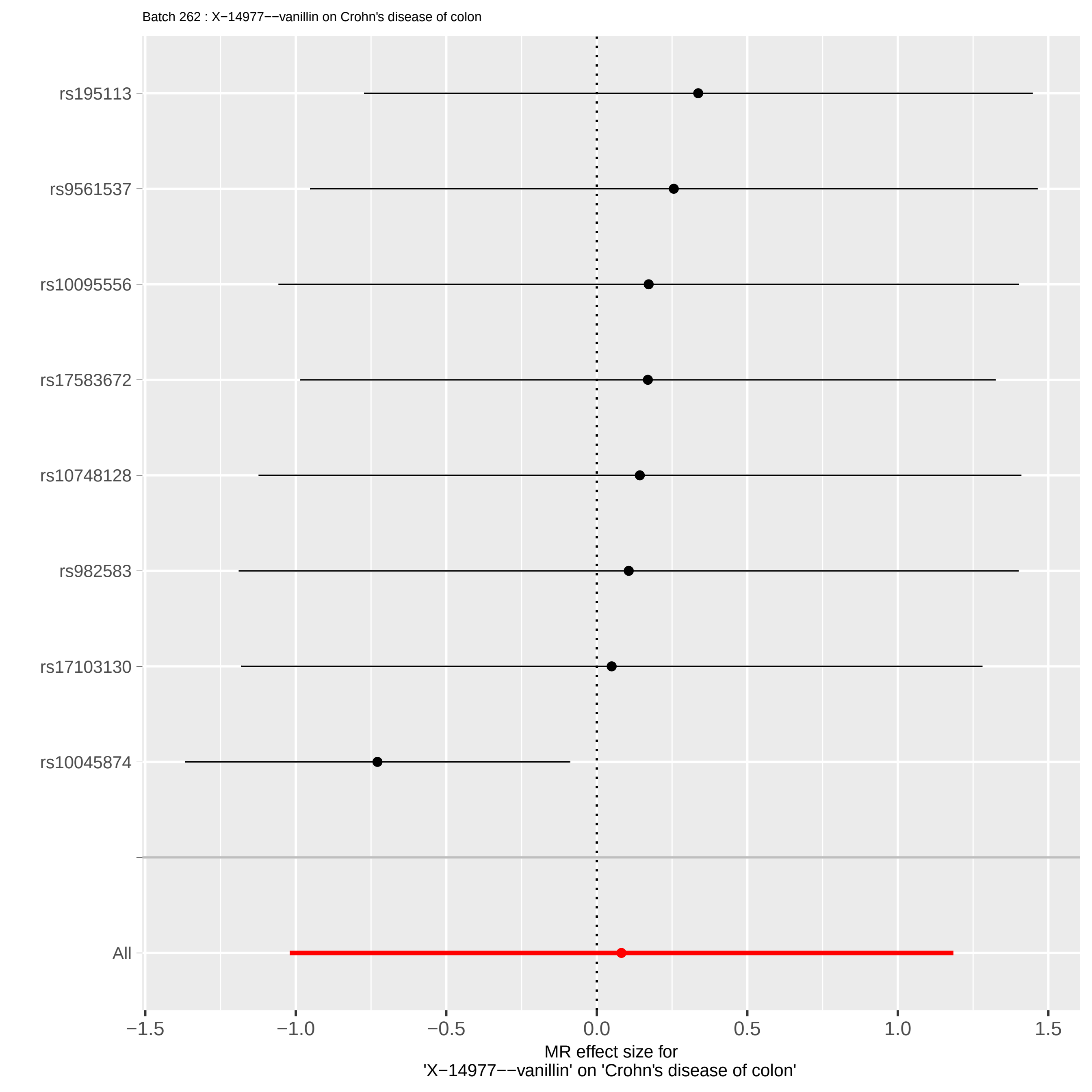

Batch 262 : X−14977−−vanillin on Crohn's disease of colon
rs195113
rs9561537
rs10095556
rs17583672
rs10748128
rs982583
rs17103130
rs10045874
All
−1.5
−1.0
−0.5
0.0
0.5
1.0
1.5
MR effect size for
'X−14977−−vanillin' on 'Crohn's disease of colon'

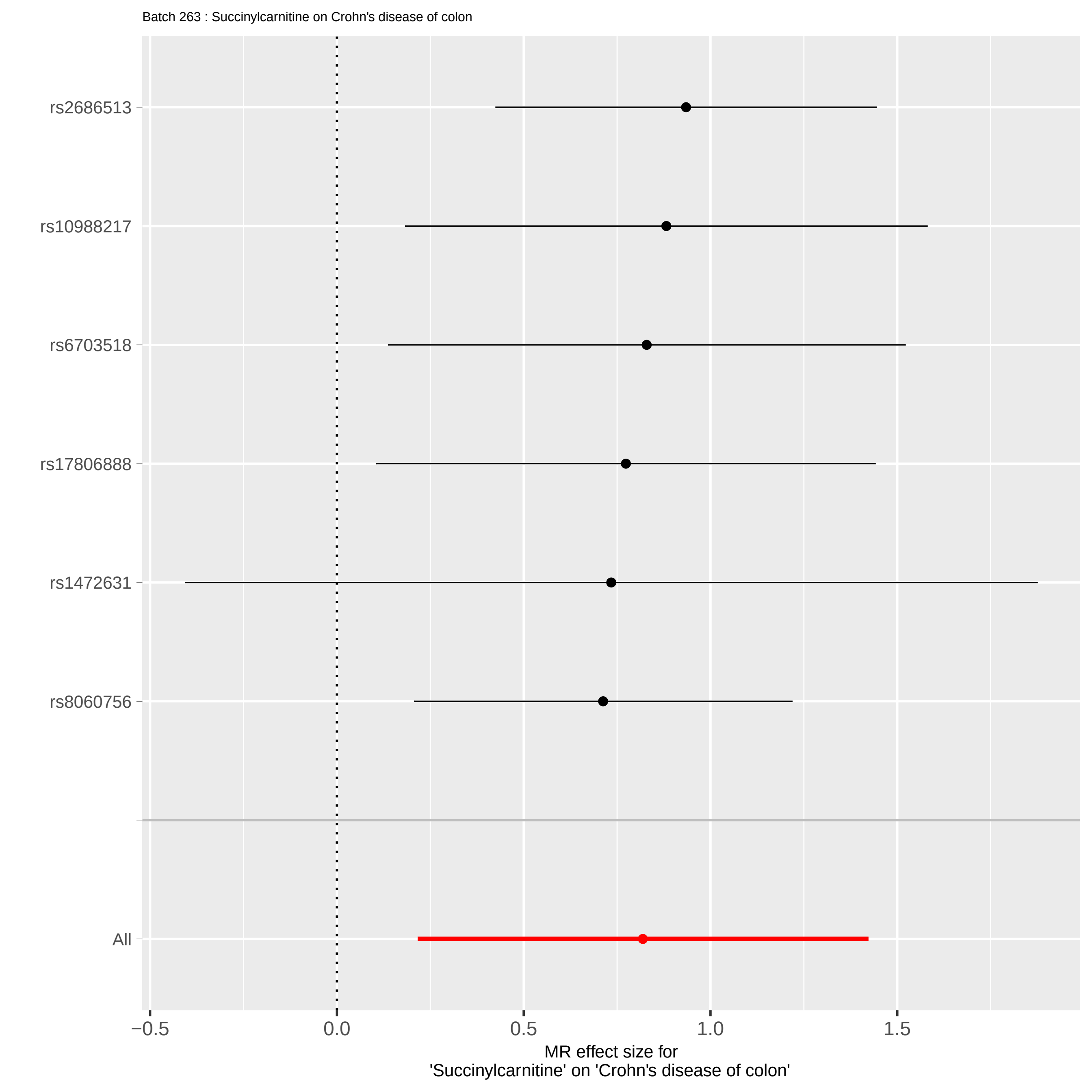
Batch 263 : Succinylcarnitine on Crohn's disease of colon
rs2686513
rs10988217
rs6703518
rs17806888
rs1472631
rs8060756
All
−0.5
0.0
0.5
1.0
1.5
MR effect size for
'Succinylcarnitine' on 'Crohn's disease of colon'

Batch 265 : Cyclo(leu−pro) on Crohn's disease of colon

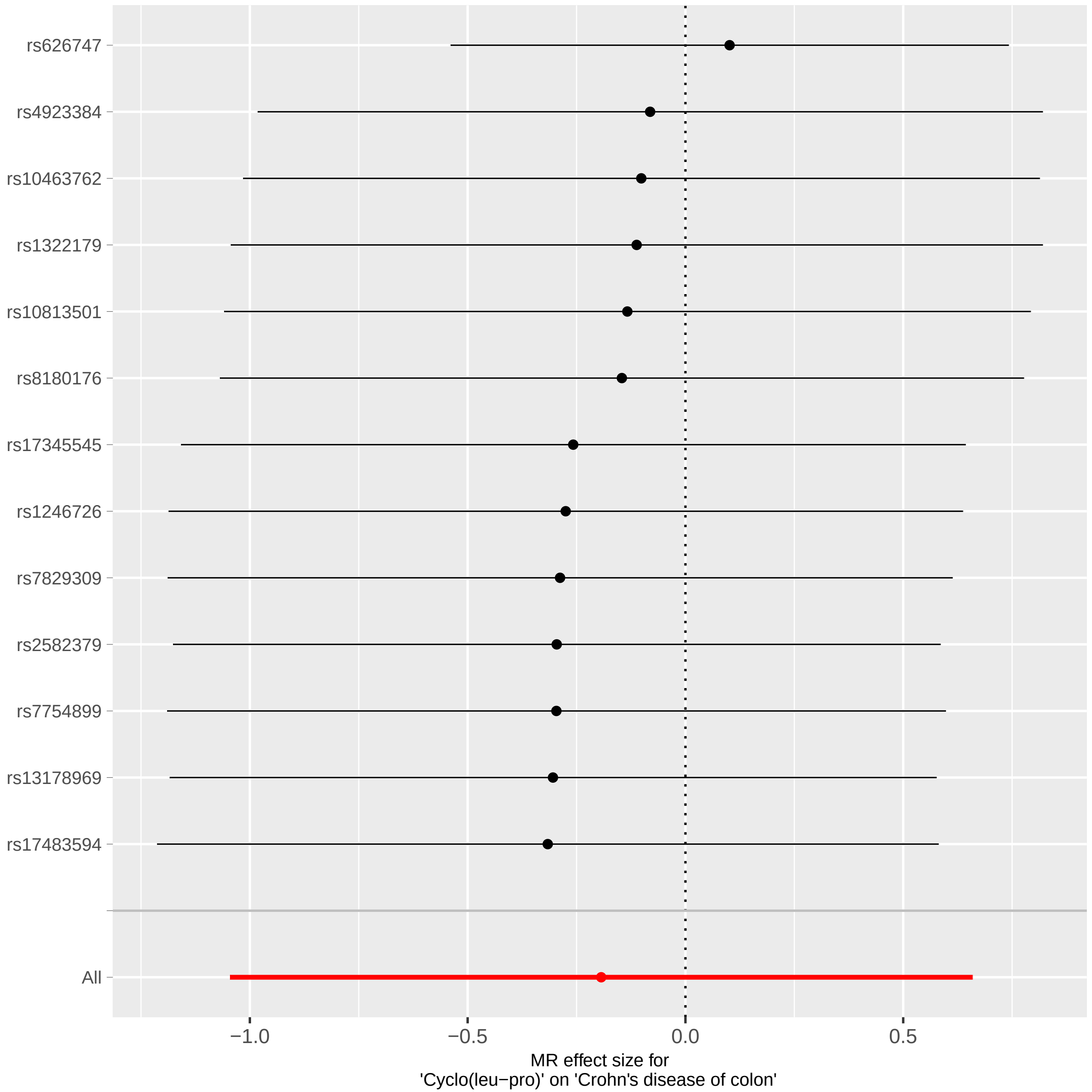

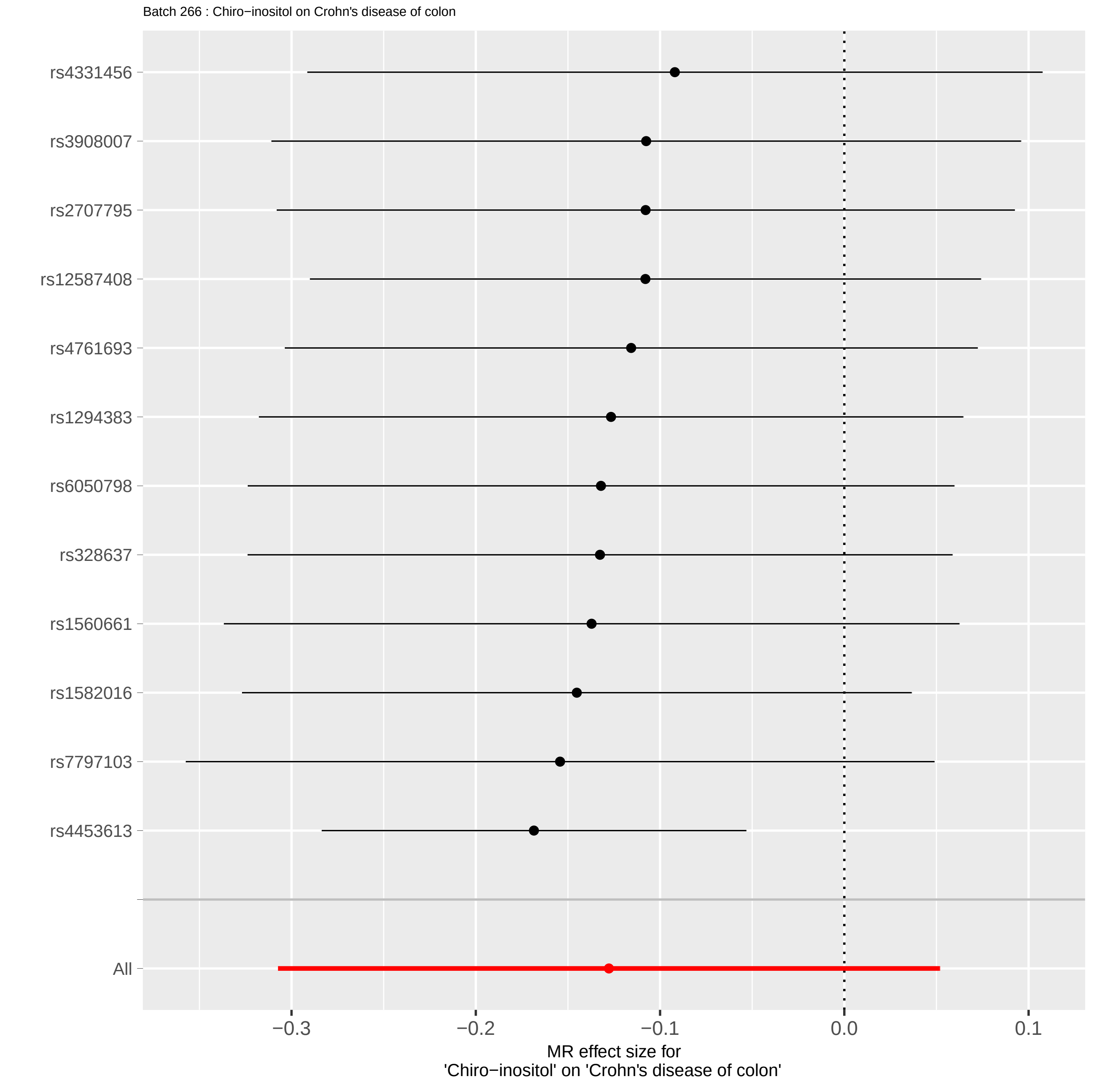

Batch 266 : Chiro−inositol on Crohn's disease of colon
rs4331456
rs3908007
rs2707795
rs12587408
rs4761693
rs1294383
rs6050798
rs328637
rs1560661
rs1582016
rs7797103
rs4453613
All
−0.3
−0.2
−0.1
0.0
0.1
MR effect size for
'Chiro−inositol' on 'Crohn's disease of colon'

Batch 270 : 2−hydroxyglutarate on Crohn's disease of colon

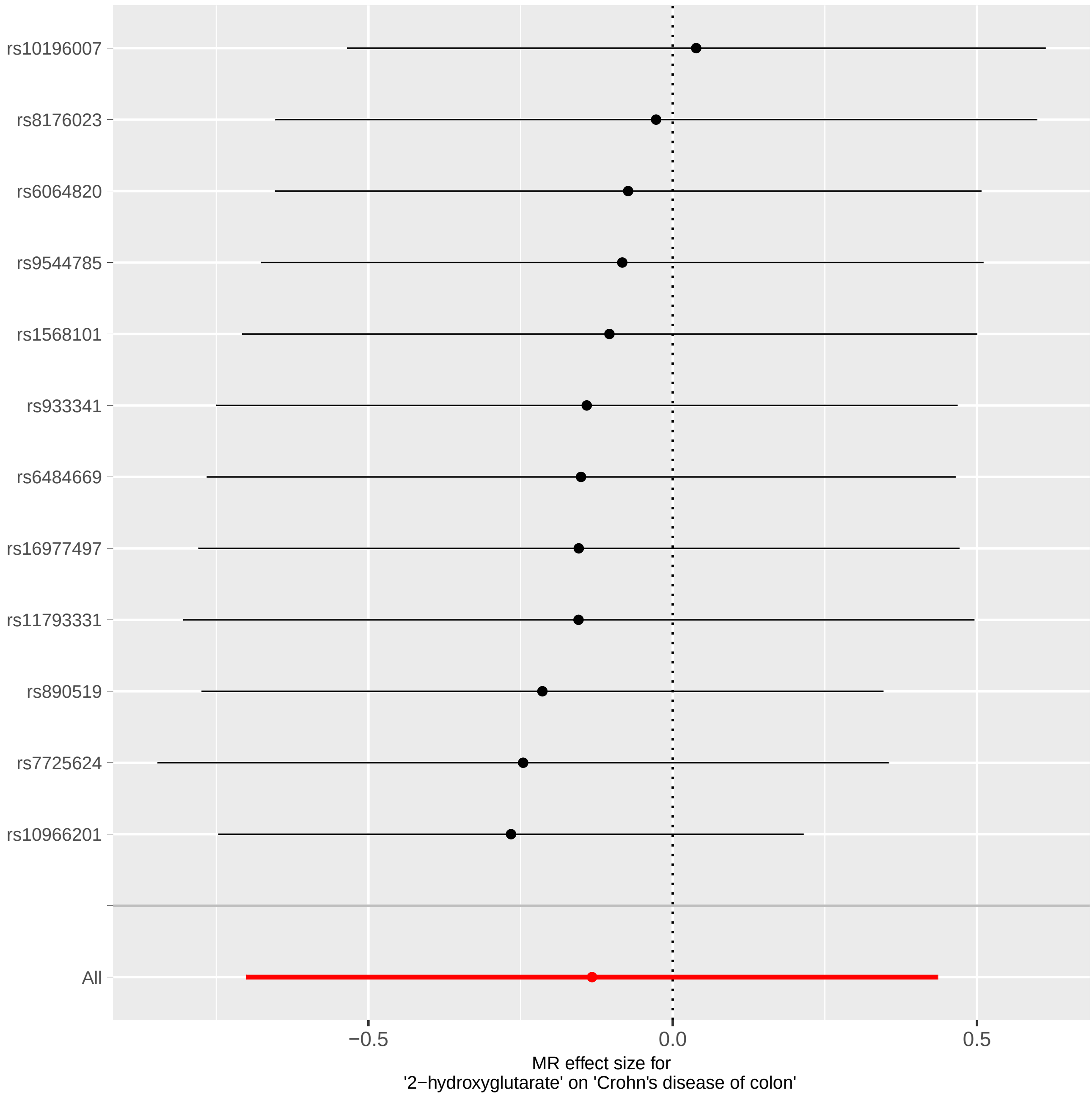

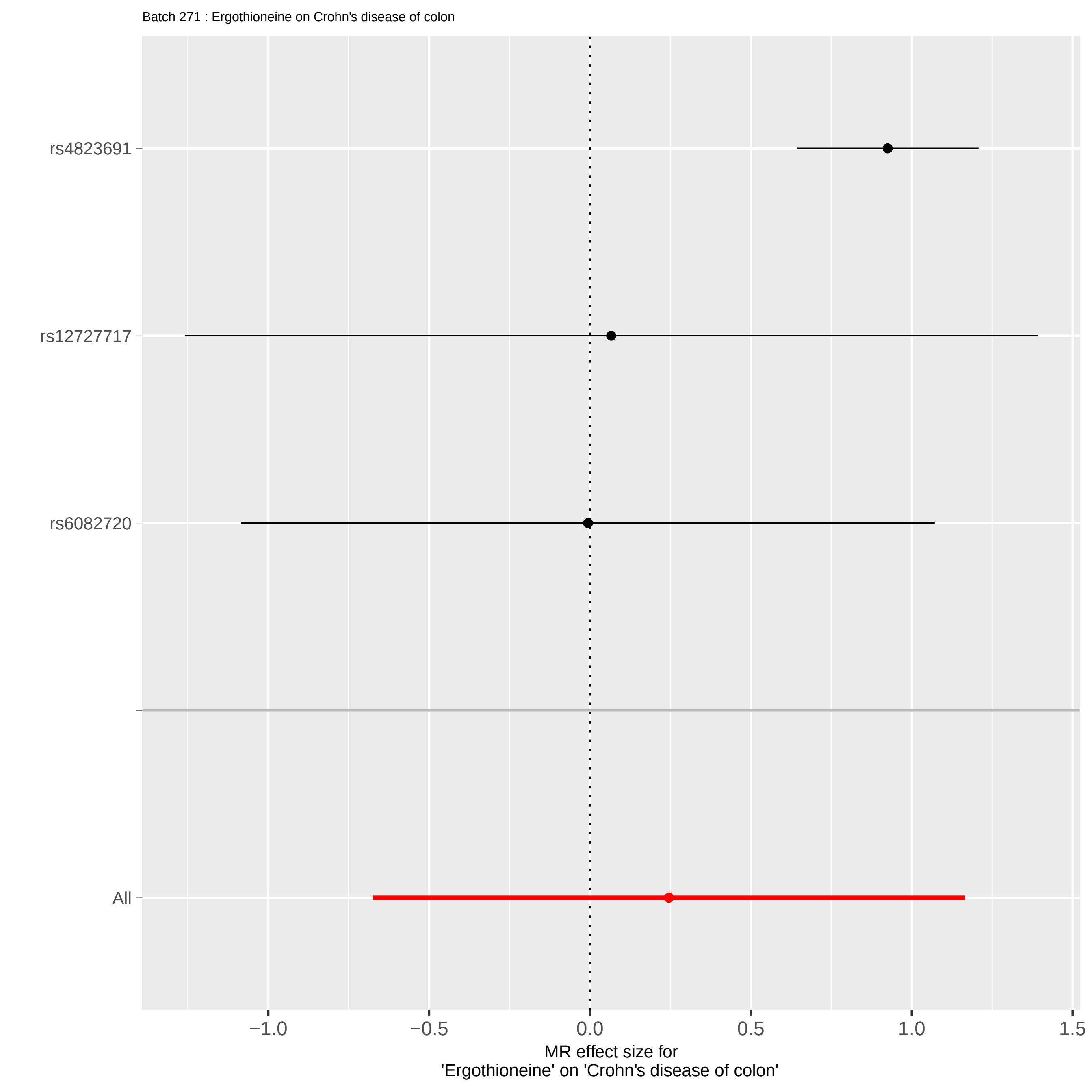
Batch 271 : Ergothioneine on Crohn's disease of colon
rs4823691
rs12727717
rs6082720
All
−1.0
−0.5
0.0
0.5
1.0
1.5
MR effect size for
'Ergothioneine' on 'Crohn's disease of colon'

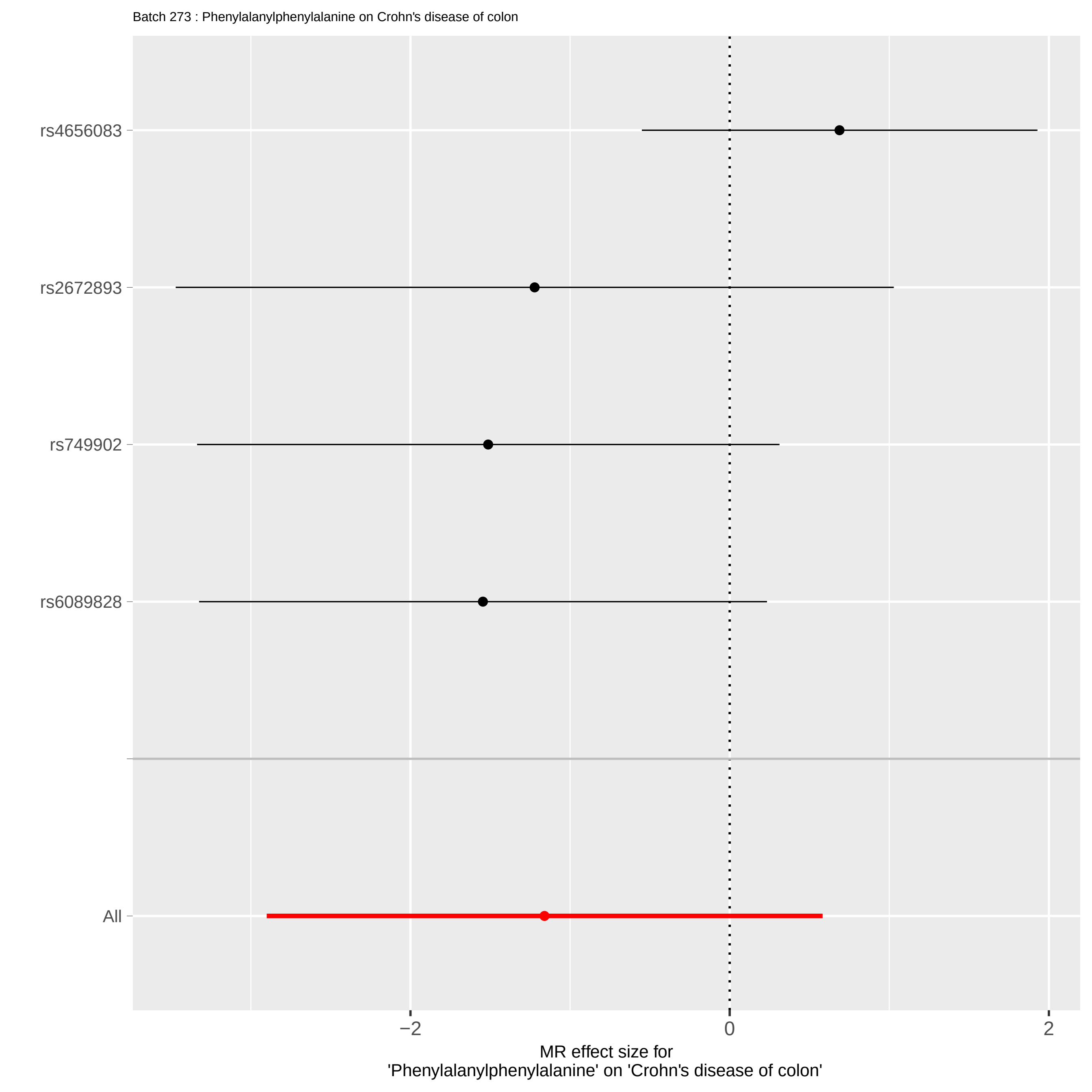
Batch 273 : Phenylalanylphenylalanine on Crohn's disease of colon
rs4656083
rs2672893
rs749902
rs6089828
All
−2
0
2
MR effect size for
'Phenylalanylphenylalanine' on 'Crohn's disease of colon'

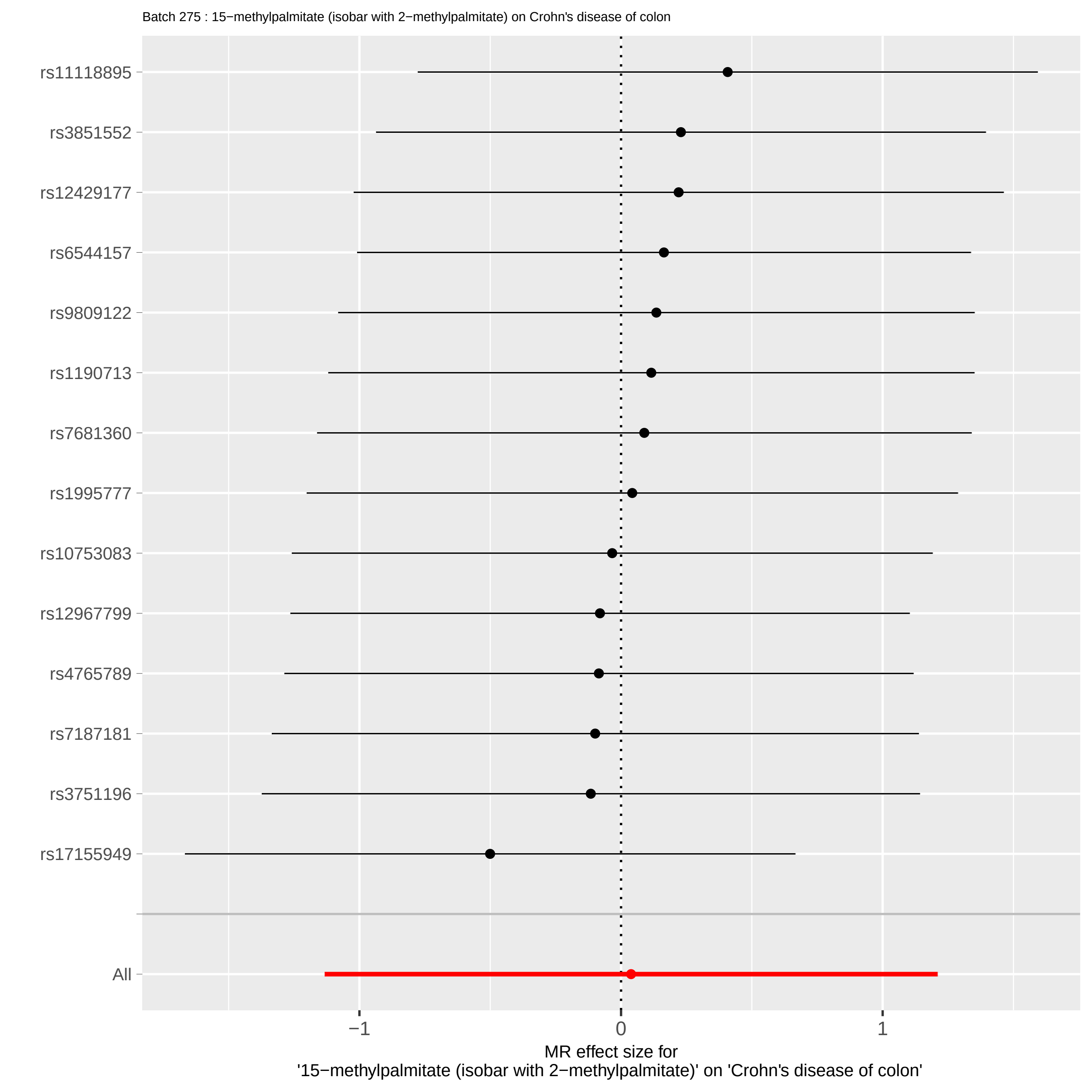
Batch 275 : 15−methylpalmitate (isobar with 2−methylpalmitate) on Crohn's disease of colon
rs11118895
rs3851552
rs12429177
rs6544157
rs9809122
rs1190713
rs7681360
rs1995777
rs10753083
rs12967799
rs4765789
rs7187181
rs3751196
rs17155949
All
−1
0
1
MR effect size for
'15−methylpalmitate (isobar with 2−methylpalmitate)' on 'Crohn's disease of colon'

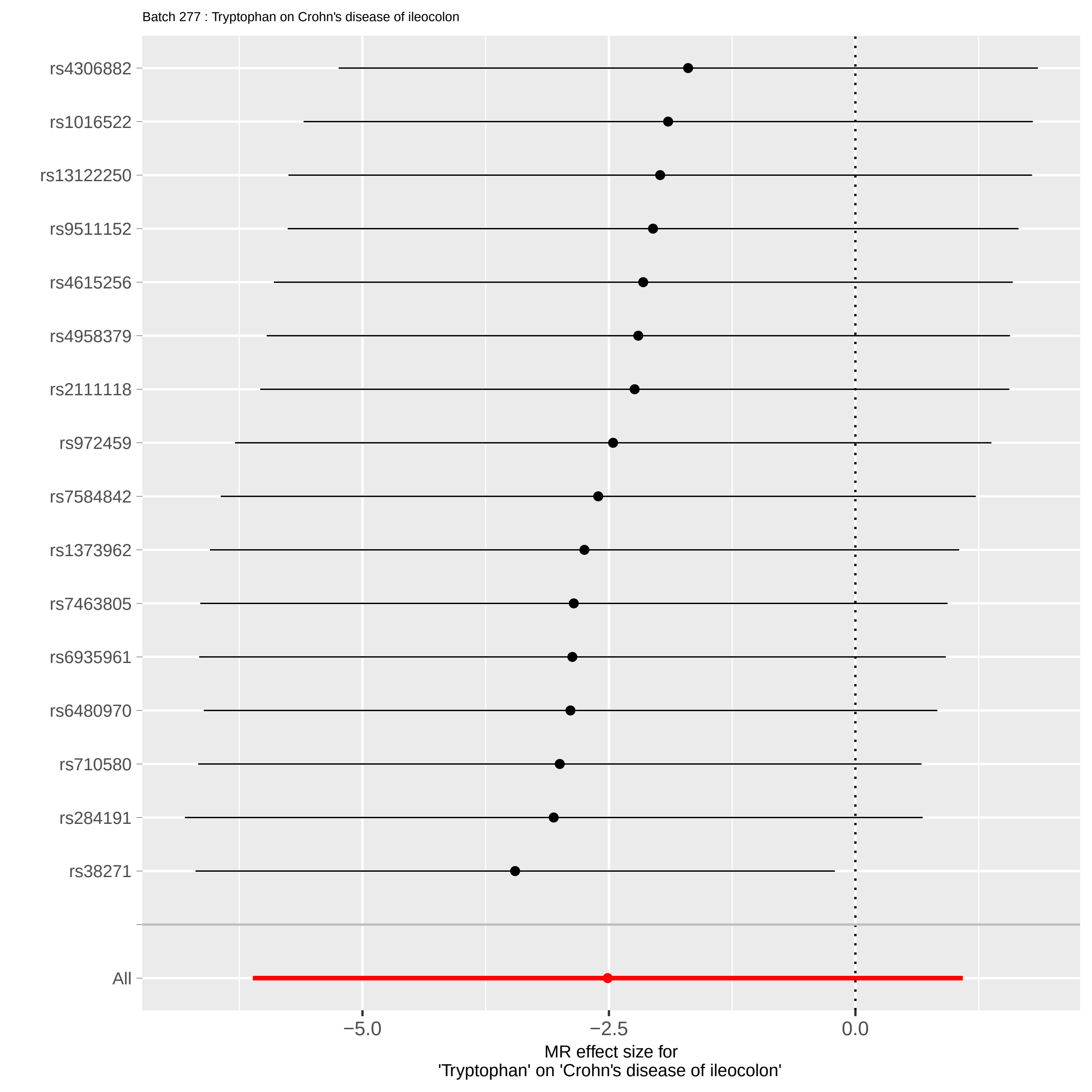

Batch 277 : Tryptophan on Crohn's disease of ileocolon
rs4306882
rs1016522
rs13122250
rs9511152
rs4615256
rs4958379
rs2111118
rs972459
rs7584842
rs1373962
rs7463805
rs6935961
rs6480970
rs710580
rs284191
rs38271
All
−5.0
−2.5
0.0
MR effect size for
'Tryptophan' on 'Crohn's disease of ileocolon'

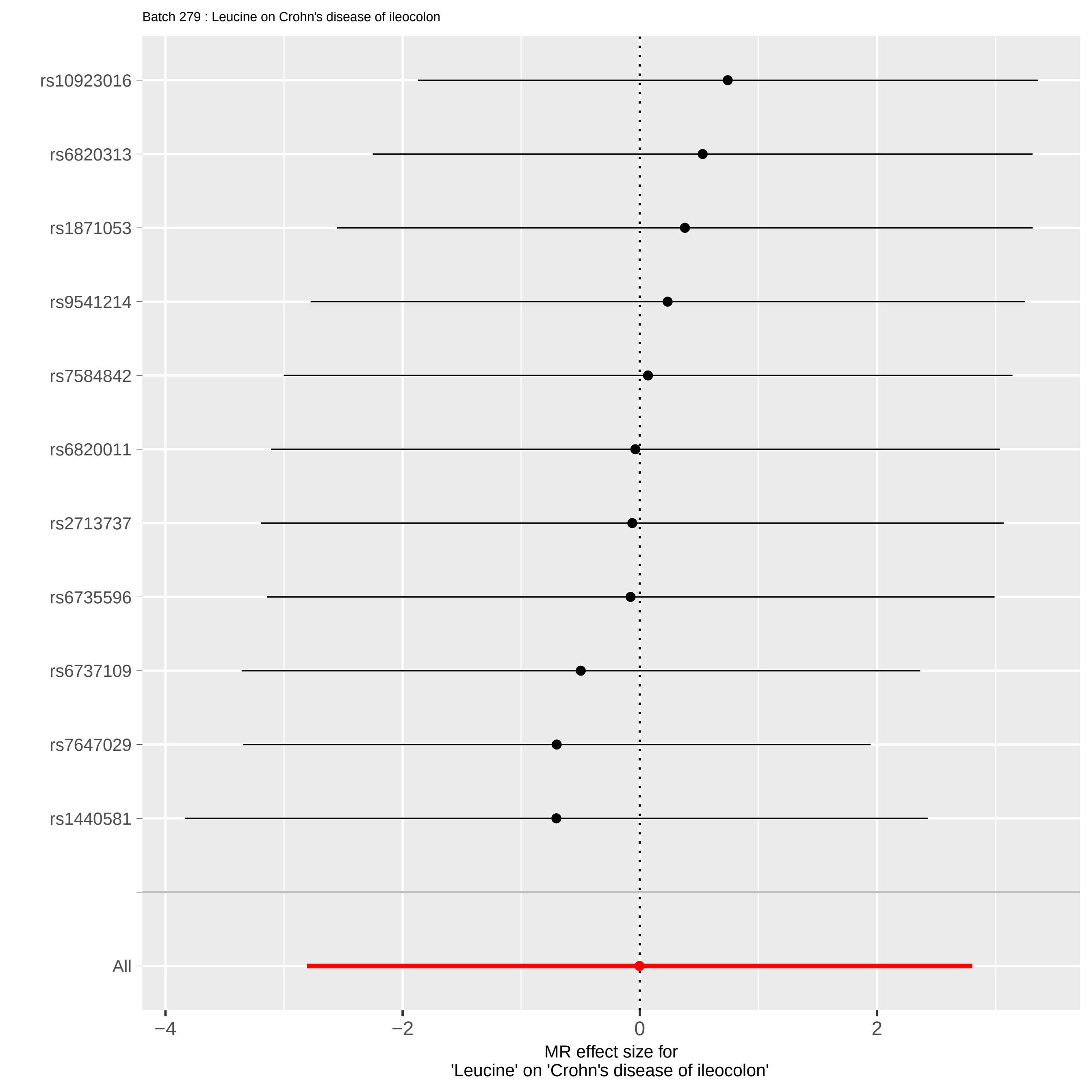
Batch 279 : Leucine on Crohn's disease of ileocolon
rs10923016
rs6820313
rs1871053
rs9541214
rs7584842
rs6820011
rs2713737
rs6735596
rs6737109
rs7647029
rs1440581
All
−4
−2
0
2
MR effect size for
'Leucine' on 'Crohn's disease of ileocolon'

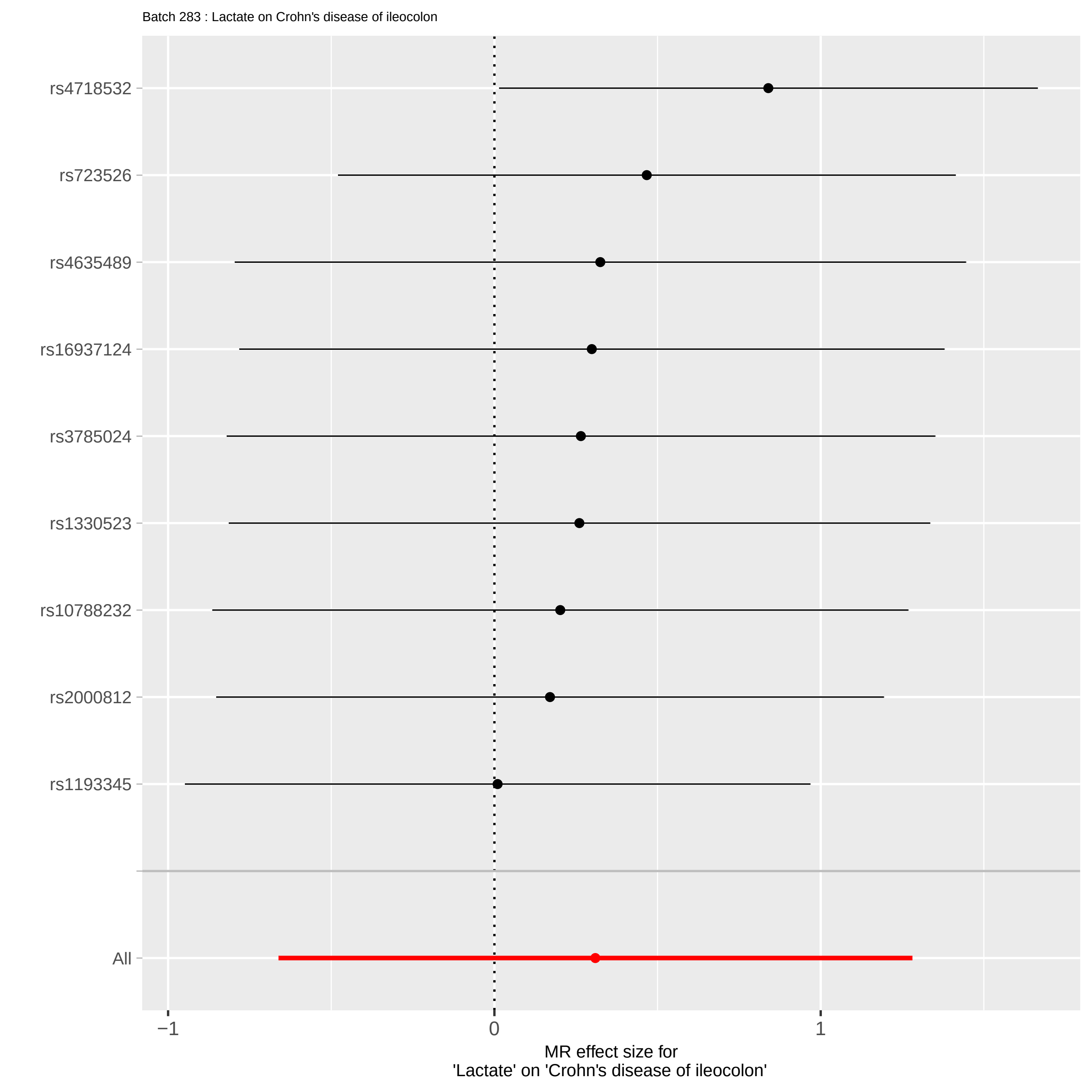
Batch 283 : Lactate on Crohn's disease of ileocolon
rs4718532
rs723526
rs4635489
rs16937124
rs3785024
rs1330523
rs10788232
rs2000812
rs1193345
All
−1
0
1
MR effect size for
'Lactate' on 'Crohn's disease of ileocolon'

Batch 284 : 3−hydroxybutyrate (BHBA) on Crohn's disease of ileocolon

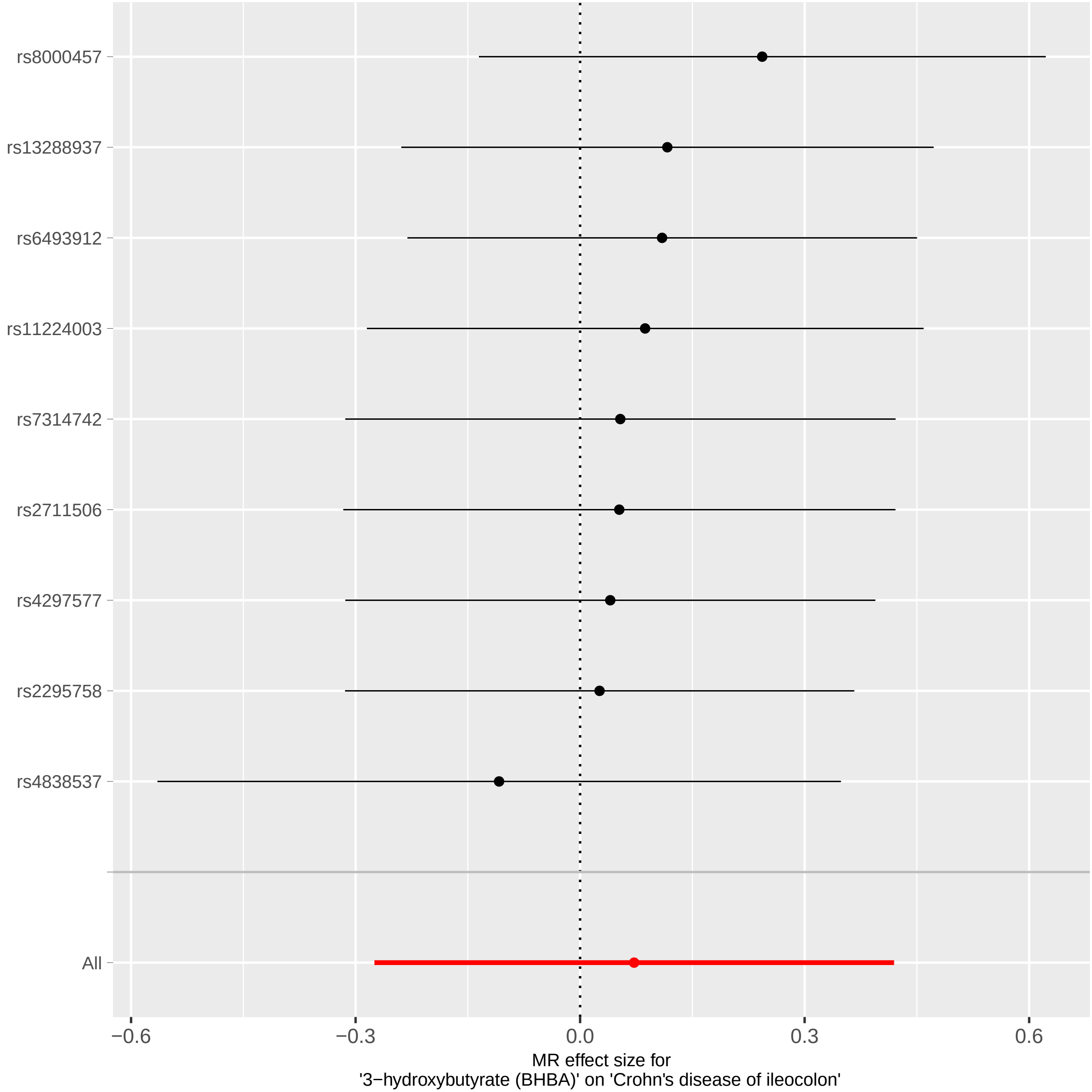

Batch 285 : Arabinose on Crohn's disease of ileocolon

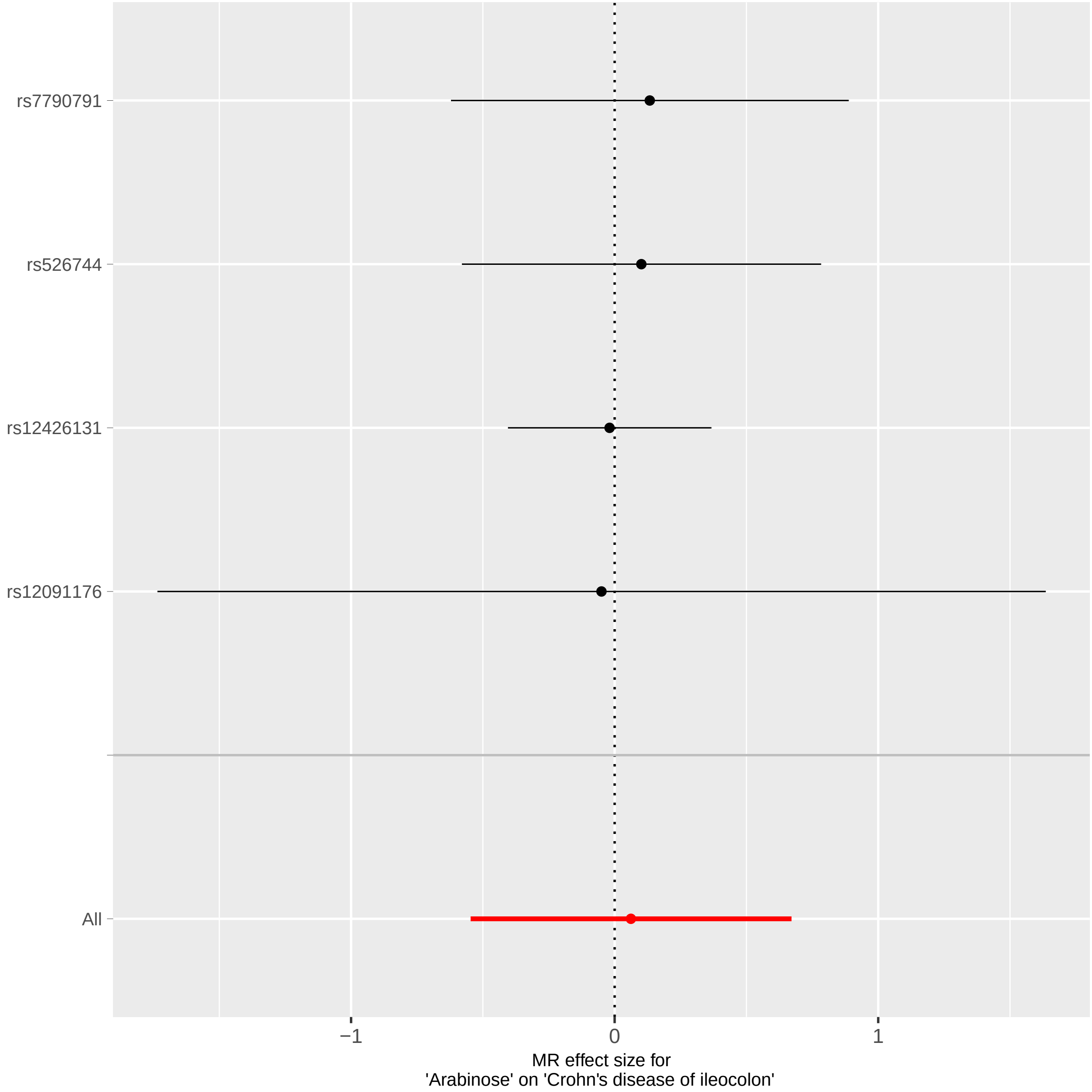

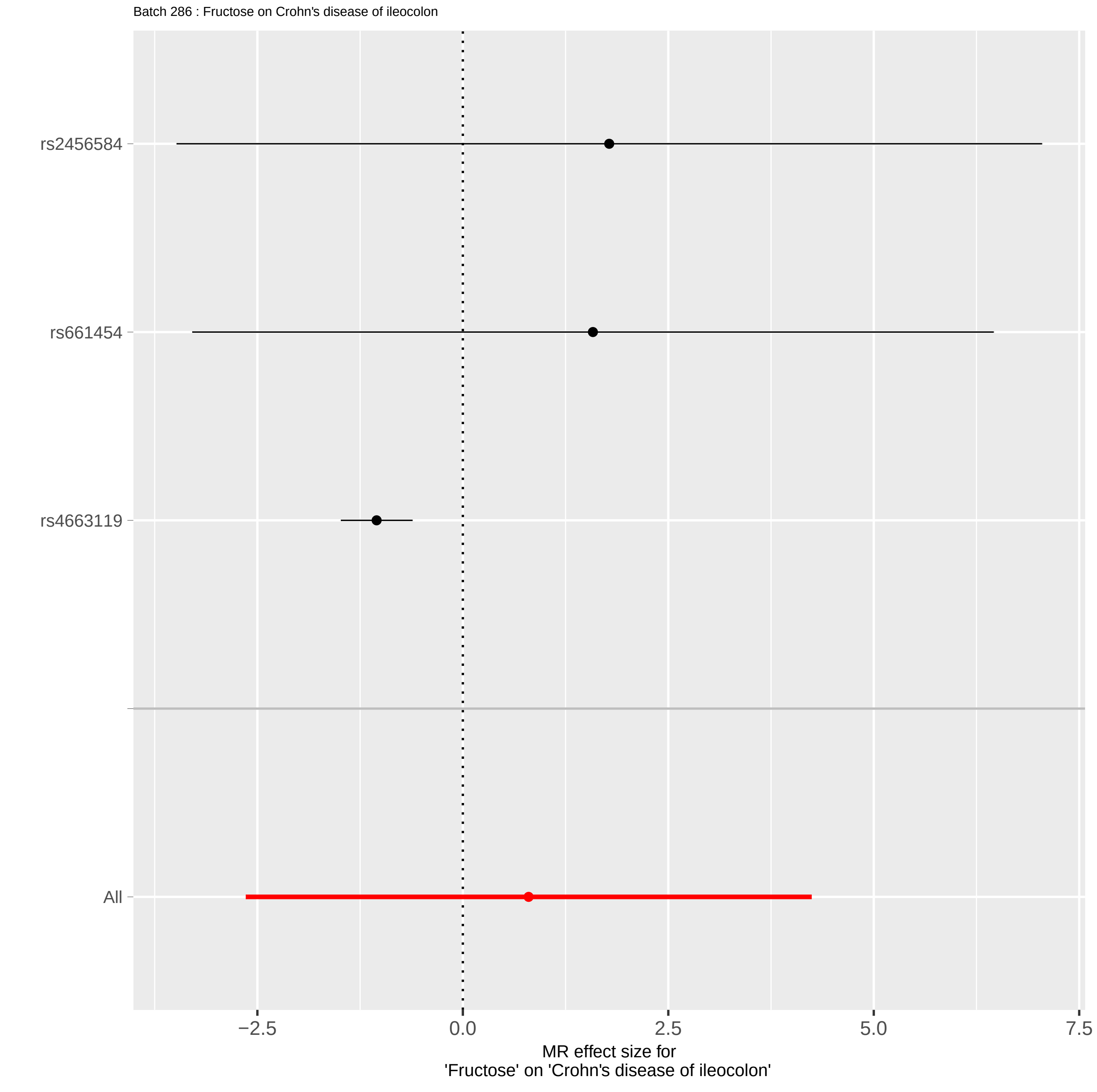
Batch 286 : Fructose on Crohn's disease of ileocolon
rs2456584
rs661454
rs4663119
All
−2.5
0.0
2.5
5.0
7.5
MR effect size for
'Fructose' on 'Crohn's disease of ileocolon'

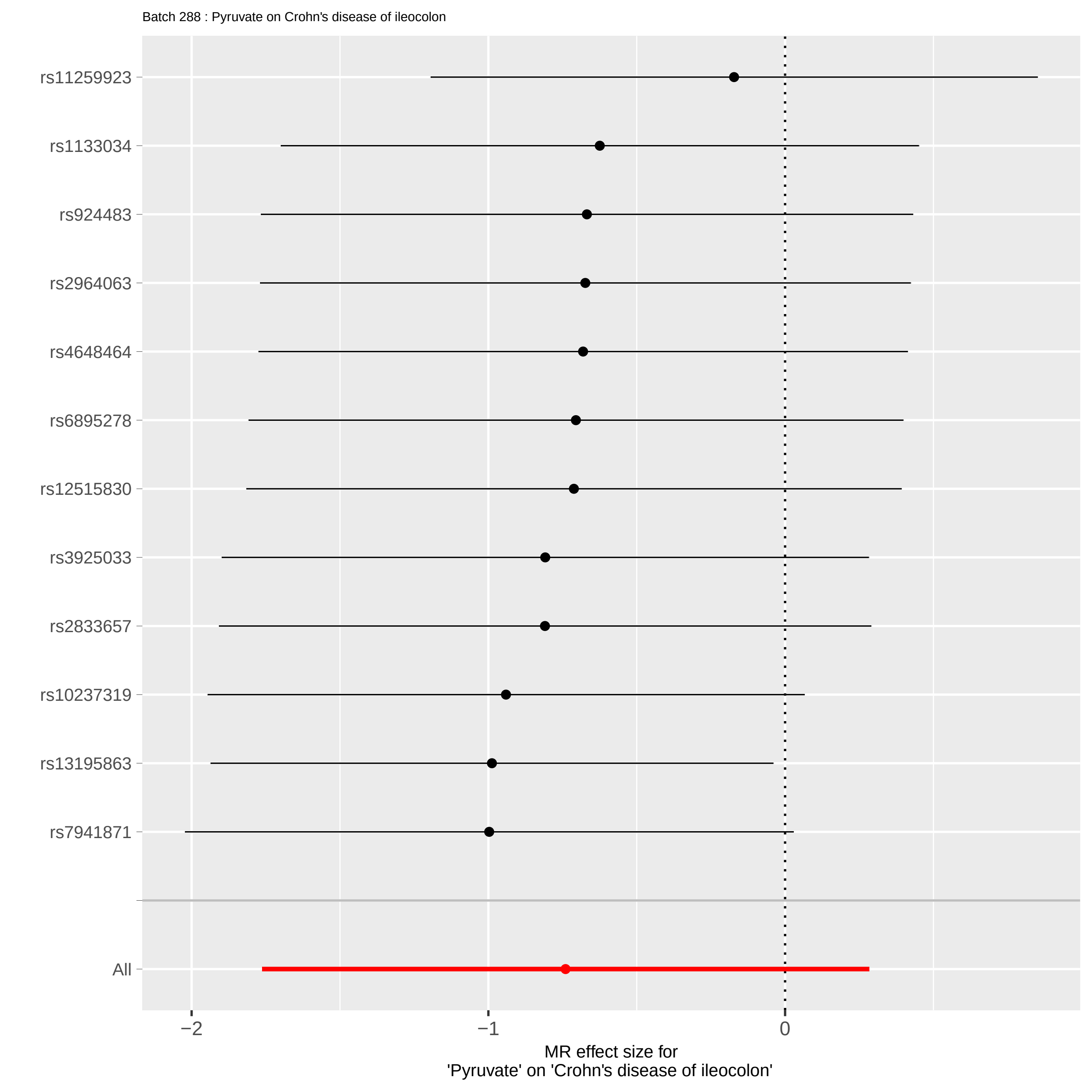

Batch 288 : Pyruvate on Crohn's disease of ileocolon
rs11259923
rs1133034
rs924483
rs2964063
rs4648464
rs6895278
rs12515830
rs3925033
rs2833657
rs10237319
rs13195863
rs7941871
All
−2
−1
0
MR effect size for
'Pyruvate' on 'Crohn's disease of ileocolon'

Batch 289 : Uridine on Crohn's disease of ileocolon

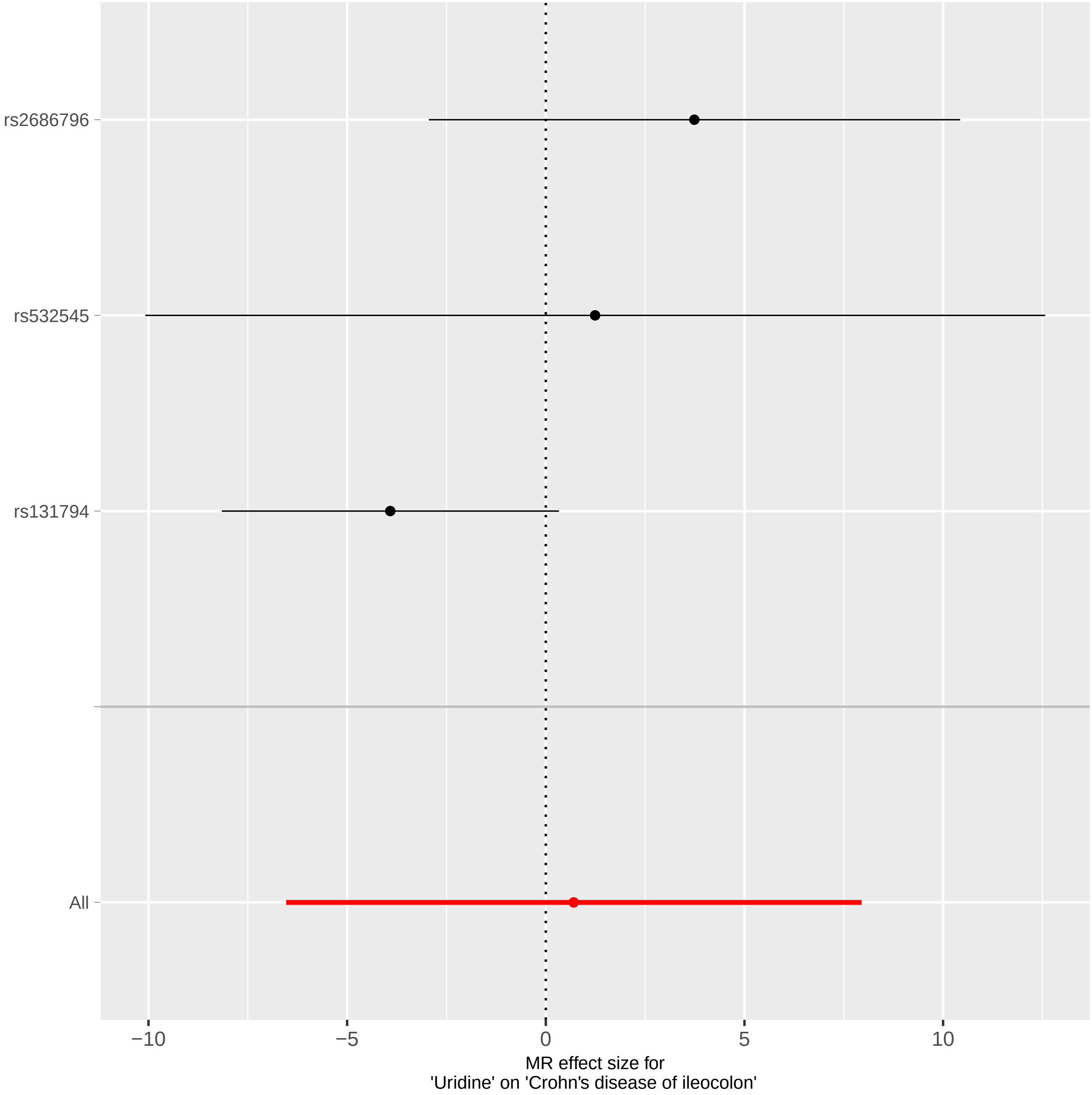

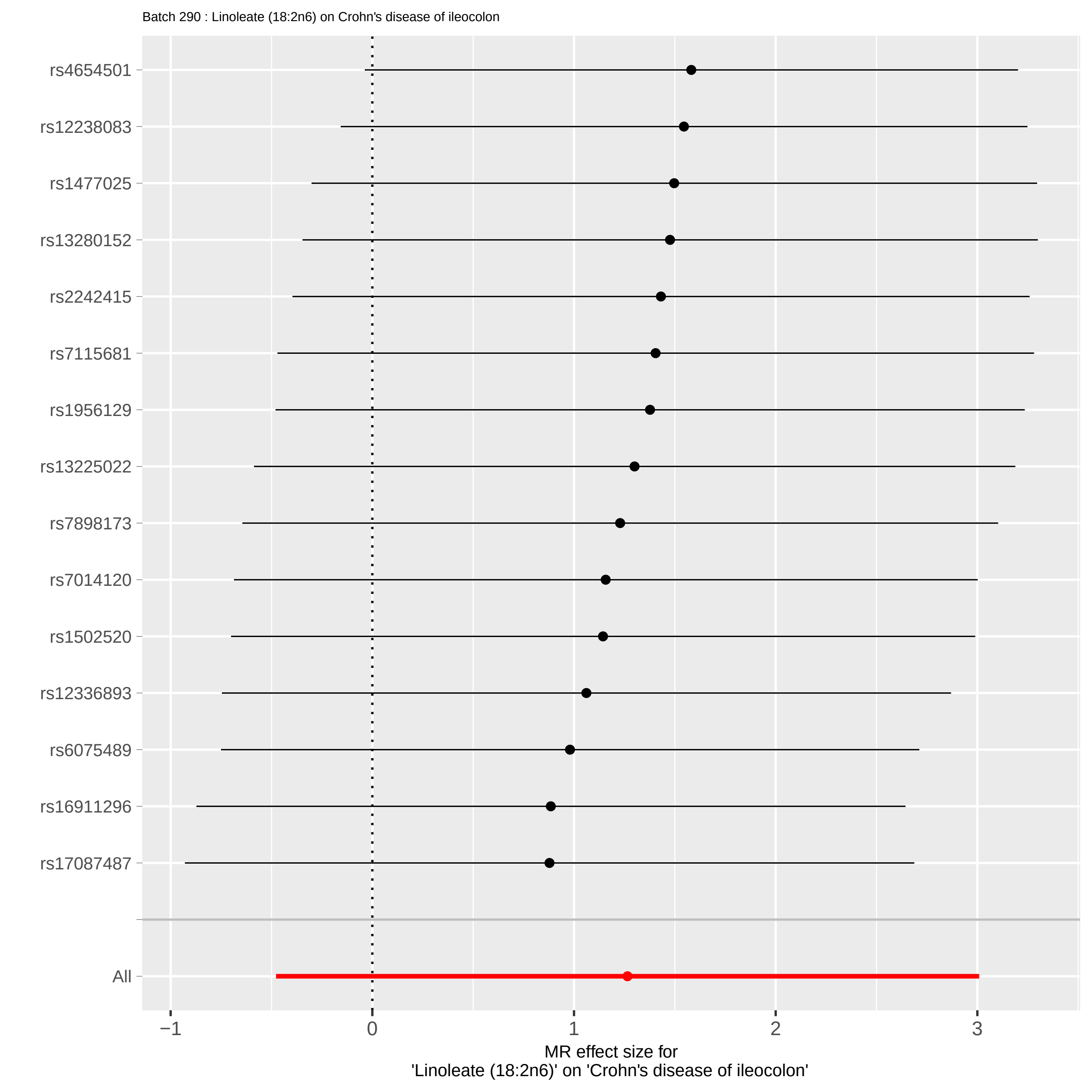
Batch 290 : Linoleate (18:2n6) on Crohn's disease of ileocolon
rs4654501
rs12238083
rs1477025
rs13280152
rs2242415
rs7115681
rs1956129
rs13225022
rs7898173
rs7014120
rs1502520
rs12336893
rs6075489
rs16911296
rs17087487
All
−1
0
1
2
3
MR effect size for
'Linoleate (18:2n6)' on 'Crohn's disease of ileocolon'

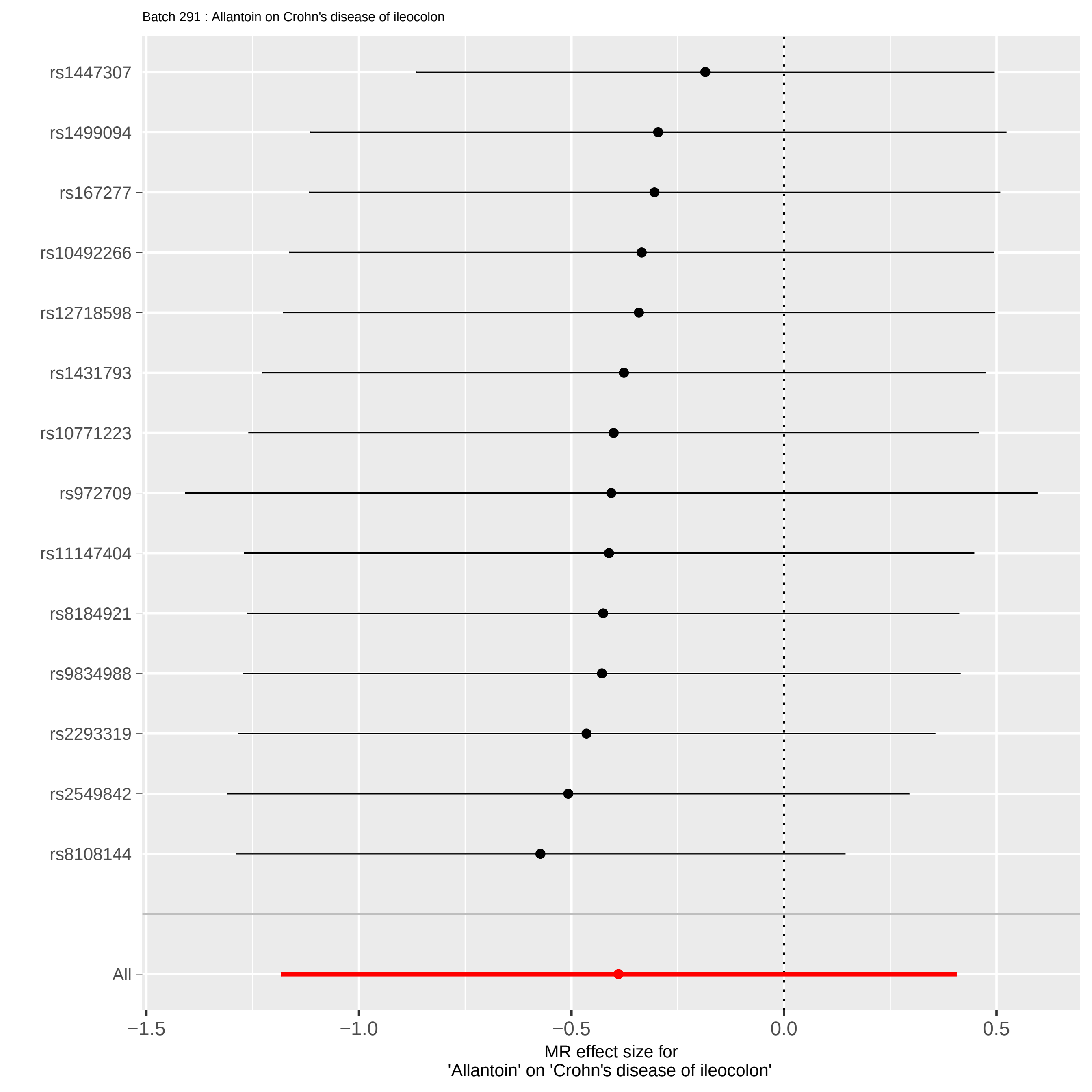

Batch 291 : Allantoin on Crohn's disease of ileocolon
rs1447307
rs1499094
rs167277
rs10492266
rs12718598
rs1431793
rs10771223
rs972709
rs11147404
rs8184921
rs9834988
rs2293319
rs2549842
rs8108144
All
−1.5
−1.0
−0.5
0.0
0.5
MR effect size for
'Allantoin' on 'Crohn's disease of ileocolon'

Batch 296 : Isoleucine on Crohn's disease of ileocolon

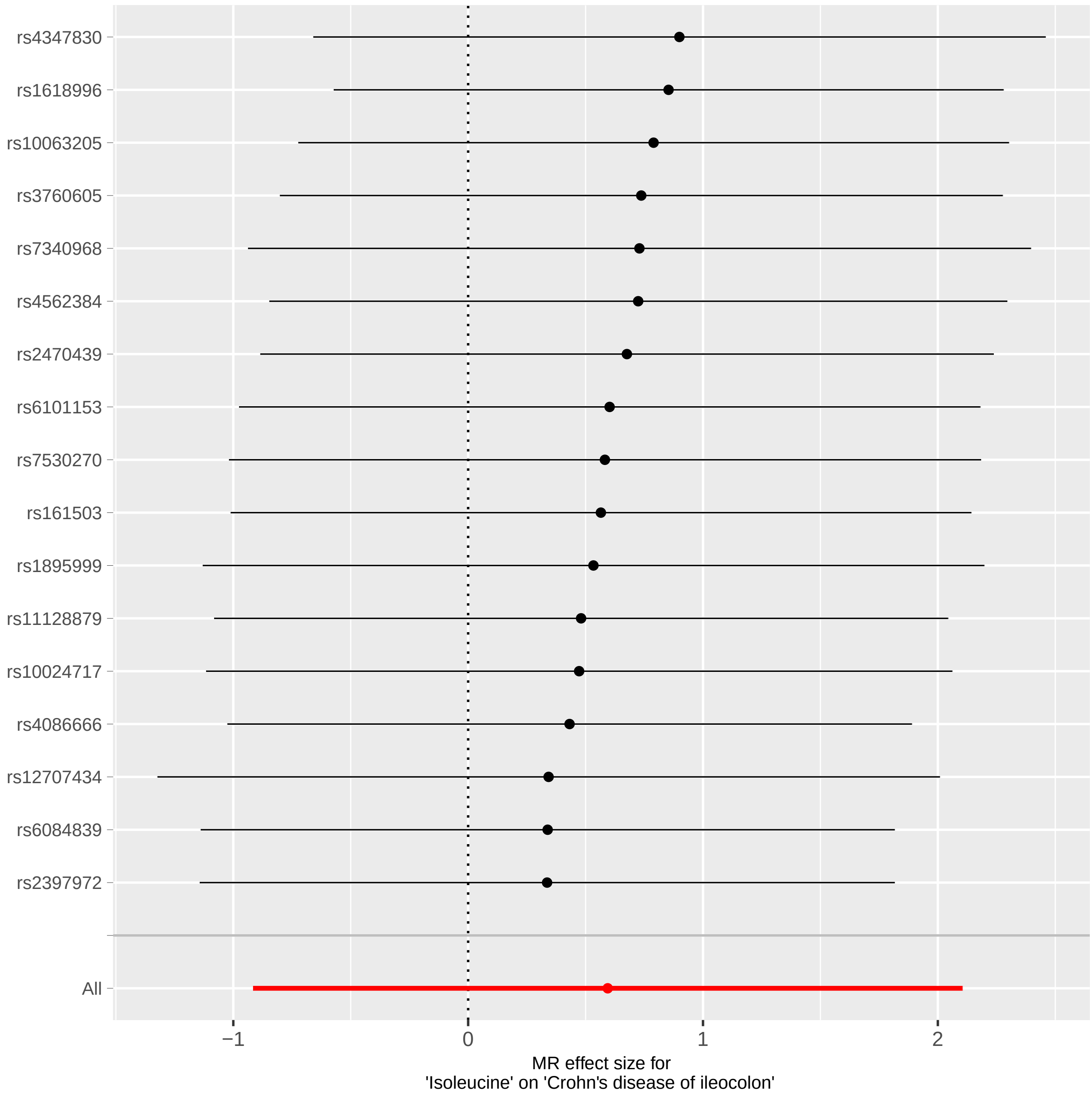

Batch 297 : Threonine on Crohn's disease of ileocolon

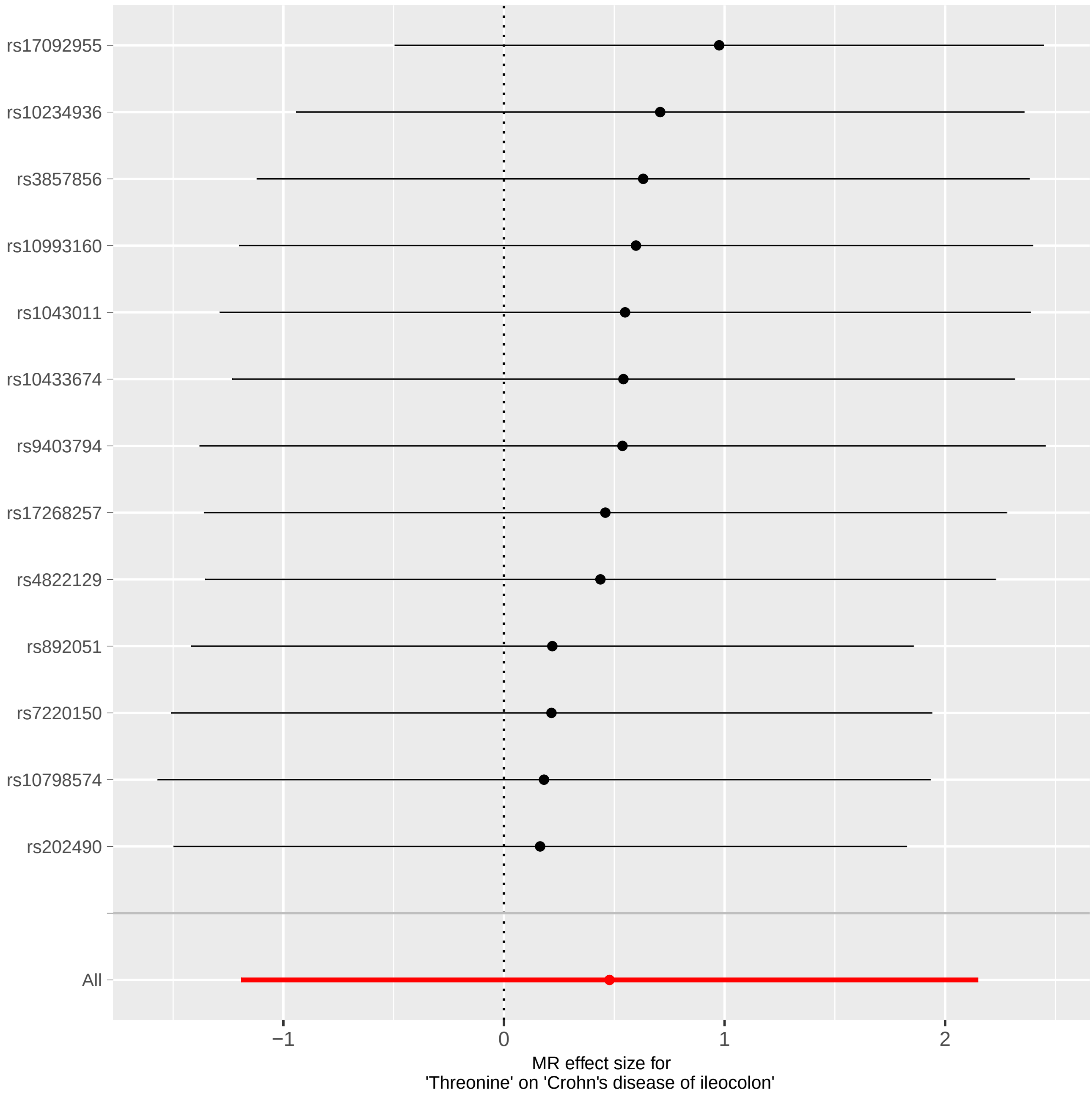

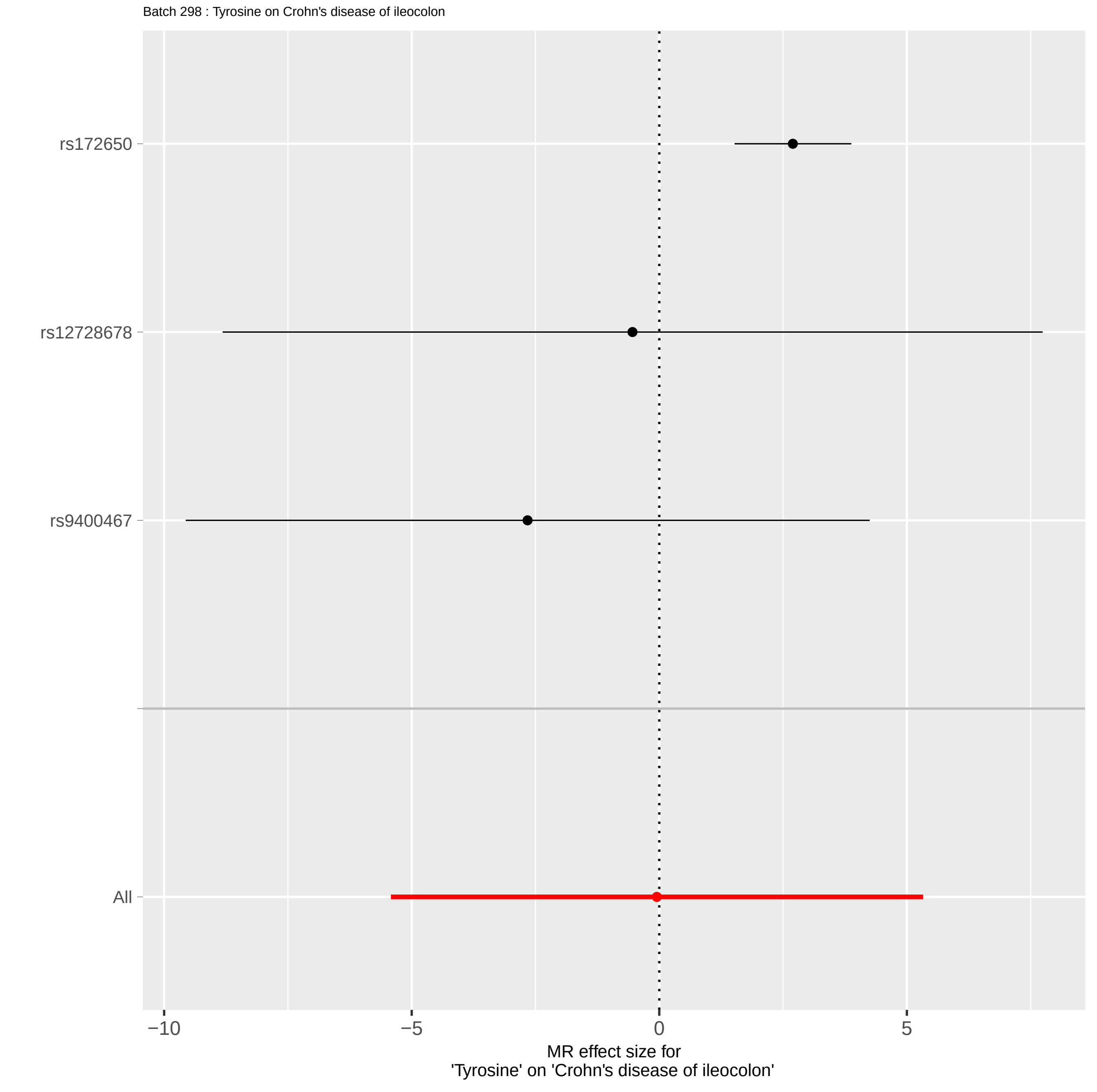
Batch 298 : Tyrosine on Crohn's disease of ileocolon
rs172650
rs12728678
rs9400467
All
−10
−5
0
5
MR effect size for
'Tyrosine' on 'Crohn's disease of ileocolon'

Batch 307 : Myristate (14:0) on Crohn's disease of ileocolon

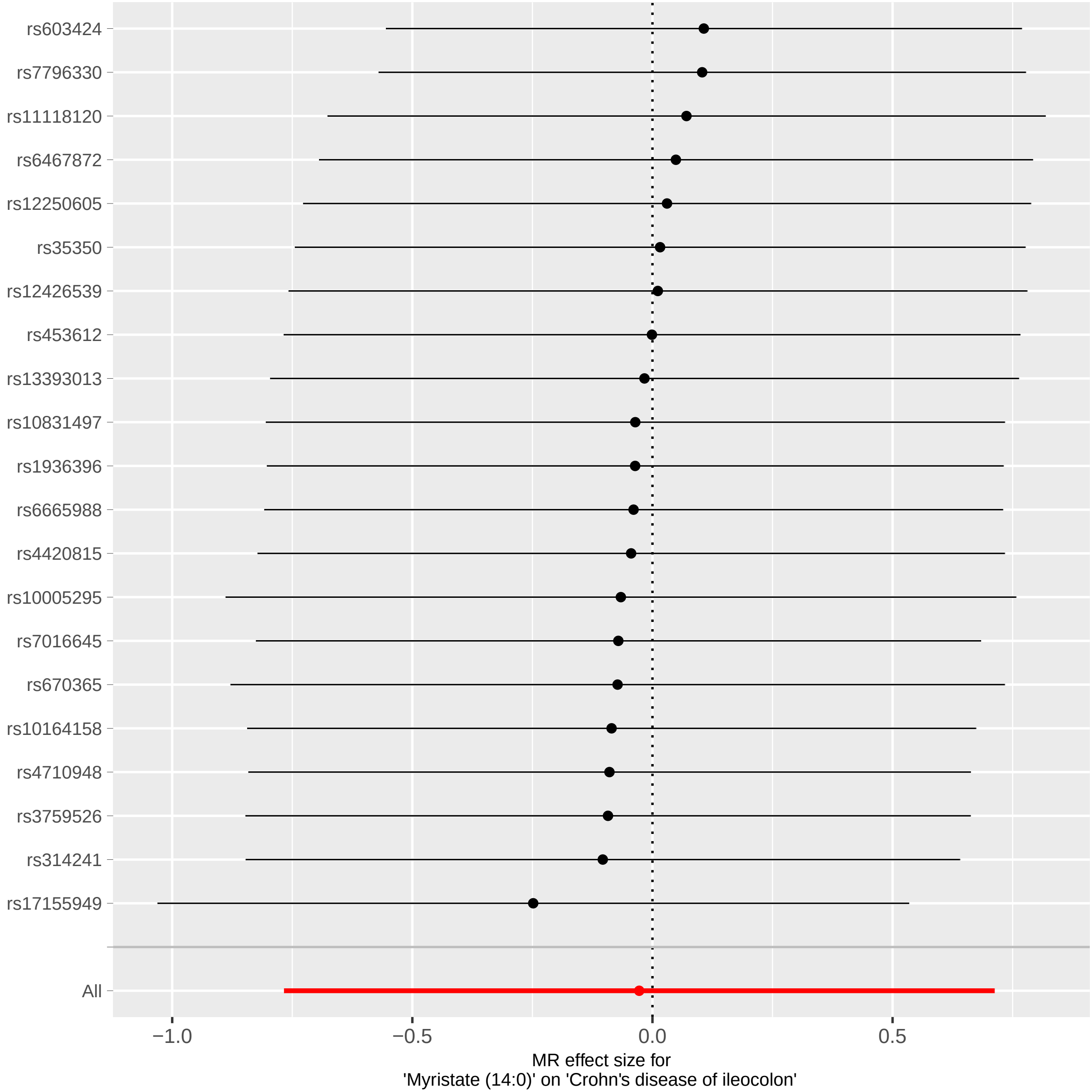

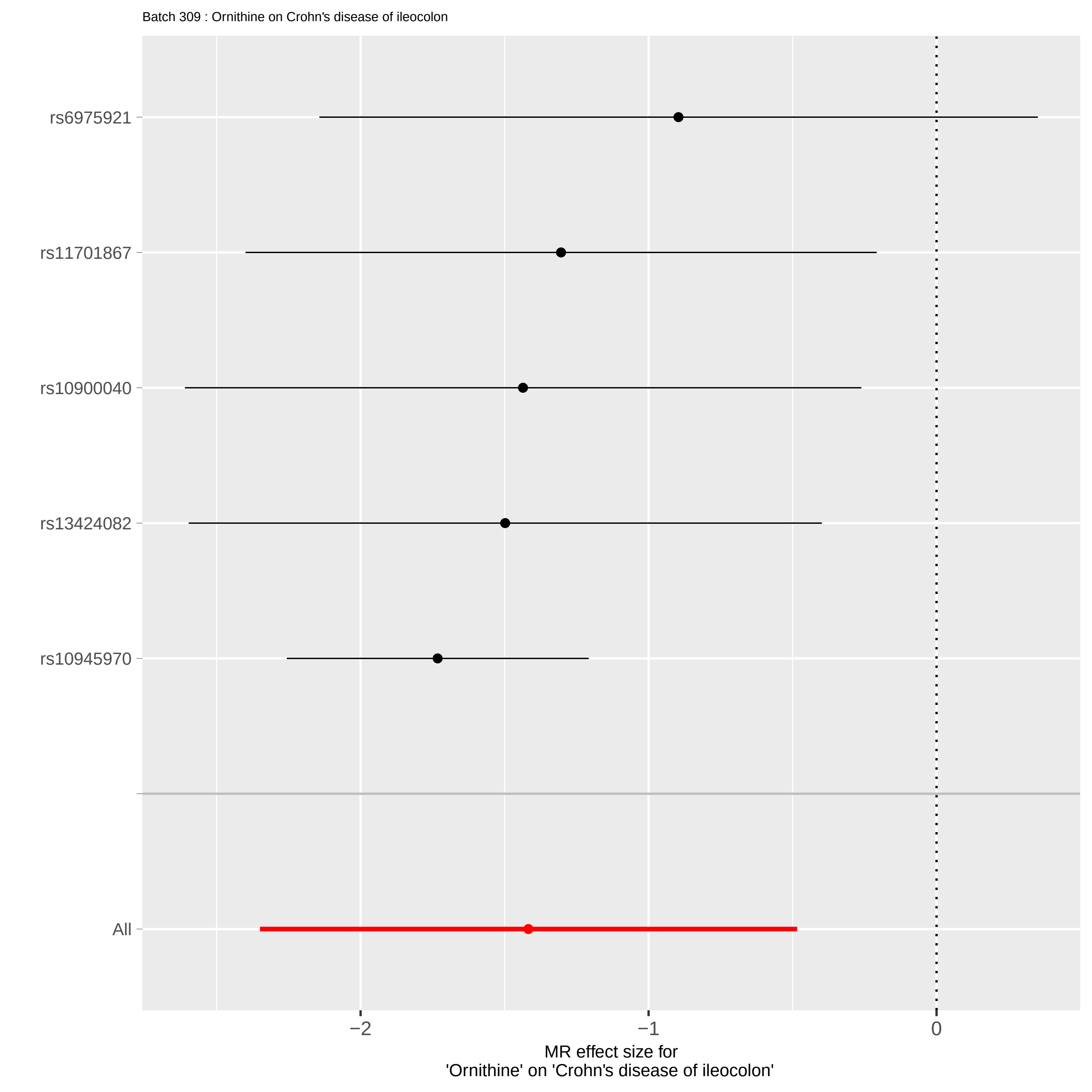

Batch 309 : Ornithine on Crohn's disease of ileocolon
rs6975921
rs11701867
rs10900040
rs13424082
rs10945970
All
−2
−1
0
MR effect size for
'Ornithine' on 'Crohn's disease of ileocolon'

Batch 313 : Alpha−tocopherol on Crohn's disease of ileocolon

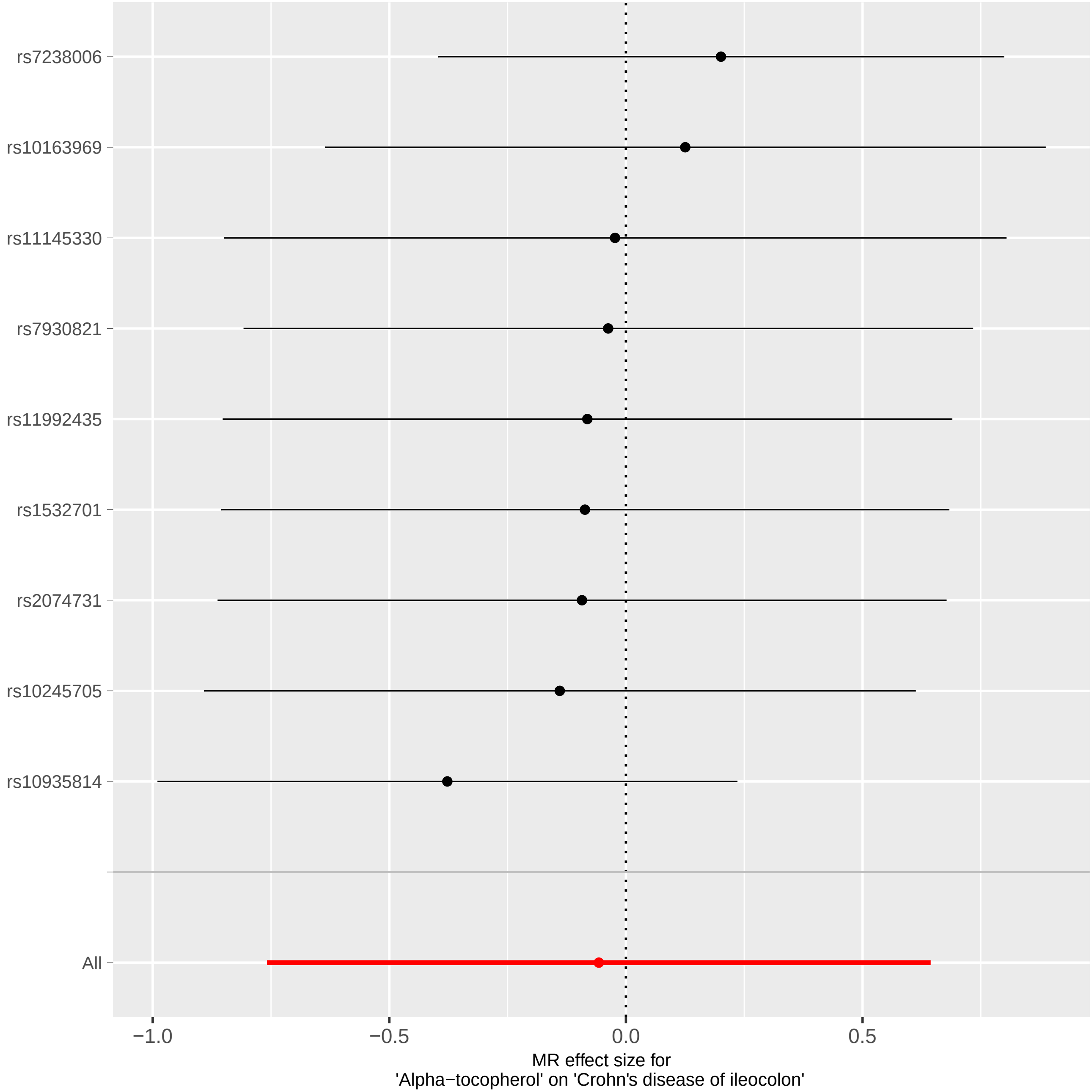

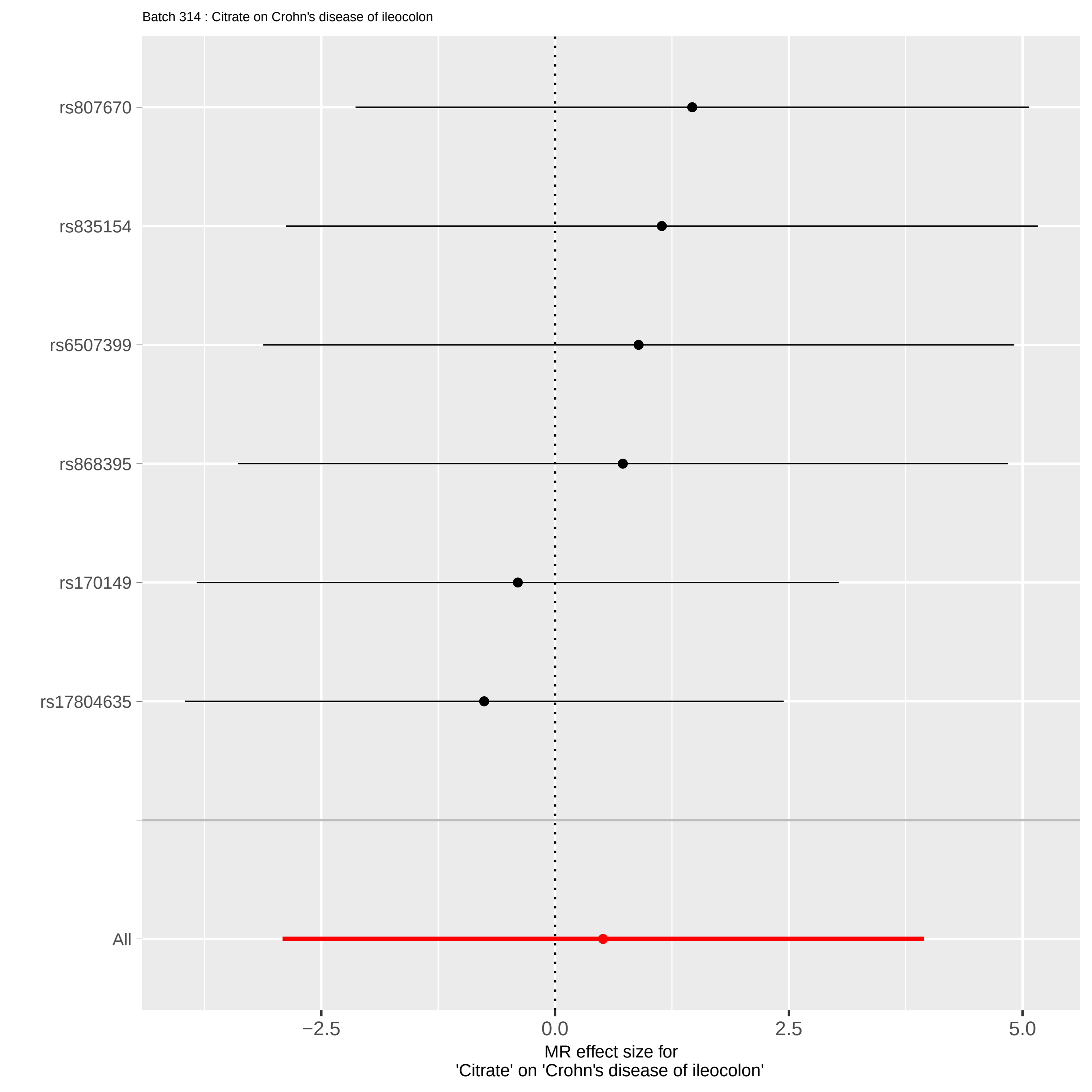
Batch 314 : Citrate on Crohn's disease of ileocolon
rs807670
rs835154
rs6507399
rs868395
rs170149
rs17804635
All
−2.5
0.0
2.5
5.0
MR effect size for
'Citrate' on 'Crohn's disease of ileocolon'

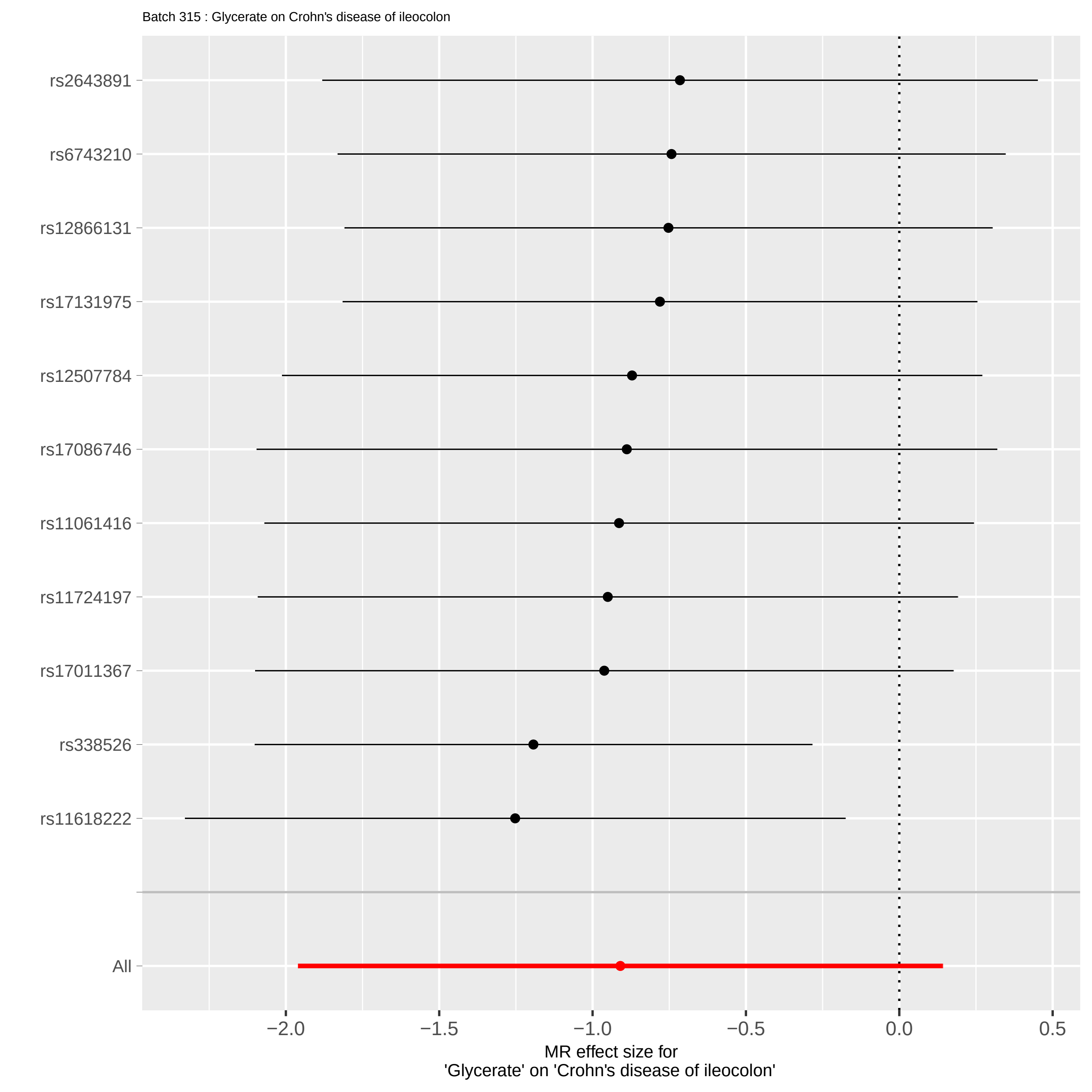

Batch 315 : Glycerate on Crohn's disease of ileocolon
rs2643891
rs6743210
rs12866131
rs17131975
rs12507784
rs17086746
rs11061416
rs11724197
rs17011367
rs338526
rs11618222
All
−2.0
−1.5
−1.0
−0.5
0.0
0.5
MR effect size for
'Glycerate' on 'Crohn's disease of ileocolon'

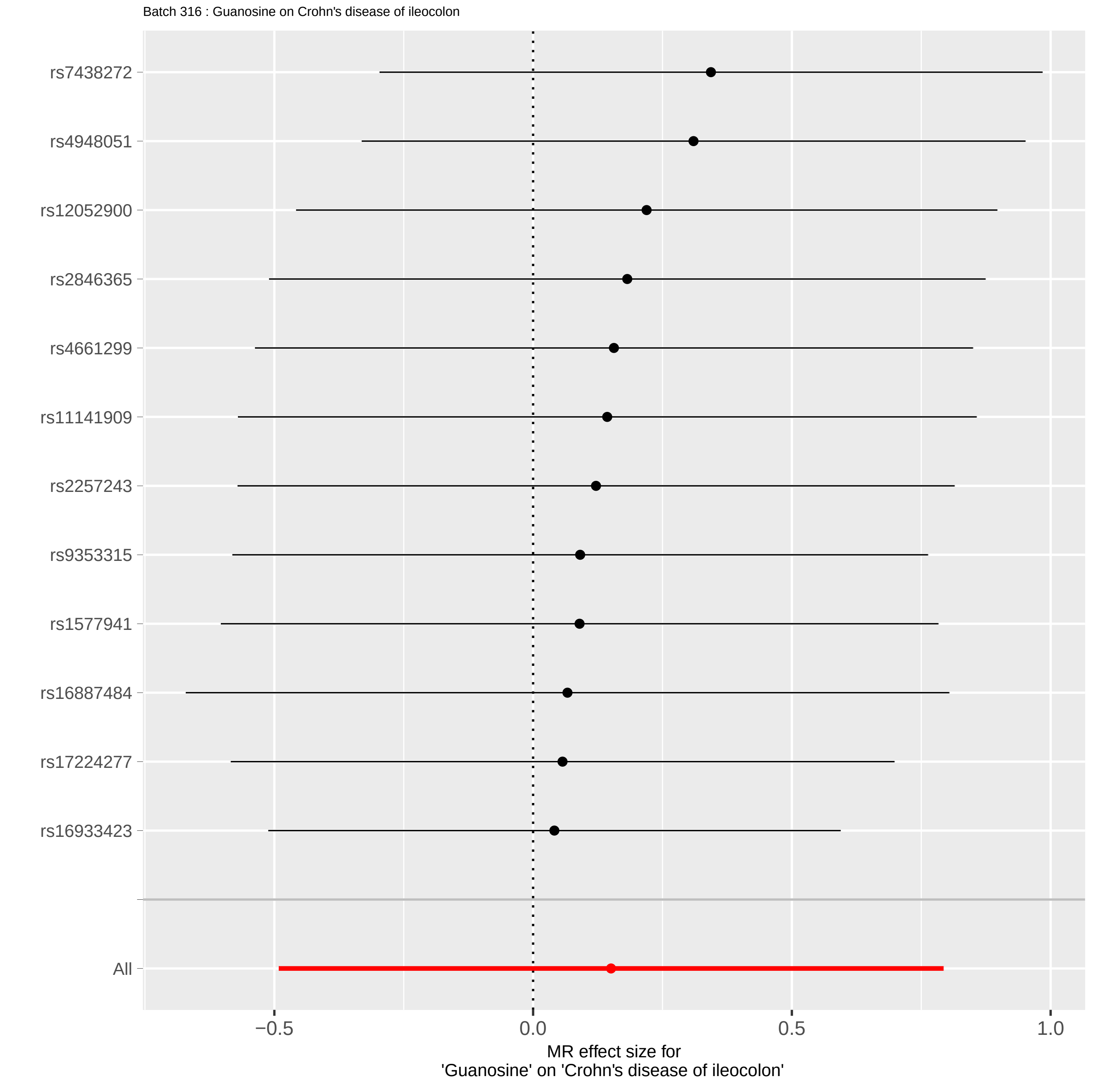
Batch 316 : Guanosine on Crohn's disease of ileocolon
rs7438272
rs4948051
rs12052900
rs2846365
rs4661299
rs11141909
rs2257243
rs9353315
rs1577941
rs16887484
rs17224277
rs16933423
All
−0.5
0.0
0.5
1.0
MR effect size for
'Guanosine' on 'Crohn's disease of ileocolon'

Batch 319 : Ursodeoxycholate on Crohn's disease of ileocolon

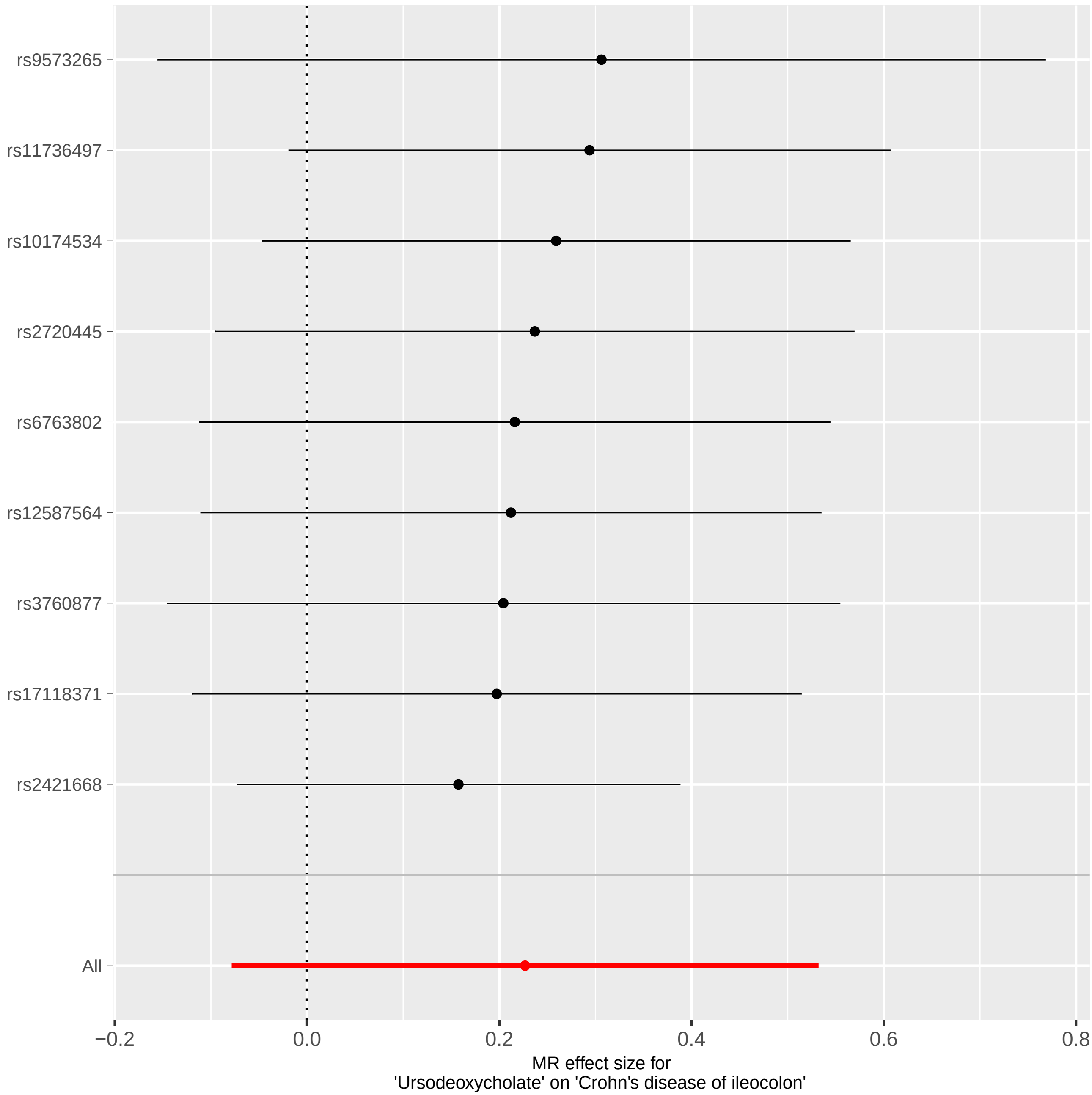

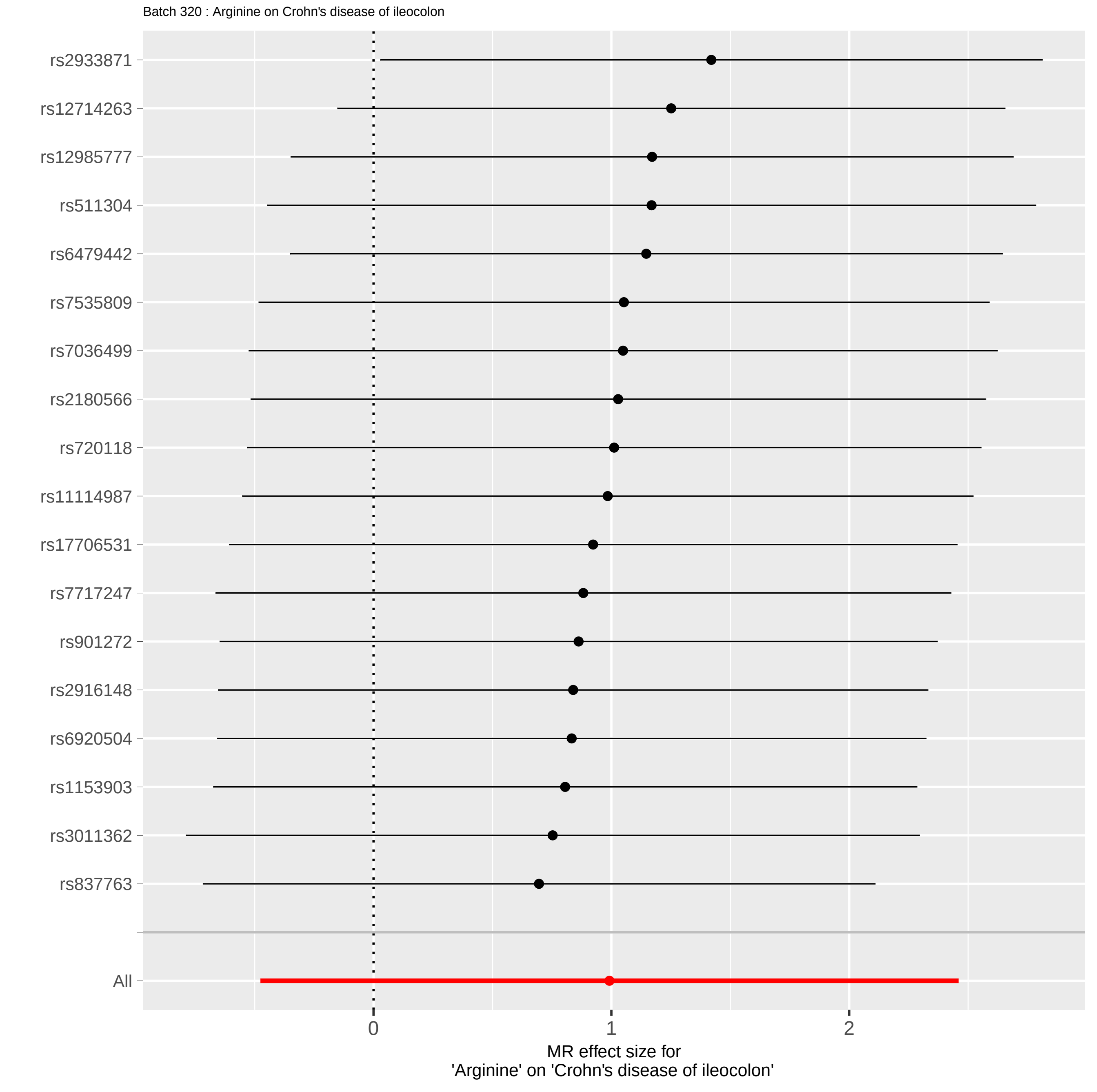

Batch 320 : Arginine on Crohn's disease of ileocolon
rs2933871
rs12714263
rs12985777
rs511304
rs6479442
rs7535809
rs7036499
rs2180566
rs720118
rs11114987
rs17706531
rs7717247
rs901272
rs2916148
rs6920504
rs1153903
rs3011362
rs837763
All
0
1
2
MR effect size for
'Arginine' on 'Crohn's disease of ileocolon'

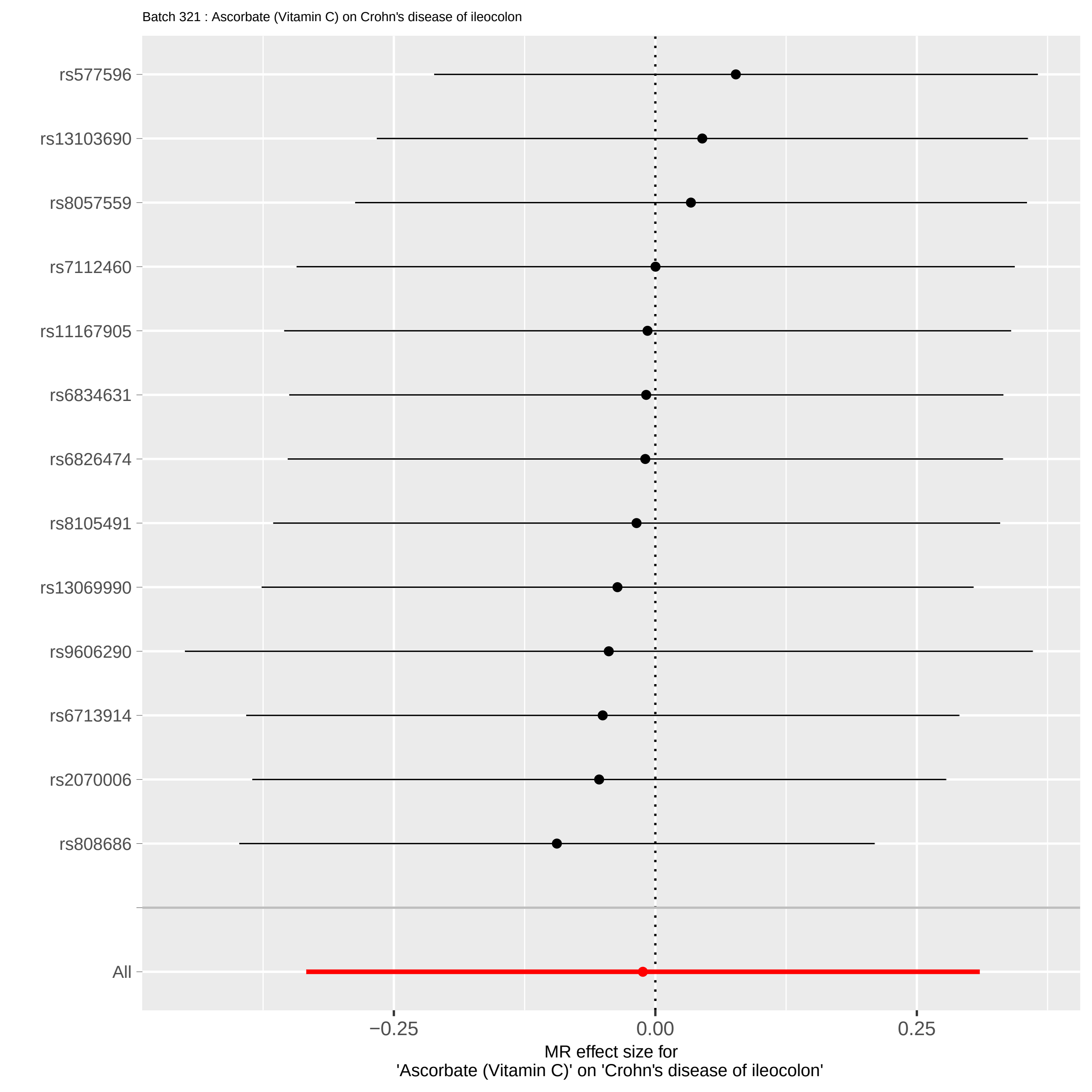

Batch 321 : Ascorbate (Vitamin C) on Crohn's disease of ileocolon
rs577596
rs13103690
rs8057559
rs7112460
rs11167905
rs6834631
rs6826474
rs8105491
rs13069990
rs9606290
rs6713914
rs2070006
rs808686
All
−0.25
0.00
0.25
MR effect size for
'Ascorbate (Vitamin C)' on 'Crohn's disease of ileocolon'

Batch 325 : Urea on Crohn's disease of ileocolon

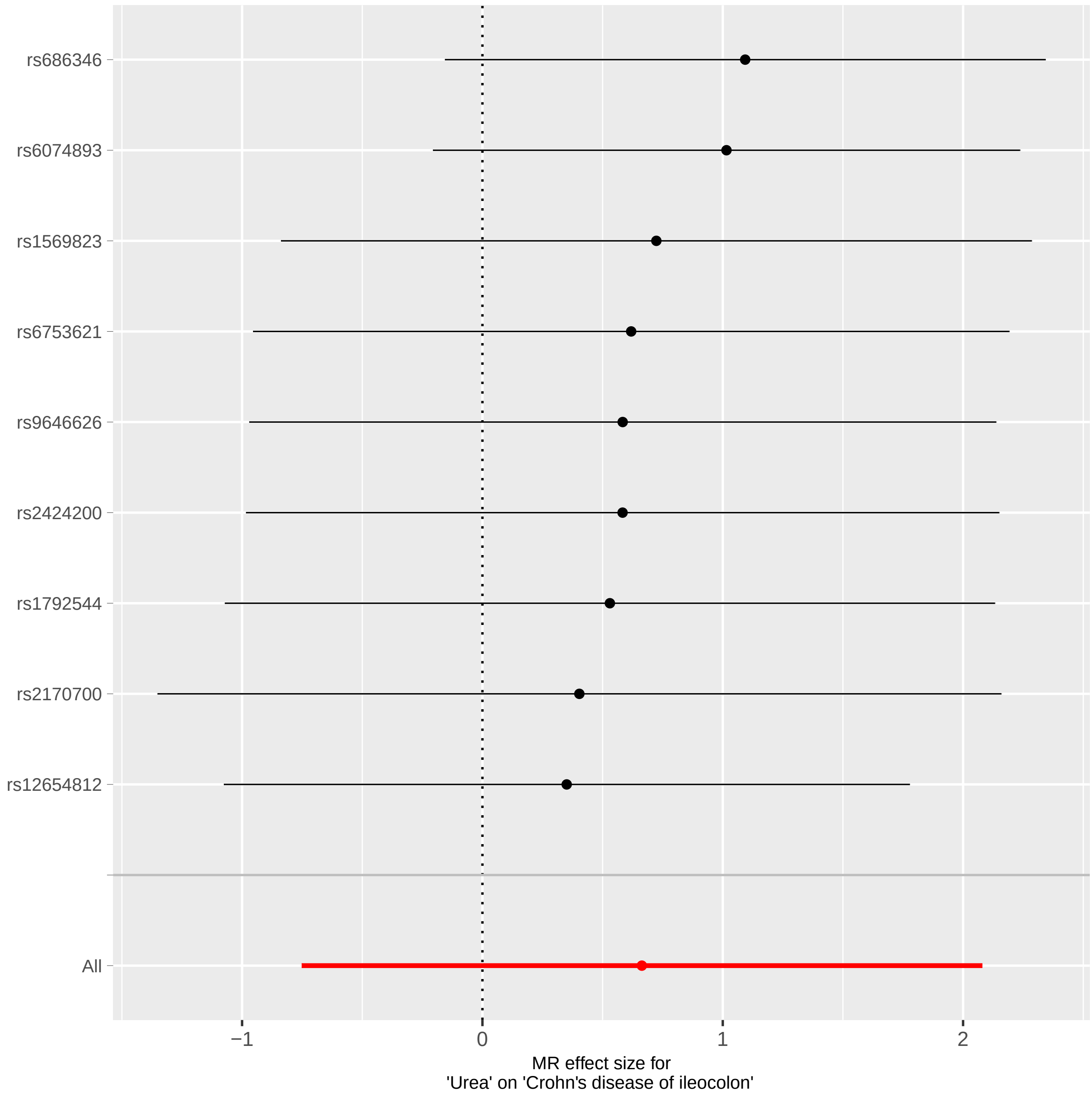

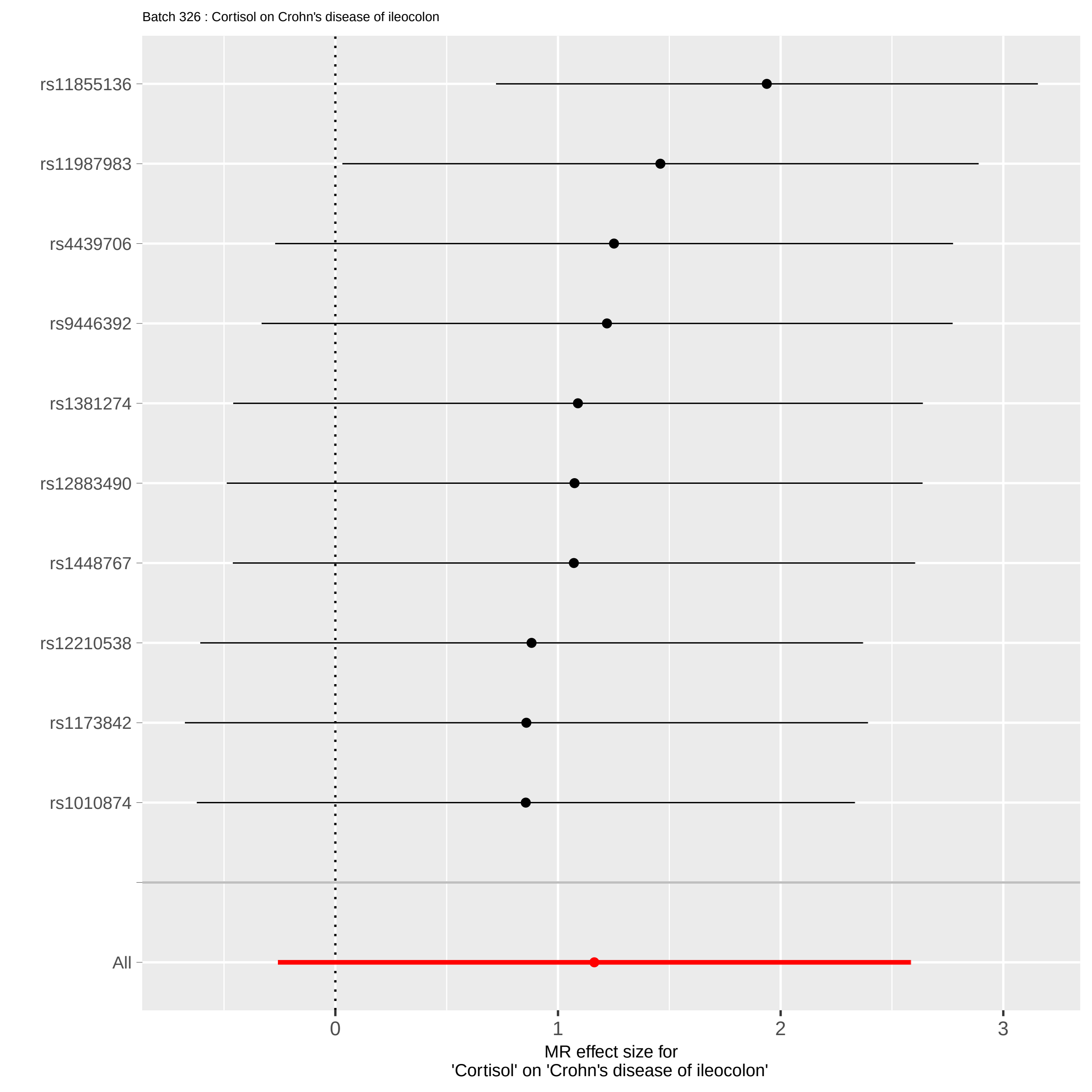
Batch 326 : Cortisol on Crohn's disease of ileocolon
rs11855136
rs11987983
rs4439706
rs9446392
rs1381274
rs12883490
rs1448767
rs12210538
rs1173842
rs1010874
All
0
1
2
3
MR effect size for
'Cortisol' on 'Crohn's disease of ileocolon'

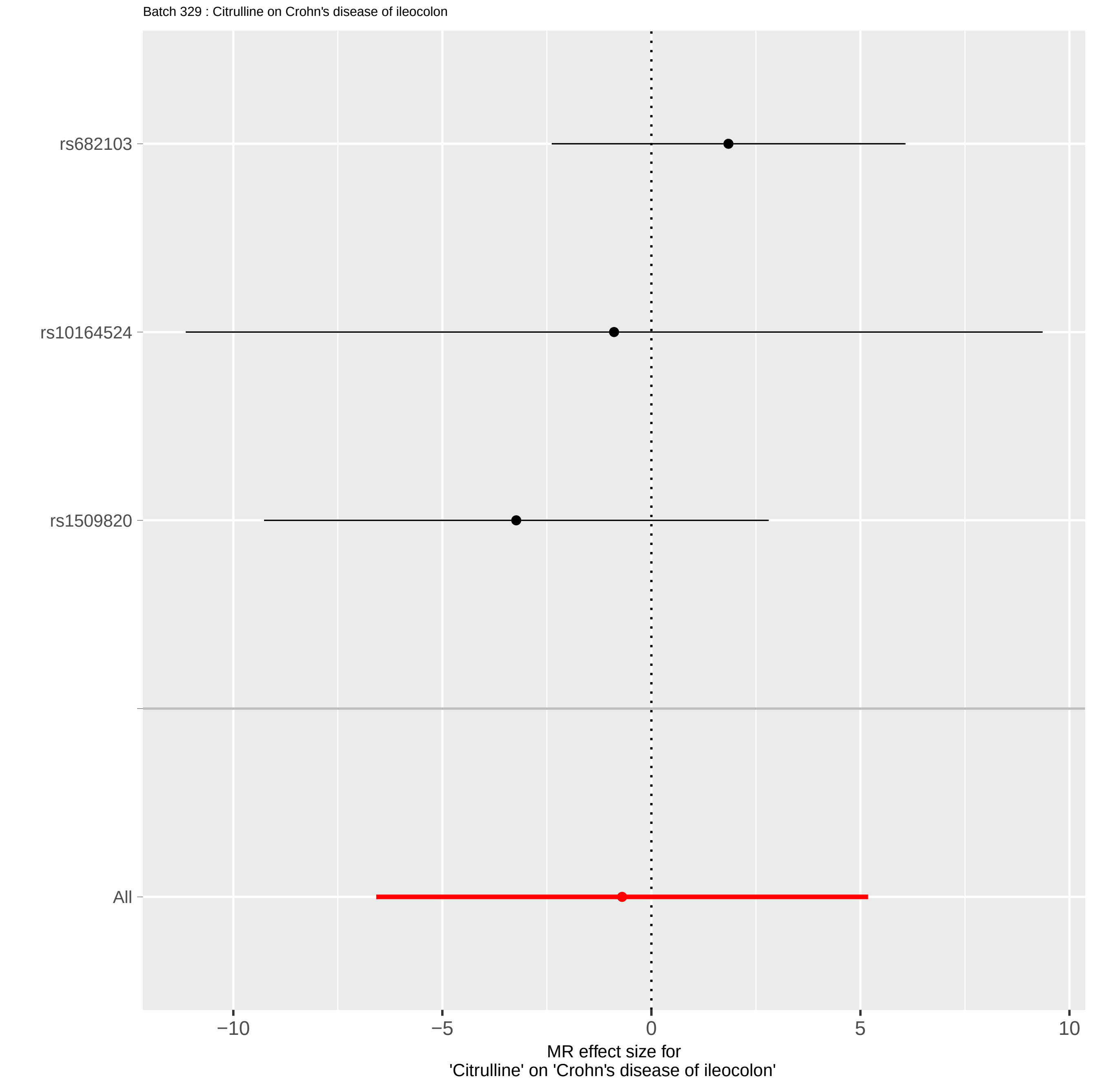

Batch 329 : Citrulline on Crohn's disease of ileocolon
rs682103
rs10164524
rs1509820
All
−10
−5
0
5
10
MR effect size for
'Citrulline' on 'Crohn's disease of ileocolon'

Batch 331 : Serotonin (5HT) on Crohn's disease of ileocolon

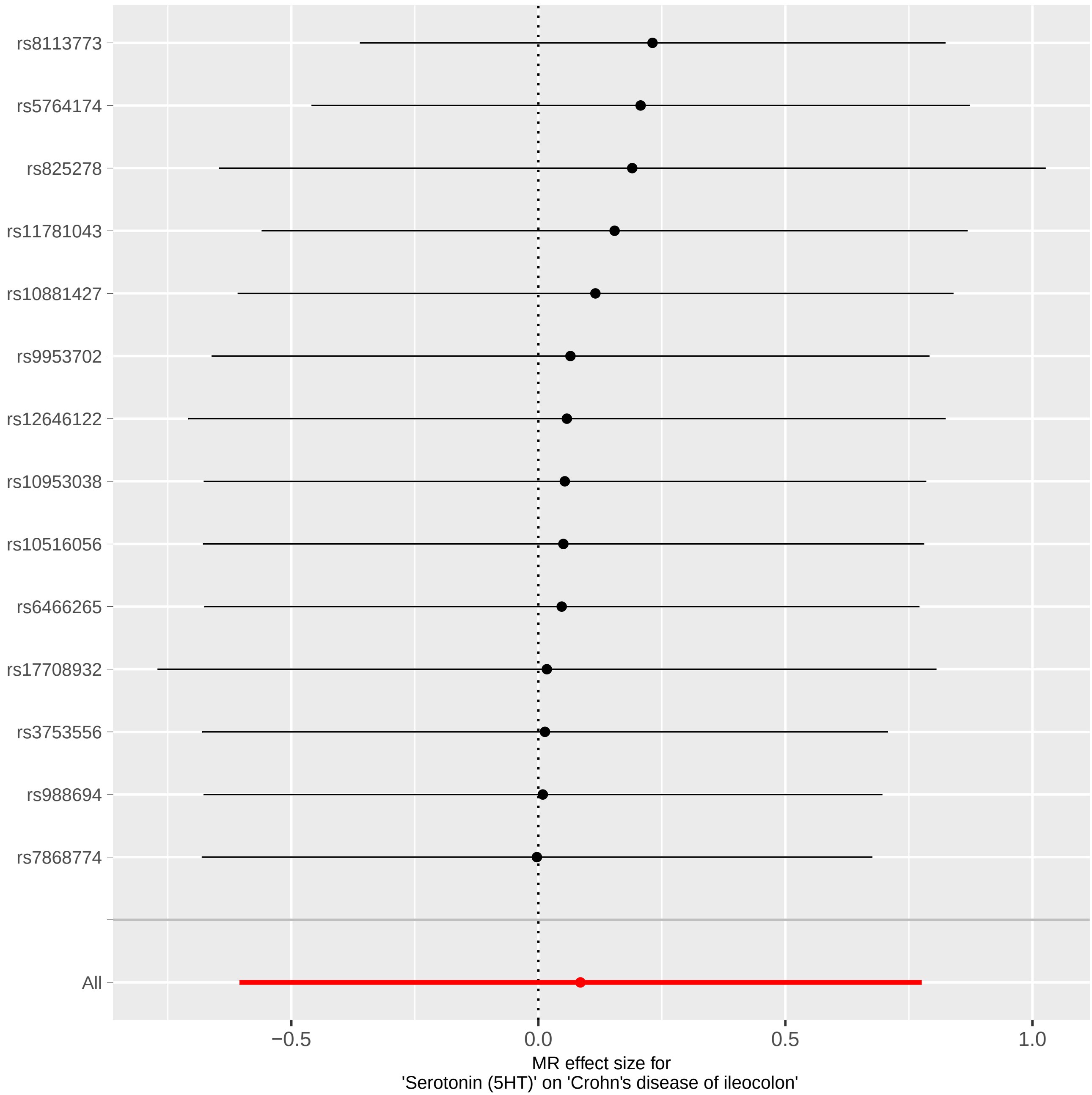

Batch 333 : Gamma−glutamyltyrosine on Crohn's disease of ileocolon

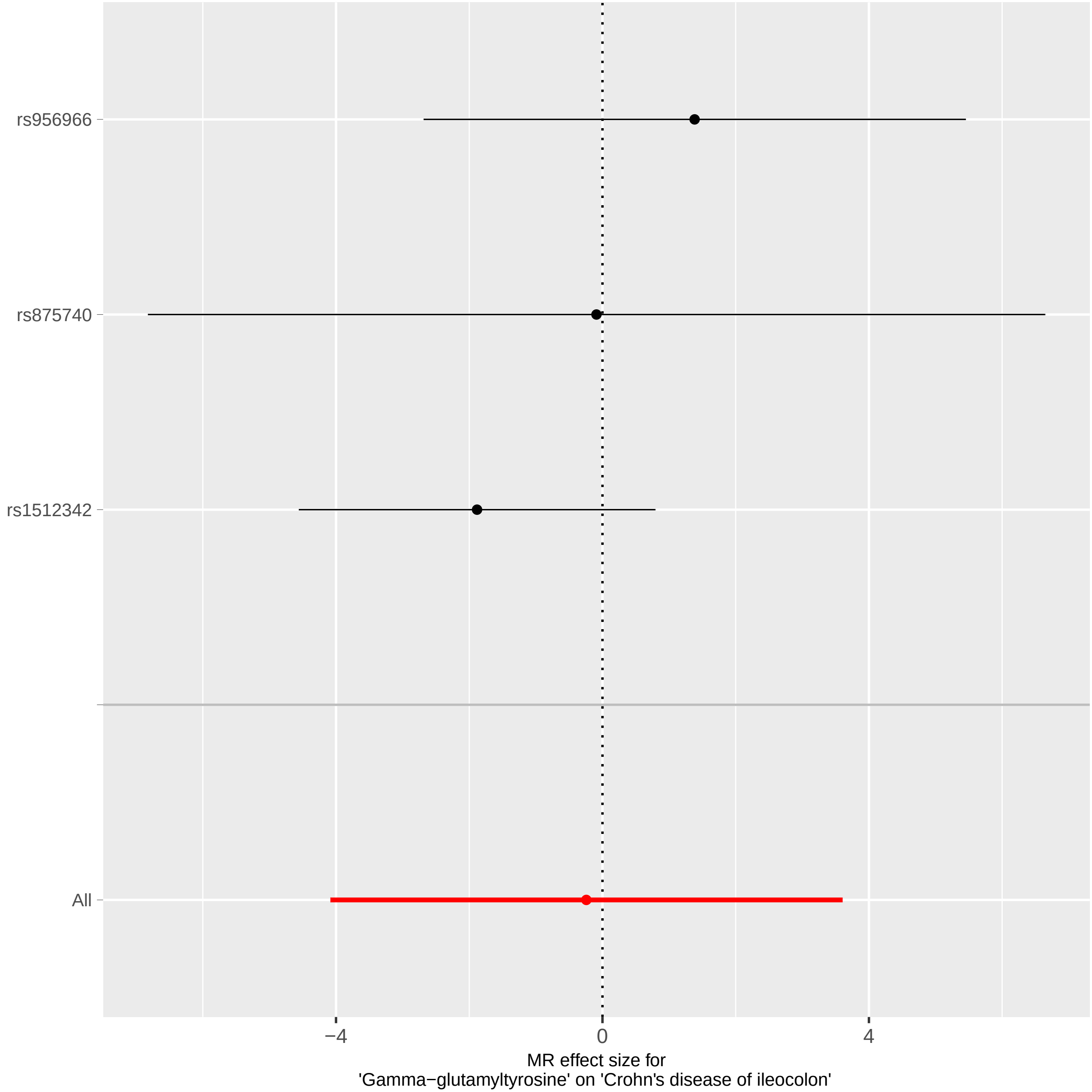

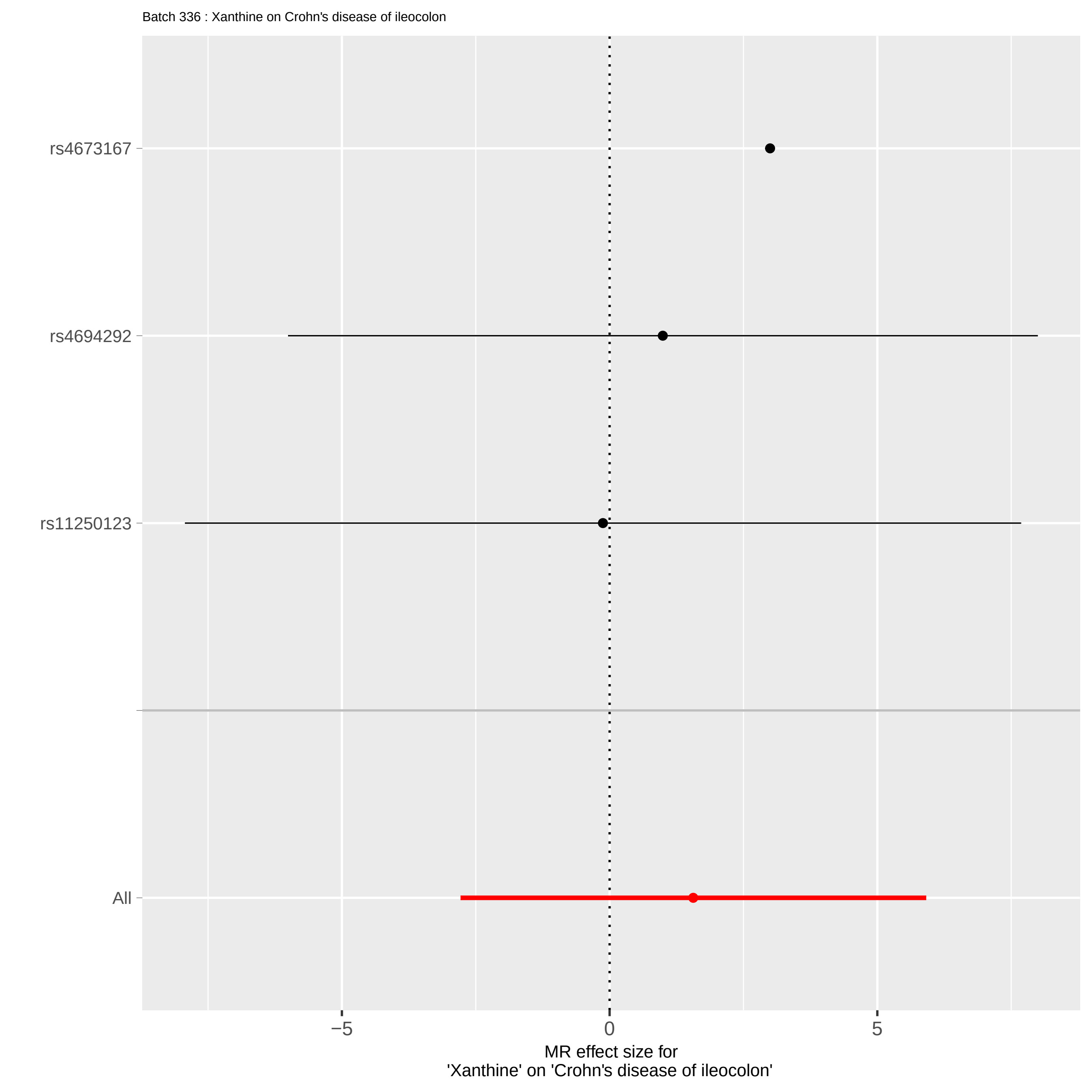
Batch 336 : Xanthine on Crohn's disease of ileocolon
rs4673167
rs4694292
rs11250123
All
−5
0
5
MR effect size for
'Xanthine' on 'Crohn's disease of ileocolon'

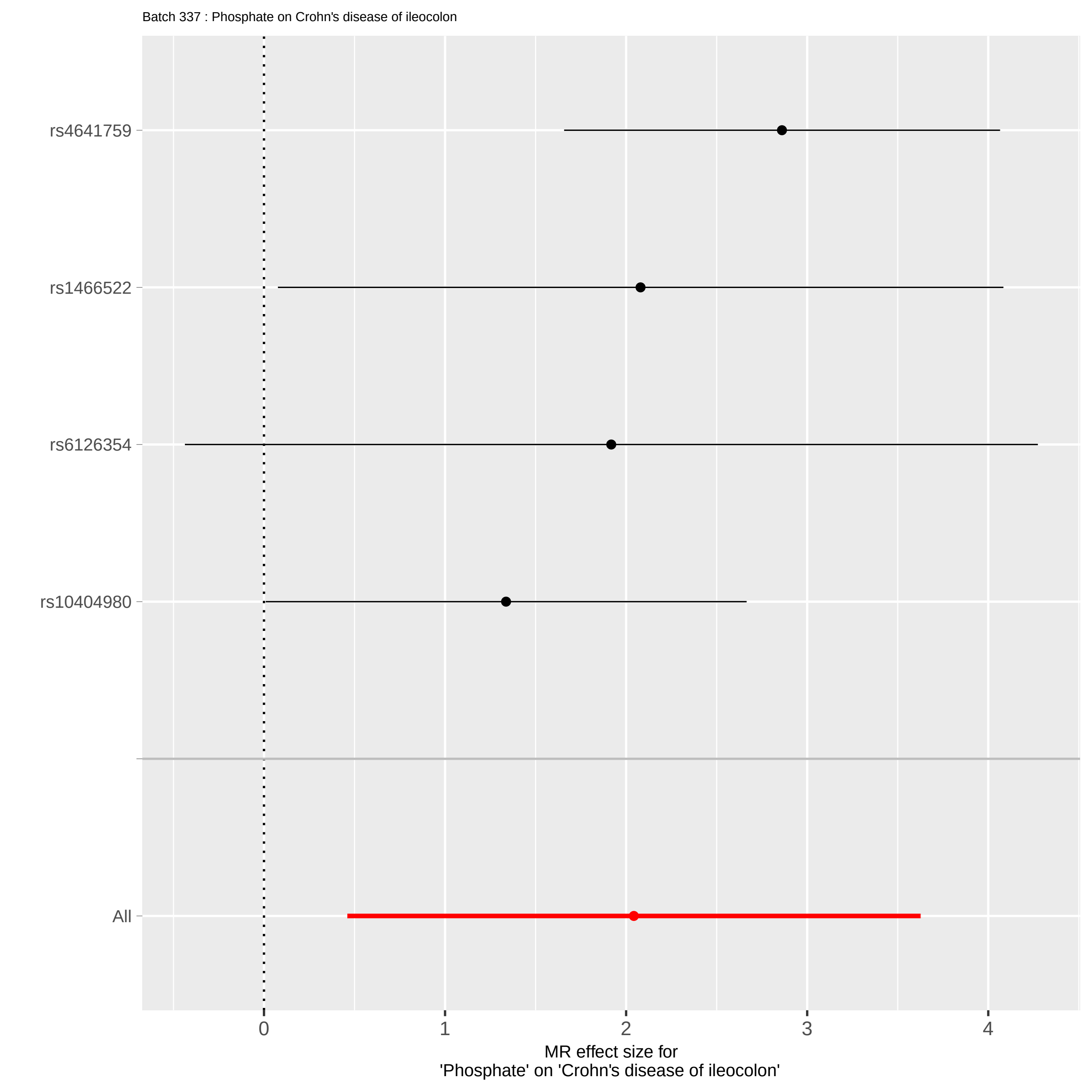

Batch 337 : Phosphate on Crohn's disease of ileocolon
rs4641759
rs1466522
rs6126354
rs10404980
All
0
1
2
3
4
MR effect size for
'Phosphate' on 'Crohn's disease of ileocolon'

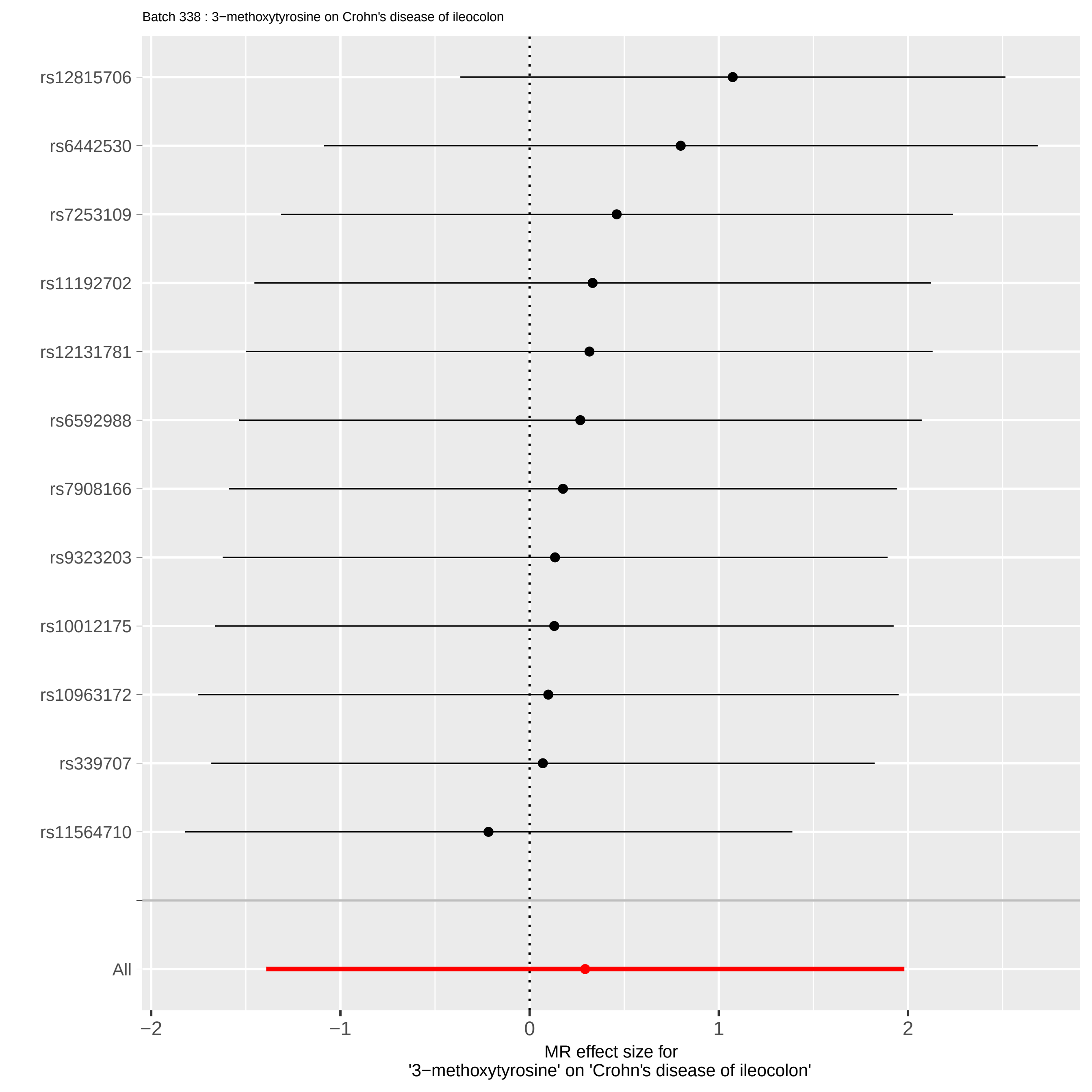

Batch 338 : 3−methoxytyrosine on Crohn's disease of ileocolon
rs12815706
rs6442530
rs7253109
rs11192702
rs12131781
rs6592988
rs7908166
rs9323203
rs10012175
rs10963172
rs339707
rs11564710
All
−2
−1
0
1
2
MR effect size for
'3−methoxytyrosine' on 'Crohn's disease of ileocolon'

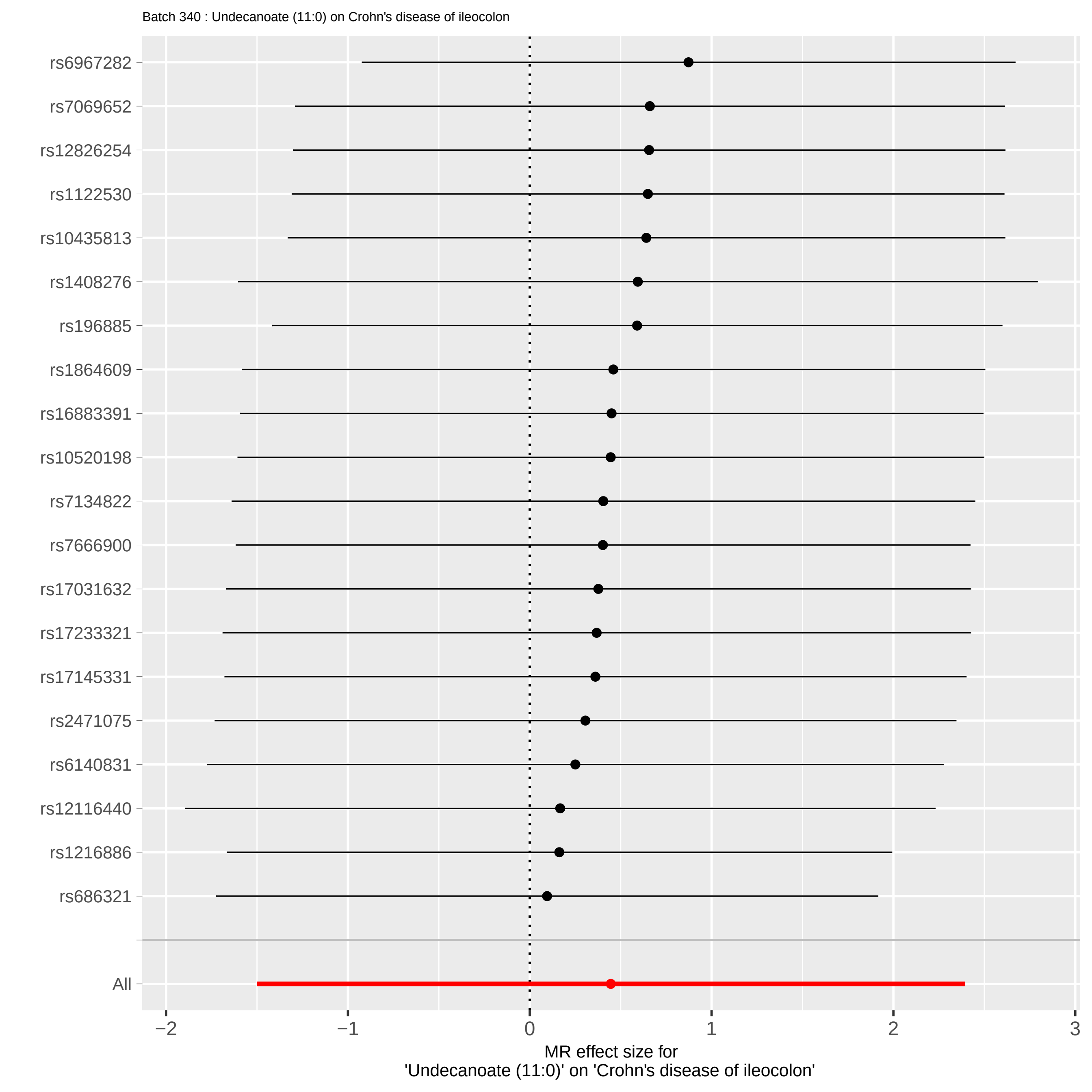

Batch 340 : Undecanoate (11:0) on Crohn's disease of ileocolon
rs6967282
rs7069652
rs12826254
rs1122530
rs10435813
rs1408276
rs196885
rs1864609
rs16883391
rs10520198
rs7134822
rs7666900
rs17031632
rs17233321
rs17145331
rs2471075
rs6140831
rs12116440
rs1216886
rs686321
All
−2
−1
0
1
2
3
MR effect size for
'Undecanoate (11:0)' on 'Crohn's disease of ileocolon'

Batch 342 : Taurodeoxycholate on Crohn's disease of ileocolon

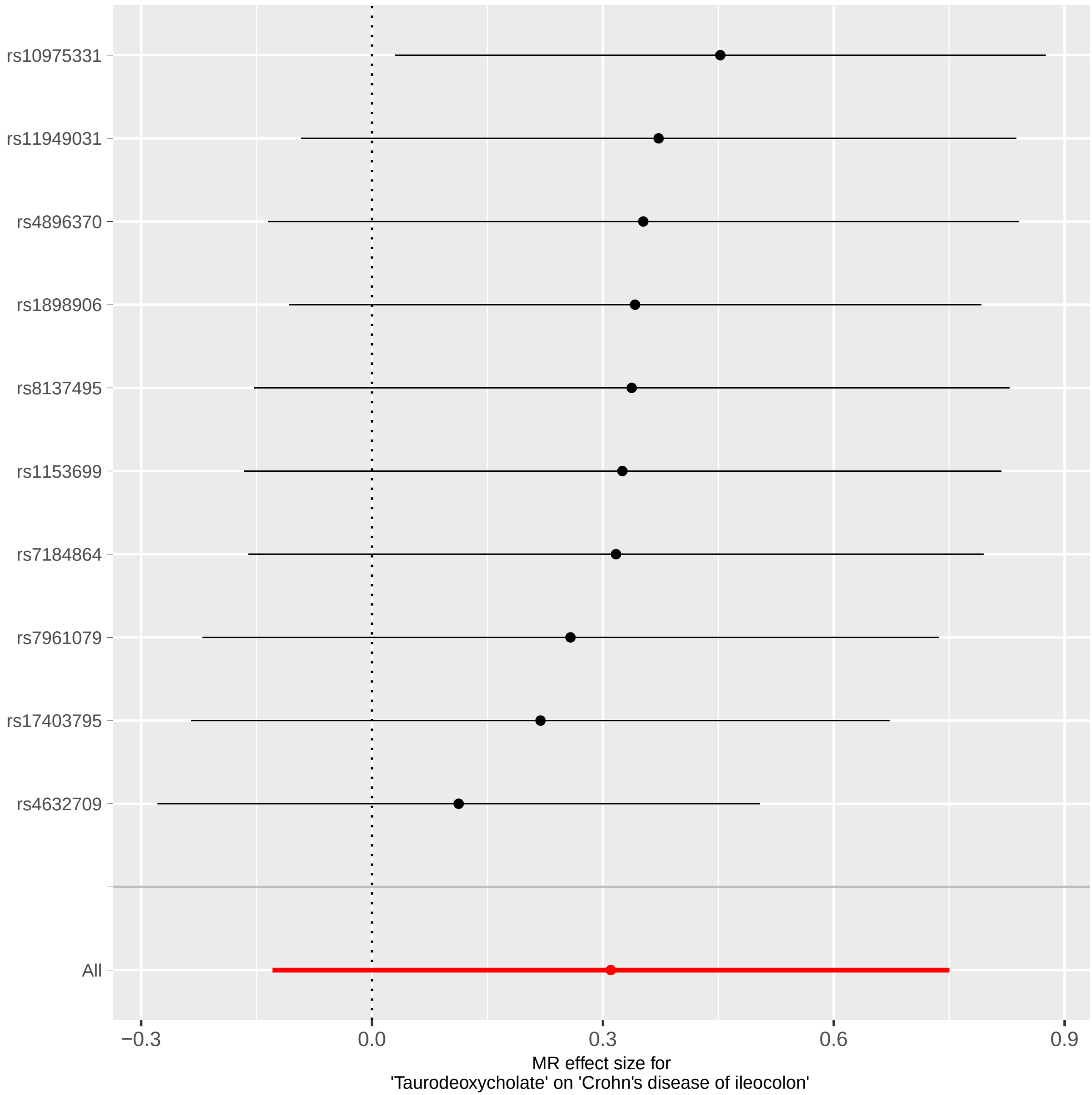

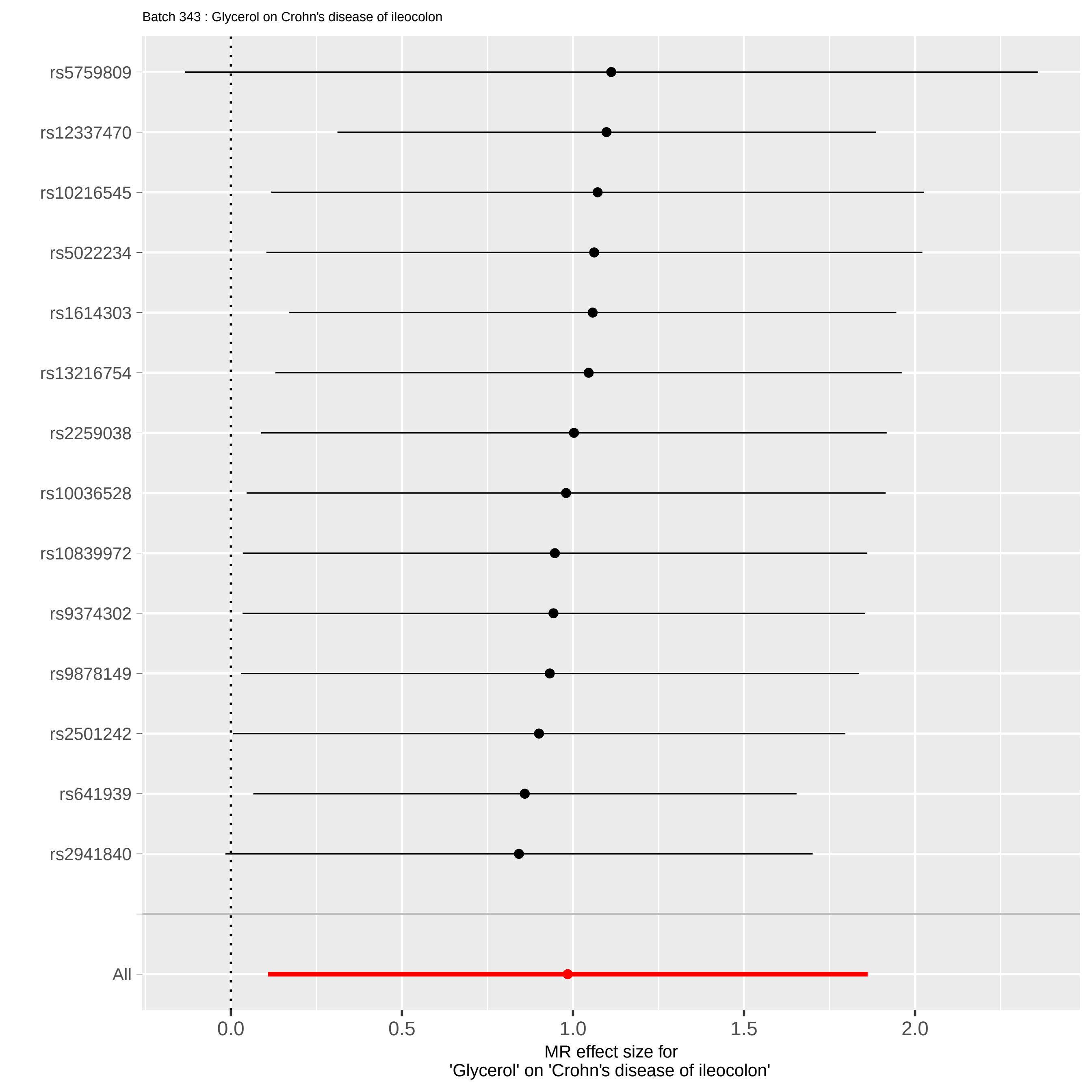

Batch 343 : Glycerol on Crohn's disease of ileocolon
rs5759809
rs12337470
rs10216545
rs5022234
rs1614303
rs13216754
rs2259038
rs10036528
rs10839972
rs9374302
rs9878149
rs2501242
rs641939
rs2941840
All
0.0
0.5
1.0
1.5
2.0
MR effect size for
'Glycerol' on 'Crohn's disease of ileocolon'

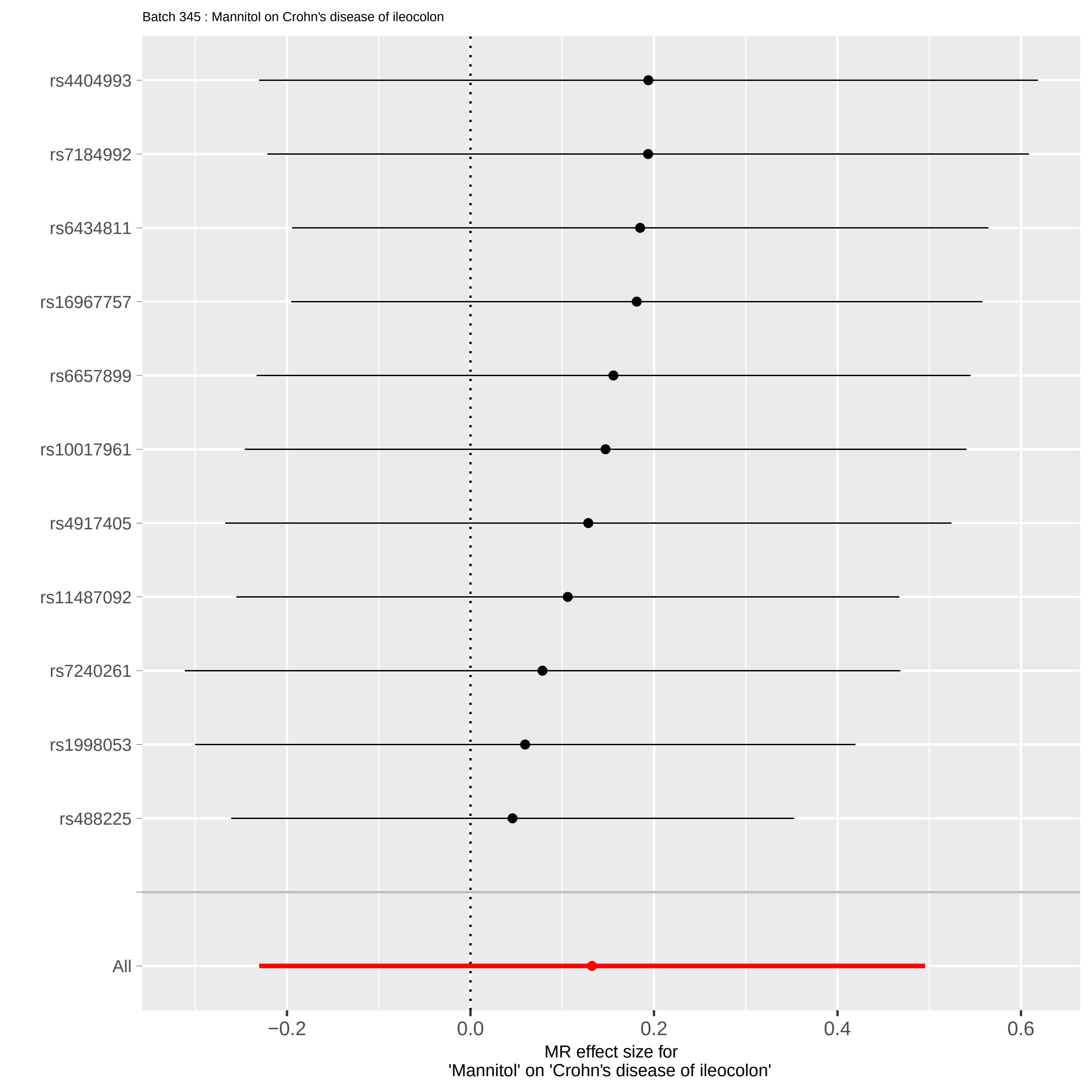
Batch 345 : Mannitol on Crohn's disease of ileocolon
rs4404993
rs7184992
rs6434811
rs16967757
rs6657899
rs10017961
rs4917405
rs11487092
rs7240261
rs1998053
rs488225
All
−0.2
0.0
0.2
0.4
0.6
MR effect size for
'Mannitol' on 'Crohn's disease of ileocolon'

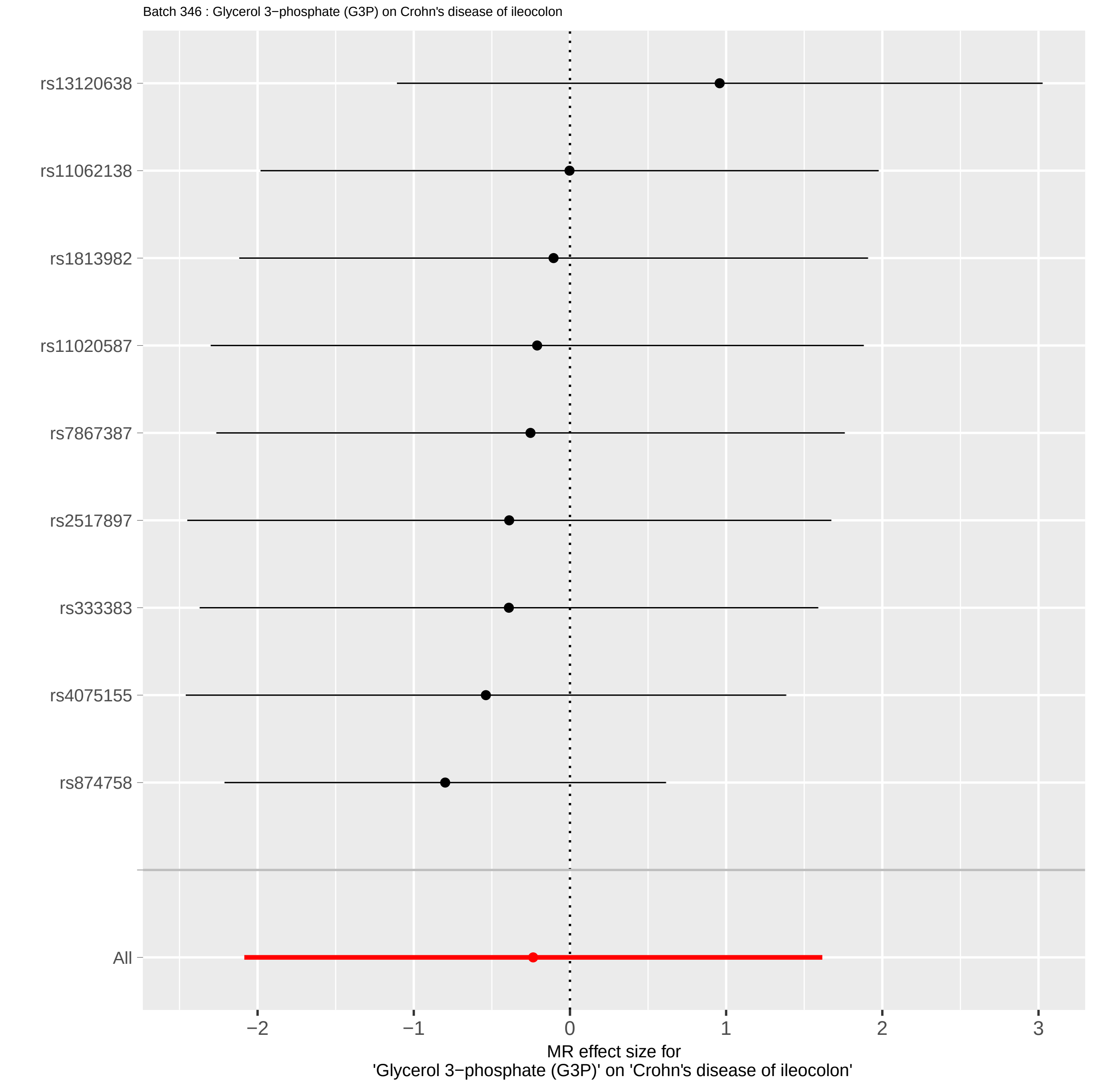

Batch 346 : Glycerol 3−phosphate (G3P) on Crohn's disease of ileocolon
rs13120638
rs11062138
rs1813982
rs11020587
rs7867387
rs2517897
rs333383
rs4075155
rs874758
All
−2
−1
0
1
2
3
MR effect size for
'Glycerol 3−phosphate (G3P)' on 'Crohn's disease of ileocolon'

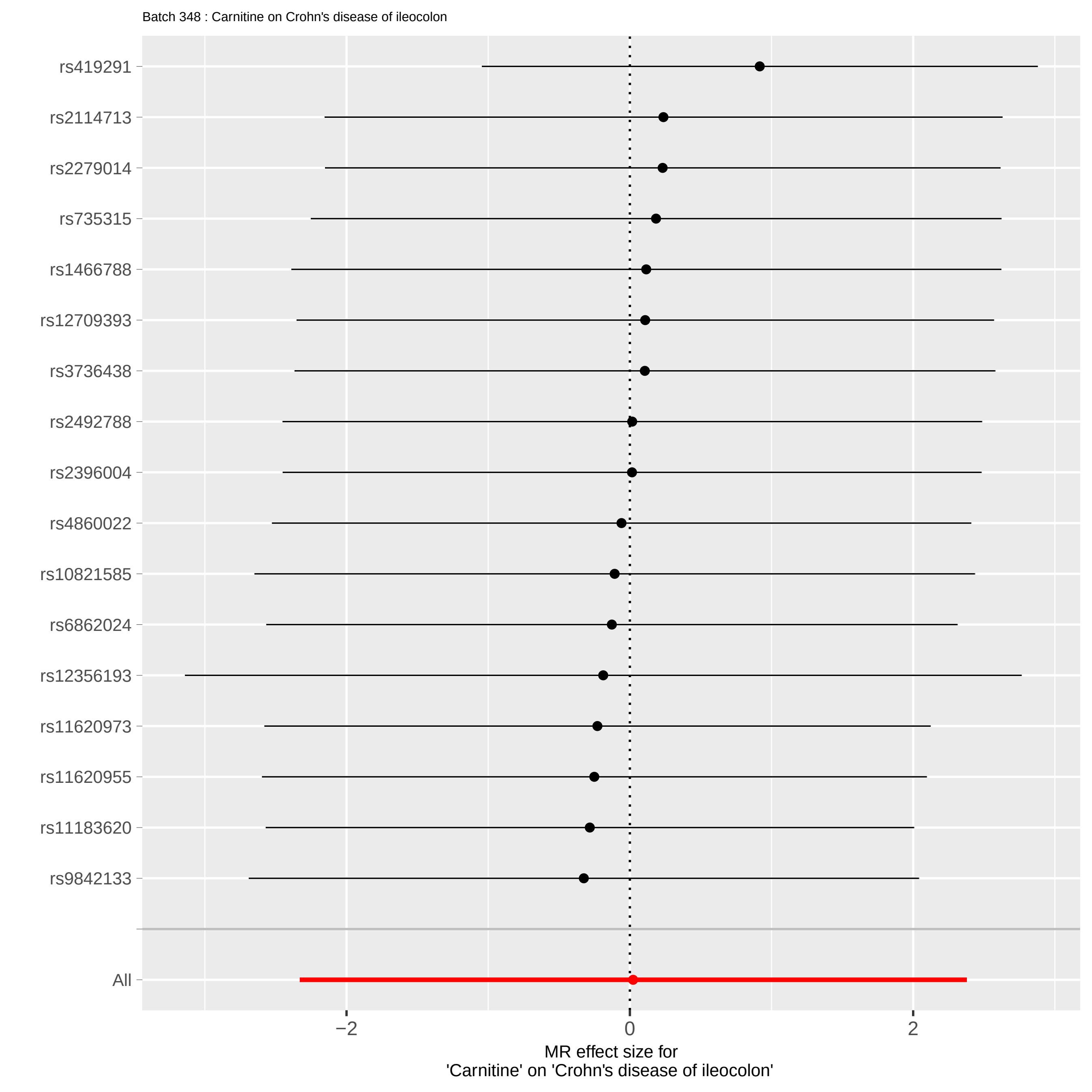
Batch 348 : Carnitine on Crohn's disease of ileocolon
rs419291
rs2114713
rs2279014
rs735315
rs1466788
rs12709393
rs3736438
rs2492788
rs2396004
rs4860022
rs10821585
rs6862024
rs12356193
rs11620973
rs11620955
rs11183620
rs9842133
All
−2
0
2
MR effect size for
'Carnitine' on 'Crohn's disease of ileocolon'

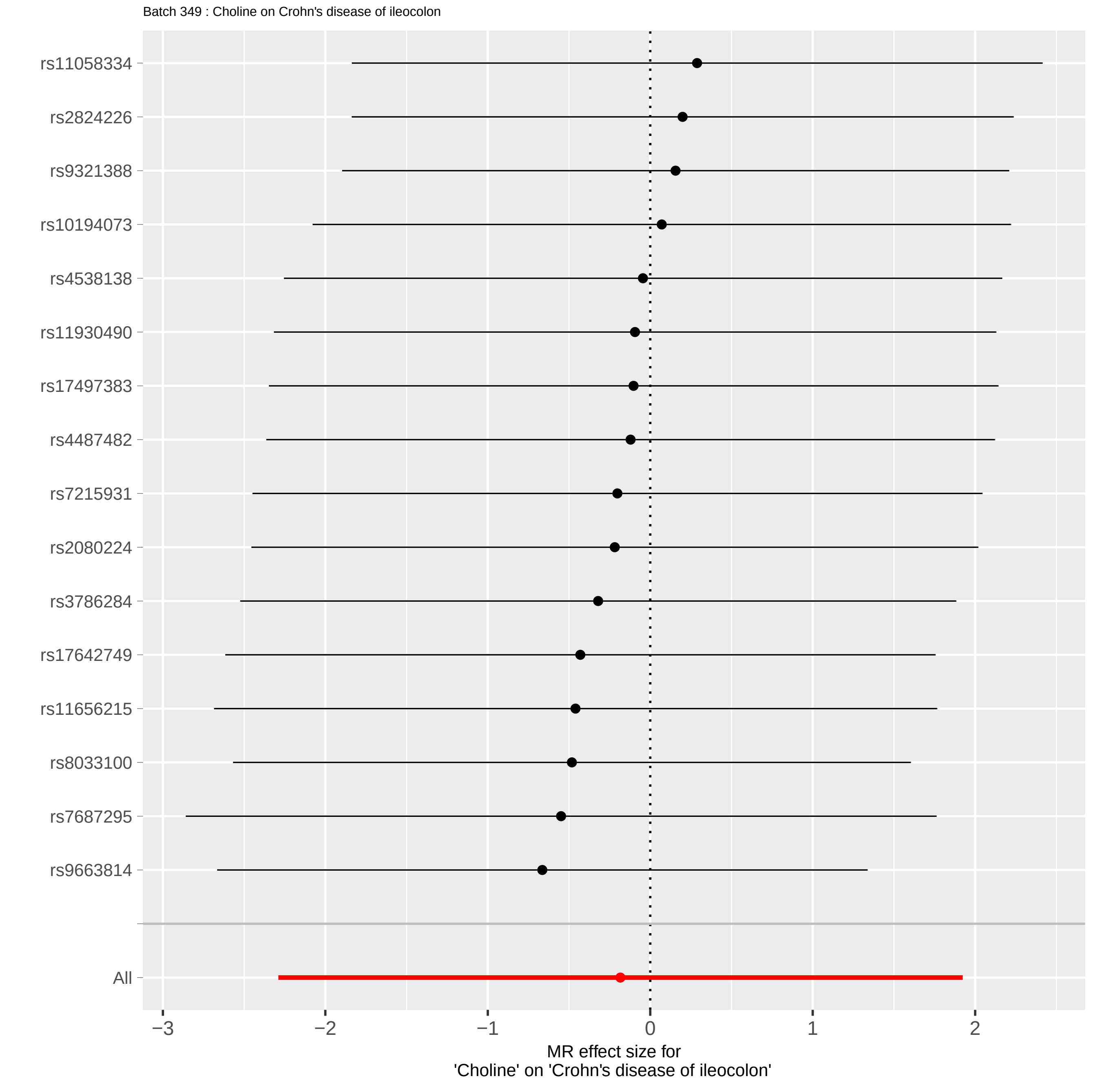

Batch 349 : Choline on Crohn's disease of ileocolon
rs11058334
rs2824226
rs9321388
rs10194073
rs4538138
rs11930490
rs17497383
rs4487482
rs7215931
rs2080224
rs3786284
rs17642749
rs11656215
rs8033100
rs7687295
rs9663814
All
−3
−2
−1
0
1
2
MR effect size for
'Choline' on 'Crohn's disease of ileocolon'

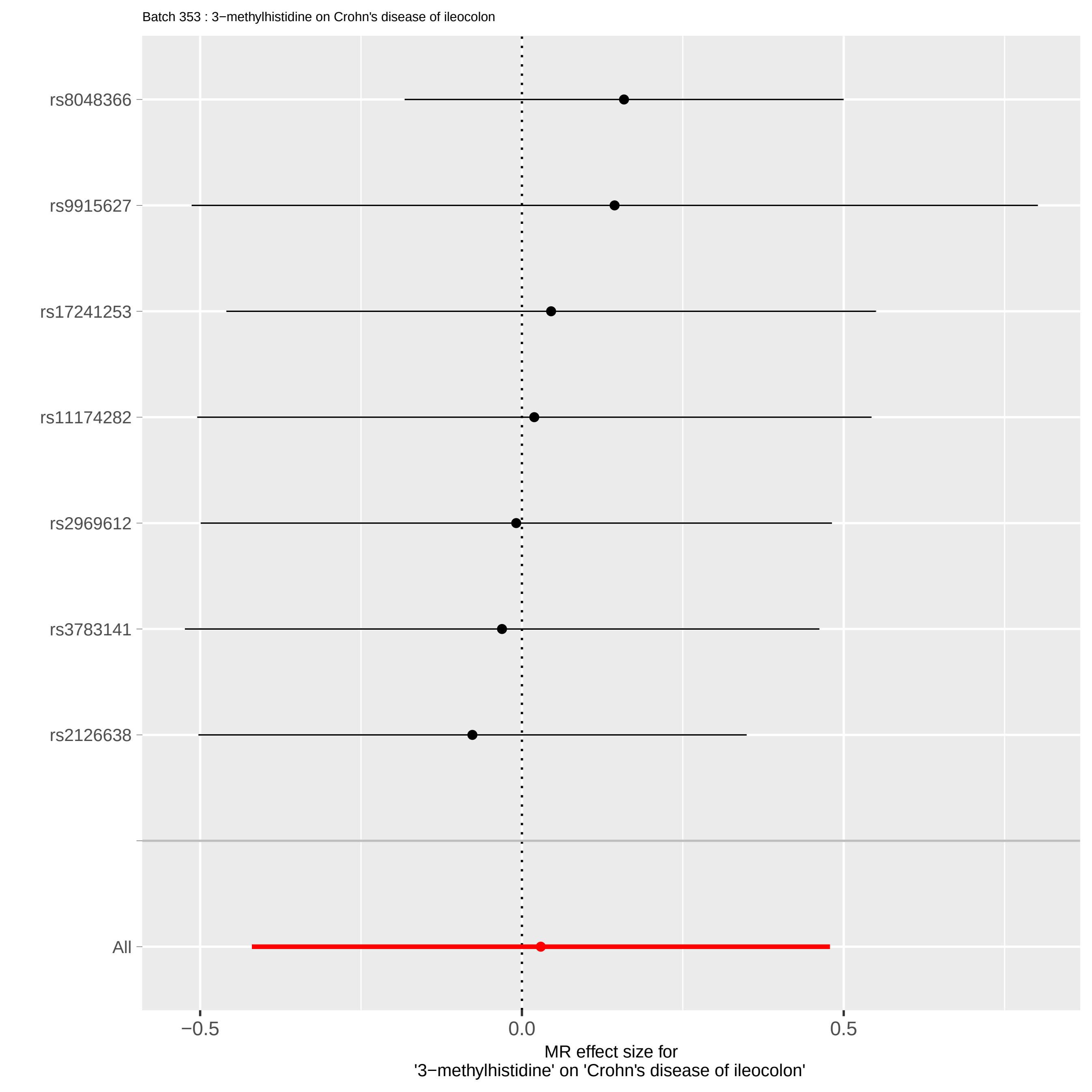
Batch 353 : 3−methylhistidine on Crohn's disease of ileocolon
rs8048366
rs9915627
rs17241253
rs11174282
rs2969612
rs3783141
rs2126638
All
−0.5
0.0
0.5
MR effect size for
'3−methylhistidine' on 'Crohn's disease of ileocolon'

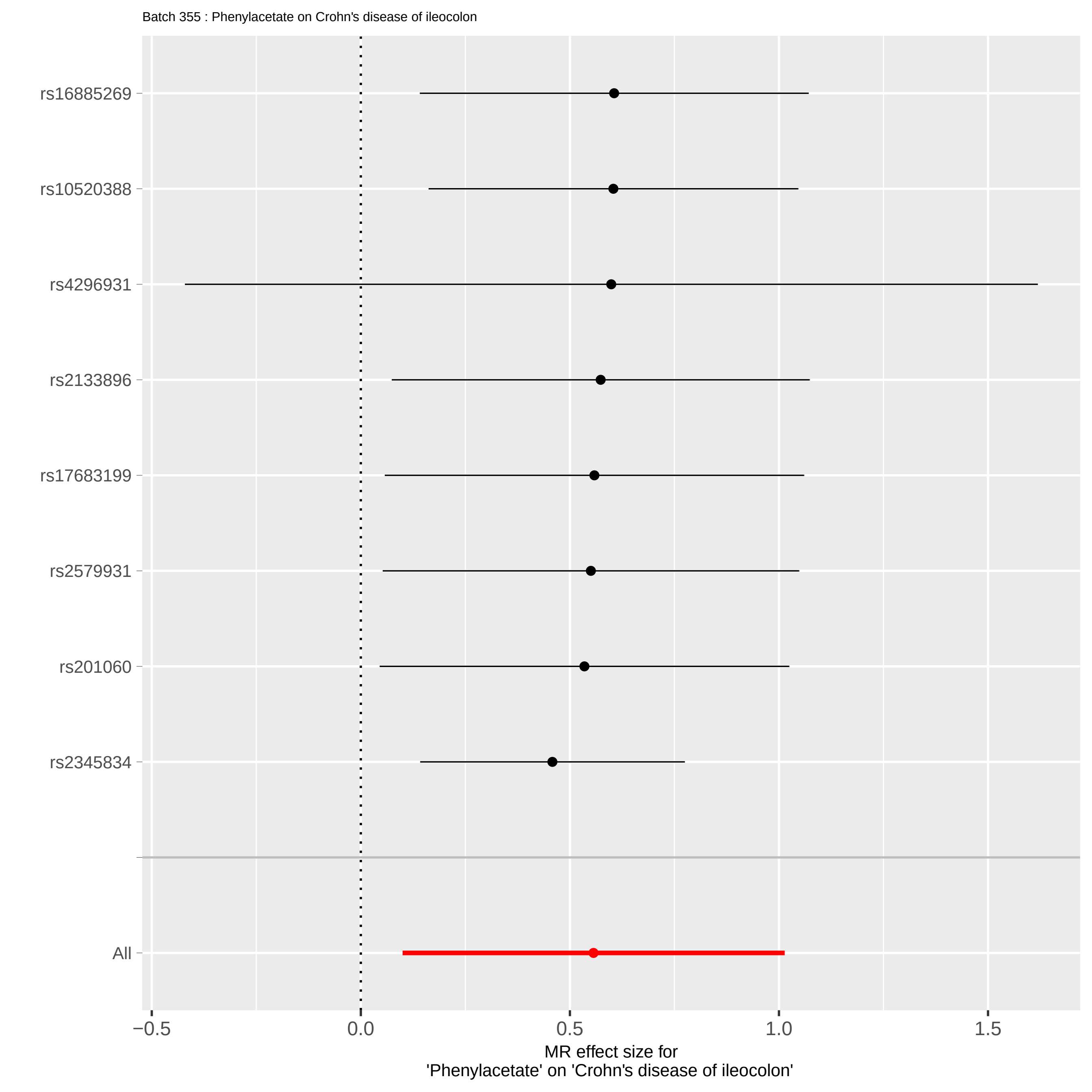

Batch 355 : Phenylacetate on Crohn's disease of ileocolon
rs16885269
rs10520388
rs4296931
rs2133896
rs17683199
rs2579931
rs201060
rs2345834
All
−0.5
0.0
0.5
1.0
1.5
MR effect size for
'Phenylacetate' on 'Crohn's disease of ileocolon'

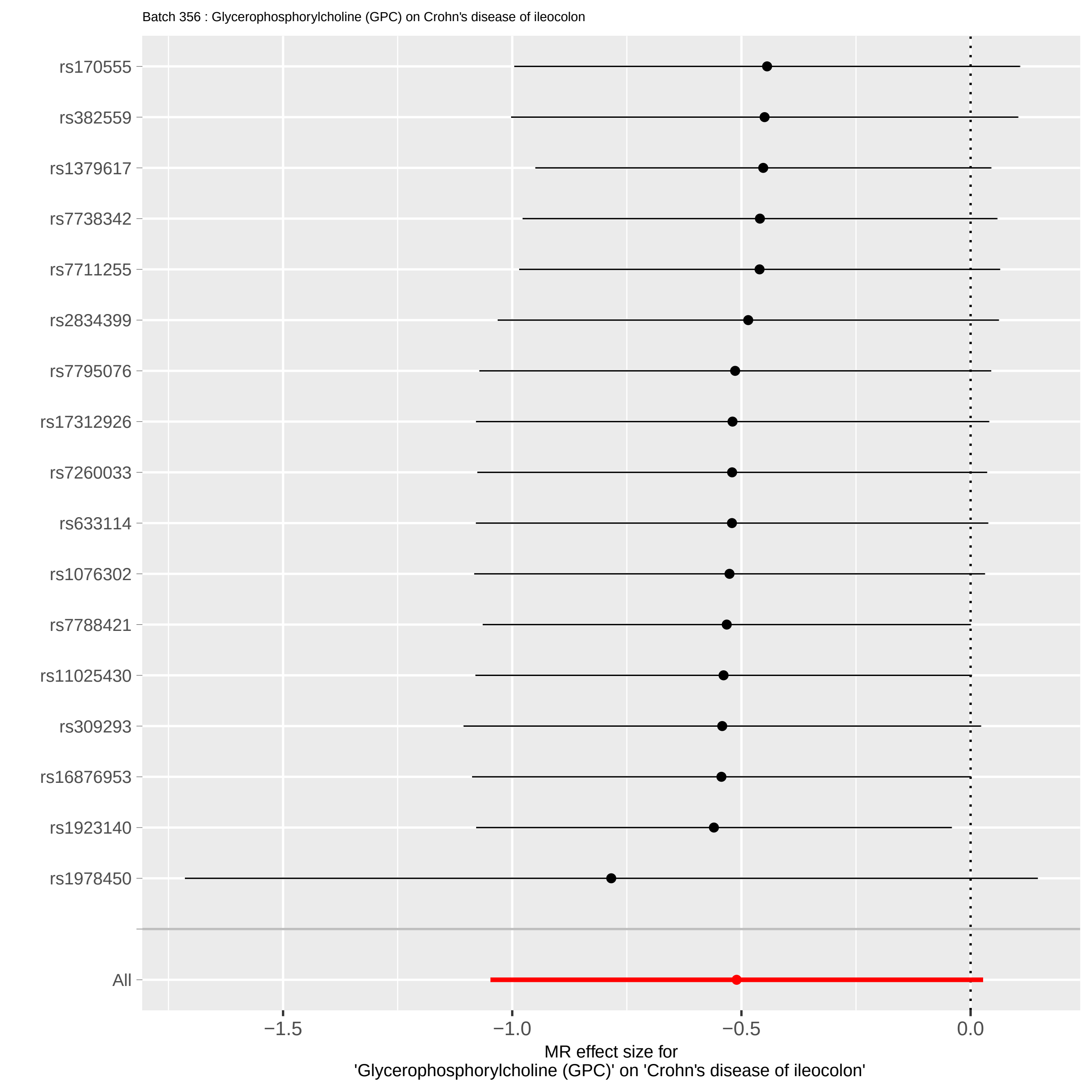

Batch 356 : Glycerophosphorylcholine (GPC) on Crohn's disease of ileocolon
rs170555
rs382559
rs1379617
rs7738342
rs7711255
rs2834399
rs7795076
rs17312926
rs7260033
rs633114
rs1076302
rs7788421
rs11025430
rs309293
rs16876953
rs1923140
rs1978450
All
−1.5
−1.0
−0.5
0.0
MR effect size for
'Glycerophosphorylcholine (GPC)' on 'Crohn's disease of ileocolon'

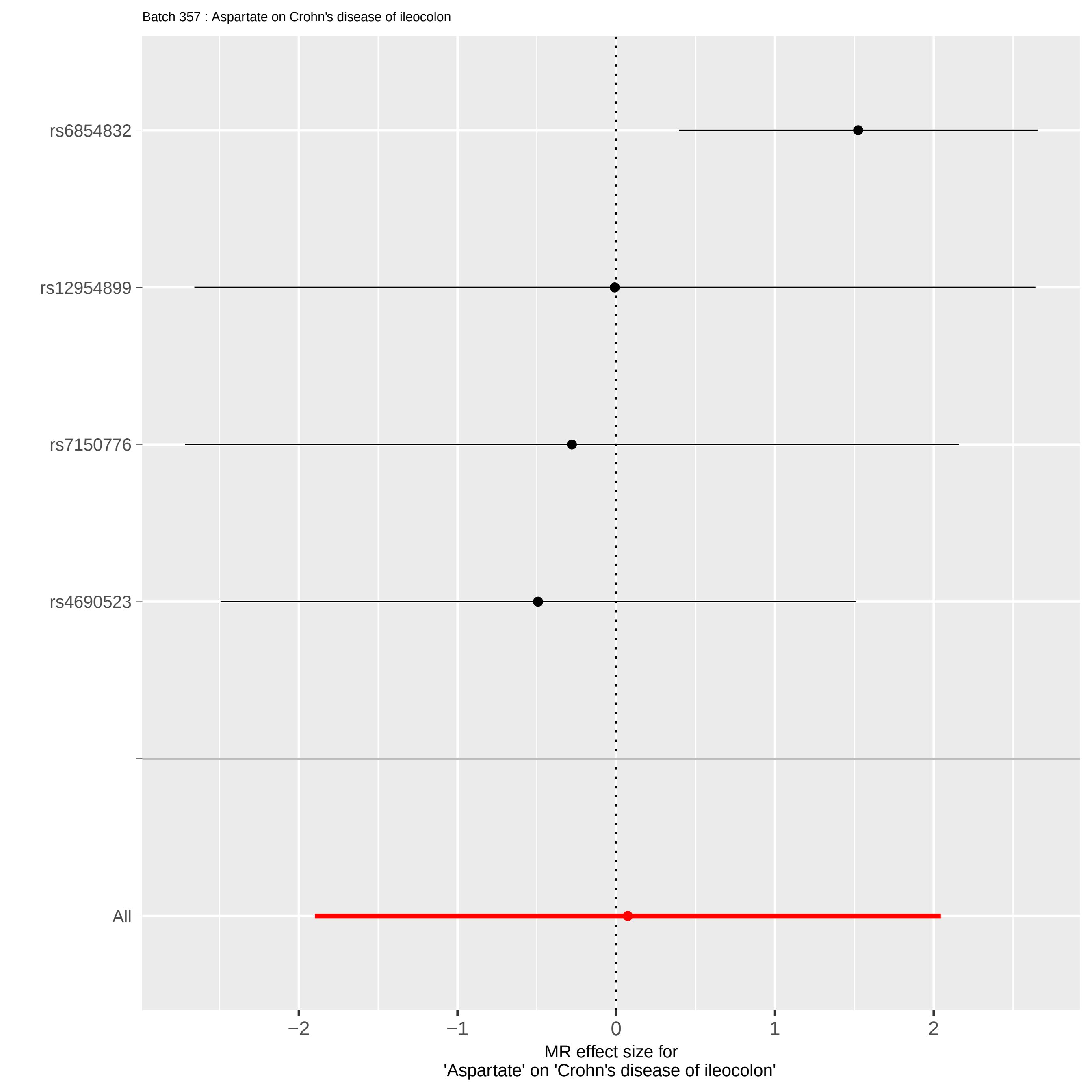

Batch 357 : Aspartate on Crohn's disease of ileocolon
rs6854832
rs12954899
rs7150776
rs4690523
All
−2
−1
0
1
2
MR effect size for
'Aspartate' on 'Crohn's disease of ileocolon'

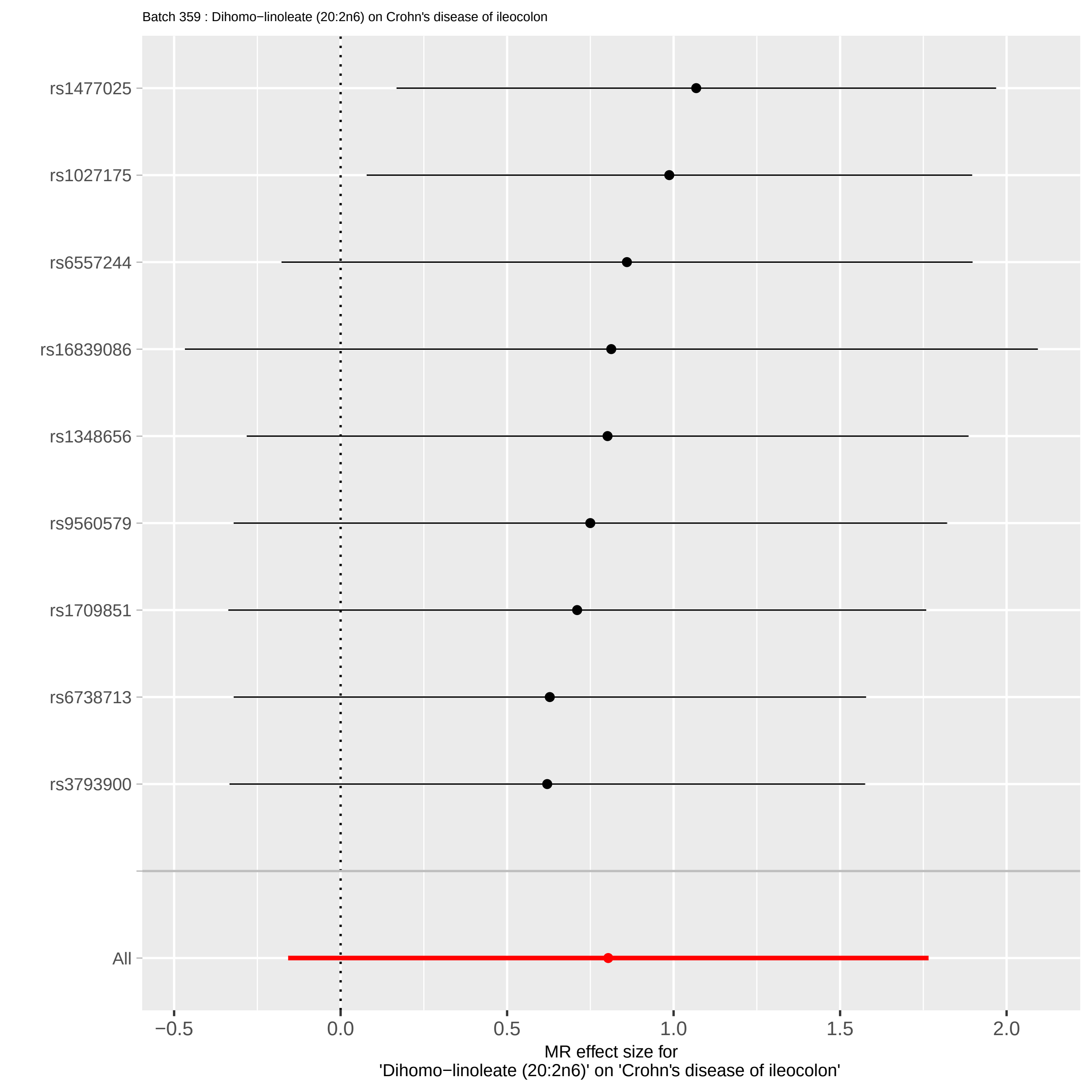
Batch 359 : Dihomo−linoleate (20:2n6) on Crohn's disease of ileocolon
rs1477025
rs1027175
rs6557244
rs16839086
rs1348656
rs9560579
rs1709851
rs6738713
rs3793900
All
−0.5
0.0
0.5
1.0
1.5
2.0
MR effect size for
'Dihomo−linoleate (20:2n6)' on 'Crohn's disease of ileocolon'

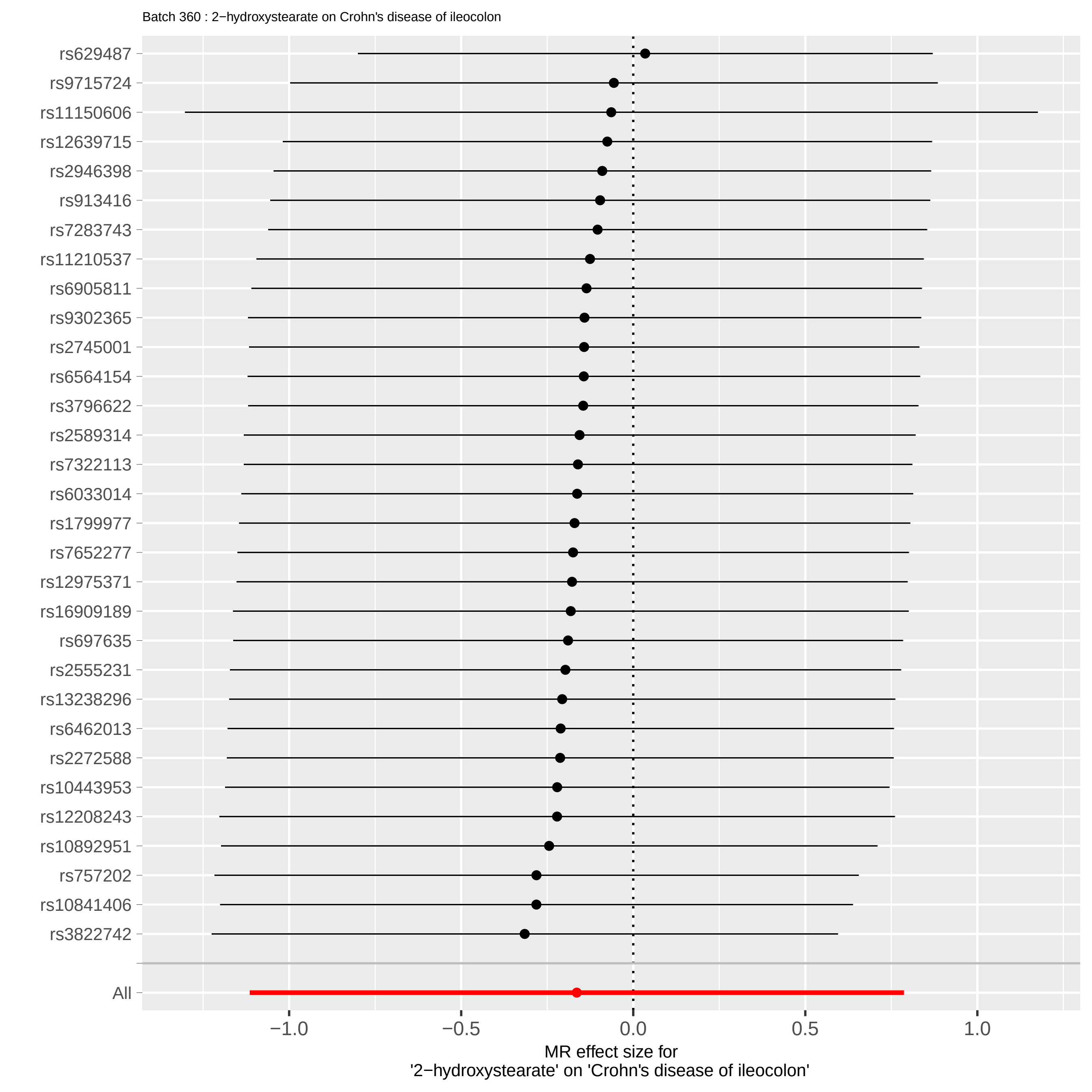

Batch 360 : 2−hydroxystearate on Crohn's disease of ileocolon
rs629487
rs9715724
rs11150606
rs12639715
rs2946398
rs913416
rs7283743
rs11210537
rs6905811
rs9302365
rs2745001
rs6564154
rs3796622
rs2589314
rs7322113
rs6033014
rs1799977
rs7652277
rs12975371
rs16909189
rs697635
rs2555231
rs13238296
rs6462013
rs2272588
rs10443953
rs12208243
rs10892951
rs757202
rs10841406
rs3822742
All
−1.0
−0.5
0.0
0.5
1.0
MR effect size for
'2−hydroxystearate' on 'Crohn's disease of ileocolon'

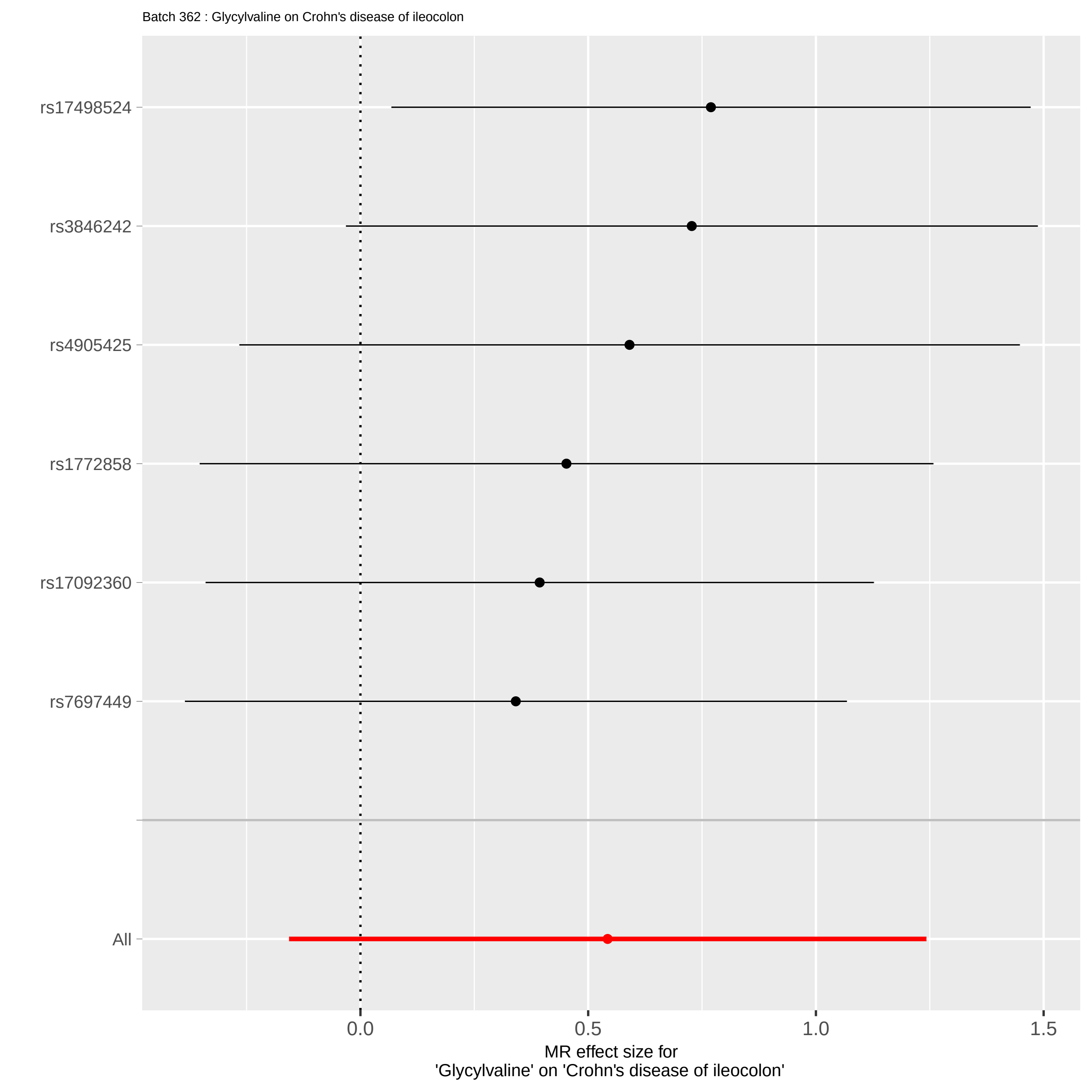

Batch 362 : Glycylvaline on Crohn's disease of ileocolon
rs17498524
rs3846242
rs4905425
rs1772858
rs17092360
rs7697449
All
0.0
0.5
1.0
1.5
MR effect size for
'Glycylvaline' on 'Crohn's disease of ileocolon'

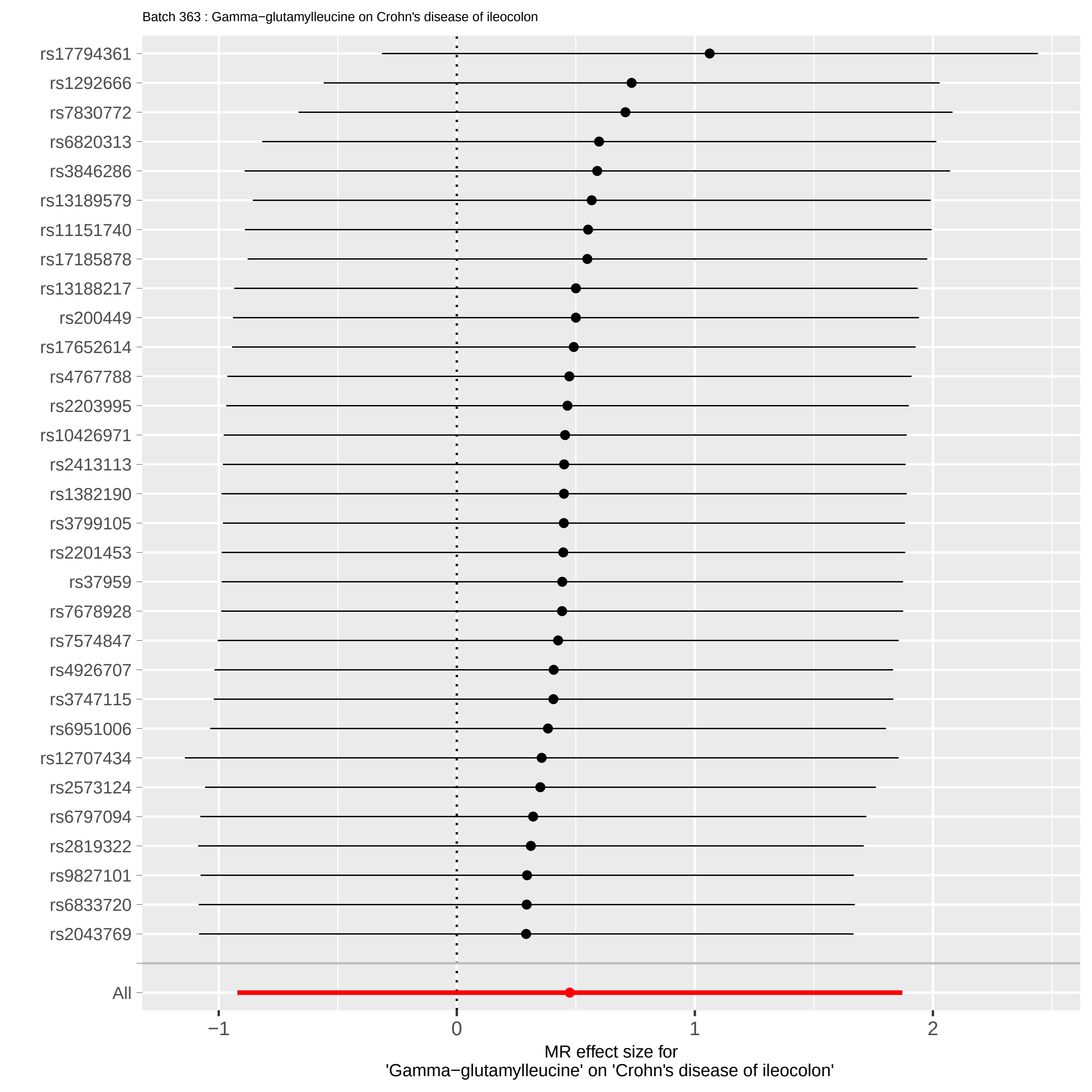

Batch 363 : Gamma−glutamylleucine on Crohn's disease of ileocolon
rs17794361
rs1292666
rs7830772
rs6820313
rs3846286
rs13189579
rs11151740
rs17185878
rs13188217
rs200449
rs17652614
rs4767788
rs2203995
rs10426971
rs2413113
rs1382190
rs3799105
rs2201453
rs37959
rs7678928
rs7574847
rs4926707
rs3747115
rs6951006
rs12707434
rs2573124
rs6797094
rs2819322
rs9827101
rs6833720
rs2043769
All
−1
0
1
2
MR effect size for
'Gamma−glutamylleucine' on 'Crohn's disease of ileocolon'

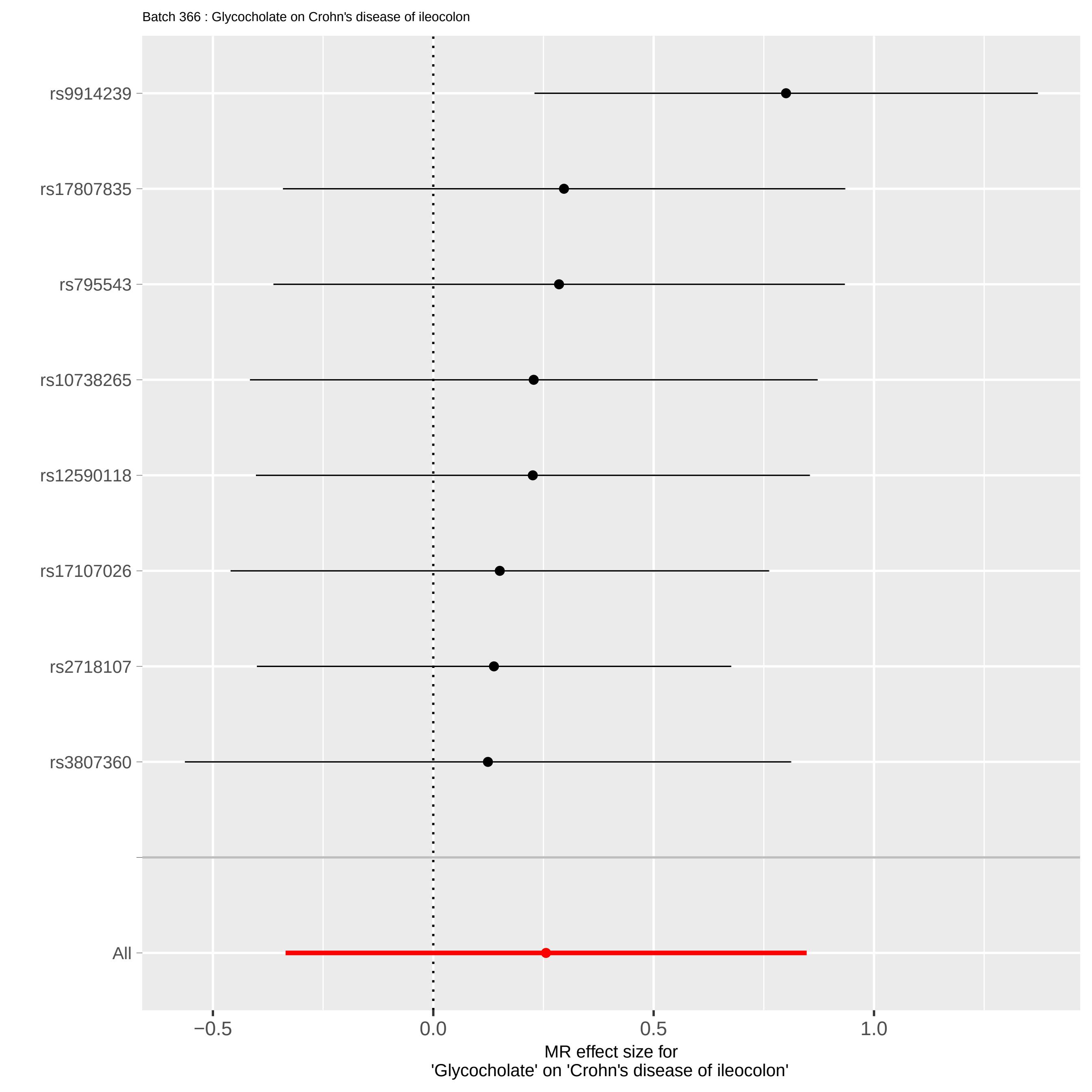
Batch 366 : Glycocholate on Crohn's disease of ileocolon
rs9914239
rs17807835
rs795543
rs10738265
rs12590118
rs17107026
rs2718107
rs3807360
All
−0.5
0.0
0.5
1.0
MR effect size for
'Glycocholate' on 'Crohn's disease of ileocolon'

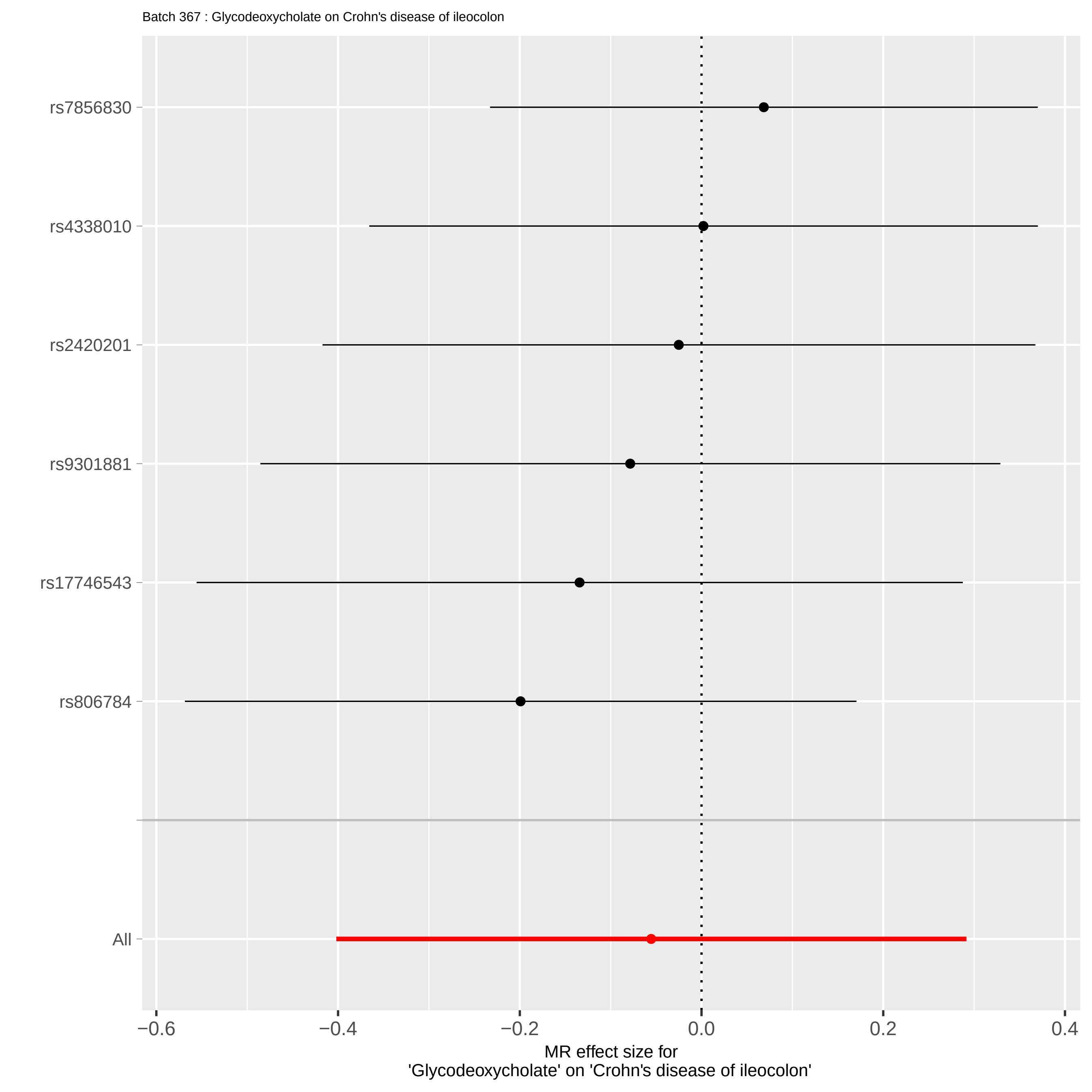

Batch 367 : Glycodeoxycholate on Crohn's disease of ileocolon
rs7856830
rs4338010
rs2420201
rs9301881
rs17746543
rs806784
All
−0.6
−0.4
−0.2
0.0
0.2
0.4
MR effect size for
'Glycodeoxycholate' on 'Crohn's disease of ileocolon'

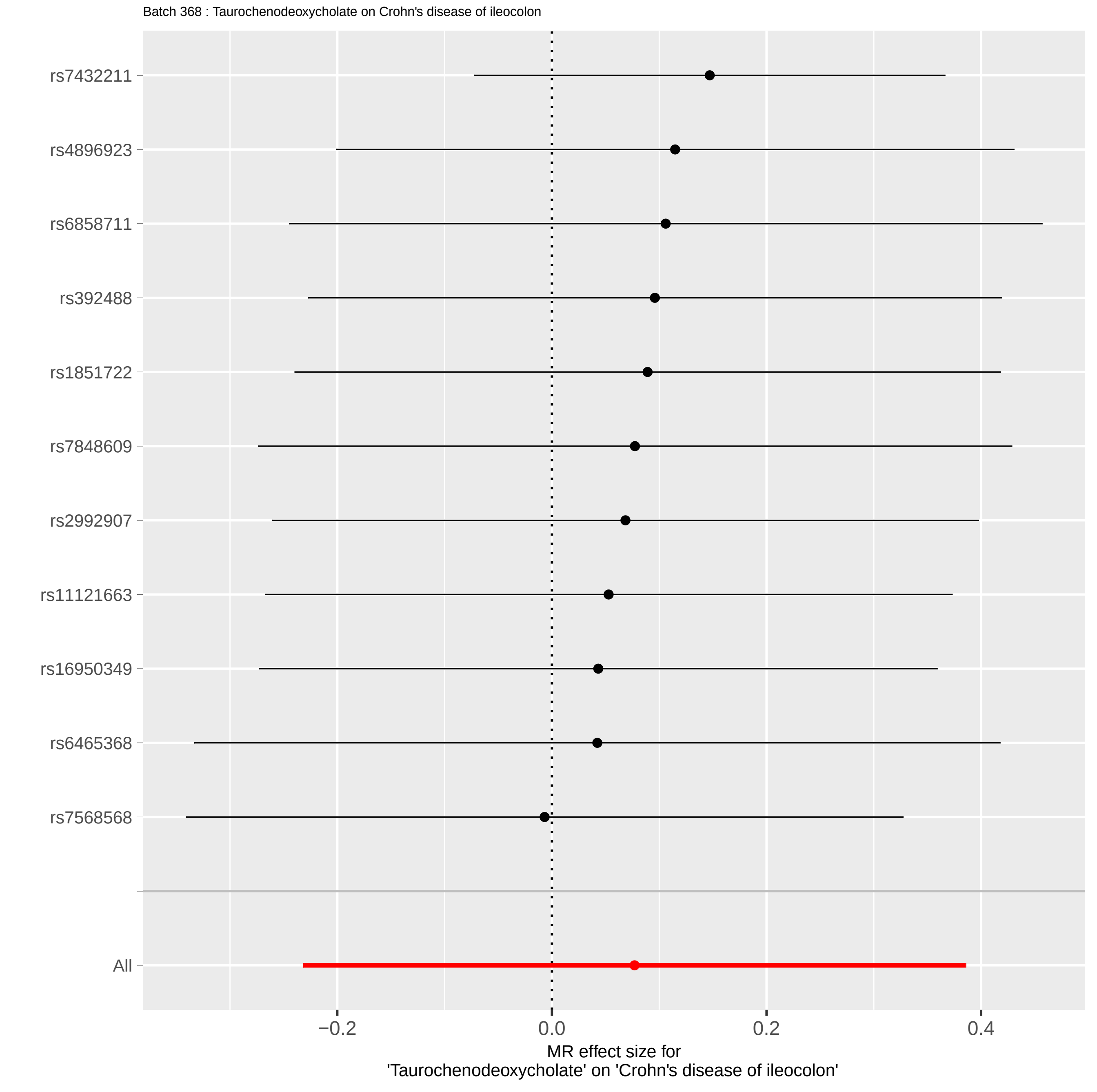

Batch 368 : Taurochenodeoxycholate on Crohn's disease of ileocolon
rs7432211
rs4896923
rs6858711
rs392488
rs1851722
rs7848609
rs2992907
rs11121663
rs16950349
rs6465368
rs7568568
All
−0.2
0.0
0.2
0.4
MR effect size for
'Taurochenodeoxycholate' on 'Crohn's disease of ileocolon'

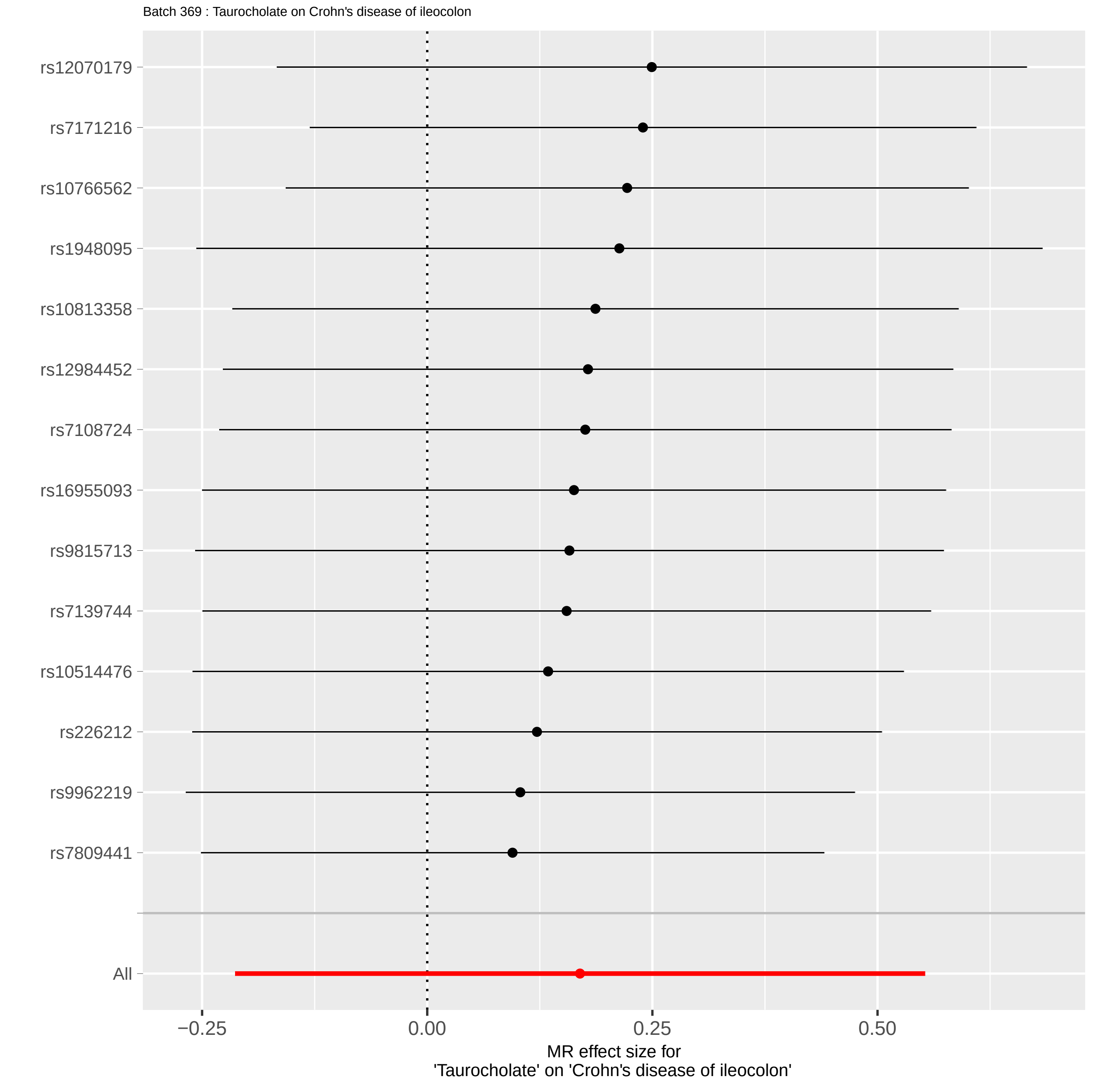

Batch 369 : Taurocholate on Crohn's disease of ileocolon
rs12070179
rs7171216
rs10766562
rs1948095
rs10813358
rs12984452
rs7108724
rs16955093
rs9815713
rs7139744
rs10514476
rs226212
rs9962219
rs7809441
All
−0.25
0.00
0.25
0.50
MR effect size for
'Taurocholate' on 'Crohn's disease of ileocolon'

Batch 371 : 1−stearoylglycerophosphoinositol on Crohn's disease of ileocolon

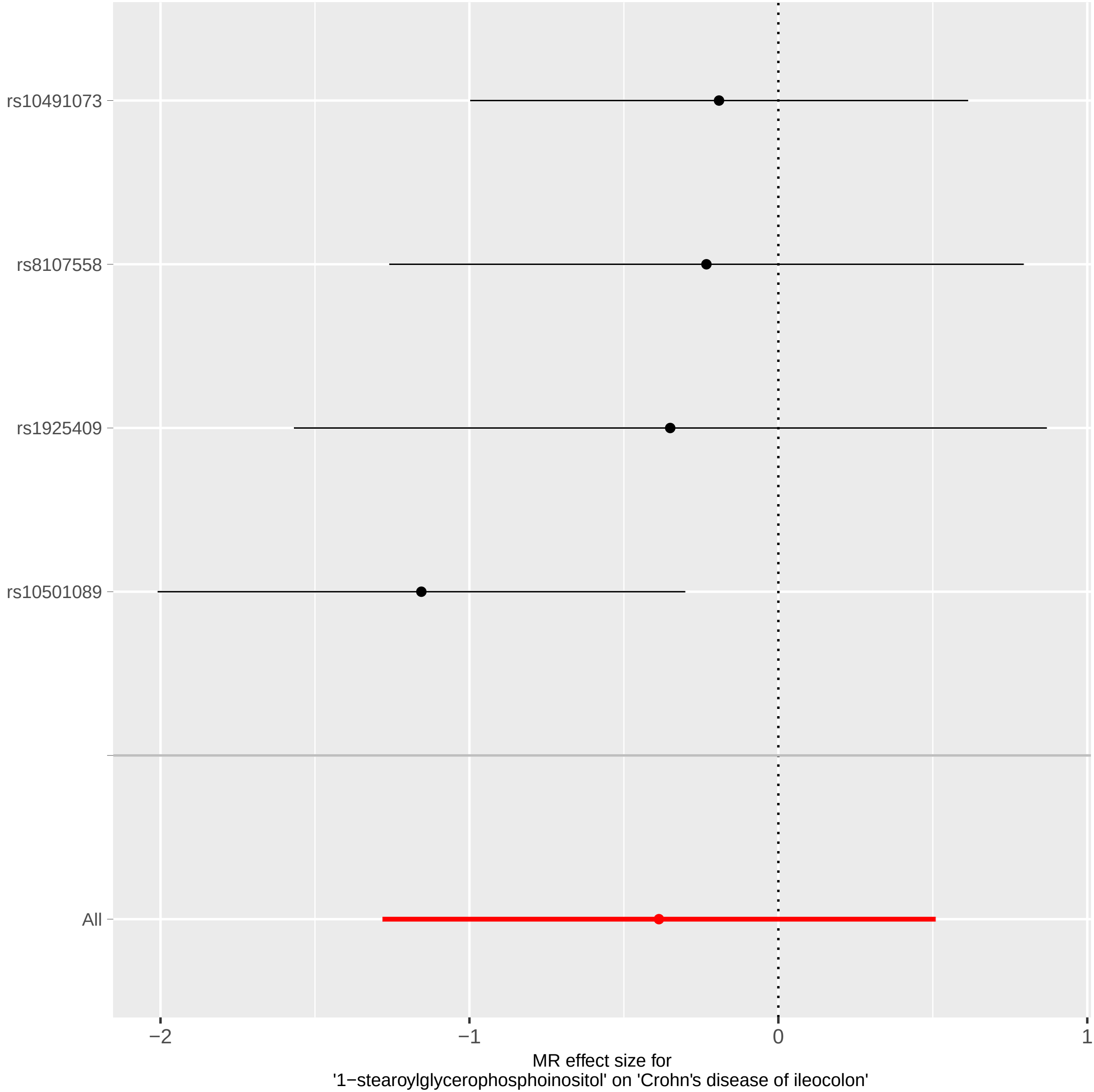

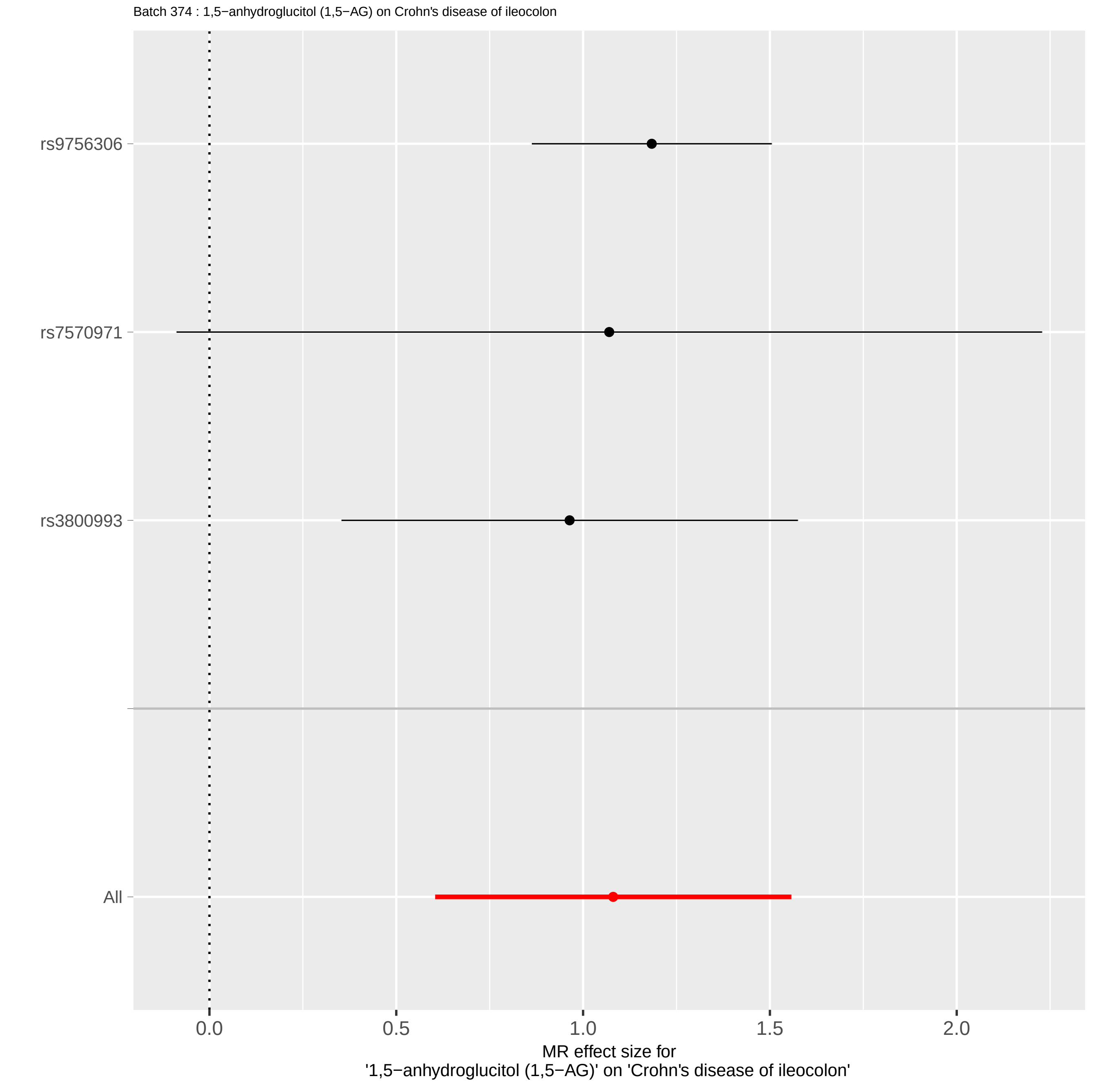

Batch 374 : 1,5−anhydroglucitol (1,5−AG) on Crohn's disease of ileocolon
rs9756306
rs7570971
rs3800993
All
0.0
0.5
1.0
1.5
2.0
MR effect size for
'1,5−anhydroglucitol (1,5−AG)' on 'Crohn's disease of ileocolon'

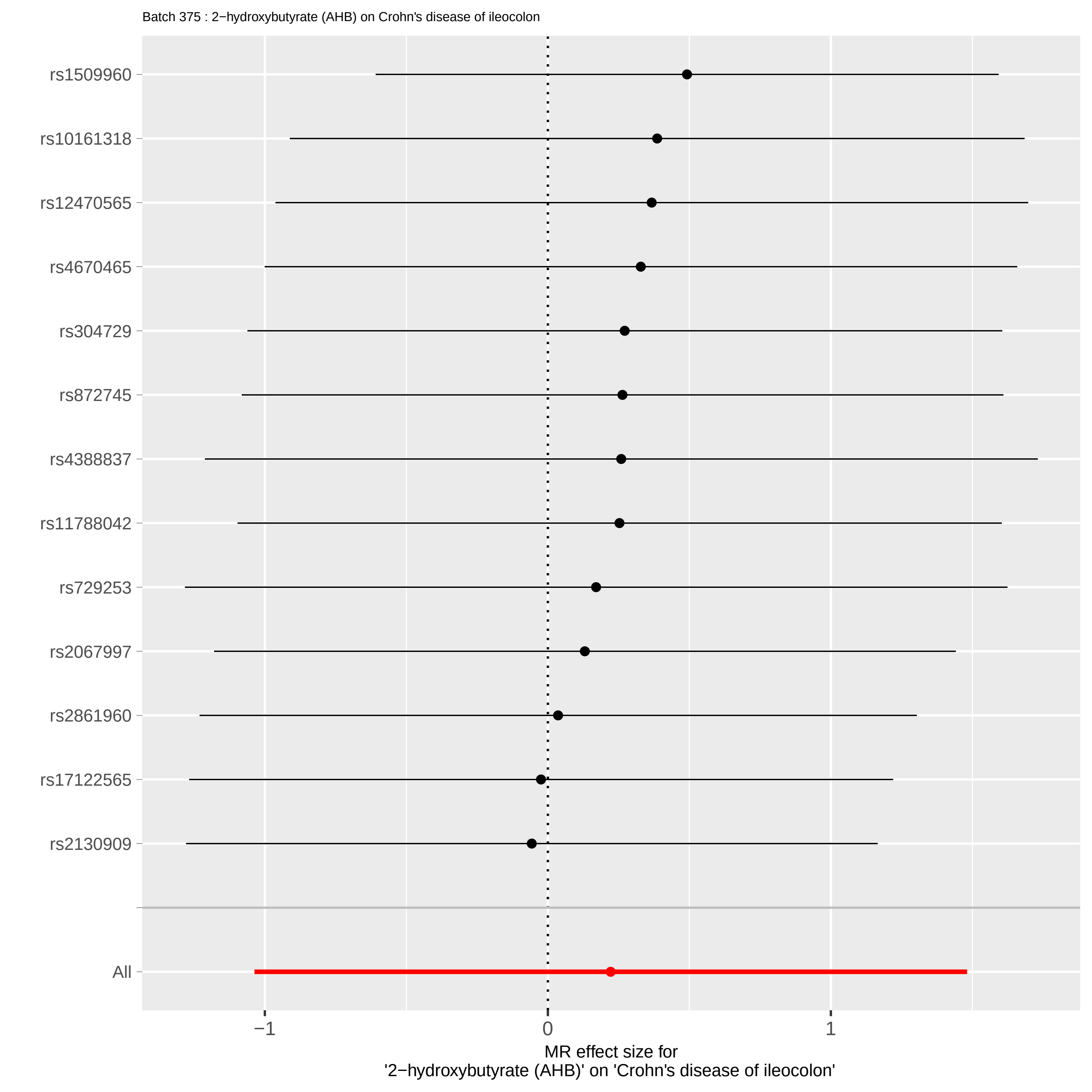
Batch 375 : 2−hydroxybutyrate (AHB) on Crohn's disease of ileocolon
rs1509960
rs10161318
rs12470565
rs4670465
rs304729
rs872745
rs4388837
rs11788042
rs729253
rs2067997
rs2861960
rs17122565
rs2130909
All
−1
0
1
MR effect size for
'2−hydroxybutyrate (AHB)' on 'Crohn's disease of ileocolon'

Batch 378 : 1−palmitoylglycerol (1−monopalmitin) on Crohn's disease of ileocolon

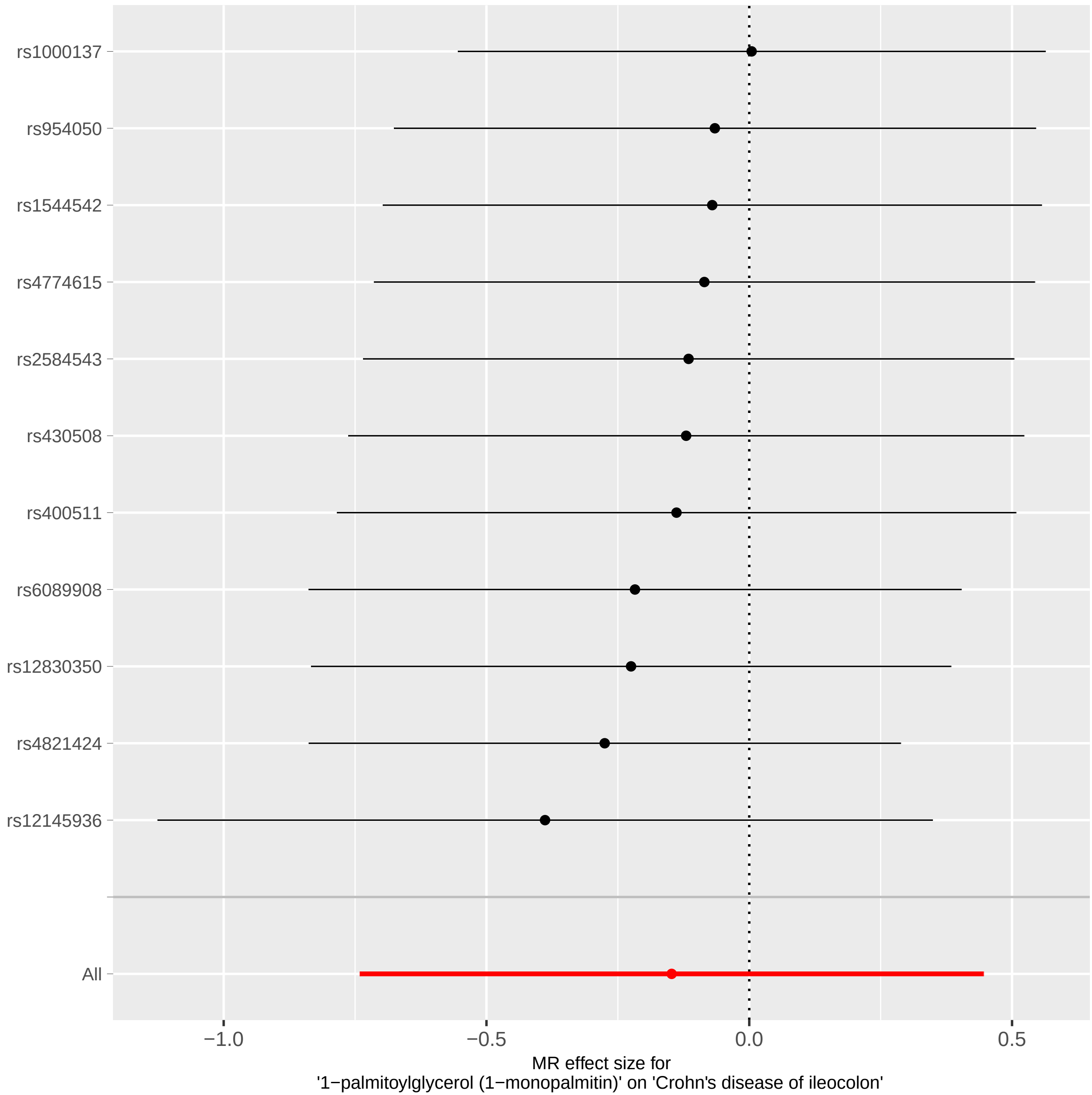

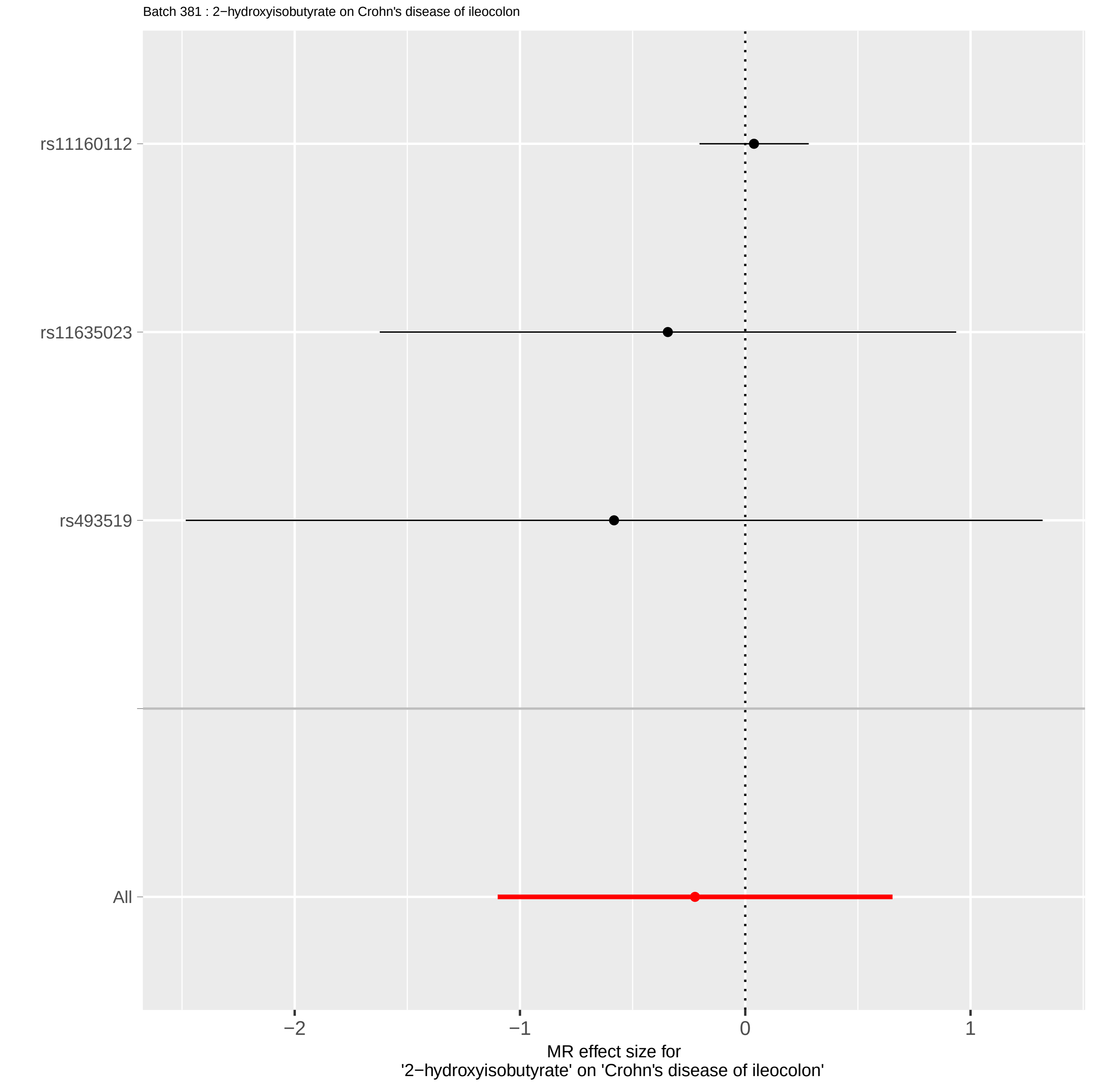

Batch 381 : 2−hydroxyisobutyrate on Crohn's disease of ileocolon
rs11160112
rs11635023
rs493519
All
−2
−1
0
1
MR effect size for
'2−hydroxyisobutyrate' on 'Crohn's disease of ileocolon'

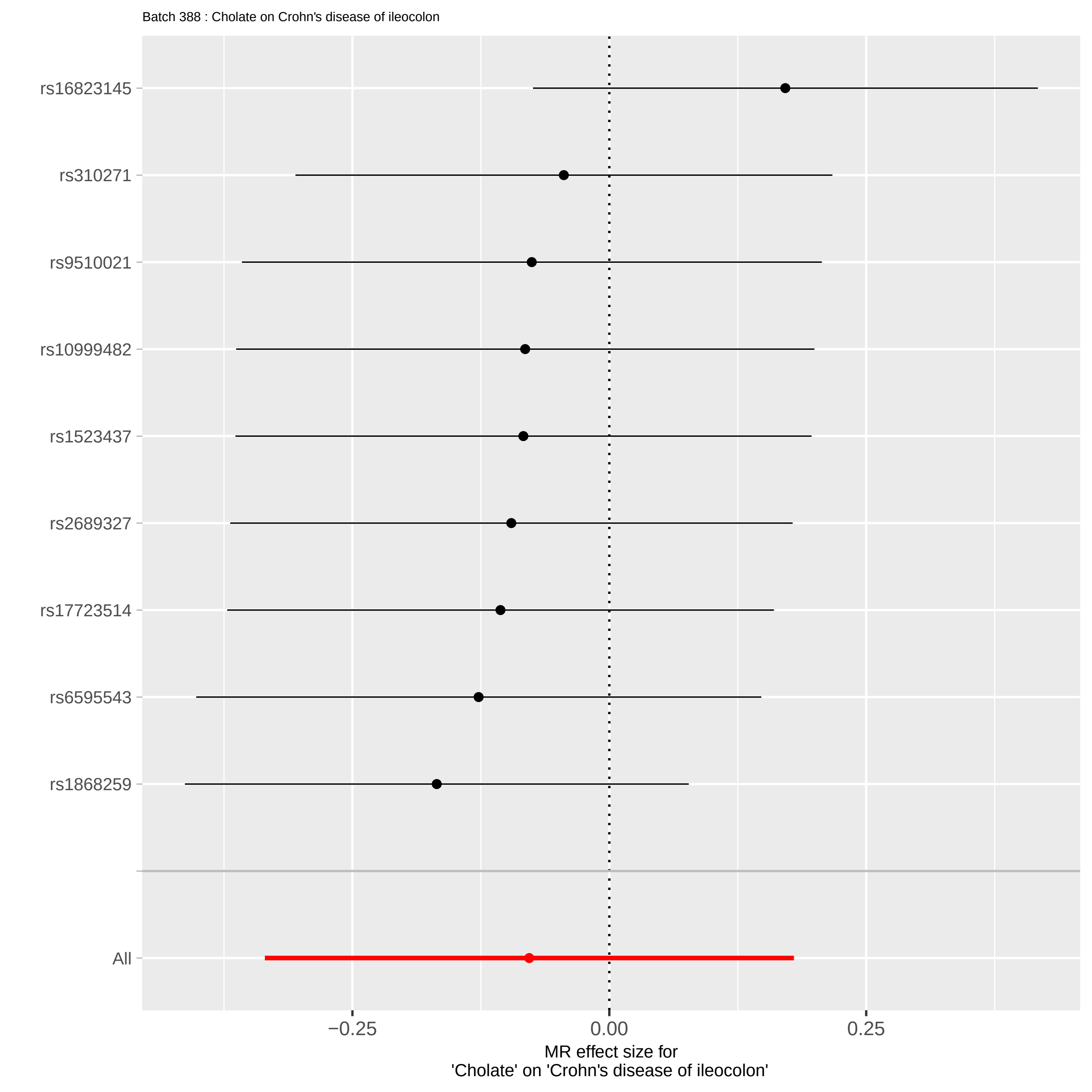

Batch 388 : Cholate on Crohn's disease of ileocolon
rs16823145
rs310271
rs9510021
rs10999482
rs1523437
rs2689327
rs17723514
rs6595543
rs1868259
All
−0.25
0.00
0.25
MR effect size for
'Cholate' on 'Crohn's disease of ileocolon'

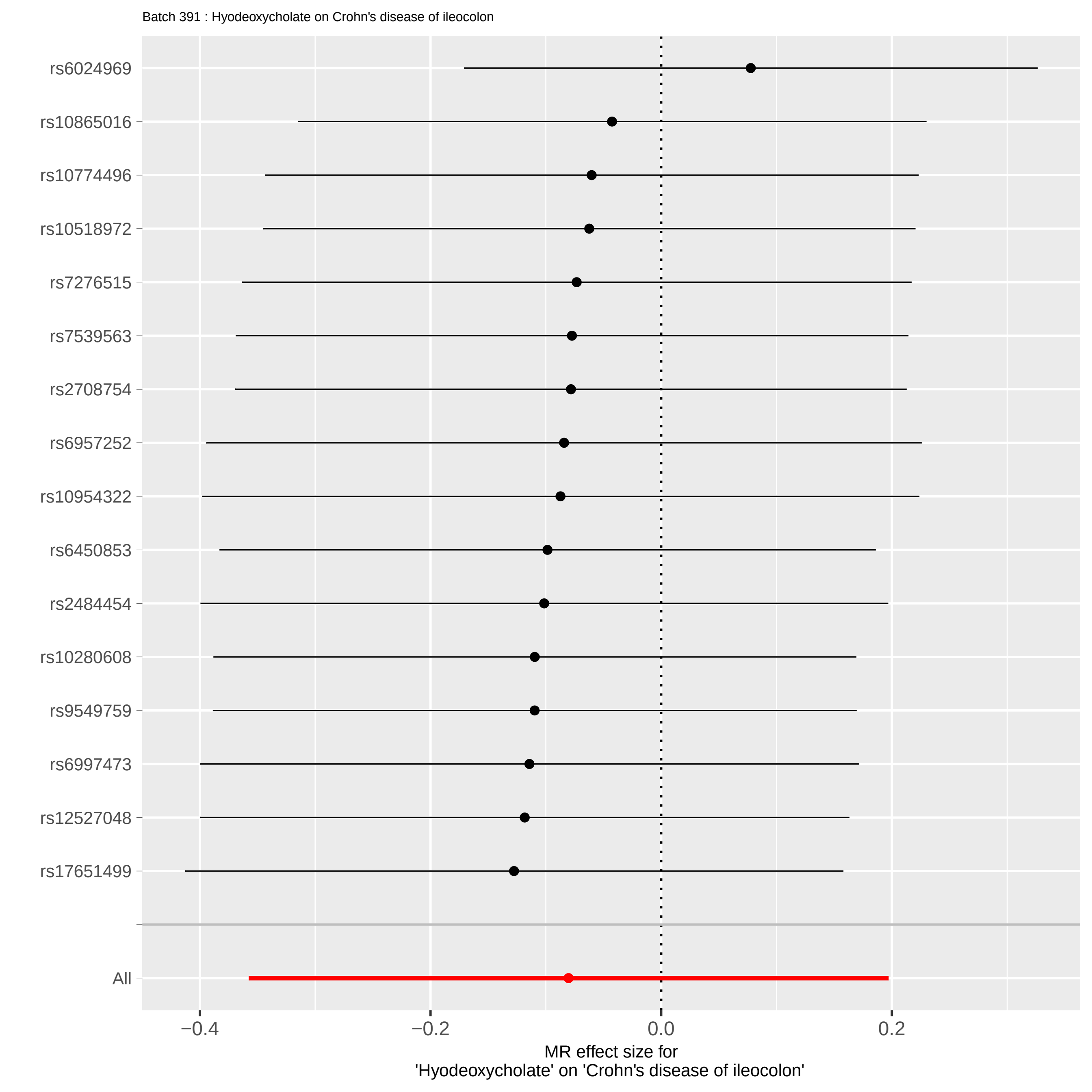

Batch 391 : Hyodeoxycholate on Crohn's disease of ileocolon
rs6024969
rs10865016
rs10774496
rs10518972
rs7276515
rs7539563
rs2708754
rs6957252
rs10954322
rs6450853
rs2484454
rs10280608
rs9549759
rs6997473
rs12527048
rs17651499
All
−0.4
−0.2
0.0
0.2
MR effect size for
'Hyodeoxycholate' on 'Crohn's disease of ileocolon'

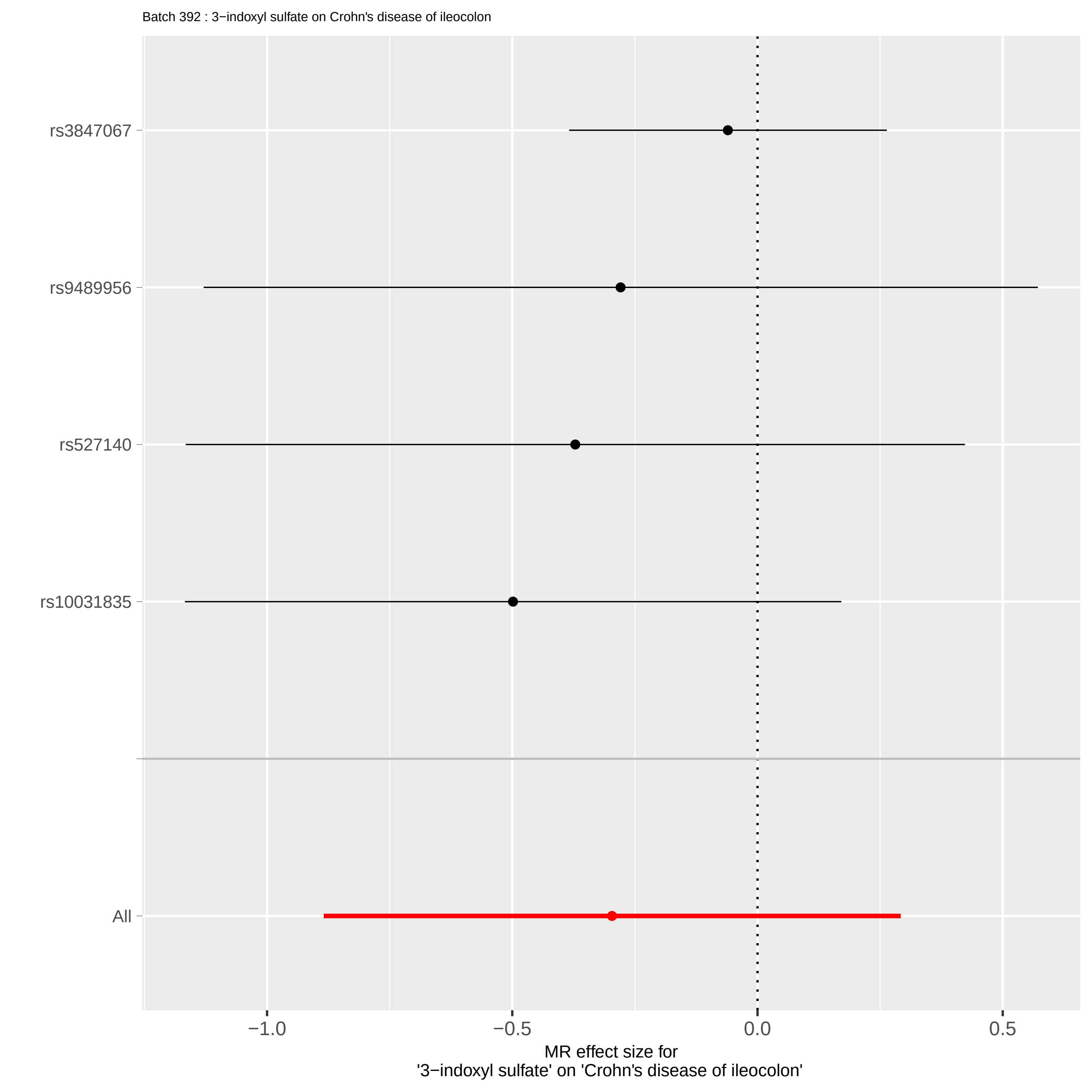

Batch 392 : 3−indoxyl sulfate on Crohn's disease of ileocolon
rs3847067
rs9489956
rs527140
rs10031835
All
−1.0
−0.5
0.0
0.5
MR effect size for
'3−indoxyl sulfate' on 'Crohn's disease of ileocolon'

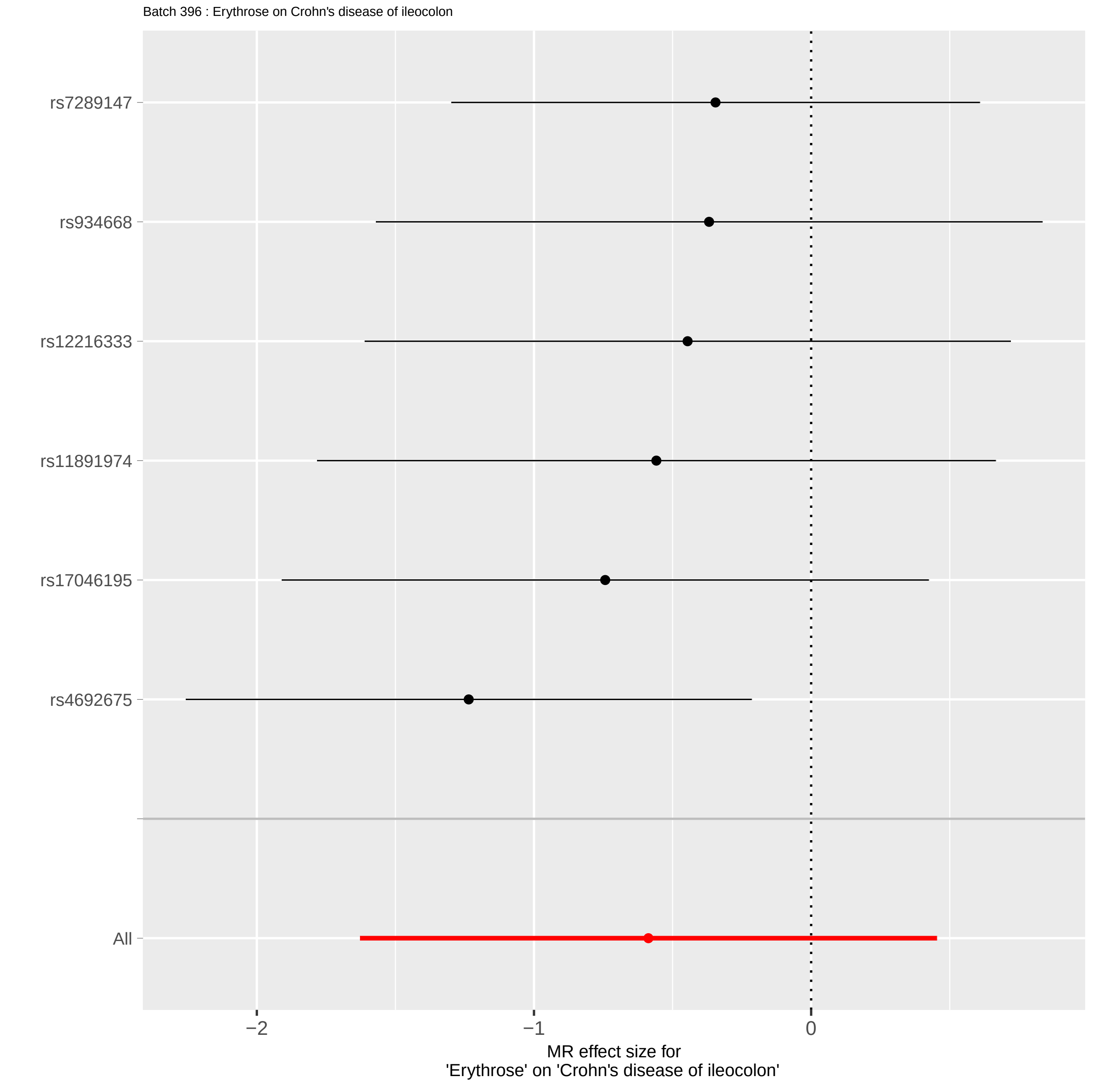
Batch 396 : Erythrose on Crohn's disease of ileocolon
rs7289147
rs934668
rs12216333
rs11891974
rs17046195
rs4692675
All
−2
−1
0
MR effect size for
'Erythrose' on 'Crohn's disease of ileocolon'

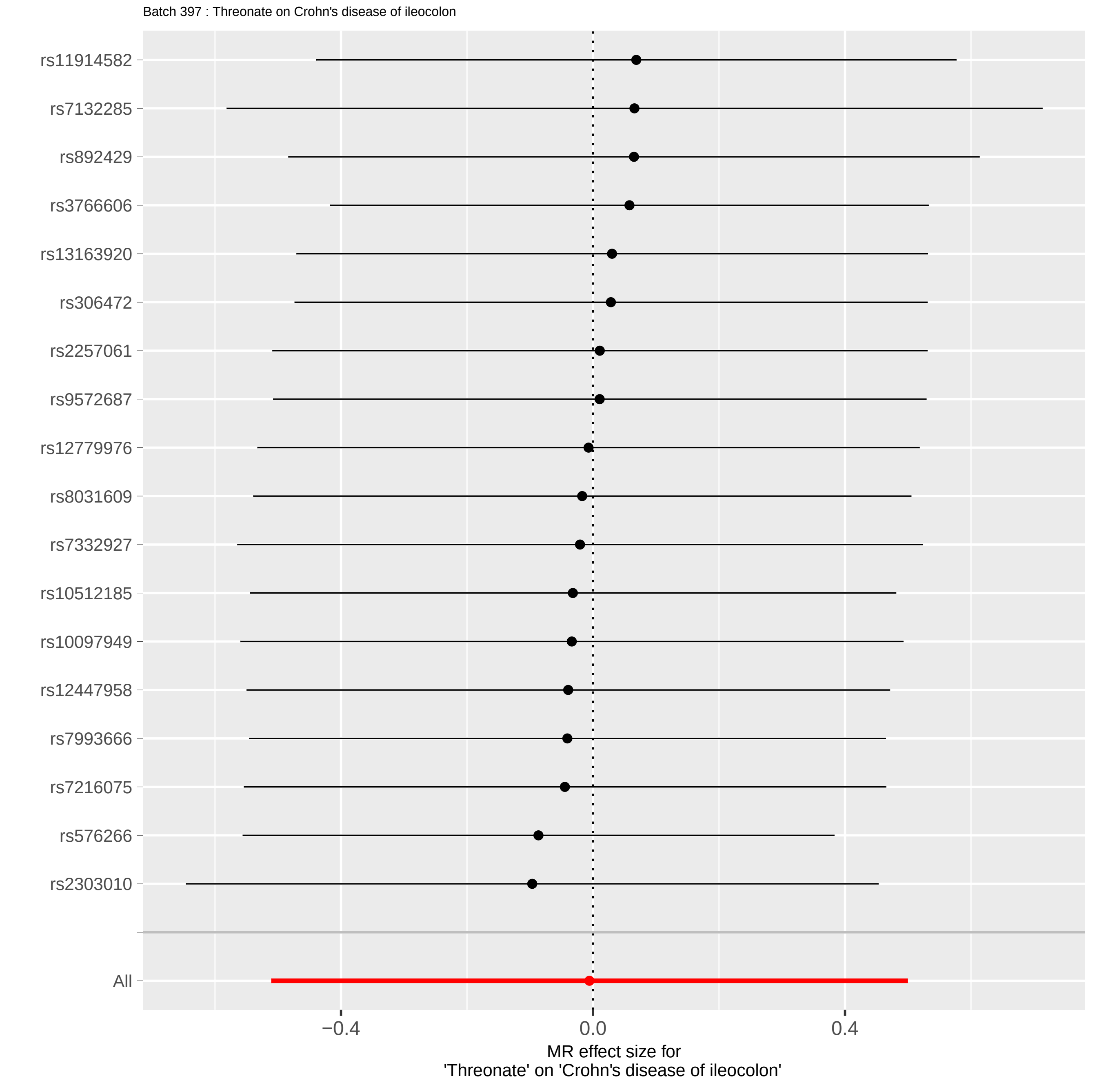

Batch 397 : Threonate on Crohn's disease of ileocolon
rs11914582
rs7132285
rs892429
rs3766606
rs13163920
rs306472
rs2257061
rs9572687
rs12779976
rs8031609
rs7332927
rs10512185
rs10097949
rs12447958
rs7993666
rs7216075
rs576266
rs2303010
All
−0.4
0.0
0.4
MR effect size for
'Threonate' on 'Crohn's disease of ileocolon'

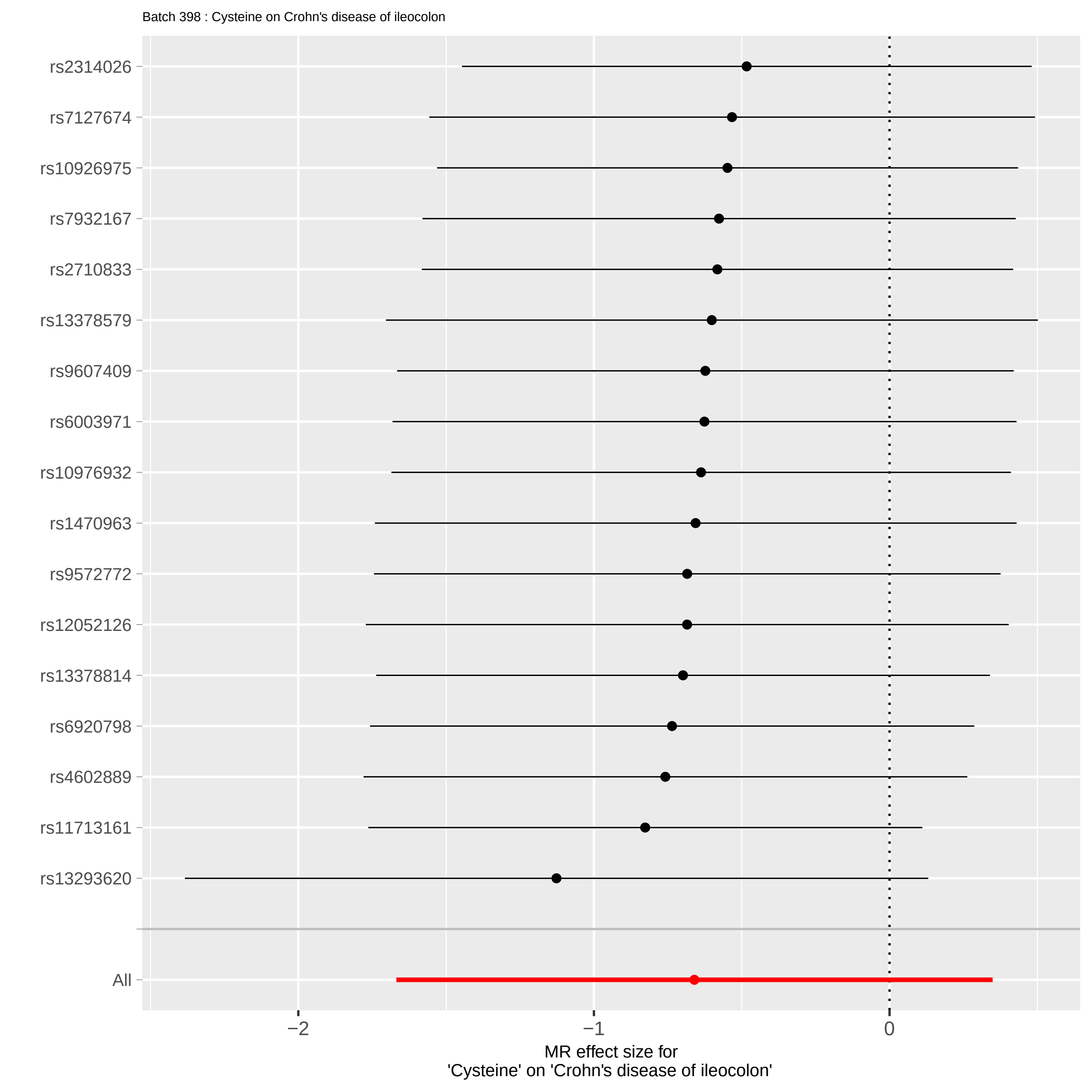
Batch 398 : Cysteine on Crohn's disease of ileocolon
rs2314026
rs7127674
rs10926975
rs7932167
rs2710833
rs13378579
rs9607409
rs6003971
rs10976932
rs1470963
rs9572772
rs12052126
rs13378814
rs6920798
rs4602889
rs11713161
rs13293620
All
−2
−1
0
MR effect size for
'Cysteine' on 'Crohn's disease of ileocolon'

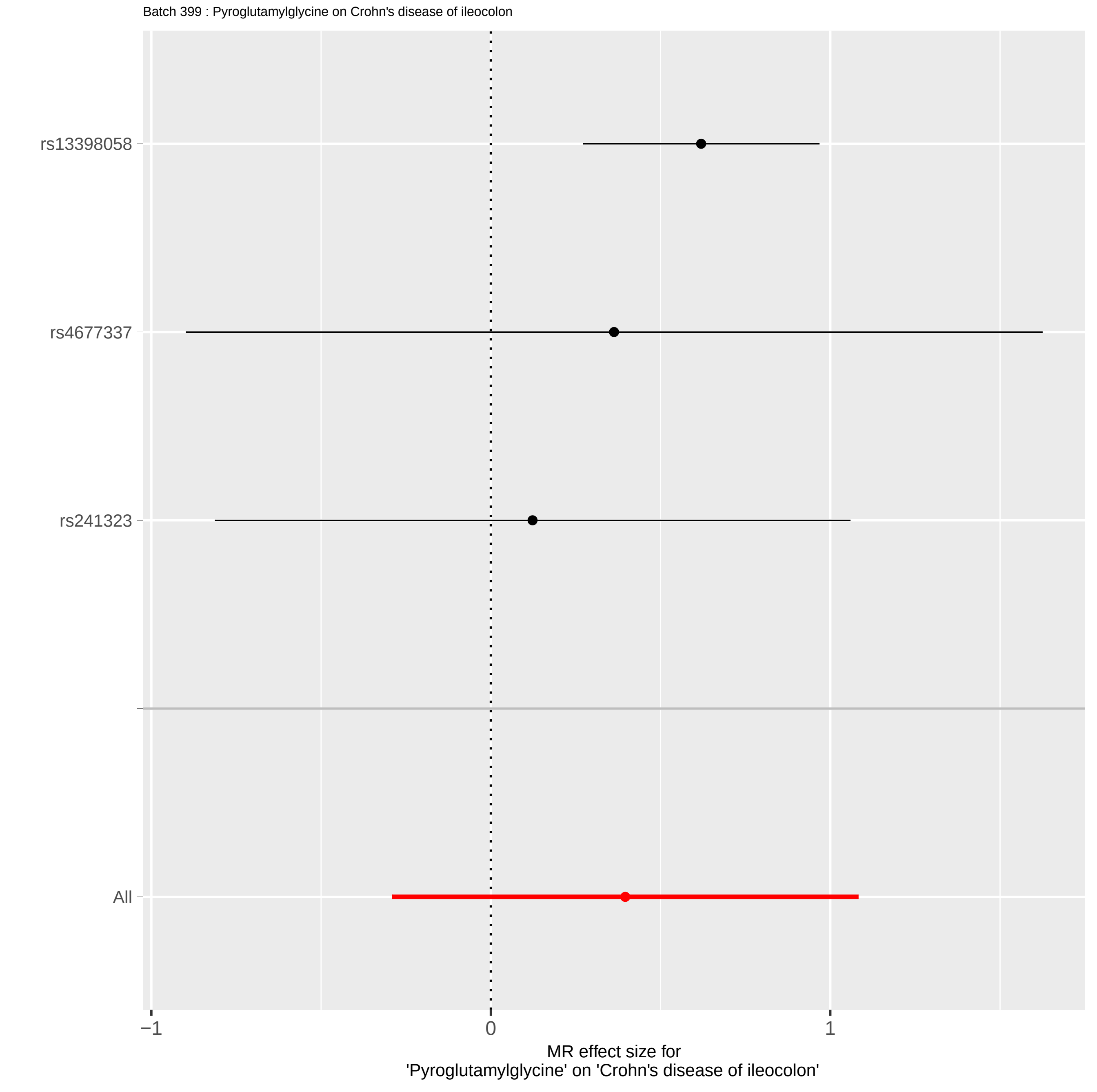
Batch 399 : Pyroglutamylglycine on Crohn's disease of ileocolon
rs13398058
rs4677337
rs241323
All
−1
0
1
MR effect size for
'Pyroglutamylglycine' on 'Crohn's disease of ileocolon'

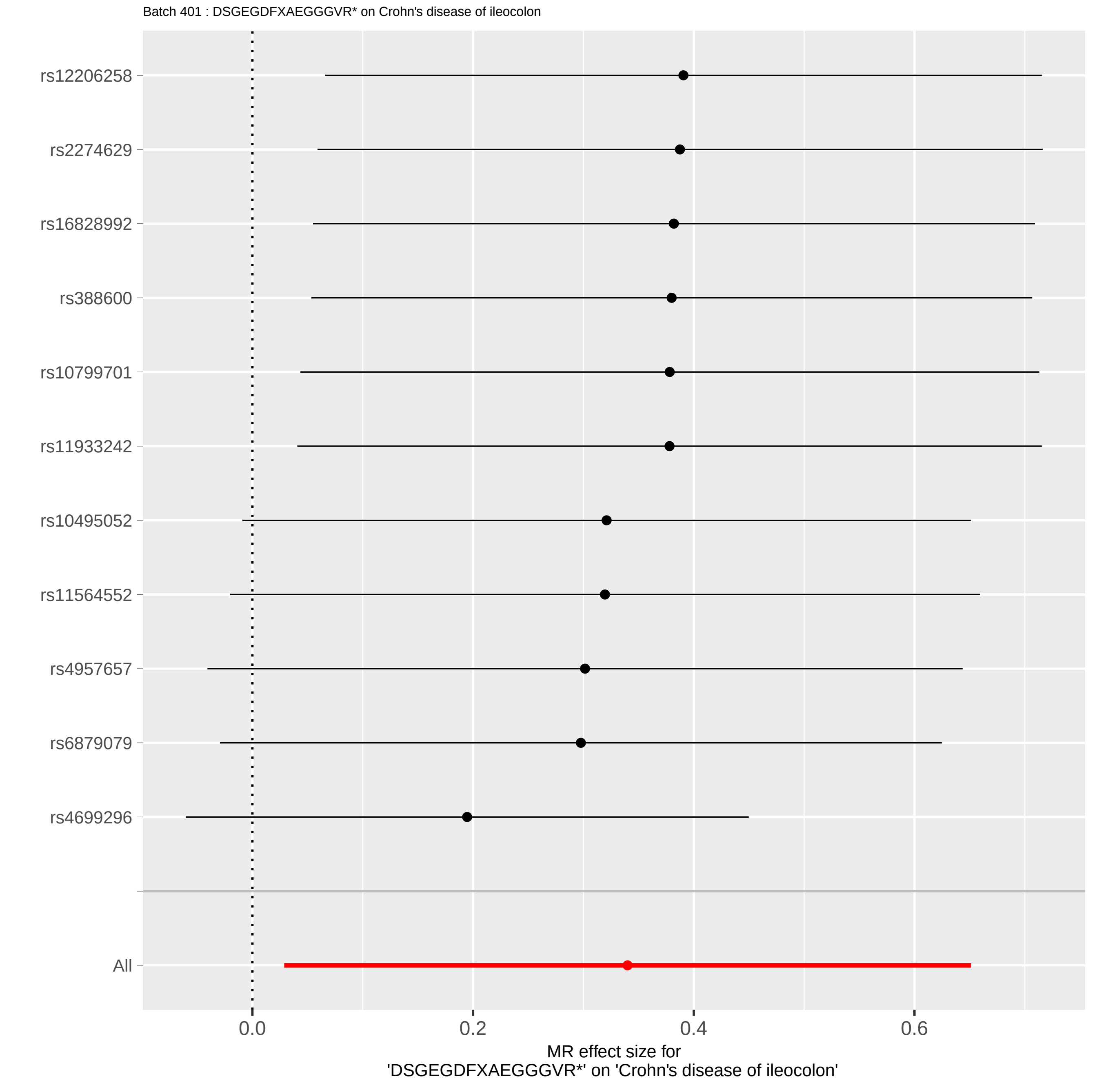

Batch 401 : DSGEGDFXAEGGGVR* on Crohn's disease of ileocolon
rs12206258
rs2274629
rs16828992
rs388600
rs10799701
rs11933242
rs10495052
rs11564552
rs4957657
rs6879079
rs4699296
All
0.0
0.2
0.4
0.6
MR effect size for
'DSGEGDFXAEGGGVR*' on 'Crohn's disease of ileocolon'

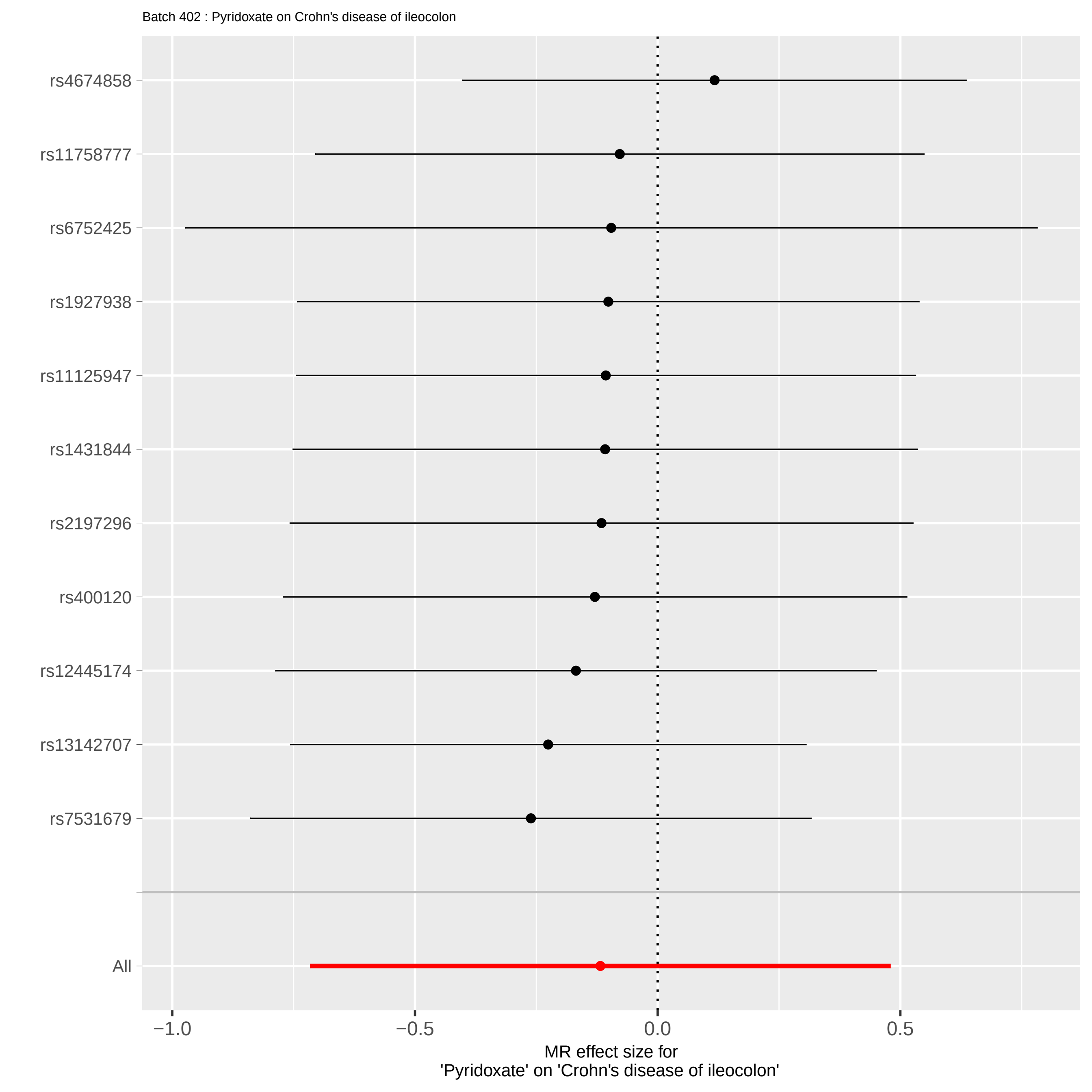

Batch 402 : Pyridoxate on Crohn's disease of ileocolon
rs4674858
rs11758777
rs6752425
rs1927938
rs11125947
rs1431844
rs2197296
rs400120
rs12445174
rs13142707
rs7531679
All
−1.0
−0.5
0.0
0.5
MR effect size for
'Pyridoxate' on 'Crohn's disease of ileocolon'

Batch 408 : Trans−4−hydroxyproline on Crohn's disease of ileocolon

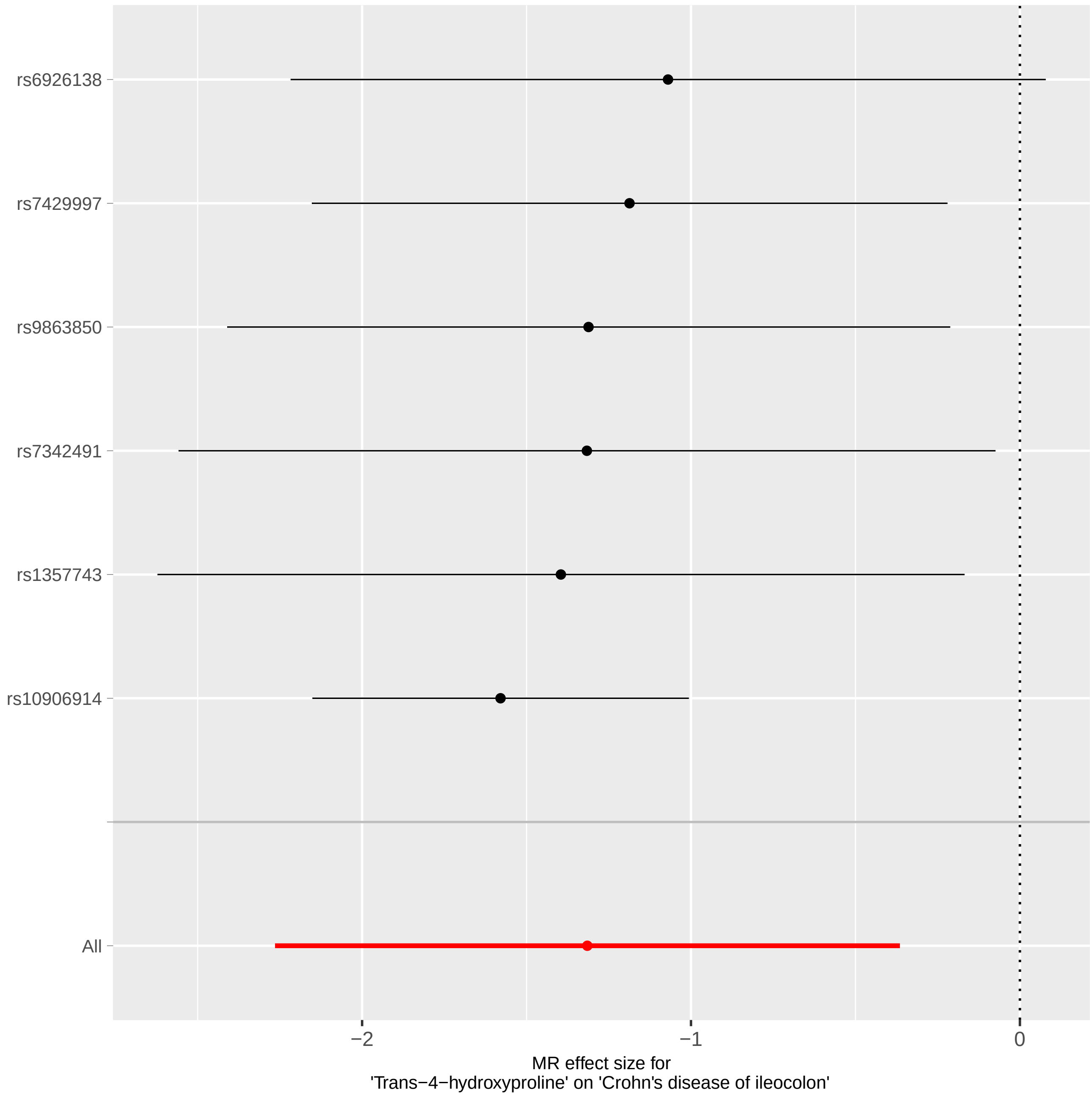

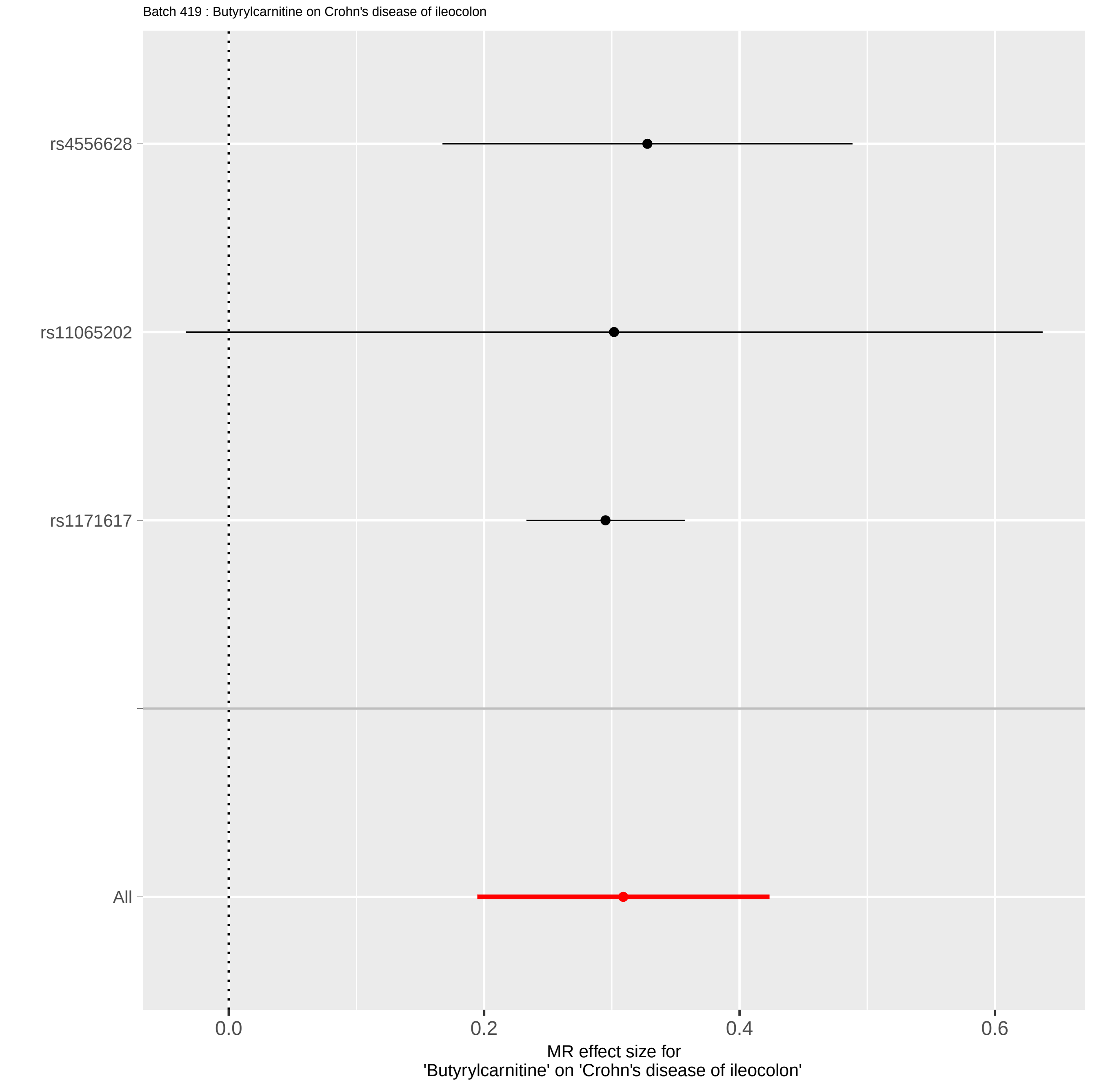

Batch 419 : Butyrylcarnitine on Crohn's disease of ileocolon
rs4556628
rs11065202
rs1171617
All
0.0
0.2
0.4
0.6
MR effect size for
'Butyrylcarnitine' on 'Crohn's disease of ileocolon'

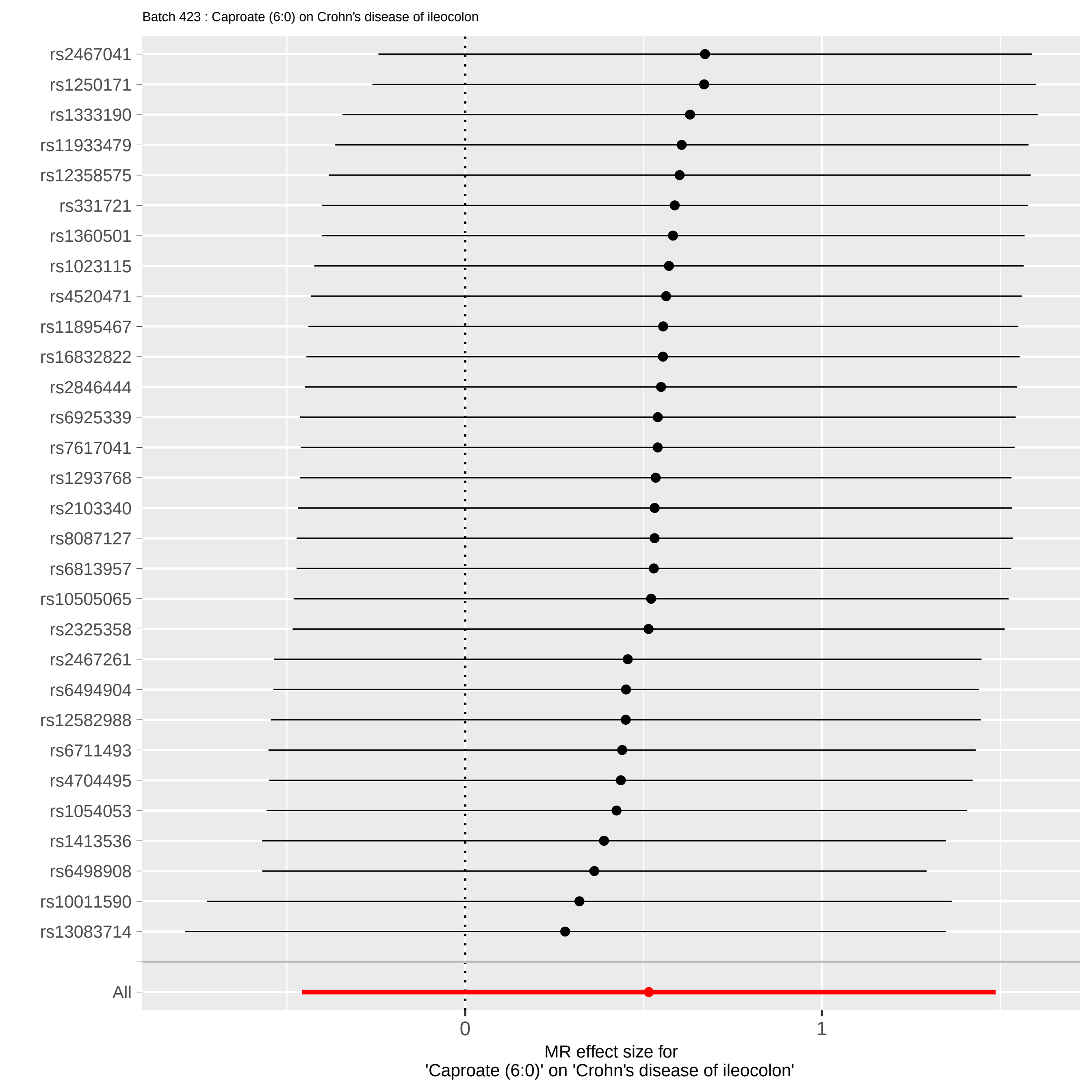
Batch 423 : Caproate (6:0) on Crohn's disease of ileocolon
rs2467041
rs1250171
rs1333190
rs11933479
rs12358575
rs331721
rs1360501
rs1023115
rs4520471
rs11895467
rs16832822
rs2846444
rs6925339
rs7617041
rs1293768
rs2103340
rs8087127
rs6813957
rs10505065
rs2325358
rs2467261
rs6494904
rs12582988
rs6711493
rs4704495
rs1054053
rs1413536
rs6498908
rs10011590
rs13083714
All
0
1
MR effect size for
'Caproate (6:0)' on 'Crohn's disease of ileocolon'

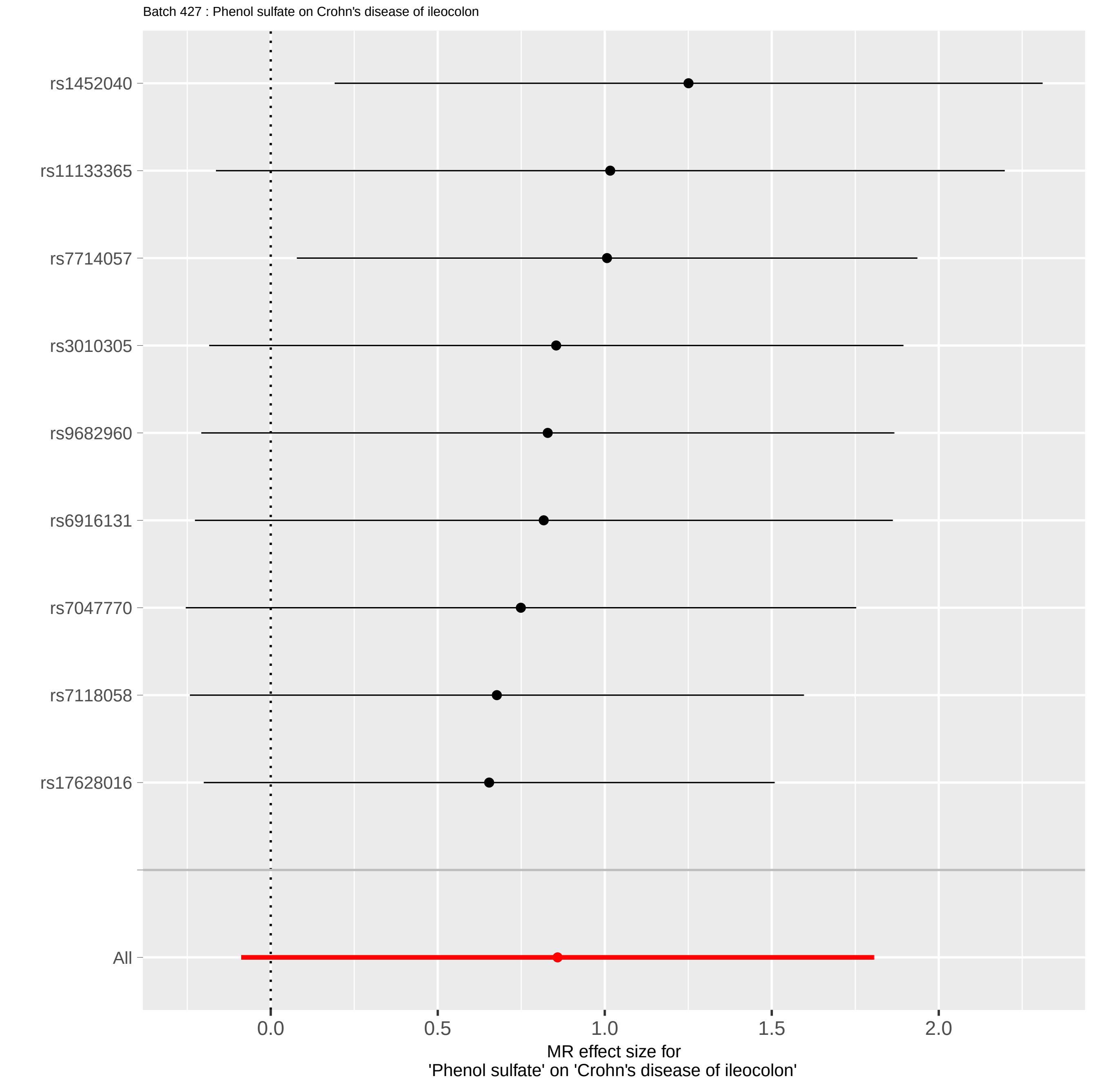

Batch 427 : Phenol sulfate on Crohn's disease of ileocolon
rs1452040
rs11133365
rs7714057
rs3010305
rs9682960
rs6916131
rs7047770
rs7118058
rs17628016
All
0.0
0.5
1.0
1.5
2.0
MR effect size for
'Phenol sulfate' on 'Crohn's disease of ileocolon'

Batch 429 : Heme* on Crohn's disease of ileocolon

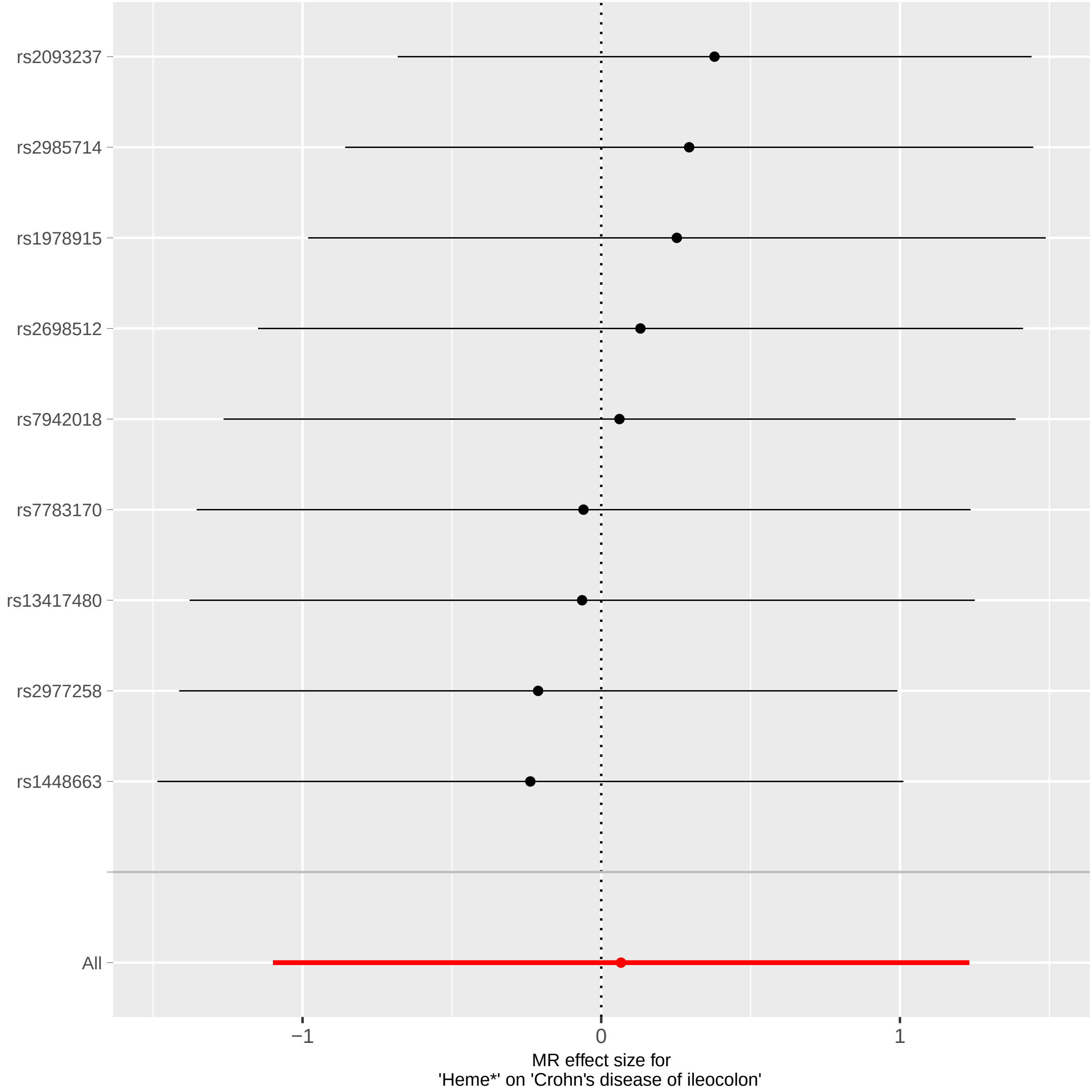

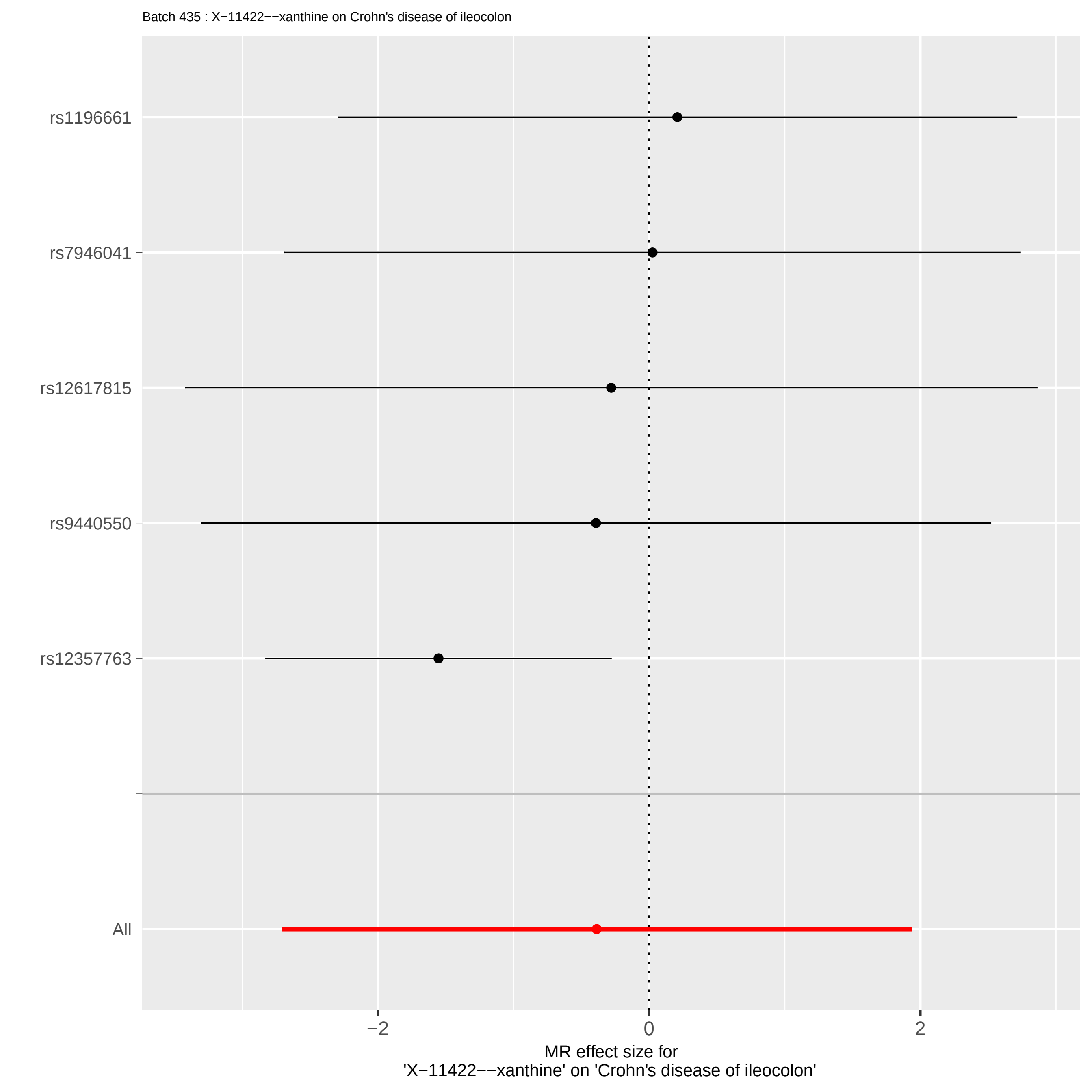

Batch 435 : X−11422−−xanthine on Crohn's disease of ileocolon
rs1196661
rs7946041
rs12617815
rs9440550
rs12357763
All
−2
0
2
MR effect size for
'X−11422−−xanthine' on 'Crohn's disease of ileocolon'

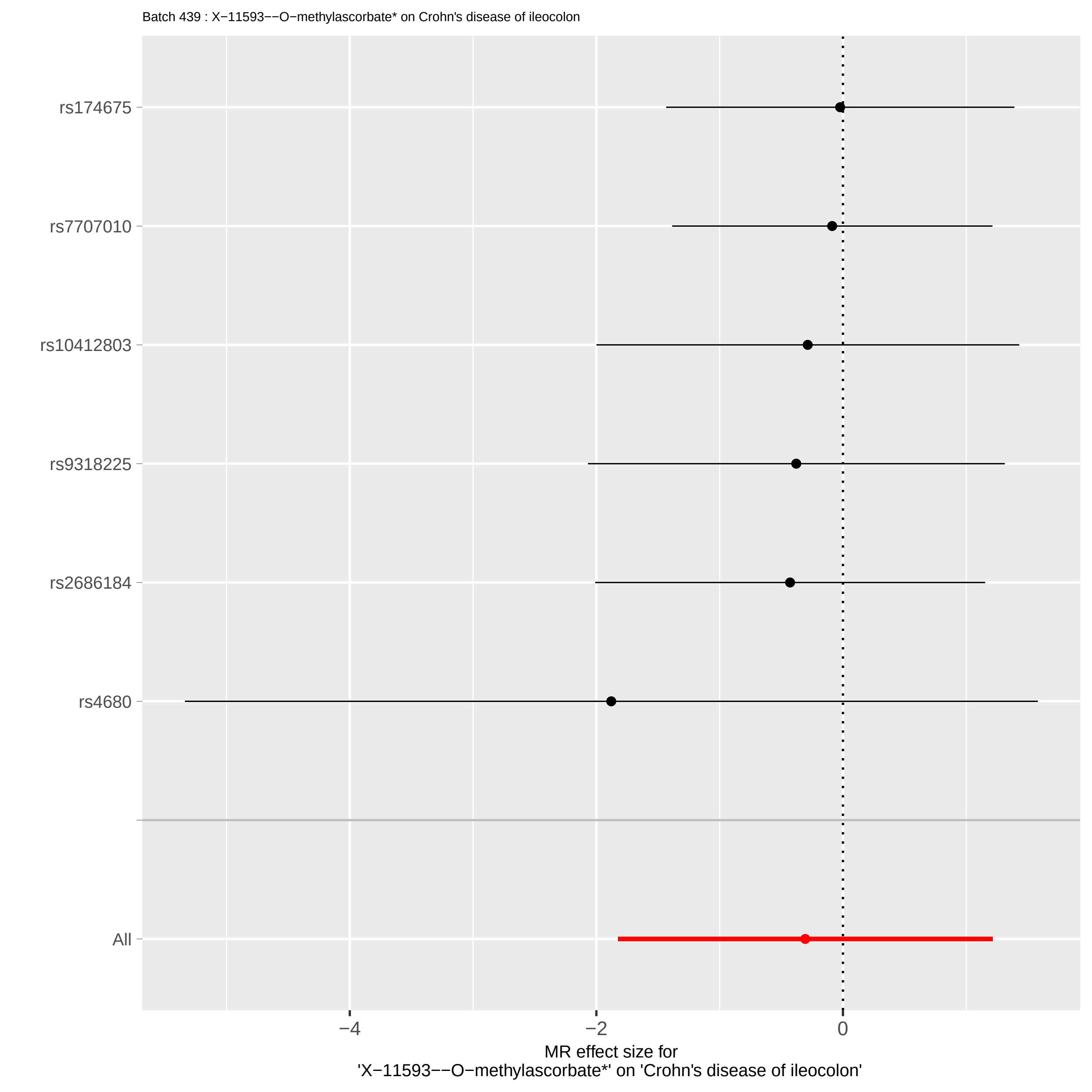

Batch 439 : X−11593−−O−methylascorbate* on Crohn's disease of ileocolon
rs174675
rs7707010
rs10412803
rs9318225
rs2686184
rs4680
All
−4
−2
0
MR effect size for
'X−11593−−O−methylascorbate*' on 'Crohn's disease of ileocolon'

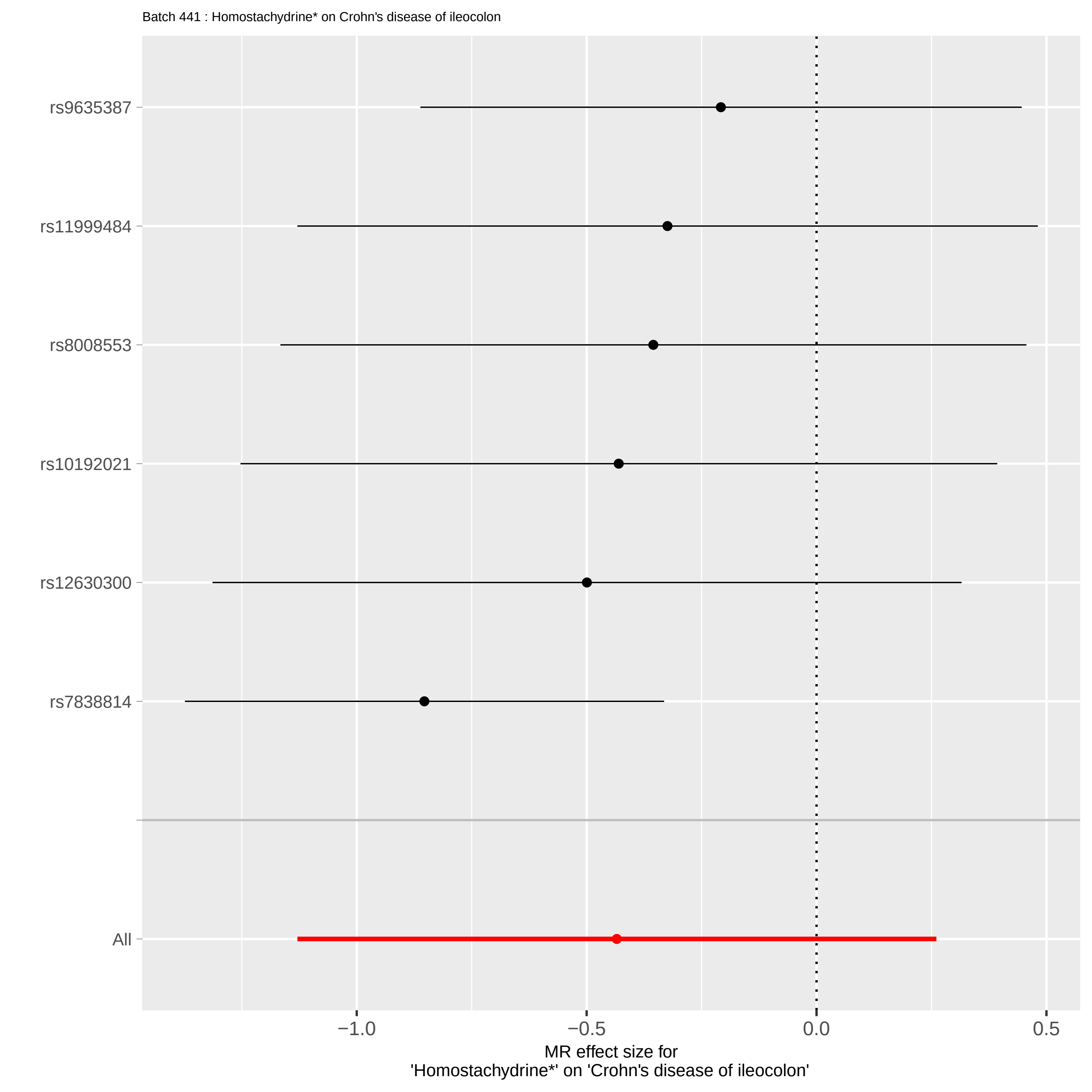
Batch 441 : Homostachydrine* on Crohn's disease of ileocolon
rs9635387
rs11999484
rs8008553
rs10192021
rs12630300
rs7838814
All
−1.0
−0.5
0.0
0.5
MR effect size for
'Homostachydrine*' on 'Crohn's disease of ileocolon'

Batch 445 : 1−arachidonoylglycerophosphocholine* on Crohn's disease of ileocolon

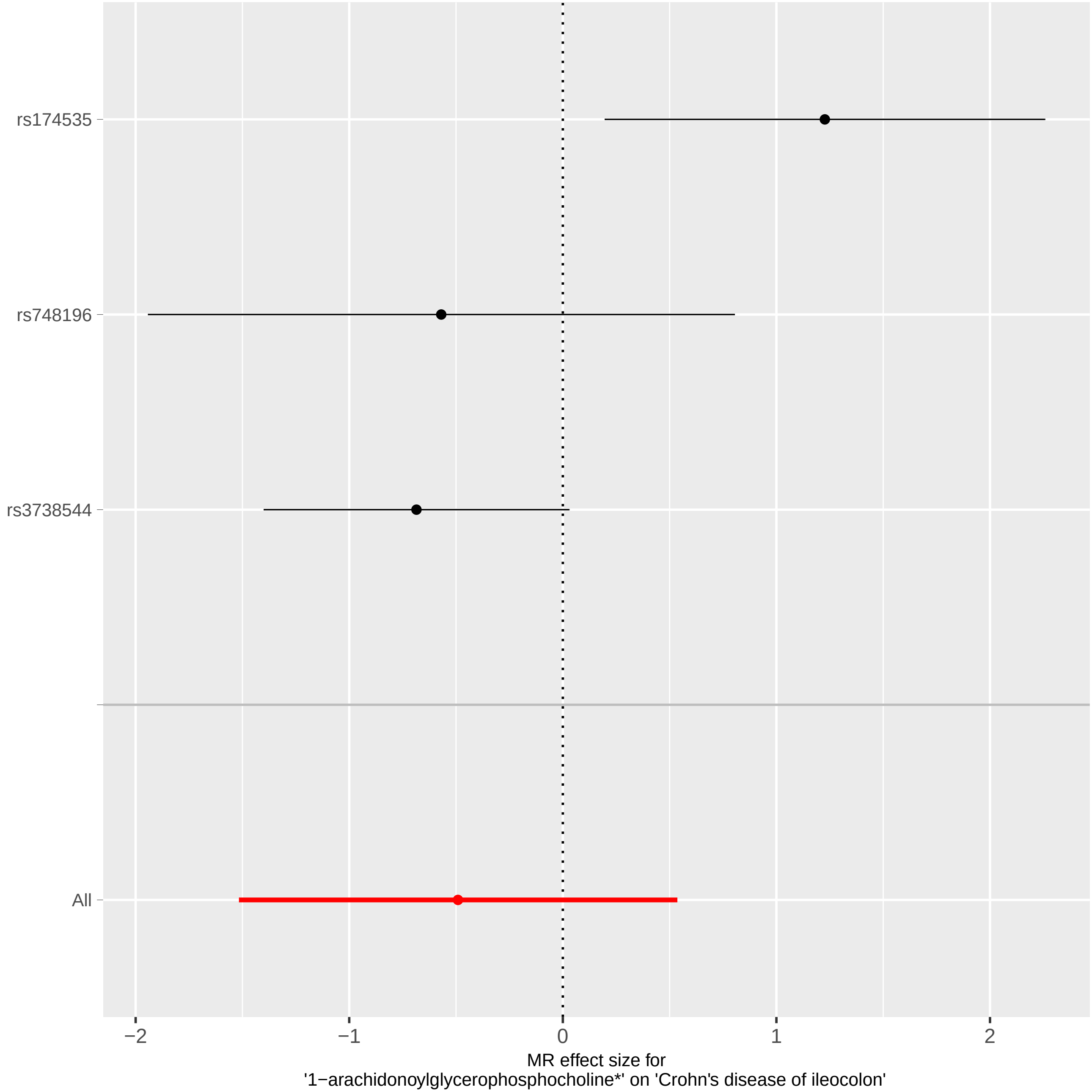

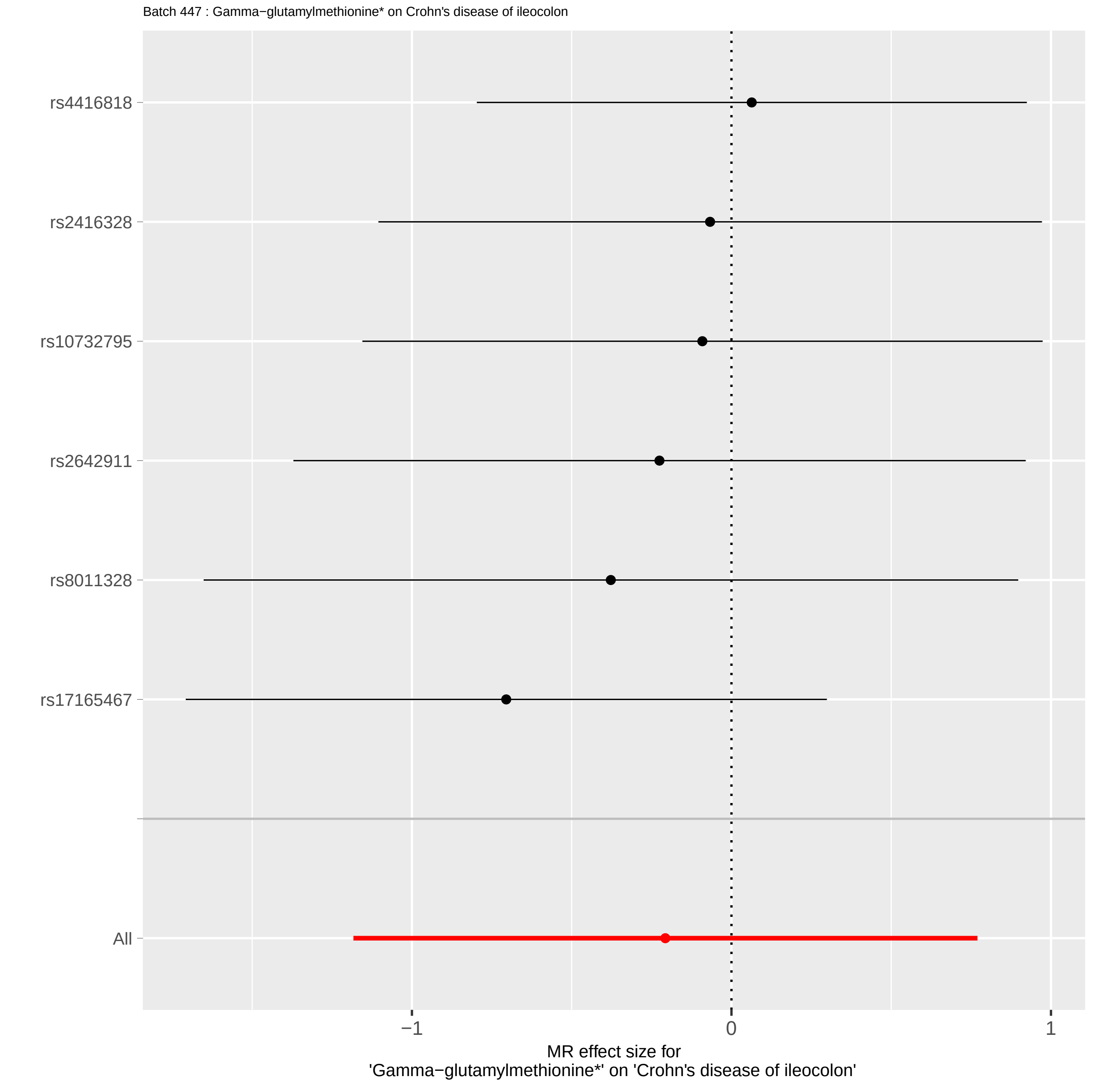

Batch 447 : Gamma−glutamylmethionine* on Crohn's disease of ileocolon
rs4416818
rs2416328
rs10732795
rs2642911
rs8011328
rs17165467
All
−1
0
1
MR effect size for
'Gamma−glutamylmethionine*' on 'Crohn's disease of ileocolon'

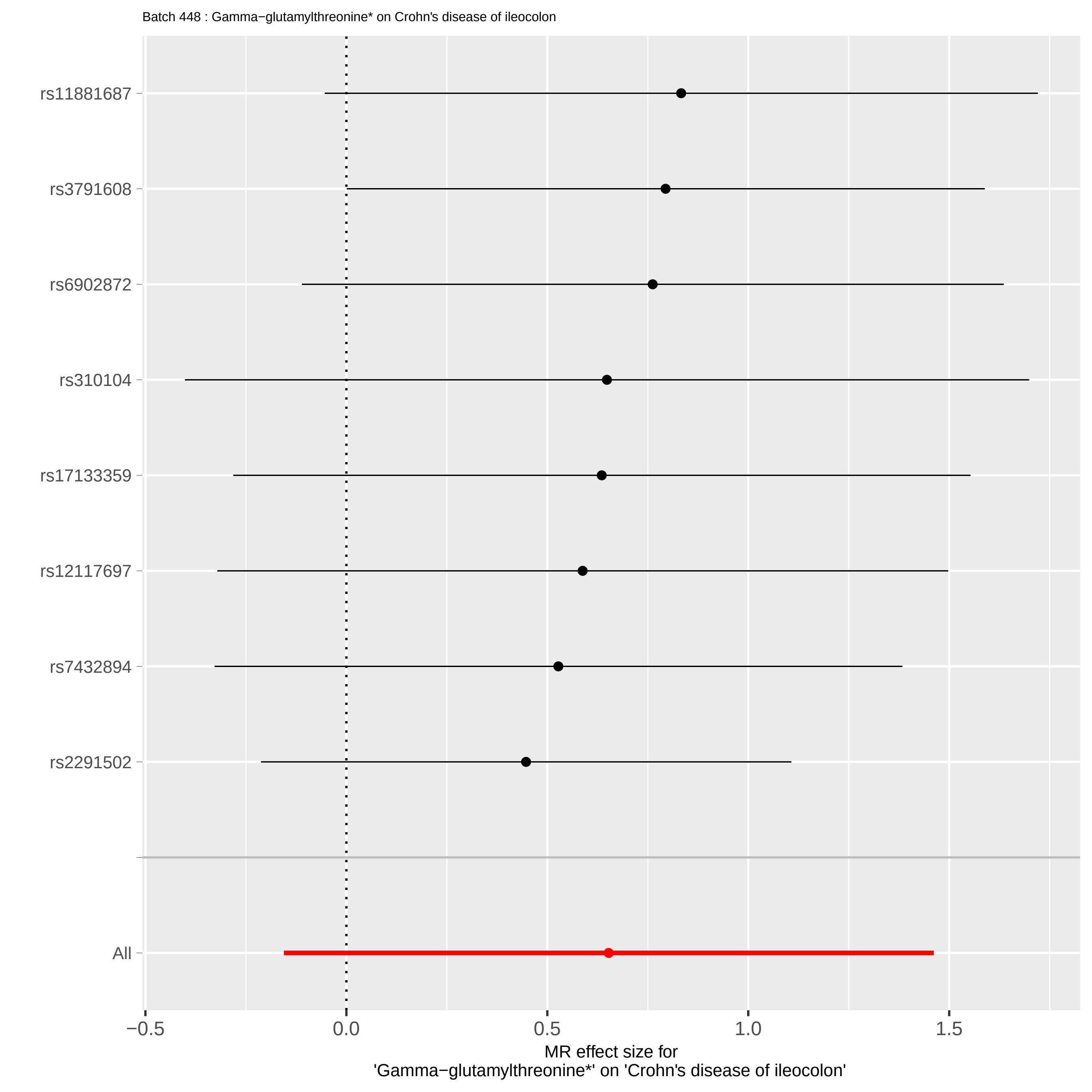

Batch 448 : Gamma−glutamylthreonine* on Crohn's disease of ileocolon
rs11881687
rs3791608
rs6902872
rs310104
rs17133359
rs12117697
rs7432894
rs2291502
All
−0.5
0.0
0.5
1.0
1.5
MR effect size for
'Gamma−glutamylthreonine*' on 'Crohn's disease of ileocolon'

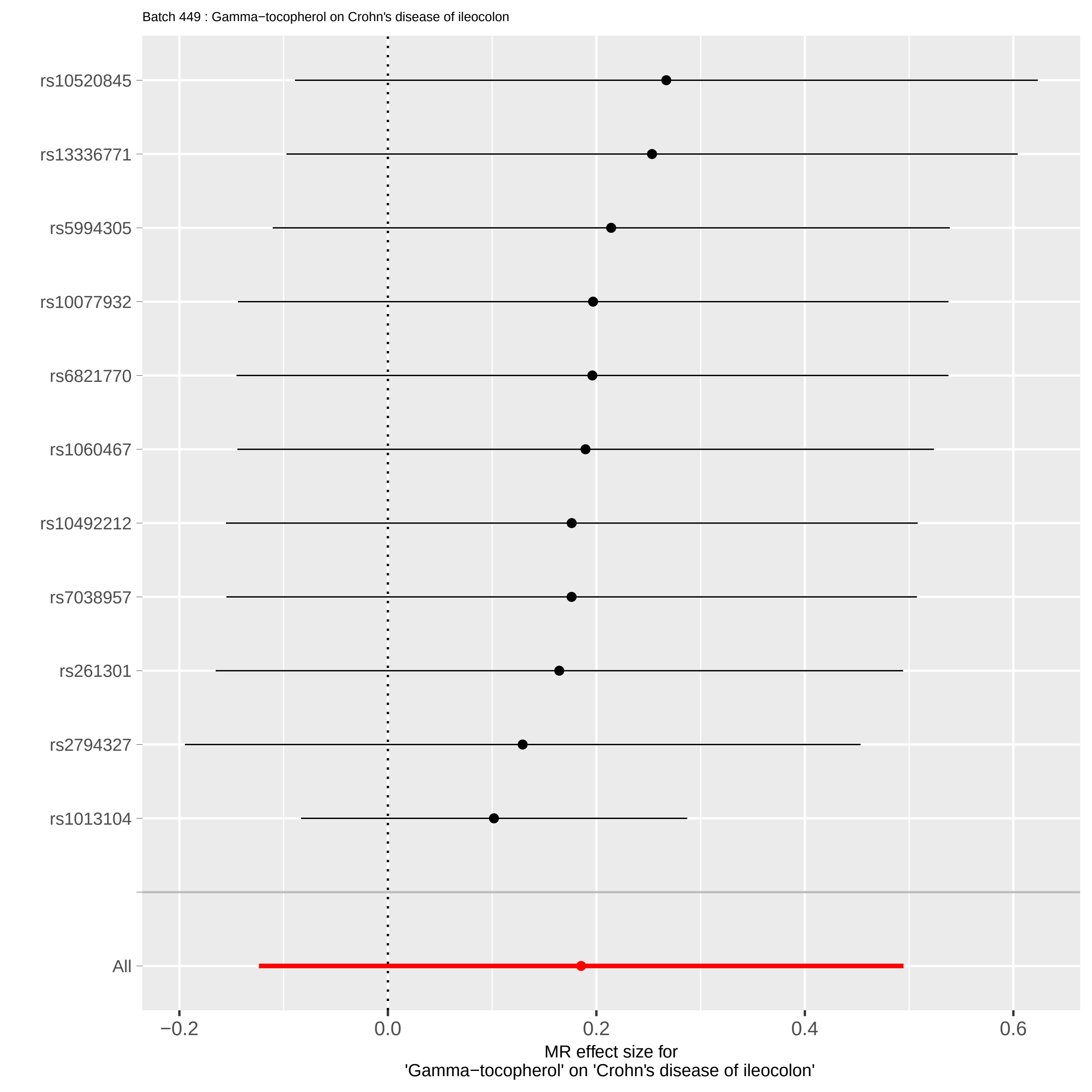
Batch 449 : Gamma−tocopherol on Crohn's disease of ileocolon
rs10520845
rs13336771
rs5994305
rs10077932
rs6821770
rs1060467
rs10492212
rs7038957
rs261301
rs2794327
rs1013104
All
−0.2
0.0
0.2
0.4
0.6
MR effect size for
'Gamma−tocopherol' on 'Crohn's disease of ileocolon'

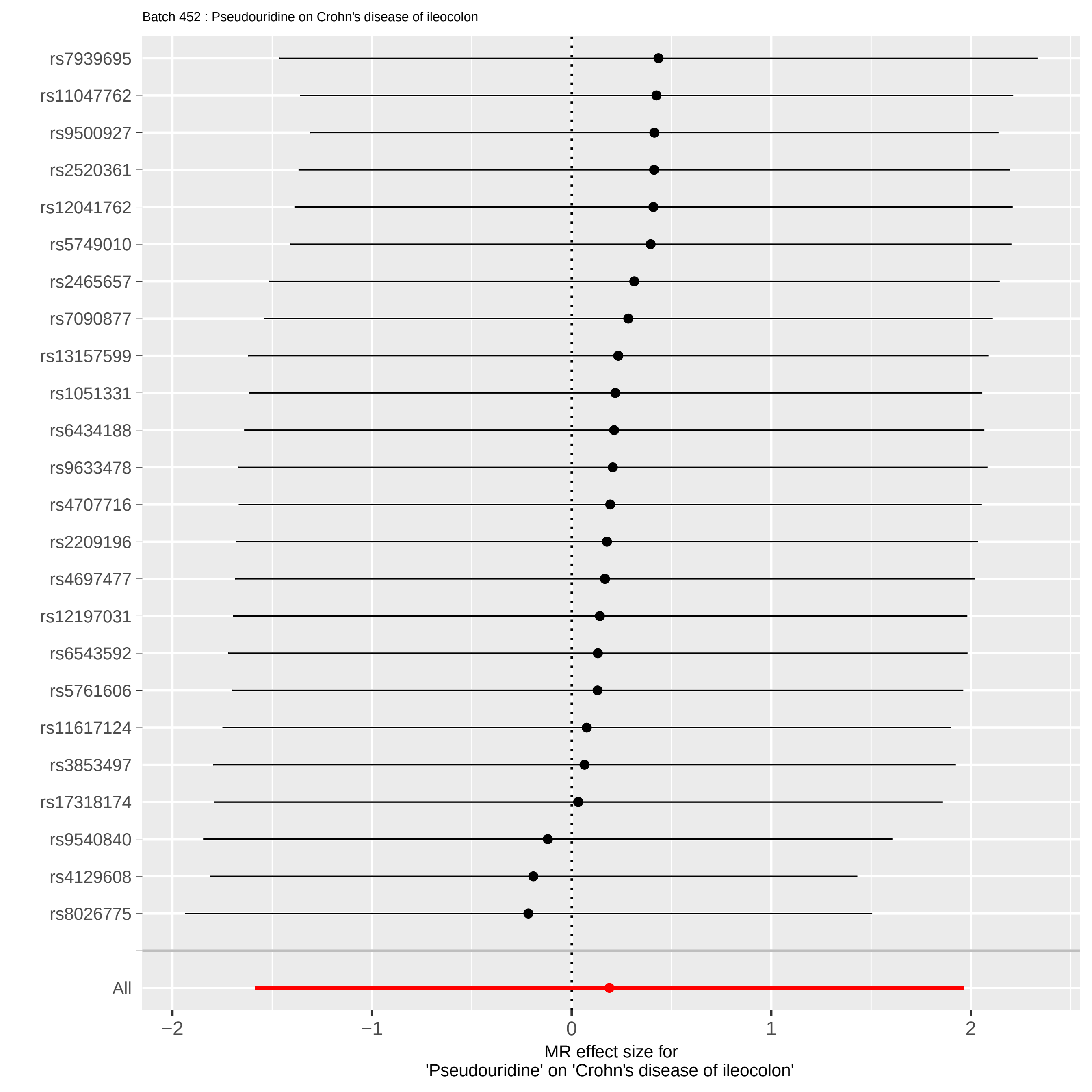
Batch 452 : Pseudouridine on Crohn's disease of ileocolon
rs7939695
rs11047762
rs9500927
rs2520361
rs12041762
rs5749010
rs2465657
rs7090877
rs13157599
rs1051331
rs6434188
rs9633478
rs4707716
rs2209196
rs4697477
rs12197031
rs6543592
rs5761606
rs11617124
rs3853497
rs17318174
rs9540840
rs4129608
rs8026775
All
−2
−1
0
1
2
MR effect size for
'Pseudouridine' on 'Crohn's disease of ileocolon'

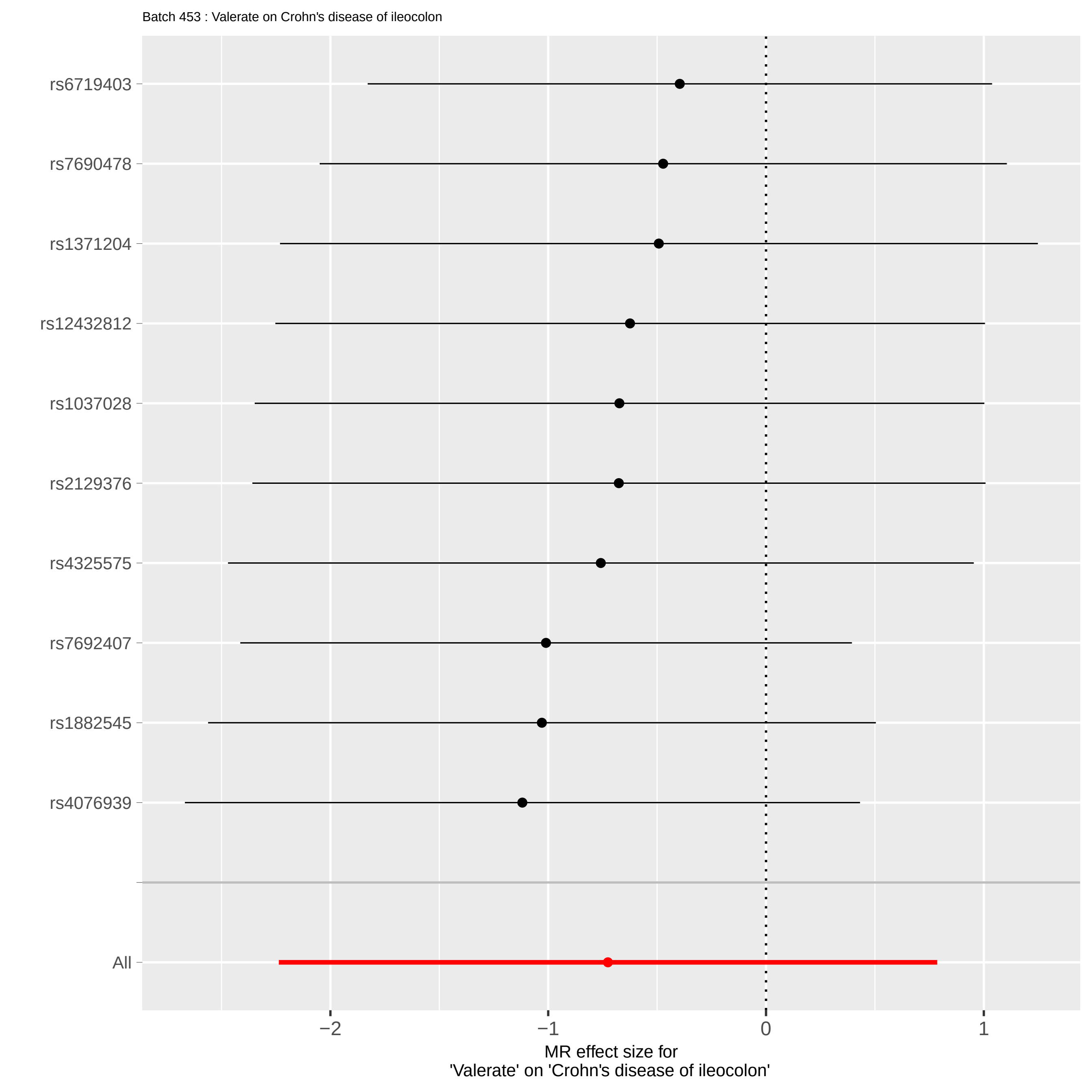
Batch 453 : Valerate on Crohn's disease of ileocolon
rs6719403
rs7690478
rs1371204
rs12432812
rs1037028
rs2129376
rs4325575
rs7692407
rs1882545
rs4076939
All
−2
−1
0
1
MR effect size for
'Valerate' on 'Crohn's disease of ileocolon'

Batch 456 : Erythronate* on Crohn's disease of ileocolon

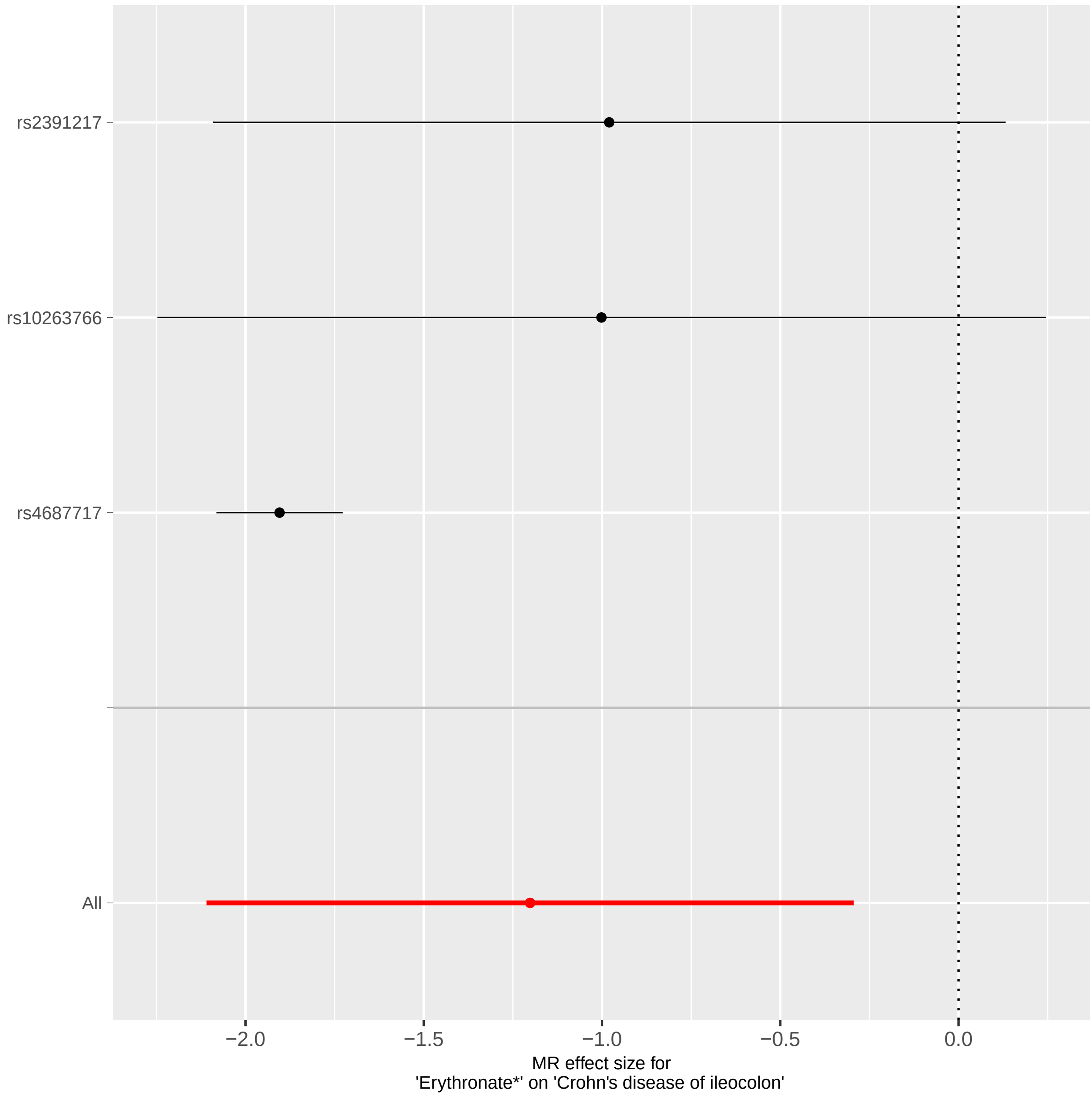

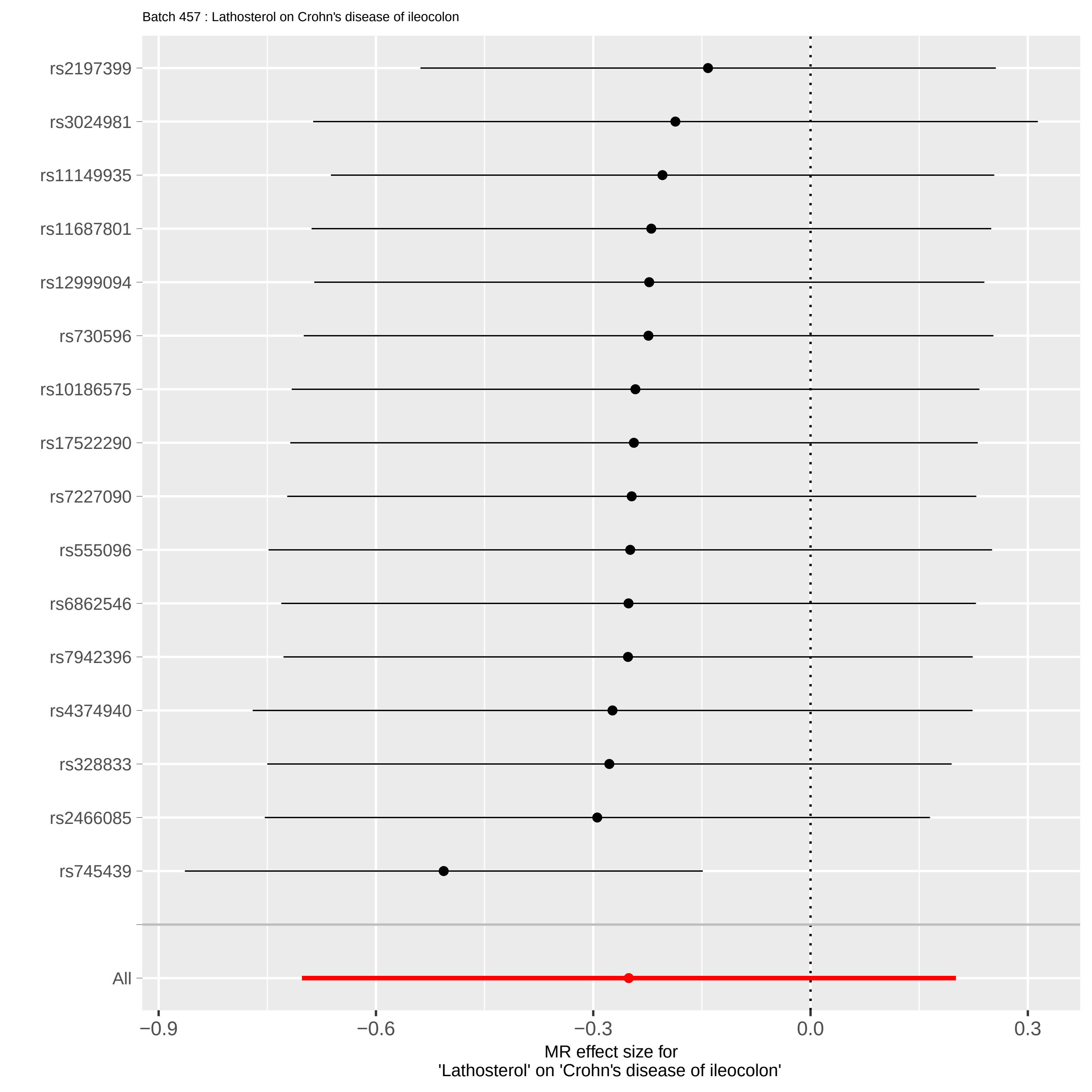
Batch 457 : Lathosterol on Crohn's disease of ileocolon
rs2197399
rs3024981
rs11149935
rs11687801
rs12999094
rs730596
rs10186575
rs17522290
rs7227090
rs555096
rs6862546
rs7942396
rs4374940
rs328833
rs2466085
rs745439
All
−0.9
−0.6
−0.3
0.0
0.3
MR effect size for
'Lathosterol' on 'Crohn's disease of ileocolon'

Batch 460 : Eicosenoate (20:1n9 or 11) on Crohn's disease of ileocolon

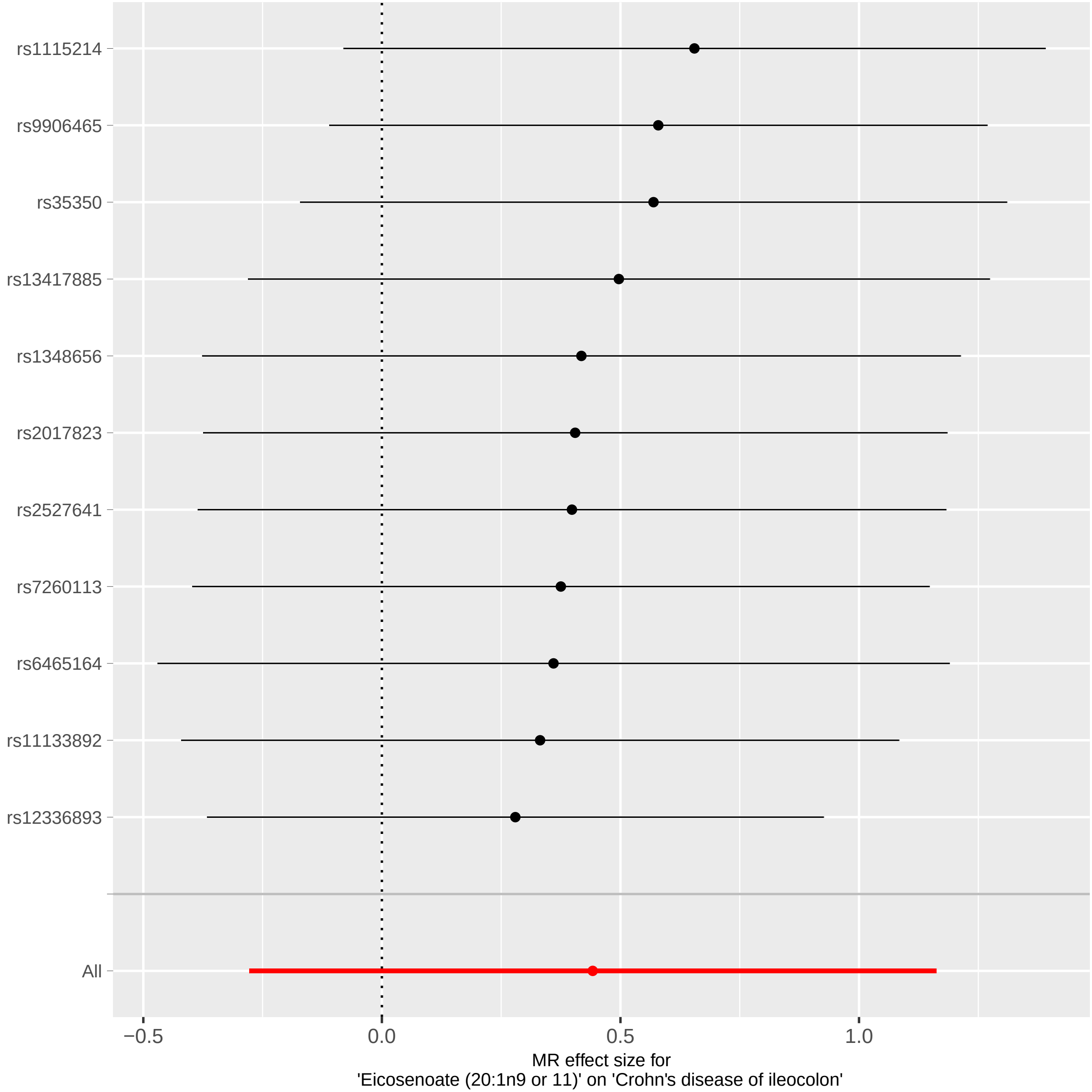

Batch 461 : X−12244−−N−acetylcarnosine on Crohn's disease of ileocolon

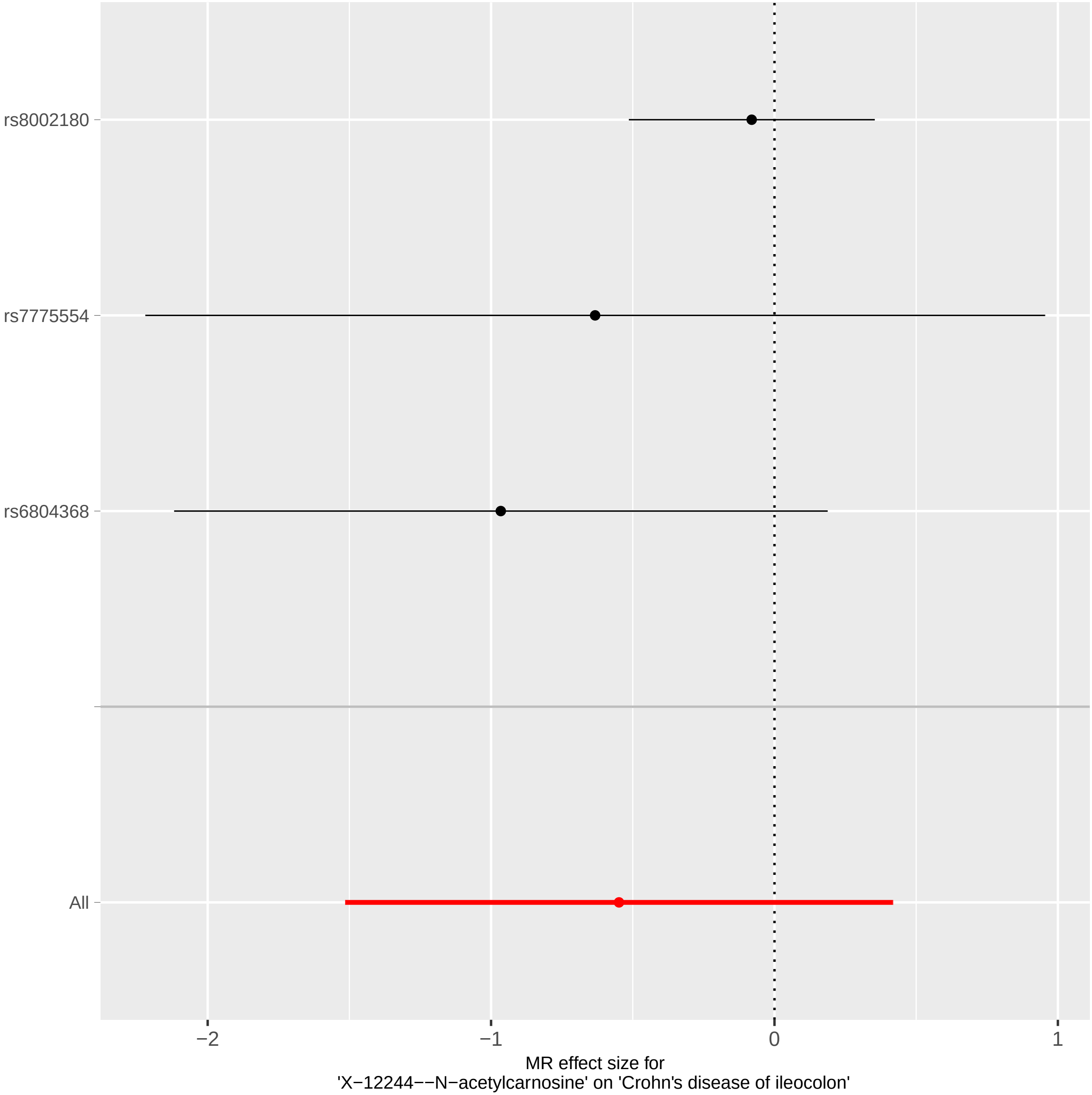

Batch 464 : 1−docosahexaenoylglycerophosphocholine* on Crohn's disease of ileocolon

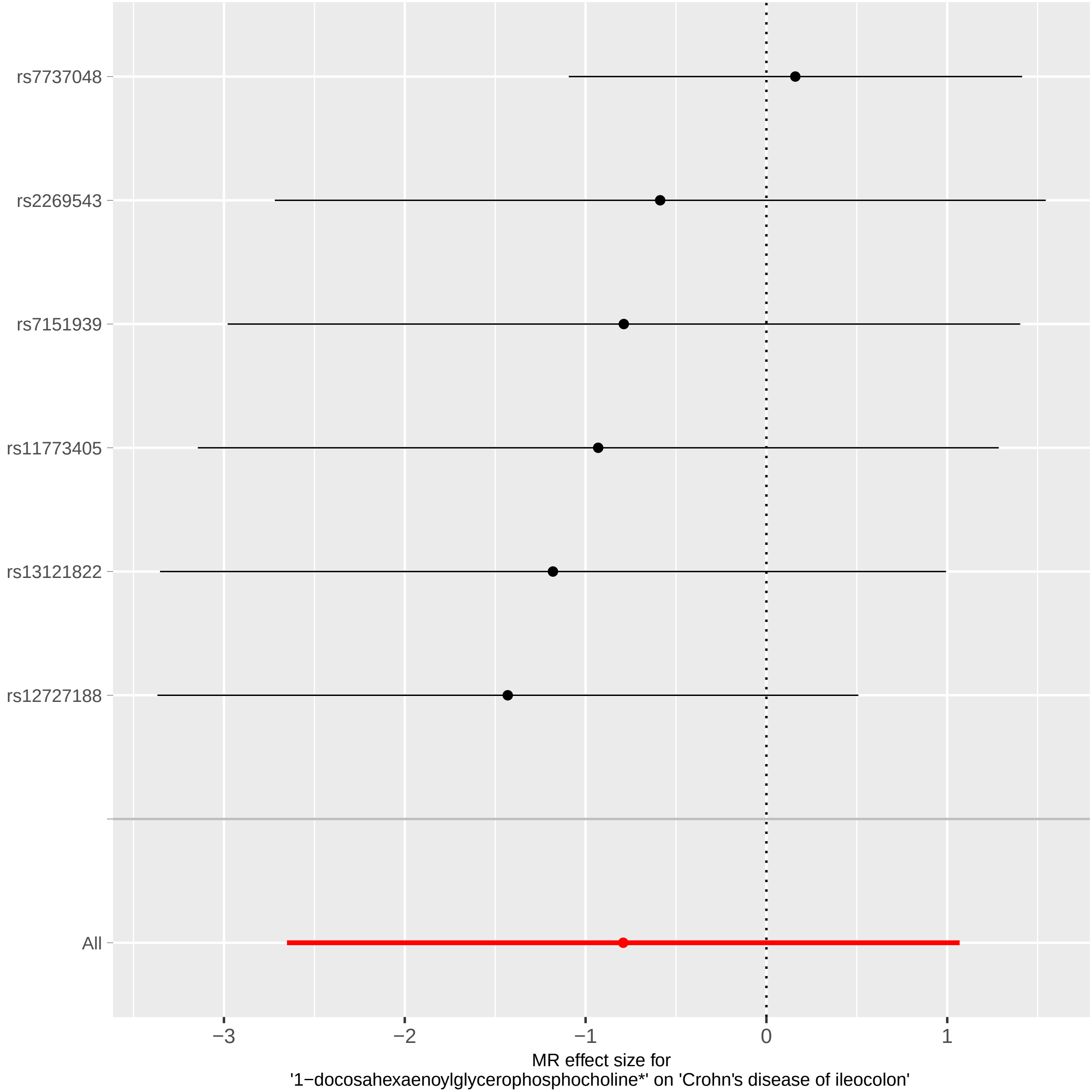

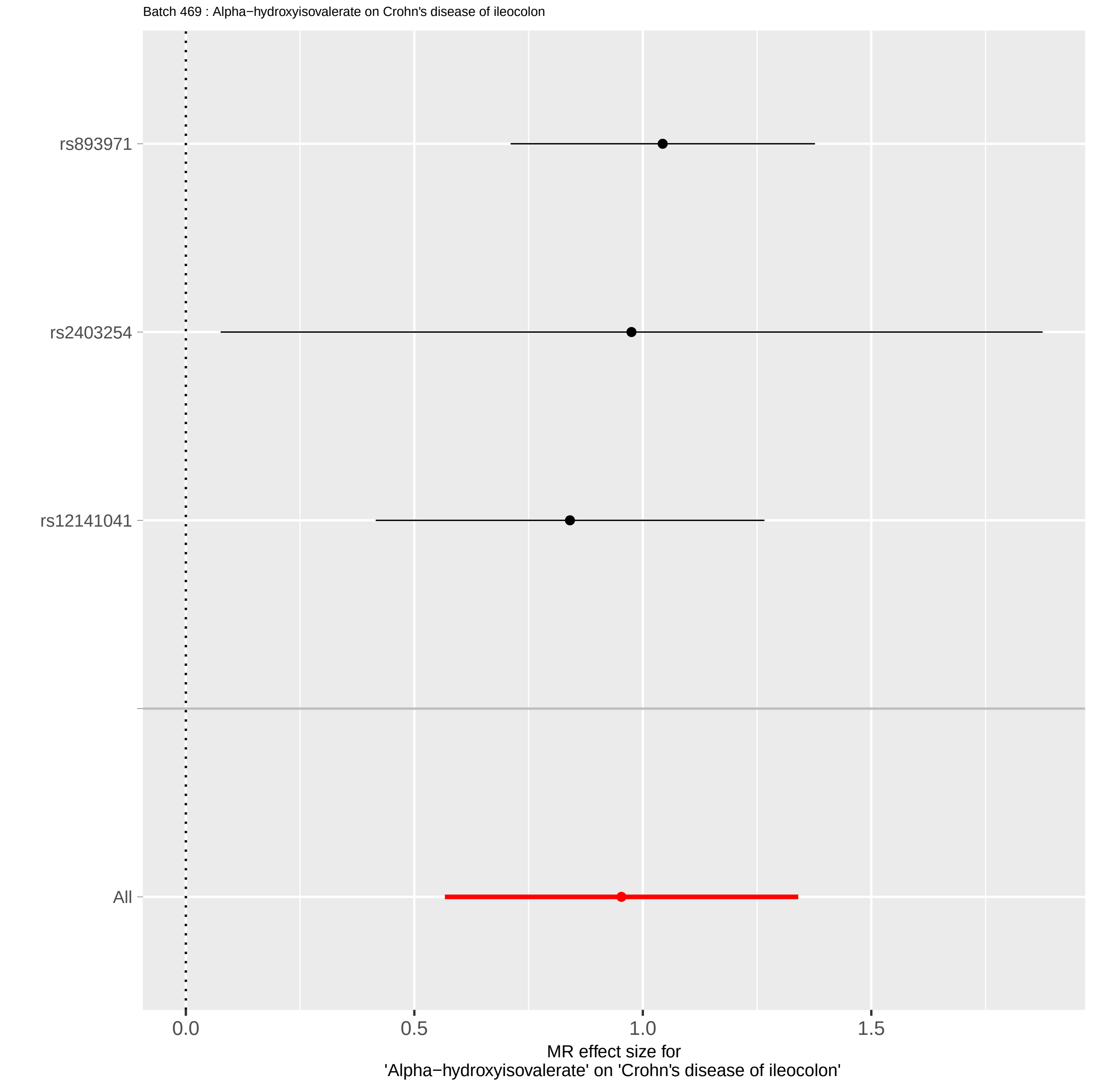

Batch 469 : Alpha−hydroxyisovalerate on Crohn's disease of ileocolon
rs893971
rs2403254
rs12141041
All
0.0
0.5
1.0
1.5
MR effect size for
'Alpha−hydroxyisovalerate' on 'Crohn's disease of ileocolon'

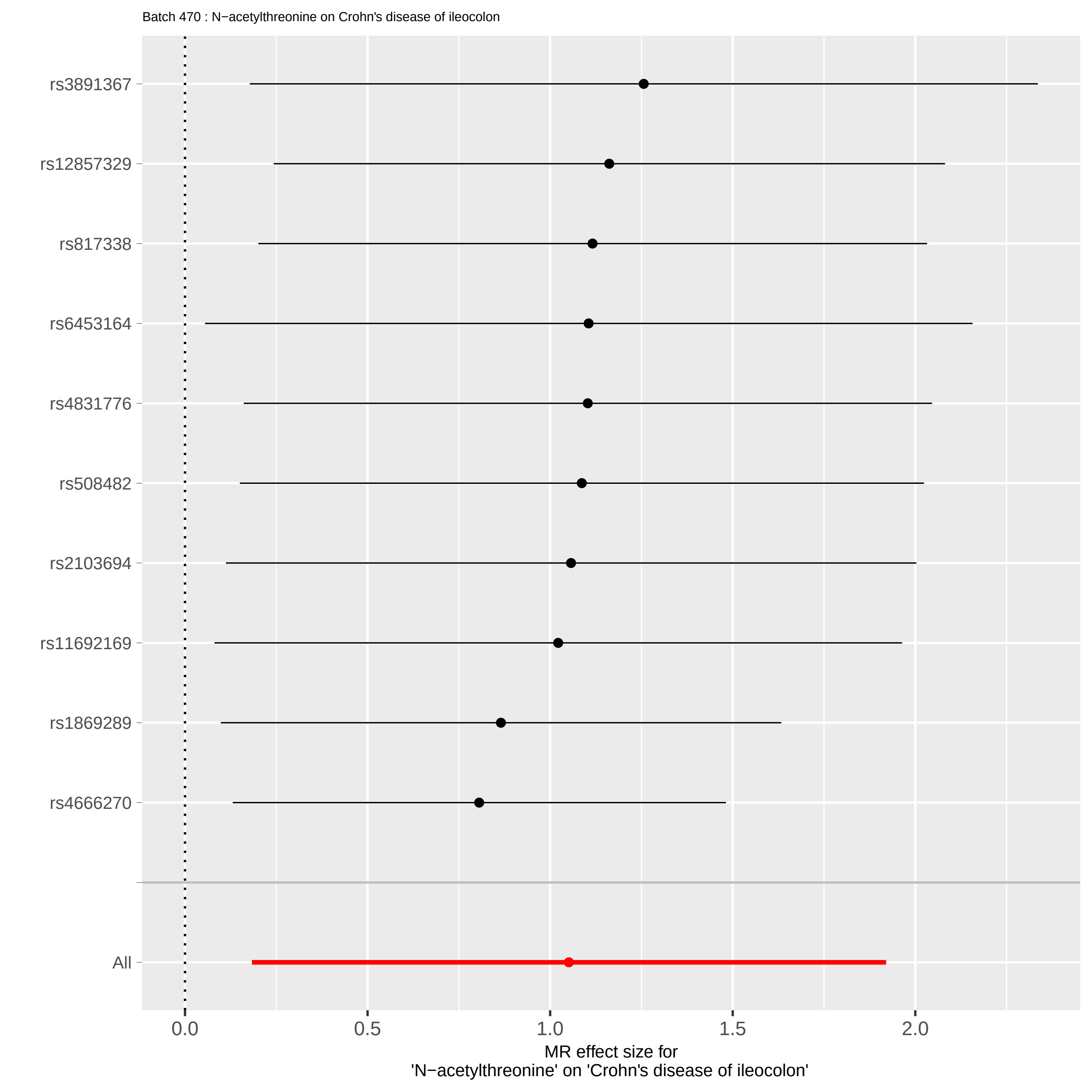

Batch 470 : N−acetylthreonine on Crohn's disease of ileocolon
rs3891367
rs12857329
rs817338
rs6453164
rs4831776
rs508482
rs2103694
rs11692169
rs1869289
rs4666270
All
0.0
0.5
1.0
1.5
2.0
MR effect size for
'N−acetylthreonine' on 'Crohn's disease of ileocolon'

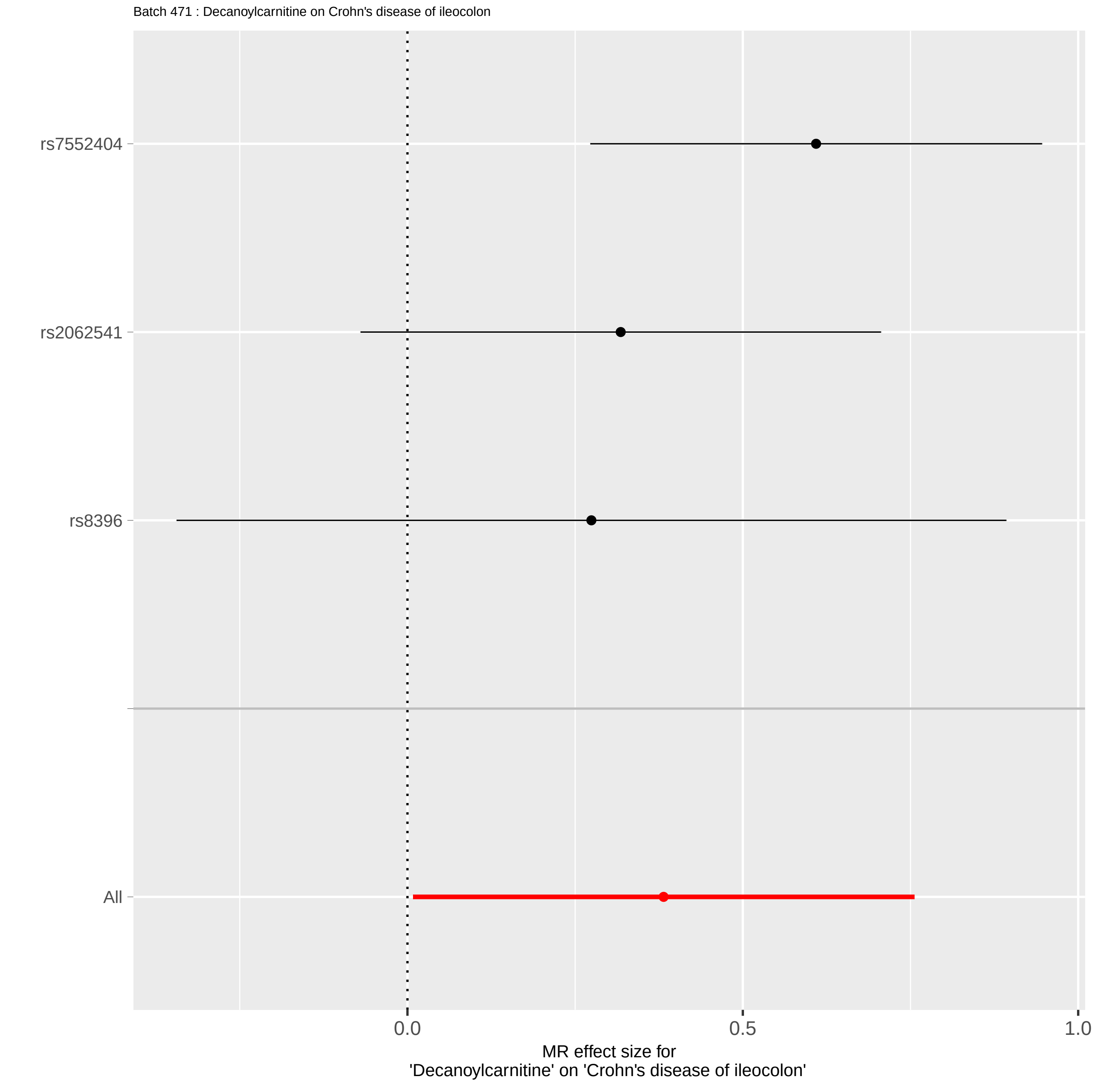

Batch 471 : Decanoylcarnitine on Crohn's disease of ileocolon
rs7552404
rs2062541
rs8396
All
0.0
0.5
1.0
MR effect size for
'Decanoylcarnitine' on 'Crohn's disease of ileocolon'

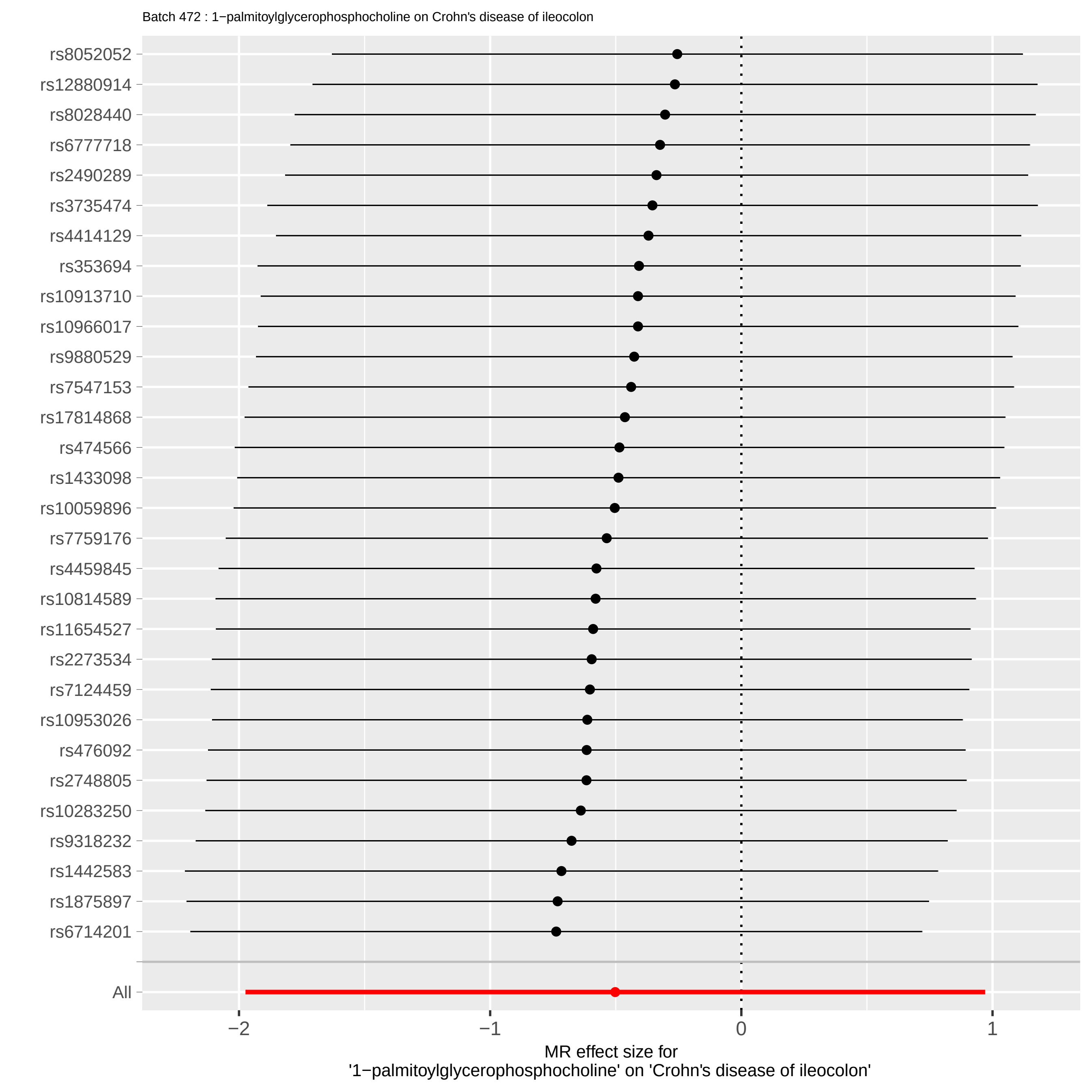
Batch 472 : 1−palmitoylglycerophosphocholine on Crohn's disease of ileocolon
rs8052052
rs12880914
rs8028440
rs6777718
rs2490289
rs3735474
rs4414129
rs353694
rs10913710
rs10966017
rs9880529
rs7547153
rs17814868
rs474566
rs1433098
rs10059896
rs7759176
rs4459845
rs10814589
rs11654527
rs2273534
rs7124459
rs10953026
rs476092
rs2748805
rs10283250
rs9318232
rs1442583
rs1875897
rs6714201
All
−2
−1
0
1
MR effect size for
'1−palmitoylglycerophosphocholine' on 'Crohn's disease of ileocolon'

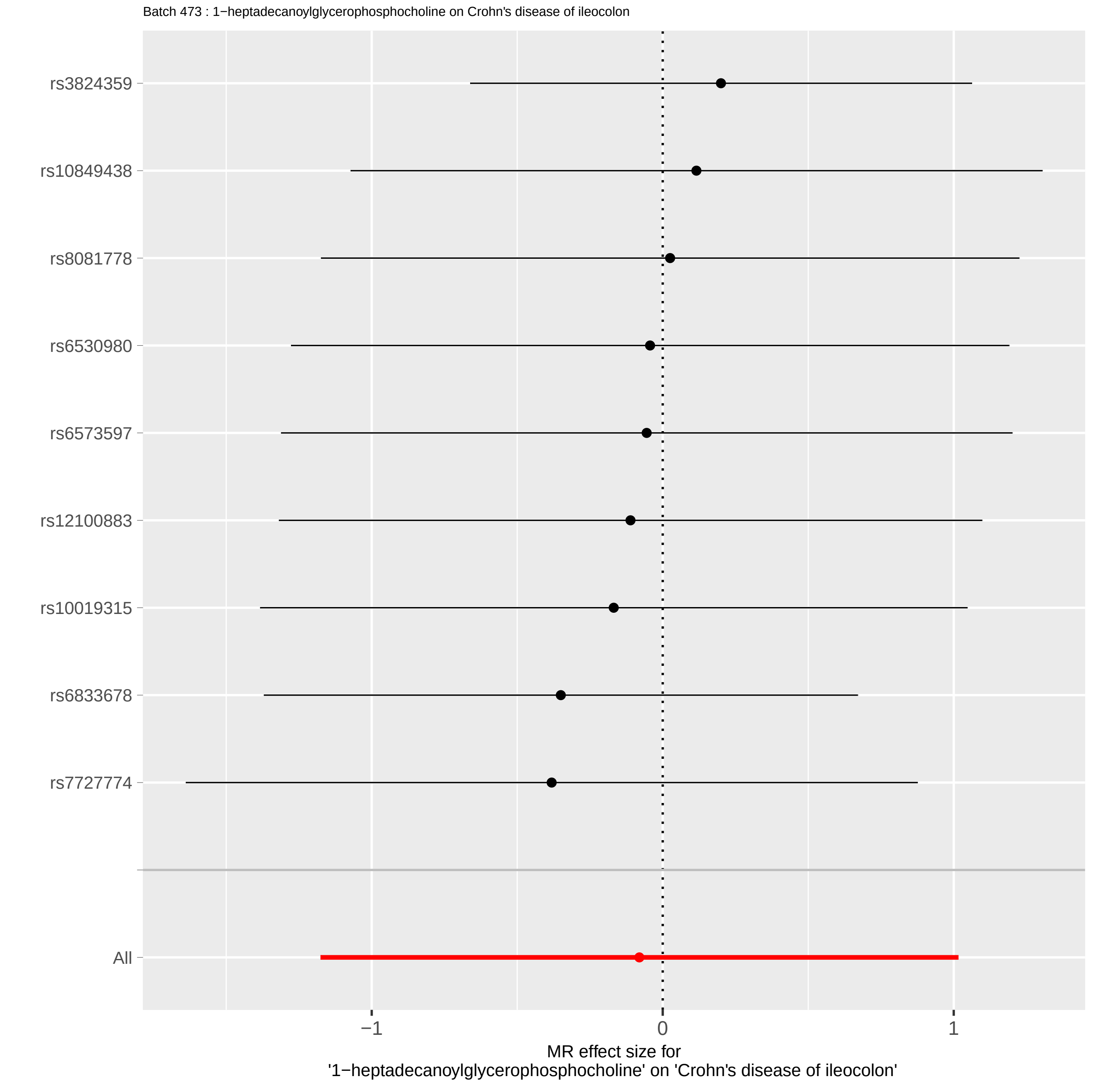

Batch 473 : 1−heptadecanoylglycerophosphocholine on Crohn's disease of ileocolon
rs3824359
rs10849438
rs8081778
rs6530980
rs6573597
rs12100883
rs10019315
rs6833678
rs7727774
All
−1
0
1
MR effect size for
'1−heptadecanoylglycerophosphocholine' on 'Crohn's disease of ileocolon'

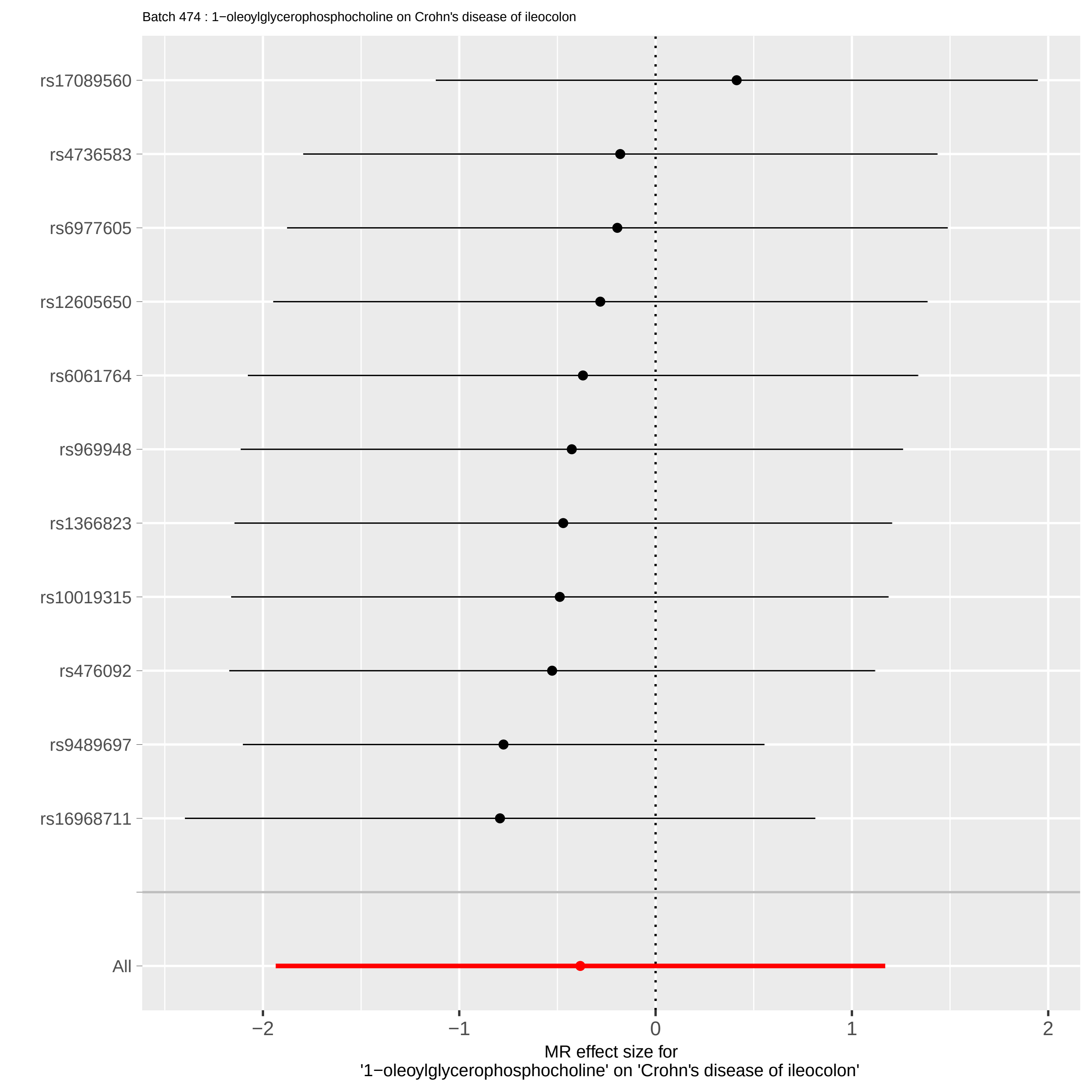

Batch 474 : 1−oleoylglycerophosphocholine on Crohn's disease of ileocolon
rs17089560
rs4736583
rs6977605
rs12605650
rs6061764
rs969948
rs1366823
rs10019315
rs476092
rs9489697
rs16968711
All
−2
−1
0
1
2
MR effect size for
'1−oleoylglycerophosphocholine' on 'Crohn's disease of ileocolon'

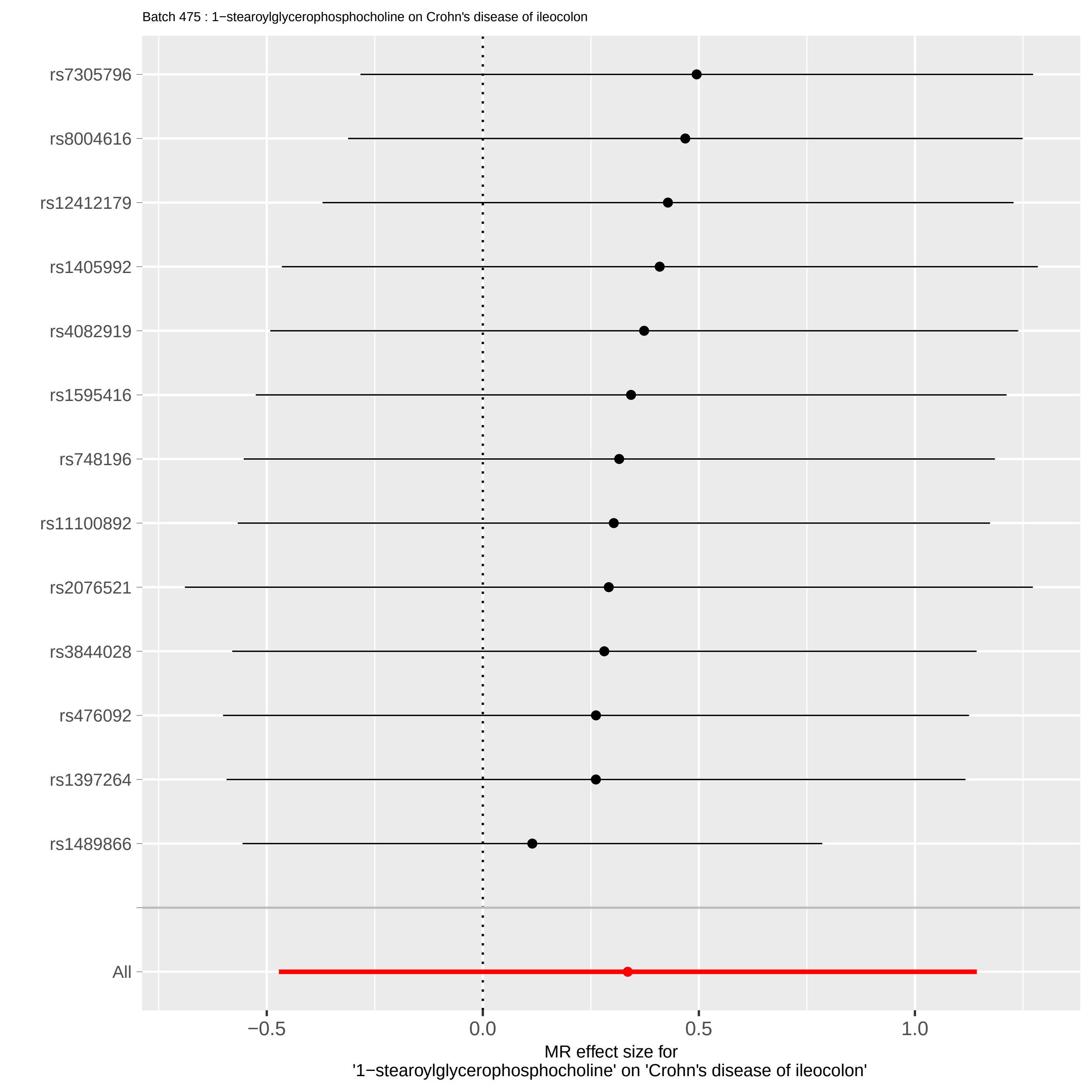

Batch 475 : 1−stearoylglycerophosphocholine on Crohn's disease of ileocolon
rs7305796
rs8004616
rs12412179
rs1405992
rs4082919
rs1595416
rs748196
rs11100892
rs2076521
rs3844028
rs476092
rs1397264
rs1489866
All
−0.5
0.0
0.5
1.0
MR effect size for
'1−stearoylglycerophosphocholine' on 'Crohn's disease of ileocolon'

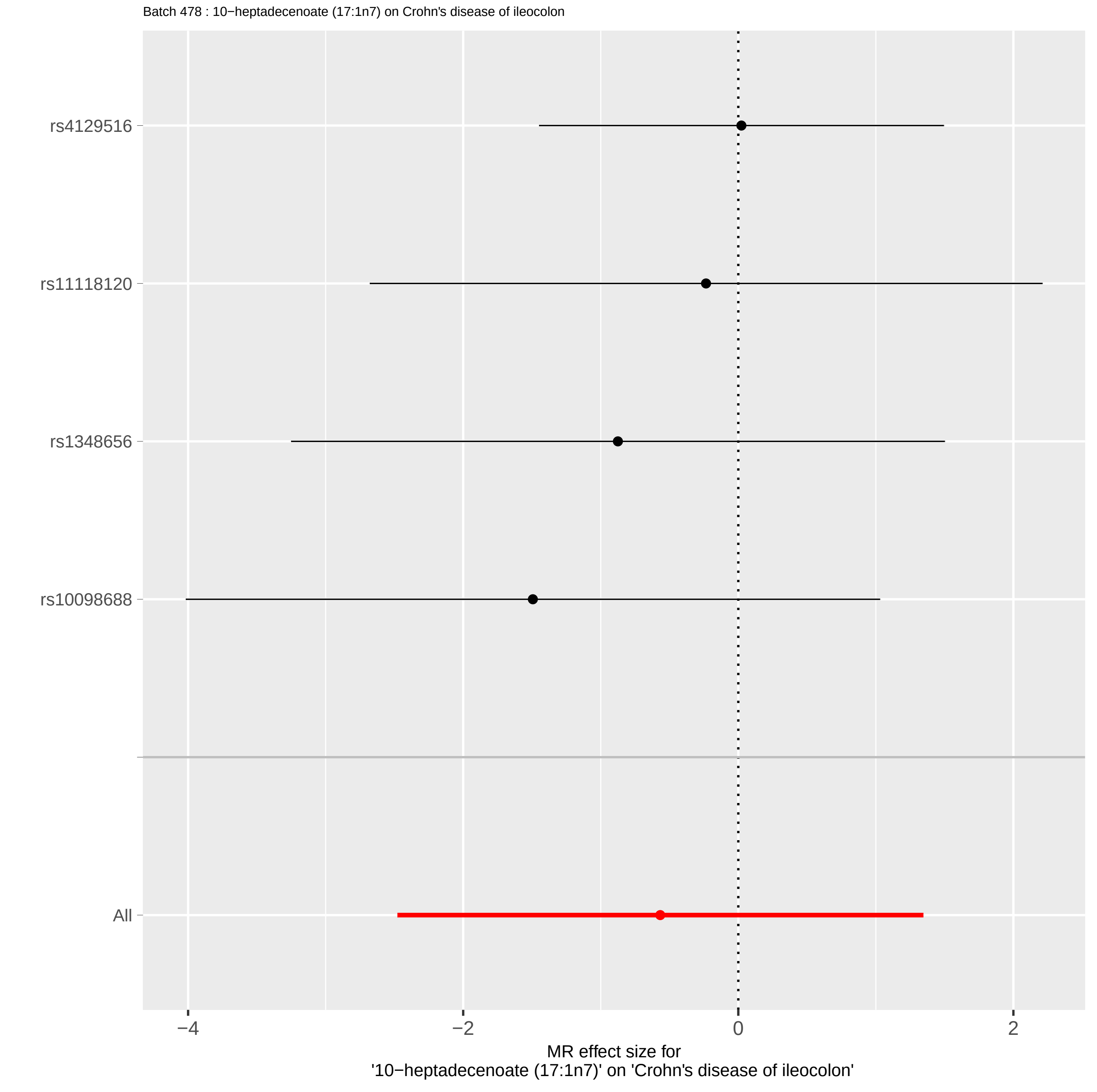

Batch 478 : 10−heptadecenoate (17:1n7) on Crohn's disease of ileocolon
rs4129516
rs11118120
rs1348656
rs10098688
All
−4
−2
0
2
MR effect size for
'10−heptadecenoate (17:1n7)' on 'Crohn's disease of ileocolon'

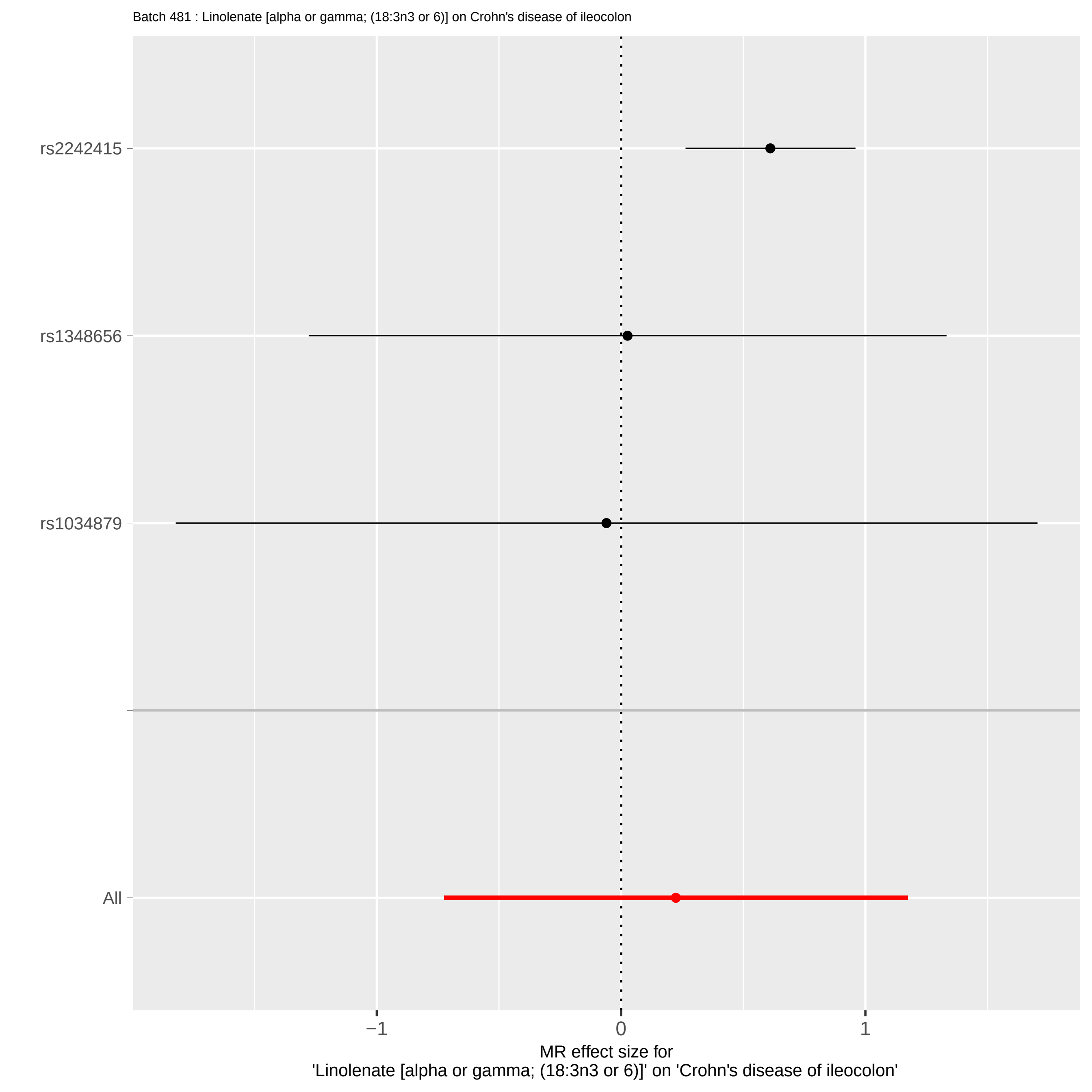

Batch 481 : Linolenate [alpha or gamma; (18:3n3 or 6)] on Crohn's disease of ileocolon
rs2242415
rs1348656
rs1034879
All
−1
0
1
MR effect size for
'Linolenate [alpha or gamma; (18:3n3 or 6)]' on 'Crohn's disease of ileocolon'

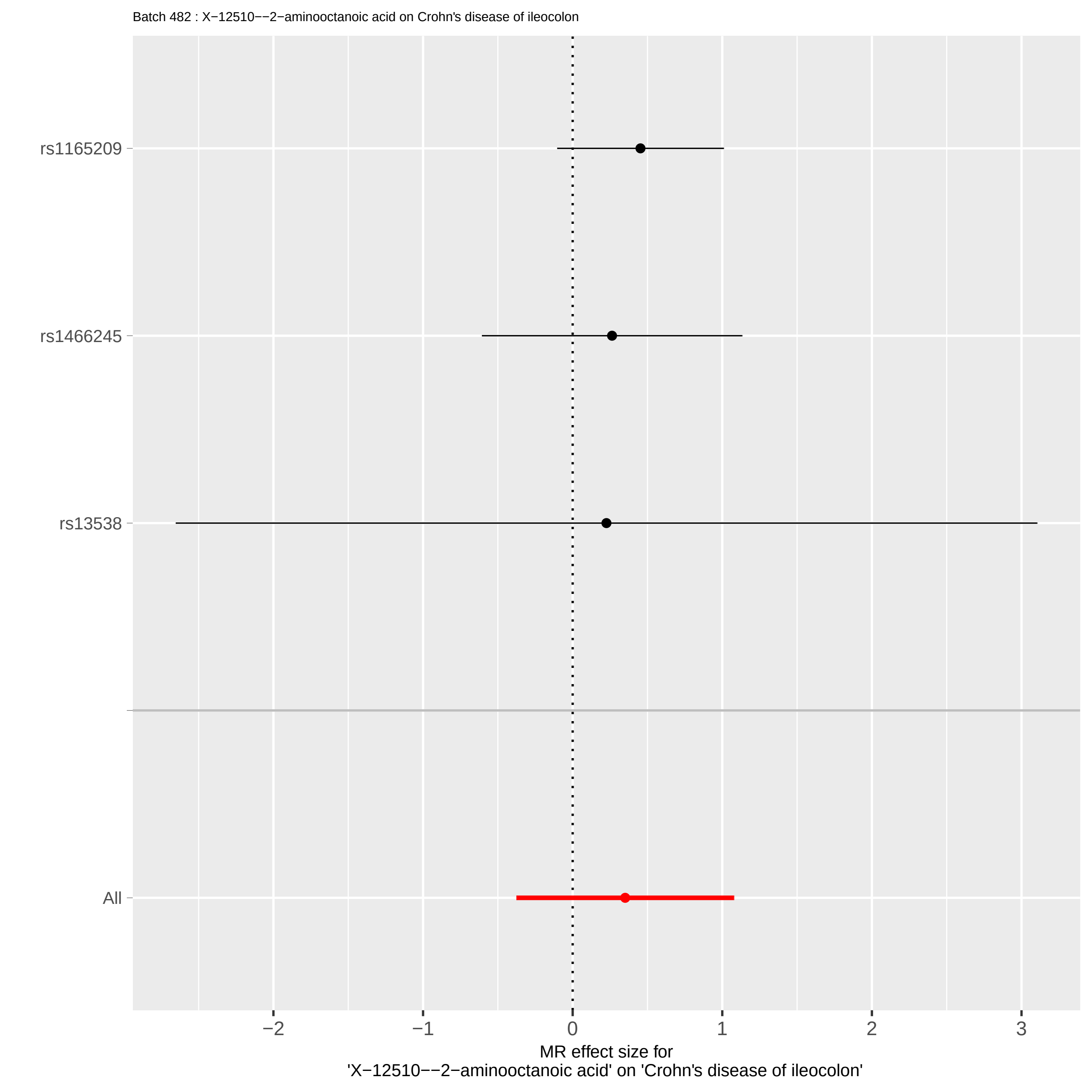
Batch 482 : X−12510−−2−aminooctanoic acid on Crohn's disease of ileocolon
rs1165209
rs1466245
rs13538
All
−2
−1
0
1
2
3
MR effect size for
'X−12510−−2−aminooctanoic acid' on 'Crohn's disease of ileocolon'

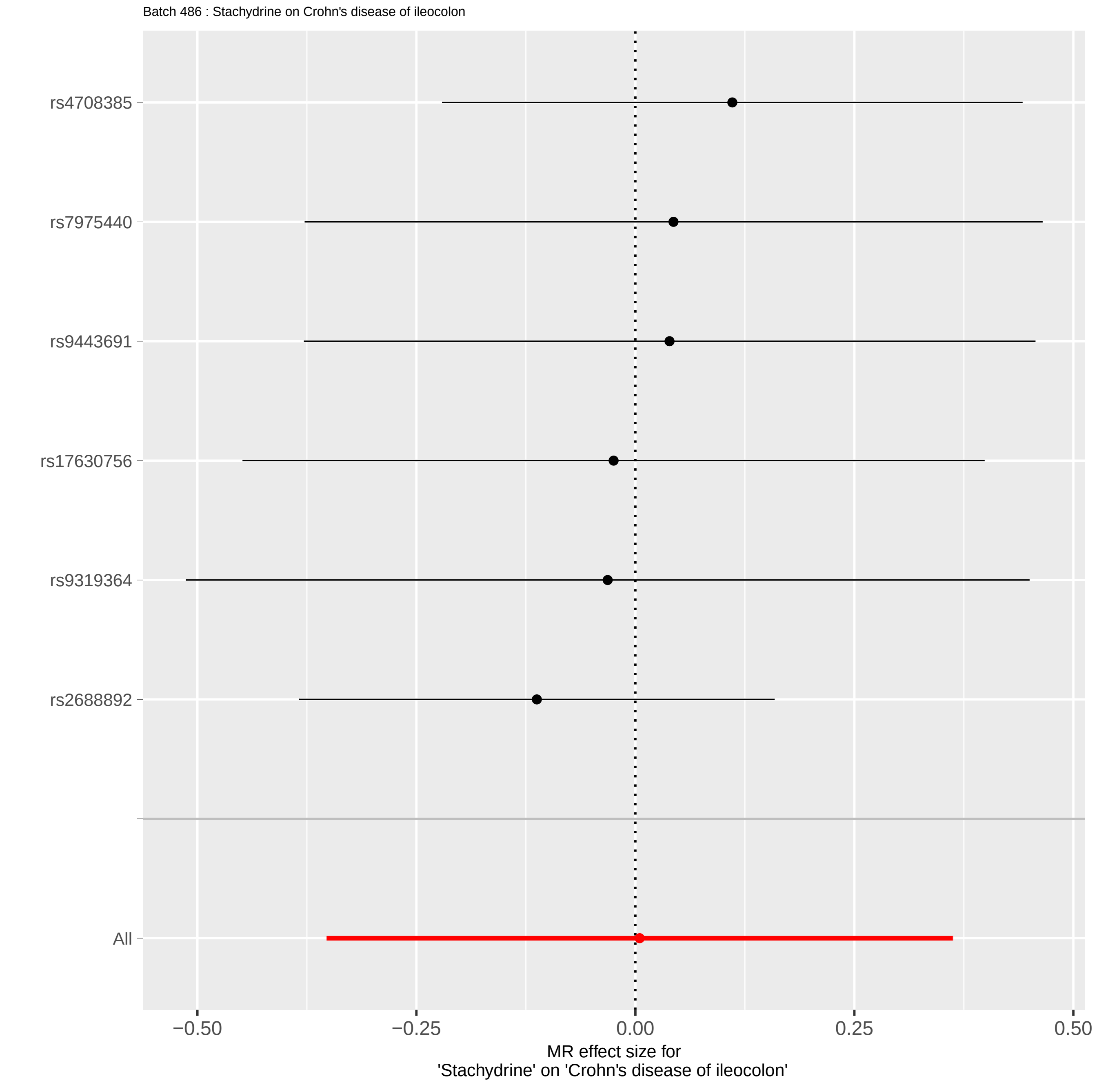
Batch 486 : Stachydrine on Crohn's disease of ileocolon
rs4708385
rs7975440
rs9443691
rs17630756
rs9319364
rs2688892
All
−0.50
−0.25
0.00
0.25
0.50
MR effect size for
'Stachydrine' on 'Crohn's disease of ileocolon'

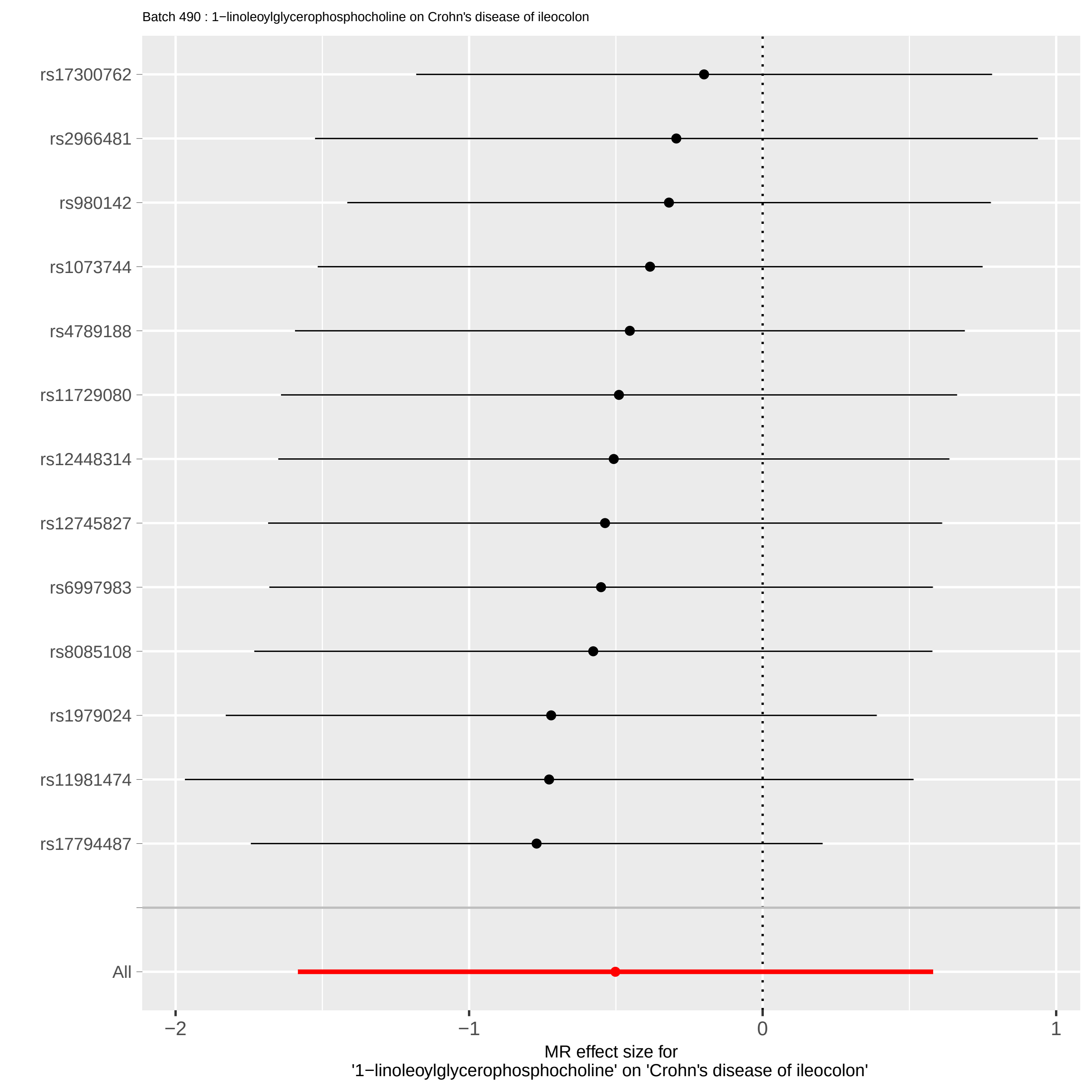
Batch 490 : 1−linoleoylglycerophosphocholine on Crohn's disease of ileocolon
rs17300762
rs2966481
rs980142
rs1073744
rs4789188
rs11729080
rs12448314
rs12745827
rs6997983
rs8085108
rs1979024
rs11981474
rs17794487
All
−2
−1
0
1
MR effect size for
'1−linoleoylglycerophosphocholine' on 'Crohn's disease of ileocolon'

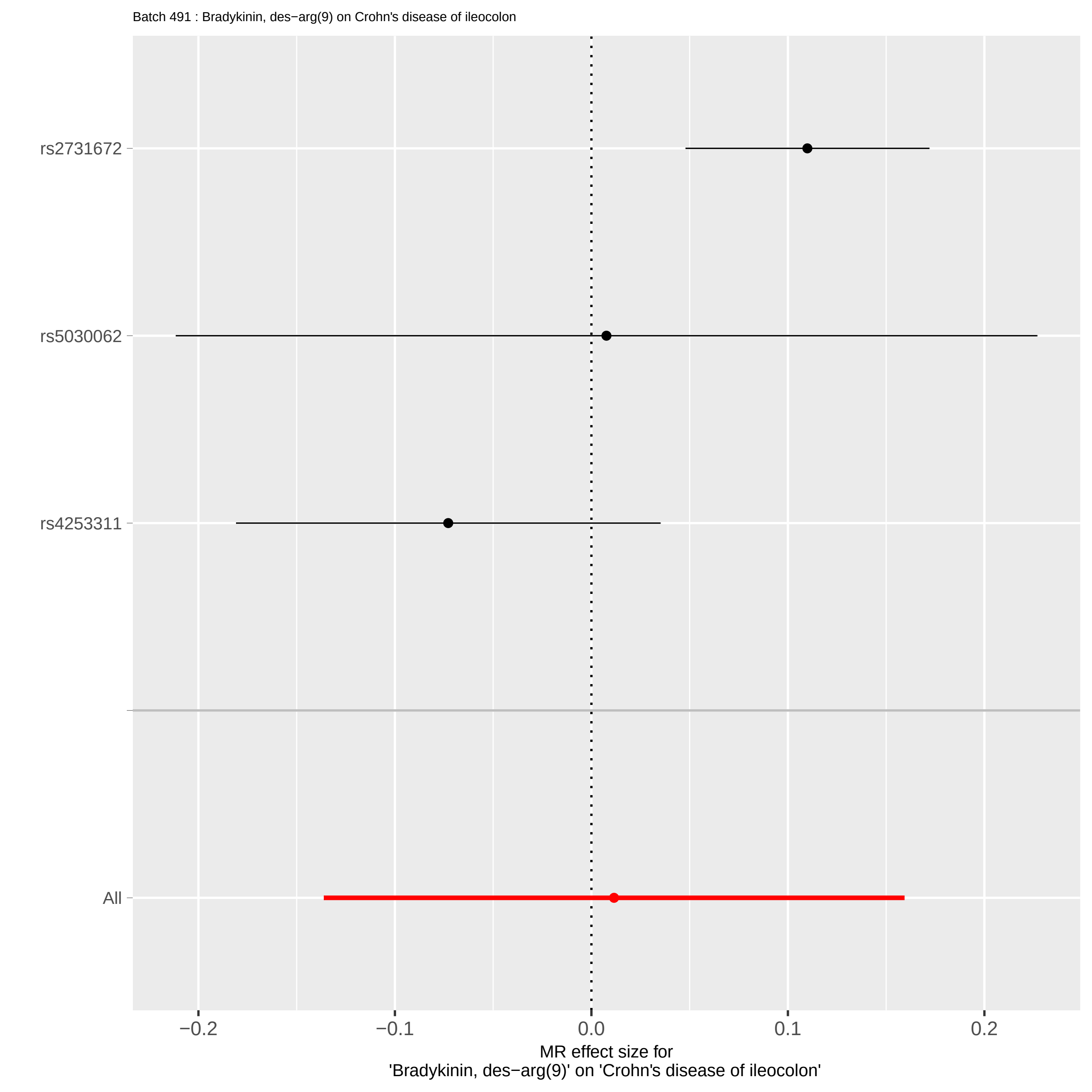

Batch 491 : Bradykinin, des−arg(9) on Crohn's disease of ileocolon
rs2731672
rs5030062
rs4253311
All
−0.2
−0.1
0.0
0.1
0.2
MR effect size for
'Bradykinin, des−arg(9)' on 'Crohn's disease of ileocolon'

Batch 492 : Gamma−glutamylisoleucine* on Crohn's disease of ileocolon

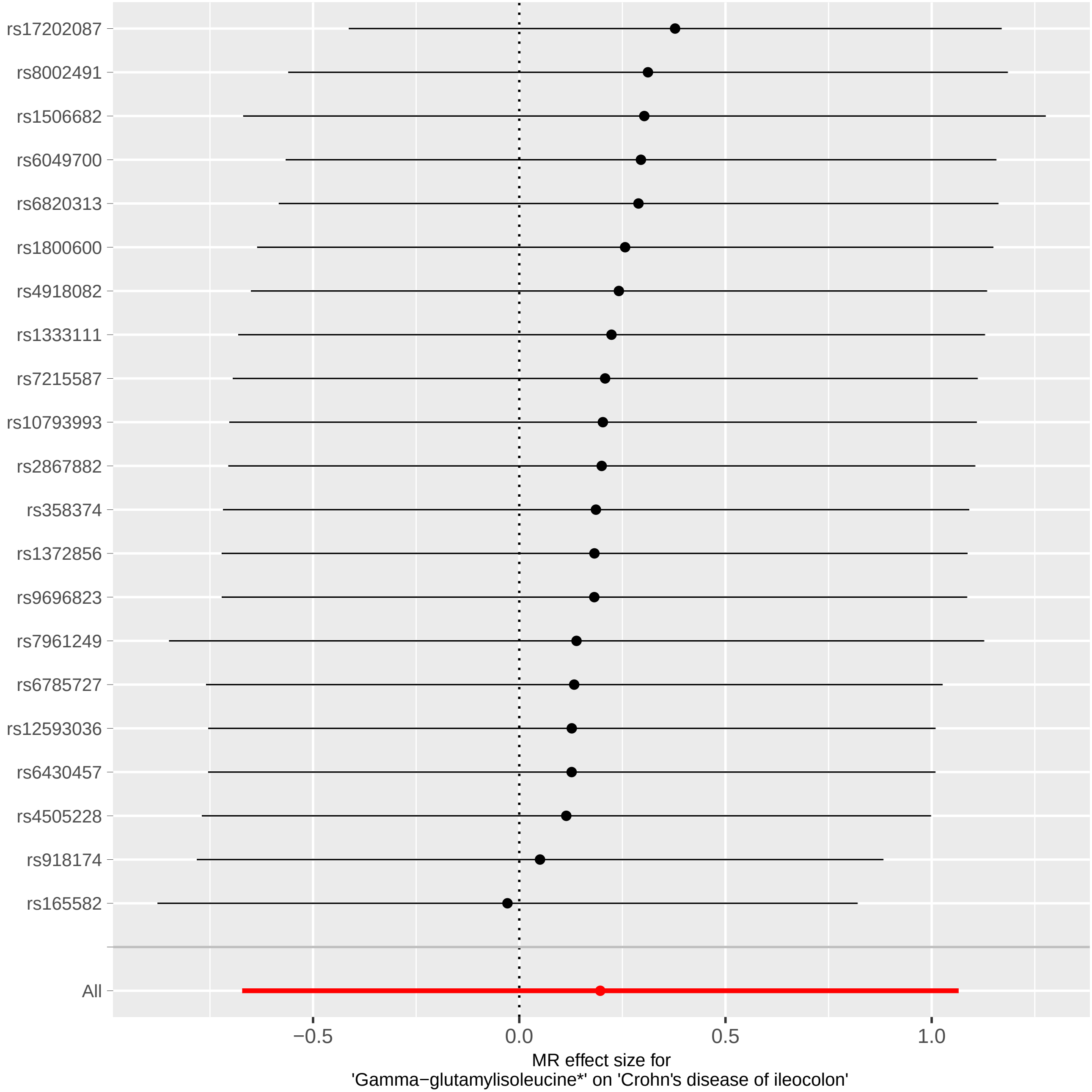

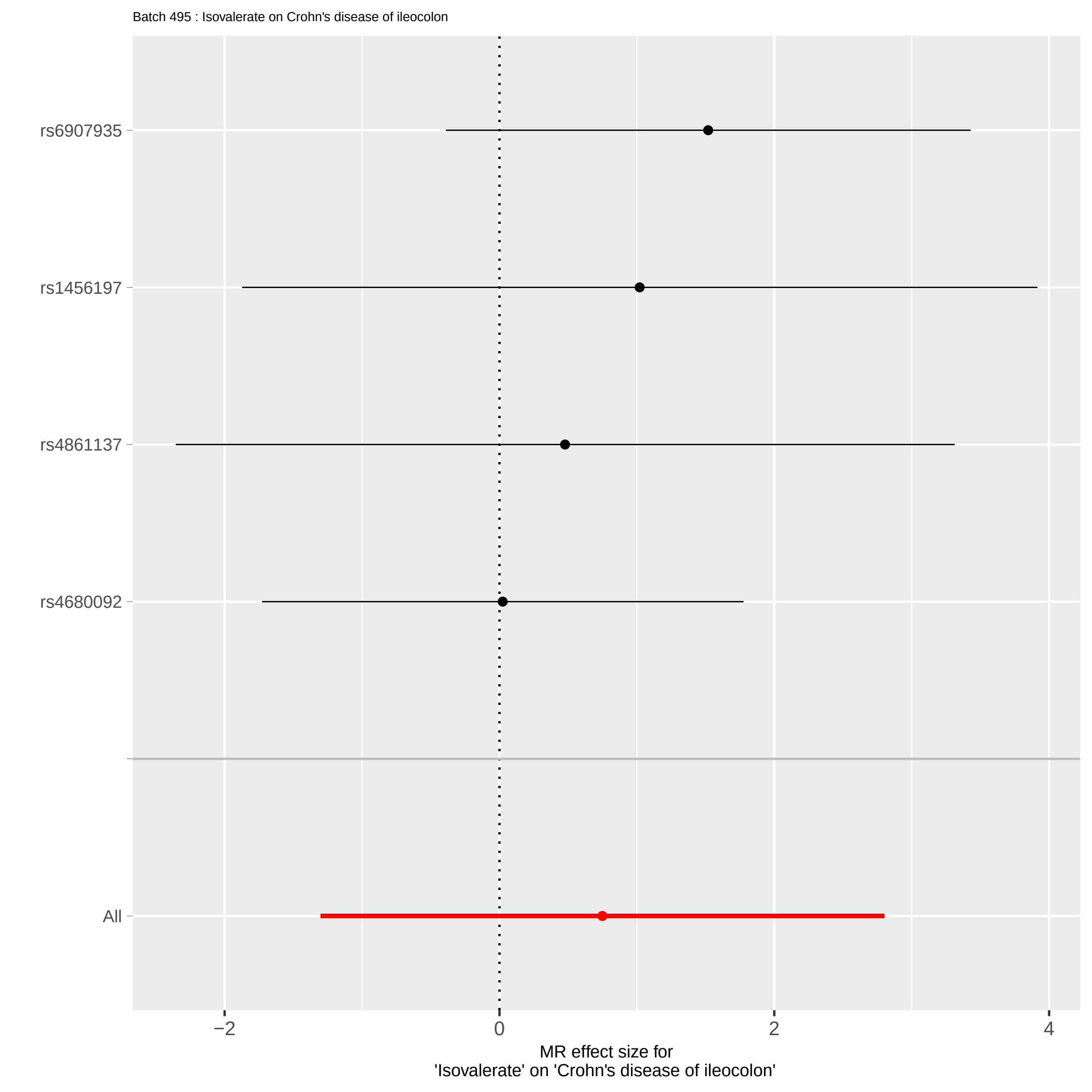

Batch 495 : Isovalerate on Crohn's disease of ileocolon
rs6907935
rs1456197
rs4861137
rs4680092
All
−2
0
2
4
MR effect size for
'Isovalerate' on 'Crohn's disease of ileocolon'

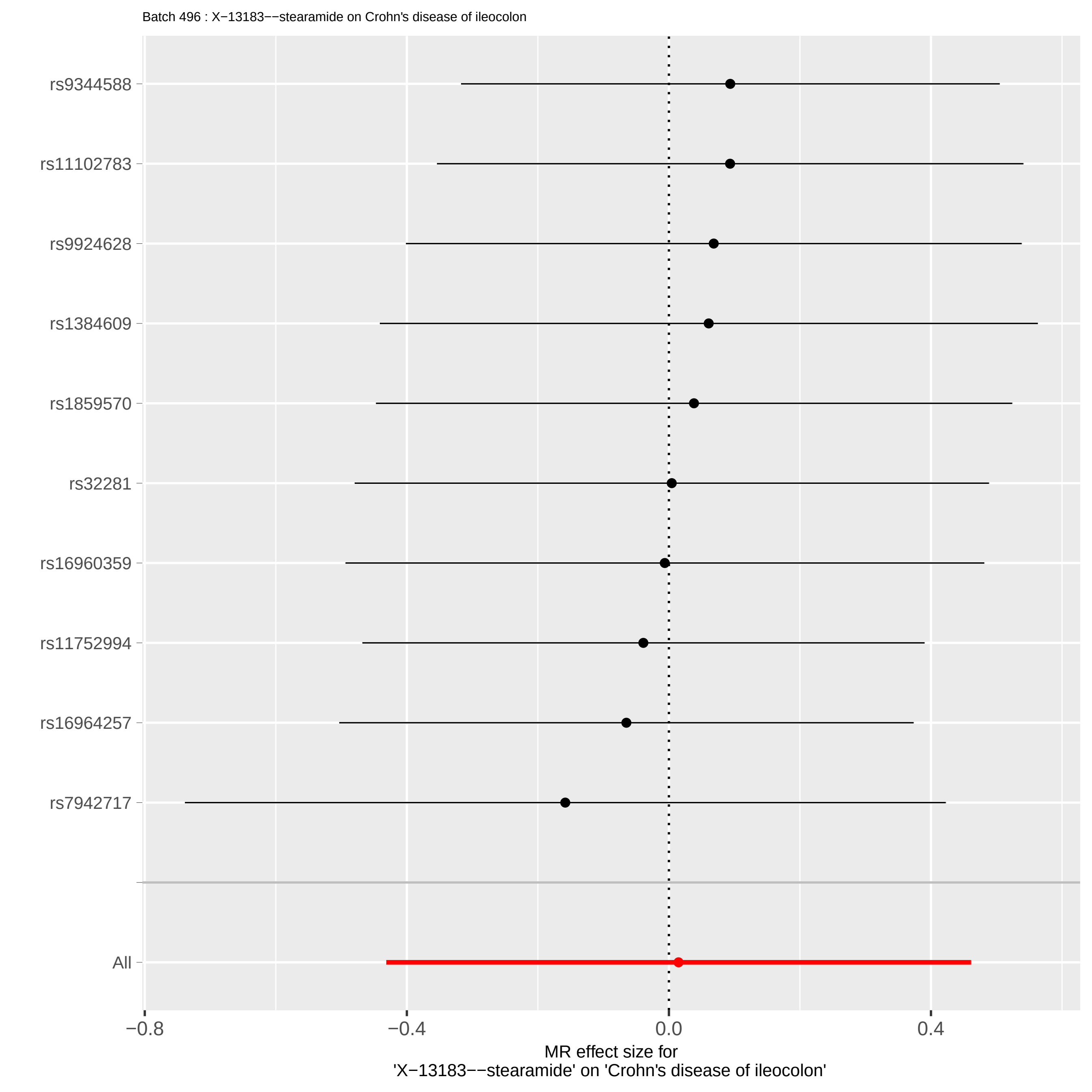

Batch 496 : X−13183−−stearamide on Crohn's disease of ileocolon
rs9344588
rs11102783
rs9924628
rs1384609
rs1859570
rs32281
rs16960359
rs11752994
rs16964257
rs7942717
All
−0.8
−0.4
0.0
0.4
MR effect size for
'X−13183−−stearamide' on 'Crohn's disease of ileocolon'

Batch 497 : 7−methylguanine on Crohn's disease of ileocolon

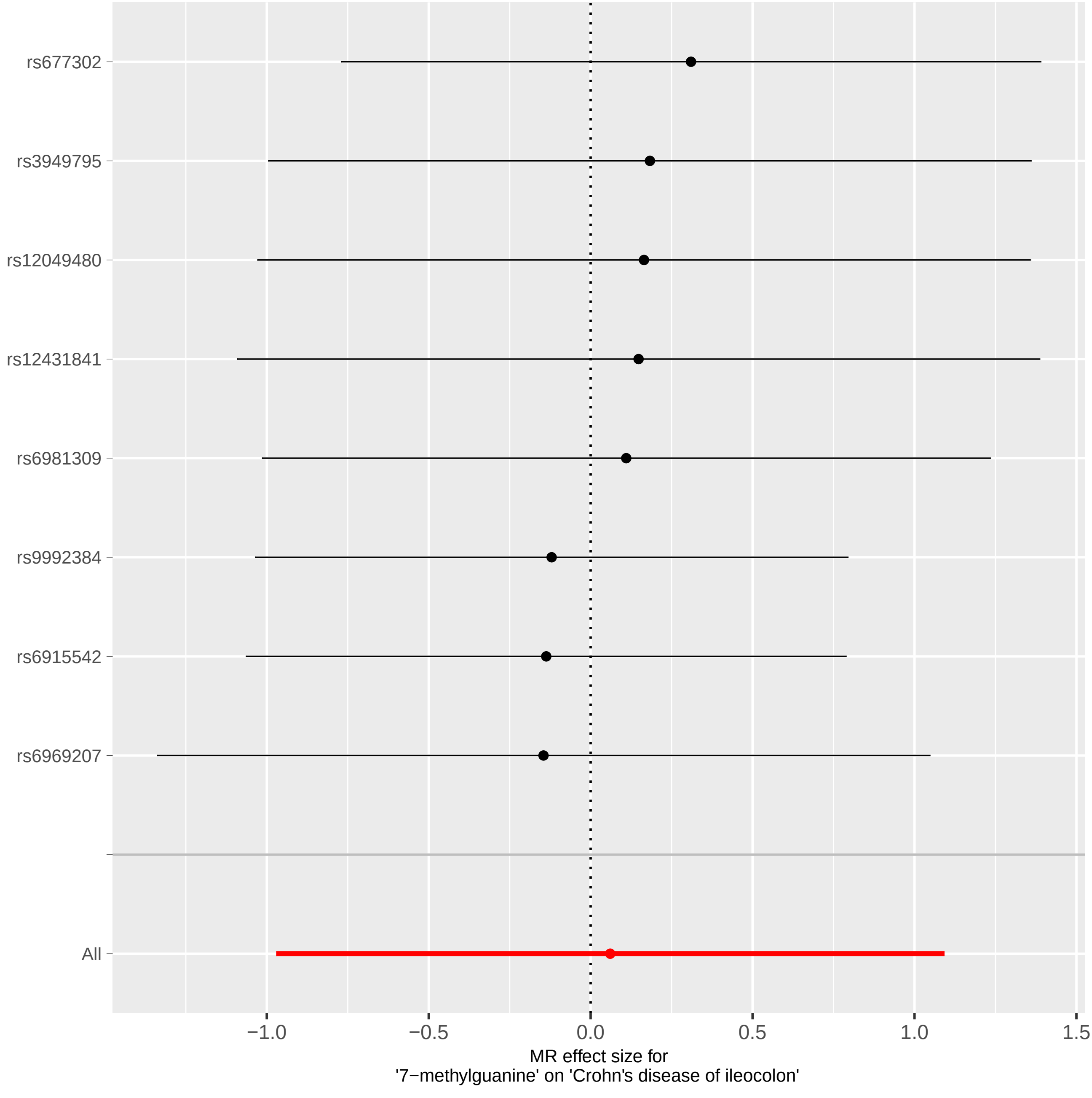

Batch 505 : 2−palmitoylglycerophosphocholine* on Crohn's disease of ileocolon

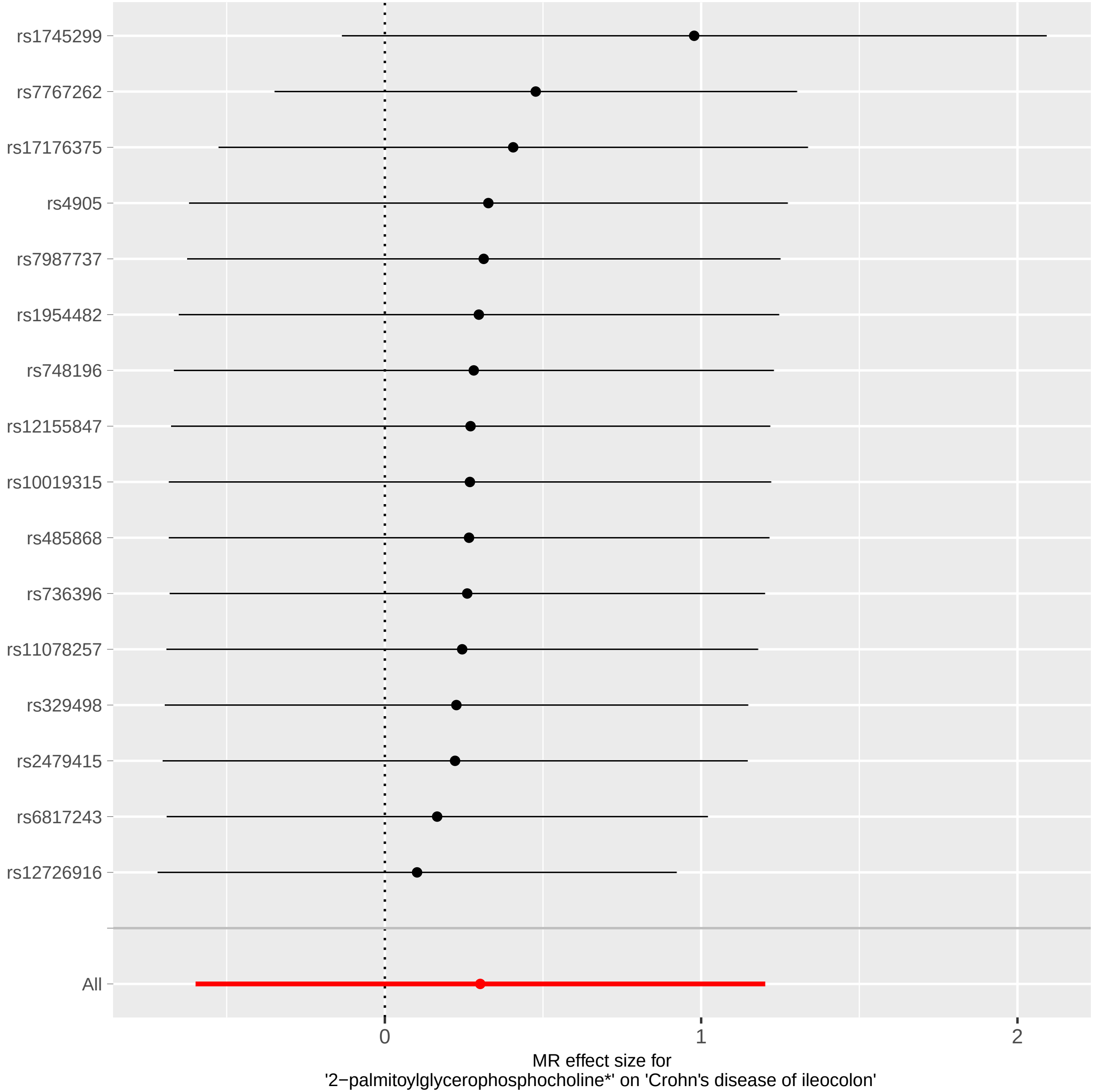

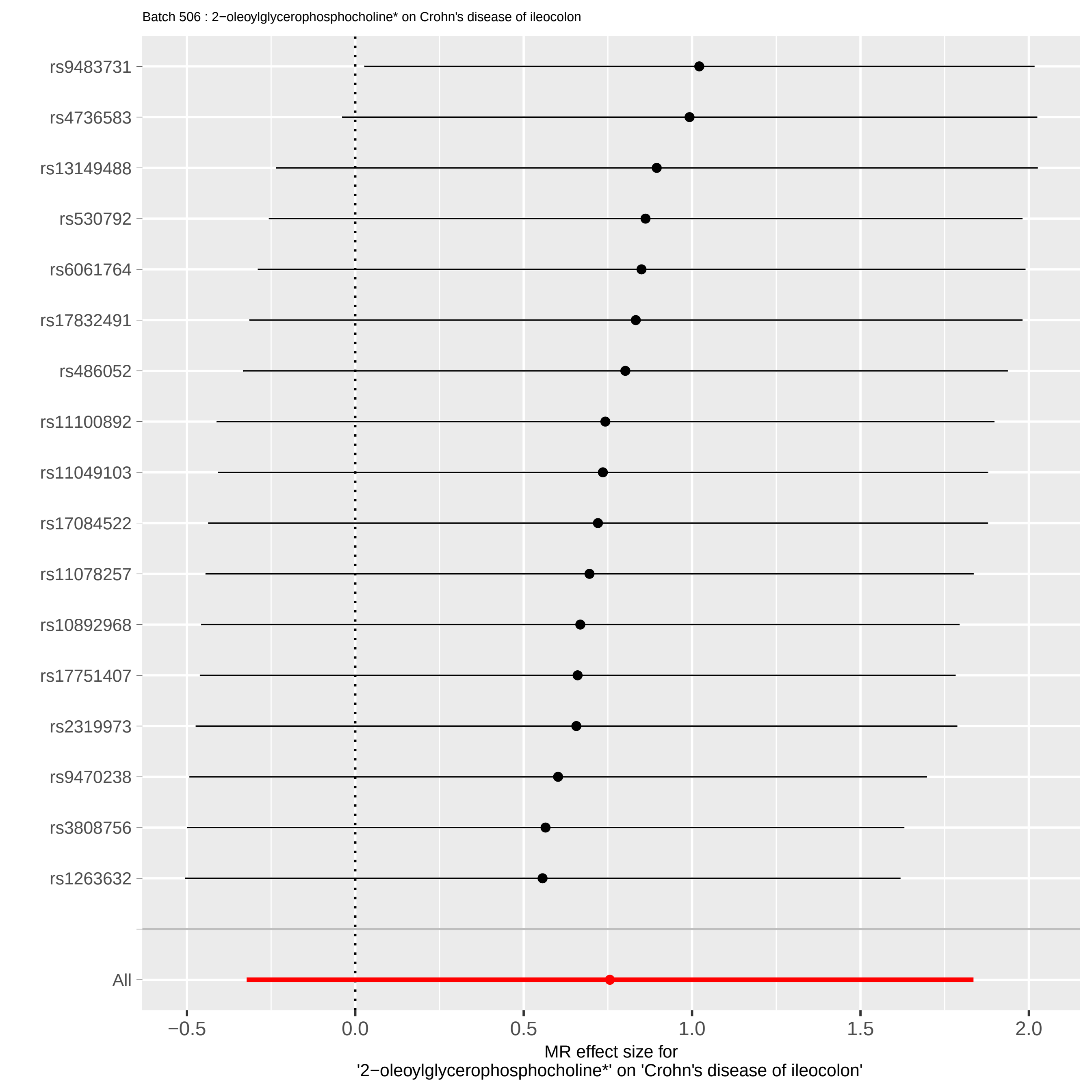

Batch 506 : 2−oleoylglycerophosphocholine* on Crohn's disease of ileocolon
rs9483731
rs4736583
rs13149488
rs530792
rs6061764
rs17832491
rs486052
rs11100892
rs11049103
rs17084522
rs11078257
rs10892968
rs17751407
rs2319973
rs9470238
rs3808756
rs1263632
All
−0.5
0.0
0.5
1.0
1.5
2.0
MR effect size for
'2−oleoylglycerophosphocholine*' on 'Crohn's disease of ileocolon'

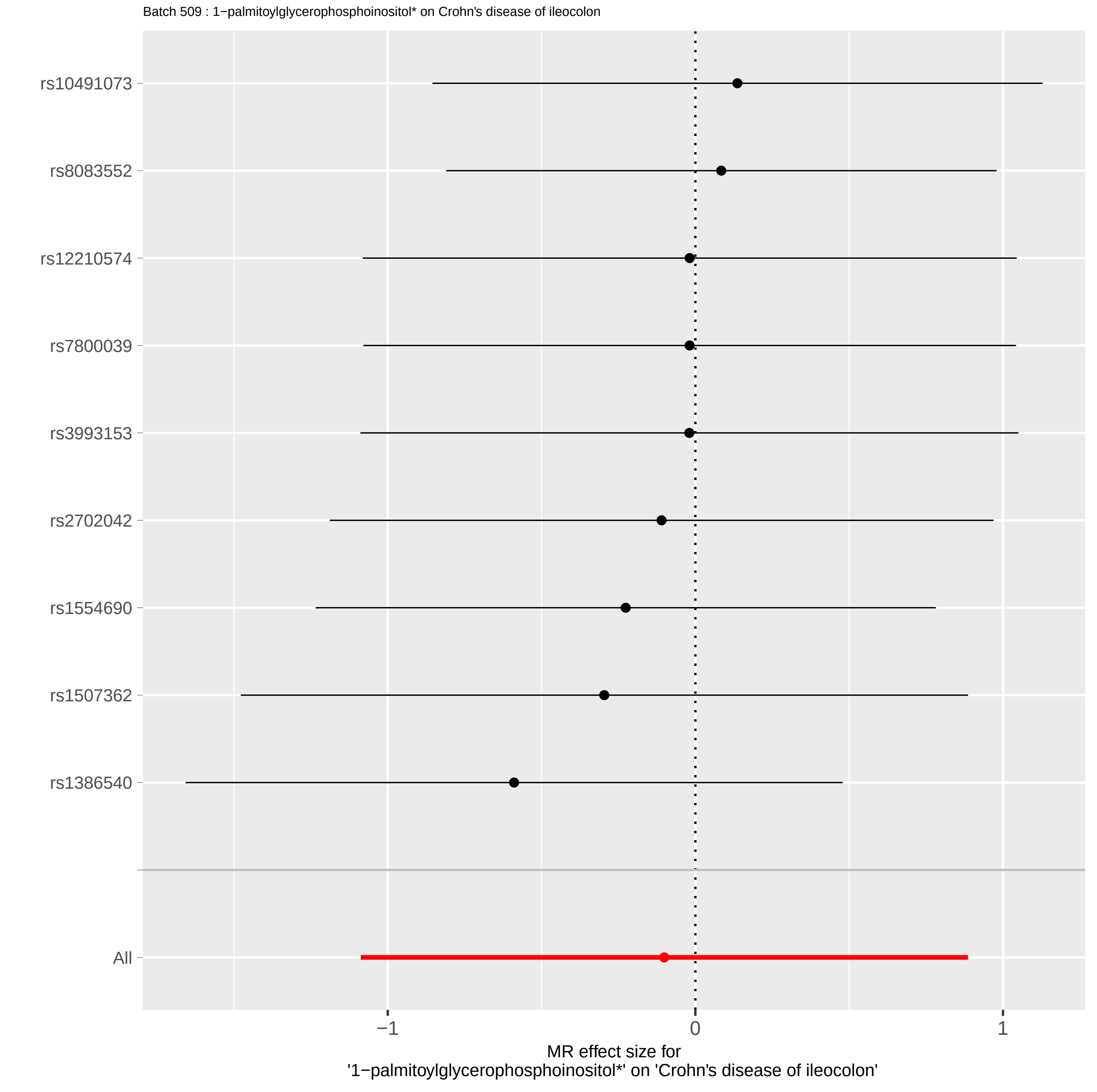

Batch 509 : 1−palmitoylglycerophosphoinositol* on Crohn's disease of ileocolon
rs10491073
rs8083552
rs12210574
rs7800039
rs3993153
rs2702042
rs1554690
rs1507362
rs1386540
All
−1
0
1
MR effect size for
'1−palmitoylglycerophosphoinositol*' on 'Crohn's disease of ileocolon'

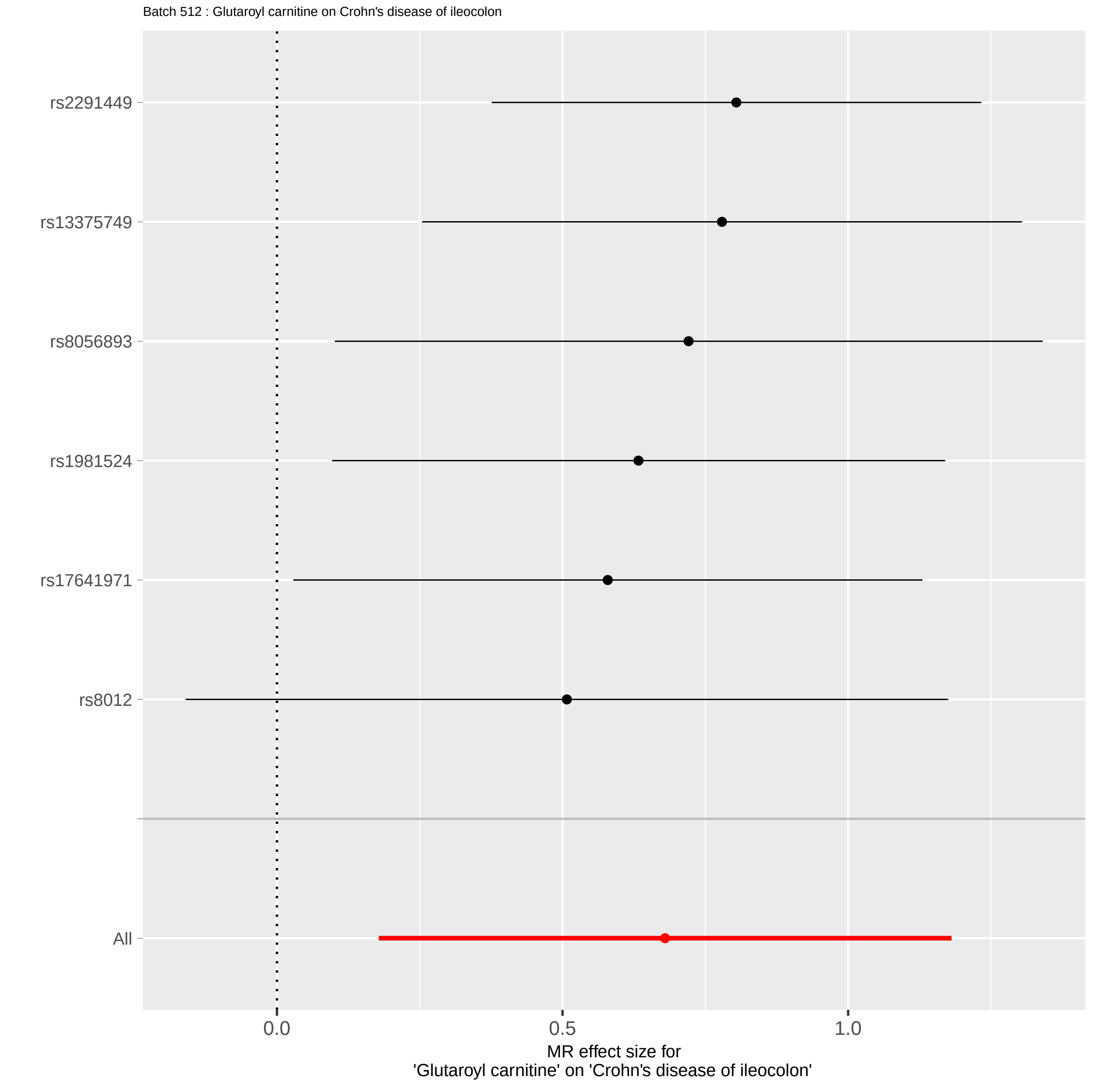

Batch 512 : Glutaroyl carnitine on Crohn's disease of ileocolon
rs2291449
rs13375749
rs8056893
rs1981524
rs17641971
rs8012
All
0.0
0.5
1.0
MR effect size for
'Glutaroyl carnitine' on 'Crohn's disease of ileocolon'

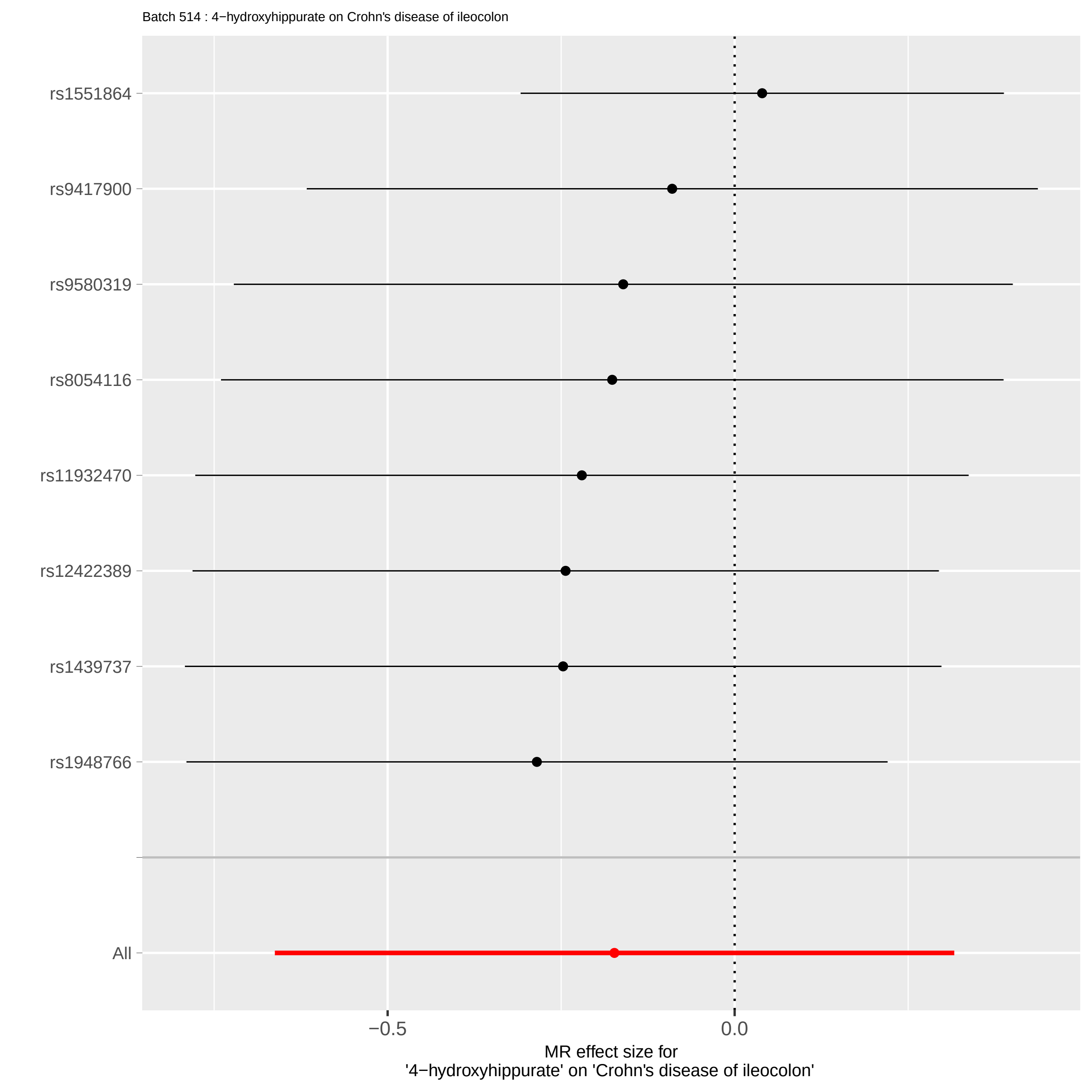

Batch 514 : 4−hydroxyhippurate on Crohn's disease of ileocolon
rs1551864
rs9417900
rs9580319
rs8054116
rs11932470
rs12422389
rs1439737
rs1948766
All
−0.5
0.0
MR effect size for
'4−hydroxyhippurate' on 'Crohn's disease of ileocolon'

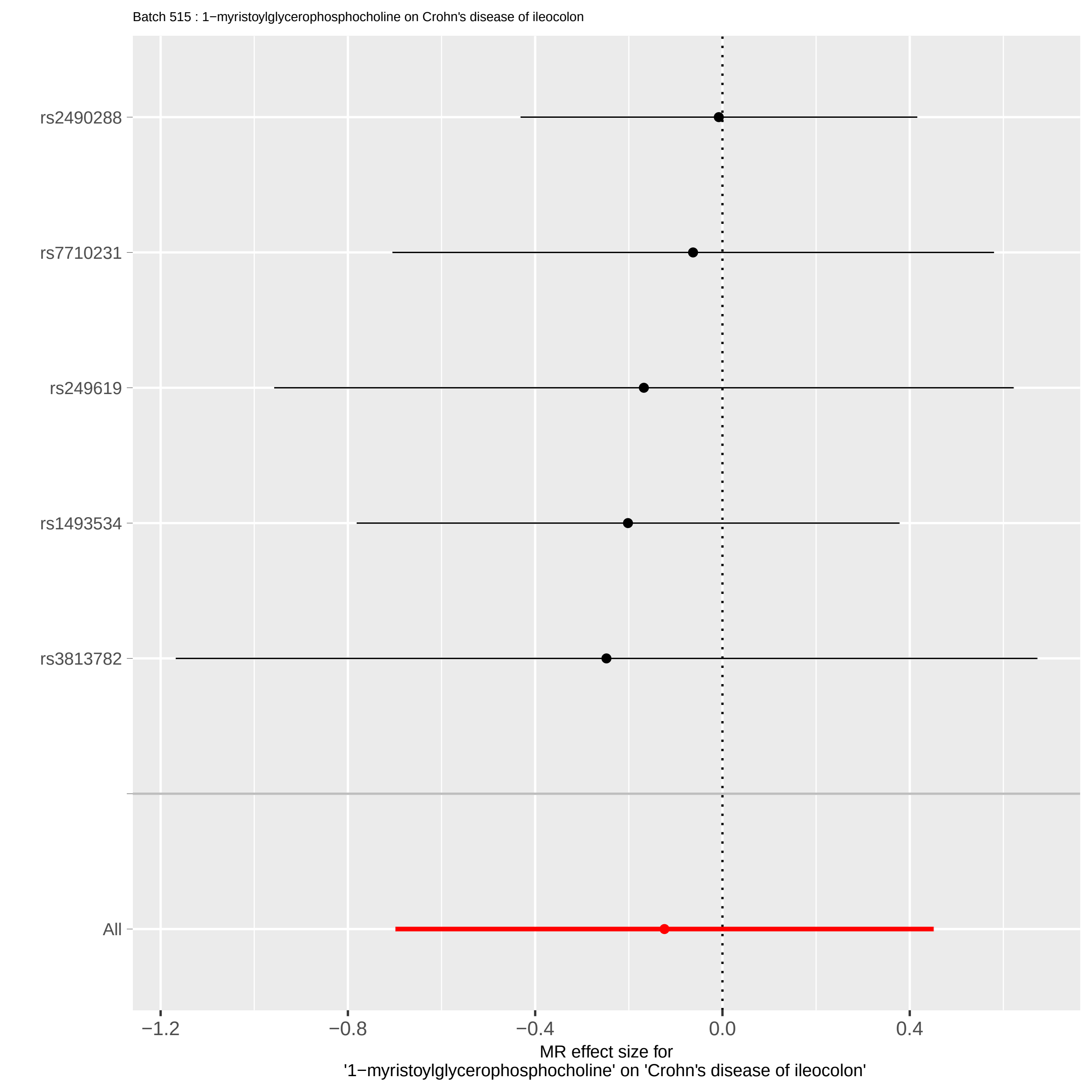

Batch 515 : 1−myristoylglycerophosphocholine on Crohn's disease of ileocolon
rs2490288
rs7710231
rs249619
rs1493534
rs3813782
All
−1.2
−0.8
−0.4
0.0
0.4
MR effect size for
'1−myristoylglycerophosphocholine' on 'Crohn's disease of ileocolon'

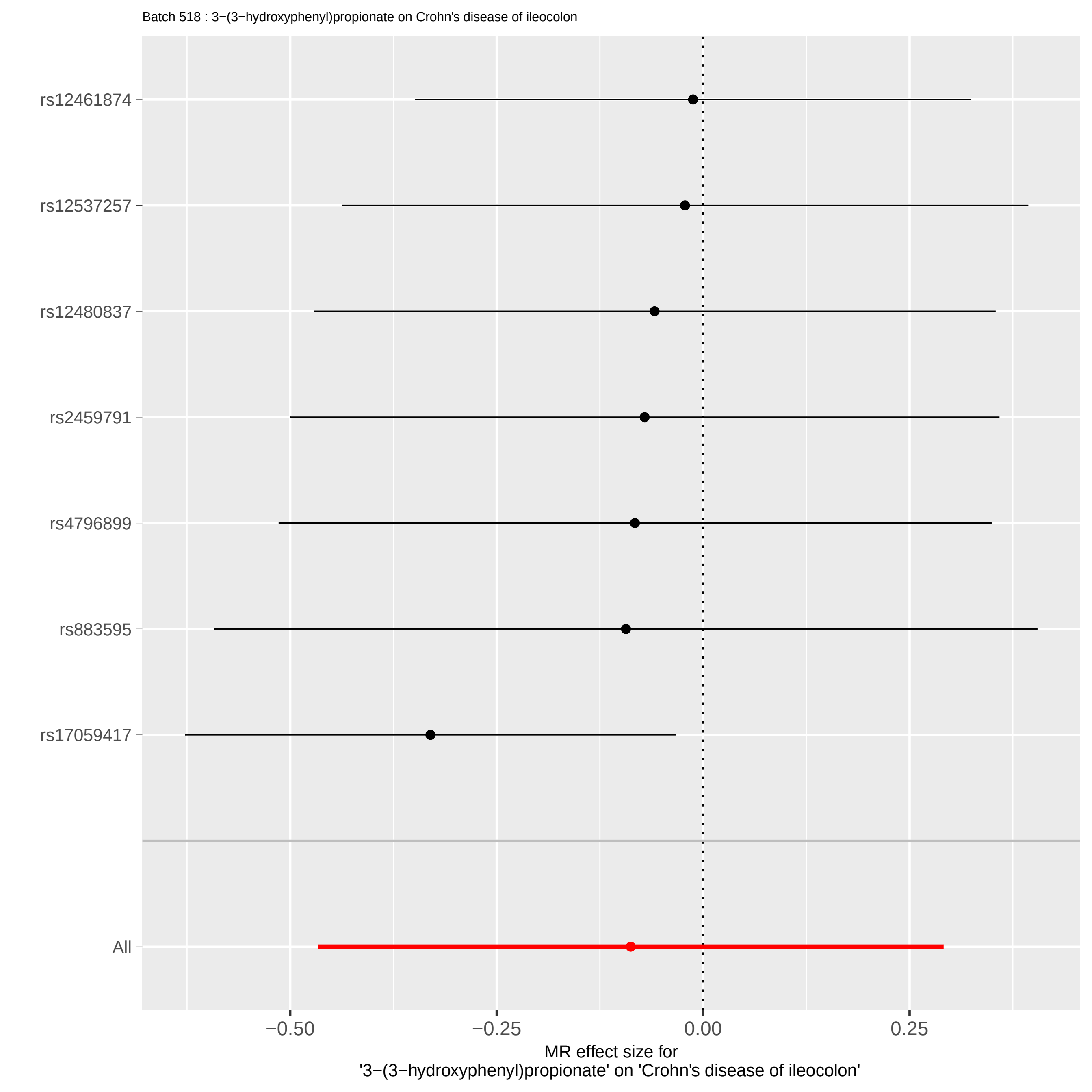
Batch 518 : 3−(3−hydroxyphenyl)propionate on Crohn's disease of ileocolon
rs12461874
rs12537257
rs12480837
rs2459791
rs4796899
rs883595
rs17059417
All
−0.50
−0.25
0.00
0.25
MR effect size for
'3−(3−hydroxyphenyl)propionate' on 'Crohn's disease of ileocolon'

Batch 523 : Threitol on Crohn's disease of ileocolon

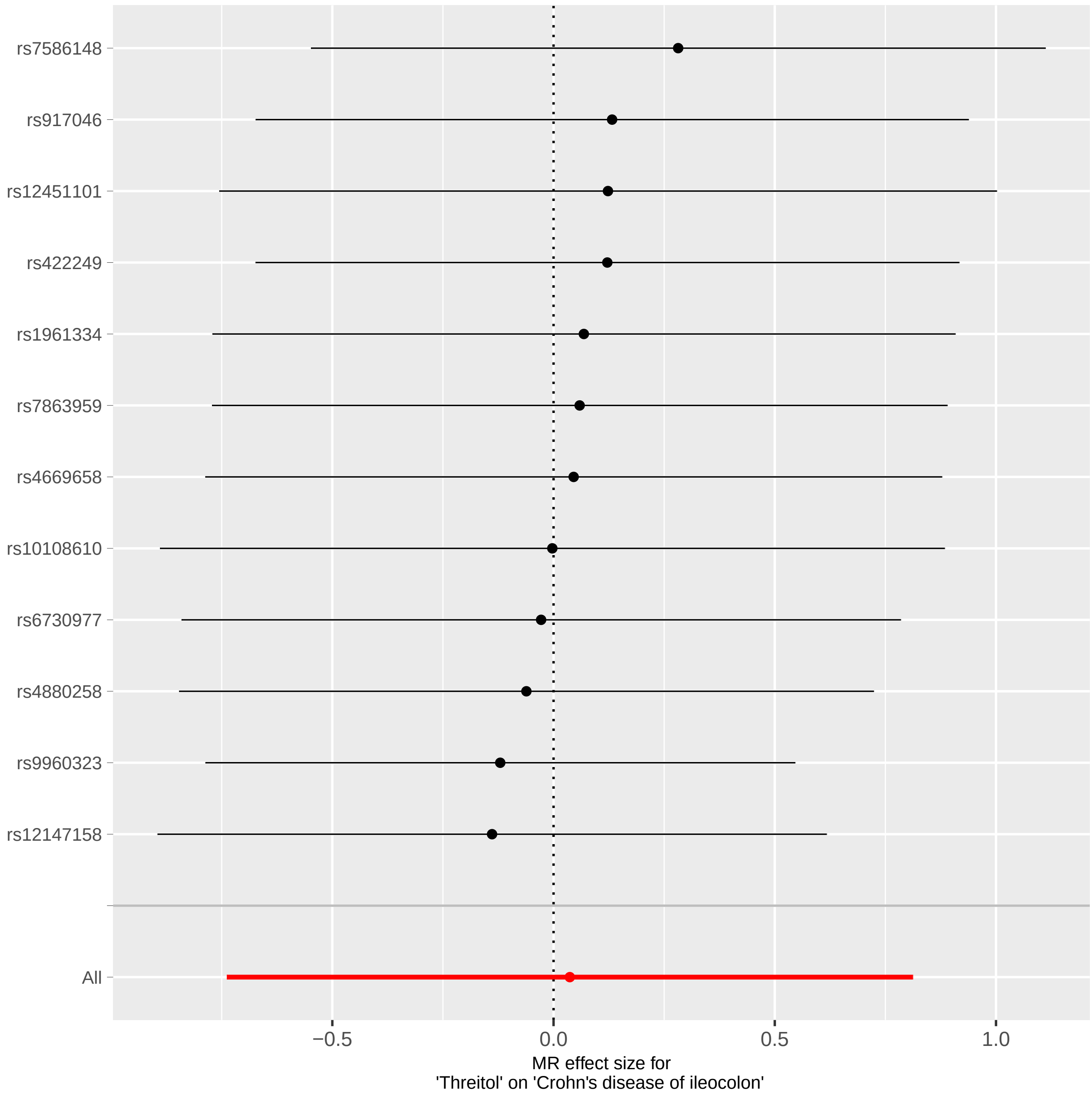

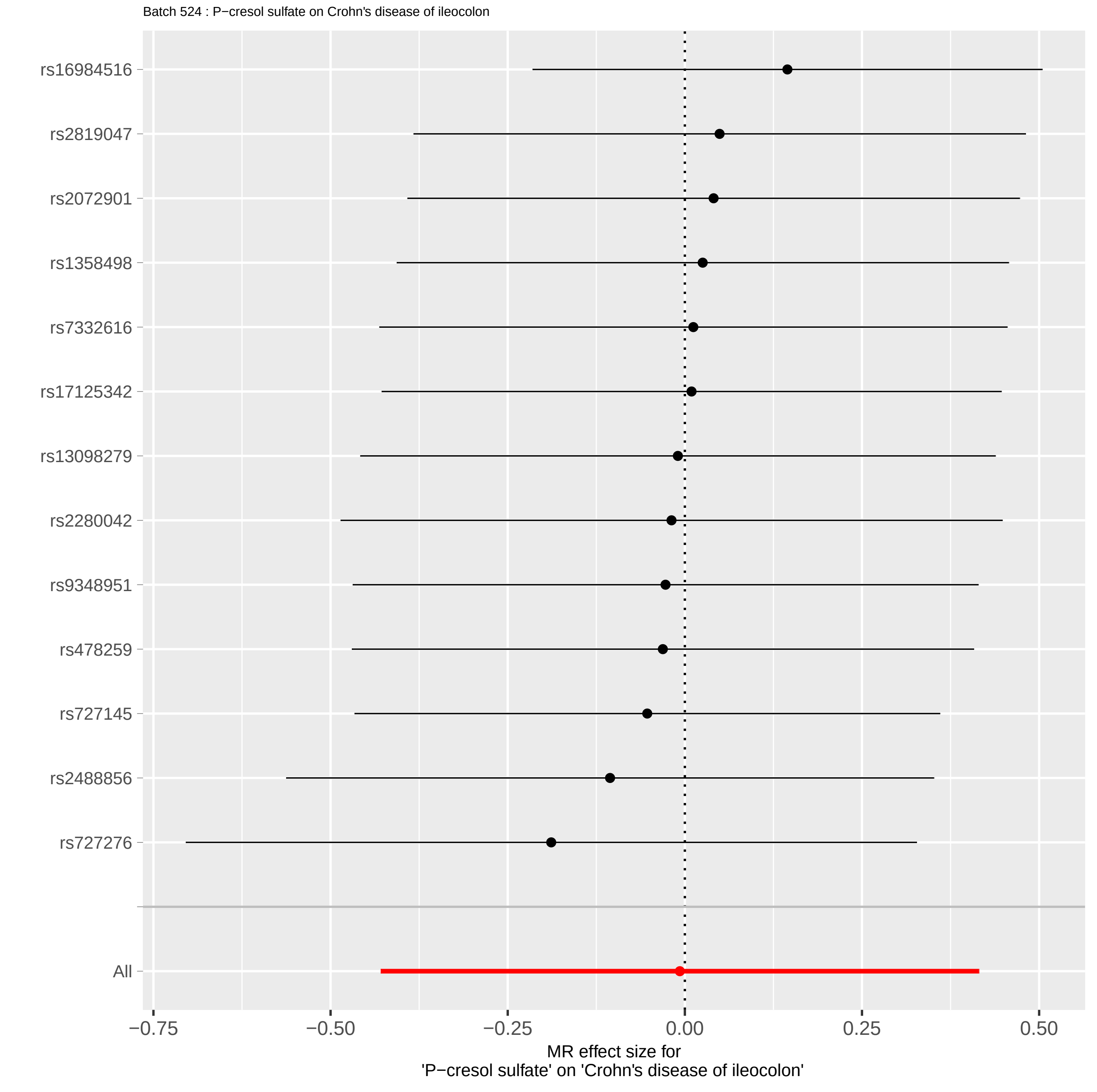

Batch 524 : P−cresol sulfate on Crohn's disease of ileocolon
rs16984516
rs2819047
rs2072901
rs1358498
rs7332616
rs17125342
rs13098279
rs2280042
rs9348951
rs478259
rs727145
rs2488856
rs727276
All
−0.75
−0.50
−0.25
0.00
0.25
0.50
MR effect size for
'P−cresol sulfate' on 'Crohn's disease of ileocolon'

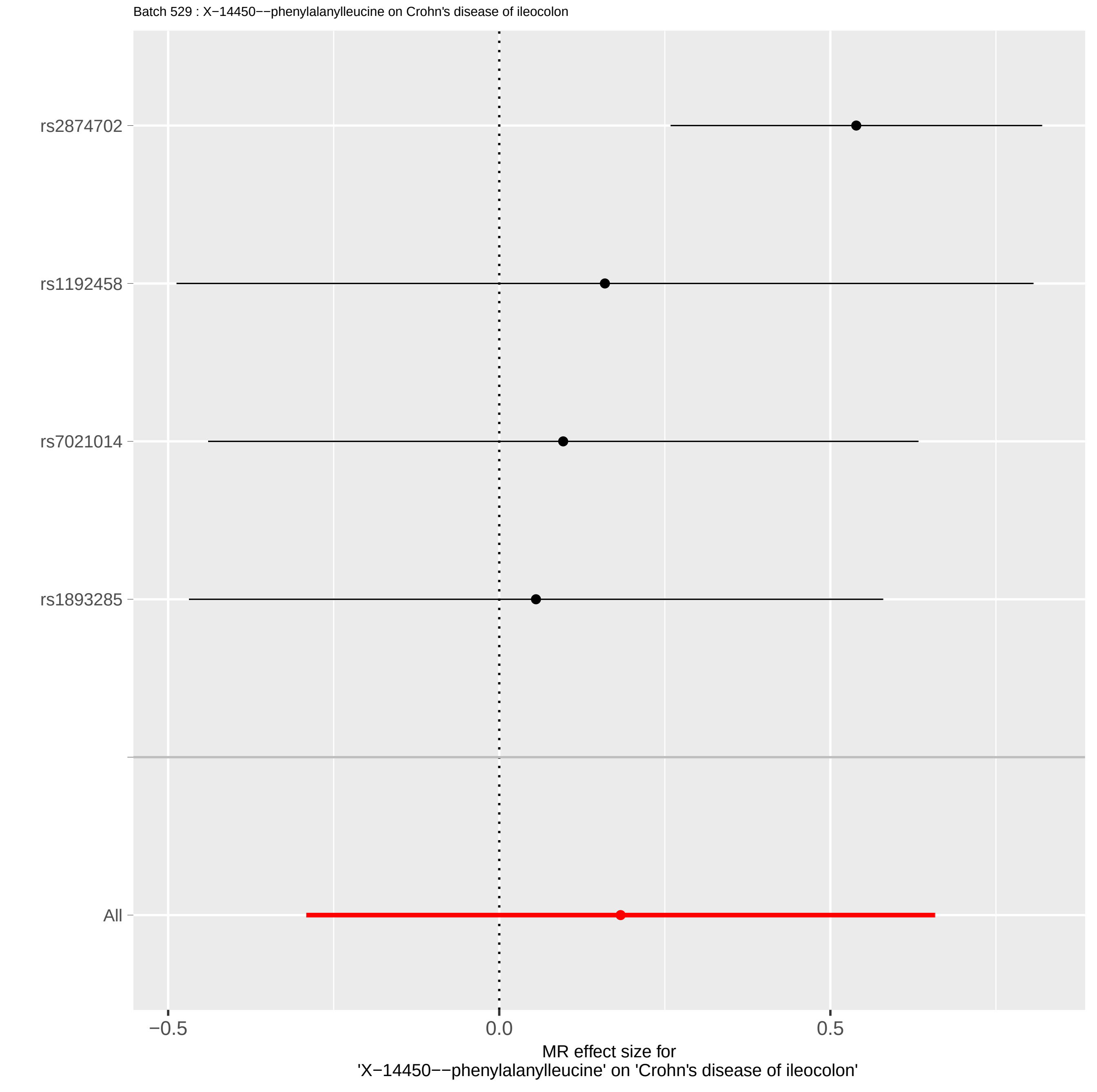
Batch 529 : X−14450−−phenylalanylleucine on Crohn's disease of ileocolon
rs2874702
rs1192458
rs7021014
rs1893285
All
−0.5
0.0
0.5
MR effect size for
'X−14450−−phenylalanylleucine' on 'Crohn's disease of ileocolon'

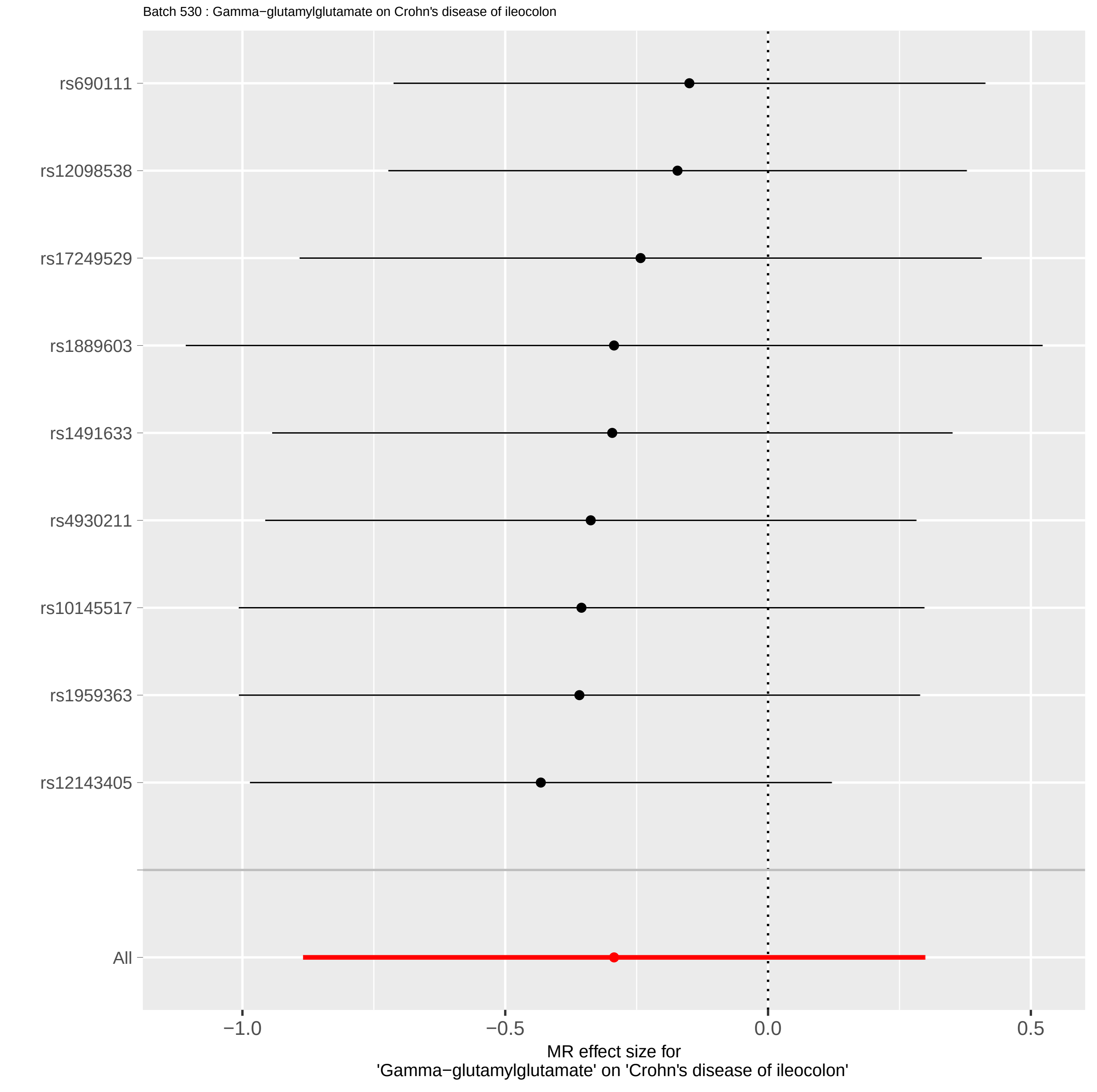
Batch 530 : Gamma−glutamylglutamate on Crohn's disease of ileocolon
rs690111
rs12098538
rs17249529
rs1889603
rs1491633
rs4930211
rs10145517
rs1959363
rs12143405
All
−1.0
−0.5
0.0
0.5
MR effect size for
'Gamma−glutamylglutamate' on 'Crohn's disease of ileocolon'

Batch 532 : Leucylleucine on Crohn's disease of ileocolon

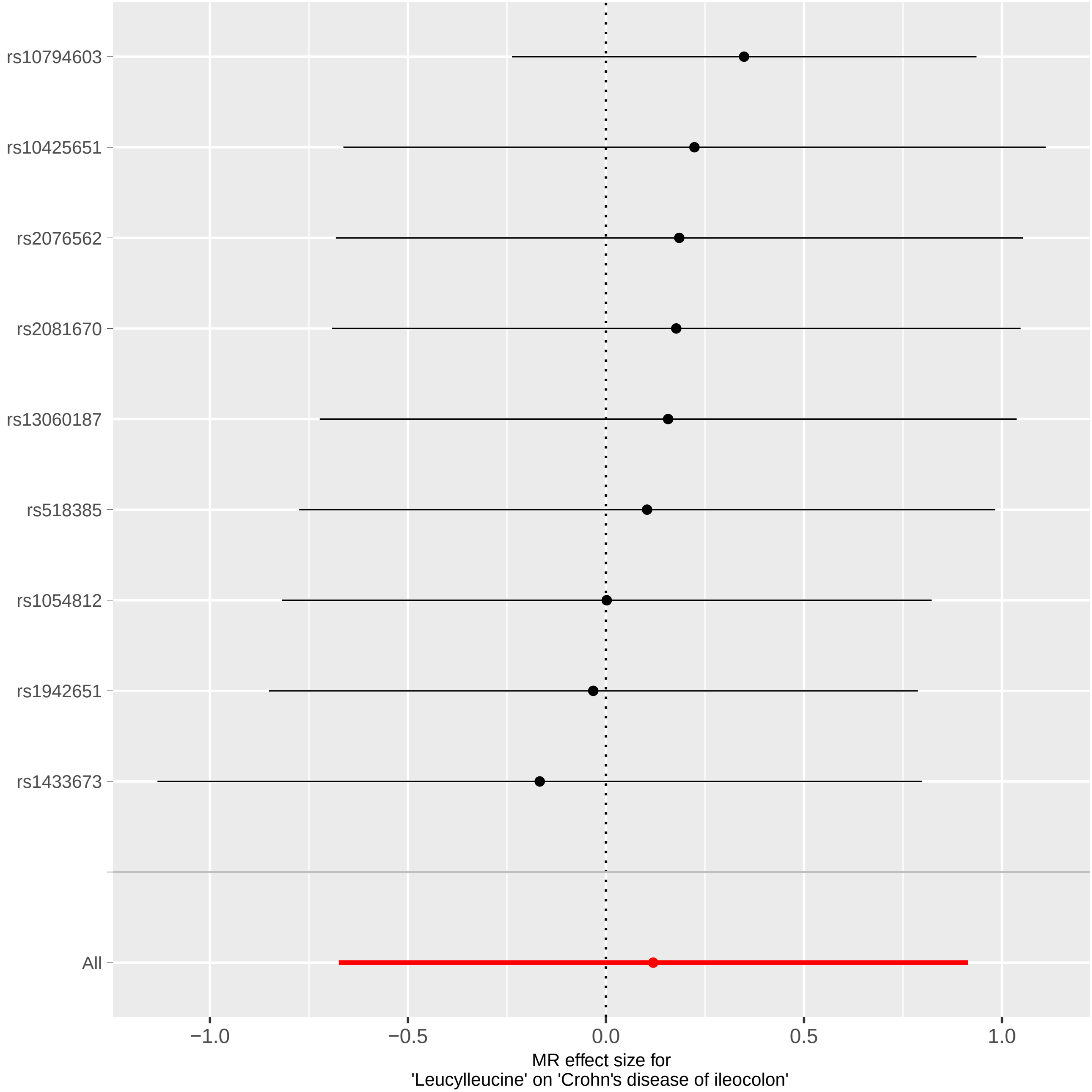

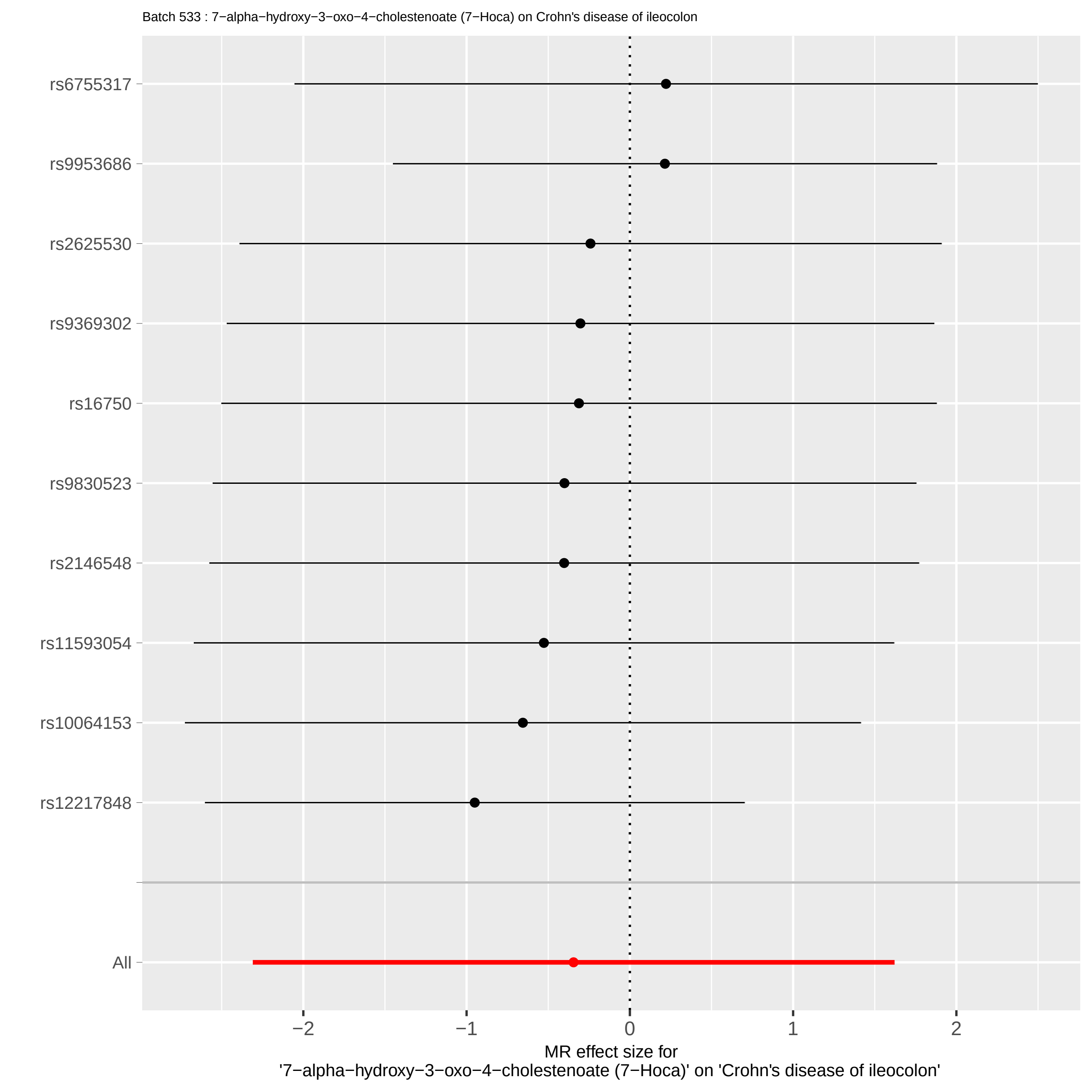

Batch 533 : 7−alpha−hydroxy−3−oxo−4−cholestenoate (7−Hoca) on Crohn's disease of ileocolon
rs6755317
rs9953686
rs2625530
rs9369302
rs16750
rs9830523
rs2146548
rs11593054
rs10064153
rs12217848
All
−2
−1
0
1
2
MR effect size for
'7−alpha−hydroxy−3−oxo−4−cholestenoate (7−Hoca)' on 'Crohn's disease of ileocolon'

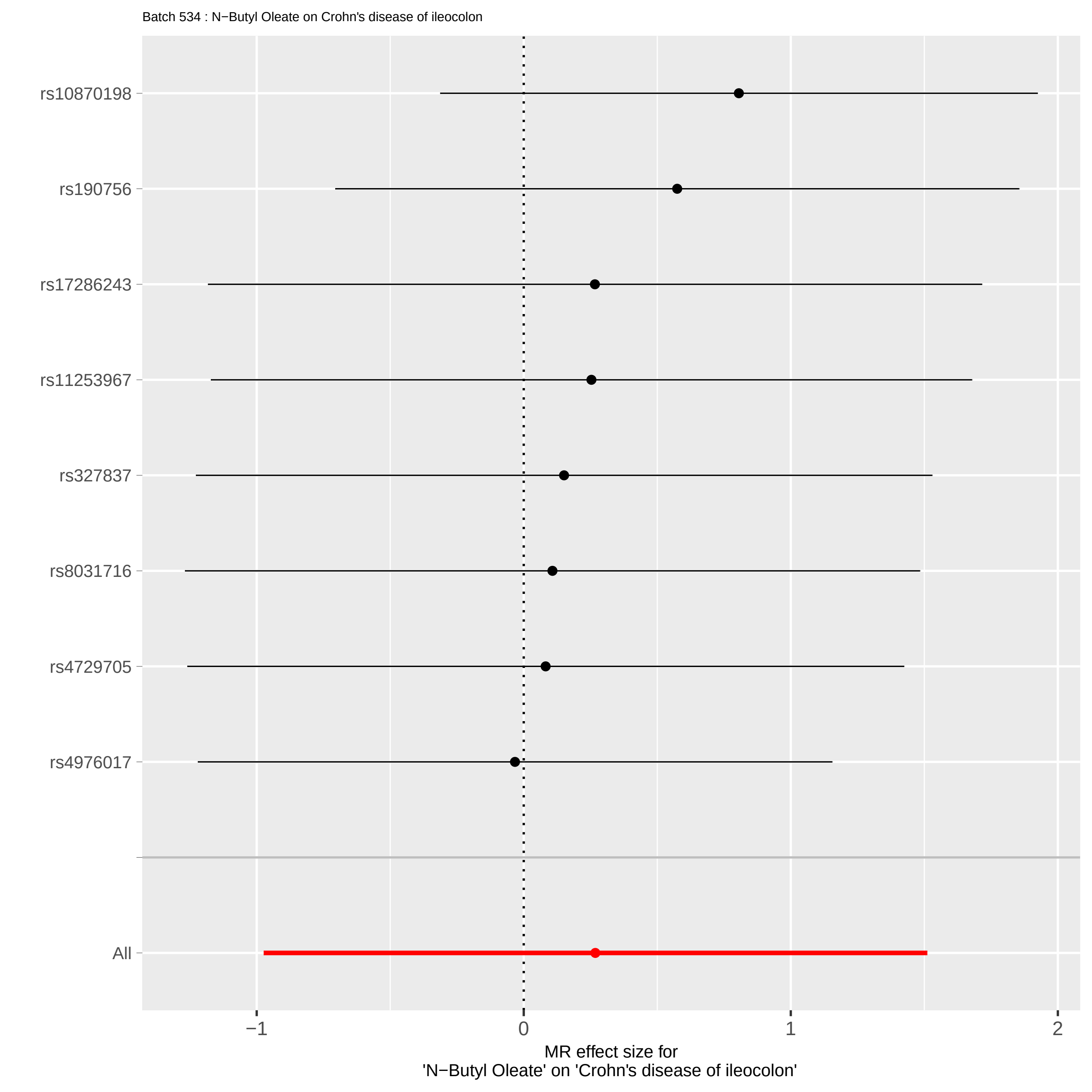
Batch 534 : N−Butyl Oleate on Crohn's disease of ileocolon
rs10870198
rs190756
rs17286243
rs11253967
rs327837
rs8031716
rs4729705
rs4976017
All
−1
0
1
2
MR effect size for
'N−Butyl Oleate' on 'Crohn's disease of ileocolon'

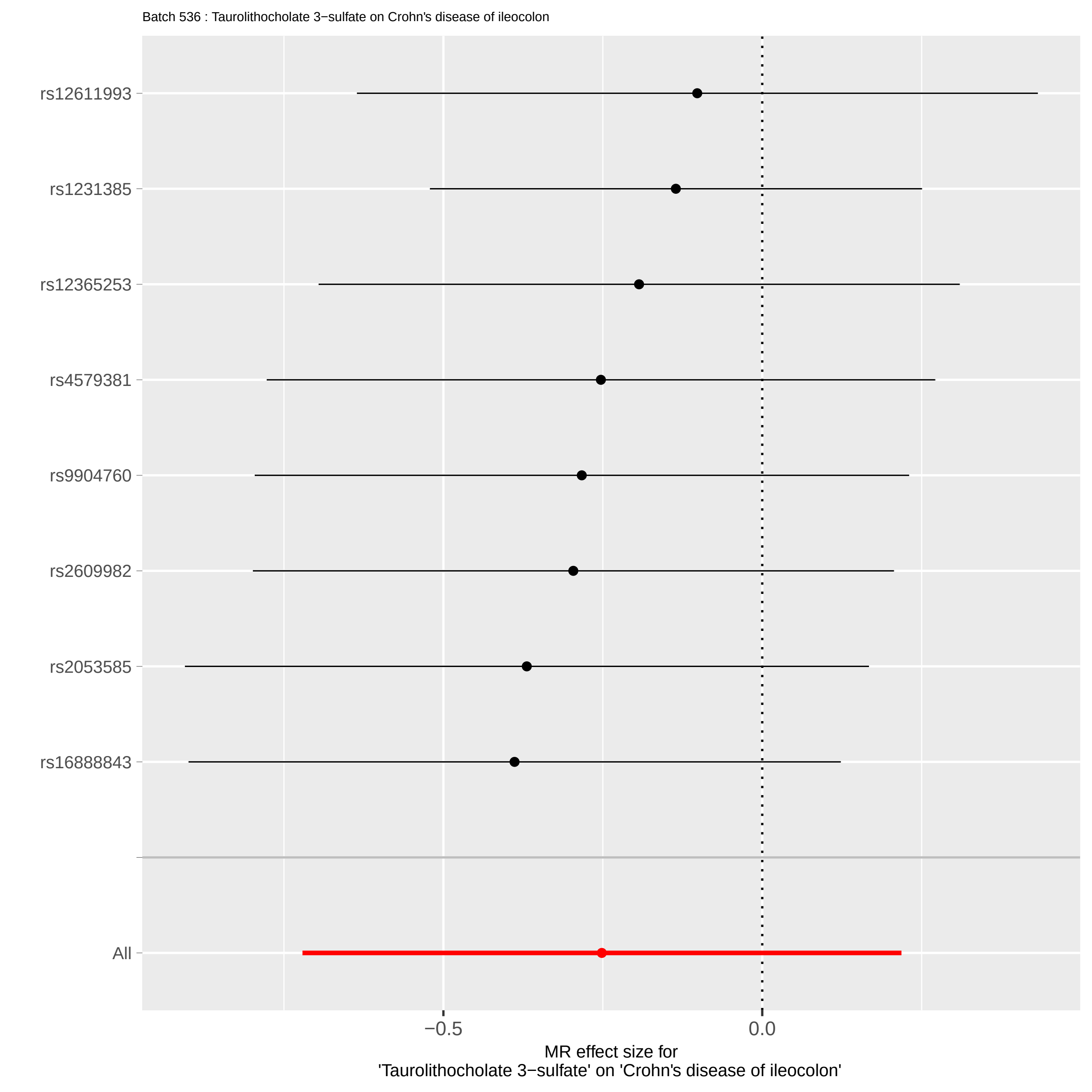
Batch 536 : Taurolithocholate 3−sulfate on Crohn's disease of ileocolon
rs12611993
rs1231385
rs12365253
rs4579381
rs9904760
rs2609982
rs2053585
rs16888843
All
−0.5
0.0
MR effect size for
'Taurolithocholate 3−sulfate' on 'Crohn's disease of ileocolon'

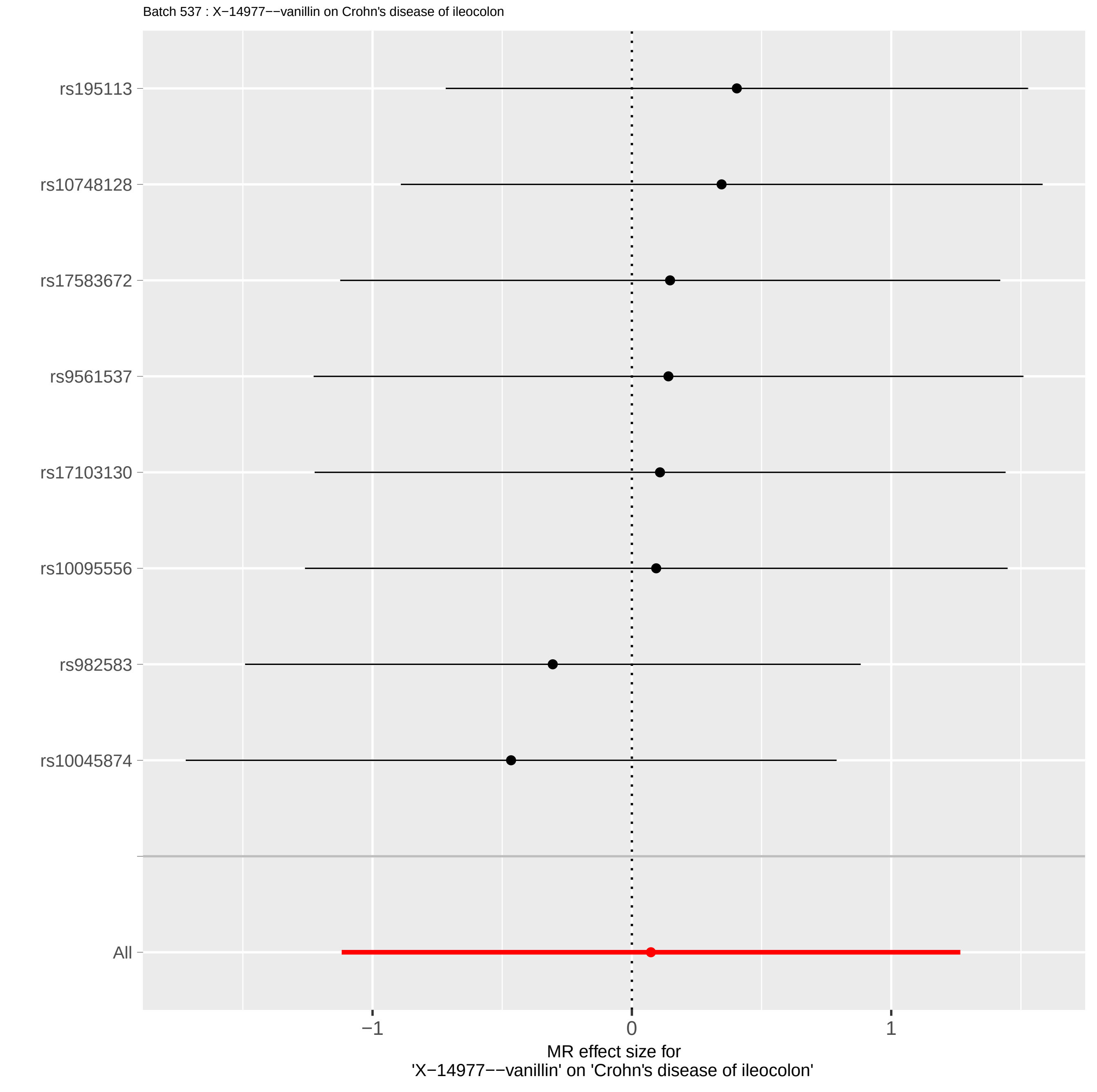

Batch 537 : X−14977−−vanillin on Crohn's disease of ileocolon
rs195113
rs10748128
rs17583672
rs9561537
rs17103130
rs10095556
rs982583
rs10045874
All
−1
0
1
MR effect size for
'X−14977−−vanillin' on 'Crohn's disease of ileocolon'

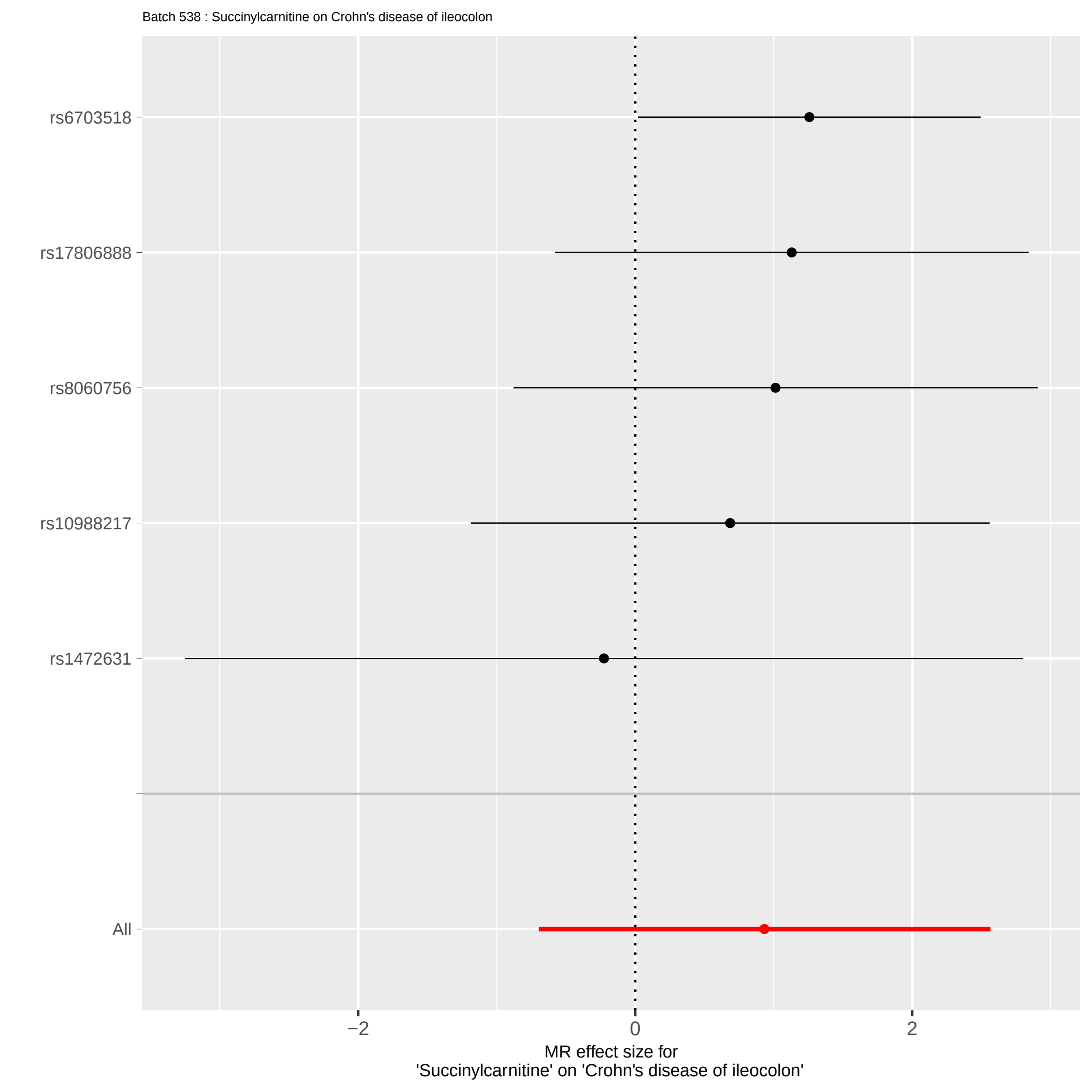
Batch 538 : Succinylcarnitine on Crohn's disease of ileocolon
rs6703518
rs17806888
rs8060756
rs10988217
rs1472631
All
−2
0
2
MR effect size for
'Succinylcarnitine' on 'Crohn's disease of ileocolon'

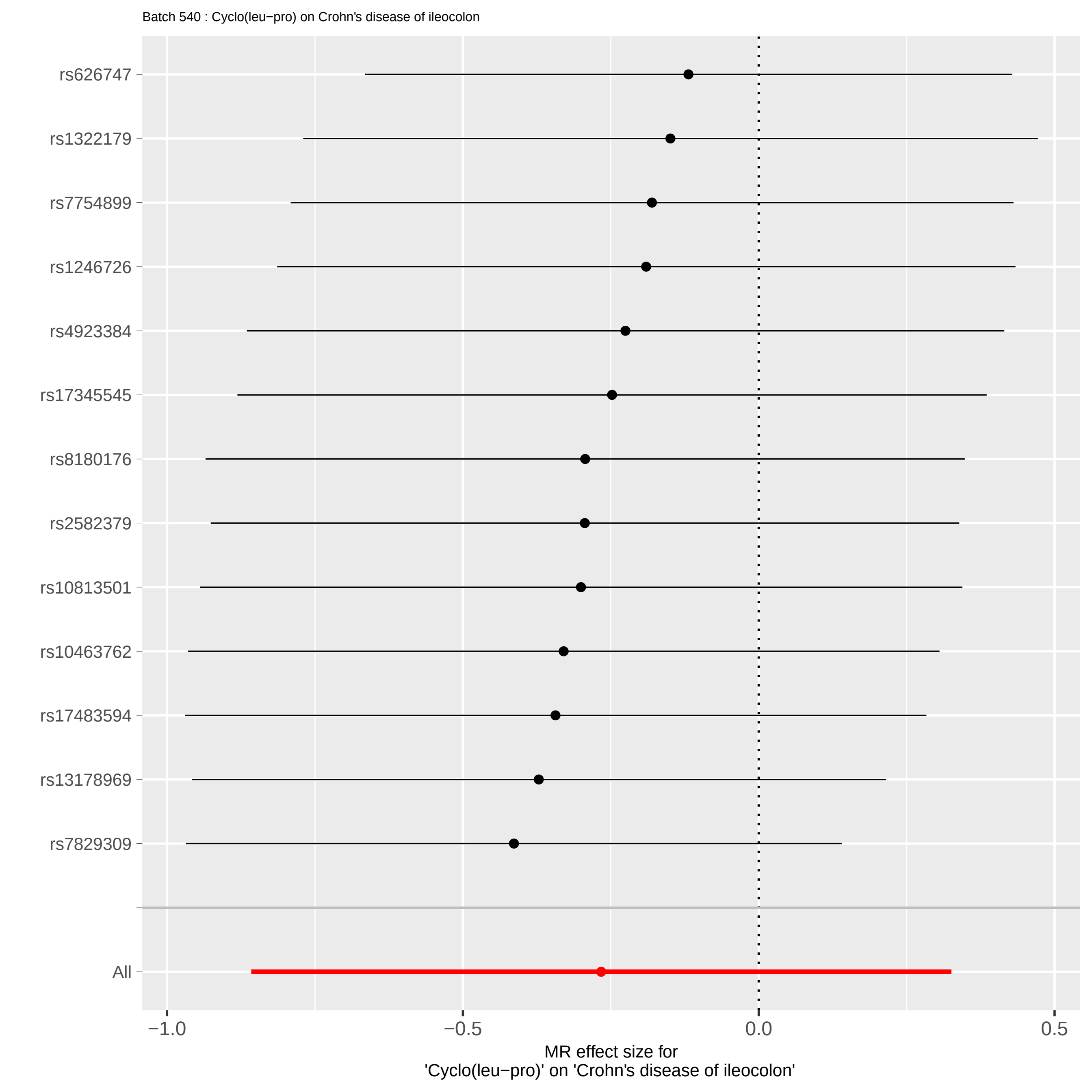

Batch 540 : Cyclo(leu−pro) on Crohn's disease of ileocolon
rs626747
rs1322179
rs7754899
rs1246726
rs4923384
rs17345545
rs8180176
rs2582379
rs10813501
rs10463762
rs17483594
rs13178969
rs7829309
All
−1.0
−0.5
0.0
0.5
MR effect size for
'Cyclo(leu−pro)' on 'Crohn's disease of ileocolon'

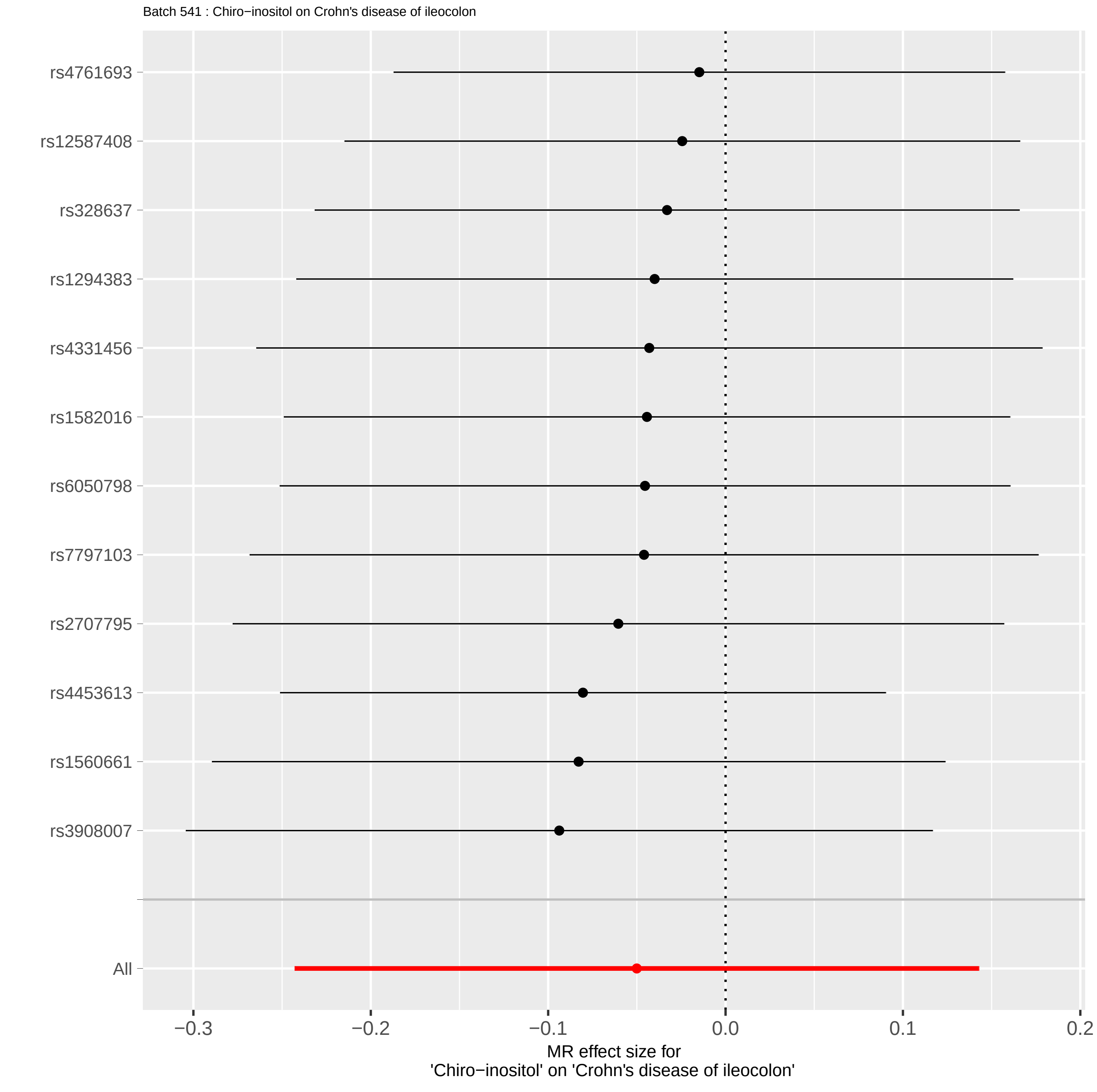
Batch 541 : Chiro−inositol on Crohn's disease of ileocolon
rs4761693
rs12587408
rs328637
rs1294383
rs4331456
rs1582016
rs6050798
rs7797103
rs2707795
rs4453613
rs1560661
rs3908007
All
−0.3
−0.2
−0.1
0.0
0.1
0.2
MR effect size for
'Chiro−inositol' on 'Crohn's disease of ileocolon'

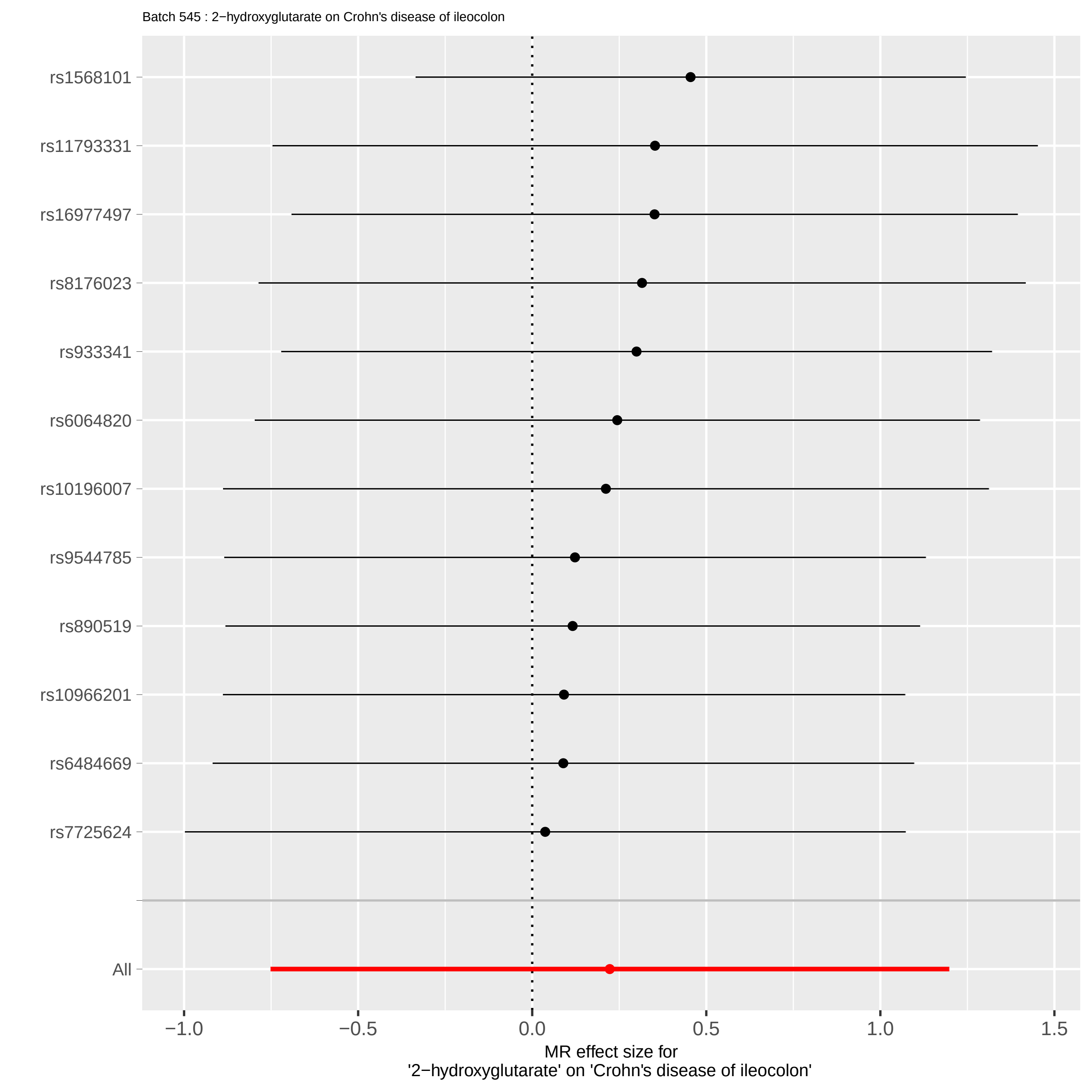

Batch 545 : 2−hydroxyglutarate on Crohn's disease of ileocolon
rs1568101
rs11793331
rs16977497
rs8176023
rs933341
rs6064820
rs10196007
rs9544785
rs890519
rs10966201
rs6484669
rs7725624
All
−1.0
−0.5
0.0
0.5
1.0
1.5
MR effect size for
'2−hydroxyglutarate' on 'Crohn's disease of ileocolon'

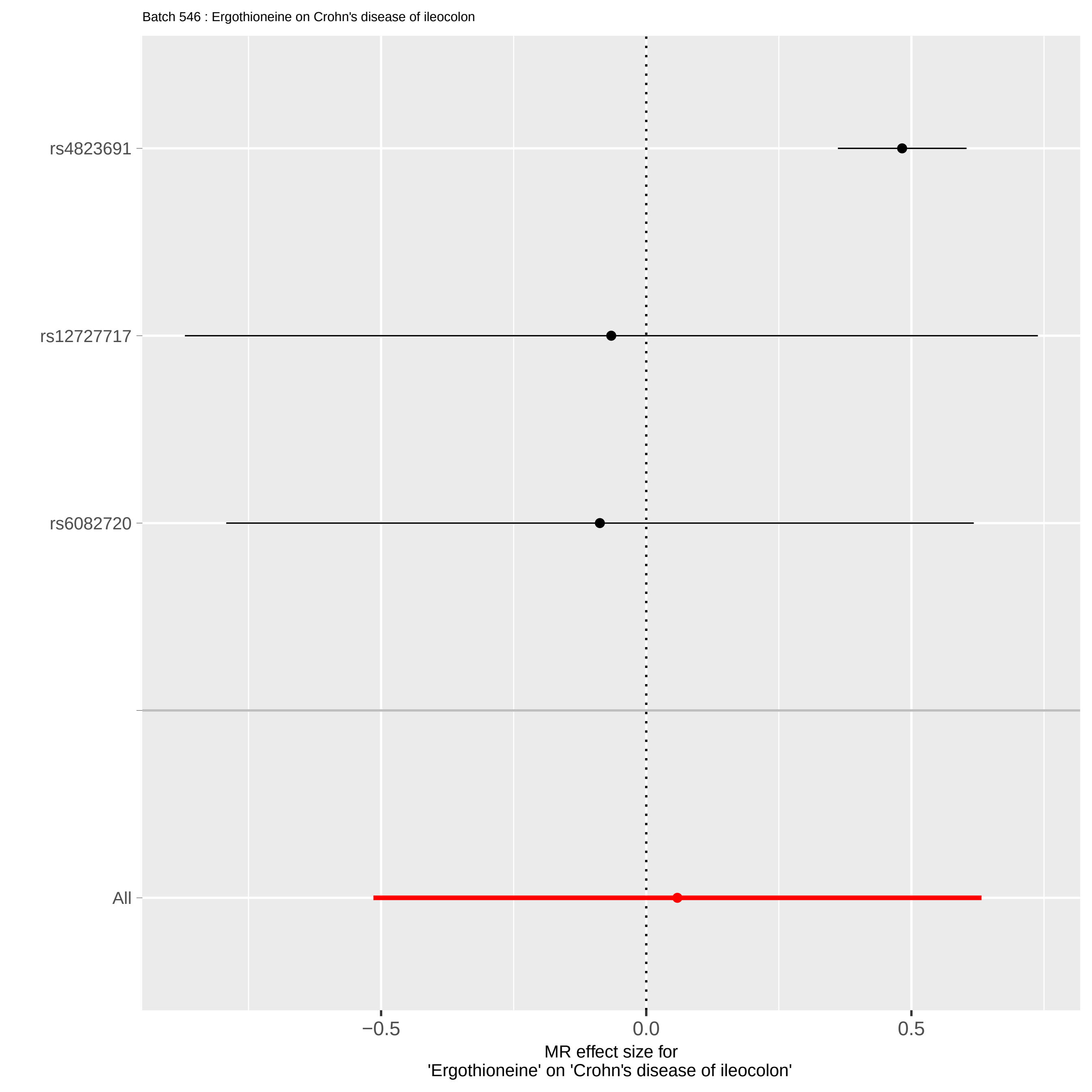
Batch 546 : Ergothioneine on Crohn's disease of ileocolon
rs4823691
rs12727717
rs6082720
All
−0.5
0.0
0.5
MR effect size for
'Ergothioneine' on 'Crohn's disease of ileocolon'

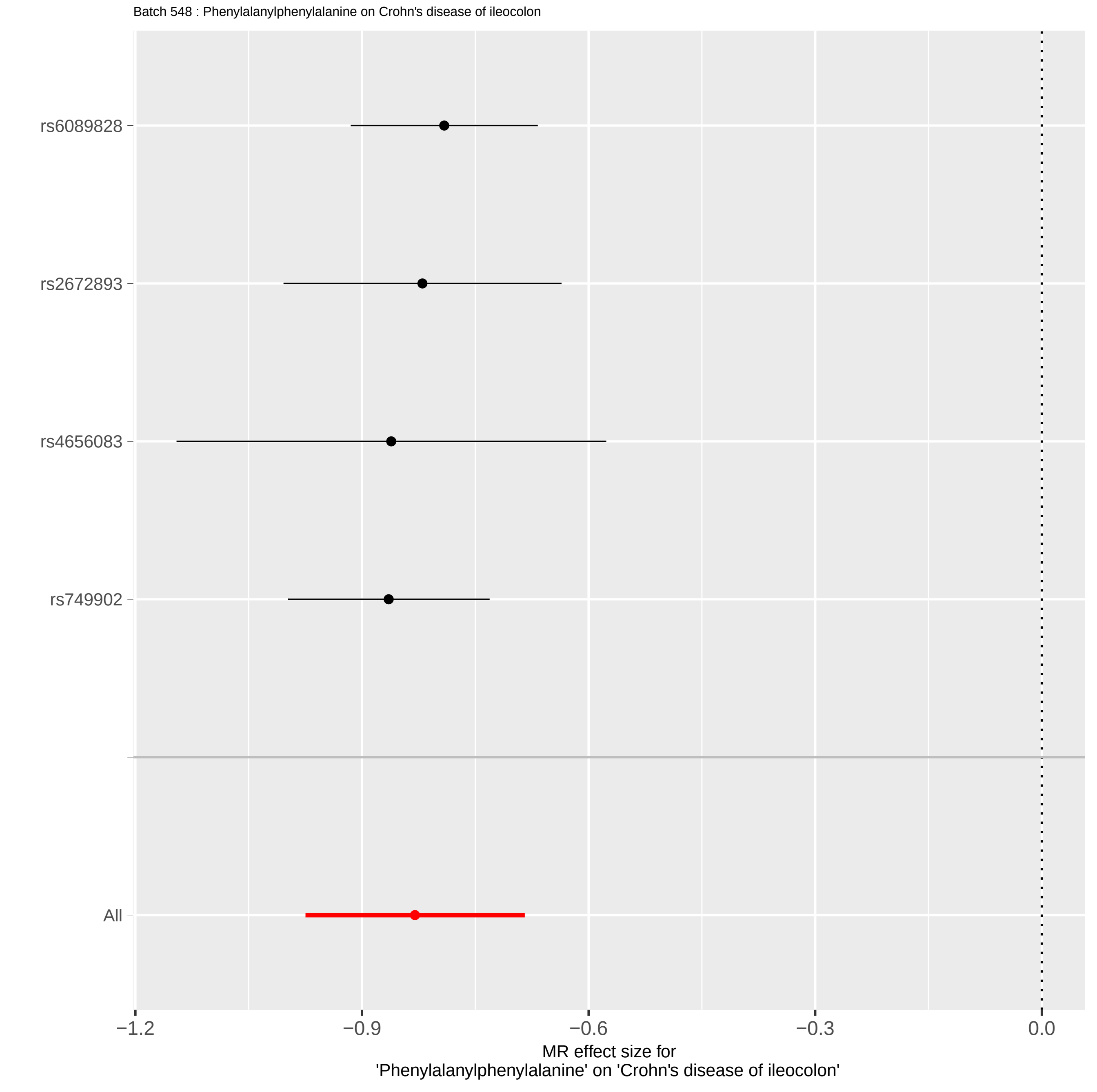
Batch 548 : Phenylalanylphenylalanine on Crohn's disease of ileocolon
rs6089828
rs2672893
rs4656083
rs749902
All
−1.2
−0.9
−0.6
−0.3
0.0
MR effect size for
'Phenylalanylphenylalanine' on 'Crohn's disease of ileocolon'

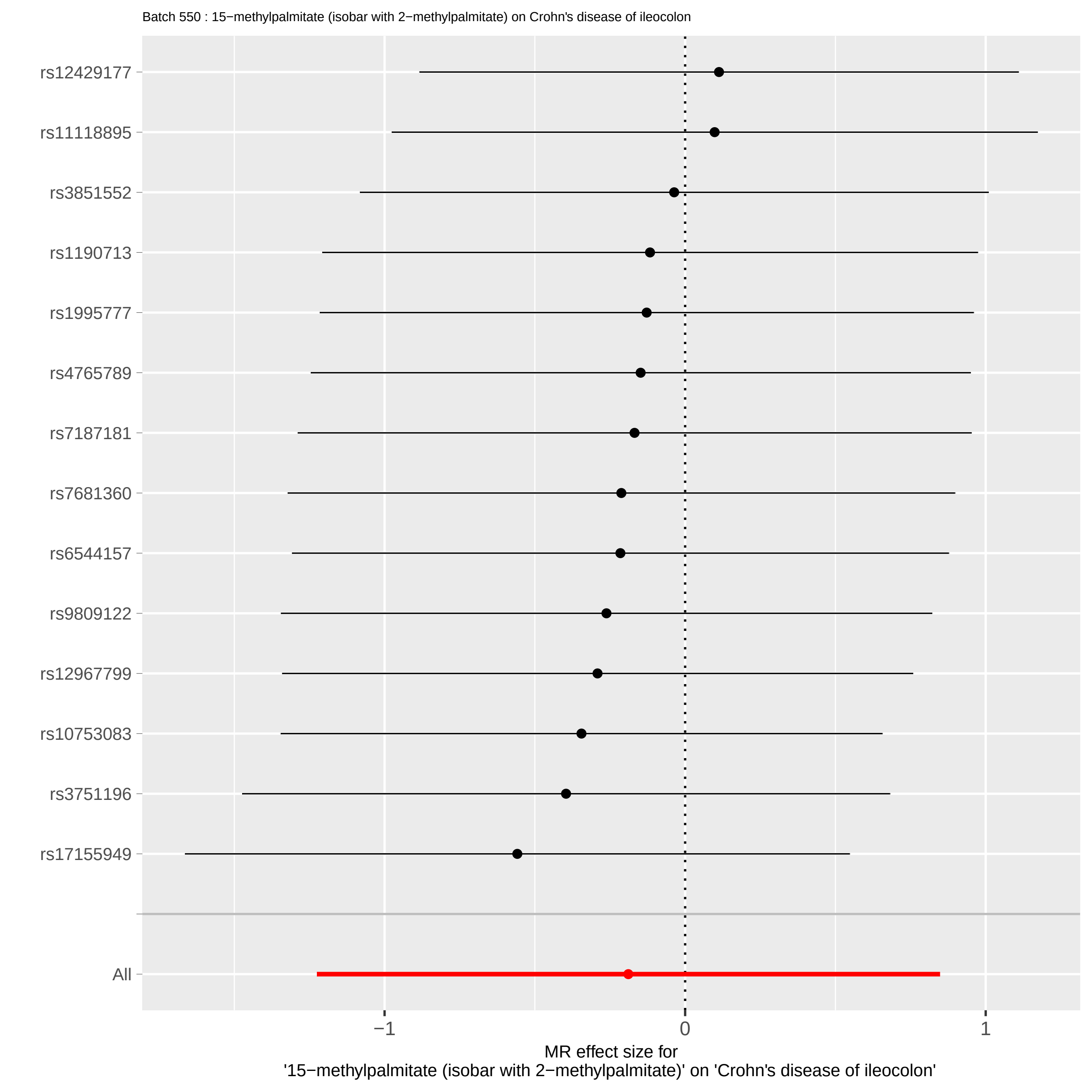

Batch 550 : 15−methylpalmitate (isobar with 2−methylpalmitate) on Crohn's disease of ileocolon
rs12429177
rs11118895
rs3851552
rs1190713
rs1995777
rs4765789
rs7187181
rs7681360
rs6544157
rs9809122
rs12967799
rs10753083
rs3751196
rs17155949
All
−1
0
1
MR effect size for
'15−methylpalmitate (isobar with 2−methylpalmitate)' on 'Crohn's disease of ileocolon'

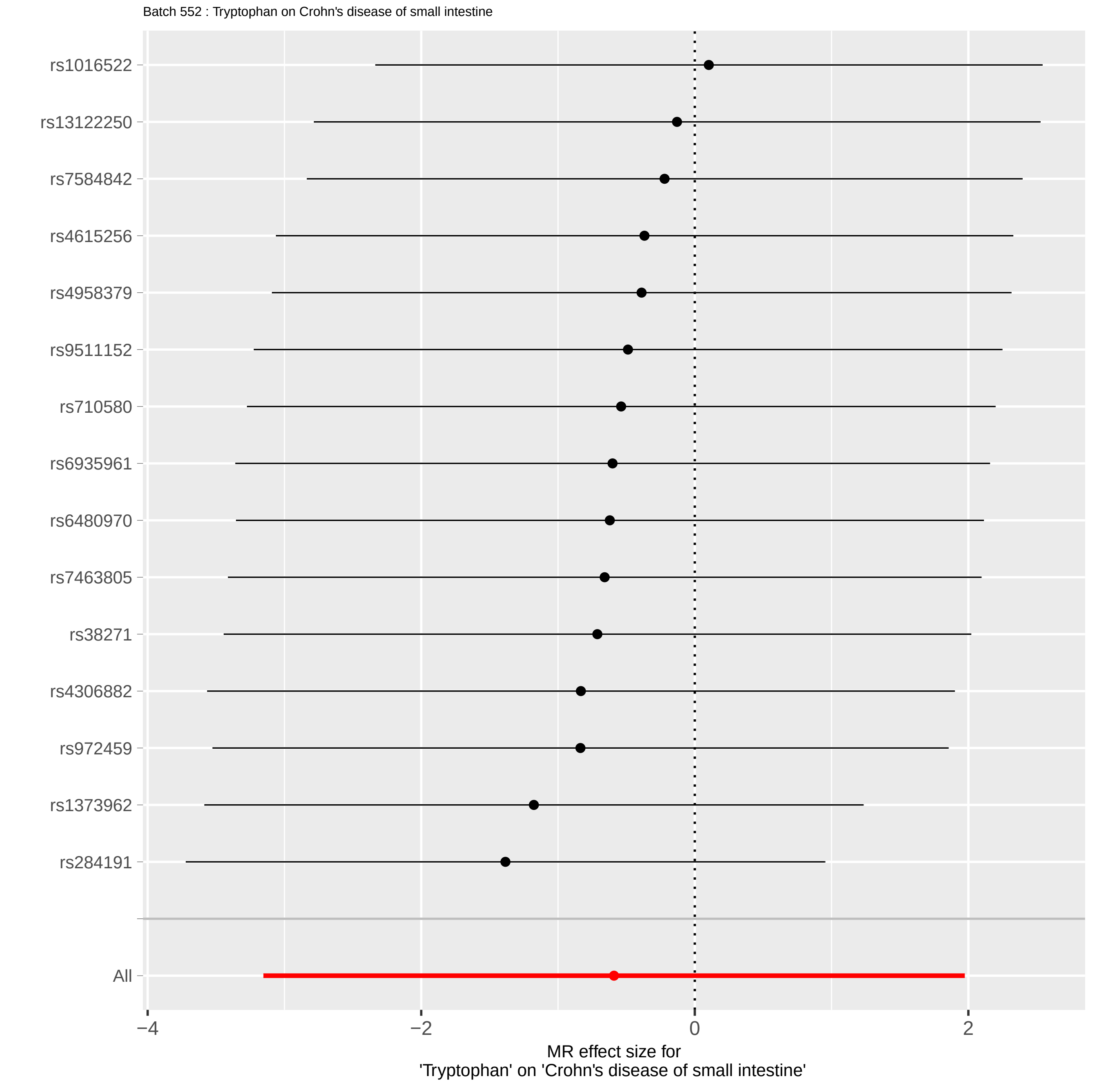

Batch 552 : Tryptophan on Crohn's disease of small intestine
rs1016522
rs13122250
rs7584842
rs4615256
rs4958379
rs9511152
rs710580
rs6935961
rs6480970
rs7463805
rs38271
rs4306882
rs972459
rs1373962
rs284191
All
−4
−2
0
2
MR effect size for
'Tryptophan' on 'Crohn's disease of small intestine'

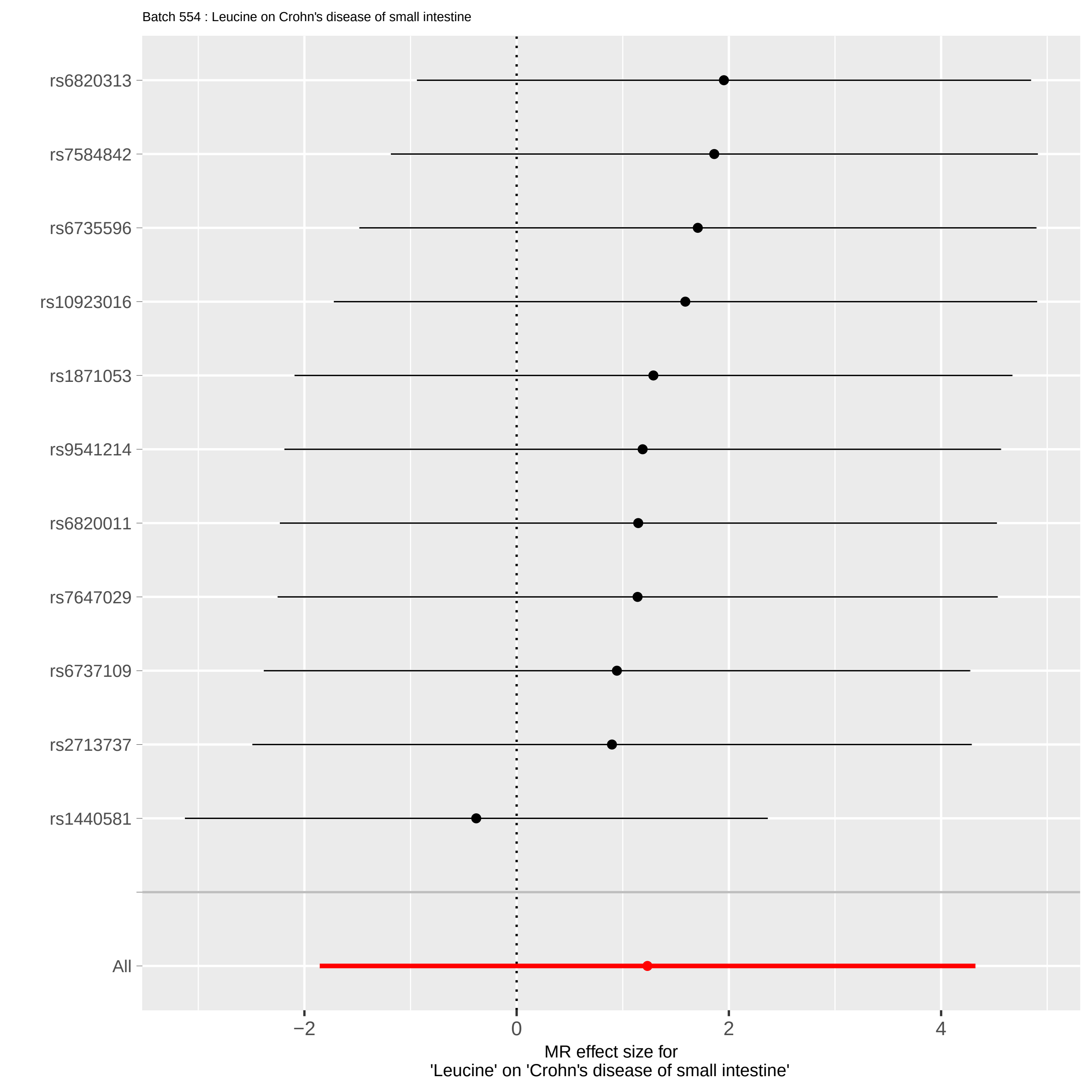
Batch 554 : Leucine on Crohn's disease of small intestine
rs6820313
rs7584842
rs6735596
rs10923016
rs1871053
rs9541214
rs6820011
rs7647029
rs6737109
rs2713737
rs1440581
All
−2
0
2
4
MR effect size for
'Leucine' on 'Crohn's disease of small intestine'

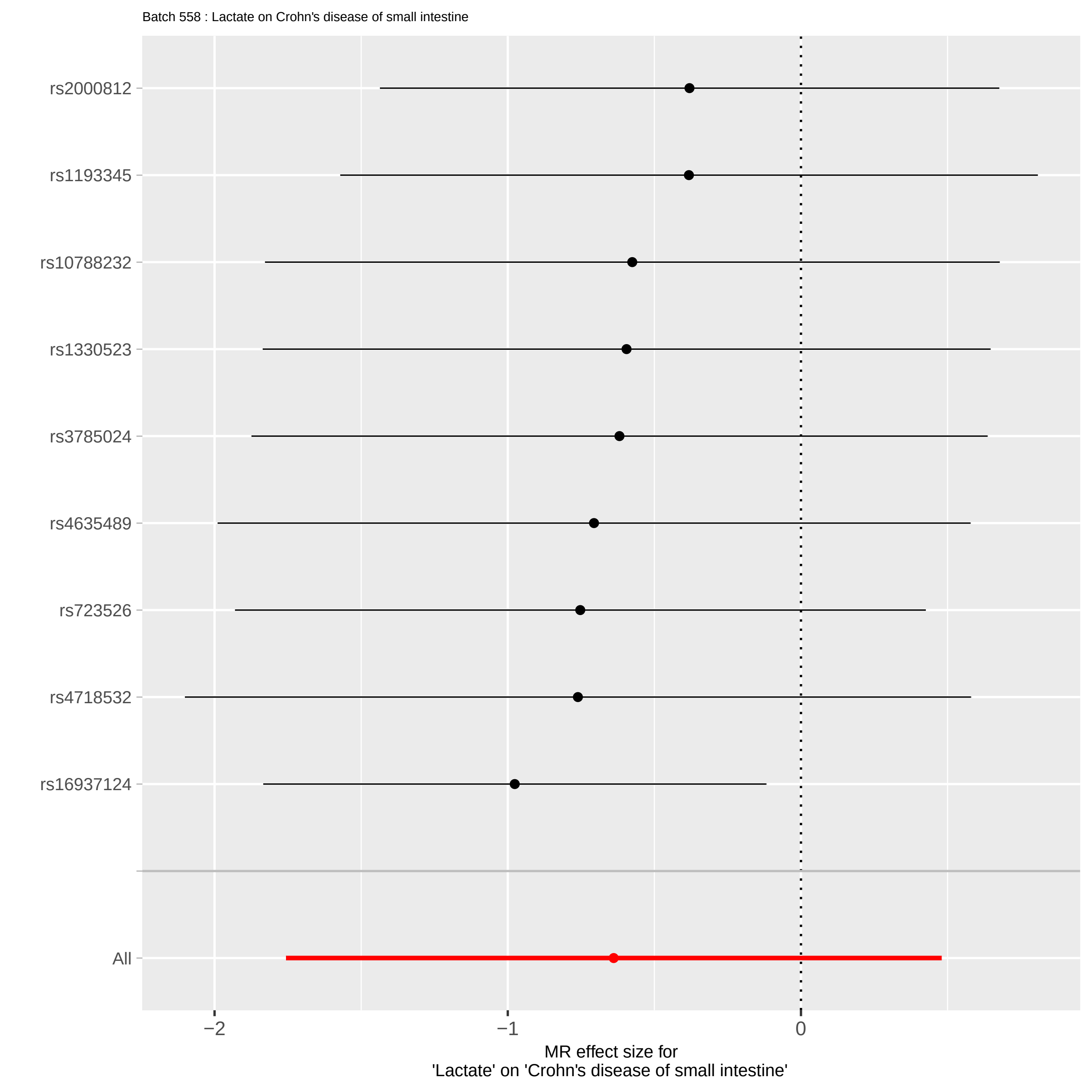
Batch 558 : Lactate on Crohn's disease of small intestine
rs2000812
rs1193345
rs10788232
rs1330523
rs3785024
rs4635489
rs723526
rs4718532
rs16937124
All
−2
−1
0
MR effect size for
'Lactate' on 'Crohn's disease of small intestine'

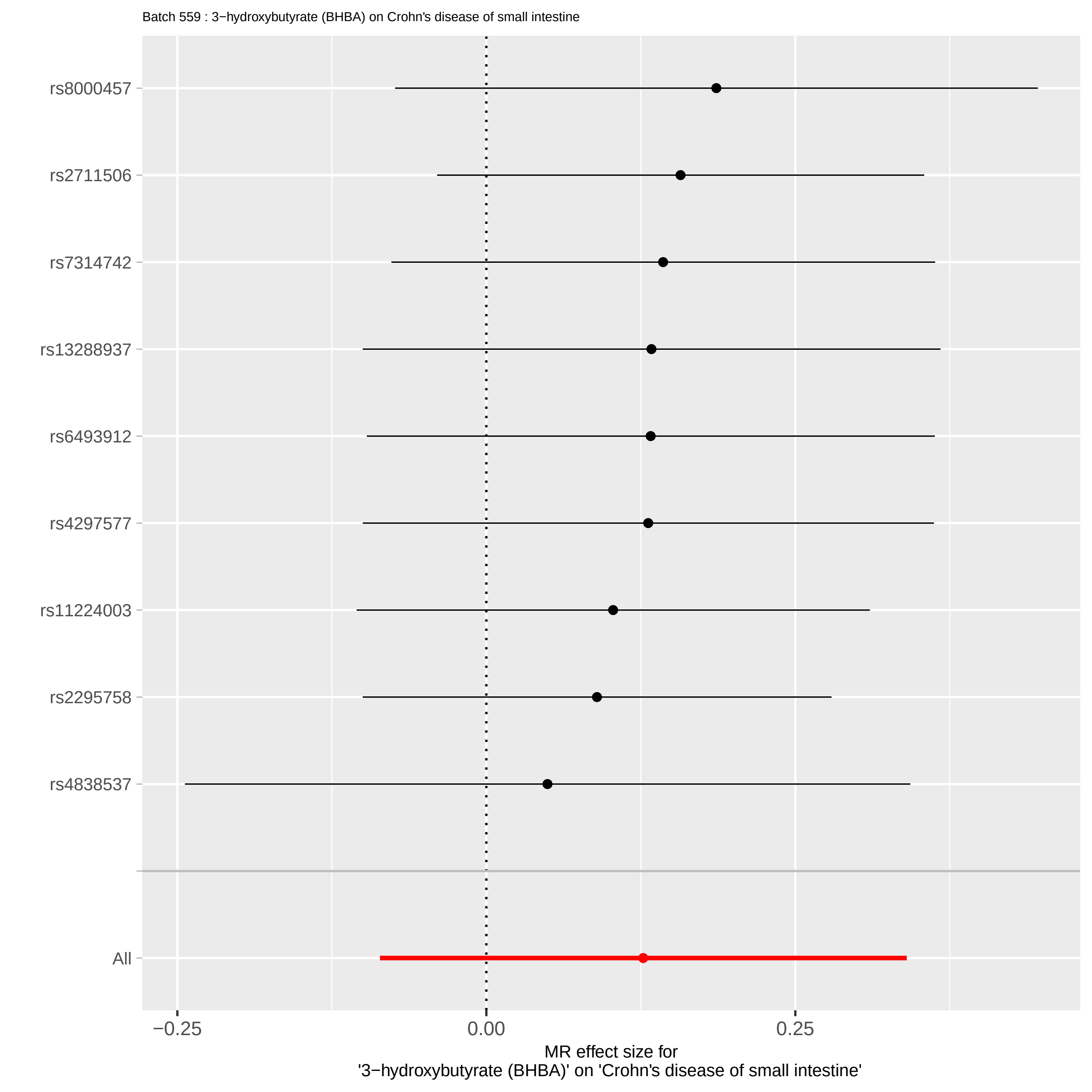
Batch 559 : 3−hydroxybutyrate (BHBA) on Crohn's disease of small intestine
rs8000457
rs2711506
rs7314742
rs13288937
rs6493912
rs4297577
rs11224003
rs2295758
rs4838537
All
−0.25
0.00
0.25
MR effect size for
'3−hydroxybutyrate (BHBA)' on 'Crohn's disease of small intestine'

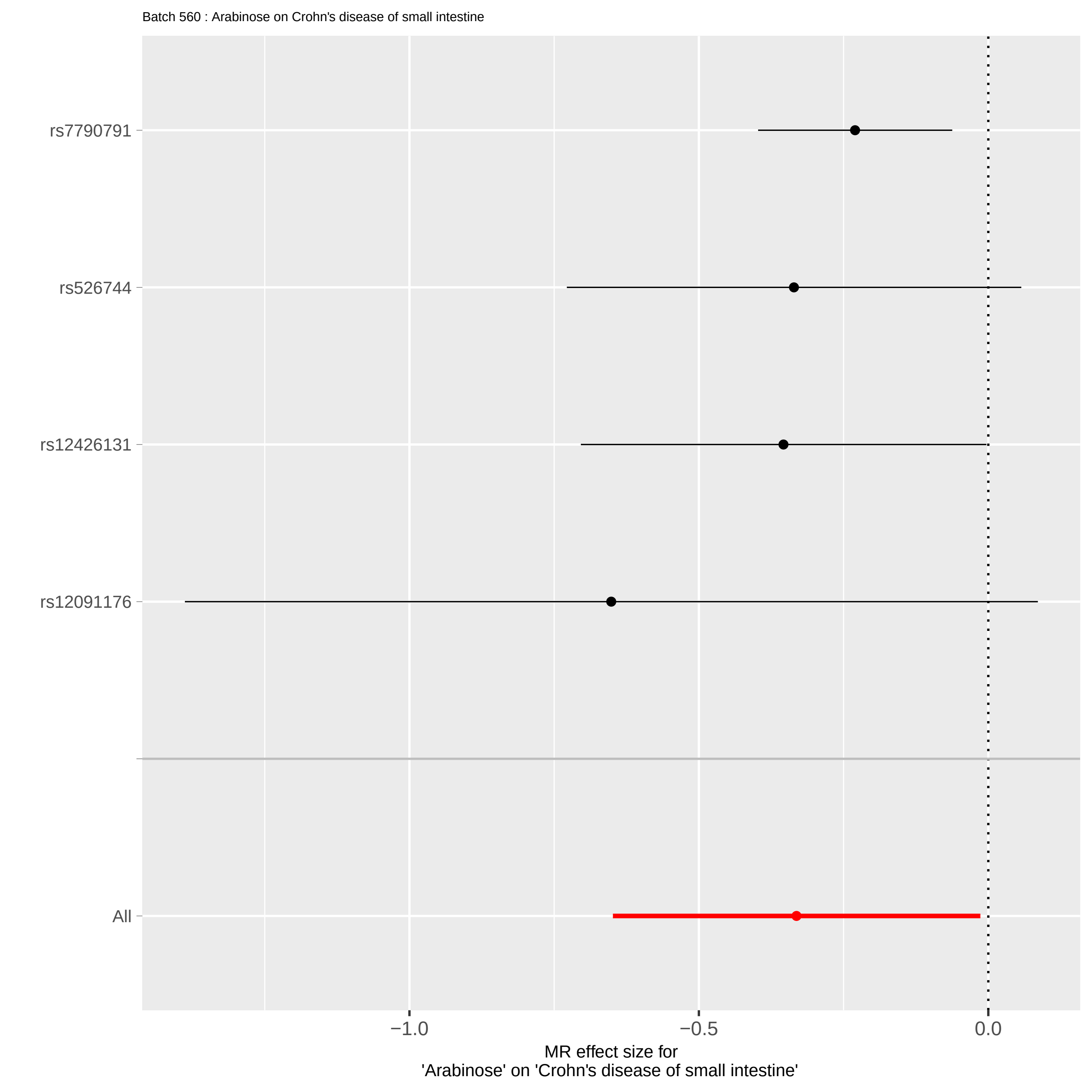
Batch 560 : Arabinose on Crohn's disease of small intestine
rs7790791
rs526744
rs12426131
rs12091176
All
−1.0
−0.5
0.0
MR effect size for
'Arabinose' on 'Crohn's disease of small intestine'

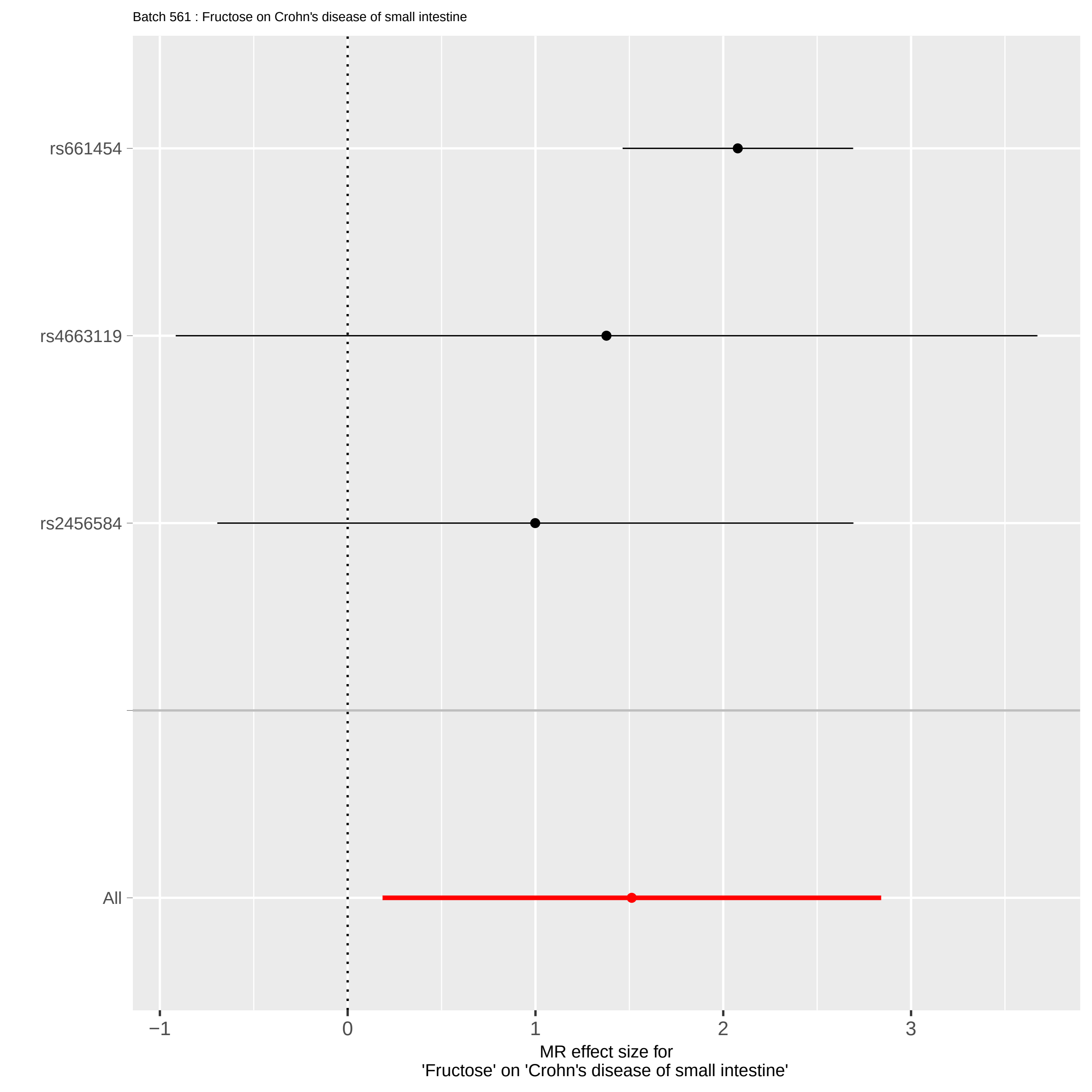
Batch 561 : Fructose on Crohn's disease of small intestine
rs661454
rs4663119
rs2456584
All
−1
0
1
2
3
MR effect size for
'Fructose' on 'Crohn's disease of small intestine'

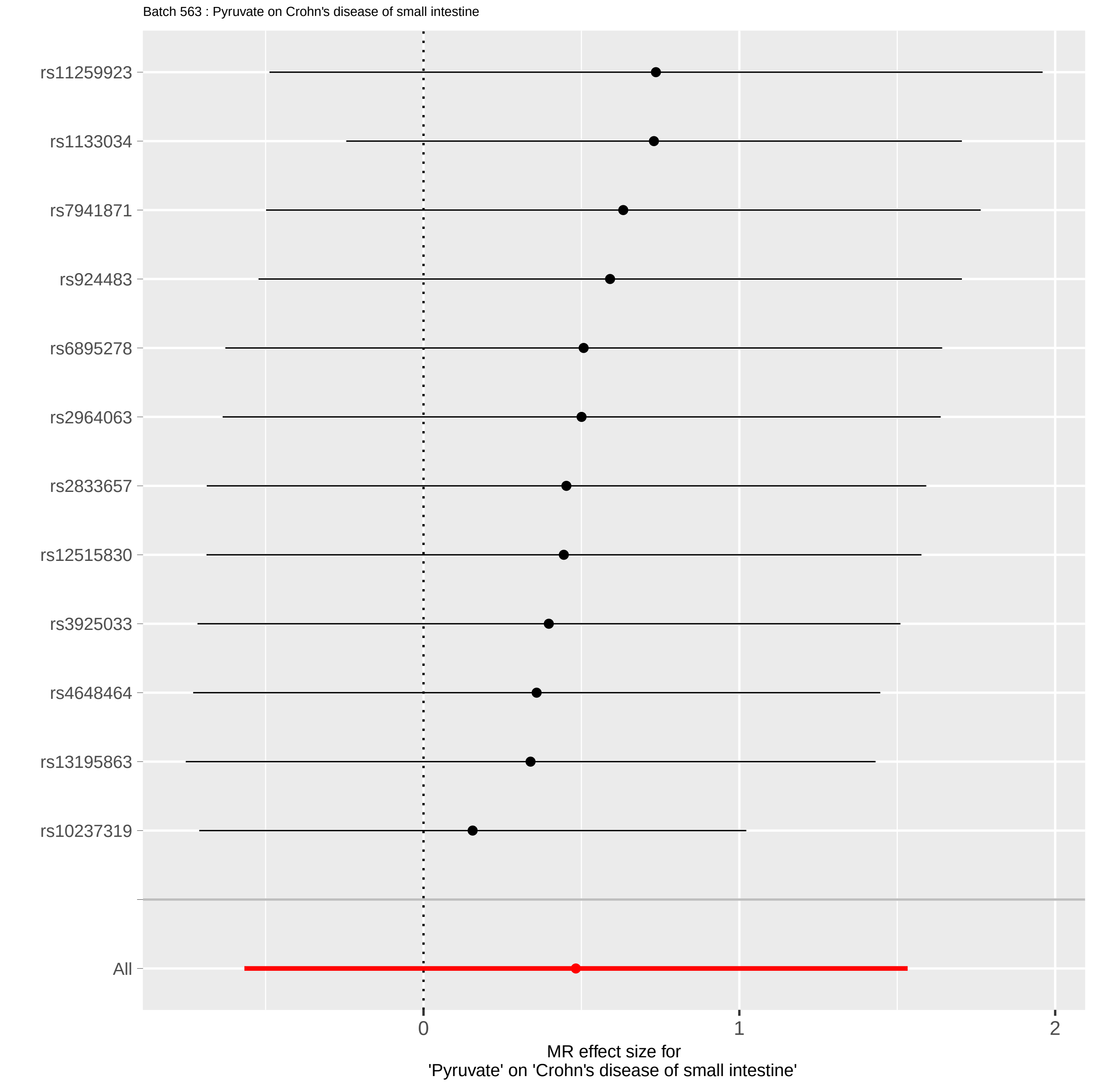
Batch 563 : Pyruvate on Crohn's disease of small intestine
rs11259923
rs1133034
rs7941871
rs924483
rs6895278
rs2964063
rs2833657
rs12515830
rs3925033
rs4648464
rs13195863
rs10237319
All
0
1
2
MR effect size for
'Pyruvate' on 'Crohn's disease of small intestine'

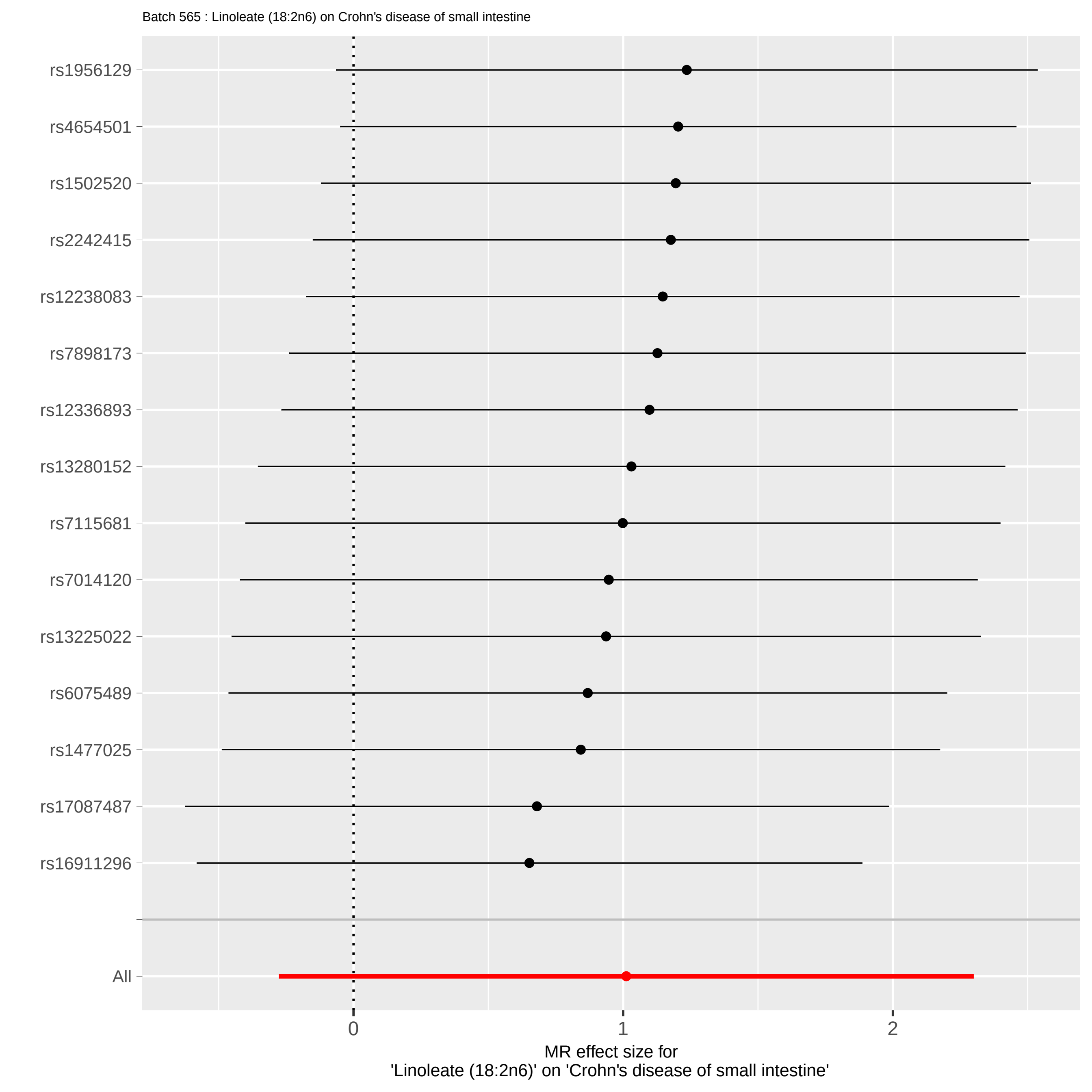

Batch 565 : Linoleate (18:2n6) on Crohn's disease of small intestine
rs1956129
rs4654501
rs1502520
rs2242415
rs12238083
rs7898173
rs12336893
rs13280152
rs7115681
rs7014120
rs13225022
rs6075489
rs1477025
rs17087487
rs16911296
All
0
1
2
MR effect size for
'Linoleate (18:2n6)' on 'Crohn's disease of small intestine'

Batch 566 : Allantoin on Crohn's disease of small intestine

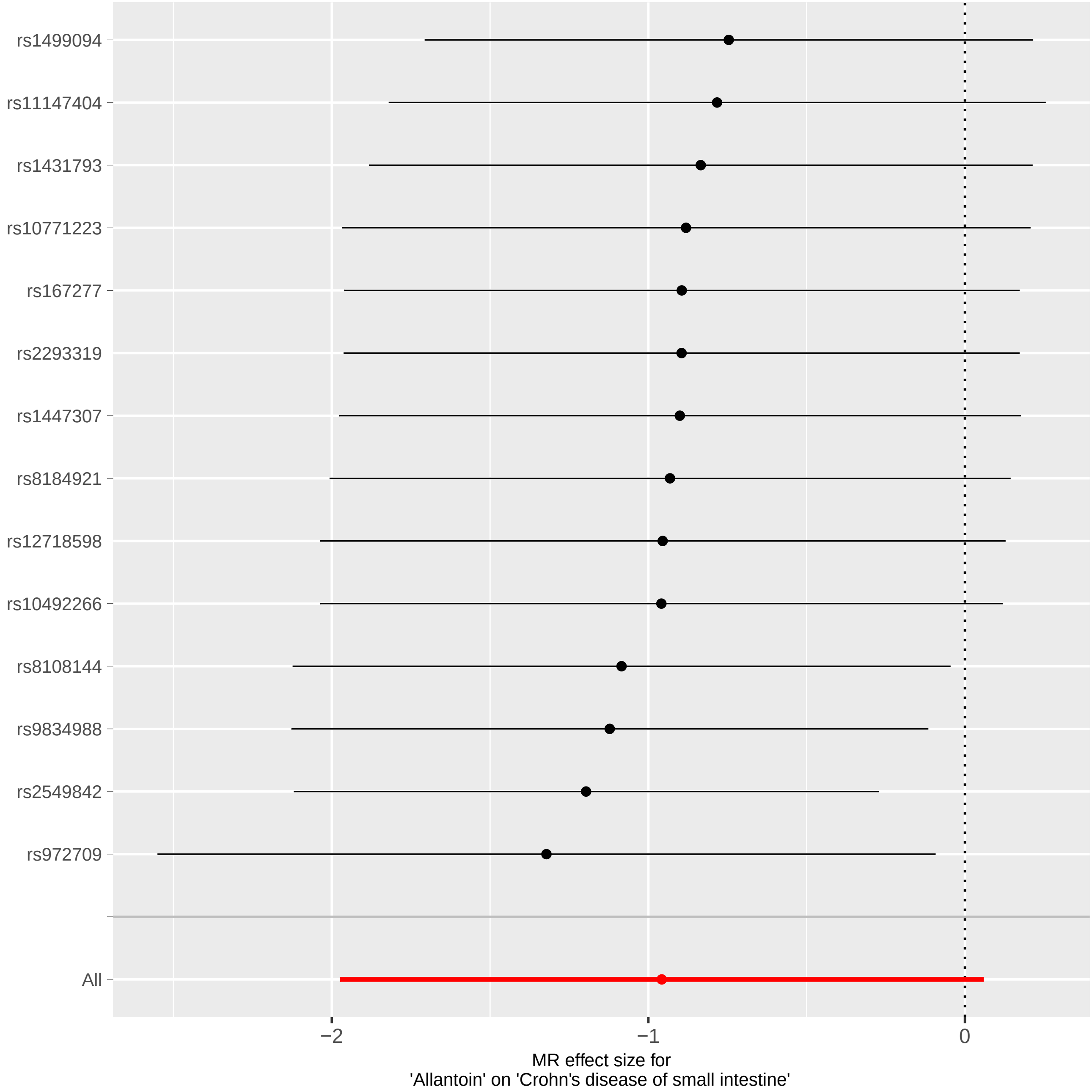

Batch 571 : Isoleucine on Crohn's disease of small intestine

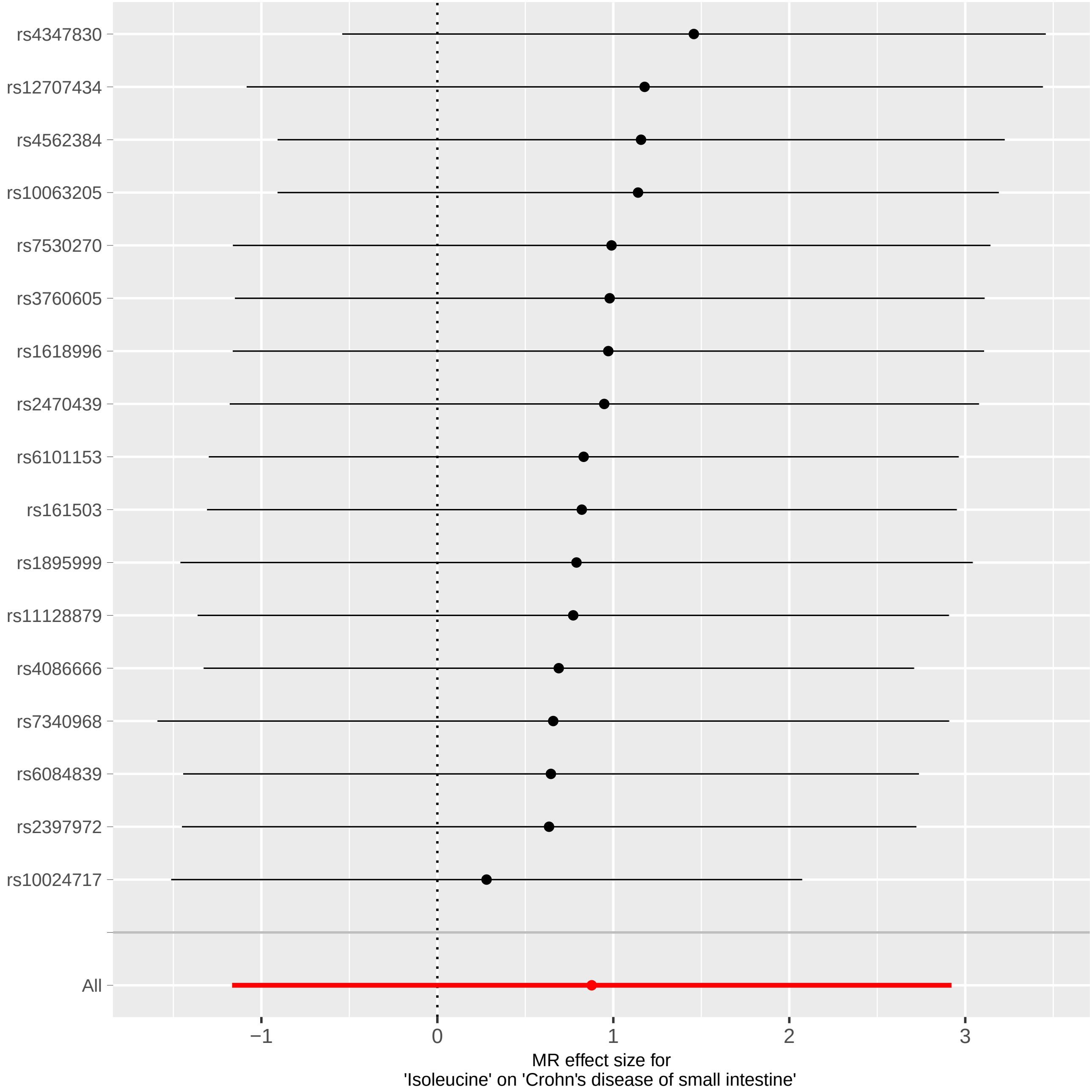

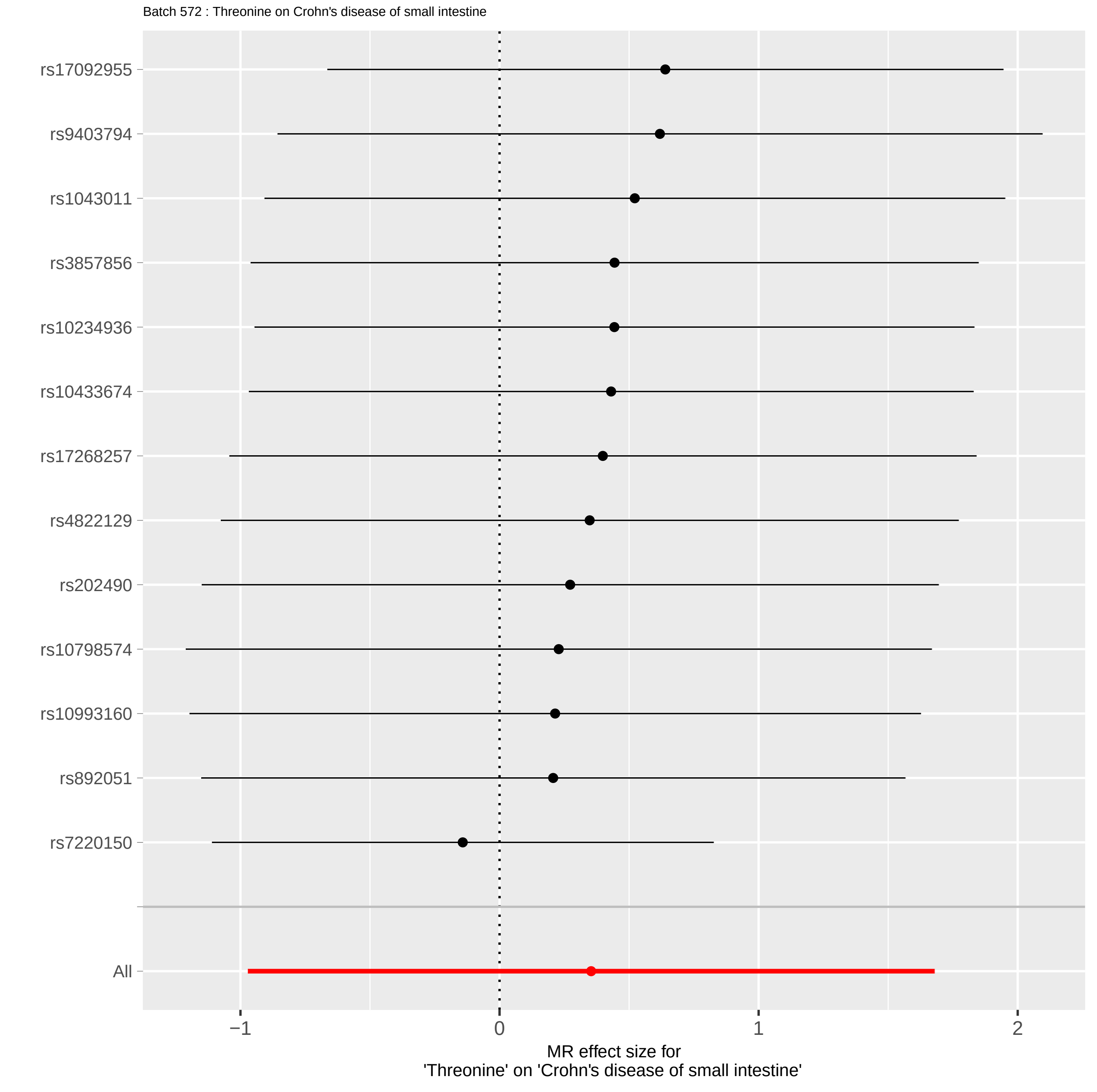
Batch 572 : Threonine on Crohn's disease of small intestine
rs17092955
rs9403794
rs1043011
rs3857856
rs10234936
rs10433674
rs17268257
rs4822129
rs202490
rs10798574
rs10993160
rs892051
rs7220150
All
−1
0
1
2
MR effect size for
'Threonine' on 'Crohn's disease of small intestine'

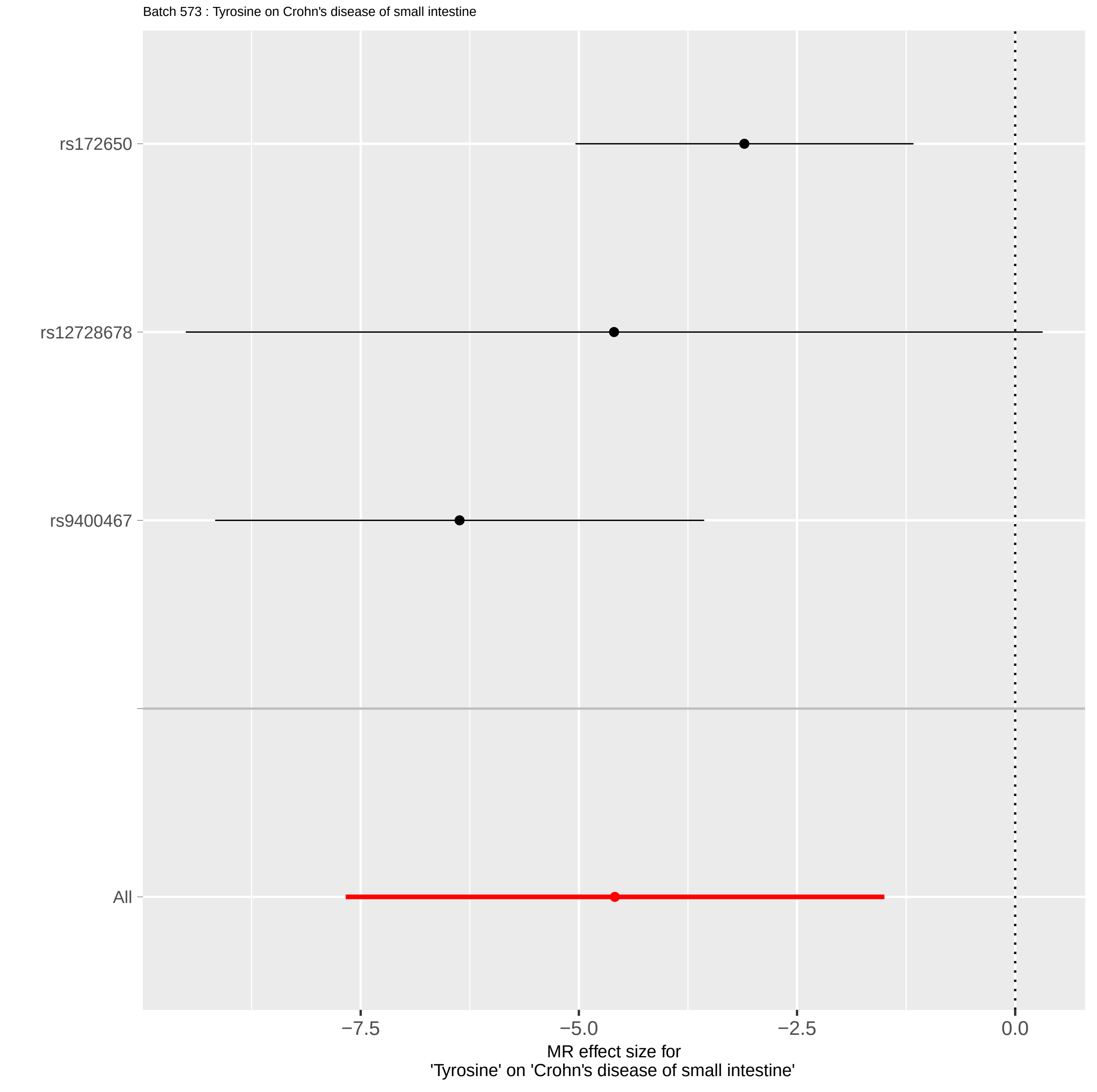

Batch 573 : Tyrosine on Crohn's disease of small intestine
rs172650
rs12728678
rs9400467
All
−7.5
−5.0
−2.5
0.0
MR effect size for
'Tyrosine' on 'Crohn's disease of small intestine'

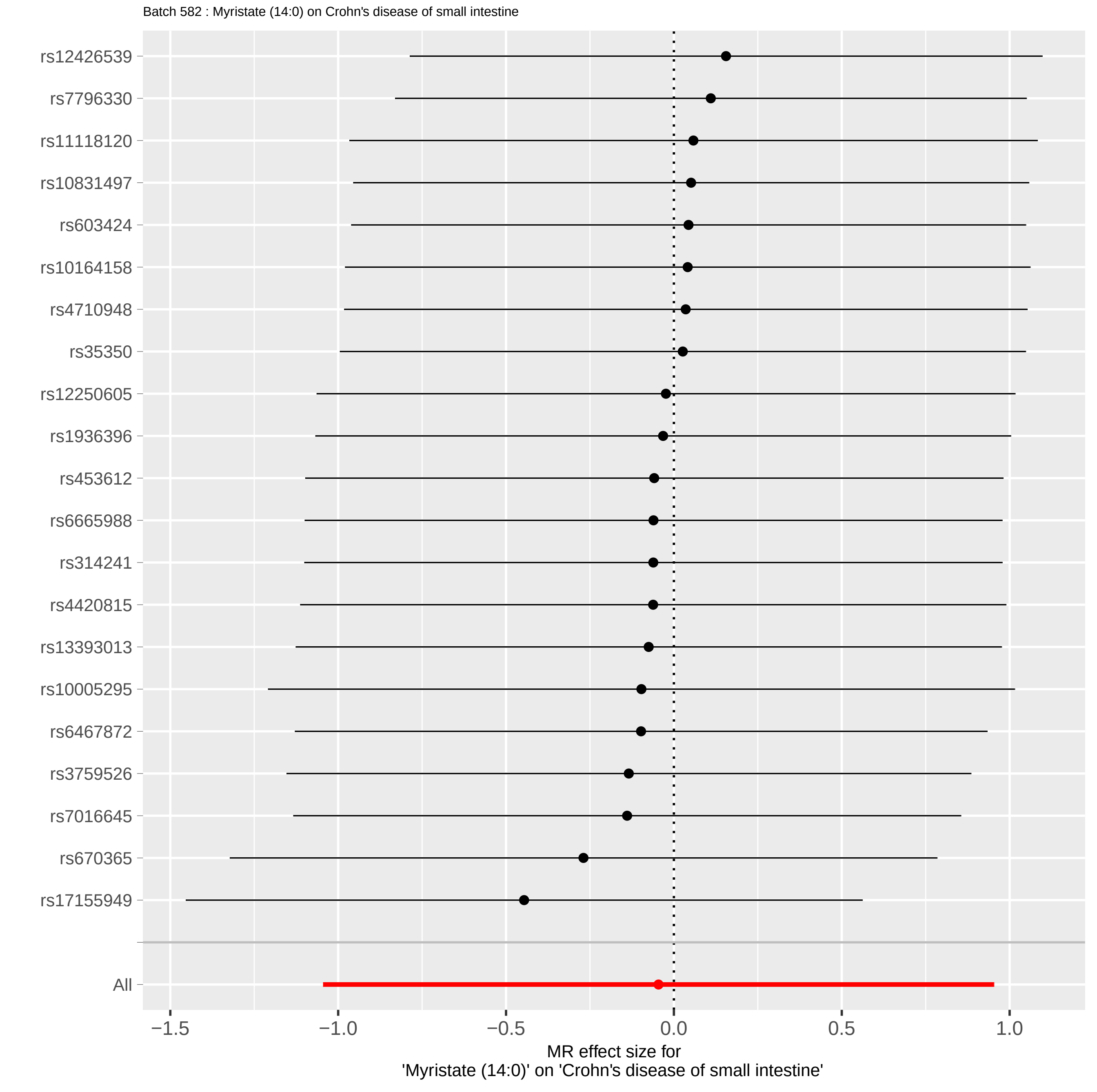
Batch 582 : Myristate (14:0) on Crohn's disease of small intestine
rs12426539
rs7796330
rs11118120
rs10831497
rs603424
rs10164158
rs4710948
rs35350
rs12250605
rs1936396
rs453612
rs6665988
rs314241
rs4420815
rs13393013
rs10005295
rs6467872
rs3759526
rs7016645
rs670365
rs17155949
All
−1.5
−1.0
−0.5
0.0
0.5
1.0
MR effect size for
'Myristate (14:0)' on 'Crohn's disease of small intestine'

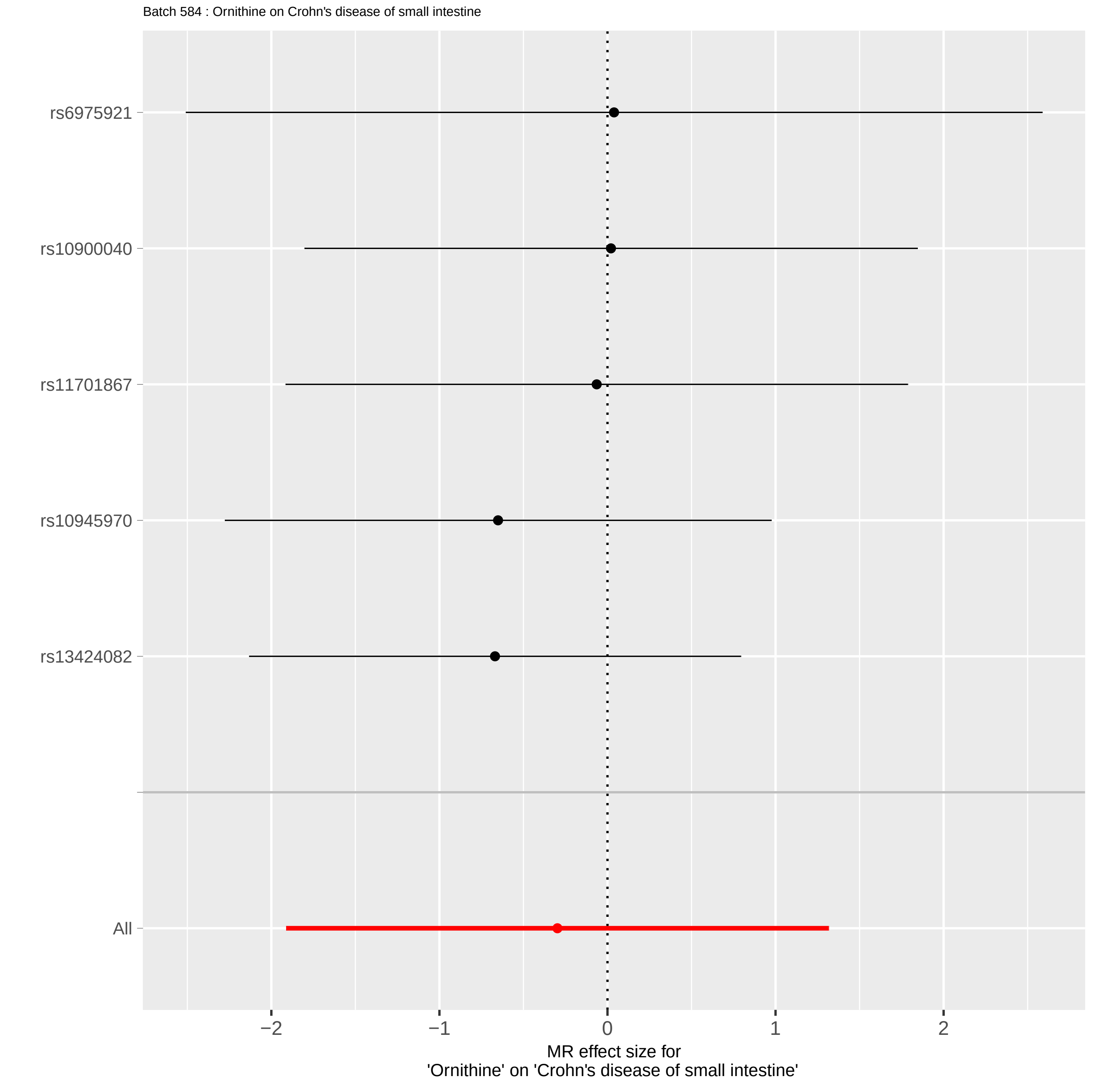

Batch 584 : Ornithine on Crohn's disease of small intestine
rs6975921
rs10900040
rs11701867
rs10945970
rs13424082
All
−2
−1
0
1
2
MR effect size for
'Ornithine' on 'Crohn's disease of small intestine'

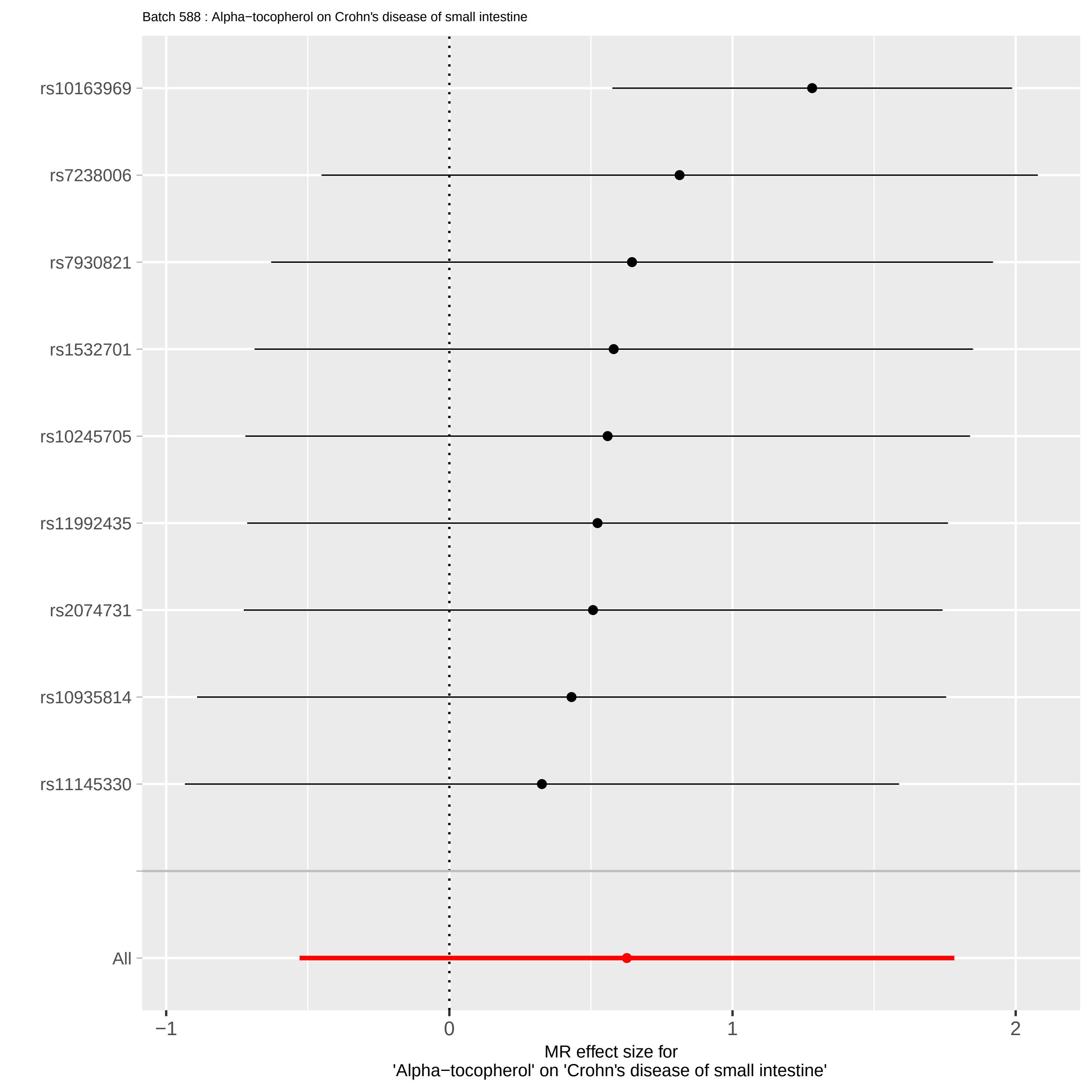

Batch 588 : Alpha−tocopherol on Crohn's disease of small intestine
rs10163969
rs7238006
rs7930821
rs1532701
rs10245705
rs11992435
rs2074731
rs10935814
rs11145330
All
−1
0
1
2
MR effect size for
'Alpha−tocopherol' on 'Crohn's disease of small intestine'

Batch 589 : Citrate on Crohn's disease of small intestine

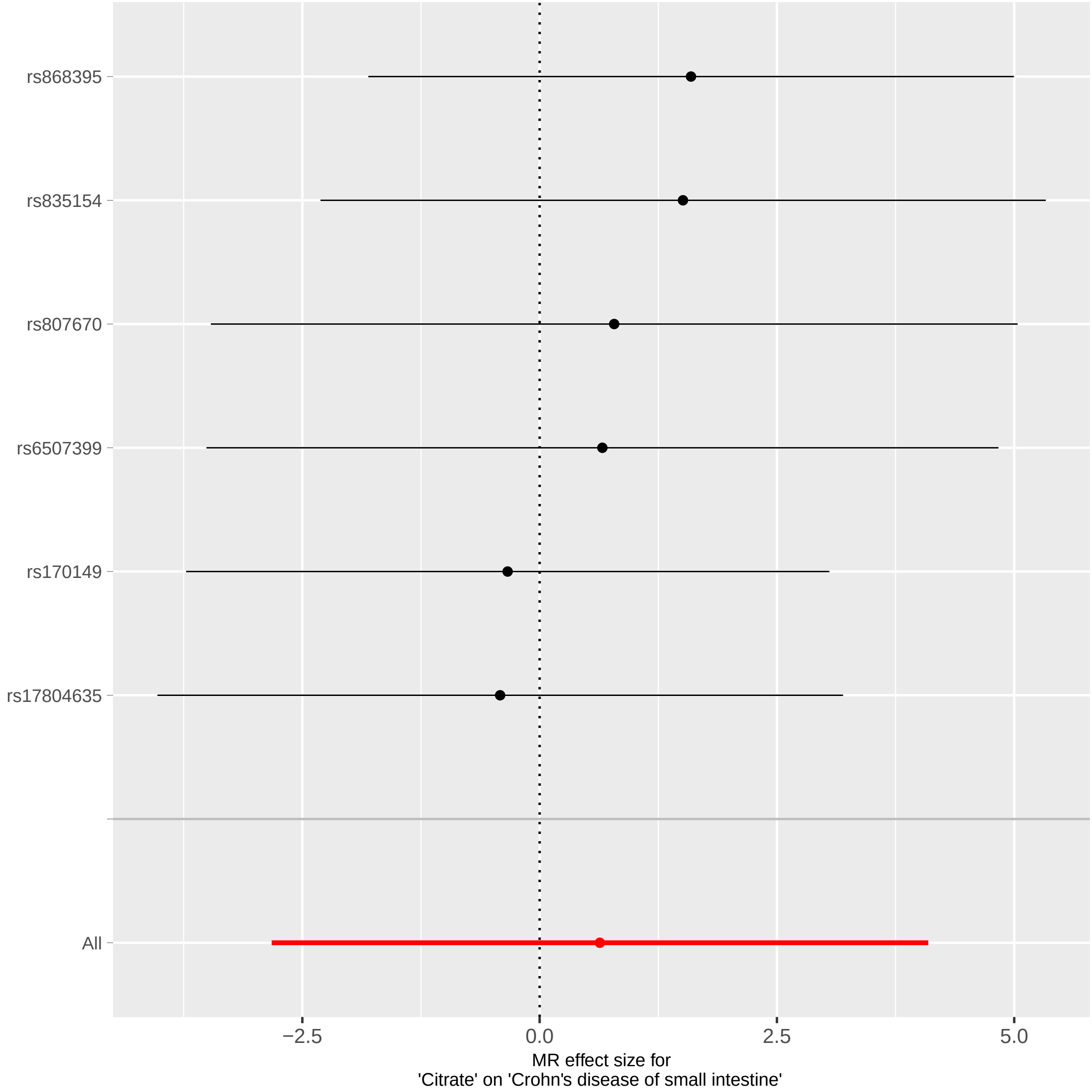

Batch 590 : Glycerate on Crohn's disease of small intestine

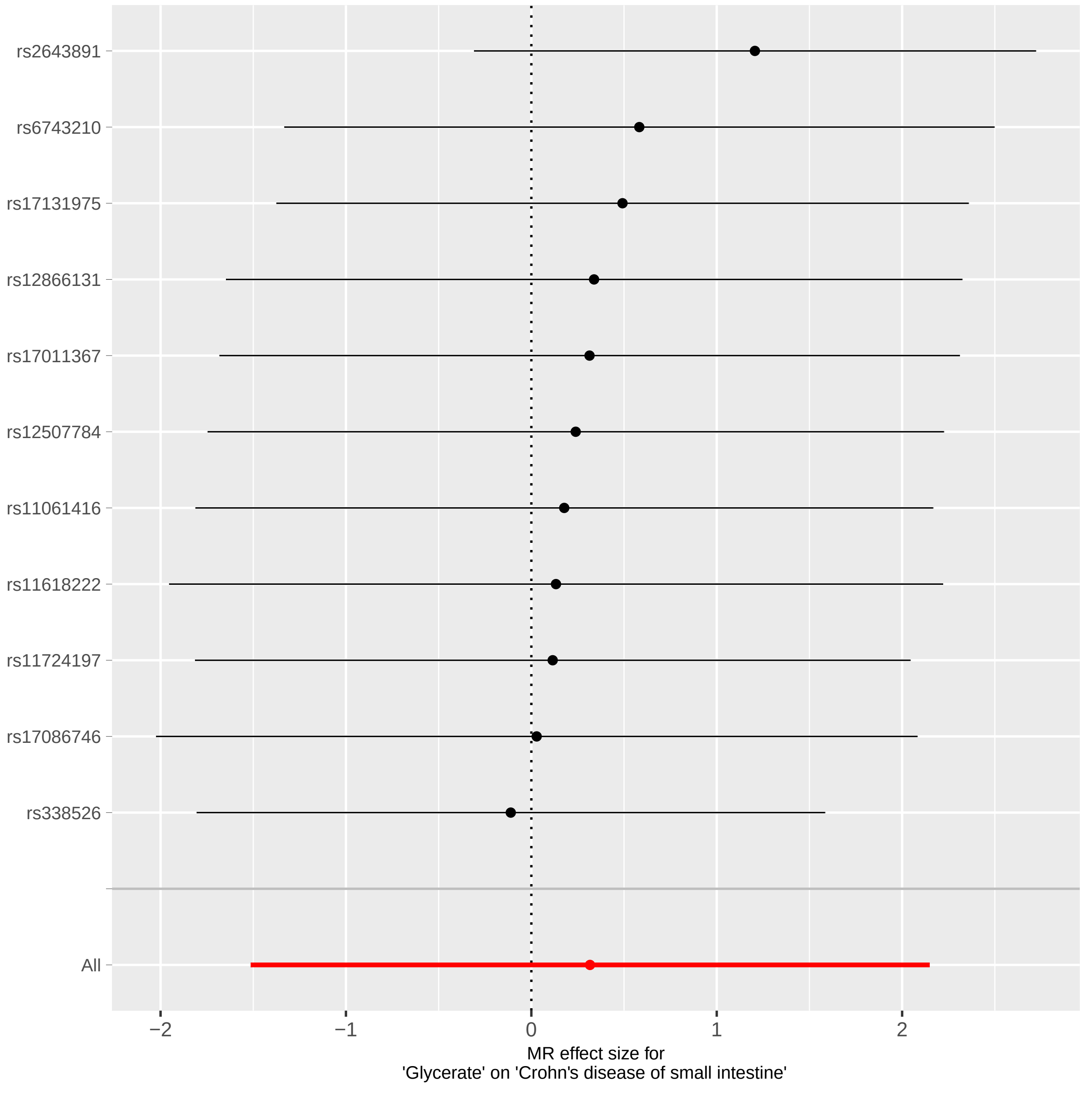

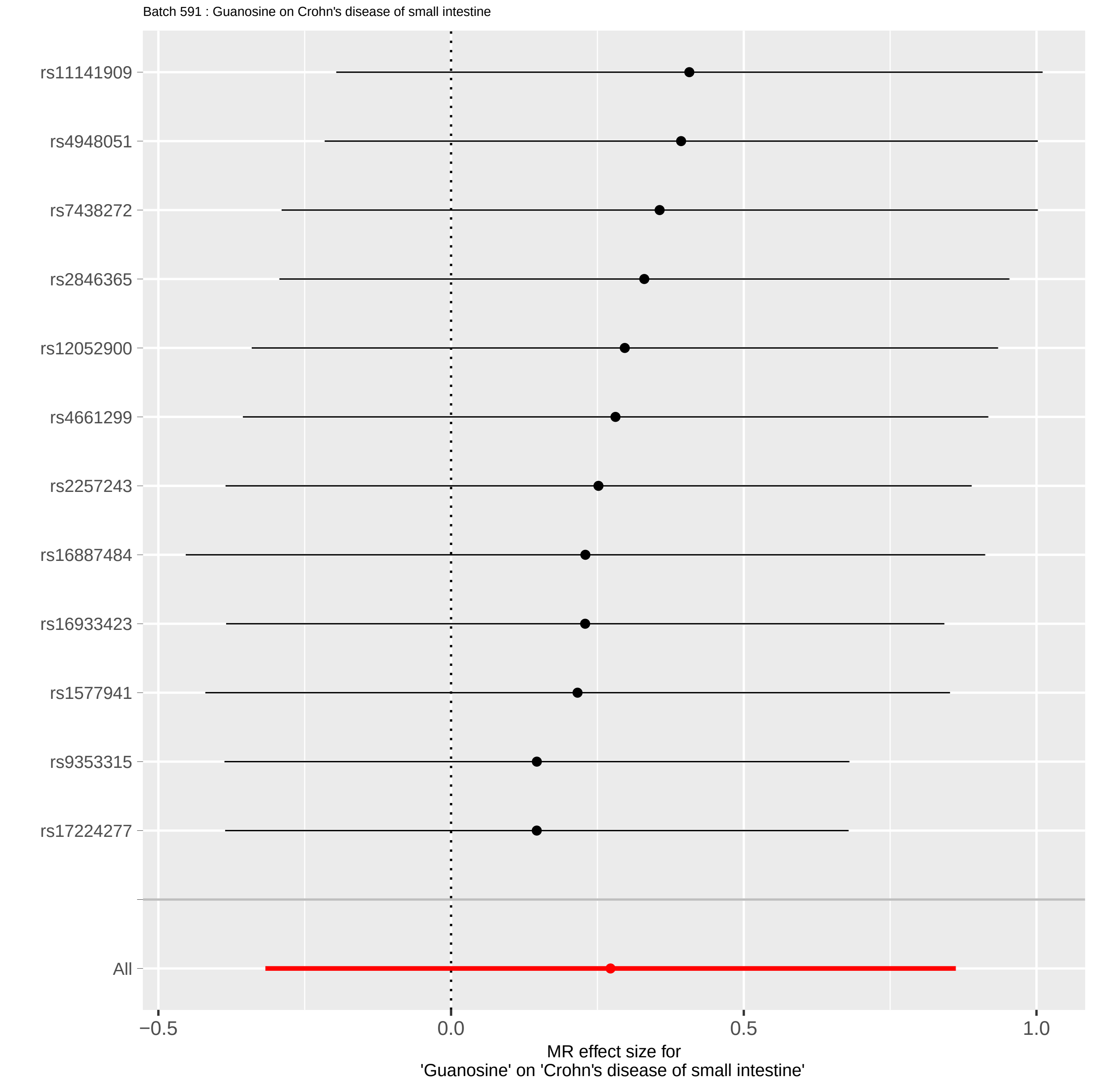
Batch 591 : Guanosine on Crohn's disease of small intestine
rs11141909
rs4948051
rs7438272
rs2846365
rs12052900
rs4661299
rs2257243
rs16887484
rs16933423
rs1577941
rs9353315
rs17224277
All
−0.5
0.0
0.5
1.0
MR effect size for
'Guanosine' on 'Crohn's disease of small intestine'

Batch 594 : Ursodeoxycholate on Crohn's disease of small intestine

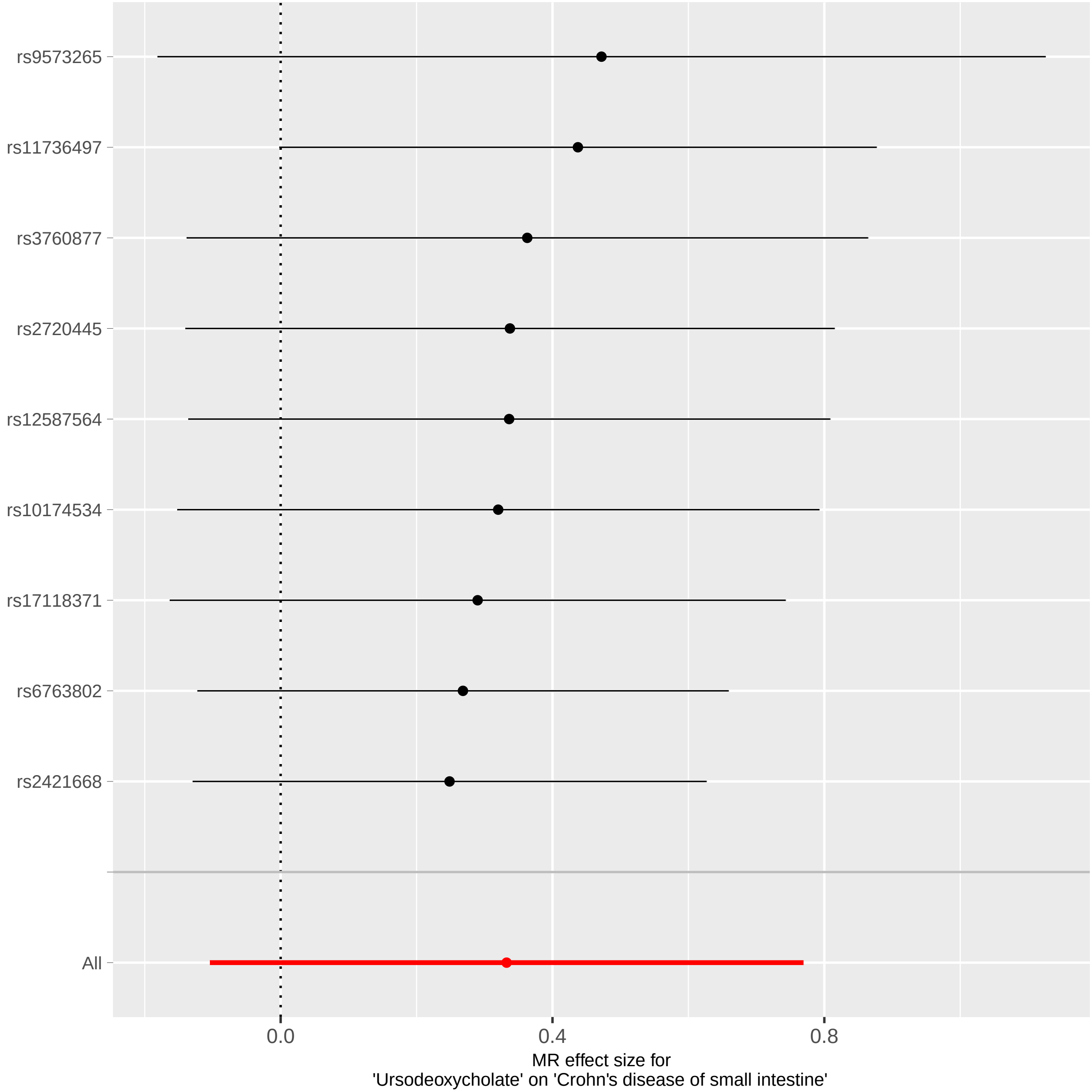

Batch 595 : Arginine on Crohn's disease of small intestine

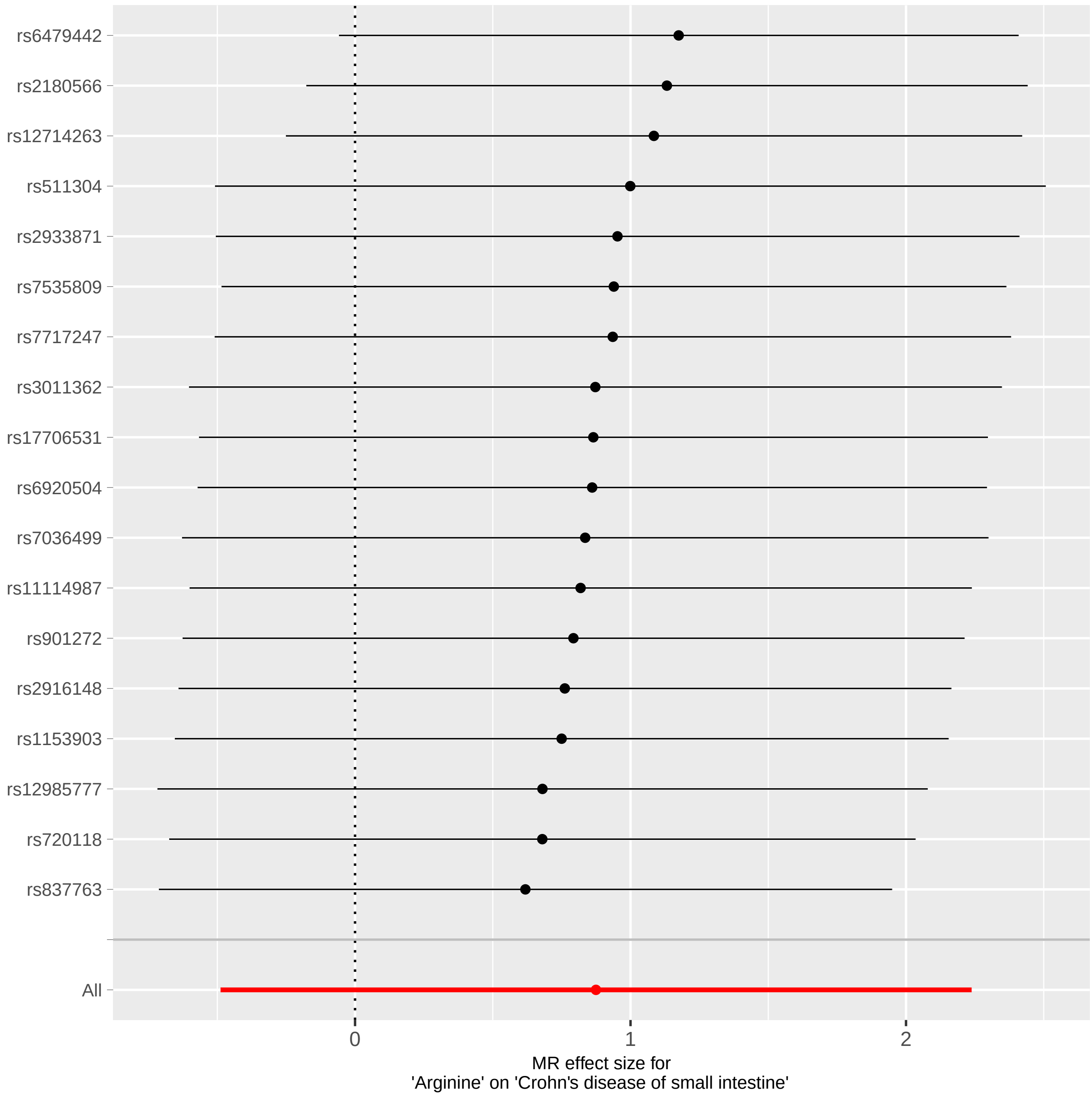

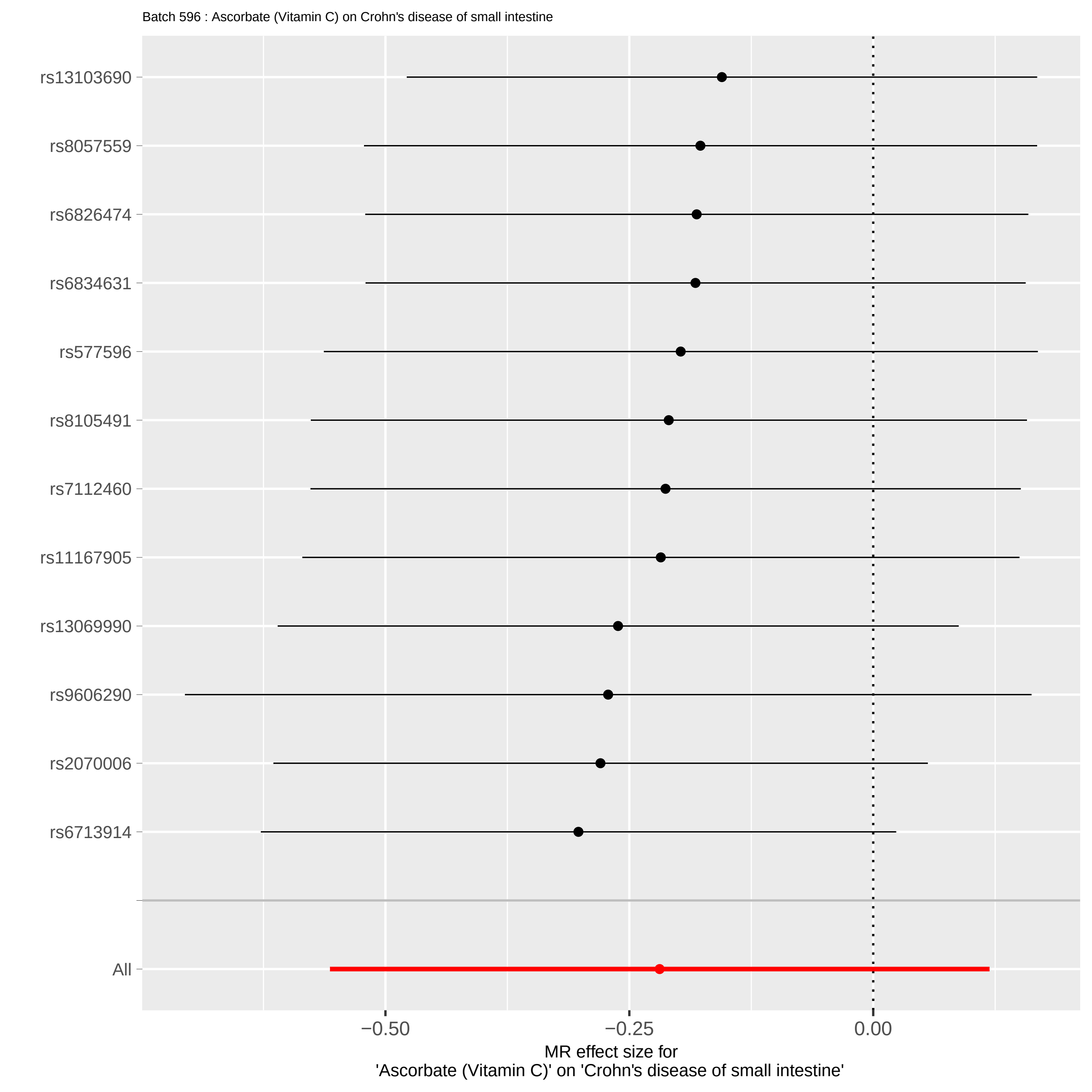
Batch 596 : Ascorbate (Vitamin C) on Crohn's disease of small intestine
rs13103690
rs8057559
rs6826474
rs6834631
rs577596
rs8105491
rs7112460
rs11167905
rs13069990
rs9606290
rs2070006
rs6713914
All
−0.50
−0.25
0.00
MR effect size for
'Ascorbate (Vitamin C)' on 'Crohn's disease of small intestine'

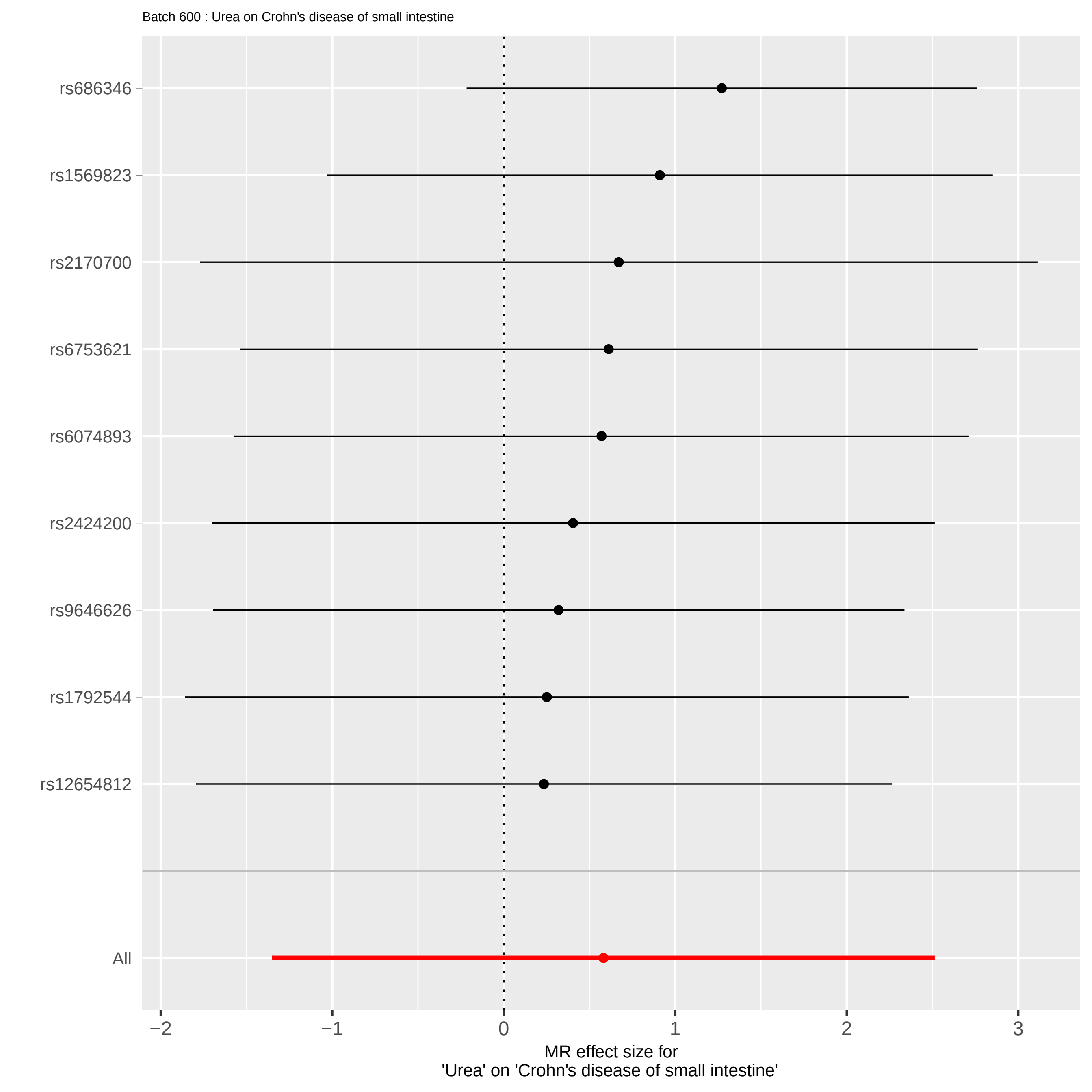

Batch 600 : Urea on Crohn's disease of small intestine
rs686346
rs1569823
rs2170700
rs6753621
rs6074893
rs2424200
rs9646626
rs1792544
rs12654812
All
−2
−1
0
1
2
3
MR effect size for
'Urea' on 'Crohn's disease of small intestine'

Batch 601 : Cortisol on Crohn's disease of small intestine

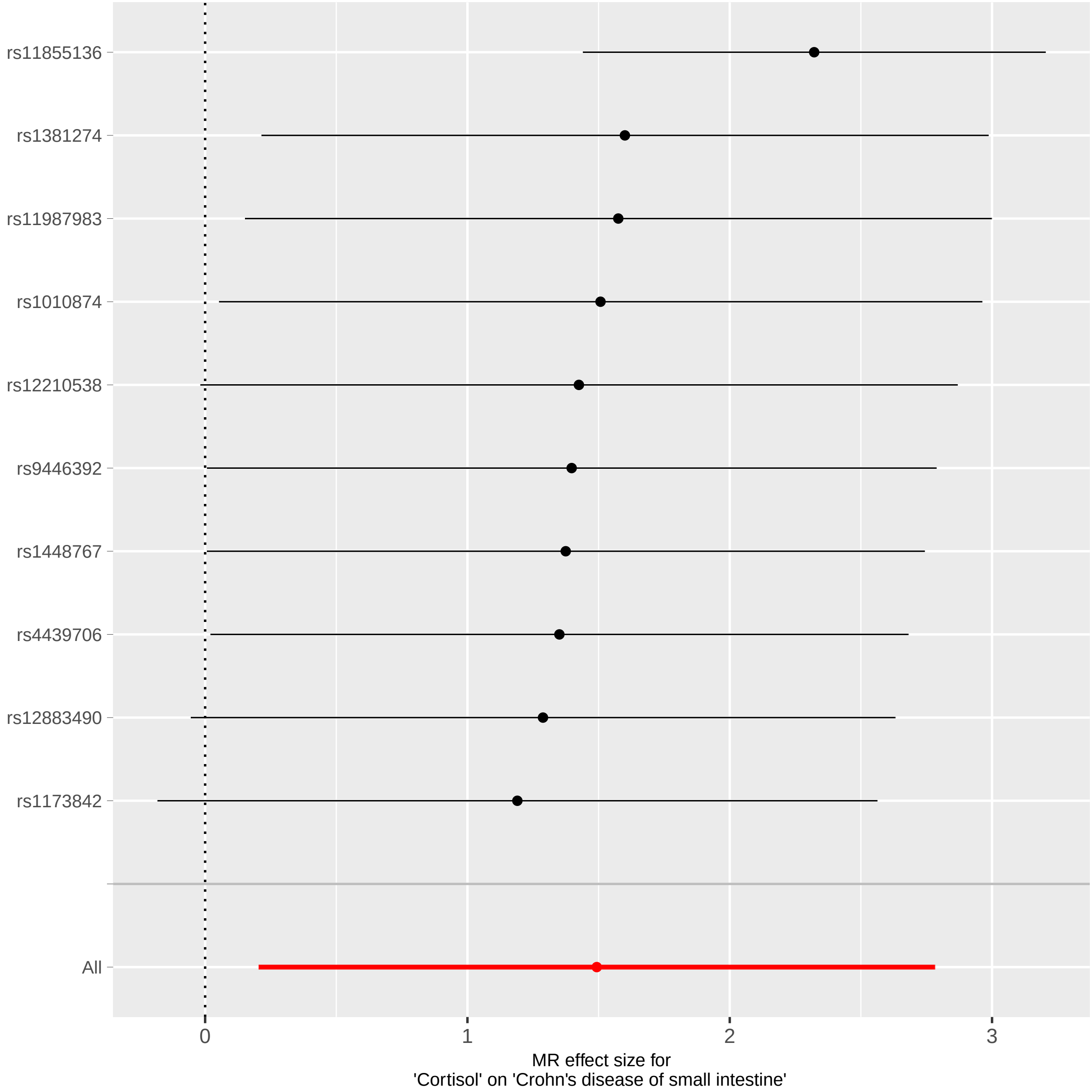

Batch 604 : Citrulline on Crohn's disease of small intestine

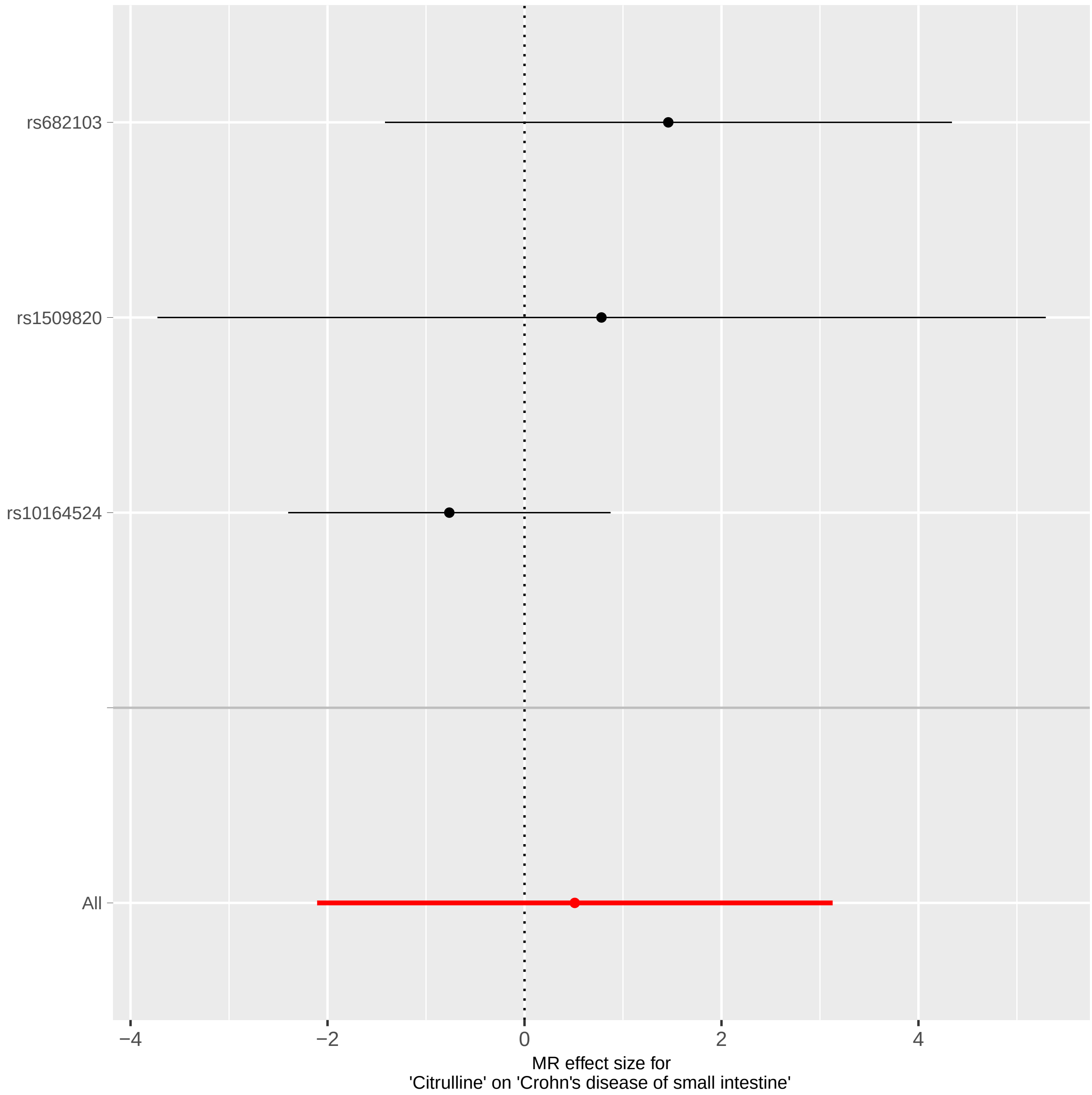

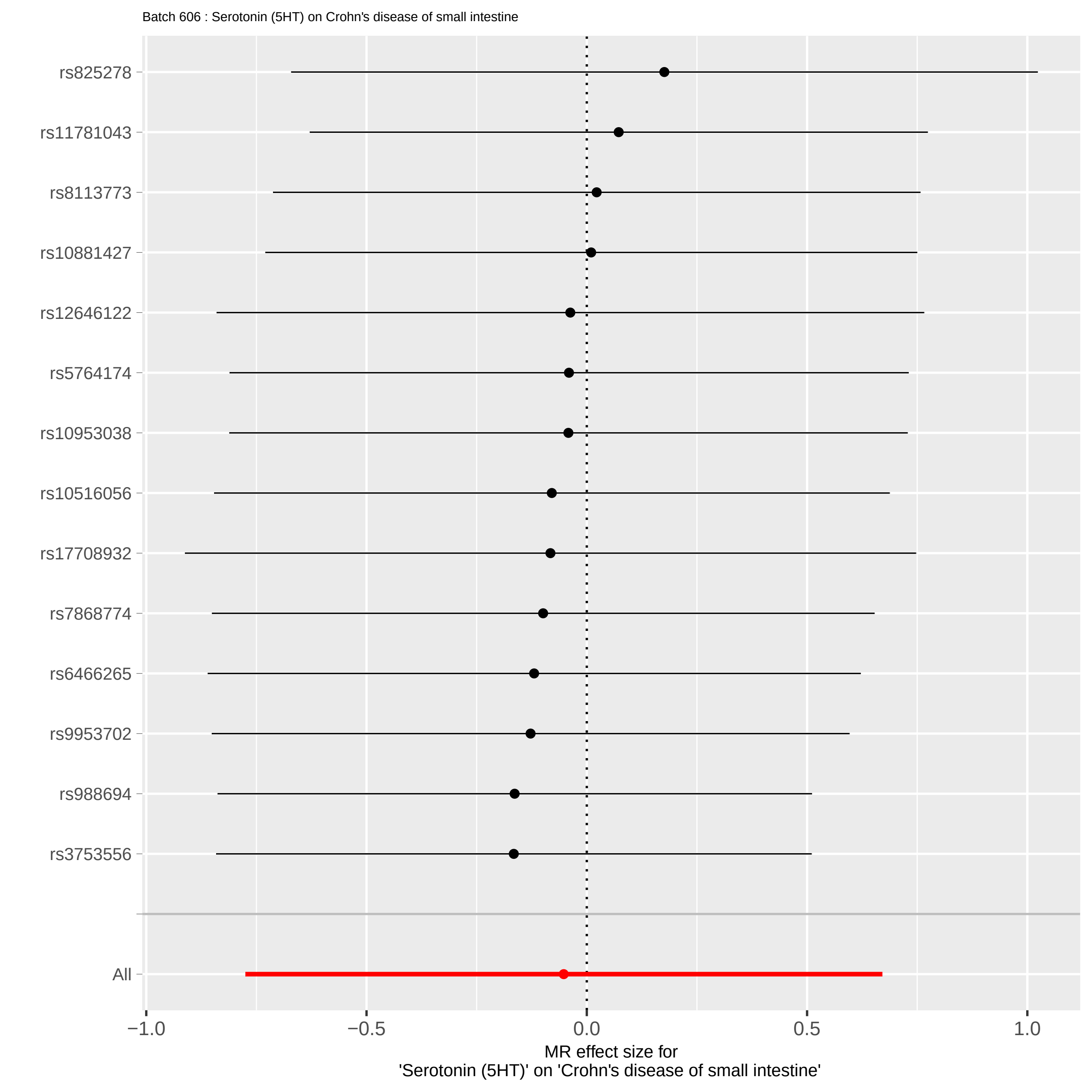

Batch 606 : Serotonin (5HT) on Crohn's disease of small intestine
rs825278
rs11781043
rs8113773
rs10881427
rs12646122
rs5764174
rs10953038
rs10516056
rs17708932
rs7868774
rs6466265
rs9953702
rs988694
rs3753556
All
−1.0
−0.5
0.0
0.5
1.0
MR effect size for
'Serotonin (5HT)' on 'Crohn's disease of small intestine'

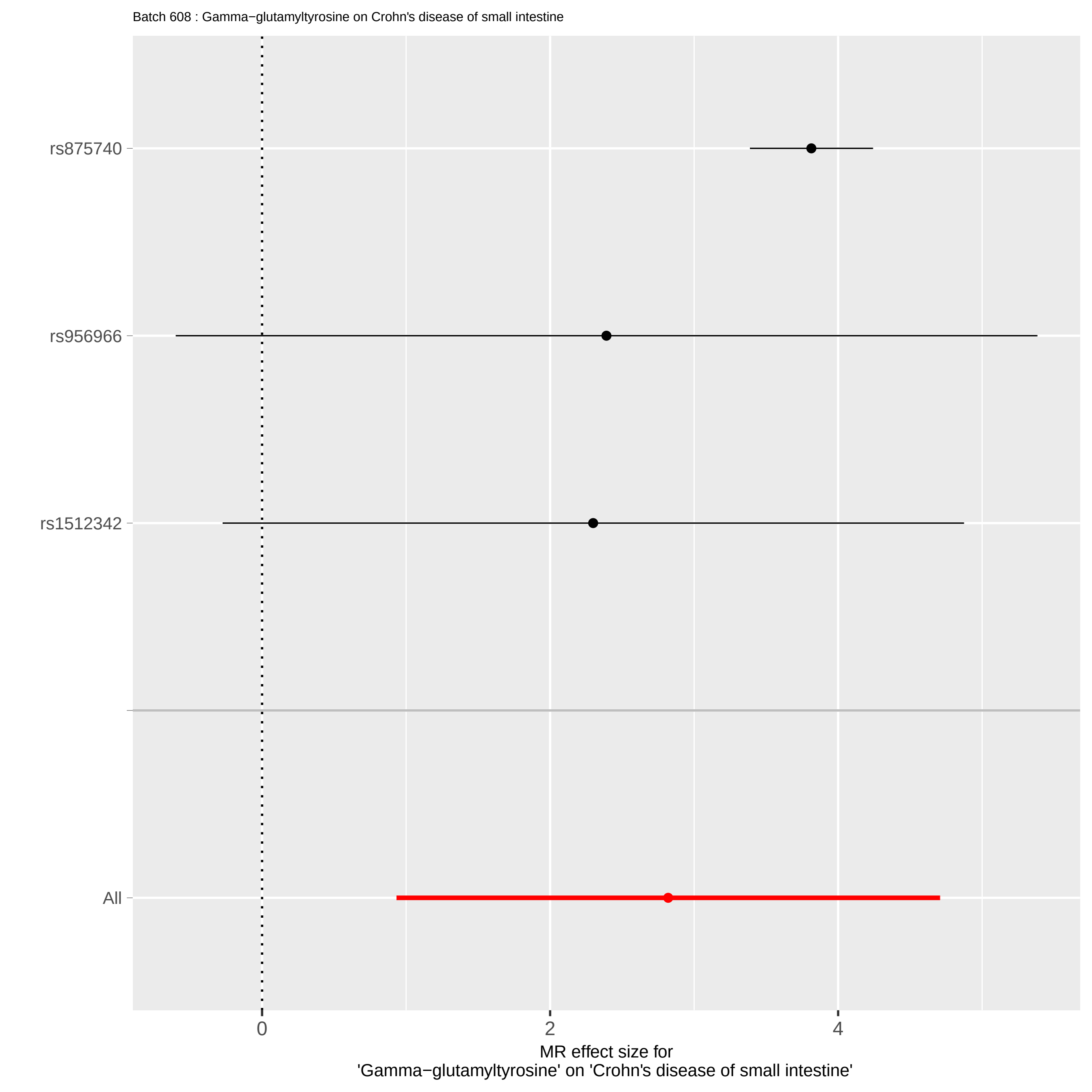
Batch 608 : Gamma−glutamyltyrosine on Crohn's disease of small intestine
rs875740
rs956966
rs1512342
All
0
2
4
MR effect size for
'Gamma−glutamyltyrosine' on 'Crohn's disease of small intestine'

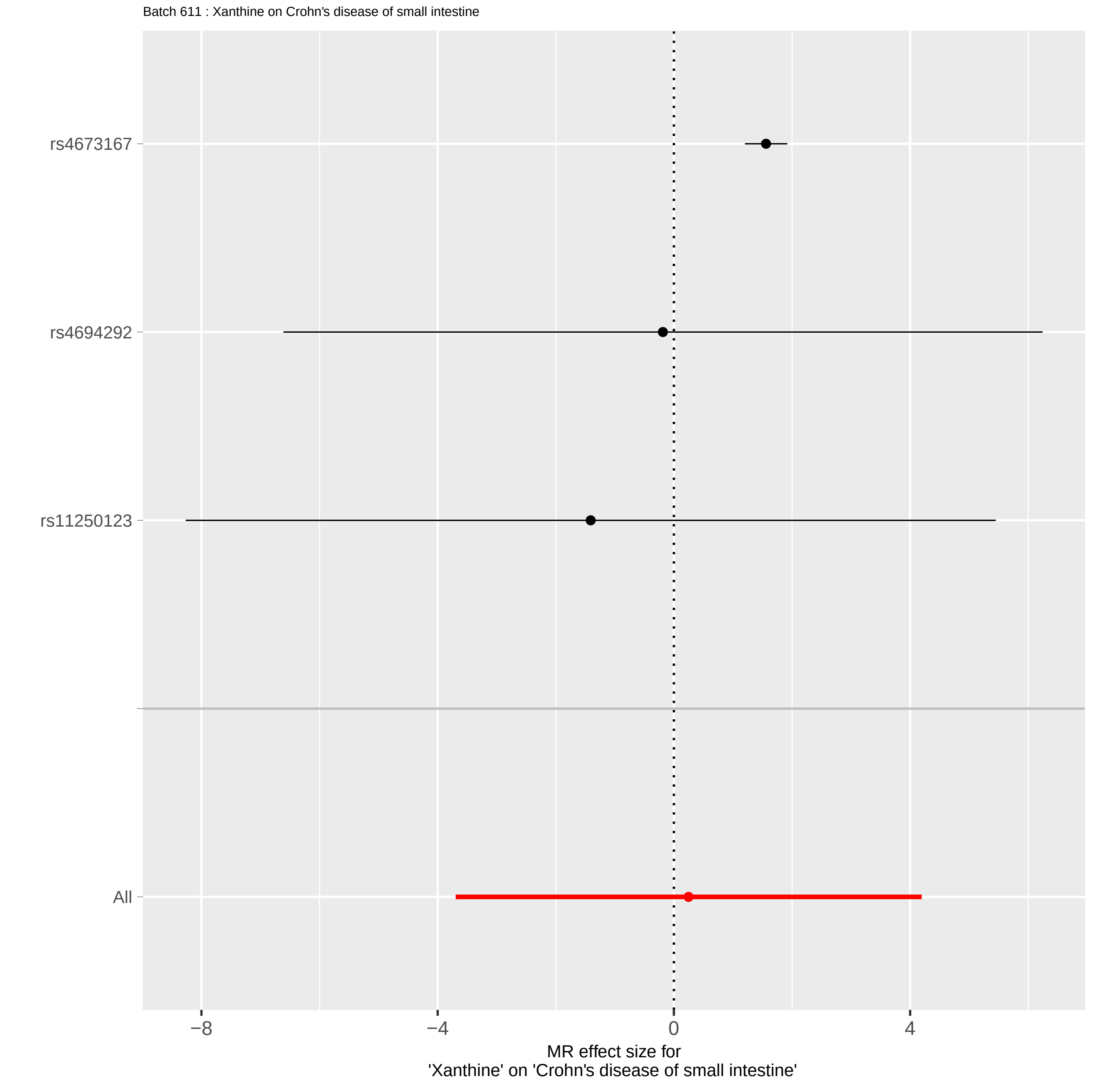

Batch 611 : Xanthine on Crohn's disease of small intestine
rs4673167
rs4694292
rs11250123
All
−8
−4
0
4
MR effect size for
'Xanthine' on 'Crohn's disease of small intestine'

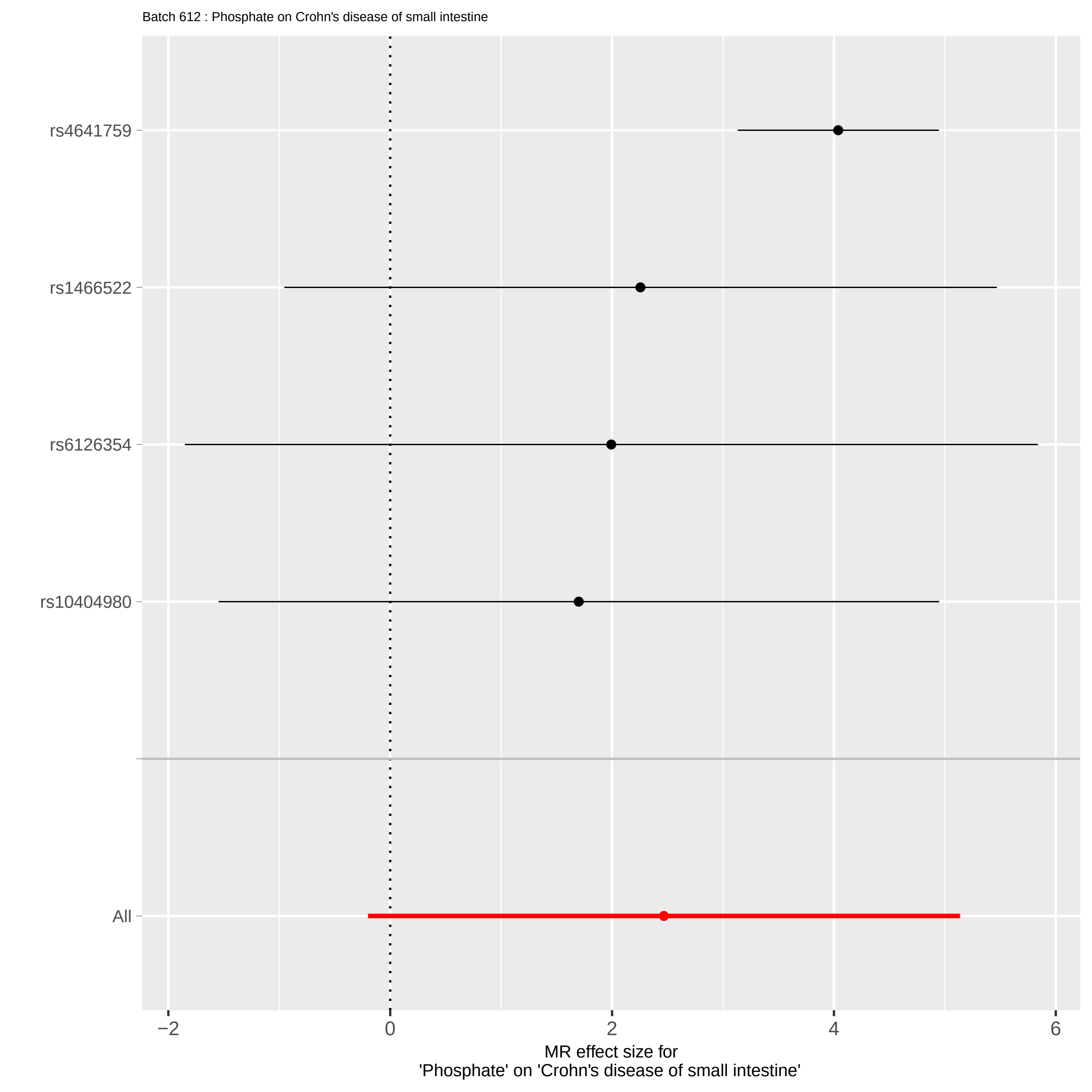
Batch 612 : Phosphate on Crohn's disease of small intestine
rs4641759
rs1466522
rs6126354
rs10404980
All
−2
0
2
4
6
MR effect size for
'Phosphate' on 'Crohn's disease of small intestine'

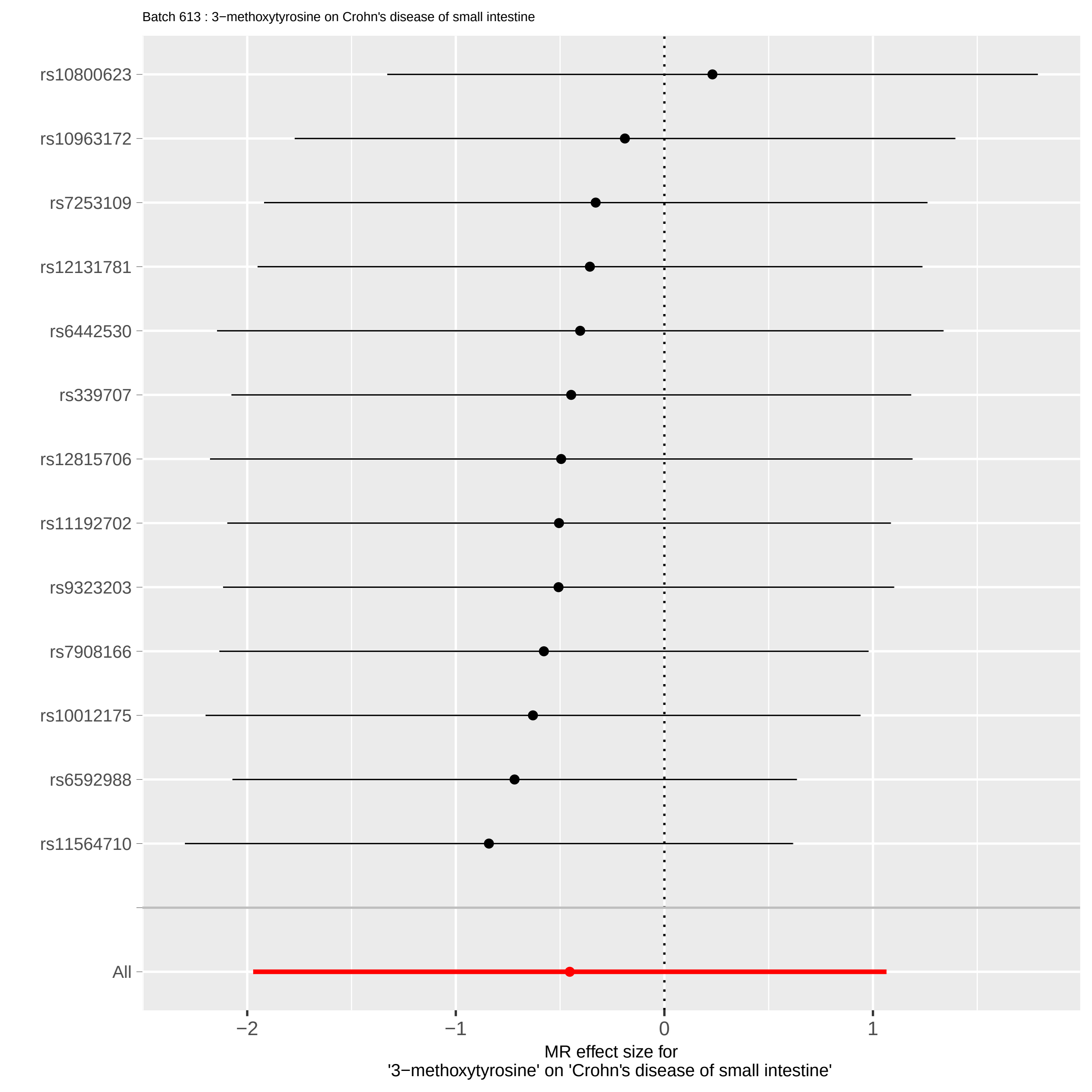

Batch 613 : 3−methoxytyrosine on Crohn's disease of small intestine
rs10800623
rs10963172
rs7253109
rs12131781
rs6442530
rs339707
rs12815706
rs11192702
rs9323203
rs7908166
rs10012175
rs6592988
rs11564710
All
−2
−1
0
1
MR effect size for
'3−methoxytyrosine' on 'Crohn's disease of small intestine'

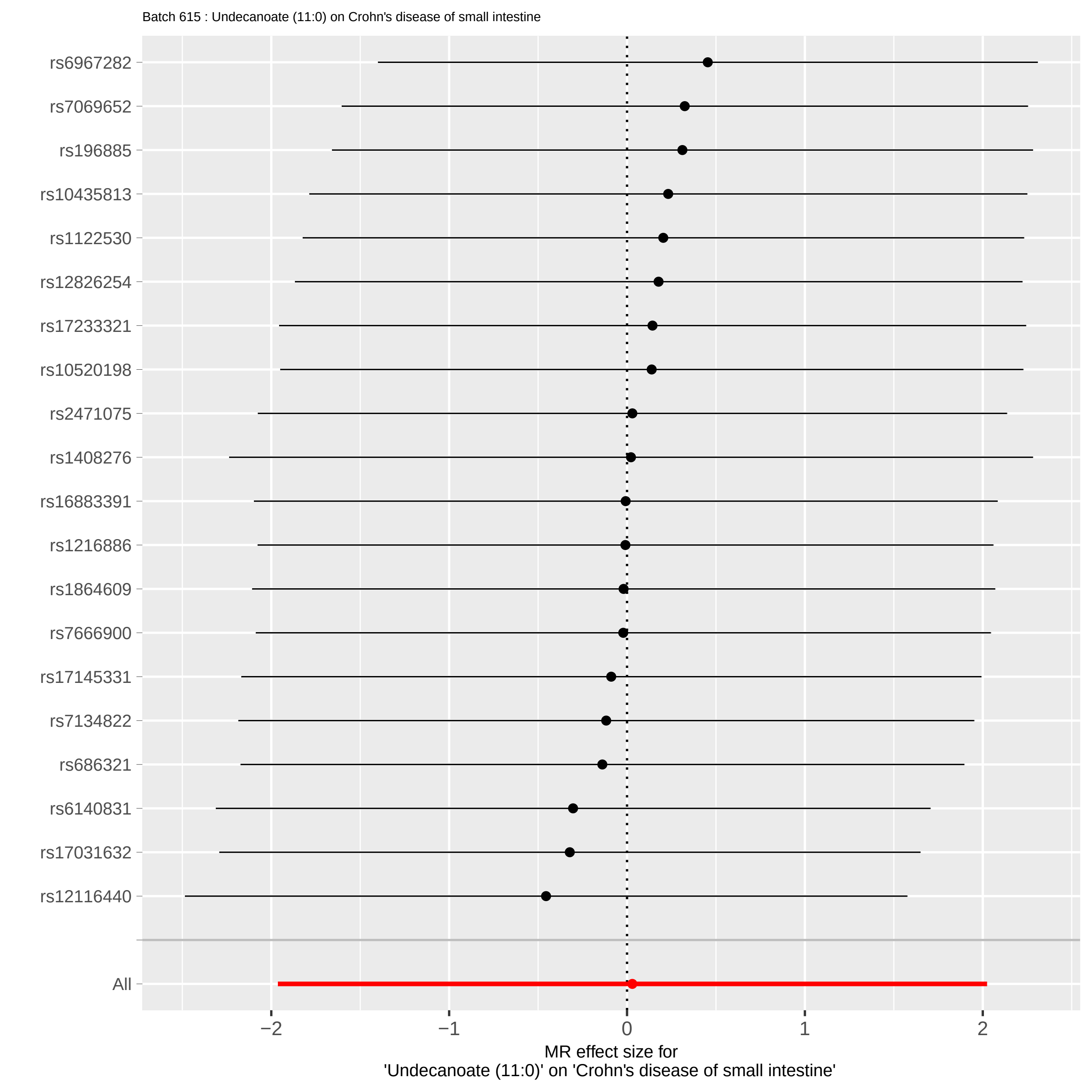
Batch 615 : Undecanoate (11:0) on Crohn's disease of small intestine
rs6967282
rs7069652
rs196885
rs10435813
rs1122530
rs12826254
rs17233321
rs10520198
rs2471075
rs1408276
rs16883391
rs1216886
rs1864609
rs7666900
rs17145331
rs7134822
rs686321
rs6140831
rs17031632
rs12116440
All
−2
−1
0
1
2
MR effect size for
'Undecanoate (11:0)' on 'Crohn's disease of small intestine'

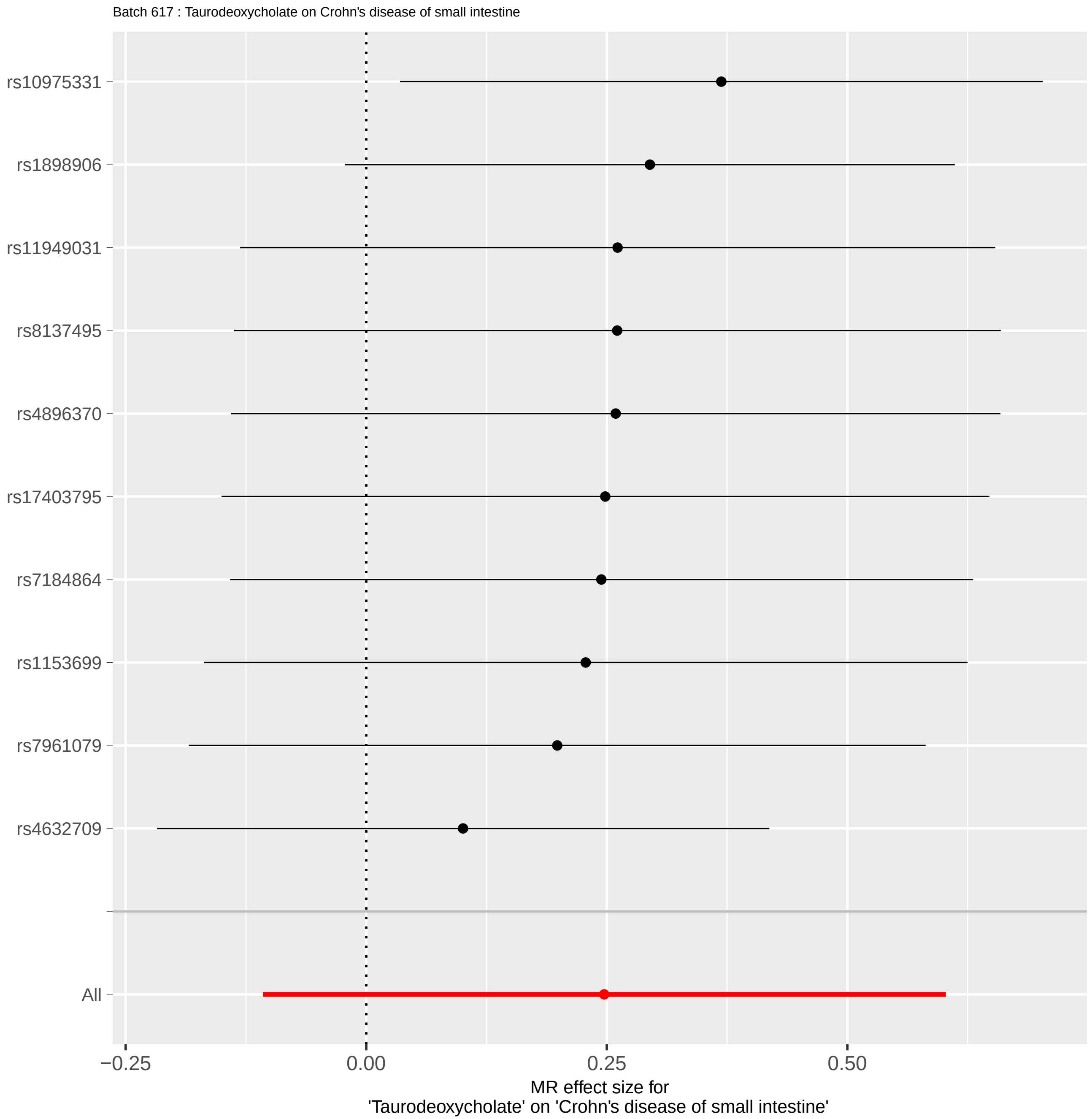
Batch 617 : Taurodeoxycholate on Crohn's disease of small intestine
rs10975331
rs1898906
rs11949031
rs8137495
rs4896370
rs17403795
rs7184864
rs1153699
rs7961079
rs4632709
All
−0.25
0.00
0.25
0.50
MR effect size for
'Taurodeoxycholate' on 'Crohn's disease of small intestine'

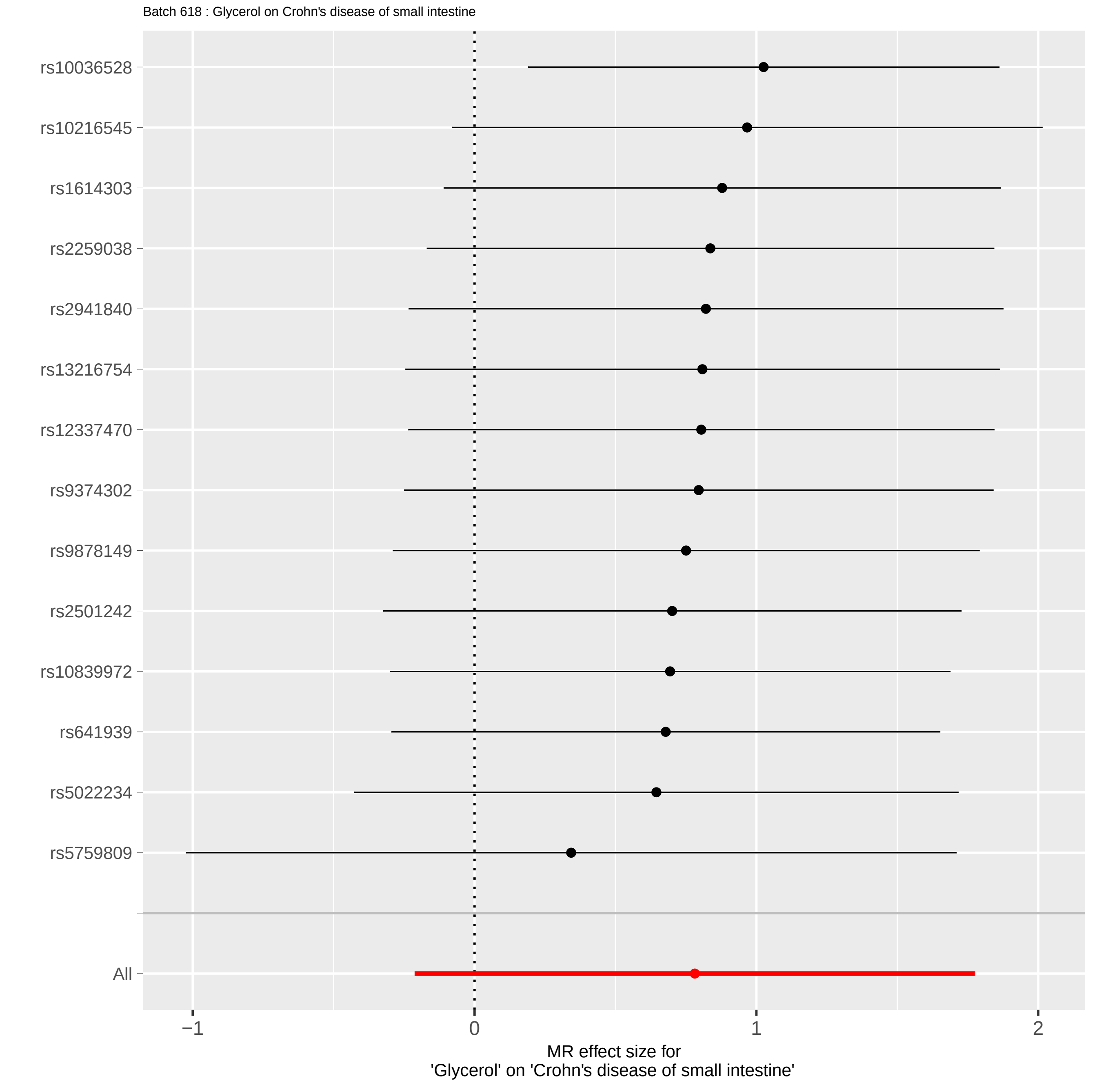

Batch 618 : Glycerol on Crohn's disease of small intestine
rs10036528
rs10216545
rs1614303
rs2259038
rs2941840
rs13216754
rs12337470
rs9374302
rs9878149
rs2501242
rs10839972
rs641939
rs5022234
rs5759809
All
−1
0
1
2
MR effect size for
'Glycerol' on 'Crohn's disease of small intestine'

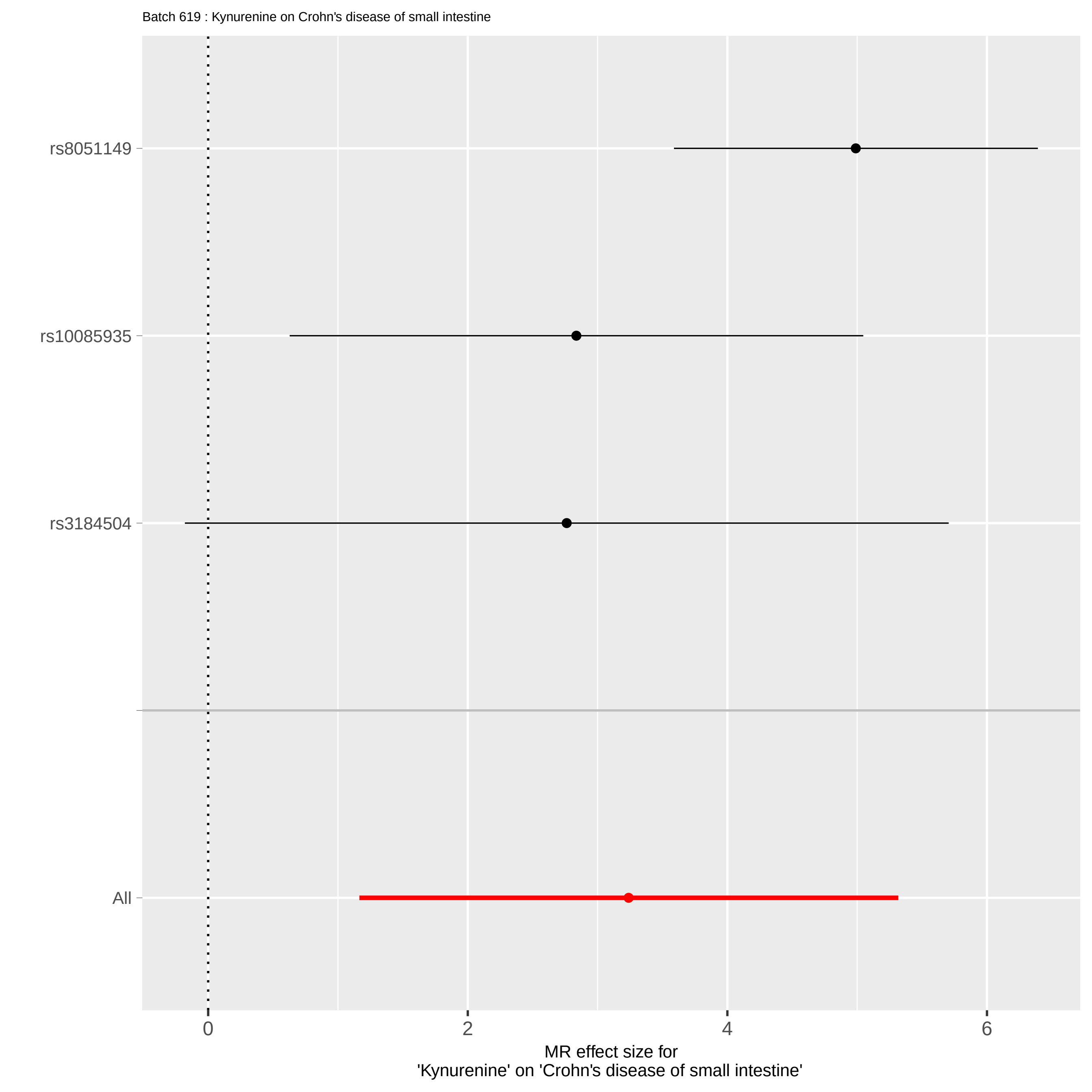

Batch 619 : Kynurenine on Crohn's disease of small intestine
rs8051149
rs10085935
rs3184504
All
0
2
4
6
MR effect size for
'Kynurenine' on 'Crohn's disease of small intestine'

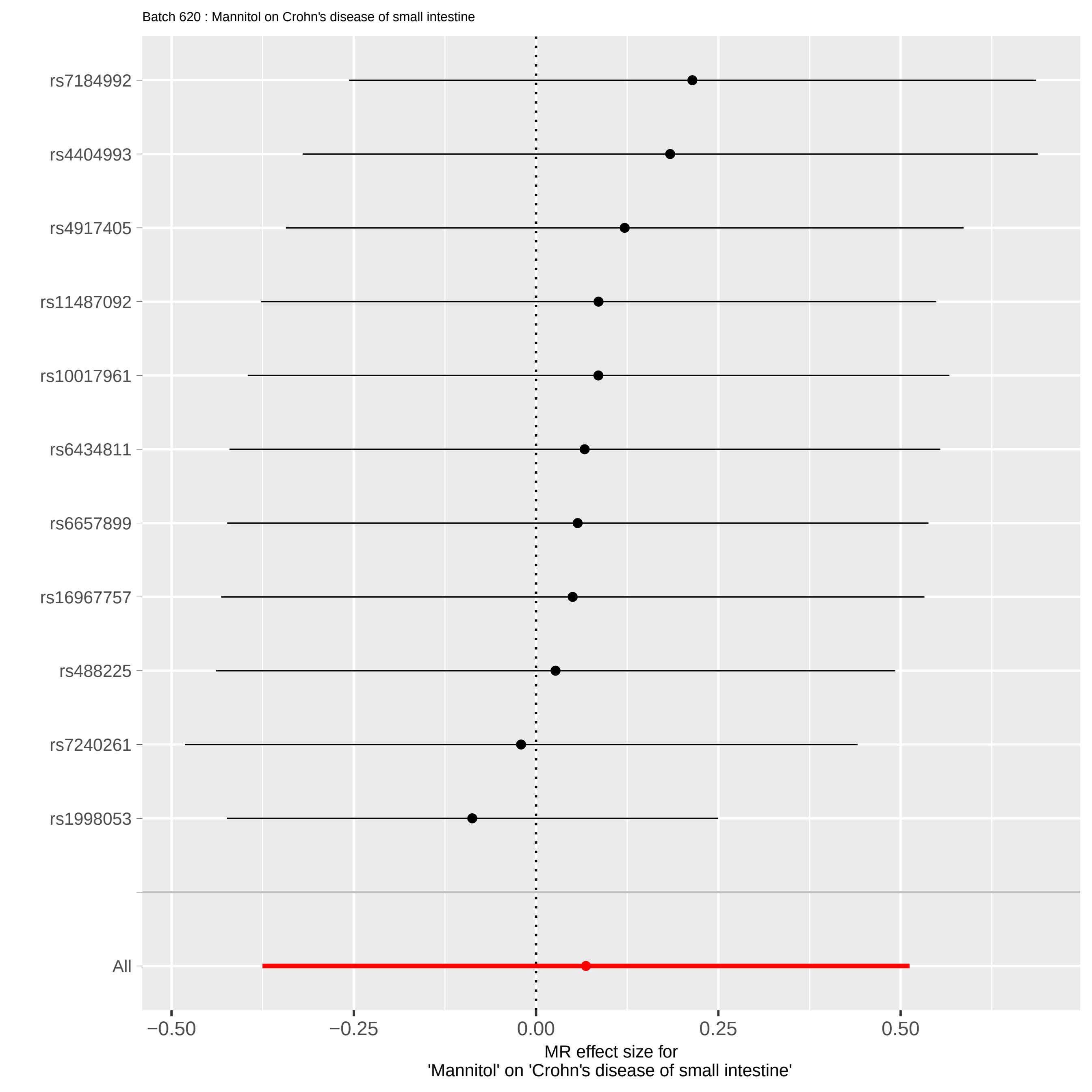

Batch 620 : Mannitol on Crohn's disease of small intestine
rs7184992
rs4404993
rs4917405
rs11487092
rs10017961
rs6434811
rs6657899
rs16967757
rs488225
rs7240261
rs1998053
All
−0.50
−0.25
0.00
0.25
0.50
MR effect size for
'Mannitol' on 'Crohn's disease of small intestine'

Batch 621 : Glycerol 3−phosphate (G3P) on Crohn's disease of small intestine

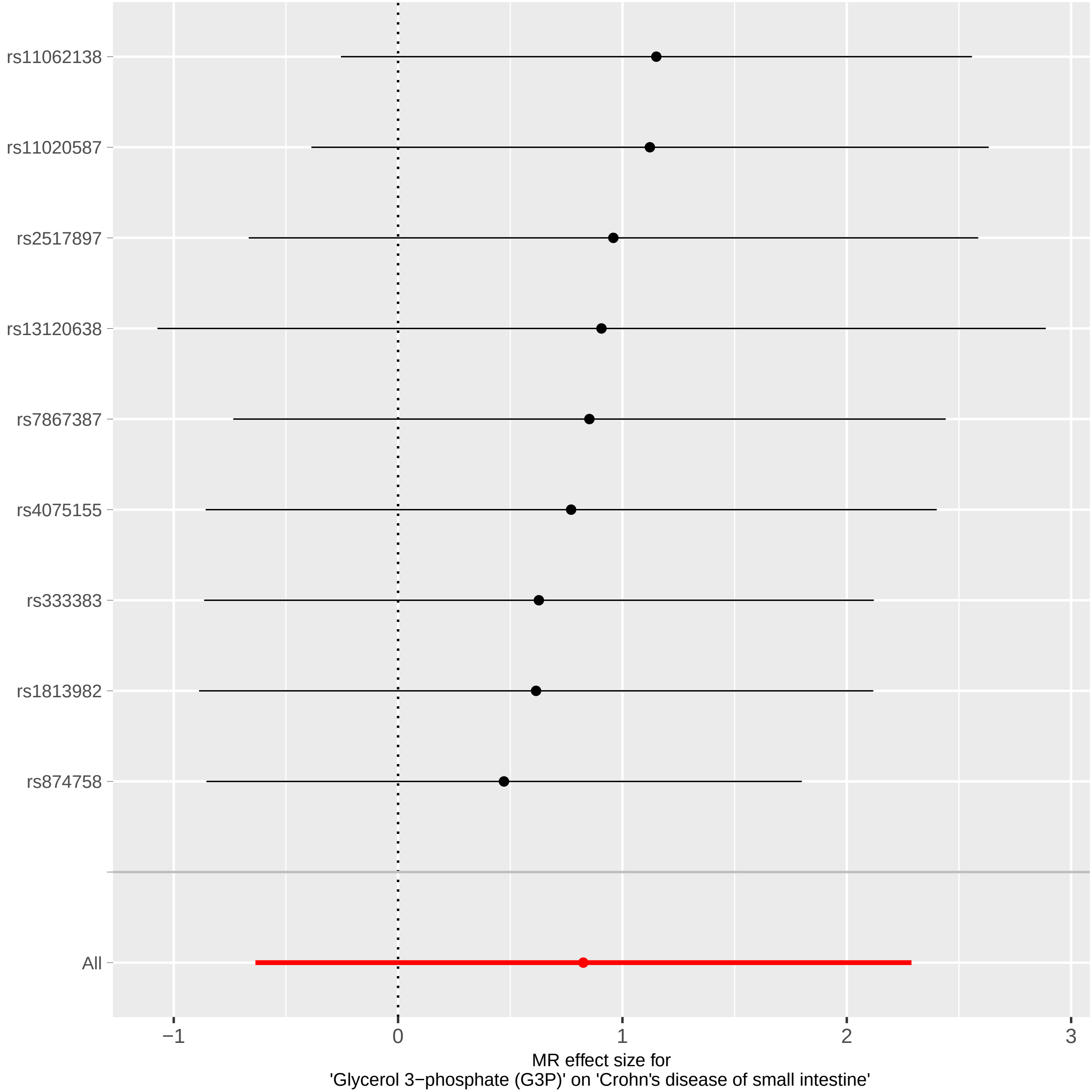

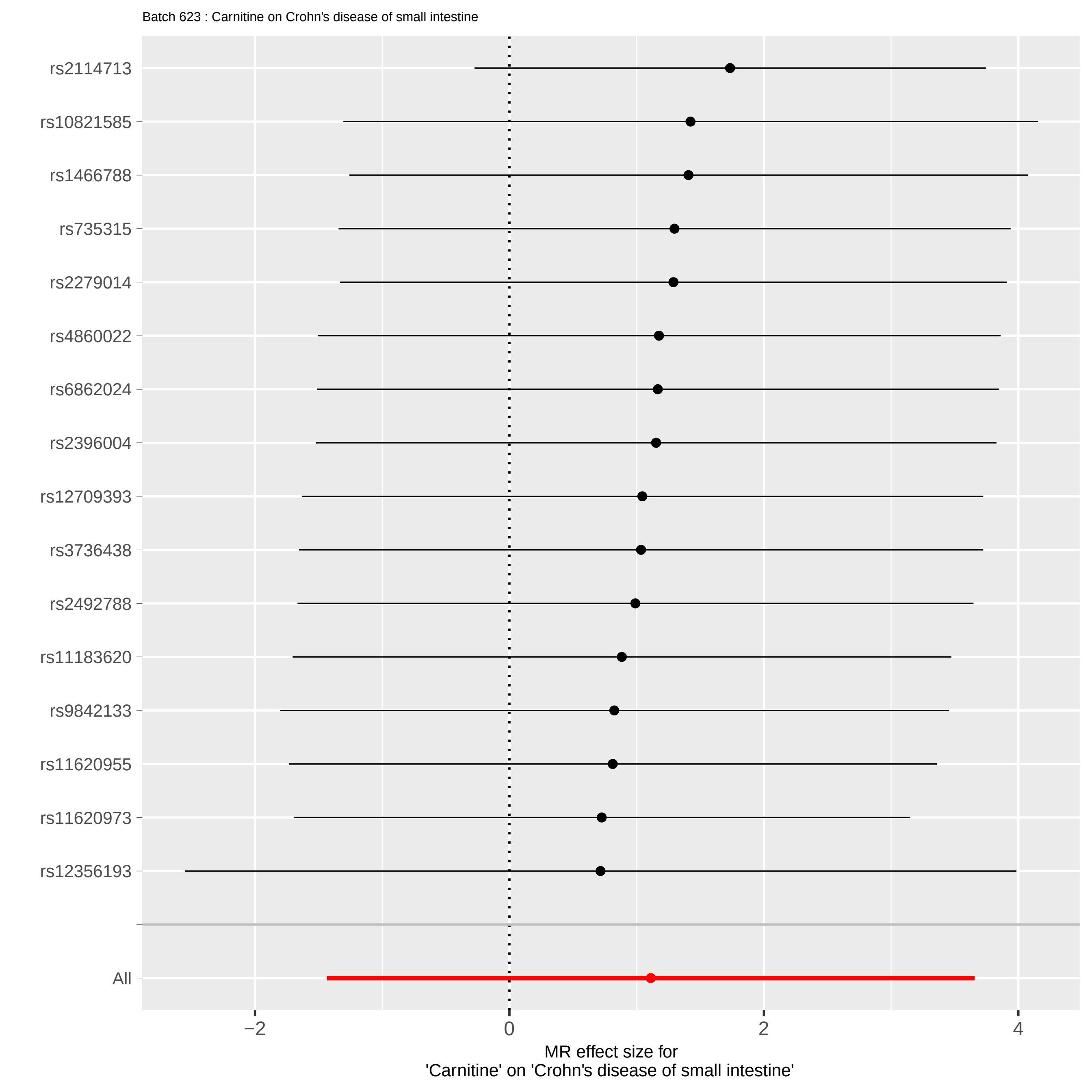
Batch 623 : Carnitine on Crohn's disease of small intestine
rs2114713
rs10821585
rs1466788
rs735315
rs2279014
rs4860022
rs6862024
rs2396004
rs12709393
rs3736438
rs2492788
rs11183620
rs9842133
rs11620955
rs11620973
rs12356193
All
−2
0
2
4
MR effect size for
'Carnitine' on 'Crohn's disease of small intestine'

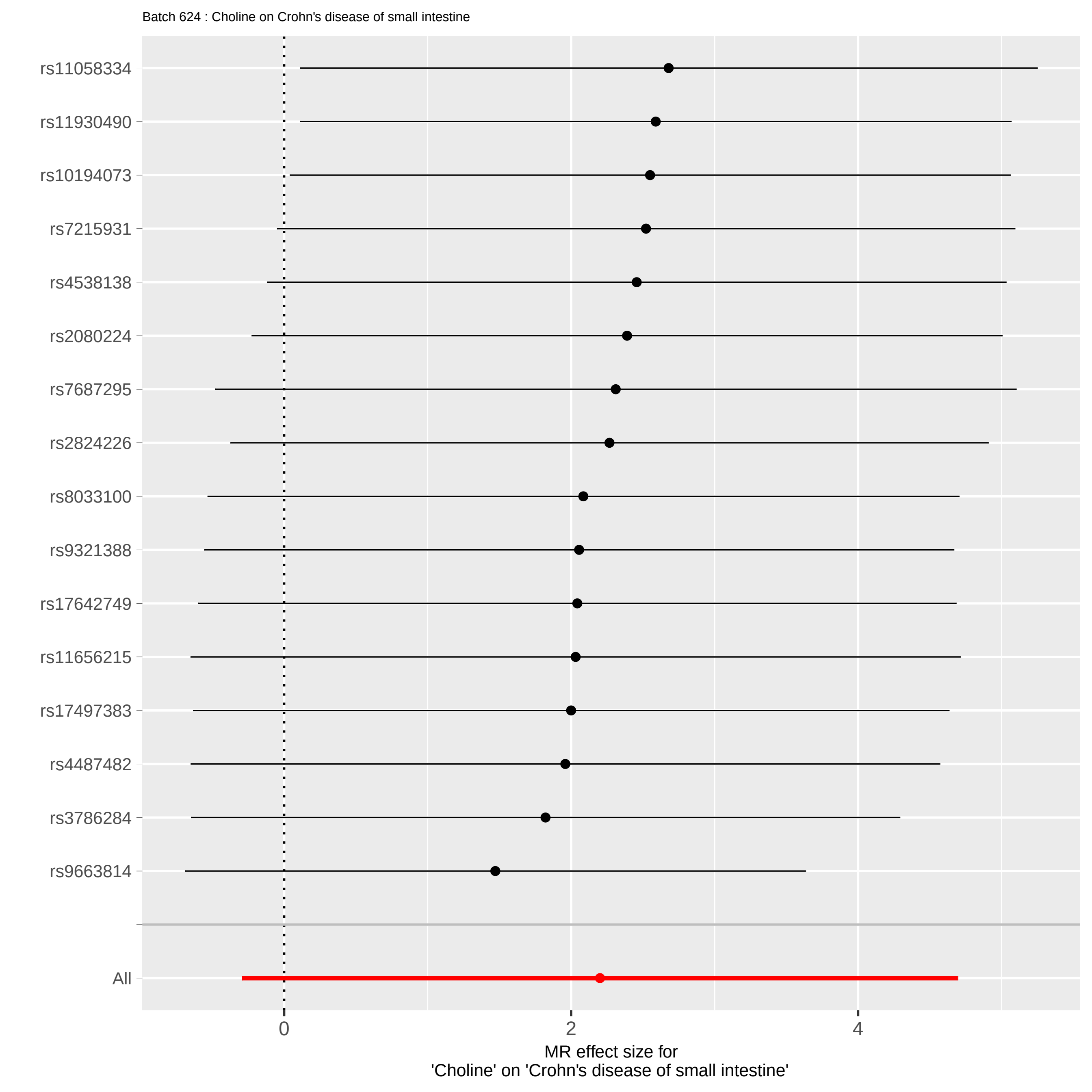

Batch 624 : Choline on Crohn's disease of small intestine
rs11058334
rs11930490
rs10194073
rs7215931
rs4538138
rs2080224
rs7687295
rs2824226
rs8033100
rs9321388
rs17642749
rs11656215
rs17497383
rs4487482
rs3786284
rs9663814
All
0
2
4
MR effect size for
'Choline' on 'Crohn's disease of small intestine'

Batch 628 : 3−methylhistidine on Crohn's disease of small intestine

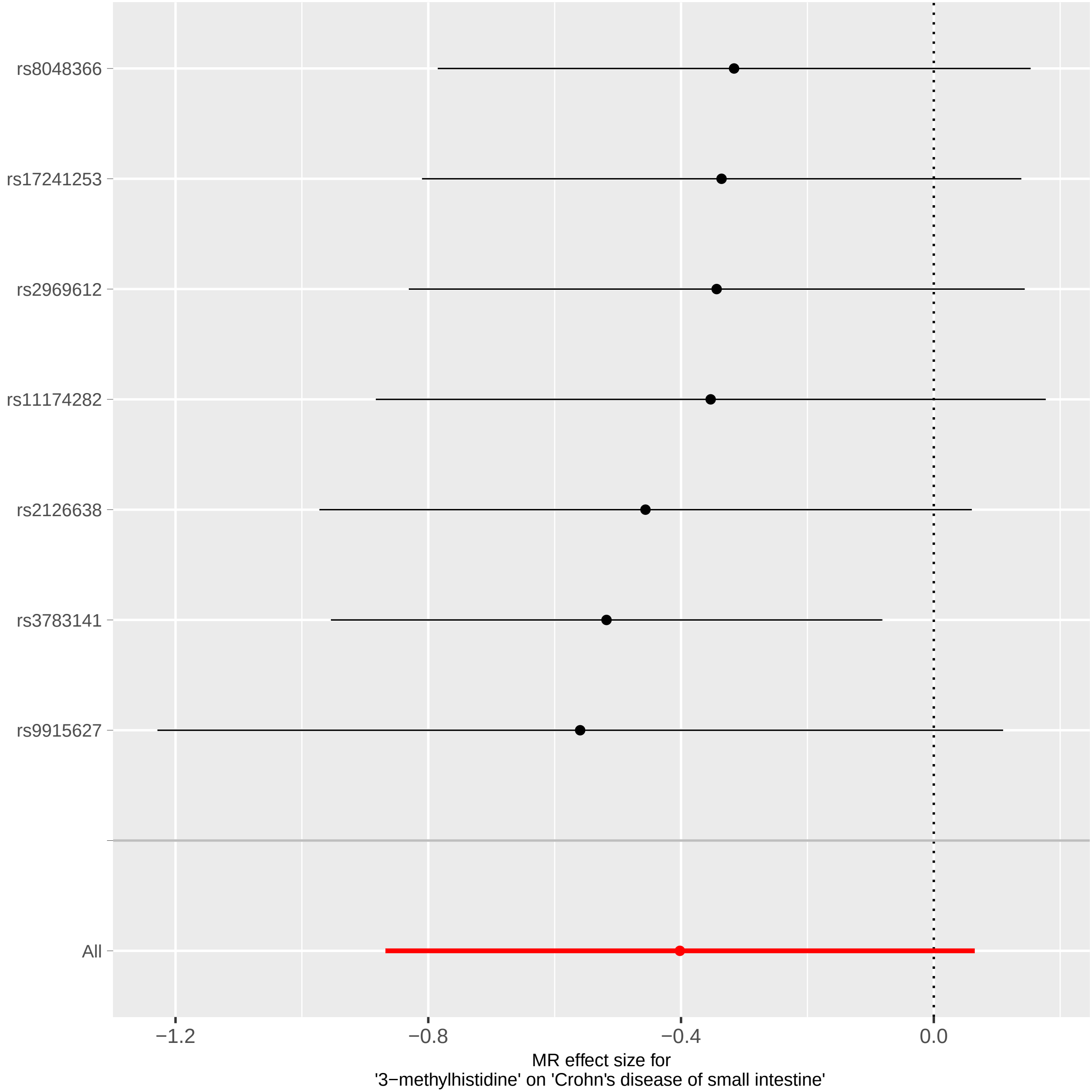

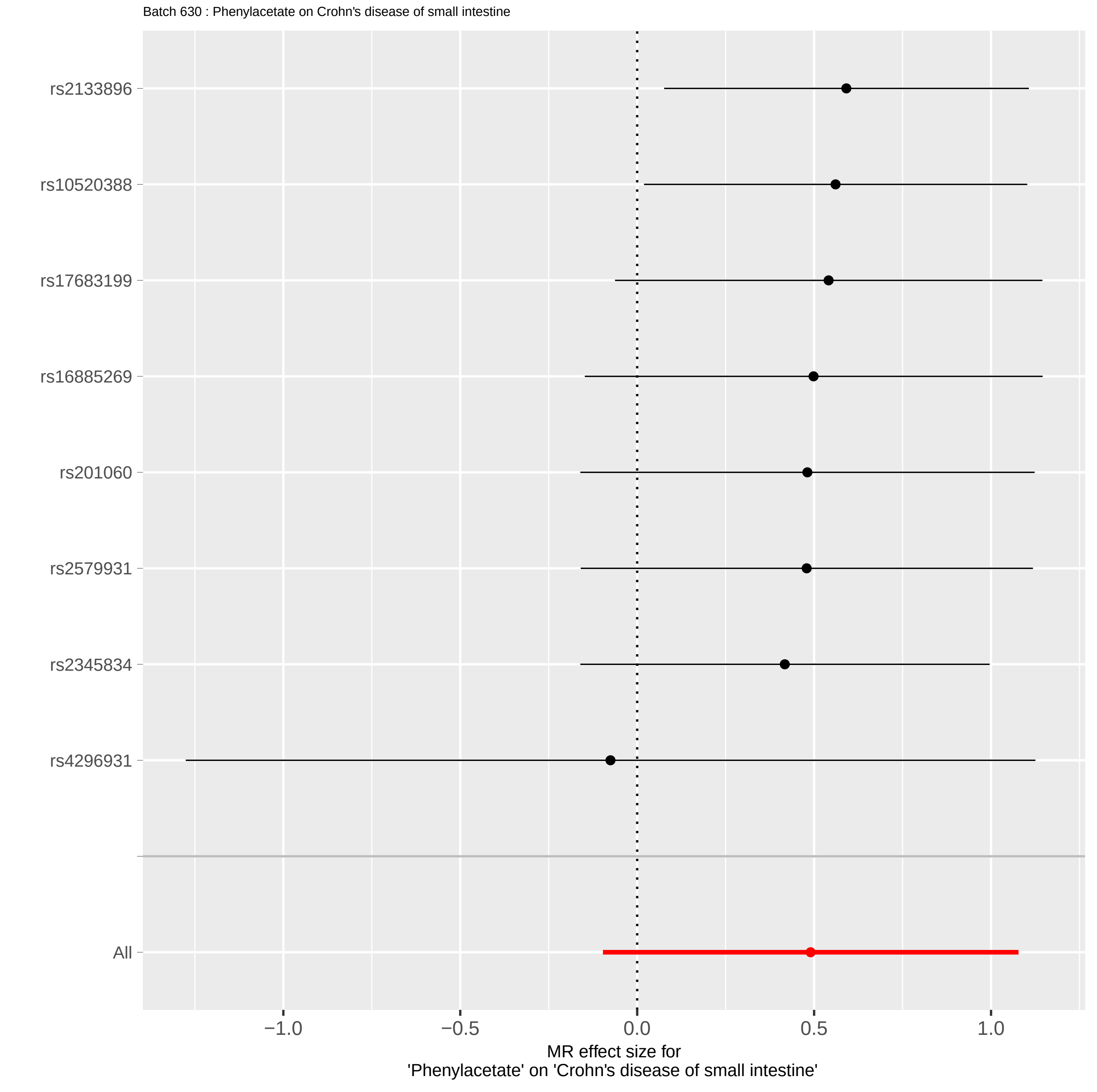

Batch 630 : Phenylacetate on Crohn's disease of small intestine
rs2133896
rs10520388
rs17683199
rs16885269
rs201060
rs2579931
rs2345834
rs4296931
All
−1.0
−0.5
0.0
0.5
1.0
MR effect size for
'Phenylacetate' on 'Crohn's disease of small intestine'

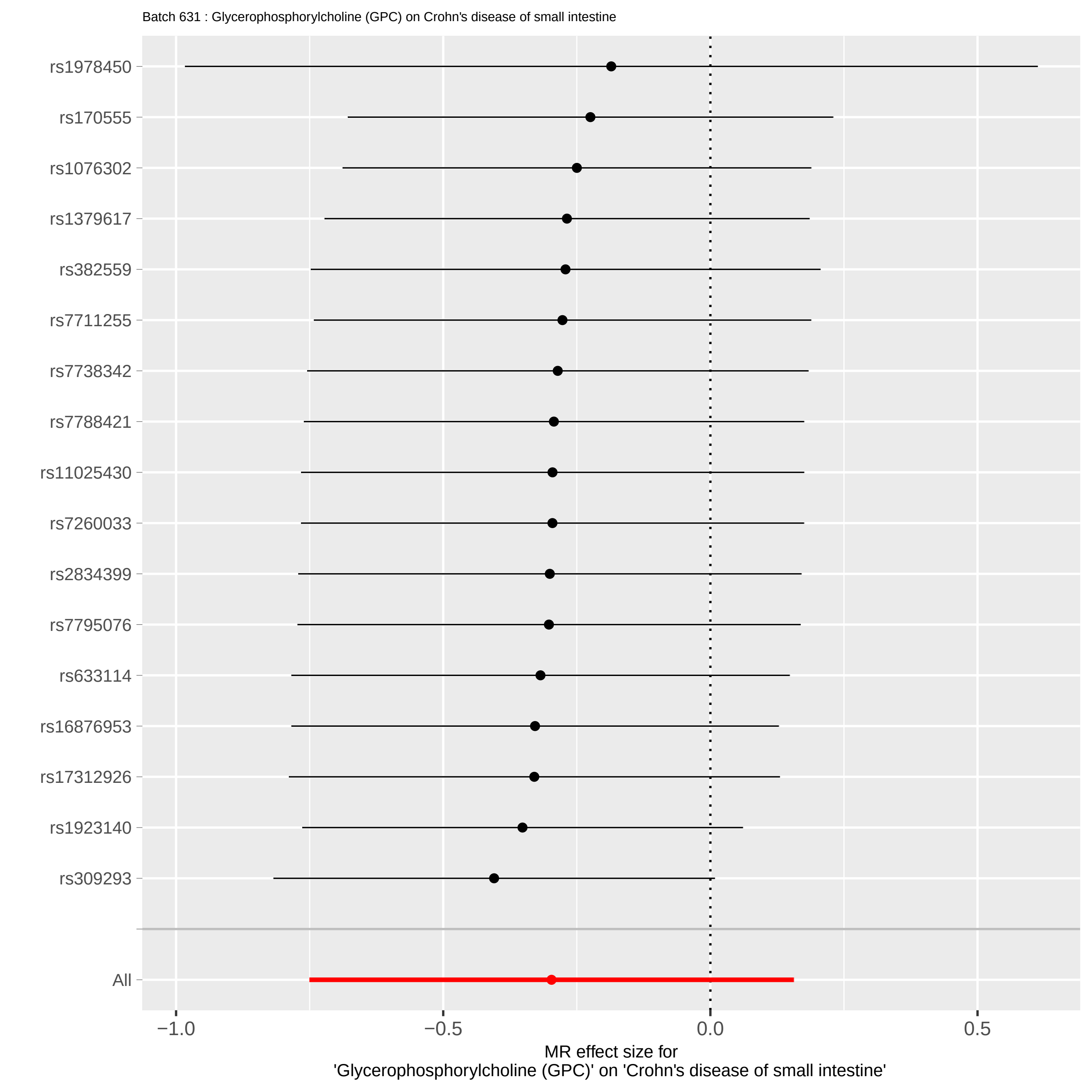

Batch 631 : Glycerophosphorylcholine (GPC) on Crohn's disease of small intestine
rs1978450
rs170555
rs1076302
rs1379617
rs382559
rs7711255
rs7738342
rs7788421
rs11025430
rs7260033
rs2834399
rs7795076
rs633114
rs16876953
rs17312926
rs1923140
rs309293
All
−1.0
−0.5
0.0
0.5
MR effect size for
'Glycerophosphorylcholine (GPC)' on 'Crohn's disease of small intestine'

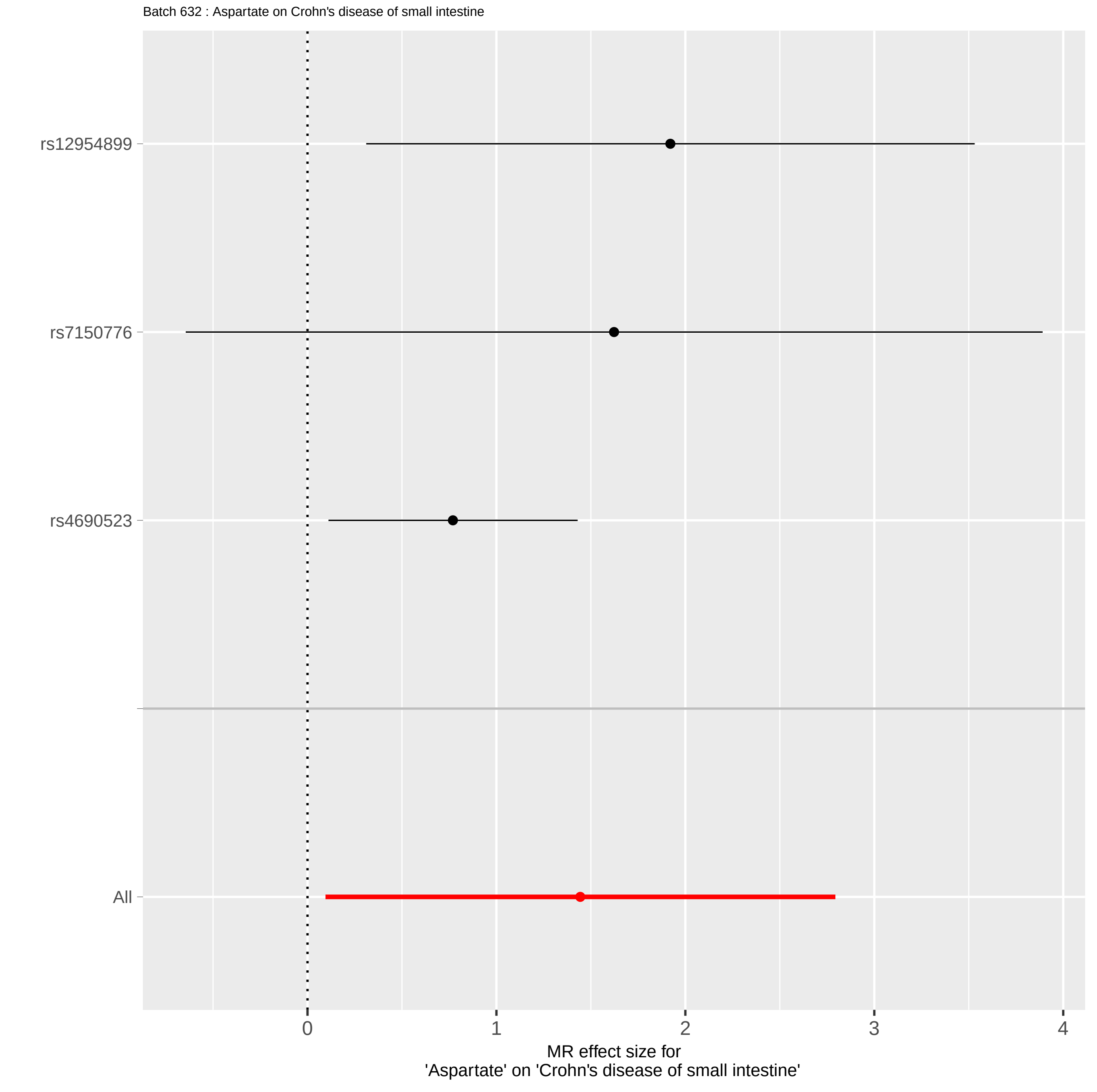

Batch 632 : Aspartate on Crohn's disease of small intestine
rs12954899
rs7150776
rs4690523
All
0
1
2
3
4
MR effect size for
'Aspartate' on 'Crohn's disease of small intestine'

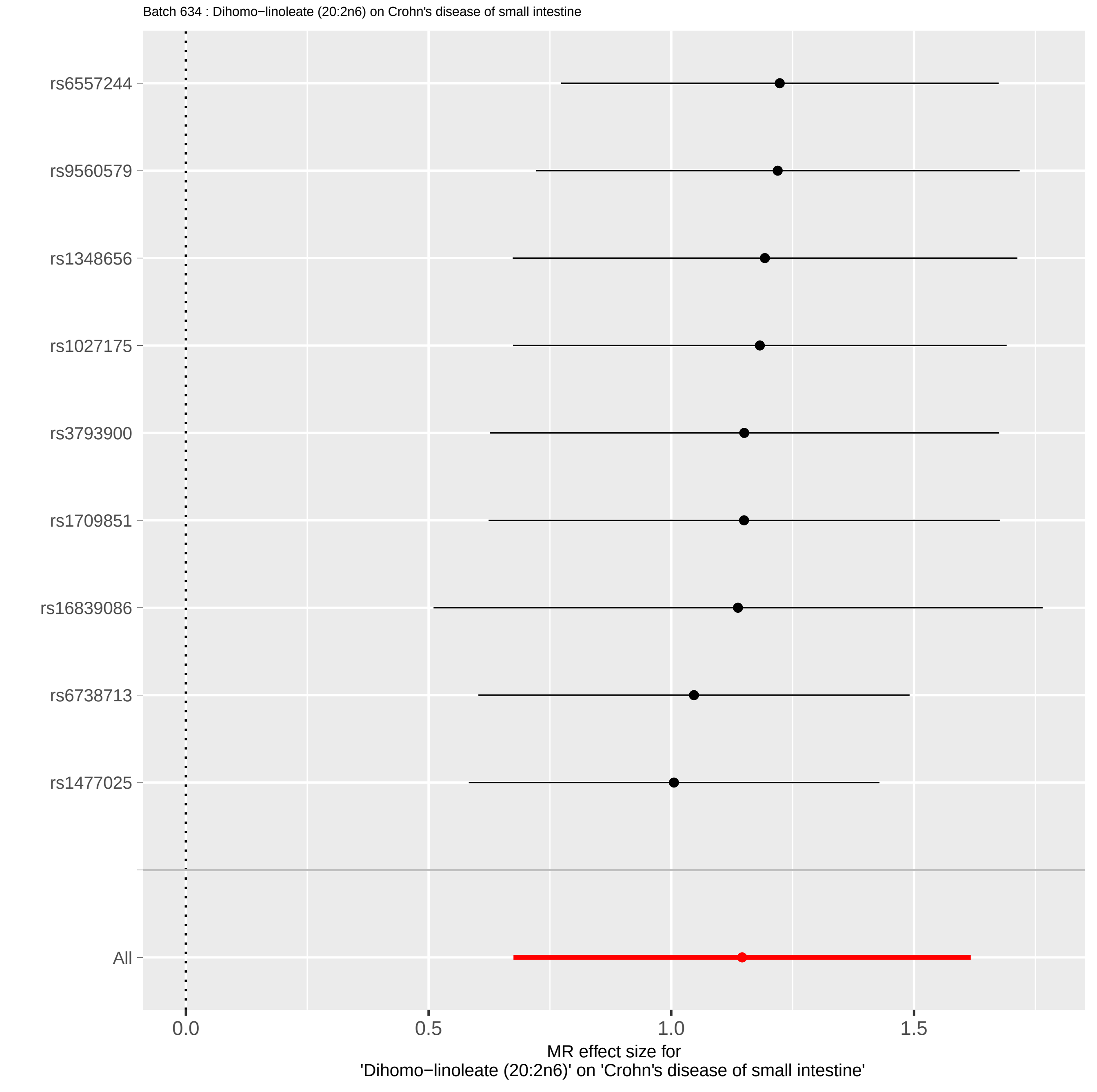

Batch 634 : Dihomo−linoleate (20:2n6) on Crohn's disease of small intestine
rs6557244
rs9560579
rs1348656
rs1027175
rs3793900
rs1709851
rs16839086
rs6738713
rs1477025
All
0.0
0.5
1.0
1.5
MR effect size for
'Dihomo−linoleate (20:2n6)' on 'Crohn's disease of small intestine'

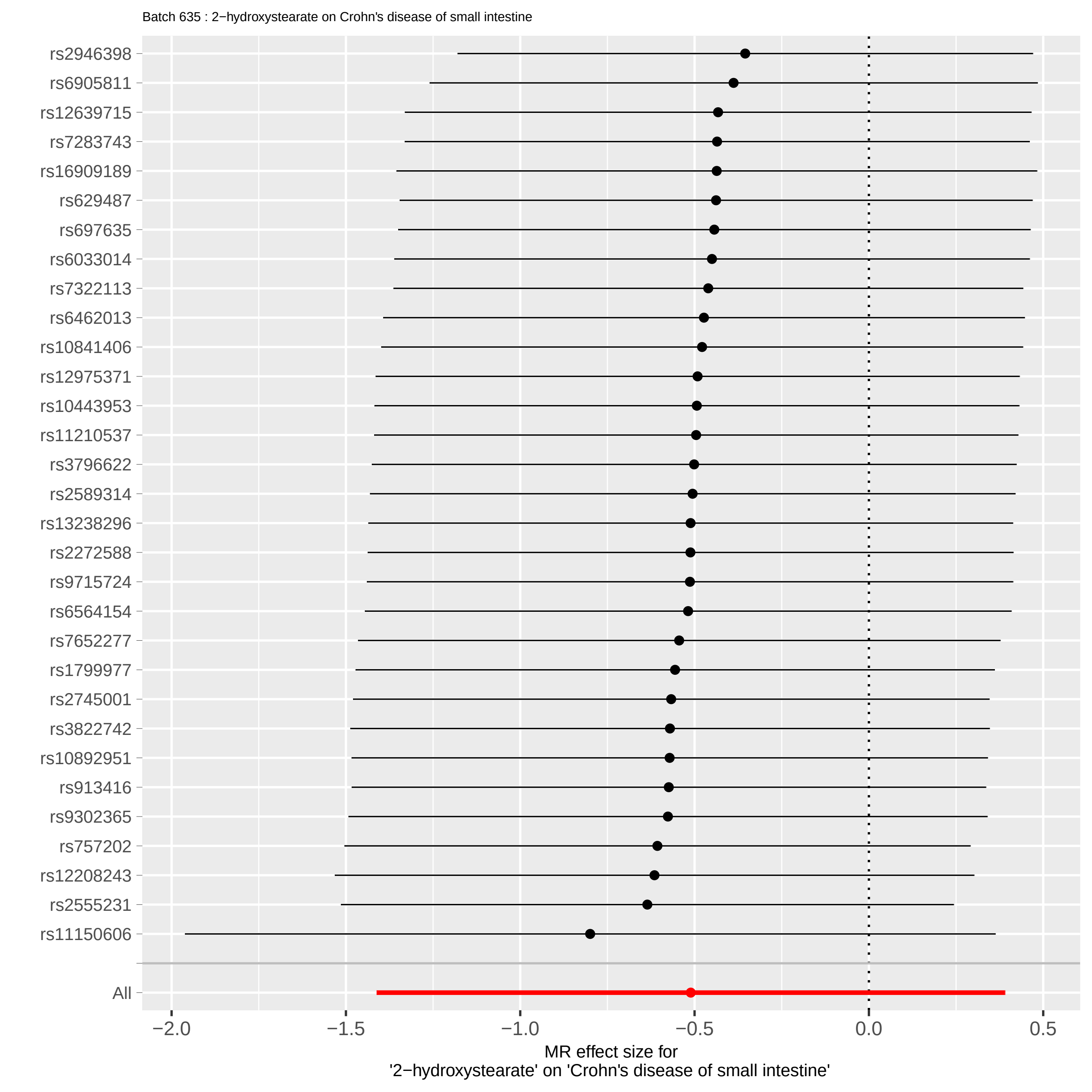
Batch 635 : 2−hydroxystearate on Crohn's disease of small intestine
rs2946398
rs6905811
rs12639715
rs7283743
rs16909189
rs629487
rs697635
rs6033014
rs7322113
rs6462013
rs10841406
rs12975371
rs10443953
rs11210537
rs3796622
rs2589314
rs13238296
rs2272588
rs9715724
rs6564154
rs7652277
rs1799977
rs2745001
rs3822742
rs10892951
rs913416
rs9302365
rs757202
rs12208243
rs2555231
rs11150606
All
−2.0
−1.5
−1.0
−0.5
0.0
0.5
MR effect size for
'2−hydroxystearate' on 'Crohn's disease of small intestine'

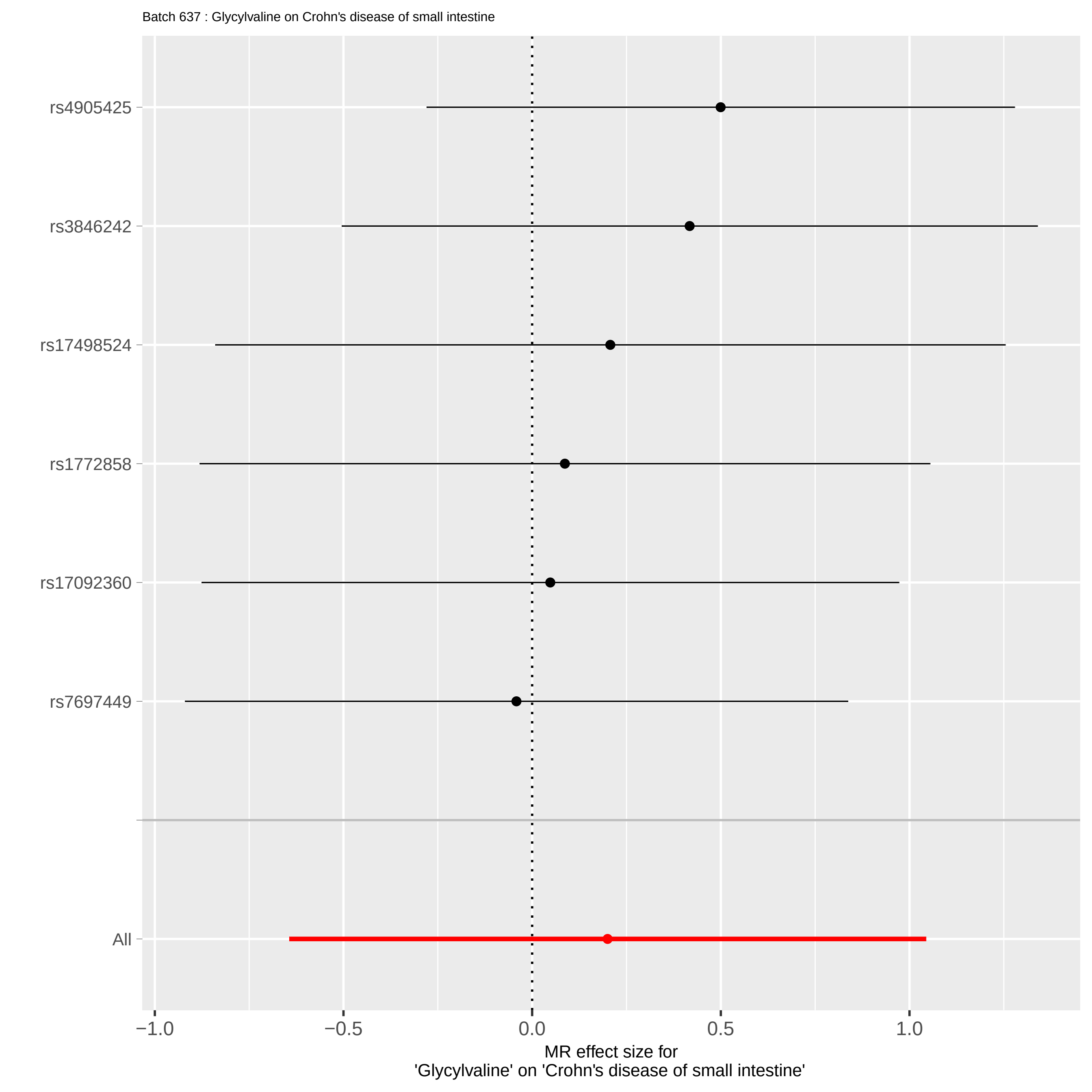
Batch 637 : Glycylvaline on Crohn's disease of small intestine
rs4905425
rs3846242
rs17498524
rs1772858
rs17092360
rs7697449
All
−1.0
−0.5
0.0
0.5
1.0
MR effect size for
'Glycylvaline' on 'Crohn's disease of small intestine'

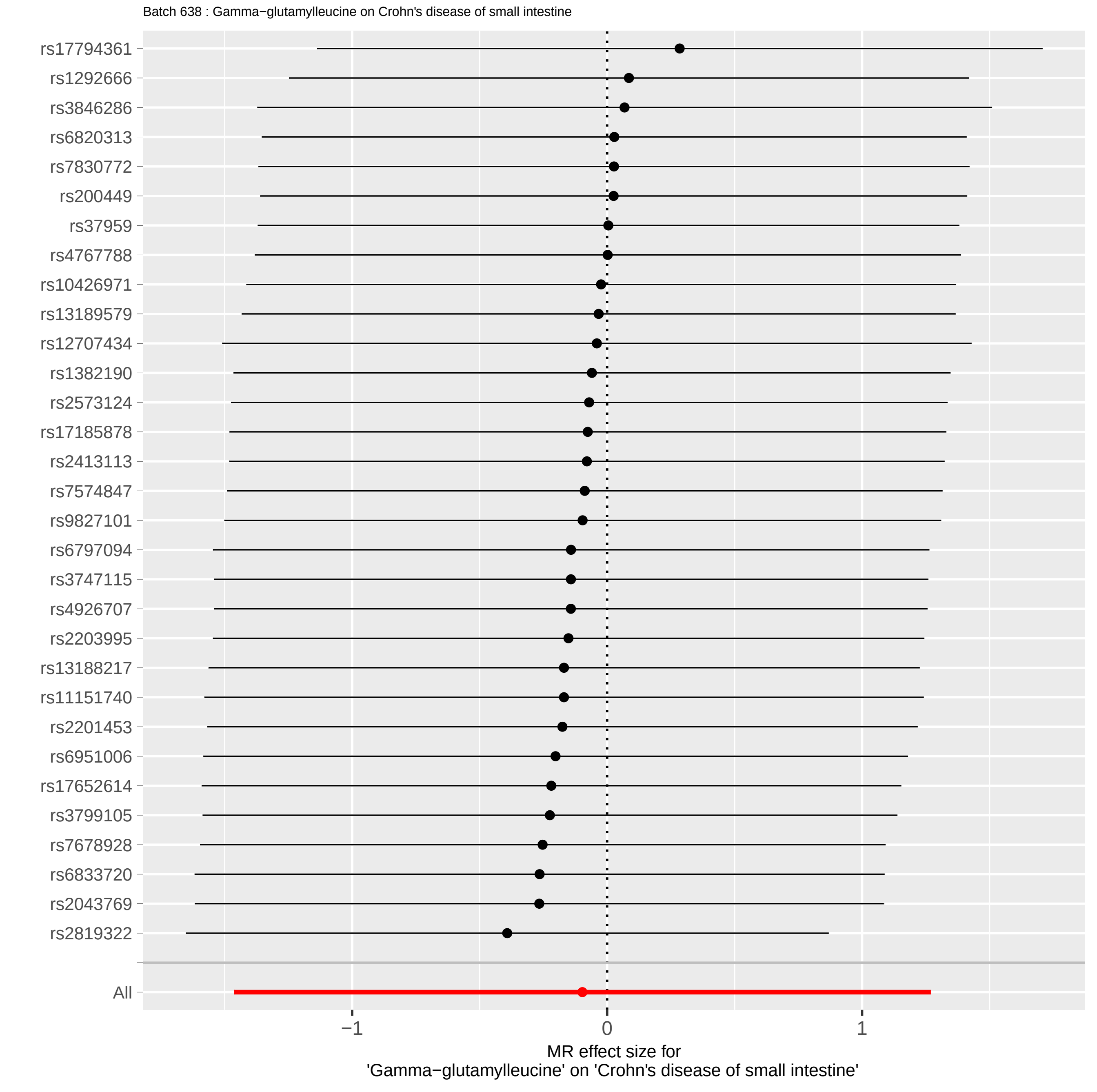

Batch 638 : Gamma−glutamylleucine on Crohn's disease of small intestine
rs17794361
rs1292666
rs3846286
rs6820313
rs7830772
rs200449
rs37959
rs4767788
rs10426971
rs13189579
rs12707434
rs1382190
rs2573124
rs17185878
rs2413113
rs7574847
rs9827101
rs6797094
rs3747115
rs4926707
rs2203995
rs13188217
rs11151740
rs2201453
rs6951006
rs17652614
rs3799105
rs7678928
rs6833720
rs2043769
rs2819322
All
−1
0
1
MR effect size for
'Gamma−glutamylleucine' on 'Crohn's disease of small intestine'

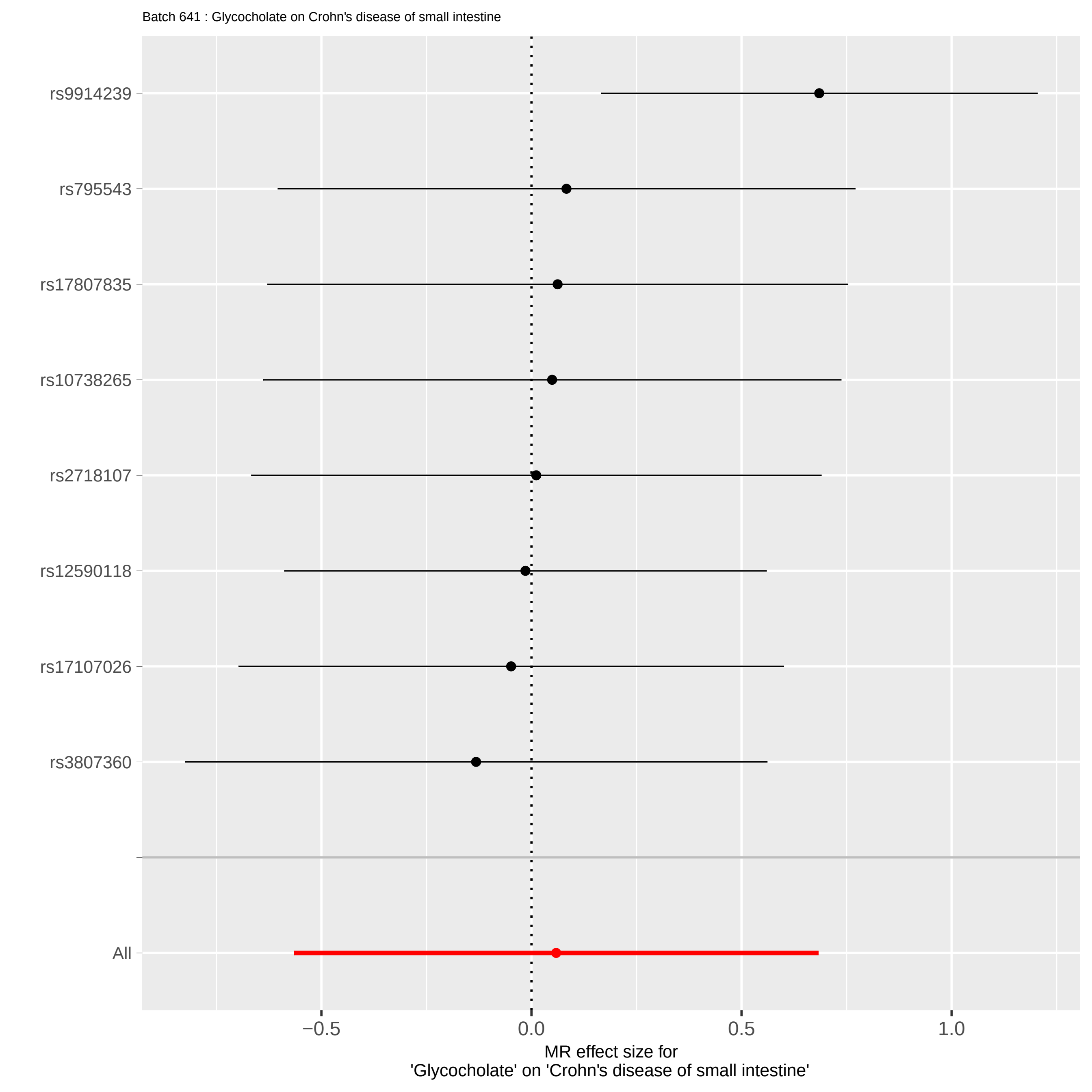

Batch 641 : Glycocholate on Crohn's disease of small intestine
rs9914239
rs795543
rs17807835
rs10738265
rs2718107
rs12590118
rs17107026
rs3807360
All
−0.5
0.0
0.5
1.0
MR effect size for
'Glycocholate' on 'Crohn's disease of small intestine'

Batch 642 : Glycodeoxycholate on Crohn's disease of small intestine

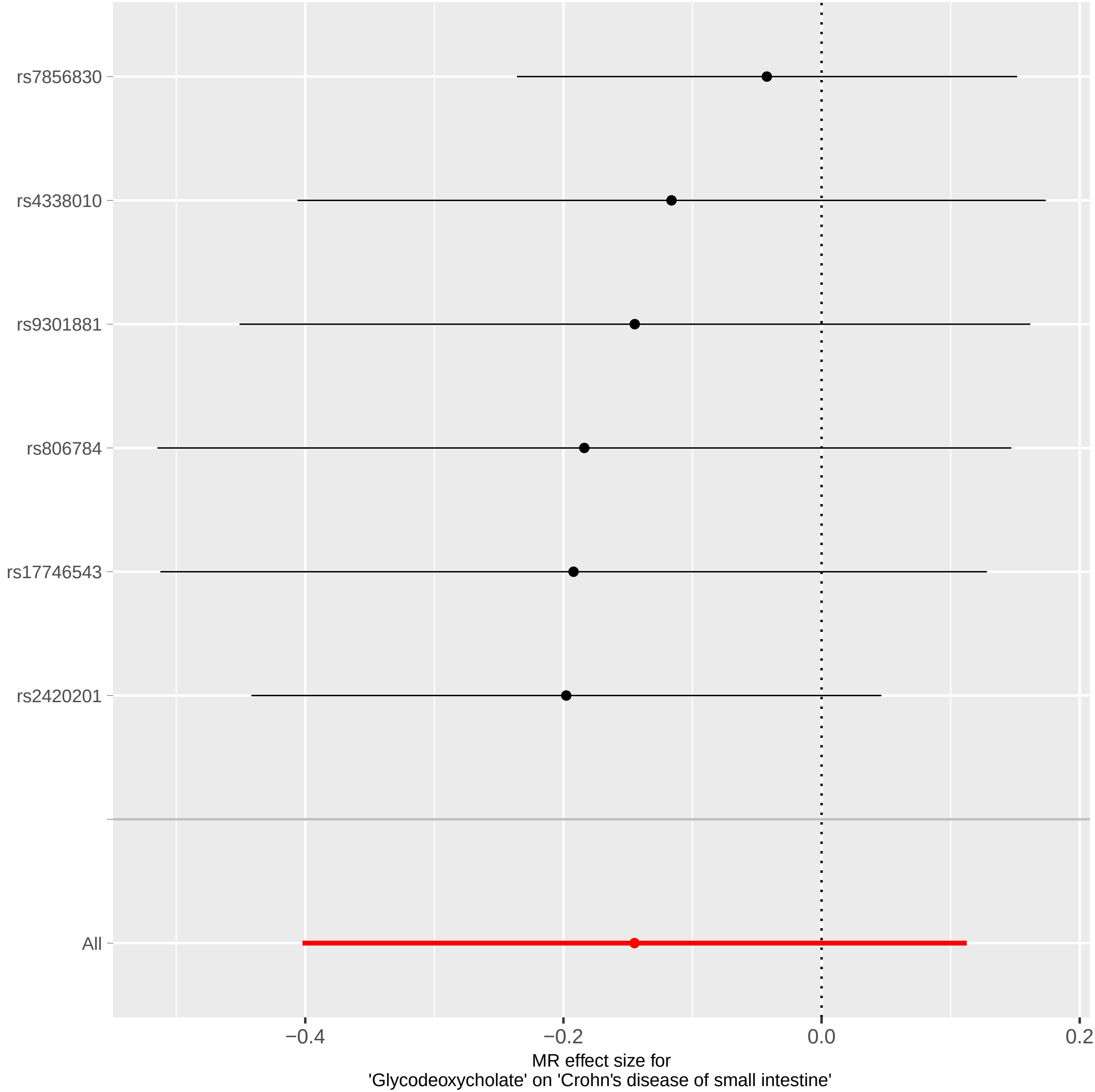

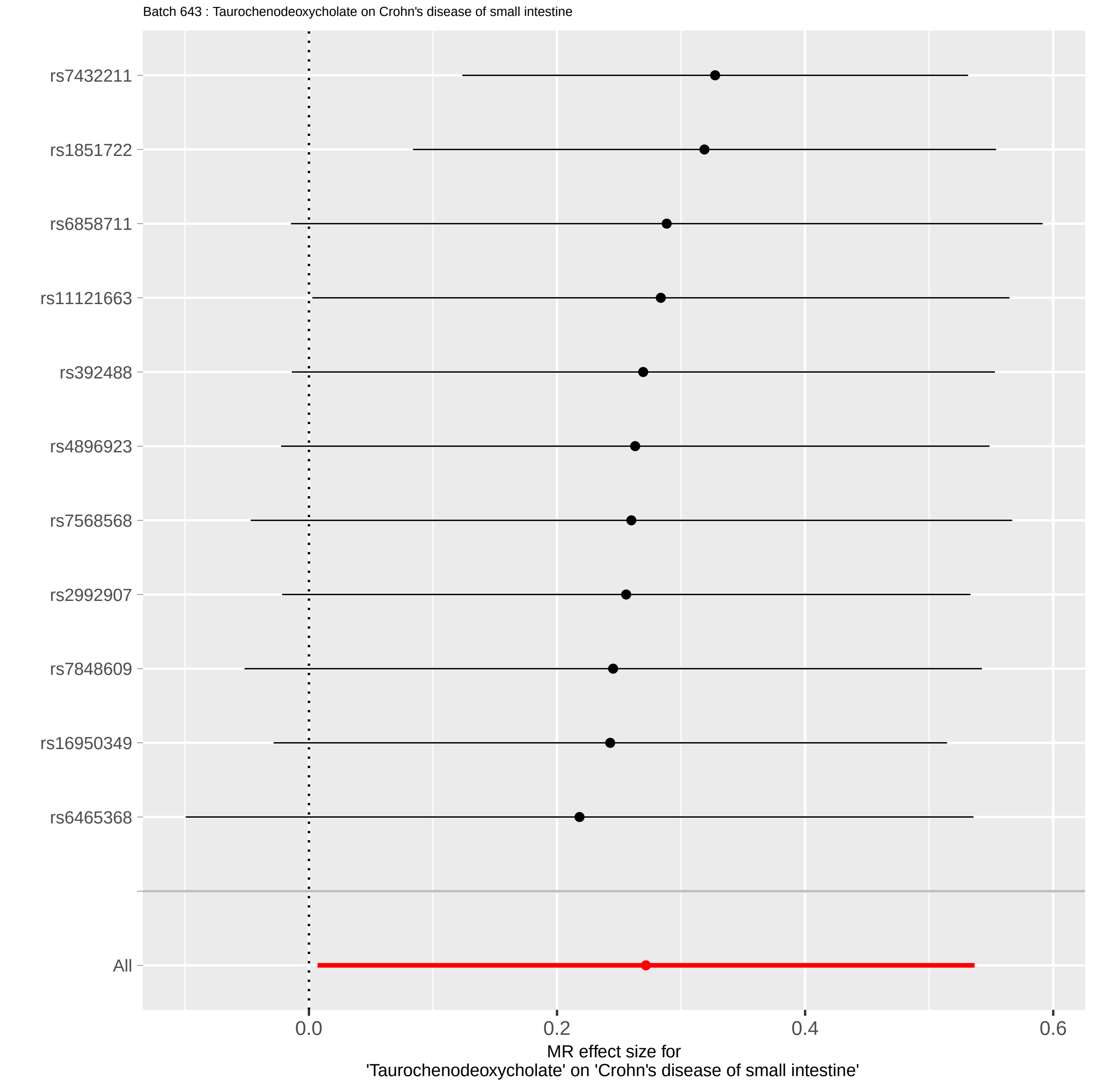
Batch 643 : Taurochenodeoxycholate on Crohn's disease of small intestine
rs7432211
rs1851722
rs6858711
rs11121663
rs392488
rs4896923
rs7568568
rs2992907
rs7848609
rs16950349
rs6465368
All
0.0
0.2
0.4
0.6
MR effect size for
'Taurochenodeoxycholate' on 'Crohn's disease of small intestine'

Batch 644 : Taurocholate on Crohn's disease of small intestine

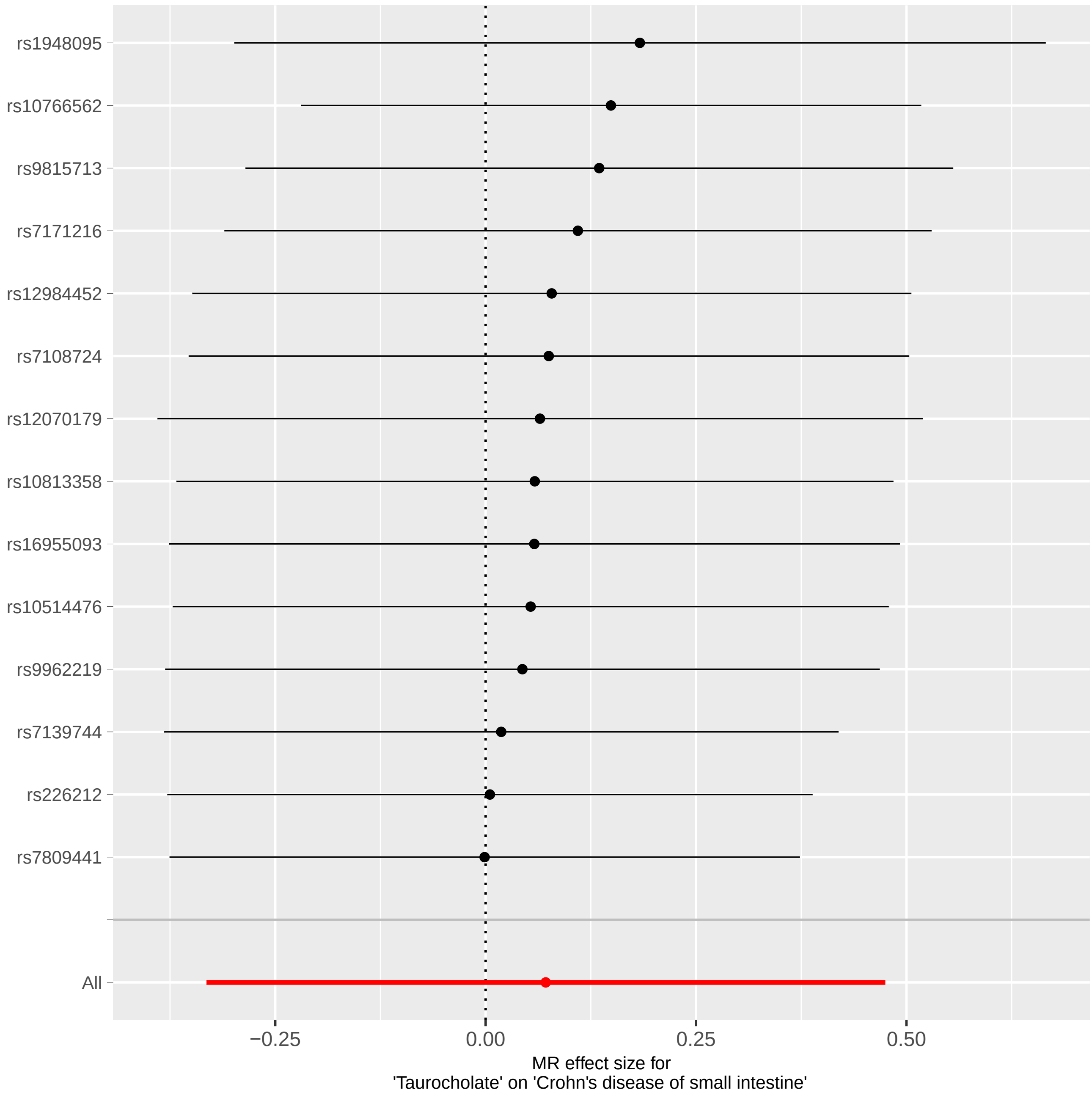

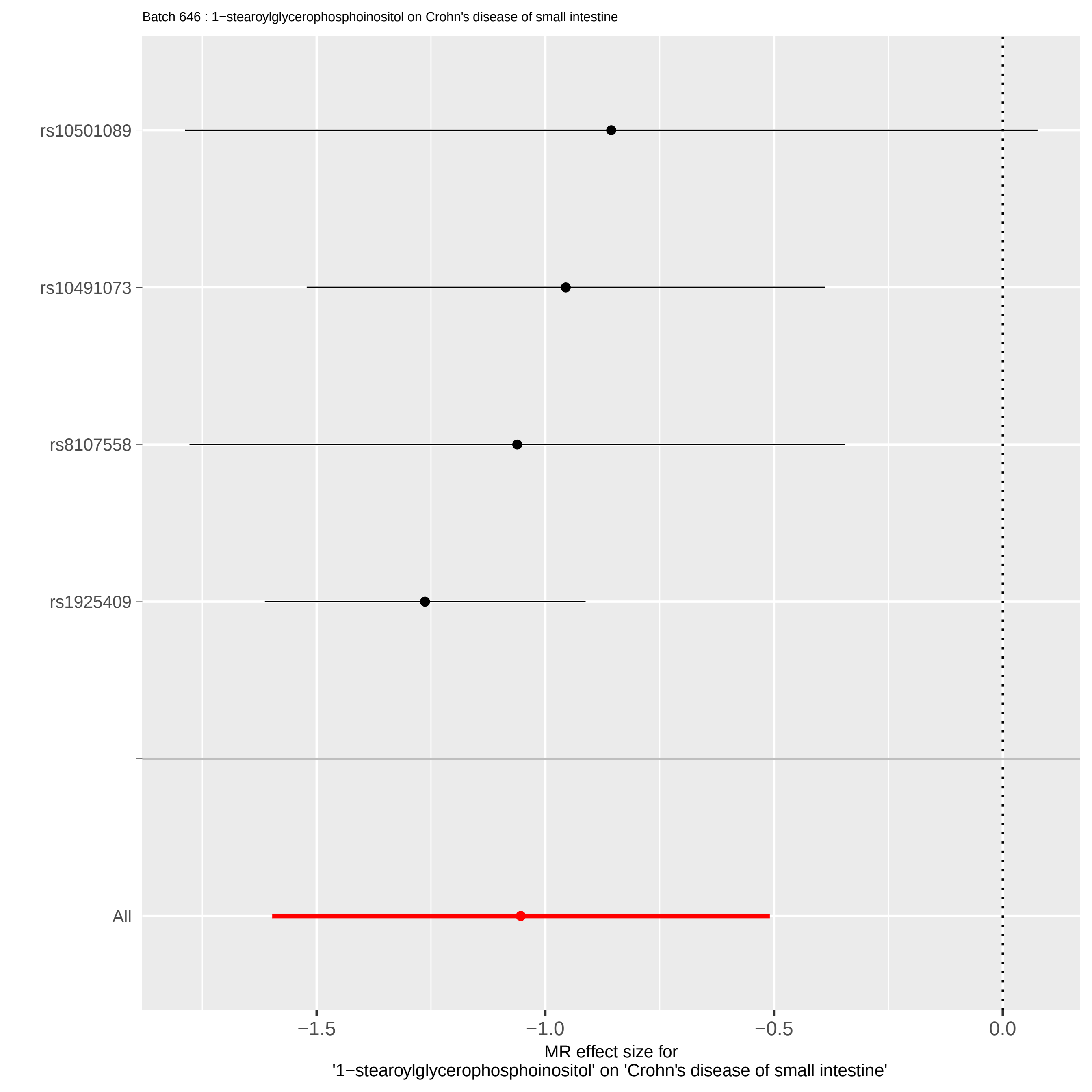

Batch 646 : 1−stearoylglycerophosphoinositol on Crohn's disease of small intestine
rs10501089
rs10491073
rs8107558
rs1925409
All
−1.5
−1.0
−0.5
0.0
MR effect size for
'1−stearoylglycerophosphoinositol' on 'Crohn's disease of small intestine'

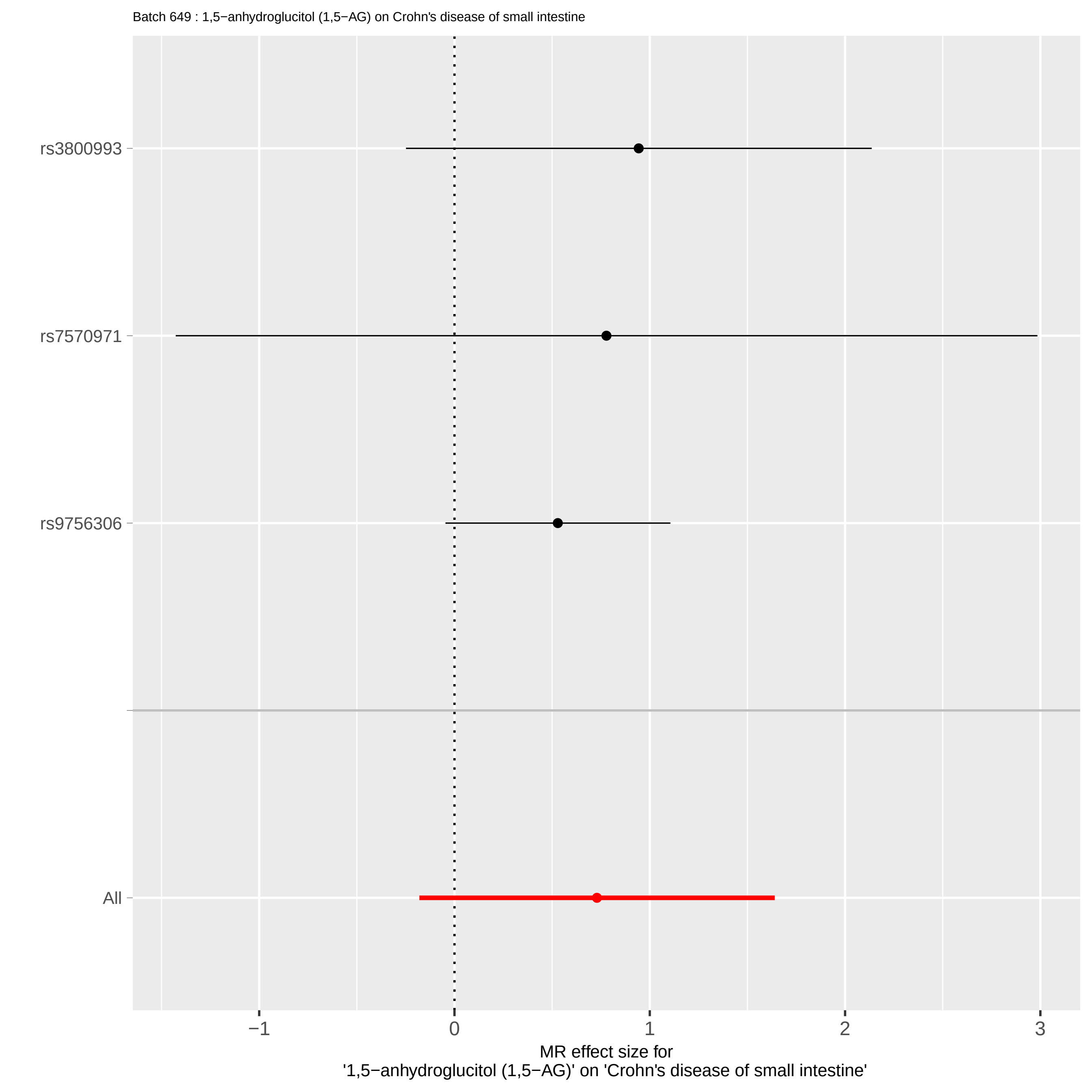
Batch 649 : 1,5−anhydroglucitol (1,5−AG) on Crohn's disease of small intestine
rs3800993
rs7570971
rs9756306
All
−1
0
1
2
3
MR effect size for
'1,5−anhydroglucitol (1,5−AG)' on 'Crohn's disease of small intestine'

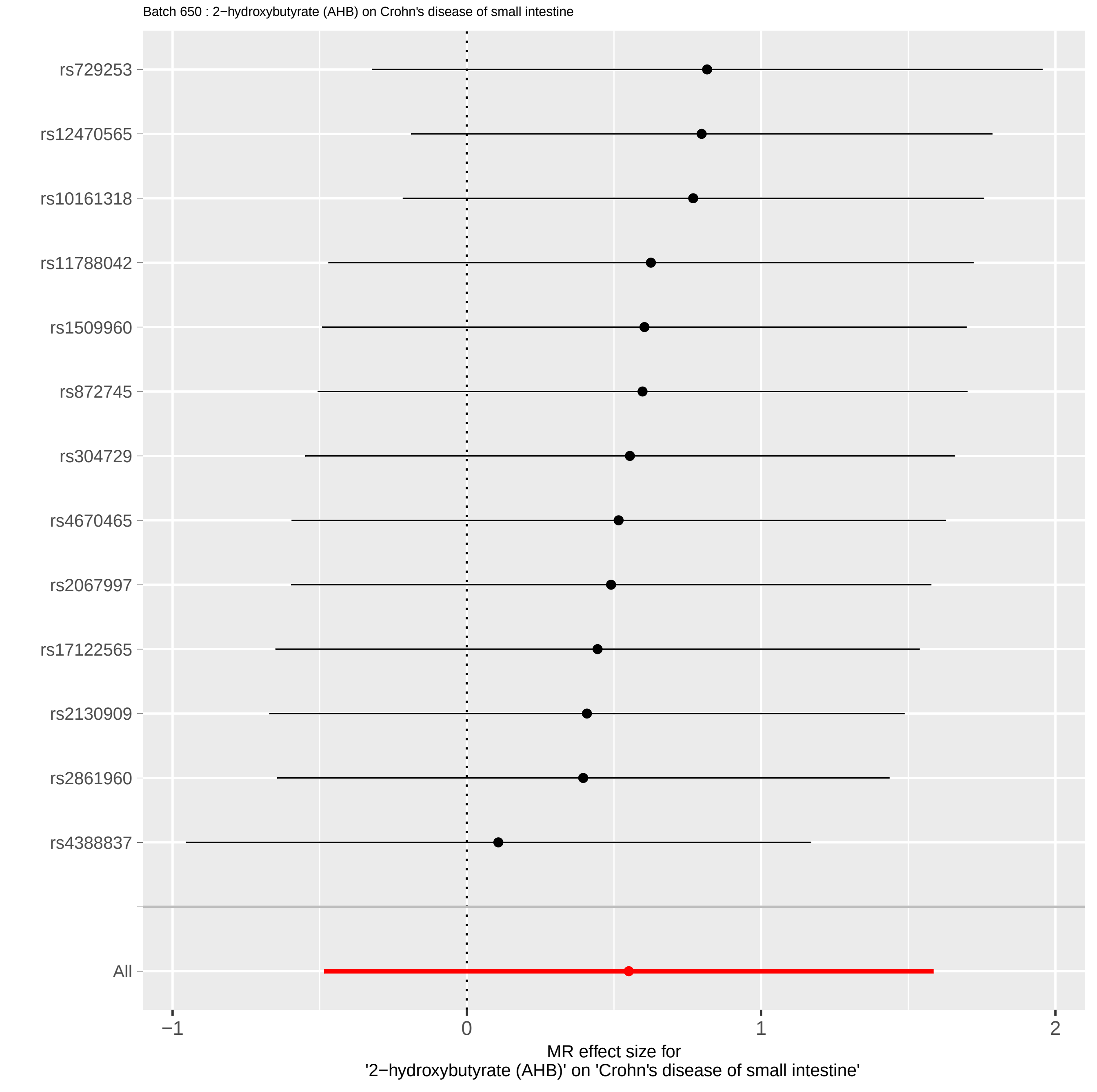
Batch 650 : 2−hydroxybutyrate (AHB) on Crohn's disease of small intestine
rs729253
rs12470565
rs10161318
rs11788042
rs1509960
rs872745
rs304729
rs4670465
rs2067997
rs17122565
rs2130909
rs2861960
rs4388837
All
−1
0
1
2
MR effect size for
'2−hydroxybutyrate (AHB)' on 'Crohn's disease of small intestine'

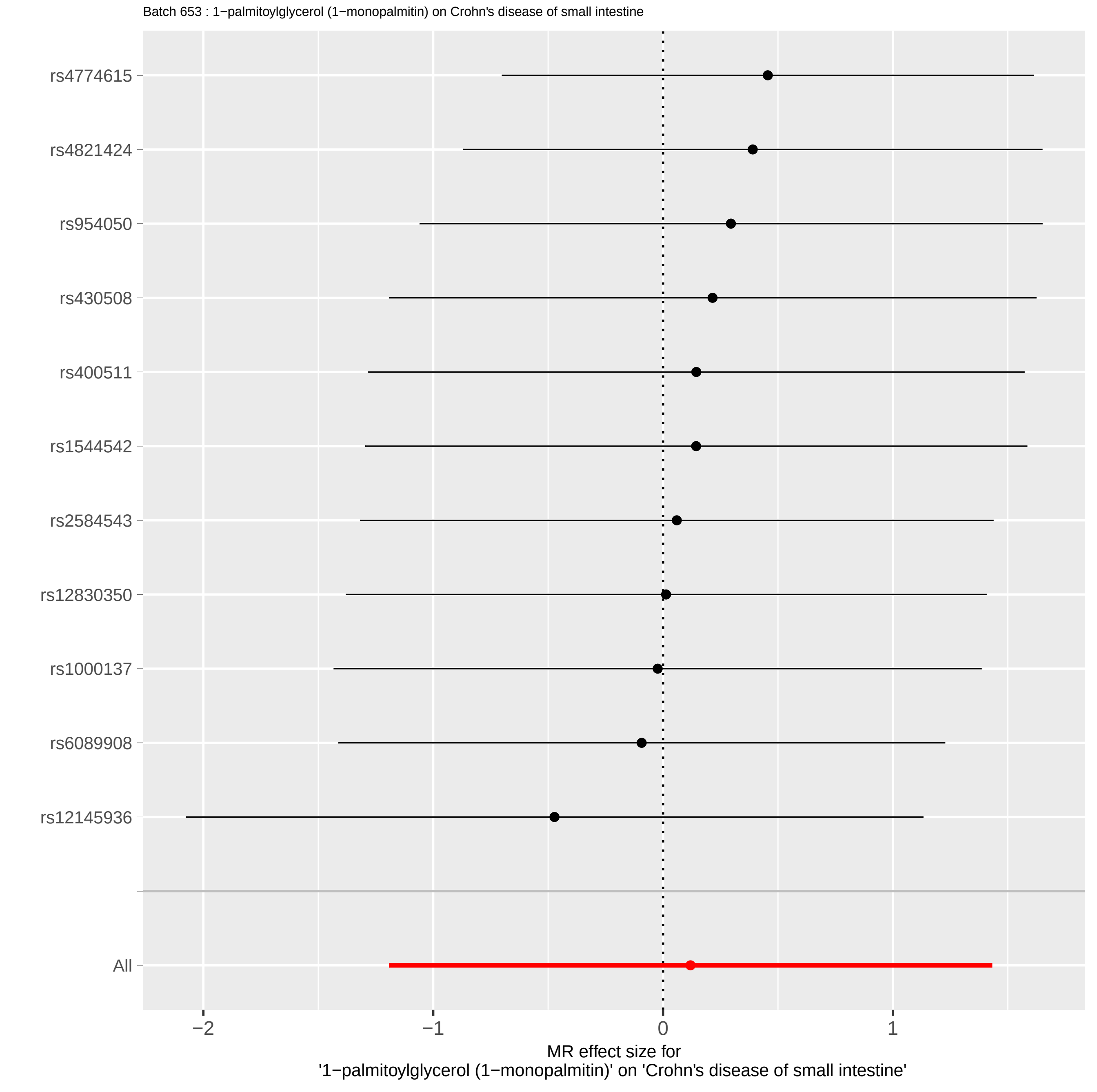

Batch 653 : 1−palmitoylglycerol (1−monopalmitin) on Crohn's disease of small intestine
rs4774615
rs4821424
rs954050
rs430508
rs400511
rs1544542
rs2584543
rs12830350
rs1000137
rs6089908
rs12145936
All
−2
−1
0
1
MR effect size for
'1−palmitoylglycerol (1−monopalmitin)' on 'Crohn's disease of small intestine'

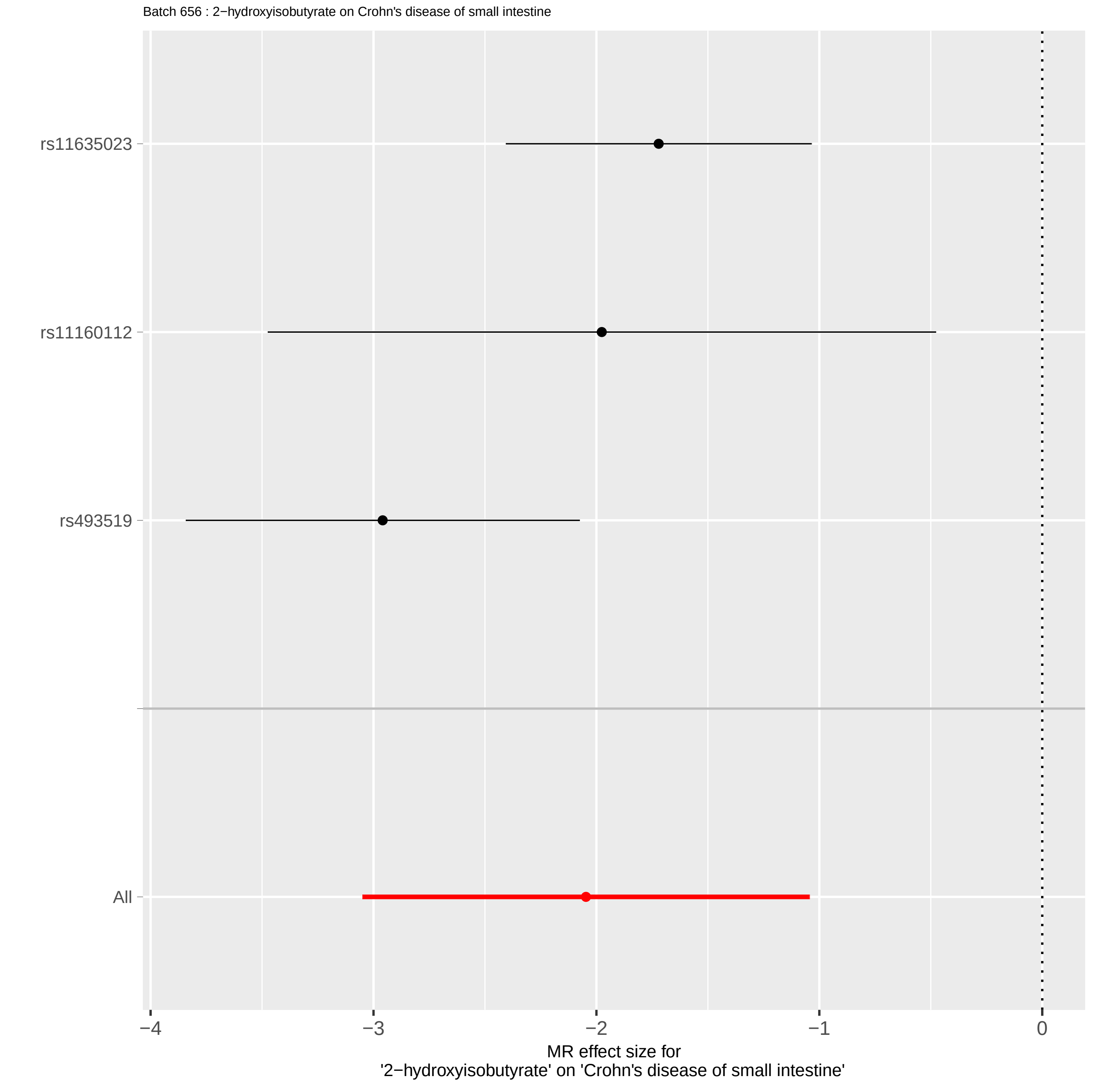

Batch 656 : 2−hydroxyisobutyrate on Crohn's disease of small intestine
rs11635023
rs11160112
rs493519
All
−4
−3
−2
−1
0
MR effect size for
'2−hydroxyisobutyrate' on 'Crohn's disease of small intestine'

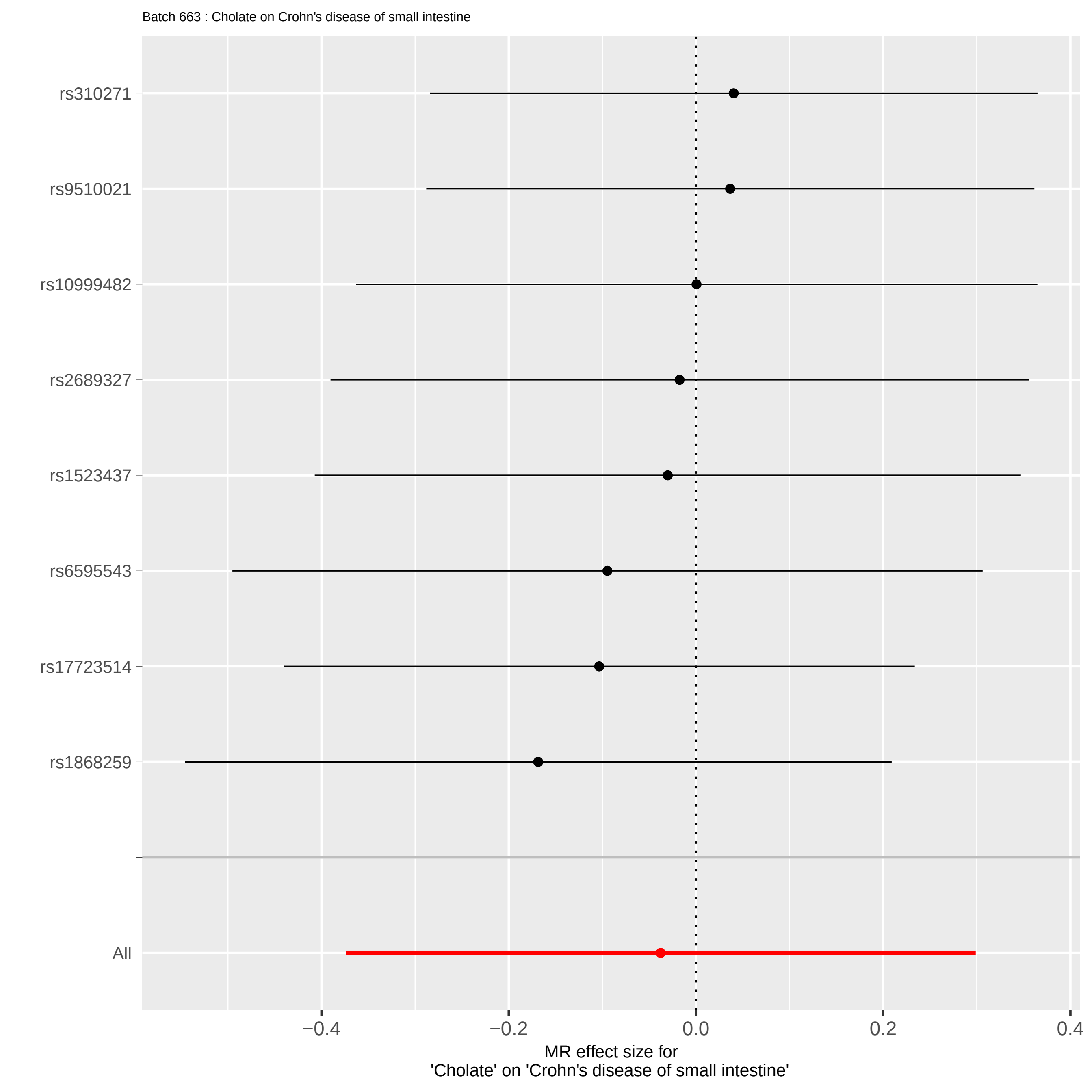

Batch 663 : Cholate on Crohn's disease of small intestine
rs310271
rs9510021
rs10999482
rs2689327
rs1523437
rs6595543
rs17723514
rs1868259
All
−0.4
−0.2
0.0
0.2
0.4
MR effect size for
'Cholate' on 'Crohn's disease of small intestine'

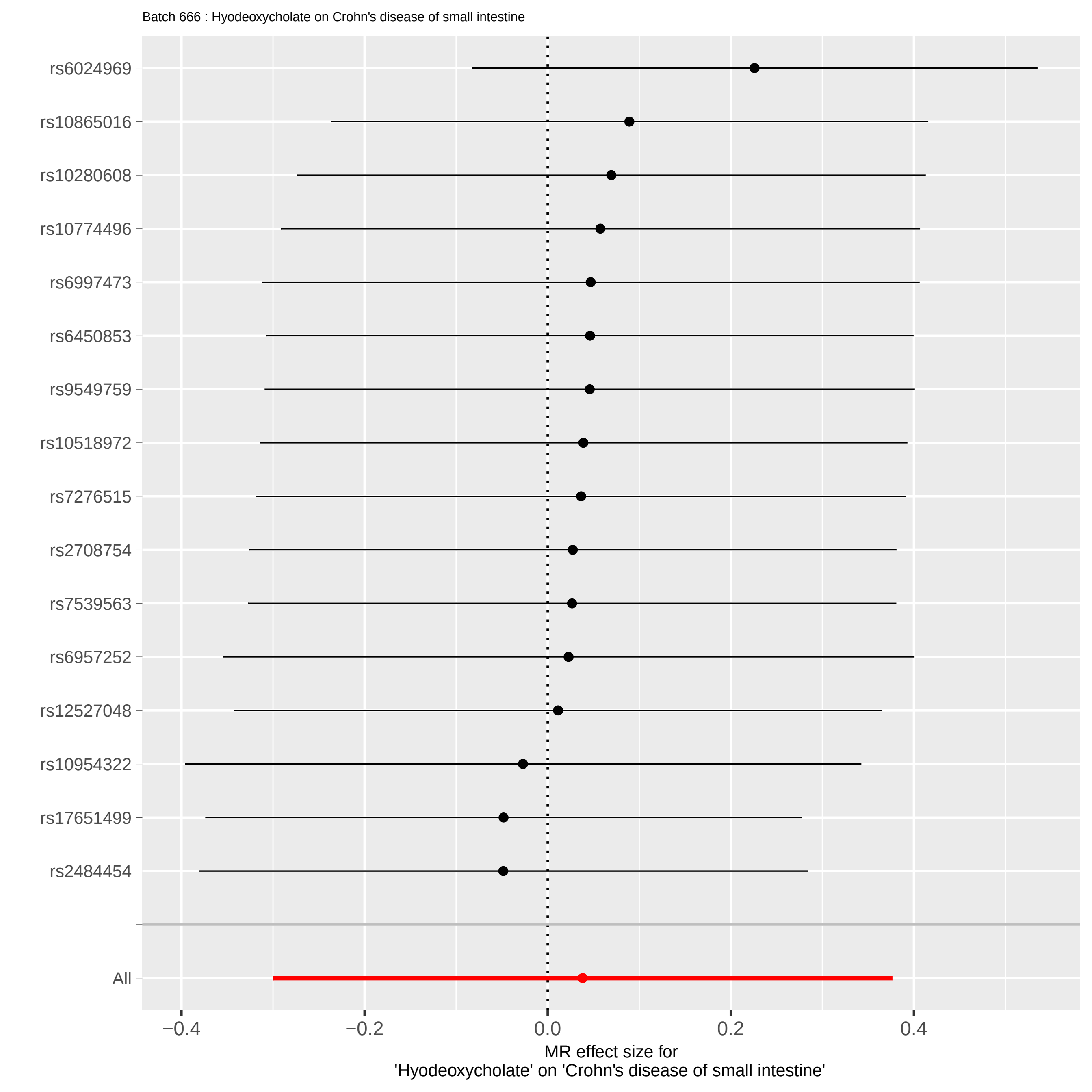

Batch 666 : Hyodeoxycholate on Crohn's disease of small intestine
rs6024969
rs10865016
rs10280608
rs10774496
rs6997473
rs6450853
rs9549759
rs10518972
rs7276515
rs2708754
rs7539563
rs6957252
rs12527048
rs10954322
rs17651499
rs2484454
All
−0.4
−0.2
0.0
0.2
0.4
MR effect size for
'Hyodeoxycholate' on 'Crohn's disease of small intestine'

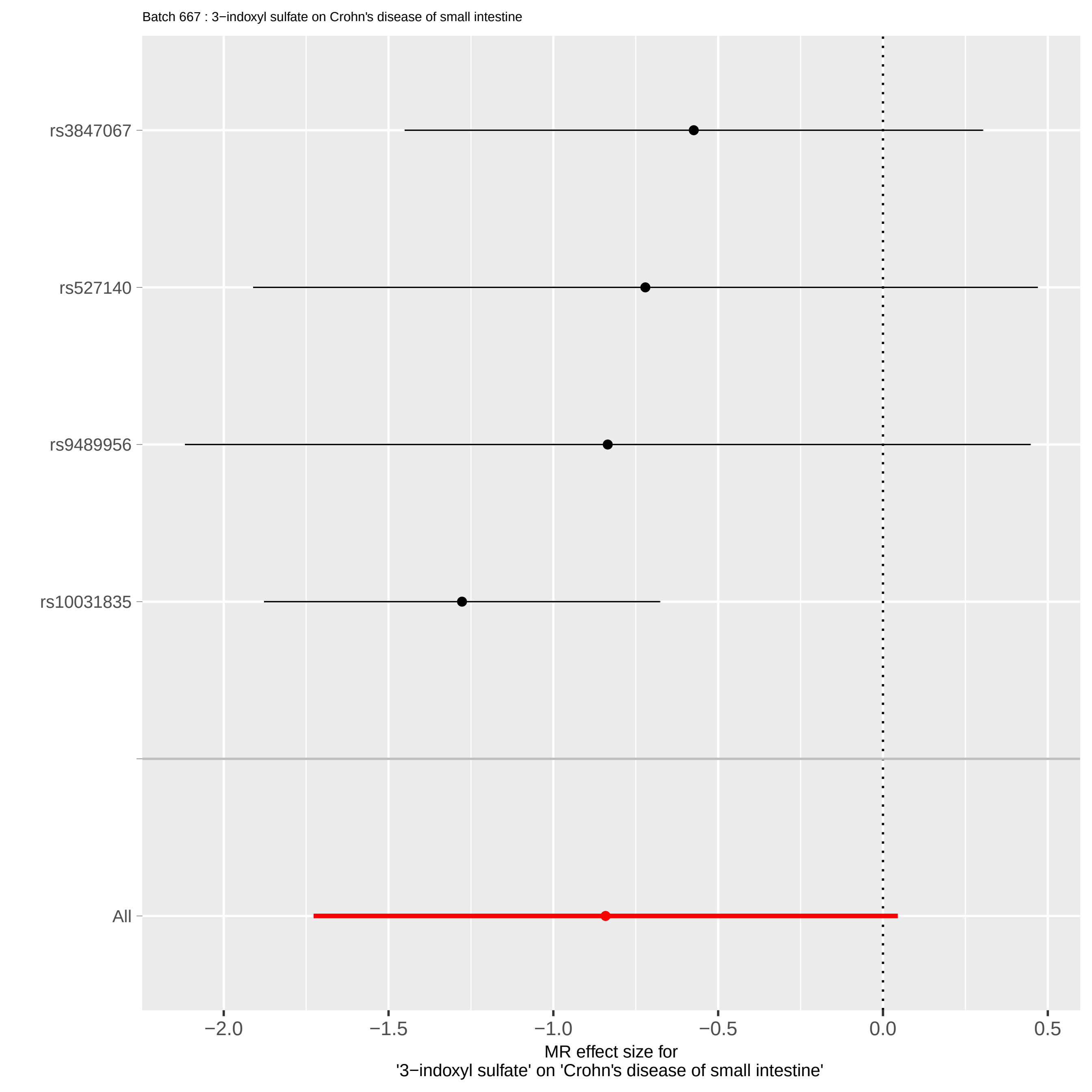

Batch 667 : 3−indoxyl sulfate on Crohn's disease of small intestine
rs3847067
rs527140
rs9489956
rs10031835
All
−2.0
−1.5
−1.0
−0.5
0.0
0.5
MR effect size for
'3−indoxyl sulfate' on 'Crohn's disease of small intestine'

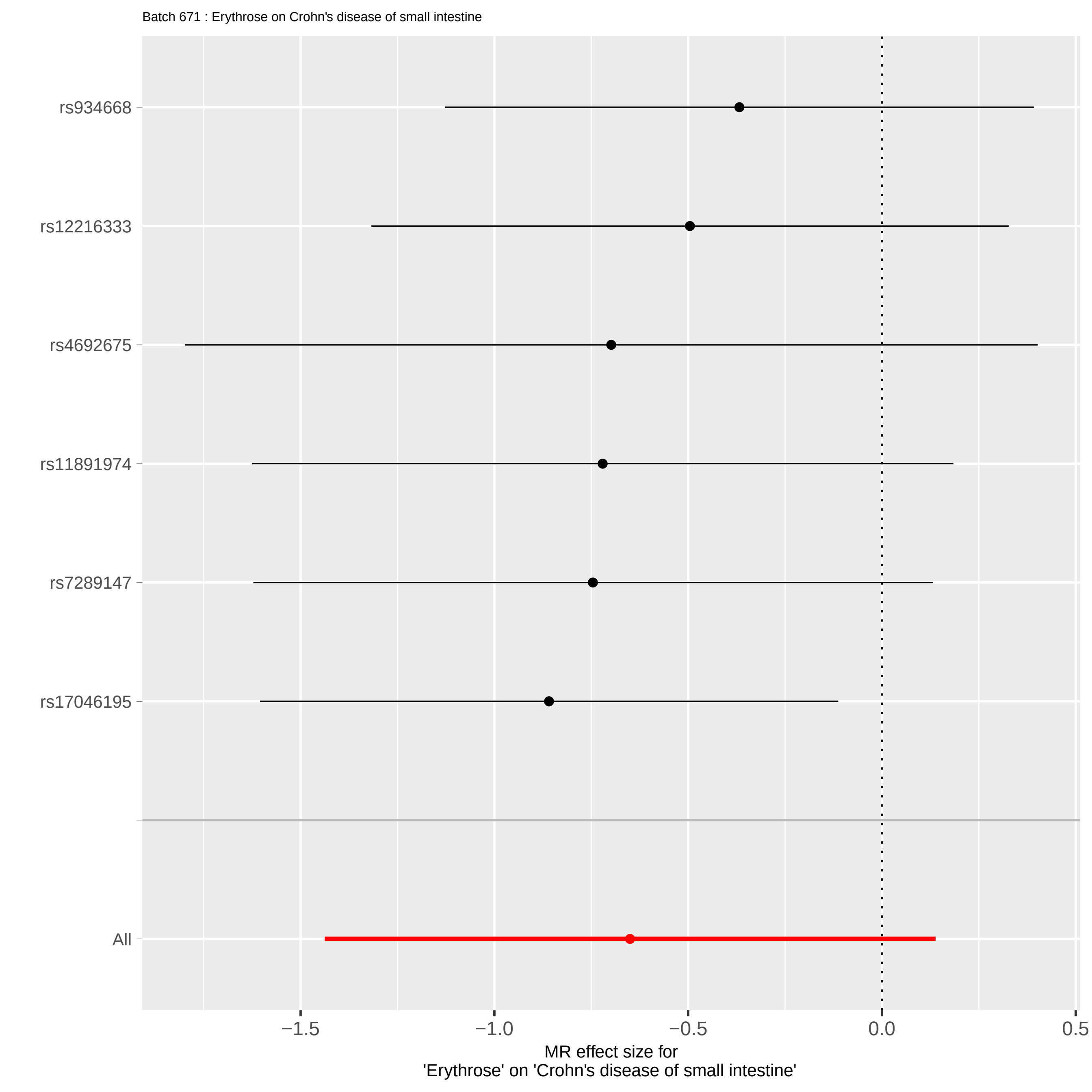

Batch 671 : Erythrose on Crohn's disease of small intestine
rs934668
rs12216333
rs4692675
rs11891974
rs7289147
rs17046195
All
−1.5
−1.0
−0.5
0.0
0.5
MR effect size for
'Erythrose' on 'Crohn's disease of small intestine'

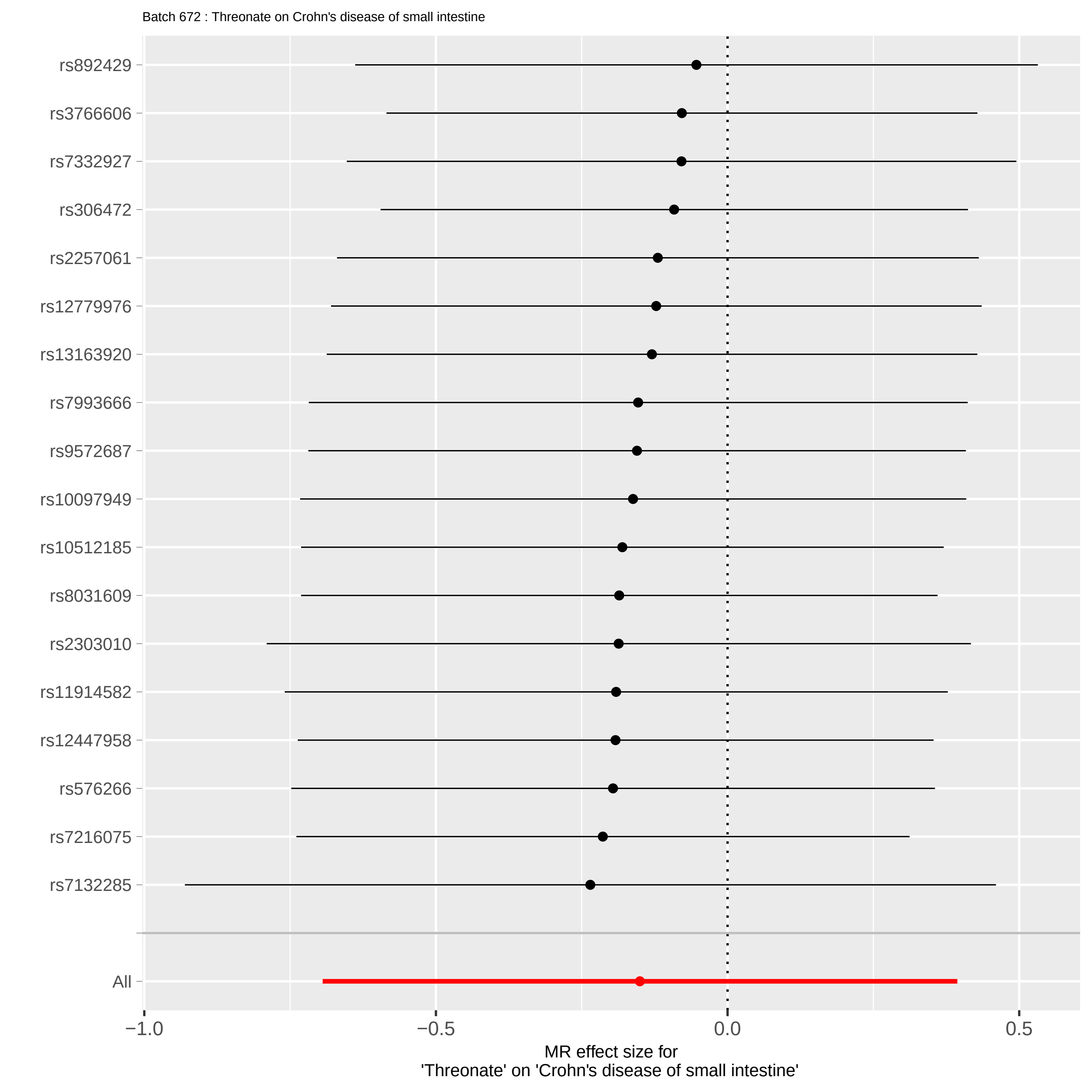

Batch 672 : Threonate on Crohn's disease of small intestine
rs892429
rs3766606
rs7332927
rs306472
rs2257061
rs12779976
rs13163920
rs7993666
rs9572687
rs10097949
rs10512185
rs8031609
rs2303010
rs11914582
rs12447958
rs576266
rs7216075
rs7132285
All
−1.0
−0.5
0.0
0.5
MR effect size for
'Threonate' on 'Crohn's disease of small intestine'

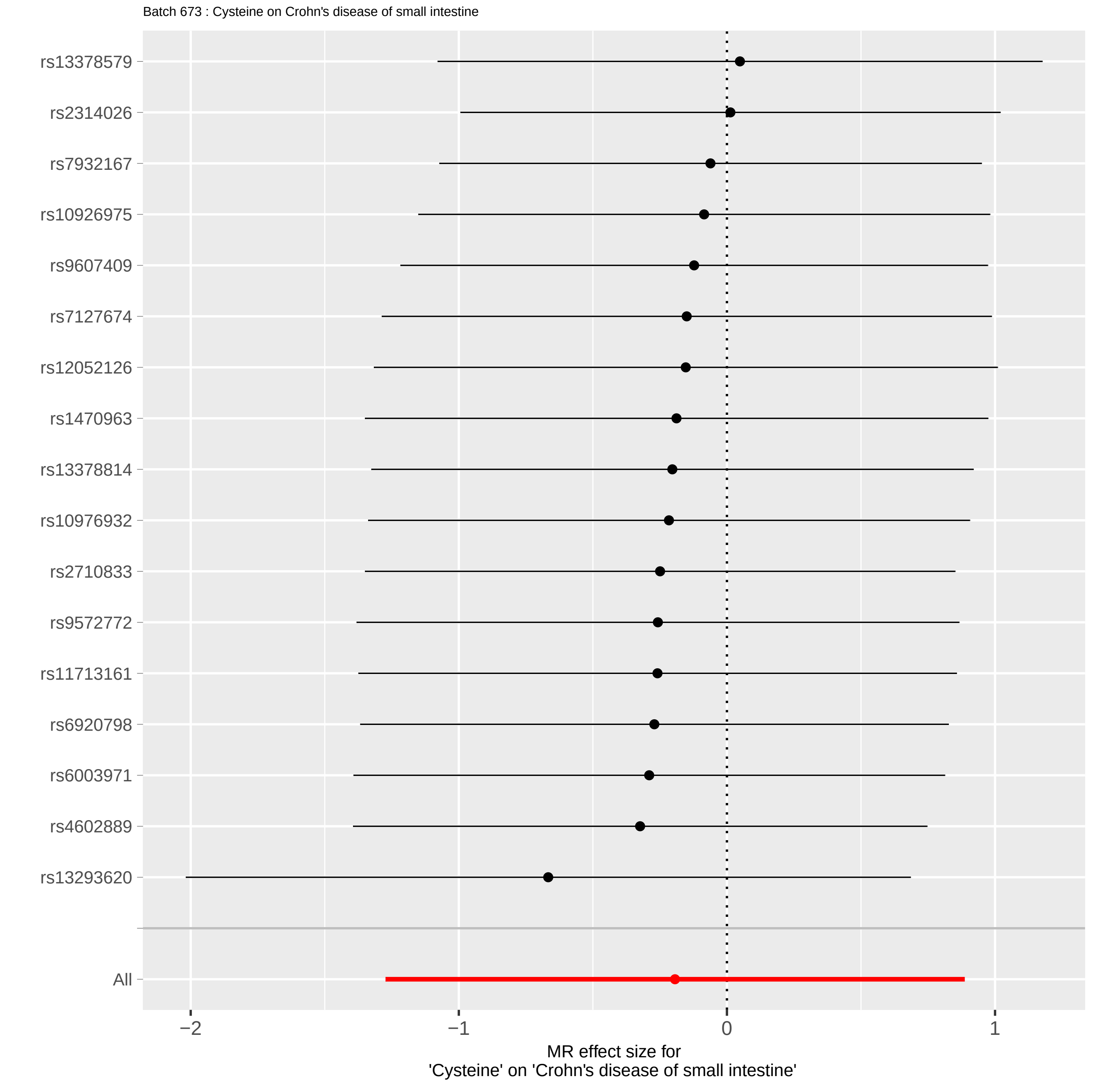

Batch 673 : Cysteine on Crohn's disease of small intestine
rs13378579
rs2314026
rs7932167
rs10926975
rs9607409
rs7127674
rs12052126
rs1470963
rs13378814
rs10976932
rs2710833
rs9572772
rs11713161
rs6920798
rs6003971
rs4602889
rs13293620
All
−2
−1
0
1
MR effect size for
'Cysteine' on 'Crohn's disease of small intestine'

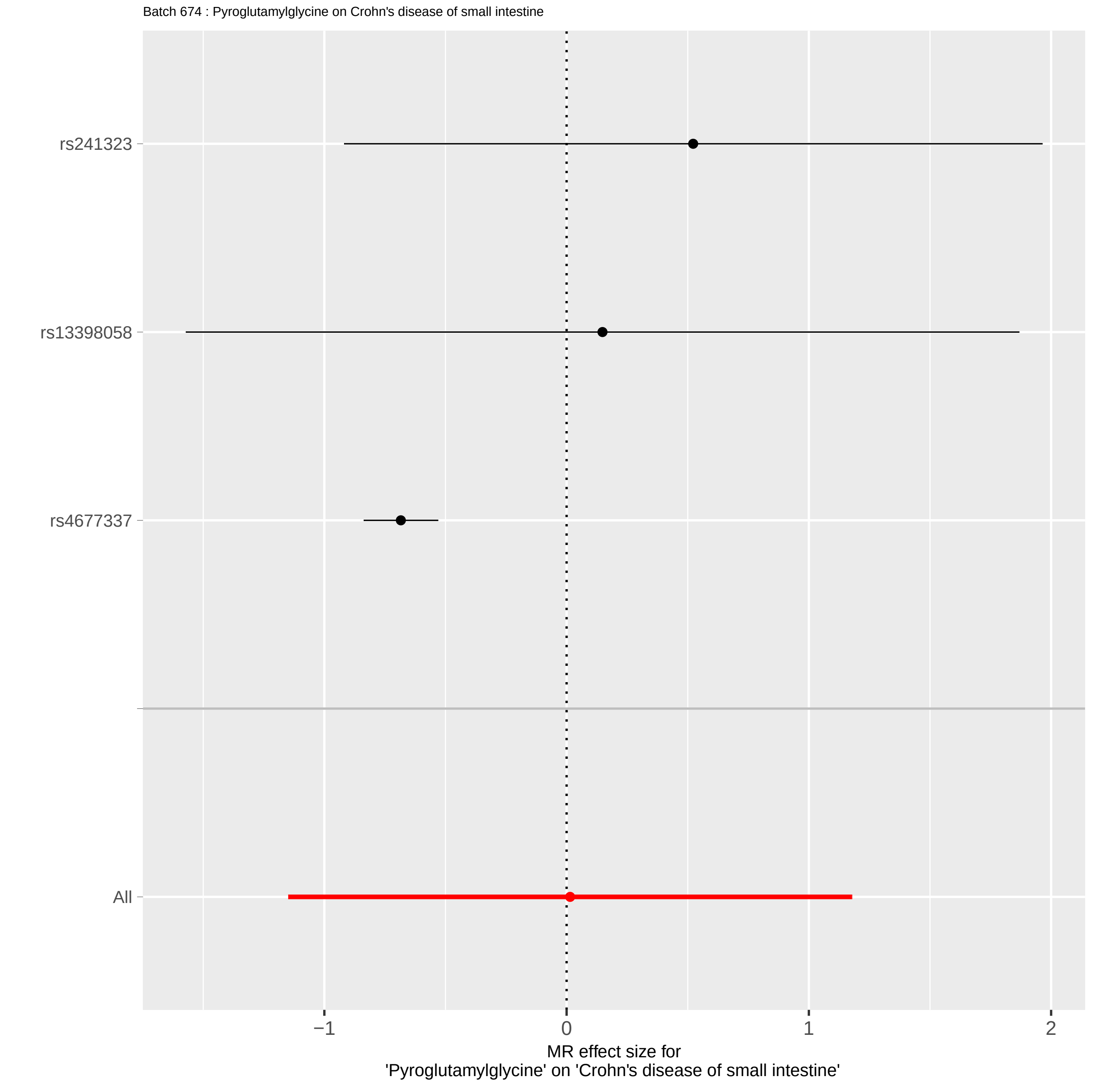
Batch 674 : Pyroglutamylglycine on Crohn's disease of small intestine
rs241323
rs13398058
rs4677337
All
−1
0
1
2
MR effect size for
'Pyroglutamylglycine' on 'Crohn's disease of small intestine'

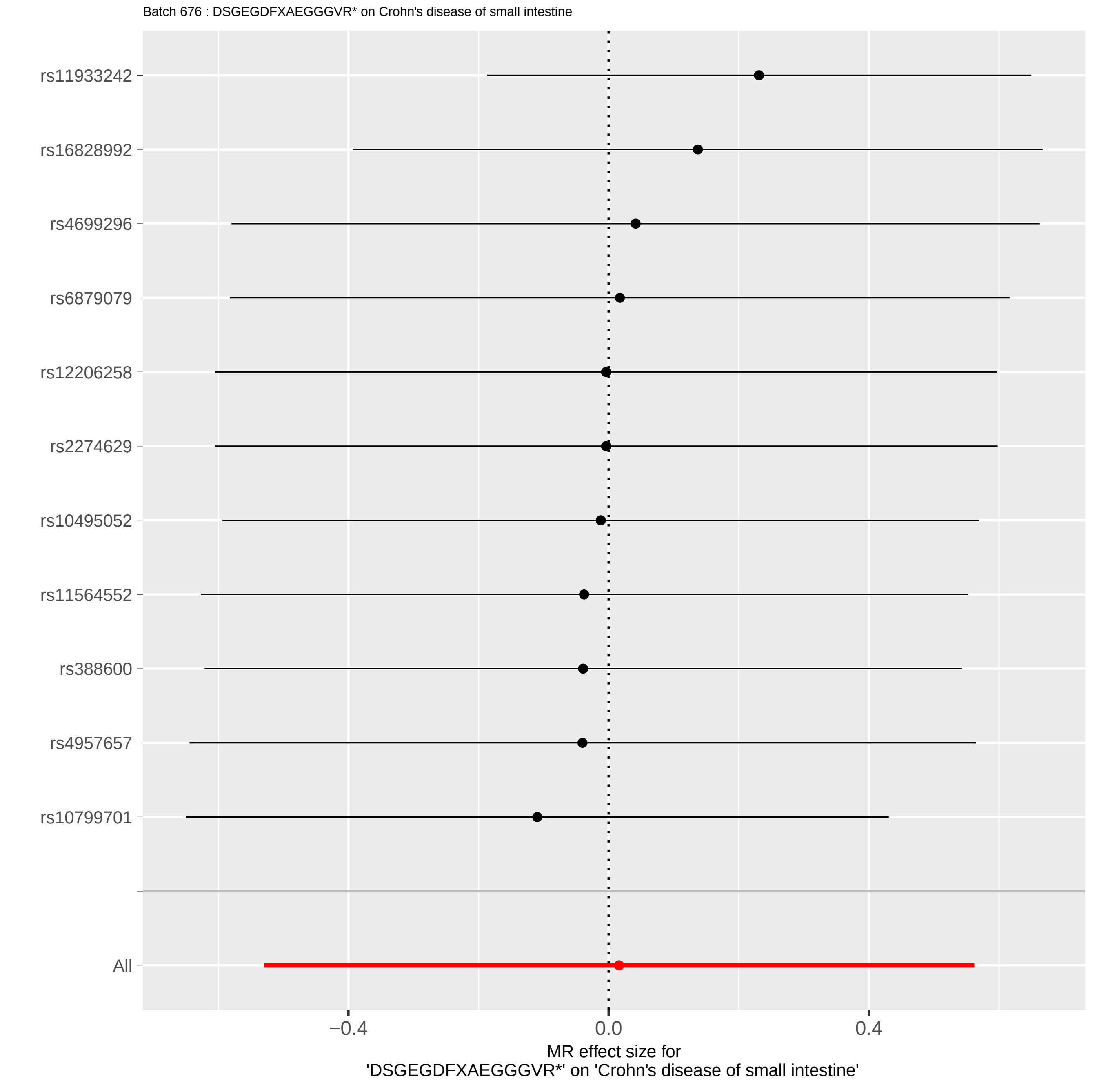

Batch 676 : DSGEGDFXAEGGGVR* on Crohn's disease of small intestine
rs11933242
rs16828992
rs4699296
rs6879079
rs12206258
rs2274629
rs10495052
rs11564552
rs388600
rs4957657
rs10799701
All
−0.4
0.0
0.4
MR effect size for
'DSGEGDFXAEGGGVR*' on 'Crohn's disease of small intestine'

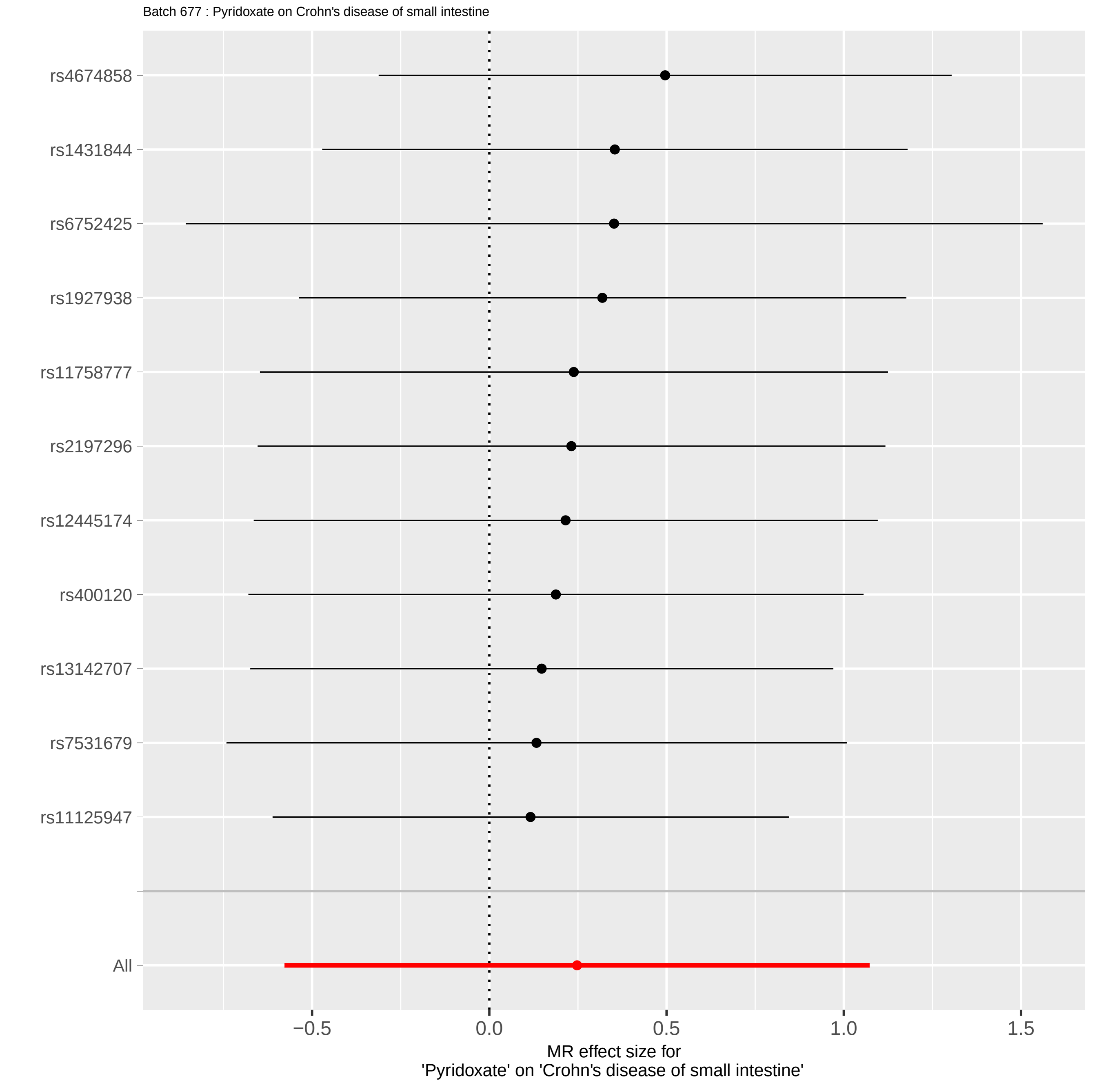
Batch 677 : Pyridoxate on Crohn's disease of small intestine
rs4674858
rs1431844
rs6752425
rs1927938
rs11758777
rs2197296
rs12445174
rs400120
rs13142707
rs7531679
rs11125947
All
−0.5
0.0
0.5
1.0
1.5
MR effect size for
'Pyridoxate' on 'Crohn's disease of small intestine'

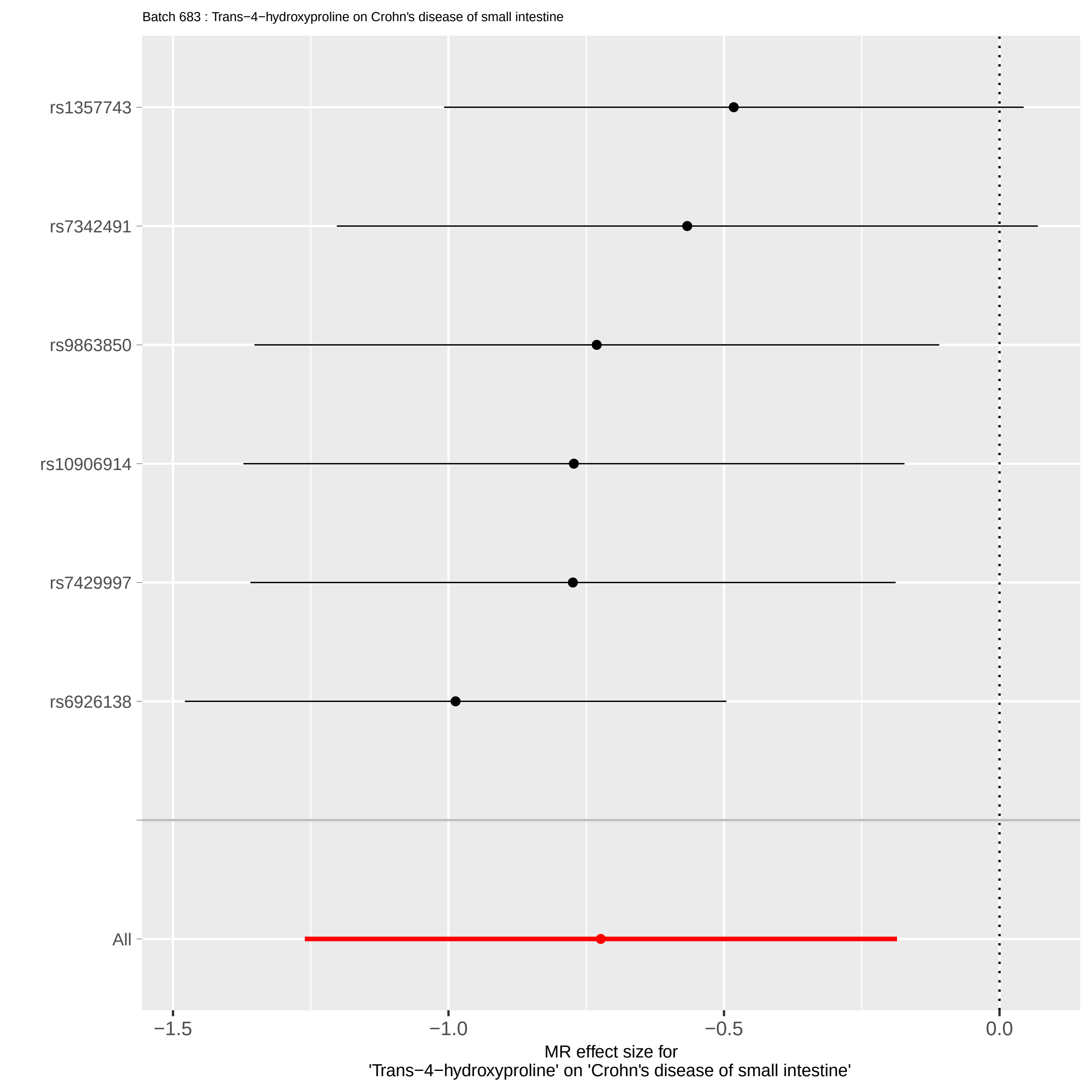

Batch 683 : Trans−4−hydroxyproline on Crohn's disease of small intestine
rs1357743
rs7342491
rs9863850
rs10906914
rs7429997
rs6926138
All
−1.5
−1.0
−0.5
0.0
MR effect size for
'Trans−4−hydroxyproline' on 'Crohn's disease of small intestine'

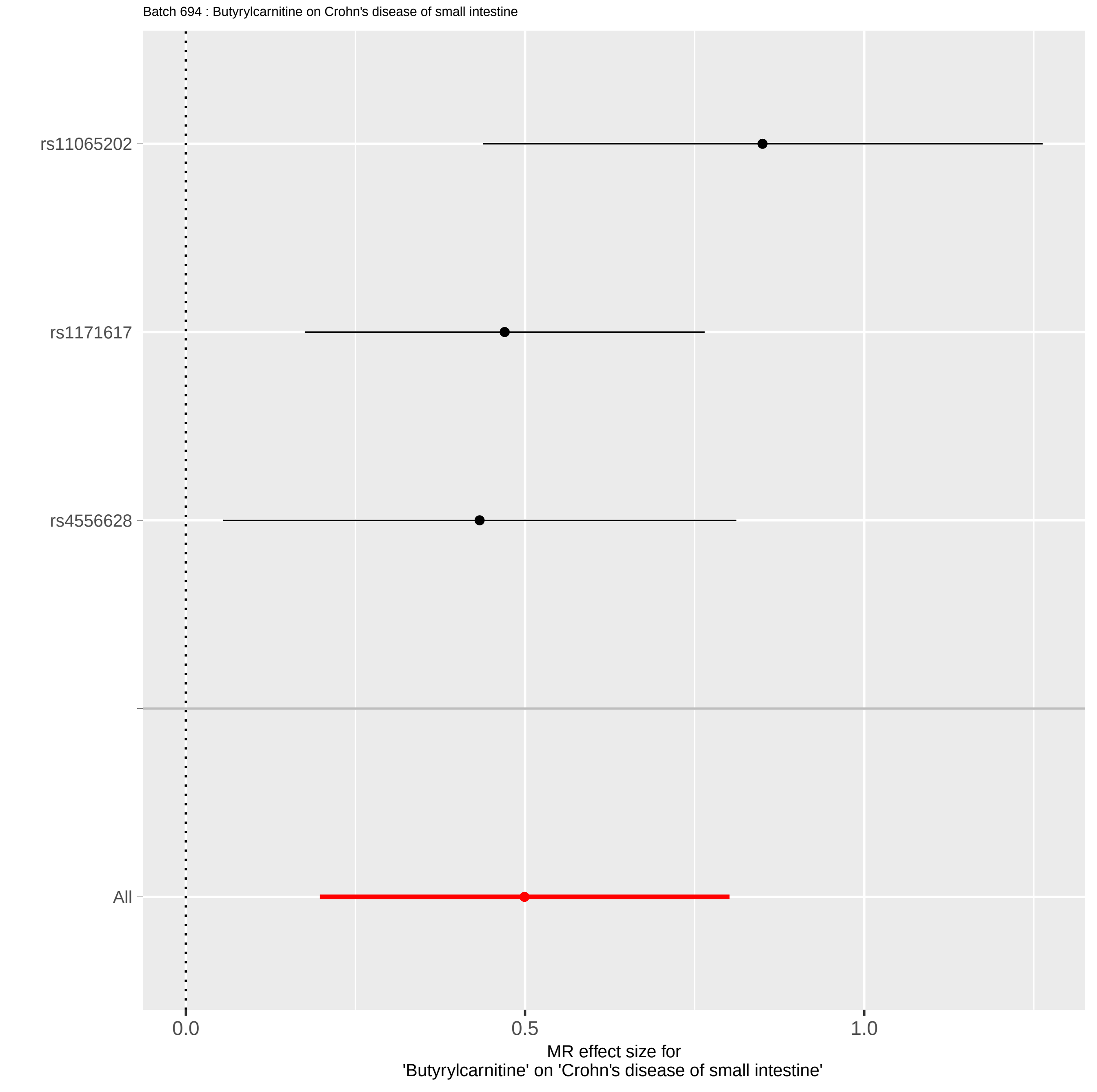
Batch 694 : Butyrylcarnitine on Crohn's disease of small intestine
rs11065202
rs1171617
rs4556628
All
0.0
0.5
1.0
MR effect size for
'Butyrylcarnitine' on 'Crohn's disease of small intestine'

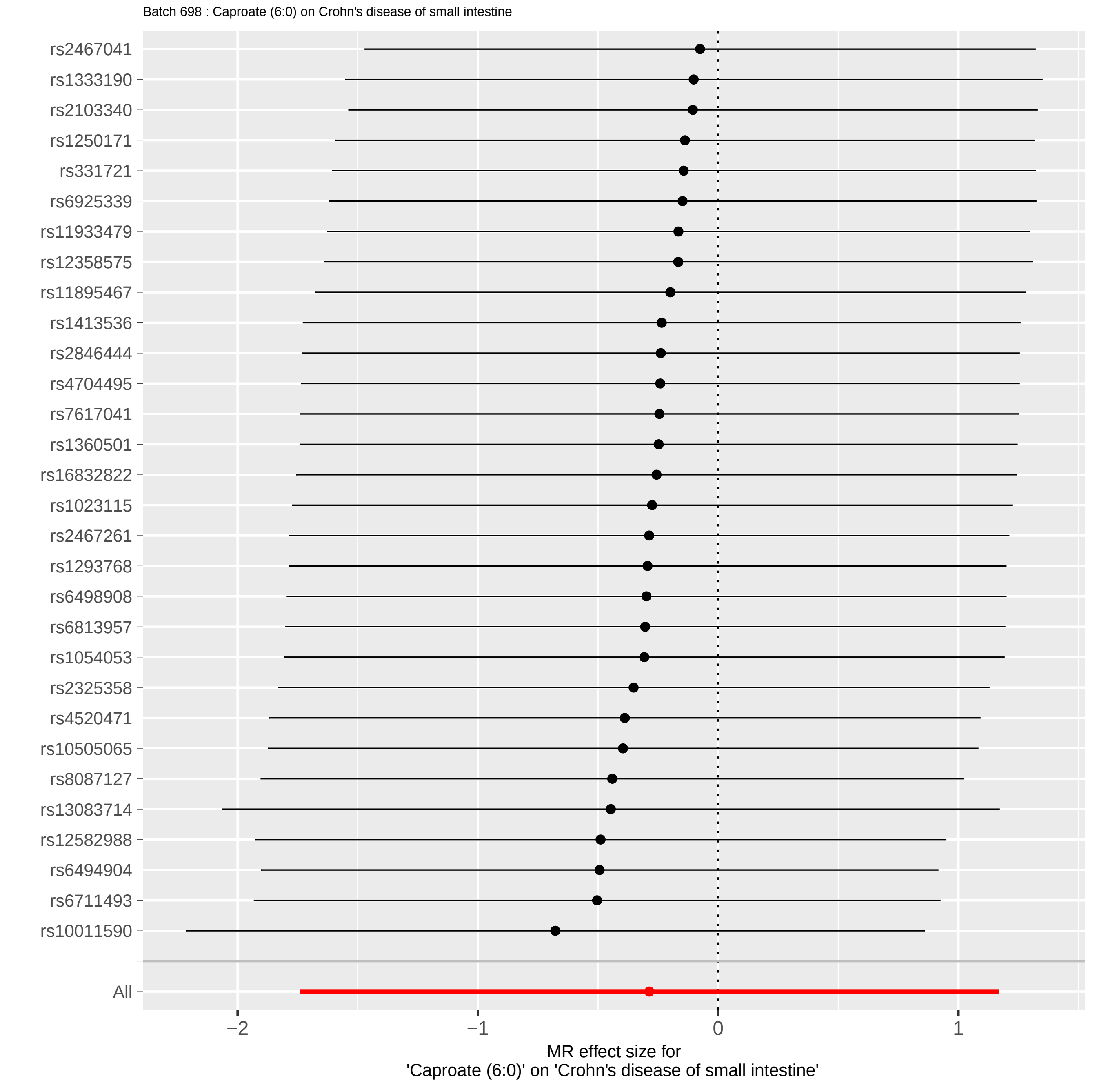
Batch 698 : Caproate (6:0) on Crohn's disease of small intestine
rs2467041
rs1333190
rs2103340
rs1250171
rs331721
rs6925339
rs11933479
rs12358575
rs11895467
rs1413536
rs2846444
rs4704495
rs7617041
rs1360501
rs16832822
rs1023115
rs2467261
rs1293768
rs6498908
rs6813957
rs1054053
rs2325358
rs4520471
rs10505065
rs8087127
rs13083714
rs12582988
rs6494904
rs6711493
rs10011590
All
−2
−1
0
1
MR effect size for
'Caproate (6:0)' on 'Crohn's disease of small intestine'

Batch 702 : Phenol sulfate on Crohn's disease of small intestine

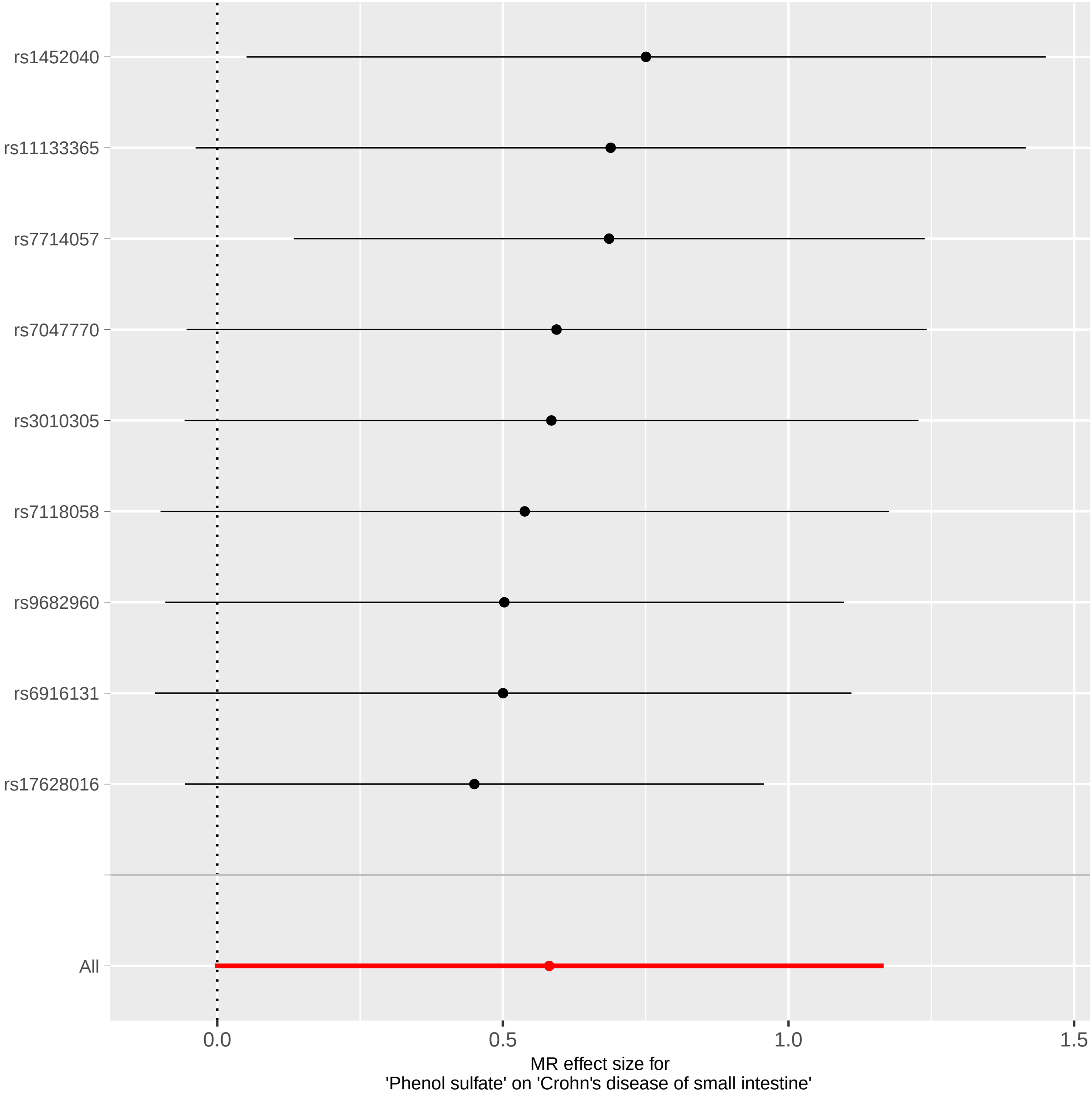

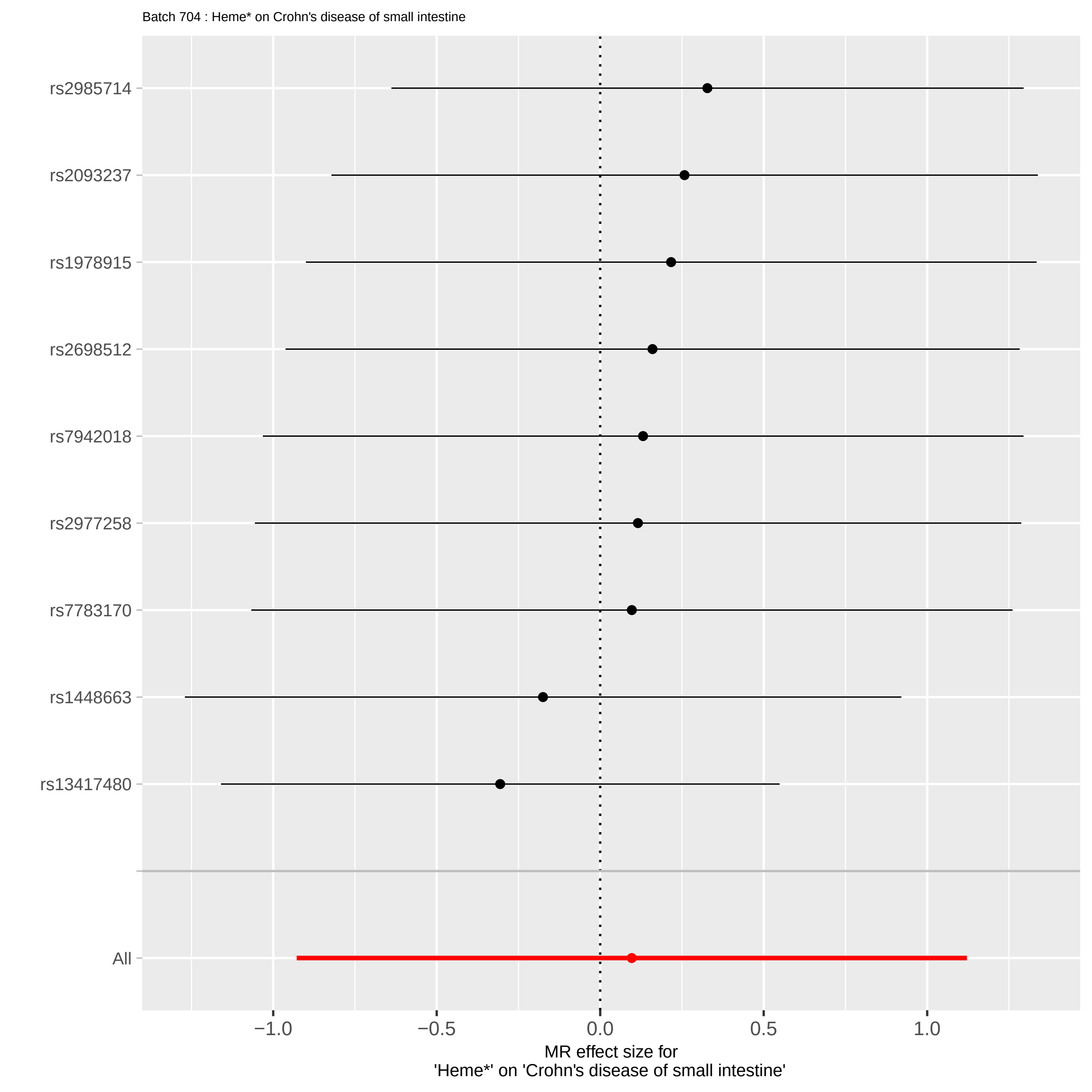

Batch 704 : Heme* on Crohn's disease of small intestine
rs2985714
rs2093237
rs1978915
rs2698512
rs7942018
rs2977258
rs7783170
rs1448663
rs13417480
All
−1.0
−0.5
0.0
0.5
1.0
MR effect size for
'Heme*' on 'Crohn's disease of small intestine'

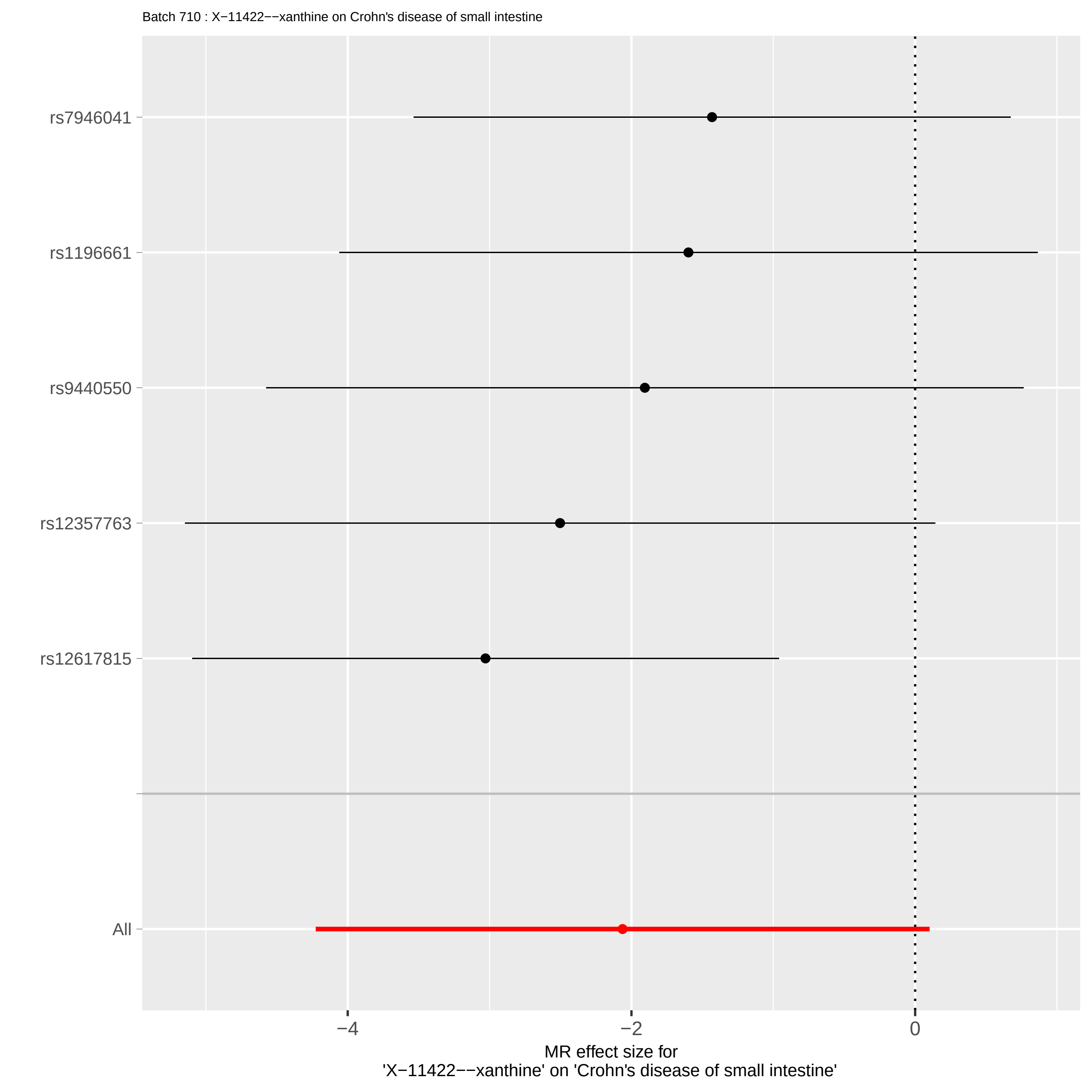
Batch 710 : X−11422−−xanthine on Crohn's disease of small intestine
rs7946041
rs1196661
rs9440550
rs12357763
rs12617815
All
−4
−2
0
MR effect size for
'X−11422−−xanthine' on 'Crohn's disease of small intestine'

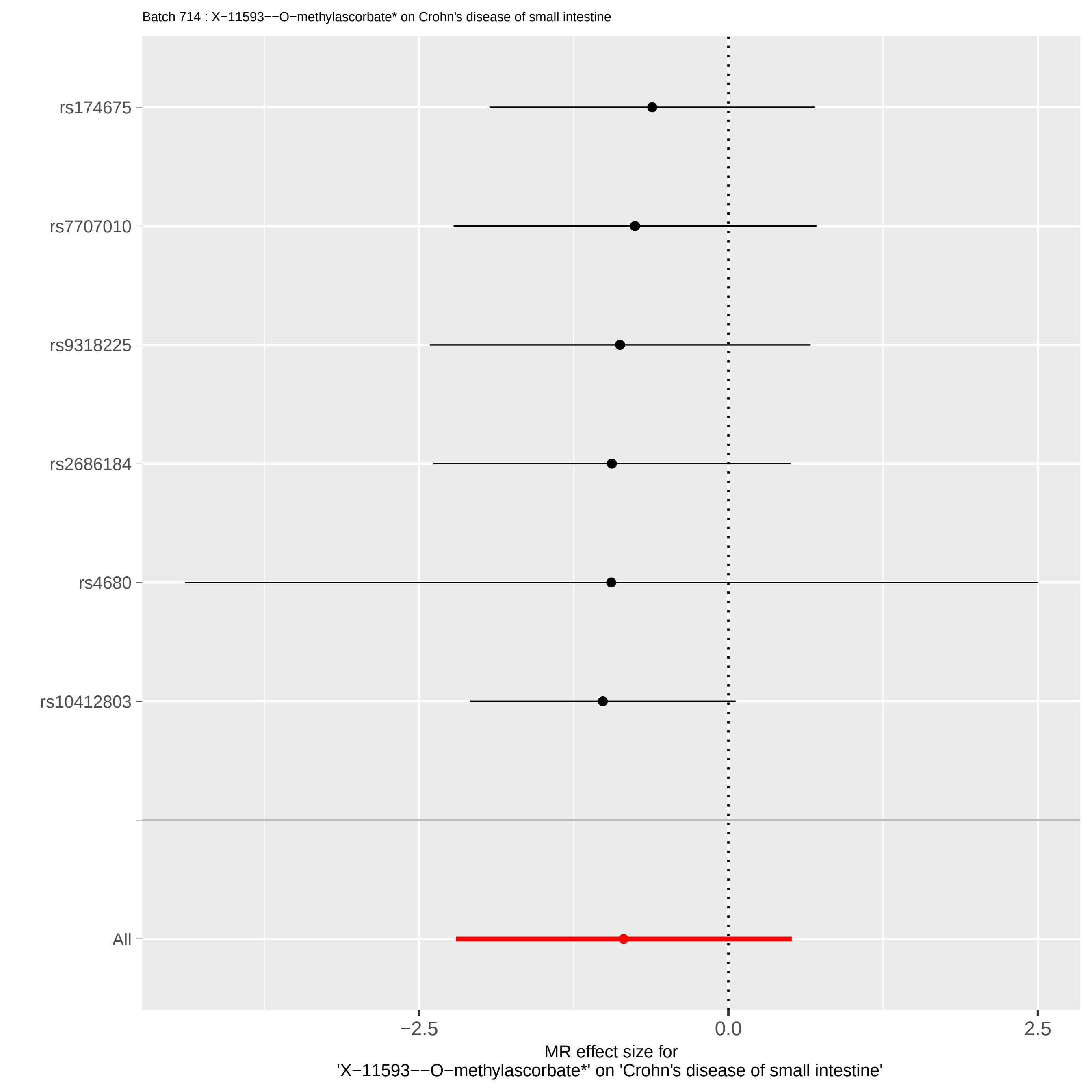

Batch 714 : X−11593−−O−methylascorbate* on Crohn's disease of small intestine
rs174675
rs7707010
rs9318225
rs2686184
rs4680
rs10412803
All
−2.5
0.0
2.5
MR effect size for
'X−11593−−O−methylascorbate*' on 'Crohn's disease of small intestine'

Batch 716 : Homostachydrine* on Crohn's disease of small intestine

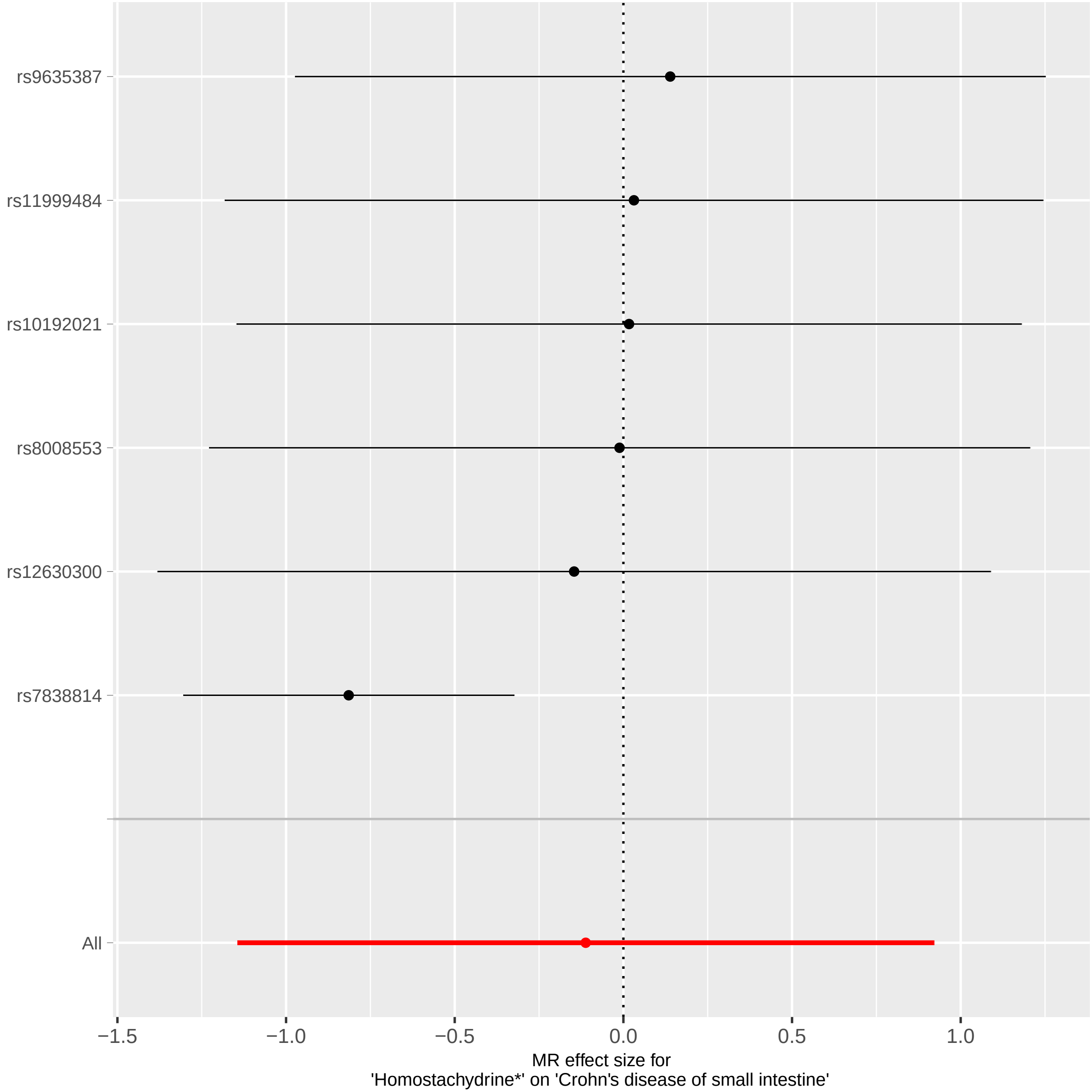

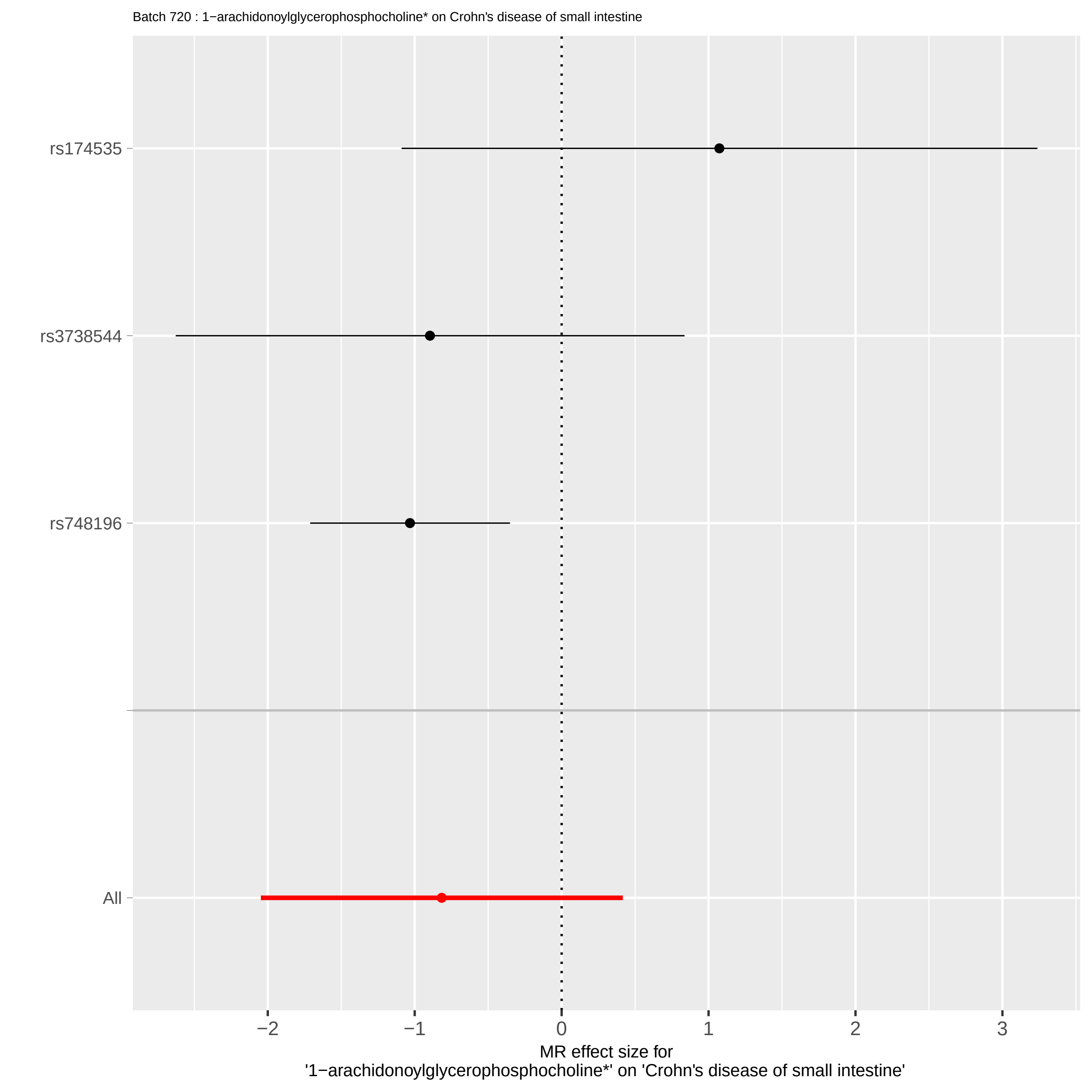
Batch 720 : 1−arachidonoylglycerophosphocholine* on Crohn's disease of small intestine
rs174535
rs3738544
rs748196
All
−2
−1
0
1
2
3
MR effect size for
'1−arachidonoylglycerophosphocholine*' on 'Crohn's disease of small intestine'

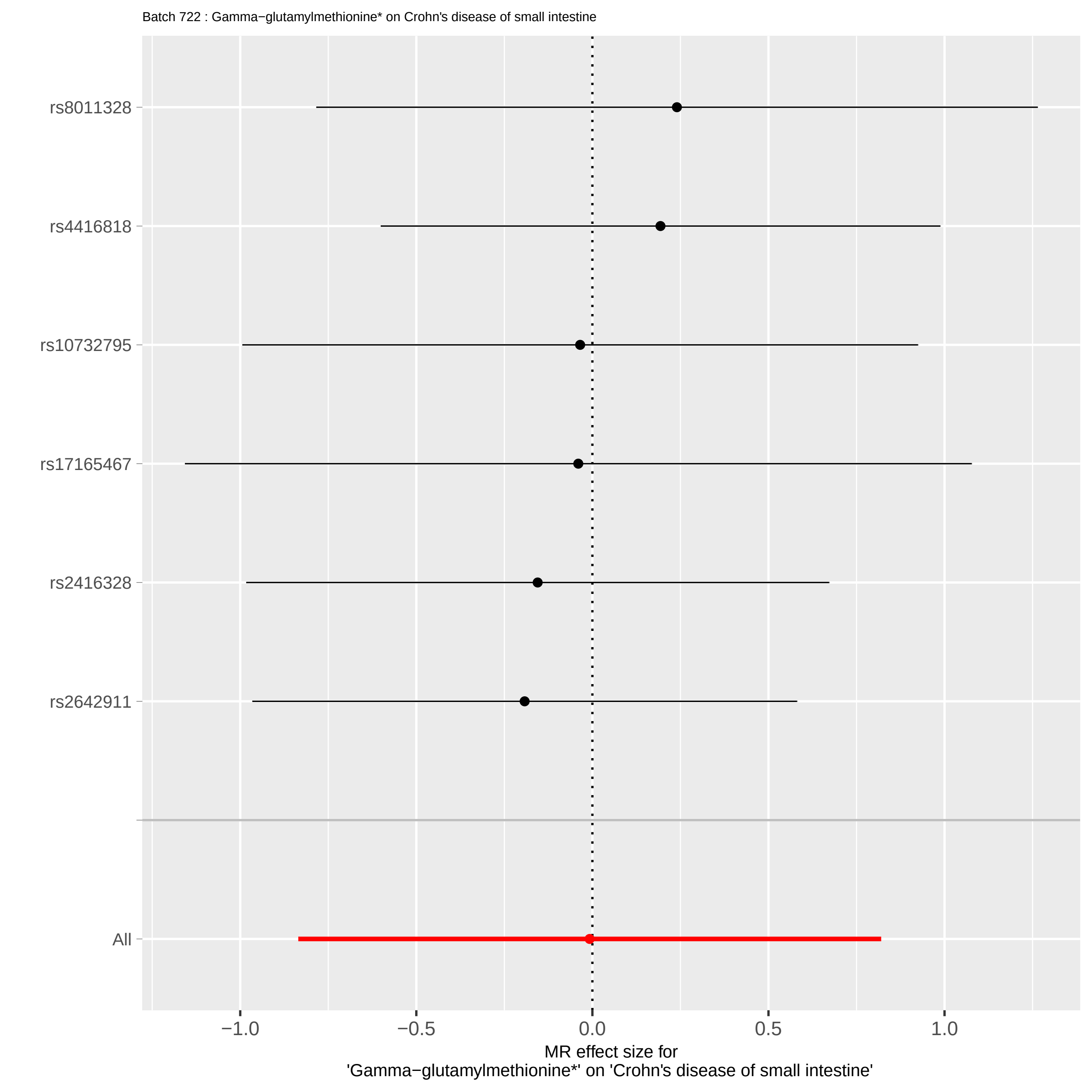

Batch 722 : Gamma−glutamylmethionine* on Crohn's disease of small intestine
rs8011328
rs4416818
rs10732795
rs17165467
rs2416328
rs2642911
All
−1.0
−0.5
0.0
0.5
1.0
MR effect size for
'Gamma−glutamylmethionine*' on 'Crohn's disease of small intestine'

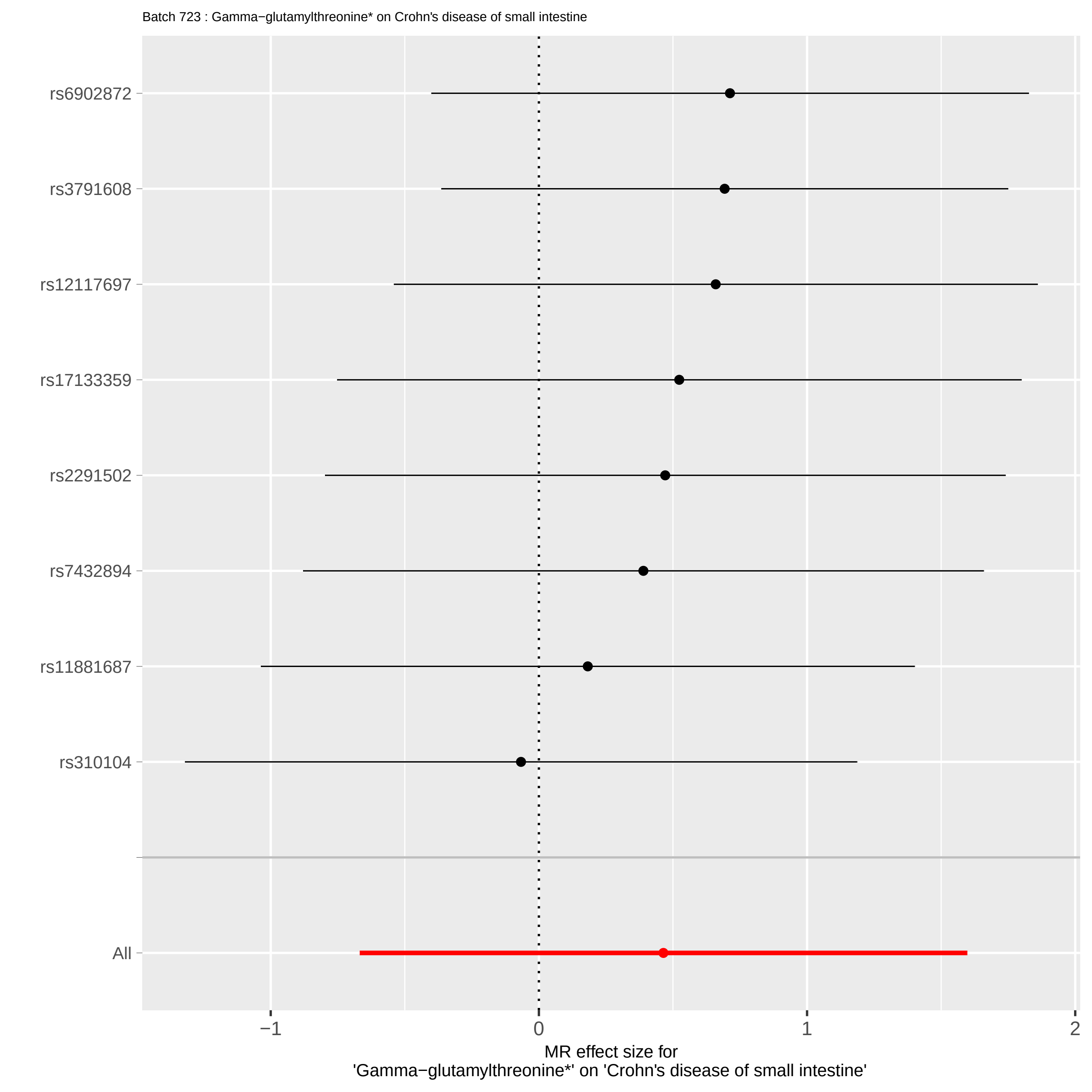
Batch 723 : Gamma−glutamylthreonine* on Crohn's disease of small intestine
rs6902872
rs3791608
rs12117697
rs17133359
rs2291502
rs7432894
rs11881687
rs310104
All
−1
0
1
2
MR effect size for
'Gamma−glutamylthreonine*' on 'Crohn's disease of small intestine'

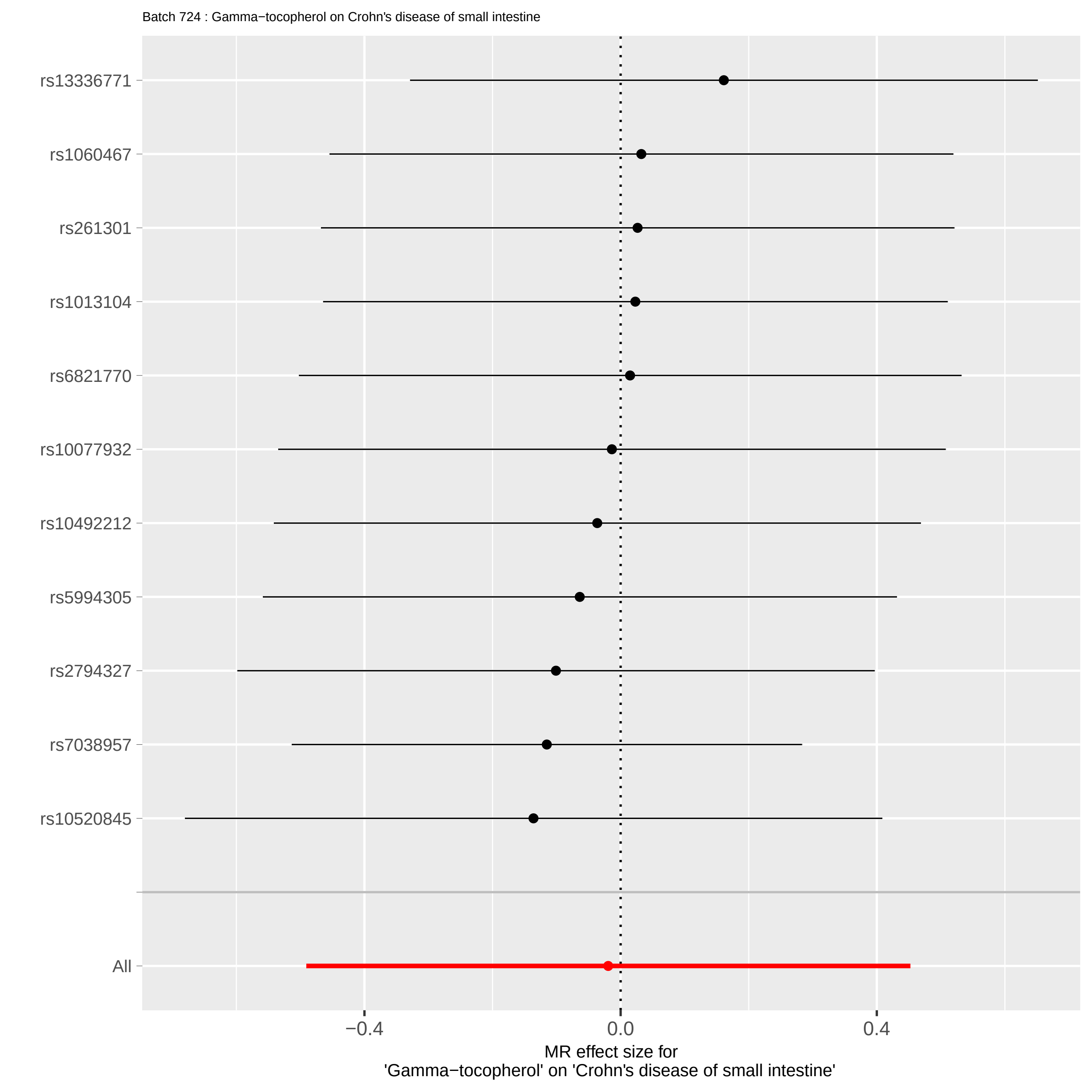

Batch 724 : Gamma−tocopherol on Crohn's disease of small intestine
rs13336771
rs1060467
rs261301
rs1013104
rs6821770
rs10077932
rs10492212
rs5994305
rs2794327
rs7038957
rs10520845
All
−0.4
0.0
0.4
MR effect size for
'Gamma−tocopherol' on 'Crohn's disease of small intestine'

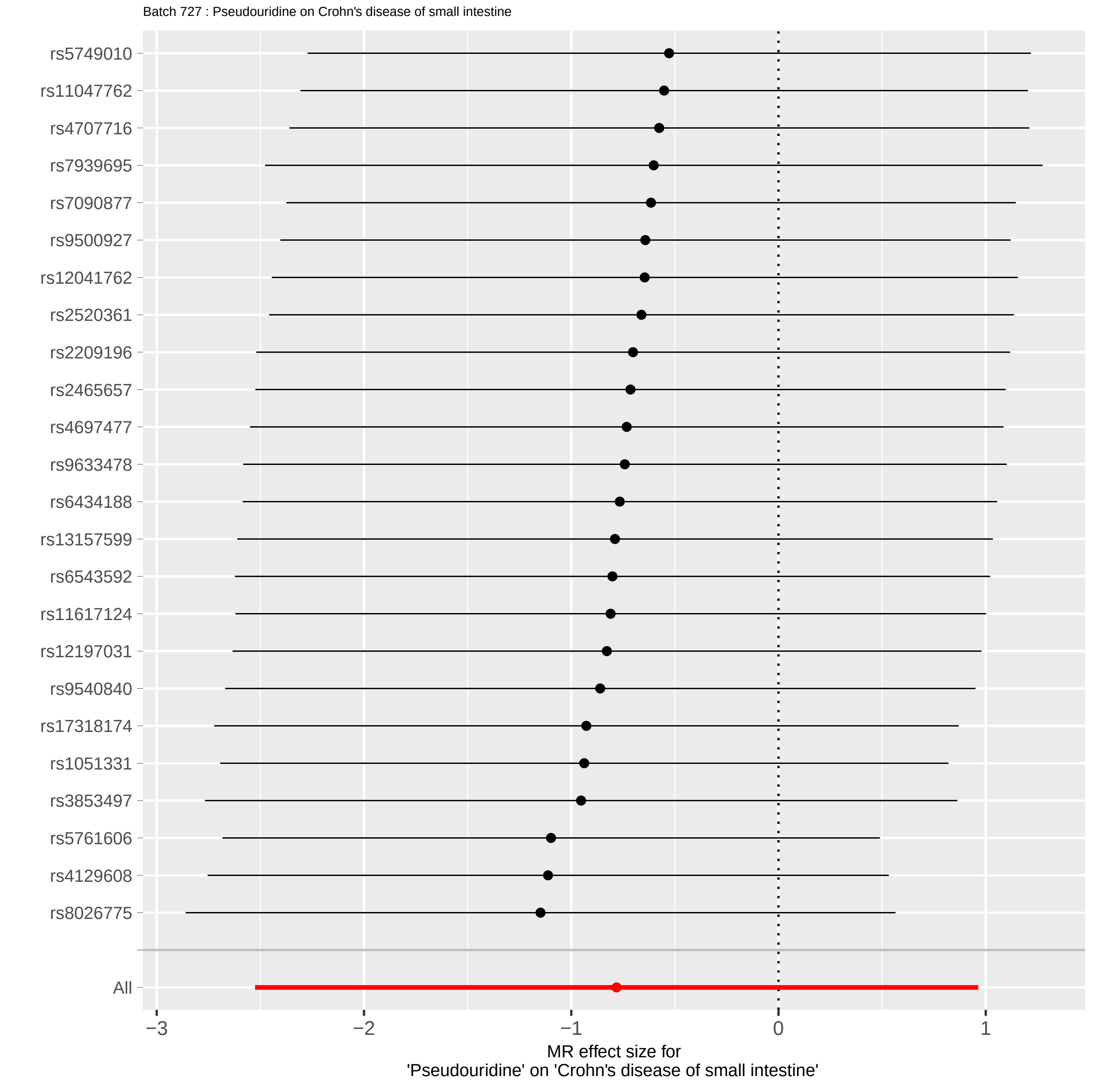

Batch 727 : Pseudouridine on Crohn's disease of small intestine
rs5749010
rs11047762
rs4707716
rs7939695
rs7090877
rs9500927
rs12041762
rs2520361
rs2209196
rs2465657
rs4697477
rs9633478
rs6434188
rs13157599
rs6543592
rs11617124
rs12197031
rs9540840
rs17318174
rs1051331
rs3853497
rs5761606
rs4129608
rs8026775
All
−3
−2
−1
0
1
MR effect size for
'Pseudouridine' on 'Crohn's disease of small intestine'

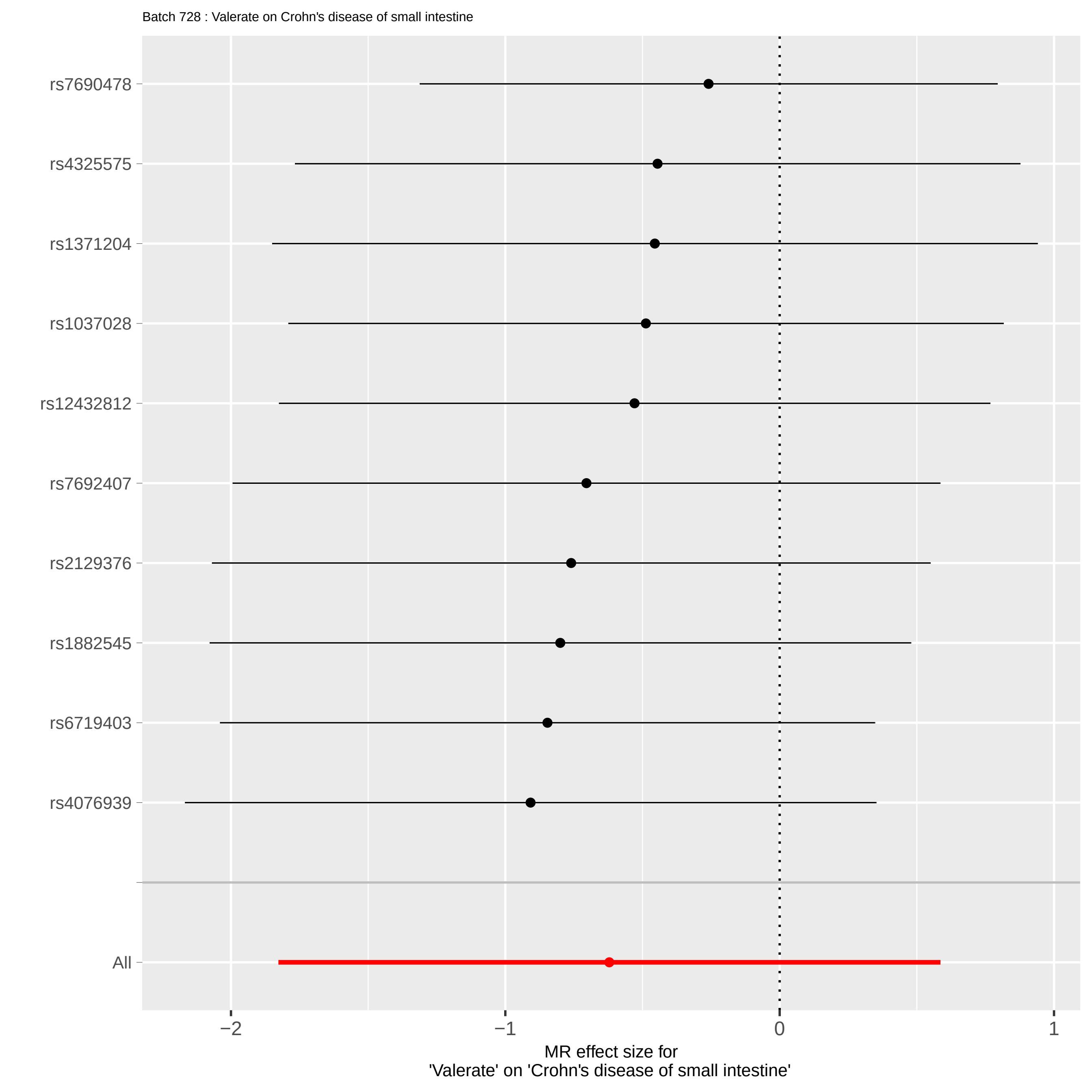
Batch 728 : Valerate on Crohn's disease of small intestine
rs7690478
rs4325575
rs1371204
rs1037028
rs12432812
rs7692407
rs2129376
rs1882545
rs6719403
rs4076939
All
−2
−1
0
1
MR effect size for
'Valerate' on 'Crohn's disease of small intestine'

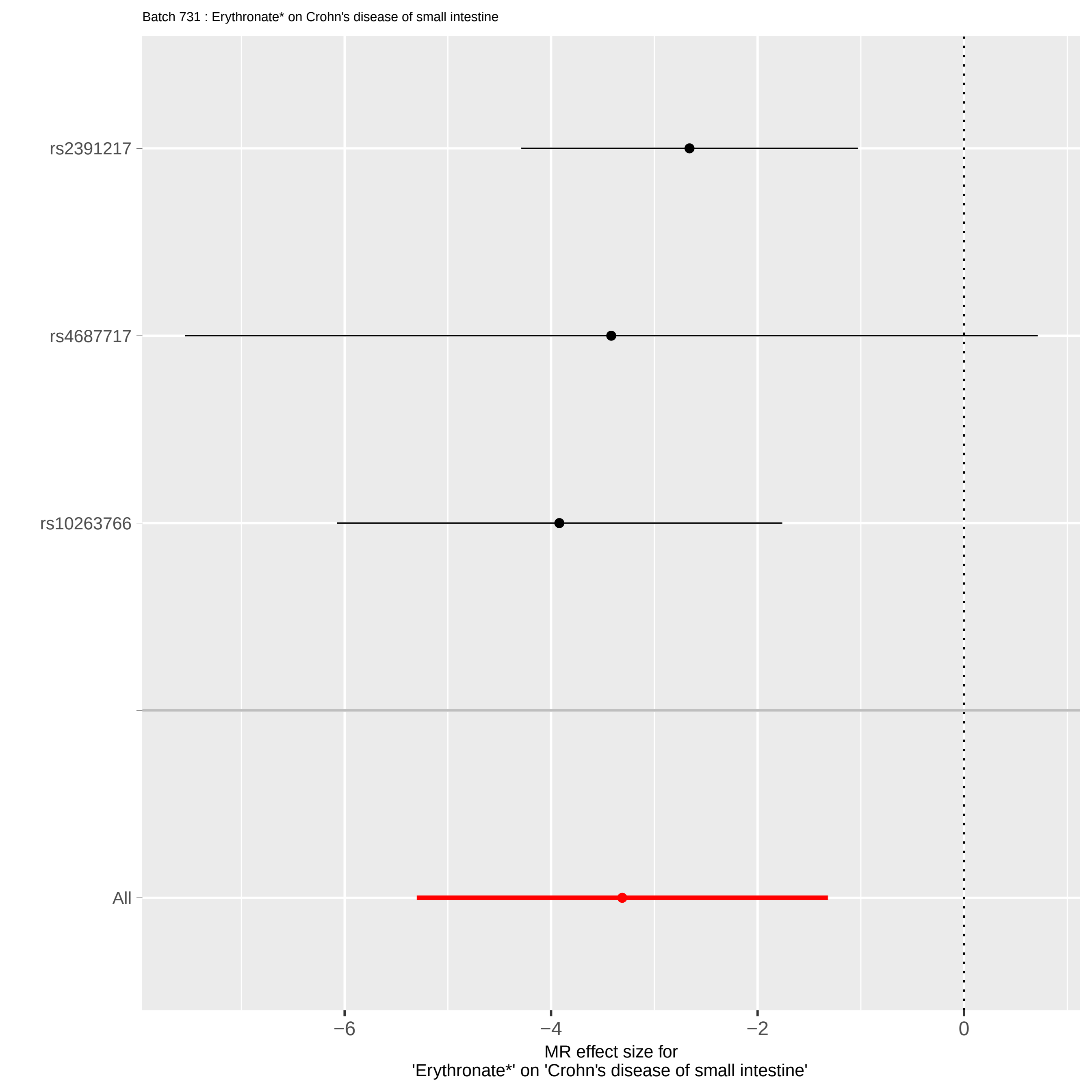

Batch 731 : Erythronate* on Crohn's disease of small intestine
rs2391217
rs4687717
rs10263766
All
−6
−4
−2
0
MR effect size for
'Erythronate*' on 'Crohn's disease of small intestine'

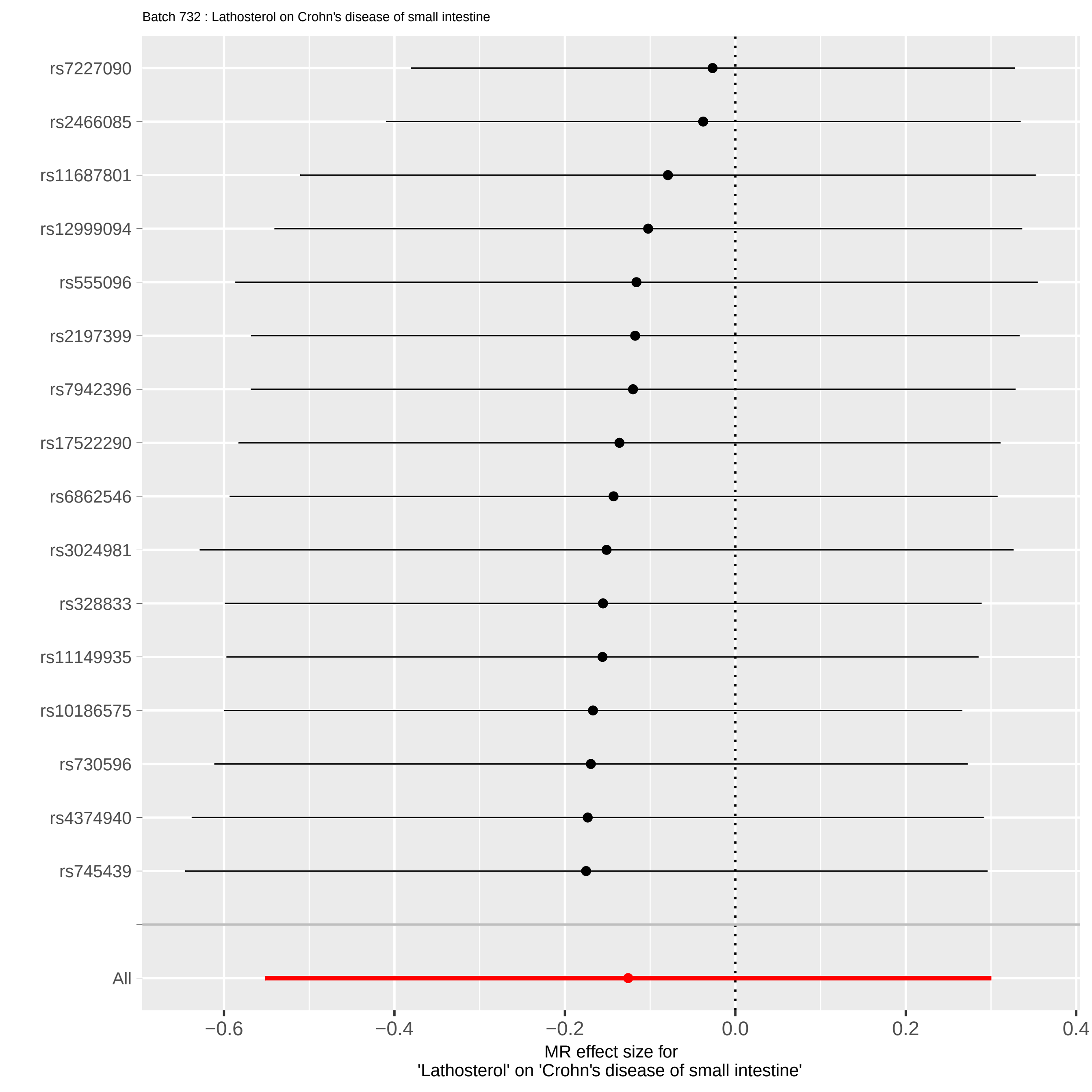

Batch 732 : Lathosterol on Crohn's disease of small intestine
rs7227090
rs2466085
rs11687801
rs12999094
rs555096
rs2197399
rs7942396
rs17522290
rs6862546
rs3024981
rs328833
rs11149935
rs10186575
rs730596
rs4374940
rs745439
All
−0.6
−0.4
−0.2
0.0
0.2
0.4
MR effect size for
'Lathosterol' on 'Crohn's disease of small intestine'

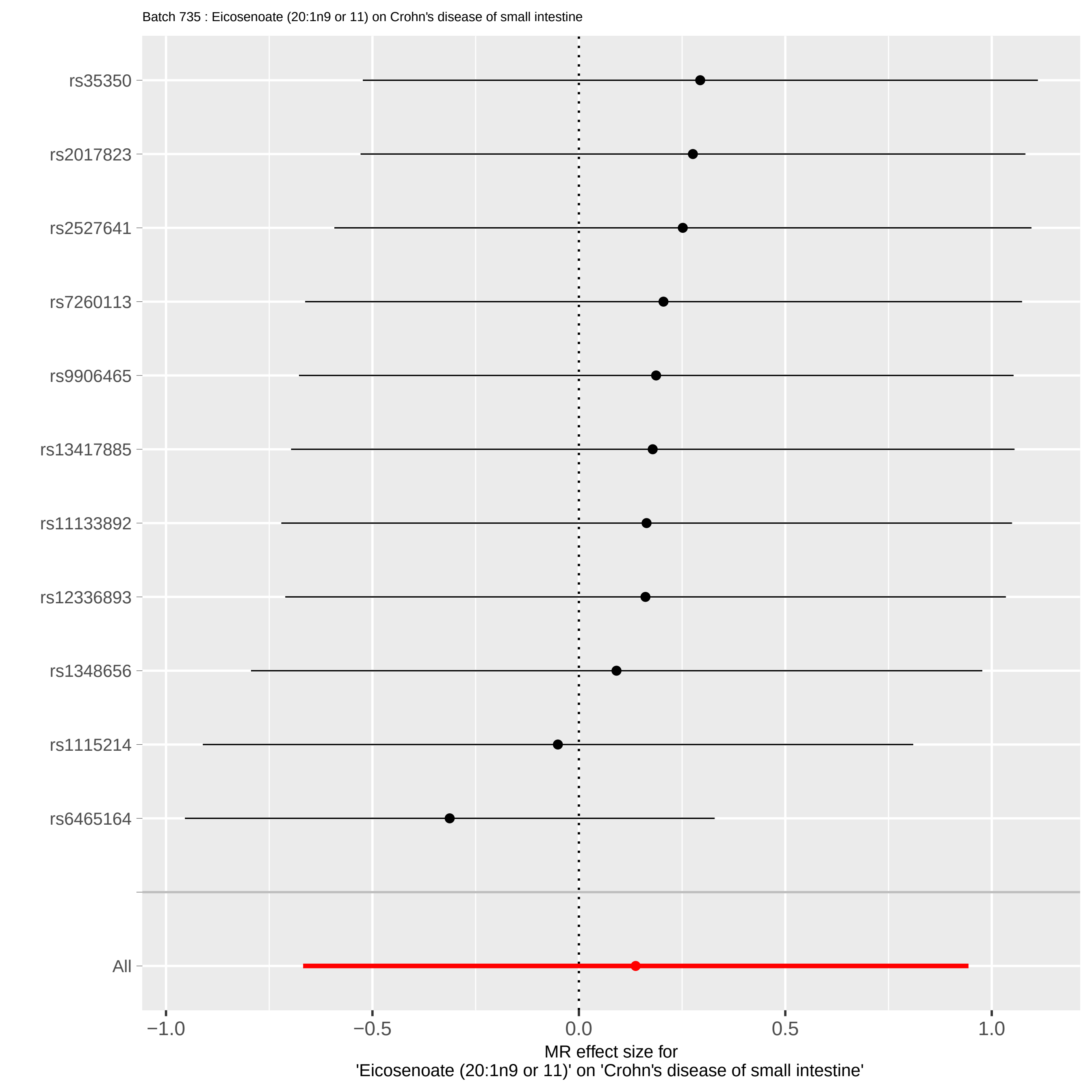
Batch 735 : Eicosenoate (20:1n9 or 11) on Crohn's disease of small intestine
rs35350
rs2017823
rs2527641
rs7260113
rs9906465
rs13417885
rs11133892
rs12336893
rs1348656
rs1115214
rs6465164
All
−1.0
−0.5
0.0
0.5
1.0
MR effect size for
'Eicosenoate (20:1n9 or 11)' on 'Crohn's disease of small intestine'

Batch 736 : X−12244−−N−acetylcarnosine on Crohn's disease of small intestine

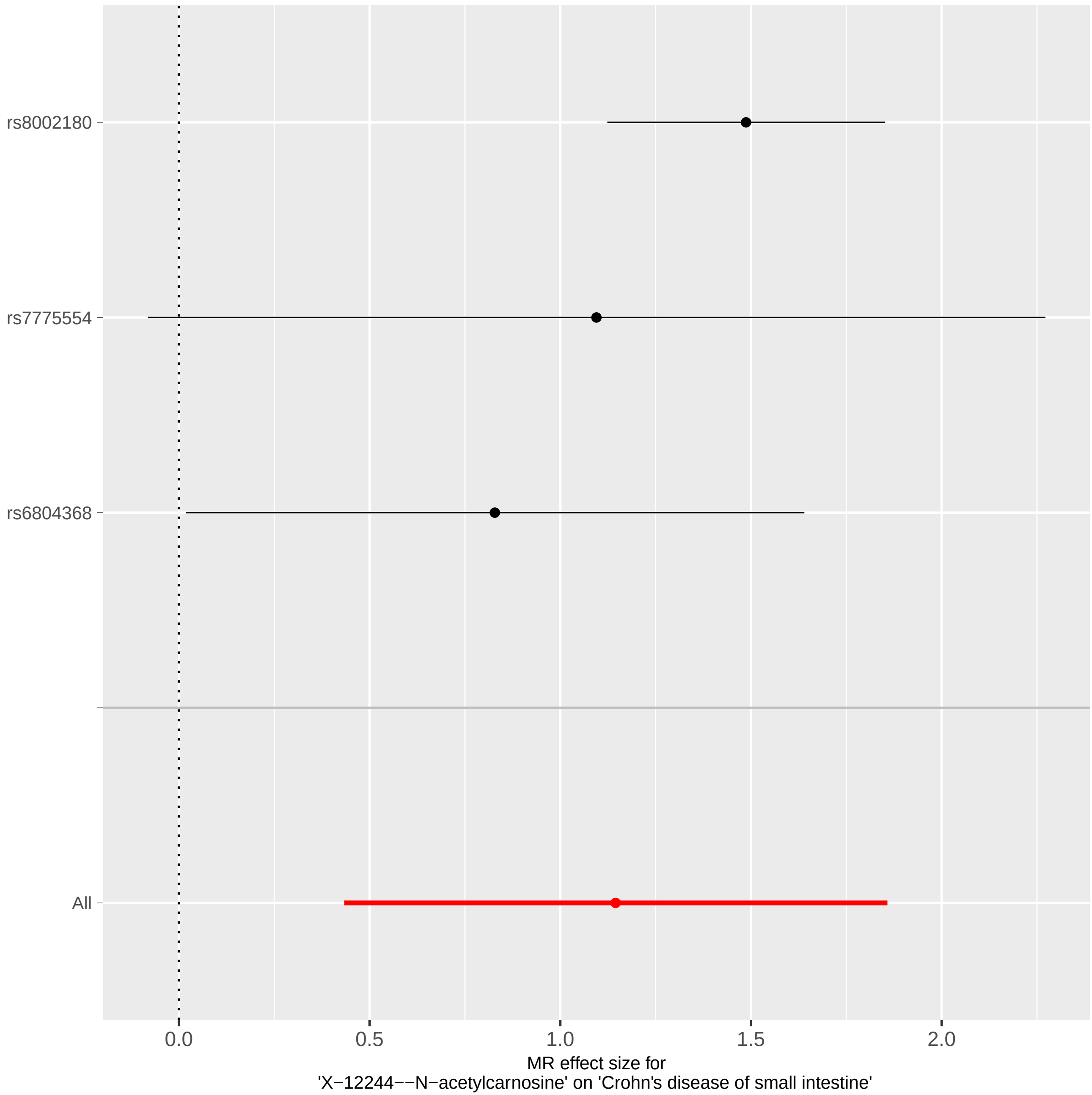

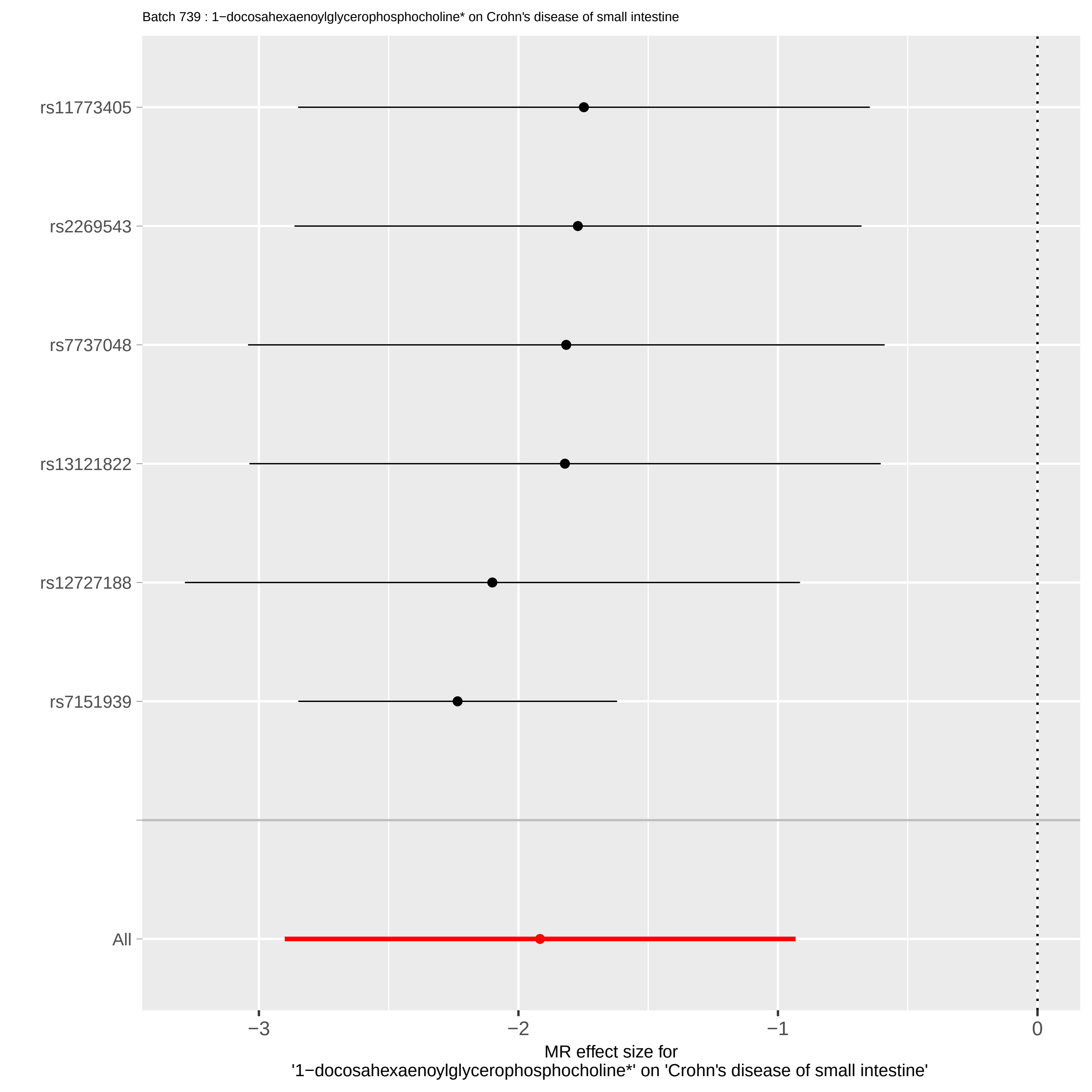
Batch 739 : 1−docosahexaenoylglycerophosphocholine* on Crohn's disease of small intestine
rs11773405
rs2269543
rs7737048
rs13121822
rs12727188
rs7151939
All
−3
−2
−1
0
MR effect size for
'1−docosahexaenoylglycerophosphocholine*' on 'Crohn's disease of small intestine'

Batch 744 : Alpha−hydroxyisovalerate on Crohn's disease of small intestine

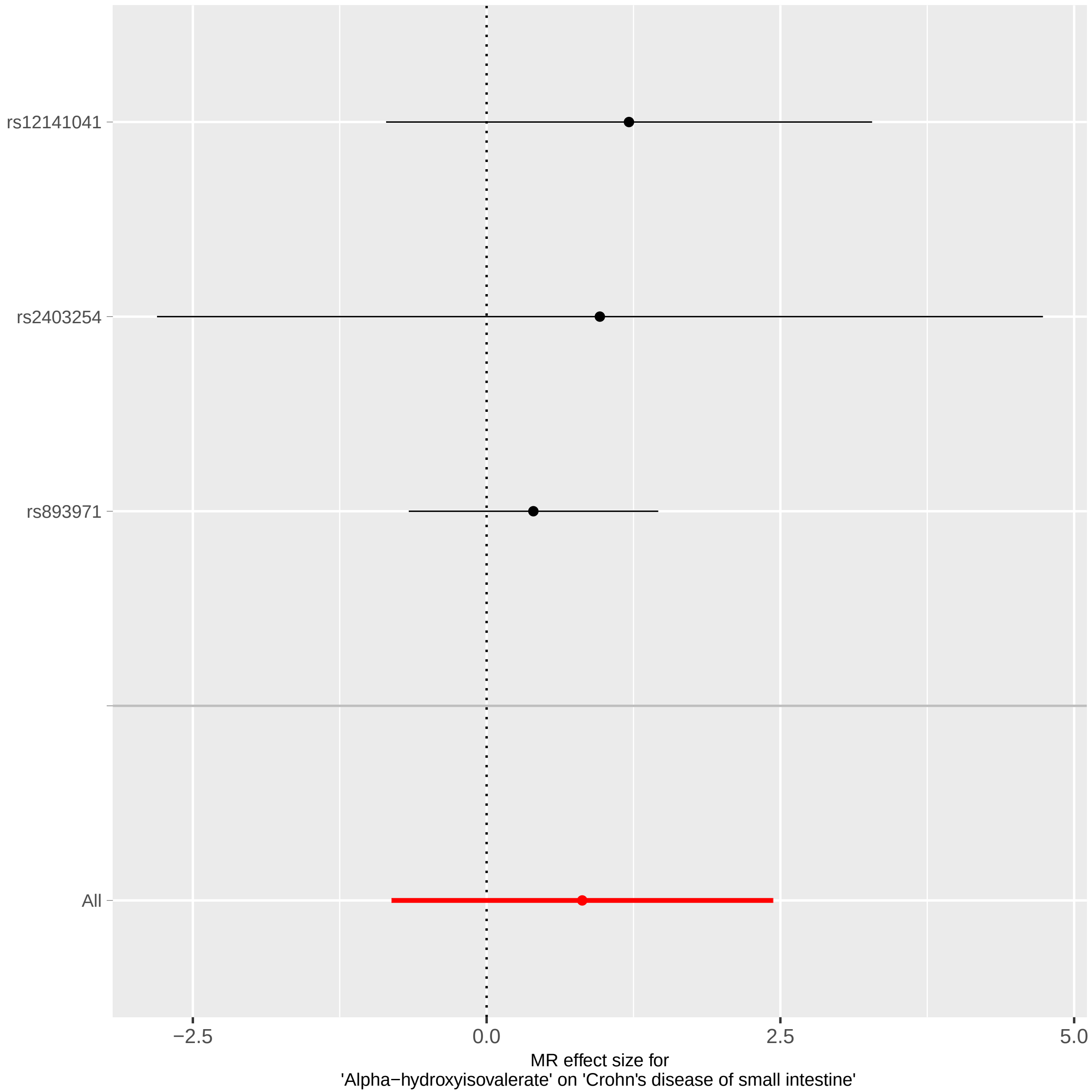

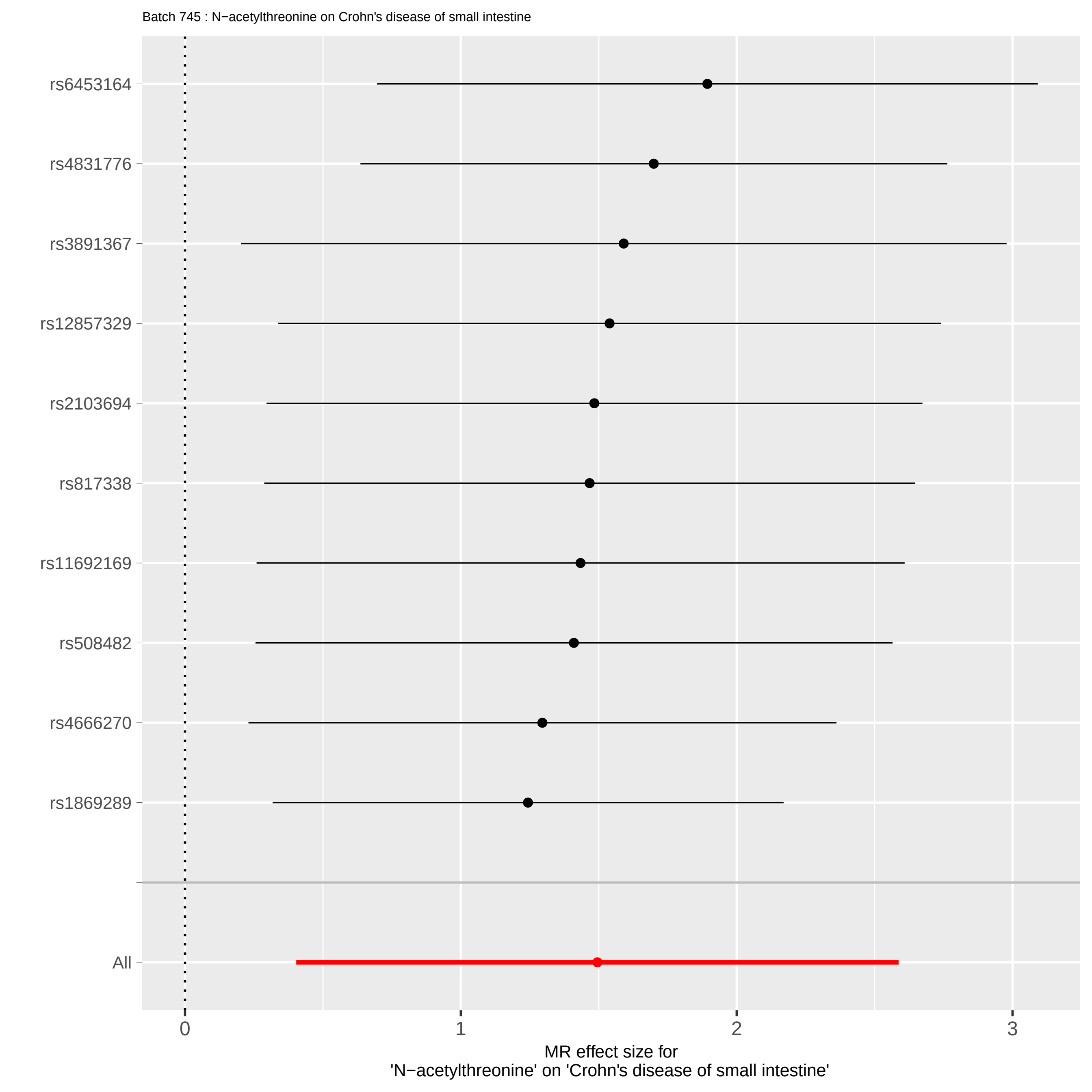

Batch 745 : N−acetylthreonine on Crohn's disease of small intestine
rs6453164
rs4831776
rs3891367
rs12857329
rs2103694
rs817338
rs11692169
rs508482
rs4666270
rs1869289
All
0
1
2
3
MR effect size for
'N−acetylthreonine' on 'Crohn's disease of small intestine'

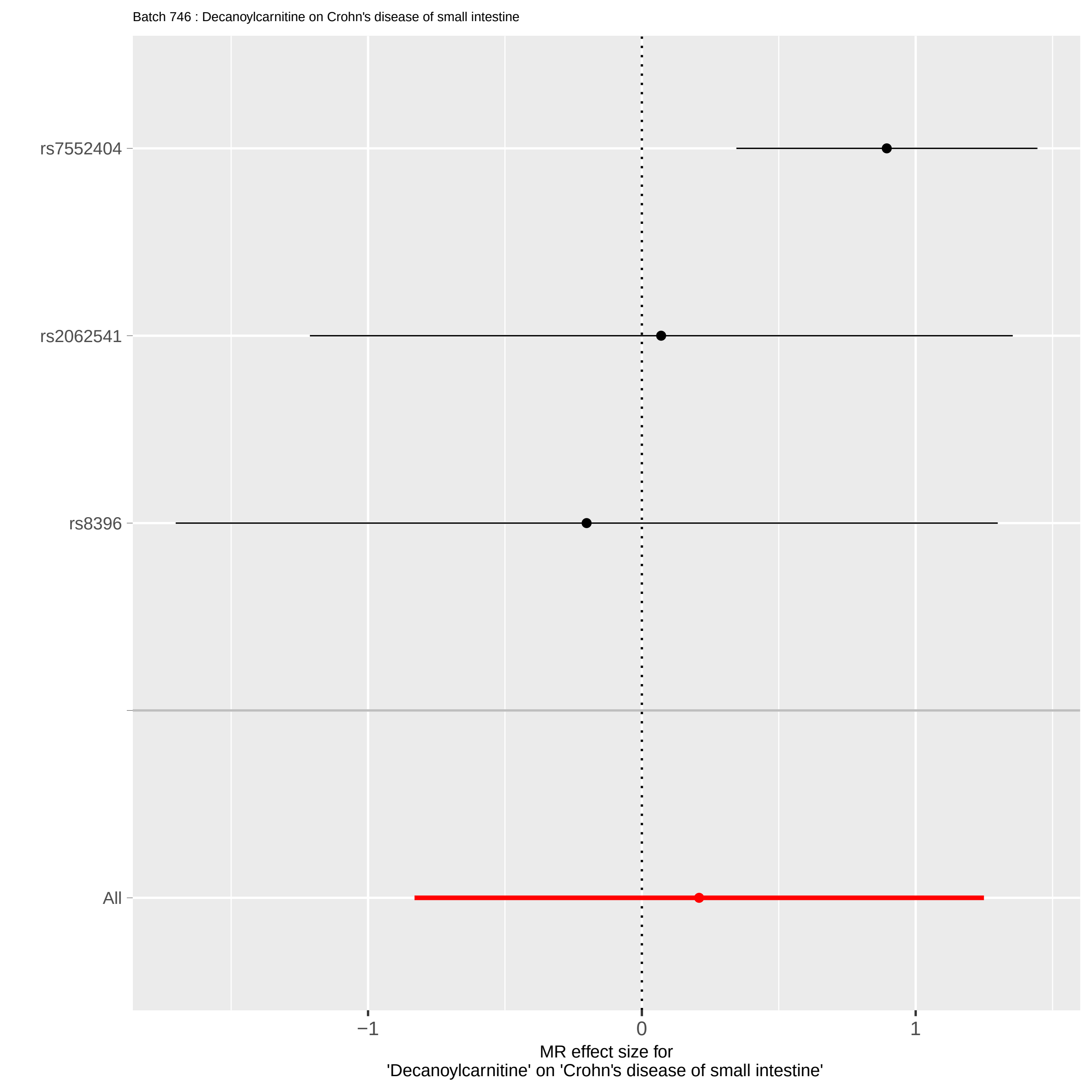

Batch 746 : Decanoylcarnitine on Crohn's disease of small intestine
rs7552404
rs2062541
rs8396
All
−1
0
1
MR effect size for
'Decanoylcarnitine' on 'Crohn's disease of small intestine'

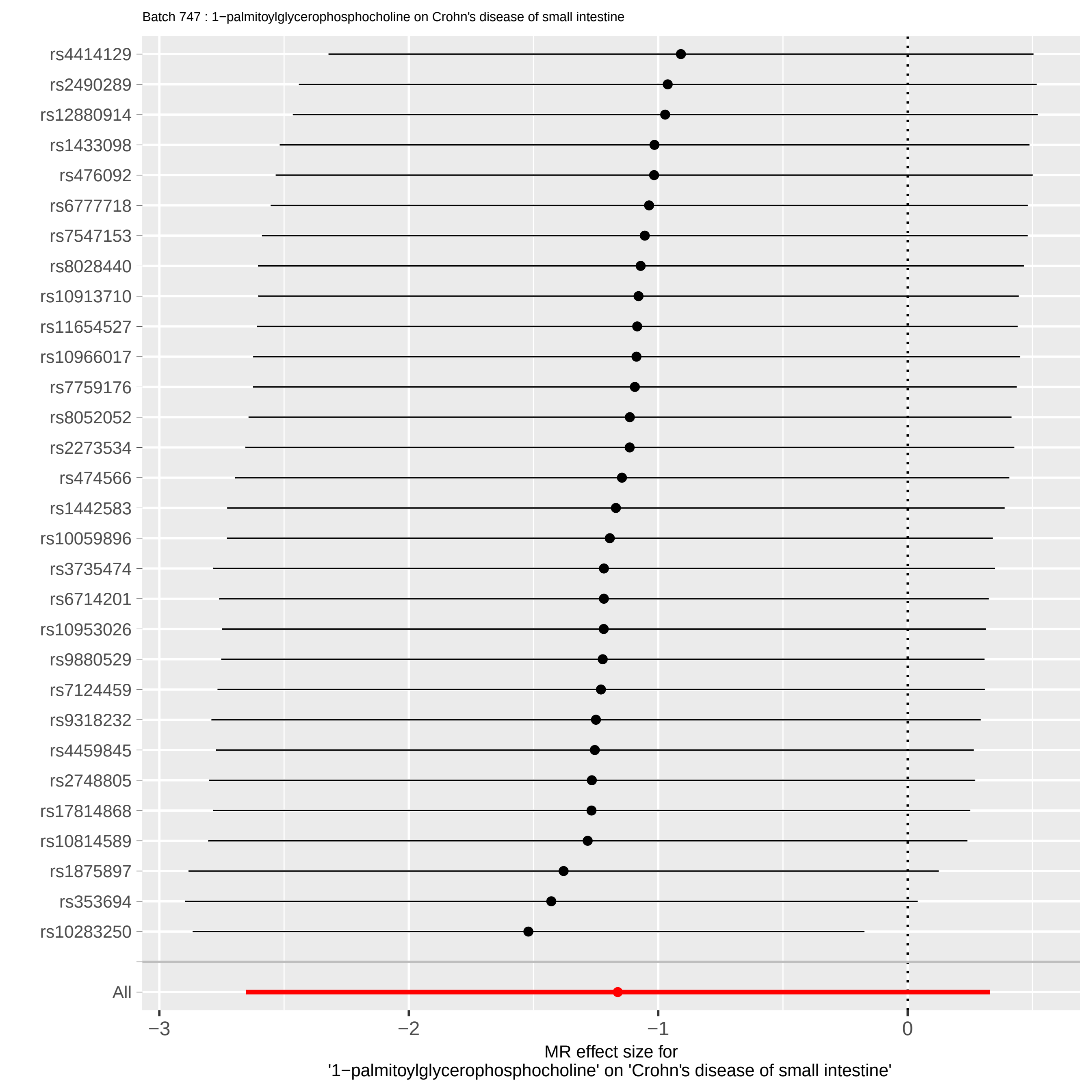

Batch 747 : 1−palmitoylglycerophosphocholine on Crohn's disease of small intestine
rs4414129
rs2490289
rs12880914
rs1433098
rs476092
rs6777718
rs7547153
rs8028440
rs10913710
rs11654527
rs10966017
rs7759176
rs8052052
rs2273534
rs474566
rs1442583
rs10059896
rs3735474
rs6714201
rs10953026
rs9880529
rs7124459
rs9318232
rs4459845
rs2748805
rs17814868
rs10814589
rs1875897
rs353694
rs10283250
All
−3
−2
−1
0
MR effect size for
'1−palmitoylglycerophosphocholine' on 'Crohn's disease of small intestine'

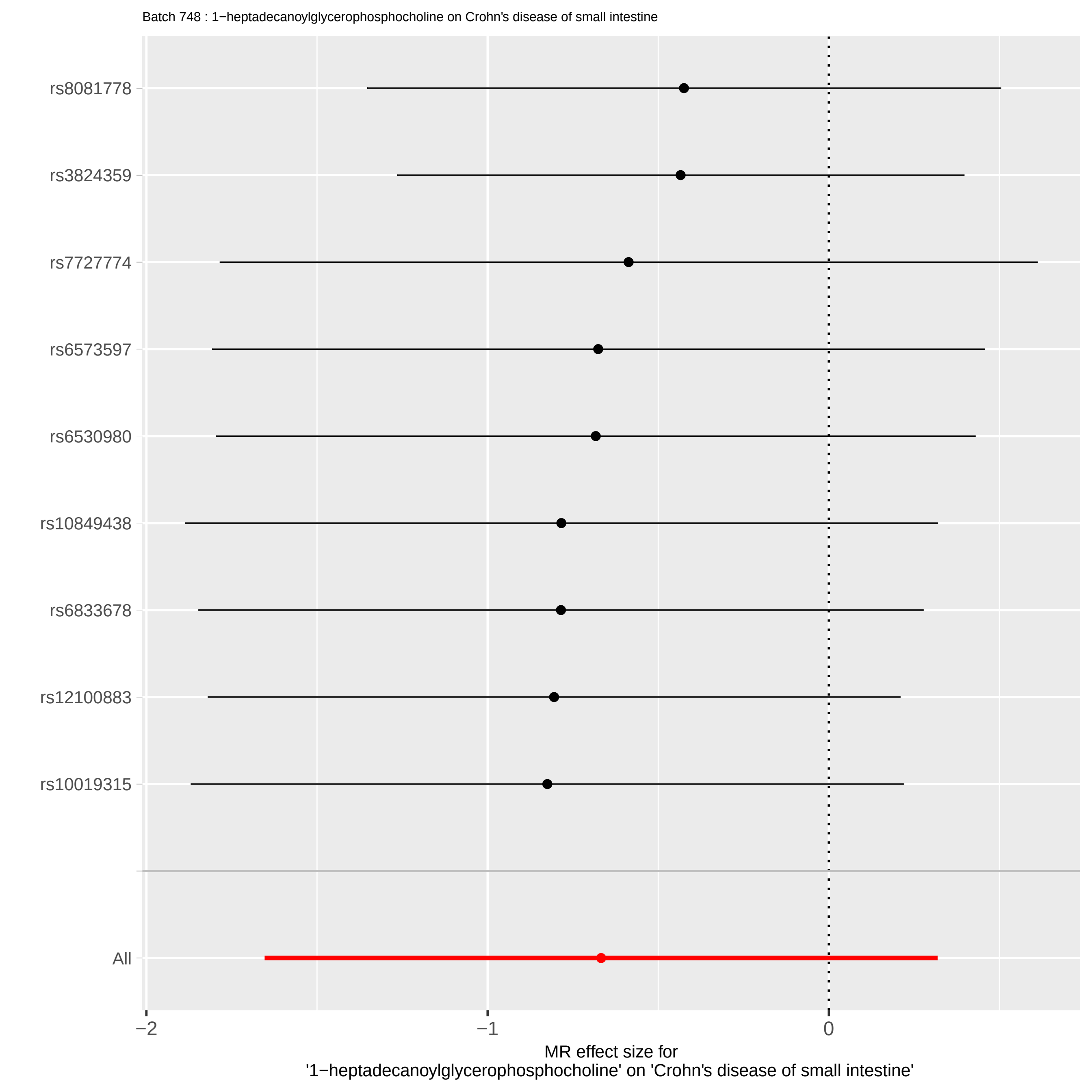
Batch 748 : 1−heptadecanoylglycerophosphocholine on Crohn's disease of small intestine
rs8081778
rs3824359
rs7727774
rs6573597
rs6530980
rs10849438
rs6833678
rs12100883
rs10019315
All
−2
−1
0
MR effect size for
'1−heptadecanoylglycerophosphocholine' on 'Crohn's disease of small intestine'

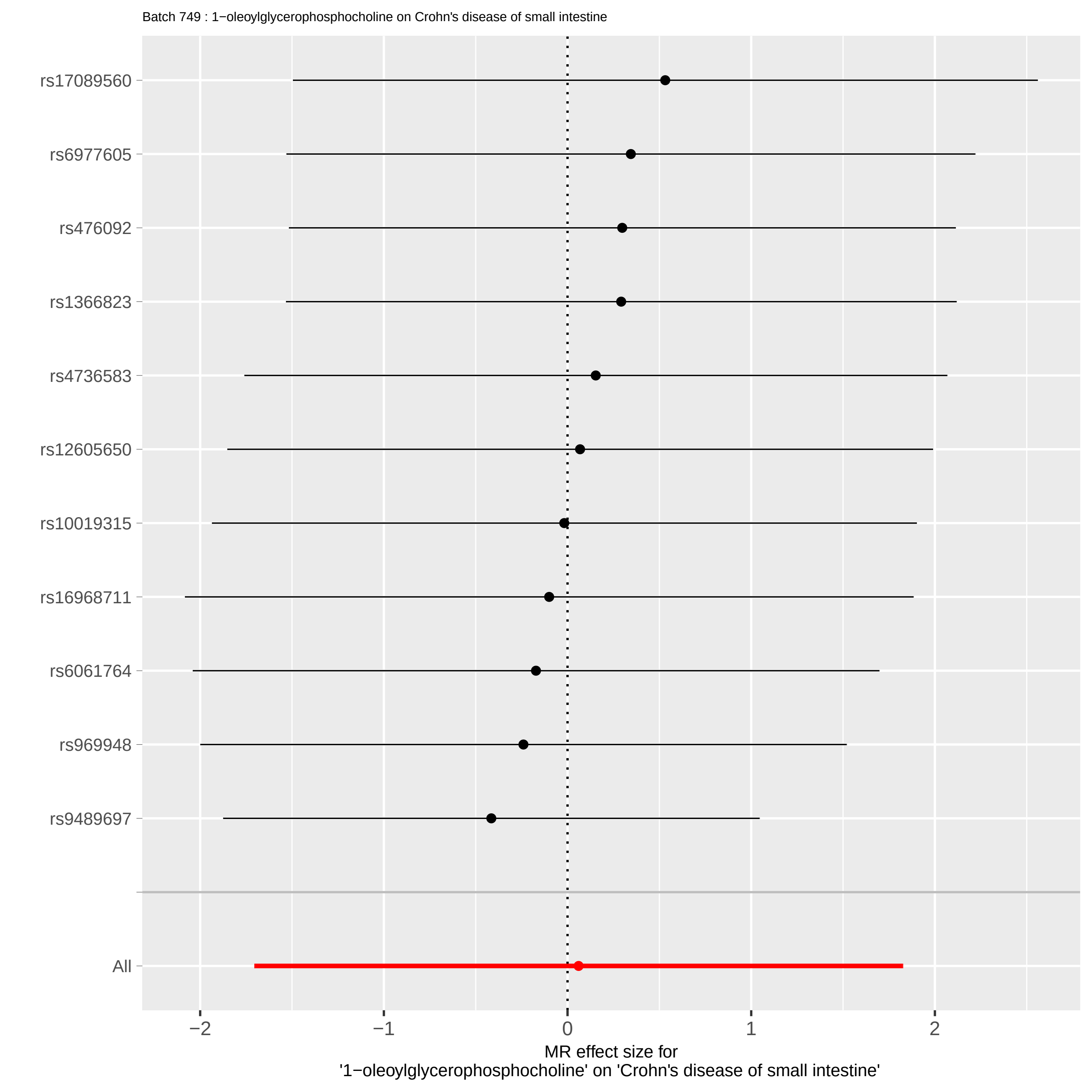
Batch 749 : 1−oleoylglycerophosphocholine on Crohn's disease of small intestine
rs17089560
rs6977605
rs476092
rs1366823
rs4736583
rs12605650
rs10019315
rs16968711
rs6061764
rs969948
rs9489697
All
−2
−1
0
1
2
MR effect size for
'1−oleoylglycerophosphocholine' on 'Crohn's disease of small intestine'

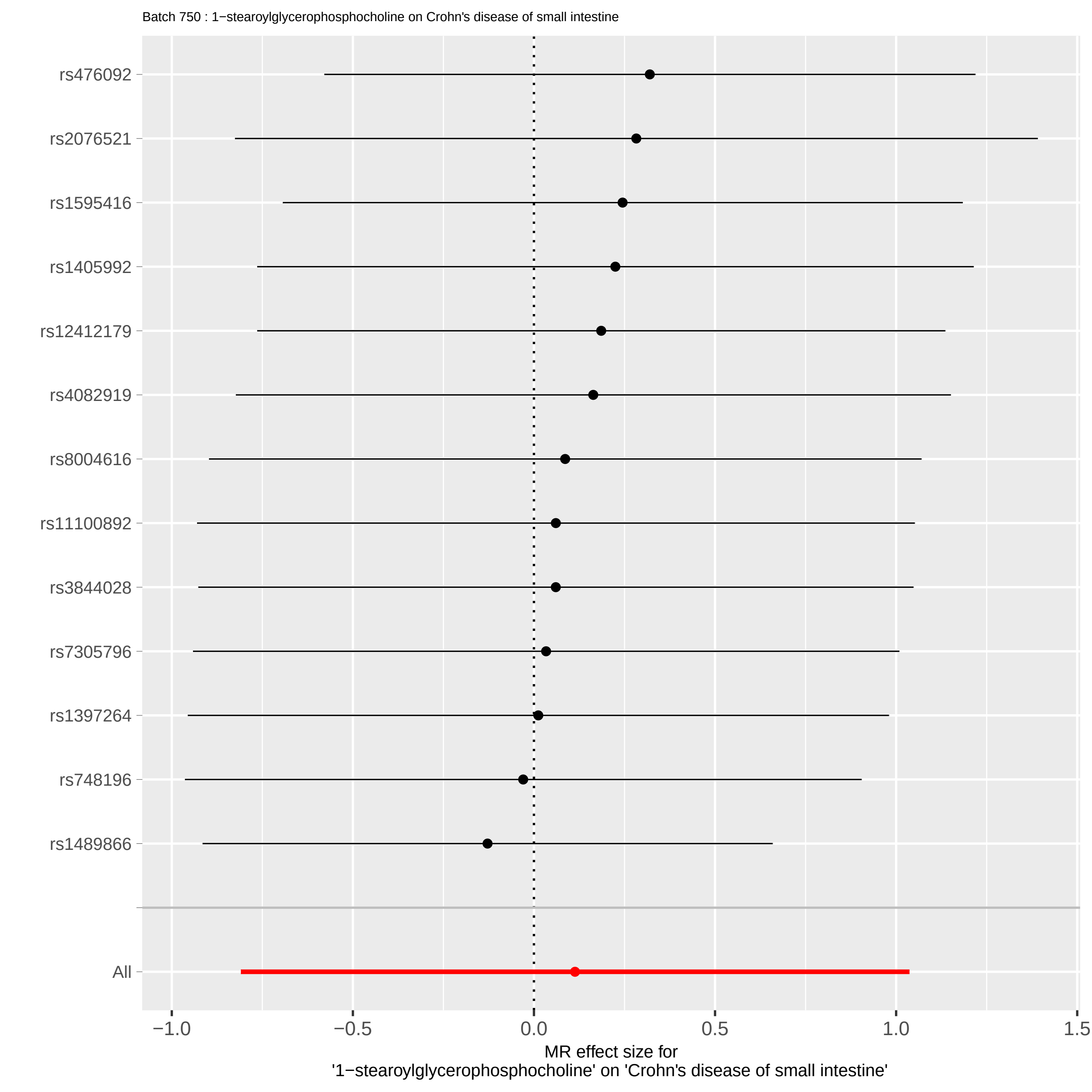
Batch 750 : 1−stearoylglycerophosphocholine on Crohn's disease of small intestine
rs476092
rs2076521
rs1595416
rs1405992
rs12412179
rs4082919
rs8004616
rs11100892
rs3844028
rs7305796
rs1397264
rs748196
rs1489866
All
−1.0
−0.5
0.0
0.5
1.0
1.5
MR effect size for
'1−stearoylglycerophosphocholine' on 'Crohn's disease of small intestine'

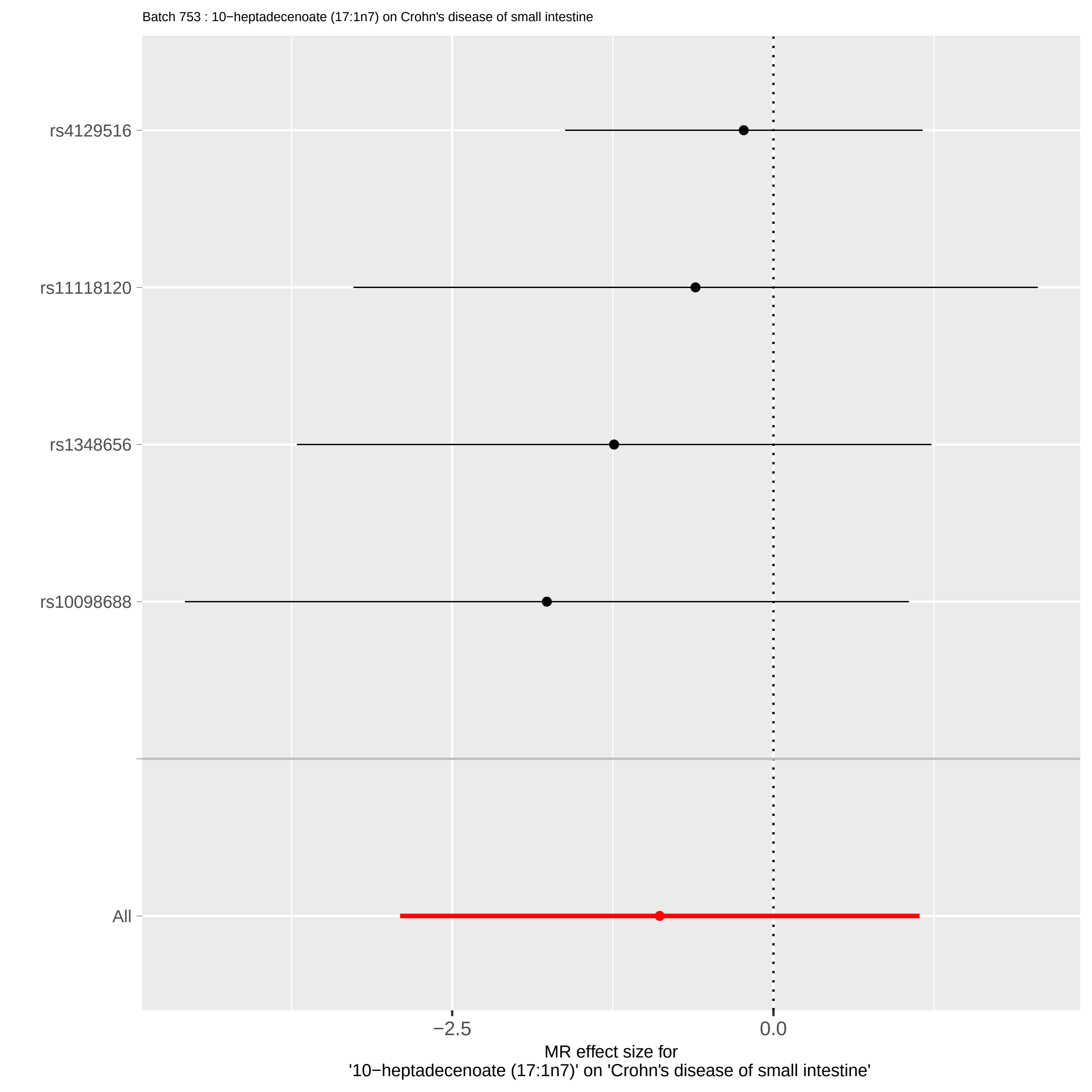
Batch 753 : 10−heptadecenoate (17:1n7) on Crohn's disease of small intestine
rs4129516
rs11118120
rs1348656
rs10098688
All
−2.5
0.0
MR effect size for
'10−heptadecenoate (17:1n7)' on 'Crohn's disease of small intestine'

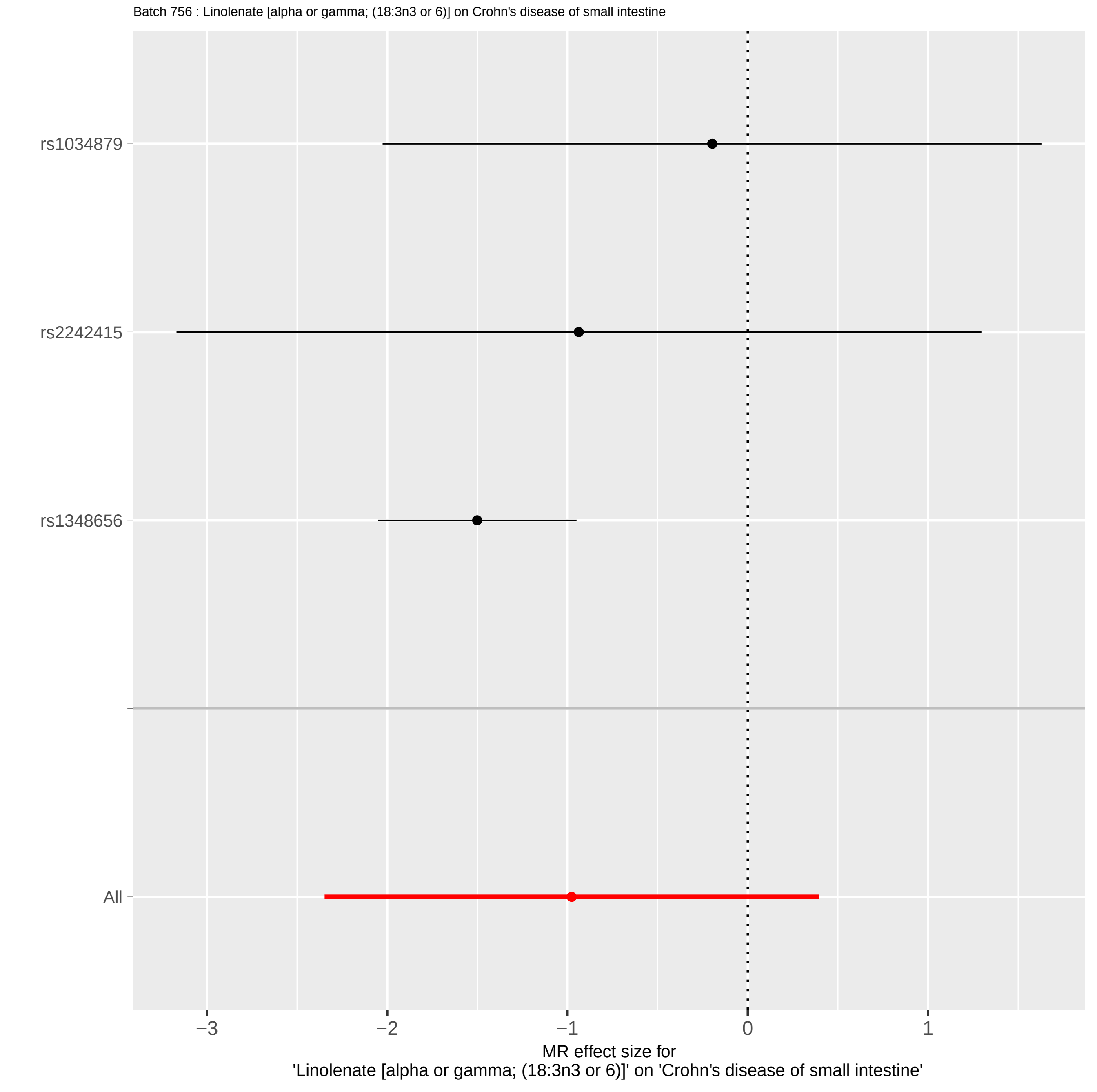
Batch 756 : Linolenate [alpha or gamma; (18:3n3 or 6)] on Crohn's disease of small intestine
rs1034879
rs2242415
rs1348656
All
−3
−2
−1
0
1
MR effect size for
'Linolenate [alpha or gamma; (18:3n3 or 6)]' on 'Crohn's disease of small intestine'

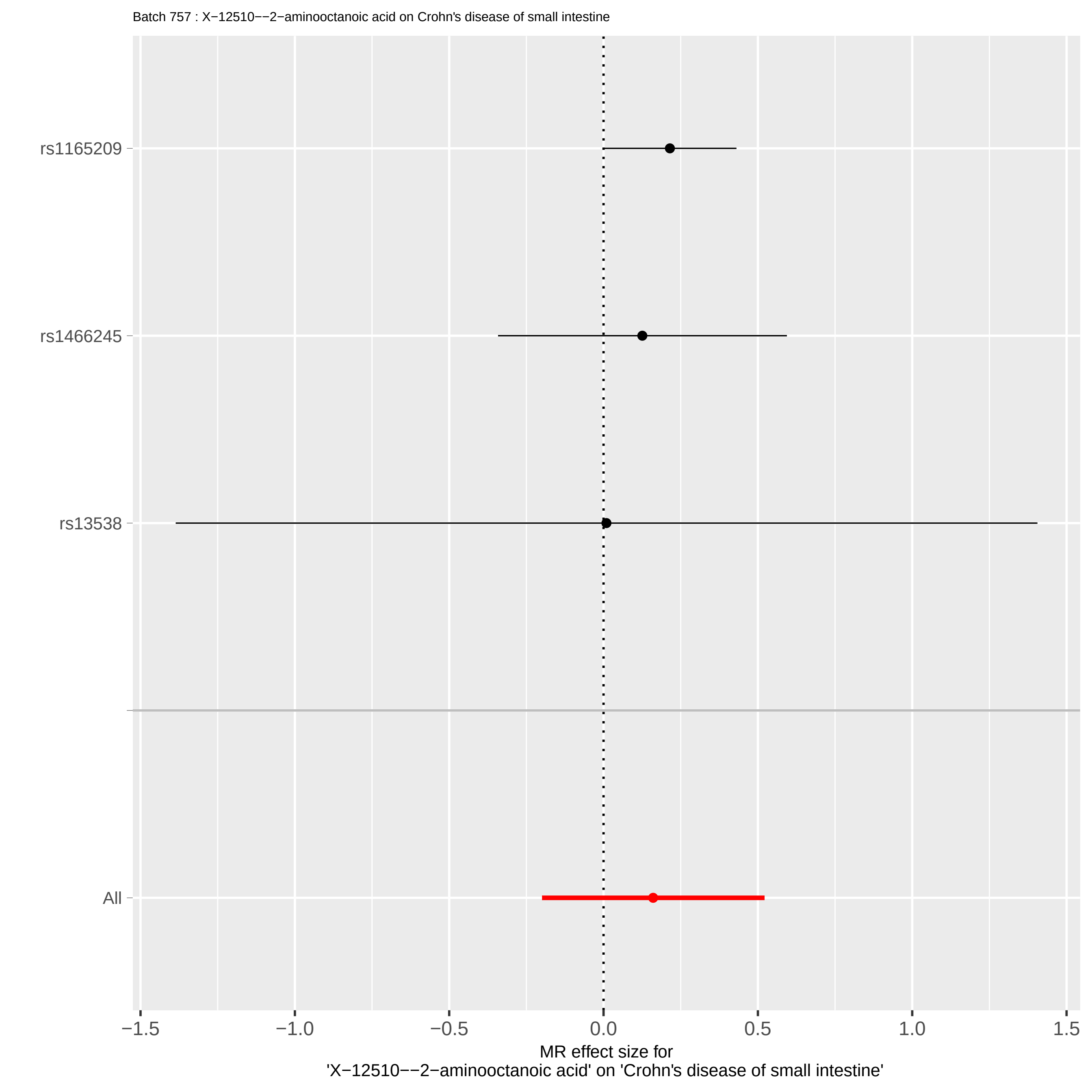

Batch 757 : X−12510−−2−aminooctanoic acid on Crohn's disease of small intestine
rs1165209
rs1466245
rs13538
All
−1.5
−1.0
−0.5
0.0
0.5
1.0
1.5
MR effect size for
'X−12510−−2−aminooctanoic acid' on 'Crohn's disease of small intestine'

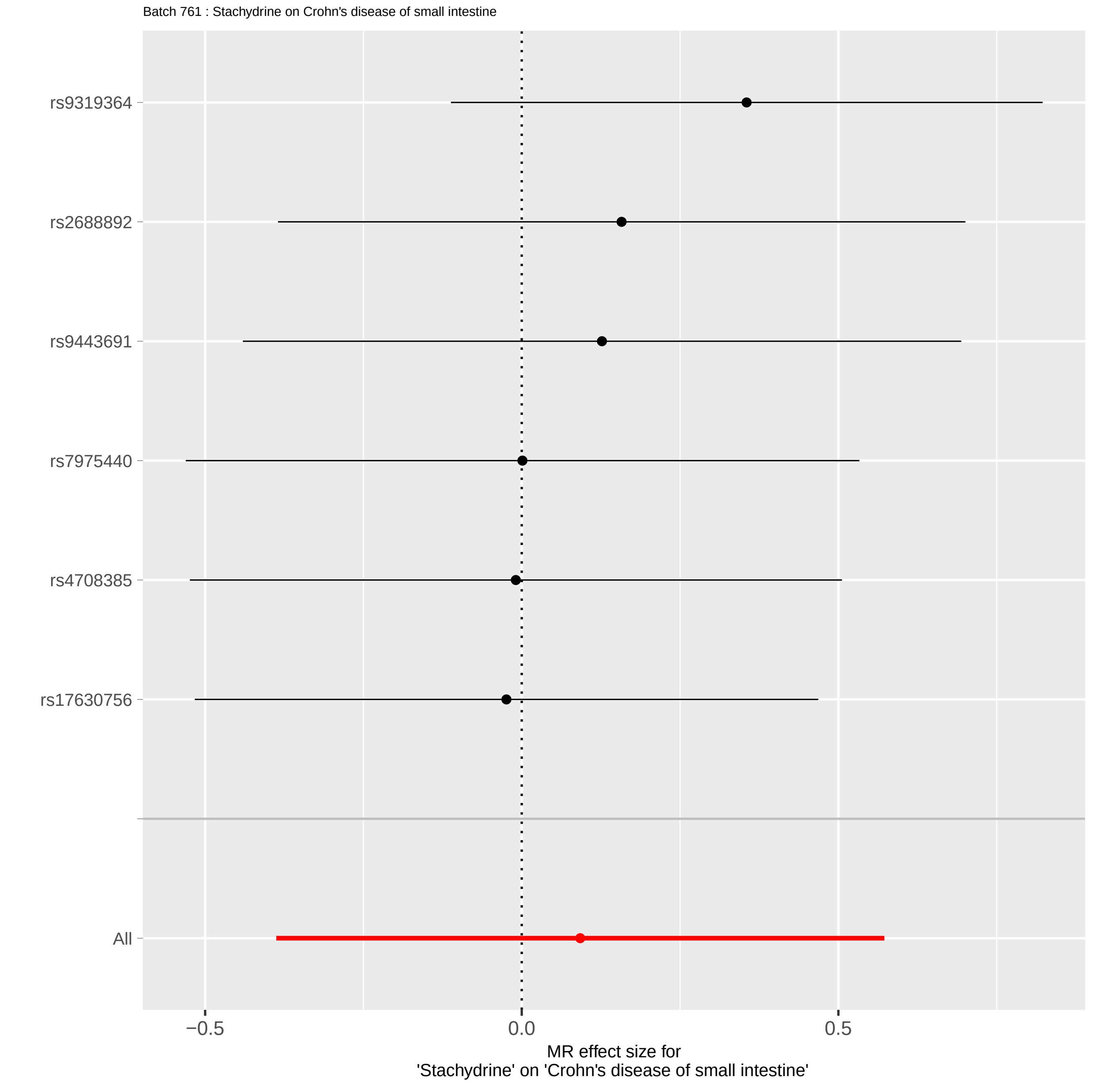

Batch 761 : Stachydrine on Crohn's disease of small intestine
rs9319364
rs2688892
rs9443691
rs7975440
rs4708385
rs17630756
All
−0.5
0.0
0.5
MR effect size for
'Stachydrine' on 'Crohn's disease of small intestine'

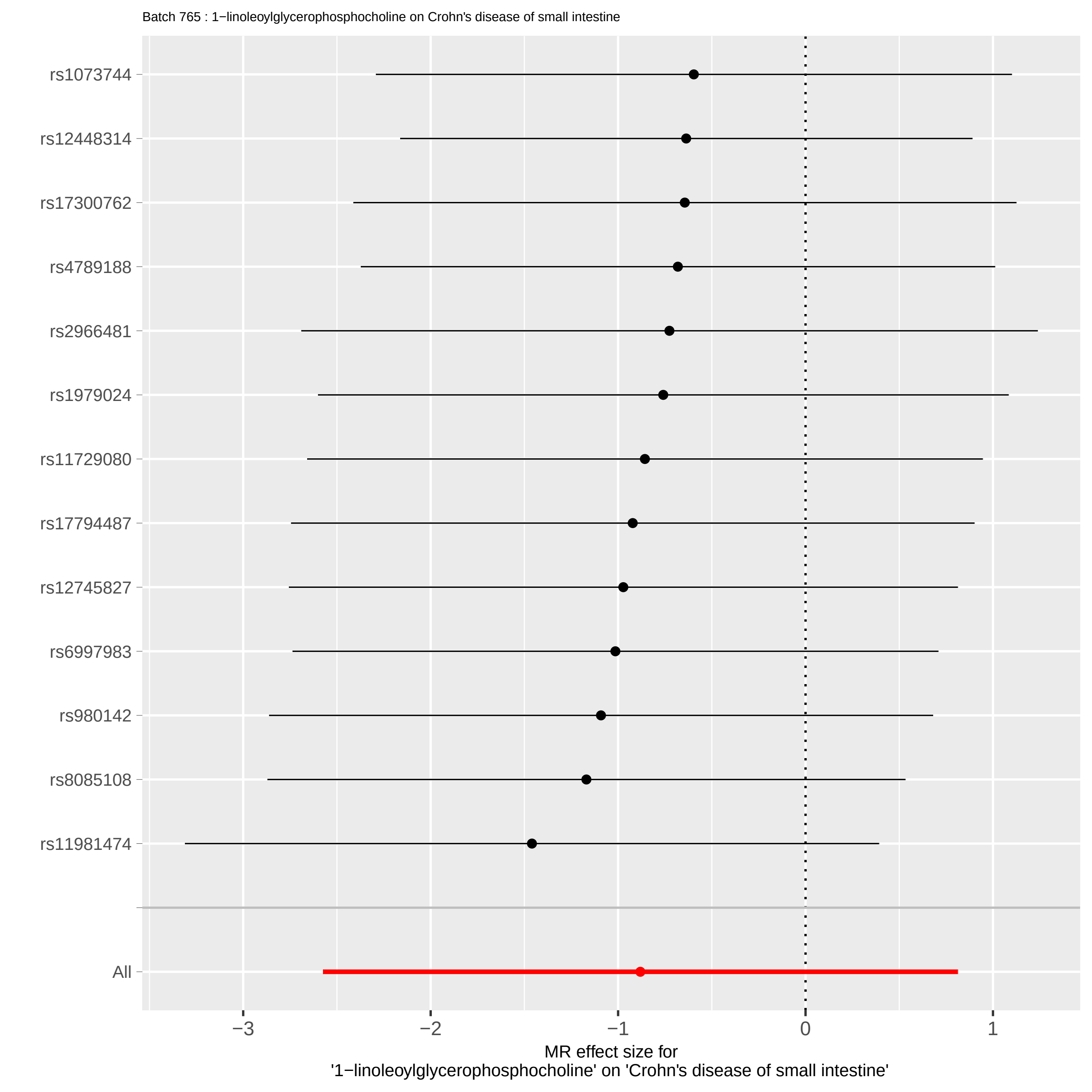

Batch 765 : 1−linoleoylglycerophosphocholine on Crohn's disease of small intestine
rs1073744
rs12448314
rs17300762
rs4789188
rs2966481
rs1979024
rs11729080
rs17794487
rs12745827
rs6997983
rs980142
rs8085108
rs11981474
All
−3
−2
−1
0
1
MR effect size for
'1−linoleoylglycerophosphocholine' on 'Crohn's disease of small intestine'

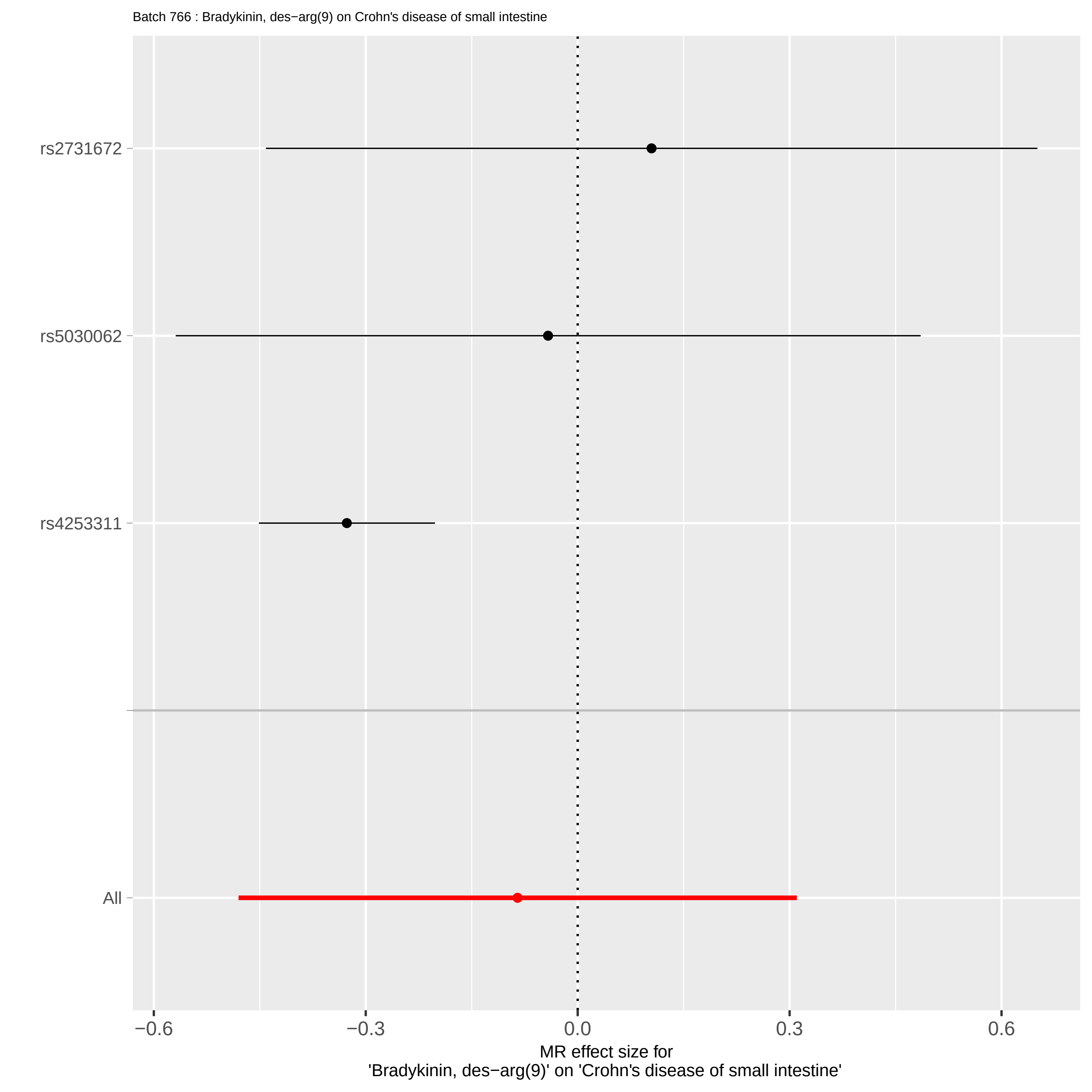
Batch 766 : Bradykinin, des−arg(9) on Crohn's disease of small intestine
rs2731672
rs5030062
rs4253311
All
−0.6
−0.3
0.0
0.3
0.6
MR effect size for
'Bradykinin, des−arg(9)' on 'Crohn's disease of small intestine'

Batch 767 : Gamma−glutamylisoleucine* on Crohn's disease of small intestine

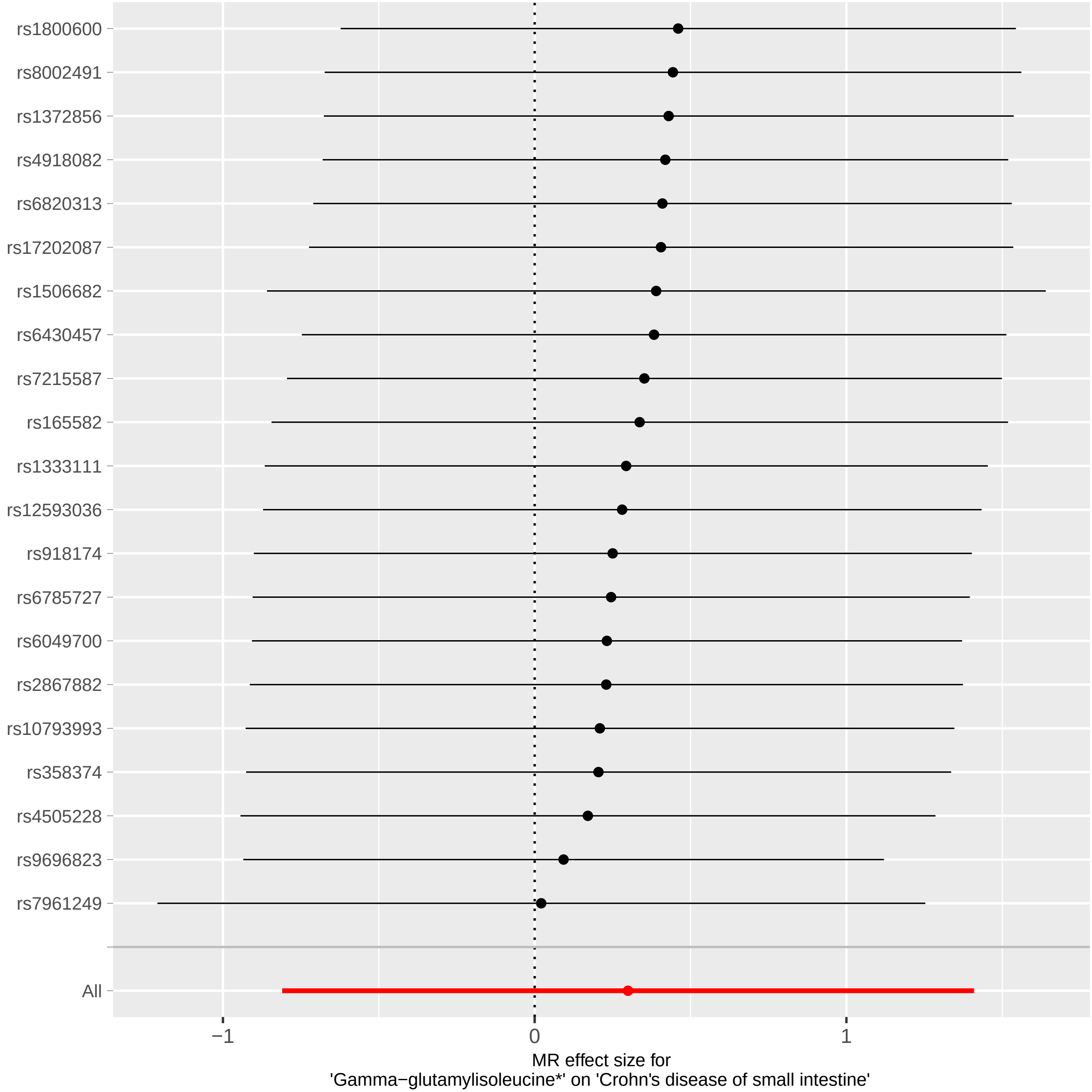

Batch 770 : Isovalerate on Crohn's disease of small intestine

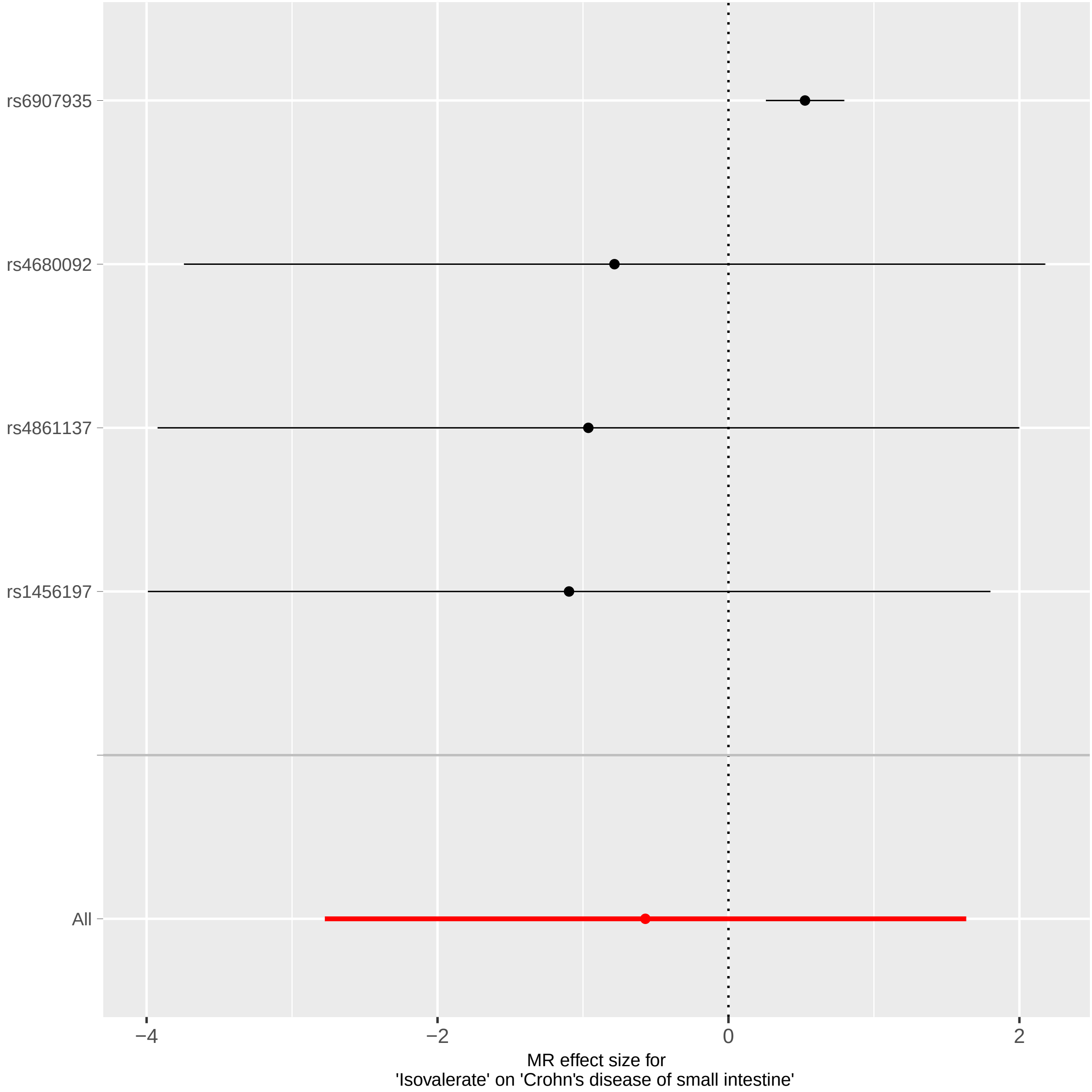

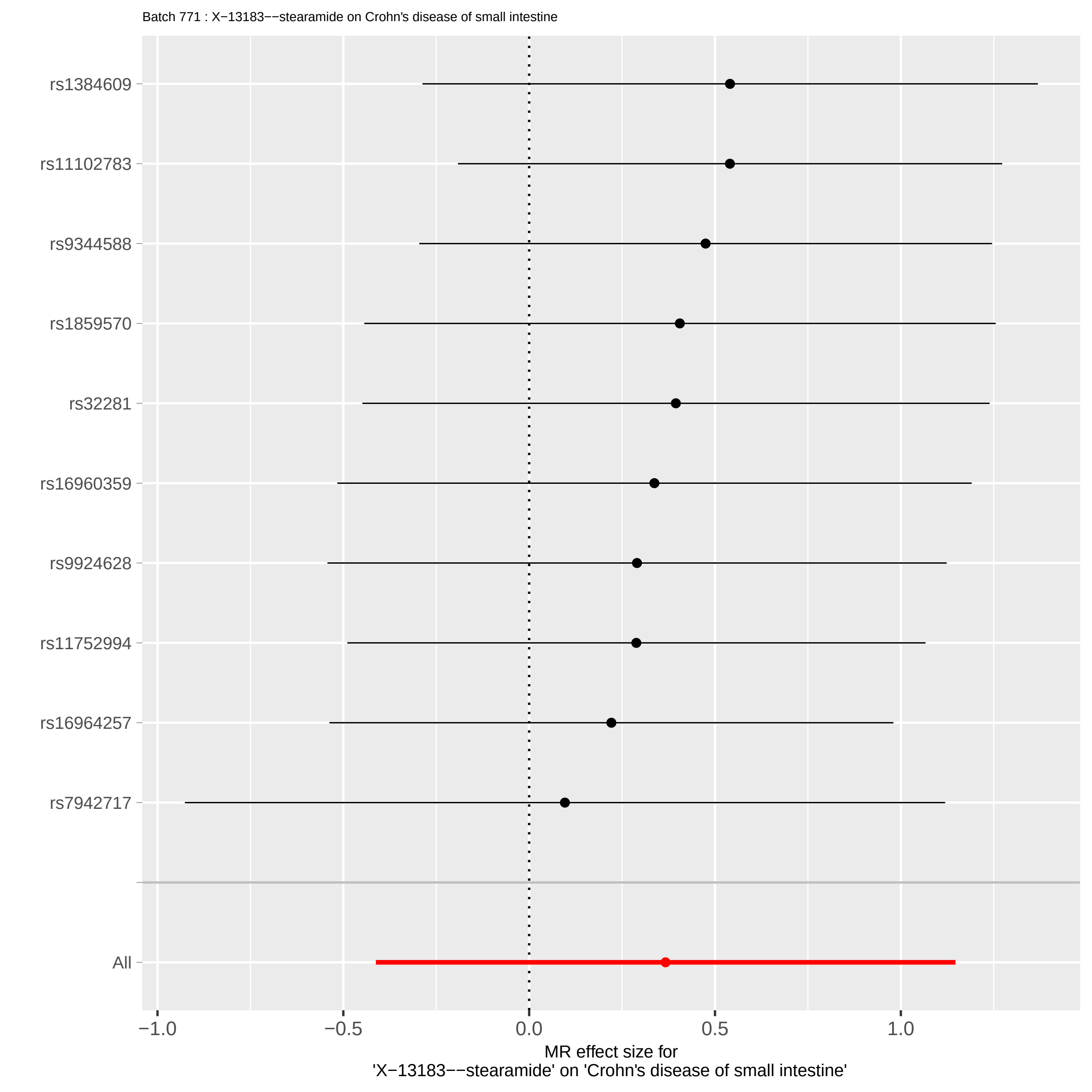

Batch 771 : X−13183−−stearamide on Crohn's disease of small intestine
rs1384609
rs11102783
rs9344588
rs1859570
rs32281
rs16960359
rs9924628
rs11752994
rs16964257
rs7942717
All
−1.0
−0.5
0.0
0.5
1.0
MR effect size for
'X−13183−−stearamide' on 'Crohn's disease of small intestine'

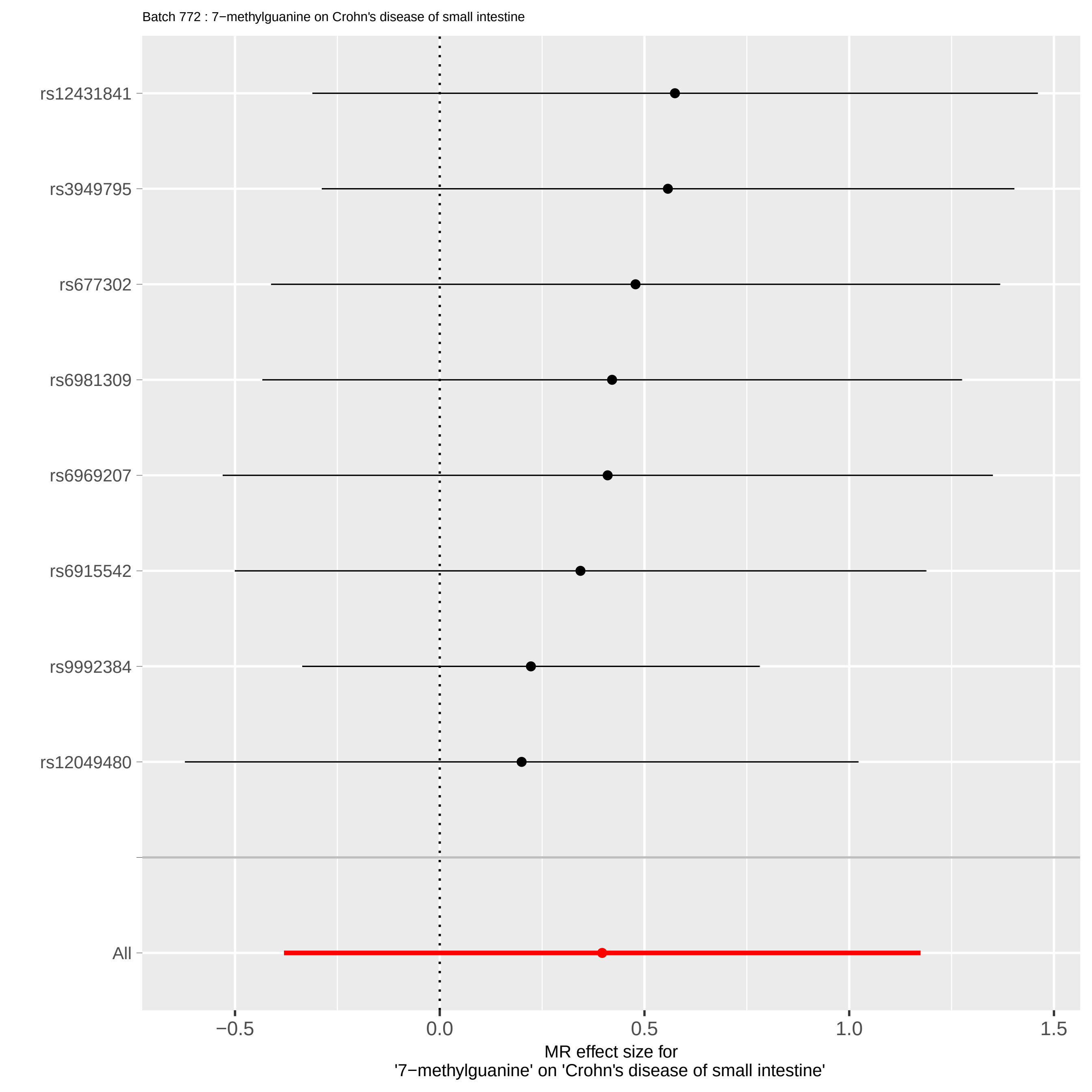

Batch 772 : 7−methylguanine on Crohn's disease of small intestine
rs12431841
rs3949795
rs677302
rs6981309
rs6969207
rs6915542
rs9992384
rs12049480
All
−0.5
0.0
0.5
1.0
1.5
MR effect size for
'7−methylguanine' on 'Crohn's disease of small intestine'

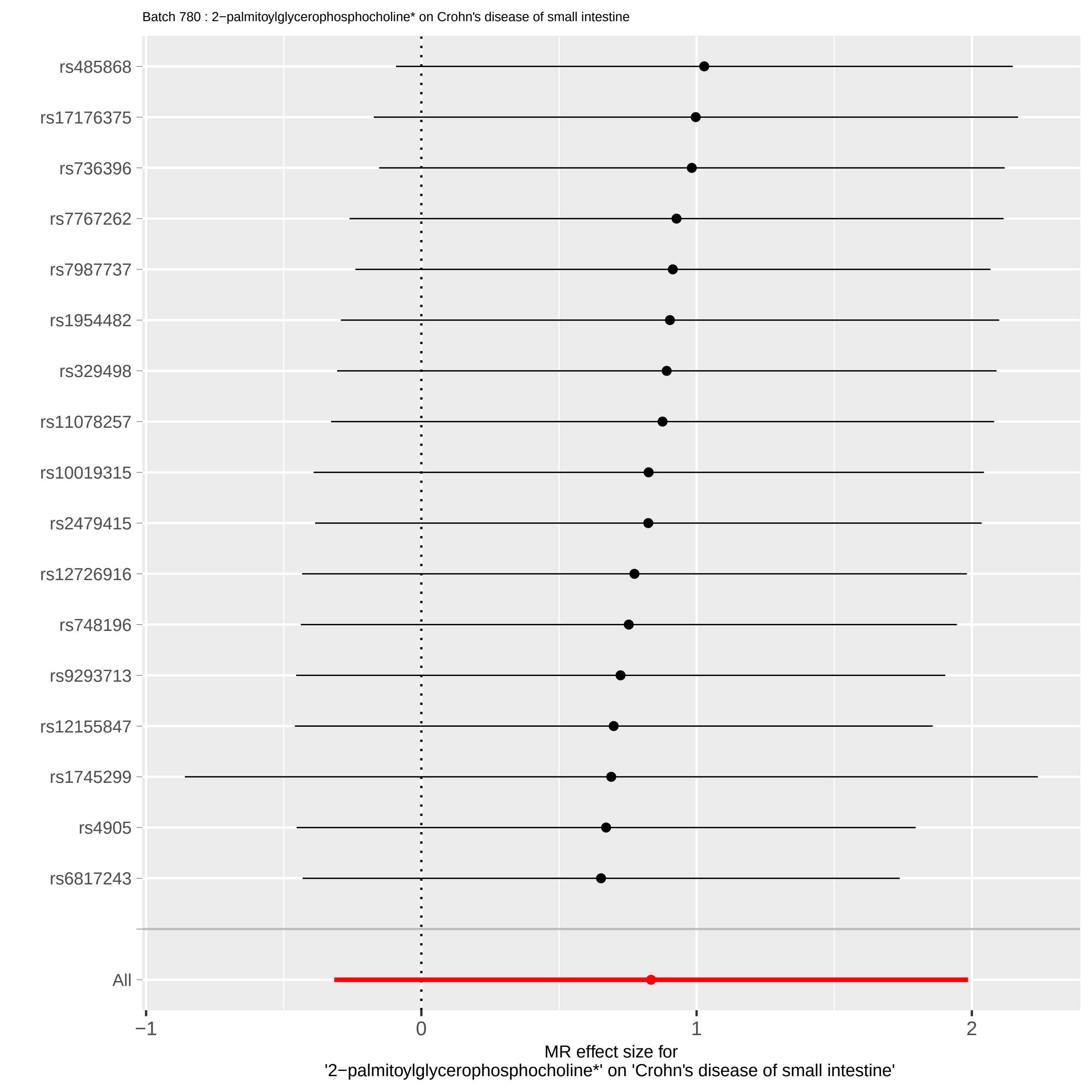

Batch 780 : 2−palmitoylglycerophosphocholine* on Crohn's disease of small intestine
rs485868
rs17176375
rs736396
rs7767262
rs7987737
rs1954482
rs329498
rs11078257
rs10019315
rs2479415
rs12726916
rs748196
rs9293713
rs12155847
rs1745299
rs4905
rs6817243
All
−1
0
1
2
MR effect size for
'2−palmitoylglycerophosphocholine*' on 'Crohn's disease of small intestine'

Batch 781 : 2−oleoylglycerophosphocholine* on Crohn's disease of small intestine

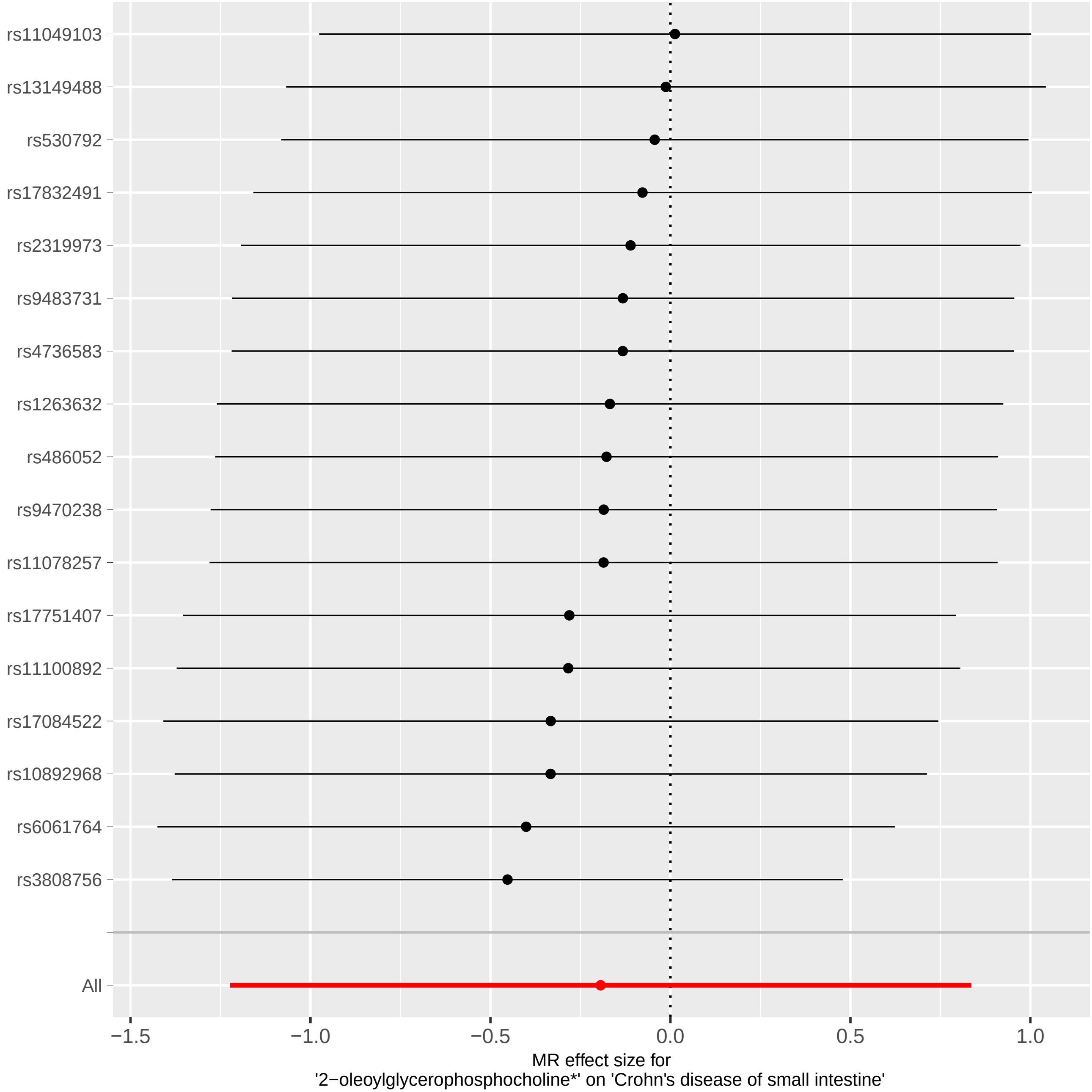

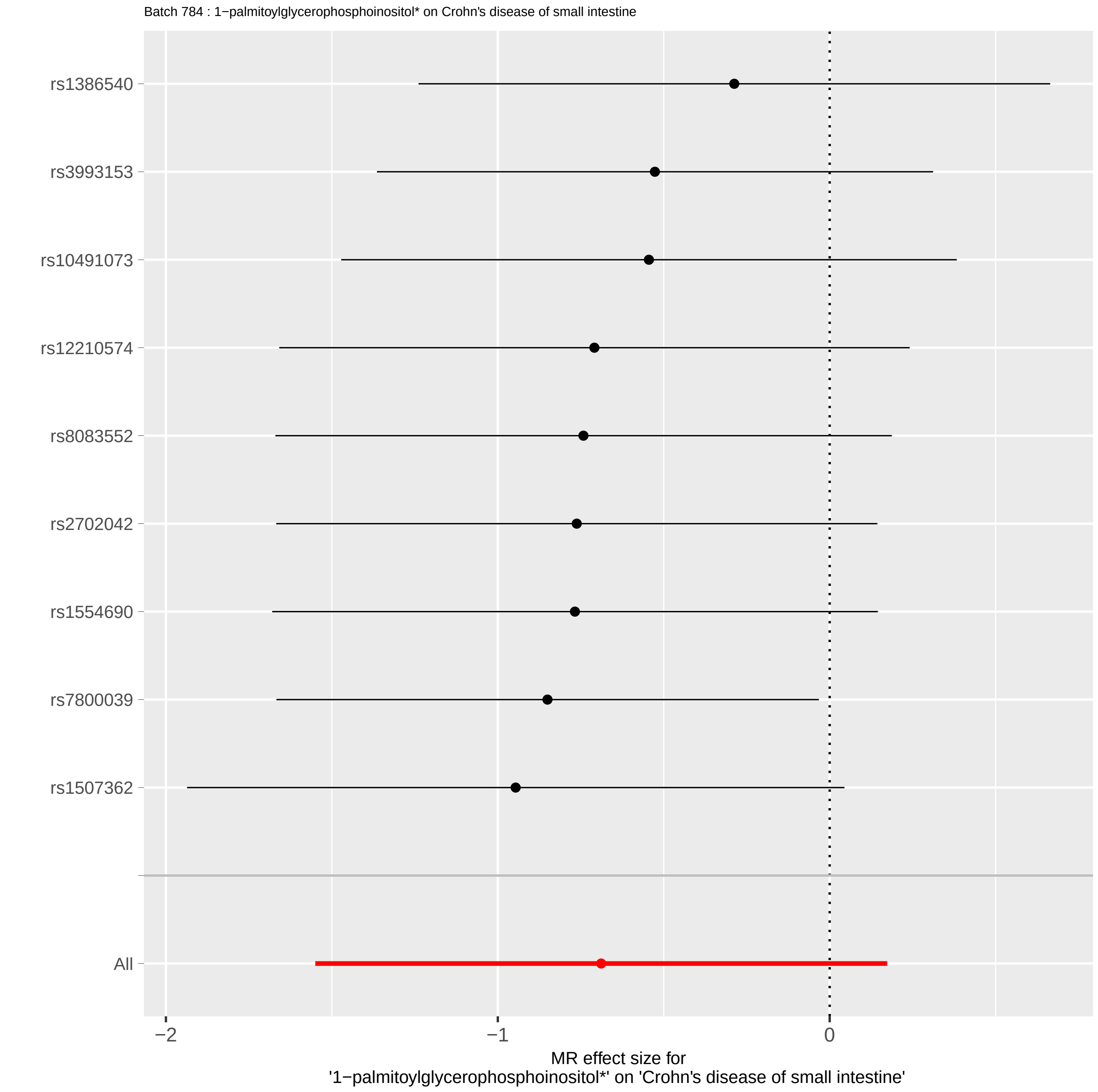

Batch 784 : 1−palmitoylglycerophosphoinositol* on Crohn's disease of small intestine
rs1386540
rs3993153
rs10491073
rs12210574
rs8083552
rs2702042
rs1554690
rs7800039
rs1507362
All
−2
−1
0
MR effect size for
'1−palmitoylglycerophosphoinositol*' on 'Crohn's disease of small intestine'

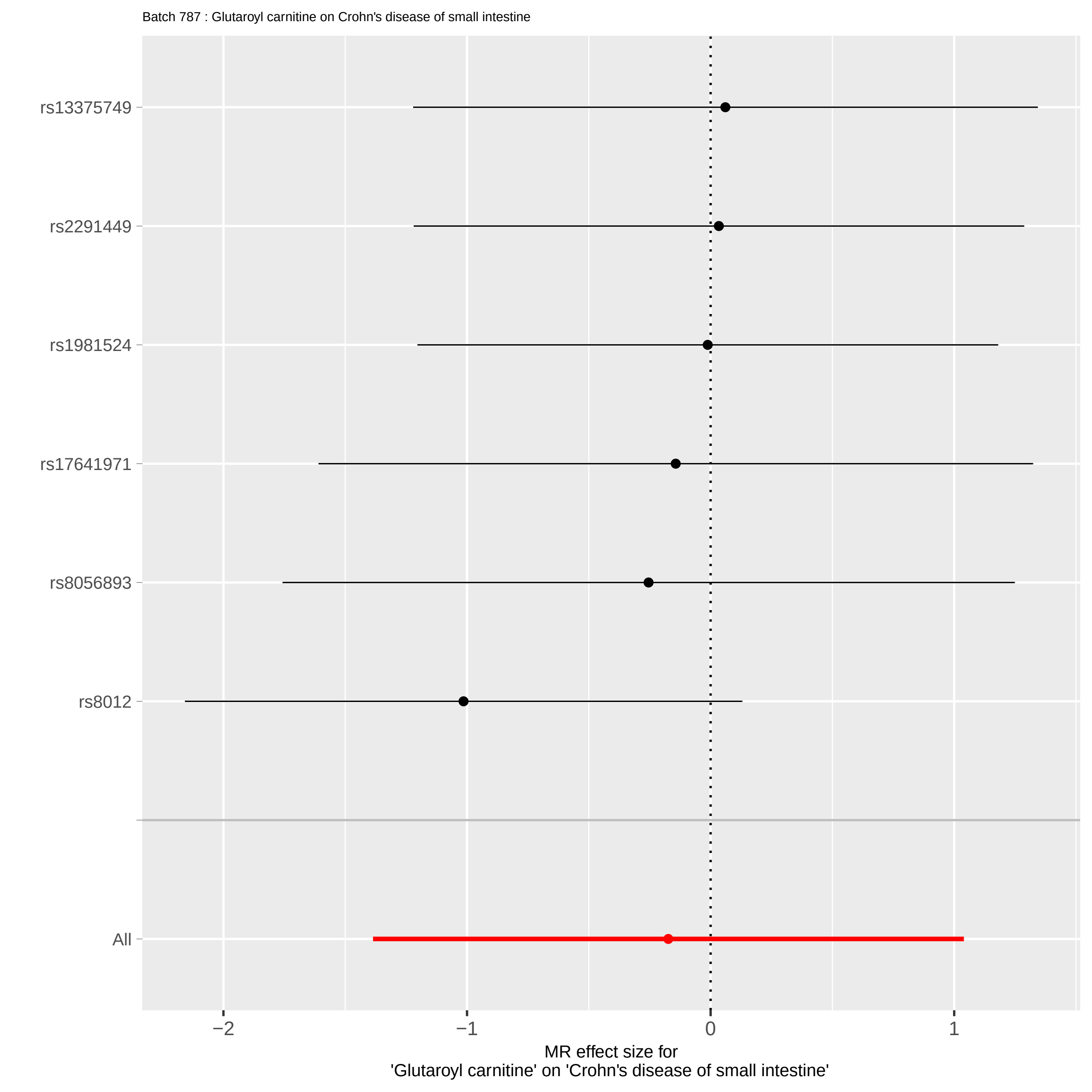

Batch 787 : Glutaroyl carnitine on Crohn's disease of small intestine
rs13375749
rs2291449
rs1981524
rs17641971
rs8056893
rs8012
All
−2
−1
0
1
MR effect size for
'Glutaroyl carnitine' on 'Crohn's disease of small intestine'

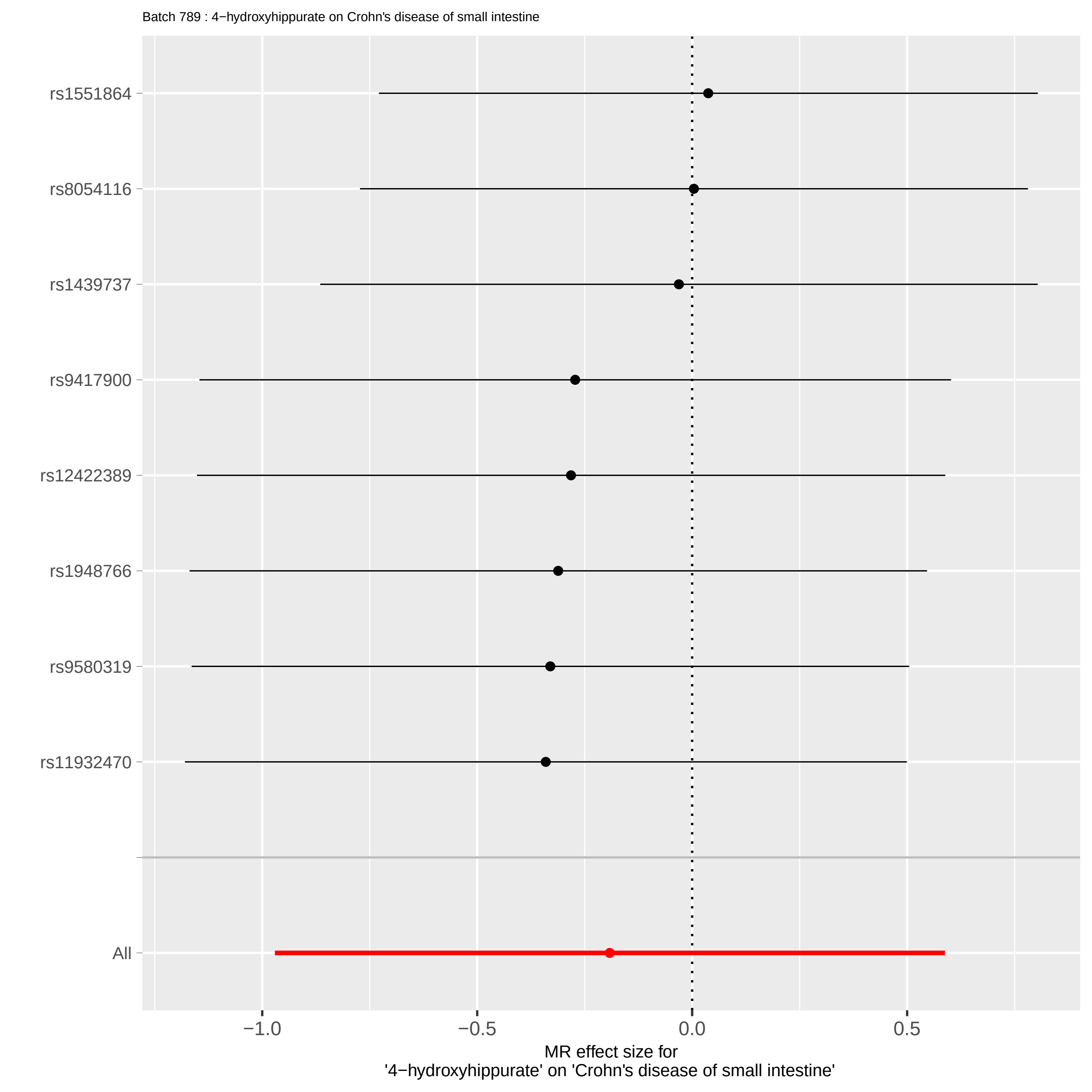
Batch 789 : 4−hydroxyhippurate on Crohn's disease of small intestine
rs1551864
rs8054116
rs1439737
rs9417900
rs12422389
rs1948766
rs9580319
rs11932470
All
−1.0
−0.5
0.0
0.5
MR effect size for
'4−hydroxyhippurate' on 'Crohn's disease of small intestine'

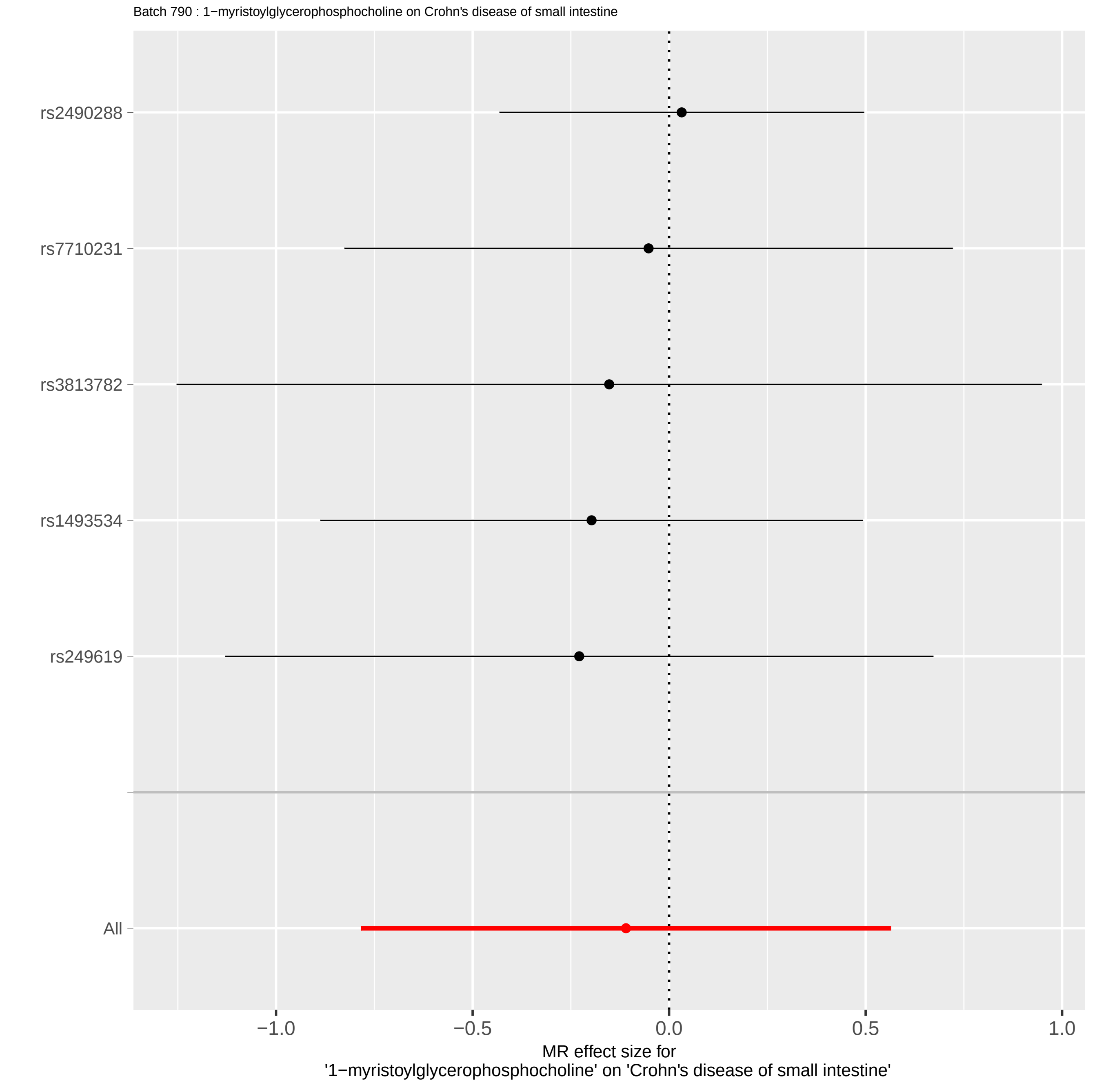

Batch 790 : 1−myristoylglycerophosphocholine on Crohn's disease of small intestine
rs2490288
rs7710231
rs3813782
rs1493534
rs249619
All
−1.0
−0.5
0.0
0.5
1.0
MR effect size for
'1−myristoylglycerophosphocholine' on 'Crohn's disease of small intestine'

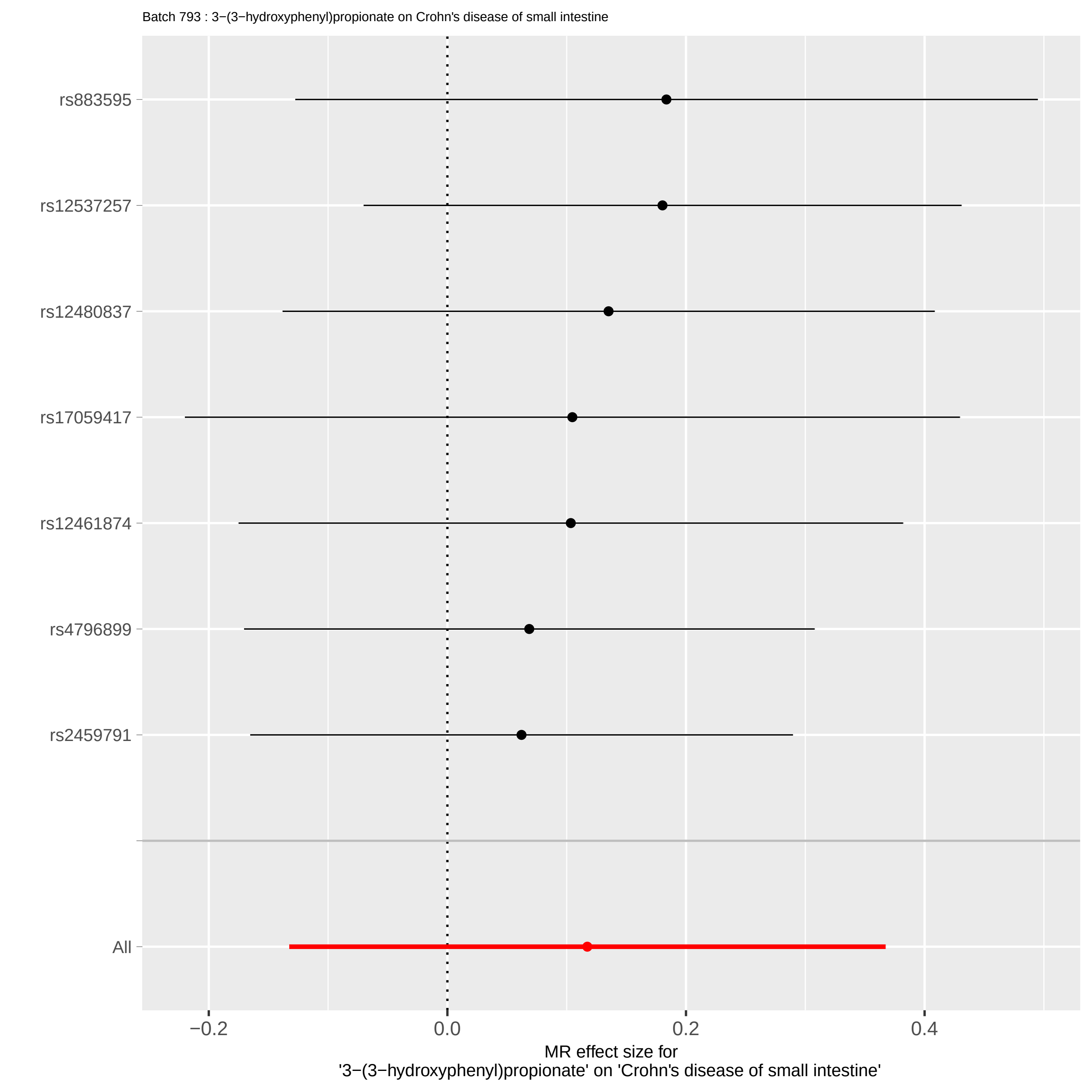

Batch 793 : 3−(3−hydroxyphenyl)propionate on Crohn's disease of small intestine
rs883595
rs12537257
rs12480837
rs17059417
rs12461874
rs4796899
rs2459791
All
−0.2
0.0
0.2
0.4
MR effect size for
'3−(3−hydroxyphenyl)propionate' on 'Crohn's disease of small intestine'

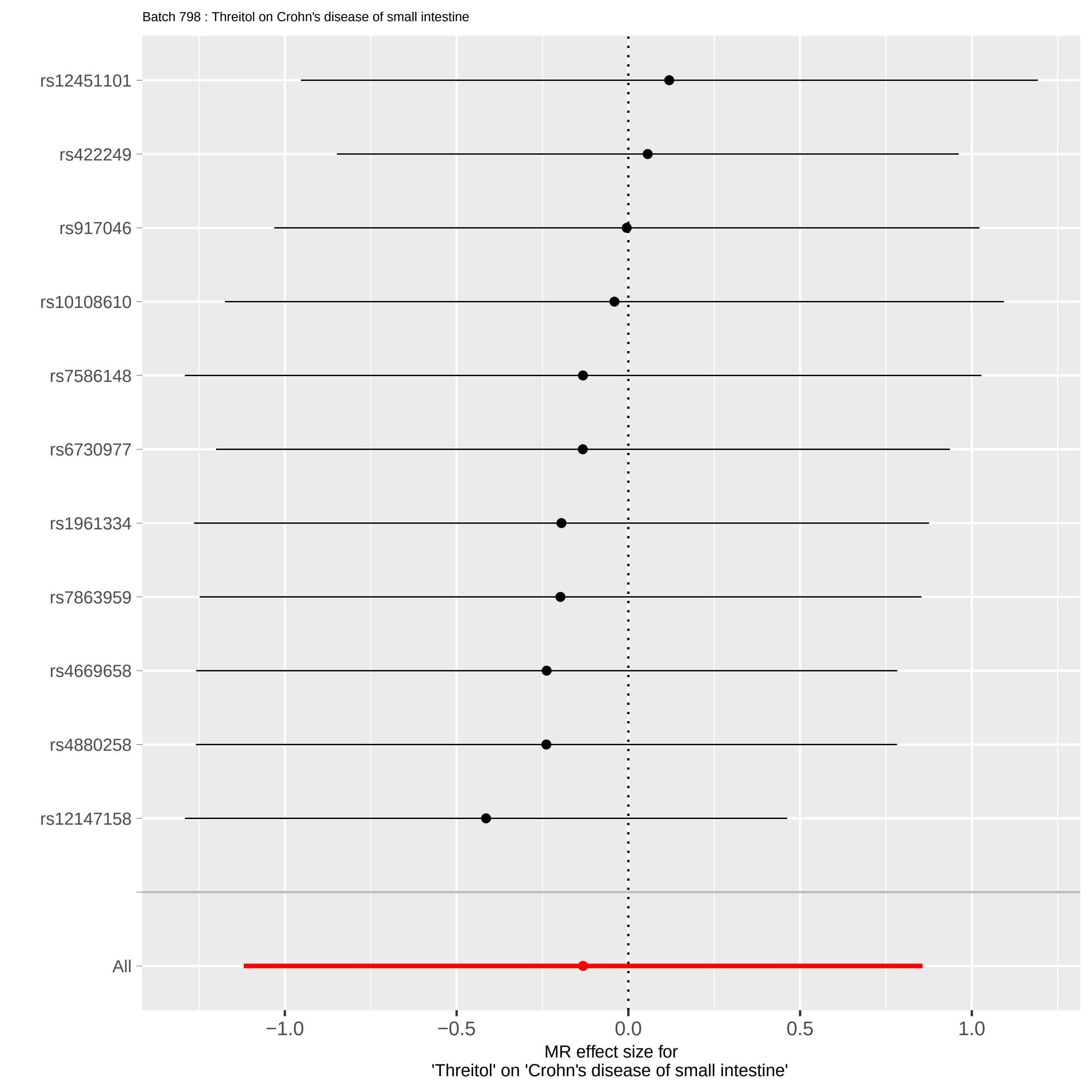

Batch 798 : Threitol on Crohn's disease of small intestine
rs12451101
rs422249
rs917046
rs10108610
rs7586148
rs6730977
rs1961334
rs7863959
rs4669658
rs4880258
rs12147158
All
−1.0
−0.5
0.0
0.5
1.0
MR effect size for
'Threitol' on 'Crohn's disease of small intestine'

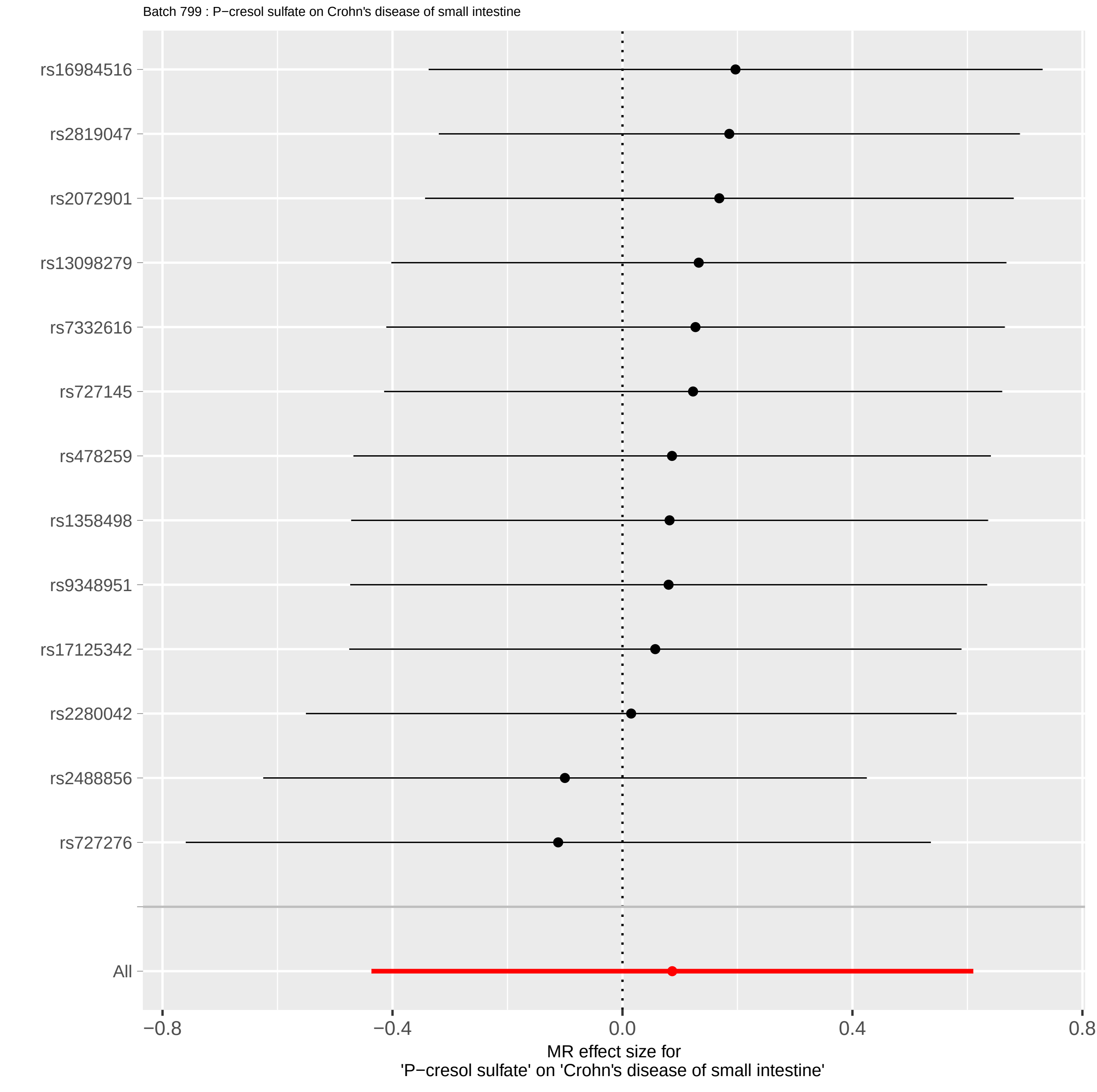

Batch 799 : P−cresol sulfate on Crohn's disease of small intestine
rs16984516
rs2819047
rs2072901
rs13098279
rs7332616
rs727145
rs478259
rs1358498
rs9348951
rs17125342
rs2280042
rs2488856
rs727276
All
−0.8
−0.4
0.0
0.4
0.8
MR effect size for
'P−cresol sulfate' on 'Crohn's disease of small intestine'

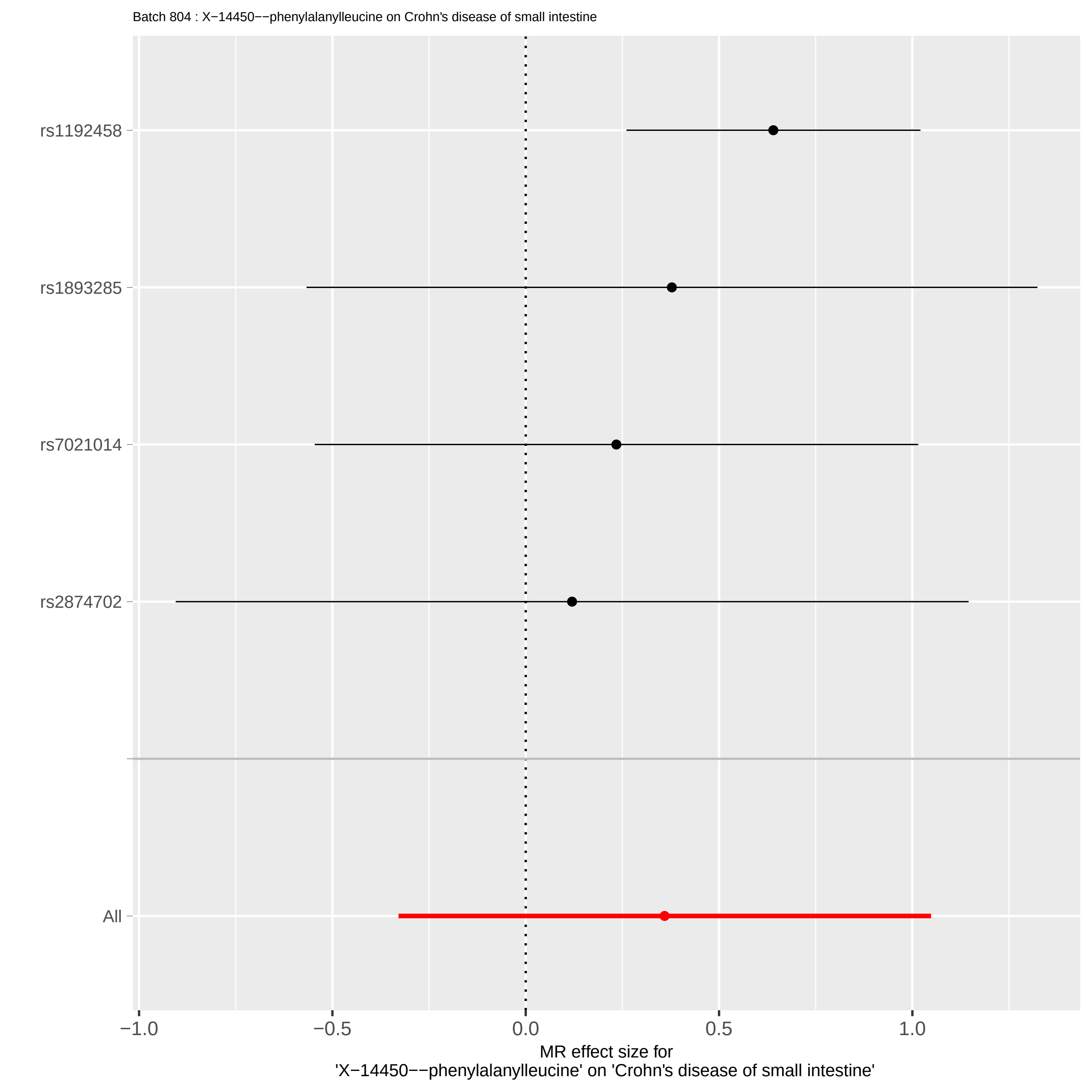

Batch 804 : X−14450−−phenylalanylleucine on Crohn's disease of small intestine
rs1192458
rs1893285
rs7021014
rs2874702
All
−1.0
−0.5
0.0
0.5
1.0
MR effect size for
'X−14450−−phenylalanylleucine' on 'Crohn's disease of small intestine'

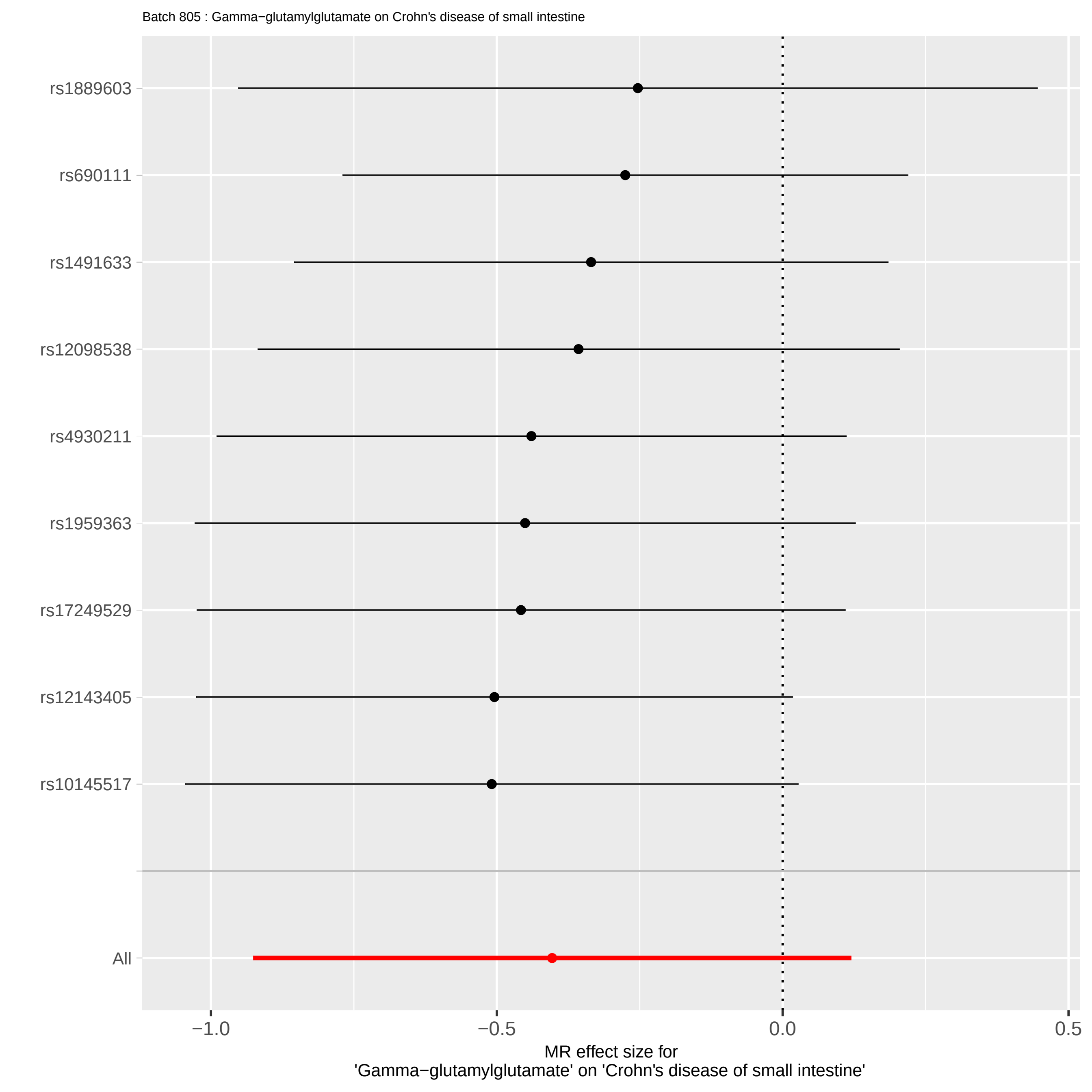
Batch 805 : Gamma−glutamylglutamate on Crohn's disease of small intestine
rs1889603
rs690111
rs1491633
rs12098538
rs4930211
rs1959363
rs17249529
rs12143405
rs10145517
All
−1.0
−0.5
0.0
0.5
MR effect size for
'Gamma−glutamylglutamate' on 'Crohn's disease of small intestine'

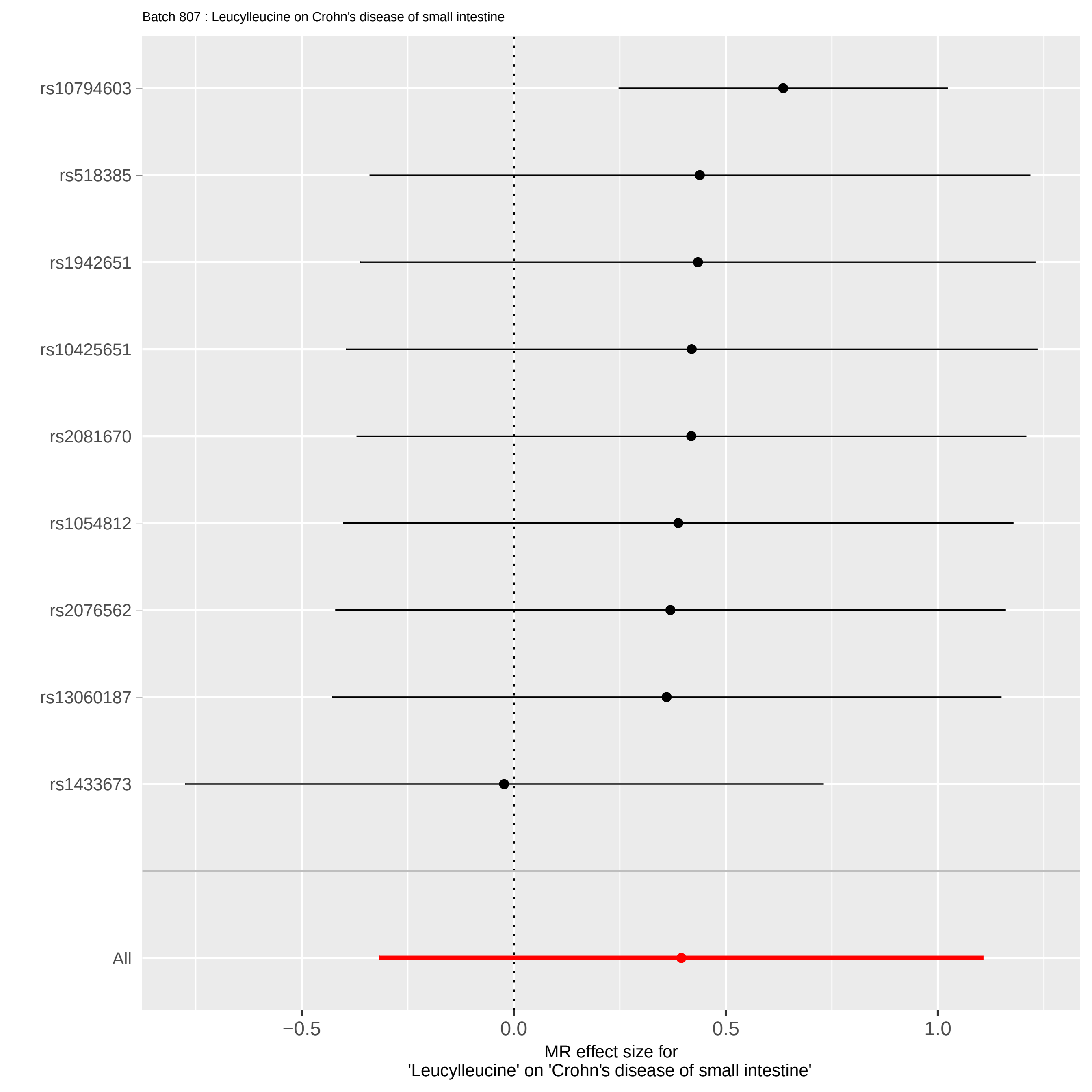

Batch 807 : Leucylleucine on Crohn's disease of small intestine
rs10794603
rs518385
rs1942651
rs10425651
rs2081670
rs1054812
rs2076562
rs13060187
rs1433673
All
−0.5
0.0
0.5
1.0
MR effect size for
'Leucylleucine' on 'Crohn's disease of small intestine'

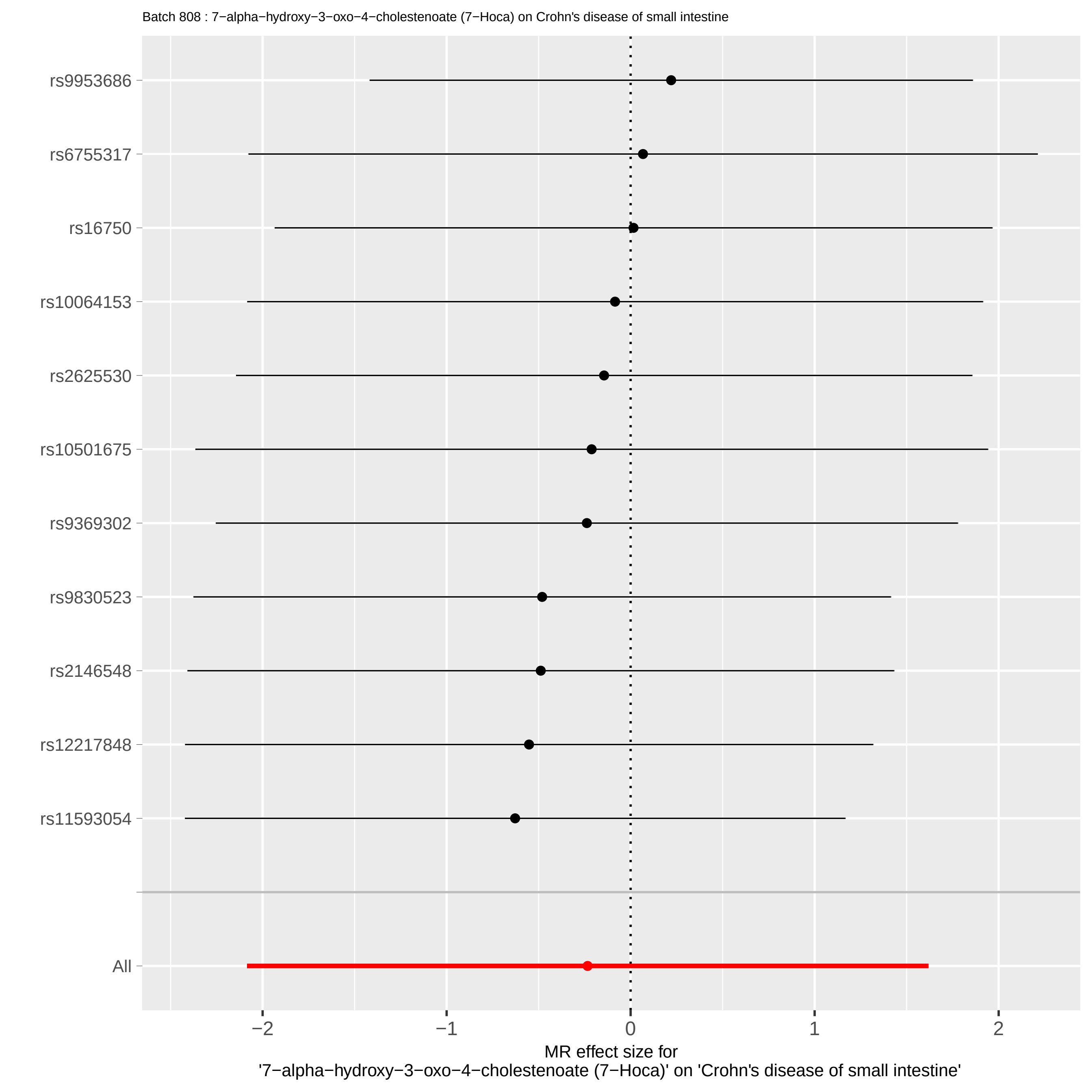

Batch 808 : 7−alpha−hydroxy−3−oxo−4−cholestenoate (7−Hoca) on Crohn's disease of small intestine
rs9953686
rs6755317
rs16750
rs10064153
rs2625530
rs10501675
rs9369302
rs9830523
rs2146548
rs12217848
rs11593054
All
−2
−1
0
1
2
MR effect size for
'7−alpha−hydroxy−3−oxo−4−cholestenoate (7−Hoca)' on 'Crohn's disease of small intestine'

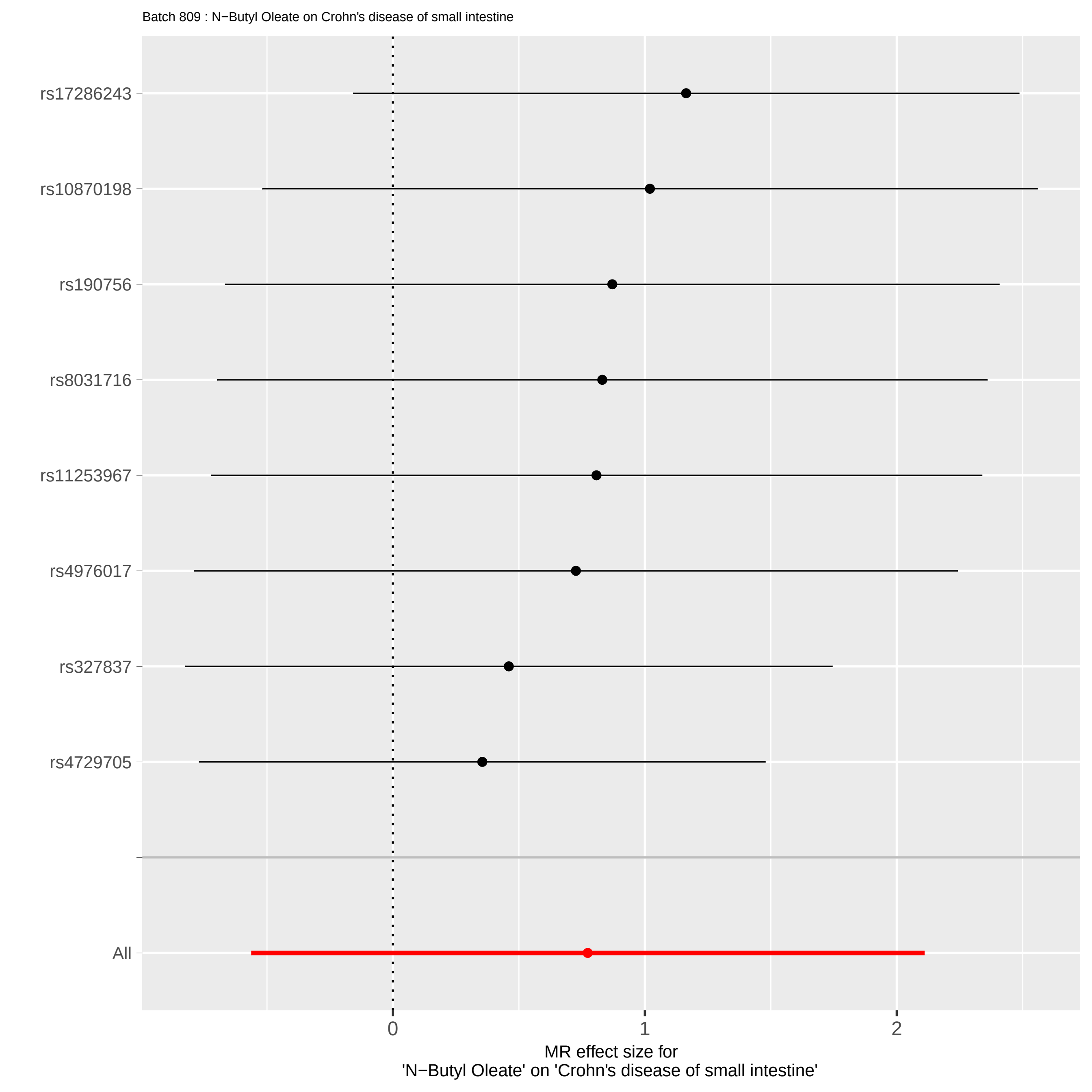

Batch 809 : N−Butyl Oleate on Crohn's disease of small intestine
rs17286243
rs10870198
rs190756
rs8031716
rs11253967
rs4976017
rs327837
rs4729705
All
0
1
2
MR effect size for
'N−Butyl Oleate' on 'Crohn's disease of small intestine'

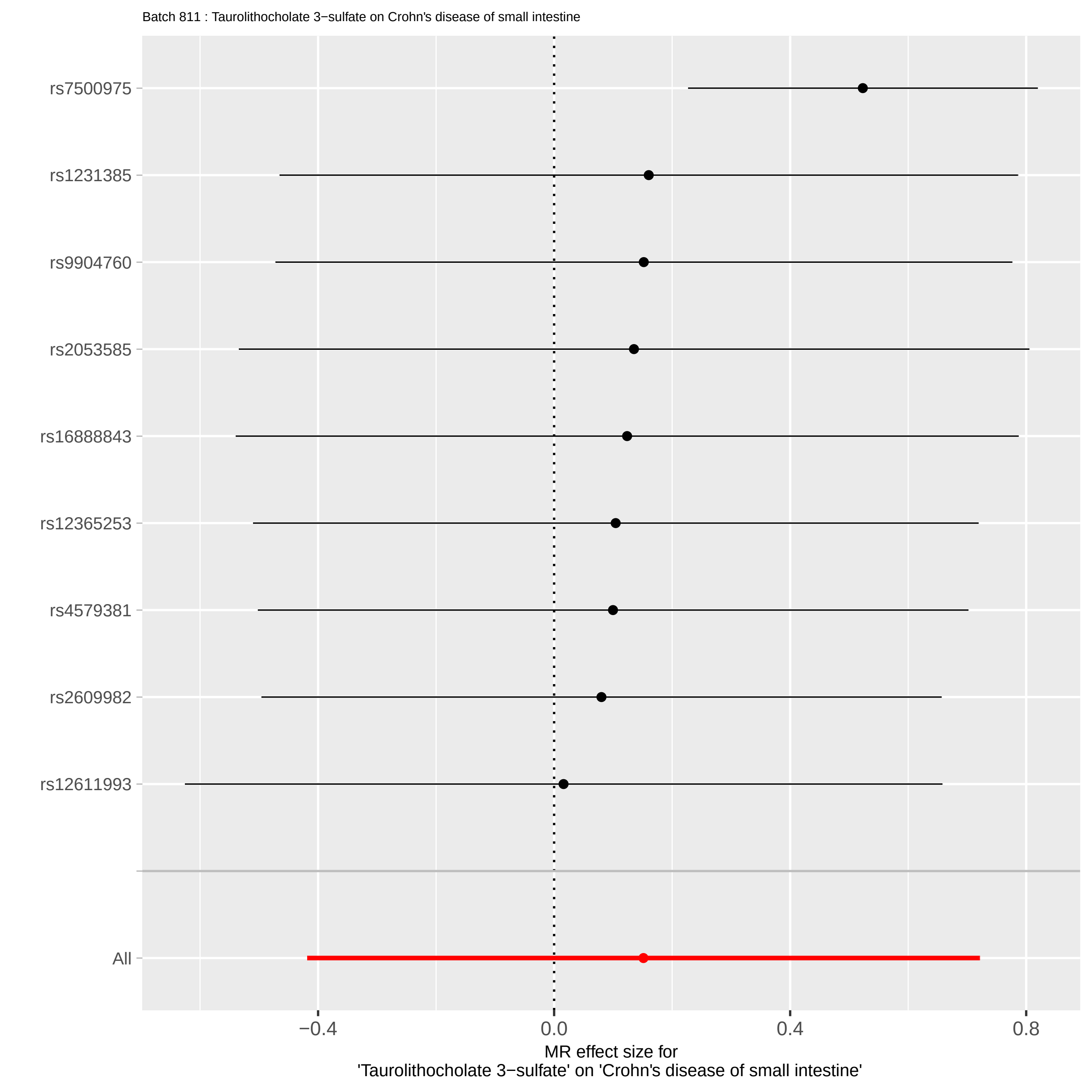
Batch 811 : Taurolithocholate 3−sulfate on Crohn's disease of small intestine
rs7500975
rs1231385
rs9904760
rs2053585
rs16888843
rs12365253
rs4579381
rs2609982
rs12611993
All
−0.4
0.0
0.4
0.8
MR effect size for
'Taurolithocholate 3−sulfate' on 'Crohn's disease of small intestine'

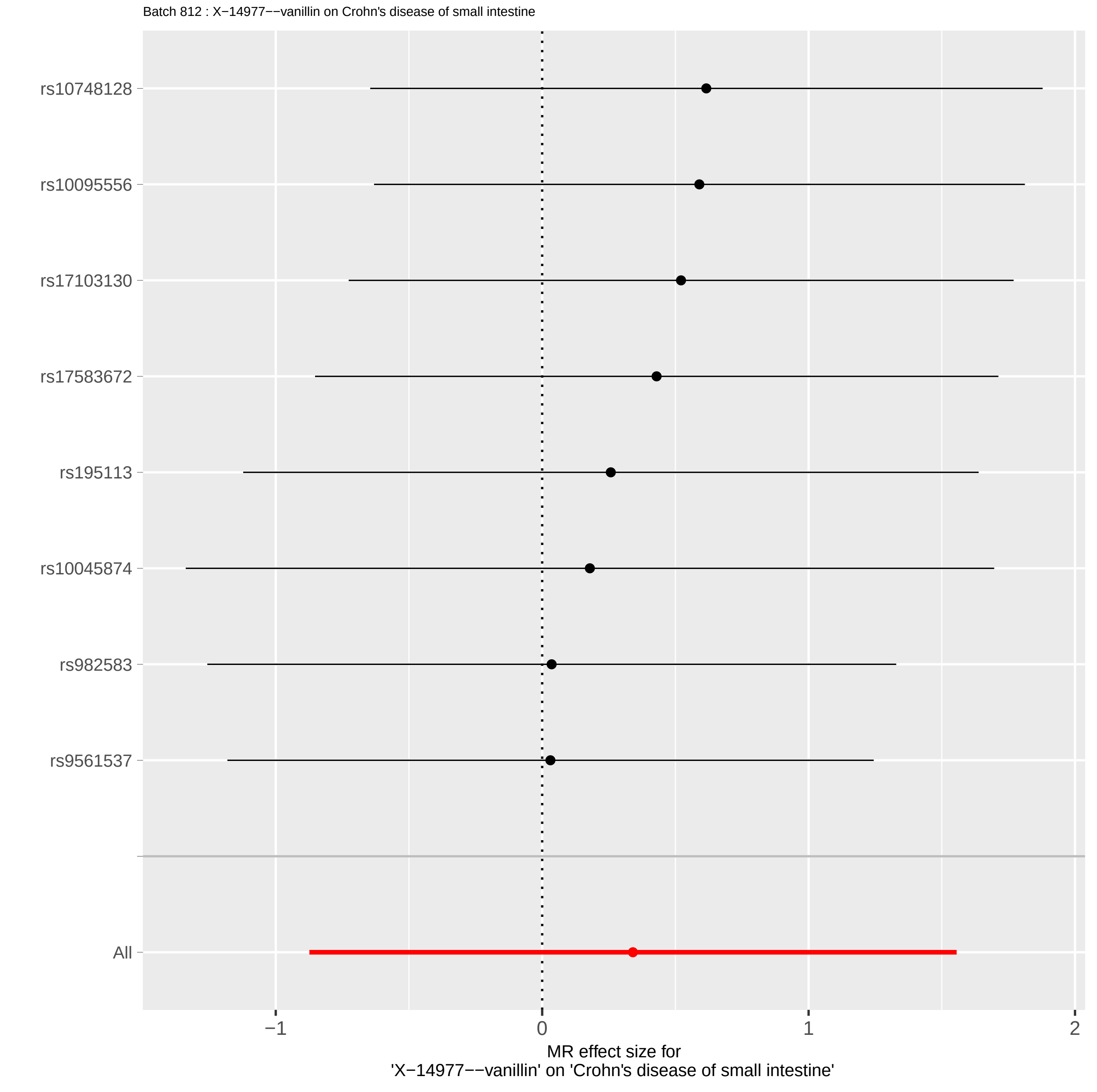

Batch 812 : X−14977−−vanillin on Crohn's disease of small intestine
rs10748128
rs10095556
rs17103130
rs17583672
rs195113
rs10045874
rs982583
rs9561537
All
−1
0
1
2
MR effect size for
'X−14977−−vanillin' on 'Crohn's disease of small intestine'

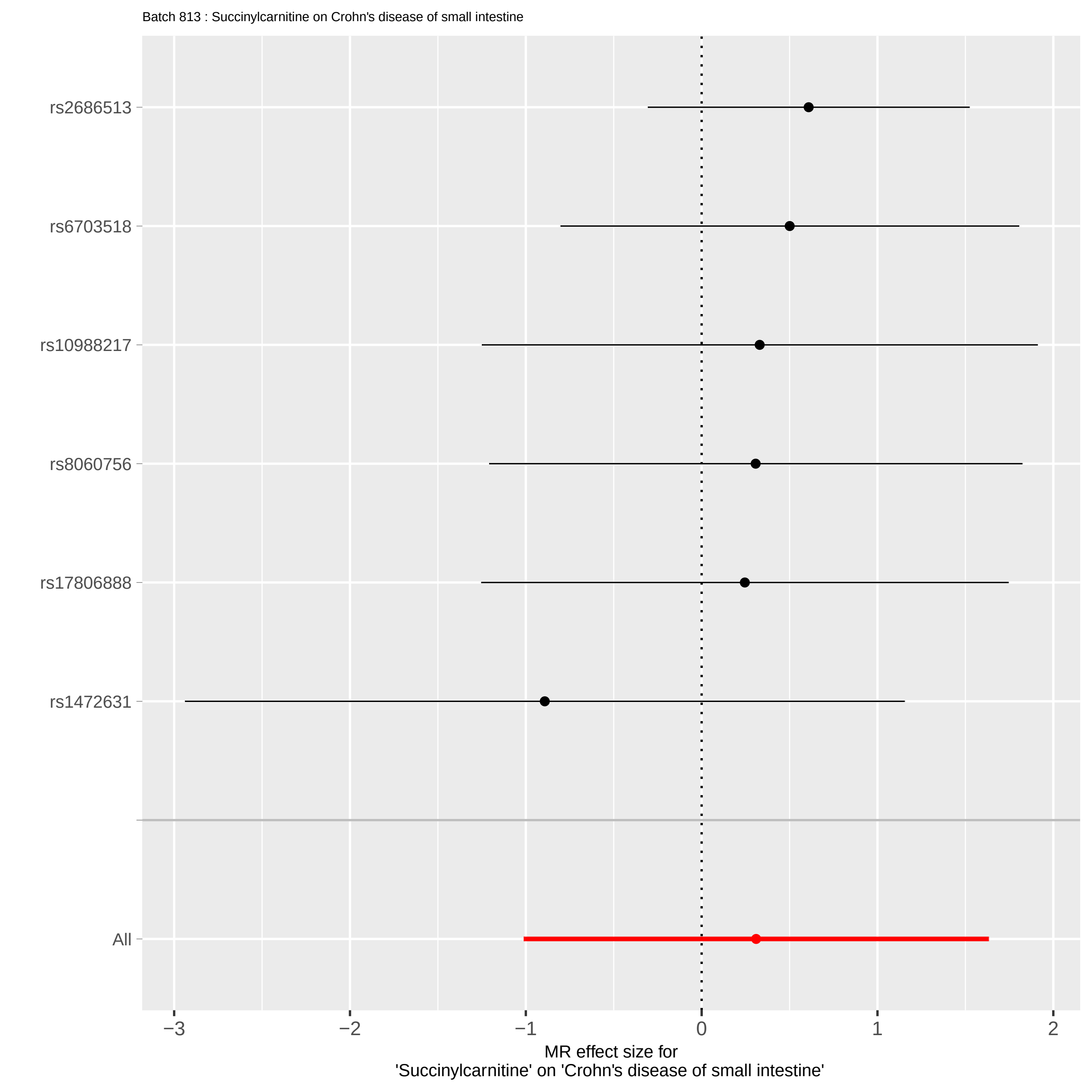
Batch 813 : Succinylcarnitine on Crohn's disease of small intestine
rs2686513
rs6703518
rs10988217
rs8060756
rs17806888
rs1472631
All
−3
−2
−1
0
1
2
MR effect size for
'Succinylcarnitine' on 'Crohn's disease of small intestine'

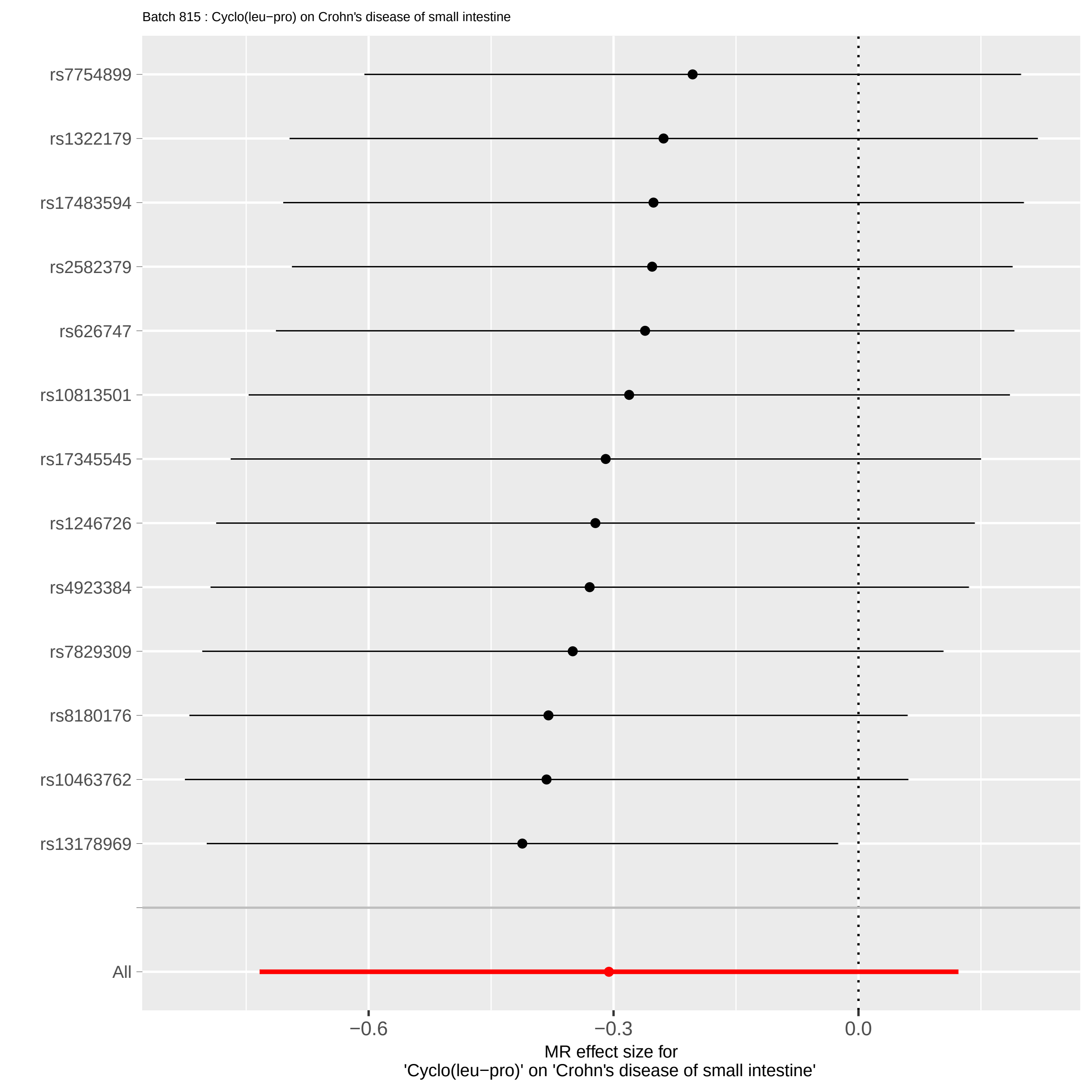

Batch 815 : Cyclo(leu−pro) on Crohn's disease of small intestine
rs7754899
rs1322179
rs17483594
rs2582379
rs626747
rs10813501
rs17345545
rs1246726
rs4923384
rs7829309
rs8180176
rs10463762
rs13178969
All
−0.6
−0.3
0.0
MR effect size for
'Cyclo(leu−pro)' on 'Crohn's disease of small intestine'

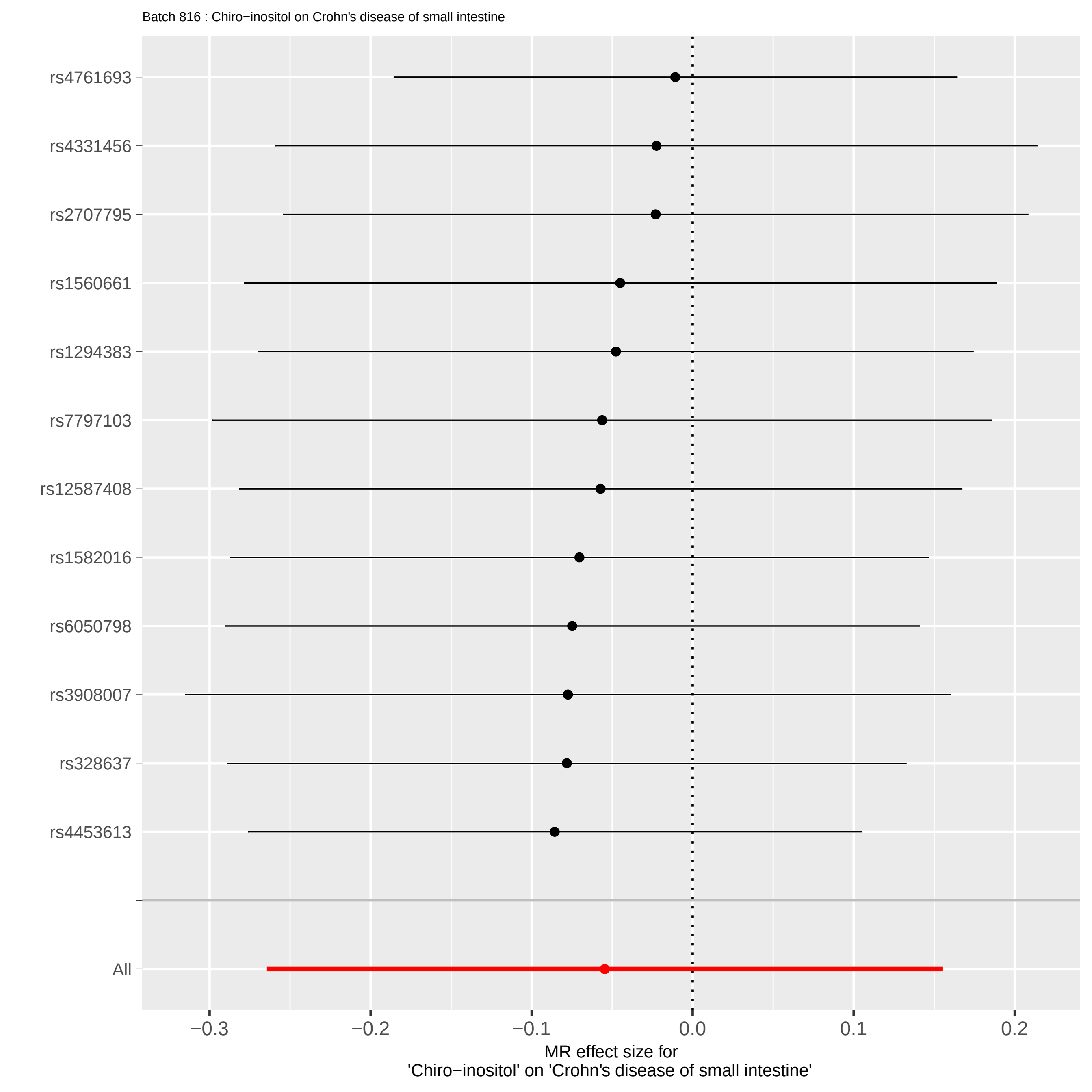

Batch 816 : Chiro−inositol on Crohn's disease of small intestine
rs4761693
rs4331456
rs2707795
rs1560661
rs1294383
rs7797103
rs12587408
rs1582016
rs6050798
rs3908007
rs328637
rs4453613
All
−0.3
−0.2
−0.1
0.0
0.1
0.2
MR effect size for
'Chiro−inositol' on 'Crohn's disease of small intestine'

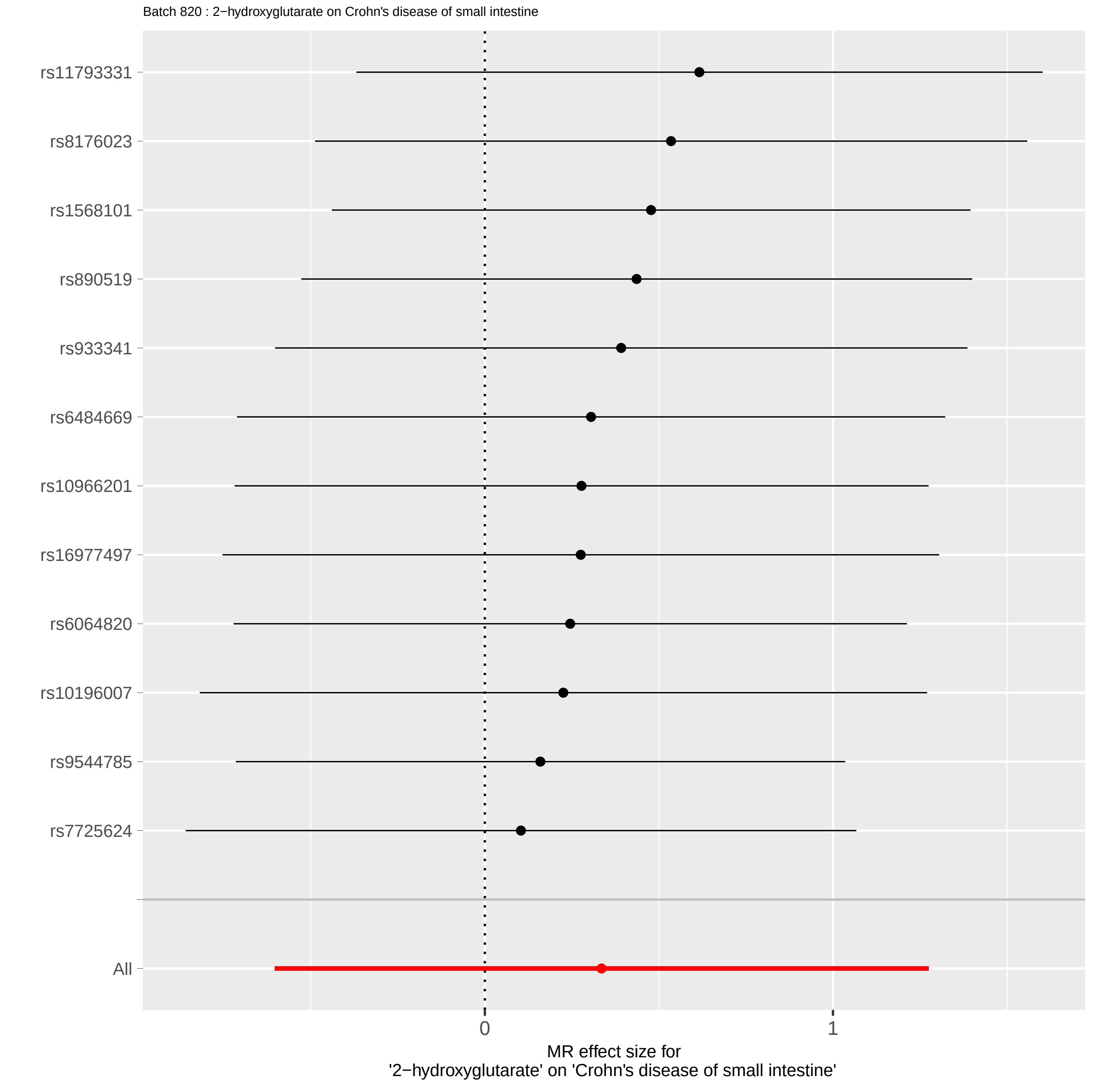

Batch 820 : 2−hydroxyglutarate on Crohn's disease of small intestine
rs11793331
rs8176023
rs1568101
rs890519
rs933341
rs6484669
rs10966201
rs16977497
rs6064820
rs10196007
rs9544785
rs7725624
All
0
1
MR effect size for
'2−hydroxyglutarate' on 'Crohn's disease of small intestine'

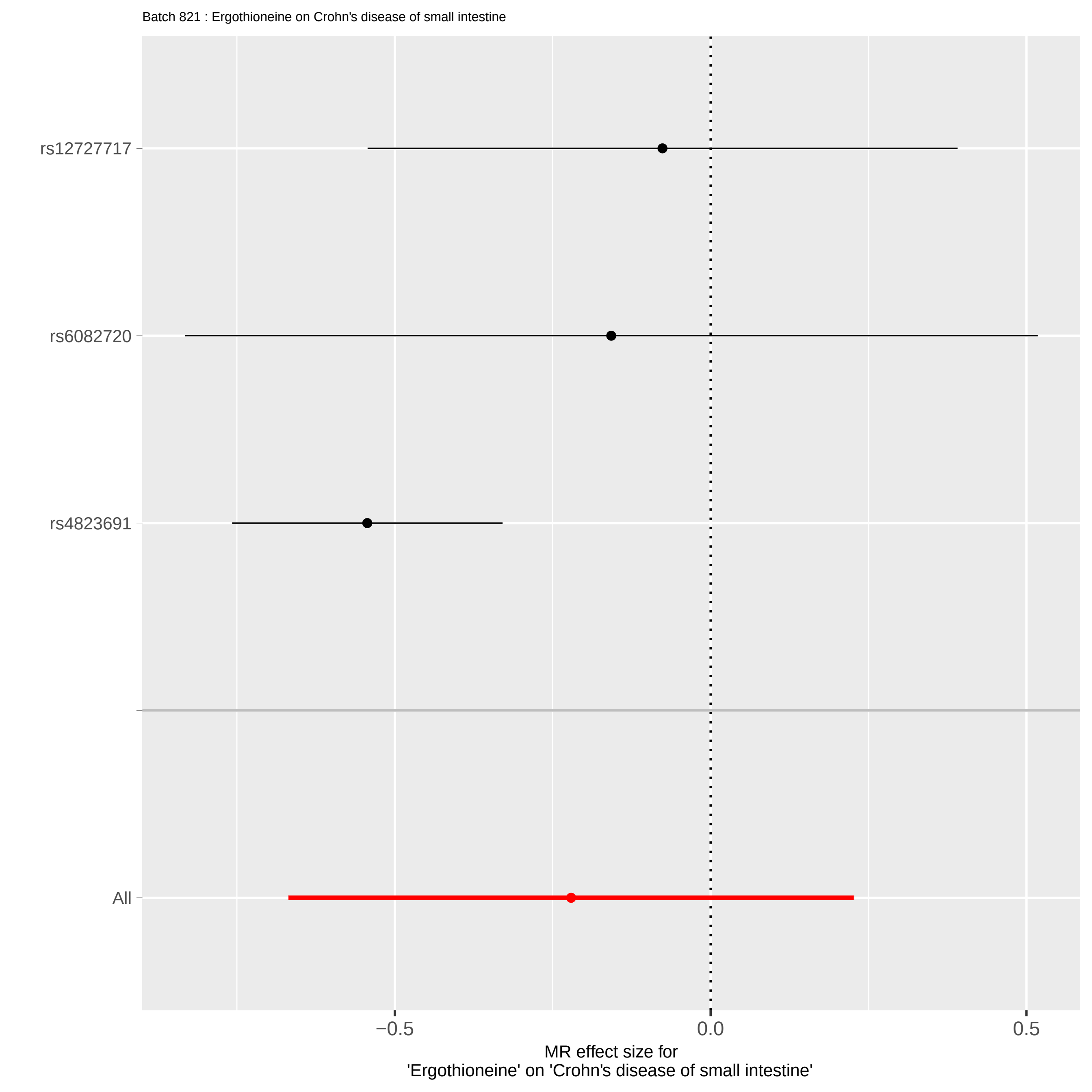

Batch 821 : Ergothioneine on Crohn's disease of small intestine
rs12727717
rs6082720
rs4823691
All
−0.5
0.0
0.5
MR effect size for
'Ergothioneine' on 'Crohn's disease of small intestine'

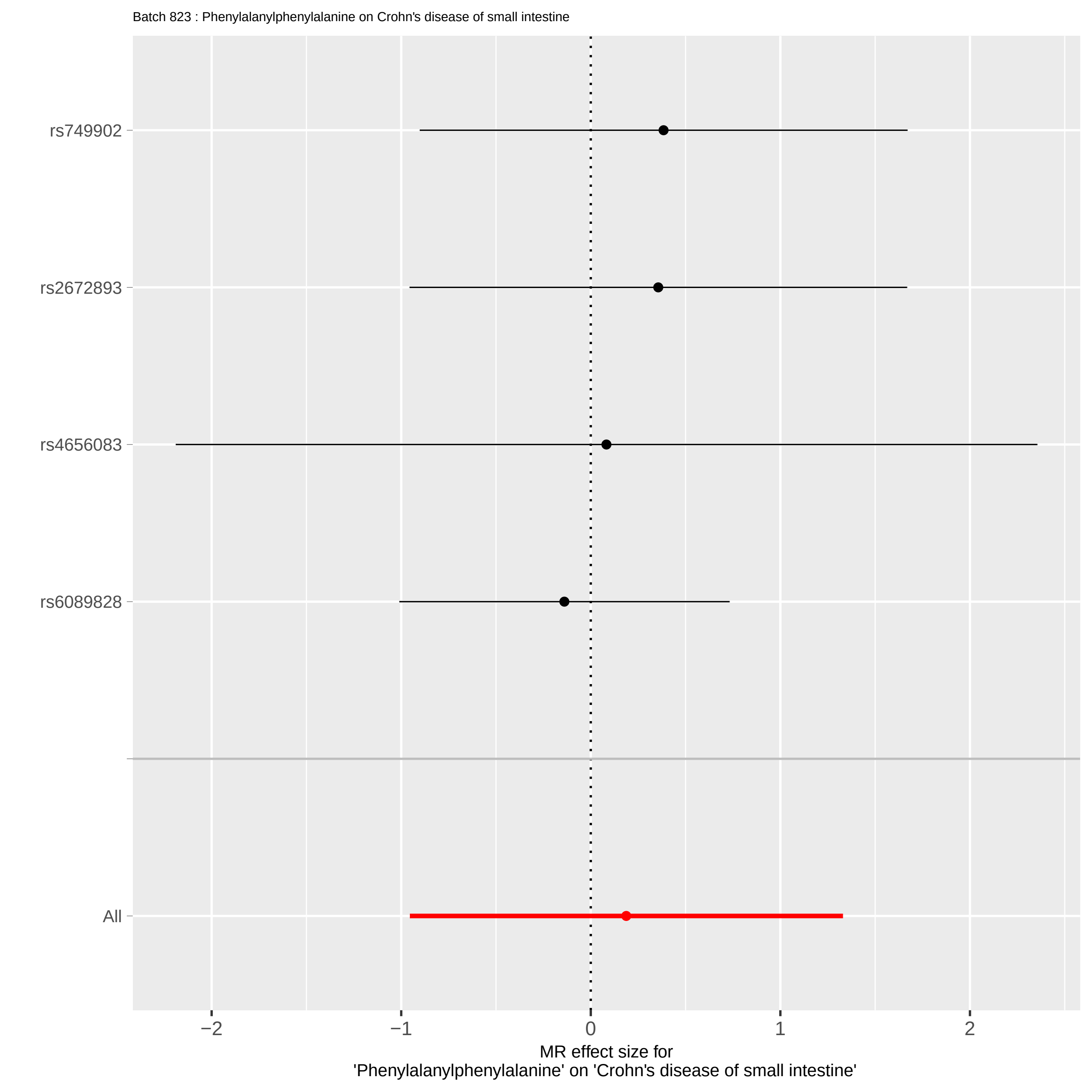

Batch 823 : Phenylalanylphenylalanine on Crohn's disease of small intestine
rs749902
rs2672893
rs4656083
rs6089828
All
−2
−1
0
1
2
MR effect size for
'Phenylalanylphenylalanine' on 'Crohn's disease of small intestine'

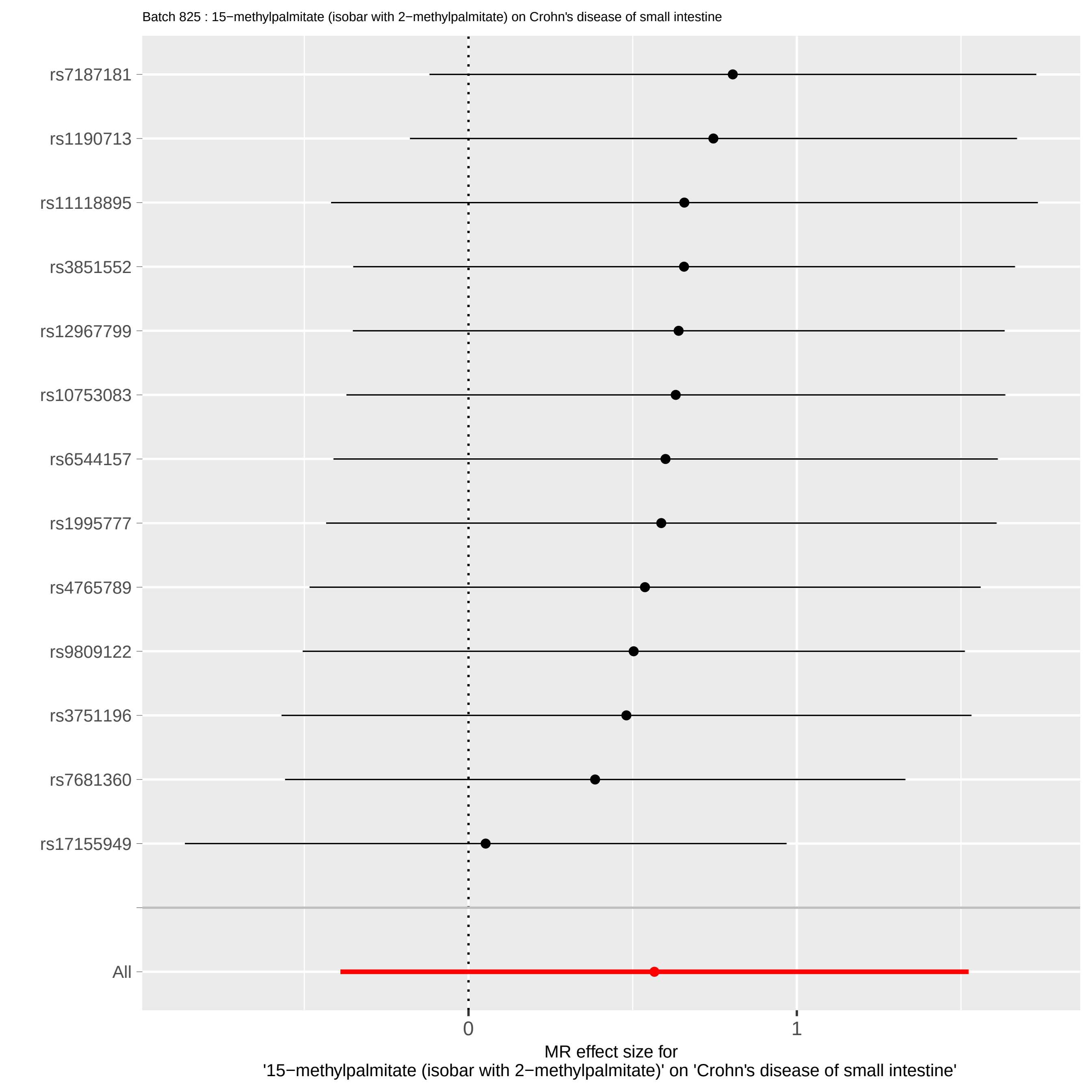

Batch 825 : 15−methylpalmitate (isobar with 2−methylpalmitate) on Crohn's disease of small intestine
rs7187181
rs1190713
rs11118895
rs3851552
rs12967799
rs10753083
rs6544157
rs1995777
rs4765789
rs9809122
rs3751196
rs7681360
rs17155949
All
0
1
MR effect size for
'15−methylpalmitate (isobar with 2−methylpalmitate)' on 'Crohn's disease of small intestine'

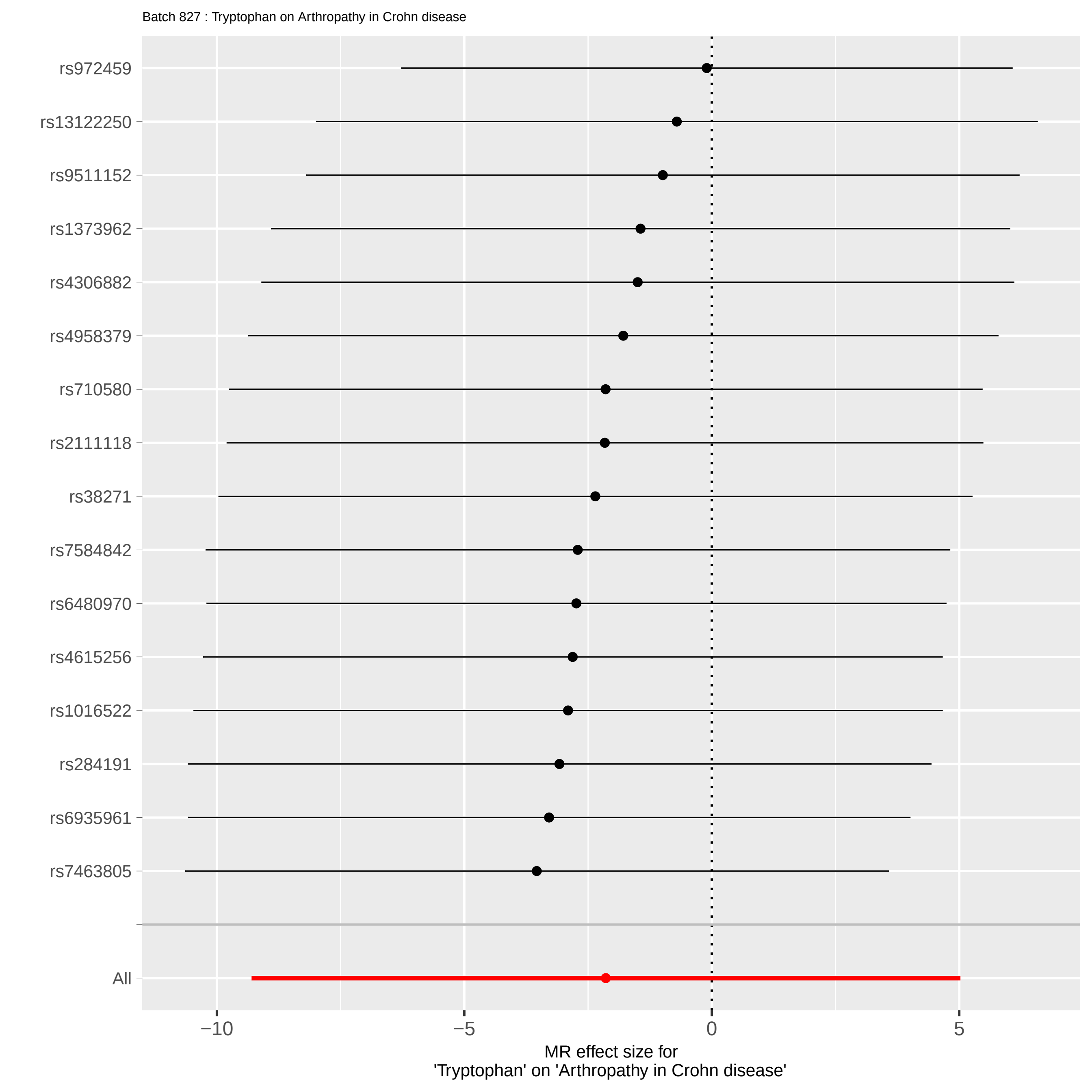

Batch 827 : Tryptophan on Arthropathy in Crohn disease
rs972459
rs13122250
rs9511152
rs1373962
rs4306882
rs4958379
rs710580
rs2111118
rs38271
rs7584842
rs6480970
rs4615256
rs1016522
rs284191
rs6935961
rs7463805
All
−10
−5
0
5
MR effect size for
'Tryptophan' on 'Arthropathy in Crohn disease'

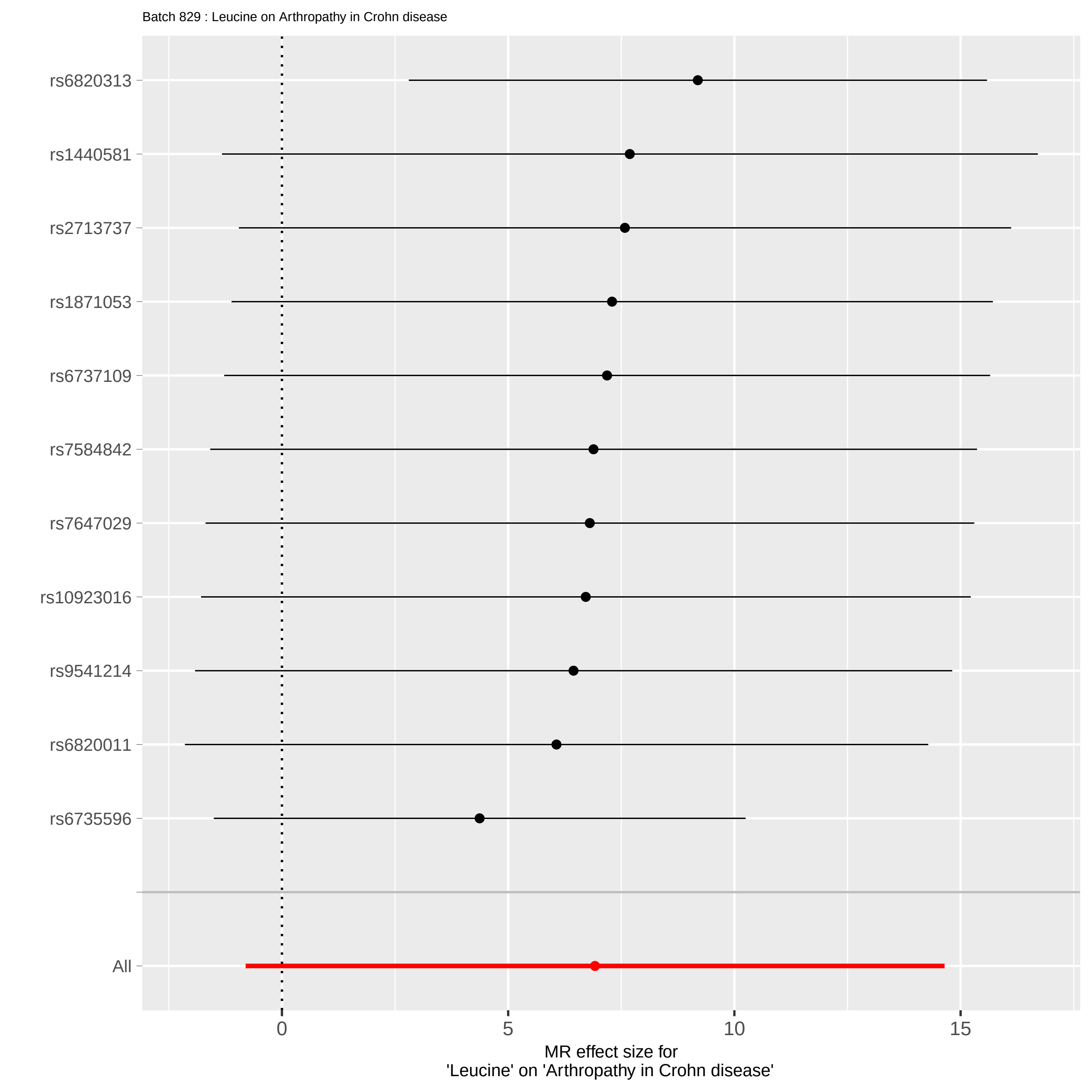

Batch 829 : Leucine on Arthropathy in Crohn disease
rs6820313
rs1440581
rs2713737
rs1871053
rs6737109
rs7584842
rs7647029
rs10923016
rs9541214
rs6820011
rs6735596
All
0
5
10
15
MR effect size for
'Leucine' on 'Arthropathy in Crohn disease'

Batch 833 : Lactate on Arthropathy in Crohn disease

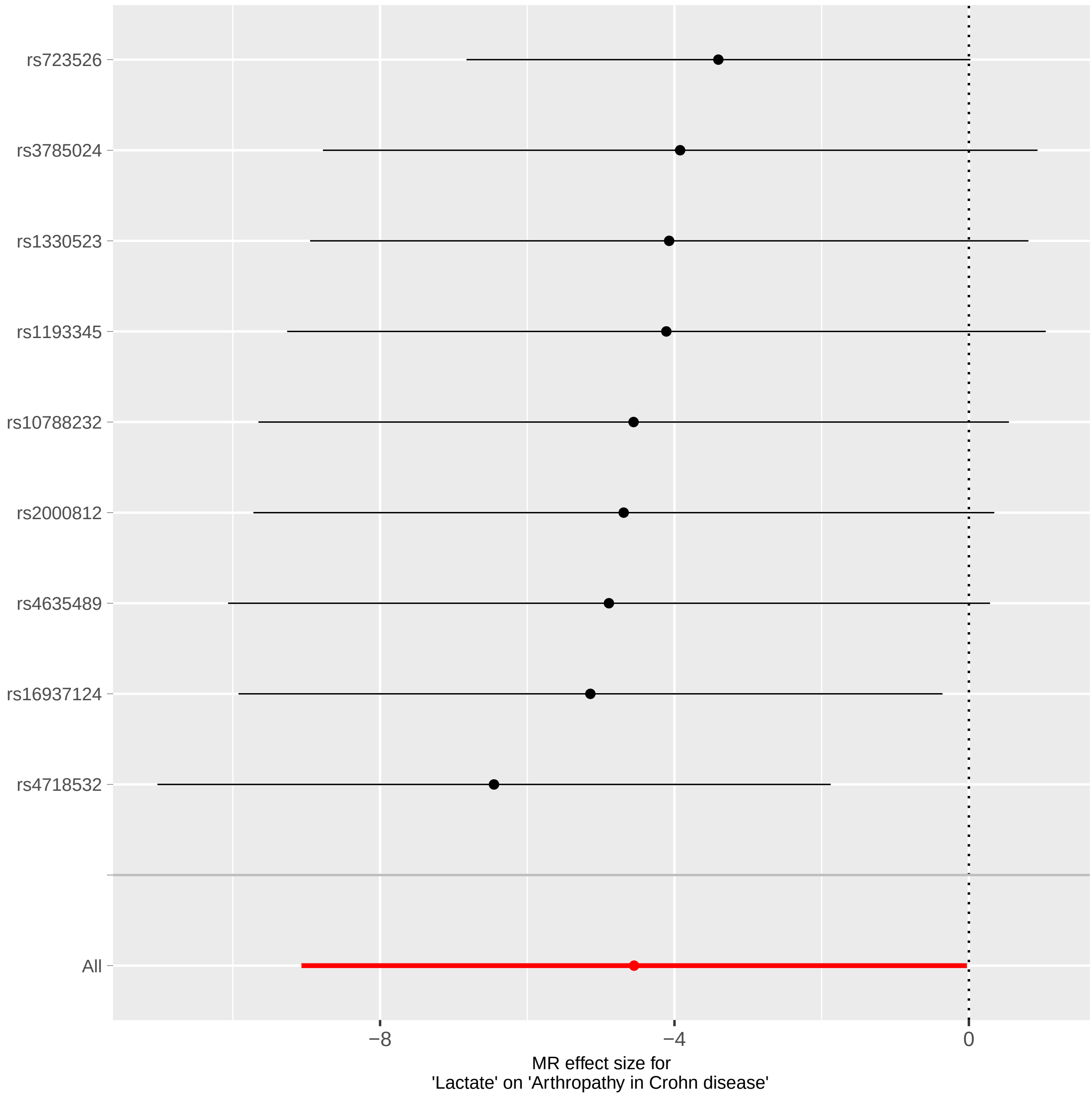

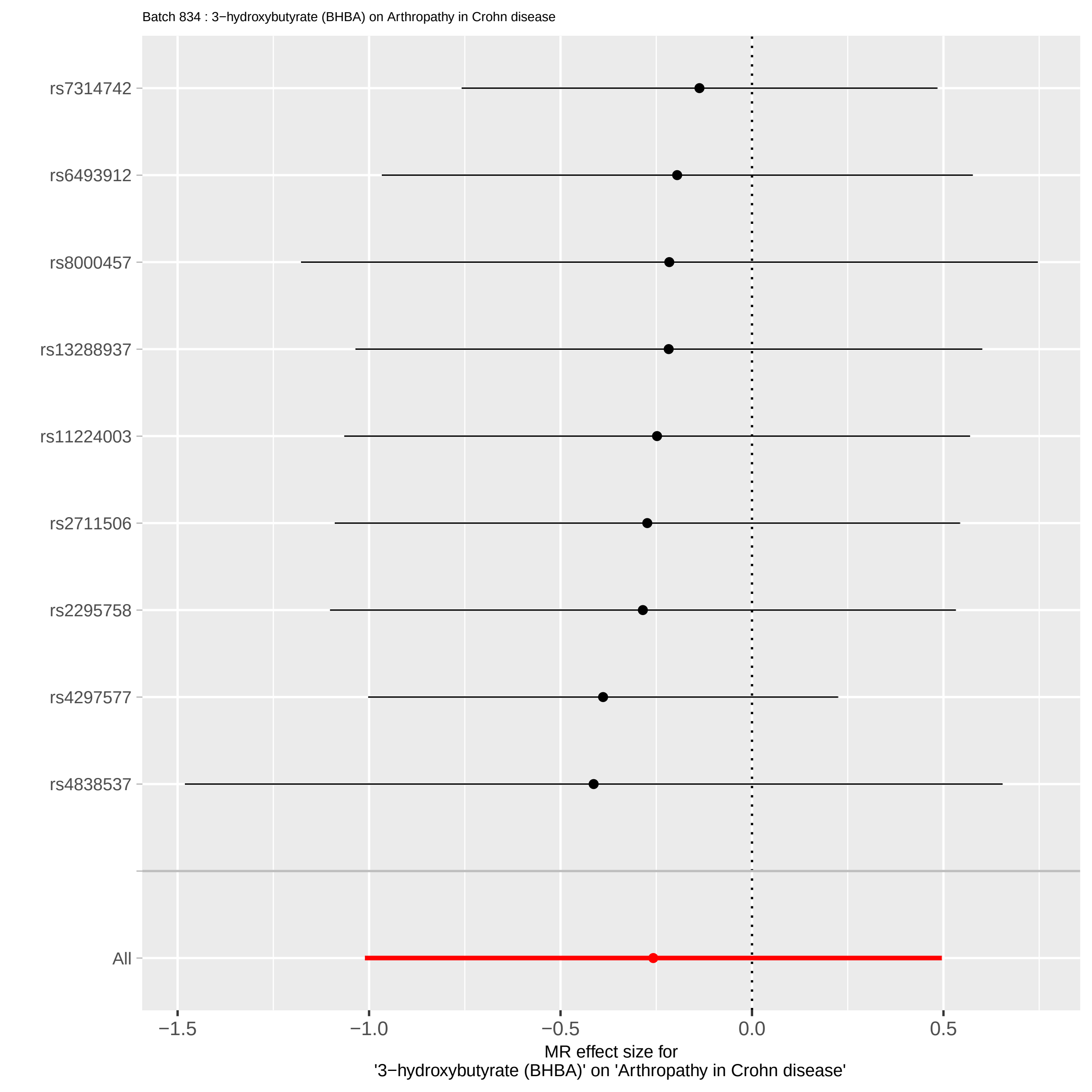

Batch 834 : 3−hydroxybutyrate (BHBA) on Arthropathy in Crohn disease
rs7314742
rs6493912
rs8000457
rs13288937
rs11224003
rs2711506
rs2295758
rs4297577
rs4838537
All
−1.5
−1.0
−0.5
0.0
0.5
MR effect size for
'3−hydroxybutyrate (BHBA)' on 'Arthropathy in Crohn disease'

Batch 835 : Arabinose on Arthropathy in Crohn disease

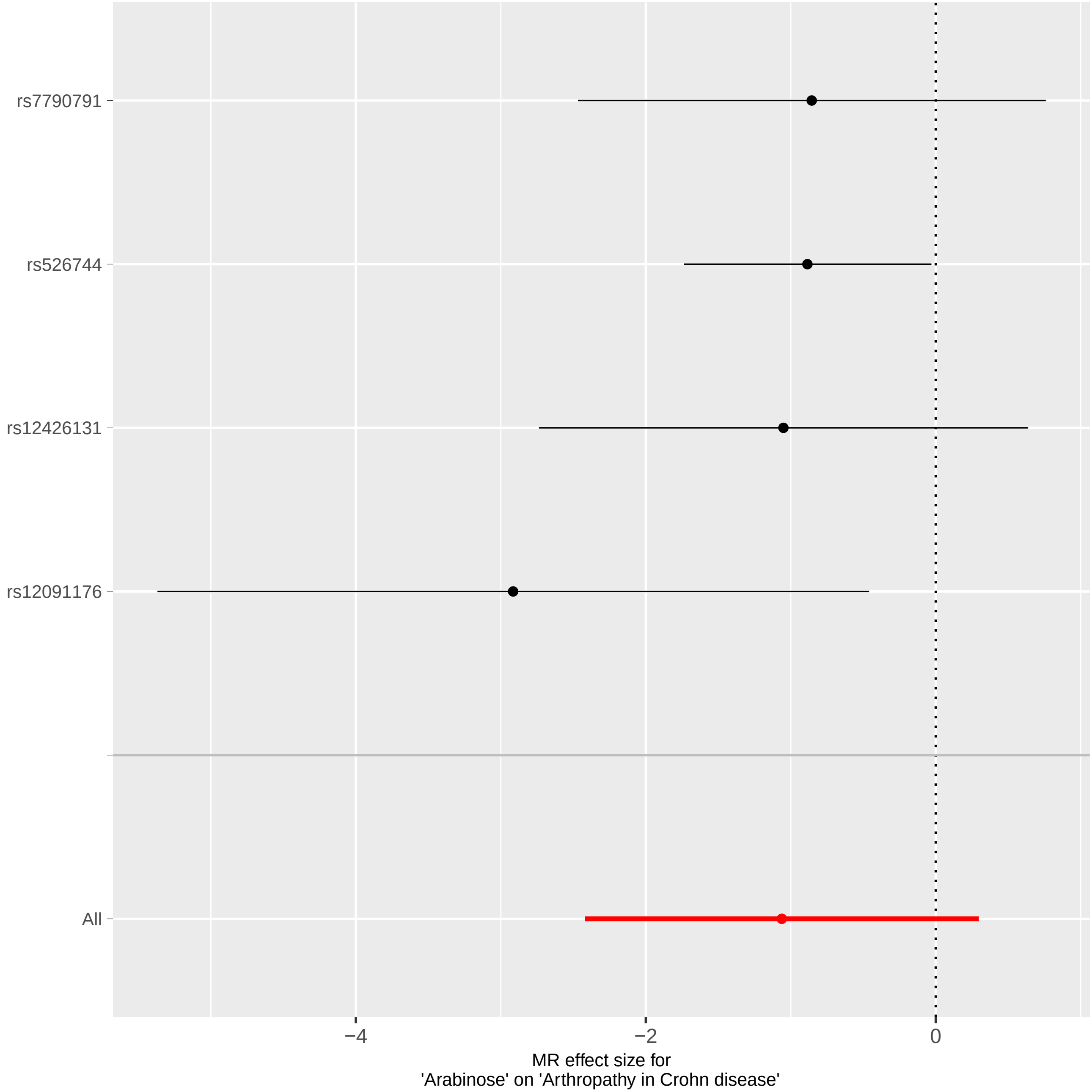

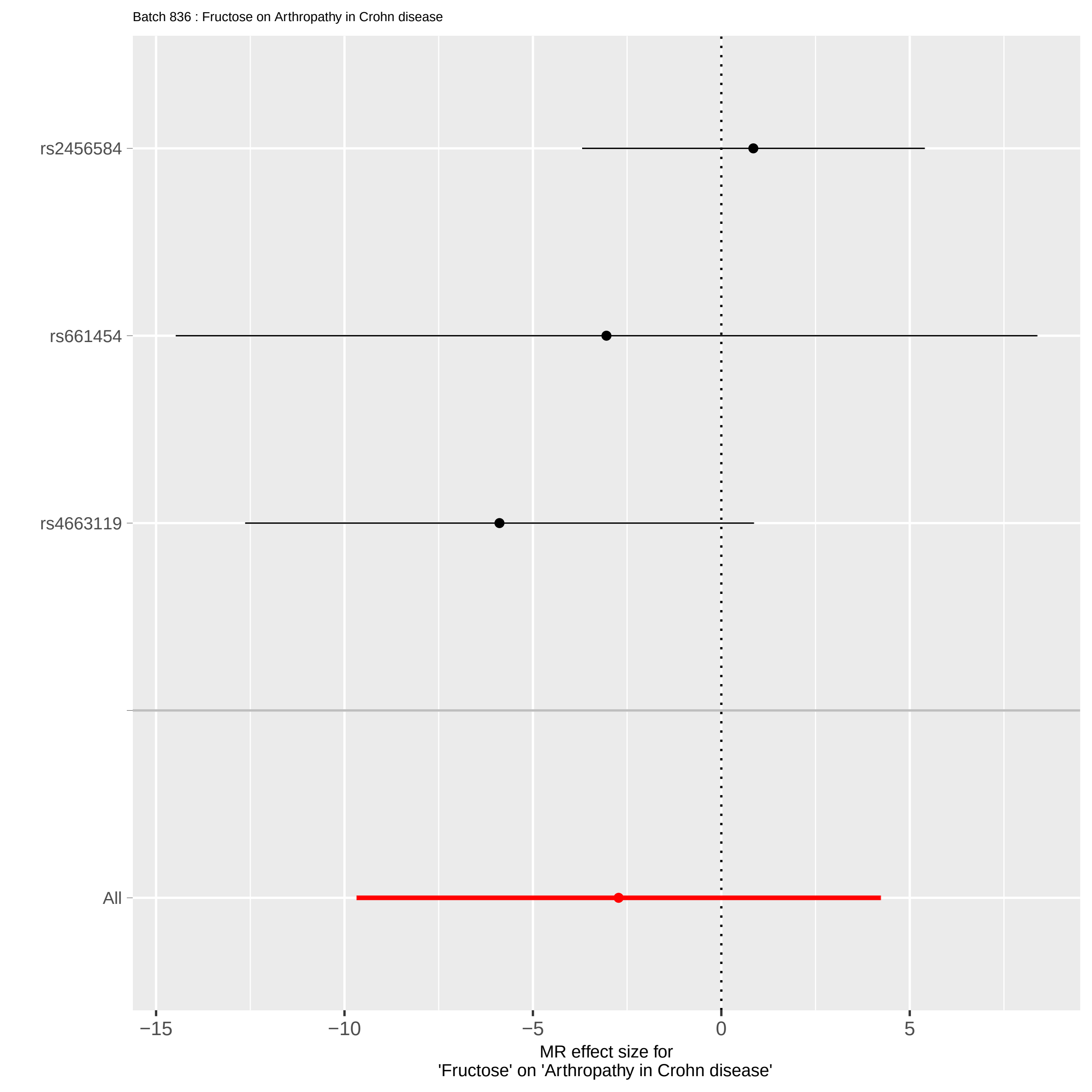

Batch 836 : Fructose on Arthropathy in Crohn disease
rs2456584
rs661454
rs4663119
All
−15
−10
−5
0
5
MR effect size for
'Fructose' on 'Arthropathy in Crohn disease'

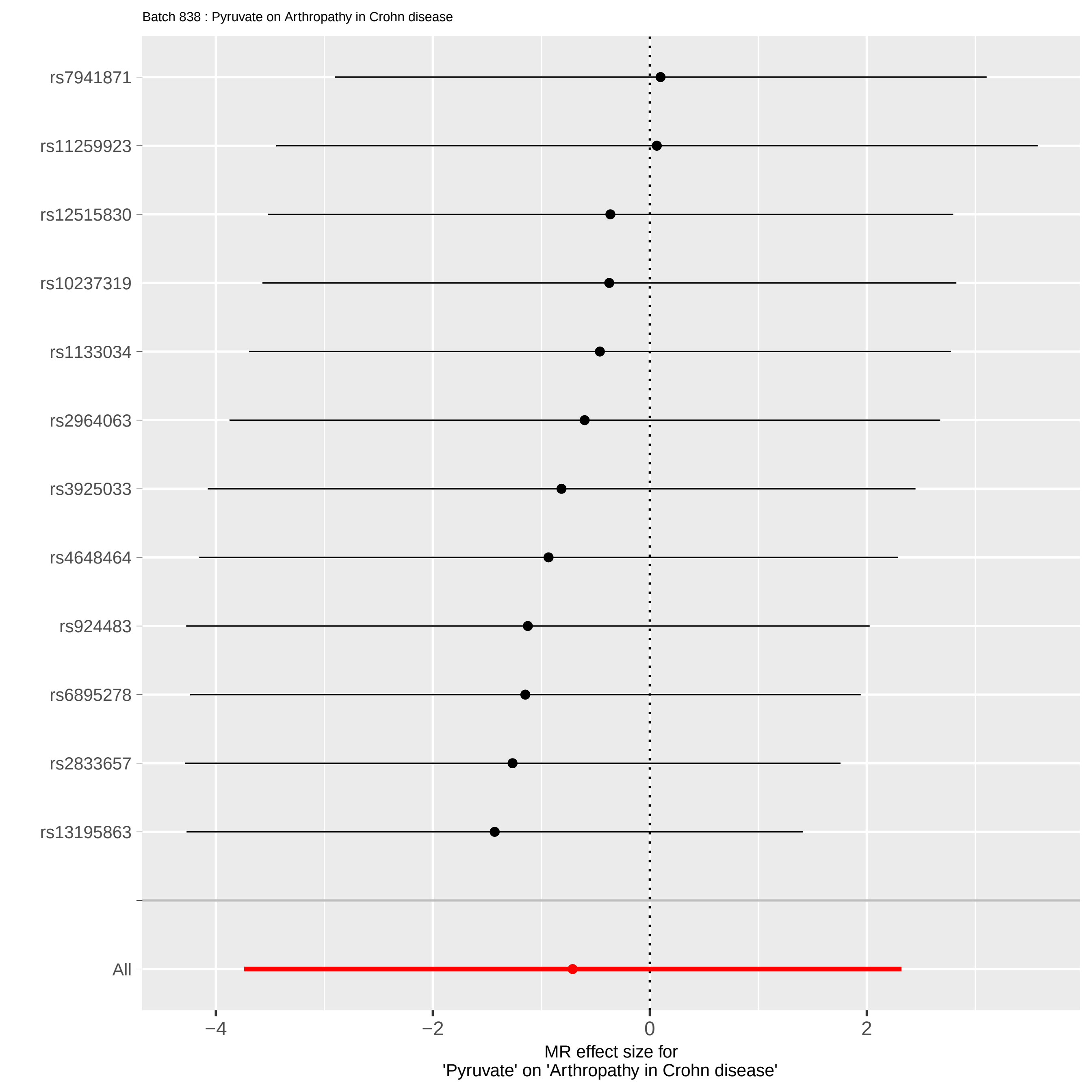

Batch 838 : Pyruvate on Arthropathy in Crohn disease
rs7941871
rs11259923
rs12515830
rs10237319
rs1133034
rs2964063
rs3925033
rs4648464
rs924483
rs6895278
rs2833657
rs13195863
All
−4
−2
0
2
MR effect size for
'Pyruvate' on 'Arthropathy in Crohn disease'

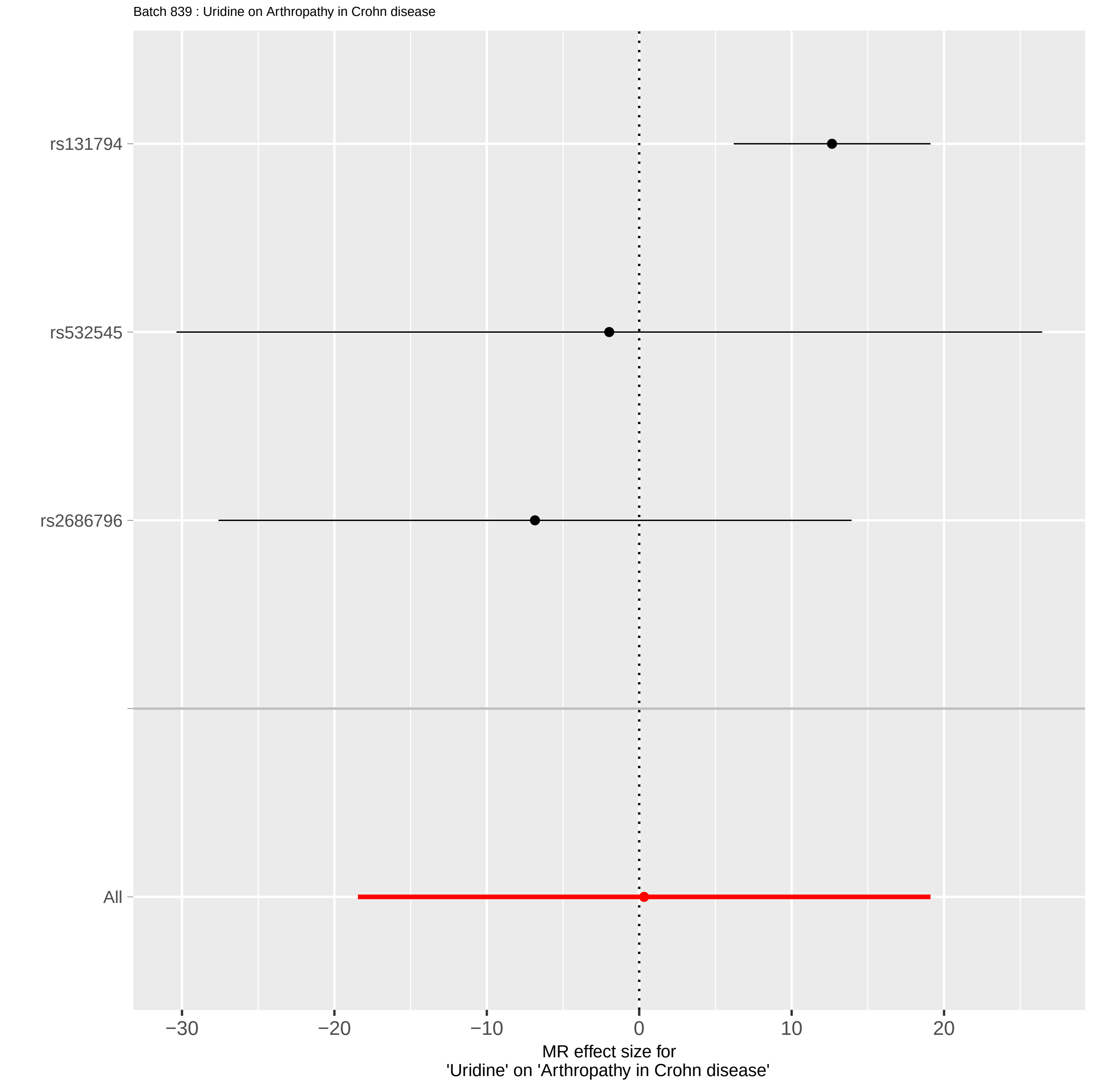
Batch 839 : Uridine on Arthropathy in Crohn disease
rs131794
rs532545
rs2686796
All
−30
−20
−10
0
10
20
MR effect size for
'Uridine' on 'Arthropathy in Crohn disease'

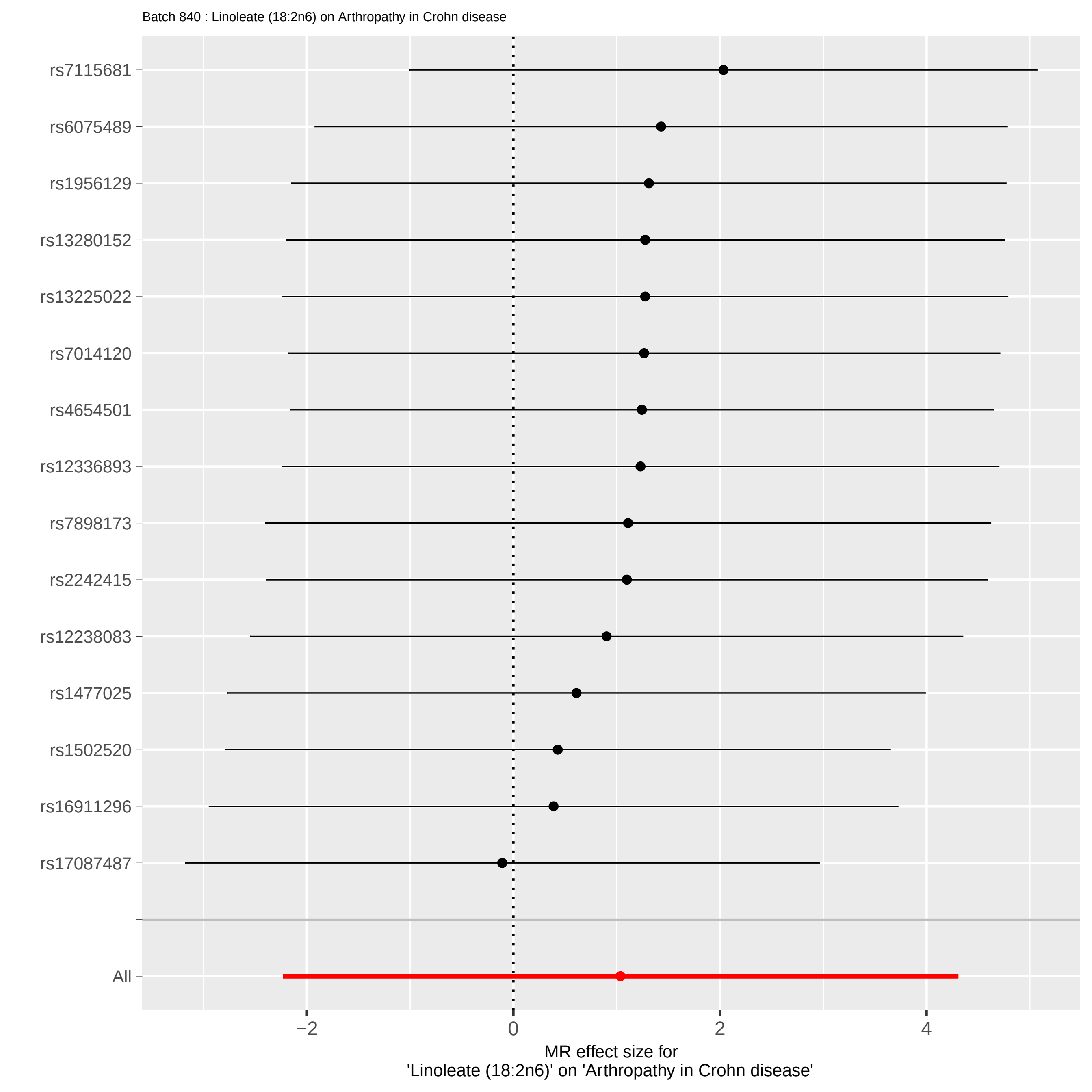

Batch 840 : Linoleate (18:2n6) on Arthropathy in Crohn disease
rs7115681
rs6075489
rs1956129
rs13280152
rs13225022
rs7014120
rs4654501
rs12336893
rs7898173
rs2242415
rs12238083
rs1477025
rs1502520
rs16911296
rs17087487
All
−2
0
2
4
MR effect size for
'Linoleate (18:2n6)' on 'Arthropathy in Crohn disease'

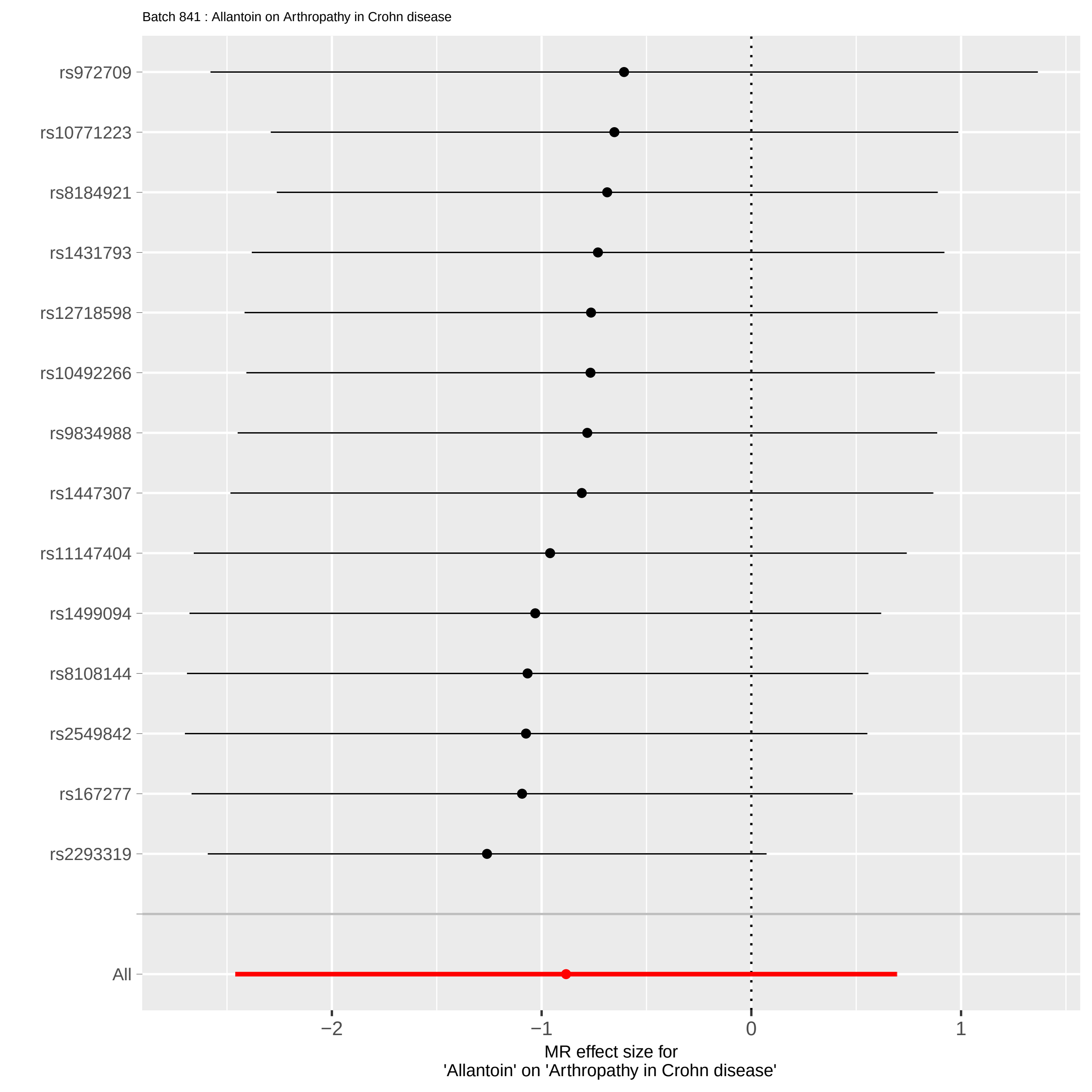
Batch 841 : Allantoin on Arthropathy in Crohn disease
rs972709
rs10771223
rs8184921
rs1431793
rs12718598
rs10492266
rs9834988
rs1447307
rs11147404
rs1499094
rs8108144
rs2549842
rs167277
rs2293319
All
−2
−1
0
1
MR effect size for
'Allantoin' on 'Arthropathy in Crohn disease'

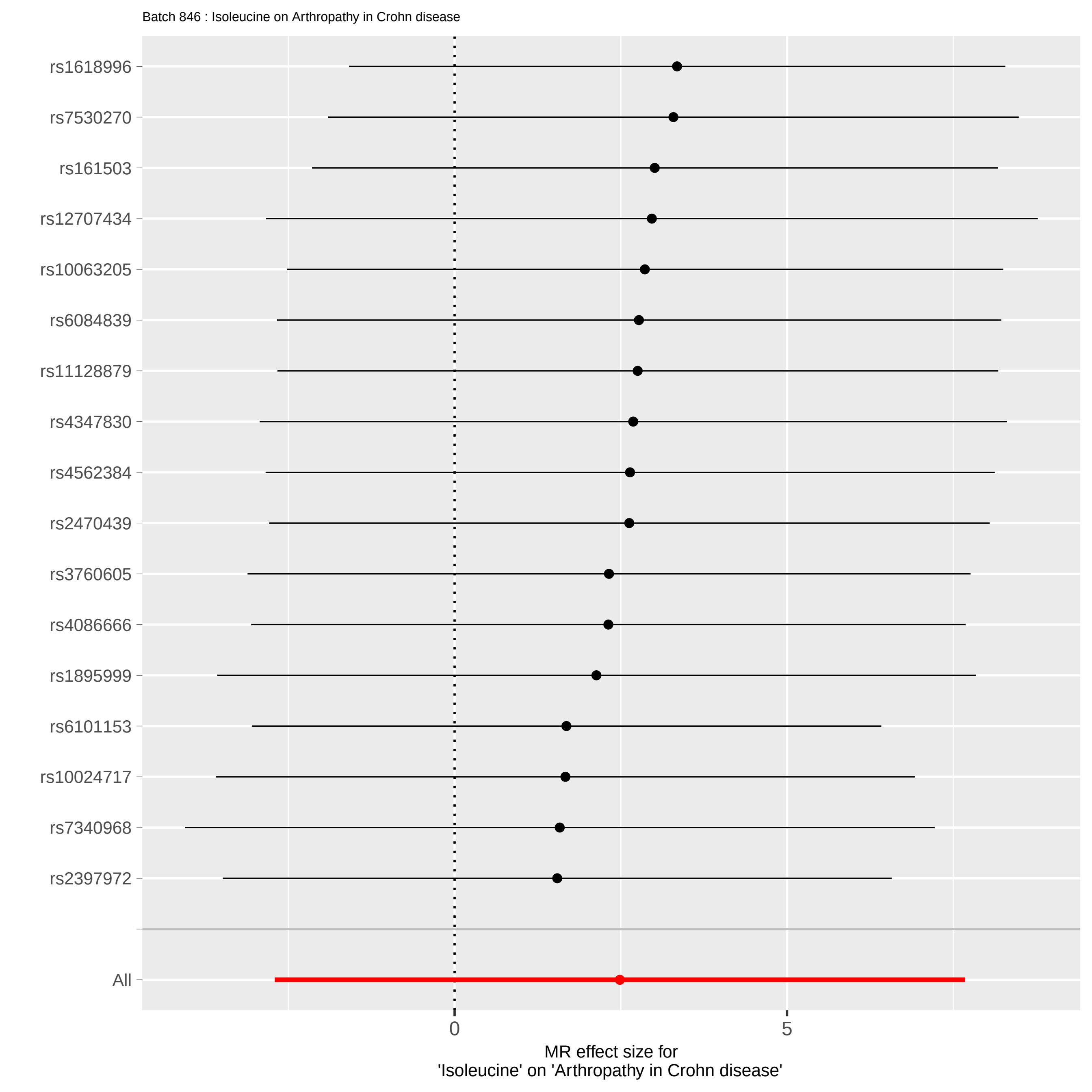

Batch 846 : Isoleucine on Arthropathy in Crohn disease
rs1618996
rs7530270
rs161503
rs12707434
rs10063205
rs6084839
rs11128879
rs4347830
rs4562384
rs2470439
rs3760605
rs4086666
rs1895999
rs6101153
rs10024717
rs7340968
rs2397972
All
0
5
MR effect size for
'Isoleucine' on 'Arthropathy in Crohn disease'

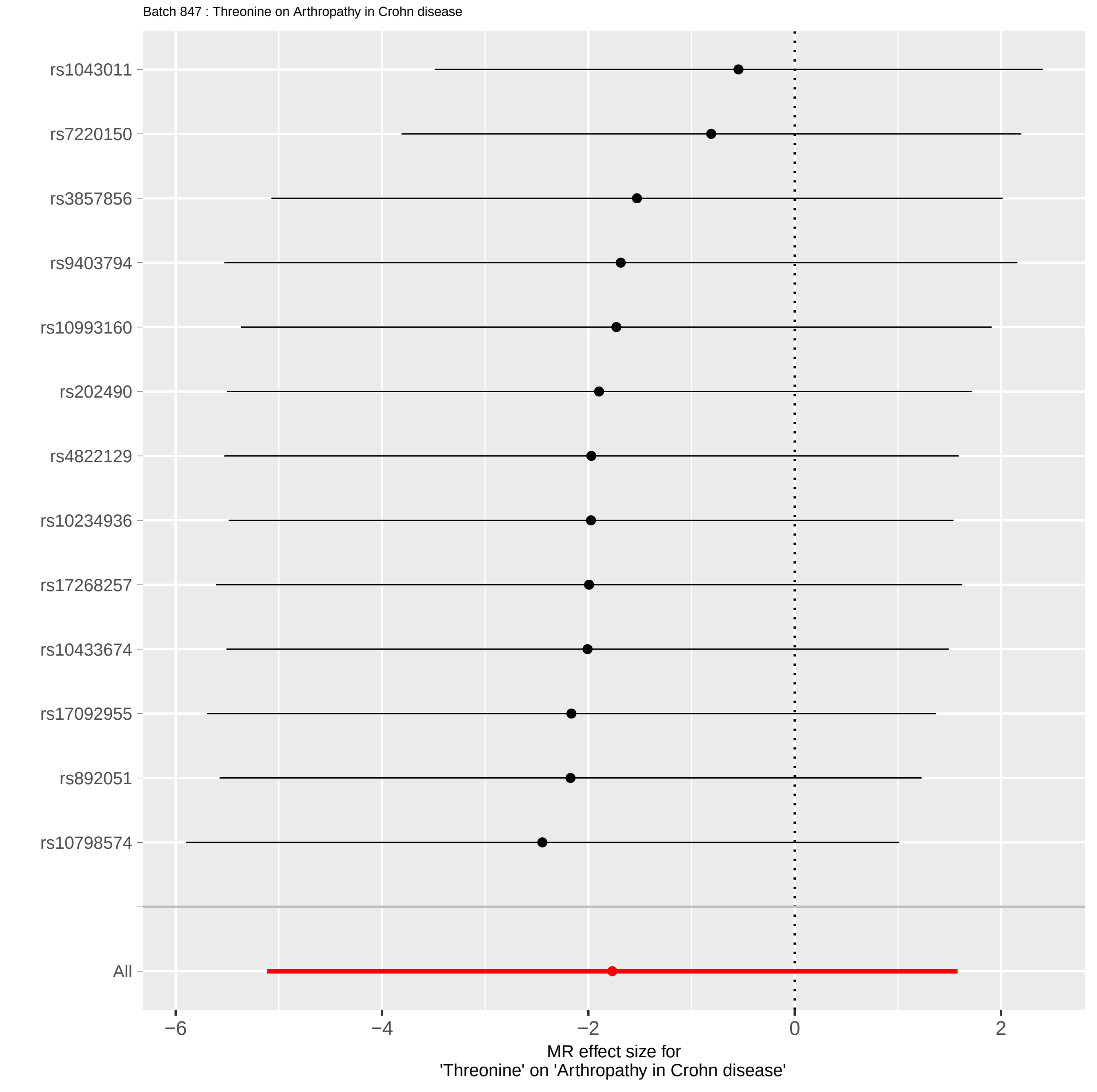

Batch 847 : Threonine on Arthropathy in Crohn disease
rs1043011
rs7220150
rs3857856
rs9403794
rs10993160
rs202490
rs4822129
rs10234936
rs17268257
rs10433674
rs17092955
rs892051
rs10798574
All
−6
−4
−2
0
2
MR effect size for
'Threonine' on 'Arthropathy in Crohn disease'

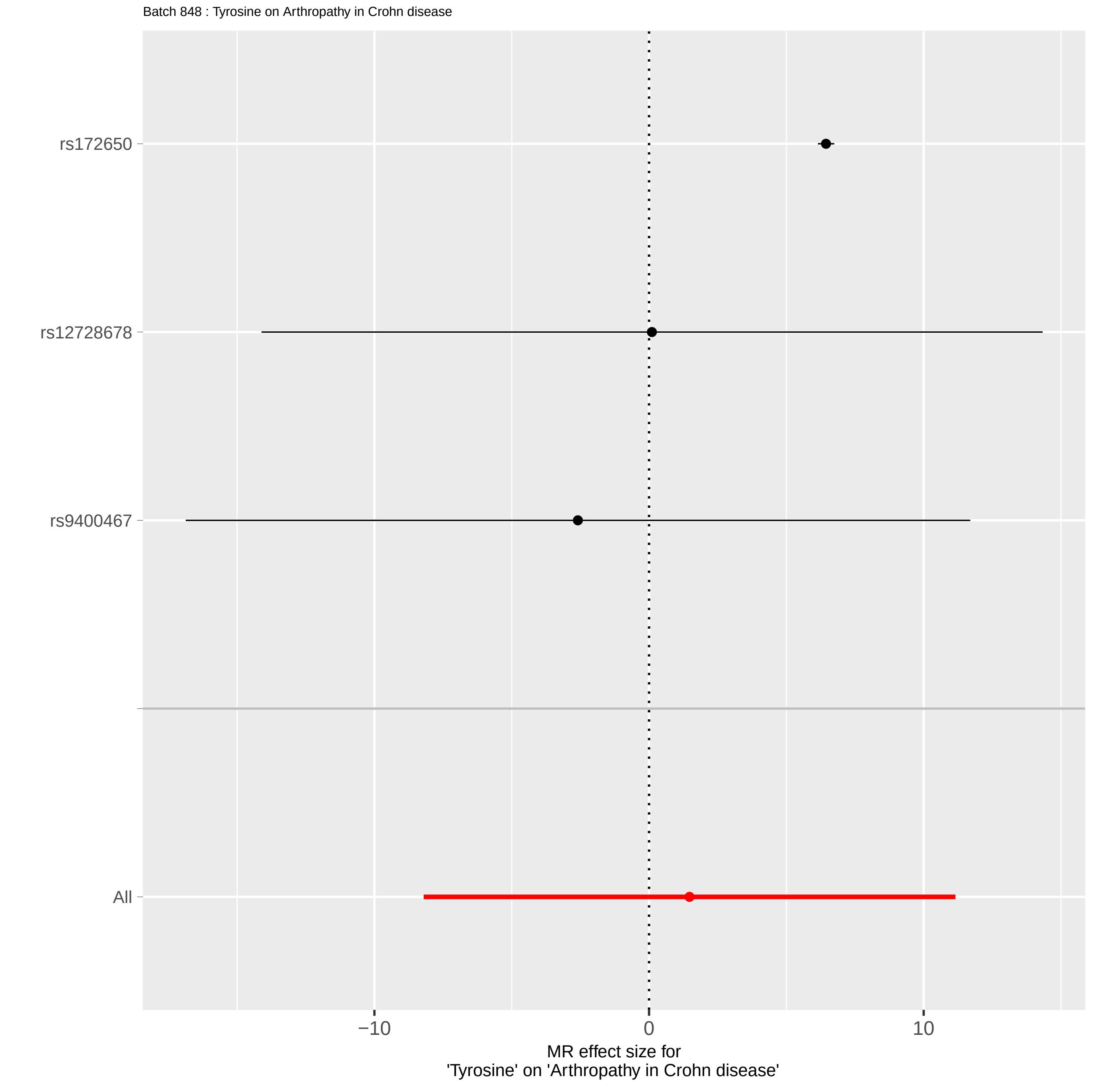

Batch 848 : Tyrosine on Arthropathy in Crohn disease
rs172650
rs12728678
rs9400467
All
−10
0
10
MR effect size for
'Tyrosine' on 'Arthropathy in Crohn disease'

Batch 857 : Myristate (14:0) on Arthropathy in Crohn disease

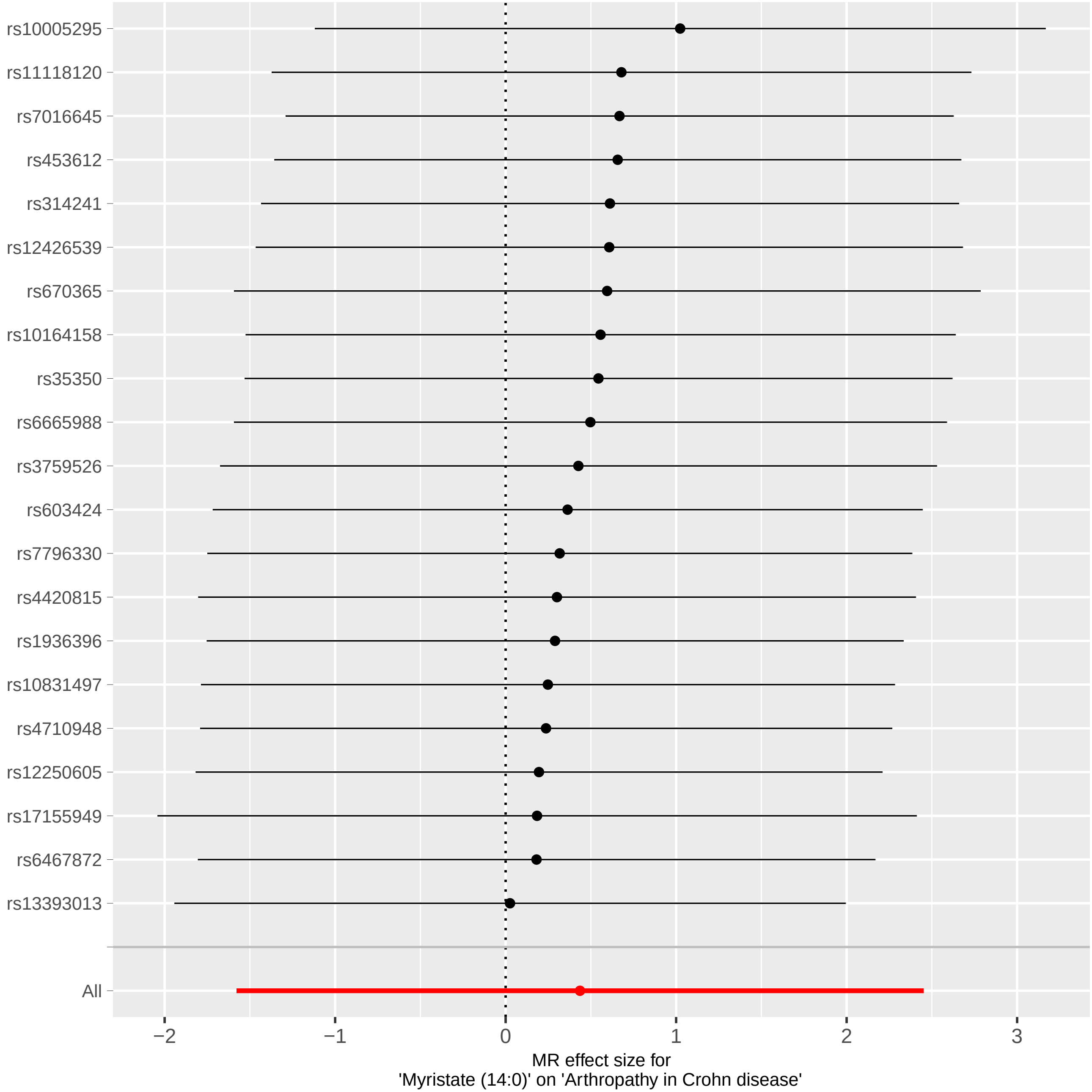

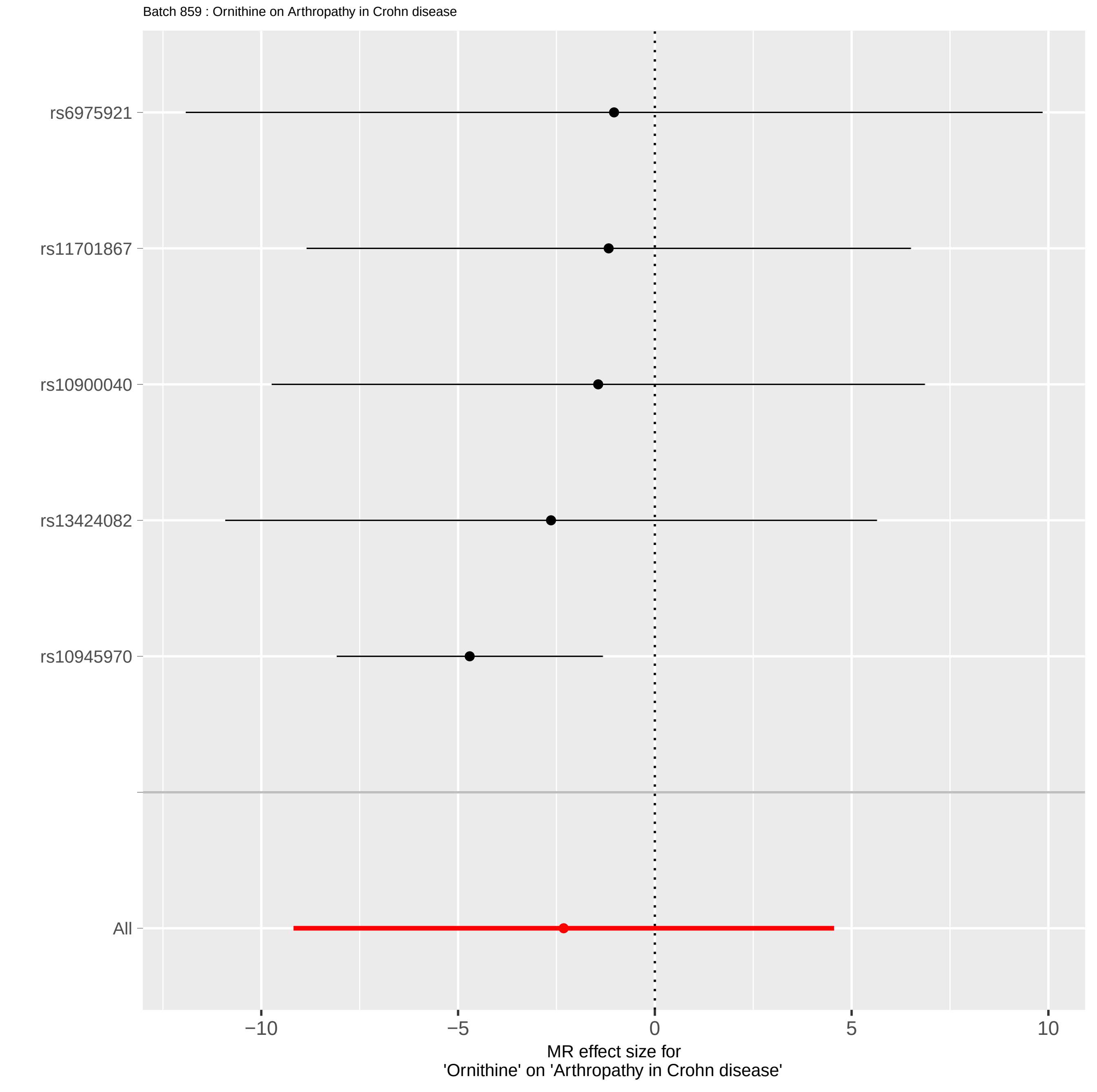
Batch 859 : Ornithine on Arthropathy in Crohn disease
rs6975921
rs11701867
rs10900040
rs13424082
rs10945970
All
−10
−5
0
5
10
MR effect size for
'Ornithine' on 'Arthropathy in Crohn disease'

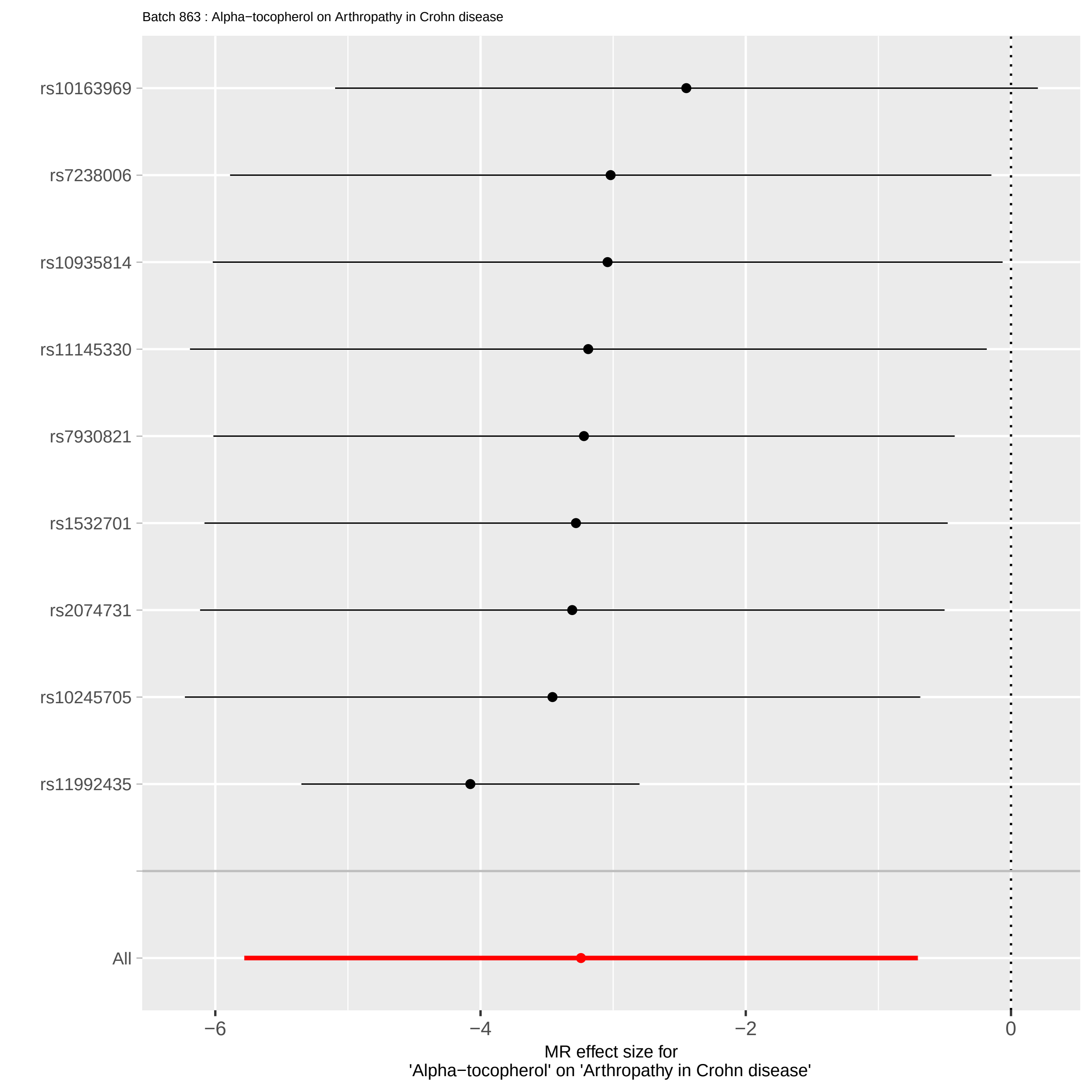

Batch 863 : Alpha−tocopherol on Arthropathy in Crohn disease
rs10163969
rs7238006
rs10935814
rs11145330
rs7930821
rs1532701
rs2074731
rs10245705
rs11992435
All
−6
−4
−2
0
MR effect size for
'Alpha−tocopherol' on 'Arthropathy in Crohn disease'

Batch 864 : Citrate on Arthropathy in Crohn disease

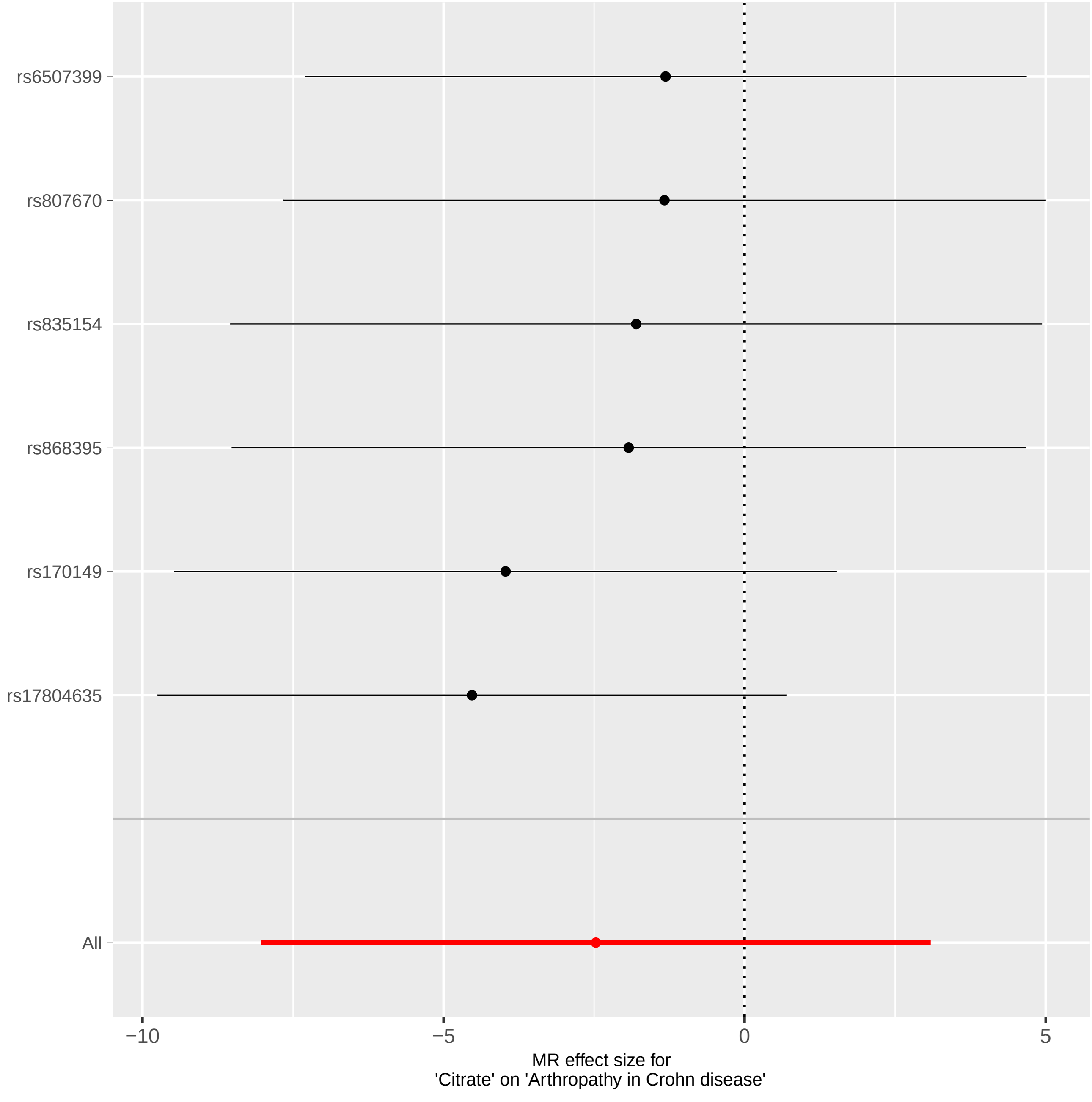

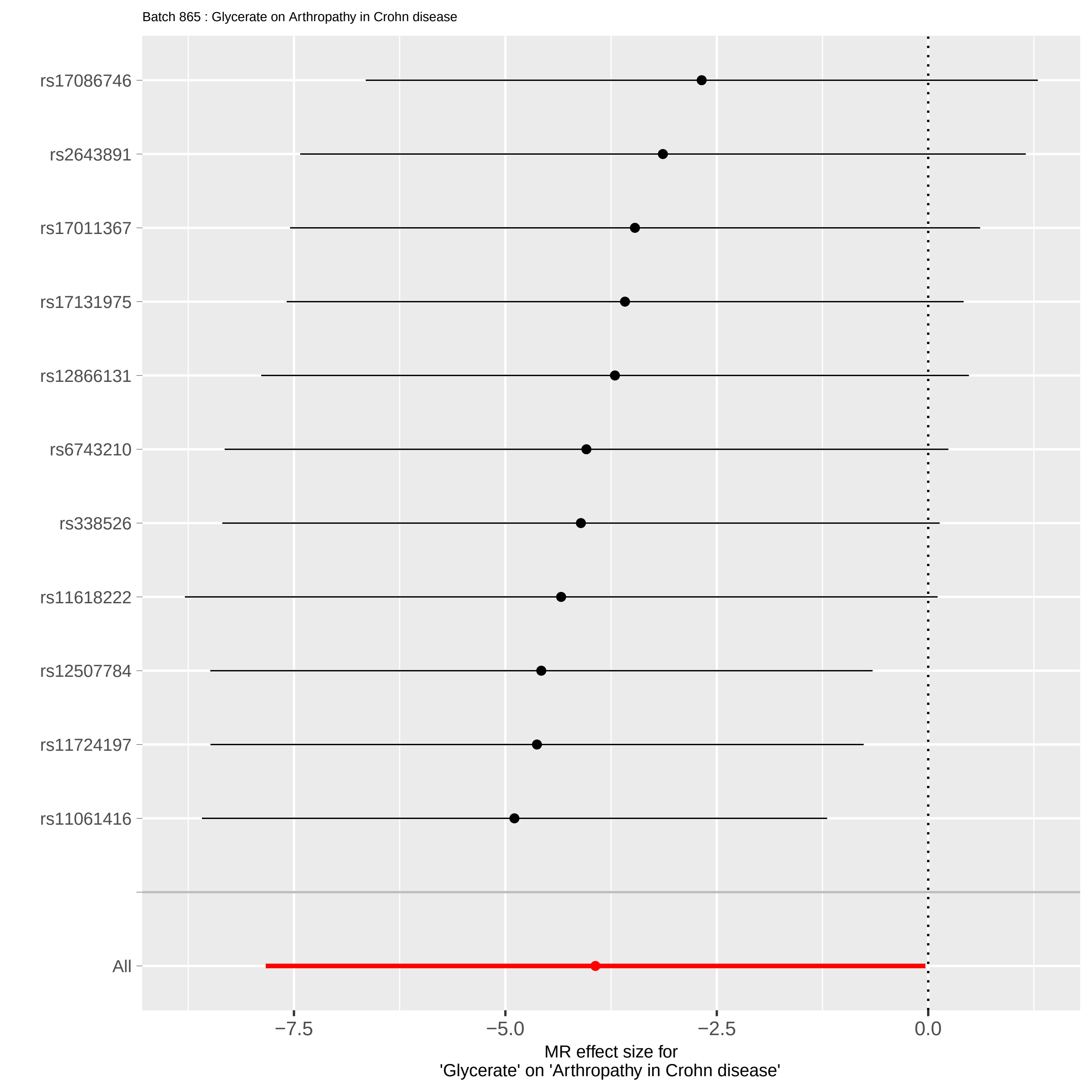

Batch 865 : Glycerate on Arthropathy in Crohn disease
rs17086746
rs2643891
rs17011367
rs17131975
rs12866131
rs6743210
rs338526
rs11618222
rs12507784
rs11724197
rs11061416
All
−7.5
−5.0
−2.5
0.0
MR effect size for
'Glycerate' on 'Arthropathy in Crohn disease'

Batch 866 : Guanosine on Arthropathy in Crohn disease

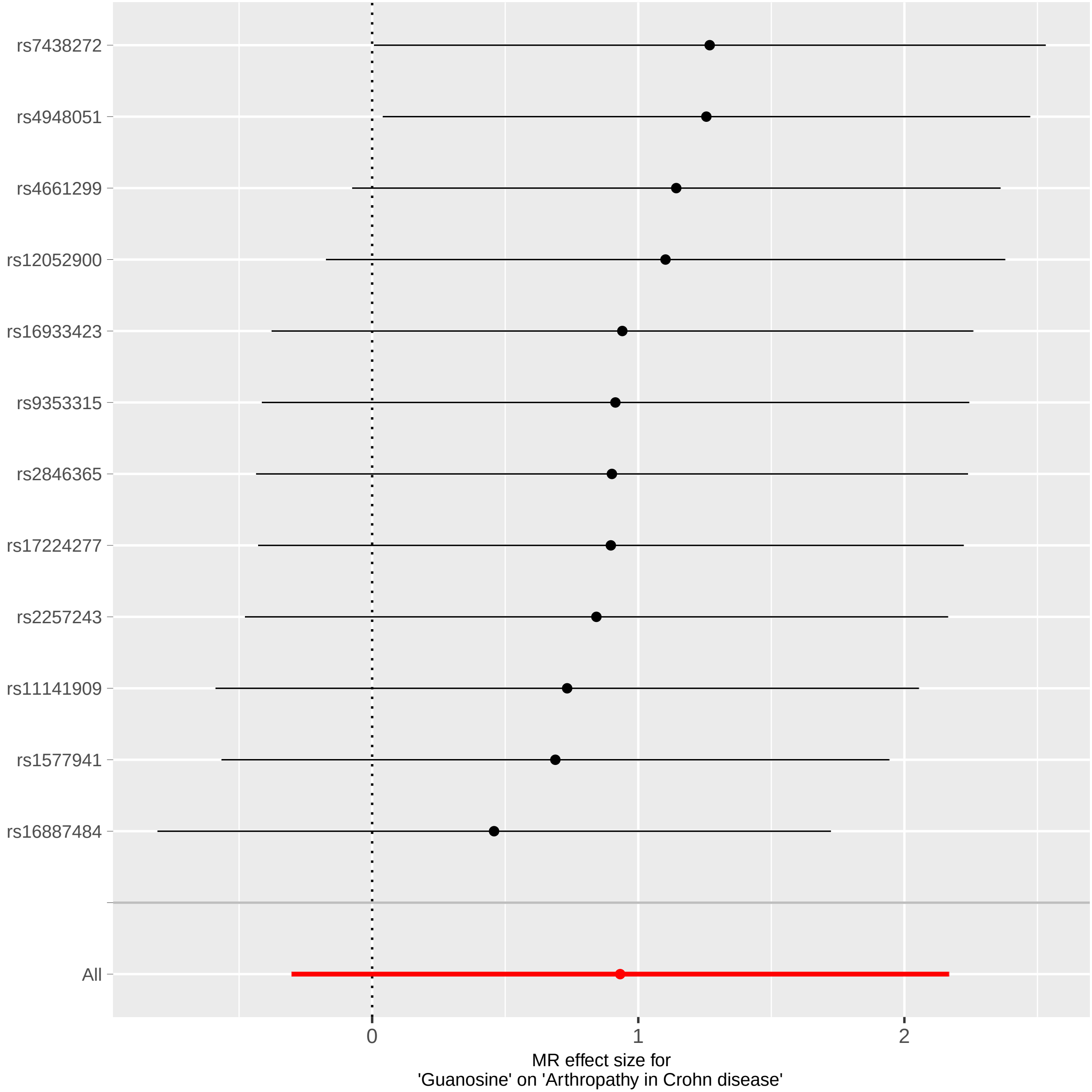

Batch 869 : Ursodeoxycholate on Arthropathy in Crohn disease

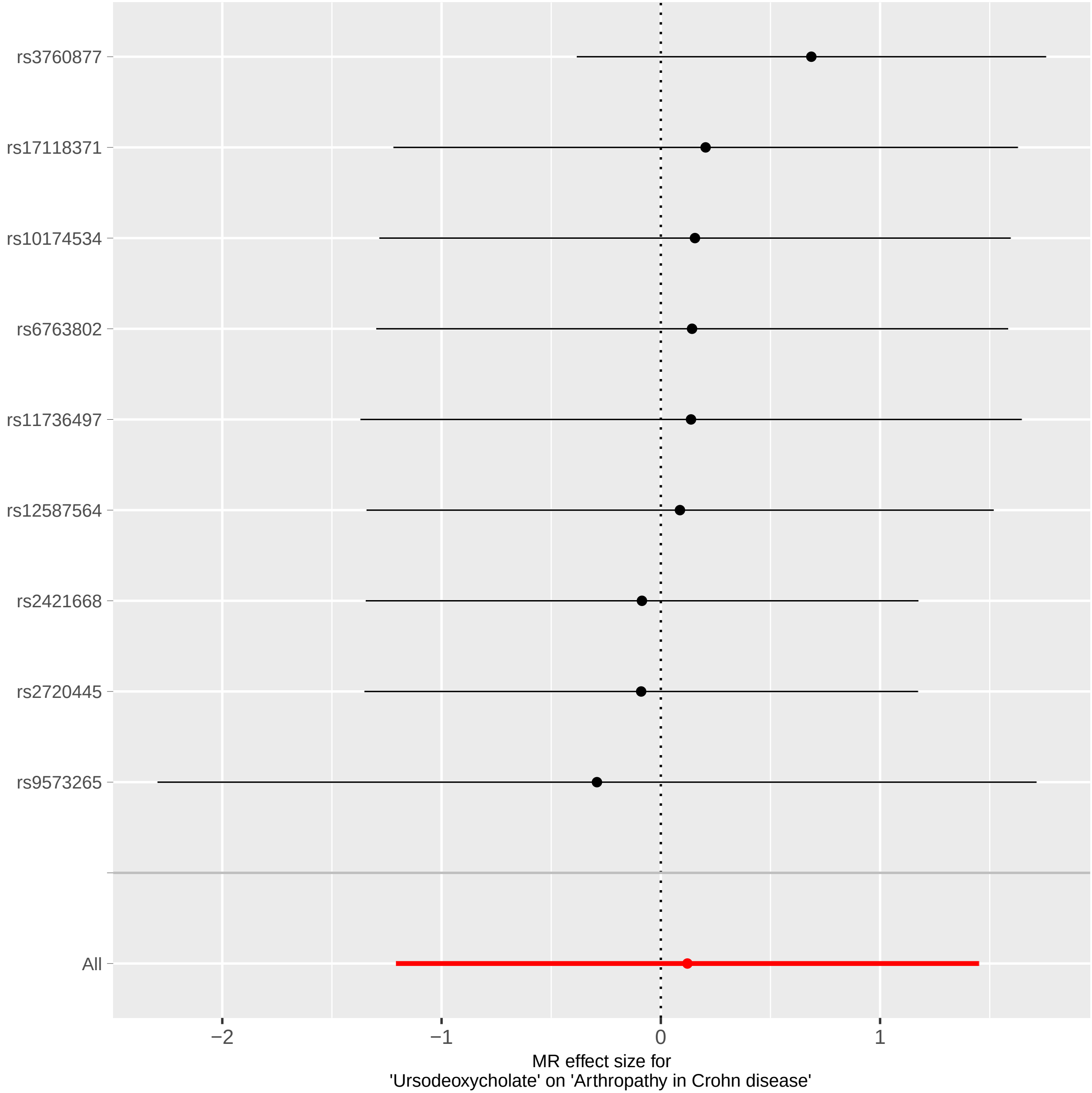

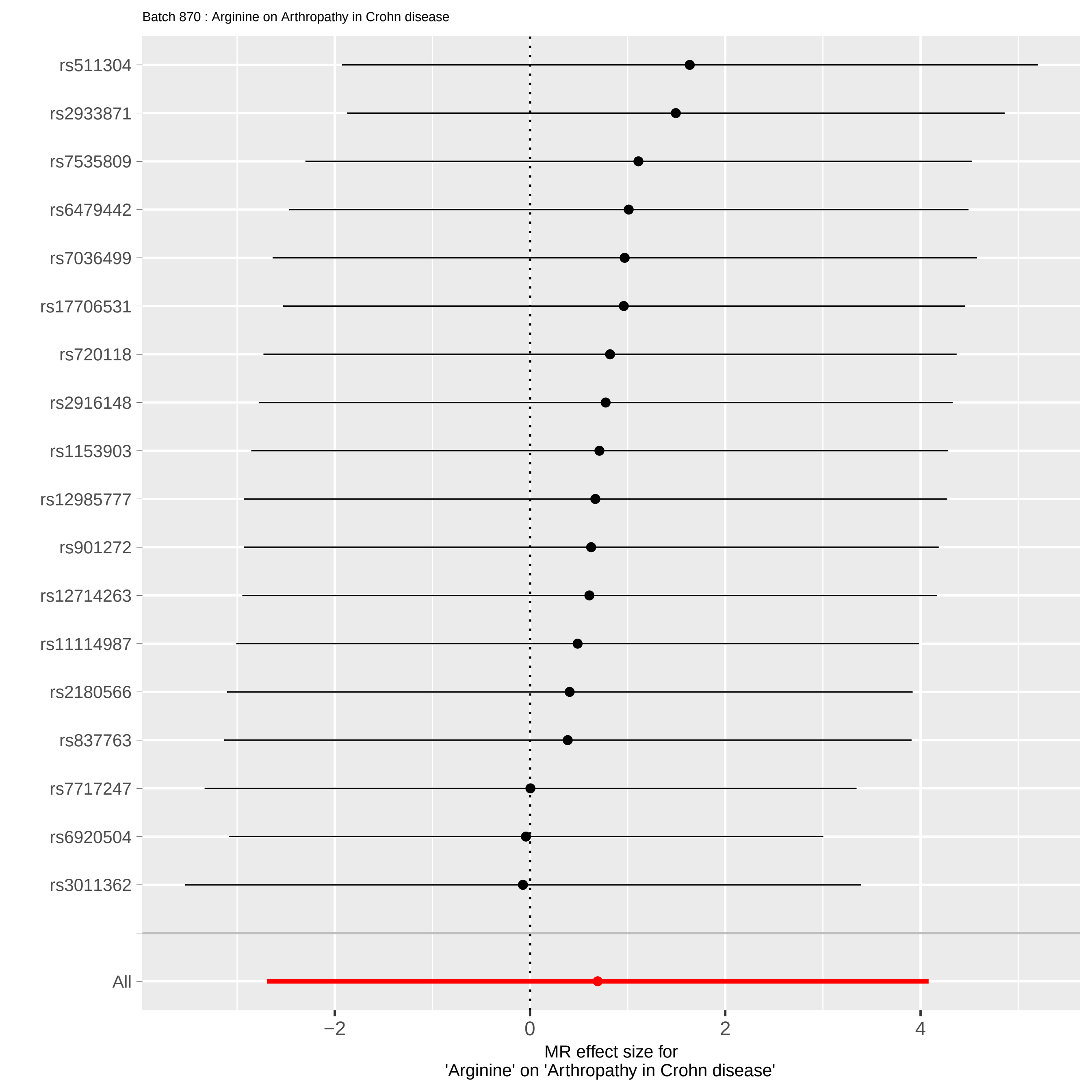

Batch 870 : Arginine on Arthropathy in Crohn disease
rs511304
rs2933871
rs7535809
rs6479442
rs7036499
rs17706531
rs720118
rs2916148
rs1153903
rs12985777
rs901272
rs12714263
rs11114987
rs2180566
rs837763
rs7717247
rs6920504
rs3011362
All
−2
0
2
4
MR effect size for
'Arginine' on 'Arthropathy in Crohn disease'

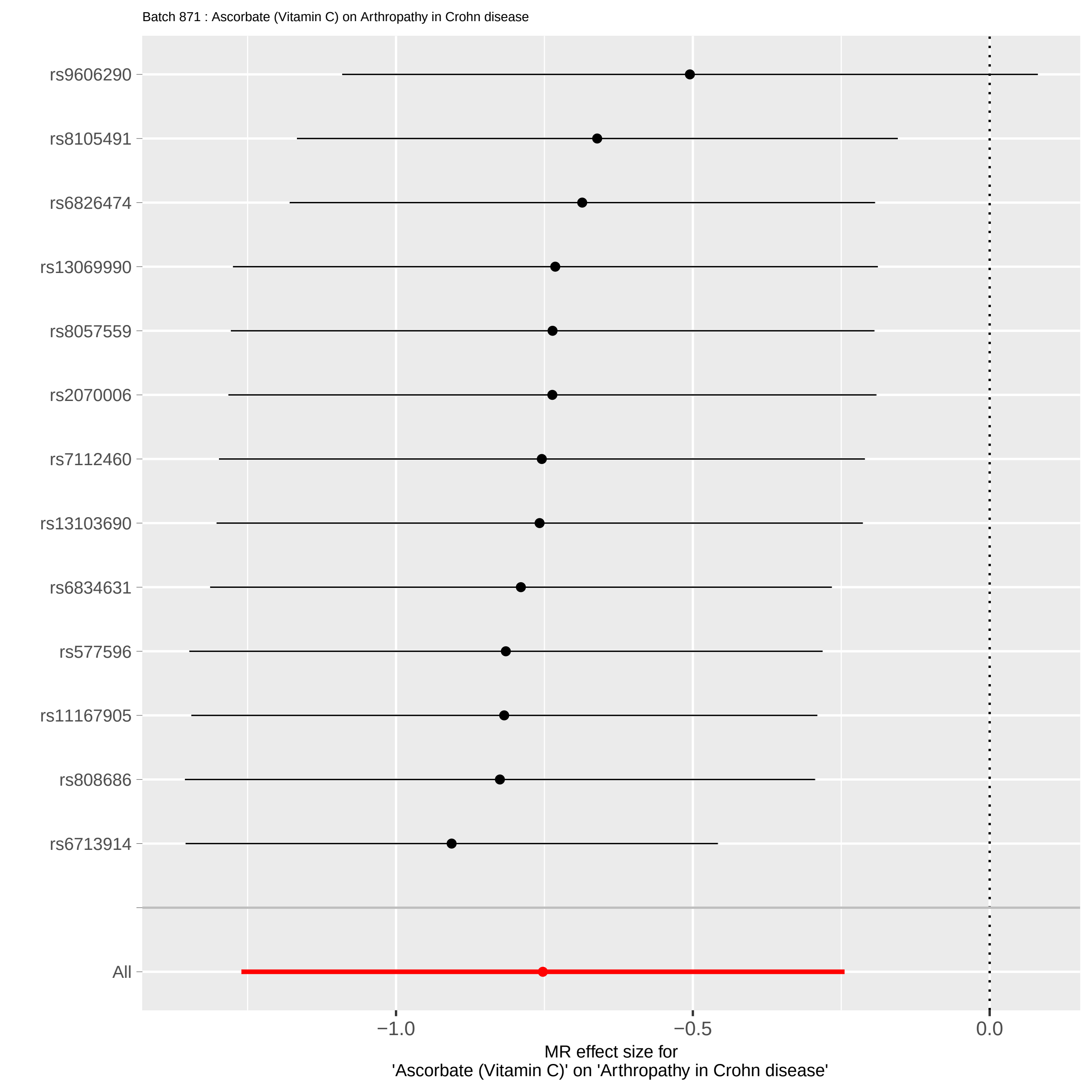
Batch 871 : Ascorbate (Vitamin C) on Arthropathy in Crohn disease
rs9606290
rs8105491
rs6826474
rs13069990
rs8057559
rs2070006
rs7112460
rs13103690
rs6834631
rs577596
rs11167905
rs808686
rs6713914
All
−1.0
−0.5
0.0
MR effect size for
'Ascorbate (Vitamin C)' on 'Arthropathy in Crohn disease'

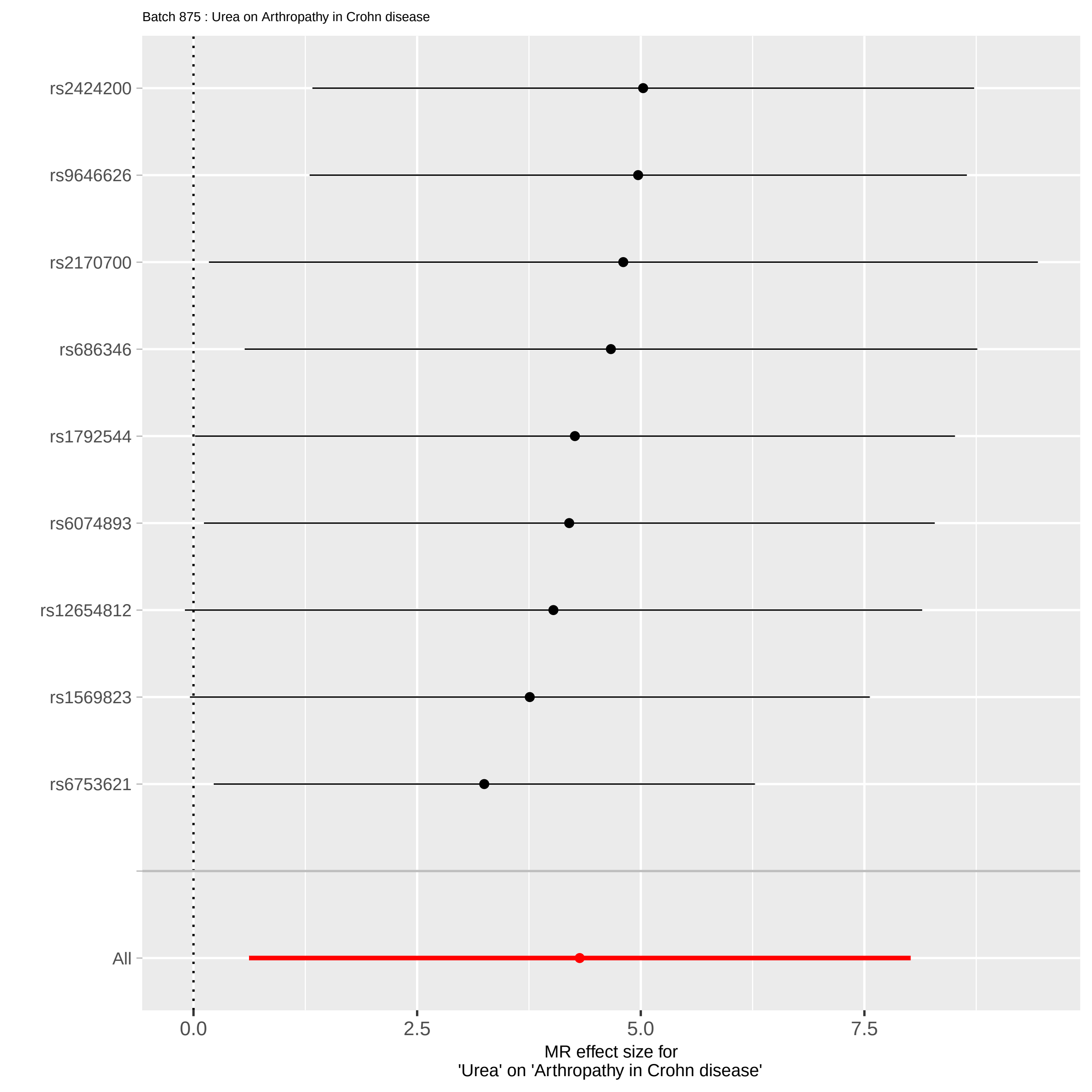

Batch 875 : Urea on Arthropathy in Crohn disease
rs2424200
rs9646626
rs2170700
rs686346
rs1792544
rs6074893
rs12654812
rs1569823
rs6753621
All
0.0
2.5
5.0
7.5
MR effect size for
'Urea' on 'Arthropathy in Crohn disease'

Batch 876 : Cortisol on Arthropathy in Crohn disease

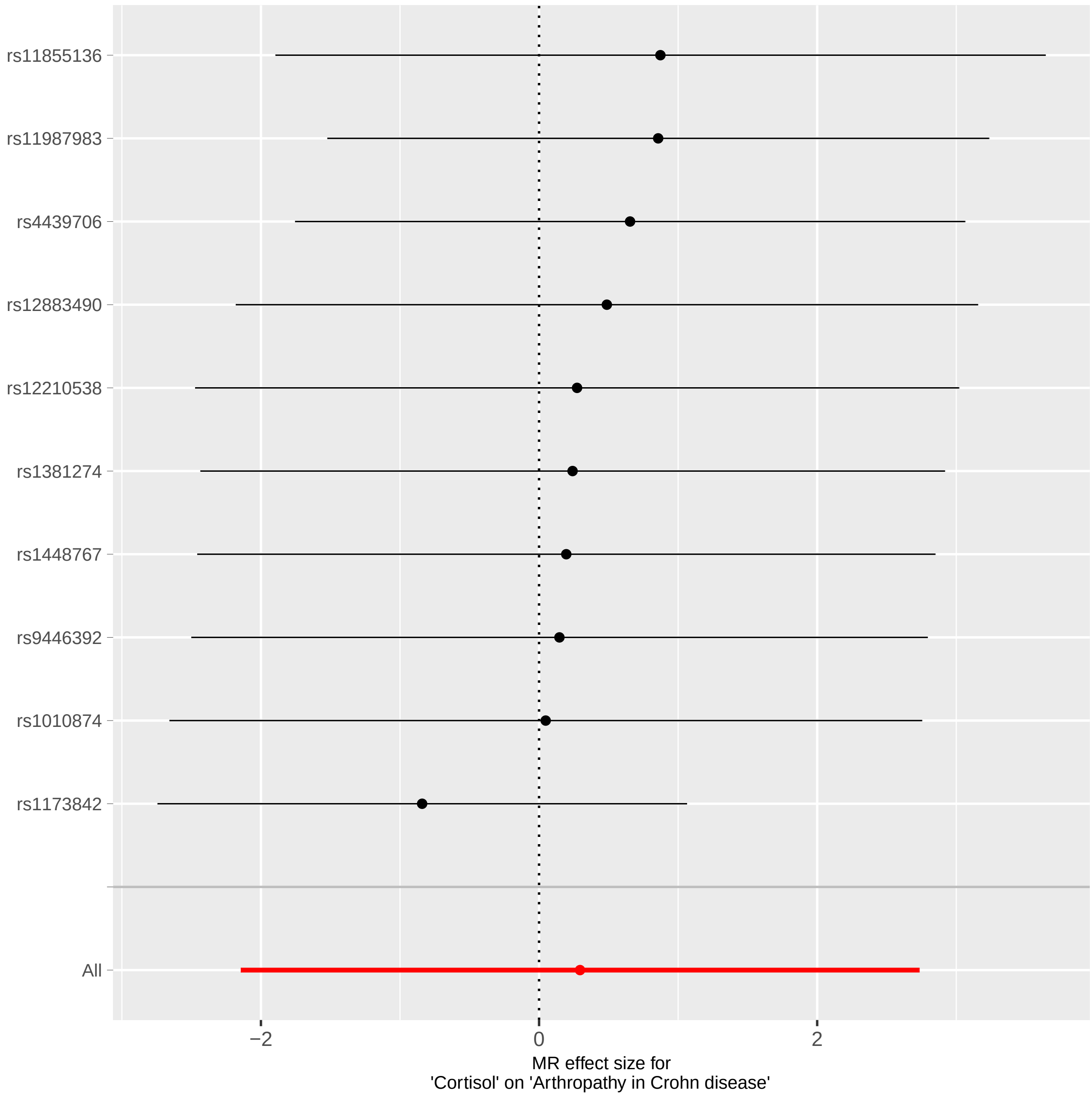

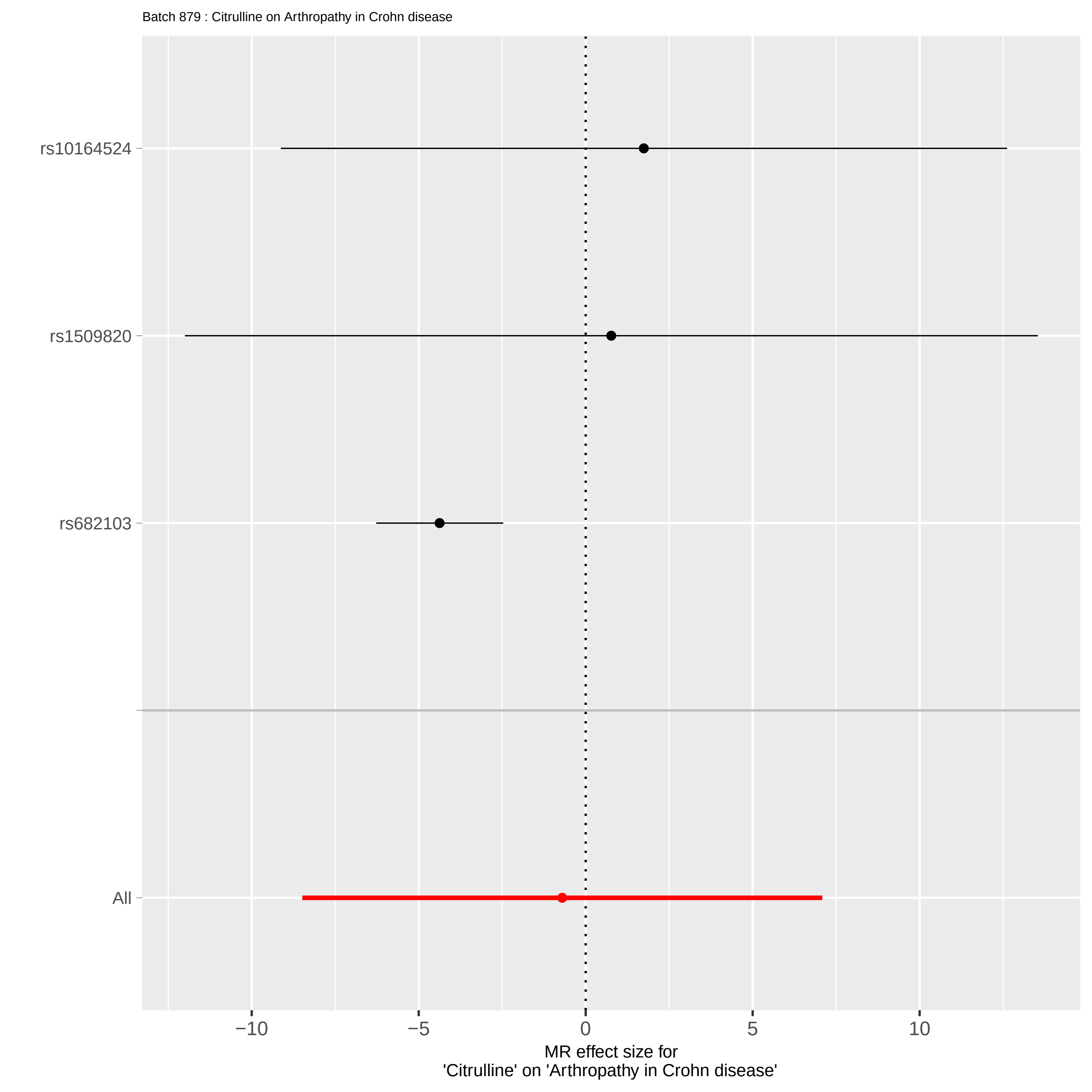
Batch 879 : Citrulline on Arthropathy in Crohn disease
rs10164524
rs1509820
rs682103
All
−10
−5
0
5
10
MR effect size for
'Citrulline' on 'Arthropathy in Crohn disease'

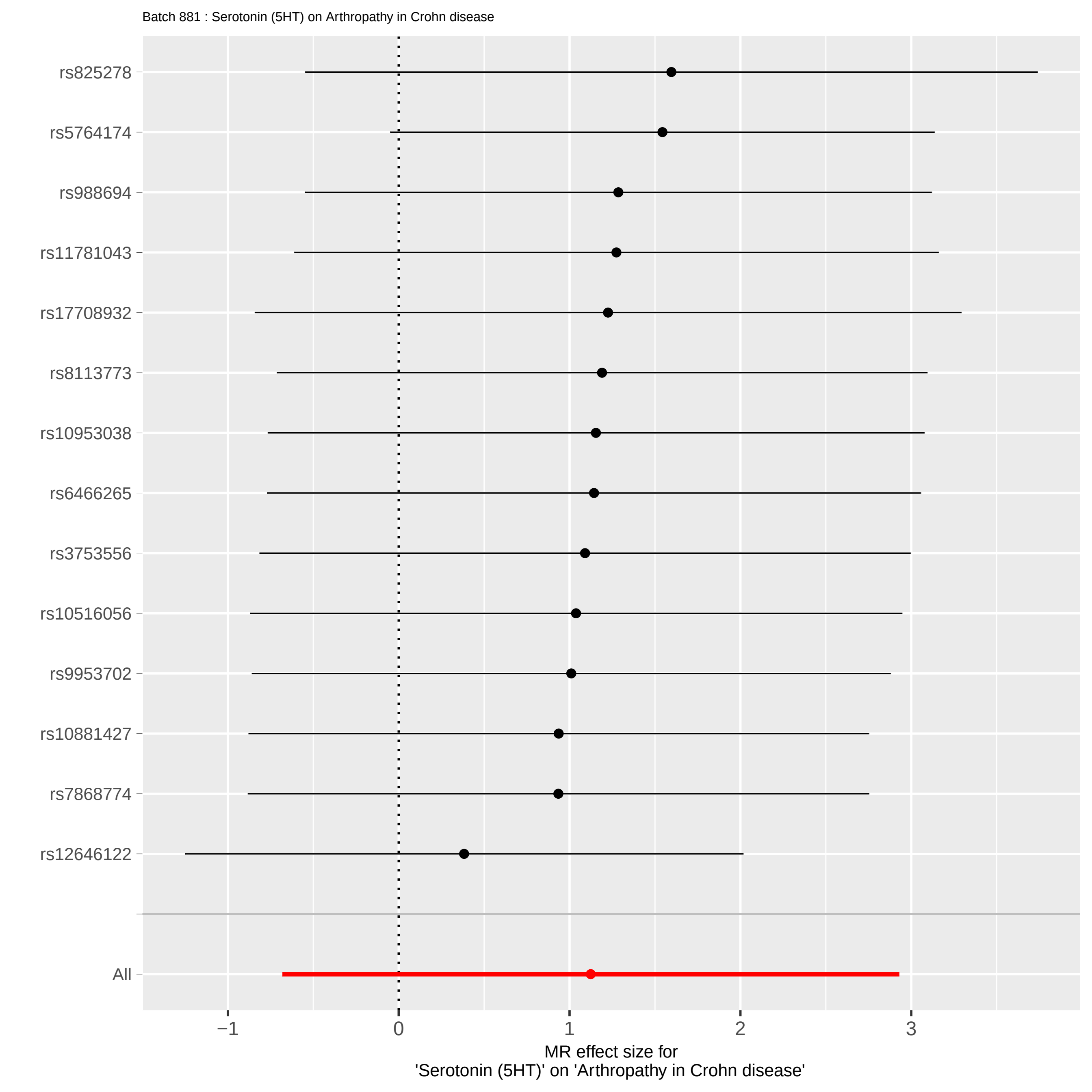
Batch 881 : Serotonin (5HT) on Arthropathy in Crohn disease
rs825278
rs5764174
rs988694
rs11781043
rs17708932
rs8113773
rs10953038
rs6466265
rs3753556
rs10516056
rs9953702
rs10881427
rs7868774
rs12646122
All
−1
0
1
2
3
MR effect size for
'Serotonin (5HT)' on 'Arthropathy in Crohn disease'

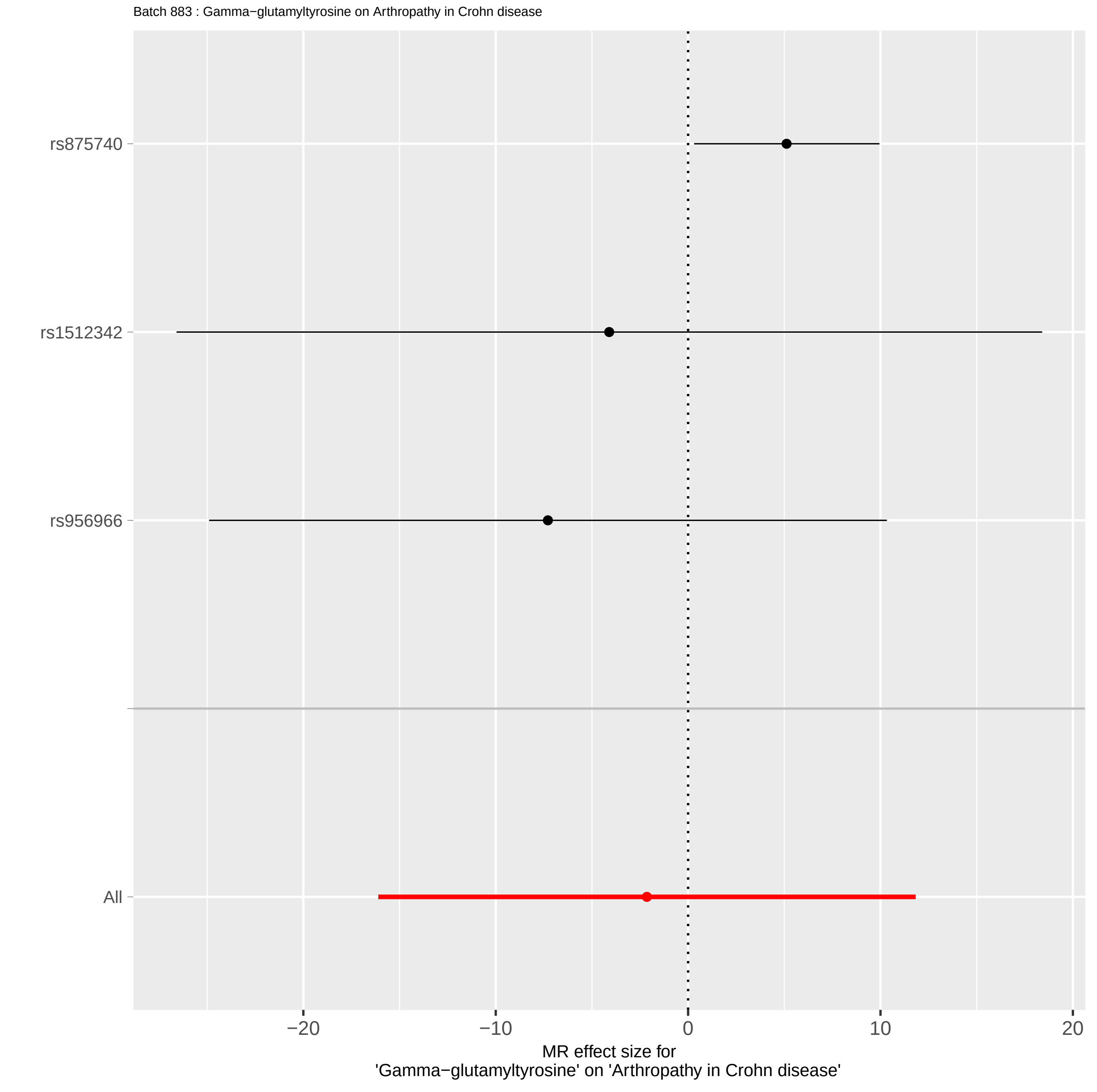
Batch 883 : Gamma−glutamyltyrosine on Arthropathy in Crohn disease
rs875740
rs1512342
rs956966
All
−20
−10
0
10
20
MR effect size for
'Gamma−glutamyltyrosine' on 'Arthropathy in Crohn disease'

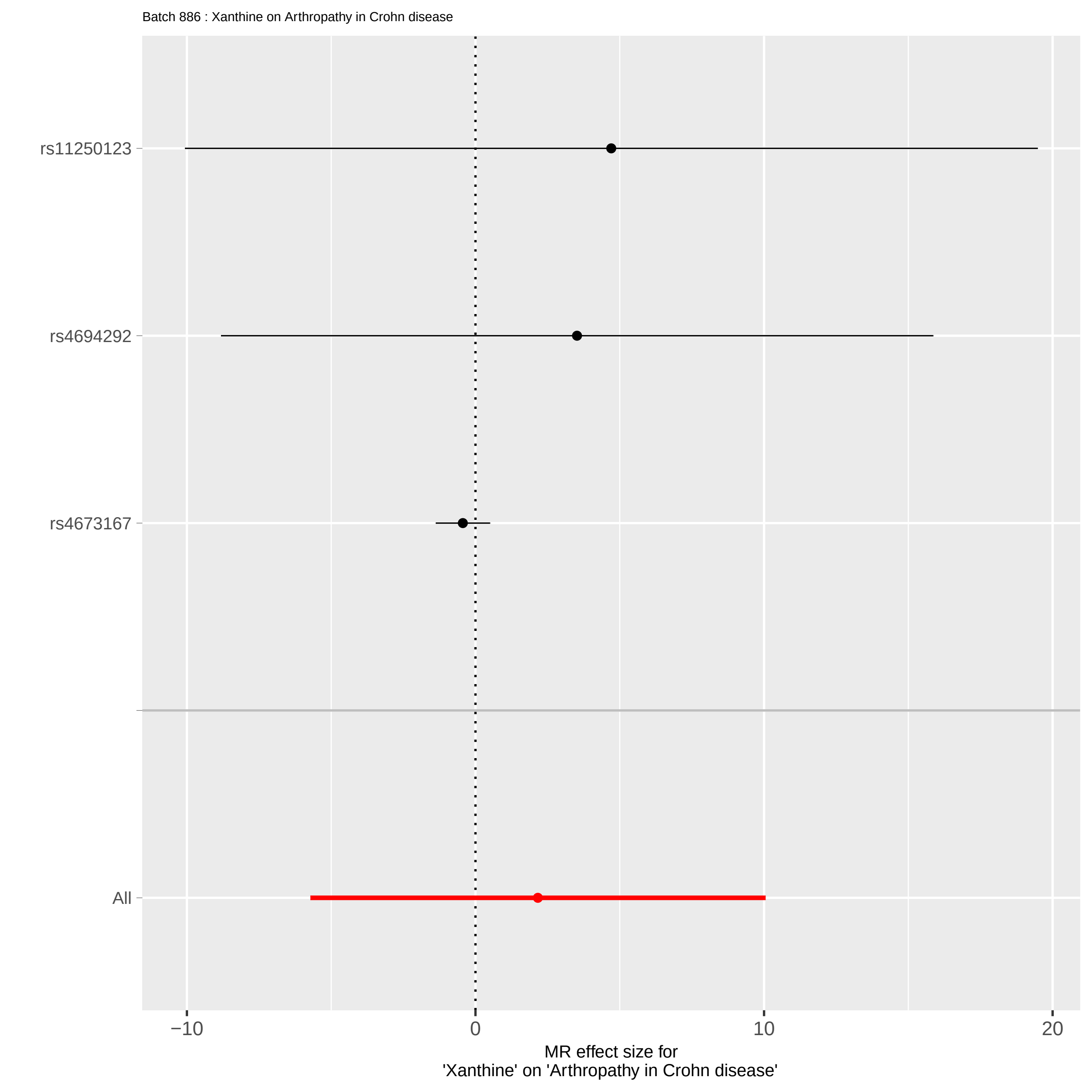

Batch 886 : Xanthine on Arthropathy in Crohn disease
rs11250123
rs4694292
rs4673167
All
−10
0
10
20
MR effect size for
'Xanthine' on 'Arthropathy in Crohn disease'

Batch 887 : Phosphate on Arthropathy in Crohn disease

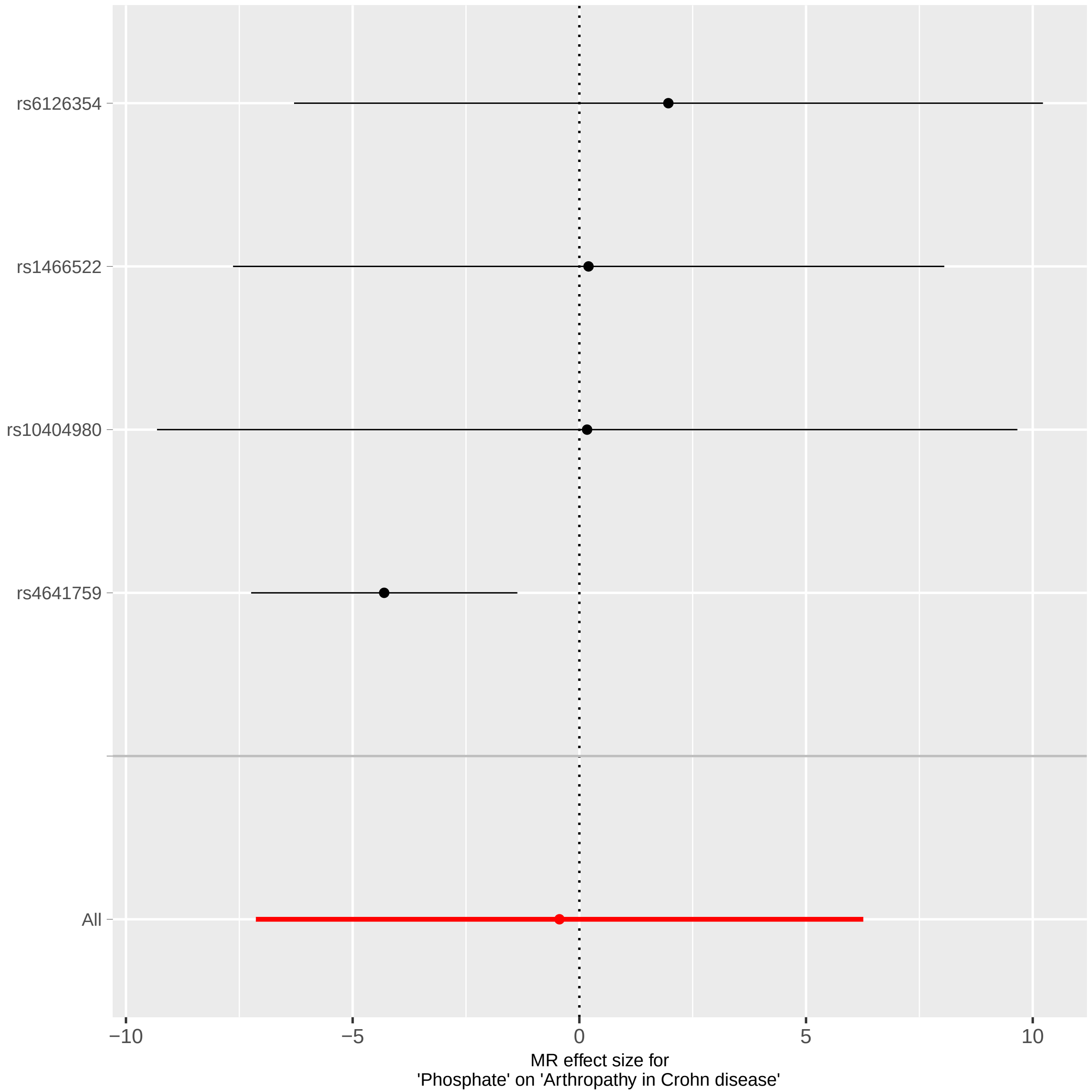

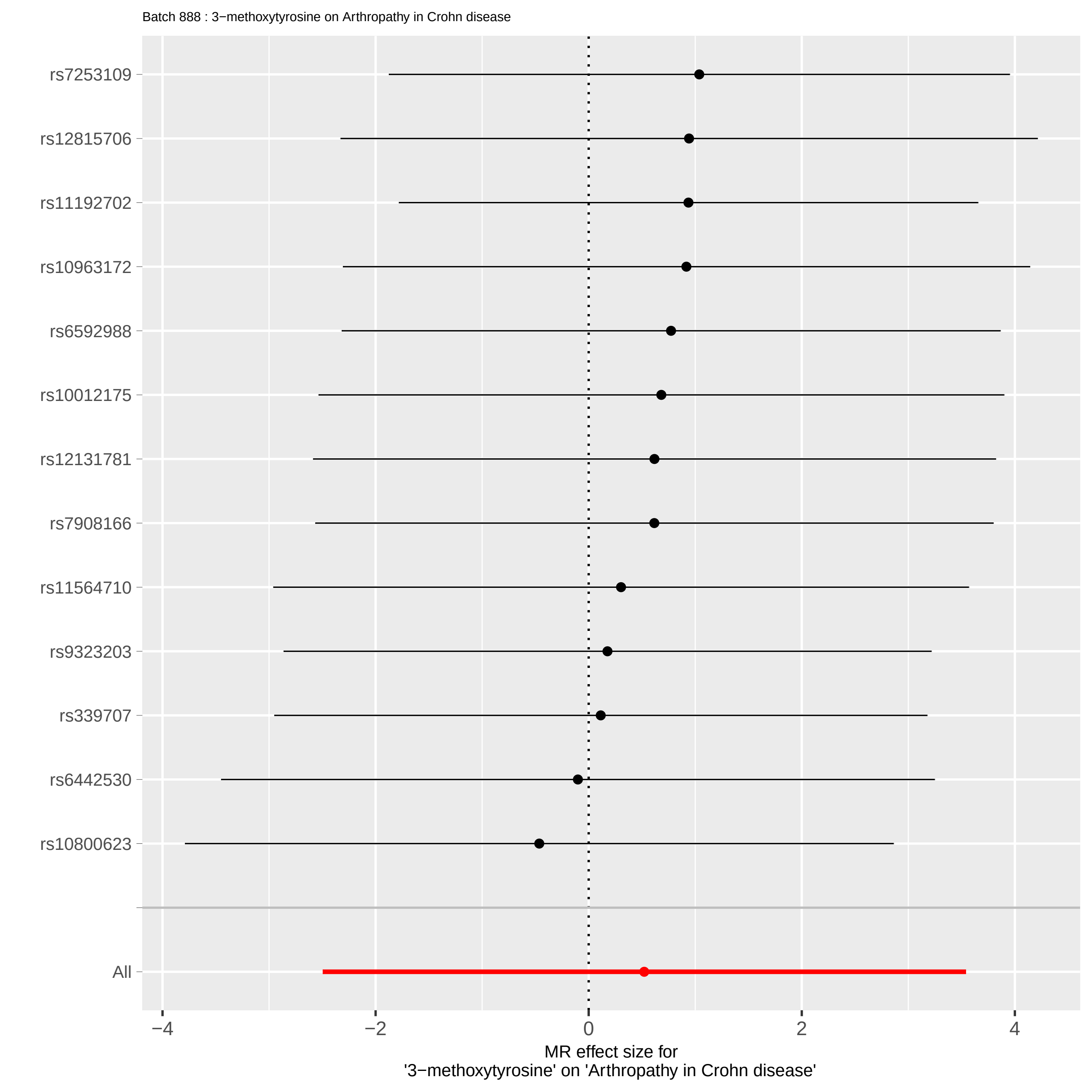

Batch 888 : 3−methoxytyrosine on Arthropathy in Crohn disease
rs7253109
rs12815706
rs11192702
rs10963172
rs6592988
rs10012175
rs12131781
rs7908166
rs11564710
rs9323203
rs339707
rs6442530
rs10800623
All
−4
−2
0
2
4
MR effect size for
'3−methoxytyrosine' on 'Arthropathy in Crohn disease'

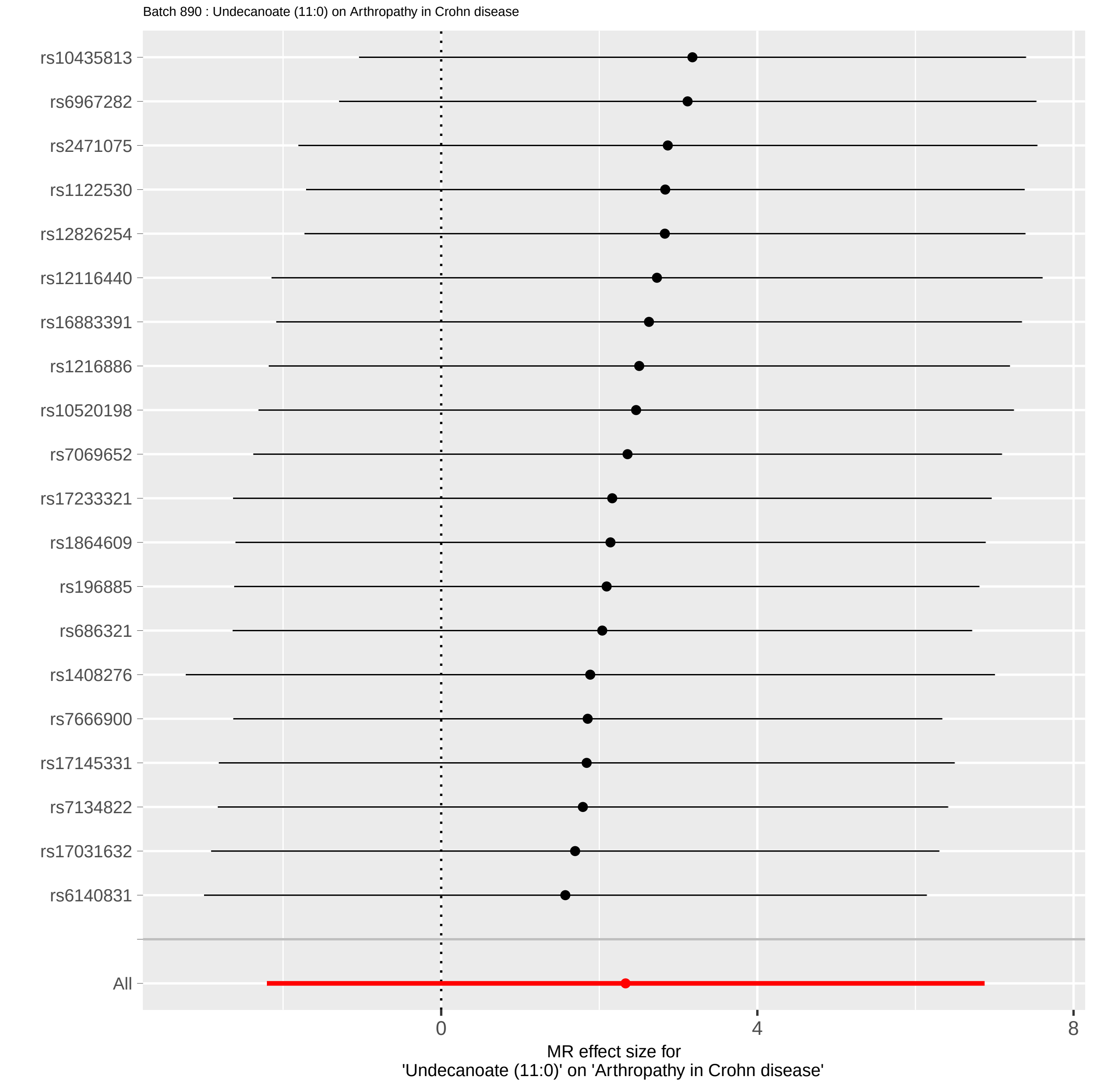

Batch 890 : Undecanoate (11:0) on Arthropathy in Crohn disease
rs10435813
rs6967282
rs2471075
rs1122530
rs12826254
rs12116440
rs16883391
rs1216886
rs10520198
rs7069652
rs17233321
rs1864609
rs196885
rs686321
rs1408276
rs7666900
rs17145331
rs7134822
rs17031632
rs6140831
All
0
4
8
MR effect size for
'Undecanoate (11:0)' on 'Arthropathy in Crohn disease'

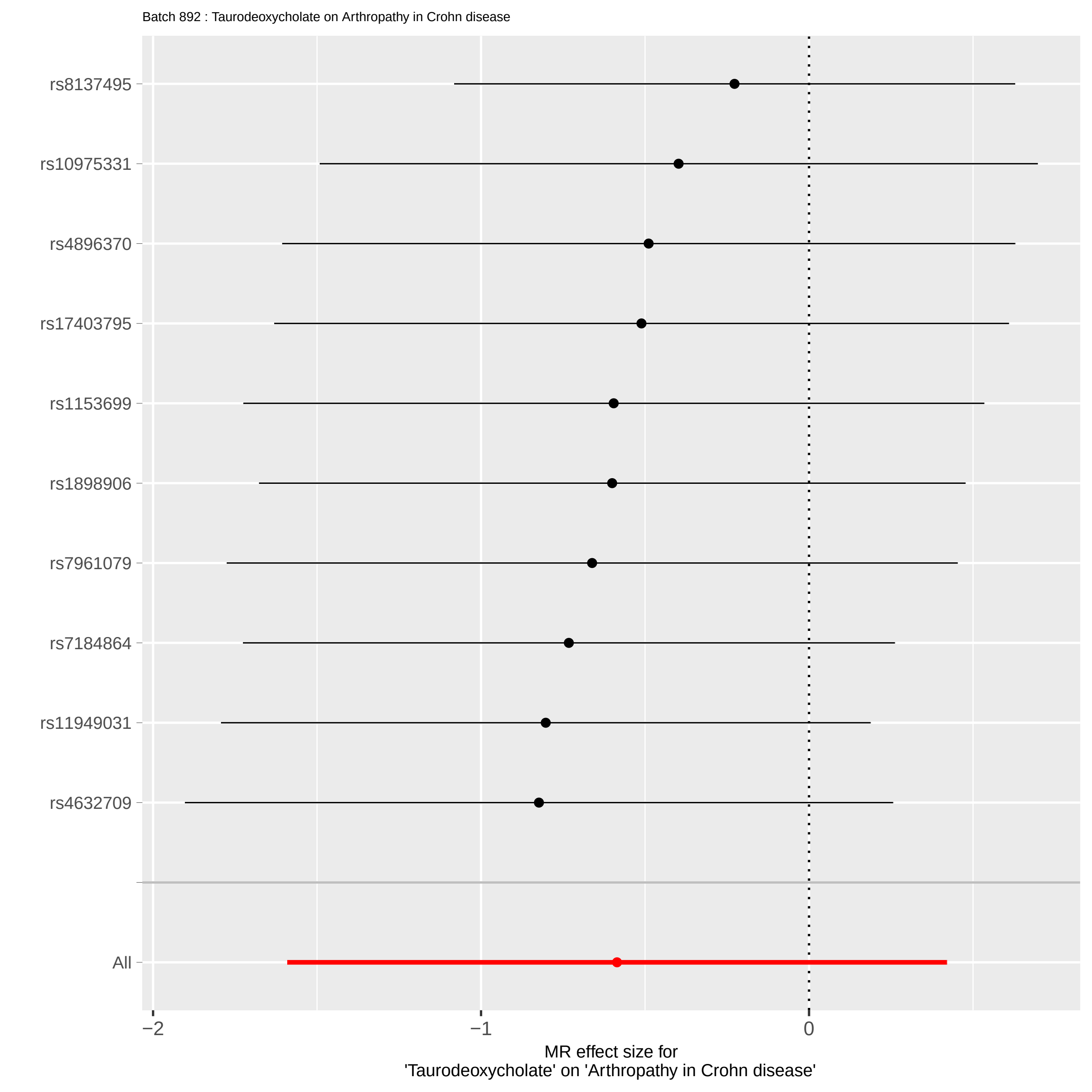
Batch 892 : Taurodeoxycholate on Arthropathy in Crohn disease
rs8137495
rs10975331
rs4896370
rs17403795
rs1153699
rs1898906
rs7961079
rs7184864
rs11949031
rs4632709
All
−2
−1
0
MR effect size for
'Taurodeoxycholate' on 'Arthropathy in Crohn disease'

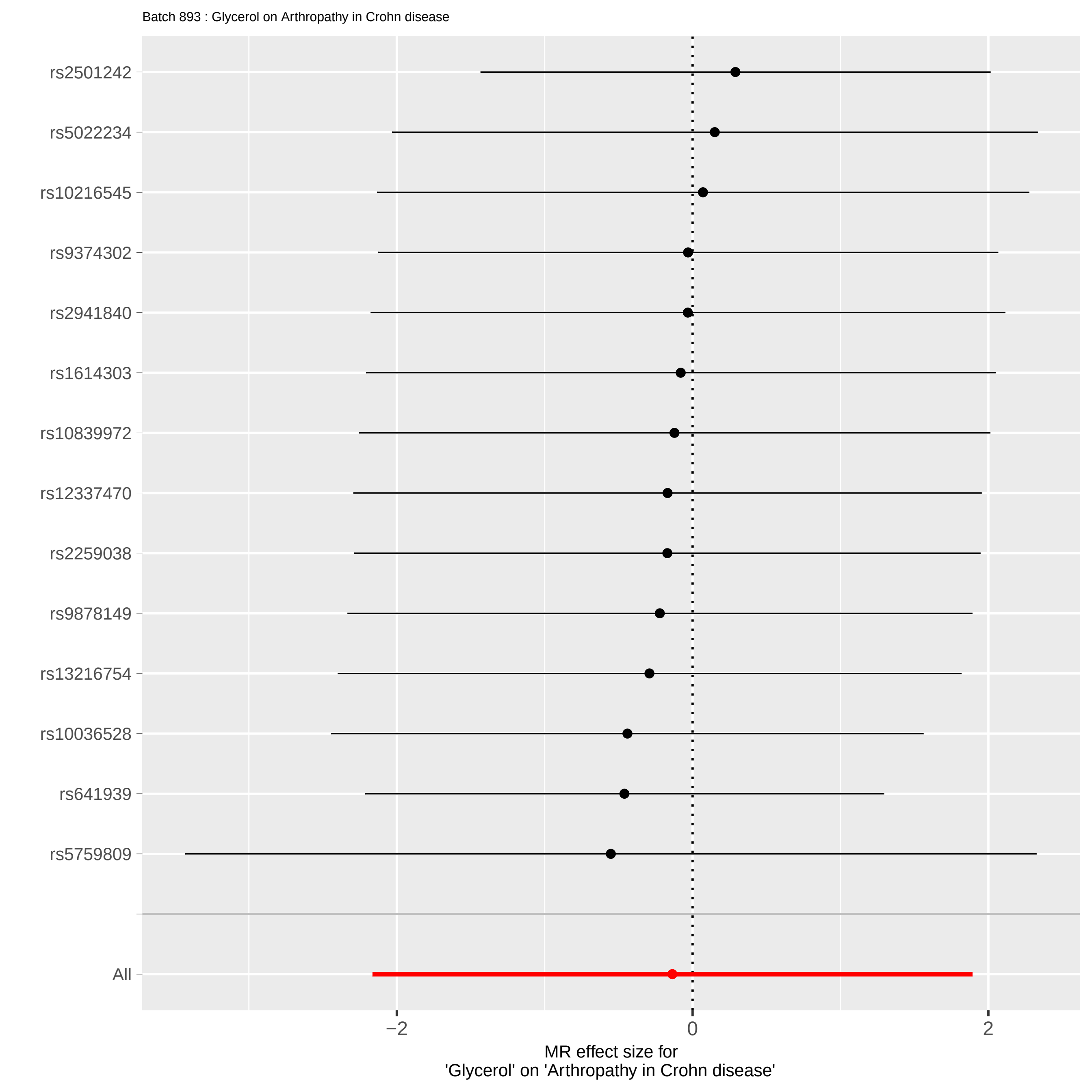

Batch 893 : Glycerol on Arthropathy in Crohn disease
rs2501242
rs5022234
rs10216545
rs9374302
rs2941840
rs1614303
rs10839972
rs12337470
rs2259038
rs9878149
rs13216754
rs10036528
rs641939
rs5759809
All
−2
0
2
MR effect size for
'Glycerol' on 'Arthropathy in Crohn disease'

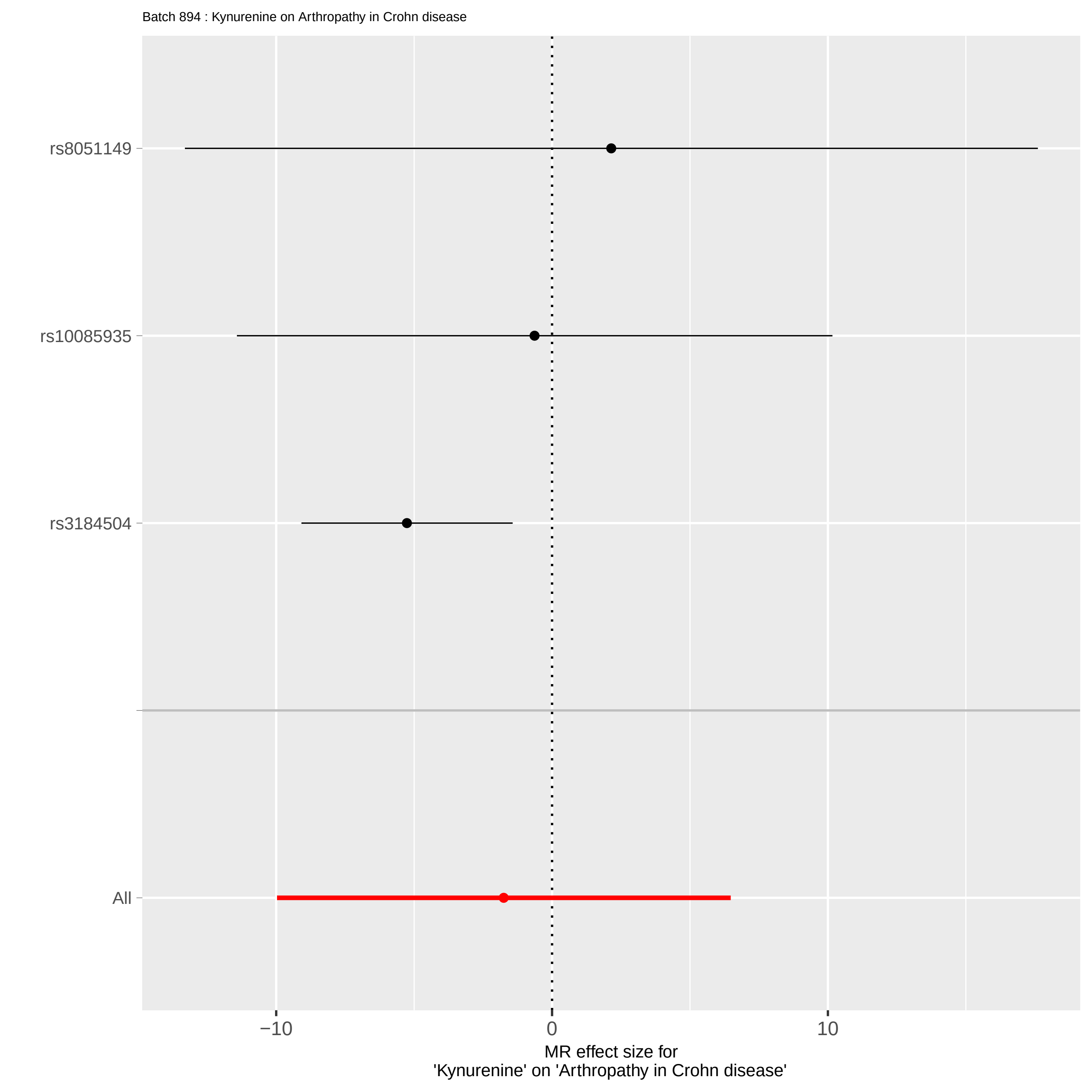

Batch 894 : Kynurenine on Arthropathy in Crohn disease
rs8051149
rs10085935
rs3184504
All
−10
0
10
MR effect size for
'Kynurenine' on 'Arthropathy in Crohn disease'

Batch 895 : Mannitol on Arthropathy in Crohn disease

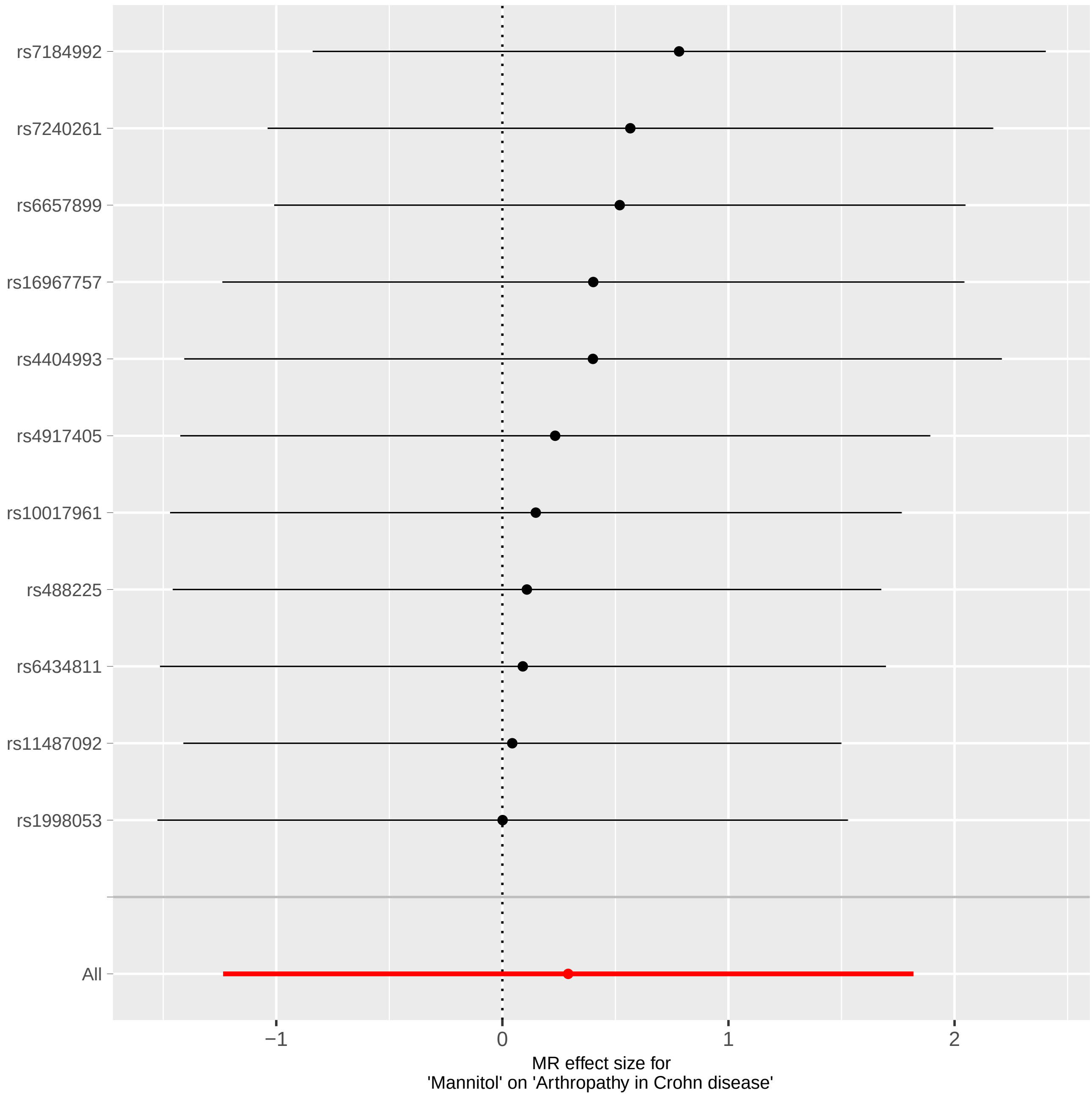

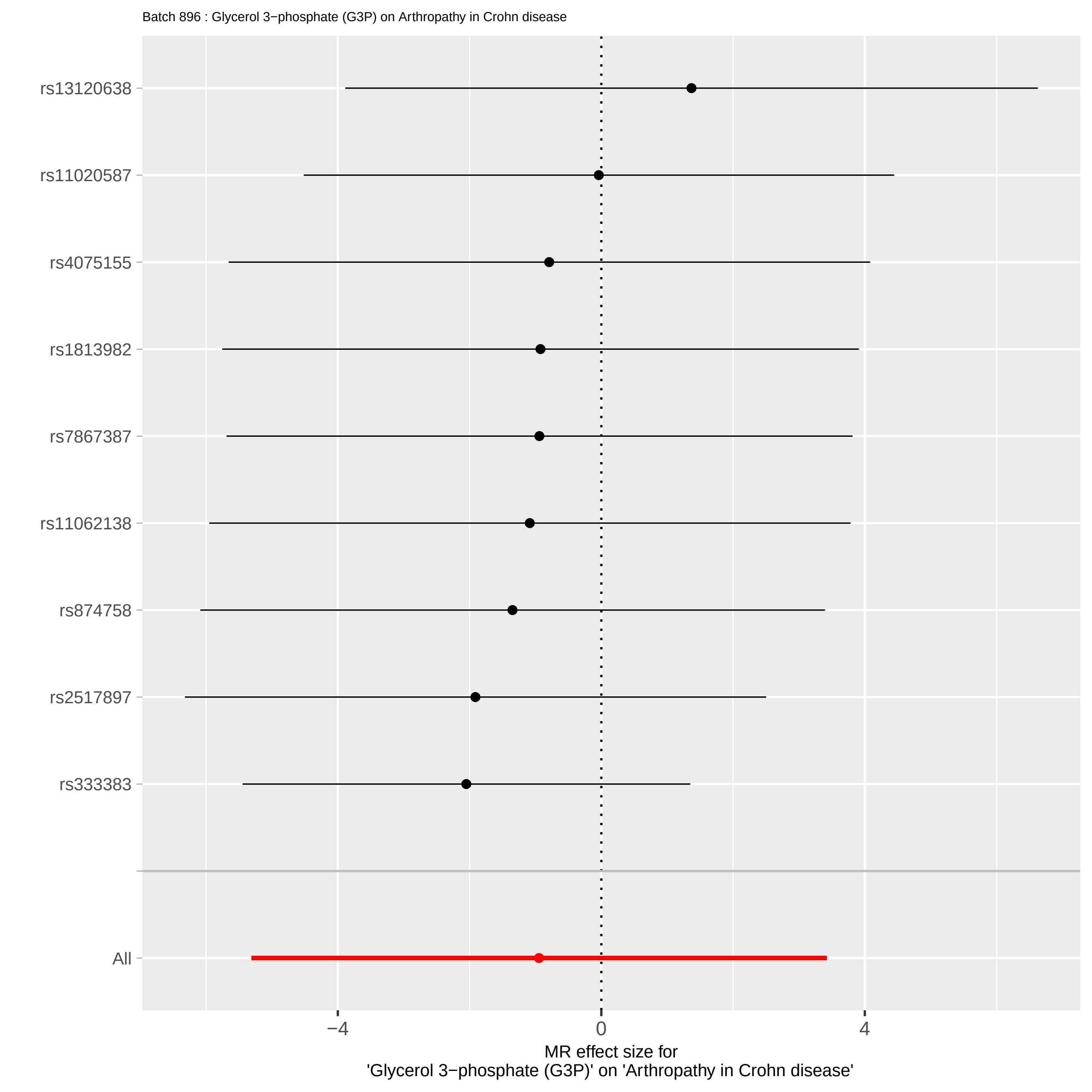

Batch 896 : Glycerol 3−phosphate (G3P) on Arthropathy in Crohn disease
rs13120638
rs11020587
rs4075155
rs1813982
rs7867387
rs11062138
rs874758
rs2517897
rs333383
All
−4
0
4
MR effect size for
'Glycerol 3−phosphate (G3P)' on 'Arthropathy in Crohn disease'

Batch 898 : Carnitine on Arthropathy in Crohn disease

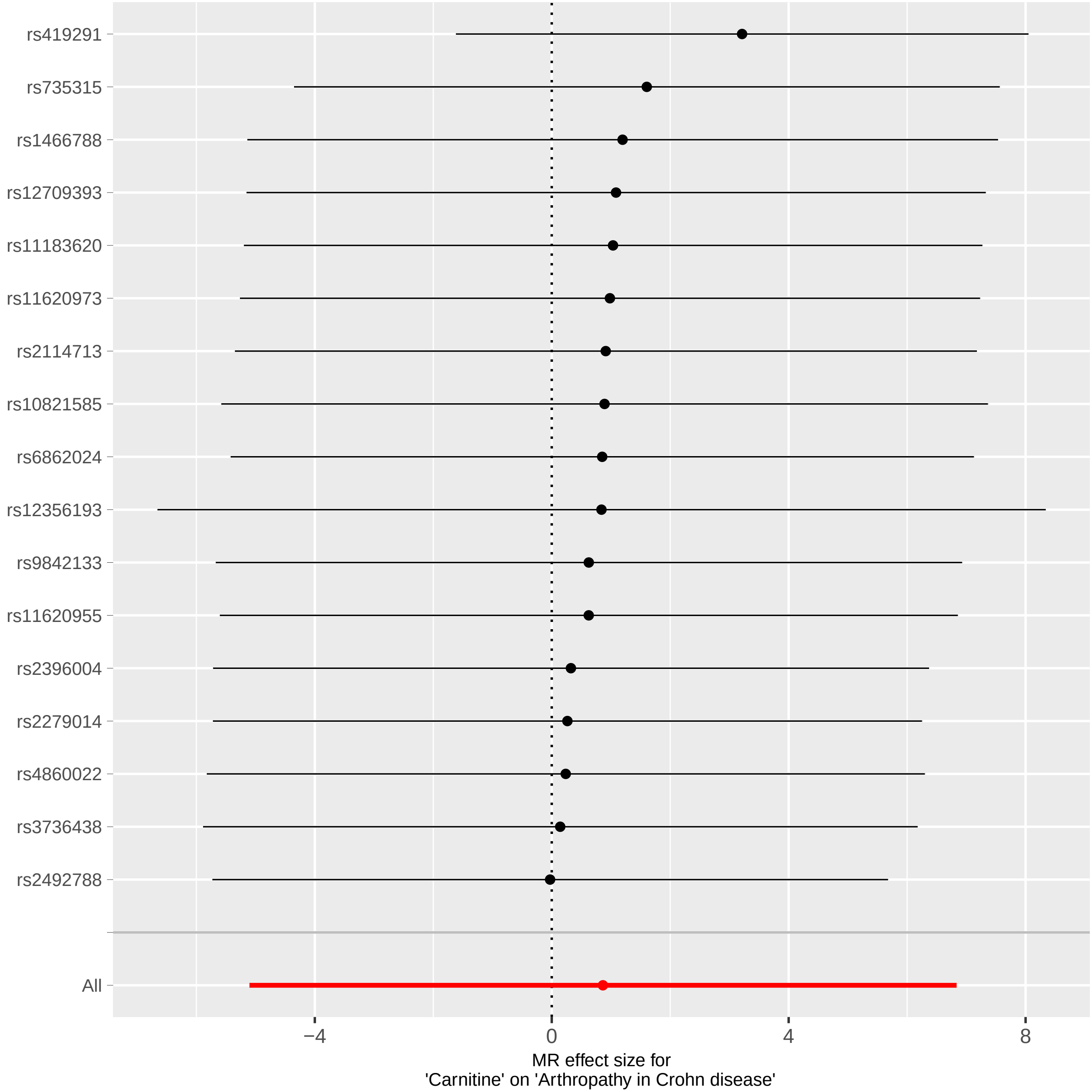

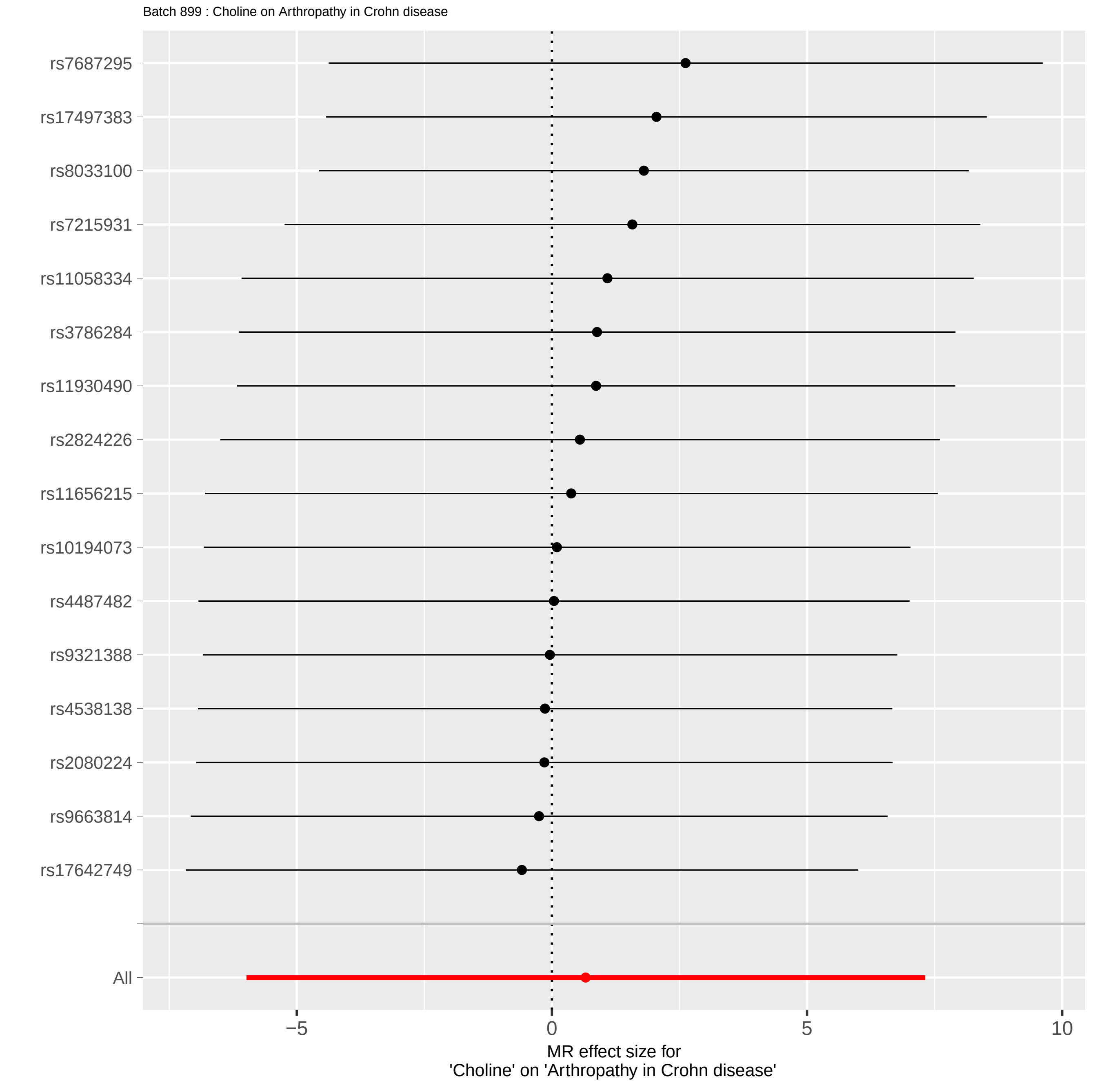

Batch 899 : Choline on Arthropathy in Crohn disease
rs7687295
rs17497383
rs8033100
rs7215931
rs11058334
rs3786284
rs11930490
rs2824226
rs11656215
rs10194073
rs4487482
rs9321388
rs4538138
rs2080224
rs9663814
rs17642749
All
−5
0
5
10
MR effect size for
'Choline' on 'Arthropathy in Crohn disease'

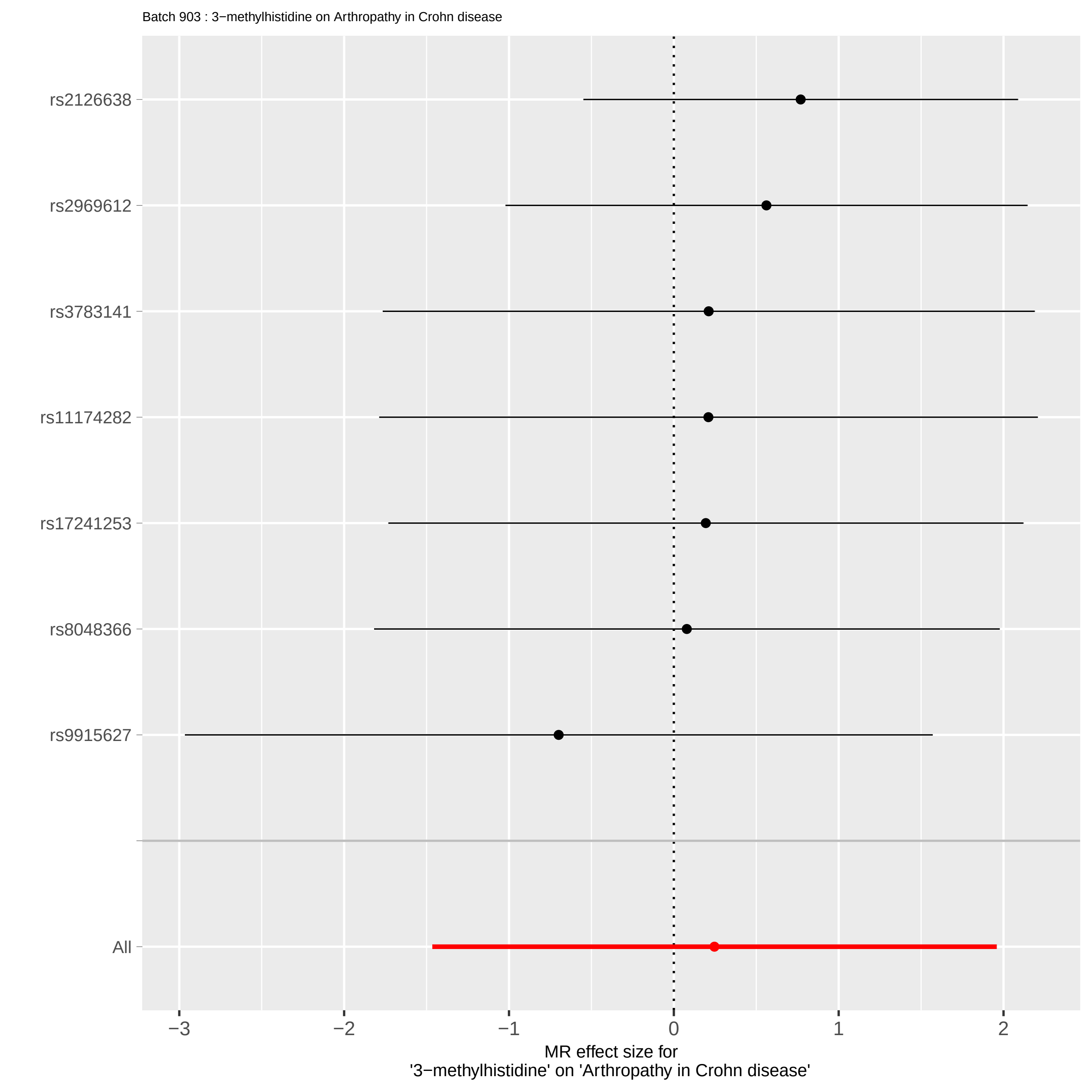
Batch 903 : 3−methylhistidine on Arthropathy in Crohn disease
rs2126638
rs2969612
rs3783141
rs11174282
rs17241253
rs8048366
rs9915627
All
−3
−2
−1
0
1
2
MR effect size for
'3−methylhistidine' on 'Arthropathy in Crohn disease'

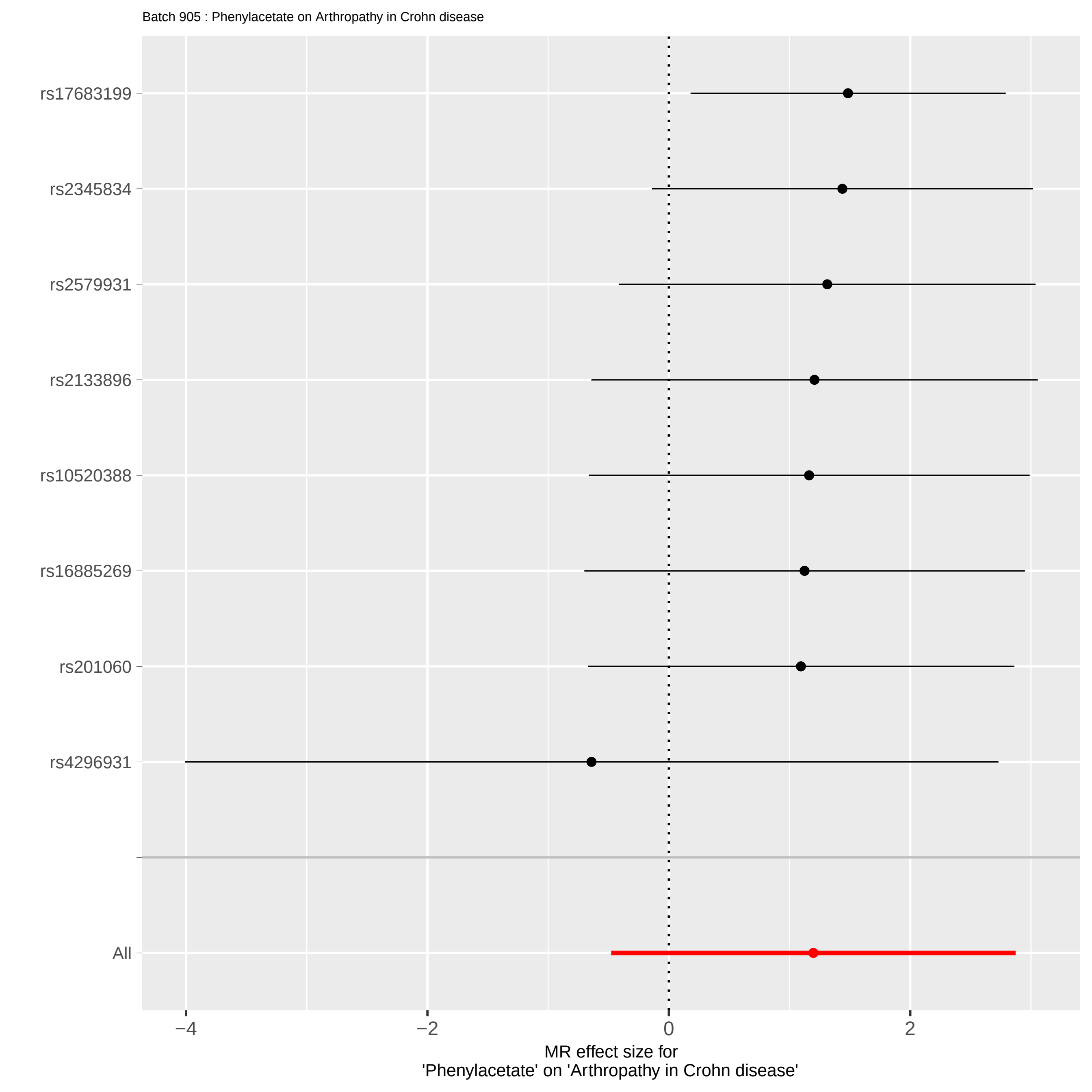
Batch 905 : Phenylacetate on Arthropathy in Crohn disease
rs17683199
rs2345834
rs2579931
rs2133896
rs10520388
rs16885269
rs201060
rs4296931
All
−4
−2
0
2
MR effect size for
'Phenylacetate' on 'Arthropathy in Crohn disease'

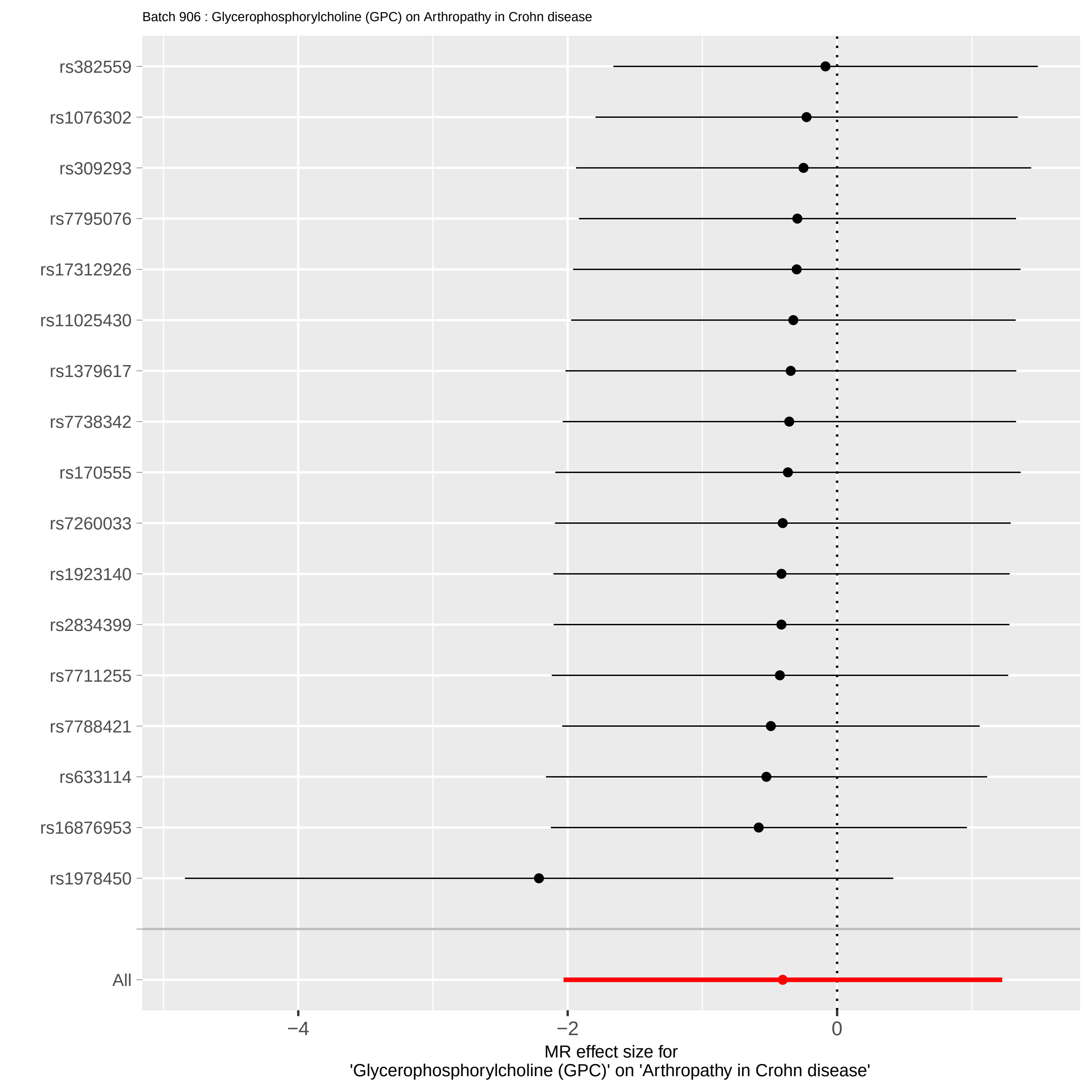
Batch 906 : Glycerophosphorylcholine (GPC) on Arthropathy in Crohn disease
rs382559
rs1076302
rs309293
rs7795076
rs17312926
rs11025430
rs1379617
rs7738342
rs170555
rs7260033
rs1923140
rs2834399
rs7711255
rs7788421
rs633114
rs16876953
rs1978450
All
−4
−2
0
MR effect size for
'Glycerophosphorylcholine (GPC)' on 'Arthropathy in Crohn disease'

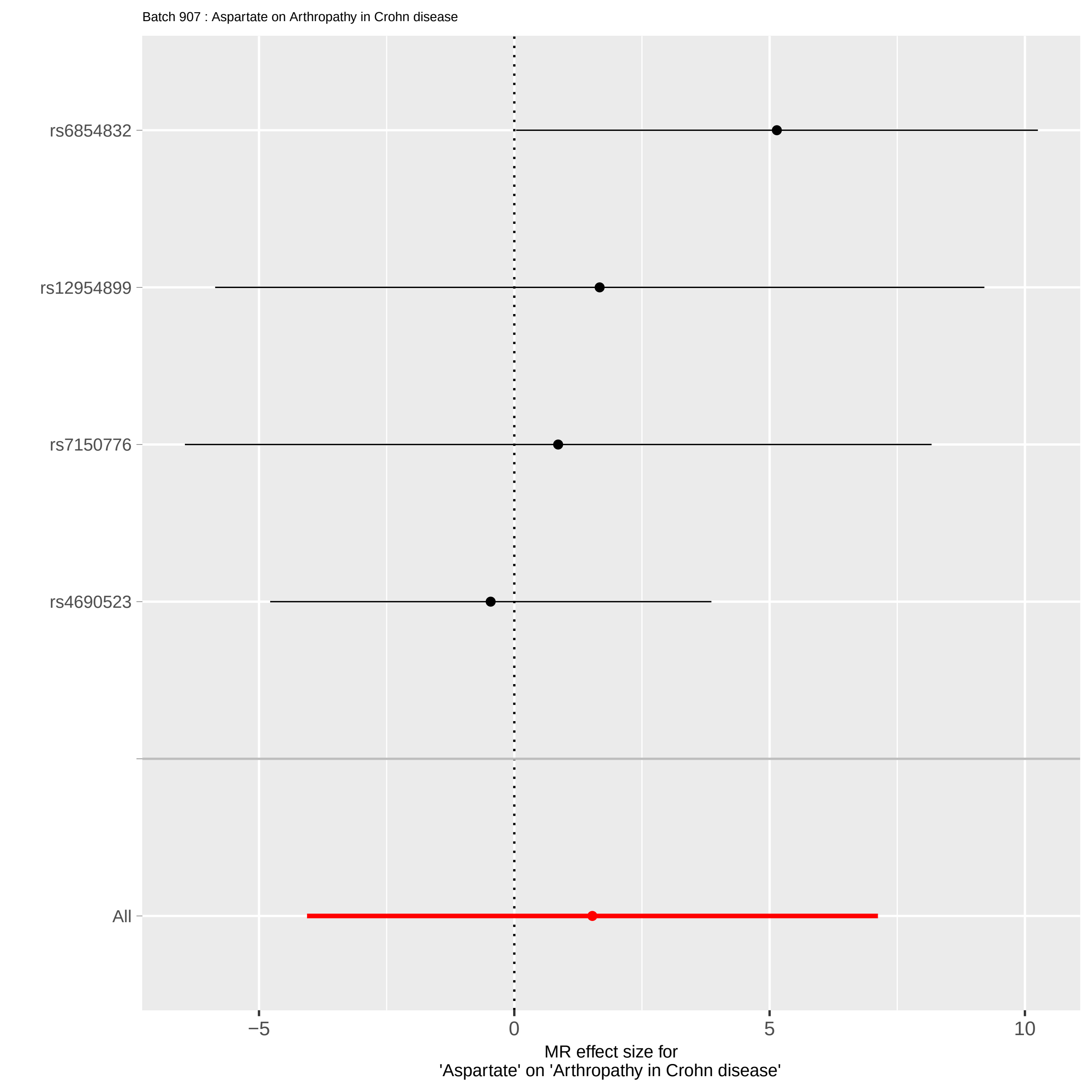

Batch 907 : Aspartate on Arthropathy in Crohn disease
rs6854832
rs12954899
rs7150776
rs4690523
All
−5
0
5
10
MR effect size for
'Aspartate' on 'Arthropathy in Crohn disease'

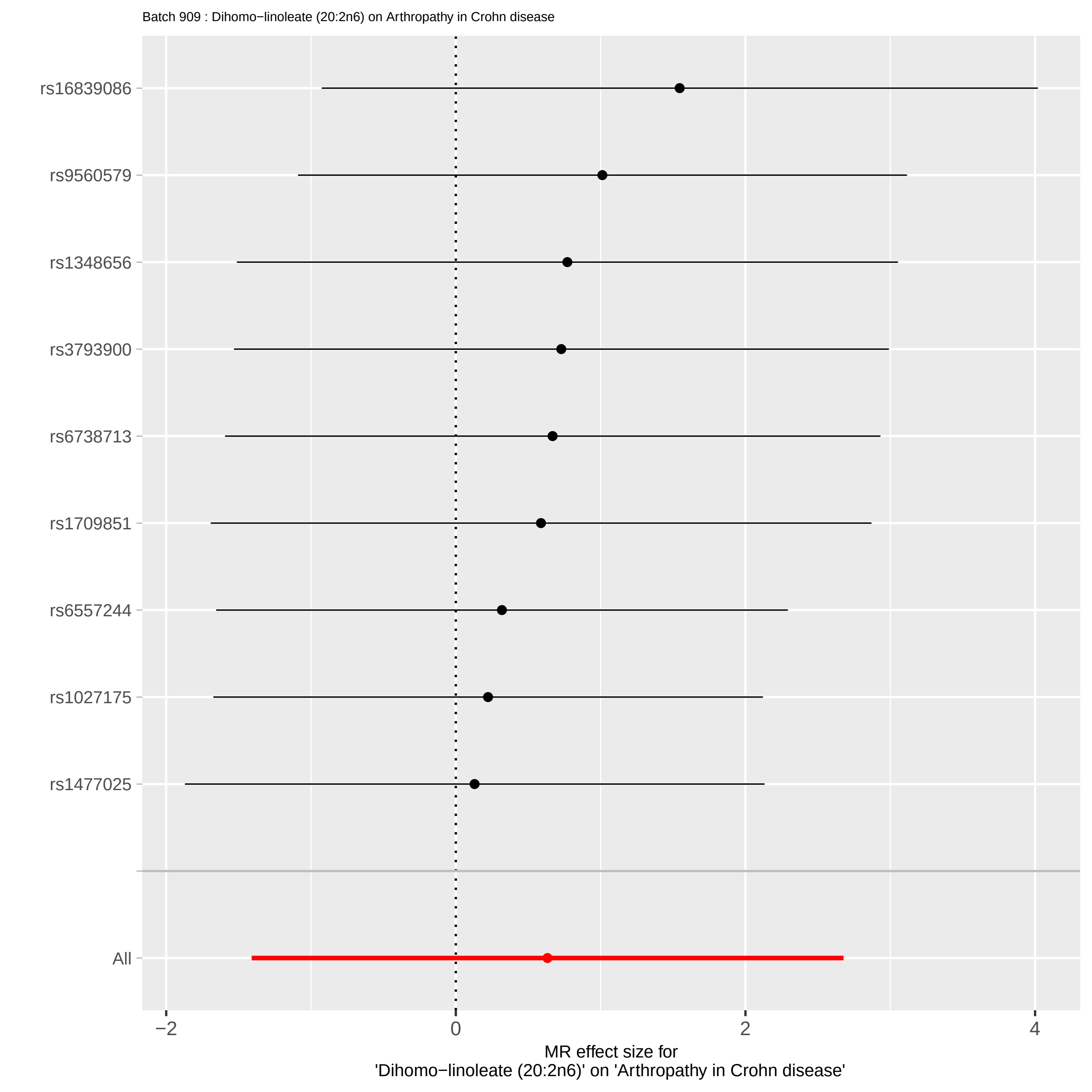

Batch 909 : Dihomo−linoleate (20:2n6) on Arthropathy in Crohn disease
rs16839086
rs9560579
rs1348656
rs3793900
rs6738713
rs1709851
rs6557244
rs1027175
rs1477025
All
−2
0
2
4
MR effect size for
'Dihomo−linoleate (20:2n6)' on 'Arthropathy in Crohn disease'

Batch 910 : 2−hydroxystearate on Arthropathy in Crohn disease

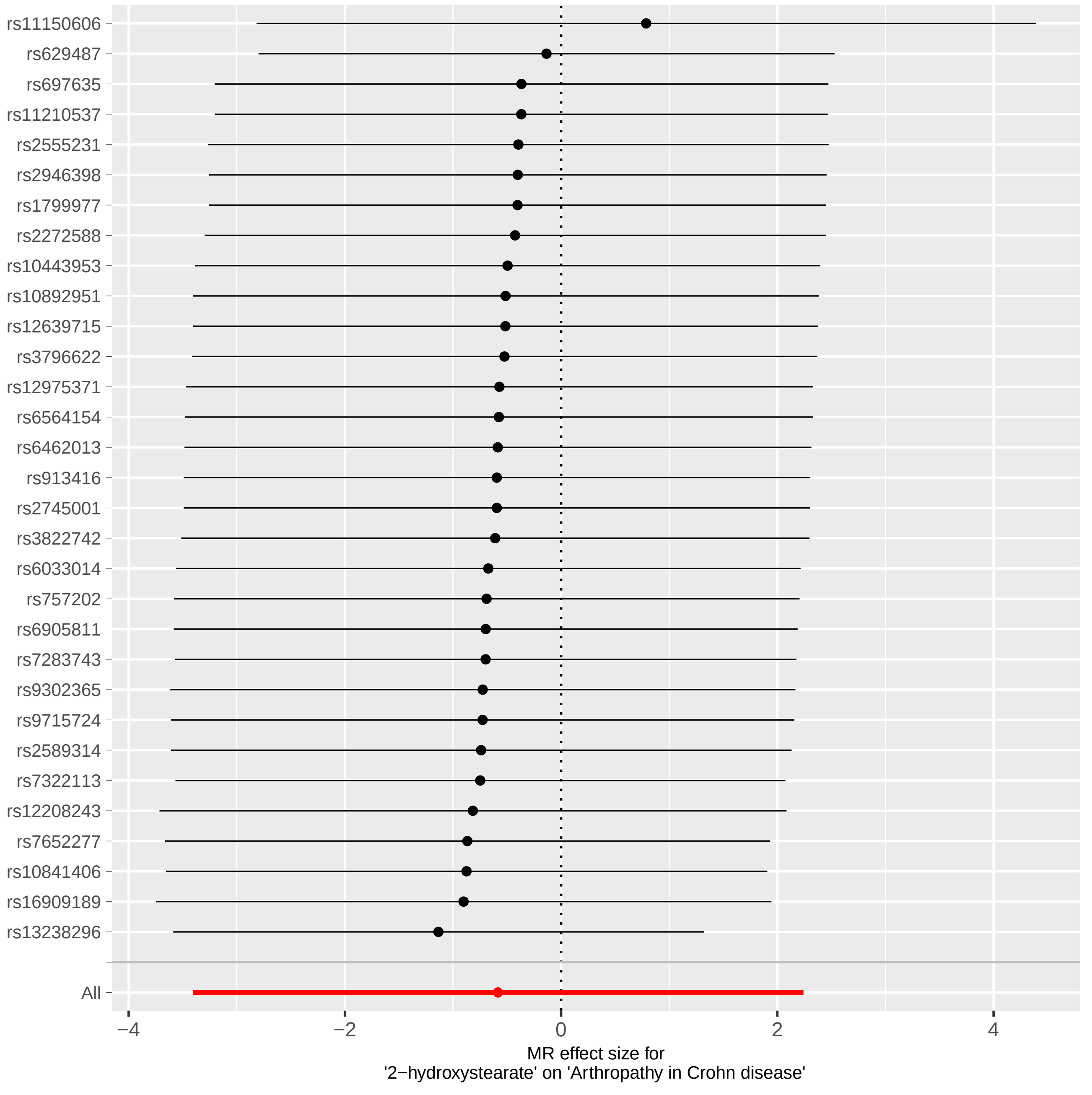

Batch 912 : Glycylvaline on Arthropathy in Crohn disease

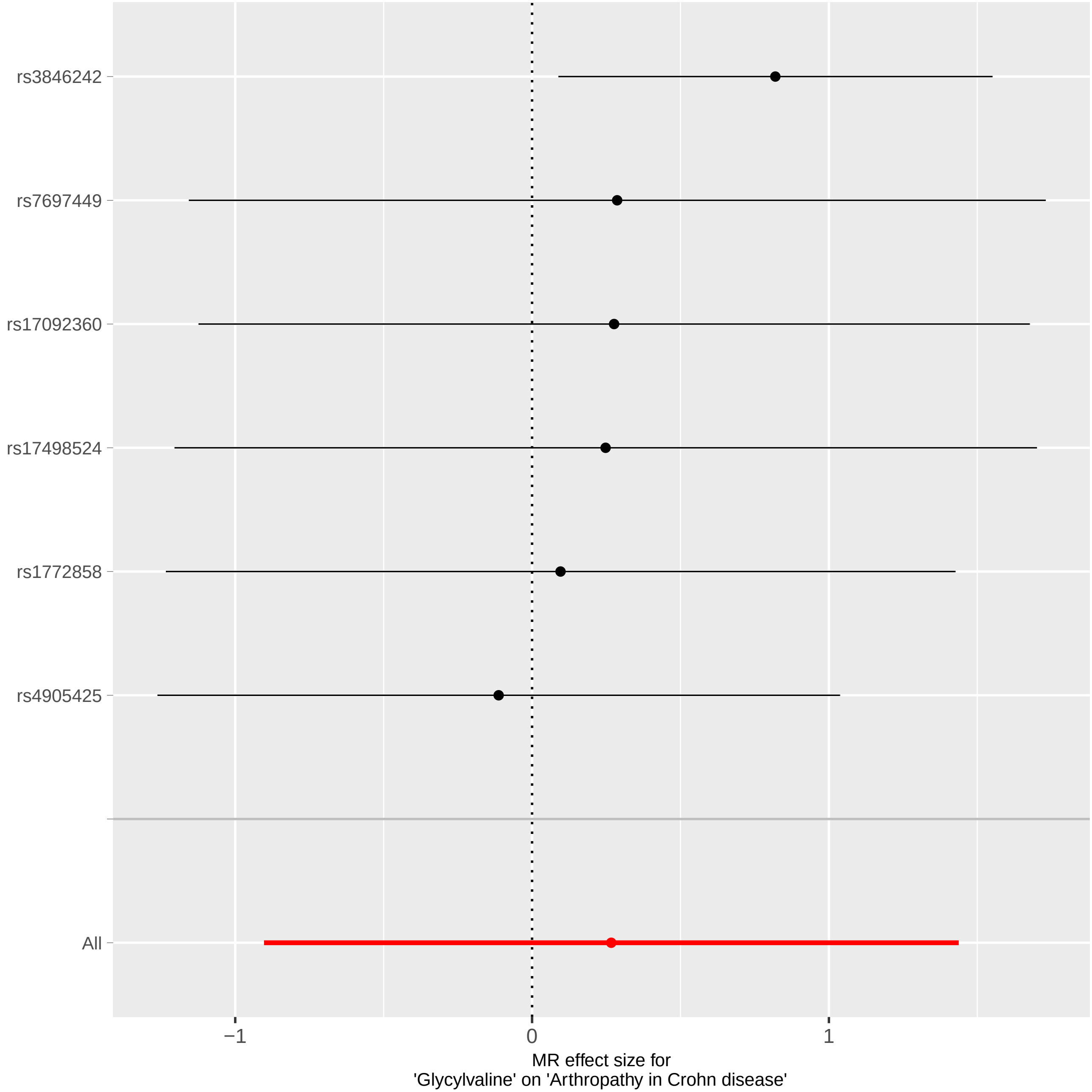

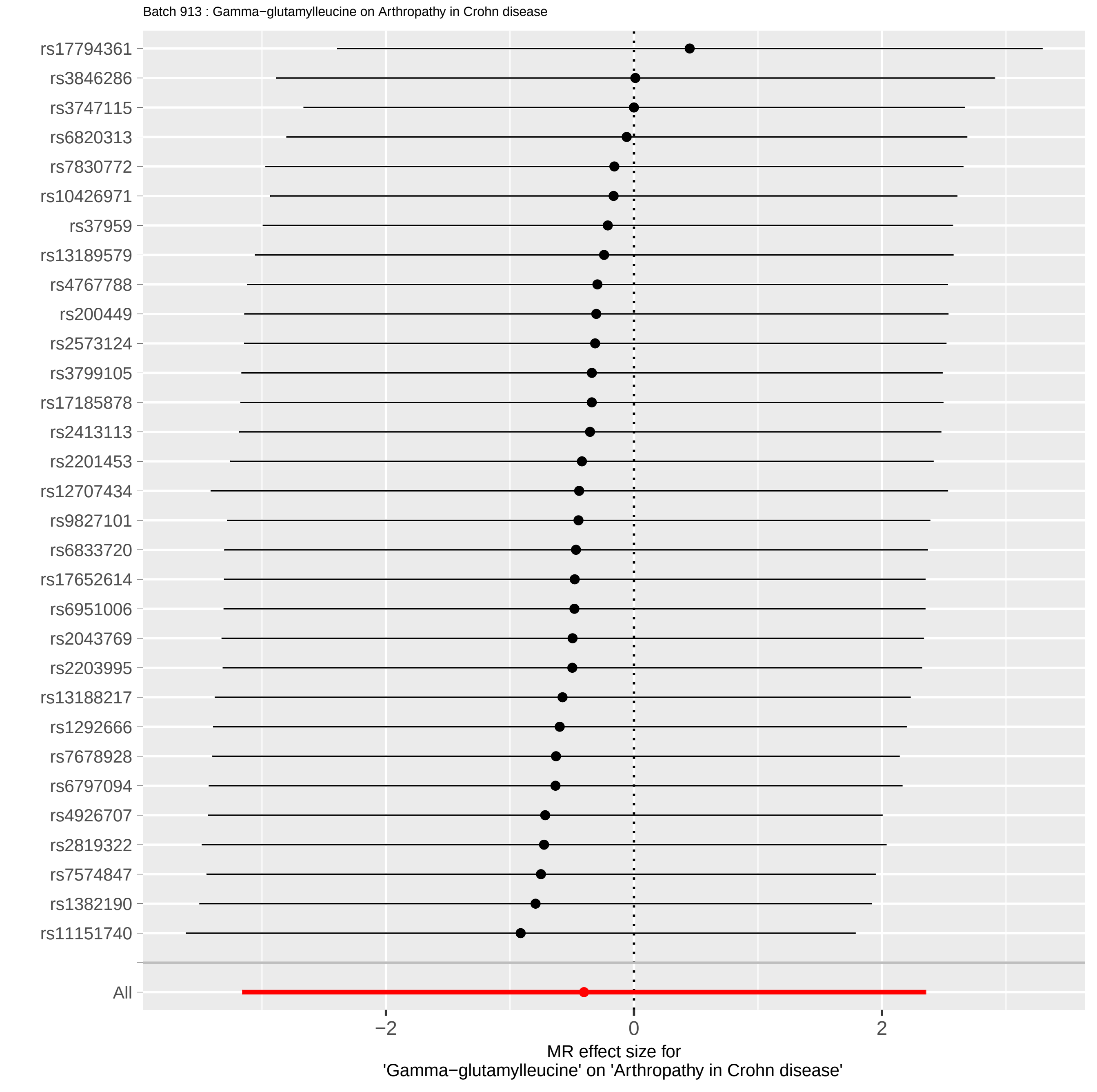

Batch 913 : Gamma−glutamylleucine on Arthropathy in Crohn disease
rs17794361
rs3846286
rs3747115
rs6820313
rs7830772
rs10426971
rs37959
rs13189579
rs4767788
rs200449
rs2573124
rs3799105
rs17185878
rs2413113
rs2201453
rs12707434
rs9827101
rs6833720
rs17652614
rs6951006
rs2043769
rs2203995
rs13188217
rs1292666
rs7678928
rs6797094
rs4926707
rs2819322
rs7574847
rs1382190
rs11151740
All
−2
0
2
MR effect size for
'Gamma−glutamylleucine' on 'Arthropathy in Crohn disease'

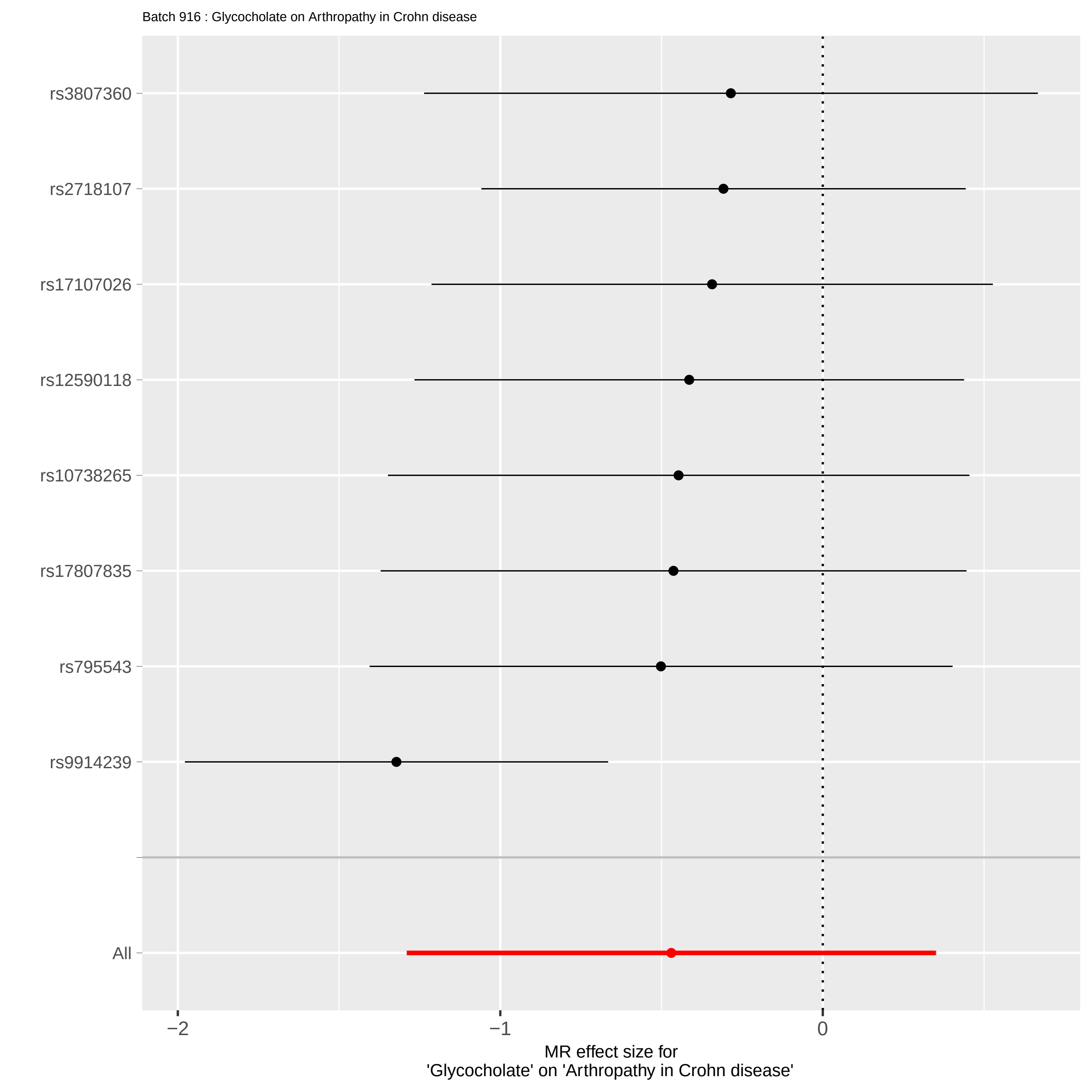
Batch 916 : Glycocholate on Arthropathy in Crohn disease
rs3807360
rs2718107
rs17107026
rs12590118
rs10738265
rs17807835
rs795543
rs9914239
All
−2
−1
0
MR effect size for
'Glycocholate' on 'Arthropathy in Crohn disease'

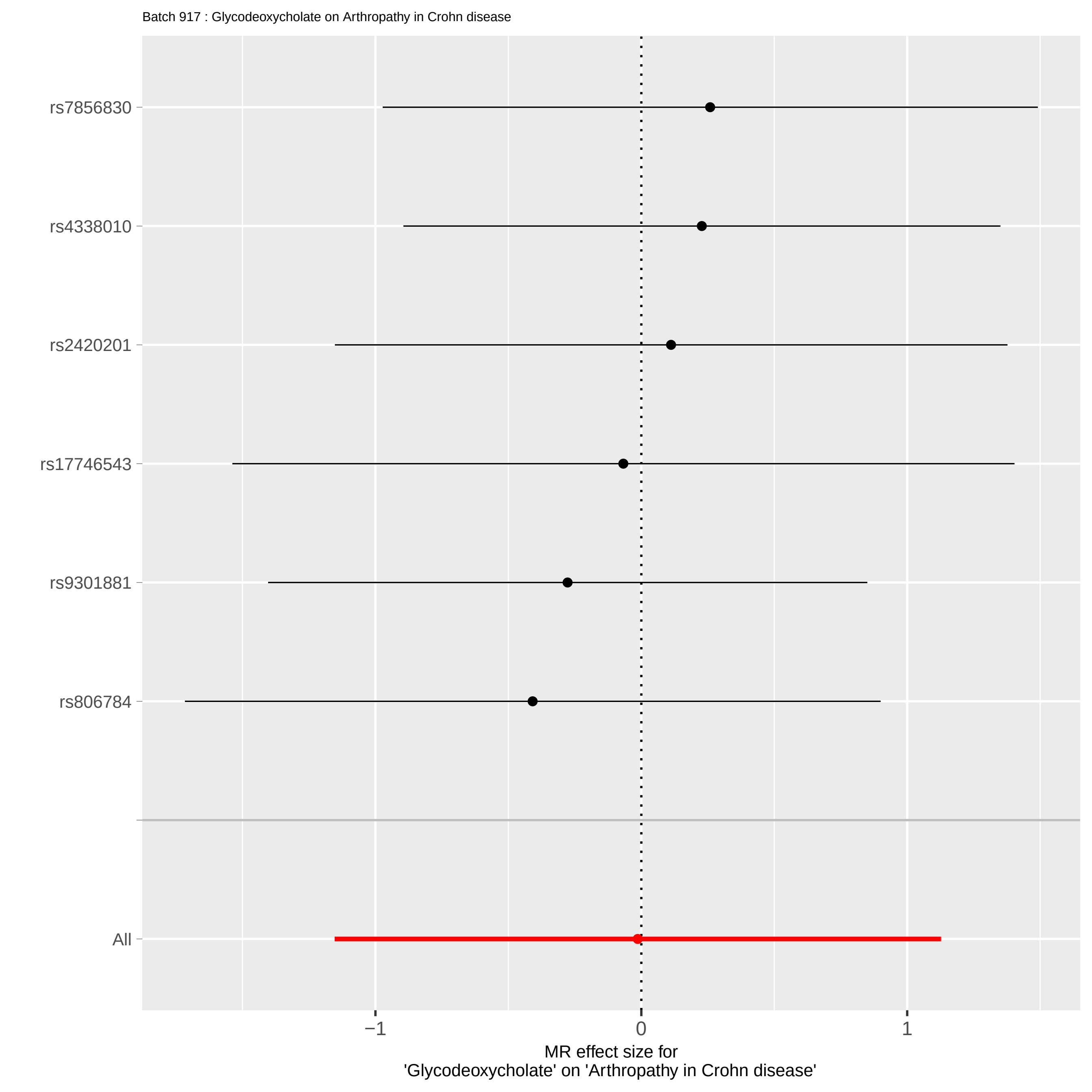

Batch 917 : Glycodeoxycholate on Arthropathy in Crohn disease
rs7856830
rs4338010
rs2420201
rs17746543
rs9301881
rs806784
All
−1
0
1
MR effect size for
'Glycodeoxycholate' on 'Arthropathy in Crohn disease'

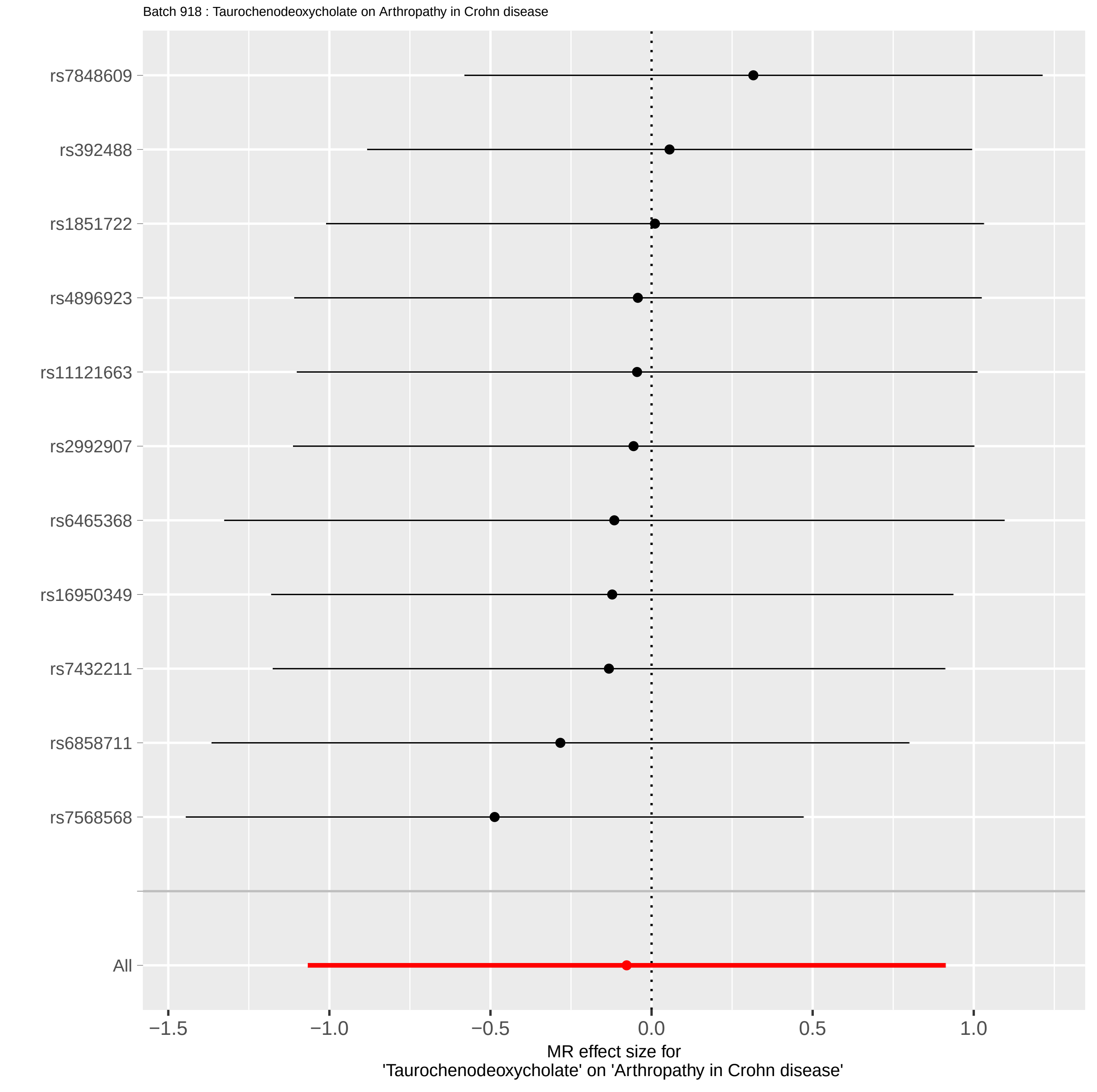

Batch 918 : Taurochenodeoxycholate on Arthropathy in Crohn disease
rs7848609
rs392488
rs1851722
rs4896923
rs11121663
rs2992907
rs6465368
rs16950349
rs7432211
rs6858711
rs7568568
All
−1.5
−1.0
−0.5
0.0
0.5
1.0
MR effect size for
'Taurochenodeoxycholate' on 'Arthropathy in Crohn disease'

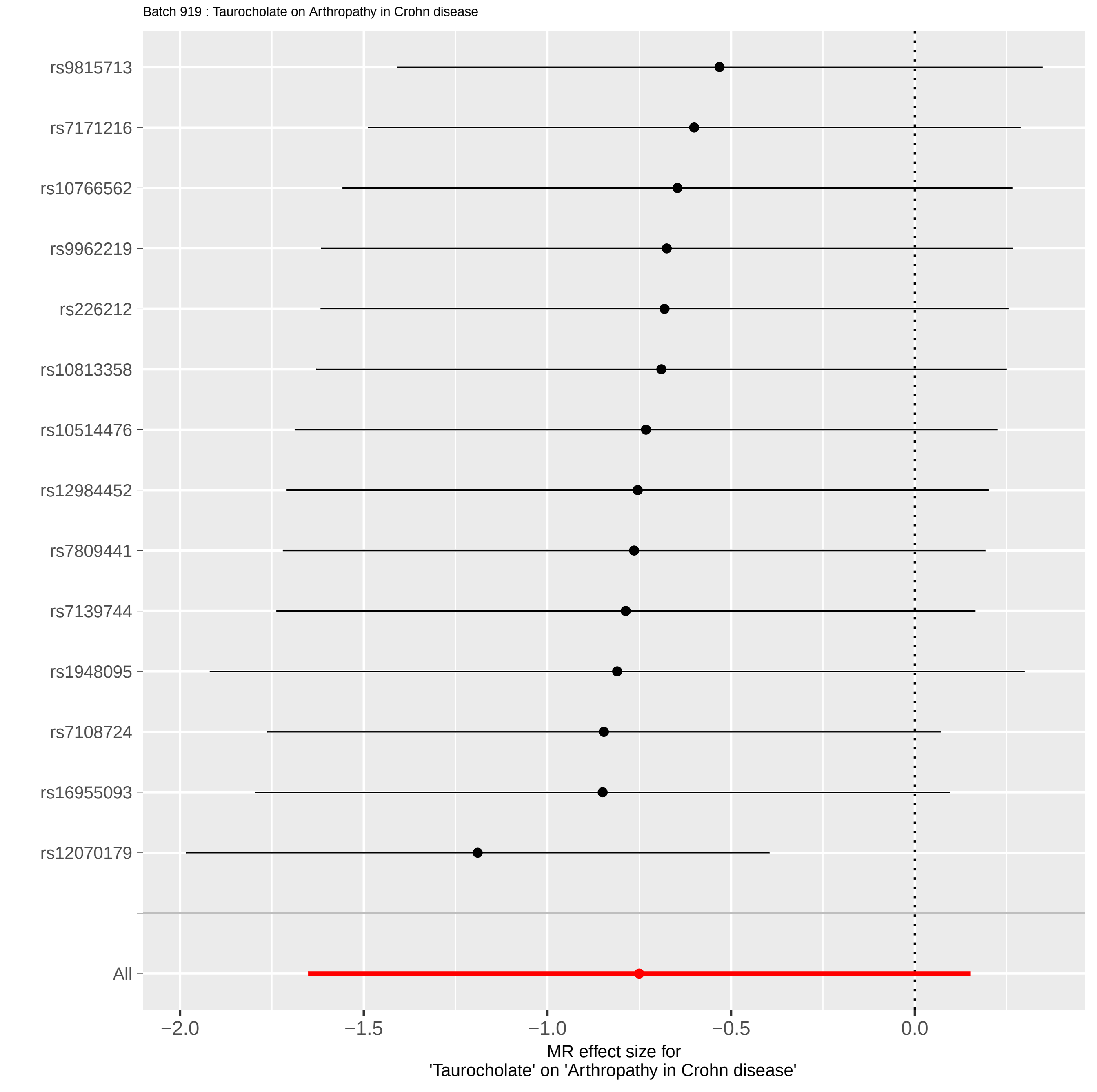
Batch 919 : Taurocholate on Arthropathy in Crohn disease
rs9815713
rs7171216
rs10766562
rs9962219
rs226212
rs10813358
rs10514476
rs12984452
rs7809441
rs7139744
rs1948095
rs7108724
rs16955093
rs12070179
All
−2.0
−1.5
−1.0
−0.5
0.0
MR effect size for
'Taurocholate' on 'Arthropathy in Crohn disease'

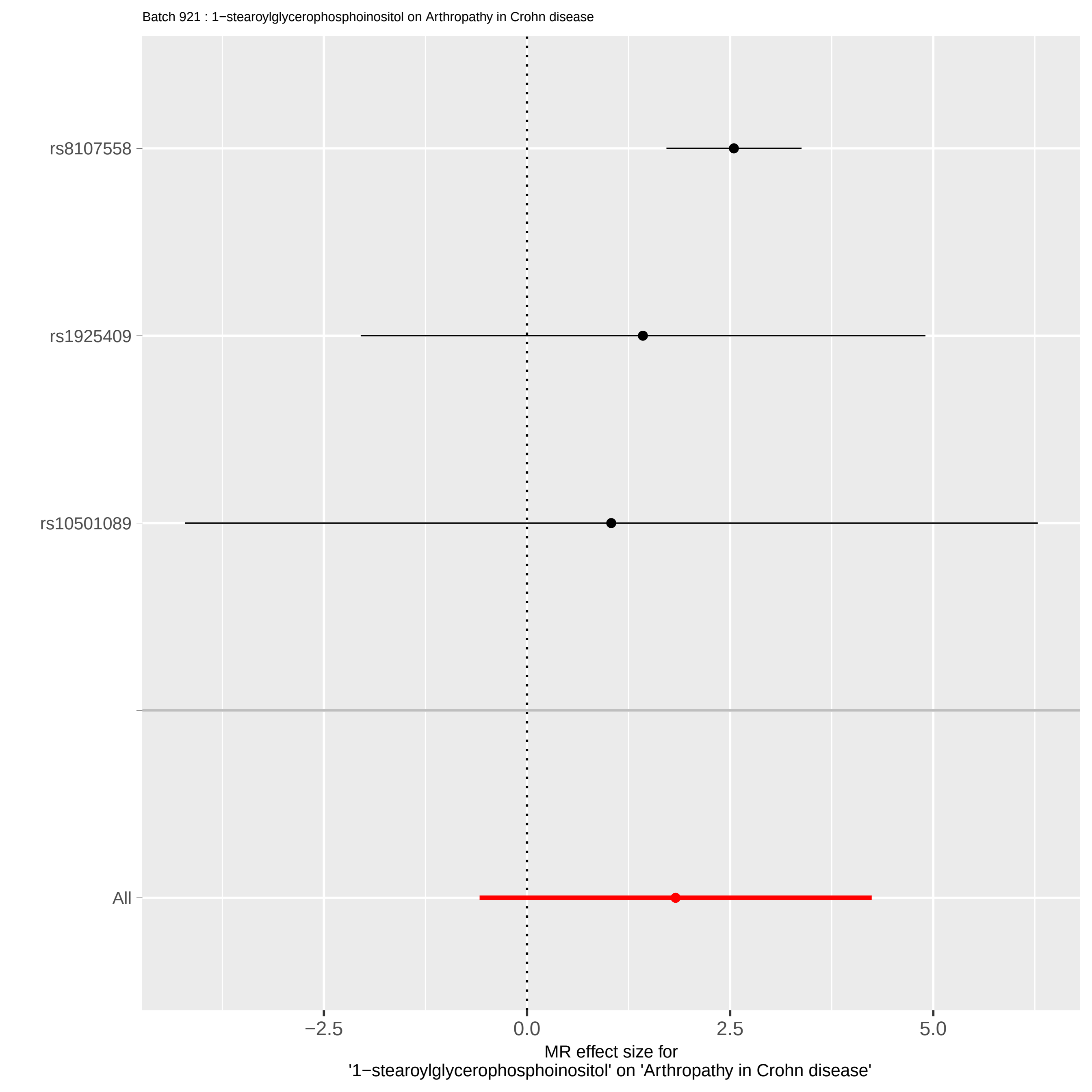

Batch 921 : 1−stearoylglycerophosphoinositol on Arthropathy in Crohn disease
rs8107558
rs1925409
rs10501089
All
−2.5
0.0
2.5
5.0
MR effect size for
'1−stearoylglycerophosphoinositol' on 'Arthropathy in Crohn disease'

Batch 924 : 1,5−anhydroglucitol (1,5−AG) on Arthropathy in Crohn disease

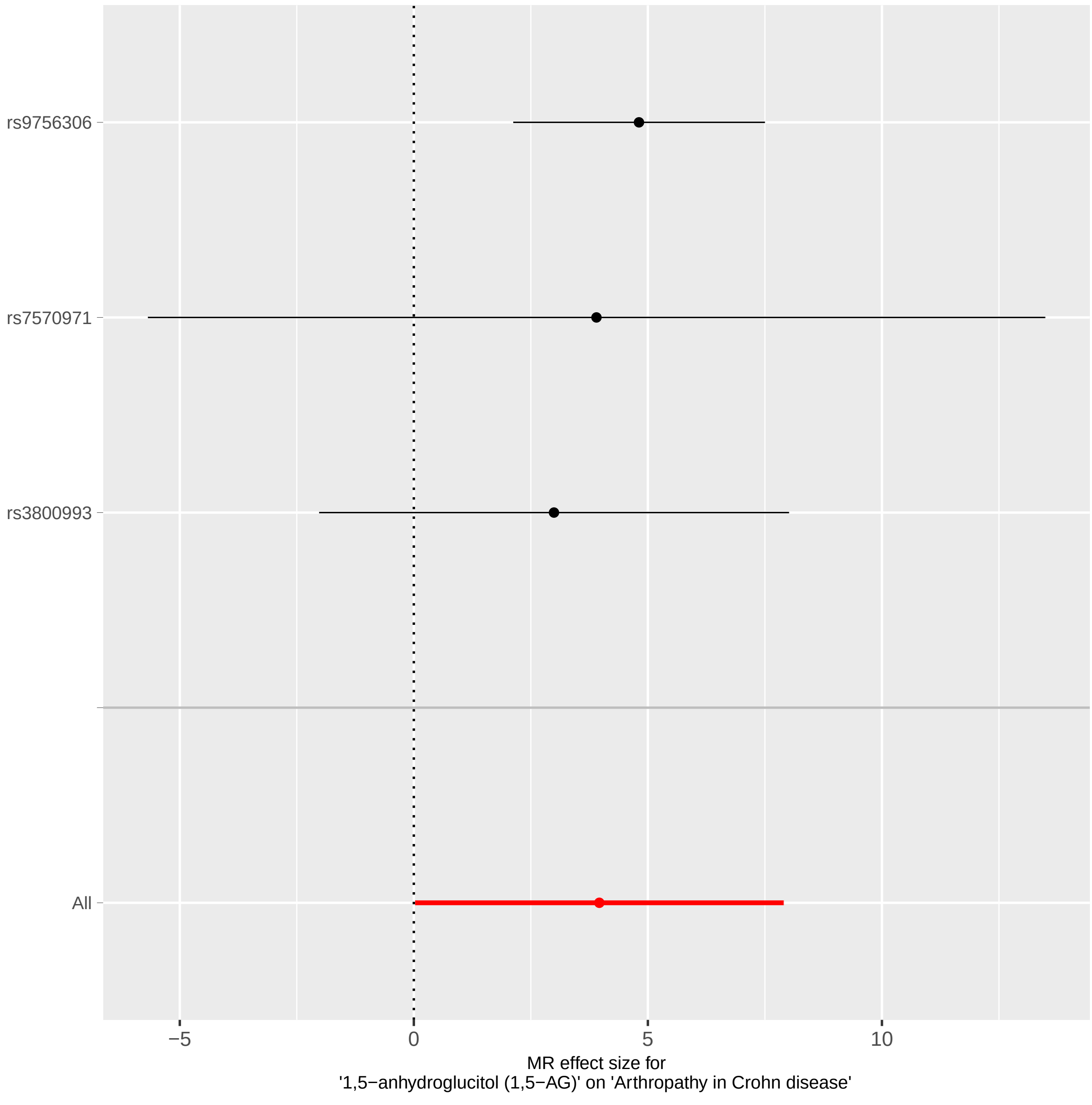

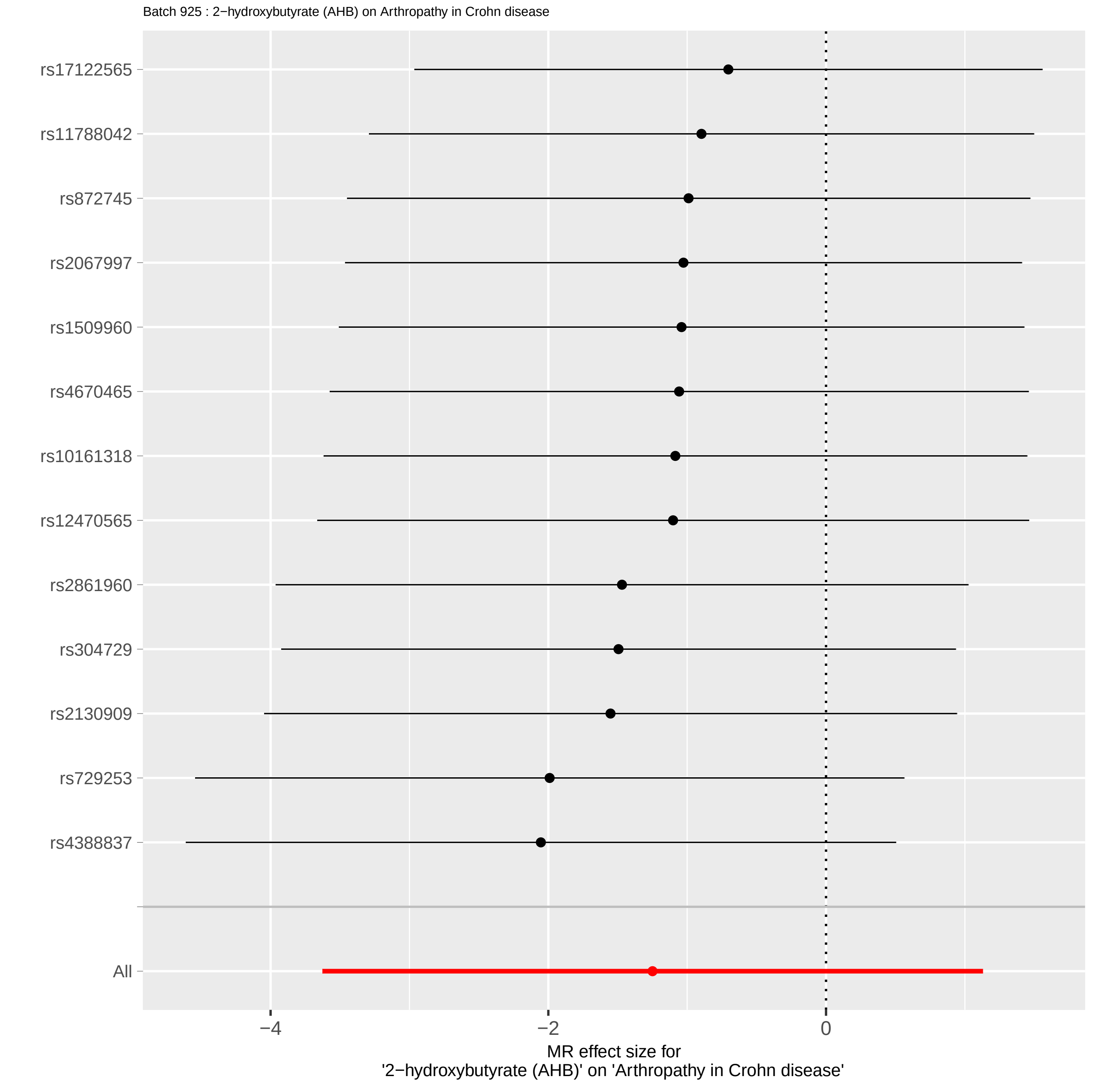

Batch 925 : 2−hydroxybutyrate (AHB) on Arthropathy in Crohn disease
rs17122565
rs11788042
rs872745
rs2067997
rs1509960
rs4670465
rs10161318
rs12470565
rs2861960
rs304729
rs2130909
rs729253
rs4388837
All
−4
−2
0
MR effect size for
'2−hydroxybutyrate (AHB)' on 'Arthropathy in Crohn disease'

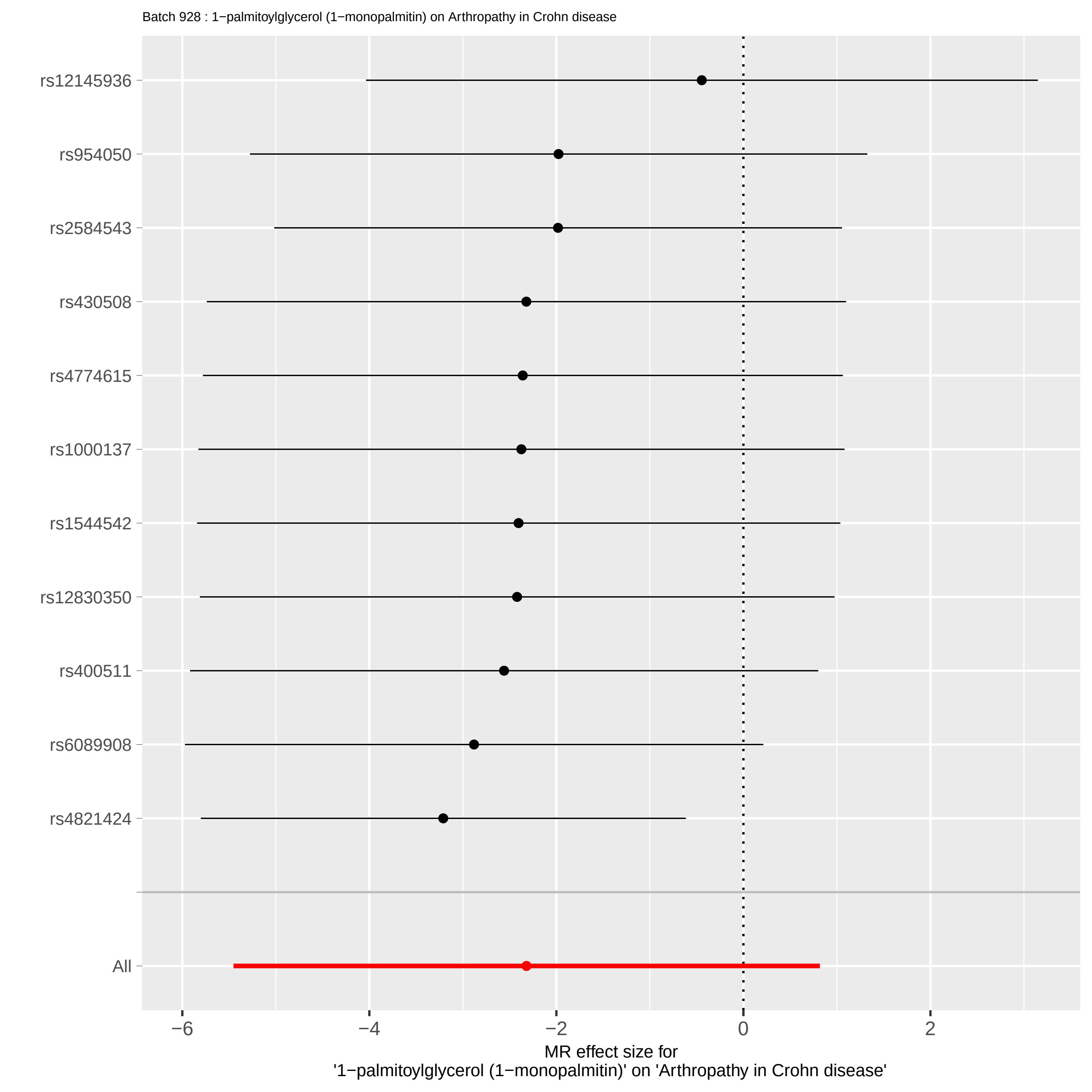

Batch 928 : 1−palmitoylglycerol (1−monopalmitin) on Arthropathy in Crohn disease
rs12145936
rs954050
rs2584543
rs430508
rs4774615
rs1000137
rs1544542
rs12830350
rs400511
rs6089908
rs4821424
All
−6
−4
−2
0
2
MR effect size for
'1−palmitoylglycerol (1−monopalmitin)' on 'Arthropathy in Crohn disease'

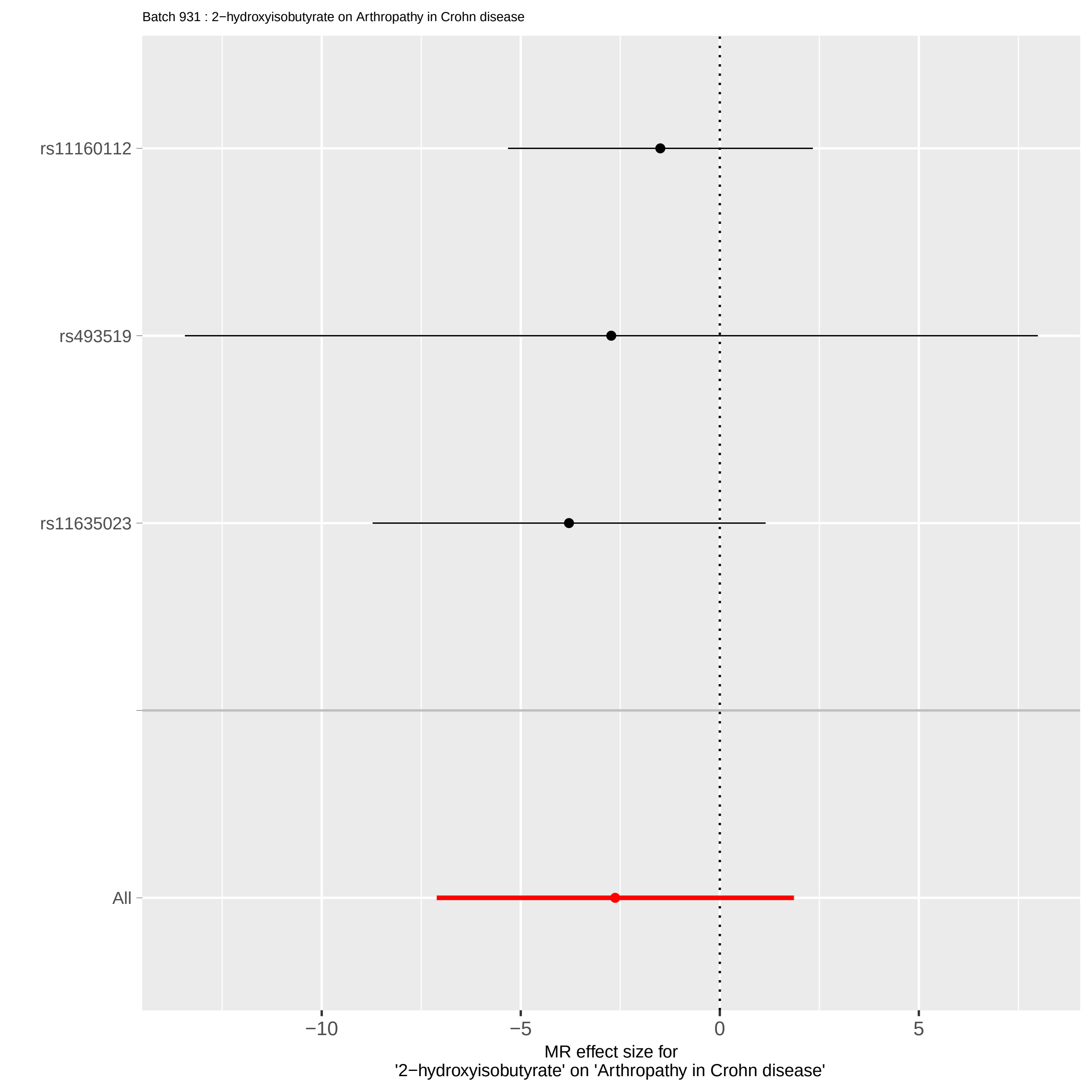

Batch 931 : 2−hydroxyisobutyrate on Arthropathy in Crohn disease
rs11160112
rs493519
rs11635023
All
−10
−5
0
5
MR effect size for
'2−hydroxyisobutyrate' on 'Arthropathy in Crohn disease'

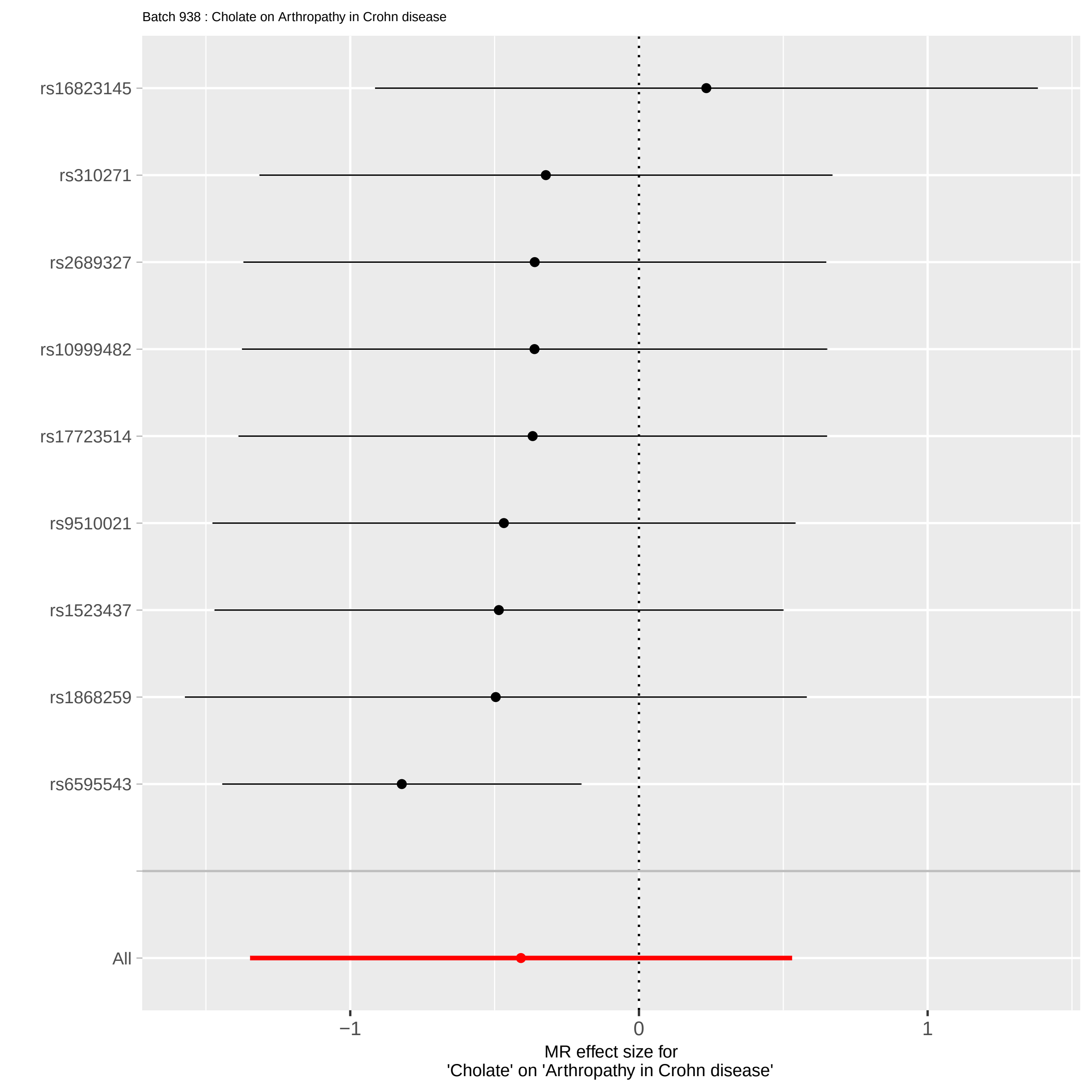
Batch 938 : Cholate on Arthropathy in Crohn disease
rs16823145
rs310271
rs2689327
rs10999482
rs17723514
rs9510021
rs1523437
rs1868259
rs6595543
All
−1
0
1
MR effect size for
'Cholate' on 'Arthropathy in Crohn disease'

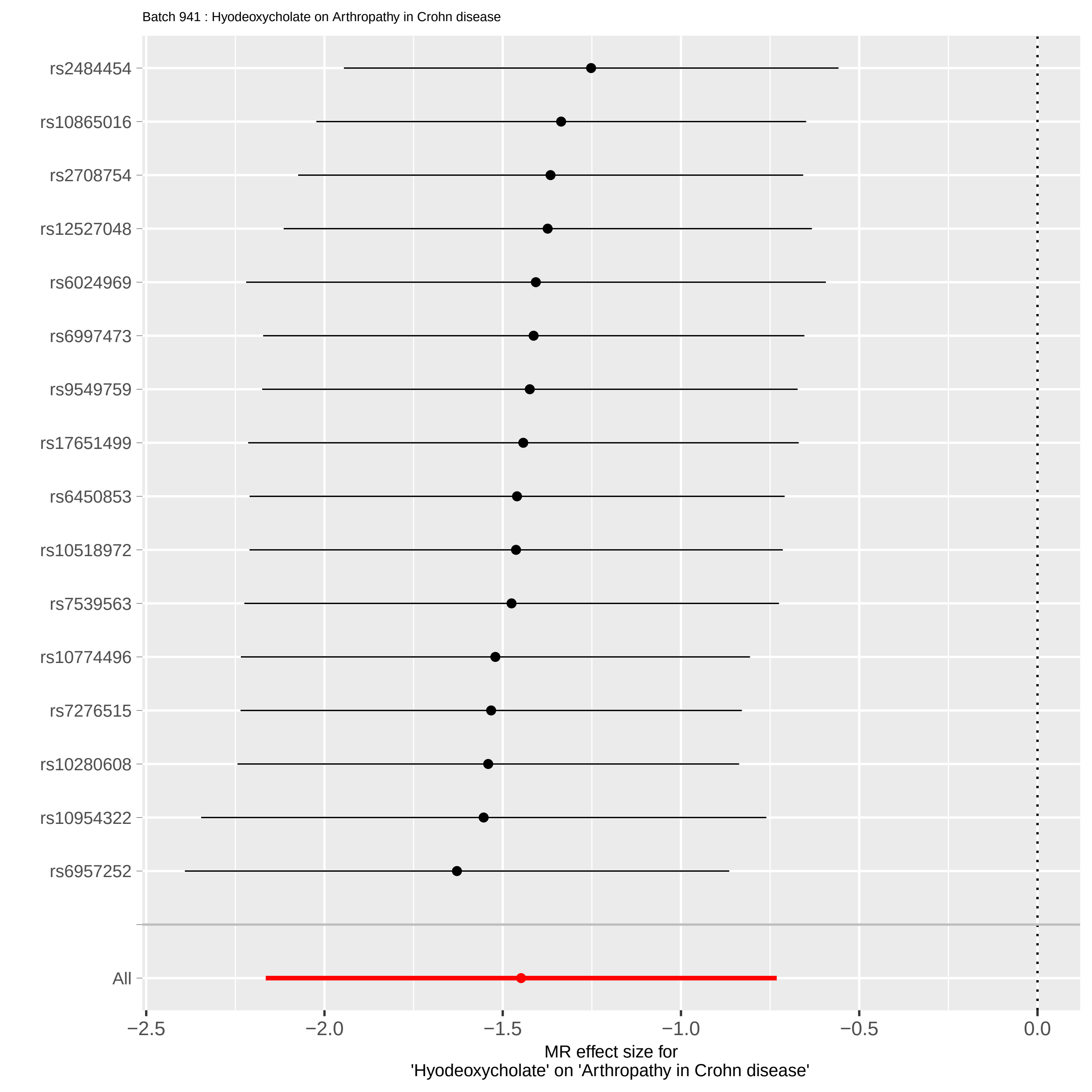

Batch 941 : Hyodeoxycholate on Arthropathy in Crohn disease
rs2484454
rs10865016
rs2708754
rs12527048
rs6024969
rs6997473
rs9549759
rs17651499
rs6450853
rs10518972
rs7539563
rs10774496
rs7276515
rs10280608
rs10954322
rs6957252
All
−2.5
−2.0
−1.5
−1.0
−0.5
0.0
MR effect size for
'Hyodeoxycholate' on 'Arthropathy in Crohn disease'

Batch 942 : 3−indoxyl sulfate on Arthropathy in Crohn disease

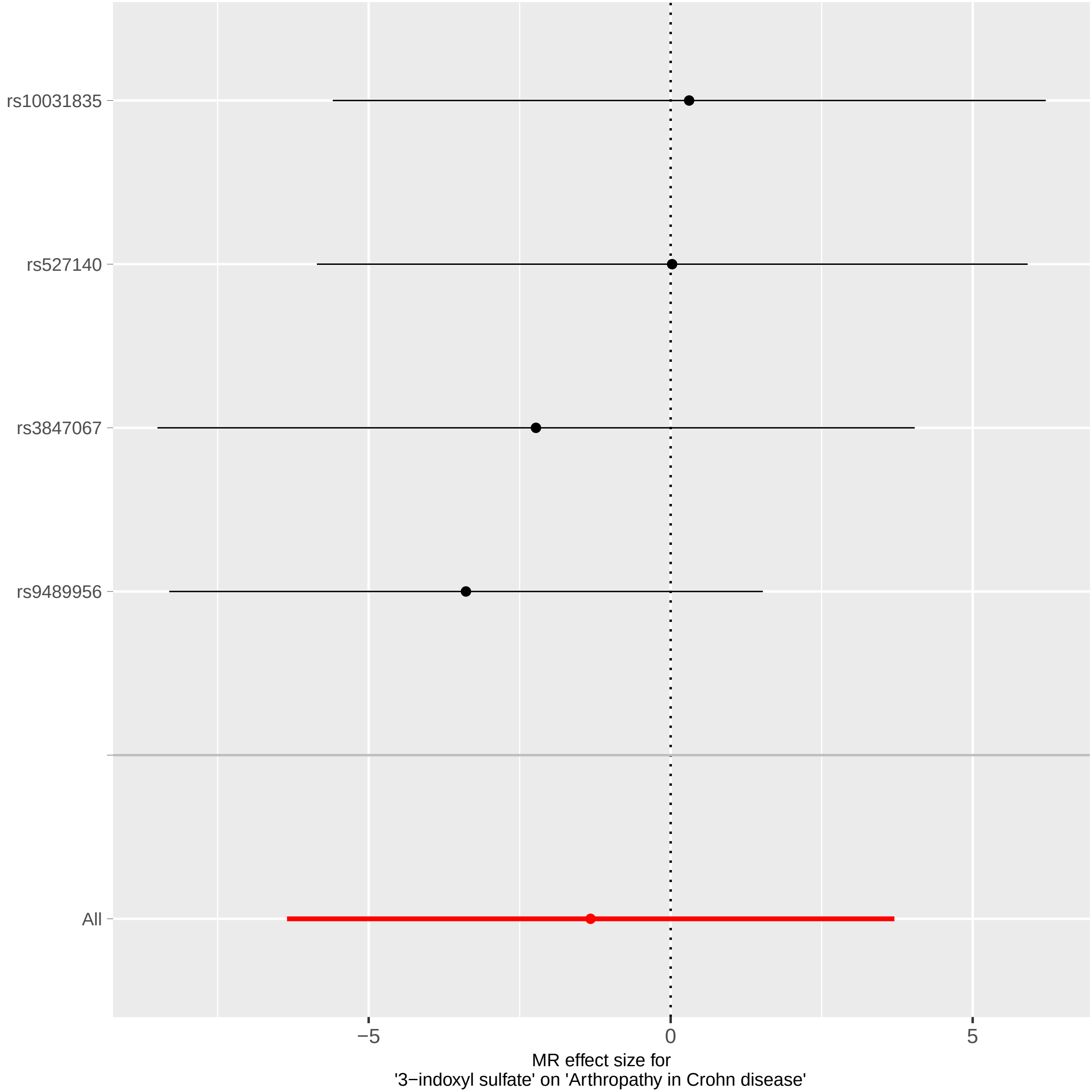

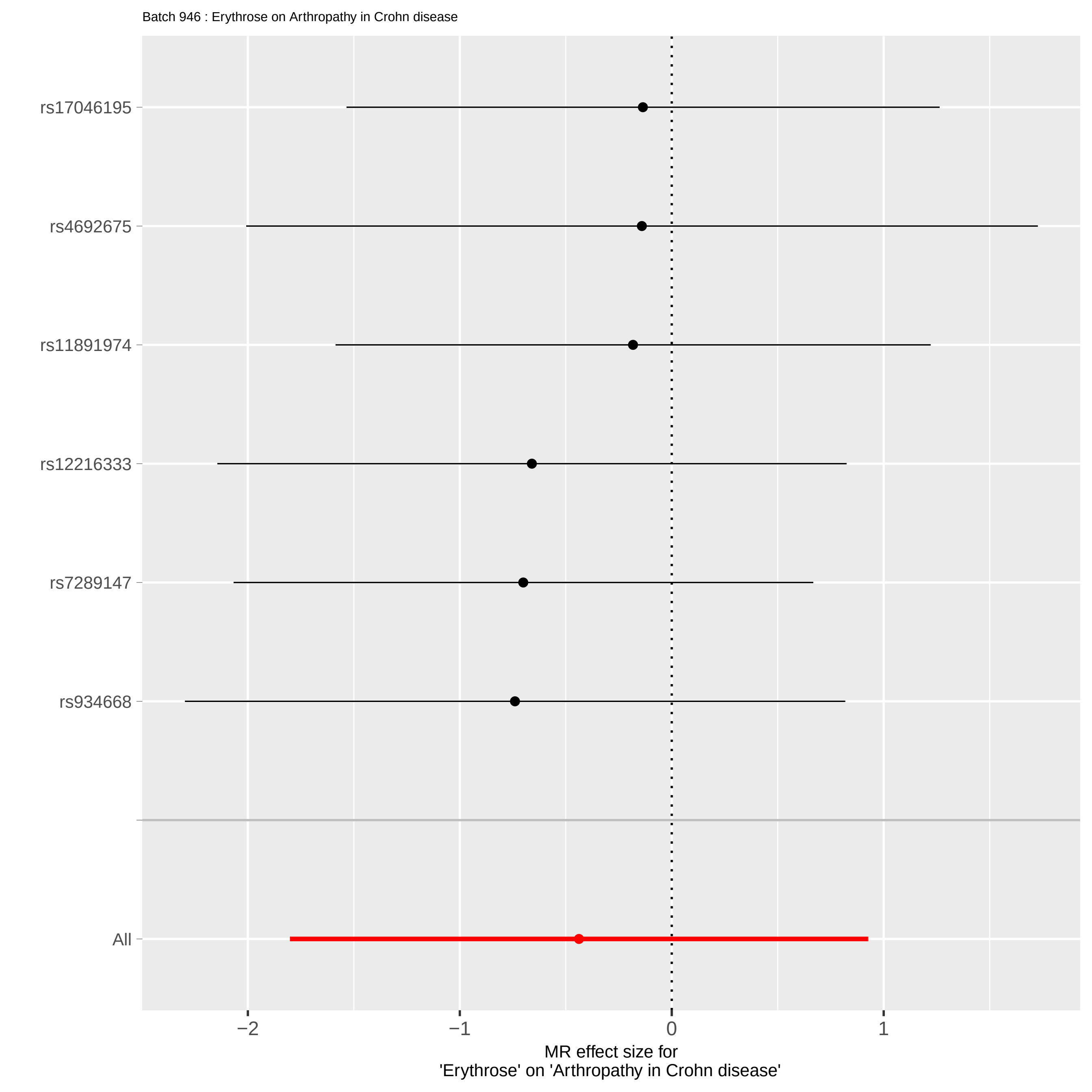

Batch 946 : Erythrose on Arthropathy in Crohn disease
rs17046195
rs4692675
rs11891974
rs12216333
rs7289147
rs934668
All
−2
−1
0
1
MR effect size for
'Erythrose' on 'Arthropathy in Crohn disease'

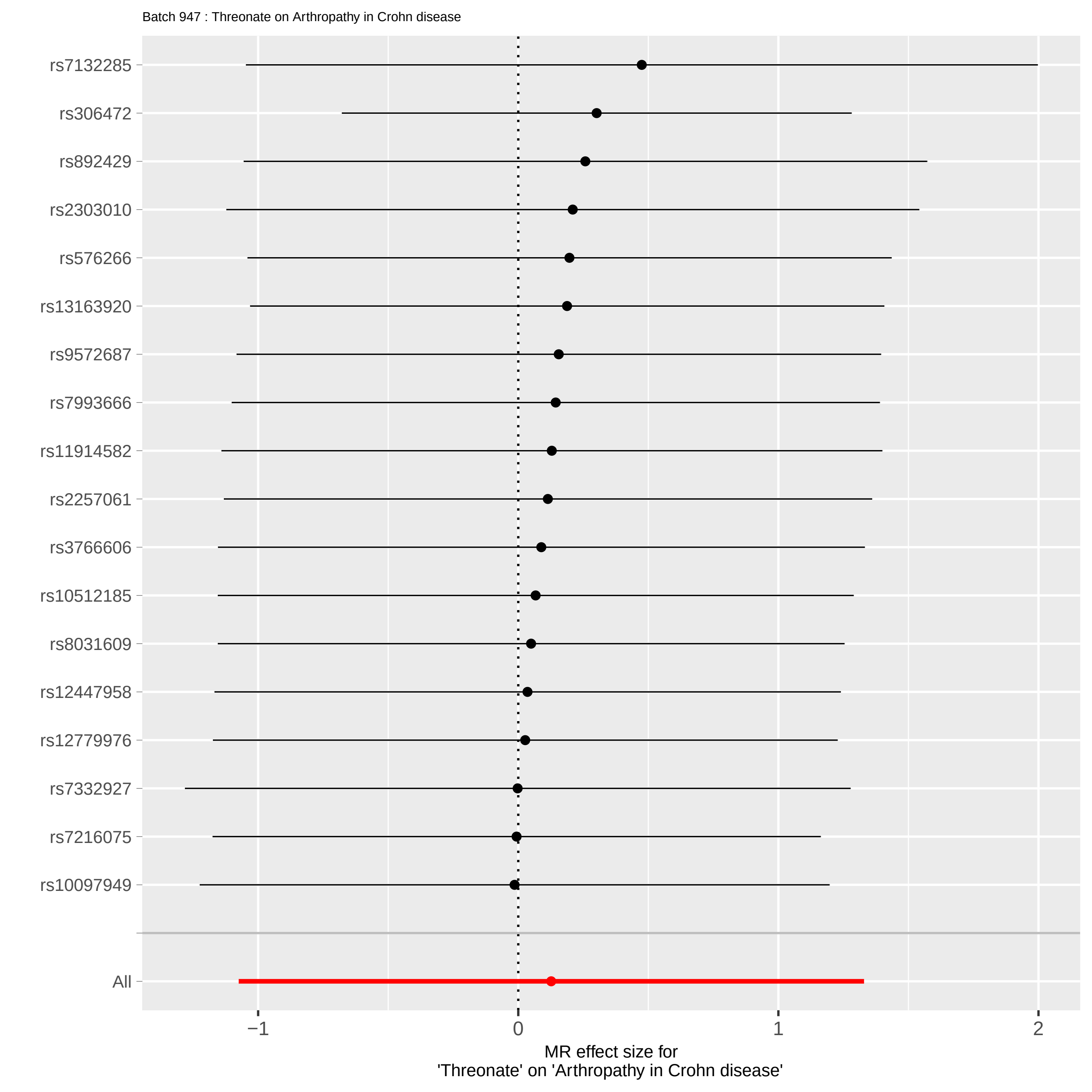

Batch 947 : Threonate on Arthropathy in Crohn disease
rs7132285
rs306472
rs892429
rs2303010
rs576266
rs13163920
rs9572687
rs7993666
rs11914582
rs2257061
rs3766606
rs10512185
rs8031609
rs12447958
rs12779976
rs7332927
rs7216075
rs10097949
All
−1
0
1
2
MR effect size for
'Threonate' on 'Arthropathy in Crohn disease'

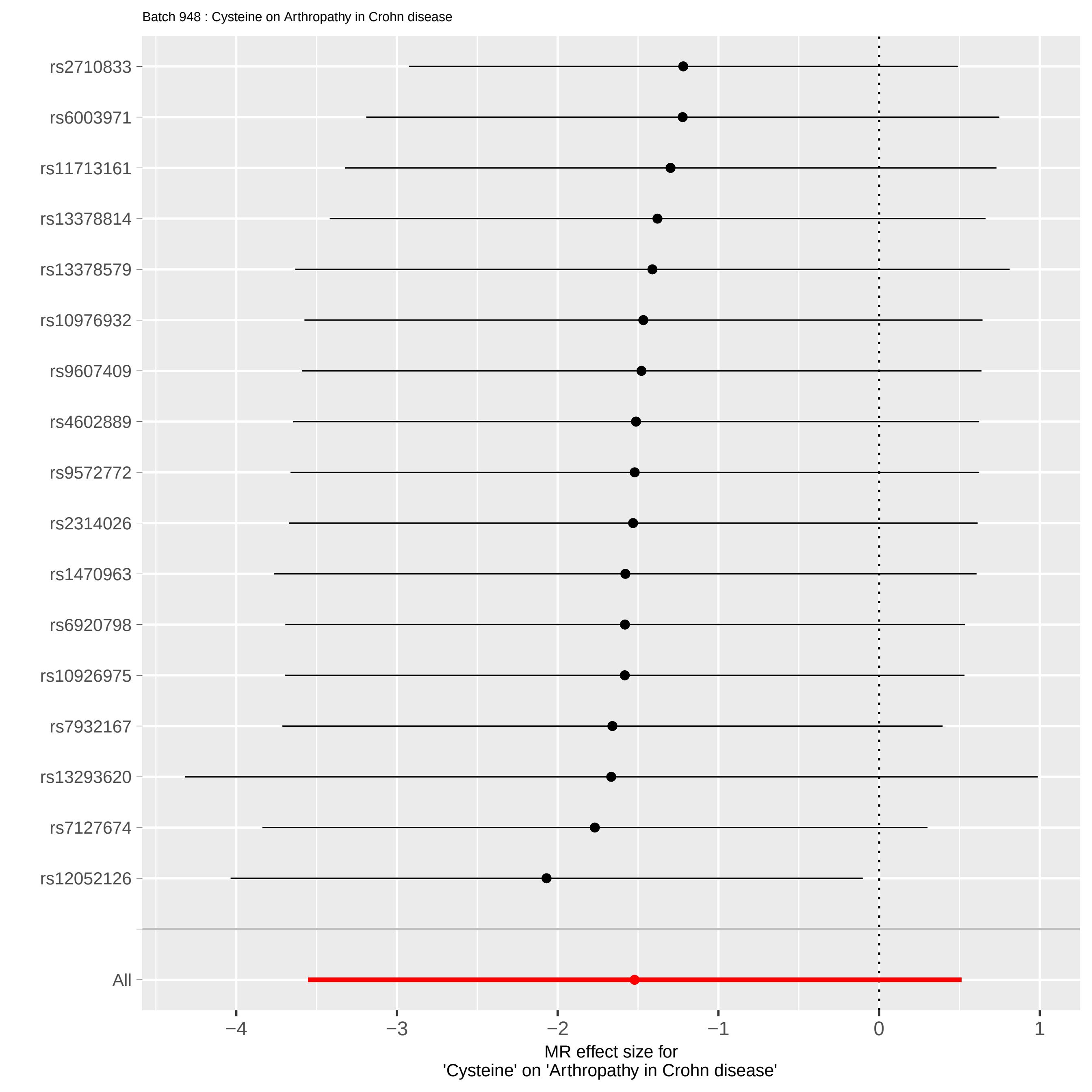
Batch 948 : Cysteine on Arthropathy in Crohn disease
rs2710833
rs6003971
rs11713161
rs13378814
rs13378579
rs10976932
rs9607409
rs4602889
rs9572772
rs2314026
rs1470963
rs6920798
rs10926975
rs7932167
rs13293620
rs7127674
rs12052126
All
−4
−3
−2
−1
0
1
MR effect size for
'Cysteine' on 'Arthropathy in Crohn disease'

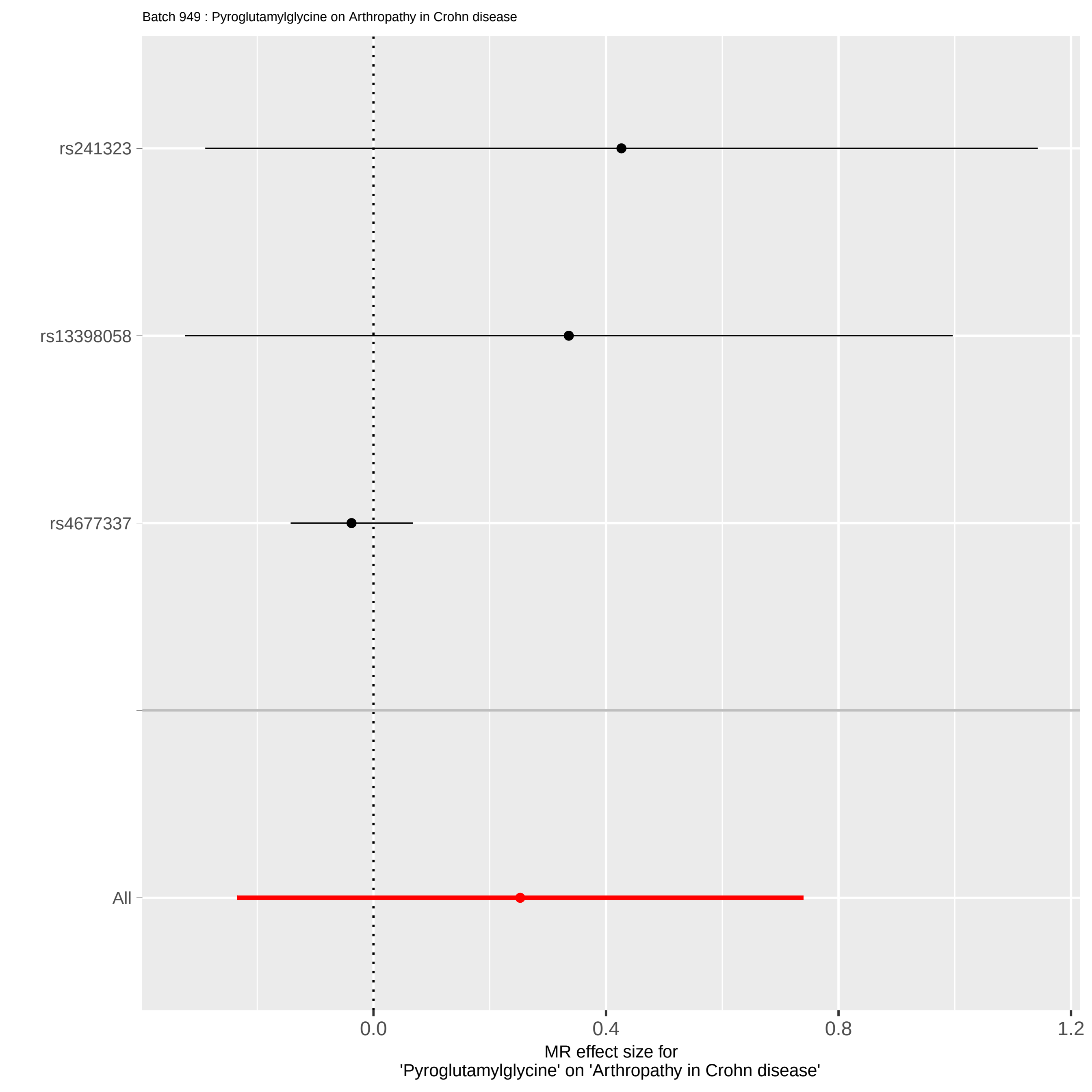

Batch 949 : Pyroglutamylglycine on Arthropathy in Crohn disease
rs241323
rs13398058
rs4677337
All
0.0
0.4
0.8
1.2
MR effect size for
'Pyroglutamylglycine' on 'Arthropathy in Crohn disease'

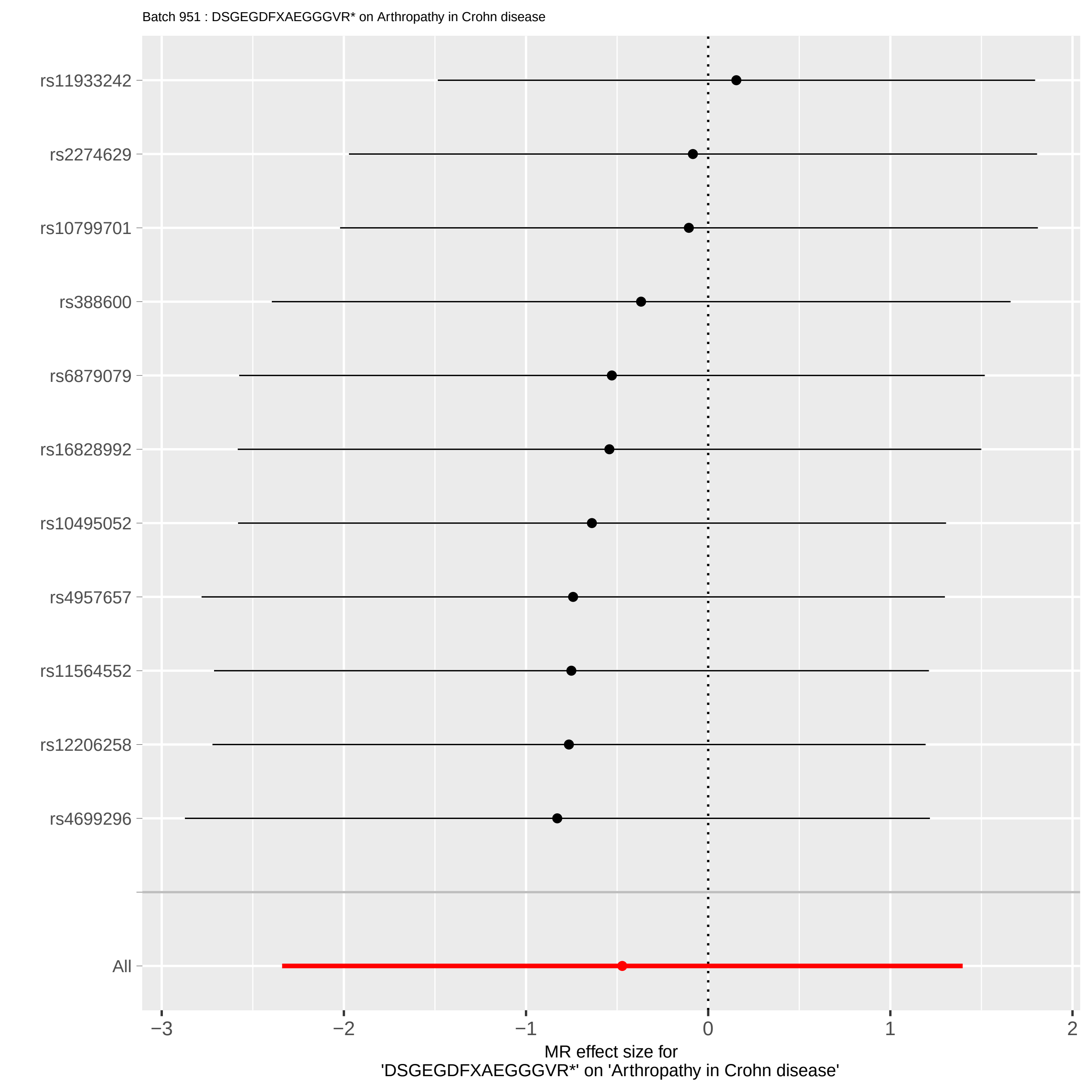

Batch 951 : DSGEGDFXAEGGGVR* on Arthropathy in Crohn disease
rs11933242
rs2274629
rs10799701
rs388600
rs6879079
rs16828992
rs10495052
rs4957657
rs11564552
rs12206258
rs4699296
All
−3
−2
−1
0
1
2
MR effect size for
'DSGEGDFXAEGGGVR*' on 'Arthropathy in Crohn disease'

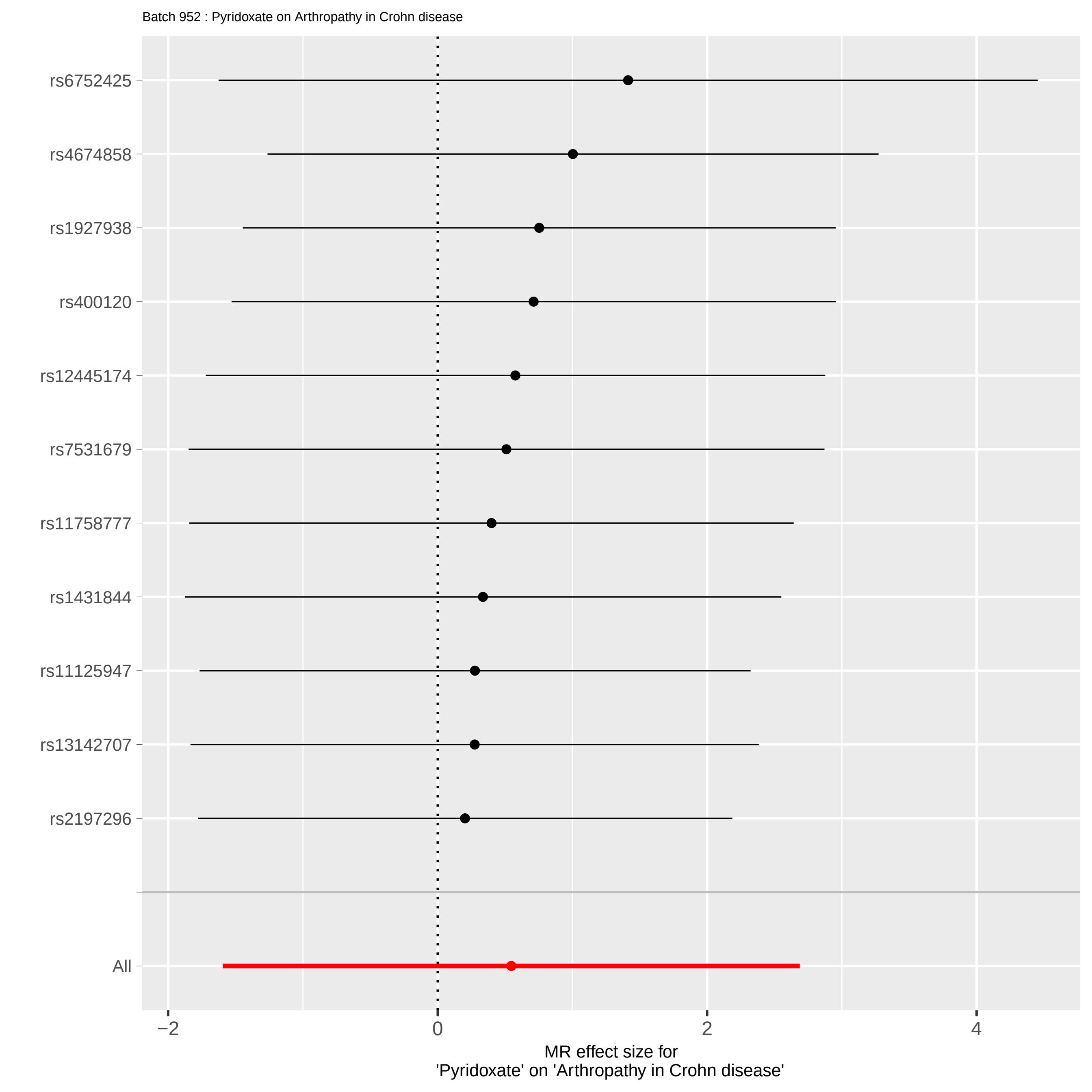
Batch 952 : Pyridoxate on Arthropathy in Crohn disease
rs6752425
rs4674858
rs1927938
rs400120
rs12445174
rs7531679
rs11758777
rs1431844
rs11125947
rs13142707
rs2197296
All
−2
0
2
4
MR effect size for
'Pyridoxate' on 'Arthropathy in Crohn disease'

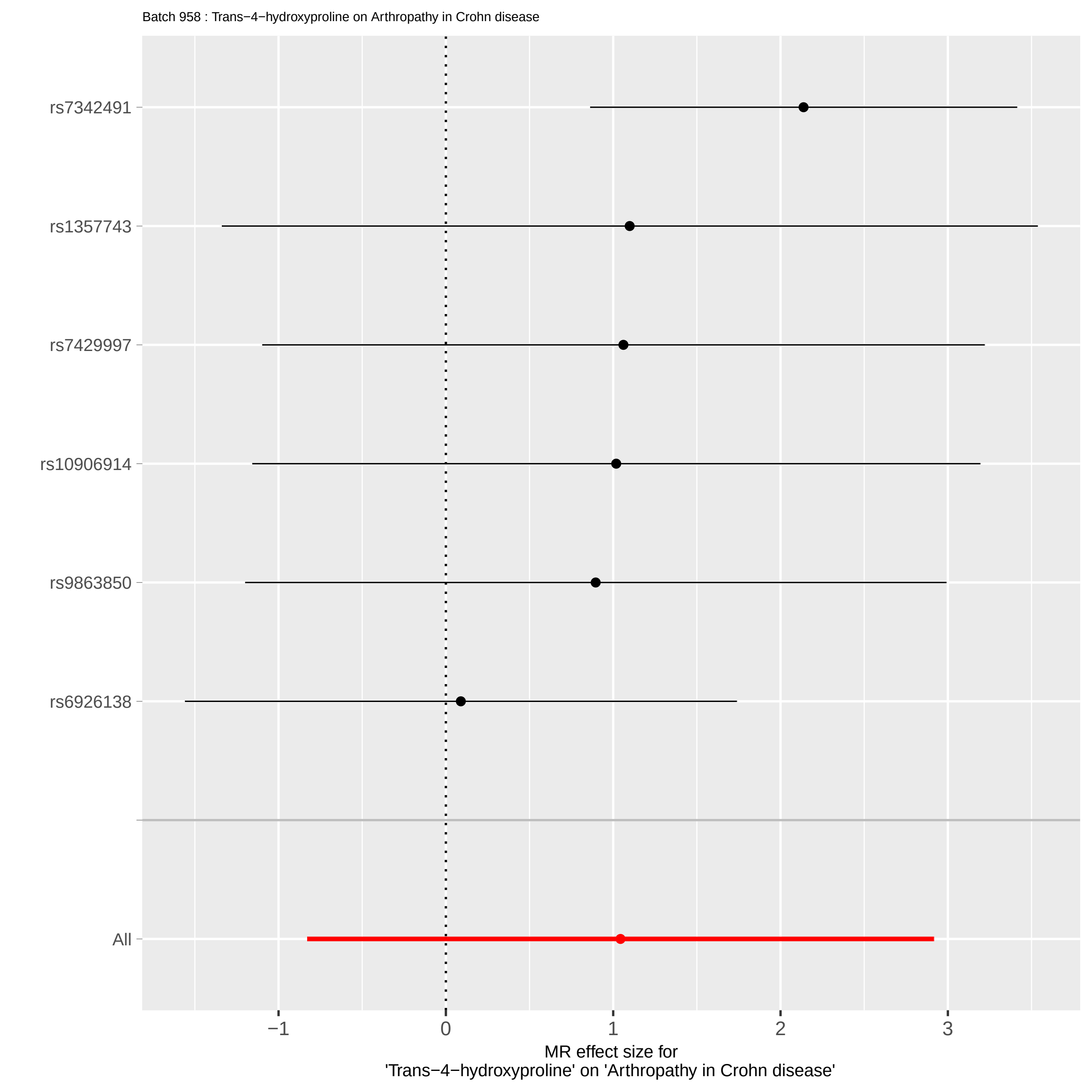

Batch 958 : Trans−4−hydroxyproline on Arthropathy in Crohn disease
rs7342491
rs1357743
rs7429997
rs10906914
rs9863850
rs6926138
All
−1
0
1
2
3
MR effect size for
'Trans−4−hydroxyproline' on 'Arthropathy in Crohn disease'

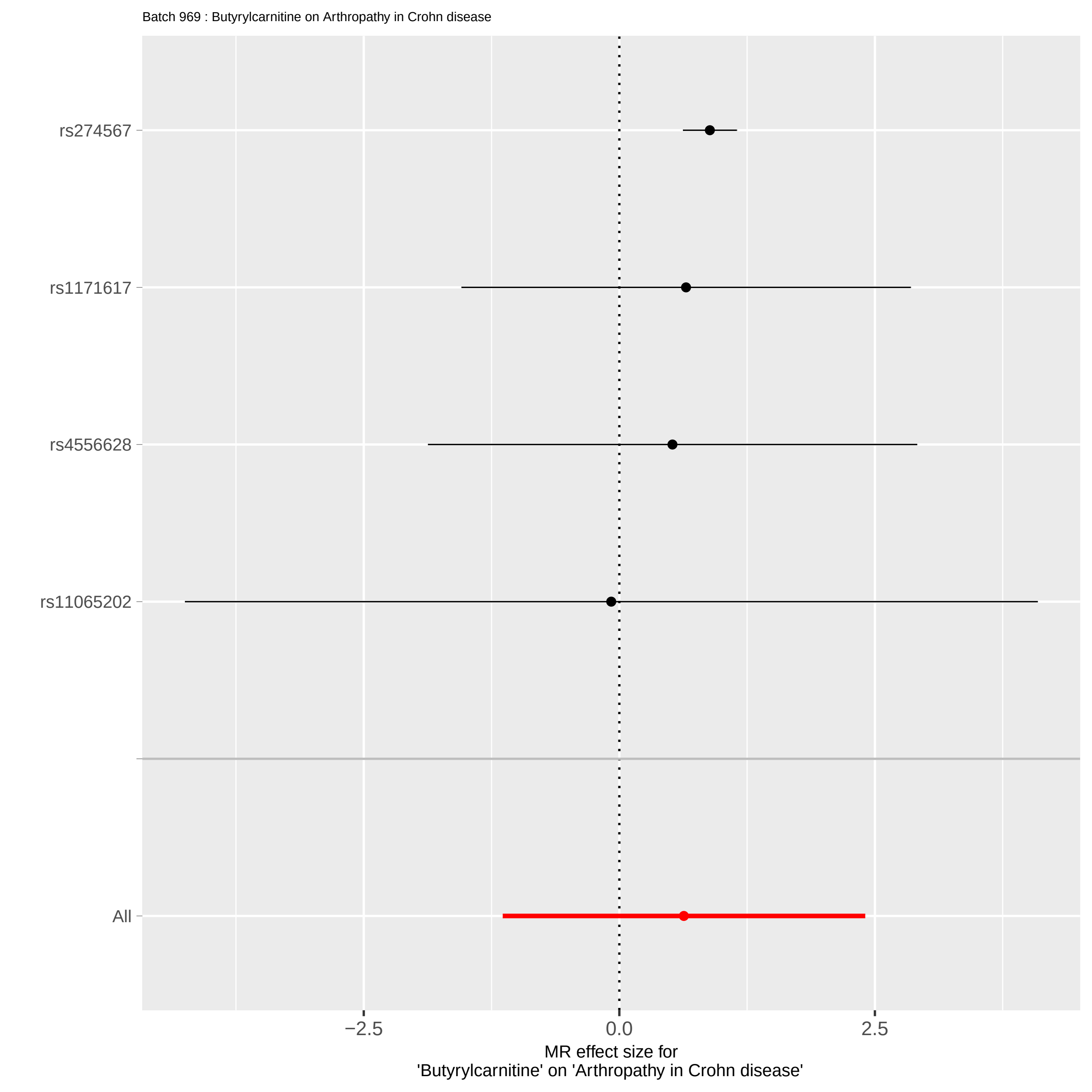

Batch 969 : Butyrylcarnitine on Arthropathy in Crohn disease
rs274567
rs1171617
rs4556628
rs11065202
All
−2.5
0.0
2.5
MR effect size for
'Butyrylcarnitine' on 'Arthropathy in Crohn disease'

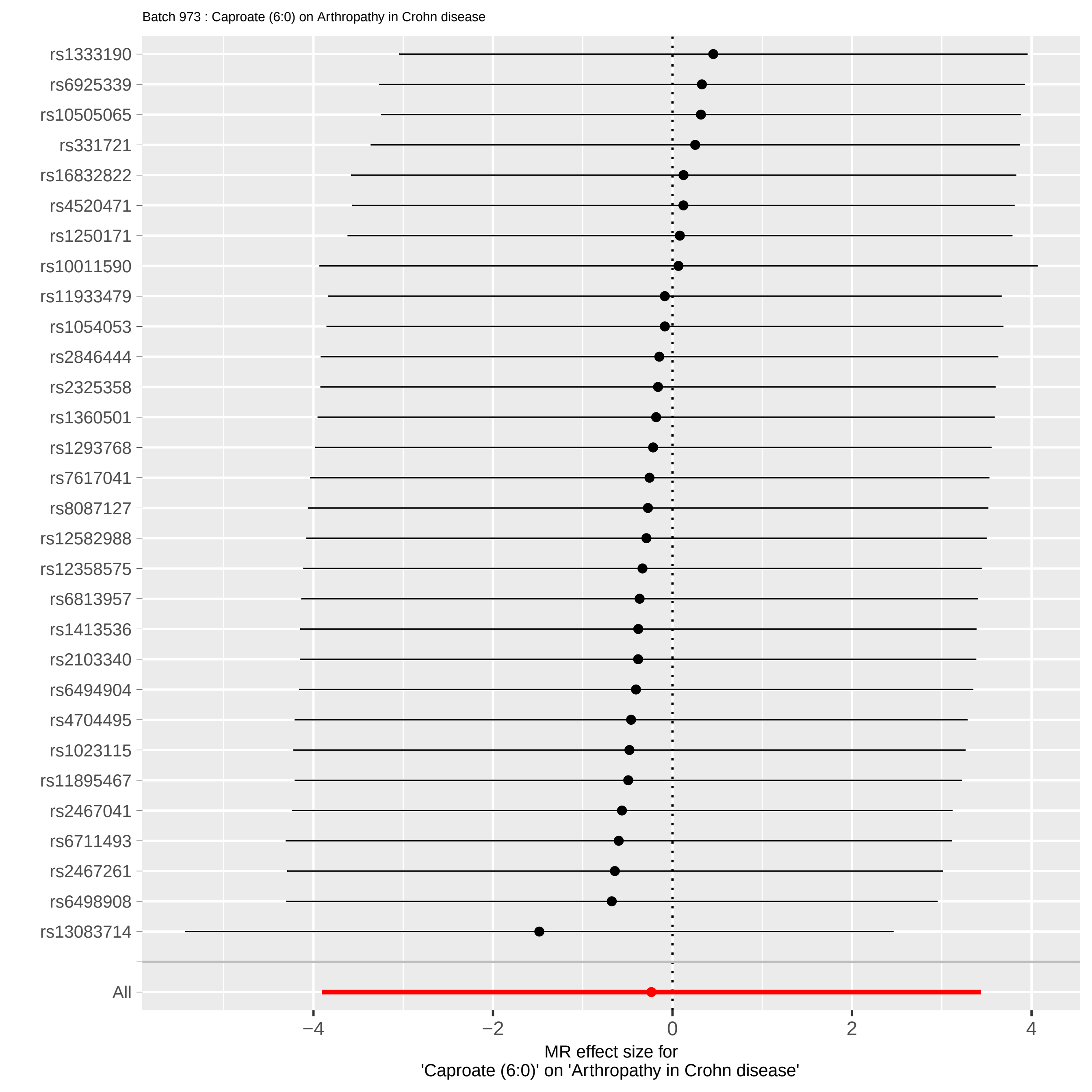
Batch 973 : Caproate (6:0) on Arthropathy in Crohn disease
rs1333190
rs6925339
rs10505065
rs331721
rs16832822
rs4520471
rs1250171
rs10011590
rs11933479
rs1054053
rs2846444
rs2325358
rs1360501
rs1293768
rs7617041
rs8087127
rs12582988
rs12358575
rs6813957
rs1413536
rs2103340
rs6494904
rs4704495
rs1023115
rs11895467
rs2467041
rs6711493
rs2467261
rs6498908
rs13083714
All
−4
−2
0
2
4
MR effect size for
'Caproate (6:0)' on 'Arthropathy in Crohn disease'

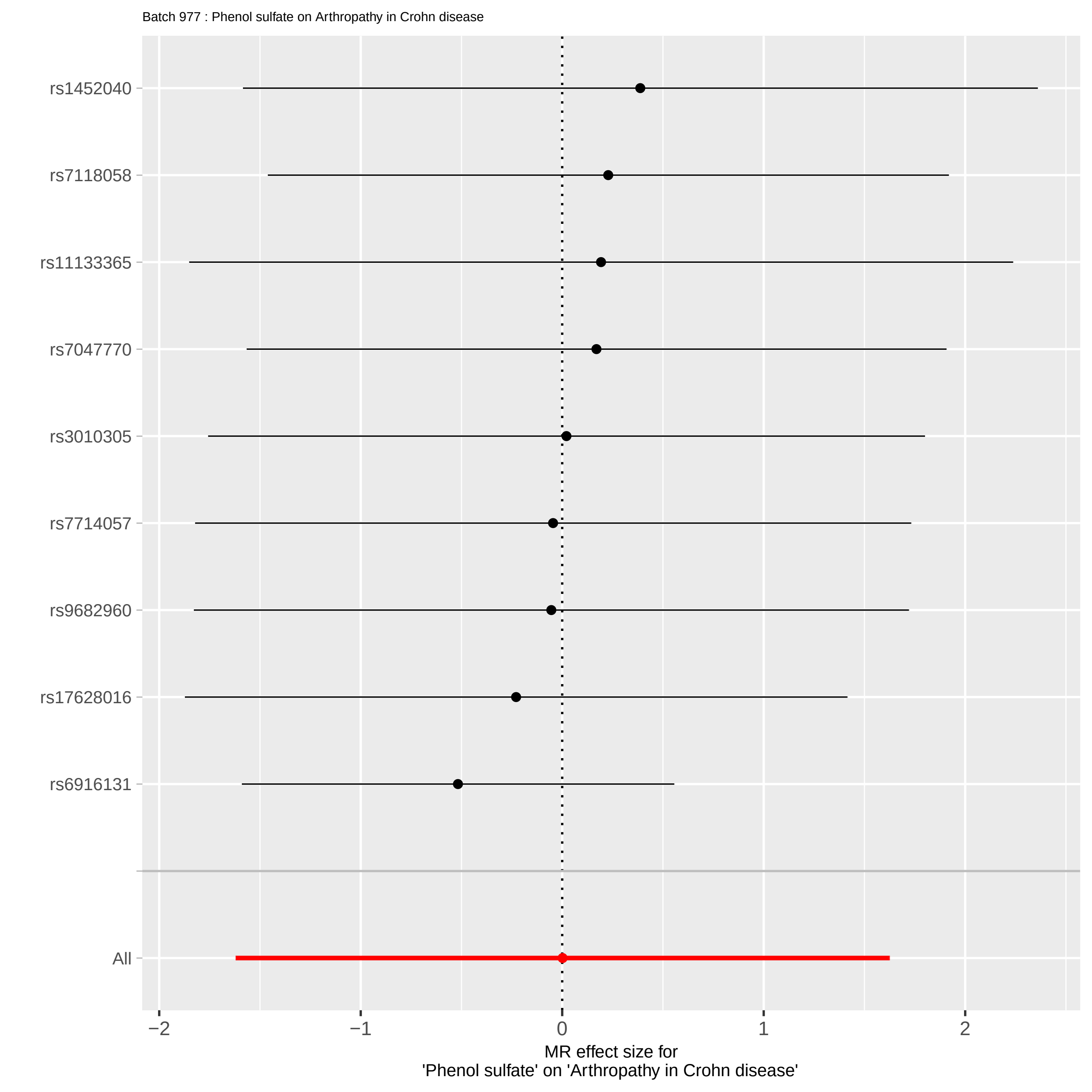

Batch 977 : Phenol sulfate on Arthropathy in Crohn disease
rs1452040
rs7118058
rs11133365
rs7047770
rs3010305
rs7714057
rs9682960
rs17628016
rs6916131
All
−2
−1
0
1
2
MR effect size for
'Phenol sulfate' on 'Arthropathy in Crohn disease'

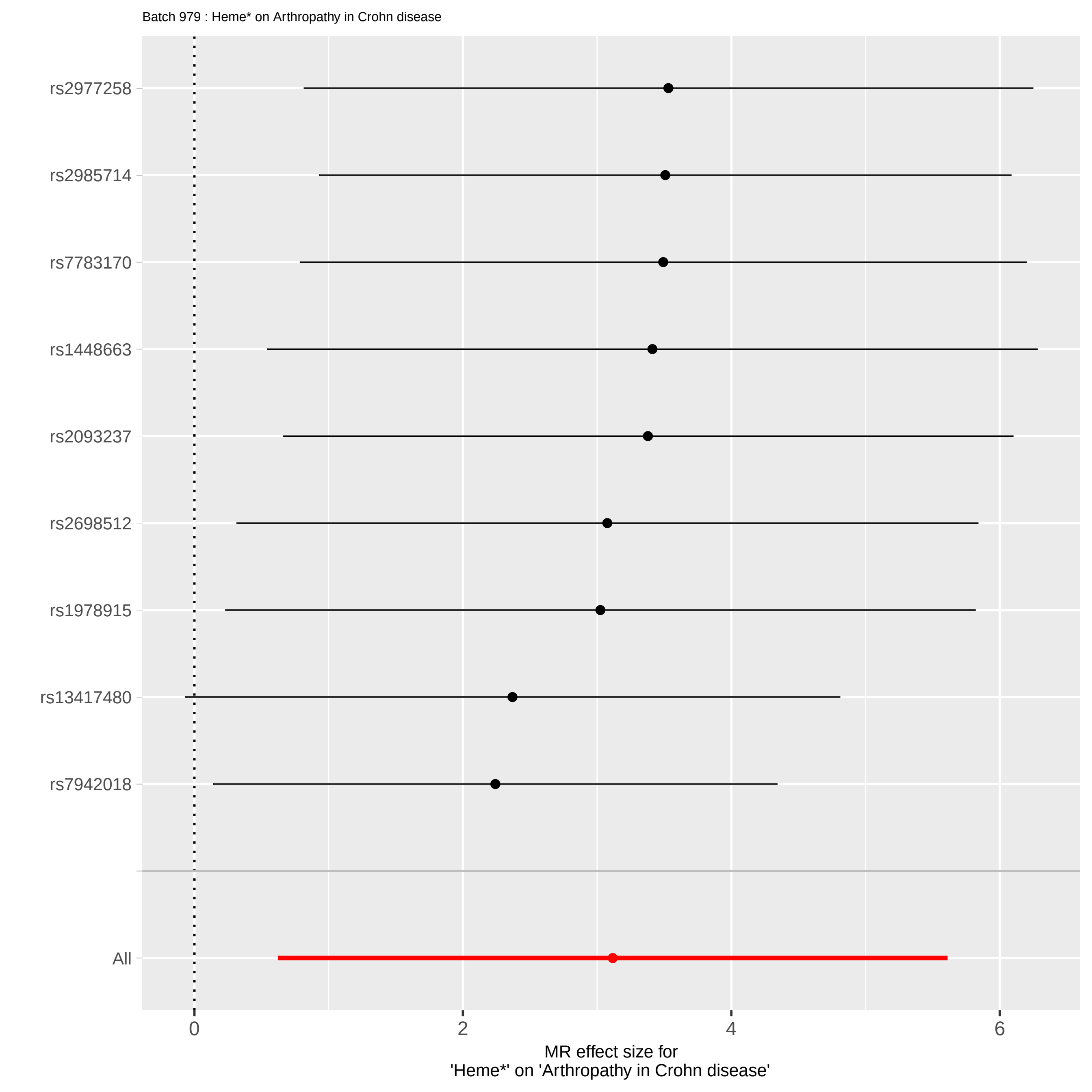

Batch 979 : Heme* on Arthropathy in Crohn disease
rs2977258
rs2985714
rs7783170
rs1448663
rs2093237
rs2698512
rs1978915
rs13417480
rs7942018
All
0
2
4
6
MR effect size for
'Heme*' on 'Arthropathy in Crohn disease'

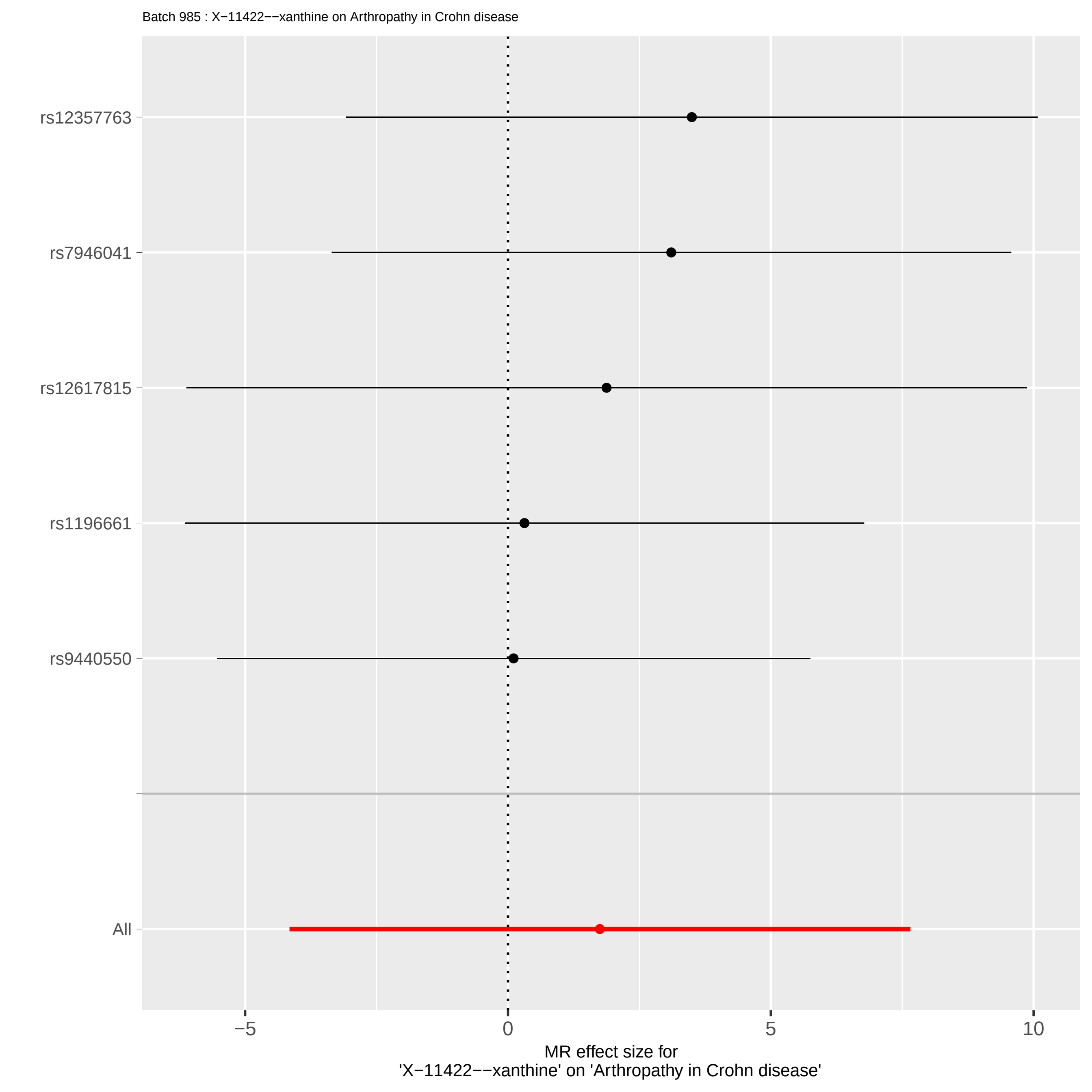

Batch 985 : X−11422−−xanthine on Arthropathy in Crohn disease
rs12357763
rs7946041
rs12617815
rs1196661
rs9440550
All
−5
0
5
10
MR effect size for
'X−11422−−xanthine' on 'Arthropathy in Crohn disease'

Batch 989 : X−11593−−O−methylascorbate* on Arthropathy in Crohn disease

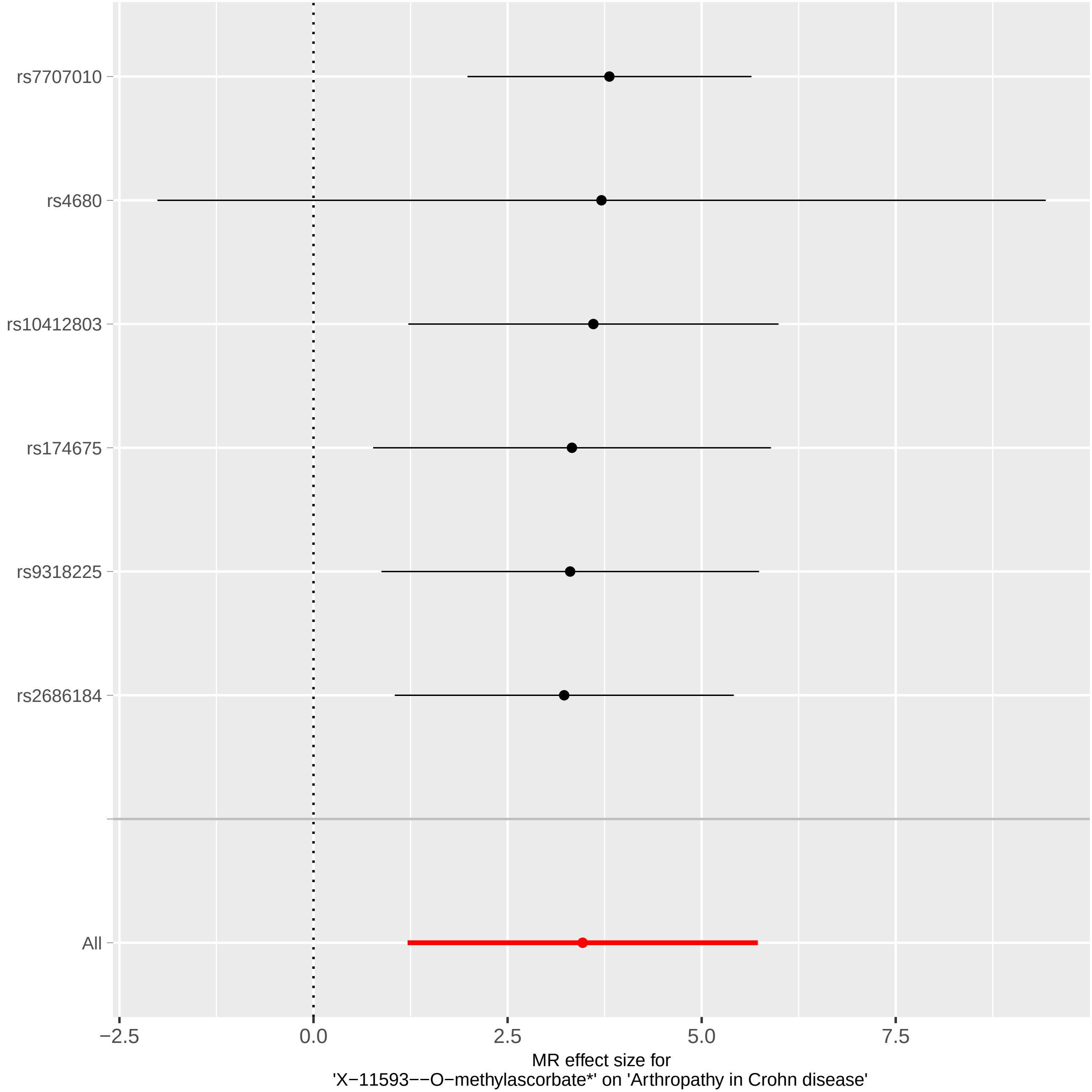

Batch 991 : Homostachydrine* on Arthropathy in Crohn disease

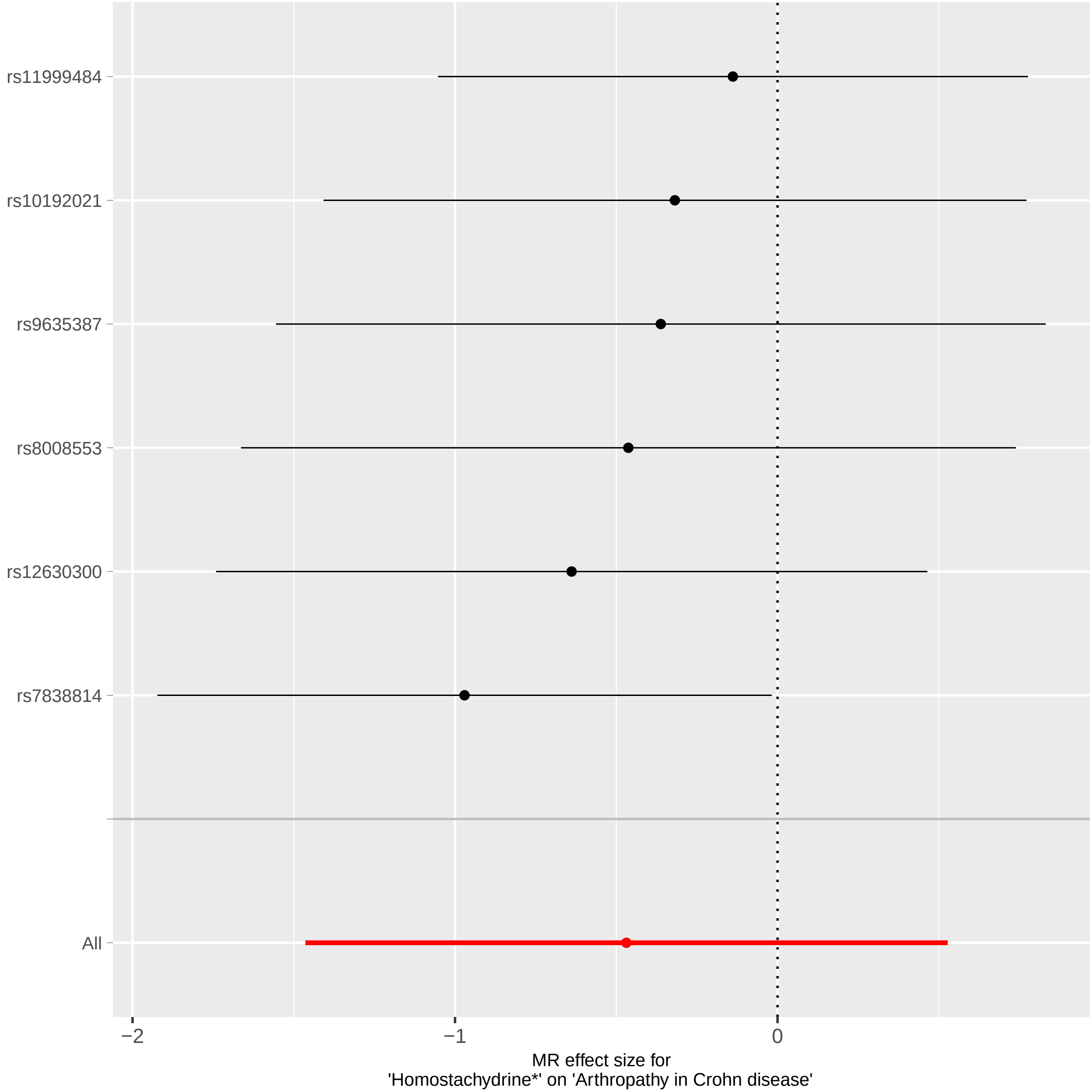

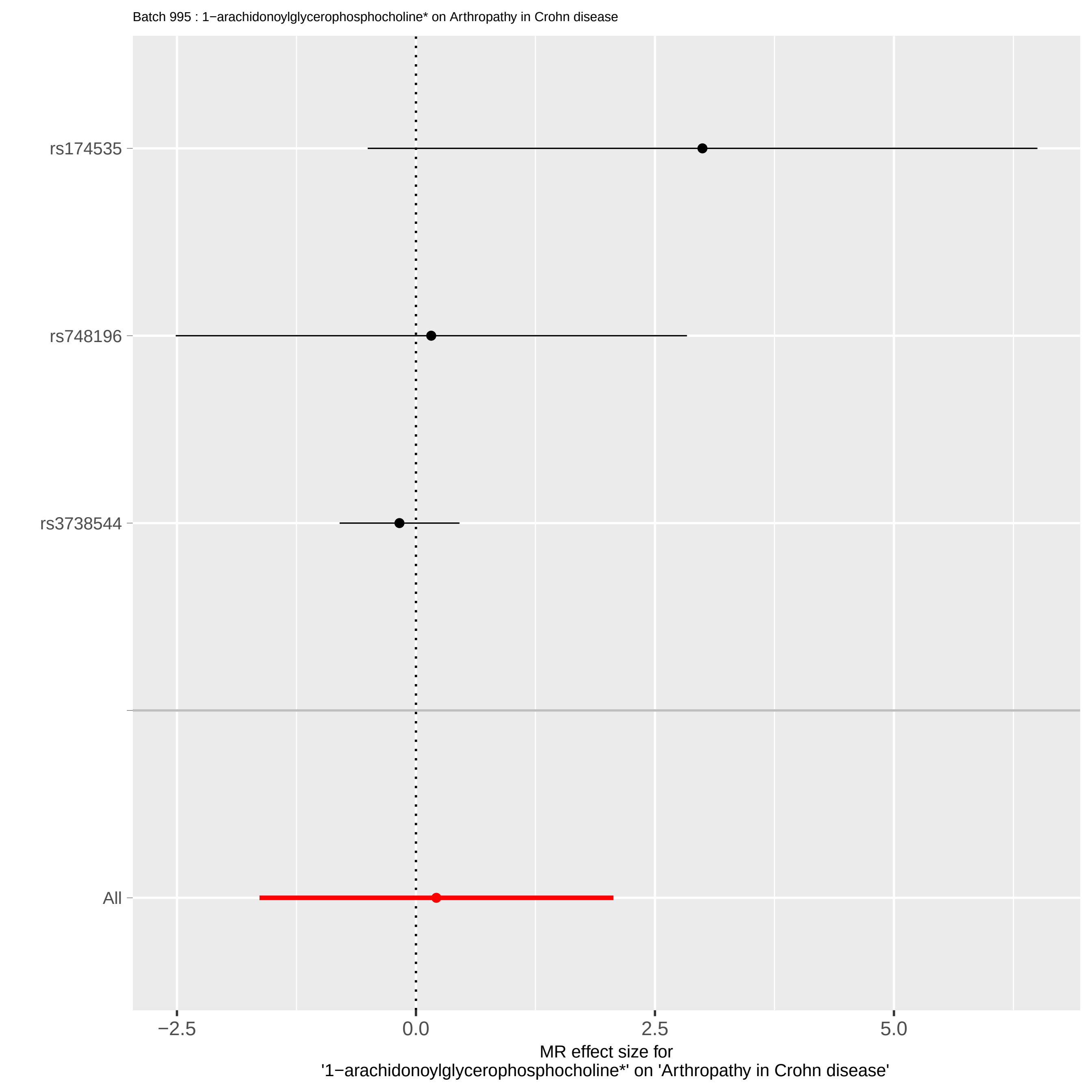

Batch 995 : 1−arachidonoylglycerophosphocholine* on Arthropathy in Crohn disease
rs174535
rs748196
rs3738544
All
−2.5
0.0
2.5
5.0
MR effect size for
'1−arachidonoylglycerophosphocholine*' on 'Arthropathy in Crohn disease'

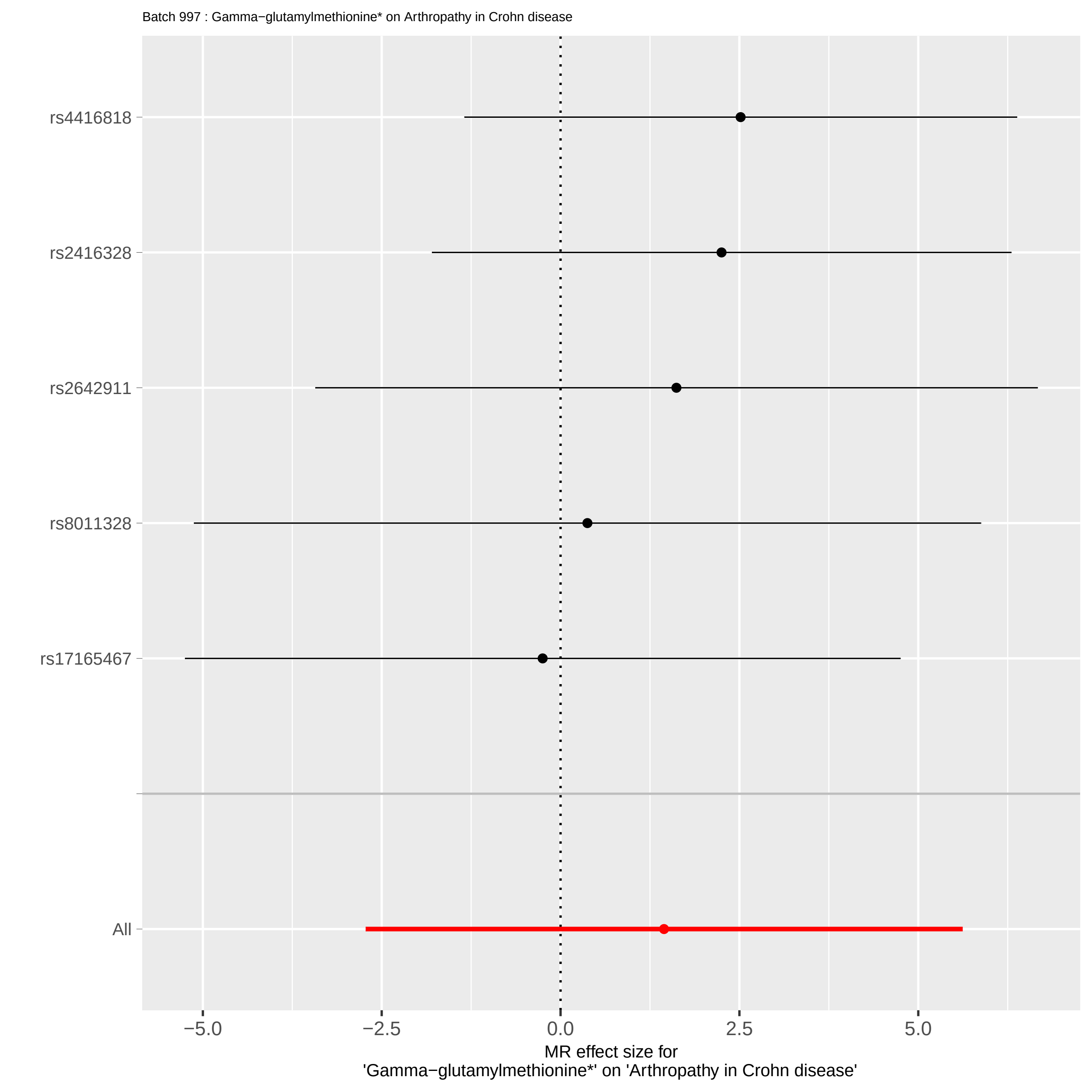
Batch 997 : Gamma−glutamylmethionine* on Arthropathy in Crohn disease
rs4416818
rs2416328
rs2642911
rs8011328
rs17165467
All
−5.0
−2.5
0.0
2.5
5.0
MR effect size for
'Gamma−glutamylmethionine*' on 'Arthropathy in Crohn disease'

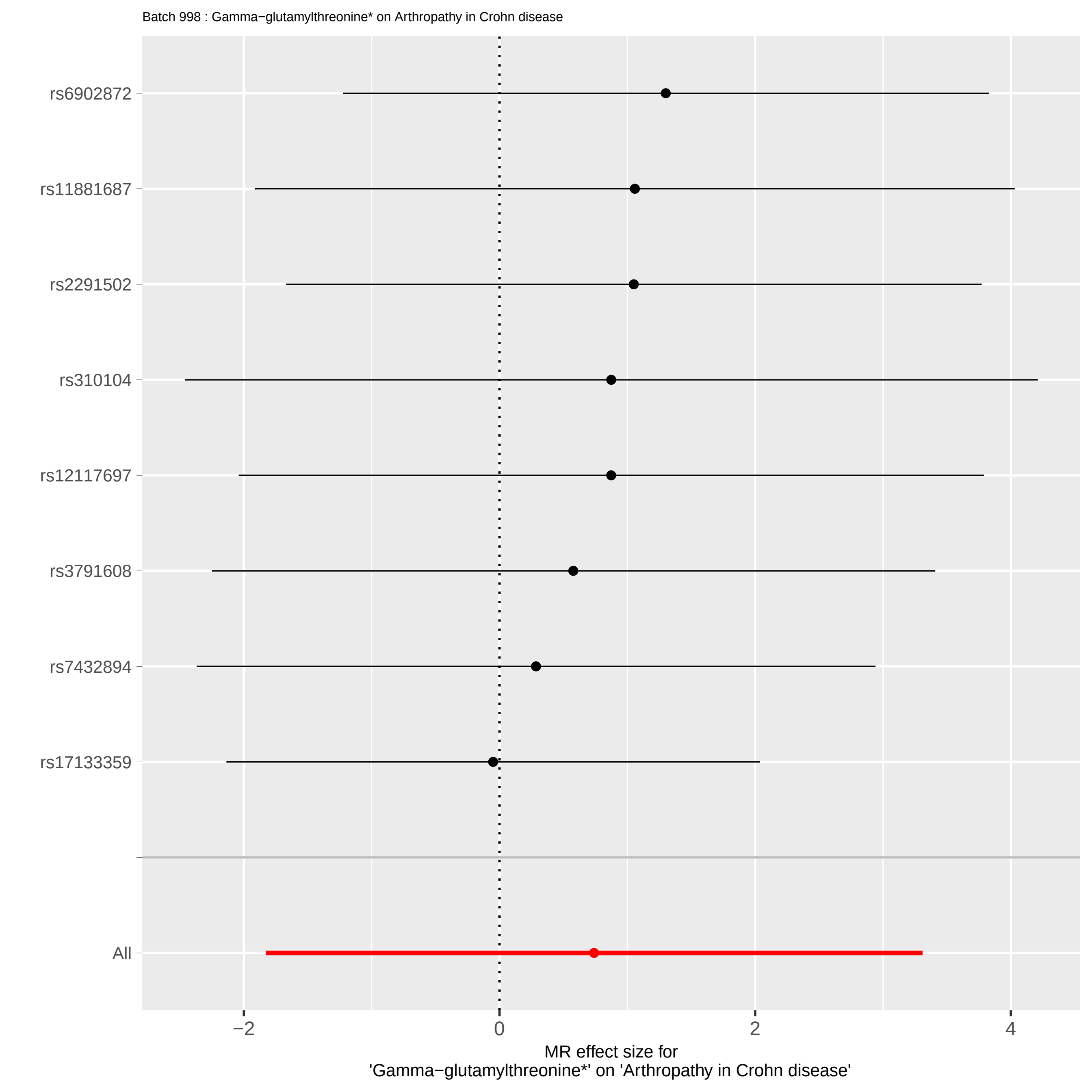

Batch 998 : Gamma−glutamylthreonine* on Arthropathy in Crohn disease
rs6902872
rs11881687
rs2291502
rs310104
rs12117697
rs3791608
rs7432894
rs17133359
All
−2
0
2
4
MR effect size for
'Gamma−glutamylthreonine*' on 'Arthropathy in Crohn disease'

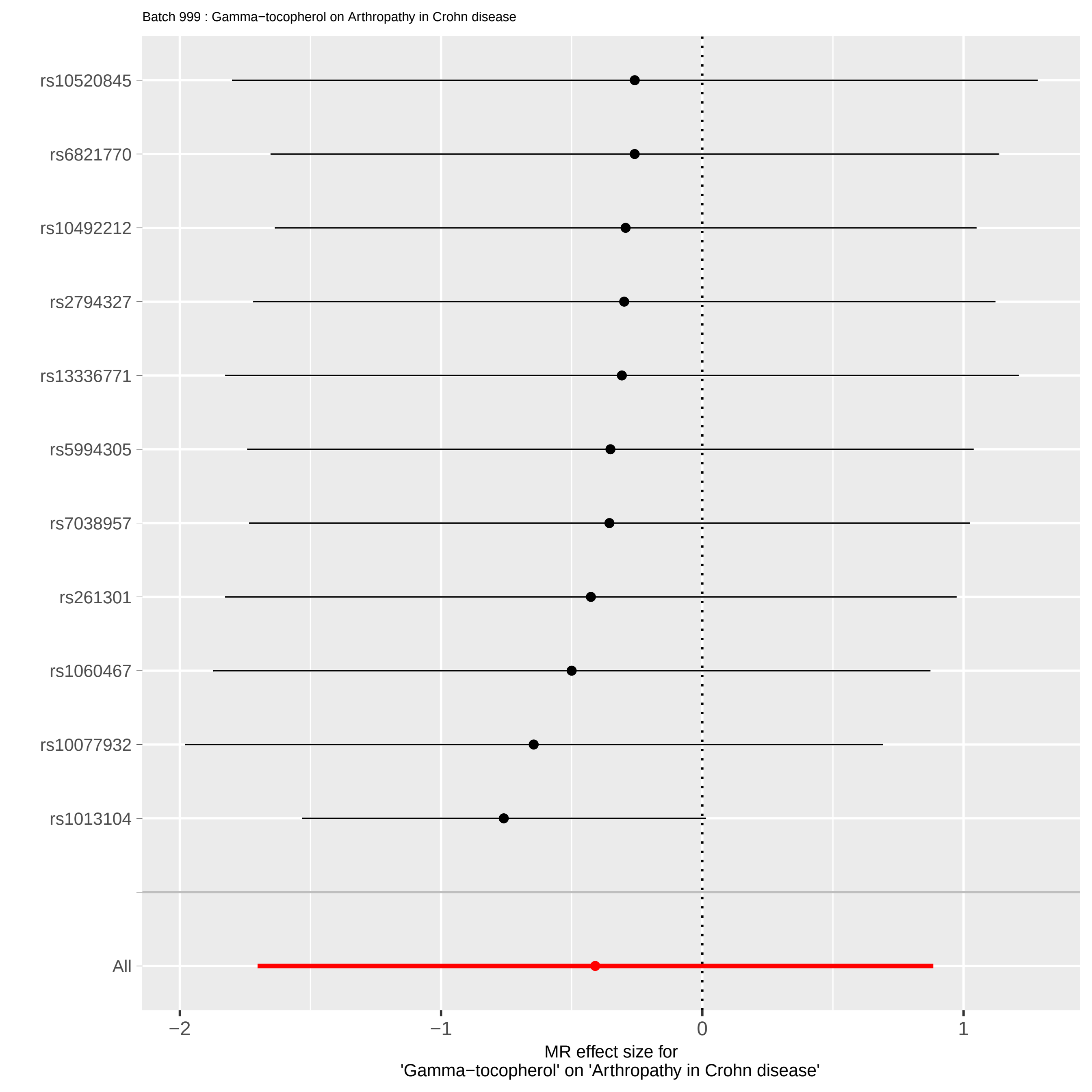

Batch 999 : Gamma−tocopherol on Arthropathy in Crohn disease
rs10520845
rs6821770
rs10492212
rs2794327
rs13336771
rs5994305
rs7038957
rs261301
rs1060467
rs10077932
rs1013104
All
−2
−1
0
1
MR effect size for
'Gamma−tocopherol' on 'Arthropathy in Crohn disease'

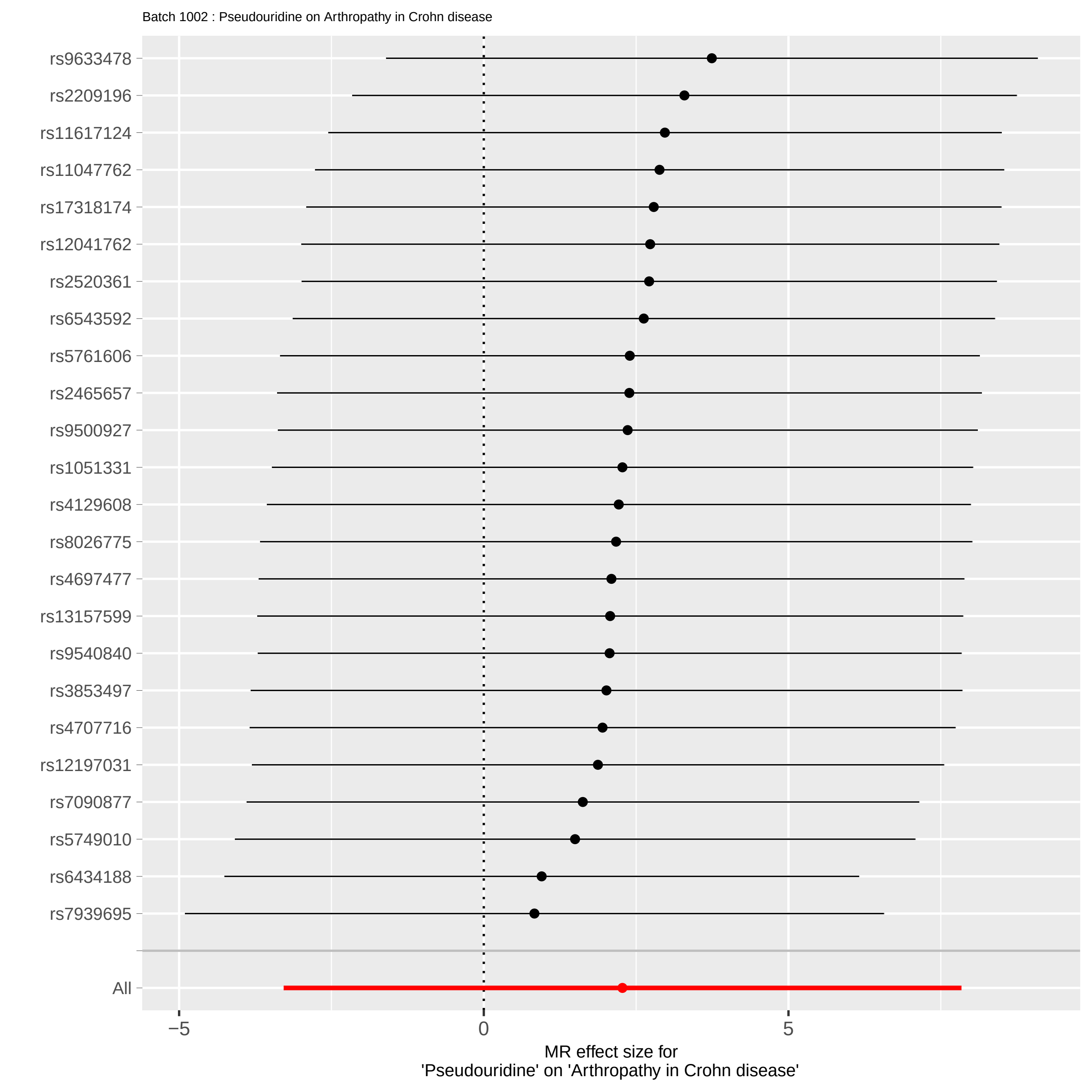
Batch 1002 : Pseudouridine on Arthropathy in Crohn disease
rs9633478
rs2209196
rs11617124
rs11047762
rs17318174
rs12041762
rs2520361
rs6543592
rs5761606
rs2465657
rs9500927
rs1051331
rs4129608
rs8026775
rs4697477
rs13157599
rs9540840
rs3853497
rs4707716
rs12197031
rs7090877
rs5749010
rs6434188
rs7939695
All
−5
0
5
MR effect size for
'Pseudouridine' on 'Arthropathy in Crohn disease'

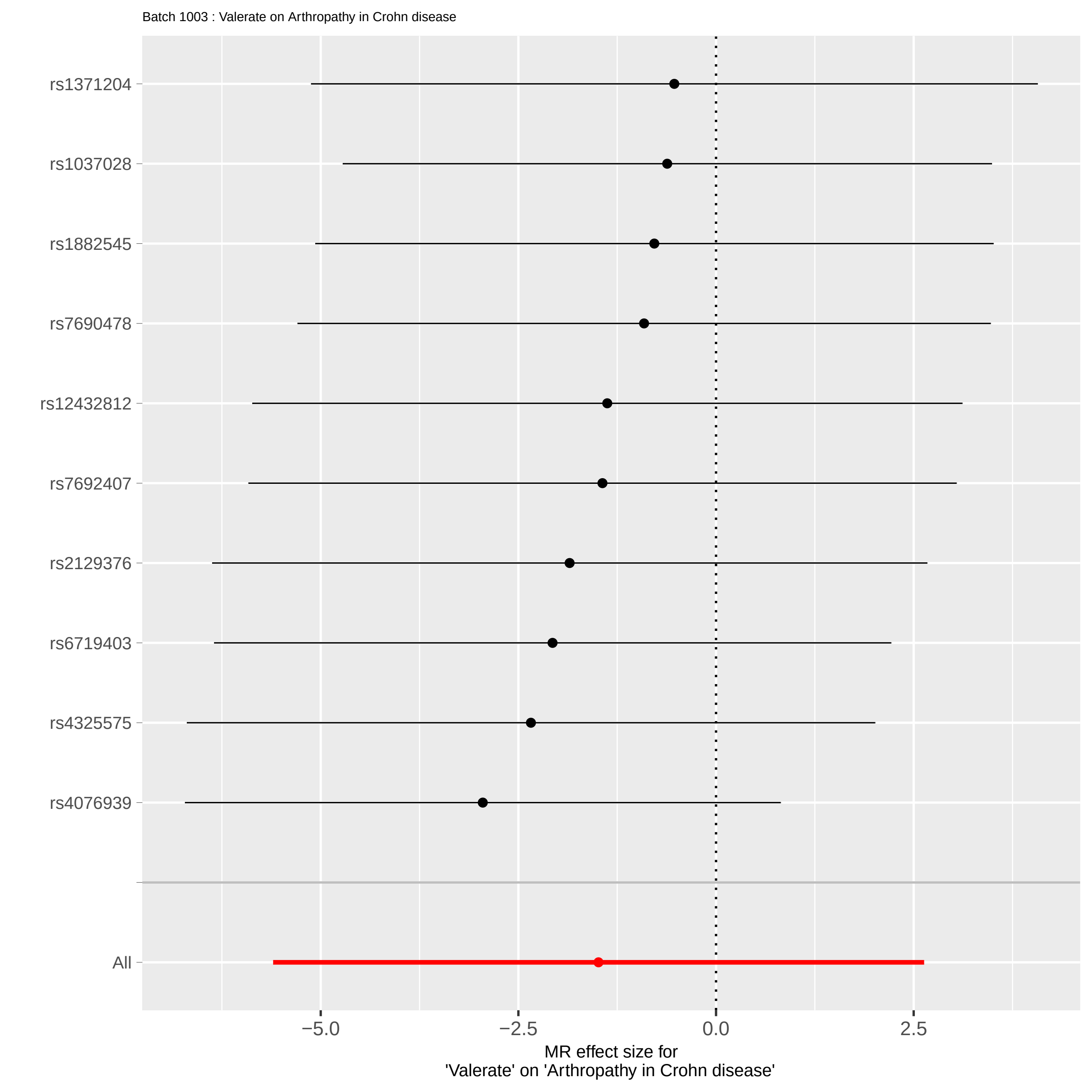
Batch 1003 : Valerate on Arthropathy in Crohn disease
rs1371204
rs1037028
rs1882545
rs7690478
rs12432812
rs7692407
rs2129376
rs6719403
rs4325575
rs4076939
All
−5.0
−2.5
0.0
2.5
MR effect size for
'Valerate' on 'Arthropathy in Crohn disease'

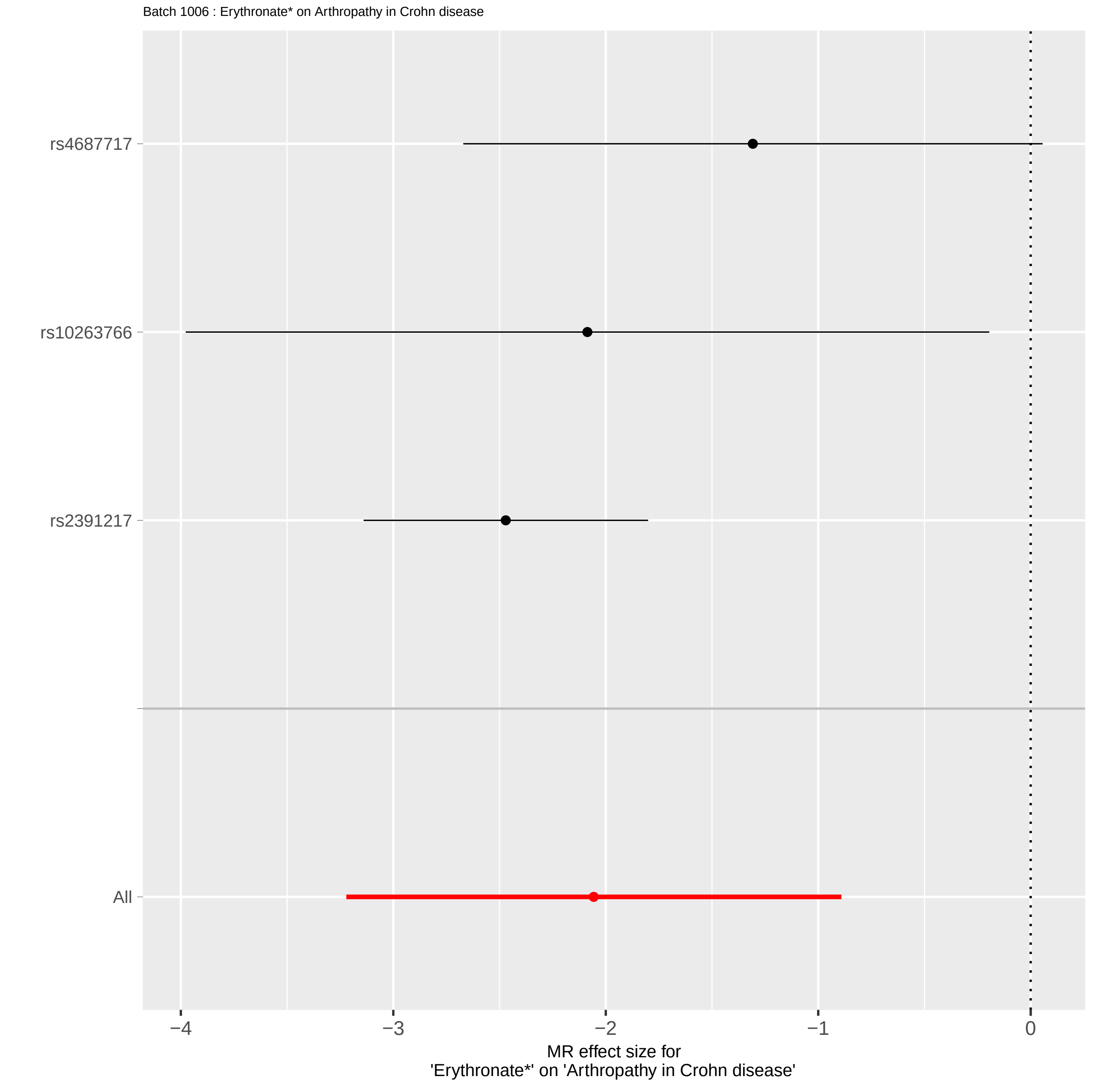

Batch 1006 : Erythronate* on Arthropathy in Crohn disease
rs4687717
rs10263766
rs2391217
All
−4
−3
−2
−1
0
MR effect size for
'Erythronate*' on 'Arthropathy in Crohn disease'

Batch 1007 : Lathosterol on Arthropathy in Crohn disease

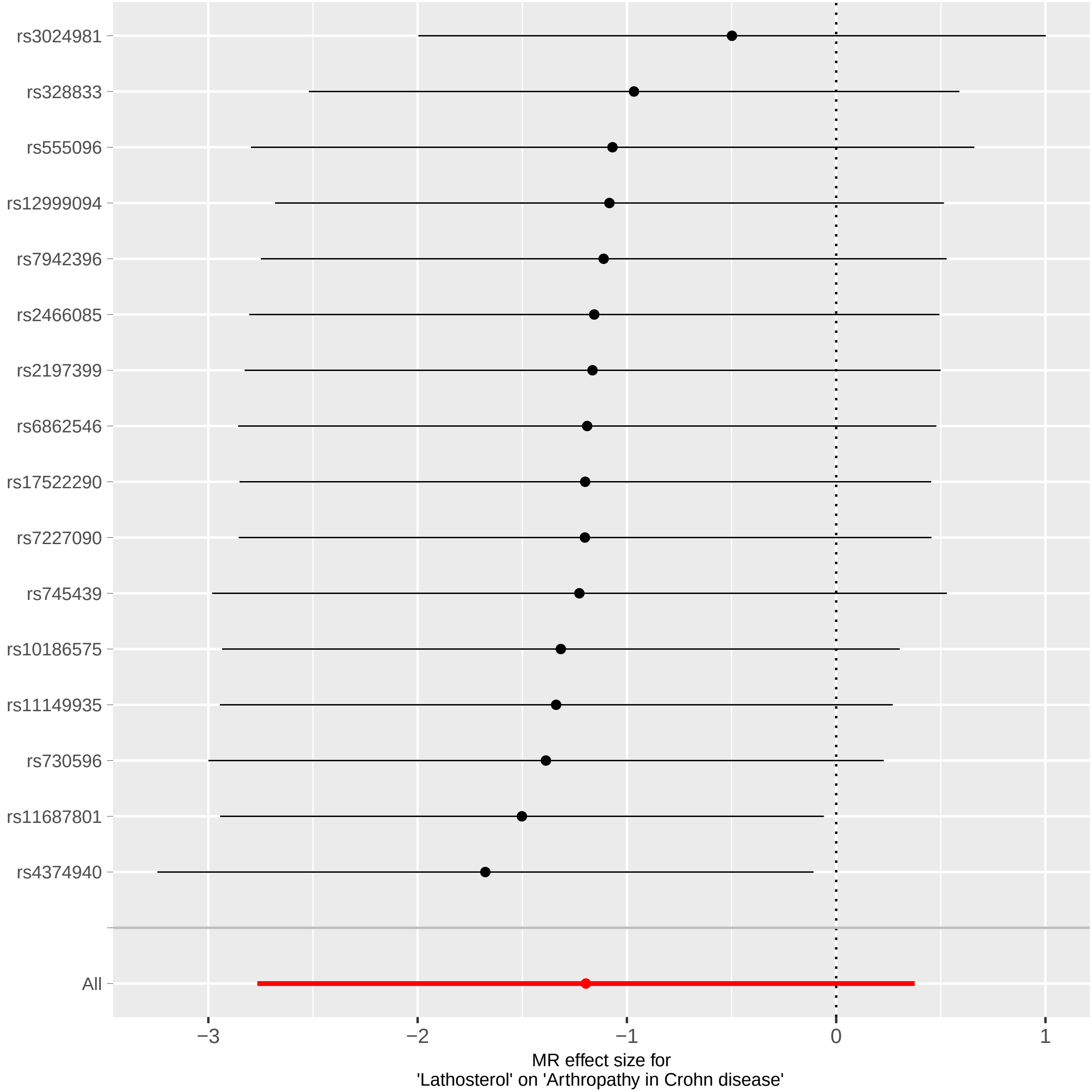

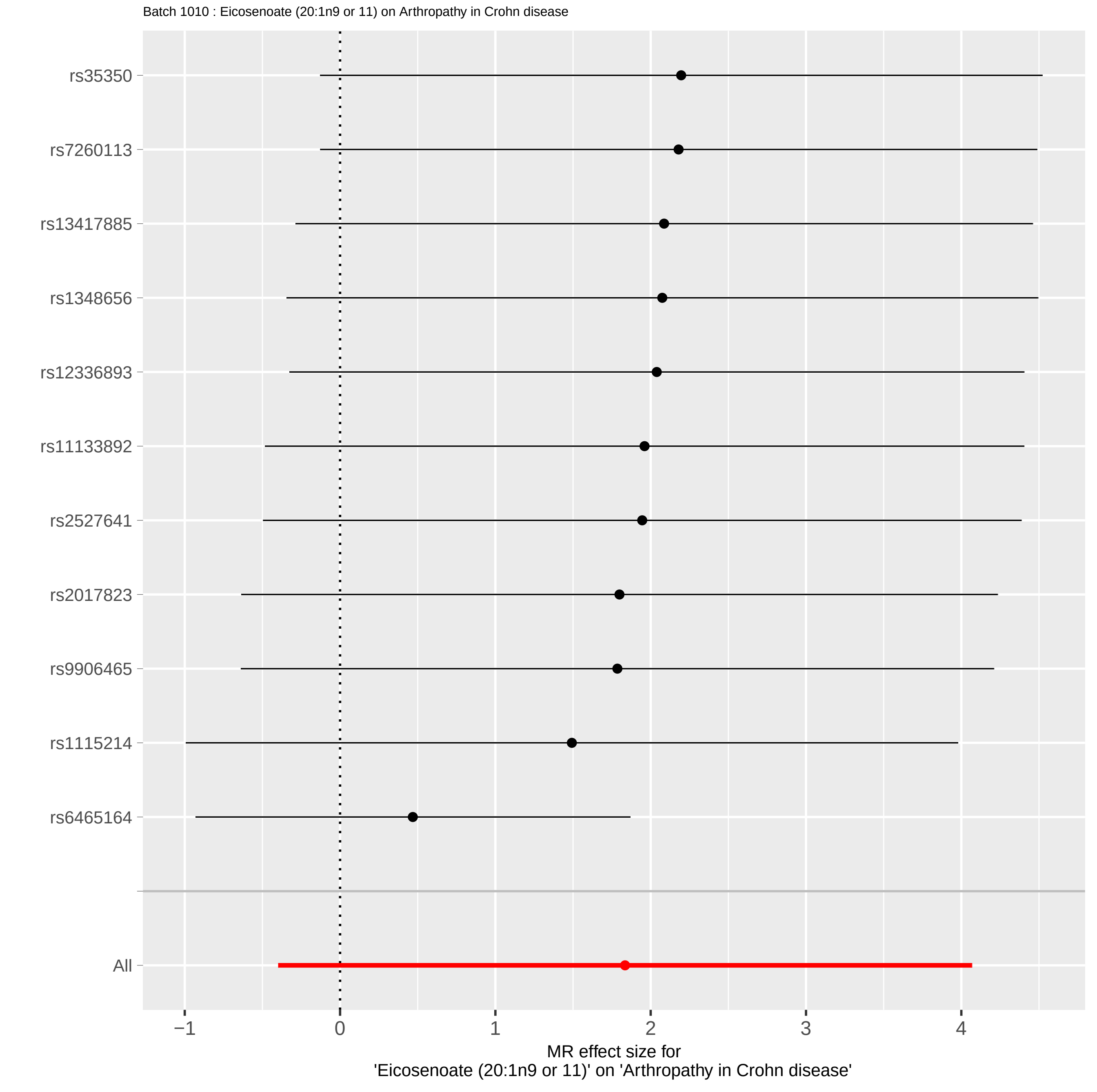

Batch 1010 : Eicosenoate (20:1n9 or 11) on Arthropathy in Crohn disease
rs35350
rs7260113
rs13417885
rs1348656
rs12336893
rs11133892
rs2527641
rs2017823
rs9906465
rs1115214
rs6465164
All
−1
0
1
2
3
4
MR effect size for
'Eicosenoate (20:1n9 or 11)' on 'Arthropathy in Crohn disease'

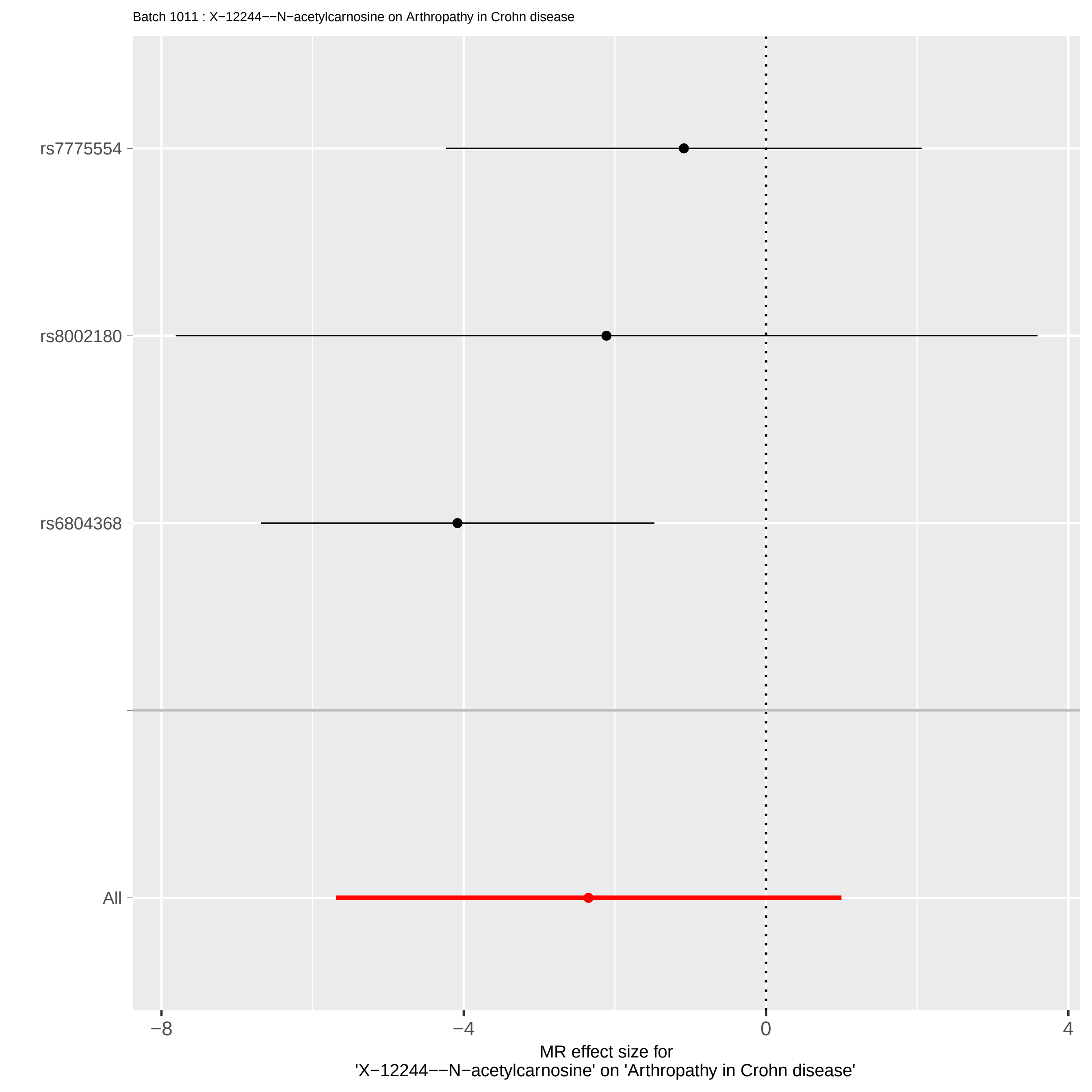

Batch 1011 : X−12244−−N−acetylcarnosine on Arthropathy in Crohn disease
rs7775554
rs8002180
rs6804368
All
−8
−4
0
4
MR effect size for
'X−12244−−N−acetylcarnosine' on 'Arthropathy in Crohn disease'

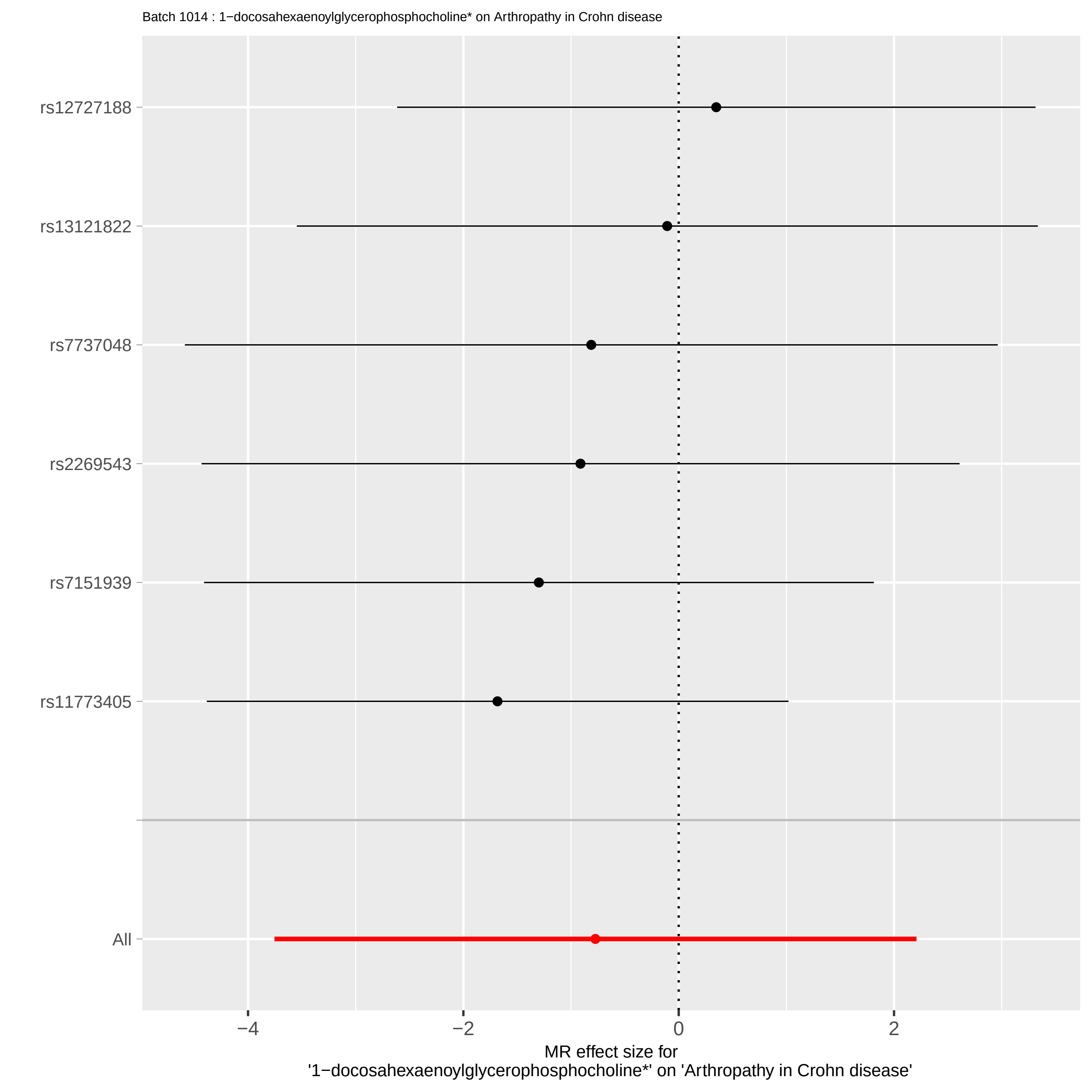

Batch 1014 : 1−docosahexaenoylglycerophosphocholine* on Arthropathy in Crohn disease
rs12727188
rs13121822
rs7737048
rs2269543
rs7151939
rs11773405
All
−4
−2
0
2
MR effect size for
'1−docosahexaenoylglycerophosphocholine*' on 'Arthropathy in Crohn disease'

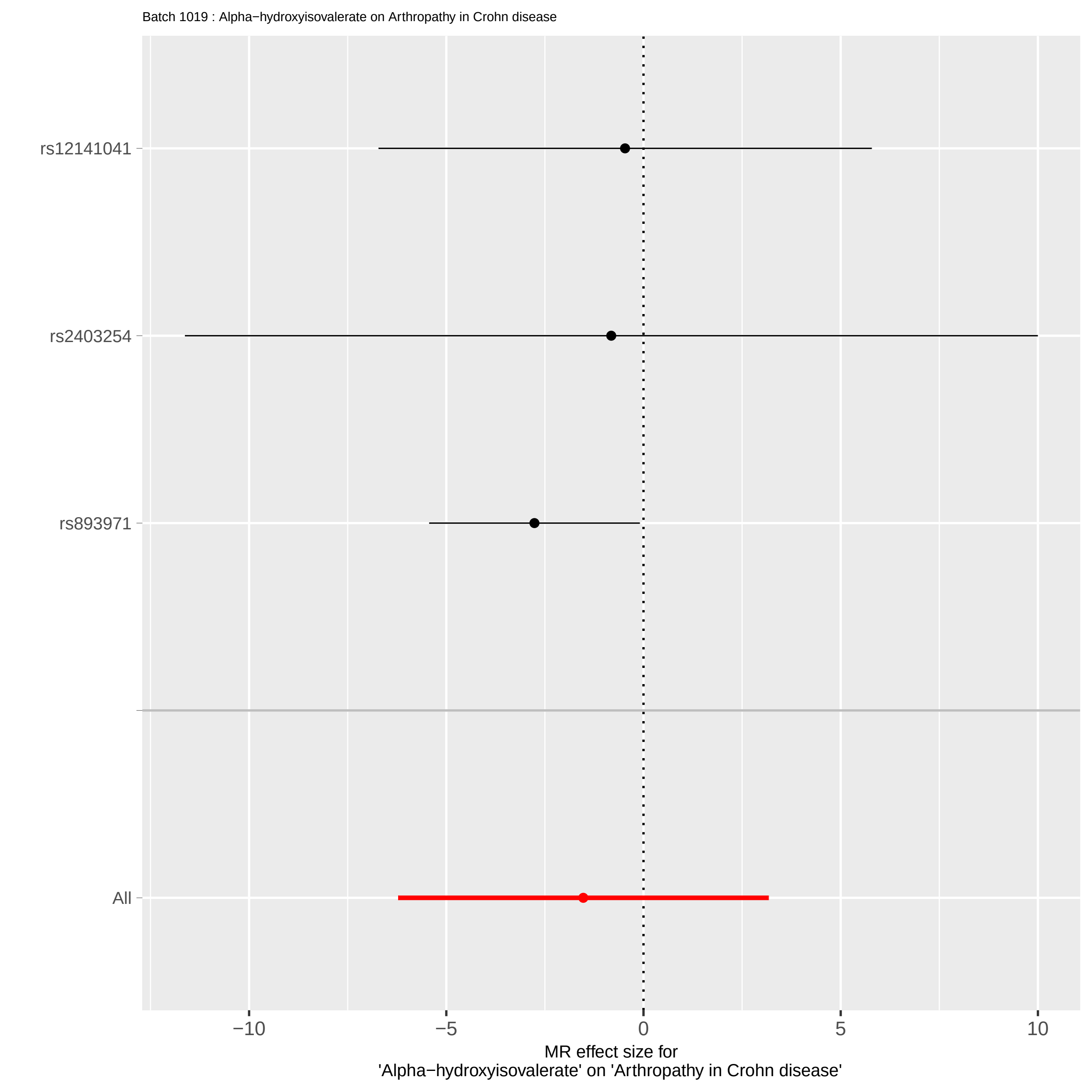

Batch 1019 : Alpha−hydroxyisovalerate on Arthropathy in Crohn disease
rs12141041
rs2403254
rs893971
All
−10
−5
0
5
10
MR effect size for
'Alpha−hydroxyisovalerate' on 'Arthropathy in Crohn disease'

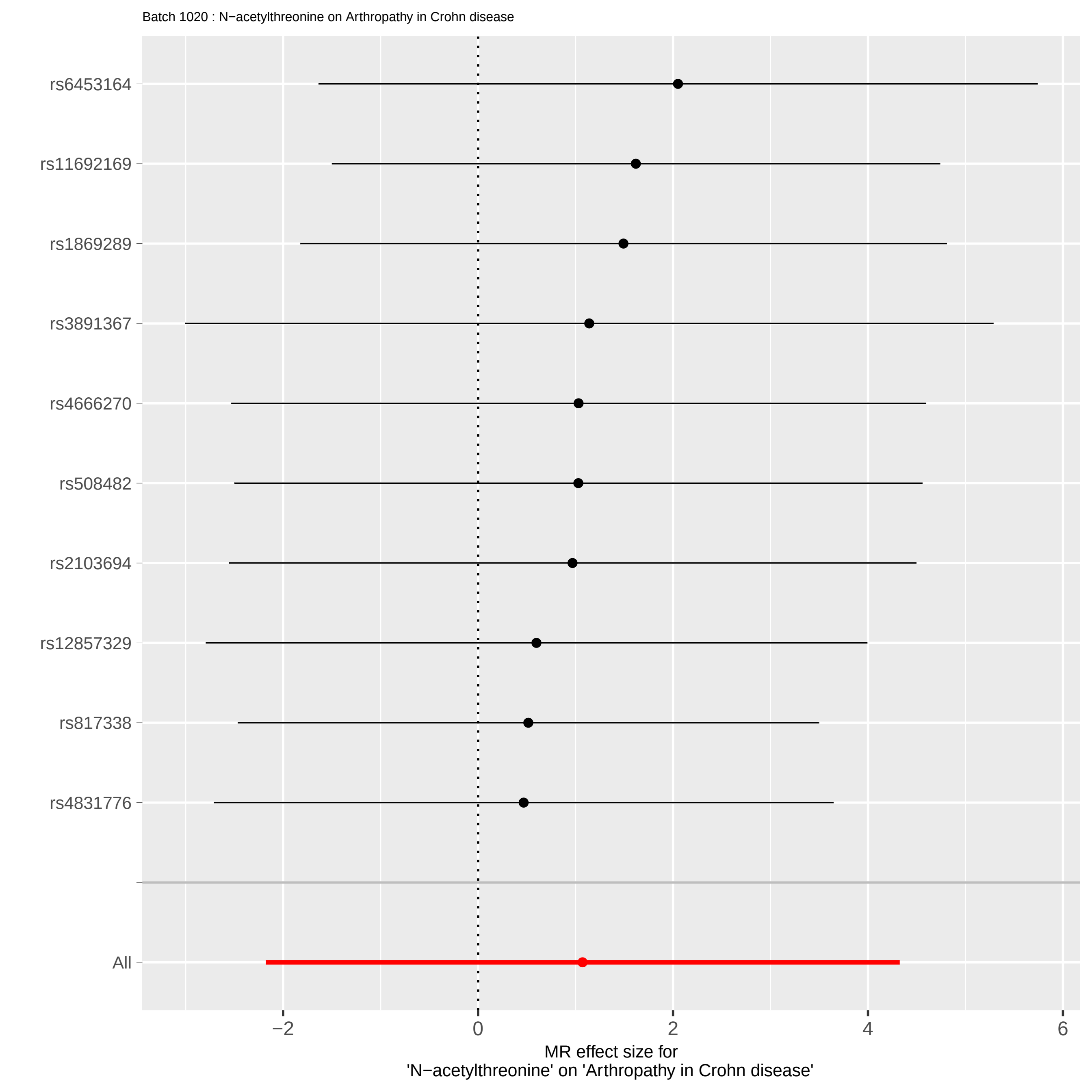

Batch 1020 : N−acetylthreonine on Arthropathy in Crohn disease
rs6453164
rs11692169
rs1869289
rs3891367
rs4666270
rs508482
rs2103694
rs12857329
rs817338
rs4831776
All
−2
0
2
4
6
MR effect size for
'N−acetylthreonine' on 'Arthropathy in Crohn disease'

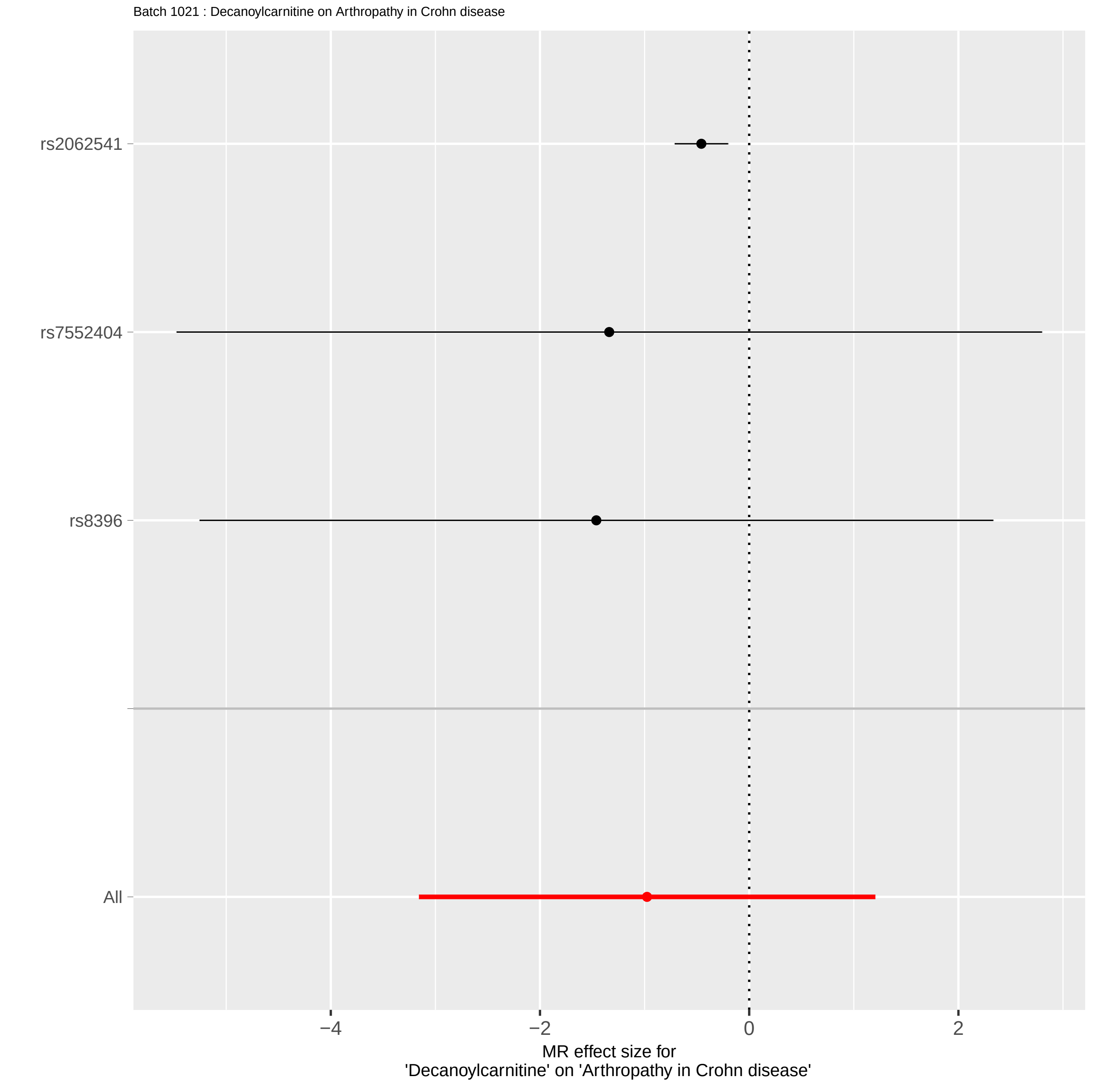
Batch 1021 : Decanoylcarnitine on Arthropathy in Crohn disease
rs2062541
rs7552404
rs8396
All
−4
−2
0
2
MR effect size for
'Decanoylcarnitine' on 'Arthropathy in Crohn disease'

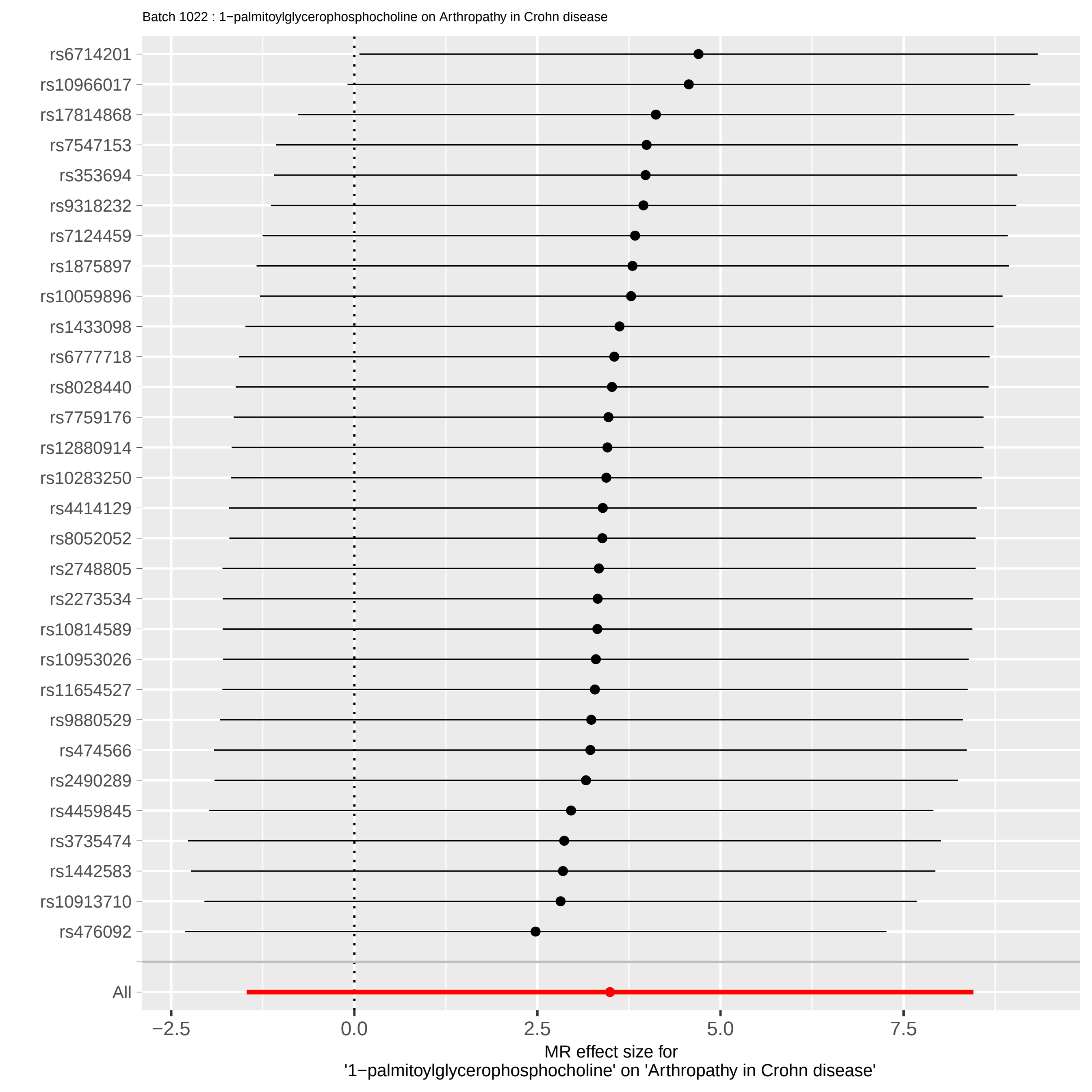

Batch 1022 : 1−palmitoylglycerophosphocholine on Arthropathy in Crohn disease
rs6714201
rs10966017
rs17814868
rs7547153
rs353694
rs9318232
rs7124459
rs1875897
rs10059896
rs1433098
rs6777718
rs8028440
rs7759176
rs12880914
rs10283250
rs4414129
rs8052052
rs2748805
rs2273534
rs10814589
rs10953026
rs11654527
rs9880529
rs474566
rs2490289
rs4459845
rs3735474
rs1442583
rs10913710
rs476092
All
−2.5
0.0
2.5
5.0
7.5
MR effect size for
'1−palmitoylglycerophosphocholine' on 'Arthropathy in Crohn disease'

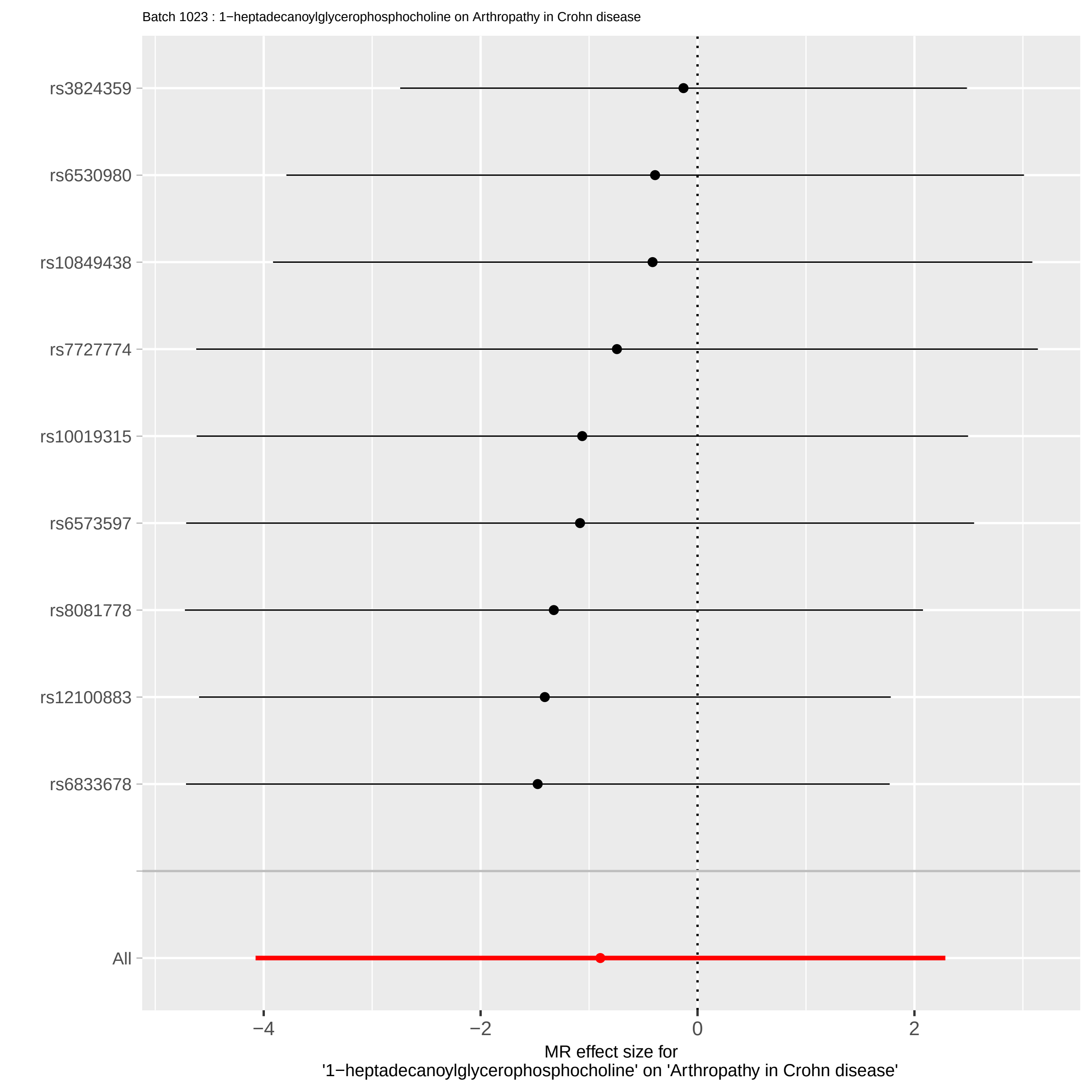

Batch 1023 : 1−heptadecanoylglycerophosphocholine on Arthropathy in Crohn disease
rs3824359
rs6530980
rs10849438
rs7727774
rs10019315
rs6573597
rs8081778
rs12100883
rs6833678
All
−4
−2
0
2
MR effect size for
'1−heptadecanoylglycerophosphocholine' on 'Arthropathy in Crohn disease'

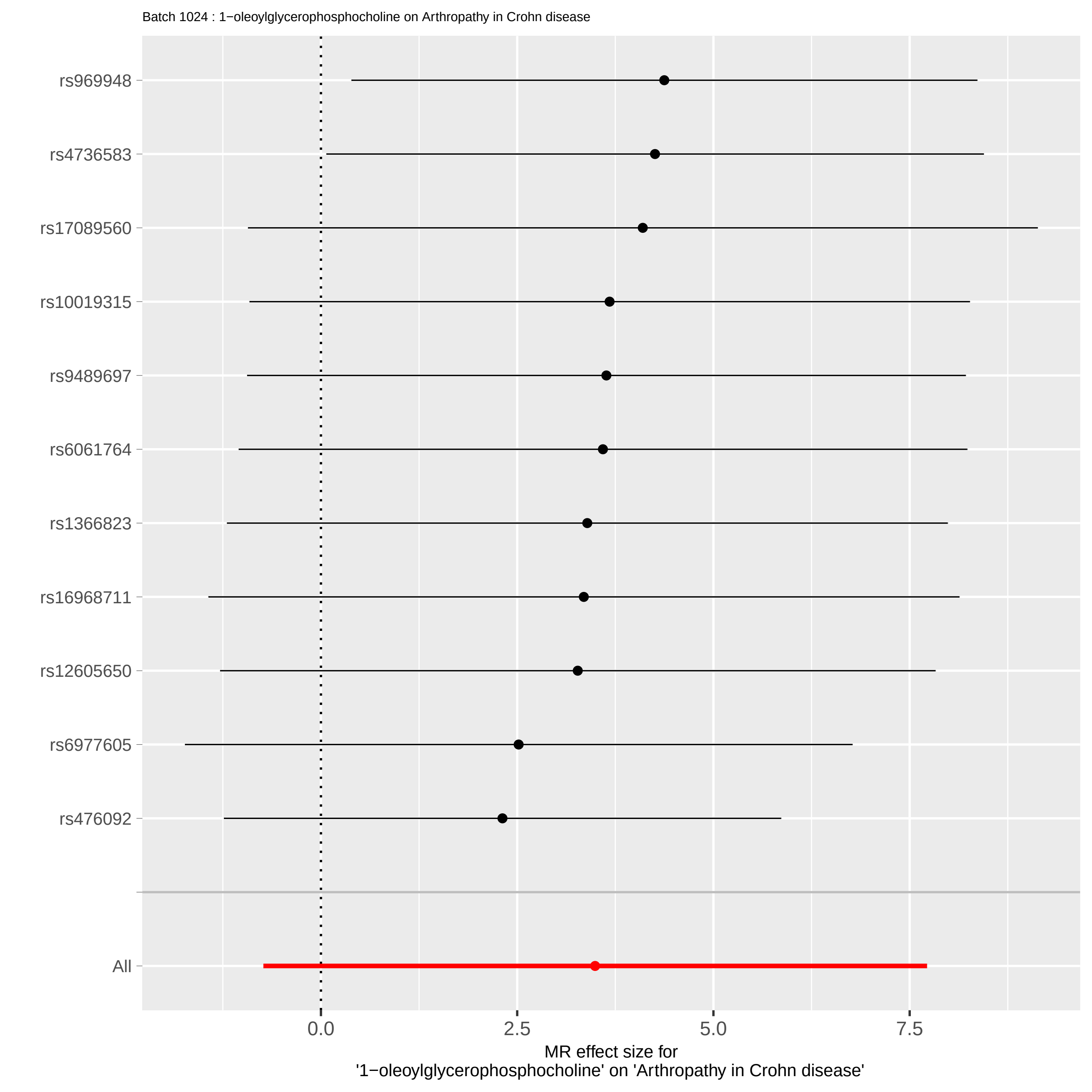

Batch 1024 : 1−oleoylglycerophosphocholine on Arthropathy in Crohn disease
rs969948
rs4736583
rs17089560
rs10019315
rs9489697
rs6061764
rs1366823
rs16968711
rs12605650
rs6977605
rs476092
All
0.0
2.5
5.0
7.5
MR effect size for
'1−oleoylglycerophosphocholine' on 'Arthropathy in Crohn disease'

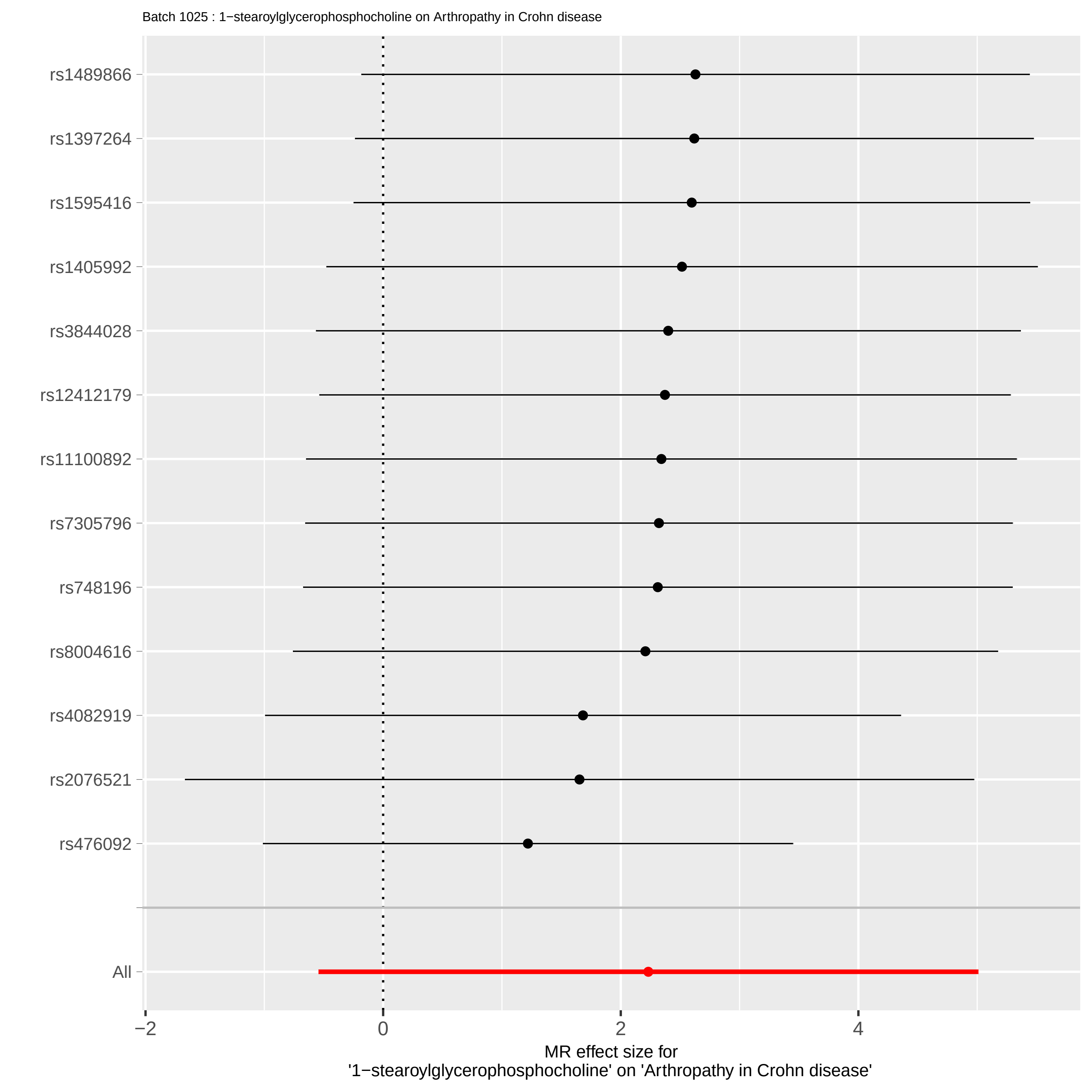
Batch 1025 : 1−stearoylglycerophosphocholine on Arthropathy in Crohn disease
rs1489866
rs1397264
rs1595416
rs1405992
rs3844028
rs12412179
rs11100892
rs7305796
rs748196
rs8004616
rs4082919
rs2076521
rs476092
All
−2
0
2
4
MR effect size for
'1−stearoylglycerophosphocholine' on 'Arthropathy in Crohn disease'

Batch 1028 : 10−heptadecenoate (17:1n7) on Arthropathy in Crohn disease

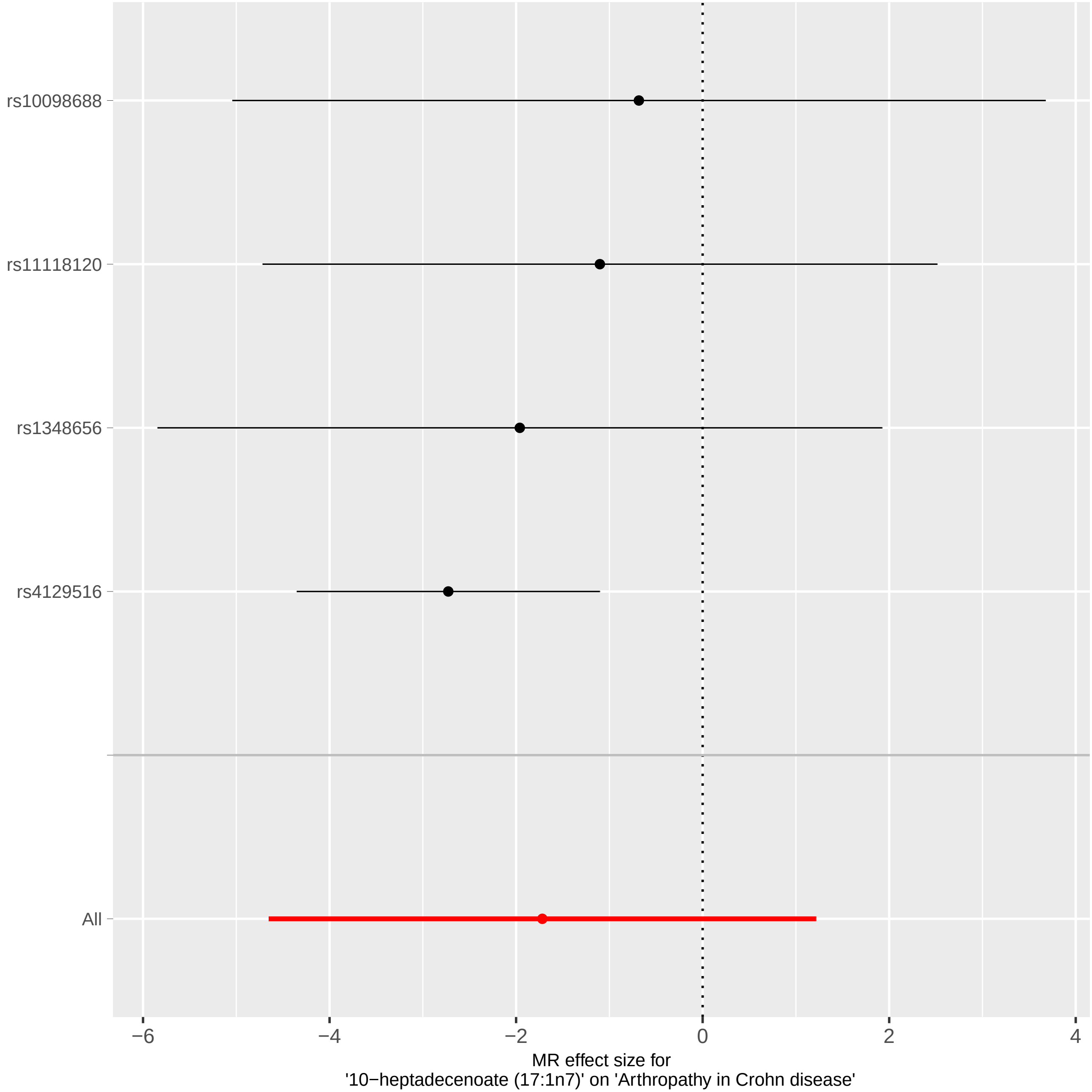

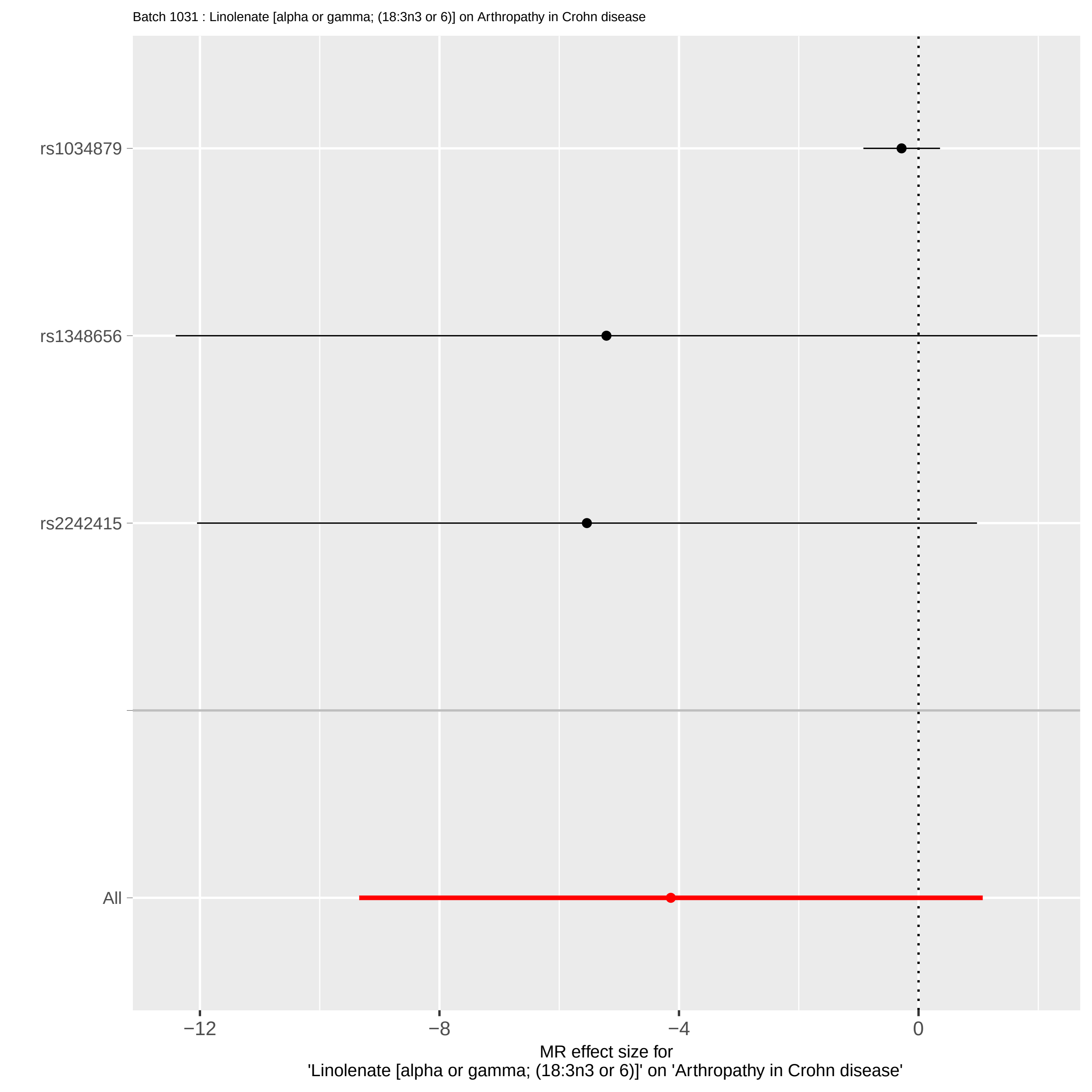
Batch 1031 : Linolenate [alpha or gamma; (18:3n3 or 6)] on Arthropathy in Crohn disease
rs1034879
rs1348656
rs2242415
All
−12
−8
−4
0
MR effect size for
'Linolenate [alpha or gamma; (18:3n3 or 6)]' on 'Arthropathy in Crohn disease'

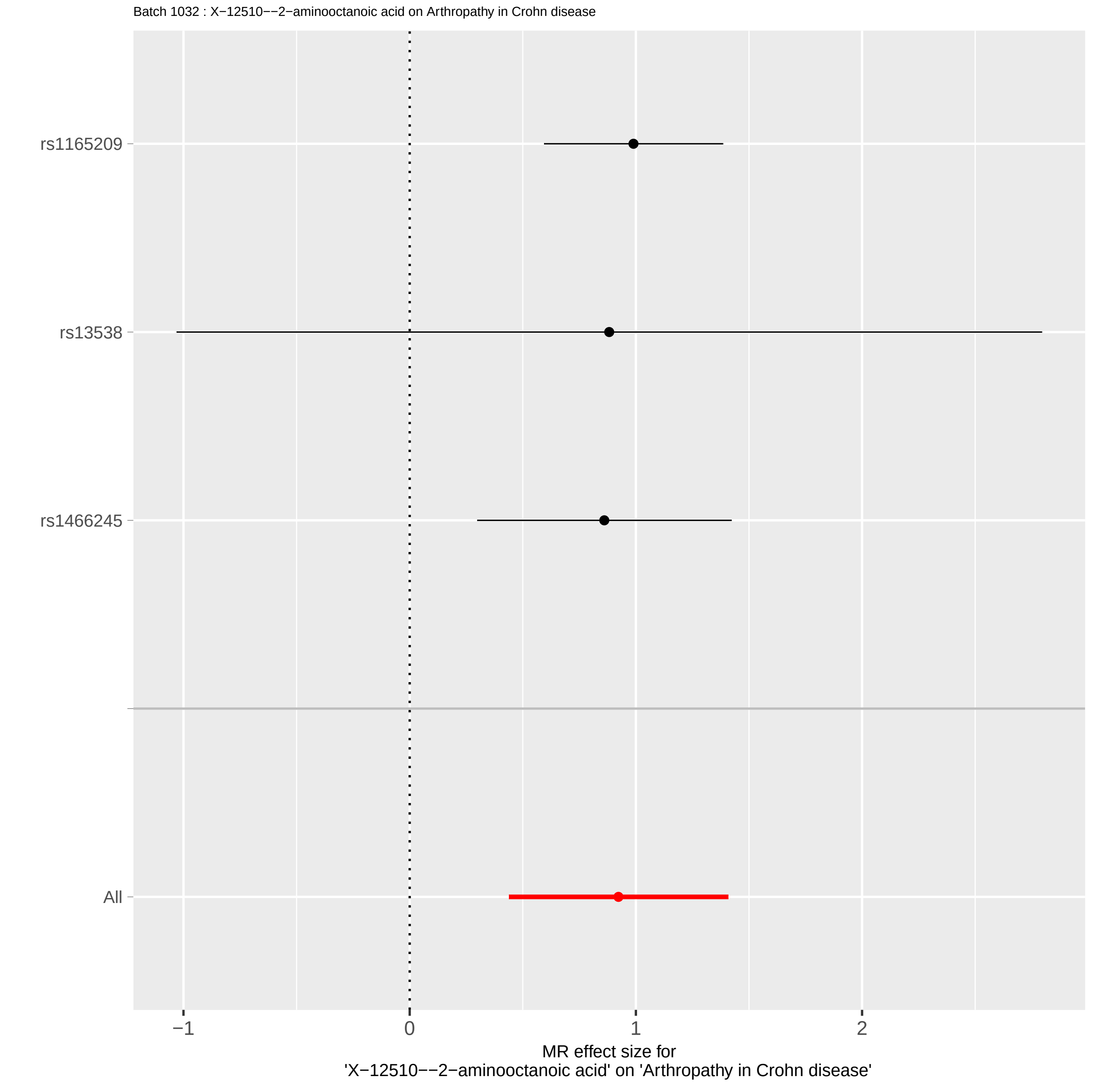
Batch 1032 : X−12510−−2−aminooctanoic acid on Arthropathy in Crohn disease
rs1165209
rs13538
rs1466245
All
−1
0
1
2
MR effect size for
'X−12510−−2−aminooctanoic acid' on 'Arthropathy in Crohn disease'

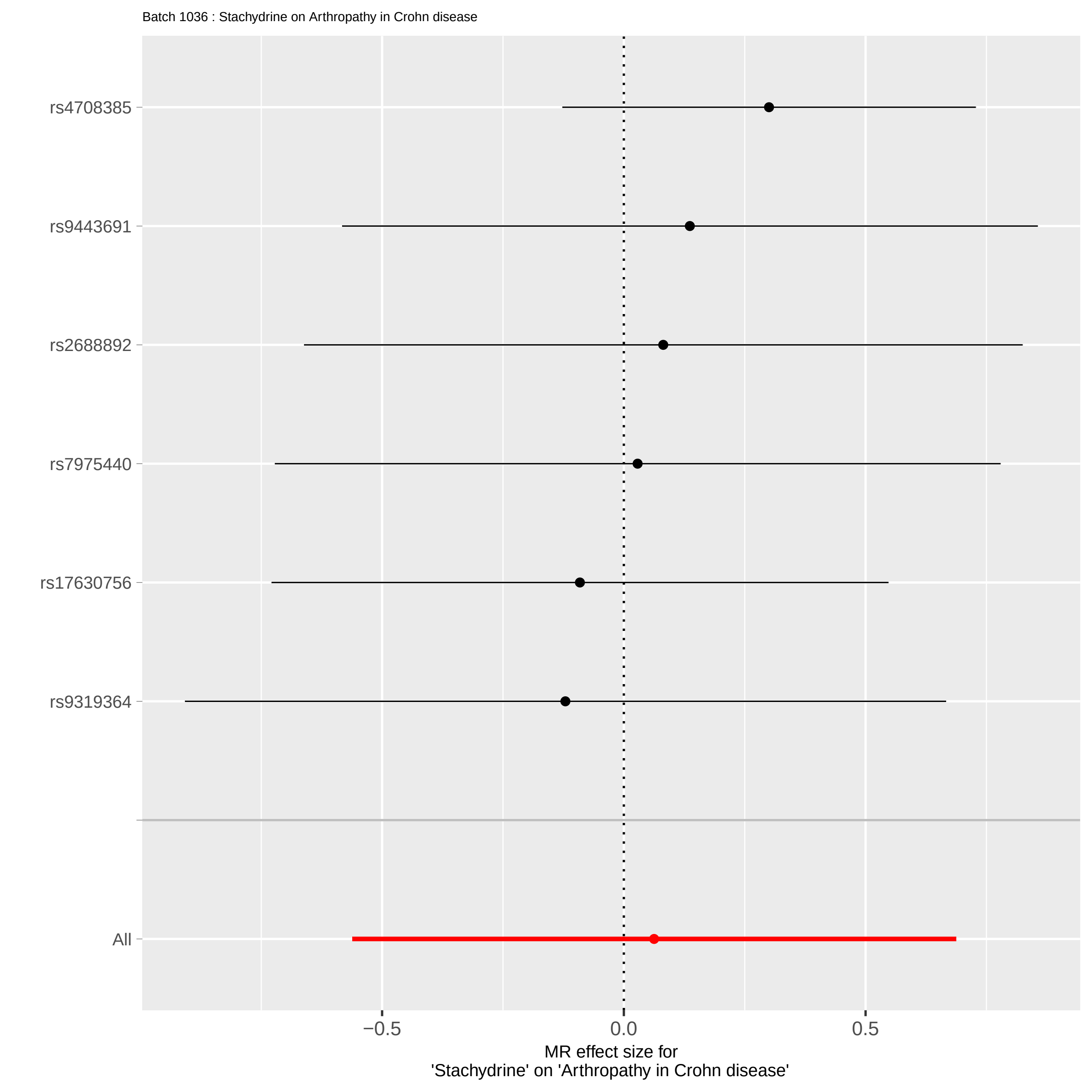
Batch 1036 : Stachydrine on Arthropathy in Crohn disease
rs4708385
rs9443691
rs2688892
rs7975440
rs17630756
rs9319364
All
−0.5
0.0
0.5
MR effect size for
'Stachydrine' on 'Arthropathy in Crohn disease'

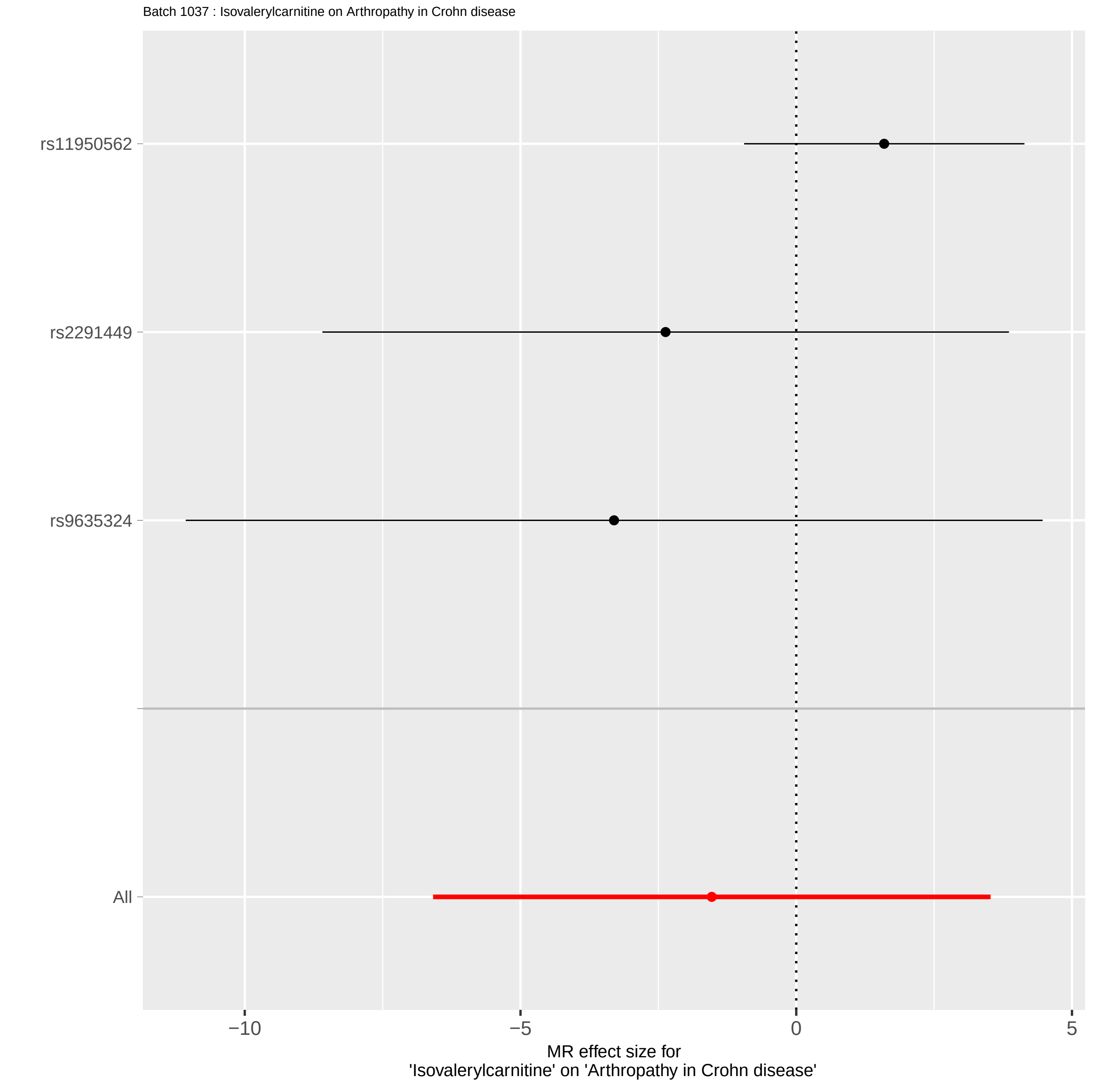

Batch 1037 : Isovalerylcarnitine on Arthropathy in Crohn disease
rs11950562
rs2291449
rs9635324
All
−10
−5
0
5
MR effect size for
'Isovalerylcarnitine' on 'Arthropathy in Crohn disease'

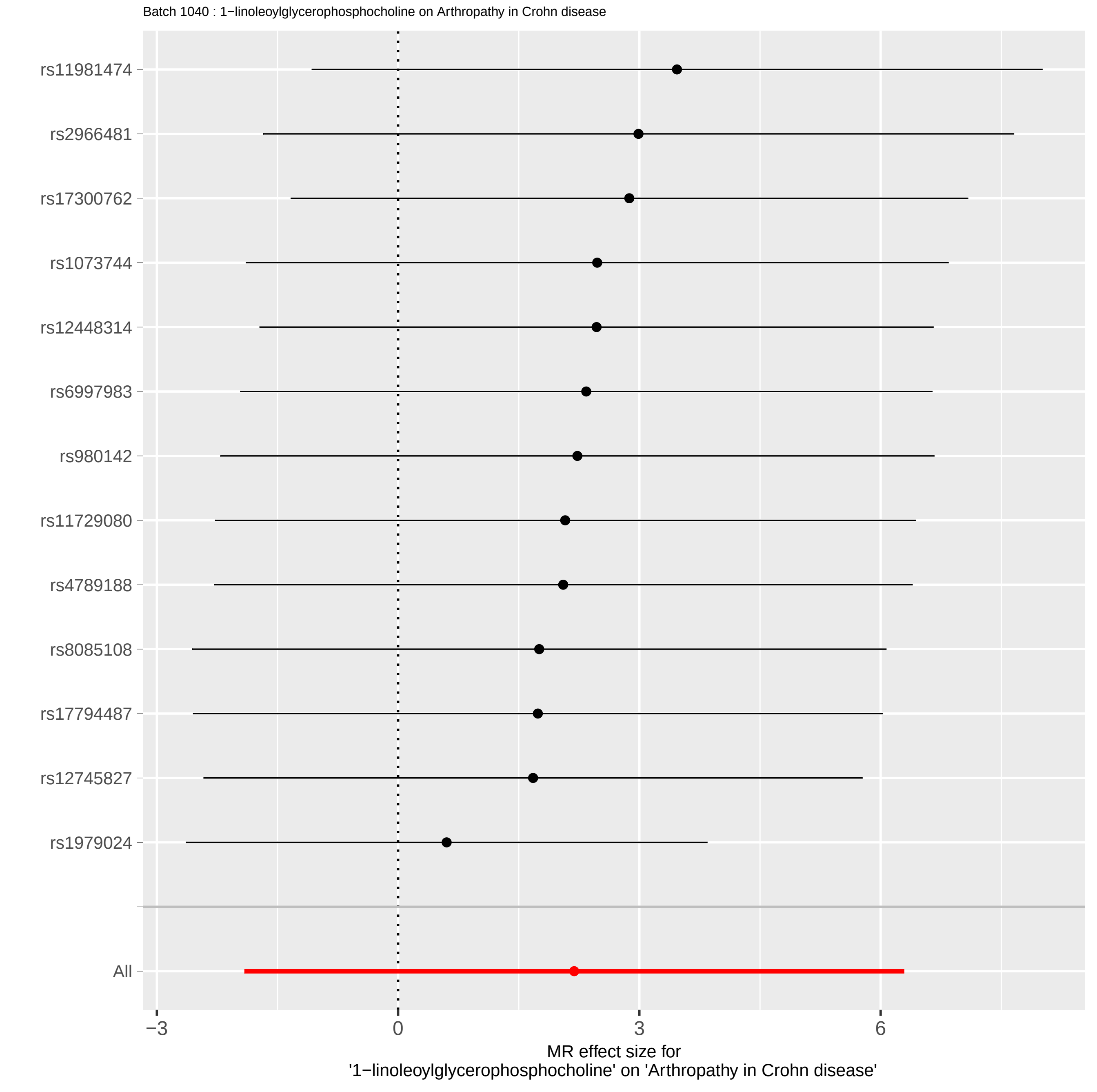

Batch 1040 : 1−linoleoylglycerophosphocholine on Arthropathy in Crohn disease
rs11981474
rs2966481
rs17300762
rs1073744
rs12448314
rs6997983
rs980142
rs11729080
rs4789188
rs8085108
rs17794487
rs12745827
rs1979024
All
−3
0
3
6
MR effect size for
'1−linoleoylglycerophosphocholine' on 'Arthropathy in Crohn disease'

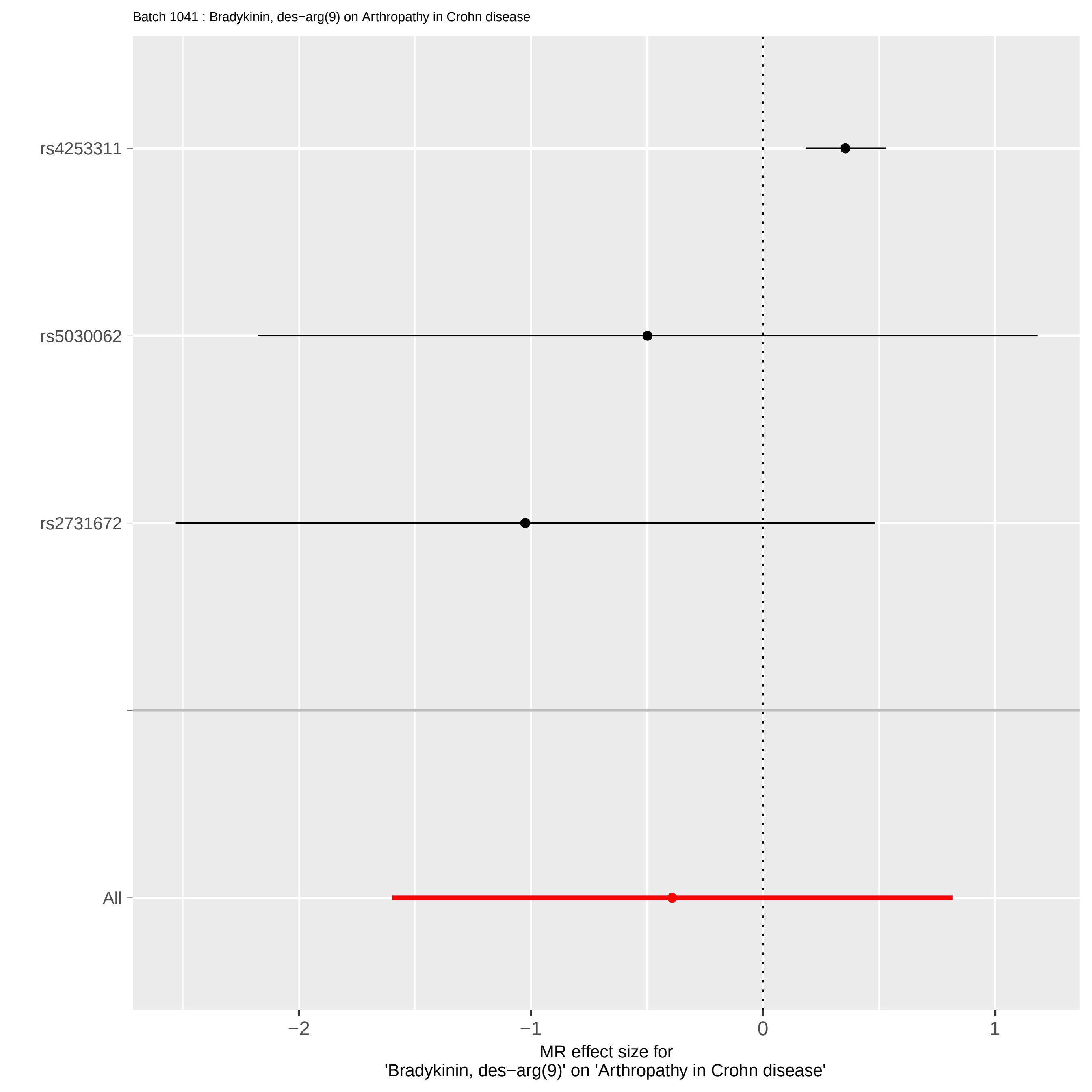

Batch 1041 : Bradykinin, des−arg(9) on Arthropathy in Crohn disease
rs4253311
rs5030062
rs2731672
All
−2
−1
0
1
MR effect size for
'Bradykinin, des−arg(9)' on 'Arthropathy in Crohn disease'

Batch 1042 : Gamma−glutamylisoleucine* on Arthropathy in Crohn disease

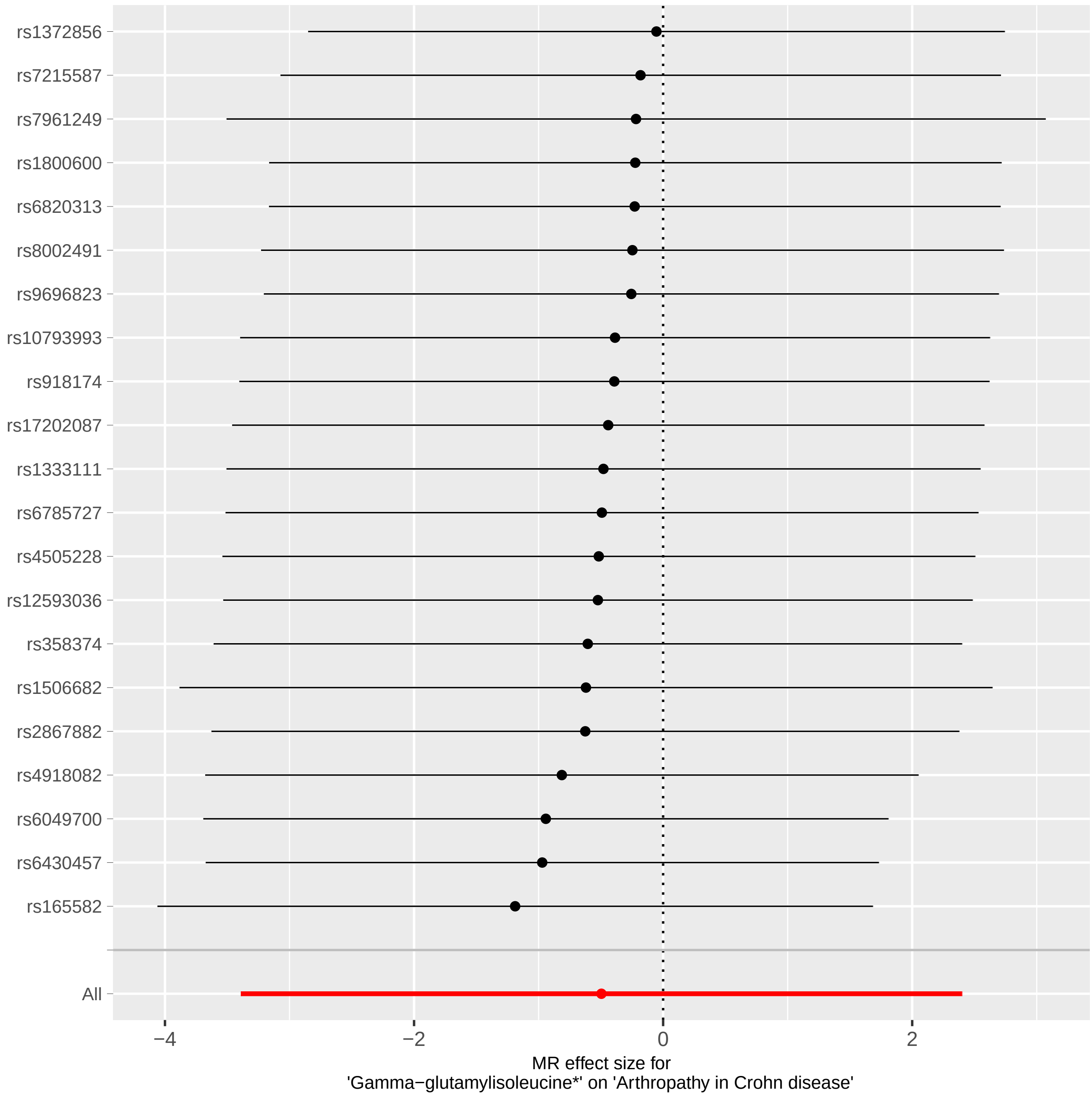

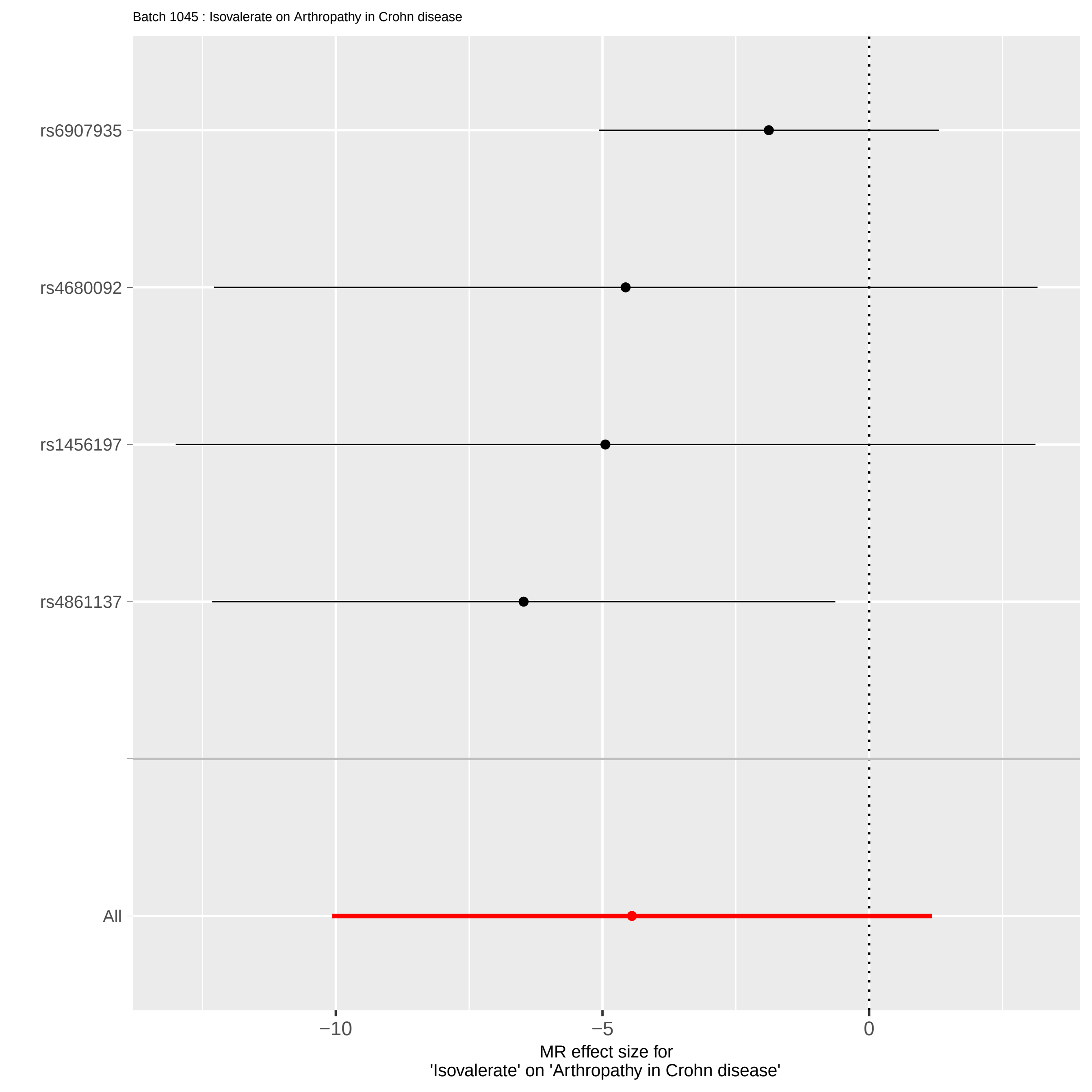

Batch 1045 : Isovalerate on Arthropathy in Crohn disease
rs6907935
rs4680092
rs1456197
rs4861137
All
−10
−5
0
MR effect size for
'Isovalerate' on 'Arthropathy in Crohn disease'

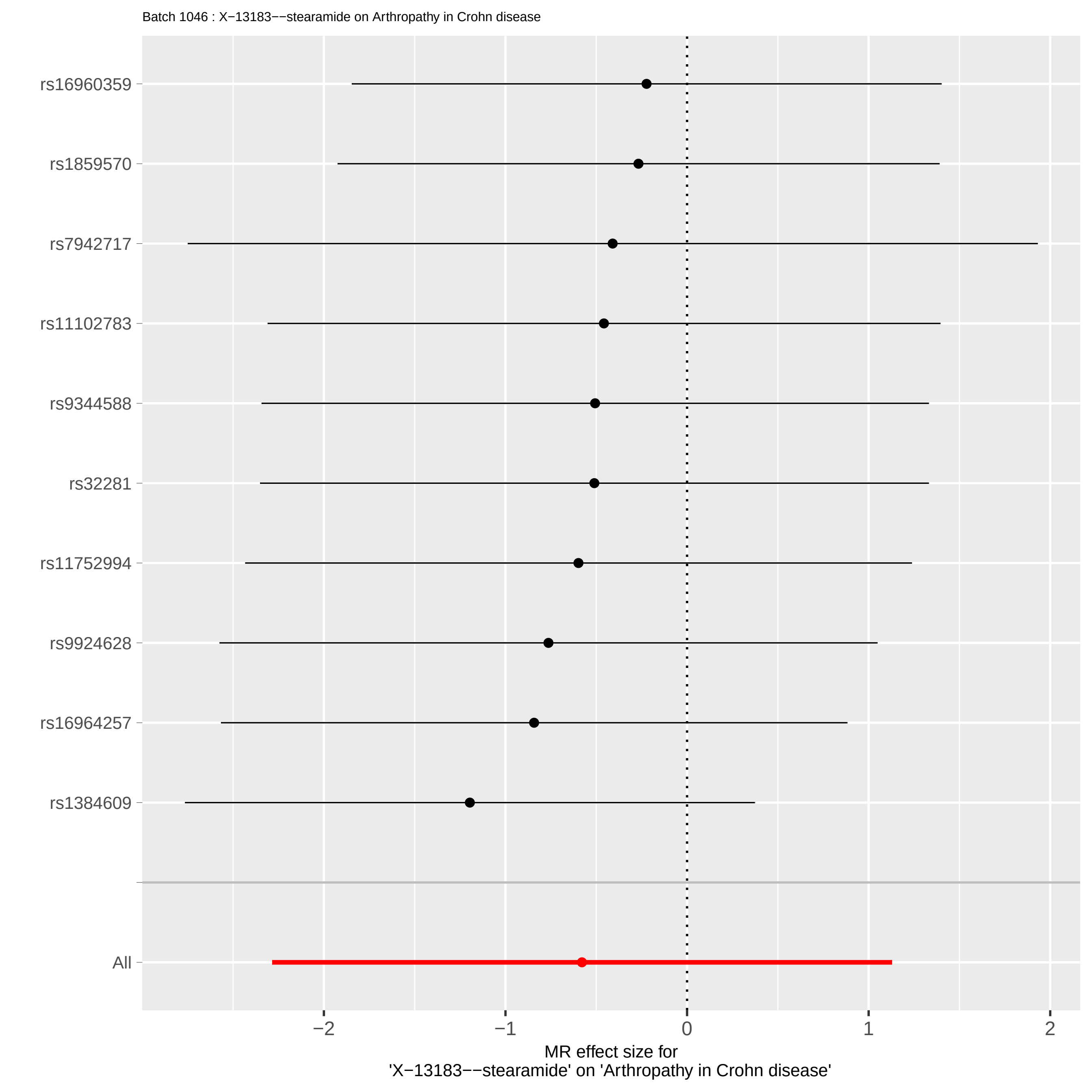
Batch 1046 : X−13183−−stearamide on Arthropathy in Crohn disease
rs16960359
rs1859570
rs7942717
rs11102783
rs9344588
rs32281
rs11752994
rs9924628
rs16964257
rs1384609
All
−2
−1
0
1
2
MR effect size for
'X−13183−−stearamide' on 'Arthropathy in Crohn disease'

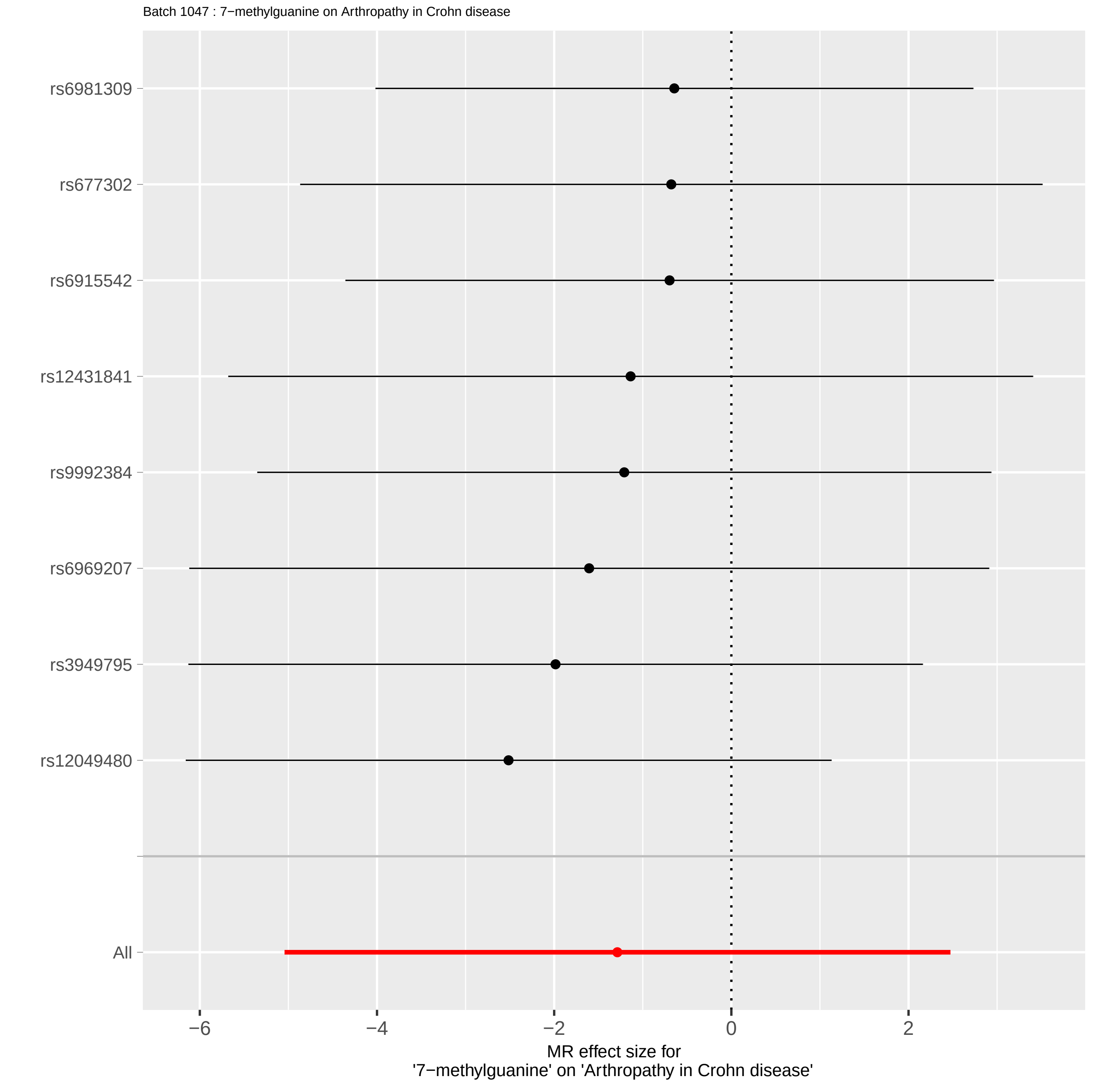

Batch 1047 : 7−methylguanine on Arthropathy in Crohn disease
rs6981309
rs677302
rs6915542
rs12431841
rs9992384
rs6969207
rs3949795
rs12049480
All
−6
−4
−2
0
2
MR effect size for
'7−methylguanine' on 'Arthropathy in Crohn disease'

Batch 1055 : 2−palmitoylglycerophosphocholine* on Arthropathy in Crohn disease

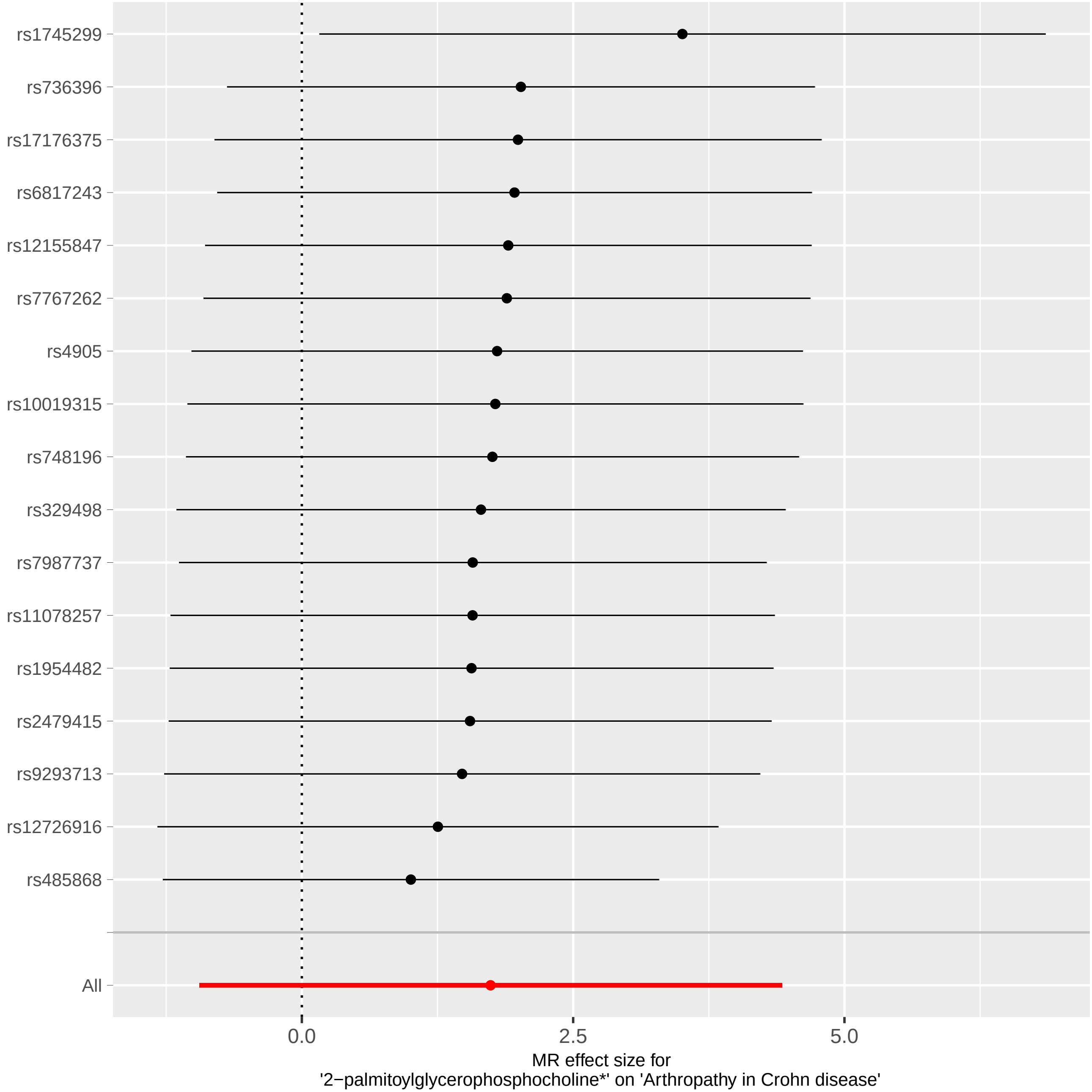

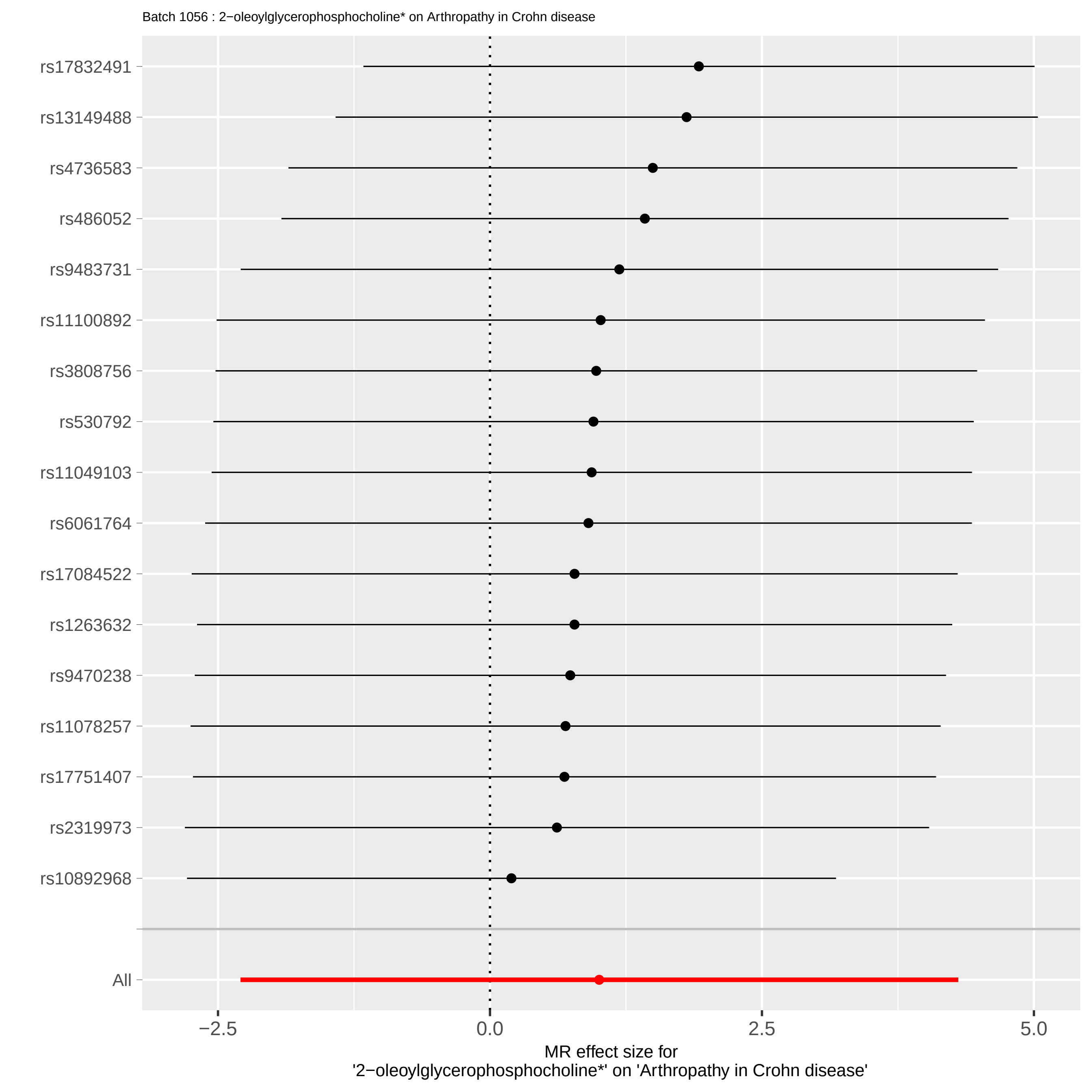

Batch 1056 : 2−oleoylglycerophosphocholine* on Arthropathy in Crohn disease
rs17832491
rs13149488
rs4736583
rs486052
rs9483731
rs11100892
rs3808756
rs530792
rs11049103
rs6061764
rs17084522
rs1263632
rs9470238
rs11078257
rs17751407
rs2319973
rs10892968
All
−2.5
0.0
2.5
5.0
MR effect size for
'2−oleoylglycerophosphocholine*' on 'Arthropathy in Crohn disease'

Batch 1059 : 1−palmitoylglycerophosphoinositol* on Arthropathy in Crohn disease

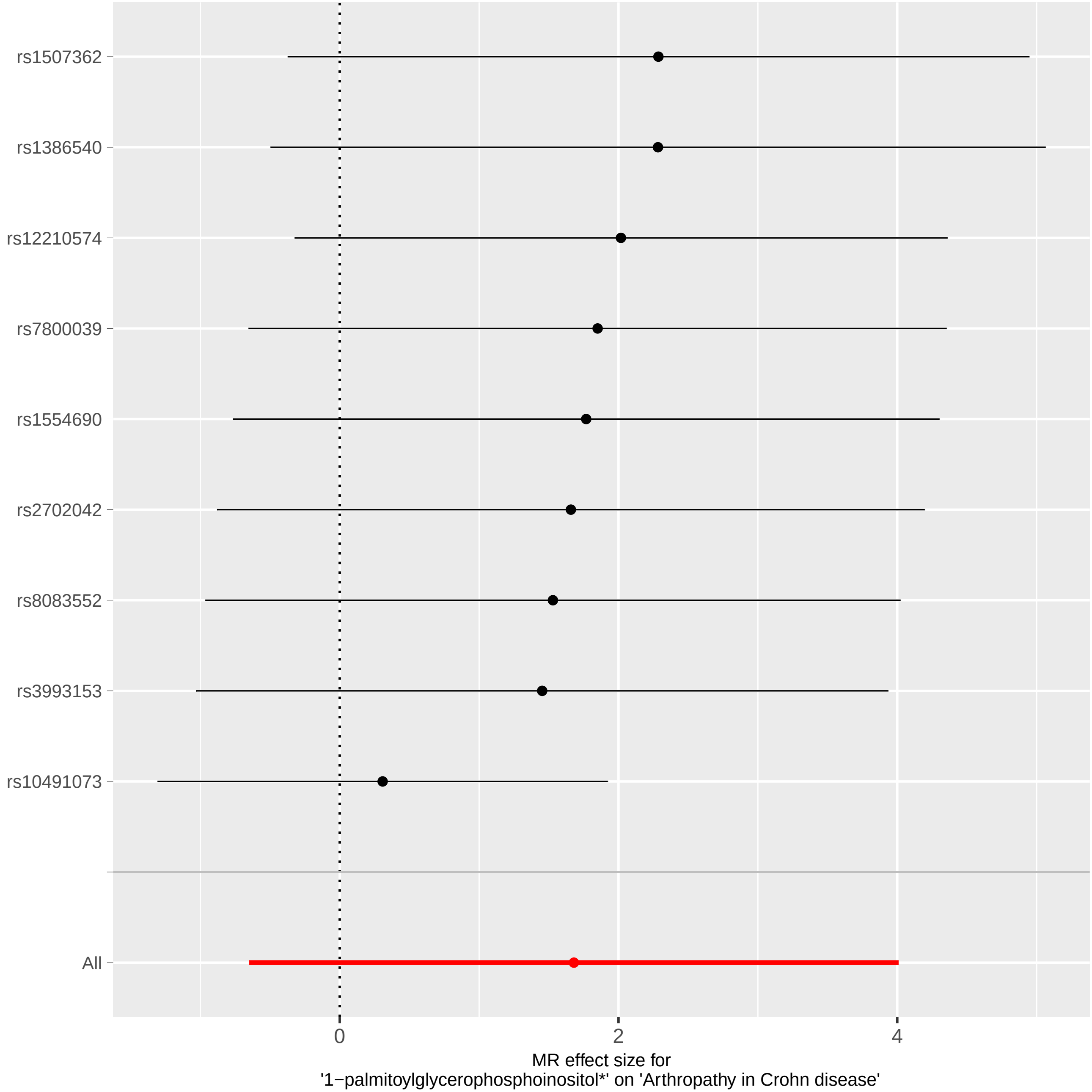

Batch 1062 : Glutaroyl carnitine on Arthropathy in Crohn disease

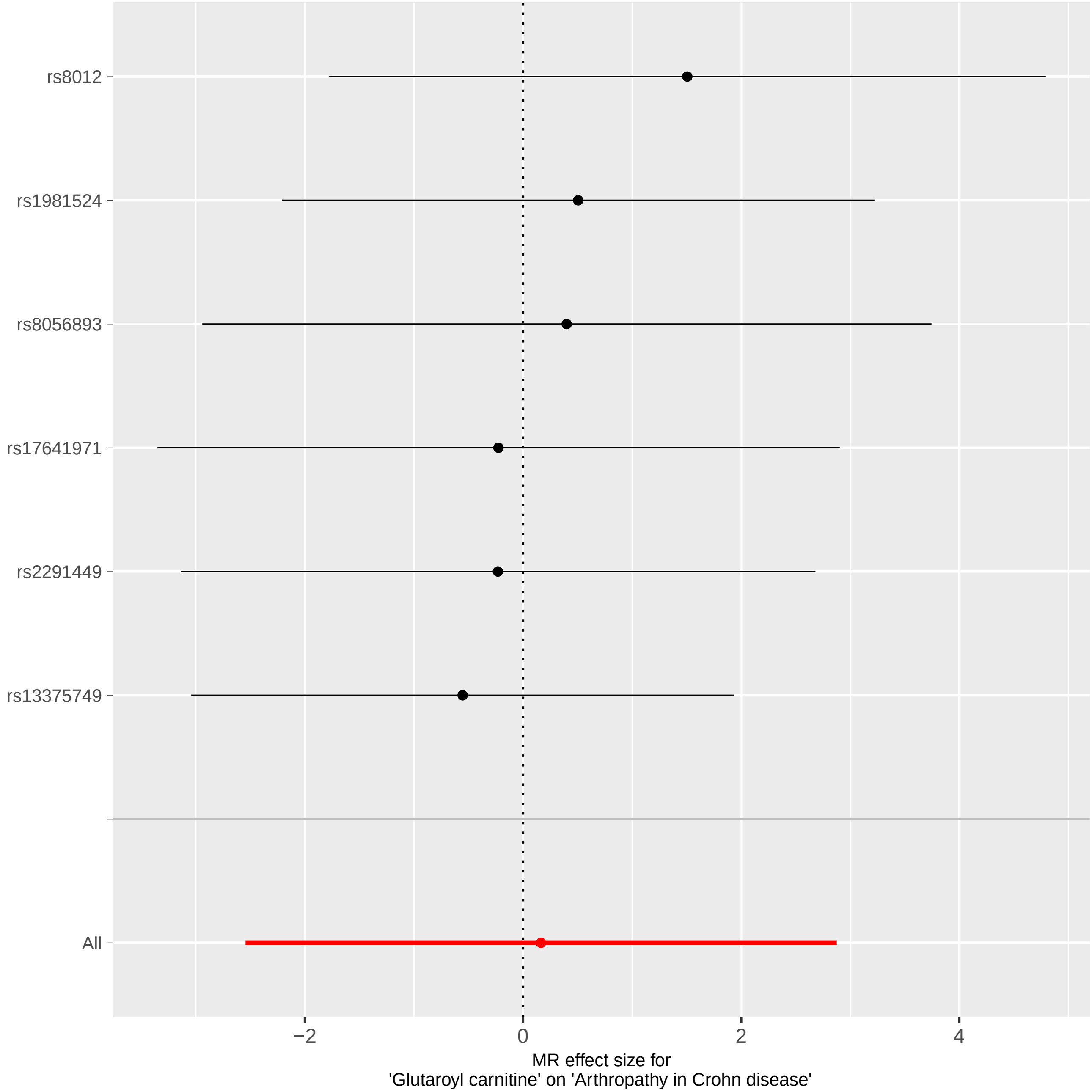

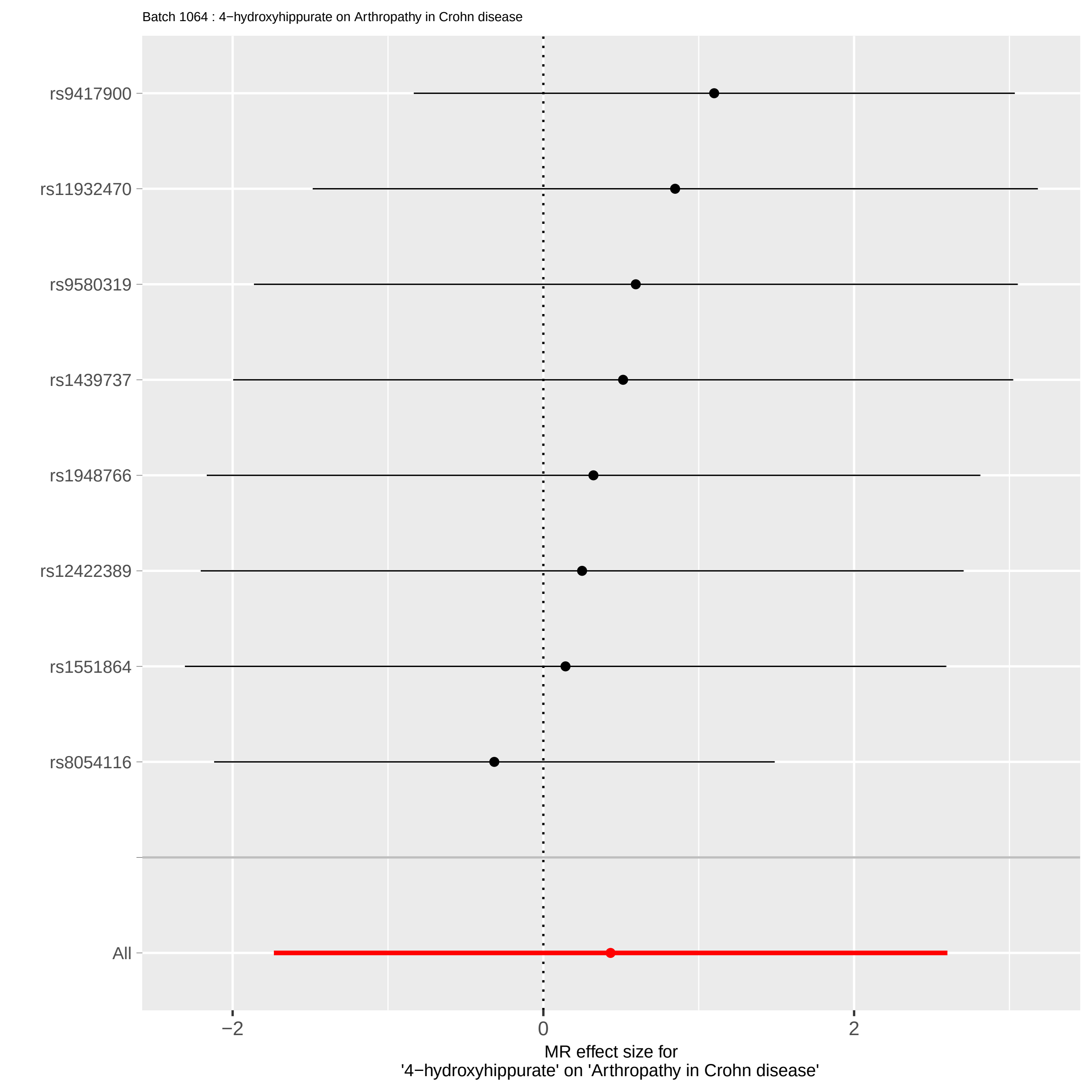

Batch 1064 : 4−hydroxyhippurate on Arthropathy in Crohn disease
rs9417900
rs11932470
rs9580319
rs1439737
rs1948766
rs12422389
rs1551864
rs8054116
All
−2
0
2
MR effect size for
'4−hydroxyhippurate' on 'Arthropathy in Crohn disease'

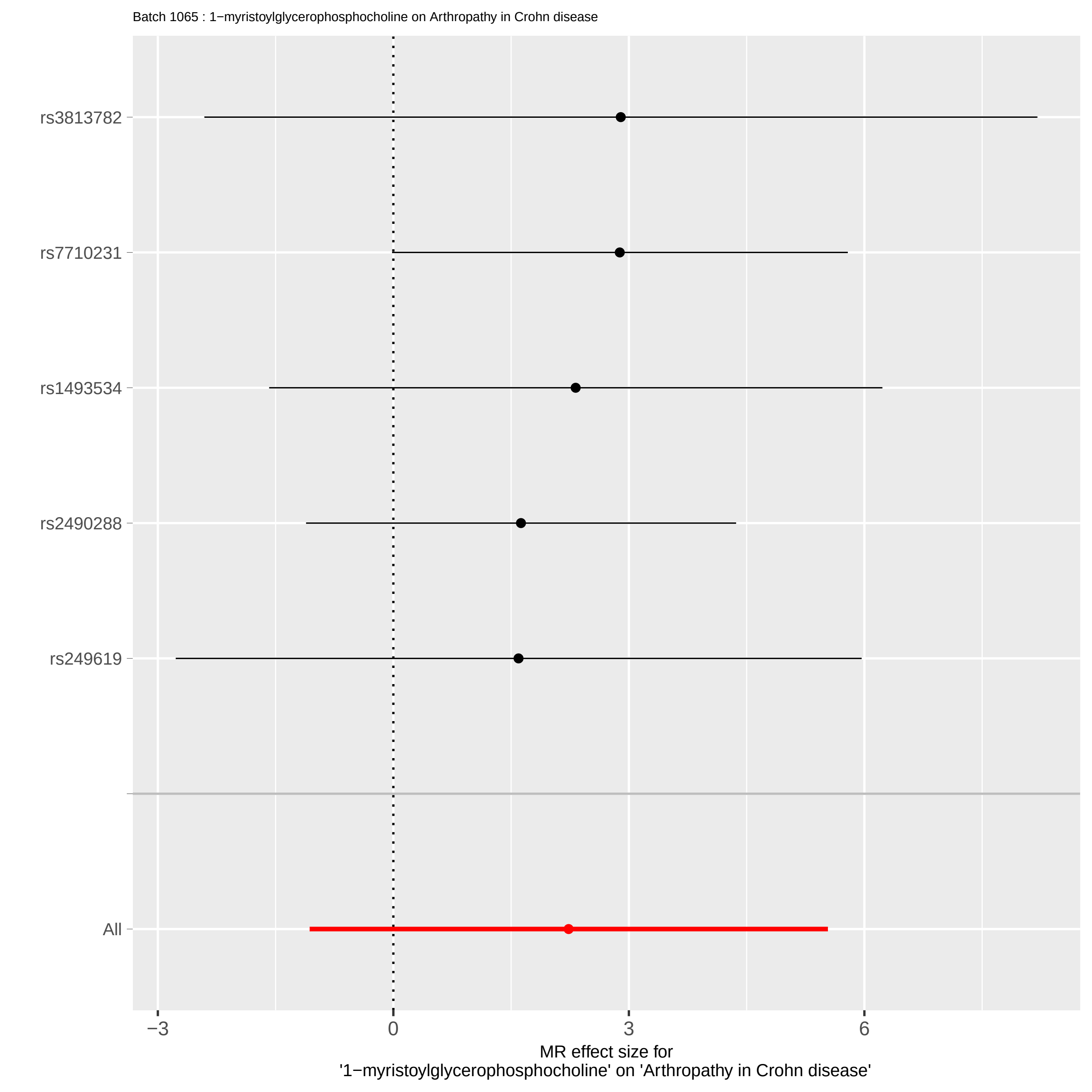

Batch 1065 : 1−myristoylglycerophosphocholine on Arthropathy in Crohn disease
rs3813782
rs7710231
rs1493534
rs2490288
rs249619
All
−3
0
3
6
MR effect size for
'1−myristoylglycerophosphocholine' on 'Arthropathy in Crohn disease'

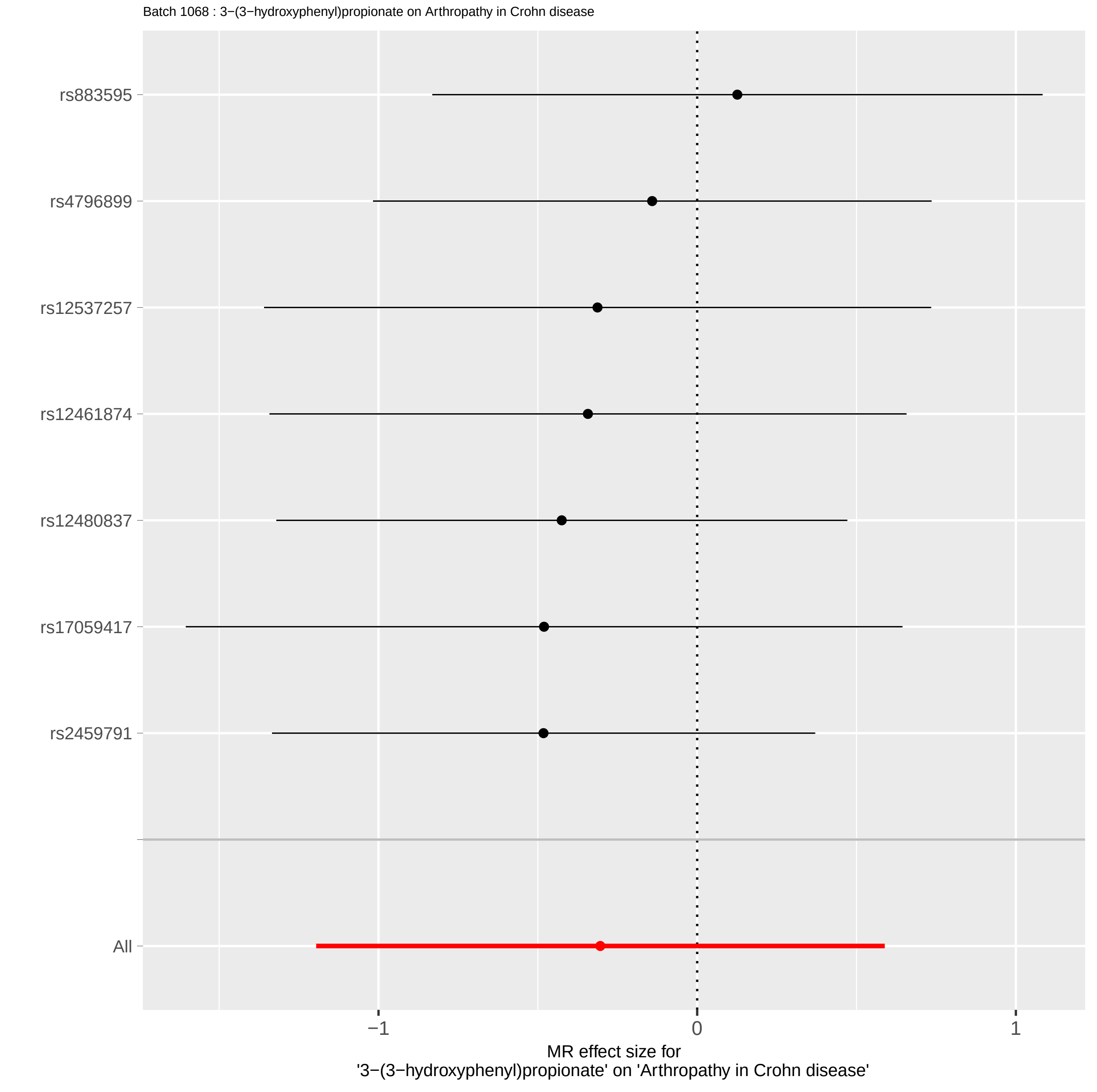

Batch 1068 : 3−(3−hydroxyphenyl)propionate on Arthropathy in Crohn disease
rs883595
rs4796899
rs12537257
rs12461874
rs12480837
rs17059417
rs2459791
All
−1
0
1
MR effect size for
'3−(3−hydroxyphenyl)propionate' on 'Arthropathy in Crohn disease'

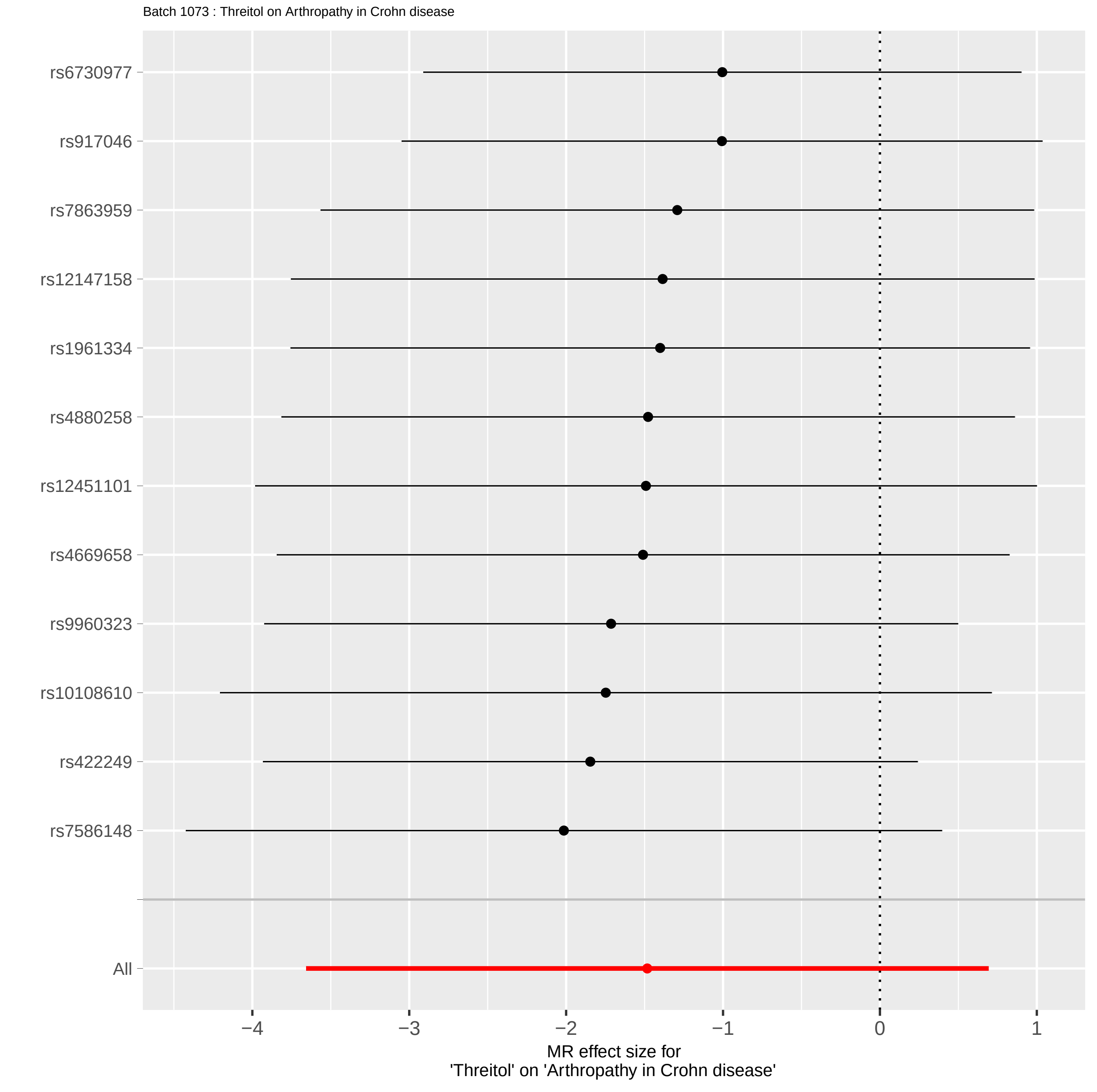
Batch 1073 : Threitol on Arthropathy in Crohn disease
rs6730977
rs917046
rs7863959
rs12147158
rs1961334
rs4880258
rs12451101
rs4669658
rs9960323
rs10108610
rs422249
rs7586148
All
−4
−3
−2
−1
0
1
MR effect size for
'Threitol' on 'Arthropathy in Crohn disease'

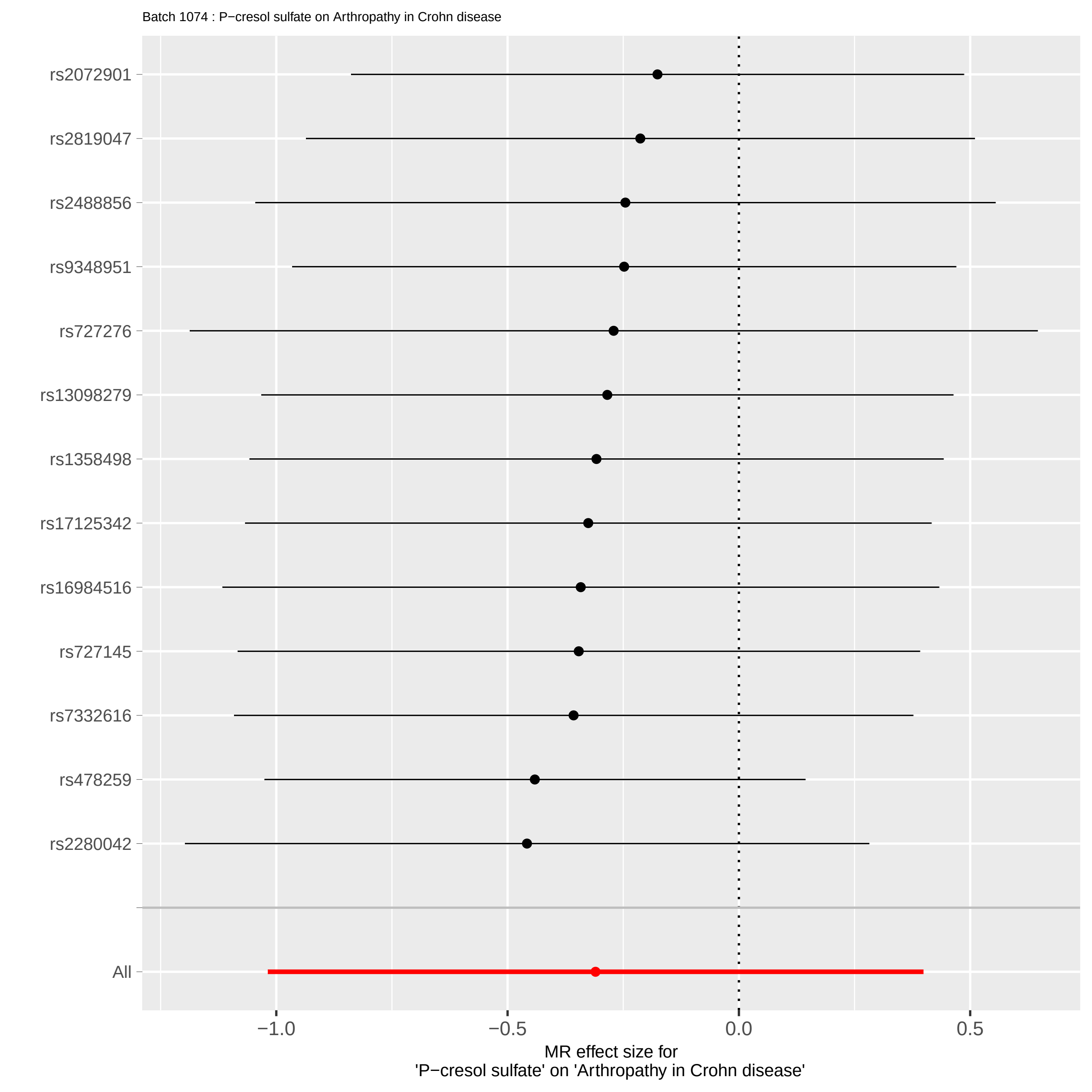

Batch 1074 : P−cresol sulfate on Arthropathy in Crohn disease
rs2072901
rs2819047
rs2488856
rs9348951
rs727276
rs13098279
rs1358498
rs17125342
rs16984516
rs727145
rs7332616
rs478259
rs2280042
All
−1.0
−0.5
0.0
0.5
MR effect size for
'P−cresol sulfate' on 'Arthropathy in Crohn disease'

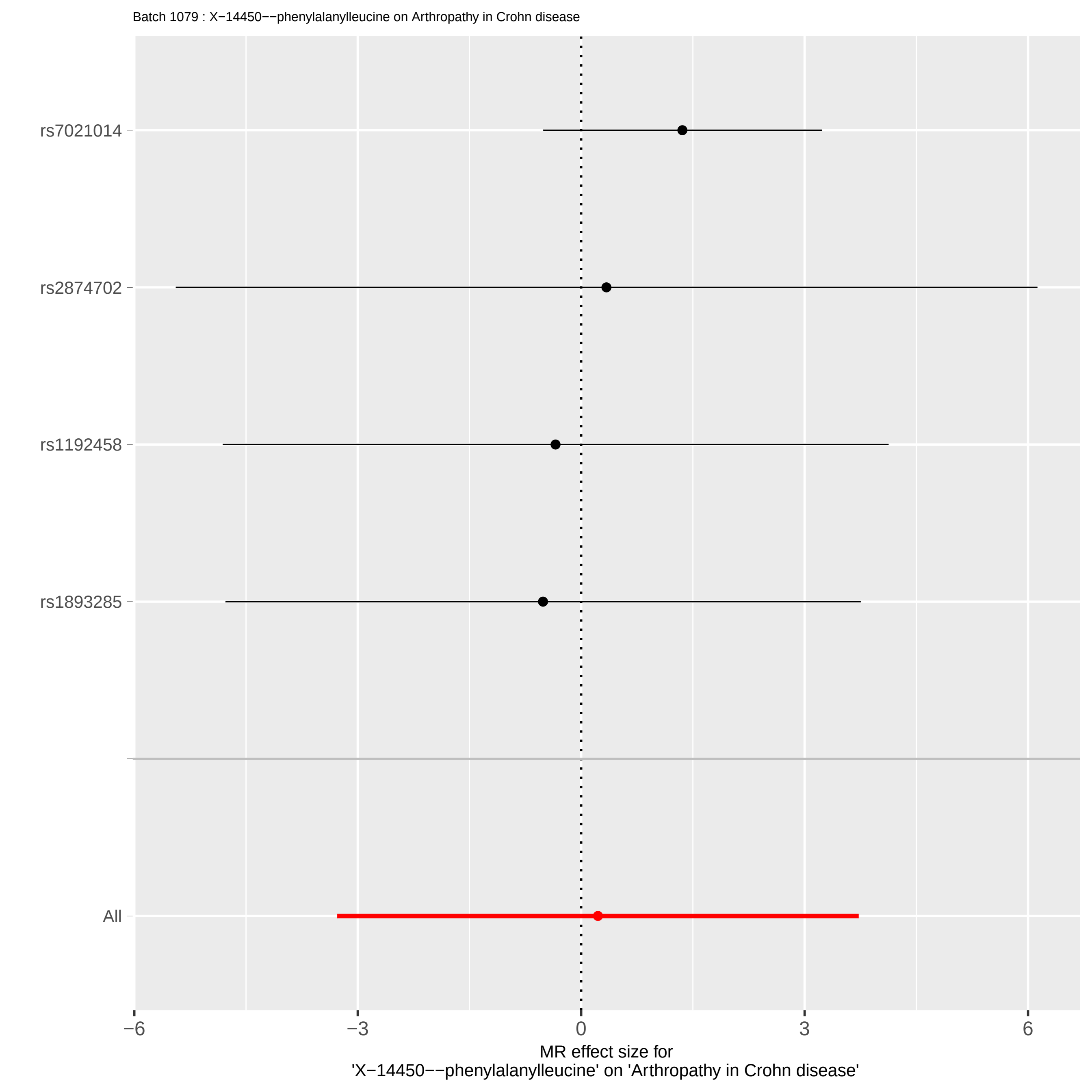

Batch 1079 : X−14450−−phenylalanylleucine on Arthropathy in Crohn disease
rs7021014
rs2874702
rs1192458
rs1893285
All
−6
−3
0
3
6
MR effect size for
'X−14450−−phenylalanylleucine' on 'Arthropathy in Crohn disease'

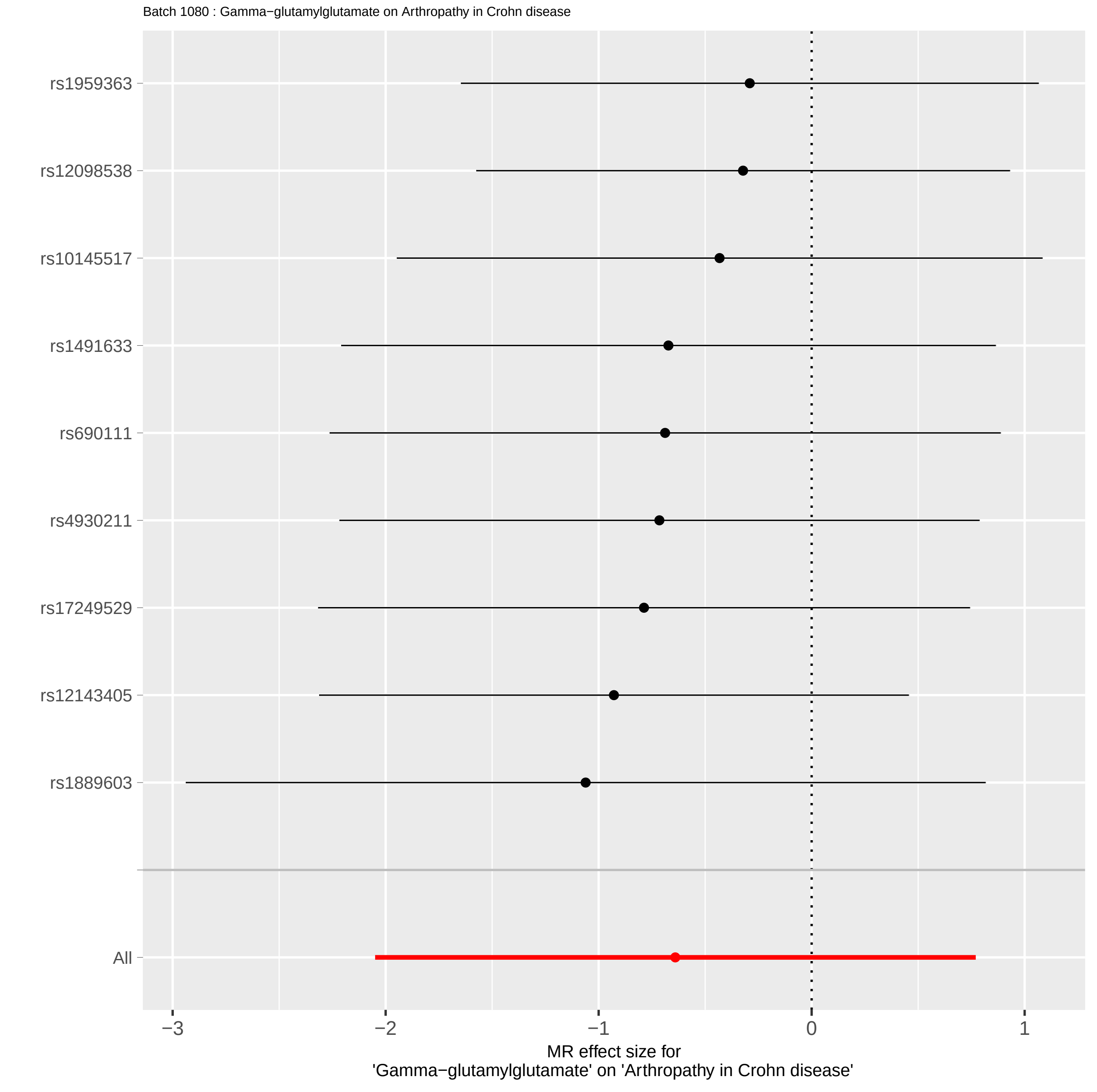
Batch 1080 : Gamma−glutamylglutamate on Arthropathy in Crohn disease
rs1959363
rs12098538
rs10145517
rs1491633
rs690111
rs4930211
rs17249529
rs12143405
rs1889603
All
−3
−2
−1
0
1
MR effect size for
'Gamma−glutamylglutamate' on 'Arthropathy in Crohn disease'

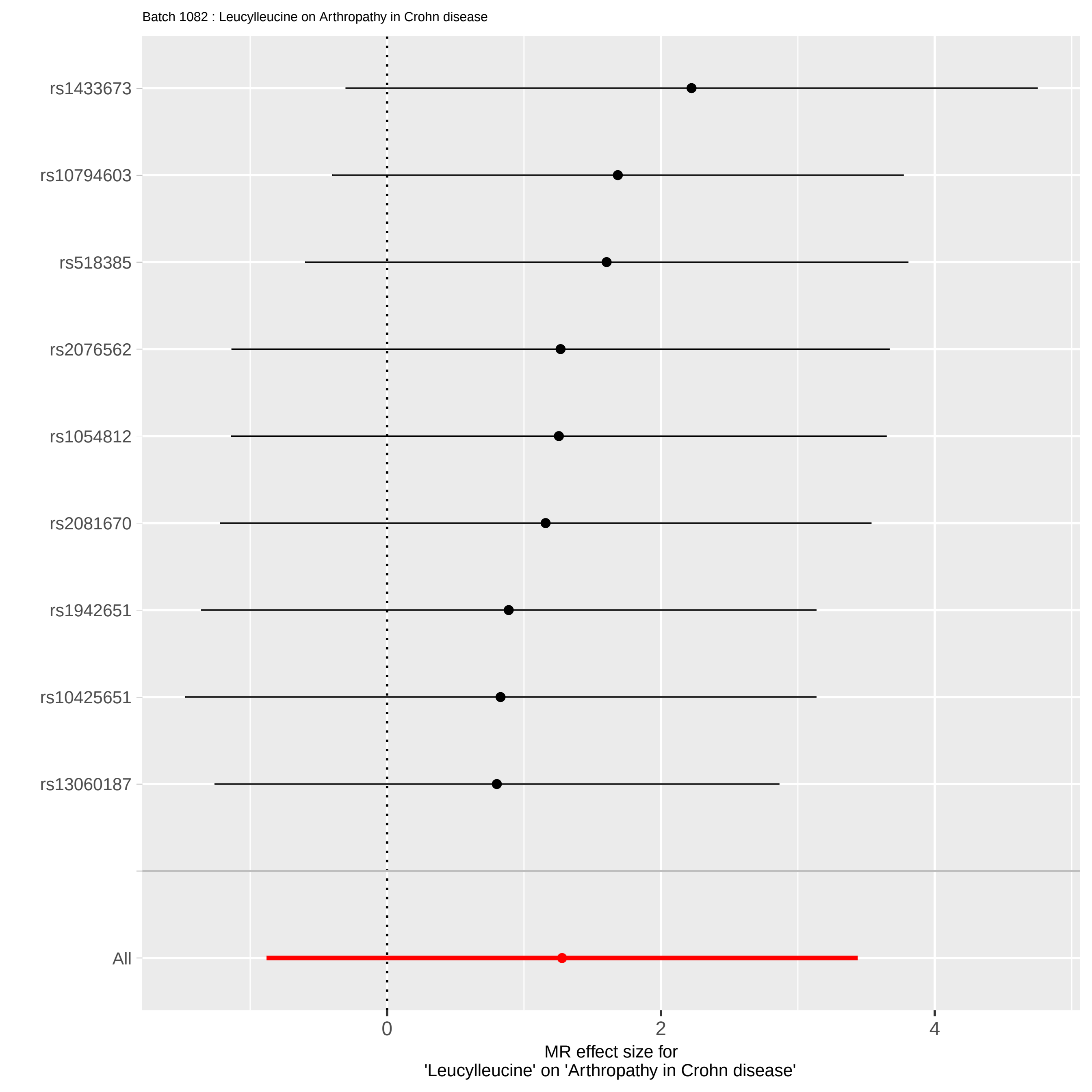

Batch 1082 : Leucylleucine on Arthropathy in Crohn disease
rs1433673
rs10794603
rs518385
rs2076562
rs1054812
rs2081670
rs1942651
rs10425651
rs13060187
All
0
2
4
MR effect size for
'Leucylleucine' on 'Arthropathy in Crohn disease'

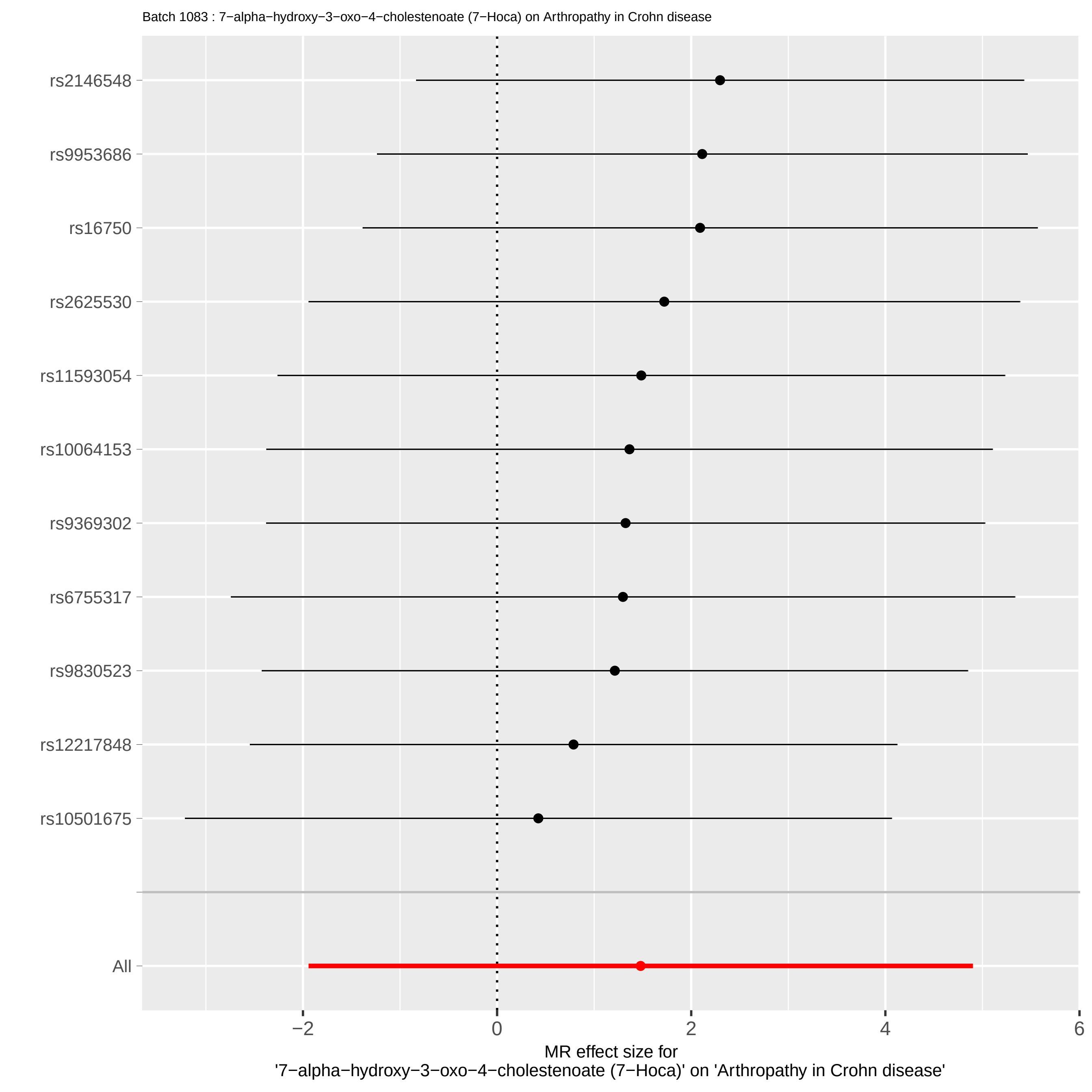
Batch 1083 : 7−alpha−hydroxy−3−oxo−4−cholestenoate (7−Hoca) on Arthropathy in Crohn disease
rs2146548
rs9953686
rs16750
rs2625530
rs11593054
rs10064153
rs9369302
rs6755317
rs9830523
rs12217848
rs10501675
All
−2
0
2
4
6
MR effect size for
'7−alpha−hydroxy−3−oxo−4−cholestenoate (7−Hoca)' on 'Arthropathy in Crohn disease'

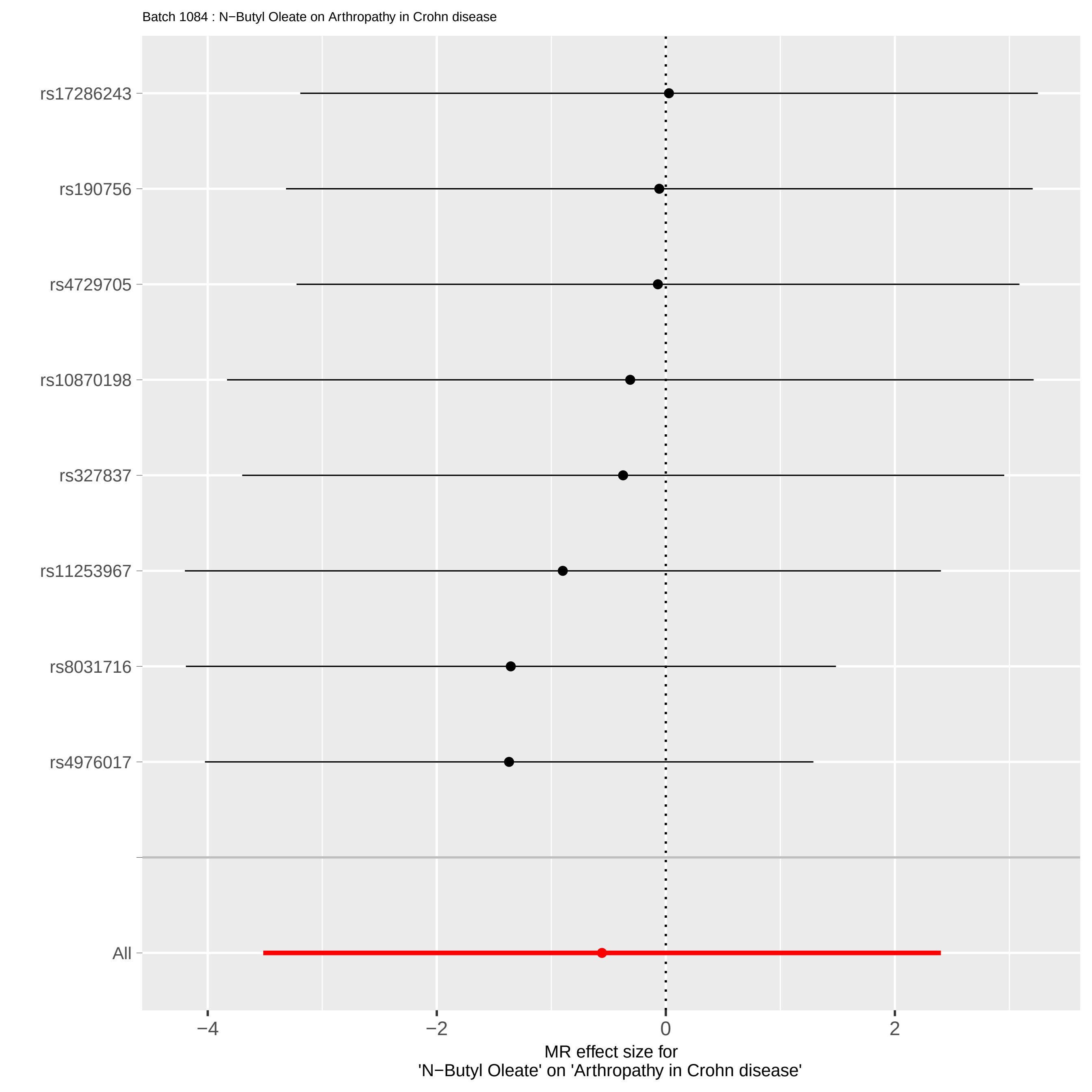
Batch 1084 : N−Butyl Oleate on Arthropathy in Crohn disease
rs17286243
rs190756
rs4729705
rs10870198
rs327837
rs11253967
rs8031716
rs4976017
All
−4
−2
0
2
MR effect size for
'N−Butyl Oleate' on 'Arthropathy in Crohn disease'

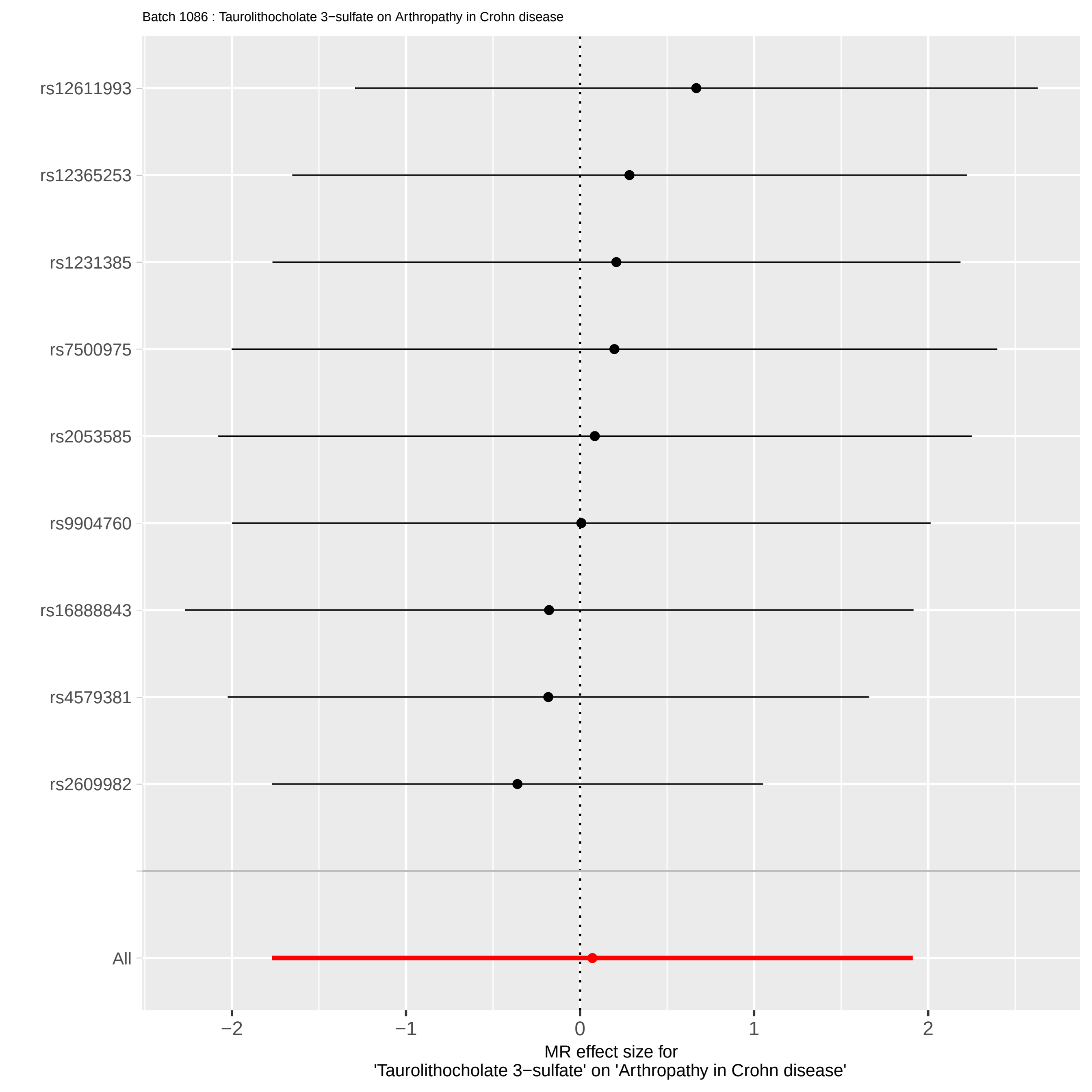

Batch 1086 : Taurolithocholate 3−sulfate on Arthropathy in Crohn disease
rs12611993
rs12365253
rs1231385
rs7500975
rs2053585
rs9904760
rs16888843
rs4579381
rs2609982
All
−2
−1
0
1
2
MR effect size for
'Taurolithocholate 3−sulfate' on 'Arthropathy in Crohn disease'

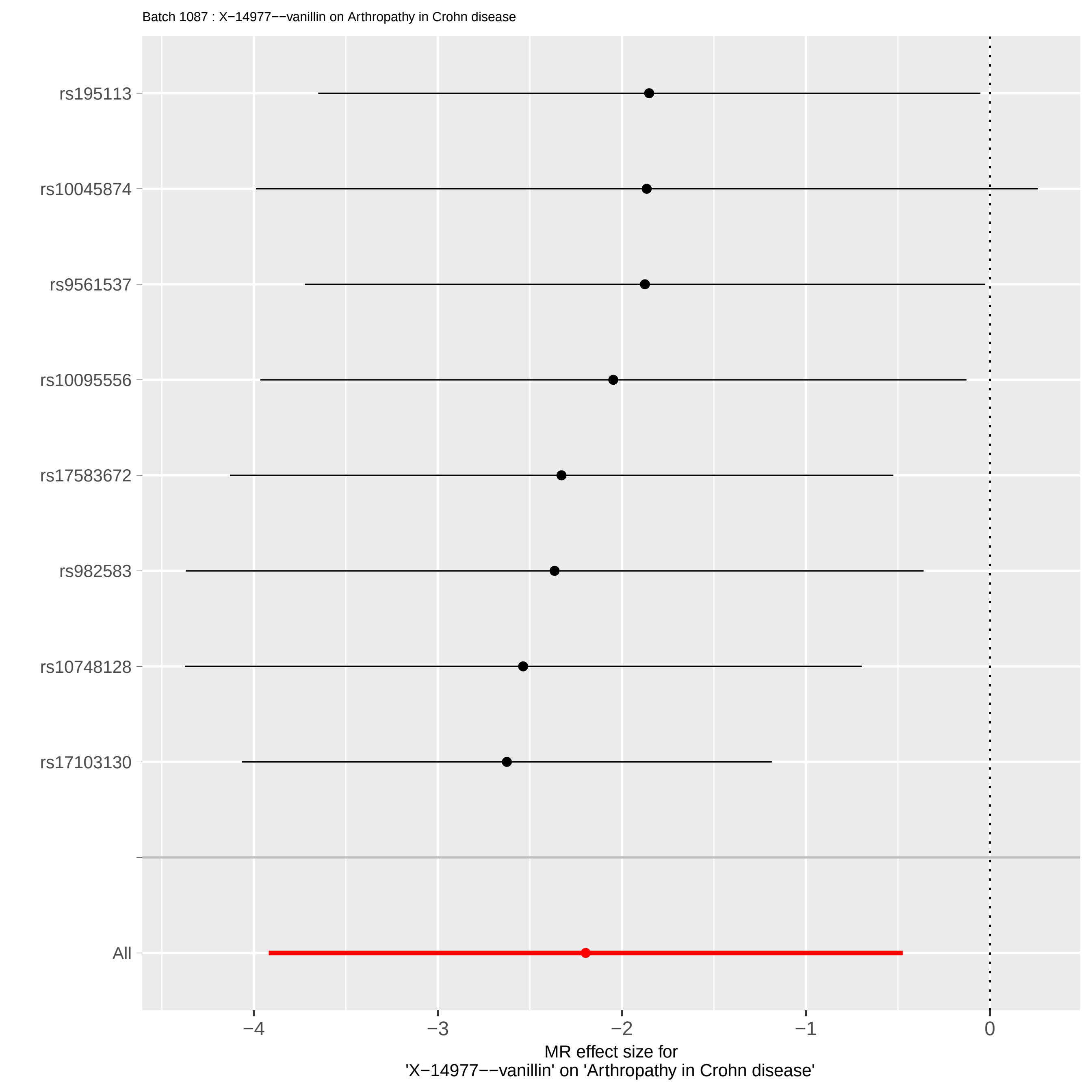

Batch 1087 : X−14977−−vanillin on Arthropathy in Crohn disease
rs195113
rs10045874
rs9561537
rs10095556
rs17583672
rs982583
rs10748128
rs17103130
All
−4
−3
−2
−1
0
MR effect size for
'X−14977−−vanillin' on 'Arthropathy in Crohn disease'

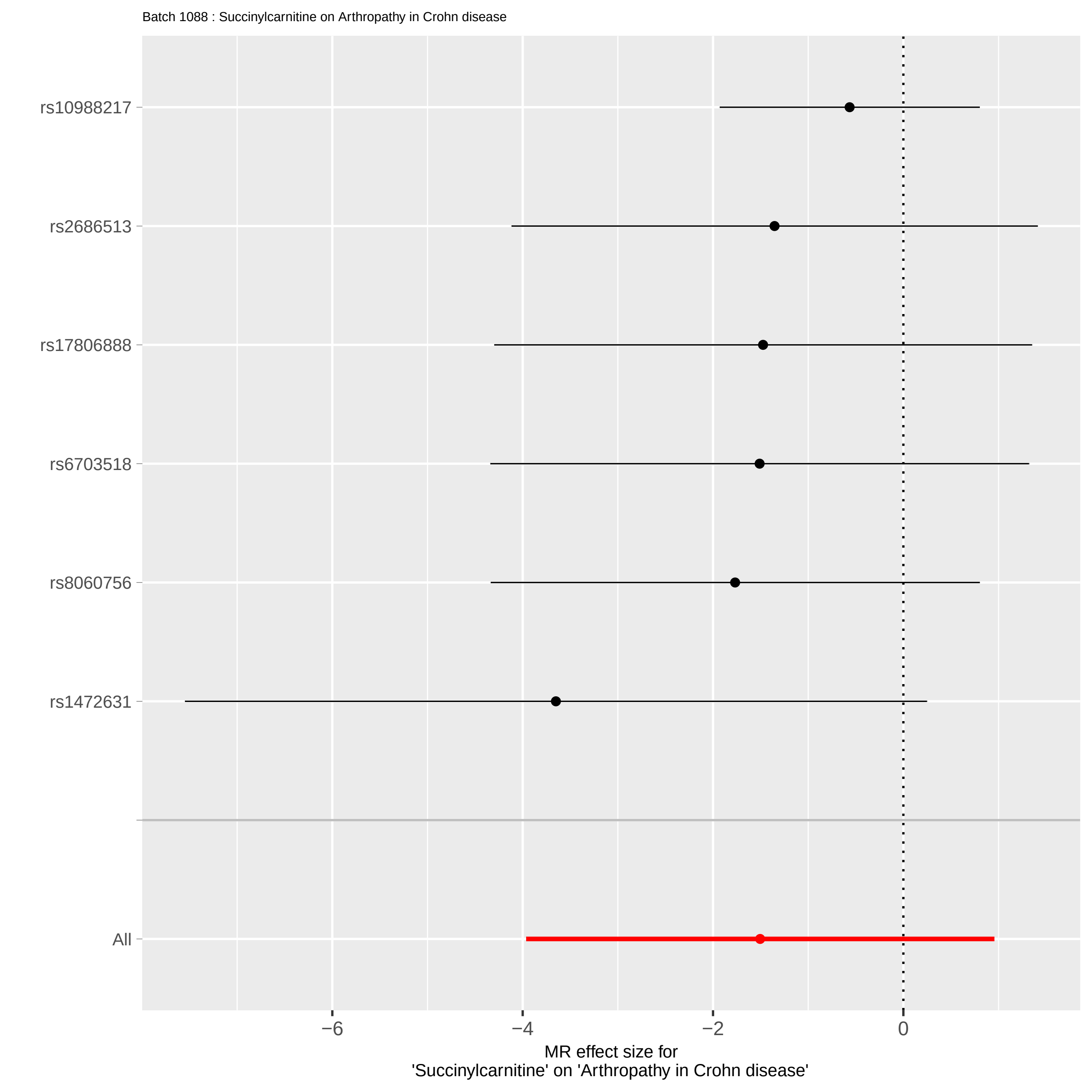
Batch 1088 : Succinylcarnitine on Arthropathy in Crohn disease
rs10988217
rs2686513
rs17806888
rs6703518
rs8060756
rs1472631
All
−6
−4
−2
0
MR effect size for
'Succinylcarnitine' on 'Arthropathy in Crohn disease'

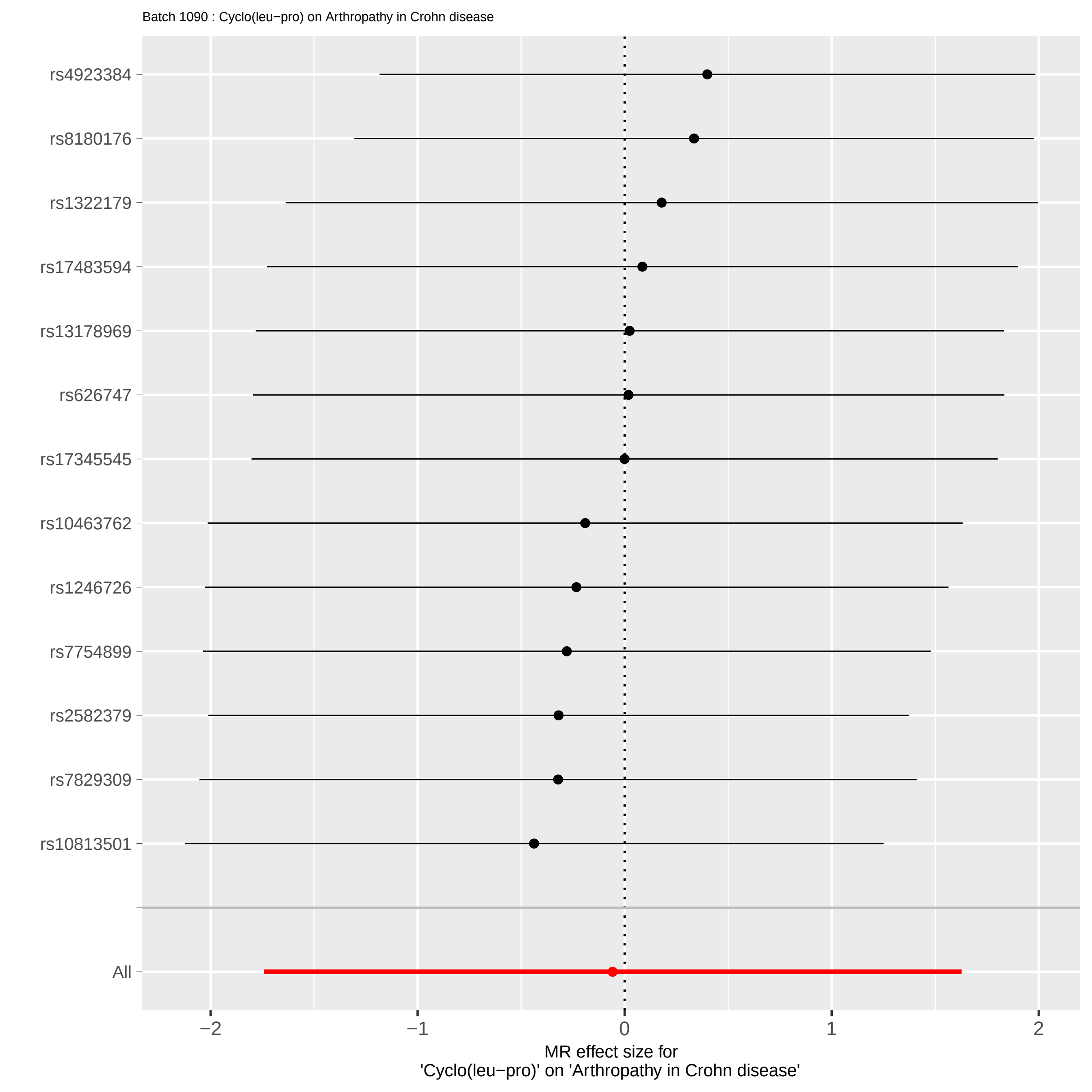

Batch 1090 : Cyclo(leu−pro) on Arthropathy in Crohn disease
rs4923384
rs8180176
rs1322179
rs17483594
rs13178969
rs626747
rs17345545
rs10463762
rs1246726
rs7754899
rs2582379
rs7829309
rs10813501
All
−2
−1
0
1
2
MR effect size for
'Cyclo(leu−pro)' on 'Arthropathy in Crohn disease'

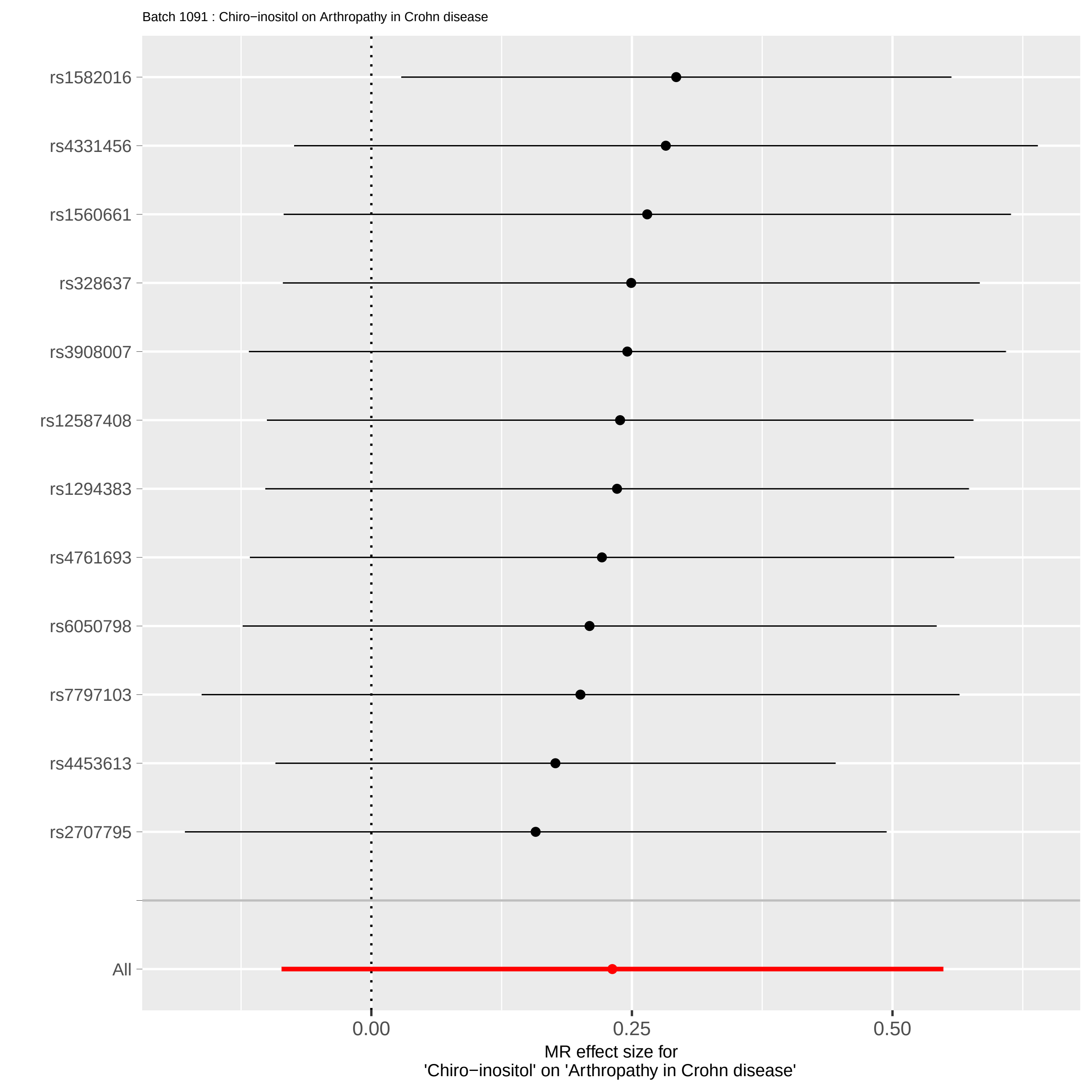
Batch 1091 : Chiro−inositol on Arthropathy in Crohn disease
rs1582016
rs4331456
rs1560661
rs328637
rs3908007
rs12587408
rs1294383
rs4761693
rs6050798
rs7797103
rs4453613
rs2707795
All
0.00
0.25
0.50
MR effect size for
'Chiro−inositol' on 'Arthropathy in Crohn disease'

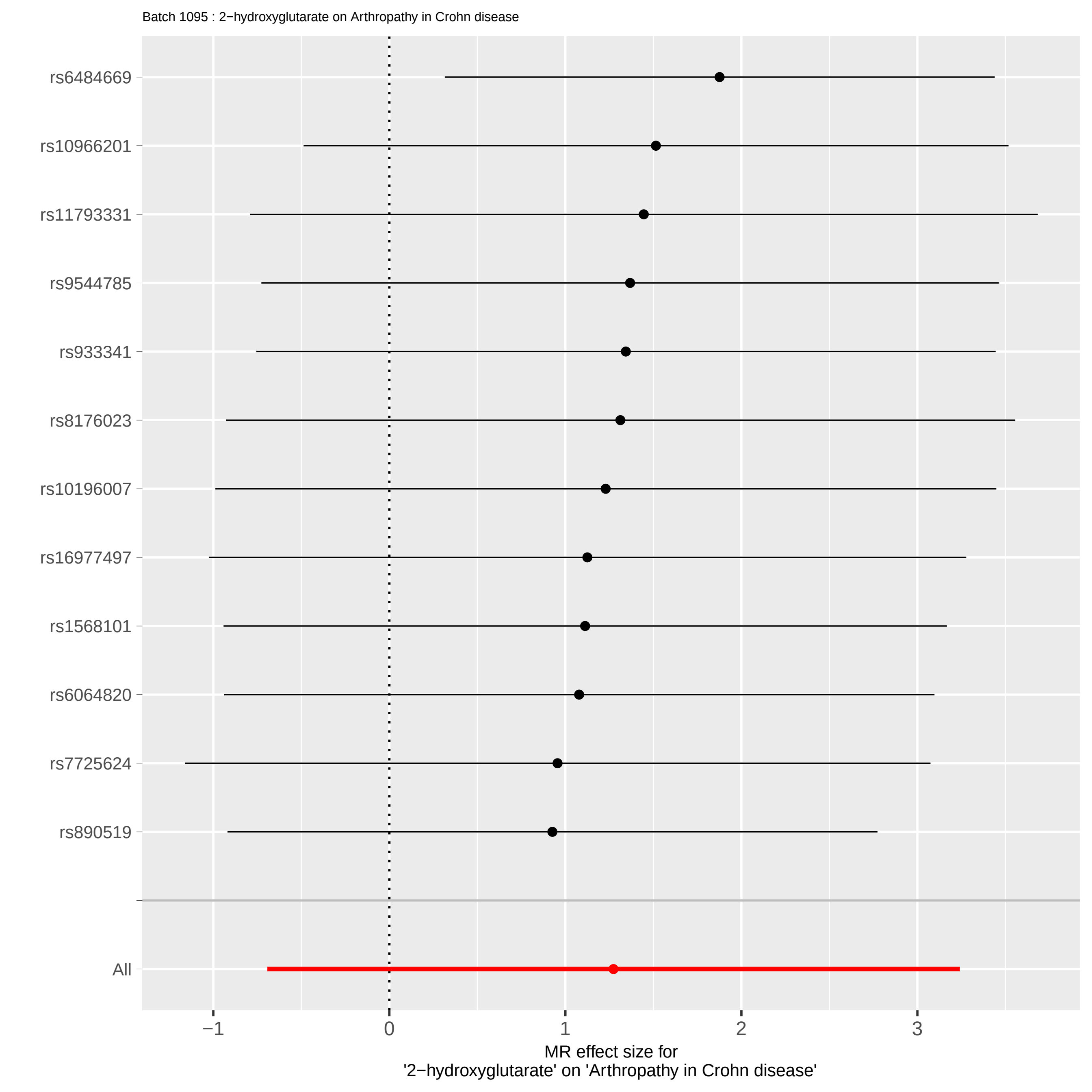

Batch 1095 : 2−hydroxyglutarate on Arthropathy in Crohn disease
rs6484669
rs10966201
rs11793331
rs9544785
rs933341
rs8176023
rs10196007
rs16977497
rs1568101
rs6064820
rs7725624
rs890519
All
−1
0
1
2
3
MR effect size for
'2−hydroxyglutarate' on 'Arthropathy in Crohn disease'

Batch 1096 : Ergothioneine on Arthropathy in Crohn disease

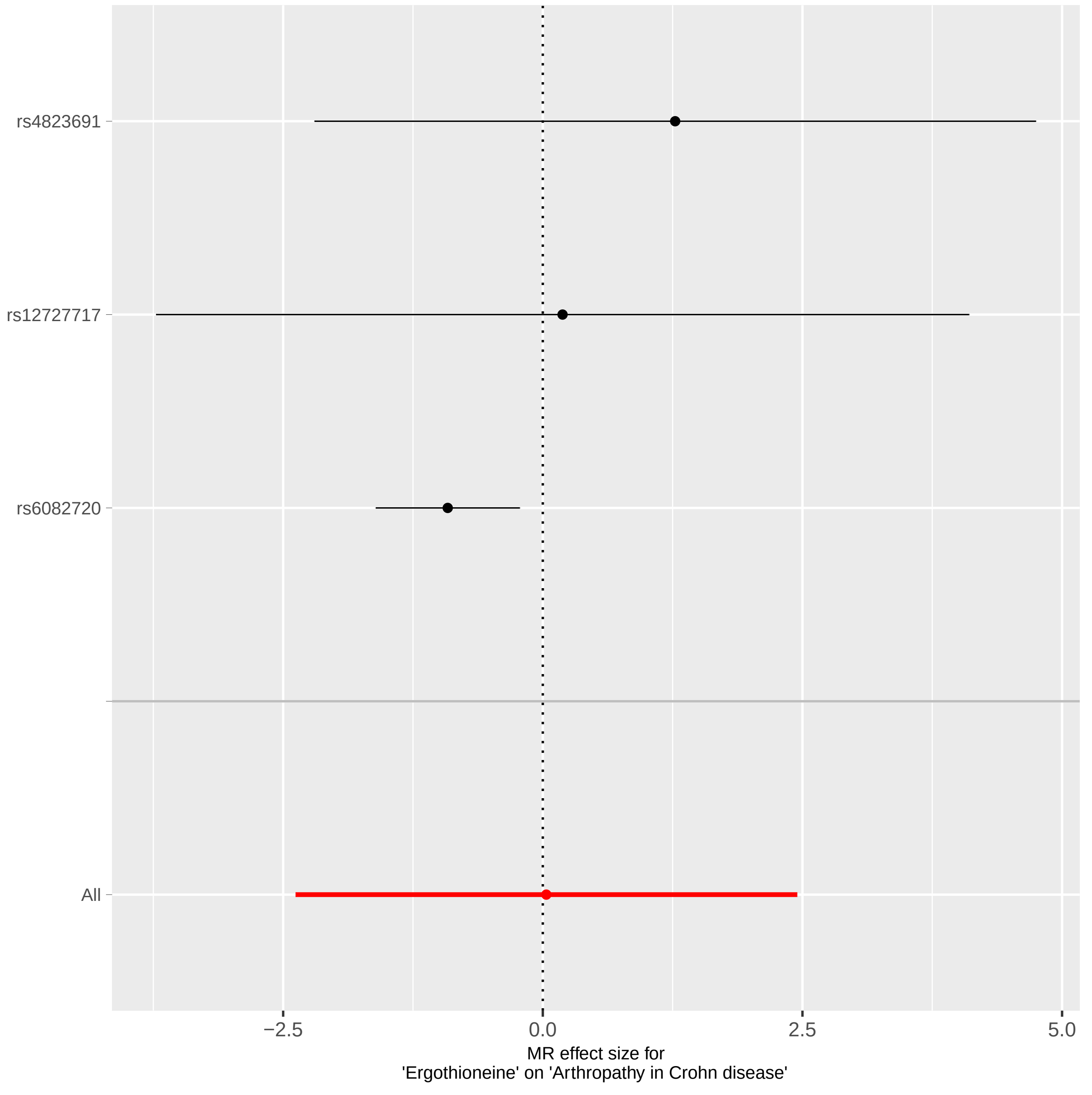

Batch 1098 : Phenylalanylphenylalanine on Arthropathy in Crohn disease

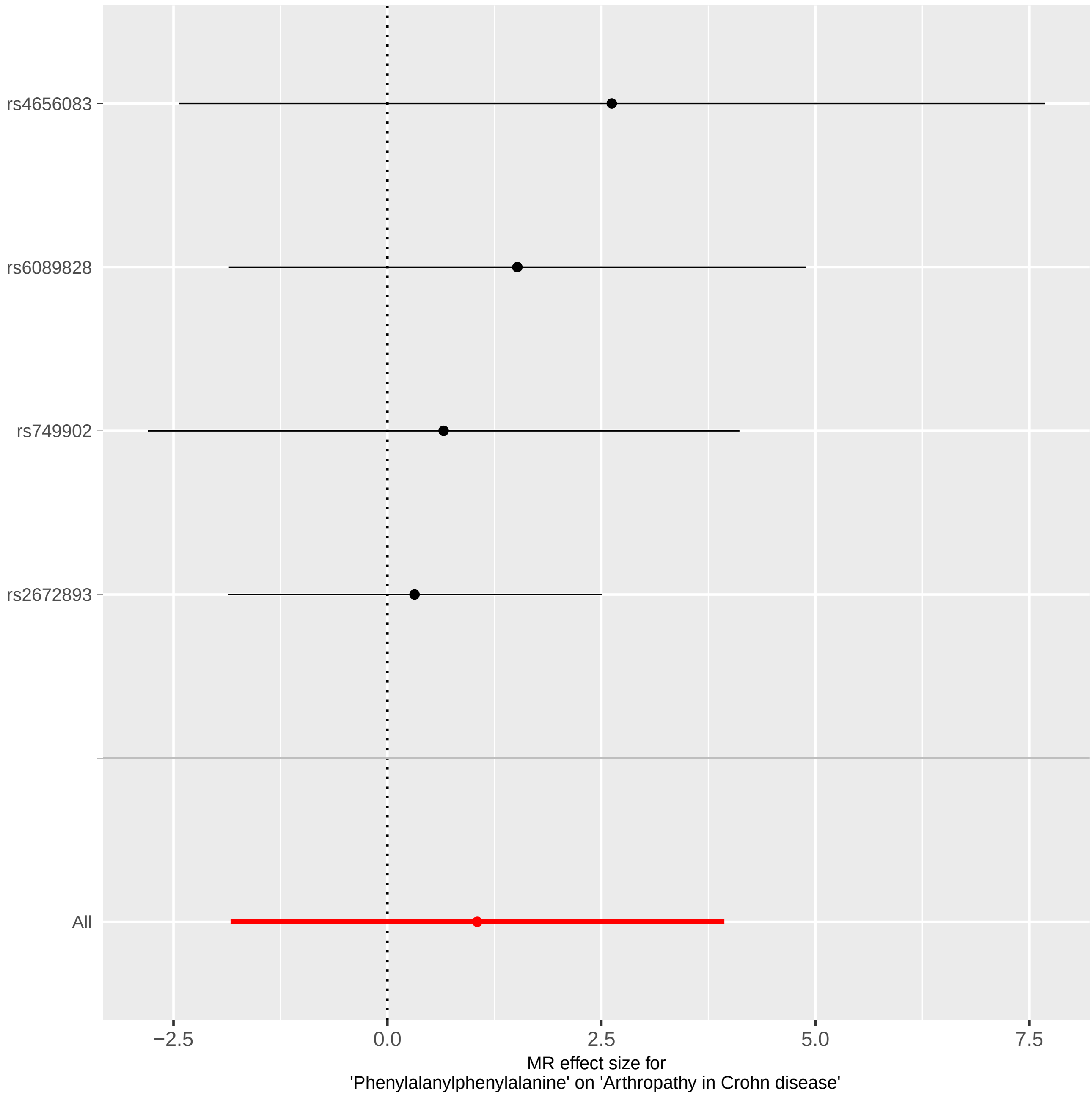

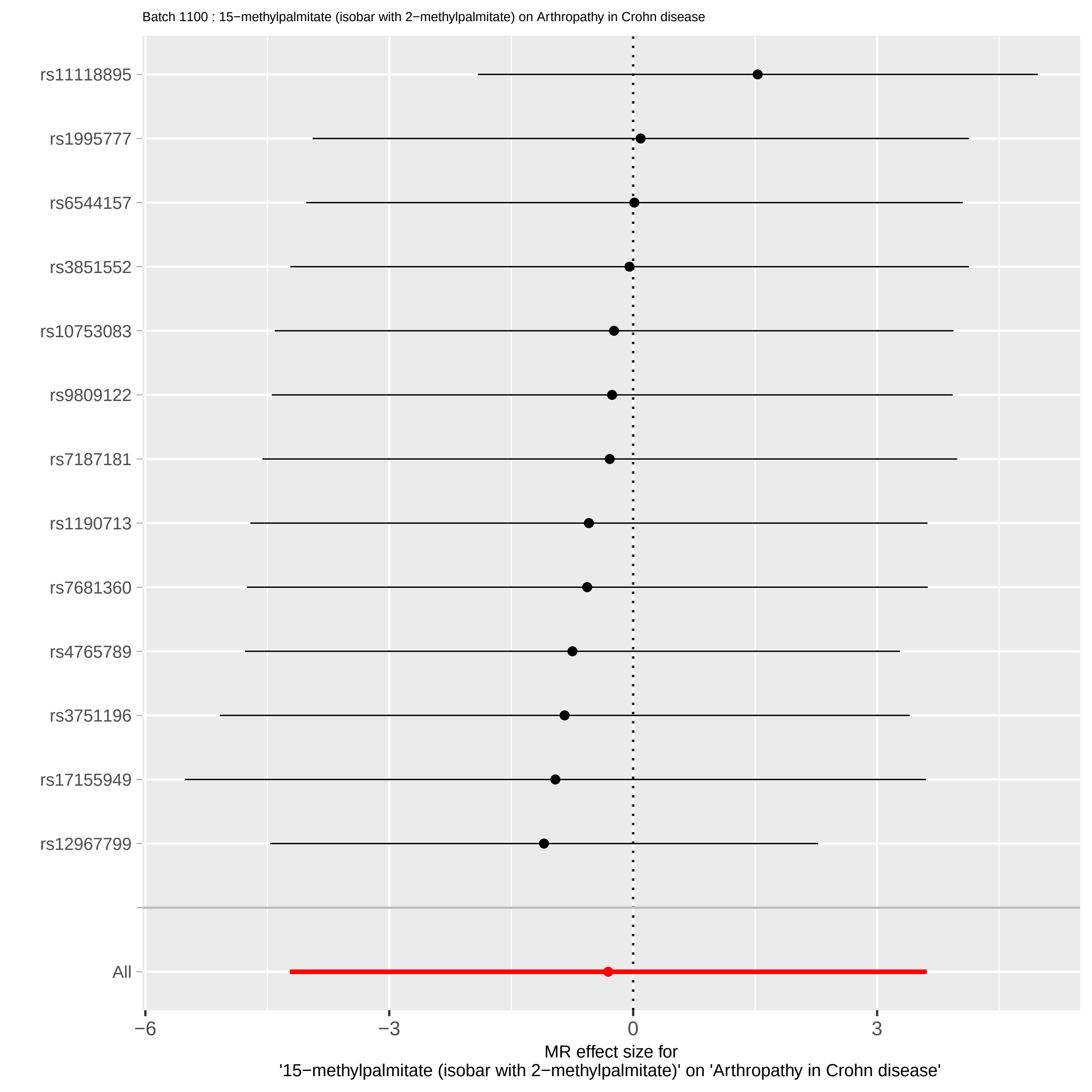
Batch 1100 : 15−methylpalmitate (isobar with 2−methylpalmitate) on Arthropathy in Crohn disease
rs11118895
rs1995777
rs6544157
rs3851552
rs10753083
rs9809122
rs7187181
rs1190713
rs7681360
rs4765789
rs3751196
rs17155949
rs12967799
All
−6
−3
0
3
MR effect size for
'15−methylpalmitate (isobar with 2−methylpalmitate)' on 'Arthropathy in Crohn disease'

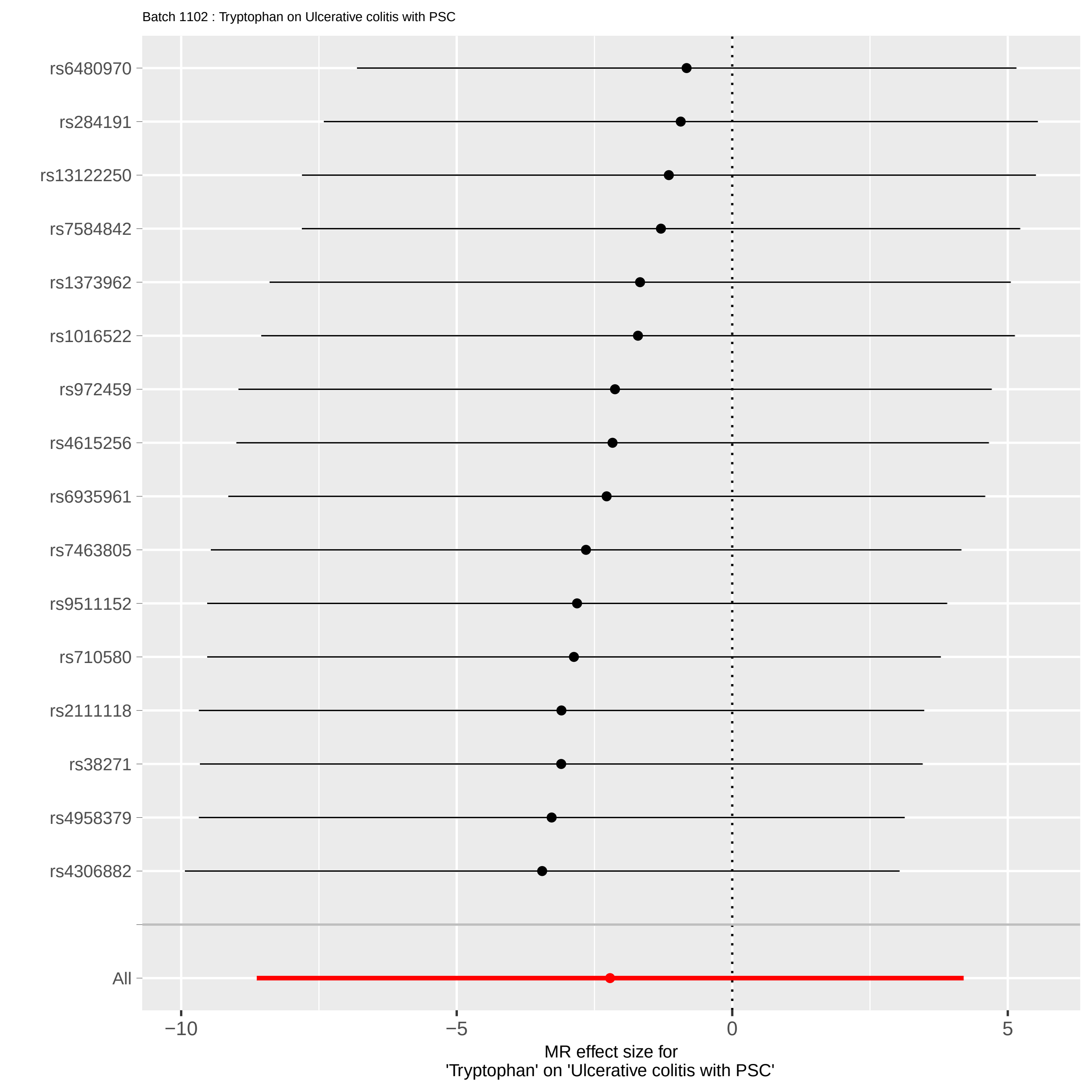

Batch 1102 : Tryptophan on Ulcerative colitis with PSC
rs6480970
rs284191
rs13122250
rs7584842
rs1373962
rs1016522
rs972459
rs4615256
rs6935961
rs7463805
rs9511152
rs710580
rs2111118
rs38271
rs4958379
rs4306882
All
−10
−5
0
5
MR effect size for
'Tryptophan' on 'Ulcerative colitis with PSC'

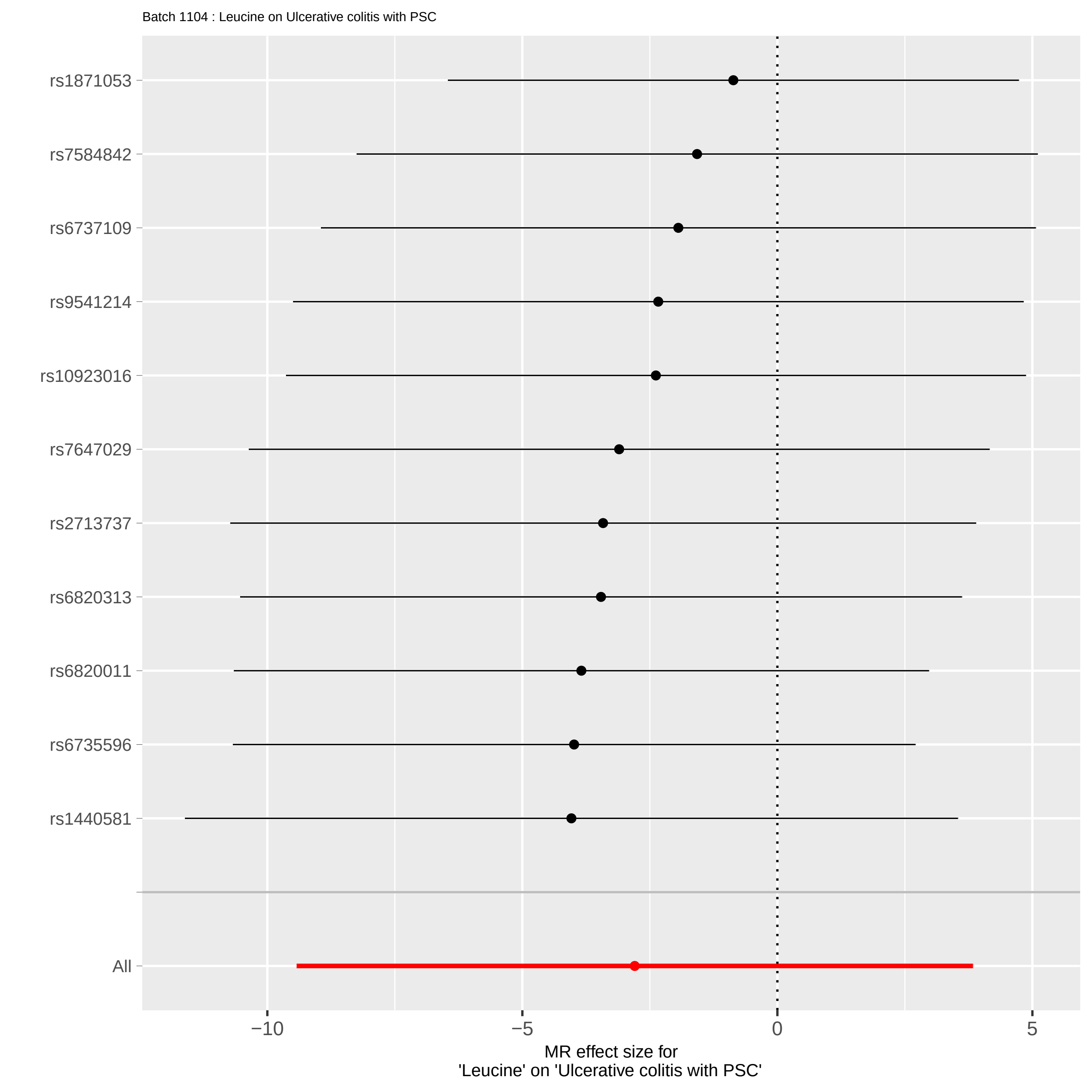

Batch 1104 : Leucine on Ulcerative colitis with PSC
rs1871053
rs7584842
rs6737109
rs9541214
rs10923016
rs7647029
rs2713737
rs6820313
rs6820011
rs6735596
rs1440581
All
−10
−5
0
5
MR effect size for
'Leucine' on 'Ulcerative colitis with PSC'

Batch 1108 : Lactate on Ulcerative colitis with PSC

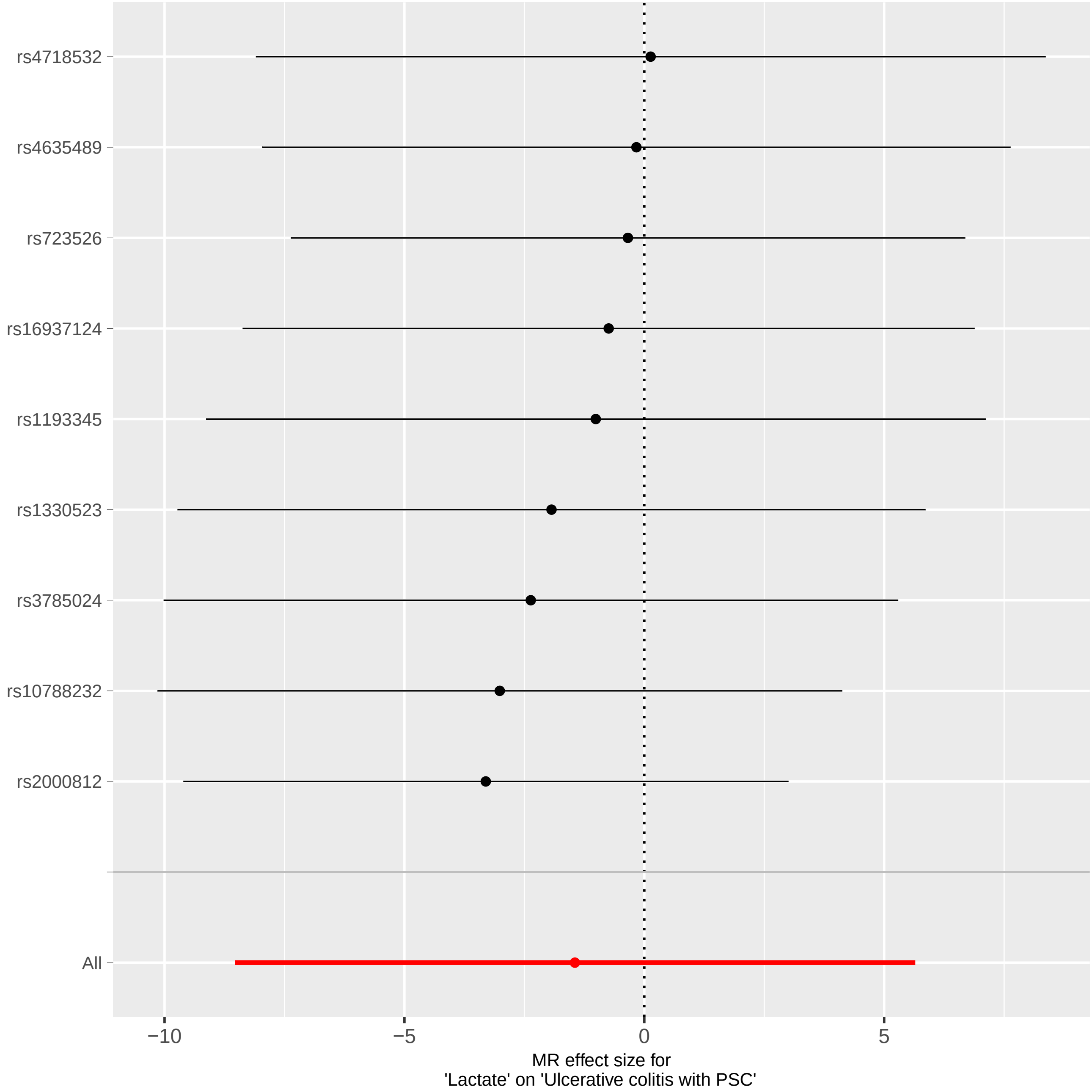

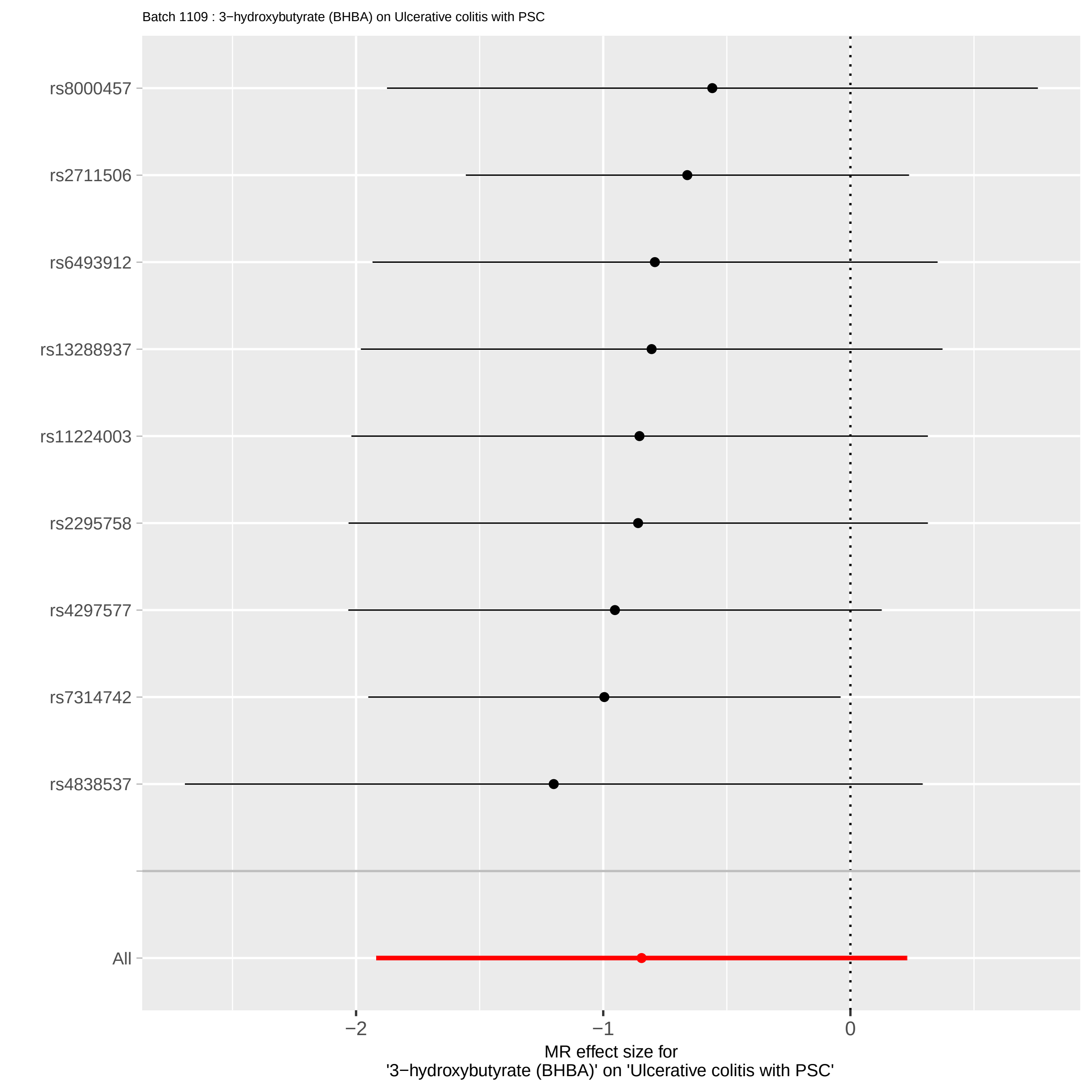

Batch 1109 : 3−hydroxybutyrate (BHBA) on Ulcerative colitis with PSC
rs8000457
rs2711506
rs6493912
rs13288937
rs11224003
rs2295758
rs4297577
rs7314742
rs4838537
All
−2
−1
0
MR effect size for
'3−hydroxybutyrate (BHBA)' on 'Ulcerative colitis with PSC'

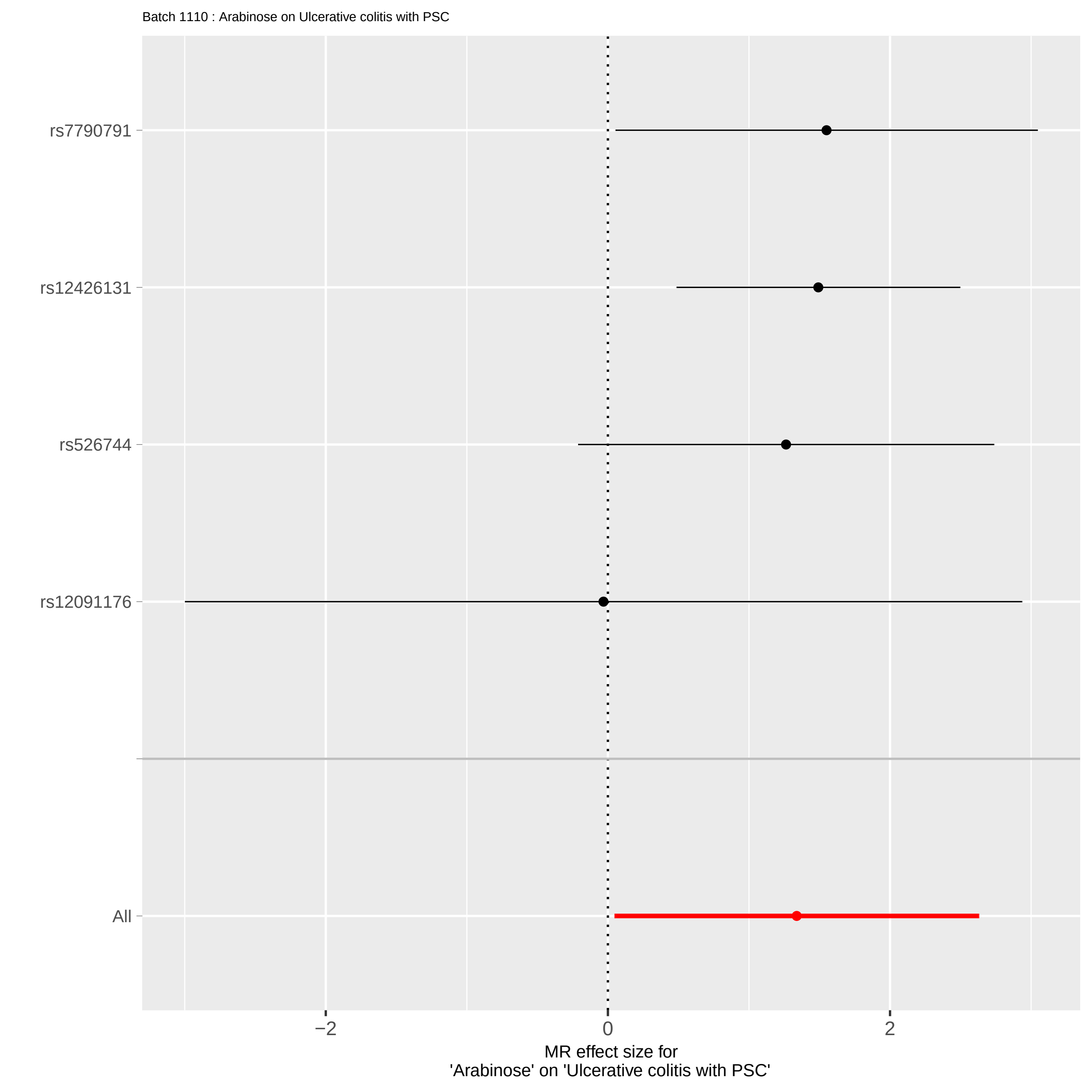

Batch 1110 : Arabinose on Ulcerative colitis with PSC
rs7790791
rs12426131
rs526744
rs12091176
All
−2
0
2
MR effect size for
'Arabinose' on 'Ulcerative colitis with PSC'

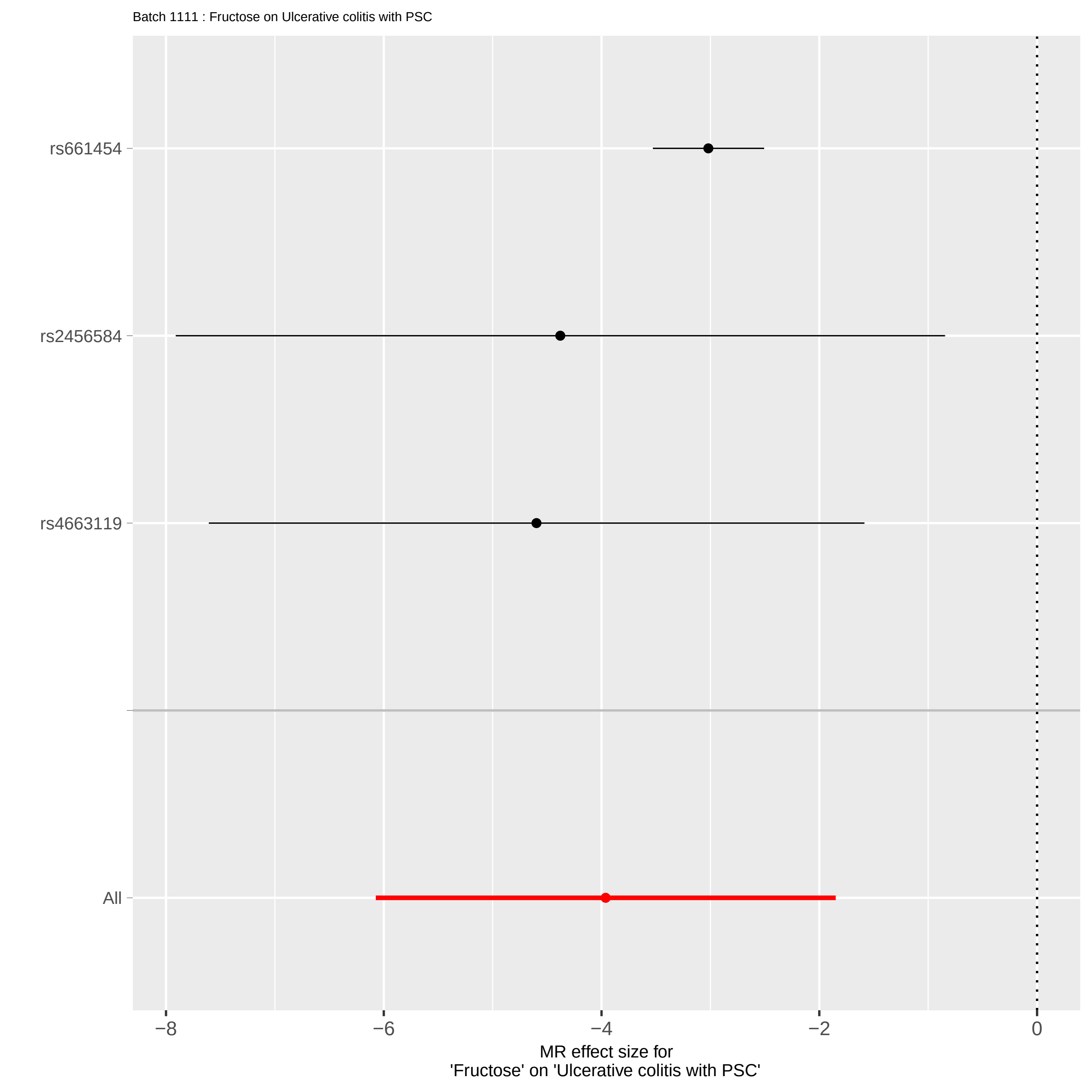

Batch 1111 : Fructose on Ulcerative colitis with PSC
rs661454
rs2456584
rs4663119
All
−8
−6
−4
−2
0
MR effect size for
'Fructose' on 'Ulcerative colitis with PSC'

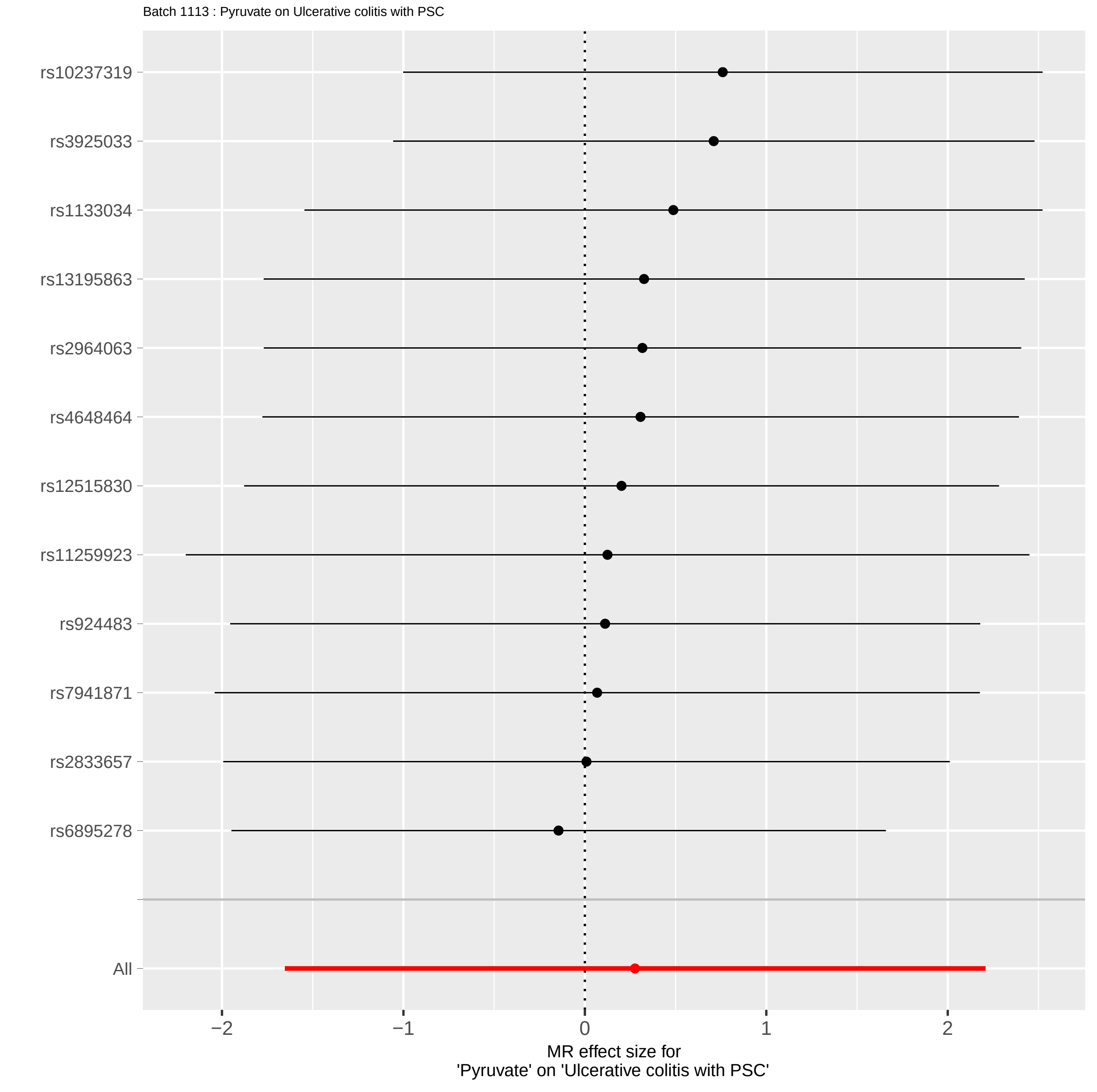
Batch 1113 : Pyruvate on Ulcerative colitis with PSC
rs10237319
rs3925033
rs1133034
rs13195863
rs2964063
rs4648464
rs12515830
rs11259923
rs924483
rs7941871
rs2833657
rs6895278
All
−2
−1
0
1
2
MR effect size for
'Pyruvate' on 'Ulcerative colitis with PSC'

Batch 1114 : Uridine on Ulcerative colitis with PSC

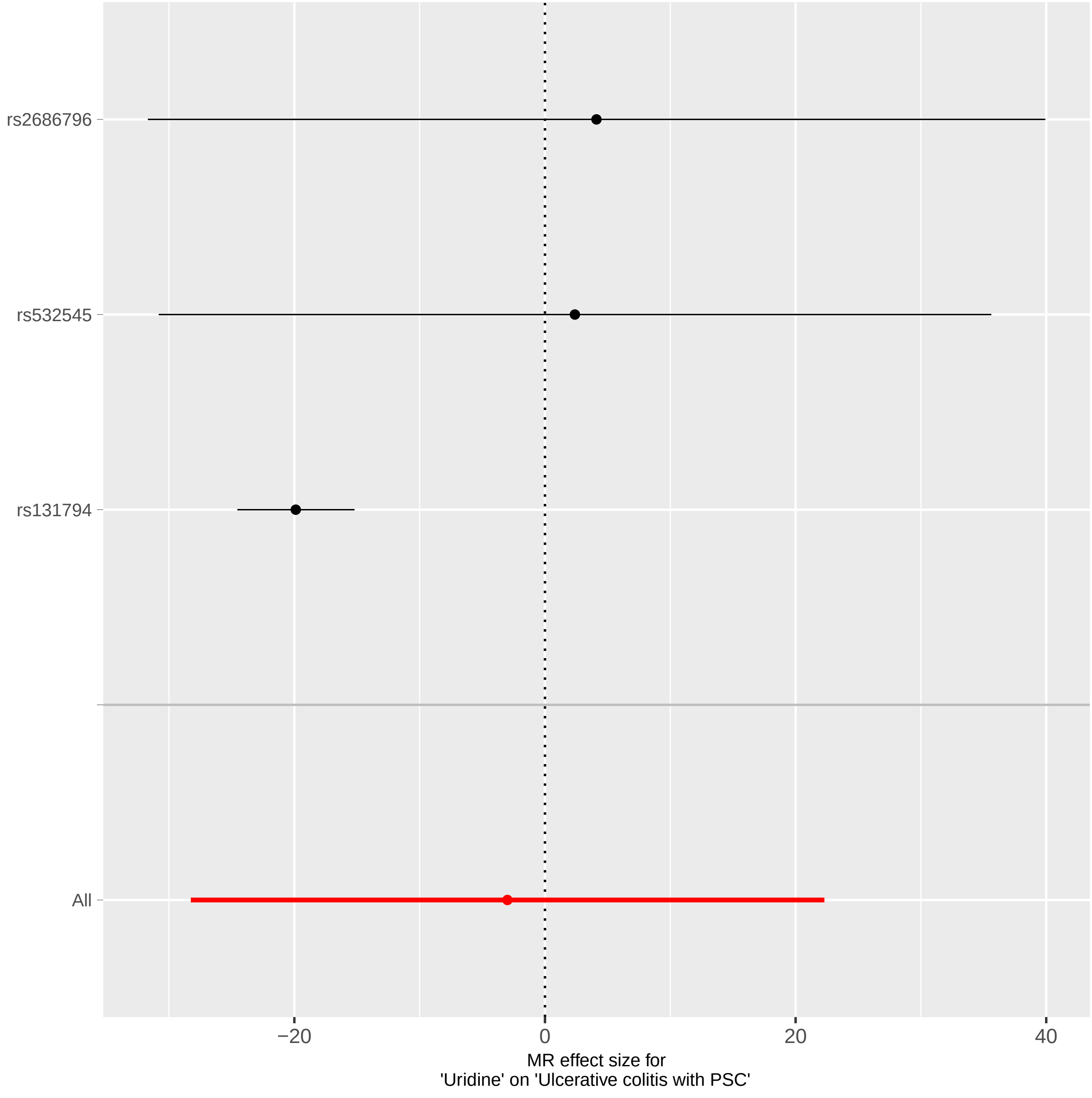

Batch 1115 : Linoleate (18:2n6) on Ulcerative colitis with PSC

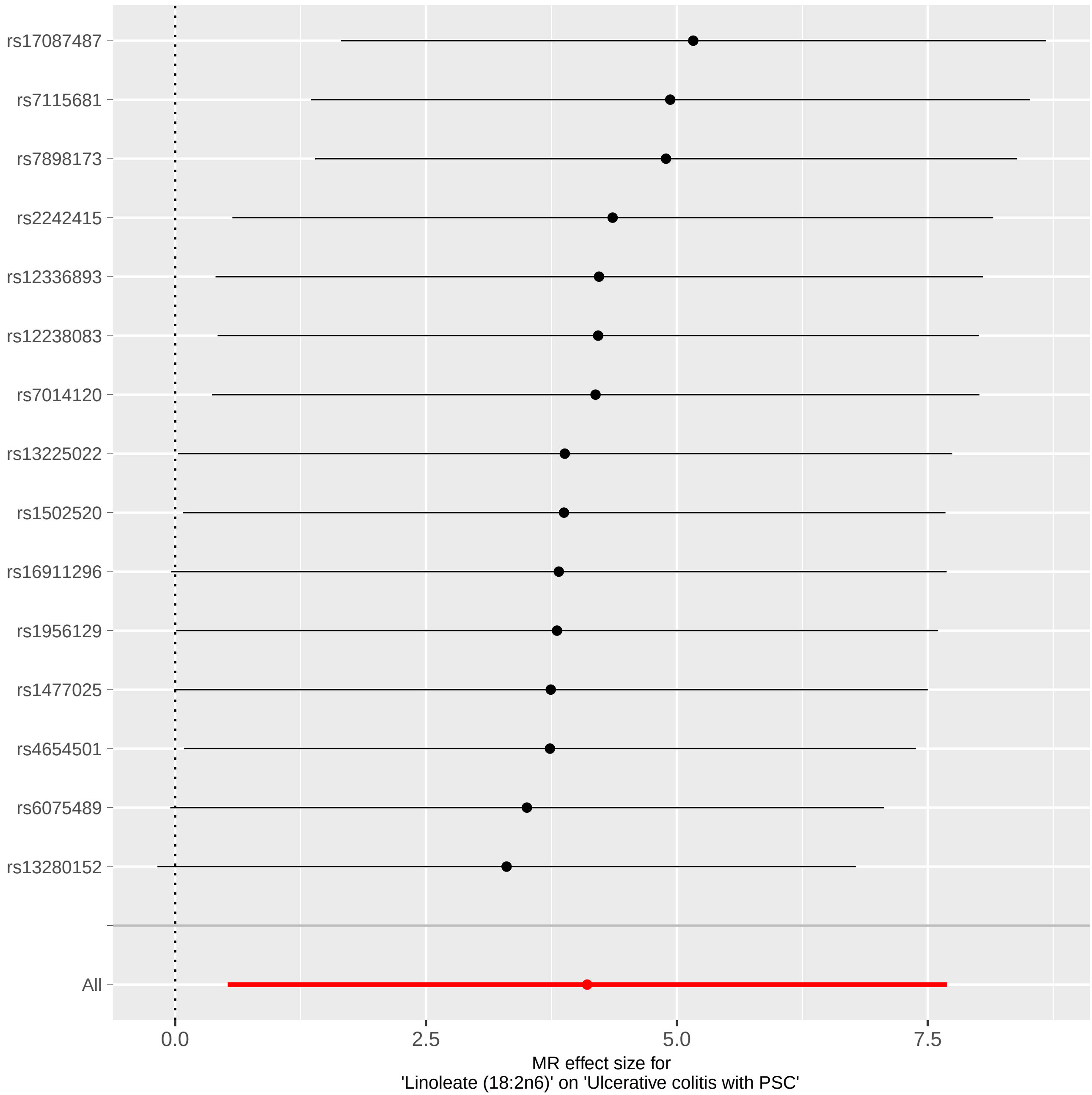

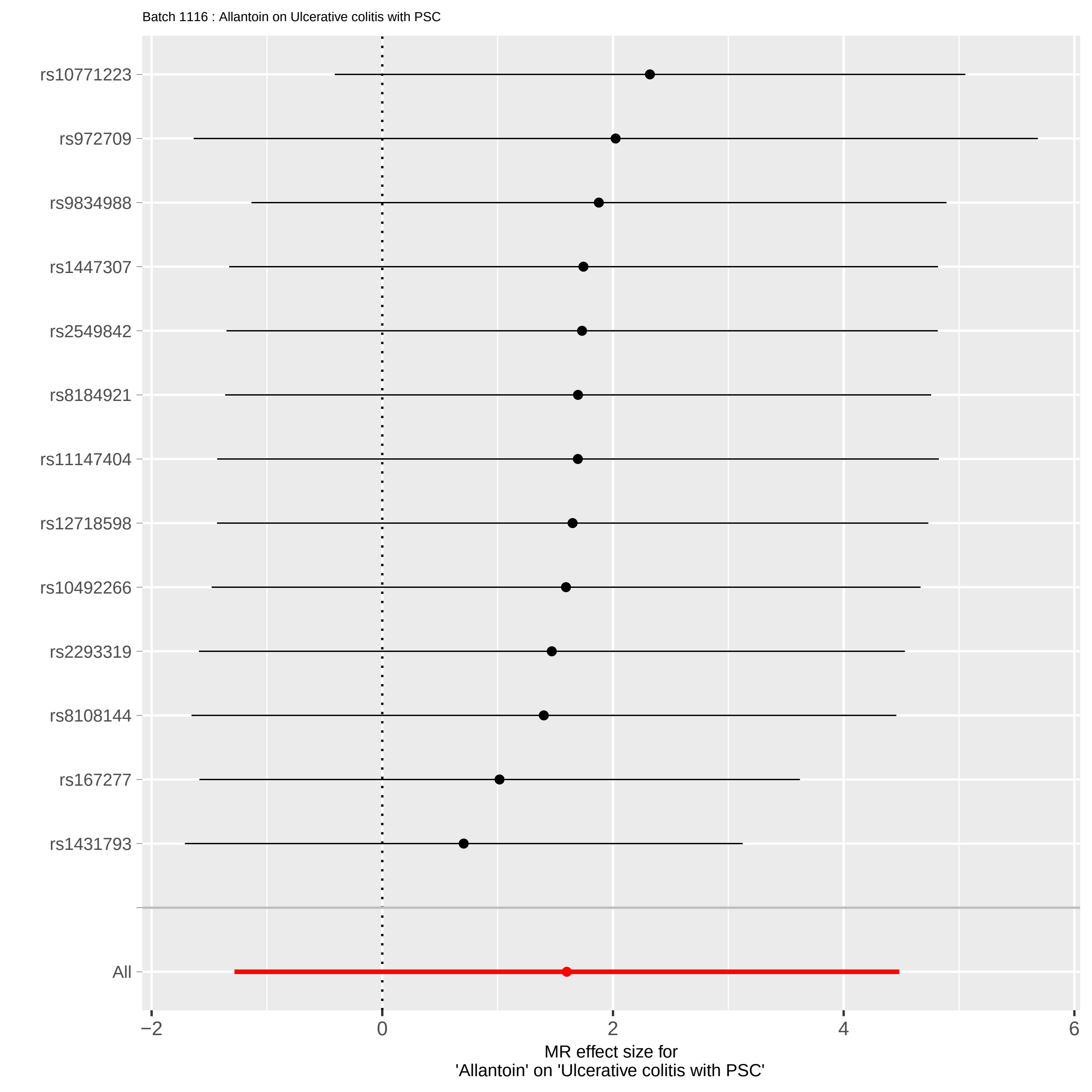

Batch 1116 : Allantoin on Ulcerative colitis with PSC
rs10771223
rs972709
rs9834988
rs1447307
rs2549842
rs8184921
rs11147404
rs12718598
rs10492266
rs2293319
rs8108144
rs167277
rs1431793
All
−2
0
2
4
6
MR effect size for
'Allantoin' on 'Ulcerative colitis with PSC'

Batch 1121 : Isoleucine on Ulcerative colitis with PSC

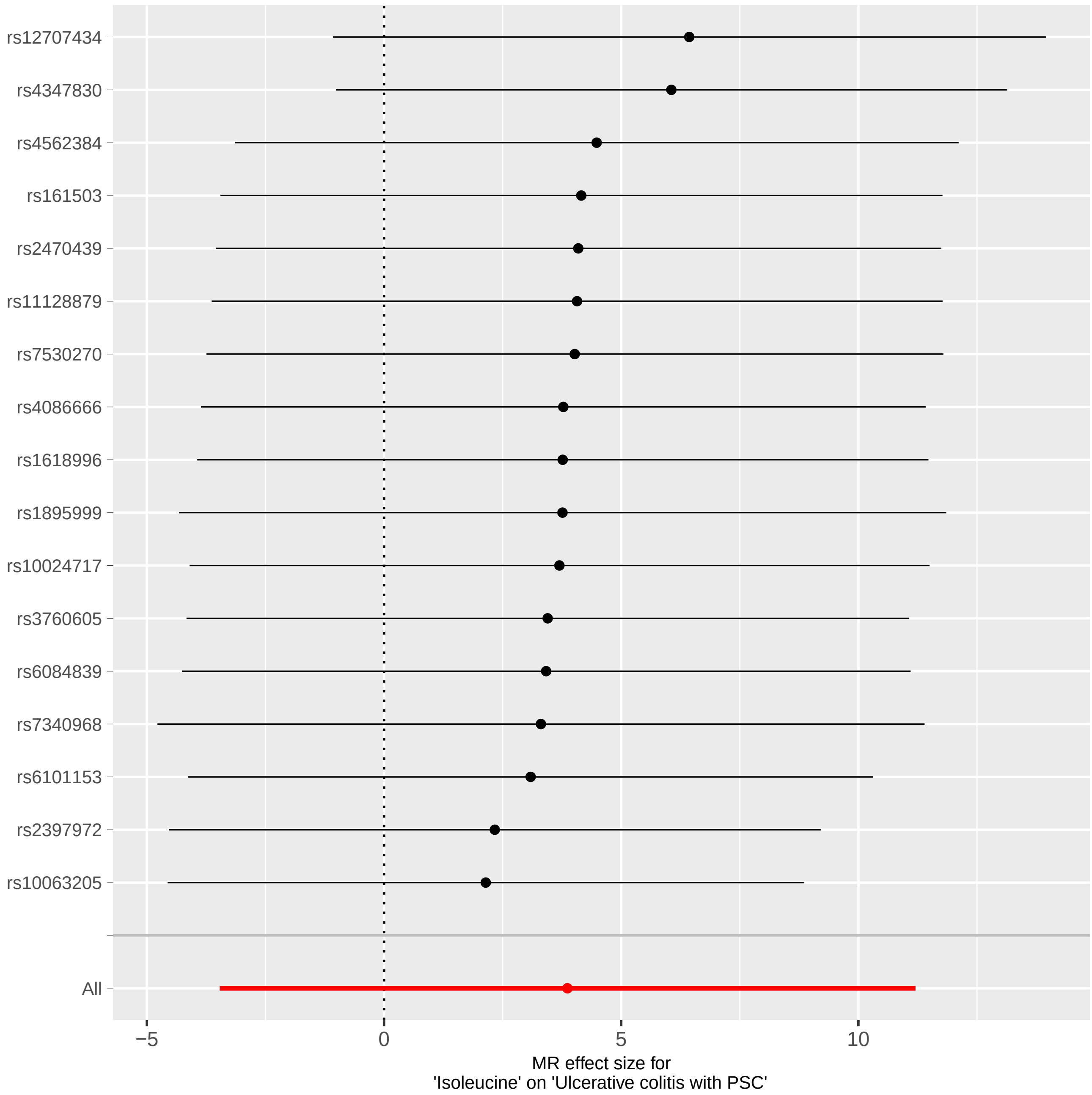

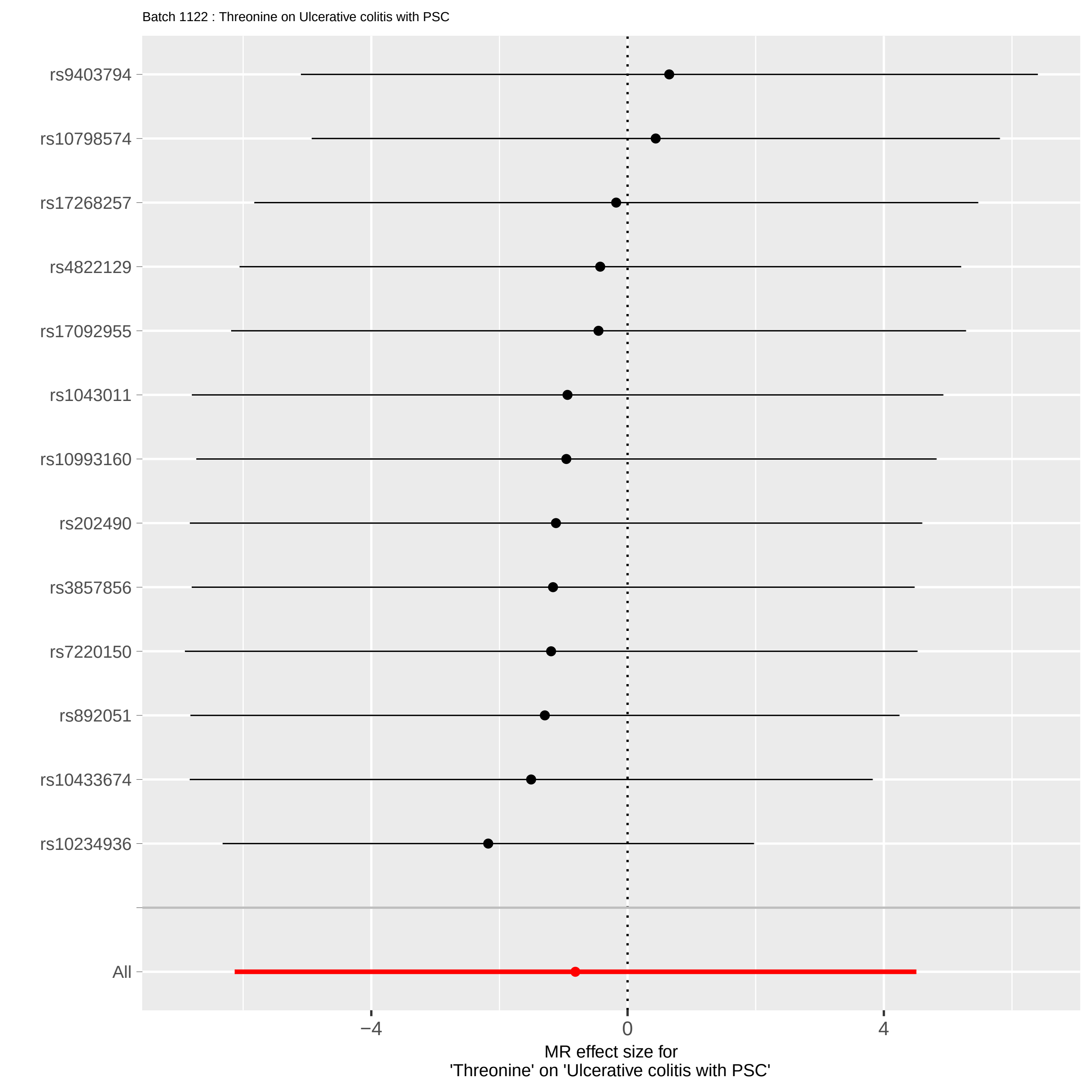
Batch 1122 : Threonine on Ulcerative colitis with PSC
rs9403794
rs10798574
rs17268257
rs4822129
rs17092955
rs1043011
rs10993160
rs202490
rs3857856
rs7220150
rs892051
rs10433674
rs10234936
All
−4
0
4
MR effect size for
'Threonine' on 'Ulcerative colitis with PSC'

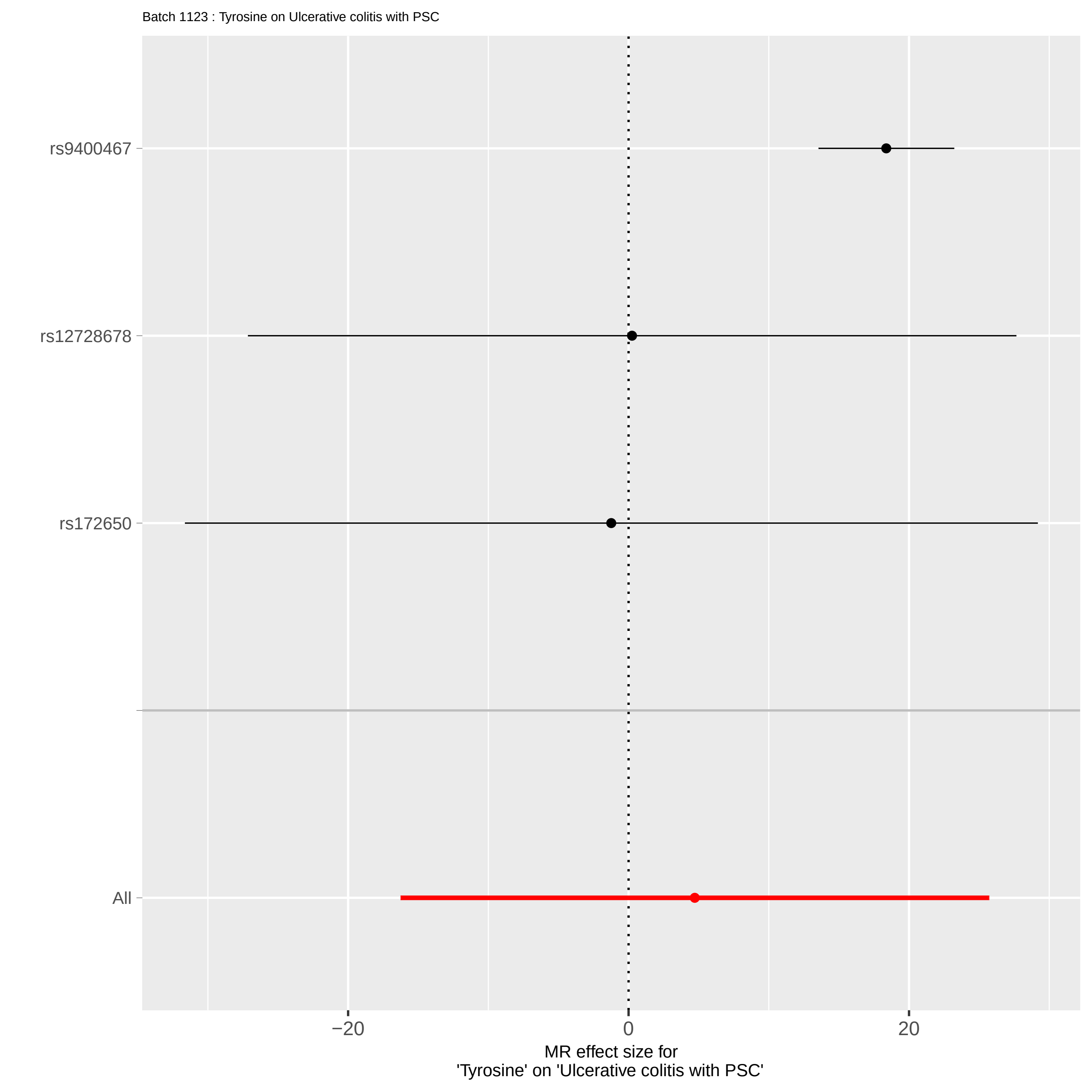
Batch 1123 : Tyrosine on Ulcerative colitis with PSC
rs9400467
rs12728678
rs172650
All
−20
0
20
MR effect size for
'Tyrosine' on 'Ulcerative colitis with PSC'

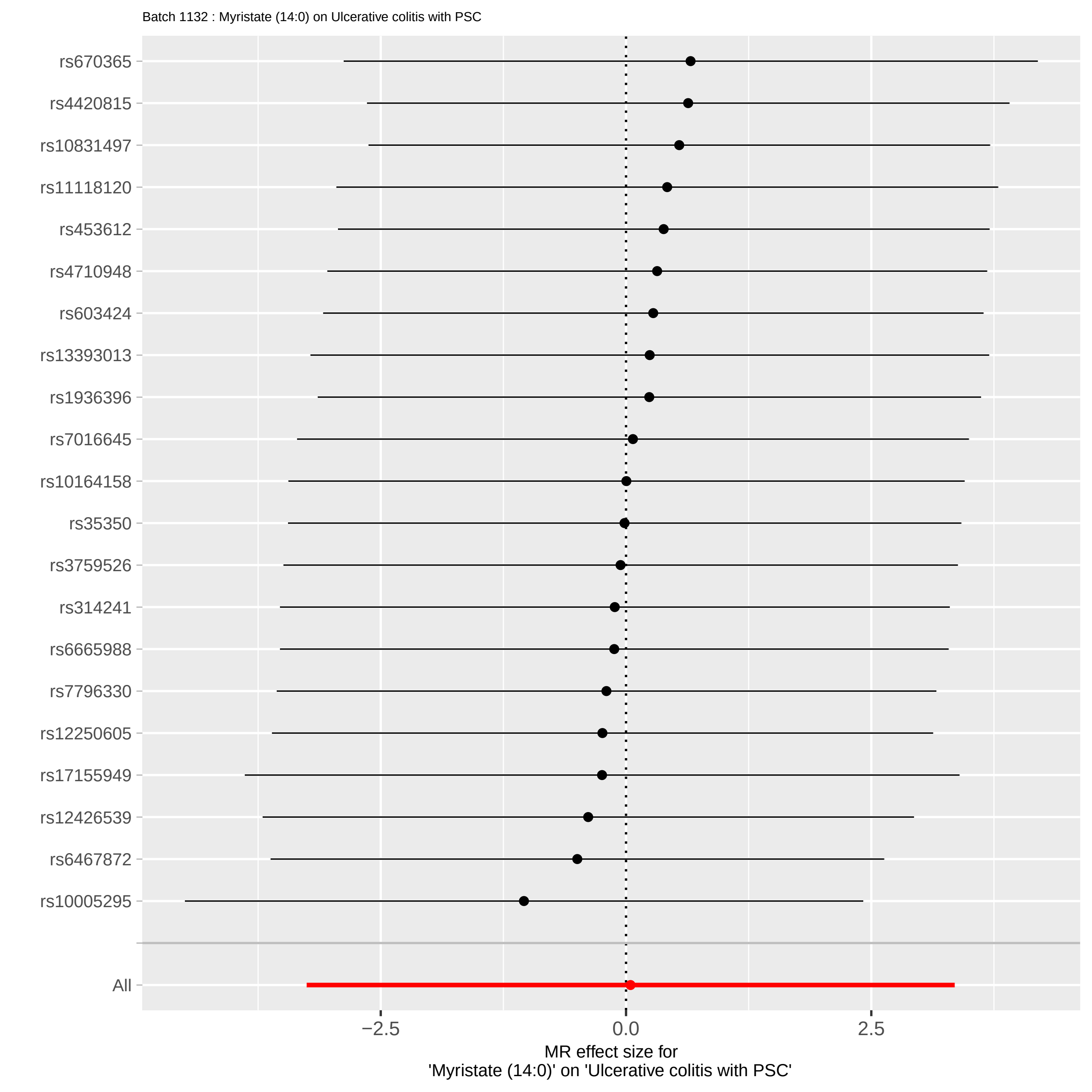

Batch 1132 : Myristate (14:0) on Ulcerative colitis with PSC
rs670365
rs4420815
rs10831497
rs11118120
rs453612
rs4710948
rs603424
rs13393013
rs1936396
rs7016645
rs10164158
rs35350
rs3759526
rs314241
rs6665988
rs7796330
rs12250605
rs17155949
rs12426539
rs6467872
rs10005295
All
−2.5
0.0
2.5
MR effect size for
'Myristate (14:0)' on 'Ulcerative colitis with PSC'

Batch 1134 : Ornithine on Ulcerative colitis with PSC

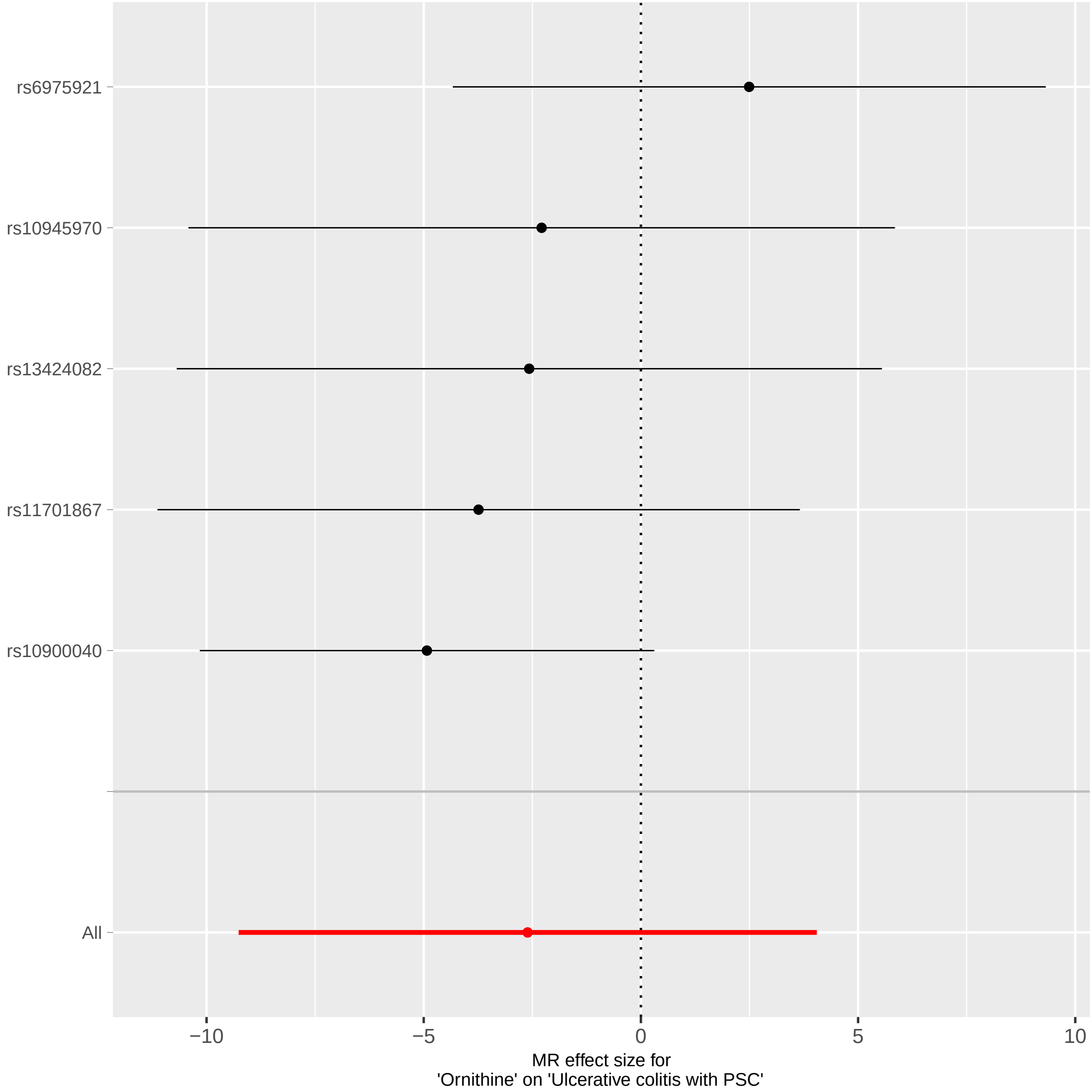

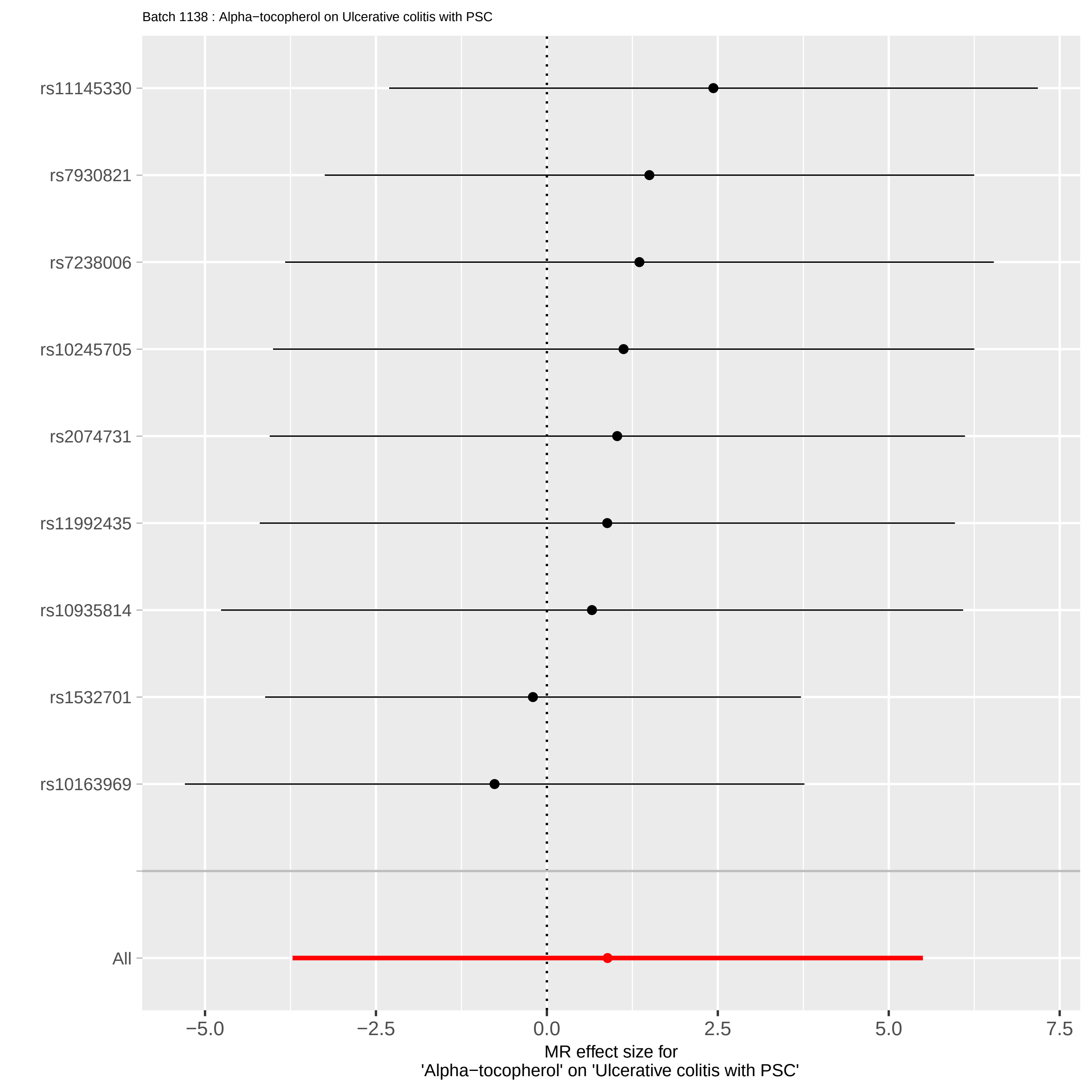
Batch 1138 : Alpha−tocopherol on Ulcerative colitis with PSC
rs11145330
rs7930821
rs7238006
rs10245705
rs2074731
rs11992435
rs10935814
rs1532701
rs10163969
All
−5.0
−2.5
0.0
2.5
5.0
7.5
MR effect size for
'Alpha−tocopherol' on 'Ulcerative colitis with PSC'

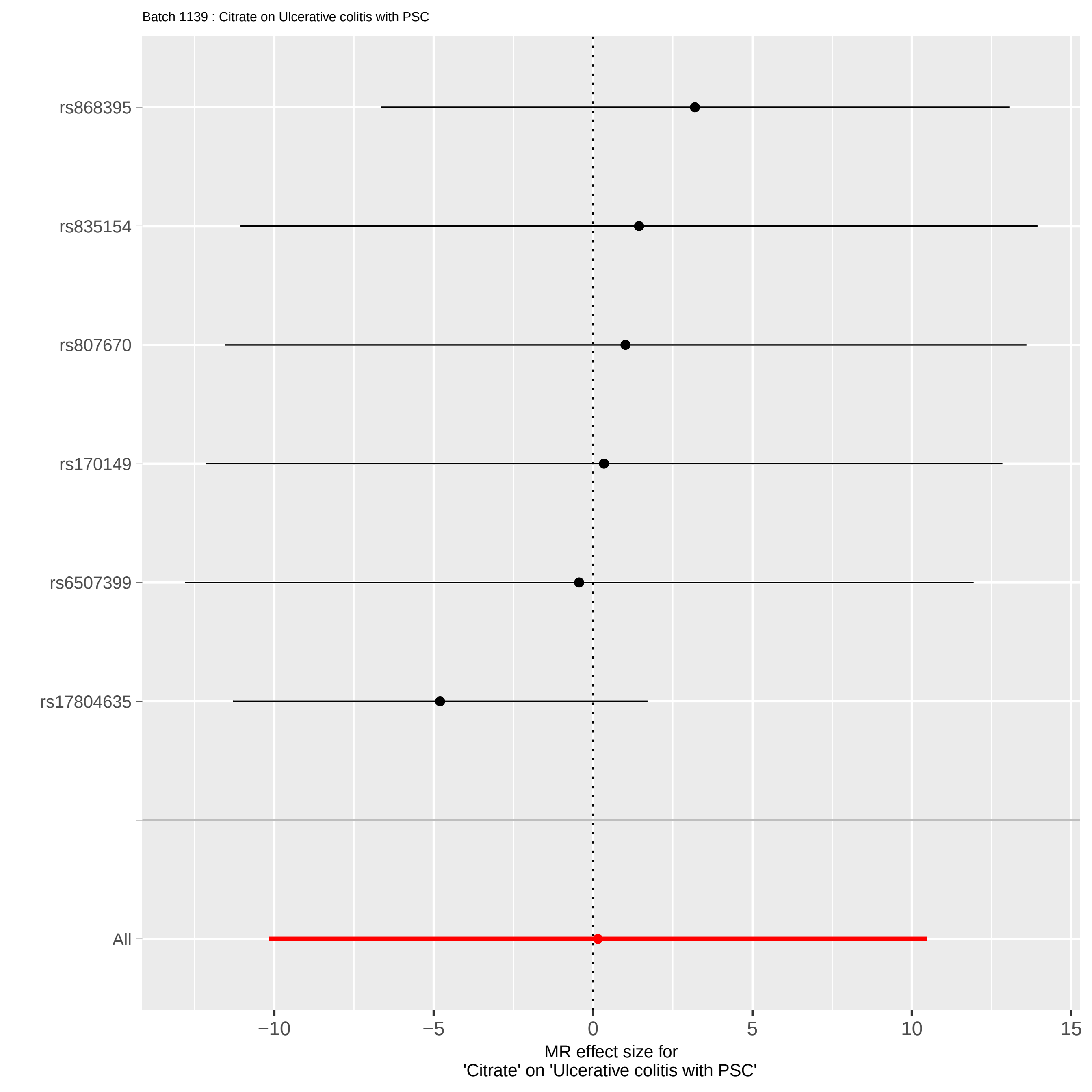
Batch 1139 : Citrate on Ulcerative colitis with PSC
rs868395
rs835154
rs807670
rs170149
rs6507399
rs17804635
All
−10
−5
0
5
10
15
MR effect size for
'Citrate' on 'Ulcerative colitis with PSC'

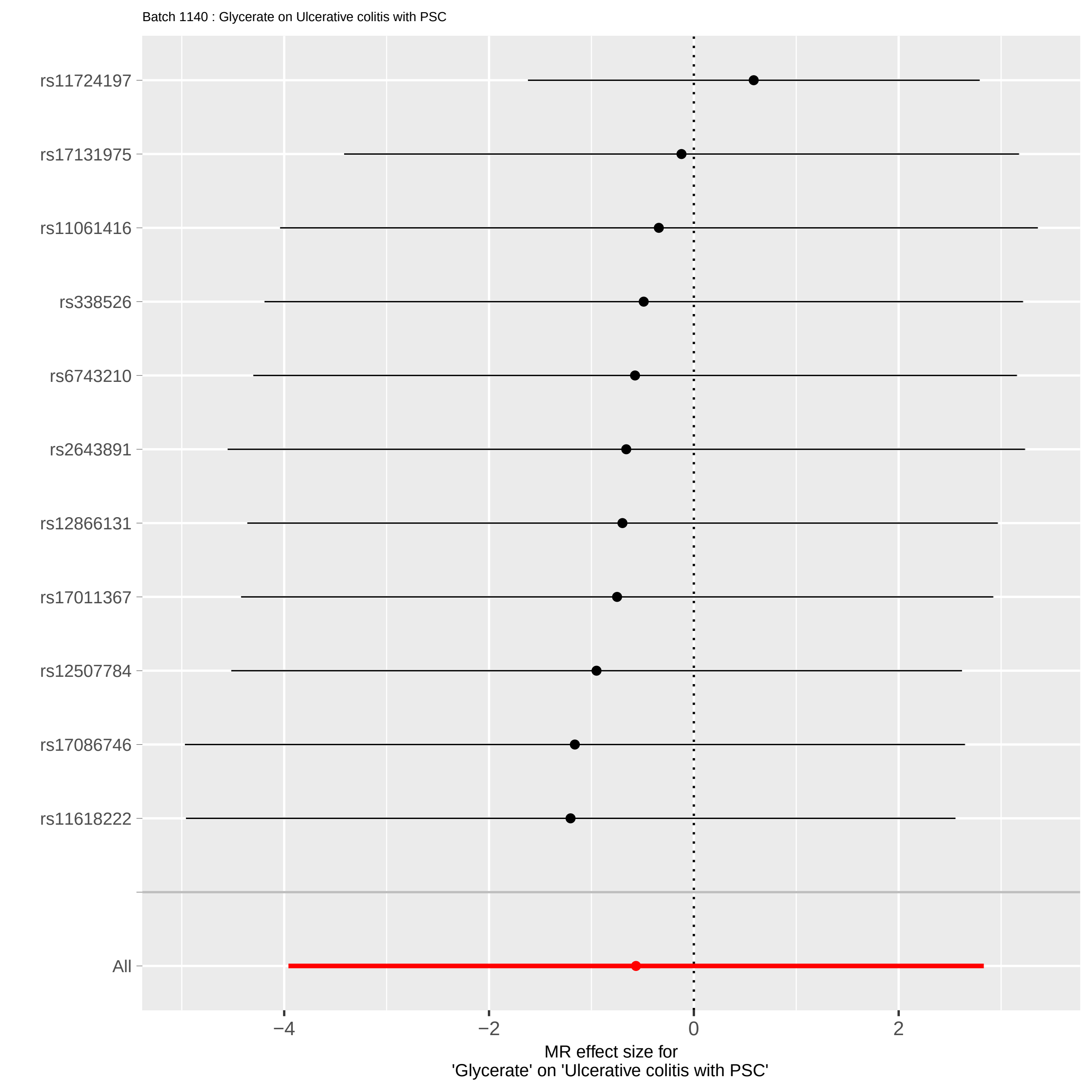

Batch 1140 : Glycerate on Ulcerative colitis with PSC
rs11724197
rs17131975
rs11061416
rs338526
rs6743210
rs2643891
rs12866131
rs17011367
rs12507784
rs17086746
rs11618222
All
−4
−2
0
2
MR effect size for
'Glycerate' on 'Ulcerative colitis with PSC'

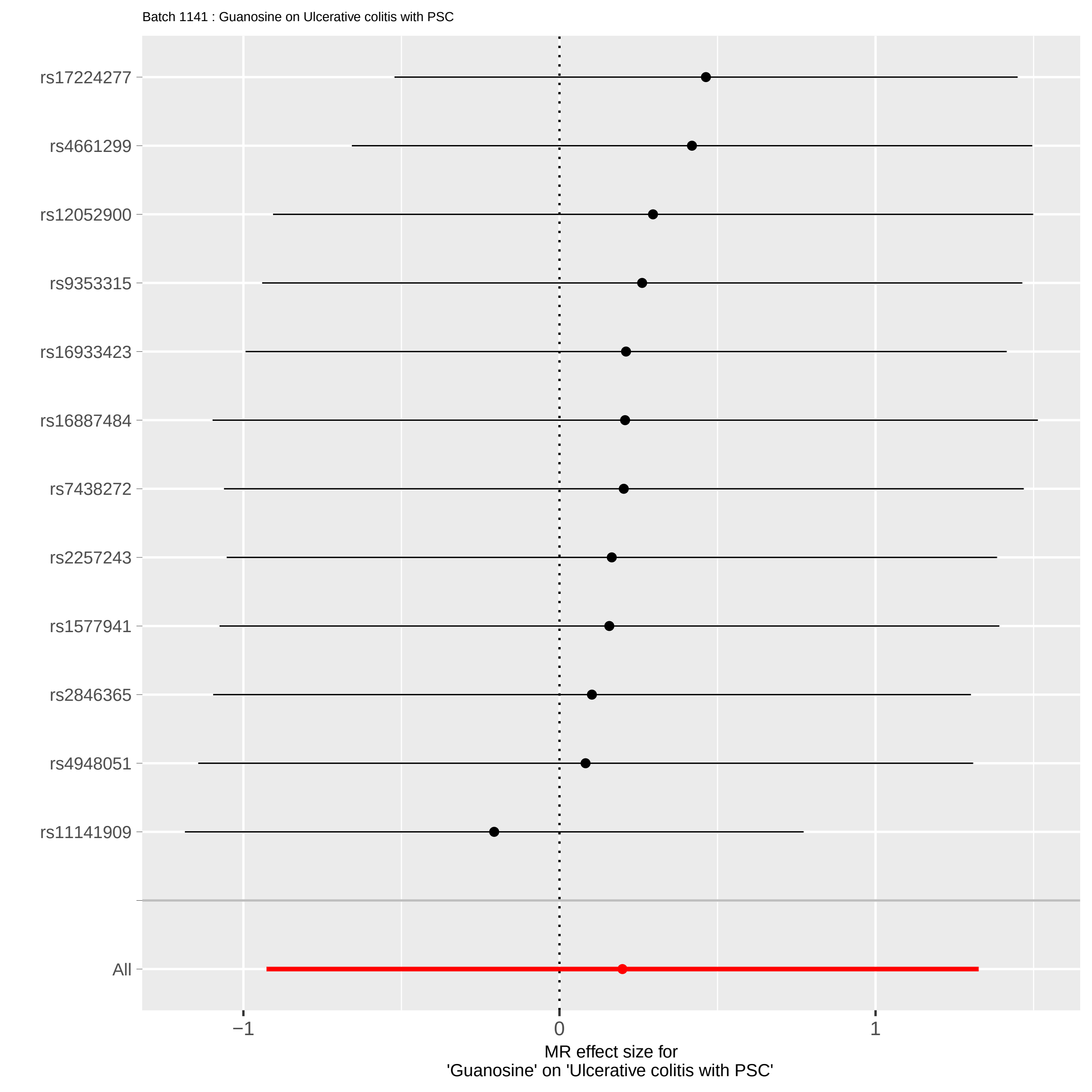
Batch 1141 : Guanosine on Ulcerative colitis with PSC
rs17224277
rs4661299
rs12052900
rs9353315
rs16933423
rs16887484
rs7438272
rs2257243
rs1577941
rs2846365
rs4948051
rs11141909
All
−1
0
1
MR effect size for
'Guanosine' on 'Ulcerative colitis with PSC'

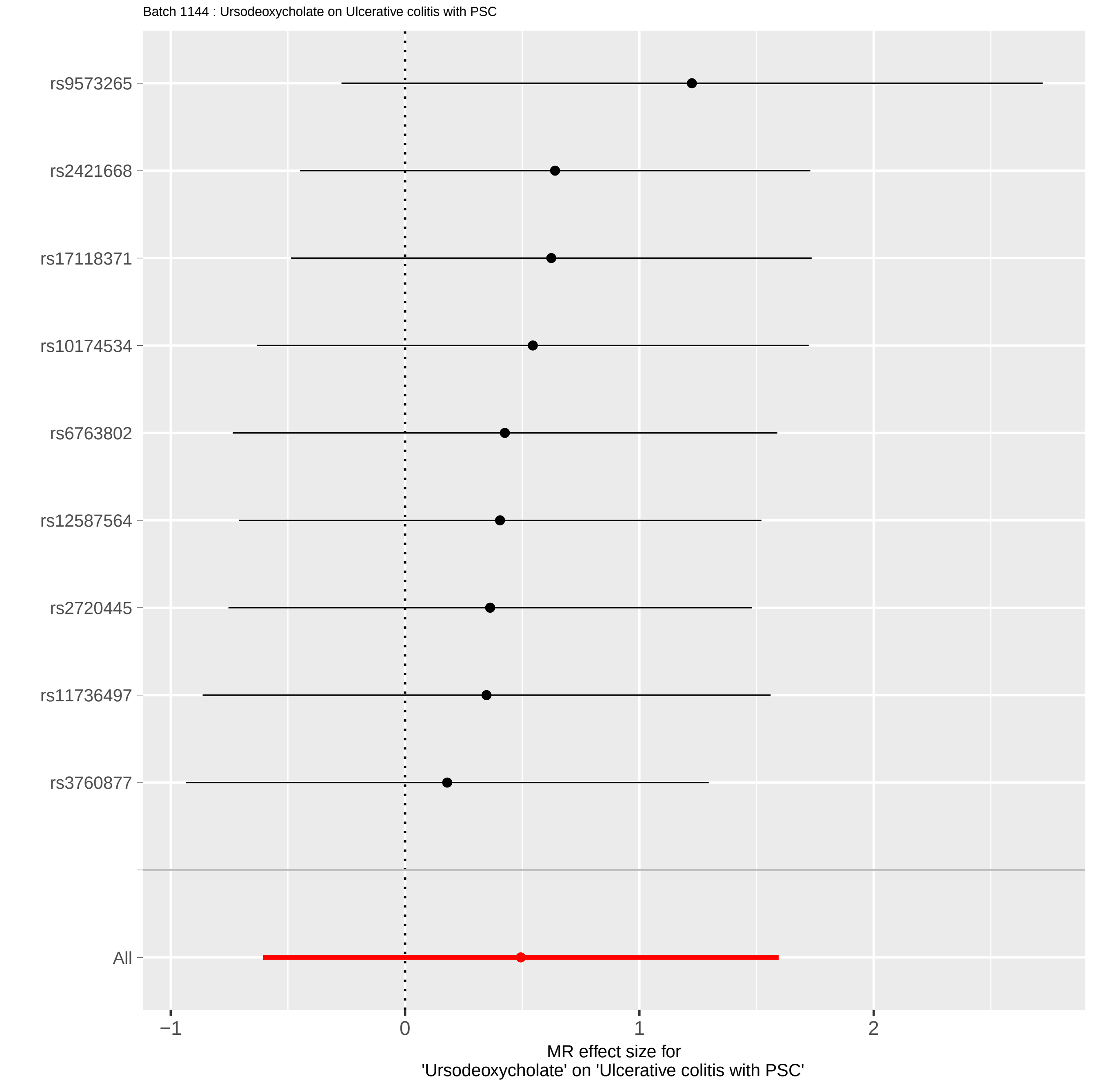

Batch 1144 : Ursodeoxycholate on Ulcerative colitis with PSC
rs9573265
rs2421668
rs17118371
rs10174534
rs6763802
rs12587564
rs2720445
rs11736497
rs3760877
All
−1
0
1
2
MR effect size for
'Ursodeoxycholate' on 'Ulcerative colitis with PSC'

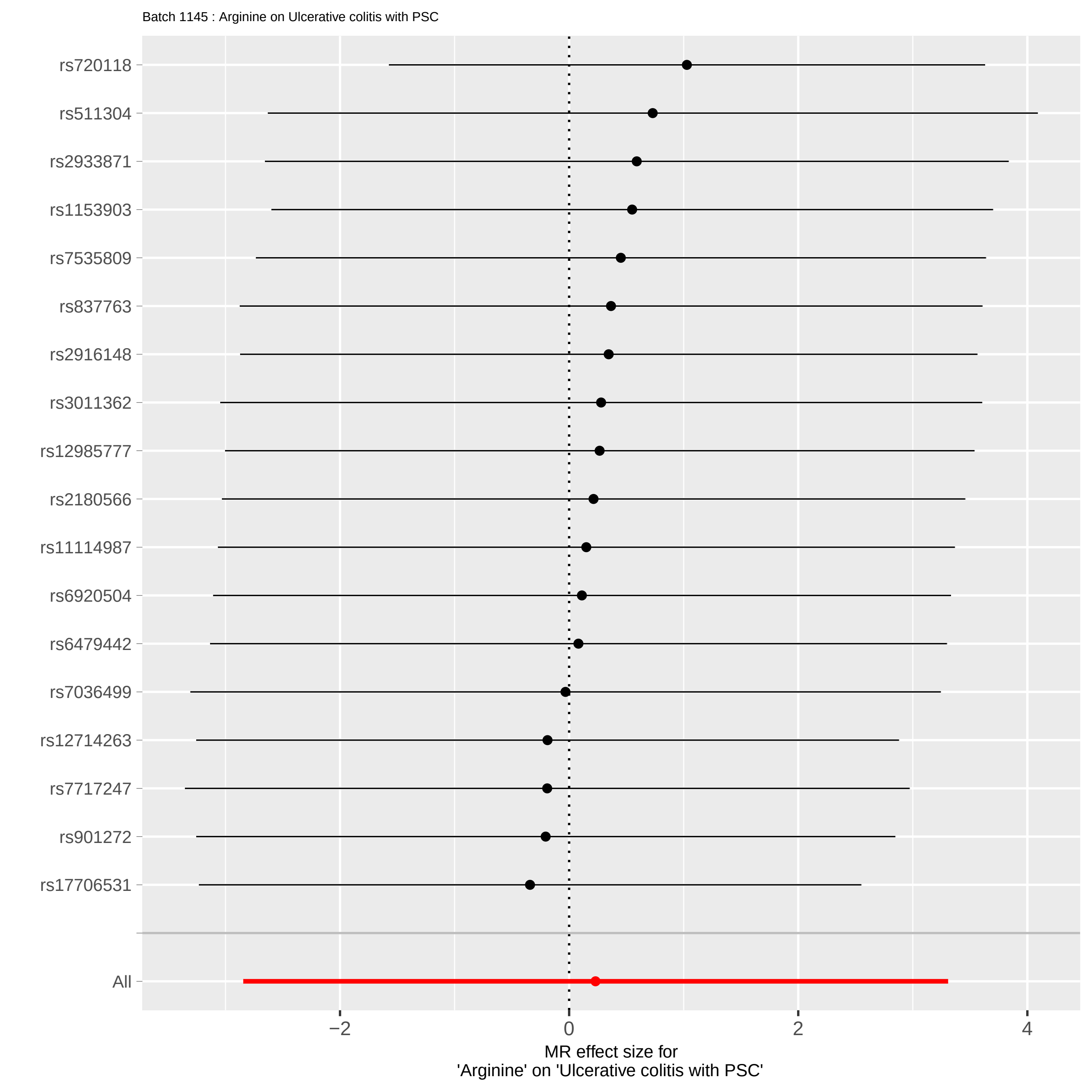

Batch 1145 : Arginine on Ulcerative colitis with PSC
rs720118
rs511304
rs2933871
rs1153903
rs7535809
rs837763
rs2916148
rs3011362
rs12985777
rs2180566
rs11114987
rs6920504
rs6479442
rs7036499
rs12714263
rs7717247
rs901272
rs17706531
All
−2
0
2
4
MR effect size for
'Arginine' on 'Ulcerative colitis with PSC'

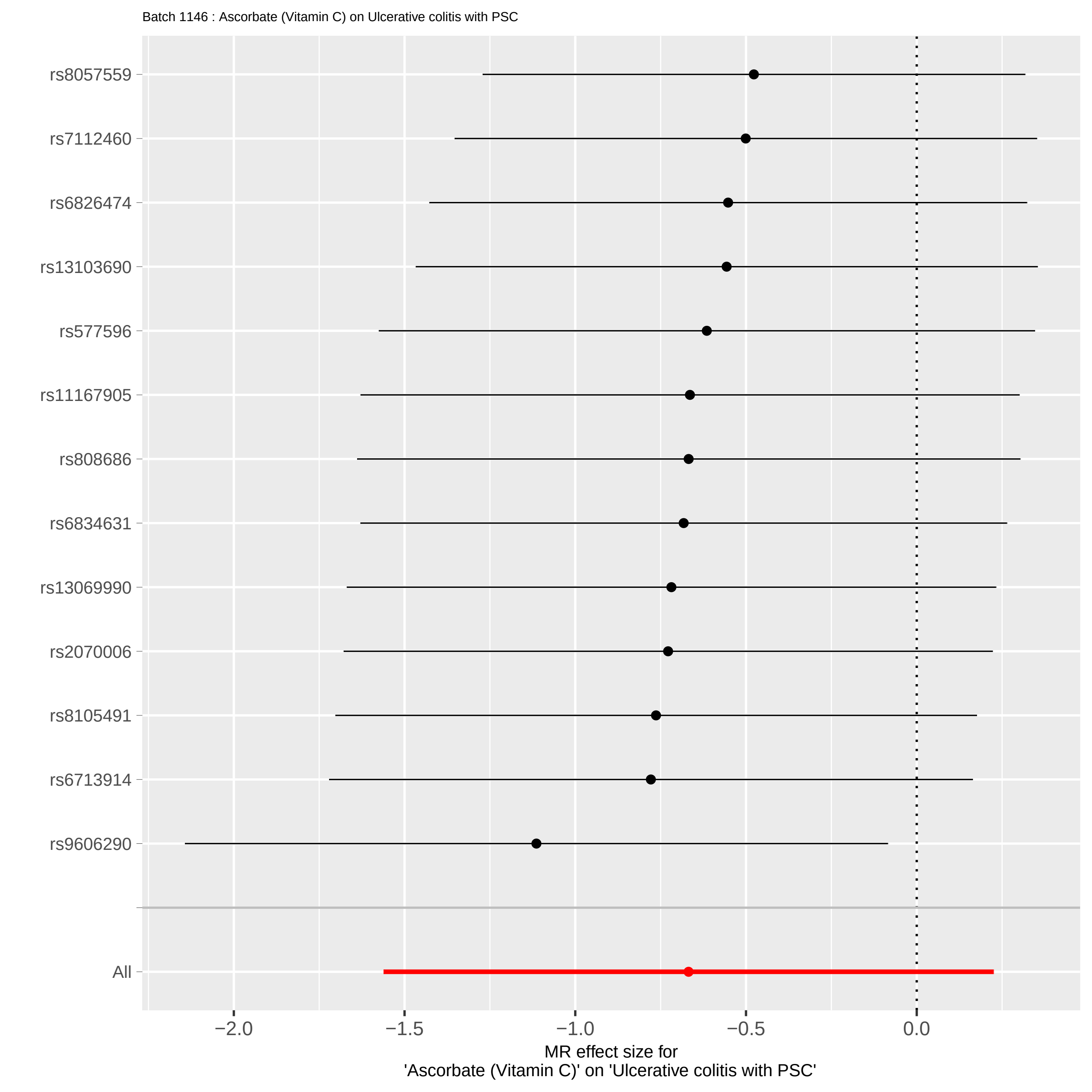
Batch 1146 : Ascorbate (Vitamin C) on Ulcerative colitis with PSC
rs8057559
rs7112460
rs6826474
rs13103690
rs577596
rs11167905
rs808686
rs6834631
rs13069990
rs2070006
rs8105491
rs6713914
rs9606290
All
−2.0
−1.5
−1.0
−0.5
0.0
MR effect size for
'Ascorbate (Vitamin C)' on 'Ulcerative colitis with PSC'

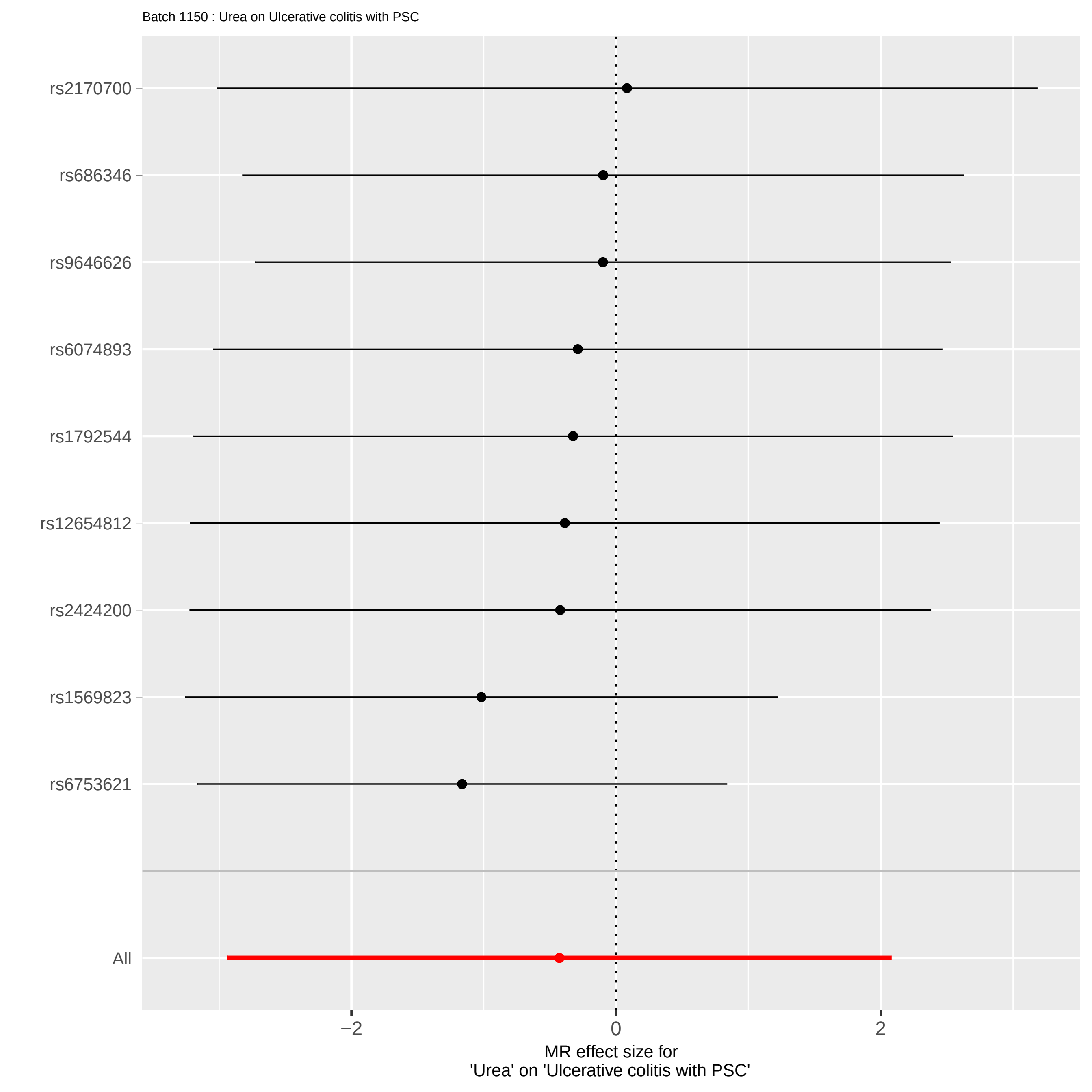

Batch 1150 : Urea on Ulcerative colitis with PSC
rs2170700
rs686346
rs9646626
rs6074893
rs1792544
rs12654812
rs2424200
rs1569823
rs6753621
All
−2
0
2
MR effect size for
'Urea' on 'Ulcerative colitis with PSC'

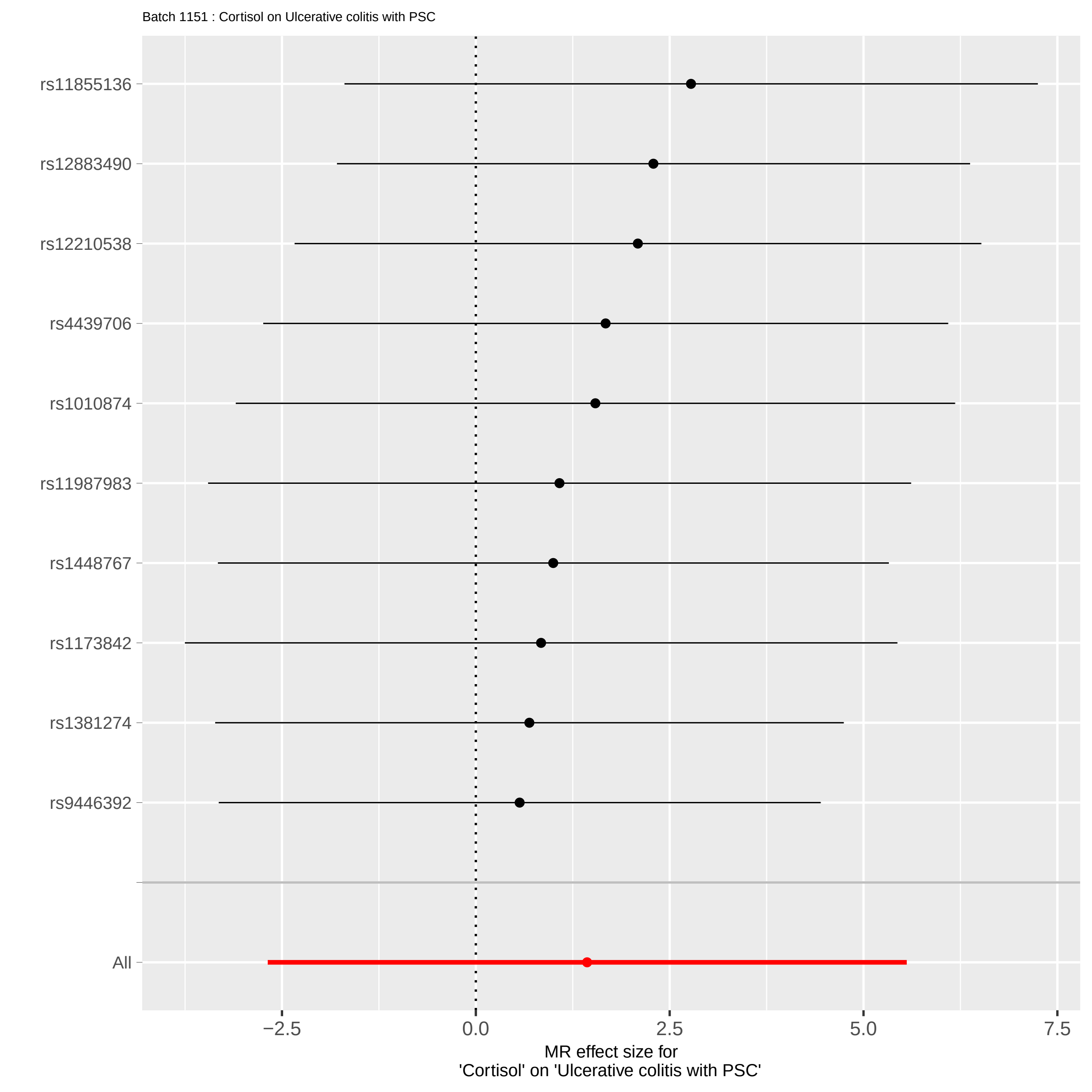

Batch 1151 : Cortisol on Ulcerative colitis with PSC
rs11855136
rs12883490
rs12210538
rs4439706
rs1010874
rs11987983
rs1448767
rs1173842
rs1381274
rs9446392
All
−2.5
0.0
2.5
5.0
7.5
MR effect size for
'Cortisol' on 'Ulcerative colitis with PSC'

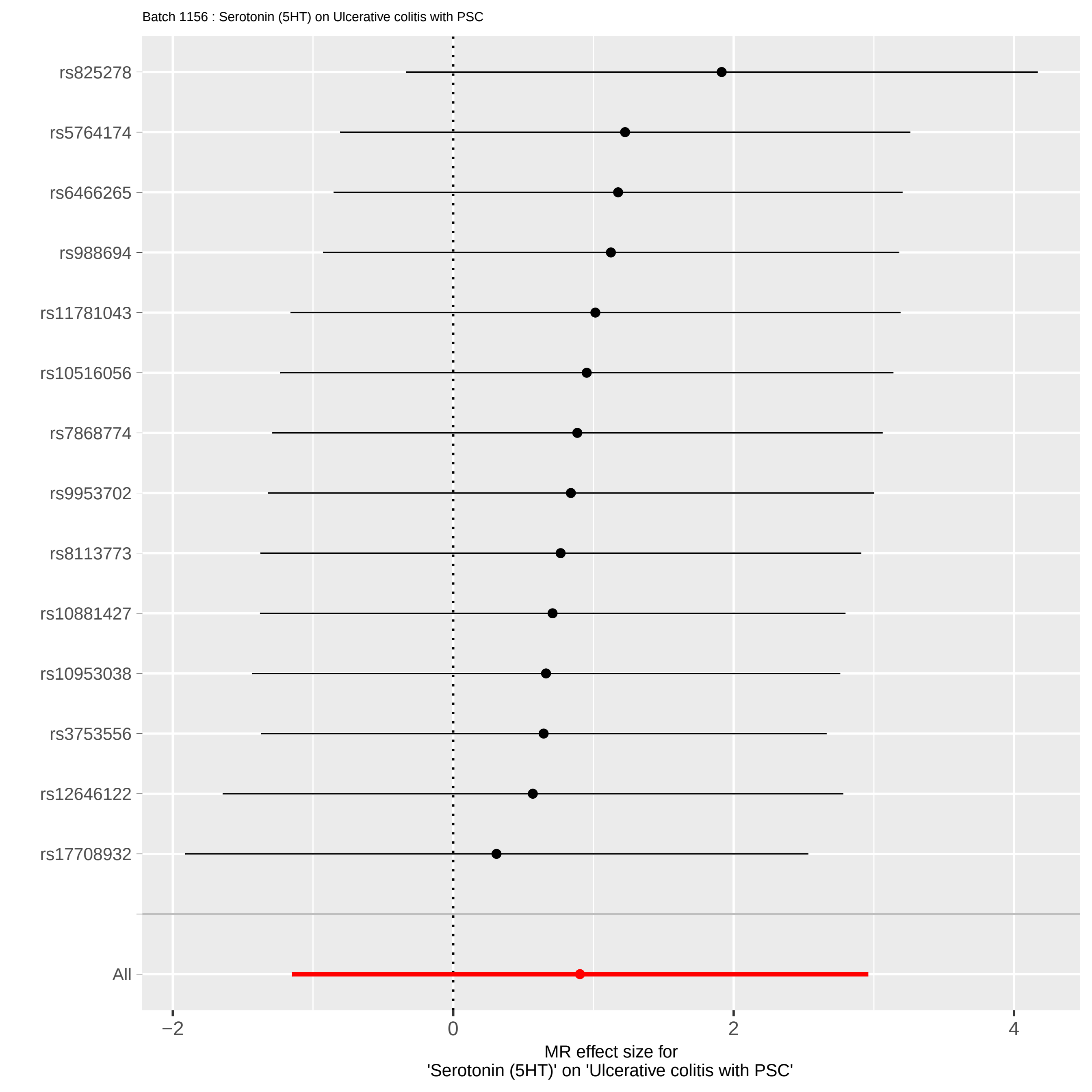

Batch 1156 : Serotonin (5HT) on Ulcerative colitis with PSC
rs825278
rs5764174
rs6466265
rs988694
rs11781043
rs10516056
rs7868774
rs9953702
rs8113773
rs10881427
rs10953038
rs3753556
rs12646122
rs17708932
All
−2
0
2
4
MR effect size for
'Serotonin (5HT)' on 'Ulcerative colitis with PSC'

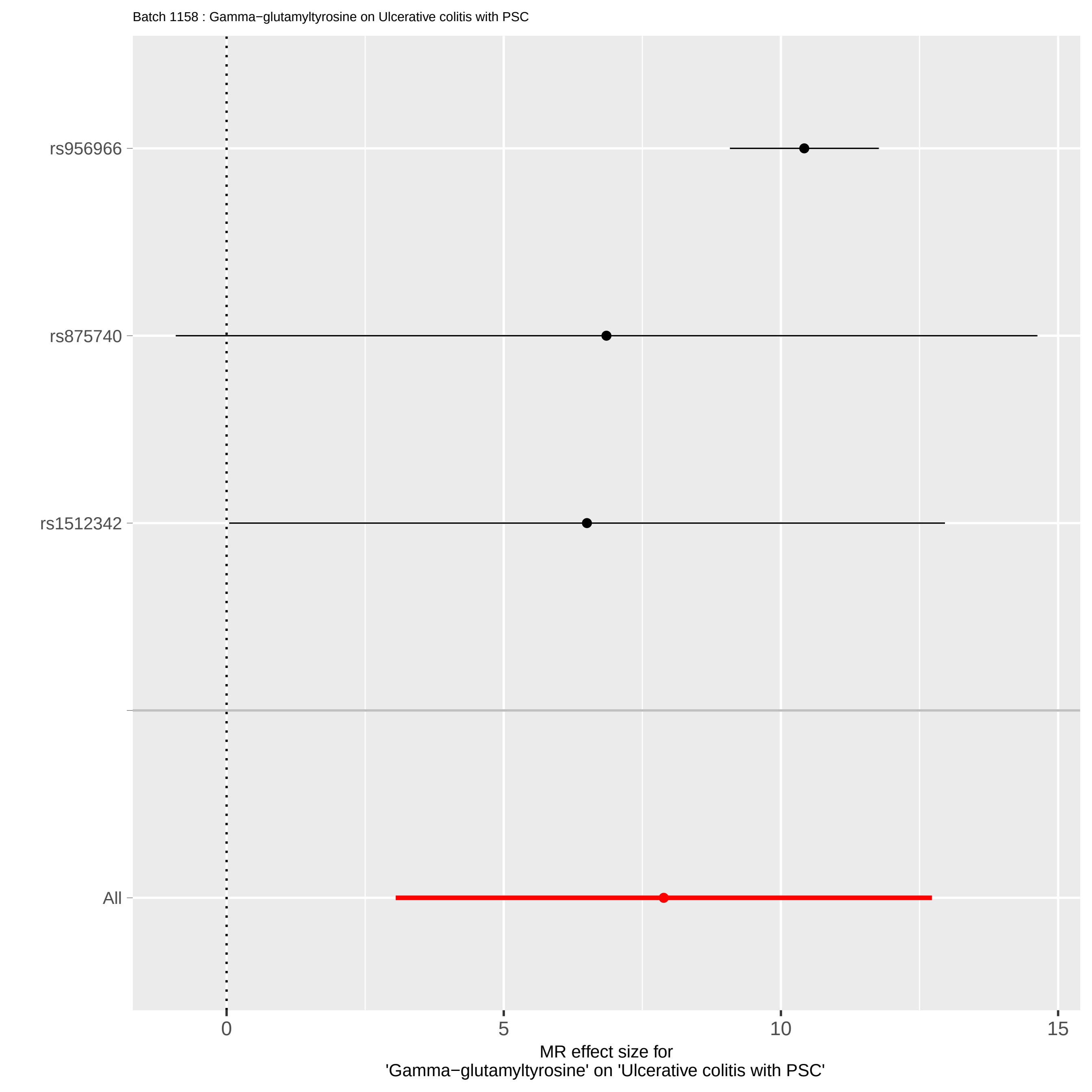

Batch 1158 : Gamma−glutamyltyrosine on Ulcerative colitis with PSC
rs956966
rs875740
rs1512342
All
0
5
10
15
MR effect size for
'Gamma−glutamyltyrosine' on 'Ulcerative colitis with PSC'

Batch 1161 : Xanthine on Ulcerative colitis with PSC

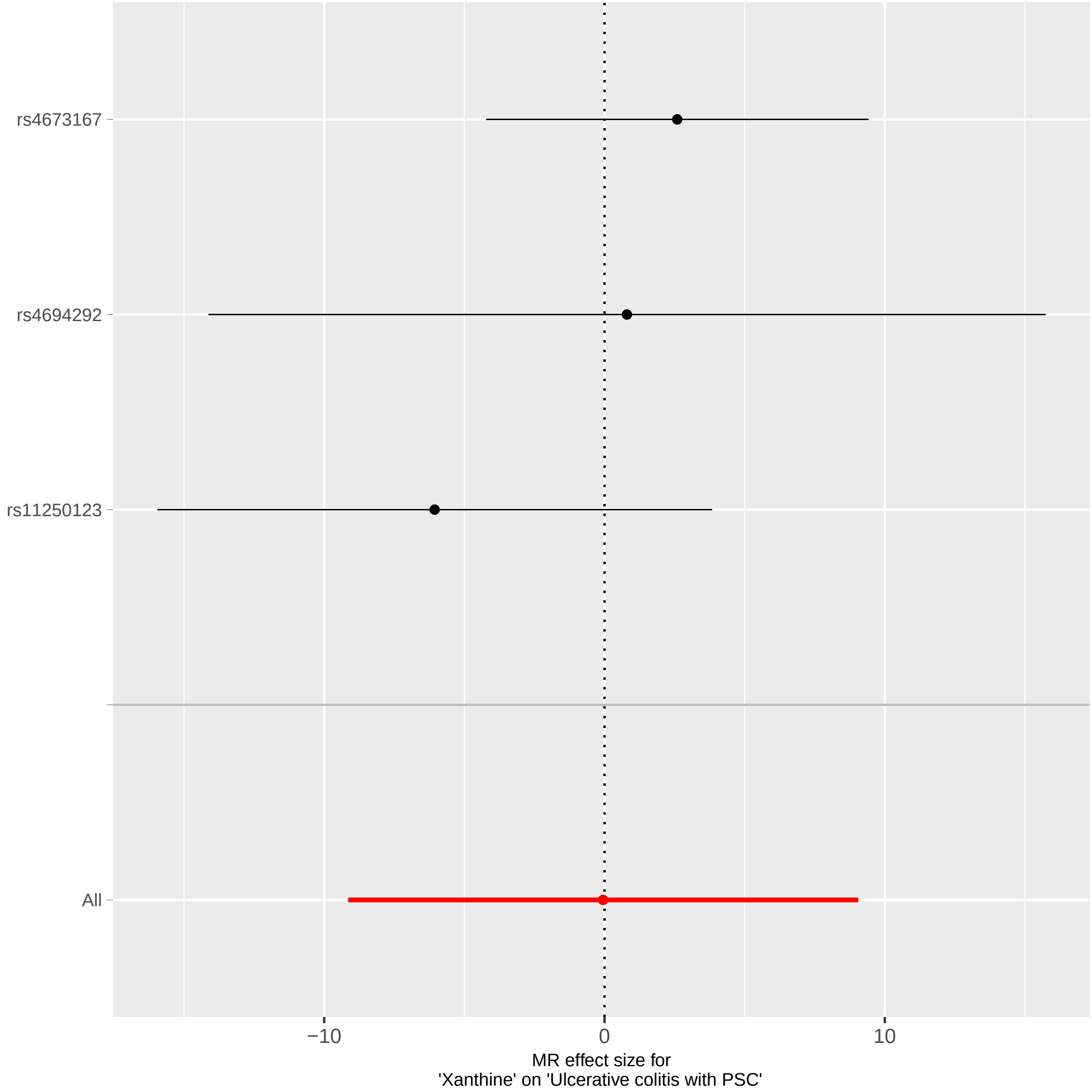

Batch 1162 : Phosphate on Ulcerative colitis with PSC

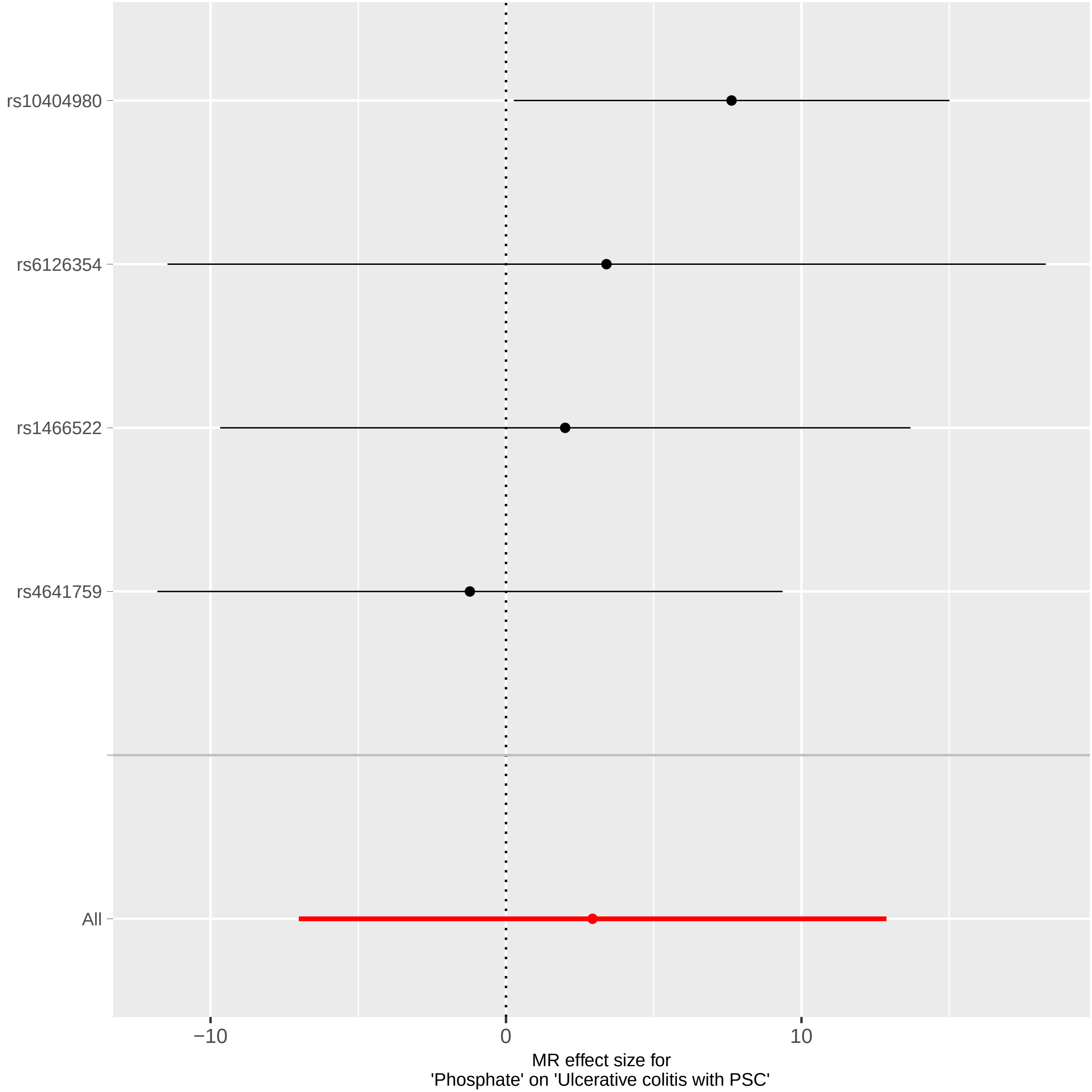

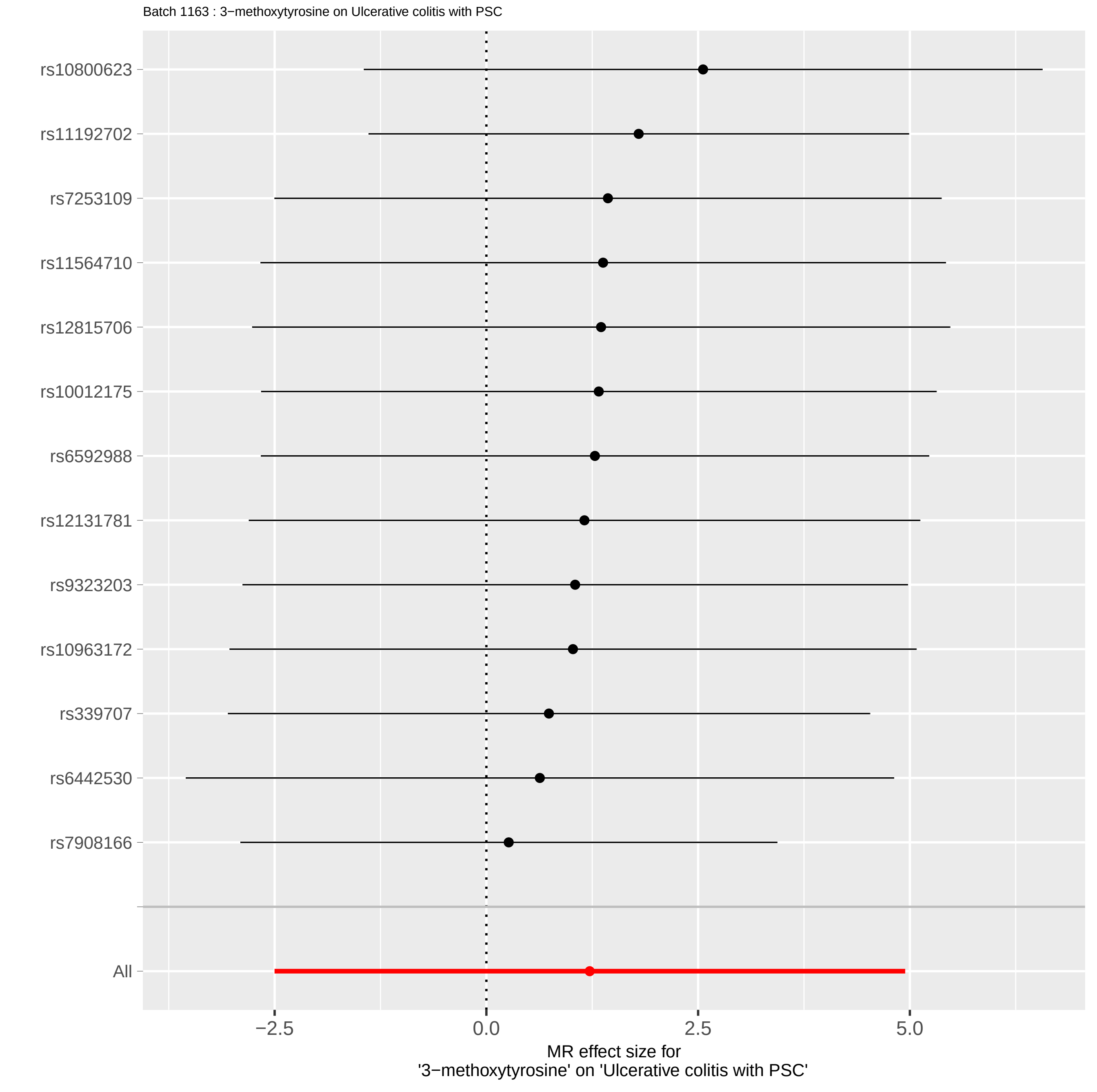
Batch 1163 : 3−methoxytyrosine on Ulcerative colitis with PSC
rs10800623
rs11192702
rs7253109
rs11564710
rs12815706
rs10012175
rs6592988
rs12131781
rs9323203
rs10963172
rs339707
rs6442530
rs7908166
All
−2.5
0.0
2.5
5.0
MR effect size for
'3−methoxytyrosine' on 'Ulcerative colitis with PSC'

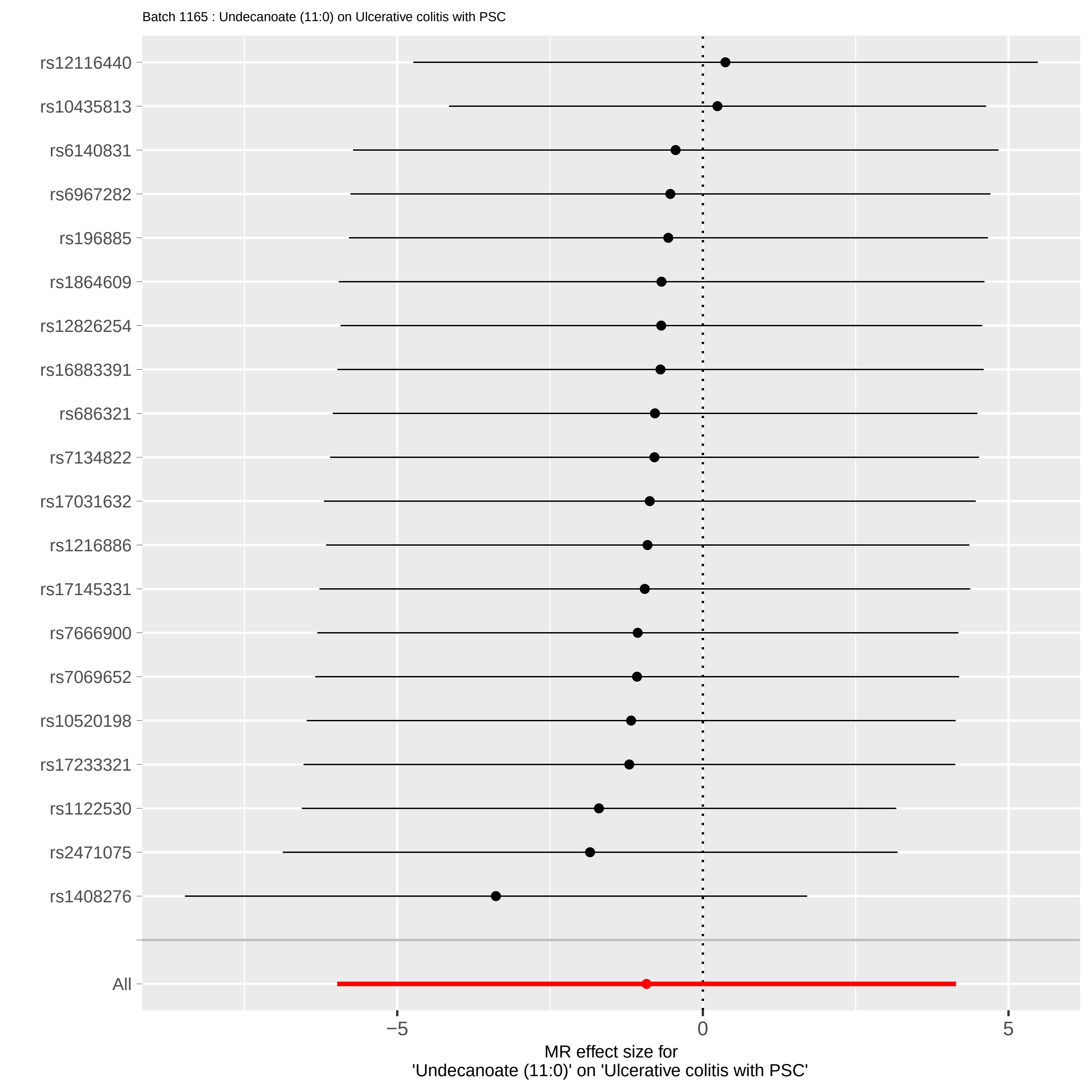

Batch 1165 : Undecanoate (11:0) on Ulcerative colitis with PSC
rs12116440
rs10435813
rs6140831
rs6967282
rs196885
rs1864609
rs12826254
rs16883391
rs686321
rs7134822
rs17031632
rs1216886
rs17145331
rs7666900
rs7069652
rs10520198
rs17233321
rs1122530
rs2471075
rs1408276
All
−5
0
5
MR effect size for
'Undecanoate (11:0)' on 'Ulcerative colitis with PSC'

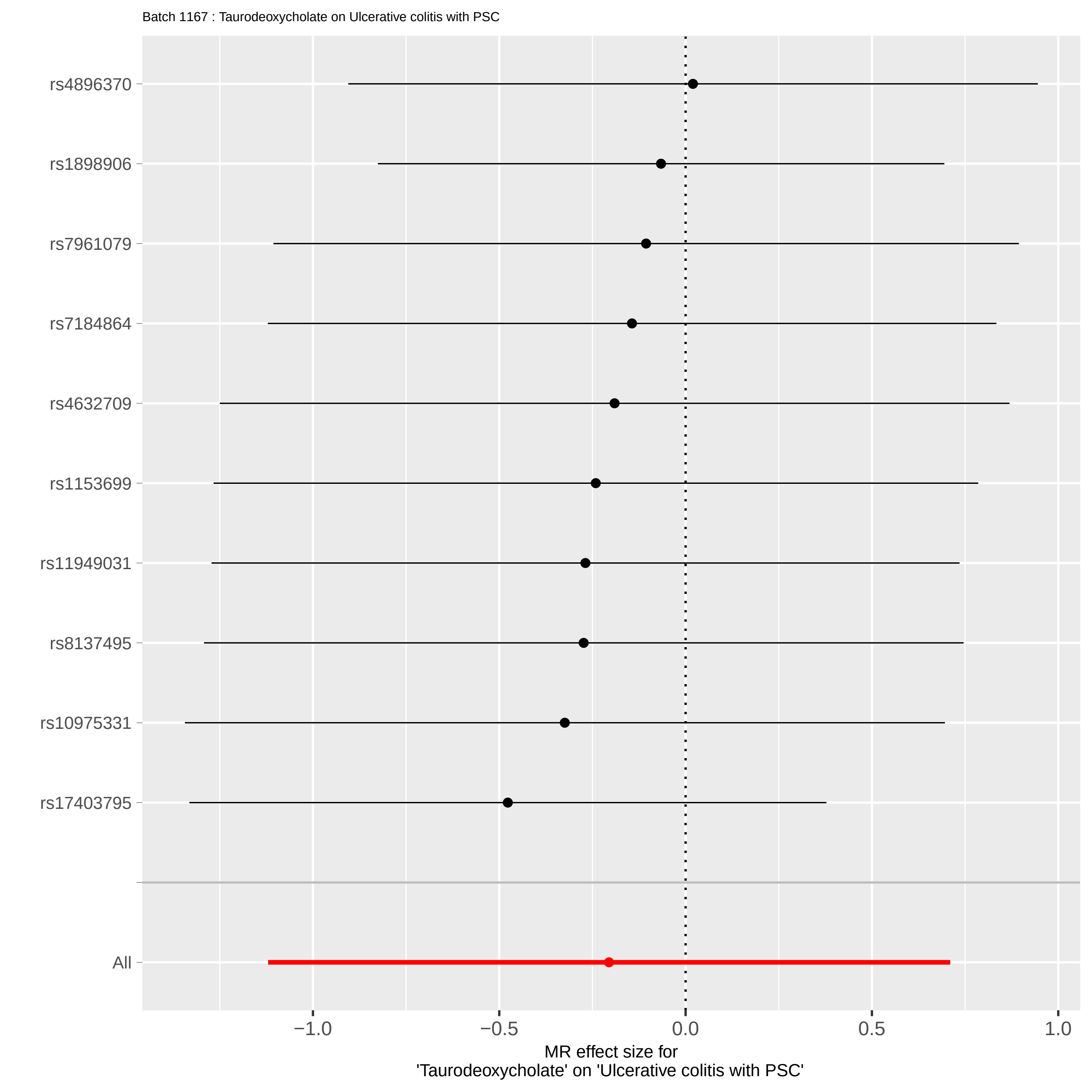
Batch 1167 : Taurodeoxycholate on Ulcerative colitis with PSC
rs4896370
rs1898906
rs7961079
rs7184864
rs4632709
rs1153699
rs11949031
rs8137495
rs10975331
rs17403795
All
−1.0
−0.5
0.0
0.5
1.0
MR effect size for
'Taurodeoxycholate' on 'Ulcerative colitis with PSC'

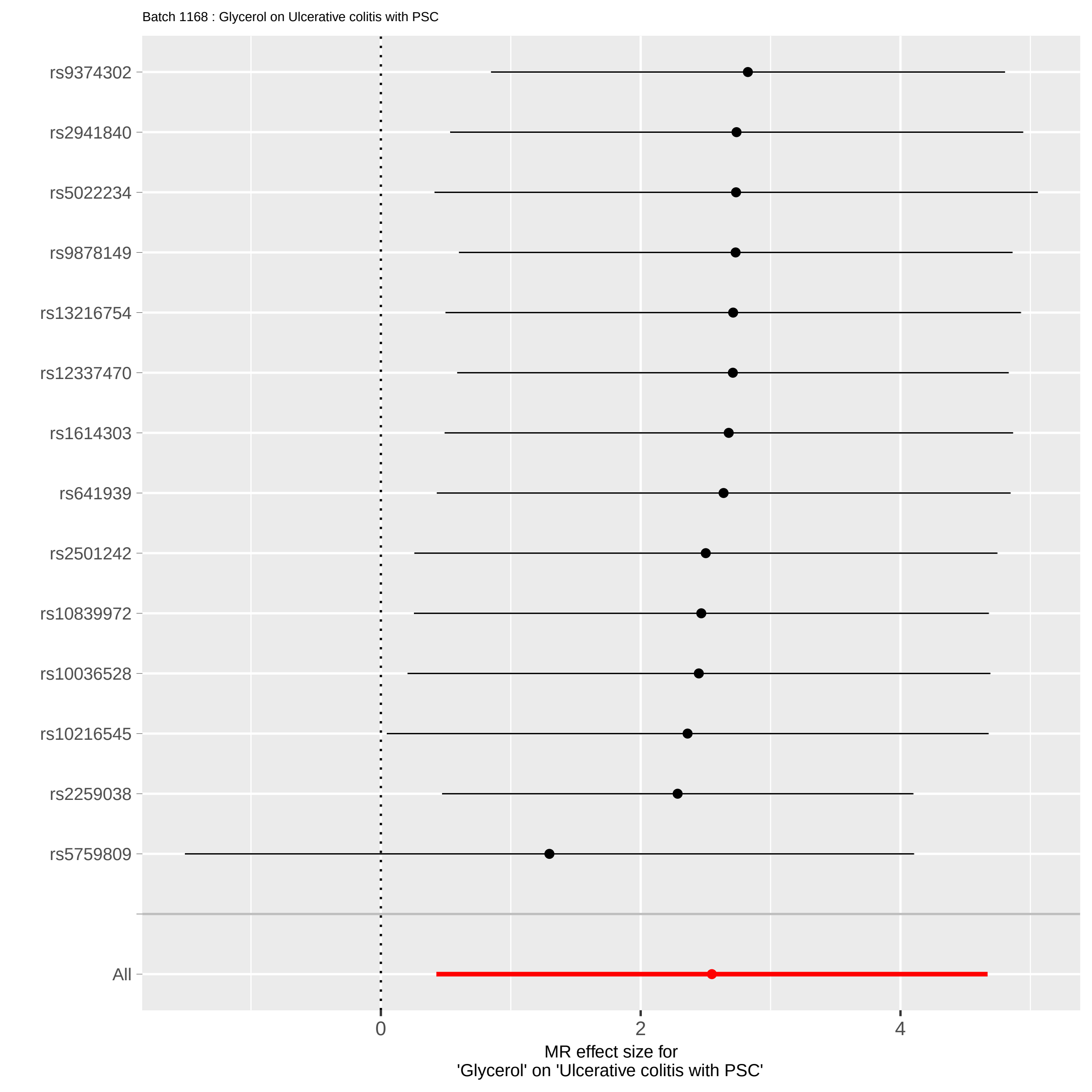
Batch 1168 : Glycerol on Ulcerative colitis with PSC
rs9374302
rs2941840
rs5022234
rs9878149
rs13216754
rs12337470
rs1614303
rs641939
rs2501242
rs10839972
rs10036528
rs10216545
rs2259038
rs5759809
All
0
2
4
MR effect size for
'Glycerol' on 'Ulcerative colitis with PSC'

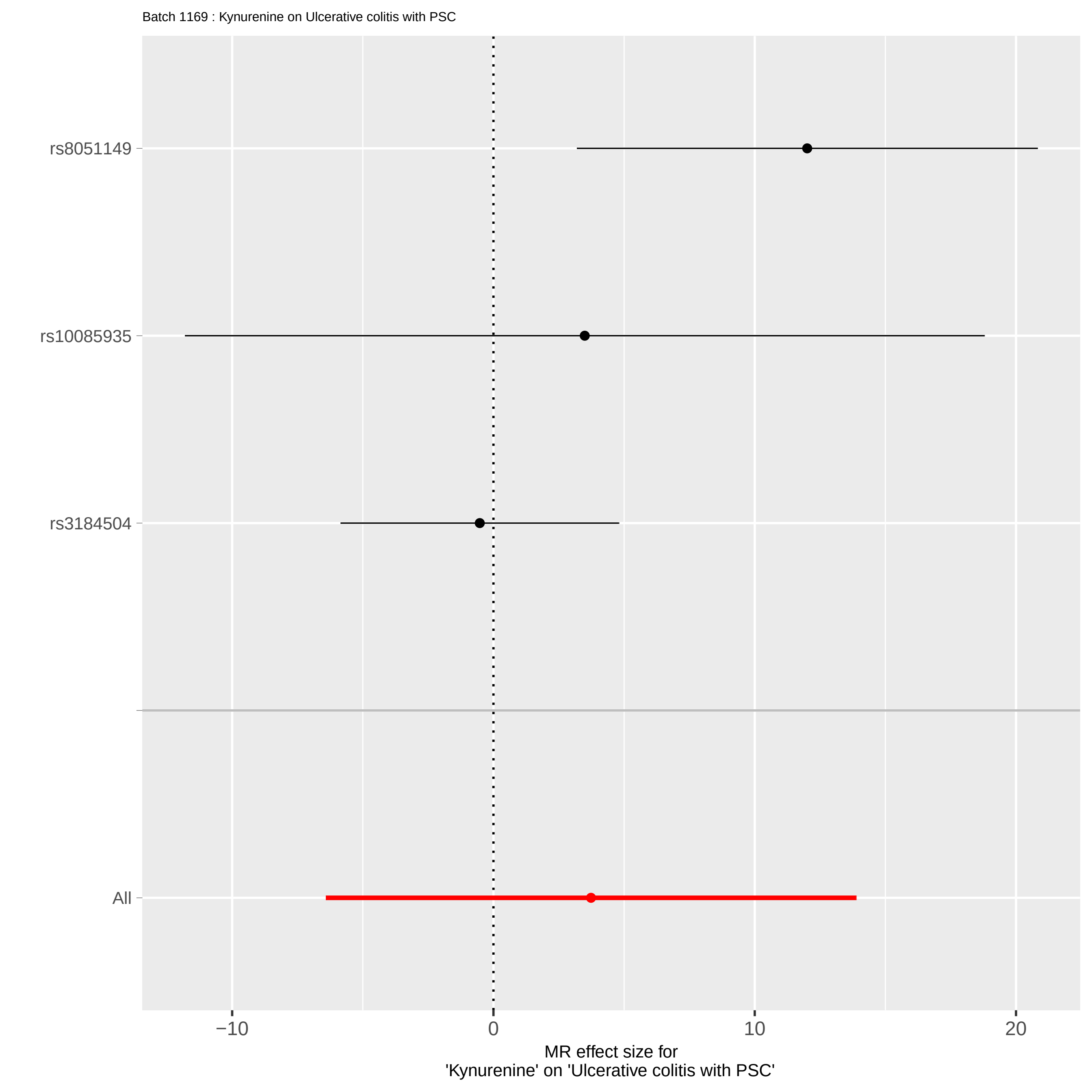
Batch 1169 : Kynurenine on Ulcerative colitis with PSC
rs8051149
rs10085935
rs3184504
All
−10
0
10
20
MR effect size for
'Kynurenine' on 'Ulcerative colitis with PSC'

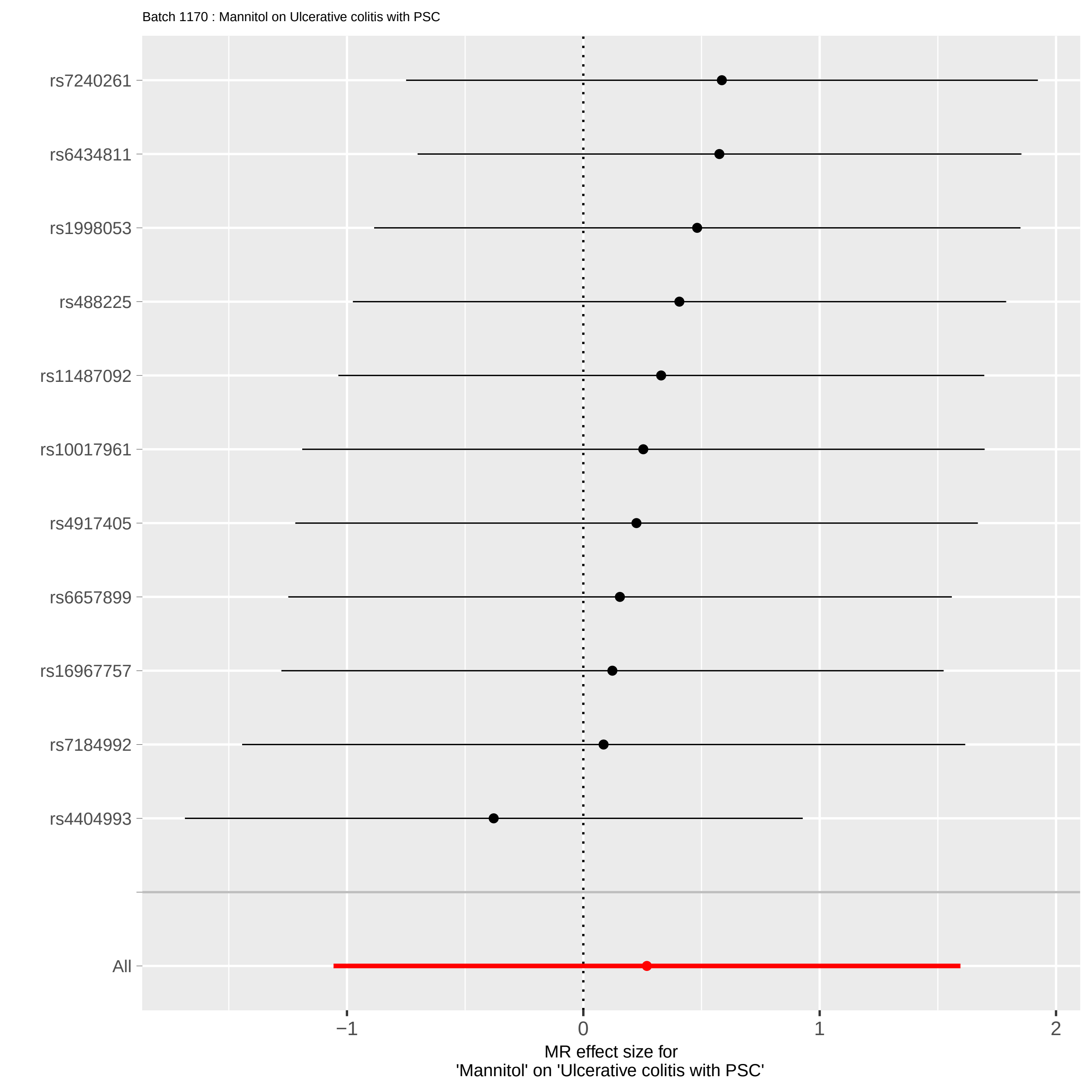
Batch 1170 : Mannitol on Ulcerative colitis with PSC
rs7240261
rs6434811
rs1998053
rs488225
rs11487092
rs10017961
rs4917405
rs6657899
rs16967757
rs7184992
rs4404993
All
−1
0
1
2
MR effect size for
'Mannitol' on 'Ulcerative colitis with PSC'

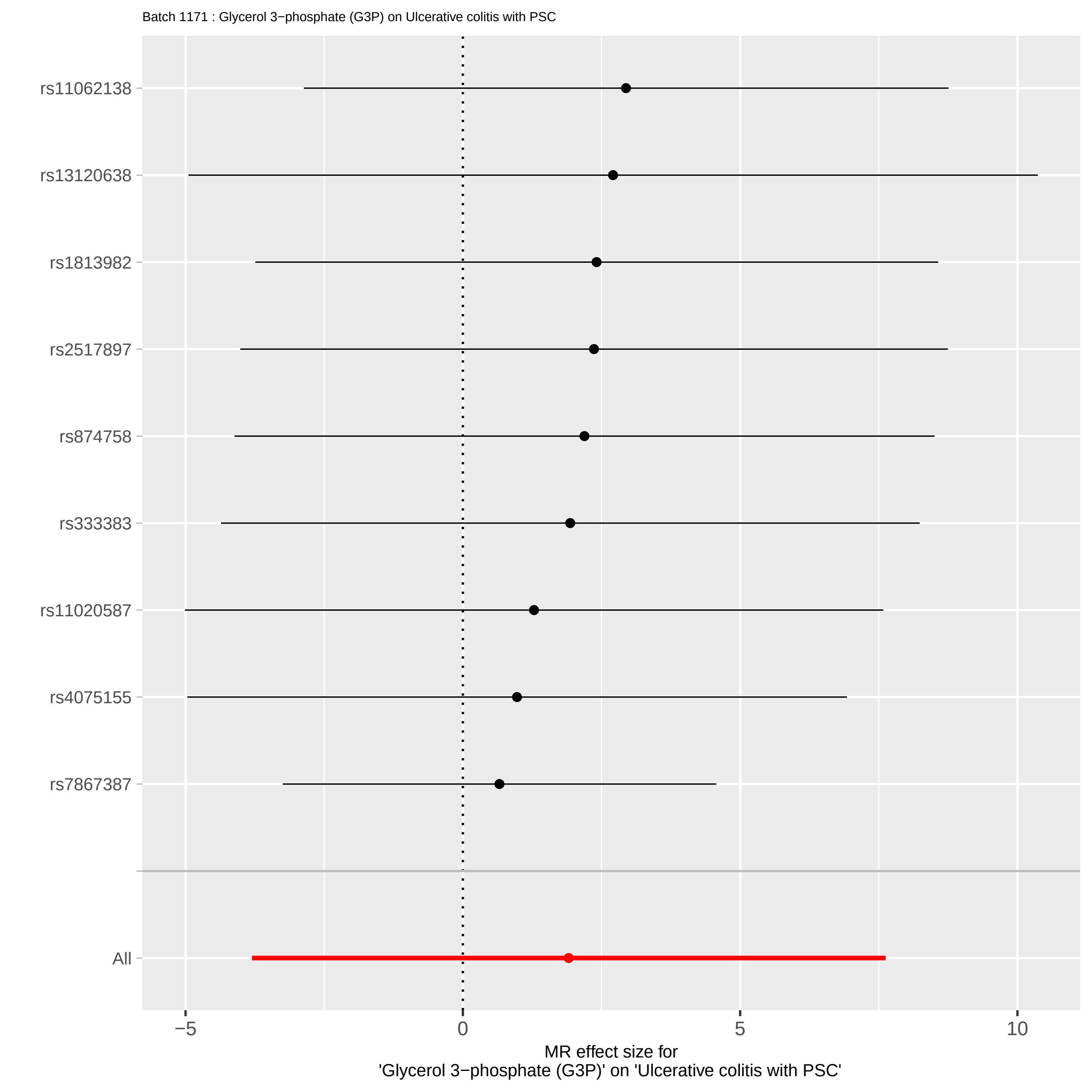
Batch 1171 : Glycerol 3−phosphate (G3P) on Ulcerative colitis with PSC
rs11062138
rs13120638
rs1813982
rs2517897
rs874758
rs333383
rs11020587
rs4075155
rs7867387
All
−5
0
5
10
MR effect size for
'Glycerol 3−phosphate (G3P)' on 'Ulcerative colitis with PSC'

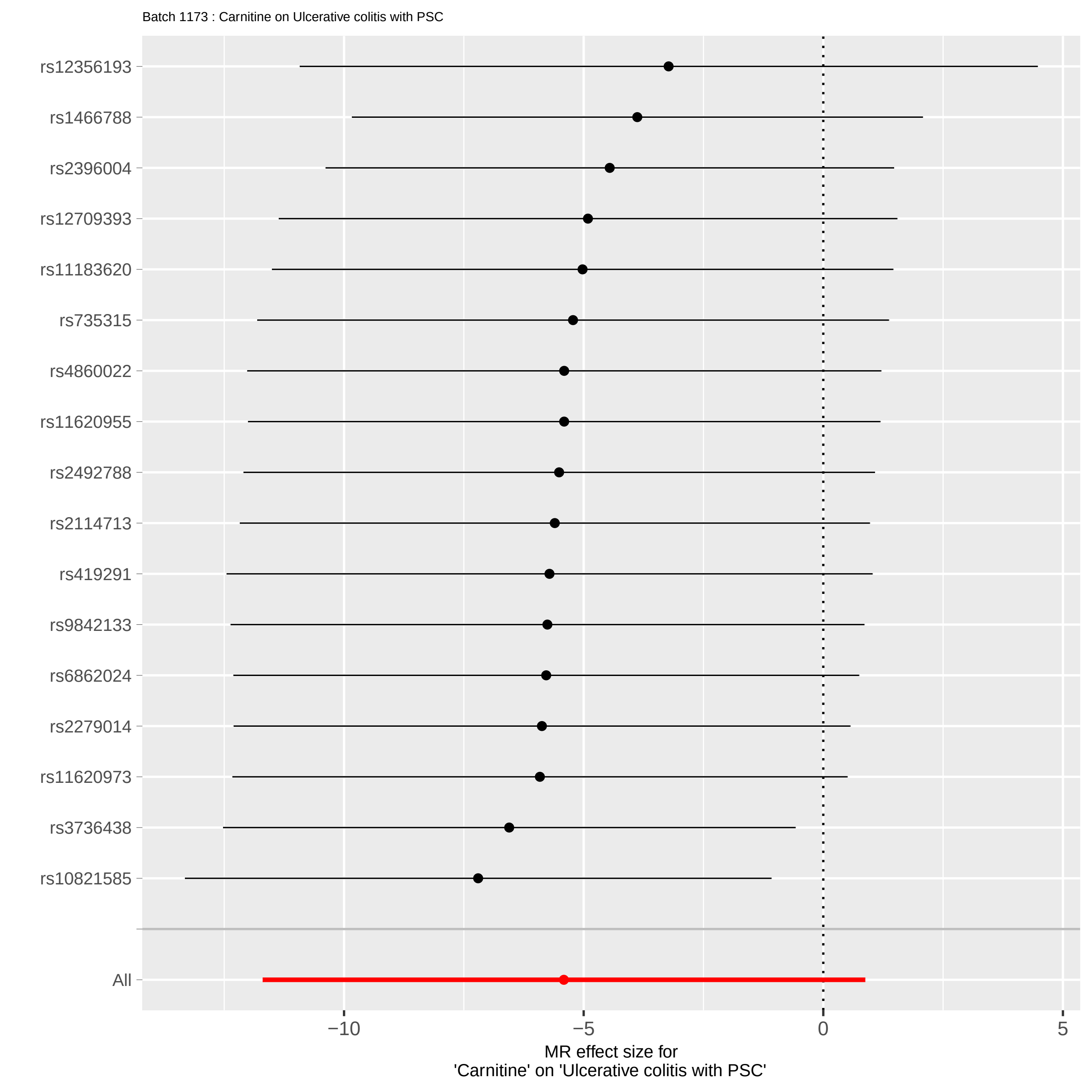

Batch 1173 : Carnitine on Ulcerative colitis with PSC
rs12356193
rs1466788
rs2396004
rs12709393
rs11183620
rs735315
rs4860022
rs11620955
rs2492788
rs2114713
rs419291
rs9842133
rs6862024
rs2279014
rs11620973
rs3736438
rs10821585
All
−10
−5
0
5
MR effect size for
'Carnitine' on 'Ulcerative colitis with PSC'

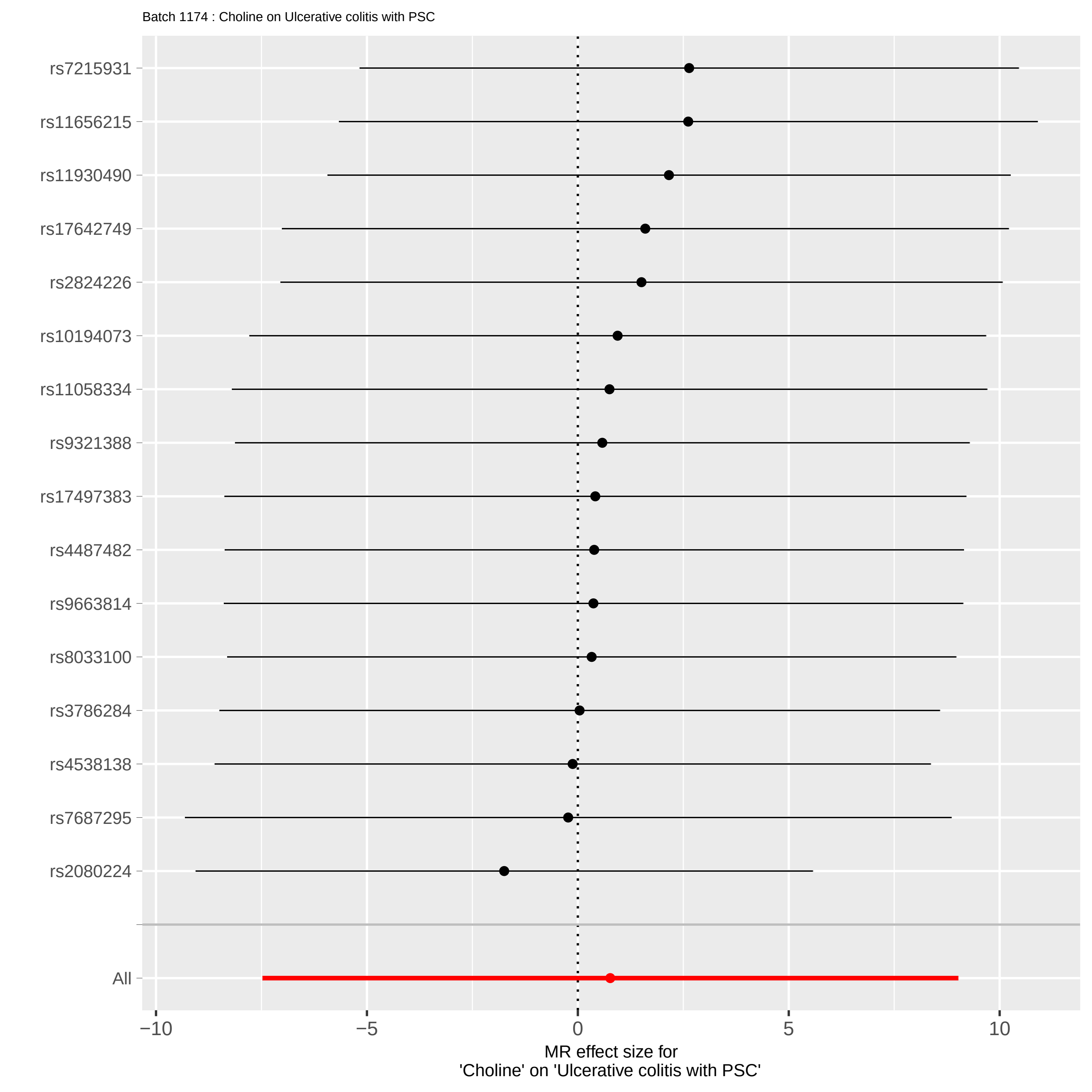
Batch 1174 : Choline on Ulcerative colitis with PSC
rs7215931
rs11656215
rs11930490
rs17642749
rs2824226
rs10194073
rs11058334
rs9321388
rs17497383
rs4487482
rs9663814
rs8033100
rs3786284
rs4538138
rs7687295
rs2080224
All
−10
−5
0
5
10
MR effect size for
'Choline' on 'Ulcerative colitis with PSC'

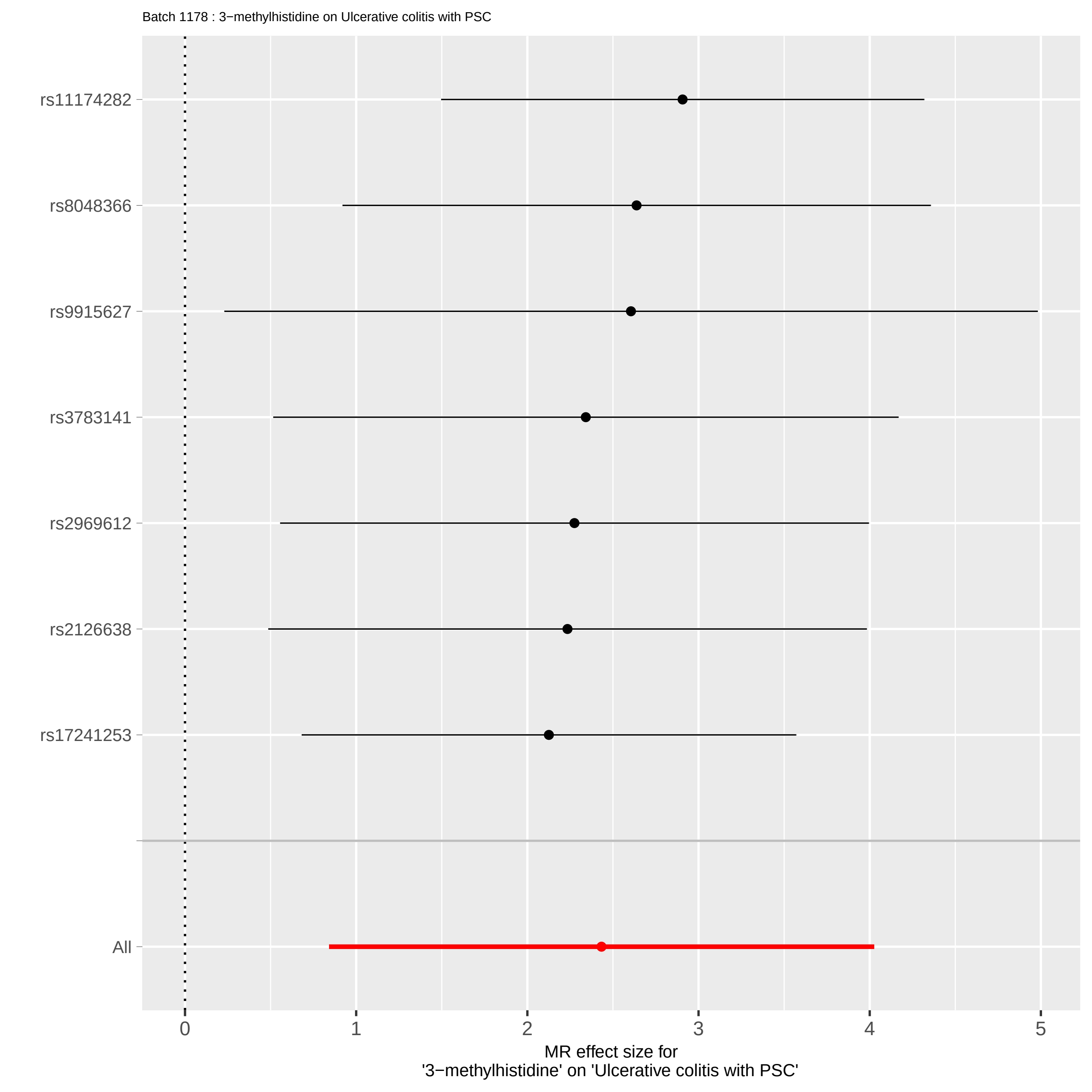

Batch 1178 : 3−methylhistidine on Ulcerative colitis with PSC
rs11174282
rs8048366
rs9915627
rs3783141
rs2969612
rs2126638
rs17241253
All
0
1
2
3
4
5
MR effect size for
'3−methylhistidine' on 'Ulcerative colitis with PSC'

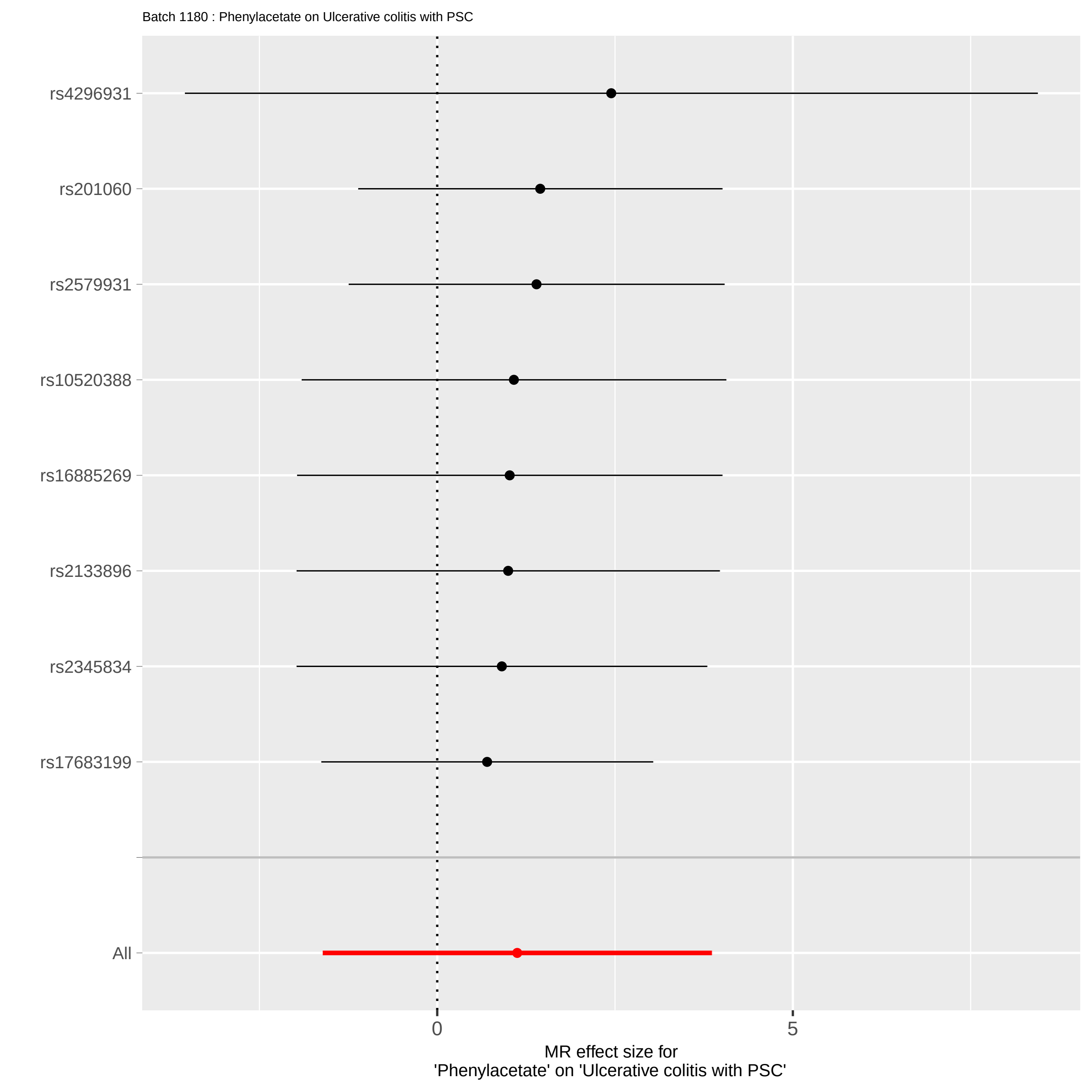
Batch 1180 : Phenylacetate on Ulcerative colitis with PSC
rs4296931
rs201060
rs2579931
rs10520388
rs16885269
rs2133896
rs2345834
rs17683199
All
0
5
MR effect size for
'Phenylacetate' on 'Ulcerative colitis with PSC'

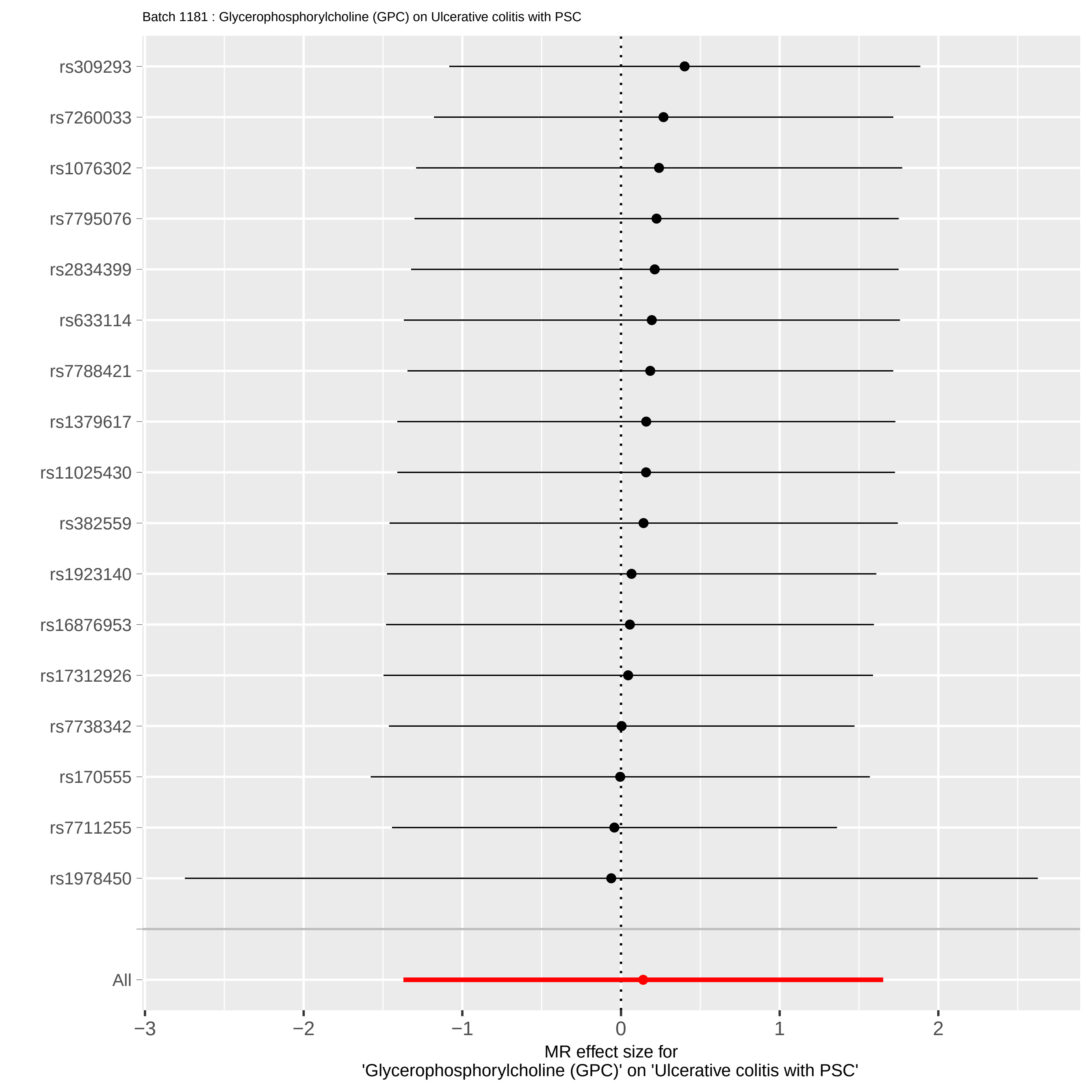
Batch 1181 : Glycerophosphorylcholine (GPC) on Ulcerative colitis with PSC
rs309293
rs7260033
rs1076302
rs7795076
rs2834399
rs633114
rs7788421
rs1379617
rs11025430
rs382559
rs1923140
rs16876953
rs17312926
rs7738342
rs170555
rs7711255
rs1978450
All
−3
−2
−1
0
1
2
MR effect size for
'Glycerophosphorylcholine (GPC)' on 'Ulcerative colitis with PSC'

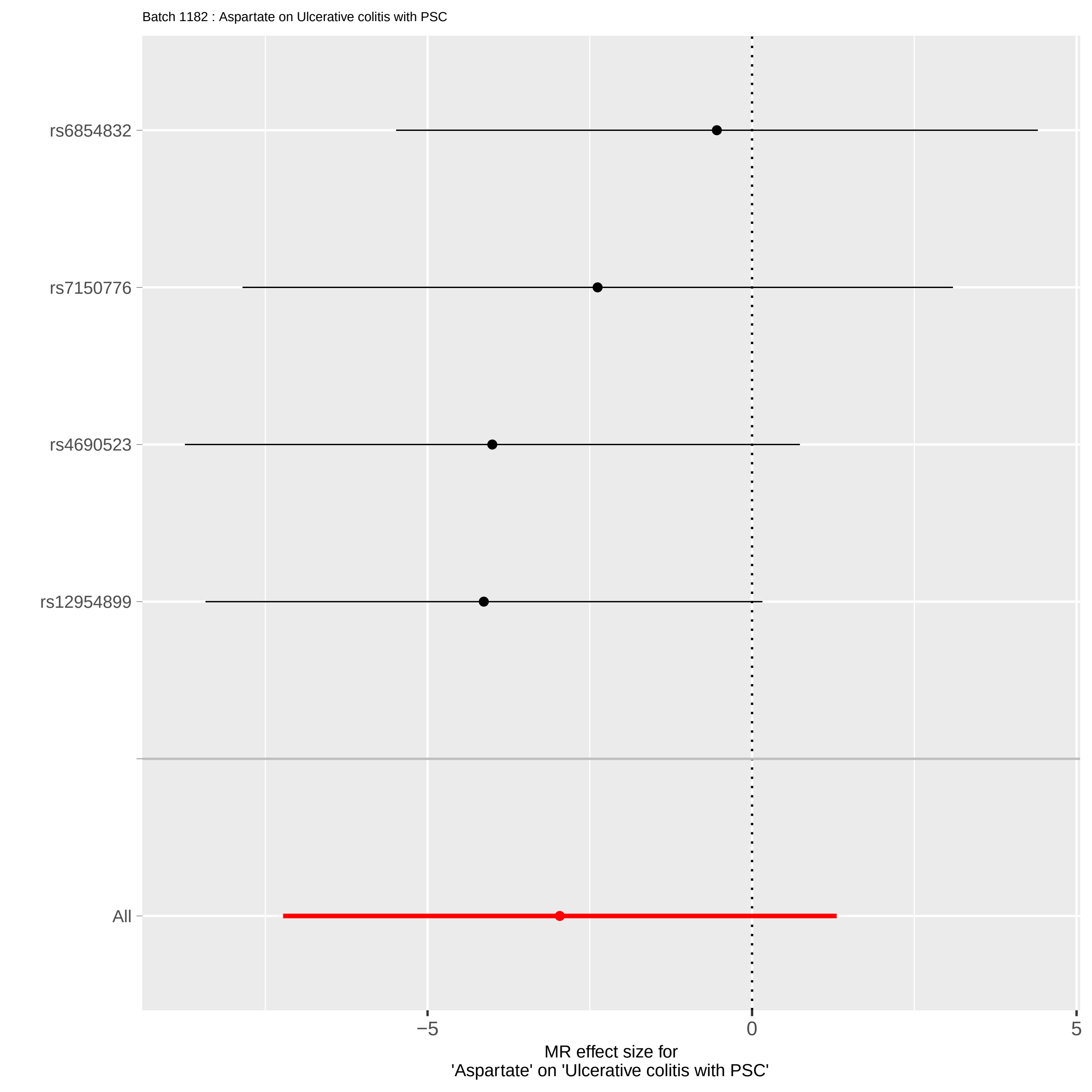

Batch 1182 : Aspartate on Ulcerative colitis with PSC
rs6854832
rs7150776
rs4690523
rs12954899
All
−5
0
5
MR effect size for
'Aspartate' on 'Ulcerative colitis with PSC'

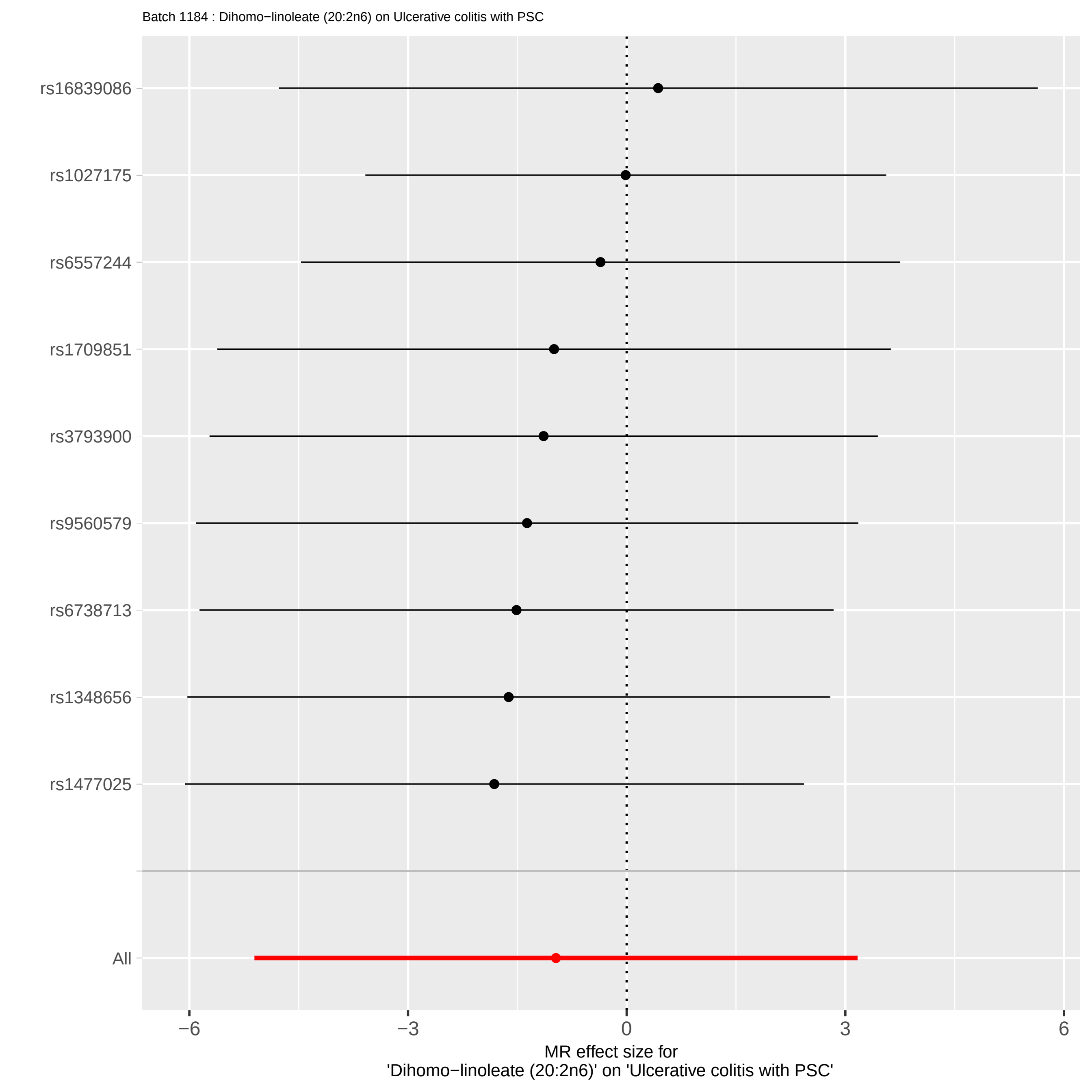

Batch 1184 : Dihomo−linoleate (20:2n6) on Ulcerative colitis with PSC
rs16839086
rs1027175
rs6557244
rs1709851
rs3793900
rs9560579
rs6738713
rs1348656
rs1477025
All
−6
−3
0
3
6
MR effect size for
'Dihomo−linoleate (20:2n6)' on 'Ulcerative colitis with PSC'

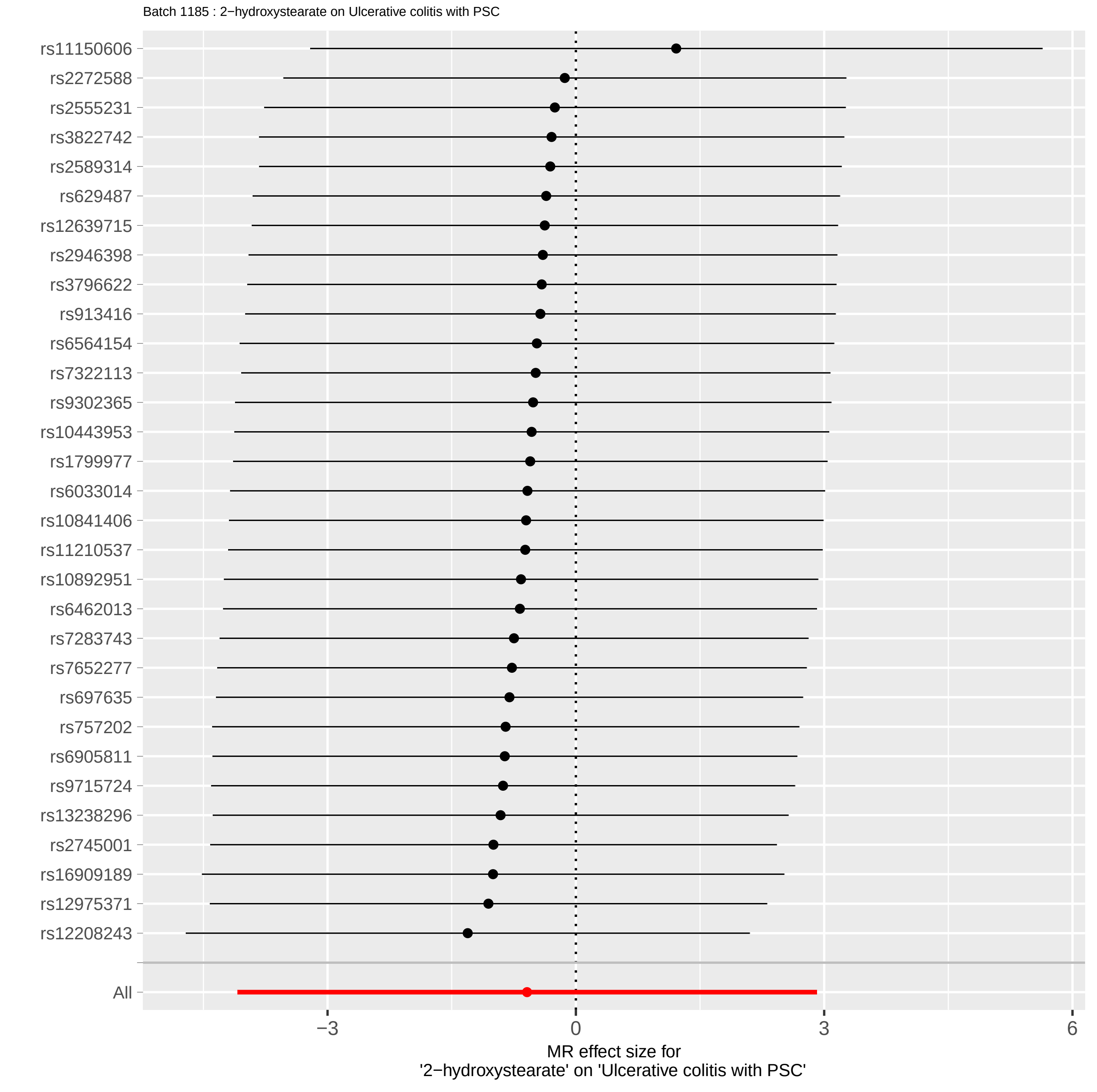
Batch 1185 : 2−hydroxystearate on Ulcerative colitis with PSC
rs11150606
rs2272588
rs2555231
rs3822742
rs2589314
rs629487
rs12639715
rs2946398
rs3796622
rs913416
rs6564154
rs7322113
rs9302365
rs10443953
rs1799977
rs6033014
rs10841406
rs11210537
rs10892951
rs6462013
rs7283743
rs7652277
rs697635
rs757202
rs6905811
rs9715724
rs13238296
rs2745001
rs16909189
rs12975371
rs12208243
All
−3
0
3
6
MR effect size for
'2−hydroxystearate' on 'Ulcerative colitis with PSC'

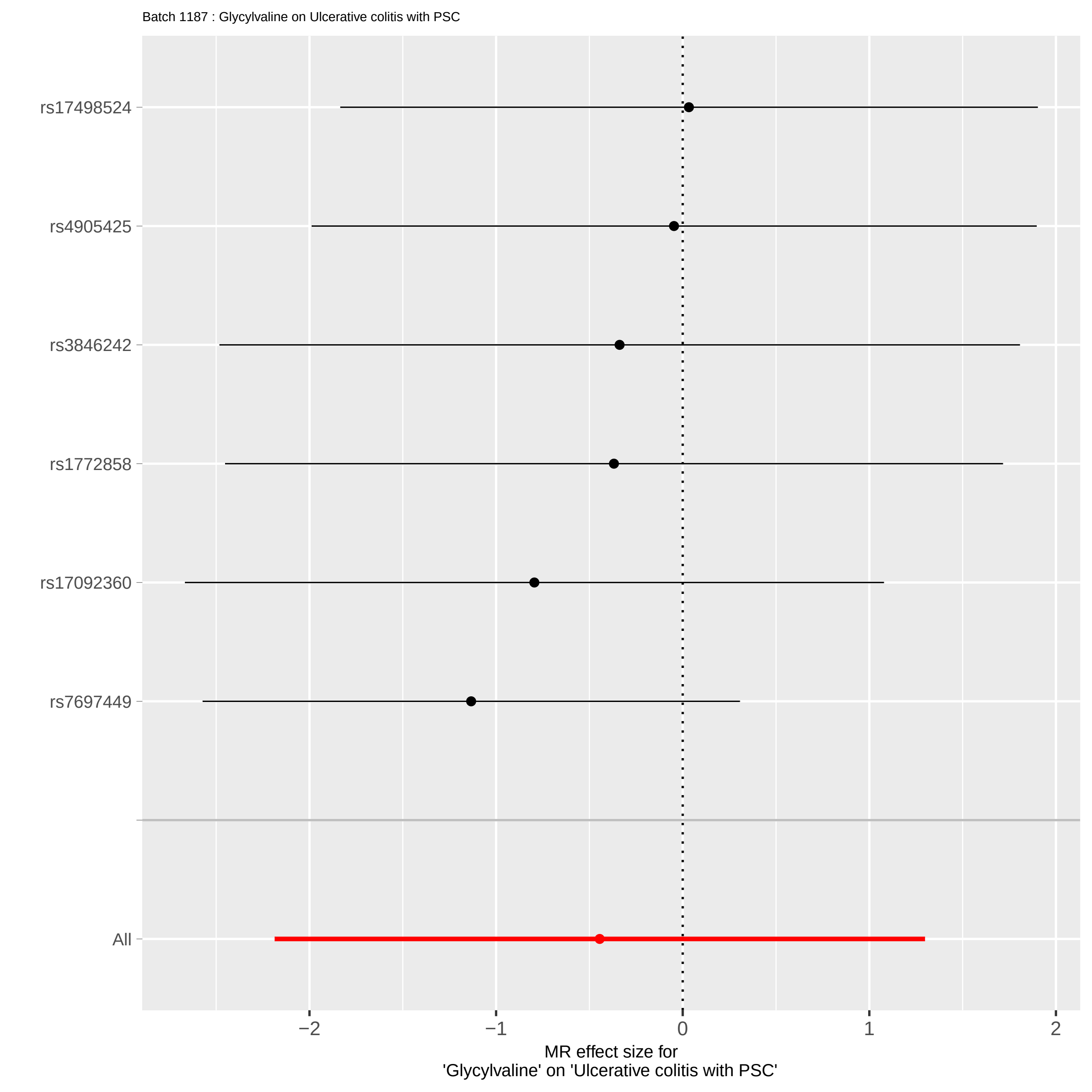

Batch 1187 : Glycylvaline on Ulcerative colitis with PSC
rs17498524
rs4905425
rs3846242
rs1772858
rs17092360
rs7697449
All
−2
−1
0
1
2
MR effect size for
'Glycylvaline' on 'Ulcerative colitis with PSC'

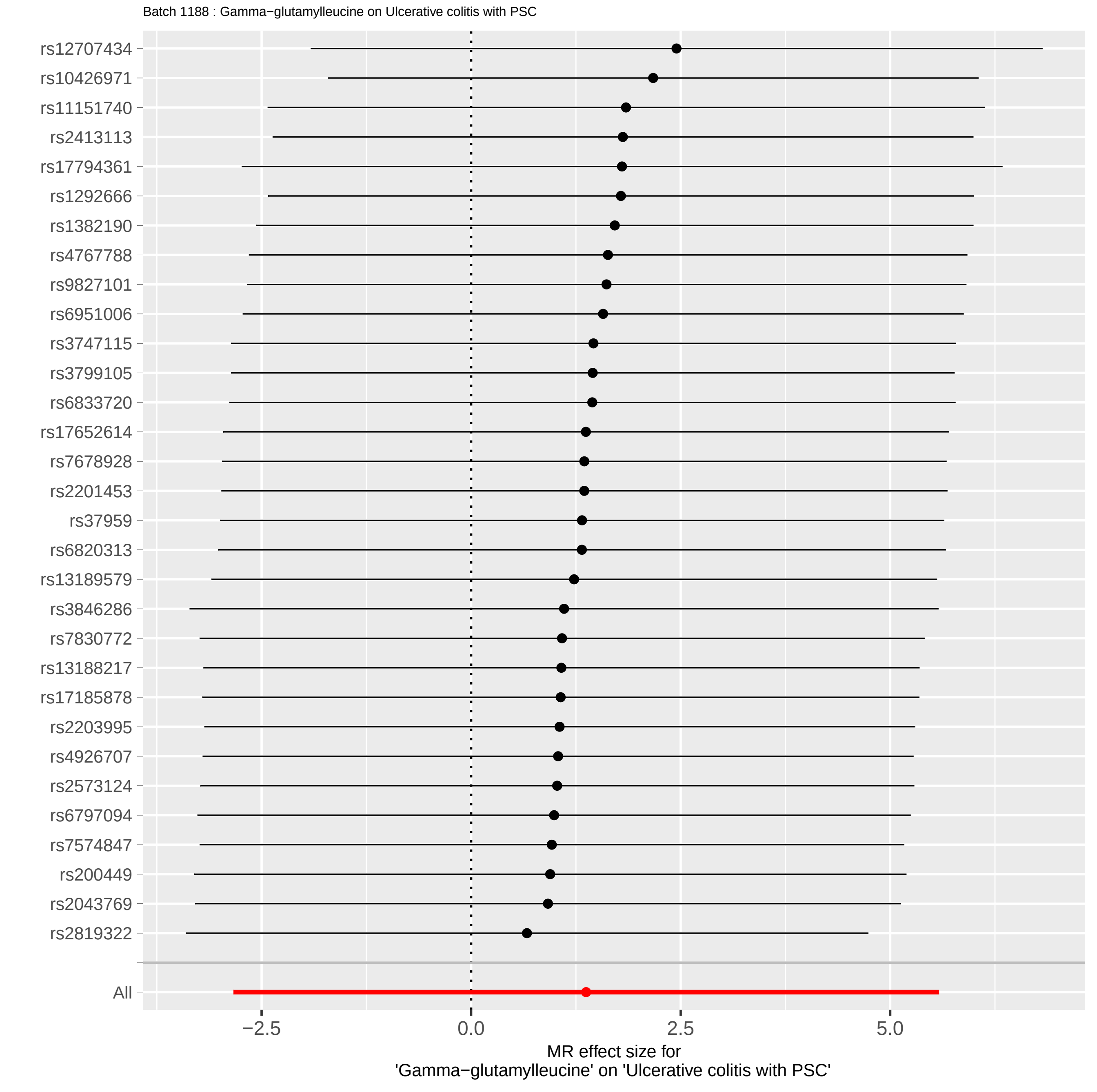

Batch 1188 : Gamma−glutamylleucine on Ulcerative colitis with PSC
rs12707434
rs10426971
rs11151740
rs2413113
rs17794361
rs1292666
rs1382190
rs4767788
rs9827101
rs6951006
rs3747115
rs3799105
rs6833720
rs17652614
rs7678928
rs2201453
rs37959
rs6820313
rs13189579
rs3846286
rs7830772
rs13188217
rs17185878
rs2203995
rs4926707
rs2573124
rs6797094
rs7574847
rs200449
rs2043769
rs2819322
All
−2.5
0.0
2.5
5.0
MR effect size for
'Gamma−glutamylleucine' on 'Ulcerative colitis with PSC'

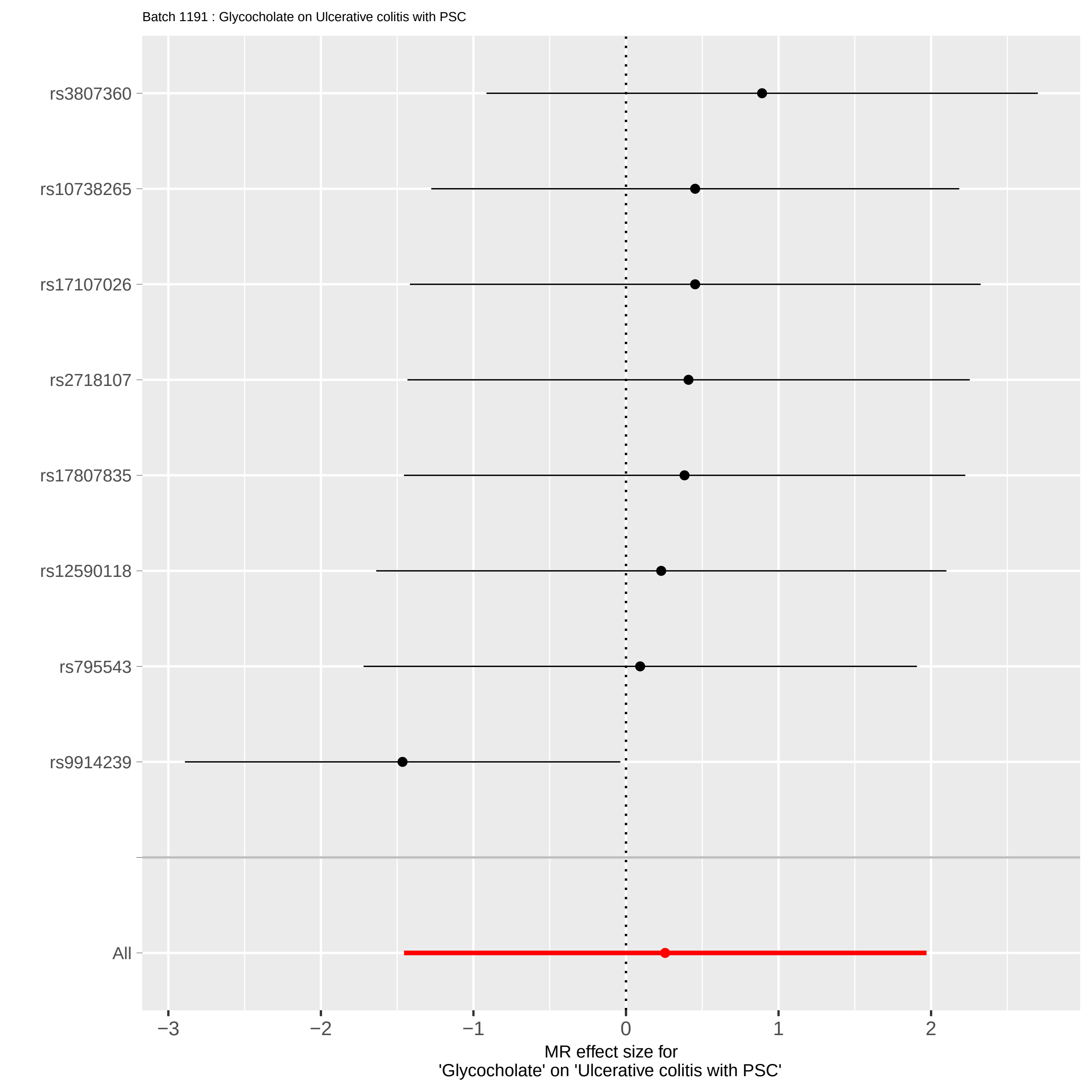

Batch 1191 : Glycocholate on Ulcerative colitis with PSC
rs3807360
rs10738265
rs17107026
rs2718107
rs17807835
rs12590118
rs795543
rs9914239
All
−3
−2
−1
0
1
2
MR effect size for
'Glycocholate' on 'Ulcerative colitis with PSC'

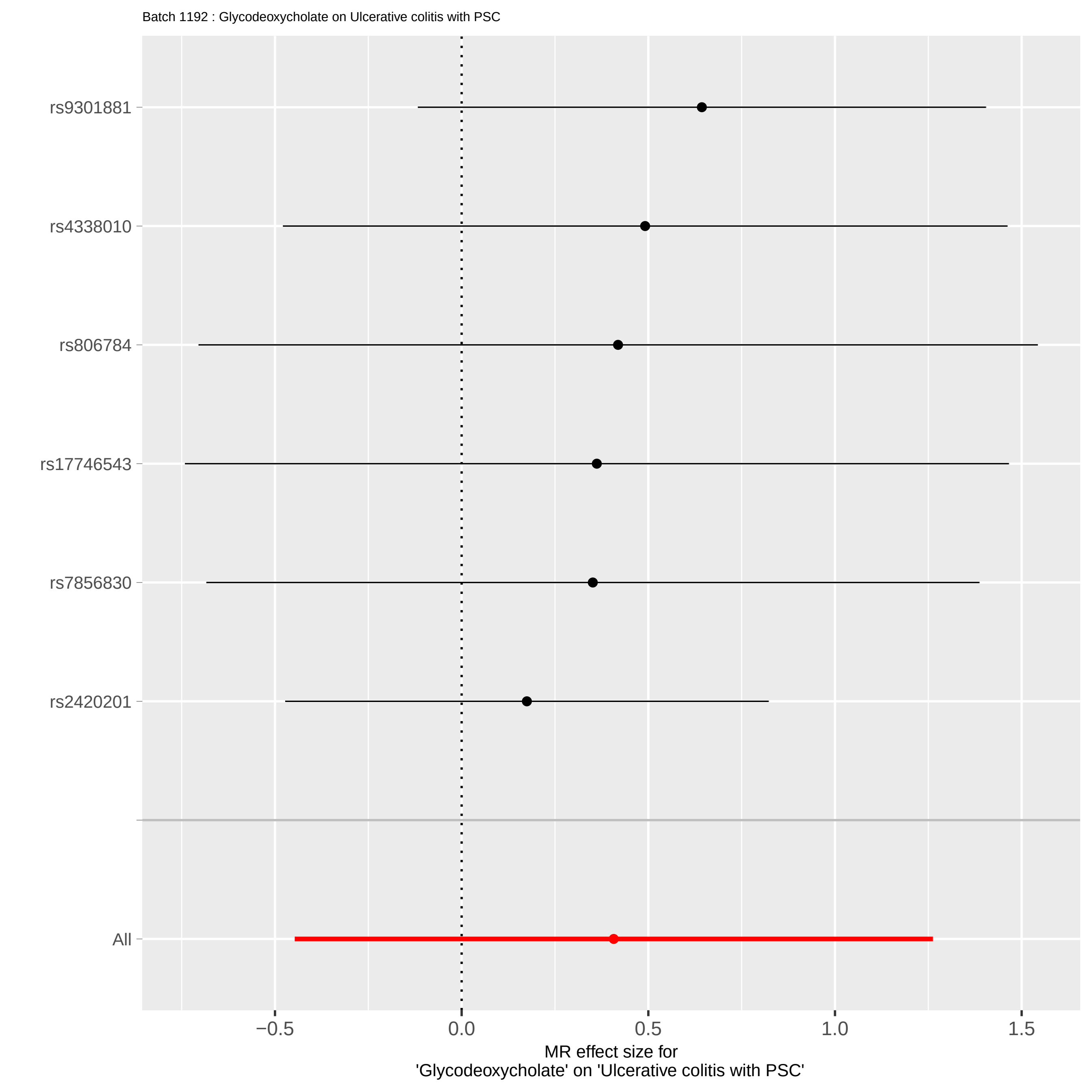

Batch 1192 : Glycodeoxycholate on Ulcerative colitis with PSC
rs9301881
rs4338010
rs806784
rs17746543
rs7856830
rs2420201
All
−0.5
0.0
0.5
1.0
1.5
MR effect size for
'Glycodeoxycholate' on 'Ulcerative colitis with PSC'

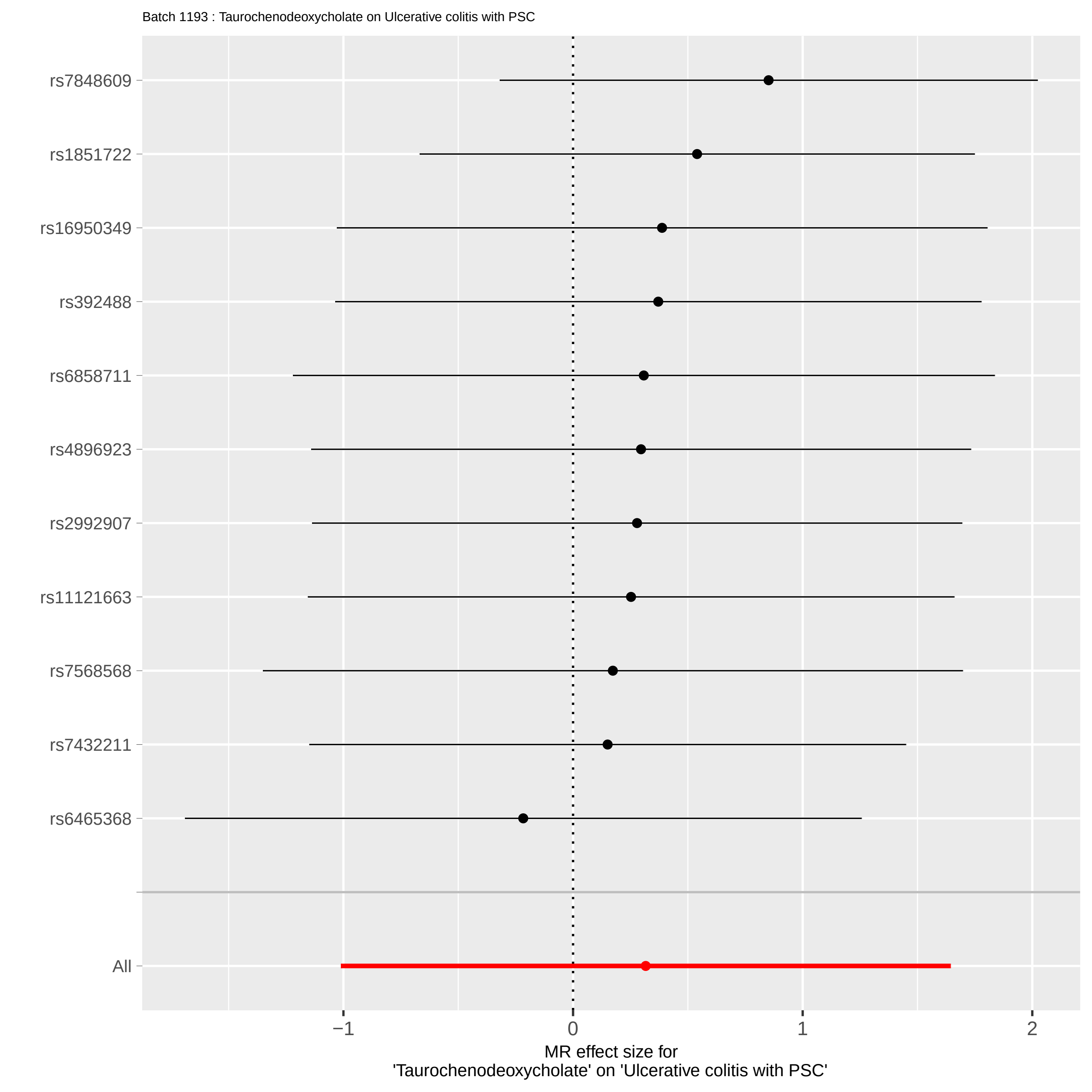
Batch 1193 : Taurochenodeoxycholate on Ulcerative colitis with PSC
rs7848609
rs1851722
rs16950349
rs392488
rs6858711
rs4896923
rs2992907
rs11121663
rs7568568
rs7432211
rs6465368
All
−1
0
1
2
MR effect size for
'Taurochenodeoxycholate' on 'Ulcerative colitis with PSC'

Batch 1194 : Taurocholate on Ulcerative colitis with PSC

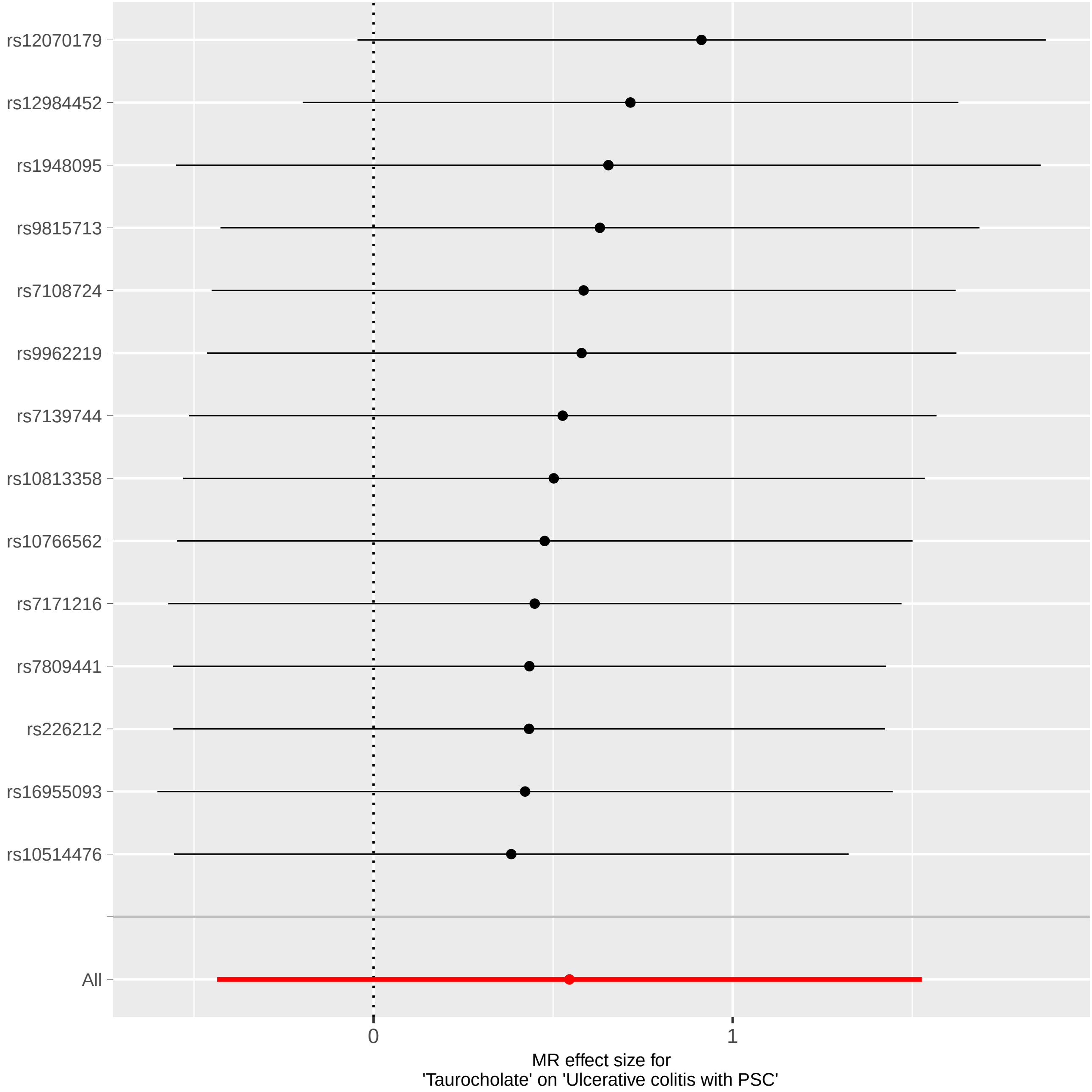

Batch 1196 : 1−stearoylglycerophosphoinositol on Ulcerative colitis with PSC

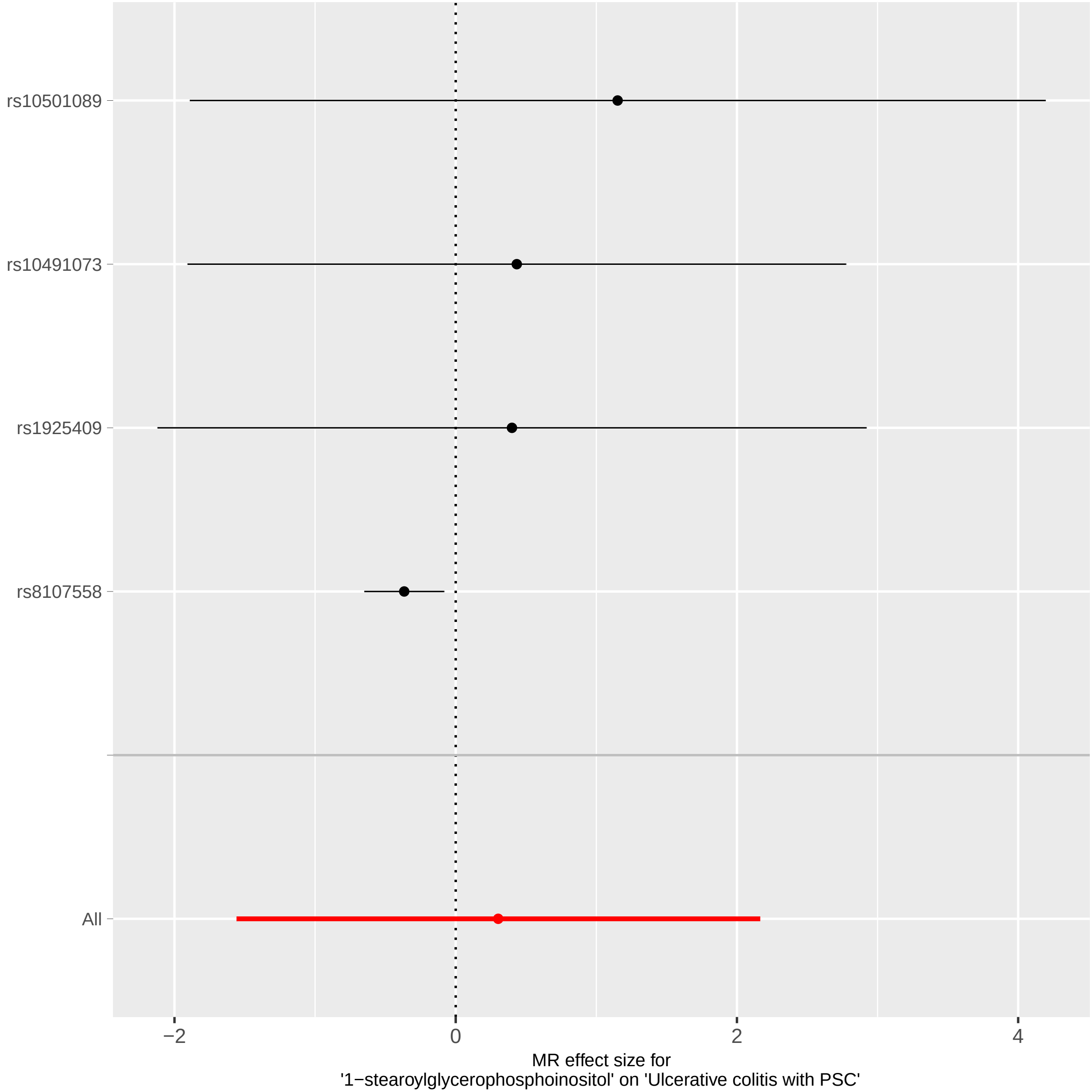

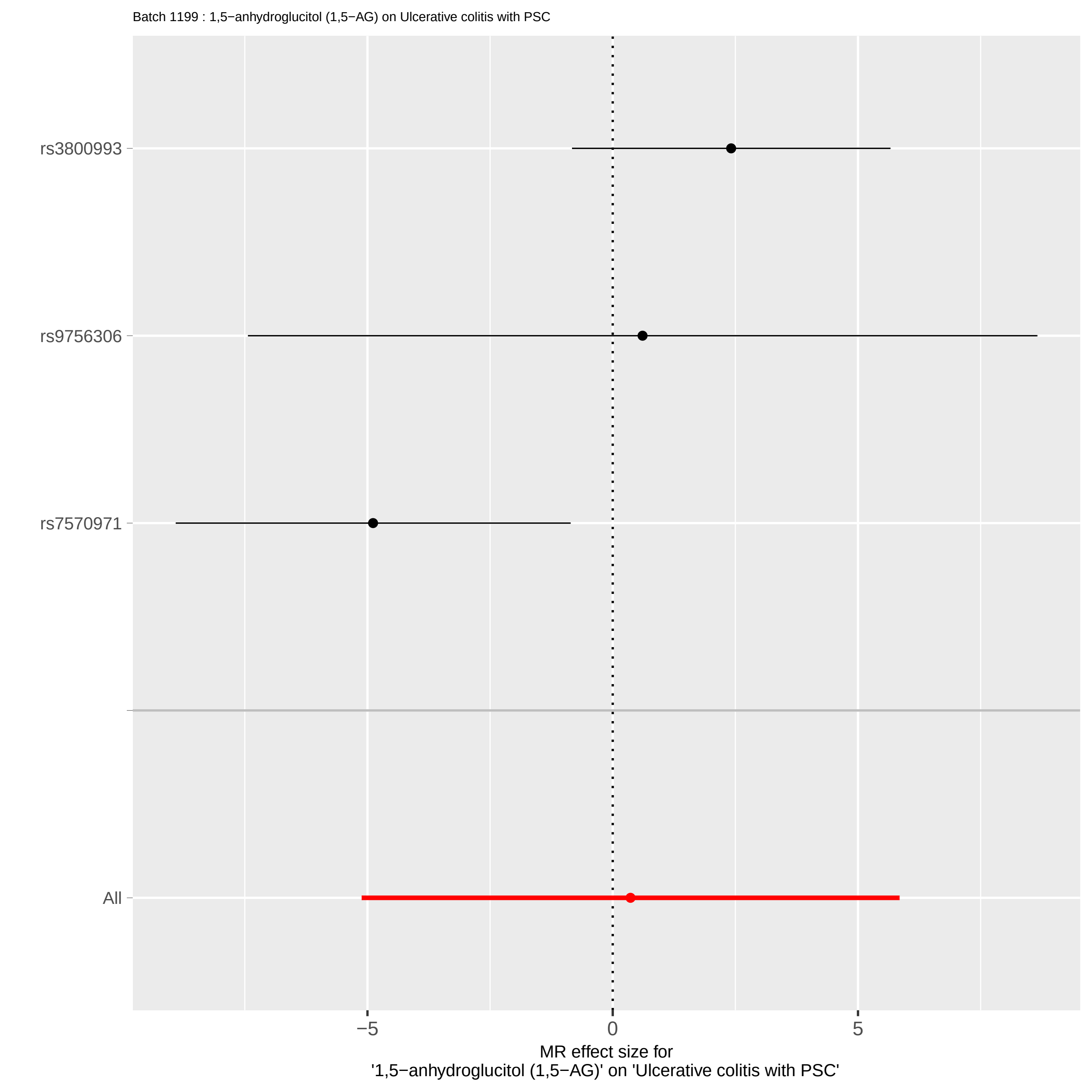
Batch 1199 : 1,5−anhydroglucitol (1,5−AG) on Ulcerative colitis with PSC
rs3800993
rs9756306
rs7570971
All
−5
0
5
MR effect size for
'1,5−anhydroglucitol (1,5−AG)' on 'Ulcerative colitis with PSC'

Batch 1200 : 2−hydroxybutyrate (AHB) on Ulcerative colitis with PSC

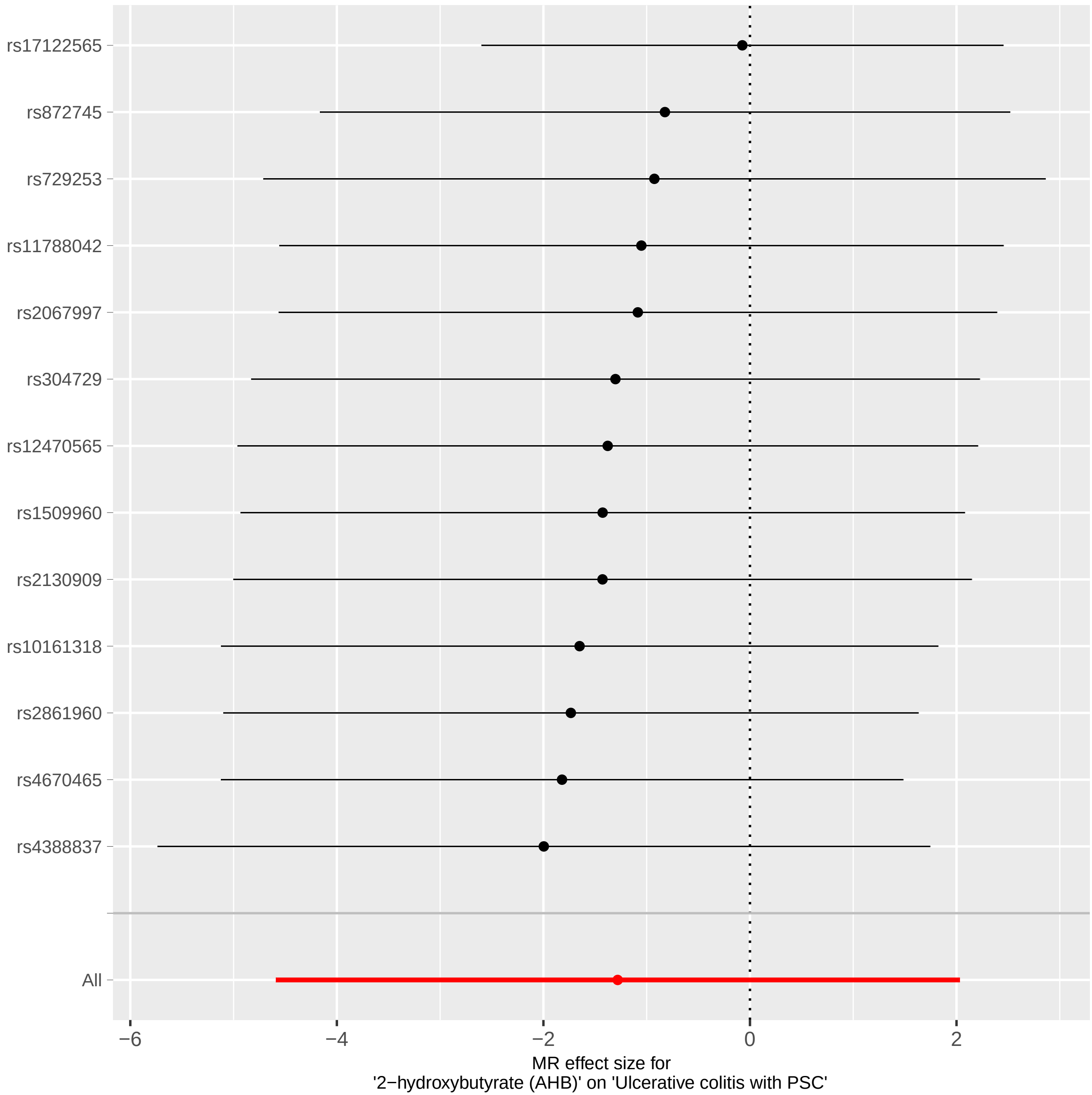

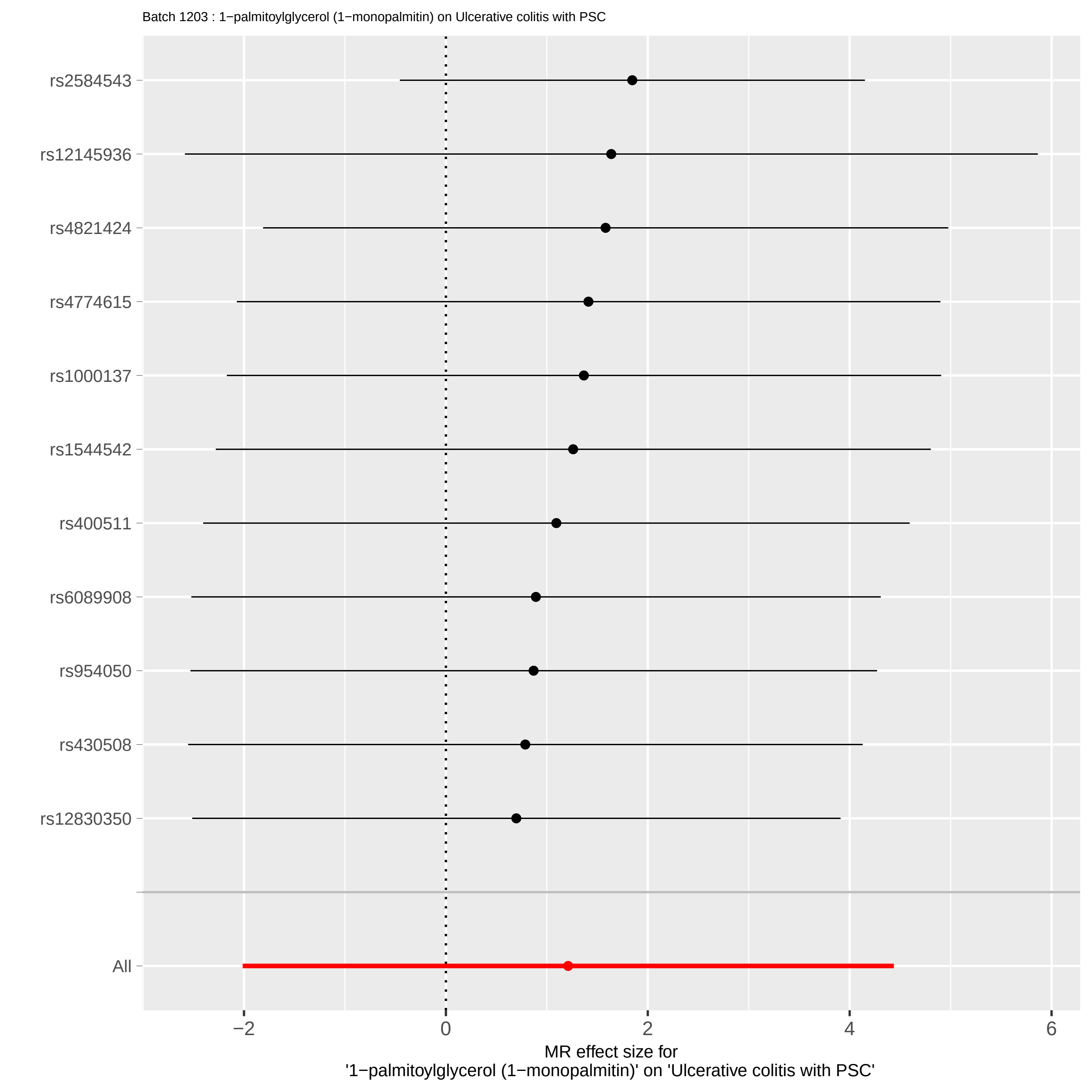
Batch 1203 : 1−palmitoylglycerol (1−monopalmitin) on Ulcerative colitis with PSC
rs2584543
rs12145936
rs4821424
rs4774615
rs1000137
rs1544542
rs400511
rs6089908
rs954050
rs430508
rs12830350
All
−2
0
2
4
6
MR effect size for
'1−palmitoylglycerol (1−monopalmitin)' on 'Ulcerative colitis with PSC'

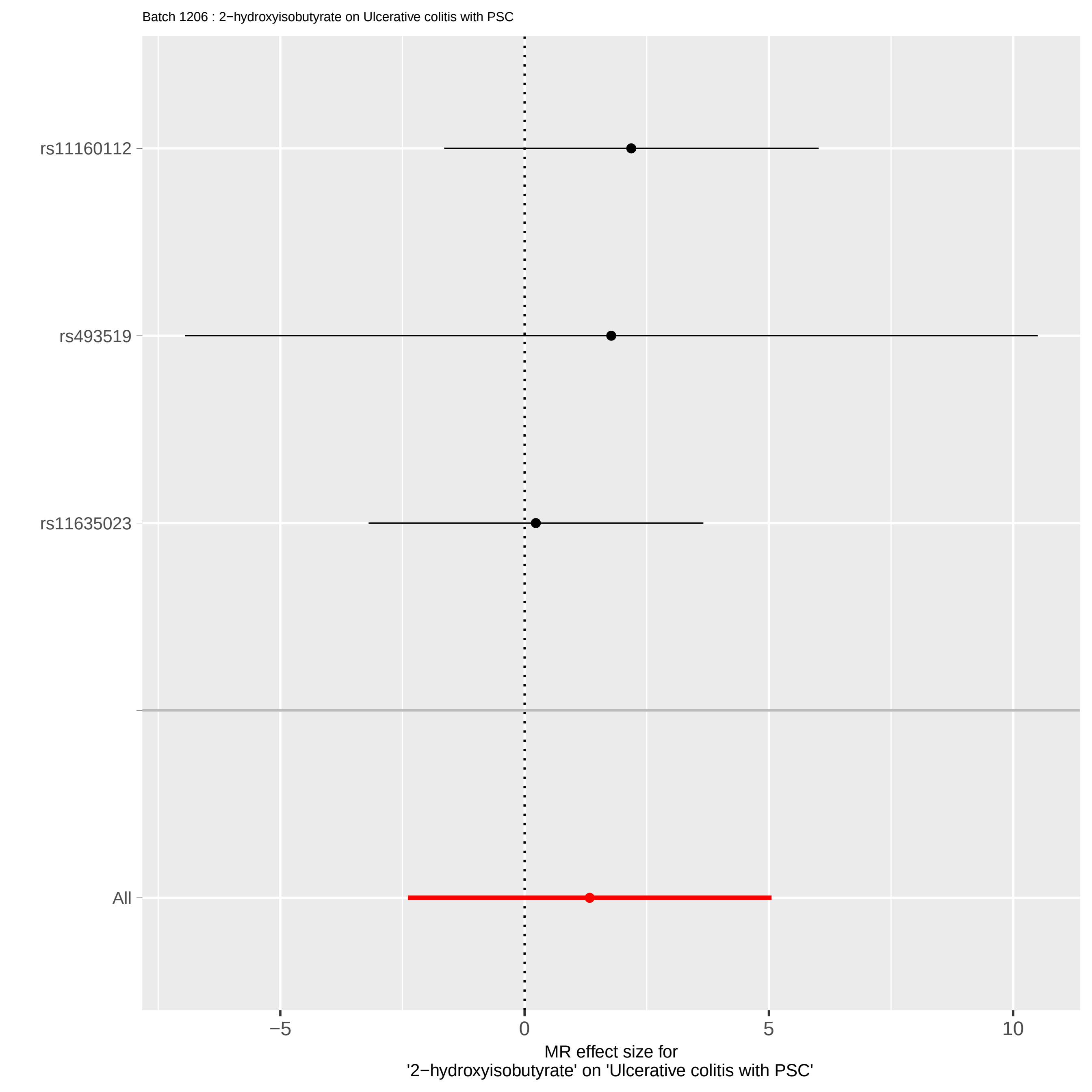

Batch 1206 : 2−hydroxyisobutyrate on Ulcerative colitis with PSC
rs11160112
rs493519
rs11635023
All
−5
0
5
10
MR effect size for
'2−hydroxyisobutyrate' on 'Ulcerative colitis with PSC'

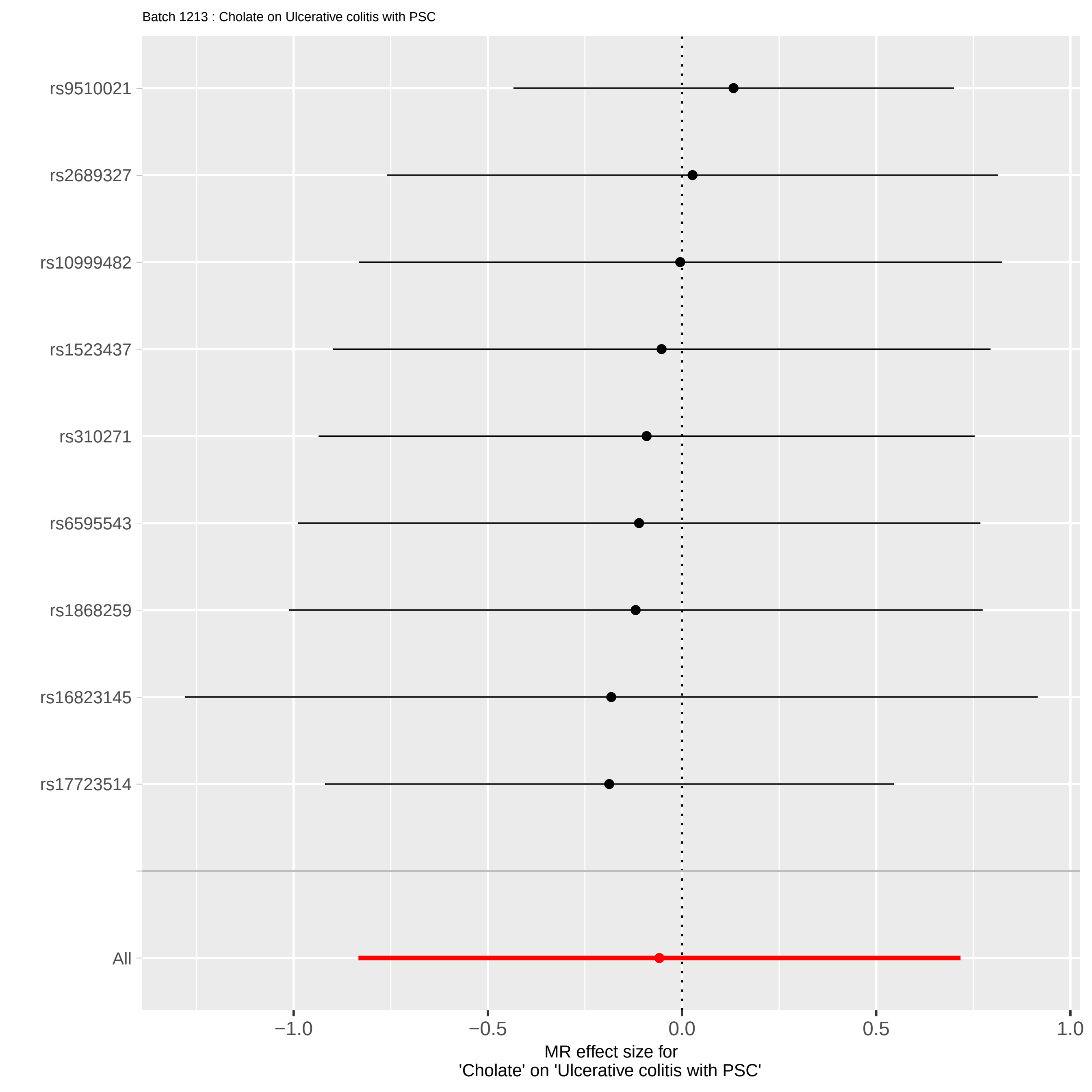

Batch 1213 : Cholate on Ulcerative colitis with PSC
rs9510021
rs2689327
rs10999482
rs1523437
rs310271
rs6595543
rs1868259
rs16823145
rs17723514
All
−1.0
−0.5
0.0
0.5
1.0
MR effect size for
'Cholate' on 'Ulcerative colitis with PSC'

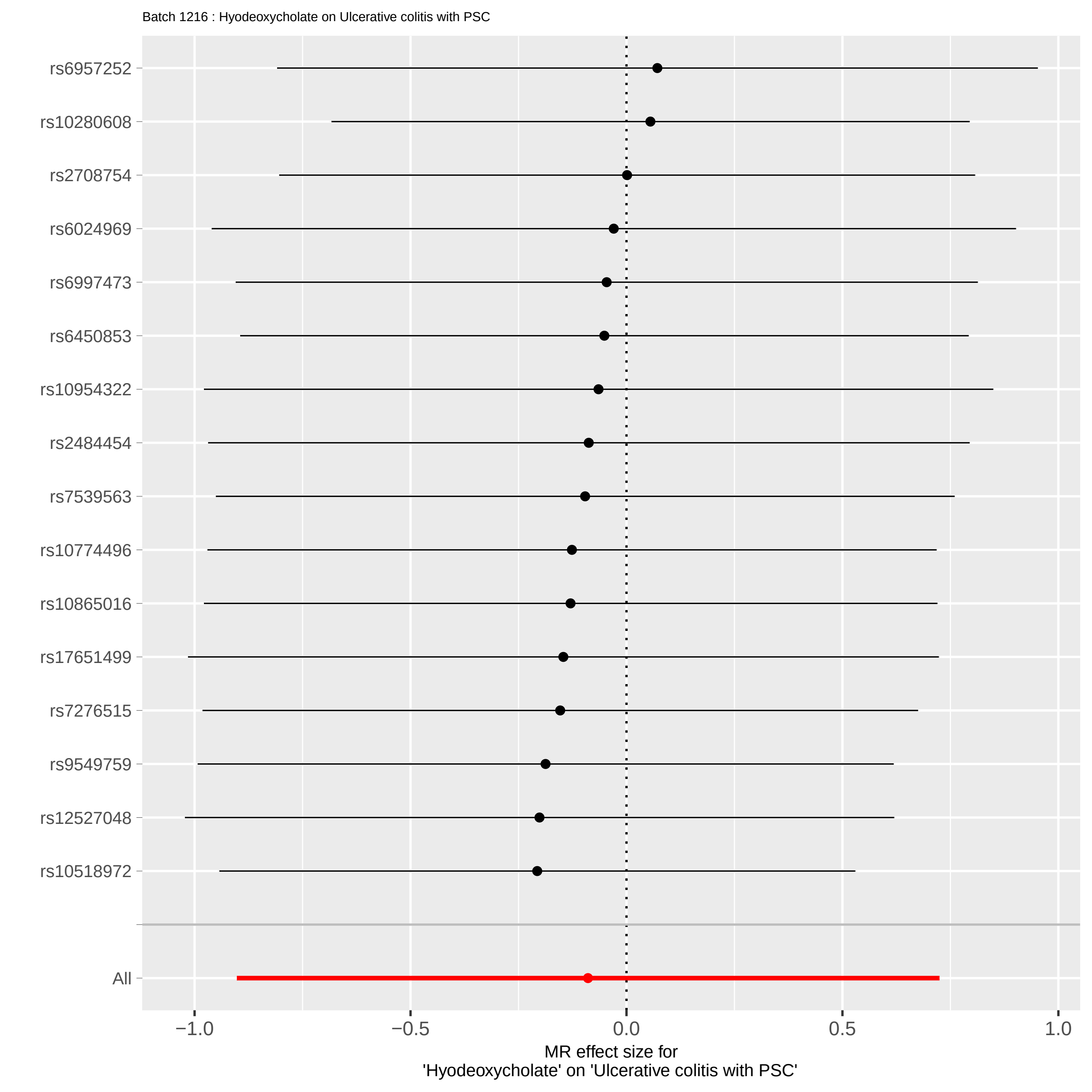
Batch 1216 : Hyodeoxycholate on Ulcerative colitis with PSC
rs6957252
rs10280608
rs2708754
rs6024969
rs6997473
rs6450853
rs10954322
rs2484454
rs7539563
rs10774496
rs10865016
rs17651499
rs7276515
rs9549759
rs12527048
rs10518972
All
−1.0
−0.5
0.0
0.5
1.0
MR effect size for
'Hyodeoxycholate' on 'Ulcerative colitis with PSC'

Batch 1217 : 3−indoxyl sulfate on Ulcerative colitis with PSC

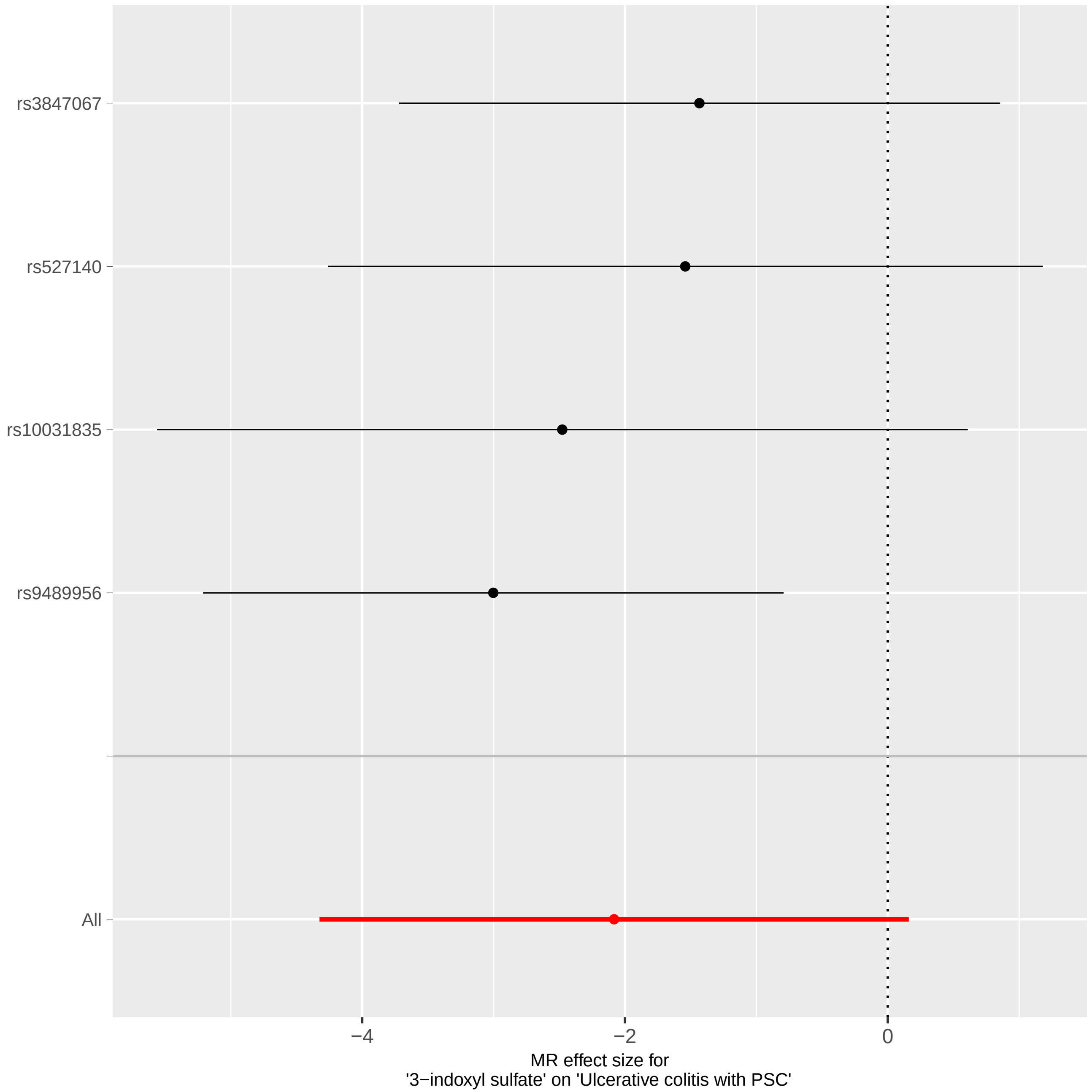

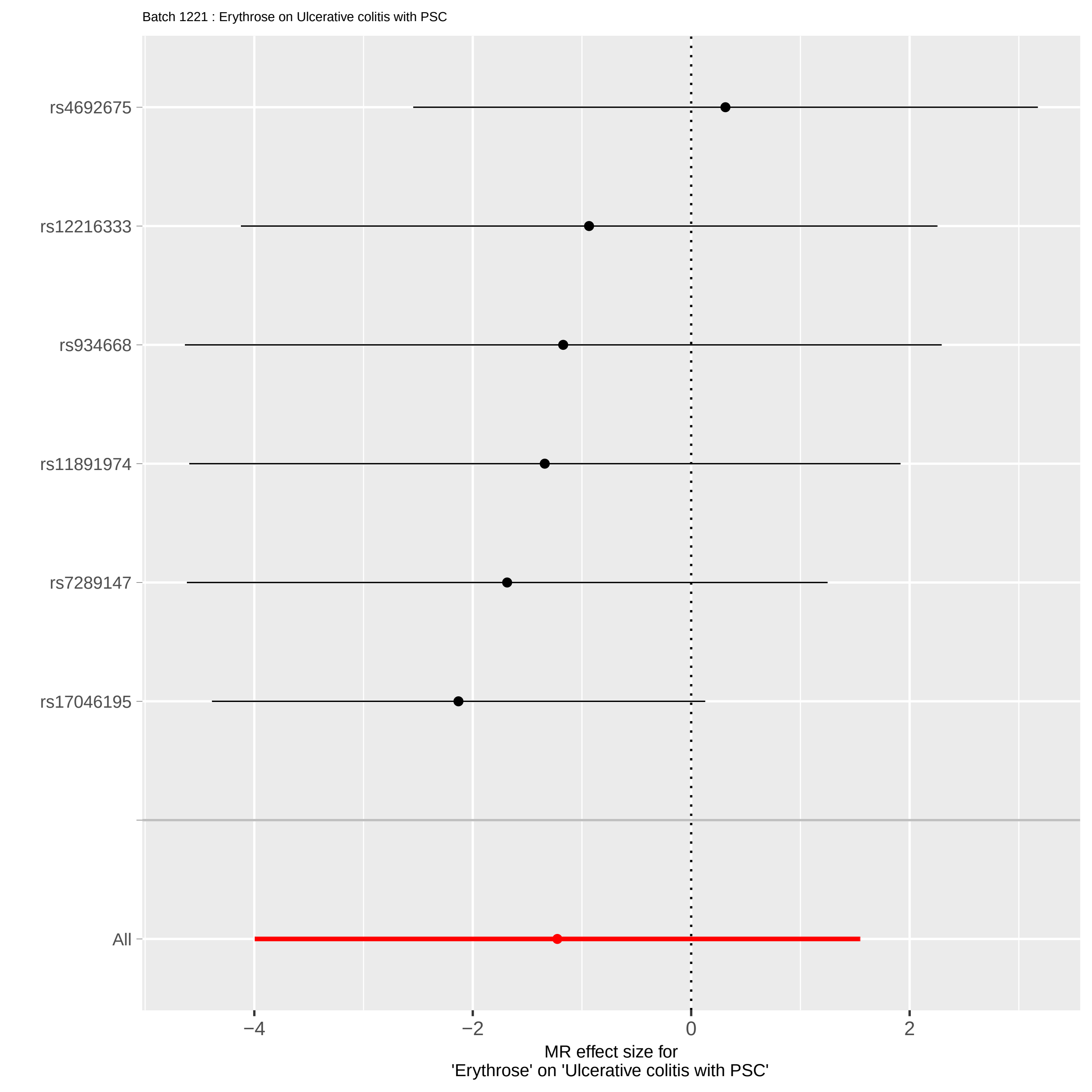

Batch 1221 : Erythrose on Ulcerative colitis with PSC
rs4692675
rs12216333
rs934668
rs11891974
rs7289147
rs17046195
All
−4
−2
0
2
MR effect size for
'Erythrose' on 'Ulcerative colitis with PSC'

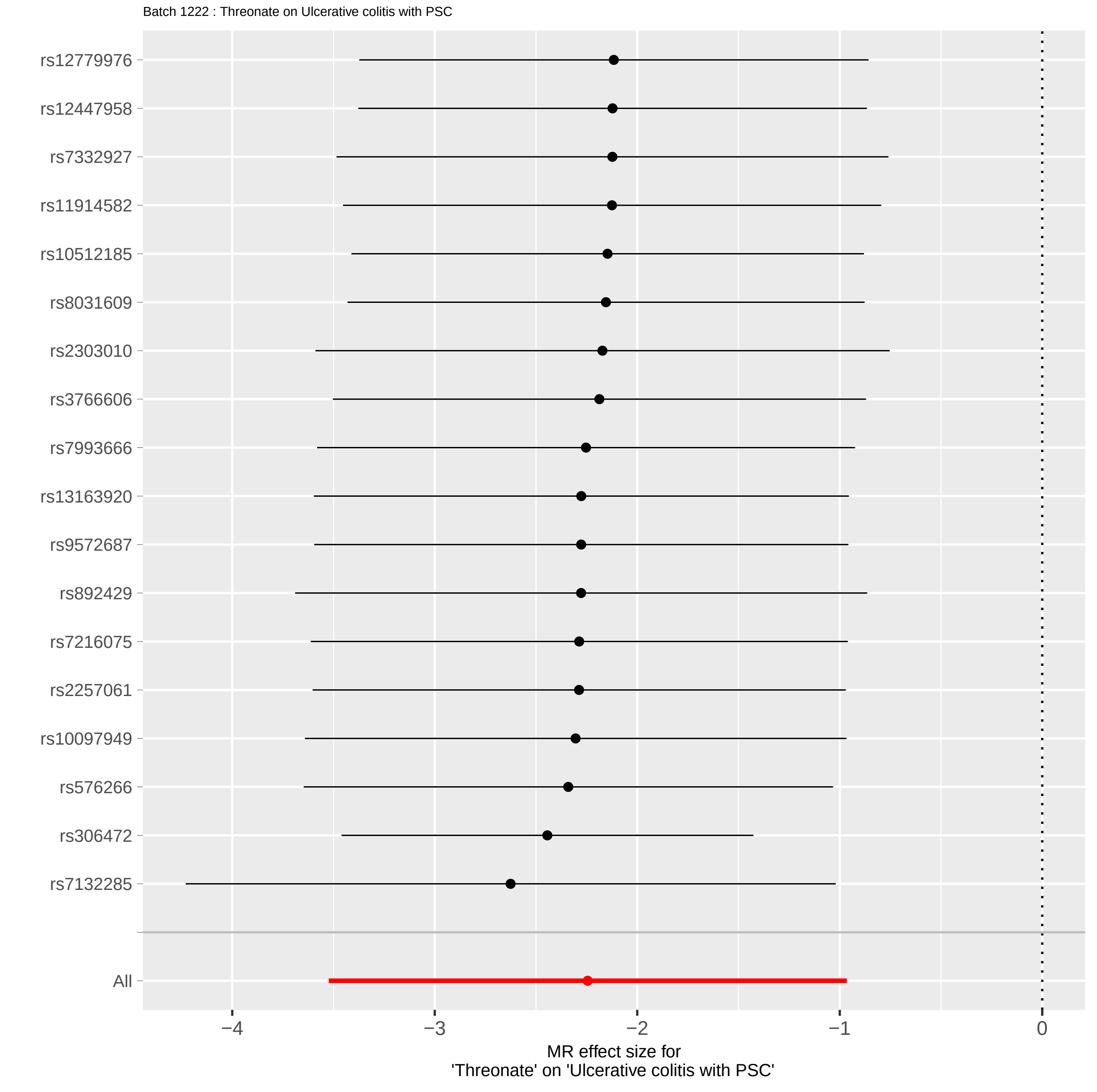

Batch 1222 : Threonate on Ulcerative colitis with PSC
rs12779976
rs12447958
rs7332927
rs11914582
rs10512185
rs8031609
rs2303010
rs3766606
rs7993666
rs13163920
rs9572687
rs892429
rs7216075
rs2257061
rs10097949
rs576266
rs306472
rs7132285
All
−4
−3
−2
−1
0
MR effect size for
'Threonate' on 'Ulcerative colitis with PSC'

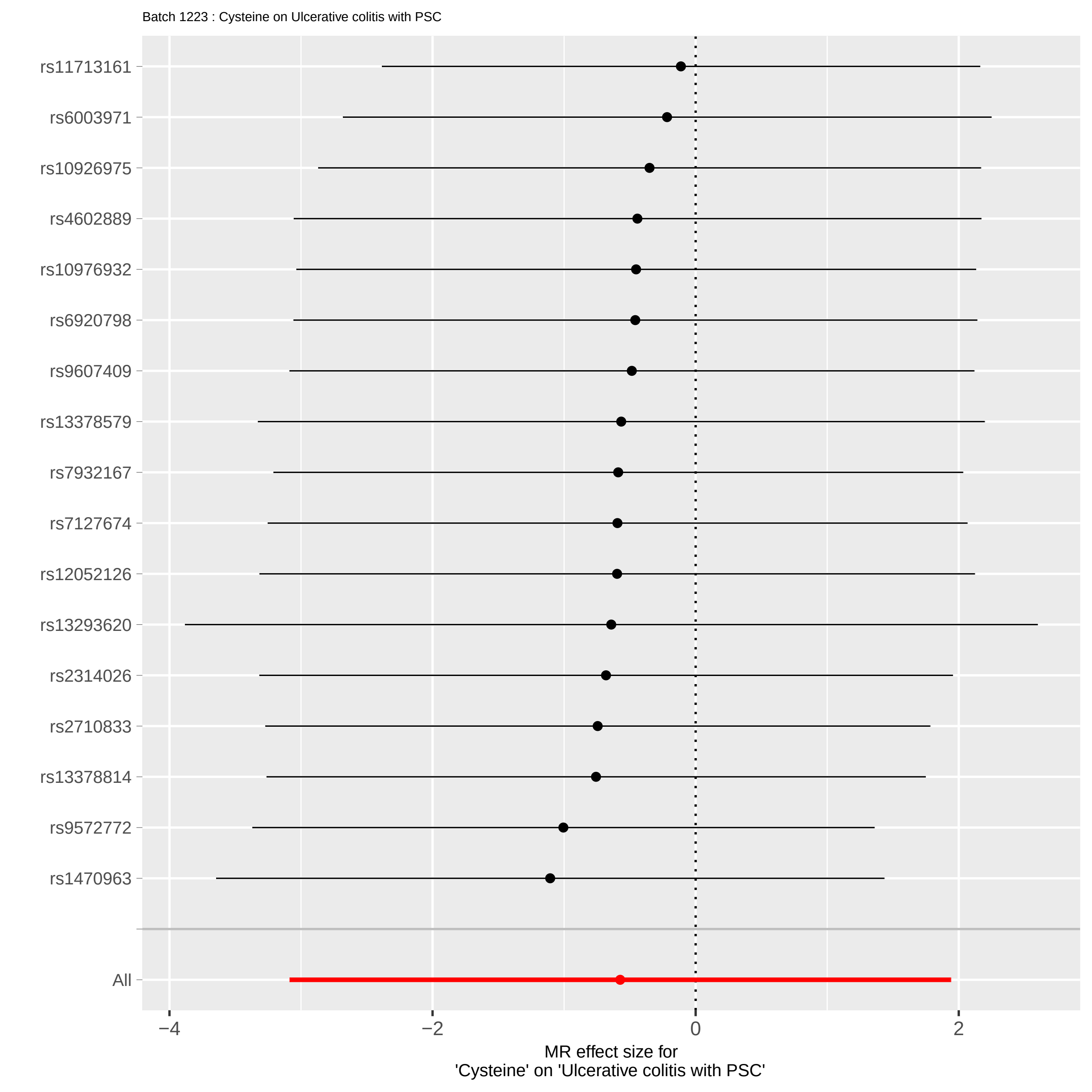
Batch 1223 : Cysteine on Ulcerative colitis with PSC
rs11713161
rs6003971
rs10926975
rs4602889
rs10976932
rs6920798
rs9607409
rs13378579
rs7932167
rs7127674
rs12052126
rs13293620
rs2314026
rs2710833
rs13378814
rs9572772
rs1470963
All
−4
−2
0
2
MR effect size for
'Cysteine' on 'Ulcerative colitis with PSC'

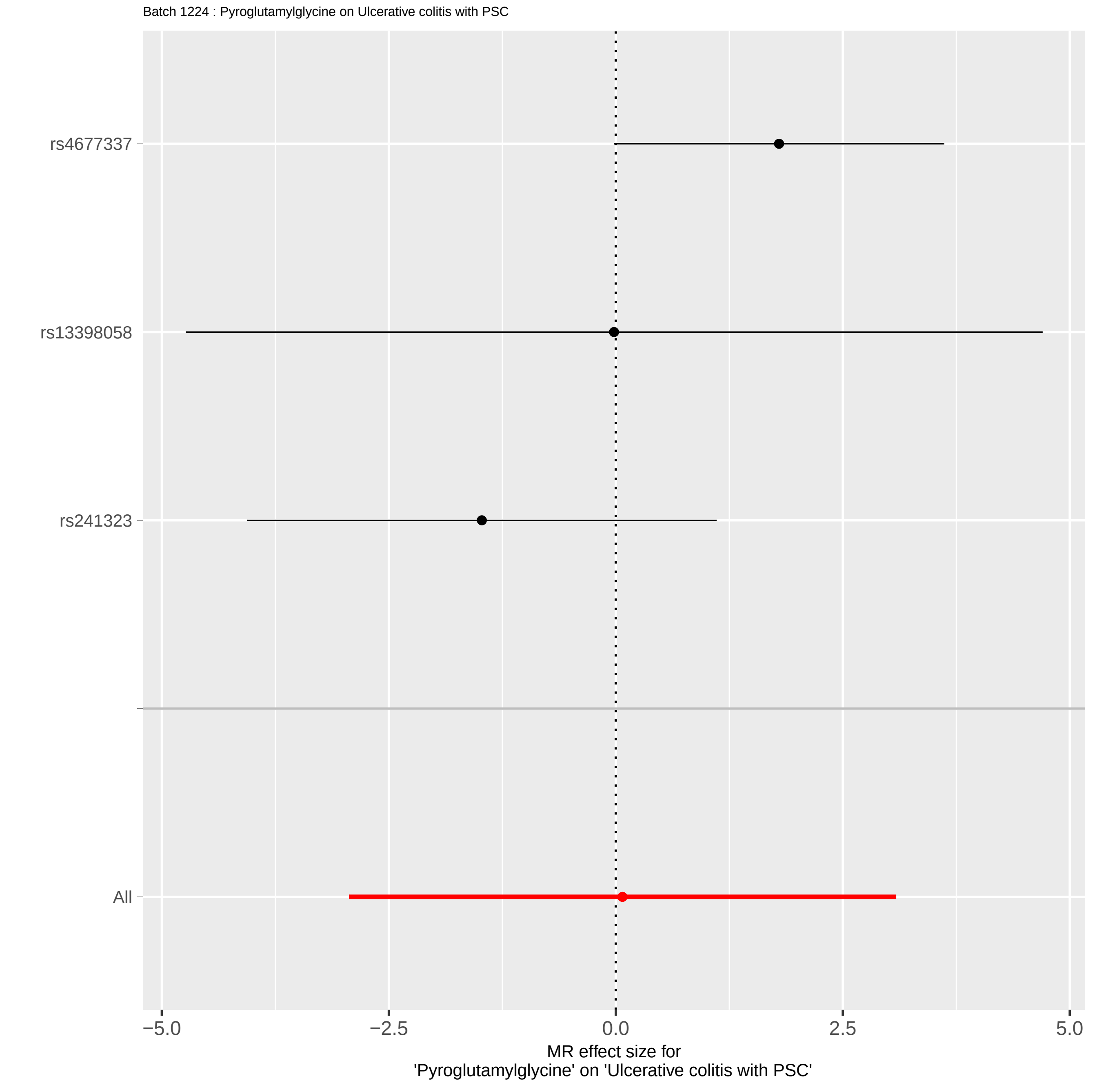
Batch 1224 : Pyroglutamylglycine on Ulcerative colitis with PSC
rs4677337
rs13398058
rs241323
All
−5.0
−2.5
0.0
2.5
5.0
MR effect size for
'Pyroglutamylglycine' on 'Ulcerative colitis with PSC'

Batch 1226 : DSGEGDFXAEGGGVR* on Ulcerative colitis with PSC

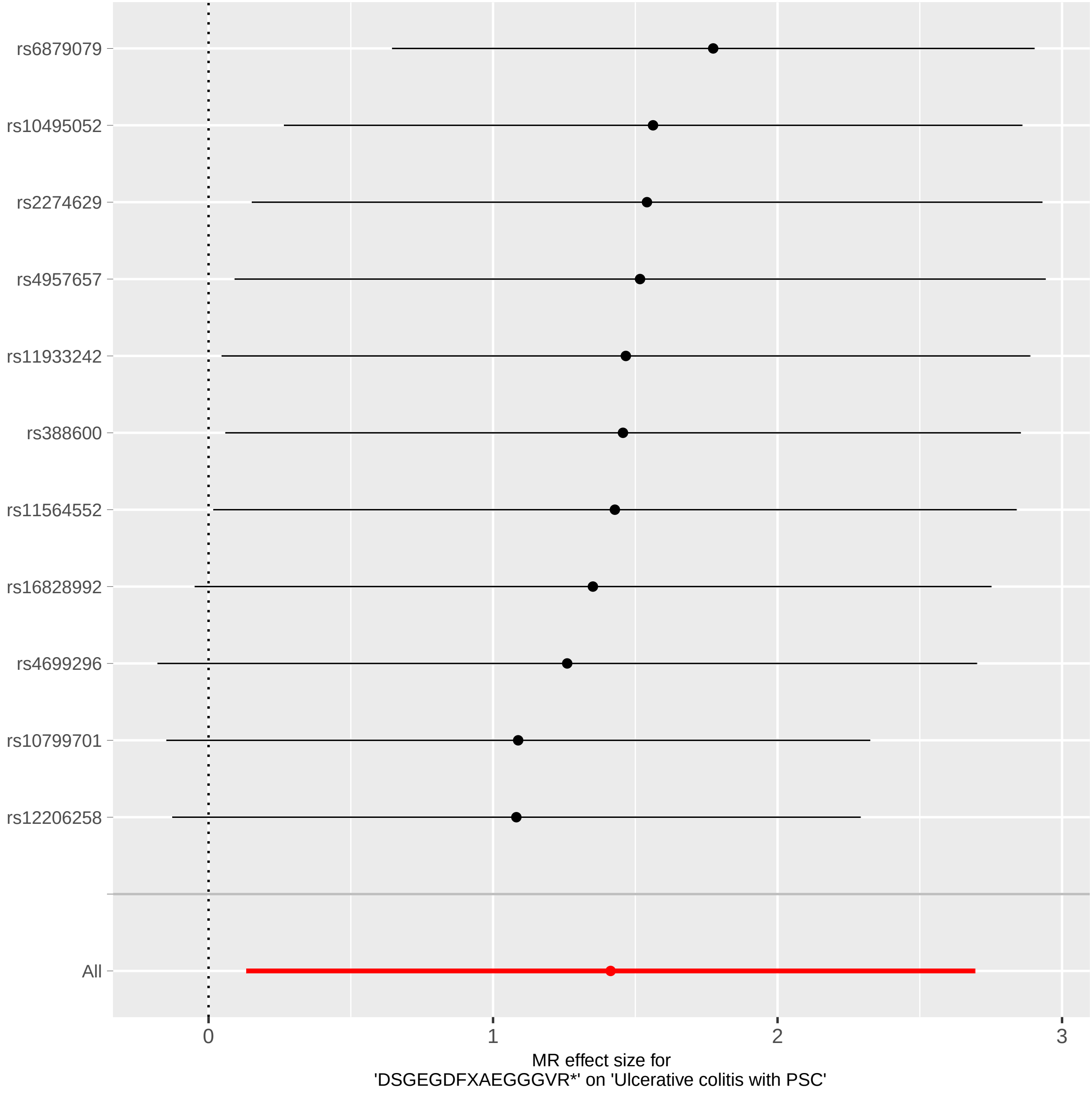

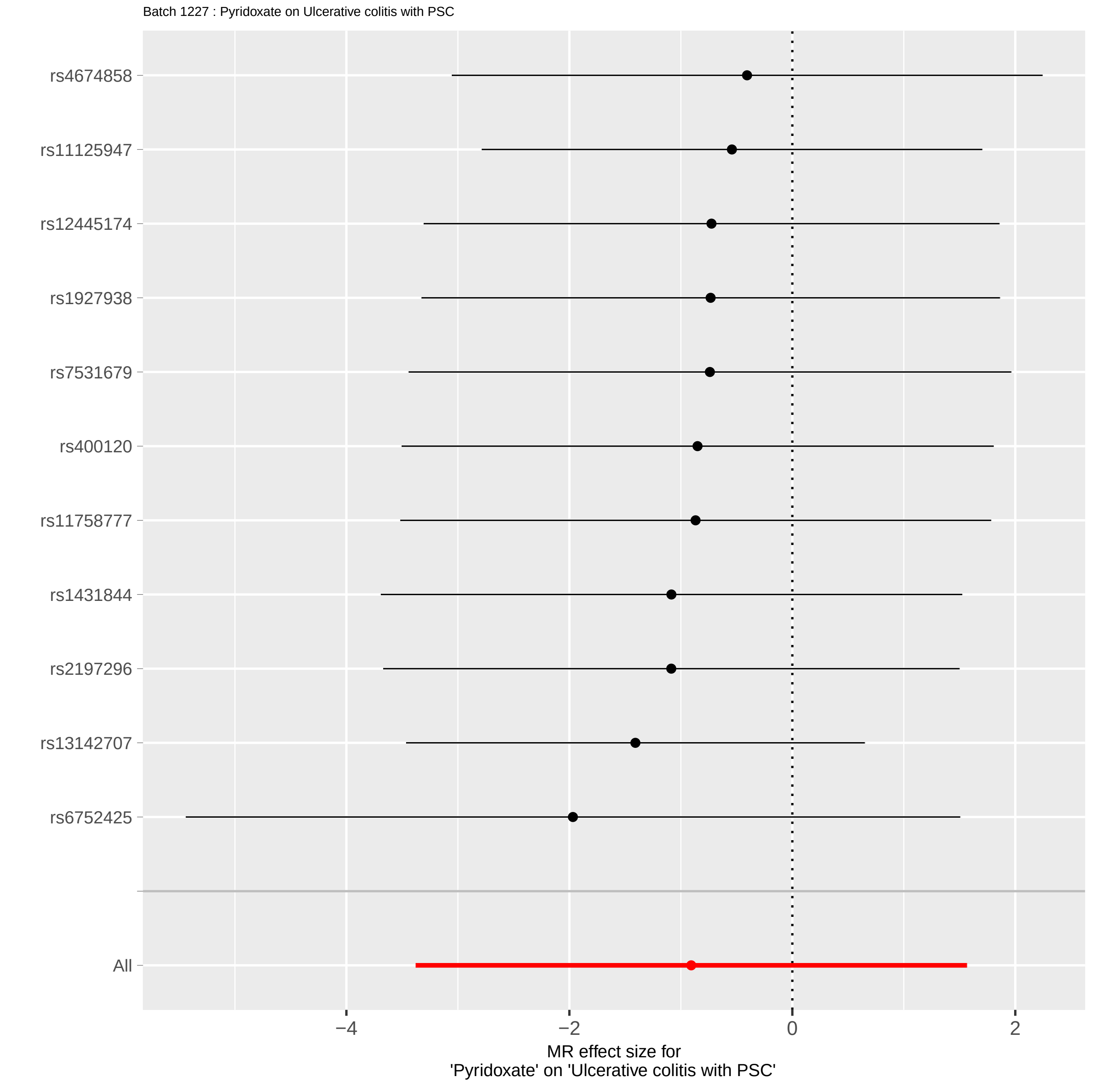

Batch 1227 : Pyridoxate on Ulcerative colitis with PSC
rs4674858
rs11125947
rs12445174
rs1927938
rs7531679
rs400120
rs11758777
rs1431844
rs2197296
rs13142707
rs6752425
All
−4
−2
0
2
MR effect size for
'Pyridoxate' on 'Ulcerative colitis with PSC'

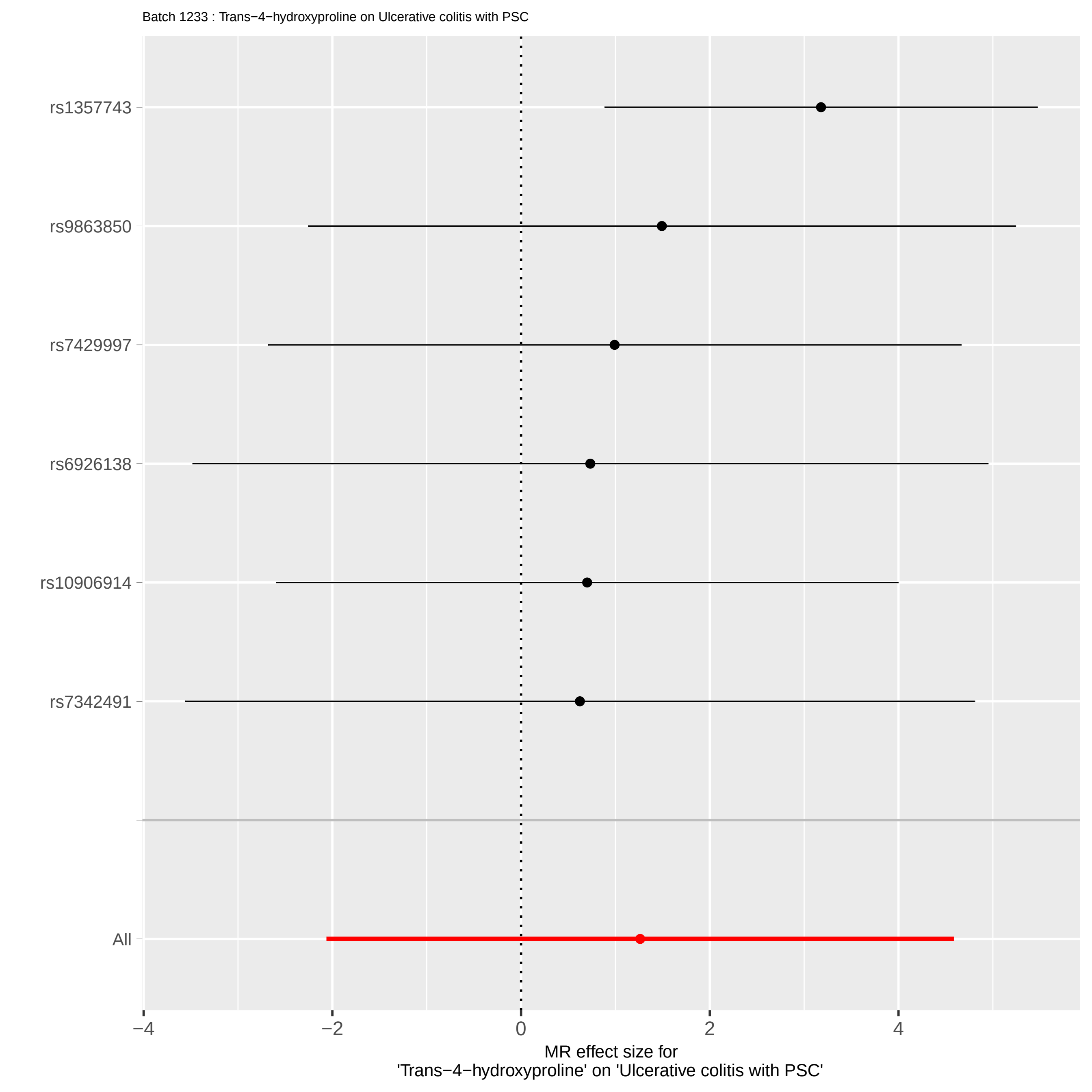

Batch 1233 : Trans−4−hydroxyproline on Ulcerative colitis with PSC
rs1357743
rs9863850
rs7429997
rs6926138
rs10906914
rs7342491
All
−4
−2
0
2
4
MR effect size for
'Trans−4−hydroxyproline' on 'Ulcerative colitis with PSC'

Batch 1235 : Hexanoylcarnitine on Ulcerative colitis with PSC

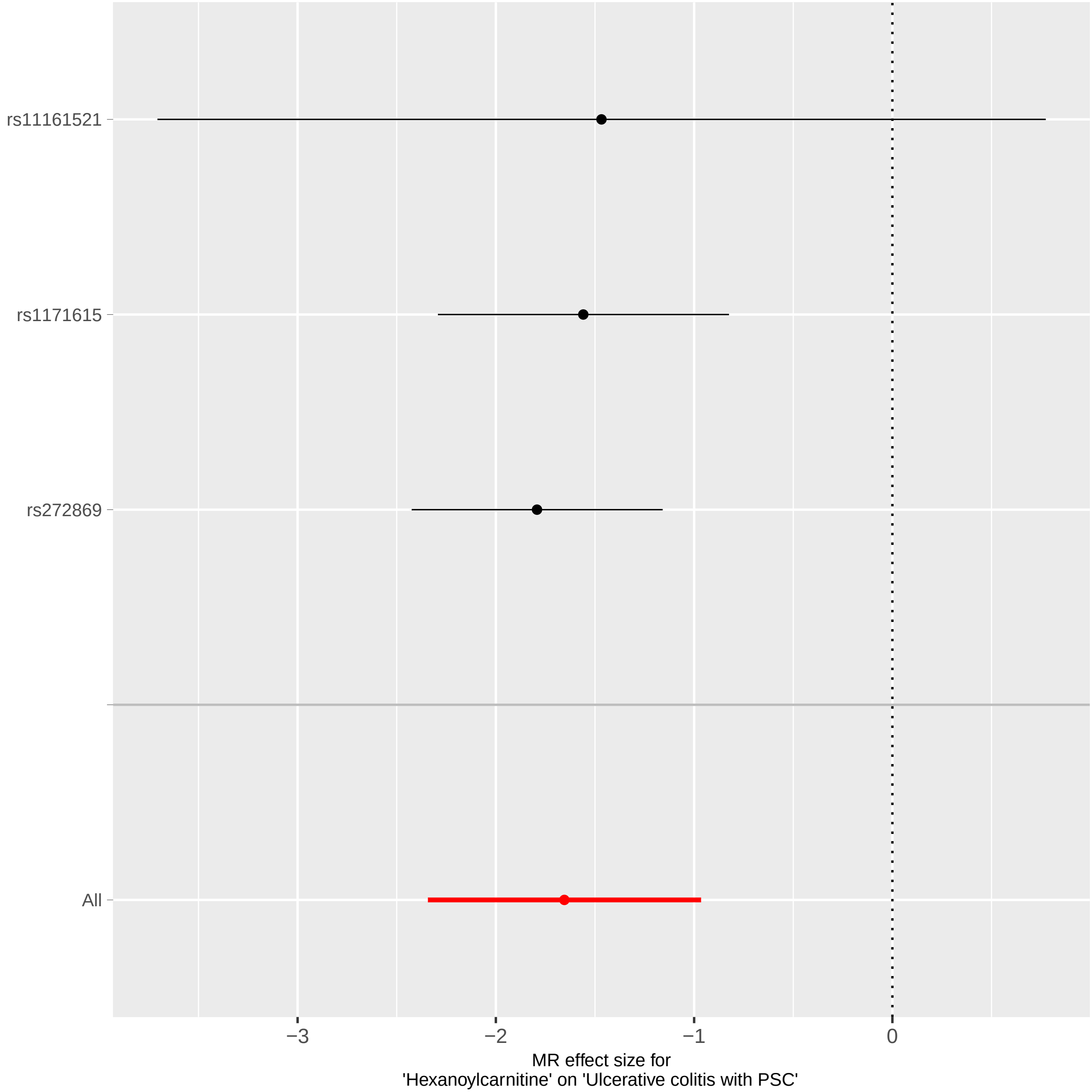

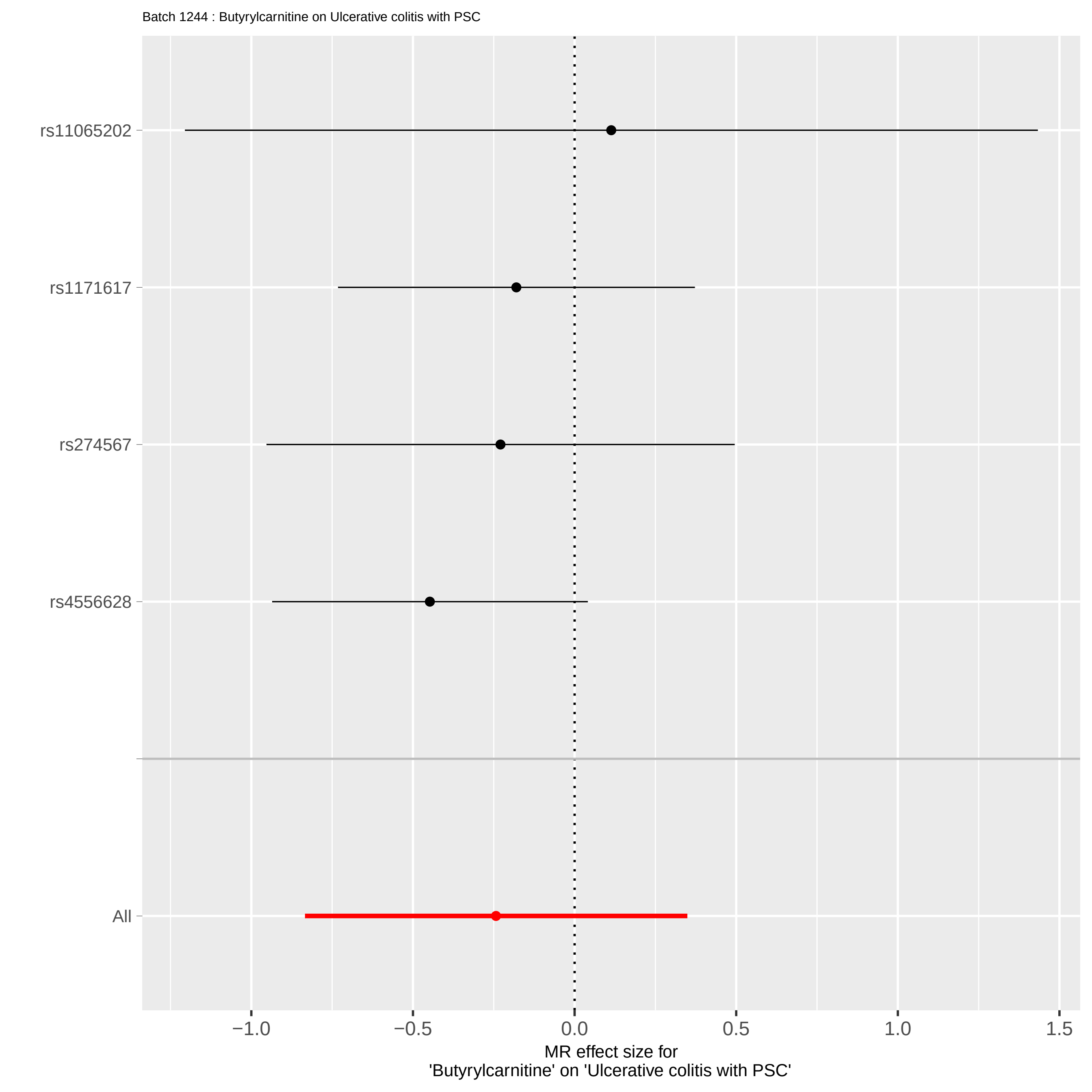

Batch 1244 : Butyrylcarnitine on Ulcerative colitis with PSC
rs11065202
rs1171617
rs274567
rs4556628
All
−1.0
−0.5
0.0
0.5
1.0
1.5
MR effect size for
'Butyrylcarnitine' on 'Ulcerative colitis with PSC'

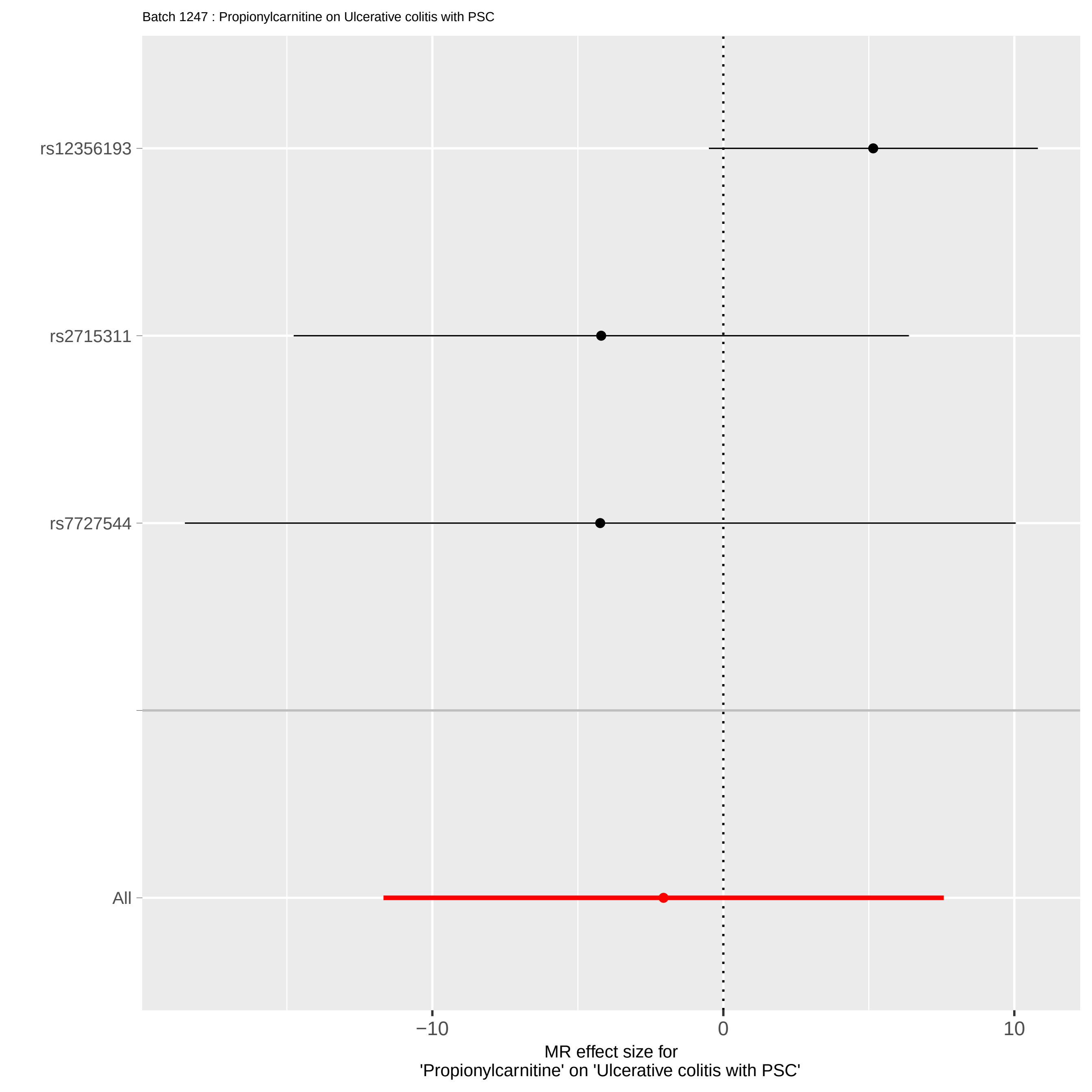
Batch 1247 : Propionylcarnitine on Ulcerative colitis with PSC
rs12356193
rs2715311
rs7727544
All
−10
0
10
MR effect size for
'Propionylcarnitine' on 'Ulcerative colitis with PSC'

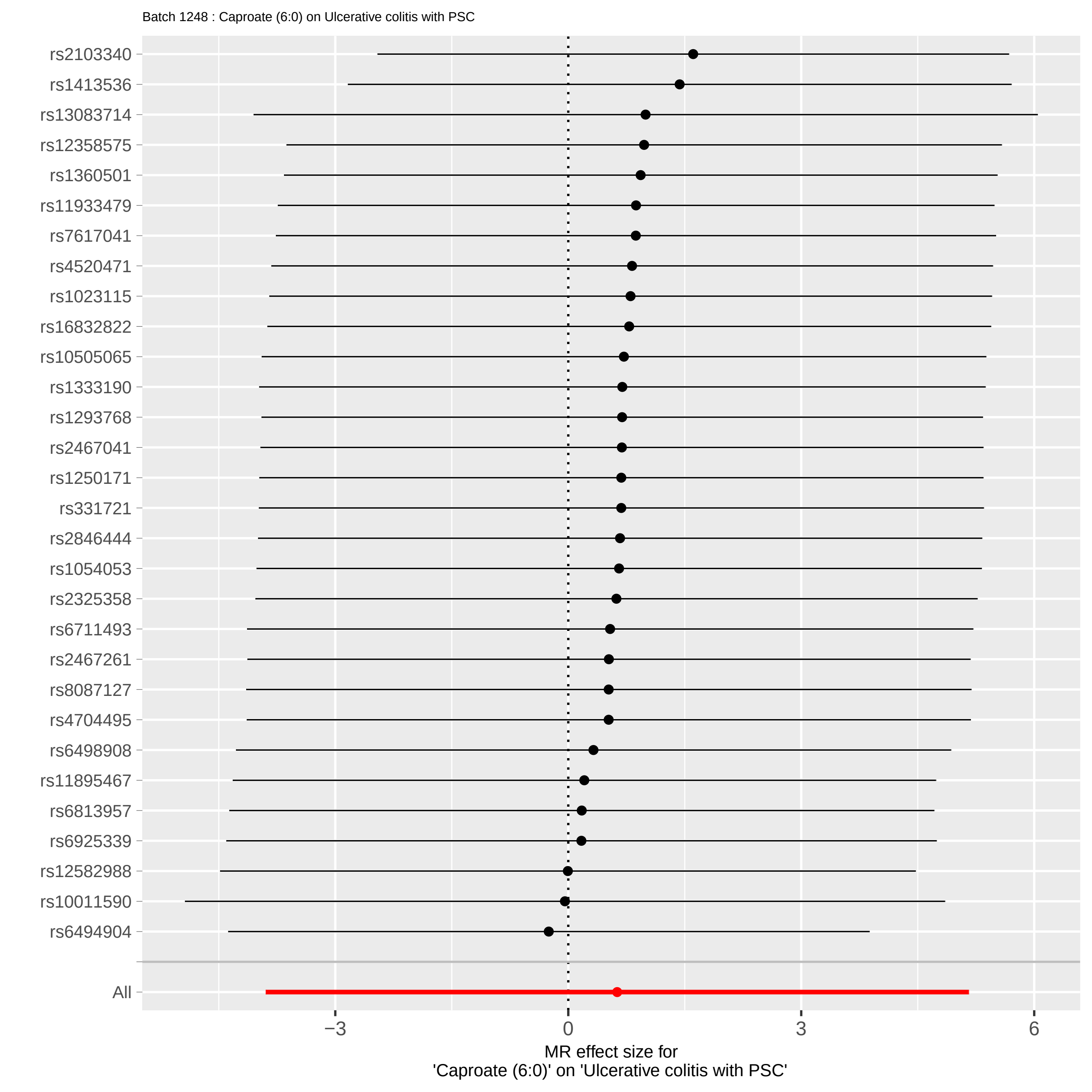
Batch 1248 : Caproate (6:0) on Ulcerative colitis with PSC
rs2103340
rs1413536
rs13083714
rs12358575
rs1360501
rs11933479
rs7617041
rs4520471
rs1023115
rs16832822
rs10505065
rs1333190
rs1293768
rs2467041
rs1250171
rs331721
rs2846444
rs1054053
rs2325358
rs6711493
rs2467261
rs8087127
rs4704495
rs6498908
rs11895467
rs6813957
rs6925339
rs12582988
rs10011590
rs6494904
All
−3
0
3
6
MR effect size for
'Caproate (6:0)' on 'Ulcerative colitis with PSC'

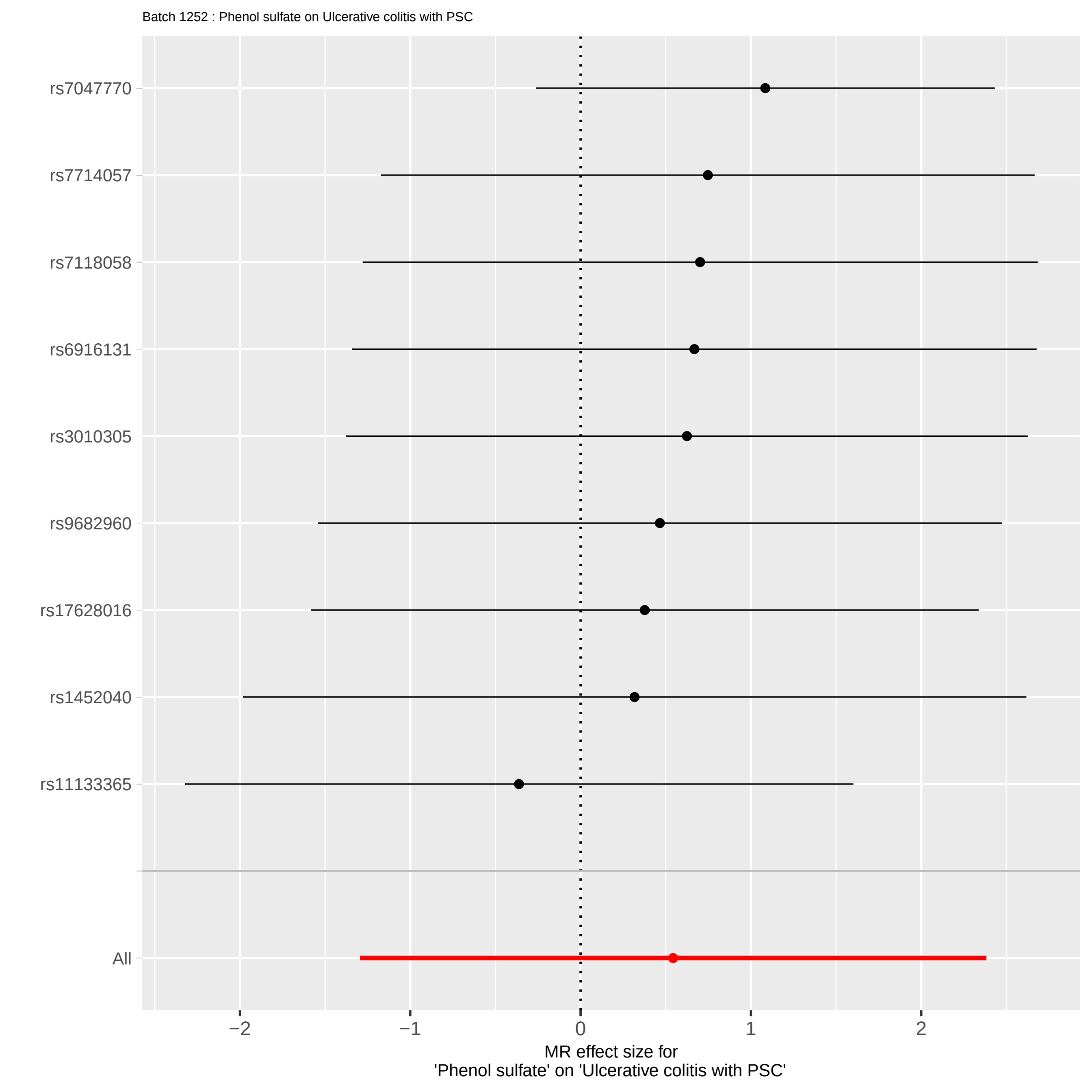

Batch 1252 : Phenol sulfate on Ulcerative colitis with PSC
rs7047770
rs7714057
rs7118058
rs6916131
rs3010305
rs9682960
rs17628016
rs1452040
rs11133365
All
−2
−1
0
1
2
MR effect size for
'Phenol sulfate' on 'Ulcerative colitis with PSC'

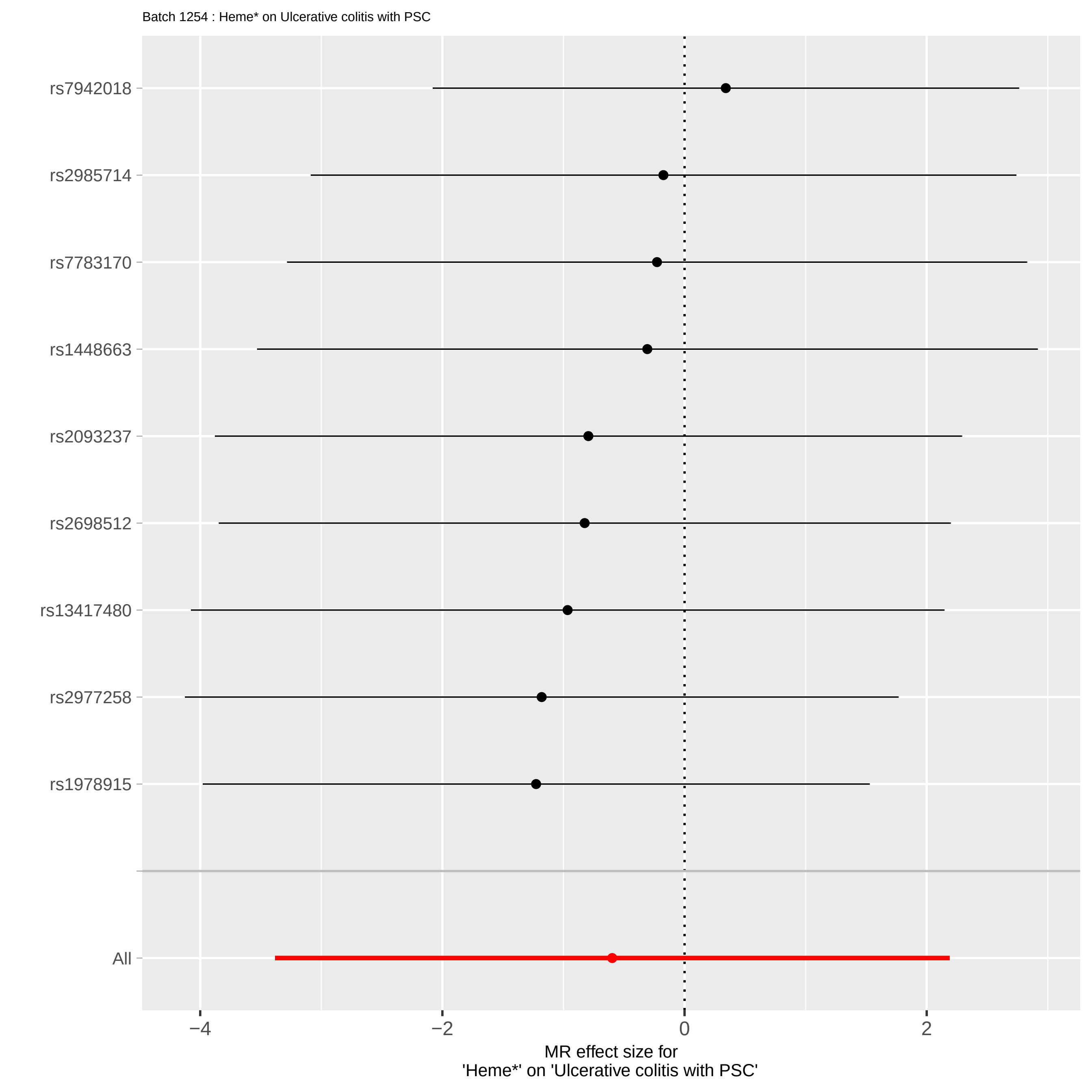

Batch 1254 : Heme* on Ulcerative colitis with PSC
rs7942018
rs2985714
rs7783170
rs1448663
rs2093237
rs2698512
rs13417480
rs2977258
rs1978915
All
−4
−2
0
2
MR effect size for
'Heme*' on 'Ulcerative colitis with PSC'

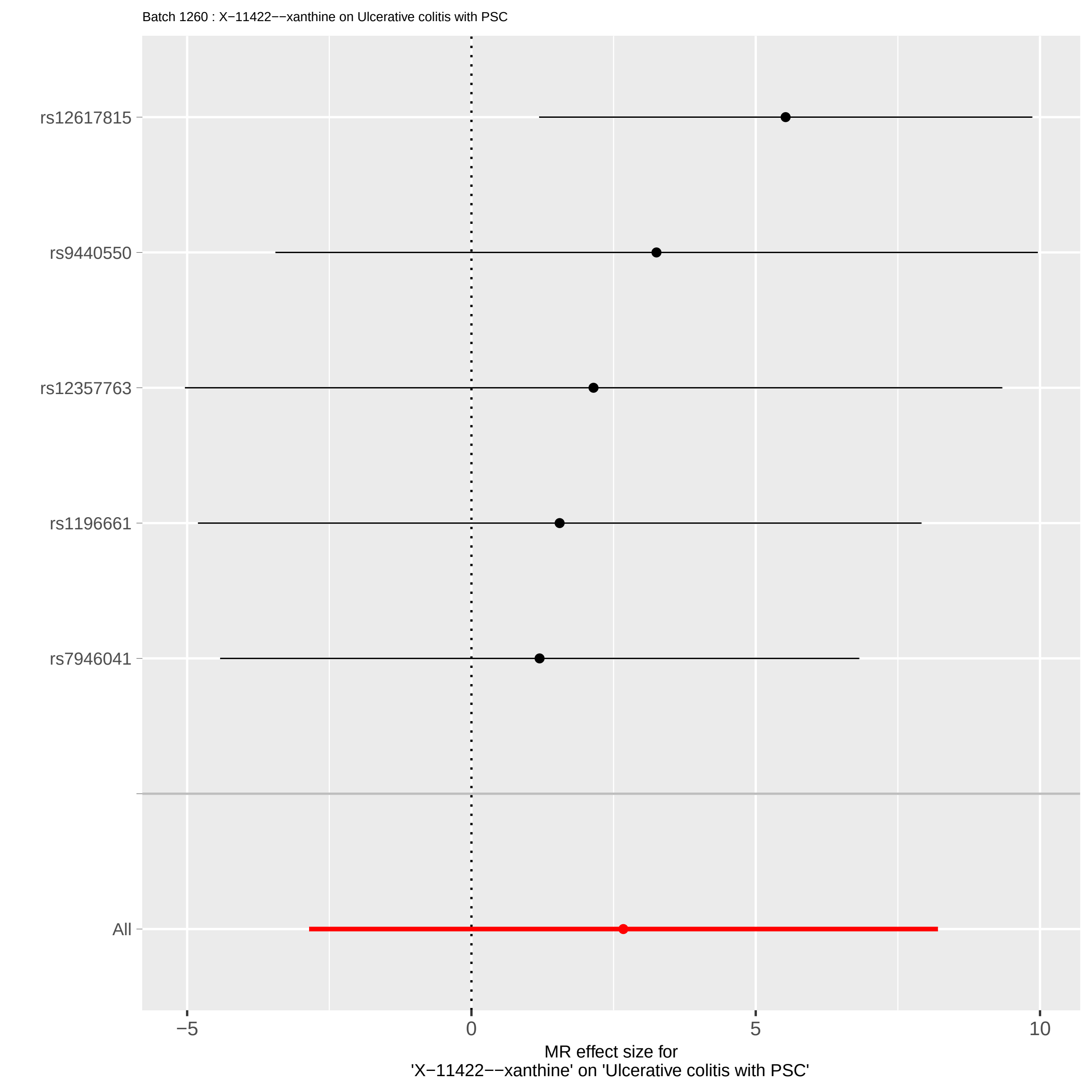

Batch 1260 : X−11422−−xanthine on Ulcerative colitis with PSC
rs12617815
rs9440550
rs12357763
rs1196661
rs7946041
All
−5
0
5
10
MR effect size for
'X−11422−−xanthine' on 'Ulcerative colitis with PSC'

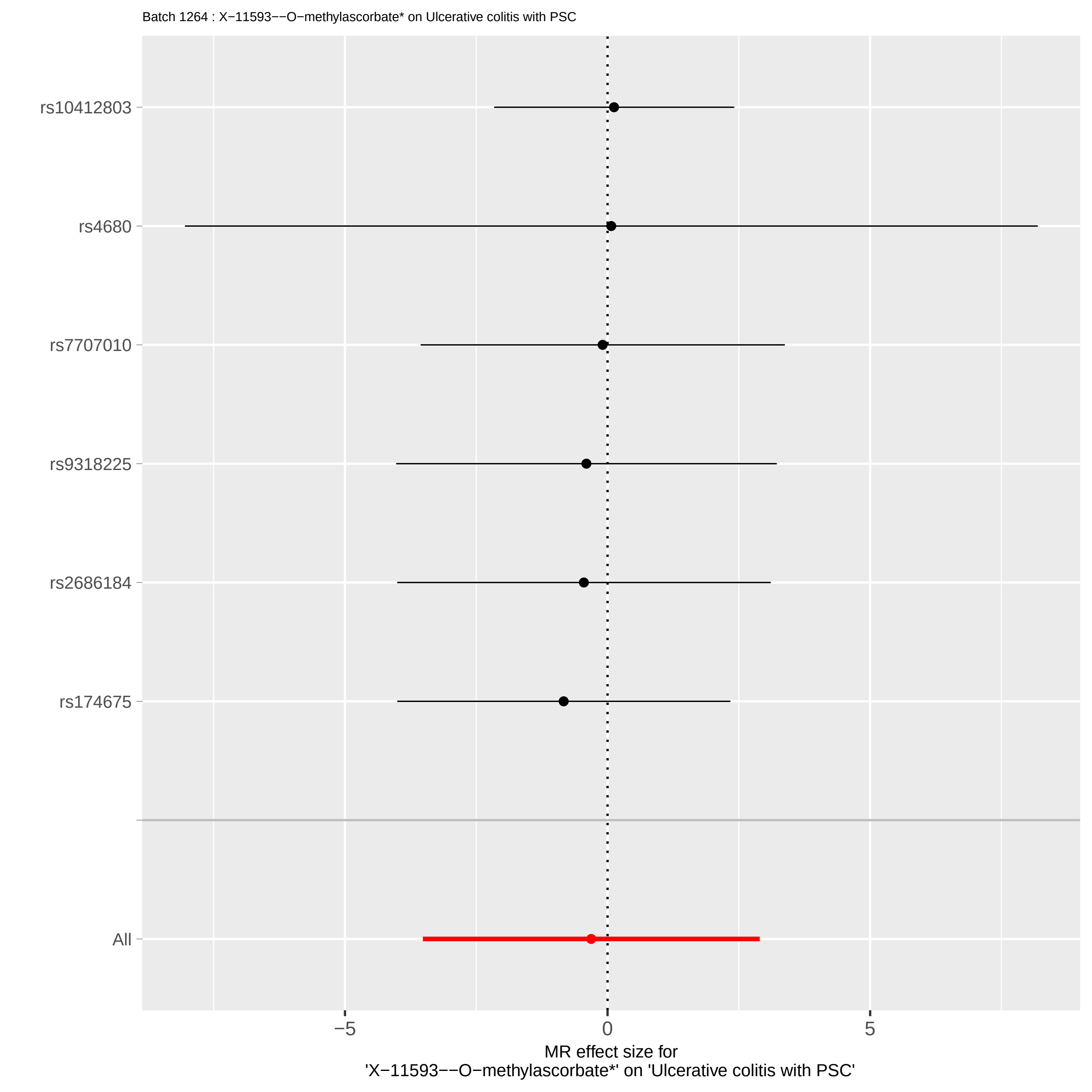

Batch 1264 : X−11593−−O−methylascorbate* on Ulcerative colitis with PSC
rs10412803
rs4680
rs7707010
rs9318225
rs2686184
rs174675
All
−5
0
5
MR effect size for
'X−11593−−O−methylascorbate*' on 'Ulcerative colitis with PSC'

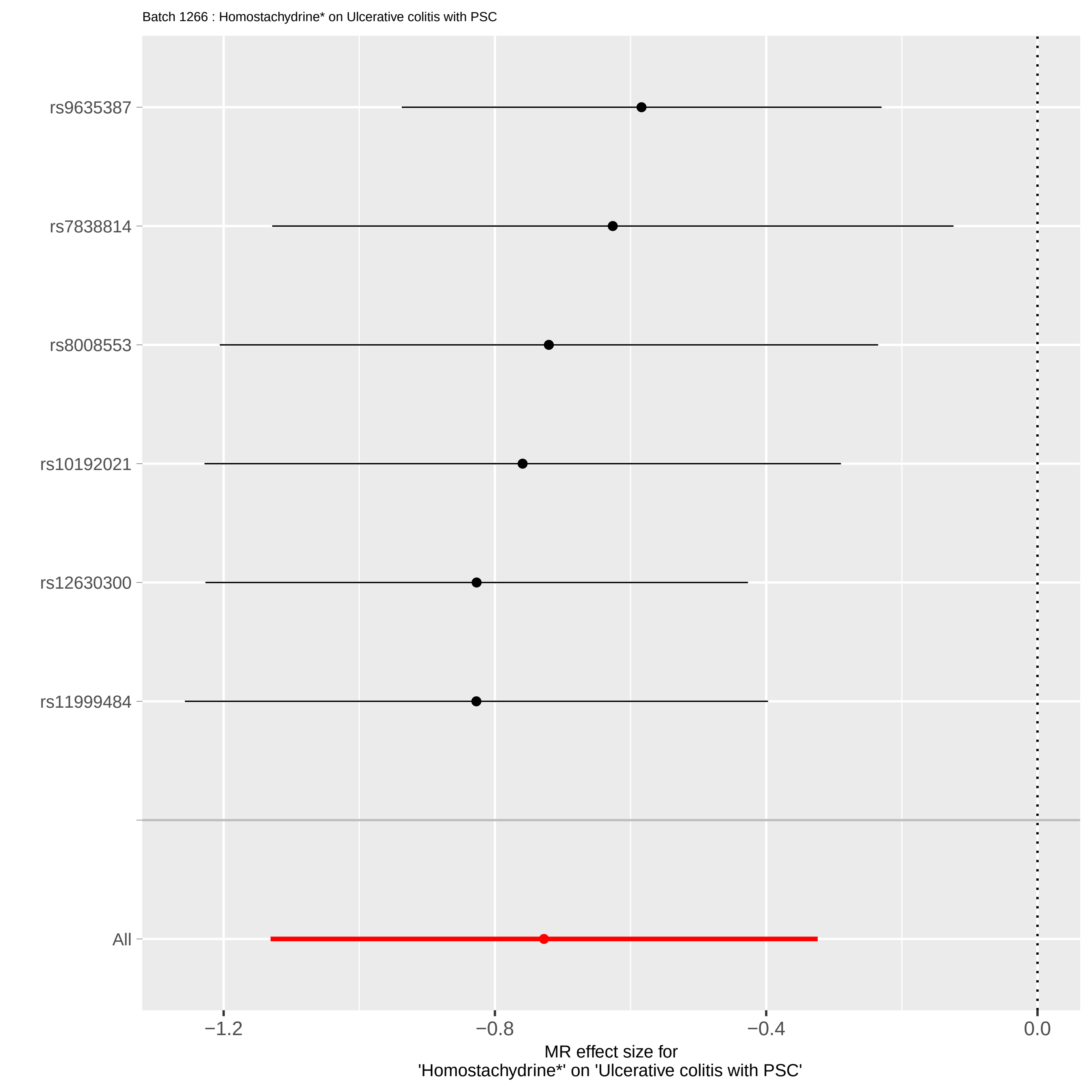

Batch 1266 : Homostachydrine* on Ulcerative colitis with PSC
rs9635387
rs7838814
rs8008553
rs10192021
rs12630300
rs11999484
All
−1.2
−0.8
−0.4
0.0
MR effect size for
'Homostachydrine*' on 'Ulcerative colitis with PSC'

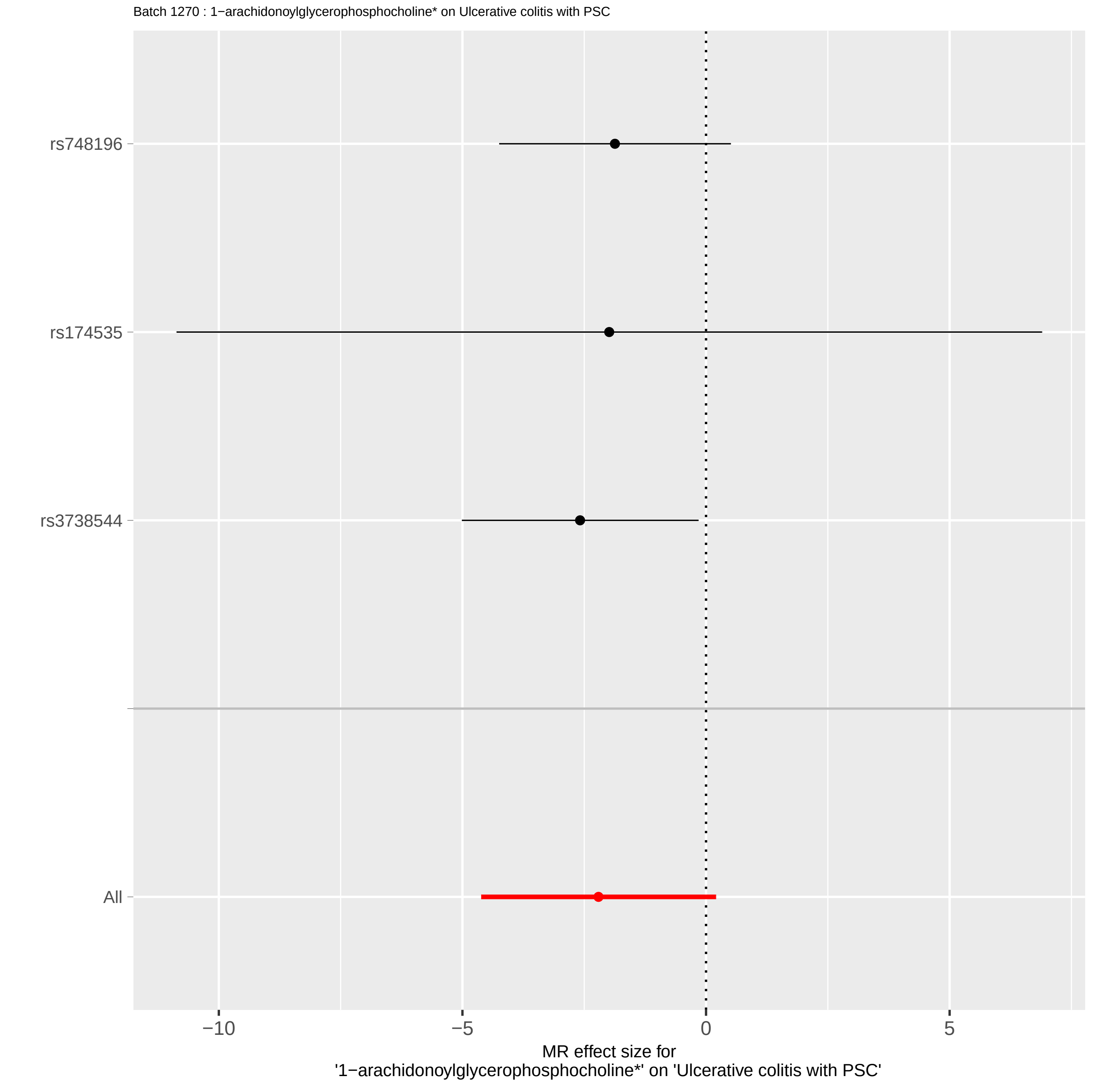
Batch 1270 : 1−arachidonoylglycerophosphocholine* on Ulcerative colitis with PSC
rs748196
rs174535
rs3738544
All
−10
−5
0
5
MR effect size for
'1−arachidonoylglycerophosphocholine*' on 'Ulcerative colitis with PSC'

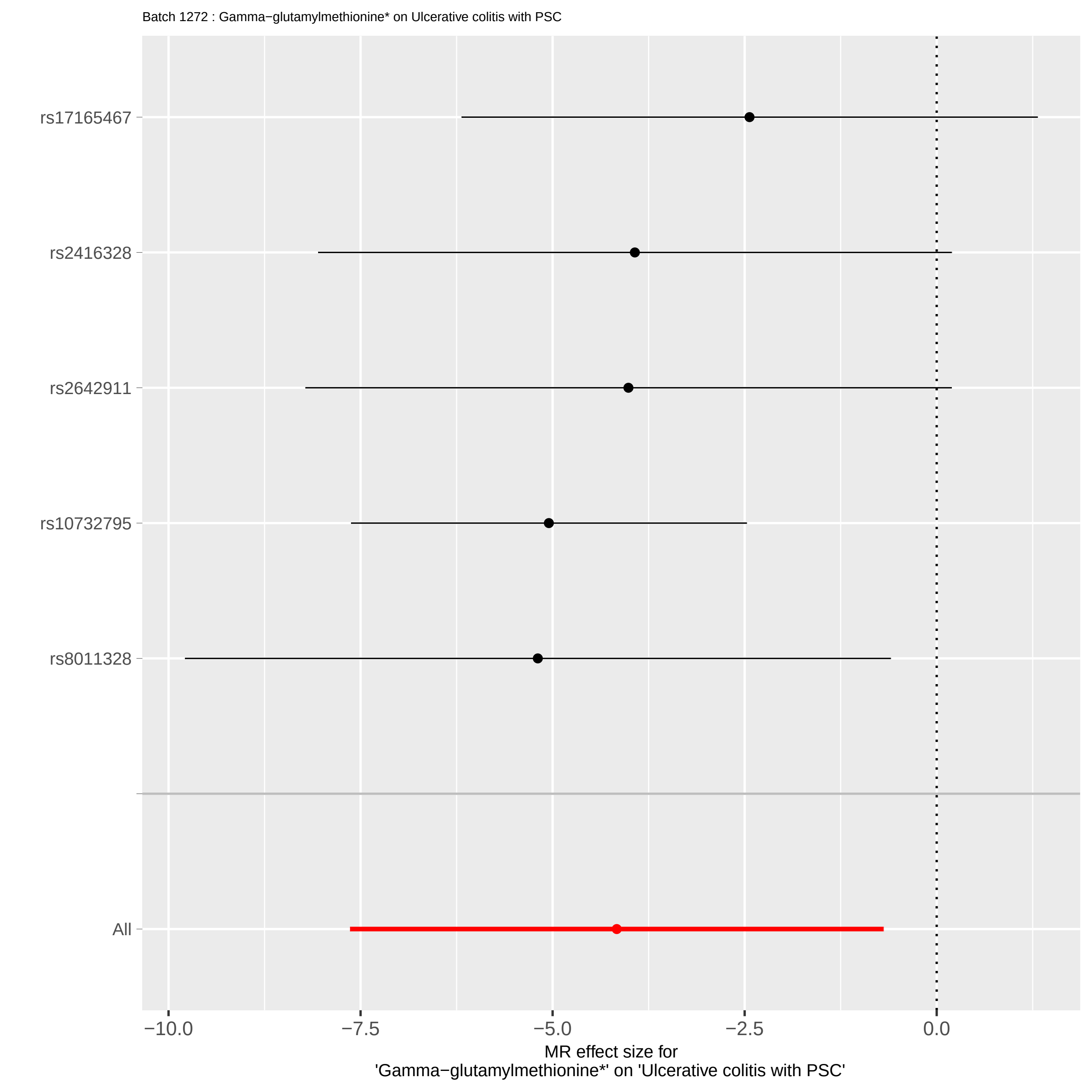
Batch 1272 : Gamma−glutamylmethionine* on Ulcerative colitis with PSC
rs17165467
rs2416328
rs2642911
rs10732795
rs8011328
All
−10.0
−7.5
−5.0
−2.5
0.0
MR effect size for
'Gamma−glutamylmethionine*' on 'Ulcerative colitis with PSC'

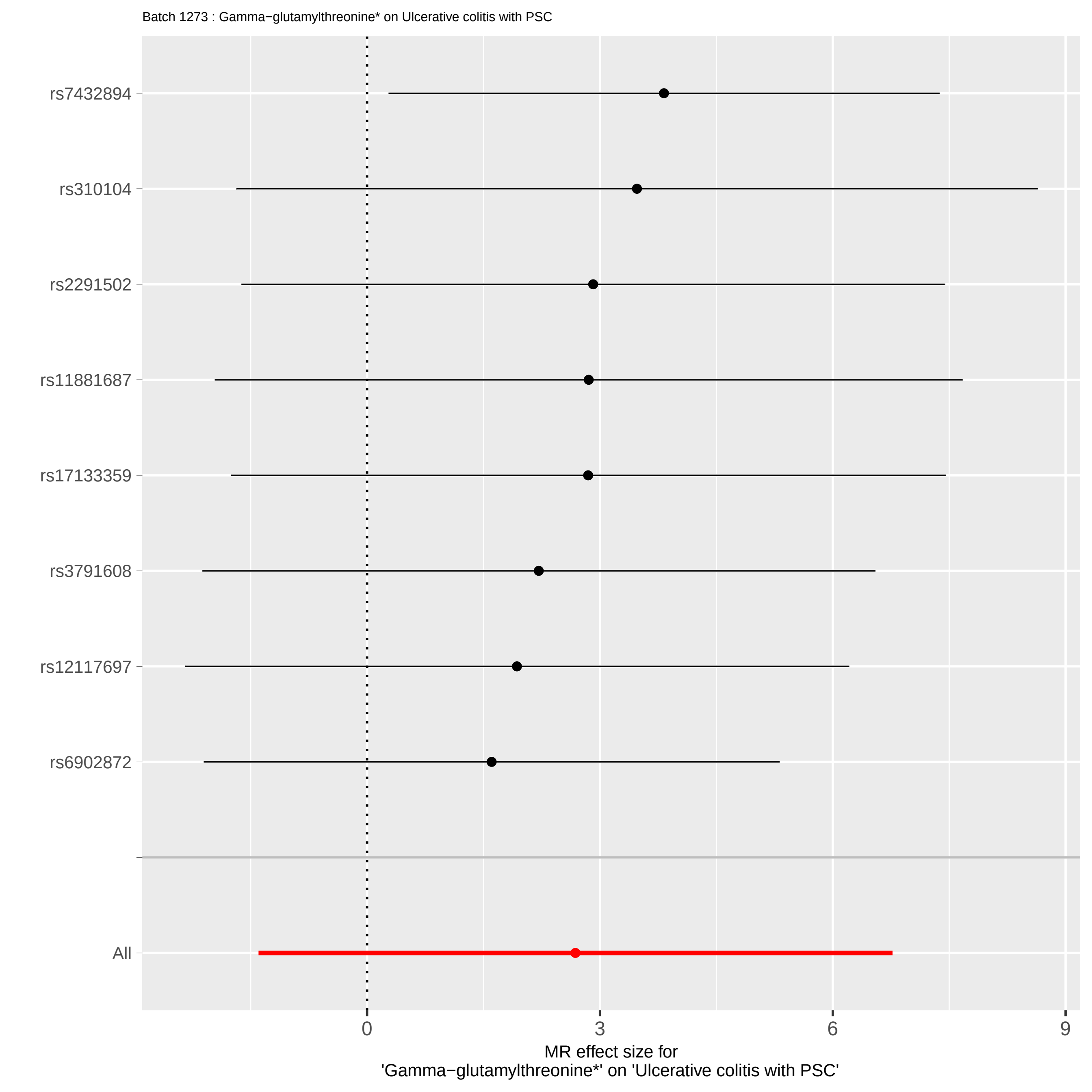

Batch 1273 : Gamma−glutamylthreonine* on Ulcerative colitis with PSC
rs7432894
rs310104
rs2291502
rs11881687
rs17133359
rs3791608
rs12117697
rs6902872
All
0
3
6
9
MR effect size for
'Gamma−glutamylthreonine*' on 'Ulcerative colitis with PSC'

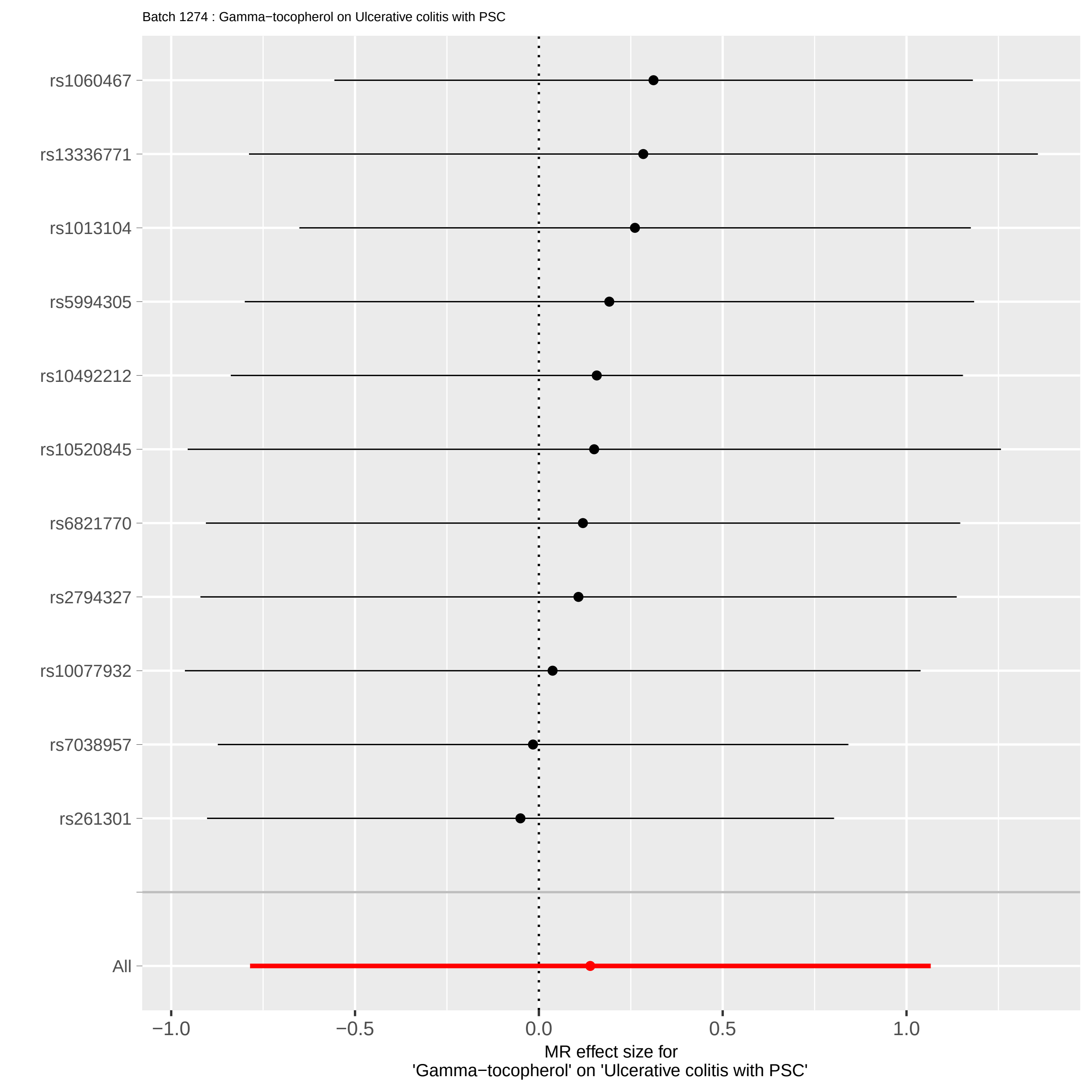

Batch 1274 : Gamma−tocopherol on Ulcerative colitis with PSC
rs1060467
rs13336771
rs1013104
rs5994305
rs10492212
rs10520845
rs6821770
rs2794327
rs10077932
rs7038957
rs261301
All
−1.0
−0.5
0.0
0.5
1.0
MR effect size for
'Gamma−tocopherol' on 'Ulcerative colitis with PSC'

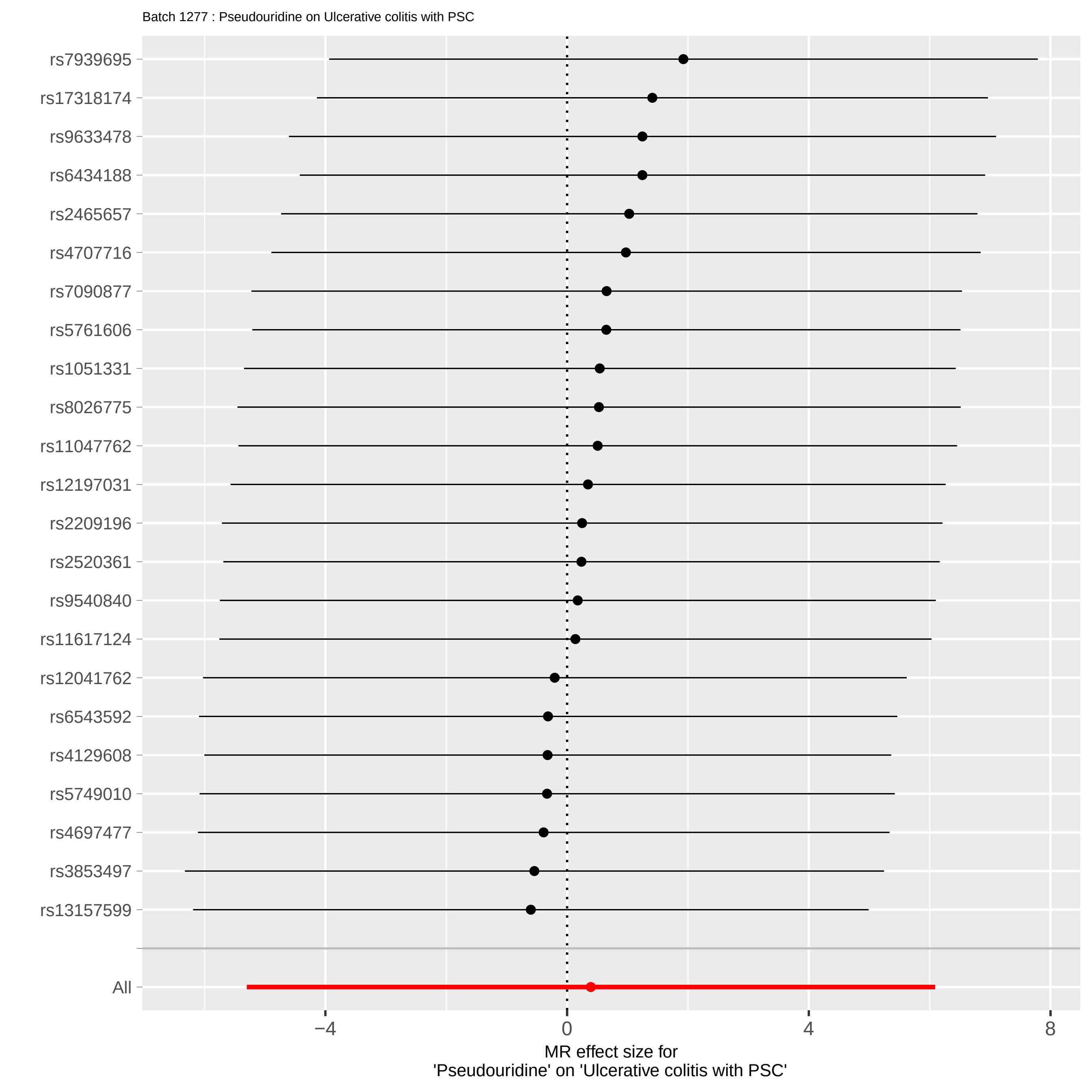

Batch 1277 : Pseudouridine on Ulcerative colitis with PSC
rs7939695
rs17318174
rs9633478
rs6434188
rs2465657
rs4707716
rs7090877
rs5761606
rs1051331
rs8026775
rs11047762
rs12197031
rs2209196
rs2520361
rs9540840
rs11617124
rs12041762
rs6543592
rs4129608
rs5749010
rs4697477
rs3853497
rs13157599
All
−4
0
4
8
MR effect size for
'Pseudouridine' on 'Ulcerative colitis with PSC'

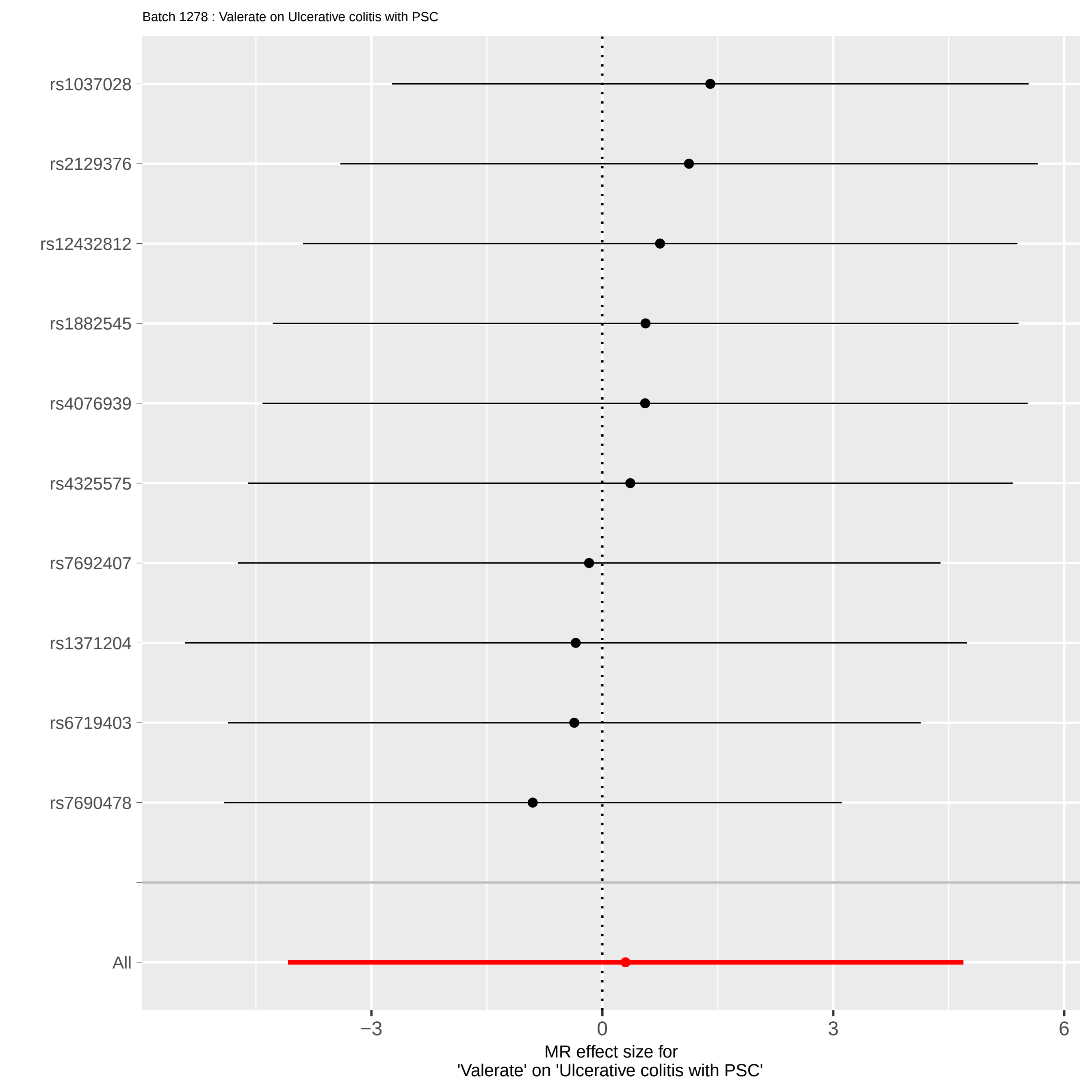
Batch 1278 : Valerate on Ulcerative colitis with PSC
rs1037028
rs2129376
rs12432812
rs1882545
rs4076939
rs4325575
rs7692407
rs1371204
rs6719403
rs7690478
All
−3
0
3
6
MR effect size for
'Valerate' on 'Ulcerative colitis with PSC'

Batch 1281 : Erythronate* on Ulcerative colitis with PSC

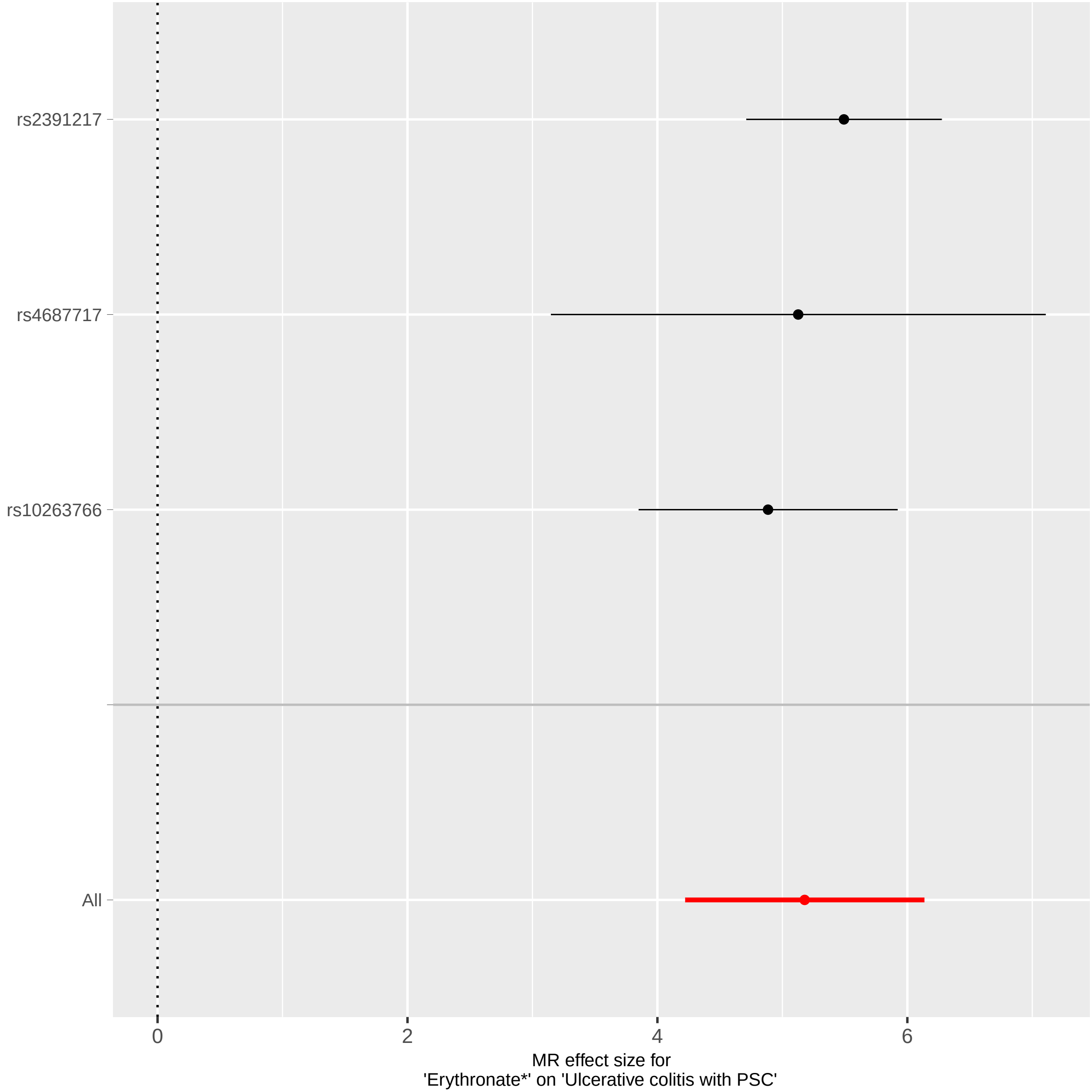

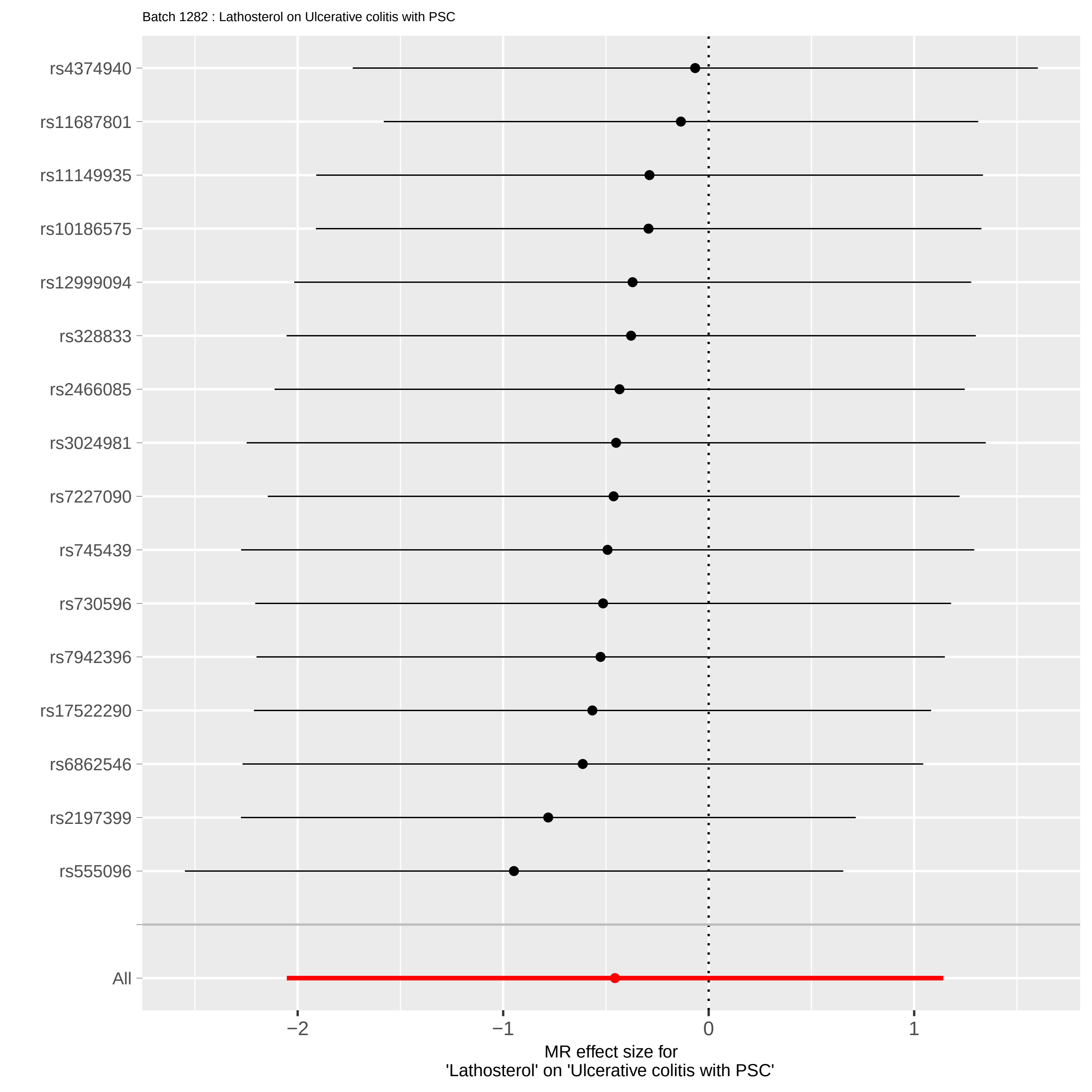

Batch 1282 : Lathosterol on Ulcerative colitis with PSC
rs4374940
rs11687801
rs11149935
rs10186575
rs12999094
rs328833
rs2466085
rs3024981
rs7227090
rs745439
rs730596
rs7942396
rs17522290
rs6862546
rs2197399
rs555096
All
−2
−1
0
1
MR effect size for
'Lathosterol' on 'Ulcerative colitis with PSC'

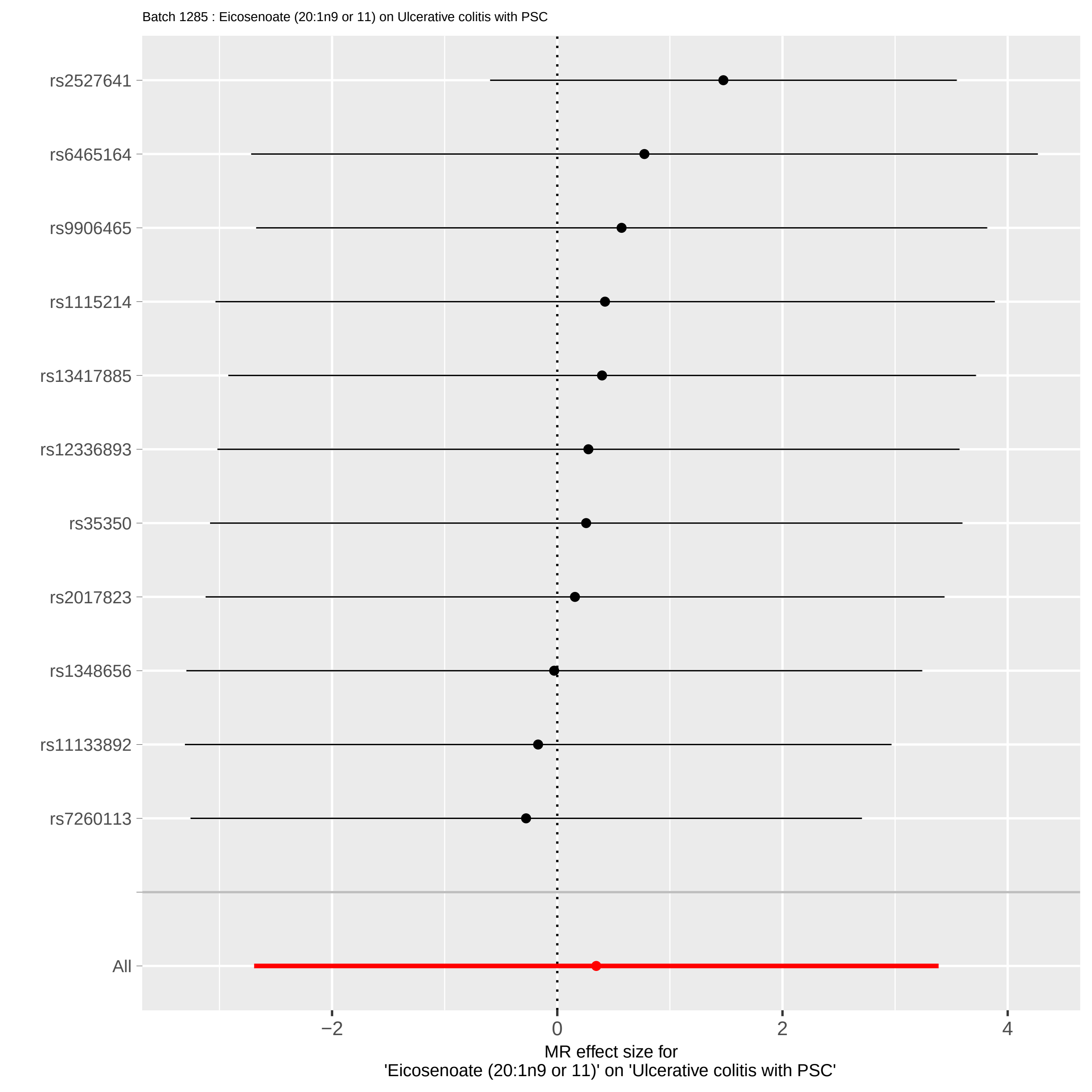

Batch 1285 : Eicosenoate (20:1n9 or 11) on Ulcerative colitis with PSC
rs2527641
rs6465164
rs9906465
rs1115214
rs13417885
rs12336893
rs35350
rs2017823
rs1348656
rs11133892
rs7260113
All
−2
0
2
4
MR effect size for
'Eicosenoate (20:1n9 or 11)' on 'Ulcerative colitis with PSC'

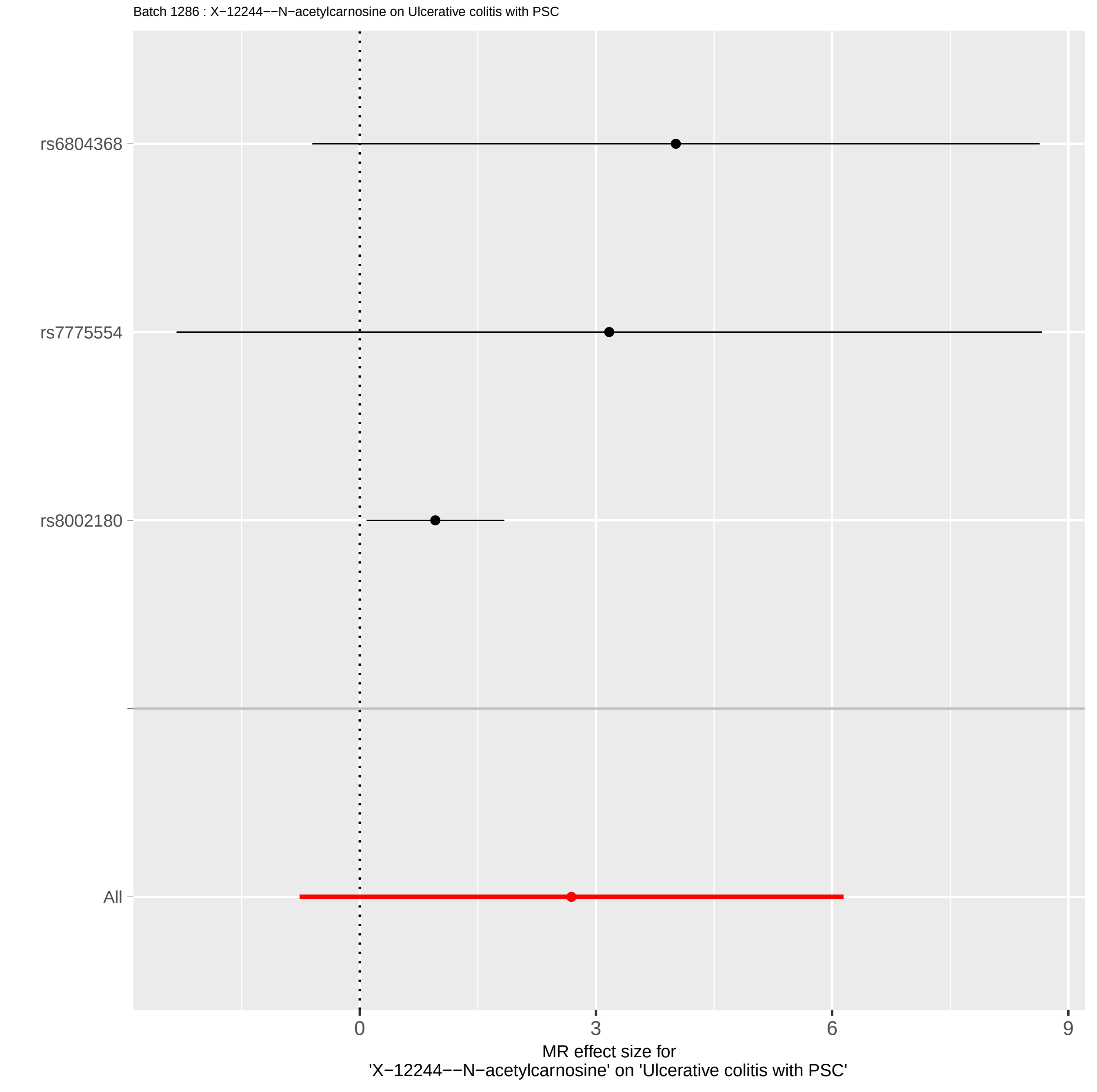
Batch 1286 : X−12244−−N−acetylcarnosine on Ulcerative colitis with PSC
rs6804368
rs7775554
rs8002180
All
0
3
6
9
MR effect size for
'X−12244−−N−acetylcarnosine' on 'Ulcerative colitis with PSC'

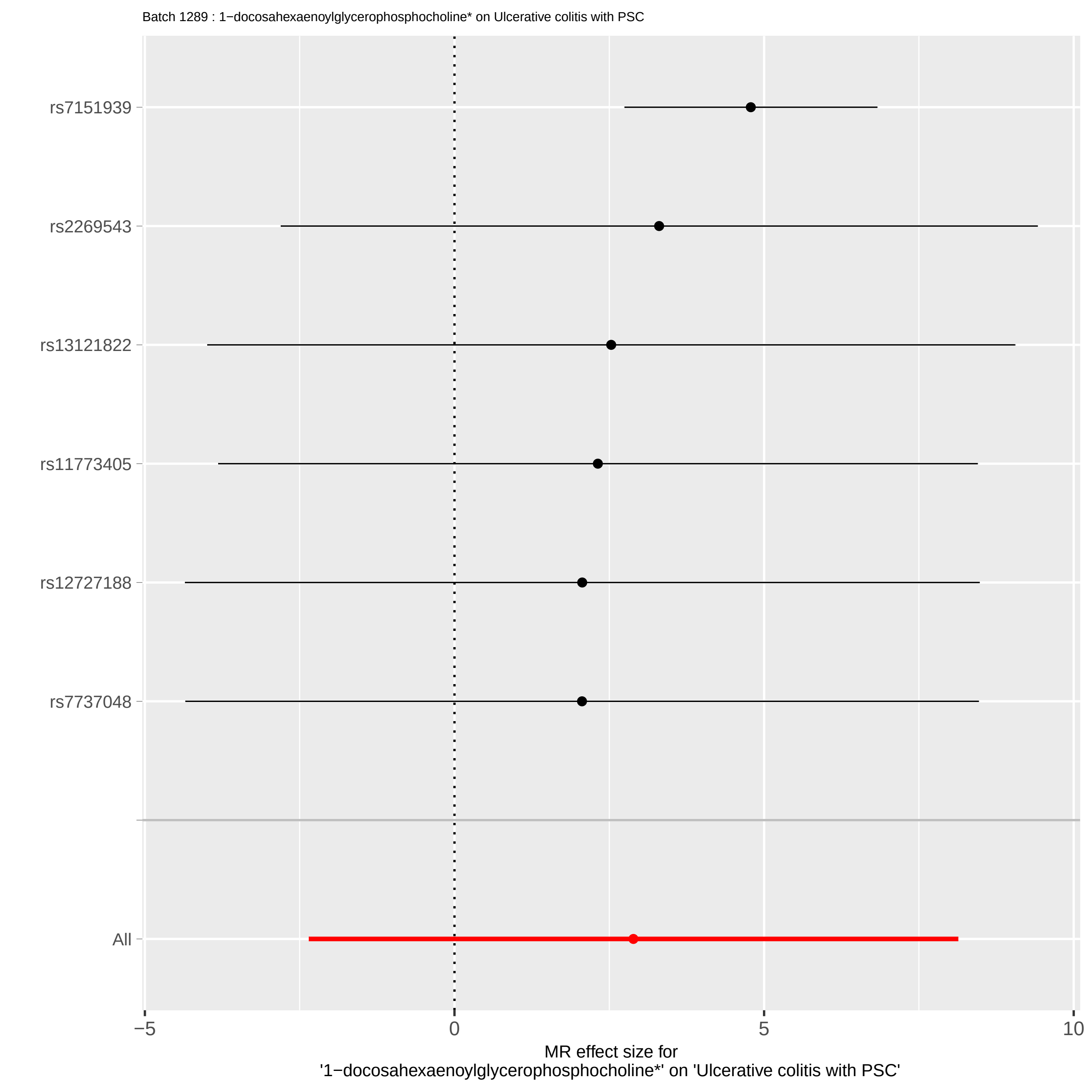

Batch 1289 : 1−docosahexaenoylglycerophosphocholine* on Ulcerative colitis with PSC
rs7151939
rs2269543
rs13121822
rs11773405
rs12727188
rs7737048
All
−5
0
5
10
MR effect size for
'1−docosahexaenoylglycerophosphocholine*' on 'Ulcerative colitis with PSC'

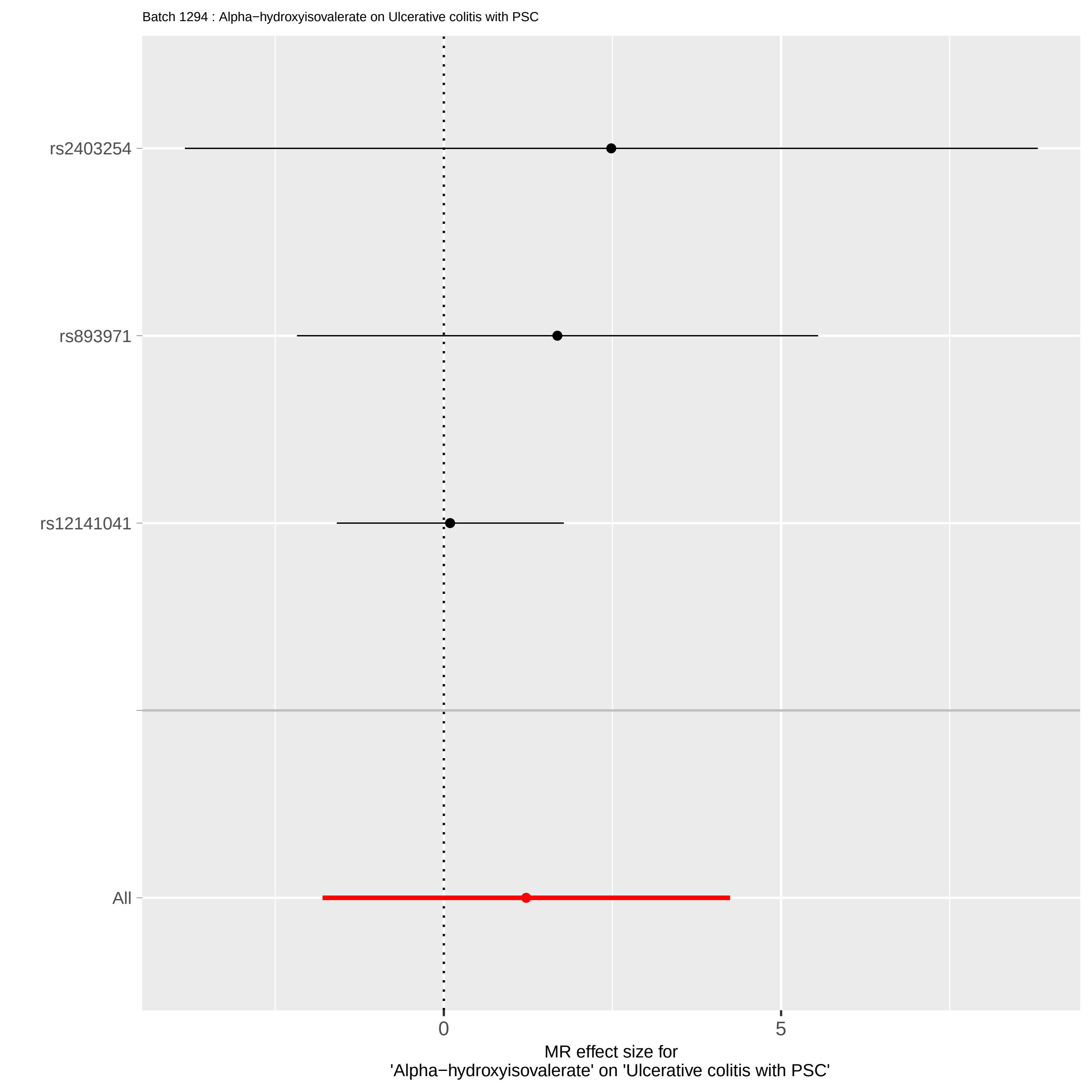
Batch 1294 : Alpha−hydroxyisovalerate on Ulcerative colitis with PSC
rs2403254
rs893971
rs12141041
All
0
5
MR effect size for
'Alpha−hydroxyisovalerate' on 'Ulcerative colitis with PSC'

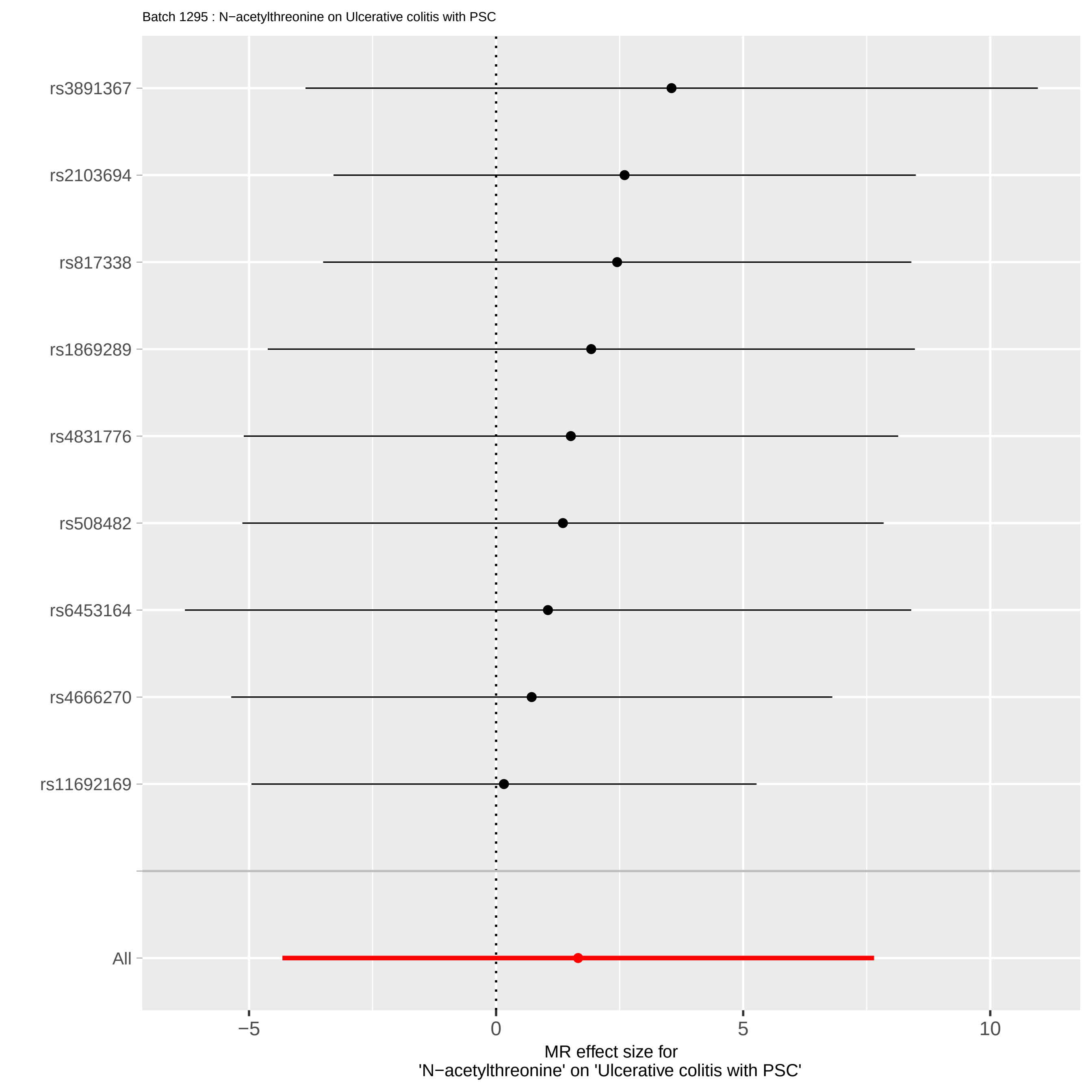
Batch 1295 : N−acetylthreonine on Ulcerative colitis with PSC
rs3891367
rs2103694
rs817338
rs1869289
rs4831776
rs508482
rs6453164
rs4666270
rs11692169
All
−5
0
5
10
MR effect size for
'N−acetylthreonine' on 'Ulcerative colitis with PSC'

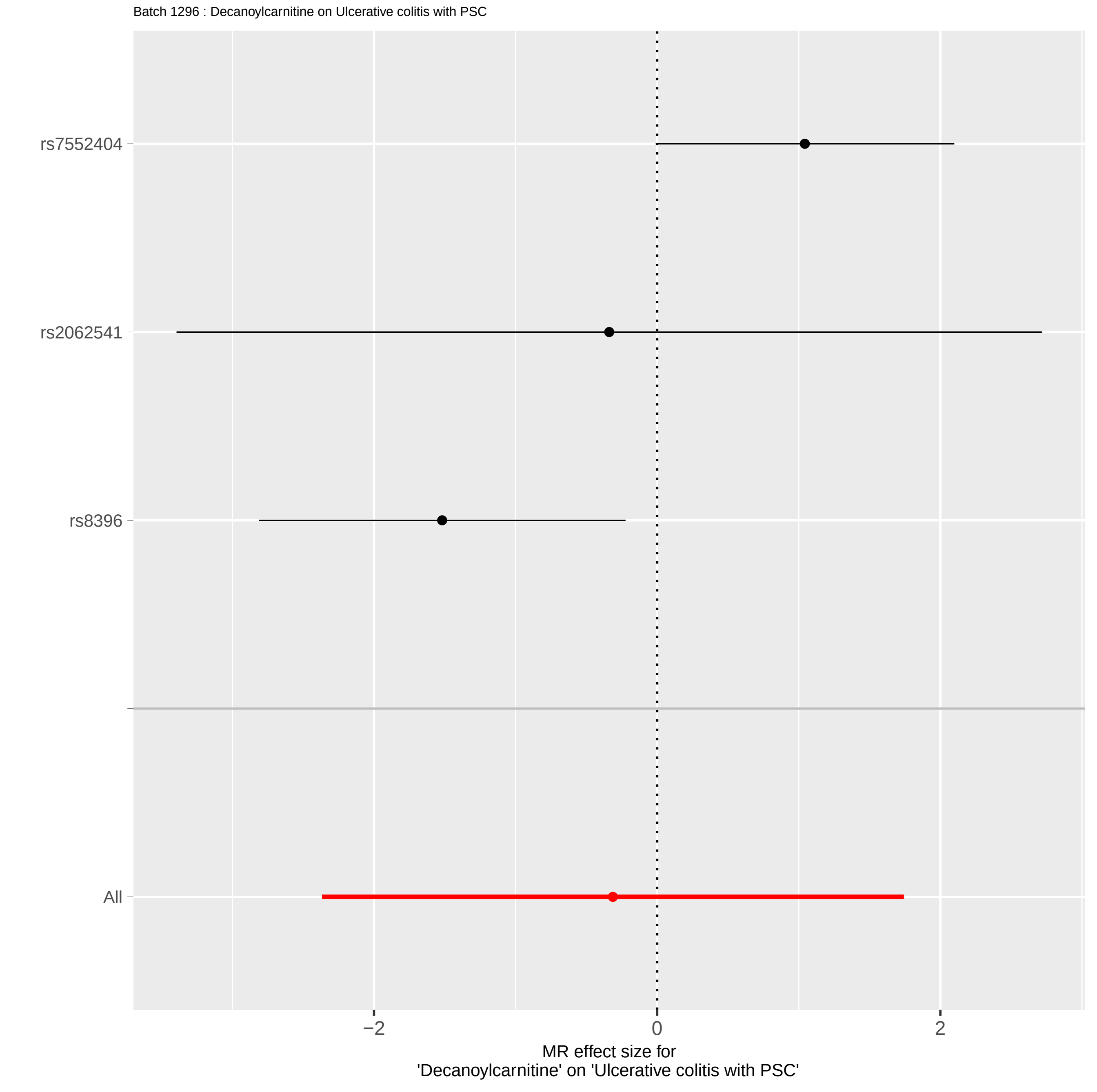

Batch 1296 : Decanoylcarnitine on Ulcerative colitis with PSC
rs7552404
rs2062541
rs8396
All
−2
0
2
MR effect size for
'Decanoylcarnitine' on 'Ulcerative colitis with PSC'

Batch 1297 : 1−palmitoylglycerophosphocholine on Ulcerative colitis with PSC

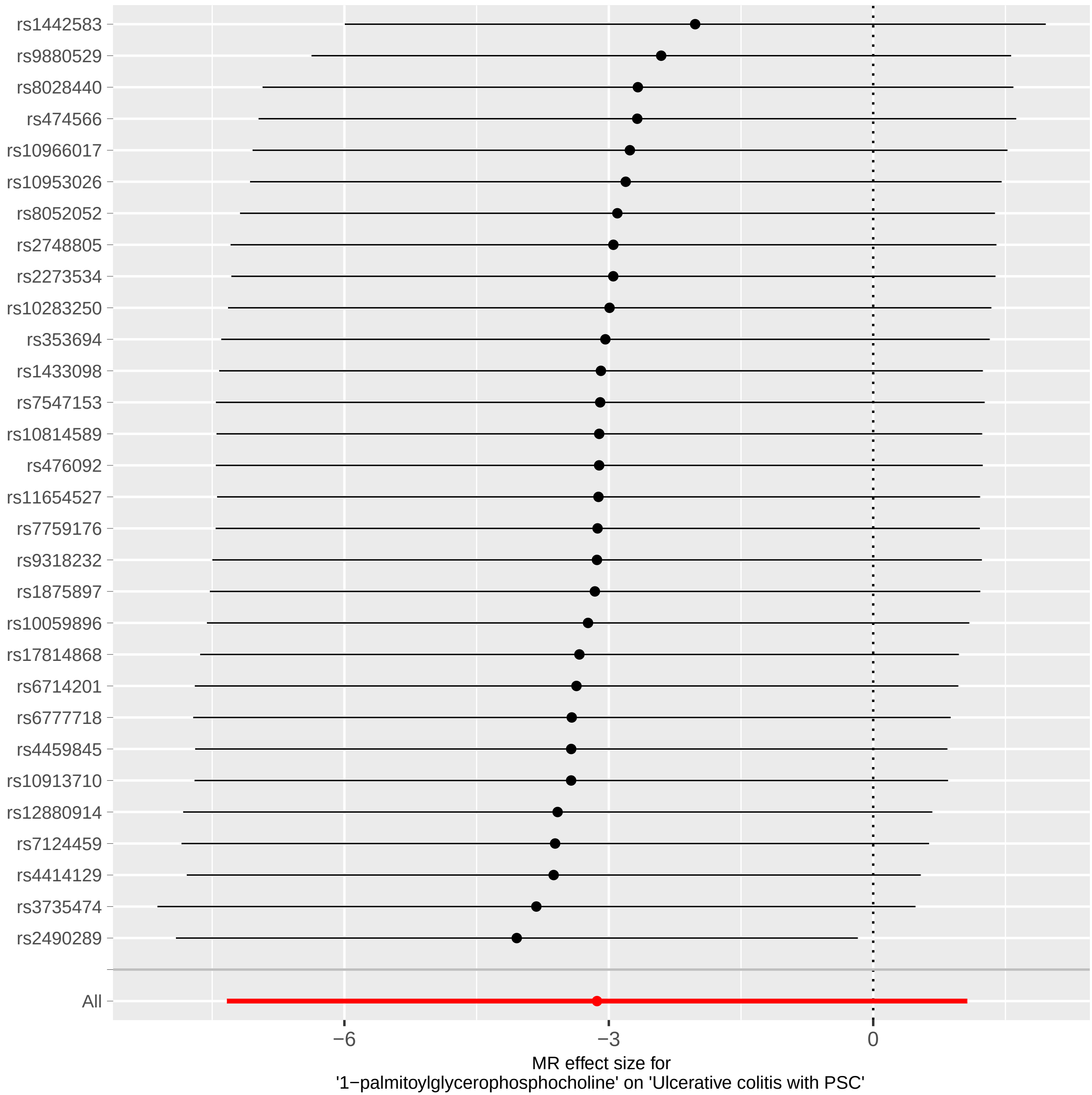

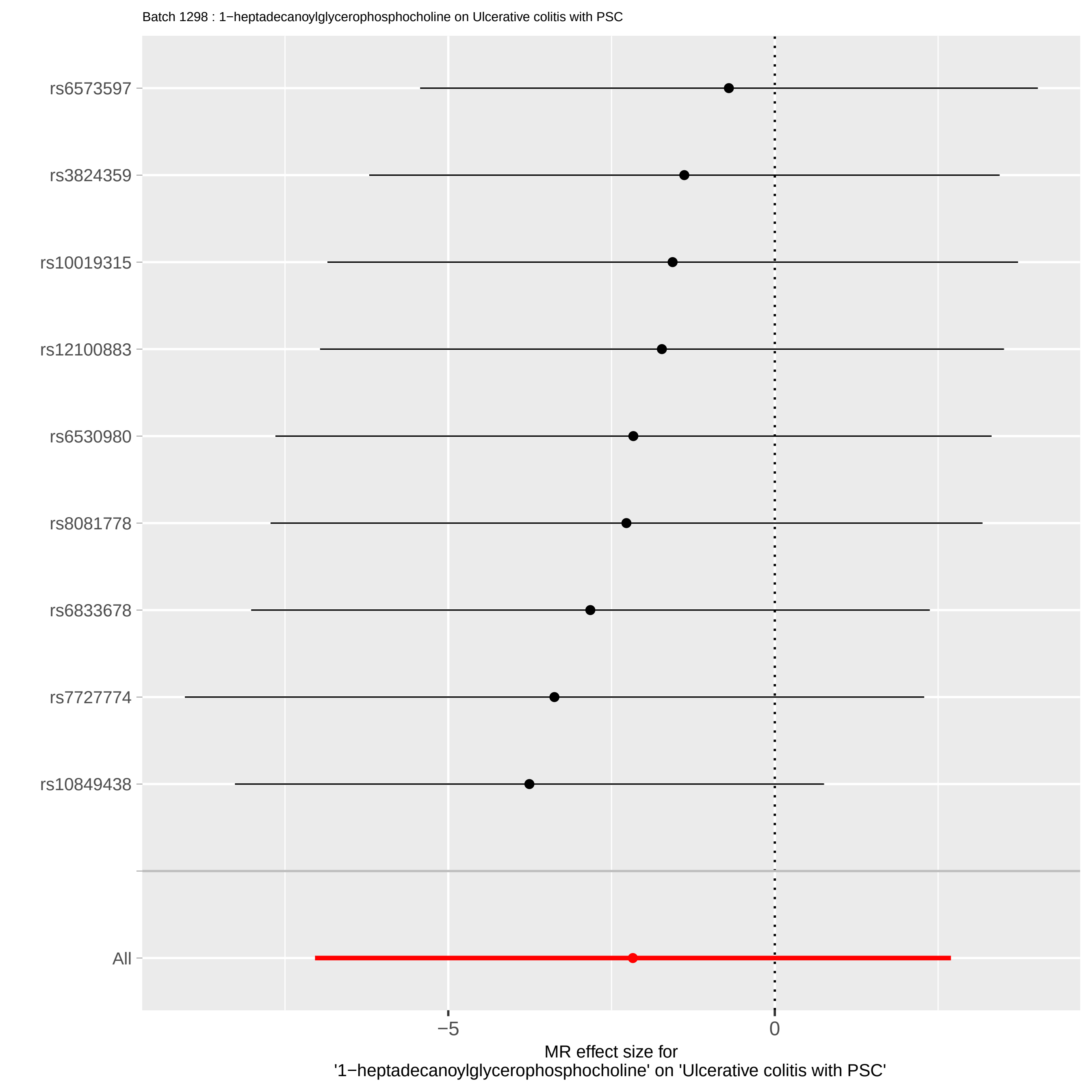

Batch 1298 : 1−heptadecanoylglycerophosphocholine on Ulcerative colitis with PSC
rs6573597
rs3824359
rs10019315
rs12100883
rs6530980
rs8081778
rs6833678
rs7727774
rs10849438
All
−5
0
MR effect size for
'1−heptadecanoylglycerophosphocholine' on 'Ulcerative colitis with PSC'

Batch 1299 : 1−oleoylglycerophosphocholine on Ulcerative colitis with PSC

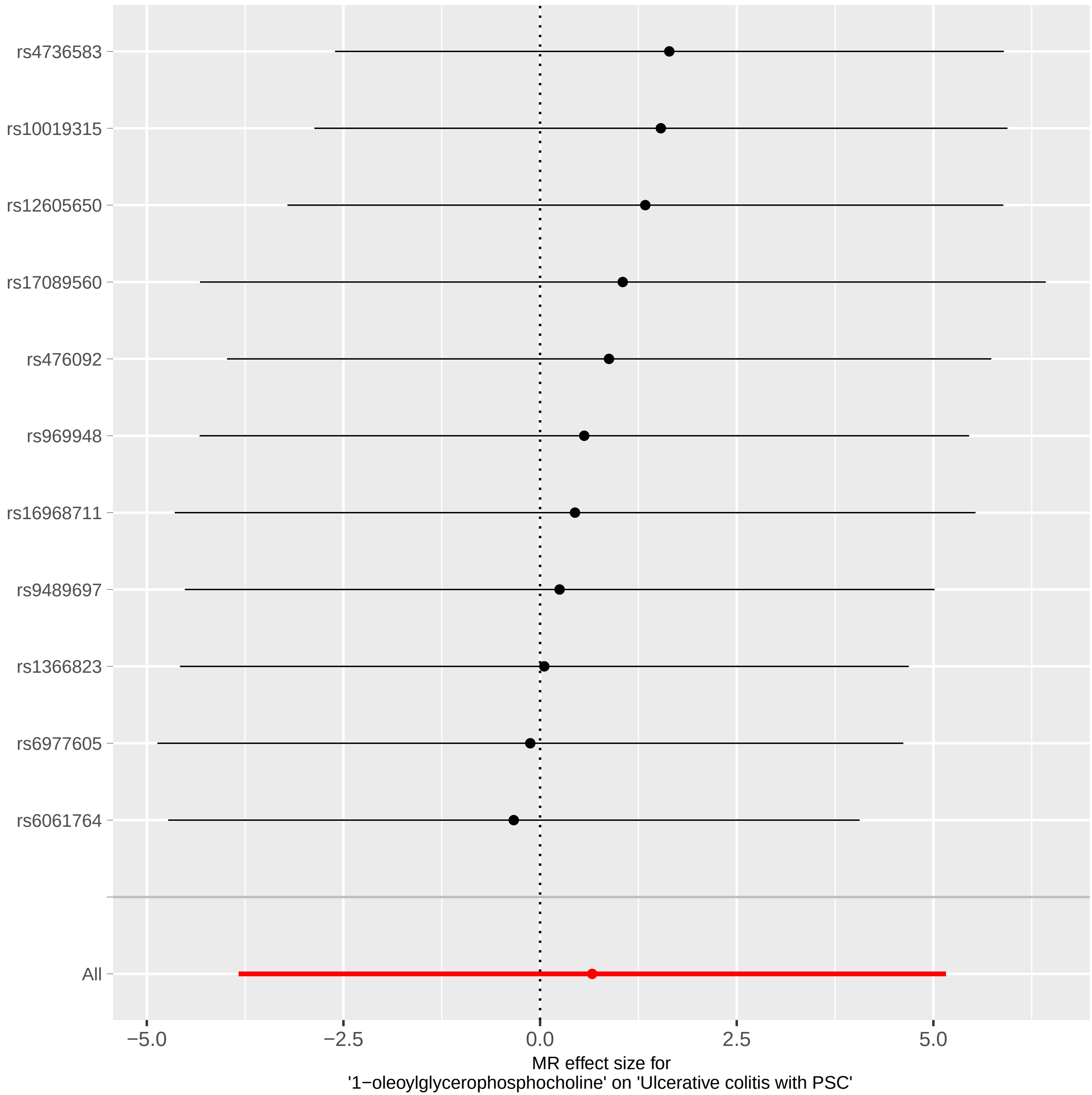

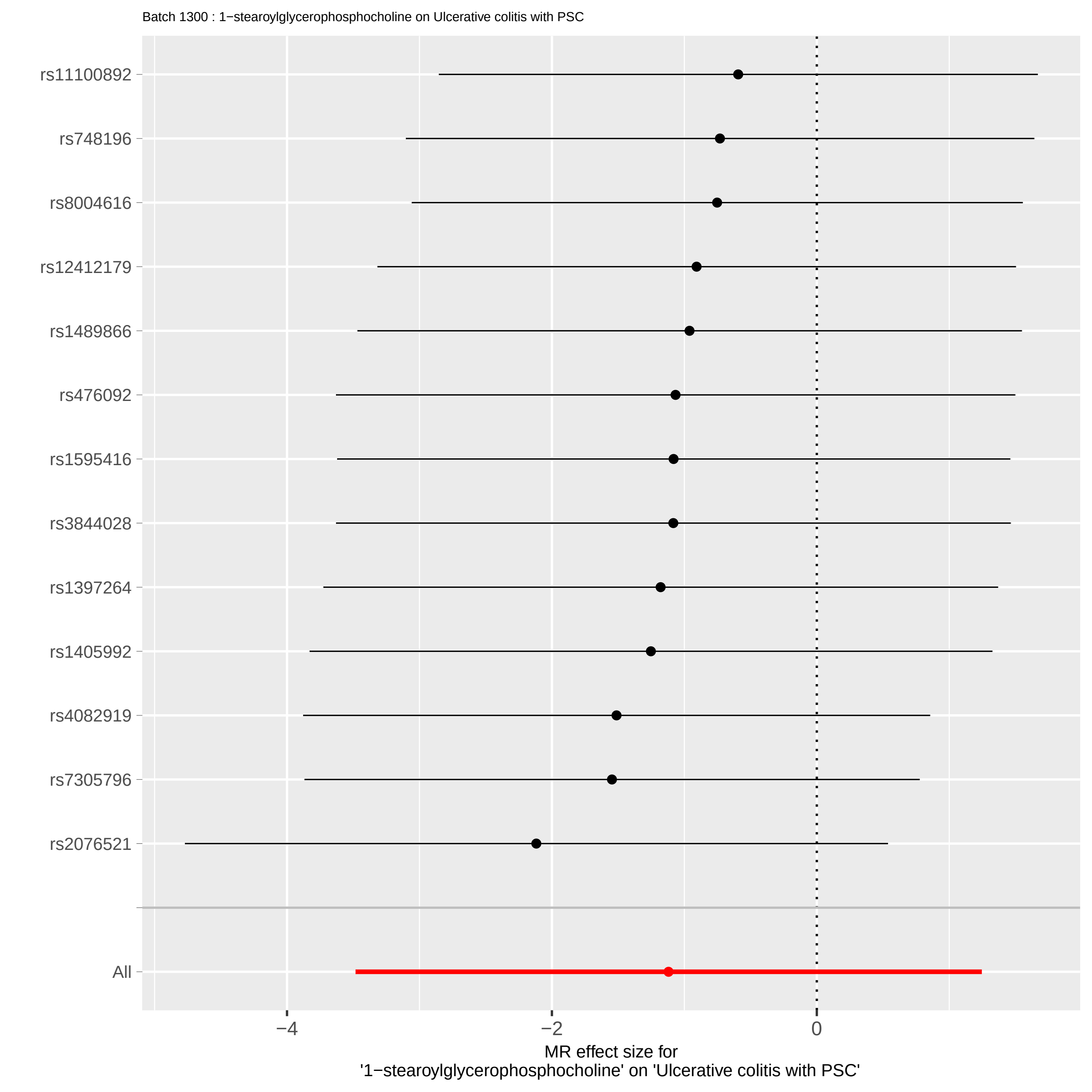
Batch 1300 : 1−stearoylglycerophosphocholine on Ulcerative colitis with PSC
rs11100892
rs748196
rs8004616
rs12412179
rs1489866
rs476092
rs1595416
rs3844028
rs1397264
rs1405992
rs4082919
rs7305796
rs2076521
All
−4
−2
0
MR effect size for
'1−stearoylglycerophosphocholine' on 'Ulcerative colitis with PSC'

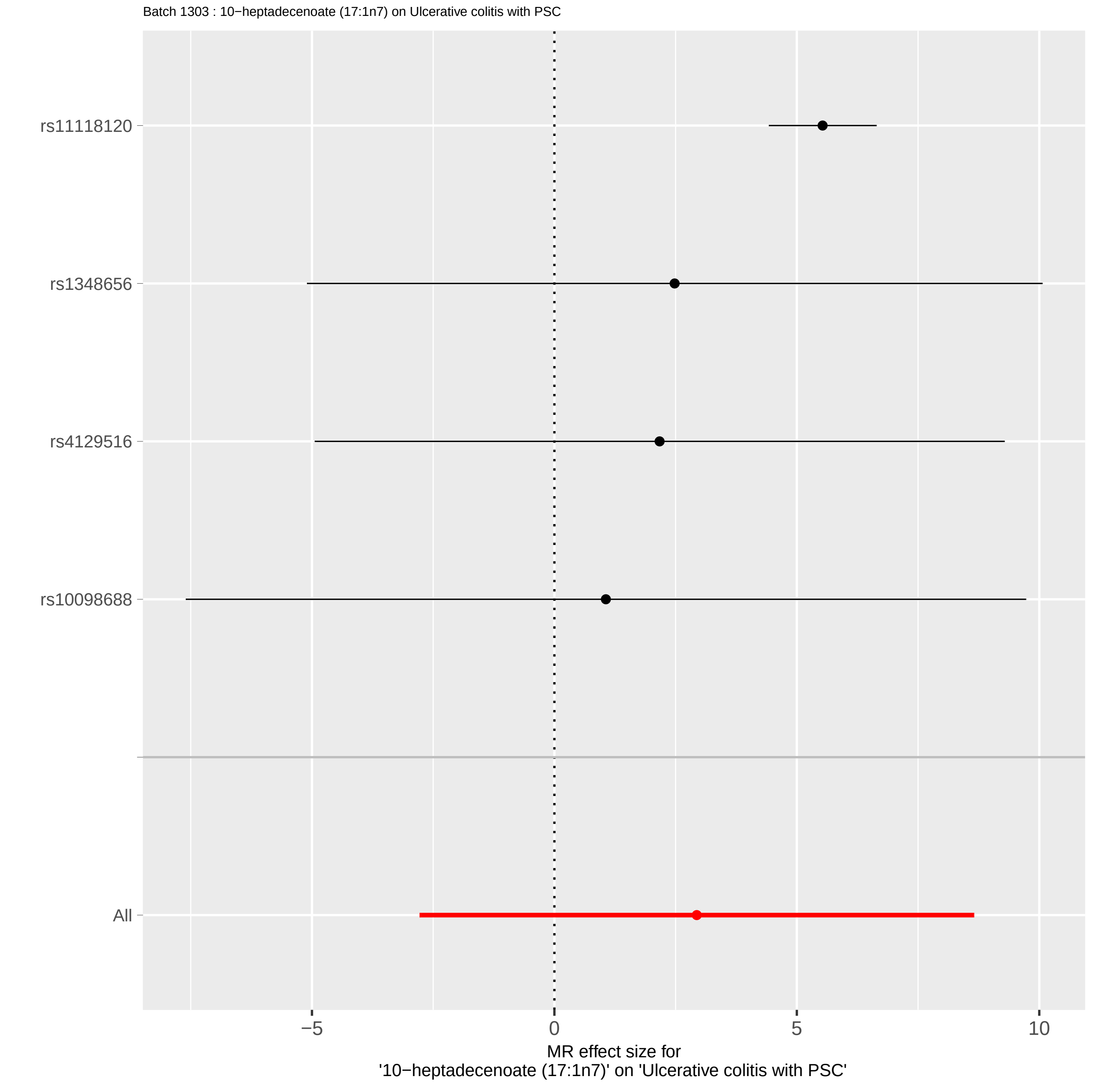
Batch 1303 : 10−heptadecenoate (17:1n7) on Ulcerative colitis with PSC
rs11118120
rs1348656
rs4129516
rs10098688
All
−5
0
5
10
MR effect size for
'10−heptadecenoate (17:1n7)' on 'Ulcerative colitis with PSC'

Batch 1306 : Linolenate [alpha or gamma; (18:3n3 or 6)] on Ulcerative colitis with PSC

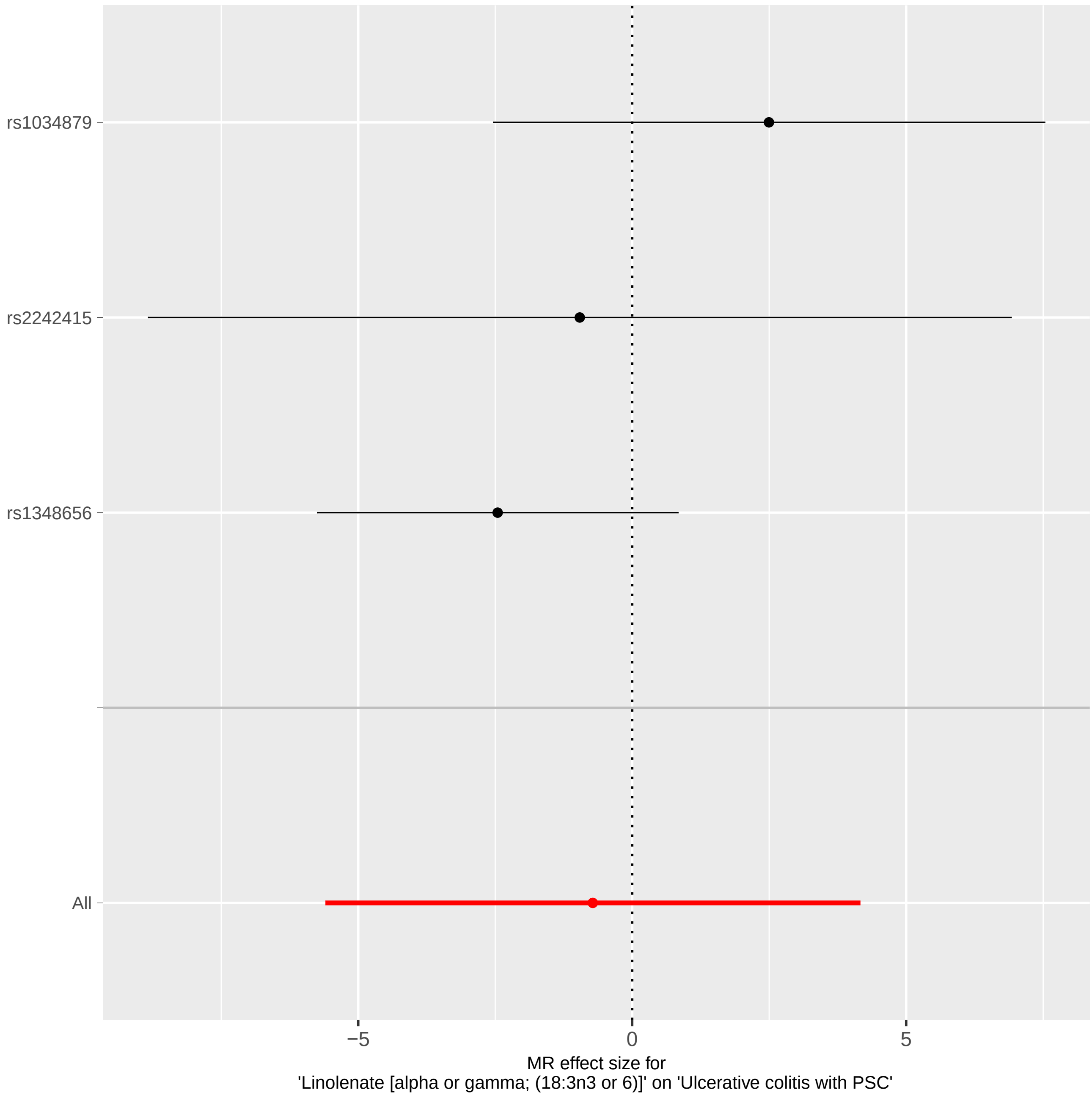

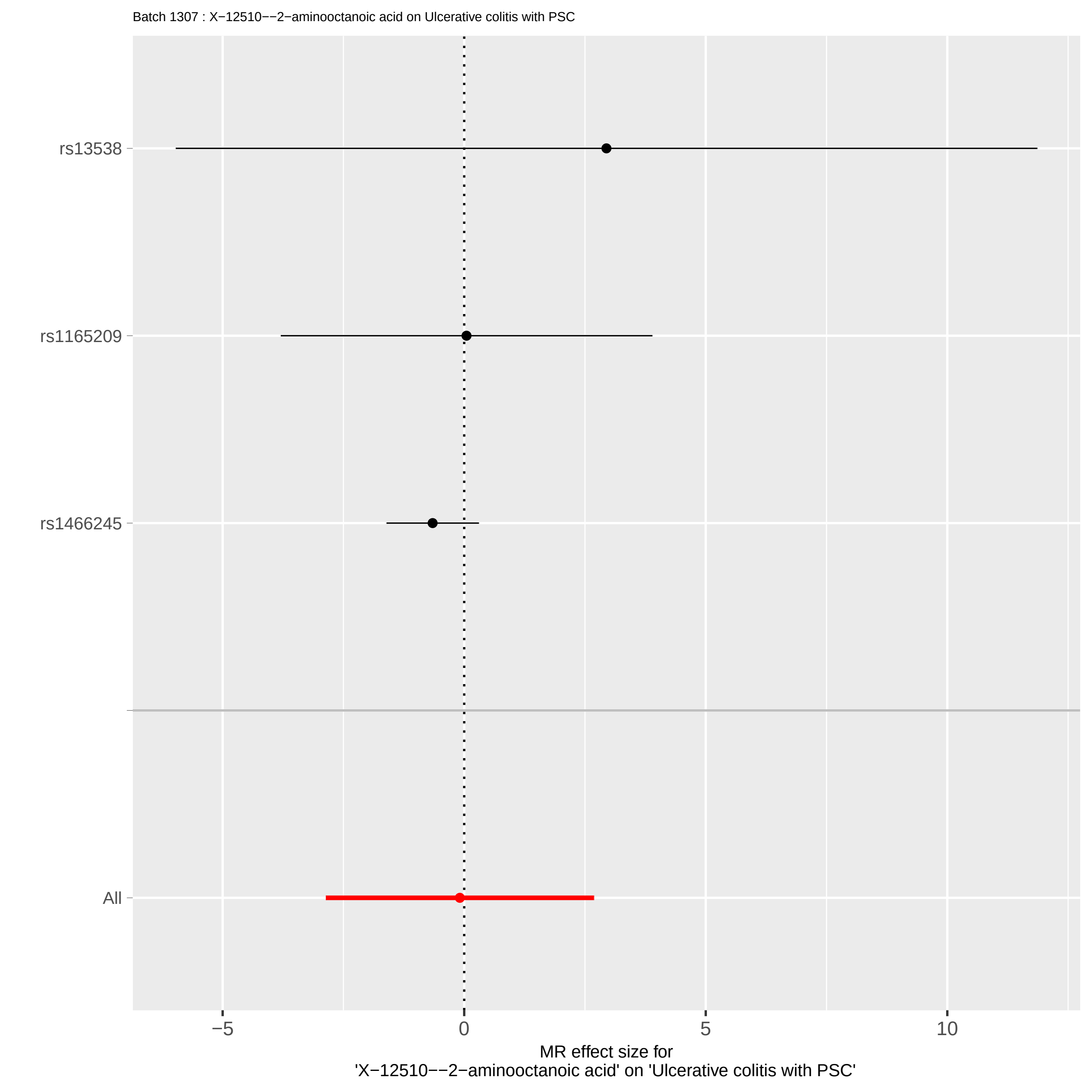
Batch 1307 : X−12510−−2−aminooctanoic acid on Ulcerative colitis with PSC
rs13538
rs1165209
rs1466245
All
−5
0
5
10
MR effect size for
'X−12510−−2−aminooctanoic acid' on 'Ulcerative colitis with PSC'

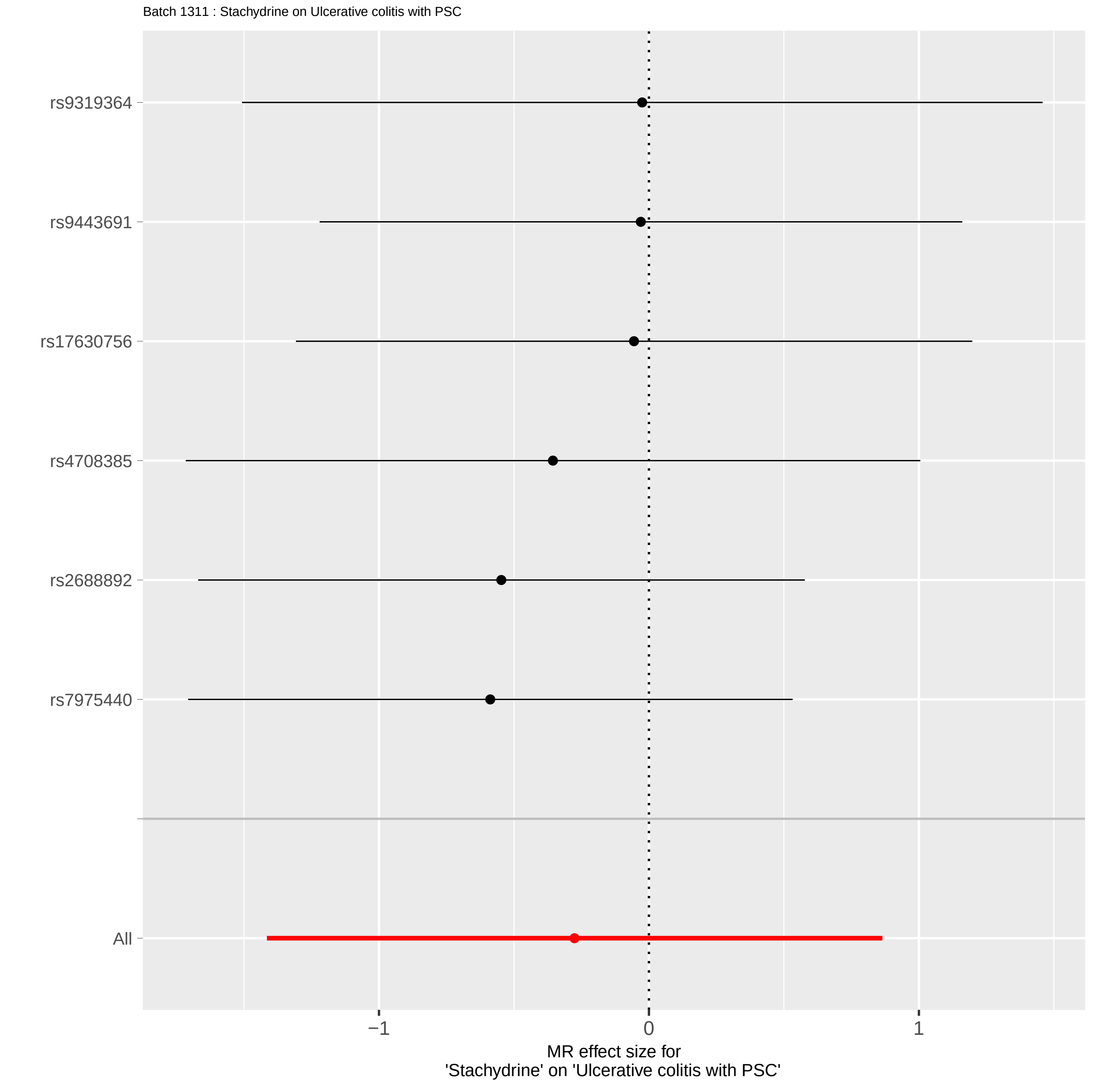

Batch 1311 : Stachydrine on Ulcerative colitis with PSC
rs9319364
rs9443691
rs17630756
rs4708385
rs2688892
rs7975440
All
−1
0
1
MR effect size for
'Stachydrine' on 'Ulcerative colitis with PSC'

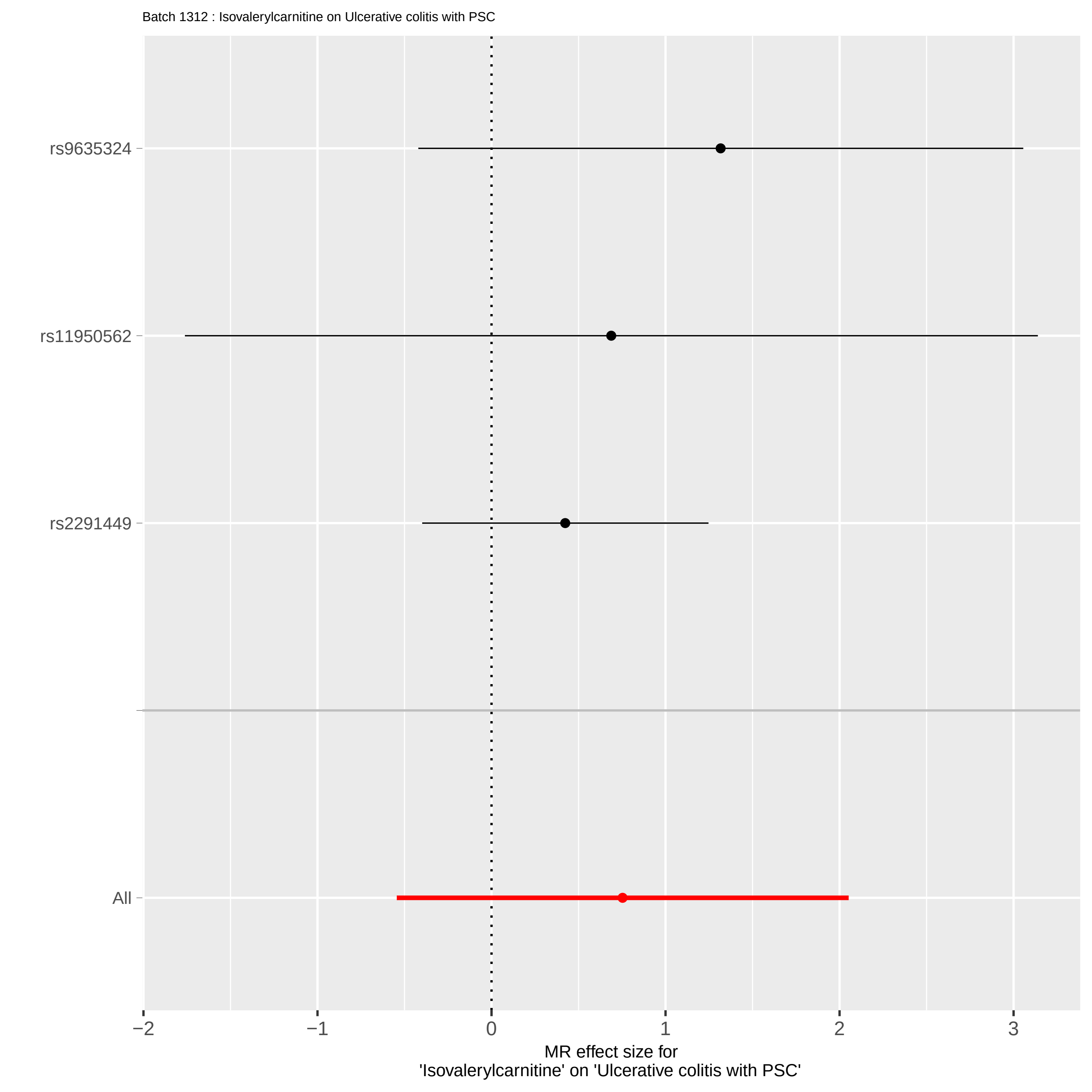

Batch 1312 : Isovalerylcarnitine on Ulcerative colitis with PSC
rs9635324
rs11950562
rs2291449
All
−2
−1
0
1
2
3
MR effect size for
'Isovalerylcarnitine' on 'Ulcerative colitis with PSC'

Batch 1315 : 1−linoleoylglycerophosphocholine on Ulcerative colitis with PSC

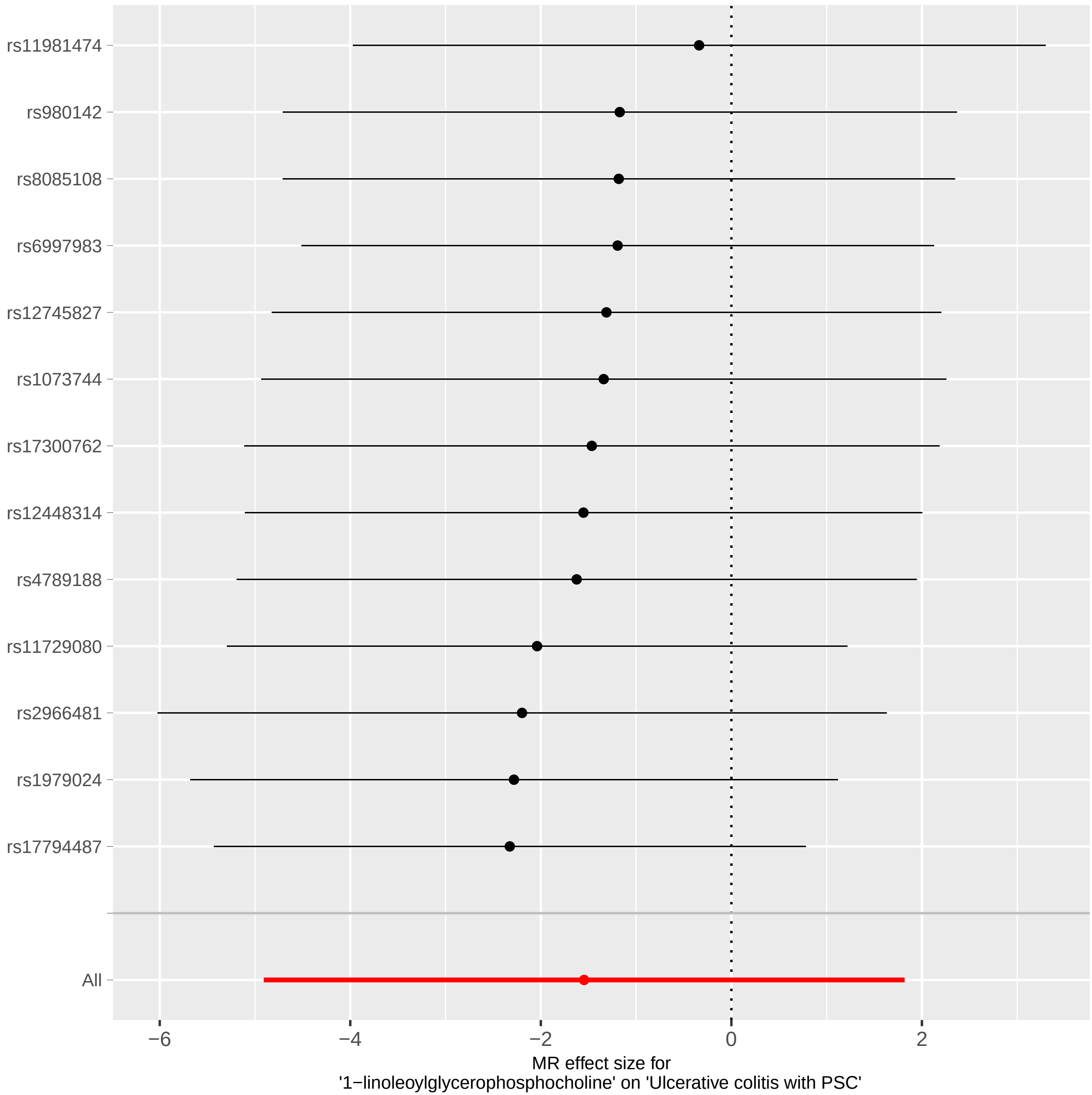

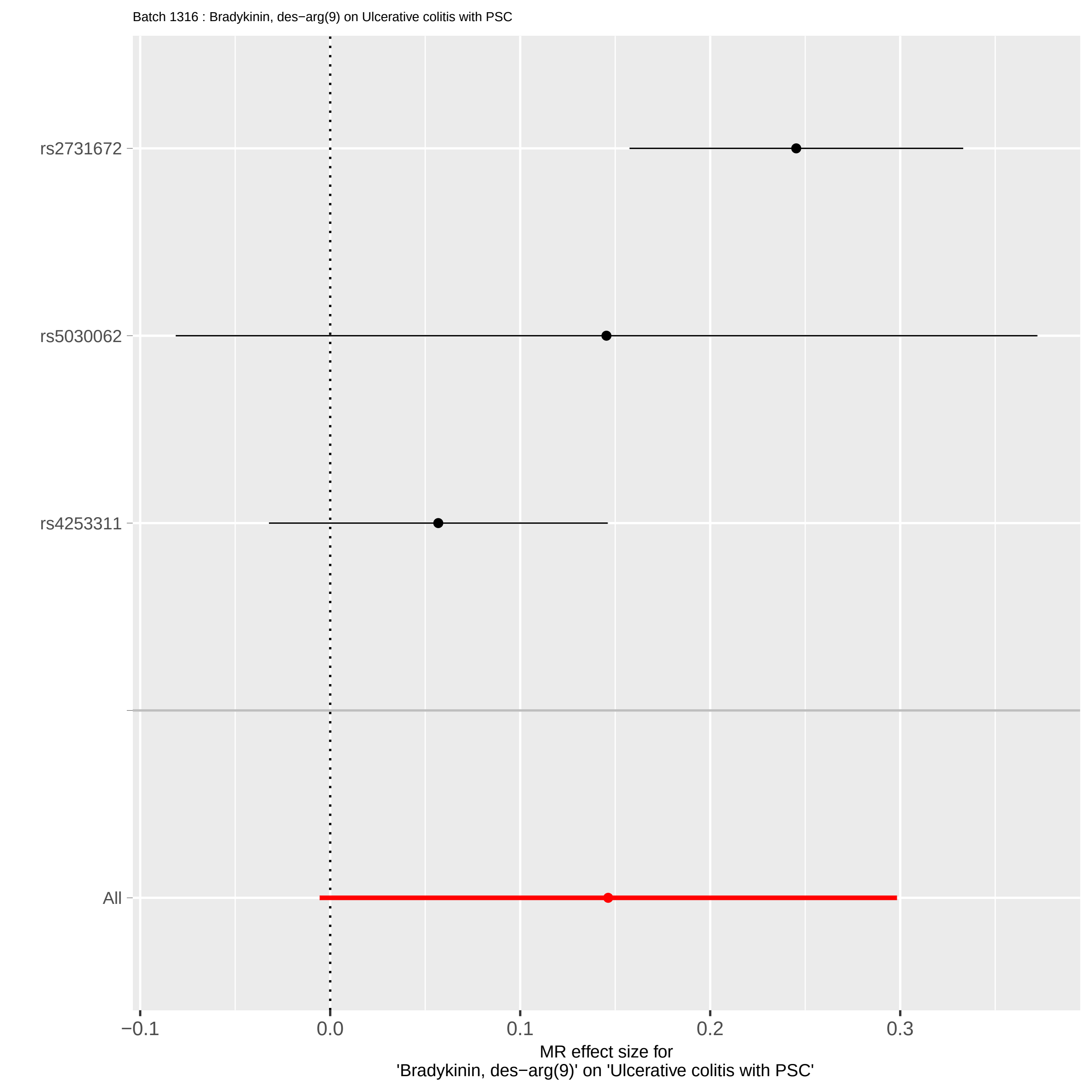

Batch 1316 : Bradykinin, des−arg(9) on Ulcerative colitis with PSC
rs2731672
rs5030062
rs4253311
All
−0.1
0.0
0.1
0.2
0.3
MR effect size for
'Bradykinin, des−arg(9)' on 'Ulcerative colitis with PSC'

Batch 1317 : Gamma−glutamylisoleucine* on Ulcerative colitis with PSC

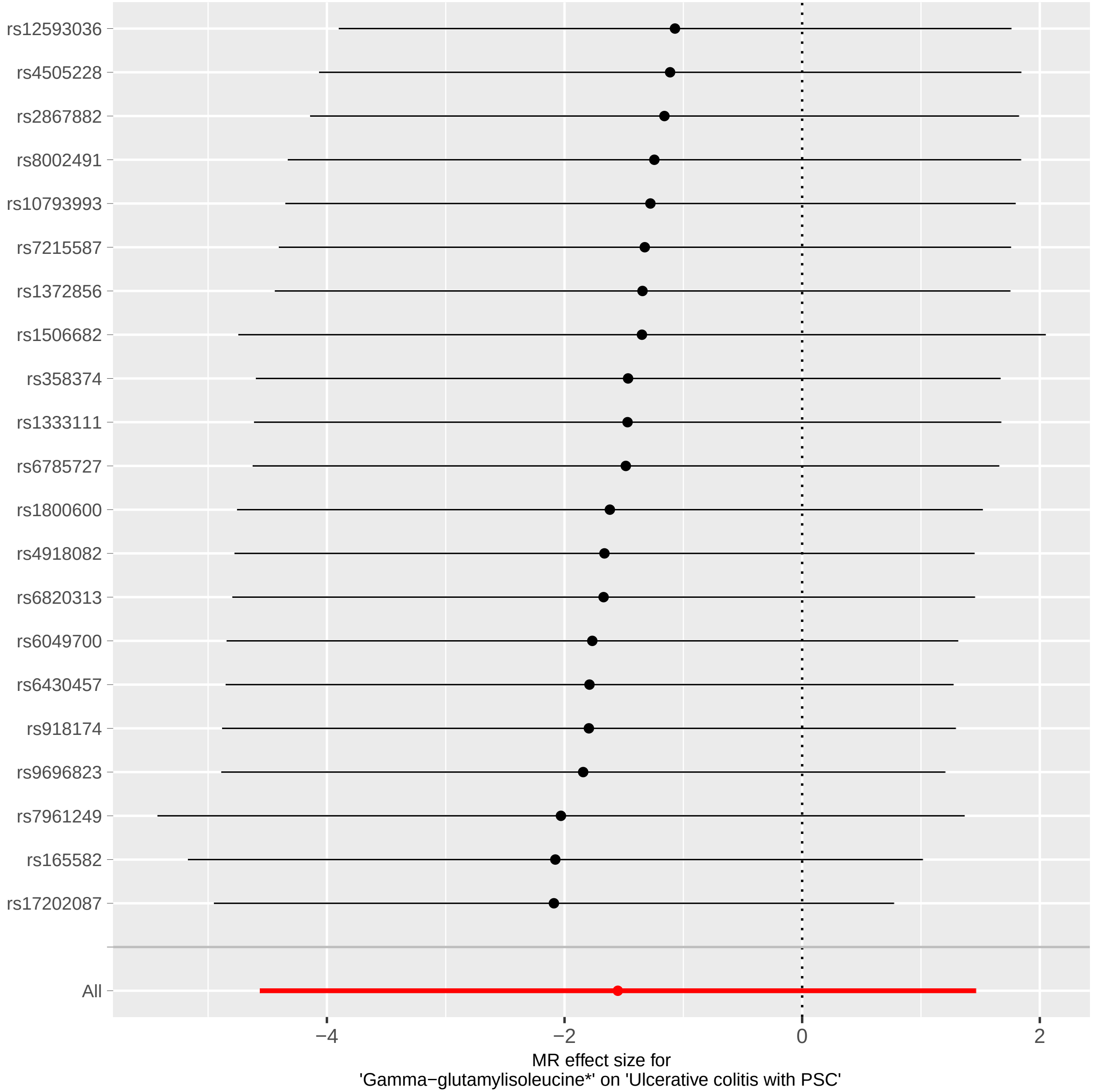

Batch 1320 : Isovalerate on Ulcerative colitis with PSC

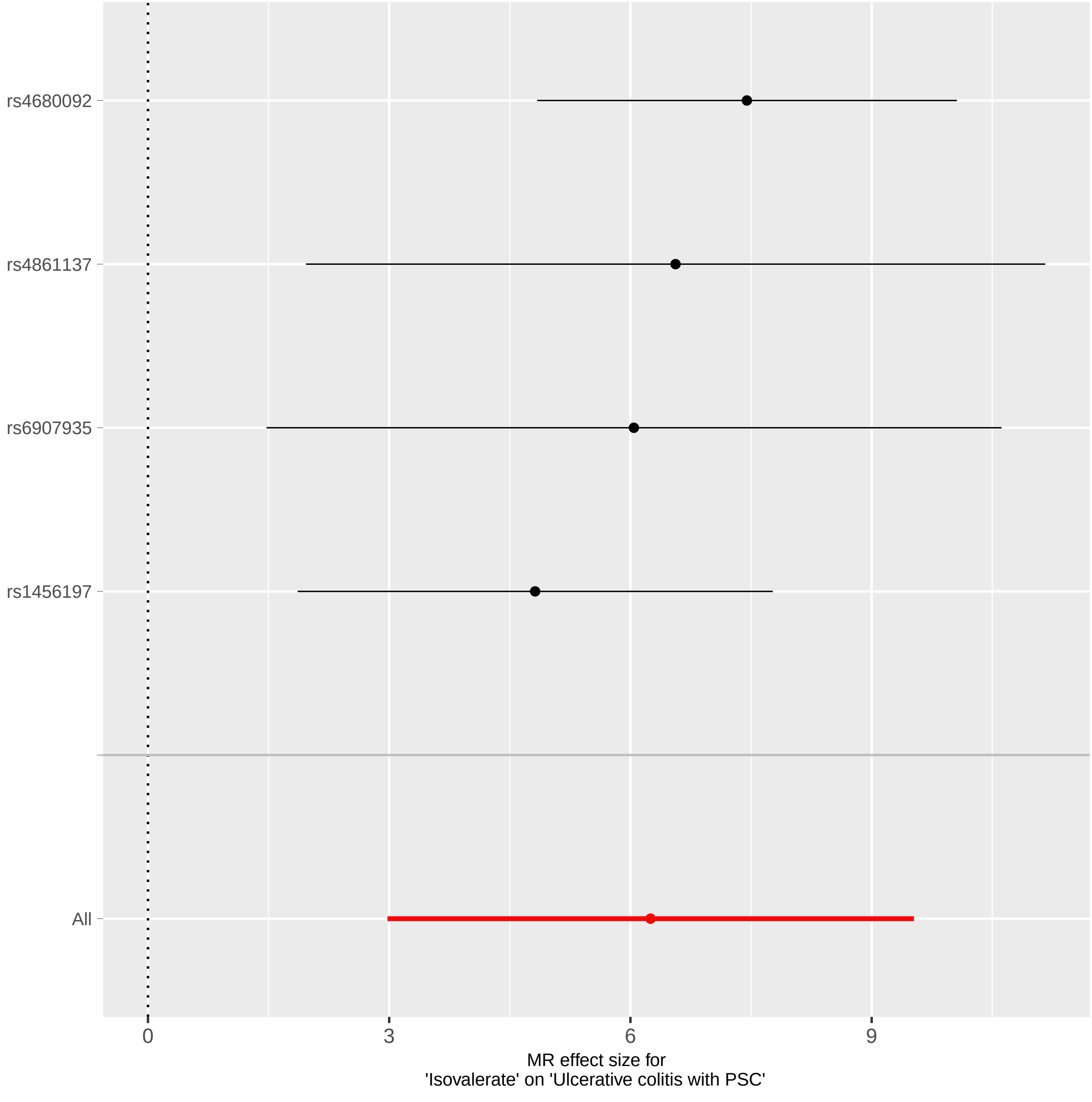

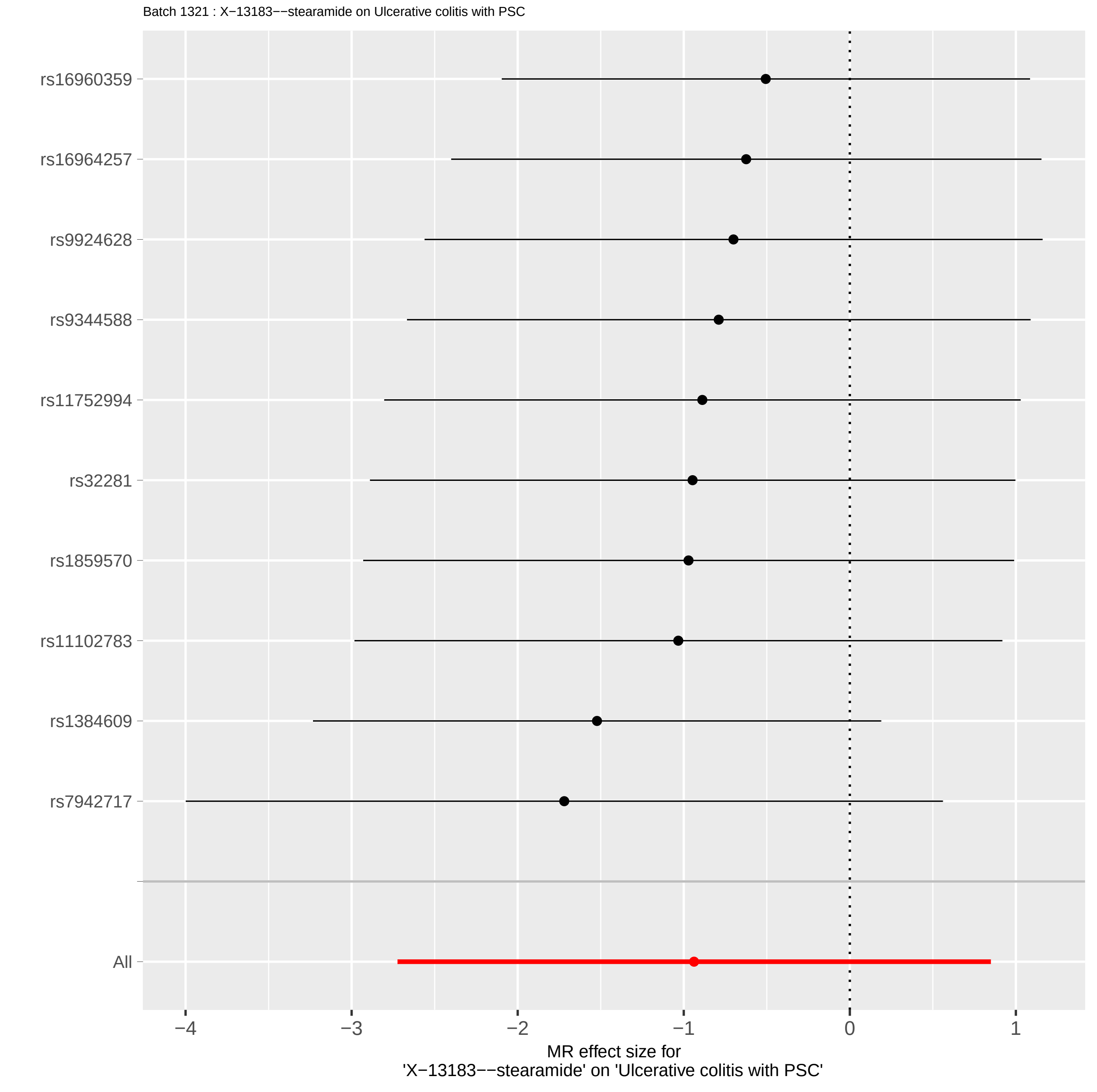

Batch 1321 : X−13183−−stearamide on Ulcerative colitis with PSC
rs16960359
rs16964257
rs9924628
rs9344588
rs11752994
rs32281
rs1859570
rs11102783
rs1384609
rs7942717
All
−4
−3
−2
−1
0
1
MR effect size for
'X−13183−−stearamide' on 'Ulcerative colitis with PSC'

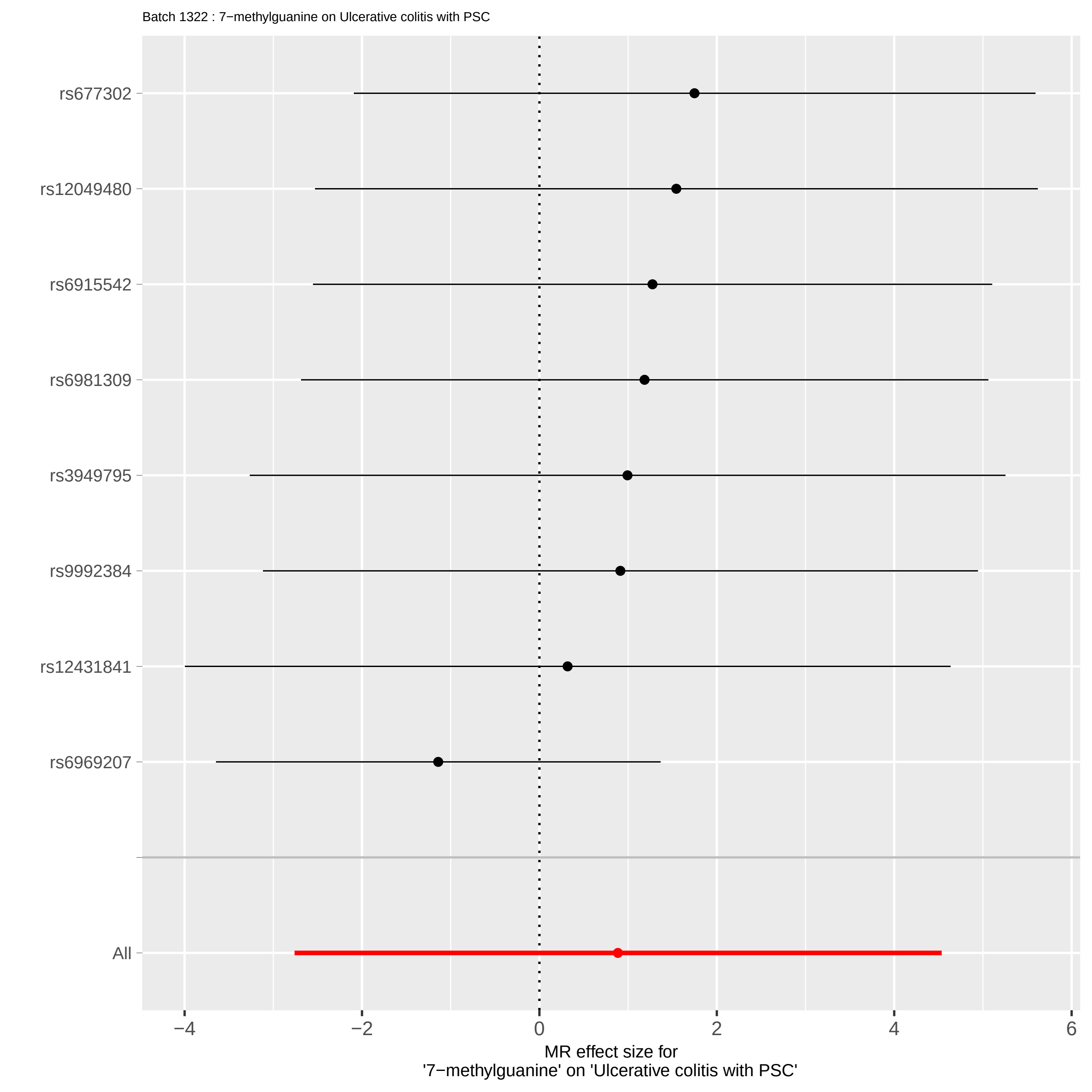

Batch 1322 : 7−methylguanine on Ulcerative colitis with PSC
rs677302
rs12049480
rs6915542
rs6981309
rs3949795
rs9992384
rs12431841
rs6969207
All
−4
−2
0
2
4
6
MR effect size for
'7−methylguanine' on 'Ulcerative colitis with PSC'

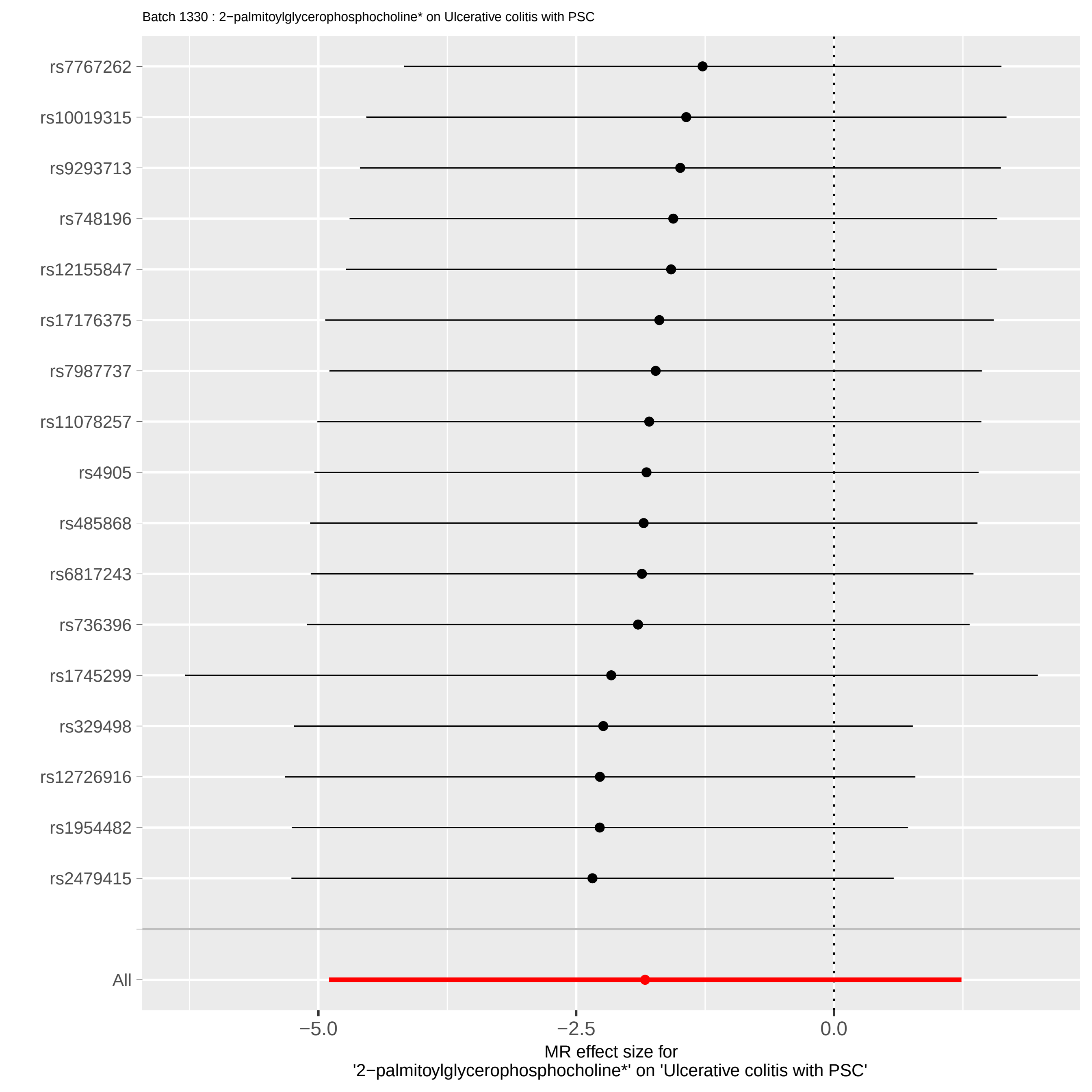

Batch 1330 : 2−palmitoylglycerophosphocholine* on Ulcerative colitis with PSC
rs7767262
rs10019315
rs9293713
rs748196
rs12155847
rs17176375
rs7987737
rs11078257
rs4905
rs485868
rs6817243
rs736396
rs1745299
rs329498
rs12726916
rs1954482
rs2479415
All
−5.0
−2.5
0.0
MR effect size for
'2−palmitoylglycerophosphocholine*' on 'Ulcerative colitis with PSC'

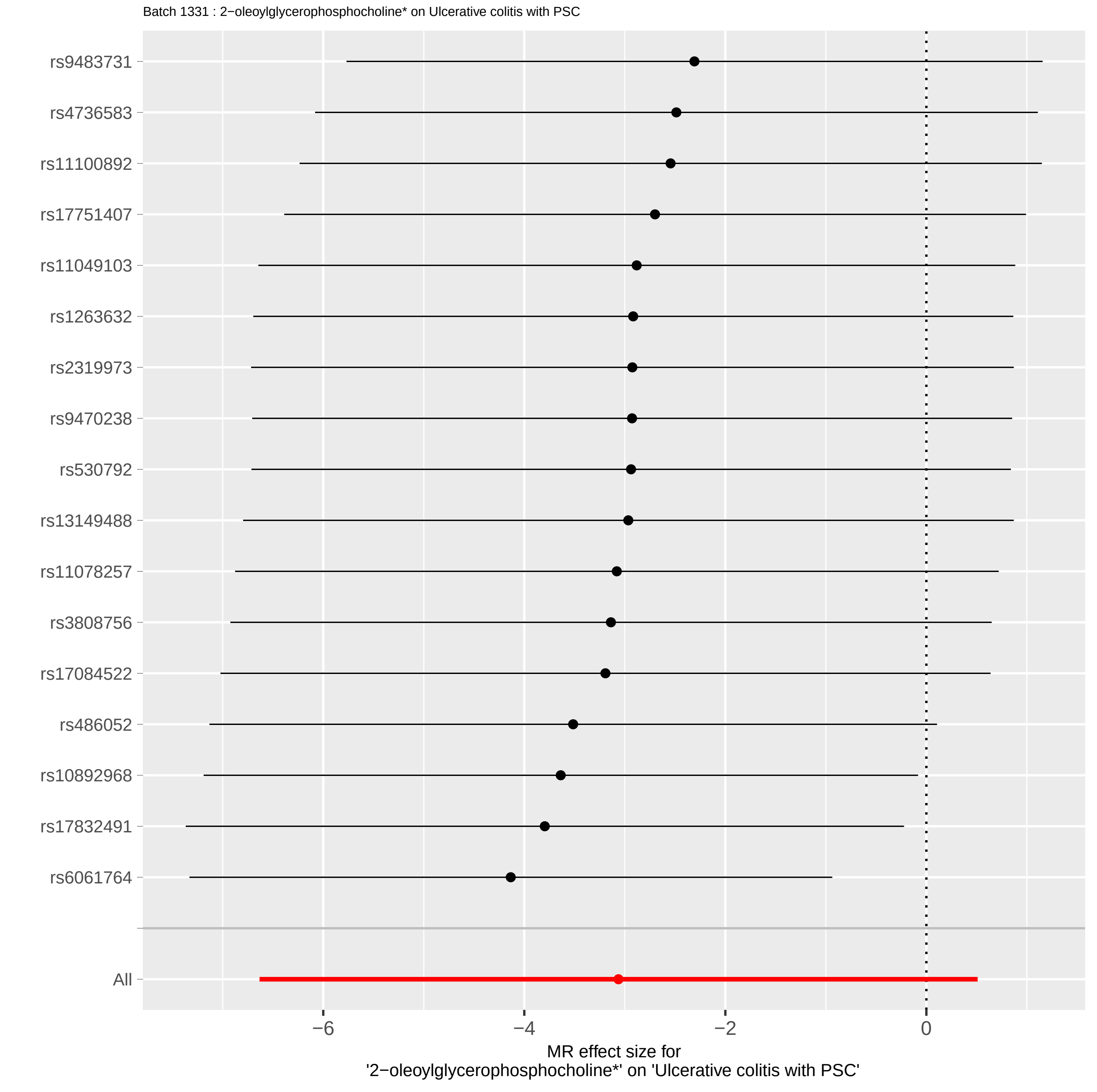

Batch 1331 : 2−oleoylglycerophosphocholine* on Ulcerative colitis with PSC
rs9483731
rs4736583
rs11100892
rs17751407
rs11049103
rs1263632
rs2319973
rs9470238
rs530792
rs13149488
rs11078257
rs3808756
rs17084522
rs486052
rs10892968
rs17832491
rs6061764
All
−6
−4
−2
0
MR effect size for
'2−oleoylglycerophosphocholine*' on 'Ulcerative colitis with PSC'

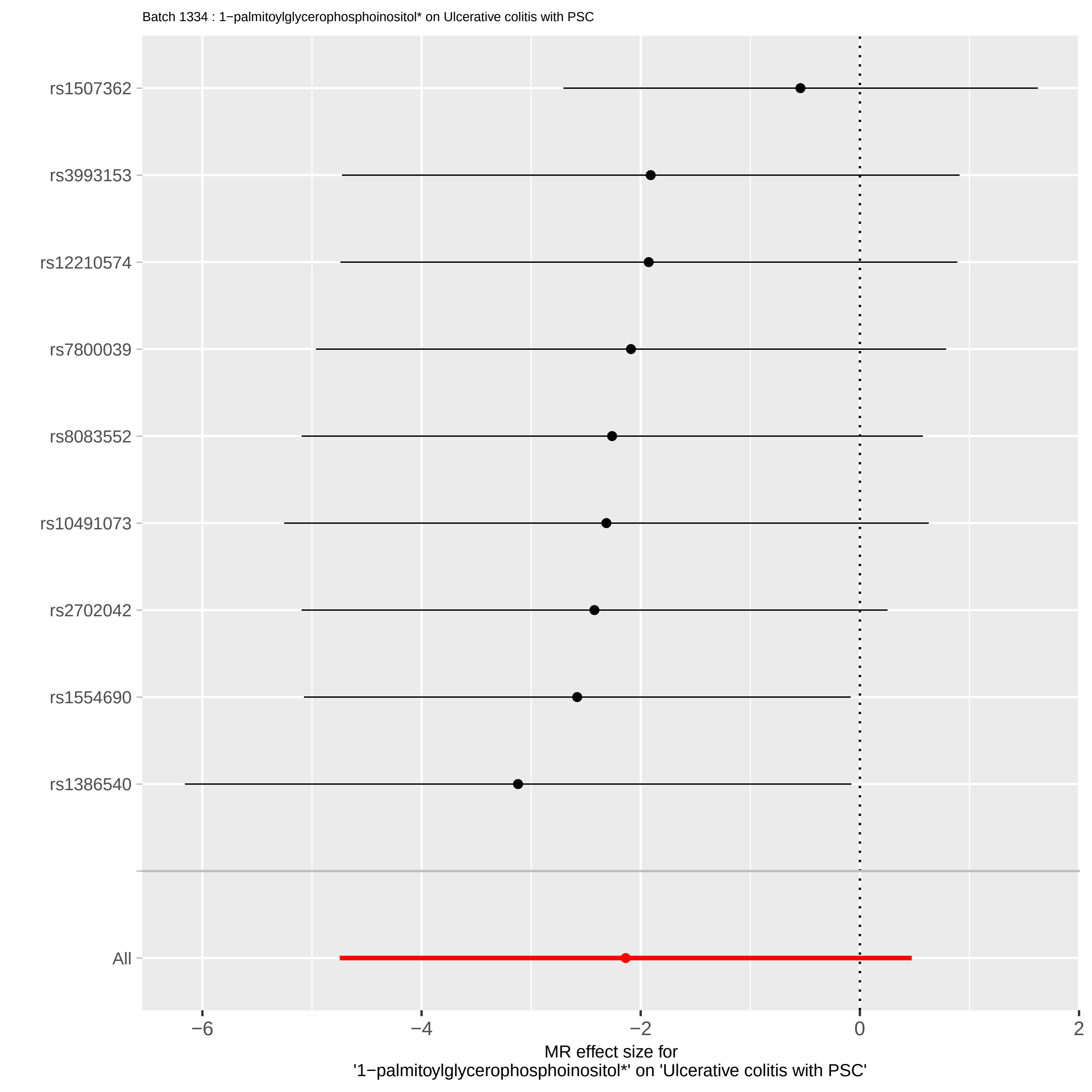

Batch 1334 : 1−palmitoylglycerophosphoinositol* on Ulcerative colitis with PSC
rs1507362
rs3993153
rs12210574
rs7800039
rs8083552
rs10491073
rs2702042
rs1554690
rs1386540
All
−6
−4
−2
0
2
MR effect size for
'1−palmitoylglycerophosphoinositol*' on 'Ulcerative colitis with PSC'

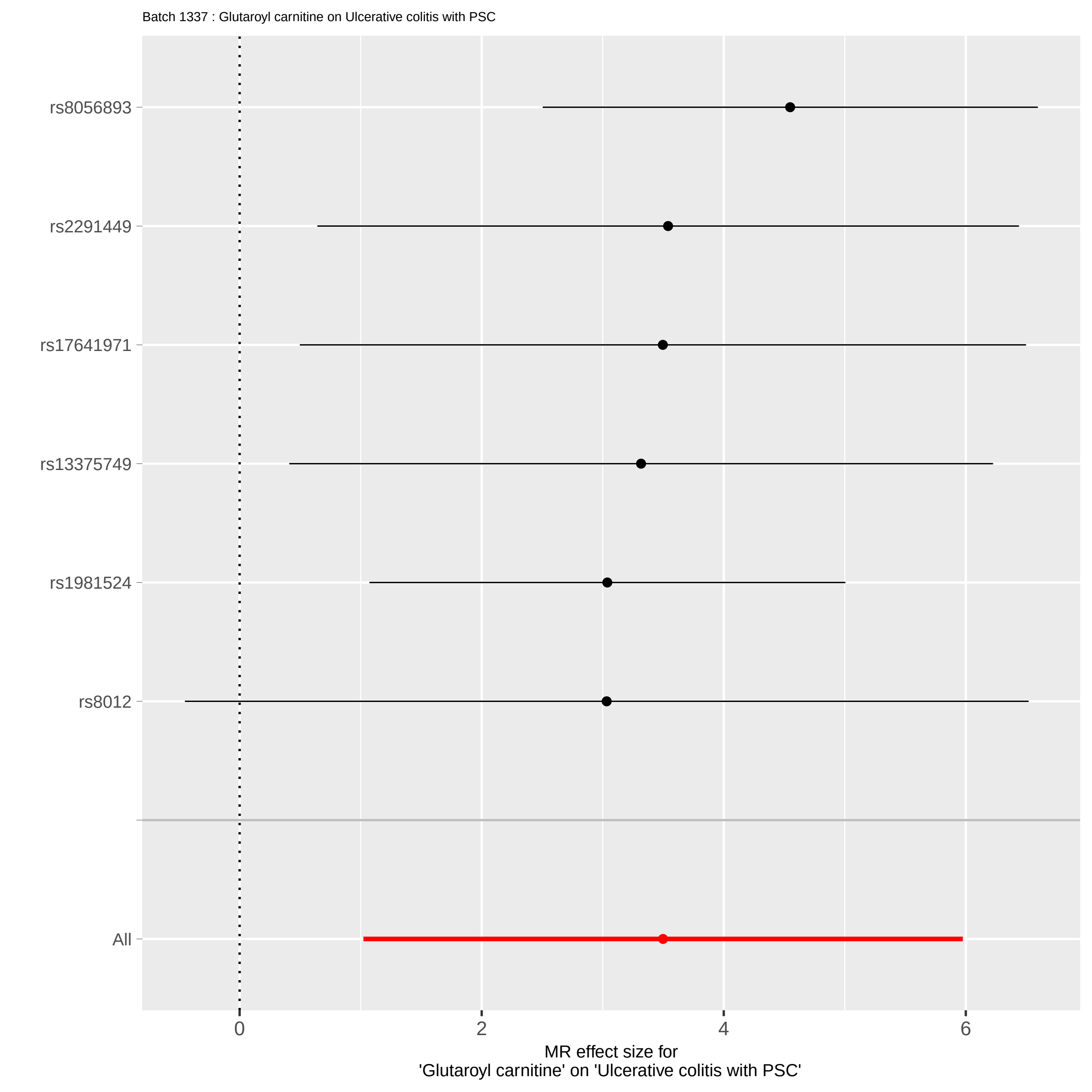

Batch 1337 : Glutaroyl carnitine on Ulcerative colitis with PSC
rs8056893
rs2291449
rs17641971
rs13375749
rs1981524
rs8012
All
0
2
4
6
MR effect size for
'Glutaroyl carnitine' on 'Ulcerative colitis with PSC'

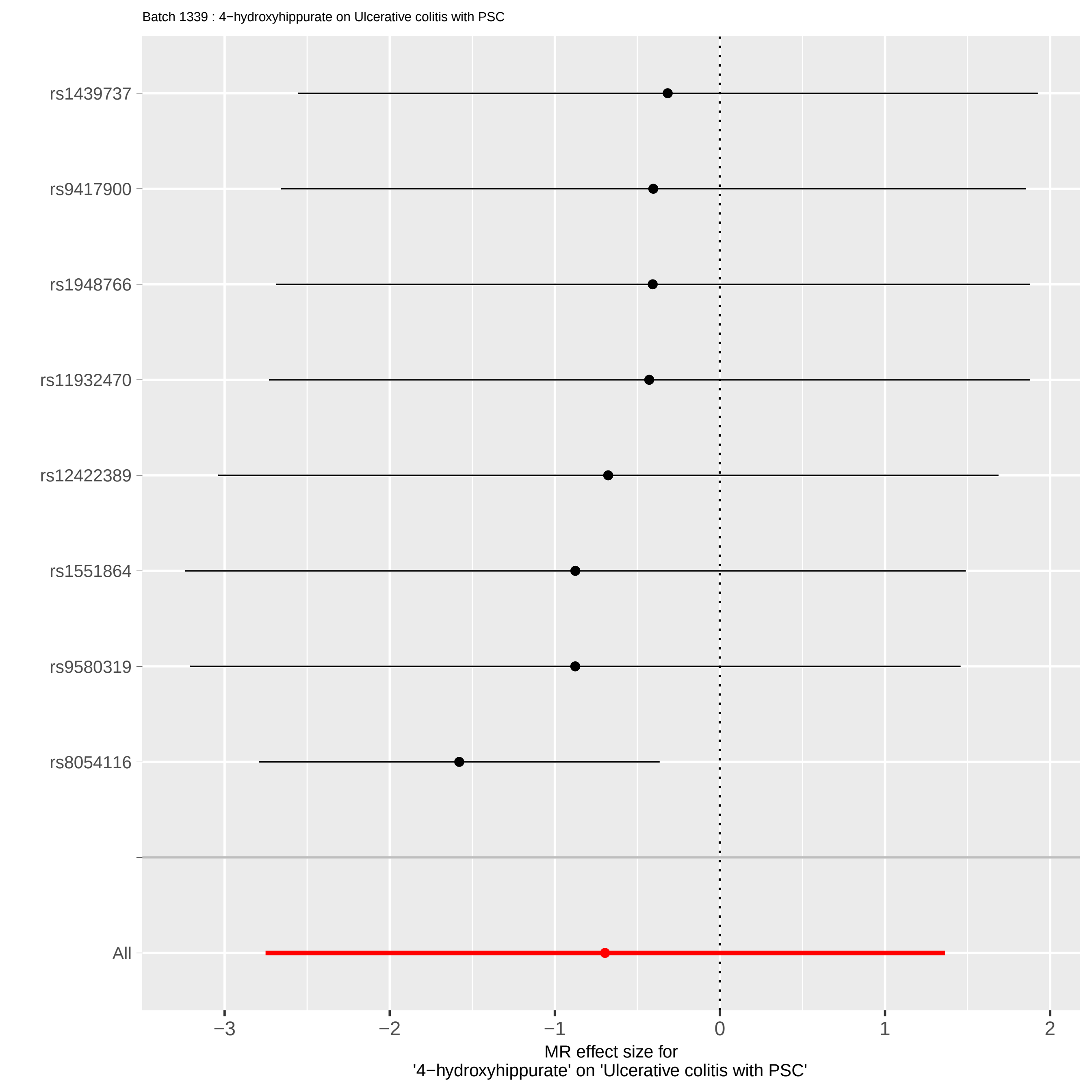

Batch 1339 : 4−hydroxyhippurate on Ulcerative colitis with PSC
rs1439737
rs9417900
rs1948766
rs11932470
rs12422389
rs1551864
rs9580319
rs8054116
All
−3
−2
−1
0
1
2
MR effect size for
'4−hydroxyhippurate' on 'Ulcerative colitis with PSC'

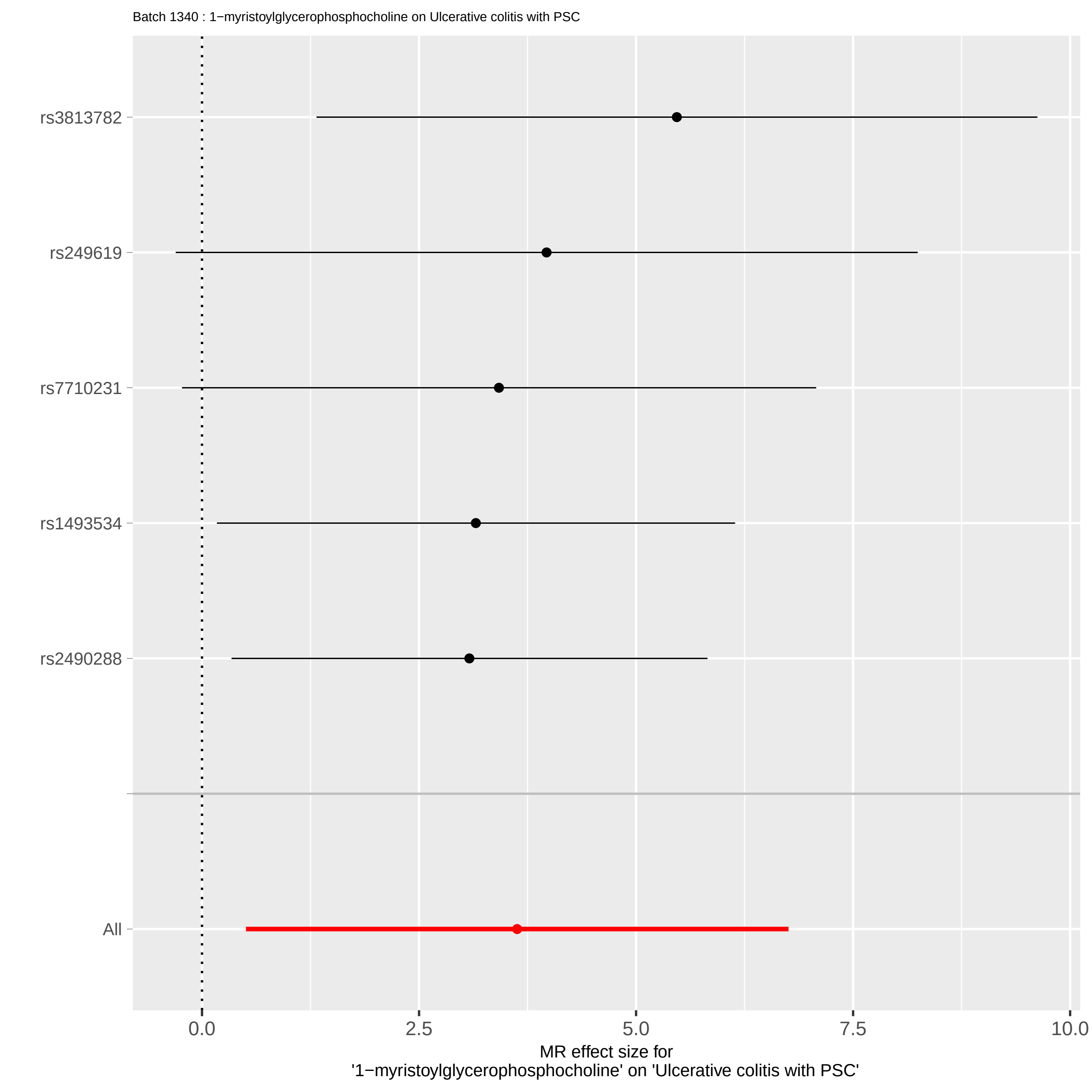

Batch 1340 : 1−myristoylglycerophosphocholine on Ulcerative colitis with PSC
rs3813782
rs249619
rs7710231
rs1493534
rs2490288
All
0.0
2.5
5.0
7.5
10.0
MR effect size for
'1−myristoylglycerophosphocholine' on 'Ulcerative colitis with PSC'

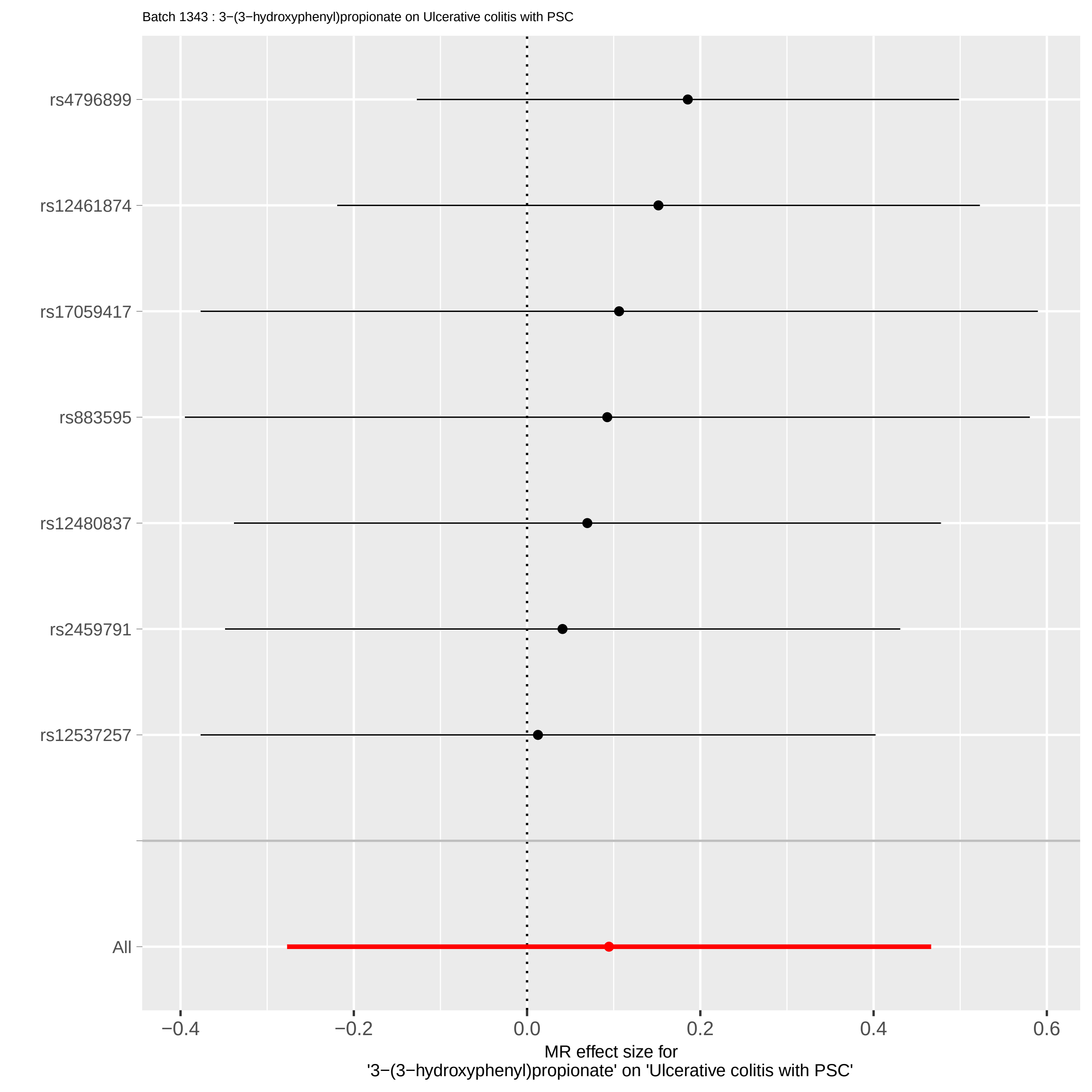

Batch 1343 : 3−(3−hydroxyphenyl)propionate on Ulcerative colitis with PSC
rs4796899
rs12461874
rs17059417
rs883595
rs12480837
rs2459791
rs12537257
All
−0.4
−0.2
0.0
0.2
0.4
0.6
MR effect size for
'3−(3−hydroxyphenyl)propionate' on 'Ulcerative colitis with PSC'

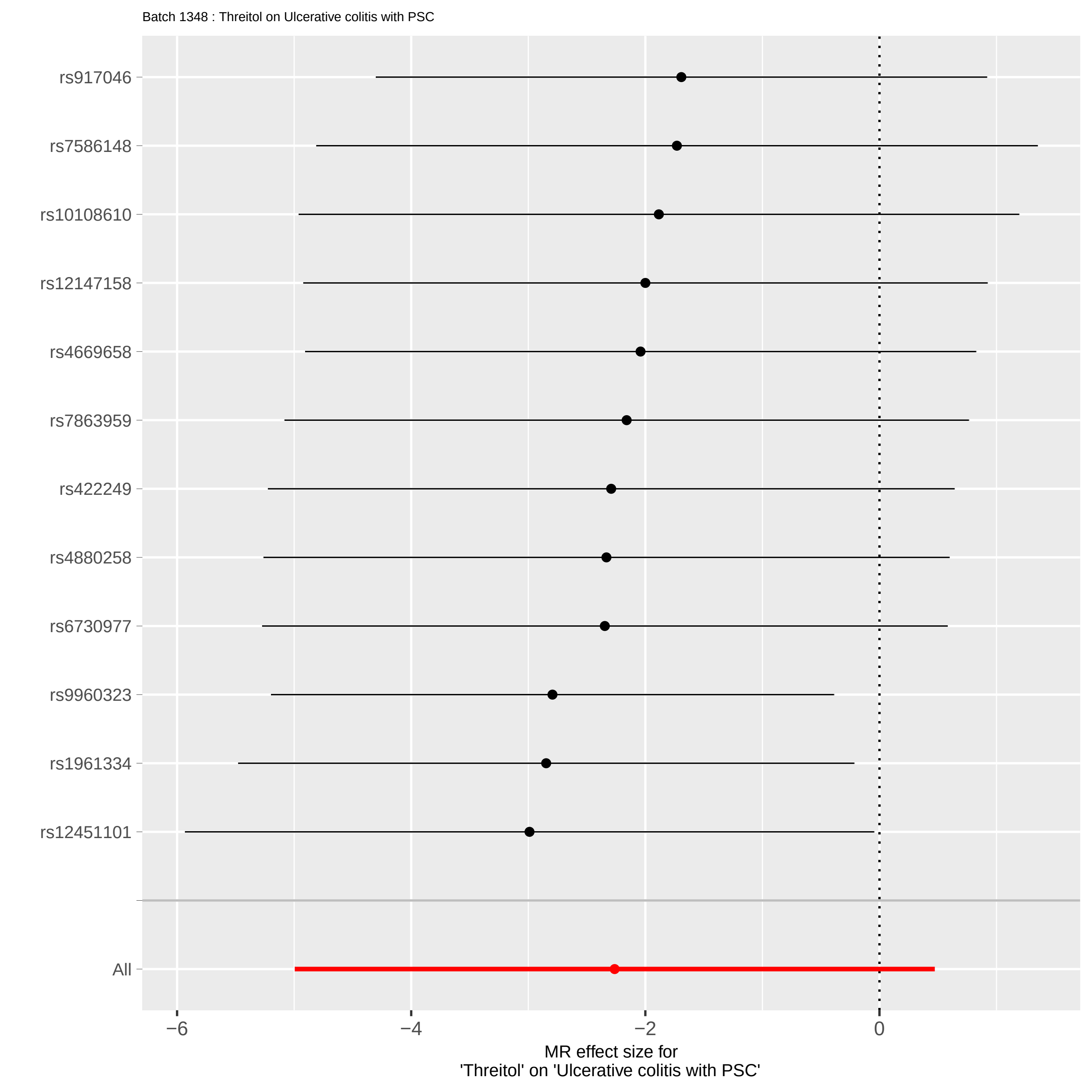
Batch 1348 : Threitol on Ulcerative colitis with PSC
rs917046
rs7586148
rs10108610
rs12147158
rs4669658
rs7863959
rs422249
rs4880258
rs6730977
rs9960323
rs1961334
rs12451101
All
−6
−4
−2
0
MR effect size for
'Threitol' on 'Ulcerative colitis with PSC'

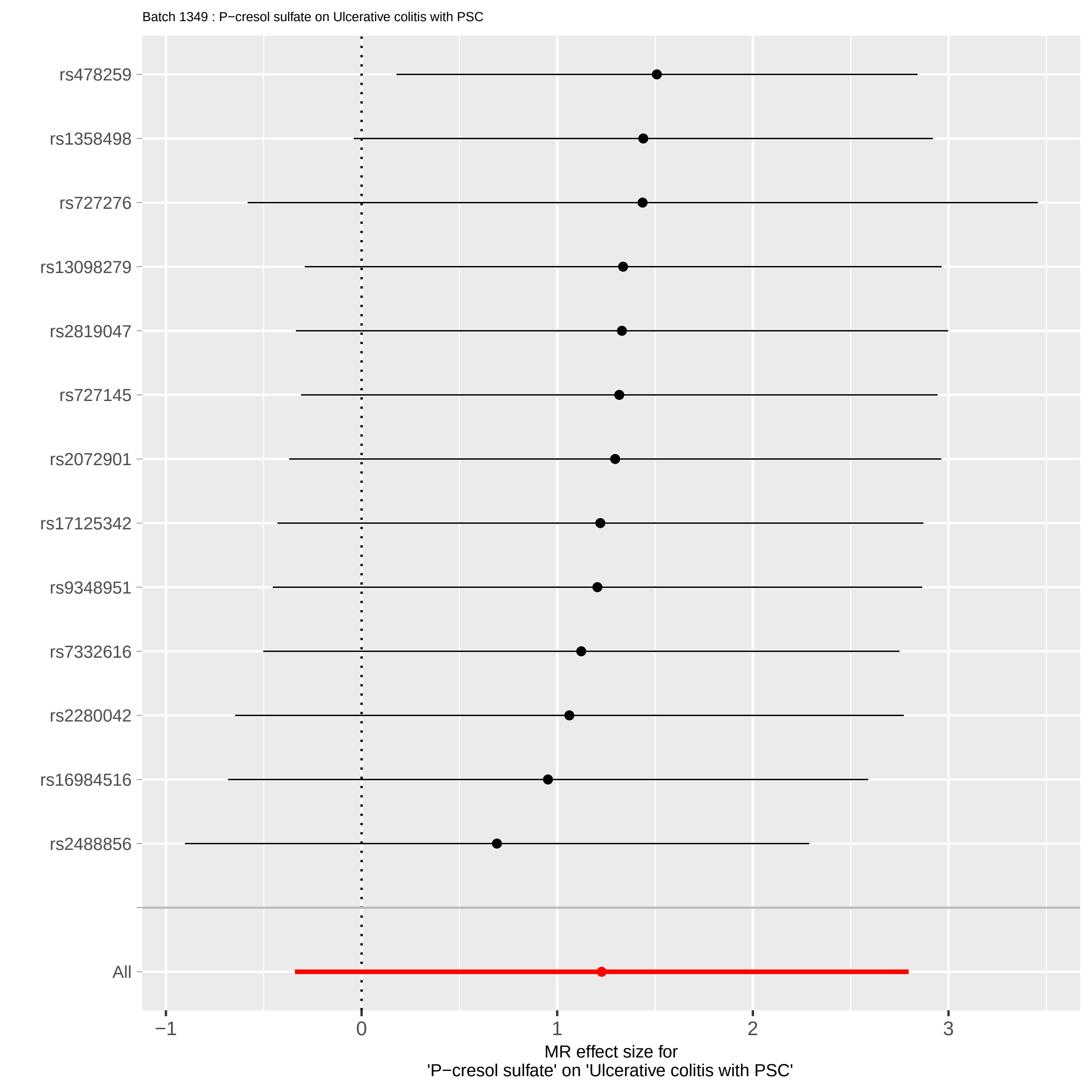
Batch 1349 : P−cresol sulfate on Ulcerative colitis with PSC
rs478259
rs1358498
rs727276
rs13098279
rs2819047
rs727145
rs2072901
rs17125342
rs9348951
rs7332616
rs2280042
rs16984516
rs2488856
All
−1
0
1
2
3
MR effect size for
'P−cresol sulfate' on 'Ulcerative colitis with PSC'

Batch 1354 : X−14450−−phenylalanylleucine on Ulcerative colitis with PSC

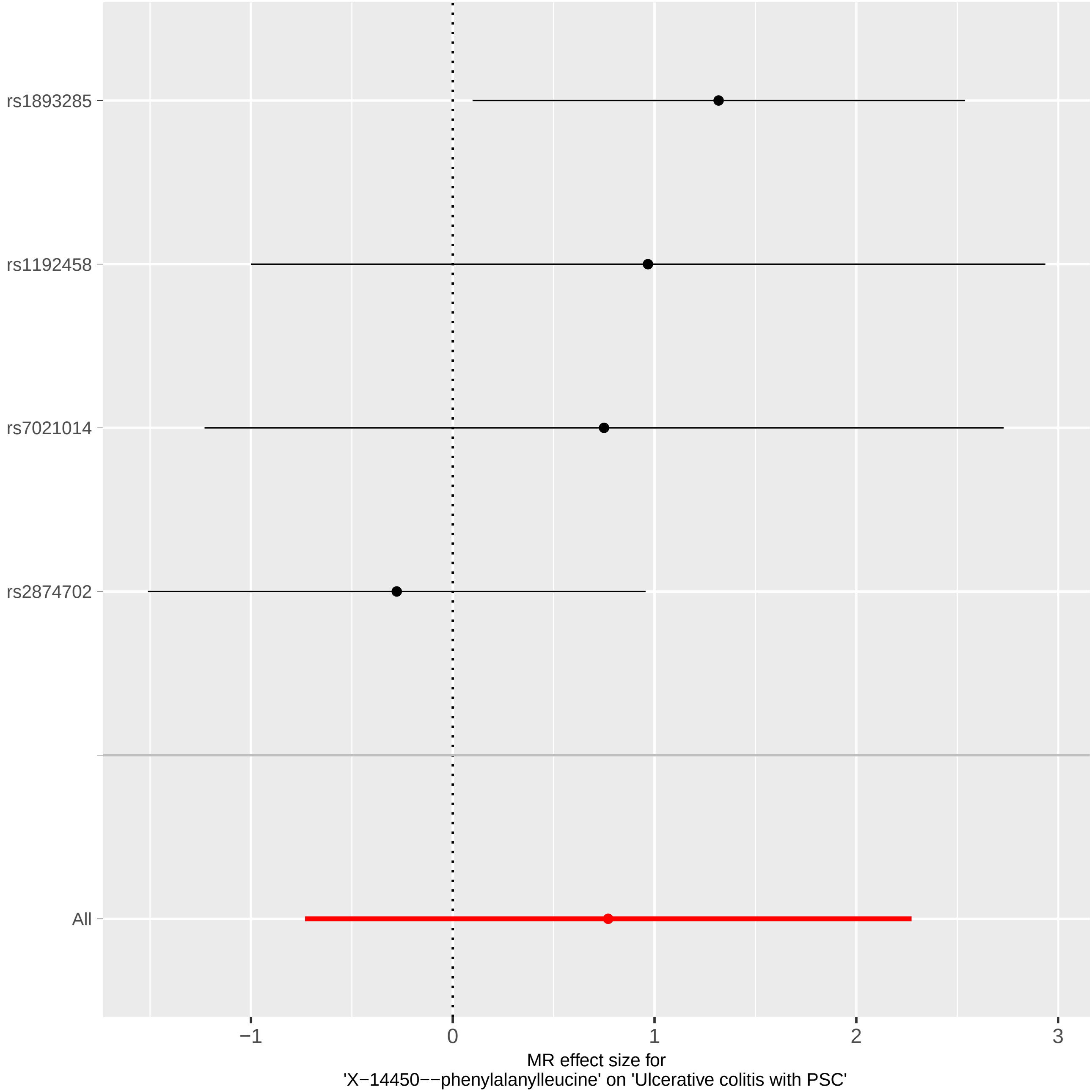

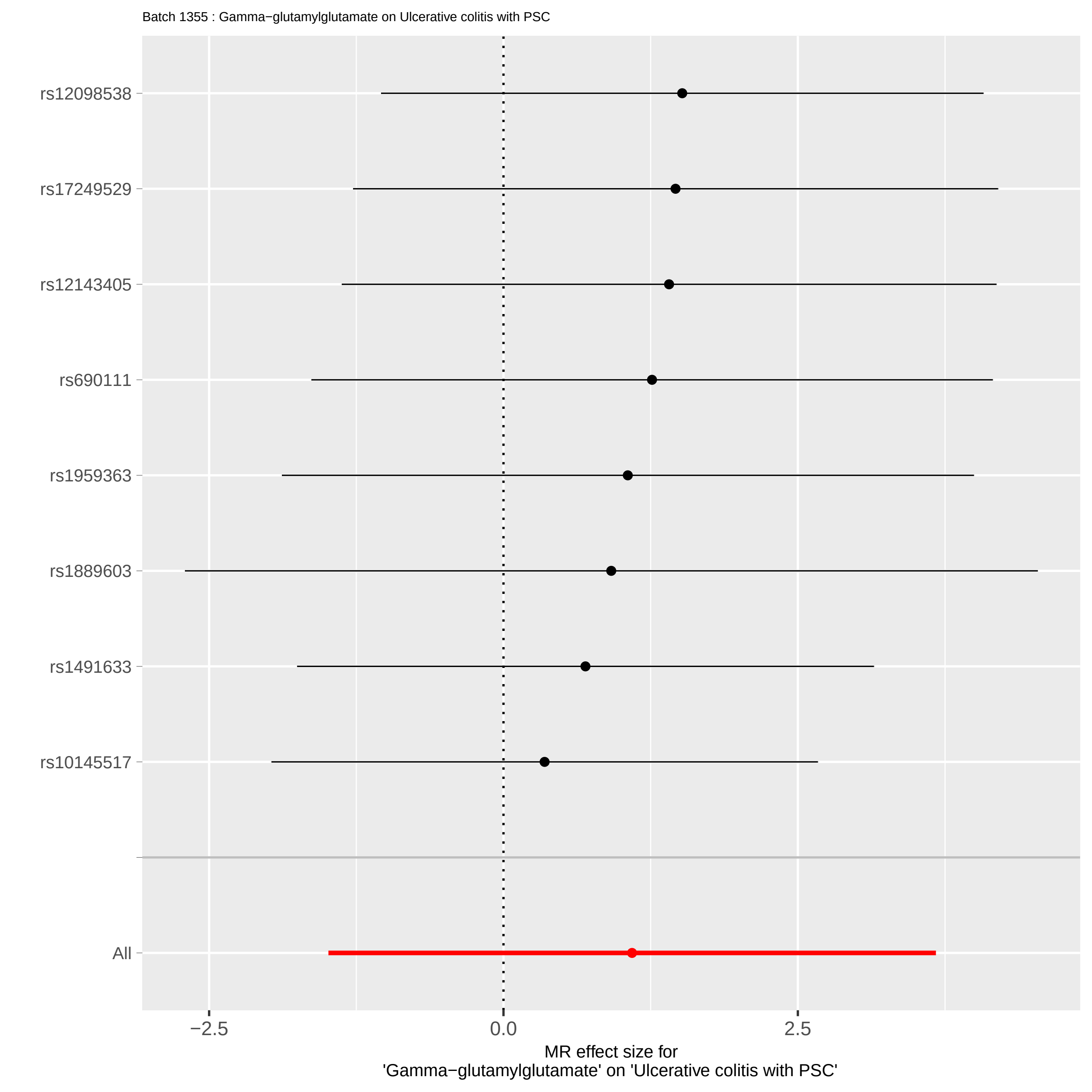

Batch 1355 : Gamma−glutamylglutamate on Ulcerative colitis with PSC
rs12098538
rs17249529
rs12143405
rs690111
rs1959363
rs1889603
rs1491633
rs10145517
All
−2.5
0.0
2.5
MR effect size for
'Gamma−glutamylglutamate' on 'Ulcerative colitis with PSC'

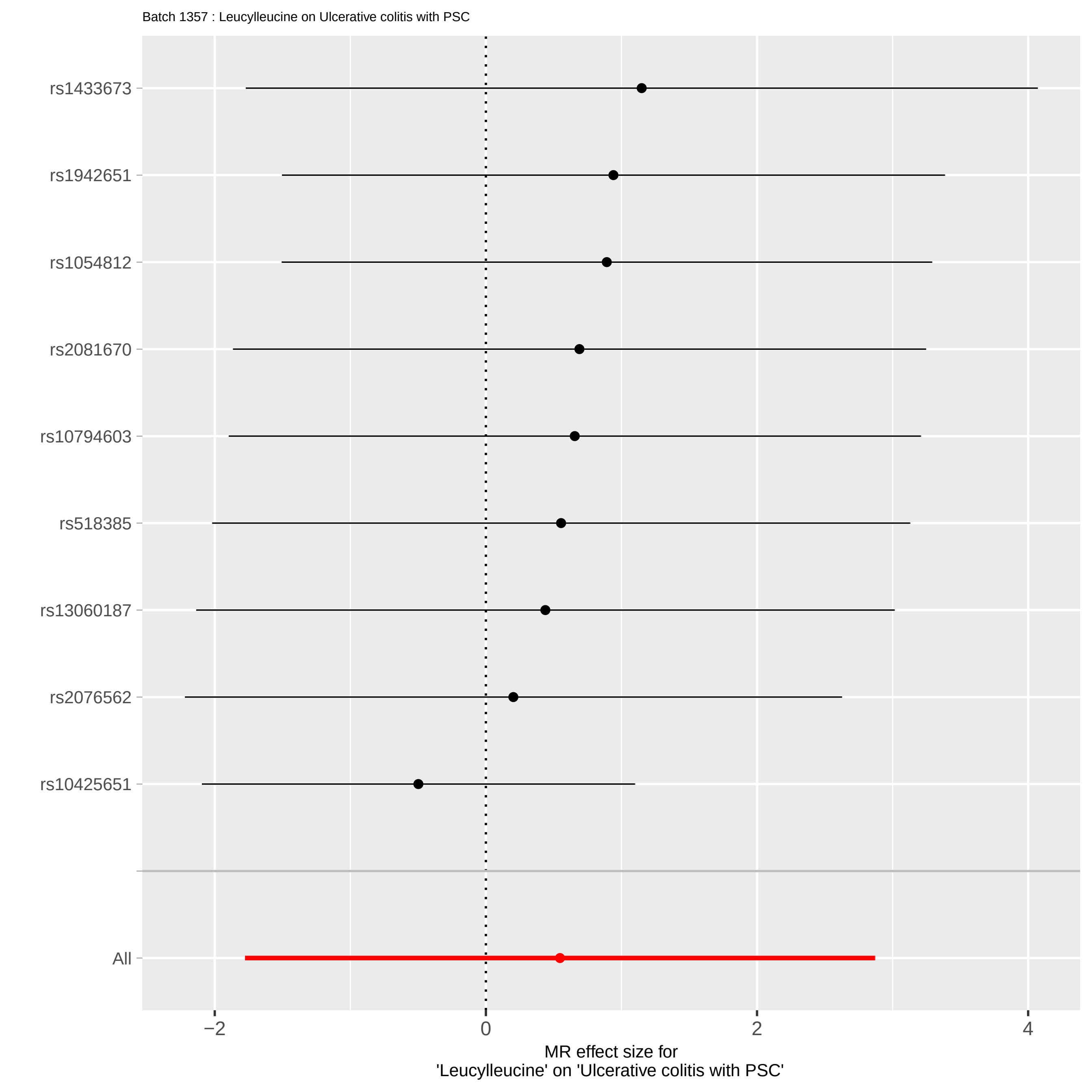
Batch 1357 : Leucylleucine on Ulcerative colitis with PSC
rs1433673
rs1942651
rs1054812
rs2081670
rs10794603
rs518385
rs13060187
rs2076562
rs10425651
All
−2
0
2
4
MR effect size for
'Leucylleucine' on 'Ulcerative colitis with PSC'

Batch 1358 : 7−alpha−hydroxy−3−oxo−4−cholestenoate (7−Hoca) on Ulcerative colitis with PSC

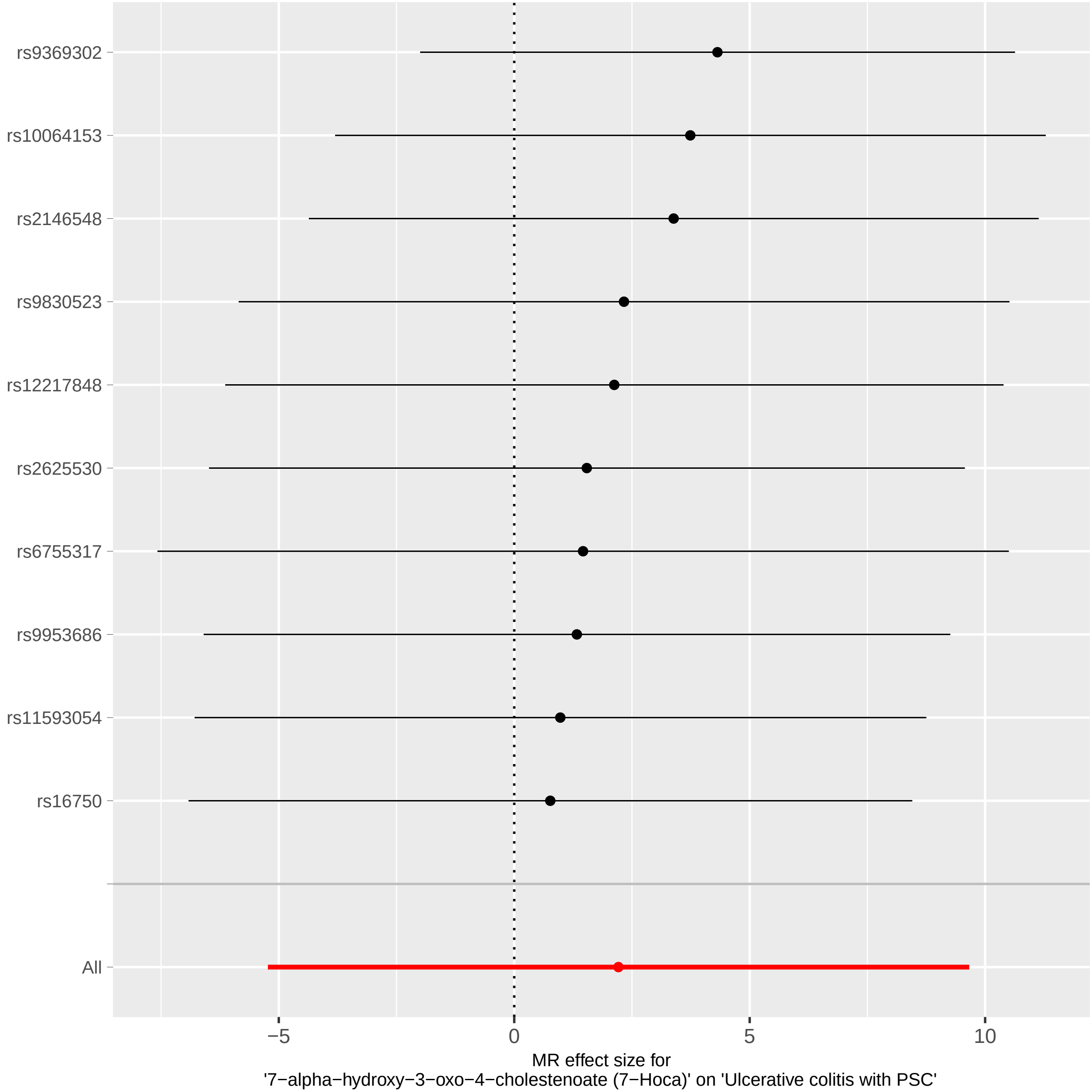

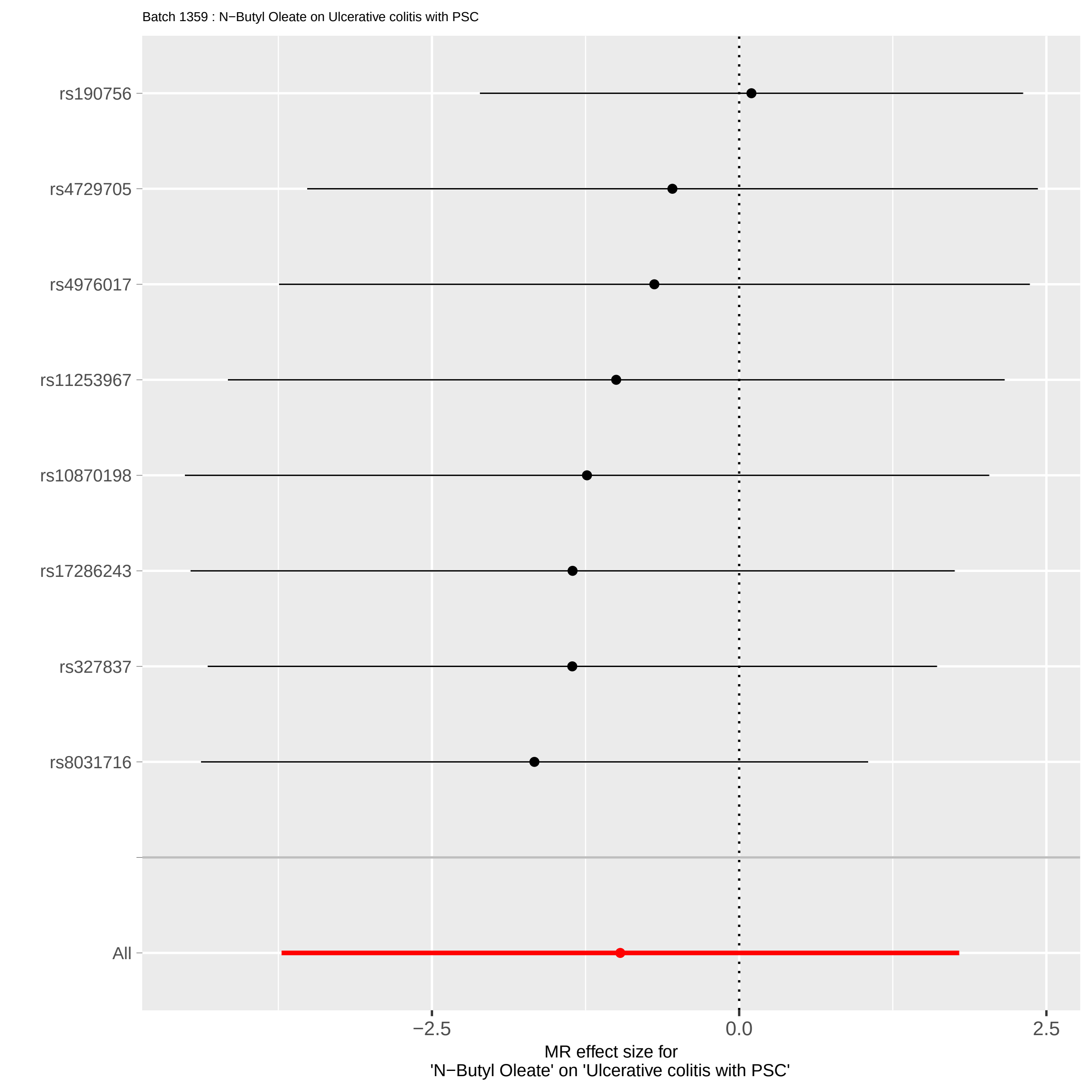

Batch 1359 : N−Butyl Oleate on Ulcerative colitis with PSC
rs190756
rs4729705
rs4976017
rs11253967
rs10870198
rs17286243
rs327837
rs8031716
All
−2.5
0.0
2.5
MR effect size for
'N−Butyl Oleate' on 'Ulcerative colitis with PSC'

Batch 1361 : Taurolithocholate 3−sulfate on Ulcerative colitis with PSC

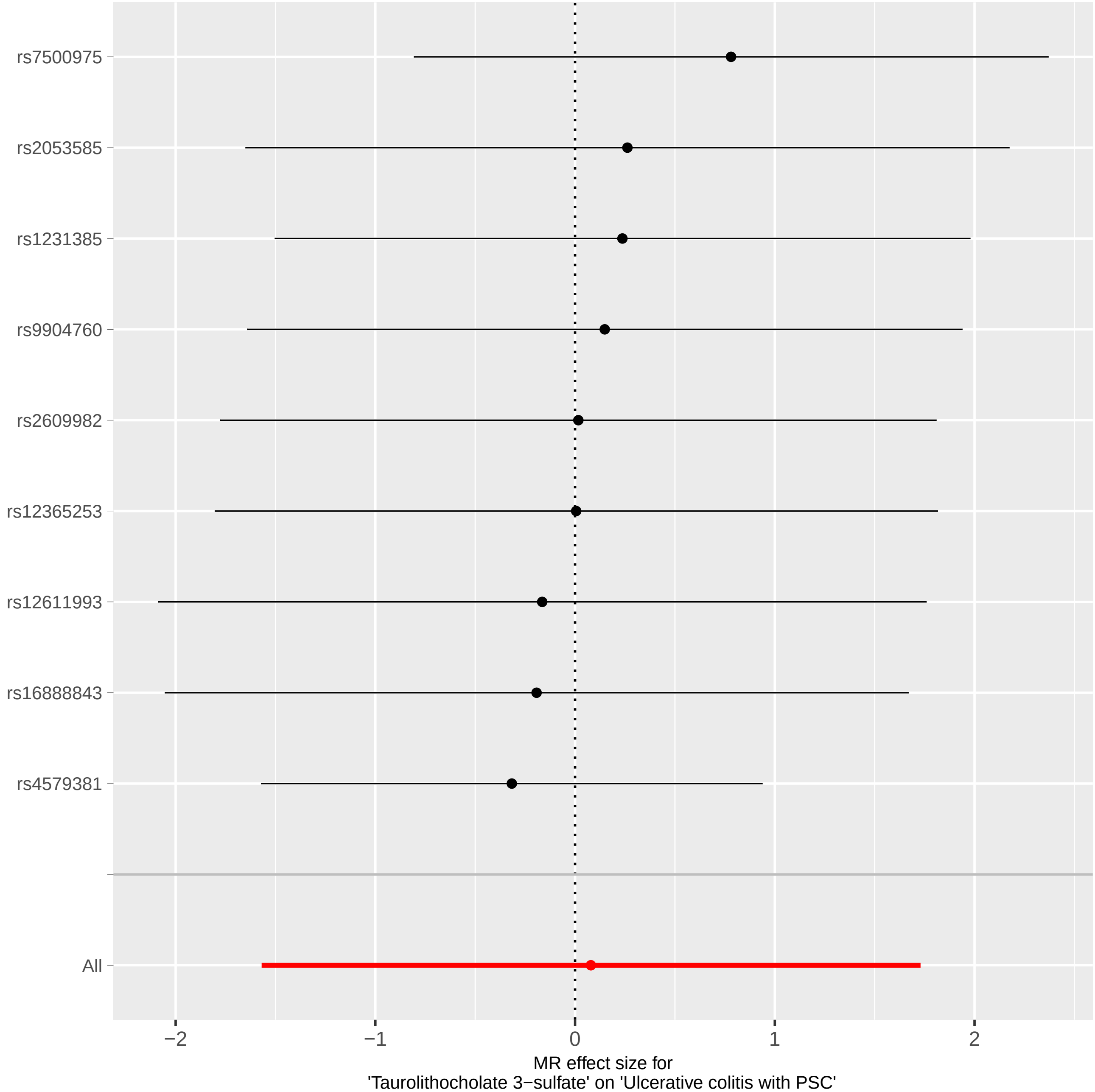

Batch 1362 : X−14977−−vanillin on Ulcerative colitis with PSC

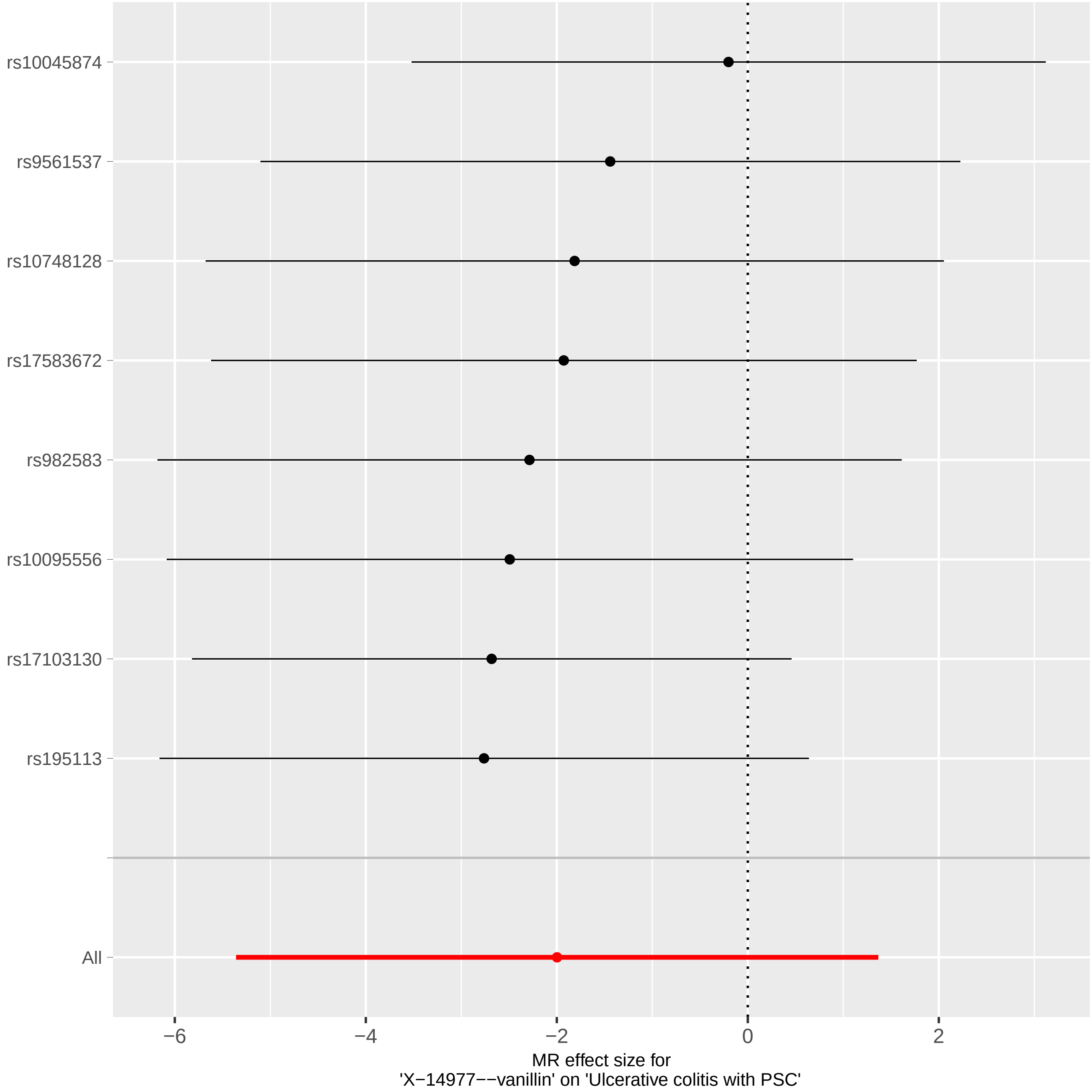

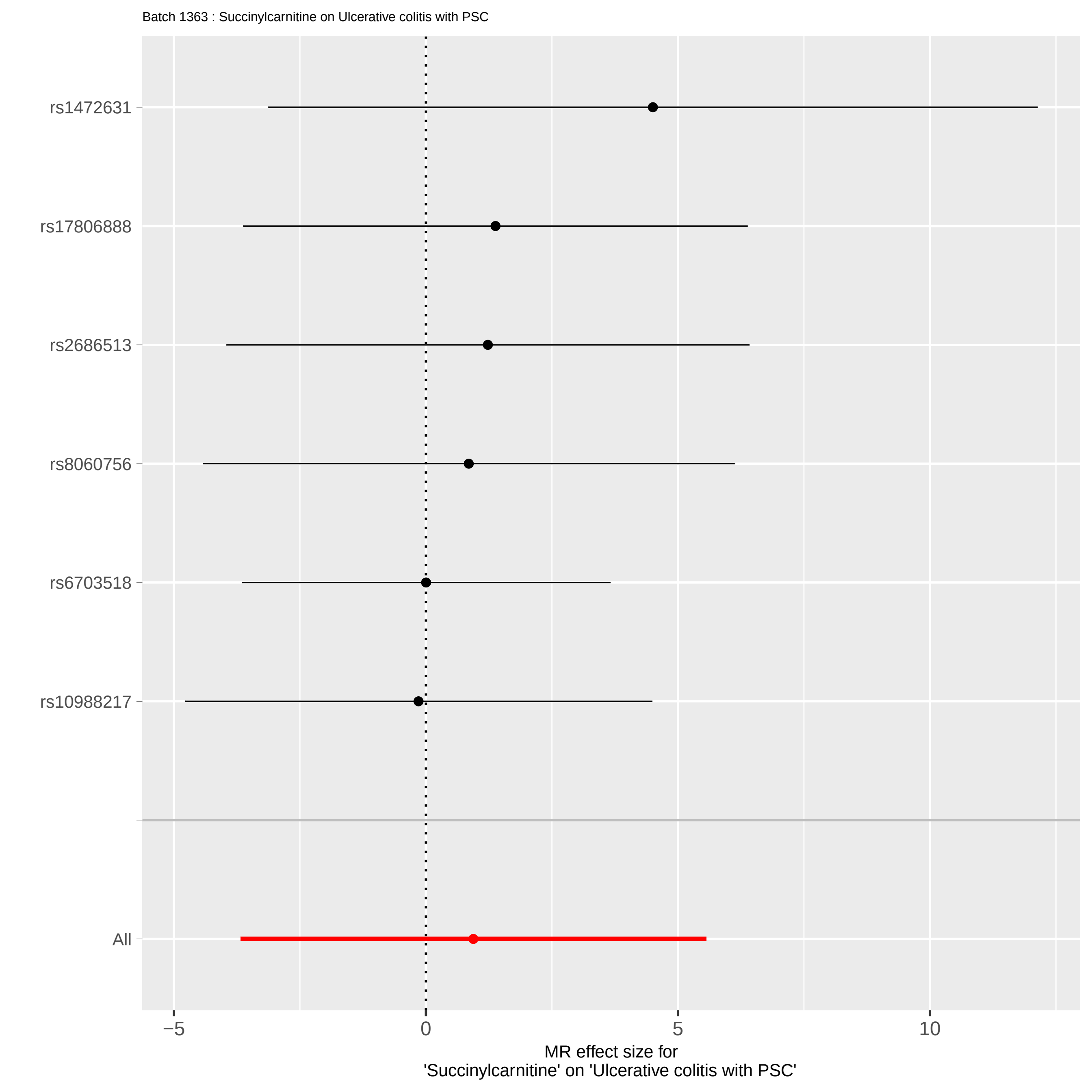
Batch 1363 : Succinylcarnitine on Ulcerative colitis with PSC
rs1472631
rs17806888
rs2686513
rs8060756
rs6703518
rs10988217
All
−5
0
5
10
MR effect size for
'Succinylcarnitine' on 'Ulcerative colitis with PSC'

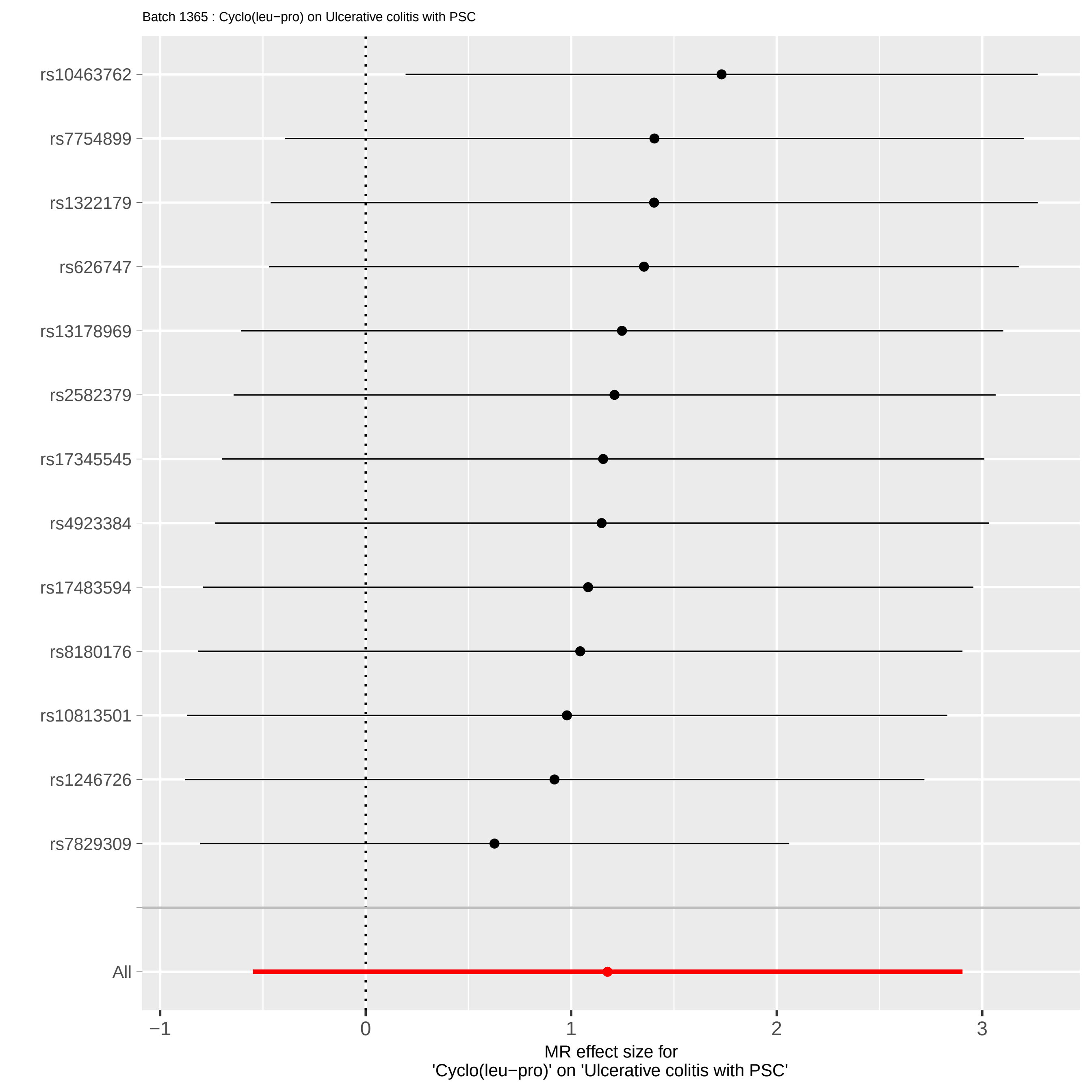
Batch 1365 : Cyclo(leu−pro) on Ulcerative colitis with PSC
rs10463762
rs7754899
rs1322179
rs626747
rs13178969
rs2582379
rs17345545
rs4923384
rs17483594
rs8180176
rs10813501
rs1246726
rs7829309
All
−1
0
1
2
3
MR effect size for
'Cyclo(leu−pro)' on 'Ulcerative colitis with PSC'

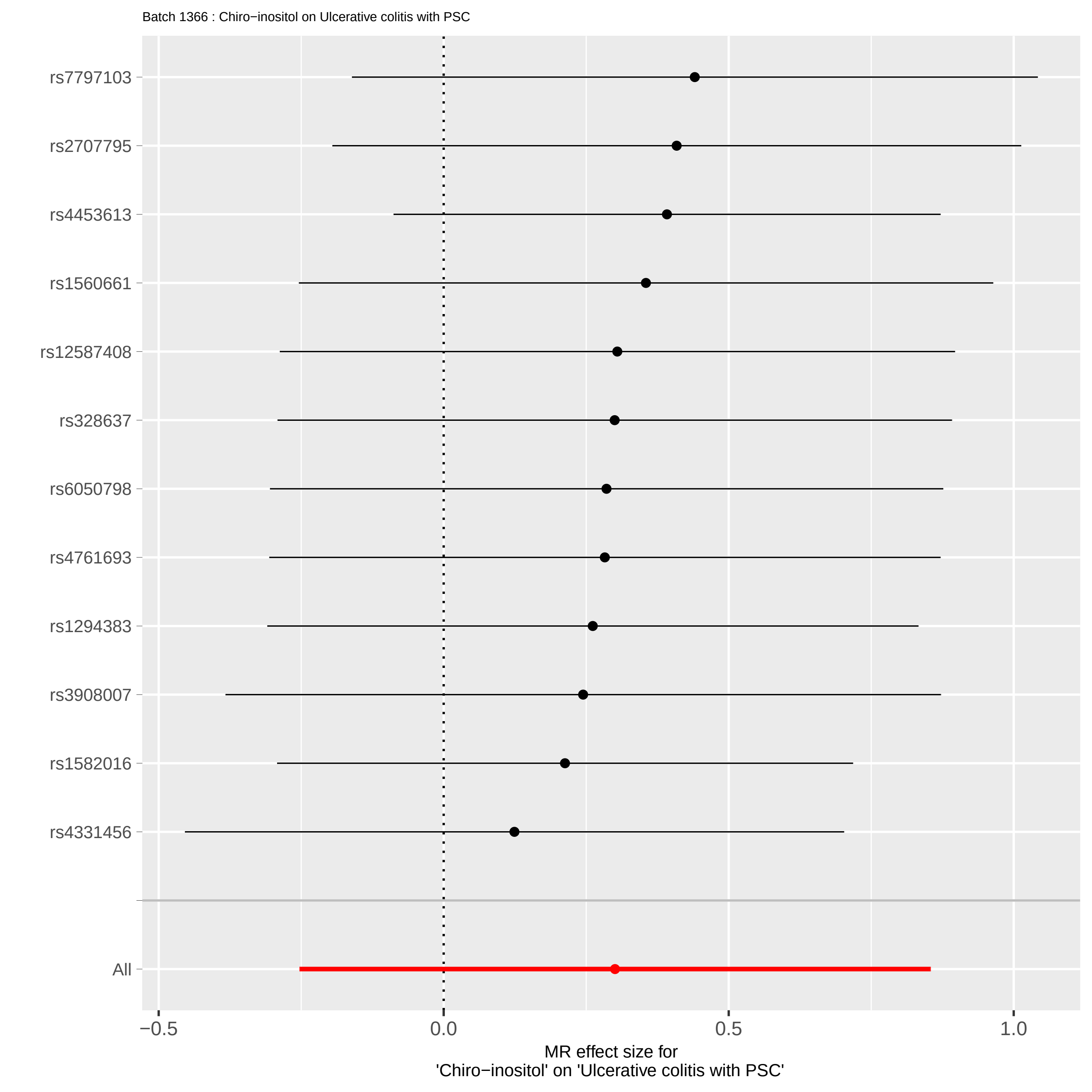
Batch 1366 : Chiro−inositol on Ulcerative colitis with PSC
rs7797103
rs2707795
rs4453613
rs1560661
rs12587408
rs328637
rs6050798
rs4761693
rs1294383
rs3908007
rs1582016
rs4331456
All
−0.5
0.0
0.5
1.0
MR effect size for
'Chiro−inositol' on 'Ulcerative colitis with PSC'

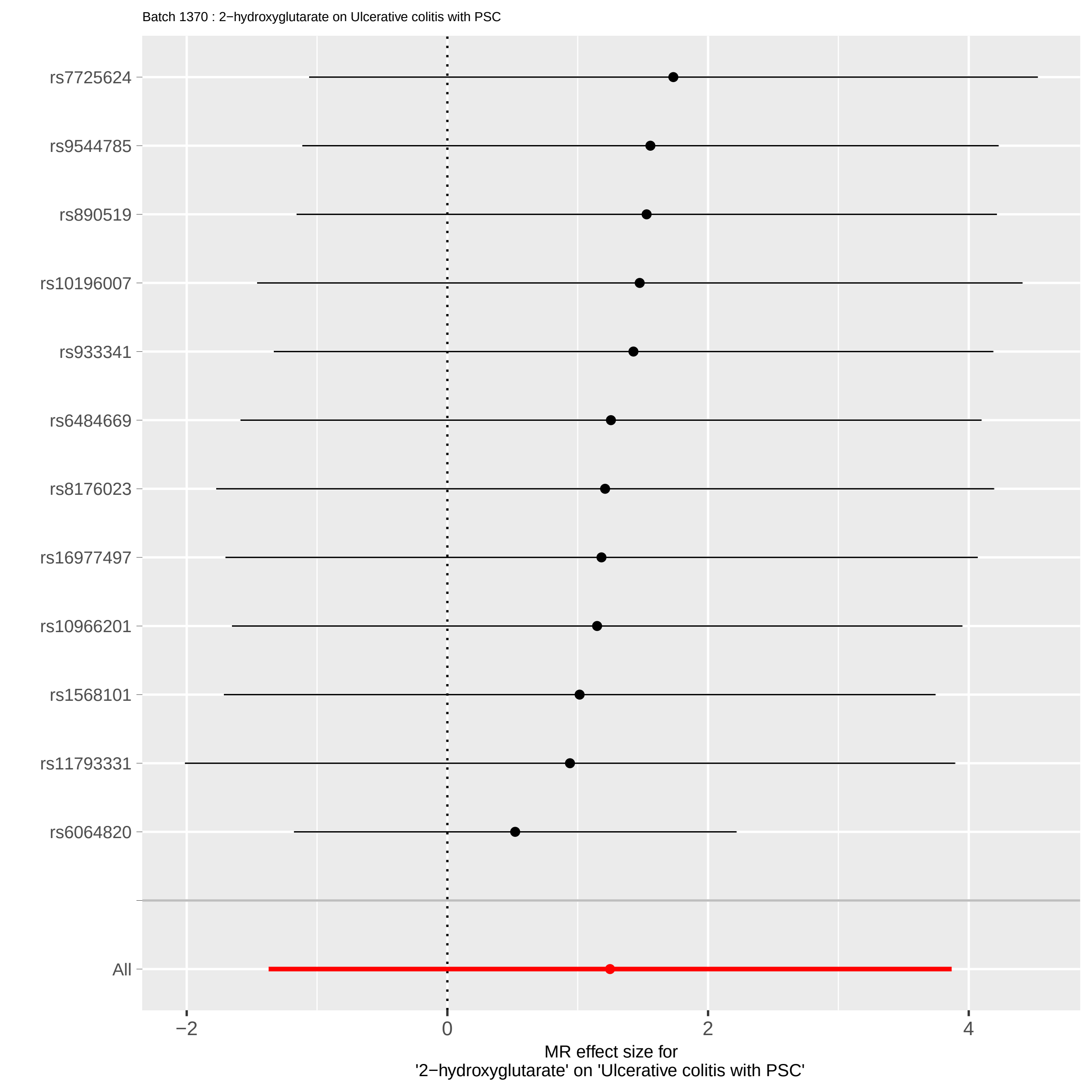

Batch 1370 : 2−hydroxyglutarate on Ulcerative colitis with PSC
rs7725624
rs9544785
rs890519
rs10196007
rs933341
rs6484669
rs8176023
rs16977497
rs10966201
rs1568101
rs11793331
rs6064820
All
−2
0
2
4
MR effect size for
'2−hydroxyglutarate' on 'Ulcerative colitis with PSC'

Batch 1371 : Ergothioneine on Ulcerative colitis with PSC

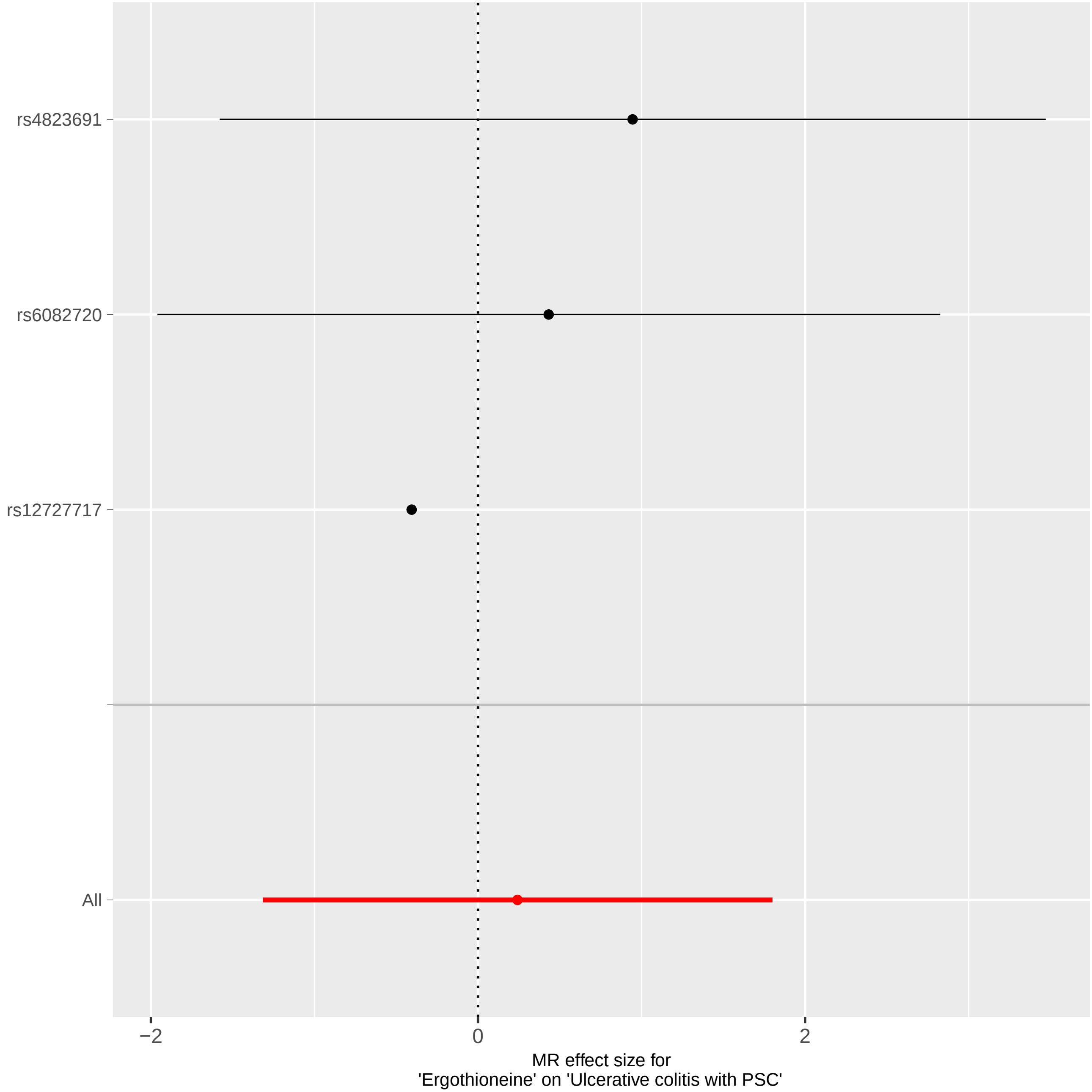

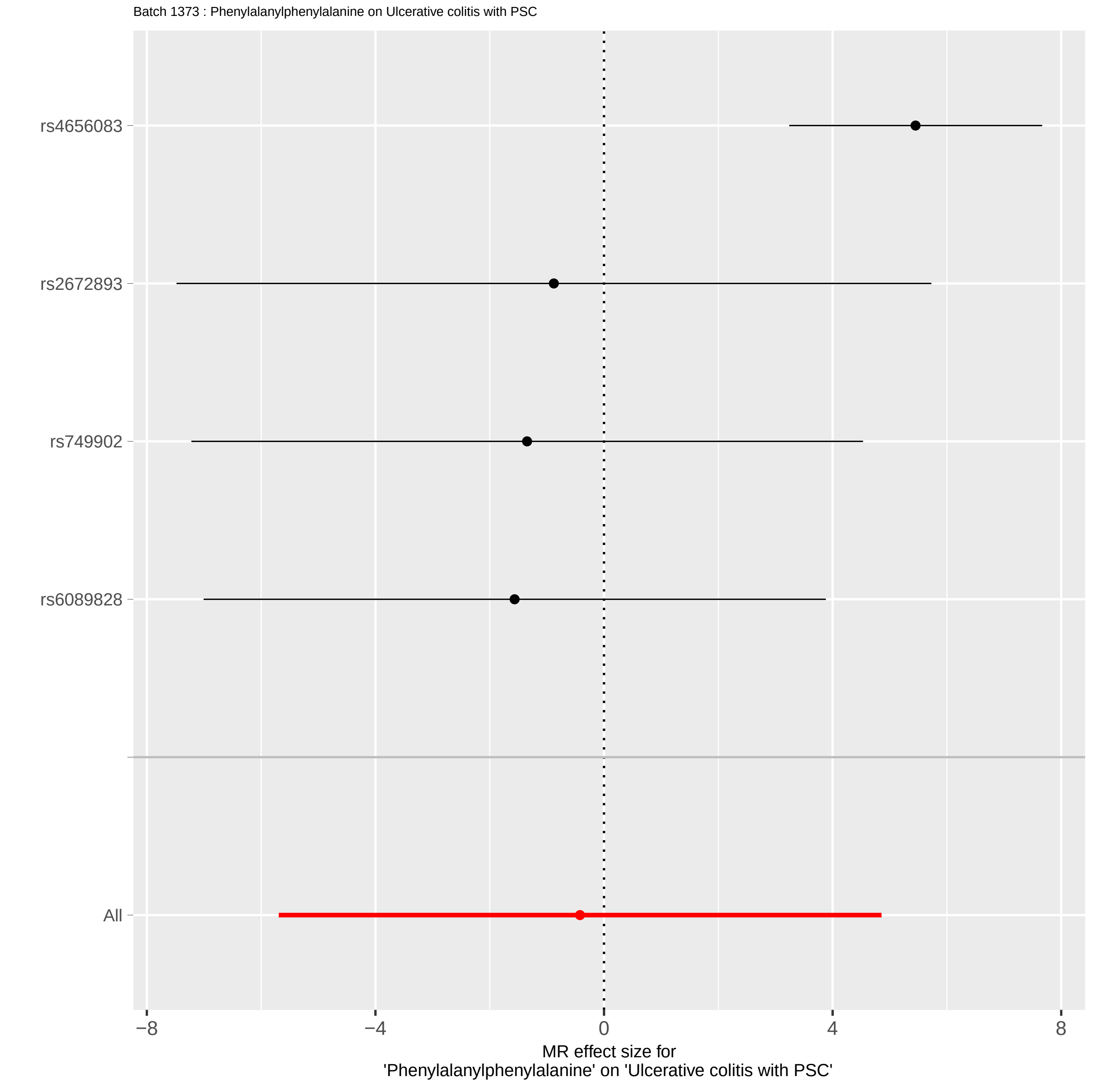

Batch 1373 : Phenylalanylphenylalanine on Ulcerative colitis with PSC
rs4656083
rs2672893
rs749902
rs6089828
All
−8
−4
0
4
8
MR effect size for
'Phenylalanylphenylalanine' on 'Ulcerative colitis with PSC'

Batch 1375 : 15−methylpalmitate (isobar with 2−methylpalmitate) on Ulcerative colitis with PSC

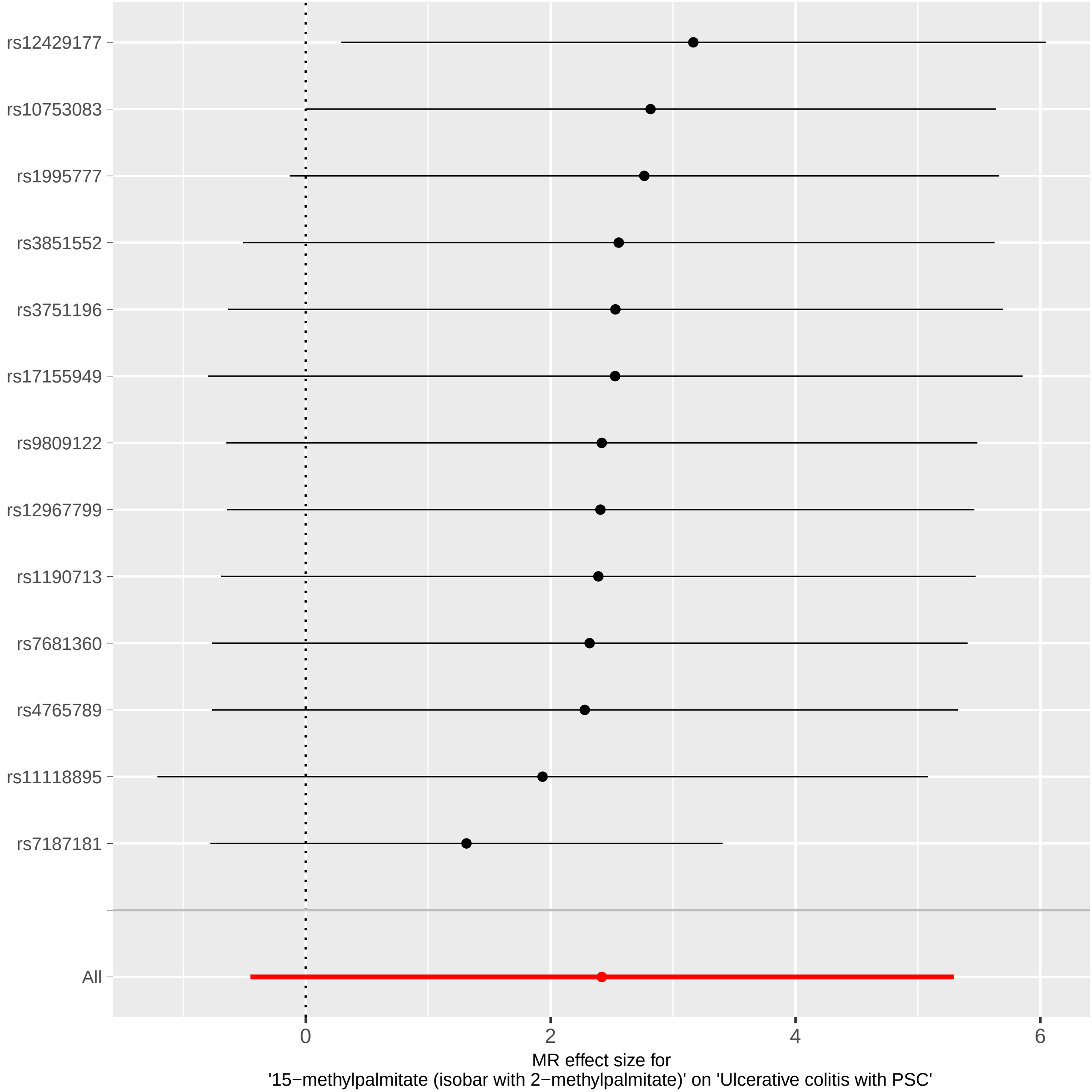

Batch 1377 : Tryptophan on Left−sided ulcerative colitis

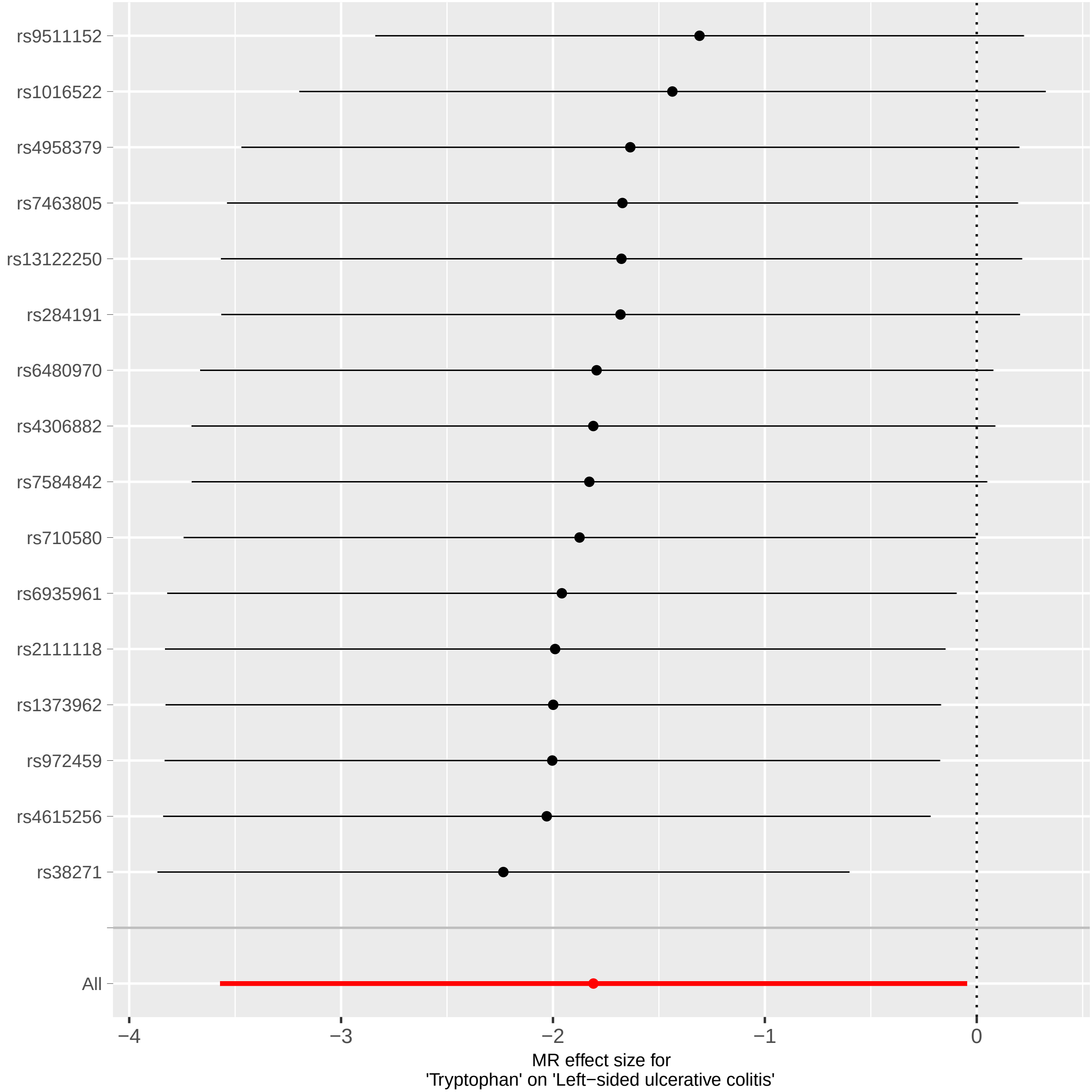

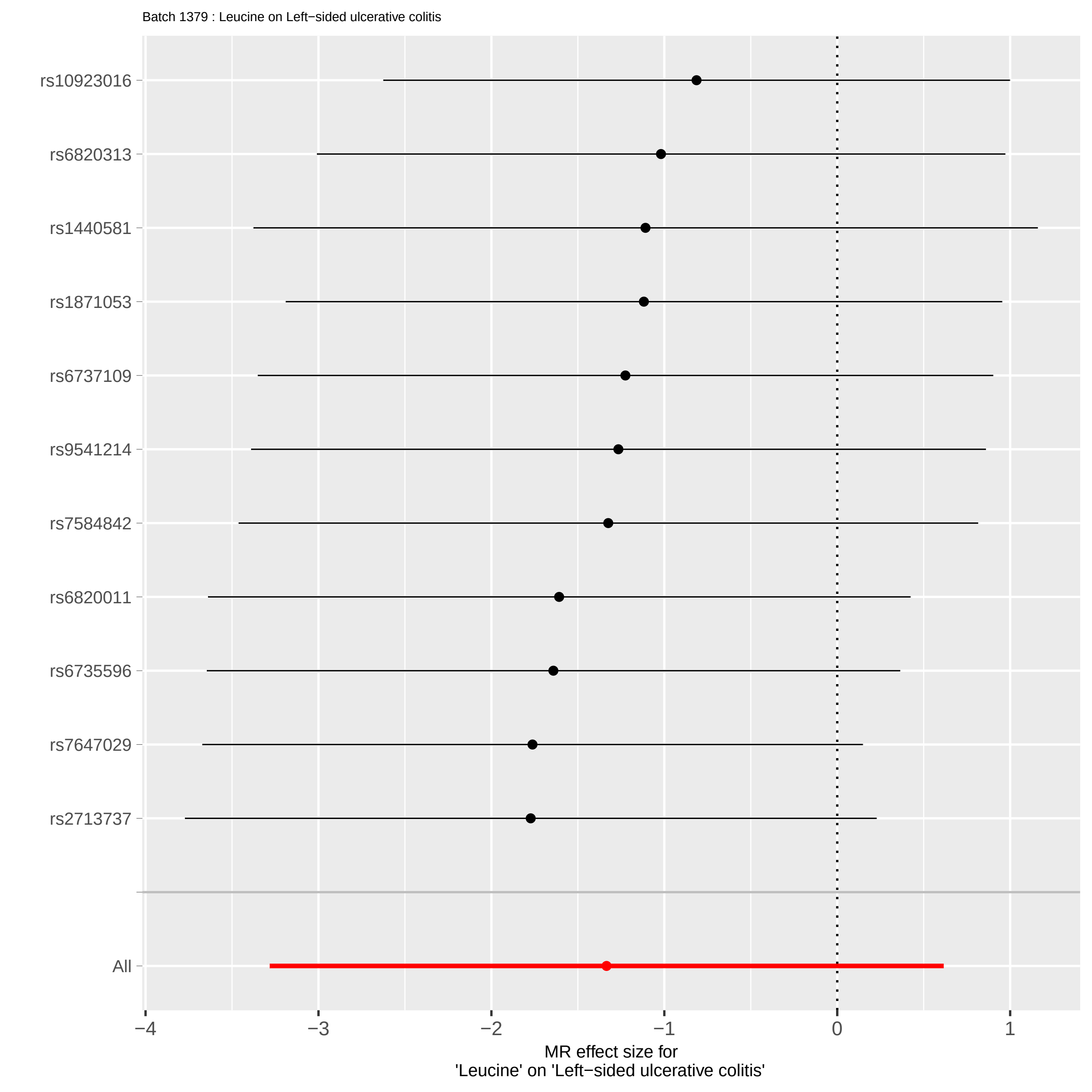
Batch 1379 : Leucine on Left−sided ulcerative colitis
rs10923016
rs6820313
rs1440581
rs1871053
rs6737109
rs9541214
rs7584842
rs6820011
rs6735596
rs7647029
rs2713737
All
−4
−3
−2
−1
0
1
MR effect size for
'Leucine' on 'Left−sided ulcerative colitis'

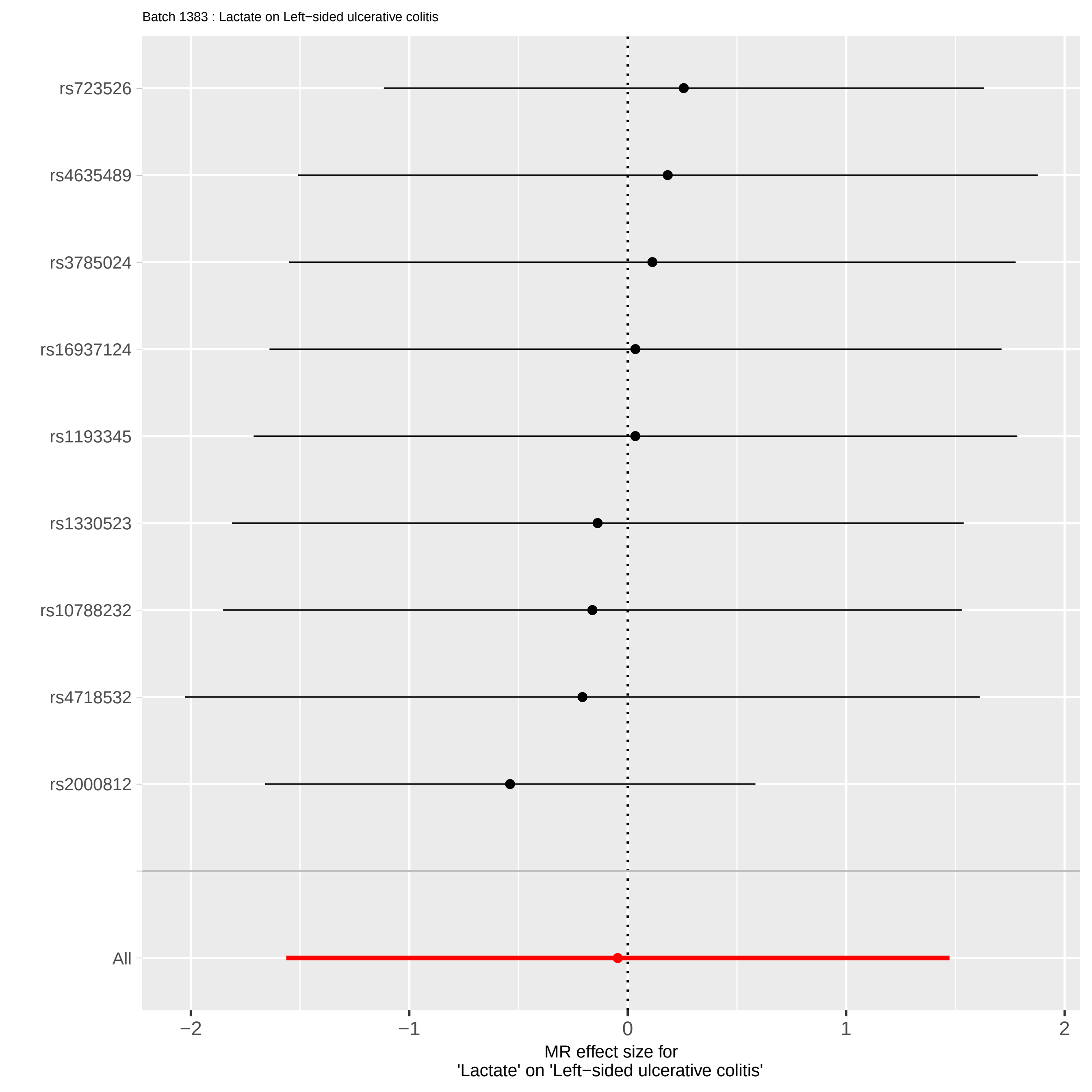

Batch 1383 : Lactate on Left−sided ulcerative colitis
rs723526
rs4635489
rs3785024
rs16937124
rs1193345
rs1330523
rs10788232
rs4718532
rs2000812
All
−2
−1
0
1
2
MR effect size for
'Lactate' on 'Left−sided ulcerative colitis'

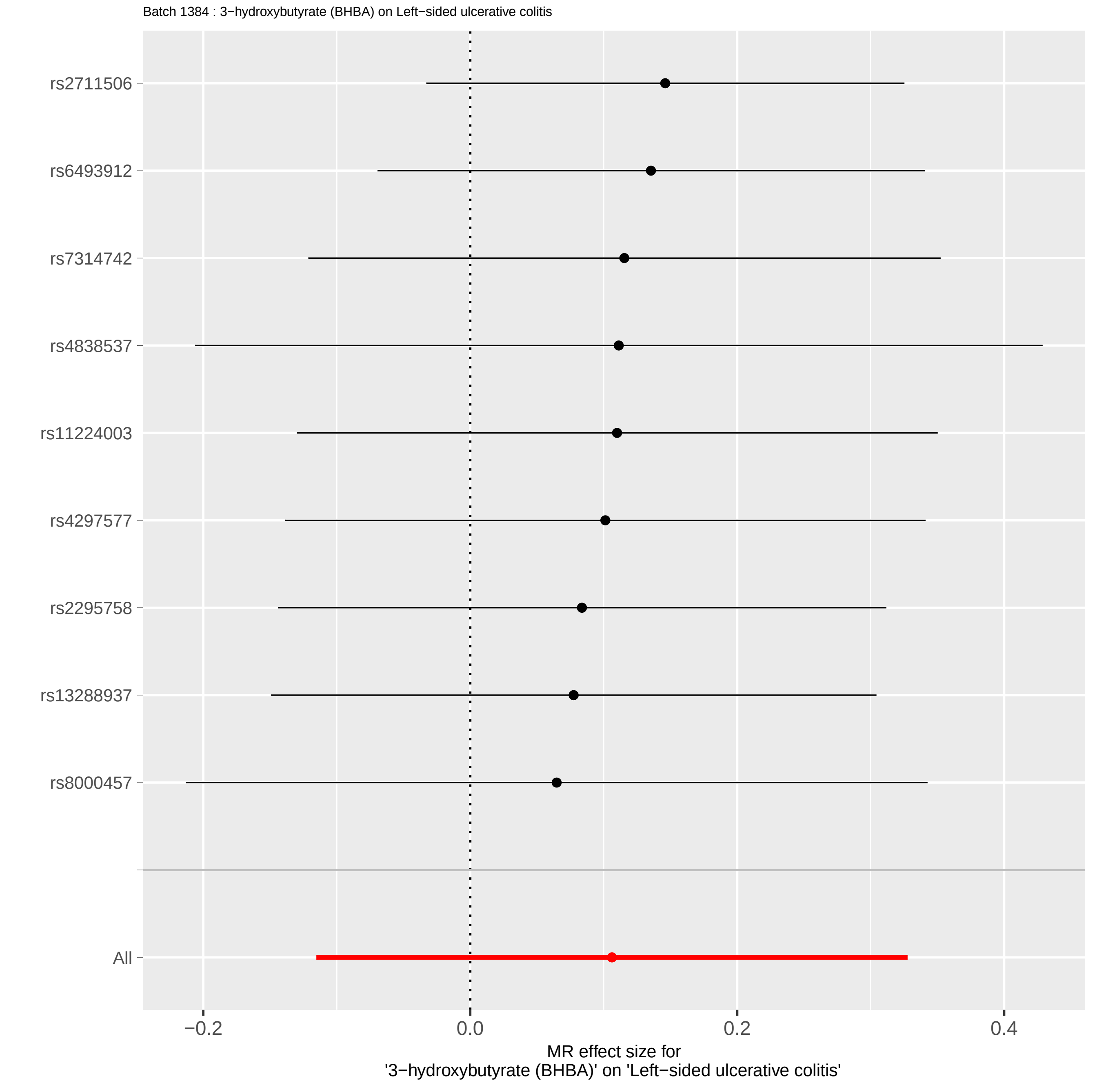

Batch 1384 : 3−hydroxybutyrate (BHBA) on Left−sided ulcerative colitis
rs2711506
rs6493912
rs7314742
rs4838537
rs11224003
rs4297577
rs2295758
rs13288937
rs8000457
All
−0.2
0.0
0.2
0.4
MR effect size for
'3−hydroxybutyrate (BHBA)' on 'Left−sided ulcerative colitis'

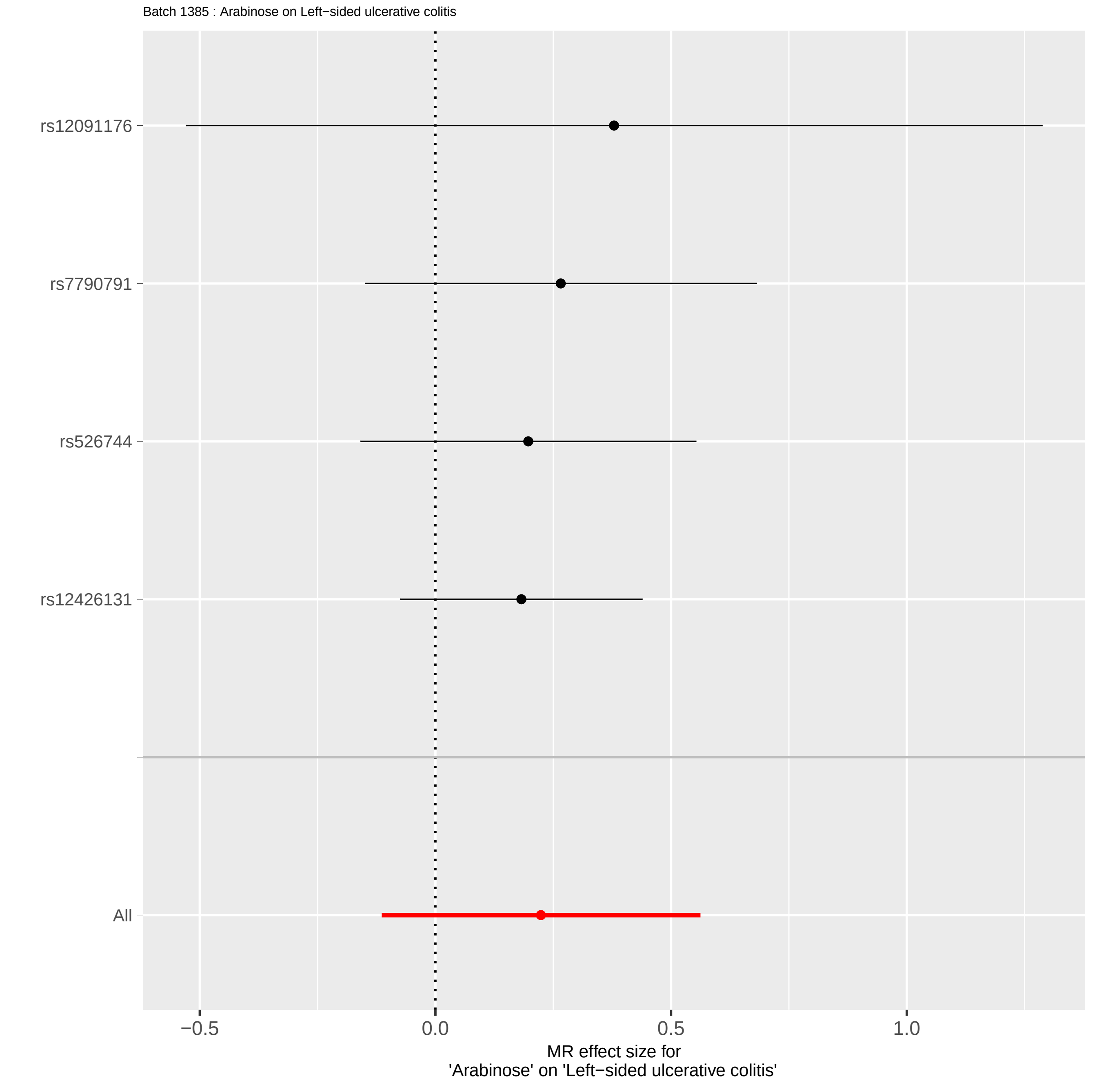

Batch 1385 : Arabinose on Left−sided ulcerative colitis
rs12091176
rs7790791
rs526744
rs12426131
All
−0.5
0.0
0.5
1.0
MR effect size for
'Arabinose' on 'Left−sided ulcerative colitis'

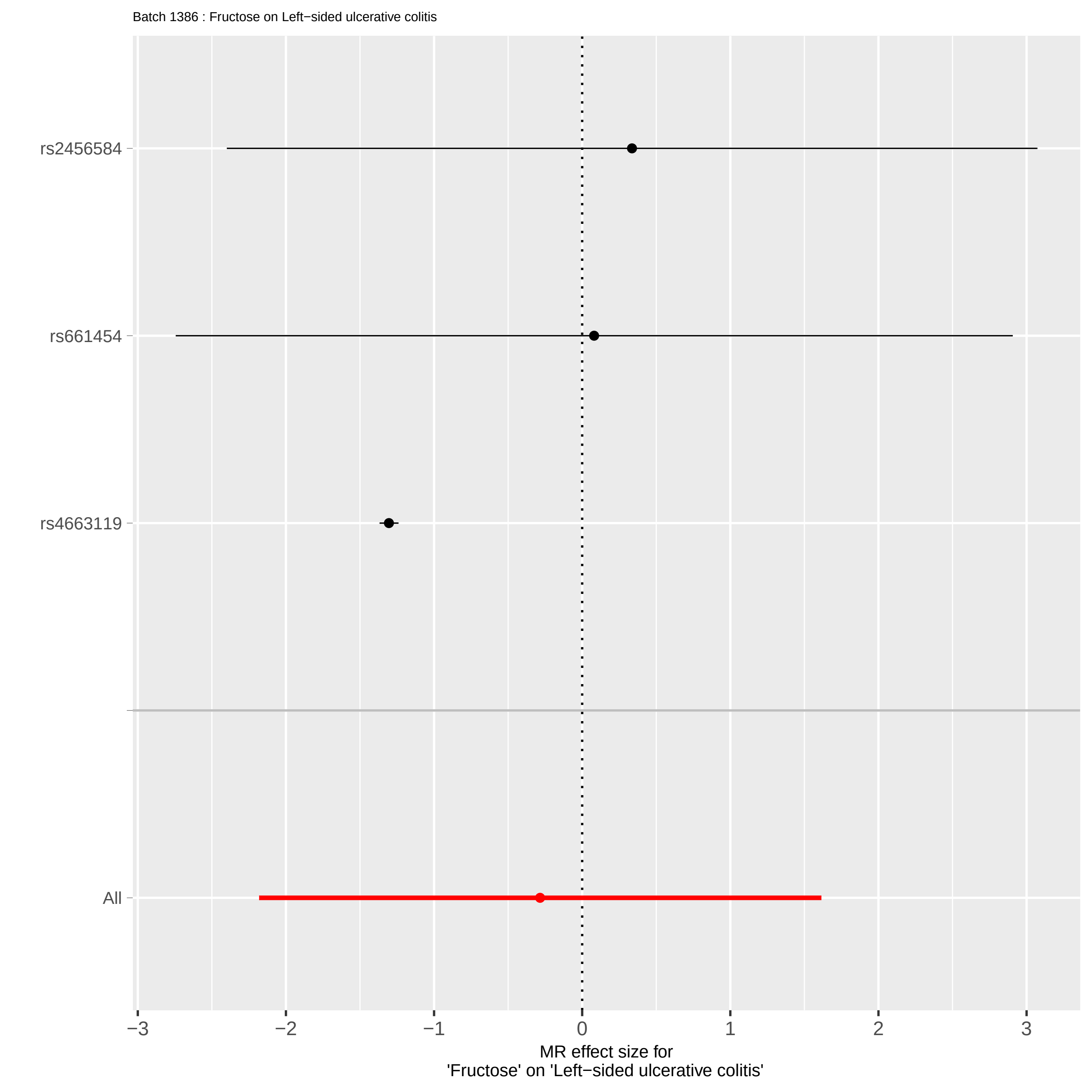
Batch 1386 : Fructose on Left−sided ulcerative colitis
rs2456584
rs661454
rs4663119
All
−3
−2
−1
0
1
2
3
MR effect size for
'Fructose' on 'Left−sided ulcerative colitis'

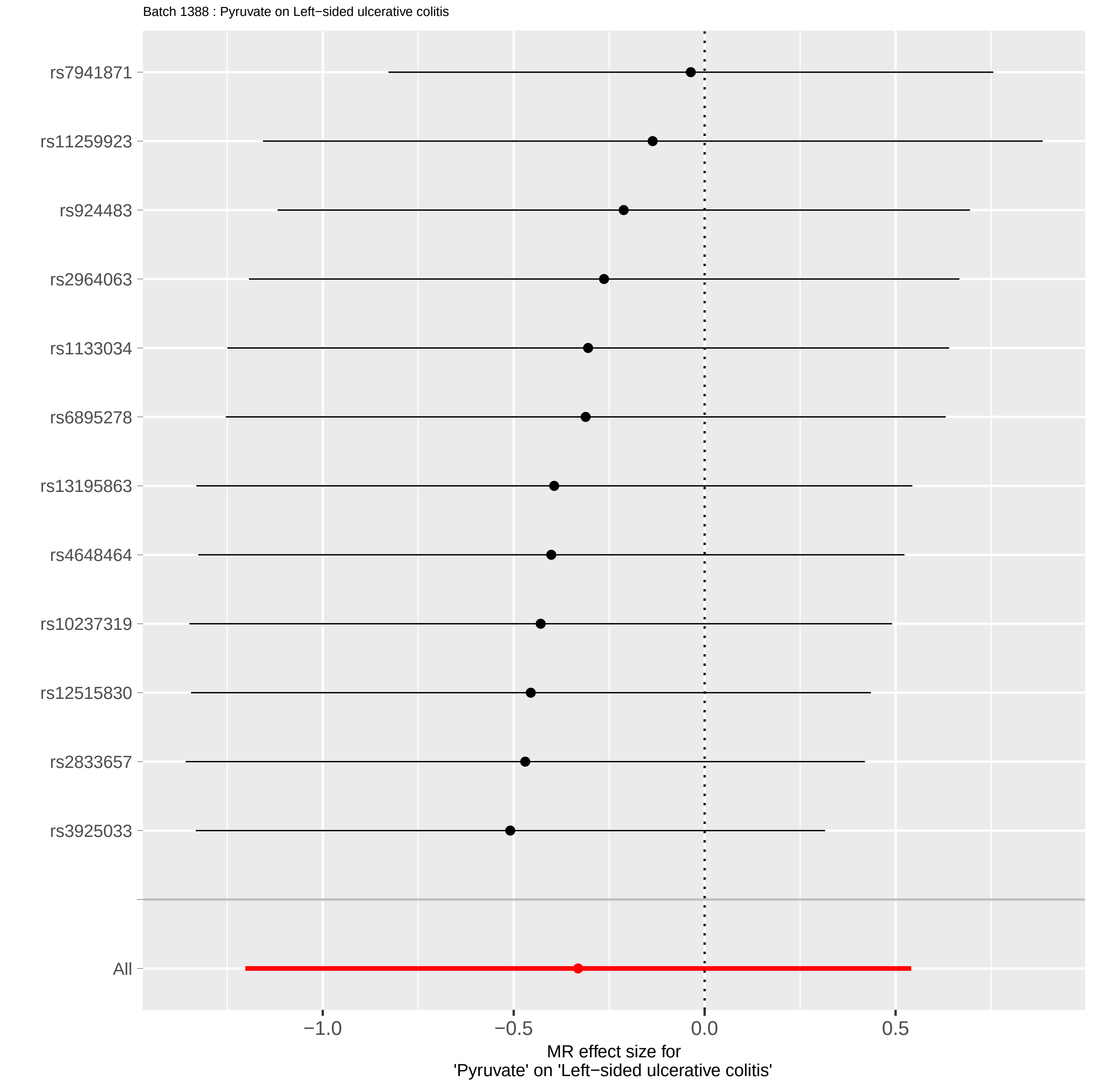

Batch 1388 : Pyruvate on Left−sided ulcerative colitis
rs7941871
rs11259923
rs924483
rs2964063
rs1133034
rs6895278
rs13195863
rs4648464
rs10237319
rs12515830
rs2833657
rs3925033
All
−1.0
−0.5
0.0
0.5
MR effect size for
'Pyruvate' on 'Left−sided ulcerative colitis'

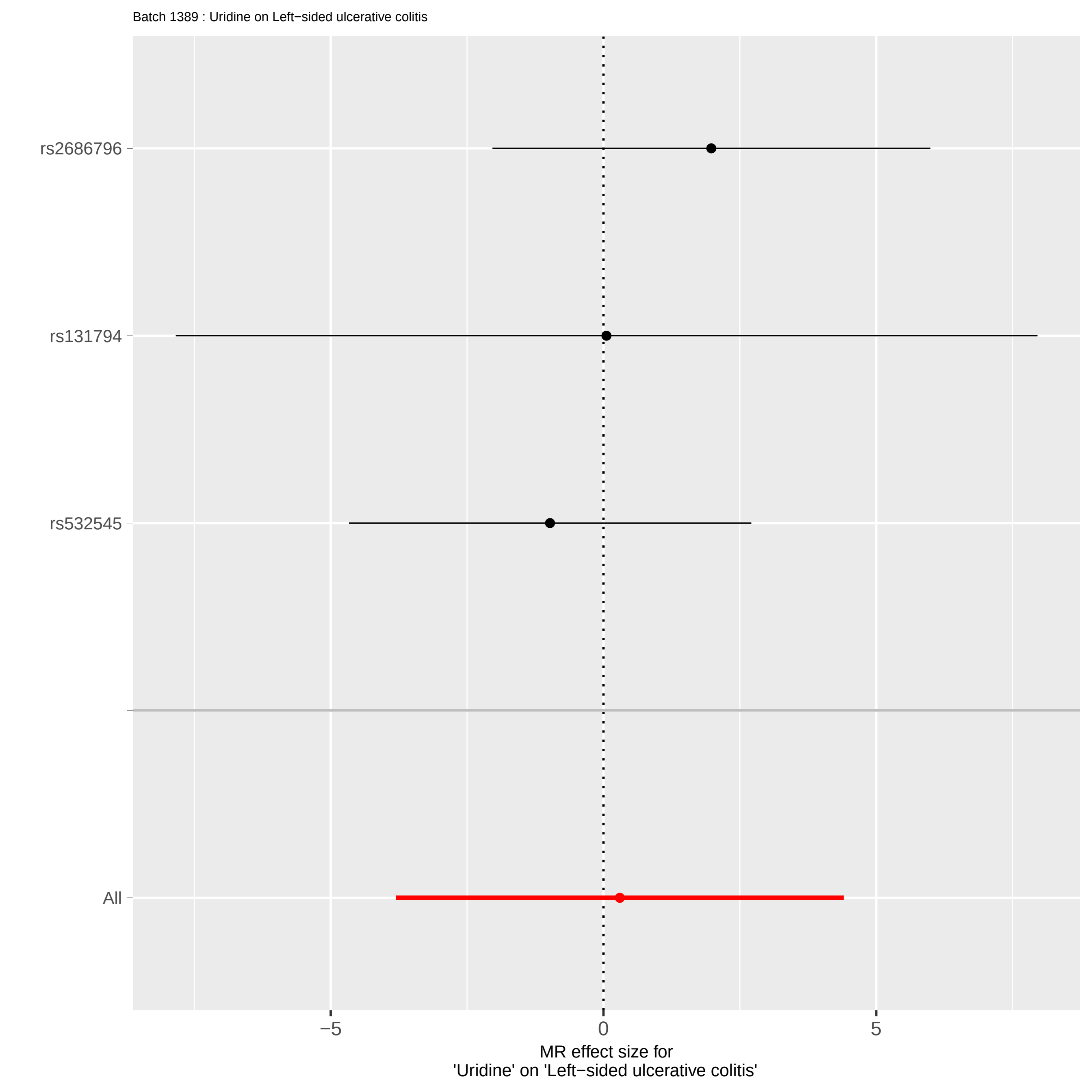

Batch 1389 : Uridine on Left−sided ulcerative colitis
rs2686796
rs131794
rs532545
All
−5
0
5
MR effect size for
'Uridine' on 'Left−sided ulcerative colitis'

Batch 1390 : Linoleate (18:2n6) on Left−sided ulcerative colitis

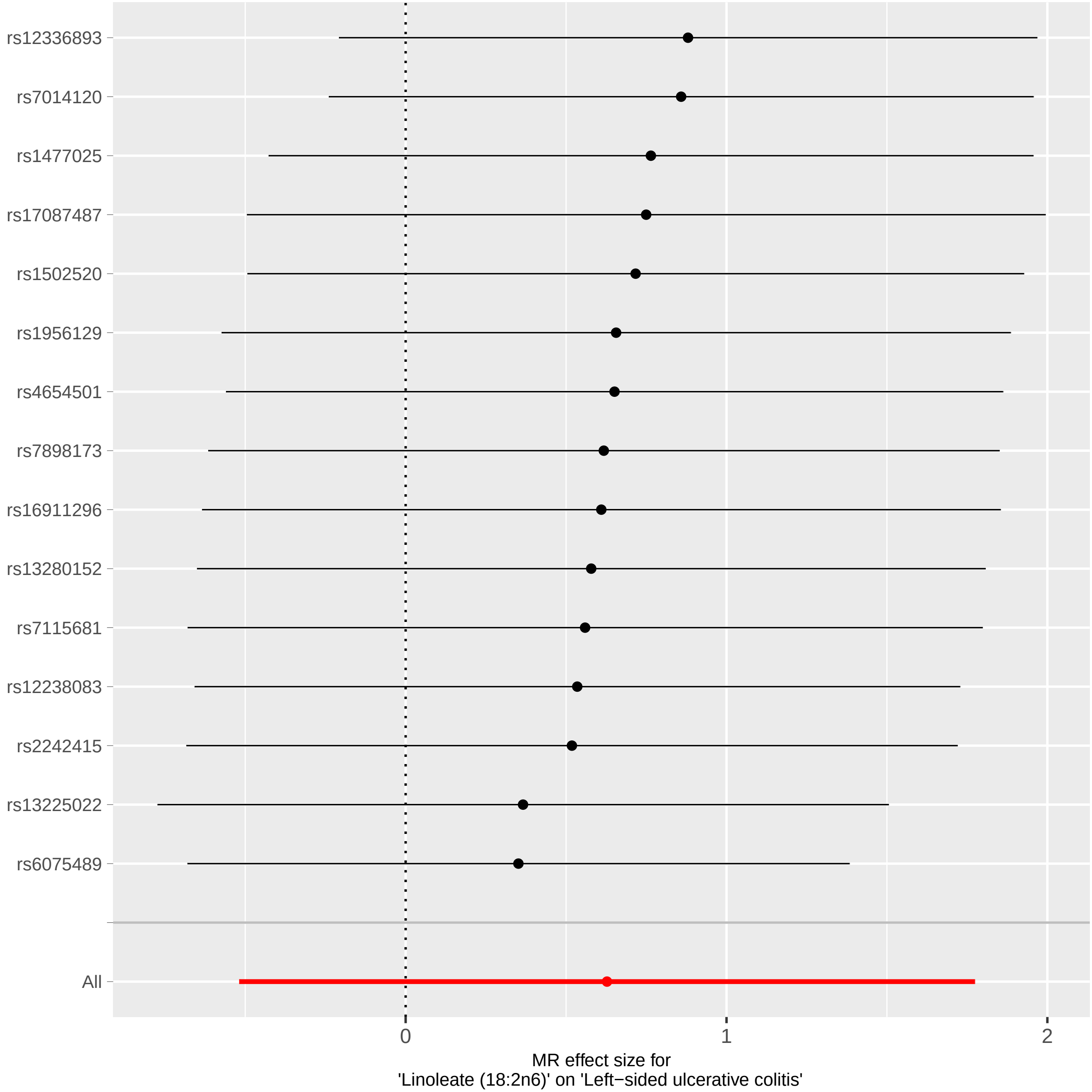

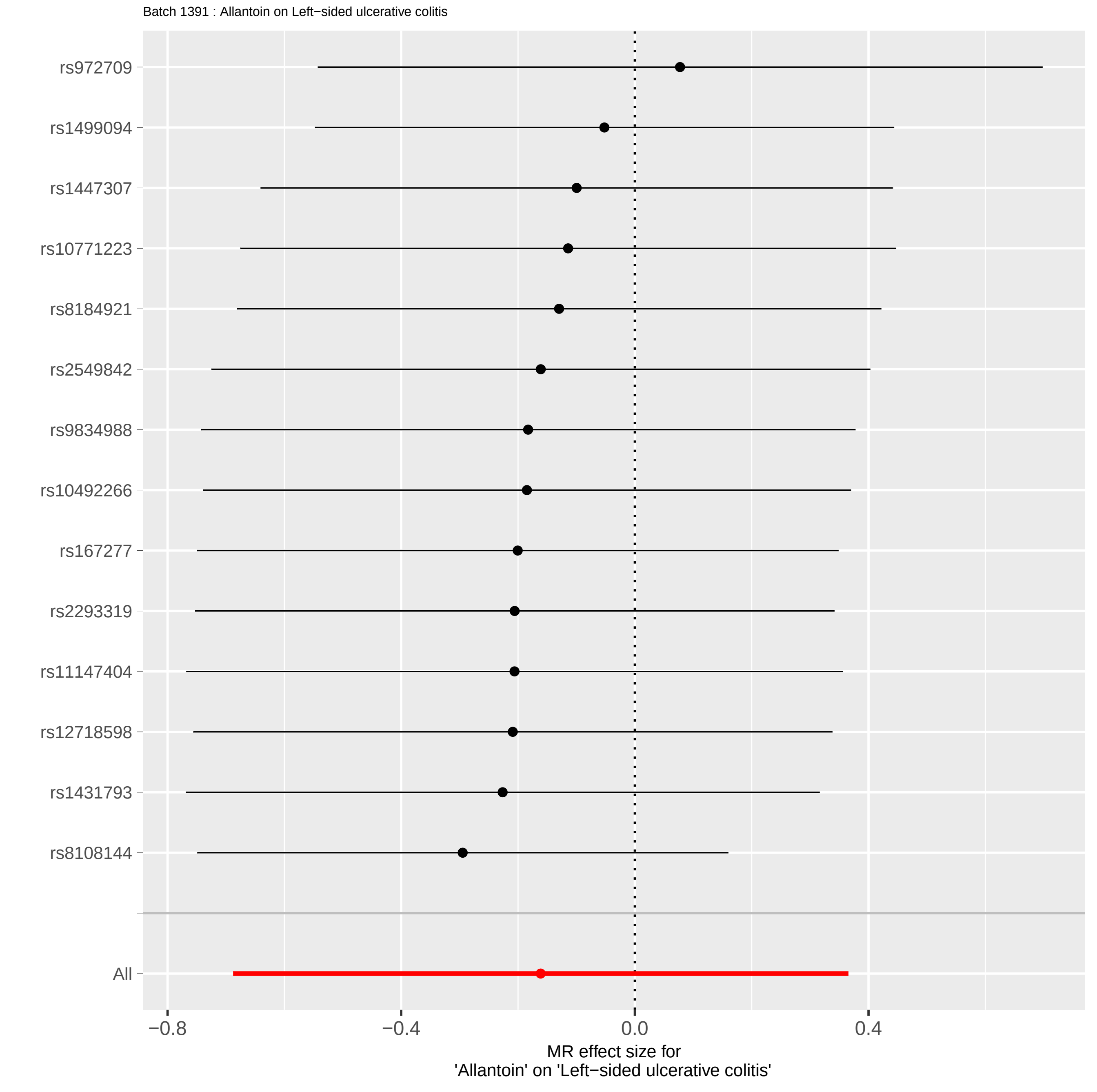
Batch 1391 : Allantoin on Left−sided ulcerative colitis
rs972709
rs1499094
rs1447307
rs10771223
rs8184921
rs2549842
rs9834988
rs10492266
rs167277
rs2293319
rs11147404
rs12718598
rs1431793
rs8108144
All
−0.8
−0.4
0.0
0.4
MR effect size for
'Allantoin' on 'Left−sided ulcerative colitis'

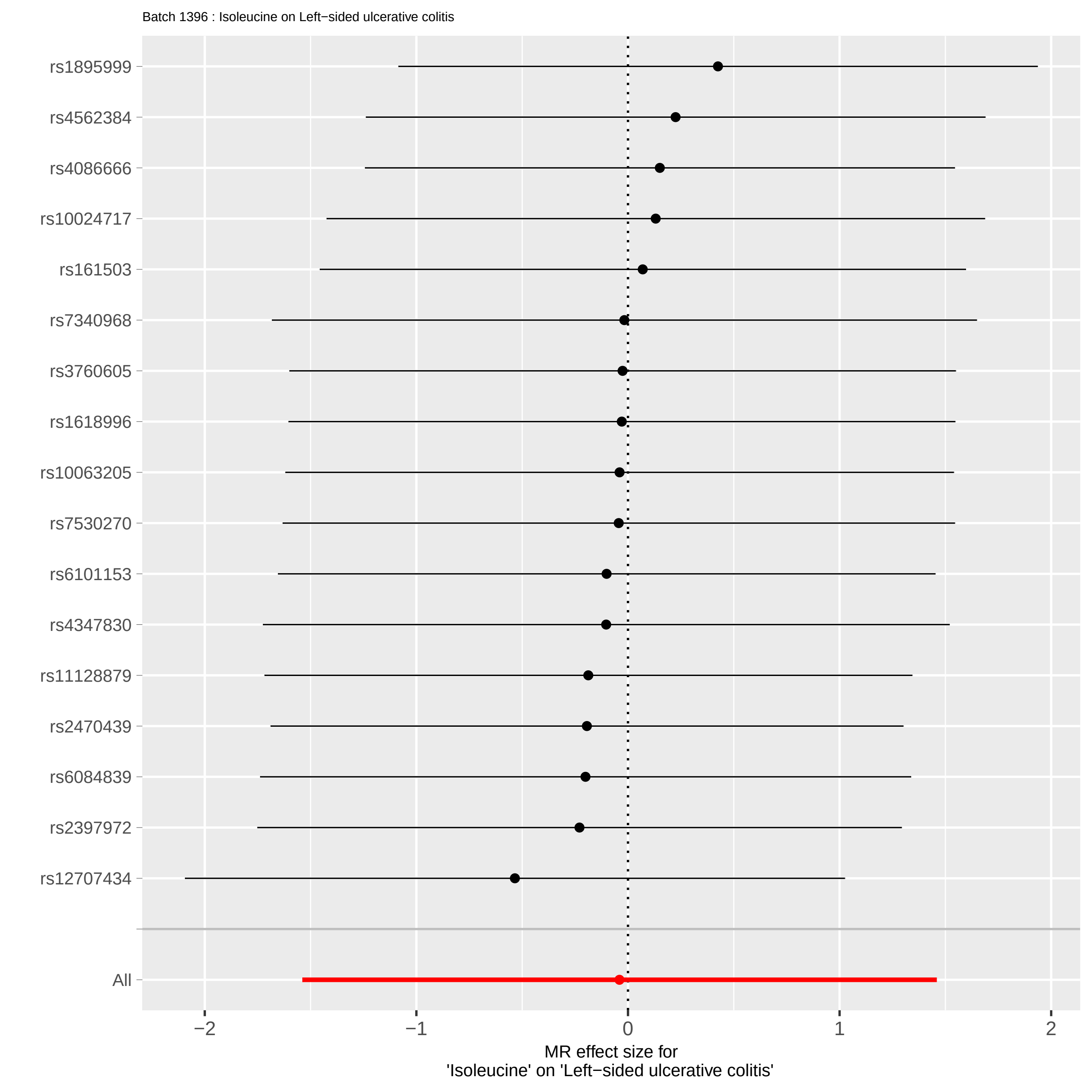

Batch 1396 : Isoleucine on Left−sided ulcerative colitis
rs1895999
rs4562384
rs4086666
rs10024717
rs161503
rs7340968
rs3760605
rs1618996
rs10063205
rs7530270
rs6101153
rs4347830
rs11128879
rs2470439
rs6084839
rs2397972
rs12707434
All
−2
−1
0
1
2
MR effect size for
'Isoleucine' on 'Left−sided ulcerative colitis'

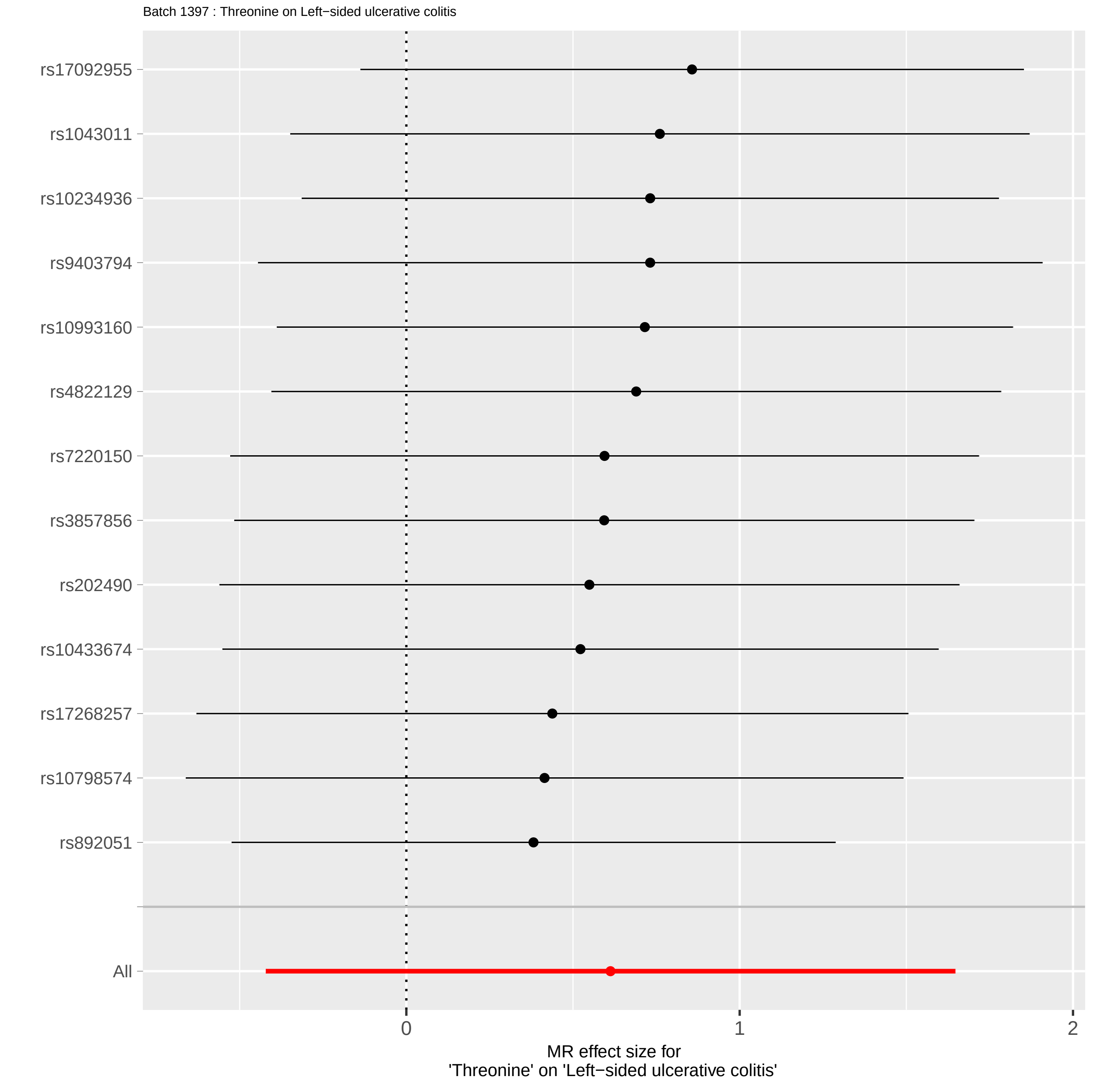

Batch 1397 : Threonine on Left−sided ulcerative colitis
rs17092955
rs1043011
rs10234936
rs9403794
rs10993160
rs4822129
rs7220150
rs3857856
rs202490
rs10433674
rs17268257
rs10798574
rs892051
All
0
1
2
MR effect size for
'Threonine' on 'Left−sided ulcerative colitis'

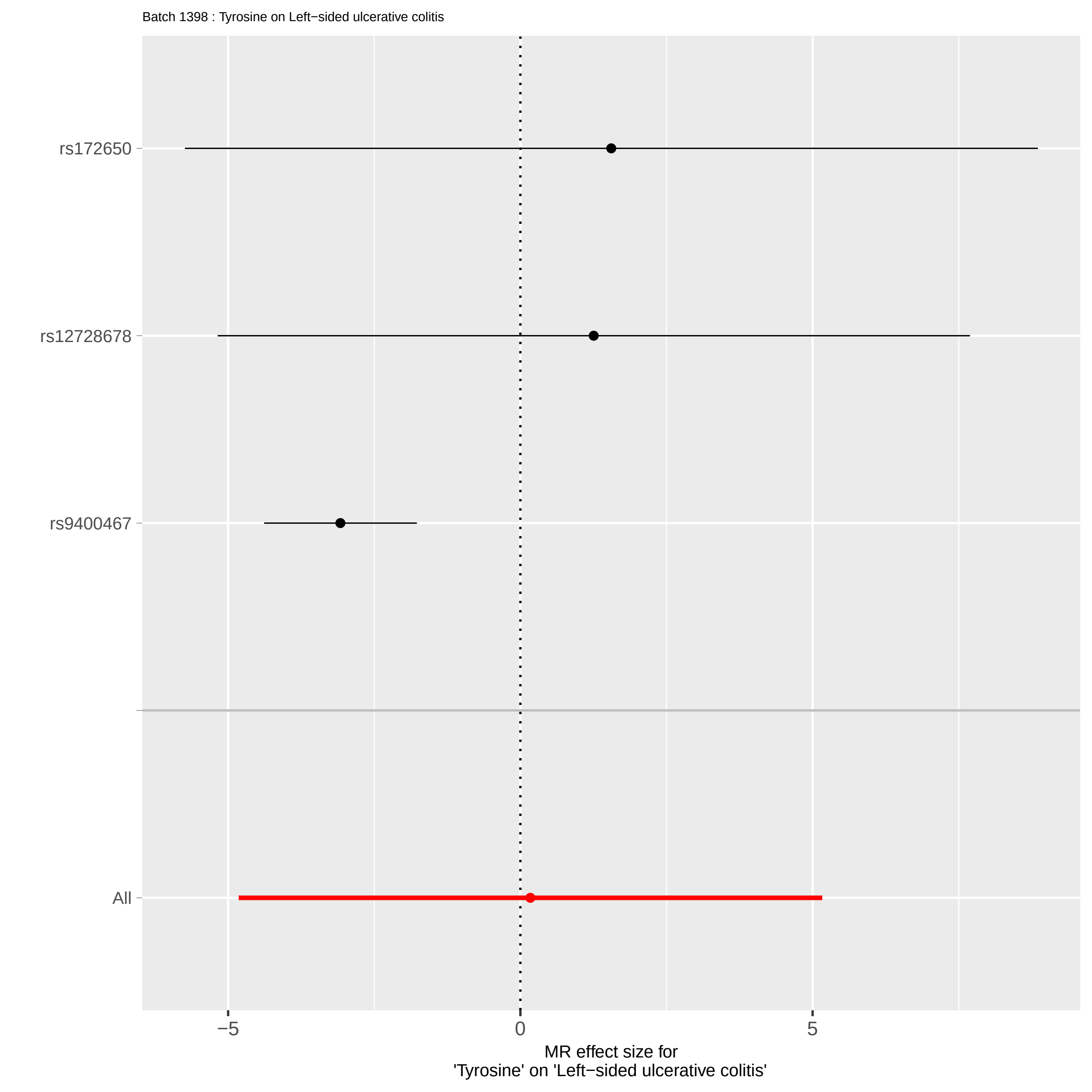
Batch 1398 : Tyrosine on Left−sided ulcerative colitis
rs172650
rs12728678
rs9400467
All
−5
0
5
MR effect size for
'Tyrosine' on 'Left−sided ulcerative colitis'

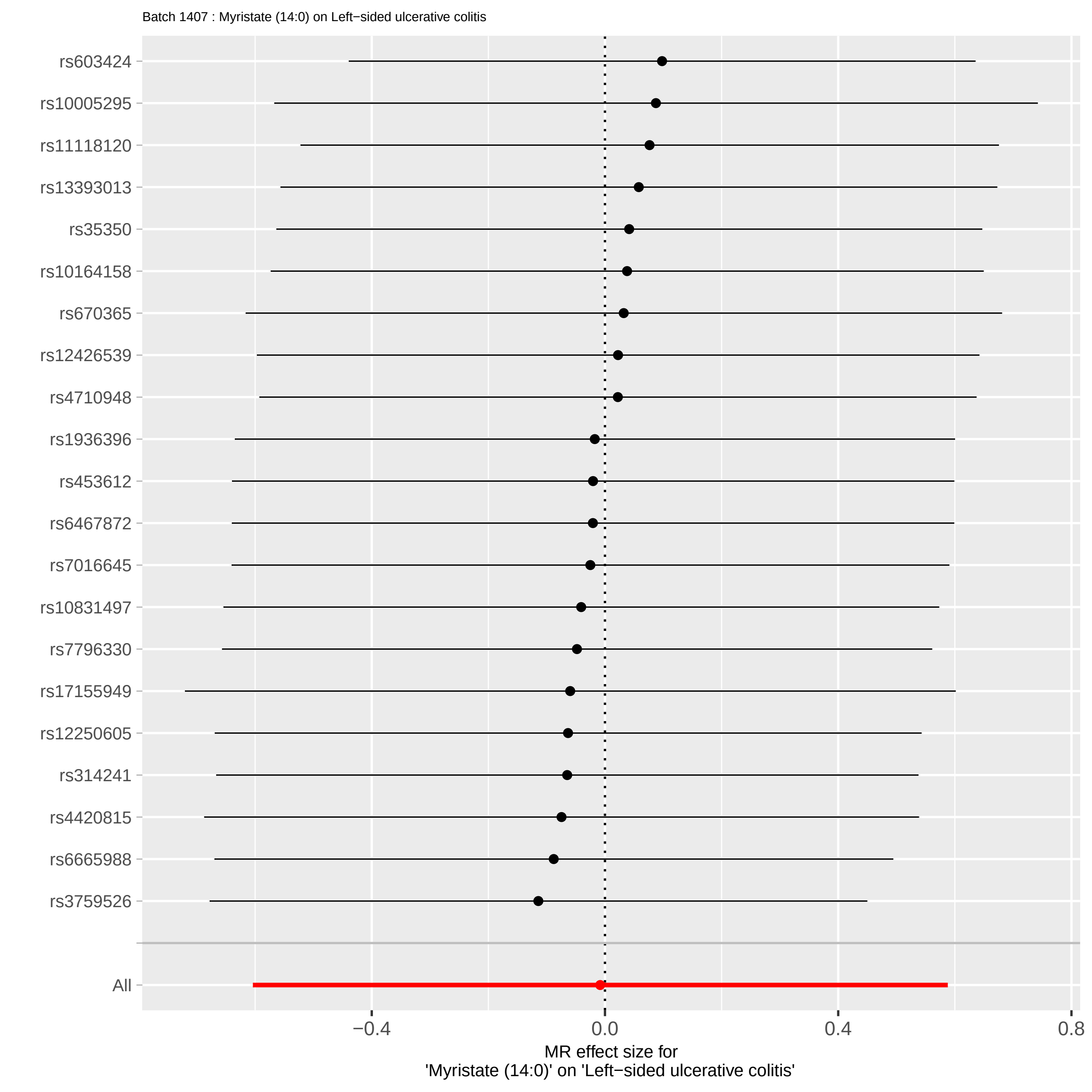

Batch 1407 : Myristate (14:0) on Left−sided ulcerative colitis
rs603424
rs10005295
rs11118120
rs13393013
rs35350
rs10164158
rs670365
rs12426539
rs4710948
rs1936396
rs453612
rs6467872
rs7016645
rs10831497
rs7796330
rs17155949
rs12250605
rs314241
rs4420815
rs6665988
rs3759526
All
−0.4
0.0
0.4
0.8
MR effect size for
'Myristate (14:0)' on 'Left−sided ulcerative colitis'

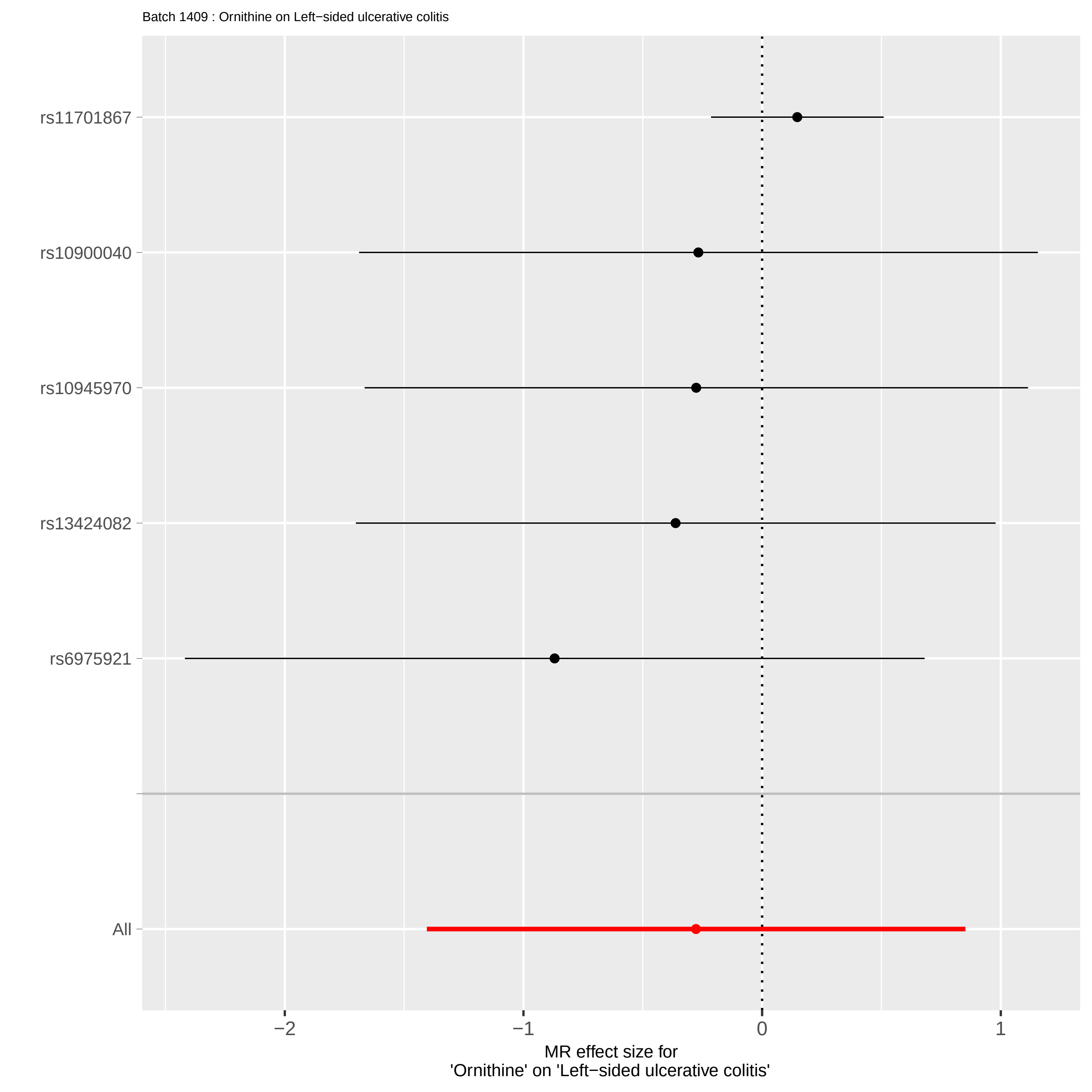

Batch 1409 : Ornithine on Left−sided ulcerative colitis
rs11701867
rs10900040
rs10945970
rs13424082
rs6975921
All
−2
−1
0
1
MR effect size for
'Ornithine' on 'Left−sided ulcerative colitis'

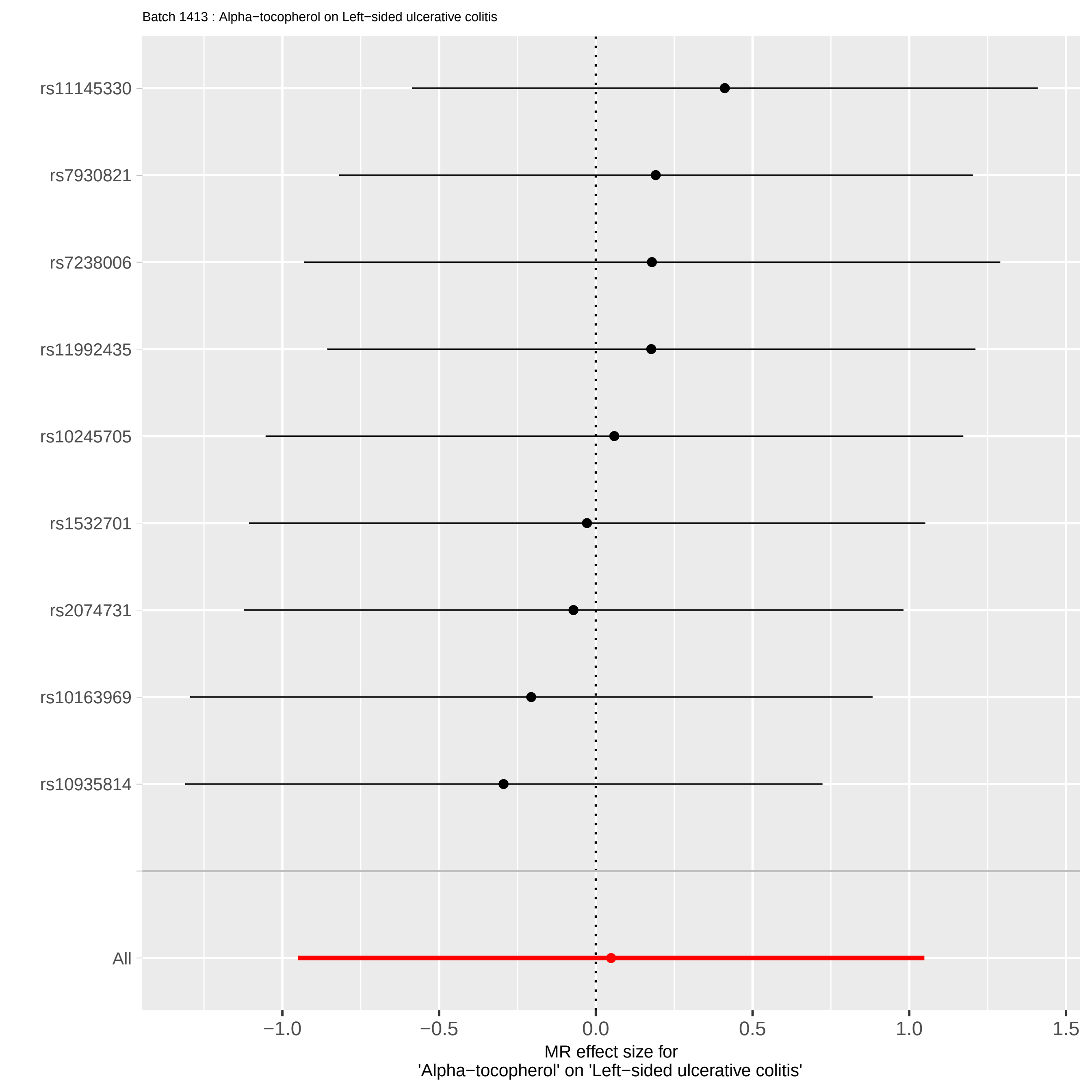
Batch 1413 : Alpha−tocopherol on Left−sided ulcerative colitis
rs11145330
rs7930821
rs7238006
rs11992435
rs10245705
rs1532701
rs2074731
rs10163969
rs10935814
All
−1.0
−0.5
0.0
0.5
1.0
1.5
MR effect size for
'Alpha−tocopherol' on 'Left−sided ulcerative colitis'

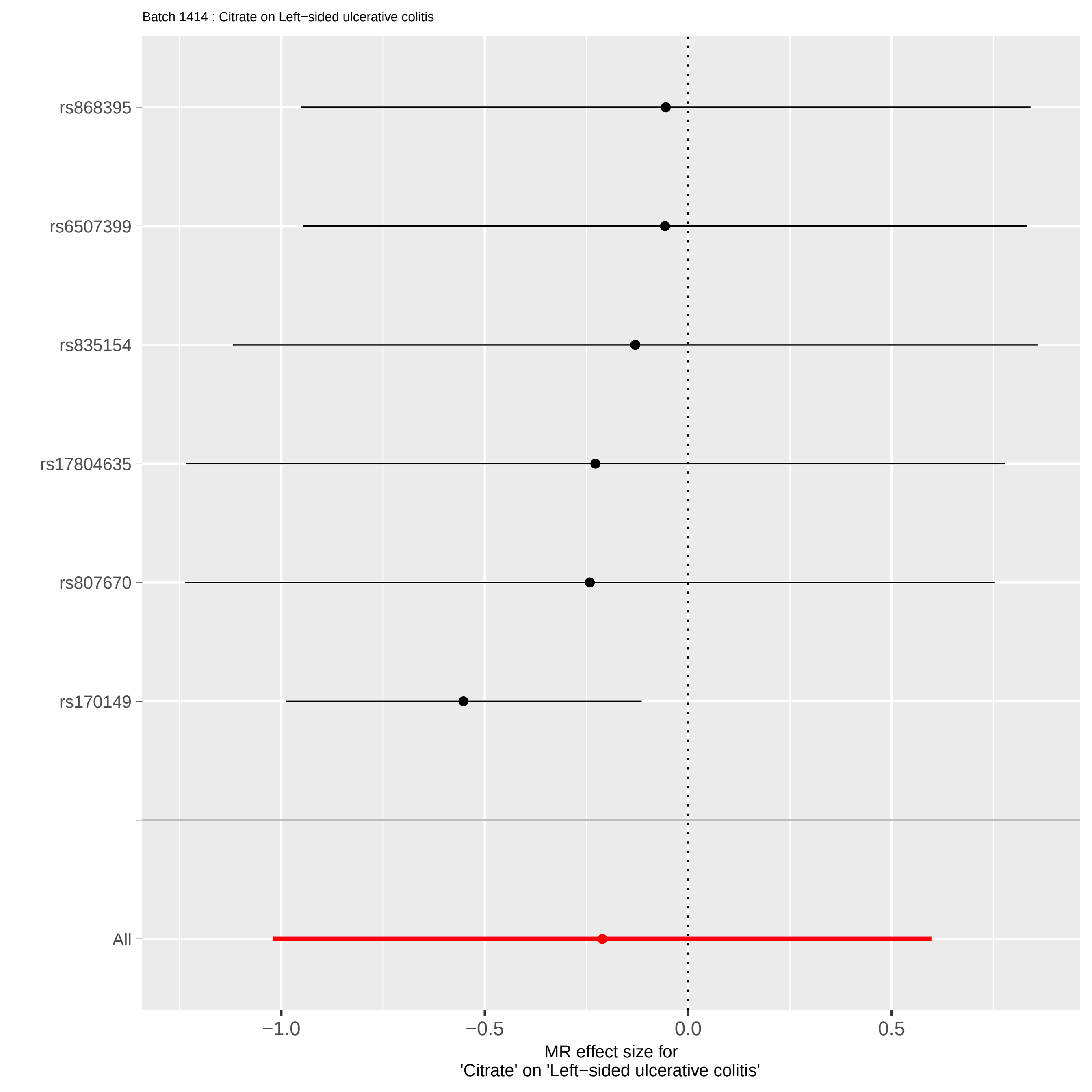

Batch 1414 : Citrate on Left−sided ulcerative colitis
rs868395
rs6507399
rs835154
rs17804635
rs807670
rs170149
All
−1.0
−0.5
0.0
0.5
MR effect size for
'Citrate' on 'Left−sided ulcerative colitis'

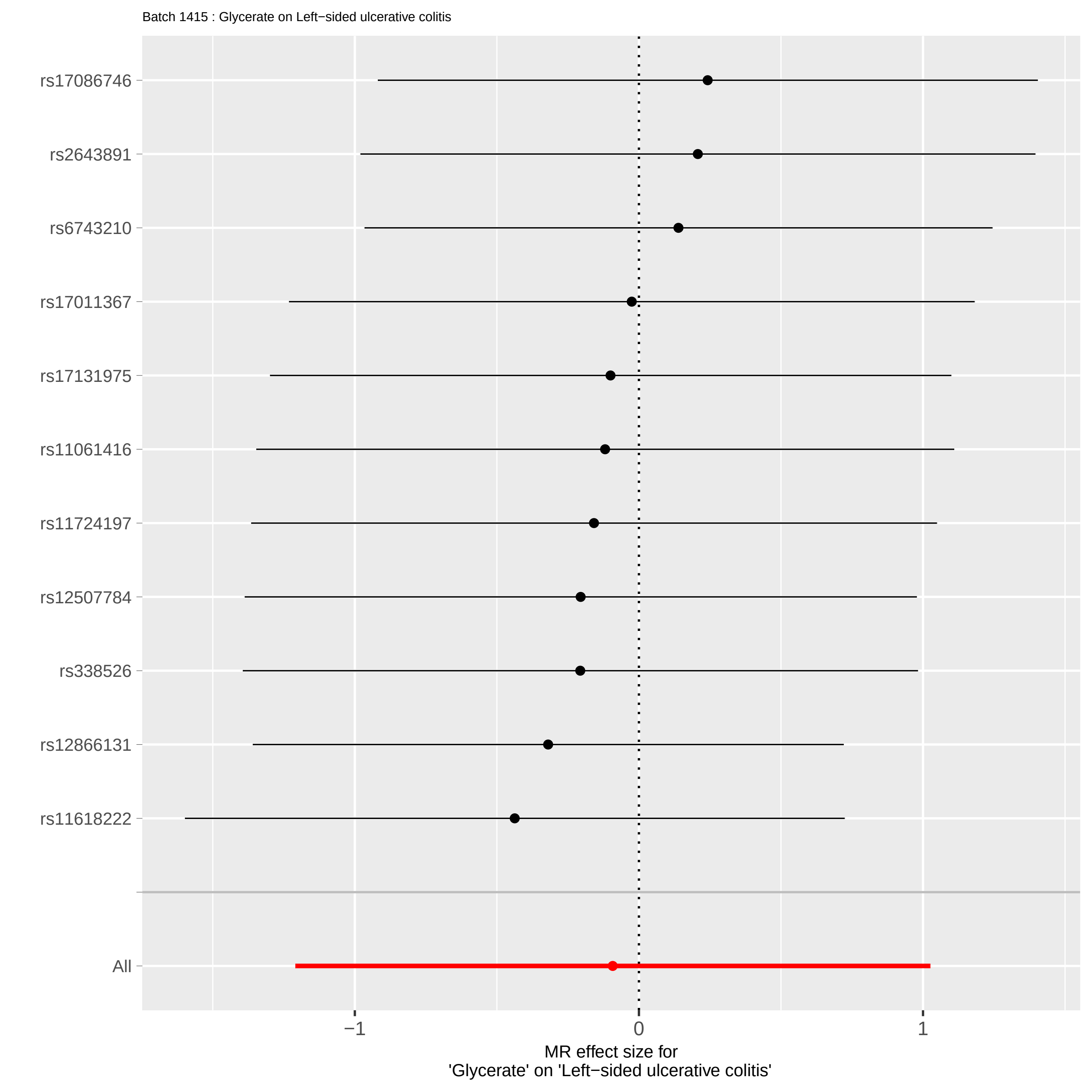
Batch 1415 : Glycerate on Left−sided ulcerative colitis
rs17086746
rs2643891
rs6743210
rs17011367
rs17131975
rs11061416
rs11724197
rs12507784
rs338526
rs12866131
rs11618222
All
−1
0
1
MR effect size for
'Glycerate' on 'Left−sided ulcerative colitis'

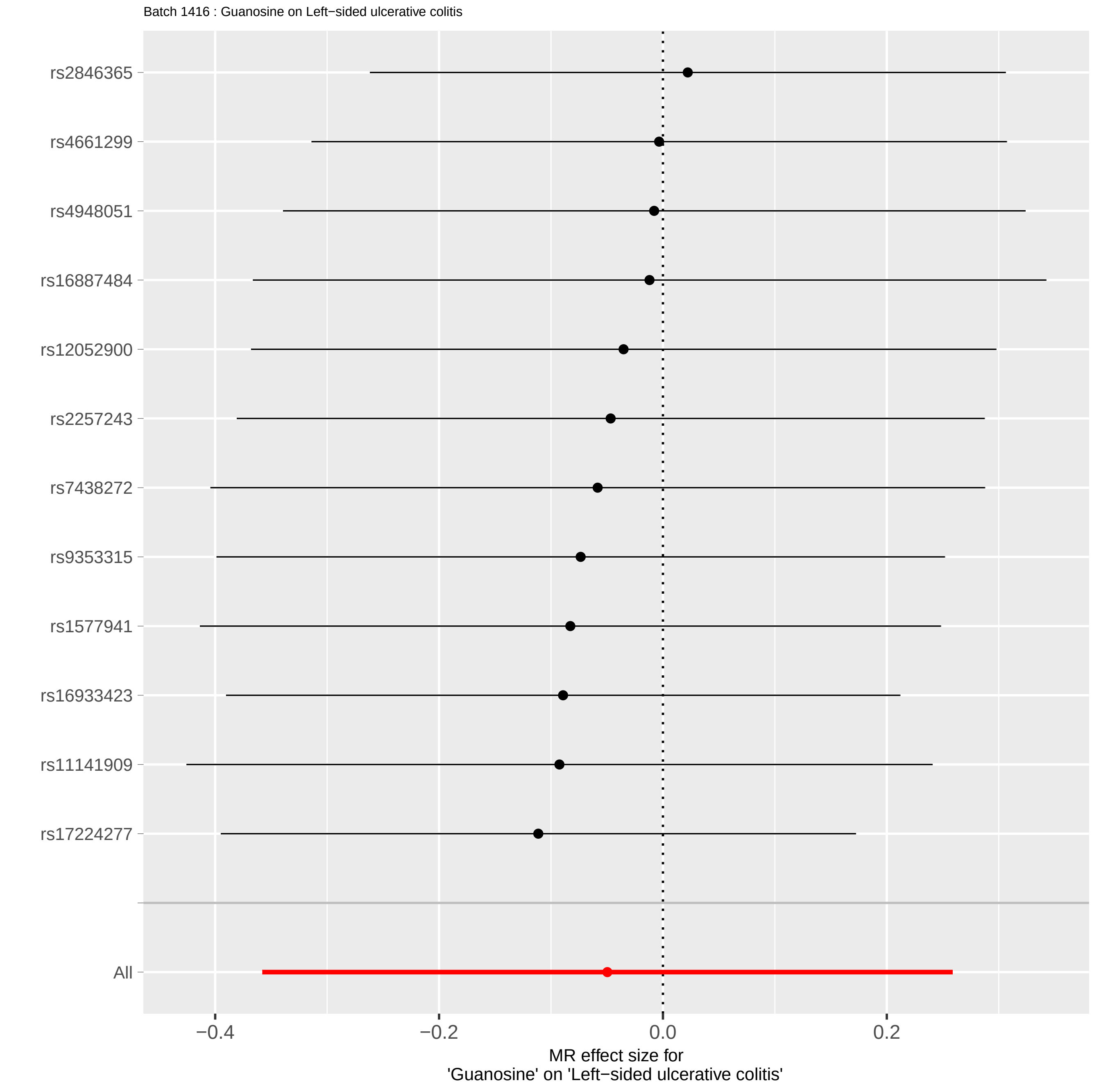

Batch 1416 : Guanosine on Left−sided ulcerative colitis
rs2846365
rs4661299
rs4948051
rs16887484
rs12052900
rs2257243
rs7438272
rs9353315
rs1577941
rs16933423
rs11141909
rs17224277
All
−0.4
−0.2
0.0
0.2
MR effect size for
'Guanosine' on 'Left−sided ulcerative colitis'

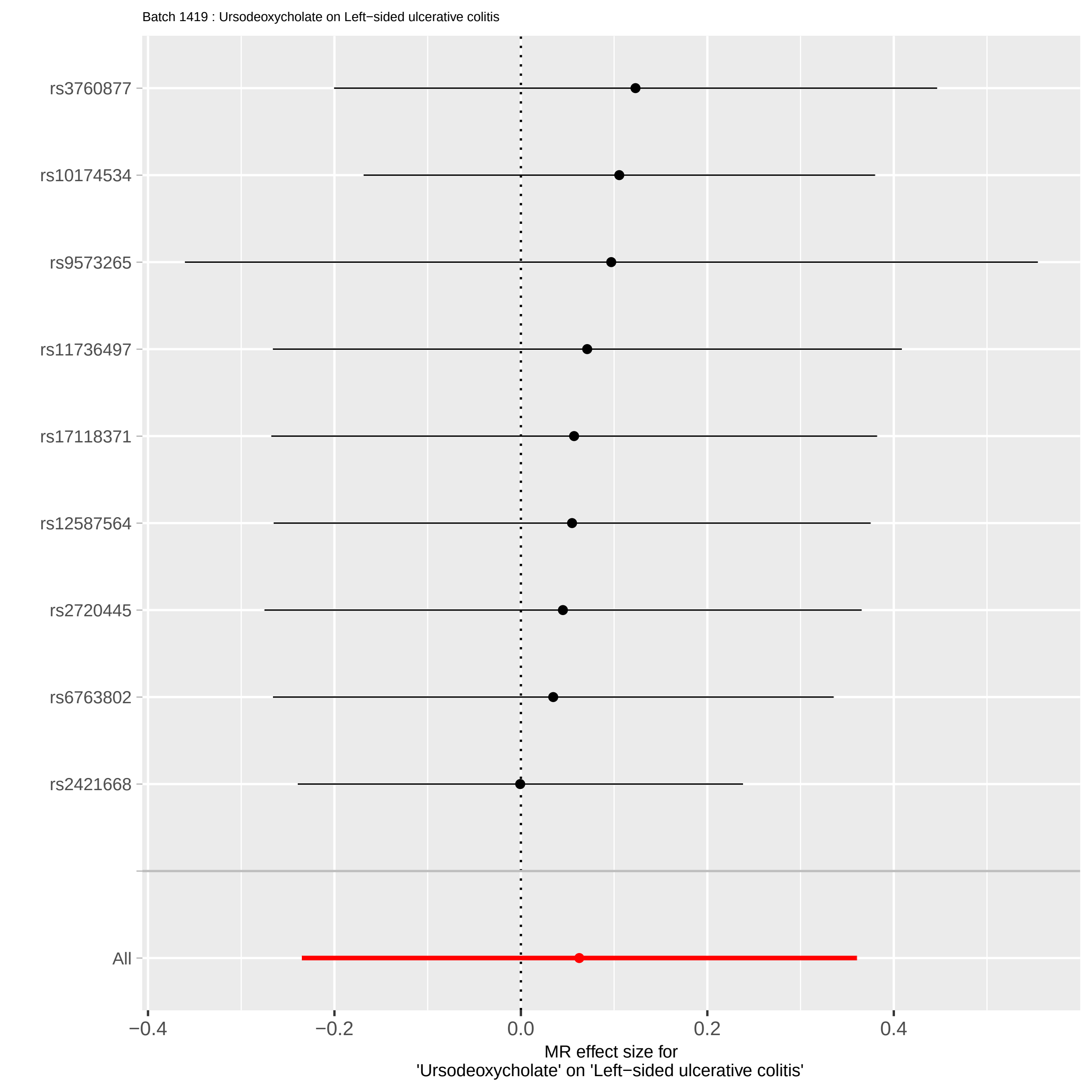

Batch 1419 : Ursodeoxycholate on Left−sided ulcerative colitis
rs3760877
rs10174534
rs9573265
rs11736497
rs17118371
rs12587564
rs2720445
rs6763802
rs2421668
All
−0.4
−0.2
0.0
0.2
0.4
MR effect size for
'Ursodeoxycholate' on 'Left−sided ulcerative colitis'

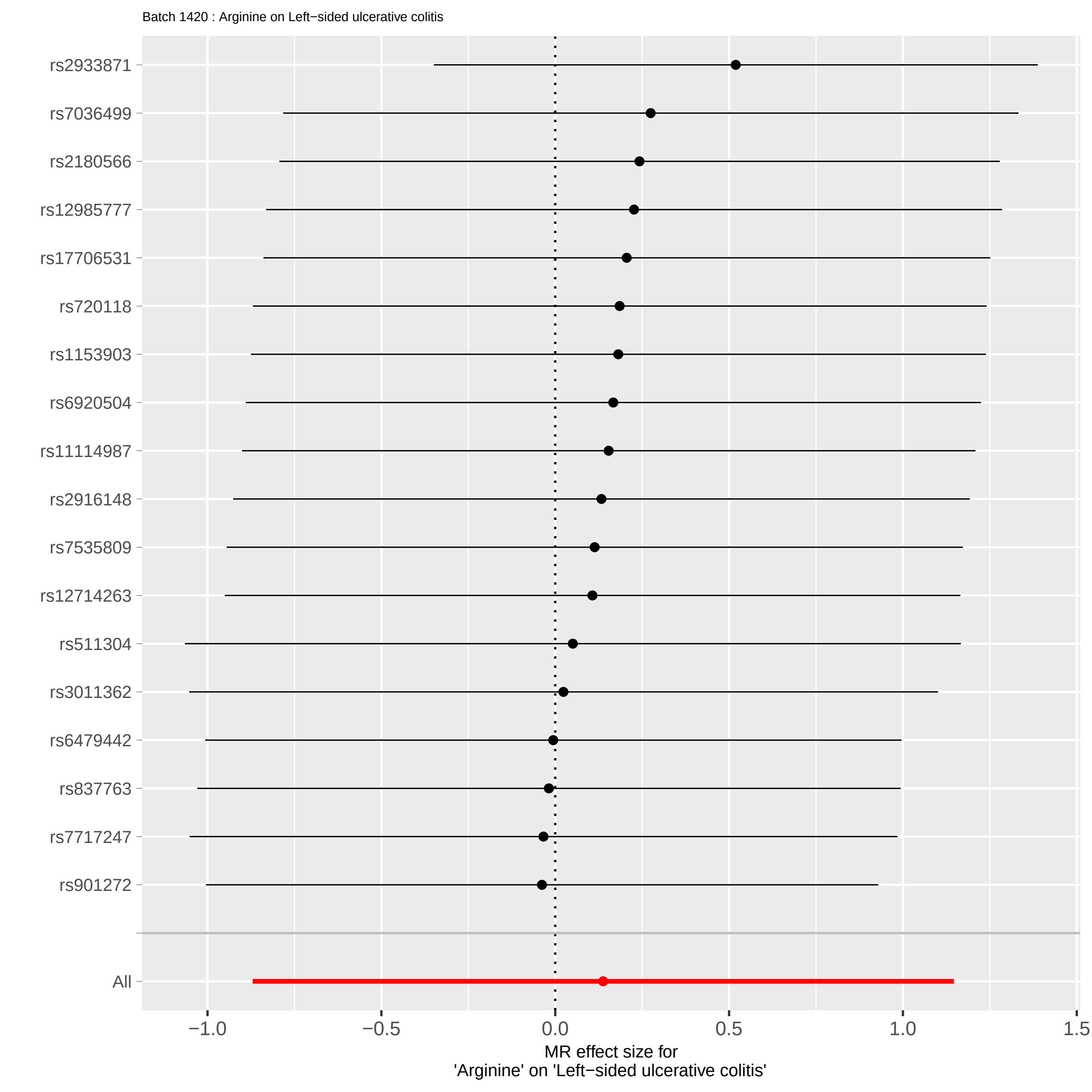

Batch 1420 : Arginine on Left−sided ulcerative colitis
rs2933871
rs7036499
rs2180566
rs12985777
rs17706531
rs720118
rs1153903
rs6920504
rs11114987
rs2916148
rs7535809
rs12714263
rs511304
rs3011362
rs6479442
rs837763
rs7717247
rs901272
All
−1.0
−0.5
0.0
0.5
1.0
1.5
MR effect size for
'Arginine' on 'Left−sided ulcerative colitis'

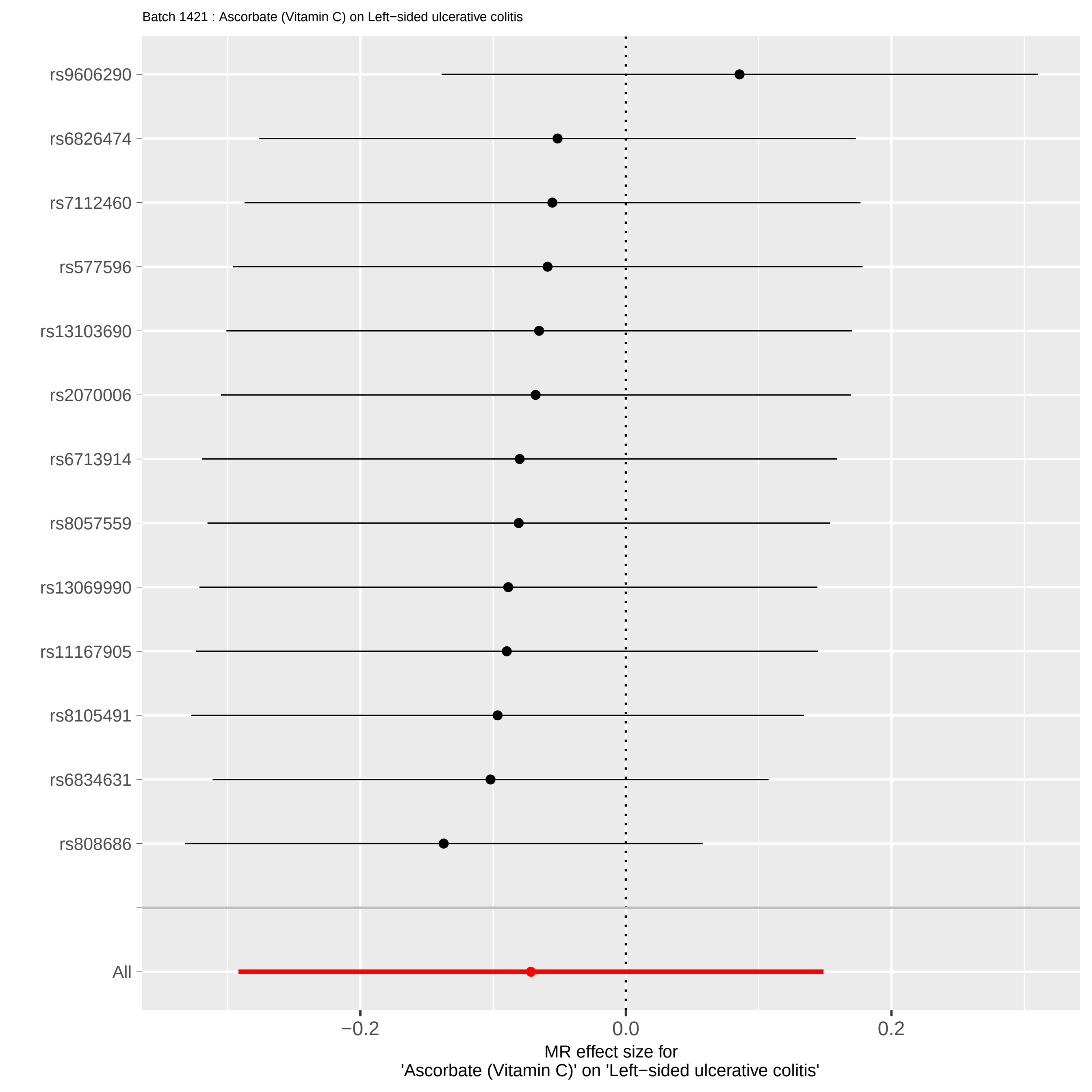
Batch 1421 : Ascorbate (Vitamin C) on Left−sided ulcerative colitis
rs9606290
rs6826474
rs7112460
rs577596
rs13103690
rs2070006
rs6713914
rs8057559
rs13069990
rs11167905
rs8105491
rs6834631
rs808686
All
−0.2
0.0
0.2
MR effect size for
'Ascorbate (Vitamin C)' on 'Left−sided ulcerative colitis'

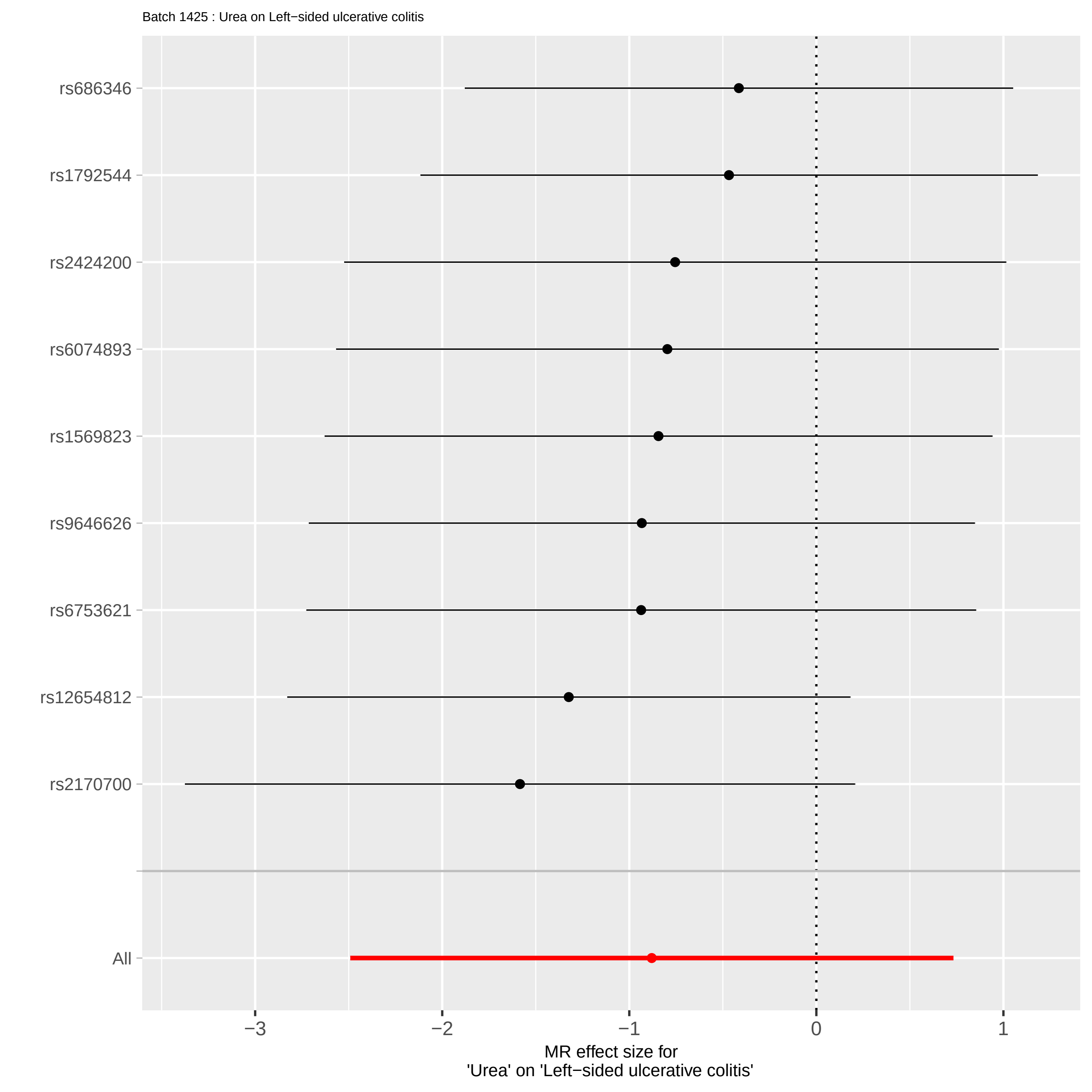
Batch 1425 : Urea on Left−sided ulcerative colitis
rs686346
rs1792544
rs2424200
rs6074893
rs1569823
rs9646626
rs6753621
rs12654812
rs2170700
All
−3
−2
−1
0
1
MR effect size for
'Urea' on 'Left−sided ulcerative colitis'

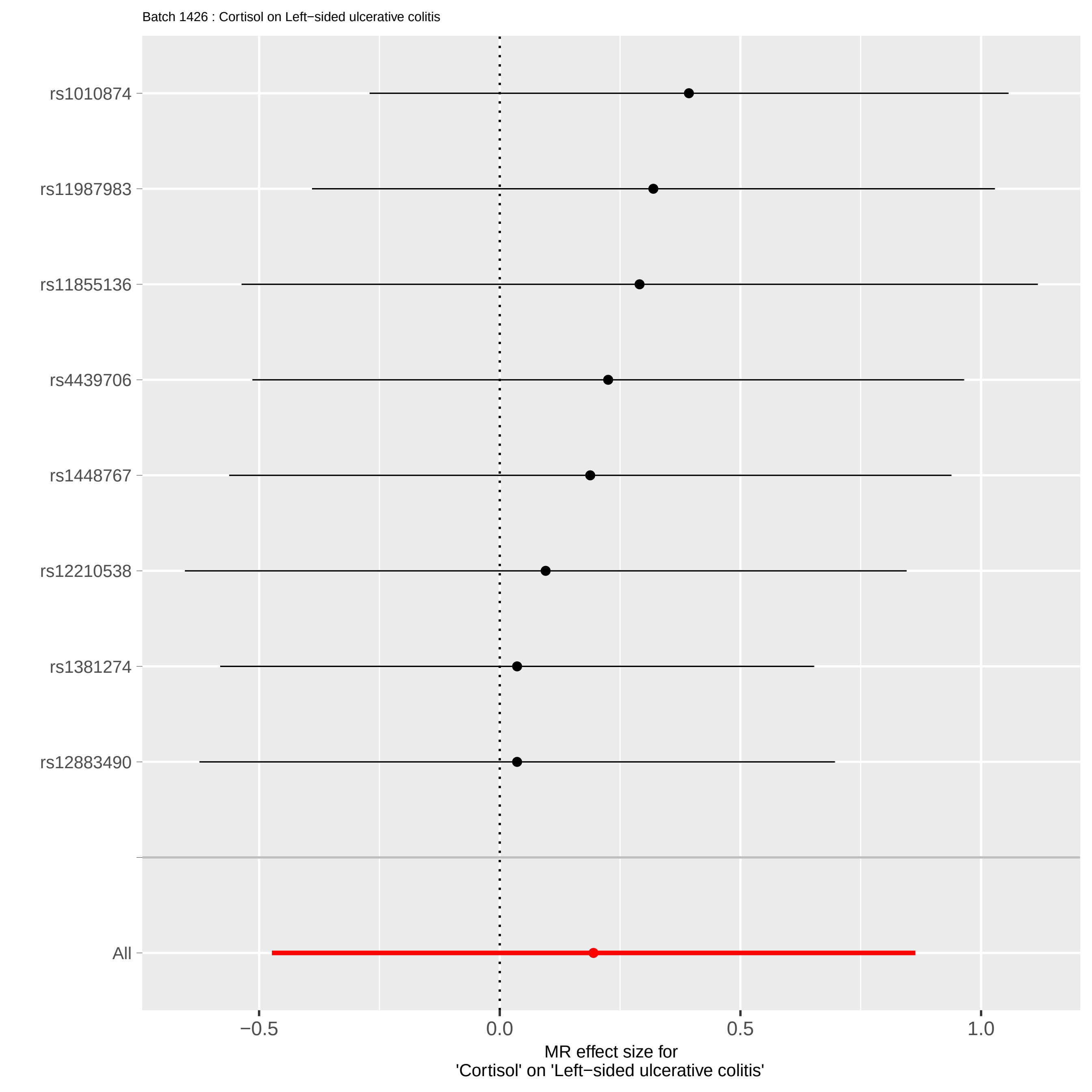

Batch 1426 : Cortisol on Left−sided ulcerative colitis
rs1010874
rs11987983
rs11855136
rs4439706
rs1448767
rs12210538
rs1381274
rs12883490
All
−0.5
0.0
0.5
1.0
MR effect size for
'Cortisol' on 'Left−sided ulcerative colitis'

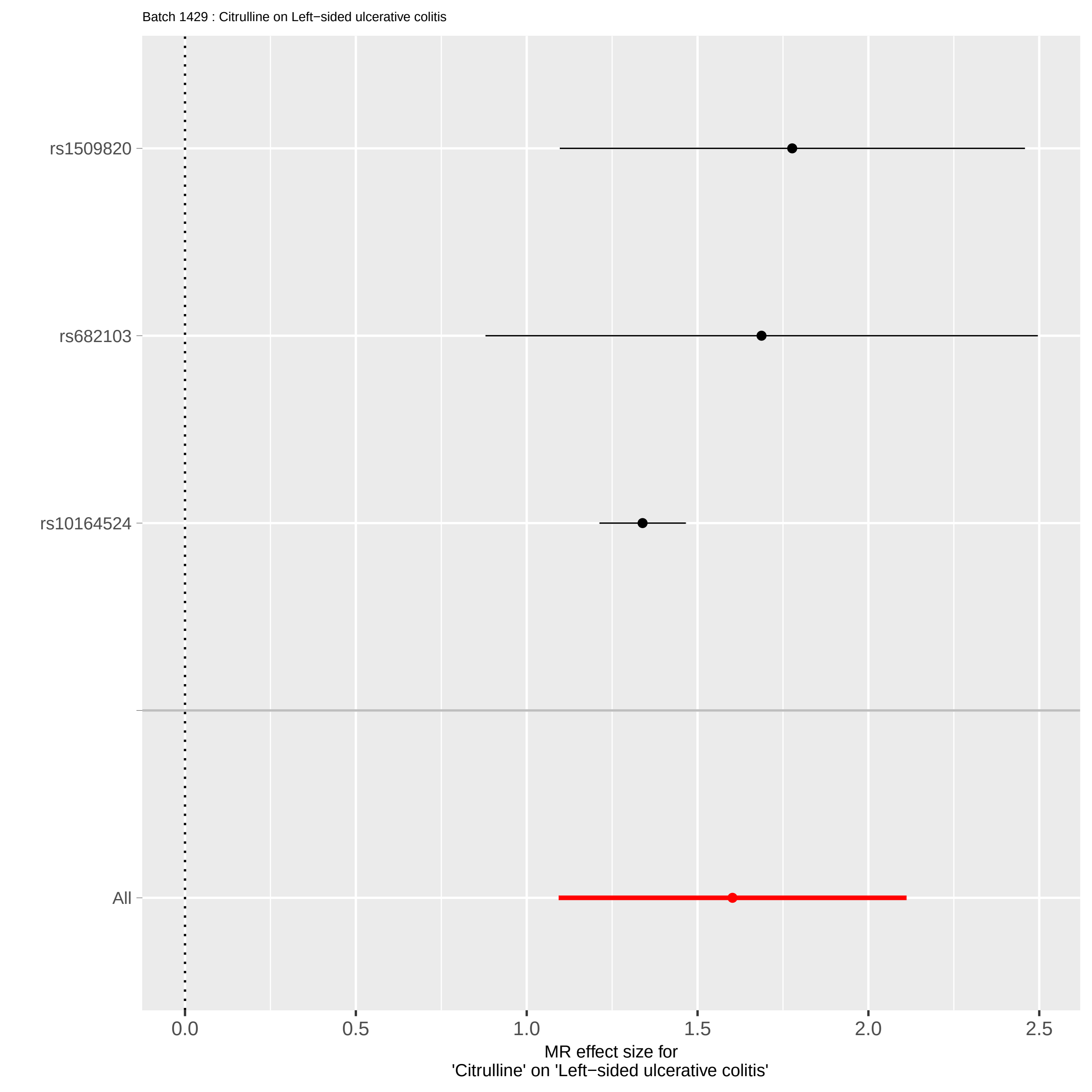

Batch 1429 : Citrulline on Left−sided ulcerative colitis
rs1509820
rs682103
rs10164524
All
0.0
0.5
1.0
1.5
2.0
2.5
MR effect size for
'Citrulline' on 'Left−sided ulcerative colitis'

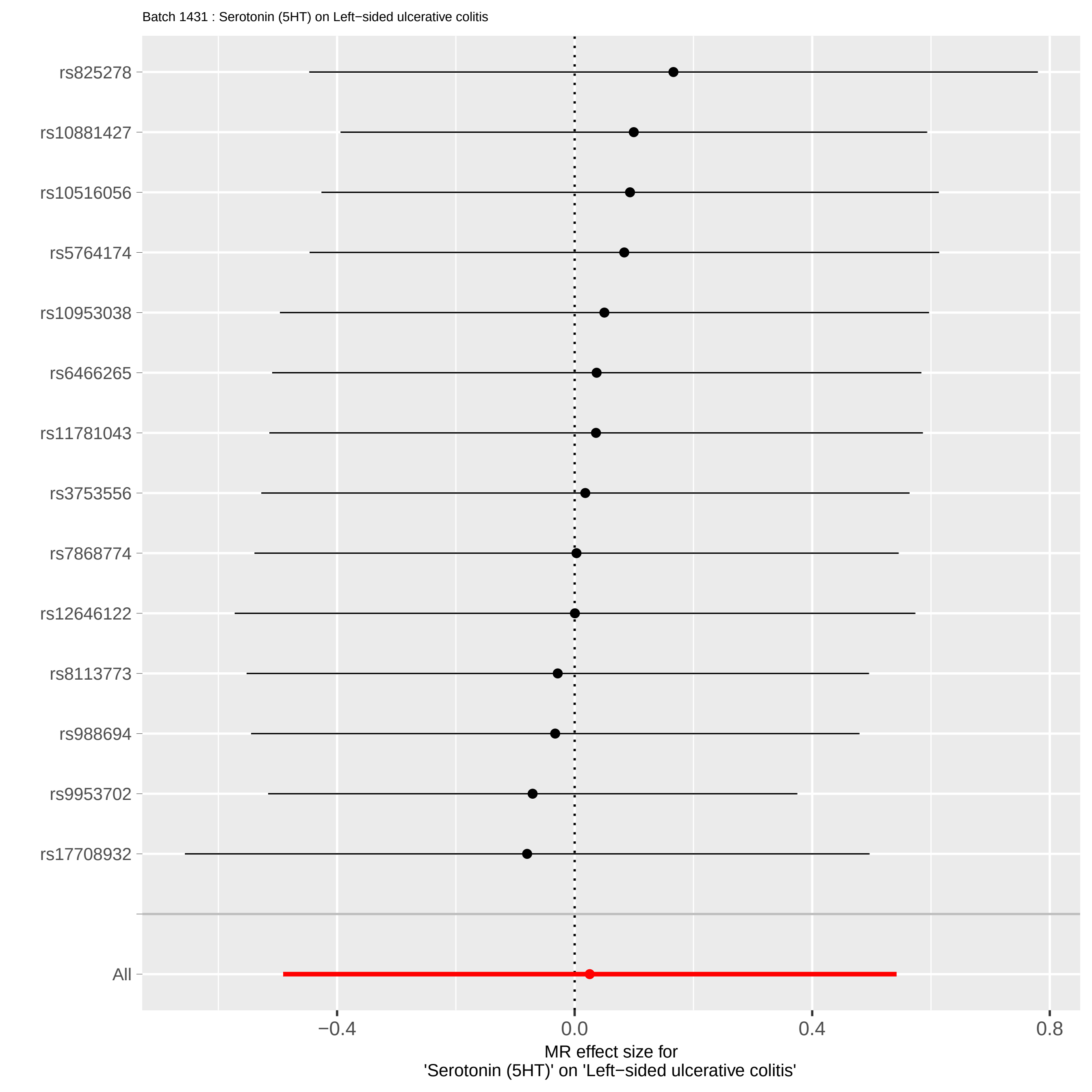

Batch 1431 : Serotonin (5HT) on Left−sided ulcerative colitis
rs825278
rs10881427
rs10516056
rs5764174
rs10953038
rs6466265
rs11781043
rs3753556
rs7868774
rs12646122
rs8113773
rs988694
rs9953702
rs17708932
All
−0.4
0.0
0.4
0.8
MR effect size for
'Serotonin (5HT)' on 'Left−sided ulcerative colitis'

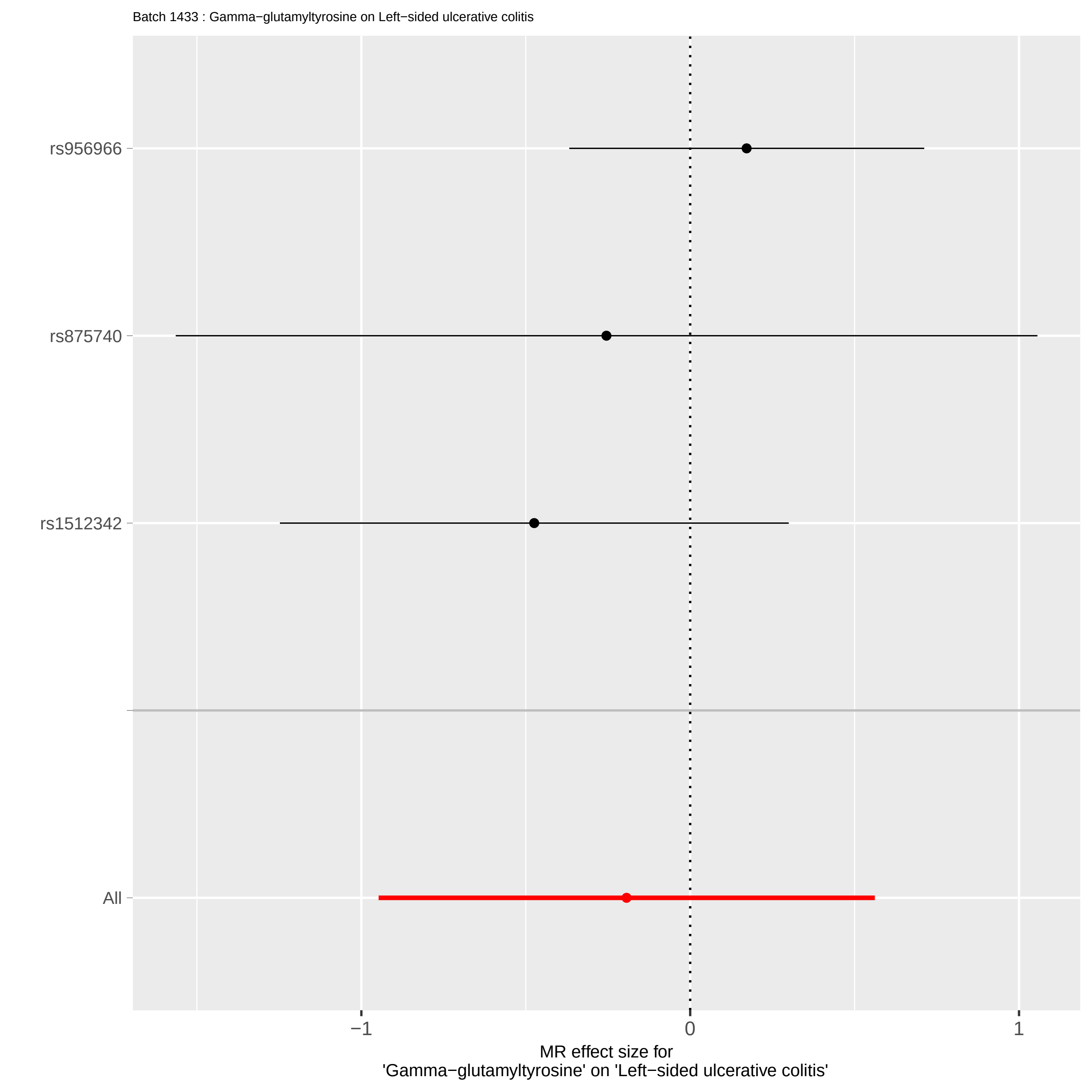

Batch 1433 : Gamma−glutamyltyrosine on Left−sided ulcerative colitis
rs956966
rs875740
rs1512342
All
−1
0
1
MR effect size for
'Gamma−glutamyltyrosine' on 'Left−sided ulcerative colitis'

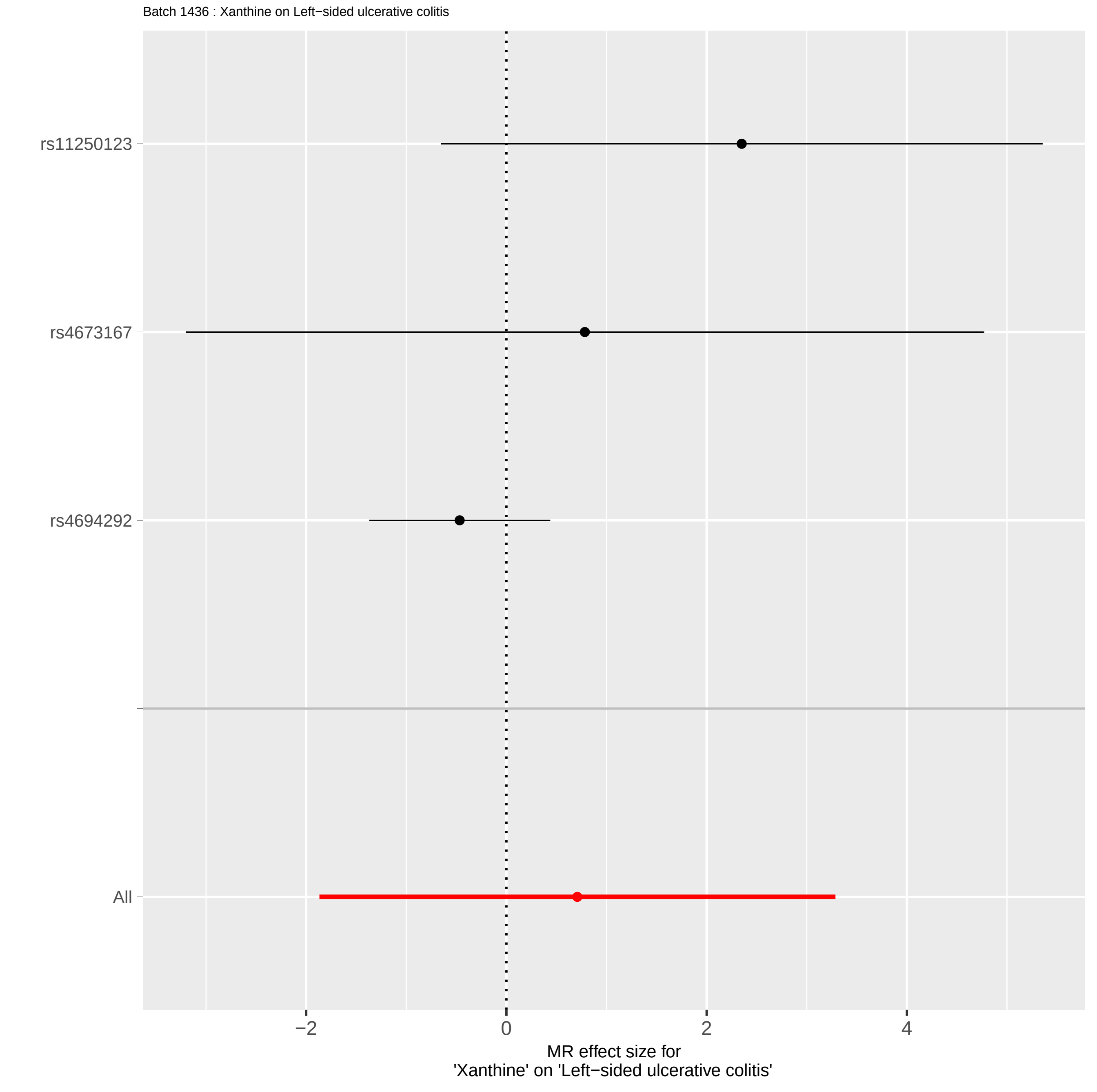

Batch 1436 : Xanthine on Left−sided ulcerative colitis
rs11250123
rs4673167
rs4694292
All
−2
0
2
4
MR effect size for
'Xanthine' on 'Left−sided ulcerative colitis'

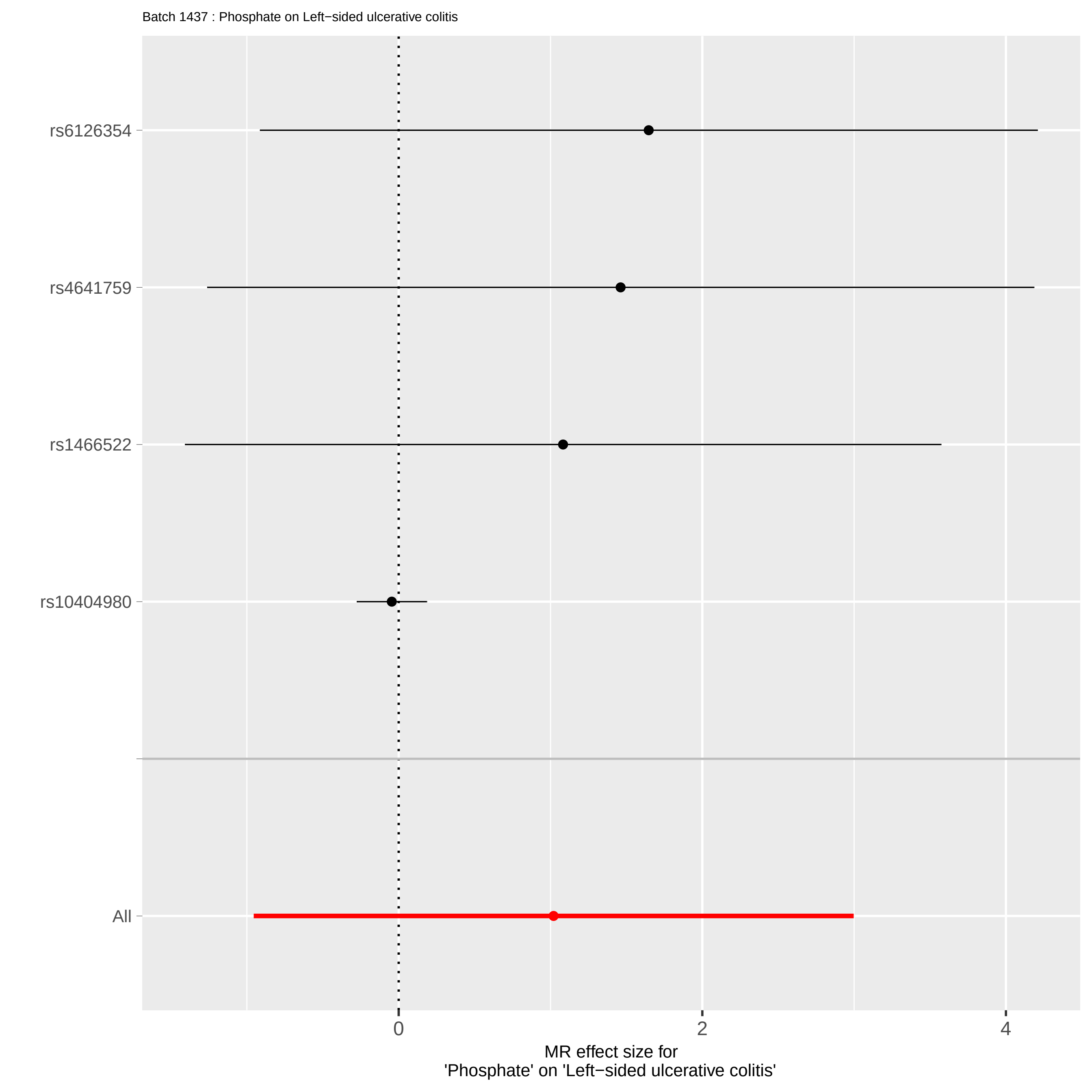

Batch 1437 : Phosphate on Left−sided ulcerative colitis
rs6126354
rs4641759
rs1466522
rs10404980
All
0
2
4
MR effect size for
'Phosphate' on 'Left−sided ulcerative colitis'

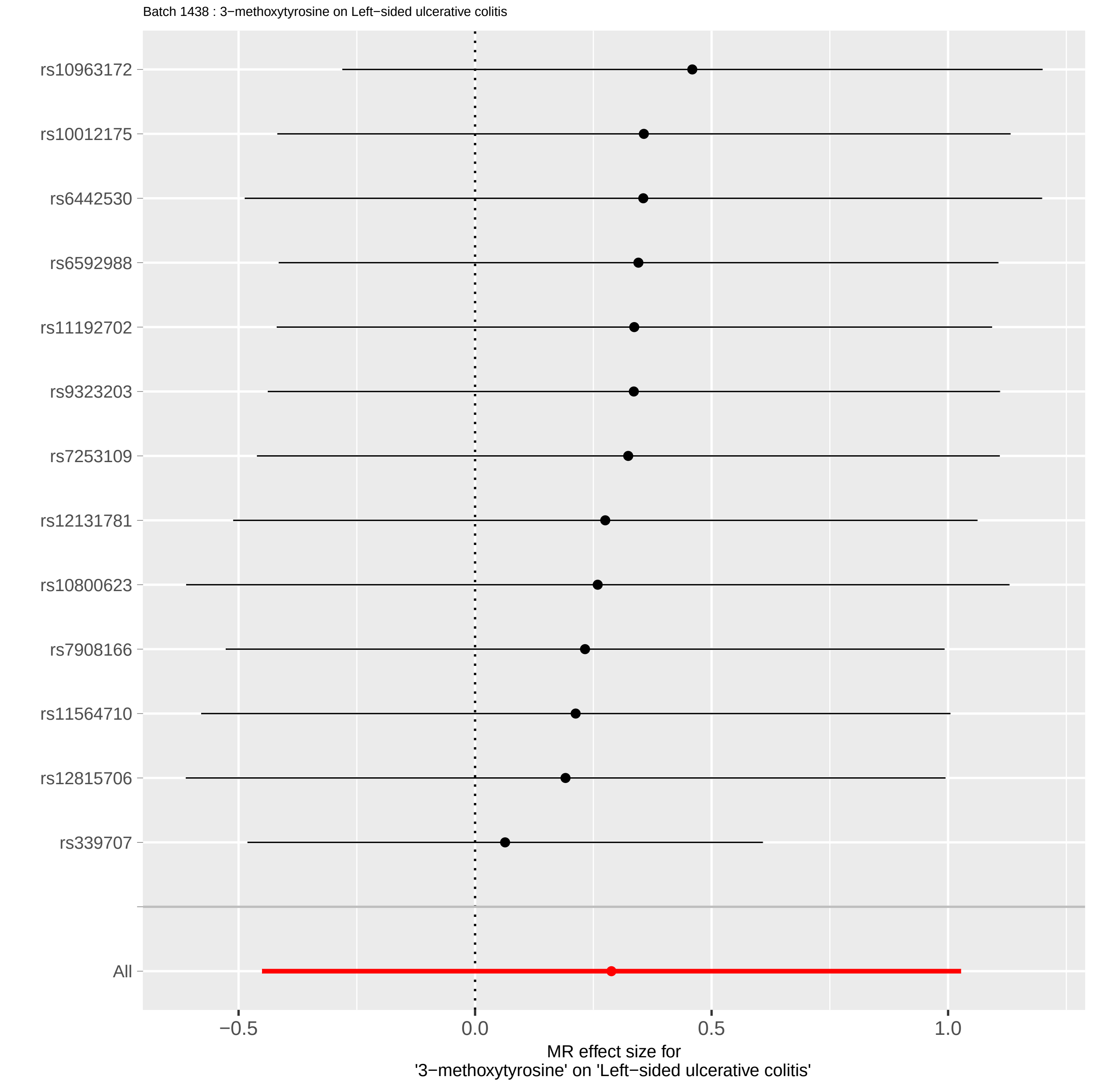

Batch 1438 : 3−methoxytyrosine on Left−sided ulcerative colitis
rs10963172
rs10012175
rs6442530
rs6592988
rs11192702
rs9323203
rs7253109
rs12131781
rs10800623
rs7908166
rs11564710
rs12815706
rs339707
All
−0.5
0.0
0.5
1.0
MR effect size for
'3−methoxytyrosine' on 'Left−sided ulcerative colitis'

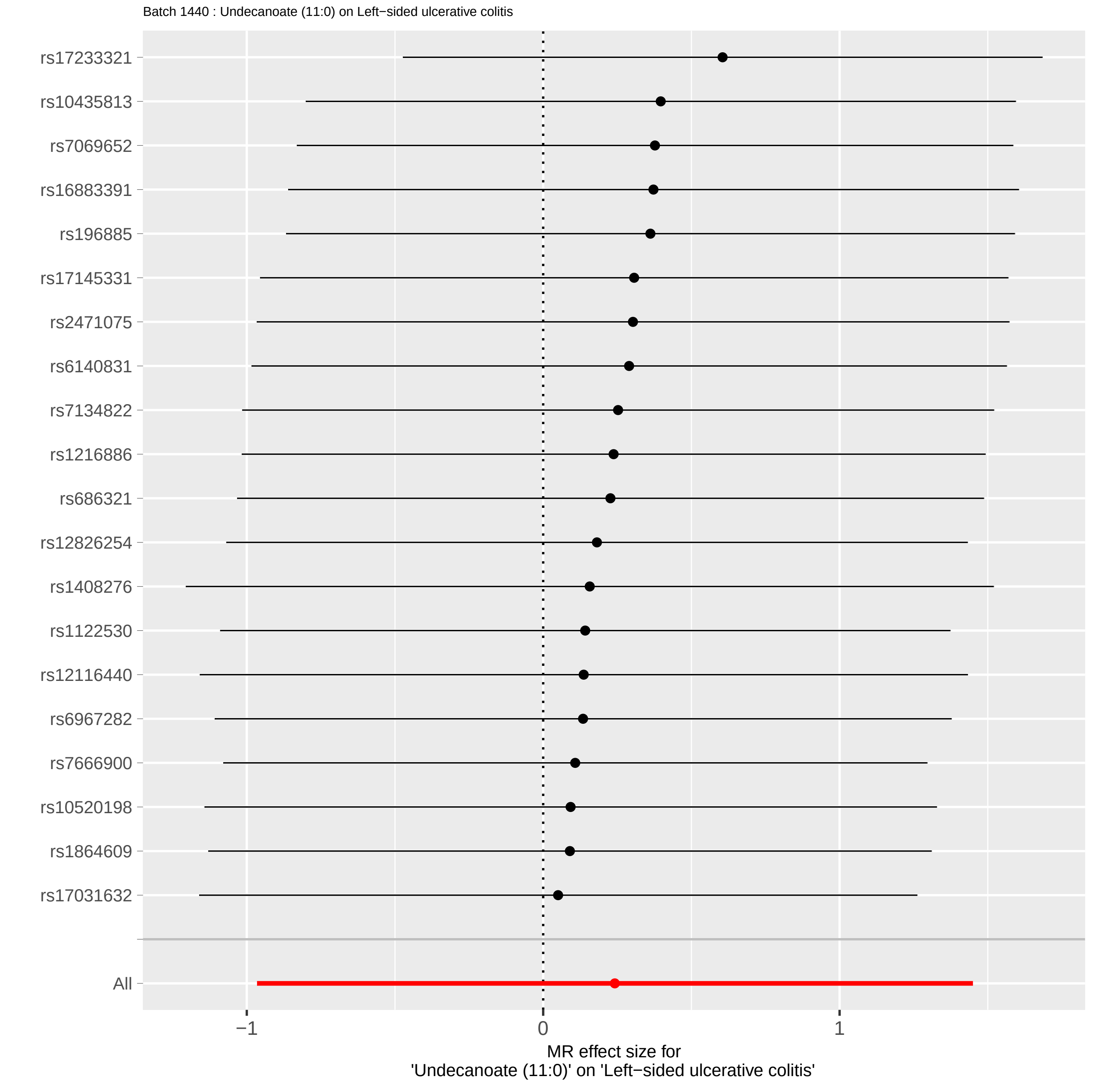

Batch 1440 : Undecanoate (11:0) on Left−sided ulcerative colitis
rs17233321
rs10435813
rs7069652
rs16883391
rs196885
rs17145331
rs2471075
rs6140831
rs7134822
rs1216886
rs686321
rs12826254
rs1408276
rs1122530
rs12116440
rs6967282
rs7666900
rs10520198
rs1864609
rs17031632
All
−1
0
1
MR effect size for
'Undecanoate (11:0)' on 'Left−sided ulcerative colitis'

Batch 1442 : Taurodeoxycholate on Left−sided ulcerative colitis

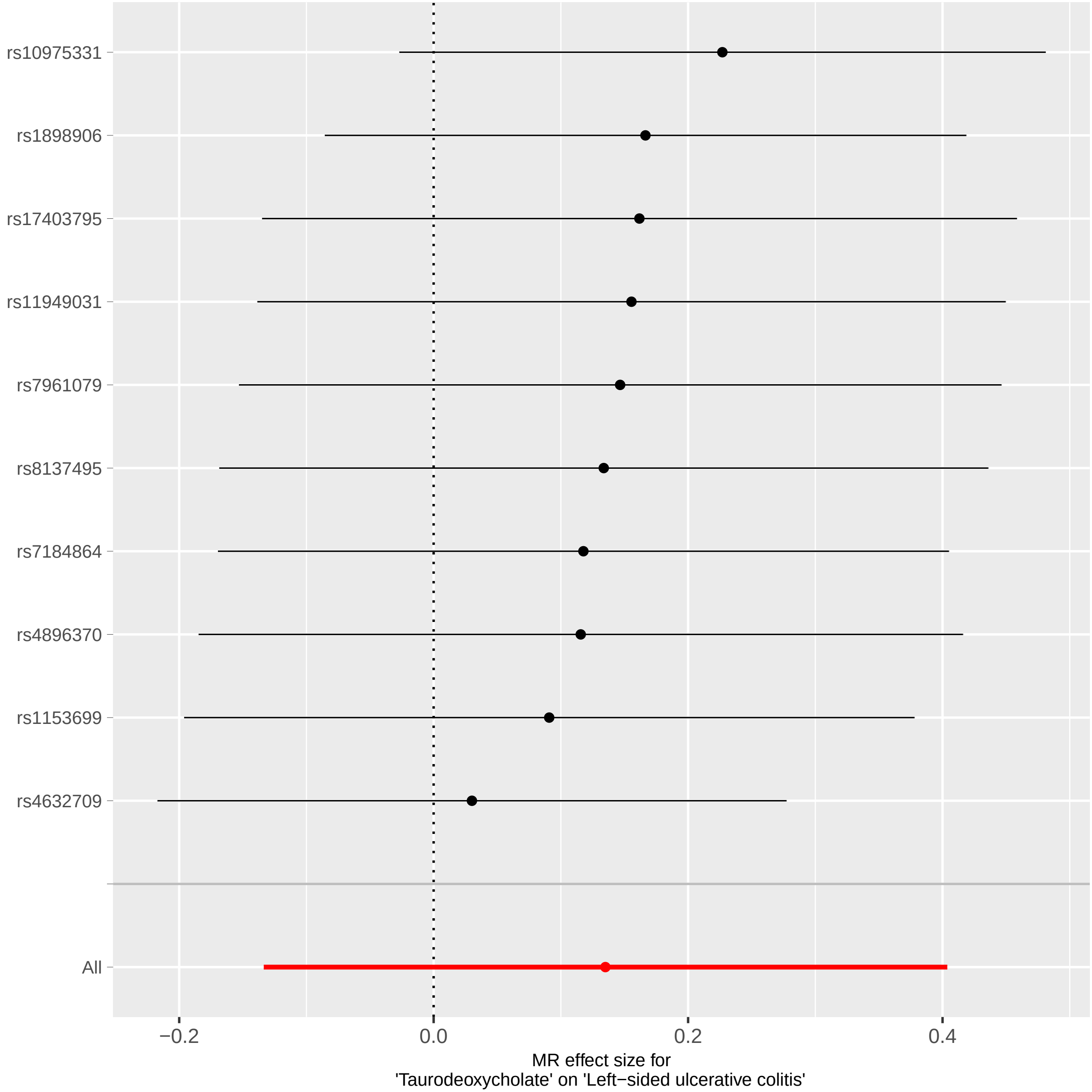

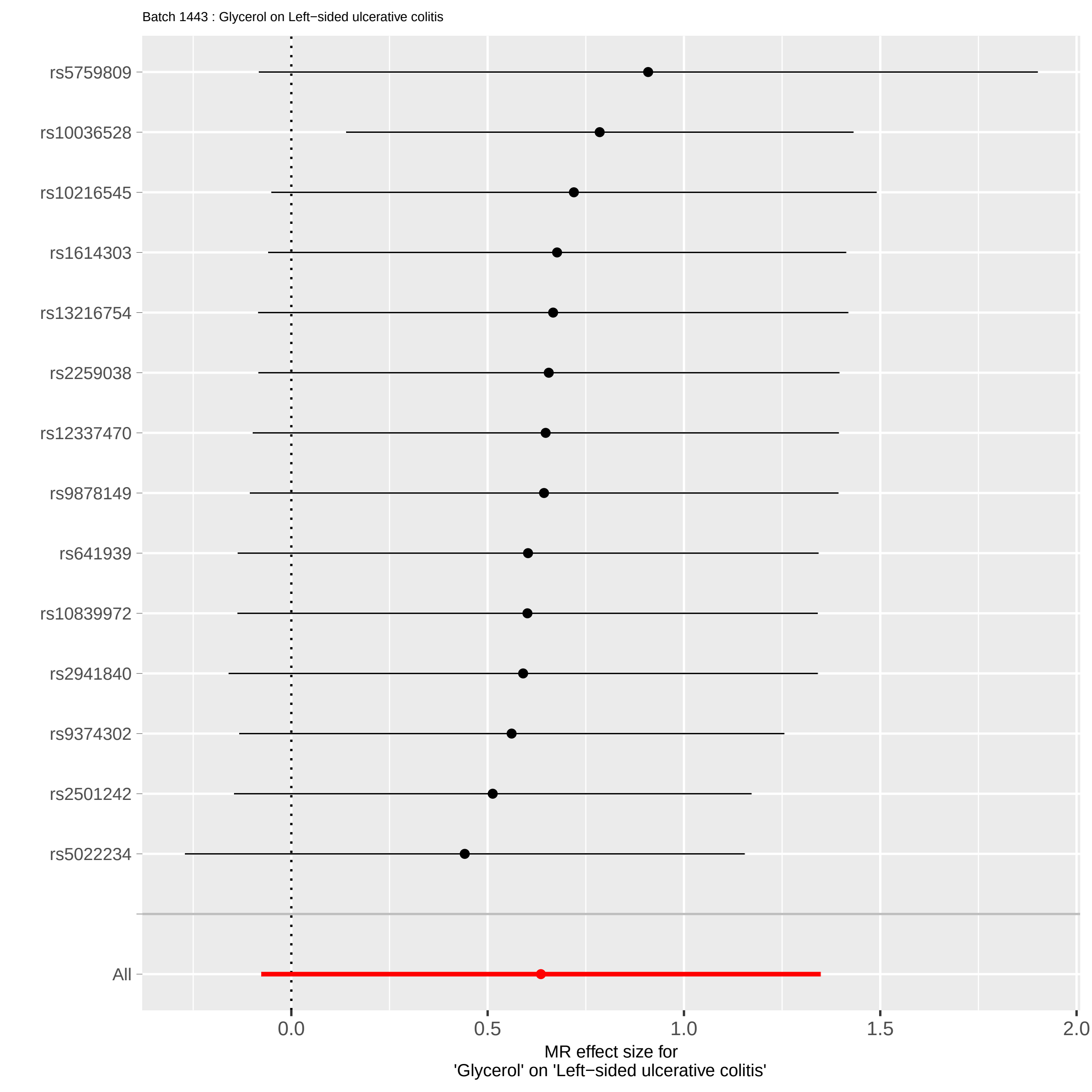

Batch 1443 : Glycerol on Left−sided ulcerative colitis
rs5759809
rs10036528
rs10216545
rs1614303
rs13216754
rs2259038
rs12337470
rs9878149
rs641939
rs10839972
rs2941840
rs9374302
rs2501242
rs5022234
All
0.0
0.5
1.0
1.5
2.0
MR effect size for
'Glycerol' on 'Left−sided ulcerative colitis'

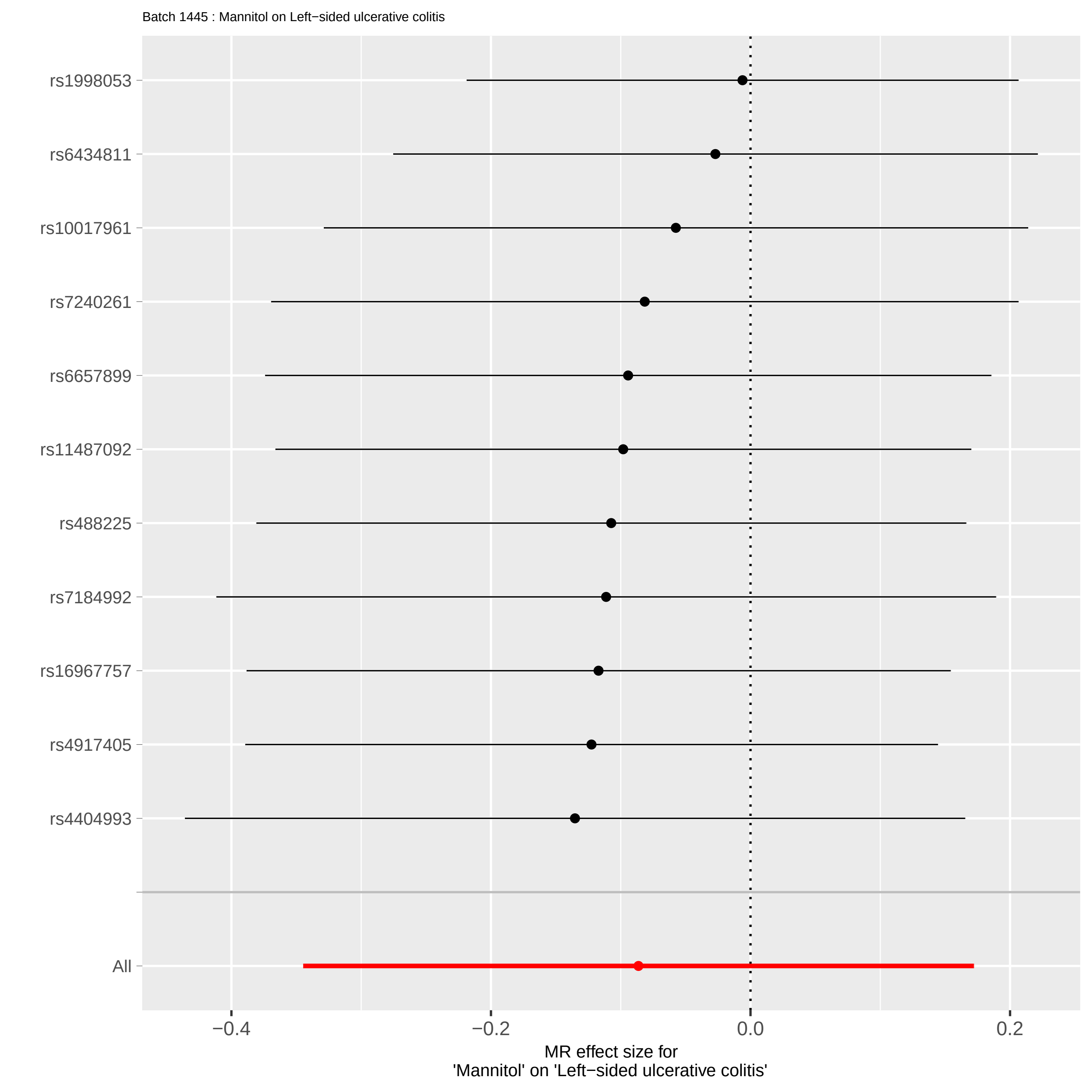
Batch 1445 : Mannitol on Left−sided ulcerative colitis
rs1998053
rs6434811
rs10017961
rs7240261
rs6657899
rs11487092
rs488225
rs7184992
rs16967757
rs4917405
rs4404993
All
−0.4
−0.2
0.0
0.2
MR effect size for
'Mannitol' on 'Left−sided ulcerative colitis'

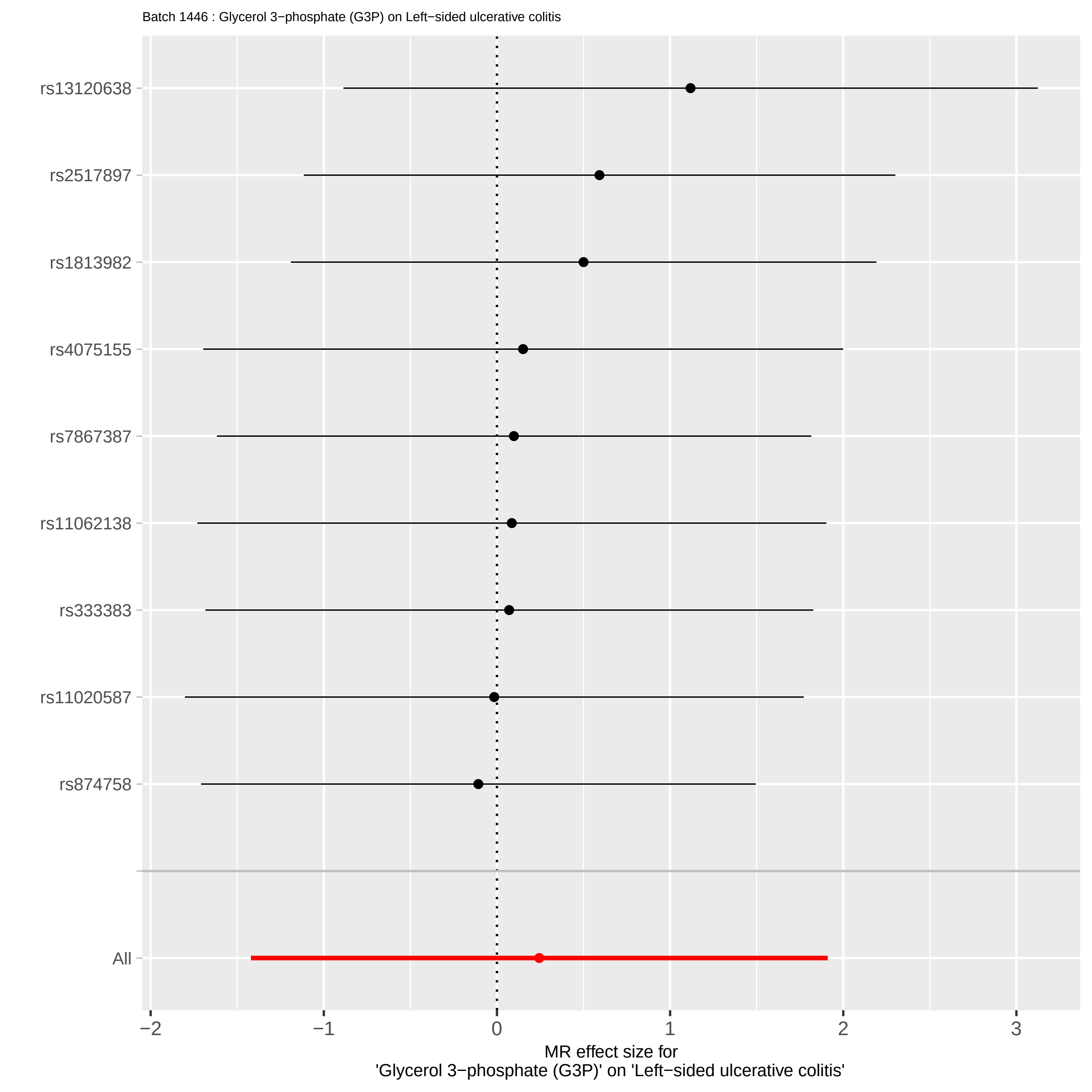

Batch 1446 : Glycerol 3−phosphate (G3P) on Left−sided ulcerative colitis
rs13120638
rs2517897
rs1813982
rs4075155
rs7867387
rs11062138
rs333383
rs11020587
rs874758
All
−2
−1
0
1
2
3
MR effect size for
'Glycerol 3−phosphate (G3P)' on 'Left−sided ulcerative colitis'

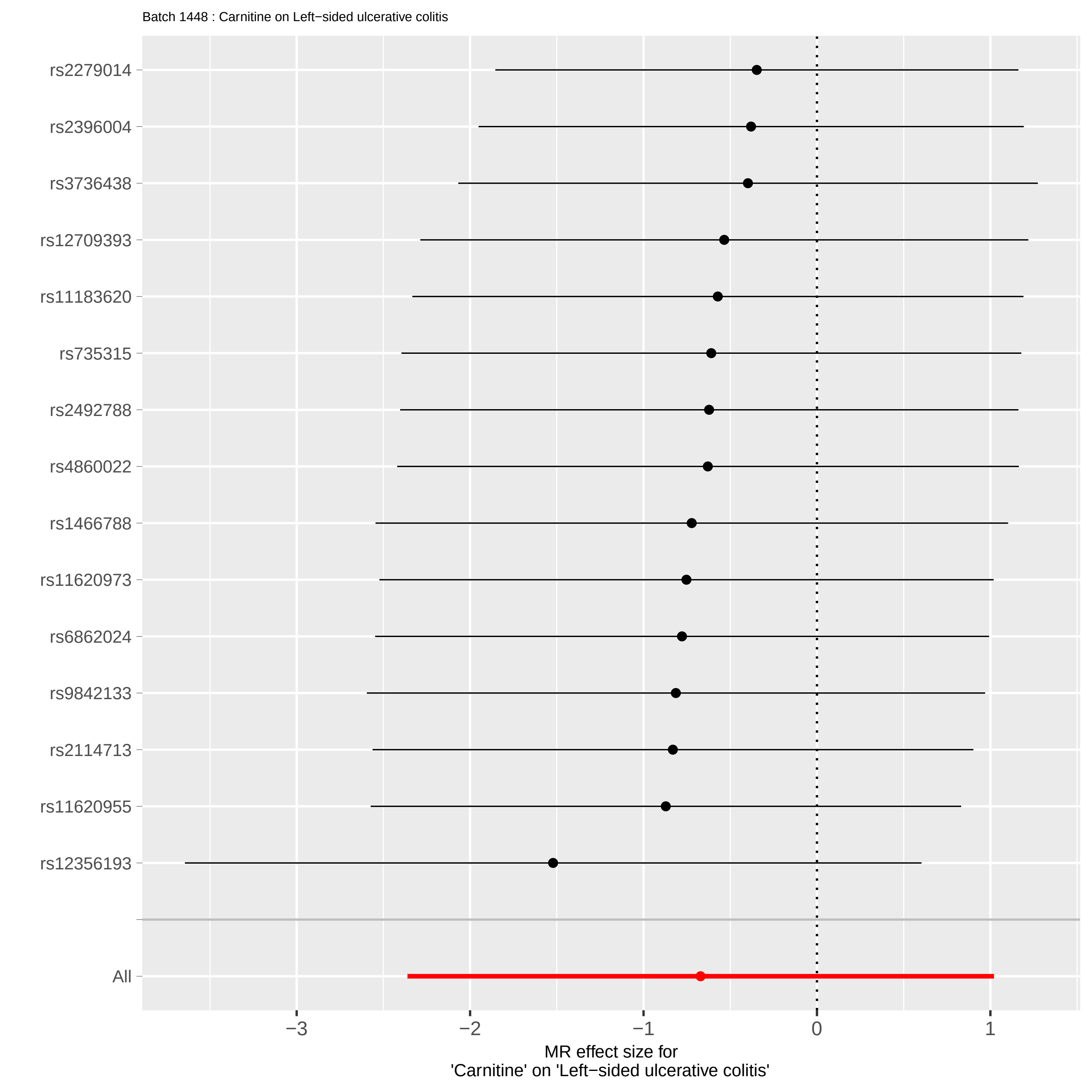

Batch 1448 : Carnitine on Left−sided ulcerative colitis
rs2279014
rs2396004
rs3736438
rs12709393
rs11183620
rs735315
rs2492788
rs4860022
rs1466788
rs11620973
rs6862024
rs9842133
rs2114713
rs11620955
rs12356193
All
−3
−2
−1
0
1
MR effect size for
'Carnitine' on 'Left−sided ulcerative colitis'

Batch 1449 : Choline on Left−sided ulcerative colitis

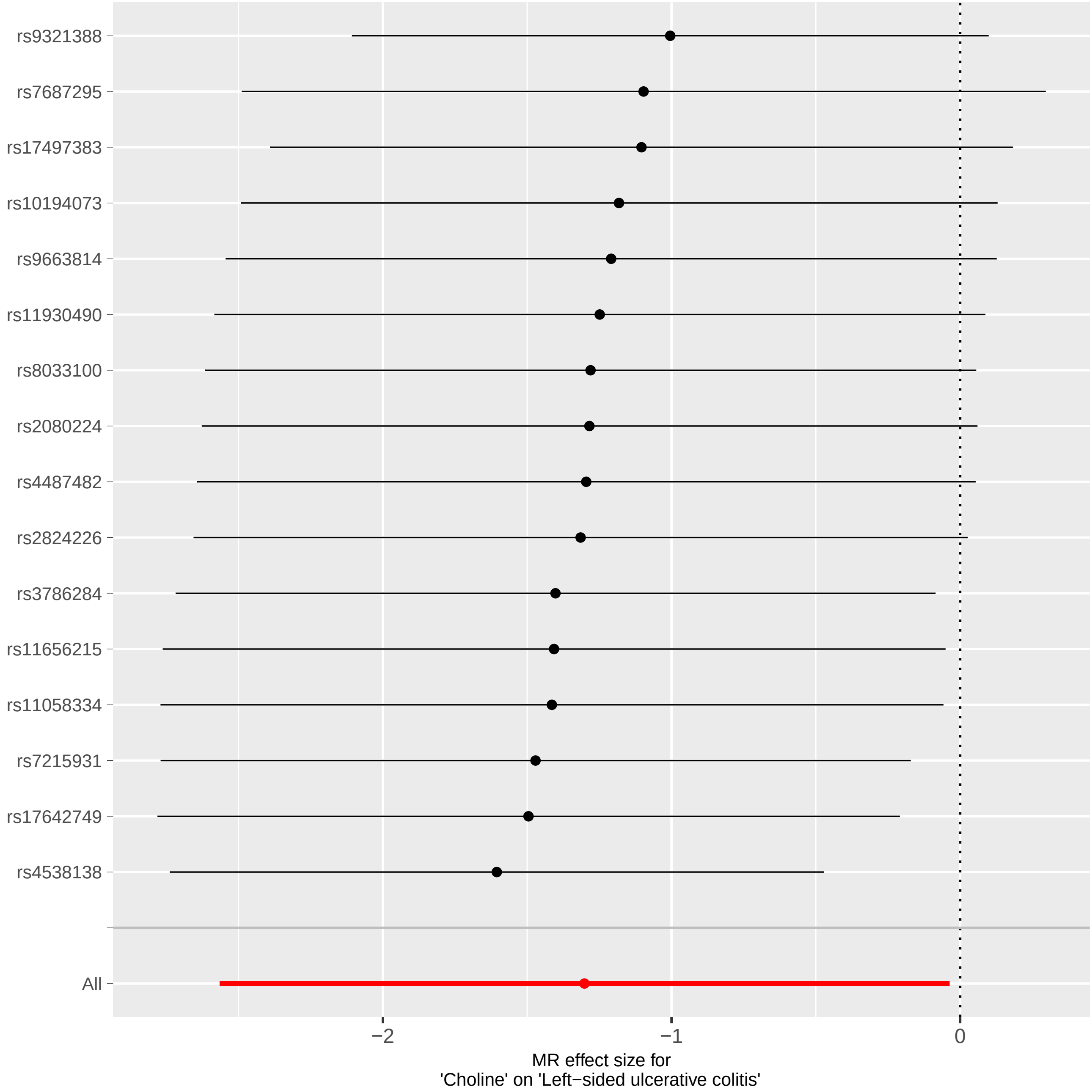

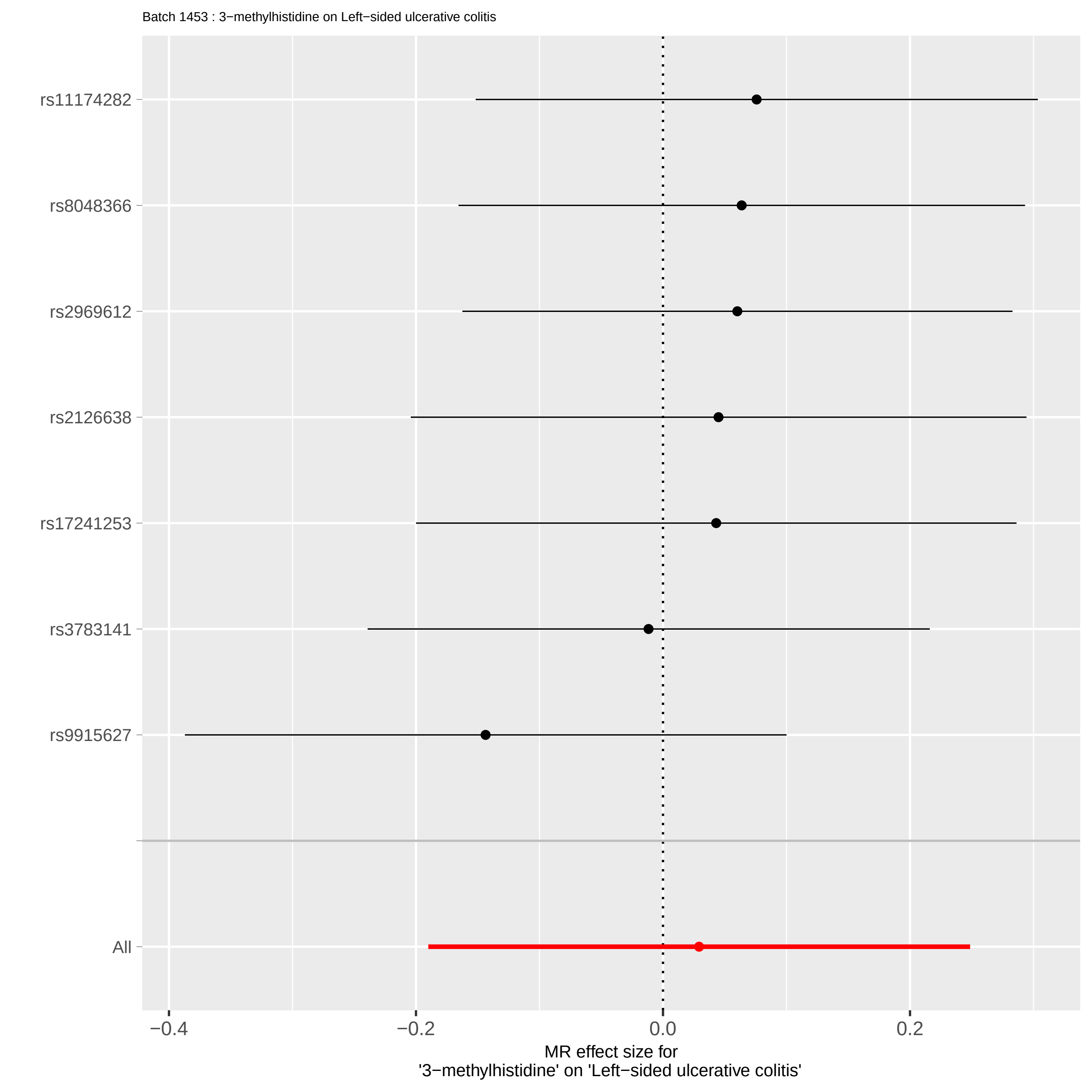

Batch 1453 : 3−methylhistidine on Left−sided ulcerative colitis
rs11174282
rs8048366
rs2969612
rs2126638
rs17241253
rs3783141
rs9915627
All
−0.4
−0.2
0.0
0.2
MR effect size for
'3−methylhistidine' on 'Left−sided ulcerative colitis'

Batch 1455 : Phenylacetate on Left−sided ulcerative colitis

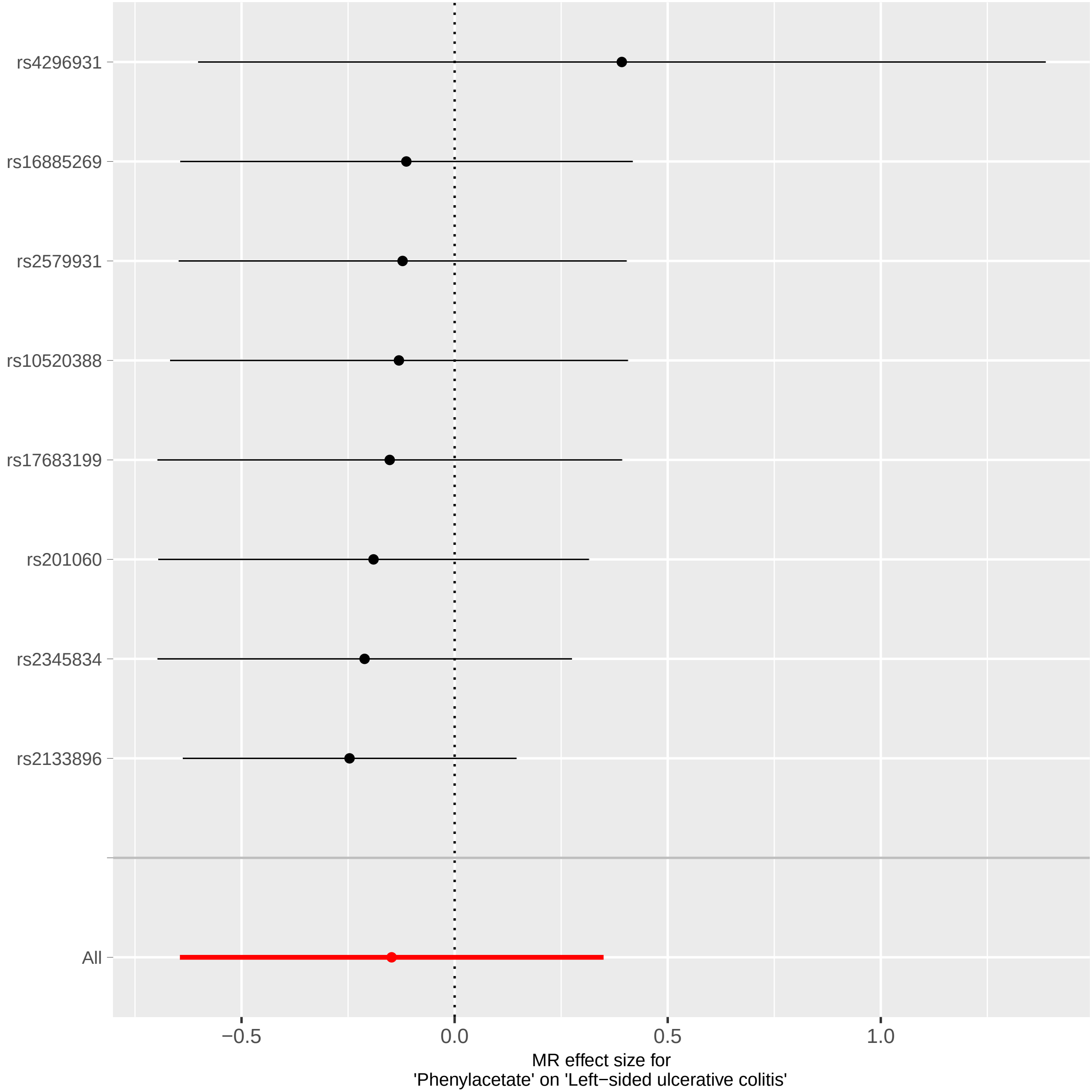

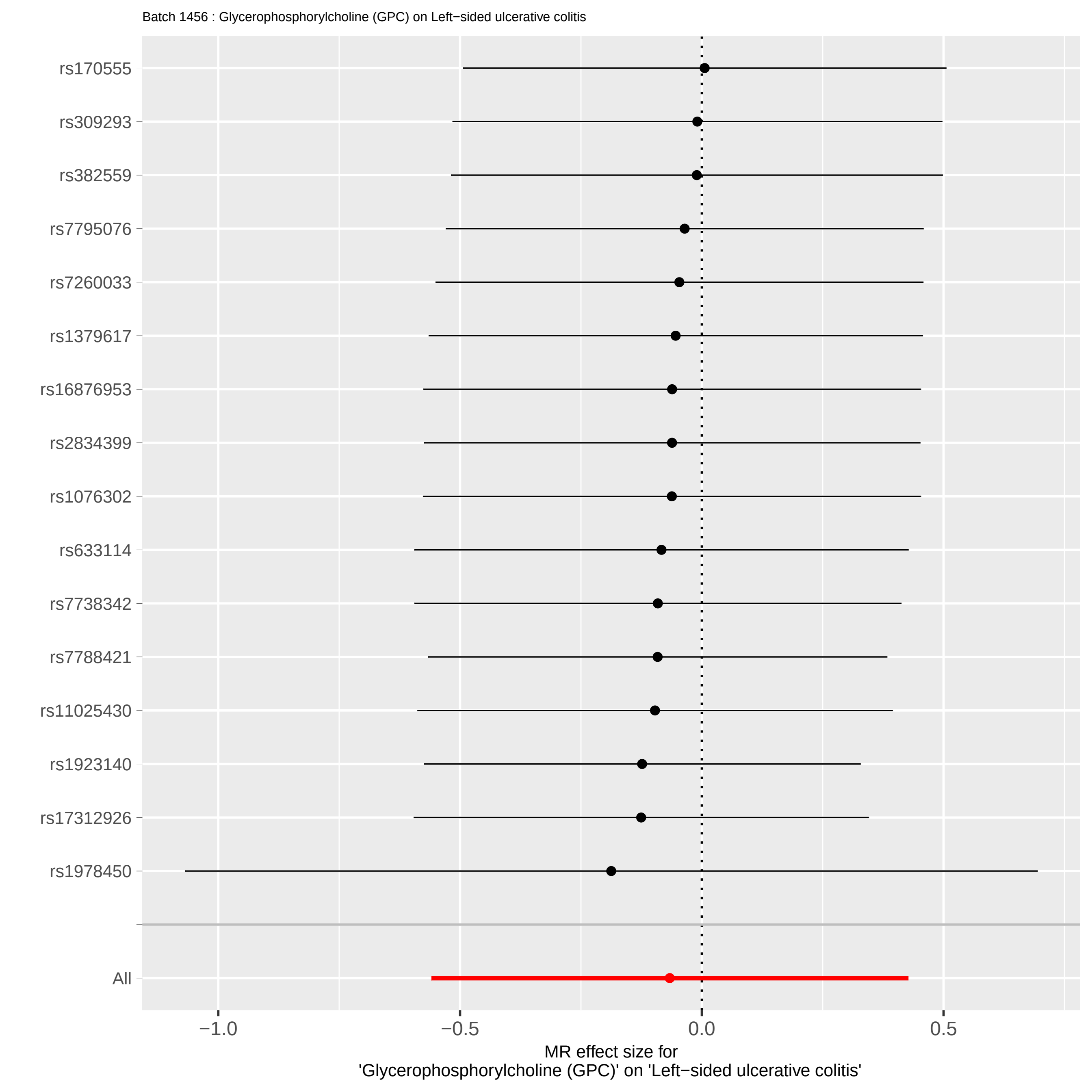
Batch 1456 : Glycerophosphorylcholine (GPC) on Left−sided ulcerative colitis
rs170555
rs309293
rs382559
rs7795076
rs7260033
rs1379617
rs16876953
rs2834399
rs1076302
rs633114
rs7738342
rs7788421
rs11025430
rs1923140
rs17312926
rs1978450
All
−1.0
−0.5
0.0
0.5
MR effect size for
'Glycerophosphorylcholine (GPC)' on 'Left−sided ulcerative colitis'

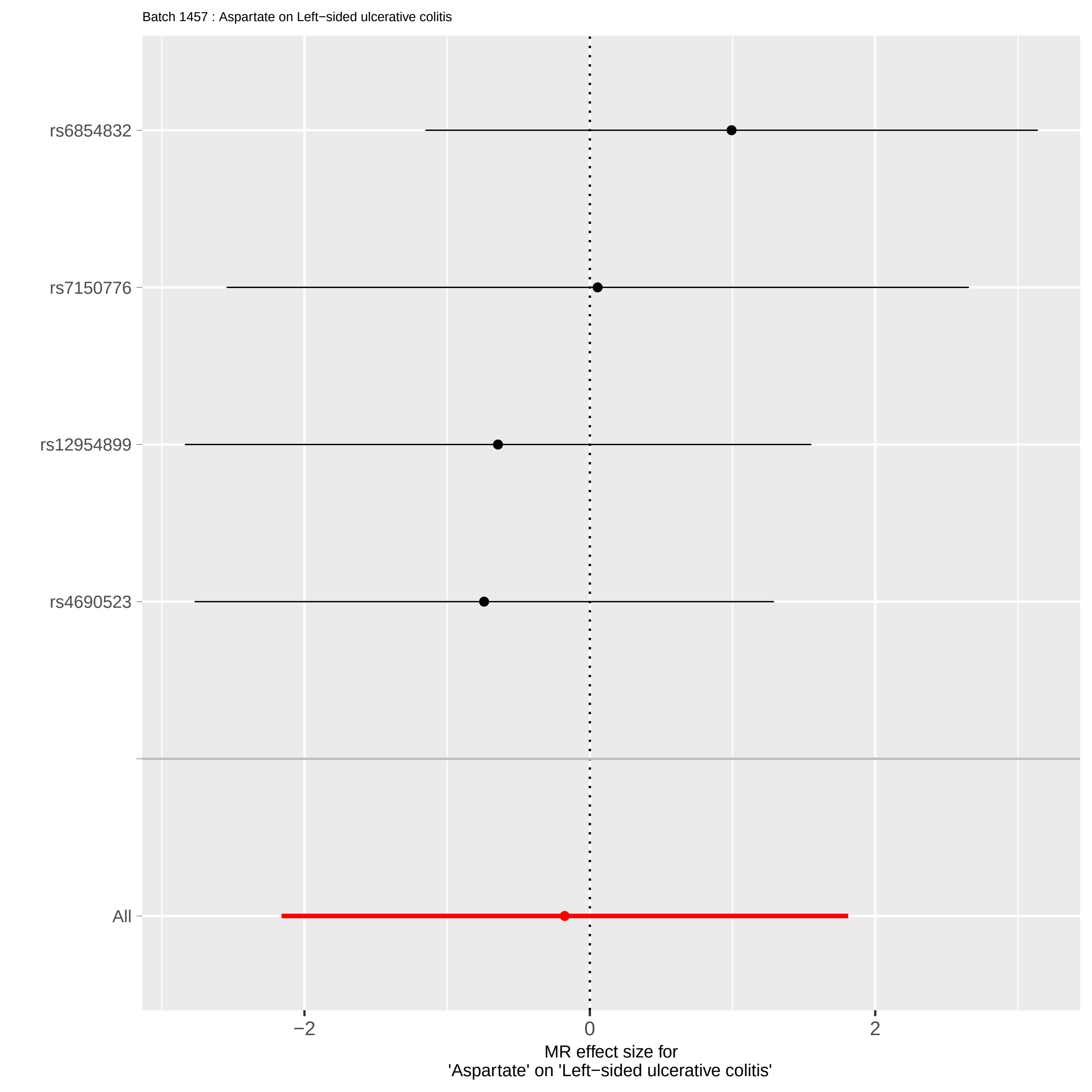

Batch 1457 : Aspartate on Left−sided ulcerative colitis
rs6854832
rs7150776
rs12954899
rs4690523
All
−2
0
2
MR effect size for
'Aspartate' on 'Left−sided ulcerative colitis'

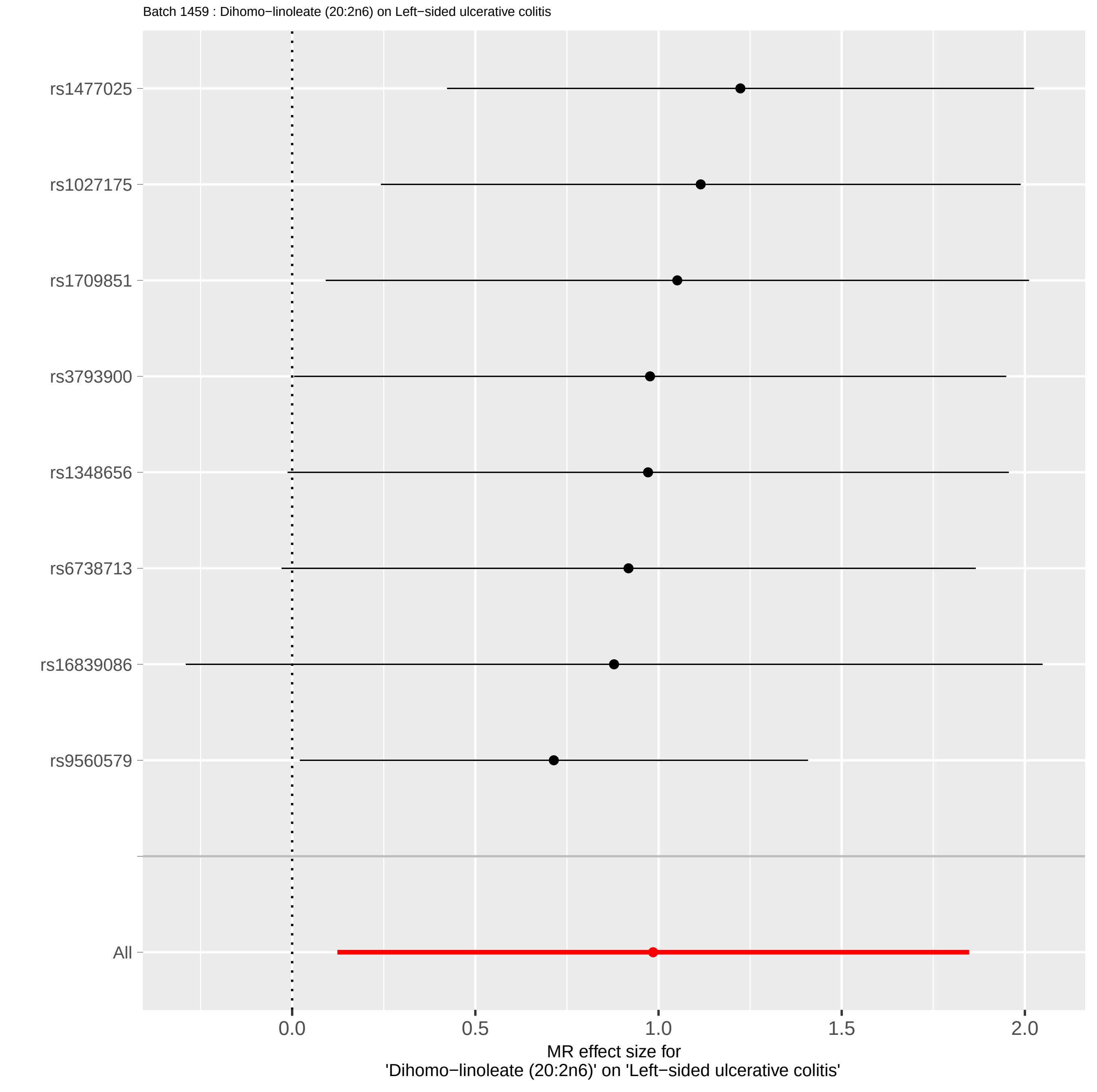

Batch 1459 : Dihomo−linoleate (20:2n6) on Left−sided ulcerative colitis
rs1477025
rs1027175
rs1709851
rs3793900
rs1348656
rs6738713
rs16839086
rs9560579
All
0.0
0.5
1.0
1.5
2.0
MR effect size for
'Dihomo−linoleate (20:2n6)' on 'Left−sided ulcerative colitis'

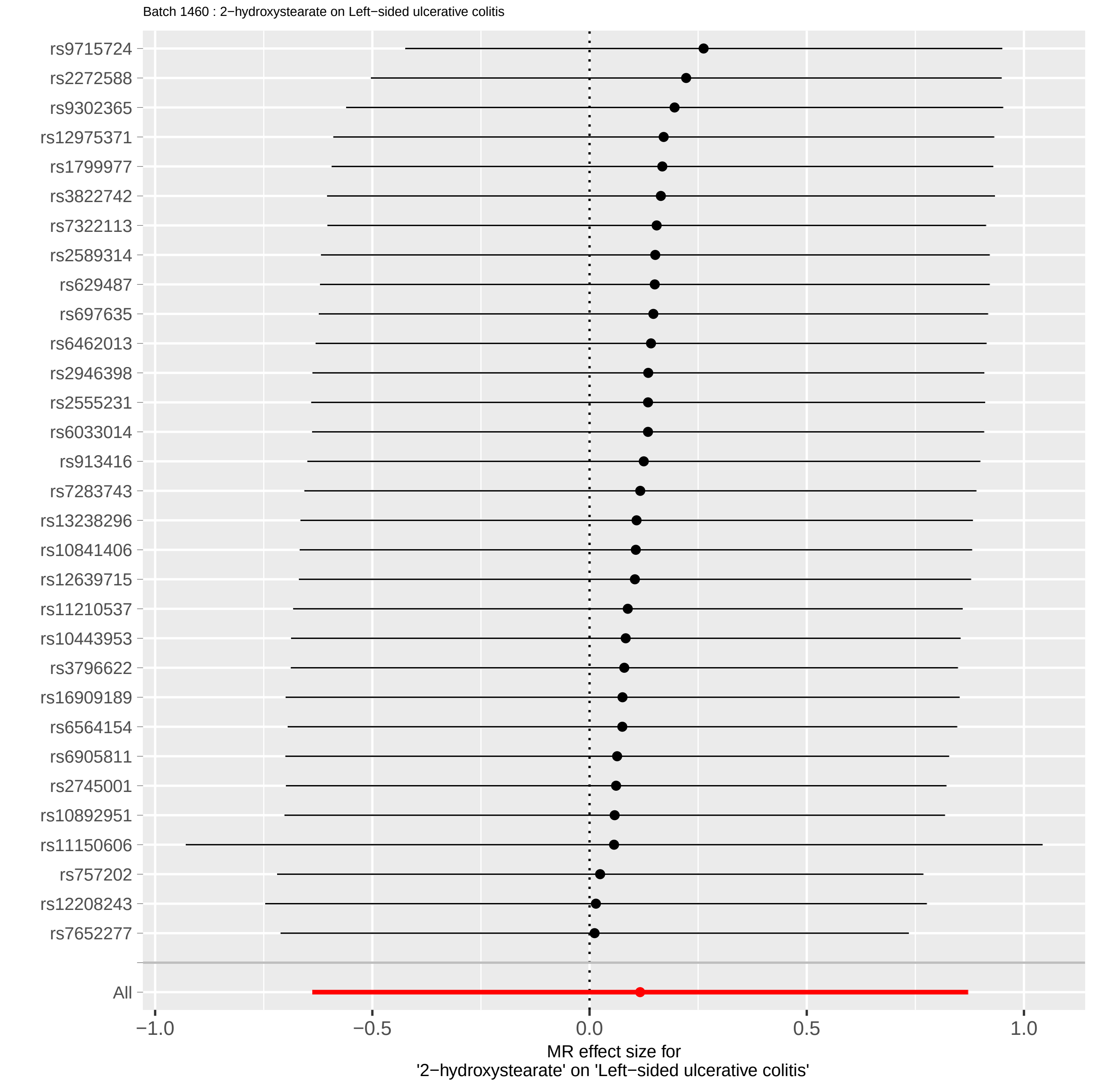

Batch 1460 : 2−hydroxystearate on Left−sided ulcerative colitis
rs9715724
rs2272588
rs9302365
rs12975371
rs1799977
rs3822742
rs7322113
rs2589314
rs629487
rs697635
rs6462013
rs2946398
rs2555231
rs6033014
rs913416
rs7283743
rs13238296
rs10841406
rs12639715
rs11210537
rs10443953
rs3796622
rs16909189
rs6564154
rs6905811
rs2745001
rs10892951
rs11150606
rs757202
rs12208243
rs7652277
All
−1.0
−0.5
0.0
0.5
1.0
MR effect size for
'2−hydroxystearate' on 'Left−sided ulcerative colitis'

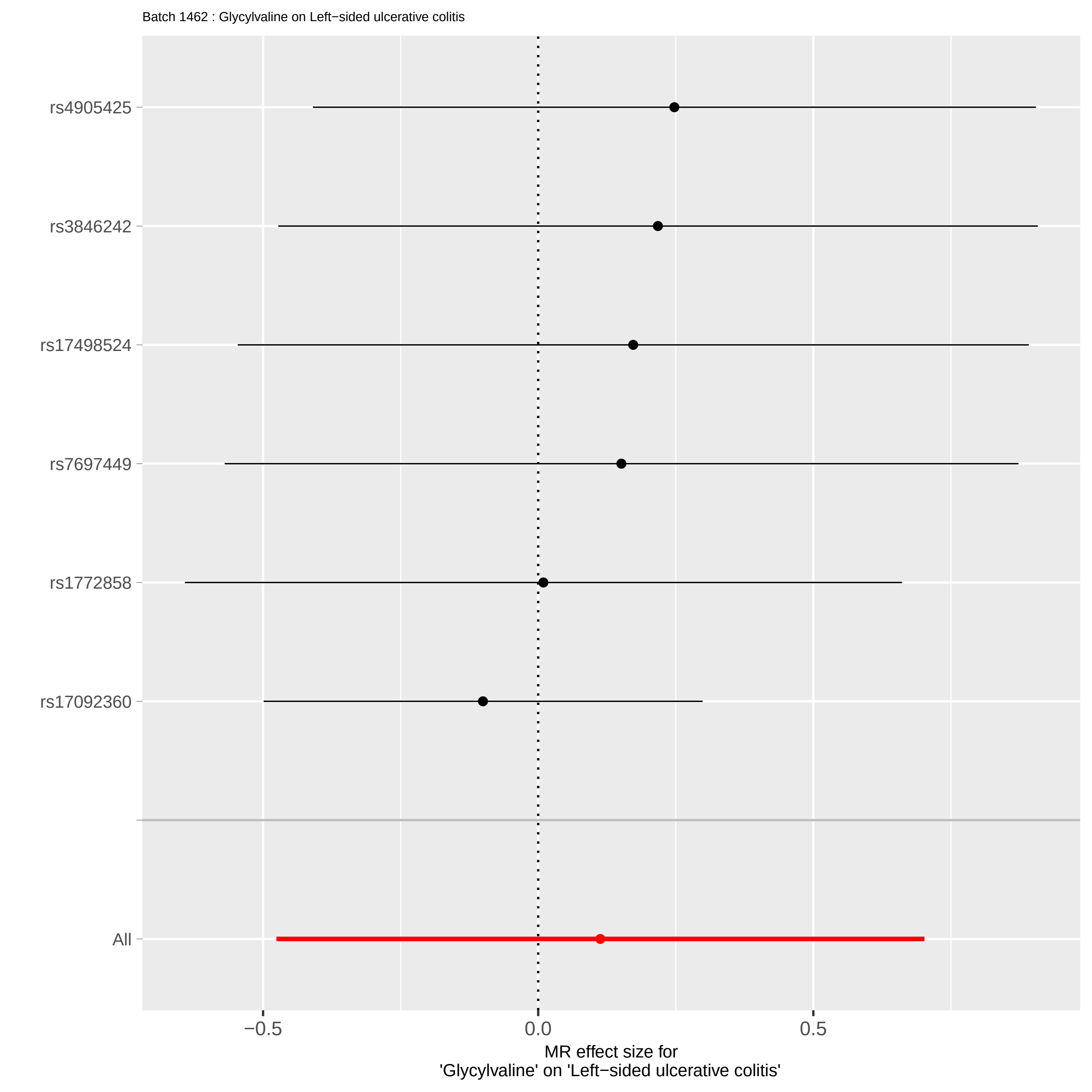

Batch 1462 : Glycylvaline on Left−sided ulcerative colitis
rs4905425
rs3846242
rs17498524
rs7697449
rs1772858
rs17092360
All
−0.5
0.0
0.5
MR effect size for
'Glycylvaline' on 'Left−sided ulcerative colitis'

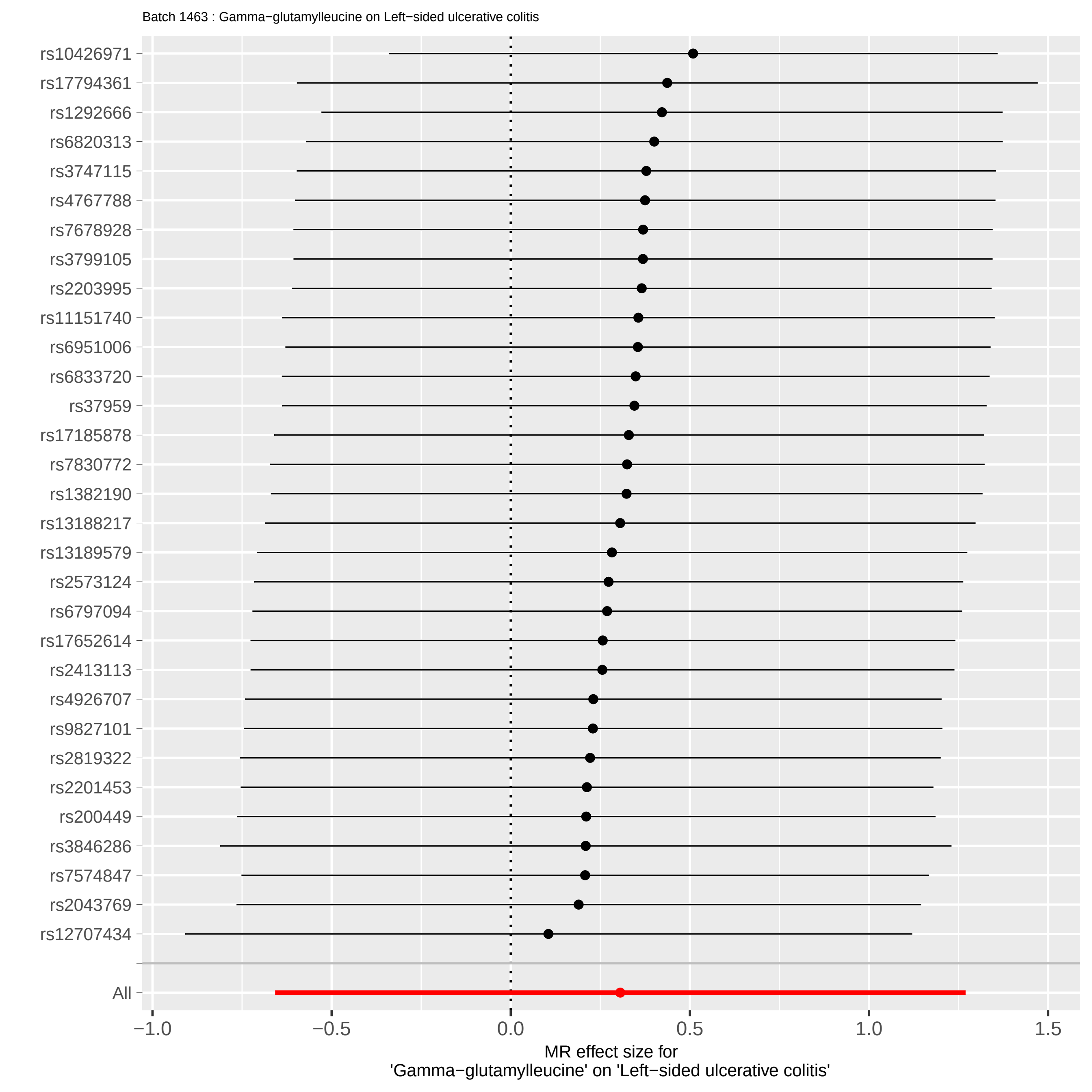
Batch 1463 : Gamma−glutamylleucine on Left−sided ulcerative colitis
rs10426971
rs17794361
rs1292666
rs6820313
rs3747115
rs4767788
rs7678928
rs3799105
rs2203995
rs11151740
rs6951006
rs6833720
rs37959
rs17185878
rs7830772
rs1382190
rs13188217
rs13189579
rs2573124
rs6797094
rs17652614
rs2413113
rs4926707
rs9827101
rs2819322
rs2201453
rs200449
rs3846286
rs7574847
rs2043769
rs12707434
All
−1.0
−0.5
0.0
0.5
1.0
1.5
MR effect size for
'Gamma−glutamylleucine' on 'Left−sided ulcerative colitis'

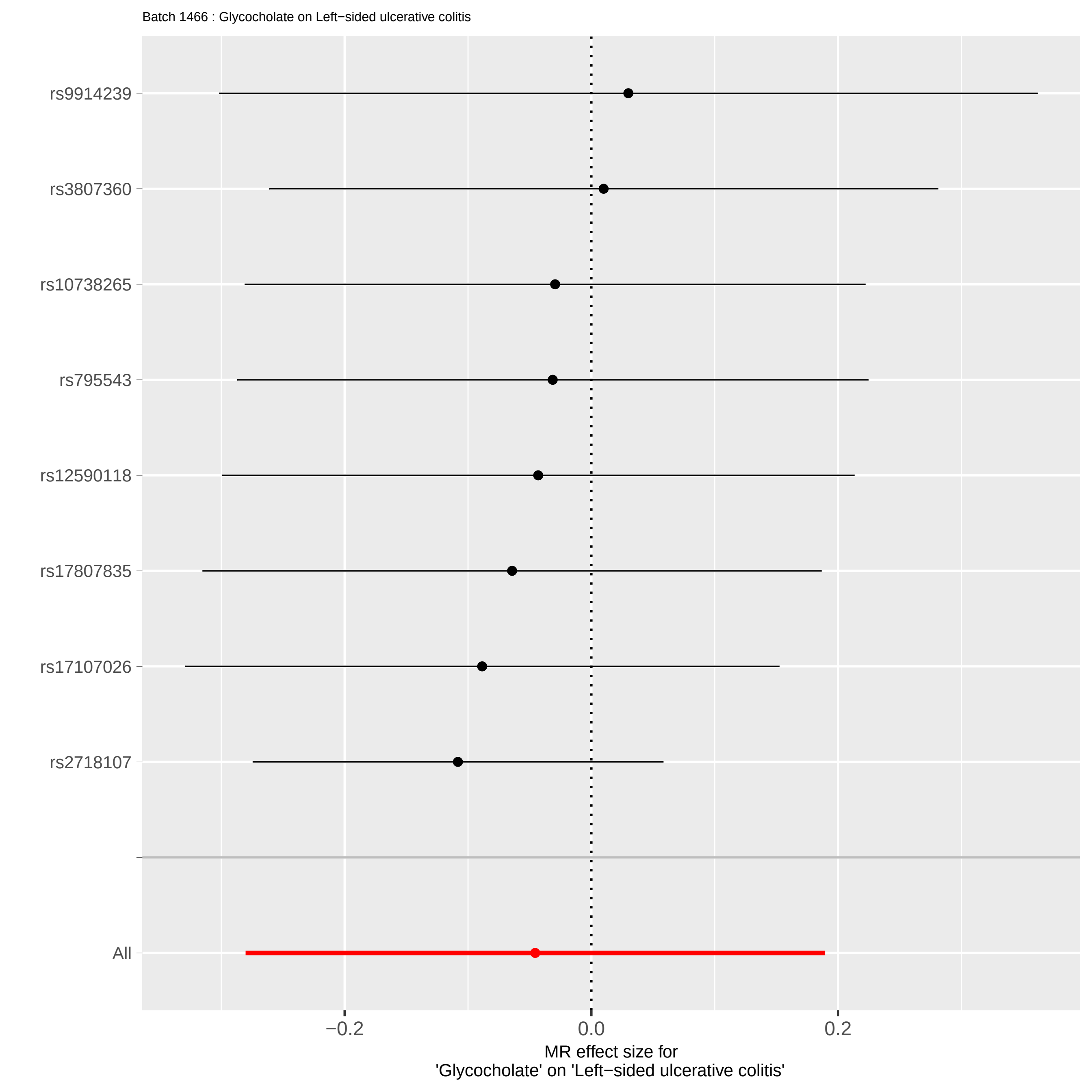

Batch 1466 : Glycocholate on Left−sided ulcerative colitis
rs9914239
rs3807360
rs10738265
rs795543
rs12590118
rs17807835
rs17107026
rs2718107
All
−0.2
0.0
0.2
MR effect size for
'Glycocholate' on 'Left−sided ulcerative colitis'

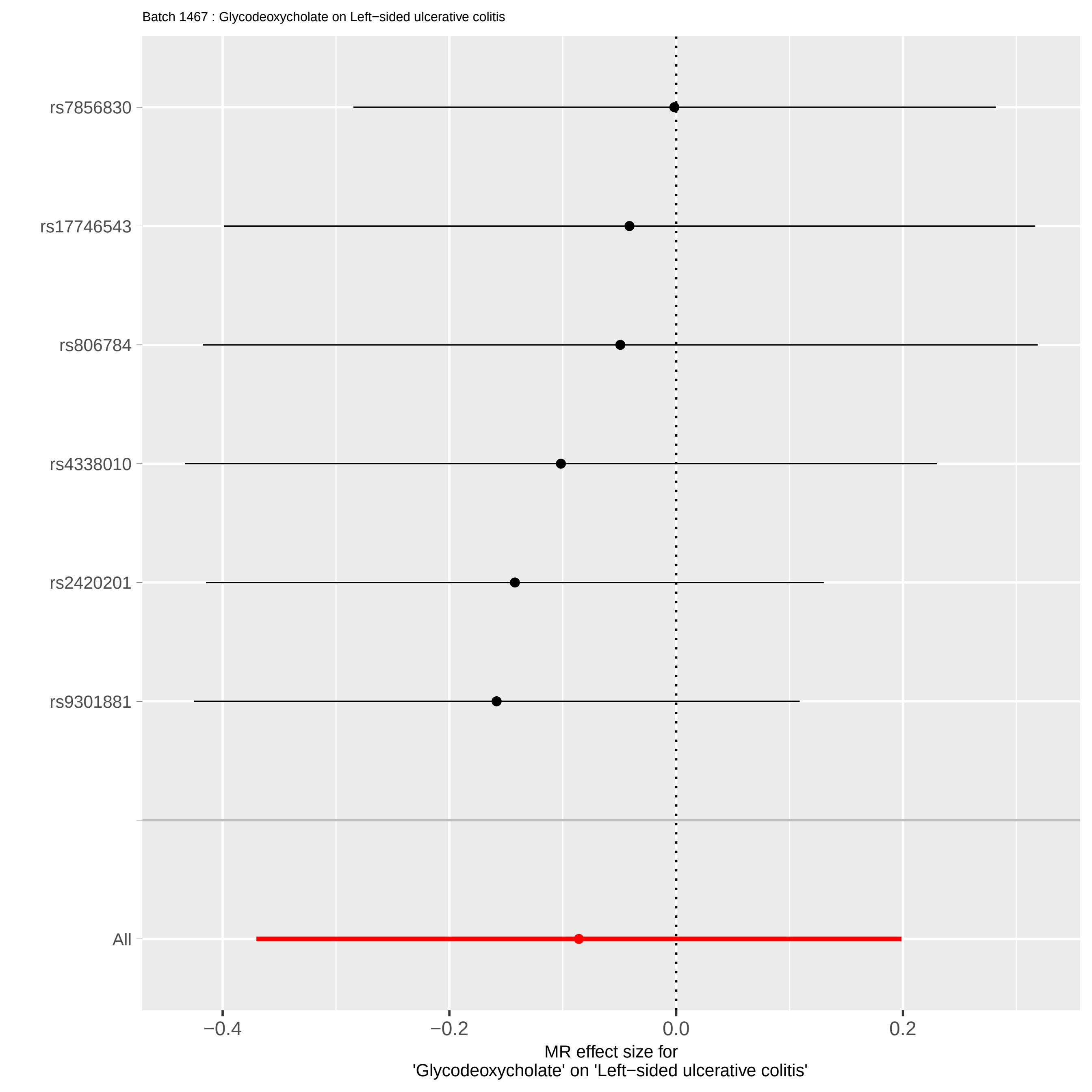

Batch 1467 : Glycodeoxycholate on Left−sided ulcerative colitis
rs7856830
rs17746543
rs806784
rs4338010
rs2420201
rs9301881
All
−0.4
−0.2
0.0
0.2
MR effect size for
'Glycodeoxycholate' on 'Left−sided ulcerative colitis'

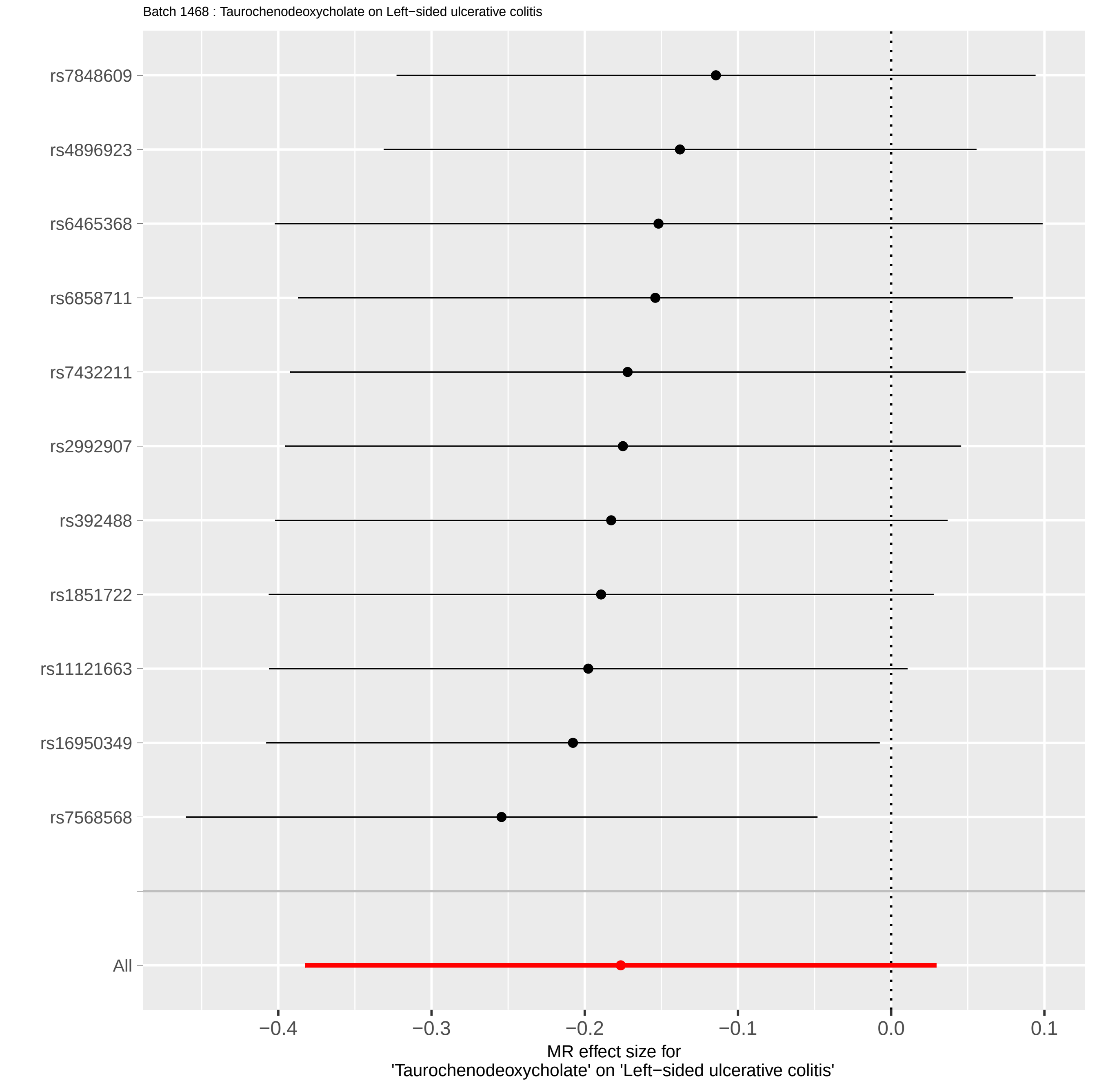
Batch 1468 : Taurochenodeoxycholate on Left−sided ulcerative colitis
rs7848609
rs4896923
rs6465368
rs6858711
rs7432211
rs2992907
rs392488
rs1851722
rs11121663
rs16950349
rs7568568
All
−0.4
−0.3
−0.2
−0.1
0.0
0.1
MR effect size for
'Taurochenodeoxycholate' on 'Left−sided ulcerative colitis'

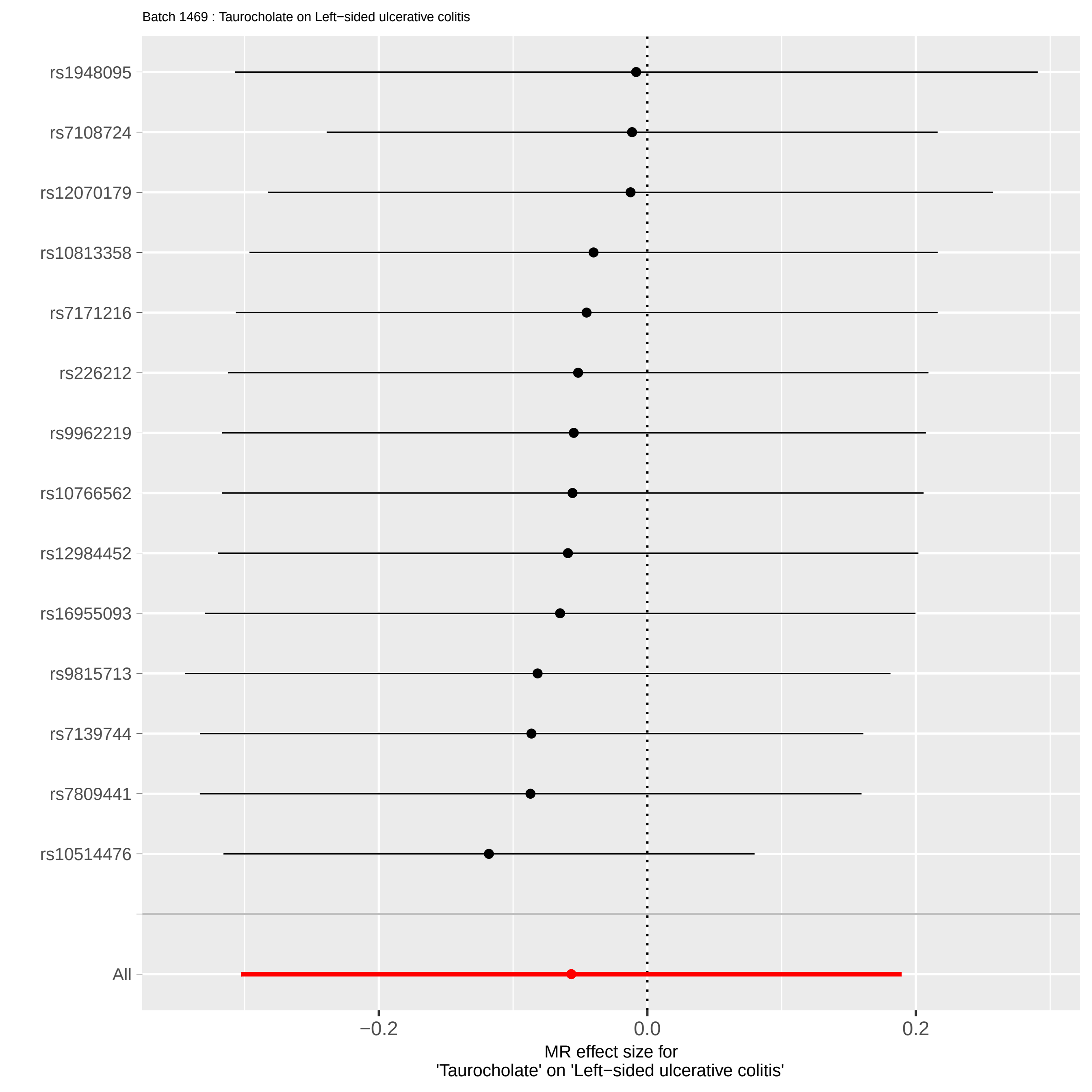

Batch 1469 : Taurocholate on Left−sided ulcerative colitis
rs1948095
rs7108724
rs12070179
rs10813358
rs7171216
rs226212
rs9962219
rs10766562
rs12984452
rs16955093
rs9815713
rs7139744
rs7809441
rs10514476
All
−0.2
0.0
0.2
MR effect size for
'Taurocholate' on 'Left−sided ulcerative colitis'

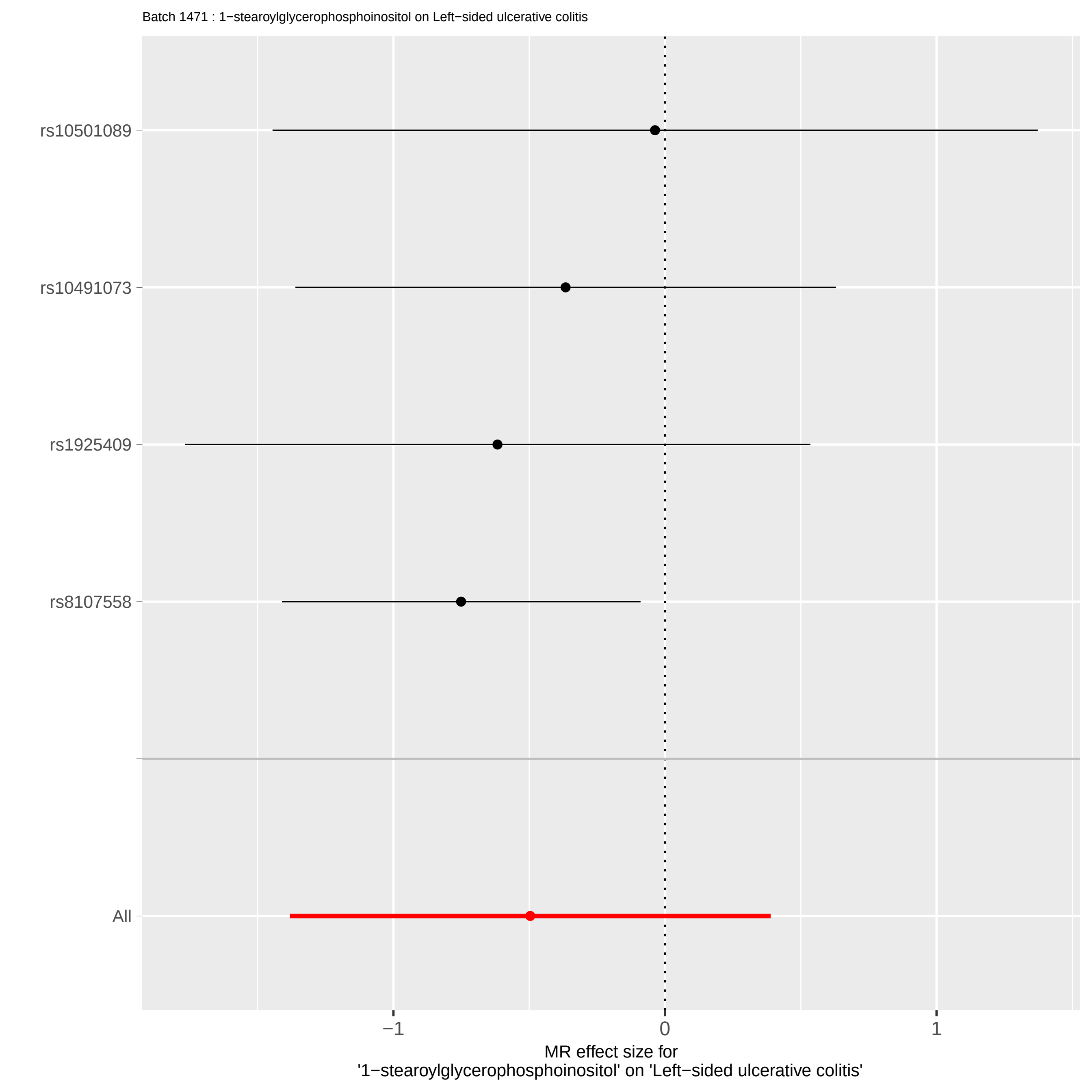
Batch 1471 : 1−stearoylglycerophosphoinositol on Left−sided ulcerative colitis
rs10501089
rs10491073
rs1925409
rs8107558
All
−1
0
1
MR effect size for
'1−stearoylglycerophosphoinositol' on 'Left−sided ulcerative colitis'

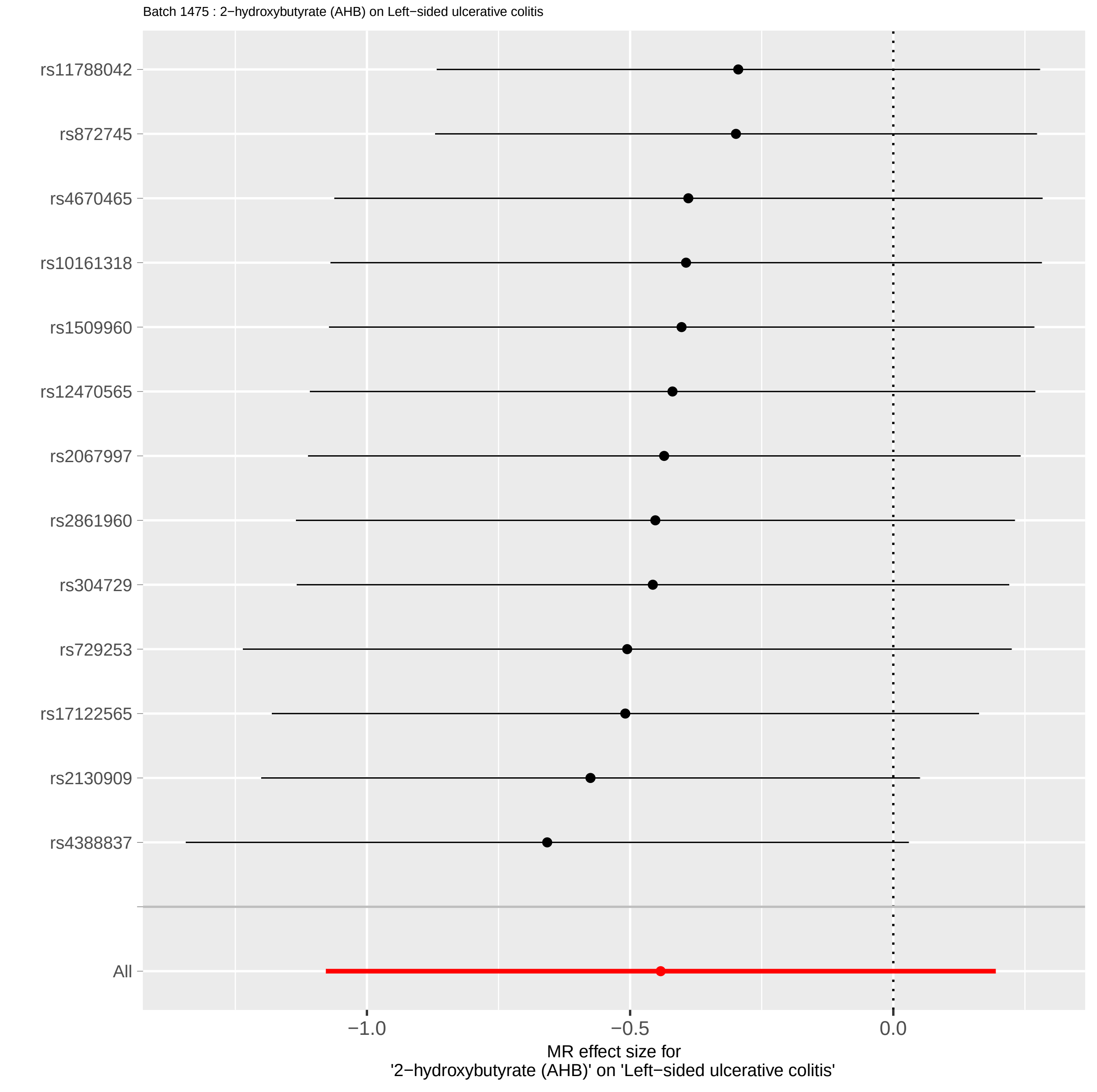
Batch 1475 : 2−hydroxybutyrate (AHB) on Left−sided ulcerative colitis
rs11788042
rs872745
rs4670465
rs10161318
rs1509960
rs12470565
rs2067997
rs2861960
rs304729
rs729253
rs17122565
rs2130909
rs4388837
All
−1.0
−0.5
0.0
MR effect size for
'2−hydroxybutyrate (AHB)' on 'Left−sided ulcerative colitis'

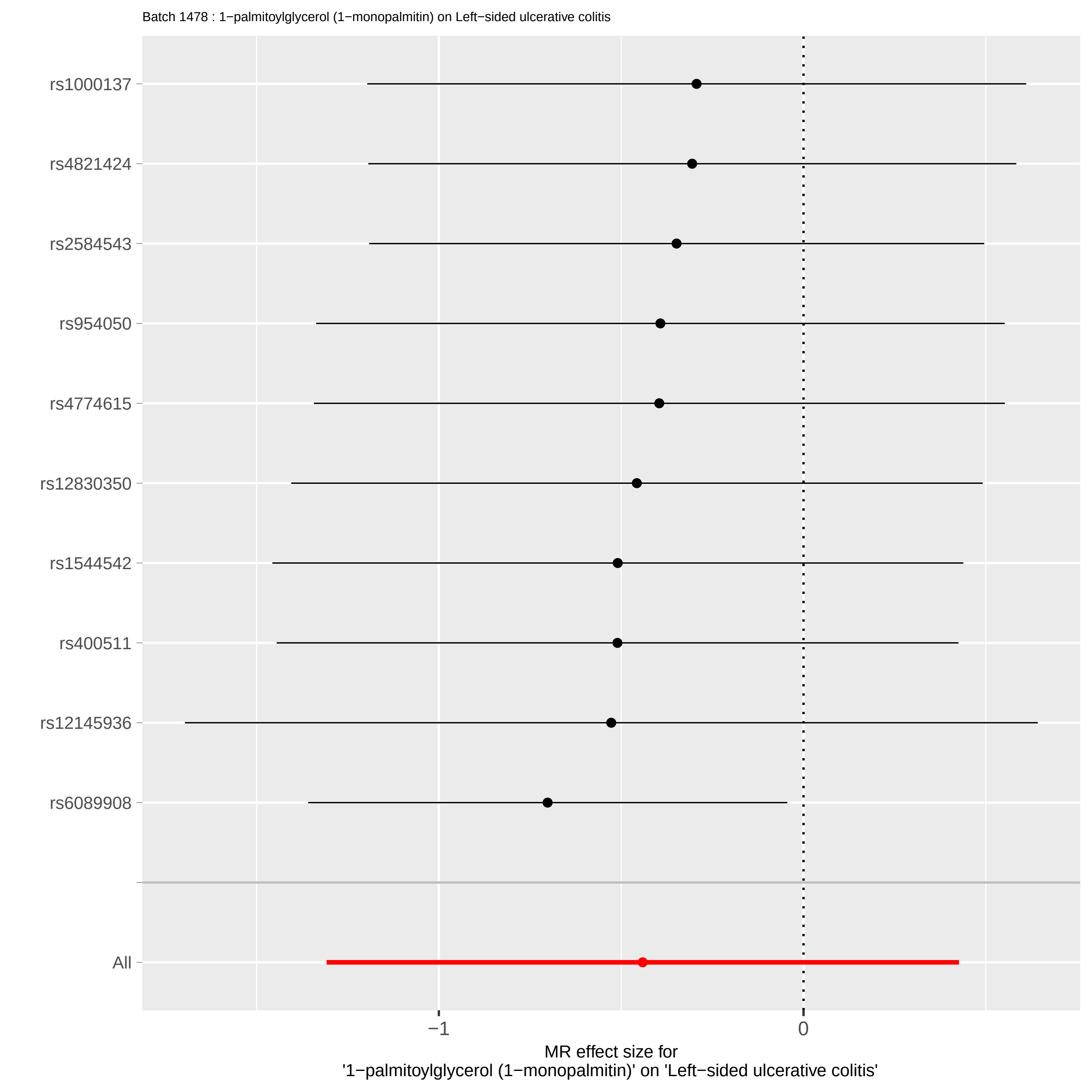

Batch 1478 : 1−palmitoylglycerol (1−monopalmitin) on Left−sided ulcerative colitis
rs1000137
rs4821424
rs2584543
rs954050
rs4774615
rs12830350
rs1544542
rs400511
rs12145936
rs6089908
All
−1
0
MR effect size for
'1−palmitoylglycerol (1−monopalmitin)' on 'Left−sided ulcerative colitis'

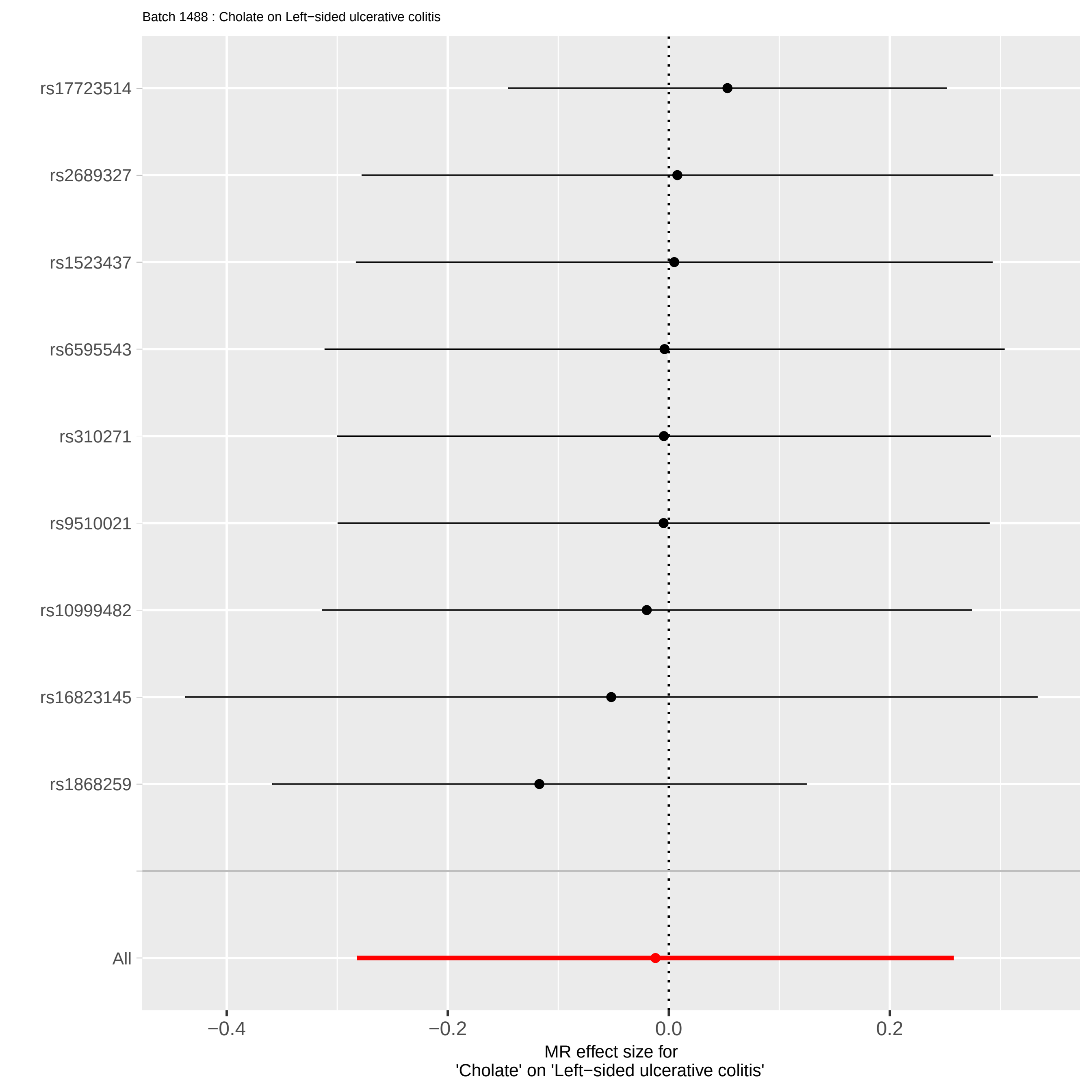

Batch 1488 : Cholate on Left−sided ulcerative colitis
rs17723514
rs2689327
rs1523437
rs6595543
rs310271
rs9510021
rs10999482
rs16823145
rs1868259
All
−0.4
−0.2
0.0
0.2
MR effect size for
'Cholate' on 'Left−sided ulcerative colitis'

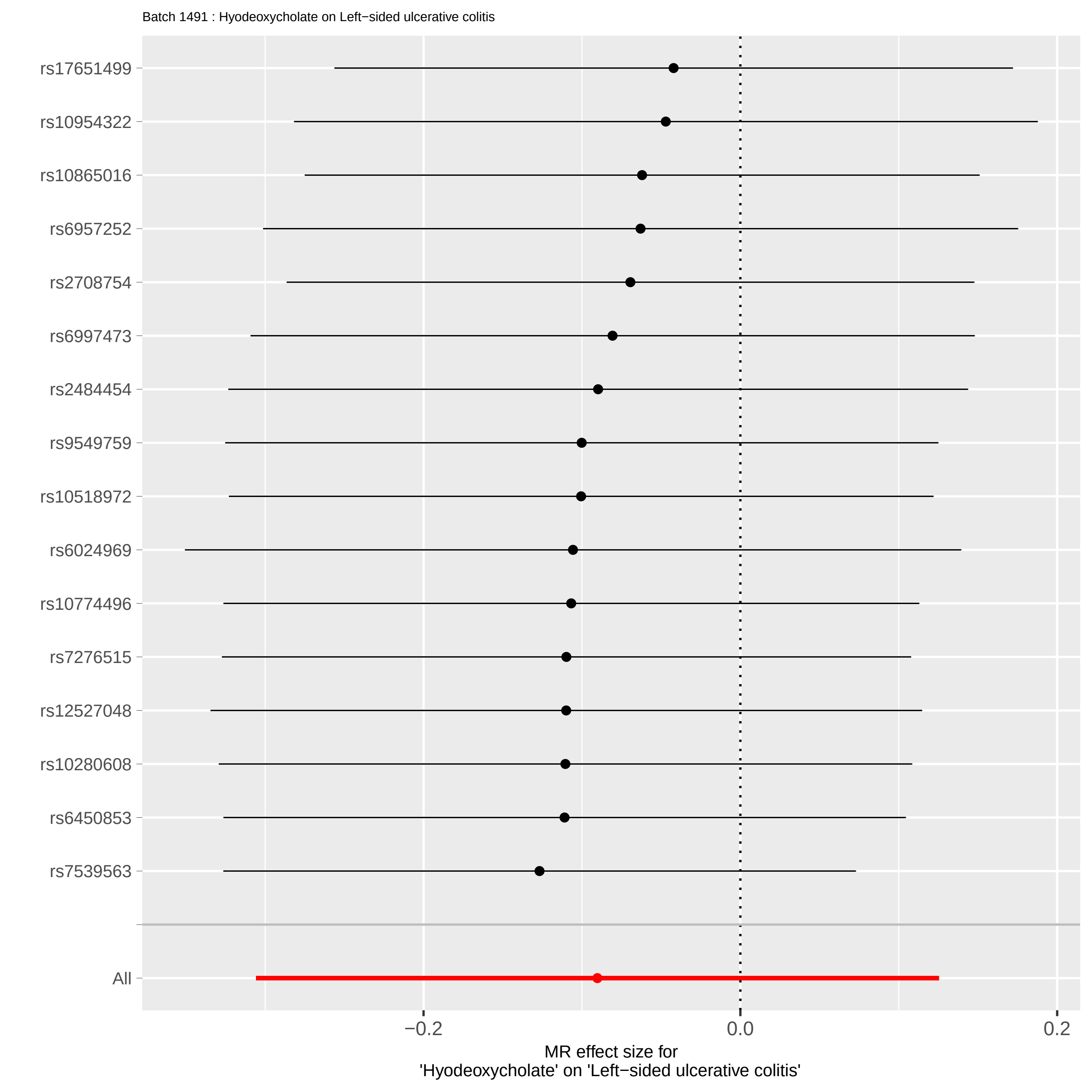

Batch 1491 : Hyodeoxycholate on Left−sided ulcerative colitis
rs17651499
rs10954322
rs10865016
rs6957252
rs2708754
rs6997473
rs2484454
rs9549759
rs10518972
rs6024969
rs10774496
rs7276515
rs12527048
rs10280608
rs6450853
rs7539563
All
−0.2
0.0
0.2
MR effect size for
'Hyodeoxycholate' on 'Left−sided ulcerative colitis'

Batch 1492 : 3−indoxyl sulfate on Left−sided ulcerative colitis

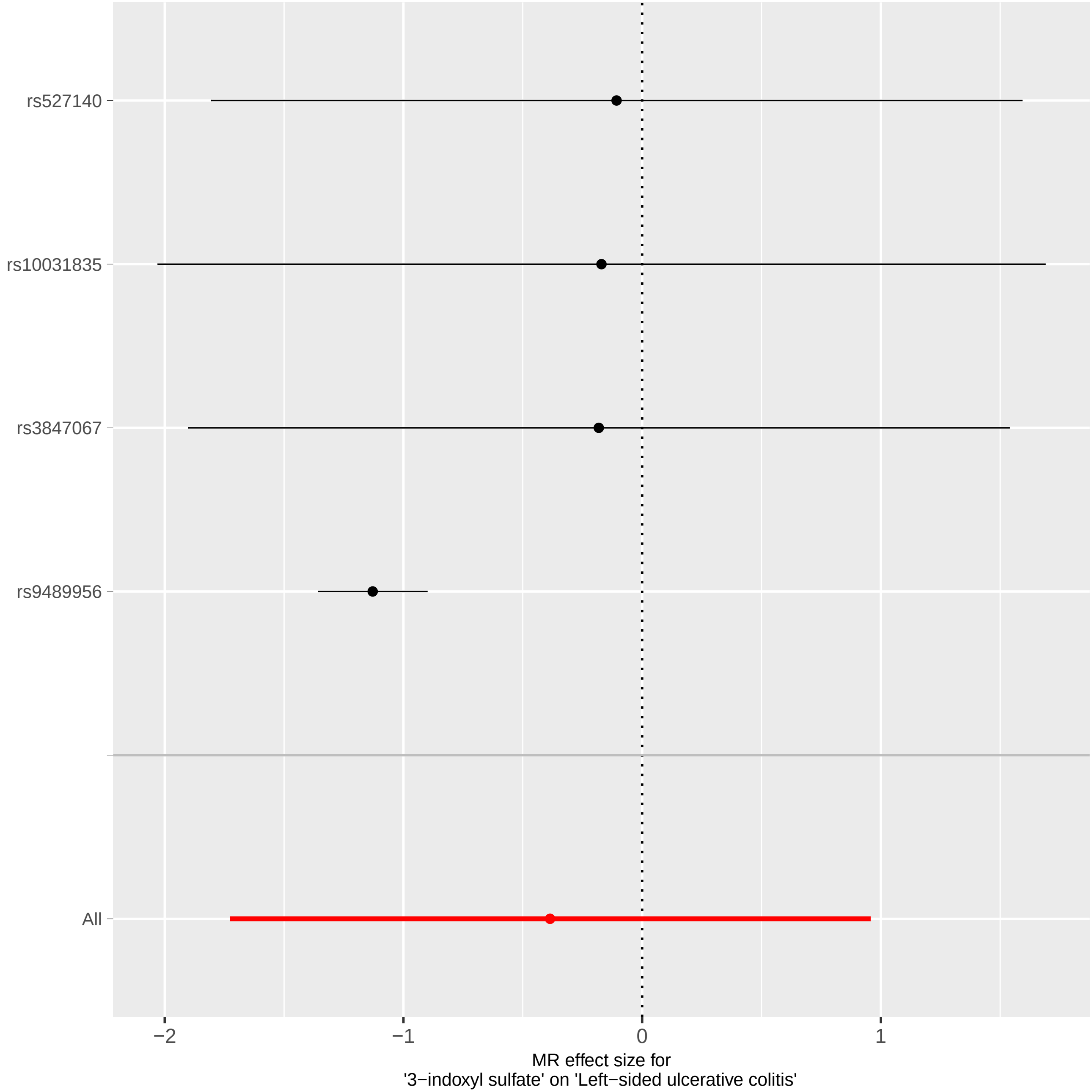

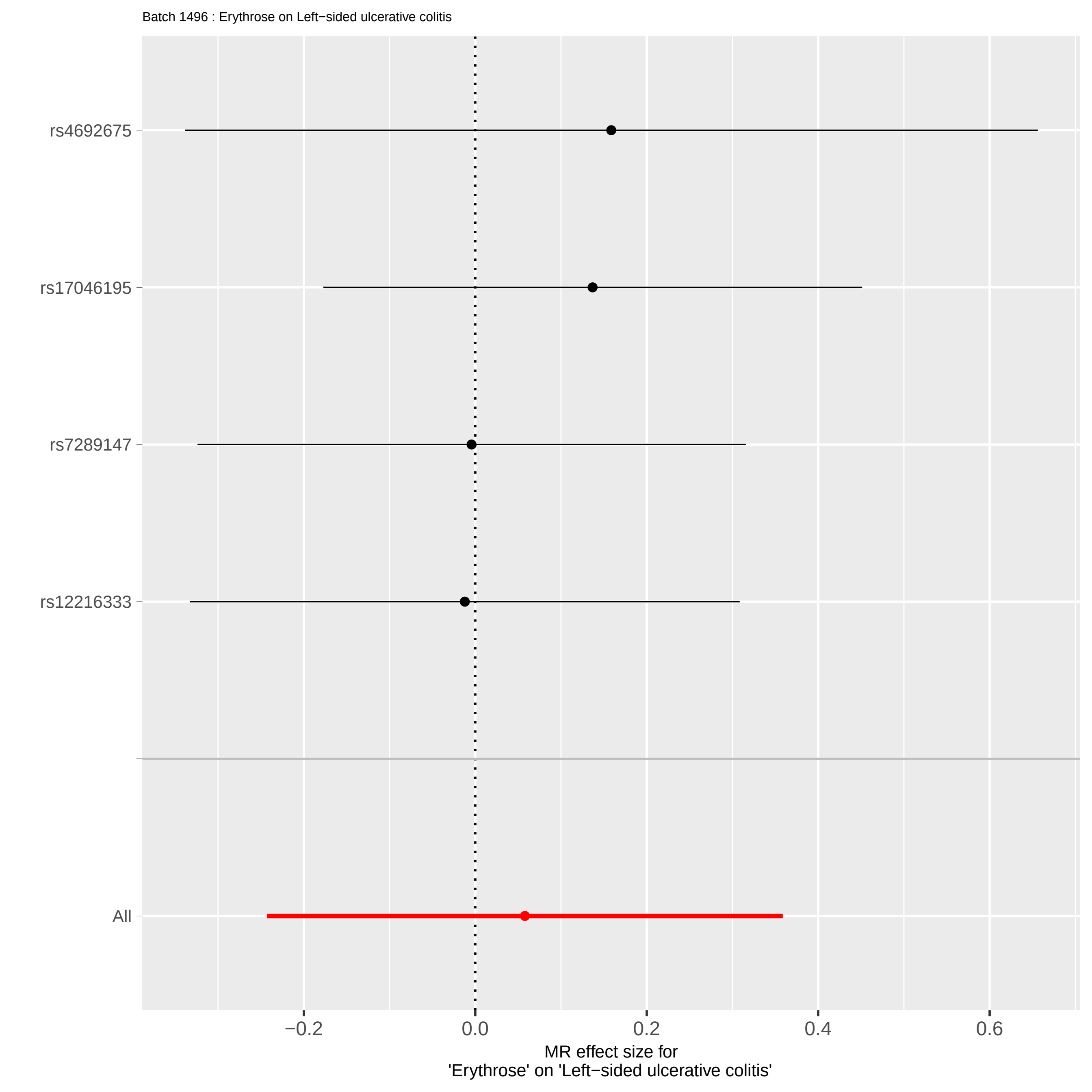

Batch 1496 : Erythrose on Left−sided ulcerative colitis
rs4692675
rs17046195
rs7289147
rs12216333
All
−0.2
0.0
0.2
0.4
0.6
MR effect size for
'Erythrose' on 'Left−sided ulcerative colitis'

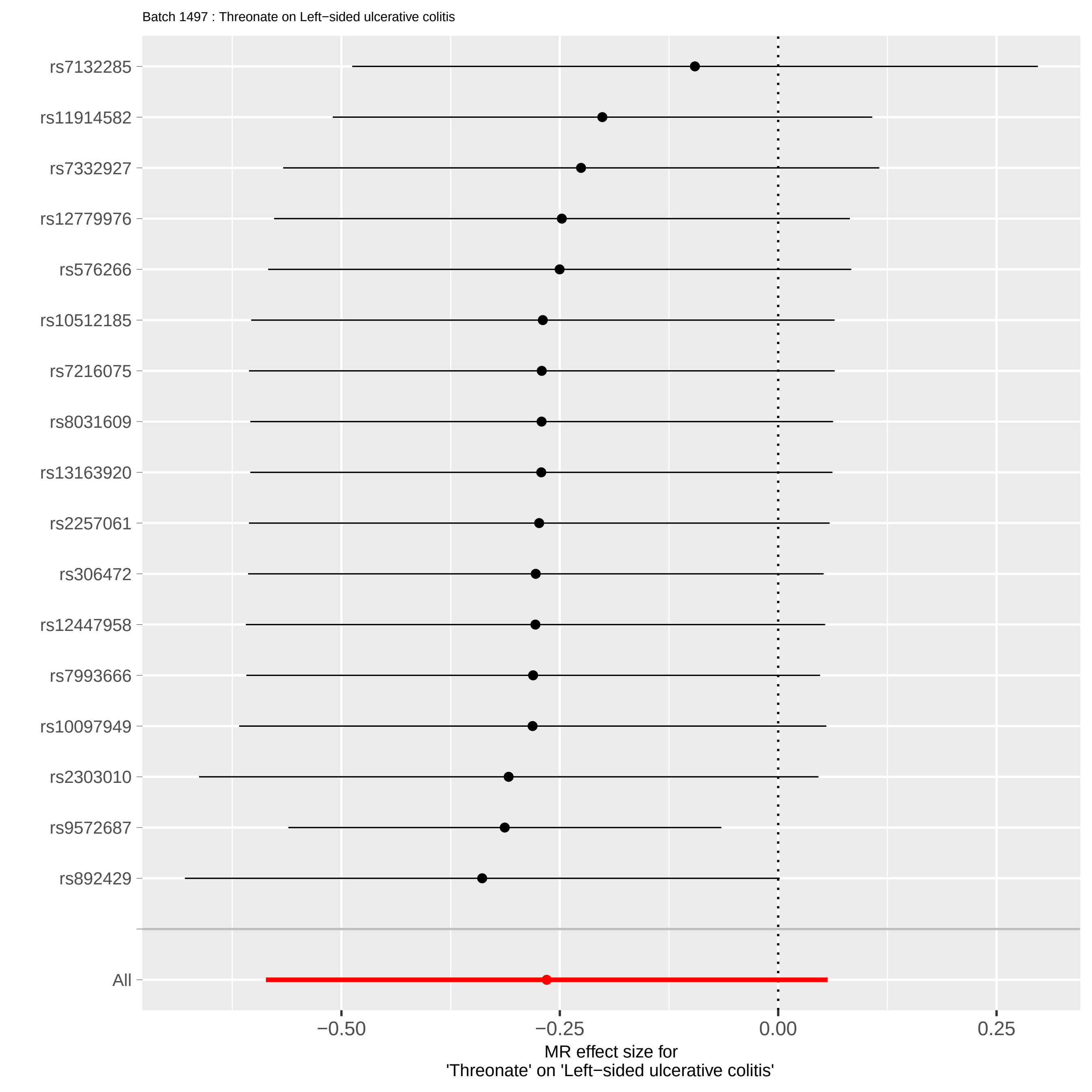

Batch 1497 : Threonate on Left−sided ulcerative colitis
rs7132285
rs11914582
rs7332927
rs12779976
rs576266
rs10512185
rs7216075
rs8031609
rs13163920
rs2257061
rs306472
rs12447958
rs7993666
rs10097949
rs2303010
rs9572687
rs892429
All
−0.50
−0.25
0.00
0.25
MR effect size for
'Threonate' on 'Left−sided ulcerative colitis'

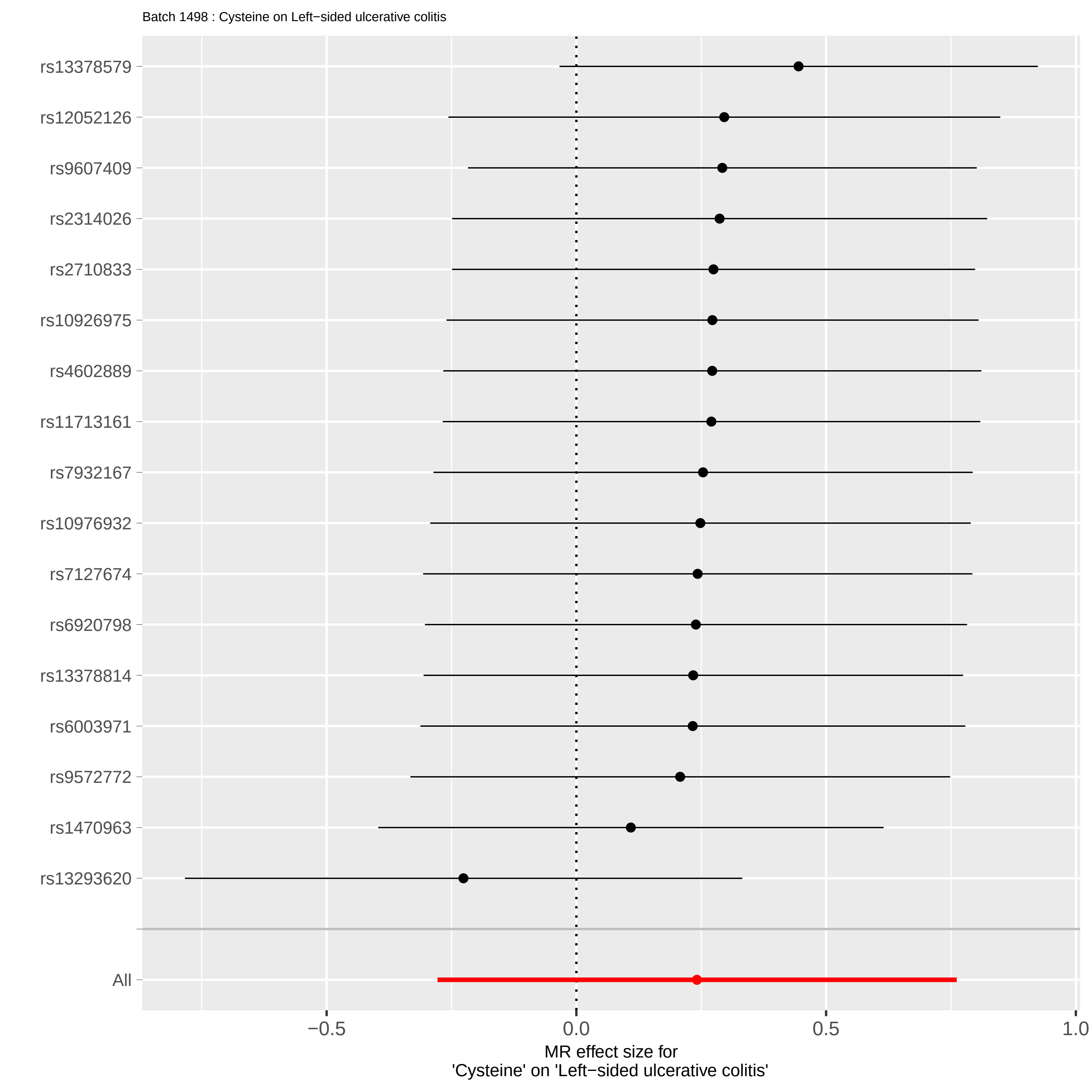

Batch 1498 : Cysteine on Left−sided ulcerative colitis
rs13378579
rs12052126
rs9607409
rs2314026
rs2710833
rs10926975
rs4602889
rs11713161
rs7932167
rs10976932
rs7127674
rs6920798
rs13378814
rs6003971
rs9572772
rs1470963
rs13293620
All
−0.5
0.0
0.5
1.0
MR effect size for
'Cysteine' on 'Left−sided ulcerative colitis'

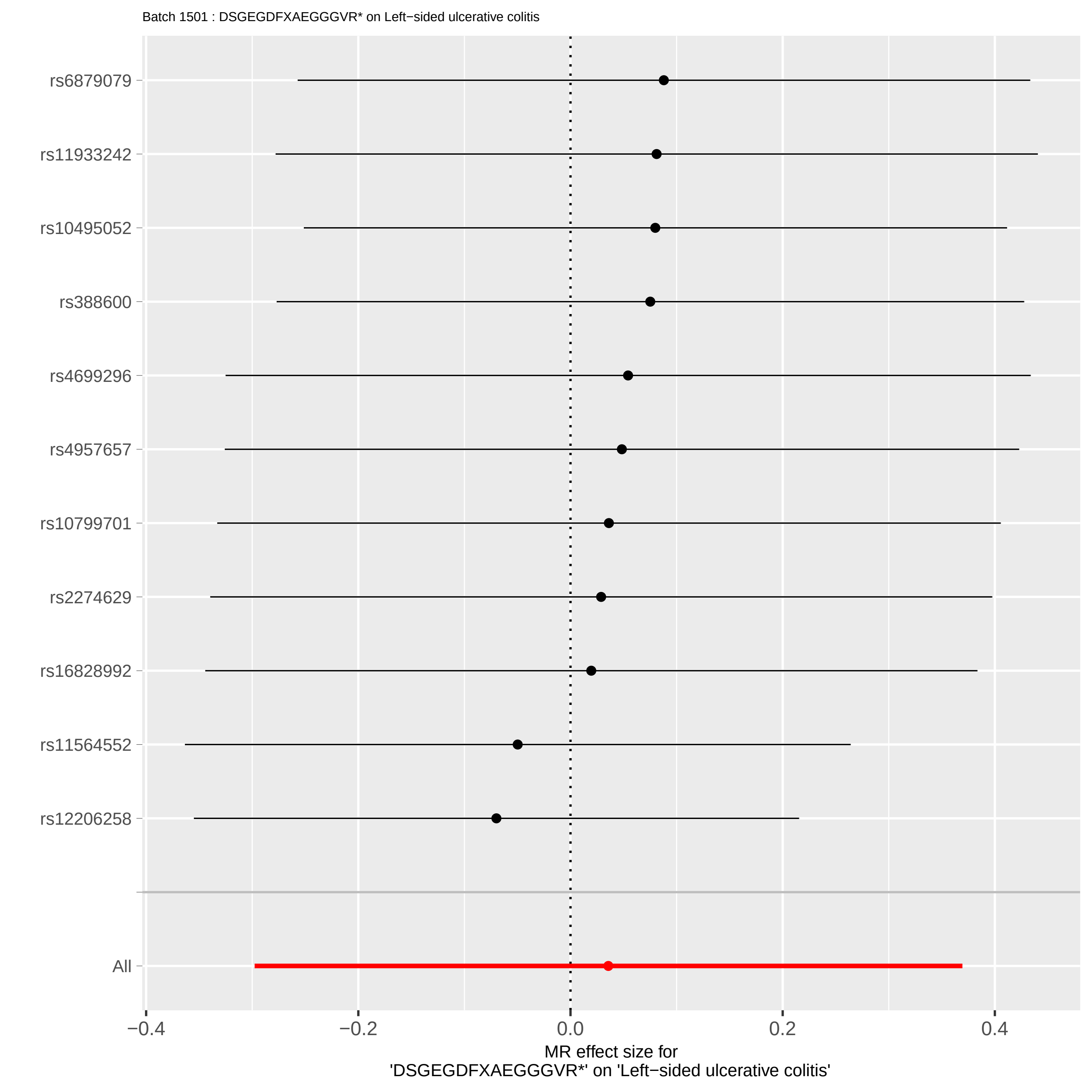

Batch 1501 : DSGEGDFXAEGGGVR* on Left−sided ulcerative colitis
rs6879079
rs11933242
rs10495052
rs388600
rs4699296
rs4957657
rs10799701
rs2274629
rs16828992
rs11564552
rs12206258
All
−0.4
−0.2
0.0
0.2
0.4
MR effect size for
'DSGEGDFXAEGGGVR*' on 'Left−sided ulcerative colitis'

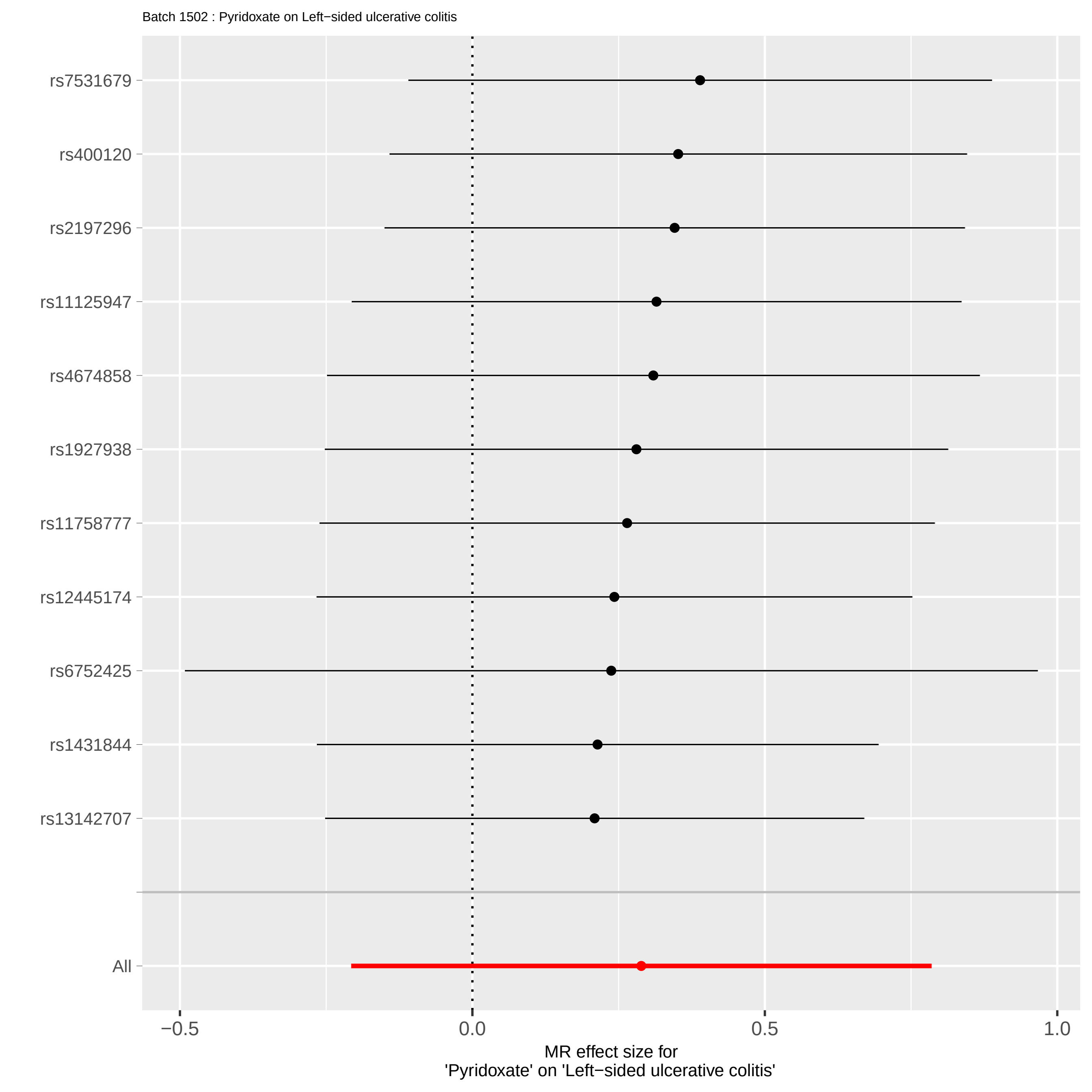

Batch 1502 : Pyridoxate on Left−sided ulcerative colitis
rs7531679
rs400120
rs2197296
rs11125947
rs4674858
rs1927938
rs11758777
rs12445174
rs6752425
rs1431844
rs13142707
All
−0.5
0.0
0.5
1.0
MR effect size for
'Pyridoxate' on 'Left−sided ulcerative colitis'

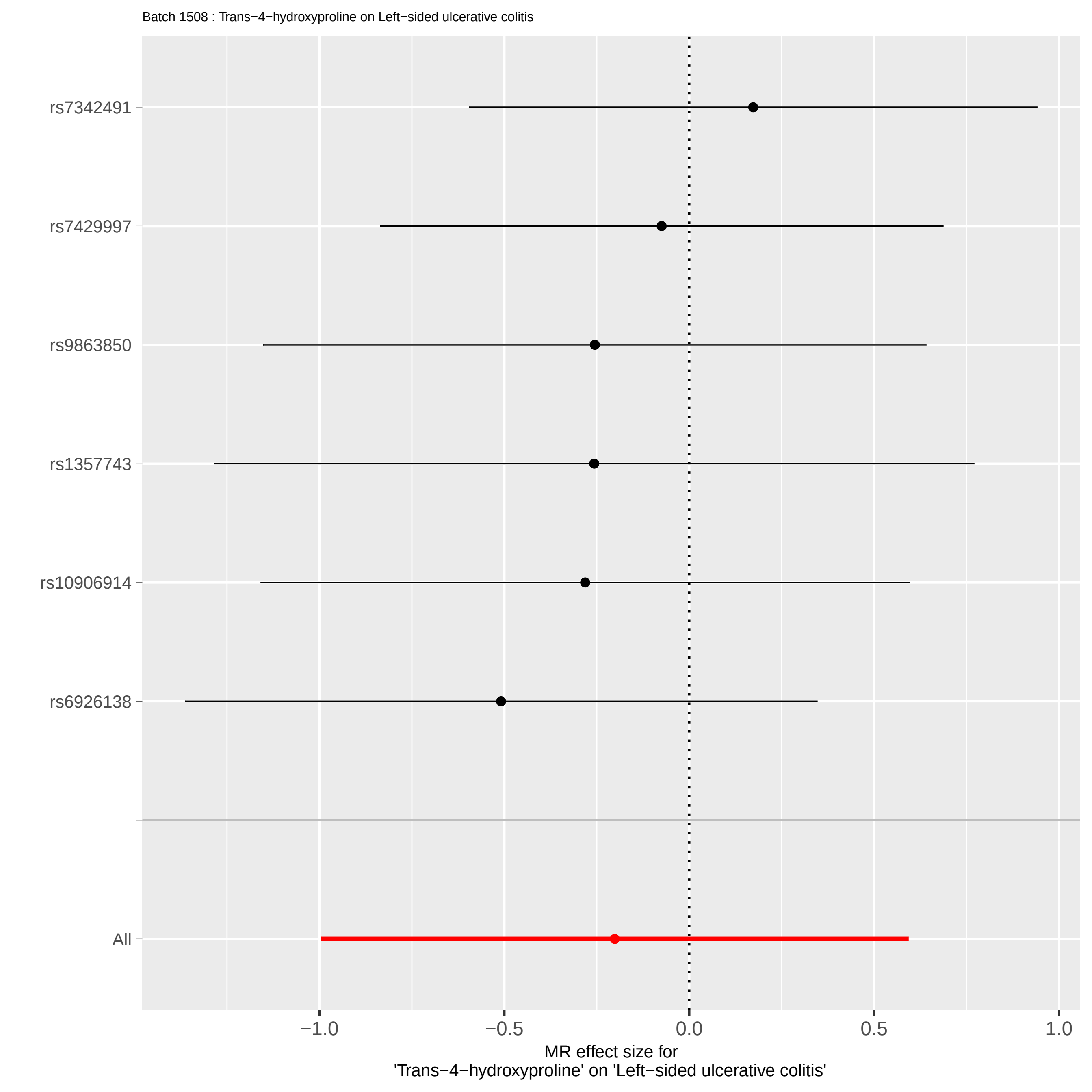
Batch 1508 : Trans−4−hydroxyproline on Left−sided ulcerative colitis
rs7342491
rs7429997
rs9863850
rs1357743
rs10906914
rs6926138
All
−1.0
−0.5
0.0
0.5
1.0
MR effect size for
'Trans−4−hydroxyproline' on 'Left−sided ulcerative colitis'

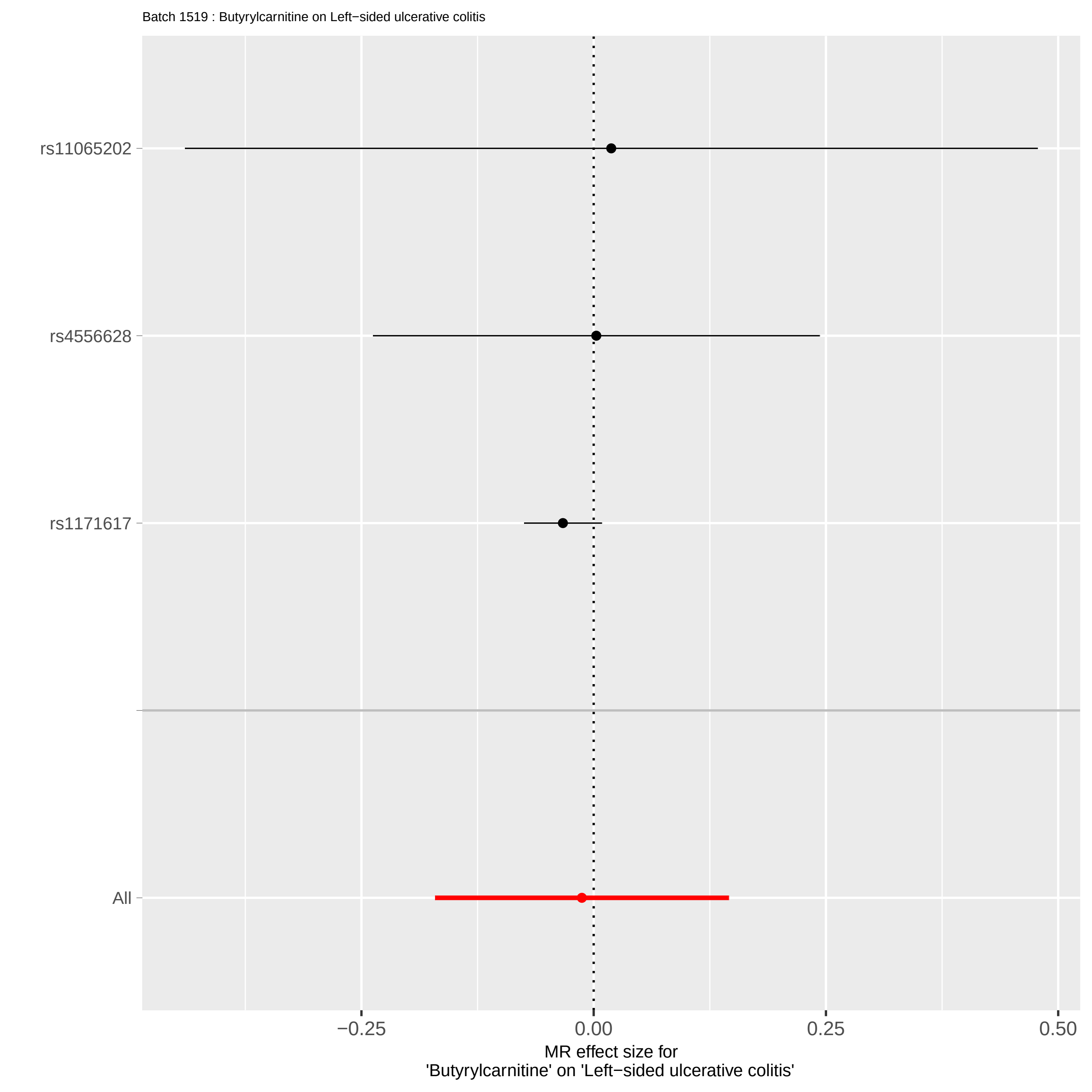
Batch 1519 : Butyrylcarnitine on Left−sided ulcerative colitis
rs11065202
rs4556628
rs1171617
All
−0.25
0.00
0.25
0.50
MR effect size for
'Butyrylcarnitine' on 'Left−sided ulcerative colitis'

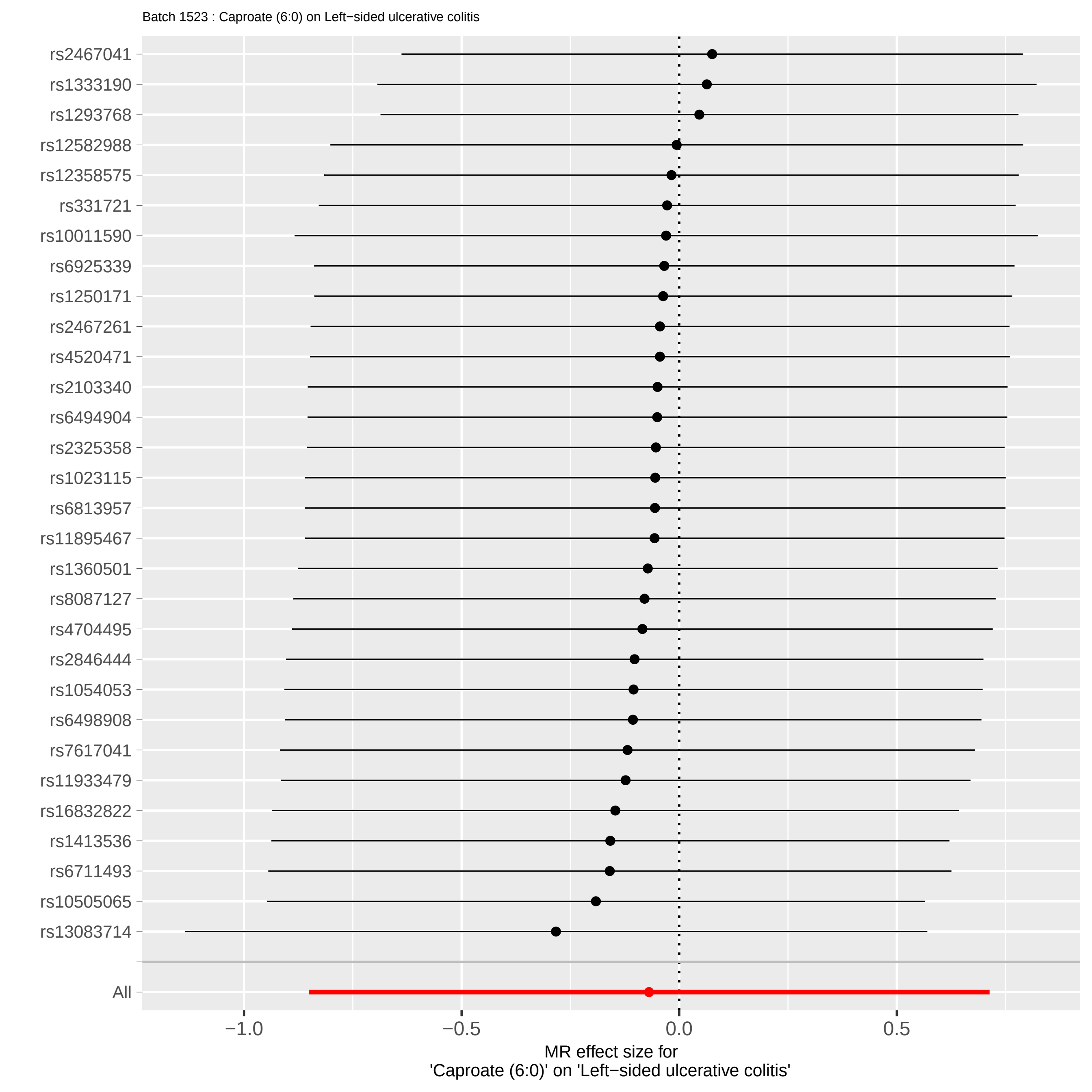
Batch 1523 : Caproate (6:0) on Left−sided ulcerative colitis
rs2467041
rs1333190
rs1293768
rs12582988
rs12358575
rs331721
rs10011590
rs6925339
rs1250171
rs2467261
rs4520471
rs2103340
rs6494904
rs2325358
rs1023115
rs6813957
rs11895467
rs1360501
rs8087127
rs4704495
rs2846444
rs1054053
rs6498908
rs7617041
rs11933479
rs16832822
rs1413536
rs6711493
rs10505065
rs13083714
All
−1.0
−0.5
0.0
0.5
MR effect size for
'Caproate (6:0)' on 'Left−sided ulcerative colitis'

Batch 1527 : Phenol sulfate on Left−sided ulcerative colitis

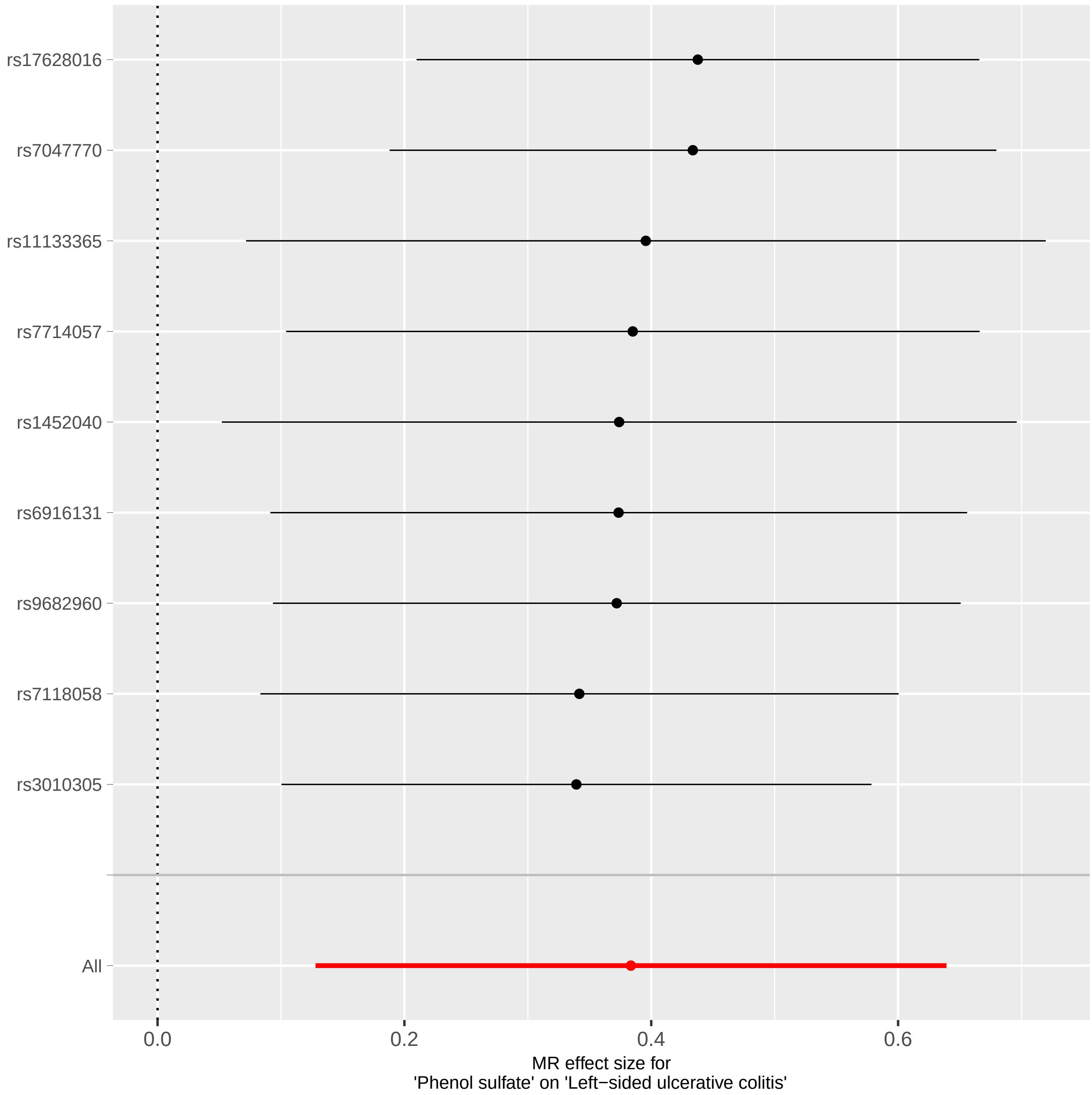

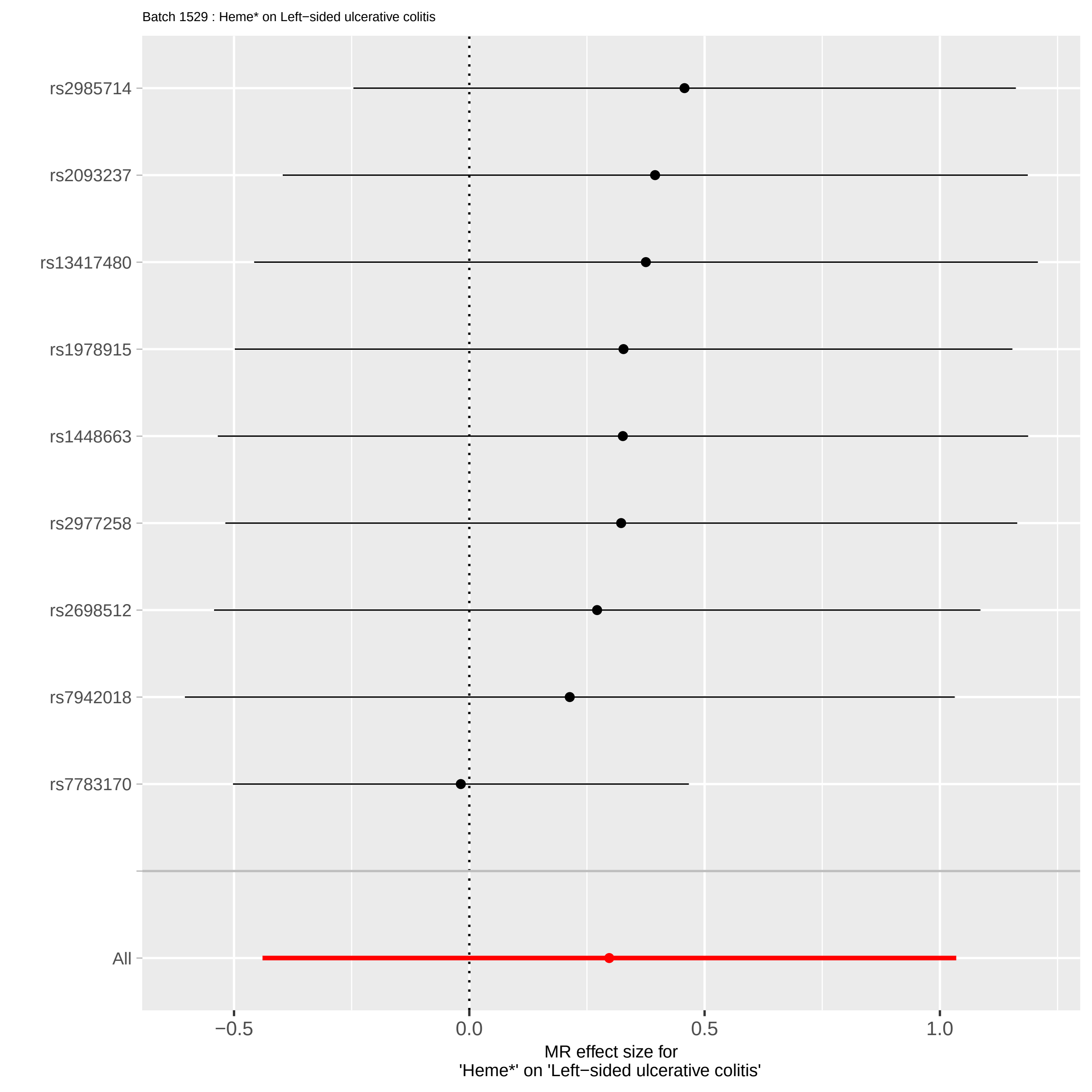

Batch 1529 : Heme* on Left−sided ulcerative colitis
rs2985714
rs2093237
rs13417480
rs1978915
rs1448663
rs2977258
rs2698512
rs7942018
rs7783170
All
−0.5
0.0
0.5
1.0
MR effect size for
'Heme*' on 'Left−sided ulcerative colitis'

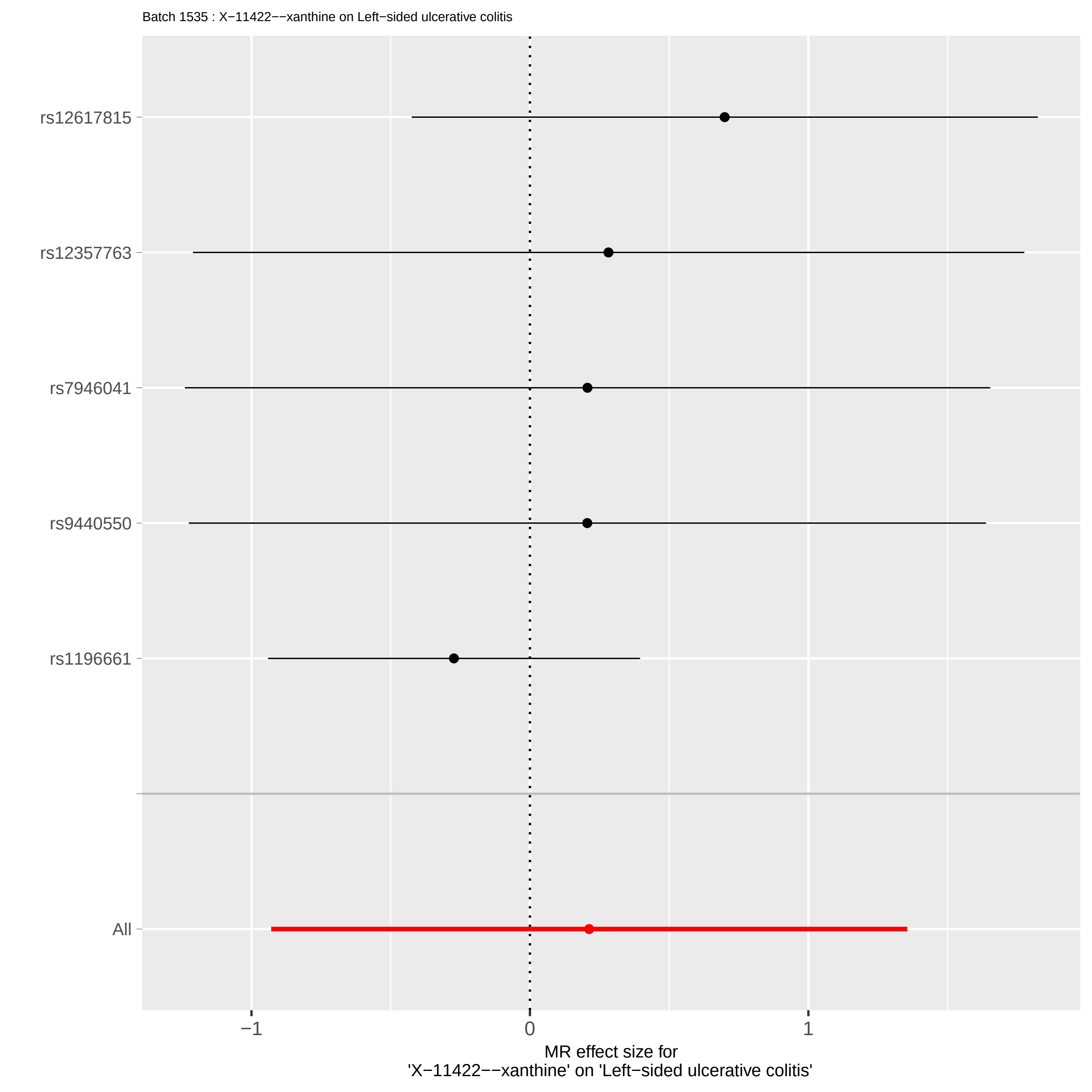
Batch 1535 : X−11422−−xanthine on Left−sided ulcerative colitis
rs12617815
rs12357763
rs7946041
rs9440550
rs1196661
All
−1
0
1
MR effect size for
'X−11422−−xanthine' on 'Left−sided ulcerative colitis'

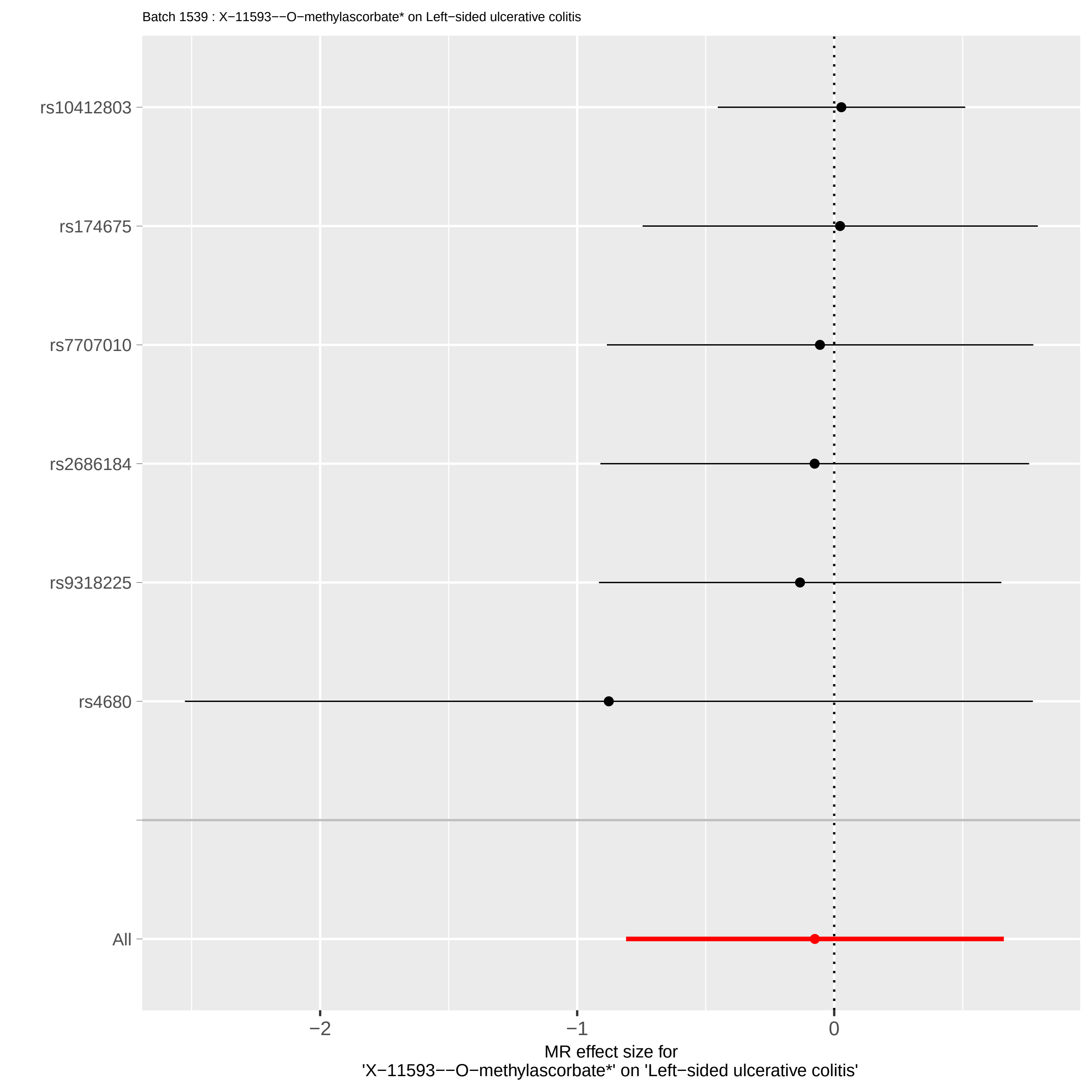

Batch 1539 : X−11593−−O−methylascorbate* on Left−sided ulcerative colitis
rs10412803
rs174675
rs7707010
rs2686184
rs9318225
rs4680
All
−2
−1
0
MR effect size for
'X−11593−−O−methylascorbate*' on 'Left−sided ulcerative colitis'

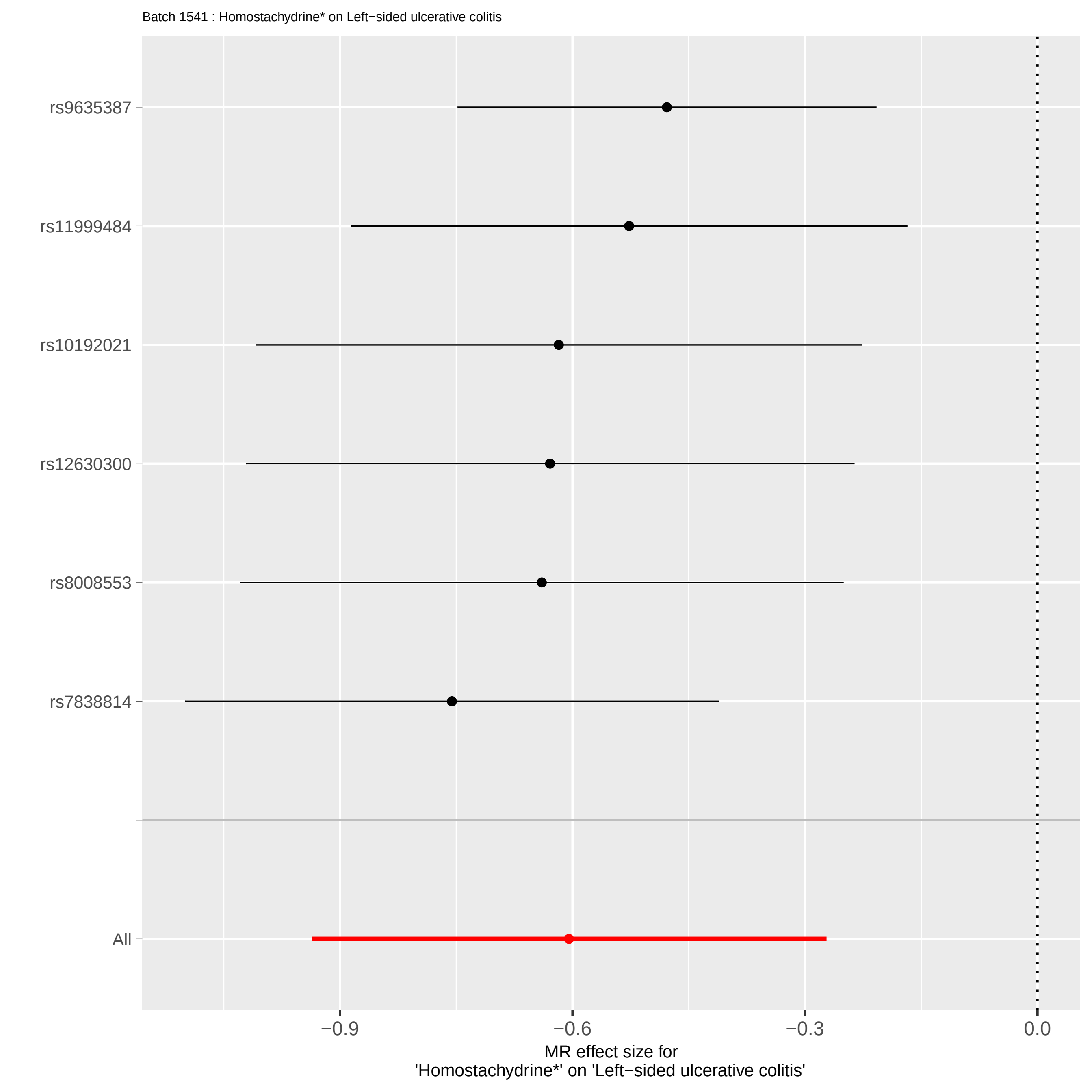

Batch 1541 : Homostachydrine* on Left−sided ulcerative colitis
rs9635387
rs11999484
rs10192021
rs12630300
rs8008553
rs7838814
All
−0.9
−0.6
−0.3
0.0
MR effect size for
'Homostachydrine*' on 'Left−sided ulcerative colitis'

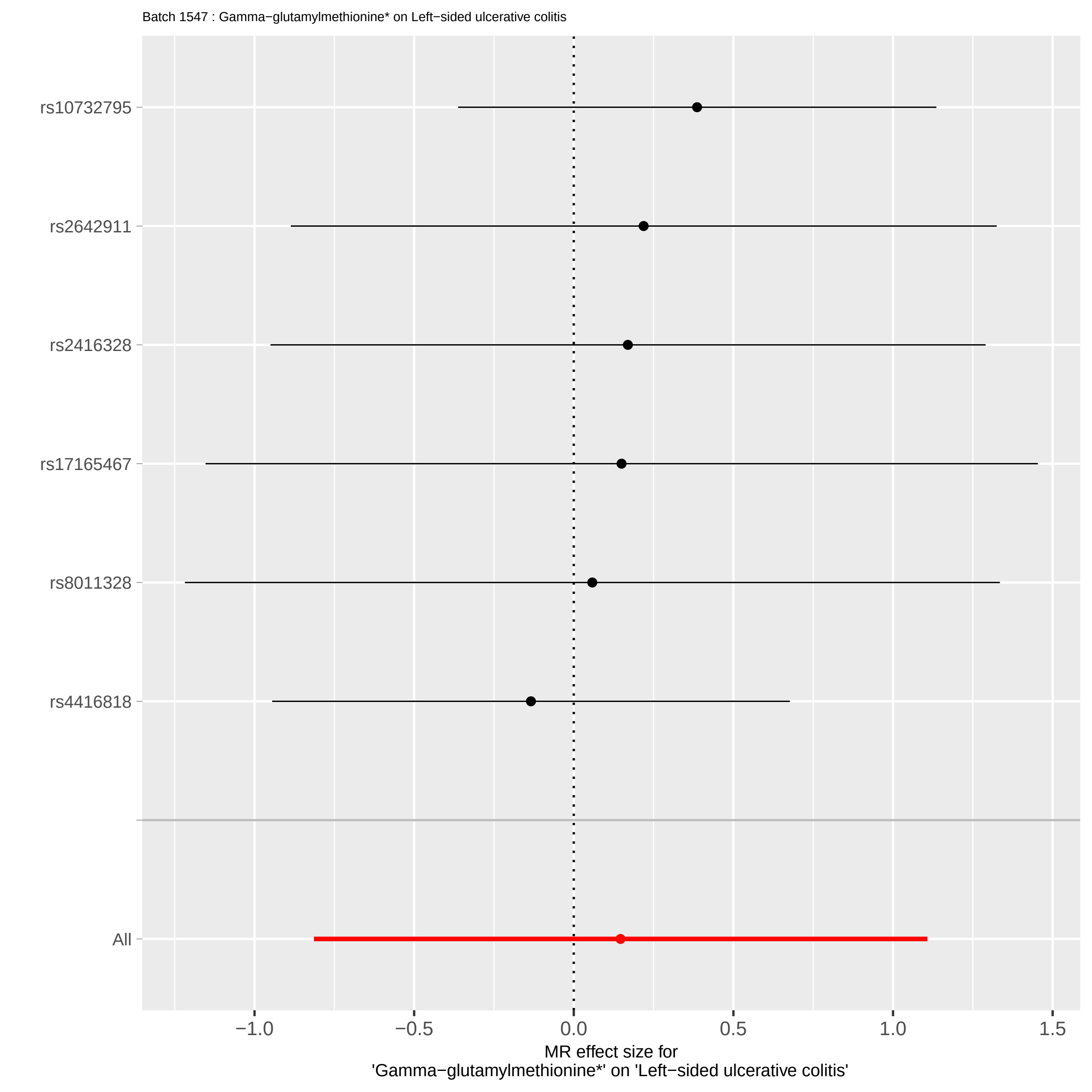

Batch 1547 : Gamma−glutamylmethionine* on Left−sided ulcerative colitis
rs10732795
rs2642911
rs2416328
rs17165467
rs8011328
rs4416818
All
−1.0
−0.5
0.0
0.5
1.0
1.5
MR effect size for
'Gamma−glutamylmethionine*' on 'Left−sided ulcerative colitis'

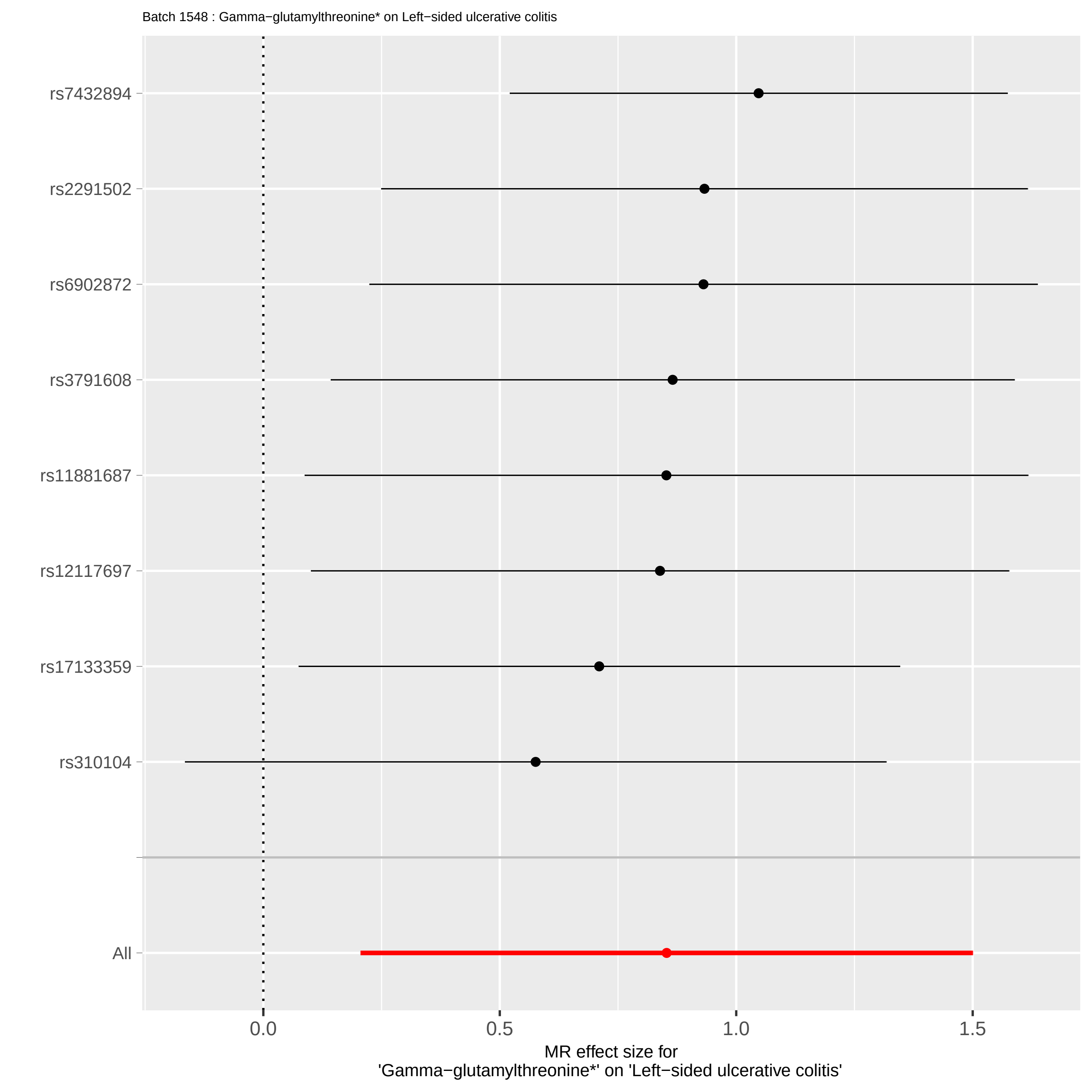
Batch 1548 : Gamma−glutamylthreonine* on Left−sided ulcerative colitis
rs7432894
rs2291502
rs6902872
rs3791608
rs11881687
rs12117697
rs17133359
rs310104
All
0.0
0.5
1.0
1.5
MR effect size for
'Gamma−glutamylthreonine*' on 'Left−sided ulcerative colitis'

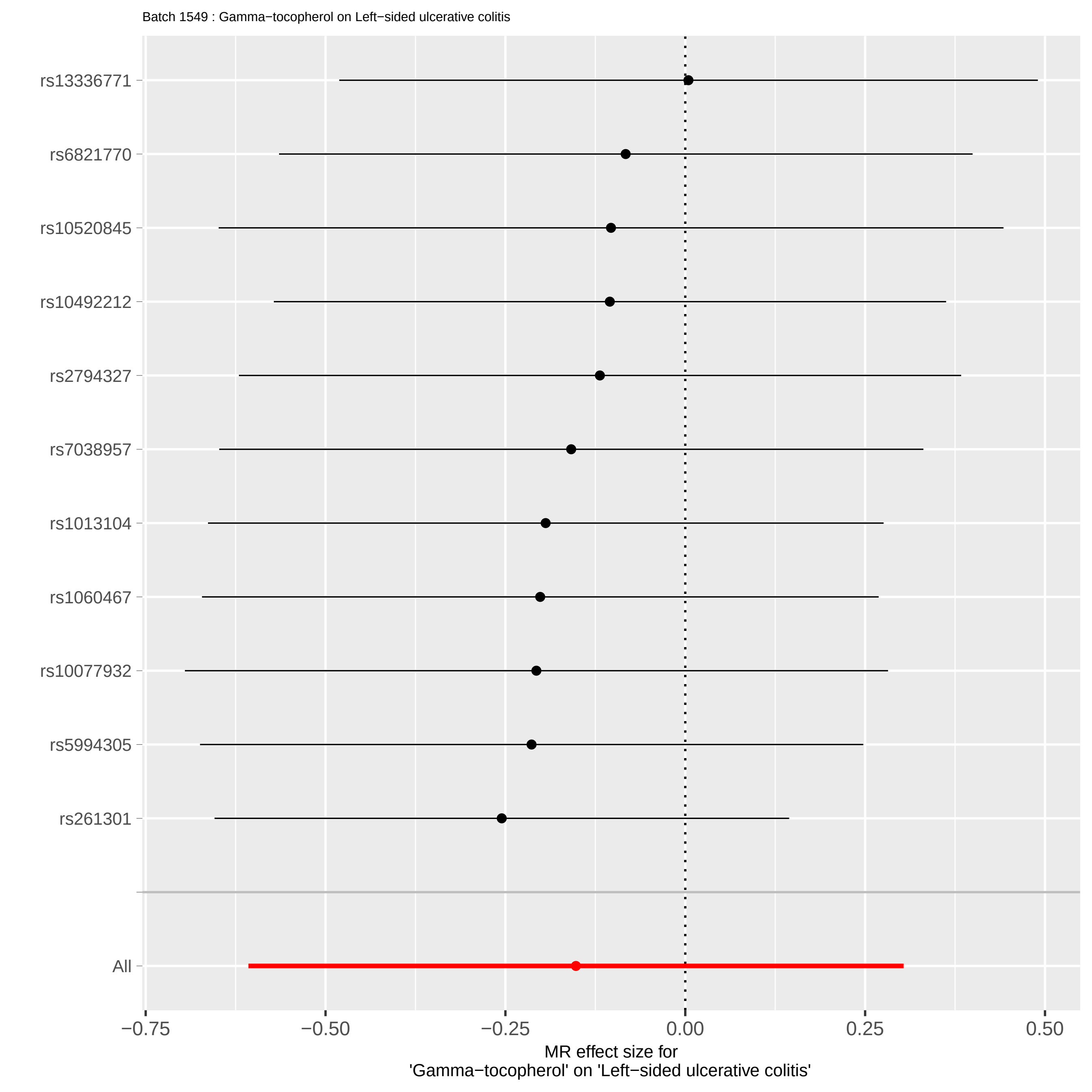

Batch 1549 : Gamma−tocopherol on Left−sided ulcerative colitis
rs13336771
rs6821770
rs10520845
rs10492212
rs2794327
rs7038957
rs1013104
rs1060467
rs10077932
rs5994305
rs261301
All
−0.75
−0.50
−0.25
0.00
0.25
0.50
MR effect size for
'Gamma−tocopherol' on 'Left−sided ulcerative colitis'

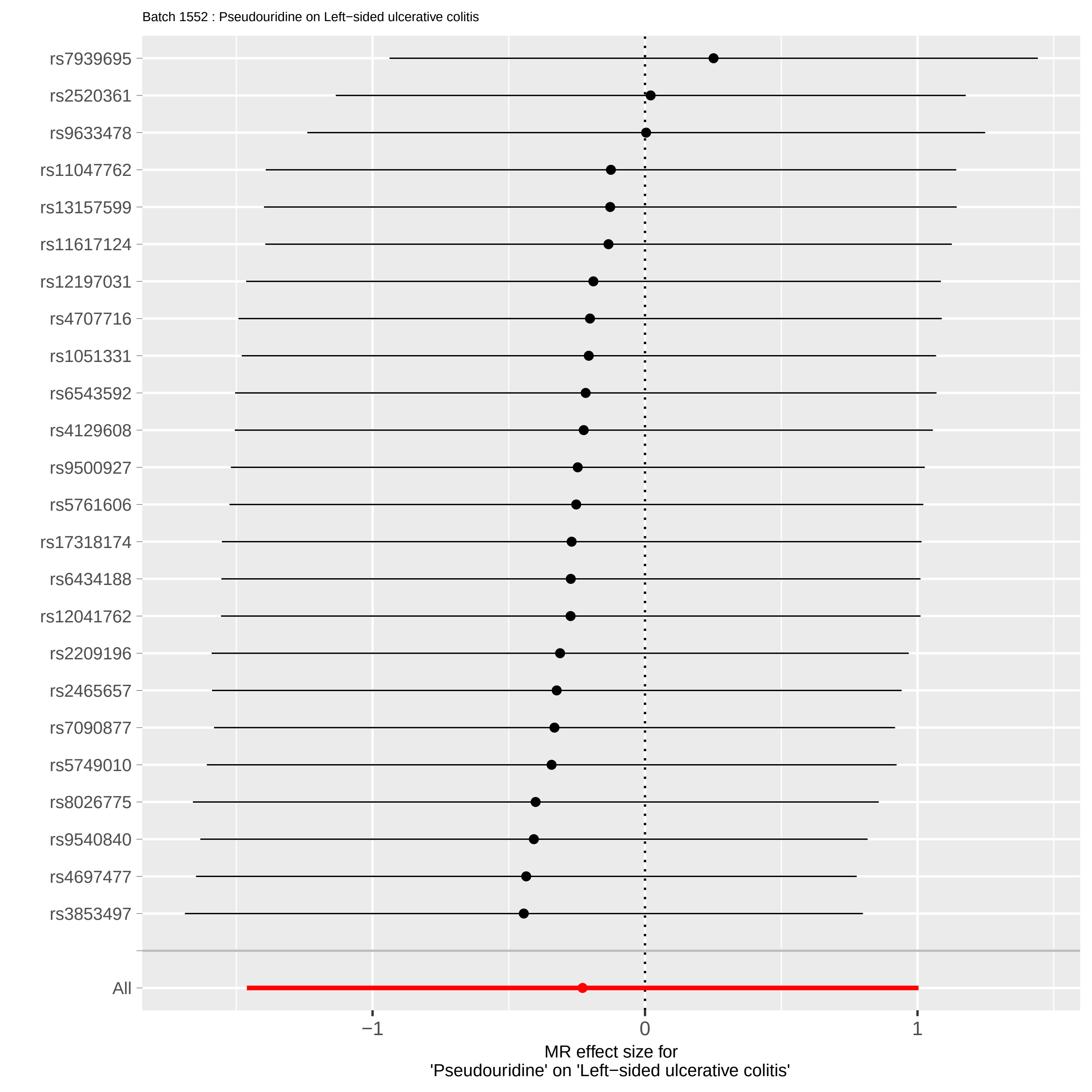

Batch 1552 : Pseudouridine on Left−sided ulcerative colitis
rs7939695
rs2520361
rs9633478
rs11047762
rs13157599
rs11617124
rs12197031
rs4707716
rs1051331
rs6543592
rs4129608
rs9500927
rs5761606
rs17318174
rs6434188
rs12041762
rs2209196
rs2465657
rs7090877
rs5749010
rs8026775
rs9540840
rs4697477
rs3853497
All
−1
0
1
MR effect size for
'Pseudouridine' on 'Left−sided ulcerative colitis'

Batch 1553 : Valerate on Left−sided ulcerative colitis

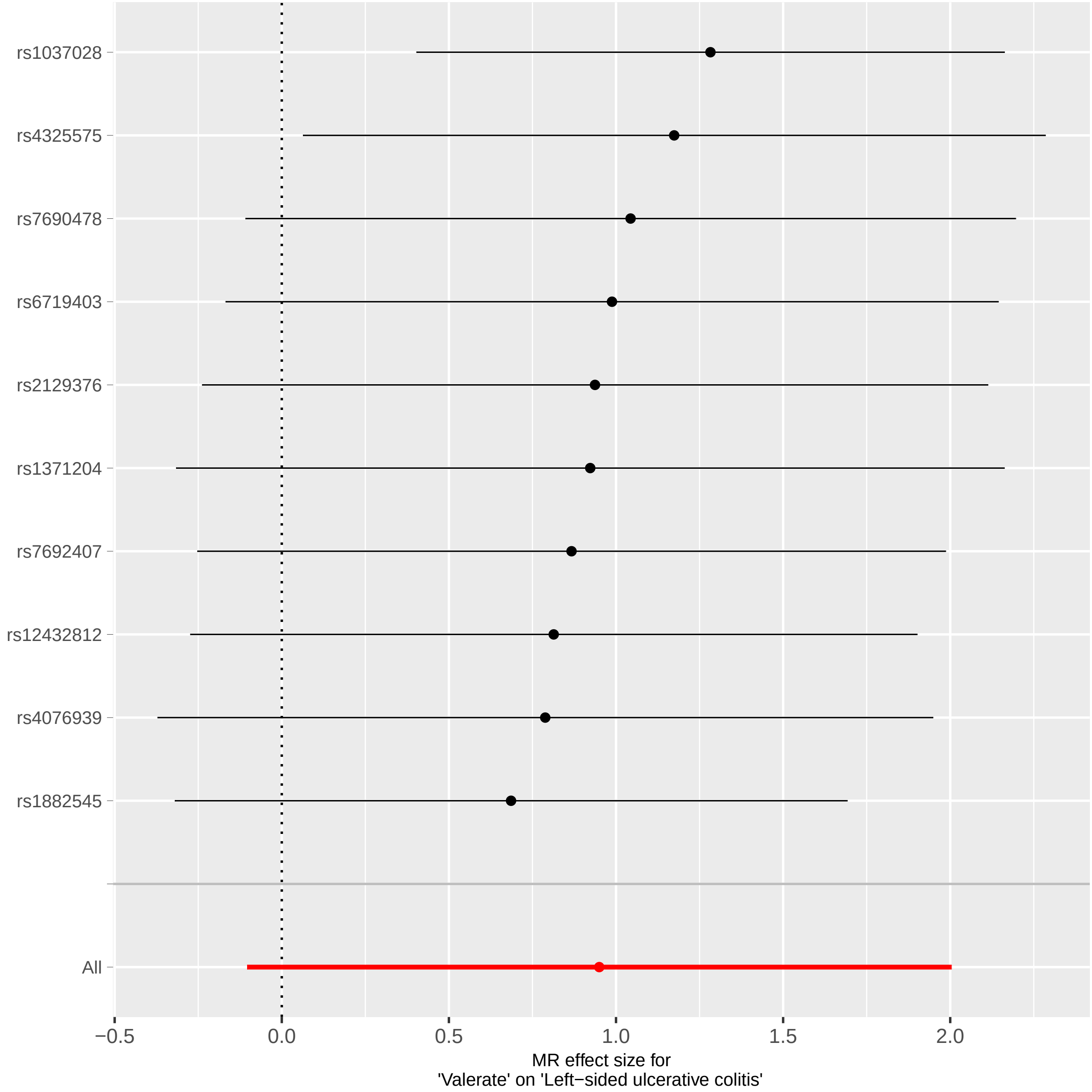

Batch 1556 : Erythronate* on Left−sided ulcerative colitis

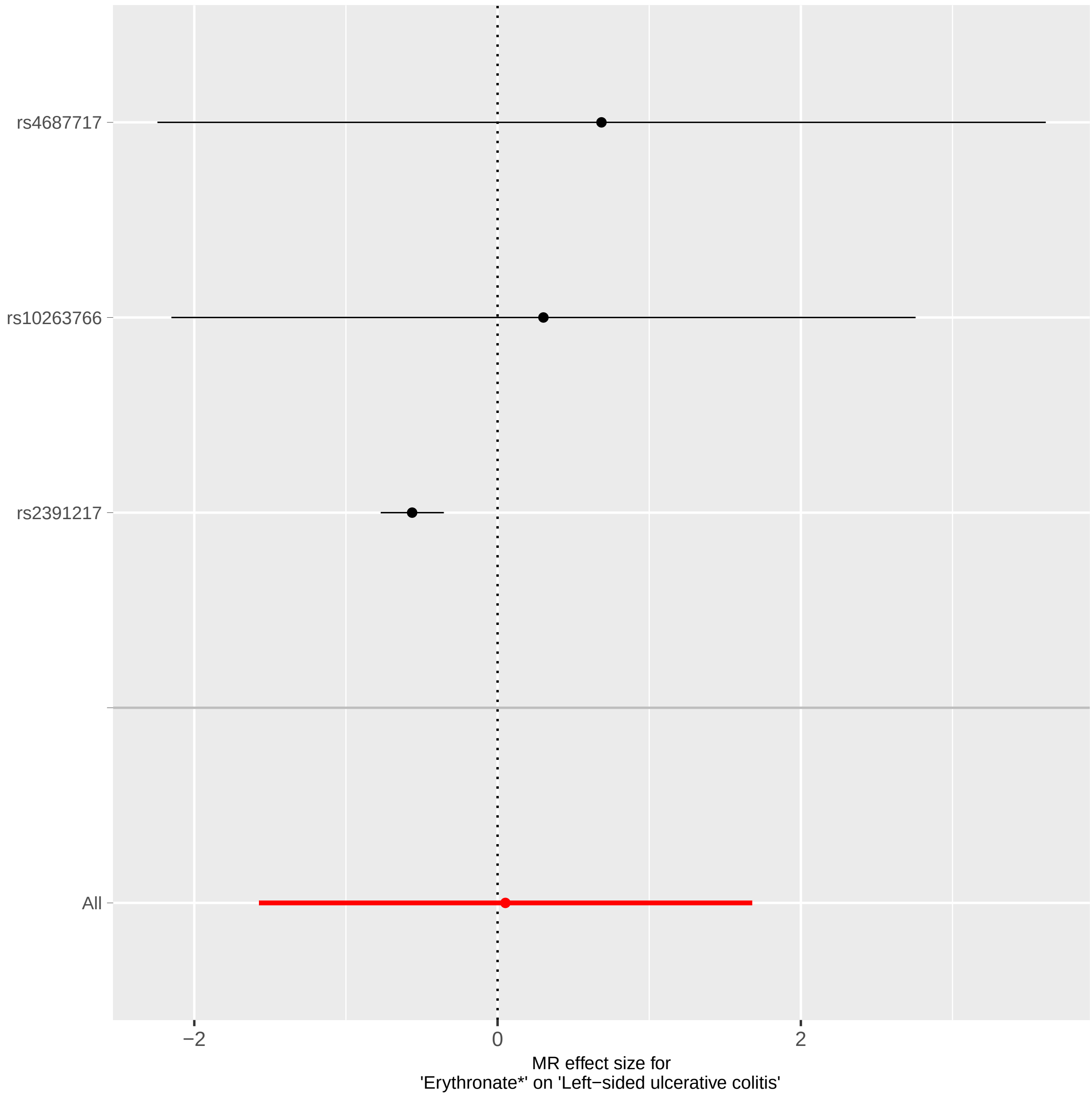

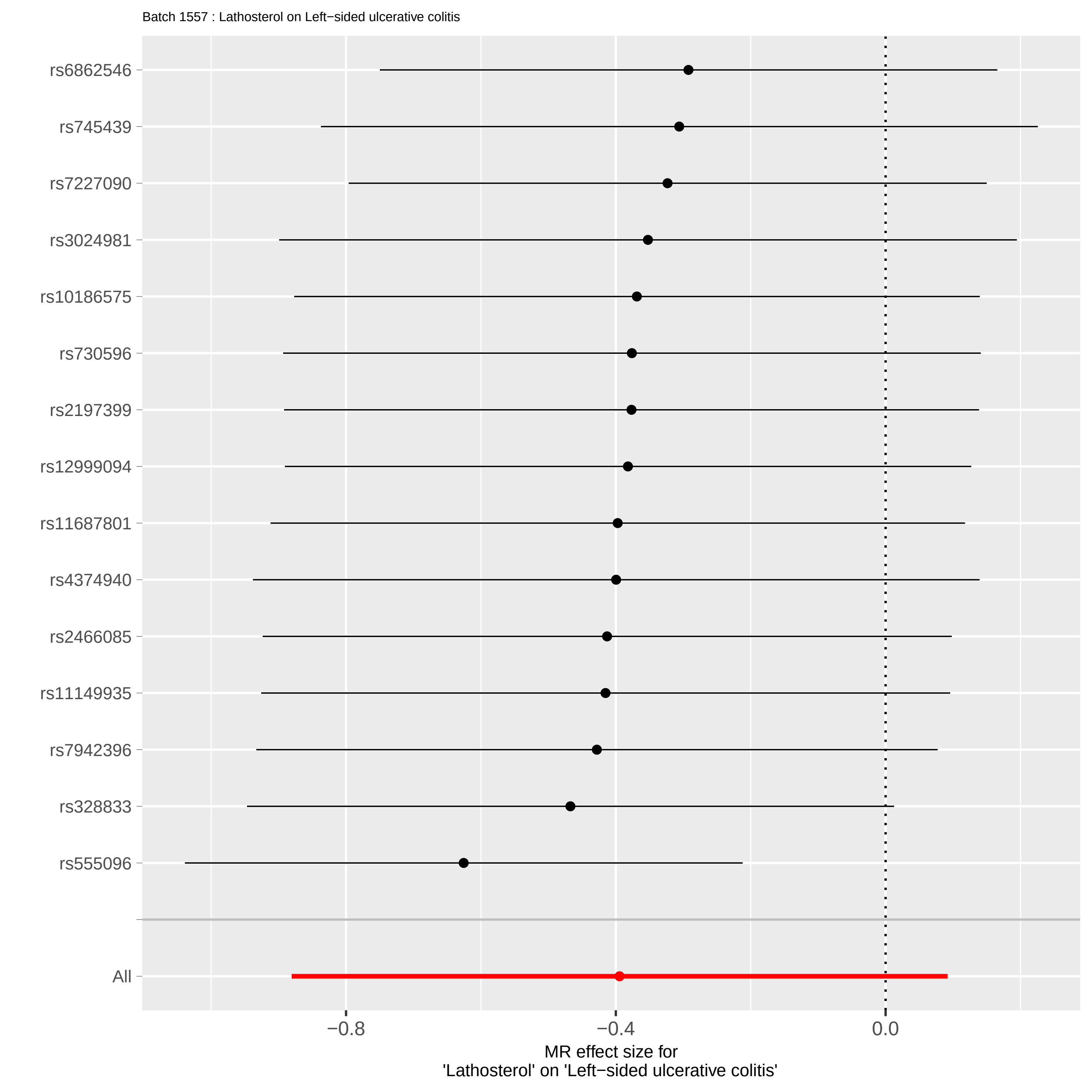
Batch 1557 : Lathosterol on Left−sided ulcerative colitis
rs6862546
rs745439
rs7227090
rs3024981
rs10186575
rs730596
rs2197399
rs12999094
rs11687801
rs4374940
rs2466085
rs11149935
rs7942396
rs328833
rs555096
All
−0.8
−0.4
0.0
MR effect size for
'Lathosterol' on 'Left−sided ulcerative colitis'

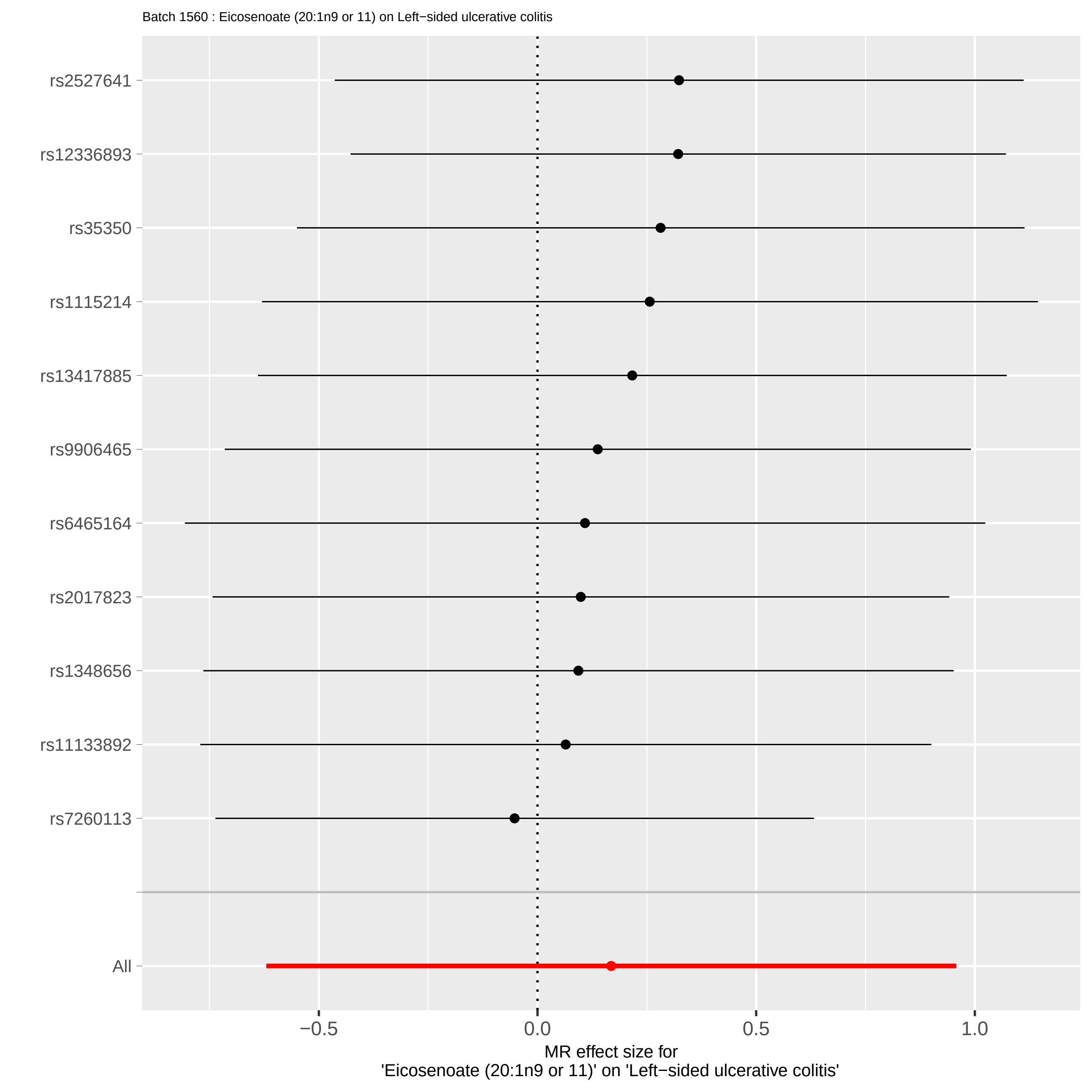

Batch 1560 : Eicosenoate (20:1n9 or 11) on Left−sided ulcerative colitis
rs2527641
rs12336893
rs35350
rs1115214
rs13417885
rs9906465
rs6465164
rs2017823
rs1348656
rs11133892
rs7260113
All
−0.5
0.0
0.5
1.0
MR effect size for
'Eicosenoate (20:1n9 or 11)' on 'Left−sided ulcerative colitis'

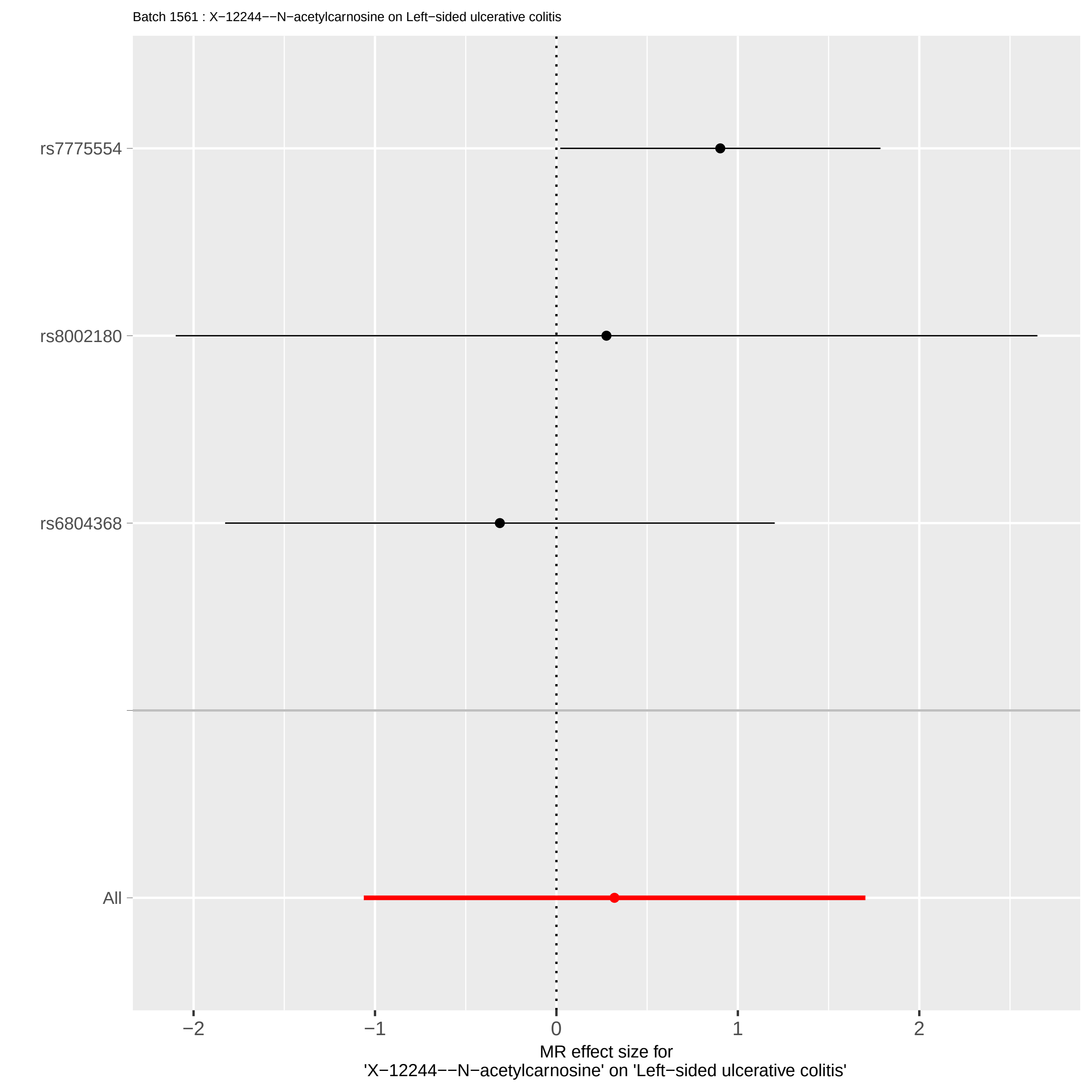

Batch 1561 : X−12244−−N−acetylcarnosine on Left−sided ulcerative colitis
rs7775554
rs8002180
rs6804368
All
−2
−1
0
1
2
MR effect size for
'X−12244−−N−acetylcarnosine' on 'Left−sided ulcerative colitis'

Batch 1564 : 1−docosahexaenoylglycerophosphocholine* on Left−sided ulcerative colitis

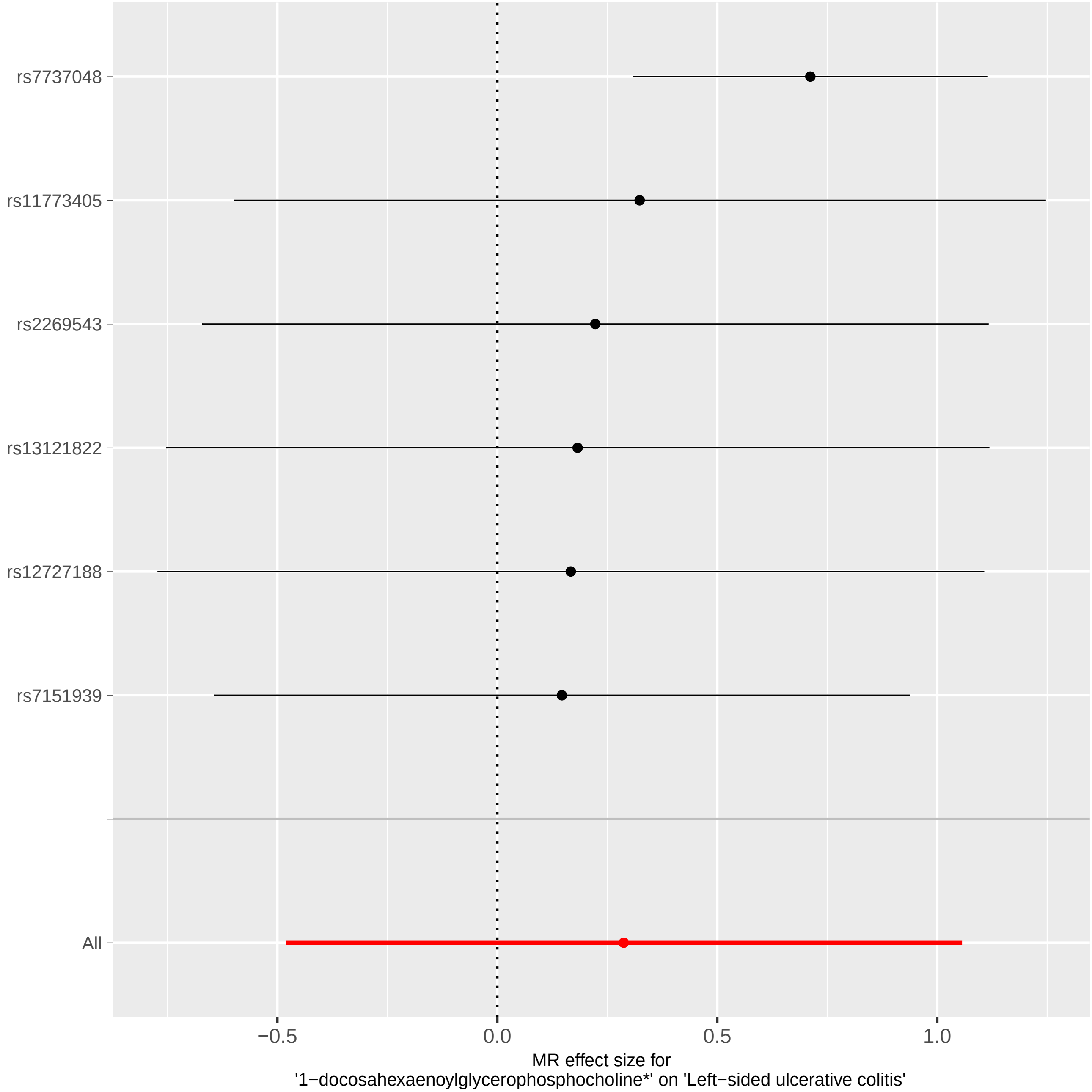

Batch 1569 : Alpha−hydroxyisovalerate on Left−sided ulcerative colitis

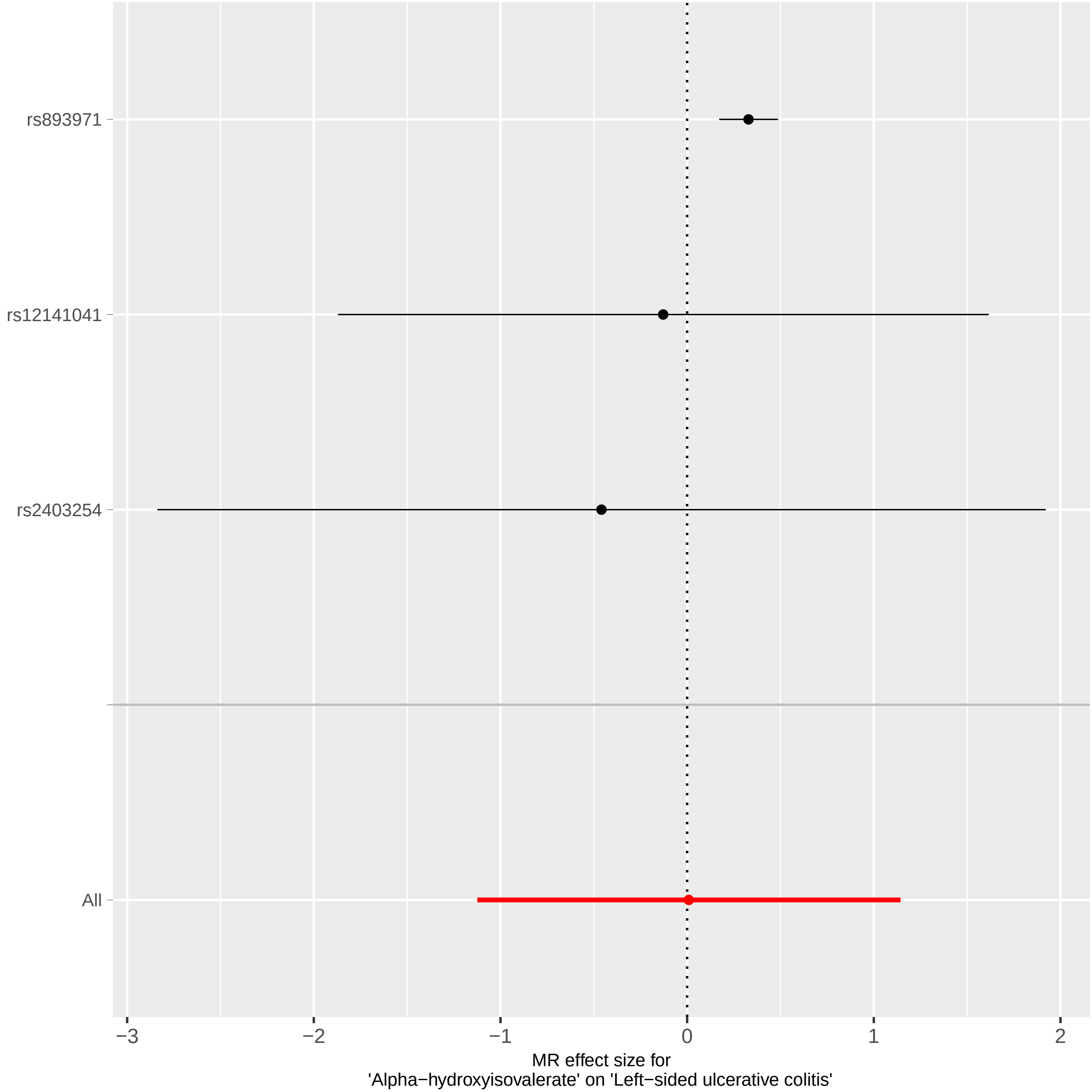

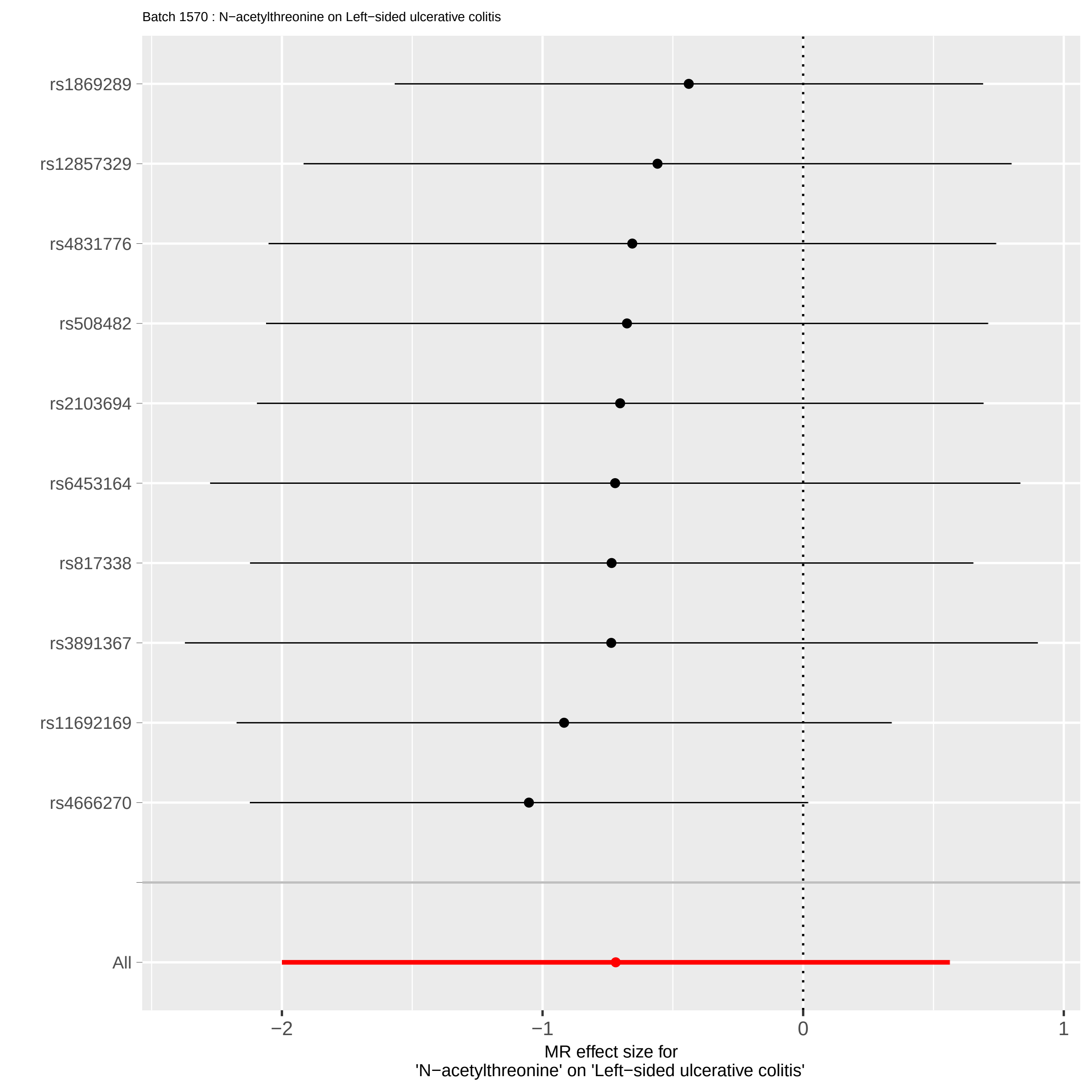

Batch 1570 : N−acetylthreonine on Left−sided ulcerative colitis
rs1869289
rs12857329
rs4831776
rs508482
rs2103694
rs6453164
rs817338
rs3891367
rs11692169
rs4666270
All
−2
−1
0
1
MR effect size for
'N−acetylthreonine' on 'Left−sided ulcerative colitis'

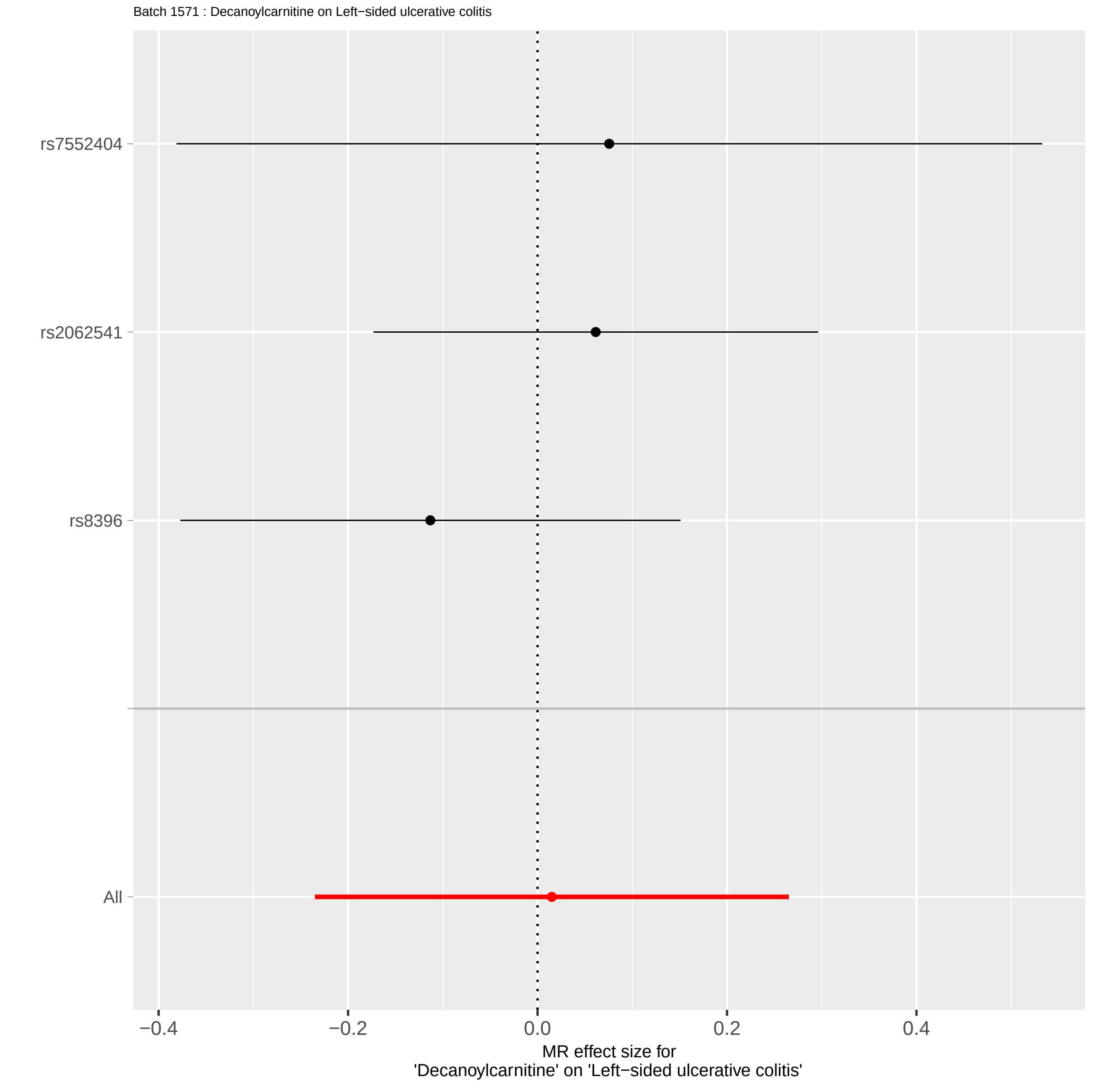
Batch 1571 : Decanoylcarnitine on Left−sided ulcerative colitis
rs7552404
rs2062541
rs8396
All
−0.4
−0.2
0.0
0.2
0.4
MR effect size for
'Decanoylcarnitine' on 'Left−sided ulcerative colitis'

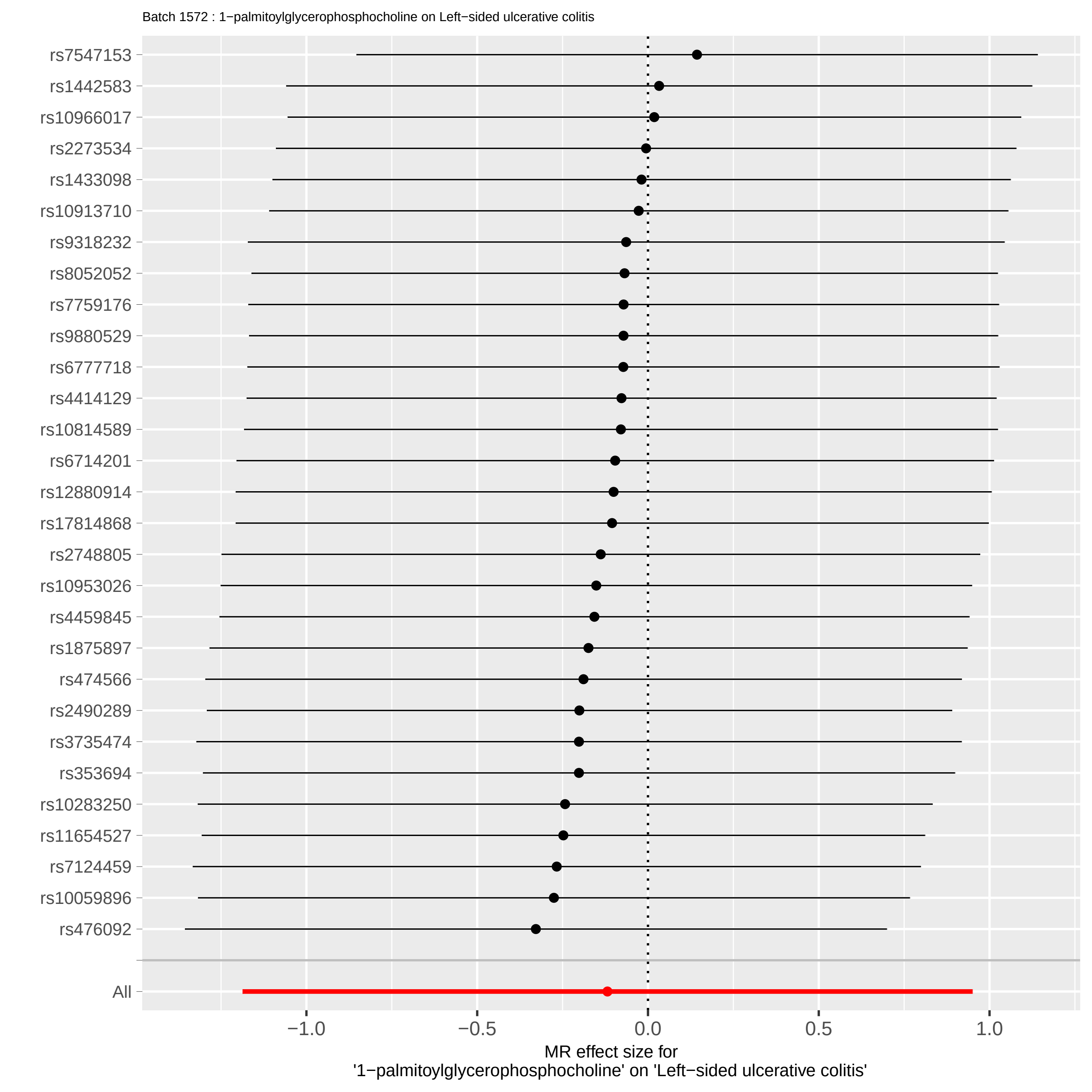

Batch 1572 : 1−palmitoylglycerophosphocholine on Left−sided ulcerative colitis
rs7547153
rs1442583
rs10966017
rs2273534
rs1433098
rs10913710
rs9318232
rs8052052
rs7759176
rs9880529
rs6777718
rs4414129
rs10814589
rs6714201
rs12880914
rs17814868
rs2748805
rs10953026
rs4459845
rs1875897
rs474566
rs2490289
rs3735474
rs353694
rs10283250
rs11654527
rs7124459
rs10059896
rs476092
All
−1.0
−0.5
0.0
0.5
1.0
MR effect size for
'1−palmitoylglycerophosphocholine' on 'Left−sided ulcerative colitis'

Batch 1573 : 1−heptadecanoylglycerophosphocholine on Left−sided ulcerative colitis

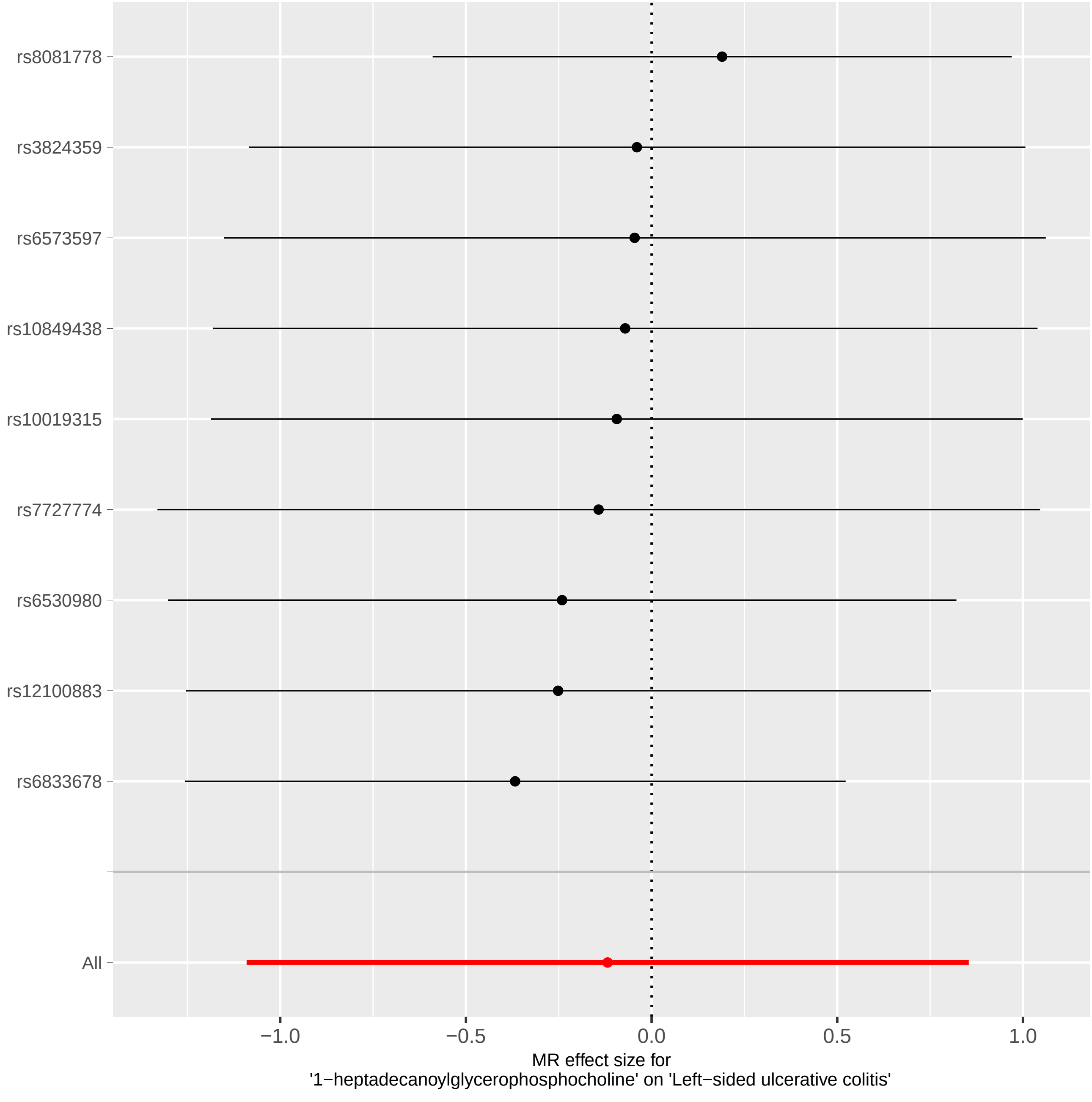

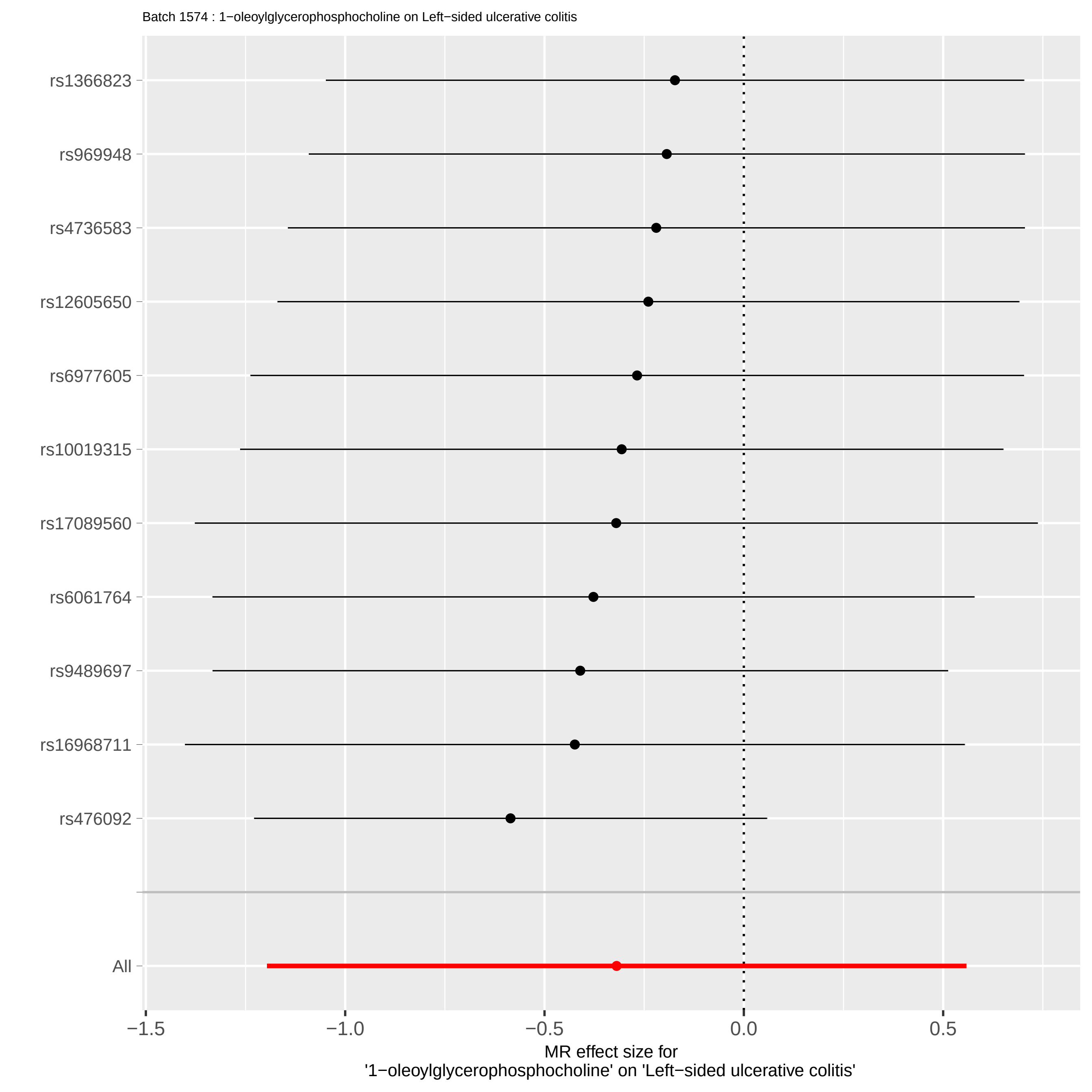

Batch 1574 : 1−oleoylglycerophosphocholine on Left−sided ulcerative colitis
rs1366823
rs969948
rs4736583
rs12605650
rs6977605
rs10019315
rs17089560
rs6061764
rs9489697
rs16968711
rs476092
All
−1.5
−1.0
−0.5
0.0
0.5
MR effect size for
'1−oleoylglycerophosphocholine' on 'Left−sided ulcerative colitis'

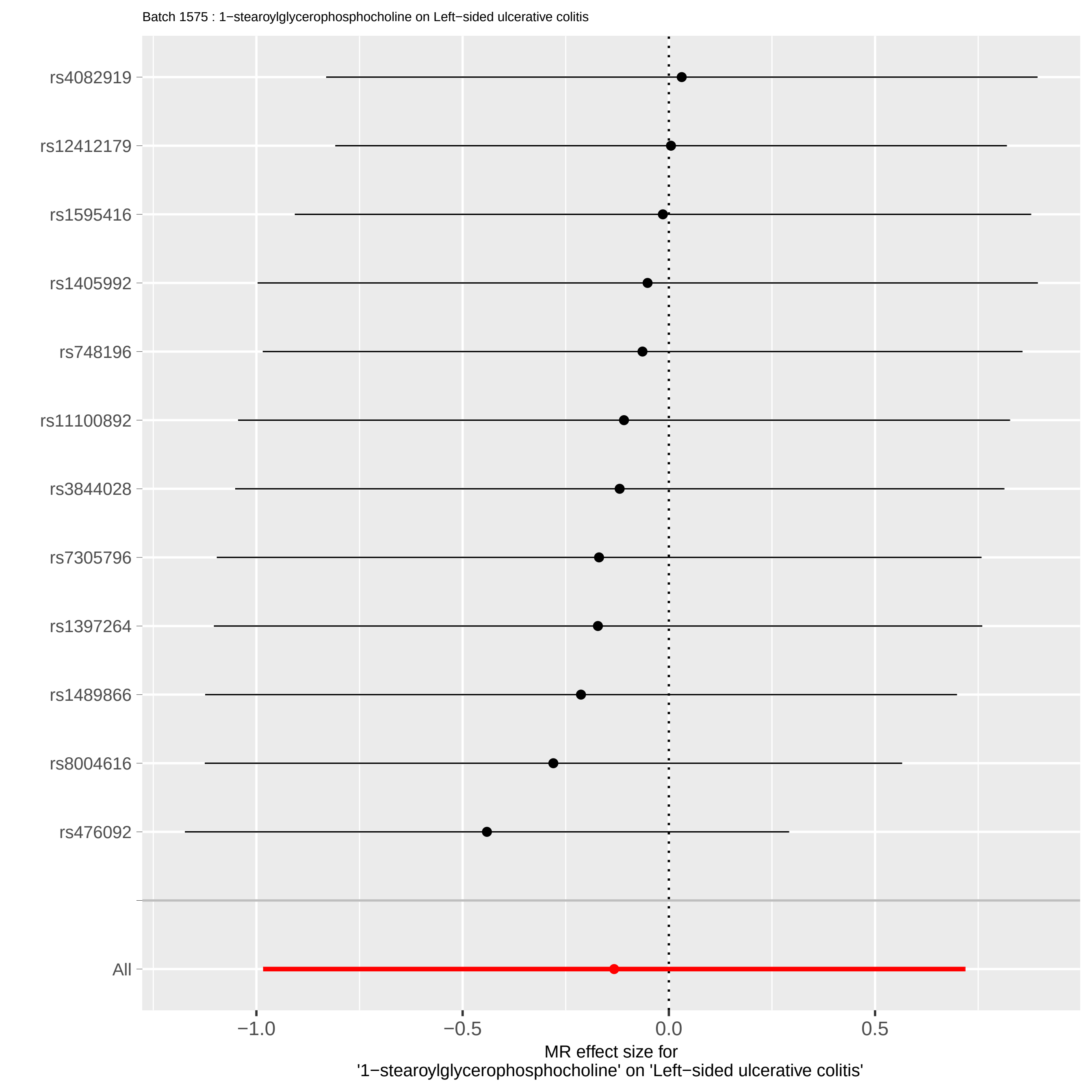

Batch 1575 : 1−stearoylglycerophosphocholine on Left−sided ulcerative colitis
rs4082919
rs12412179
rs1595416
rs1405992
rs748196
rs11100892
rs3844028
rs7305796
rs1397264
rs1489866
rs8004616
rs476092
All
−1.0
−0.5
0.0
0.5
MR effect size for
'1−stearoylglycerophosphocholine' on 'Left−sided ulcerative colitis'

Batch 1578 : 10−heptadecenoate (17:1n7) on Left−sided ulcerative colitis

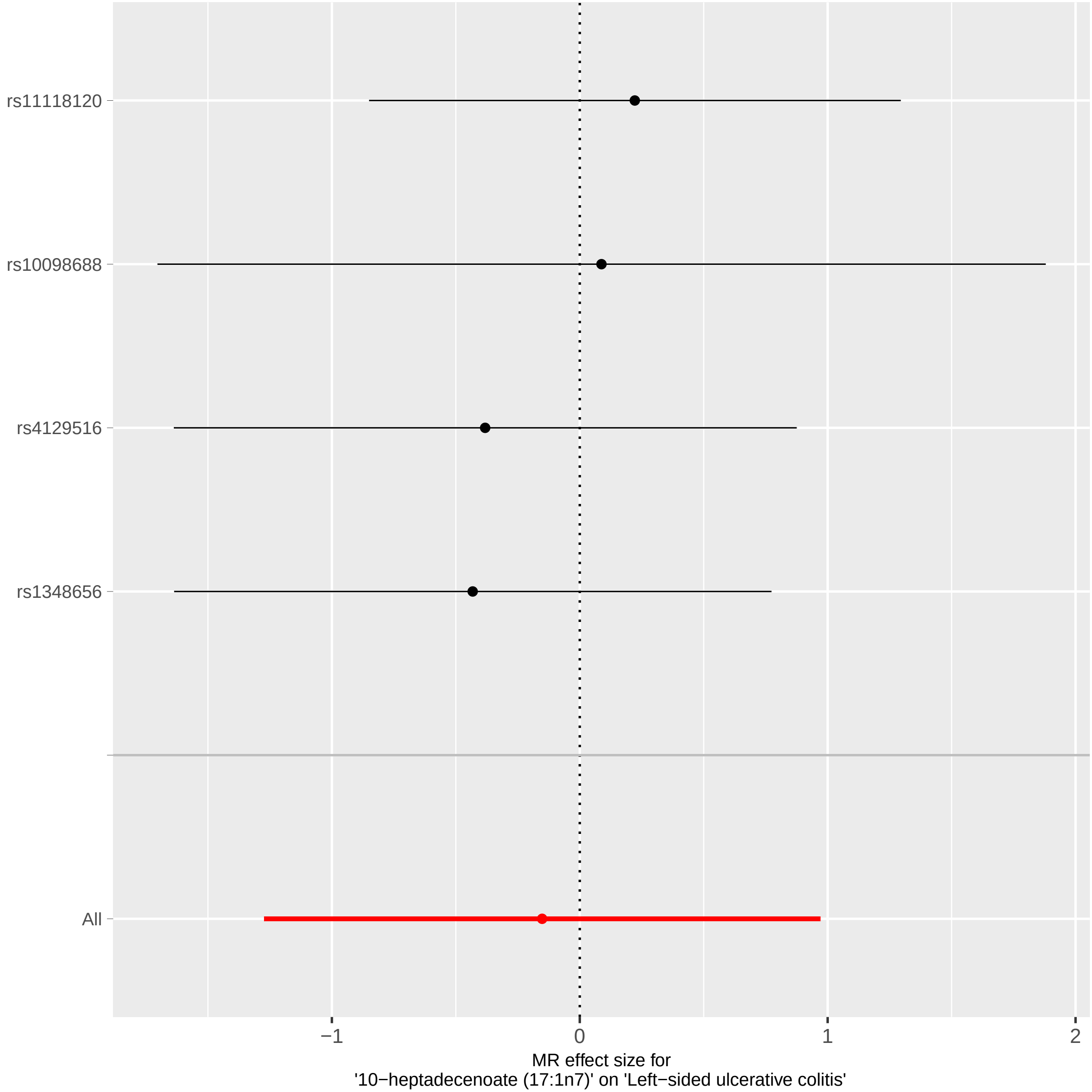

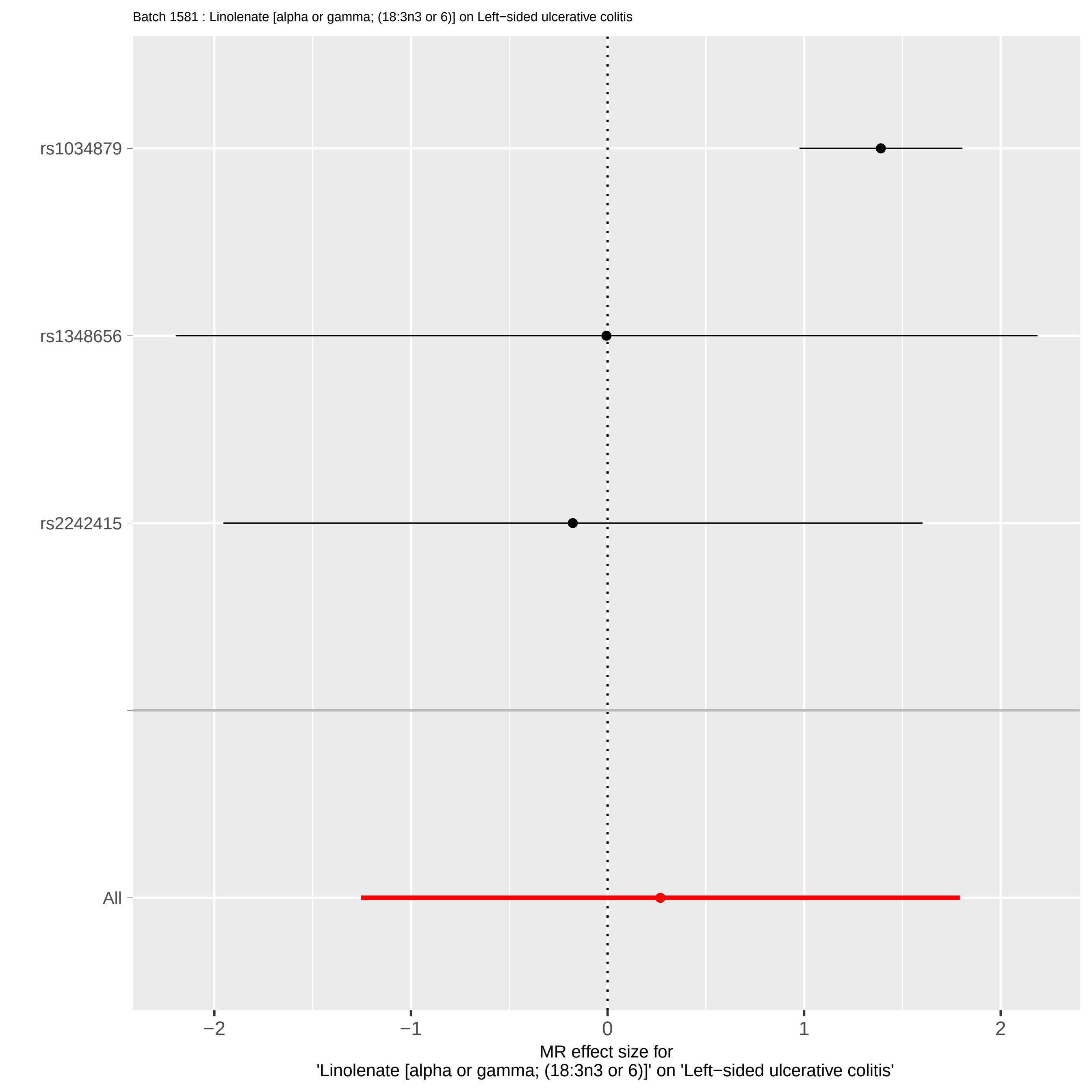
Batch 1581 : Linolenate [alpha or gamma; (18:3n3 or 6)] on Left−sided ulcerative colitis
rs1034879
rs1348656
rs2242415
All
−2
−1
0
1
2
MR effect size for
'Linolenate [alpha or gamma; (18:3n3 or 6)]' on 'Left−sided ulcerative colitis'

Batch 1582 : X−12510−−2−aminooctanoic acid on Left−sided ulcerative colitis

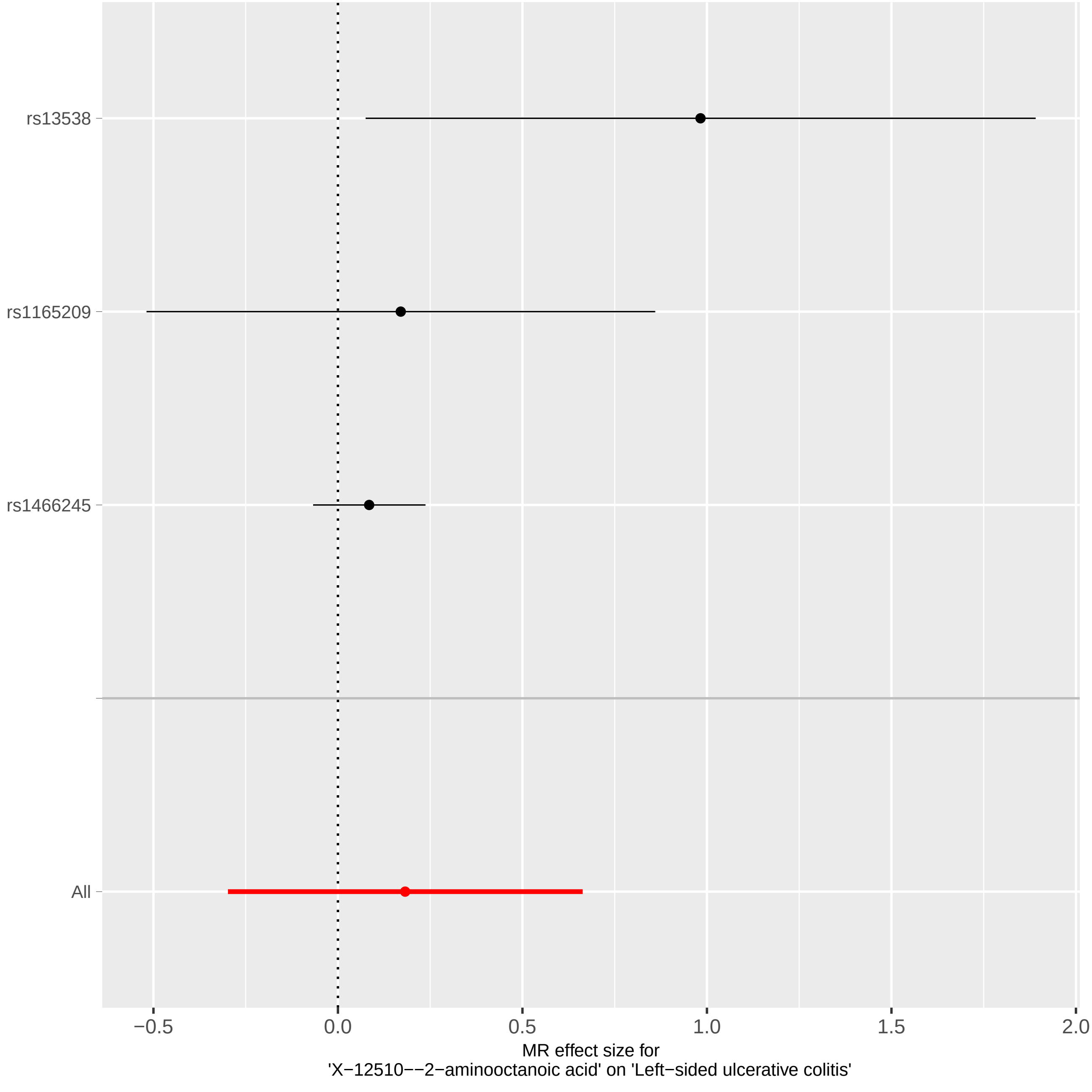

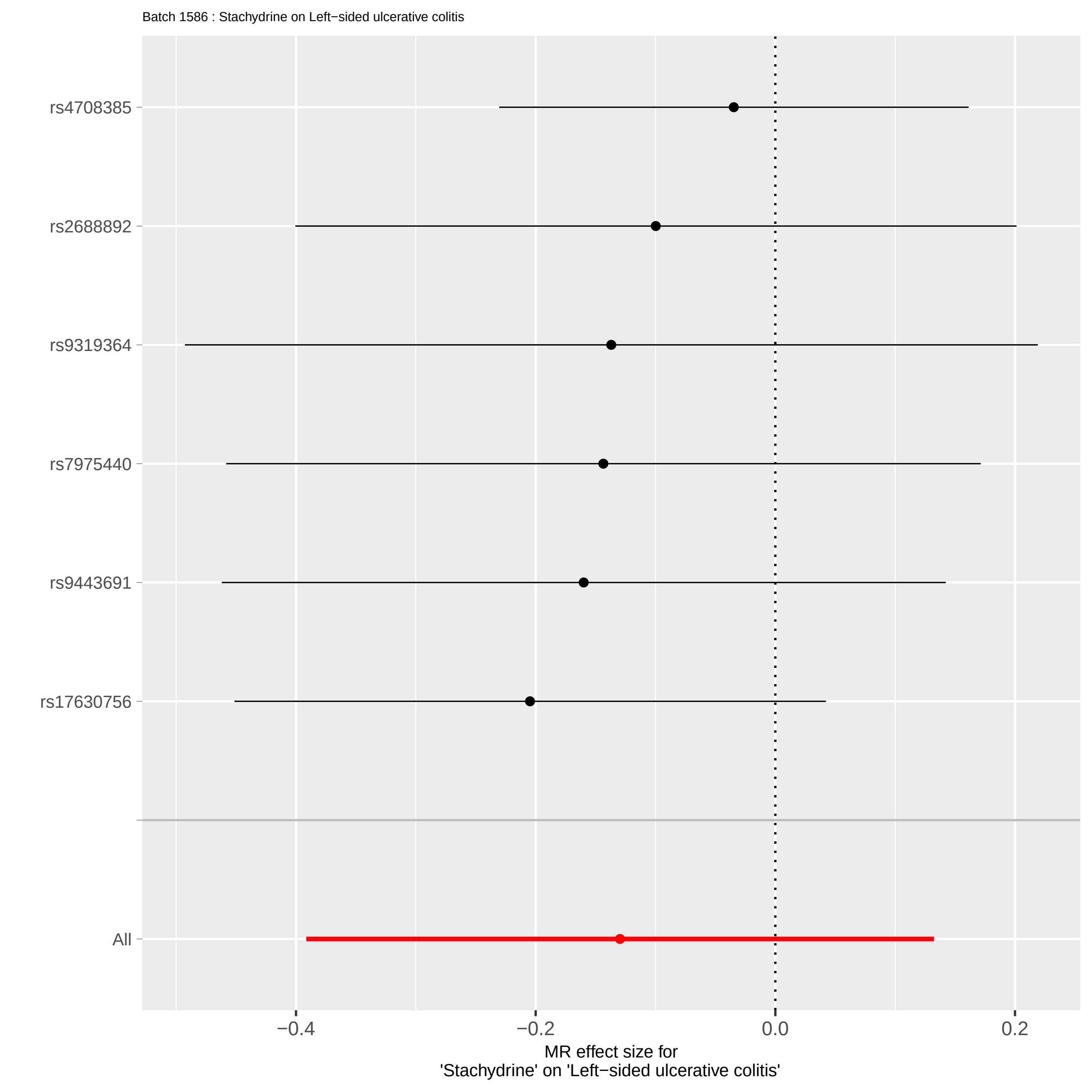

Batch 1586 : Stachydrine on Left−sided ulcerative colitis
rs4708385
rs2688892
rs9319364
rs7975440
rs9443691
rs17630756
All
−0.4
−0.2
0.0
0.2
MR effect size for
'Stachydrine' on 'Left−sided ulcerative colitis'

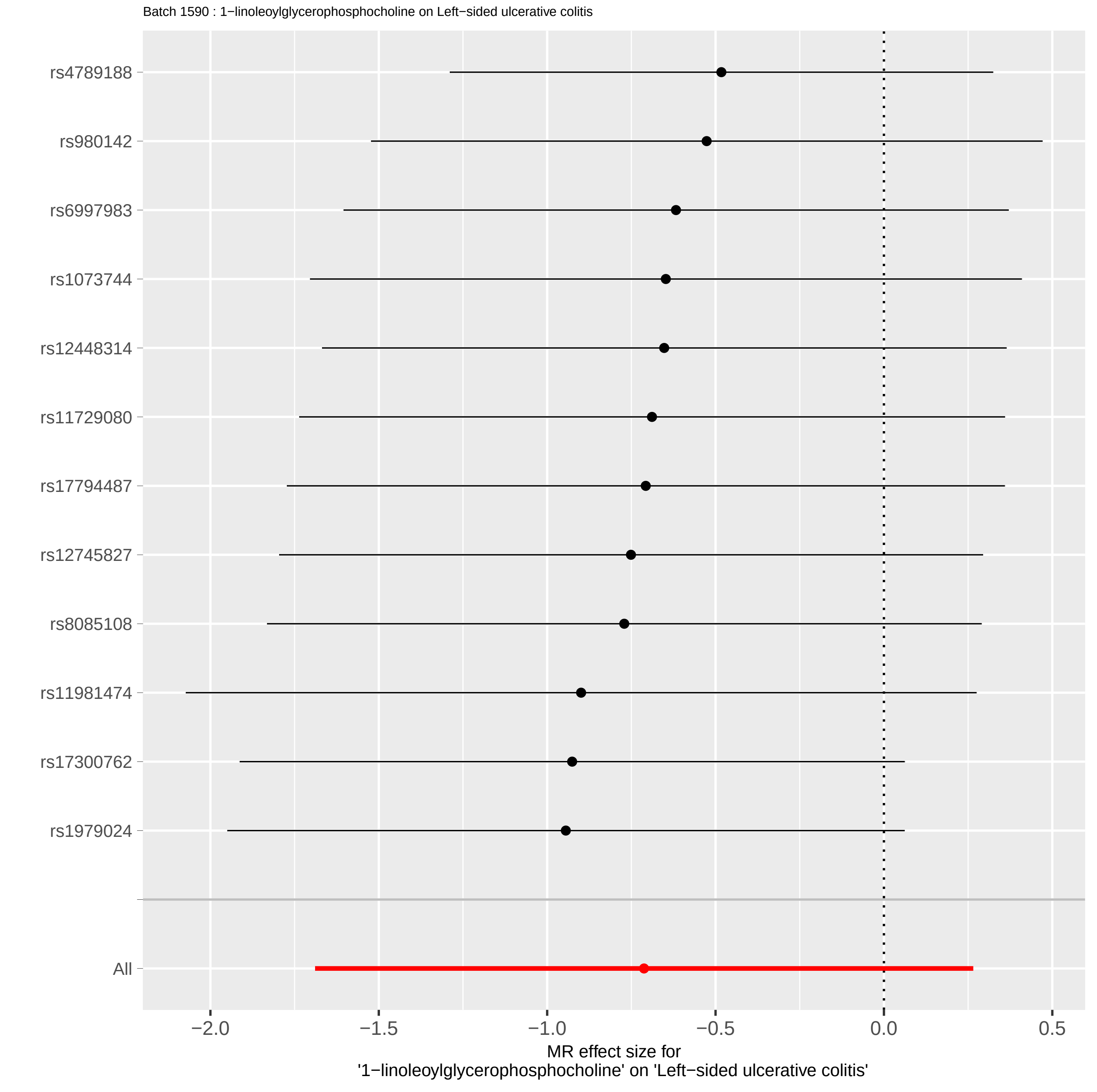

Batch 1590 : 1−linoleoylglycerophosphocholine on Left−sided ulcerative colitis
rs4789188
rs980142
rs6997983
rs1073744
rs12448314
rs11729080
rs17794487
rs12745827
rs8085108
rs11981474
rs17300762
rs1979024
All
−2.0
−1.5
−1.0
−0.5
0.0
0.5
MR effect size for
'1−linoleoylglycerophosphocholine' on 'Left−sided ulcerative colitis'

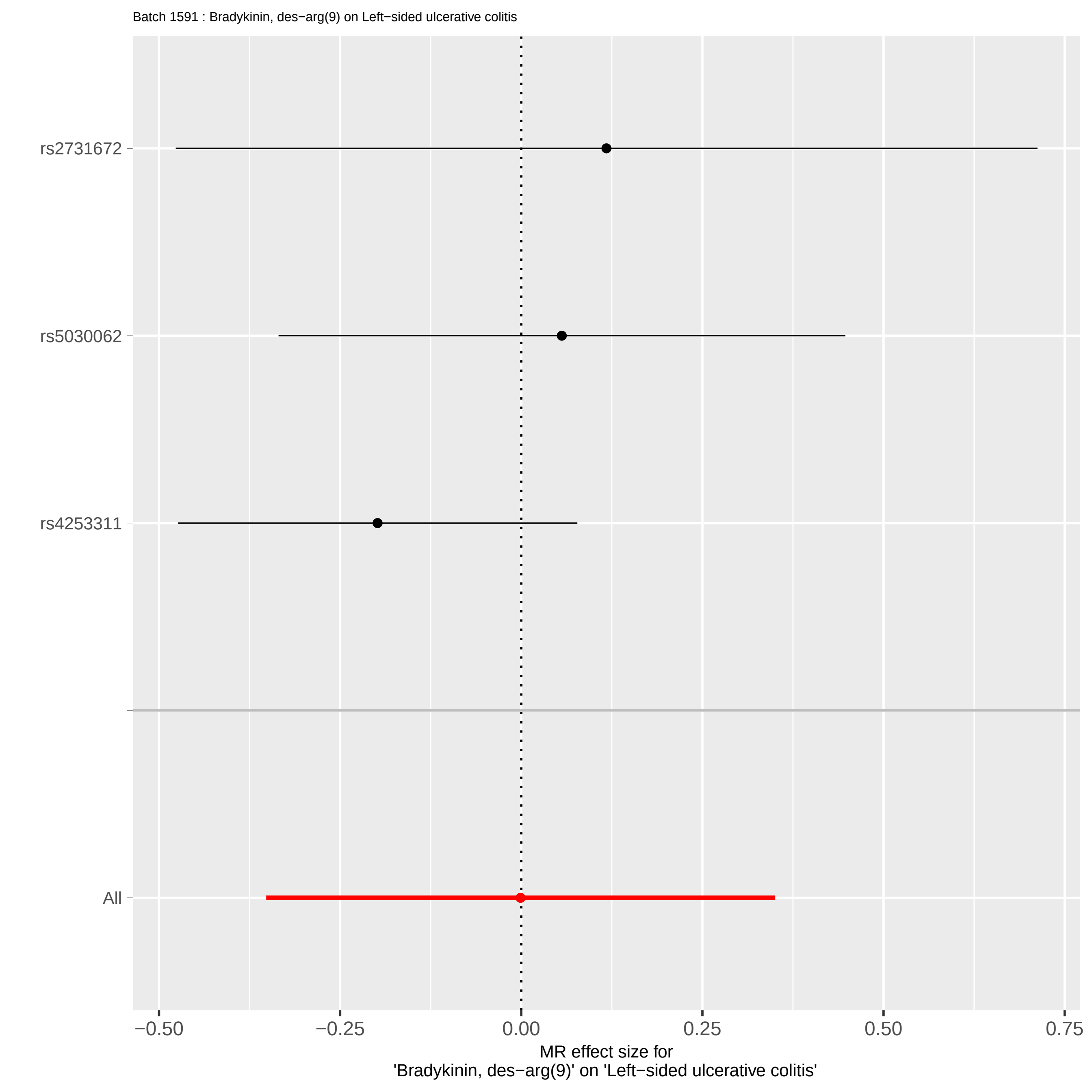

Batch 1591 : Bradykinin, des−arg(9) on Left−sided ulcerative colitis
rs2731672
rs5030062
rs4253311
All
−0.50
−0.25
0.00
0.25
0.50
0.75
MR effect size for
'Bradykinin, des−arg(9)' on 'Left−sided ulcerative colitis'

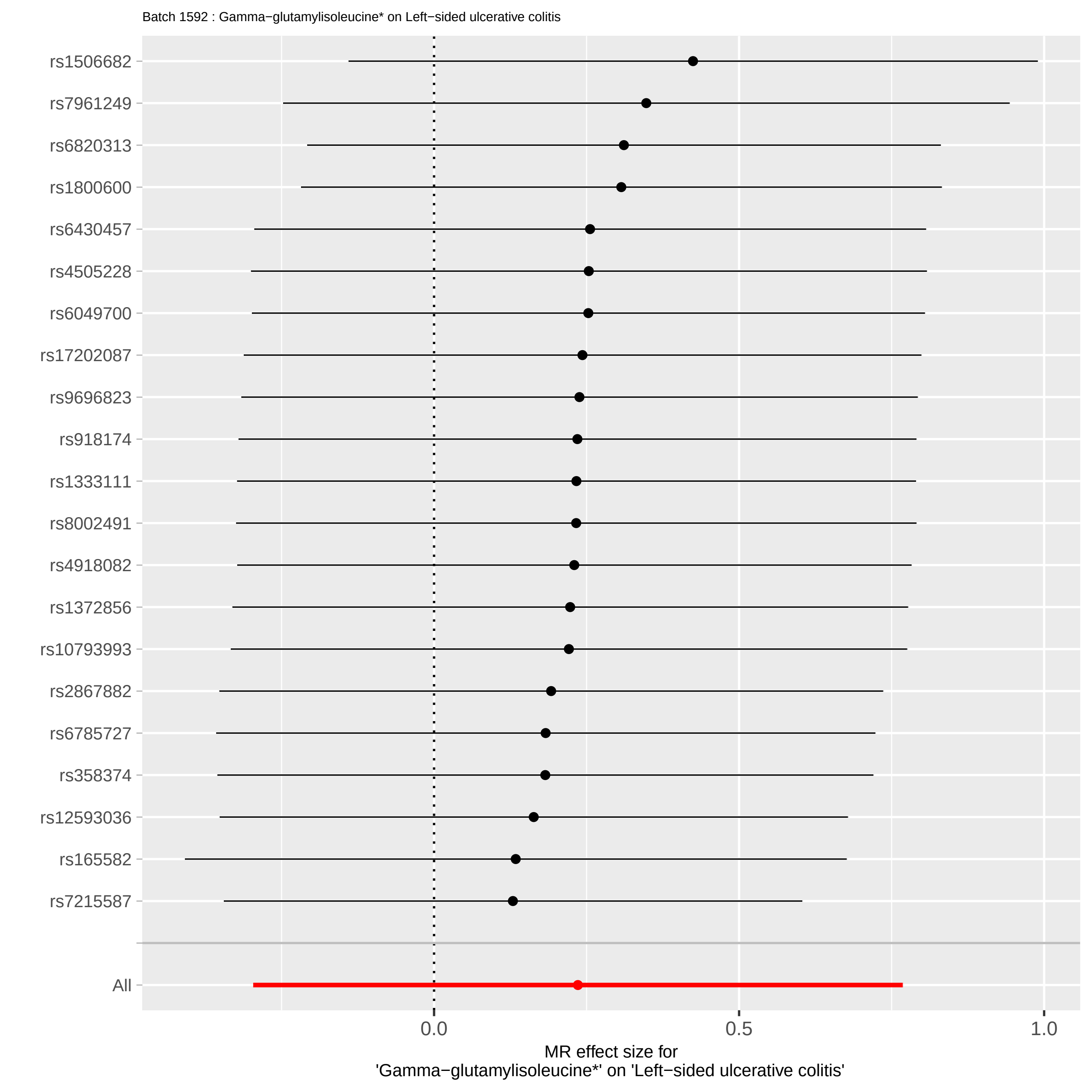
Batch 1592 : Gamma−glutamylisoleucine* on Left−sided ulcerative colitis
rs1506682
rs7961249
rs6820313
rs1800600
rs6430457
rs4505228
rs6049700
rs17202087
rs9696823
rs918174
rs1333111
rs8002491
rs4918082
rs1372856
rs10793993
rs2867882
rs6785727
rs358374
rs12593036
rs165582
rs7215587
All
0.0
0.5
1.0
MR effect size for
'Gamma−glutamylisoleucine*' on 'Left−sided ulcerative colitis'

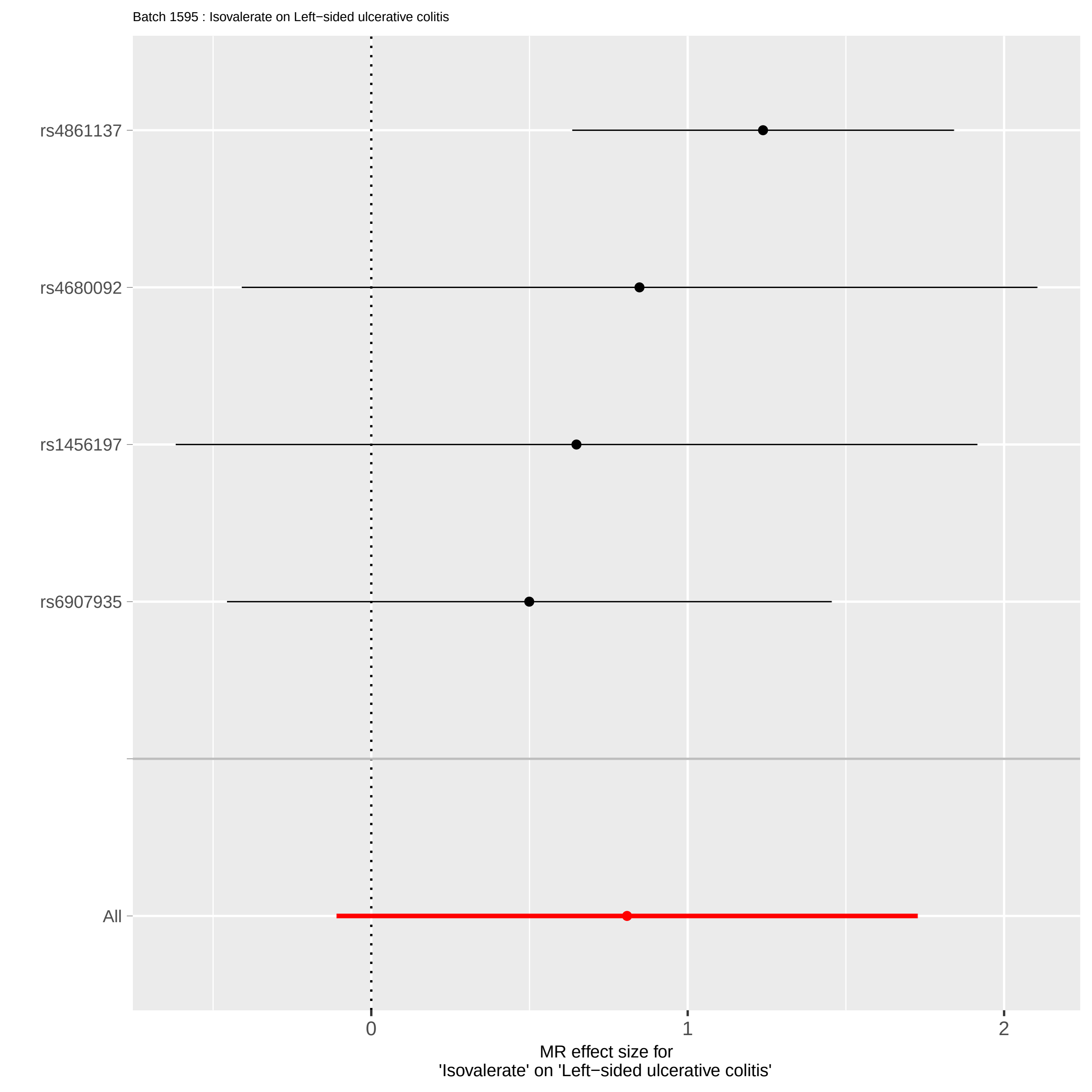
Batch 1595 : Isovalerate on Left−sided ulcerative colitis
rs4861137
rs4680092
rs1456197
rs6907935
All
0
1
2
MR effect size for
'Isovalerate' on 'Left−sided ulcerative colitis'

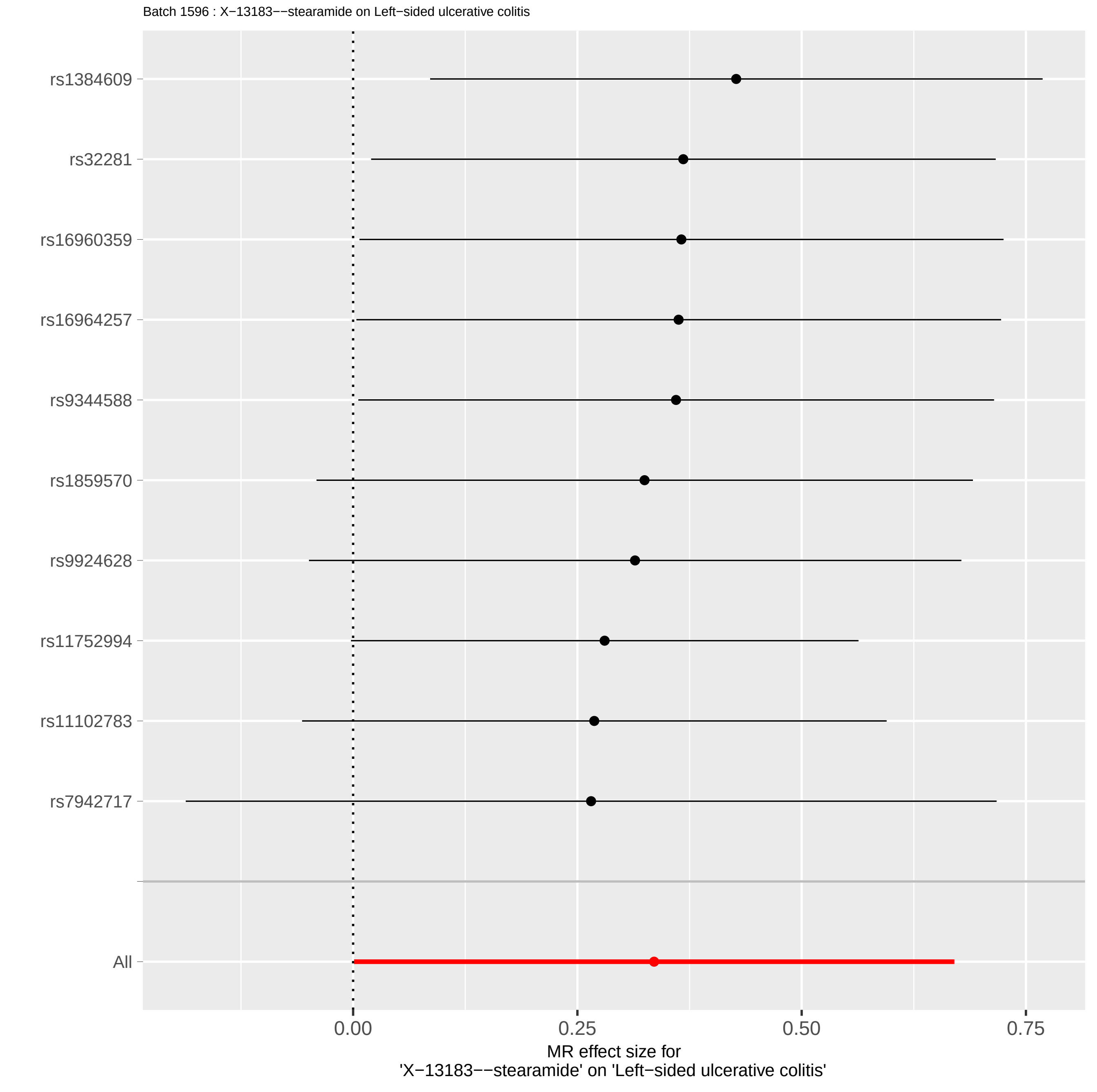
Batch 1596 : X−13183−−stearamide on Left−sided ulcerative colitis
rs1384609
rs32281
rs16960359
rs16964257
rs9344588
rs1859570
rs9924628
rs11752994
rs11102783
rs7942717
All
0.00
0.25
0.50
0.75
MR effect size for
'X−13183−−stearamide' on 'Left−sided ulcerative colitis'

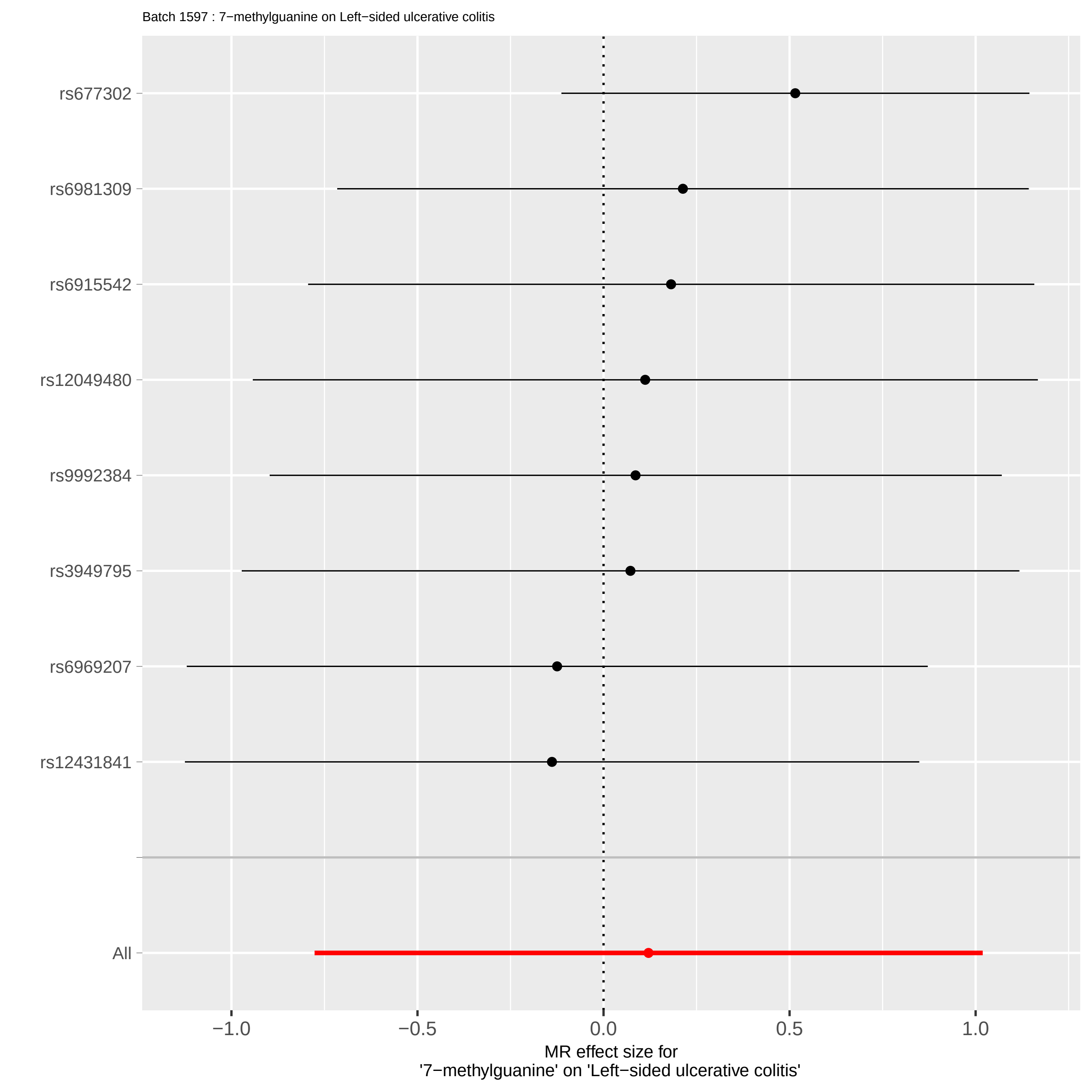
Batch 1597 : 7−methylguanine on Left−sided ulcerative colitis
rs677302
rs6981309
rs6915542
rs12049480
rs9992384
rs3949795
rs6969207
rs12431841
All
−1.0
−0.5
0.0
0.5
1.0
MR effect size for
'7−methylguanine' on 'Left−sided ulcerative colitis'

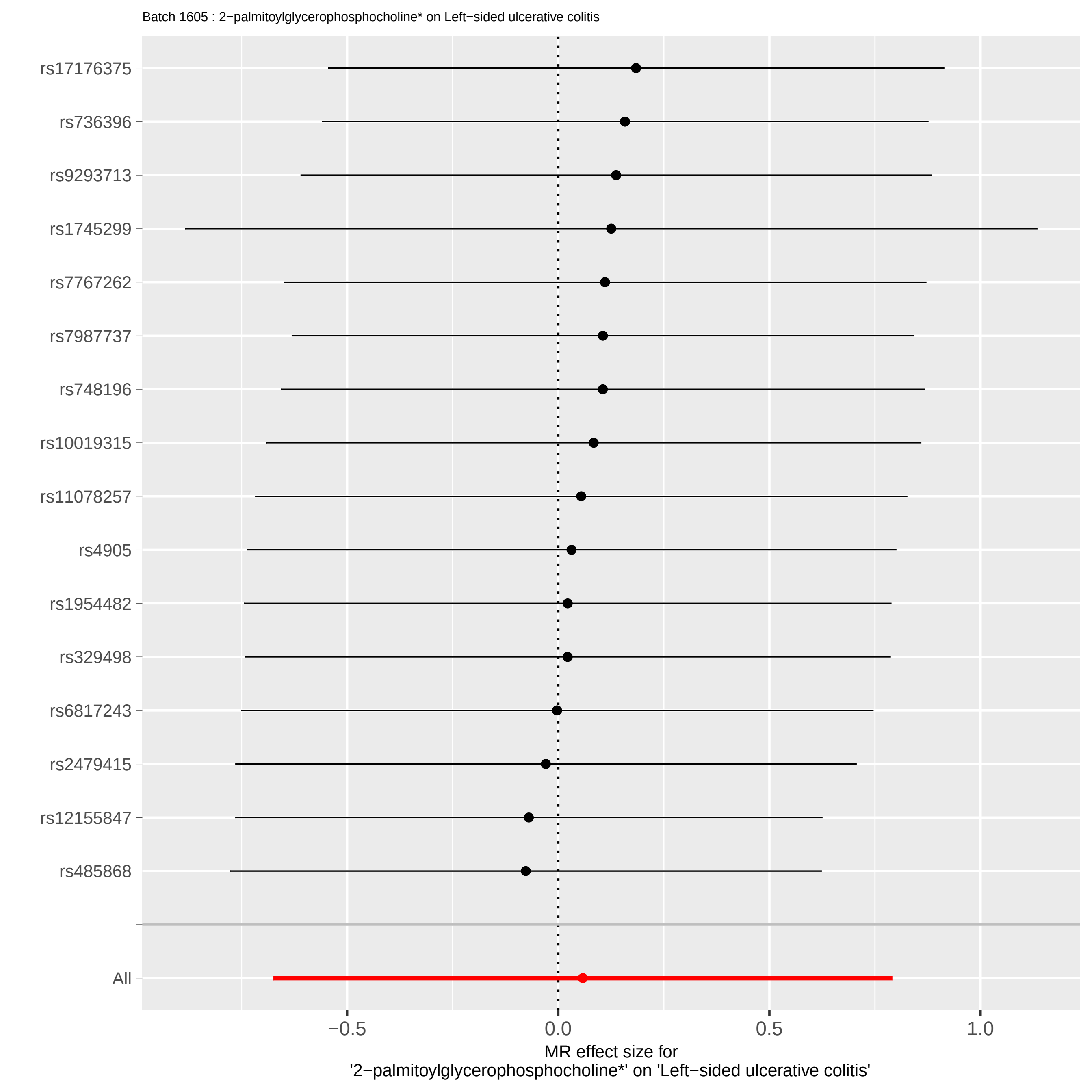

Batch 1605 : 2−palmitoylglycerophosphocholine* on Left−sided ulcerative colitis
rs17176375
rs736396
rs9293713
rs1745299
rs7767262
rs7987737
rs748196
rs10019315
rs11078257
rs4905
rs1954482
rs329498
rs6817243
rs2479415
rs12155847
rs485868
All
−0.5
0.0
0.5
1.0
MR effect size for
'2−palmitoylglycerophosphocholine*' on 'Left−sided ulcerative colitis'

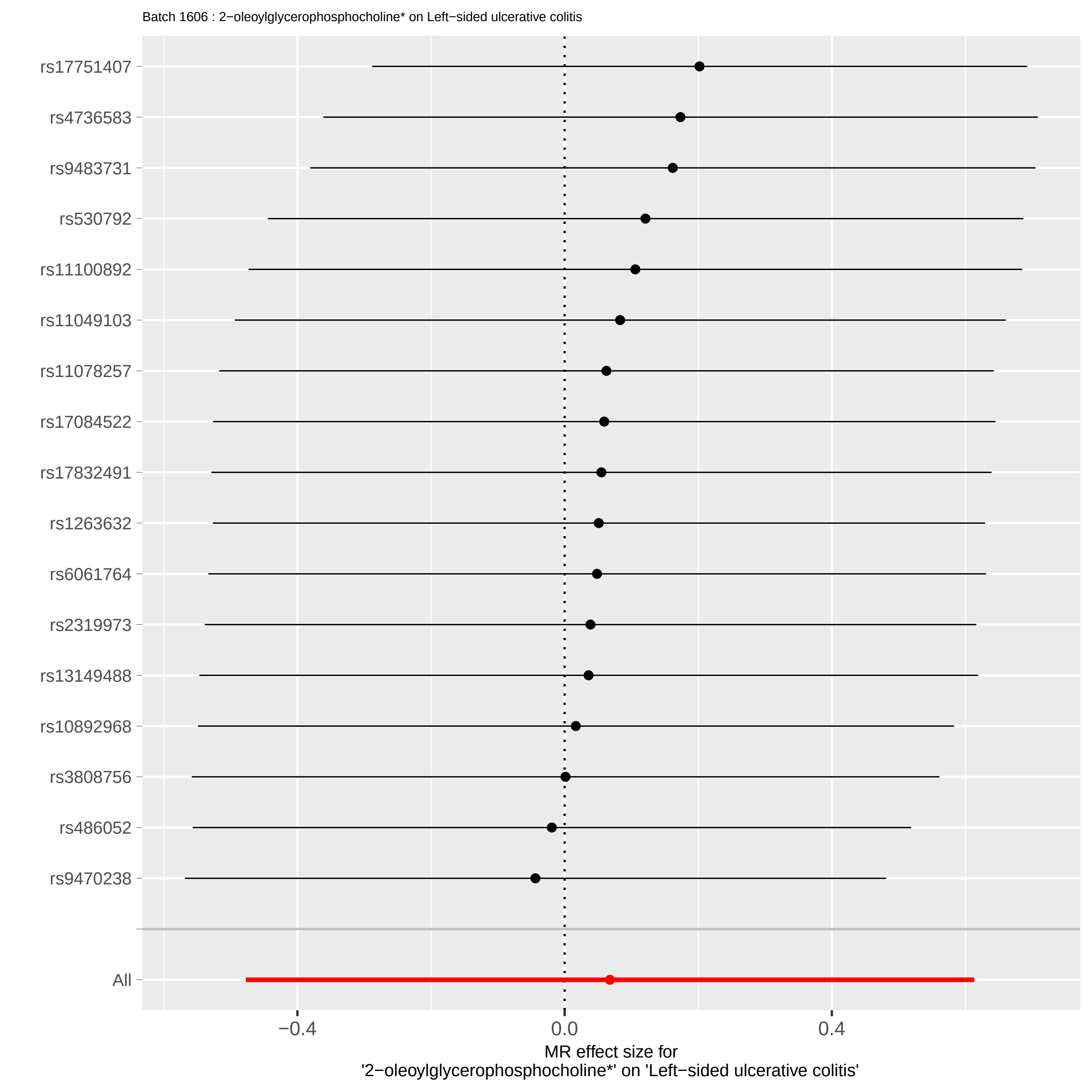

Batch 1606 : 2−oleoylglycerophosphocholine* on Left−sided ulcerative colitis
rs17751407
rs4736583
rs9483731
rs530792
rs11100892
rs11049103
rs11078257
rs17084522
rs17832491
rs1263632
rs6061764
rs2319973
rs13149488
rs10892968
rs3808756
rs486052
rs9470238
All
−0.4
0.0
0.4
MR effect size for
'2−oleoylglycerophosphocholine*' on 'Left−sided ulcerative colitis'

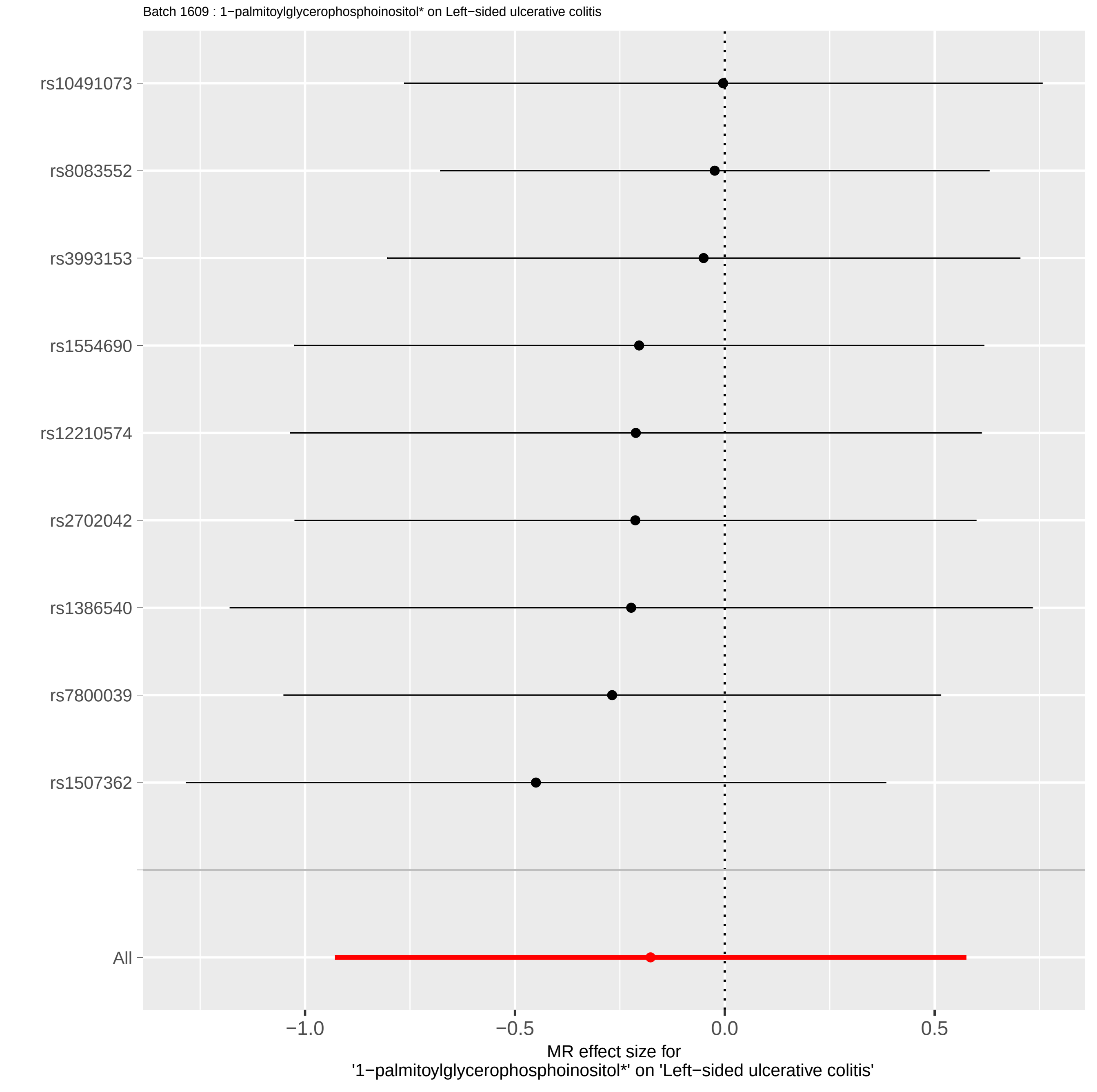

Batch 1609 : 1−palmitoylglycerophosphoinositol* on Left−sided ulcerative colitis
rs10491073
rs8083552
rs3993153
rs1554690
rs12210574
rs2702042
rs1386540
rs7800039
rs1507362
All
−1.0
−0.5
0.0
0.5
MR effect size for
'1−palmitoylglycerophosphoinositol*' on 'Left−sided ulcerative colitis'

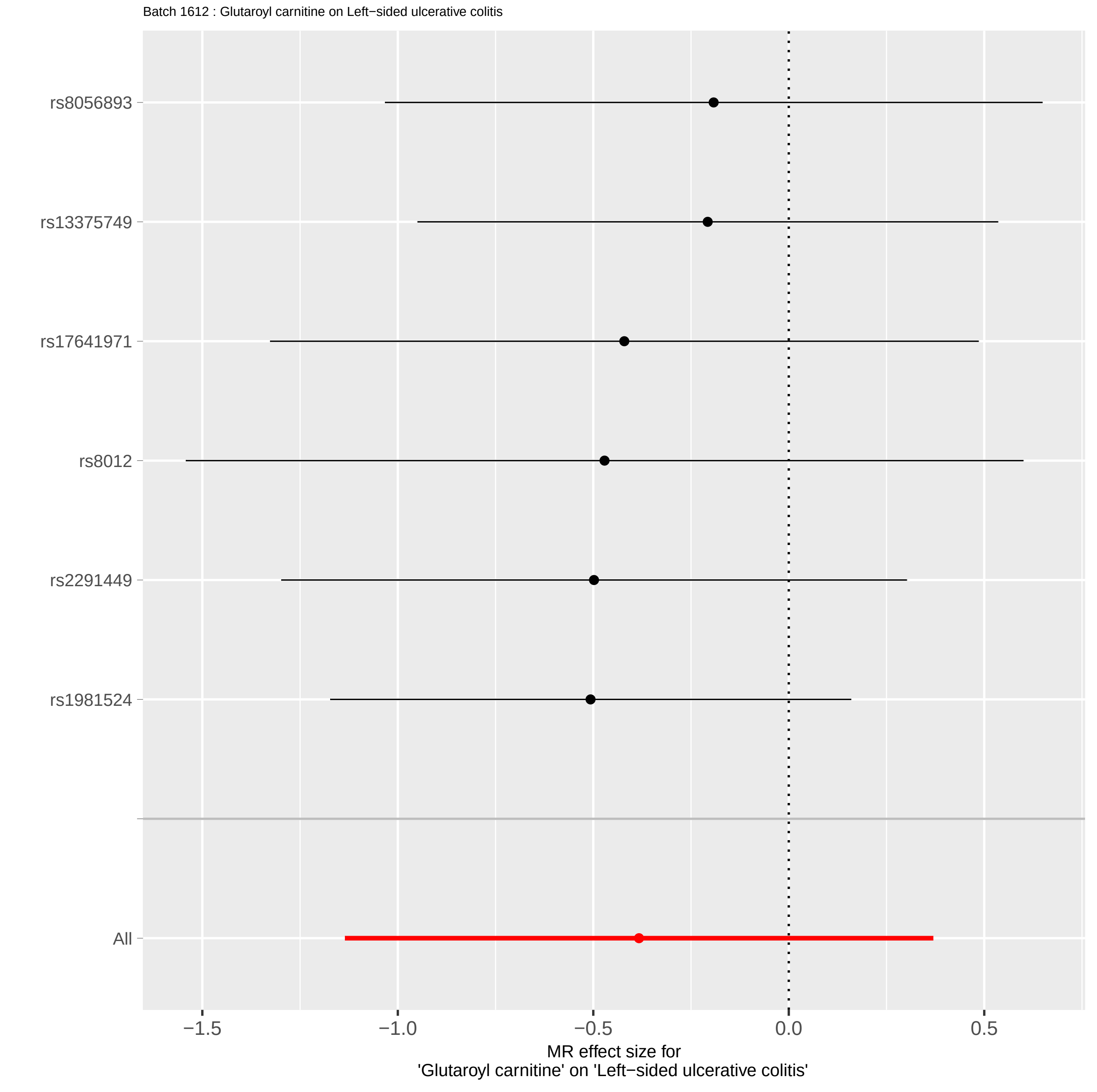

Batch 1612 : Glutaroyl carnitine on Left−sided ulcerative colitis
rs8056893
rs13375749
rs17641971
rs8012
rs2291449
rs1981524
All
−1.5
−1.0
−0.5
0.0
0.5
MR effect size for
'Glutaroyl carnitine' on 'Left−sided ulcerative colitis'

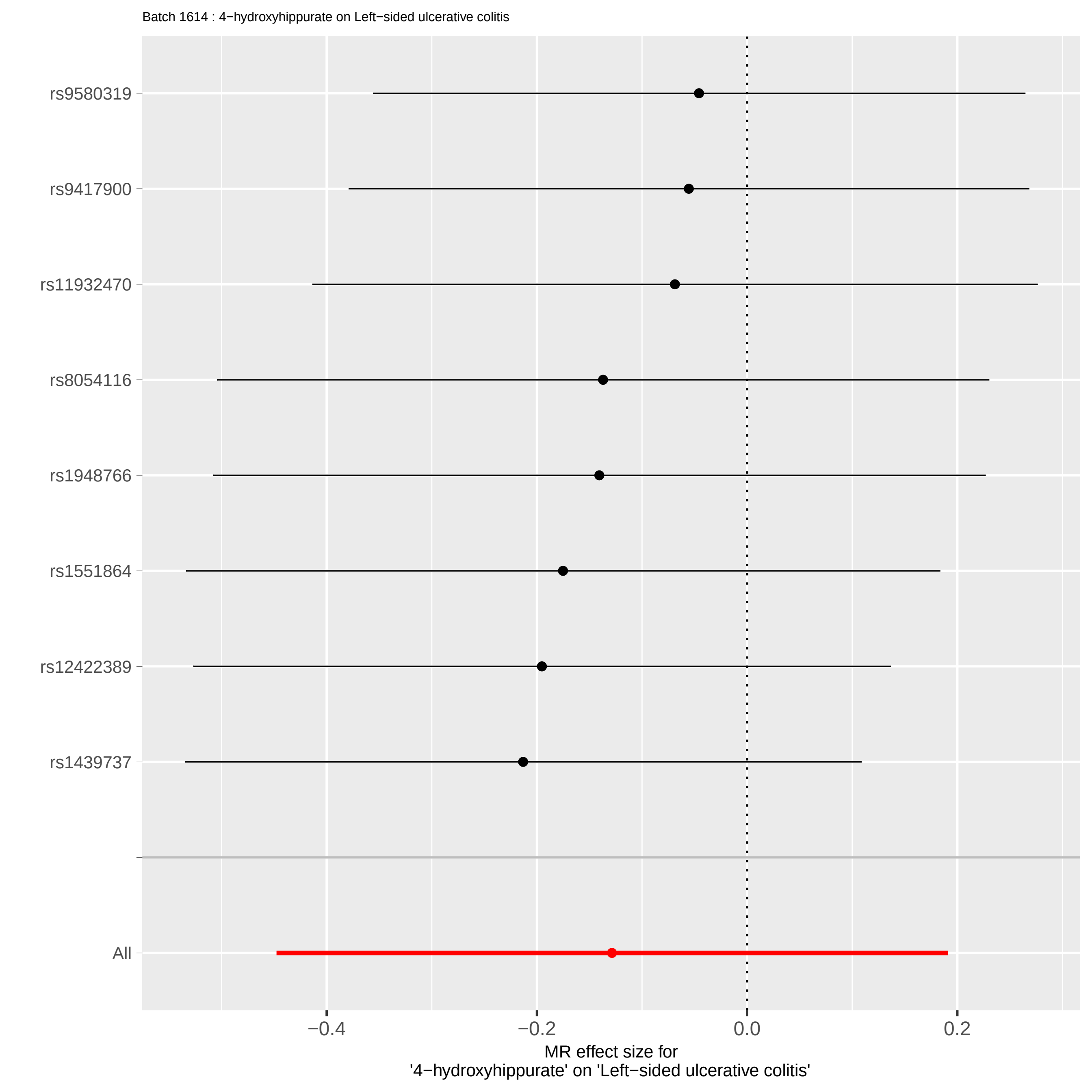
Batch 1614 : 4−hydroxyhippurate on Left−sided ulcerative colitis
rs9580319
rs9417900
rs11932470
rs8054116
rs1948766
rs1551864
rs12422389
rs1439737
All
−0.4
−0.2
0.0
0.2
MR effect size for
'4−hydroxyhippurate' on 'Left−sided ulcerative colitis'

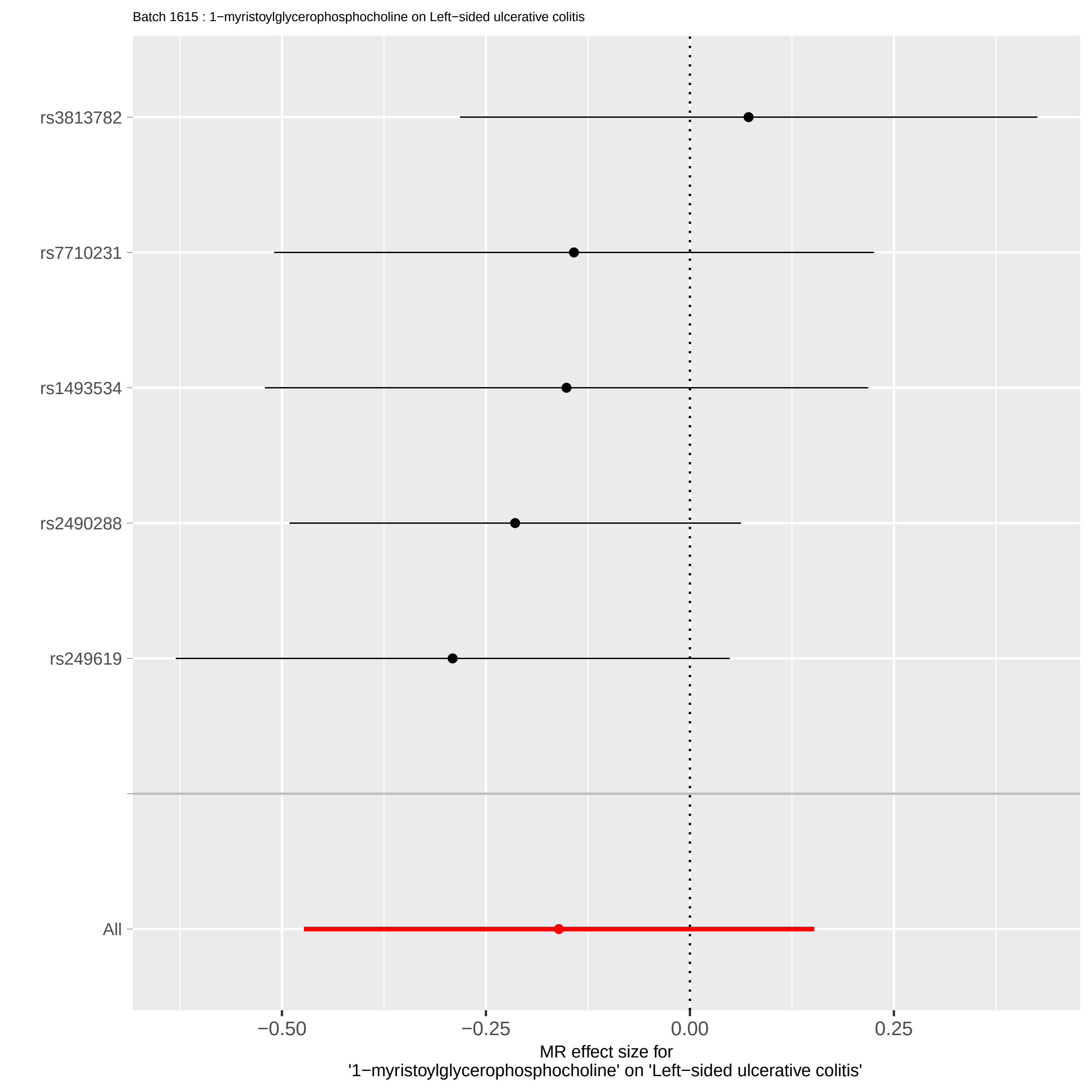

Batch 1615 : 1−myristoylglycerophosphocholine on Left−sided ulcerative colitis
rs3813782
rs7710231
rs1493534
rs2490288
rs249619
All
−0.50
−0.25
0.00
0.25
MR effect size for
'1−myristoylglycerophosphocholine' on 'Left−sided ulcerative colitis'

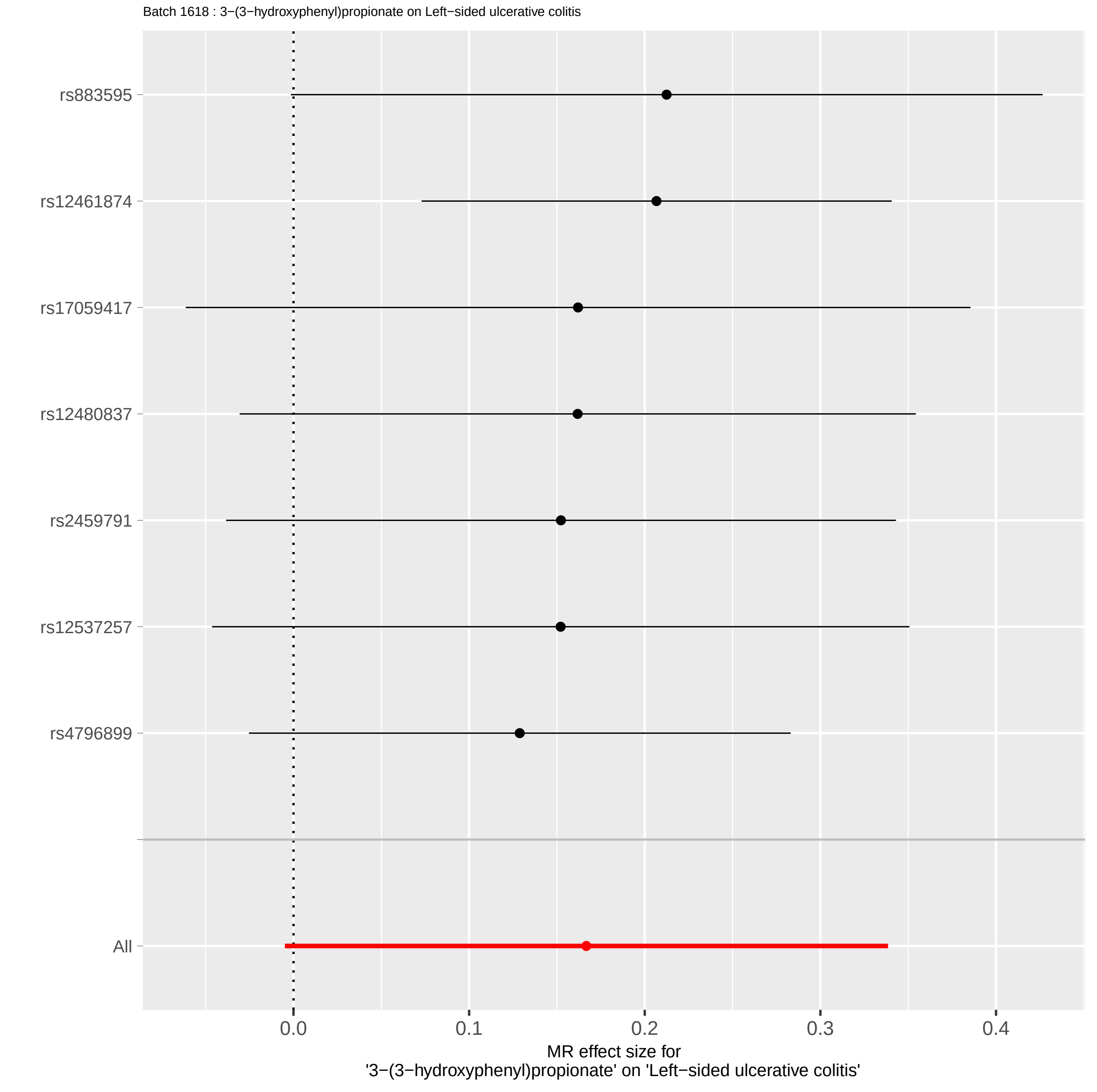

Batch 1618 : 3−(3−hydroxyphenyl)propionate on Left−sided ulcerative colitis
rs883595
rs12461874
rs17059417
rs12480837
rs2459791
rs12537257
rs4796899
All
0.0
0.1
0.2
0.3
0.4
MR effect size for
'3−(3−hydroxyphenyl)propionate' on 'Left−sided ulcerative colitis'

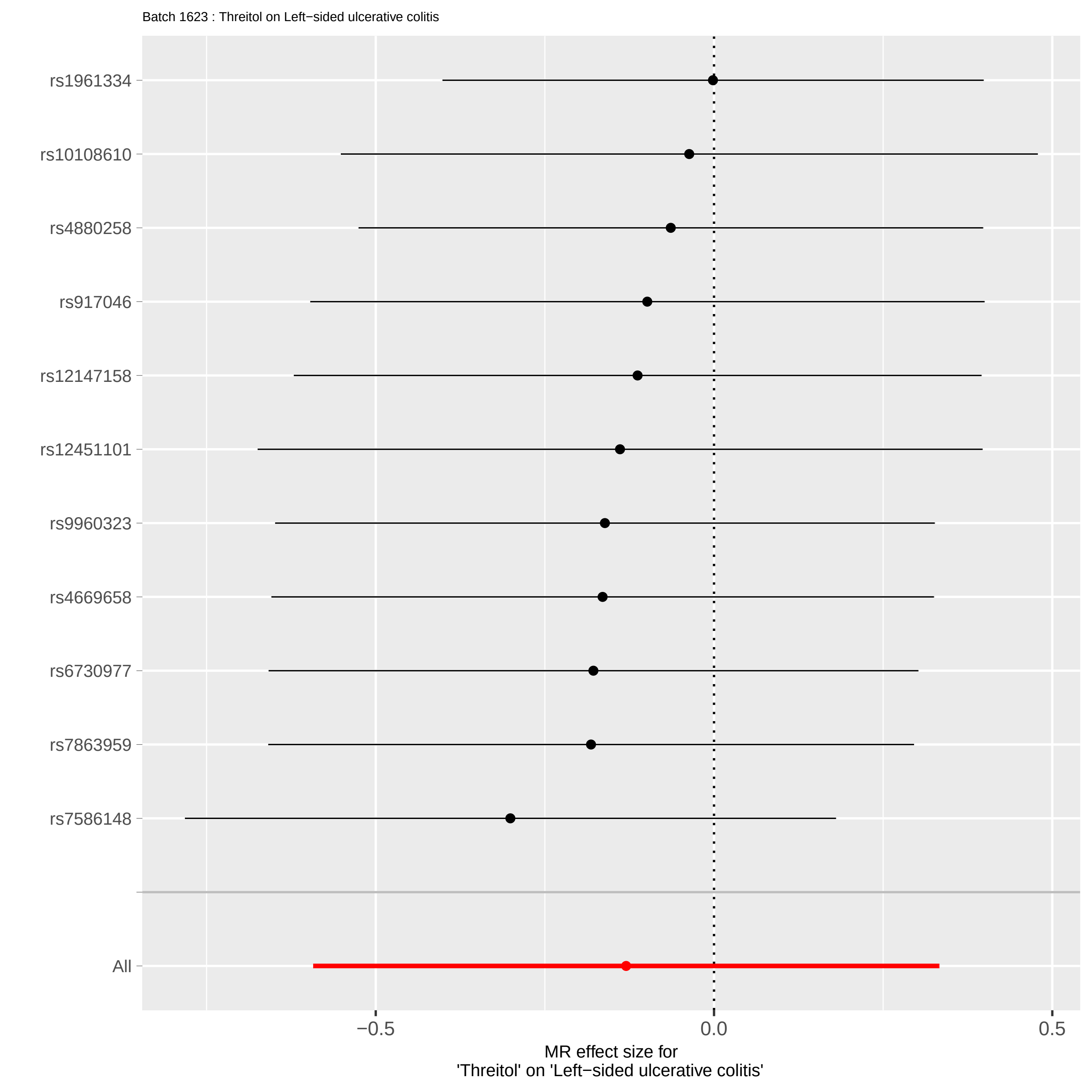
Batch 1623 : Threitol on Left−sided ulcerative colitis
rs1961334
rs10108610
rs4880258
rs917046
rs12147158
rs12451101
rs9960323
rs4669658
rs6730977
rs7863959
rs7586148
All
−0.5
0.0
0.5
MR effect size for
'Threitol' on 'Left−sided ulcerative colitis'

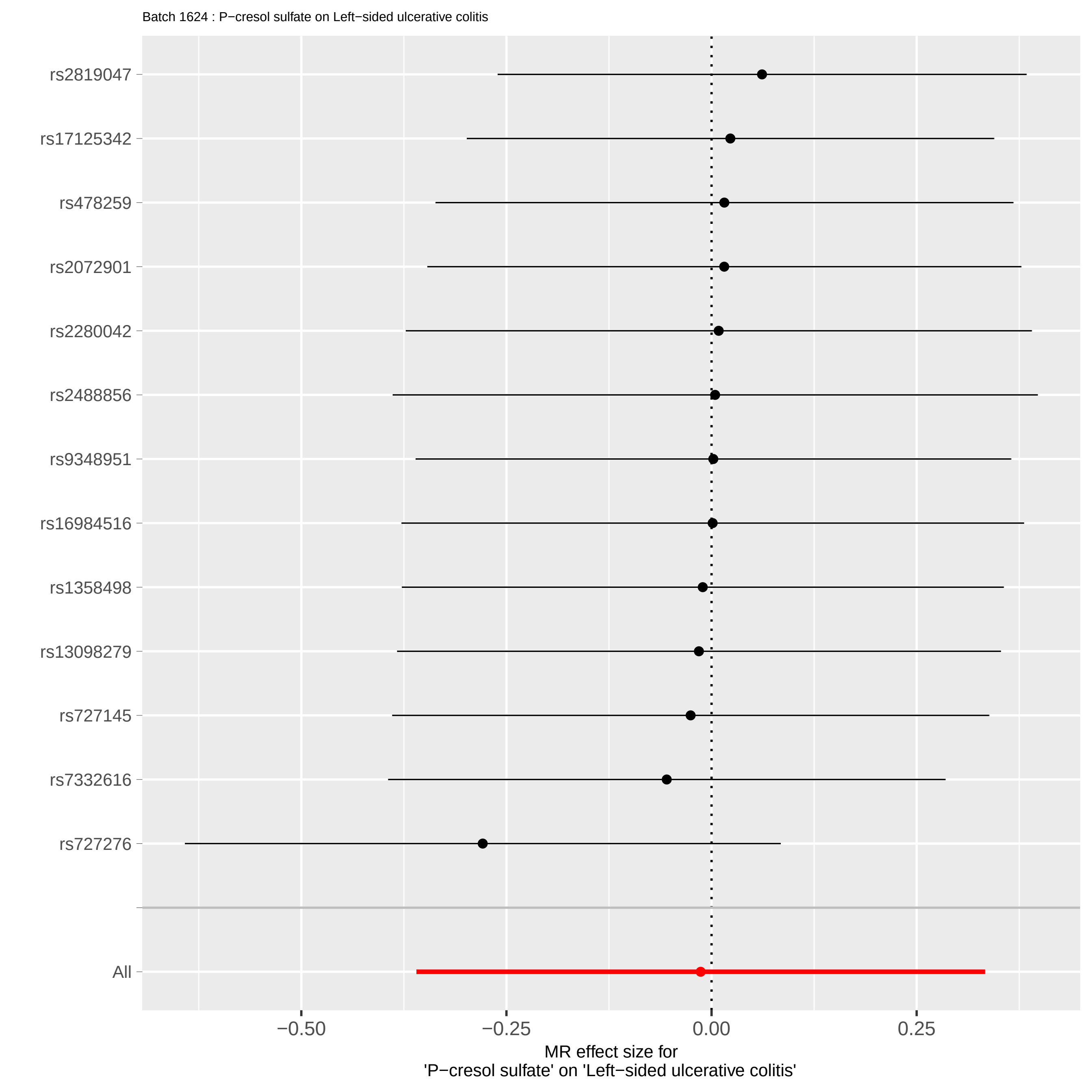

Batch 1624 : P−cresol sulfate on Left−sided ulcerative colitis
rs2819047
rs17125342
rs478259
rs2072901
rs2280042
rs2488856
rs9348951
rs16984516
rs1358498
rs13098279
rs727145
rs7332616
rs727276
All
−0.50
−0.25
0.00
0.25
MR effect size for
'P−cresol sulfate' on 'Left−sided ulcerative colitis'

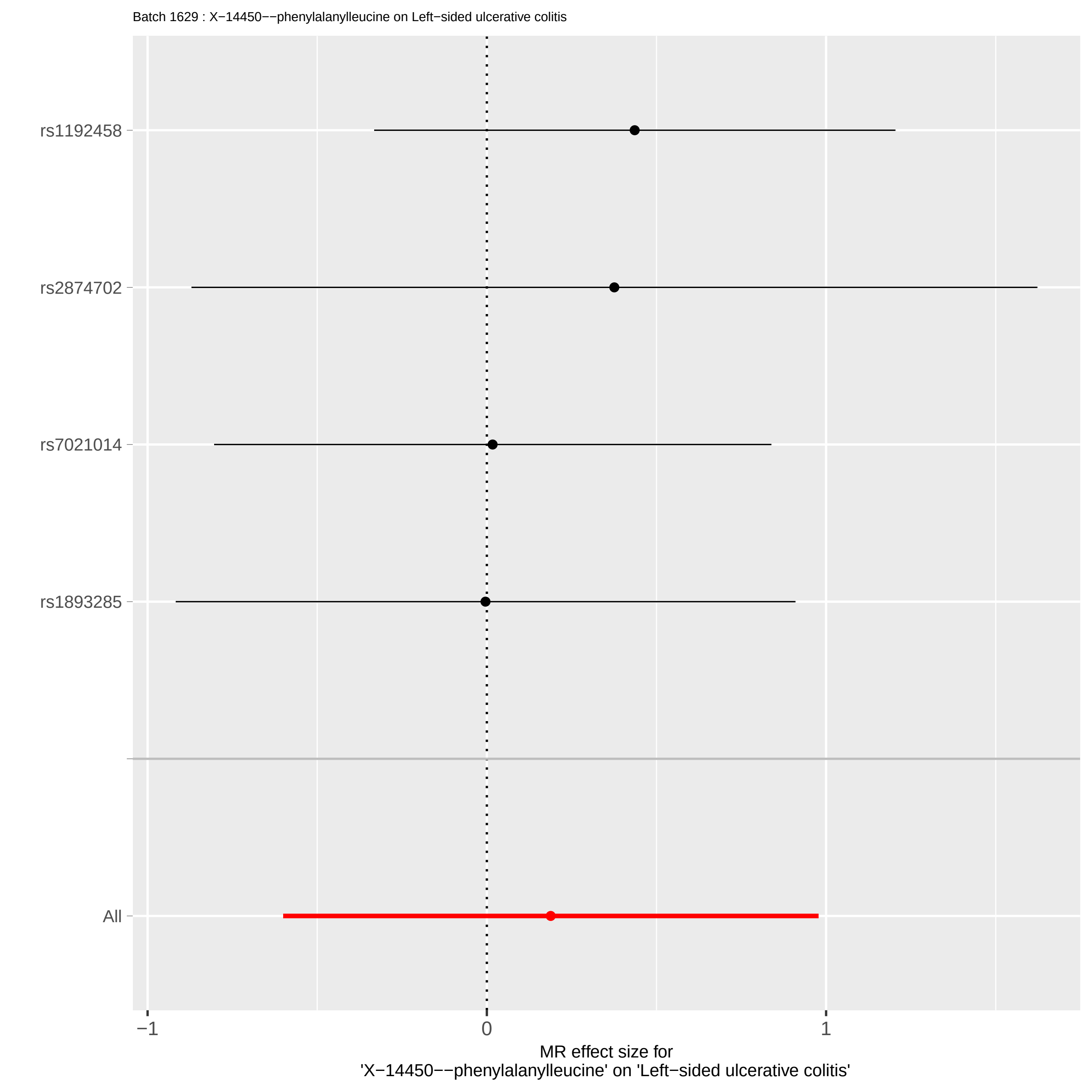
Batch 1629 : X−14450−−phenylalanylleucine on Left−sided ulcerative colitis
rs1192458
rs2874702
rs7021014
rs1893285
All
−1
0
1
MR effect size for
'X−14450−−phenylalanylleucine' on 'Left−sided ulcerative colitis'

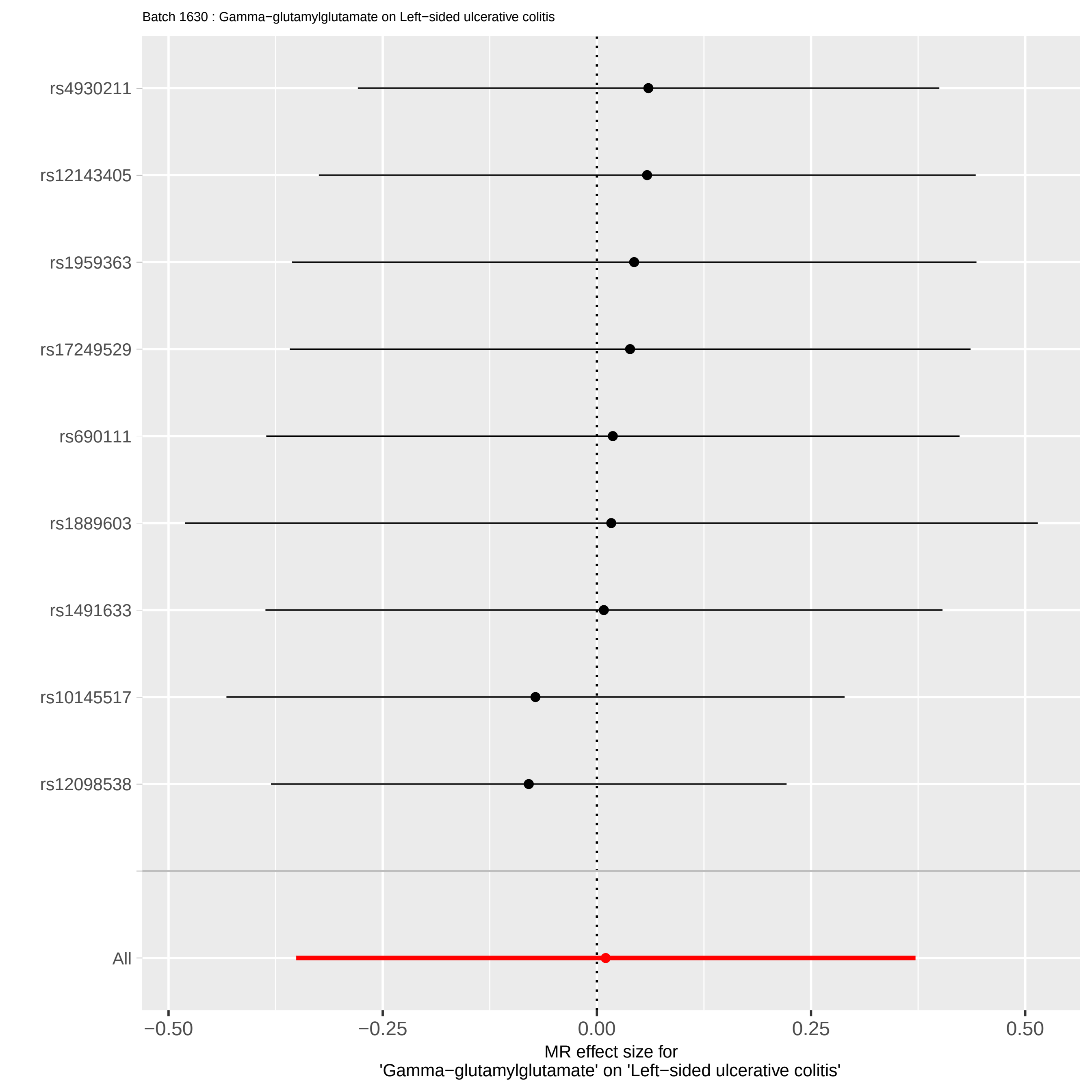
Batch 1630 : Gamma−glutamylglutamate on Left−sided ulcerative colitis
rs4930211
rs12143405
rs1959363
rs17249529
rs690111
rs1889603
rs1491633
rs10145517
rs12098538
All
−0.50
−0.25
0.00
0.25
0.50
MR effect size for
'Gamma−glutamylglutamate' on 'Left−sided ulcerative colitis'

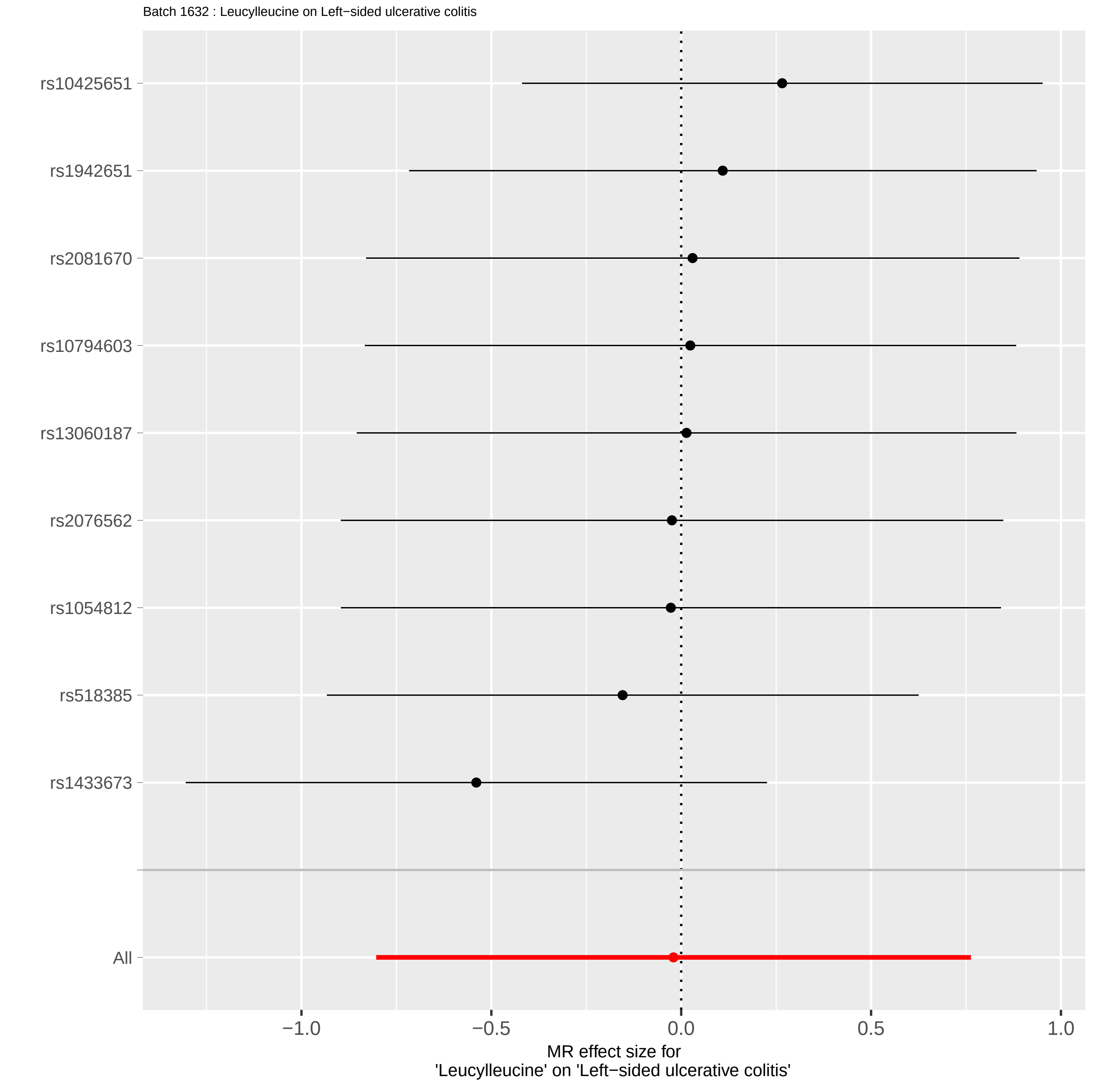

Batch 1632 : Leucylleucine on Left−sided ulcerative colitis
rs10425651
rs1942651
rs2081670
rs10794603
rs13060187
rs2076562
rs1054812
rs518385
rs1433673
All
−1.0
−0.5
0.0
0.5
1.0
MR effect size for
'Leucylleucine' on 'Left−sided ulcerative colitis'

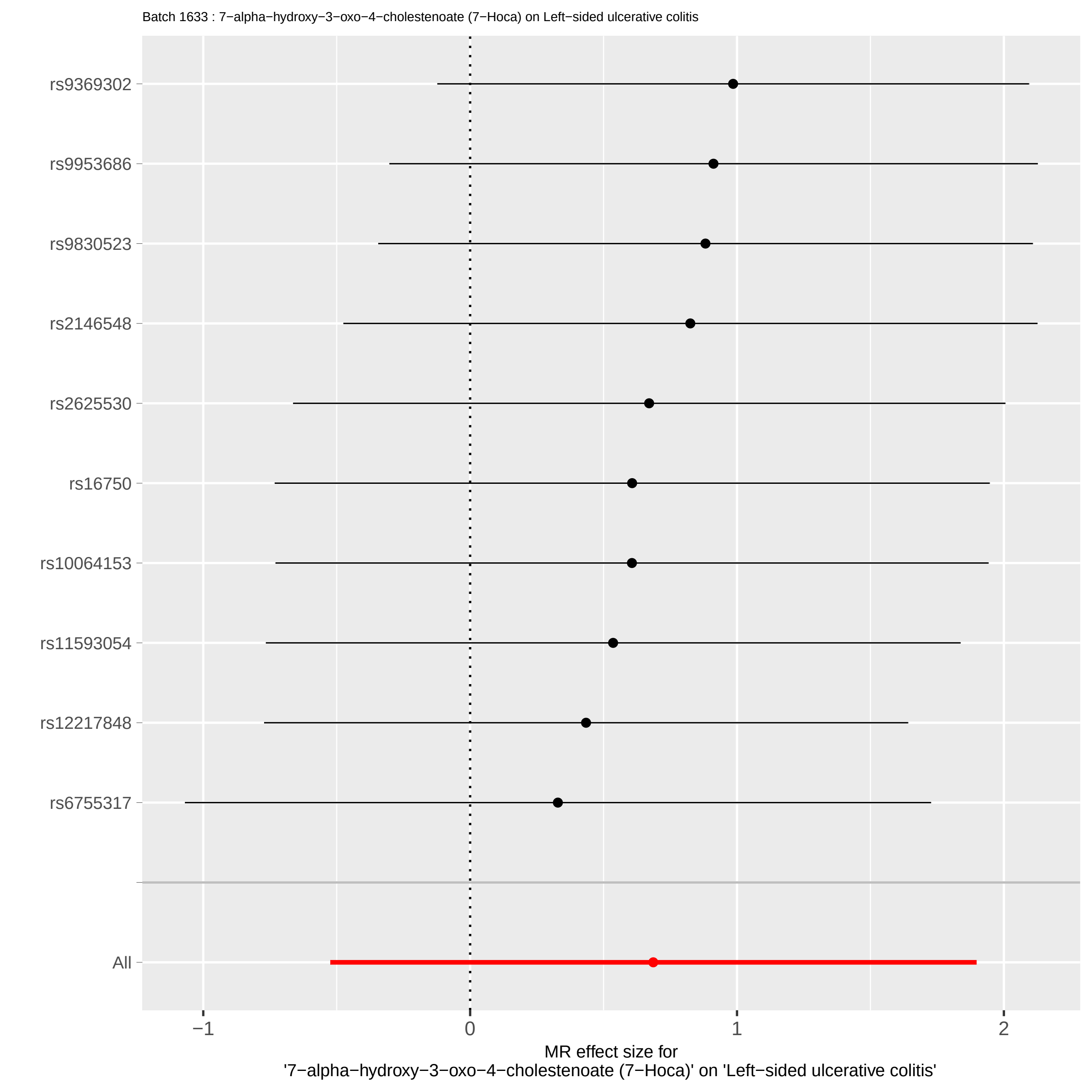
Batch 1633 : 7−alpha−hydroxy−3−oxo−4−cholestenoate (7−Hoca) on Left−sided ulcerative colitis
rs9369302
rs9953686
rs9830523
rs2146548
rs2625530
rs16750
rs10064153
rs11593054
rs12217848
rs6755317
All
−1
0
1
2
MR effect size for
'7−alpha−hydroxy−3−oxo−4−cholestenoate (7−Hoca)' on 'Left−sided ulcerative colitis'

Batch 1634 : N−Butyl Oleate on Left−sided ulcerative colitis

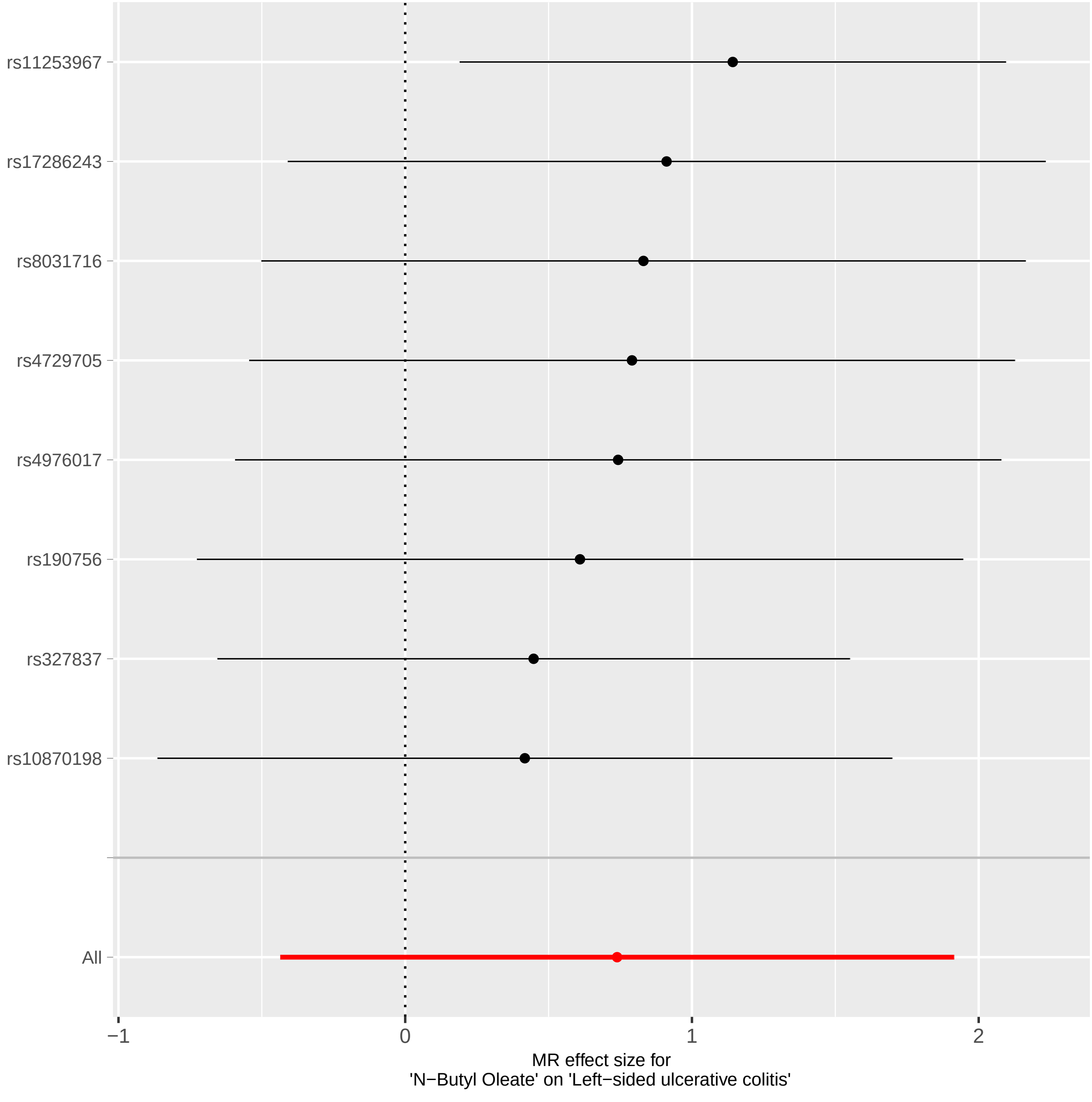

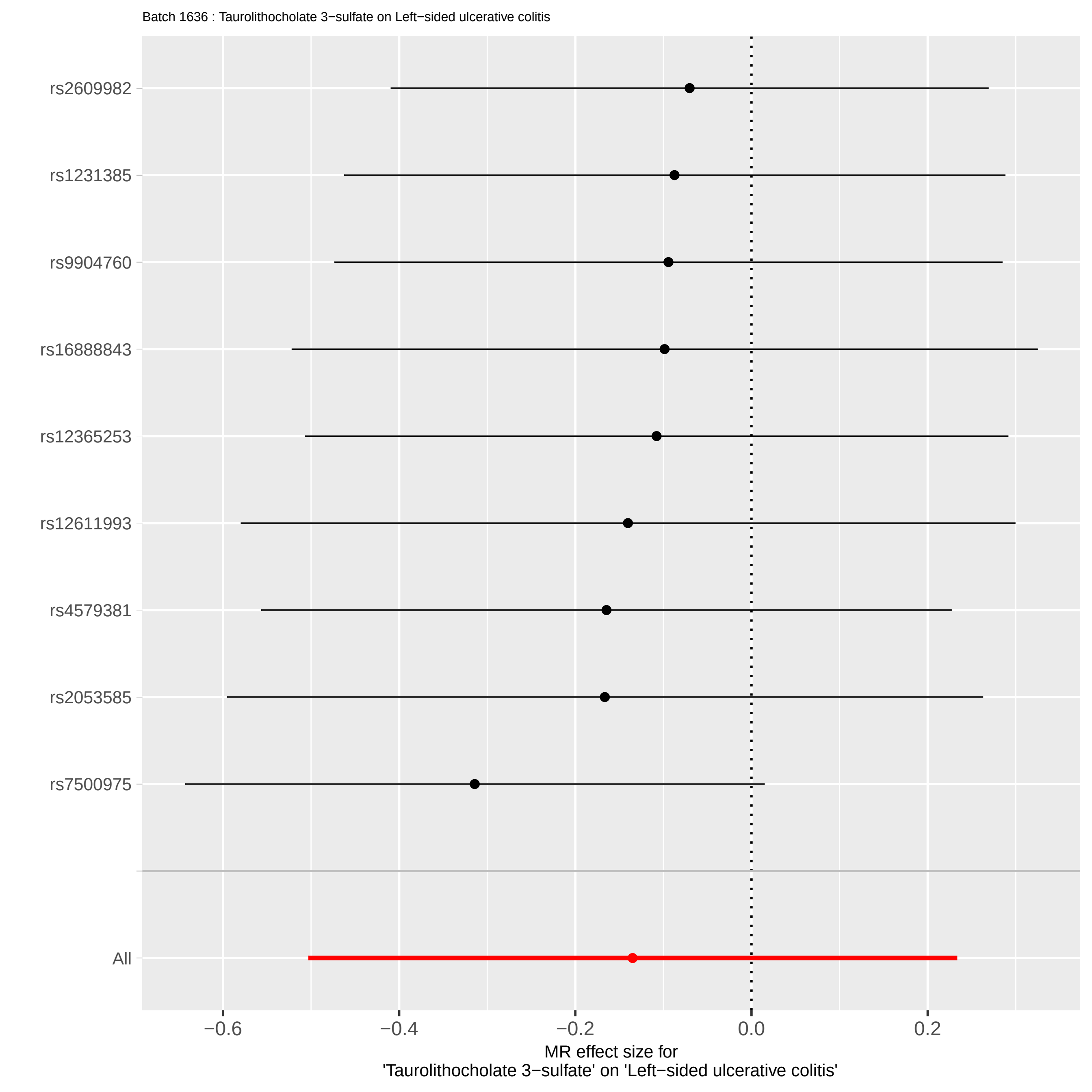

Batch 1636 : Taurolithocholate 3−sulfate on Left−sided ulcerative colitis
rs2609982
rs1231385
rs9904760
rs16888843
rs12365253
rs12611993
rs4579381
rs2053585
rs7500975
All
−0.6
−0.4
−0.2
0.0
0.2
MR effect size for
'Taurolithocholate 3−sulfate' on 'Left−sided ulcerative colitis'

Batch 1637 : X−14977−−vanillin on Left−sided ulcerative colitis

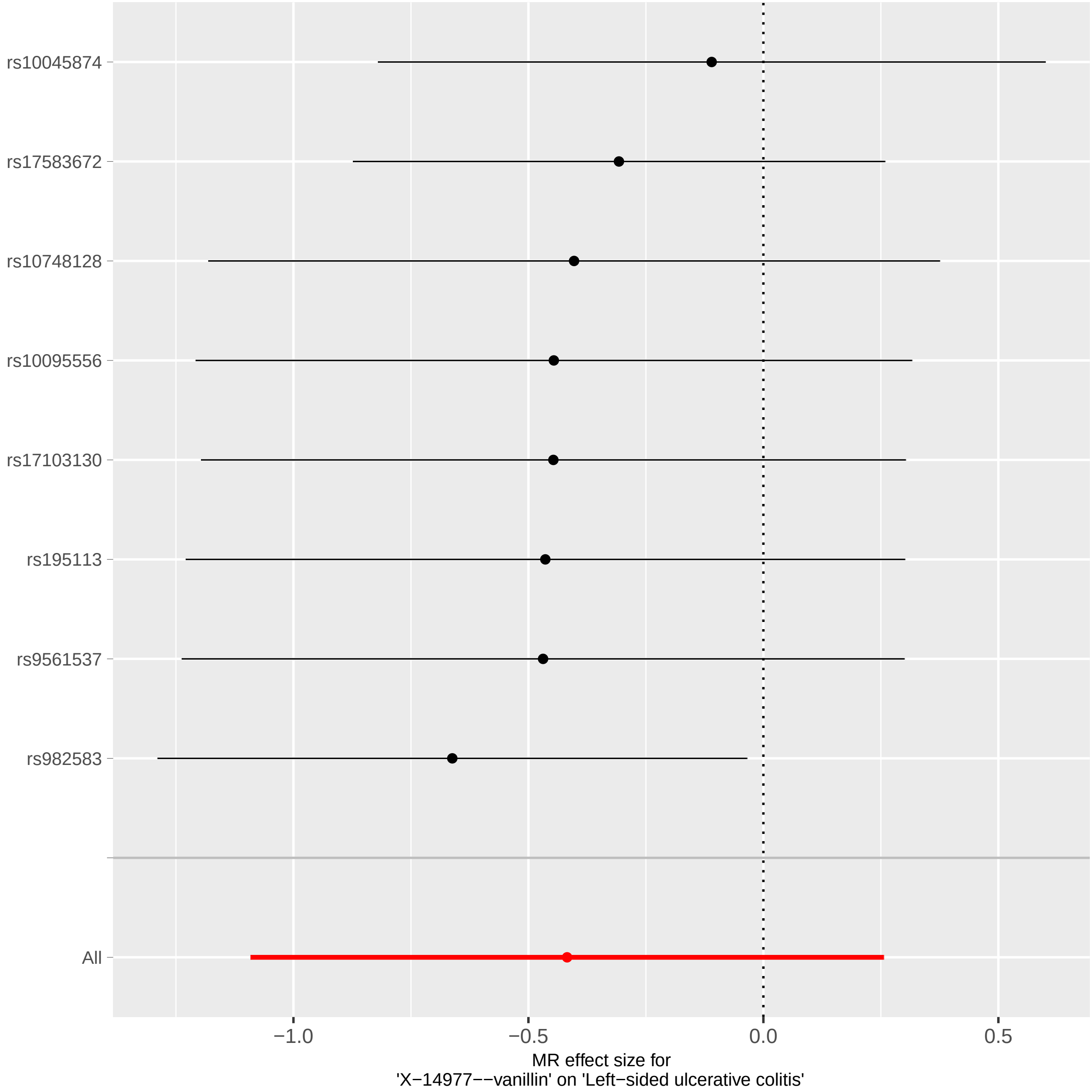

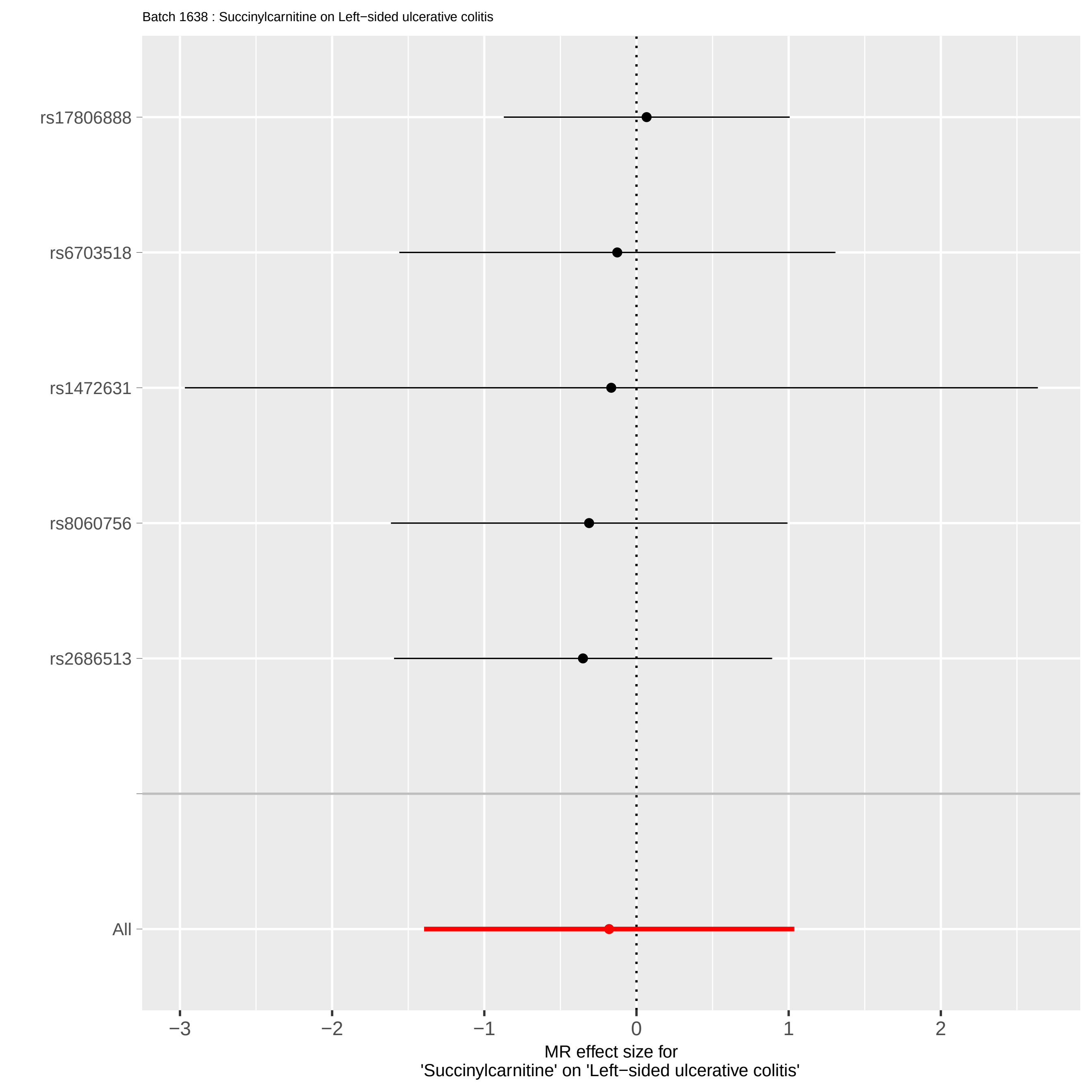

Batch 1638 : Succinylcarnitine on Left−sided ulcerative colitis
rs17806888
rs6703518
rs1472631
rs8060756
rs2686513
All
−3
−2
−1
0
1
2
MR effect size for
'Succinylcarnitine' on 'Left−sided ulcerative colitis'

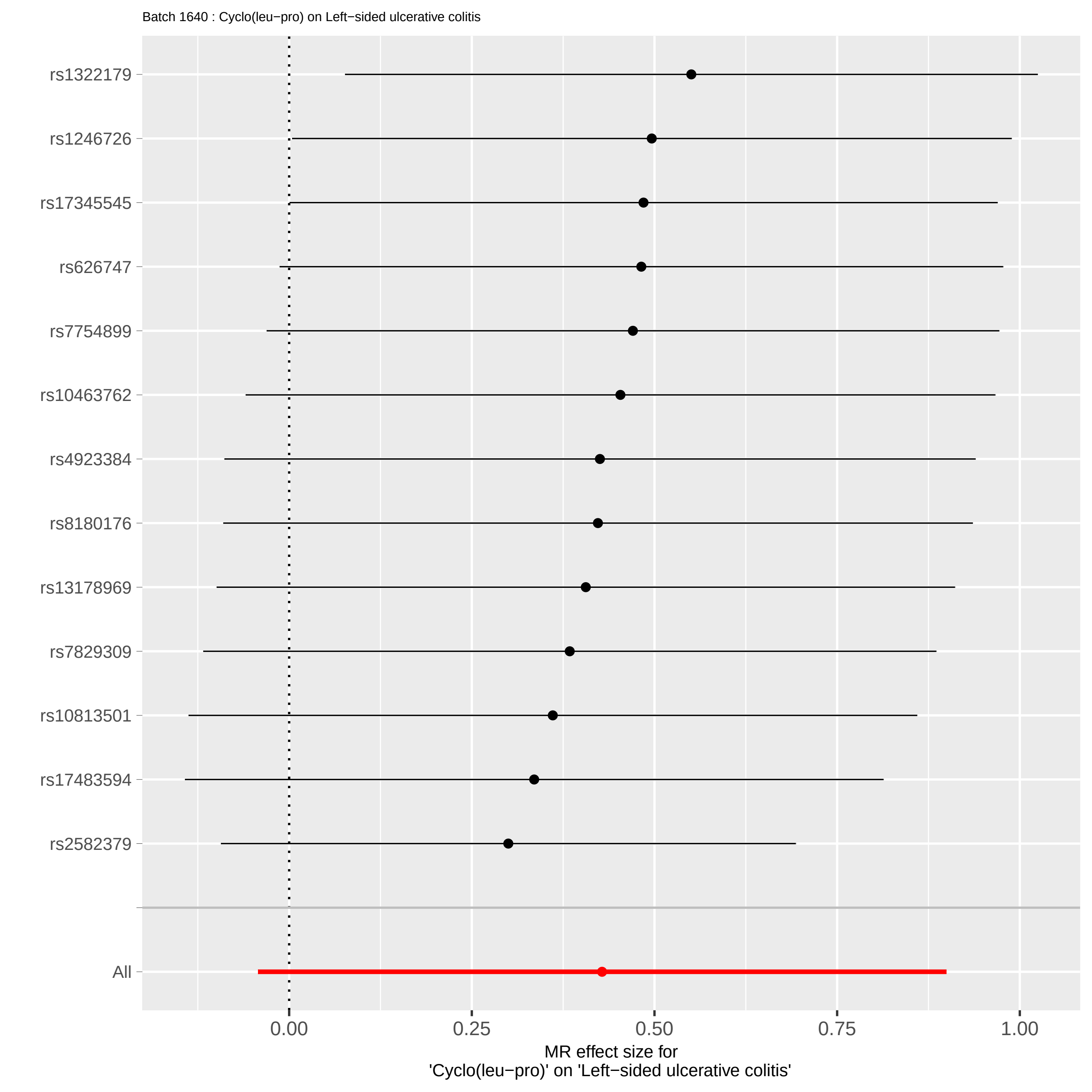

Batch 1640 : Cyclo(leu−pro) on Left−sided ulcerative colitis
rs1322179
rs1246726
rs17345545
rs626747
rs7754899
rs10463762
rs4923384
rs8180176
rs13178969
rs7829309
rs10813501
rs17483594
rs2582379
All
0.00
0.25
0.50
0.75
1.00
MR effect size for
'Cyclo(leu−pro)' on 'Left−sided ulcerative colitis'

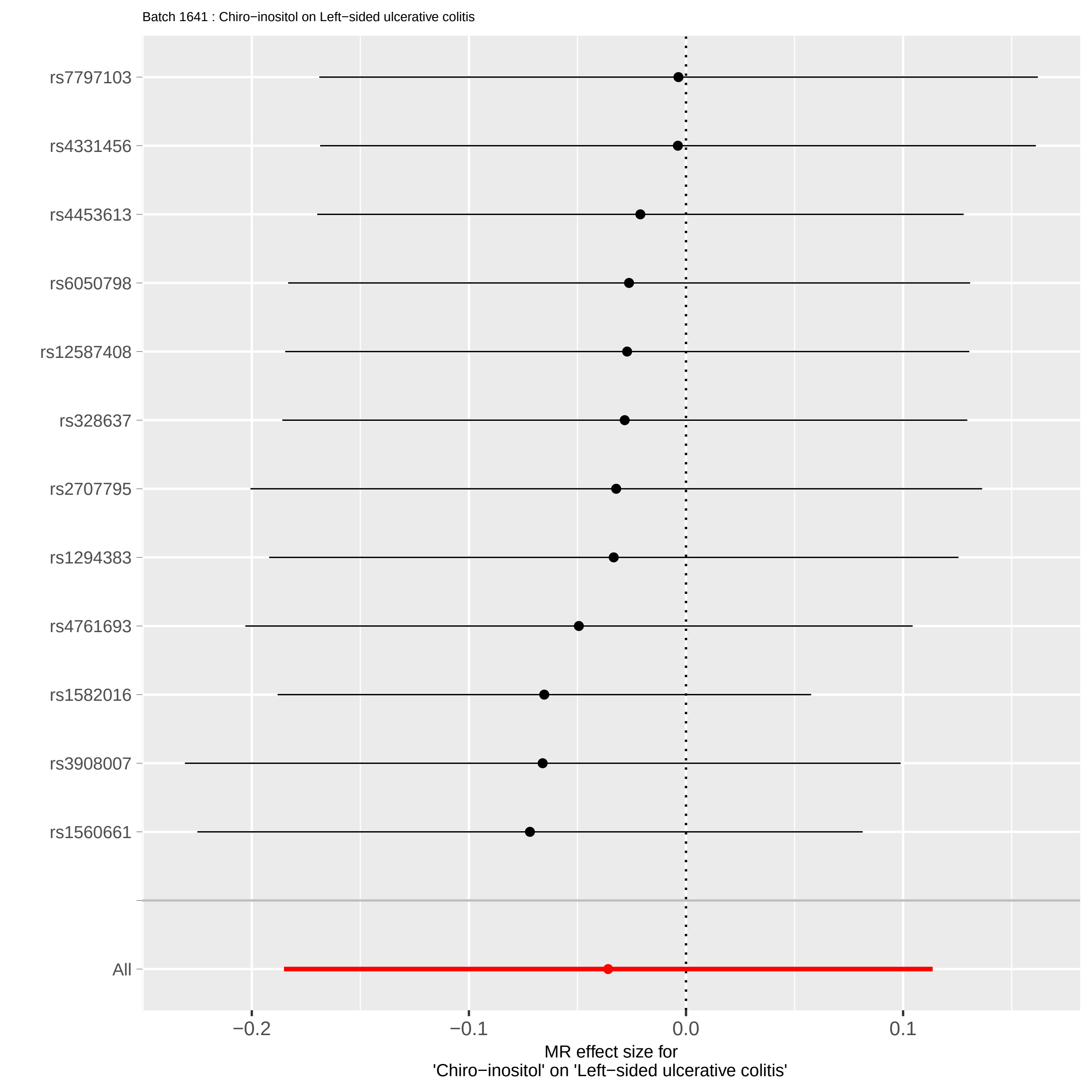

Batch 1641 : Chiro−inositol on Left−sided ulcerative colitis
rs7797103
rs4331456
rs4453613
rs6050798
rs12587408
rs328637
rs2707795
rs1294383
rs4761693
rs1582016
rs3908007
rs1560661
All
−0.2
−0.1
0.0
0.1
MR effect size for
'Chiro−inositol' on 'Left−sided ulcerative colitis'

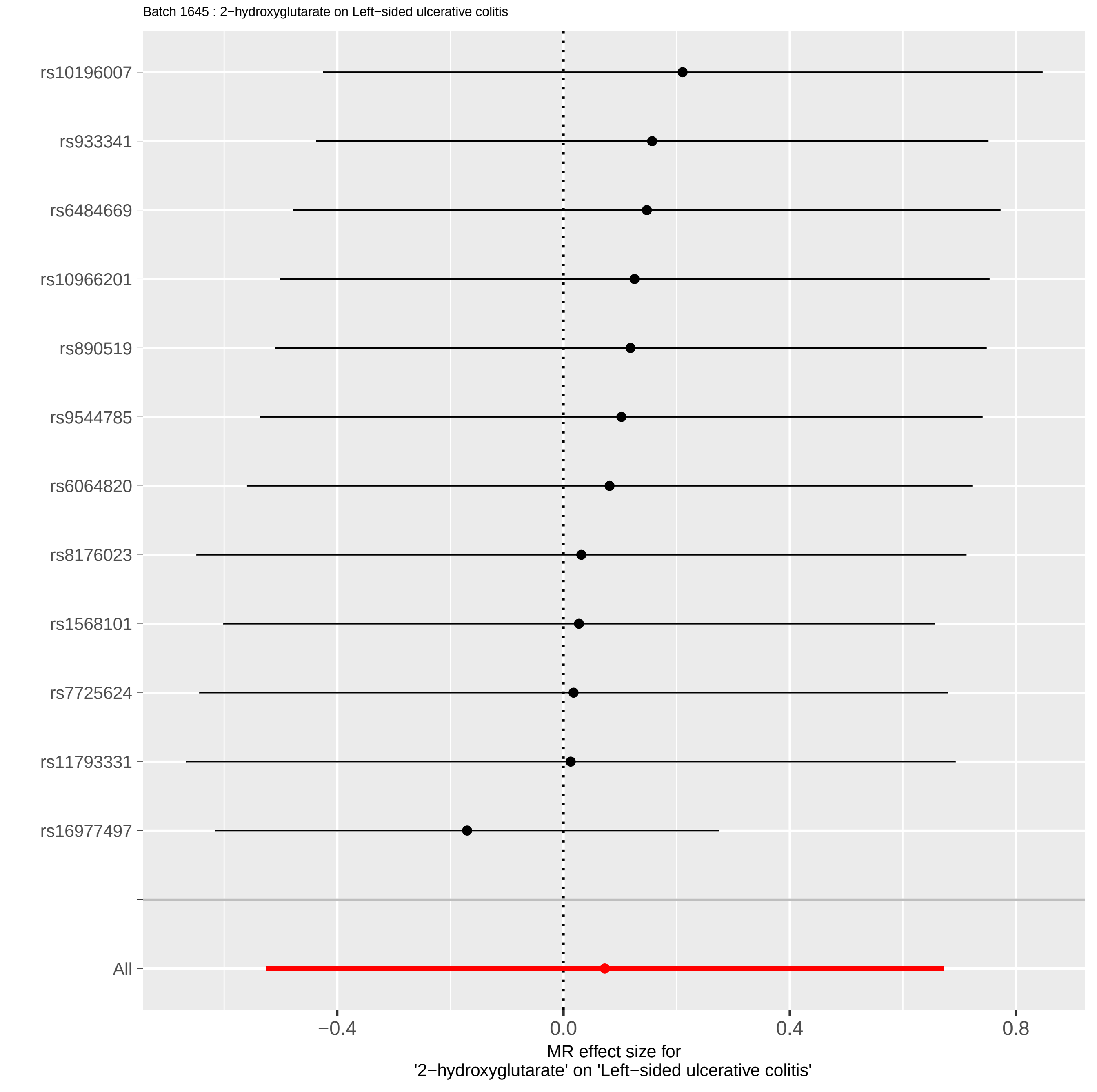

Batch 1645 : 2−hydroxyglutarate on Left−sided ulcerative colitis
rs10196007
rs933341
rs6484669
rs10966201
rs890519
rs9544785
rs6064820
rs8176023
rs1568101
rs7725624
rs11793331
rs16977497
All
−0.4
0.0
0.4
0.8
MR effect size for
'2−hydroxyglutarate' on 'Left−sided ulcerative colitis'

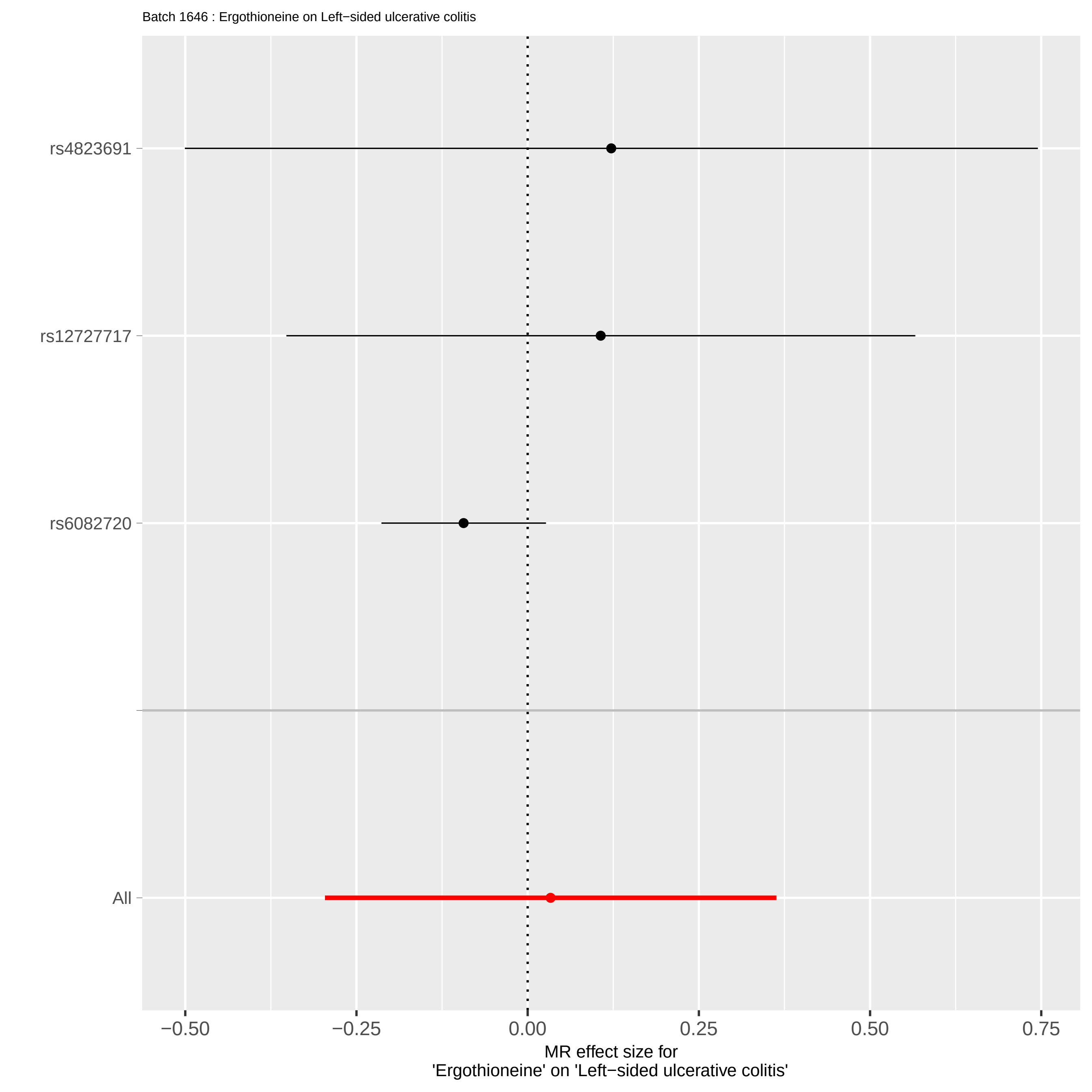

Batch 1646 : Ergothioneine on Left−sided ulcerative colitis
rs4823691
rs12727717
rs6082720
All
−0.50
−0.25
0.00
0.25
0.50
0.75
MR effect size for
'Ergothioneine' on 'Left−sided ulcerative colitis'

Batch 1648 : Phenylalanylphenylalanine on Left−sided ulcerative colitis

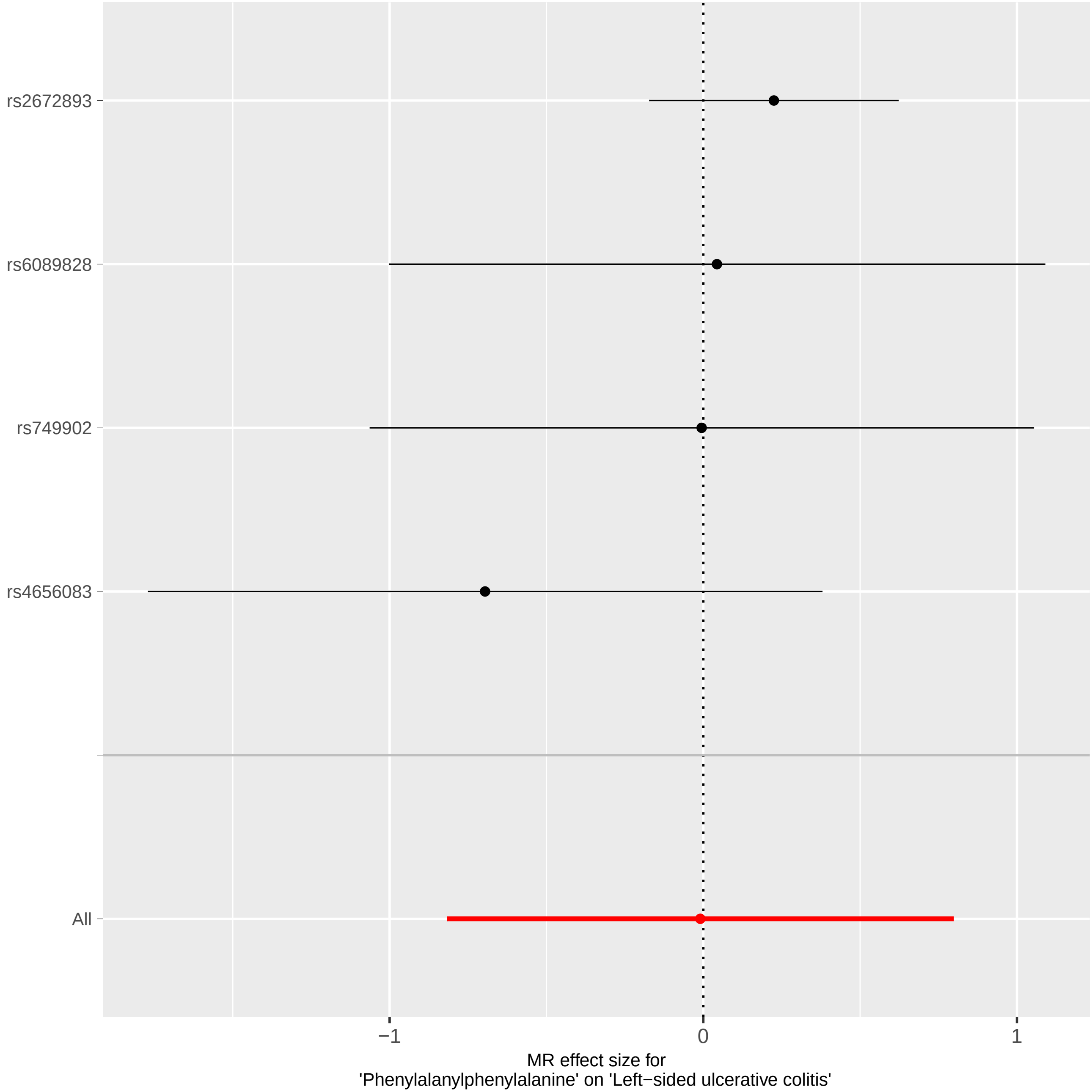

Batch 1650 : 15−methylpalmitate (isobar with 2−methylpalmitate) on Left−sided ulcerative colitis

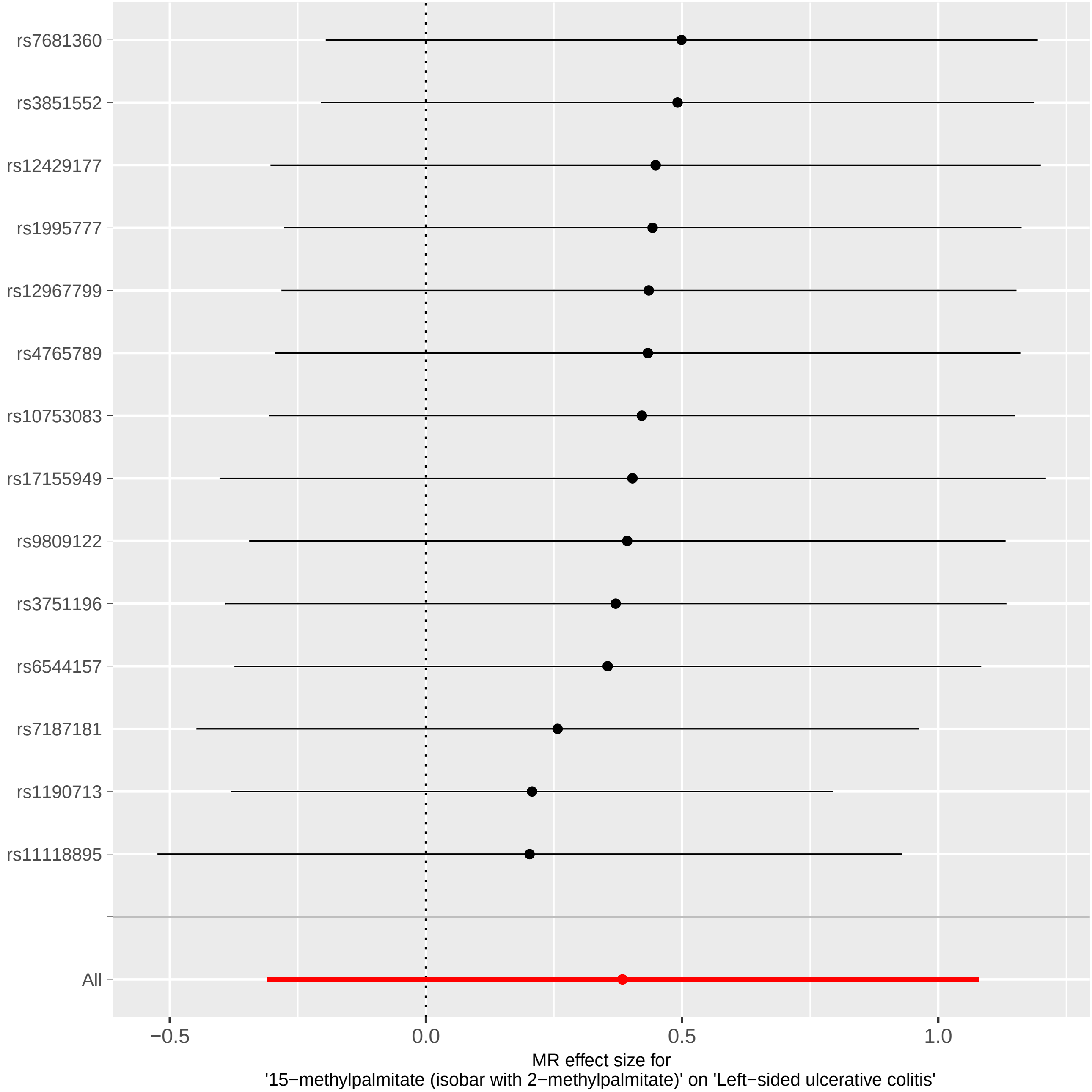

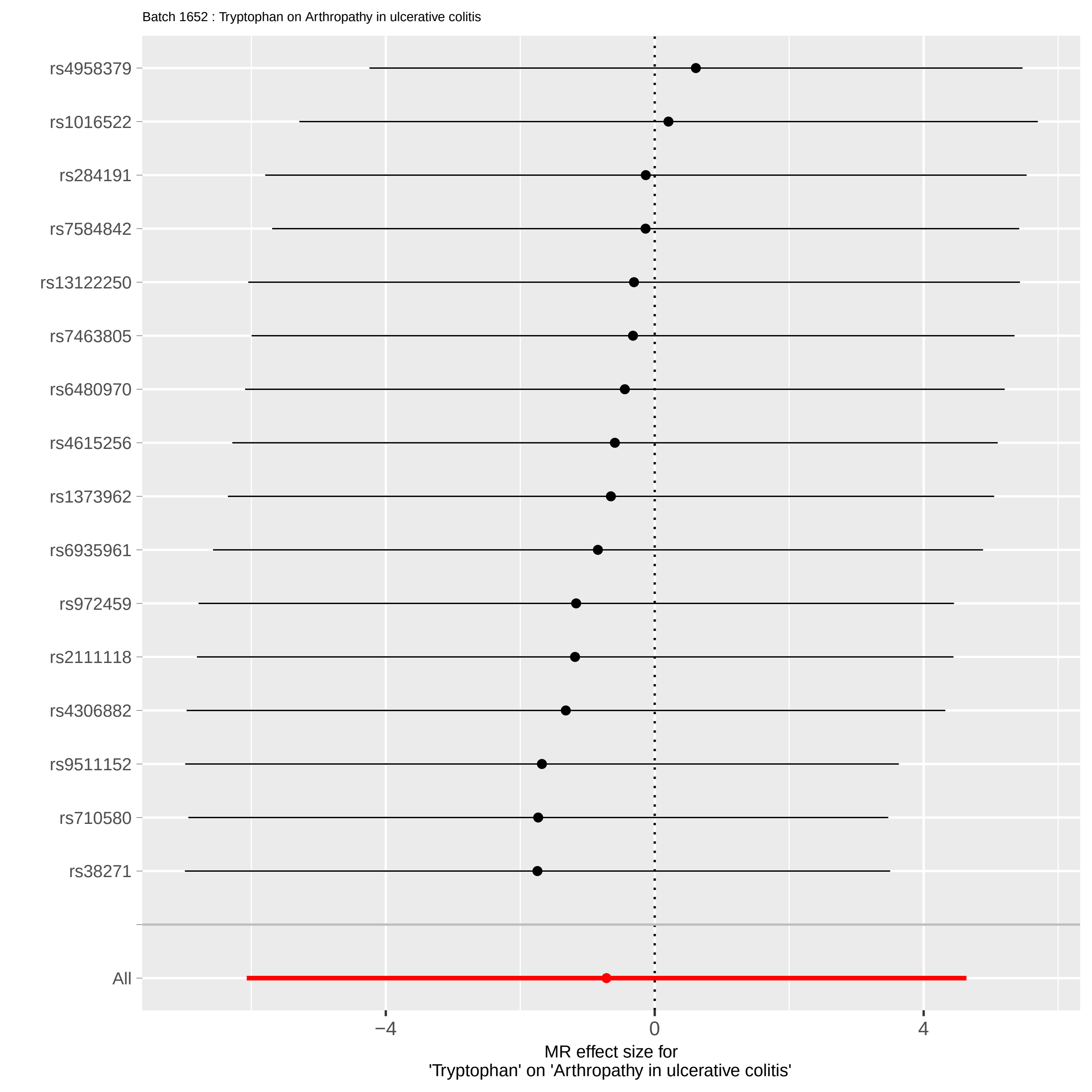

Batch 1652 : Tryptophan on Arthropathy in ulcerative colitis
rs4958379
rs1016522
rs284191
rs7584842
rs13122250
rs7463805
rs6480970
rs4615256
rs1373962
rs6935961
rs972459
rs2111118
rs4306882
rs9511152
rs710580
rs38271
All
−4
0
4
MR effect size for
'Tryptophan' on 'Arthropathy in ulcerative colitis'

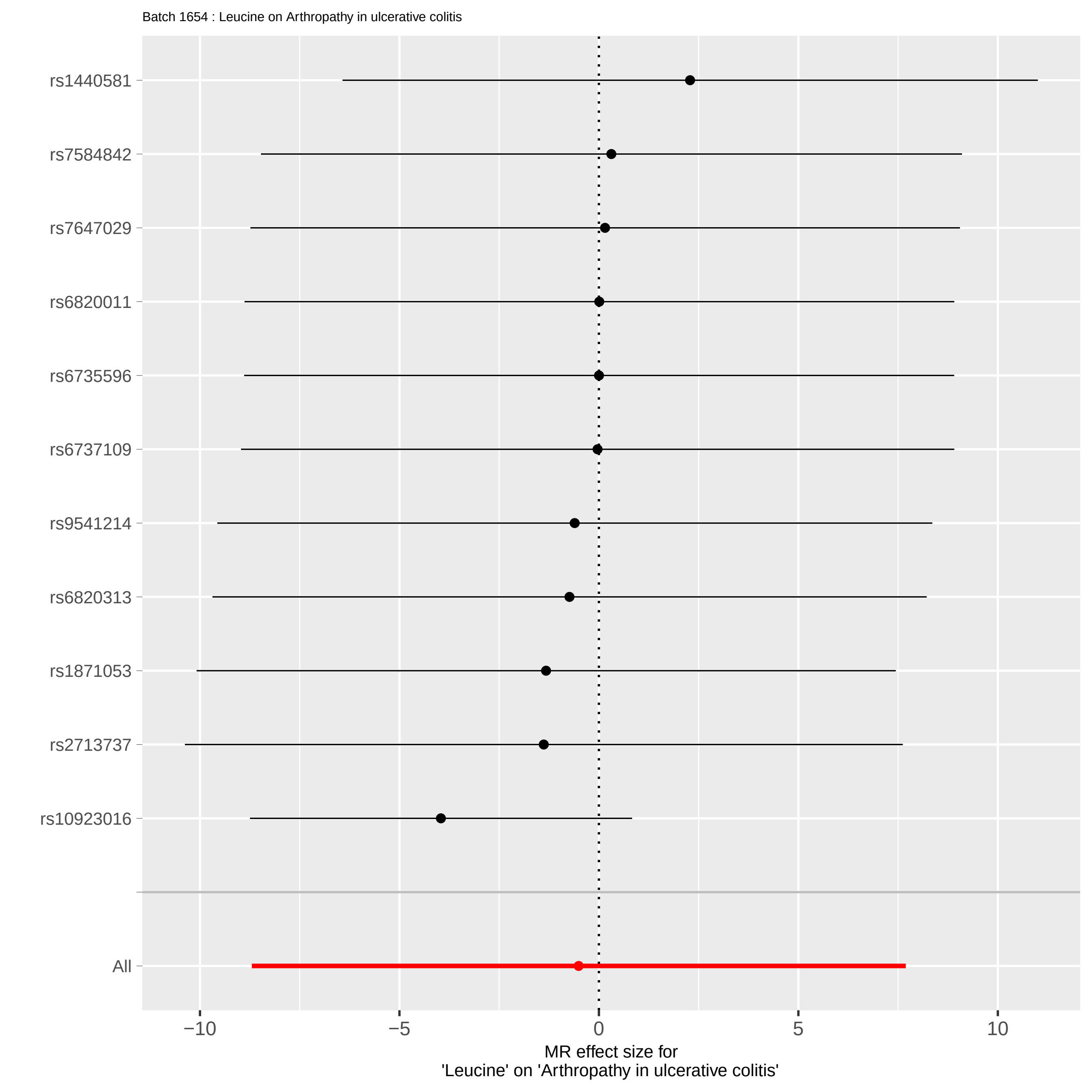

Batch 1654 : Leucine on Arthropathy in ulcerative colitis
rs1440581
rs7584842
rs7647029
rs6820011
rs6735596
rs6737109
rs9541214
rs6820313
rs1871053
rs2713737
rs10923016
All
−10
−5
0
5
10
MR effect size for
'Leucine' on 'Arthropathy in ulcerative colitis'

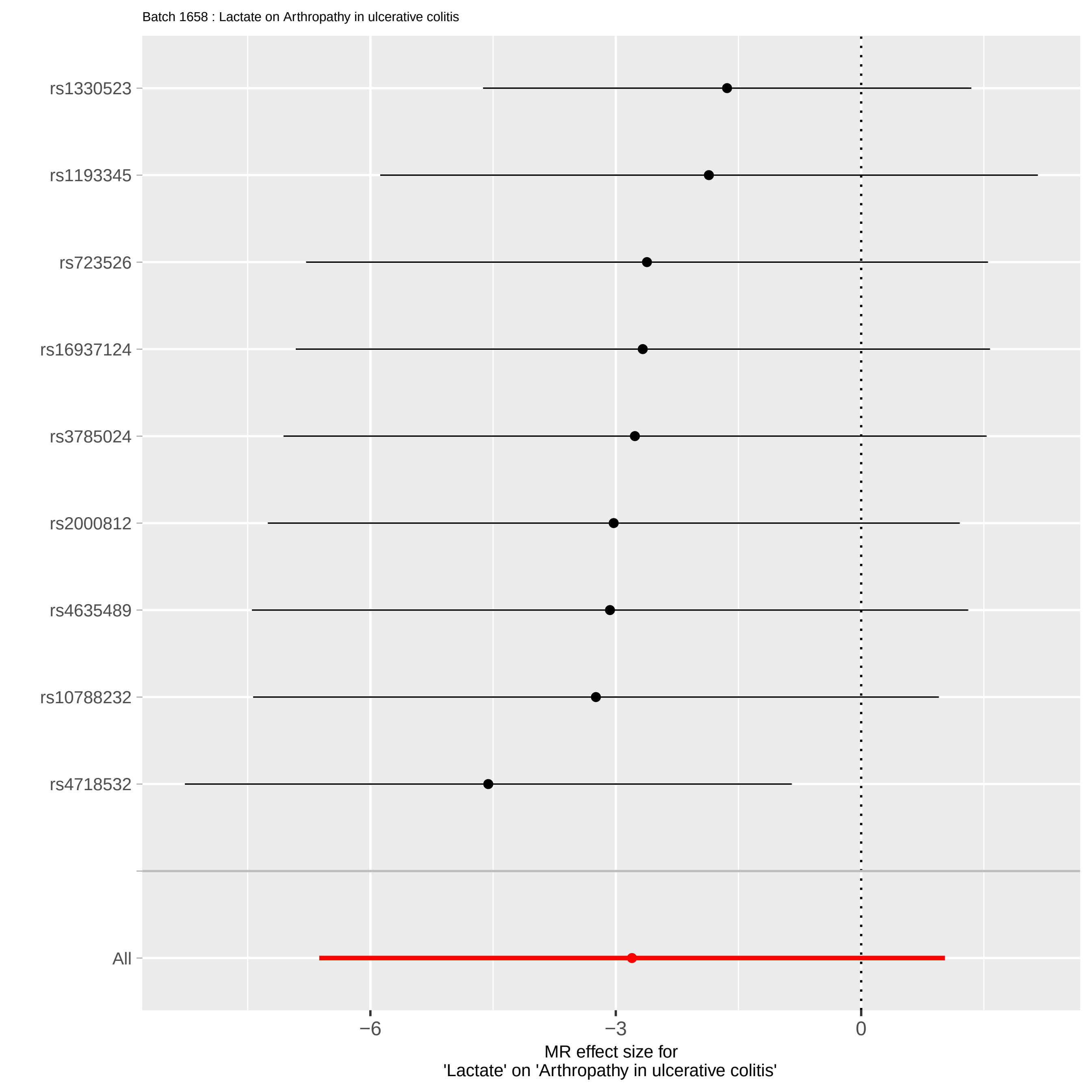

Batch 1658 : Lactate on Arthropathy in ulcerative colitis
rs1330523
rs1193345
rs723526
rs16937124
rs3785024
rs2000812
rs4635489
rs10788232
rs4718532
All
−6
−3
0
MR effect size for
'Lactate' on 'Arthropathy in ulcerative colitis'

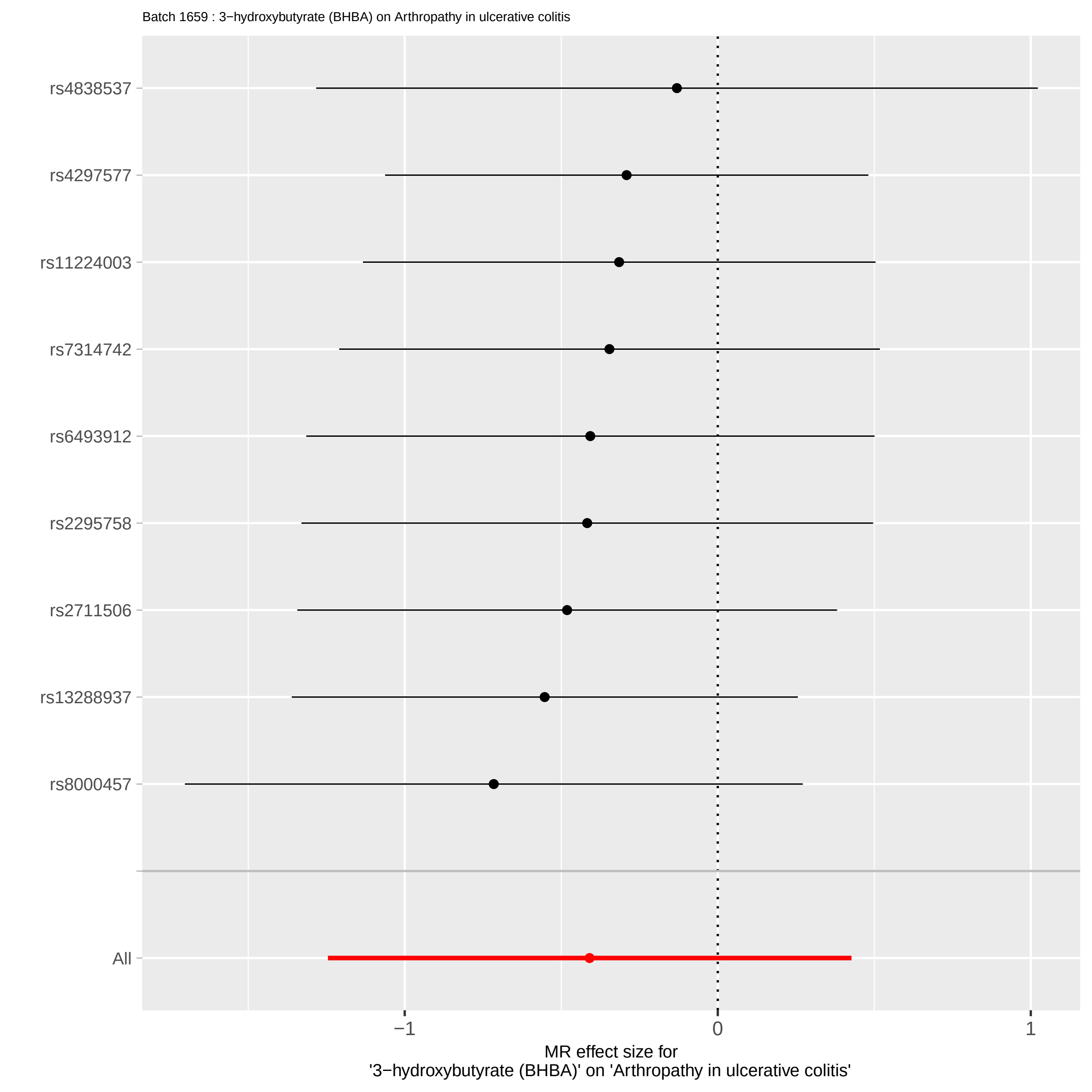

Batch 1659 : 3−hydroxybutyrate (BHBA) on Arthropathy in ulcerative colitis
rs4838537
rs4297577
rs11224003
rs7314742
rs6493912
rs2295758
rs2711506
rs13288937
rs8000457
All
−1
0
1
MR effect size for
'3−hydroxybutyrate (BHBA)' on 'Arthropathy in ulcerative colitis'

Batch 1660 : Arabinose on Arthropathy in ulcerative colitis

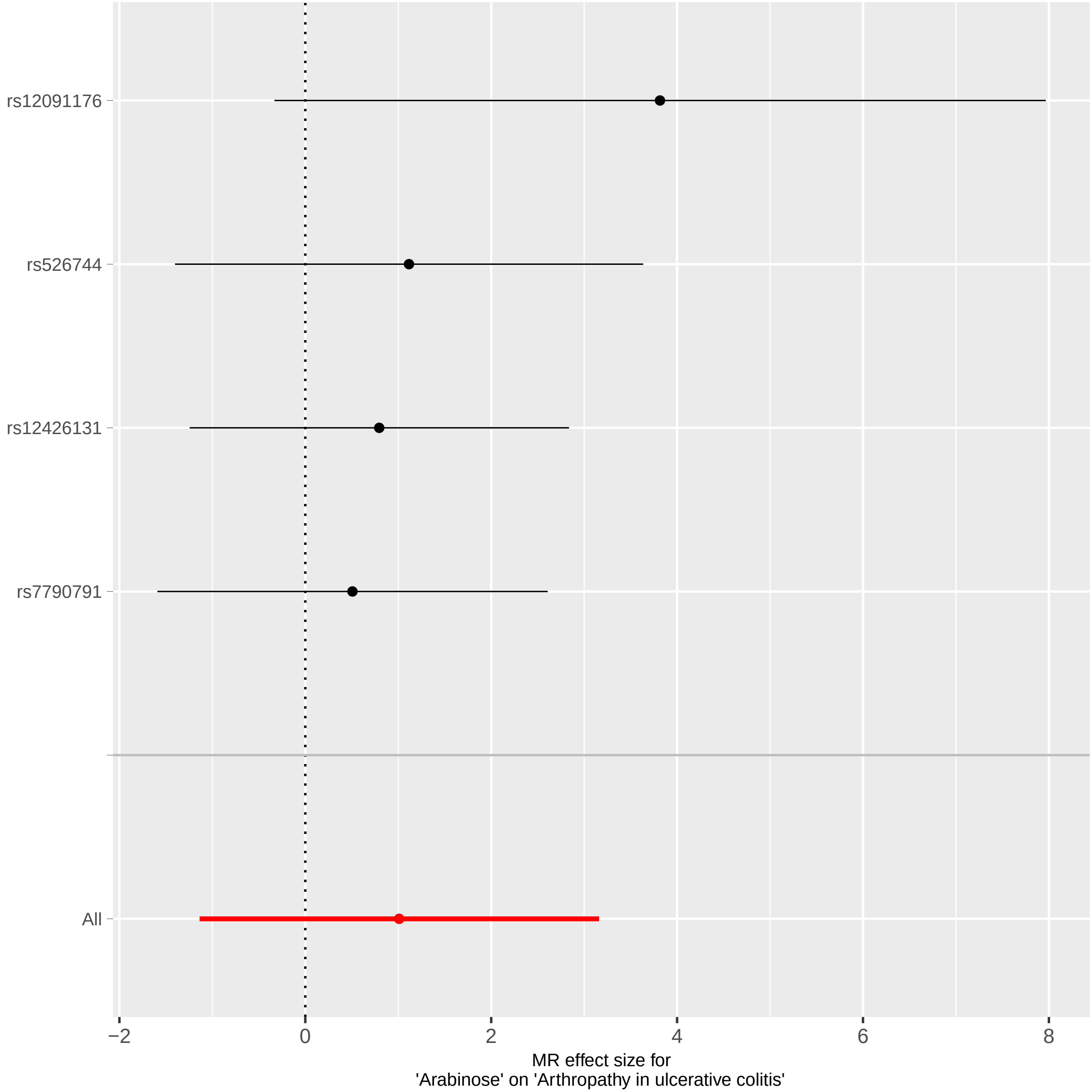

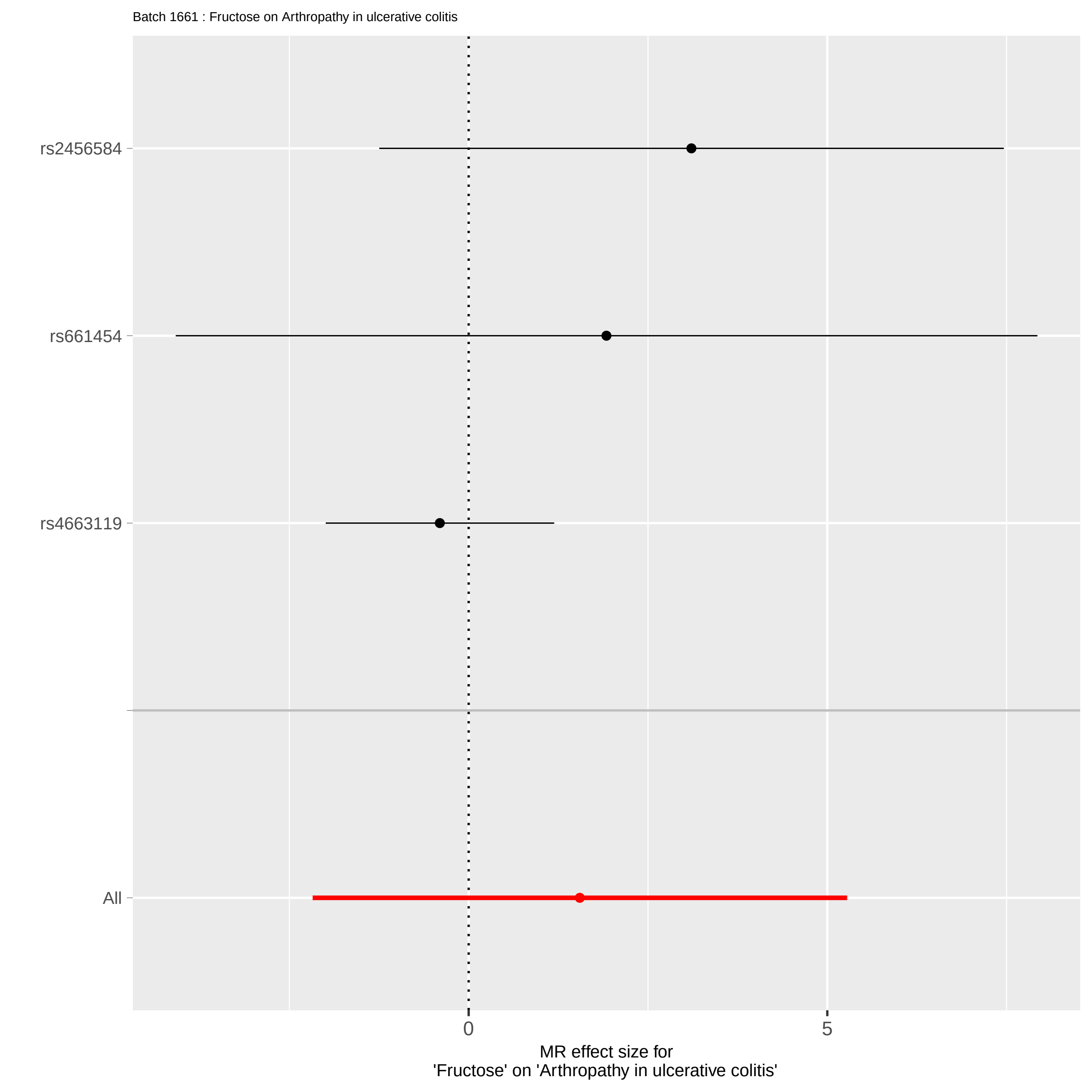

Batch 1661 : Fructose on Arthropathy in ulcerative colitis
rs2456584
rs661454
rs4663119
All
0
5
MR effect size for
'Fructose' on 'Arthropathy in ulcerative colitis'

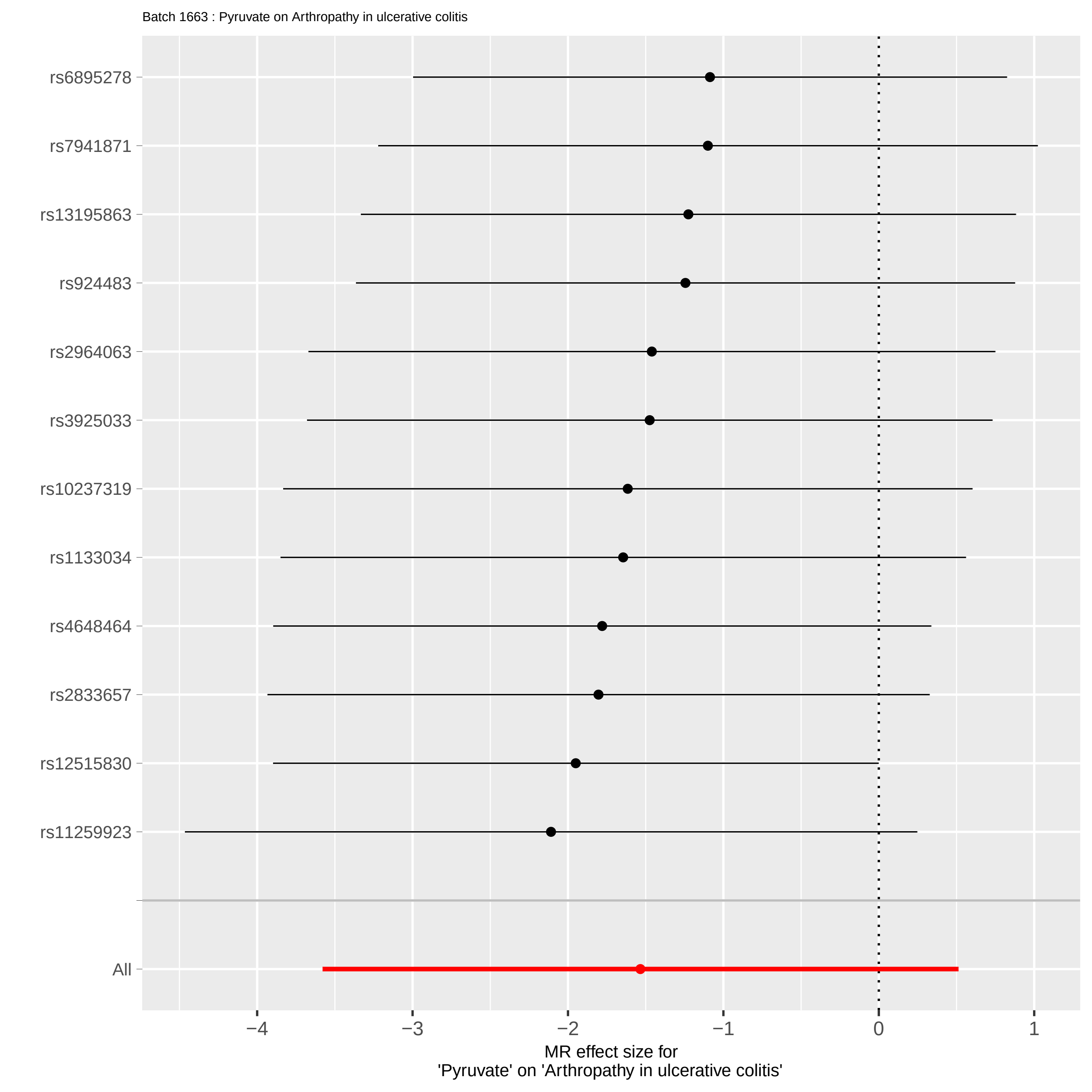
Batch 1663 : Pyruvate on Arthropathy in ulcerative colitis
rs6895278
rs7941871
rs13195863
rs924483
rs2964063
rs3925033
rs10237319
rs1133034
rs4648464
rs2833657
rs12515830
rs11259923
All
−4
−3
−2
−1
0
1
MR effect size for
'Pyruvate' on 'Arthropathy in ulcerative colitis'

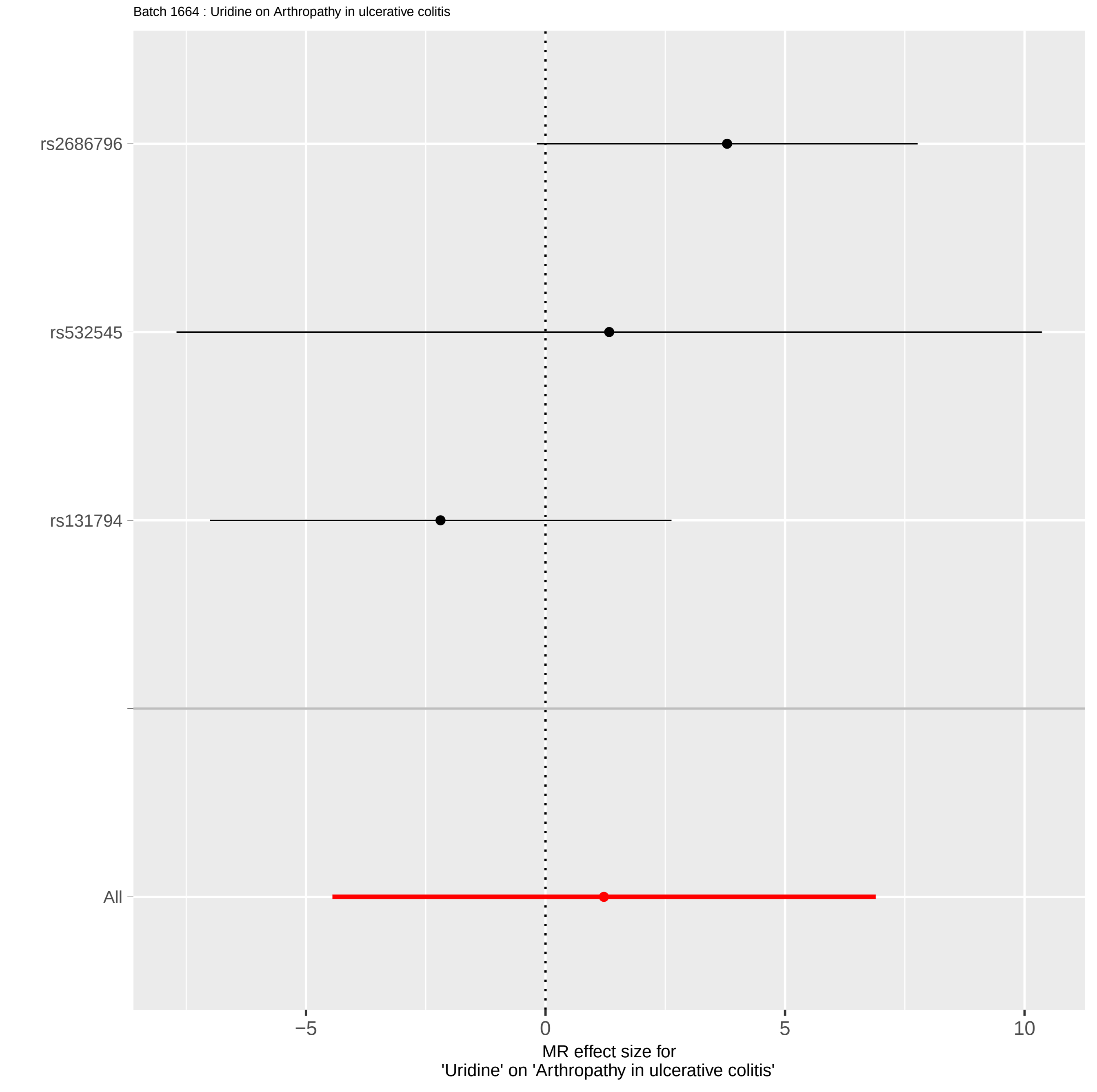

Batch 1664 : Uridine on Arthropathy in ulcerative colitis
rs2686796
rs532545
rs131794
All
−5
0
5
10
MR effect size for
'Uridine' on 'Arthropathy in ulcerative colitis'

Batch 1665 : Linoleate (18:2n6) on Arthropathy in ulcerative colitis

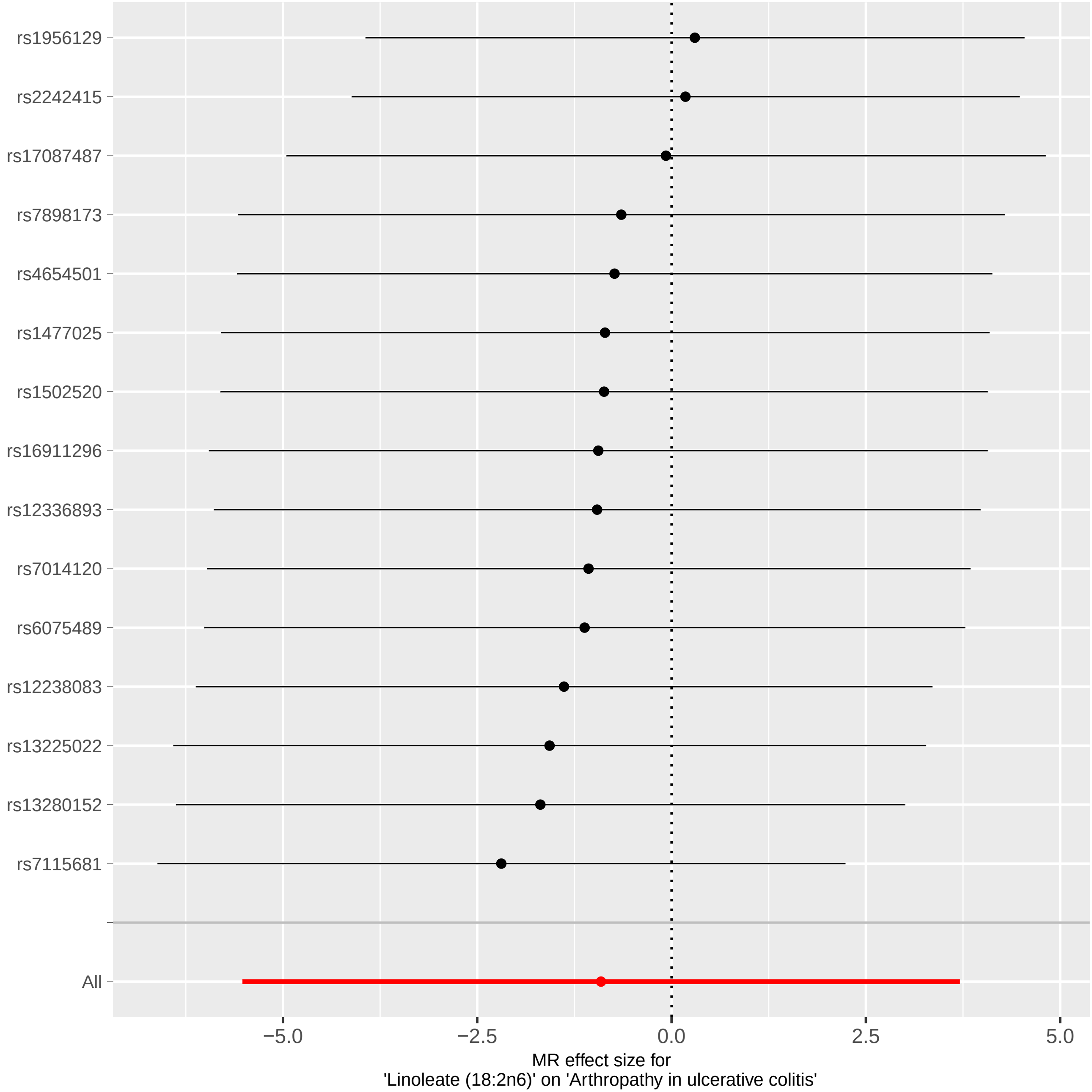

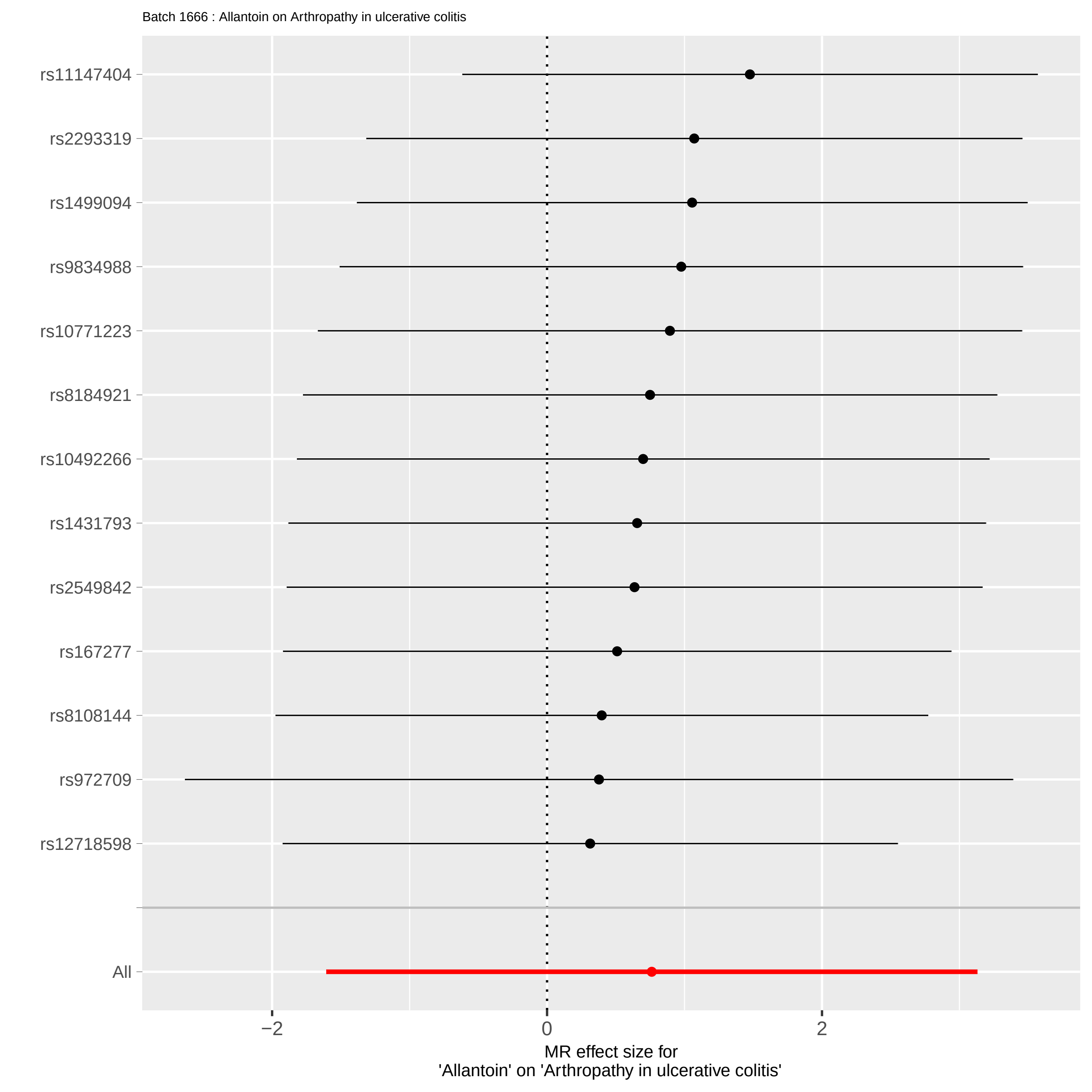

Batch 1666 : Allantoin on Arthropathy in ulcerative colitis
rs11147404
rs2293319
rs1499094
rs9834988
rs10771223
rs8184921
rs10492266
rs1431793
rs2549842
rs167277
rs8108144
rs972709
rs12718598
All
−2
0
2
MR effect size for
'Allantoin' on 'Arthropathy in ulcerative colitis'

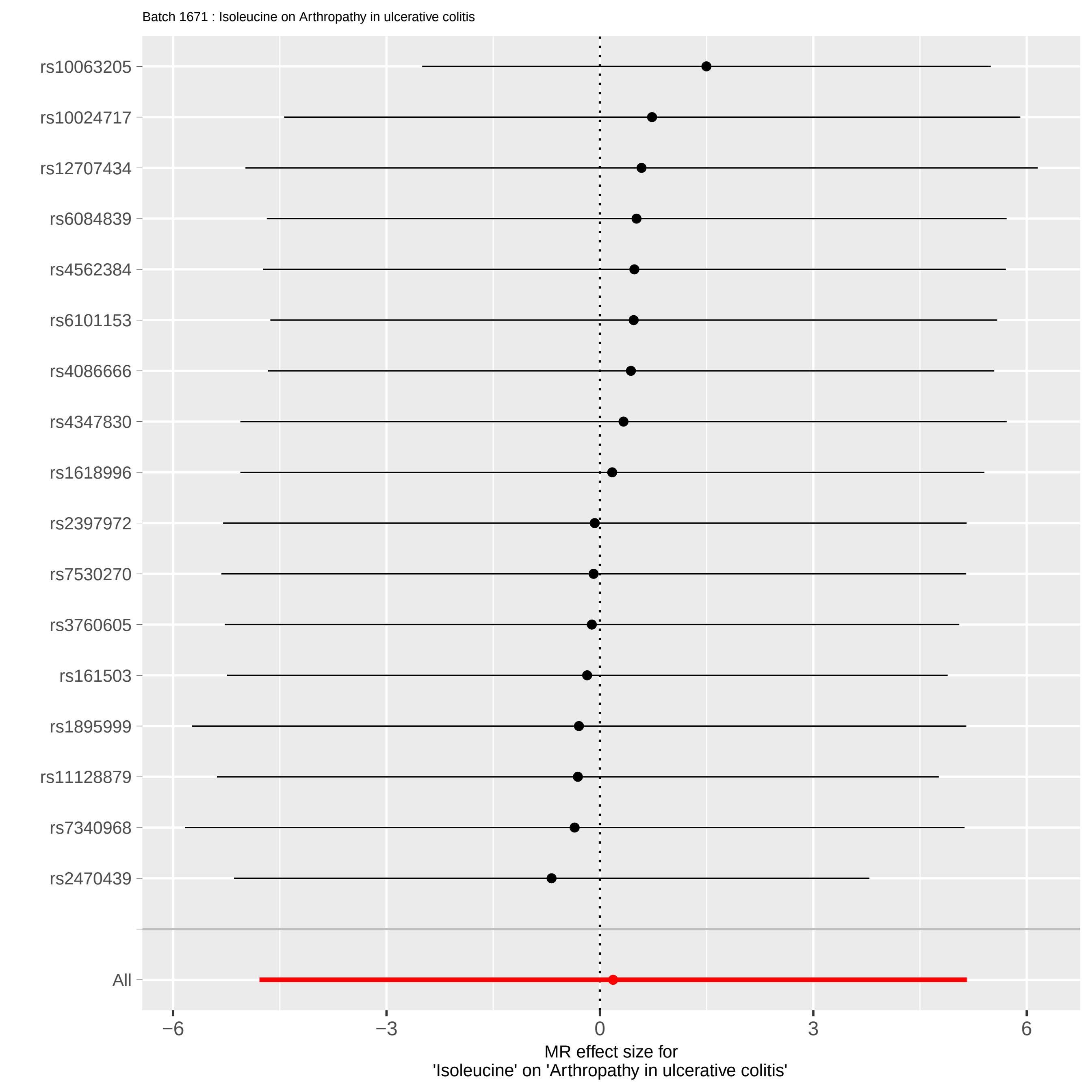
Batch 1671 : Isoleucine on Arthropathy in ulcerative colitis
rs10063205
rs10024717
rs12707434
rs6084839
rs4562384
rs6101153
rs4086666
rs4347830
rs1618996
rs2397972
rs7530270
rs3760605
rs161503
rs1895999
rs11128879
rs7340968
rs2470439
All
−6
−3
0
3
6
MR effect size for
'Isoleucine' on 'Arthropathy in ulcerative colitis'

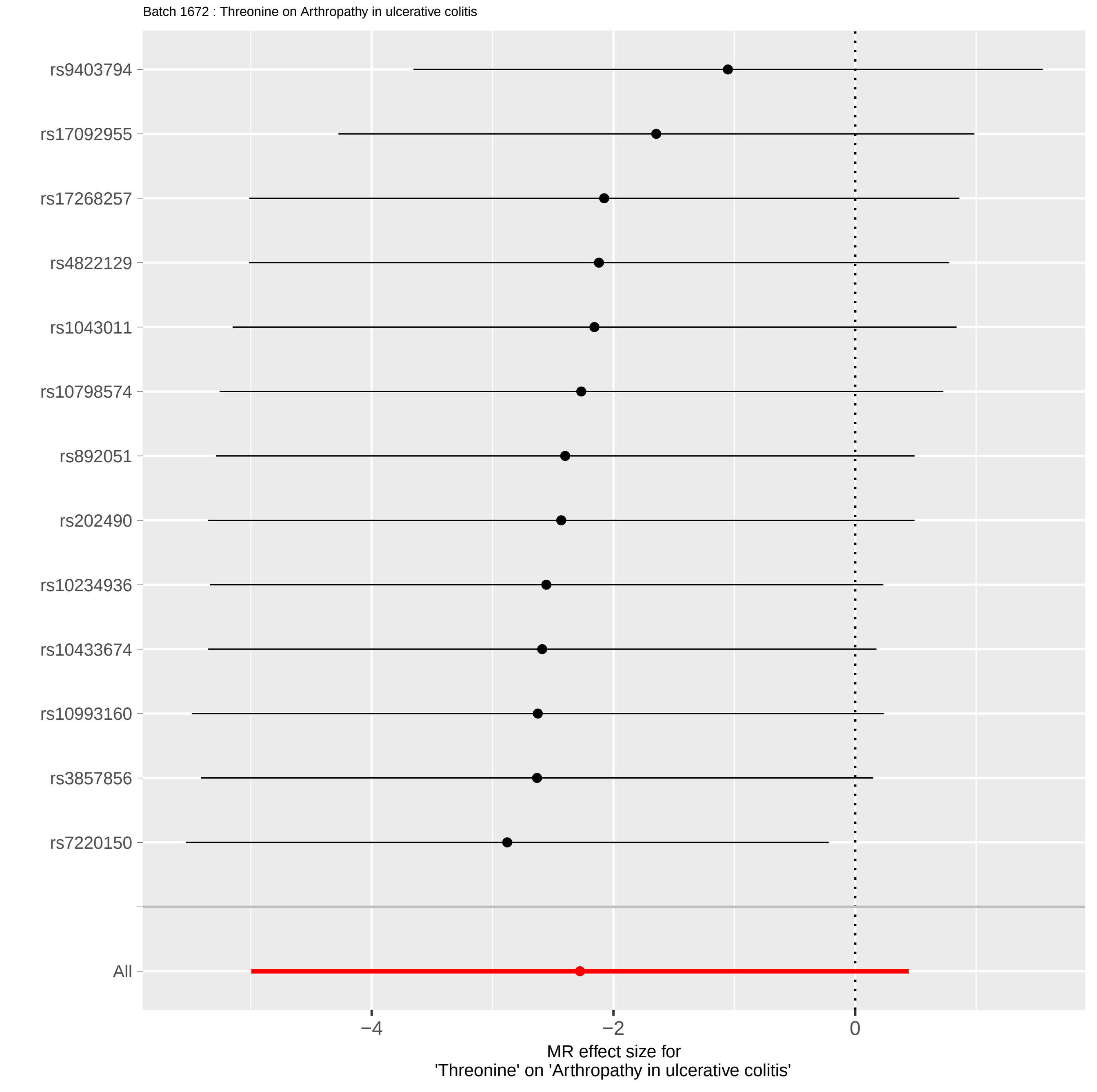

Batch 1672 : Threonine on Arthropathy in ulcerative colitis
rs9403794
rs17092955
rs17268257
rs4822129
rs1043011
rs10798574
rs892051
rs202490
rs10234936
rs10433674
rs10993160
rs3857856
rs7220150
All
−4
−2
0
MR effect size for
'Threonine' on 'Arthropathy in ulcerative colitis'

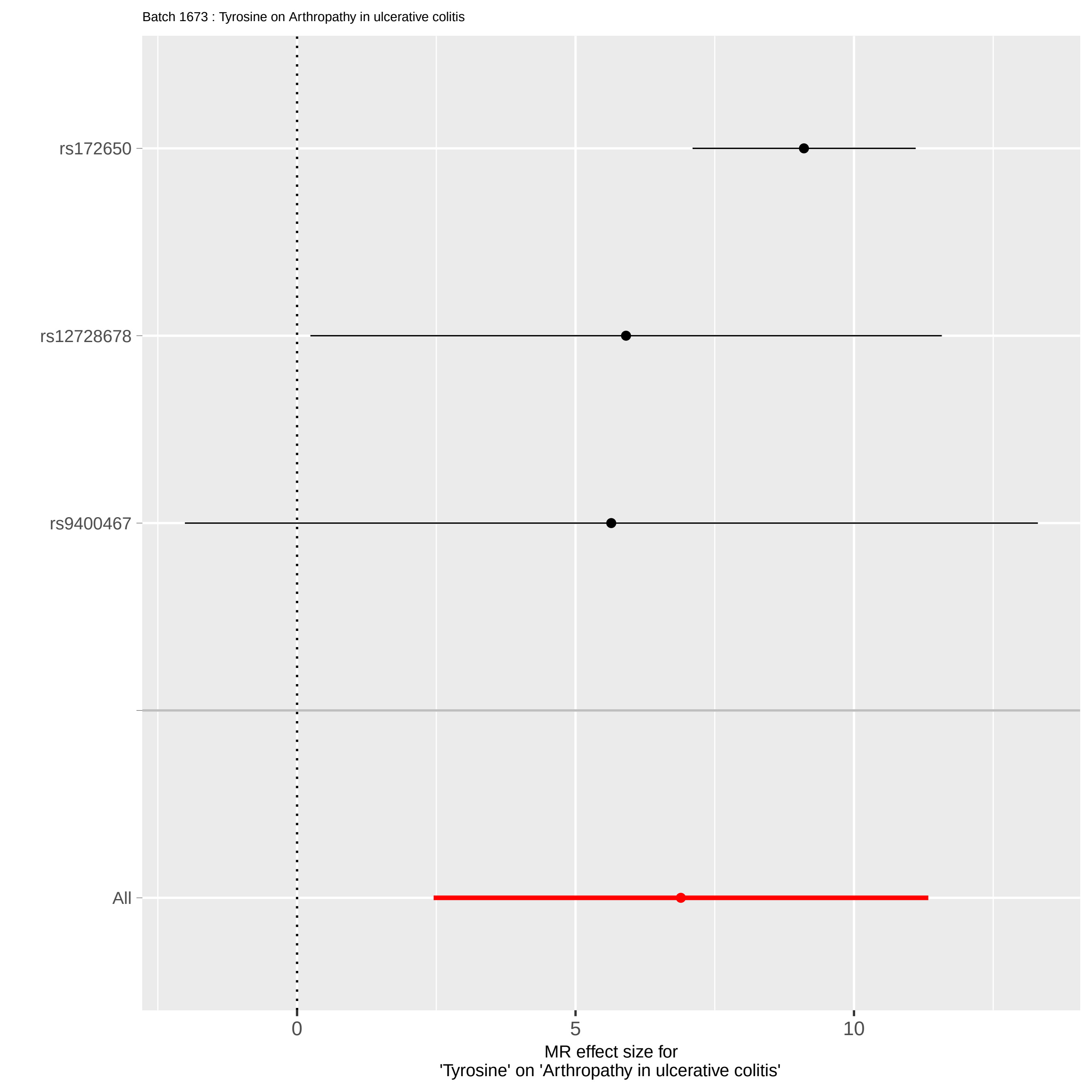
Batch 1673 : Tyrosine on Arthropathy in ulcerative colitis
rs172650
rs12728678
rs9400467
All
0
5
10
MR effect size for
'Tyrosine' on 'Arthropathy in ulcerative colitis'

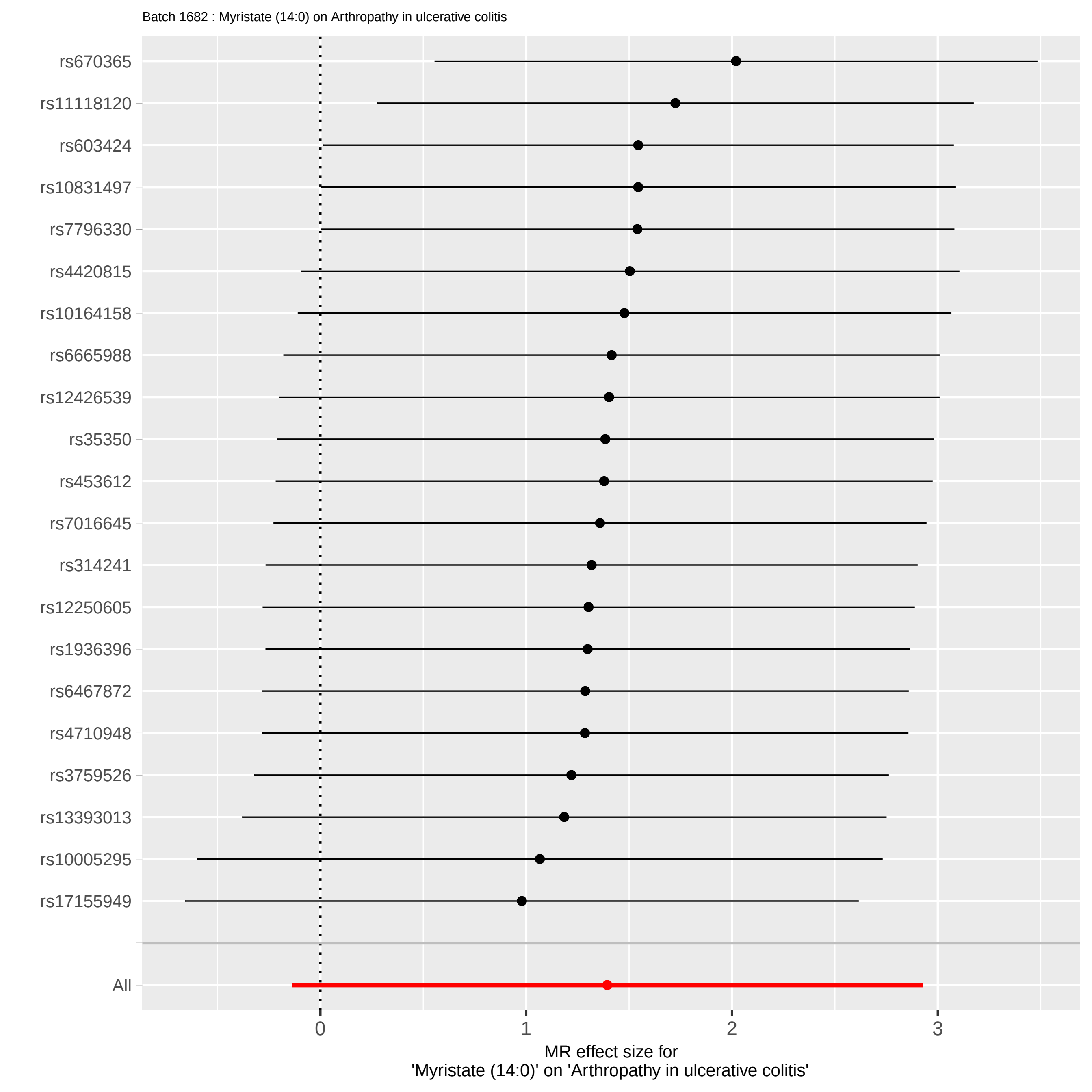

Batch 1682 : Myristate (14:0) on Arthropathy in ulcerative colitis
rs670365
rs11118120
rs603424
rs10831497
rs7796330
rs4420815
rs10164158
rs6665988
rs12426539
rs35350
rs453612
rs7016645
rs314241
rs12250605
rs1936396
rs6467872
rs4710948
rs3759526
rs13393013
rs10005295
rs17155949
All
0
1
2
3
MR effect size for
'Myristate (14:0)' on 'Arthropathy in ulcerative colitis'

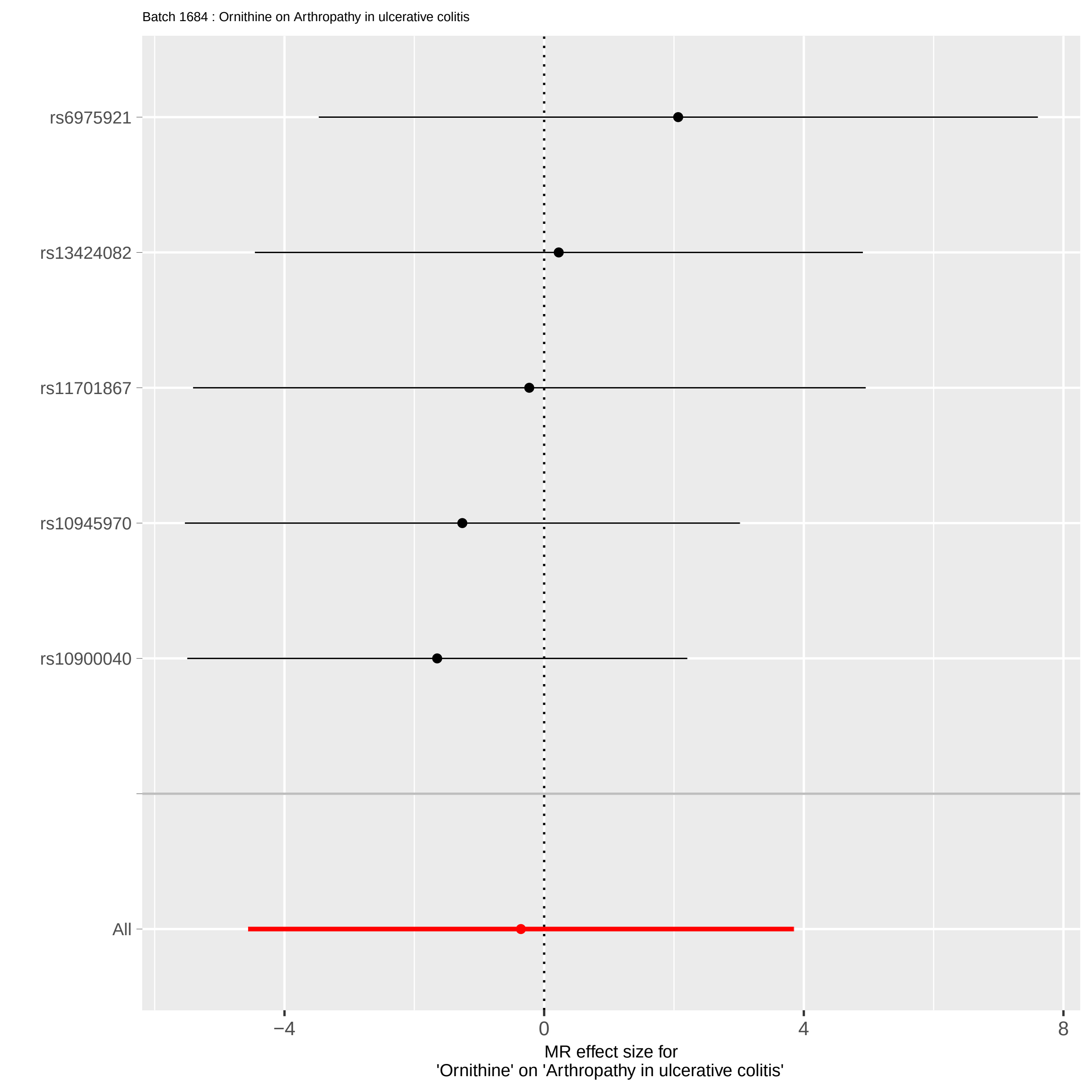

Batch 1684 : Ornithine on Arthropathy in ulcerative colitis
rs6975921
rs13424082
rs11701867
rs10945970
rs10900040
All
−4
0
4
8
MR effect size for
'Ornithine' on 'Arthropathy in ulcerative colitis'

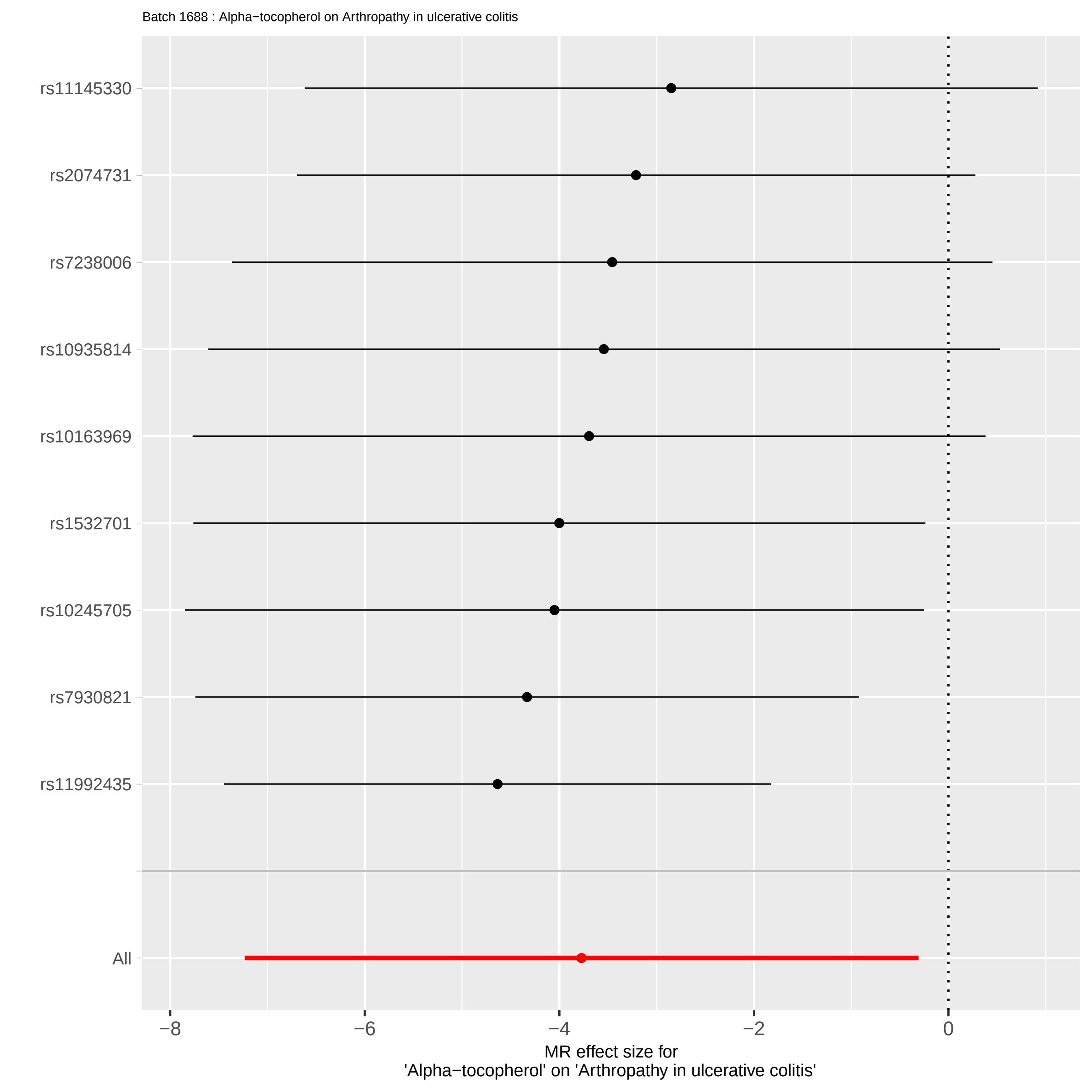

Batch 1688 : Alpha−tocopherol on Arthropathy in ulcerative colitis
rs11145330
rs2074731
rs7238006
rs10935814
rs10163969
rs1532701
rs10245705
rs7930821
rs11992435
All
−8
−6
−4
−2
0
MR effect size for
'Alpha−tocopherol' on 'Arthropathy in ulcerative colitis'

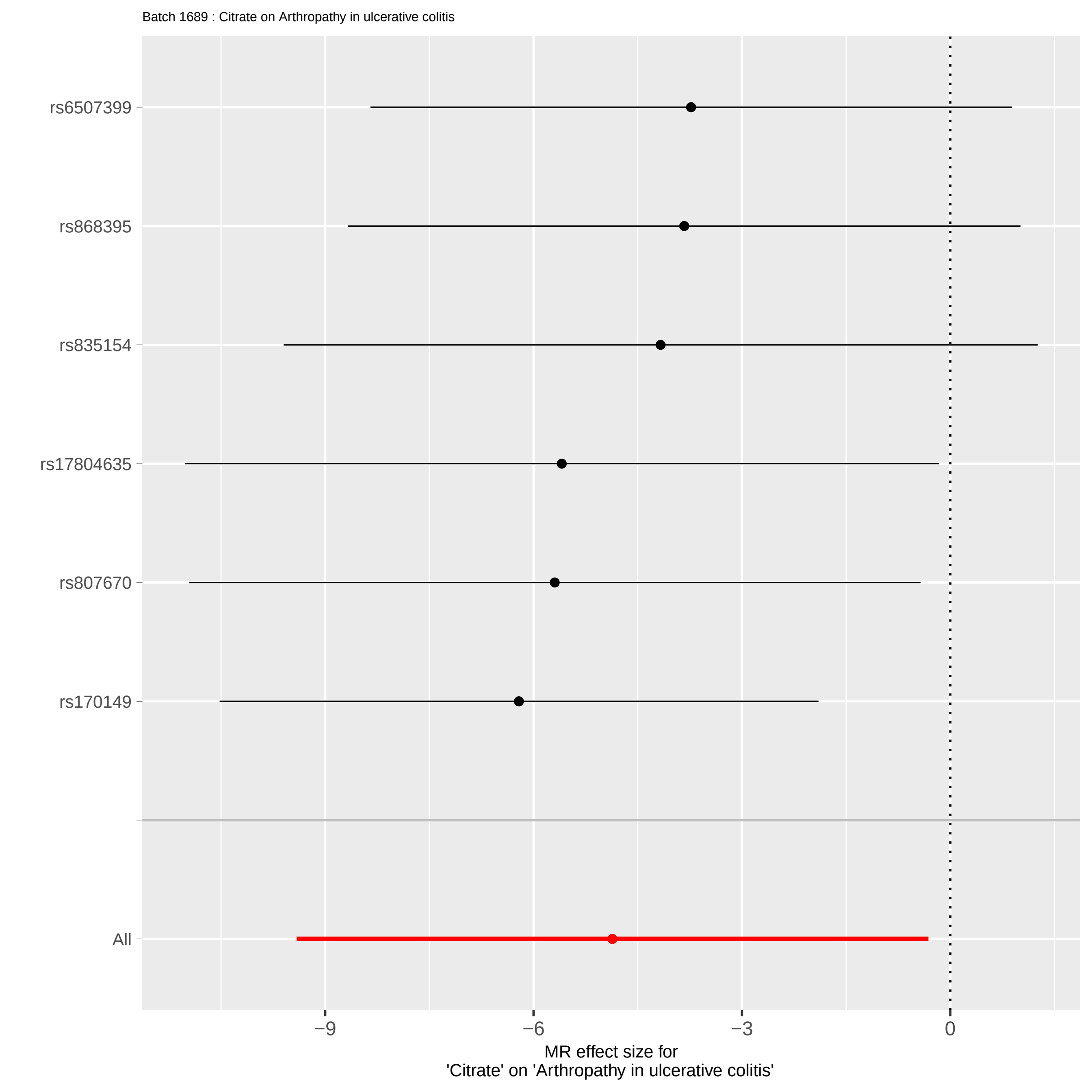

Batch 1689 : Citrate on Arthropathy in ulcerative colitis
rs6507399
rs868395
rs835154
rs17804635
rs807670
rs170149
All
−9
−6
−3
0
MR effect size for
'Citrate' on 'Arthropathy in ulcerative colitis'

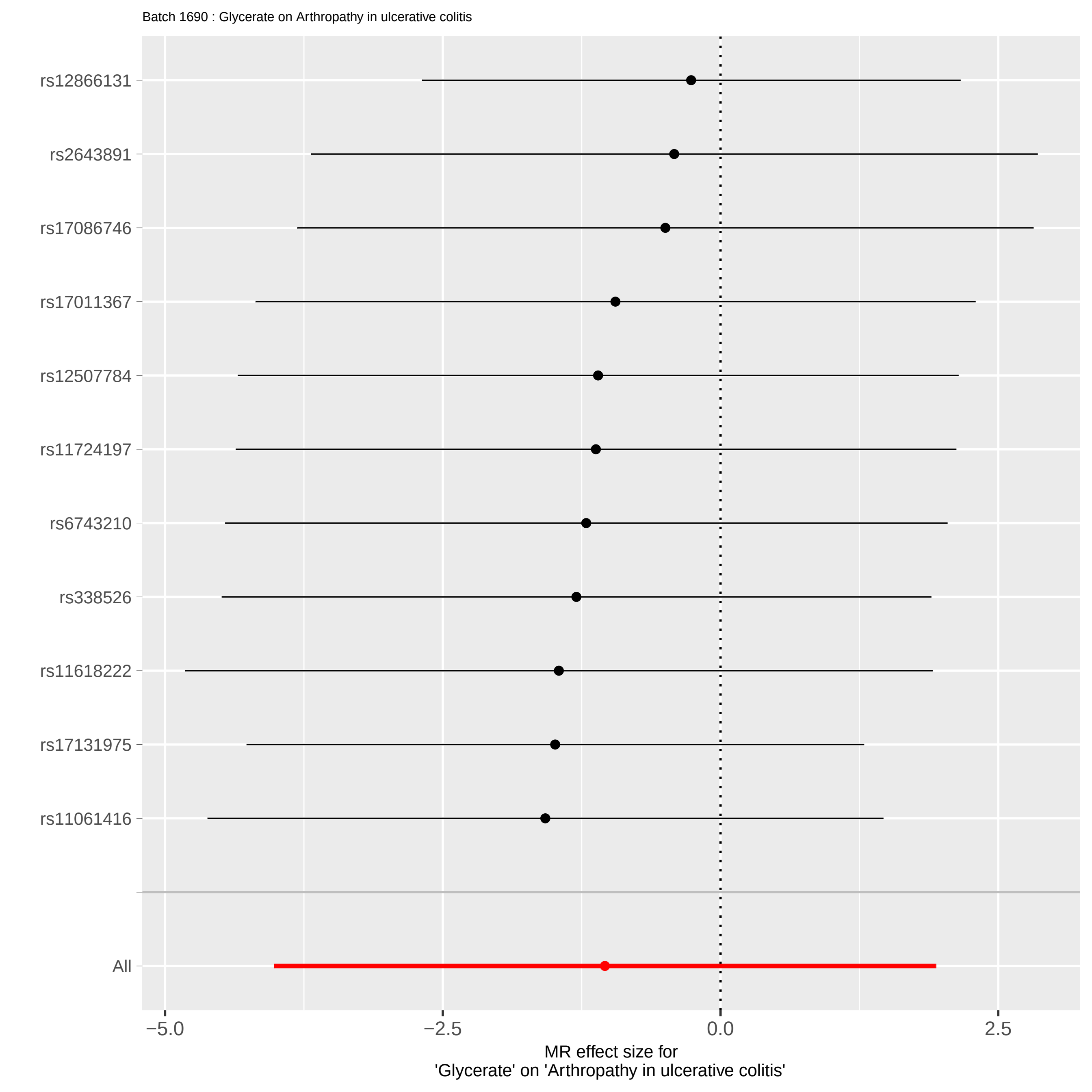

Batch 1690 : Glycerate on Arthropathy in ulcerative colitis
rs12866131
rs2643891
rs17086746
rs17011367
rs12507784
rs11724197
rs6743210
rs338526
rs11618222
rs17131975
rs11061416
All
−5.0
−2.5
0.0
2.5
MR effect size for
'Glycerate' on 'Arthropathy in ulcerative colitis'

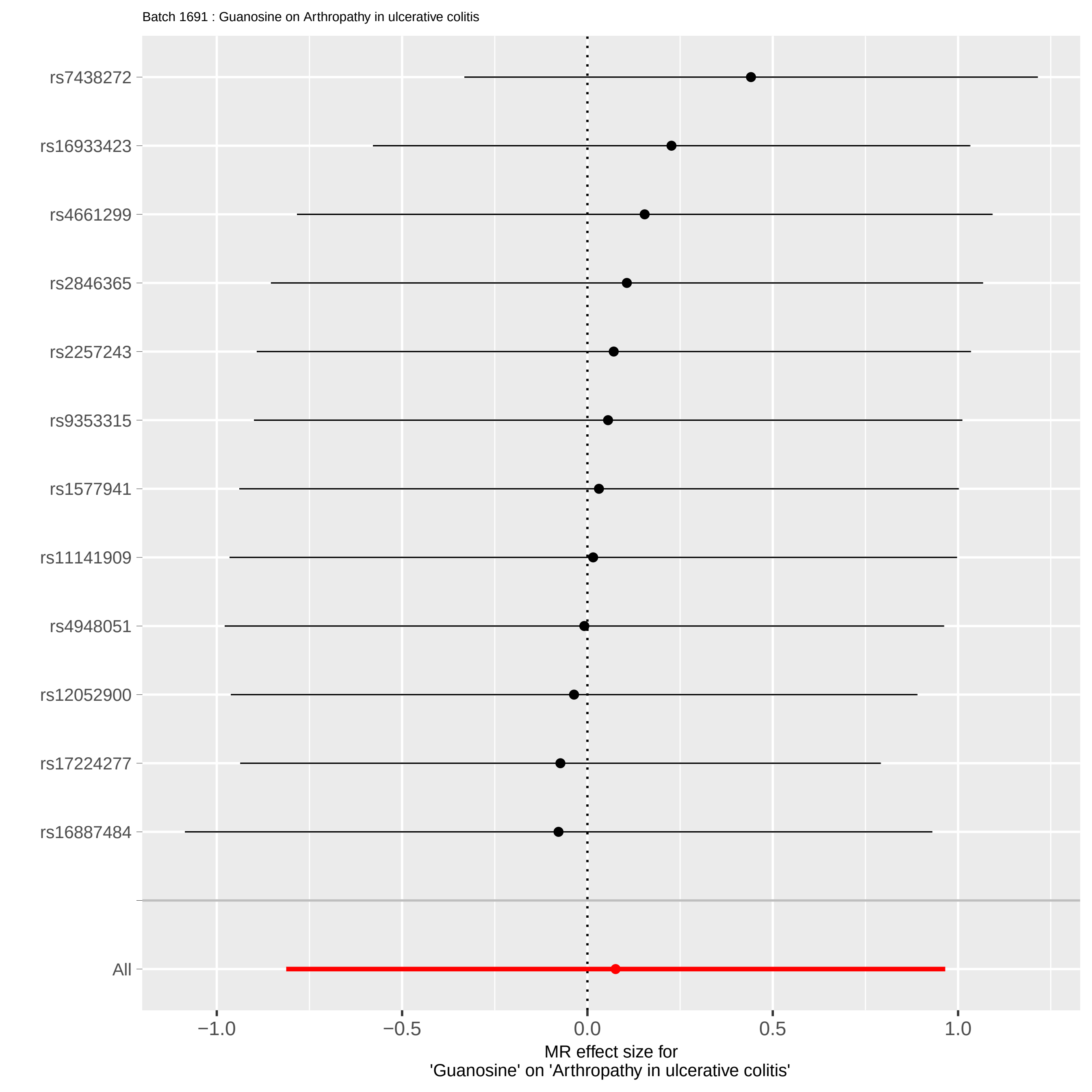
Batch 1691 : Guanosine on Arthropathy in ulcerative colitis
rs7438272
rs16933423
rs4661299
rs2846365
rs2257243
rs9353315
rs1577941
rs11141909
rs4948051
rs12052900
rs17224277
rs16887484
All
−1.0
−0.5
0.0
0.5
1.0
MR effect size for
'Guanosine' on 'Arthropathy in ulcerative colitis'

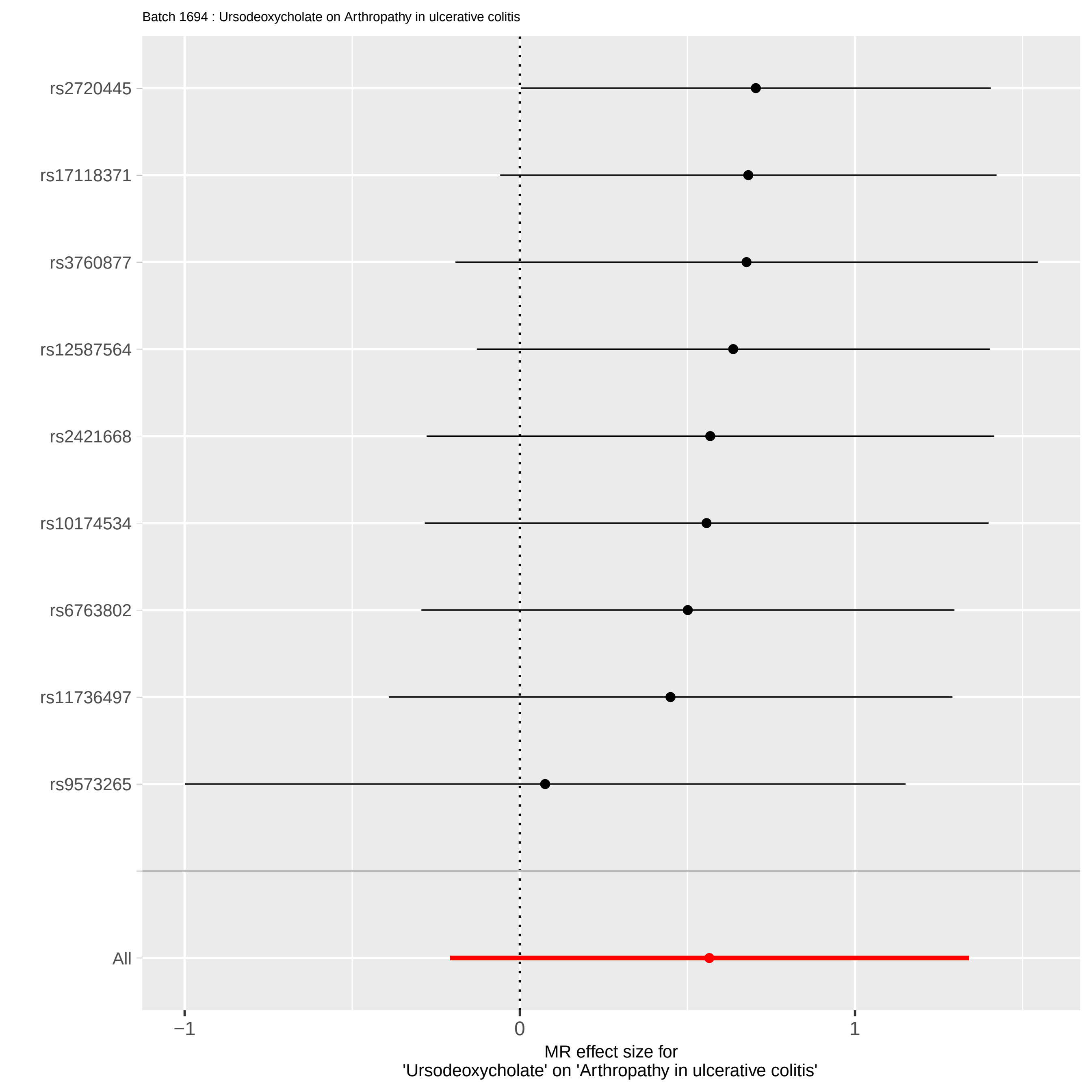

Batch 1694 : Ursodeoxycholate on Arthropathy in ulcerative colitis
rs2720445
rs17118371
rs3760877
rs12587564
rs2421668
rs10174534
rs6763802
rs11736497
rs9573265
All
−1
0
1
MR effect size for
'Ursodeoxycholate' on 'Arthropathy in ulcerative colitis'

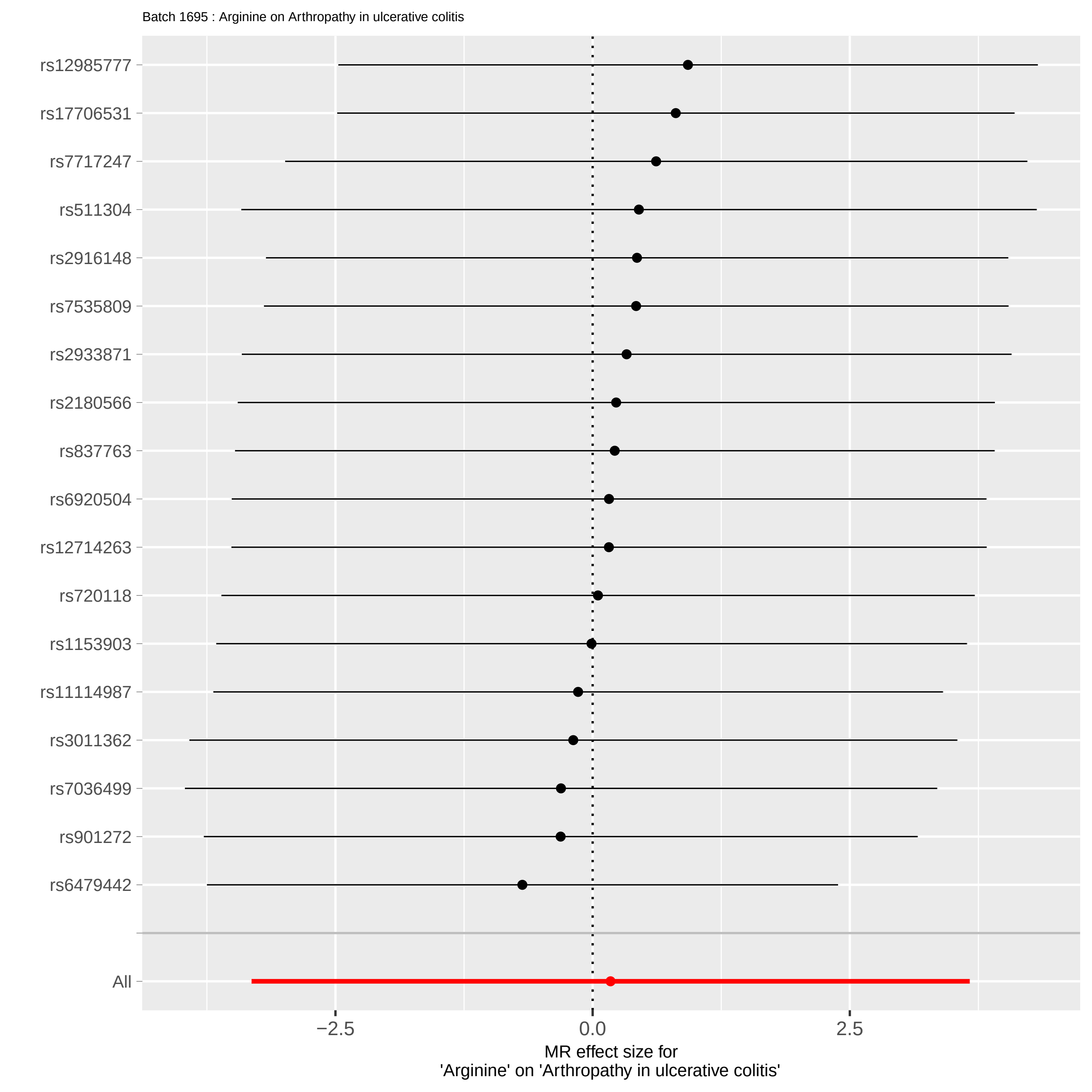
Batch 1695 : Arginine on Arthropathy in ulcerative colitis
rs12985777
rs17706531
rs7717247
rs511304
rs2916148
rs7535809
rs2933871
rs2180566
rs837763
rs6920504
rs12714263
rs720118
rs1153903
rs11114987
rs3011362
rs7036499
rs901272
rs6479442
All
−2.5
0.0
2.5
MR effect size for
'Arginine' on 'Arthropathy in ulcerative colitis'

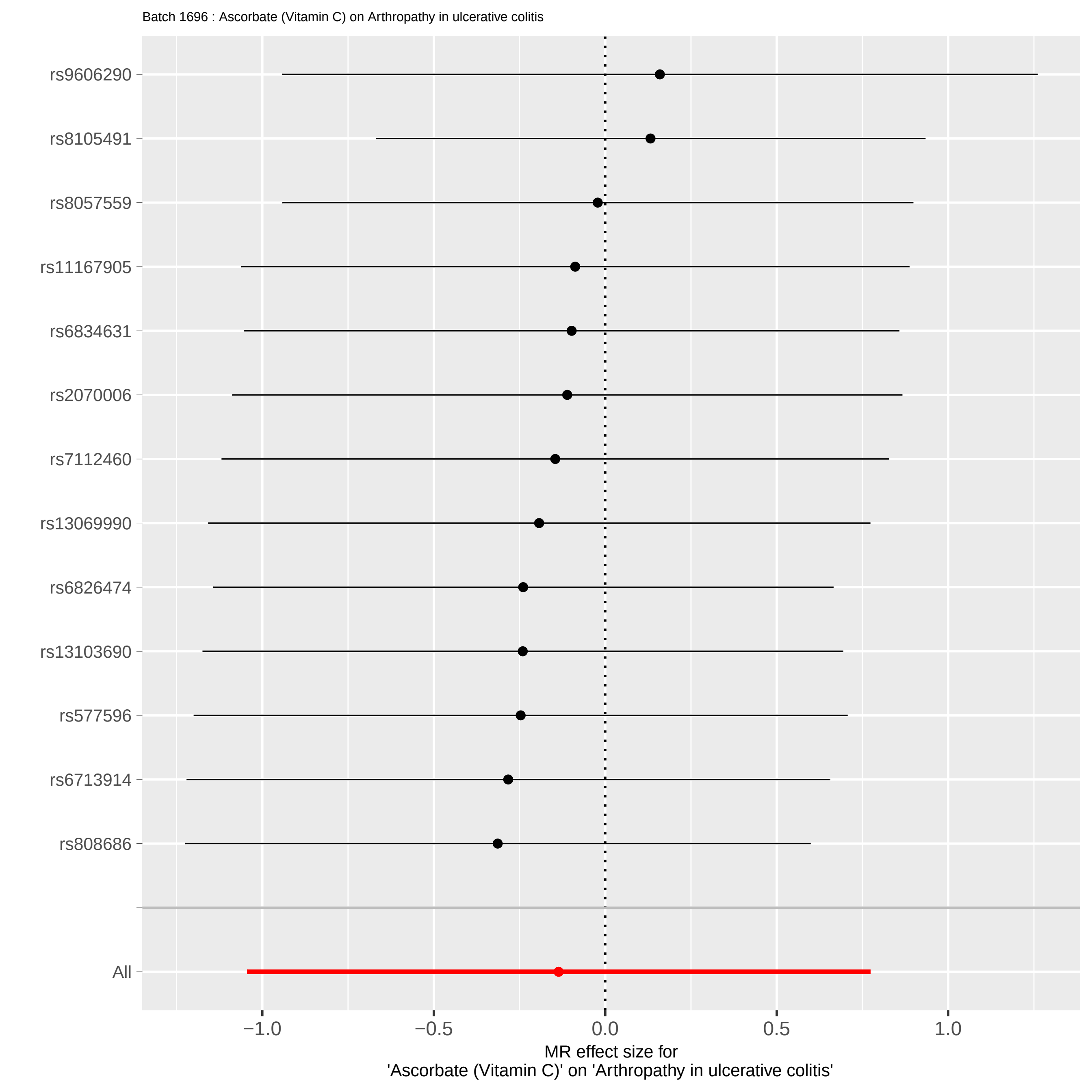
Batch 1696 : Ascorbate (Vitamin C) on Arthropathy in ulcerative colitis
rs9606290
rs8105491
rs8057559
rs11167905
rs6834631
rs2070006
rs7112460
rs13069990
rs6826474
rs13103690
rs577596
rs6713914
rs808686
All
−1.0
−0.5
0.0
0.5
1.0
MR effect size for
'Ascorbate (Vitamin C)' on 'Arthropathy in ulcerative colitis'

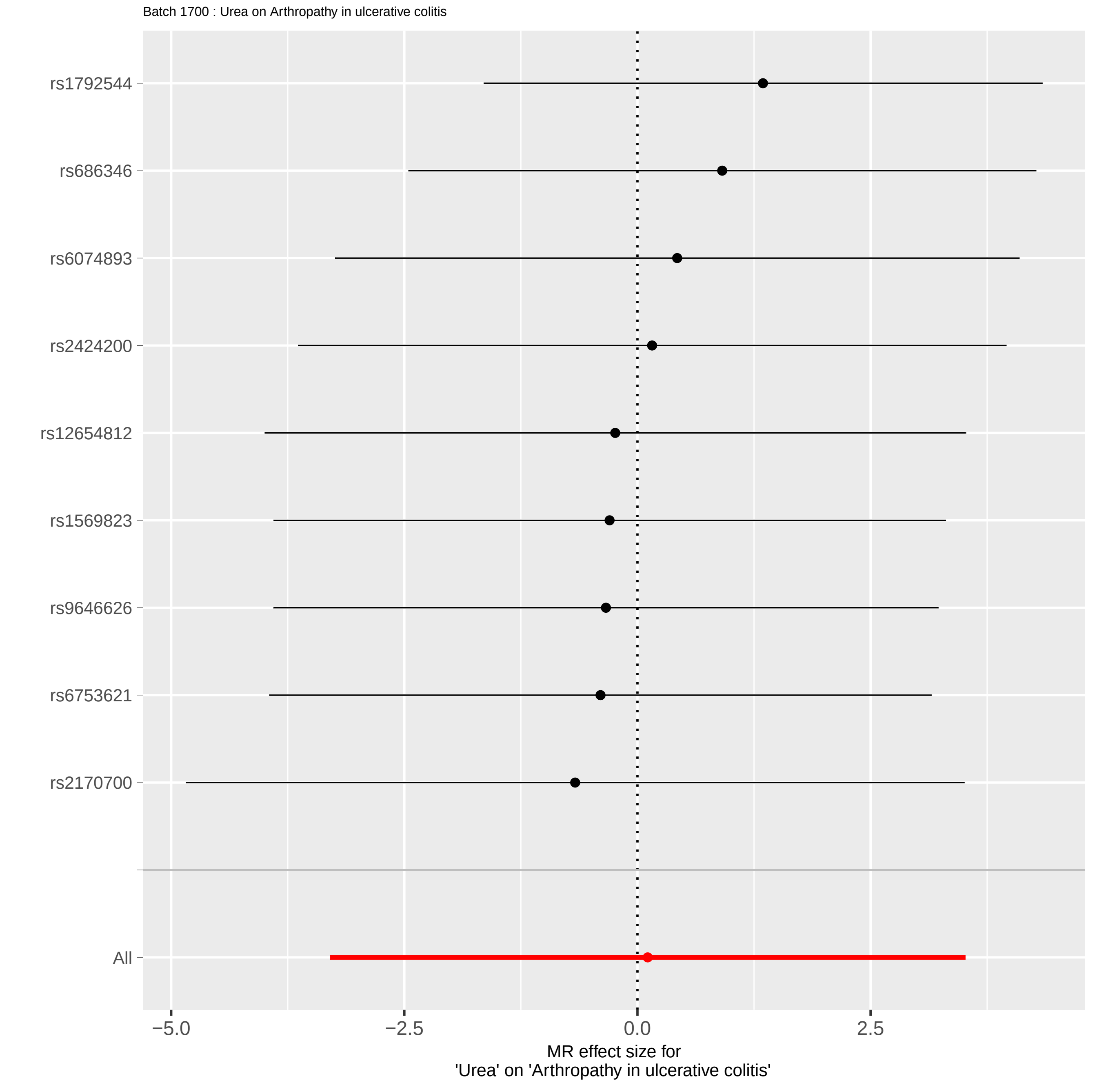
Batch 1700 : Urea on Arthropathy in ulcerative colitis
rs1792544
rs686346
rs6074893
rs2424200
rs12654812
rs1569823
rs9646626
rs6753621
rs2170700
All
−5.0
−2.5
0.0
2.5
MR effect size for
'Urea' on 'Arthropathy in ulcerative colitis'

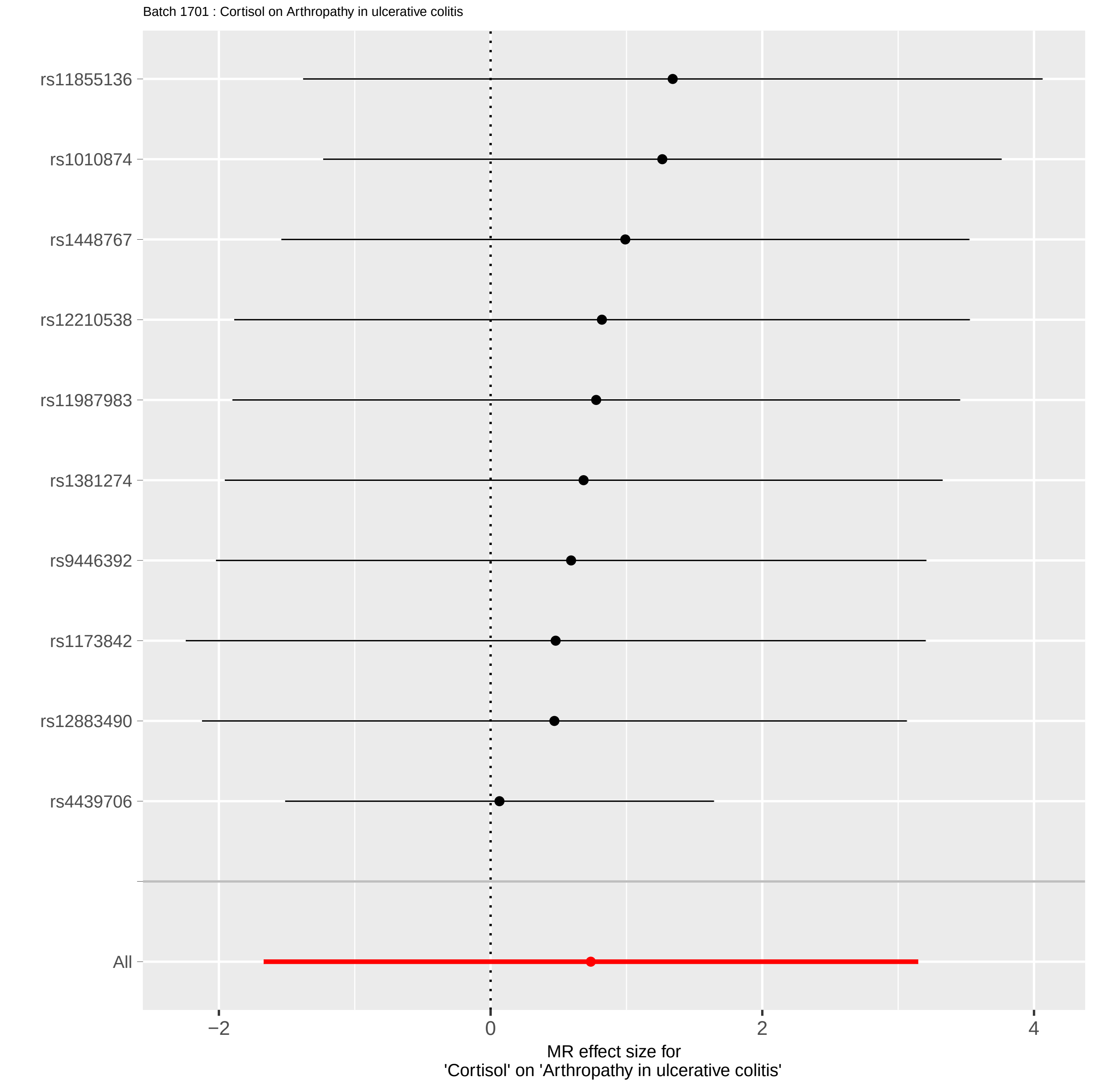

Batch 1701 : Cortisol on Arthropathy in ulcerative colitis
rs11855136
rs1010874
rs1448767
rs12210538
rs11987983
rs1381274
rs9446392
rs1173842
rs12883490
rs4439706
All
−2
0
2
4
MR effect size for
'Cortisol' on 'Arthropathy in ulcerative colitis'

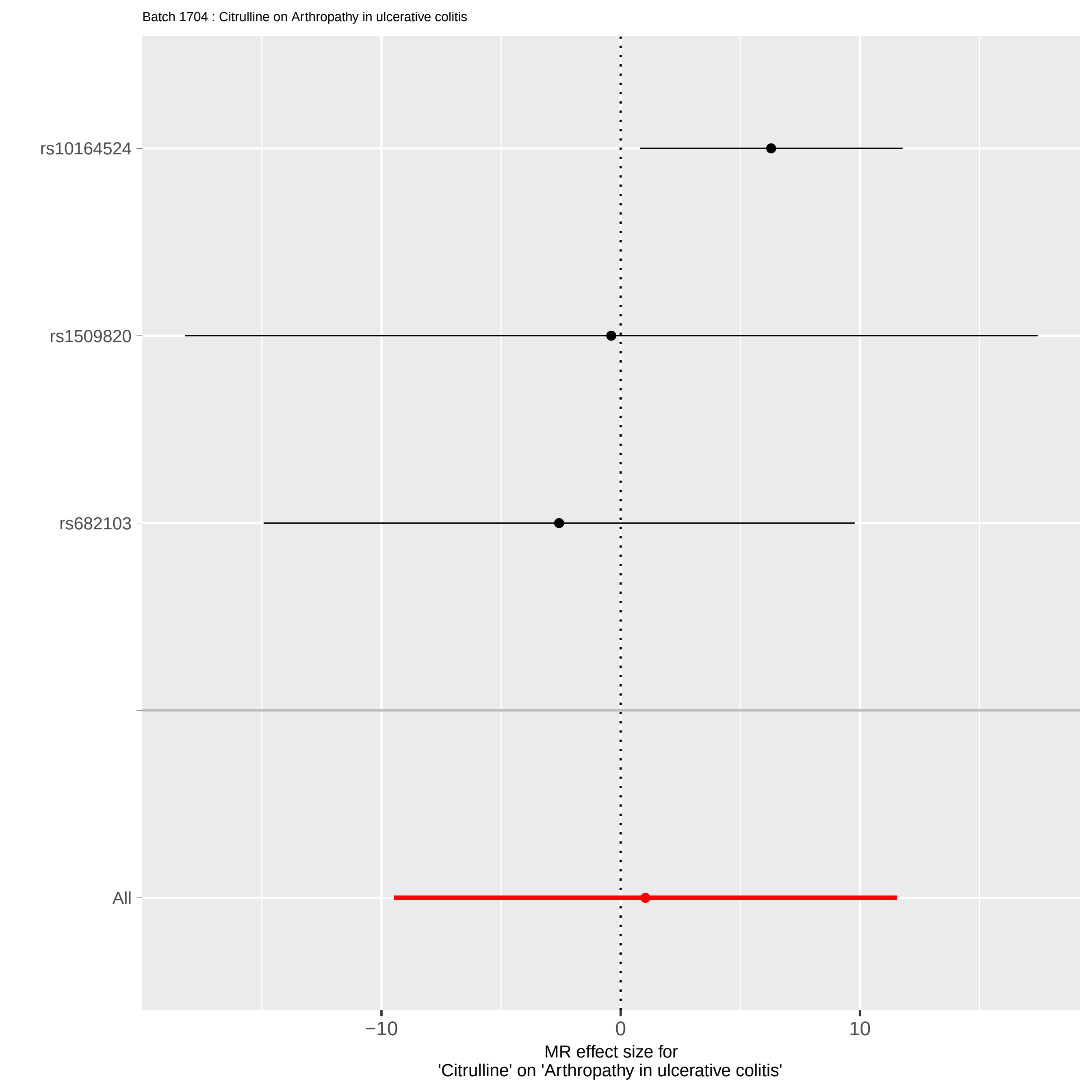

Batch 1704 : Citrulline on Arthropathy in ulcerative colitis
rs10164524
rs1509820
rs682103
All
−10
0
10
MR effect size for
'Citrulline' on 'Arthropathy in ulcerative colitis'

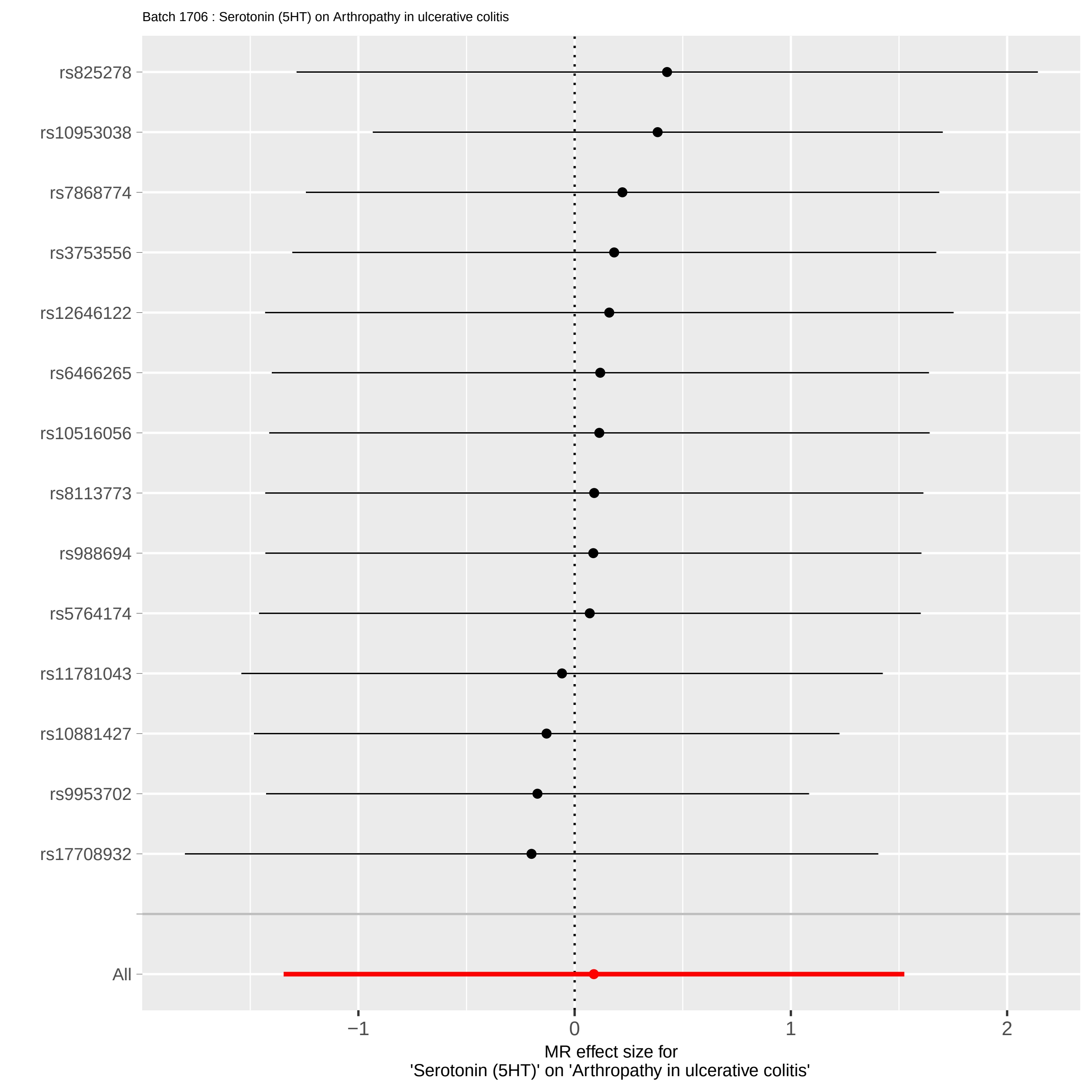
Batch 1706 : Serotonin (5HT) on Arthropathy in ulcerative colitis
rs825278
rs10953038
rs7868774
rs3753556
rs12646122
rs6466265
rs10516056
rs8113773
rs988694
rs5764174
rs11781043
rs10881427
rs9953702
rs17708932
All
−1
0
1
2
MR effect size for
'Serotonin (5HT)' on 'Arthropathy in ulcerative colitis'

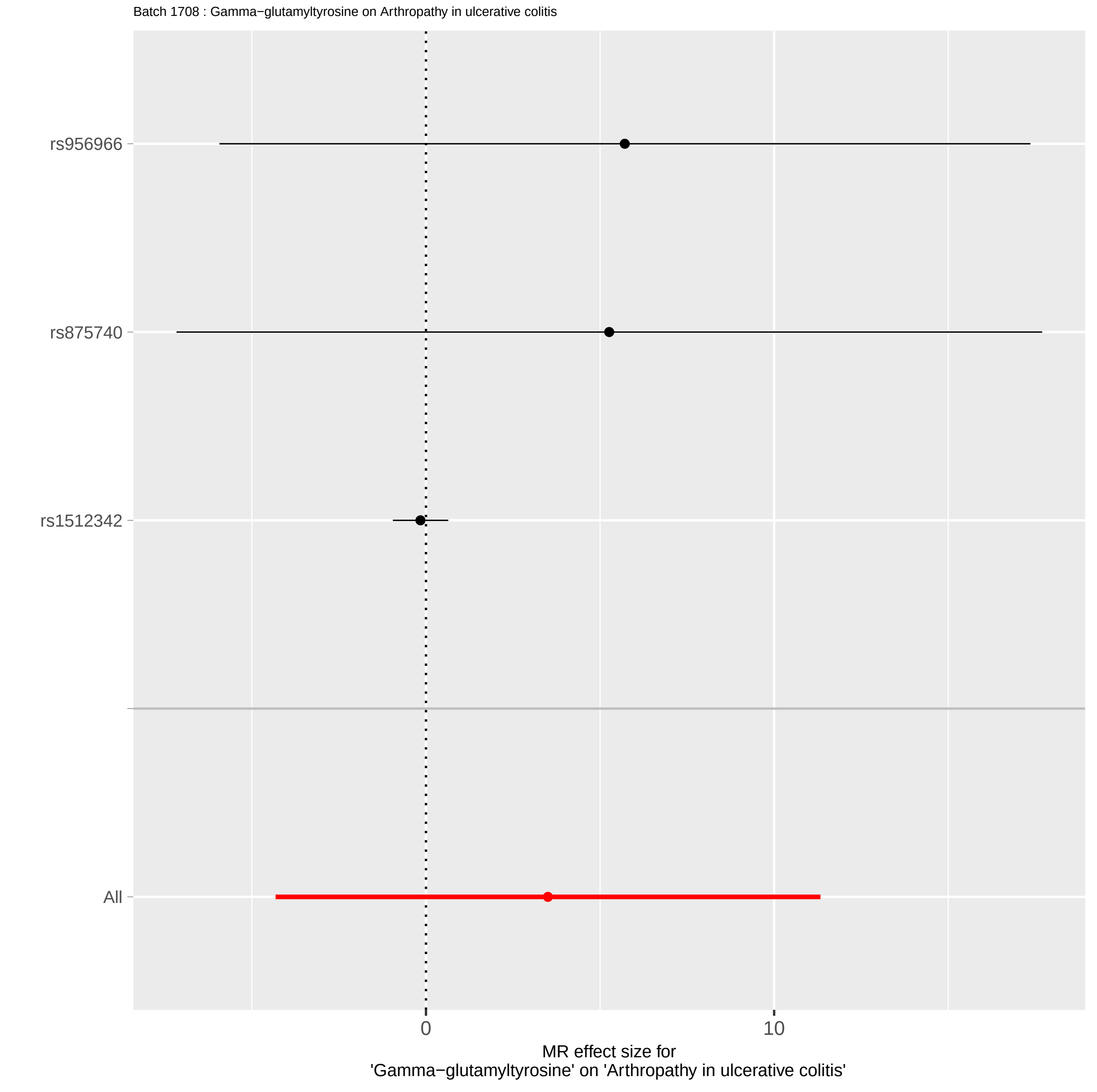

Batch 1708 : Gamma−glutamyltyrosine on Arthropathy in ulcerative colitis
rs956966
rs875740
rs1512342
All
0
10
MR effect size for
'Gamma−glutamyltyrosine' on 'Arthropathy in ulcerative colitis'

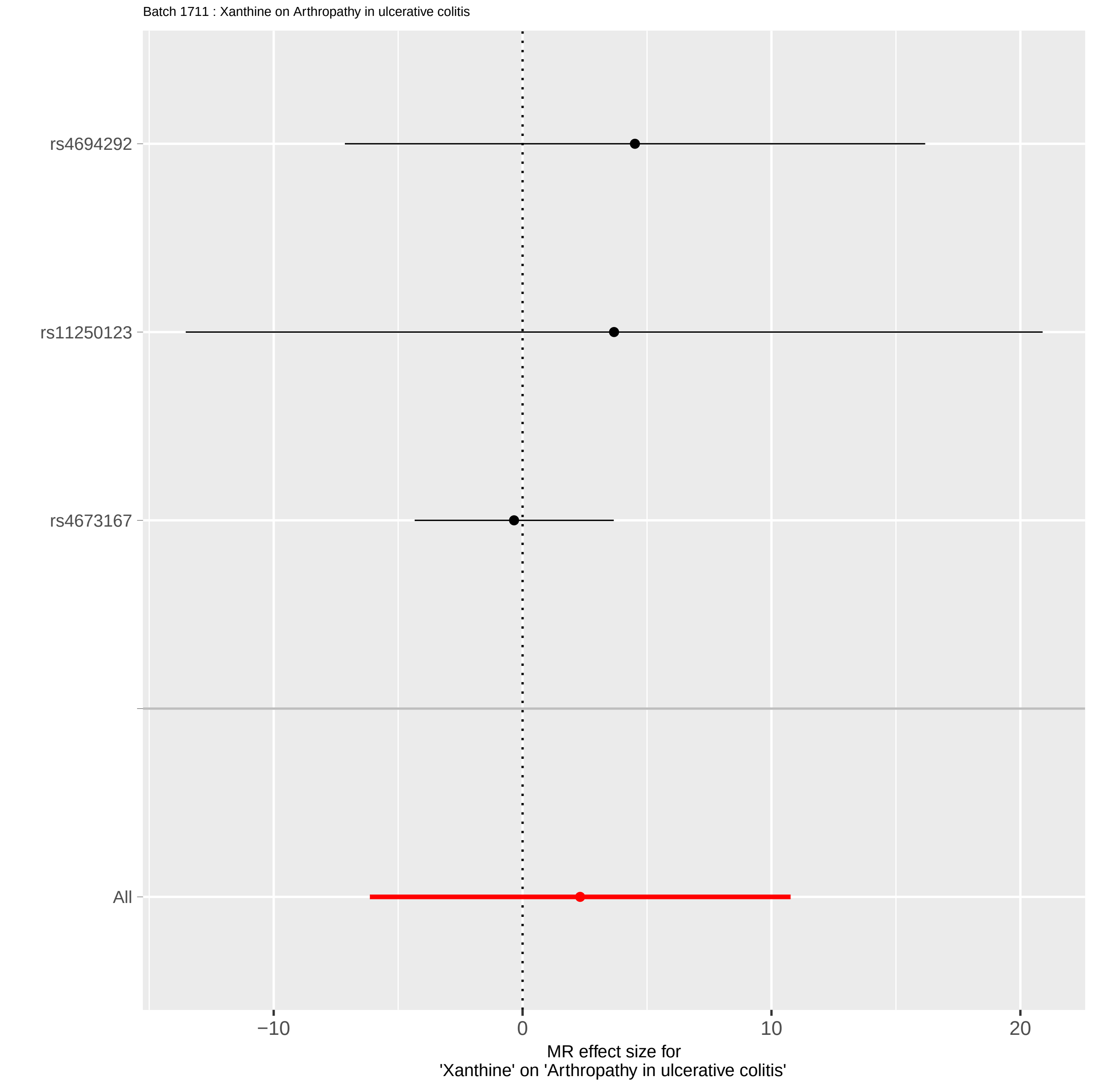

Batch 1711 : Xanthine on Arthropathy in ulcerative colitis
rs4694292
rs11250123
rs4673167
All
−10
0
10
20
MR effect size for
'Xanthine' on 'Arthropathy in ulcerative colitis'

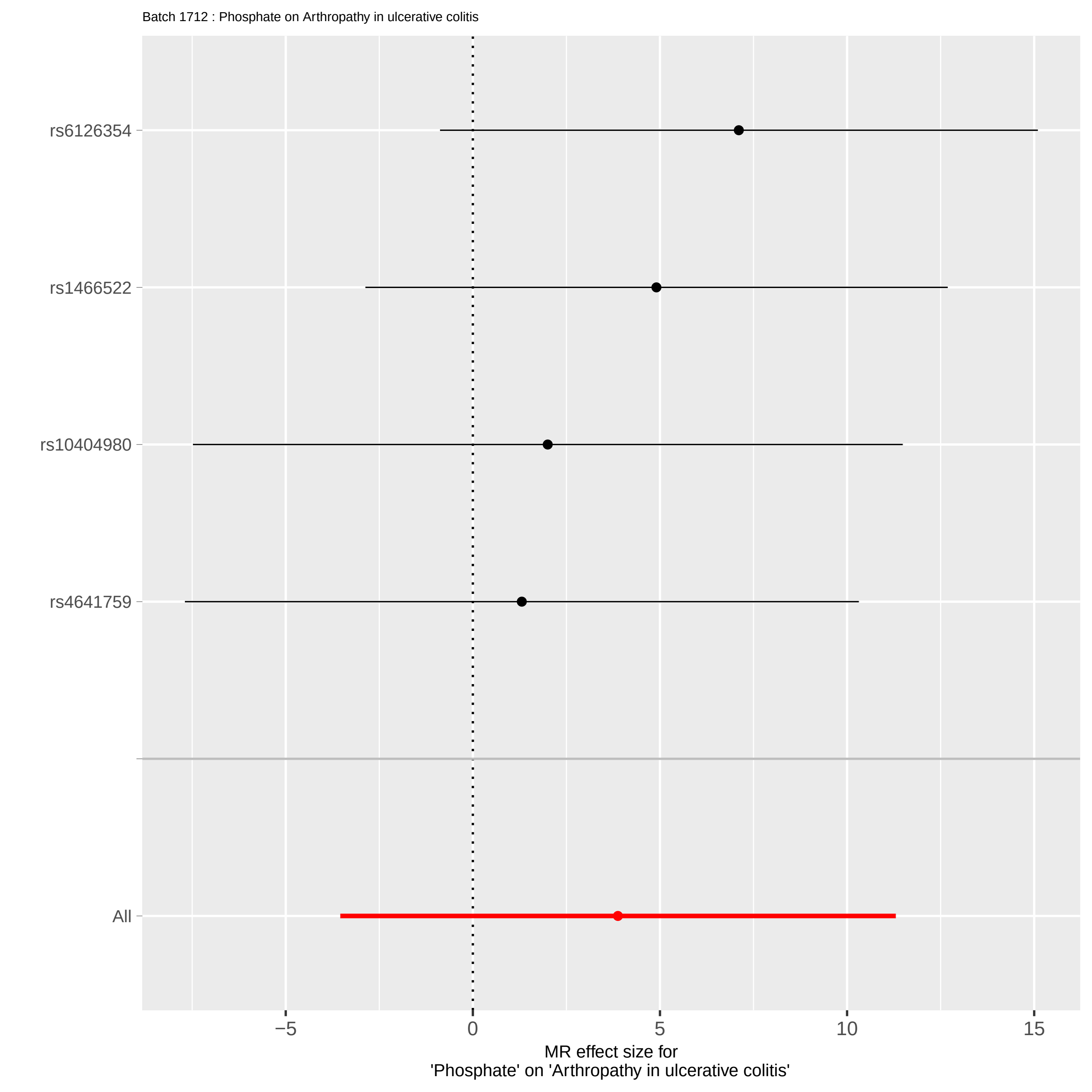

Batch 1712 : Phosphate on Arthropathy in ulcerative colitis
rs6126354
rs1466522
rs10404980
rs4641759
All
−5
0
5
10
15
MR effect size for
'Phosphate' on 'Arthropathy in ulcerative colitis'

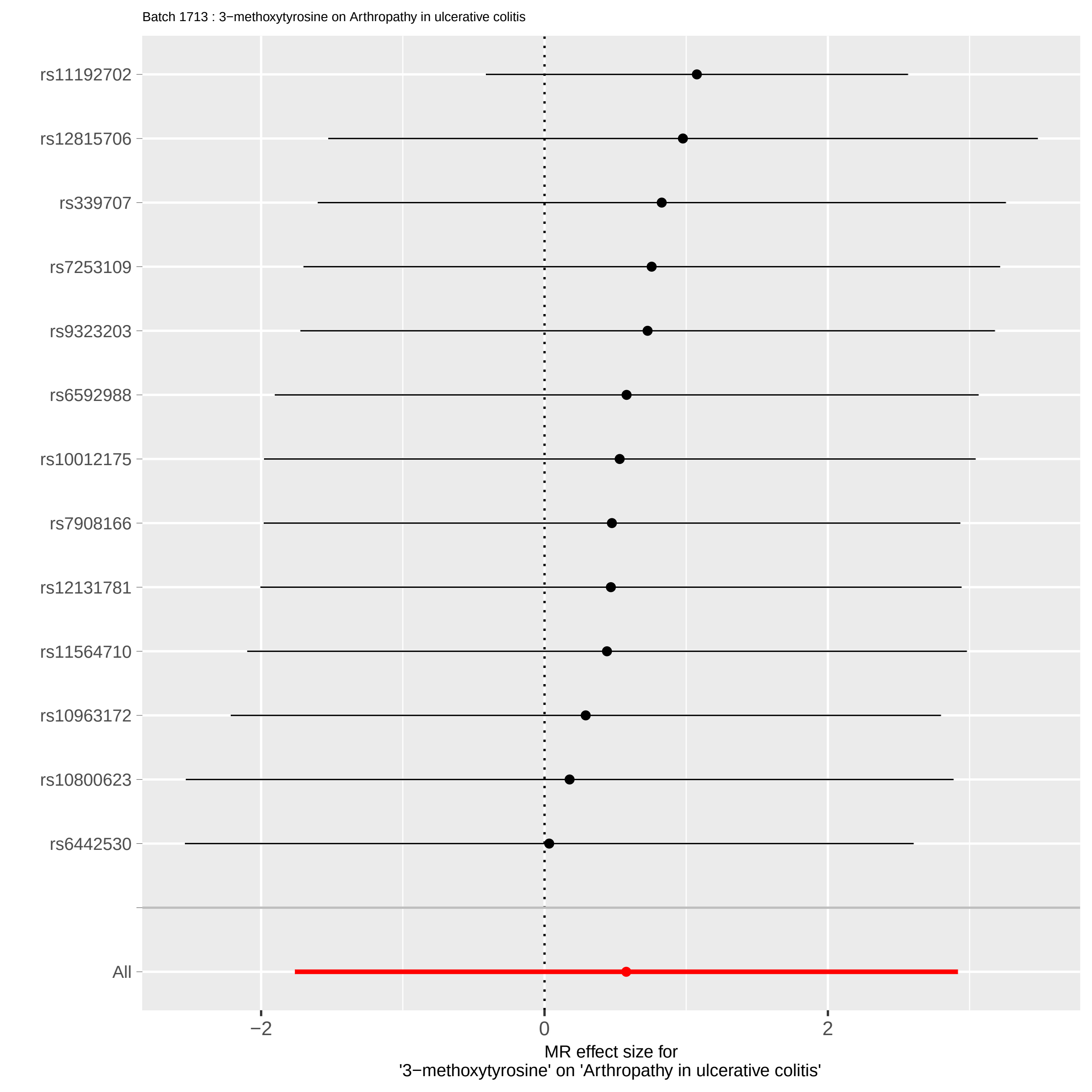

Batch 1713 : 3−methoxytyrosine on Arthropathy in ulcerative colitis
rs11192702
rs12815706
rs339707
rs7253109
rs9323203
rs6592988
rs10012175
rs7908166
rs12131781
rs11564710
rs10963172
rs10800623
rs6442530
All
−2
0
2
MR effect size for
'3−methoxytyrosine' on 'Arthropathy in ulcerative colitis'

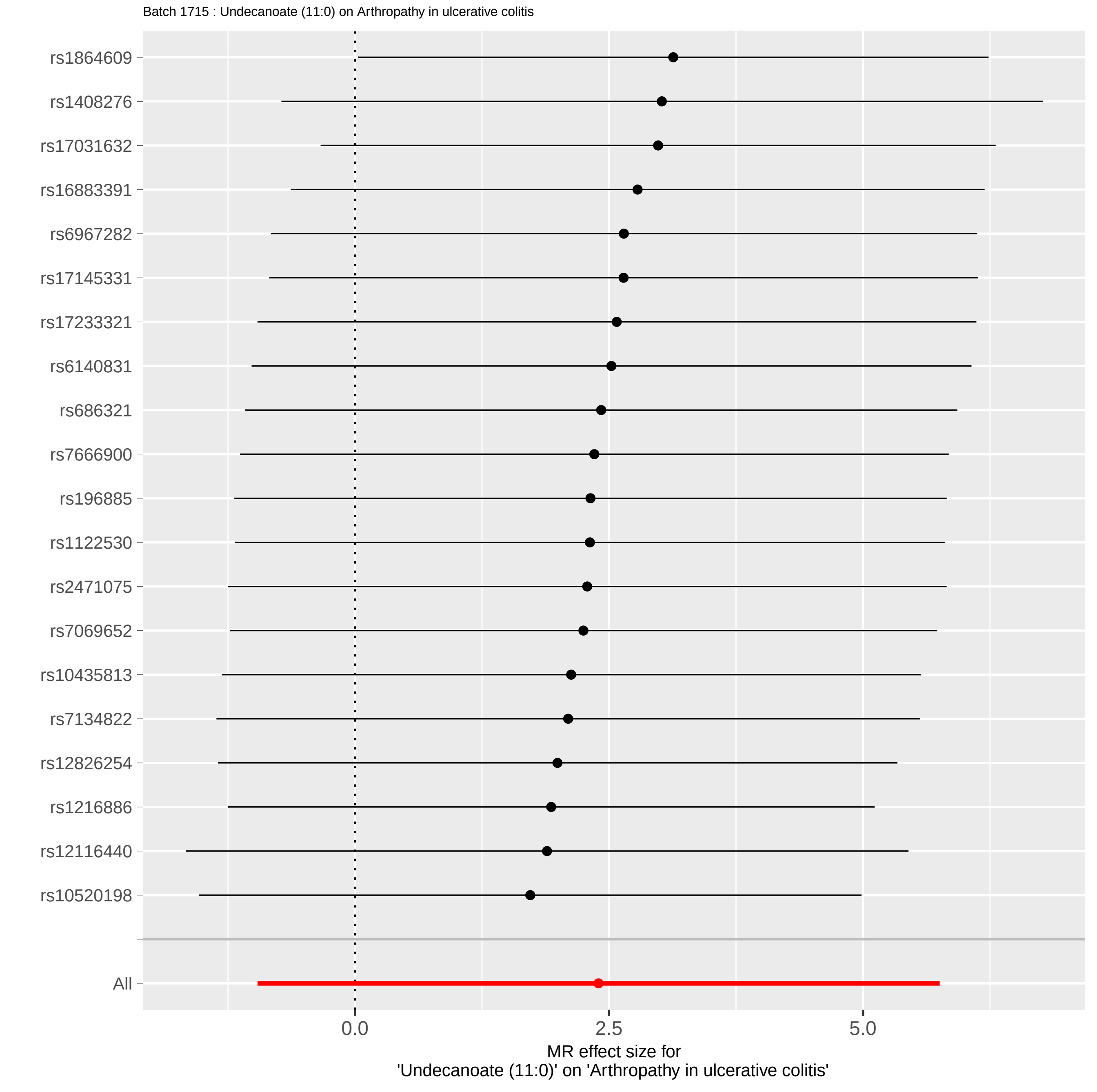

Batch 1715 : Undecanoate (11:0) on Arthropathy in ulcerative colitis
rs1864609
rs1408276
rs17031632
rs16883391
rs6967282
rs17145331
rs17233321
rs6140831
rs686321
rs7666900
rs196885
rs1122530
rs2471075
rs7069652
rs10435813
rs7134822
rs12826254
rs1216886
rs12116440
rs10520198
All
0.0
2.5
5.0
MR effect size for
'Undecanoate (11:0)' on 'Arthropathy in ulcerative colitis'

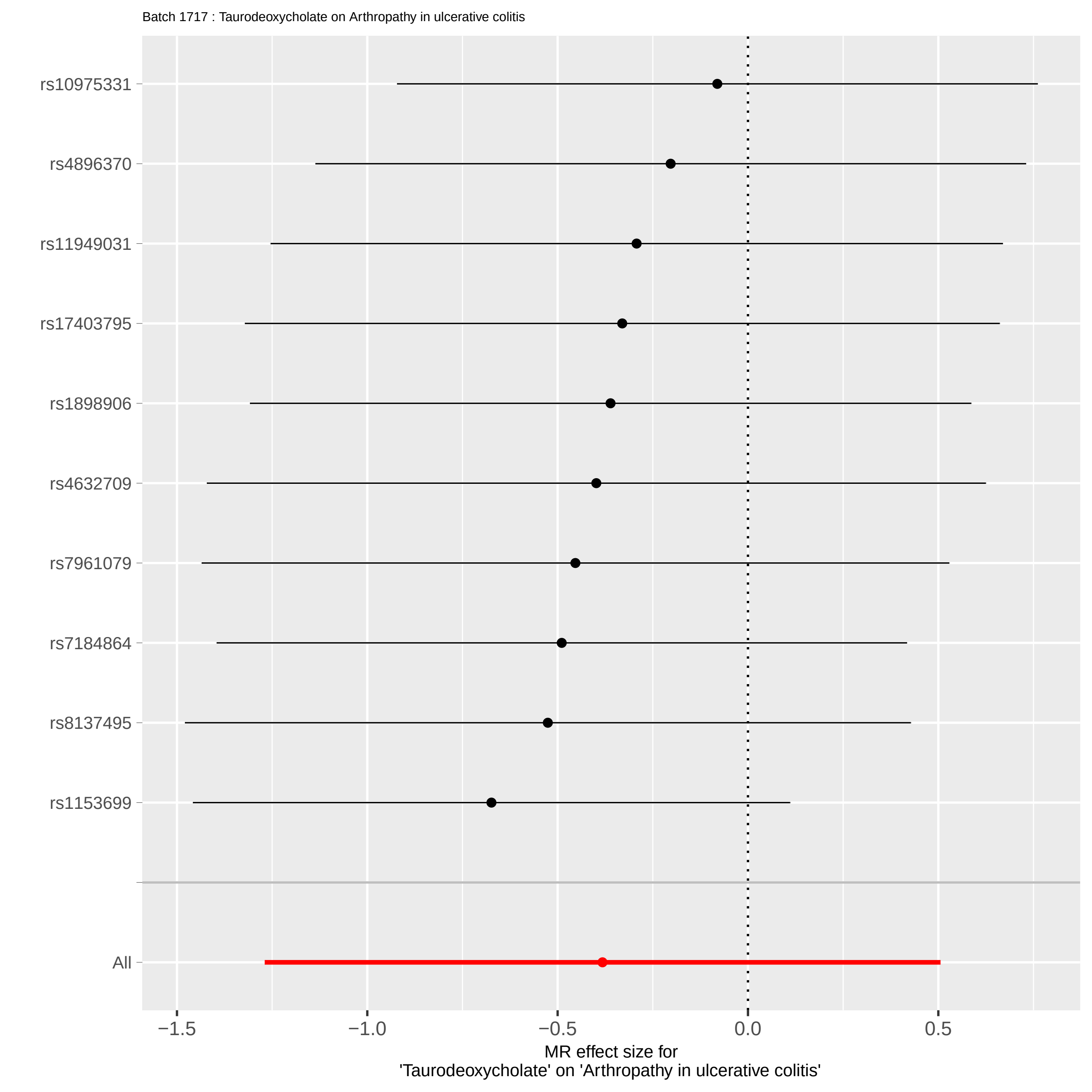

Batch 1717 : Taurodeoxycholate on Arthropathy in ulcerative colitis
rs10975331
rs4896370
rs11949031
rs17403795
rs1898906
rs4632709
rs7961079
rs7184864
rs8137495
rs1153699
All
−1.5
−1.0
−0.5
0.0
0.5
MR effect size for
'Taurodeoxycholate' on 'Arthropathy in ulcerative colitis'

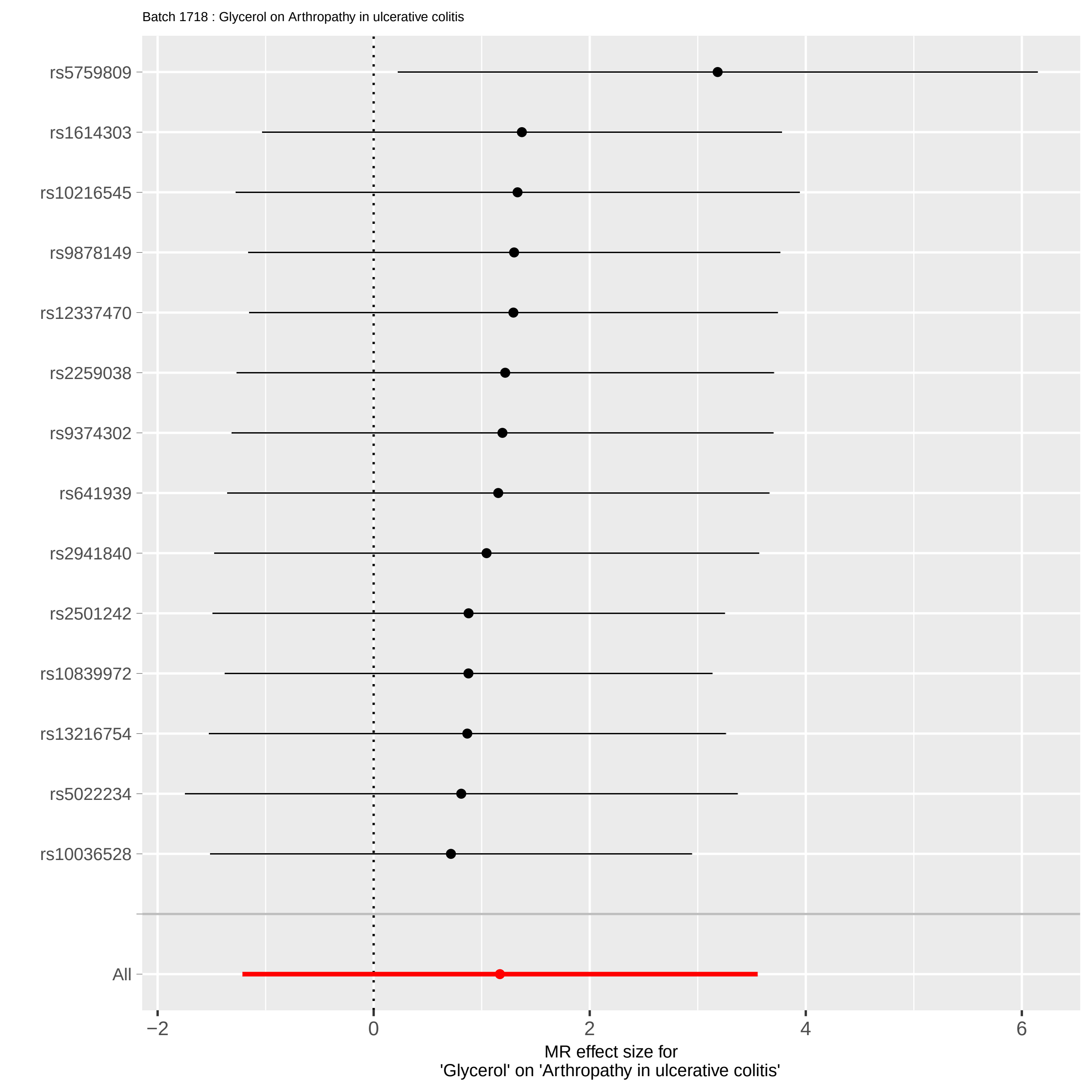

Batch 1718 : Glycerol on Arthropathy in ulcerative colitis
rs5759809
rs1614303
rs10216545
rs9878149
rs12337470
rs2259038
rs9374302
rs641939
rs2941840
rs2501242
rs10839972
rs13216754
rs5022234
rs10036528
All
−2
0
2
4
6
MR effect size for
'Glycerol' on 'Arthropathy in ulcerative colitis'

Batch 1720 : Mannitol on Arthropathy in ulcerative colitis

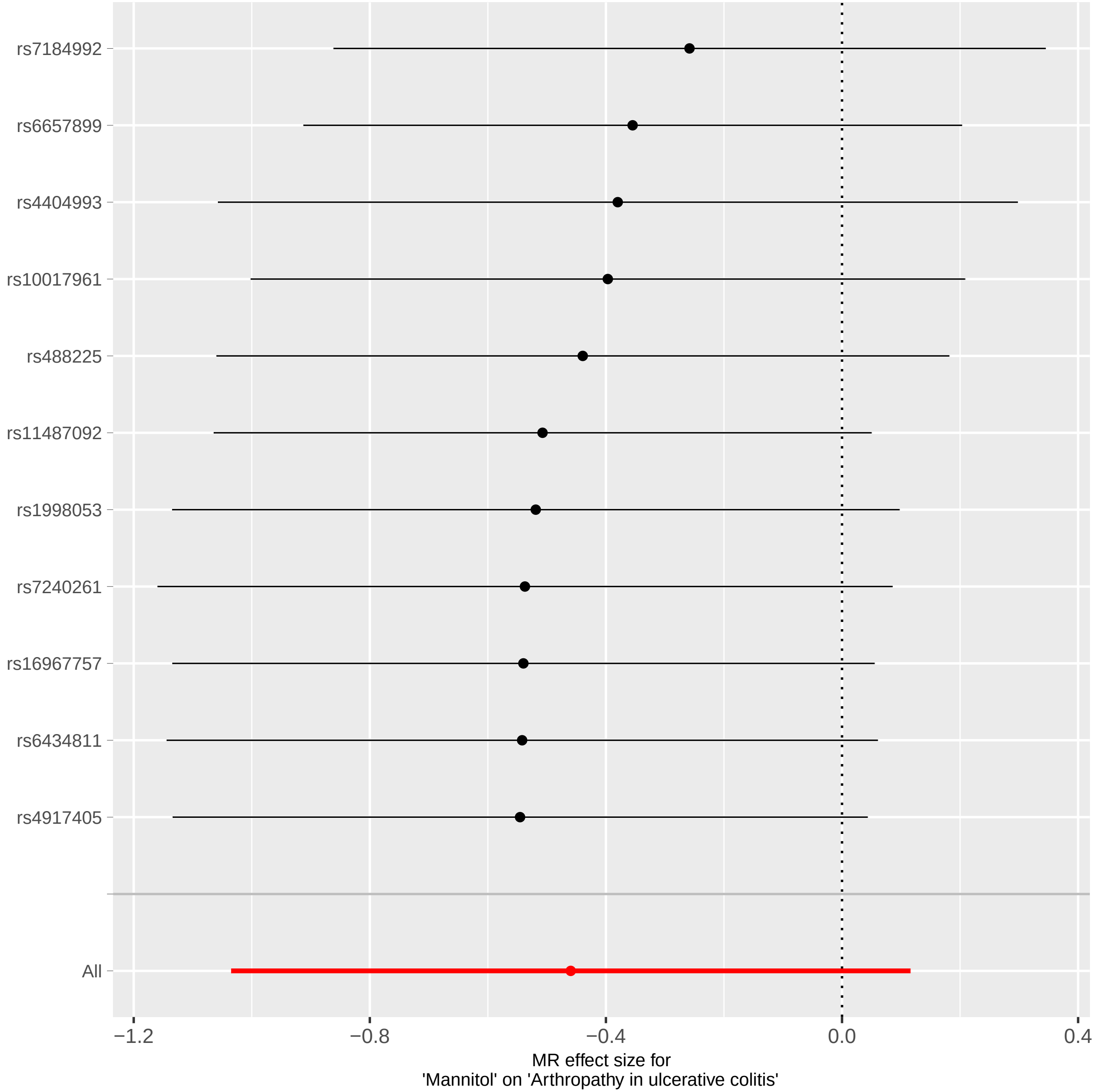

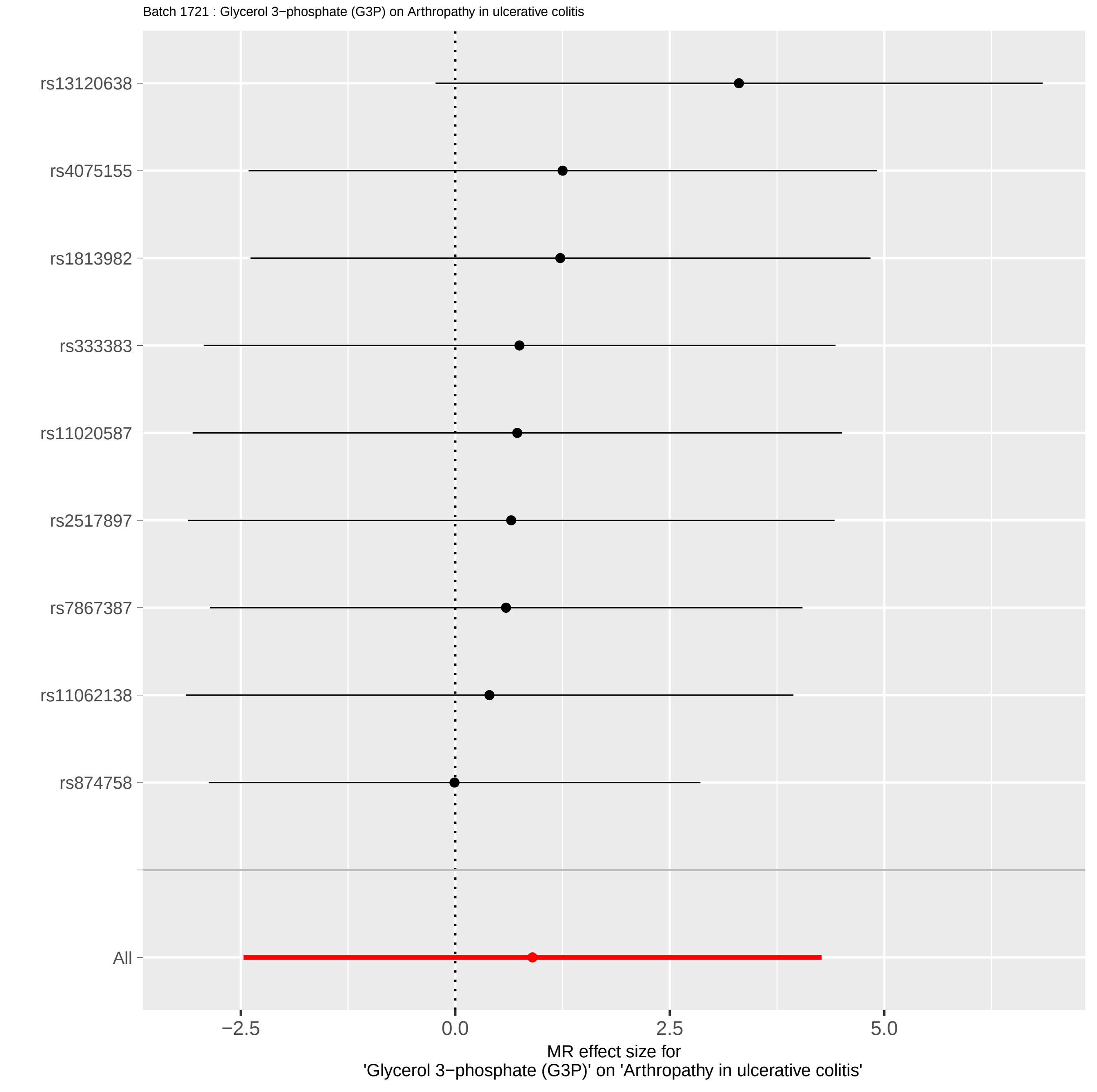

Batch 1721 : Glycerol 3−phosphate (G3P) on Arthropathy in ulcerative colitis
rs13120638
rs4075155
rs1813982
rs333383
rs11020587
rs2517897
rs7867387
rs11062138
rs874758
All
−2.5
0.0
2.5
5.0
MR effect size for
'Glycerol 3−phosphate (G3P)' on 'Arthropathy in ulcerative colitis'

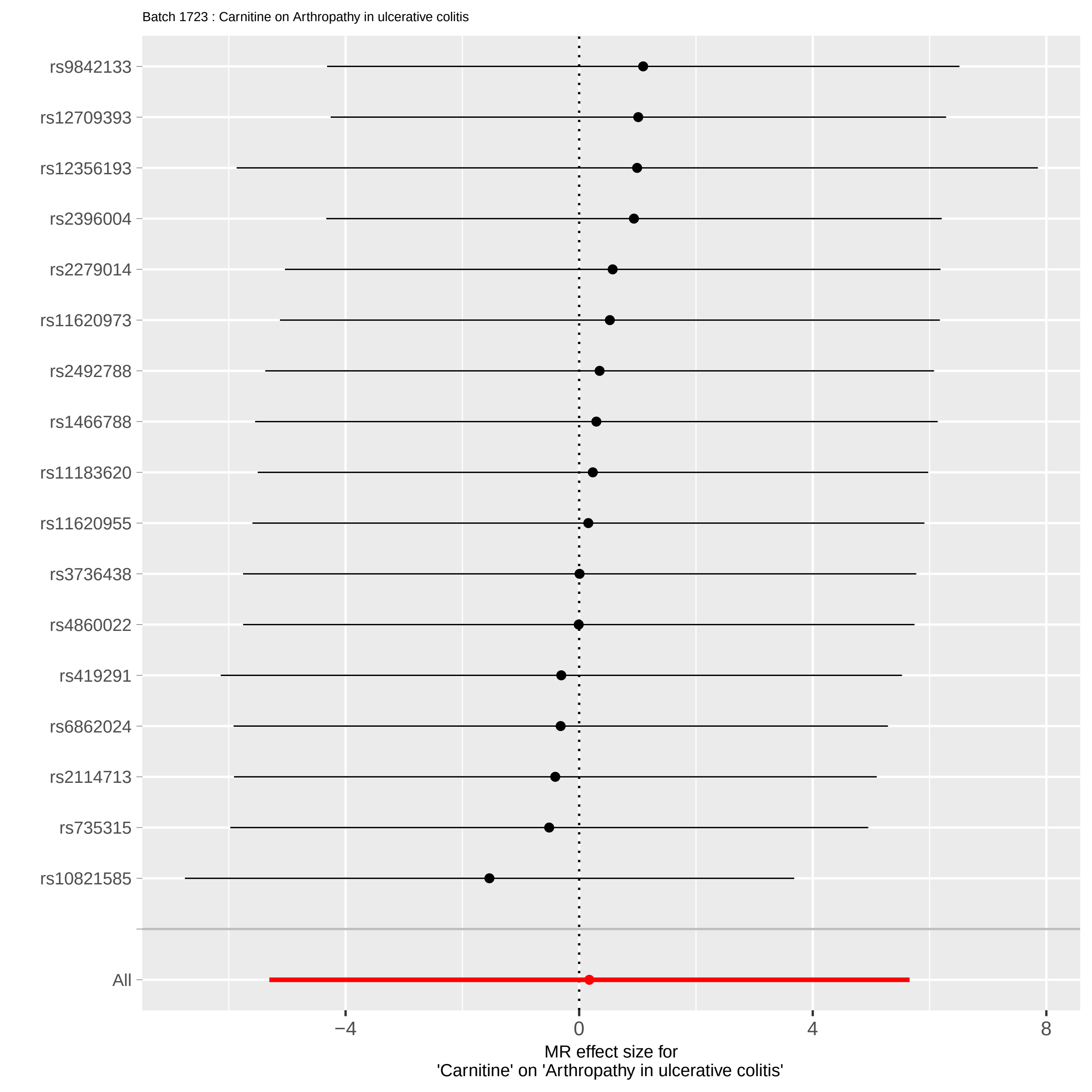

Batch 1723 : Carnitine on Arthropathy in ulcerative colitis
rs9842133
rs12709393
rs12356193
rs2396004
rs2279014
rs11620973
rs2492788
rs1466788
rs11183620
rs11620955
rs3736438
rs4860022
rs419291
rs6862024
rs2114713
rs735315
rs10821585
All
−4
0
4
8
MR effect size for
'Carnitine' on 'Arthropathy in ulcerative colitis'

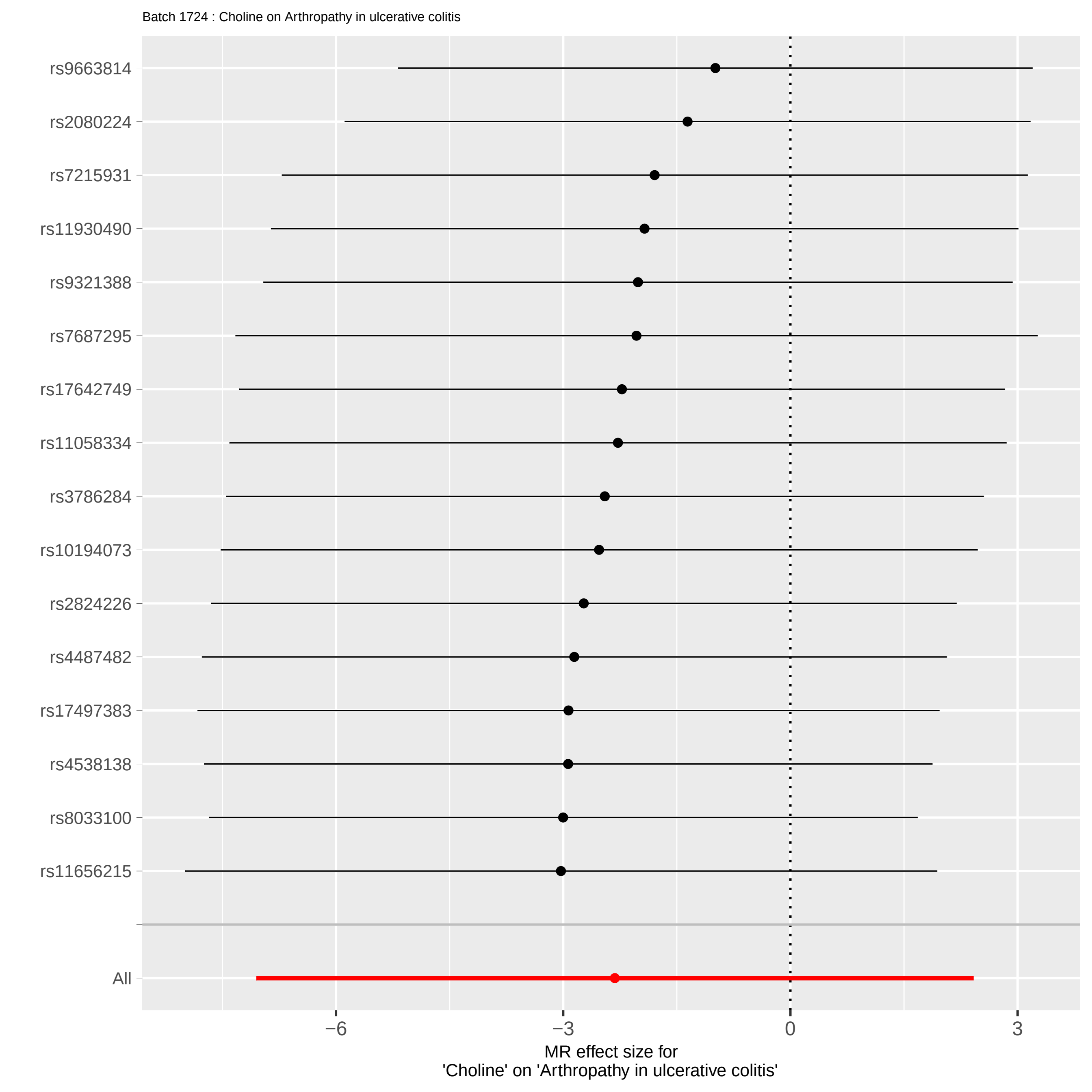
Batch 1724 : Choline on Arthropathy in ulcerative colitis
rs9663814
rs2080224
rs7215931
rs11930490
rs9321388
rs7687295
rs17642749
rs11058334
rs3786284
rs10194073
rs2824226
rs4487482
rs17497383
rs4538138
rs8033100
rs11656215
All
−6
−3
0
3
MR effect size for
'Choline' on 'Arthropathy in ulcerative colitis'

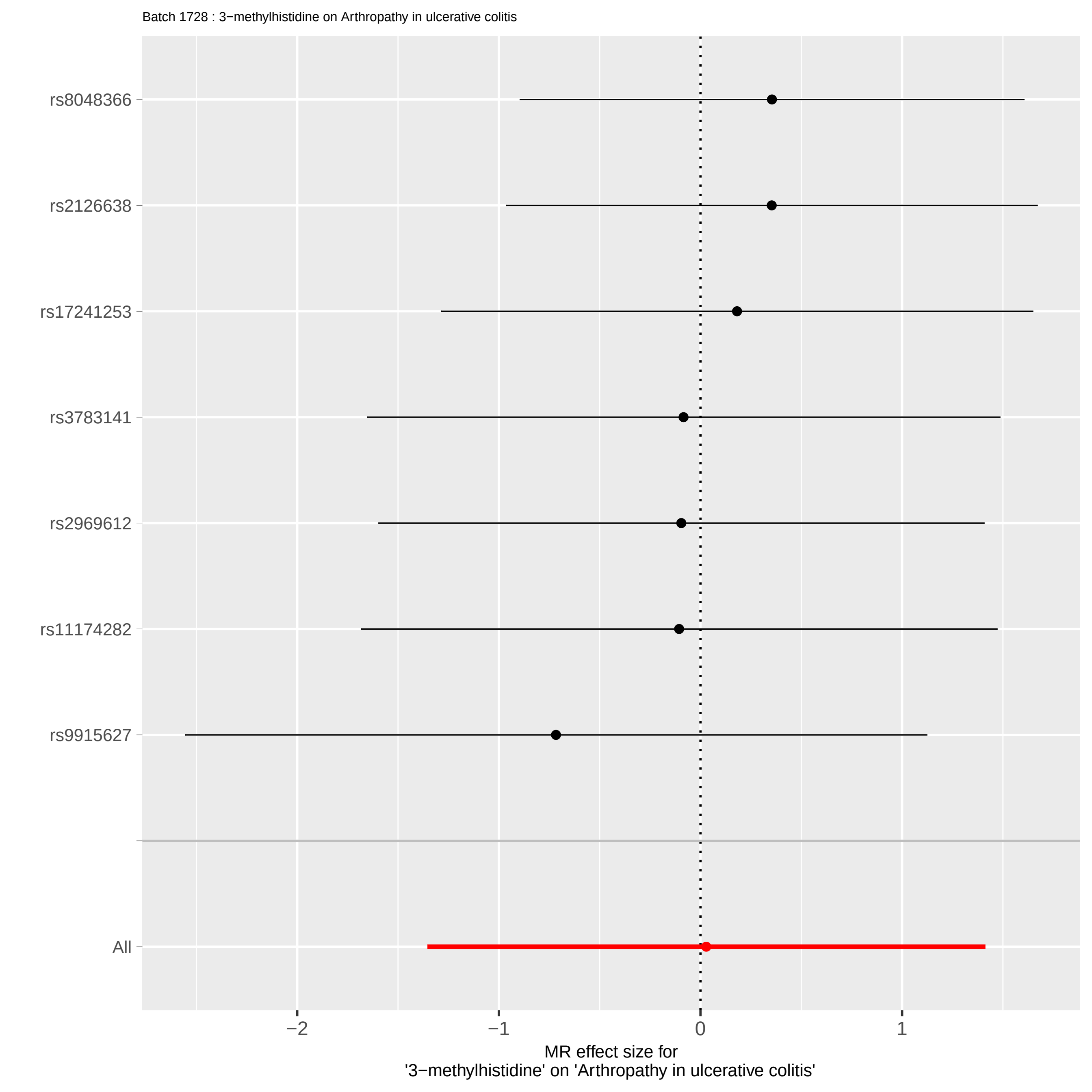

Batch 1728 : 3−methylhistidine on Arthropathy in ulcerative colitis
rs8048366
rs2126638
rs17241253
rs3783141
rs2969612
rs11174282
rs9915627
All
−2
−1
0
1
MR effect size for
'3−methylhistidine' on 'Arthropathy in ulcerative colitis'

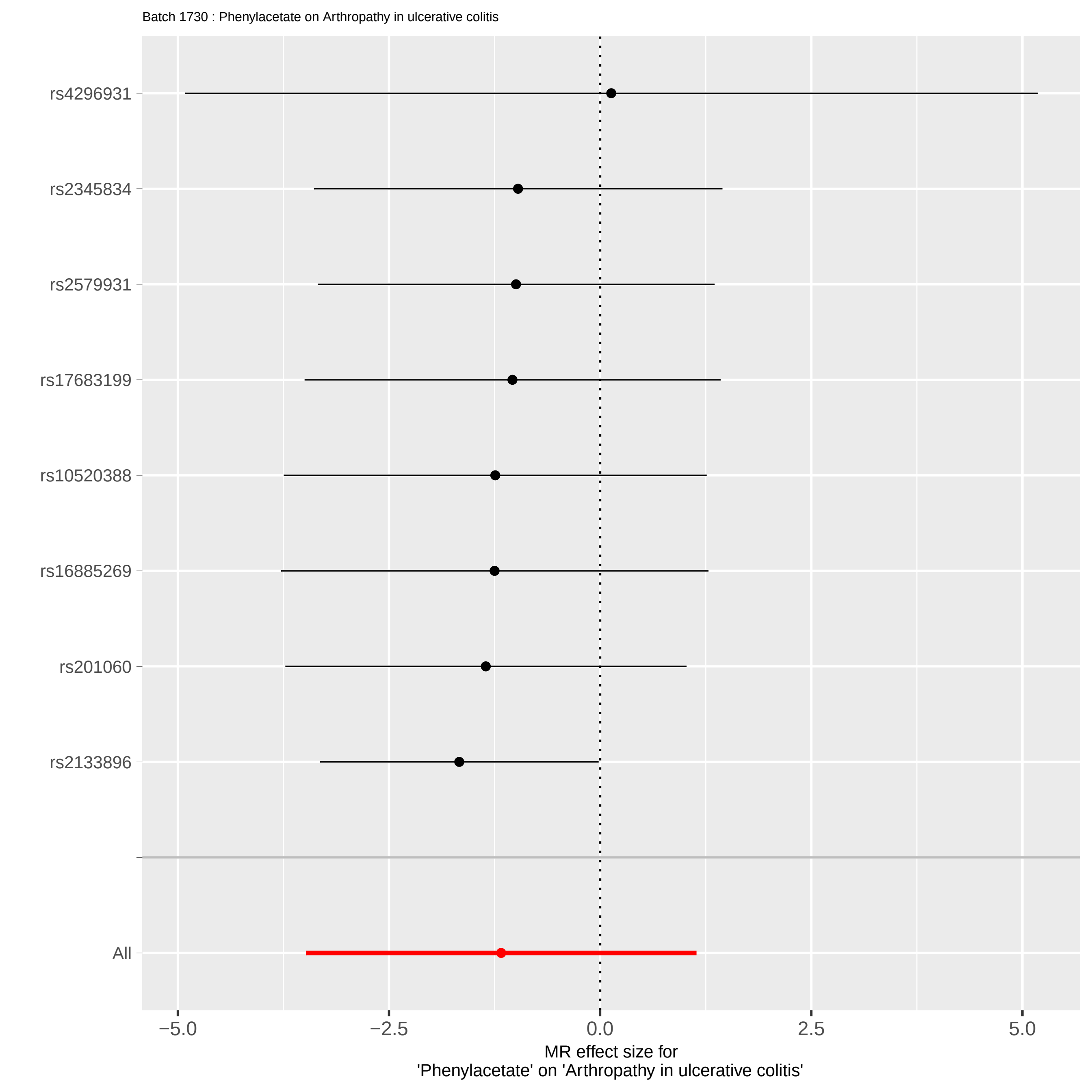

Batch 1730 : Phenylacetate on Arthropathy in ulcerative colitis
rs4296931
rs2345834
rs2579931
rs17683199
rs10520388
rs16885269
rs201060
rs2133896
All
−5.0
−2.5
0.0
2.5
5.0
MR effect size for
'Phenylacetate' on 'Arthropathy in ulcerative colitis'

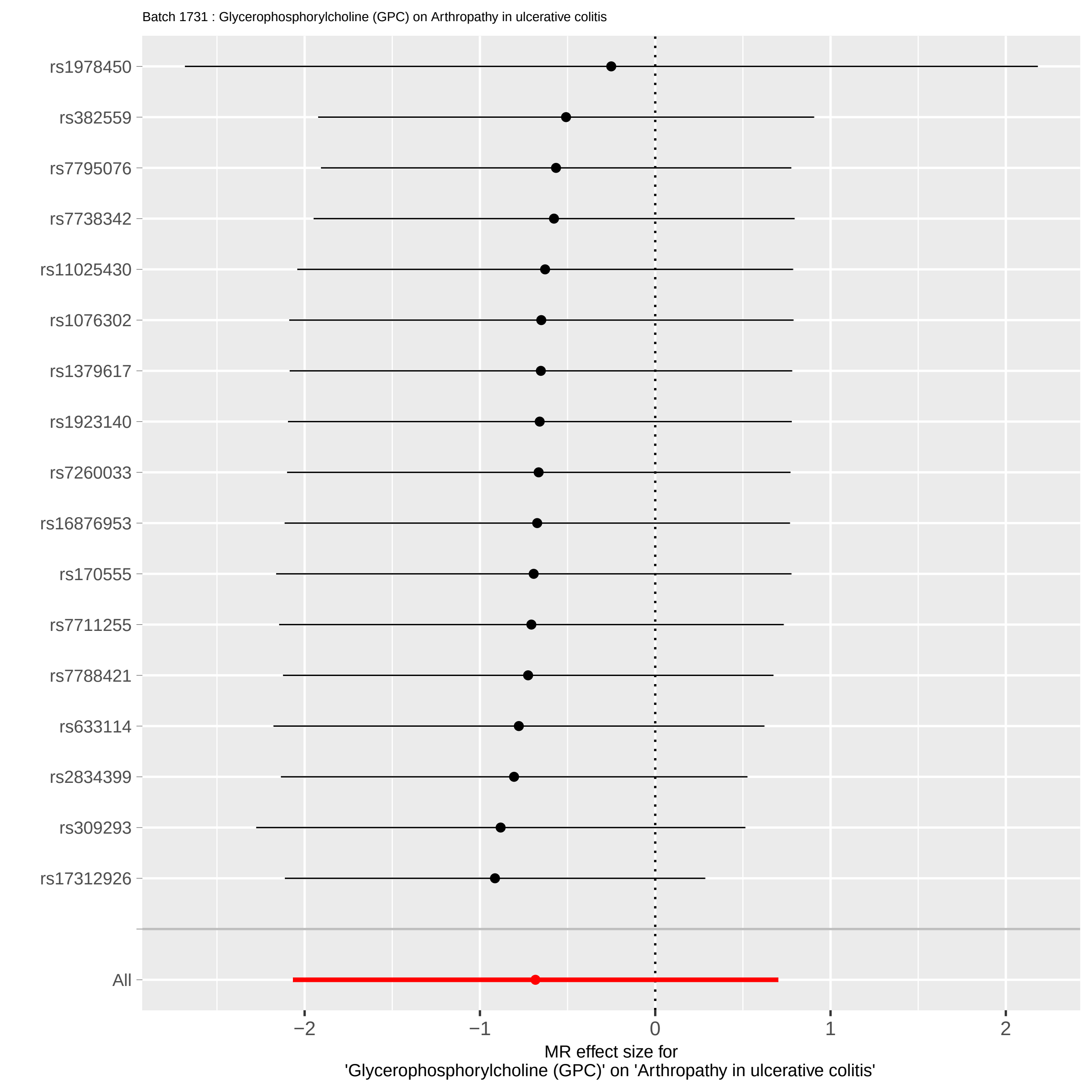
Batch 1731 : Glycerophosphorylcholine (GPC) on Arthropathy in ulcerative colitis
rs1978450
rs382559
rs7795076
rs7738342
rs11025430
rs1076302
rs1379617
rs1923140
rs7260033
rs16876953
rs170555
rs7711255
rs7788421
rs633114
rs2834399
rs309293
rs17312926
All
−2
−1
0
1
2
MR effect size for
'Glycerophosphorylcholine (GPC)' on 'Arthropathy in ulcerative colitis'

Batch 1732 : Aspartate on Arthropathy in ulcerative colitis

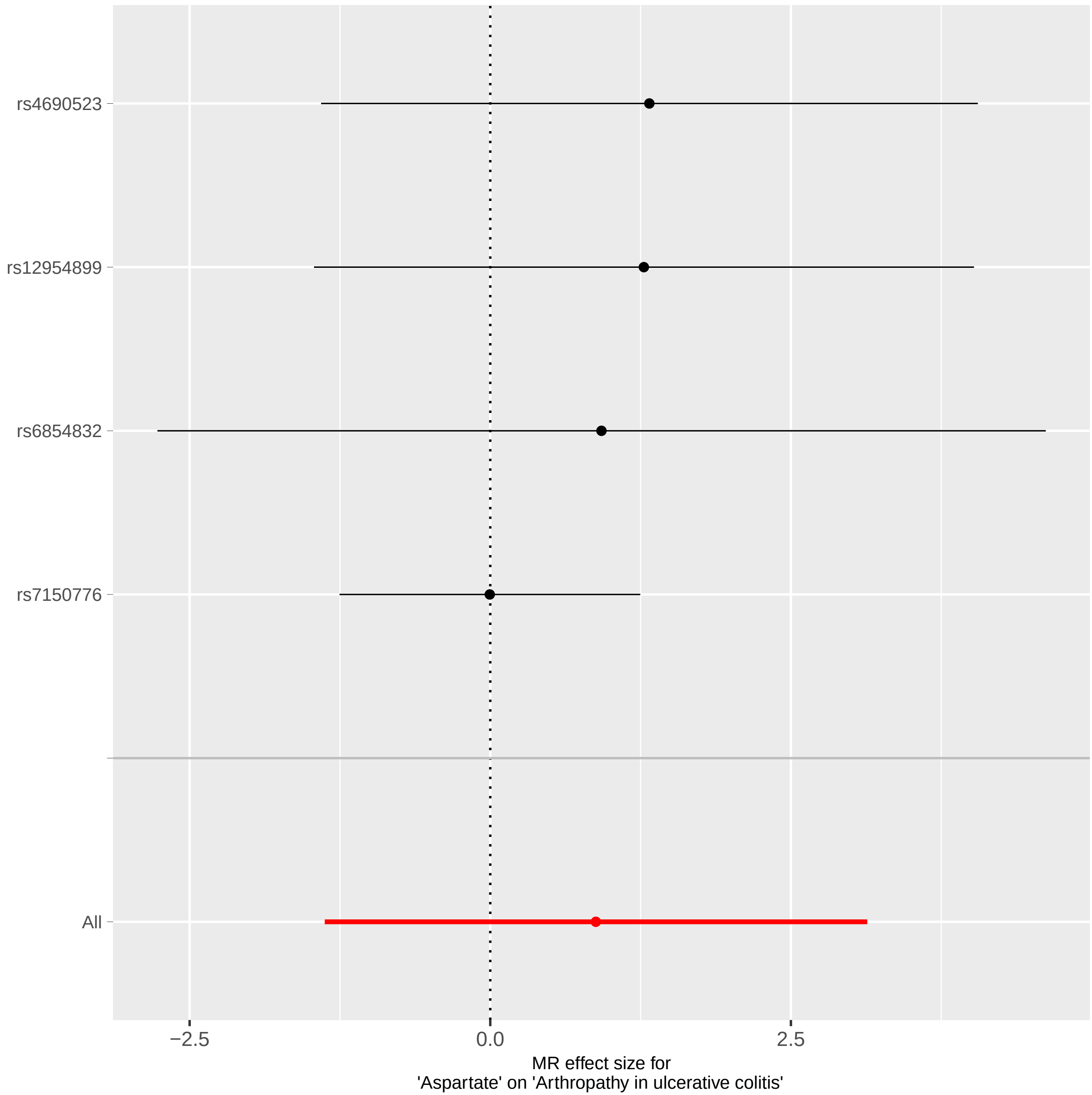

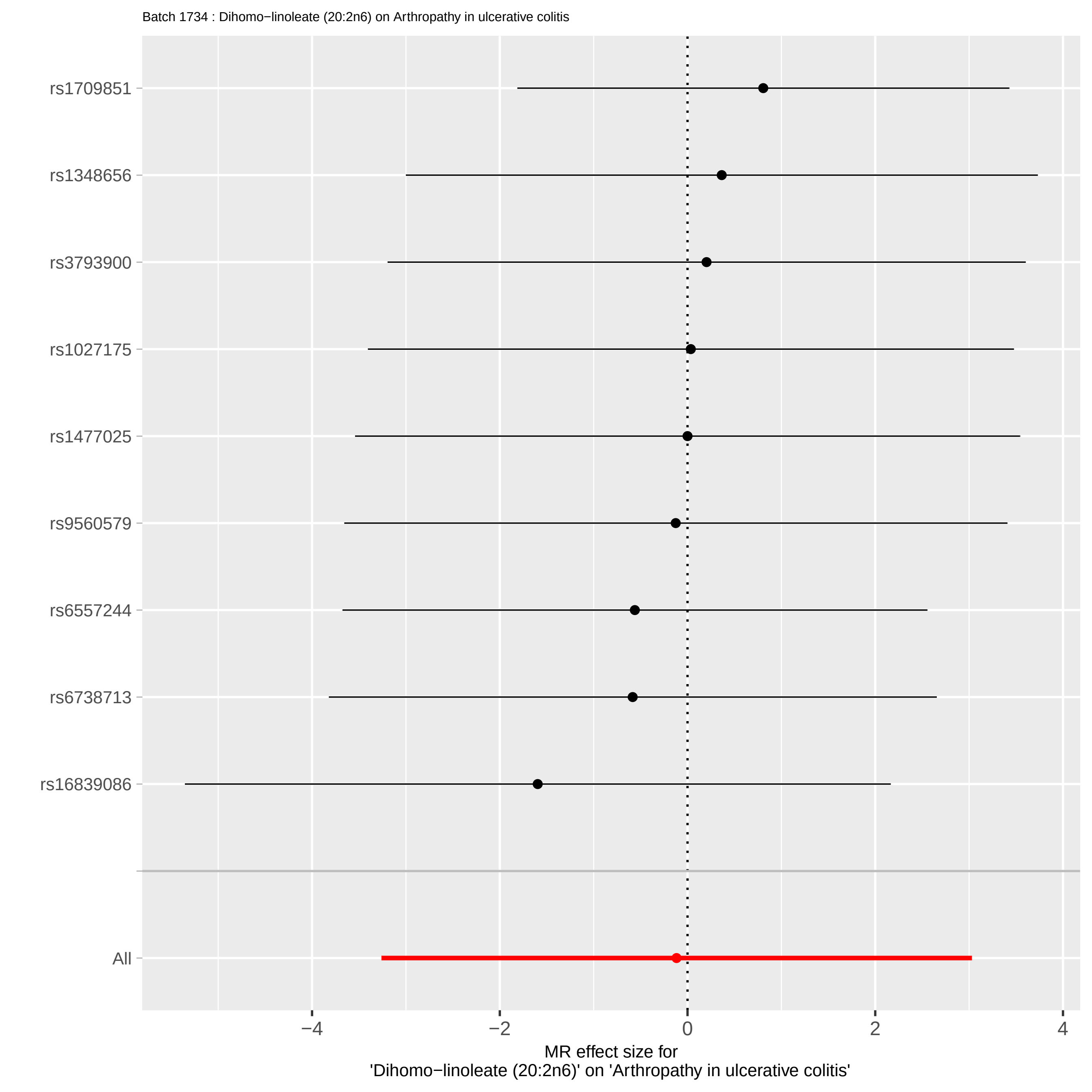

Batch 1734 : Dihomo−linoleate (20:2n6) on Arthropathy in ulcerative colitis
rs1709851
rs1348656
rs3793900
rs1027175
rs1477025
rs9560579
rs6557244
rs6738713
rs16839086
All
−4
−2
0
2
4
MR effect size for
'Dihomo−linoleate (20:2n6)' on 'Arthropathy in ulcerative colitis'

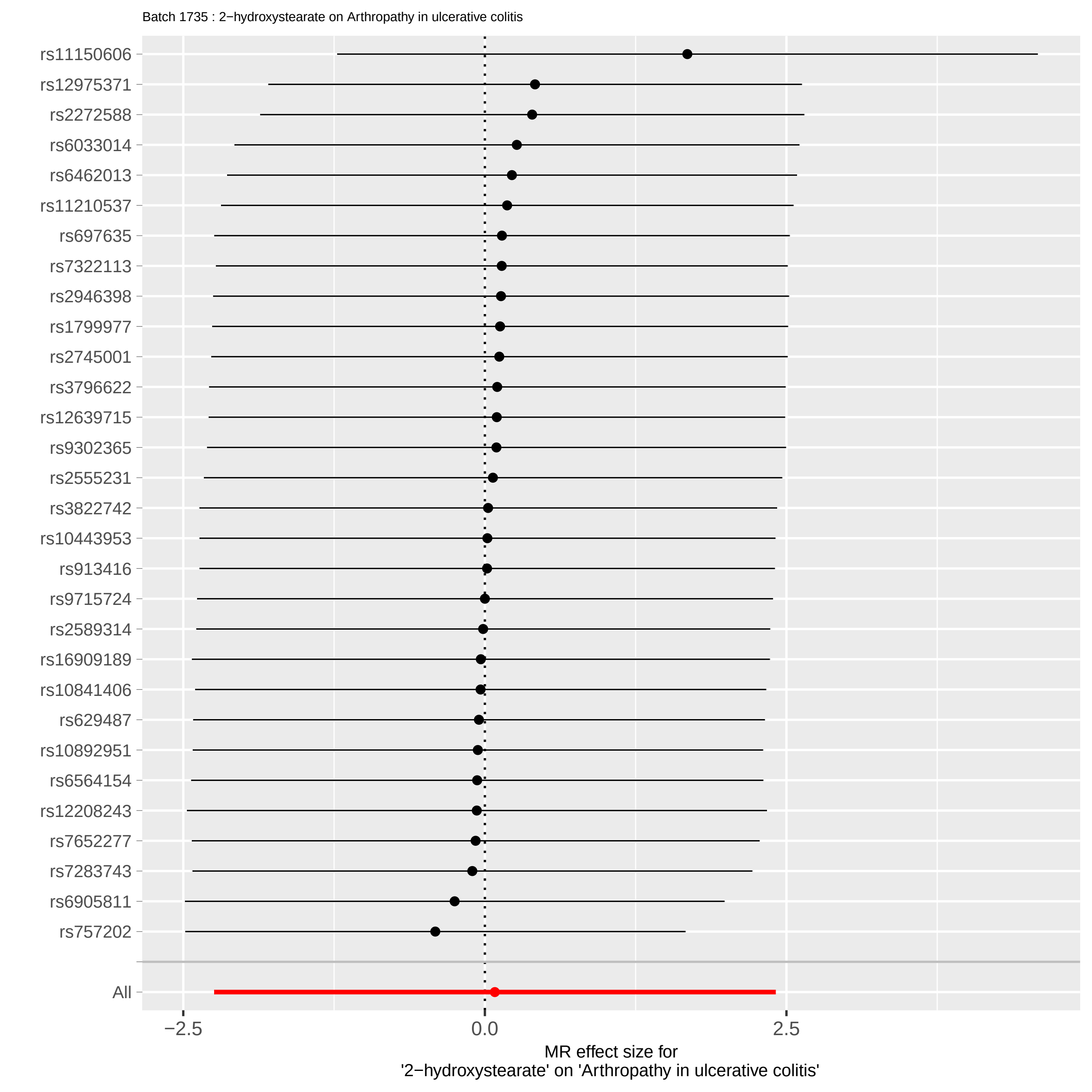
Batch 1735 : 2−hydroxystearate on Arthropathy in ulcerative colitis
rs11150606
rs12975371
rs2272588
rs6033014
rs6462013
rs11210537
rs697635
rs7322113
rs2946398
rs1799977
rs2745001
rs3796622
rs12639715
rs9302365
rs2555231
rs3822742
rs10443953
rs913416
rs9715724
rs2589314
rs16909189
rs10841406
rs629487
rs10892951
rs6564154
rs12208243
rs7652277
rs7283743
rs6905811
rs757202
All
−2.5
0.0
2.5
MR effect size for
'2−hydroxystearate' on 'Arthropathy in ulcerative colitis'

Batch 1737 : Glycylvaline on Arthropathy in ulcerative colitis

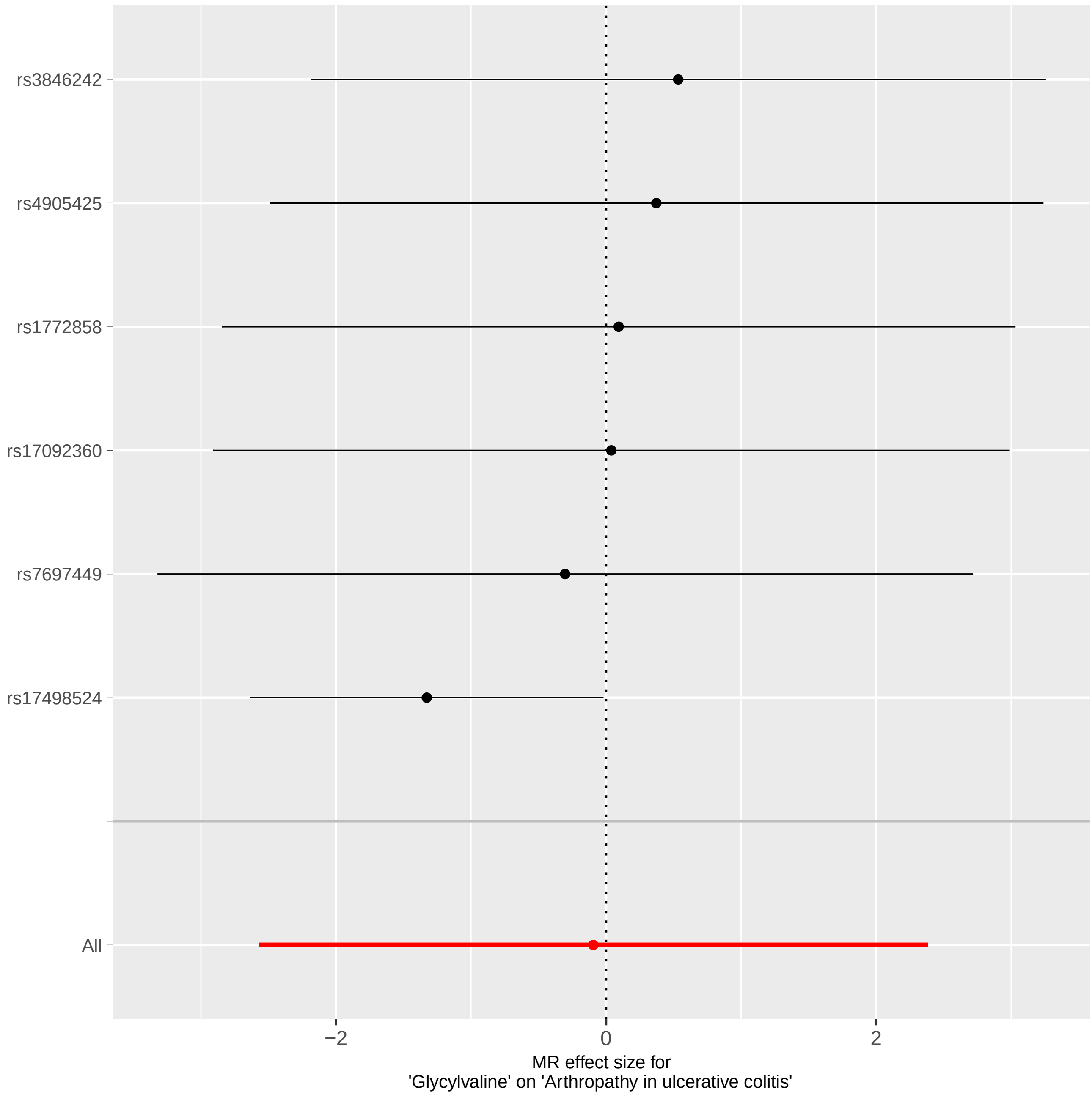

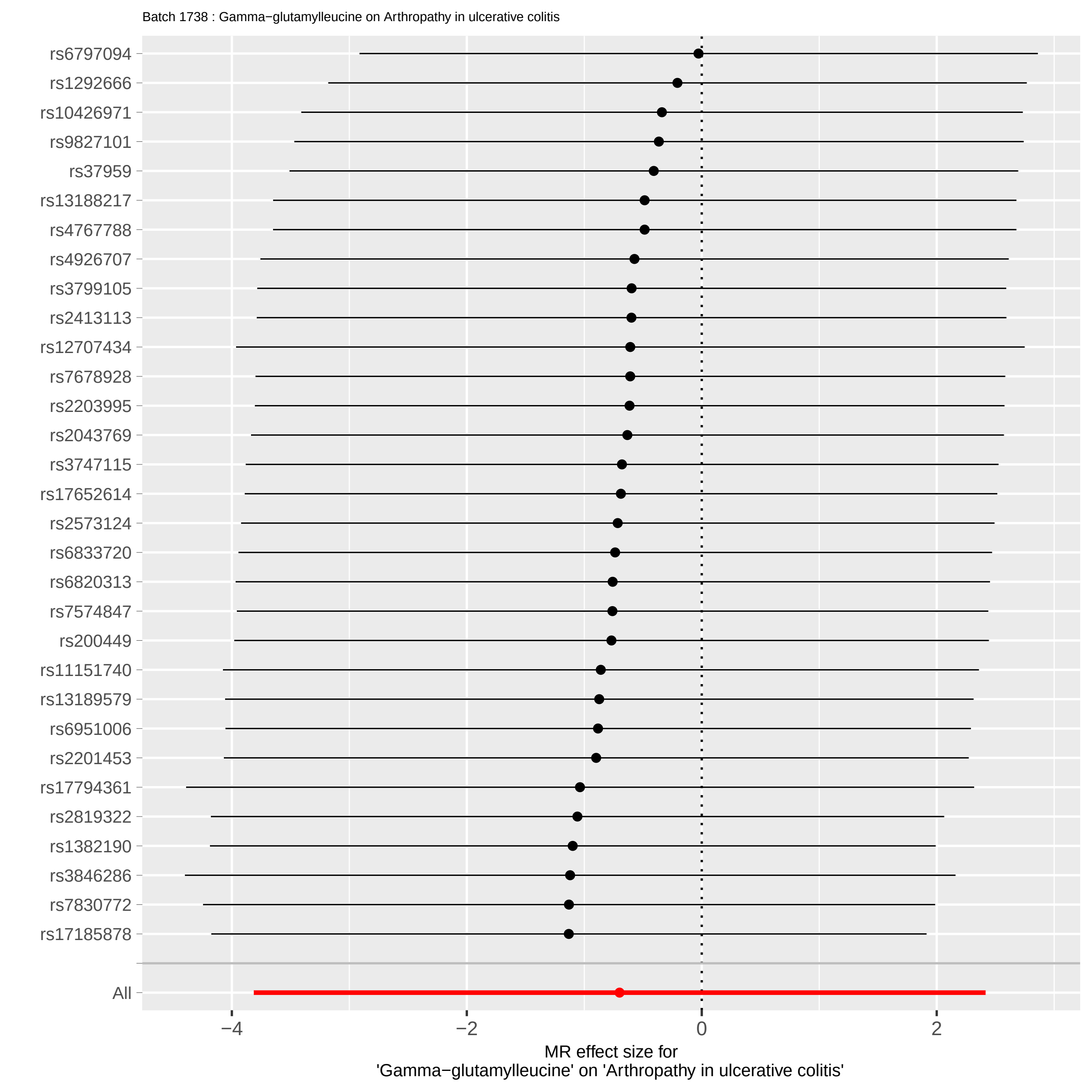

Batch 1738 : Gamma−glutamylleucine on Arthropathy in ulcerative colitis
rs6797094
rs1292666
rs10426971
rs9827101
rs37959
rs13188217
rs4767788
rs4926707
rs3799105
rs2413113
rs12707434
rs7678928
rs2203995
rs2043769
rs3747115
rs17652614
rs2573124
rs6833720
rs6820313
rs7574847
rs200449
rs11151740
rs13189579
rs6951006
rs2201453
rs17794361
rs2819322
rs1382190
rs3846286
rs7830772
rs17185878
All
−4
−2
0
2
MR effect size for
'Gamma−glutamylleucine' on 'Arthropathy in ulcerative colitis'

Batch 1741 : Glycocholate on Arthropathy in ulcerative colitis

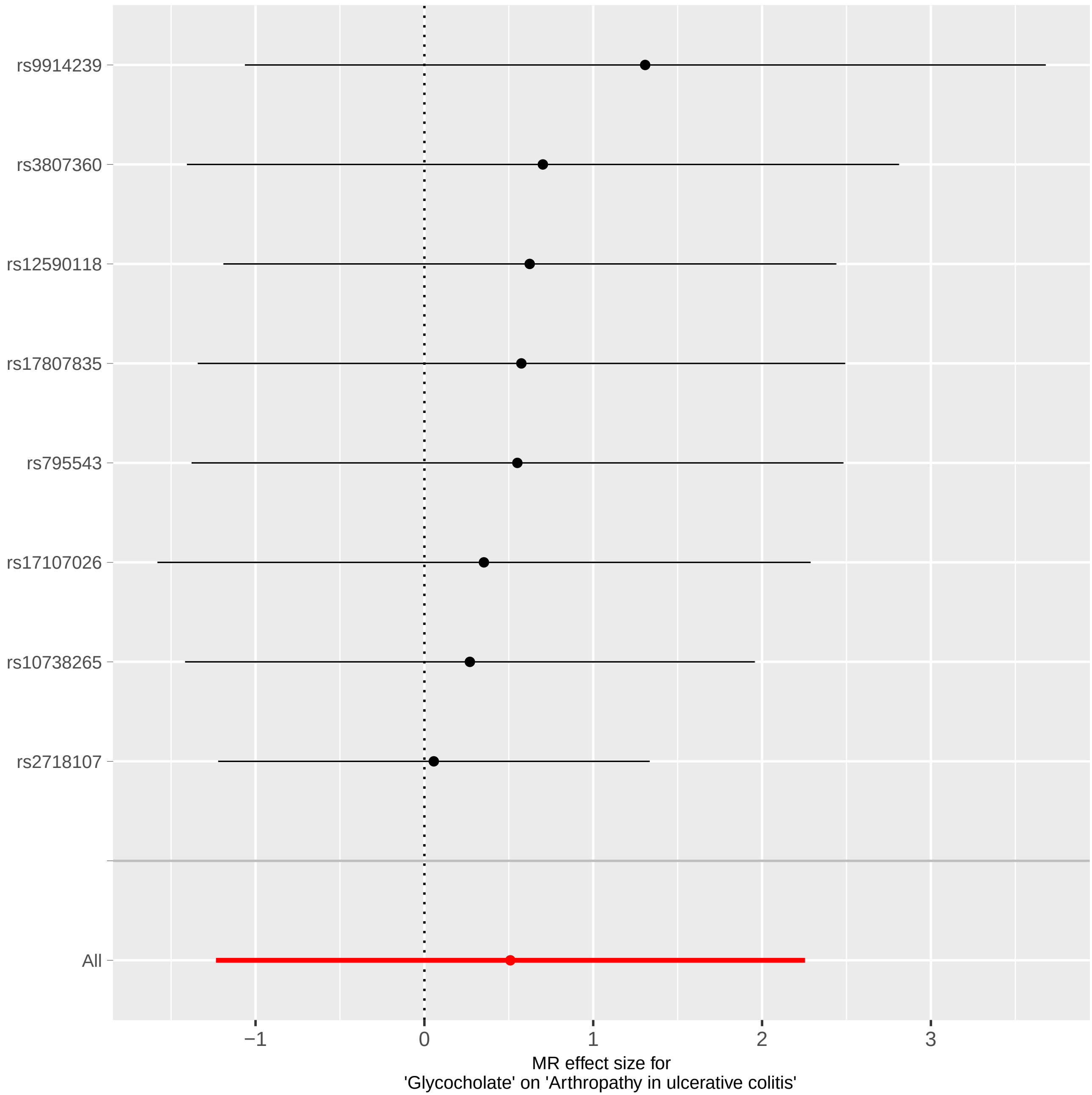

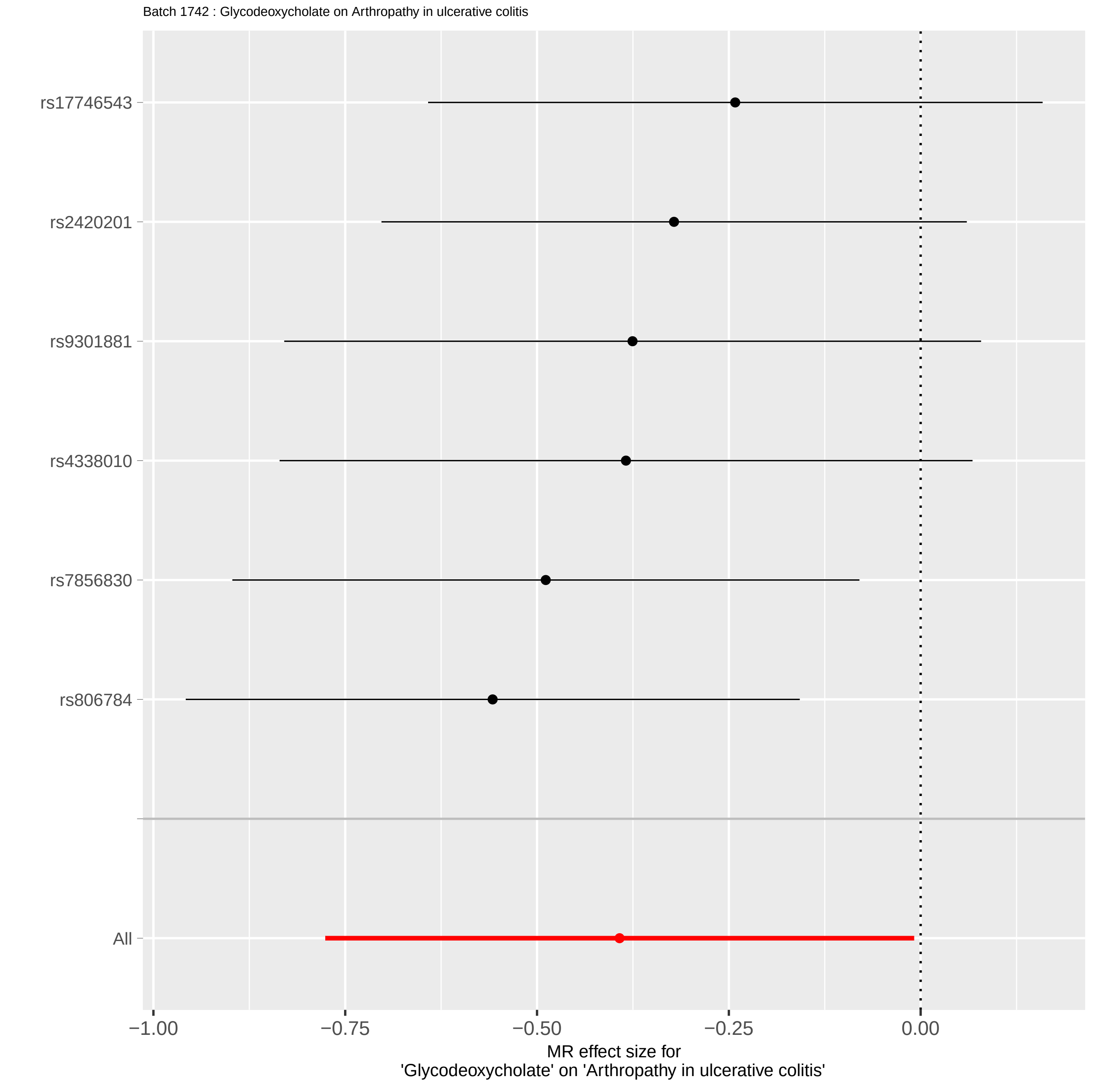
Batch 1742 : Glycodeoxycholate on Arthropathy in ulcerative colitis
rs17746543
rs2420201
rs9301881
rs4338010
rs7856830
rs806784
All
−1.00
−0.75
−0.50
−0.25
0.00
MR effect size for
'Glycodeoxycholate' on 'Arthropathy in ulcerative colitis'

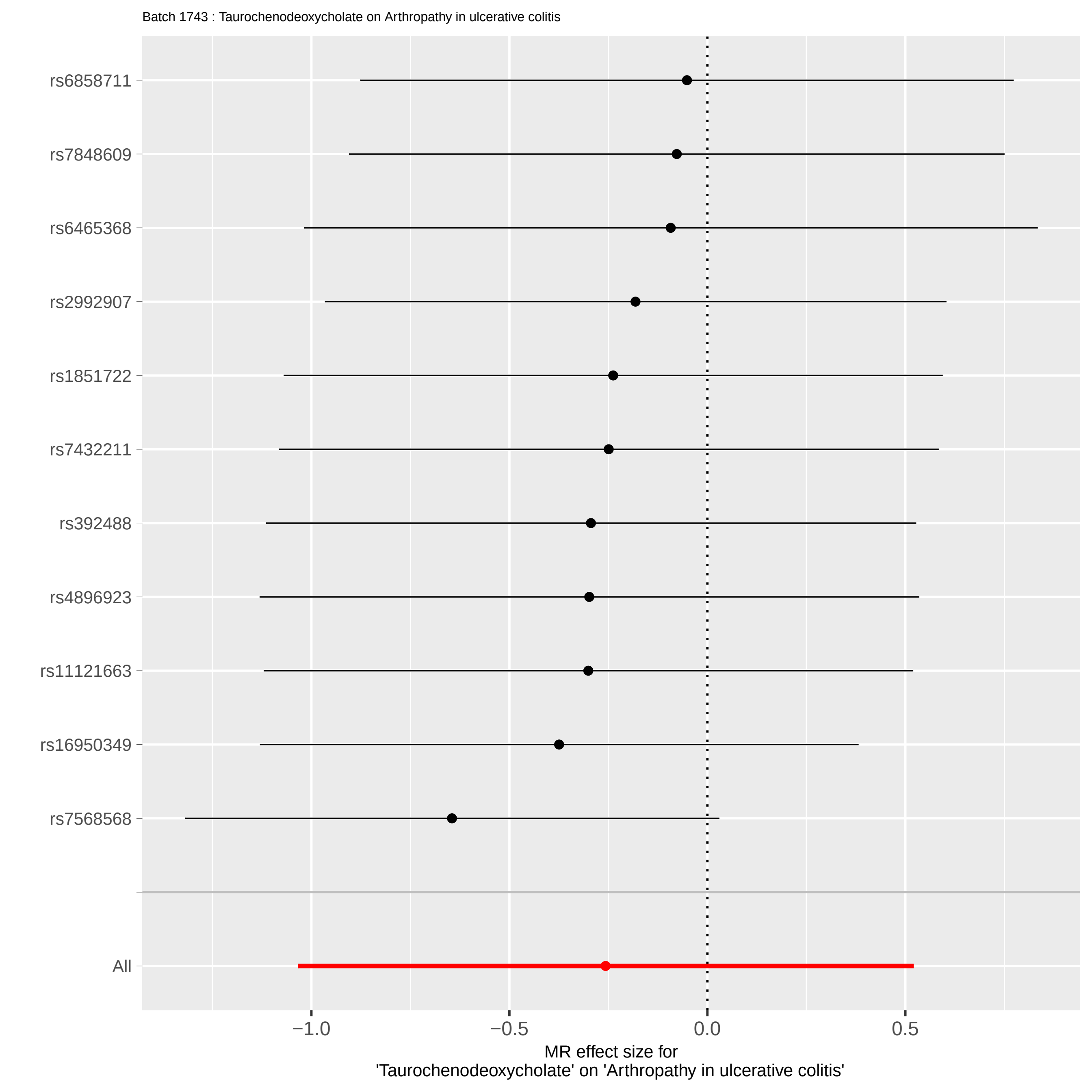

Batch 1743 : Taurochenodeoxycholate on Arthropathy in ulcerative colitis
rs6858711
rs7848609
rs6465368
rs2992907
rs1851722
rs7432211
rs392488
rs4896923
rs11121663
rs16950349
rs7568568
All
−1.0
−0.5
0.0
0.5
MR effect size for
'Taurochenodeoxycholate' on 'Arthropathy in ulcerative colitis'

Batch 1744 : Taurocholate on Arthropathy in ulcerative colitis

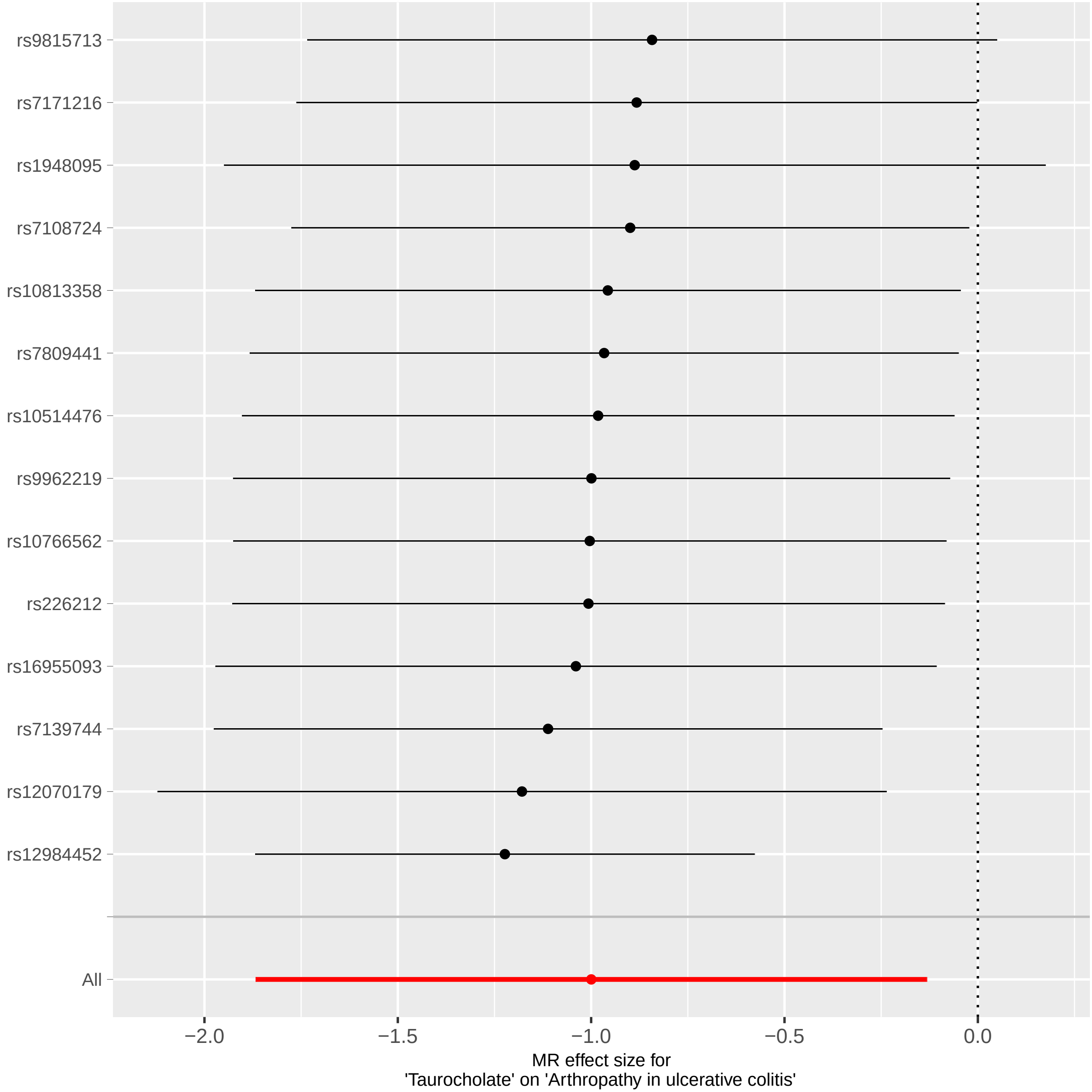

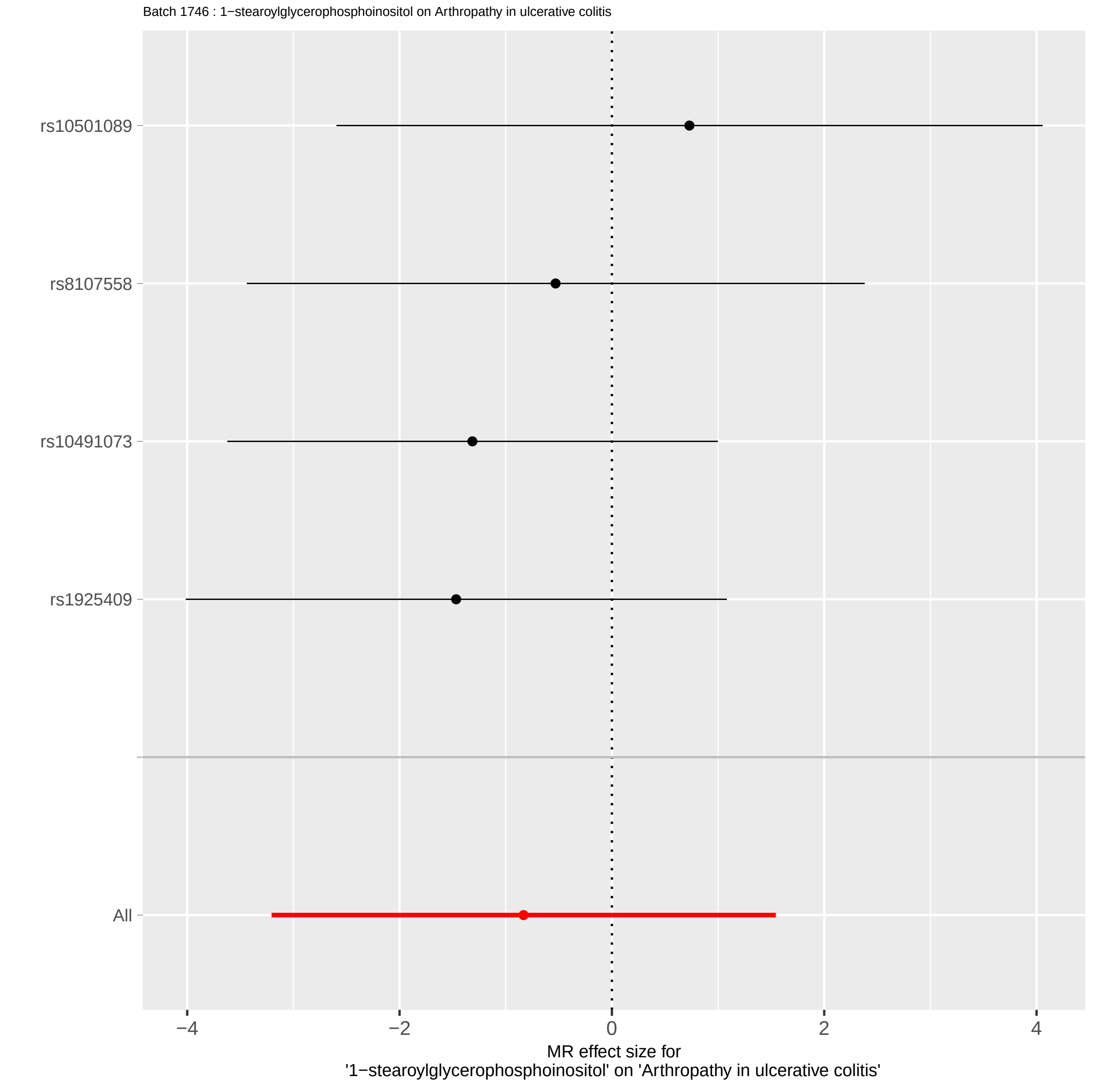
Batch 1746 : 1−stearoylglycerophosphoinositol on Arthropathy in ulcerative colitis
rs10501089
rs8107558
rs10491073
rs1925409
All
−4
−2
0
2
4
MR effect size for
'1−stearoylglycerophosphoinositol' on 'Arthropathy in ulcerative colitis'

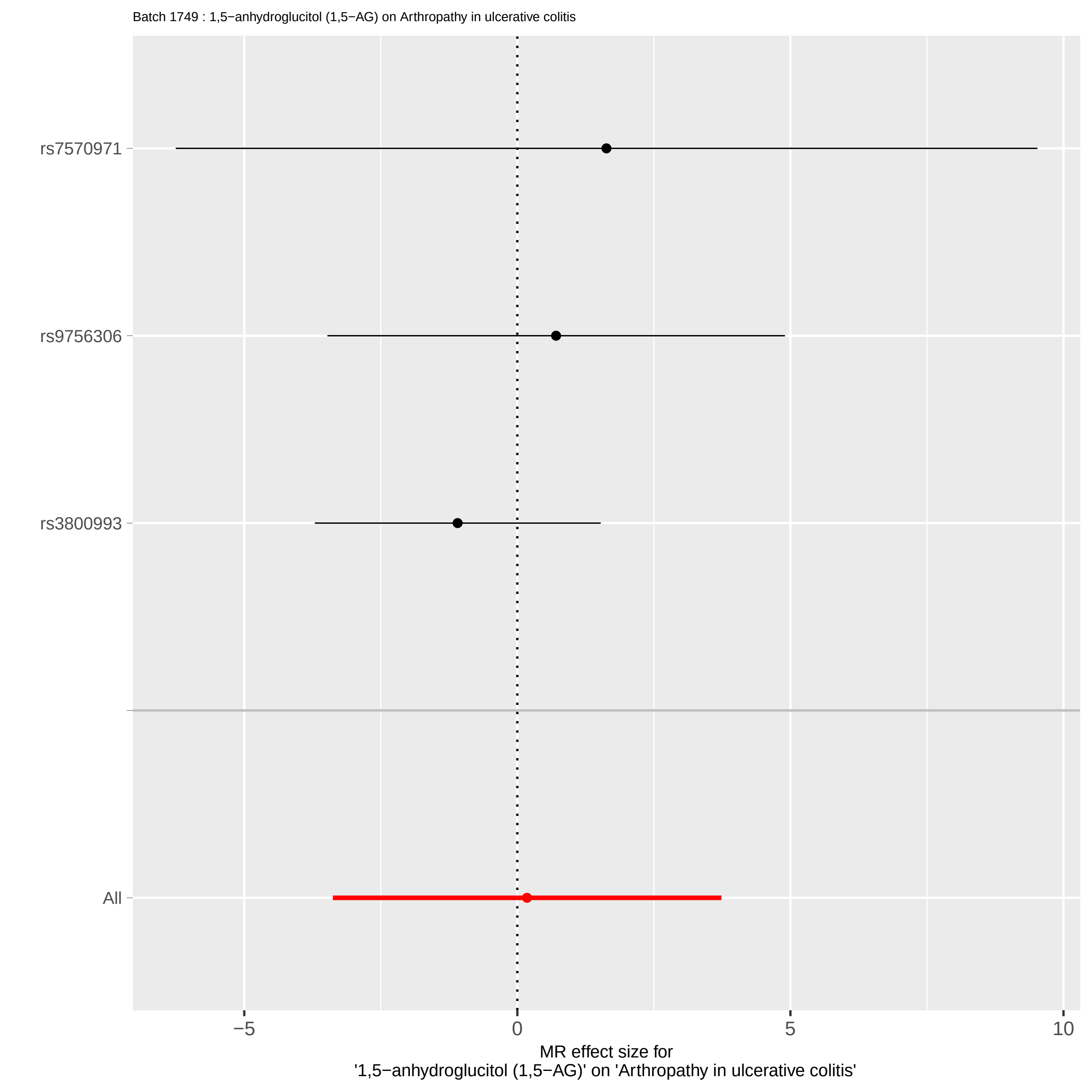

Batch 1749 : 1,5−anhydroglucitol (1,5−AG) on Arthropathy in ulcerative colitis
rs7570971
rs9756306
rs3800993
All
−5
0
5
10
MR effect size for
'1,5−anhydroglucitol (1,5−AG)' on 'Arthropathy in ulcerative colitis'

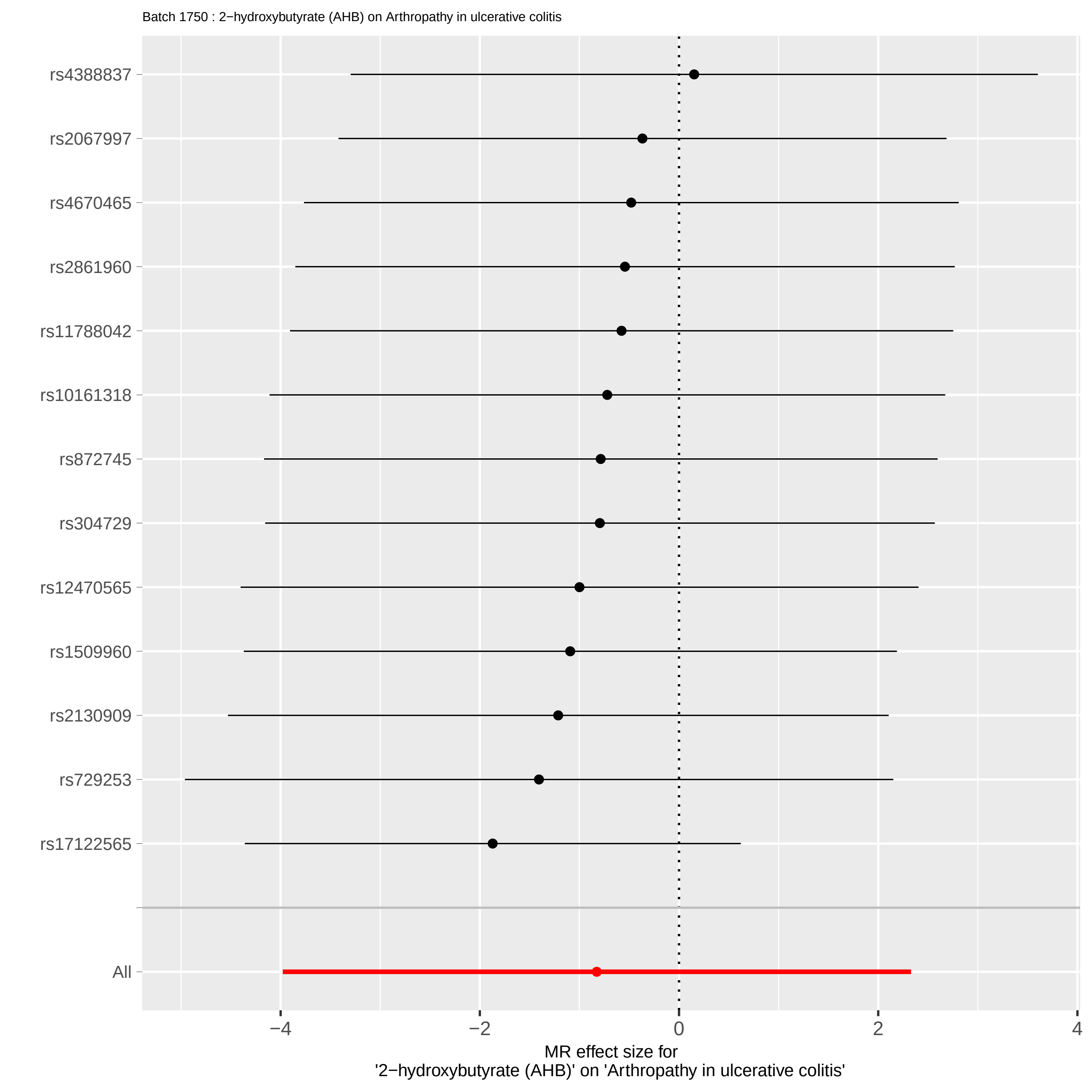
Batch 1750 : 2−hydroxybutyrate (AHB) on Arthropathy in ulcerative colitis
rs4388837
rs2067997
rs4670465
rs2861960
rs11788042
rs10161318
rs872745
rs304729
rs12470565
rs1509960
rs2130909
rs729253
rs17122565
All
−4
−2
0
2
4
MR effect size for
'2−hydroxybutyrate (AHB)' on 'Arthropathy in ulcerative colitis'

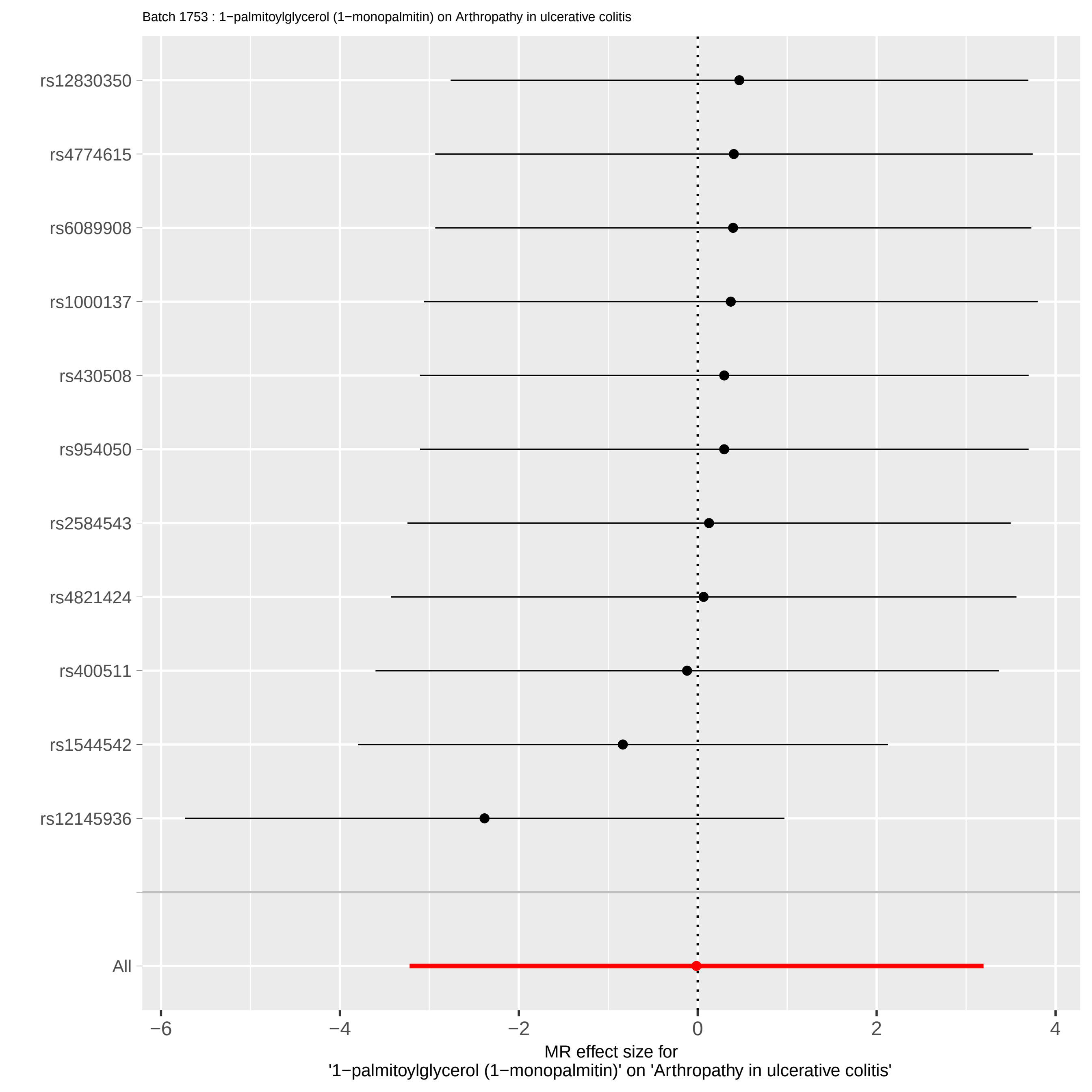

Batch 1753 : 1−palmitoylglycerol (1−monopalmitin) on Arthropathy in ulcerative colitis
rs12830350
rs4774615
rs6089908
rs1000137
rs430508
rs954050
rs2584543
rs4821424
rs400511
rs1544542
rs12145936
All
−6
−4
−2
0
2
4
MR effect size for
'1−palmitoylglycerol (1−monopalmitin)' on 'Arthropathy in ulcerative colitis'

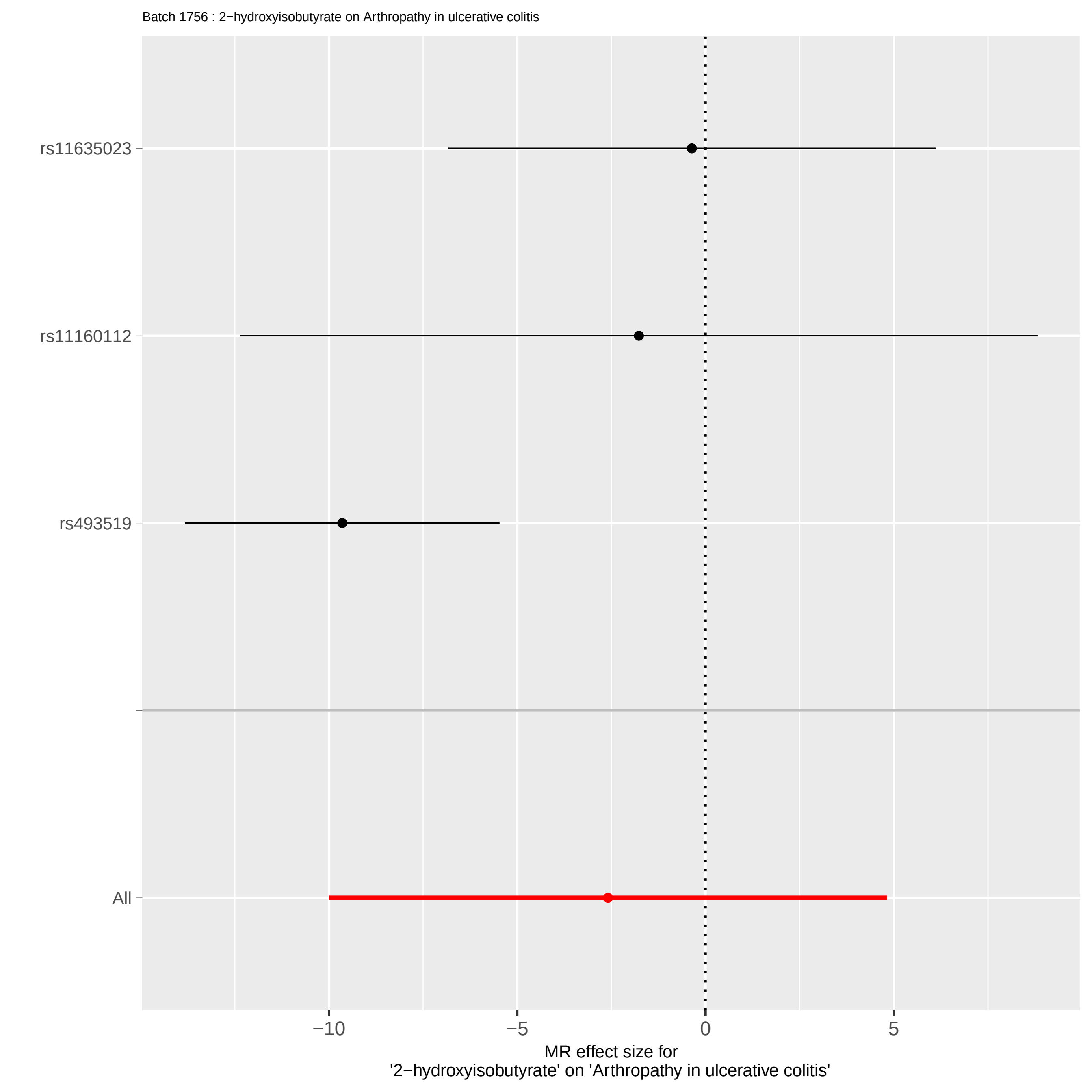

Batch 1756 : 2−hydroxyisobutyrate on Arthropathy in ulcerative colitis
rs11635023
rs11160112
rs493519
All
−10
−5
0
5
MR effect size for
'2−hydroxyisobutyrate' on 'Arthropathy in ulcerative colitis'

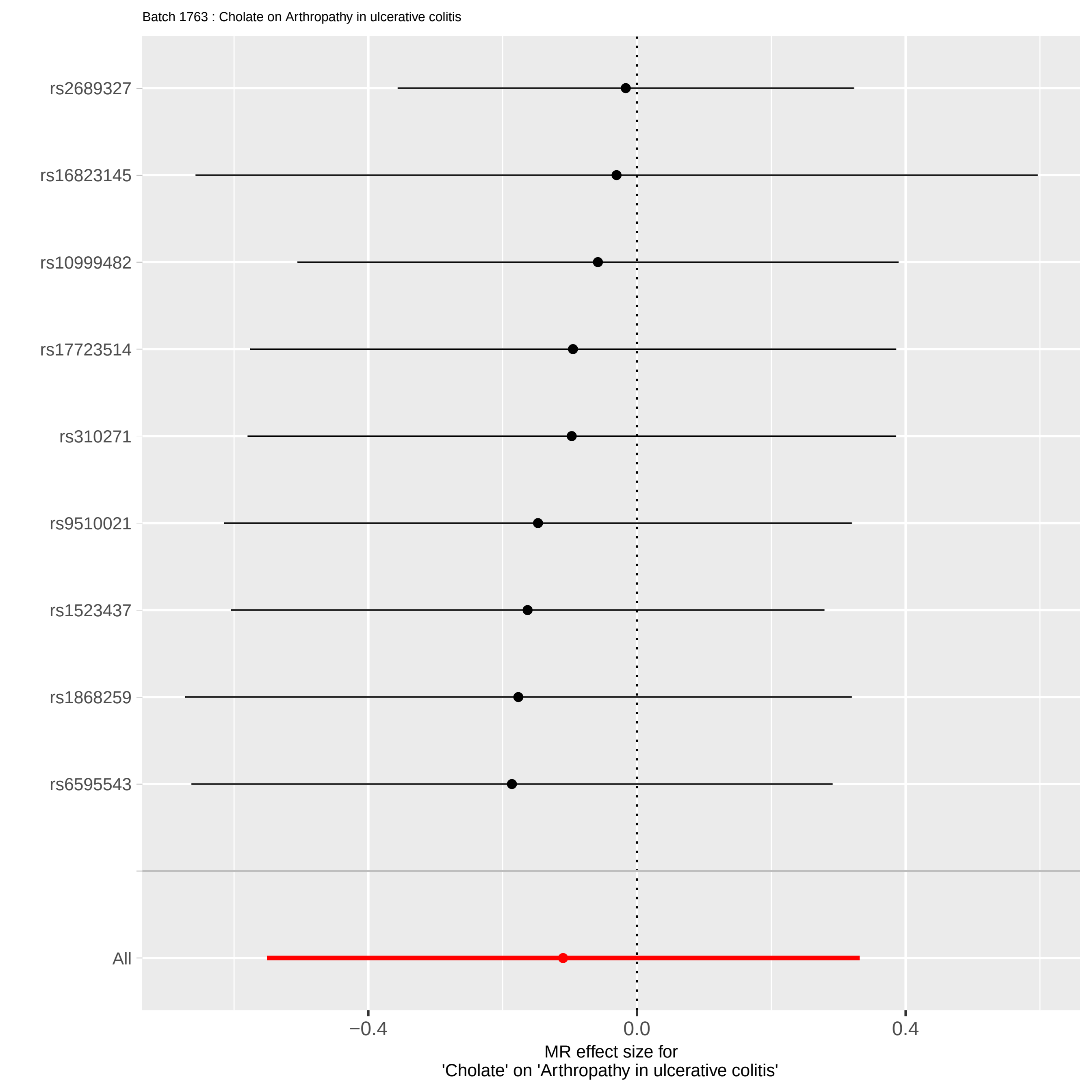
Batch 1763 : Cholate on Arthropathy in ulcerative colitis
rs2689327
rs16823145
rs10999482
rs17723514
rs310271
rs9510021
rs1523437
rs1868259
rs6595543
All
−0.4
0.0
0.4
MR effect size for
'Cholate' on 'Arthropathy in ulcerative colitis'

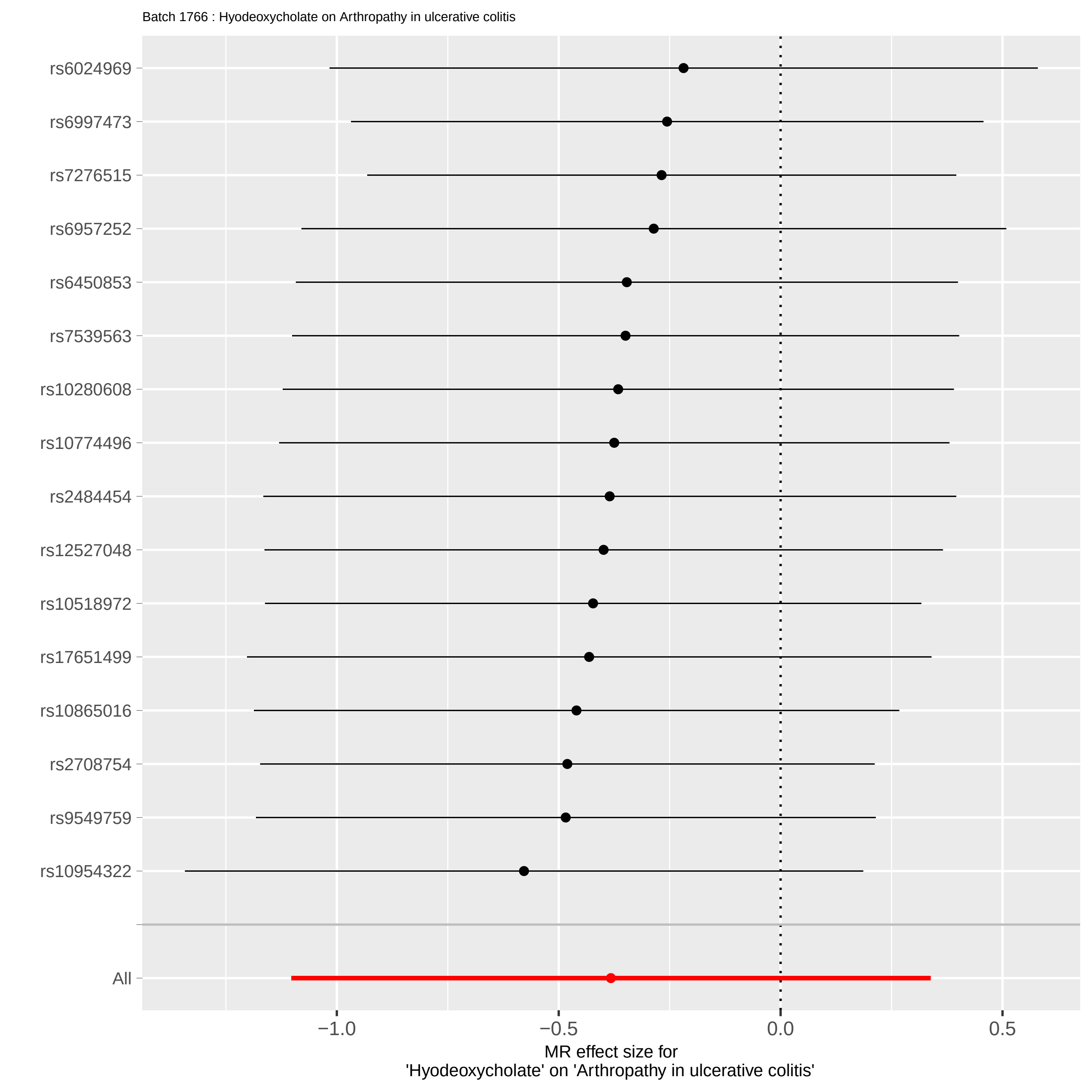

Batch 1766 : Hyodeoxycholate on Arthropathy in ulcerative colitis
rs6024969
rs6997473
rs7276515
rs6957252
rs6450853
rs7539563
rs10280608
rs10774496
rs2484454
rs12527048
rs10518972
rs17651499
rs10865016
rs2708754
rs9549759
rs10954322
All
−1.0
−0.5
0.0
0.5
MR effect size for
'Hyodeoxycholate' on 'Arthropathy in ulcerative colitis'

Batch 1767 : 3−indoxyl sulfate on Arthropathy in ulcerative colitis

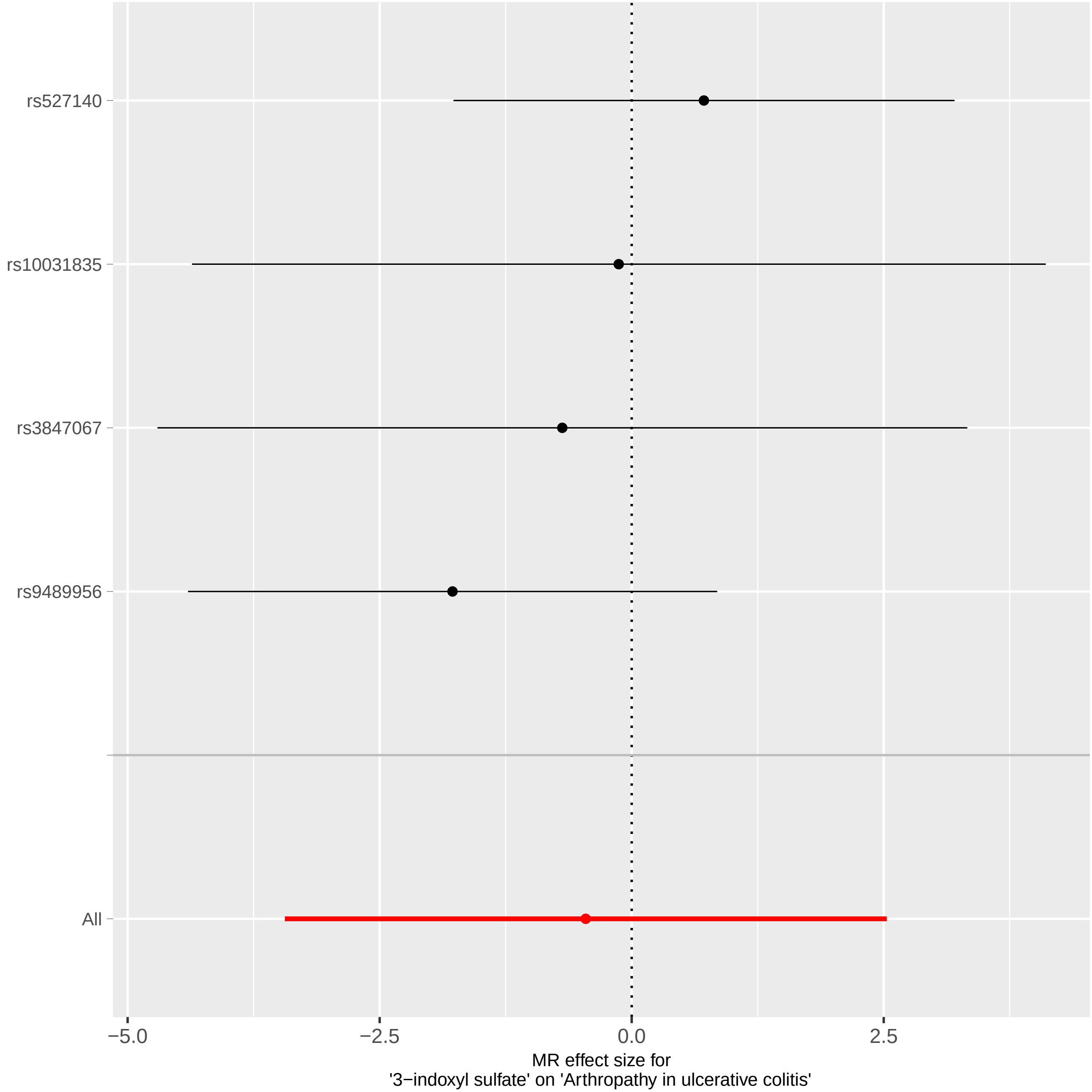

Batch 1771 : Erythrose on Arthropathy in ulcerative colitis

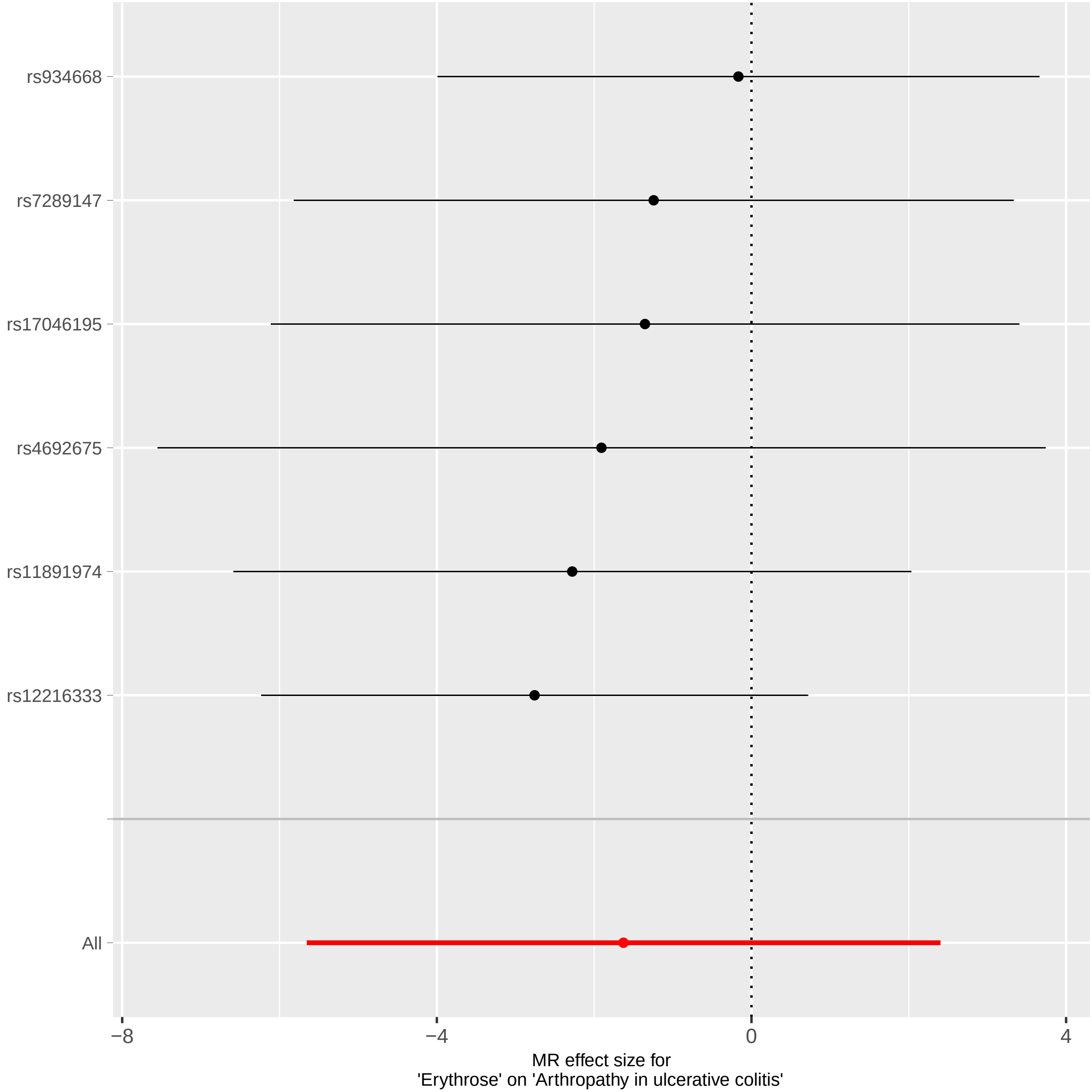

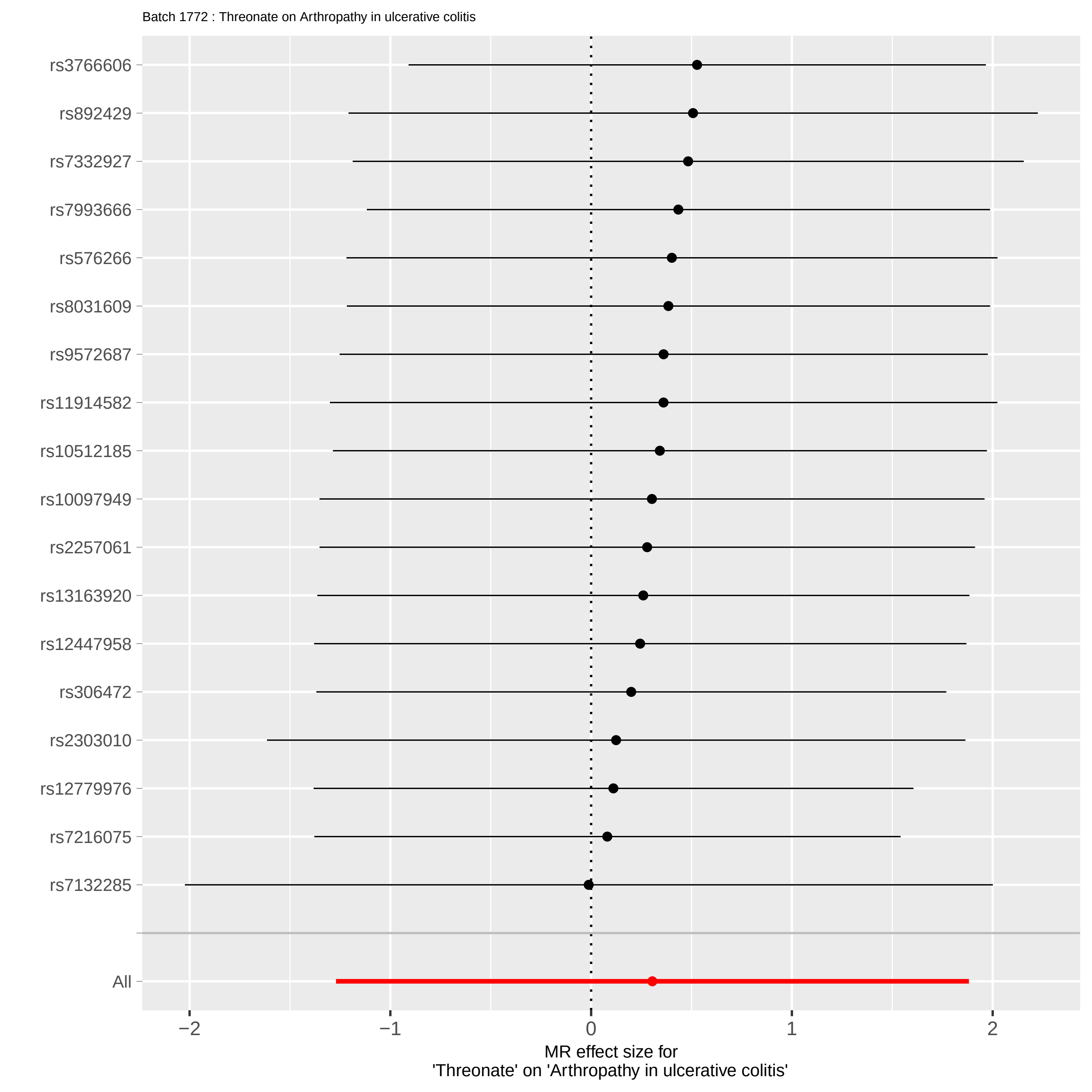
Batch 1772 : Threonate on Arthropathy in ulcerative colitis
rs3766606
rs892429
rs7332927
rs7993666
rs576266
rs8031609
rs9572687
rs11914582
rs10512185
rs10097949
rs2257061
rs13163920
rs12447958
rs306472
rs2303010
rs12779976
rs7216075
rs7132285
All
−2
−1
0
1
2
MR effect size for
'Threonate' on 'Arthropathy in ulcerative colitis'

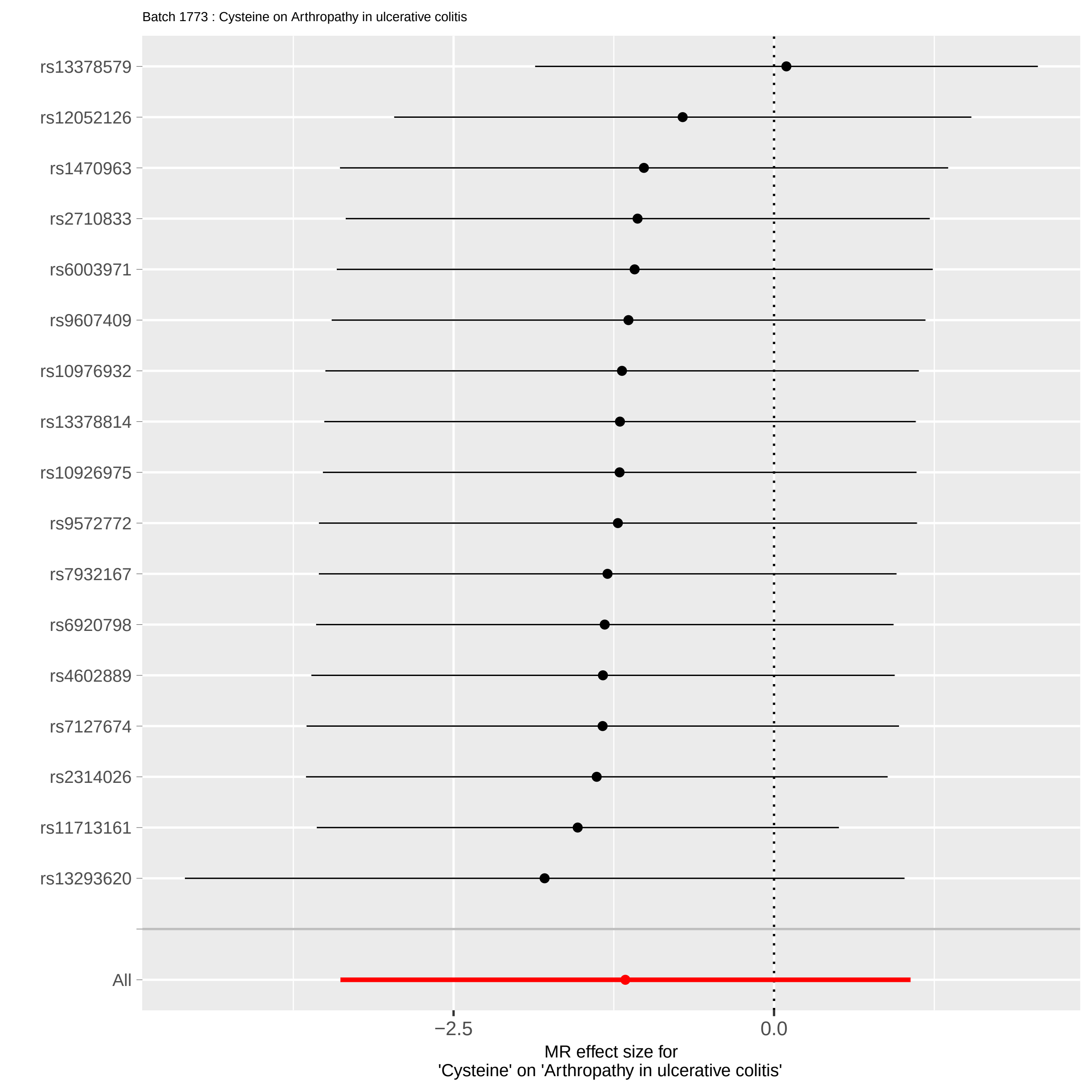

Batch 1773 : Cysteine on Arthropathy in ulcerative colitis
rs13378579
rs12052126
rs1470963
rs2710833
rs6003971
rs9607409
rs10976932
rs13378814
rs10926975
rs9572772
rs7932167
rs6920798
rs4602889
rs7127674
rs2314026
rs11713161
rs13293620
All
−2.5
0.0
MR effect size for
'Cysteine' on 'Arthropathy in ulcerative colitis'

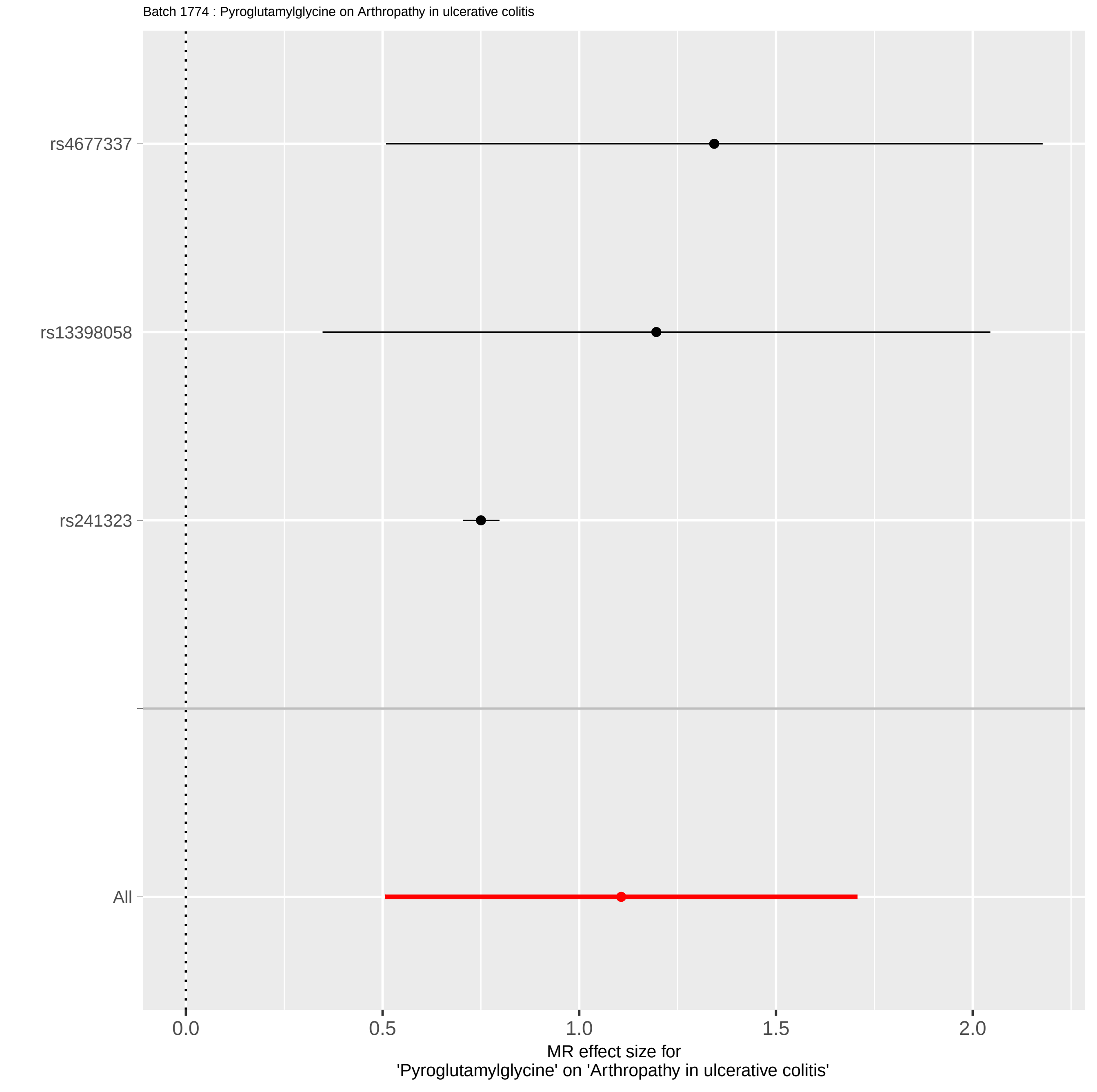
Batch 1774 : Pyroglutamylglycine on Arthropathy in ulcerative colitis
rs4677337
rs13398058
rs241323
All
0.0
0.5
1.0
1.5
2.0
MR effect size for
'Pyroglutamylglycine' on 'Arthropathy in ulcerative colitis'

Batch 1776 : DSGEGDFXAEGGGVR* on Arthropathy in ulcerative colitis

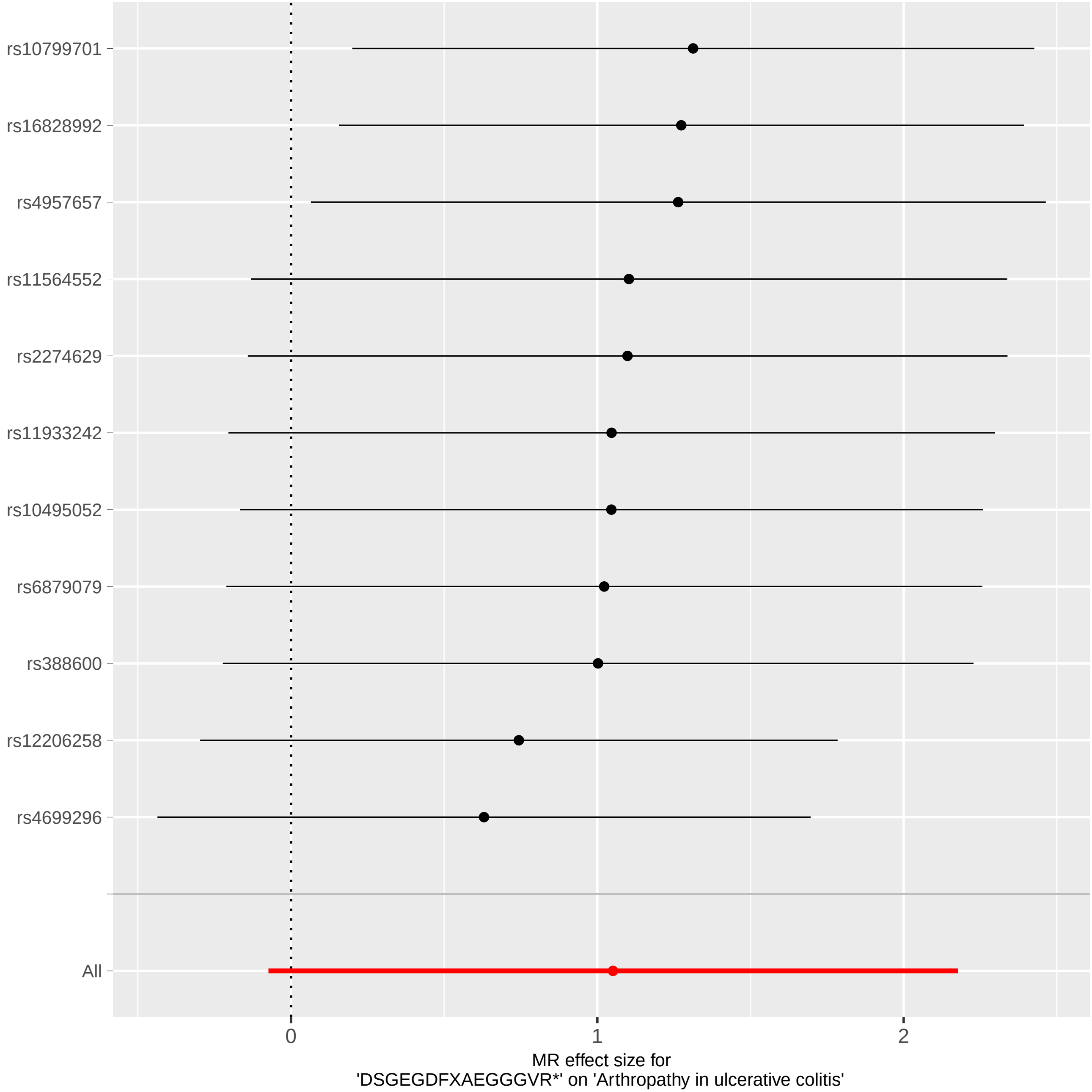

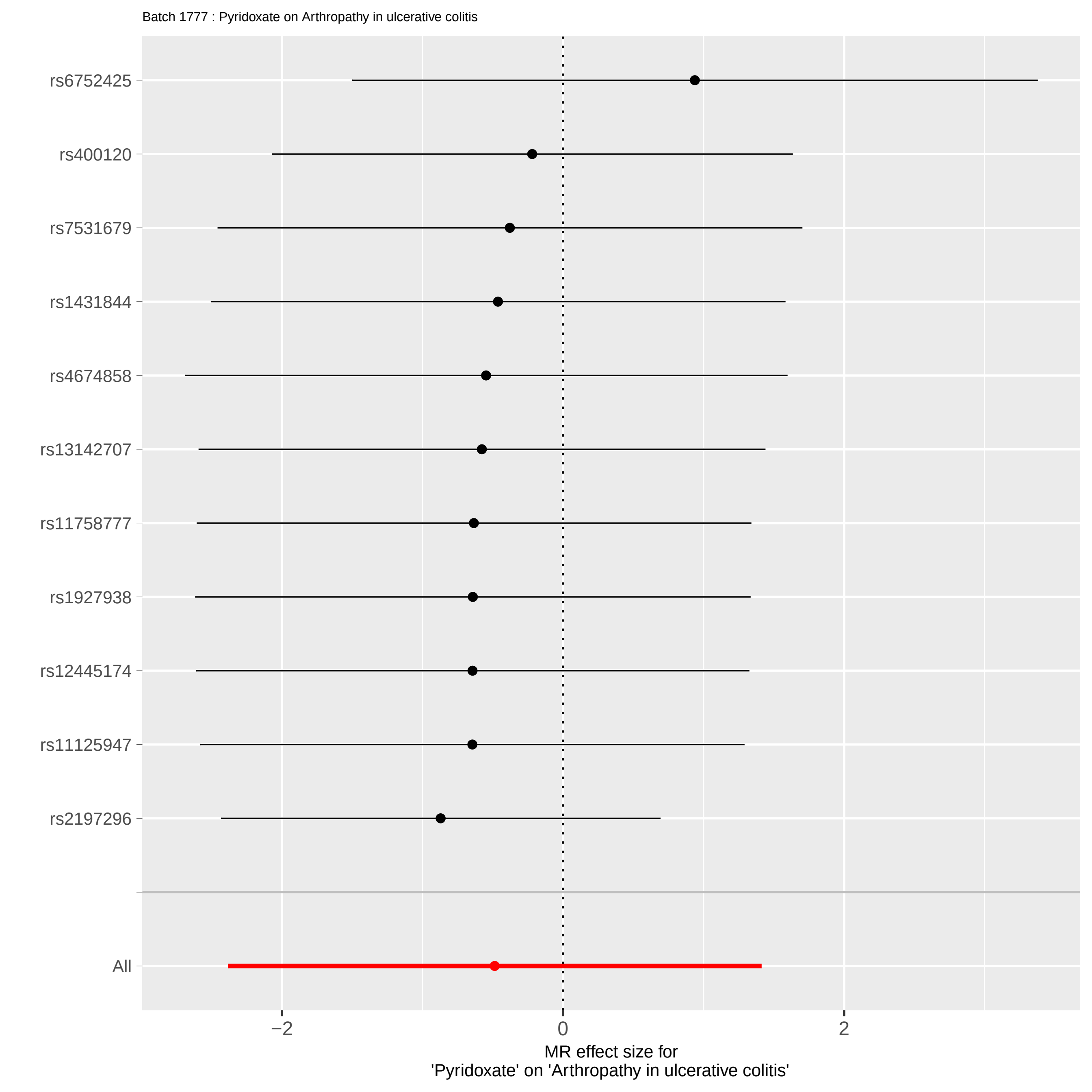

Batch 1777 : Pyridoxate on Arthropathy in ulcerative colitis
rs6752425
rs400120
rs7531679
rs1431844
rs4674858
rs13142707
rs11758777
rs1927938
rs12445174
rs11125947
rs2197296
All
−2
0
2
MR effect size for
'Pyridoxate' on 'Arthropathy in ulcerative colitis'

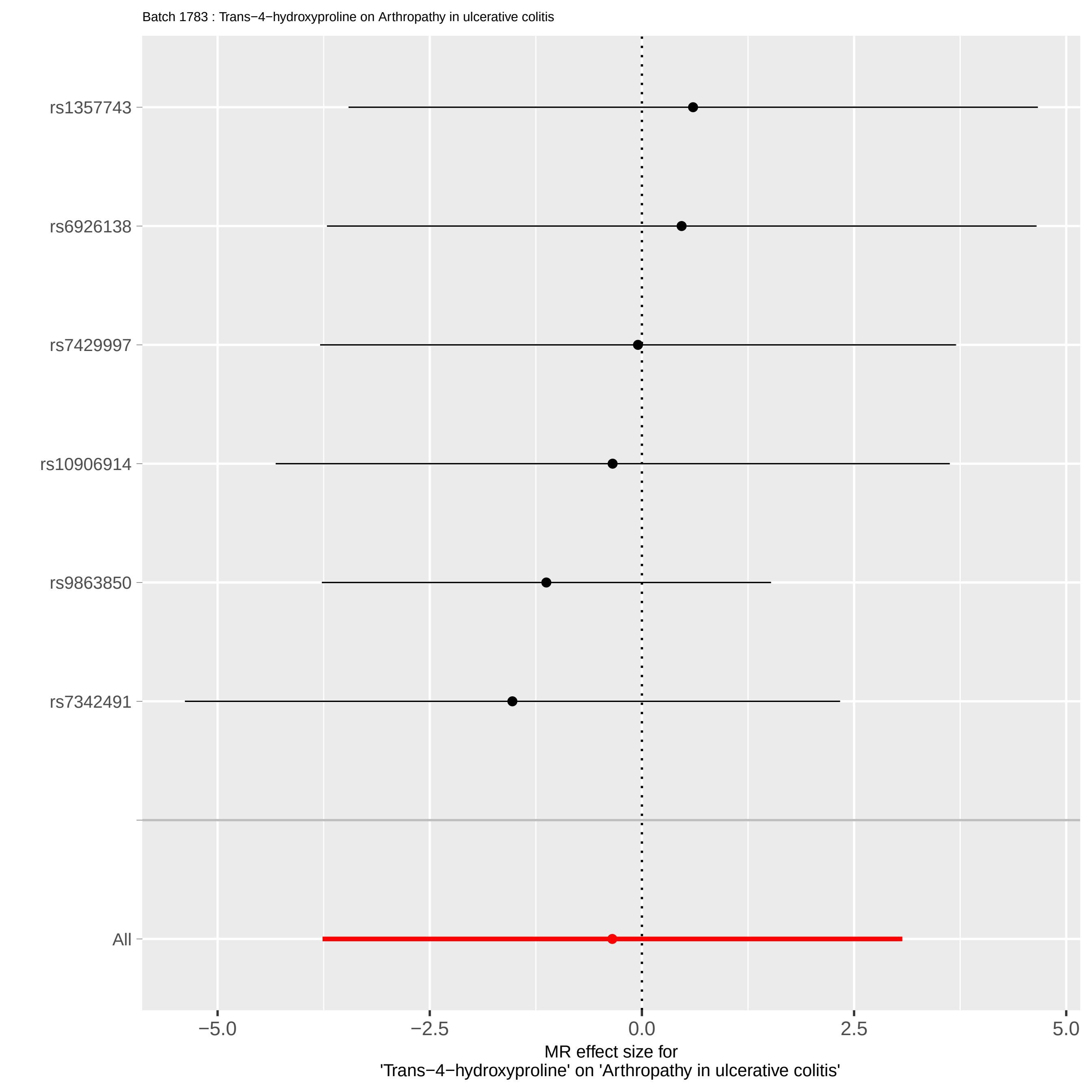

Batch 1783 : Trans−4−hydroxyproline on Arthropathy in ulcerative colitis
rs1357743
rs6926138
rs7429997
rs10906914
rs9863850
rs7342491
All
−5.0
−2.5
0.0
2.5
5.0
MR effect size for
'Trans−4−hydroxyproline' on 'Arthropathy in ulcerative colitis'

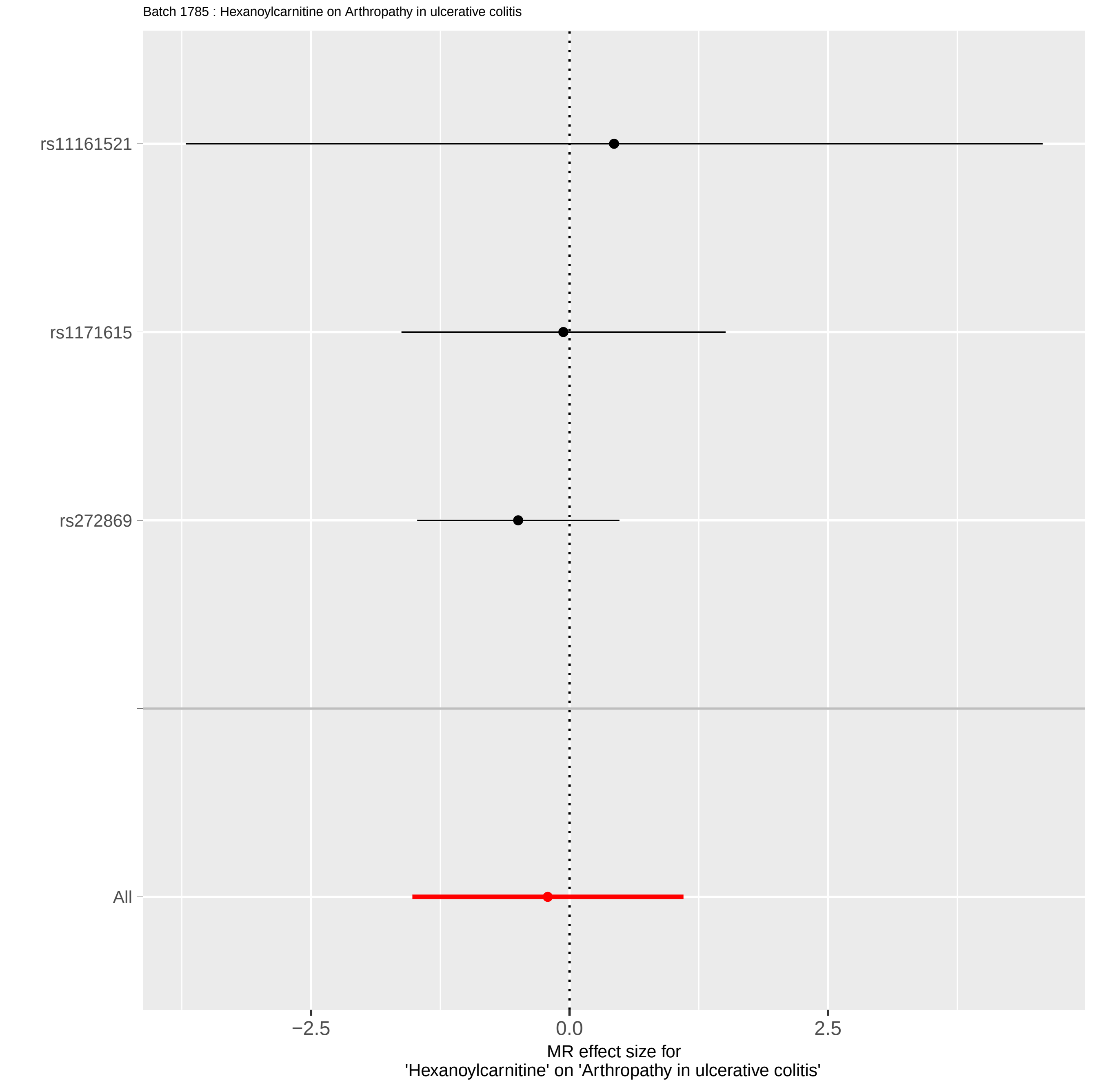

Batch 1785 : Hexanoylcarnitine on Arthropathy in ulcerative colitis
rs11161521
rs1171615
rs272869
All
−2.5
0.0
2.5
MR effect size for
'Hexanoylcarnitine' on 'Arthropathy in ulcerative colitis'

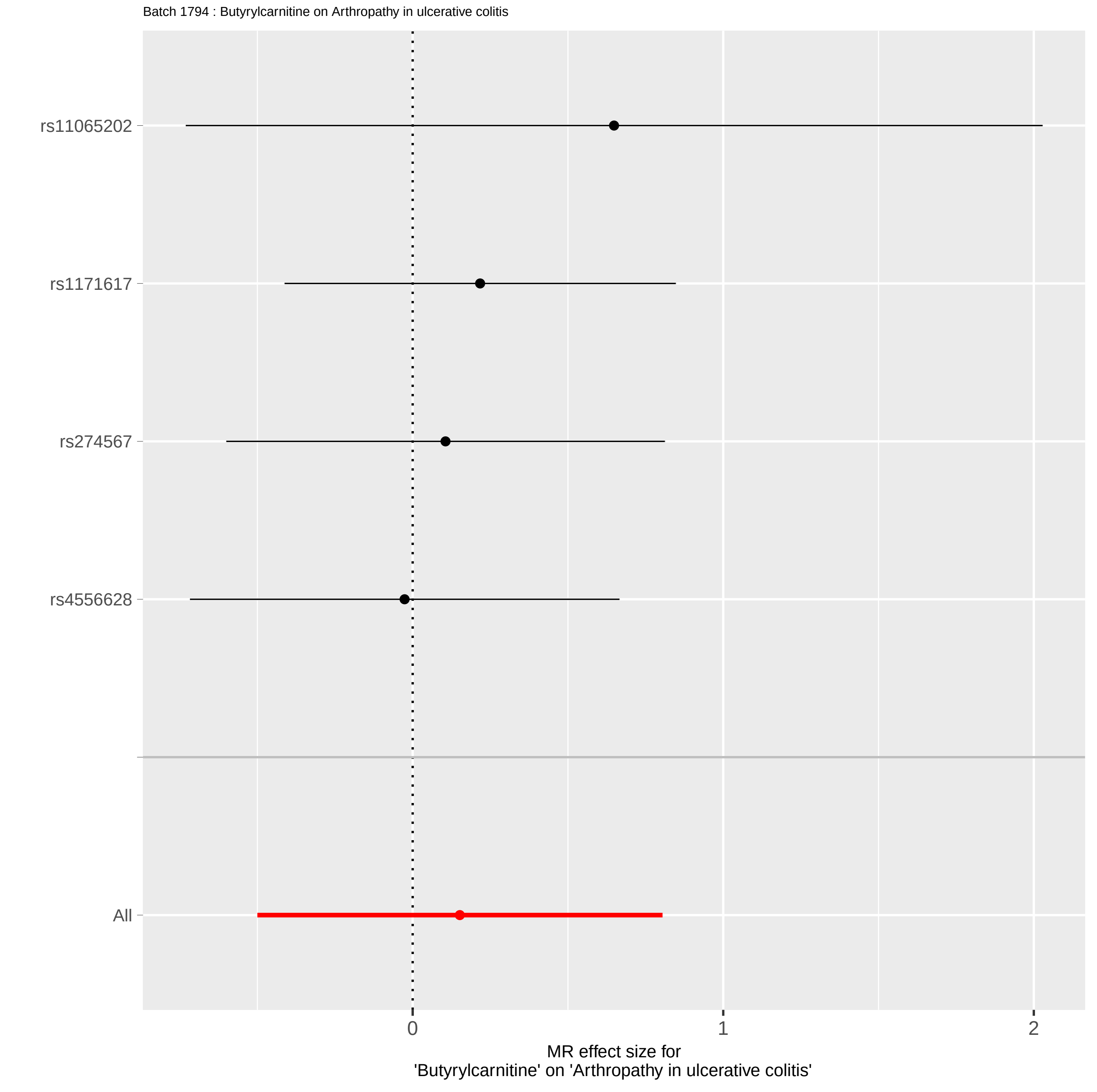

Batch 1794 : Butyrylcarnitine on Arthropathy in ulcerative colitis
rs11065202
rs1171617
rs274567
rs4556628
All
0
1
2
MR effect size for
'Butyrylcarnitine' on 'Arthropathy in ulcerative colitis'

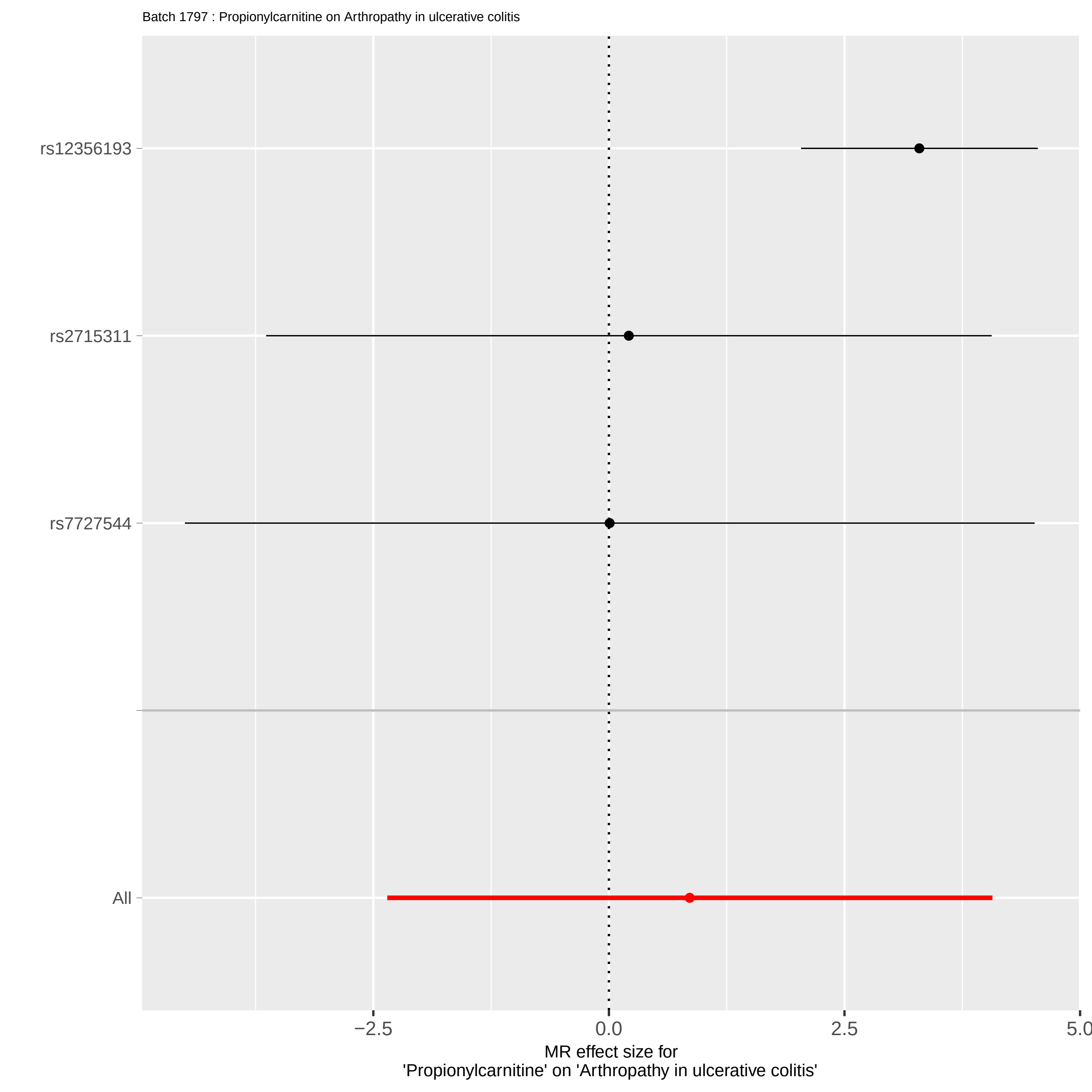
Batch 1797 : Propionylcarnitine on Arthropathy in ulcerative colitis
rs12356193
rs2715311
rs7727544
All
−2.5
0.0
2.5
5.0
MR effect size for
'Propionylcarnitine' on 'Arthropathy in ulcerative colitis'

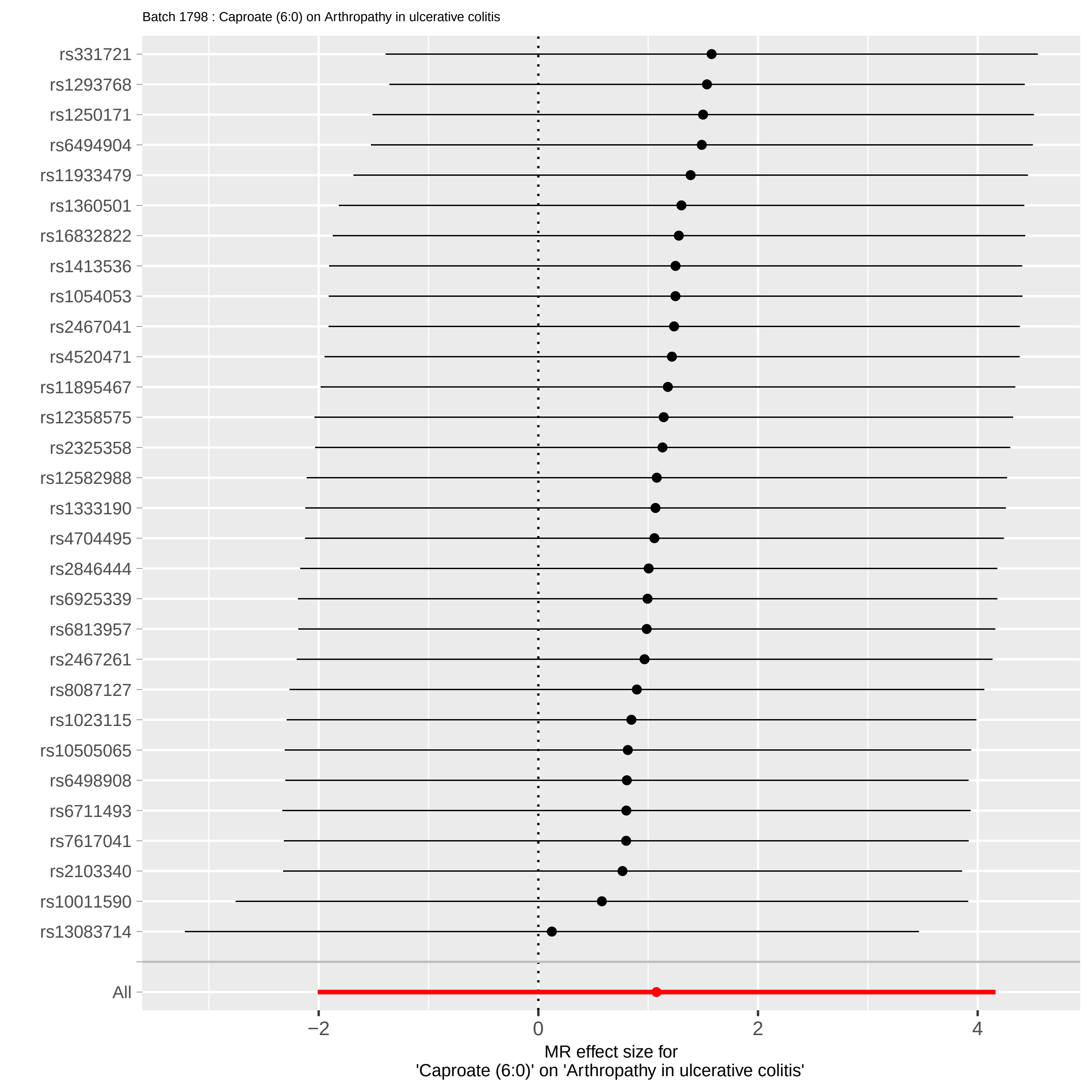

Batch 1798 : Caproate (6:0) on Arthropathy in ulcerative colitis
rs331721
rs1293768
rs1250171
rs6494904
rs11933479
rs1360501
rs16832822
rs1413536
rs1054053
rs2467041
rs4520471
rs11895467
rs12358575
rs2325358
rs12582988
rs1333190
rs4704495
rs2846444
rs6925339
rs6813957
rs2467261
rs8087127
rs1023115
rs10505065
rs6498908
rs6711493
rs7617041
rs2103340
rs10011590
rs13083714
All
−2
0
2
4
MR effect size for
'Caproate (6:0)' on 'Arthropathy in ulcerative colitis'

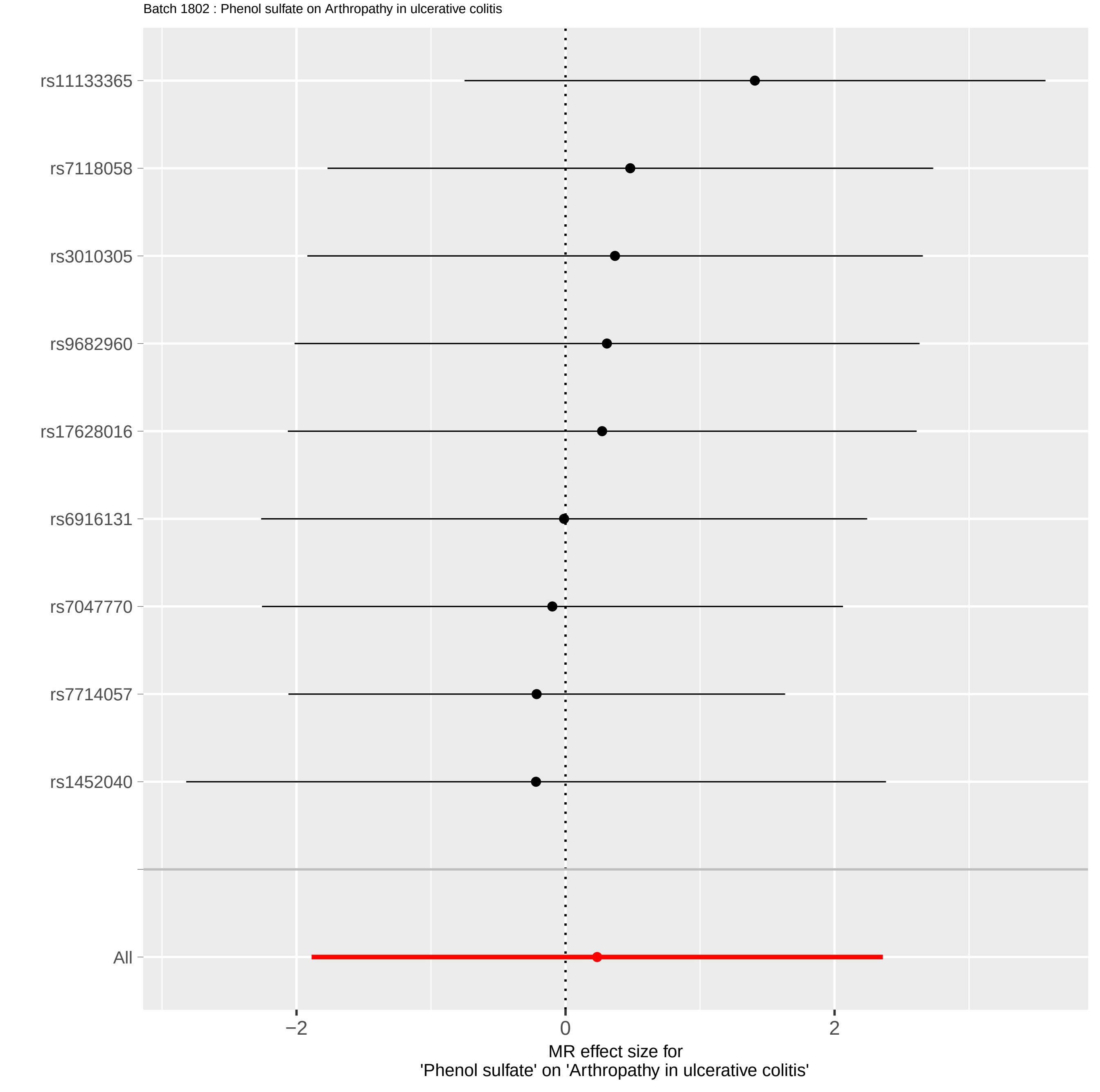
Batch 1802 : Phenol sulfate on Arthropathy in ulcerative colitis
rs11133365
rs7118058
rs3010305
rs9682960
rs17628016
rs6916131
rs7047770
rs7714057
rs1452040
All
−2
0
2
MR effect size for
'Phenol sulfate' on 'Arthropathy in ulcerative colitis'

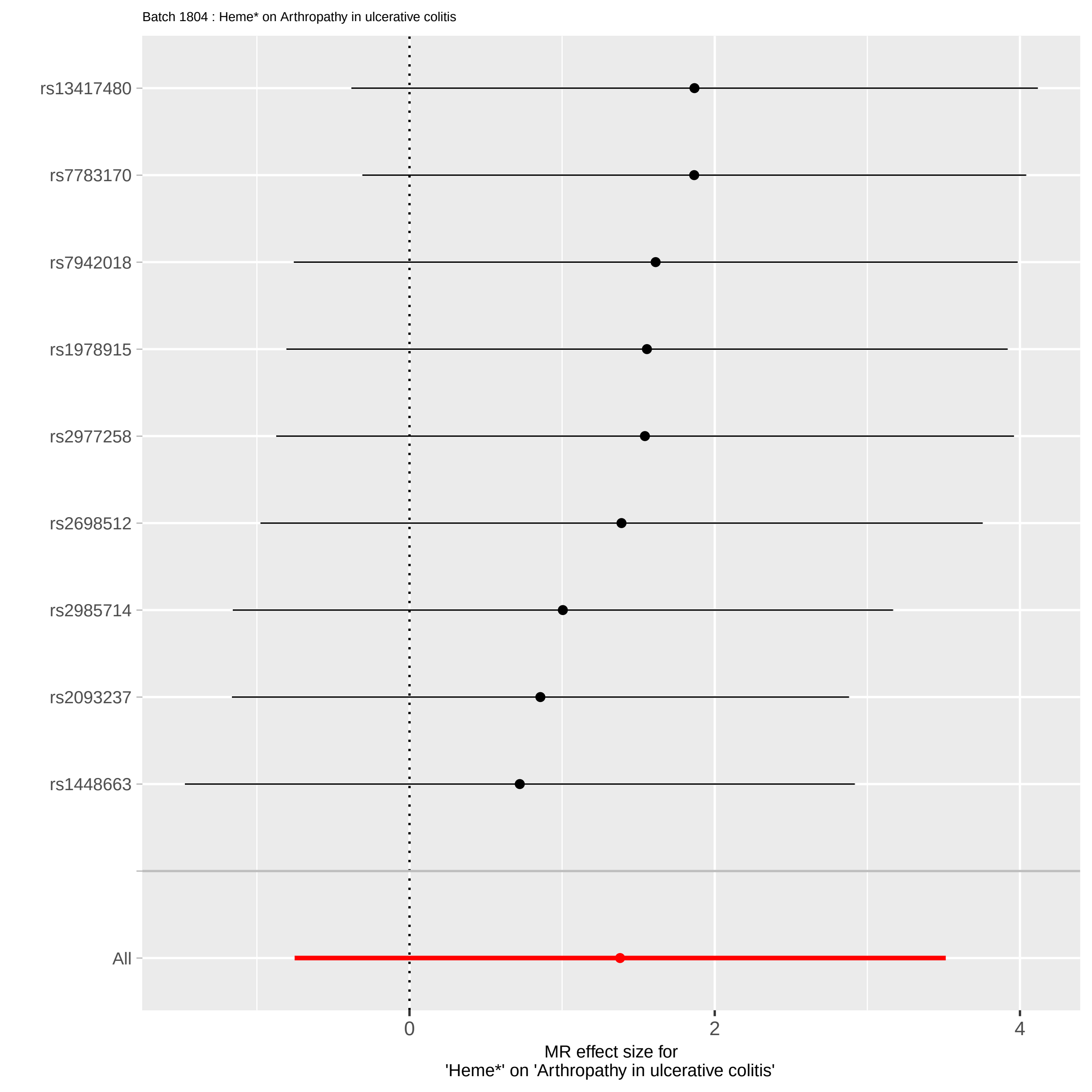
Batch 1804 : Heme* on Arthropathy in ulcerative colitis
rs13417480
rs7783170
rs7942018
rs1978915
rs2977258
rs2698512
rs2985714
rs2093237
rs1448663
All
0
2
4
MR effect size for
'Heme*' on 'Arthropathy in ulcerative colitis'

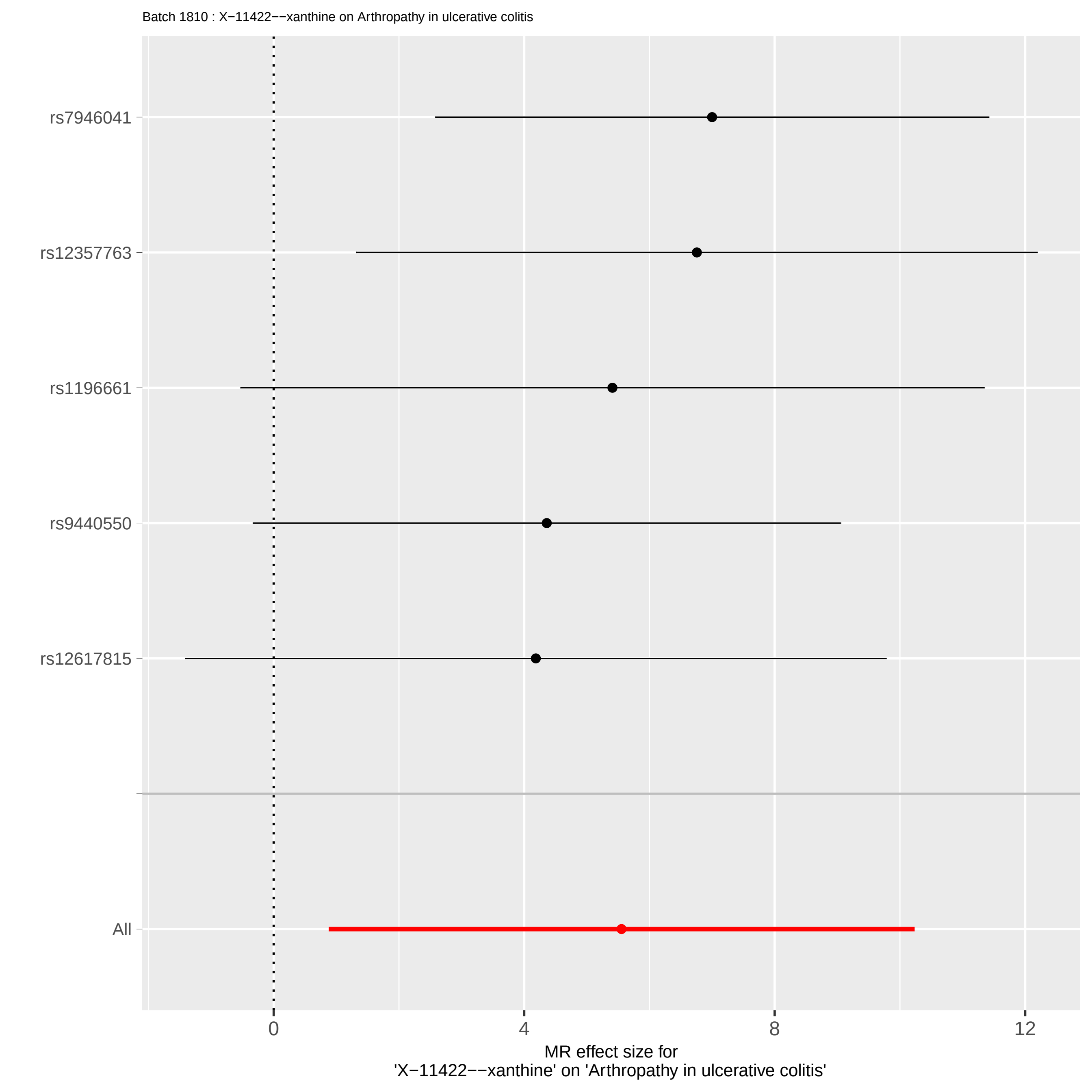

Batch 1810 : X−11422−−xanthine on Arthropathy in ulcerative colitis
rs7946041
rs12357763
rs1196661
rs9440550
rs12617815
All
0
4
8
12
MR effect size for
'X−11422−−xanthine' on 'Arthropathy in ulcerative colitis'

Batch 1814 : X−11593−−O−methylascorbate* on Arthropathy in ulcerative colitis

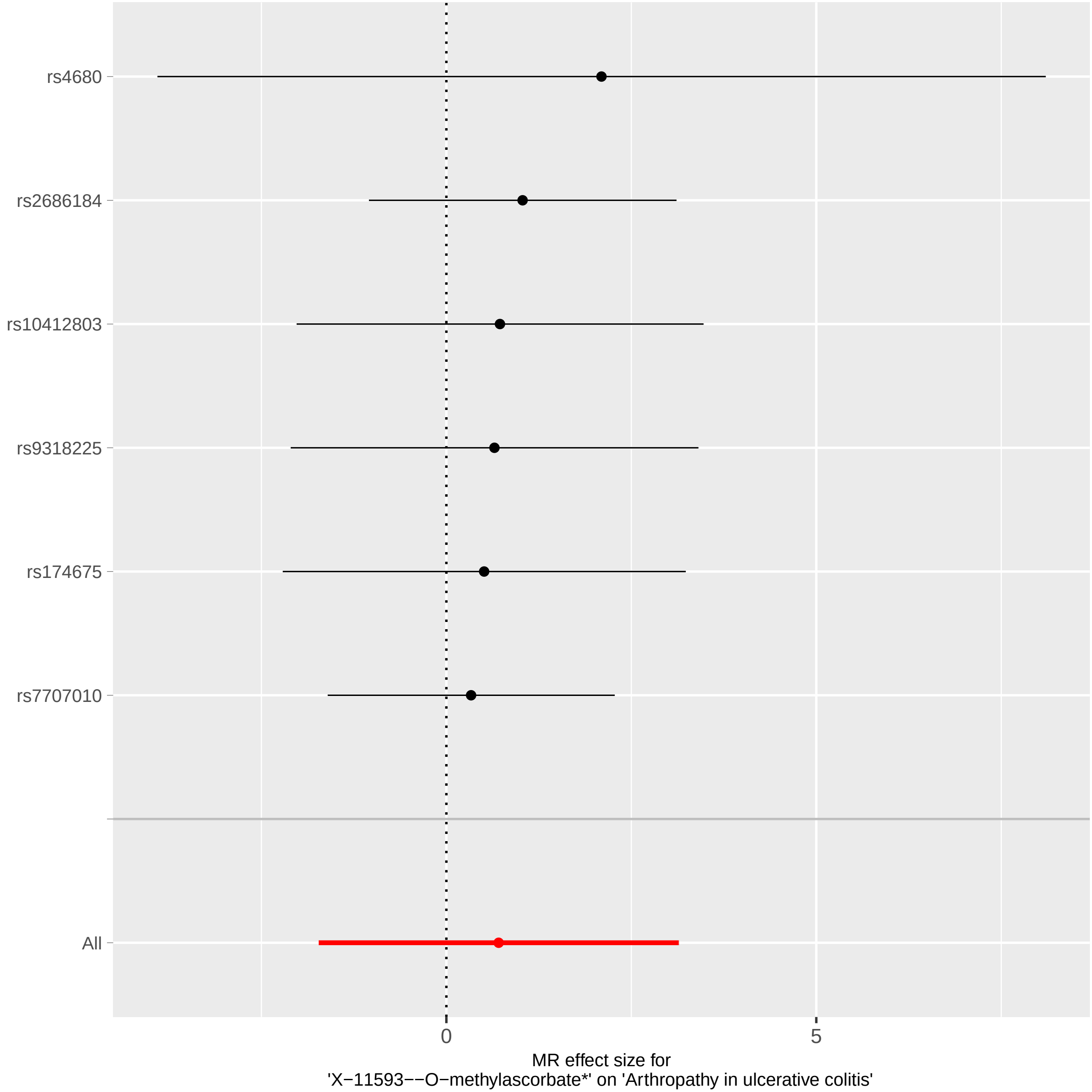

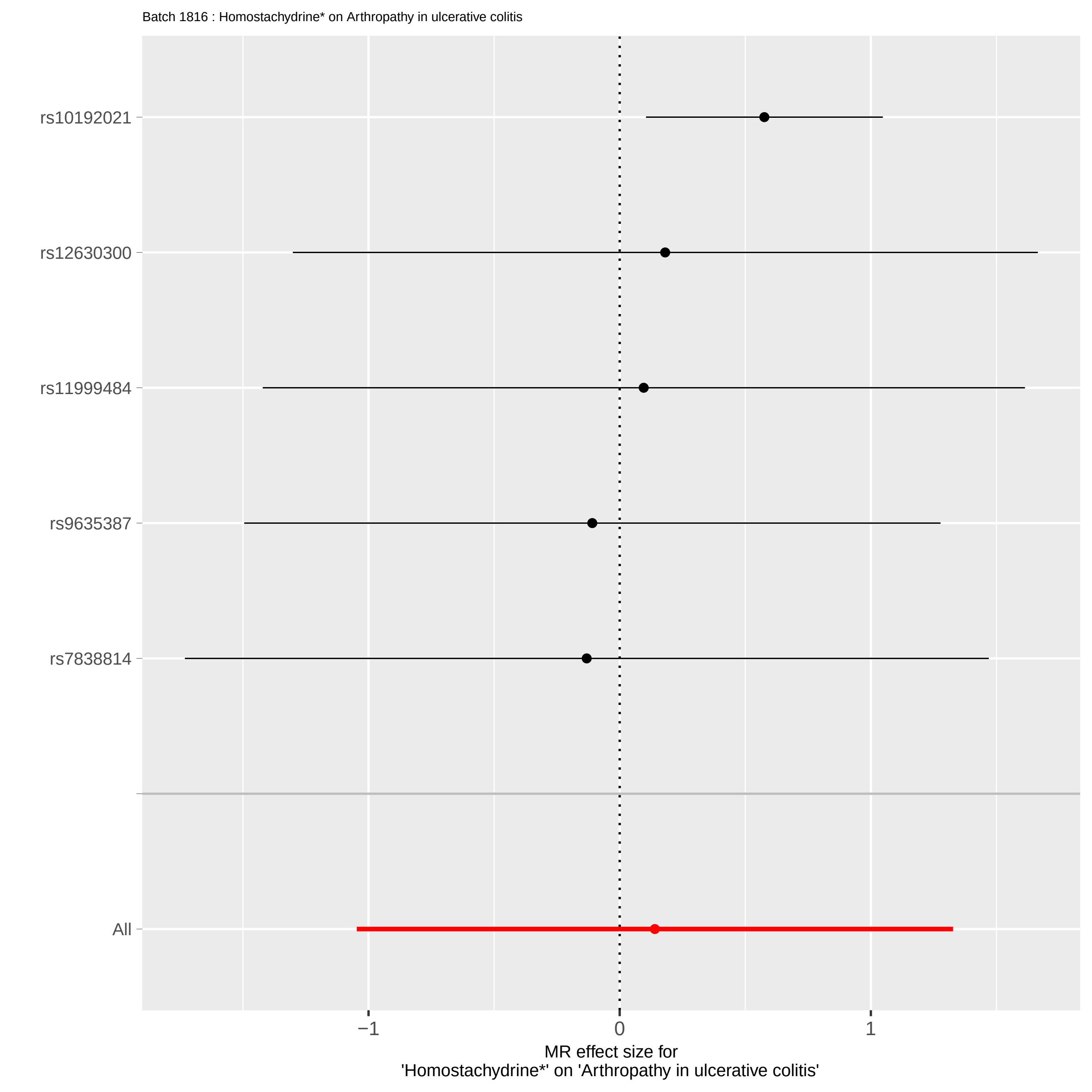
Batch 1816 : Homostachydrine* on Arthropathy in ulcerative colitis
rs10192021
rs12630300
rs11999484
rs9635387
rs7838814
All
−1
0
1
MR effect size for
'Homostachydrine*' on 'Arthropathy in ulcerative colitis'

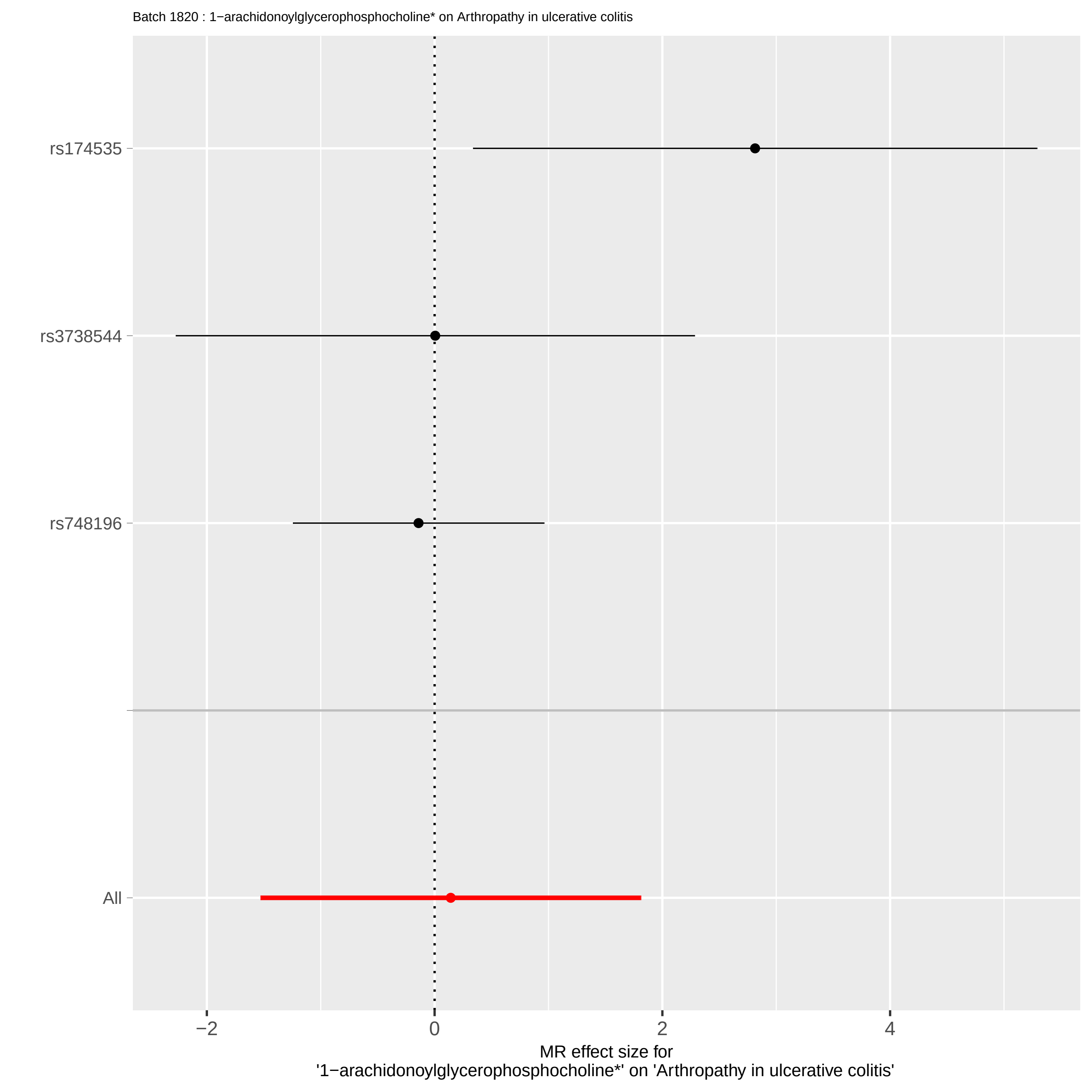

Batch 1820 : 1−arachidonoylglycerophosphocholine* on Arthropathy in ulcerative colitis
rs174535
rs3738544
rs748196
All
−2
0
2
4
MR effect size for
'1−arachidonoylglycerophosphocholine*' on 'Arthropathy in ulcerative colitis'

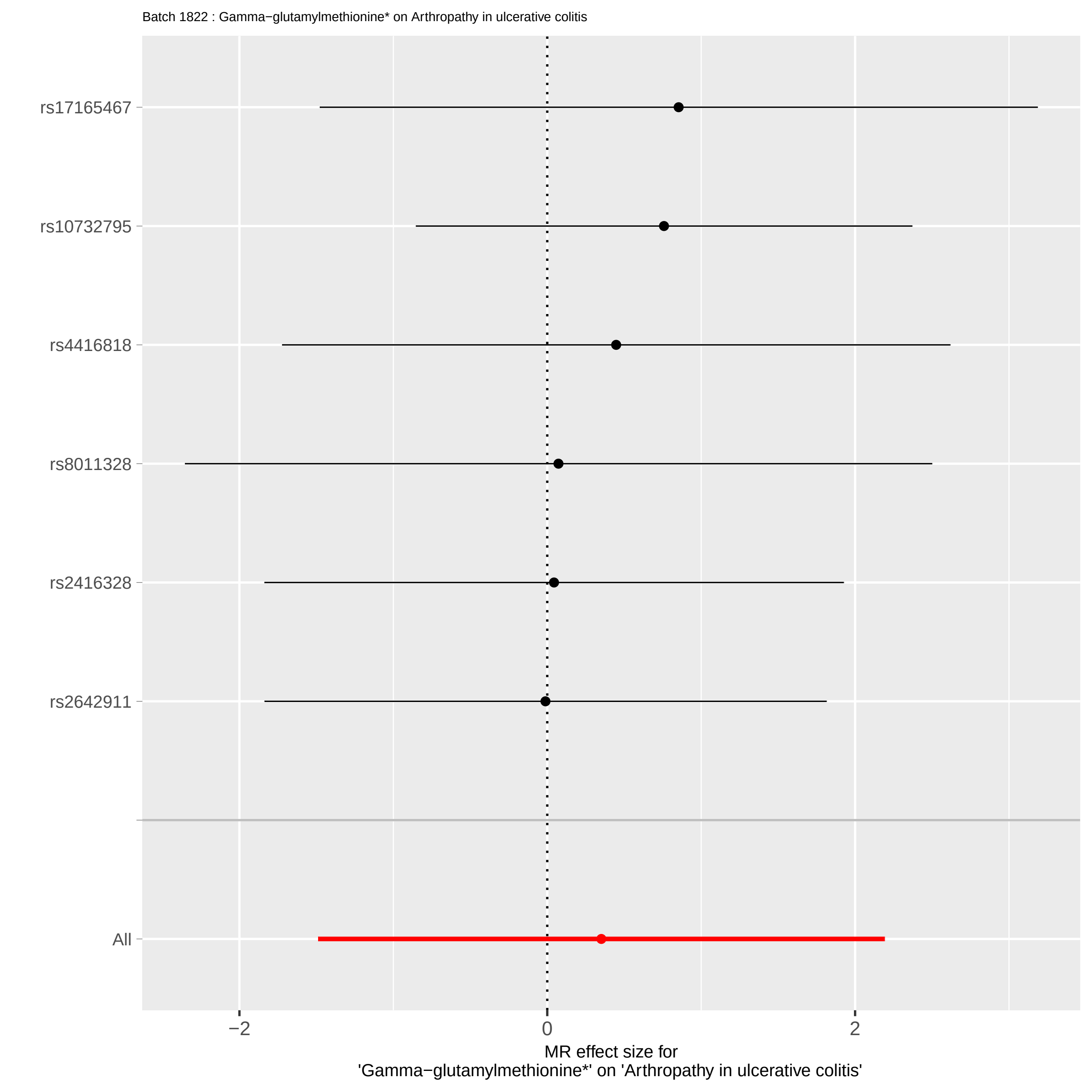
Batch 1822 : Gamma−glutamylmethionine* on Arthropathy in ulcerative colitis
rs17165467
rs10732795
rs4416818
rs8011328
rs2416328
rs2642911
All
−2
0
2
MR effect size for
'Gamma−glutamylmethionine*' on 'Arthropathy in ulcerative colitis'

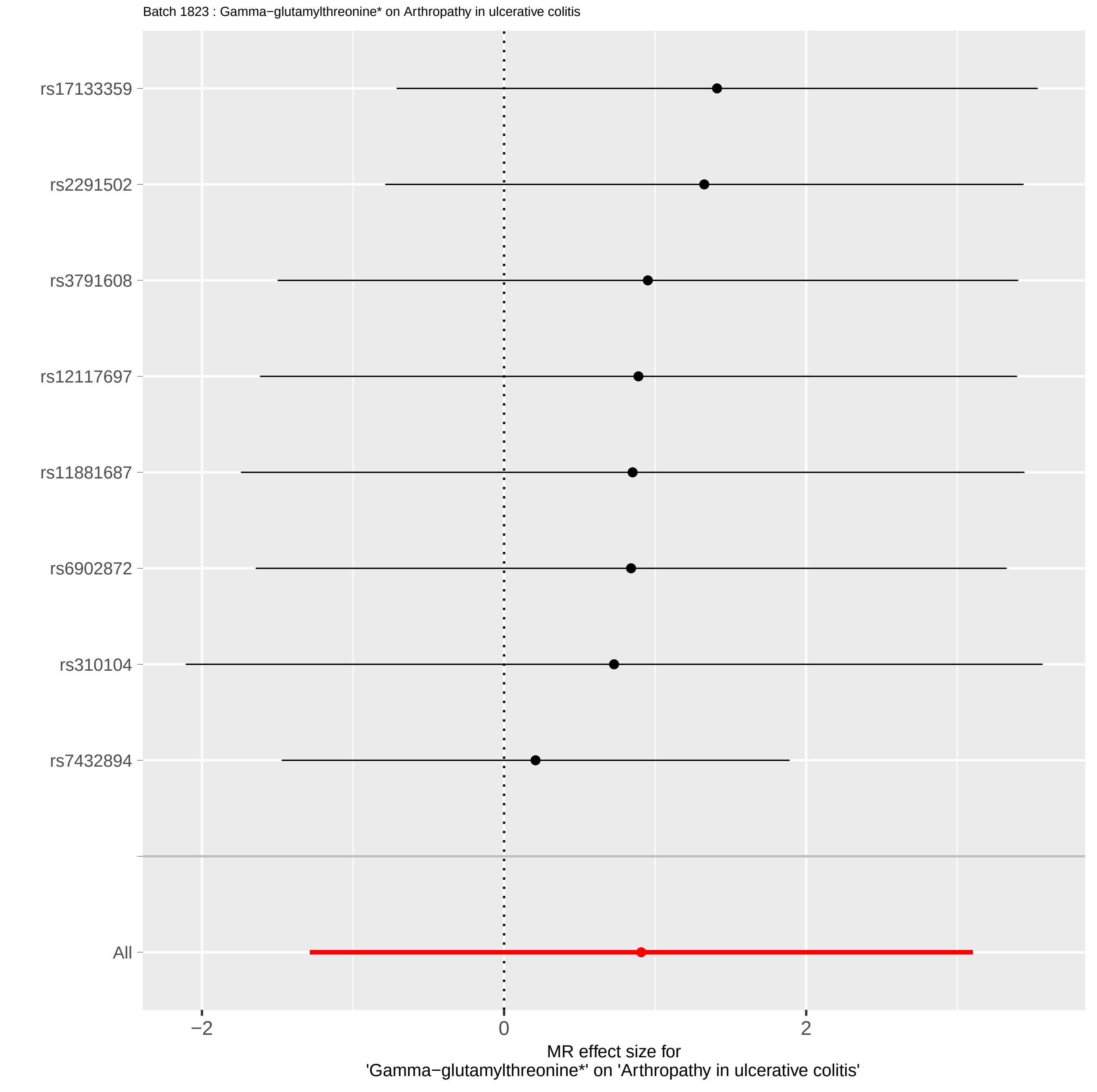

Batch 1823 : Gamma−glutamylthreonine* on Arthropathy in ulcerative colitis
rs17133359
rs2291502
rs3791608
rs12117697
rs11881687
rs6902872
rs310104
rs7432894
All
−2
0
2
MR effect size for
'Gamma−glutamylthreonine*' on 'Arthropathy in ulcerative colitis'

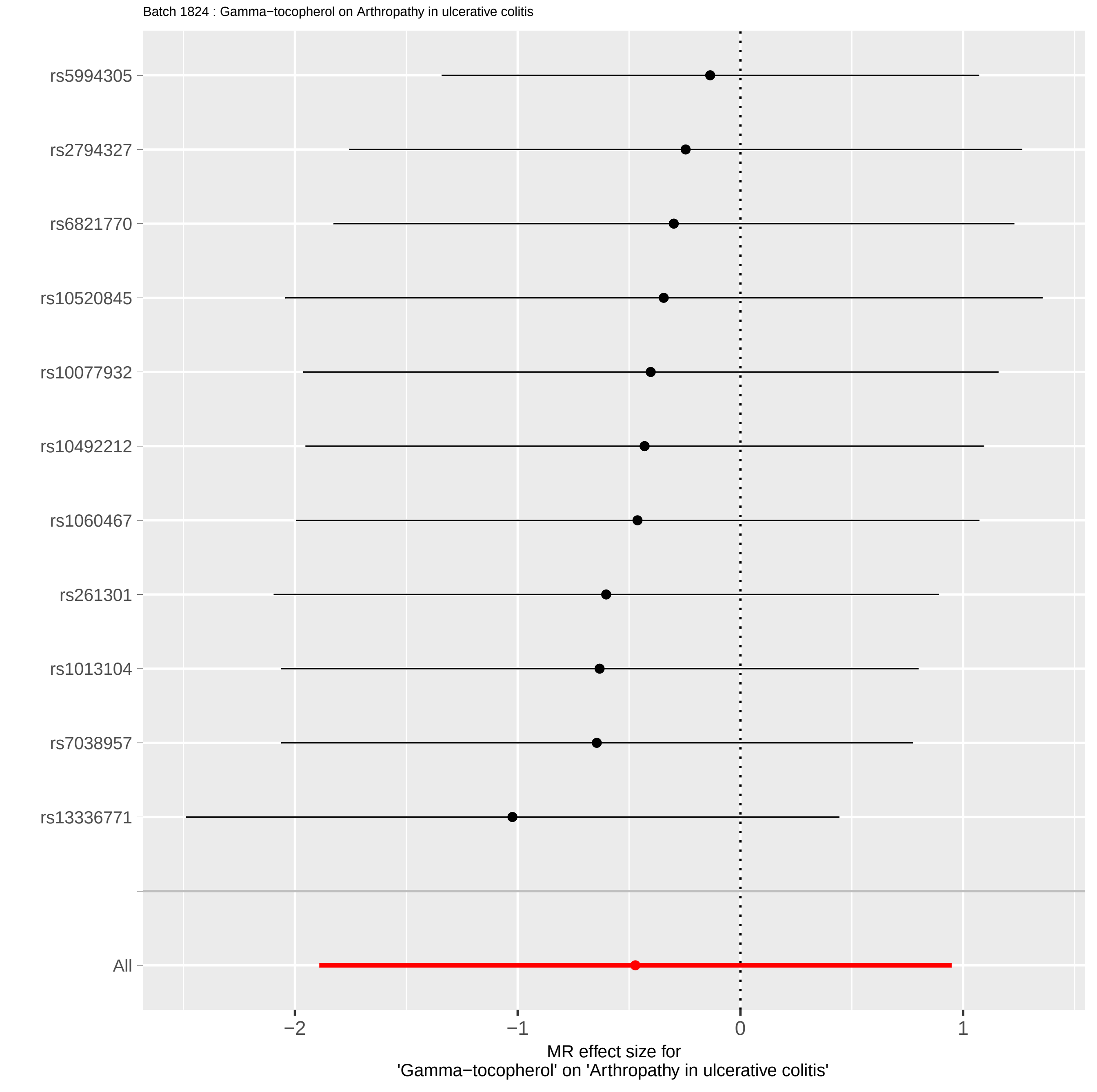

Batch 1824 : Gamma−tocopherol on Arthropathy in ulcerative colitis
rs5994305
rs2794327
rs6821770
rs10520845
rs10077932
rs10492212
rs1060467
rs261301
rs1013104
rs7038957
rs13336771
All
−2
−1
0
1
MR effect size for
'Gamma−tocopherol' on 'Arthropathy in ulcerative colitis'

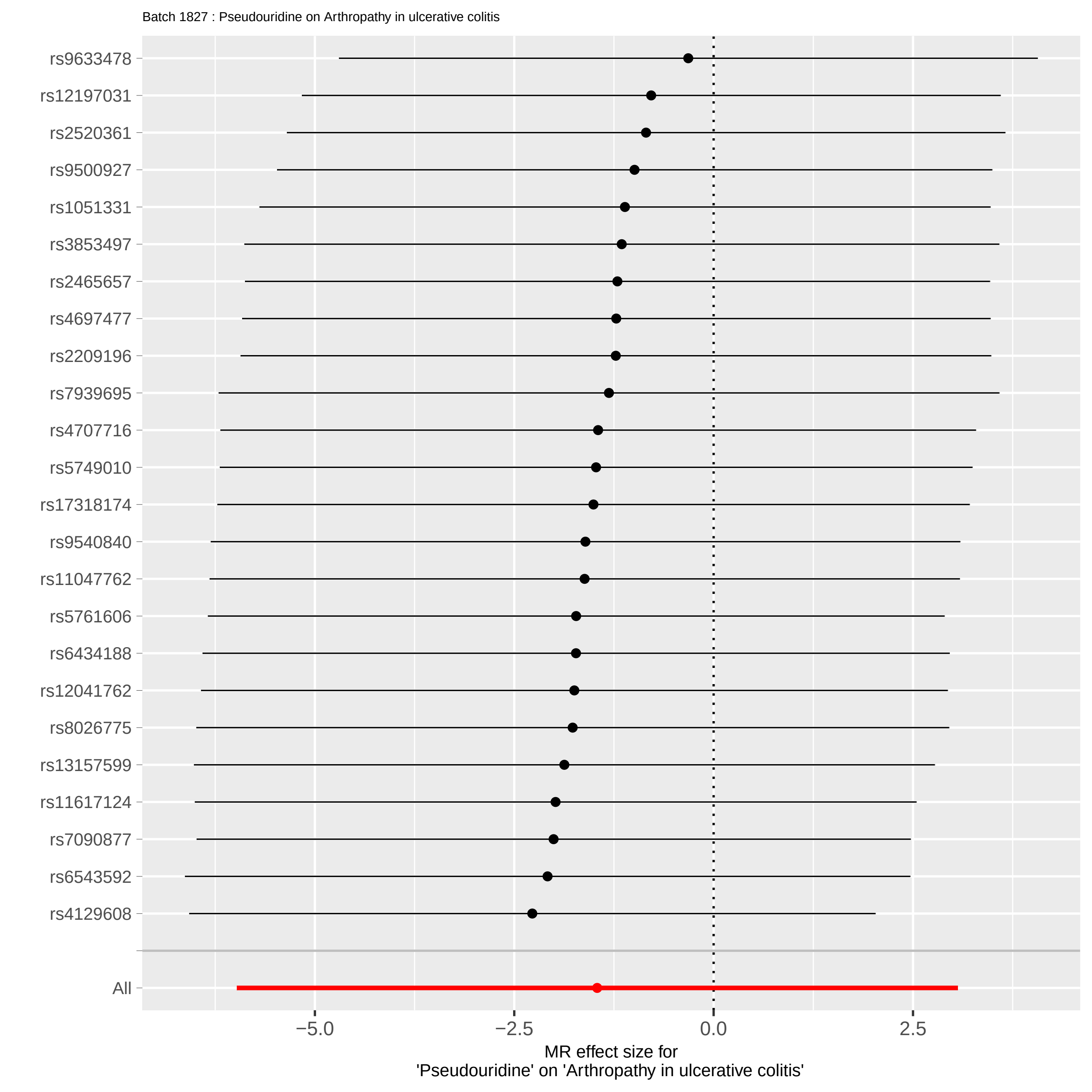

Batch 1827 : Pseudouridine on Arthropathy in ulcerative colitis
rs9633478
rs12197031
rs2520361
rs9500927
rs1051331
rs3853497
rs2465657
rs4697477
rs2209196
rs7939695
rs4707716
rs5749010
rs17318174
rs9540840
rs11047762
rs5761606
rs6434188
rs12041762
rs8026775
rs13157599
rs11617124
rs7090877
rs6543592
rs4129608
All
−5.0
−2.5
0.0
2.5
MR effect size for
'Pseudouridine' on 'Arthropathy in ulcerative colitis'

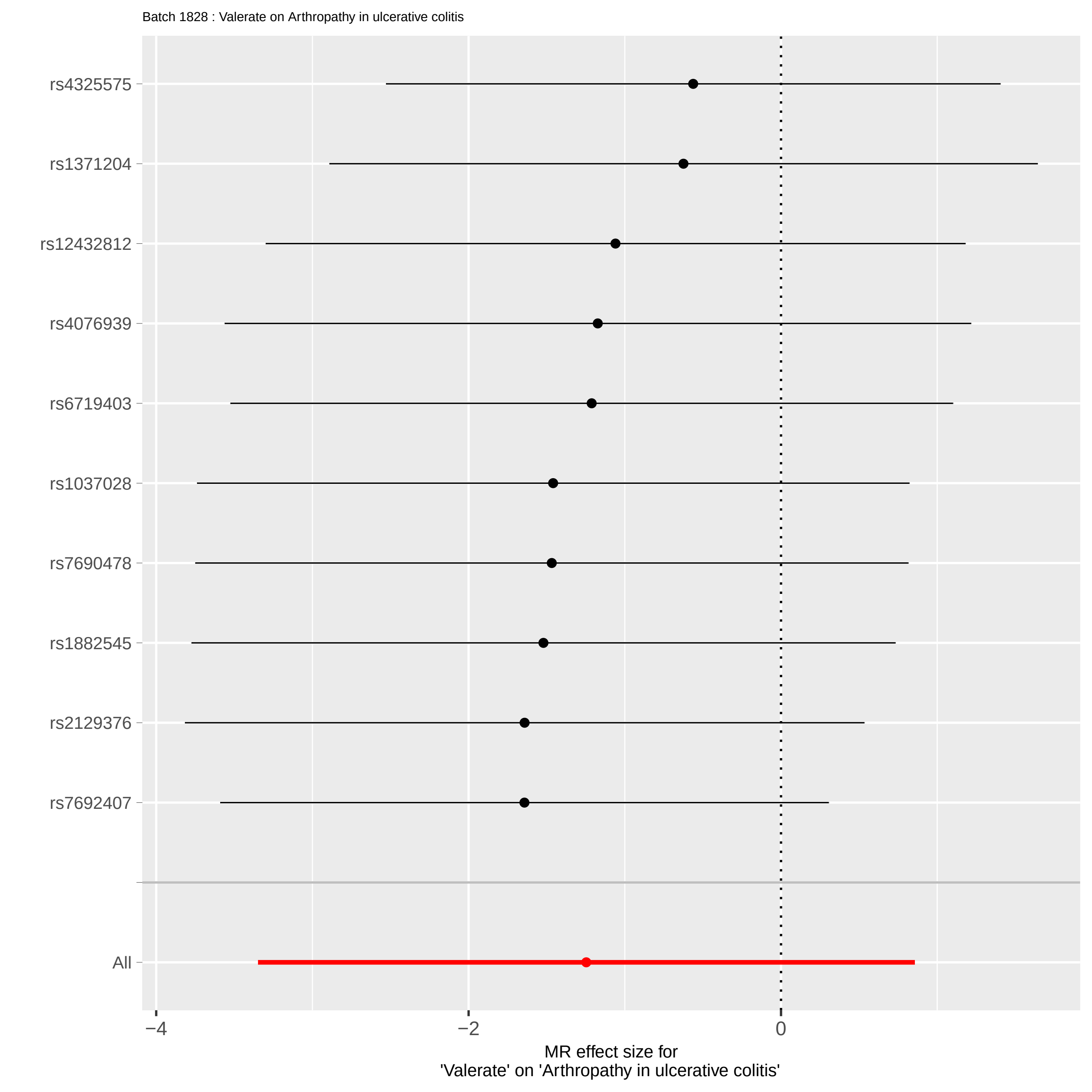

Batch 1828 : Valerate on Arthropathy in ulcerative colitis
rs4325575
rs1371204
rs12432812
rs4076939
rs6719403
rs1037028
rs7690478
rs1882545
rs2129376
rs7692407
All
−4
−2
0
MR effect size for
'Valerate' on 'Arthropathy in ulcerative colitis'

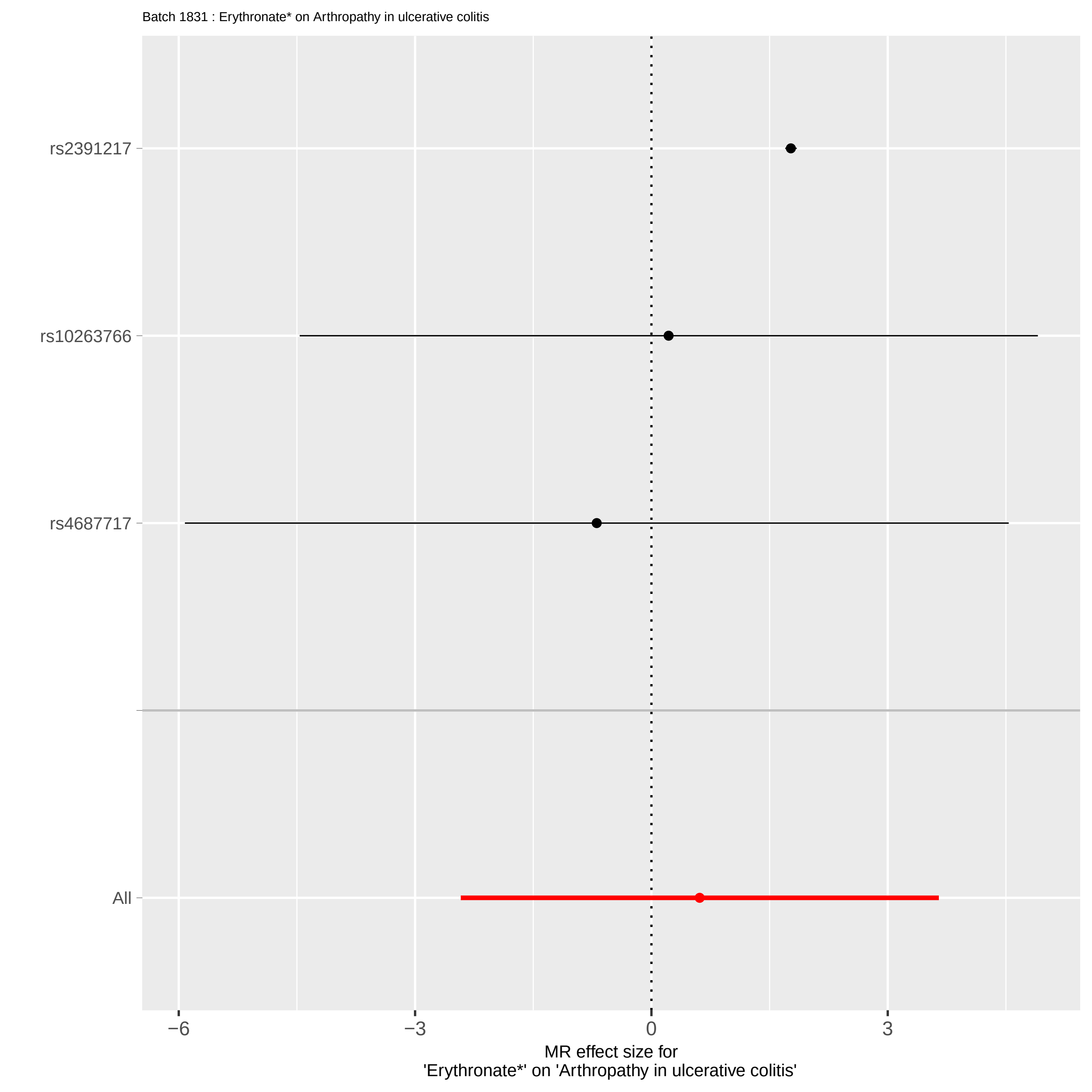

Batch 1831 : Erythronate* on Arthropathy in ulcerative colitis
rs2391217
rs10263766
rs4687717
All
−6
−3
0
3
MR effect size for
'Erythronate*' on 'Arthropathy in ulcerative colitis'

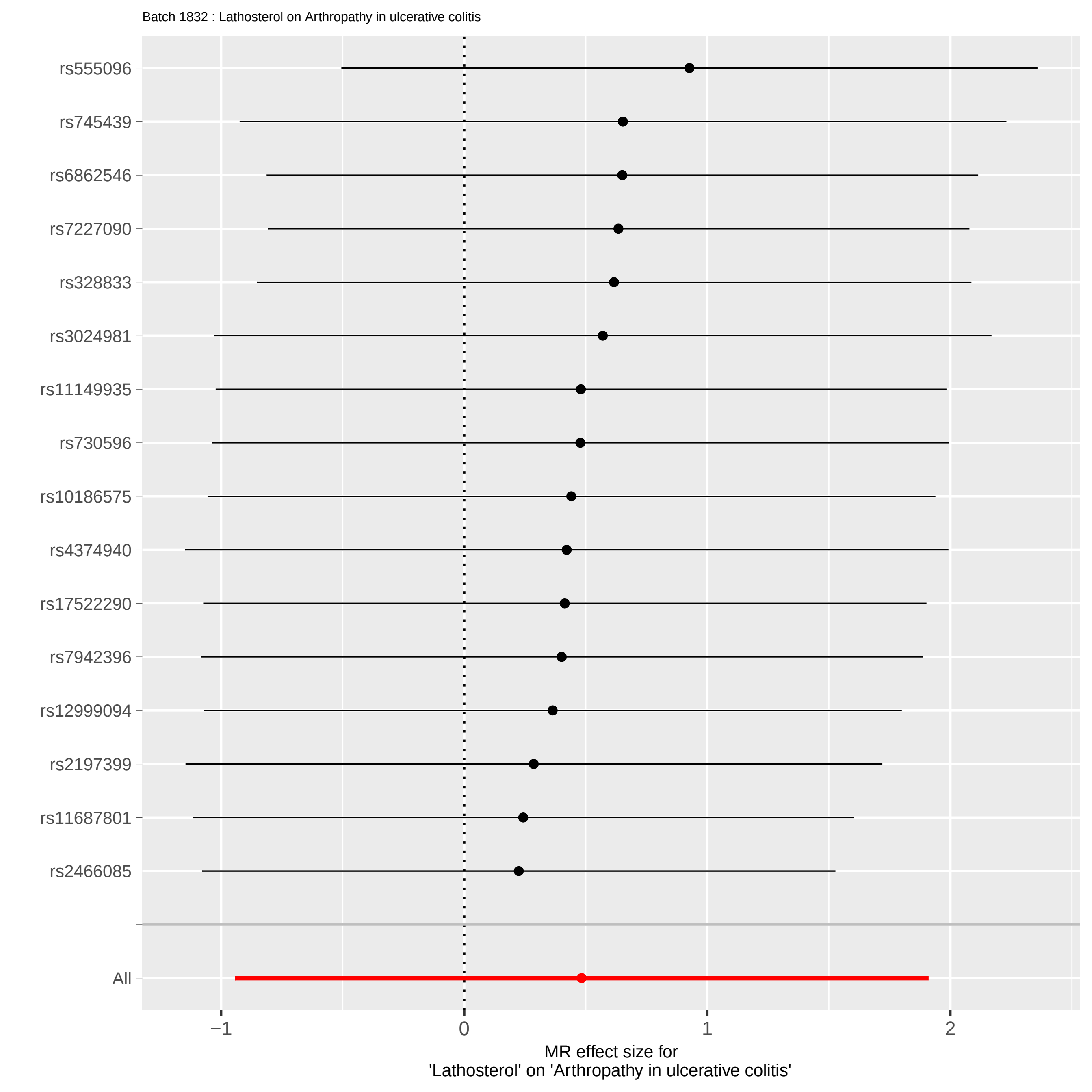

Batch 1832 : Lathosterol on Arthropathy in ulcerative colitis
rs555096
rs745439
rs6862546
rs7227090
rs328833
rs3024981
rs11149935
rs730596
rs10186575
rs4374940
rs17522290
rs7942396
rs12999094
rs2197399
rs11687801
rs2466085
All
−1
0
1
2
MR effect size for
'Lathosterol' on 'Arthropathy in ulcerative colitis'

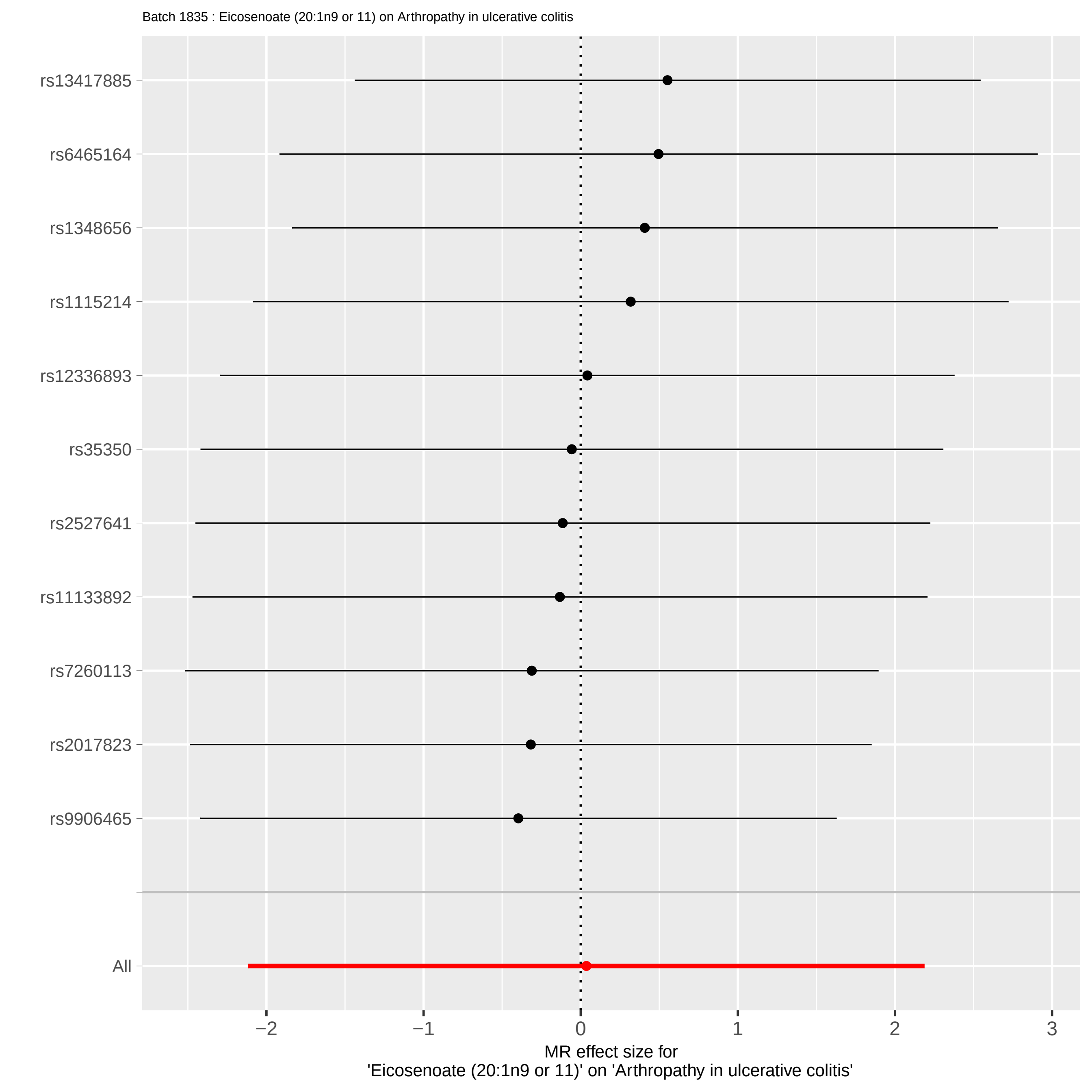
Batch 1835 : Eicosenoate (20:1n9 or 11) on Arthropathy in ulcerative colitis
rs13417885
rs6465164
rs1348656
rs1115214
rs12336893
rs35350
rs2527641
rs11133892
rs7260113
rs2017823
rs9906465
All
−2
−1
0
1
2
3
MR effect size for
'Eicosenoate (20:1n9 or 11)' on 'Arthropathy in ulcerative colitis'

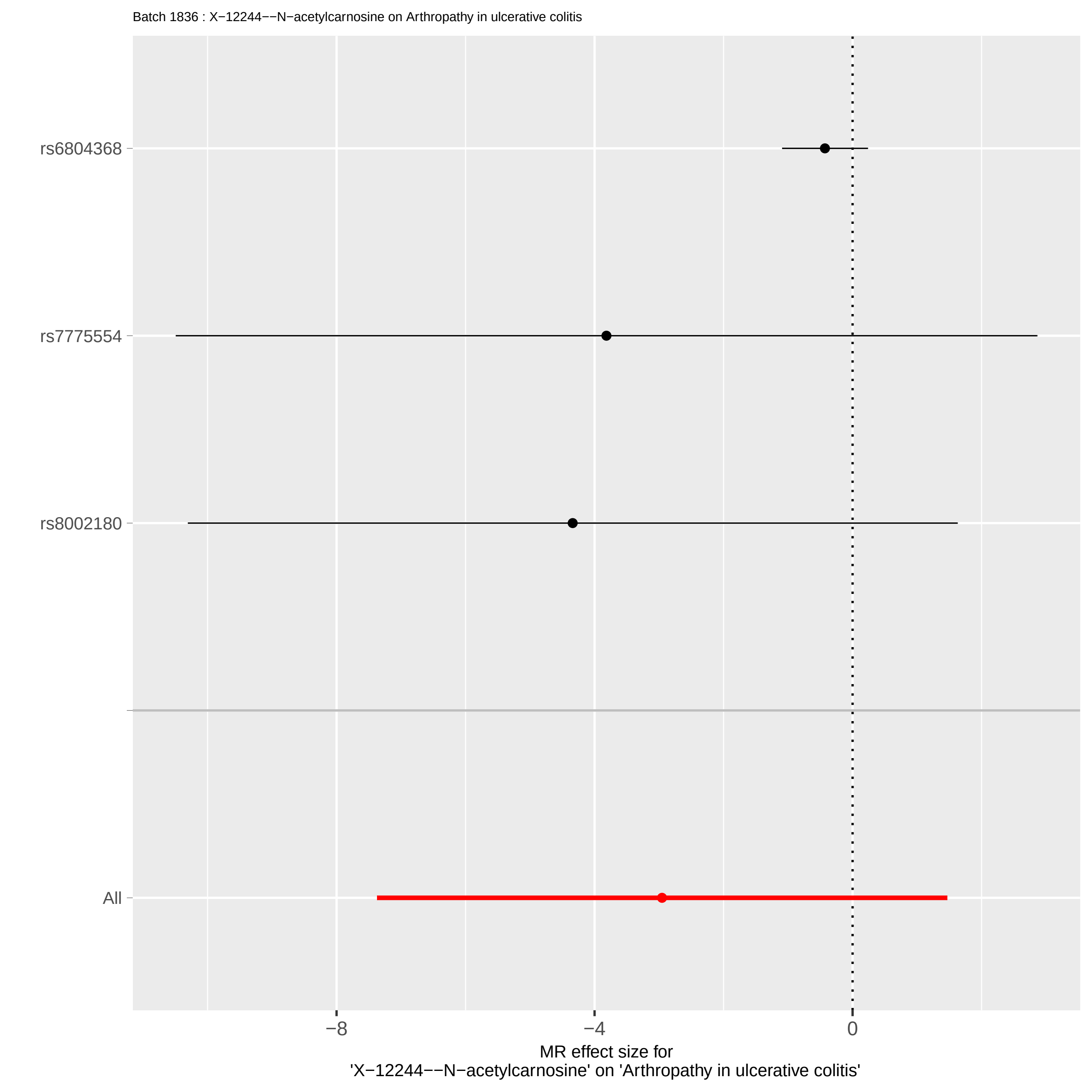

Batch 1836 : X−12244−−N−acetylcarnosine on Arthropathy in ulcerative colitis
rs6804368
rs7775554
rs8002180
All
−8
−4
0
MR effect size for
'X−12244−−N−acetylcarnosine' on 'Arthropathy in ulcerative colitis'

Batch 1839 : 1−docosahexaenoylglycerophosphocholine* on Arthropathy in ulcerative colitis

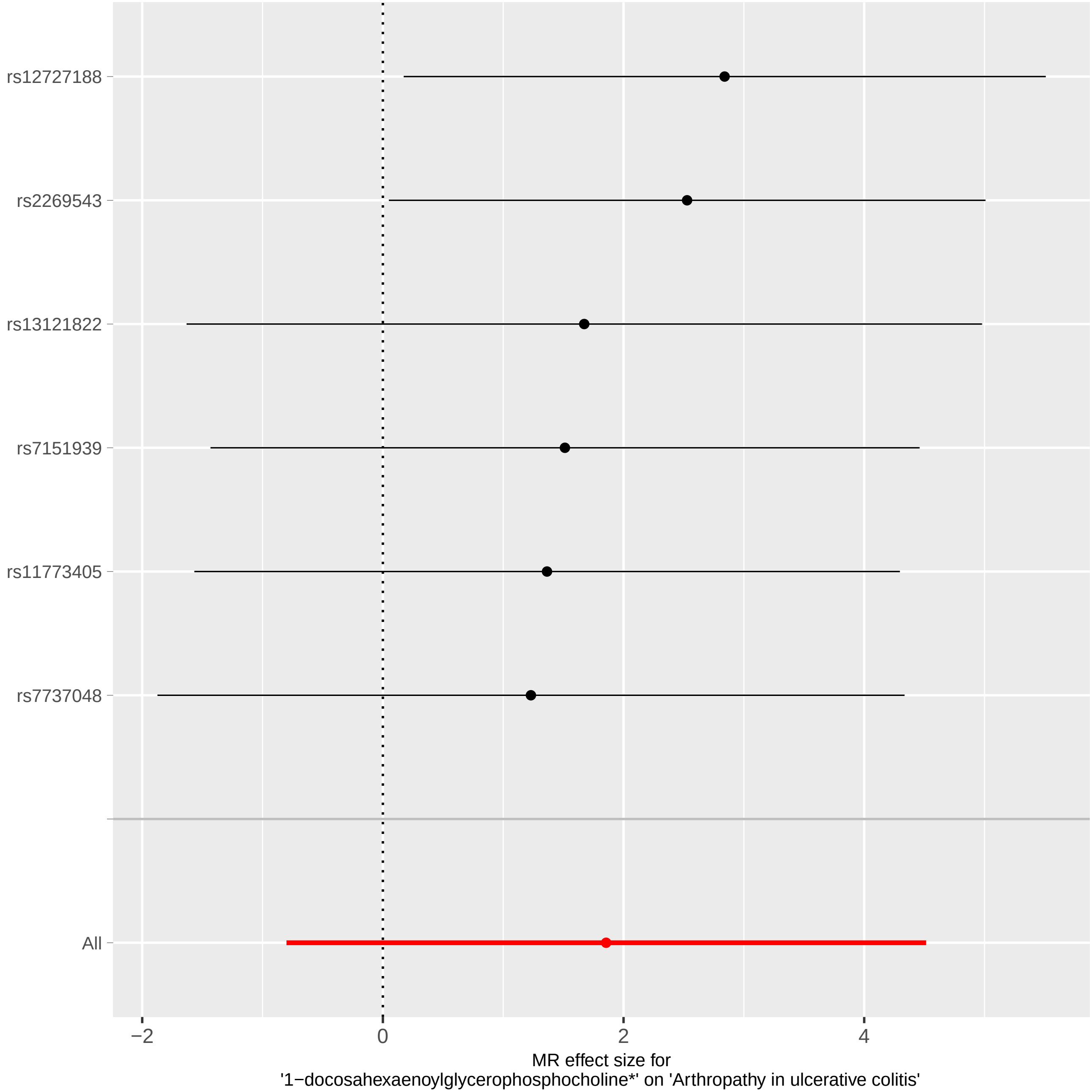

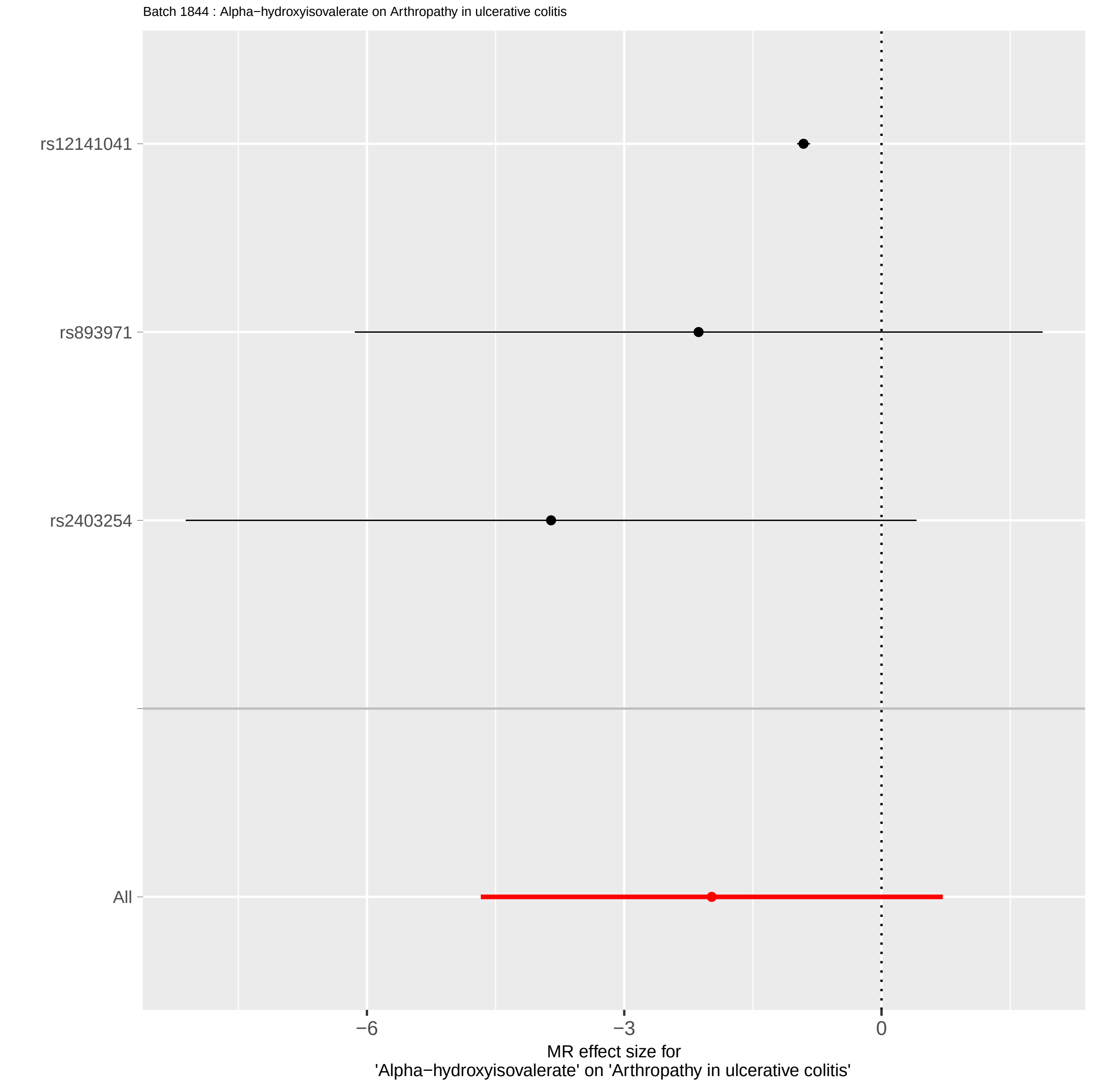
Batch 1844 : Alpha−hydroxyisovalerate on Arthropathy in ulcerative colitis
rs12141041
rs893971
rs2403254
All
−6
−3
0
MR effect size for
'Alpha−hydroxyisovalerate' on 'Arthropathy in ulcerative colitis'

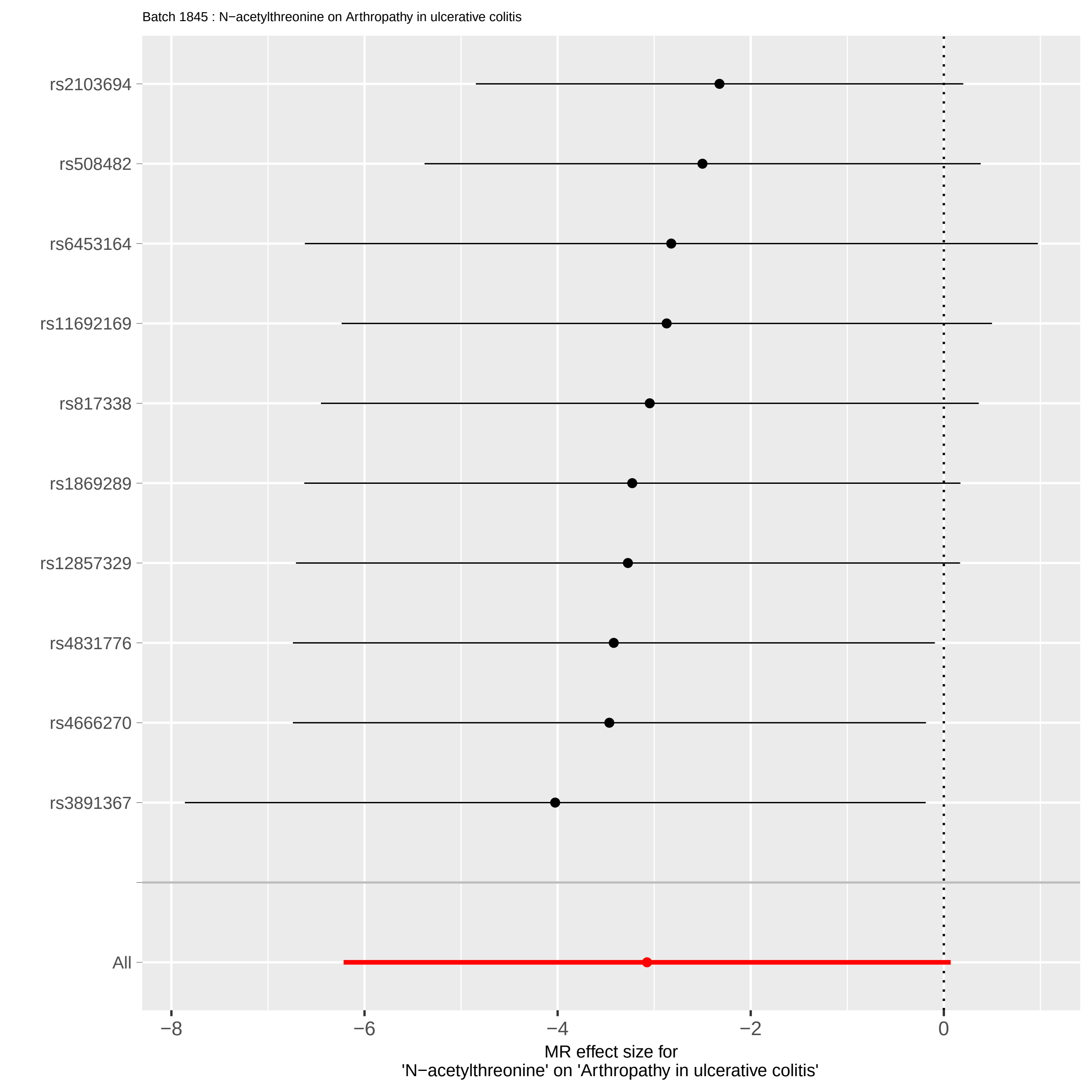

Batch 1845 : N−acetylthreonine on Arthropathy in ulcerative colitis
rs2103694
rs508482
rs6453164
rs11692169
rs817338
rs1869289
rs12857329
rs4831776
rs4666270
rs3891367
All
−8
−6
−4
−2
0
MR effect size for
'N−acetylthreonine' on 'Arthropathy in ulcerative colitis'

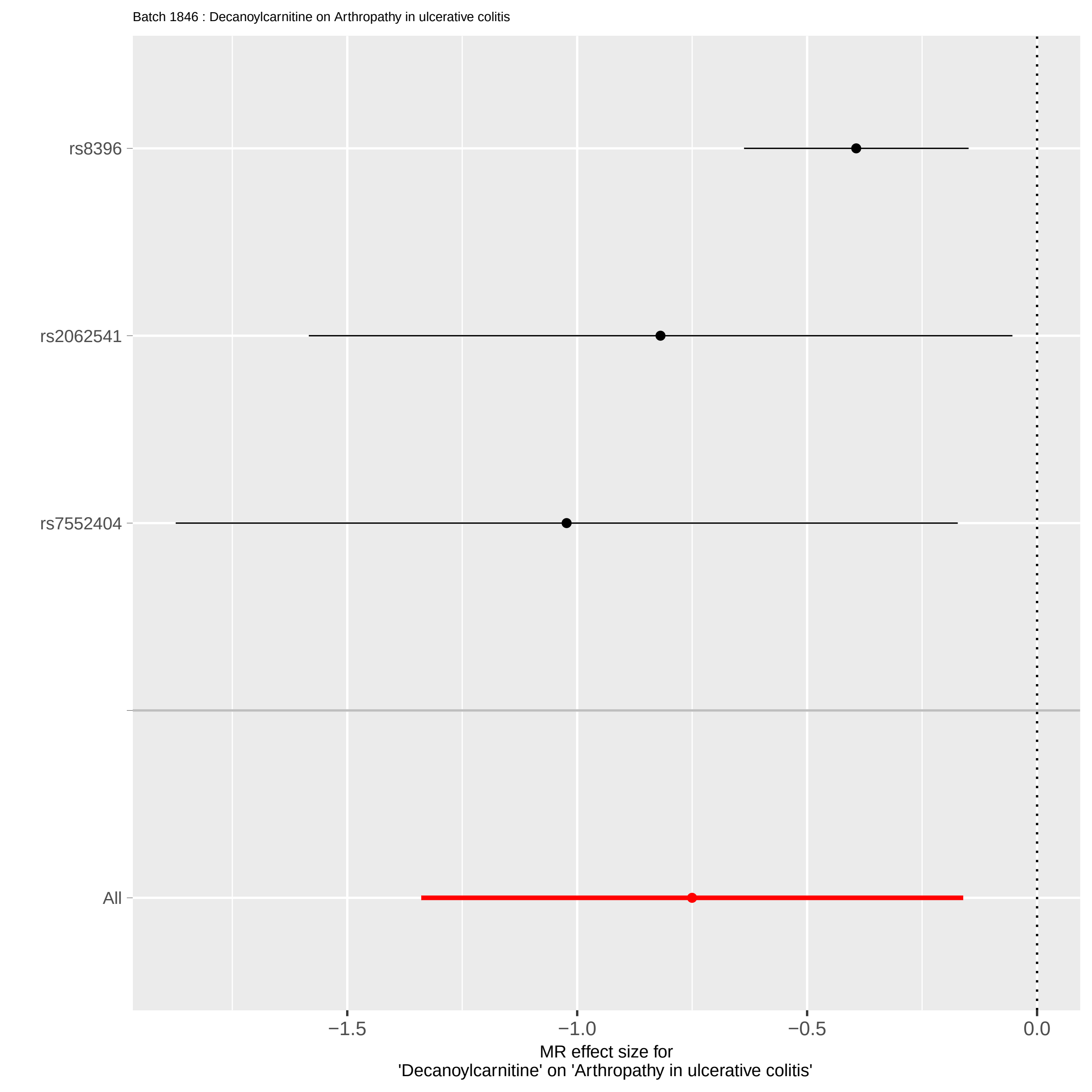
Batch 1846 : Decanoylcarnitine on Arthropathy in ulcerative colitis
rs8396
rs2062541
rs7552404
All
−1.5
−1.0
−0.5
0.0
MR effect size for
'Decanoylcarnitine' on 'Arthropathy in ulcerative colitis'

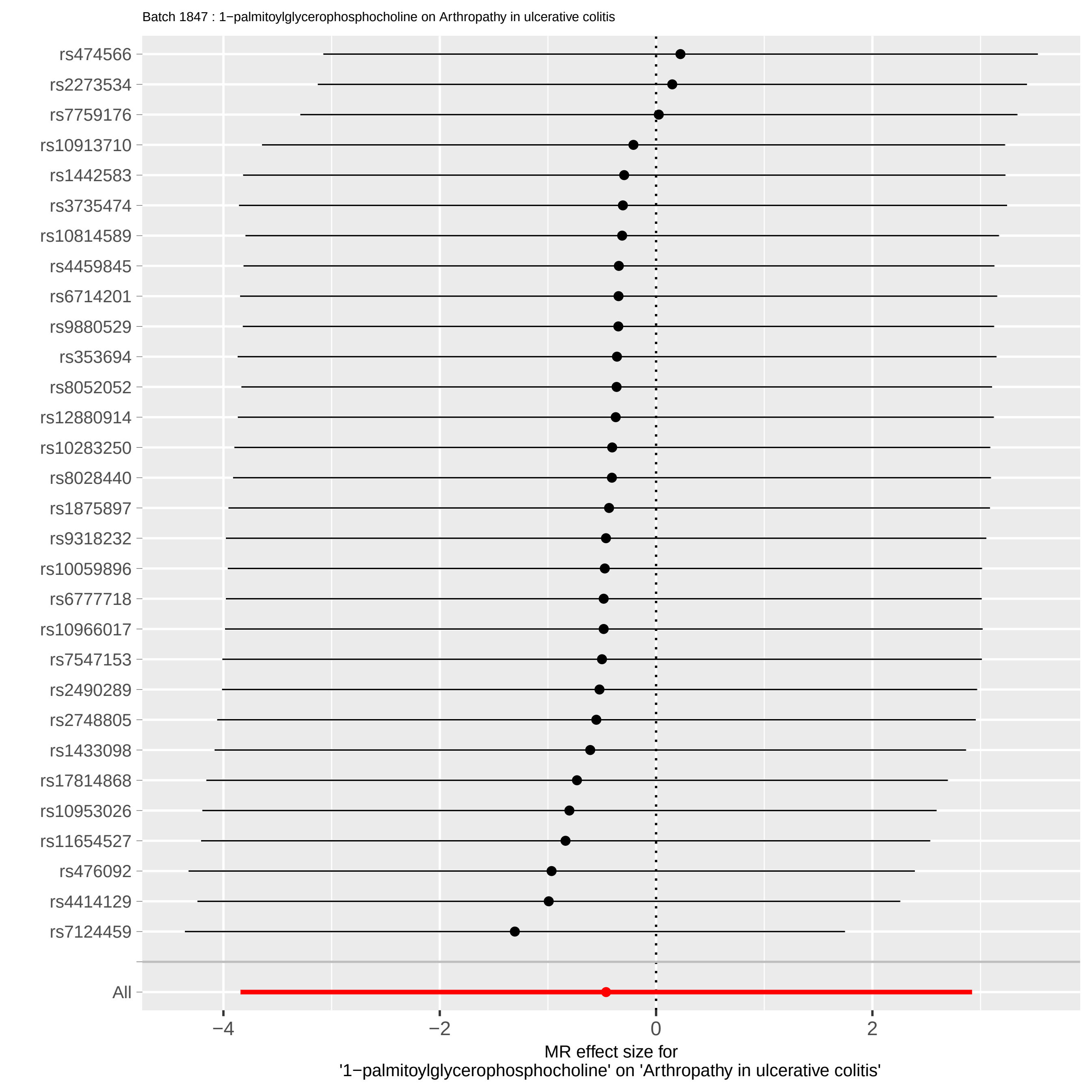

Batch 1847 : 1−palmitoylglycerophosphocholine on Arthropathy in ulcerative colitis
rs474566
rs2273534
rs7759176
rs10913710
rs1442583
rs3735474
rs10814589
rs4459845
rs6714201
rs9880529
rs353694
rs8052052
rs12880914
rs10283250
rs8028440
rs1875897
rs9318232
rs10059896
rs6777718
rs10966017
rs7547153
rs2490289
rs2748805
rs1433098
rs17814868
rs10953026
rs11654527
rs476092
rs4414129
rs7124459
All
−4
−2
0
2
MR effect size for
'1−palmitoylglycerophosphocholine' on 'Arthropathy in ulcerative colitis'

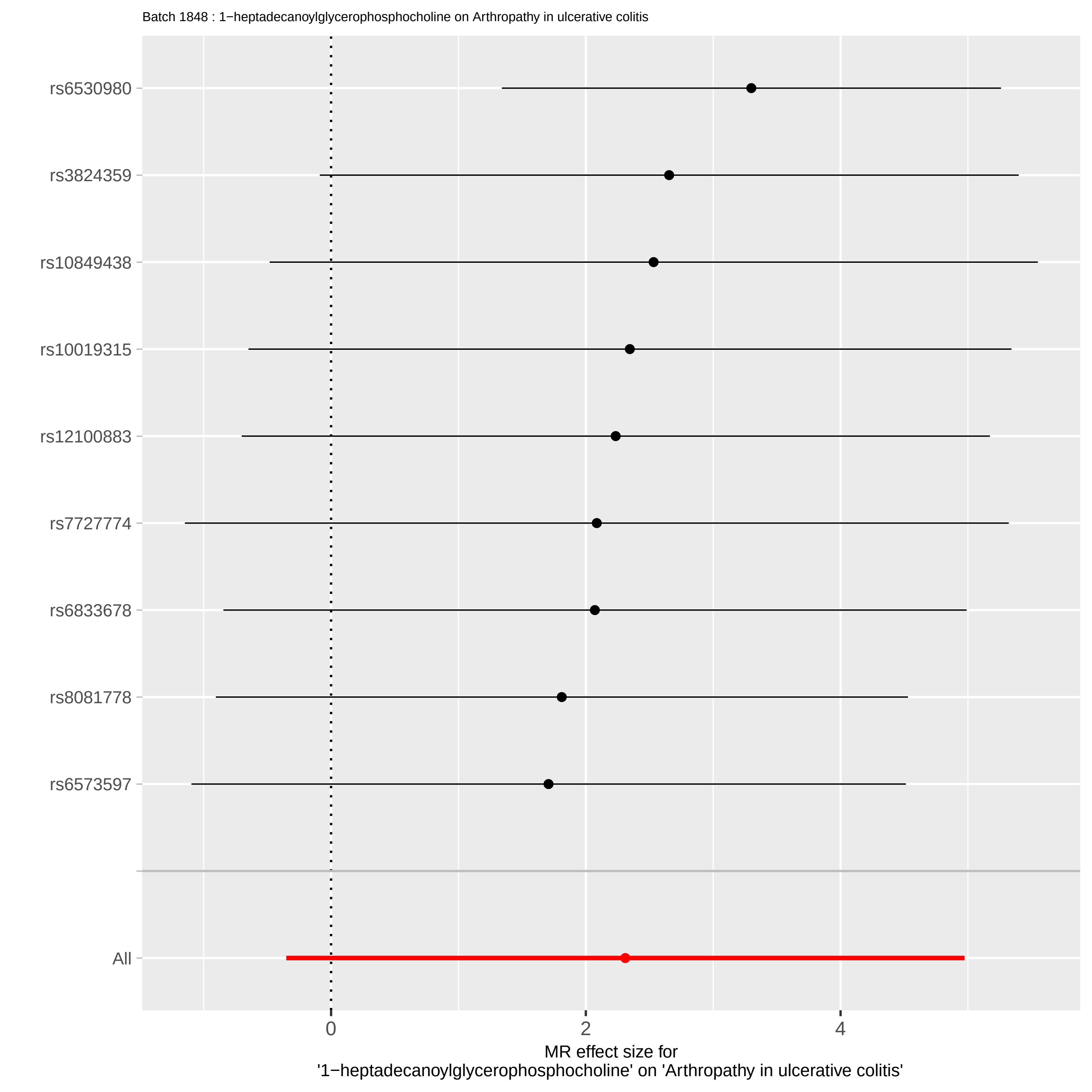

Batch 1848 : 1−heptadecanoylglycerophosphocholine on Arthropathy in ulcerative colitis
rs6530980
rs3824359
rs10849438
rs10019315
rs12100883
rs7727774
rs6833678
rs8081778
rs6573597
All
0
2
4
MR effect size for
'1−heptadecanoylglycerophosphocholine' on 'Arthropathy in ulcerative colitis'

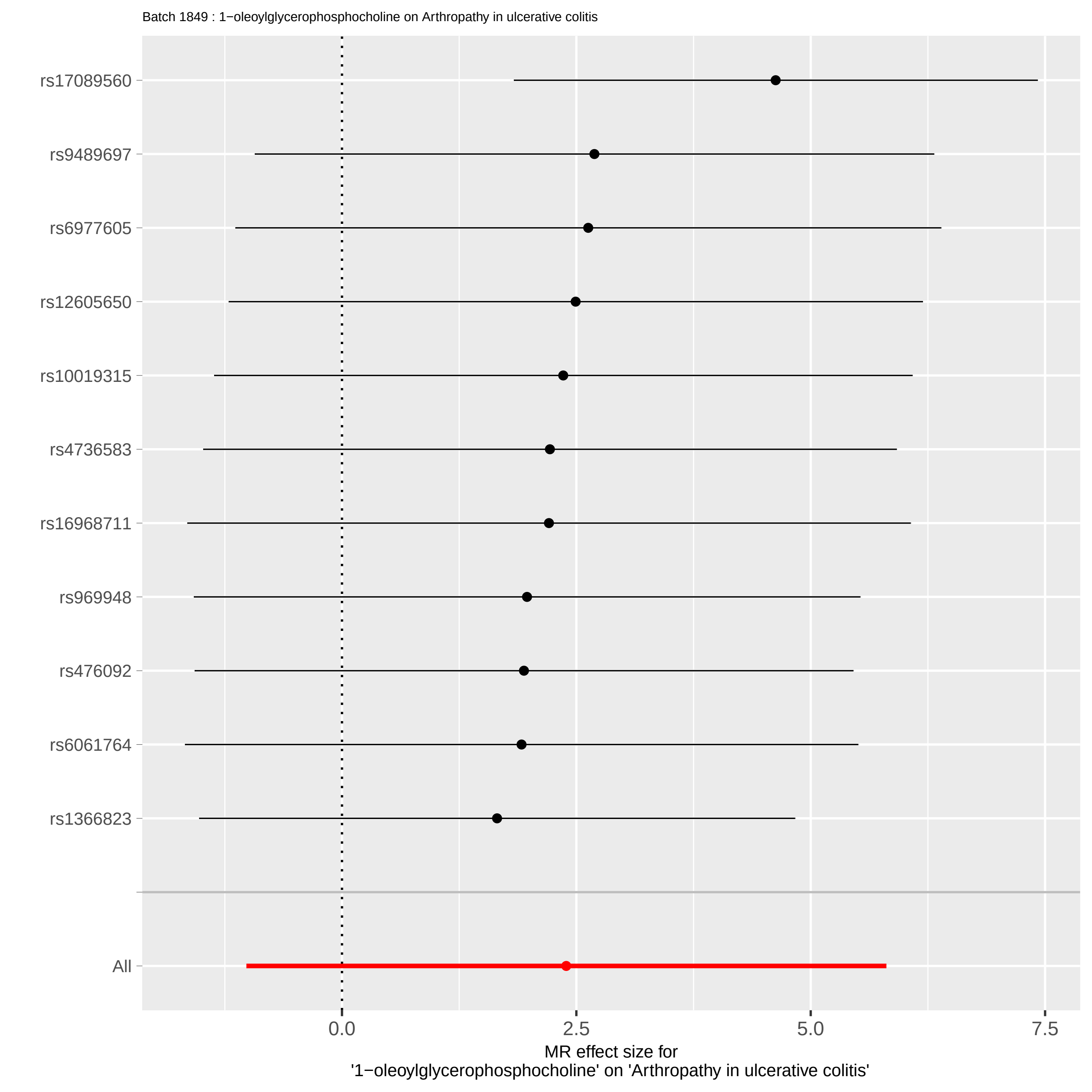

Batch 1849 : 1−oleoylglycerophosphocholine on Arthropathy in ulcerative colitis
rs17089560
rs9489697
rs6977605
rs12605650
rs10019315
rs4736583
rs16968711
rs969948
rs476092
rs6061764
rs1366823
All
0.0
2.5
5.0
7.5
MR effect size for
'1−oleoylglycerophosphocholine' on 'Arthropathy in ulcerative colitis'

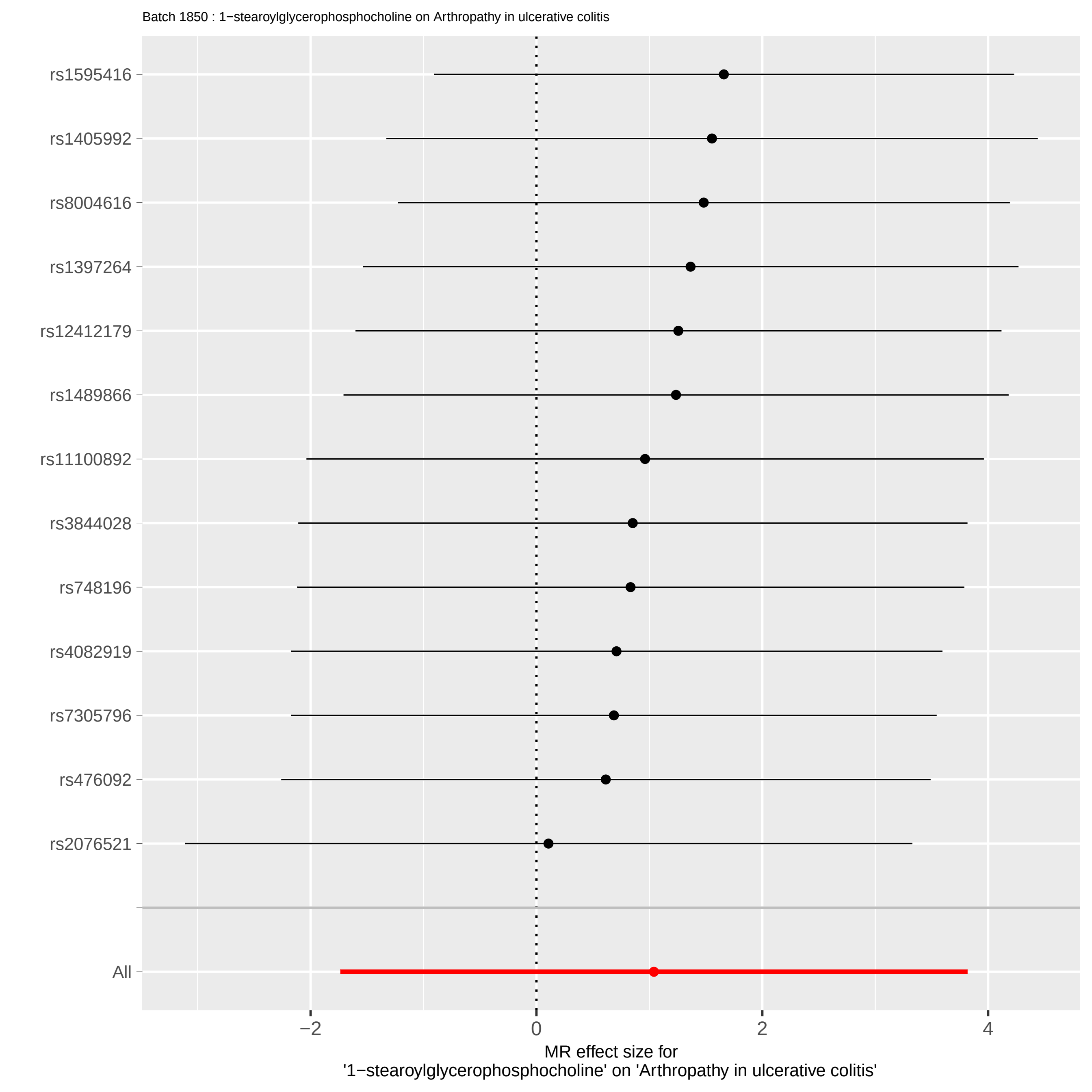

Batch 1850 : 1−stearoylglycerophosphocholine on Arthropathy in ulcerative colitis
rs1595416
rs1405992
rs8004616
rs1397264
rs12412179
rs1489866
rs11100892
rs3844028
rs748196
rs4082919
rs7305796
rs476092
rs2076521
All
−2
0
2
4
MR effect size for
'1−stearoylglycerophosphocholine' on 'Arthropathy in ulcerative colitis'

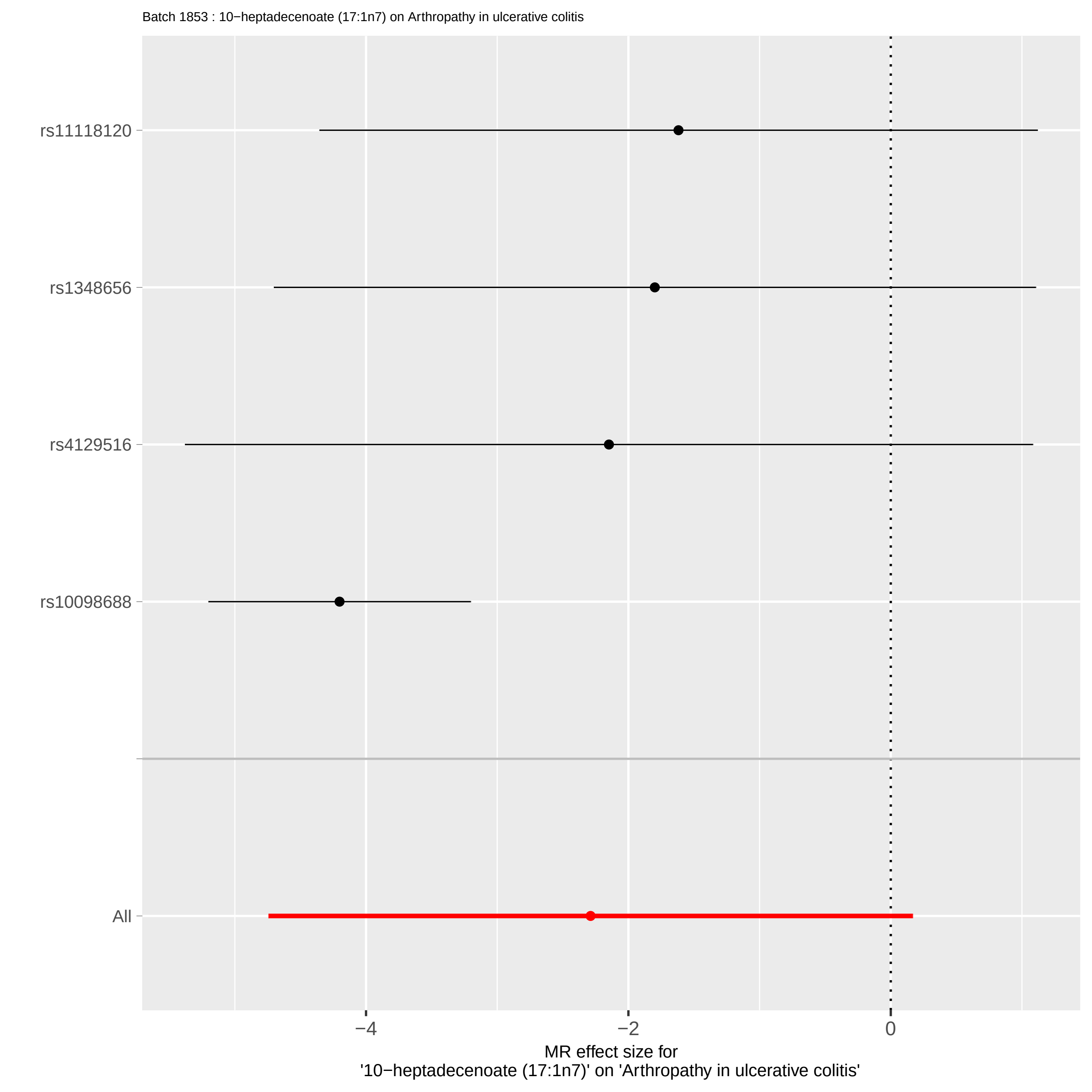

Batch 1853 : 10−heptadecenoate (17:1n7) on Arthropathy in ulcerative colitis
rs11118120
rs1348656
rs4129516
rs10098688
All
−4
−2
0
MR effect size for
'10−heptadecenoate (17:1n7)' on 'Arthropathy in ulcerative colitis'

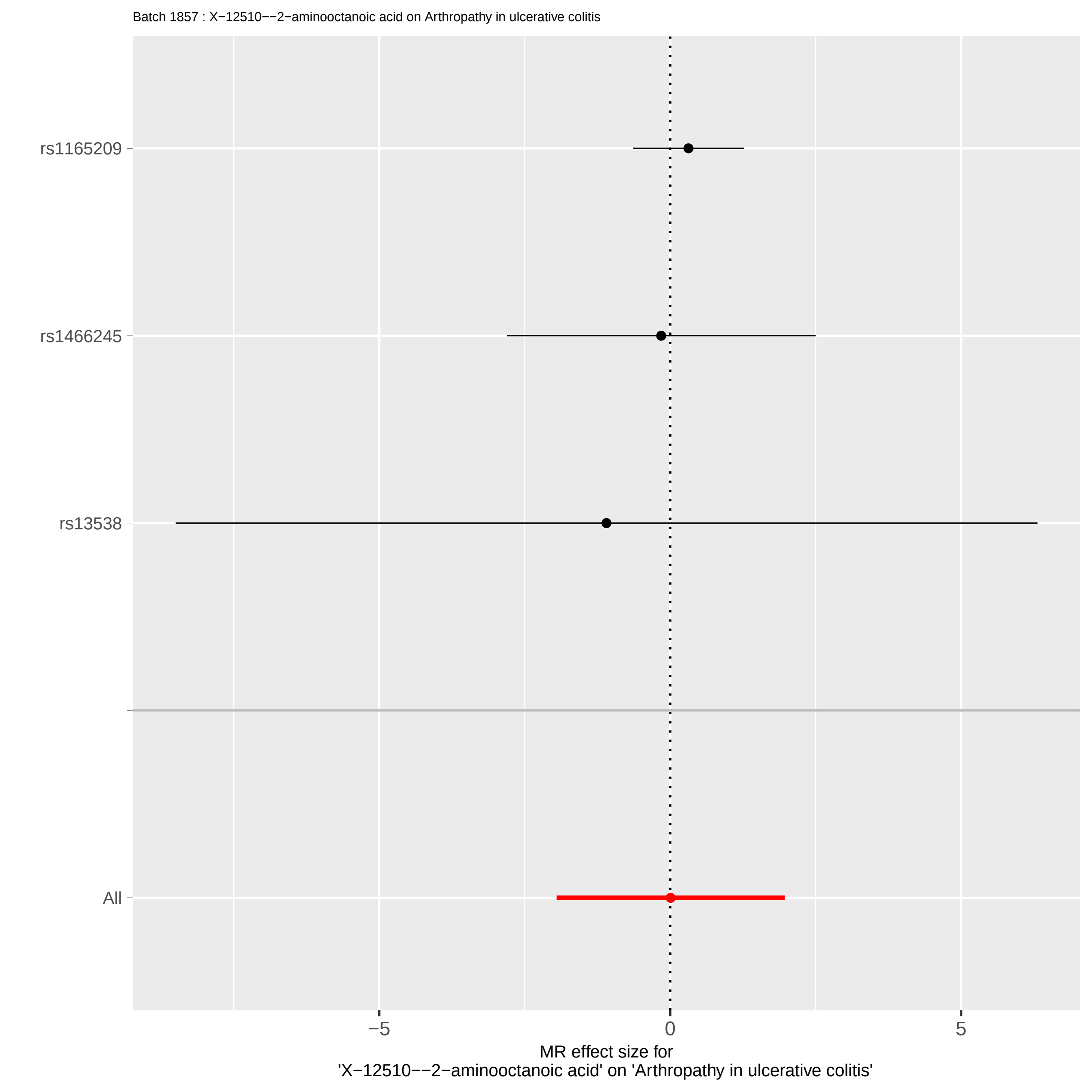

Batch 1857 : X−12510−−2−aminooctanoic acid on Arthropathy in ulcerative colitis
rs1165209
rs1466245
rs13538
All
−5
0
5
MR effect size for
'X−12510−−2−aminooctanoic acid' on 'Arthropathy in ulcerative colitis'

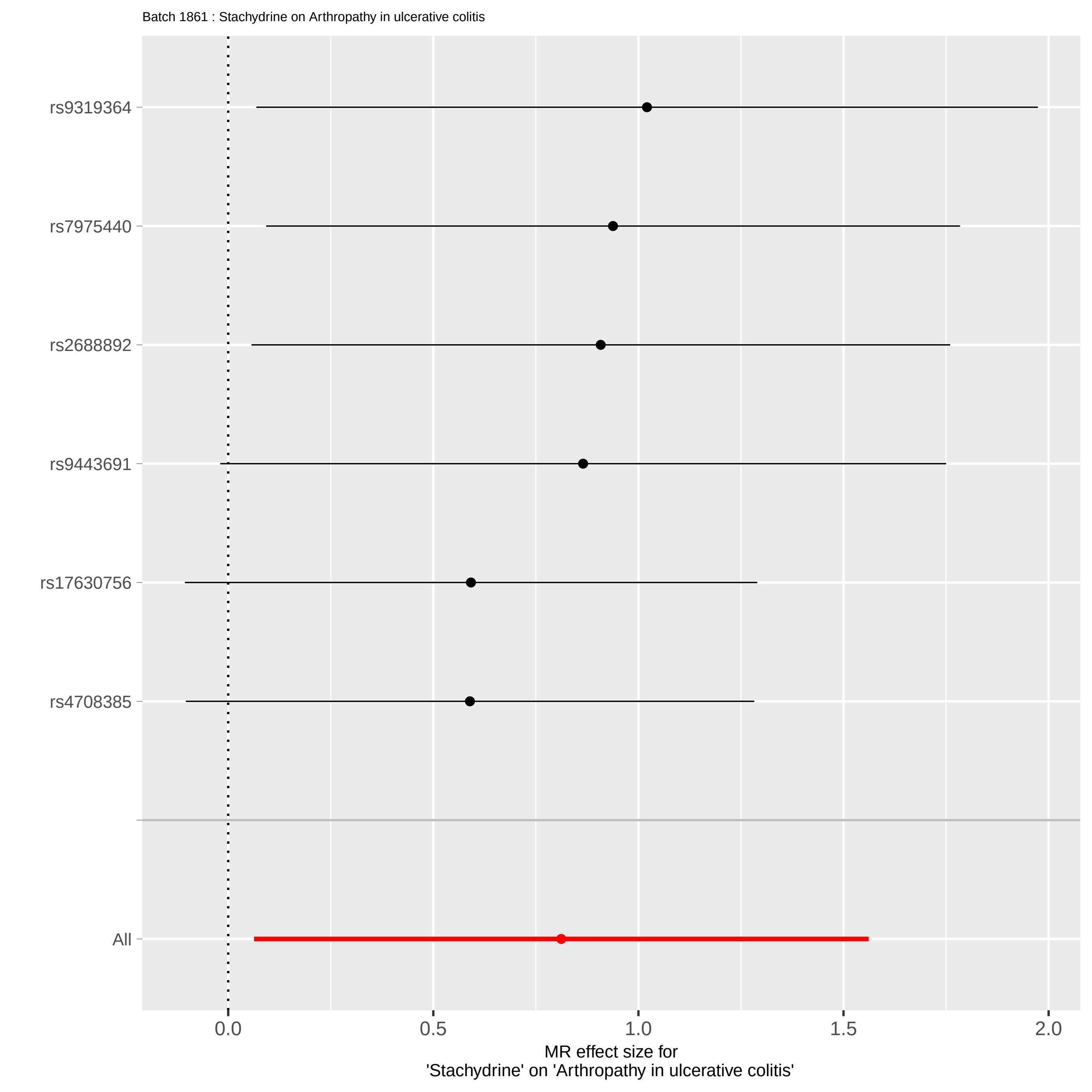
Batch 1861 : Stachydrine on Arthropathy in ulcerative colitis
rs9319364
rs7975440
rs2688892
rs9443691
rs17630756
rs4708385
All
0.0
0.5
1.0
1.5
2.0
MR effect size for
'Stachydrine' on 'Arthropathy in ulcerative colitis'

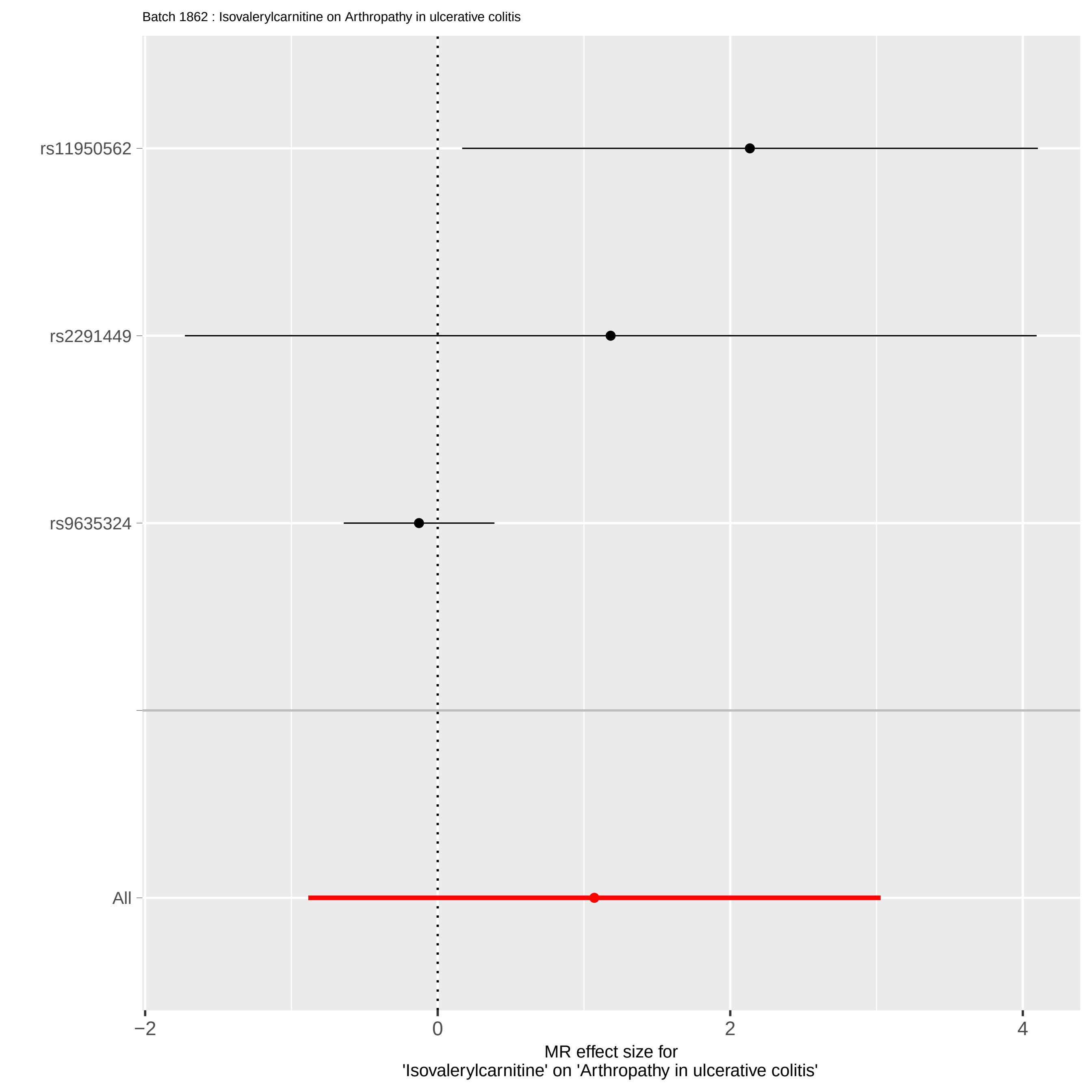

Batch 1862 : Isovalerylcarnitine on Arthropathy in ulcerative colitis
rs11950562
rs2291449
rs9635324
All
−2
0
2
4
MR effect size for
'Isovalerylcarnitine' on 'Arthropathy in ulcerative colitis'

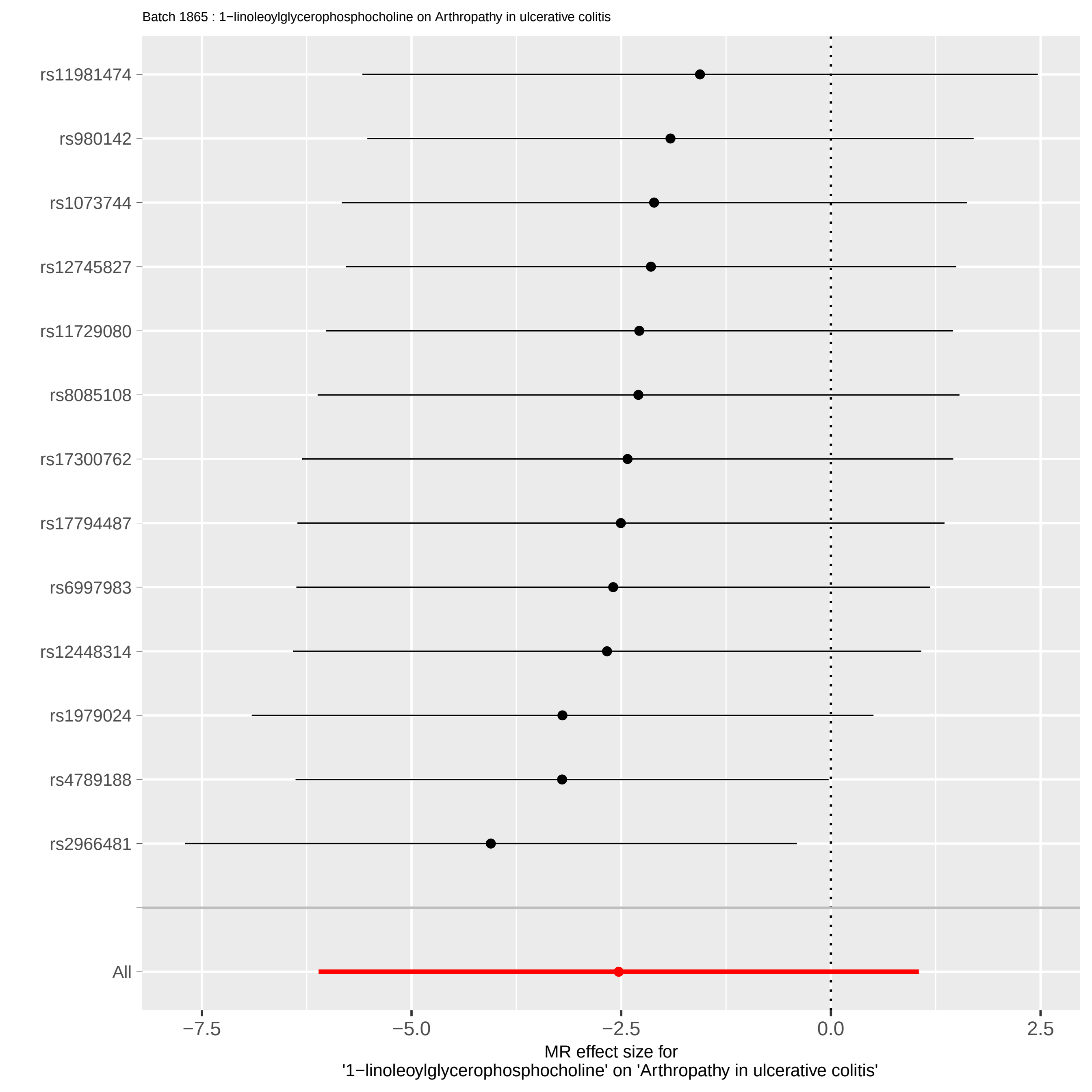
Batch 1865 : 1−linoleoylglycerophosphocholine on Arthropathy in ulcerative colitis
rs11981474
rs980142
rs1073744
rs12745827
rs11729080
rs8085108
rs17300762
rs17794487
rs6997983
rs12448314
rs1979024
rs4789188
rs2966481
All
−7.5
−5.0
−2.5
0.0
2.5
MR effect size for
'1−linoleoylglycerophosphocholine' on 'Arthropathy in ulcerative colitis'

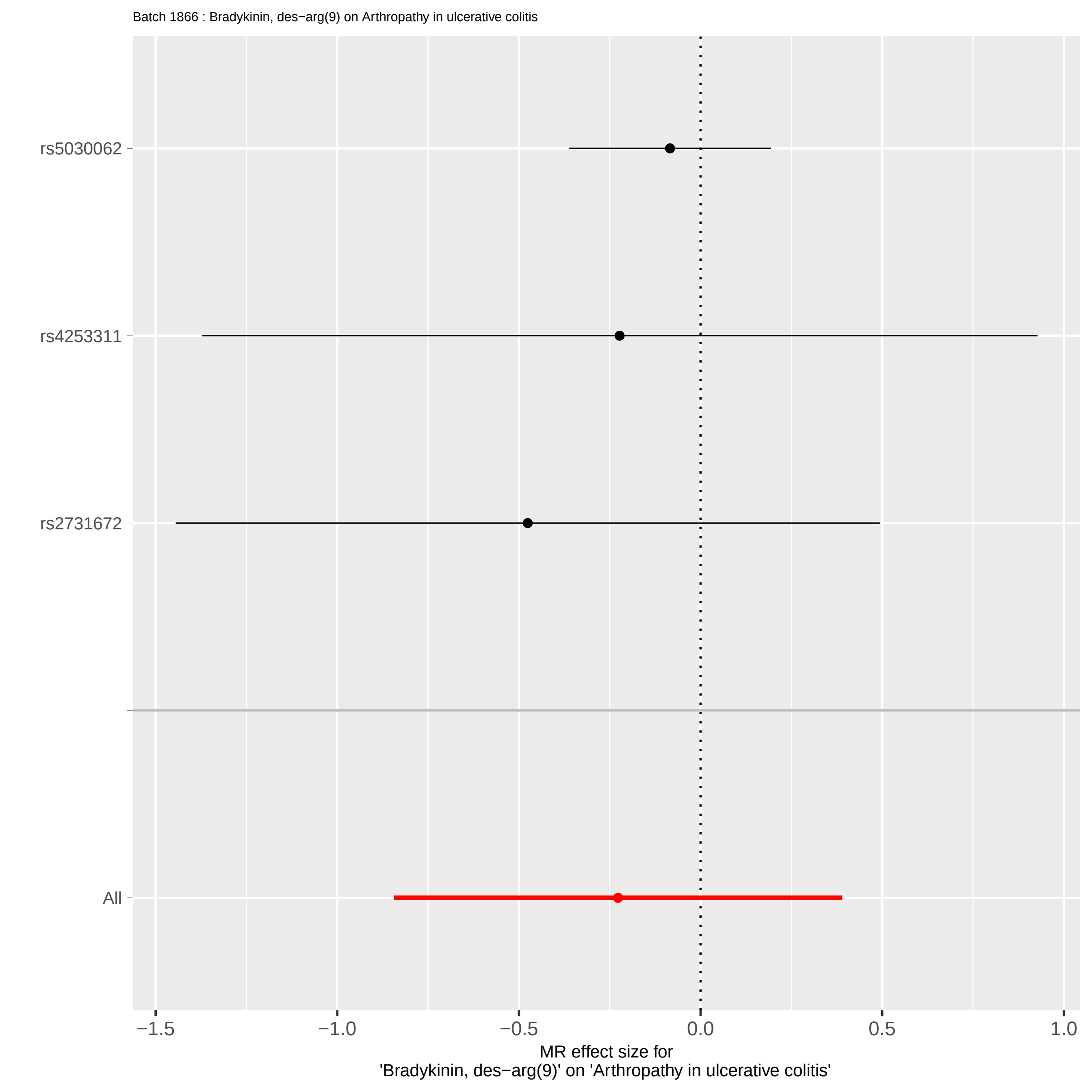

Batch 1866 : Bradykinin, des−arg(9) on Arthropathy in ulcerative colitis
rs5030062
rs4253311
rs2731672
All
−1.5
−1.0
−0.5
0.0
0.5
1.0
MR effect size for
'Bradykinin, des−arg(9)' on 'Arthropathy in ulcerative colitis'

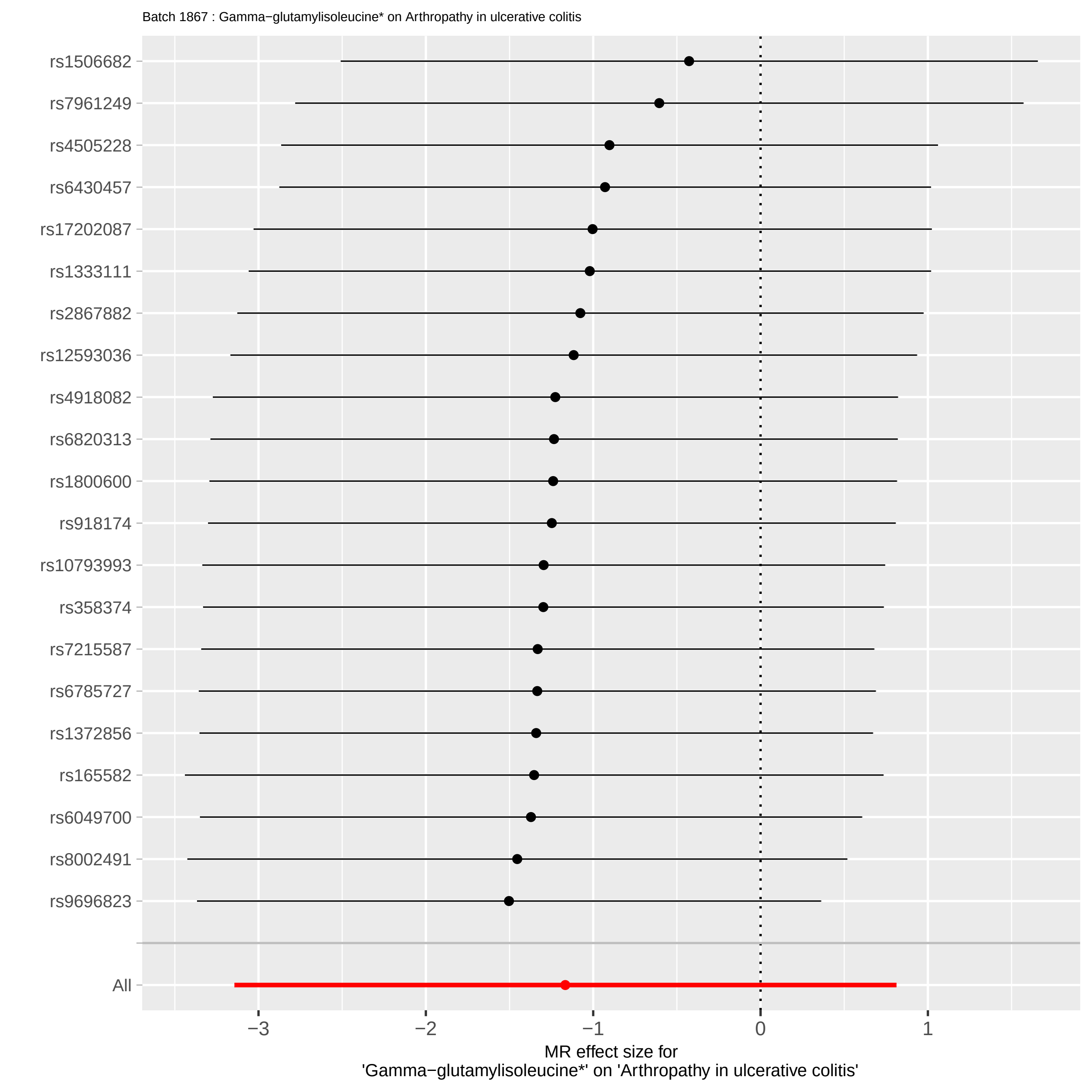

Batch 1867 : Gamma−glutamylisoleucine* on Arthropathy in ulcerative colitis
rs1506682
rs7961249
rs4505228
rs6430457
rs17202087
rs1333111
rs2867882
rs12593036
rs4918082
rs6820313
rs1800600
rs918174
rs10793993
rs358374
rs7215587
rs6785727
rs1372856
rs165582
rs6049700
rs8002491
rs9696823
All
−3
−2
−1
0
1
MR effect size for
'Gamma−glutamylisoleucine*' on 'Arthropathy in ulcerative colitis'

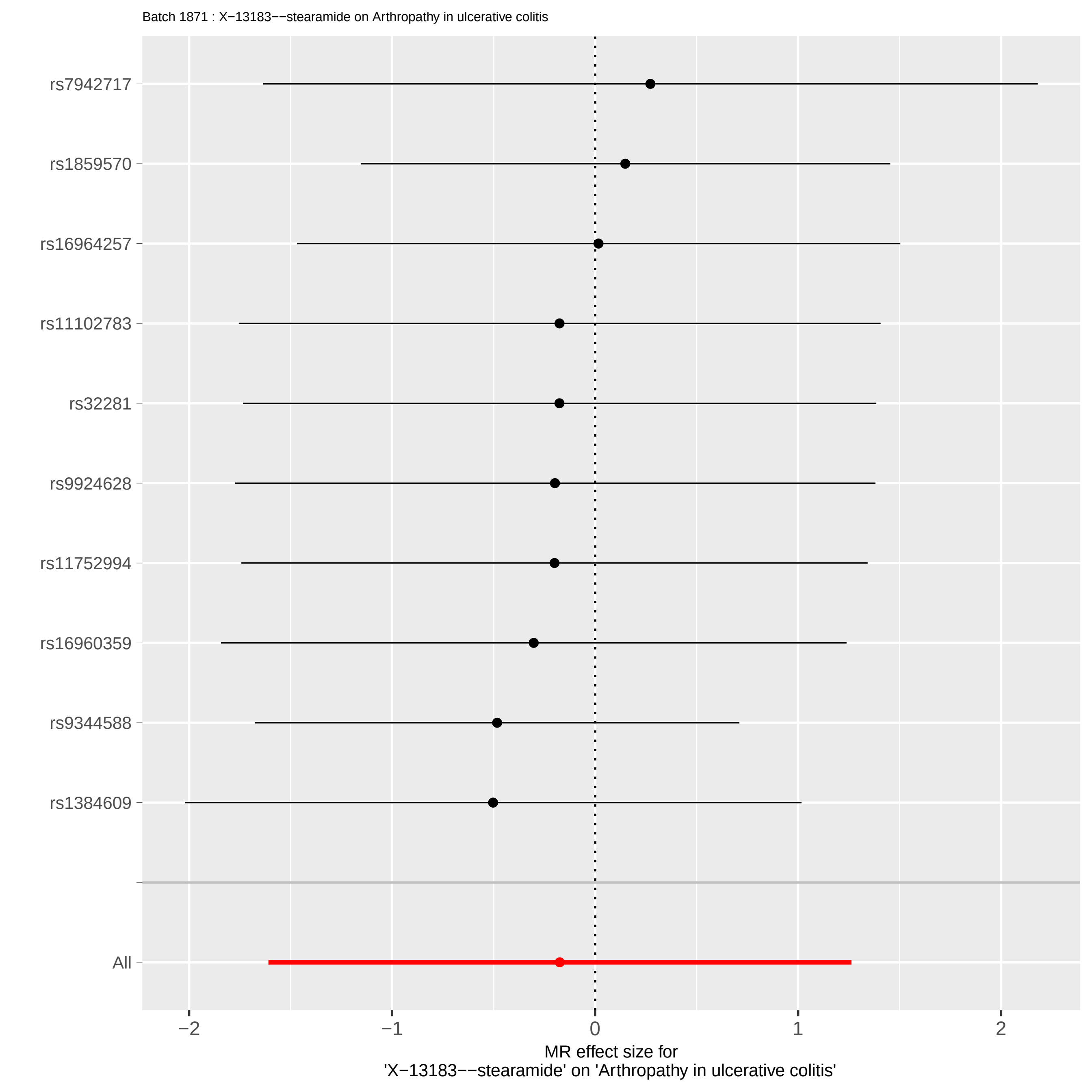

Batch 1871 : X−13183−−stearamide on Arthropathy in ulcerative colitis
rs7942717
rs1859570
rs16964257
rs11102783
rs32281
rs9924628
rs11752994
rs16960359
rs9344588
rs1384609
All
−2
−1
0
1
2
MR effect size for
'X−13183−−stearamide' on 'Arthropathy in ulcerative colitis'

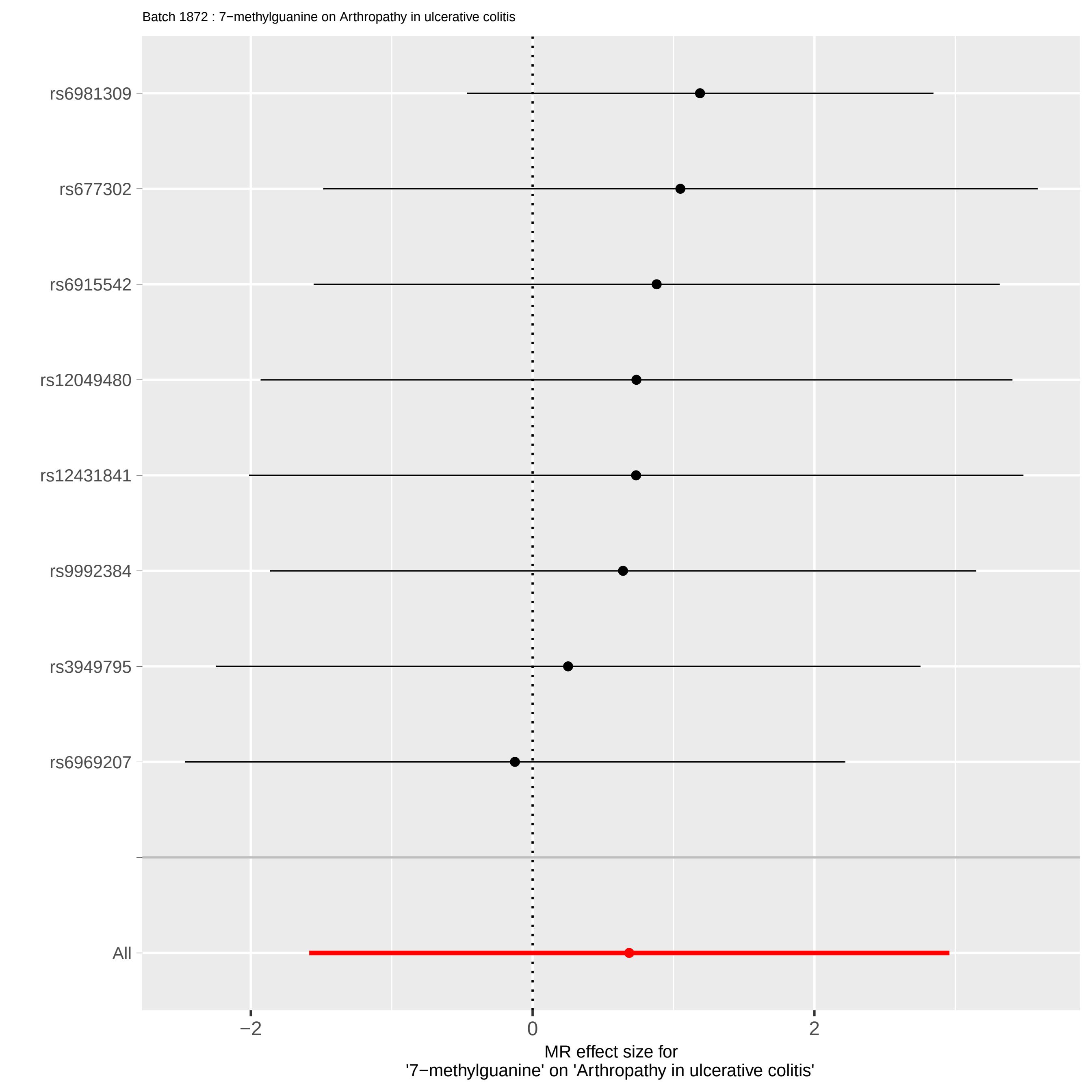

Batch 1872 : 7−methylguanine on Arthropathy in ulcerative colitis
rs6981309
rs677302
rs6915542
rs12049480
rs12431841
rs9992384
rs3949795
rs6969207
All
−2
0
2
MR effect size for
'7−methylguanine' on 'Arthropathy in ulcerative colitis'

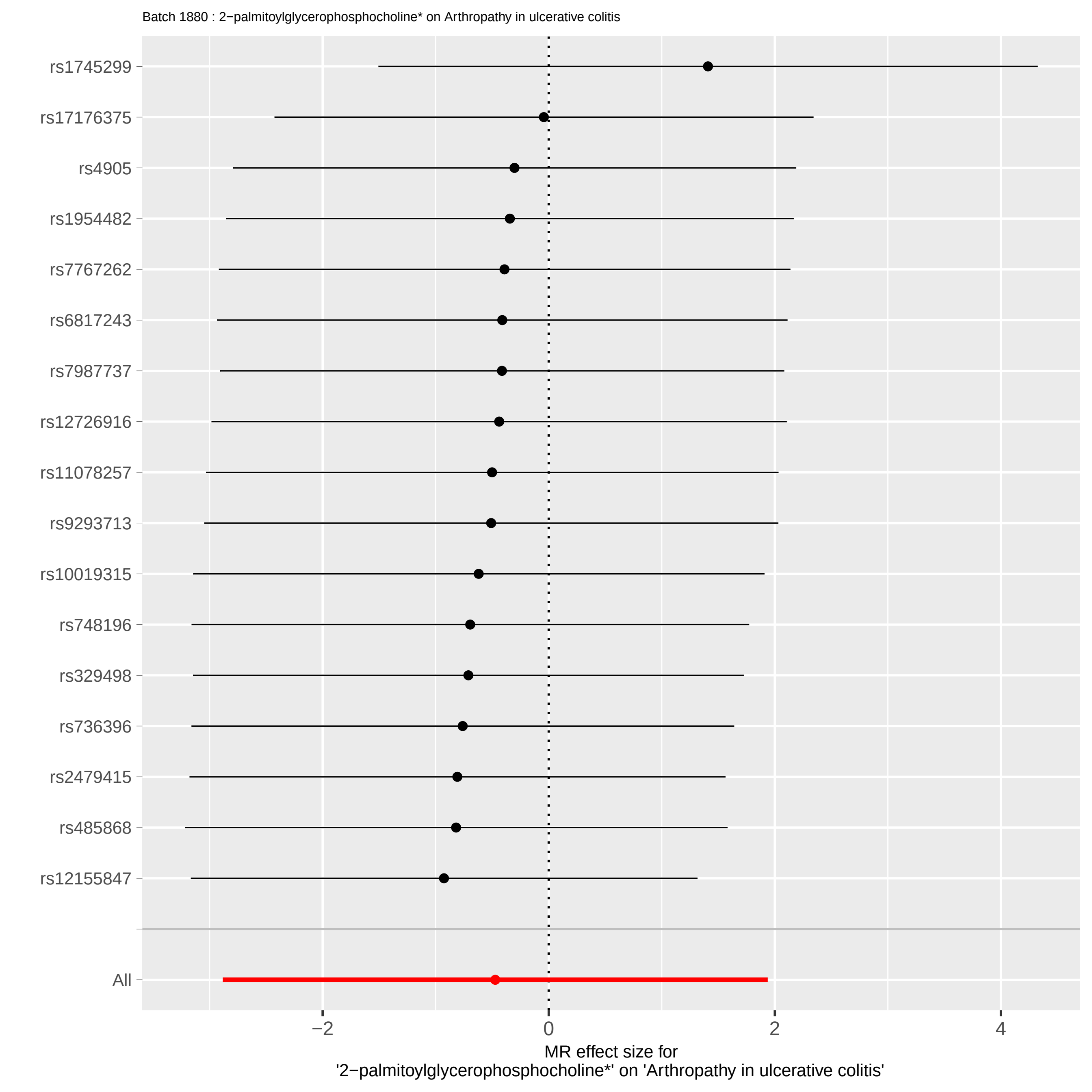

Batch 1880 : 2−palmitoylglycerophosphocholine* on Arthropathy in ulcerative colitis
rs1745299
rs17176375
rs4905
rs1954482
rs7767262
rs6817243
rs7987737
rs12726916
rs11078257
rs9293713
rs10019315
rs748196
rs329498
rs736396
rs2479415
rs485868
rs12155847
All
−2
0
2
4
MR effect size for
'2−palmitoylglycerophosphocholine*' on 'Arthropathy in ulcerative colitis'

Batch 1881 : 2−oleoylglycerophosphocholine* on Arthropathy in ulcerative colitis

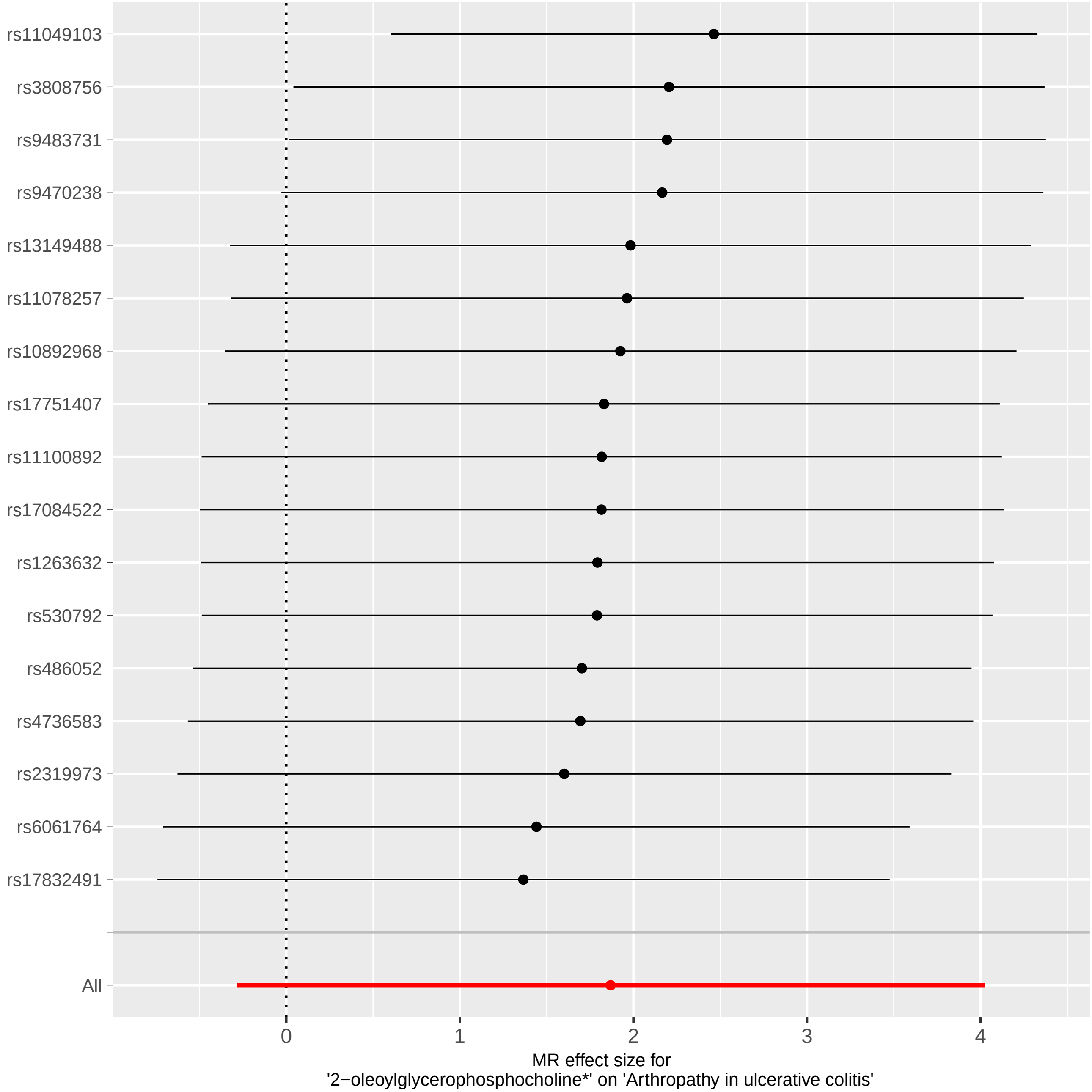

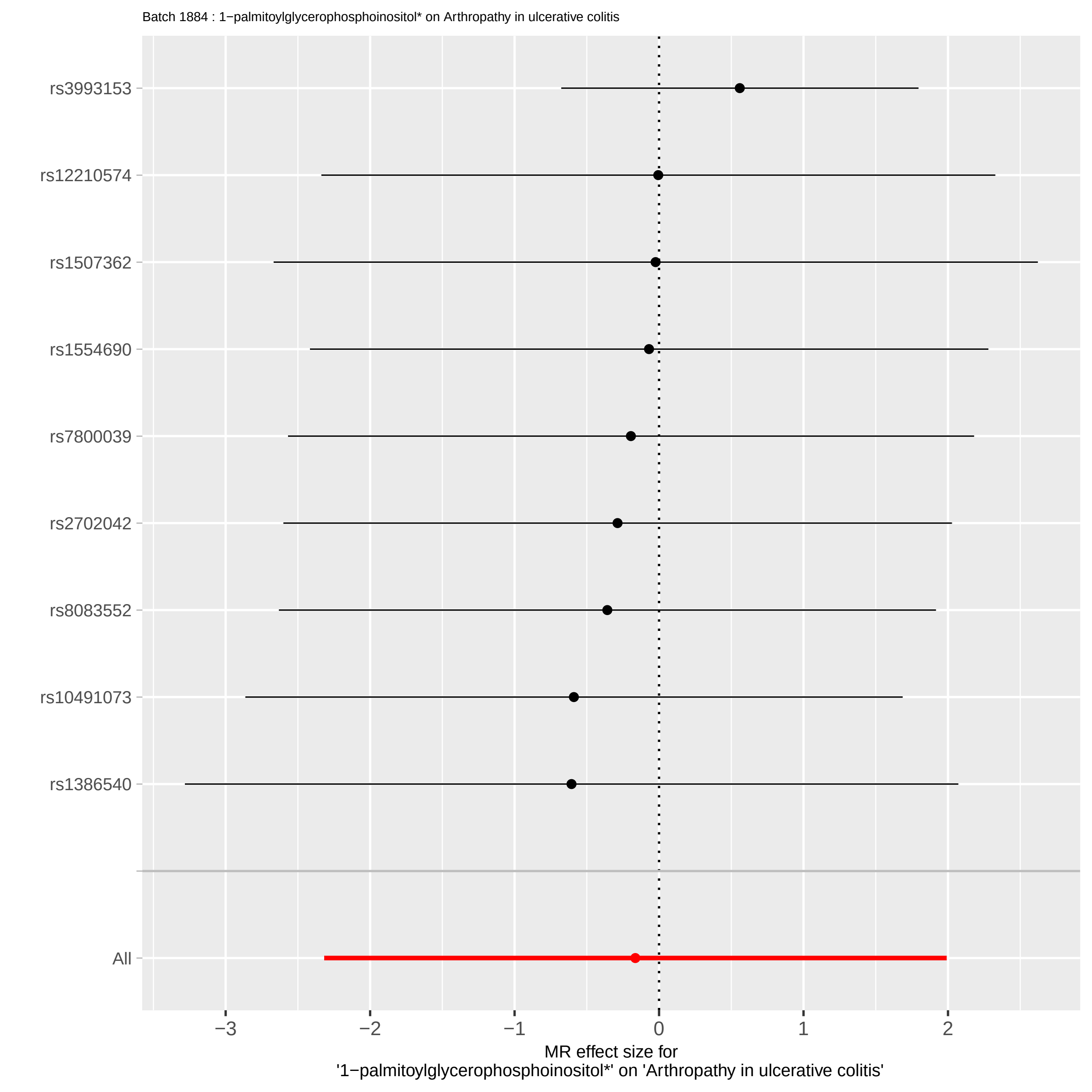

Batch 1884 : 1−palmitoylglycerophosphoinositol* on Arthropathy in ulcerative colitis
rs3993153
rs12210574
rs1507362
rs1554690
rs7800039
rs2702042
rs8083552
rs10491073
rs1386540
All
−3
−2
−1
0
1
2
MR effect size for
'1−palmitoylglycerophosphoinositol*' on 'Arthropathy in ulcerative colitis'

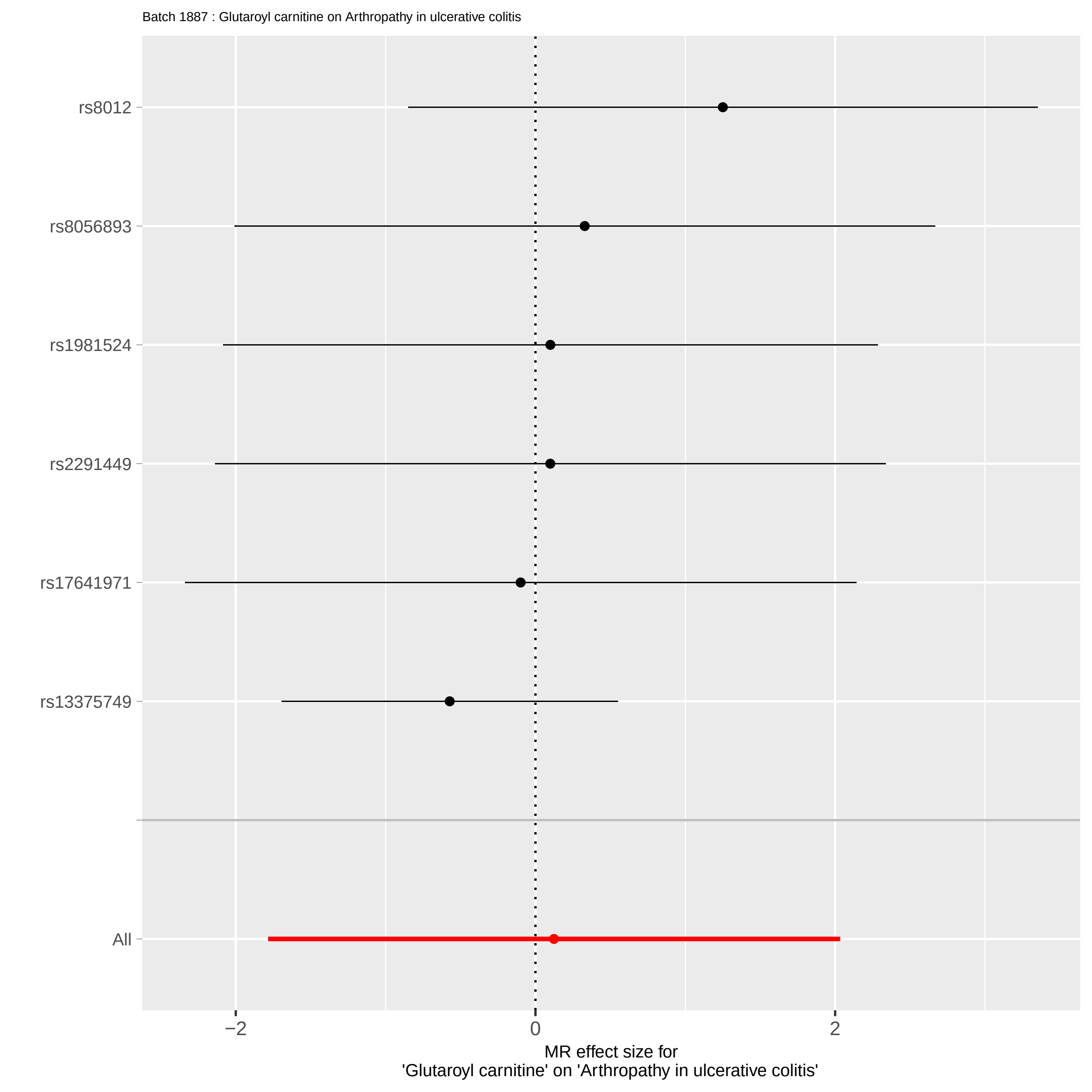

Batch 1887 : Glutaroyl carnitine on Arthropathy in ulcerative colitis
rs8012
rs8056893
rs1981524
rs2291449
rs17641971
rs13375749
All
−2
0
2
MR effect size for
'Glutaroyl carnitine' on 'Arthropathy in ulcerative colitis'

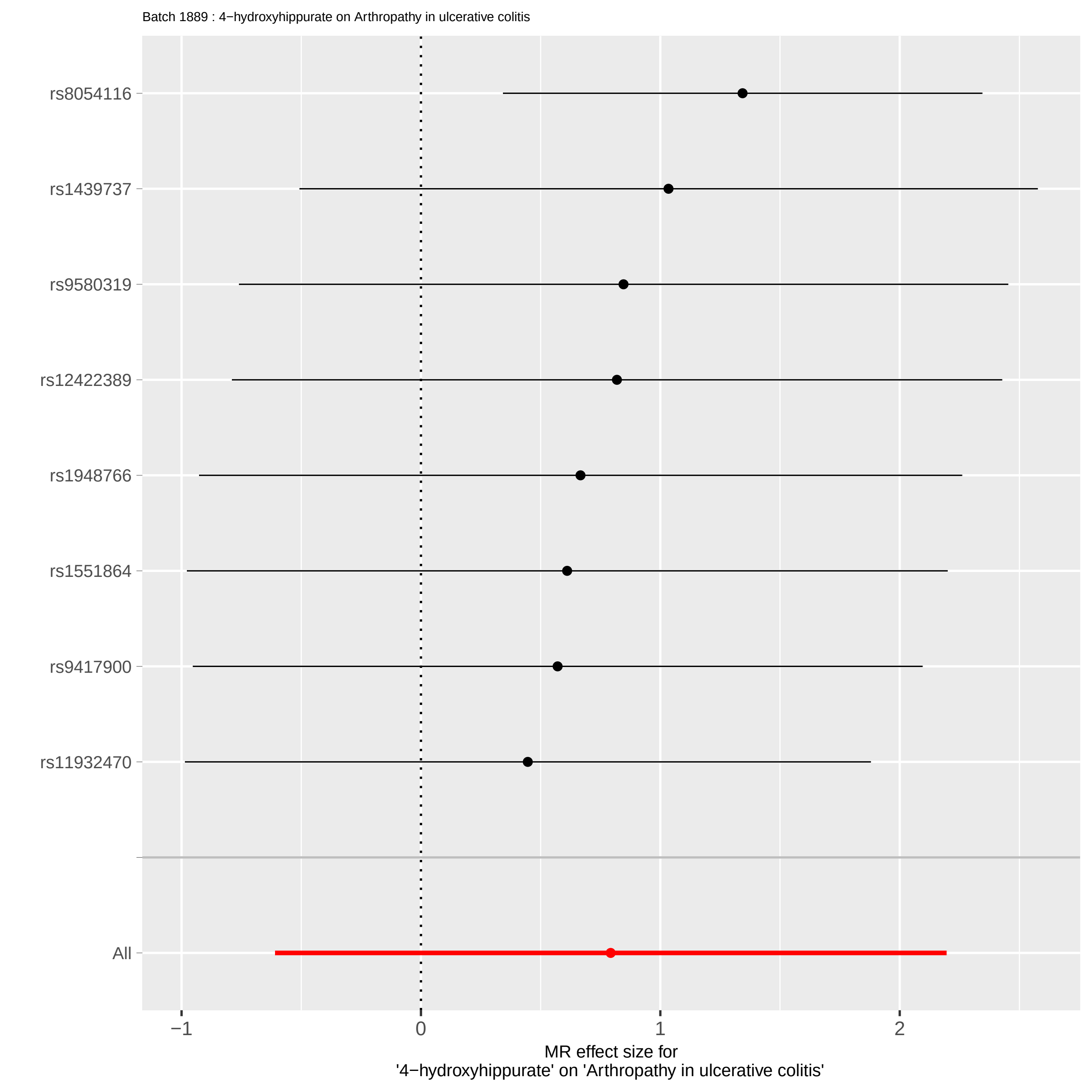
Batch 1889 : 4−hydroxyhippurate on Arthropathy in ulcerative colitis
rs8054116
rs1439737
rs9580319
rs12422389
rs1948766
rs1551864
rs9417900
rs11932470
All
−1
0
1
2
MR effect size for
'4−hydroxyhippurate' on 'Arthropathy in ulcerative colitis'

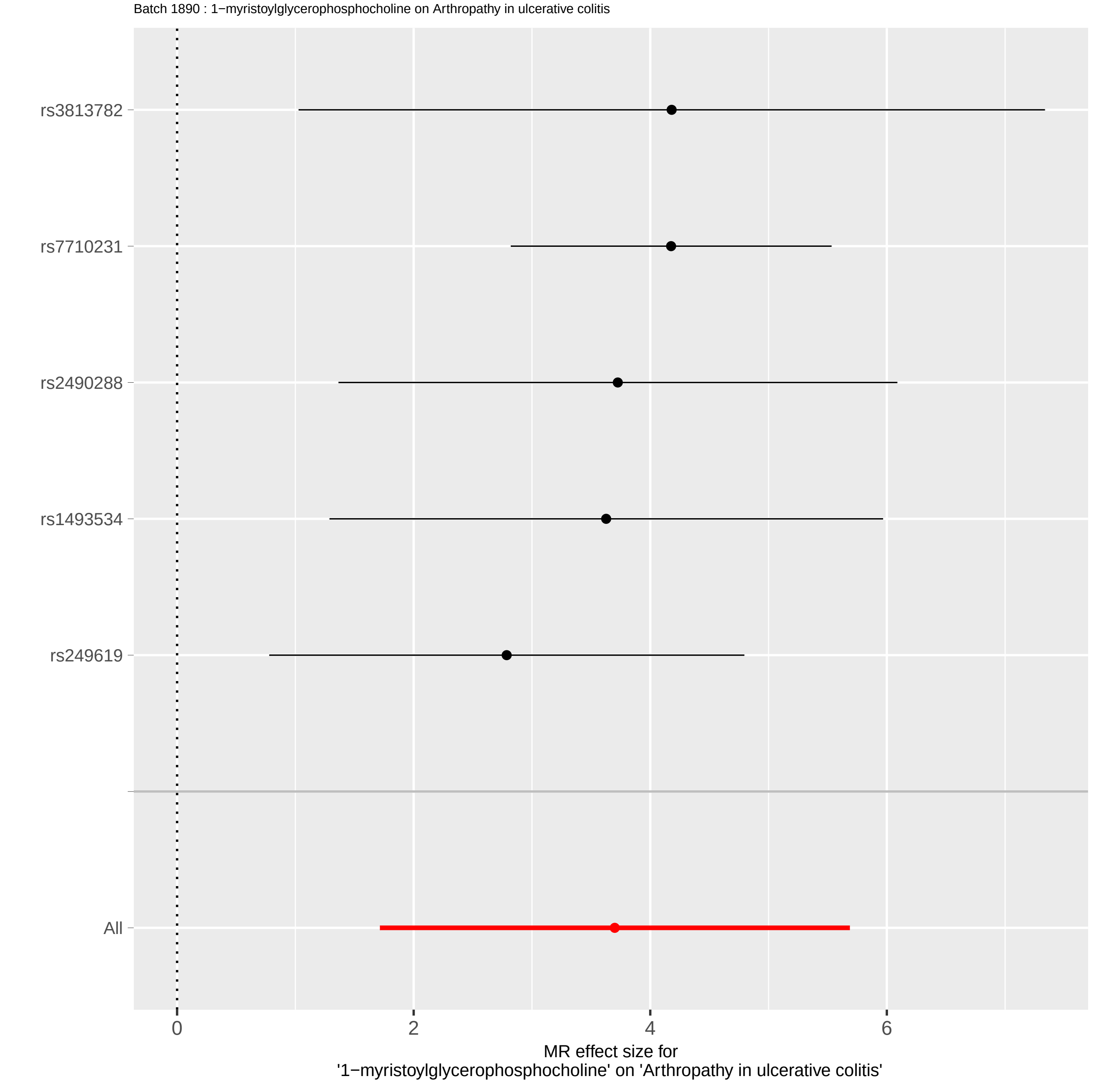

Batch 1890 : 1−myristoylglycerophosphocholine on Arthropathy in ulcerative colitis
rs3813782
rs7710231
rs2490288
rs1493534
rs249619
All
0
2
4
6
MR effect size for
'1−myristoylglycerophosphocholine' on 'Arthropathy in ulcerative colitis'

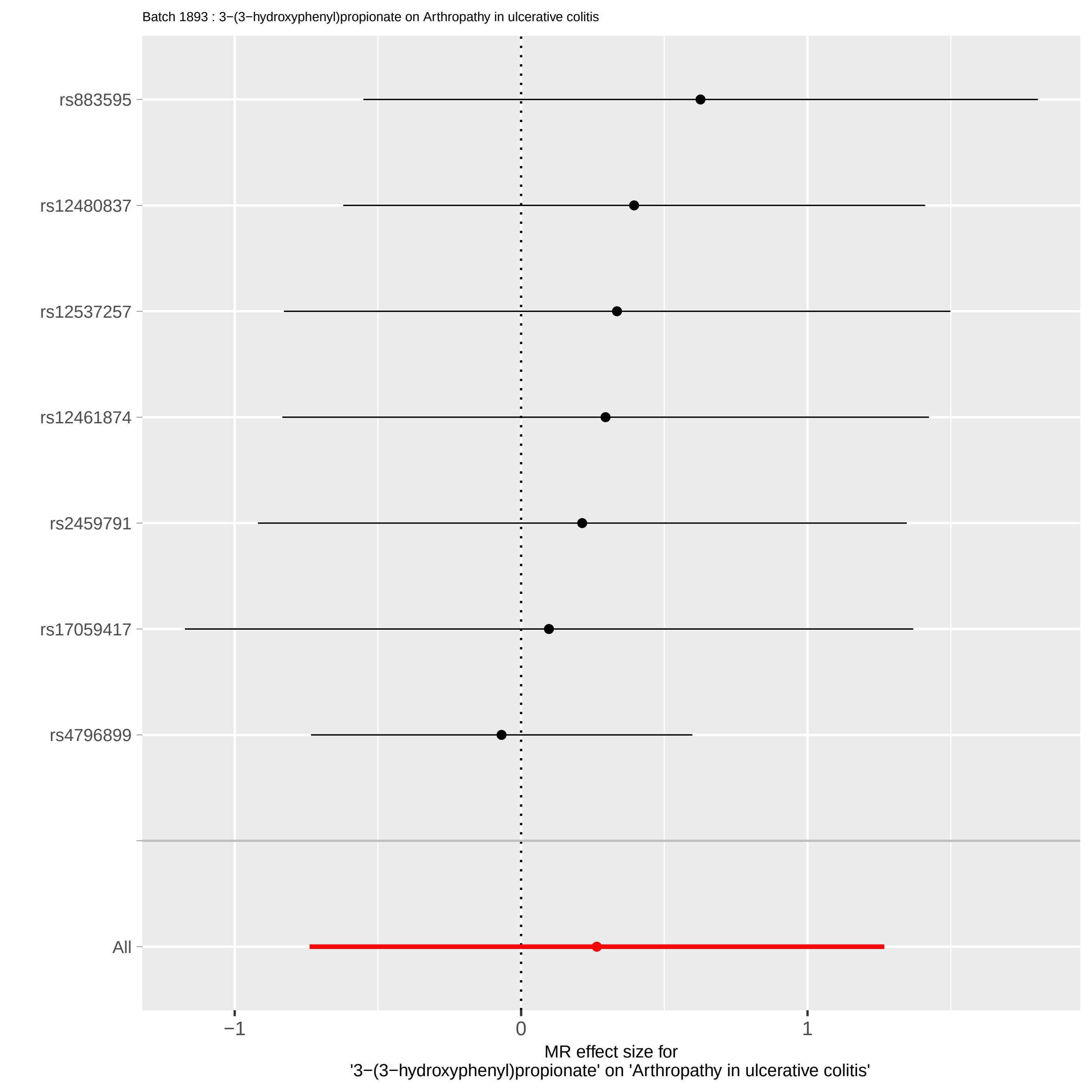

Batch 1893 : 3−(3−hydroxyphenyl)propionate on Arthropathy in ulcerative colitis
rs883595
rs12480837
rs12537257
rs12461874
rs2459791
rs17059417
rs4796899
All
−1
0
1
MR effect size for
'3−(3−hydroxyphenyl)propionate' on 'Arthropathy in ulcerative colitis'

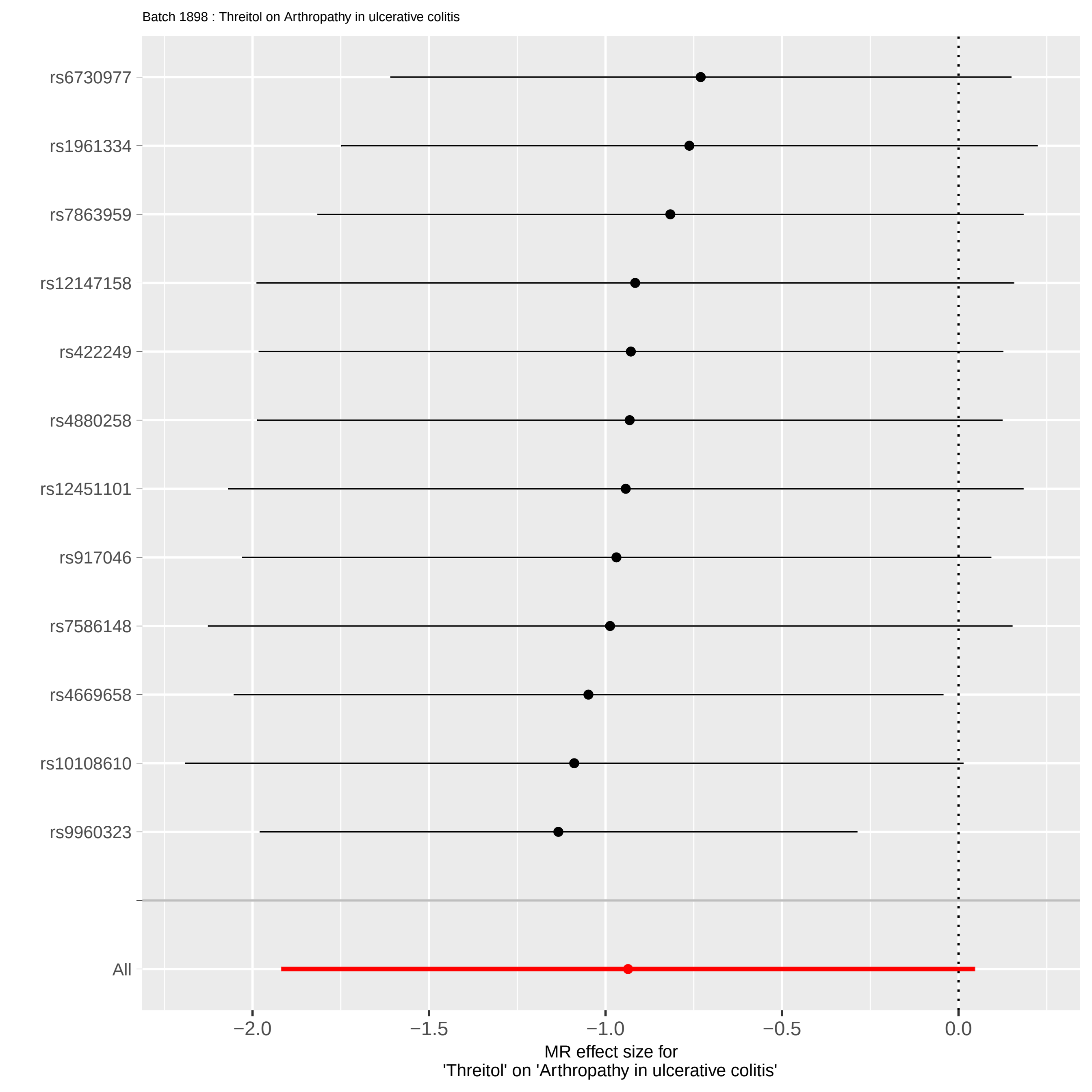
Batch 1898 : Threitol on Arthropathy in ulcerative colitis
rs6730977
rs1961334
rs7863959
rs12147158
rs422249
rs4880258
rs12451101
rs917046
rs7586148
rs4669658
rs10108610
rs9960323
All
−2.0
−1.5
−1.0
−0.5
0.0
MR effect size for
'Threitol' on 'Arthropathy in ulcerative colitis'

Batch 1899 : P−cresol sulfate on Arthropathy in ulcerative colitis

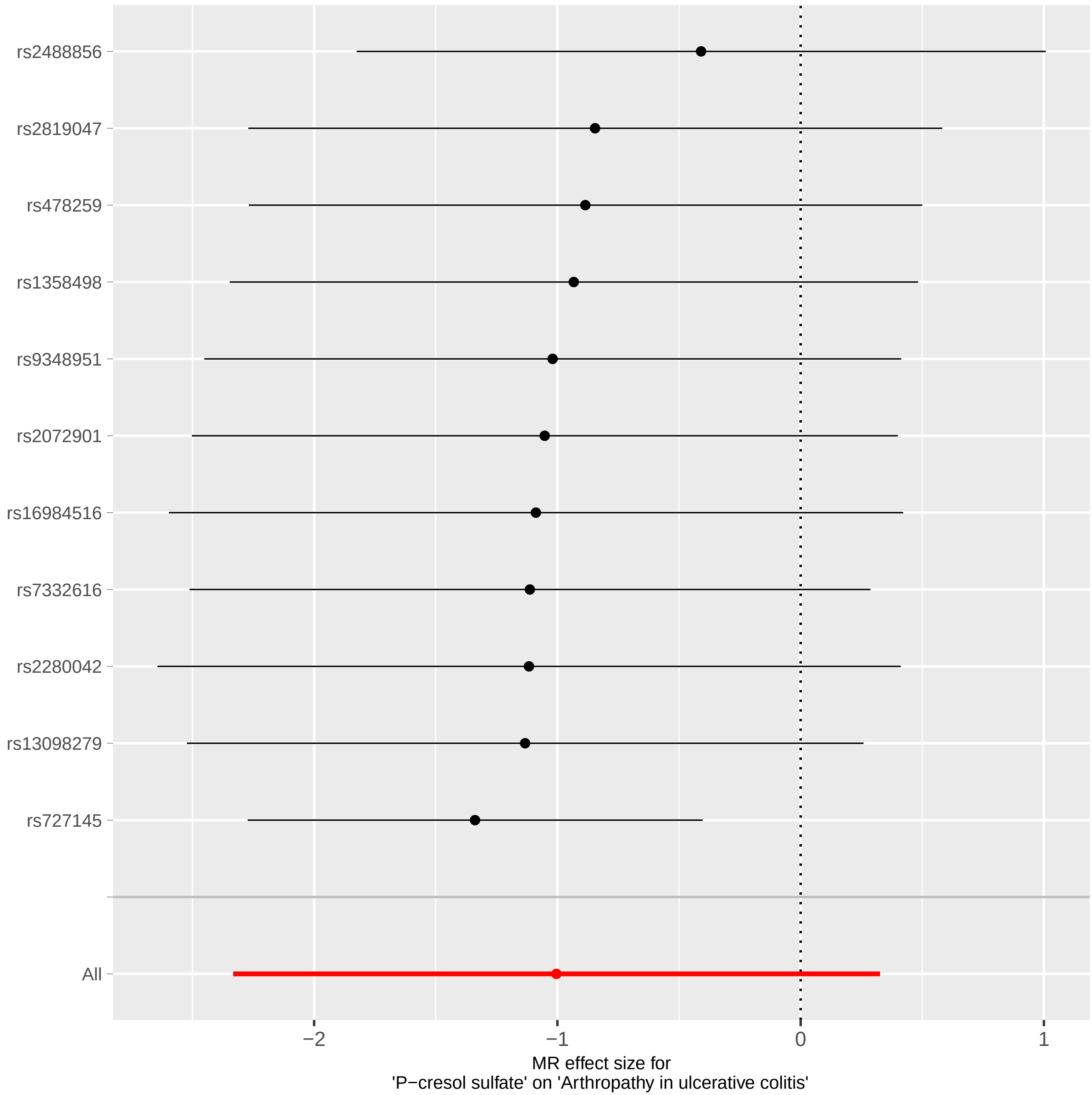

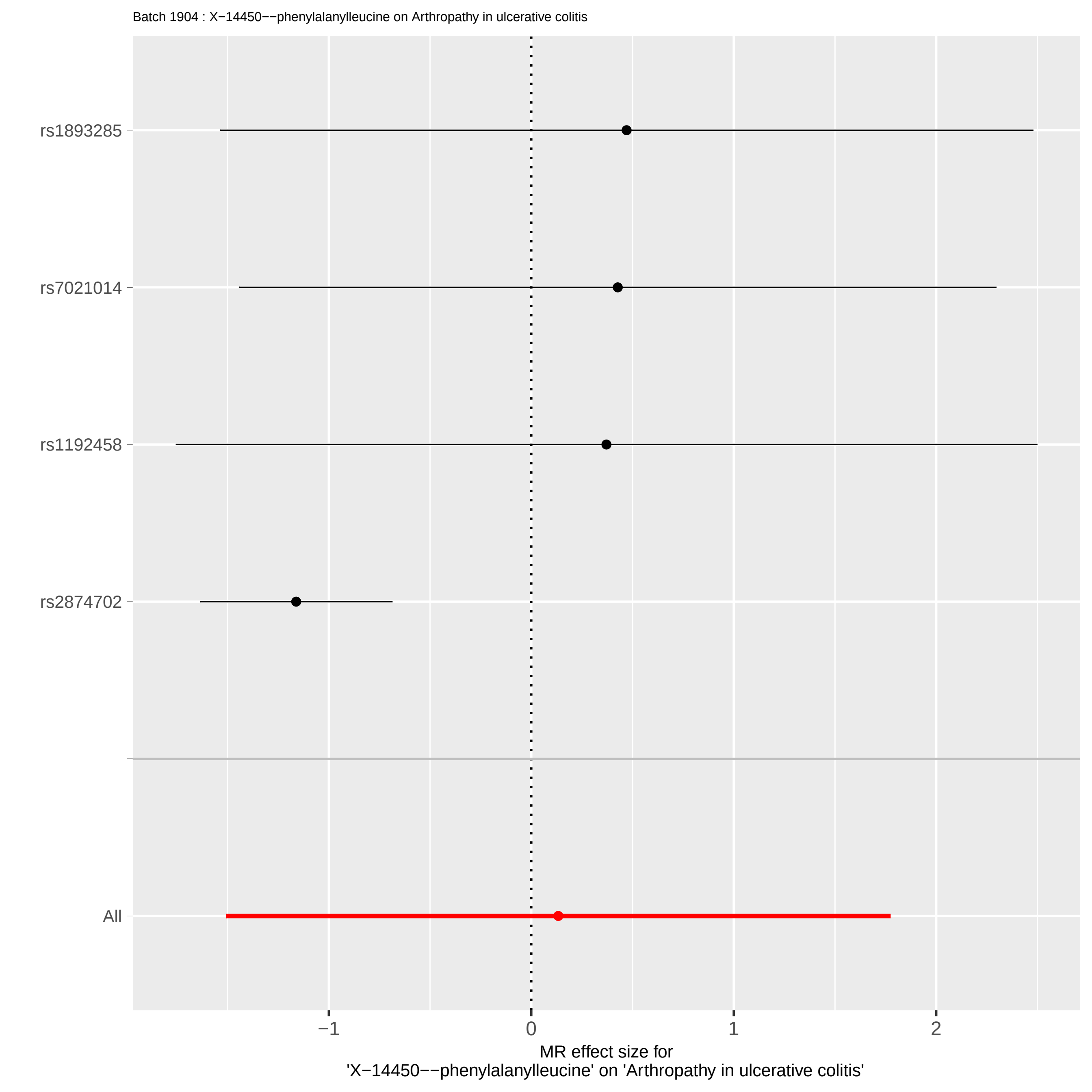
Batch 1904 : X−14450−−phenylalanylleucine on Arthropathy in ulcerative colitis
rs1893285
rs7021014
rs1192458
rs2874702
All
−1
0
1
2
MR effect size for
'X−14450−−phenylalanylleucine' on 'Arthropathy in ulcerative colitis'

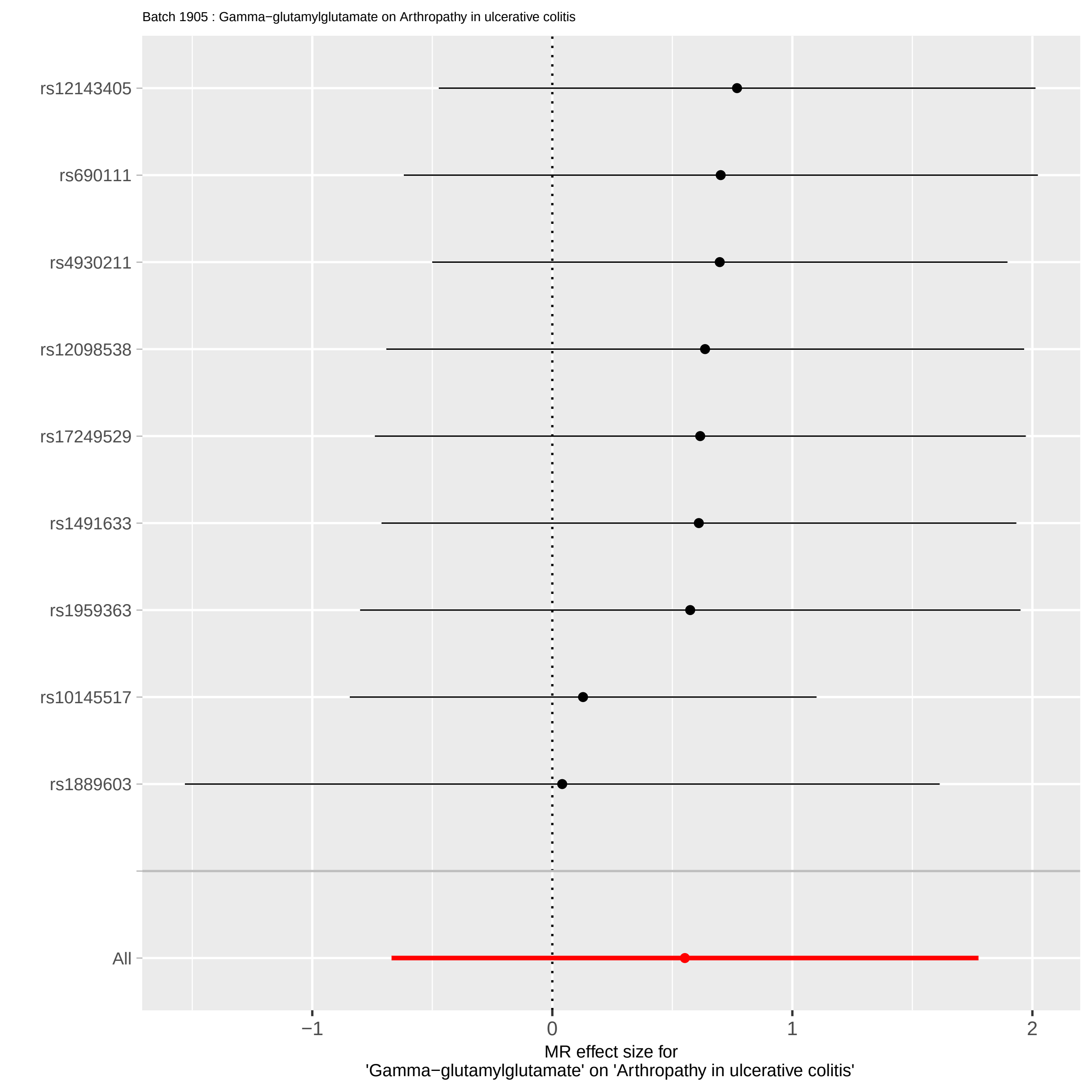

Batch 1905 : Gamma−glutamylglutamate on Arthropathy in ulcerative colitis
rs12143405
rs690111
rs4930211
rs12098538
rs17249529
rs1491633
rs1959363
rs10145517
rs1889603
All
−1
0
1
2
MR effect size for
'Gamma−glutamylglutamate' on 'Arthropathy in ulcerative colitis'

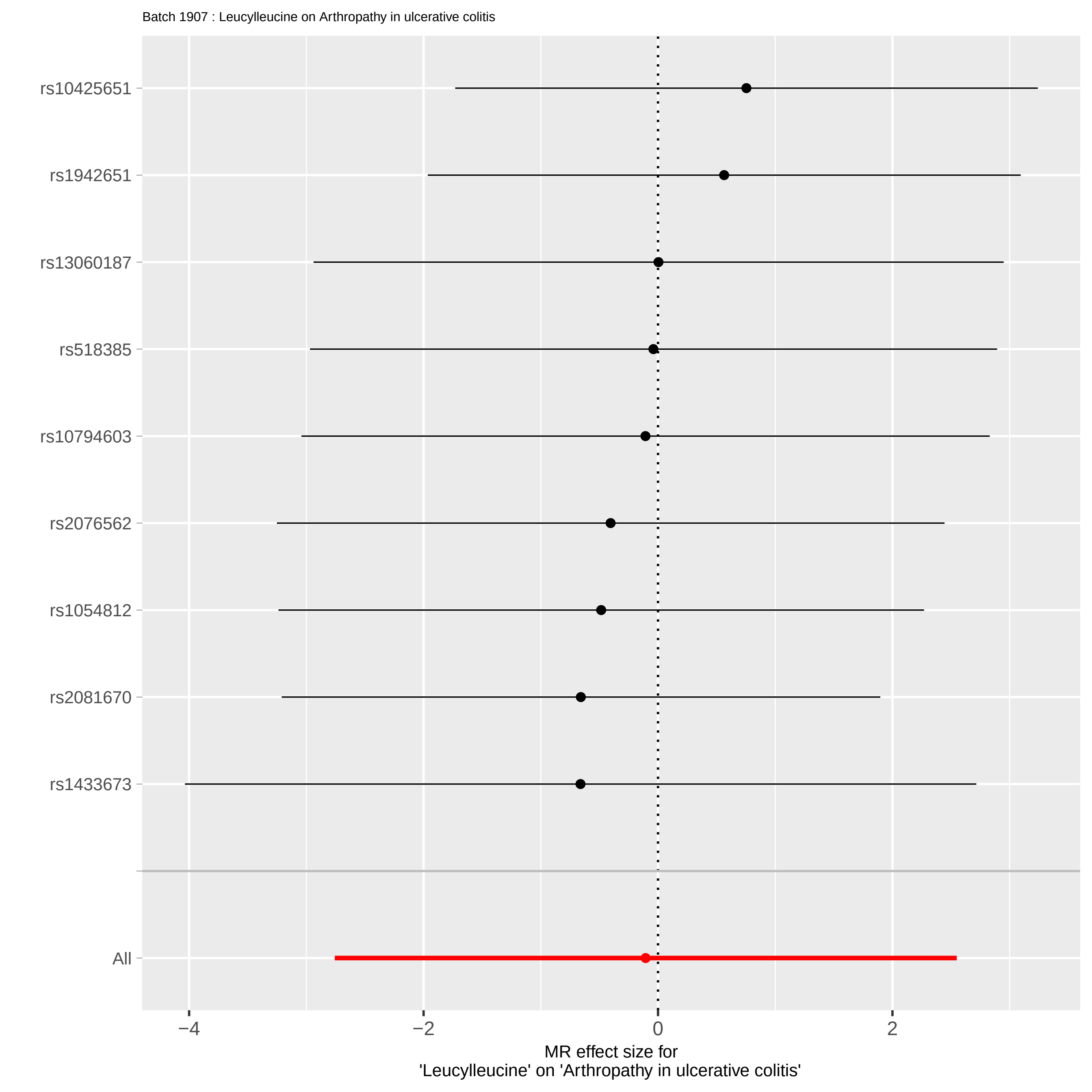

Batch 1907 : Leucylleucine on Arthropathy in ulcerative colitis
rs10425651
rs1942651
rs13060187
rs518385
rs10794603
rs2076562
rs1054812
rs2081670
rs1433673
All
−4
−2
0
2
MR effect size for
'Leucylleucine' on 'Arthropathy in ulcerative colitis'

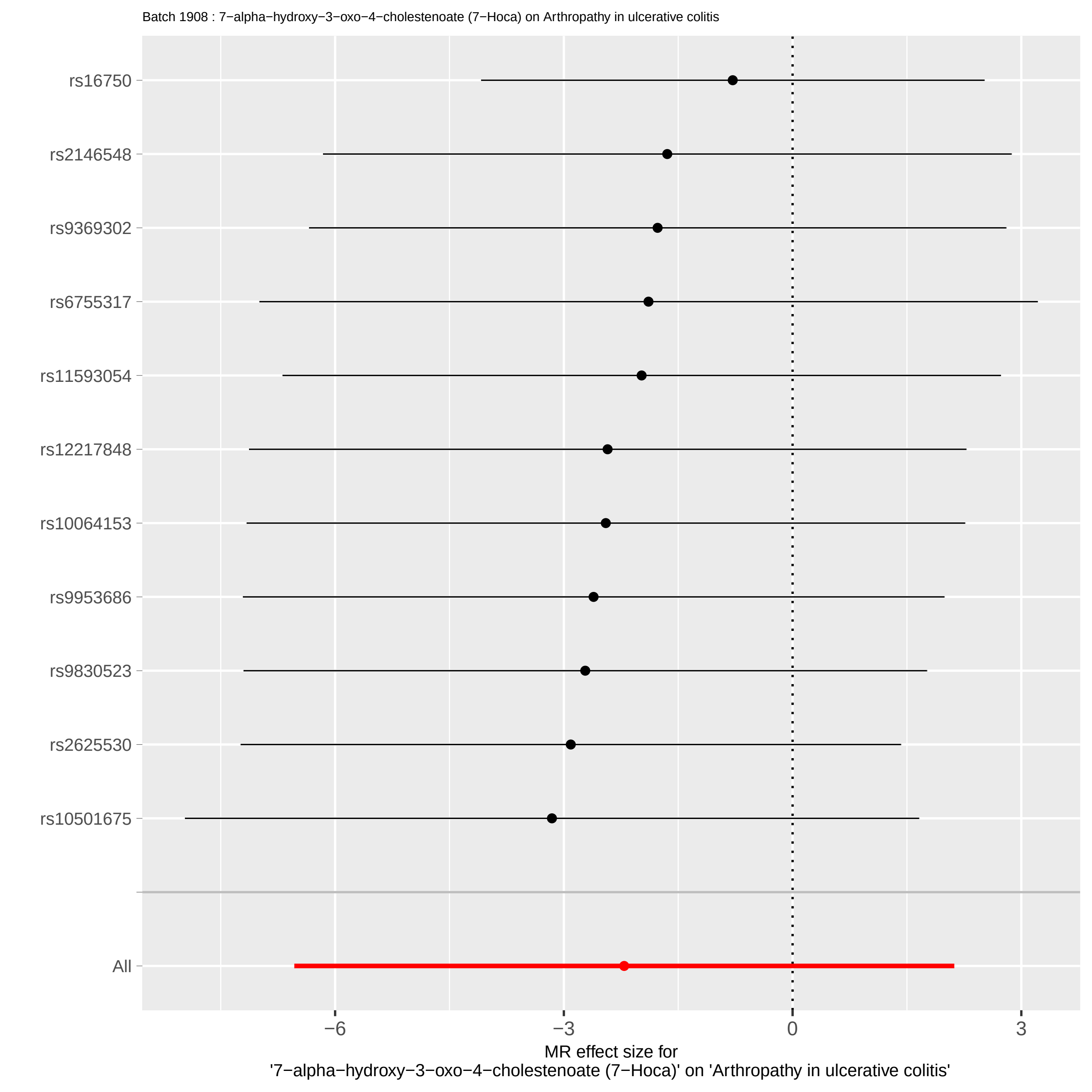

Batch 1908 : 7−alpha−hydroxy−3−oxo−4−cholestenoate (7−Hoca) on Arthropathy in ulcerative colitis
rs16750
rs2146548
rs9369302
rs6755317
rs11593054
rs12217848
rs10064153
rs9953686
rs9830523
rs2625530
rs10501675
All
−6
−3
0
3
MR effect size for
'7−alpha−hydroxy−3−oxo−4−cholestenoate (7−Hoca)' on 'Arthropathy in ulcerative colitis'

Batch 1909 : N−Butyl Oleate on Arthropathy in ulcerative colitis

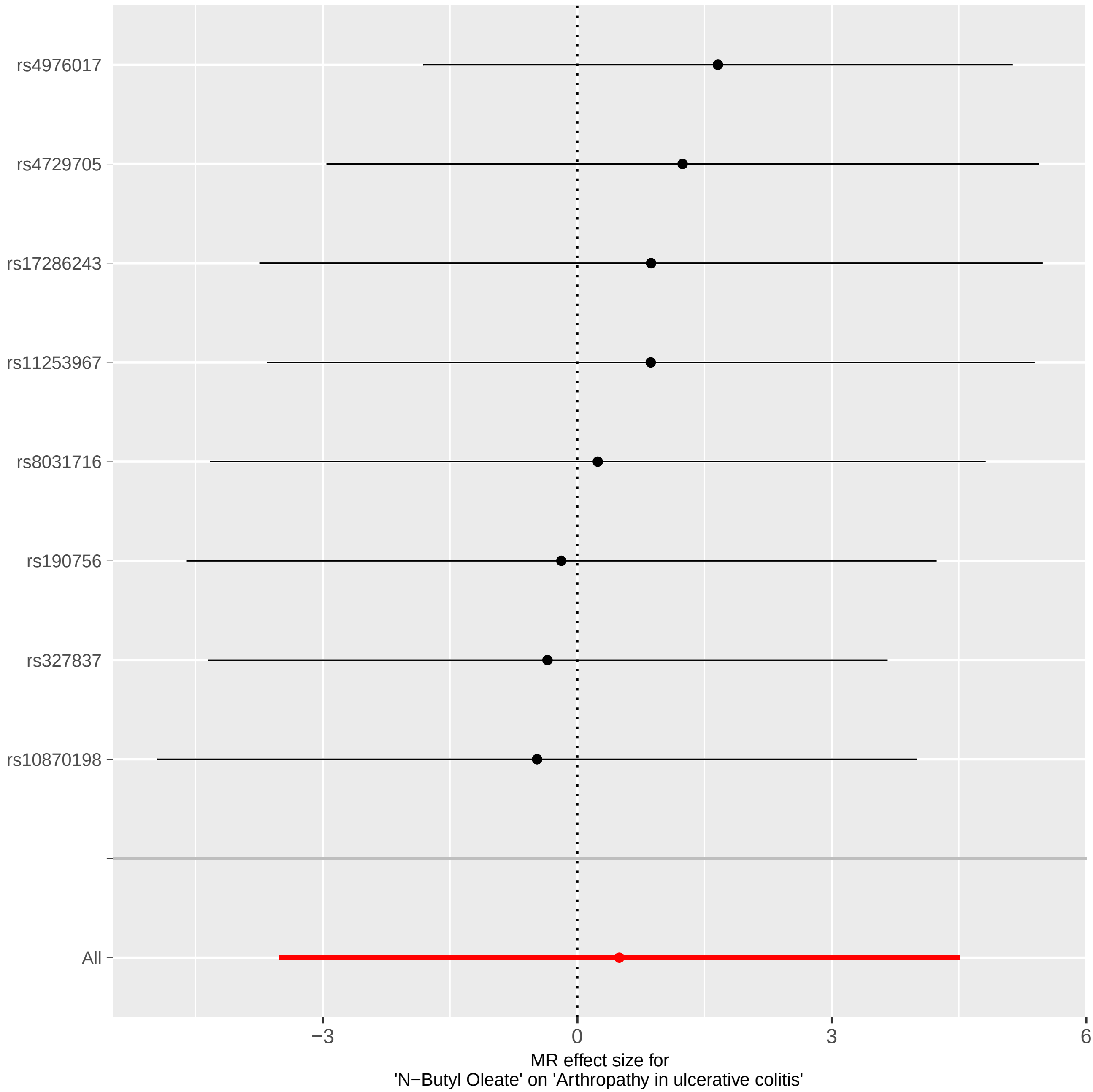

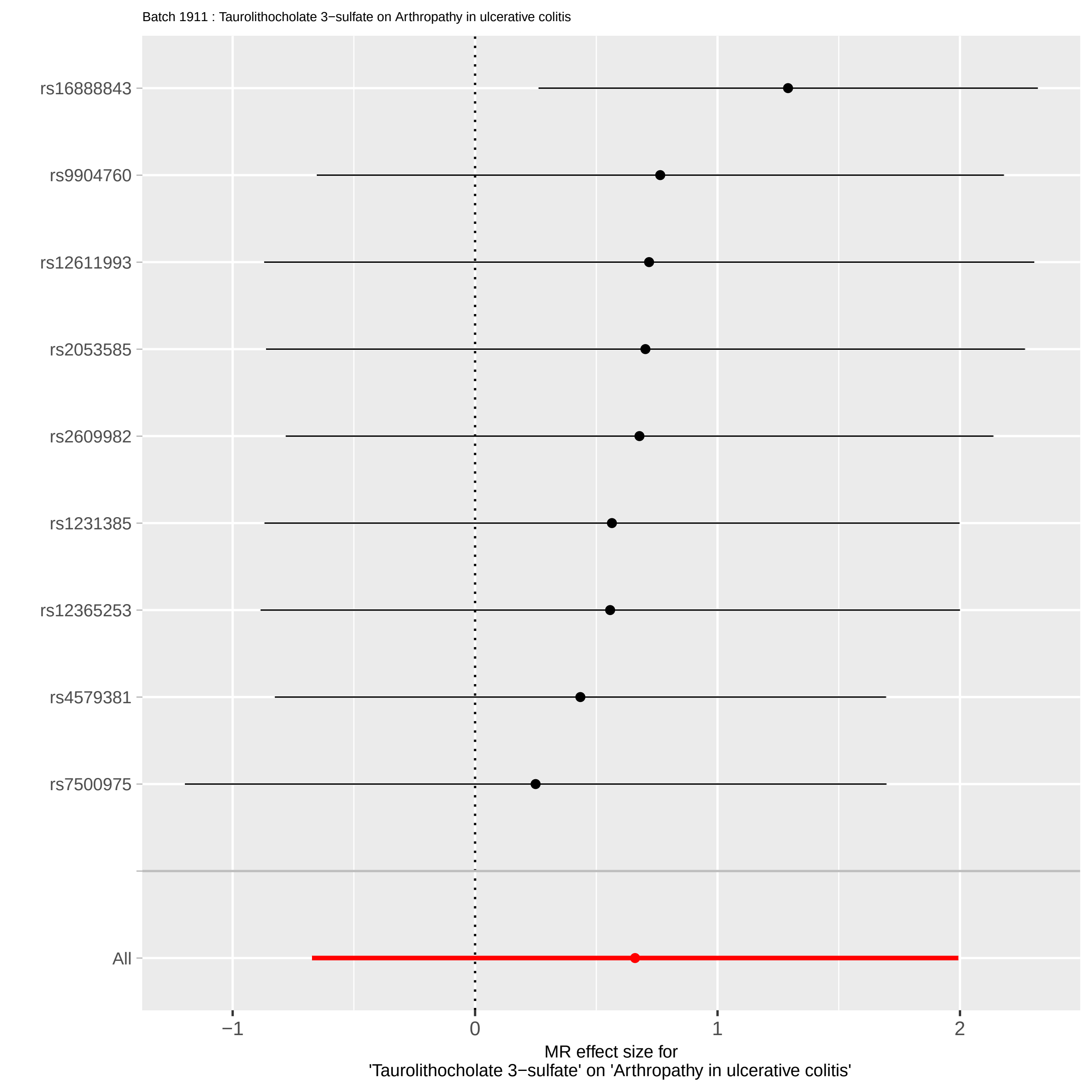

Batch 1911 : Taurolithocholate 3−sulfate on Arthropathy in ulcerative colitis
rs16888843
rs9904760
rs12611993
rs2053585
rs2609982
rs1231385
rs12365253
rs4579381
rs7500975
All
−1
0
1
2
MR effect size for
'Taurolithocholate 3−sulfate' on 'Arthropathy in ulcerative colitis'

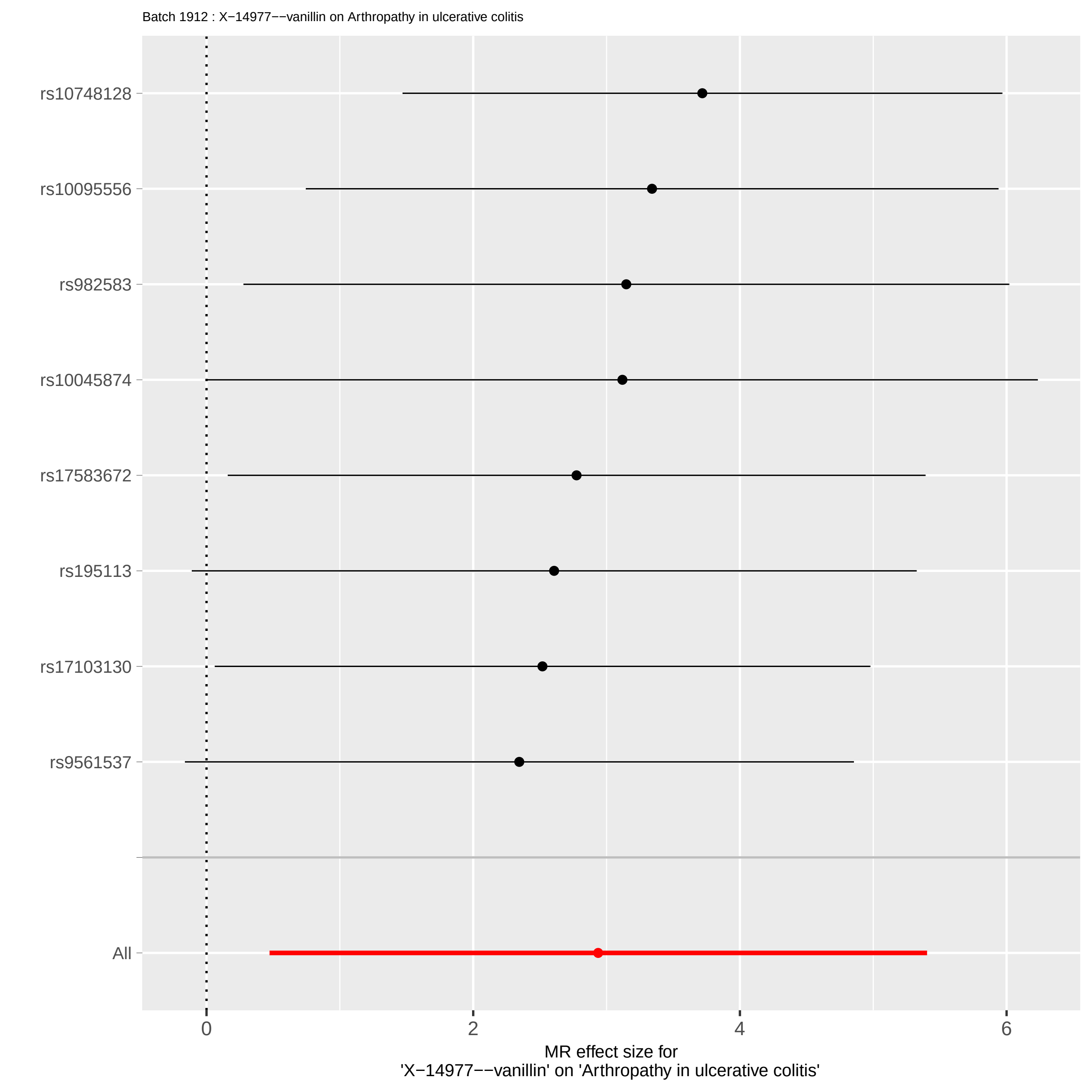
Batch 1912 : X−14977−−vanillin on Arthropathy in ulcerative colitis
rs10748128
rs10095556
rs982583
rs10045874
rs17583672
rs195113
rs17103130
rs9561537
All
0
2
4
6
MR effect size for
'X−14977−−vanillin' on 'Arthropathy in ulcerative colitis'

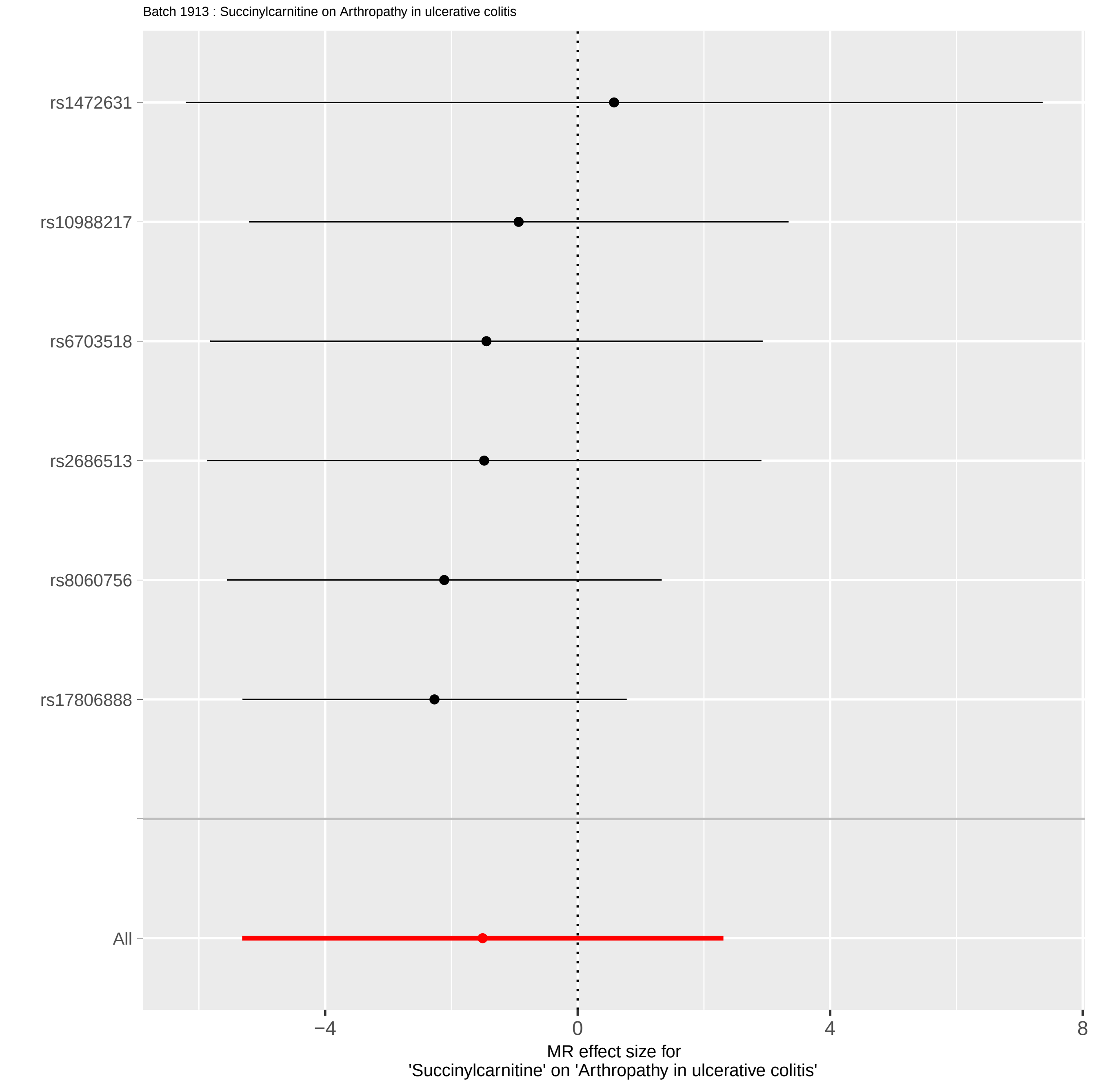
Batch 1913 : Succinylcarnitine on Arthropathy in ulcerative colitis
rs1472631
rs10988217
rs6703518
rs2686513
rs8060756
rs17806888
All
−4
0
4
8
MR effect size for
'Succinylcarnitine' on 'Arthropathy in ulcerative colitis'

Batch 1915 : Cyclo(leu−pro) on Arthropathy in ulcerative colitis

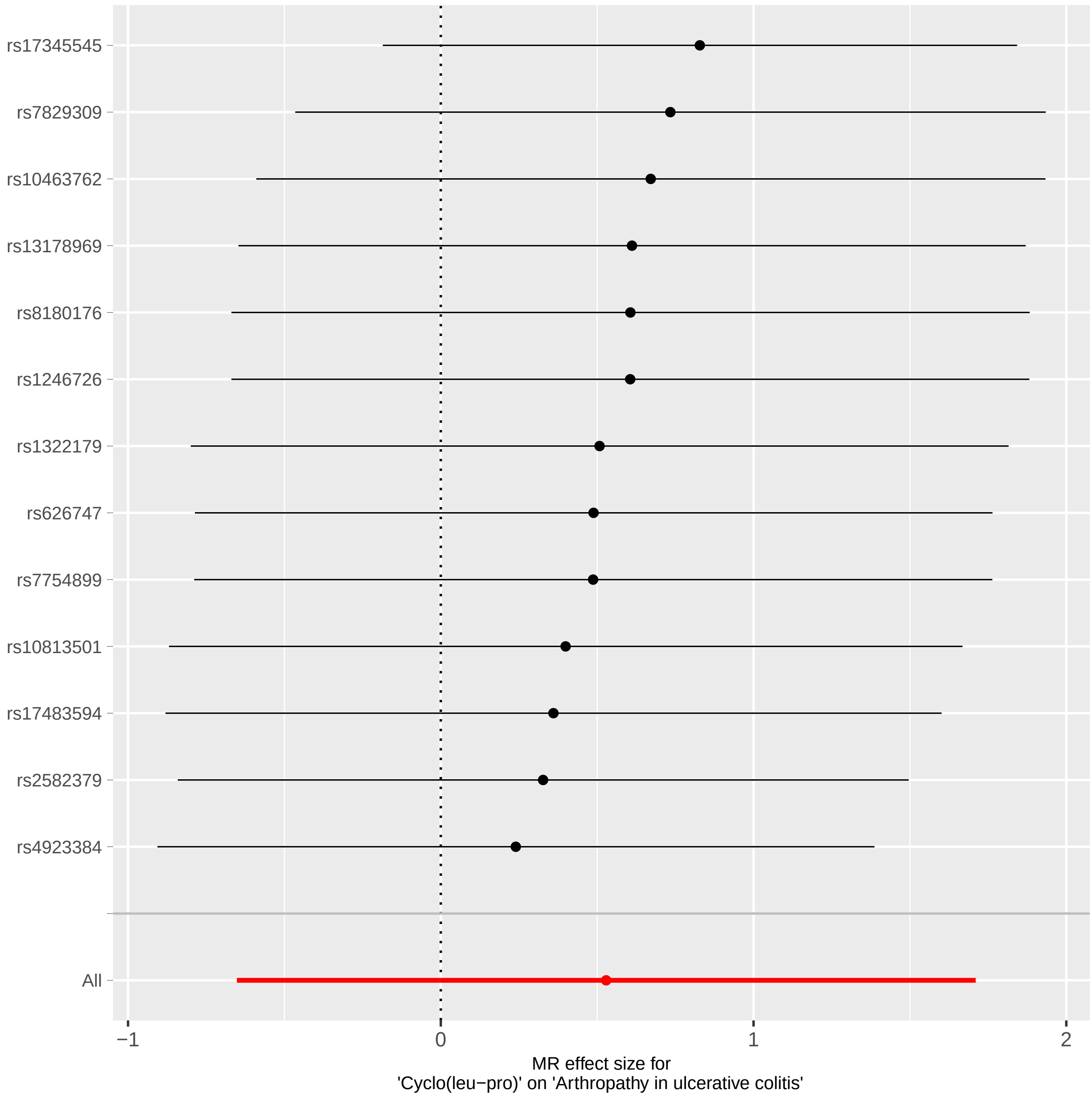

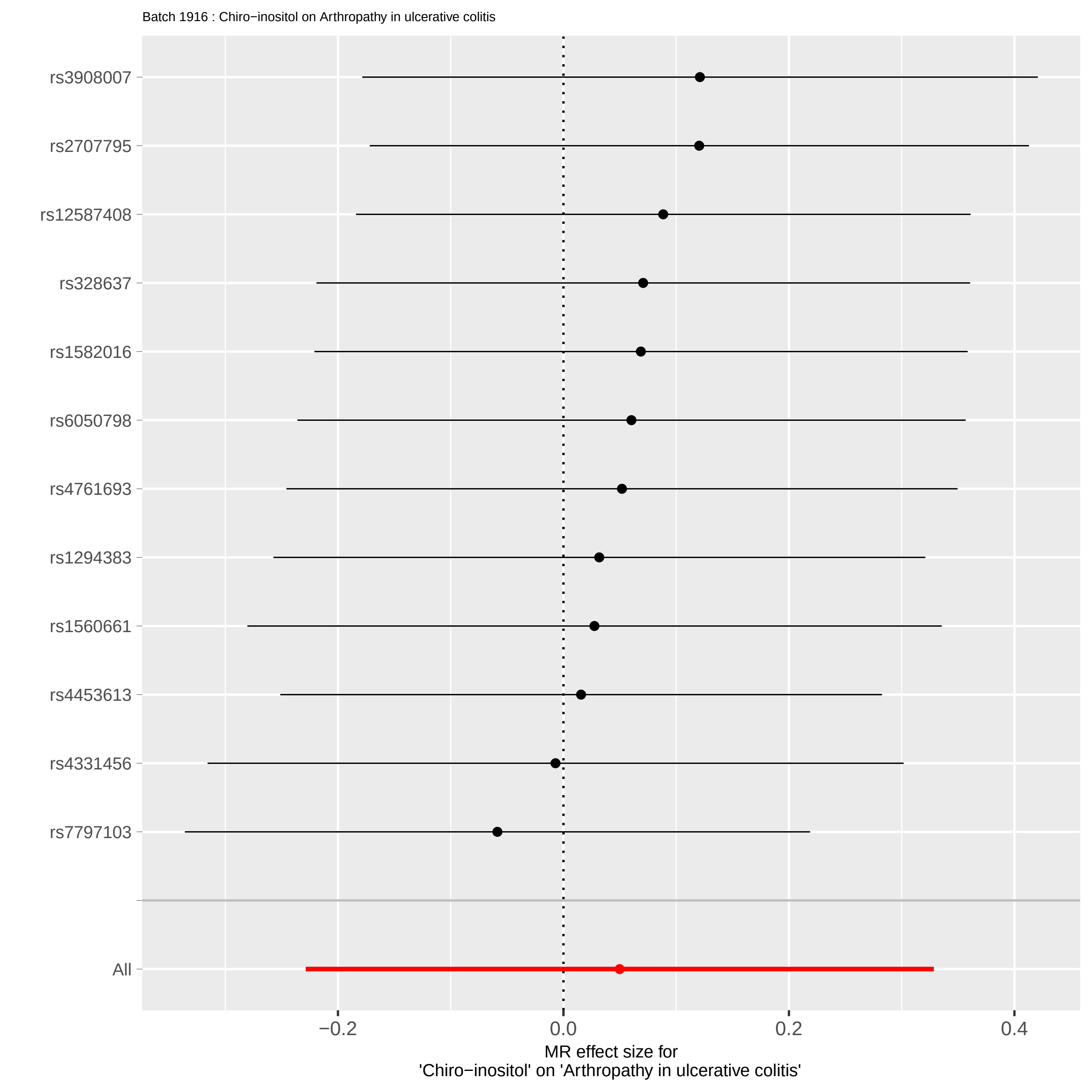

Batch 1916 : Chiro−inositol on Arthropathy in ulcerative colitis
rs3908007
rs2707795
rs12587408
rs328637
rs1582016
rs6050798
rs4761693
rs1294383
rs1560661
rs4453613
rs4331456
rs7797103
All
−0.2
0.0
0.2
0.4
MR effect size for
'Chiro−inositol' on 'Arthropathy in ulcerative colitis'

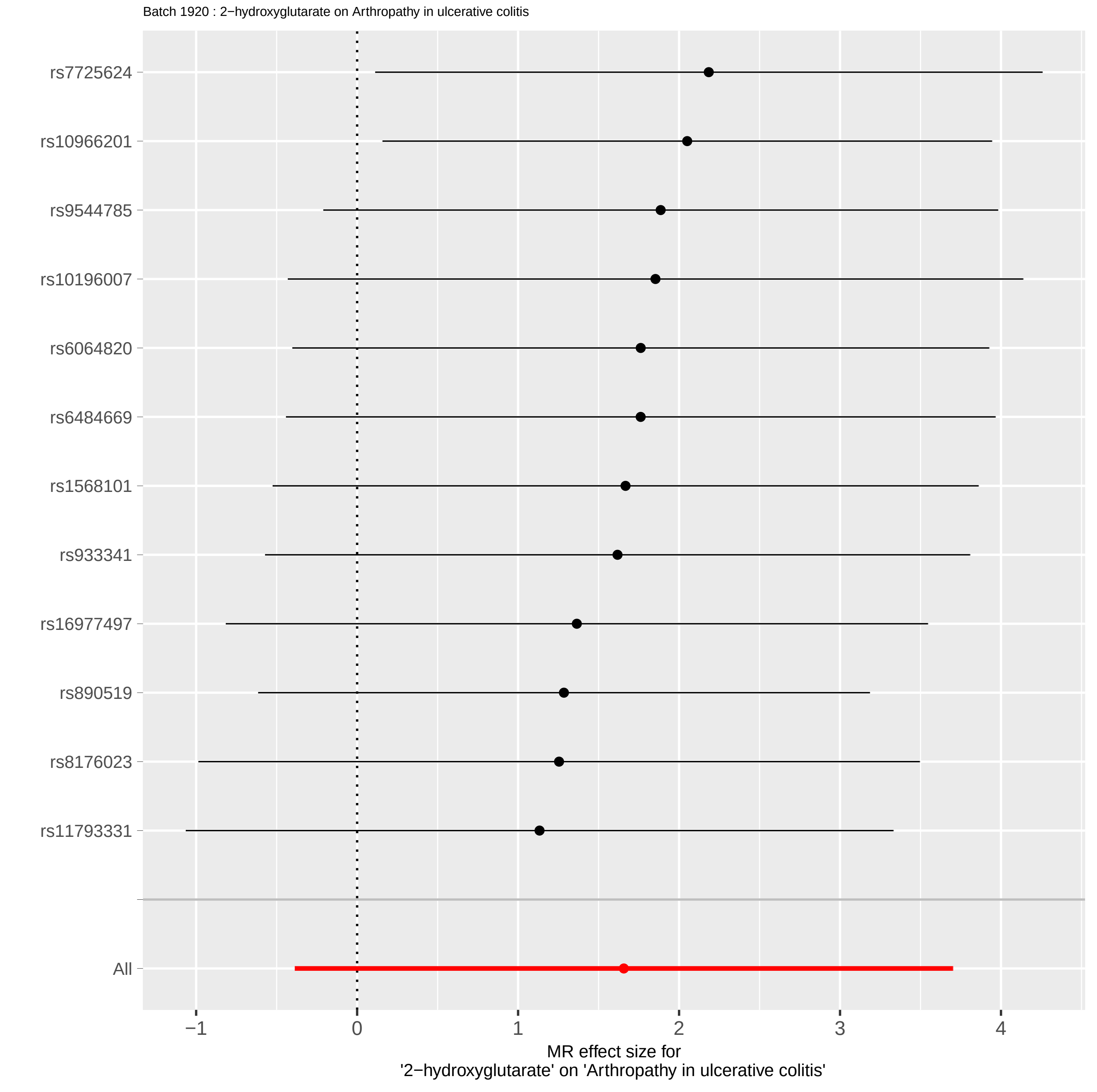

Batch 1920 : 2−hydroxyglutarate on Arthropathy in ulcerative colitis
rs7725624
rs10966201
rs9544785
rs10196007
rs6064820
rs6484669
rs1568101
rs933341
rs16977497
rs890519
rs8176023
rs11793331
All
−1
0
1
2
3
4
MR effect size for
'2−hydroxyglutarate' on 'Arthropathy in ulcerative colitis'

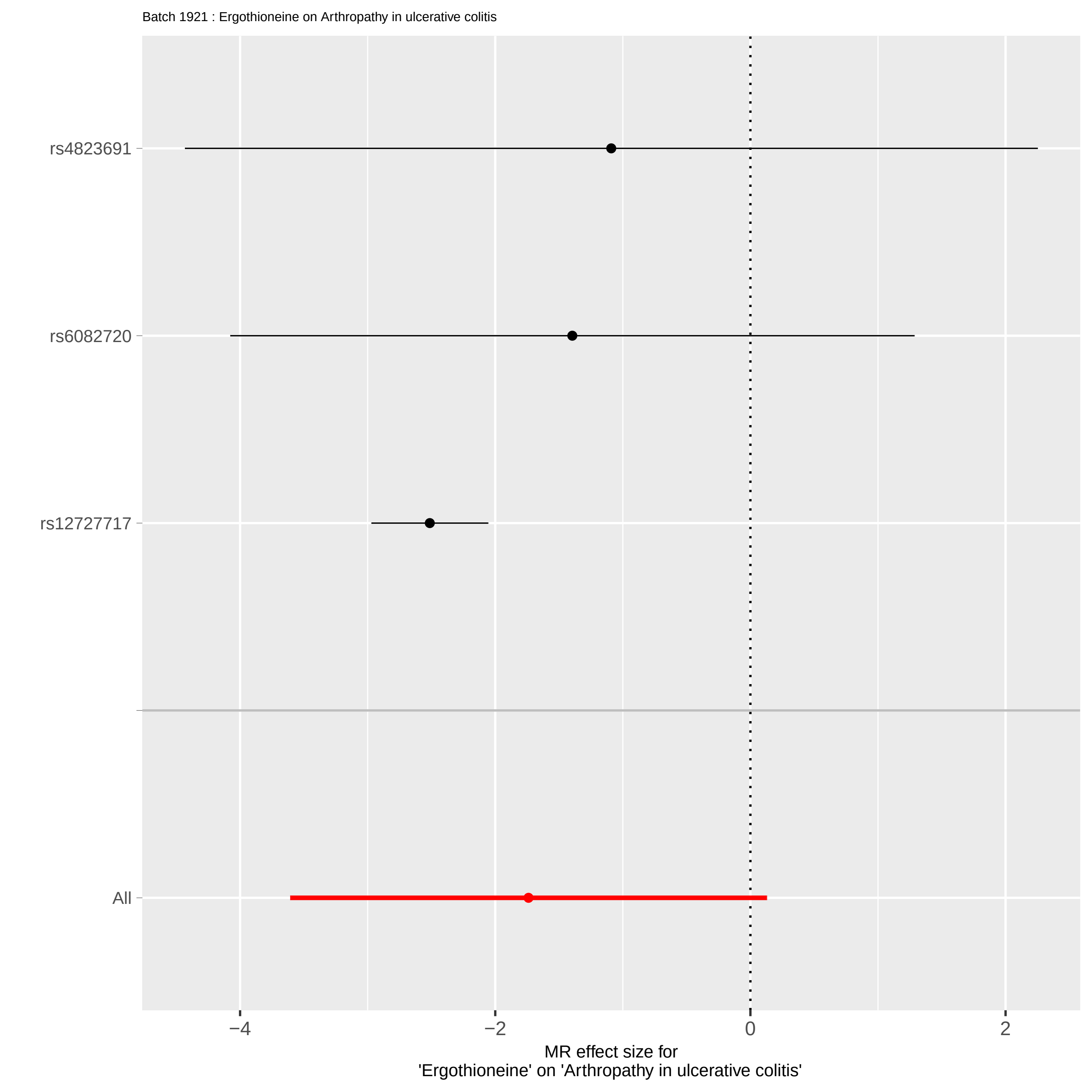
Batch 1921 : Ergothioneine on Arthropathy in ulcerative colitis
rs4823691
rs6082720
rs12727717
All
−4
−2
0
2
MR effect size for
'Ergothioneine' on 'Arthropathy in ulcerative colitis'

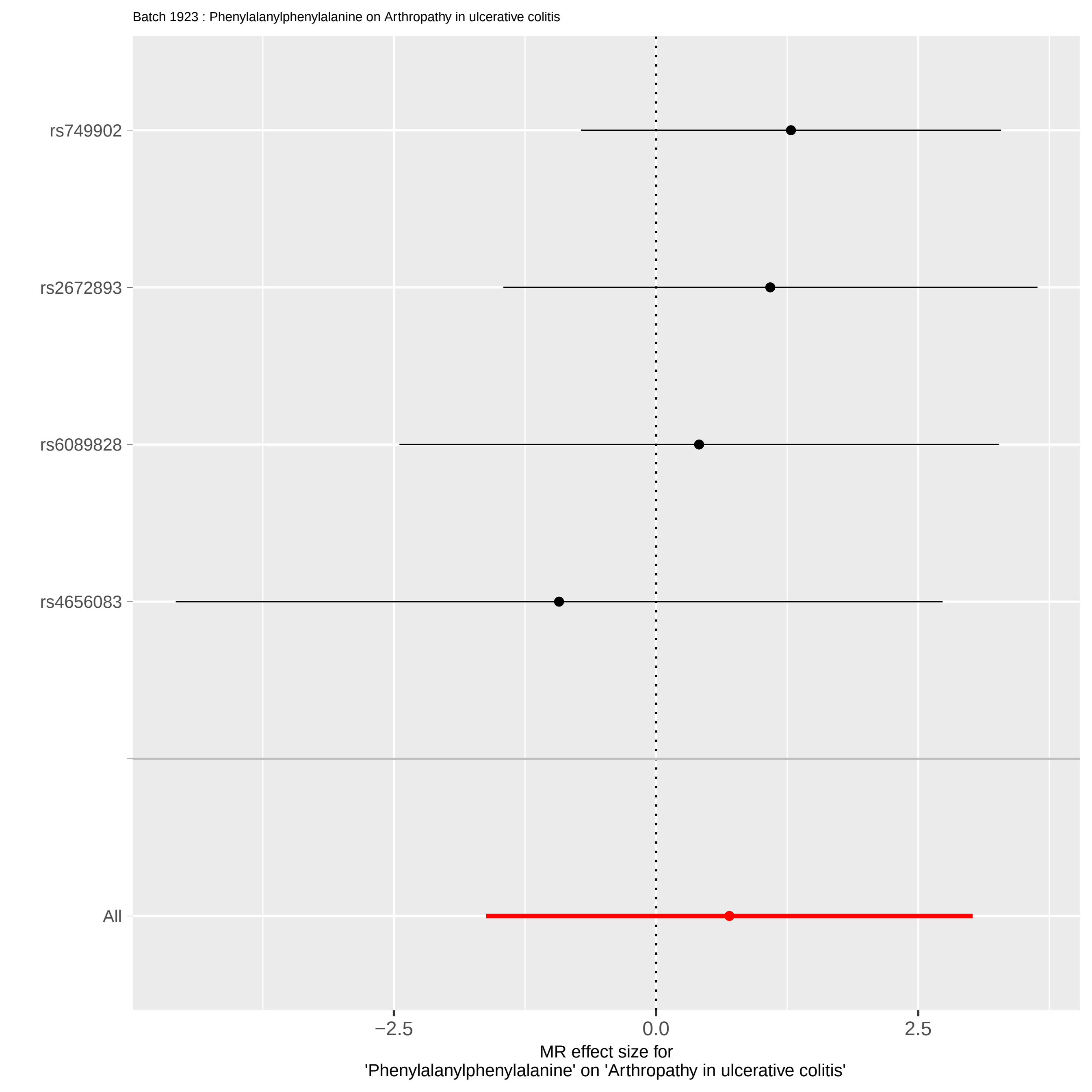

Batch 1923 : Phenylalanylphenylalanine on Arthropathy in ulcerative colitis
rs749902
rs2672893
rs6089828
rs4656083
All
−2.5
0.0
2.5
MR effect size for
'Phenylalanylphenylalanine' on 'Arthropathy in ulcerative colitis'

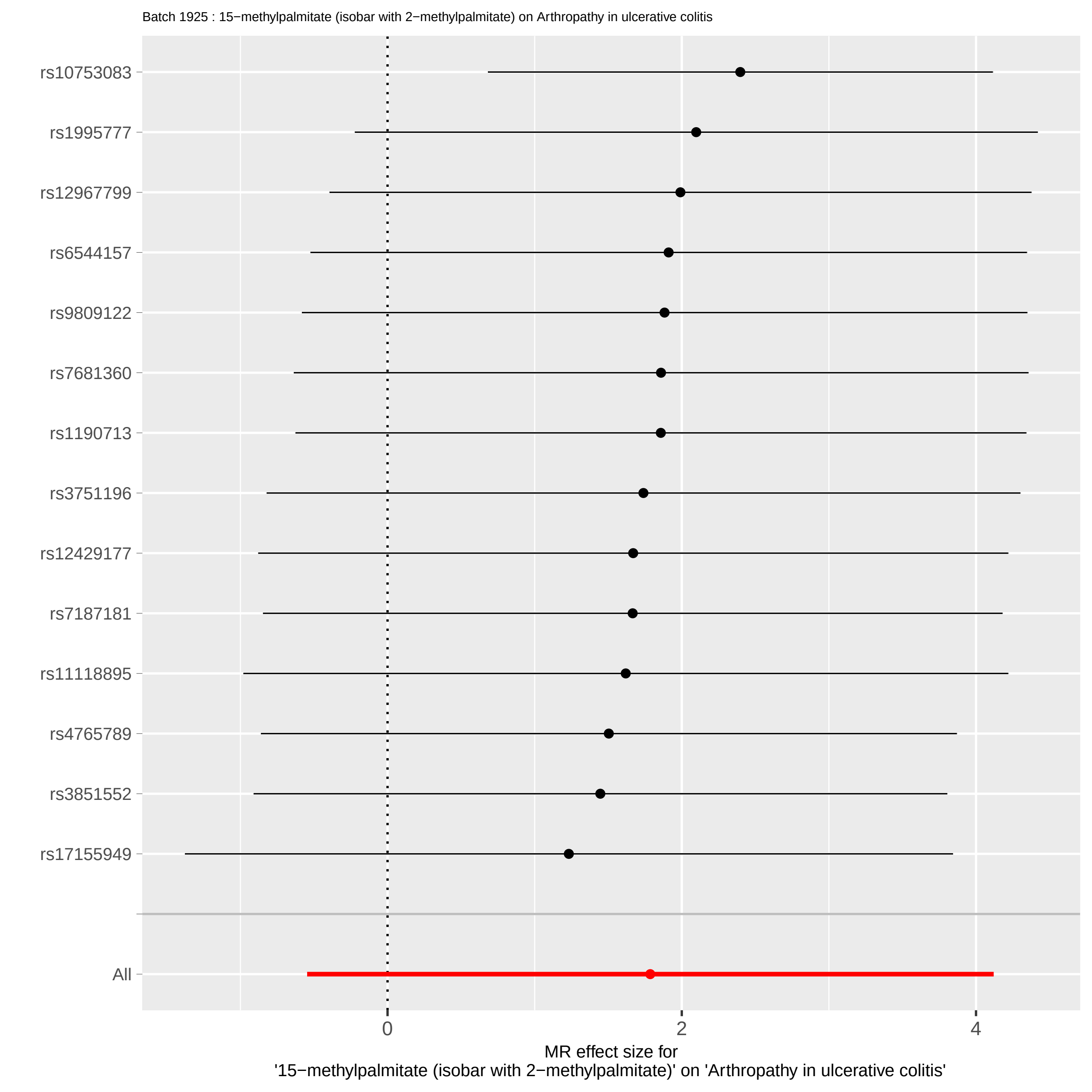

Batch 1925 : 15−methylpalmitate (isobar with 2−methylpalmitate) on Arthropathy in ulcerative colitis
rs10753083
rs1995777
rs12967799
rs6544157
rs9809122
rs7681360
rs1190713
rs3751196
rs12429177
rs7187181
rs11118895
rs4765789
rs3851552
rs17155949
All
0
2
4
MR effect size for
'15−methylpalmitate (isobar with 2−methylpalmitate)' on 'Arthropathy in ulcerative colitis'

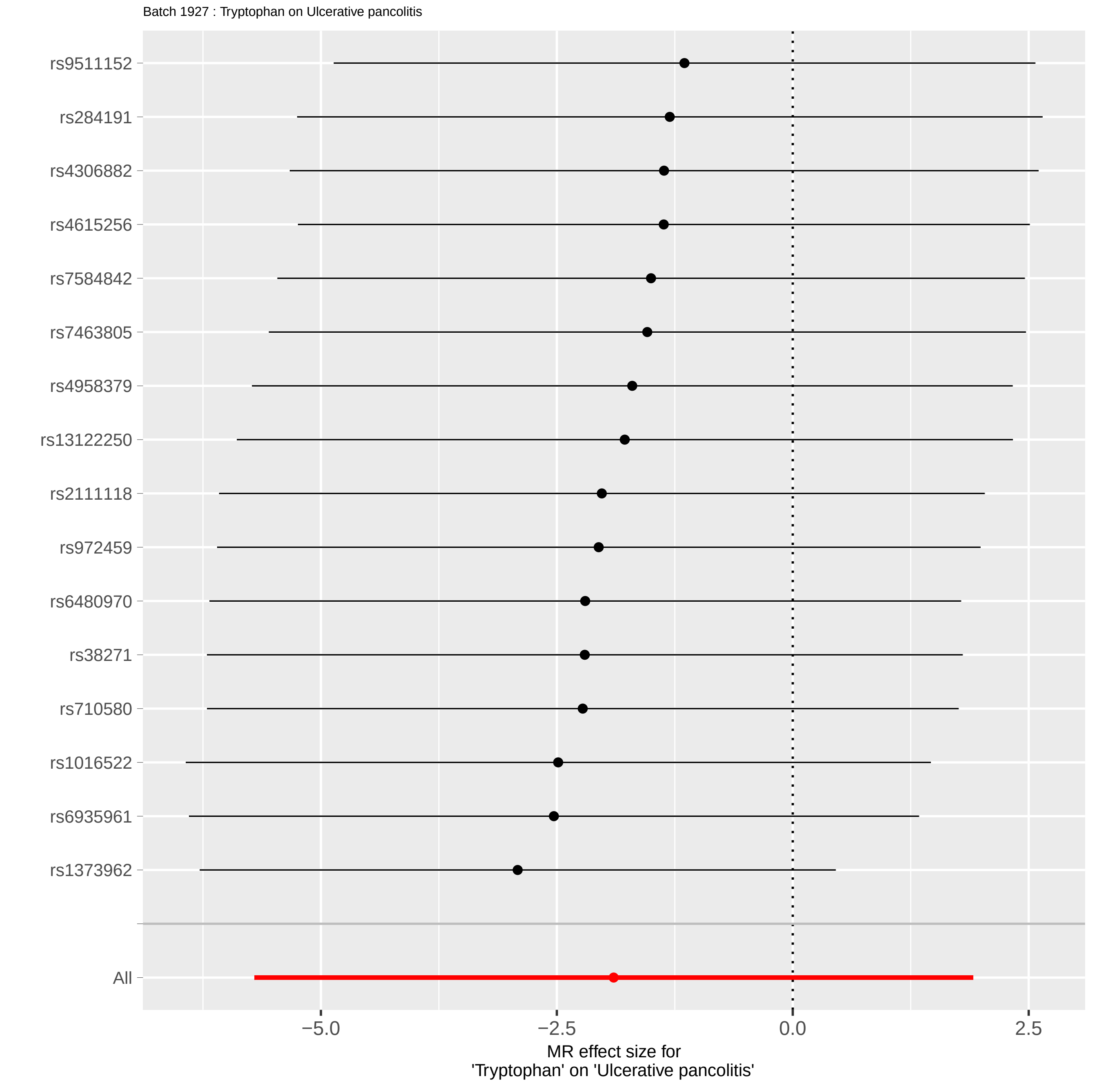

Batch 1927 : Tryptophan on Ulcerative pancolitis
rs9511152
rs284191
rs4306882
rs4615256
rs7584842
rs7463805
rs4958379
rs13122250
rs2111118
rs972459
rs6480970
rs38271
rs710580
rs1016522
rs6935961
rs1373962
All
−5.0
−2.5
0.0
2.5
MR effect size for
'Tryptophan' on 'Ulcerative pancolitis'

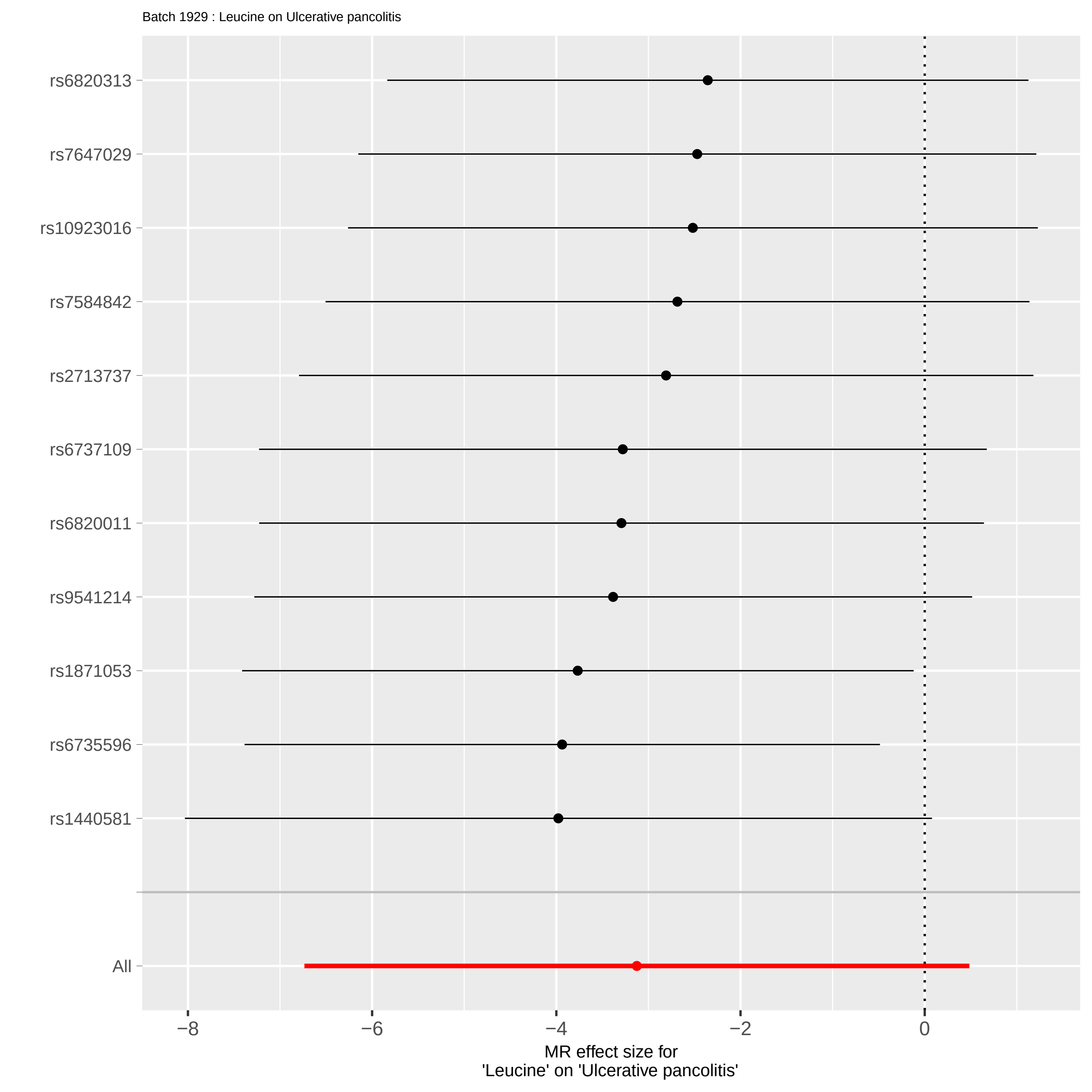

Batch 1929 : Leucine on Ulcerative pancolitis
rs6820313
rs7647029
rs10923016
rs7584842
rs2713737
rs6737109
rs6820011
rs9541214
rs1871053
rs6735596
rs1440581
All
−8
−6
−4
−2
0
MR effect size for
'Leucine' on 'Ulcerative pancolitis'

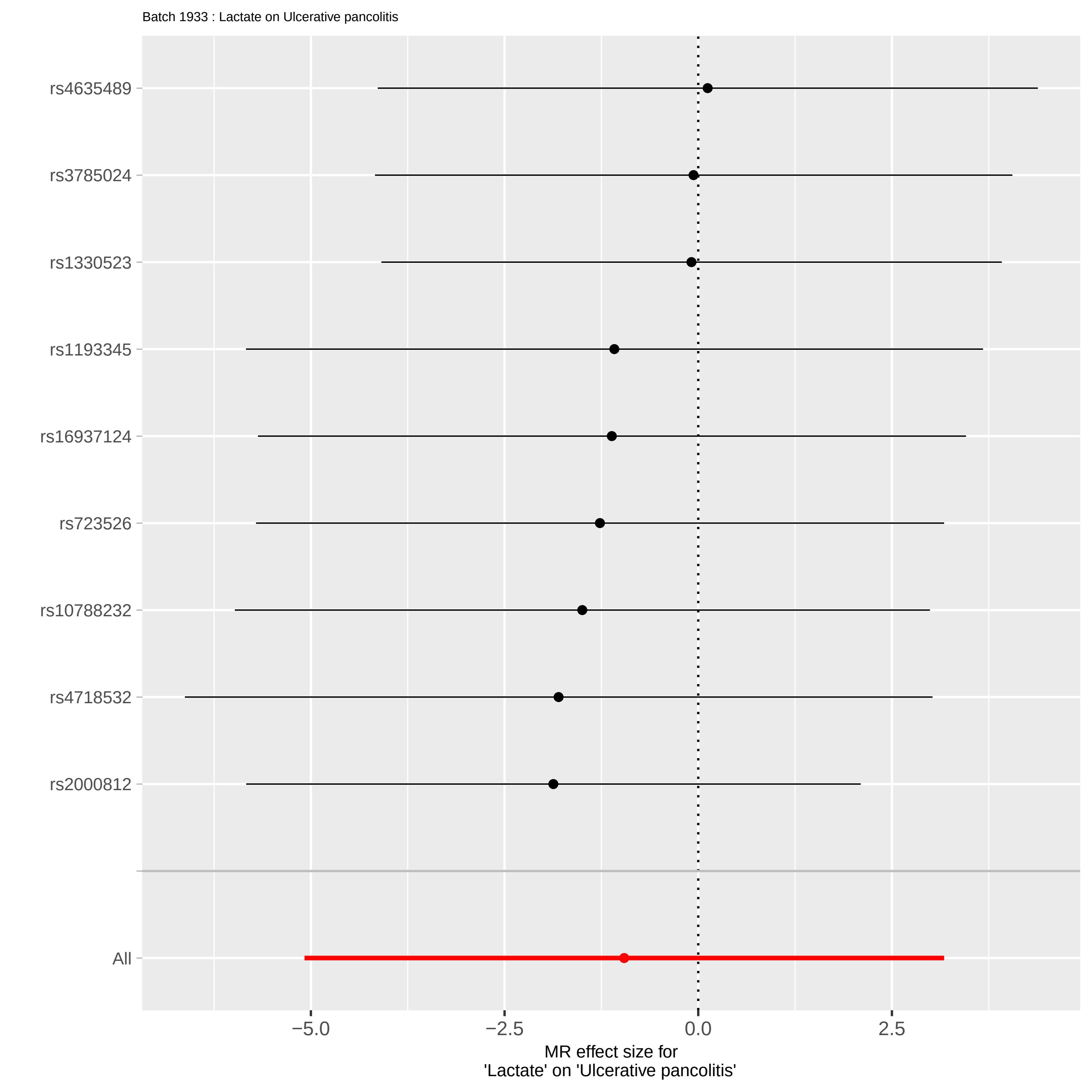

Batch 1933 : Lactate on Ulcerative pancolitis
rs4635489
rs3785024
rs1330523
rs1193345
rs16937124
rs723526
rs10788232
rs4718532
rs2000812
All
−5.0
−2.5
0.0
2.5
MR effect size for
'Lactate' on 'Ulcerative pancolitis'

Batch 1934 : 3−hydroxybutyrate (BHBA) on Ulcerative pancolitis

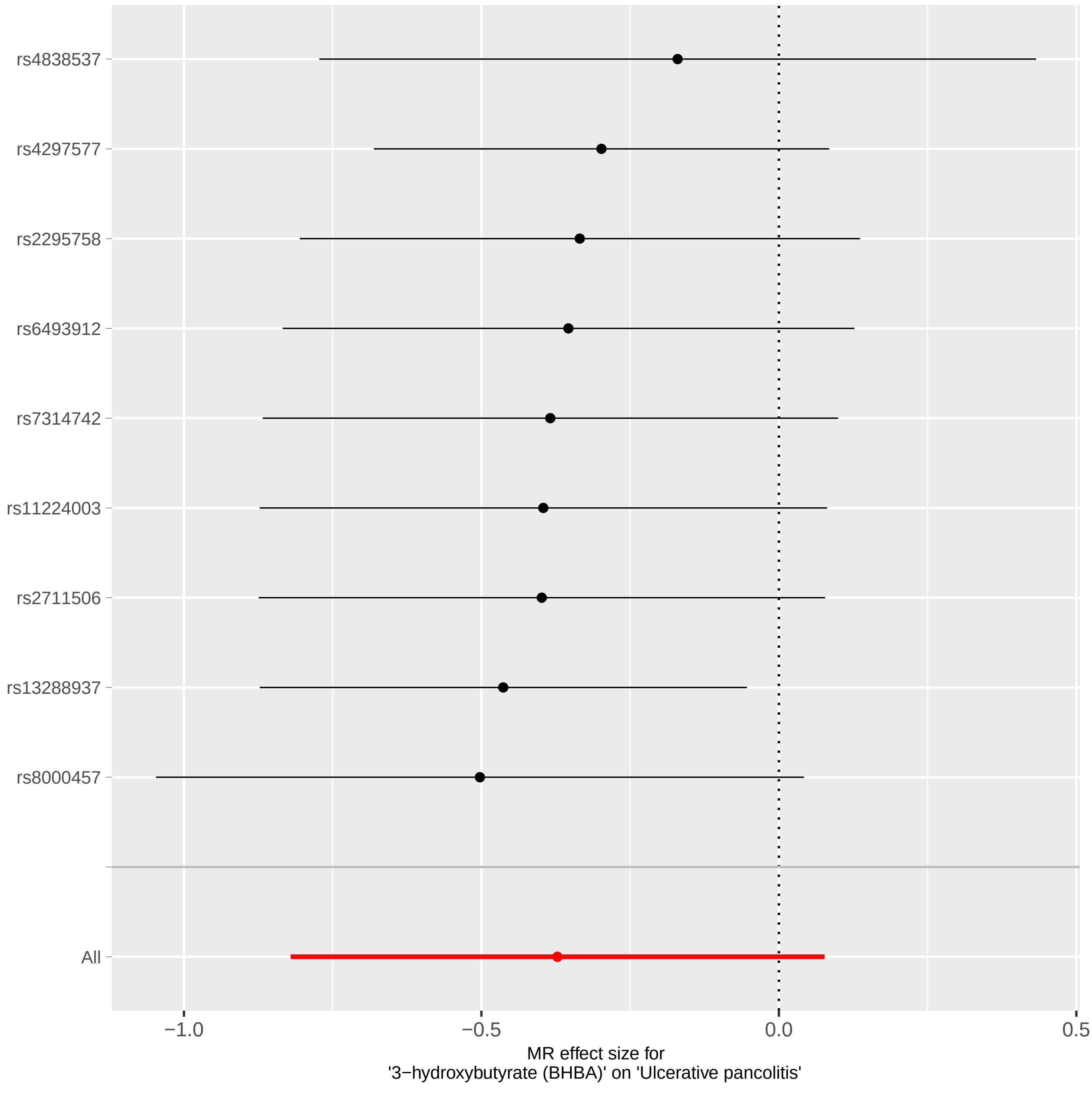

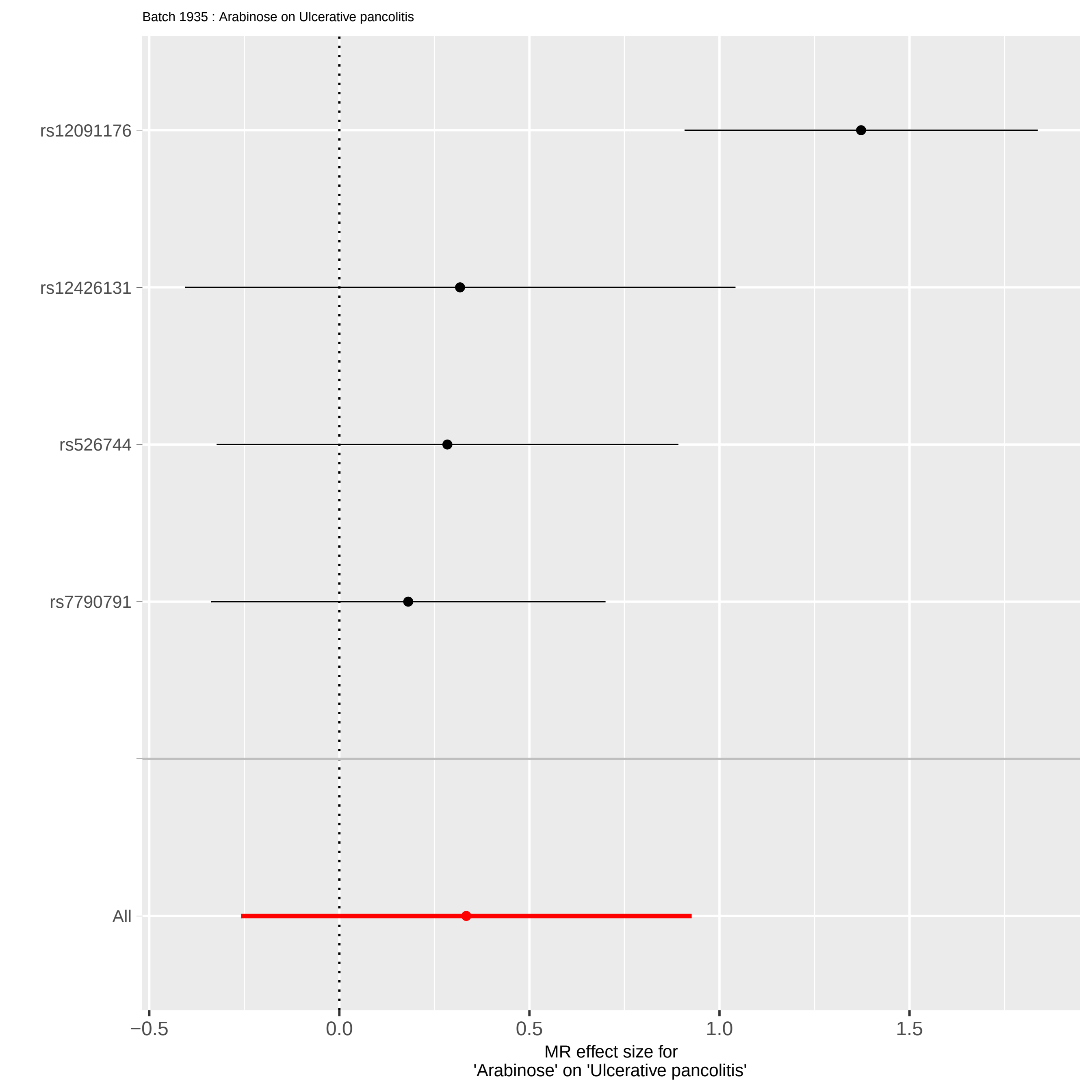

Batch 1935 : Arabinose on Ulcerative pancolitis
rs12091176
rs12426131
rs526744
rs7790791
All
−0.5
0.0
0.5
1.0
1.5
MR effect size for
'Arabinose' on 'Ulcerative pancolitis'

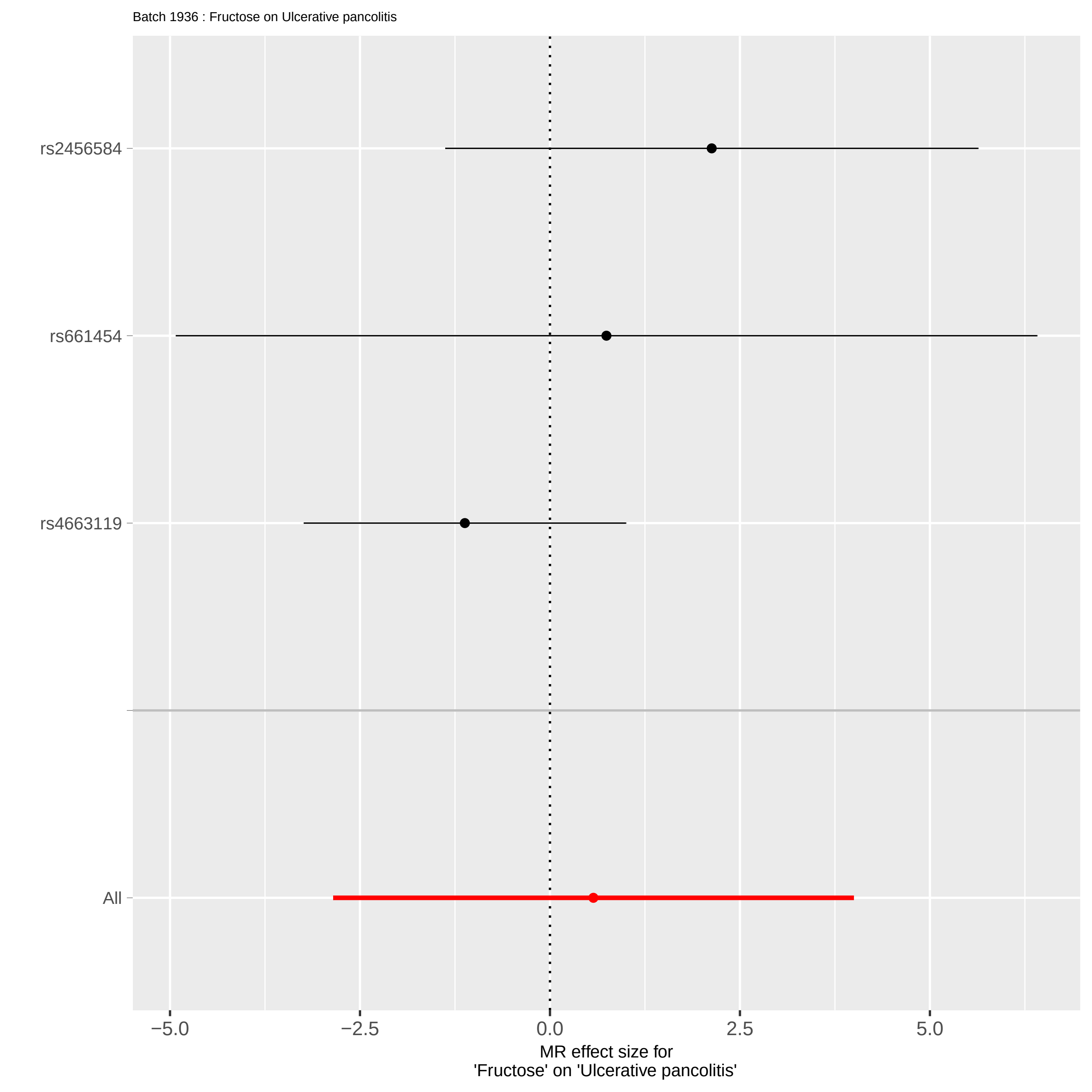

Batch 1936 : Fructose on Ulcerative pancolitis
rs2456584
rs661454
rs4663119
All
−5.0
−2.5
0.0
2.5
5.0
MR effect size for
'Fructose' on 'Ulcerative pancolitis'

Batch 1938 : Pyruvate on Ulcerative pancolitis

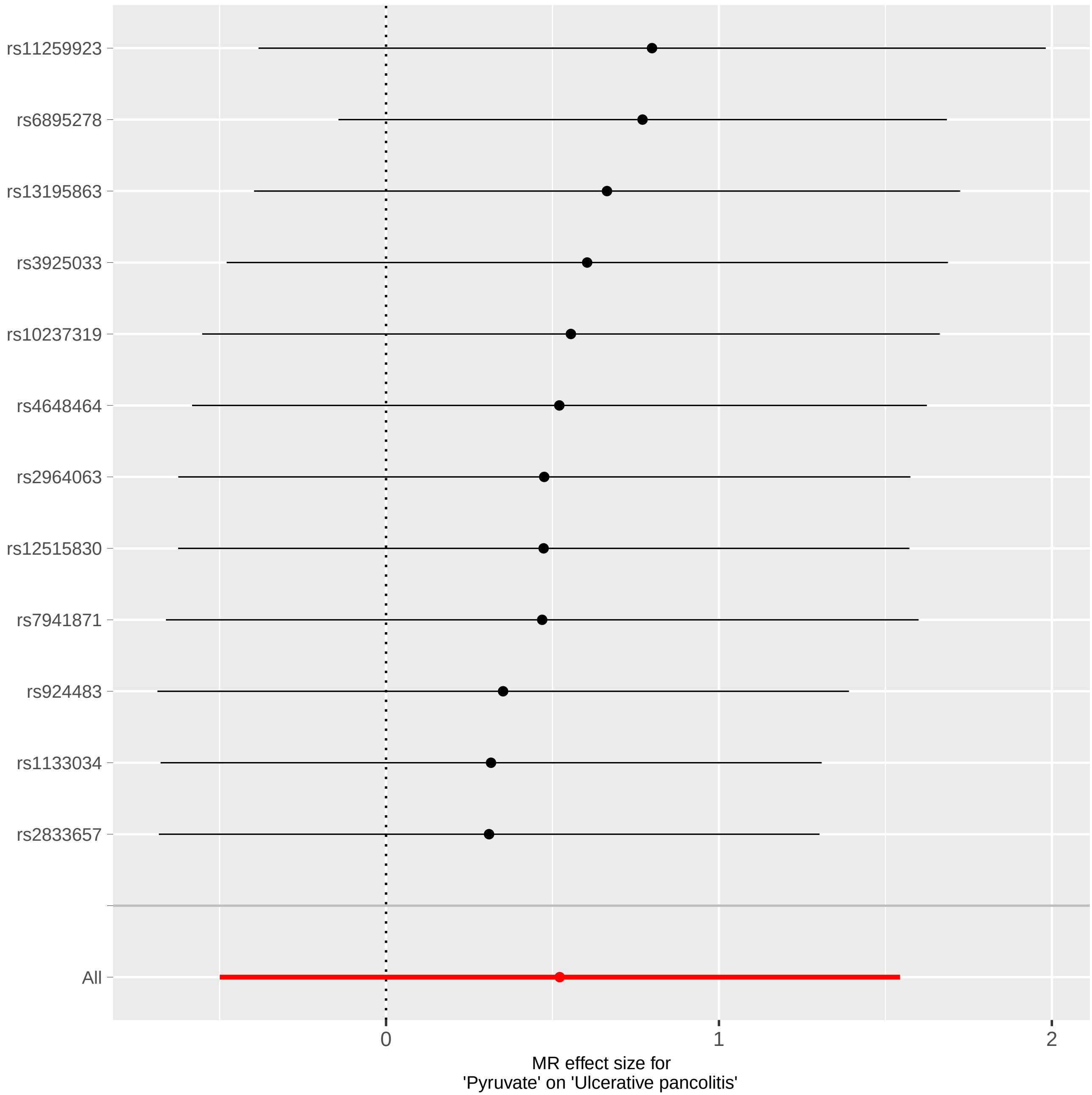

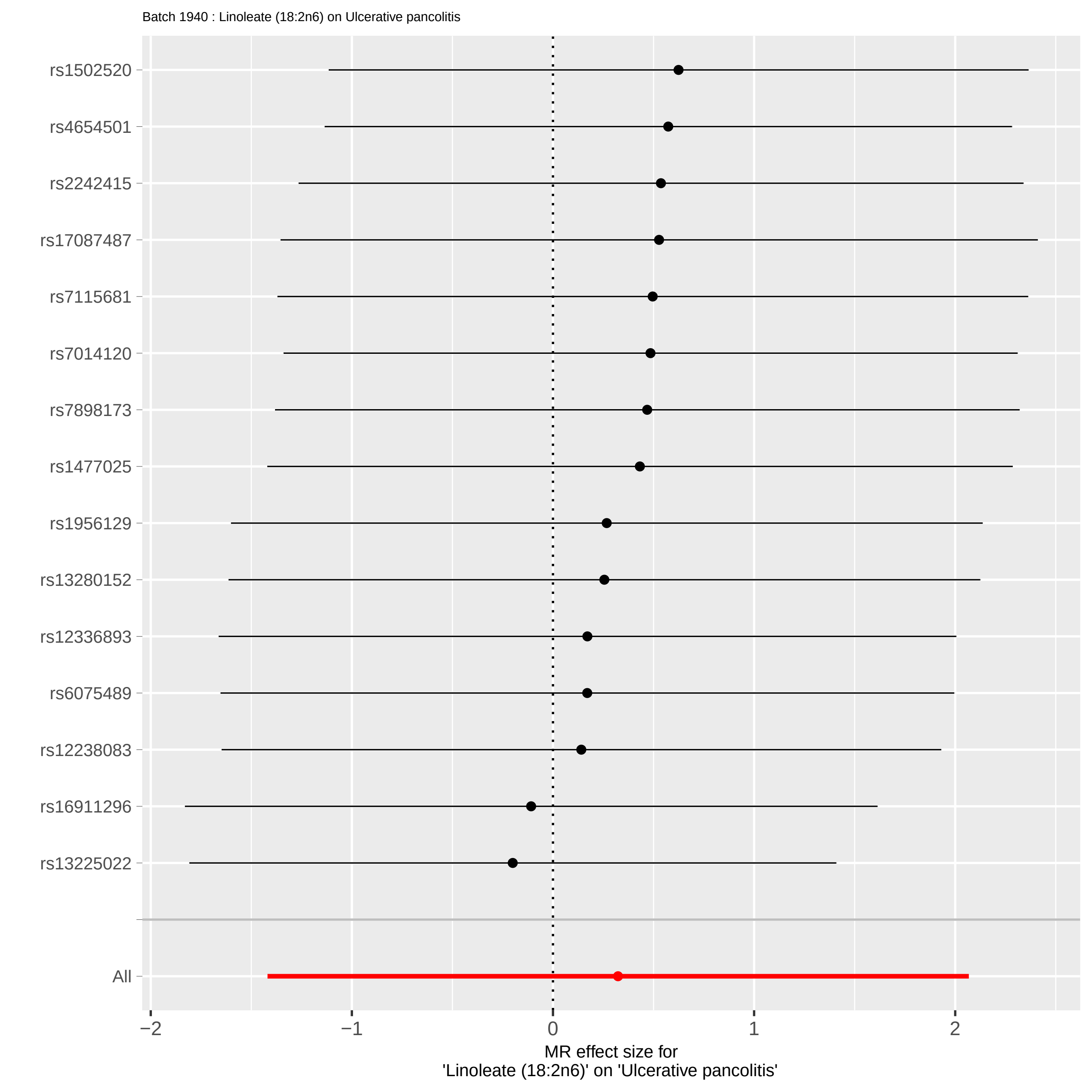

Batch 1940 : Linoleate (18:2n6) on Ulcerative pancolitis
rs1502520
rs4654501
rs2242415
rs17087487
rs7115681
rs7014120
rs7898173
rs1477025
rs1956129
rs13280152
rs12336893
rs6075489
rs12238083
rs16911296
rs13225022
All
−2
−1
0
1
2
MR effect size for
'Linoleate (18:2n6)' on 'Ulcerative pancolitis'

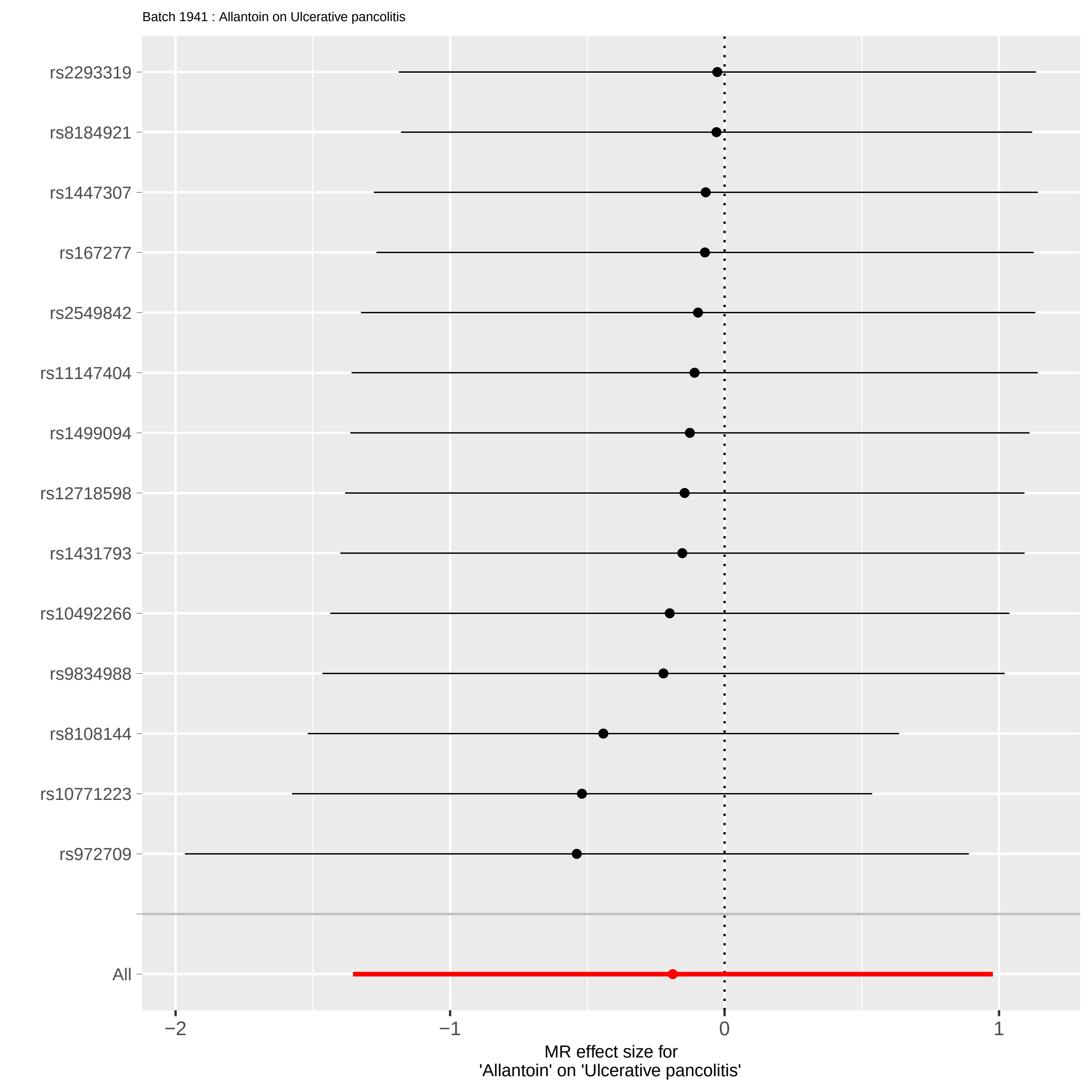
Batch 1941 : Allantoin on Ulcerative pancolitis
rs2293319
rs8184921
rs1447307
rs167277
rs2549842
rs11147404
rs1499094
rs12718598
rs1431793
rs10492266
rs9834988
rs8108144
rs10771223
rs972709
All
−2
−1
0
1
MR effect size for
'Allantoin' on 'Ulcerative pancolitis'

Batch 1946 : Isoleucine on Ulcerative pancolitis

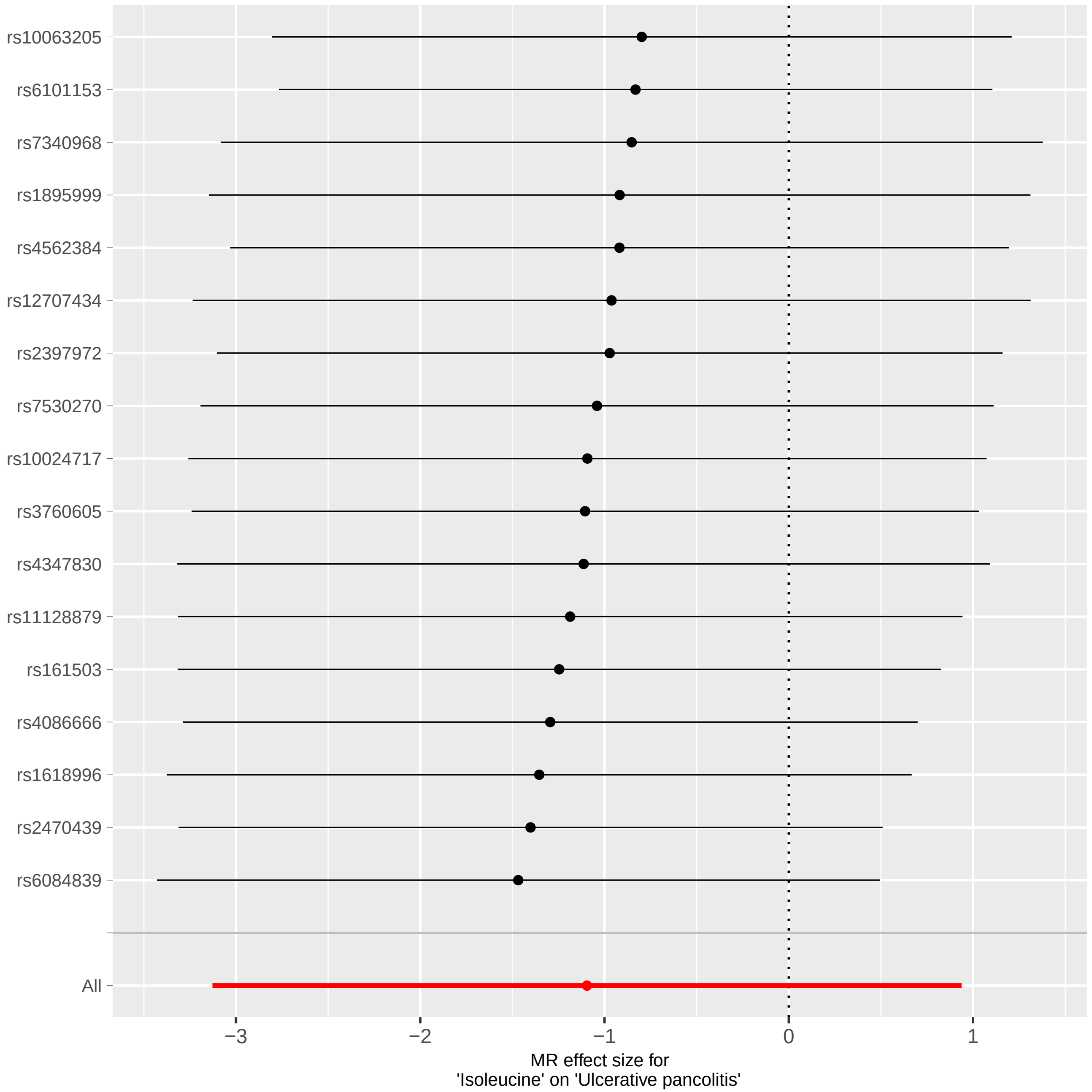

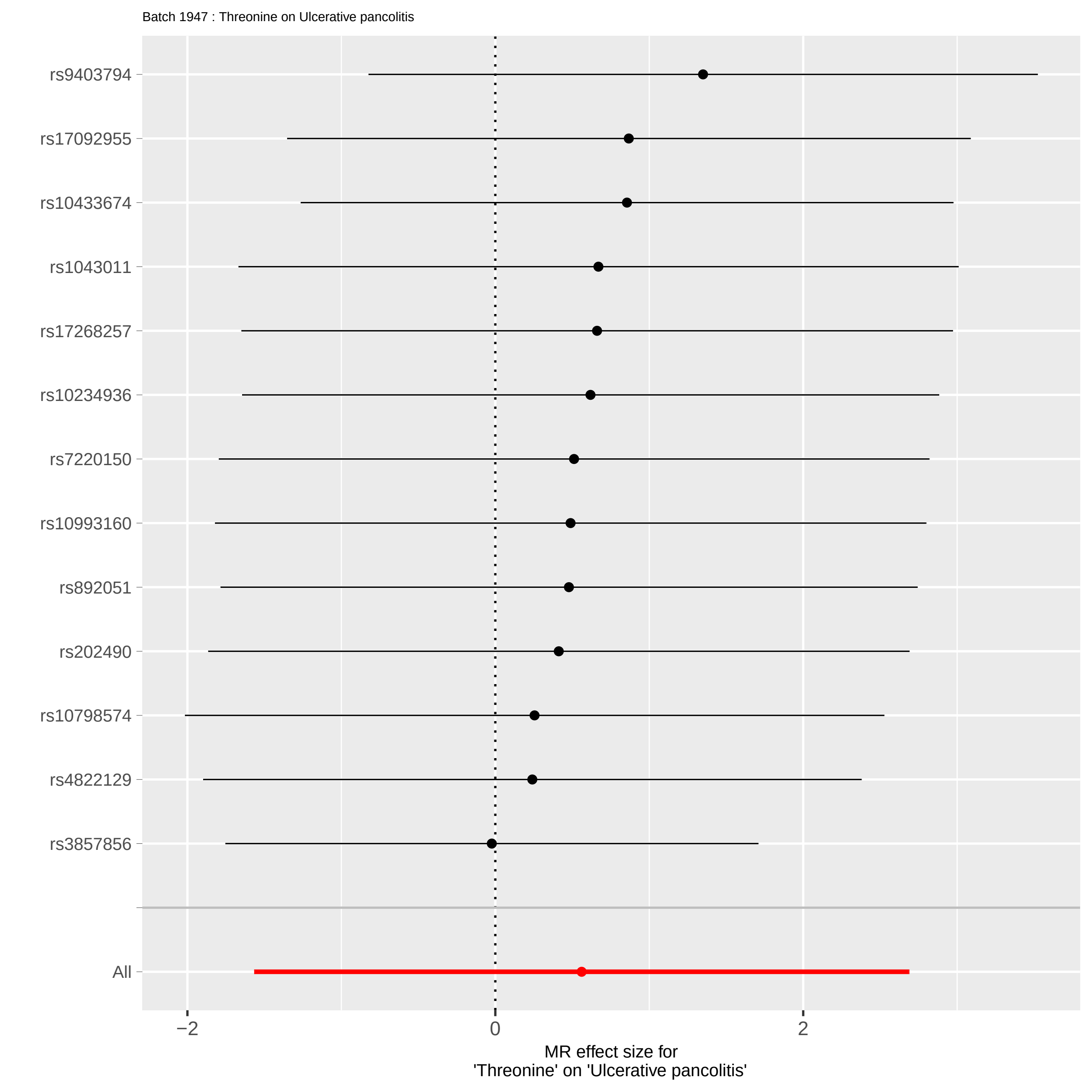

Batch 1947 : Threonine on Ulcerative pancolitis
rs9403794
rs17092955
rs10433674
rs1043011
rs17268257
rs10234936
rs7220150
rs10993160
rs892051
rs202490
rs10798574
rs4822129
rs3857856
All
−2
0
2
MR effect size for
'Threonine' on 'Ulcerative pancolitis'

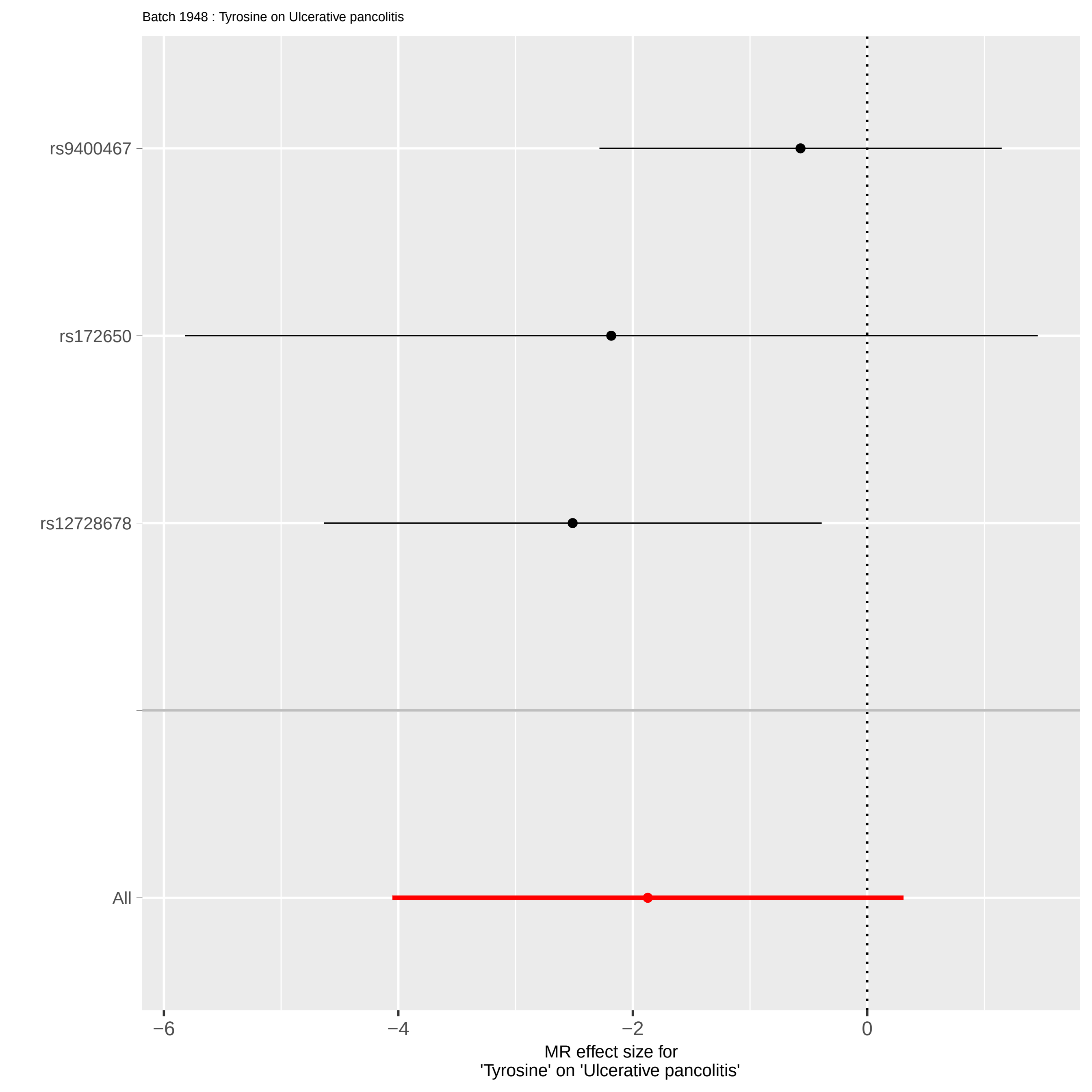
Batch 1948 : Tyrosine on Ulcerative pancolitis
rs9400467
rs172650
rs12728678
All
−6
−4
−2
0
MR effect size for
'Tyrosine' on 'Ulcerative pancolitis'

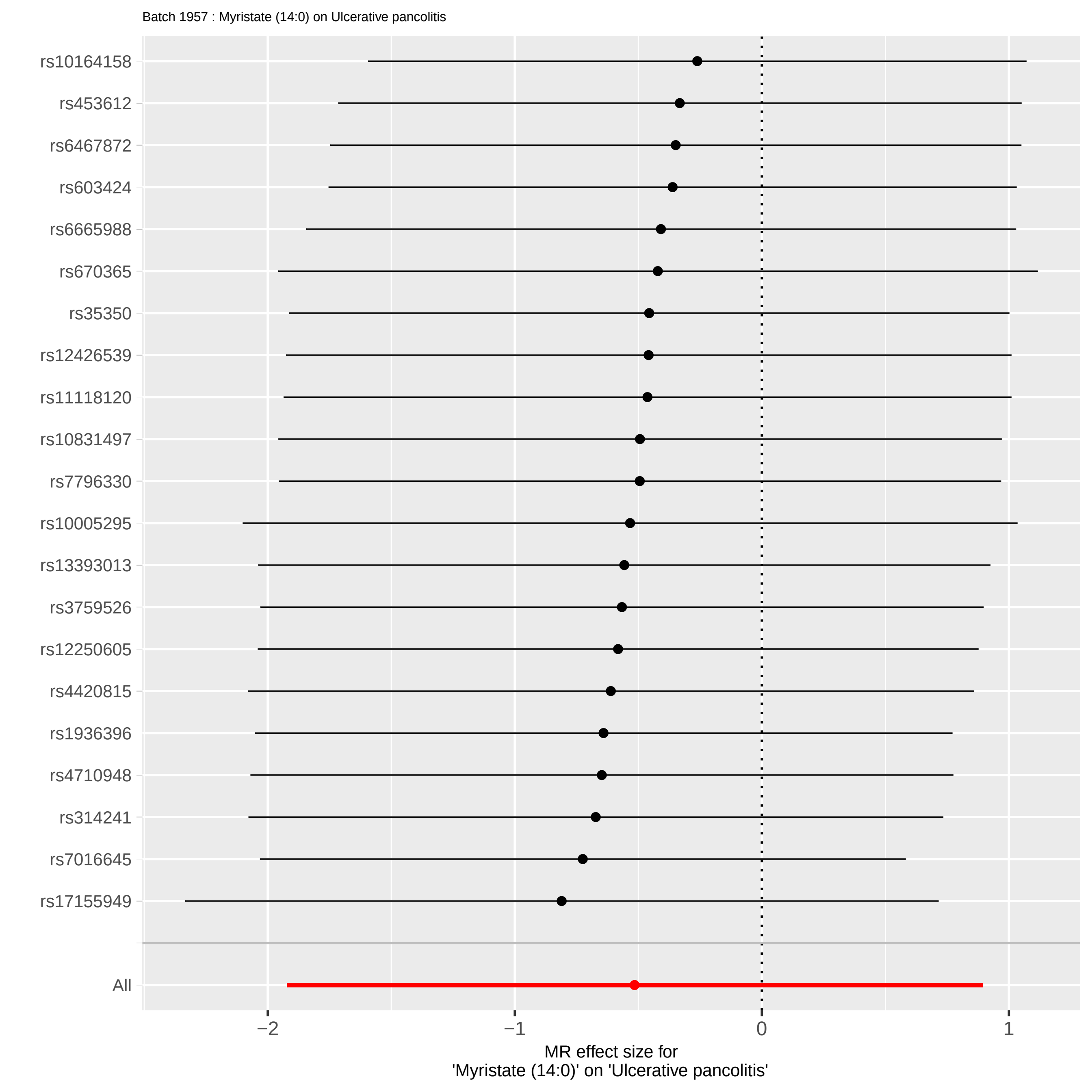

Batch 1957 : Myristate (14:0) on Ulcerative pancolitis
rs10164158
rs453612
rs6467872
rs603424
rs6665988
rs670365
rs35350
rs12426539
rs11118120
rs10831497
rs7796330
rs10005295
rs13393013
rs3759526
rs12250605
rs4420815
rs1936396
rs4710948
rs314241
rs7016645
rs17155949
All
−2
−1
0
1
MR effect size for
'Myristate (14:0)' on 'Ulcerative pancolitis'

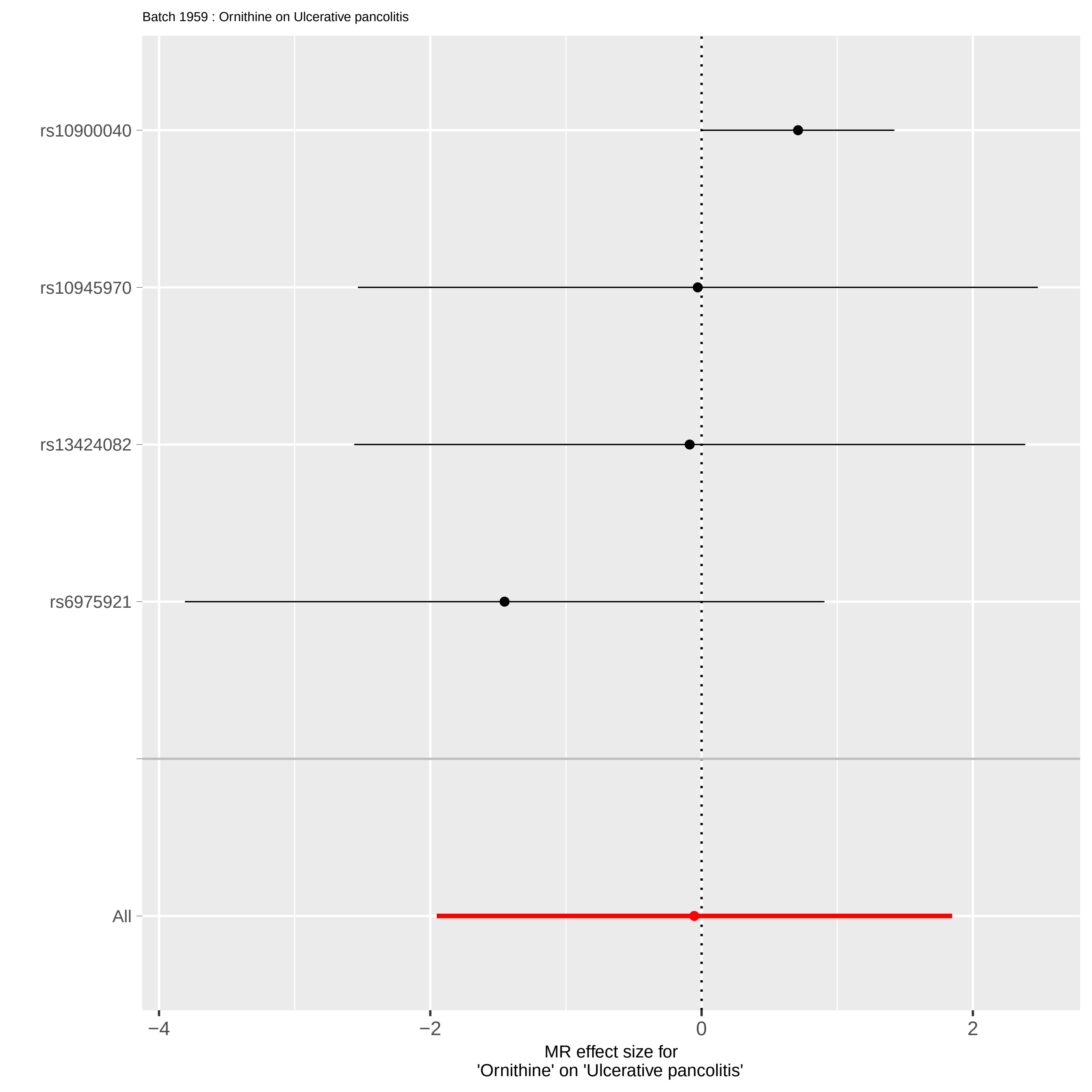
Batch 1959 : Ornithine on Ulcerative pancolitis
rs10900040
rs10945970
rs13424082
rs6975921
All
−4
−2
0
2
MR effect size for
'Ornithine' on 'Ulcerative pancolitis'

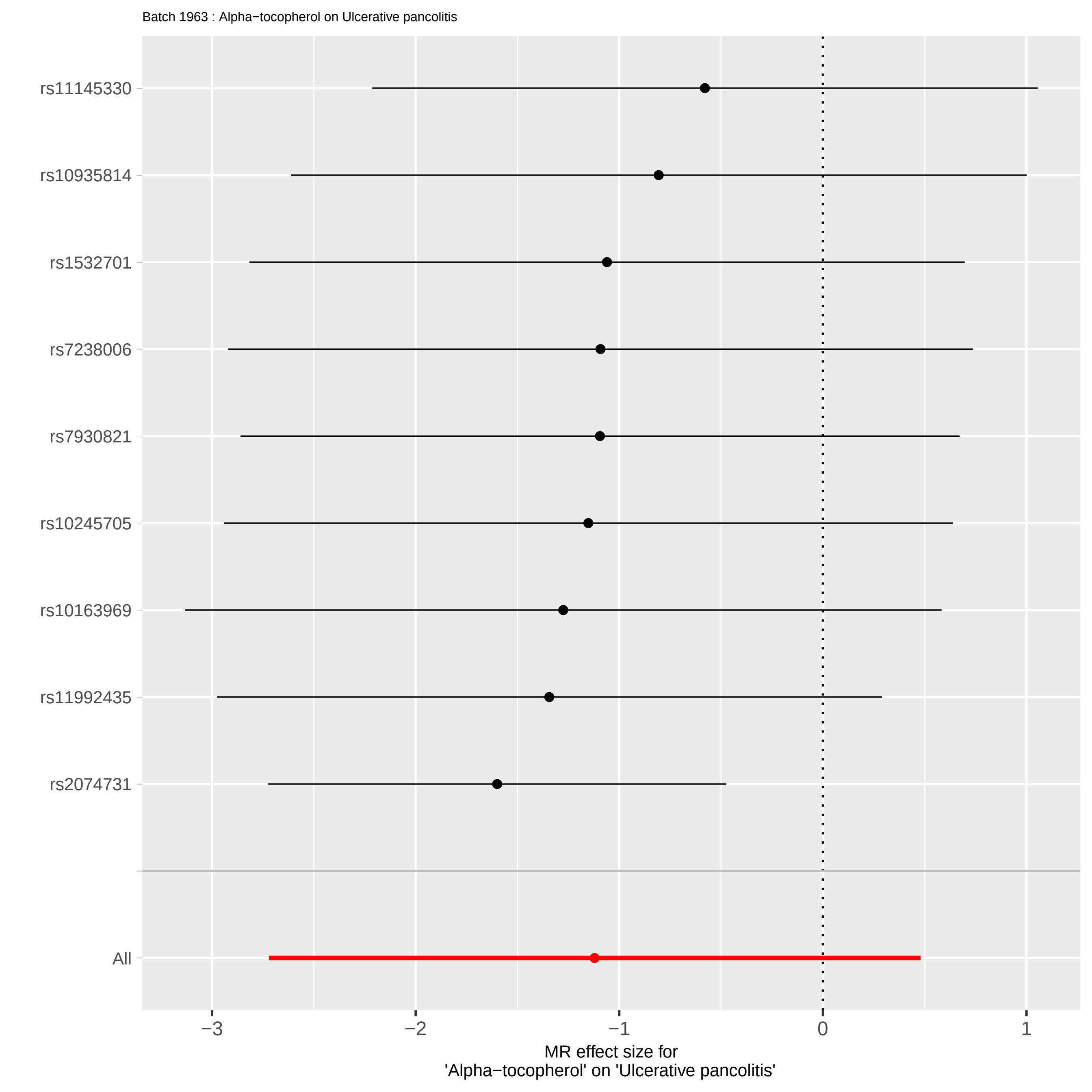
Batch 1963 : Alpha−tocopherol on Ulcerative pancolitis
rs11145330
rs10935814
rs1532701
rs7238006
rs7930821
rs10245705
rs10163969
rs11992435
rs2074731
All
−3
−2
−1
0
1
MR effect size for
'Alpha−tocopherol' on 'Ulcerative pancolitis'

Batch 1964 : Citrate on Ulcerative pancolitis

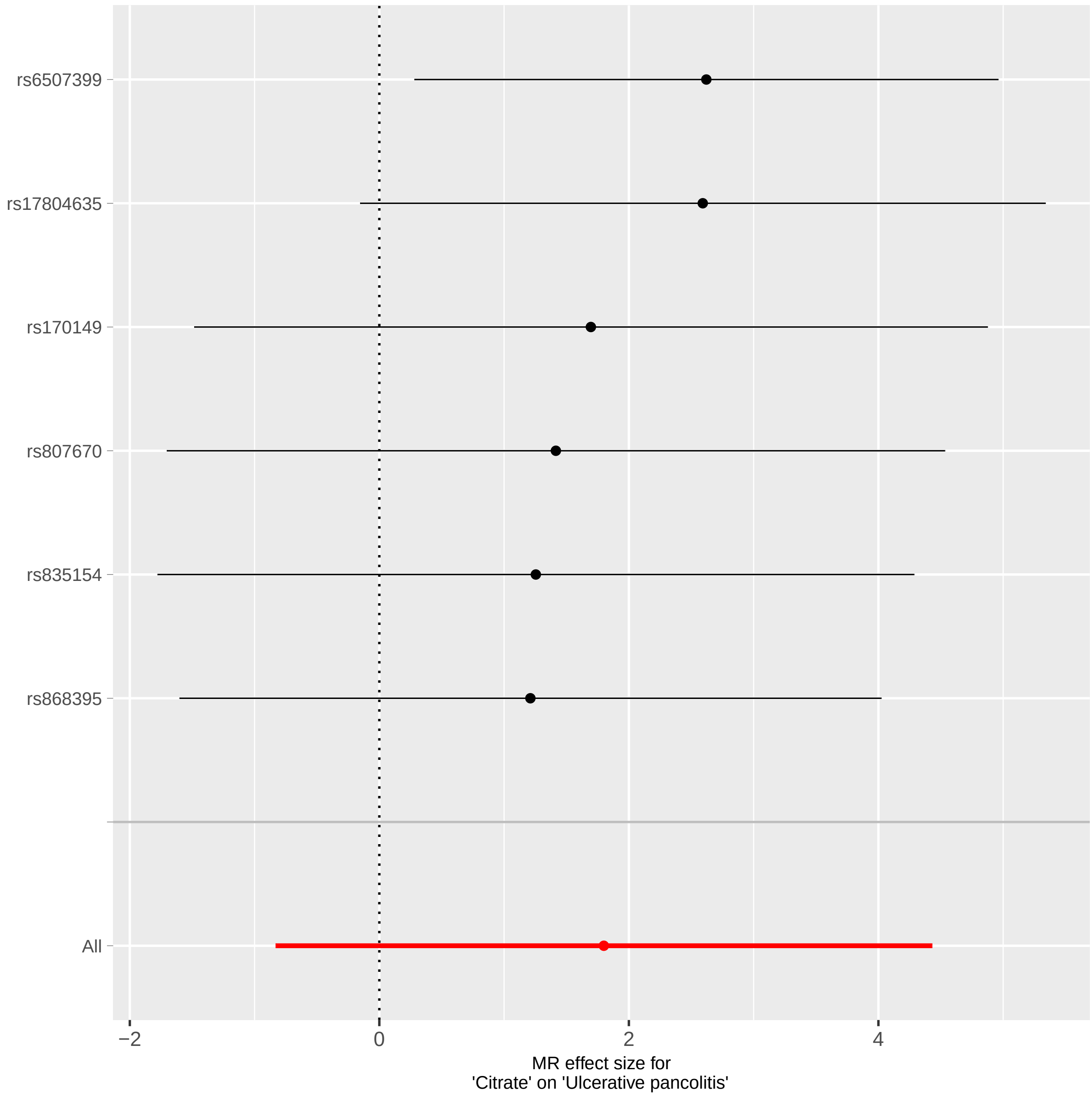

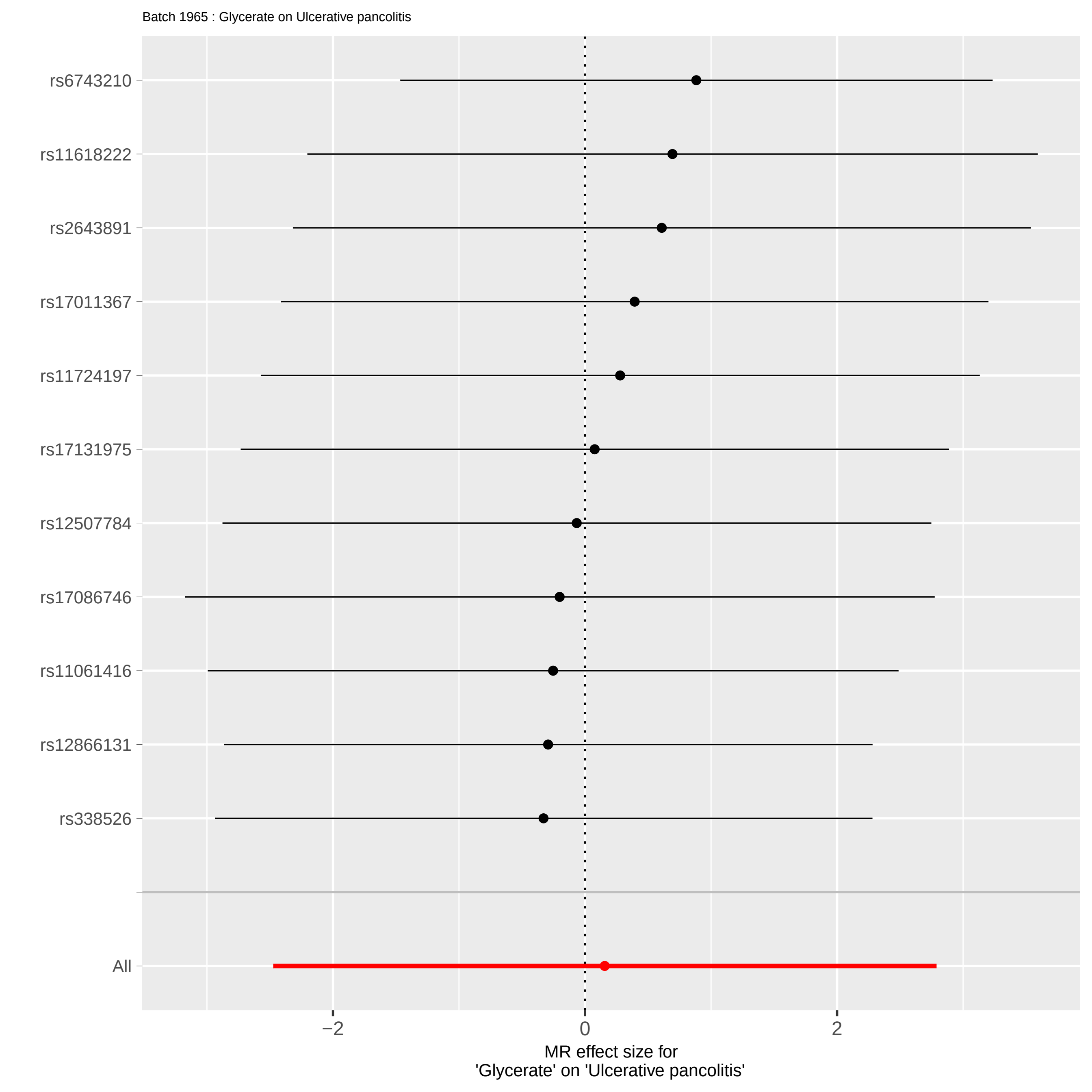

Batch 1965 : Glycerate on Ulcerative pancolitis
rs6743210
rs11618222
rs2643891
rs17011367
rs11724197
rs17131975
rs12507784
rs17086746
rs11061416
rs12866131
rs338526
All
−2
0
2
MR effect size for
'Glycerate' on 'Ulcerative pancolitis'

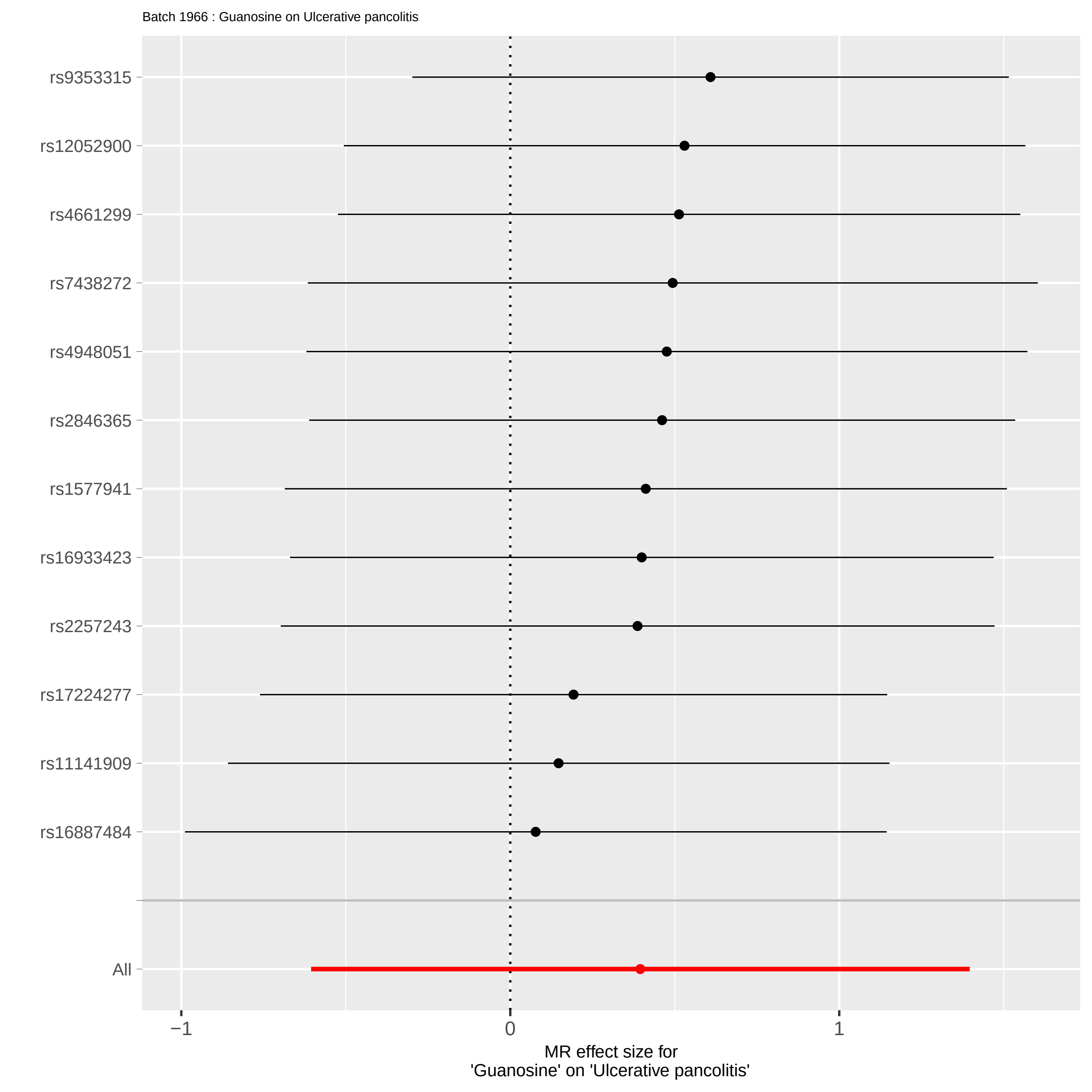

Batch 1966 : Guanosine on Ulcerative pancolitis
rs9353315
rs12052900
rs4661299
rs7438272
rs4948051
rs2846365
rs1577941
rs16933423
rs2257243
rs17224277
rs11141909
rs16887484
All
−1
0
1
MR effect size for
'Guanosine' on 'Ulcerative pancolitis'

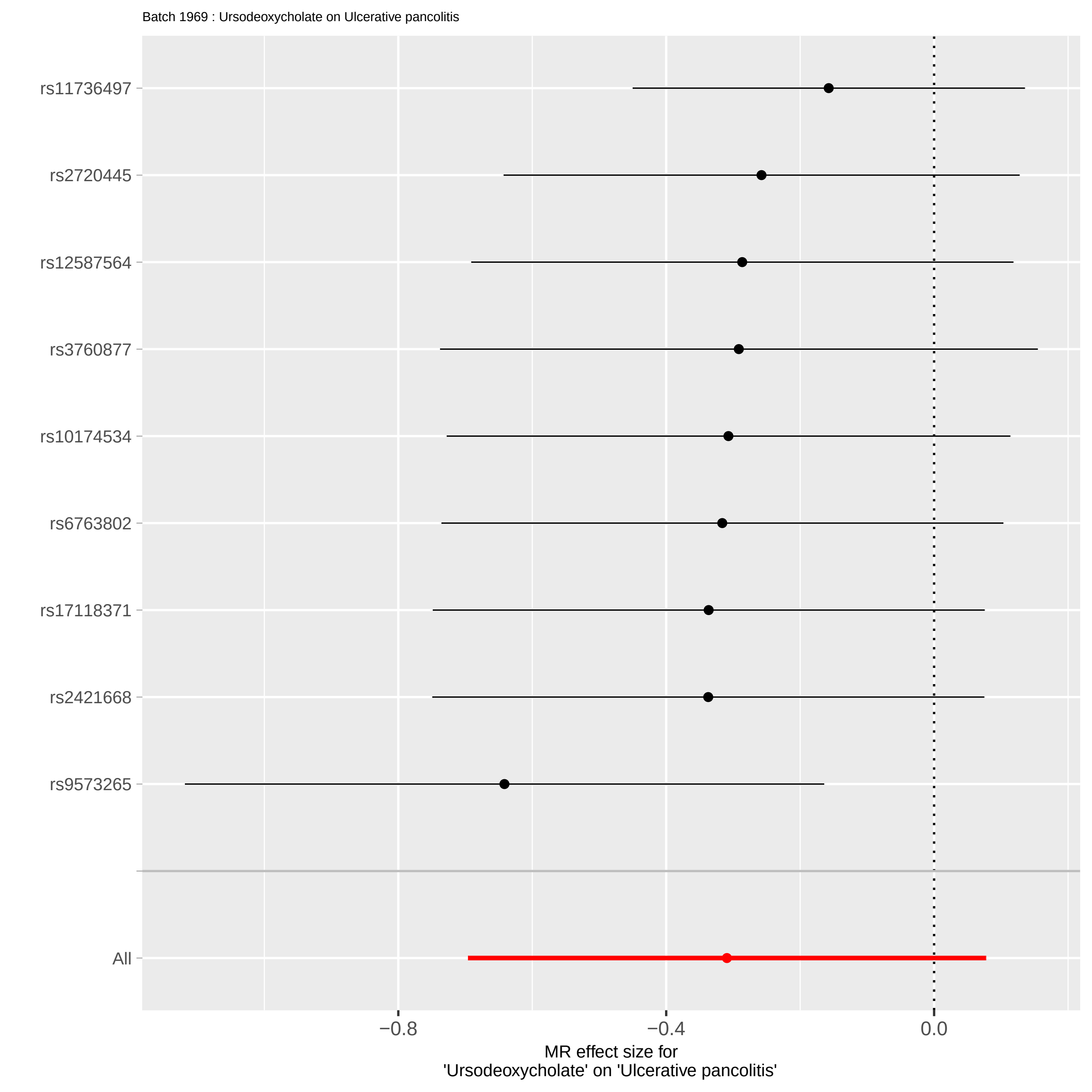

Batch 1969 : Ursodeoxycholate on Ulcerative pancolitis
rs11736497
rs2720445
rs12587564
rs3760877
rs10174534
rs6763802
rs17118371
rs2421668
rs9573265
All
−0.8
−0.4
0.0
MR effect size for
'Ursodeoxycholate' on 'Ulcerative pancolitis'

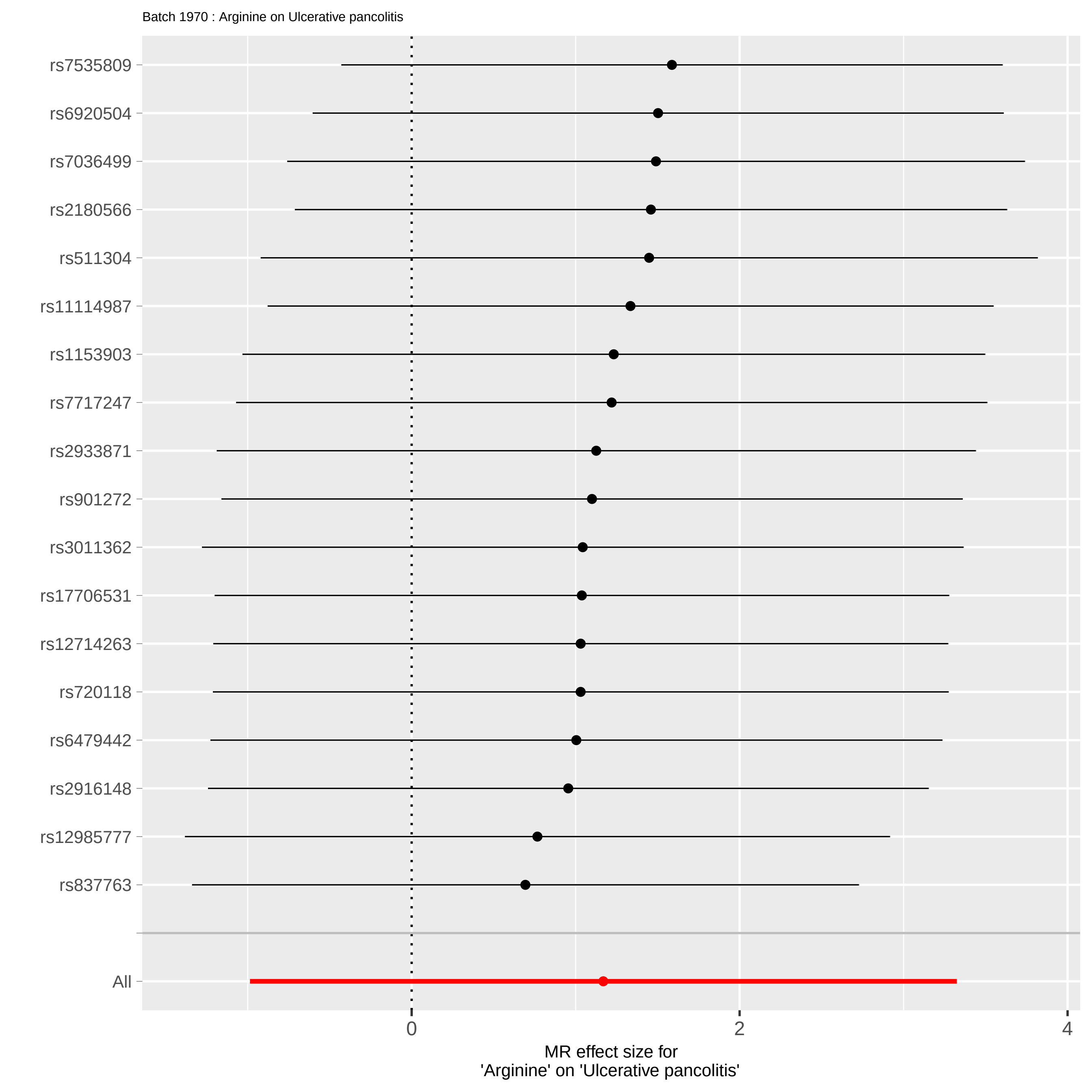

Batch 1970 : Arginine on Ulcerative pancolitis
rs7535809
rs6920504
rs7036499
rs2180566
rs511304
rs11114987
rs1153903
rs7717247
rs2933871
rs901272
rs3011362
rs17706531
rs12714263
rs720118
rs6479442
rs2916148
rs12985777
rs837763
All
0
2
4
MR effect size for
'Arginine' on 'Ulcerative pancolitis'

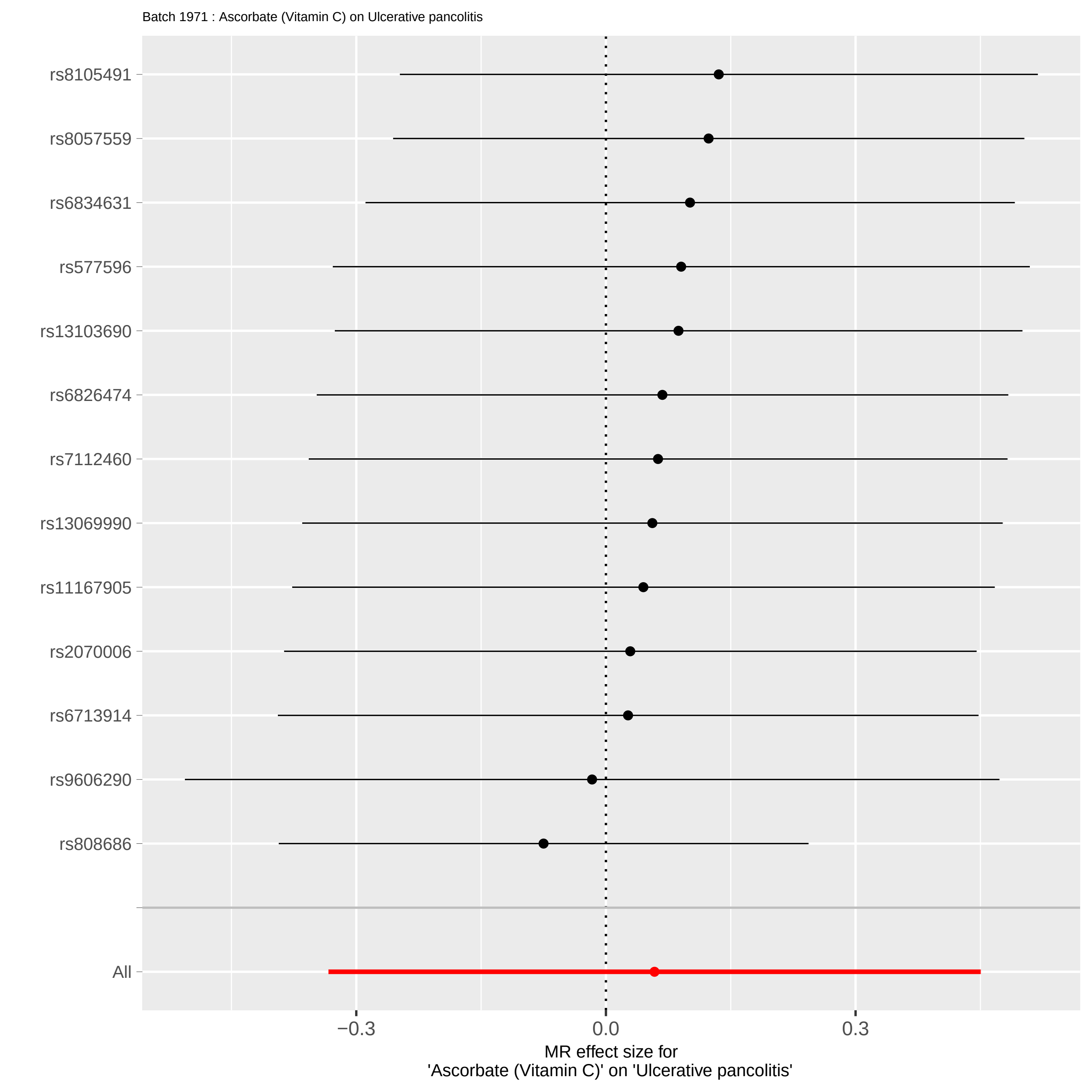

Batch 1971 : Ascorbate (Vitamin C) on Ulcerative pancolitis
rs8105491
rs8057559
rs6834631
rs577596
rs13103690
rs6826474
rs7112460
rs13069990
rs11167905
rs2070006
rs6713914
rs9606290
rs808686
All
−0.3
0.0
0.3
MR effect size for
'Ascorbate (Vitamin C)' on 'Ulcerative pancolitis'

Batch 1975 : Urea on Ulcerative pancolitis

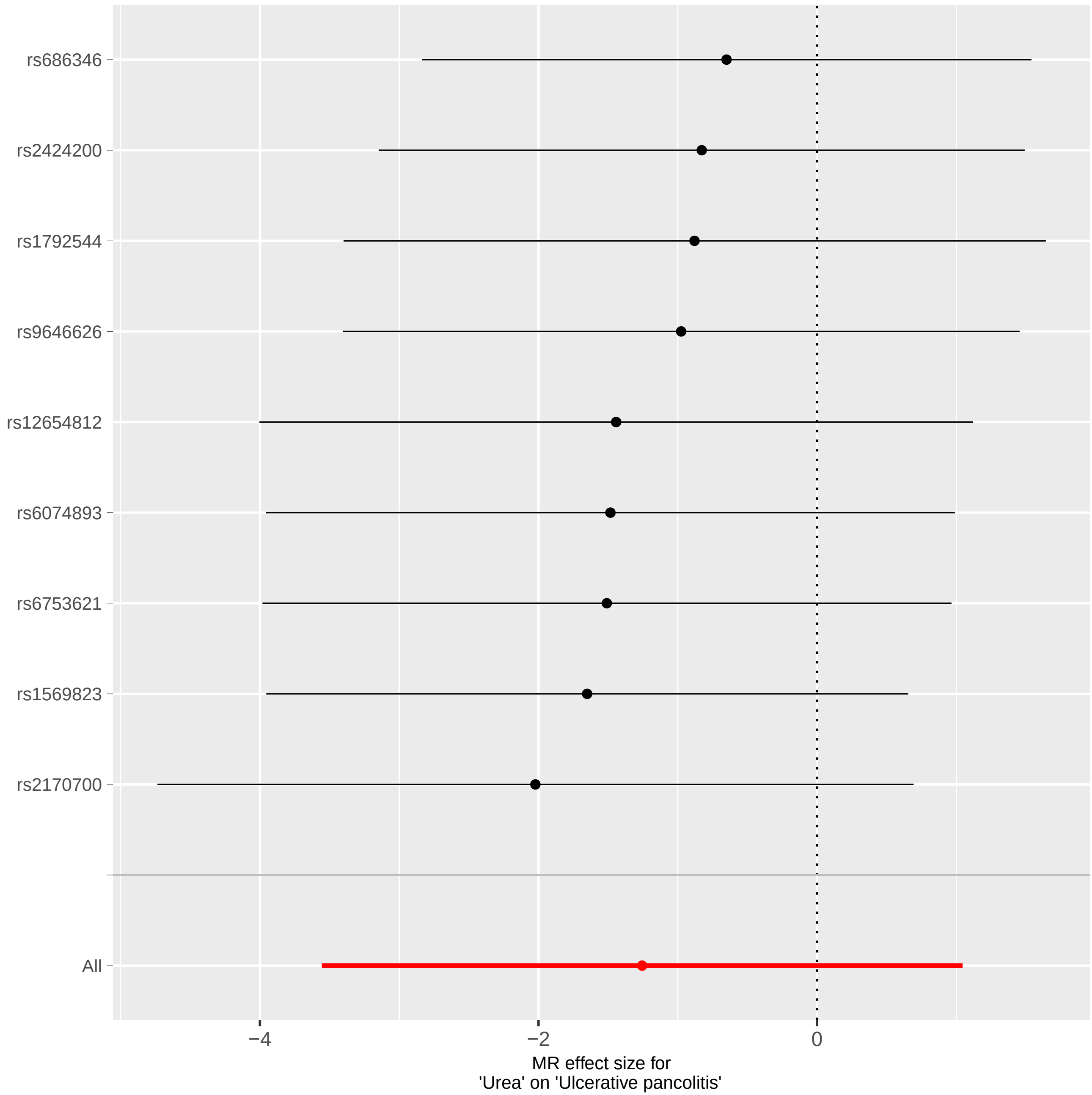

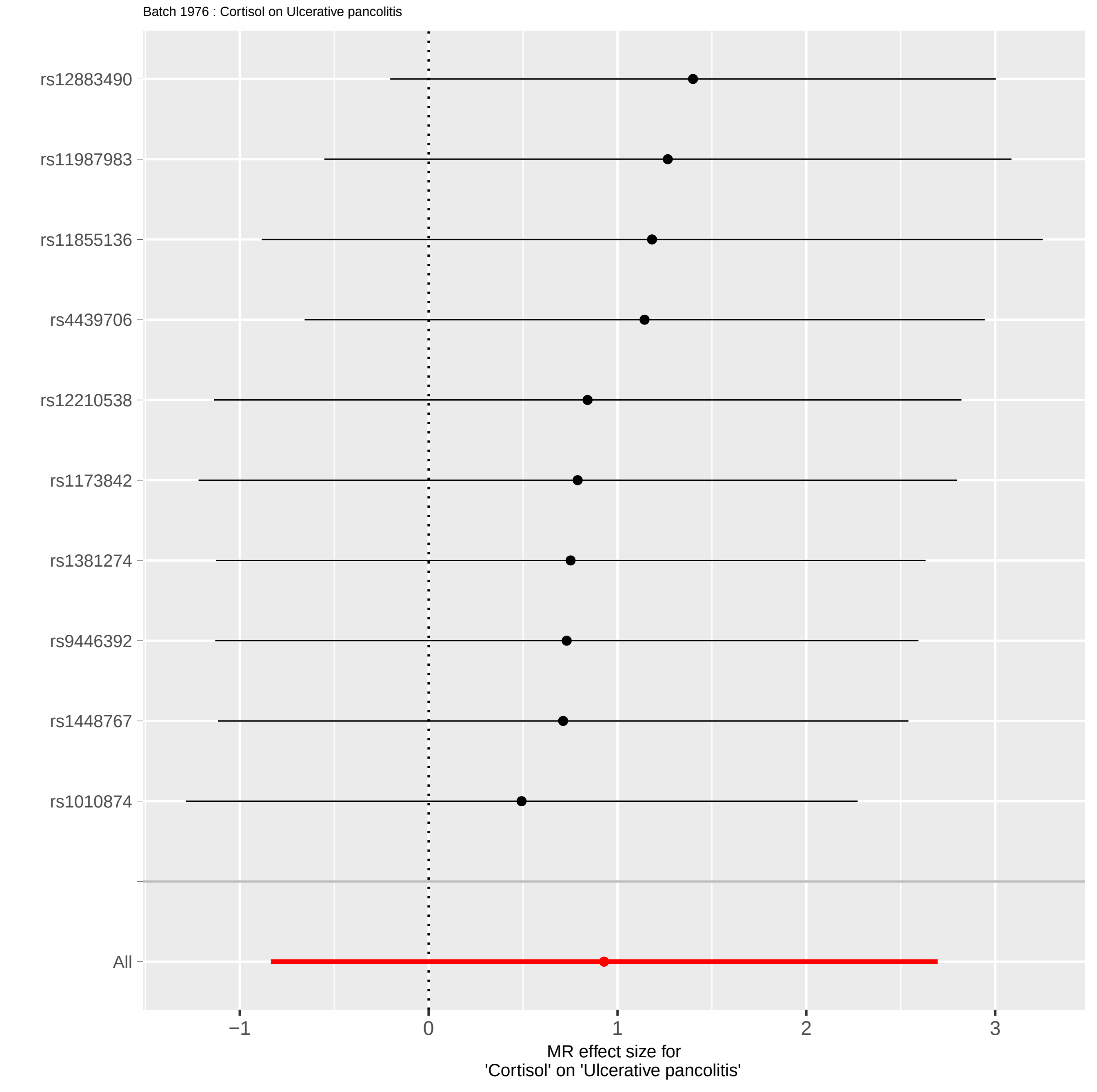

Batch 1976 : Cortisol on Ulcerative pancolitis
rs12883490
rs11987983
rs11855136
rs4439706
rs12210538
rs1173842
rs1381274
rs9446392
rs1448767
rs1010874
All
−1
0
1
2
3
MR effect size for
'Cortisol' on 'Ulcerative pancolitis'

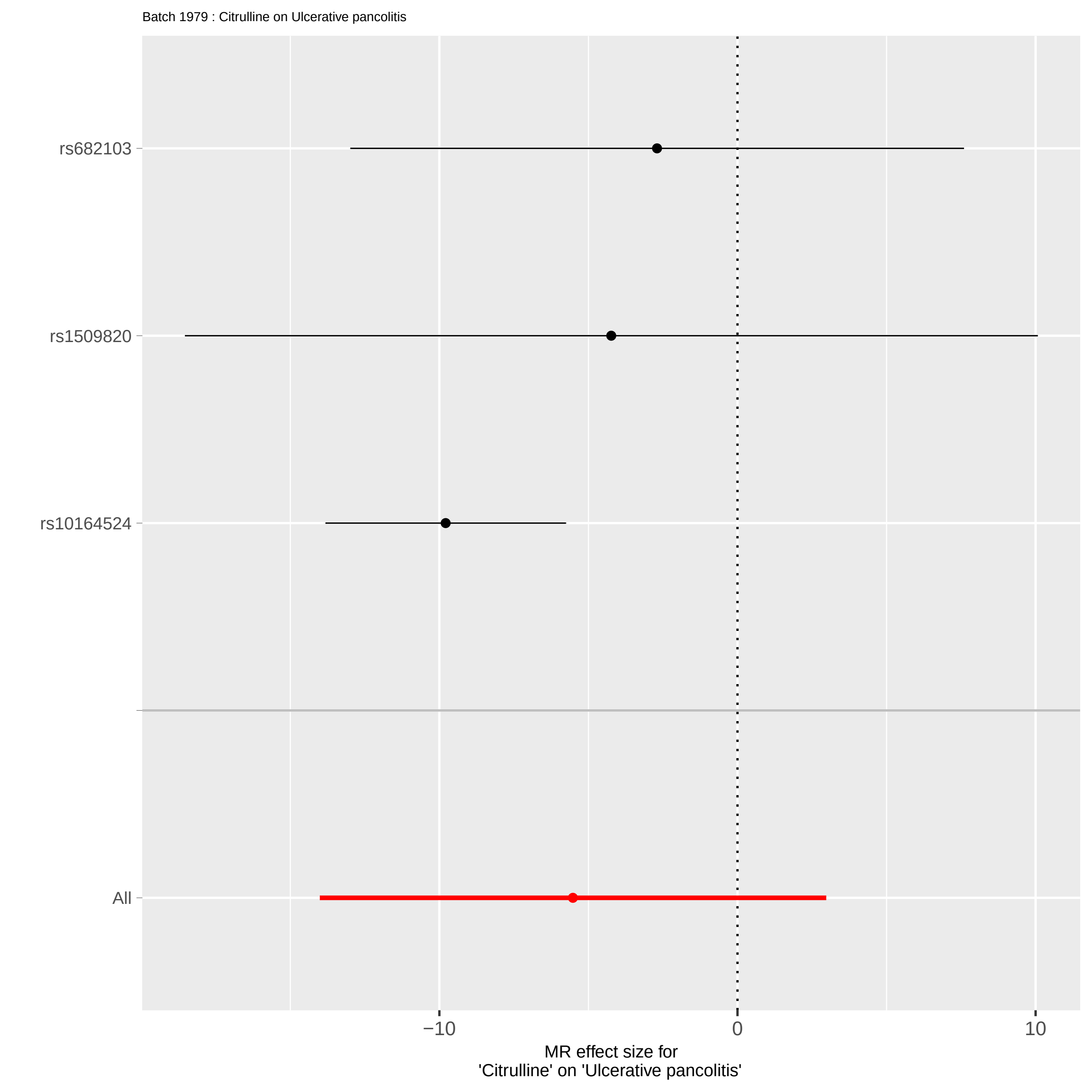
Batch 1979 : Citrulline on Ulcerative pancolitis
rs682103
rs1509820
rs10164524
All
−10
0
10
MR effect size for
'Citrulline' on 'Ulcerative pancolitis'

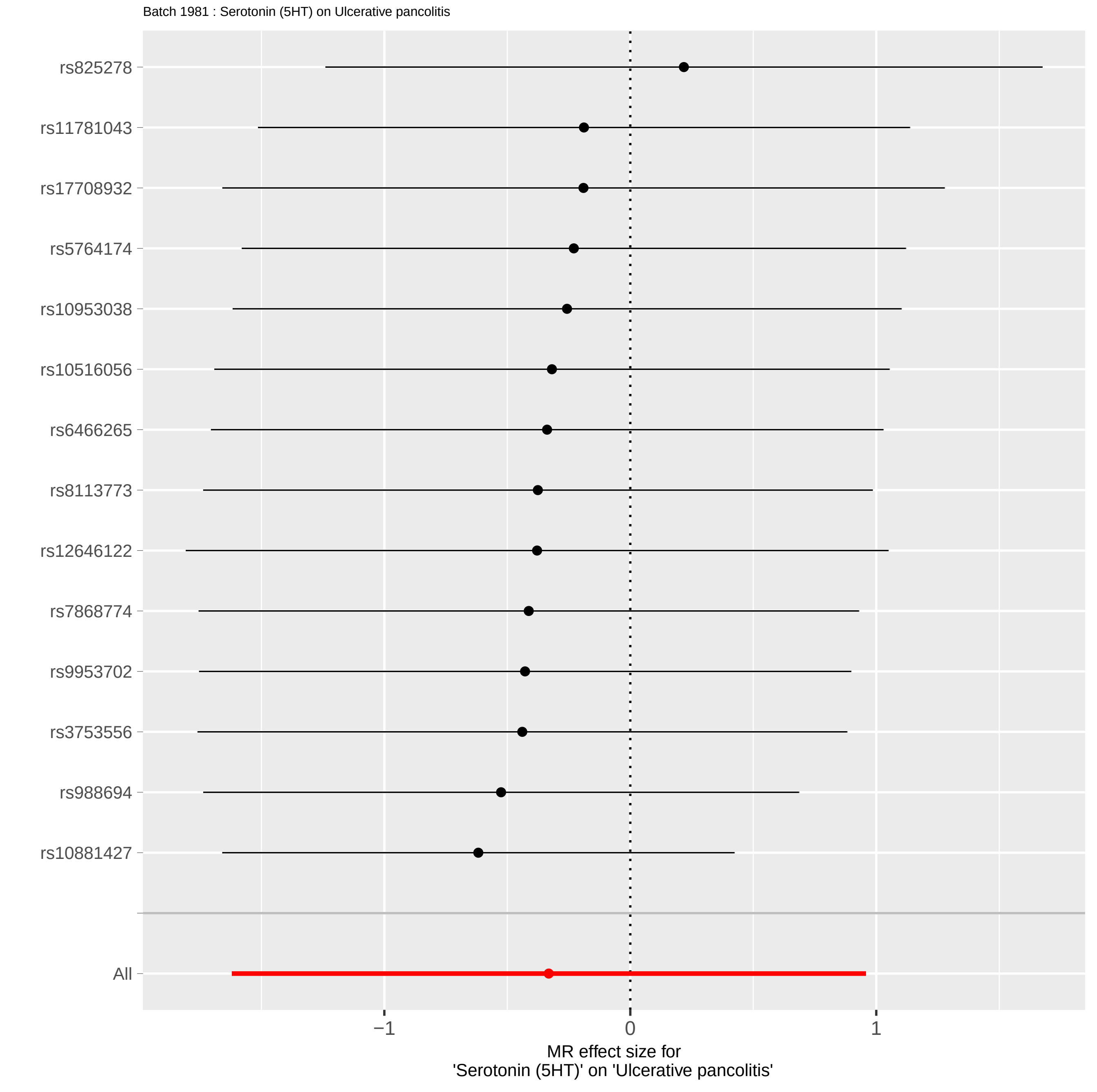

Batch 1981 : Serotonin (5HT) on Ulcerative pancolitis
rs825278
rs11781043
rs17708932
rs5764174
rs10953038
rs10516056
rs6466265
rs8113773
rs12646122
rs7868774
rs9953702
rs3753556
rs988694
rs10881427
All
−1
0
1
MR effect size for
'Serotonin (5HT)' on 'Ulcerative pancolitis'

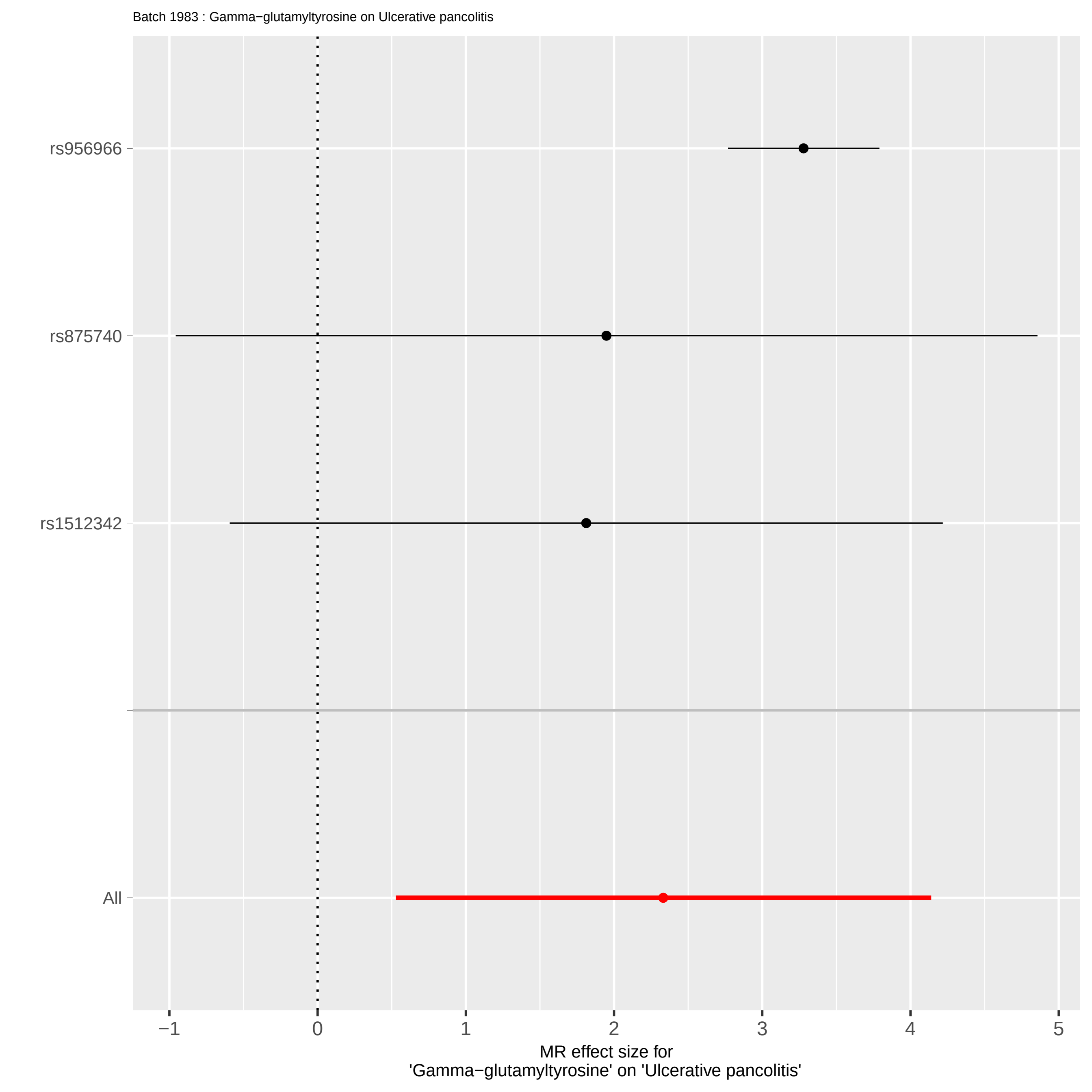
Batch 1983 : Gamma−glutamyltyrosine on Ulcerative pancolitis
rs956966
rs875740
rs1512342
All
−1
0
1
2
3
4
5
MR effect size for
'Gamma−glutamyltyrosine' on 'Ulcerative pancolitis'

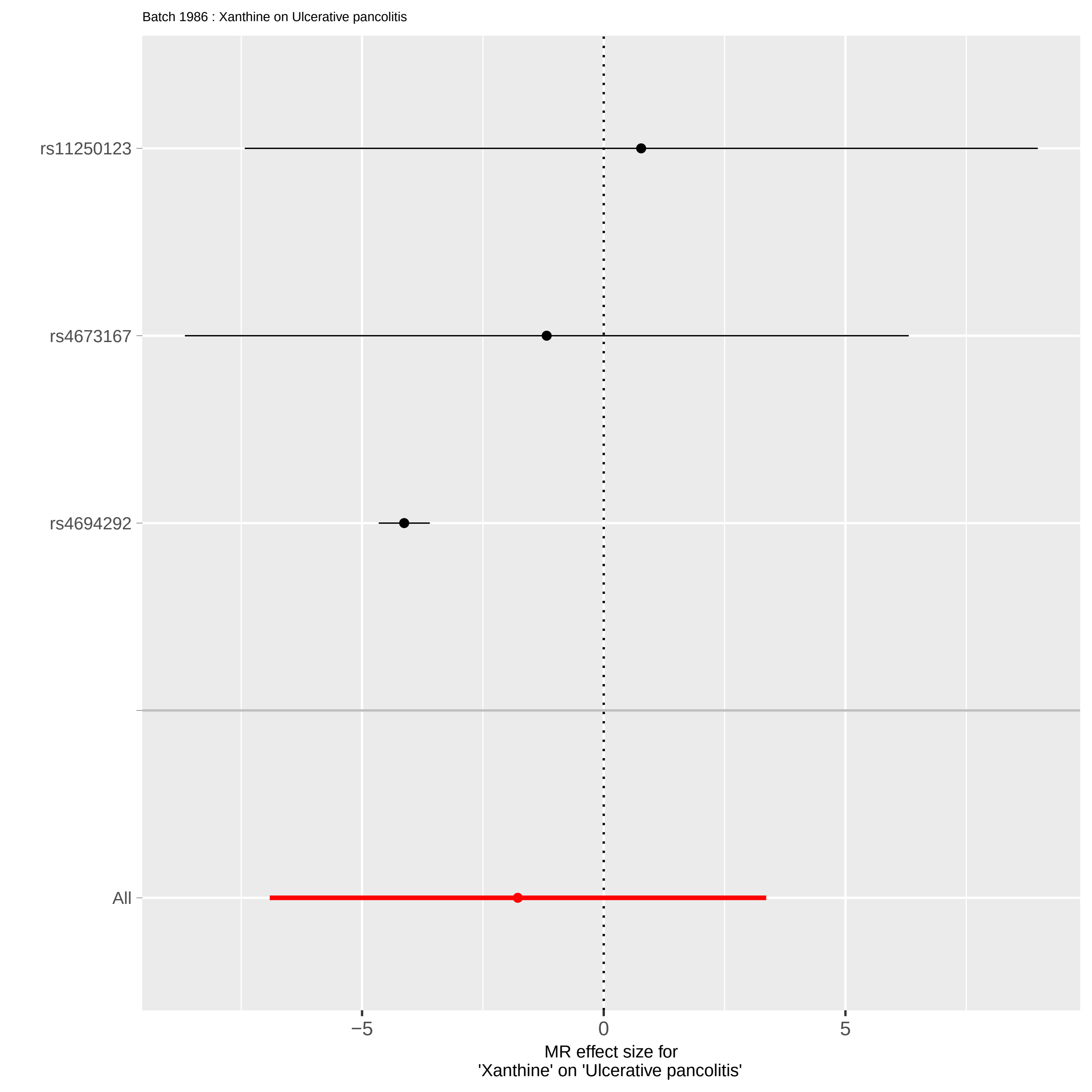
Batch 1986 : Xanthine on Ulcerative pancolitis
rs11250123
rs4673167
rs4694292
All
−5
0
5
MR effect size for
'Xanthine' on 'Ulcerative pancolitis'

Batch 1987 : Phosphate on Ulcerative pancolitis

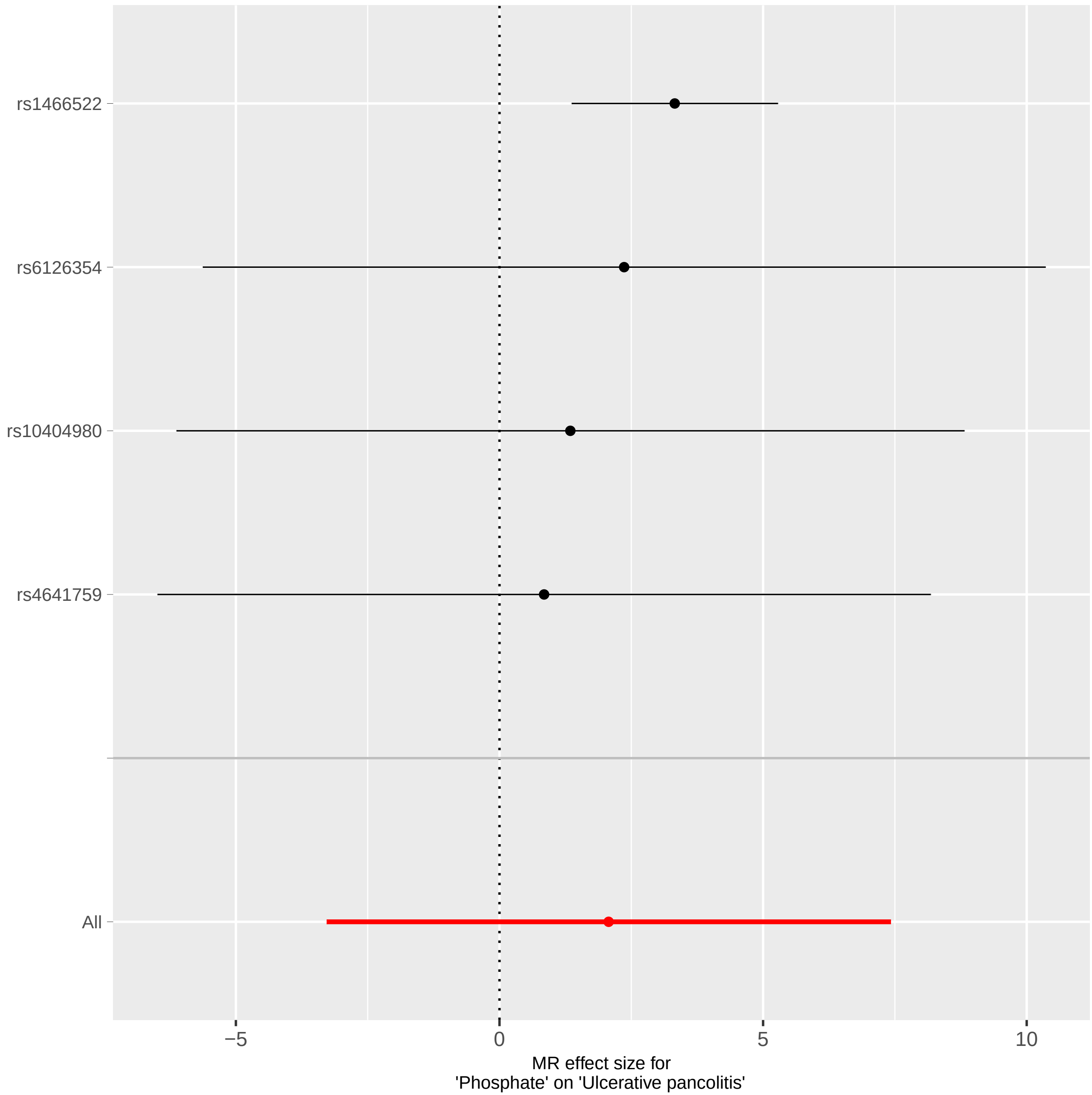

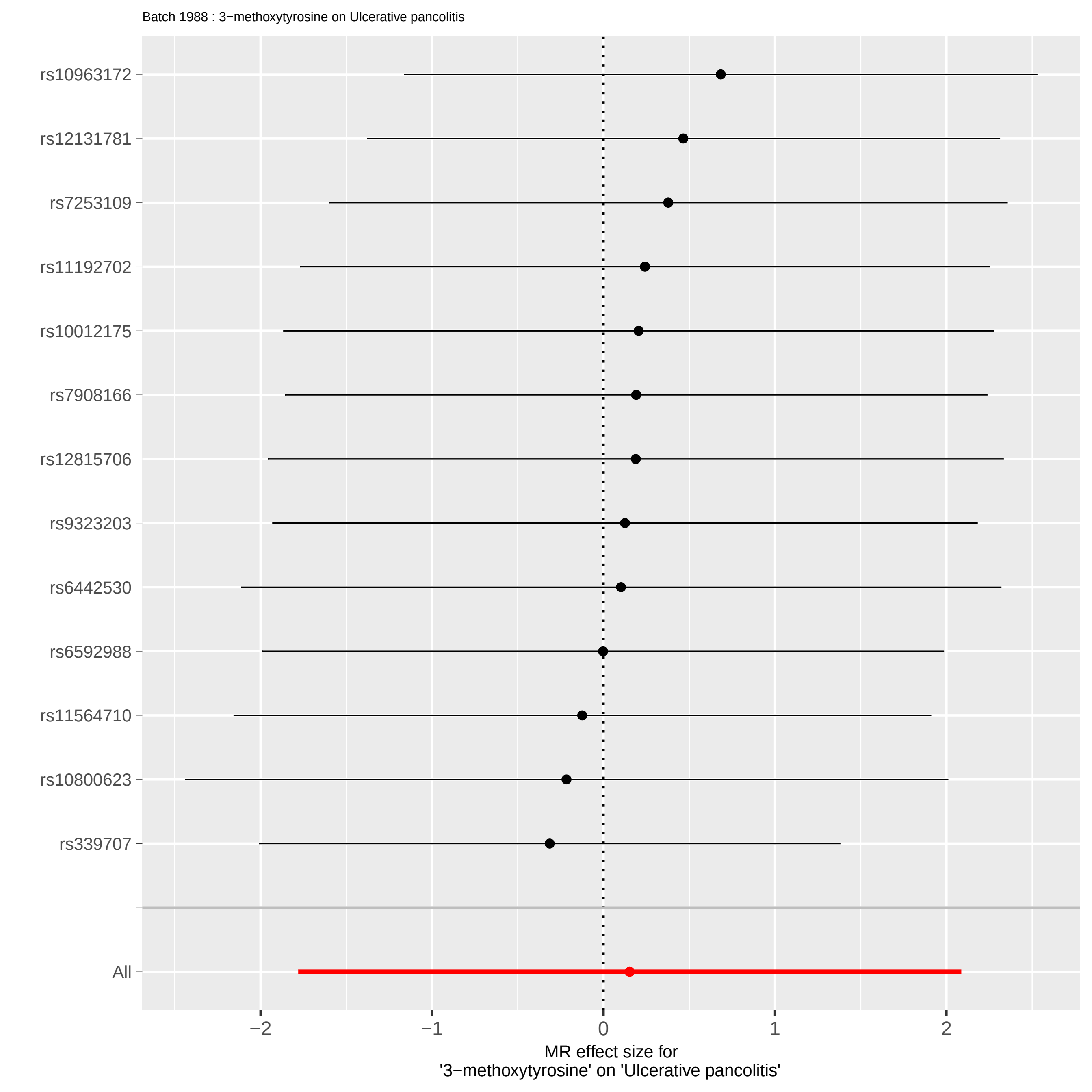
Batch 1988 : 3−methoxytyrosine on Ulcerative pancolitis
rs10963172
rs12131781
rs7253109
rs11192702
rs10012175
rs7908166
rs12815706
rs9323203
rs6442530
rs6592988
rs11564710
rs10800623
rs339707
All
−2
−1
0
1
2
MR effect size for
'3−methoxytyrosine' on 'Ulcerative pancolitis'

Batch 1990 : Undecanoate (11:0) on Ulcerative pancolitis

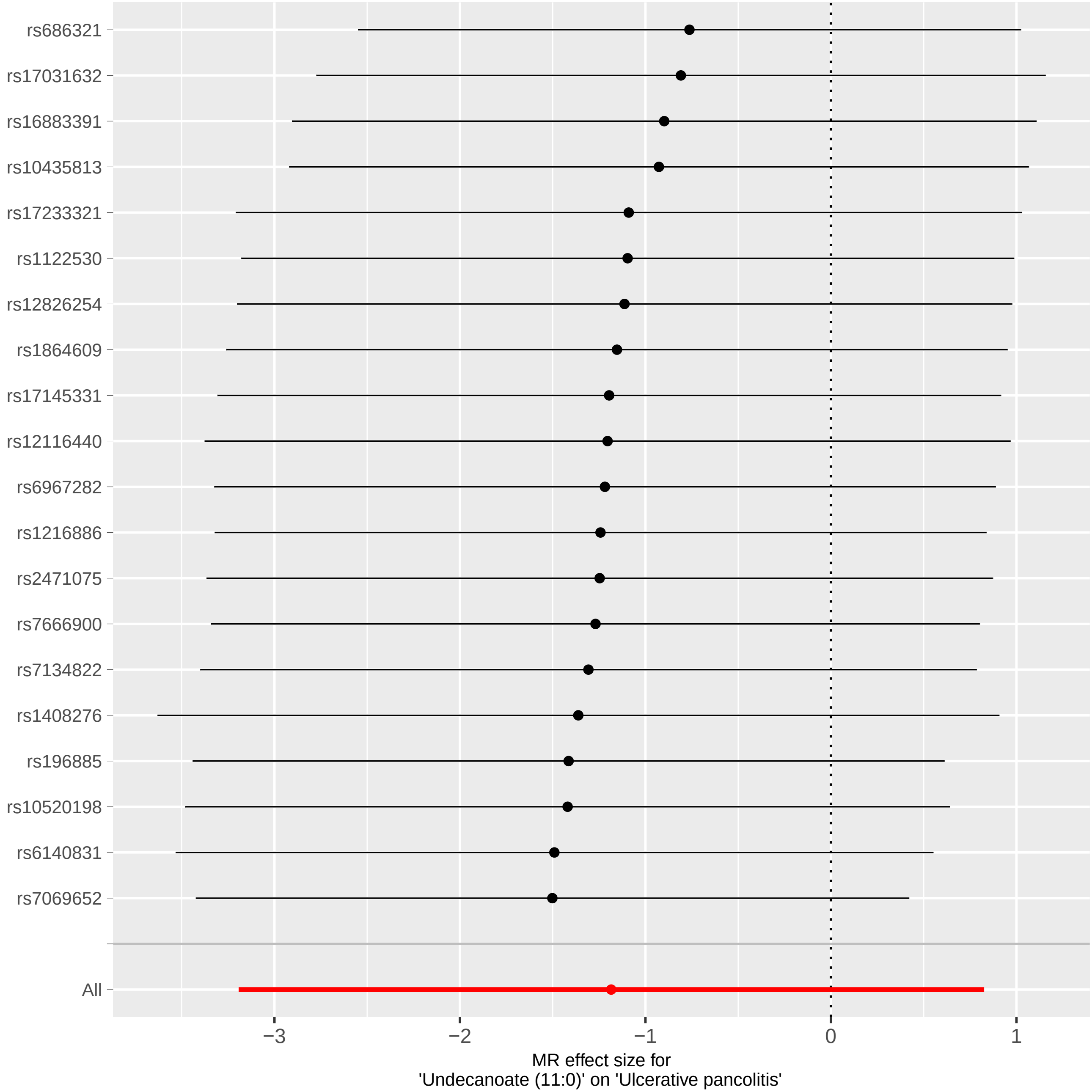

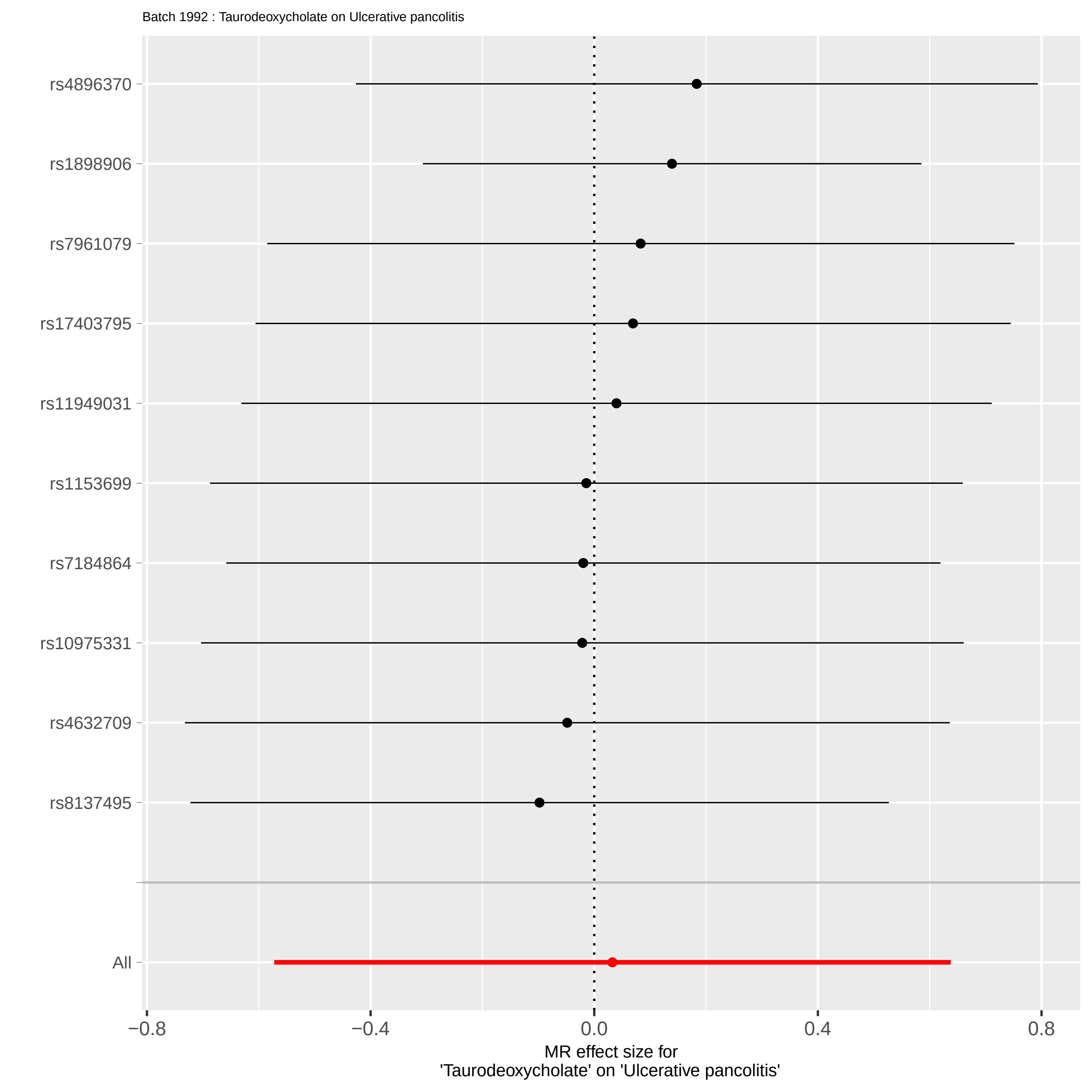

Batch 1992 : Taurodeoxycholate on Ulcerative pancolitis
rs4896370
rs1898906
rs7961079
rs17403795
rs11949031
rs1153699
rs7184864
rs10975331
rs4632709
rs8137495
All
−0.8
−0.4
0.0
0.4
0.8
MR effect size for
'Taurodeoxycholate' on 'Ulcerative pancolitis'

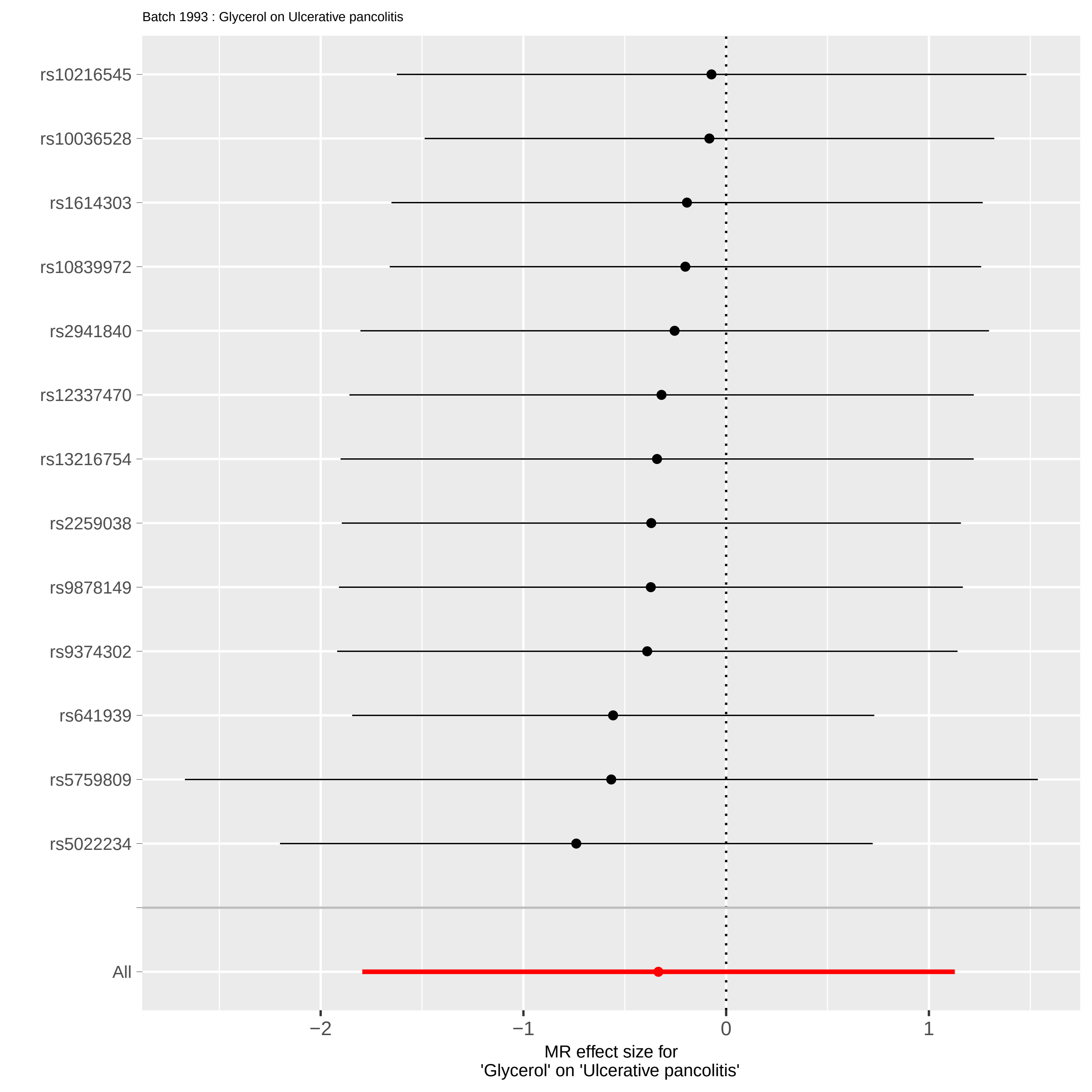
Batch 1993 : Glycerol on Ulcerative pancolitis
rs10216545
rs10036528
rs1614303
rs10839972
rs2941840
rs12337470
rs13216754
rs2259038
rs9878149
rs9374302
rs641939
rs5759809
rs5022234
All
−2
−1
0
1
MR effect size for
'Glycerol' on 'Ulcerative pancolitis'

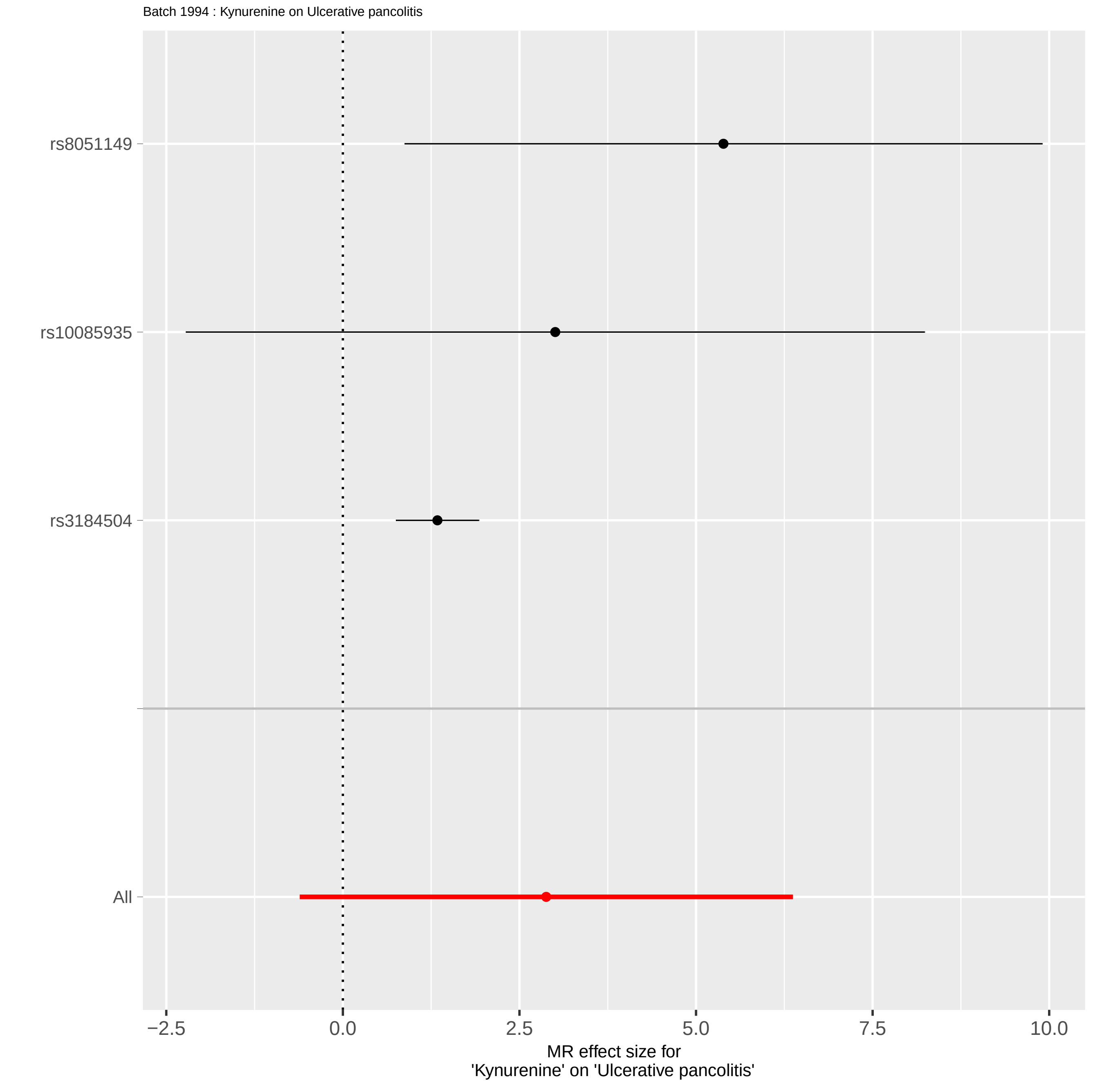

Batch 1994 : Kynurenine on Ulcerative pancolitis
rs8051149
rs10085935
rs3184504
All
−2.5
0.0
2.5
5.0
7.5
10.0
MR effect size for
'Kynurenine' on 'Ulcerative pancolitis'

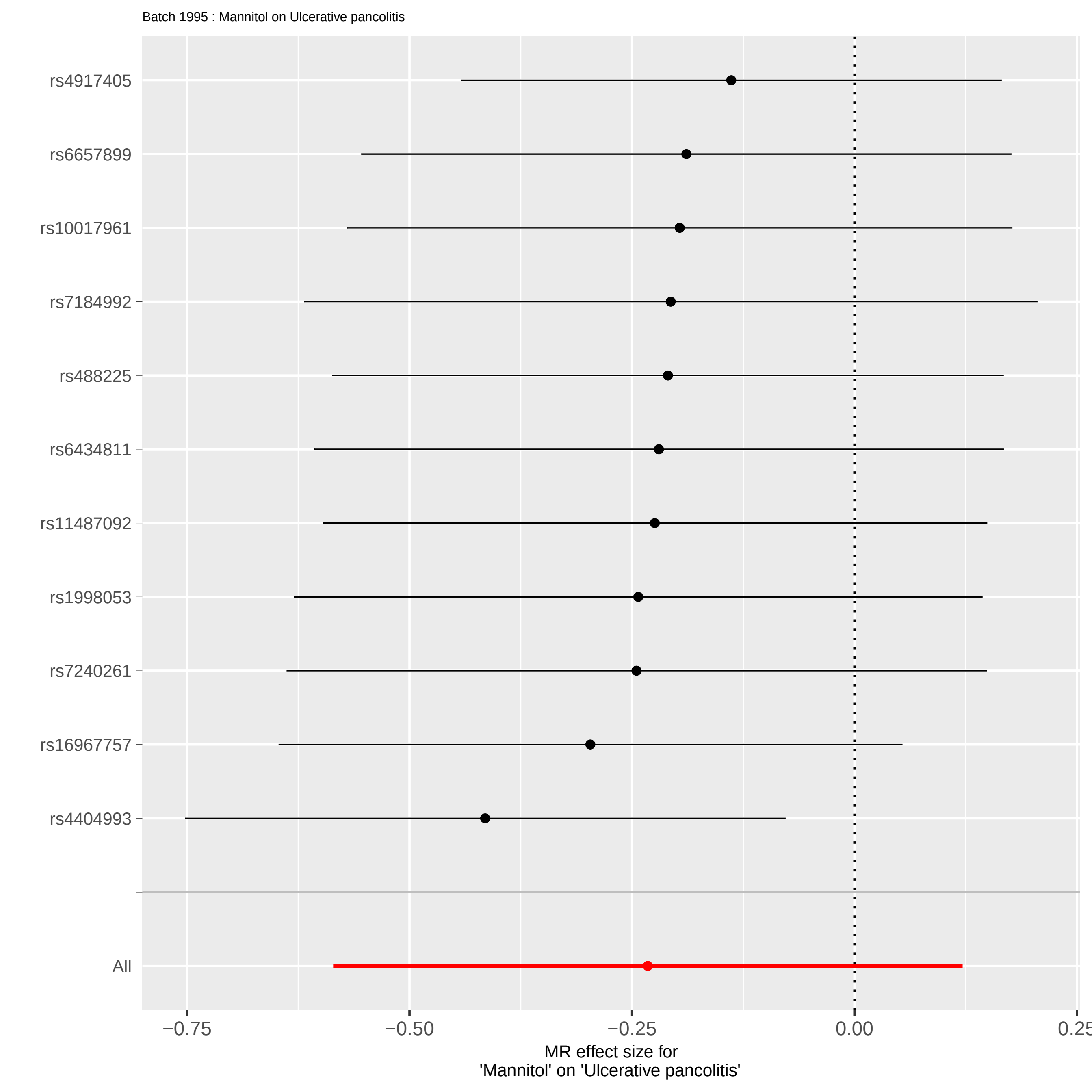
Batch 1995 : Mannitol on Ulcerative pancolitis
rs4917405
rs6657899
rs10017961
rs7184992
rs488225
rs6434811
rs11487092
rs1998053
rs7240261
rs16967757
rs4404993
All
−0.75
−0.50
−0.25
0.00
0.25
MR effect size for
'Mannitol' on 'Ulcerative pancolitis'

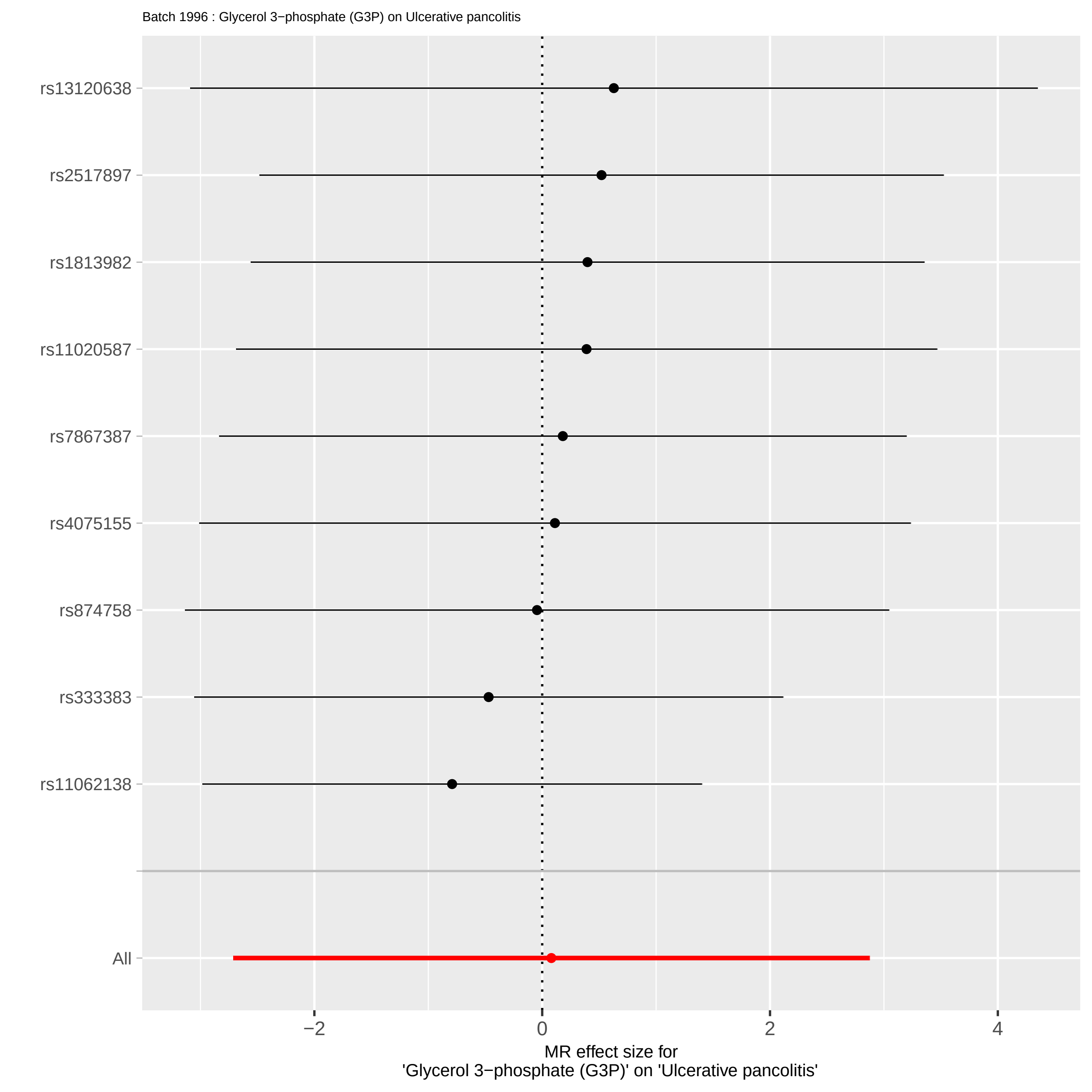

Batch 1996 : Glycerol 3−phosphate (G3P) on Ulcerative pancolitis
rs13120638
rs2517897
rs1813982
rs11020587
rs7867387
rs4075155
rs874758
rs333383
rs11062138
All
−2
0
2
4
MR effect size for
'Glycerol 3−phosphate (G3P)' on 'Ulcerative pancolitis'

Batch 1998 : Carnitine on Ulcerative pancolitis

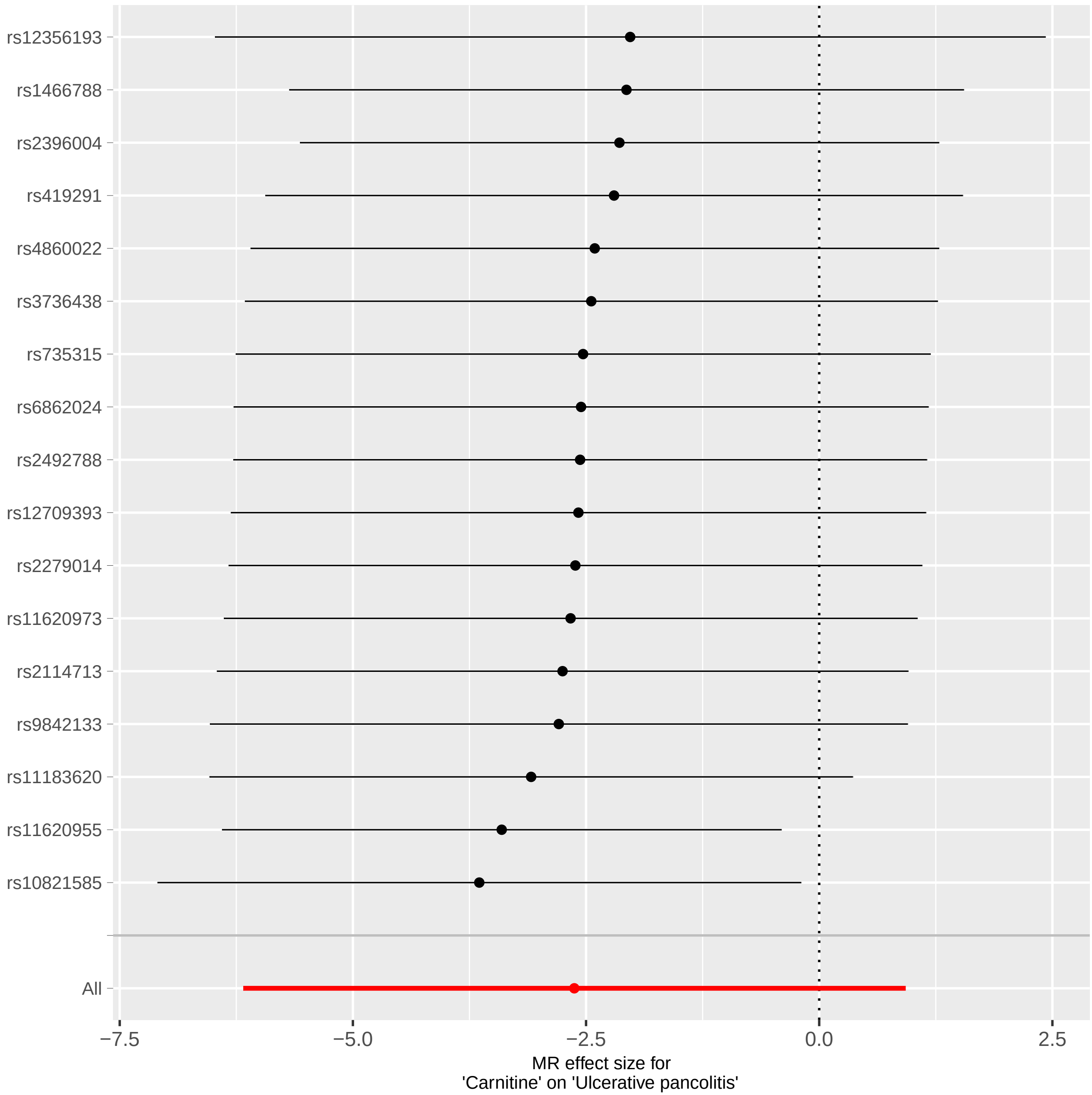

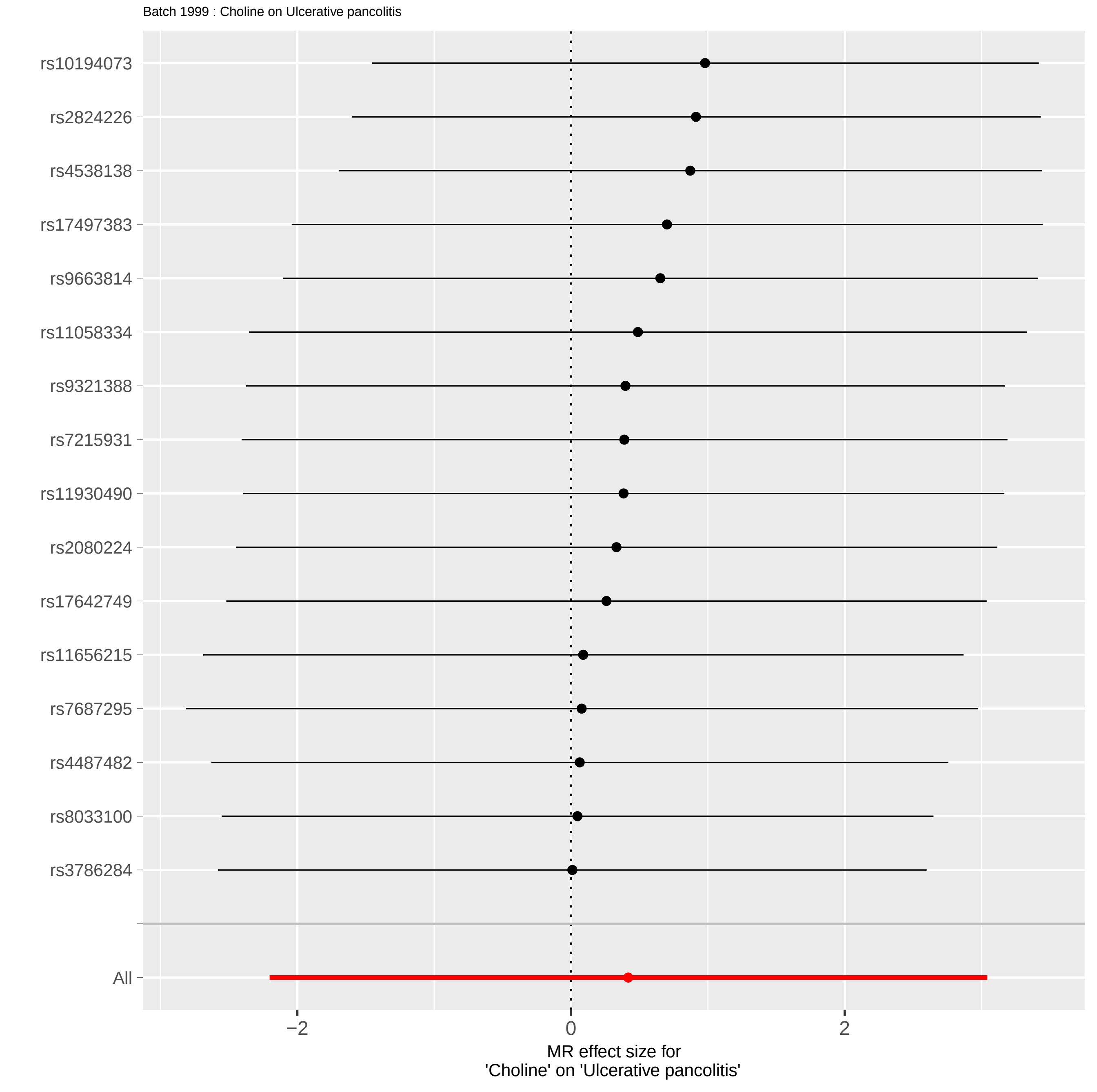

Batch 1999 : Choline on Ulcerative pancolitis
rs10194073
rs2824226
rs4538138
rs17497383
rs9663814
rs11058334
rs9321388
rs7215931
rs11930490
rs2080224
rs17642749
rs11656215
rs7687295
rs4487482
rs8033100
rs3786284
All
−2
0
2
MR effect size for
'Choline' on 'Ulcerative pancolitis'

Batch 2003 : 3−methylhistidine on Ulcerative pancolitis

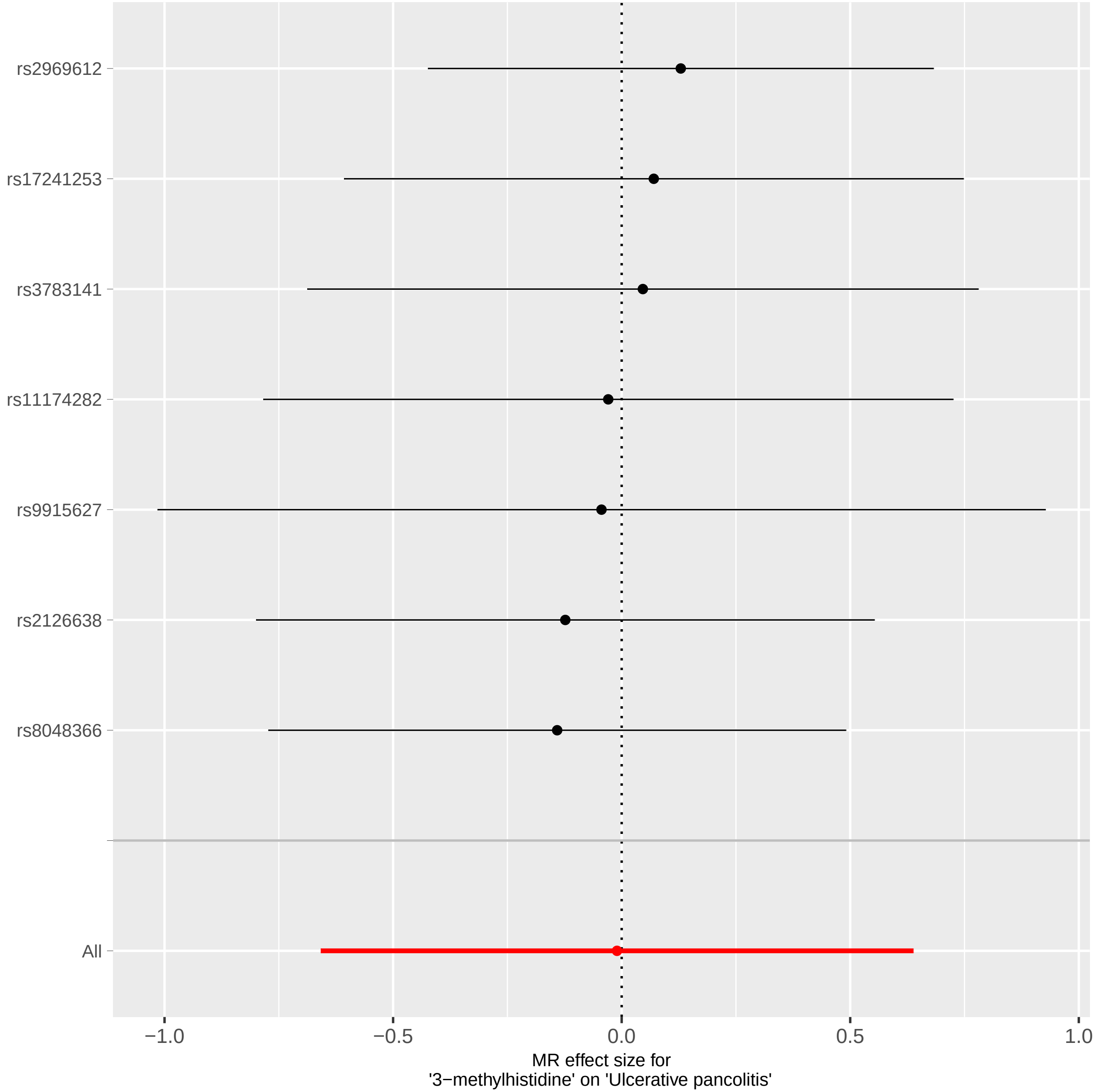

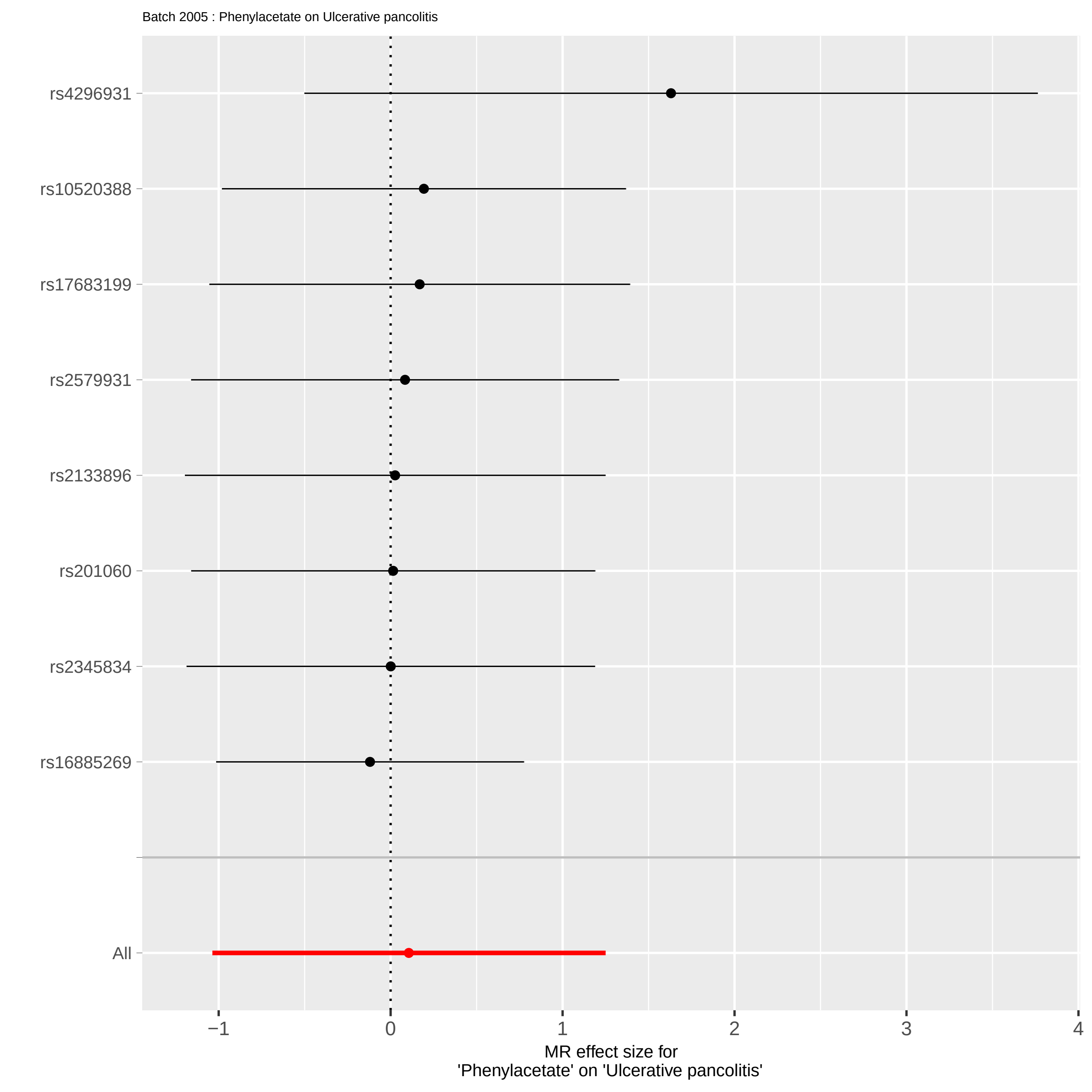

Batch 2005 : Phenylacetate on Ulcerative pancolitis
rs4296931
rs10520388
rs17683199
rs2579931
rs2133896
rs201060
rs2345834
rs16885269
All
−1
0
1
2
3
4
MR effect size for
'Phenylacetate' on 'Ulcerative pancolitis'

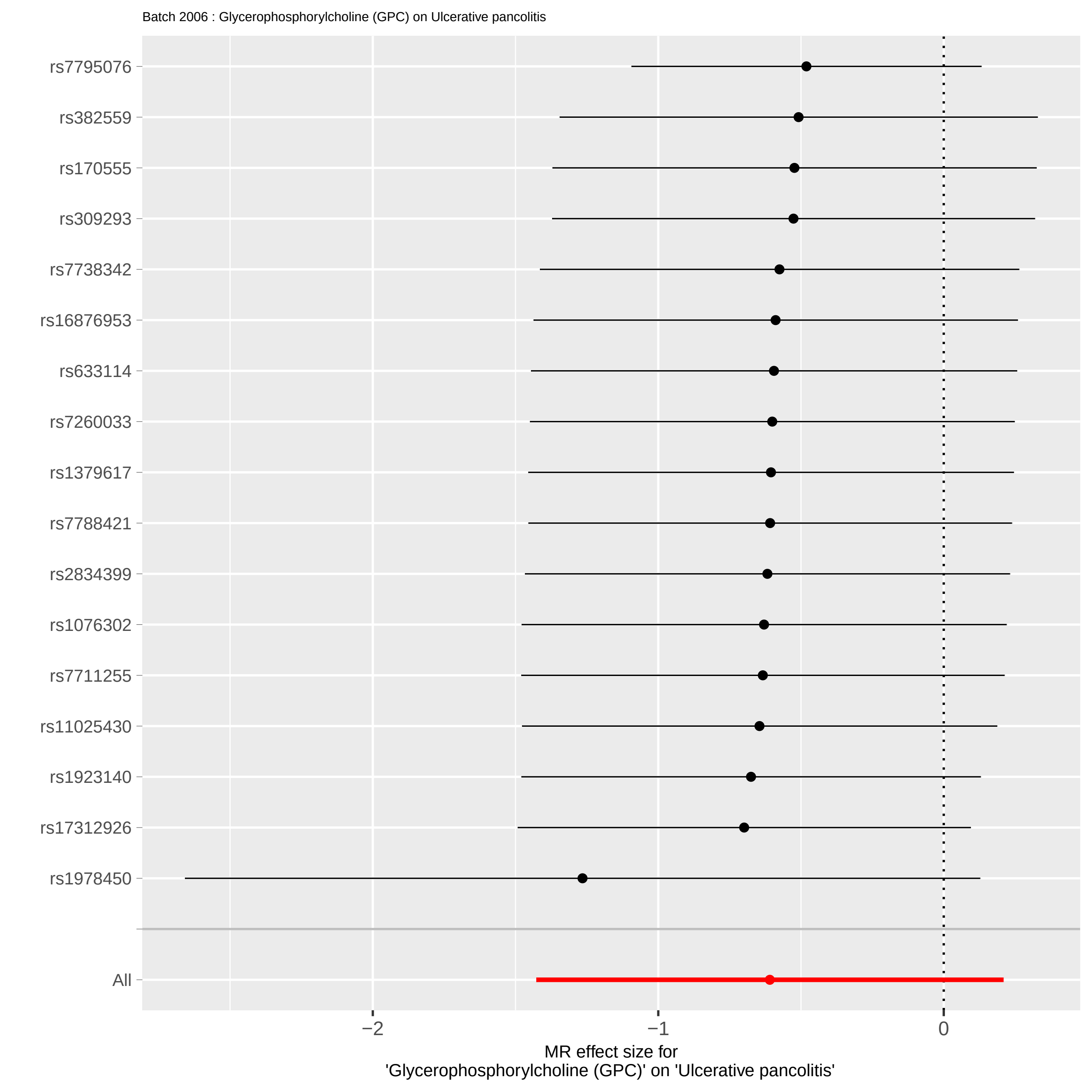
Batch 2006 : Glycerophosphorylcholine (GPC) on Ulcerative pancolitis
rs7795076
rs382559
rs170555
rs309293
rs7738342
rs16876953
rs633114
rs7260033
rs1379617
rs7788421
rs2834399
rs1076302
rs7711255
rs11025430
rs1923140
rs17312926
rs1978450
All
−2
−1
0
MR effect size for
'Glycerophosphorylcholine (GPC)' on 'Ulcerative pancolitis'

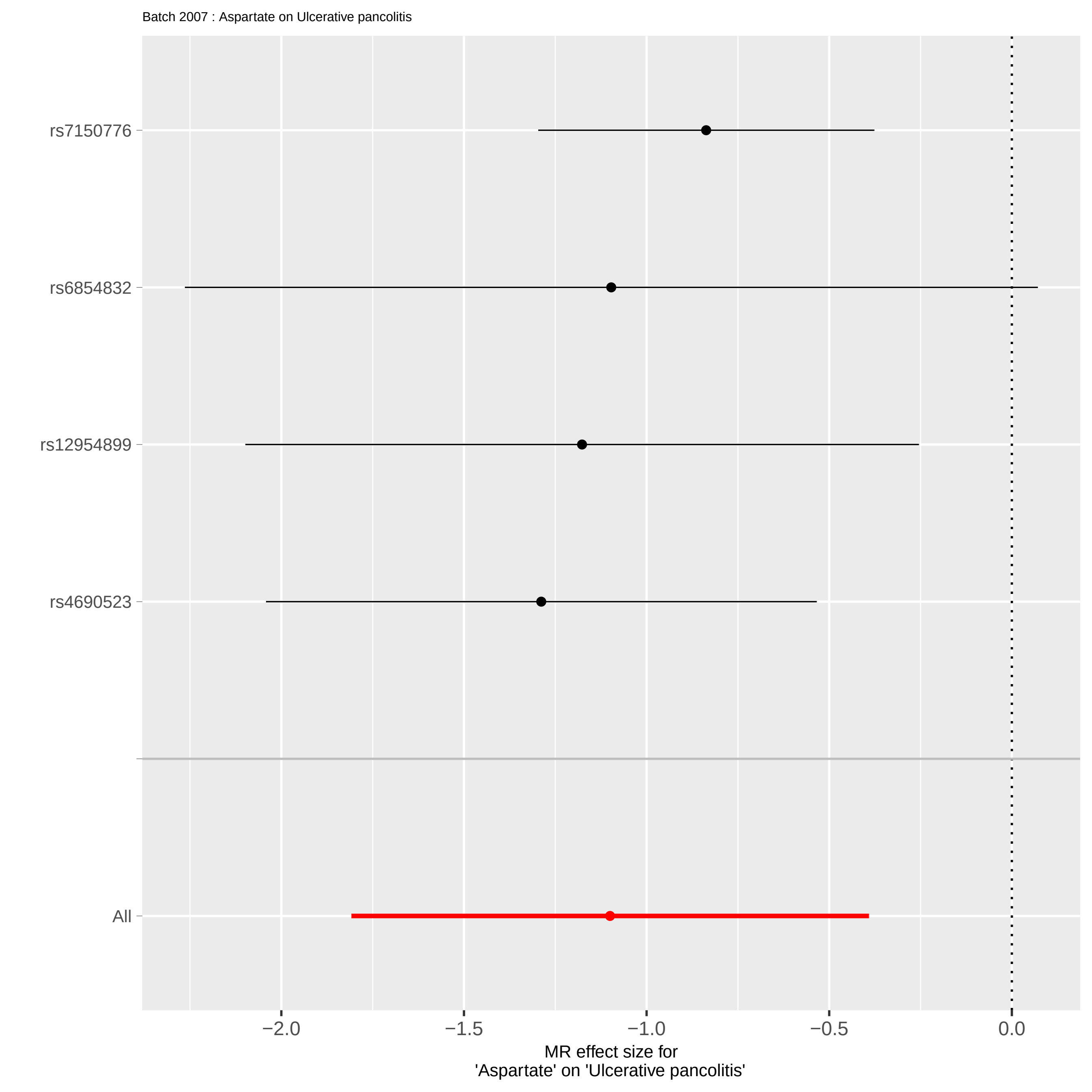
Batch 2007 : Aspartate on Ulcerative pancolitis
rs7150776
rs6854832
rs12954899
rs4690523
All
−2.0
−1.5
−1.0
−0.5
0.0
MR effect size for
'Aspartate' on 'Ulcerative pancolitis'

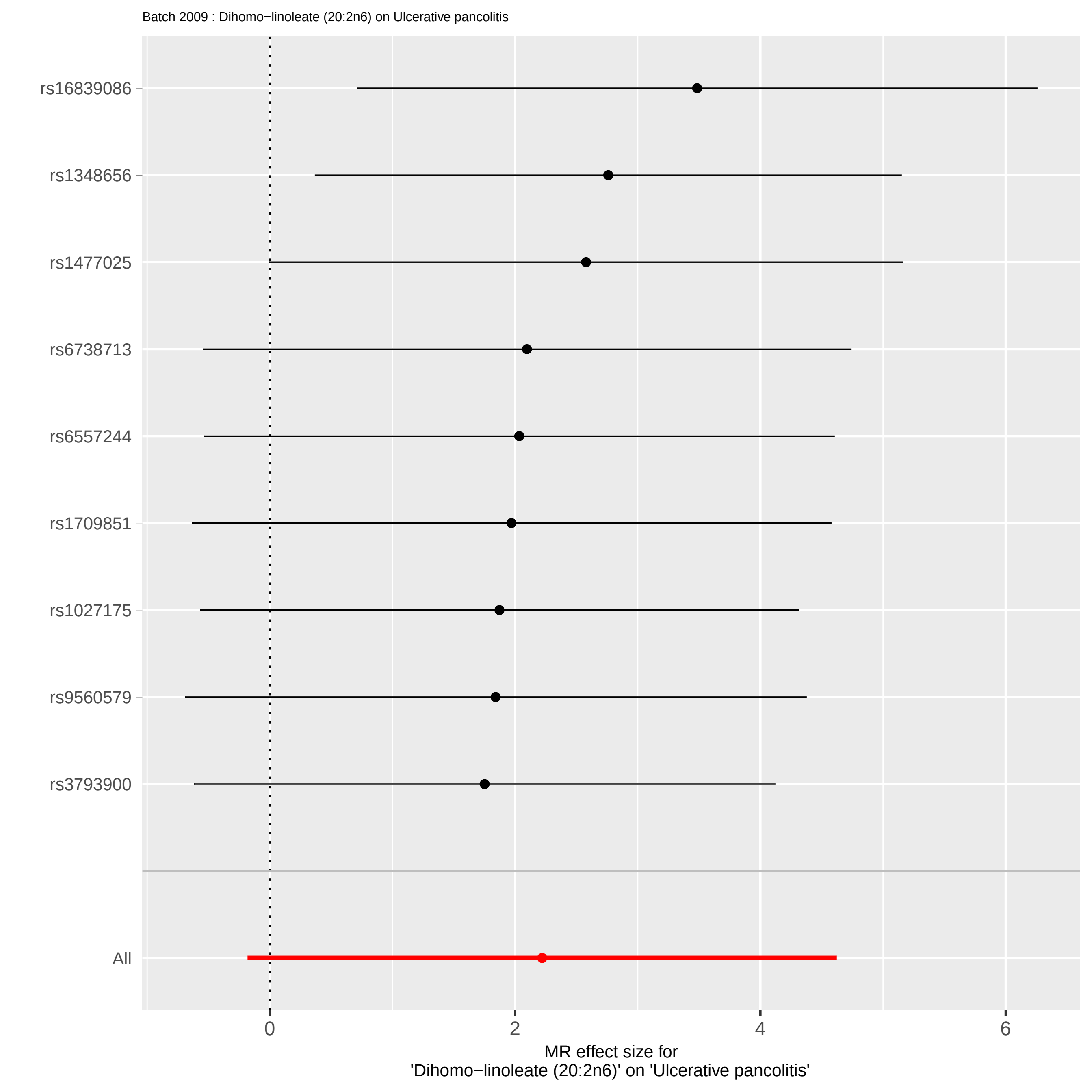

Batch 2009 : Dihomo−linoleate (20:2n6) on Ulcerative pancolitis
rs16839086
rs1348656
rs1477025
rs6738713
rs6557244
rs1709851
rs1027175
rs9560579
rs3793900
All
0
2
4
6
MR effect size for
'Dihomo−linoleate (20:2n6)' on 'Ulcerative pancolitis'

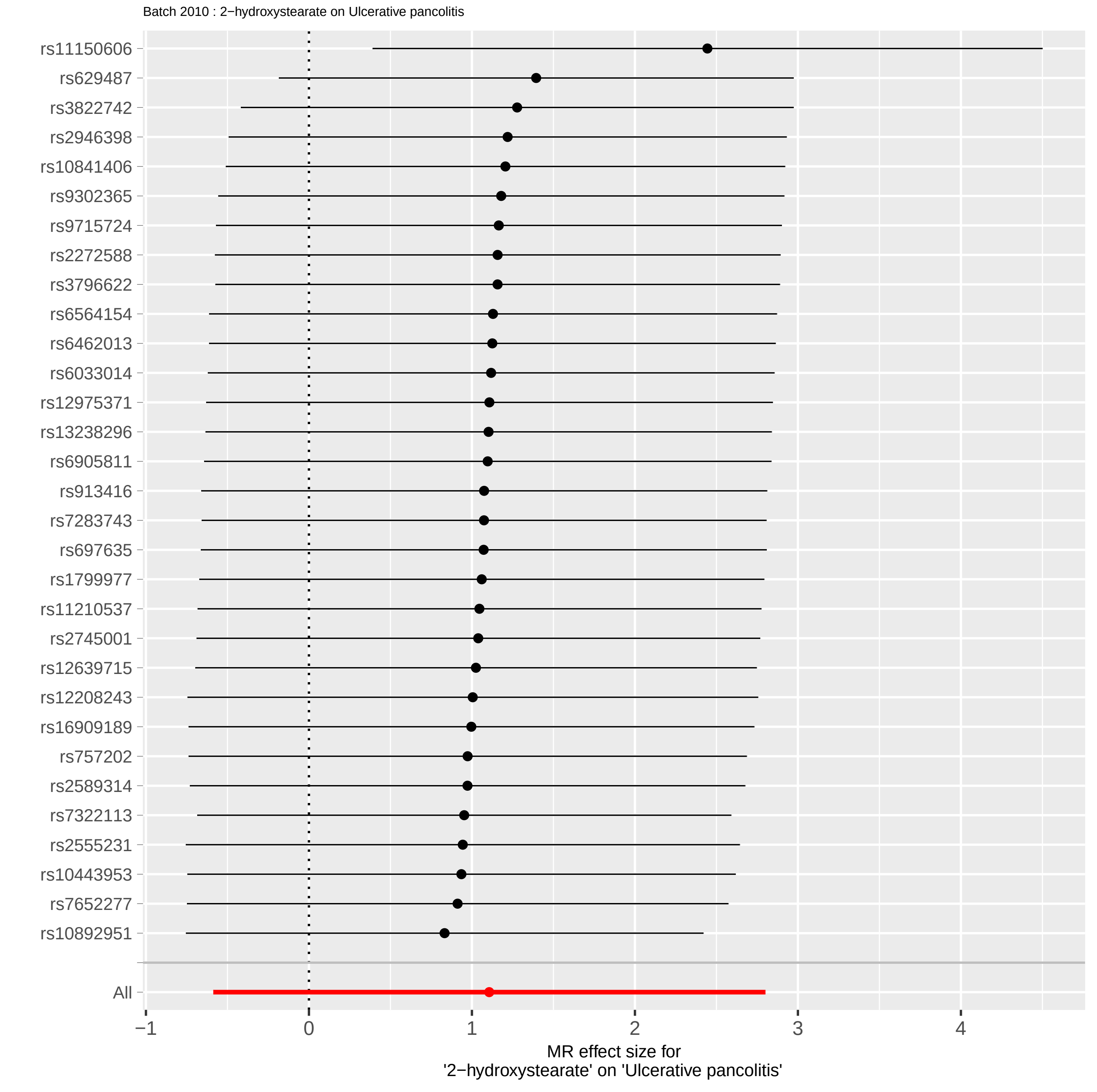
Batch 2010 : 2−hydroxystearate on Ulcerative pancolitis
rs11150606
rs629487
rs3822742
rs2946398
rs10841406
rs9302365
rs9715724
rs2272588
rs3796622
rs6564154
rs6462013
rs6033014
rs12975371
rs13238296
rs6905811
rs913416
rs7283743
rs697635
rs1799977
rs11210537
rs2745001
rs12639715
rs12208243
rs16909189
rs757202
rs2589314
rs7322113
rs2555231
rs10443953
rs7652277
rs10892951
All
−1
0
1
2
3
4
MR effect size for
'2−hydroxystearate' on 'Ulcerative pancolitis'

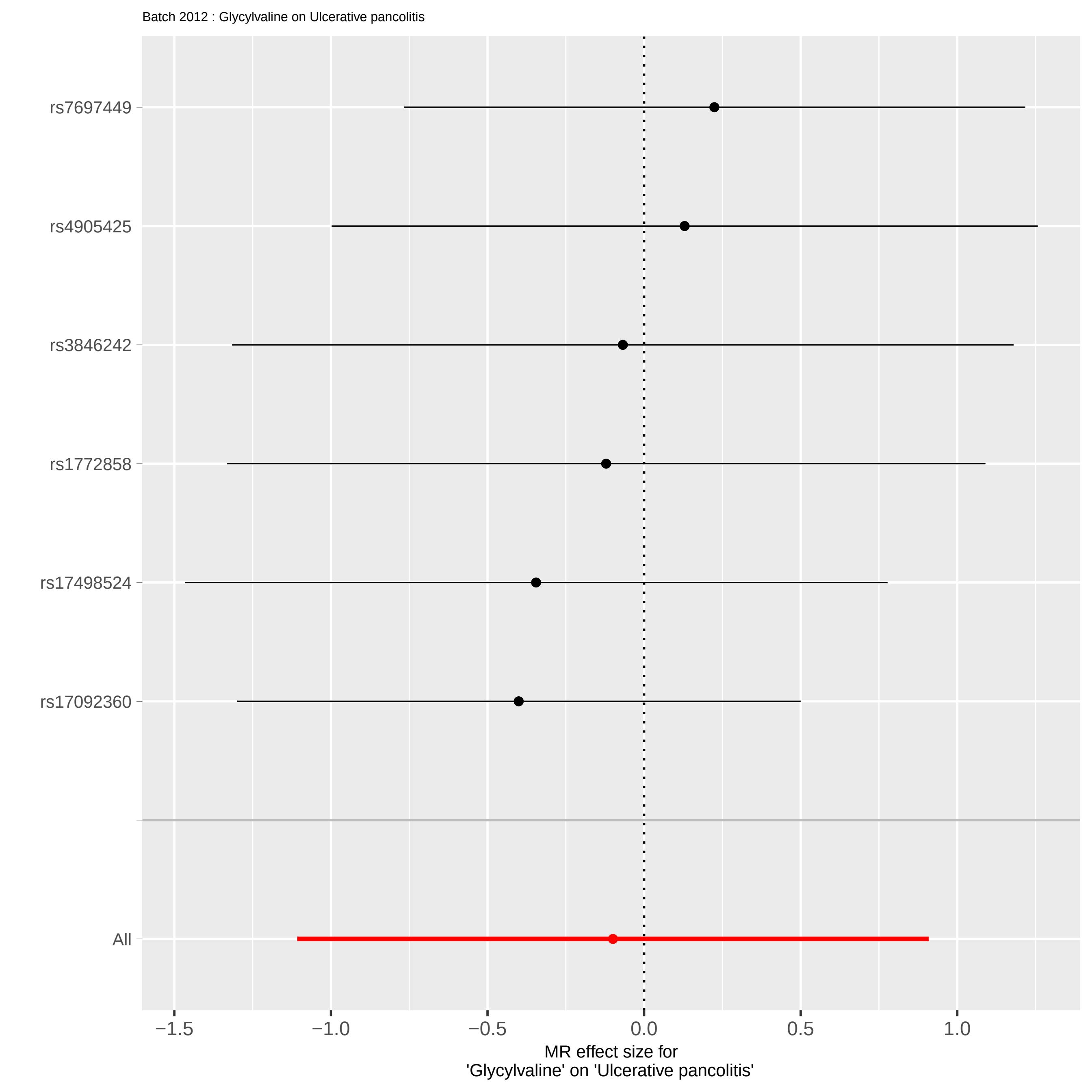

Batch 2012 : Glycylvaline on Ulcerative pancolitis
rs7697449
rs4905425
rs3846242
rs1772858
rs17498524
rs17092360
All
−1.5
−1.0
−0.5
0.0
0.5
1.0
MR effect size for
'Glycylvaline' on 'Ulcerative pancolitis'

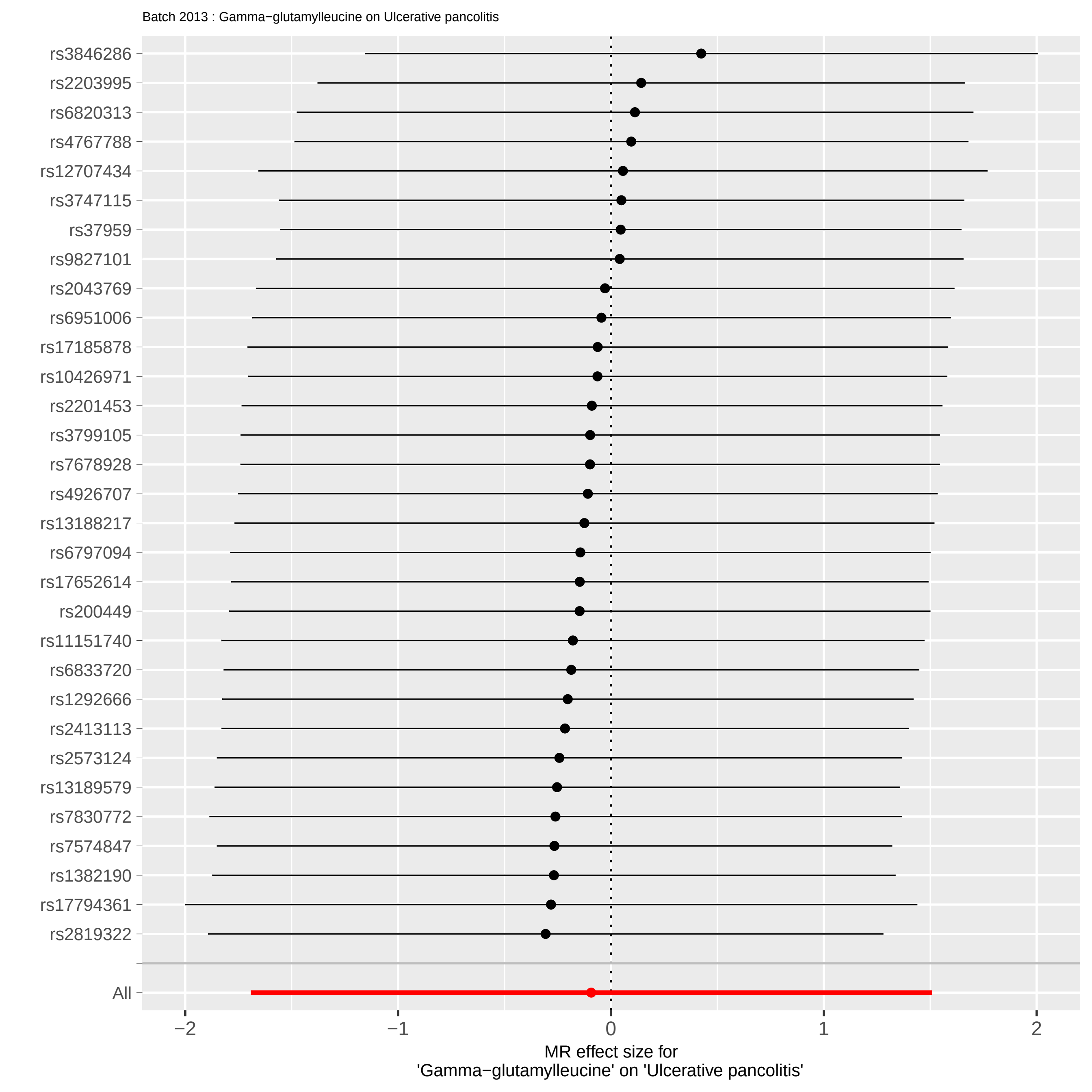

Batch 2013 : Gamma−glutamylleucine on Ulcerative pancolitis
rs3846286
rs2203995
rs6820313
rs4767788
rs12707434
rs3747115
rs37959
rs9827101
rs2043769
rs6951006
rs17185878
rs10426971
rs2201453
rs3799105
rs7678928
rs4926707
rs13188217
rs6797094
rs17652614
rs200449
rs11151740
rs6833720
rs1292666
rs2413113
rs2573124
rs13189579
rs7830772
rs7574847
rs1382190
rs17794361
rs2819322
All
−2
−1
0
1
2
MR effect size for
'Gamma−glutamylleucine' on 'Ulcerative pancolitis'

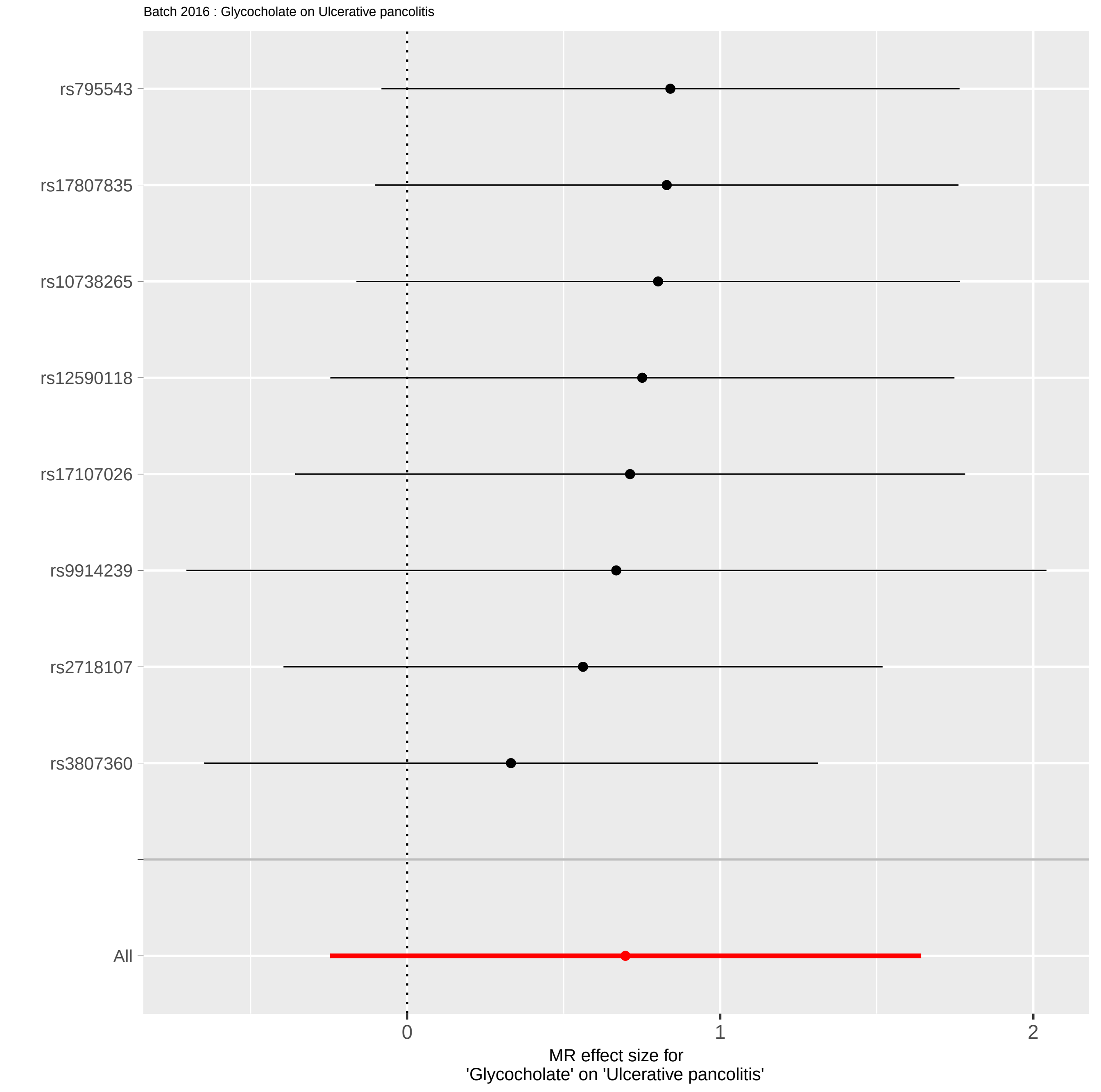

Batch 2016 : Glycocholate on Ulcerative pancolitis
rs795543
rs17807835
rs10738265
rs12590118
rs17107026
rs9914239
rs2718107
rs3807360
All
0
1
2
MR effect size for
'Glycocholate' on 'Ulcerative pancolitis'

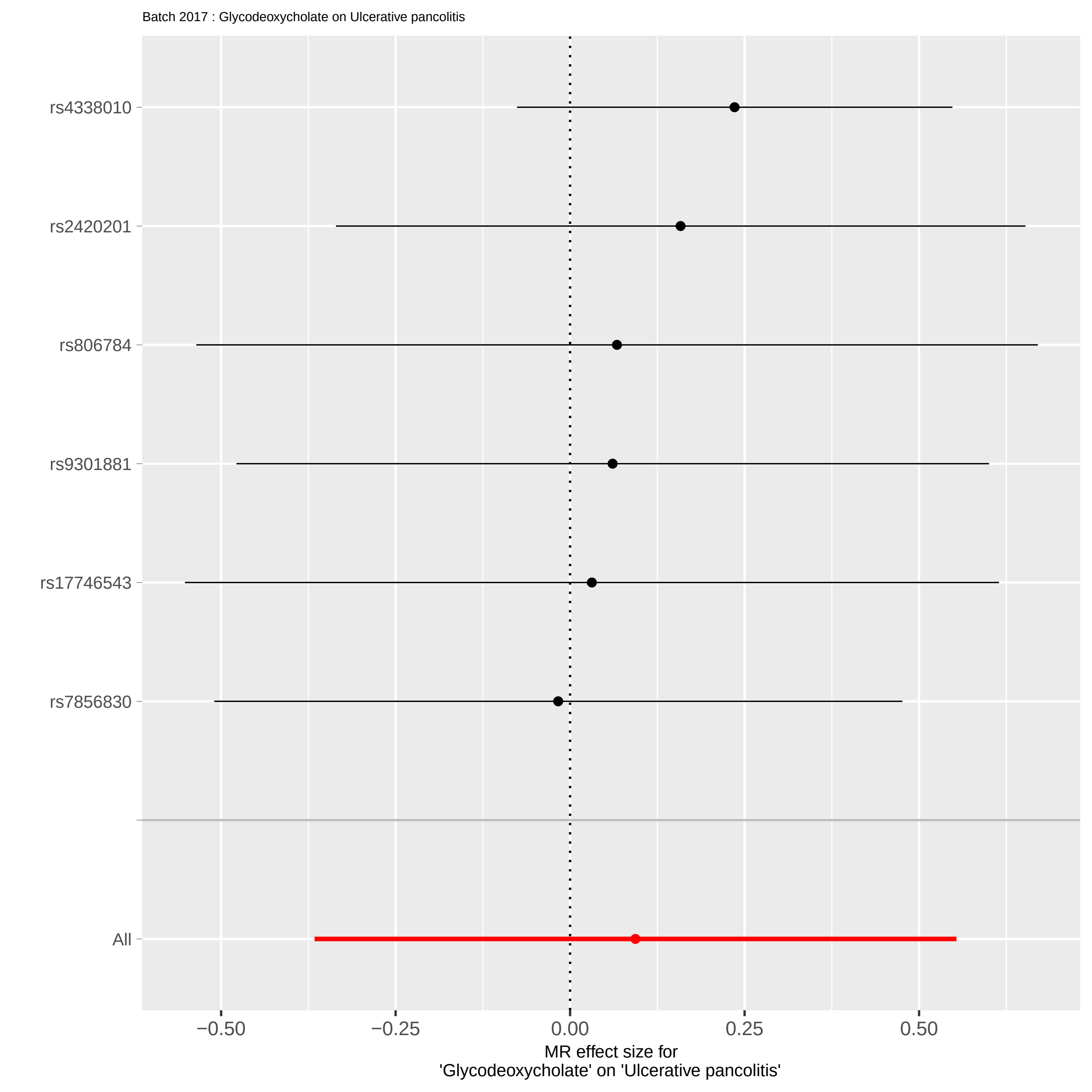
Batch 2017 : Glycodeoxycholate on Ulcerative pancolitis
rs4338010
rs2420201
rs806784
rs9301881
rs17746543
rs7856830
All
−0.50
−0.25
0.00
0.25
0.50
MR effect size for
'Glycodeoxycholate' on 'Ulcerative pancolitis'

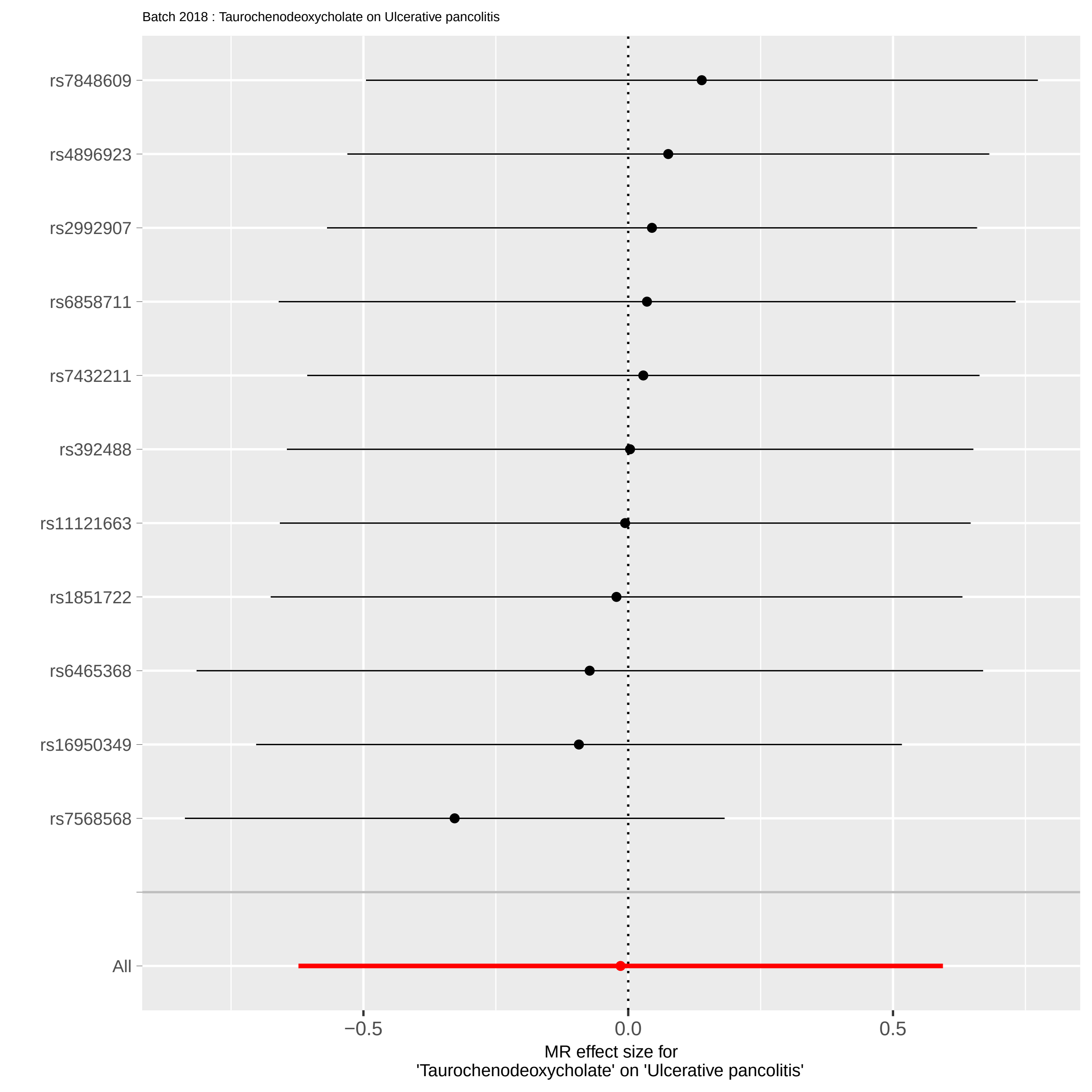
Batch 2018 : Taurochenodeoxycholate on Ulcerative pancolitis
rs7848609
rs4896923
rs2992907
rs6858711
rs7432211
rs392488
rs11121663
rs1851722
rs6465368
rs16950349
rs7568568
All
−0.5
0.0
0.5
MR effect size for
'Taurochenodeoxycholate' on 'Ulcerative pancolitis'

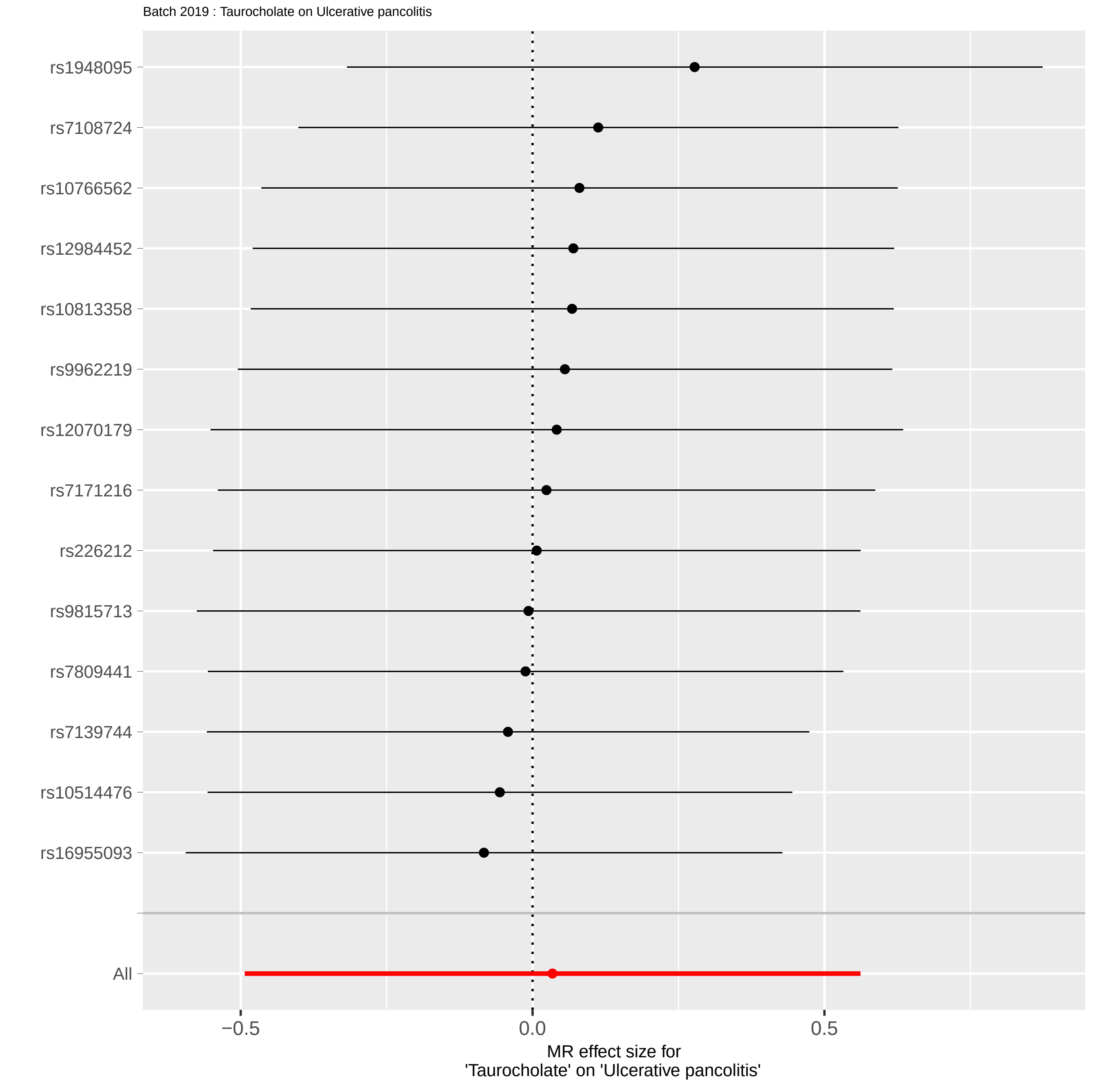

Batch 2019 : Taurocholate on Ulcerative pancolitis
rs1948095
rs7108724
rs10766562
rs12984452
rs10813358
rs9962219
rs12070179
rs7171216
rs226212
rs9815713
rs7809441
rs7139744
rs10514476
rs16955093
All
−0.5
0.0
0.5
MR effect size for
'Taurocholate' on 'Ulcerative pancolitis'

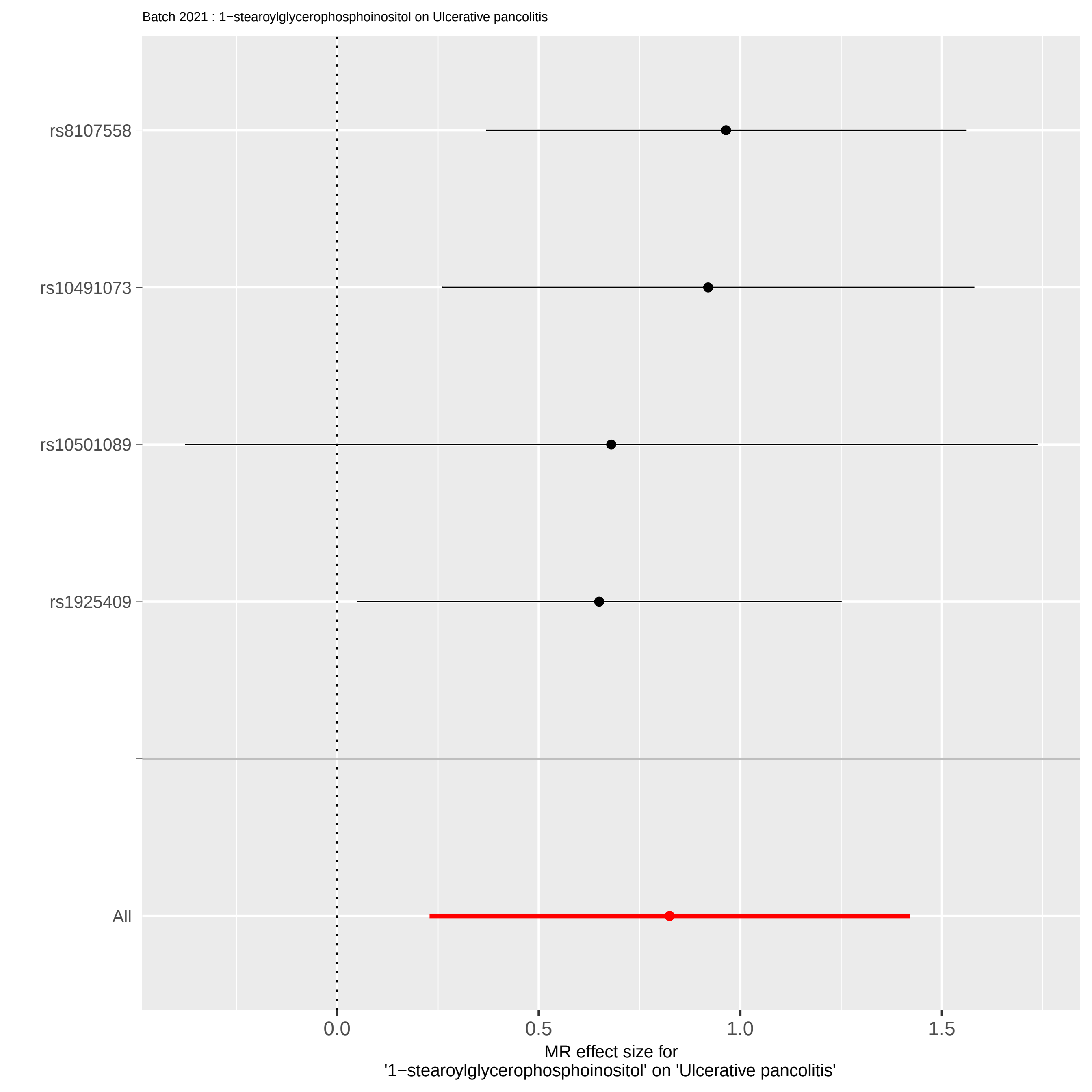

Batch 2021 : 1−stearoylglycerophosphoinositol on Ulcerative pancolitis
rs8107558
rs10491073
rs10501089
rs1925409
All
0.0
0.5
1.0
1.5
MR effect size for
'1−stearoylglycerophosphoinositol' on 'Ulcerative pancolitis'

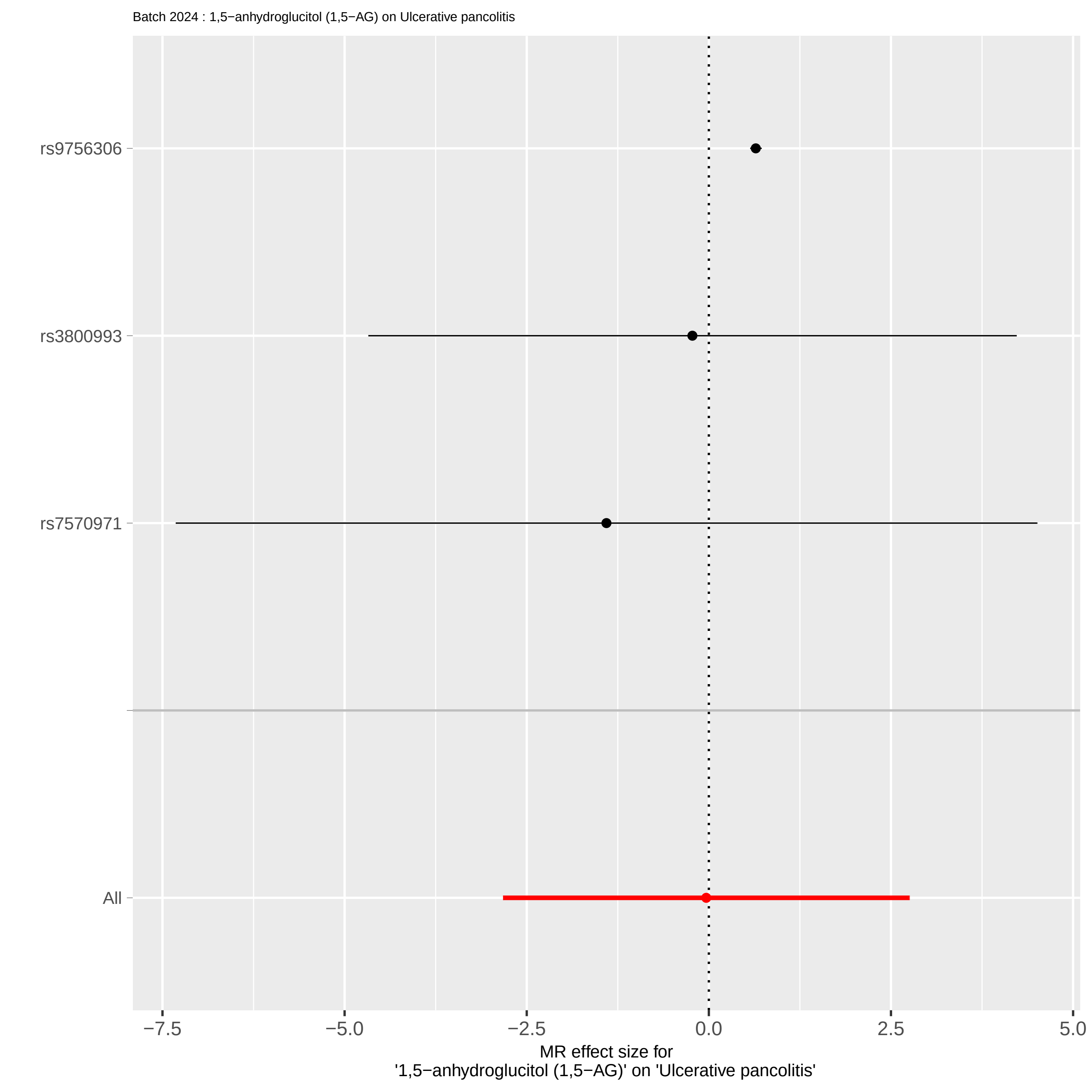
Batch 2024 : 1,5−anhydroglucitol (1,5−AG) on Ulcerative pancolitis
rs9756306
rs3800993
rs7570971
All
−7.5
−5.0
−2.5
0.0
2.5
5.0
MR effect size for
'1,5−anhydroglucitol (1,5−AG)' on 'Ulcerative pancolitis'

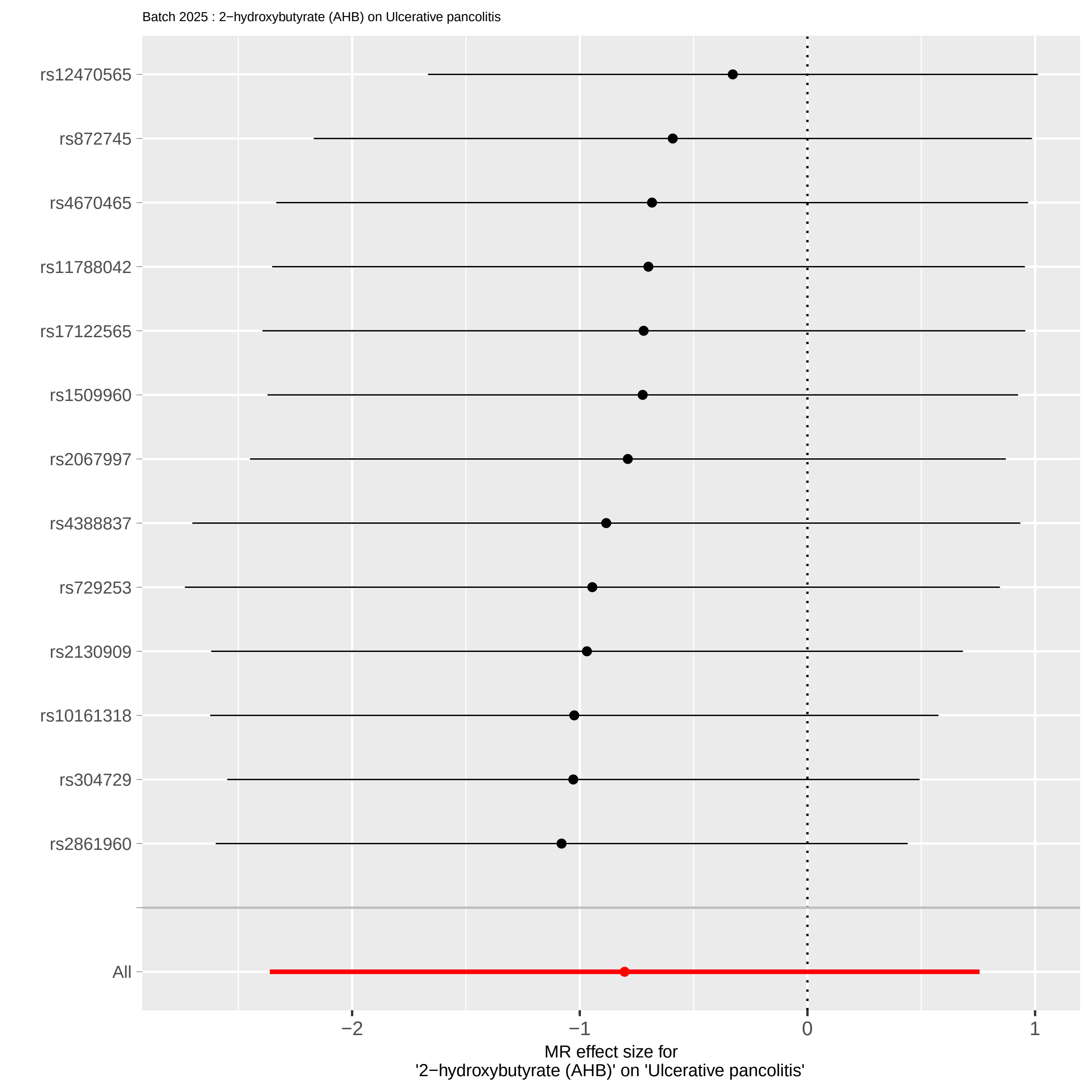

Batch 2025 : 2−hydroxybutyrate (AHB) on Ulcerative pancolitis
rs12470565
rs872745
rs4670465
rs11788042
rs17122565
rs1509960
rs2067997
rs4388837
rs729253
rs2130909
rs10161318
rs304729
rs2861960
All
−2
−1
0
1
MR effect size for
'2−hydroxybutyrate (AHB)' on 'Ulcerative pancolitis'

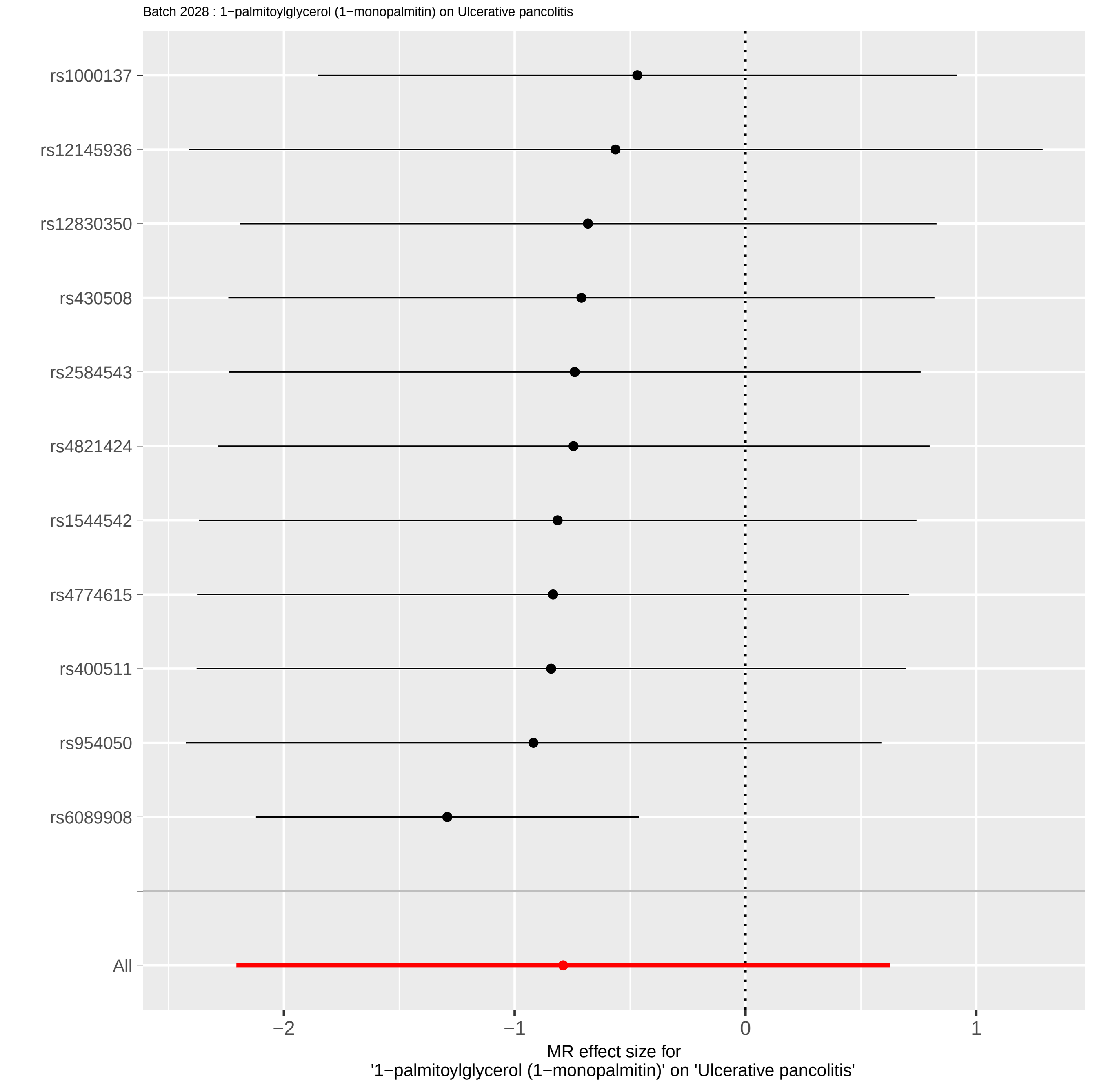
Batch 2028 : 1−palmitoylglycerol (1−monopalmitin) on Ulcerative pancolitis
rs1000137
rs12145936
rs12830350
rs430508
rs2584543
rs4821424
rs1544542
rs4774615
rs400511
rs954050
rs6089908
All
−2
−1
0
1
MR effect size for
'1−palmitoylglycerol (1−monopalmitin)' on 'Ulcerative pancolitis'

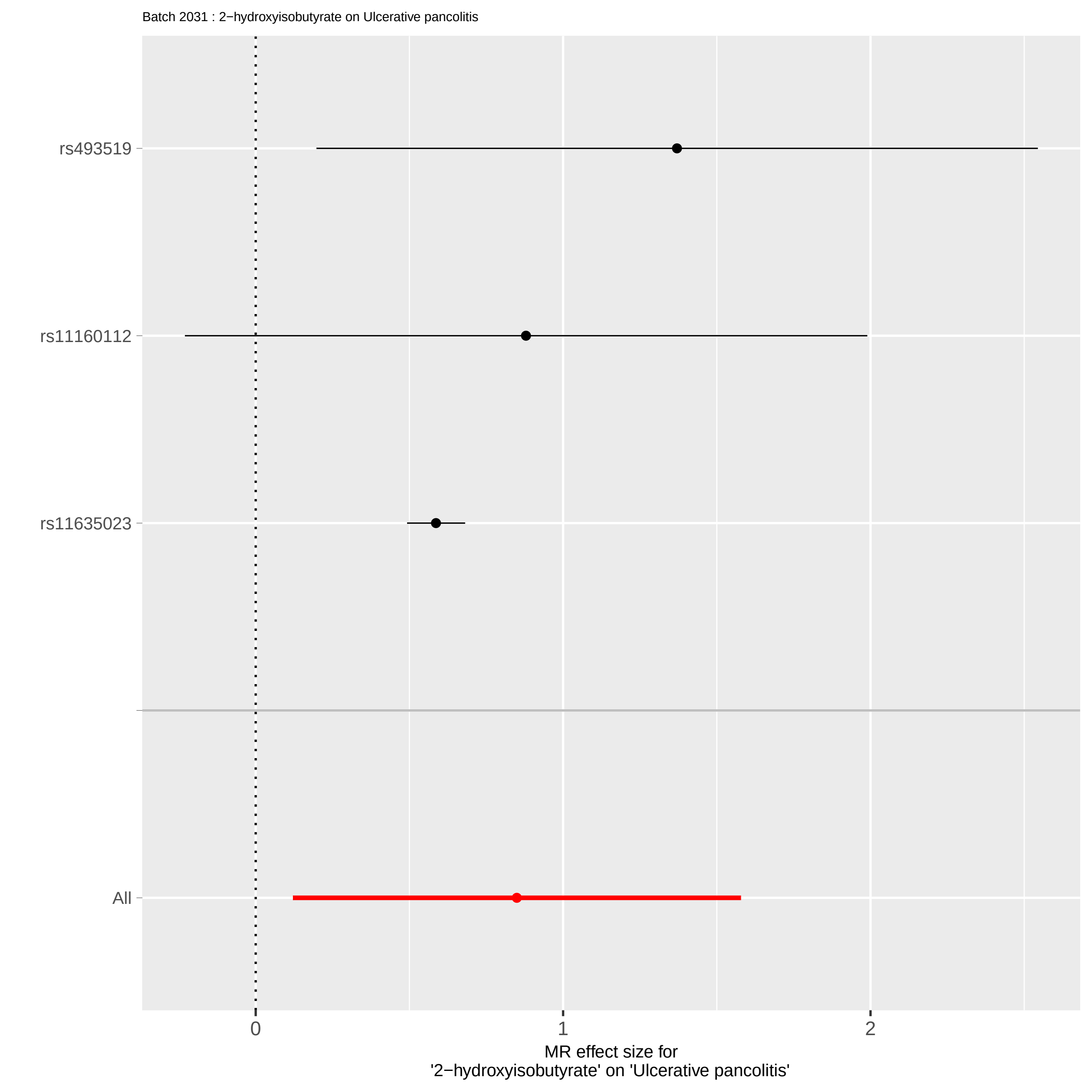
Batch 2031 : 2−hydroxyisobutyrate on Ulcerative pancolitis
rs493519
rs11160112
rs11635023
All
0
1
2
MR effect size for
'2−hydroxyisobutyrate' on 'Ulcerative pancolitis'

Batch 2038 : Cholate on Ulcerative pancolitis

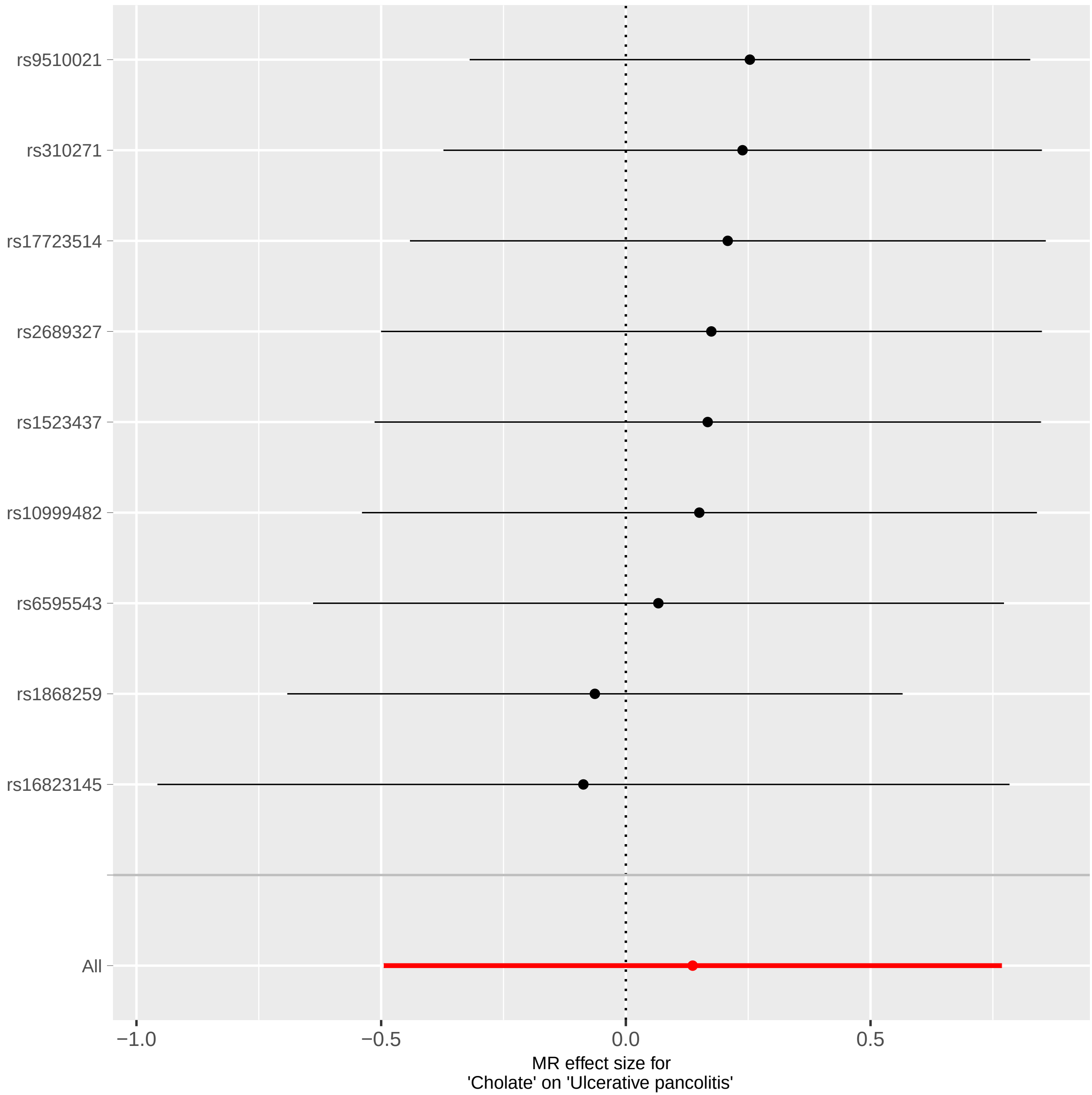

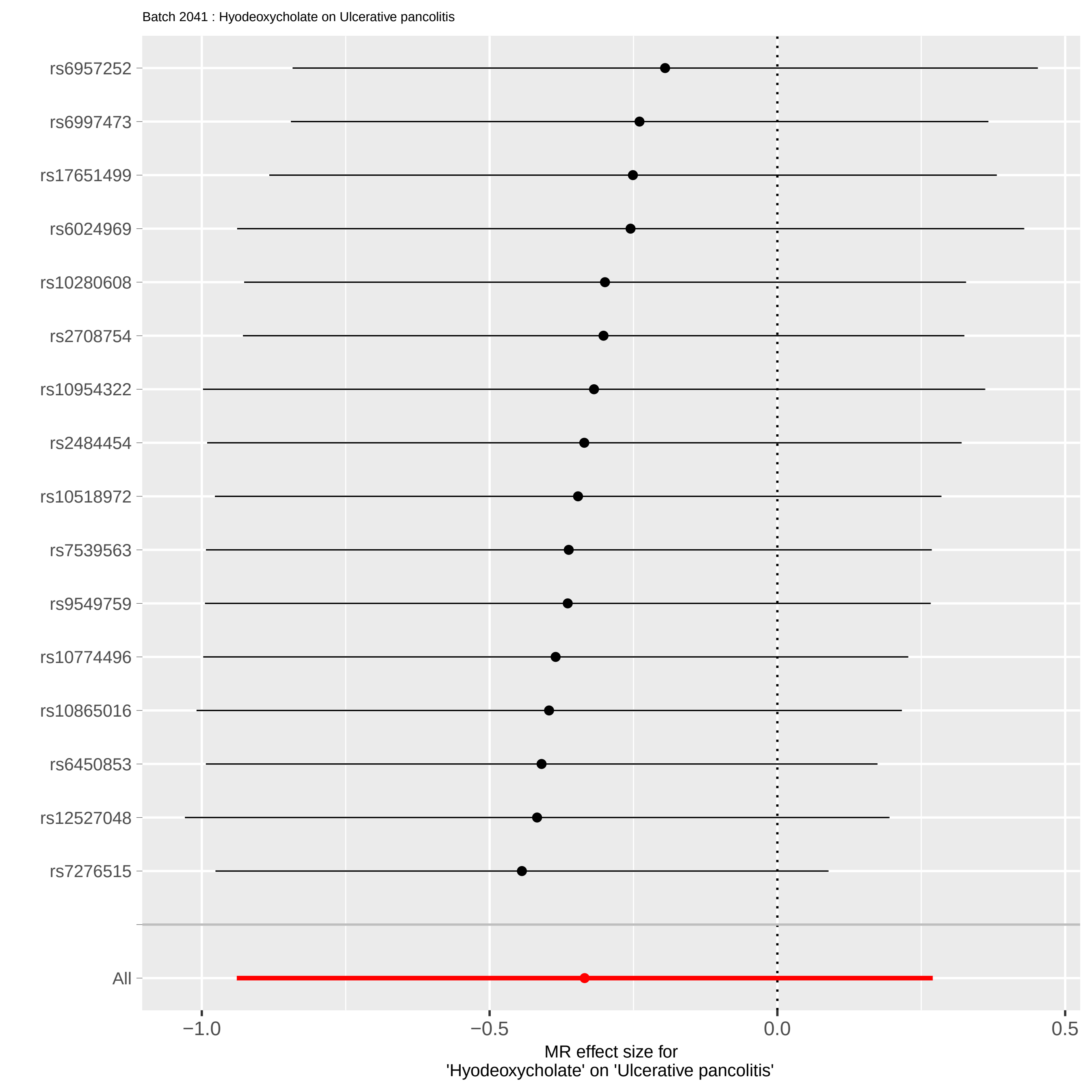

Batch 2041 : Hyodeoxycholate on Ulcerative pancolitis
rs6957252
rs6997473
rs17651499
rs6024969
rs10280608
rs2708754
rs10954322
rs2484454
rs10518972
rs7539563
rs9549759
rs10774496
rs10865016
rs6450853
rs12527048
rs7276515
All
−1.0
−0.5
0.0
0.5
MR effect size for
'Hyodeoxycholate' on 'Ulcerative pancolitis'

Batch 2042 : 3−indoxyl sulfate on Ulcerative pancolitis

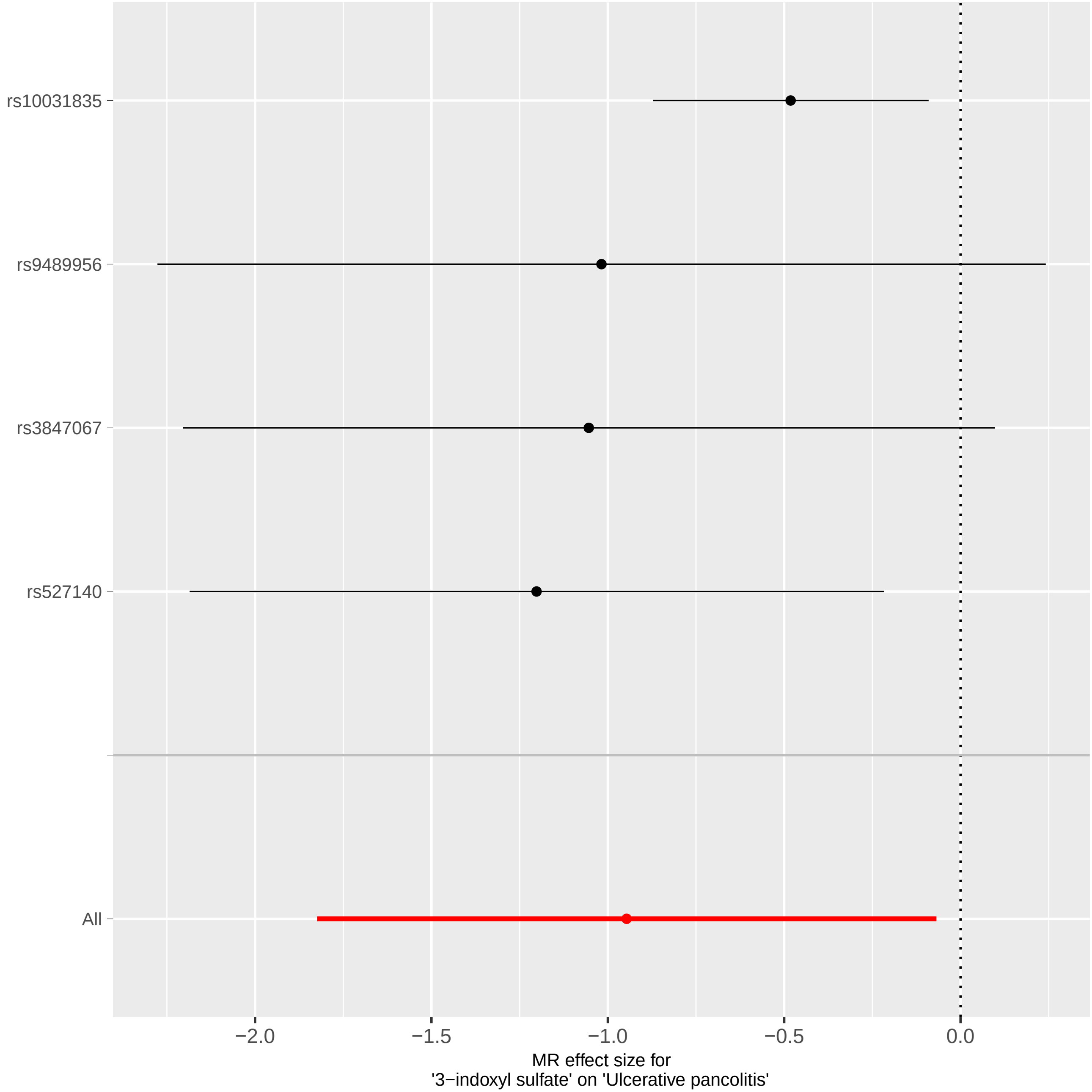

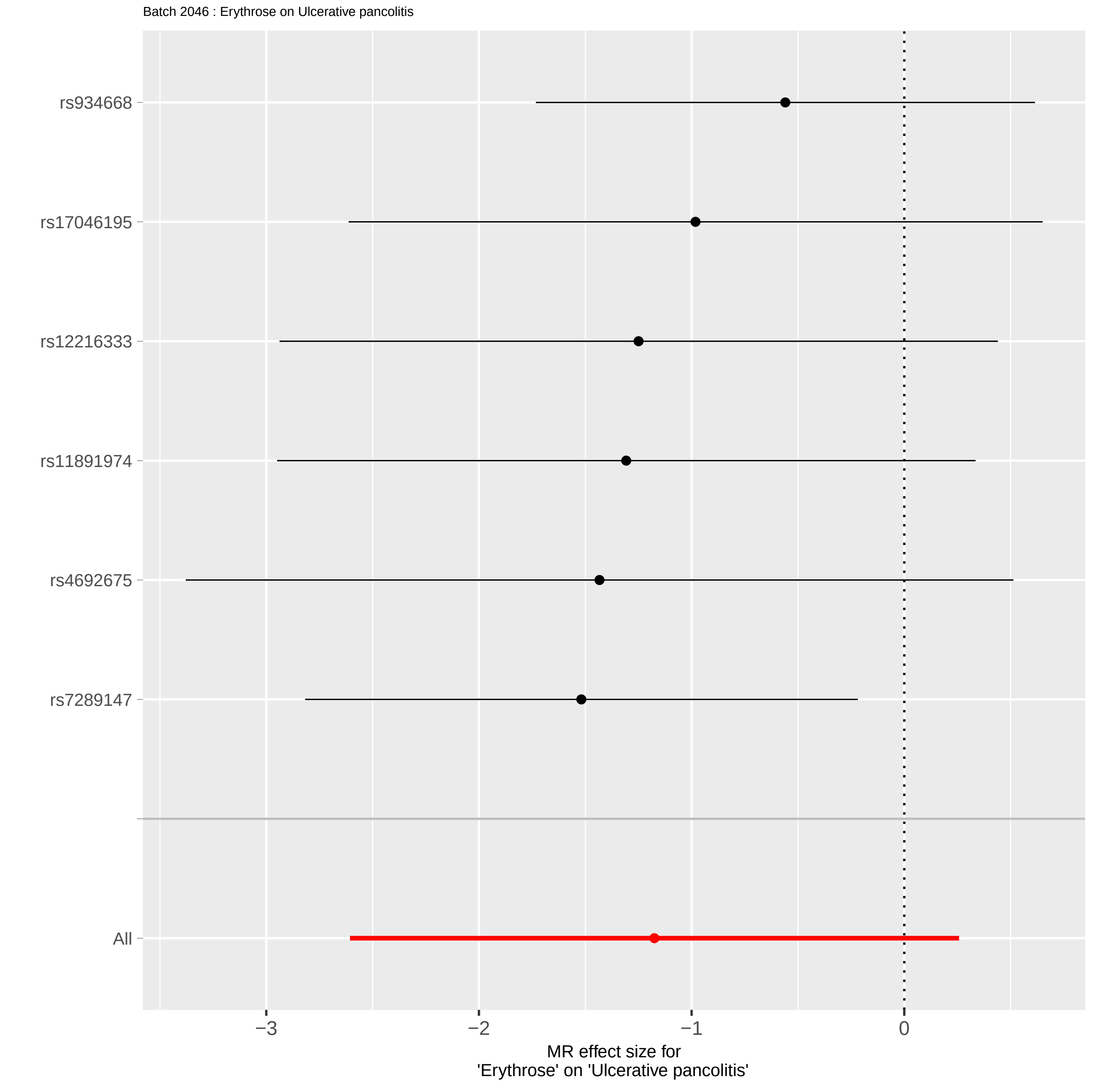
Batch 2046 : Erythrose on Ulcerative pancolitis
rs934668
rs17046195
rs12216333
rs11891974
rs4692675
rs7289147
All
−3
−2
−1
0
MR effect size for
'Erythrose' on 'Ulcerative pancolitis'

Batch 2047 : Threonate on Ulcerative pancolitis

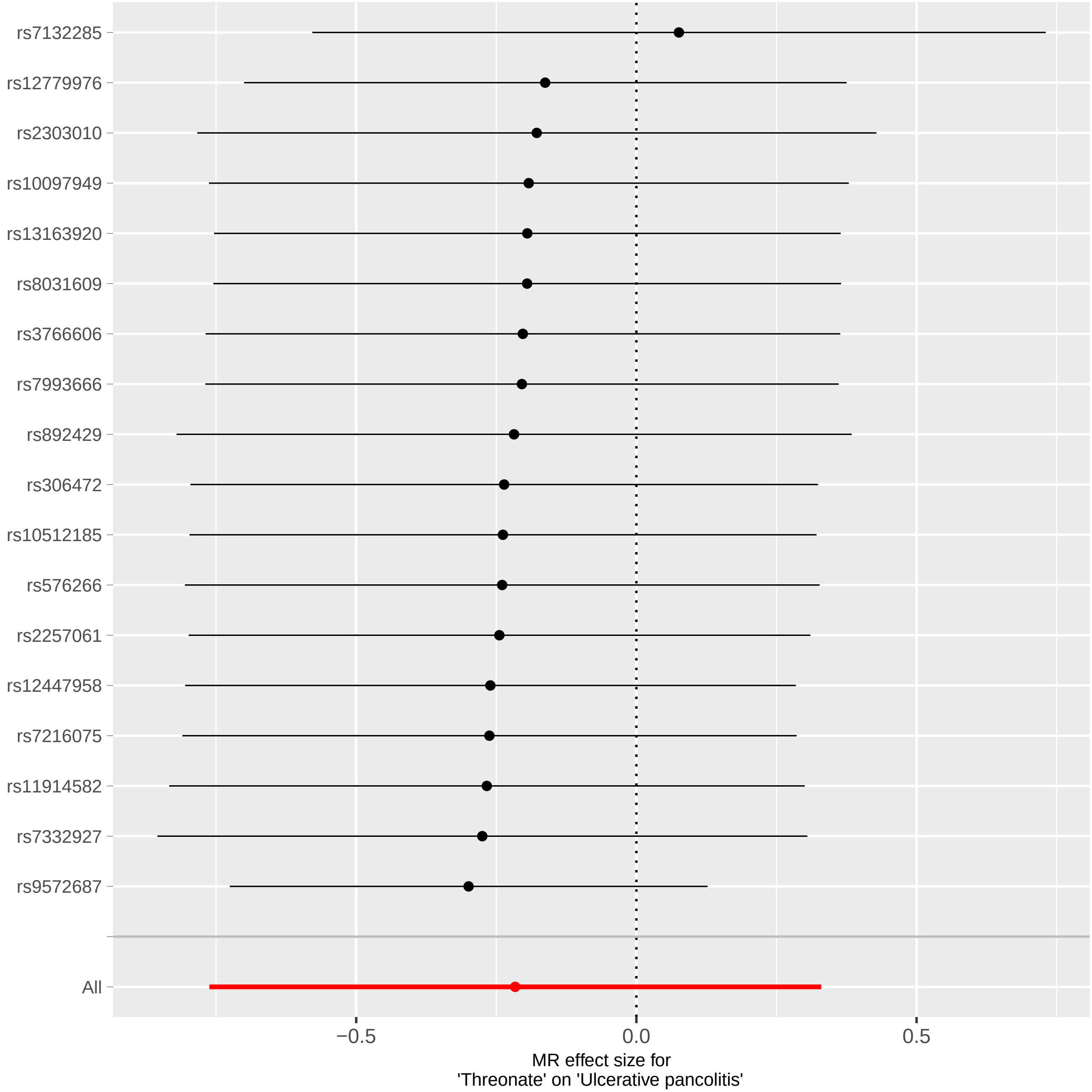

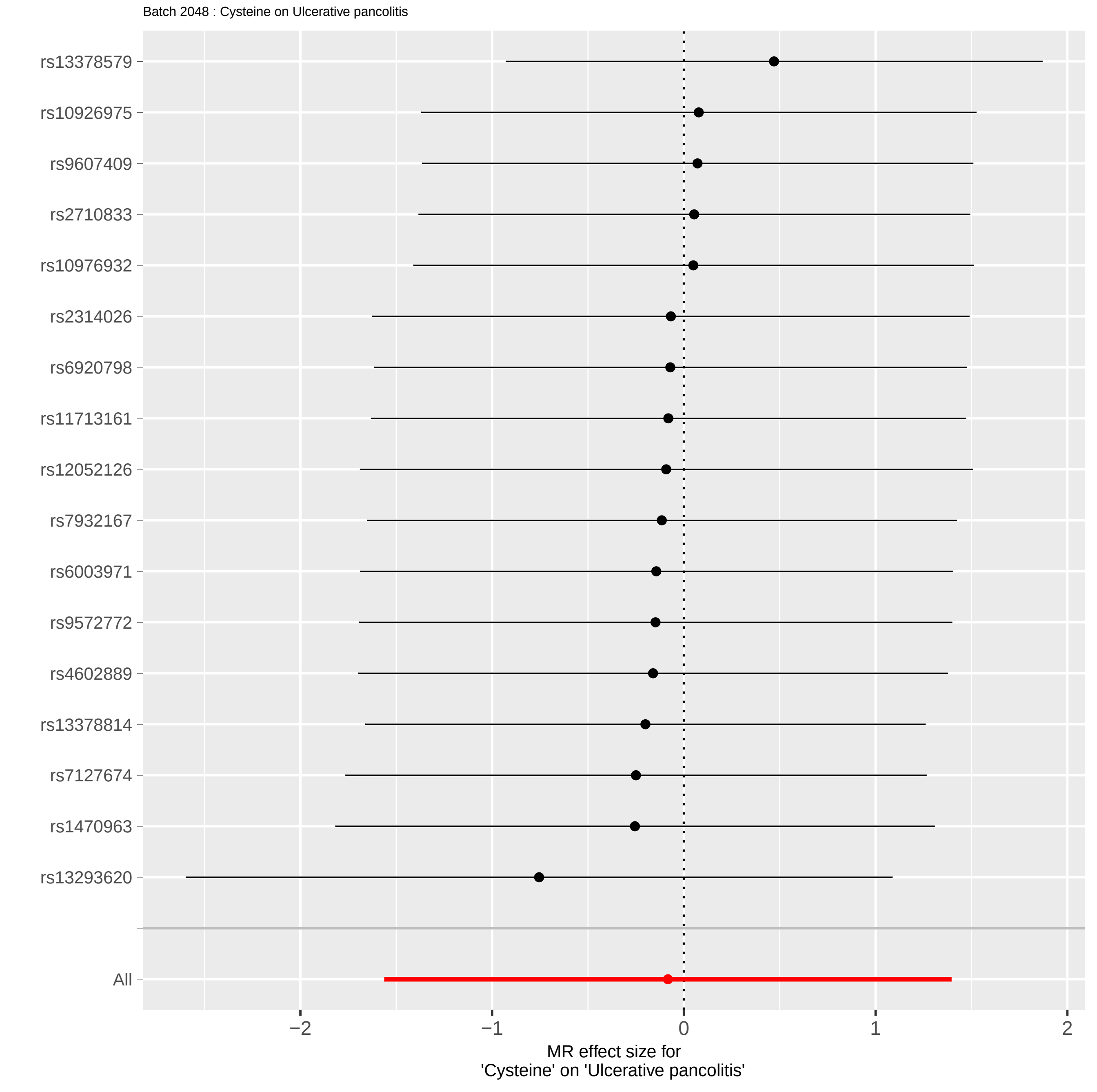

Batch 2048 : Cysteine on Ulcerative pancolitis
rs13378579
rs10926975
rs9607409
rs2710833
rs10976932
rs2314026
rs6920798
rs11713161
rs12052126
rs7932167
rs6003971
rs9572772
rs4602889
rs13378814
rs7127674
rs1470963
rs13293620
All
−2
−1
0
1
2
MR effect size for
'Cysteine' on 'Ulcerative pancolitis'

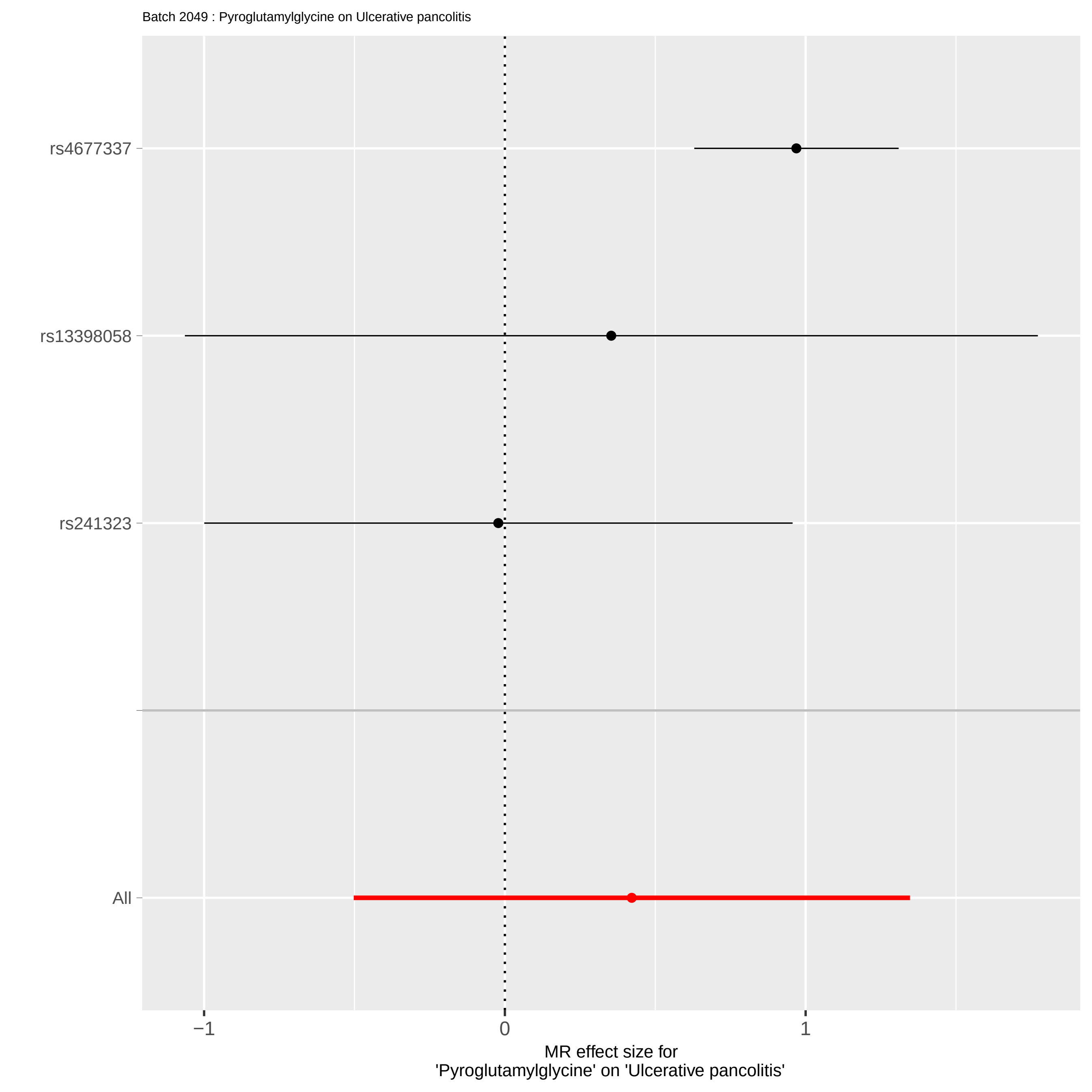

Batch 2049 : Pyroglutamylglycine on Ulcerative pancolitis
rs4677337
rs13398058
rs241323
All
−1
0
1
MR effect size for
'Pyroglutamylglycine' on 'Ulcerative pancolitis'

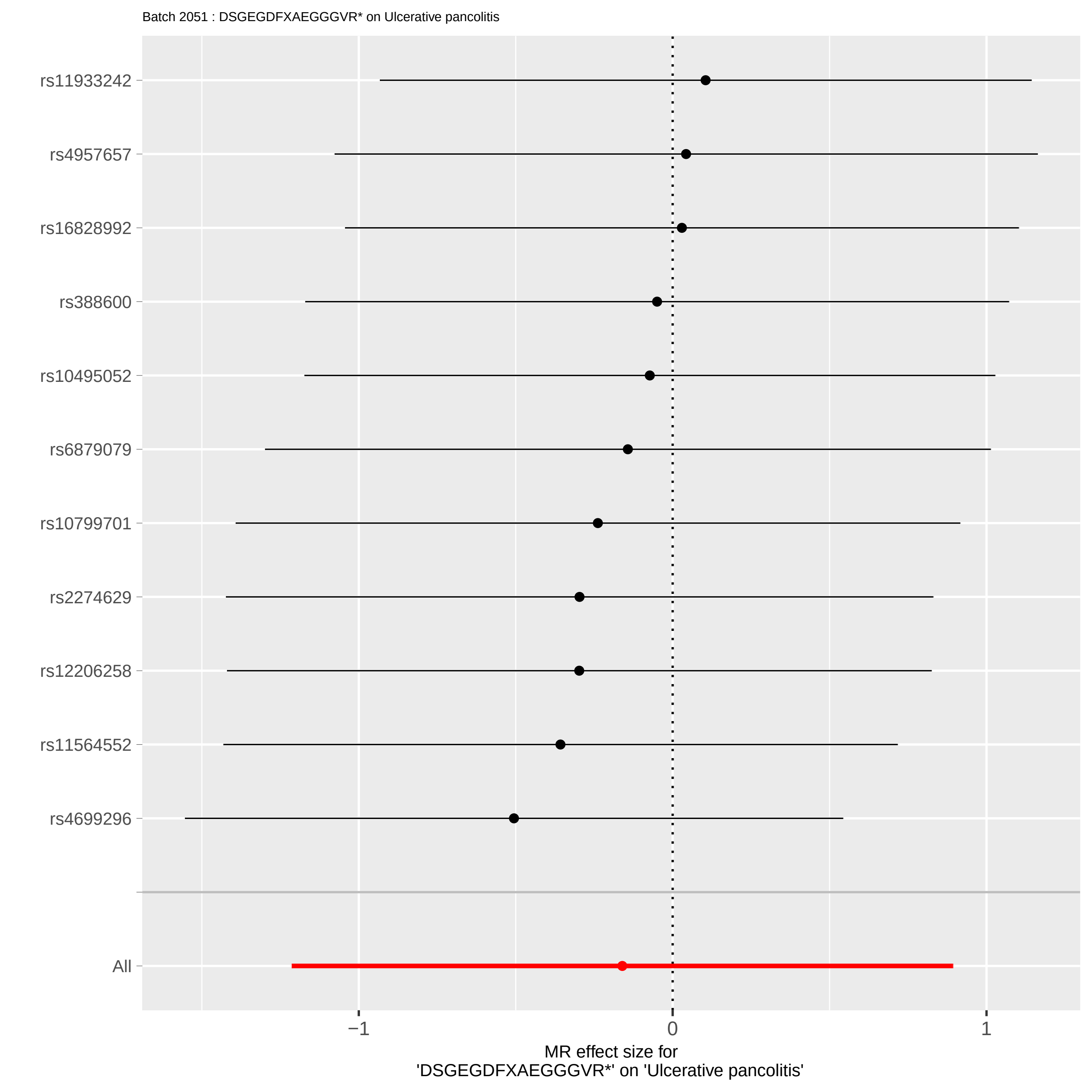

Batch 2051 : DSGEGDFXAEGGGVR* on Ulcerative pancolitis
rs11933242
rs4957657
rs16828992
rs388600
rs10495052
rs6879079
rs10799701
rs2274629
rs12206258
rs11564552
rs4699296
All
−1
0
1
MR effect size for
'DSGEGDFXAEGGGVR*' on 'Ulcerative pancolitis'

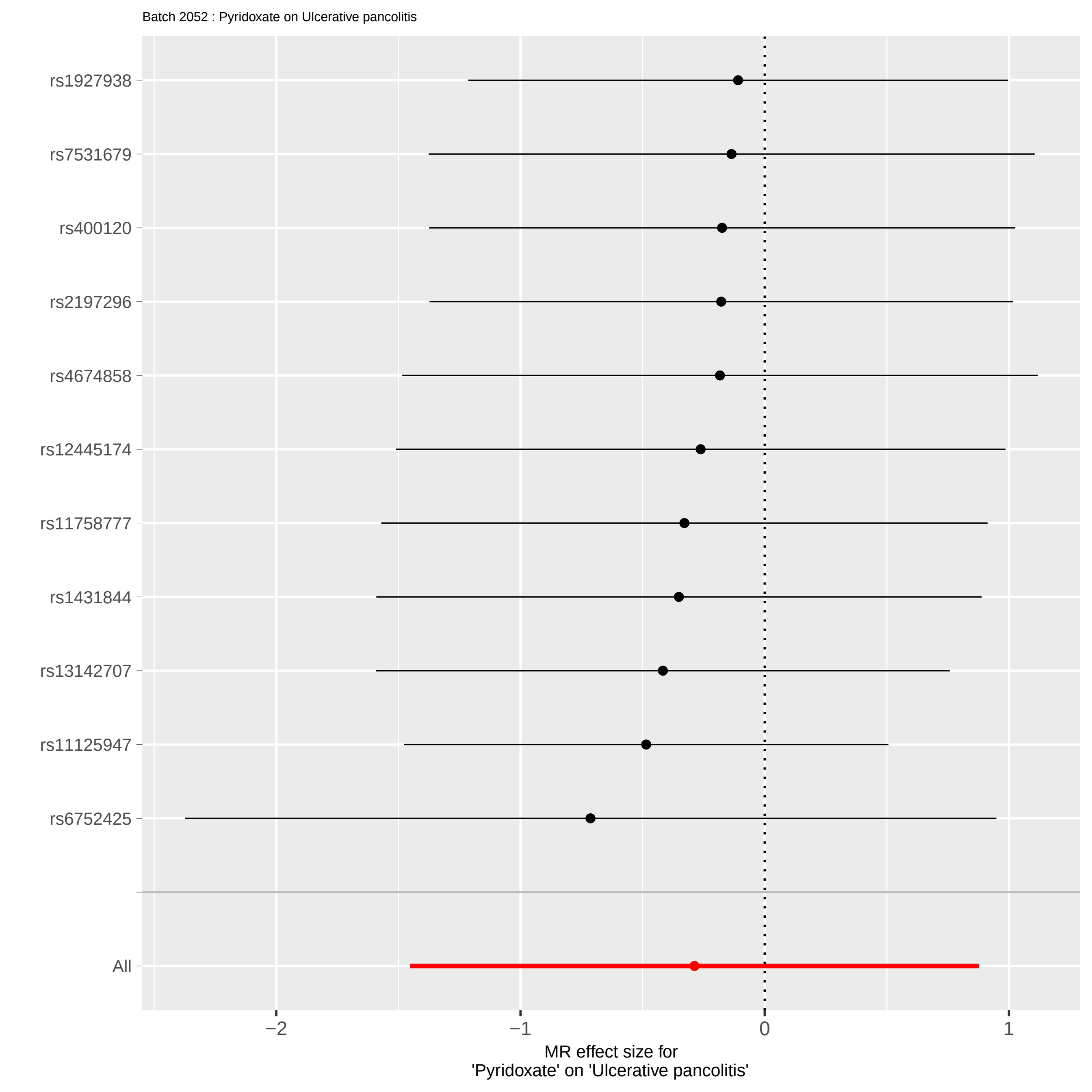
Batch 2052 : Pyridoxate on Ulcerative pancolitis
rs1927938
rs7531679
rs400120
rs2197296
rs4674858
rs12445174
rs11758777
rs1431844
rs13142707
rs11125947
rs6752425
All
−2
−1
0
1
MR effect size for
'Pyridoxate' on 'Ulcerative pancolitis'

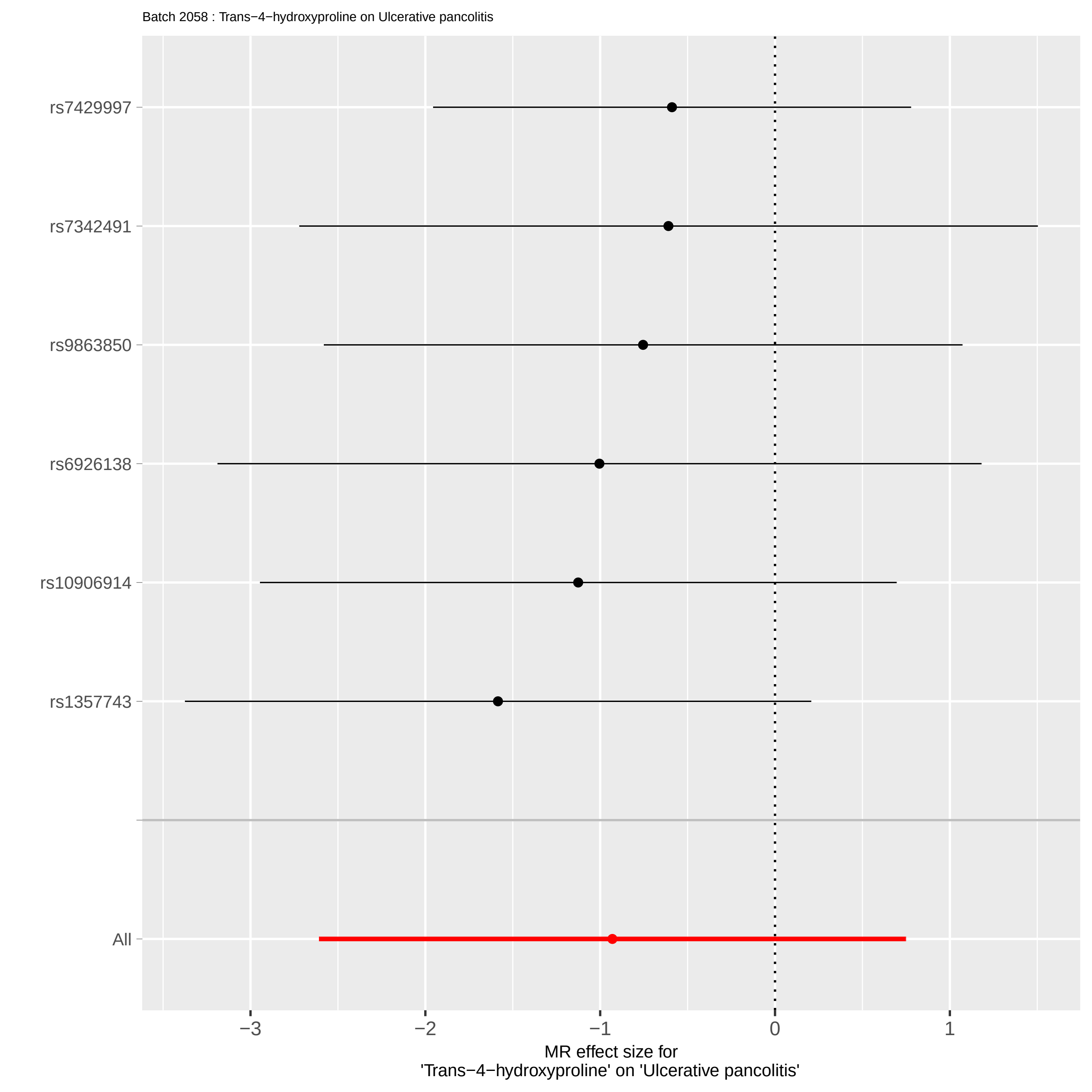

Batch 2058 : Trans−4−hydroxyproline on Ulcerative pancolitis
rs7429997
rs7342491
rs9863850
rs6926138
rs10906914
rs1357743
All
−3
−2
−1
0
1
MR effect size for
'Trans−4−hydroxyproline' on 'Ulcerative pancolitis'

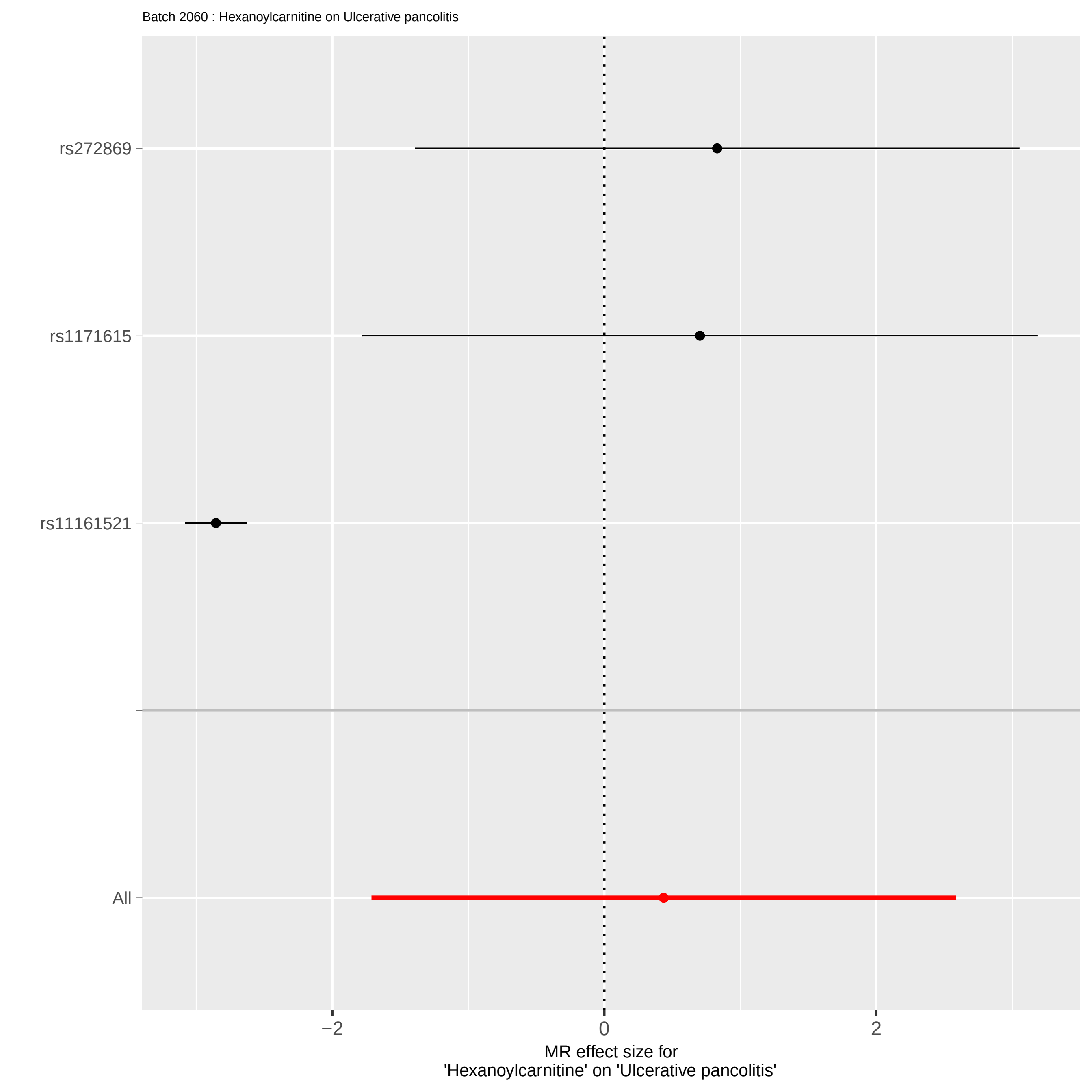

Batch 2060 : Hexanoylcarnitine on Ulcerative pancolitis
rs272869
rs1171615
rs11161521
All
−2
0
2
MR effect size for
'Hexanoylcarnitine' on 'Ulcerative pancolitis'

Batch 2069 : Butyrylcarnitine on Ulcerative pancolitis

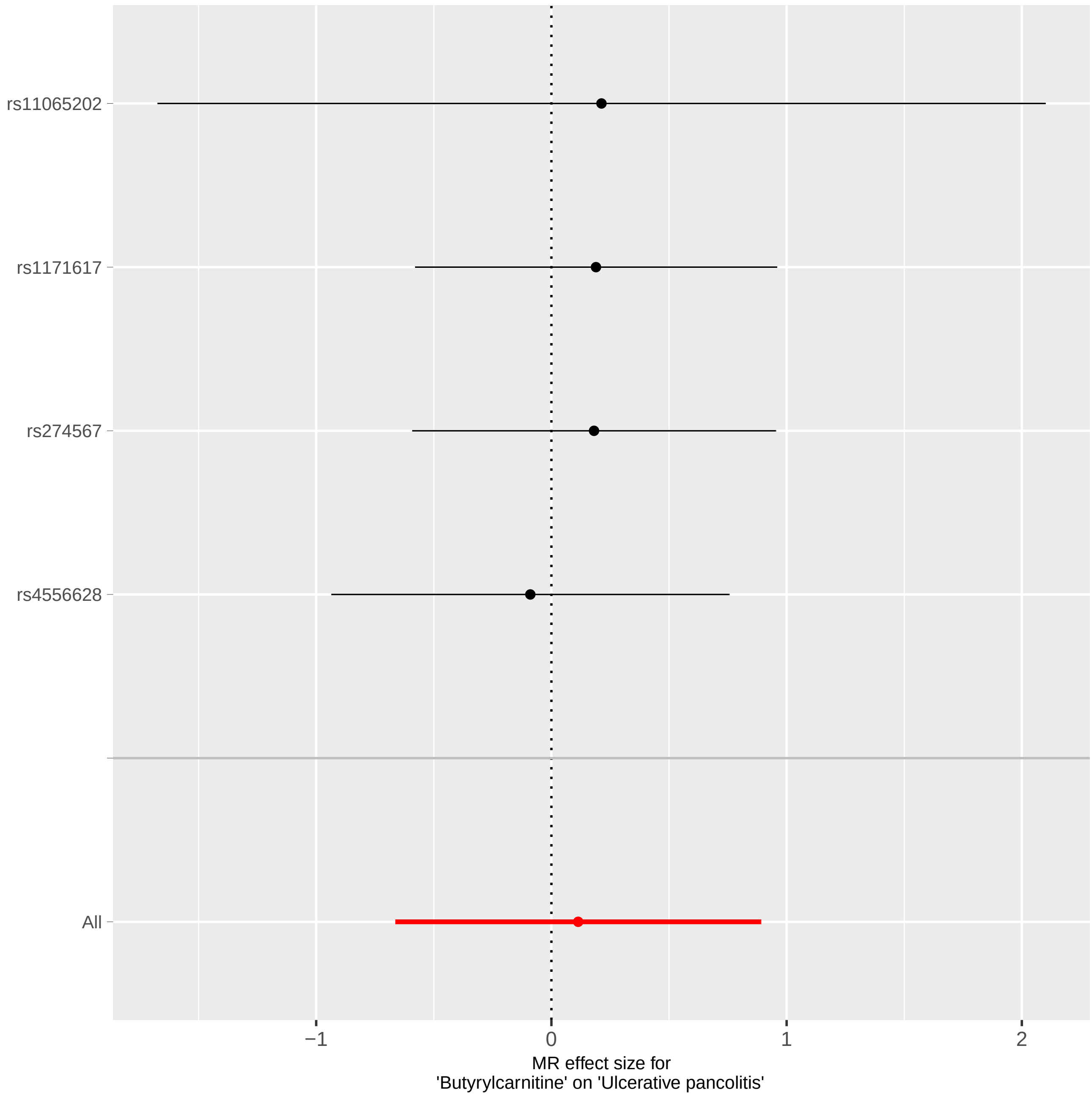

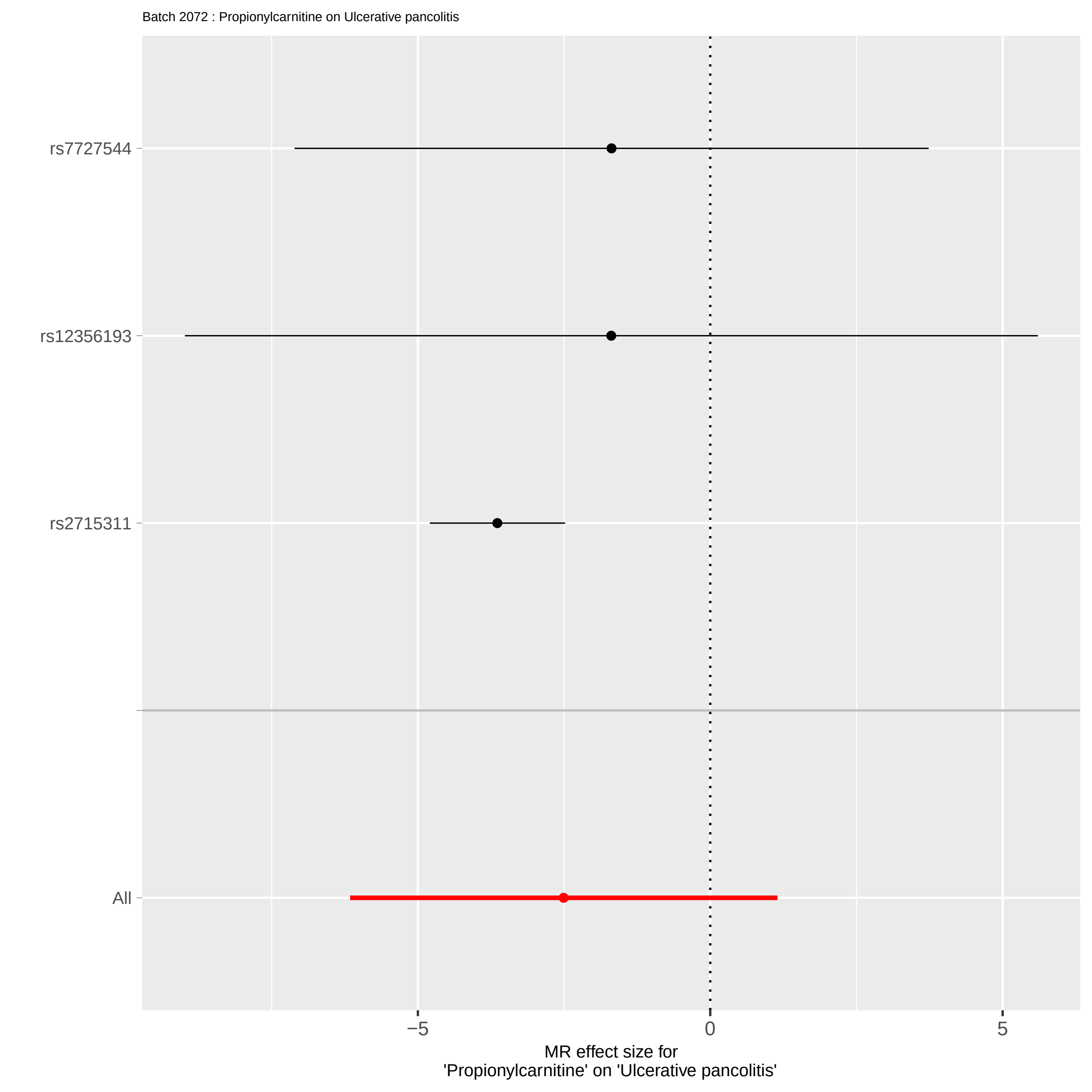

Batch 2072 : Propionylcarnitine on Ulcerative pancolitis
rs7727544
rs12356193
rs2715311
All
−5
0
5
MR effect size for
'Propionylcarnitine' on 'Ulcerative pancolitis'

Batch 2073 : Caproate (6:0) on Ulcerative pancolitis

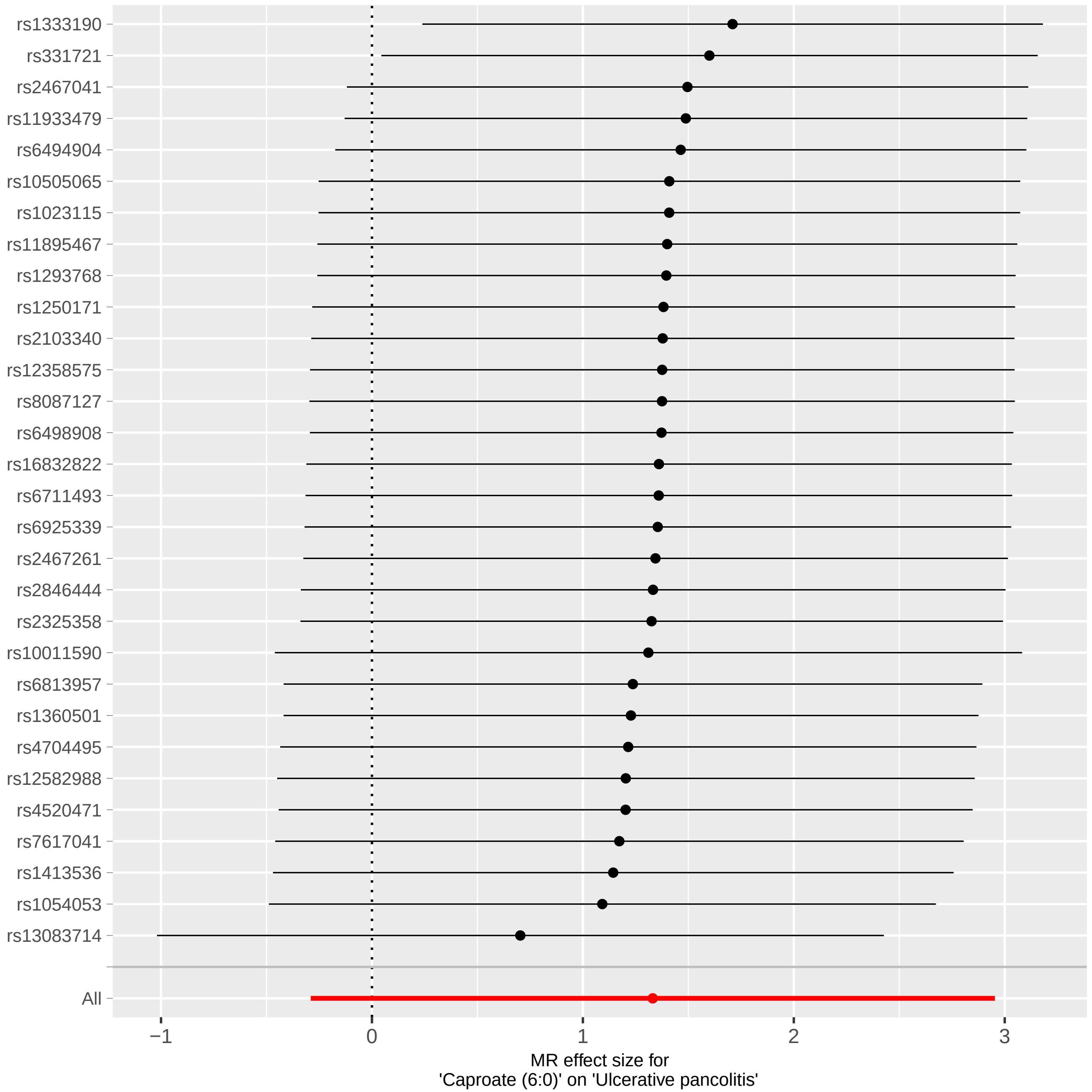

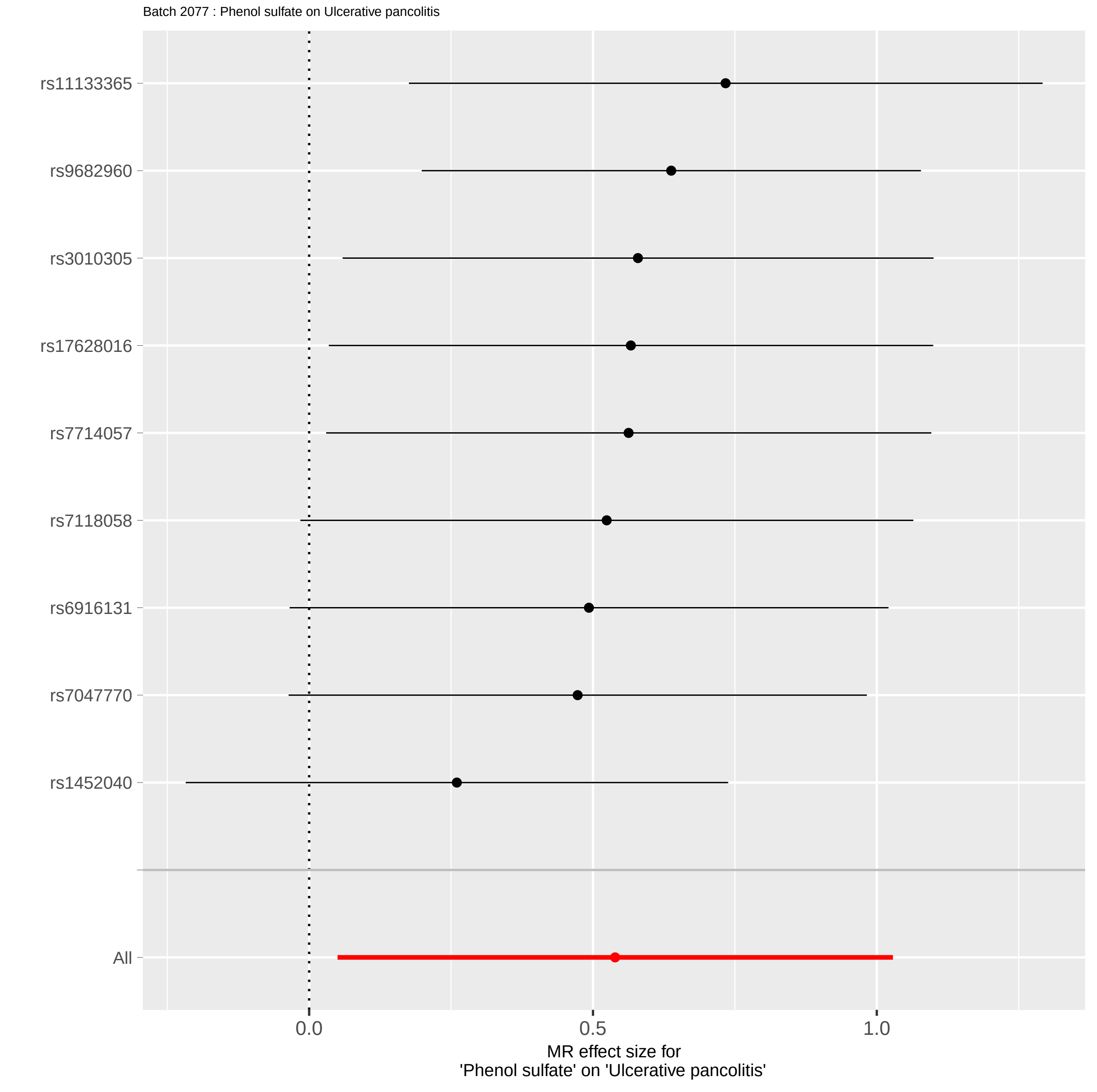
Batch 2077 : Phenol sulfate on Ulcerative pancolitis
rs11133365
rs9682960
rs3010305
rs17628016
rs7714057
rs7118058
rs6916131
rs7047770
rs1452040
All
0.0
0.5
1.0
MR effect size for
'Phenol sulfate' on 'Ulcerative pancolitis'

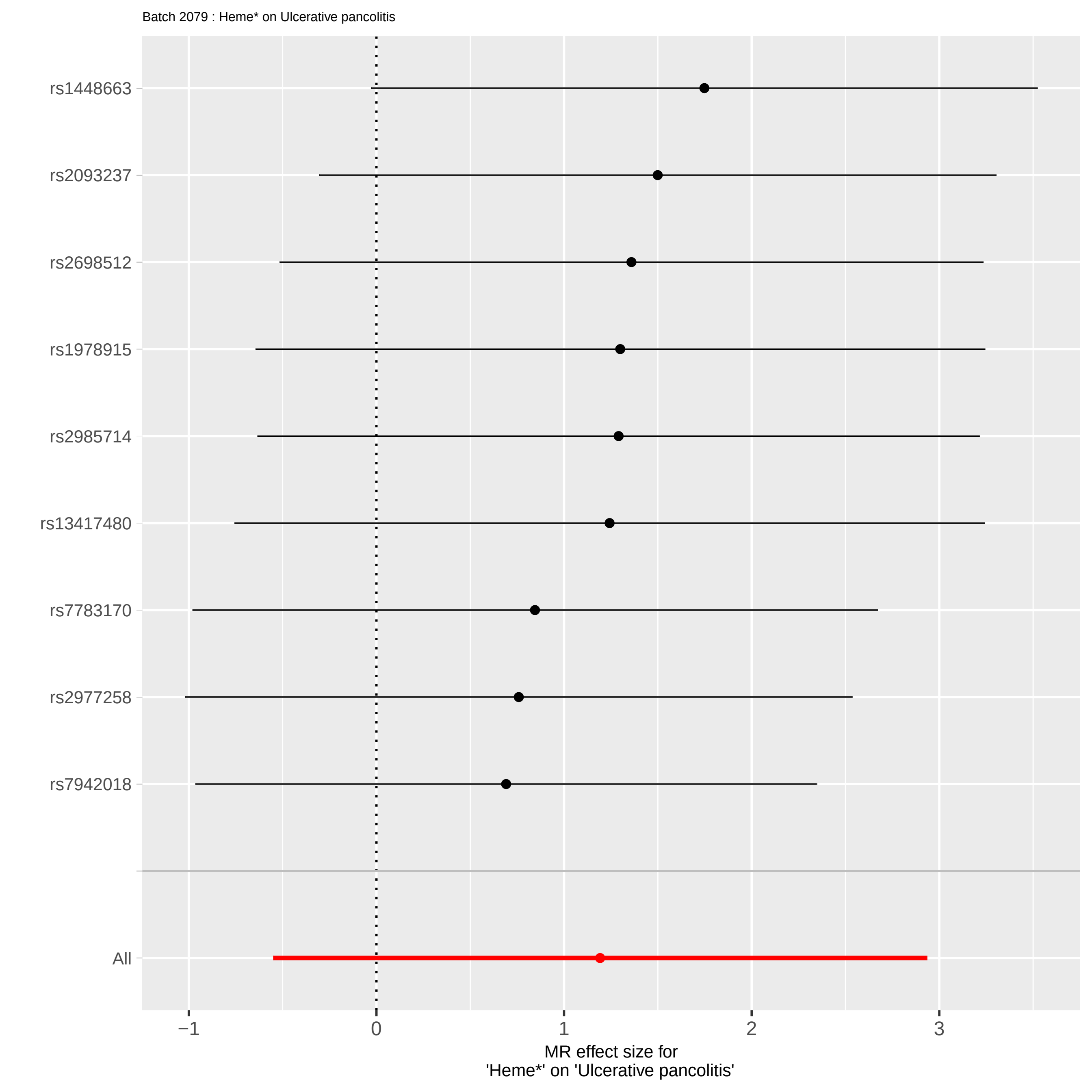

Batch 2079 : Heme* on Ulcerative pancolitis
rs1448663
rs2093237
rs2698512
rs1978915
rs2985714
rs13417480
rs7783170
rs2977258
rs7942018
All
−1
0
1
2
3
MR effect size for
'Heme*' on 'Ulcerative pancolitis'

Batch 2085 : X−11422−−xanthine on Ulcerative pancolitis

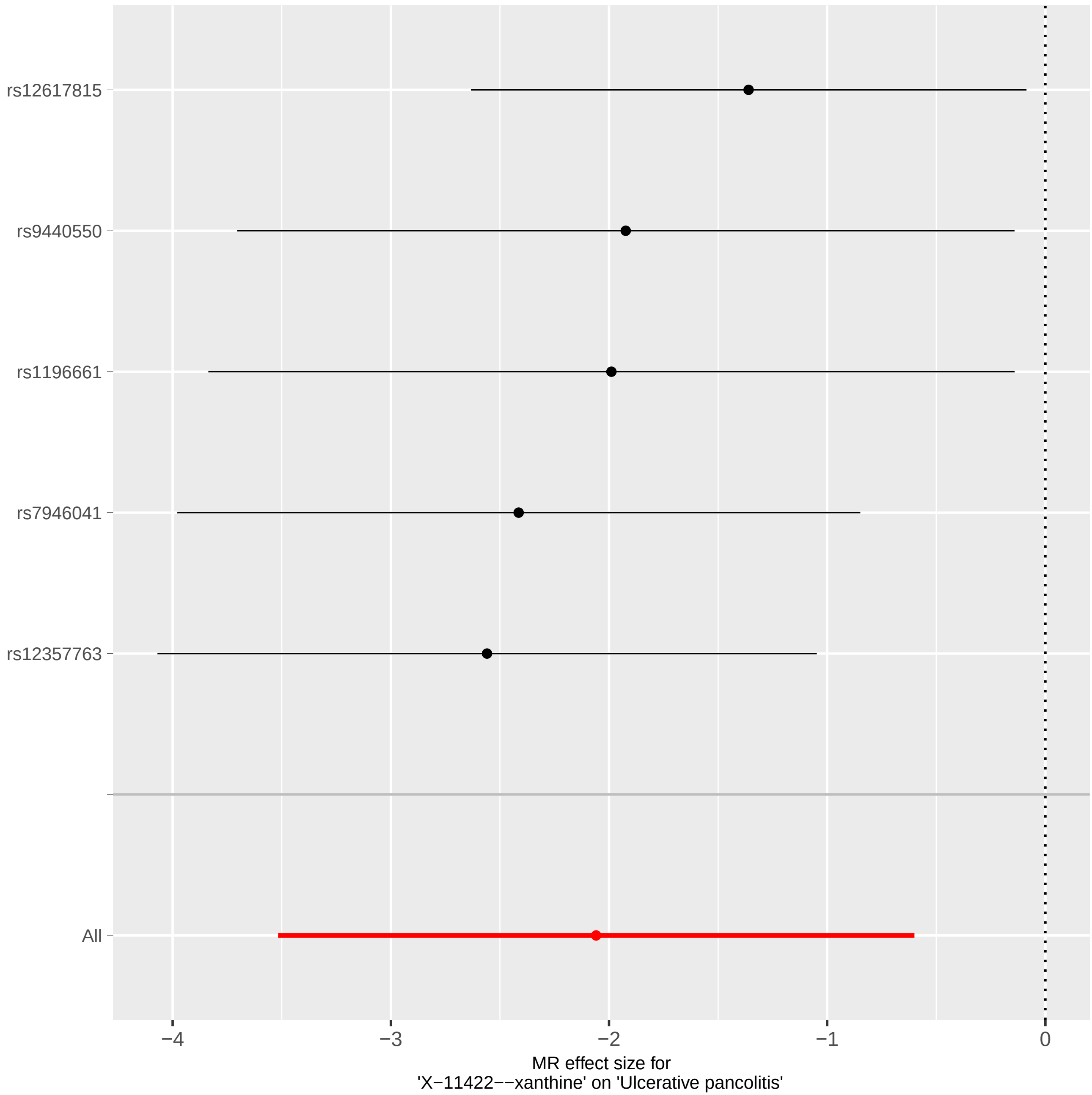

Batch 2089 : X−11593−−O−methylascorbate* on Ulcerative pancolitis

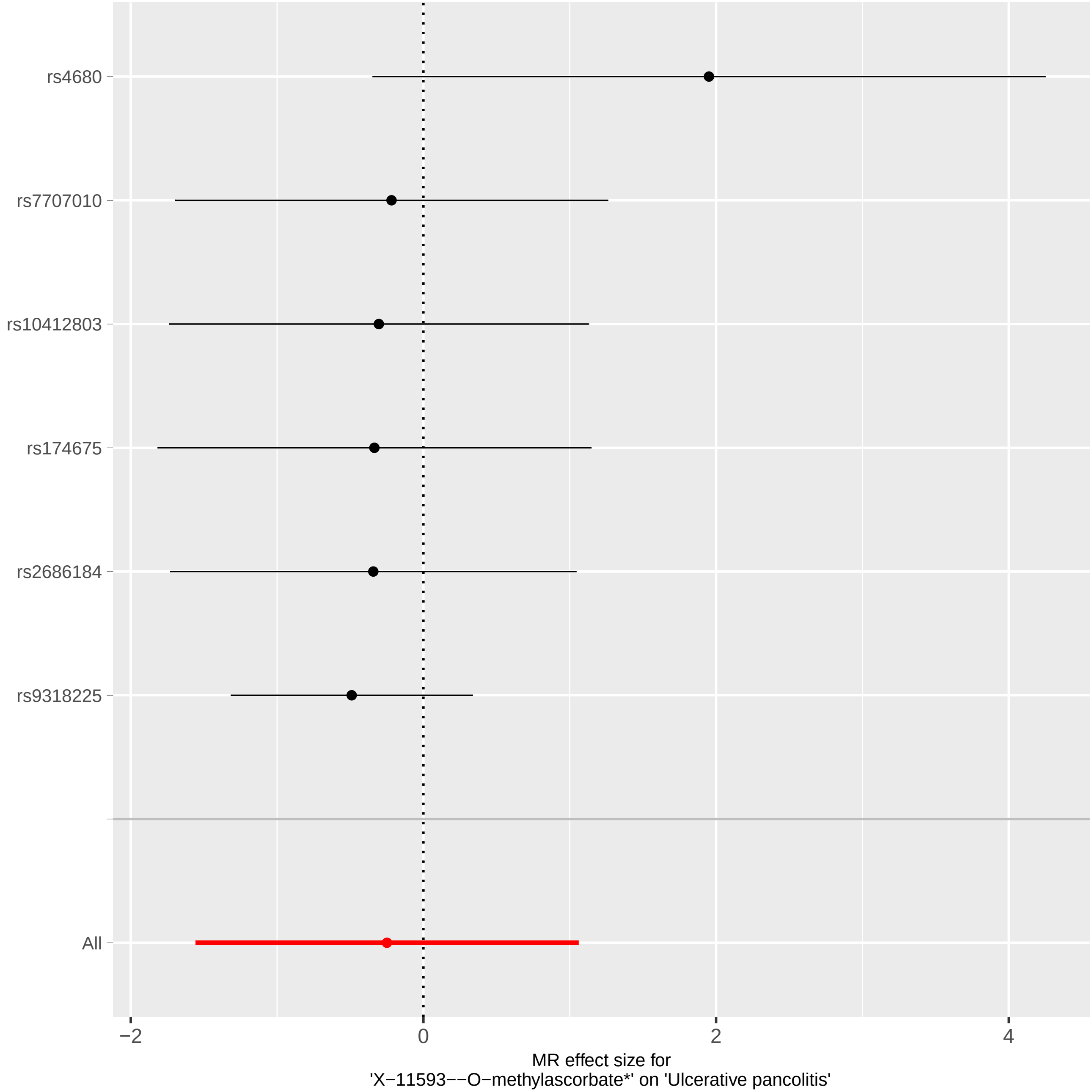

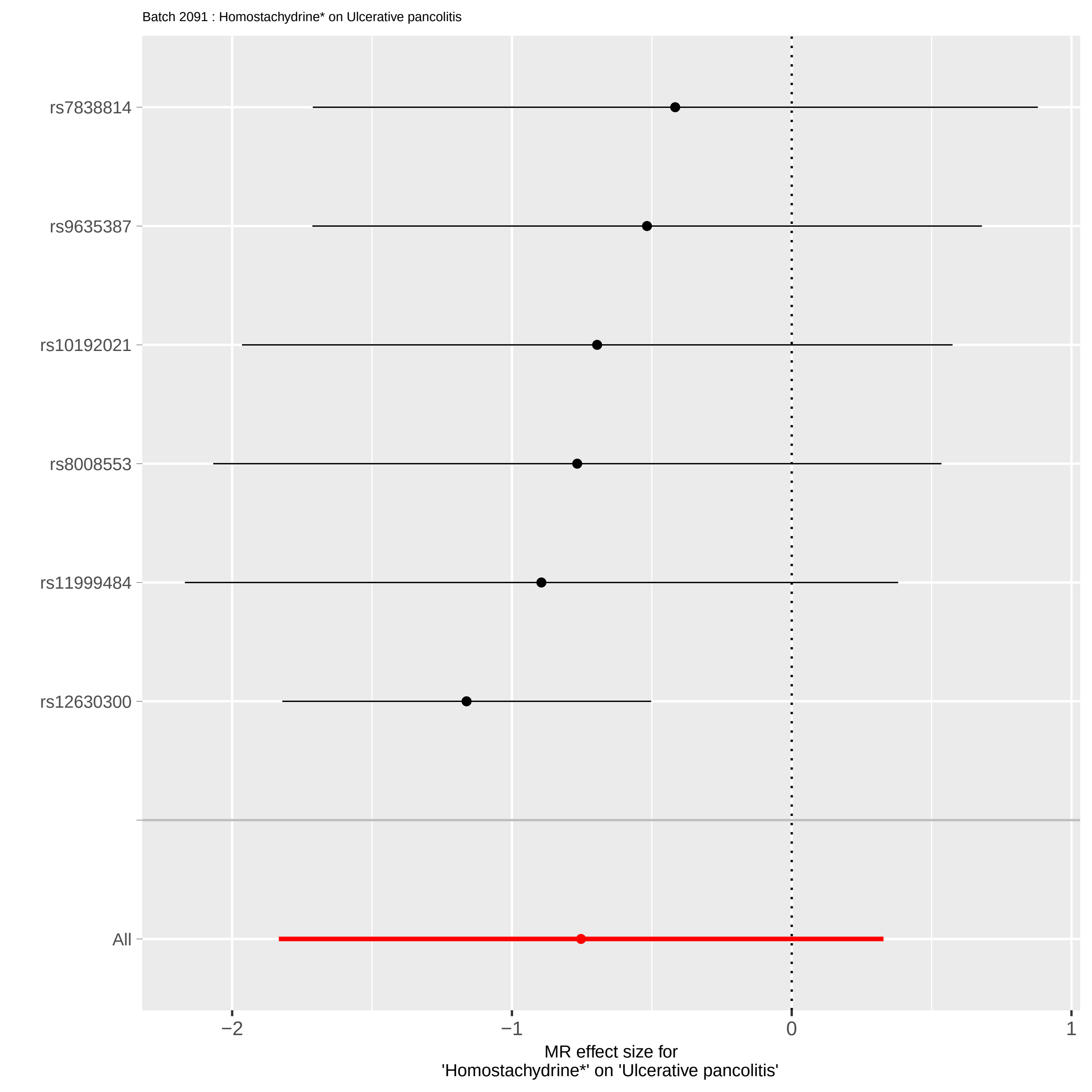

Batch 2091 : Homostachydrine* on Ulcerative pancolitis
rs7838814
rs9635387
rs10192021
rs8008553
rs11999484
rs12630300
All
−2
−1
0
1
MR effect size for
'Homostachydrine*' on 'Ulcerative pancolitis'

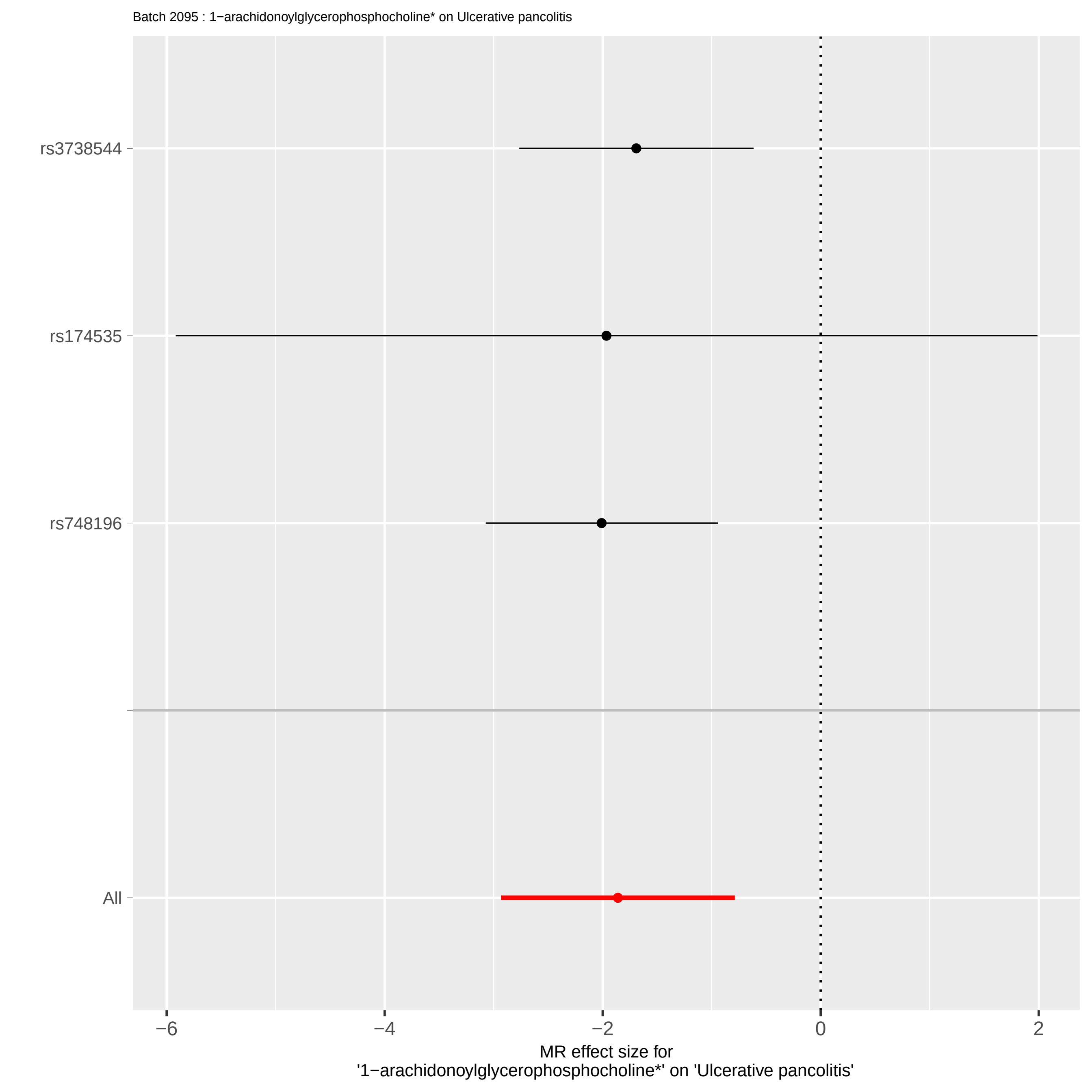
Batch 2095 : 1−arachidonoylglycerophosphocholine* on Ulcerative pancolitis
rs3738544
rs174535
rs748196
All
−6
−4
−2
0
2
MR effect size for
'1−arachidonoylglycerophosphocholine*' on 'Ulcerative pancolitis'

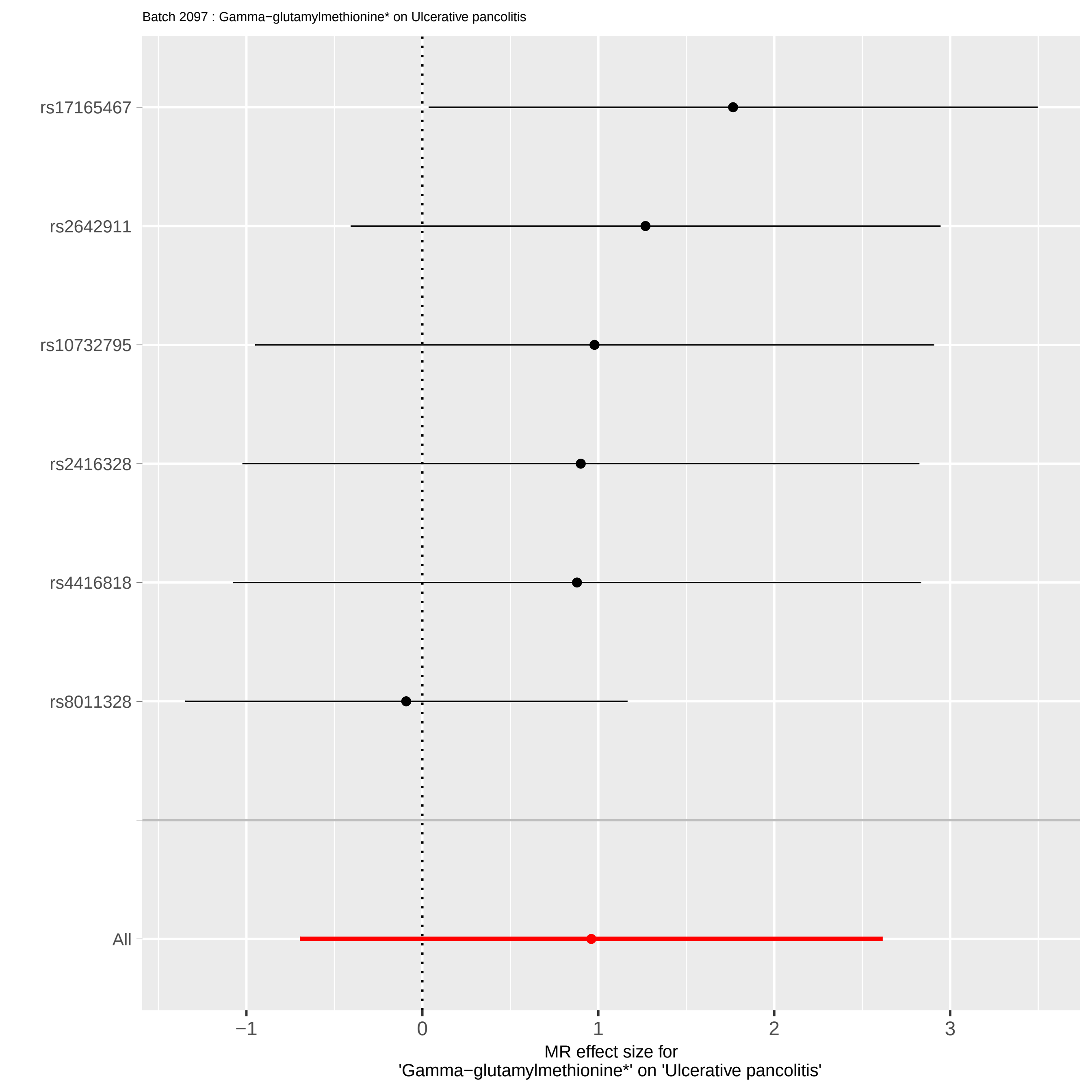

Batch 2097 : Gamma−glutamylmethionine* on Ulcerative pancolitis
rs17165467
rs2642911
rs10732795
rs2416328
rs4416818
rs8011328
All
−1
0
1
2
3
MR effect size for
'Gamma−glutamylmethionine*' on 'Ulcerative pancolitis'

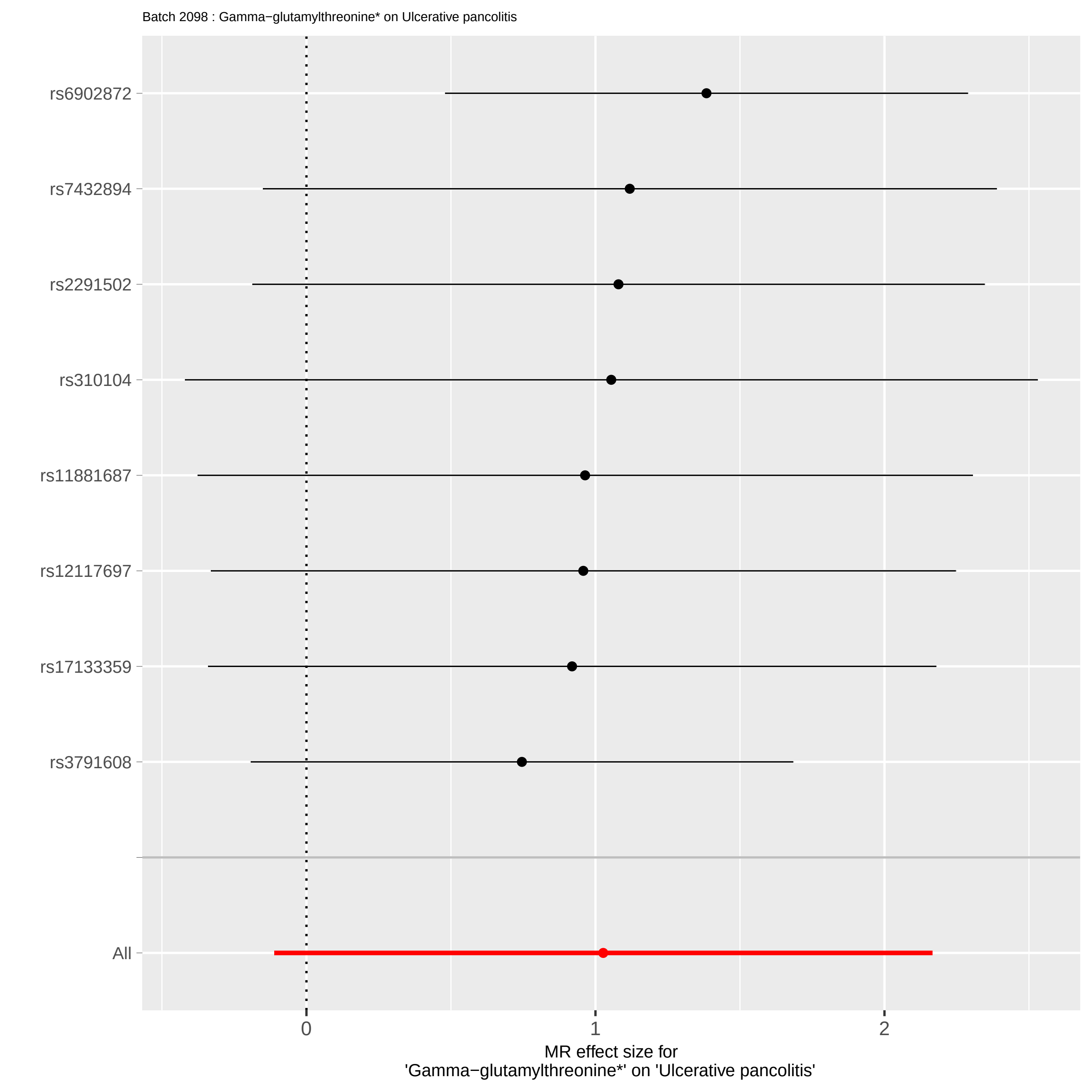

Batch 2098 : Gamma−glutamylthreonine* on Ulcerative pancolitis
rs6902872
rs7432894
rs2291502
rs310104
rs11881687
rs12117697
rs17133359
rs3791608
All
0
1
2
MR effect size for
'Gamma−glutamylthreonine*' on 'Ulcerative pancolitis'

Batch 2099 : Gamma−tocopherol on Ulcerative pancolitis

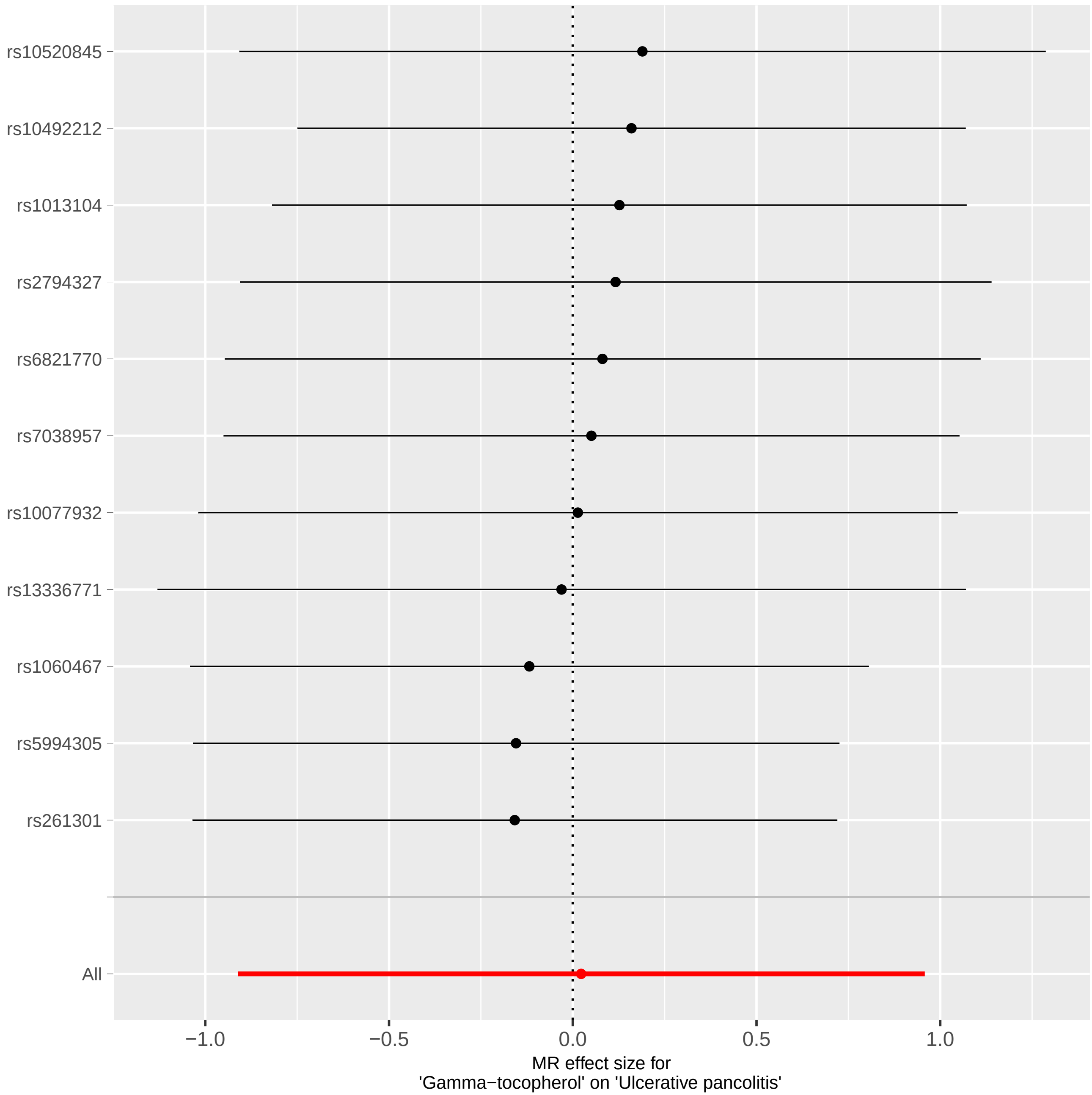

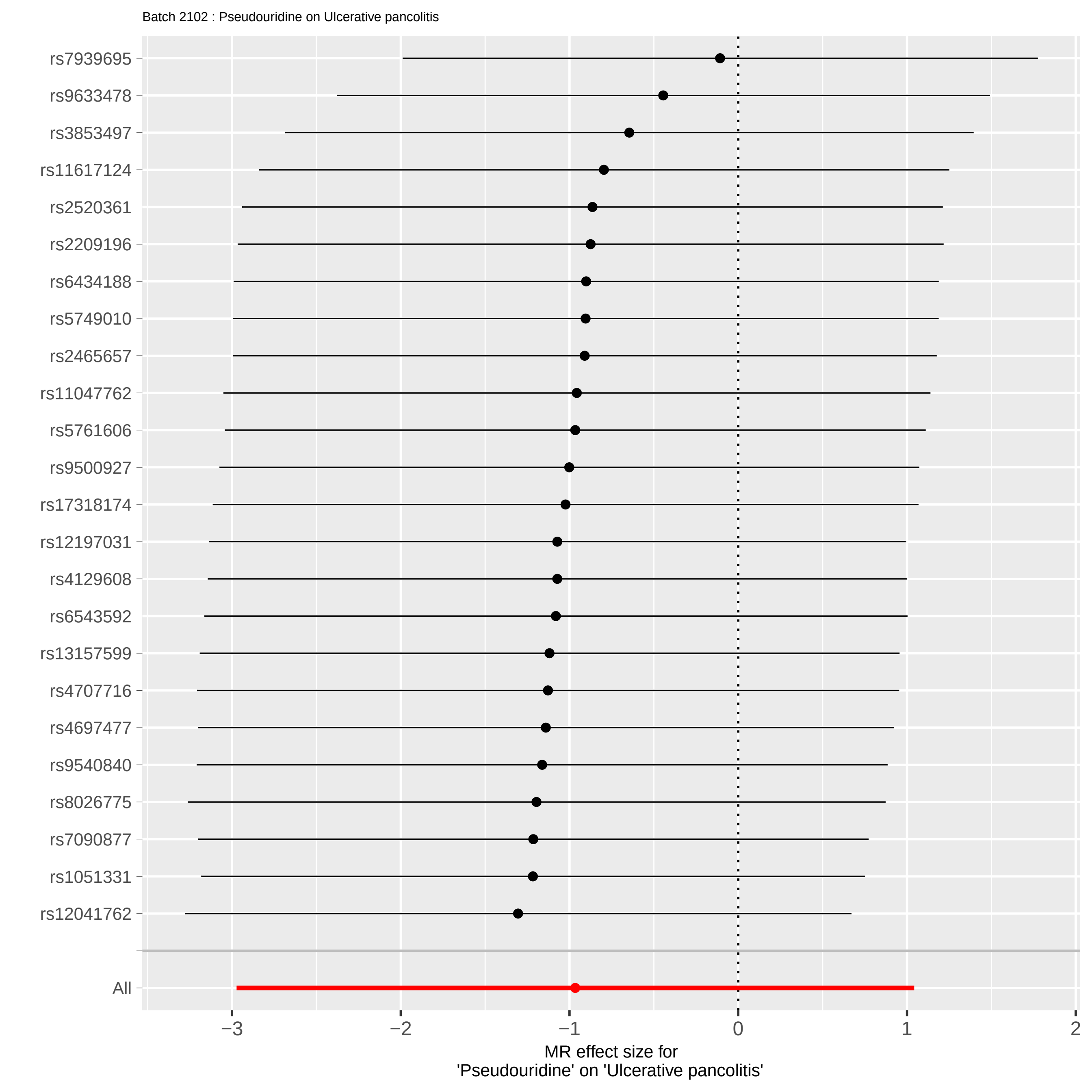
Batch 2102 : Pseudouridine on Ulcerative pancolitis
rs7939695
rs9633478
rs3853497
rs11617124
rs2520361
rs2209196
rs6434188
rs5749010
rs2465657
rs11047762
rs5761606
rs9500927
rs17318174
rs12197031
rs4129608
rs6543592
rs13157599
rs4707716
rs4697477
rs9540840
rs8026775
rs7090877
rs1051331
rs12041762
All
−3
−2
−1
0
1
2
MR effect size for
'Pseudouridine' on 'Ulcerative pancolitis'

Batch 2103 : Valerate on Ulcerative pancolitis

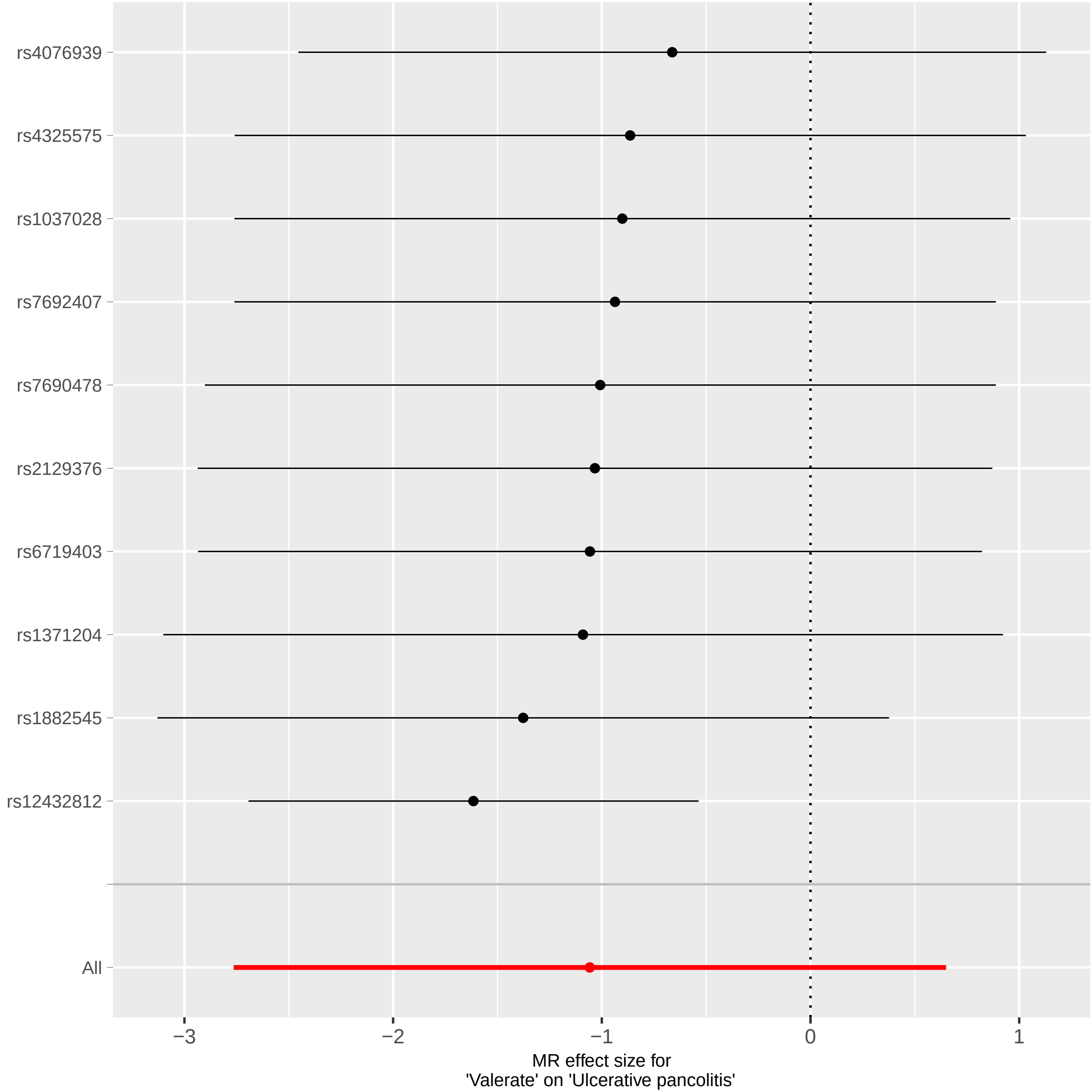

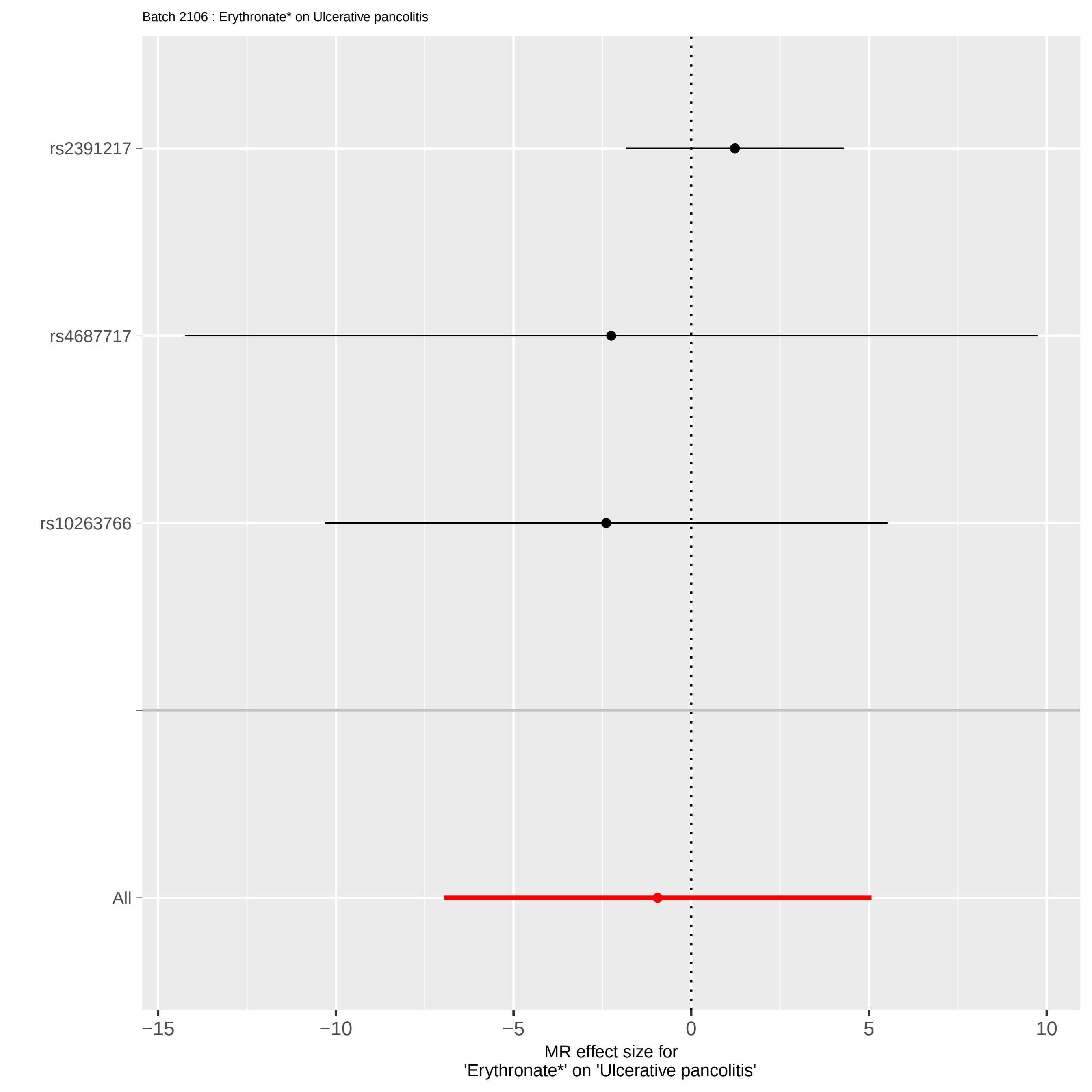
Batch 2106 : Erythronate* on Ulcerative pancolitis
rs2391217
rs4687717
rs10263766
All
−15
−10
−5
0
5
10
MR effect size for
'Erythronate*' on 'Ulcerative pancolitis'

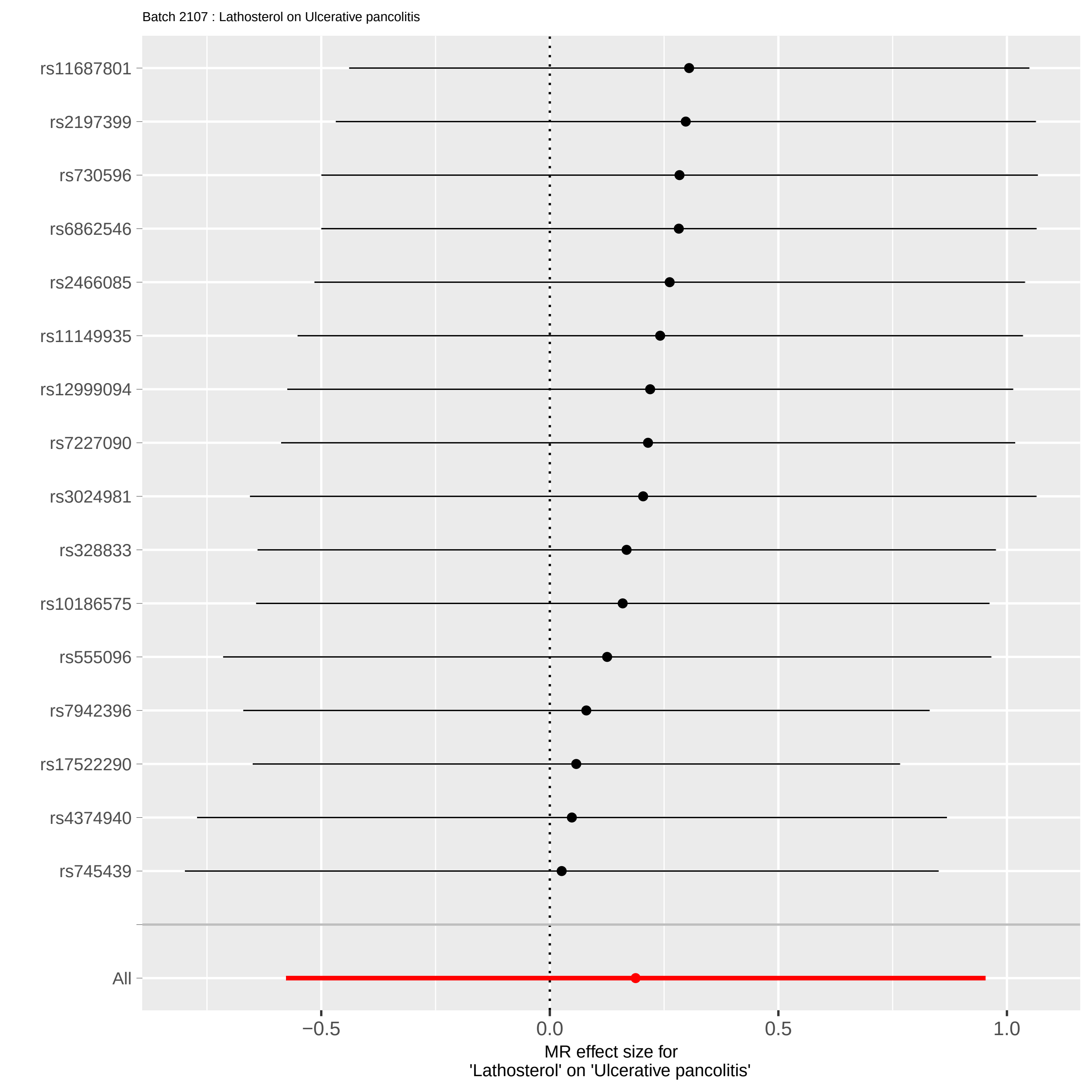

Batch 2107 : Lathosterol on Ulcerative pancolitis
rs11687801
rs2197399
rs730596
rs6862546
rs2466085
rs11149935
rs12999094
rs7227090
rs3024981
rs328833
rs10186575
rs555096
rs7942396
rs17522290
rs4374940
rs745439
All
−0.5
0.0
0.5
1.0
MR effect size for
'Lathosterol' on 'Ulcerative pancolitis'

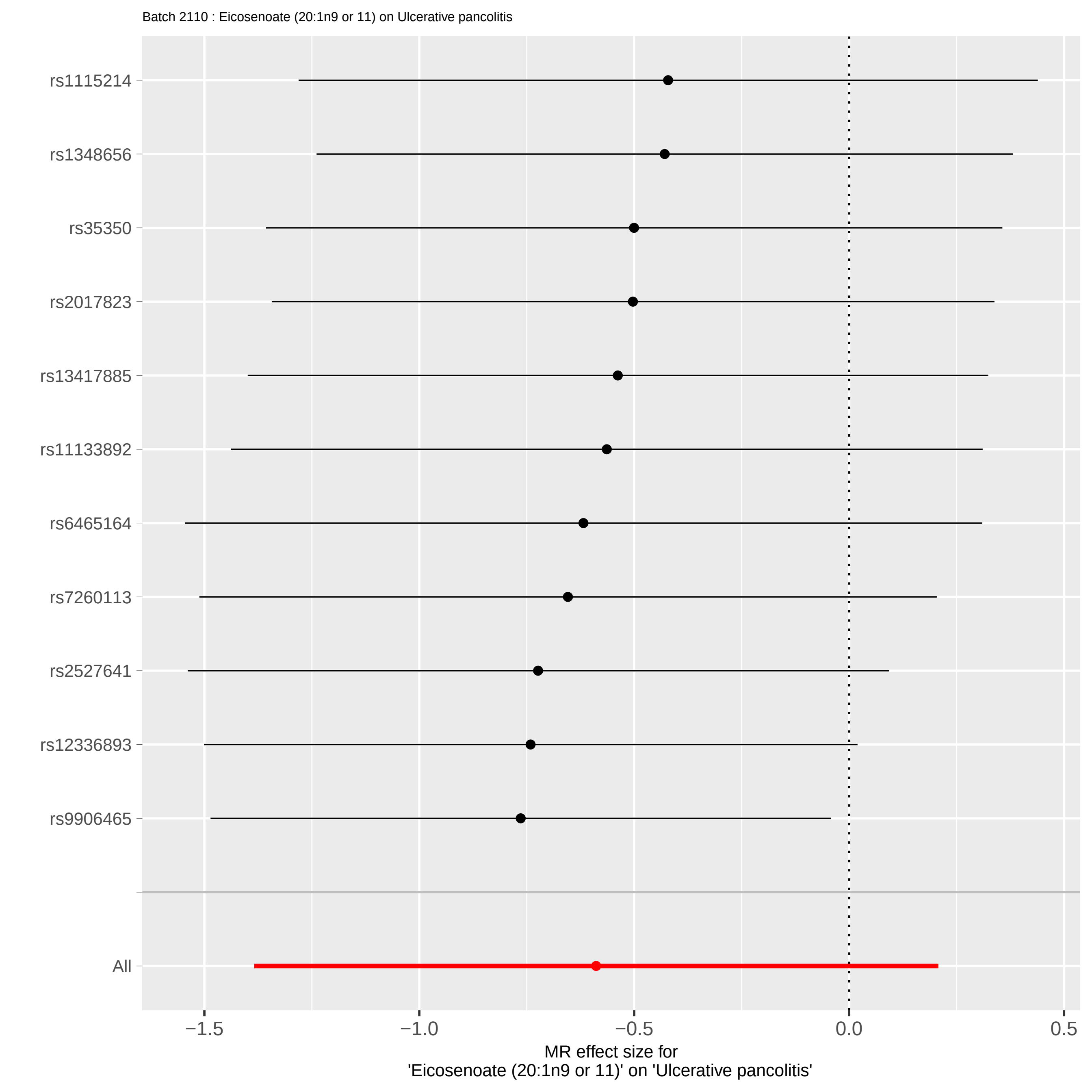
Batch 2110 : Eicosenoate (20:1n9 or 11) on Ulcerative pancolitis
rs1115214
rs1348656
rs35350
rs2017823
rs13417885
rs11133892
rs6465164
rs7260113
rs2527641
rs12336893
rs9906465
All
−1.5
−1.0
−0.5
0.0
0.5
MR effect size for
'Eicosenoate (20:1n9 or 11)' on 'Ulcerative pancolitis'

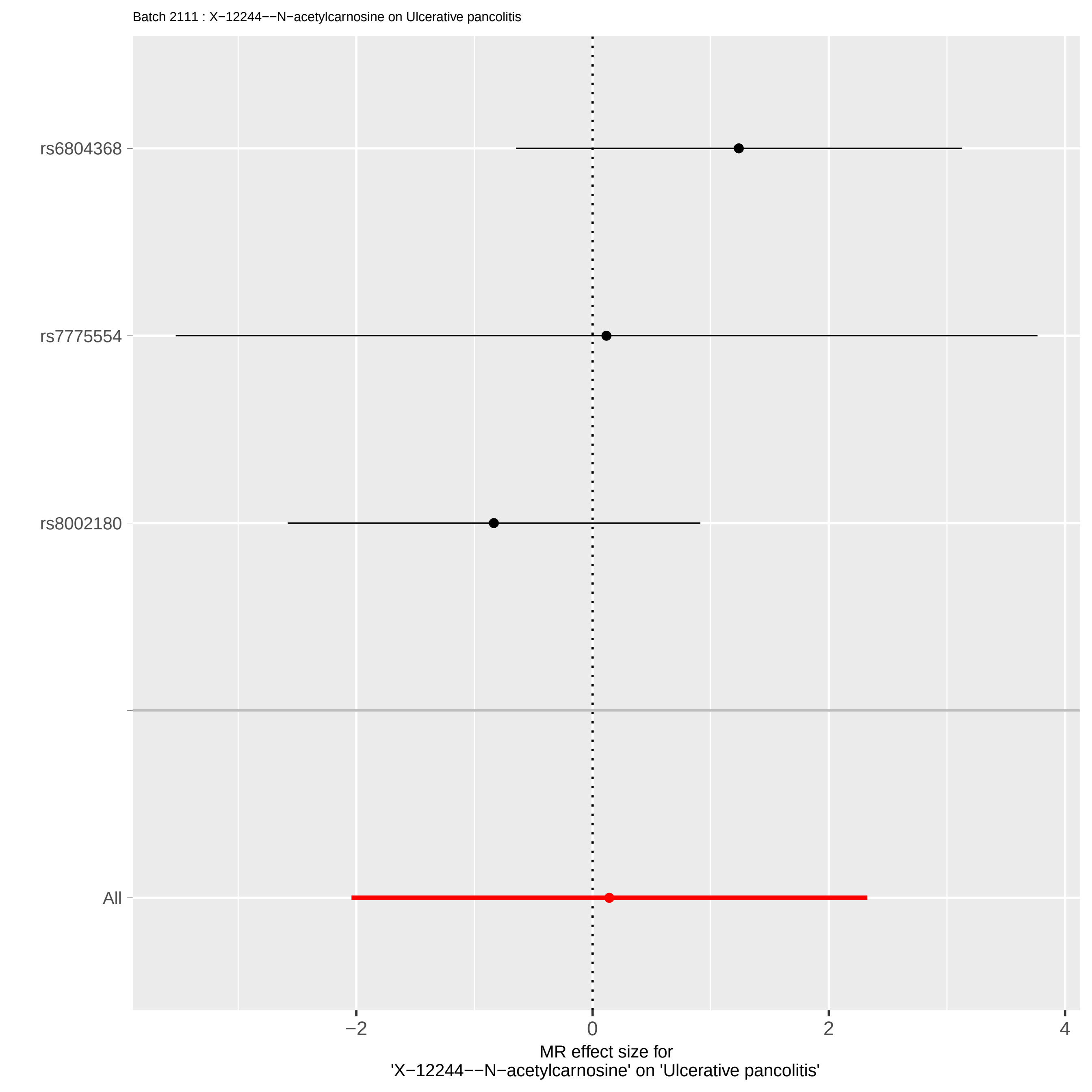
Batch 2111 : X−12244−−N−acetylcarnosine on Ulcerative pancolitis
rs6804368
rs7775554
rs8002180
All
−2
0
2
4
MR effect size for
'X−12244−−N−acetylcarnosine' on 'Ulcerative pancolitis'

Batch 2114 : 1−docosahexaenoylglycerophosphocholine* on Ulcerative pancolitis

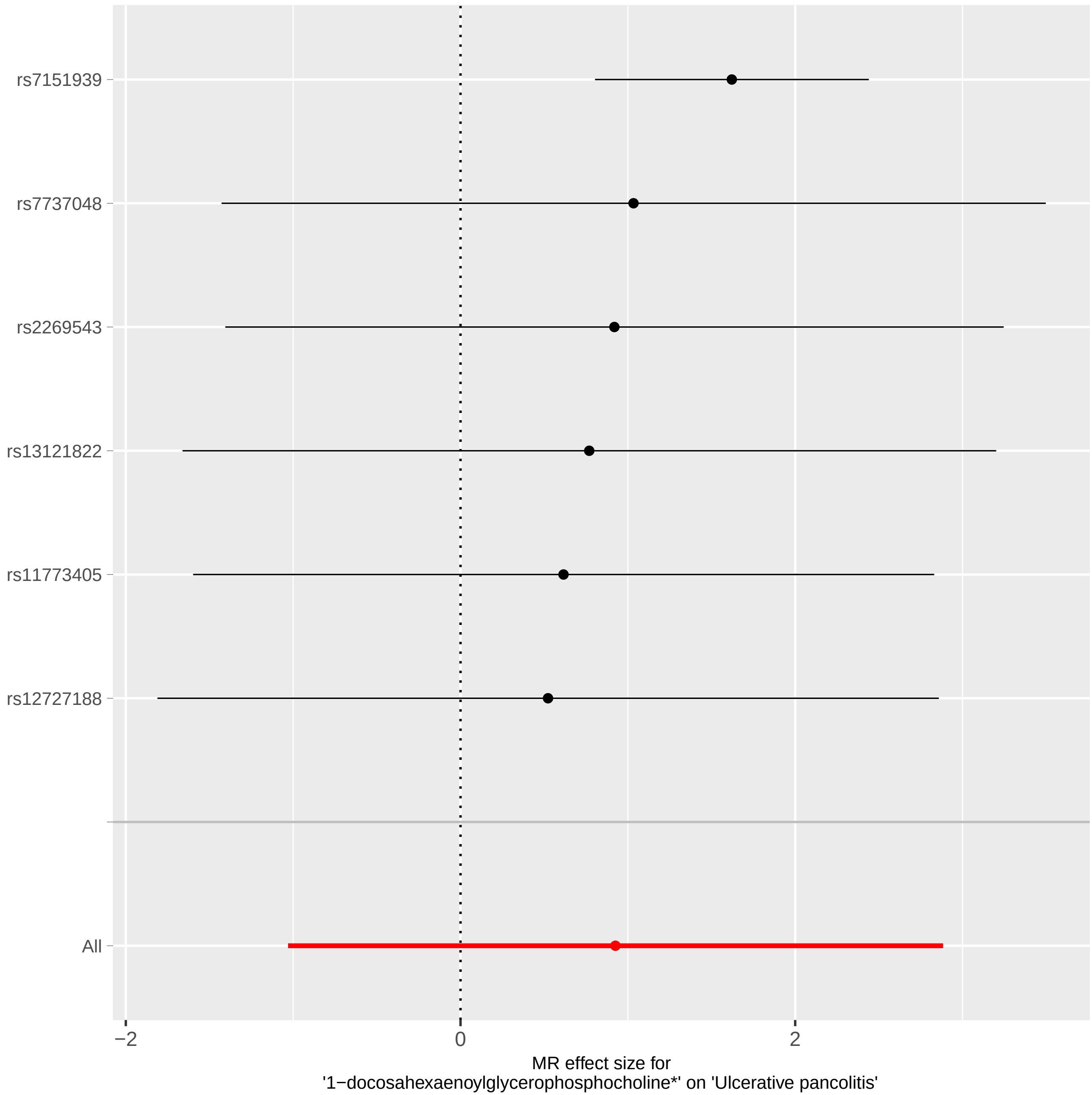

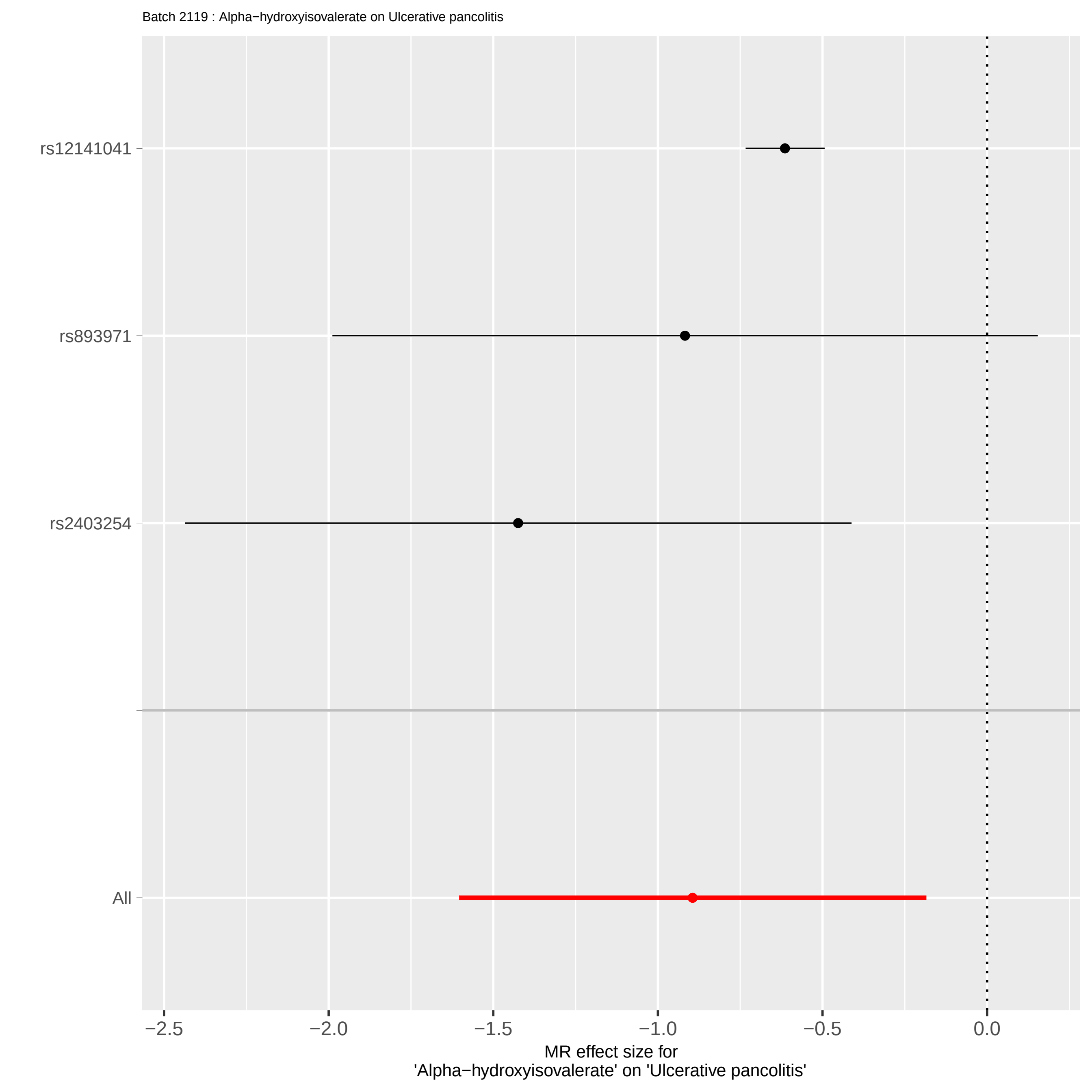
Batch 2119 : Alpha−hydroxyisovalerate on Ulcerative pancolitis
rs12141041
rs893971
rs2403254
All
−2.5
−2.0
−1.5
−1.0
−0.5
0.0
MR effect size for
'Alpha−hydroxyisovalerate' on 'Ulcerative pancolitis'

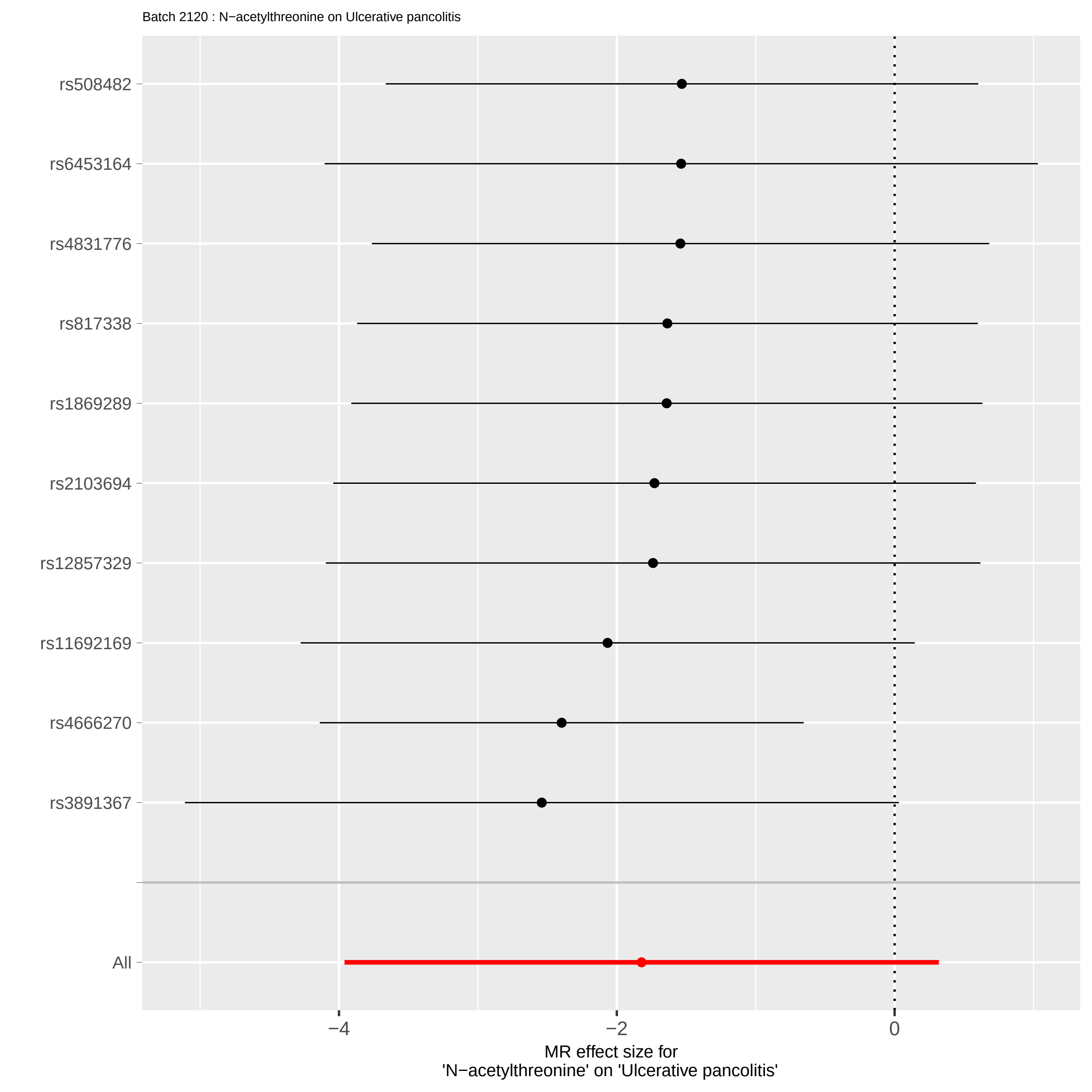

Batch 2120 : N−acetylthreonine on Ulcerative pancolitis
rs508482
rs6453164
rs4831776
rs817338
rs1869289
rs2103694
rs12857329
rs11692169
rs4666270
rs3891367
All
−4
−2
0
MR effect size for
'N−acetylthreonine' on 'Ulcerative pancolitis'

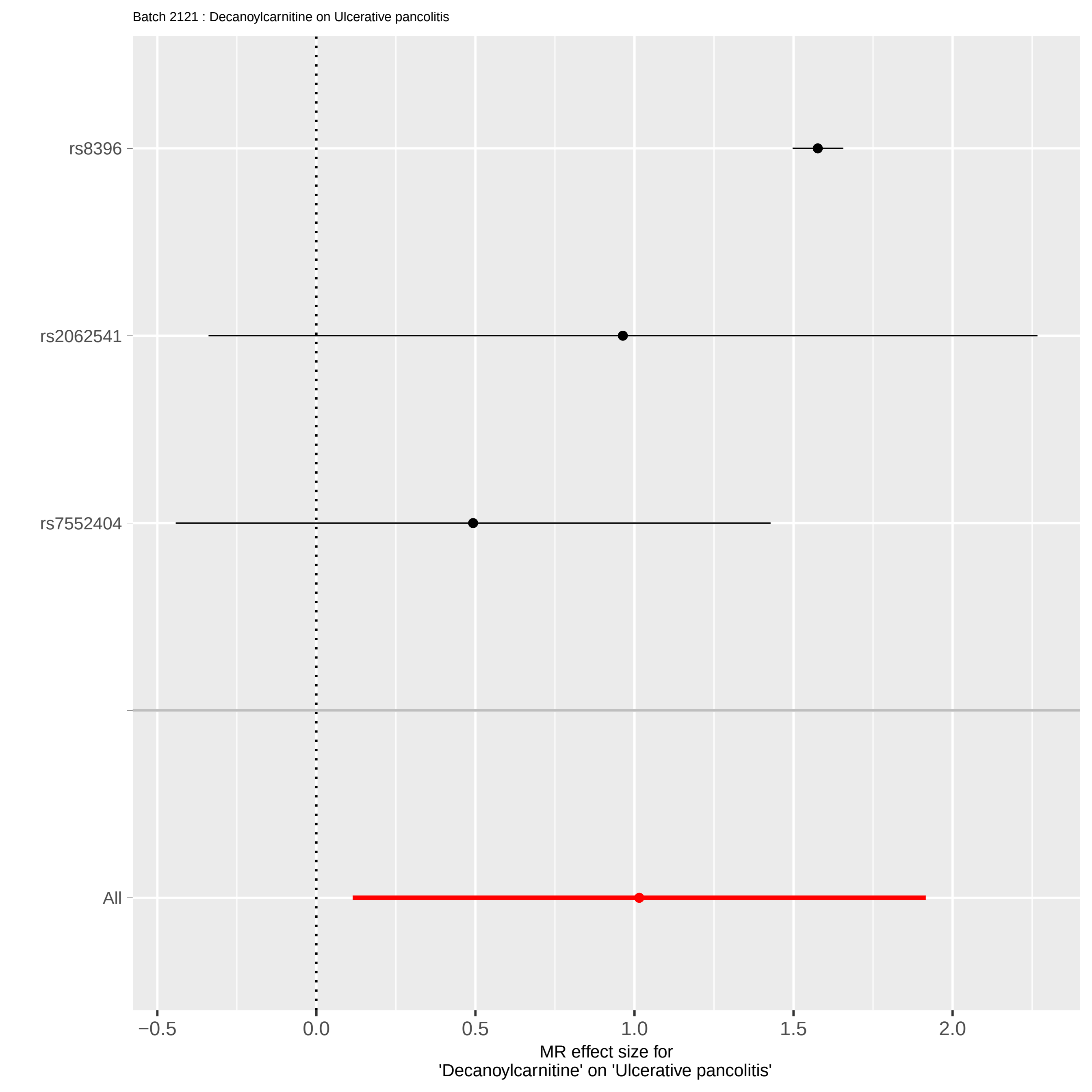
Batch 2121 : Decanoylcarnitine on Ulcerative pancolitis
rs8396
rs2062541
rs7552404
All
−0.5
0.0
0.5
1.0
1.5
2.0
MR effect size for
'Decanoylcarnitine' on 'Ulcerative pancolitis'

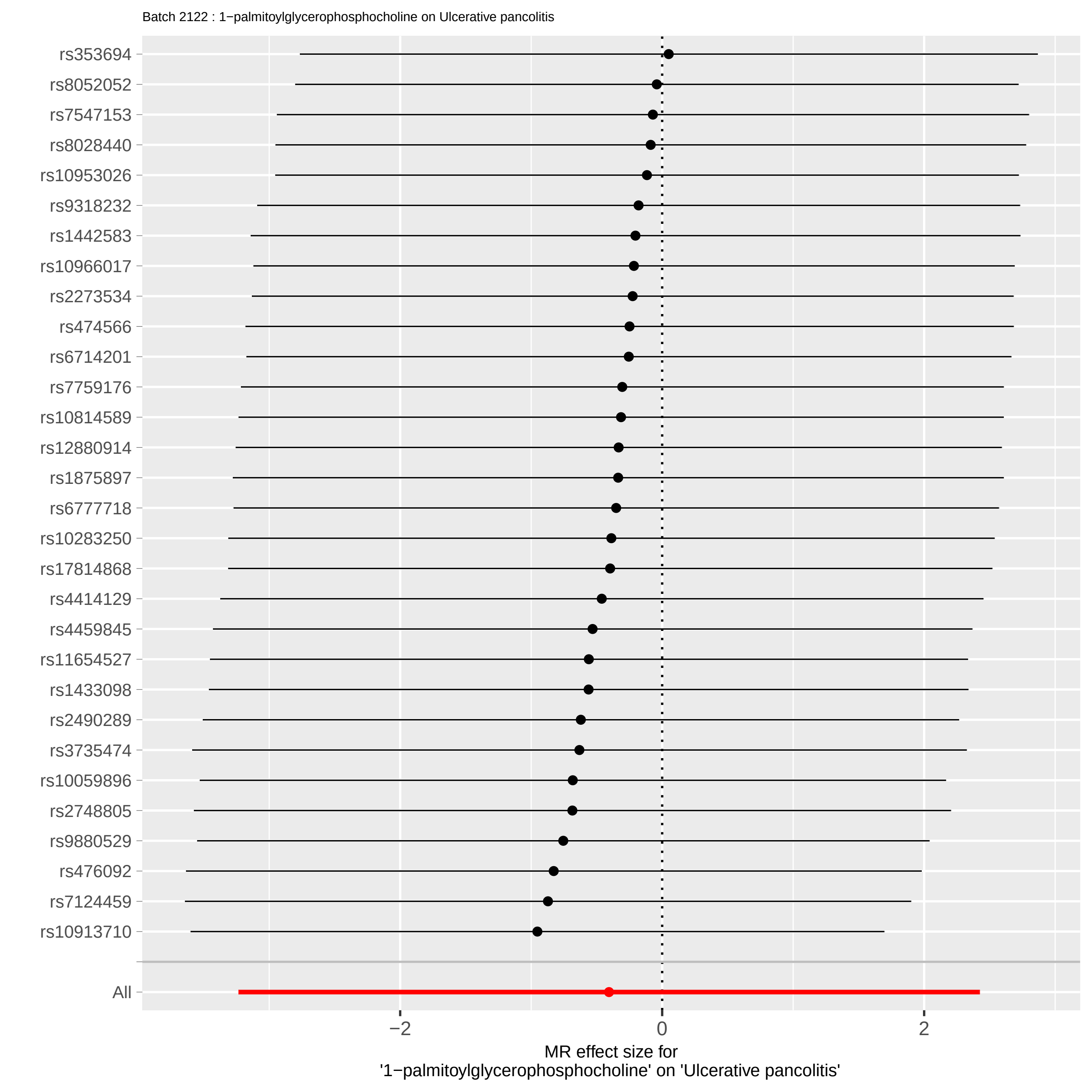

Batch 2122 : 1−palmitoylglycerophosphocholine on Ulcerative pancolitis
rs353694
rs8052052
rs7547153
rs8028440
rs10953026
rs9318232
rs1442583
rs10966017
rs2273534
rs474566
rs6714201
rs7759176
rs10814589
rs12880914
rs1875897
rs6777718
rs10283250
rs17814868
rs4414129
rs4459845
rs11654527
rs1433098
rs2490289
rs3735474
rs10059896
rs2748805
rs9880529
rs476092
rs7124459
rs10913710
All
−2
0
2
MR effect size for
'1−palmitoylglycerophosphocholine' on 'Ulcerative pancolitis'

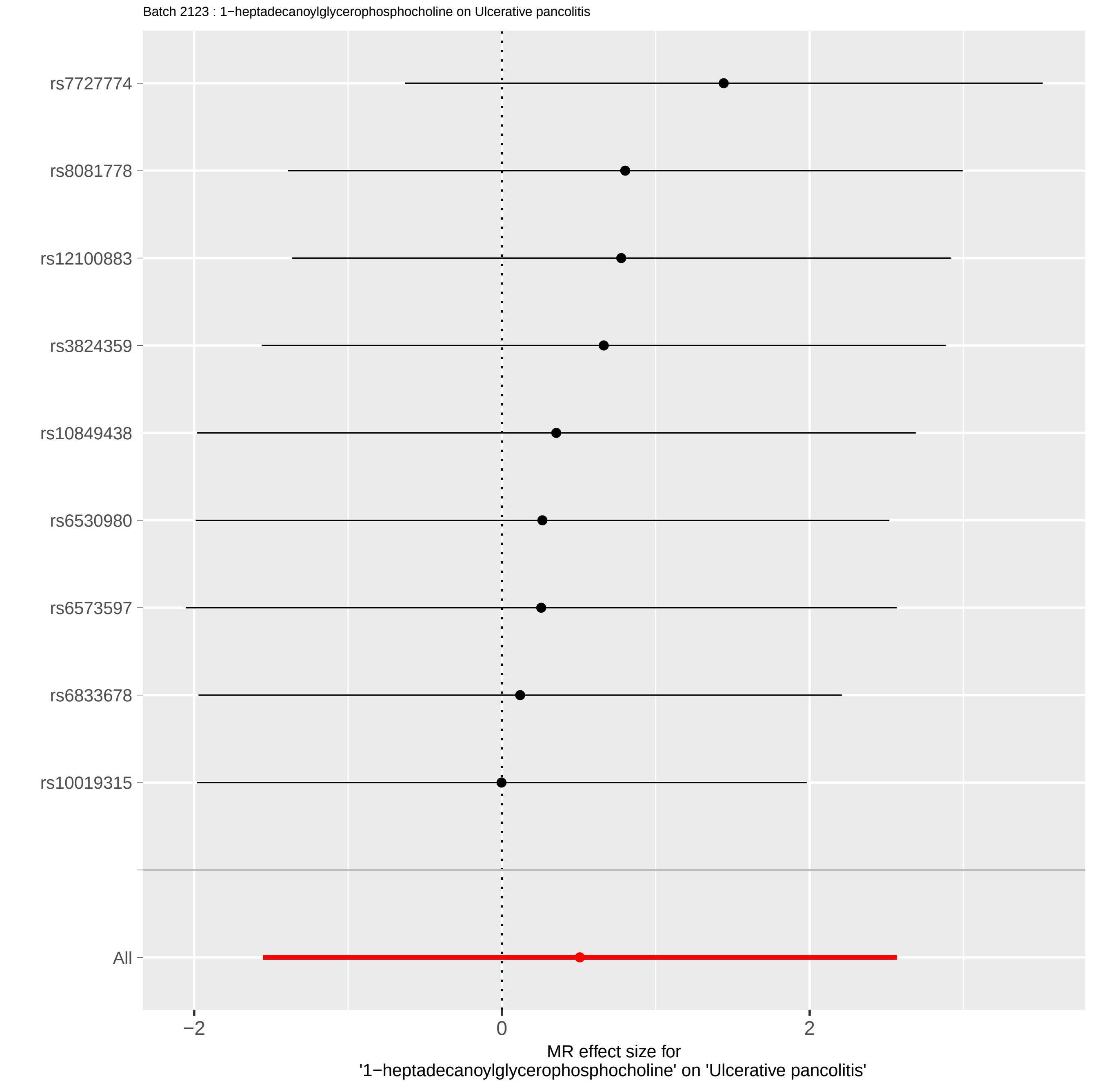

Batch 2123 : 1−heptadecanoylglycerophosphocholine on Ulcerative pancolitis
rs7727774
rs8081778
rs12100883
rs3824359
rs10849438
rs6530980
rs6573597
rs6833678
rs10019315
All
−2
0
2
MR effect size for
'1−heptadecanoylglycerophosphocholine' on 'Ulcerative pancolitis'

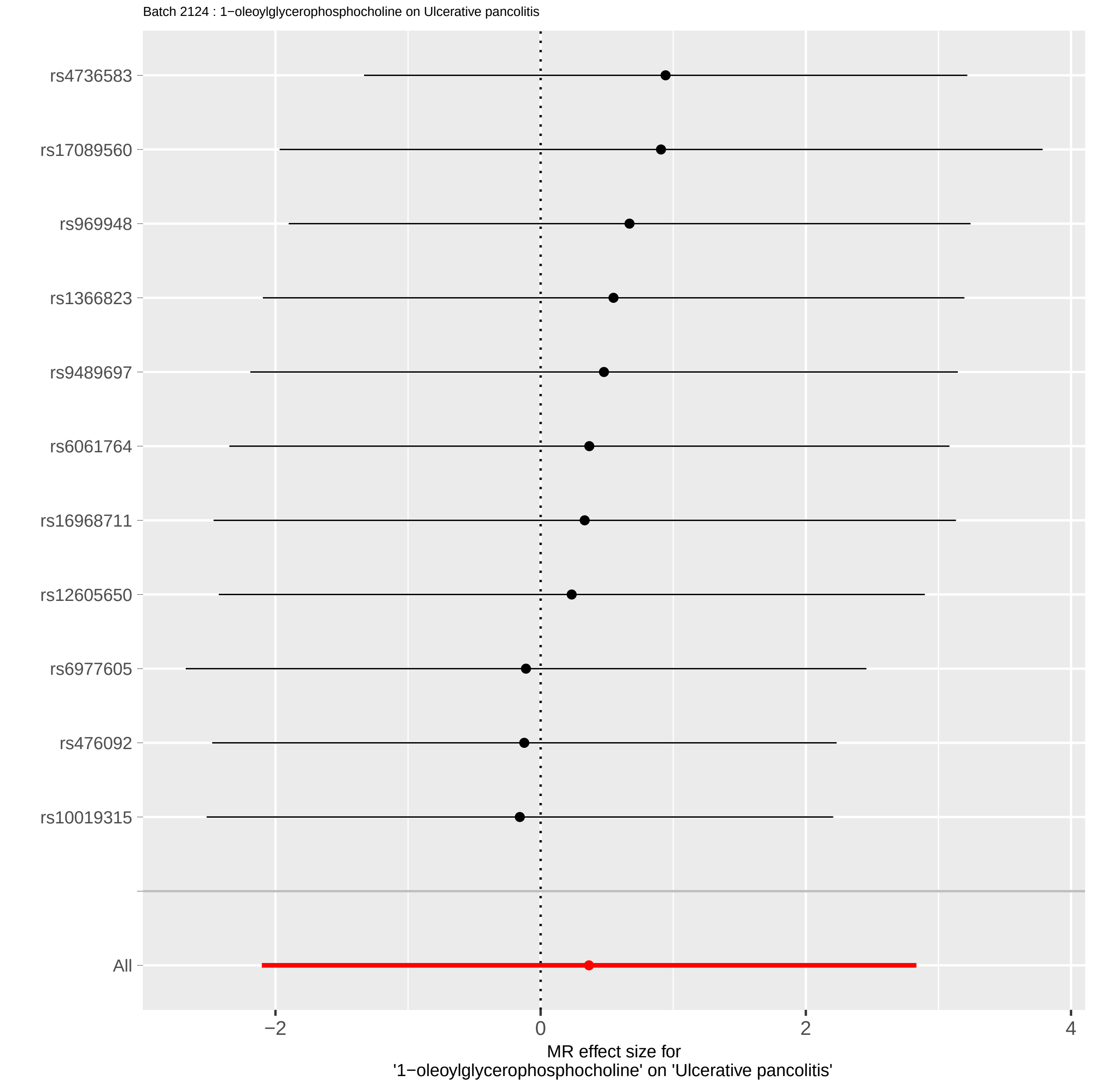

Batch 2124 : 1−oleoylglycerophosphocholine on Ulcerative pancolitis
rs4736583
rs17089560
rs969948
rs1366823
rs9489697
rs6061764
rs16968711
rs12605650
rs6977605
rs476092
rs10019315
All
−2
0
2
4
MR effect size for
'1−oleoylglycerophosphocholine' on 'Ulcerative pancolitis'

Batch 2125 : 1−stearoylglycerophosphocholine on Ulcerative pancolitis

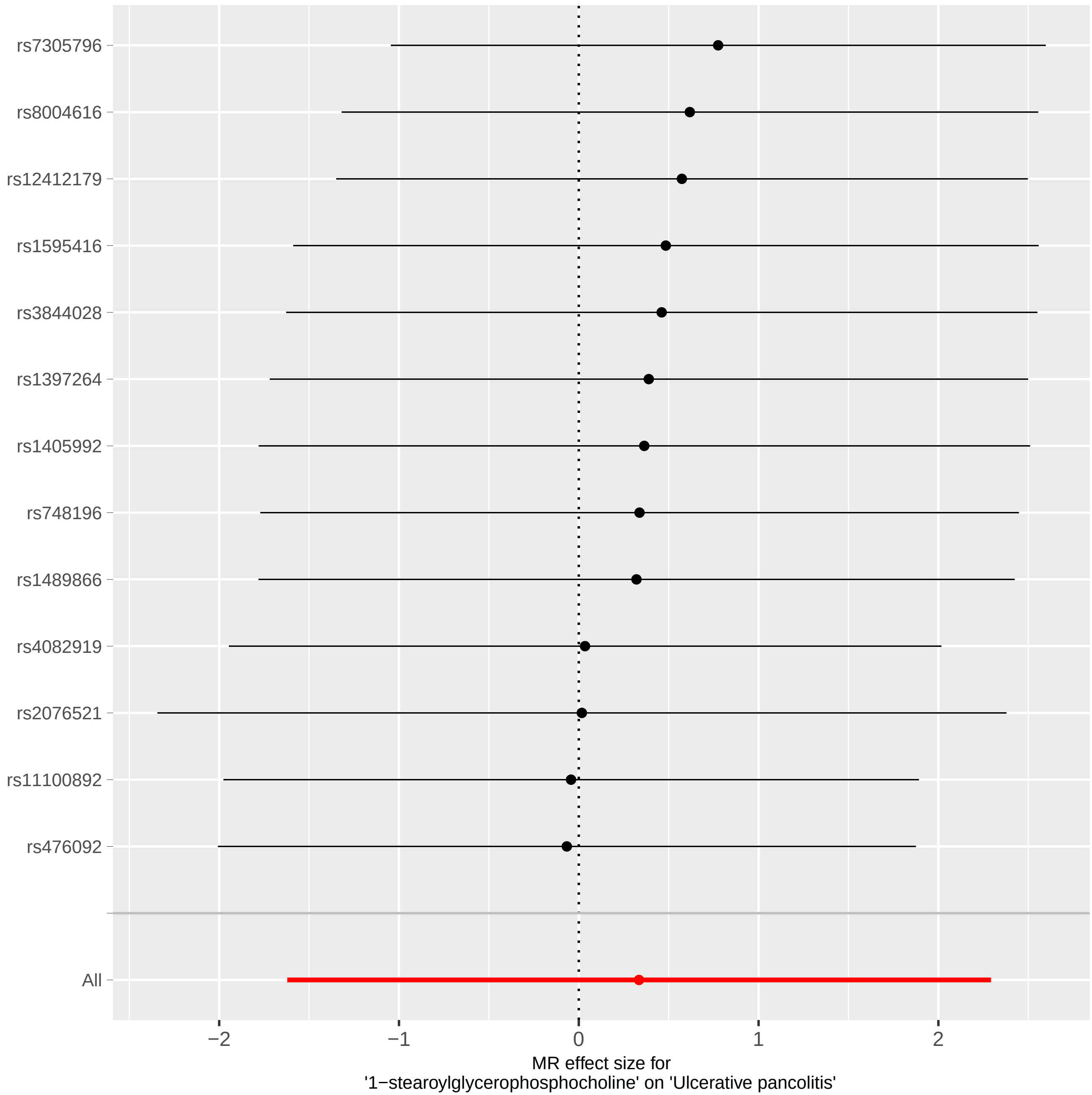

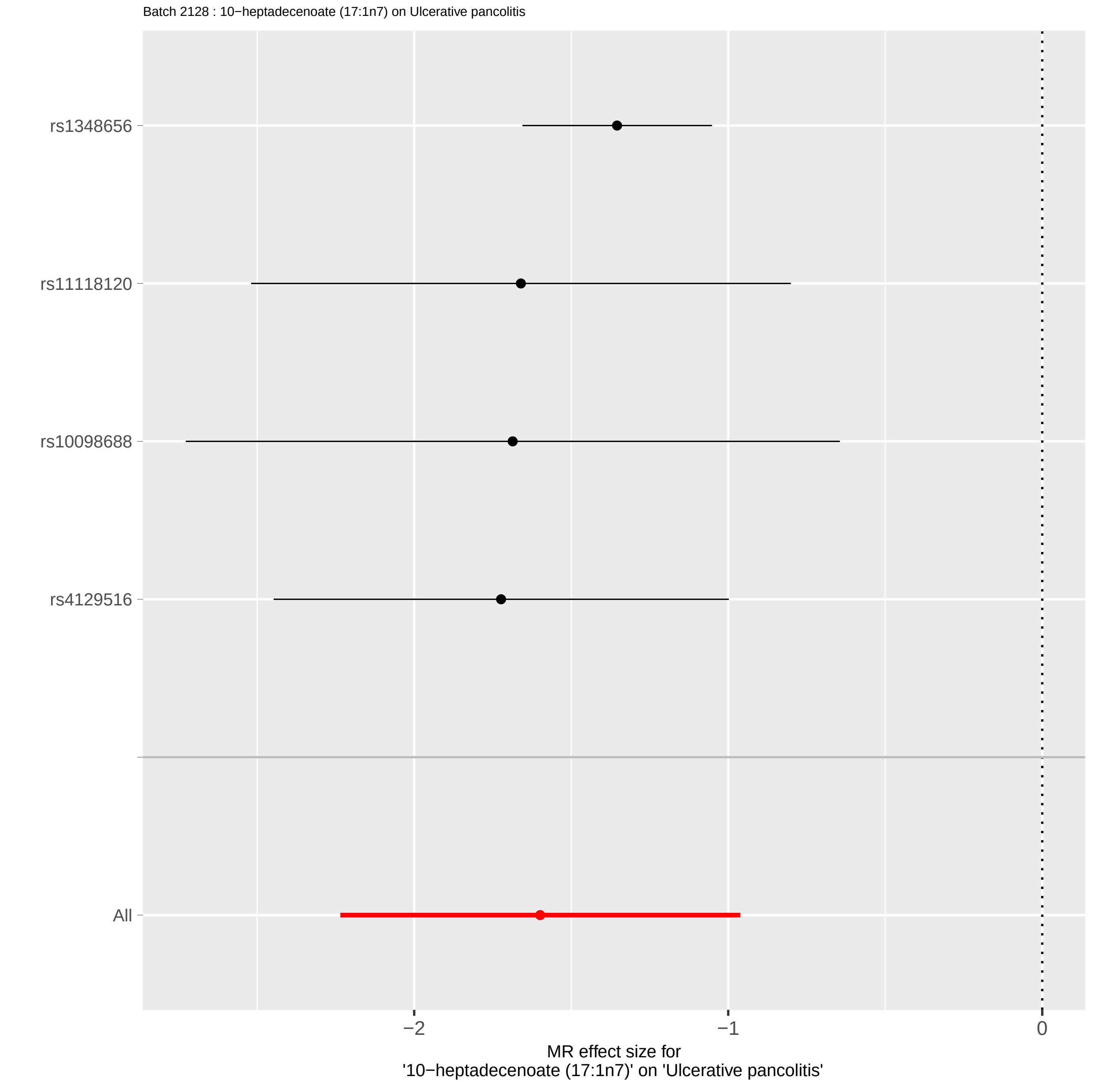
Batch 2128 : 10−heptadecenoate (17:1n7) on Ulcerative pancolitis
rs1348656
rs11118120
rs10098688
rs4129516
All
−2
−1
0
MR effect size for
'10−heptadecenoate (17:1n7)' on 'Ulcerative pancolitis'

Batch 2131 : Linolenate [alpha or gamma; (18:3n3 or 6)] on Ulcerative pancolitis

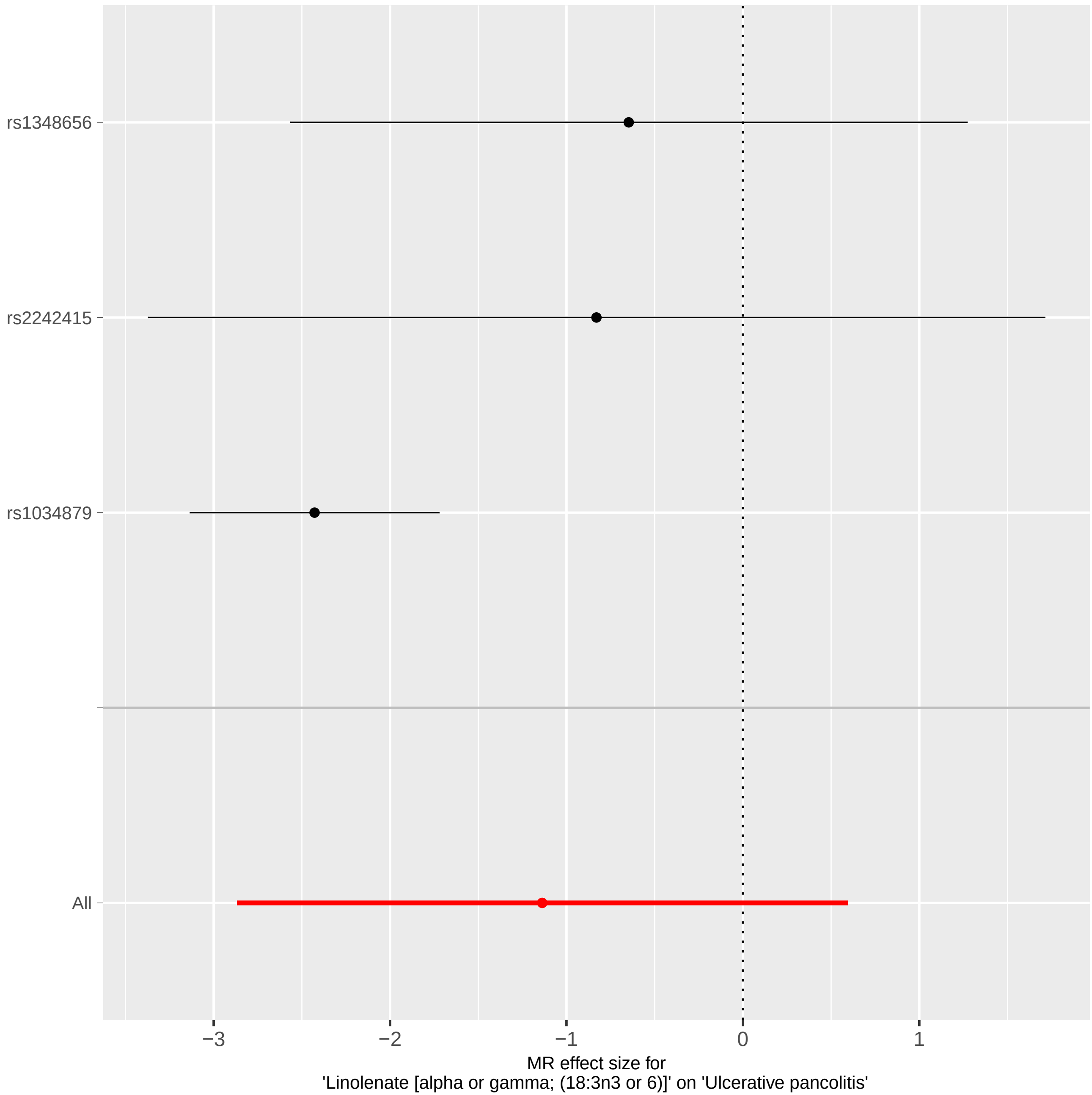

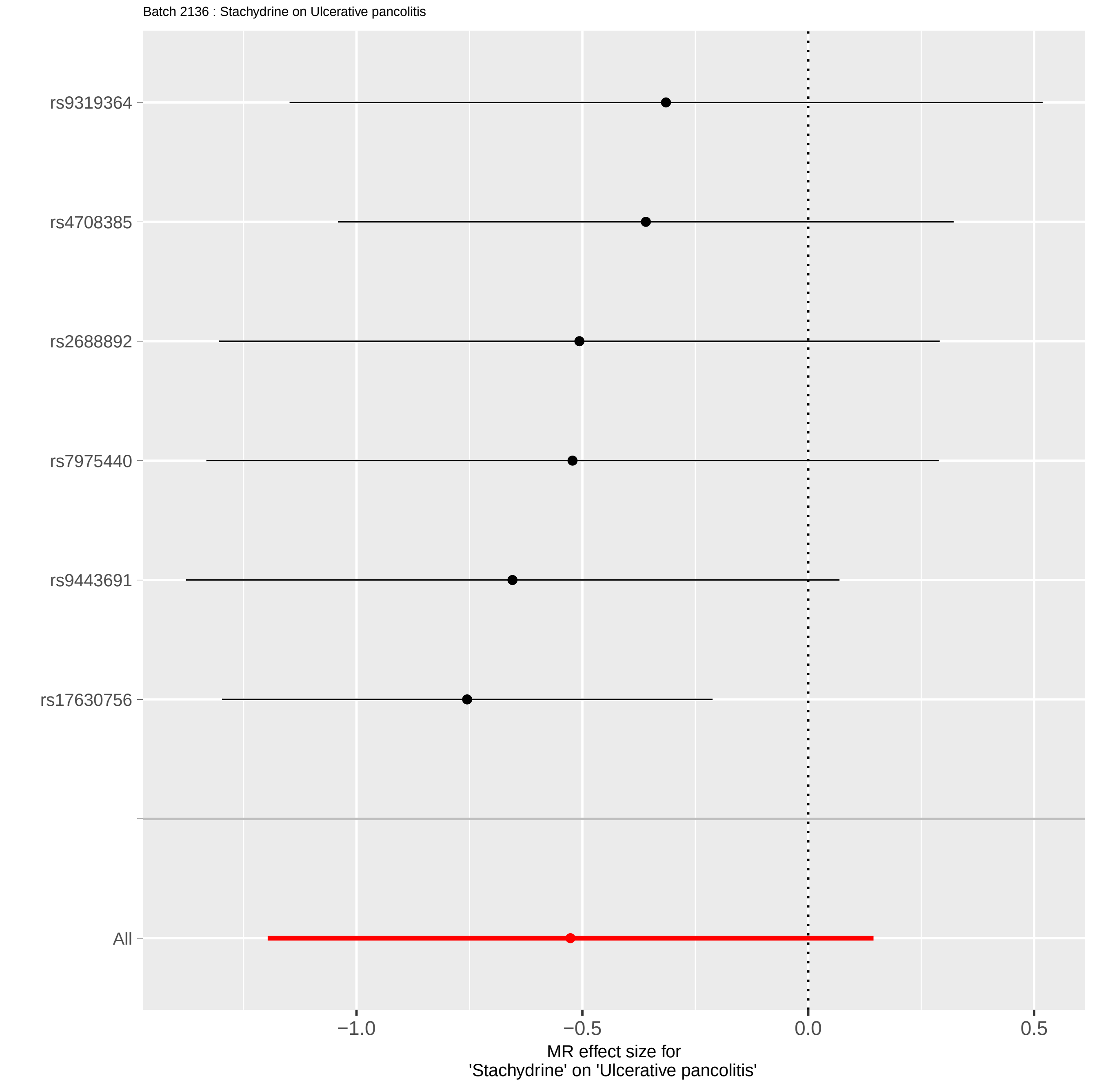

Batch 2136 : Stachydrine on Ulcerative pancolitis
rs9319364
rs4708385
rs2688892
rs7975440
rs9443691
rs17630756
All
−1.0
−0.5
0.0
0.5
MR effect size for
'Stachydrine' on 'Ulcerative pancolitis'

Batch 2137 : Isovalerylcarnitine on Ulcerative pancolitis

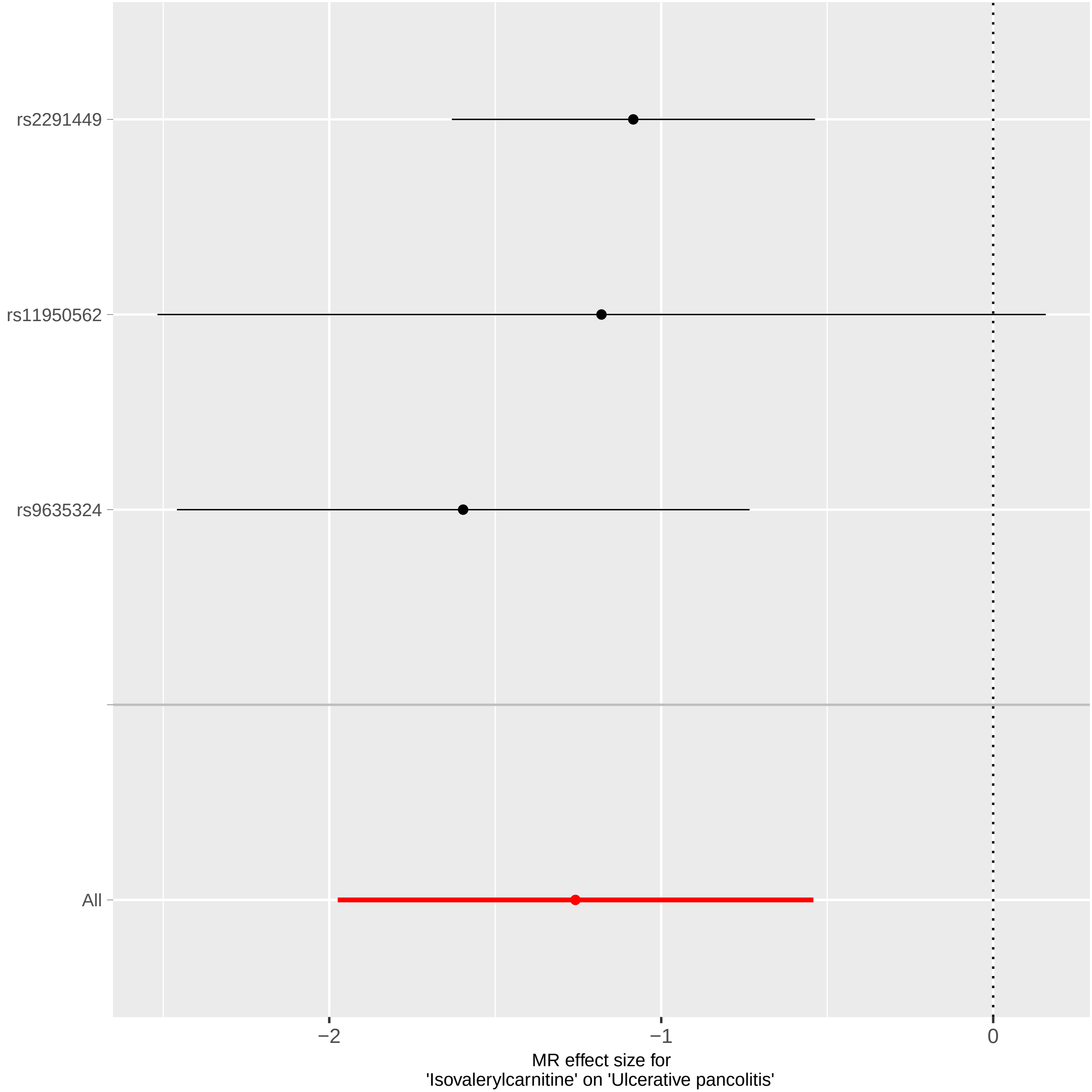

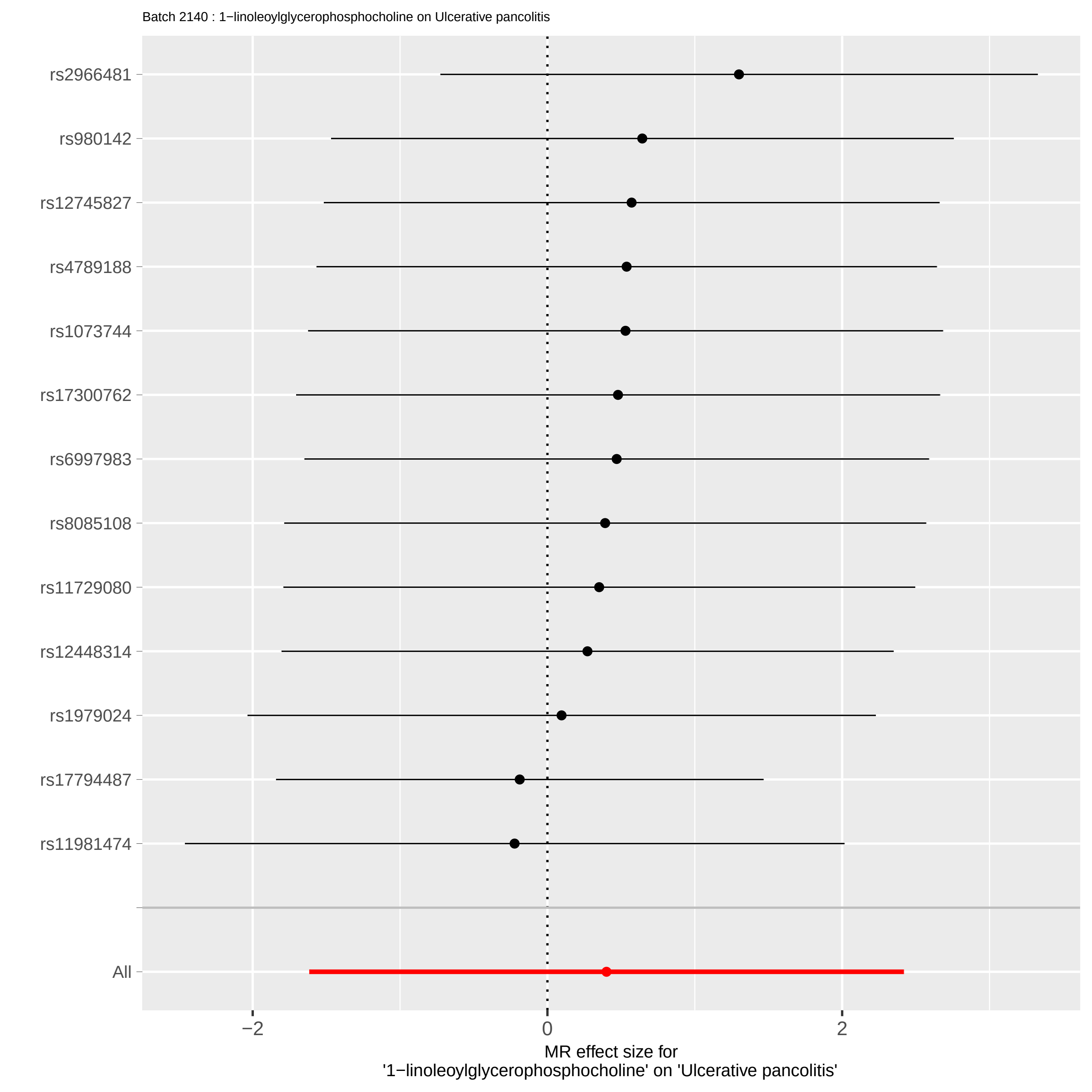
Batch 2140 : 1−linoleoylglycerophosphocholine on Ulcerative pancolitis
rs2966481
rs980142
rs12745827
rs4789188
rs1073744
rs17300762
rs6997983
rs8085108
rs11729080
rs12448314
rs1979024
rs17794487
rs11981474
All
−2
0
2
MR effect size for
'1−linoleoylglycerophosphocholine' on 'Ulcerative pancolitis'

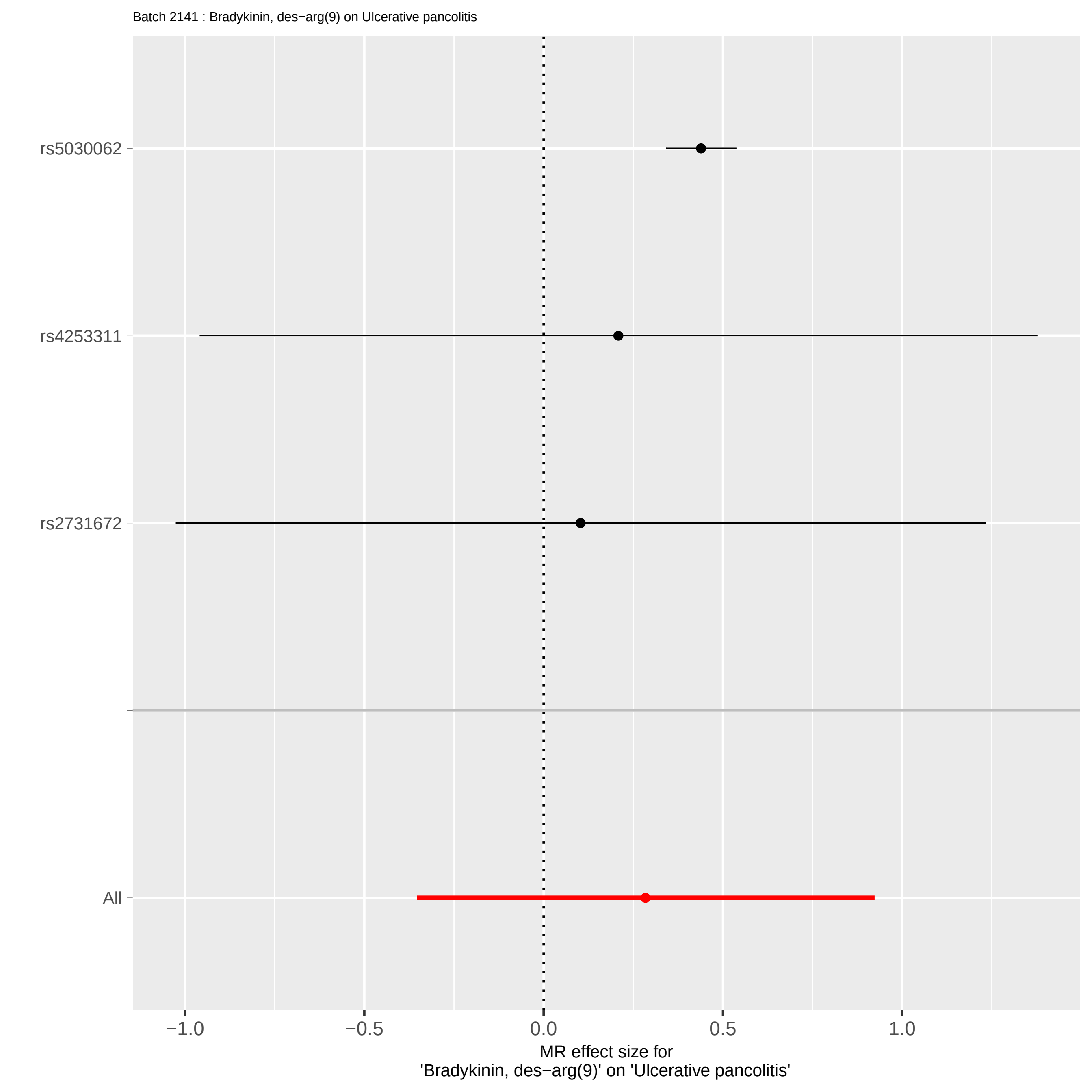

Batch 2141 : Bradykinin, des−arg(9) on Ulcerative pancolitis
rs5030062
rs4253311
rs2731672
All
−1.0
−0.5
0.0
0.5
1.0
MR effect size for
'Bradykinin, des−arg(9)' on 'Ulcerative pancolitis'

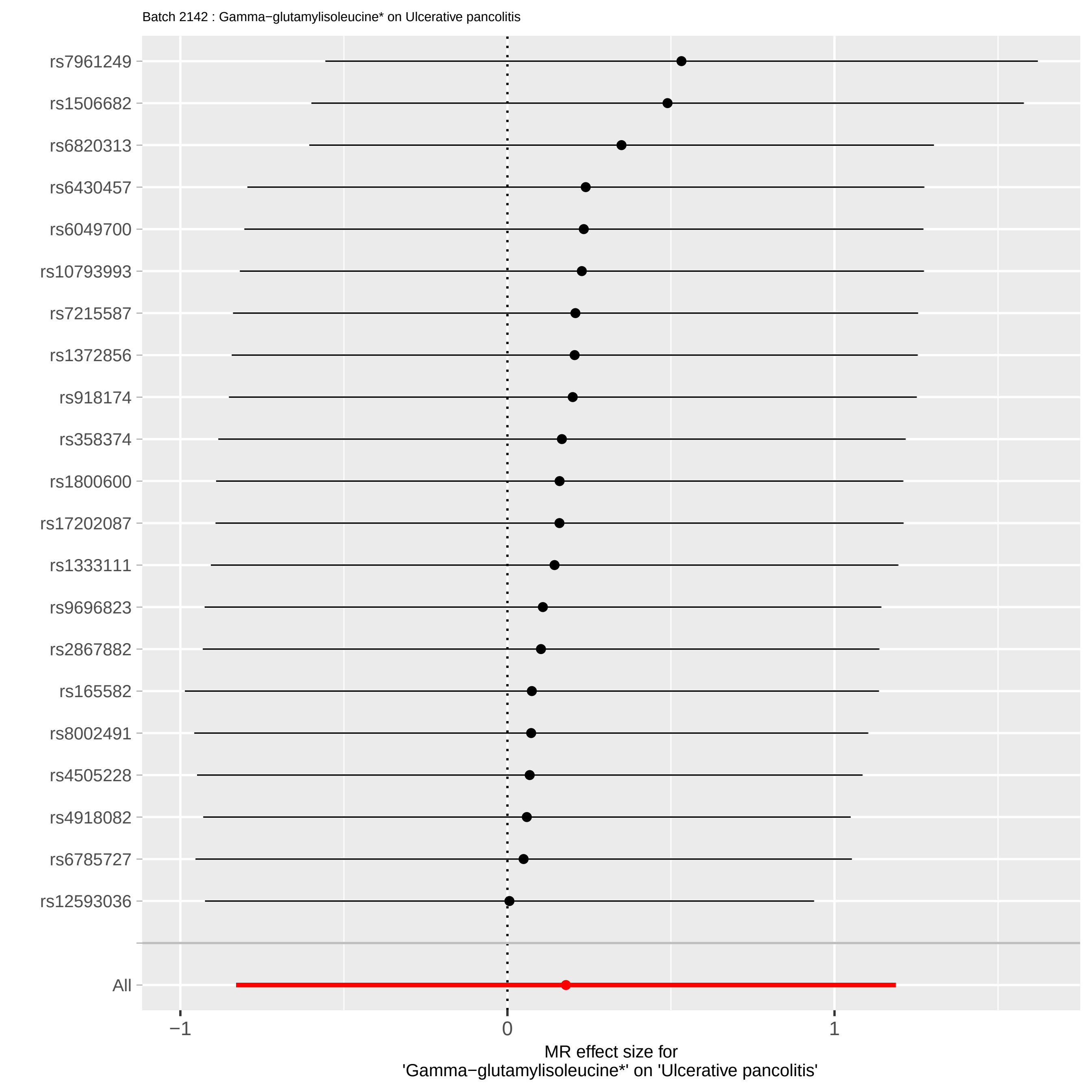

Batch 2142 : Gamma−glutamylisoleucine* on Ulcerative pancolitis
rs7961249
rs1506682
rs6820313
rs6430457
rs6049700
rs10793993
rs7215587
rs1372856
rs918174
rs358374
rs1800600
rs17202087
rs1333111
rs9696823
rs2867882
rs165582
rs8002491
rs4505228
rs4918082
rs6785727
rs12593036
All
−1
0
1
MR effect size for
'Gamma−glutamylisoleucine*' on 'Ulcerative pancolitis'

Batch 2145 : Isovalerate on Ulcerative pancolitis

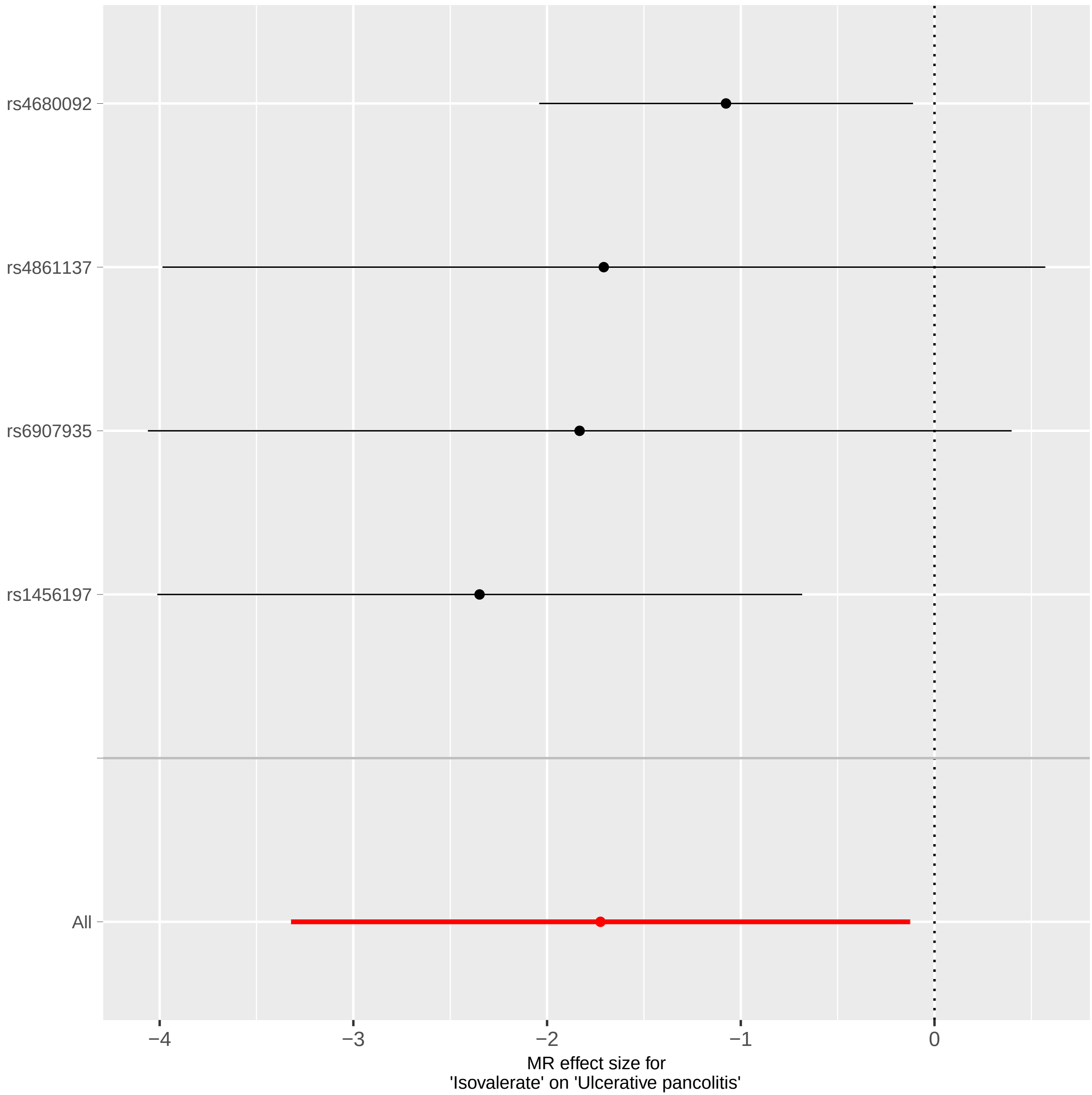

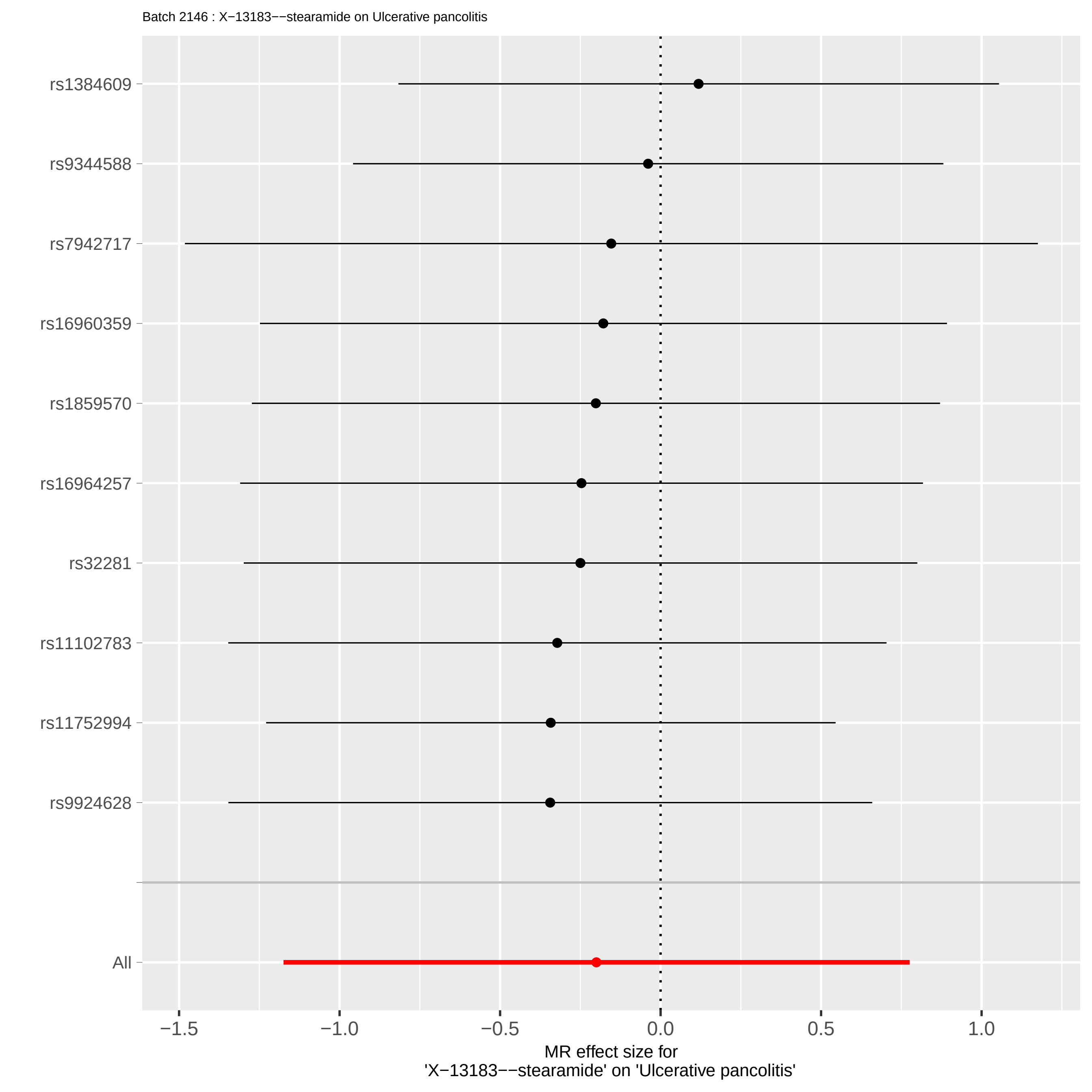

Batch 2146 : X−13183−−stearamide on Ulcerative pancolitis
rs1384609
rs9344588
rs7942717
rs16960359
rs1859570
rs16964257
rs32281
rs11102783
rs11752994
rs9924628
All
−1.5
−1.0
−0.5
0.0
0.5
1.0
MR effect size for
'X−13183−−stearamide' on 'Ulcerative pancolitis'

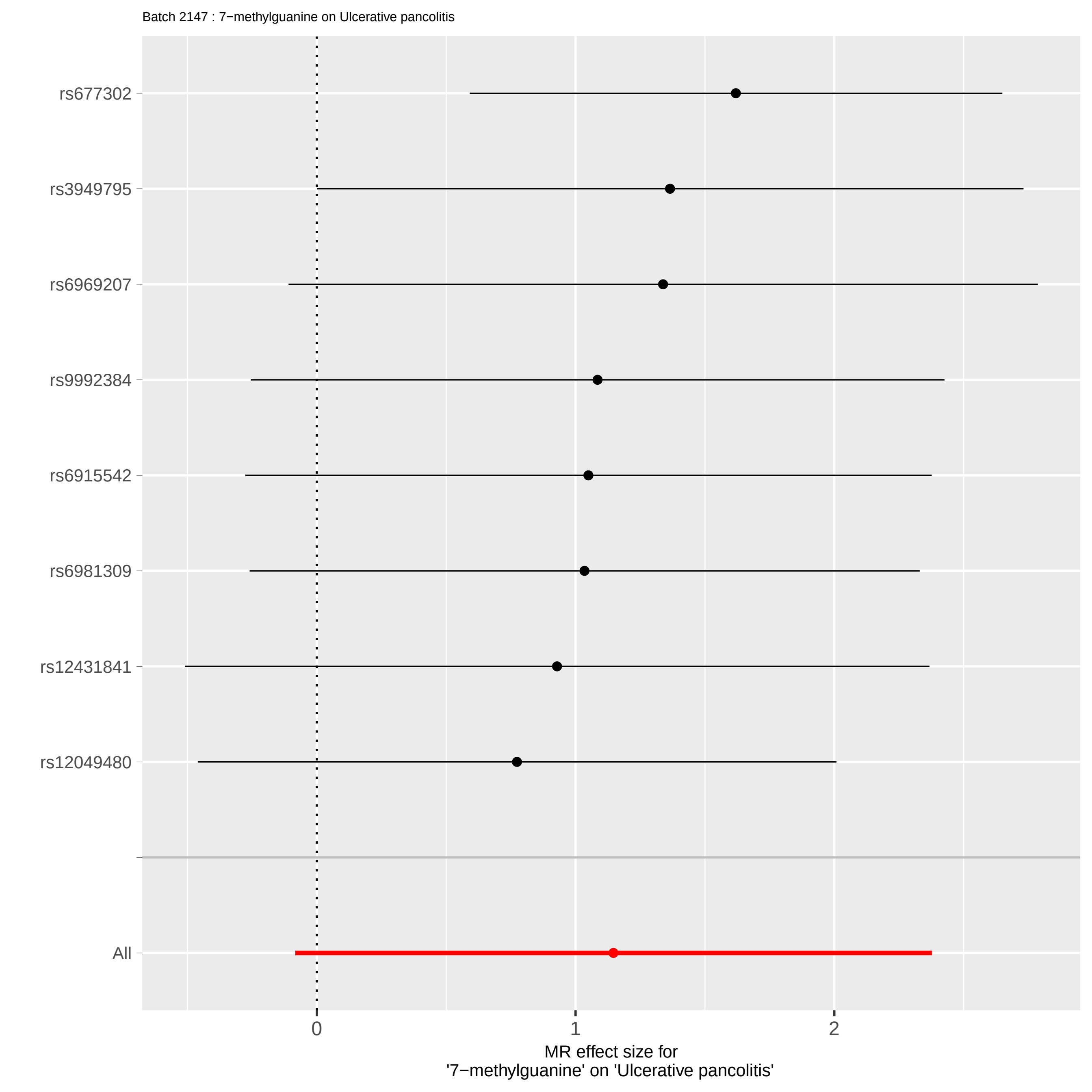
Batch 2147 : 7−methylguanine on Ulcerative pancolitis
rs677302
rs3949795
rs6969207
rs9992384
rs6915542
rs6981309
rs12431841
rs12049480
All
0
1
2
MR effect size for
'7−methylguanine' on 'Ulcerative pancolitis'

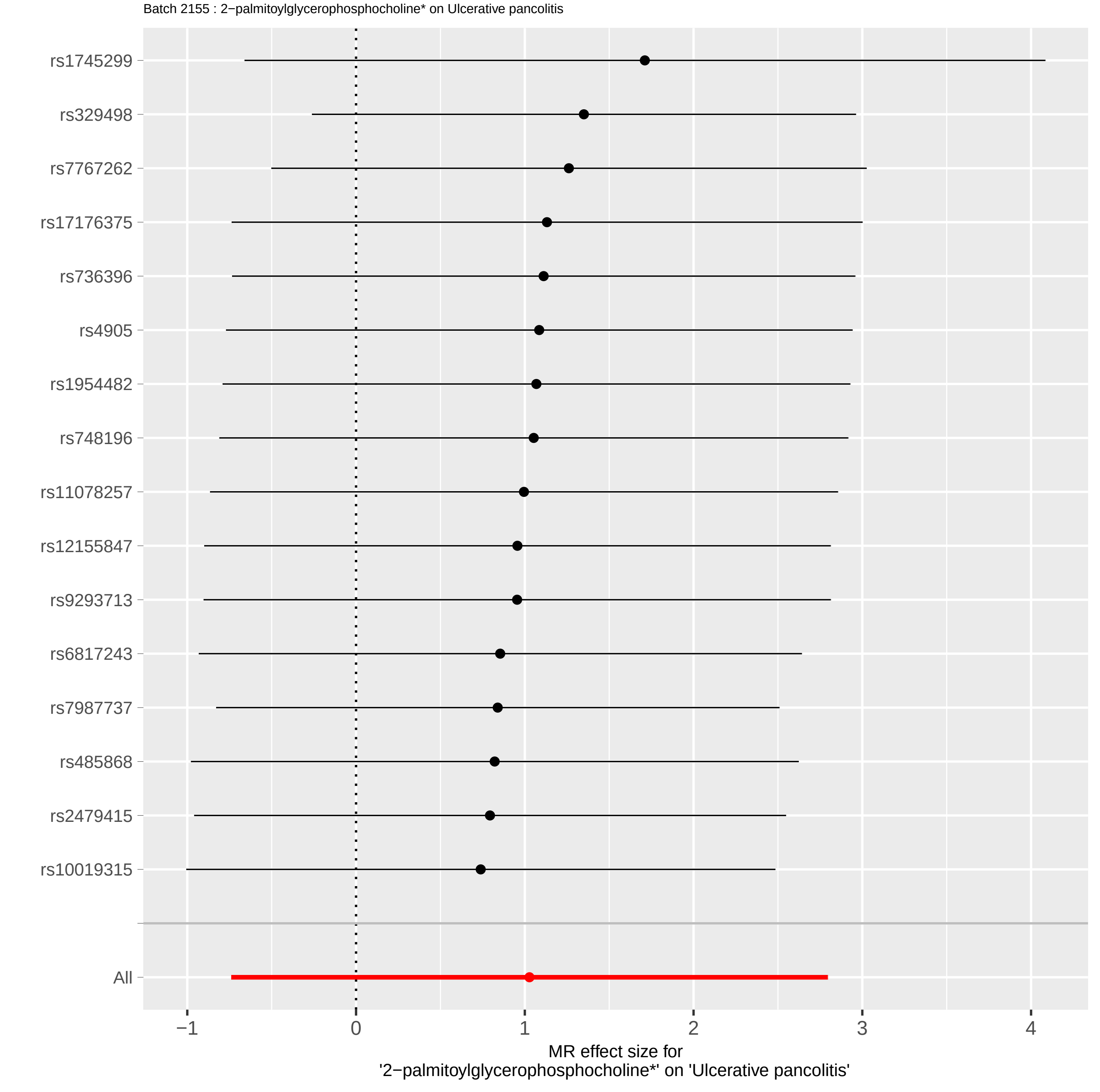

Batch 2155 : 2−palmitoylglycerophosphocholine* on Ulcerative pancolitis
rs1745299
rs329498
rs7767262
rs17176375
rs736396
rs4905
rs1954482
rs748196
rs11078257
rs12155847
rs9293713
rs6817243
rs7987737
rs485868
rs2479415
rs10019315
All
−1
0
1
2
3
4
MR effect size for
'2−palmitoylglycerophosphocholine*' on 'Ulcerative pancolitis'

Batch 2156 : 2−oleoylglycerophosphocholine* on Ulcerative pancolitis

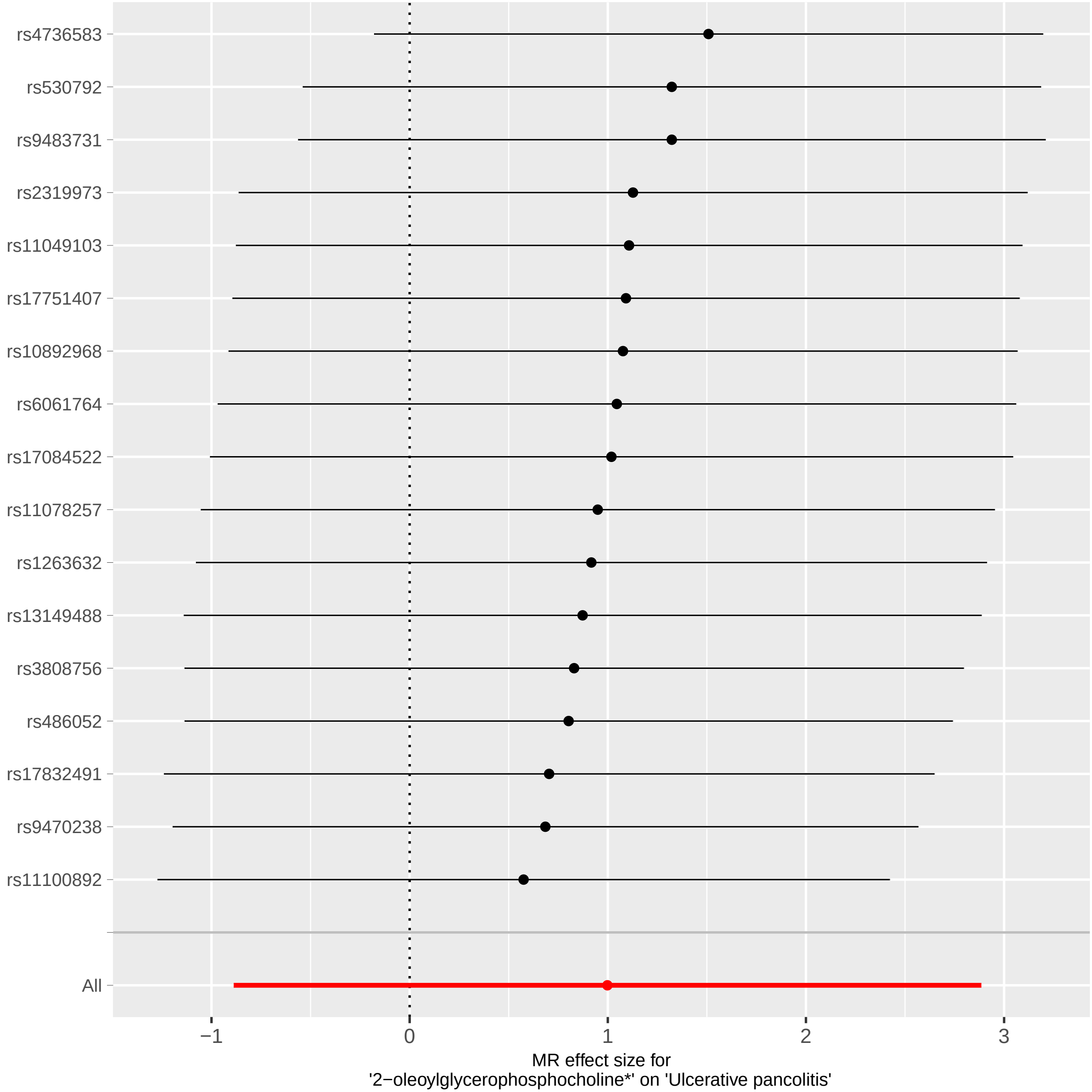

Batch 2159 : 1−palmitoylglycerophosphoinositol* on Ulcerative pancolitis

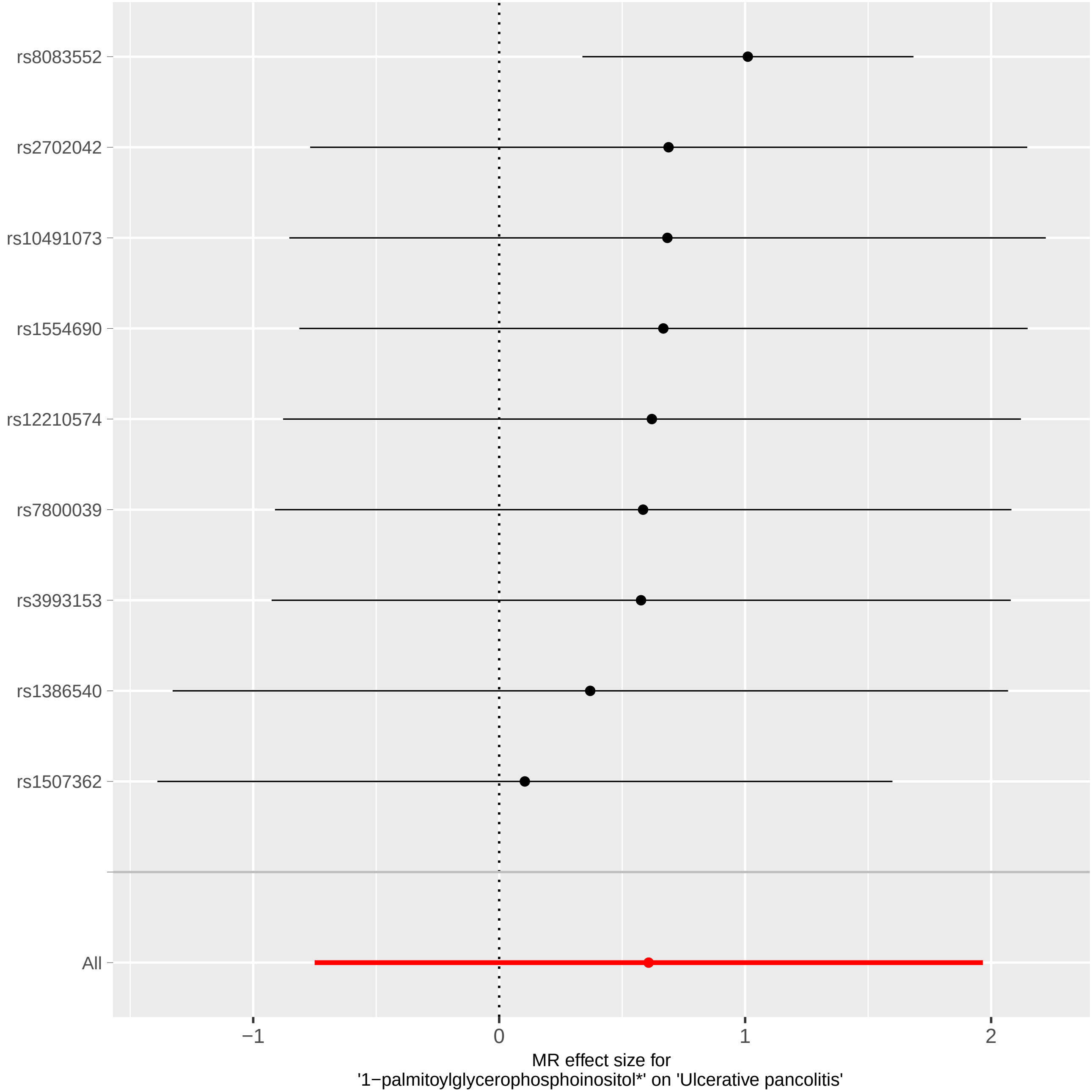

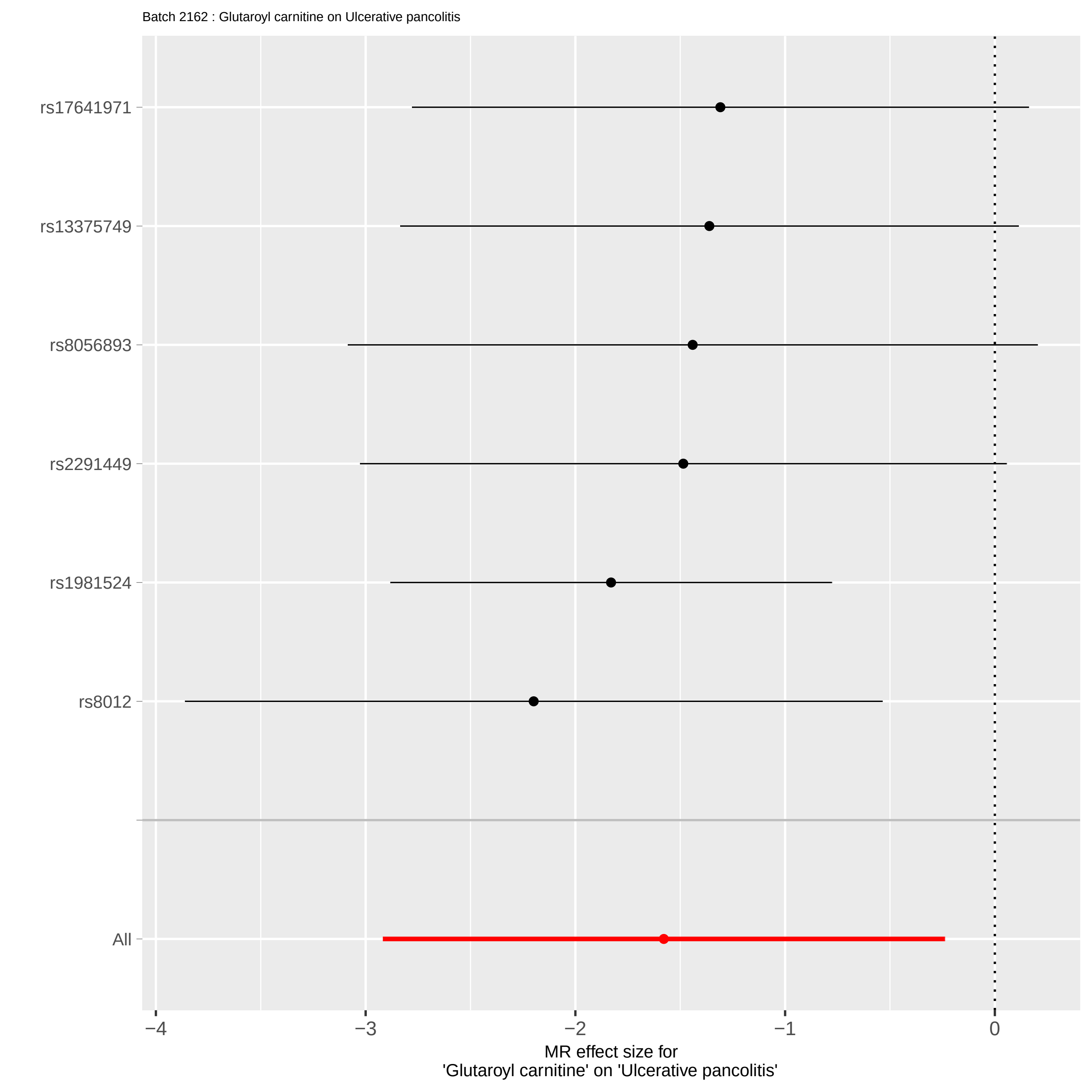
Batch 2162 : Glutaroyl carnitine on Ulcerative pancolitis
rs17641971
rs13375749
rs8056893
rs2291449
rs1981524
rs8012
All
−4
−3
−2
−1
0
MR effect size for
'Glutaroyl carnitine' on 'Ulcerative pancolitis'

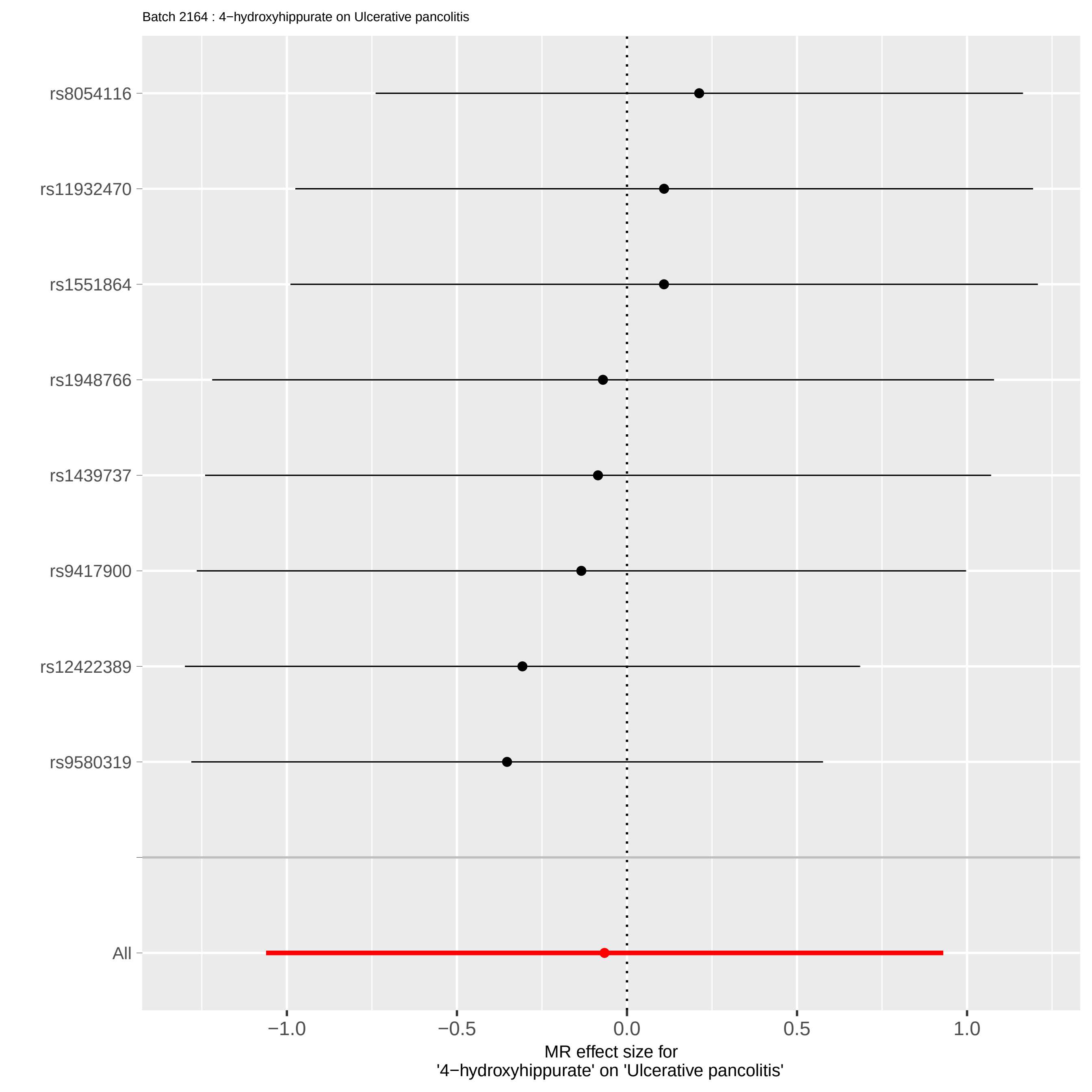

Batch 2164 : 4−hydroxyhippurate on Ulcerative pancolitis
rs8054116
rs11932470
rs1551864
rs1948766
rs1439737
rs9417900
rs12422389
rs9580319
All
−1.0
−0.5
0.0
0.5
1.0
MR effect size for
'4−hydroxyhippurate' on 'Ulcerative pancolitis'

Batch 2165 : 1−myristoylglycerophosphocholine on Ulcerative pancolitis

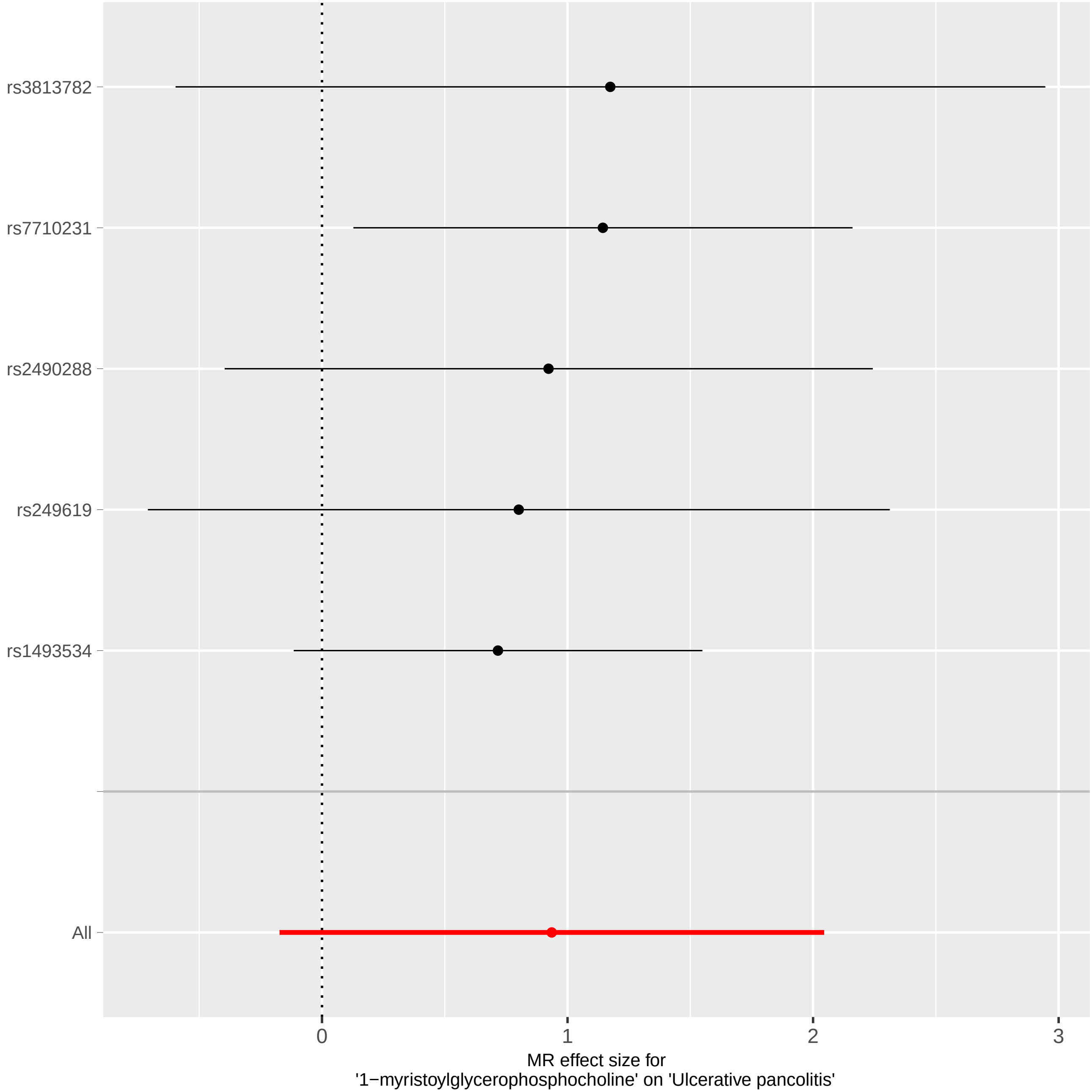

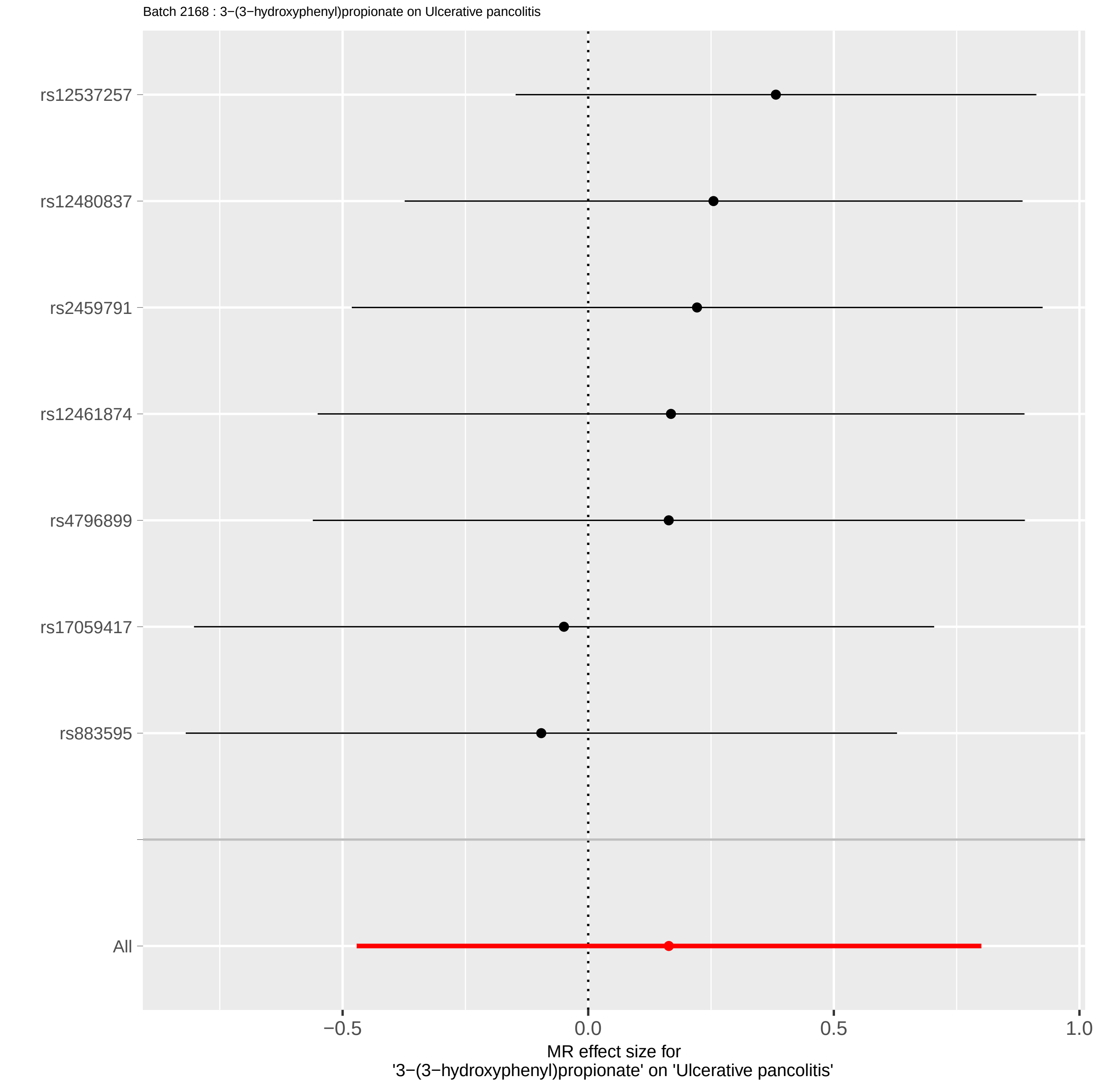

Batch 2168 : 3−(3−hydroxyphenyl)propionate on Ulcerative pancolitis
rs12537257
rs12480837
rs2459791
rs12461874
rs4796899
rs17059417
rs883595
All
−0.5
0.0
0.5
1.0
MR effect size for
'3−(3−hydroxyphenyl)propionate' on 'Ulcerative pancolitis'

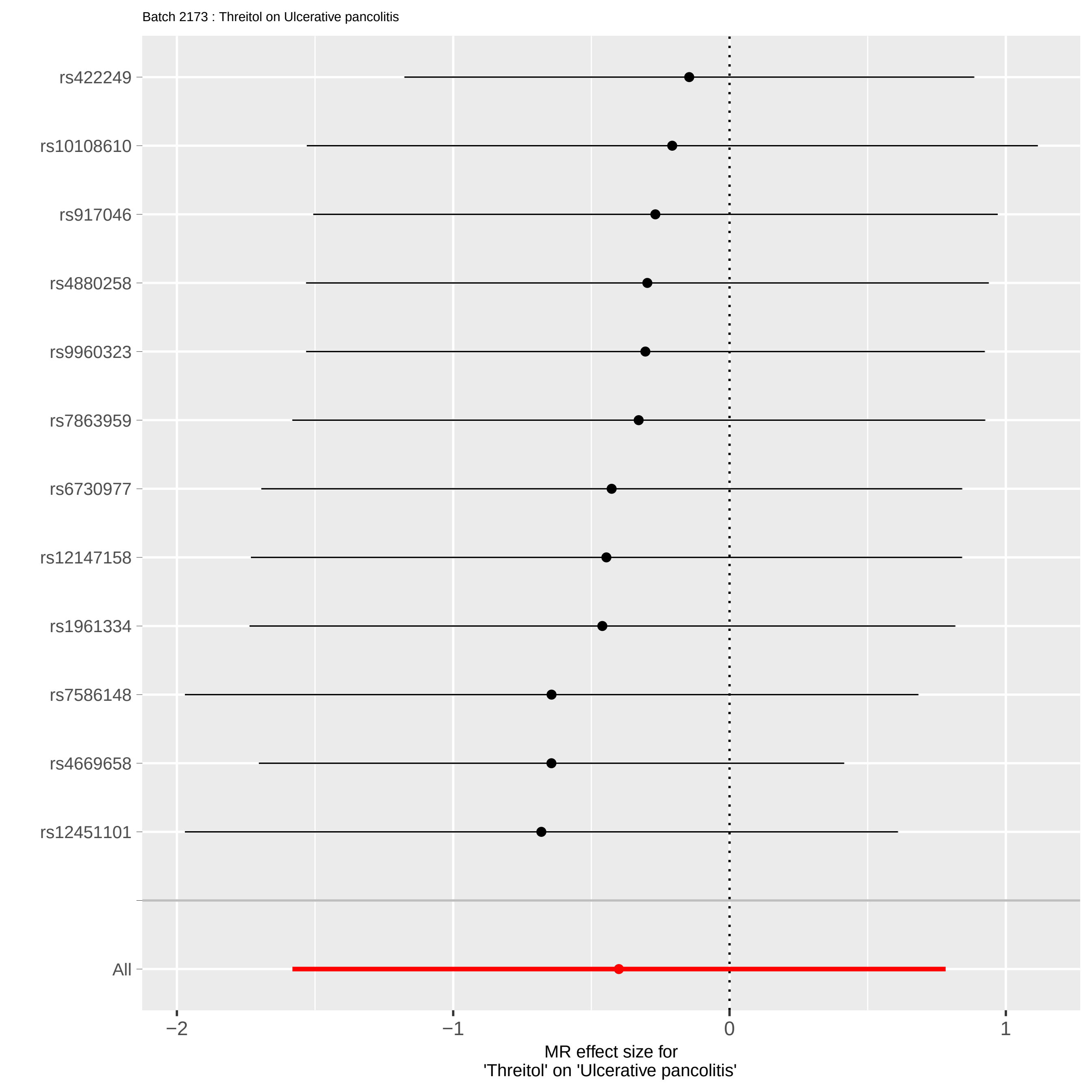
Batch 2173 : Threitol on Ulcerative pancolitis
rs422249
rs10108610
rs917046
rs4880258
rs9960323
rs7863959
rs6730977
rs12147158
rs1961334
rs7586148
rs4669658
rs12451101
All
−2
−1
0
1
MR effect size for
'Threitol' on 'Ulcerative pancolitis'

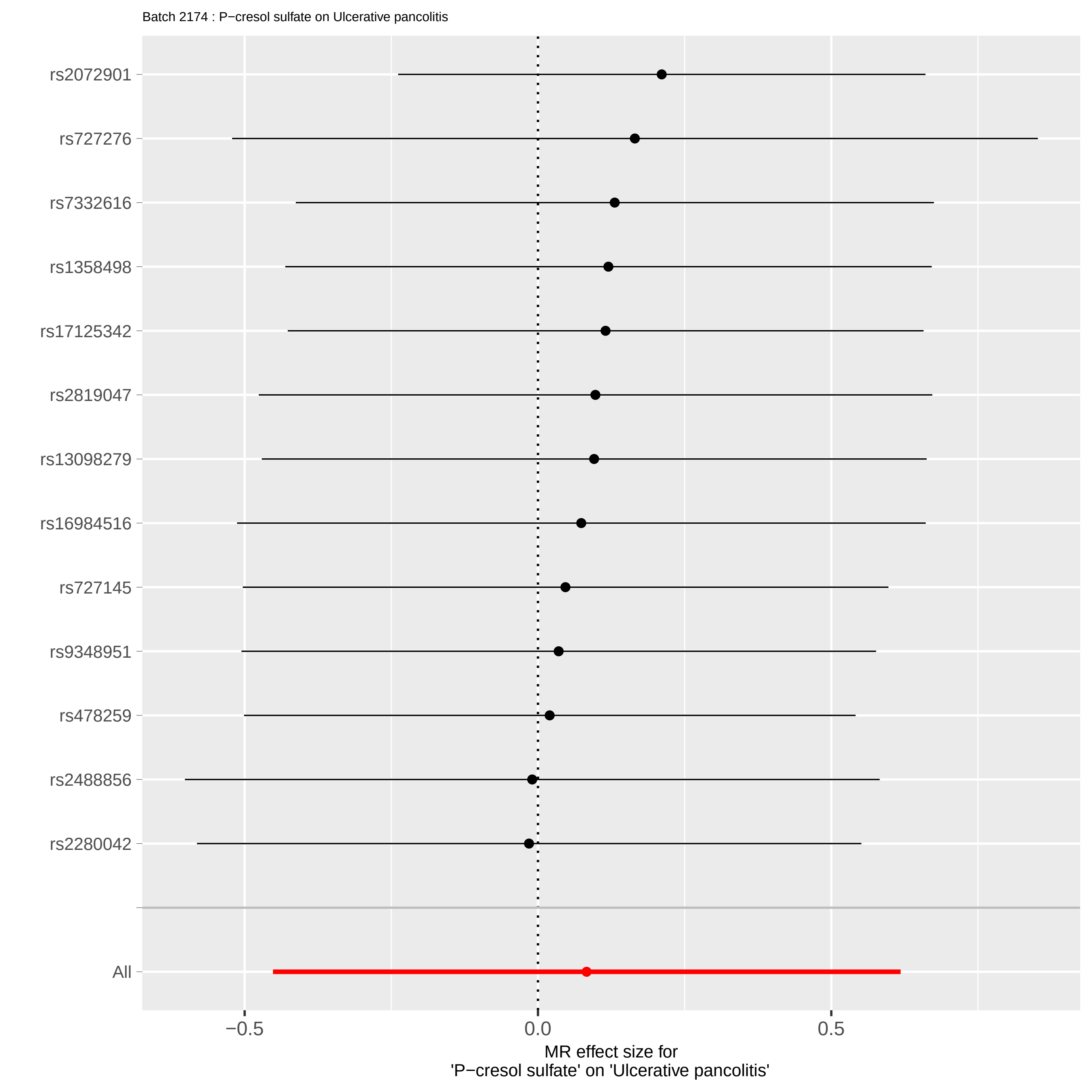

Batch 2174 : P−cresol sulfate on Ulcerative pancolitis
rs2072901
rs727276
rs7332616
rs1358498
rs17125342
rs2819047
rs13098279
rs16984516
rs727145
rs9348951
rs478259
rs2488856
rs2280042
All
−0.5
0.0
0.5
MR effect size for
'P−cresol sulfate' on 'Ulcerative pancolitis'

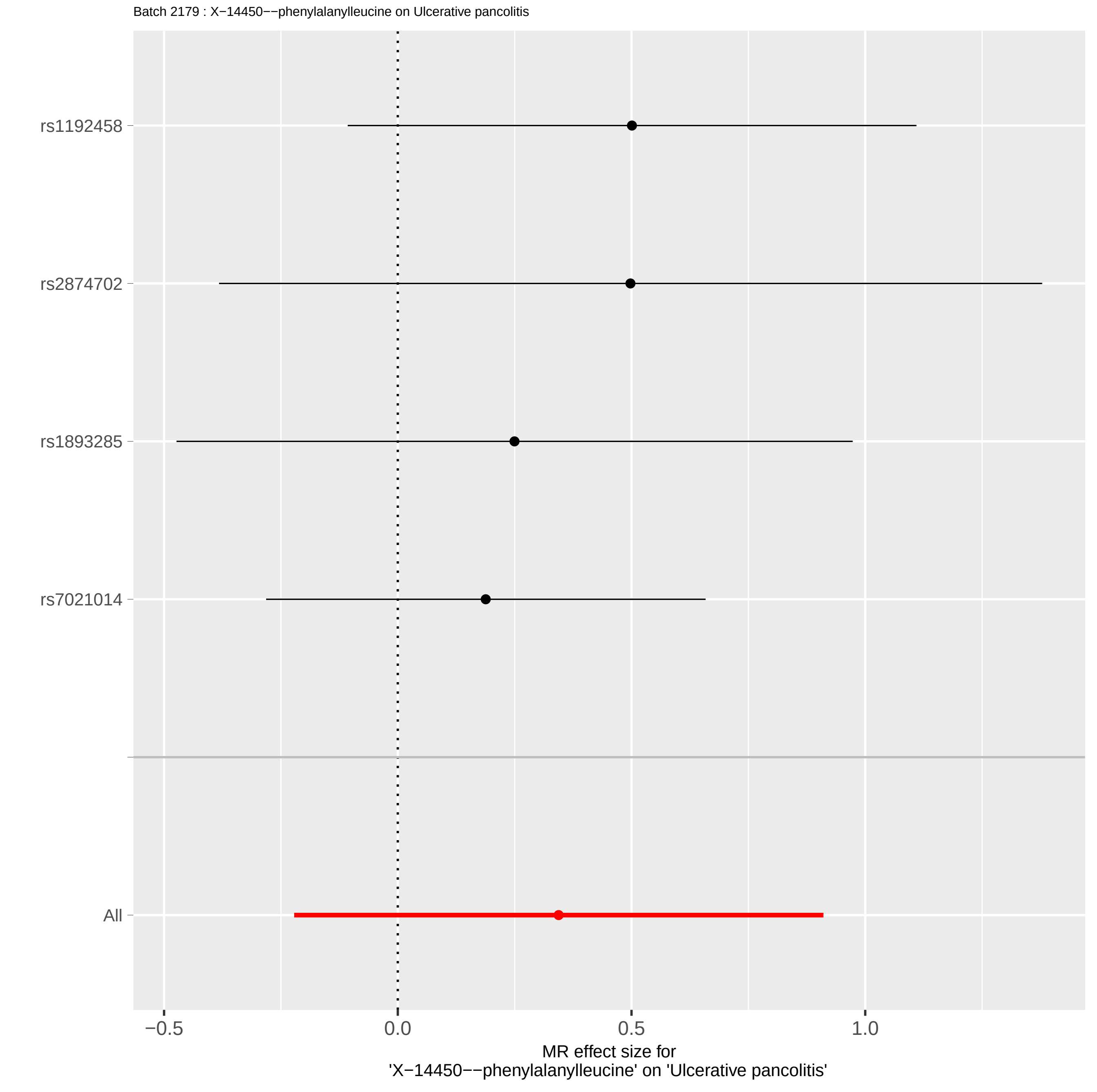
Batch 2179 : X−14450−−phenylalanylleucine on Ulcerative pancolitis
rs1192458
rs2874702
rs1893285
rs7021014
All
−0.5
0.0
0.5
1.0
MR effect size for
'X−14450−−phenylalanylleucine' on 'Ulcerative pancolitis'

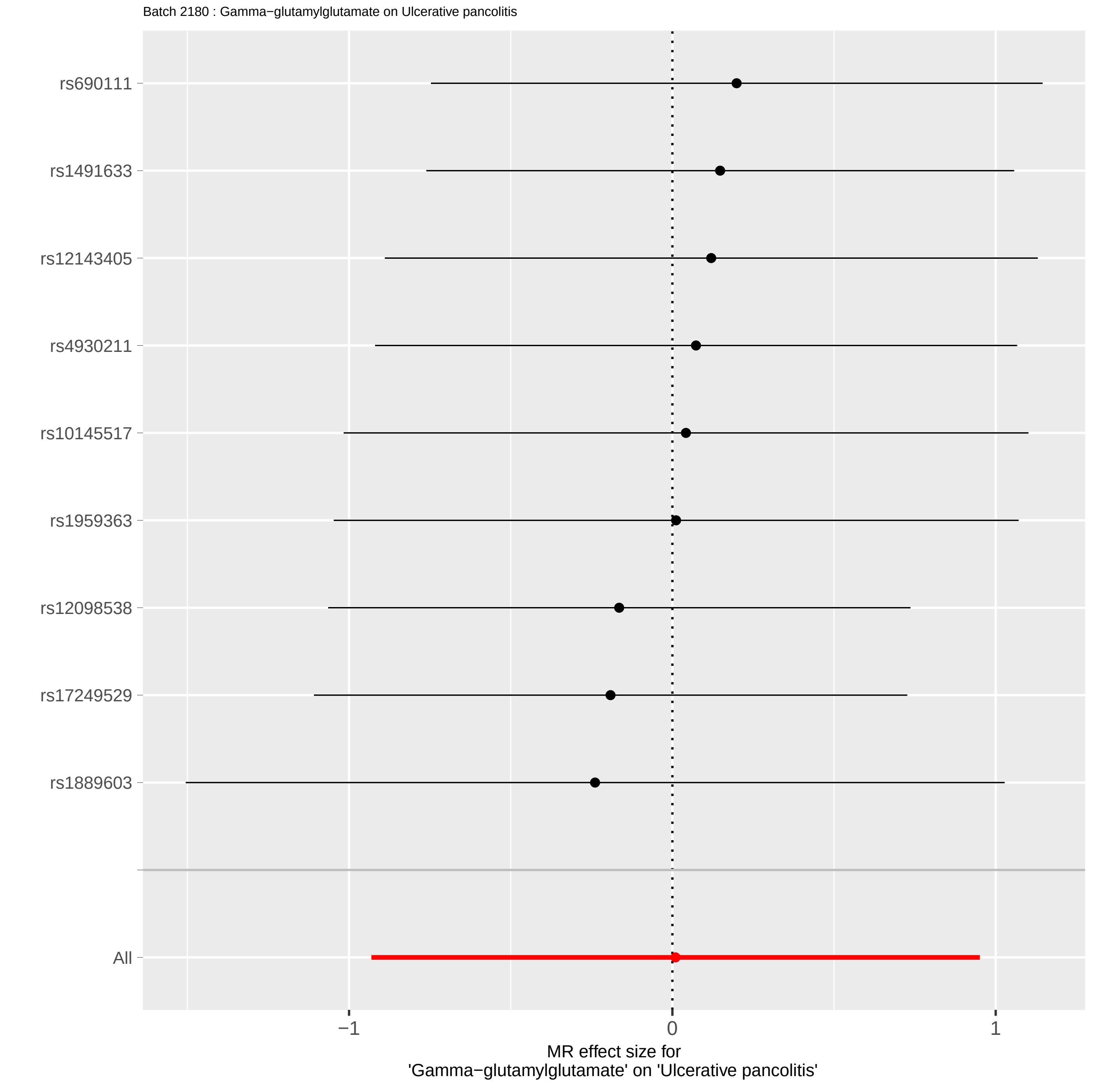
Batch 2180 : Gamma−glutamylglutamate on Ulcerative pancolitis
rs690111
rs1491633
rs12143405
rs4930211
rs10145517
rs1959363
rs12098538
rs17249529
rs1889603
All
−1
0
1
MR effect size for
'Gamma−glutamylglutamate' on 'Ulcerative pancolitis'

Batch 2182 : Leucylleucine on Ulcerative pancolitis

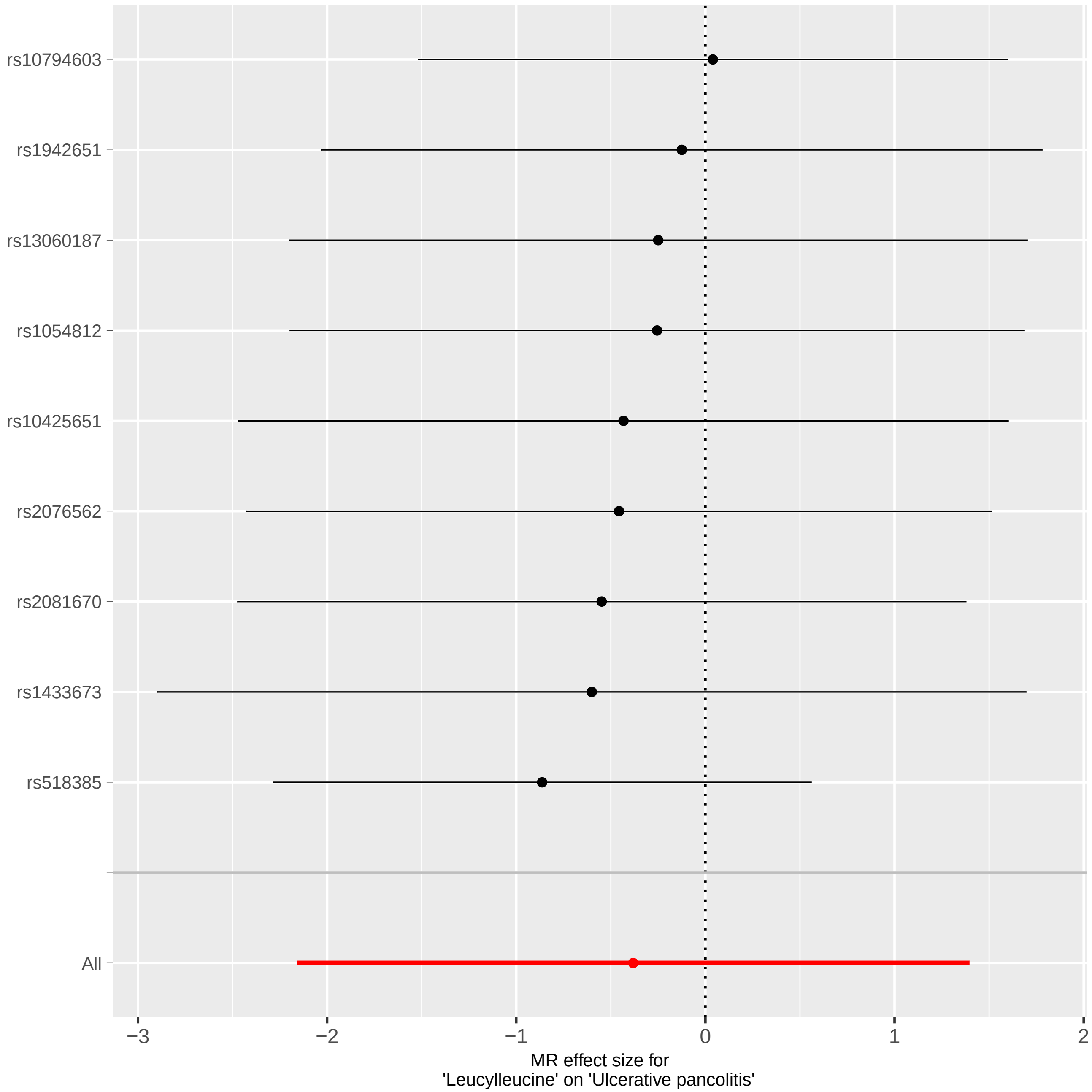

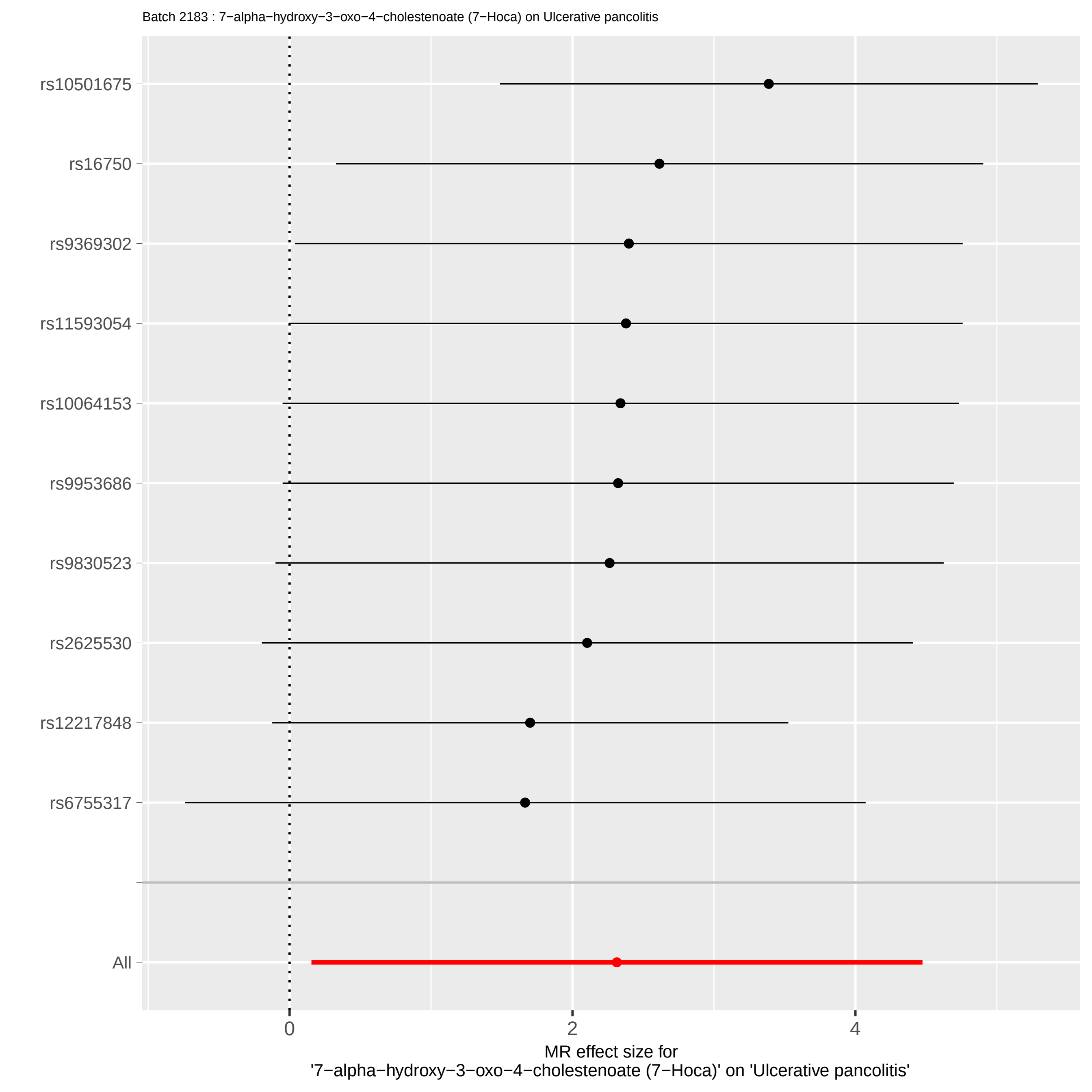
Batch 2183 : 7−alpha−hydroxy−3−oxo−4−cholestenoate (7−Hoca) on Ulcerative pancolitis
rs10501675
rs16750
rs9369302
rs11593054
rs10064153
rs9953686
rs9830523
rs2625530
rs12217848
rs6755317
All
0
2
4
MR effect size for
'7−alpha−hydroxy−3−oxo−4−cholestenoate (7−Hoca)' on 'Ulcerative pancolitis'

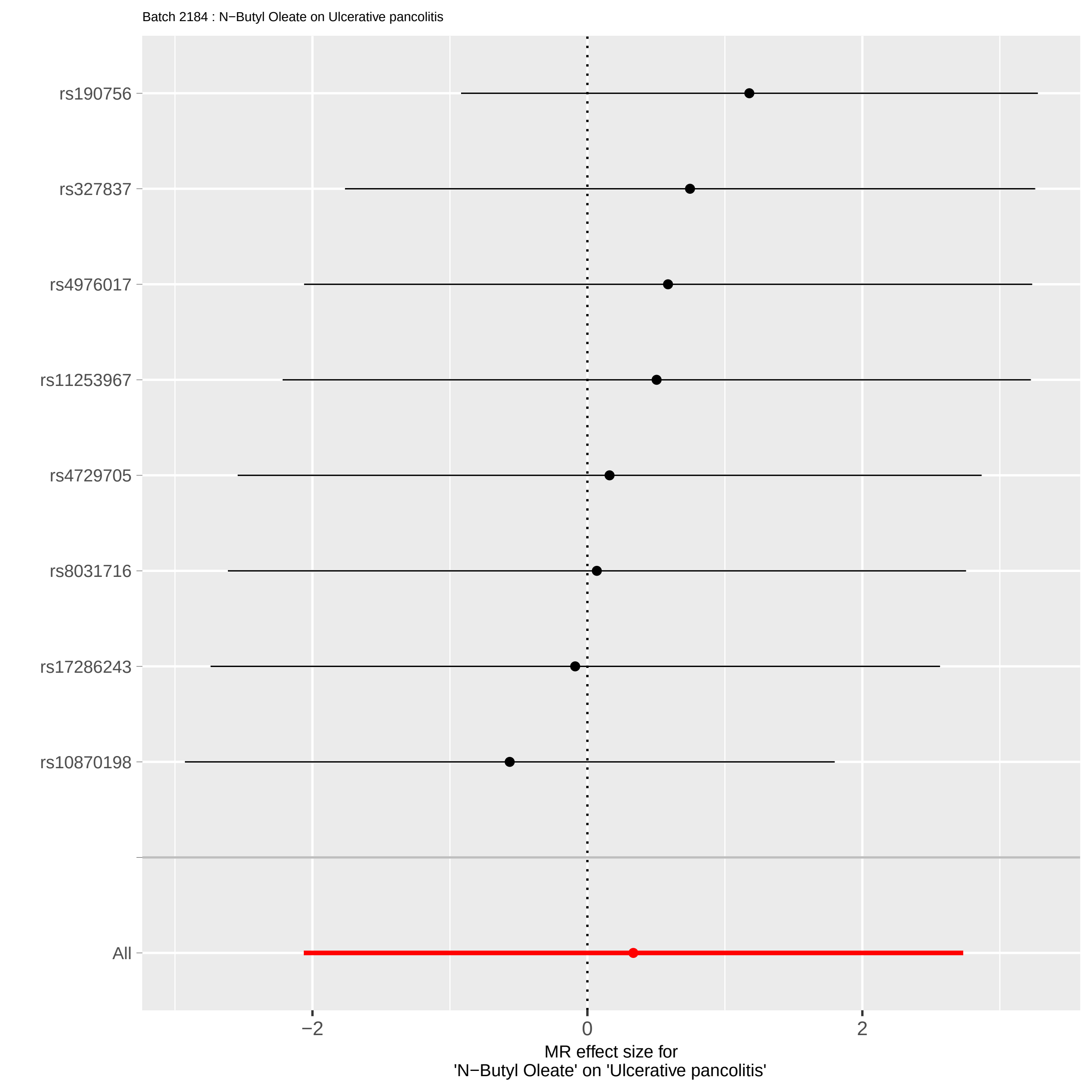

Batch 2184 : N−Butyl Oleate on Ulcerative pancolitis
rs190756
rs327837
rs4976017
rs11253967
rs4729705
rs8031716
rs17286243
rs10870198
All
−2
0
2
MR effect size for
'N−Butyl Oleate' on 'Ulcerative pancolitis'

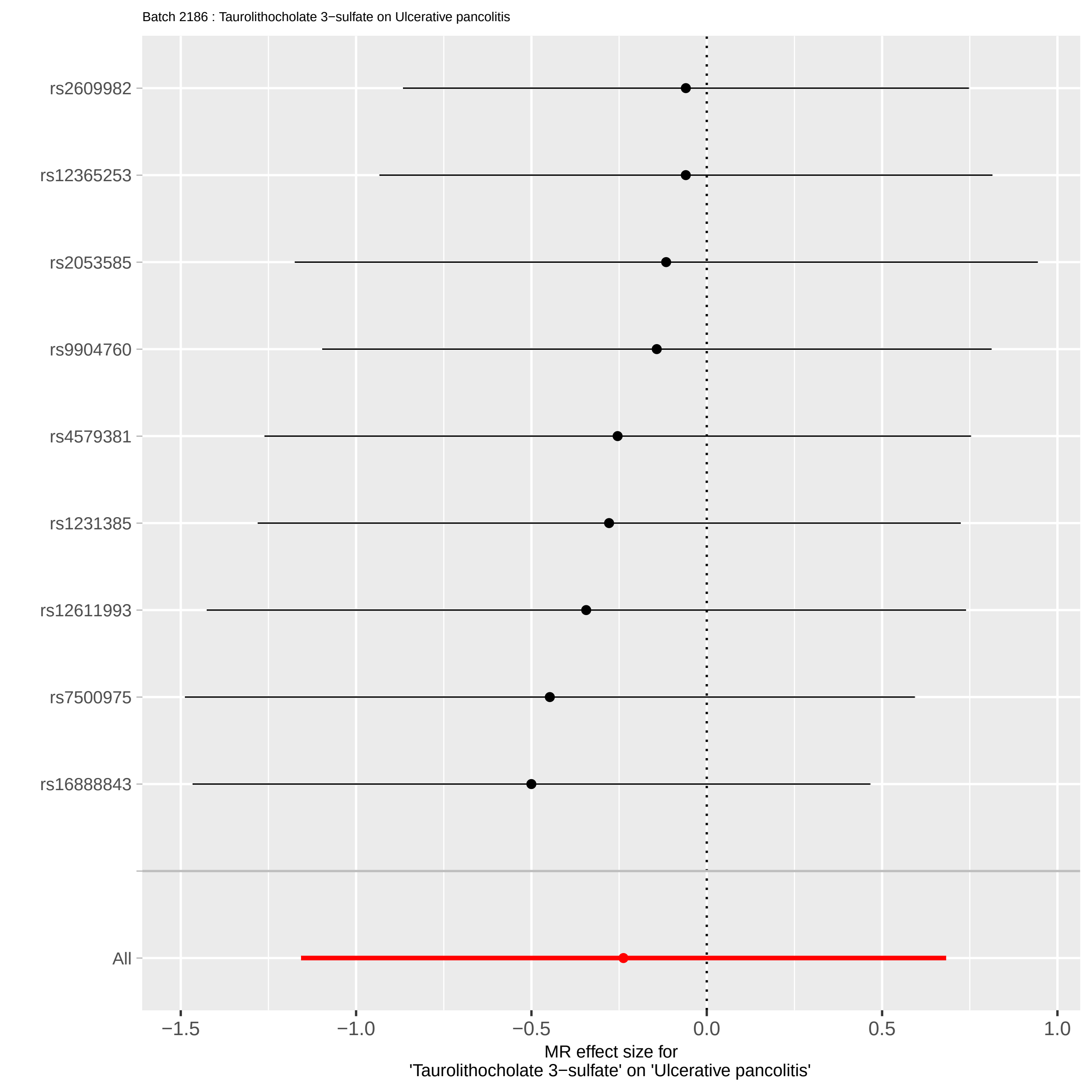
Batch 2186 : Taurolithocholate 3−sulfate on Ulcerative pancolitis
rs2609982
rs12365253
rs2053585
rs9904760
rs4579381
rs1231385
rs12611993
rs7500975
rs16888843
All
−1.5
−1.0
−0.5
0.0
0.5
1.0
MR effect size for
'Taurolithocholate 3−sulfate' on 'Ulcerative pancolitis'

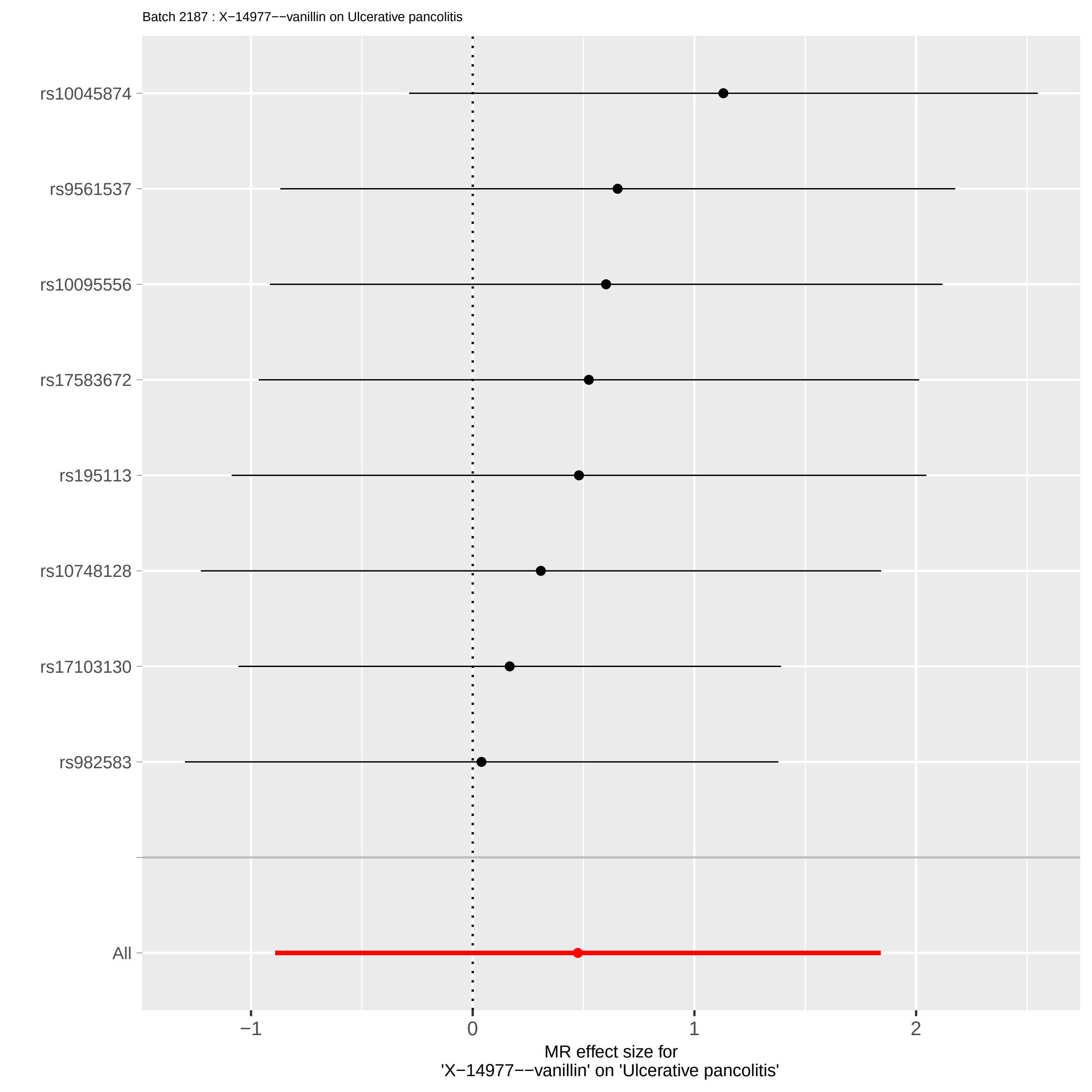

Batch 2187 : X−14977−−vanillin on Ulcerative pancolitis
rs10045874
rs9561537
rs10095556
rs17583672
rs195113
rs10748128
rs17103130
rs982583
All
−1
0
1
2
MR effect size for
'X−14977−−vanillin' on 'Ulcerative pancolitis'

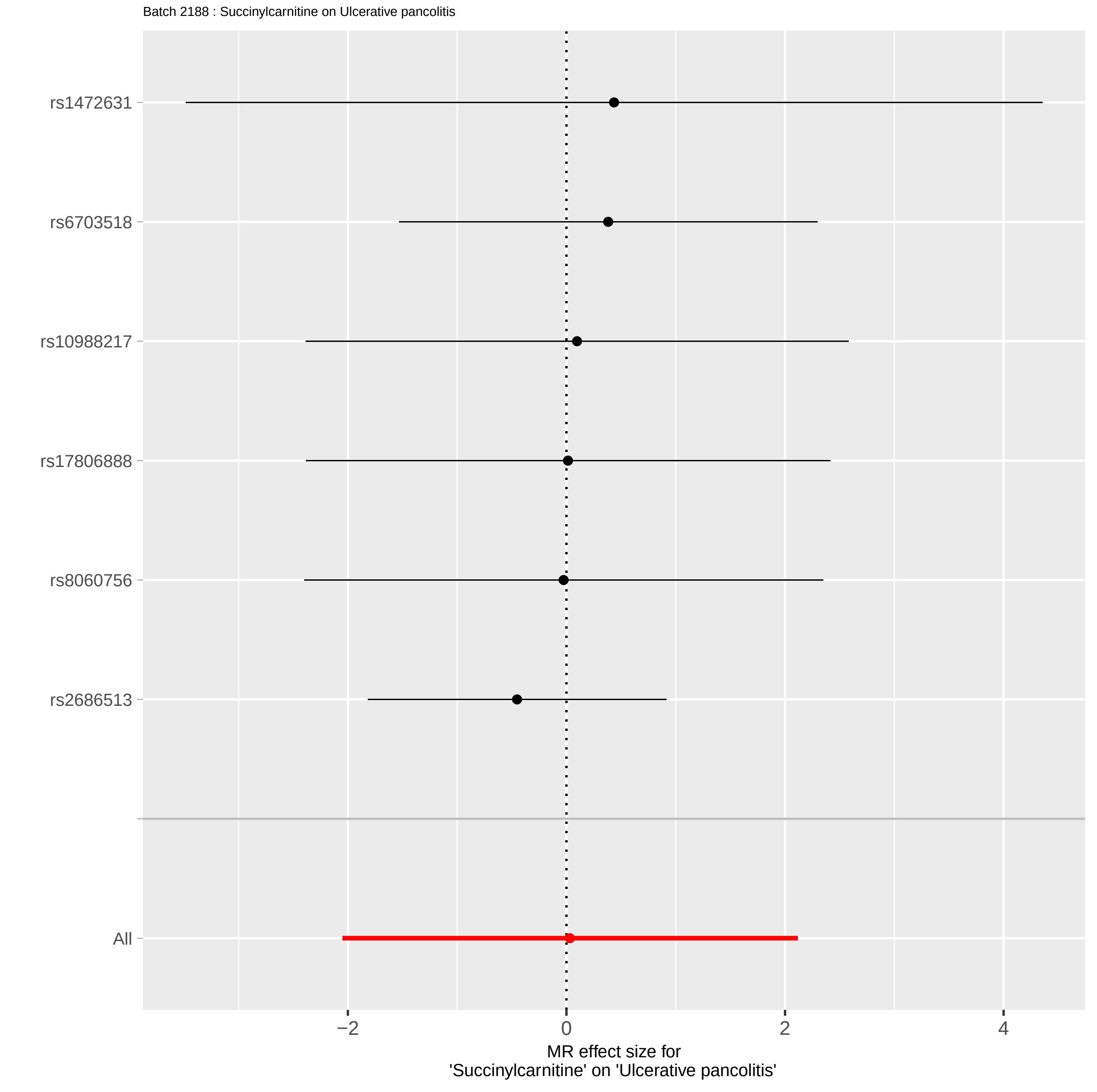
Batch 2188 : Succinylcarnitine on Ulcerative pancolitis
rs1472631
rs6703518
rs10988217
rs17806888
rs8060756
rs2686513
All
−2
0
2
4
MR effect size for
'Succinylcarnitine' on 'Ulcerative pancolitis'

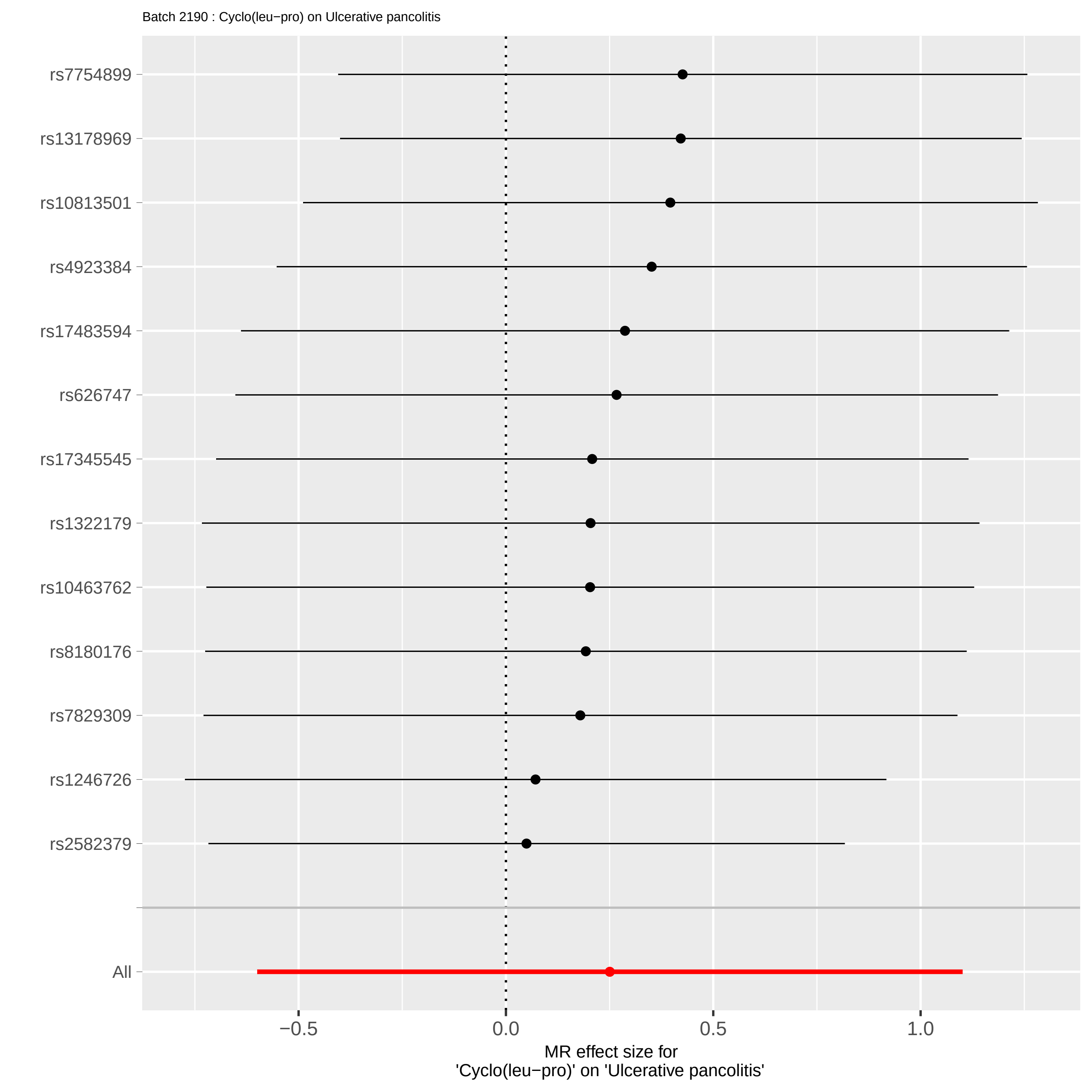
Batch 2190 : Cyclo(leu−pro) on Ulcerative pancolitis
rs7754899
rs13178969
rs10813501
rs4923384
rs17483594
rs626747
rs17345545
rs1322179
rs10463762
rs8180176
rs7829309
rs1246726
rs2582379
All
−0.5
0.0
0.5
1.0
MR effect size for
'Cyclo(leu−pro)' on 'Ulcerative pancolitis'

Batch 2191 : Chiro−inositol on Ulcerative pancolitis

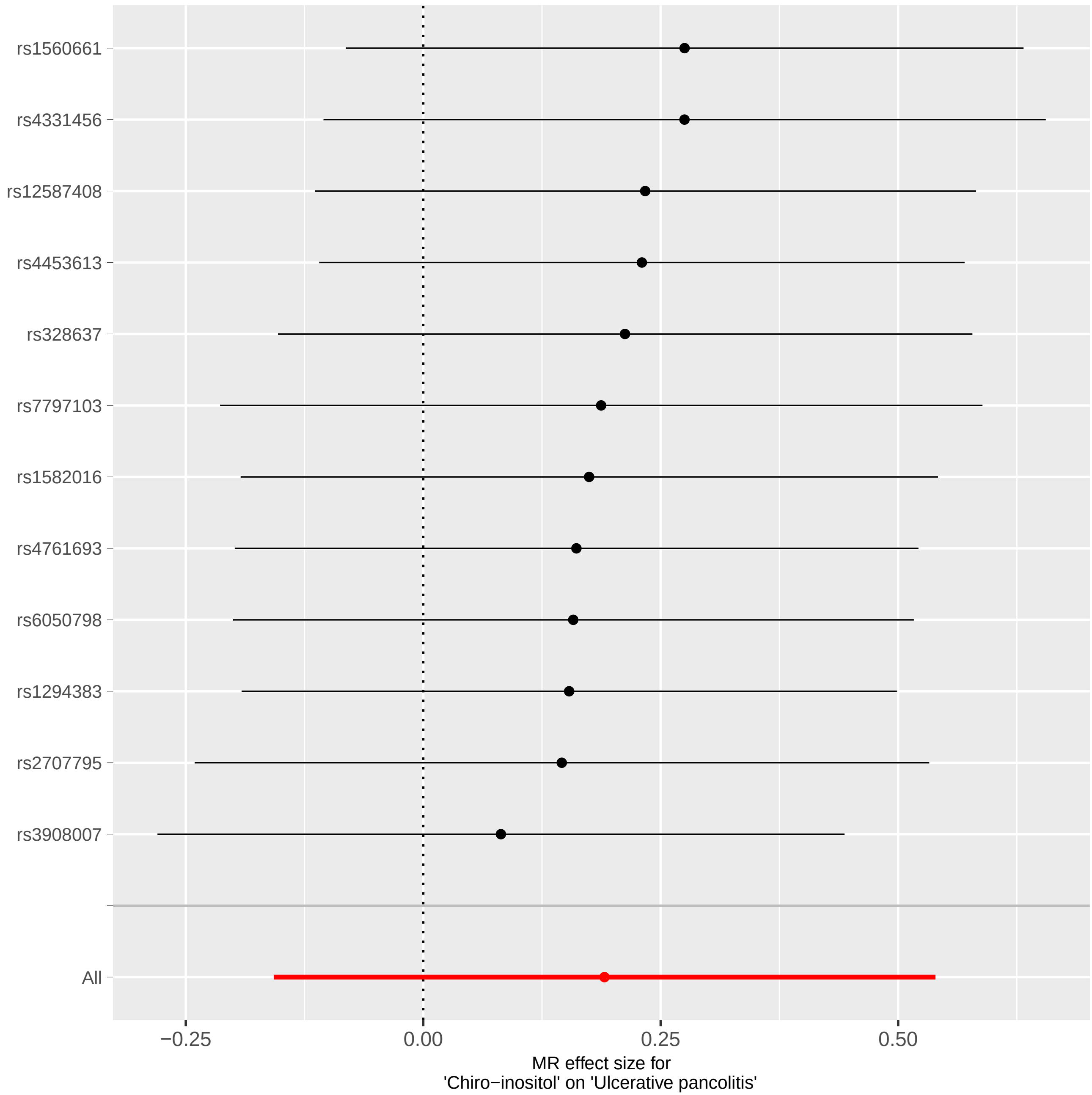

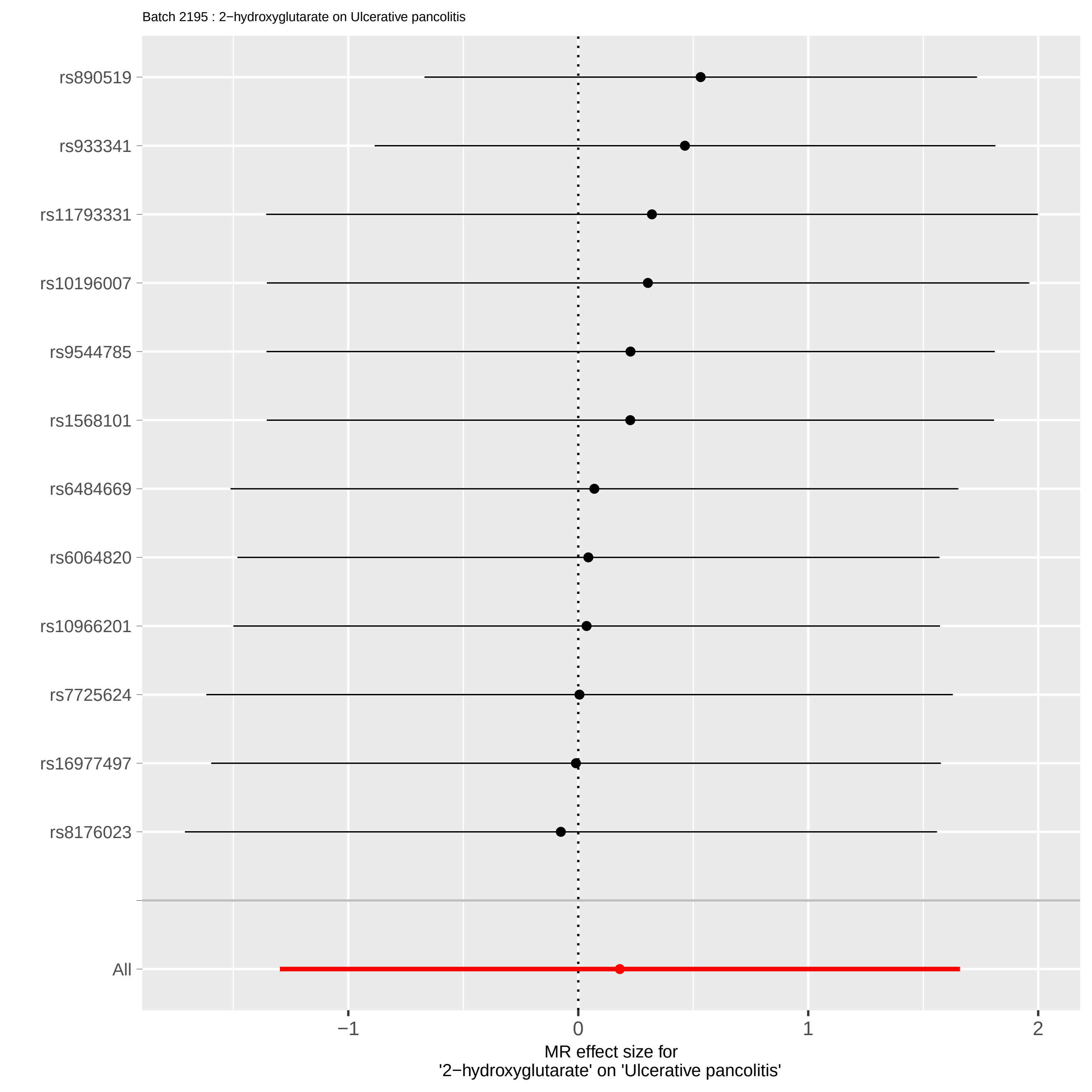
Batch 2195 : 2−hydroxyglutarate on Ulcerative pancolitis
rs890519
rs933341
rs11793331
rs10196007
rs9544785
rs1568101
rs6484669
rs6064820
rs10966201
rs7725624
rs16977497
rs8176023
All
−1
0
1
2
MR effect size for
'2−hydroxyglutarate' on 'Ulcerative pancolitis'

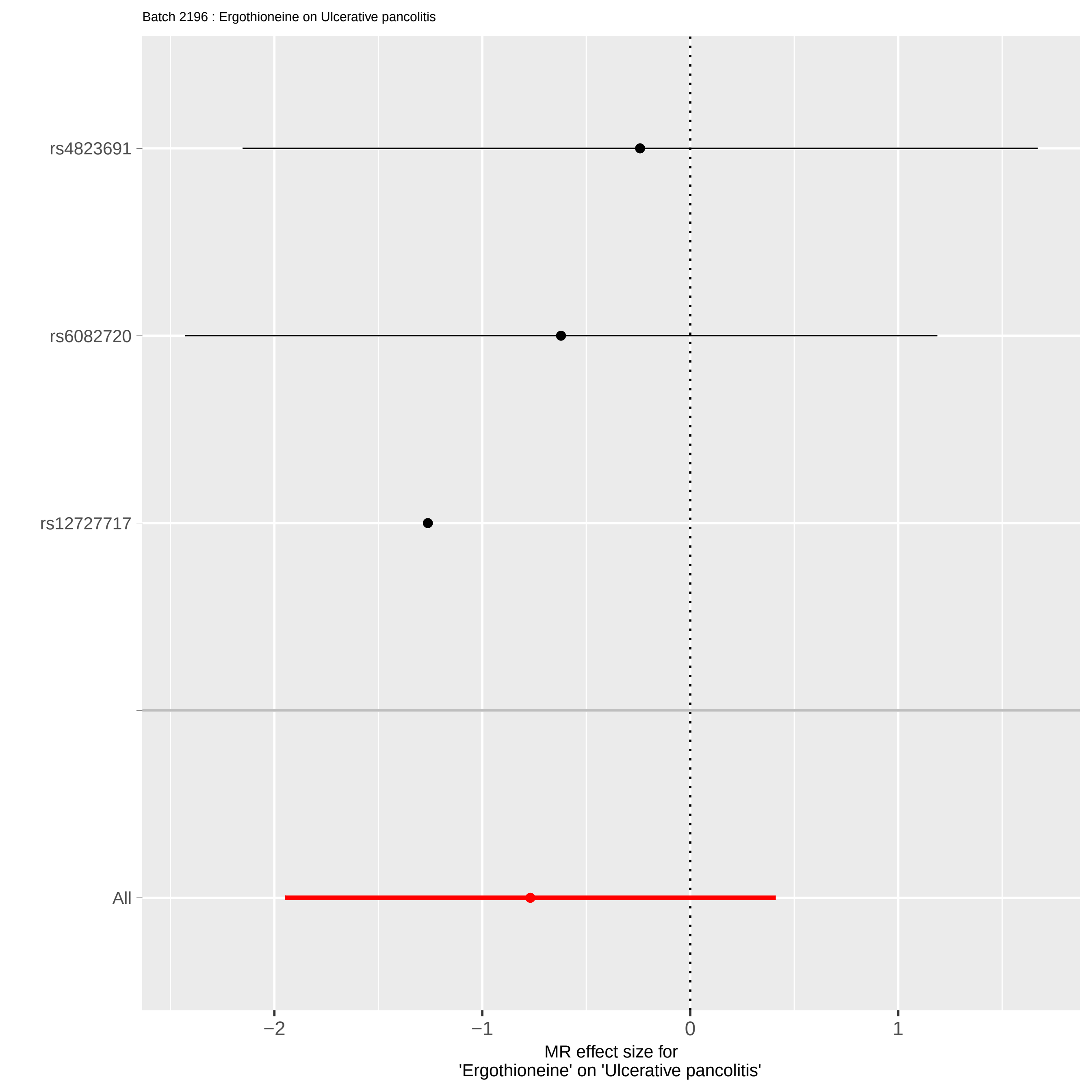
Batch 2196 : Ergothioneine on Ulcerative pancolitis
rs4823691
rs6082720
rs12727717
All
−2
−1
0
1
MR effect size for
'Ergothioneine' on 'Ulcerative pancolitis'

Batch 2198 : Phenylalanylphenylalanine on Ulcerative pancolitis

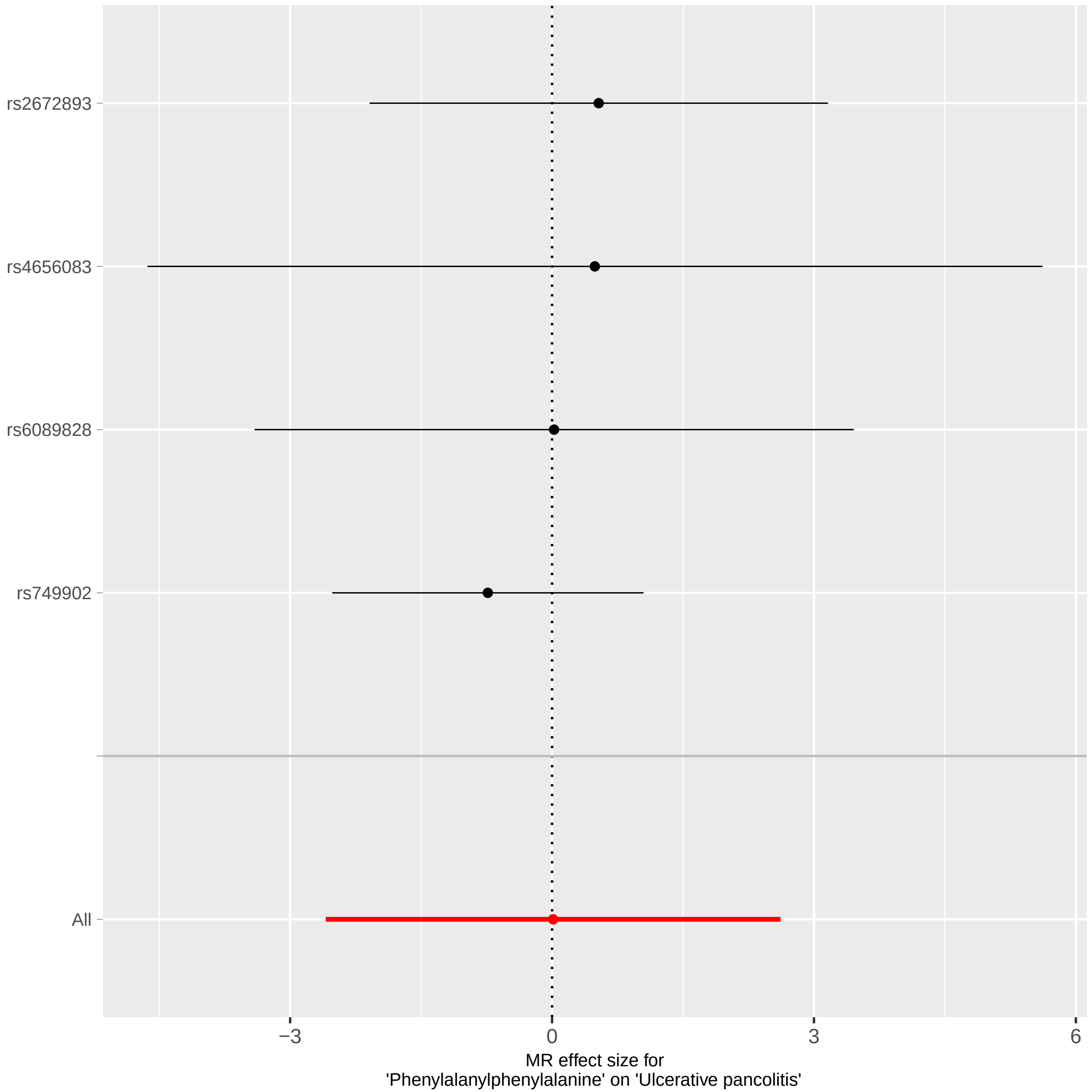

Batch 2200 : 15−methylpalmitate (isobar with 2−methylpalmitate) on Ulcerative pancolitis

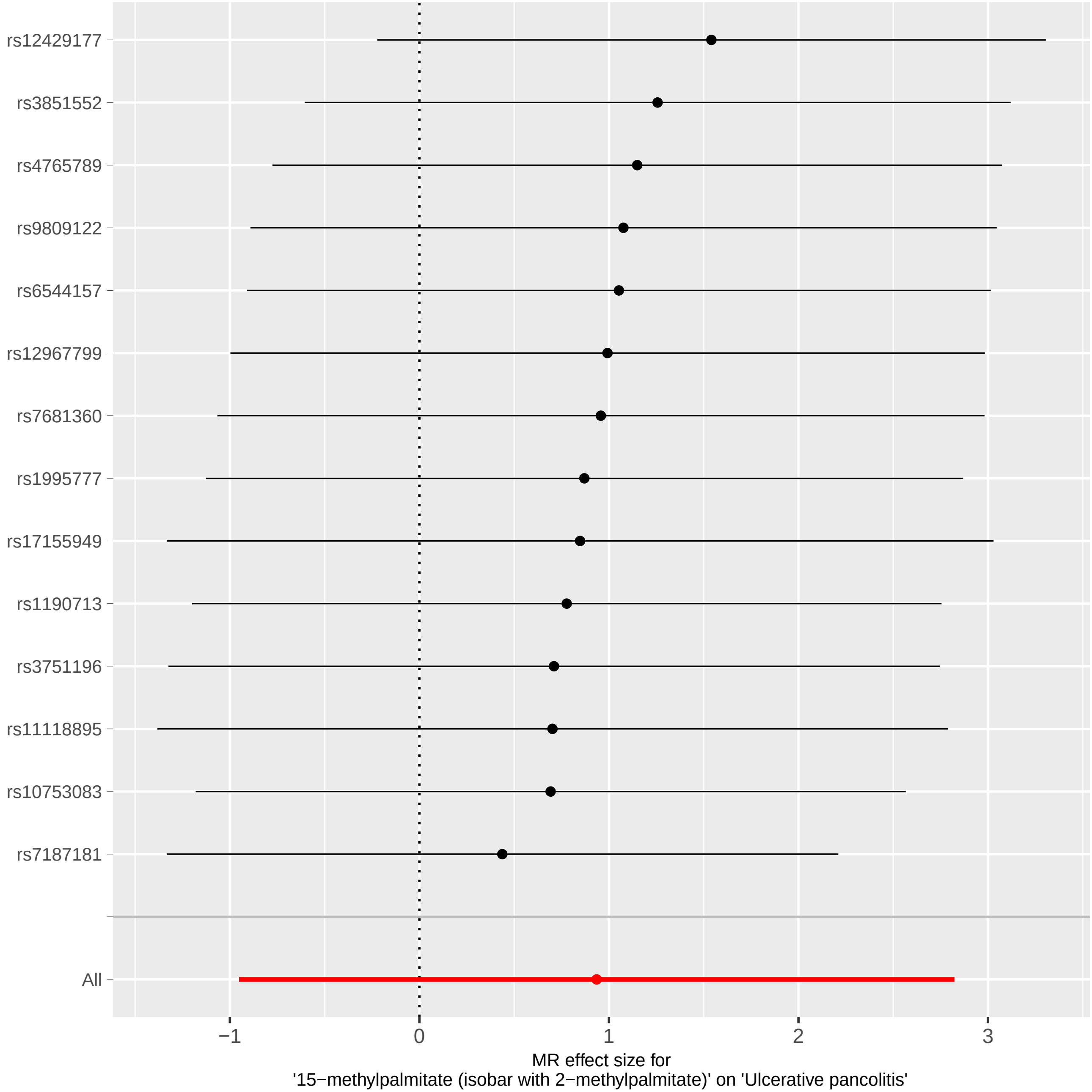

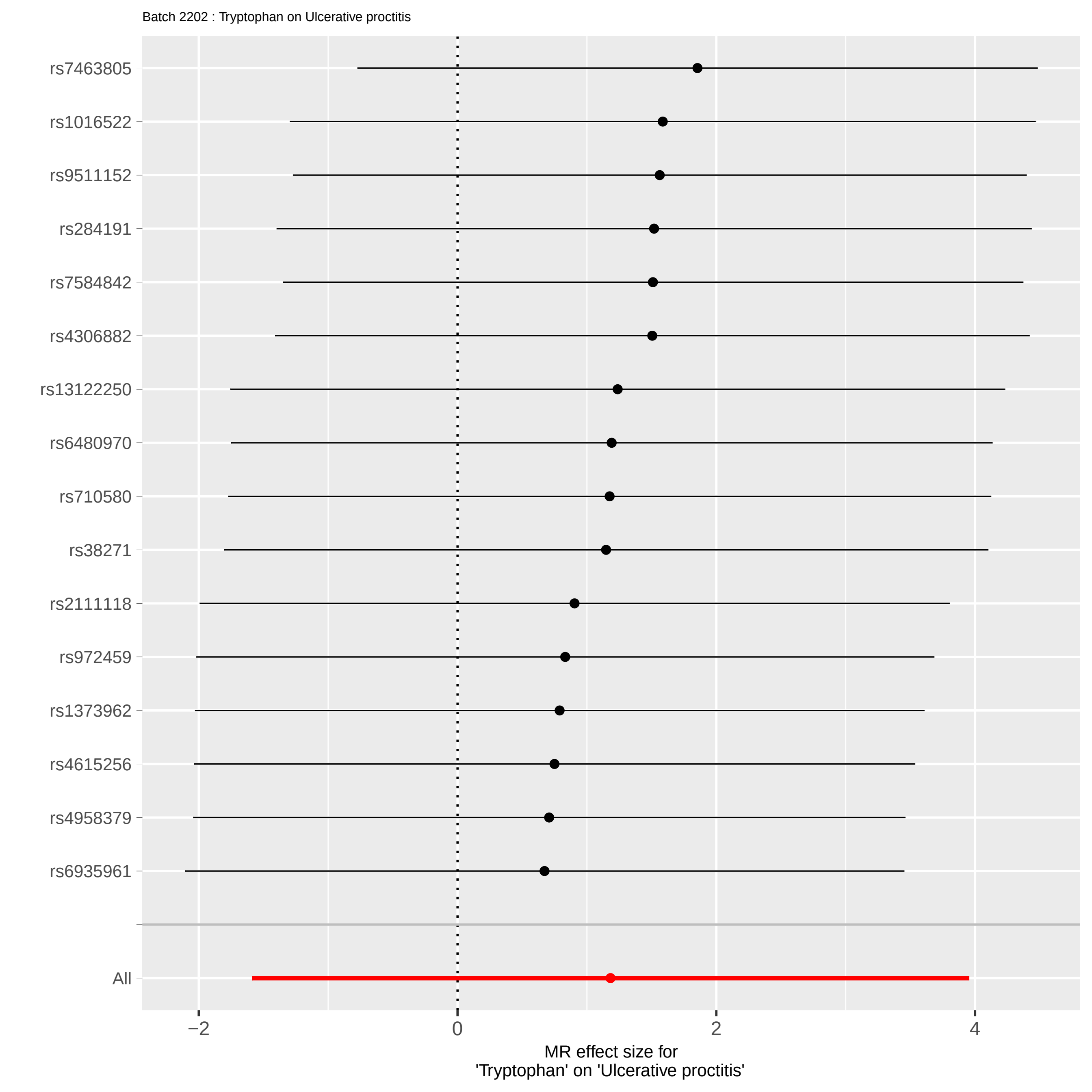
Batch 2202 : Tryptophan on Ulcerative proctitis
rs7463805
rs1016522
rs9511152
rs284191
rs7584842
rs4306882
rs13122250
rs6480970
rs710580
rs38271
rs2111118
rs972459
rs1373962
rs4615256
rs4958379
rs6935961
All
−2
0
2
4
MR effect size for
'Tryptophan' on 'Ulcerative proctitis'

Batch 2204 : Leucine on Ulcerative proctitis

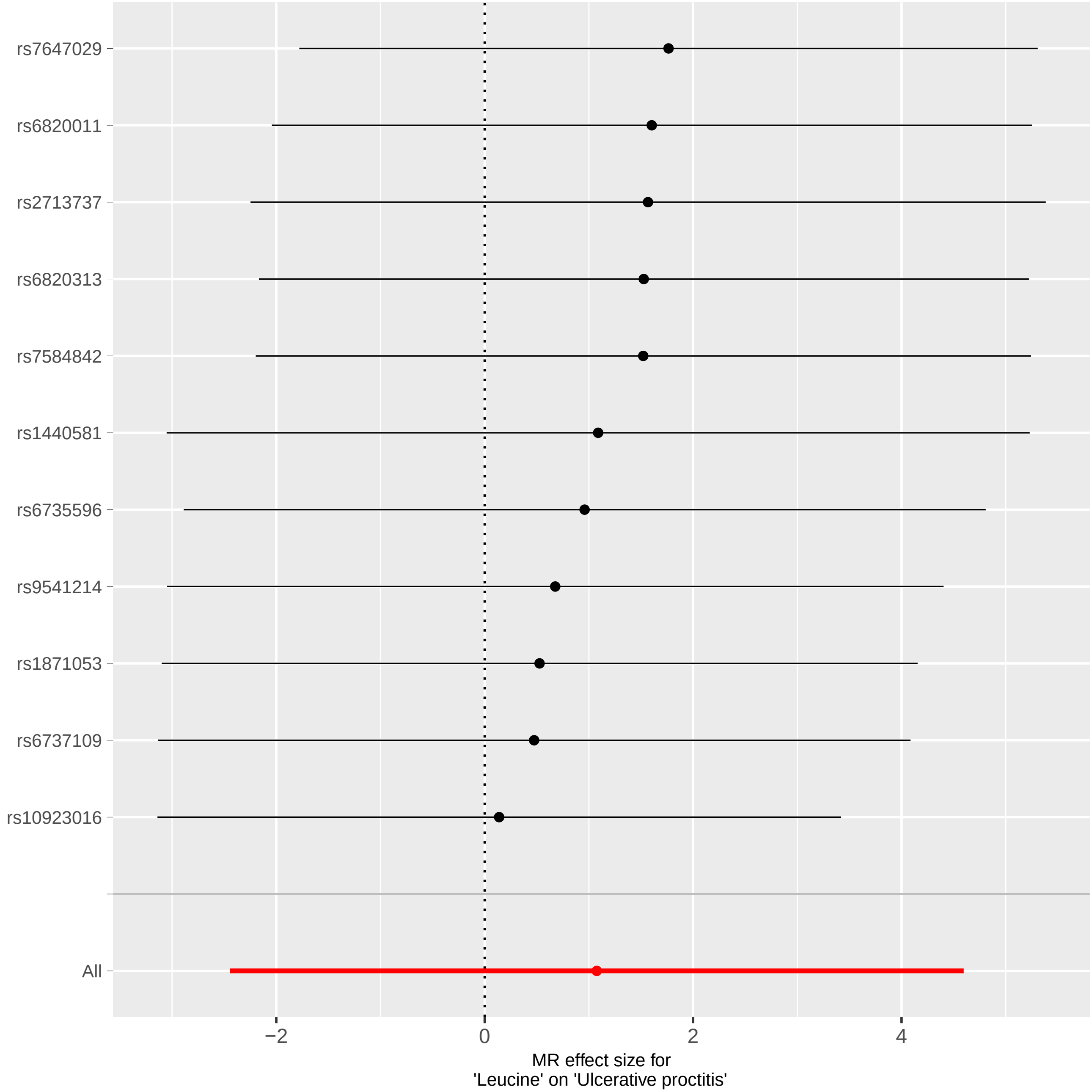

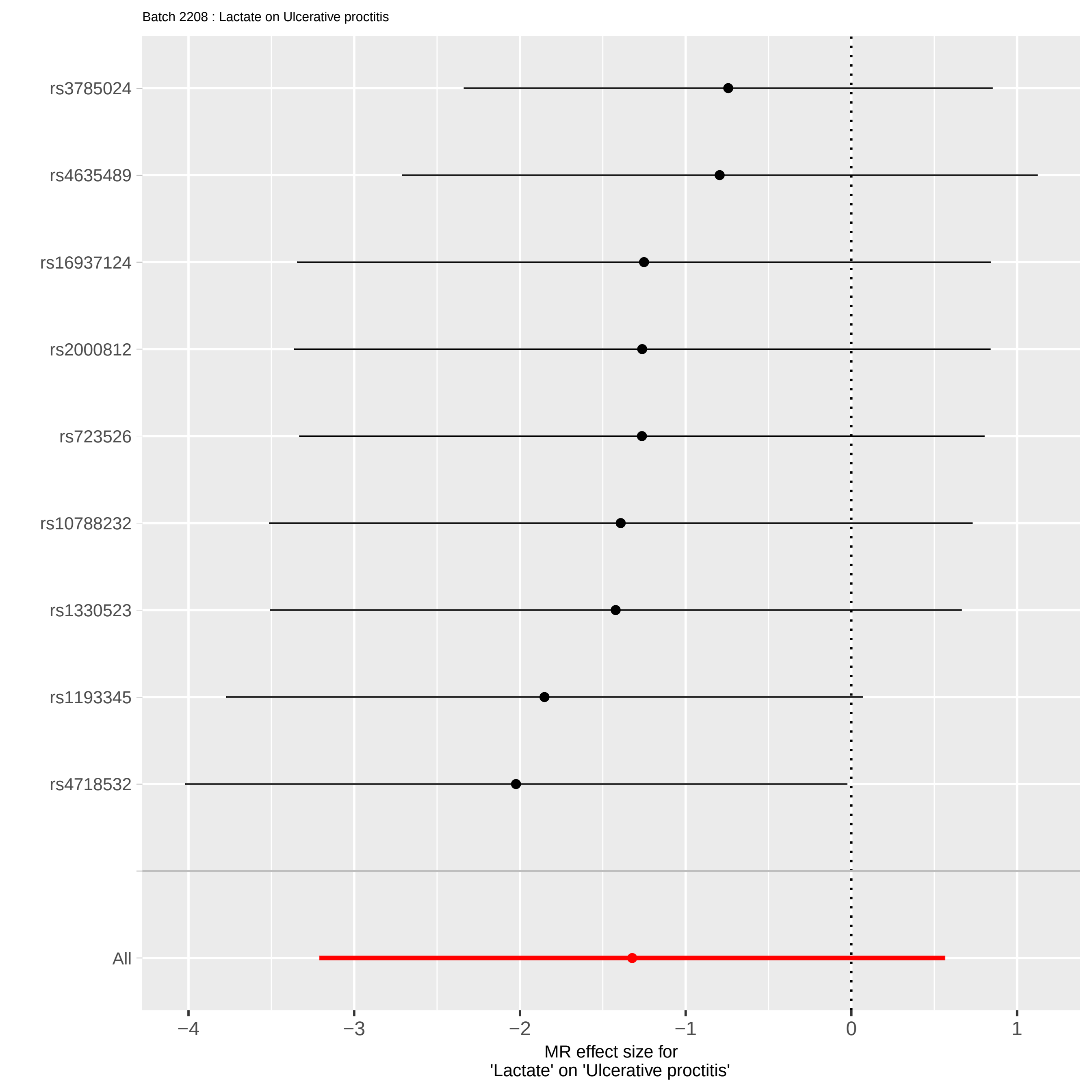
Batch 2208 : Lactate on Ulcerative proctitis
rs3785024
rs4635489
rs16937124
rs2000812
rs723526
rs10788232
rs1330523
rs1193345
rs4718532
All
−4
−3
−2
−1
0
1
MR effect size for
'Lactate' on 'Ulcerative proctitis'

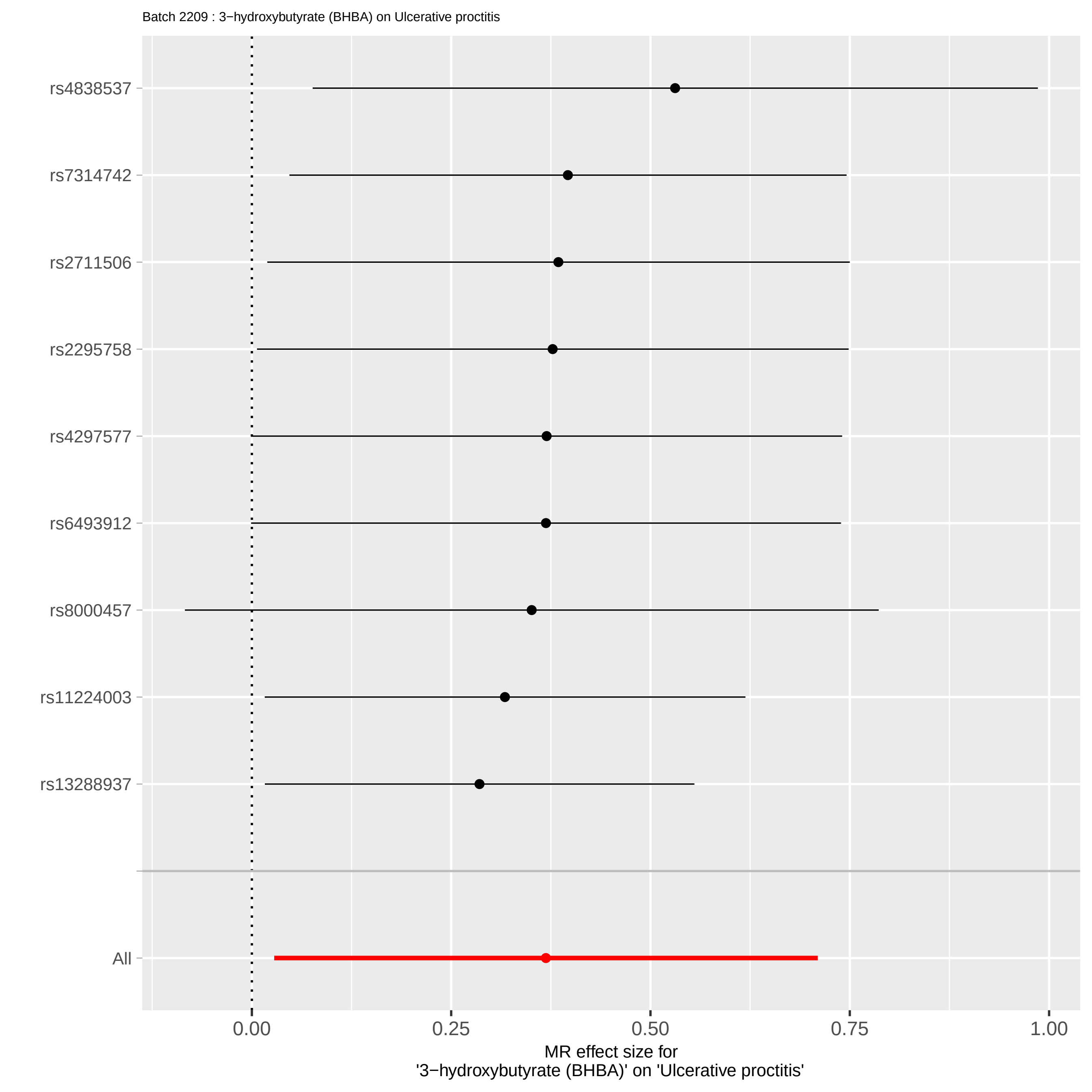
Batch 2209 : 3−hydroxybutyrate (BHBA) on Ulcerative proctitis
rs4838537
rs7314742
rs2711506
rs2295758
rs4297577
rs6493912
rs8000457
rs11224003
rs13288937
All
0.00
0.25
0.50
0.75
1.00
MR effect size for
'3−hydroxybutyrate (BHBA)' on 'Ulcerative proctitis'

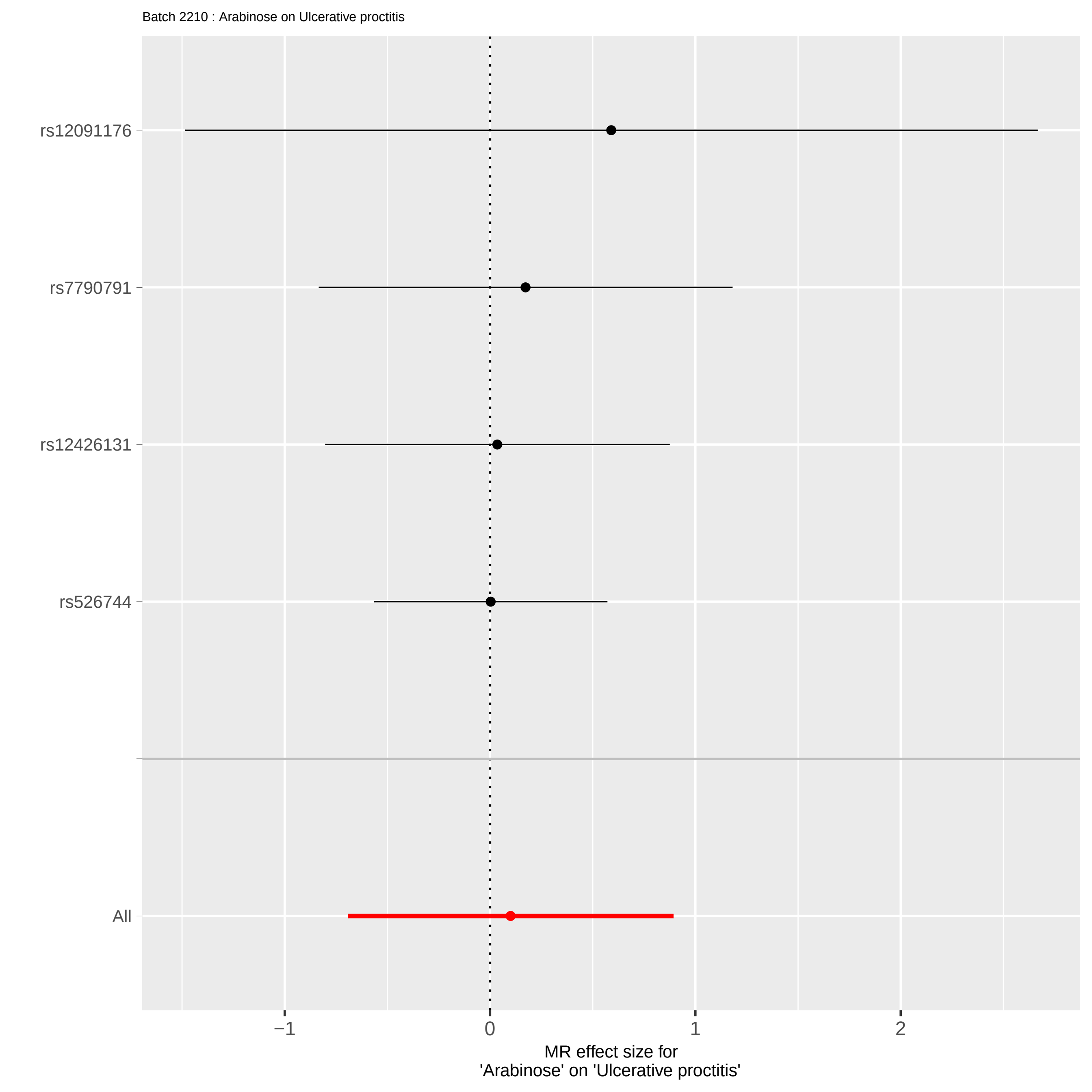

Batch 2210 : Arabinose on Ulcerative proctitis
rs12091176
rs7790791
rs12426131
rs526744
All
−1
0
1
2
MR effect size for
'Arabinose' on 'Ulcerative proctitis'

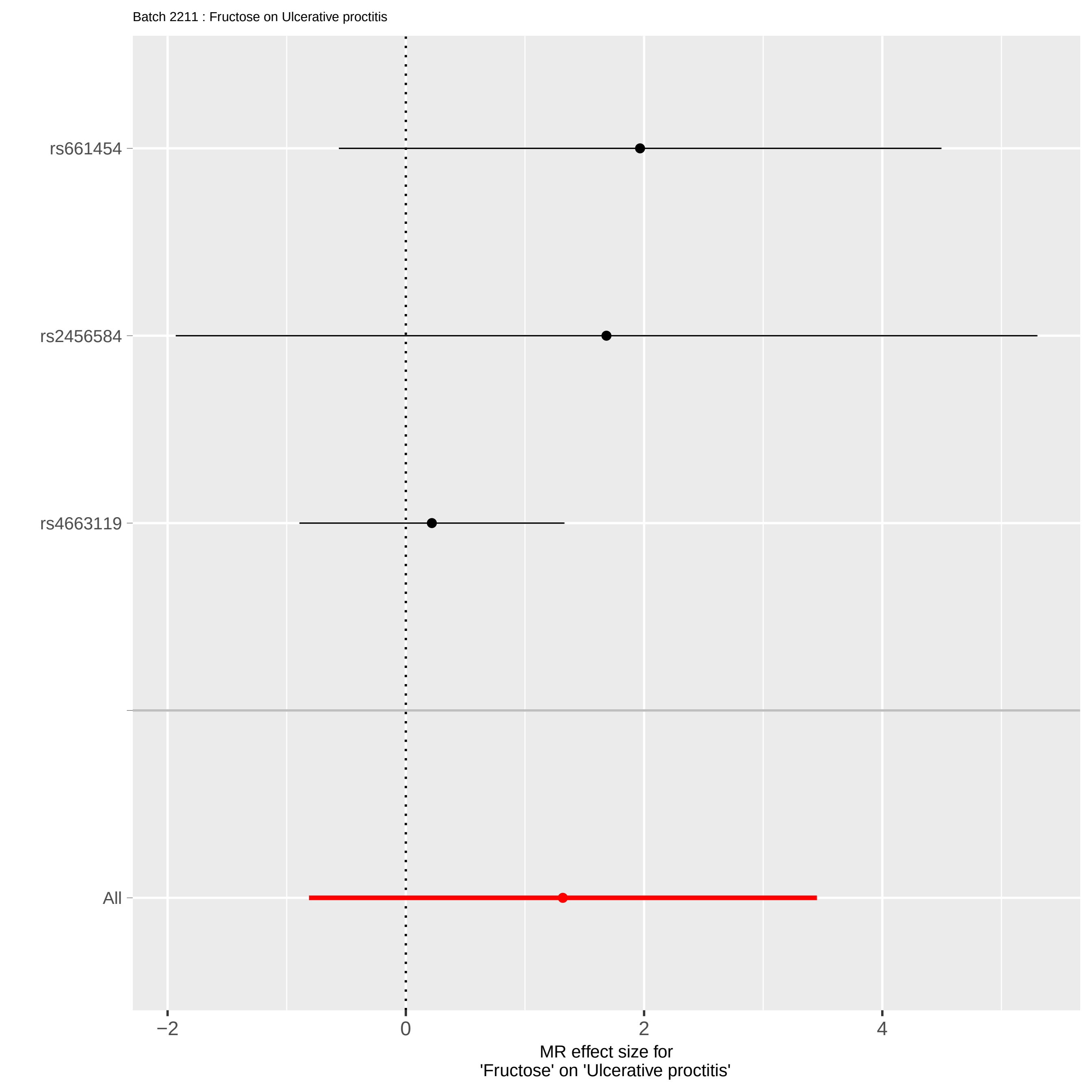

Batch 2211 : Fructose on Ulcerative proctitis
rs661454
rs2456584
rs4663119
All
−2
0
2
4
MR effect size for
'Fructose' on 'Ulcerative proctitis'

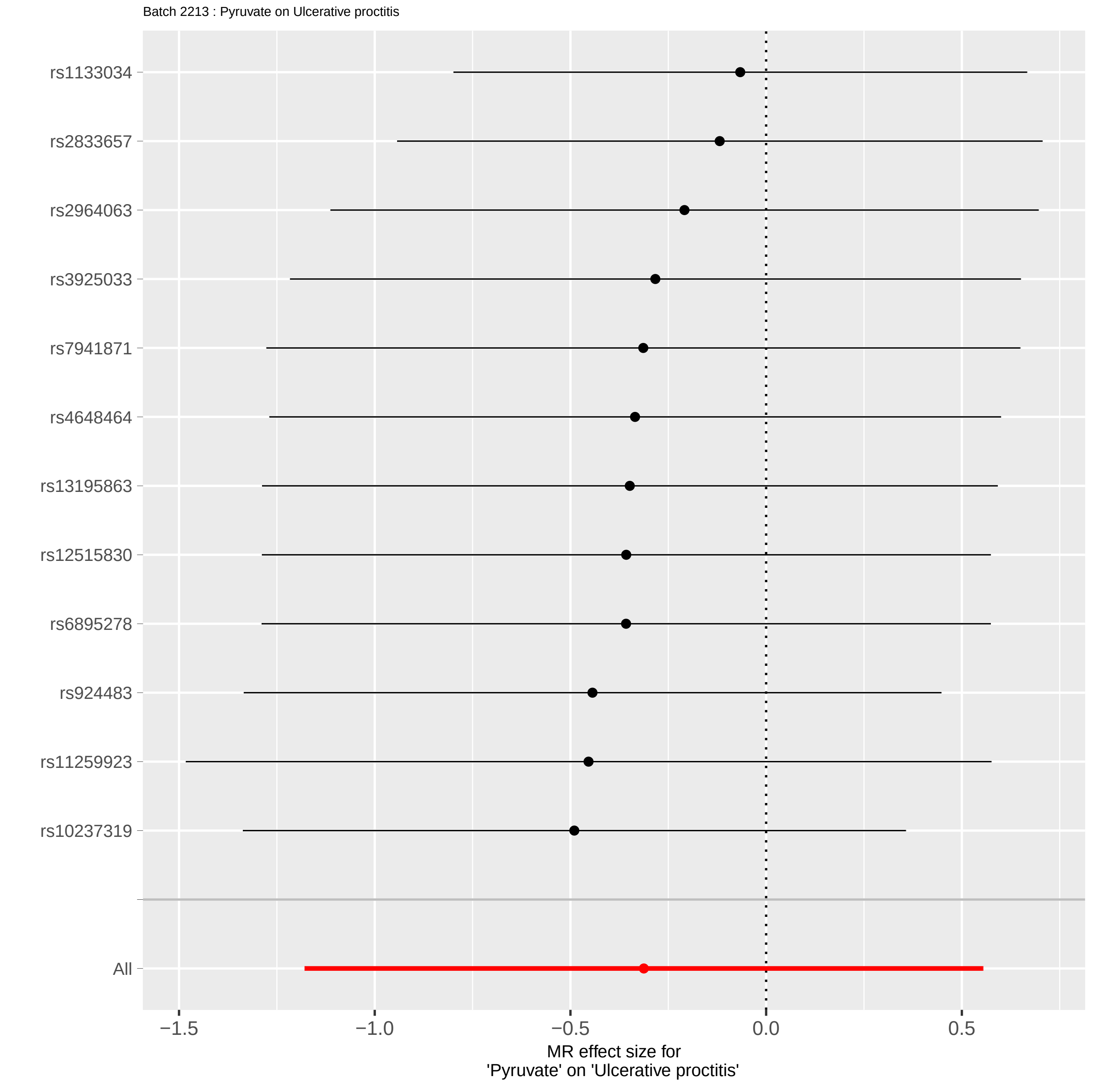
Batch 2213 : Pyruvate on Ulcerative proctitis
rs1133034
rs2833657
rs2964063
rs3925033
rs7941871
rs4648464
rs13195863
rs12515830
rs6895278
rs924483
rs11259923
rs10237319
All
−1.5
−1.0
−0.5
0.0
0.5
MR effect size for
'Pyruvate' on 'Ulcerative proctitis'

Batch 2214 : Uridine on Ulcerative proctitis

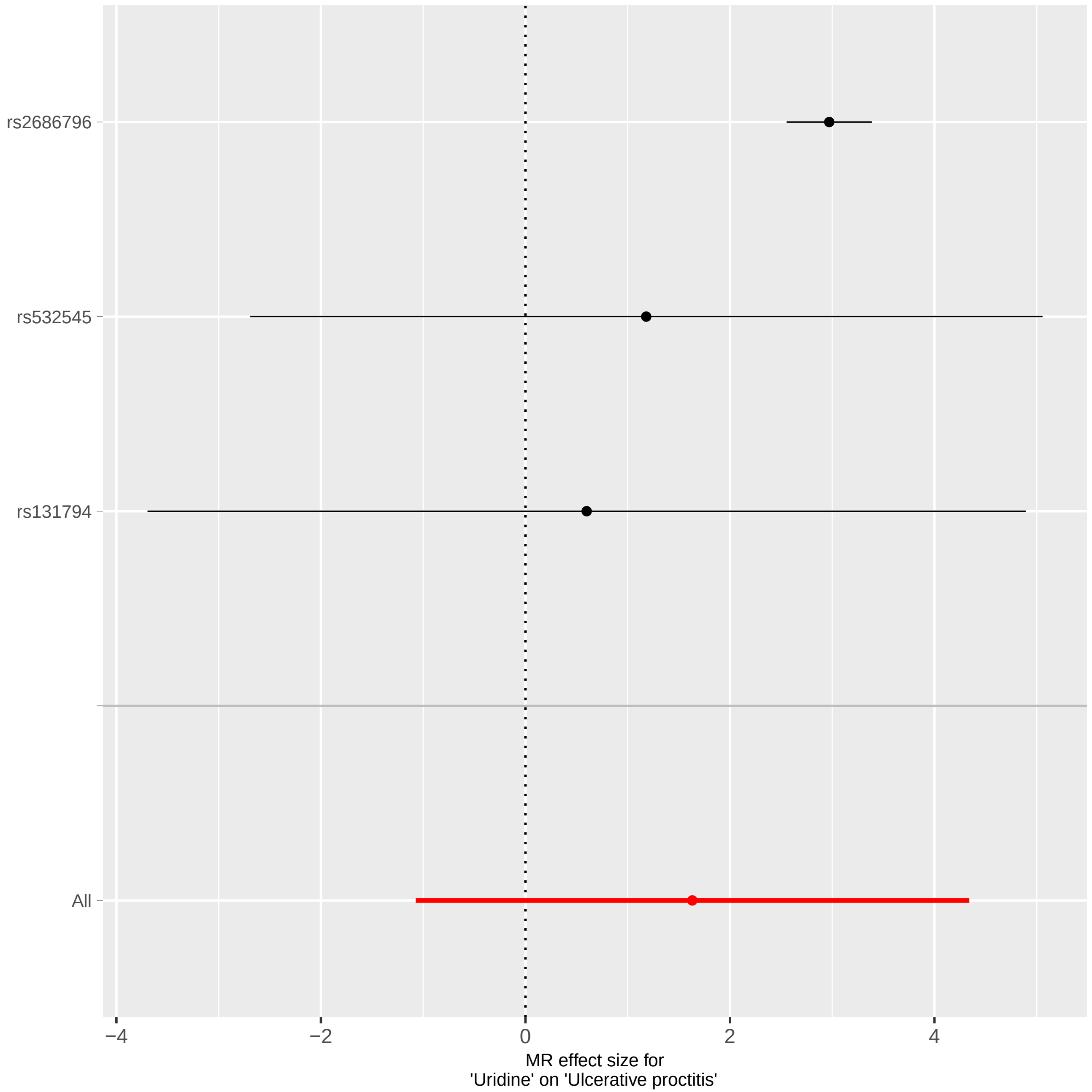

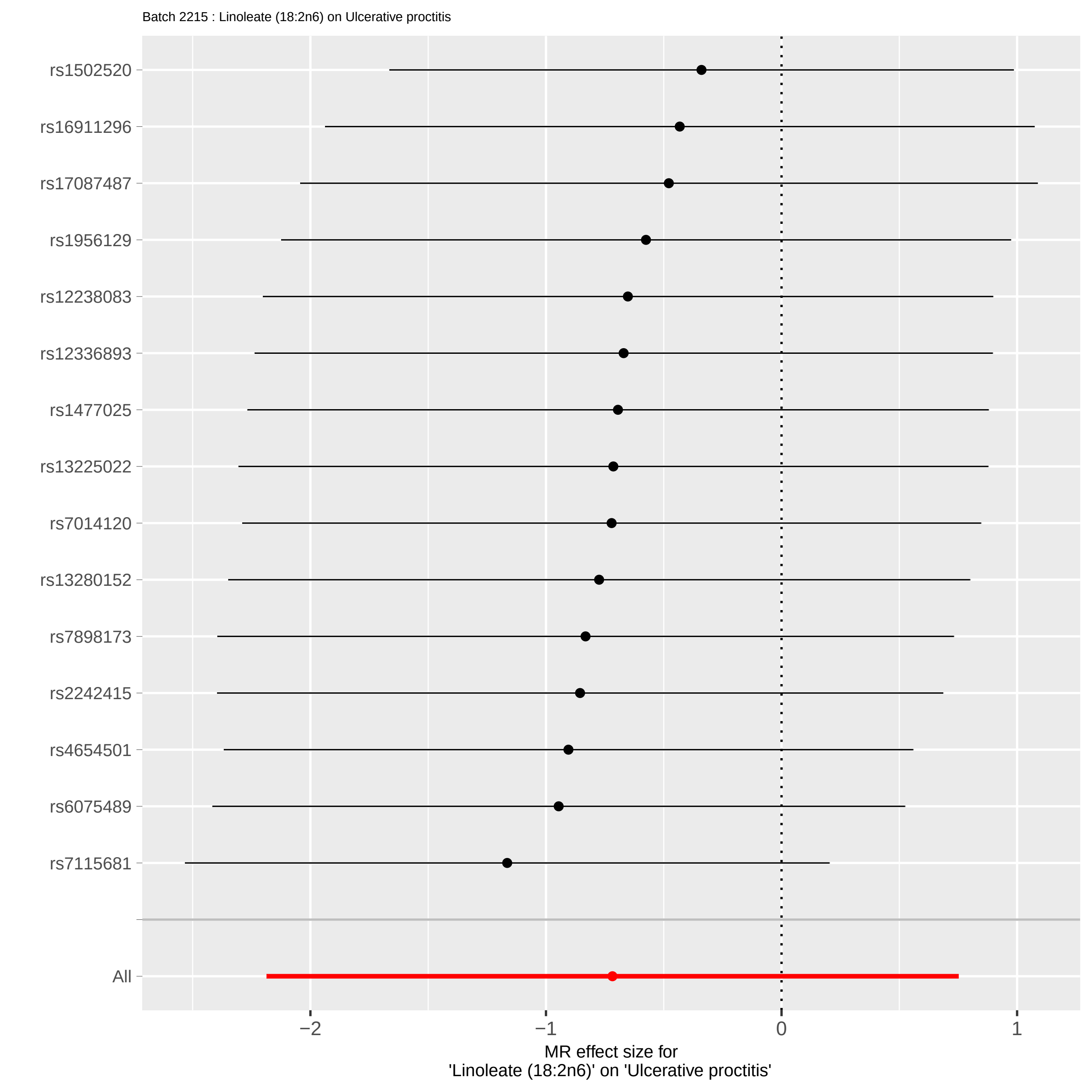
Batch 2215 : Linoleate (18:2n6) on Ulcerative proctitis
rs1502520
rs16911296
rs17087487
rs1956129
rs12238083
rs12336893
rs1477025
rs13225022
rs7014120
rs13280152
rs7898173
rs2242415
rs4654501
rs6075489
rs7115681
All
−2
−1
0
1
MR effect size for
'Linoleate (18:2n6)' on 'Ulcerative proctitis'

Batch 2216 : Allantoin on Ulcerative proctitis

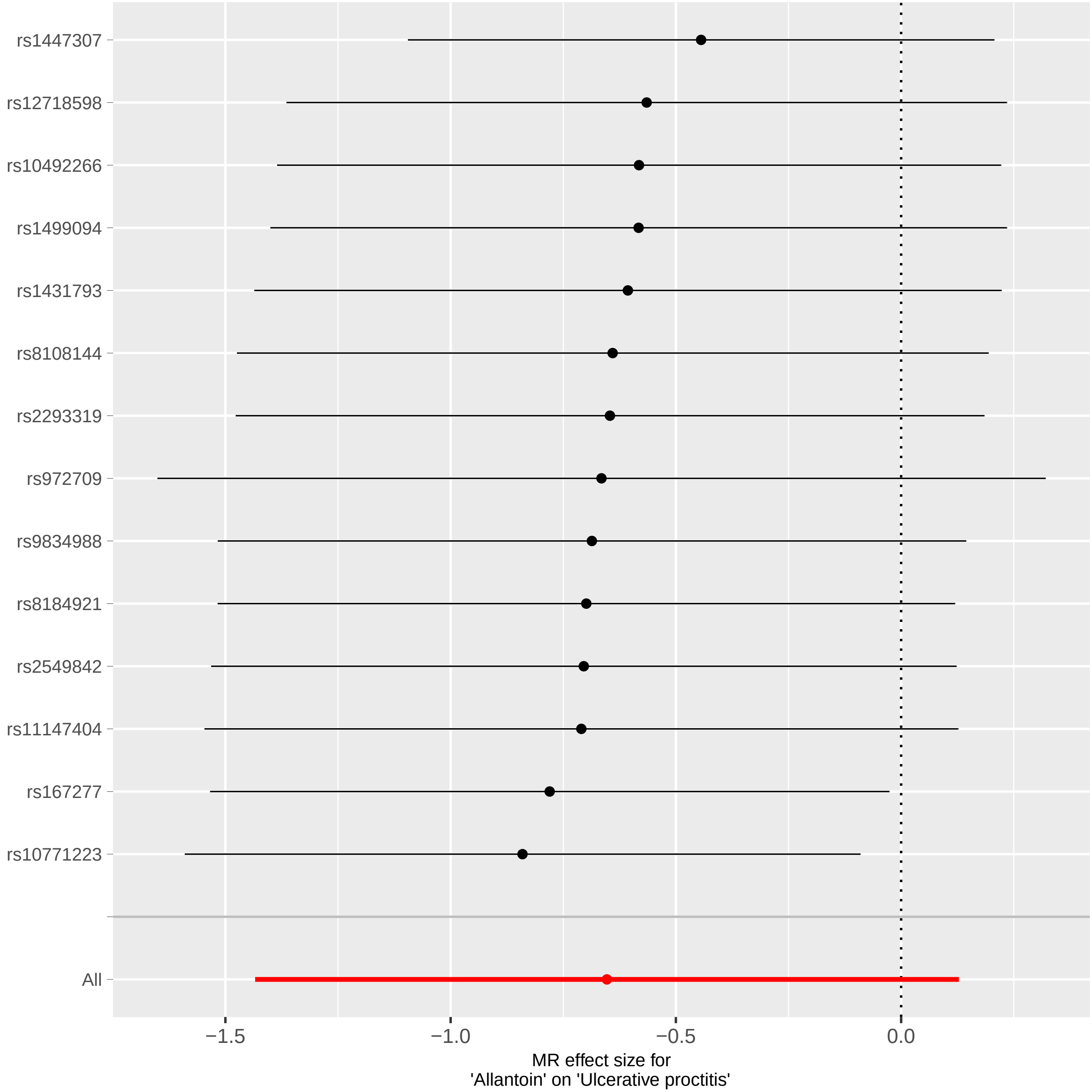

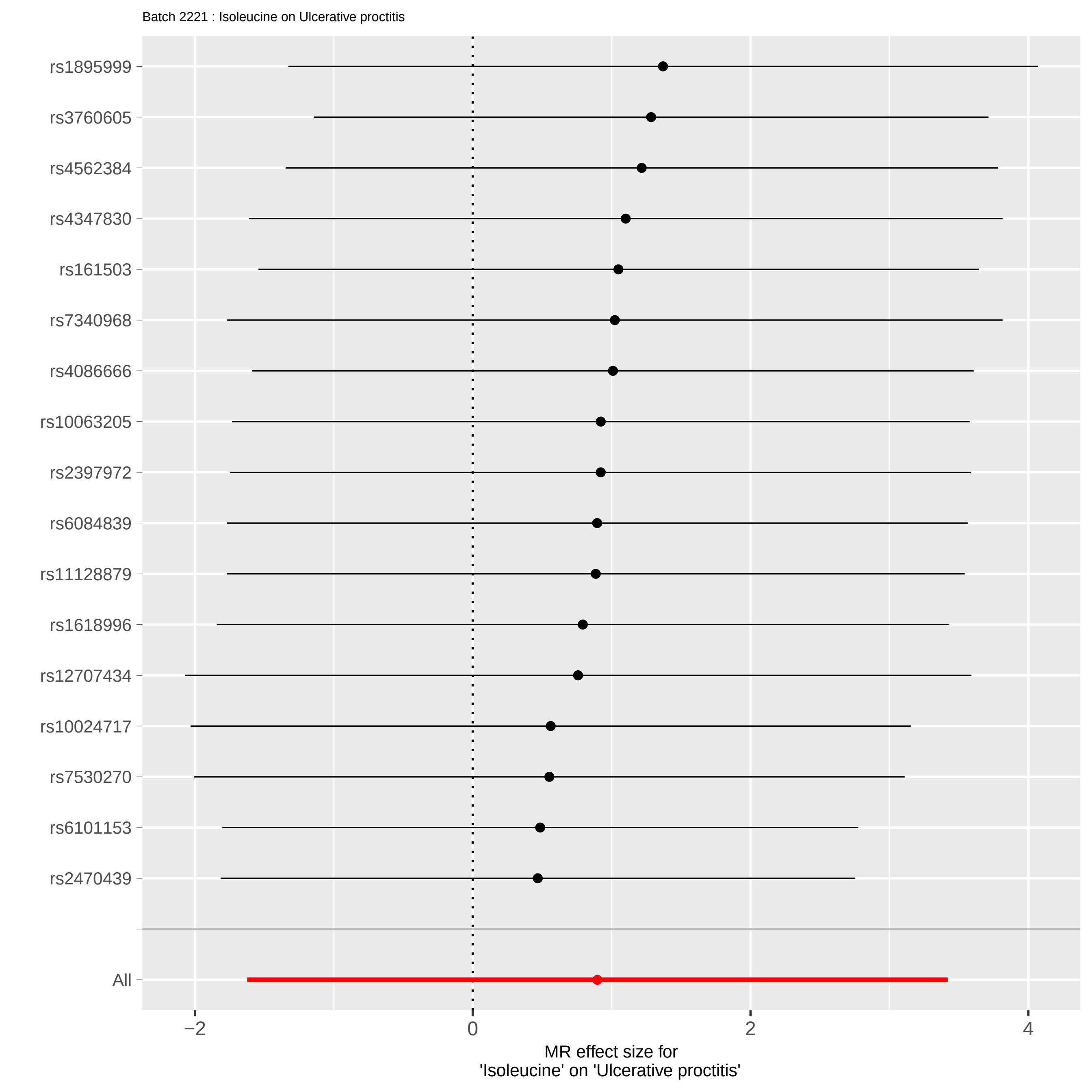
Batch 2221 : Isoleucine on Ulcerative proctitis
rs1895999
rs3760605
rs4562384
rs4347830
rs161503
rs7340968
rs4086666
rs10063205
rs2397972
rs6084839
rs11128879
rs1618996
rs12707434
rs10024717
rs7530270
rs6101153
rs2470439
All
−2
0
2
4
MR effect size for
'Isoleucine' on 'Ulcerative proctitis'

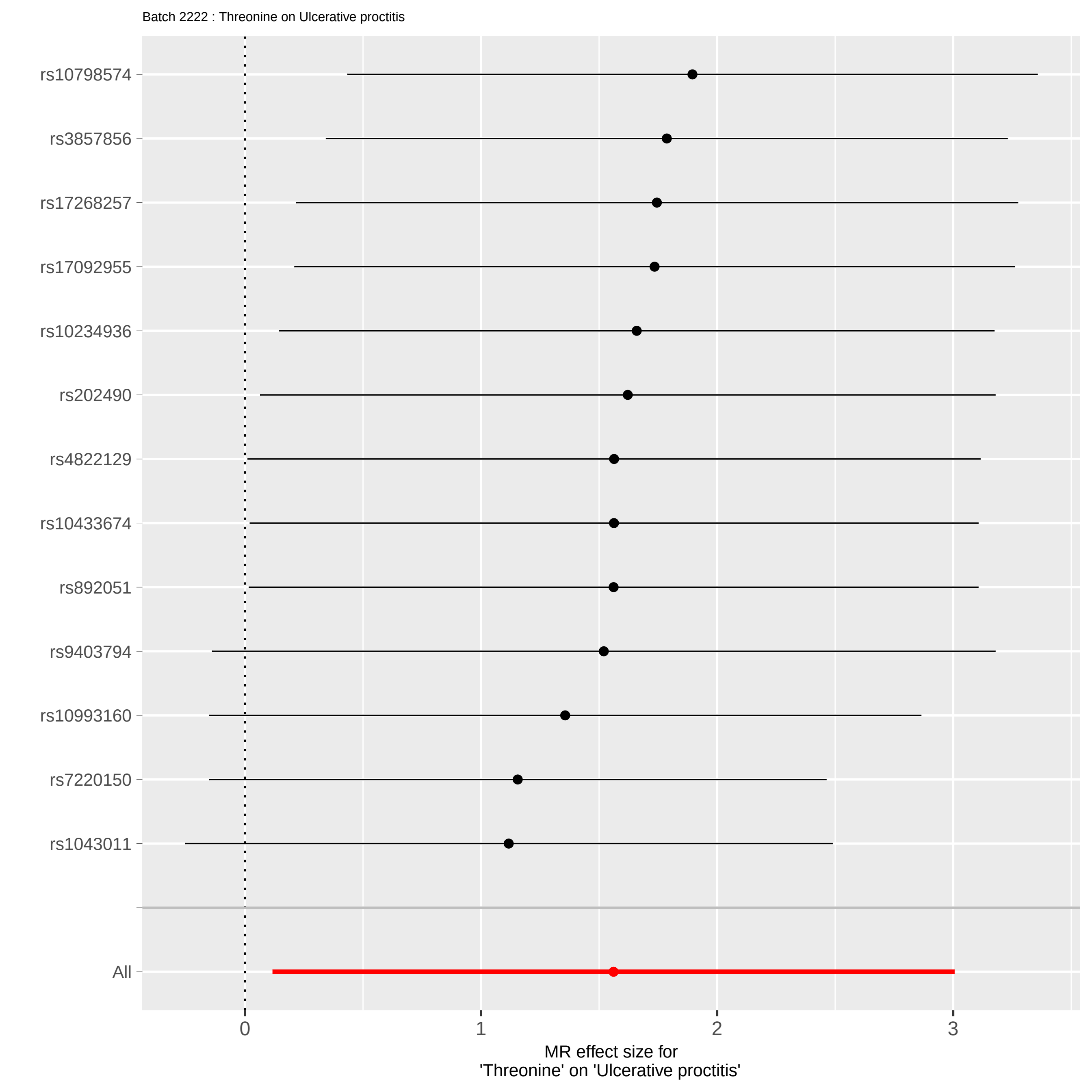

Batch 2222 : Threonine on Ulcerative proctitis
rs10798574
rs3857856
rs17268257
rs17092955
rs10234936
rs202490
rs4822129
rs10433674
rs892051
rs9403794
rs10993160
rs7220150
rs1043011
All
0
1
2
3
MR effect size for
'Threonine' on 'Ulcerative proctitis'

Batch 2223 : Tyrosine on Ulcerative proctitis

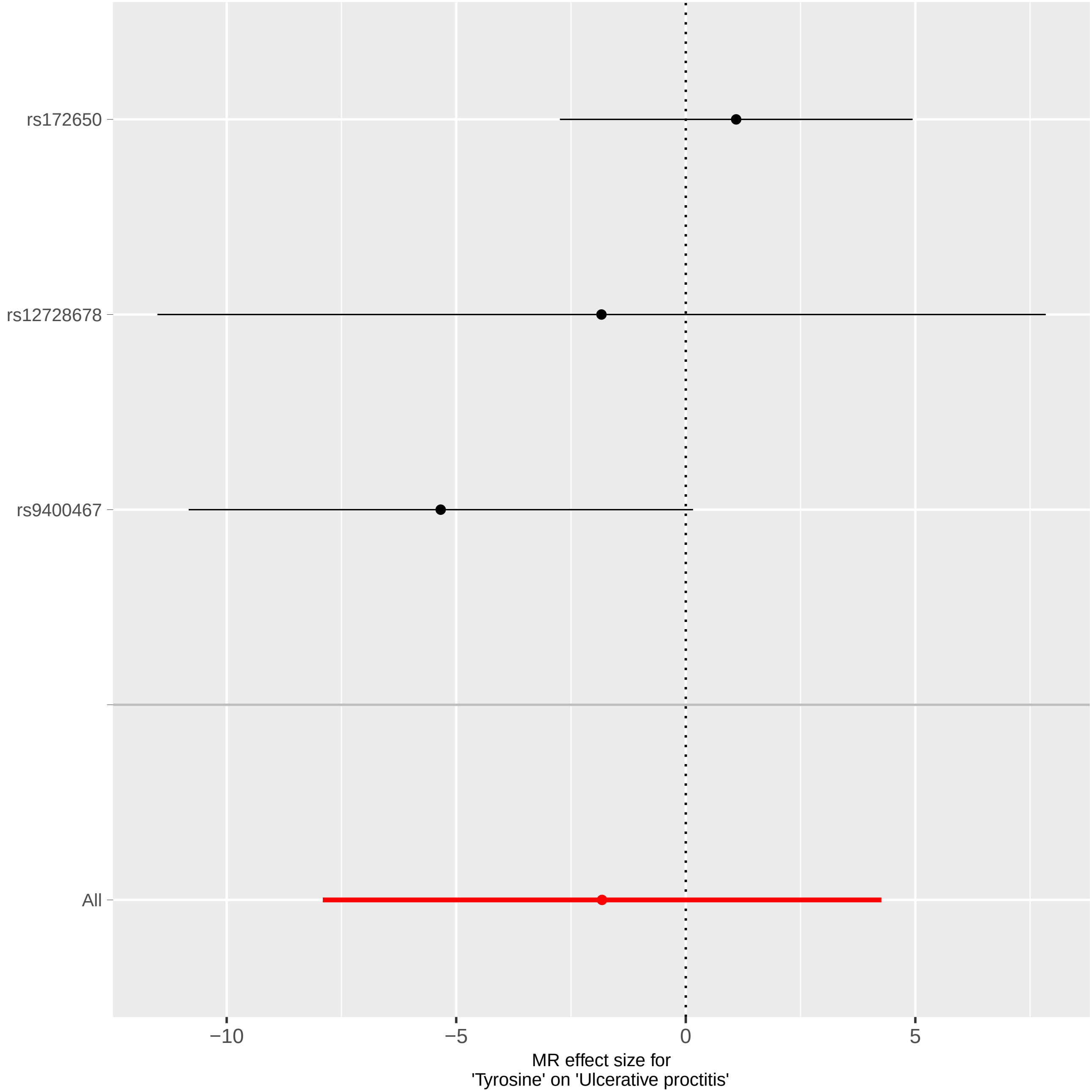

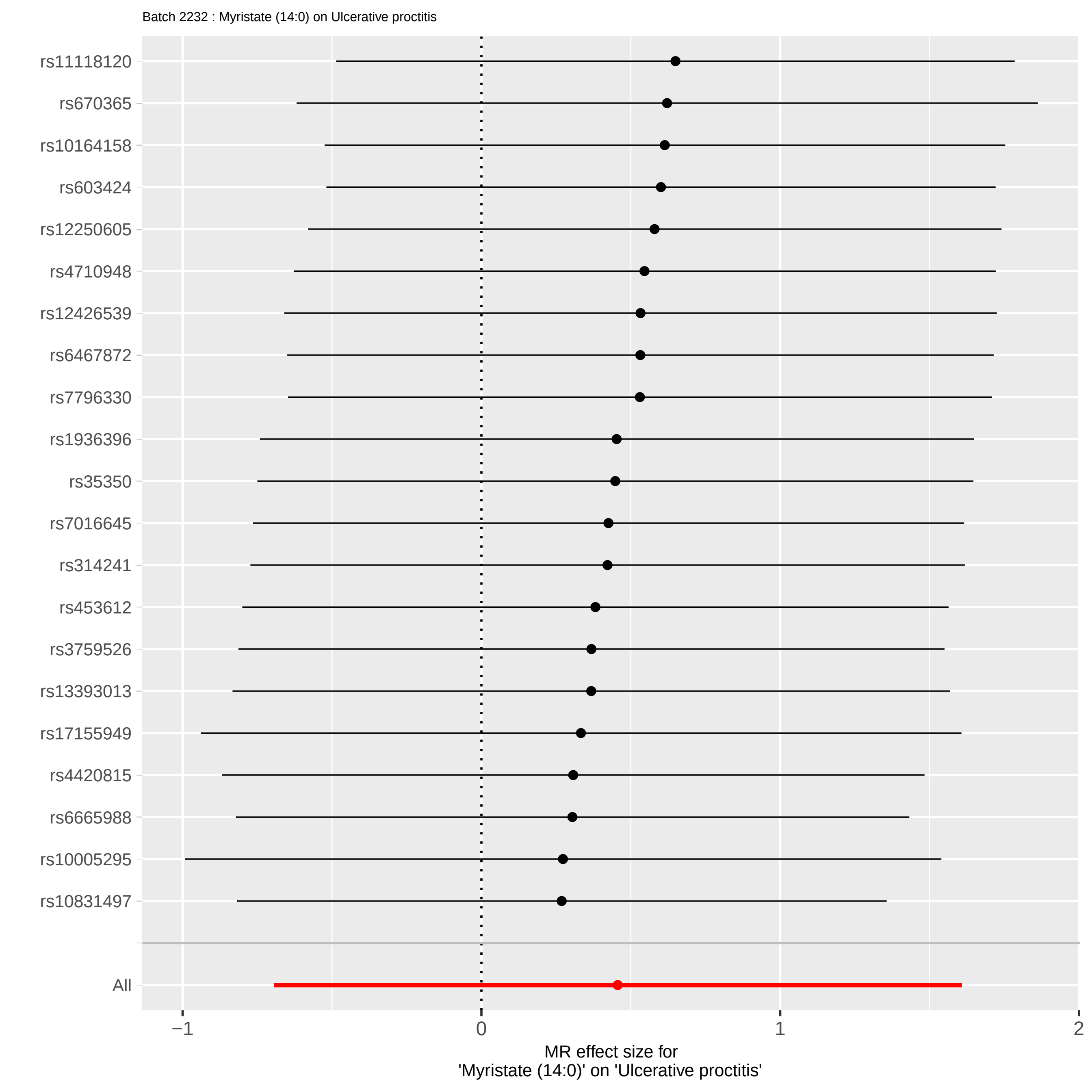

Batch 2232 : Myristate (14:0) on Ulcerative proctitis
rs11118120
rs670365
rs10164158
rs603424
rs12250605
rs4710948
rs12426539
rs6467872
rs7796330
rs1936396
rs35350
rs7016645
rs314241
rs453612
rs3759526
rs13393013
rs17155949
rs4420815
rs6665988
rs10005295
rs10831497
All
−1
0
1
2
MR effect size for
'Myristate (14:0)' on 'Ulcerative proctitis'

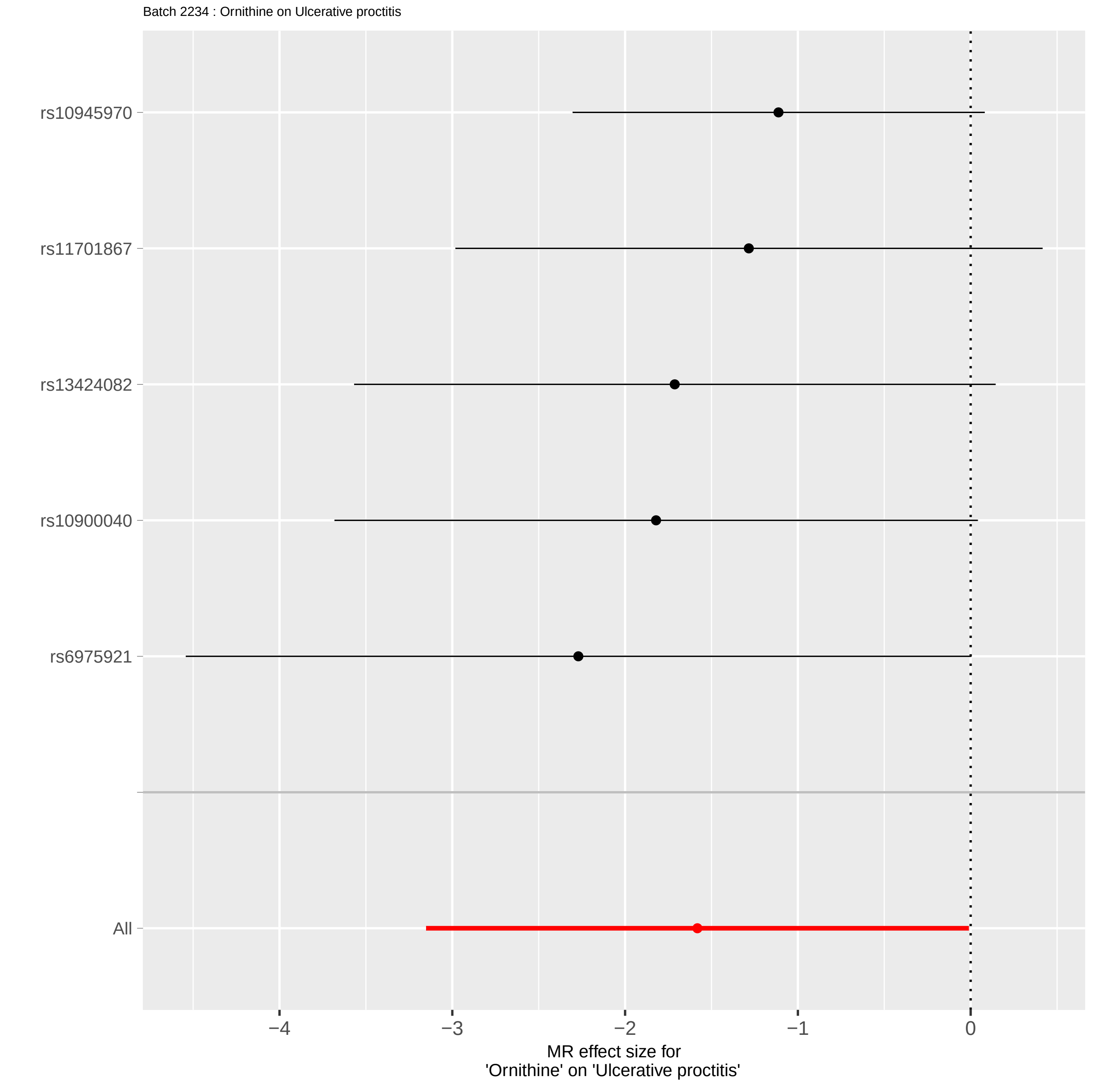

Batch 2234 : Ornithine on Ulcerative proctitis
rs10945970
rs11701867
rs13424082
rs10900040
rs6975921
All
−4
−3
−2
−1
0
MR effect size for
'Ornithine' on 'Ulcerative proctitis'

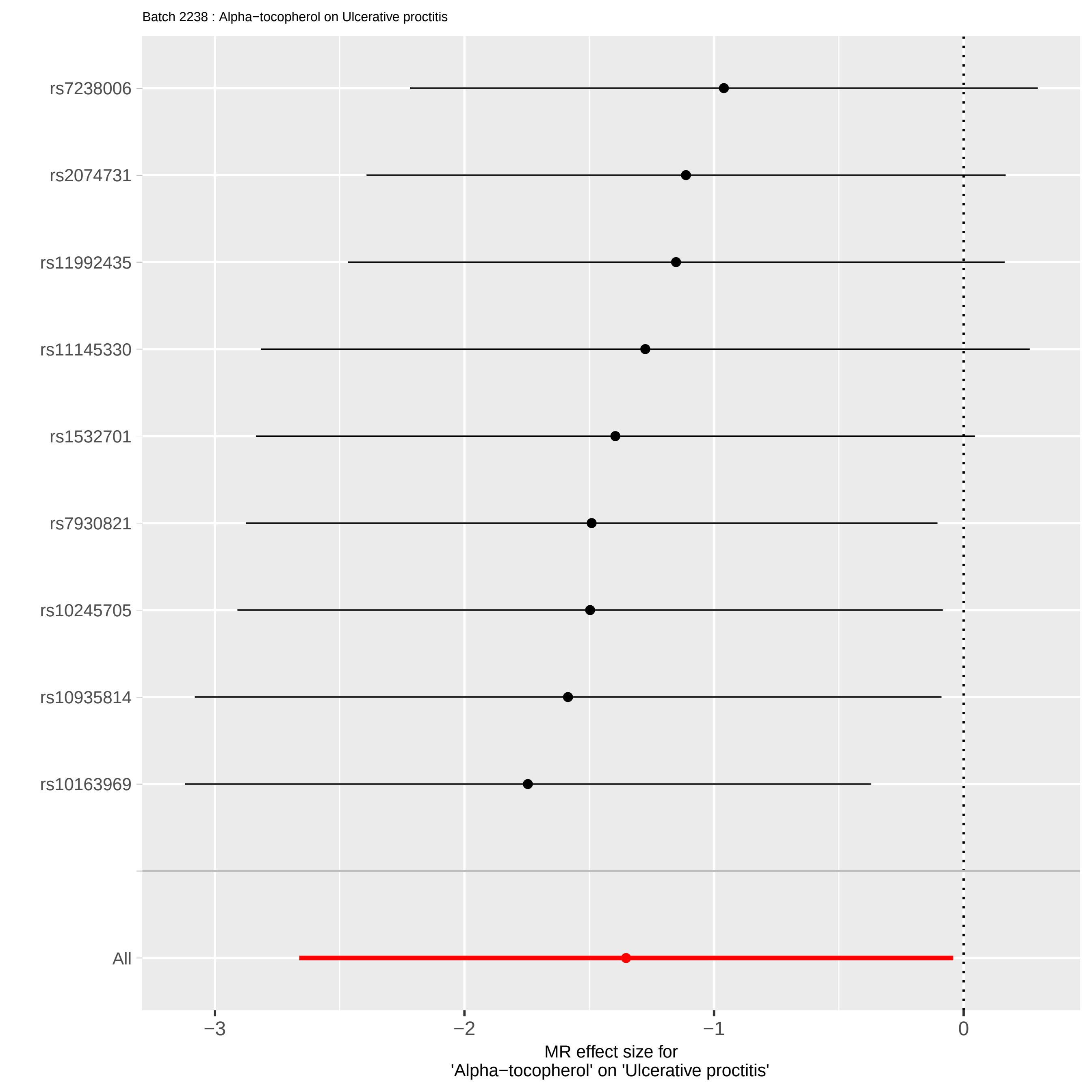
Batch 2238 : Alpha−tocopherol on Ulcerative proctitis
rs7238006
rs2074731
rs11992435
rs11145330
rs1532701
rs7930821
rs10245705
rs10935814
rs10163969
All
−3
−2
−1
0
MR effect size for
'Alpha−tocopherol' on 'Ulcerative proctitis'

Batch 2239 : Citrate on Ulcerative proctitis

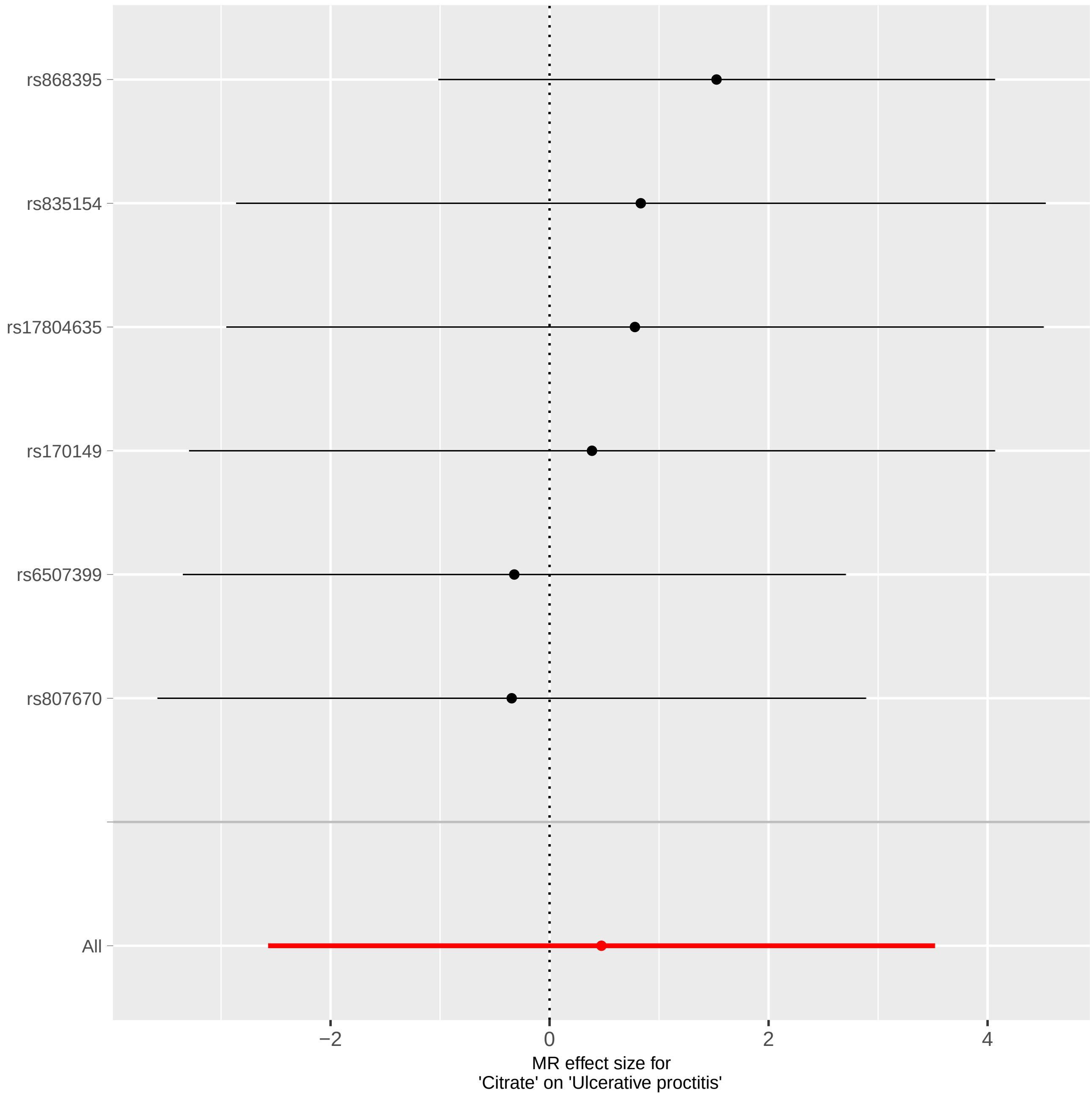

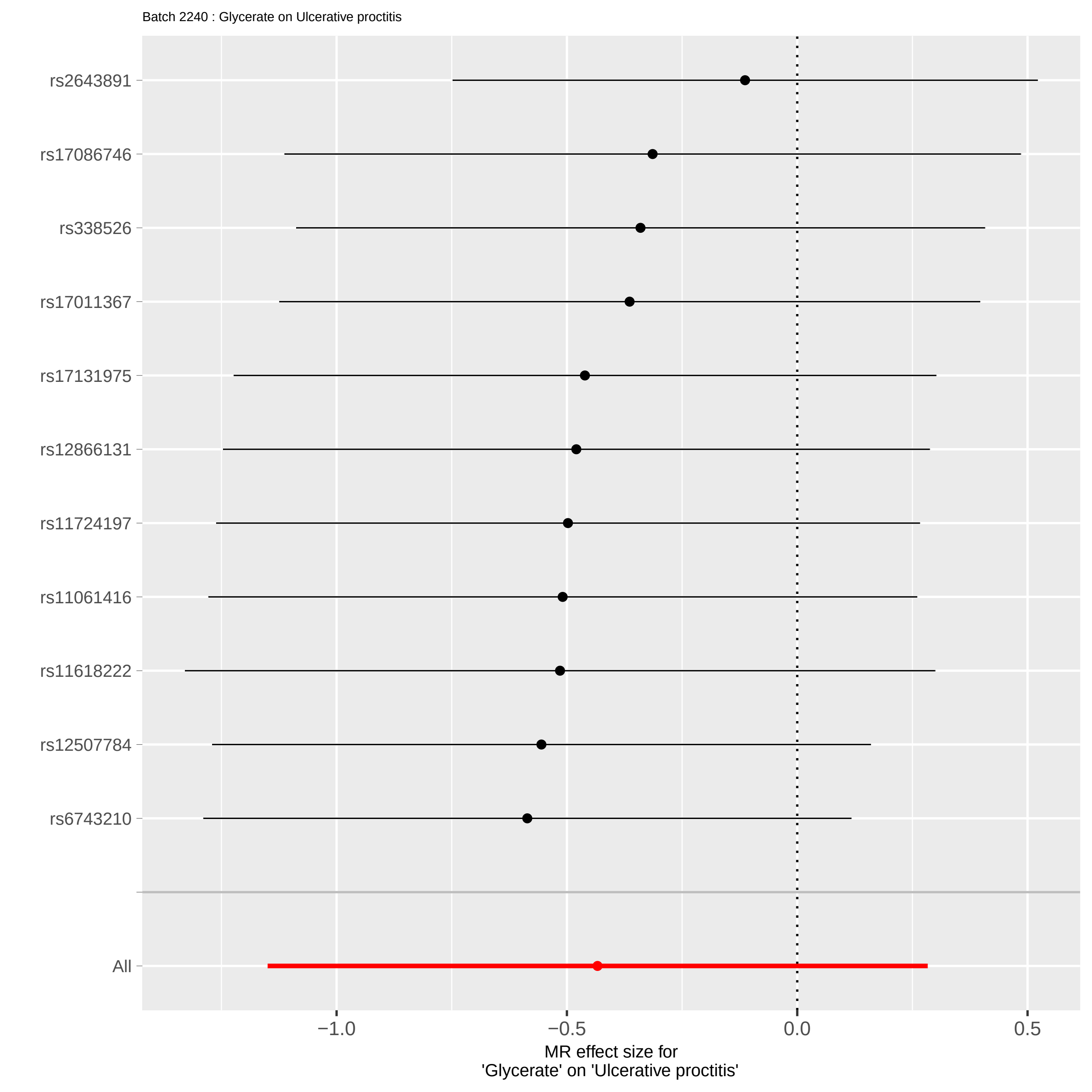
Batch 2240 : Glycerate on Ulcerative proctitis
rs2643891
rs17086746
rs338526
rs17011367
rs17131975
rs12866131
rs11724197
rs11061416
rs11618222
rs12507784
rs6743210
All
−1.0
−0.5
0.0
0.5
MR effect size for
'Glycerate' on 'Ulcerative proctitis'

Batch 2241 : Guanosine on Ulcerative proctitis

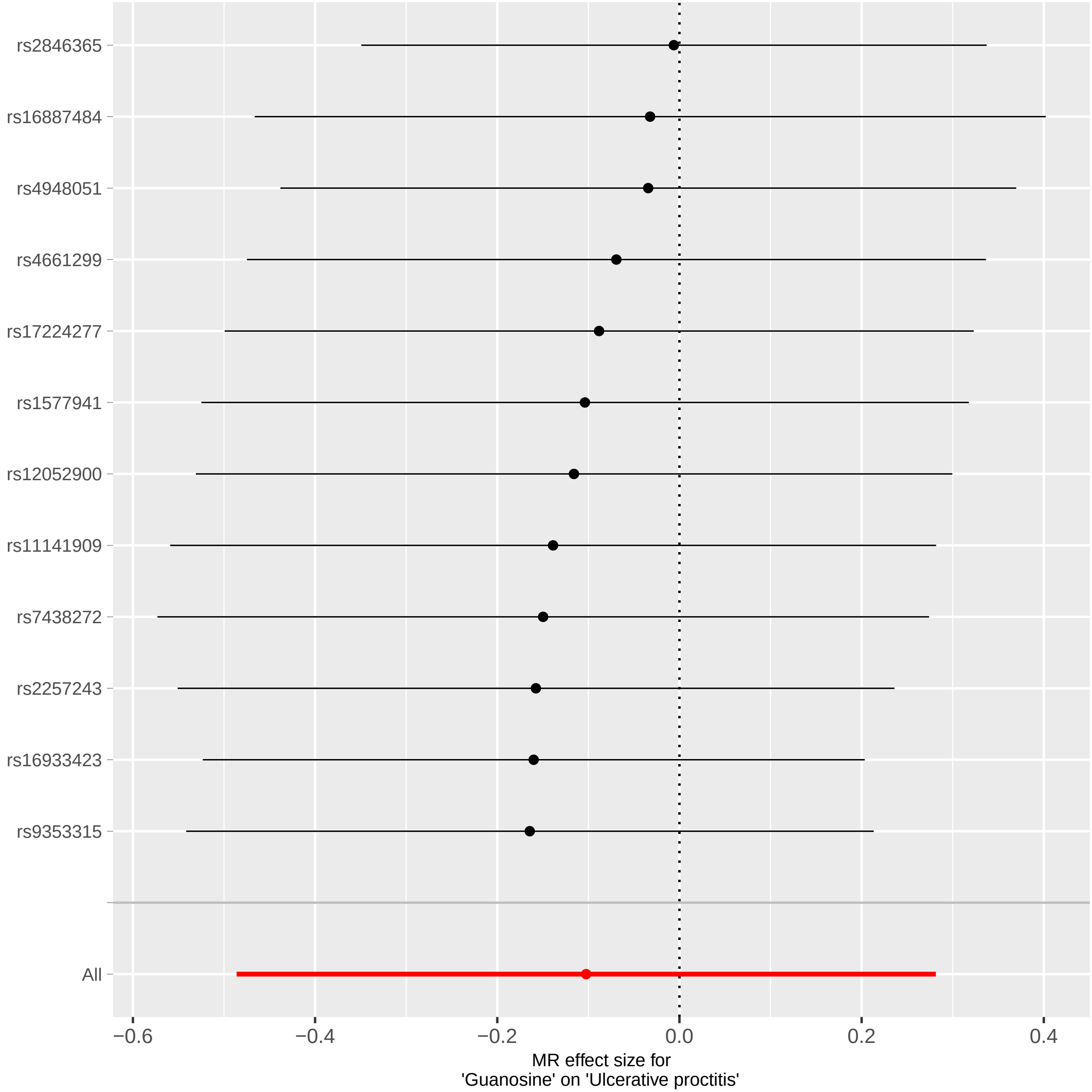

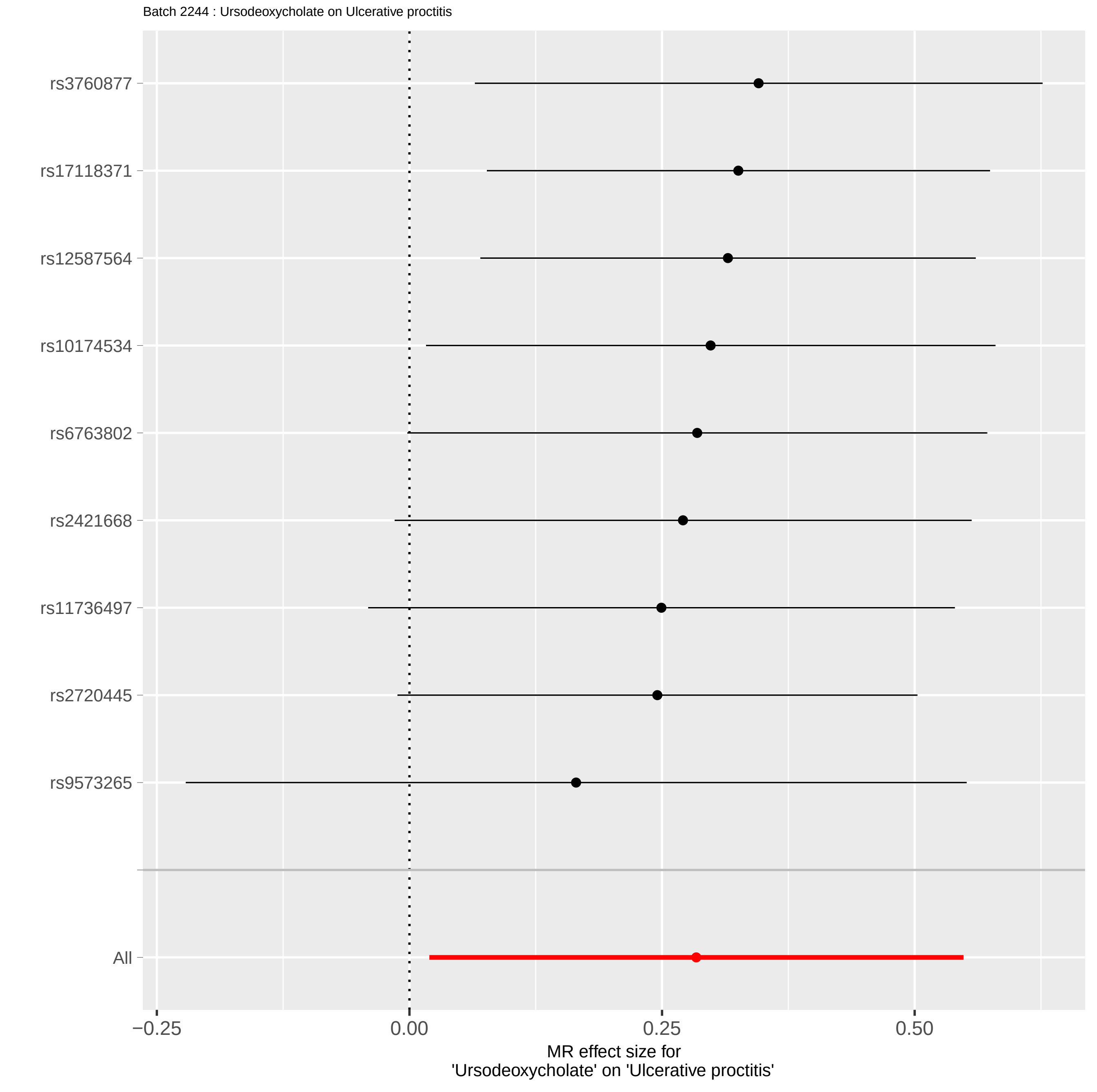
Batch 2244 : Ursodeoxycholate on Ulcerative proctitis
rs3760877
rs17118371
rs12587564
rs10174534
rs6763802
rs2421668
rs11736497
rs2720445
rs9573265
All
−0.25
0.00
0.25
0.50
MR effect size for
'Ursodeoxycholate' on 'Ulcerative proctitis'

Batch 2245 : Arginine on Ulcerative proctitis

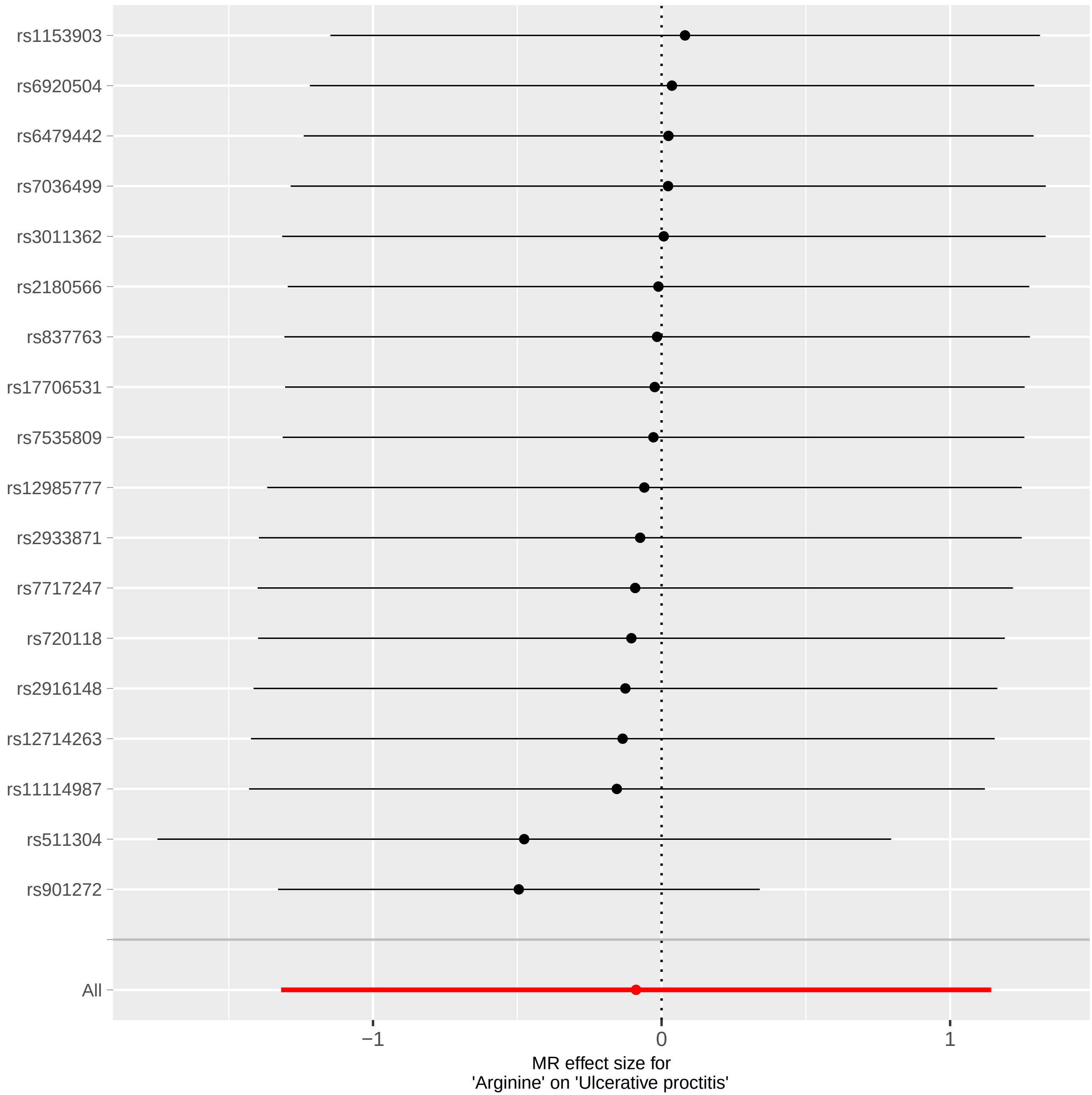

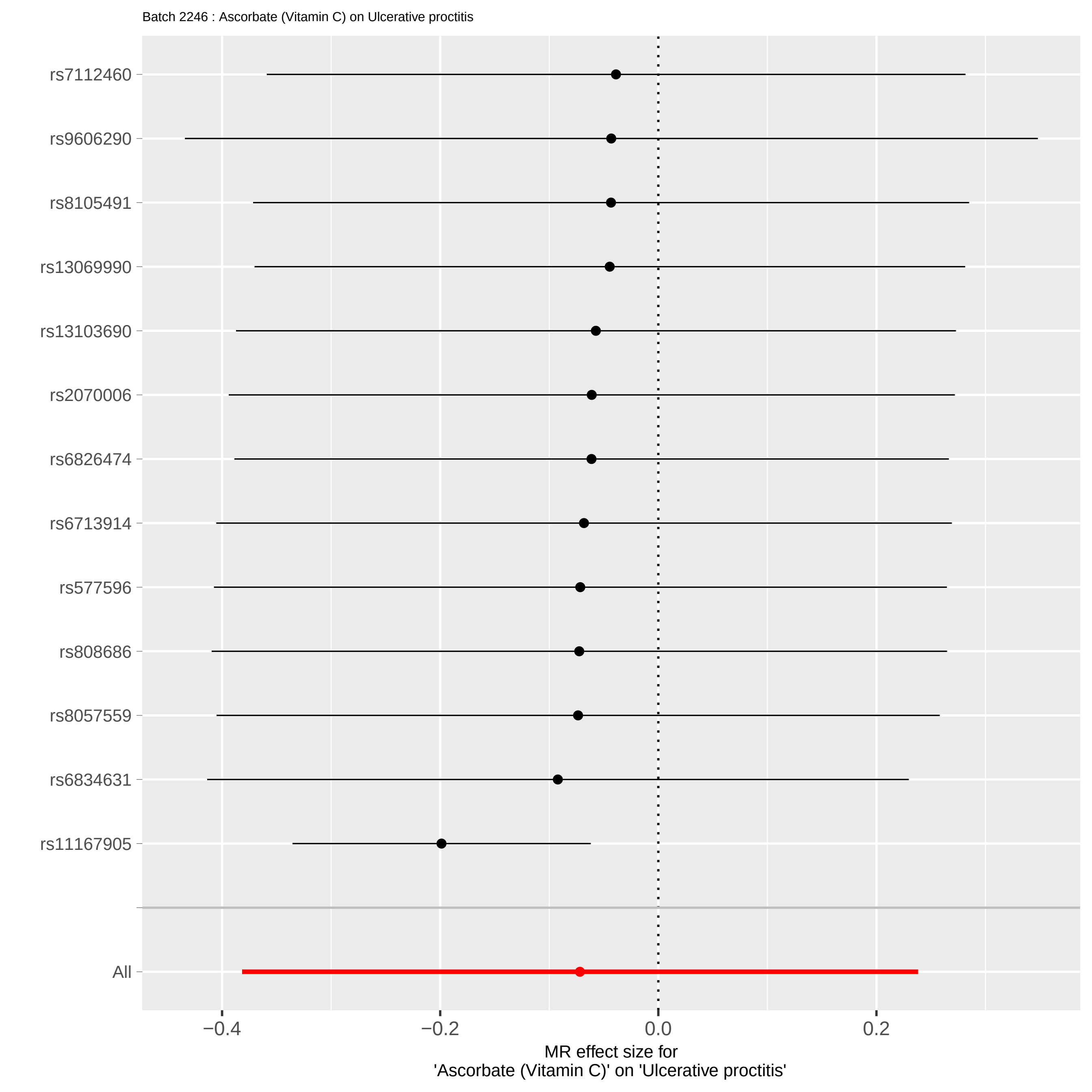
Batch 2246 : Ascorbate (Vitamin C) on Ulcerative proctitis
rs7112460
rs9606290
rs8105491
rs13069990
rs13103690
rs2070006
rs6826474
rs6713914
rs577596
rs808686
rs8057559
rs6834631
rs11167905
All
−0.4
−0.2
0.0
0.2
MR effect size for
'Ascorbate (Vitamin C)' on 'Ulcerative proctitis'

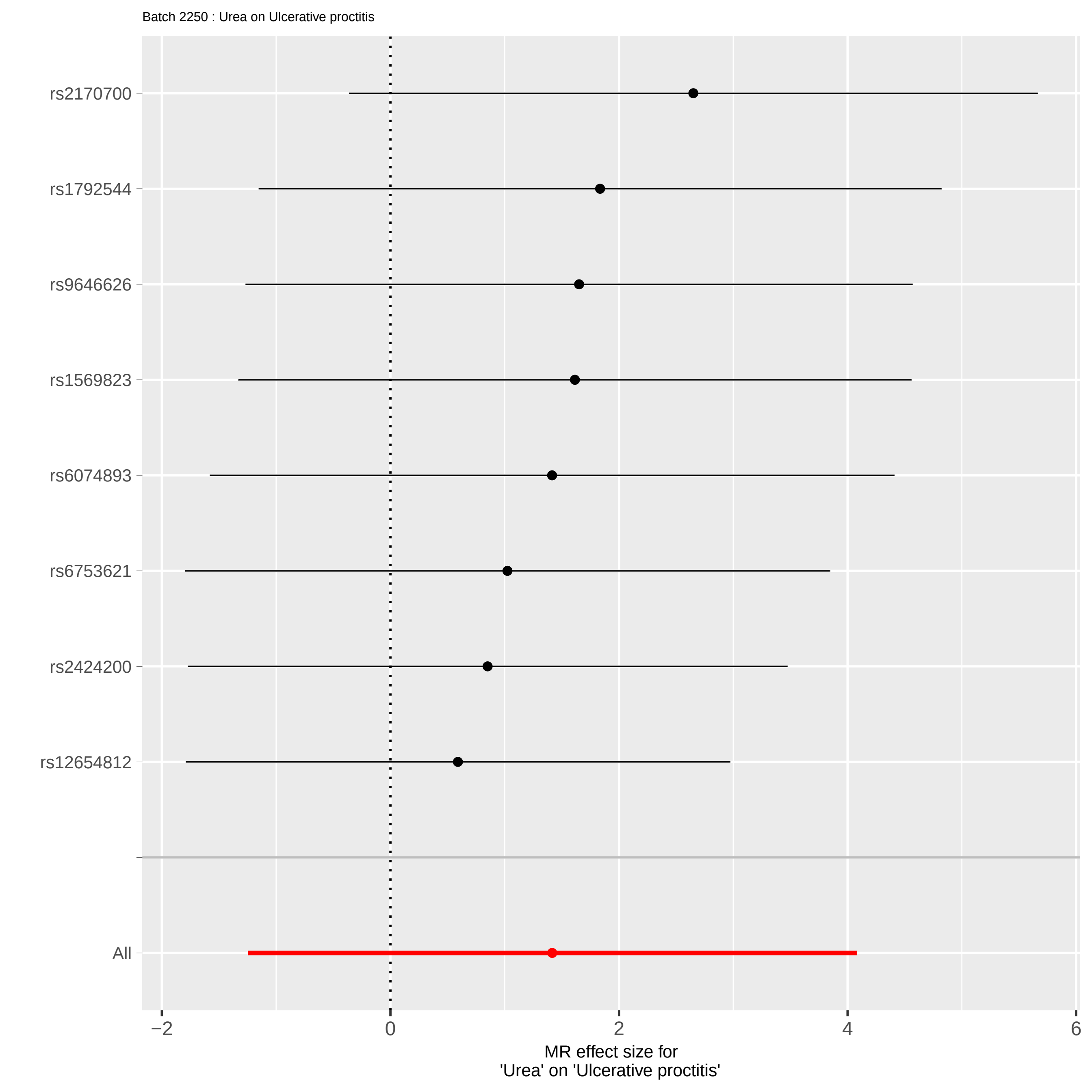
Batch 2250 : Urea on Ulcerative proctitis
rs2170700
rs1792544
rs9646626
rs1569823
rs6074893
rs6753621
rs2424200
rs12654812
All
−2
0
2
4
6
MR effect size for
'Urea' on 'Ulcerative proctitis'

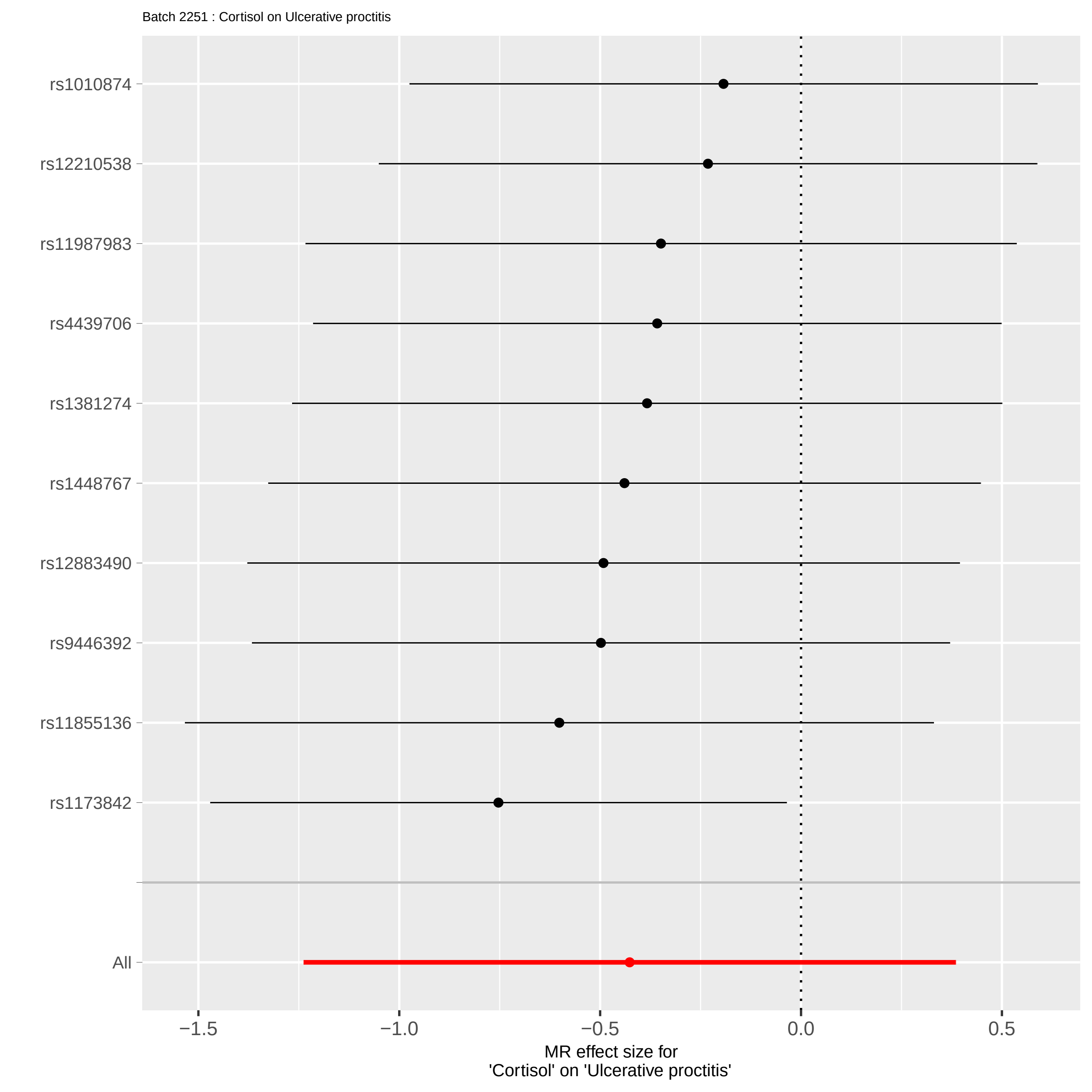
Batch 2251 : Cortisol on Ulcerative proctitis
rs1010874
rs12210538
rs11987983
rs4439706
rs1381274
rs1448767
rs12883490
rs9446392
rs11855136
rs1173842
All
−1.5
−1.0
−0.5
0.0
0.5
MR effect size for
'Cortisol' on 'Ulcerative proctitis'

Batch 2254 : Citrulline on Ulcerative proctitis

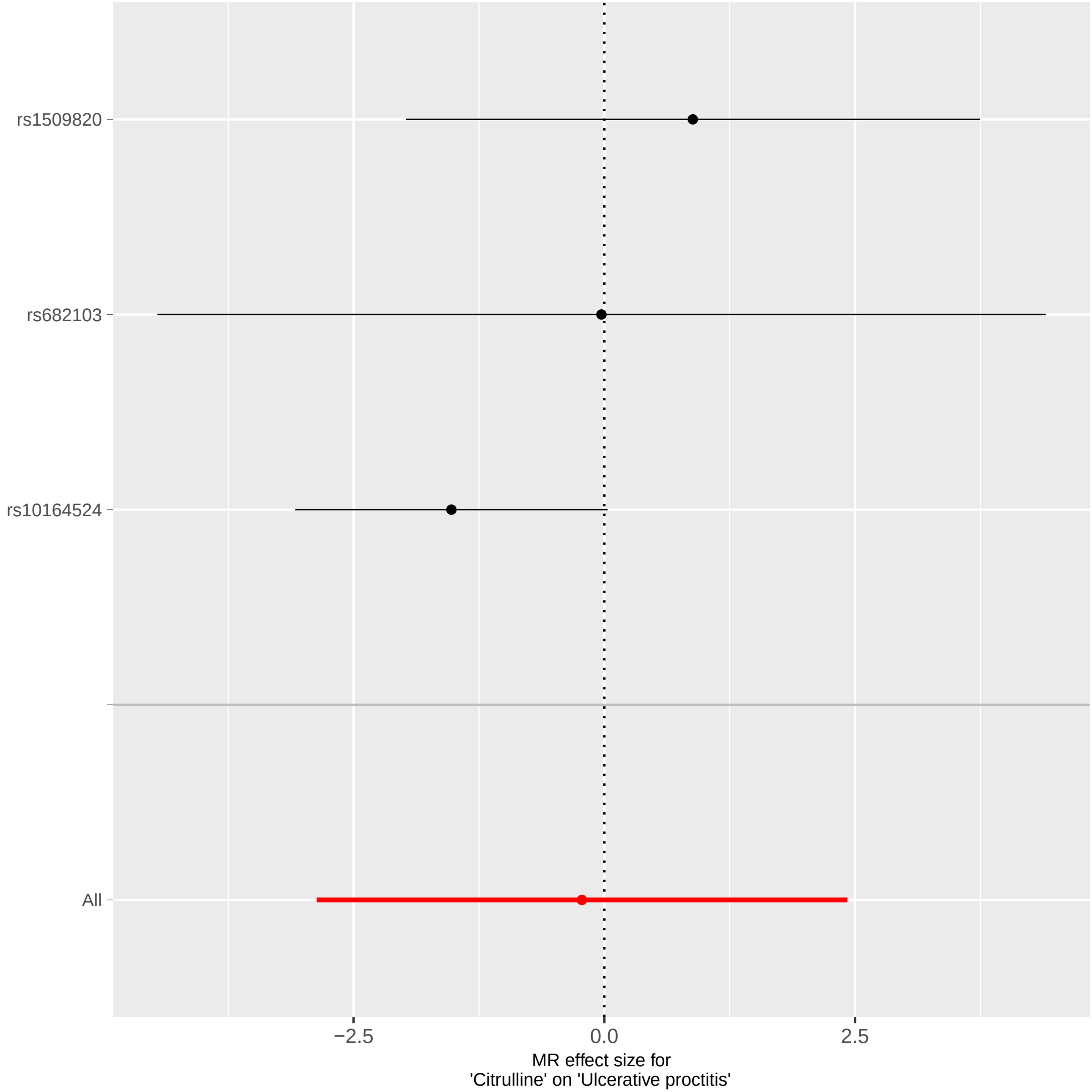

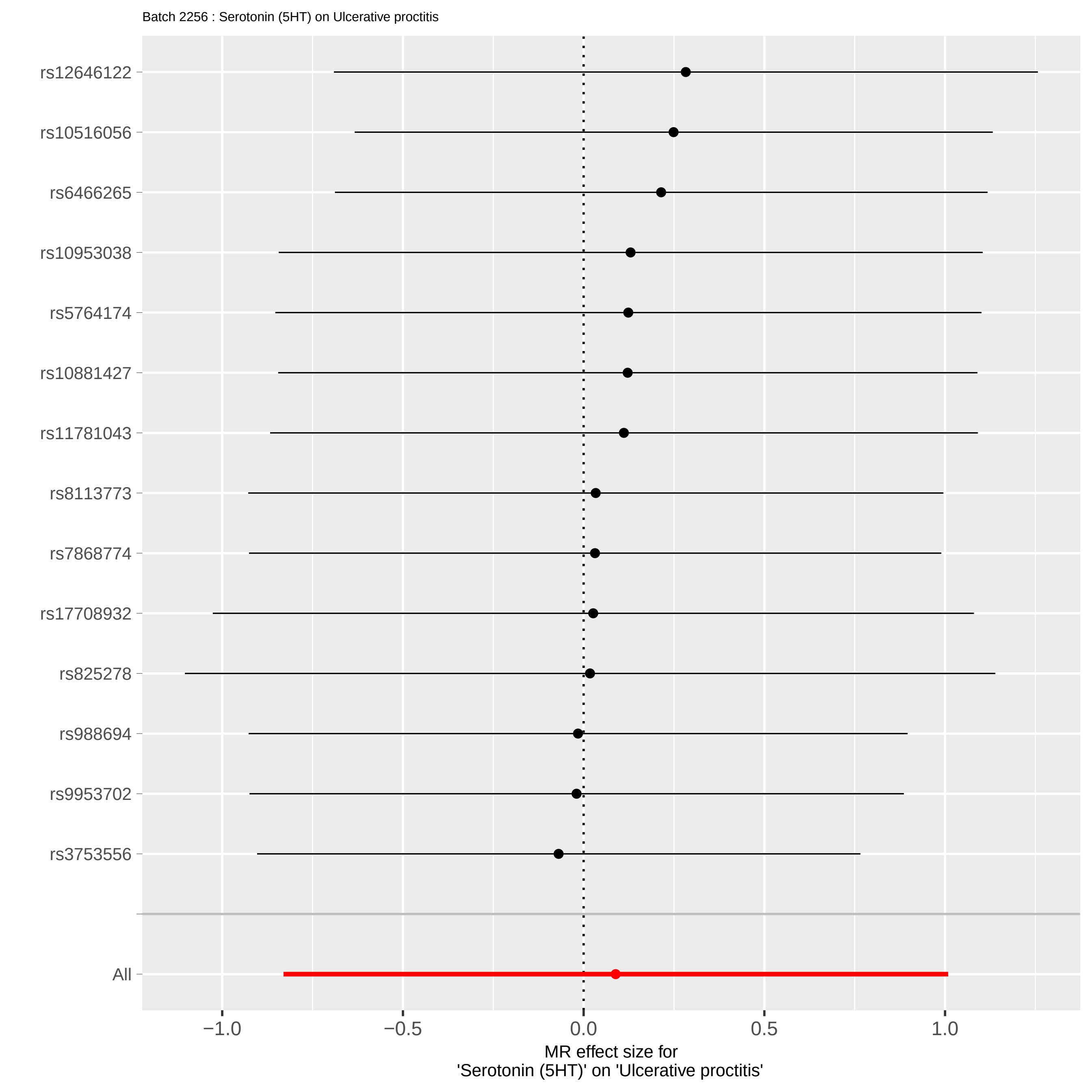

Batch 2256 : Serotonin (5HT) on Ulcerative proctitis
rs12646122
rs10516056
rs6466265
rs10953038
rs5764174
rs10881427
rs11781043
rs8113773
rs7868774
rs17708932
rs825278
rs988694
rs9953702
rs3753556
All
−1.0
−0.5
0.0
0.5
1.0
MR effect size for
'Serotonin (5HT)' on 'Ulcerative proctitis'

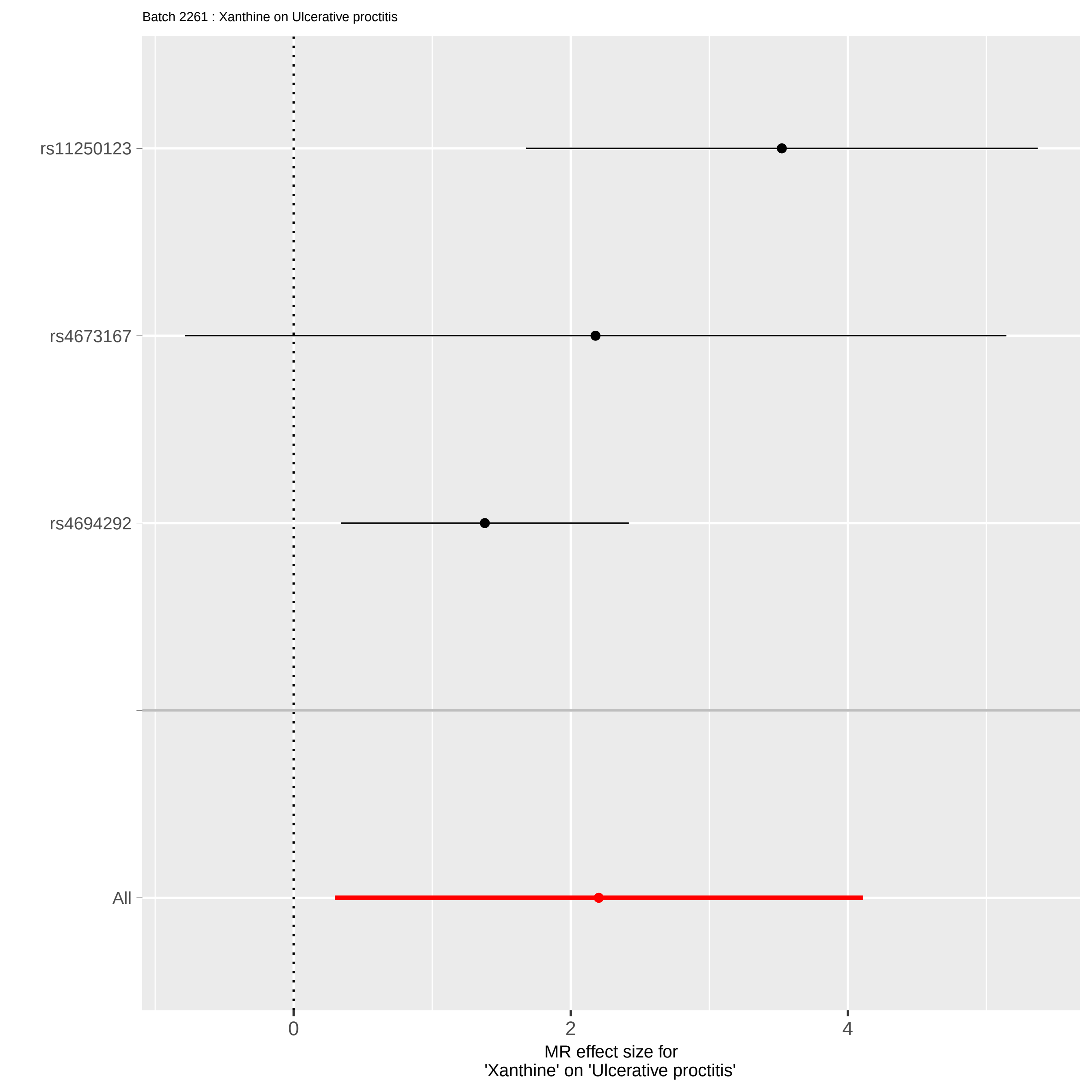
Batch 2261 : Xanthine on Ulcerative proctitis
rs11250123
rs4673167
rs4694292
All
0
2
4
MR effect size for
'Xanthine' on 'Ulcerative proctitis'

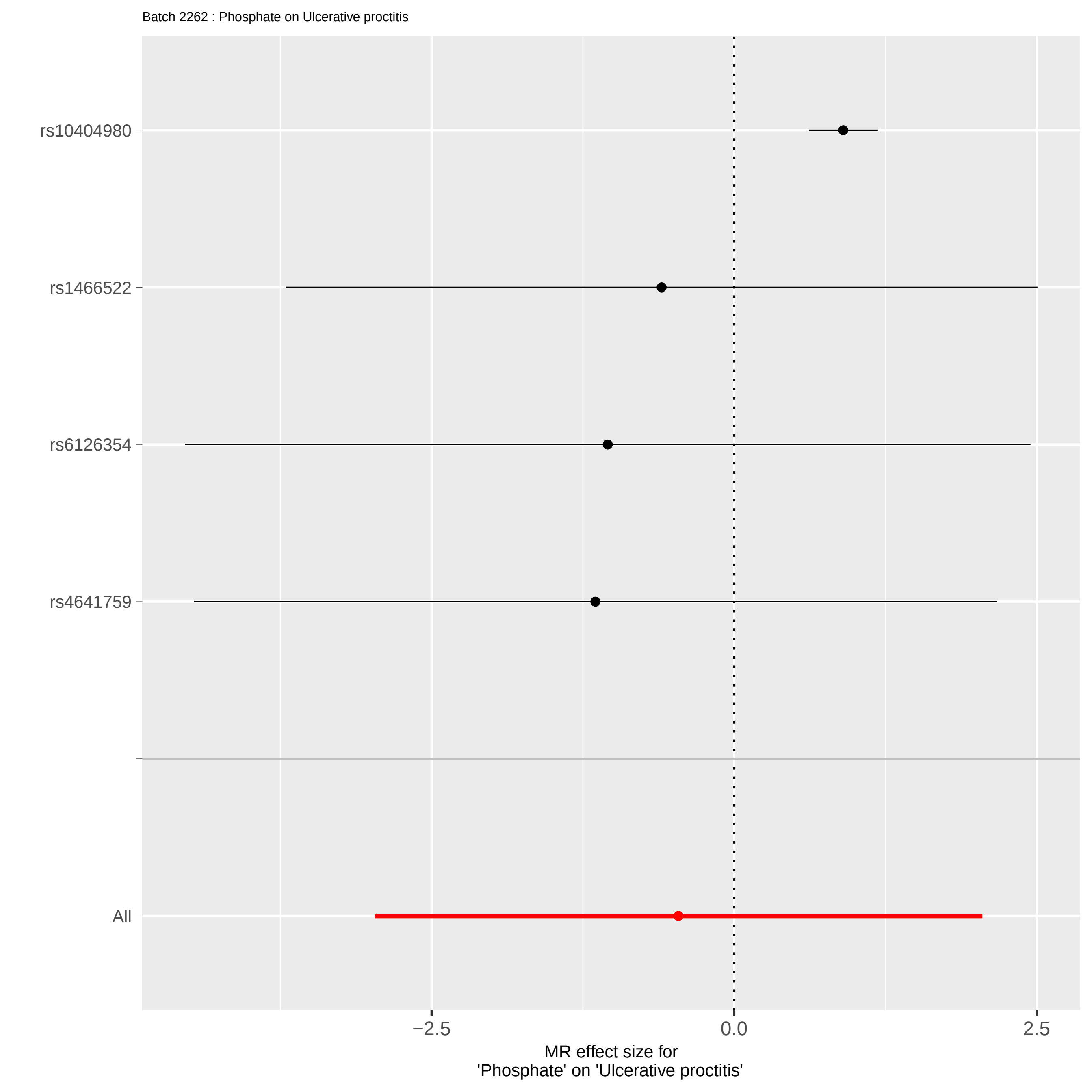
Batch 2262 : Phosphate on Ulcerative proctitis
rs10404980
rs1466522
rs6126354
rs4641759
All
−2.5
0.0
2.5
MR effect size for
'Phosphate' on 'Ulcerative proctitis'

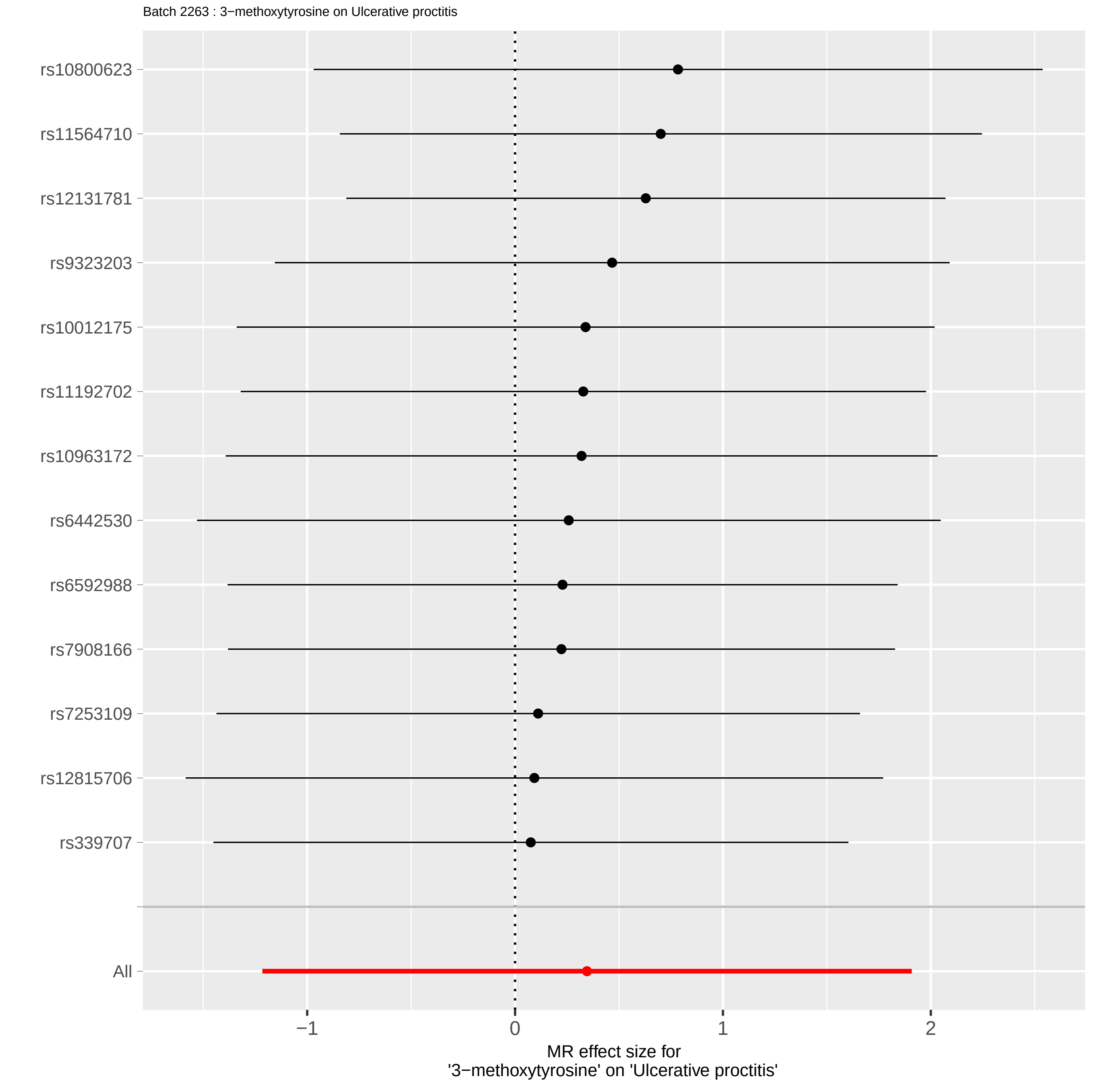
Batch 2263 : 3−methoxytyrosine on Ulcerative proctitis
rs10800623
rs11564710
rs12131781
rs9323203
rs10012175
rs11192702
rs10963172
rs6442530
rs6592988
rs7908166
rs7253109
rs12815706
rs339707
All
−1
0
1
2
MR effect size for
'3−methoxytyrosine' on 'Ulcerative proctitis'

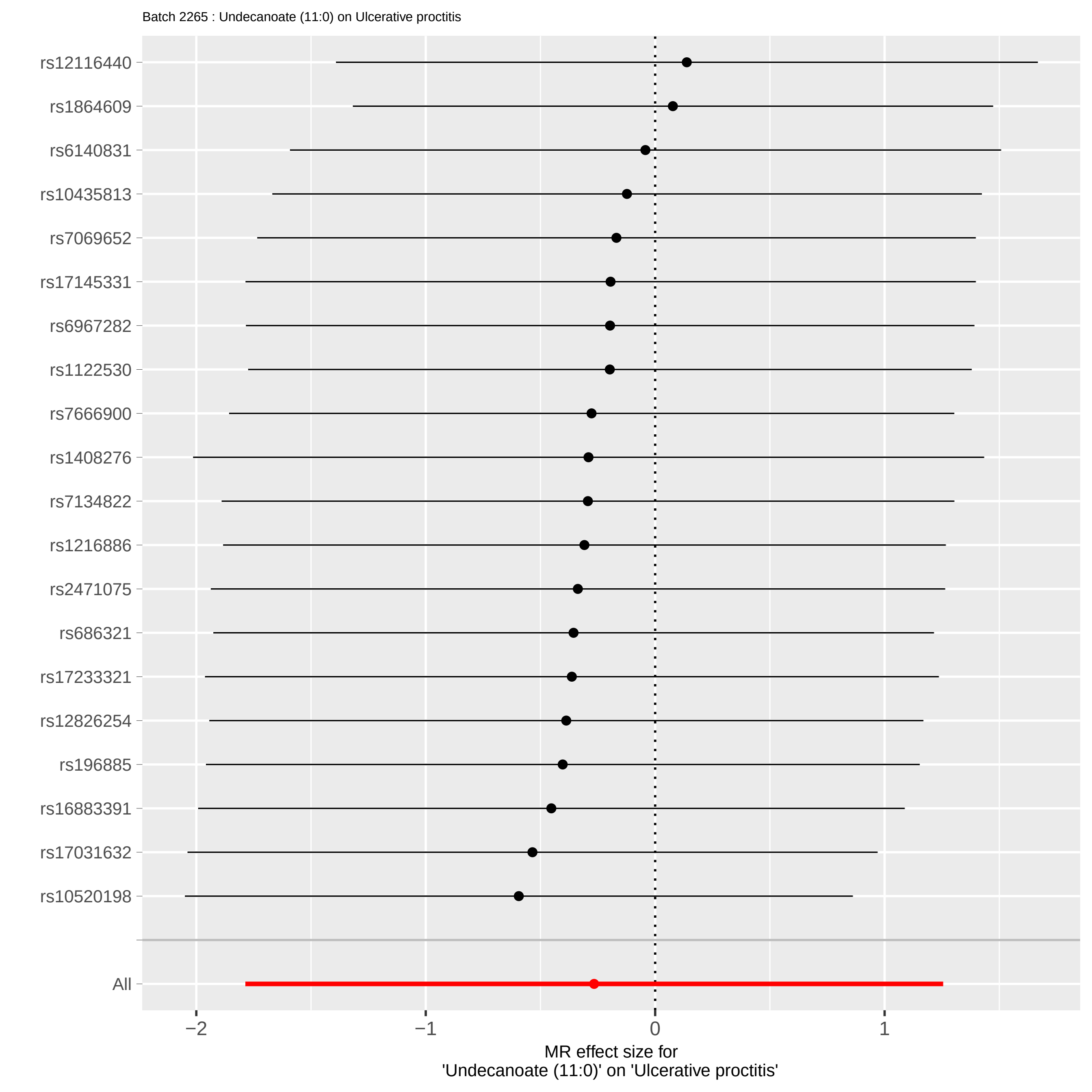

Batch 2265 : Undecanoate (11:0) on Ulcerative proctitis
rs12116440
rs1864609
rs6140831
rs10435813
rs7069652
rs17145331
rs6967282
rs1122530
rs7666900
rs1408276
rs7134822
rs1216886
rs2471075
rs686321
rs17233321
rs12826254
rs196885
rs16883391
rs17031632
rs10520198
All
−2
−1
0
1
MR effect size for
'Undecanoate (11:0)' on 'Ulcerative proctitis'

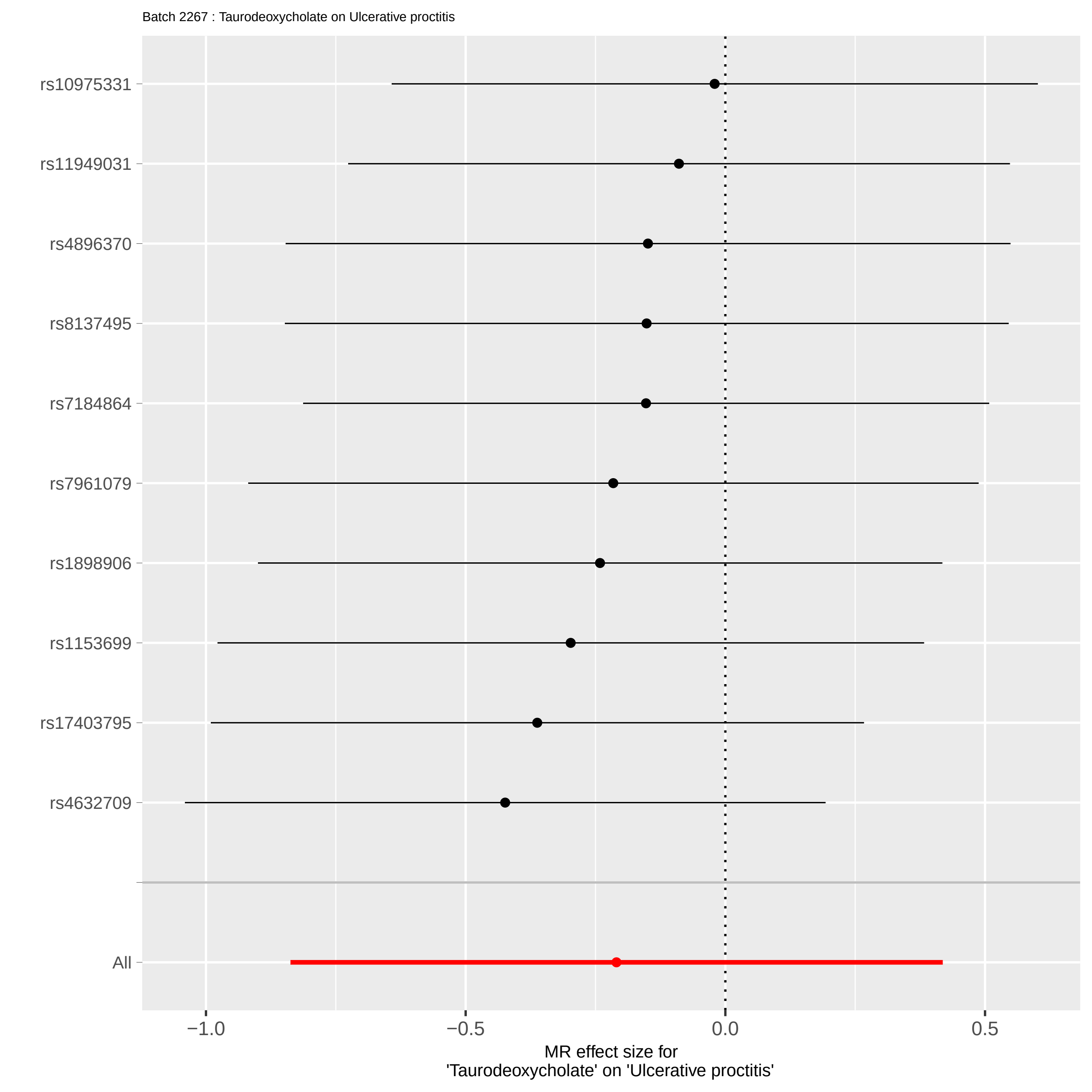
Batch 2267 : Taurodeoxycholate on Ulcerative proctitis
rs10975331
rs11949031
rs4896370
rs8137495
rs7184864
rs7961079
rs1898906
rs1153699
rs17403795
rs4632709
All
−1.0
−0.5
0.0
0.5
MR effect size for
'Taurodeoxycholate' on 'Ulcerative proctitis'

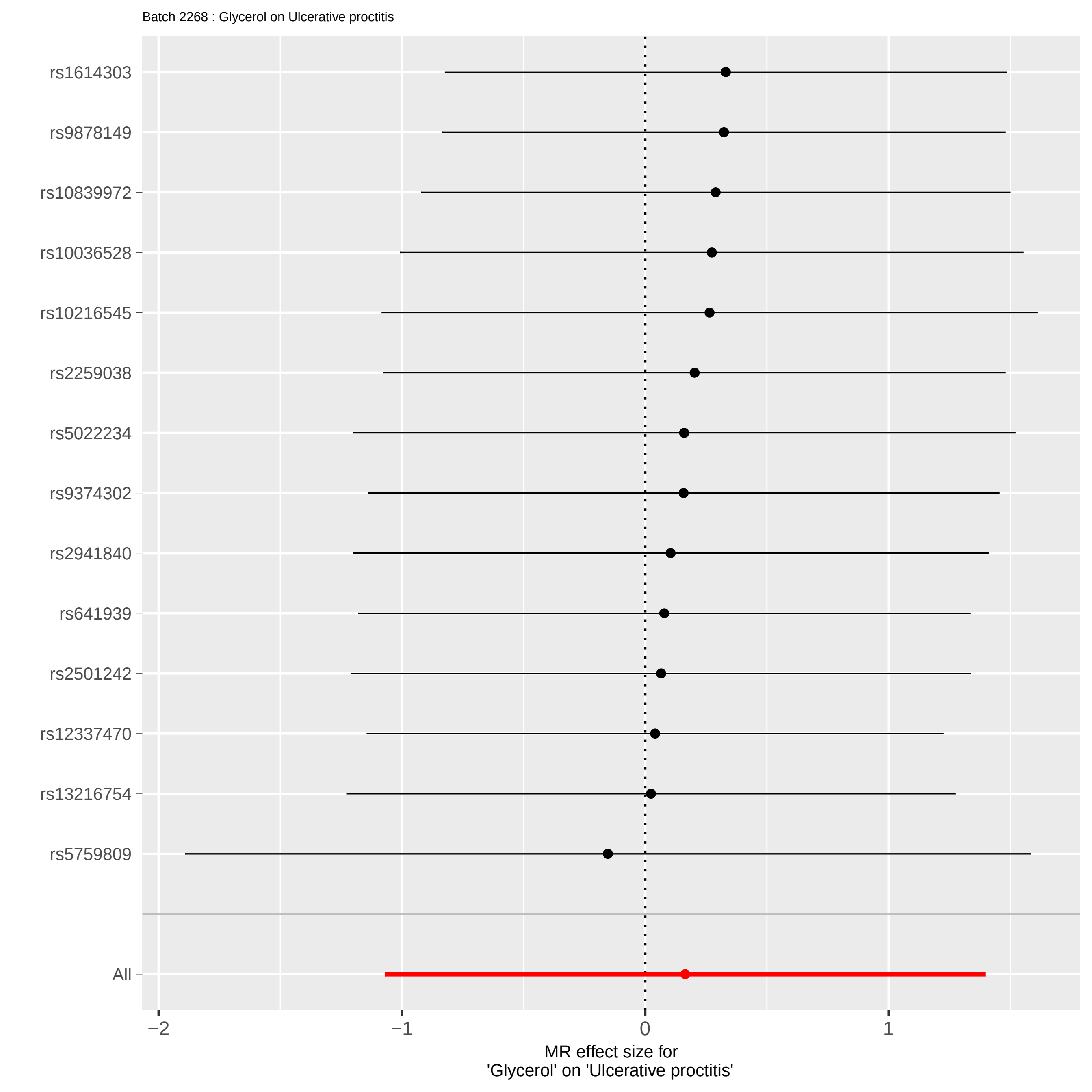
Batch 2268 : Glycerol on Ulcerative proctitis
rs1614303
rs9878149
rs10839972
rs10036528
rs10216545
rs2259038
rs5022234
rs9374302
rs2941840
rs641939
rs2501242
rs12337470
rs13216754
rs5759809
All
−2
−1
0
1
MR effect size for
'Glycerol' on 'Ulcerative proctitis'

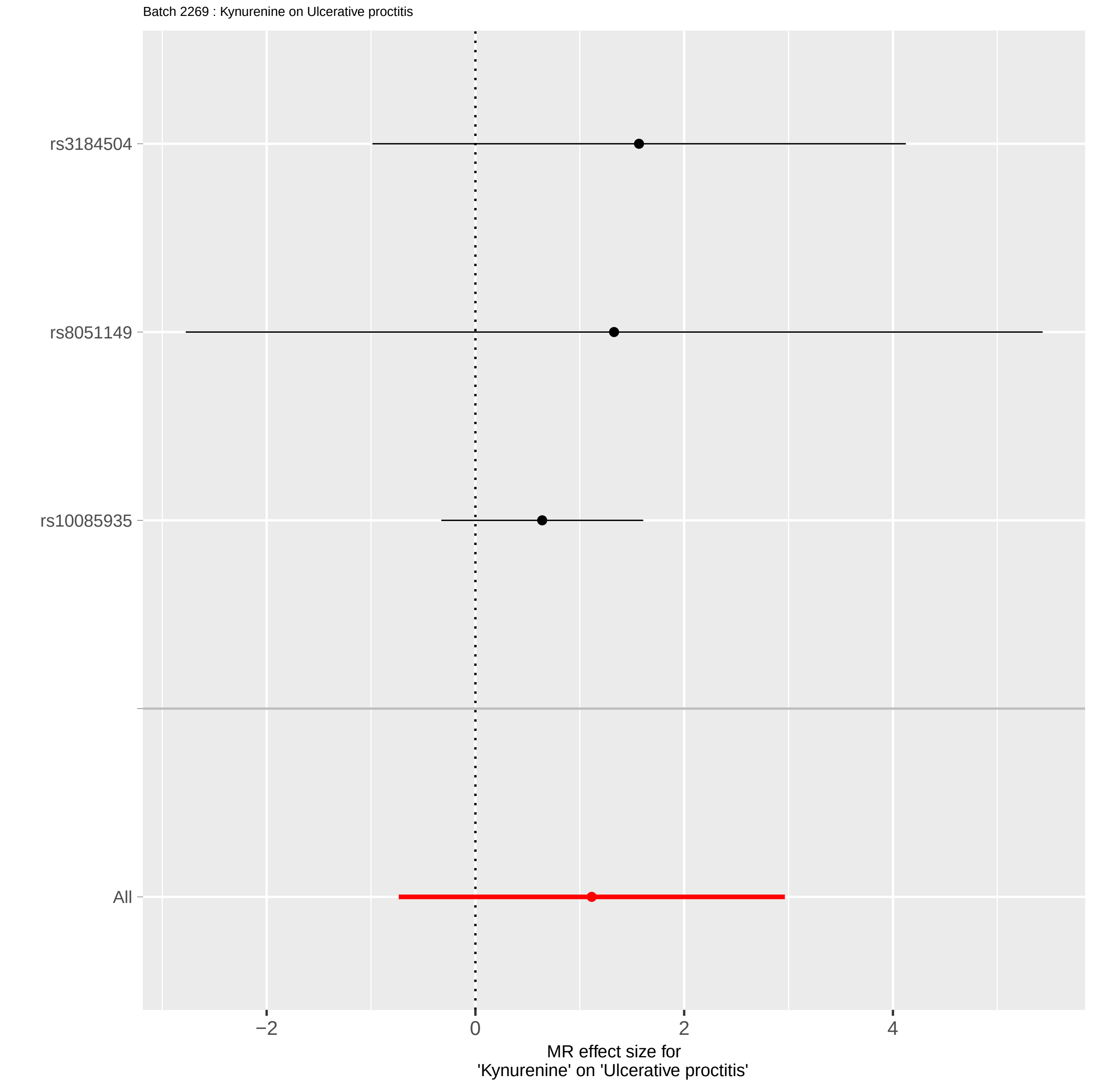
Batch 2269 : Kynurenine on Ulcerative proctitis
rs3184504
rs8051149
rs10085935
All
−2
0
2
4
MR effect size for
'Kynurenine' on 'Ulcerative proctitis'

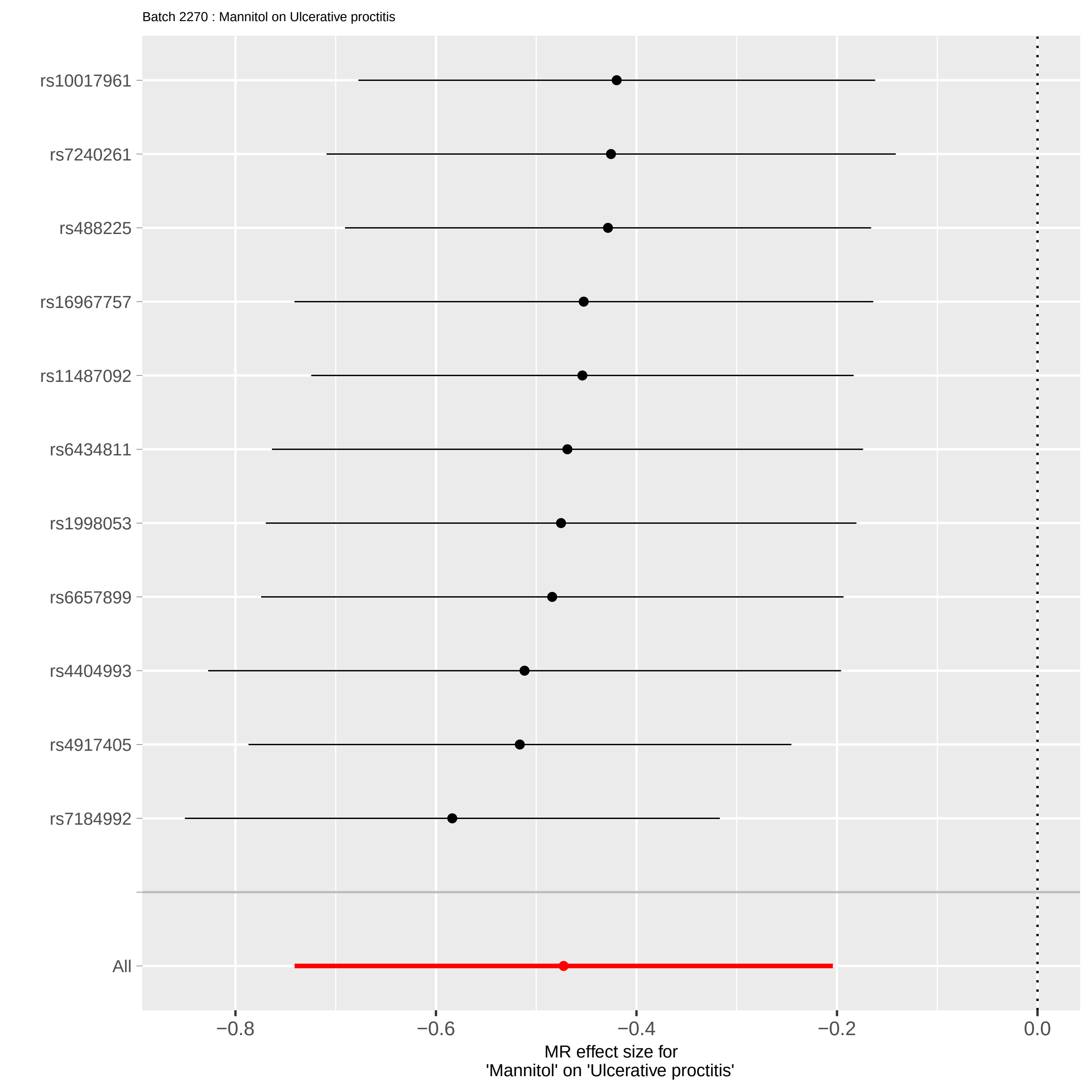

Batch 2270 : Mannitol on Ulcerative proctitis
rs10017961
rs7240261
rs488225
rs16967757
rs11487092
rs6434811
rs1998053
rs6657899
rs4404993
rs4917405
rs7184992
All
−0.8
−0.6
−0.4
−0.2
0.0
MR effect size for
'Mannitol' on 'Ulcerative proctitis'

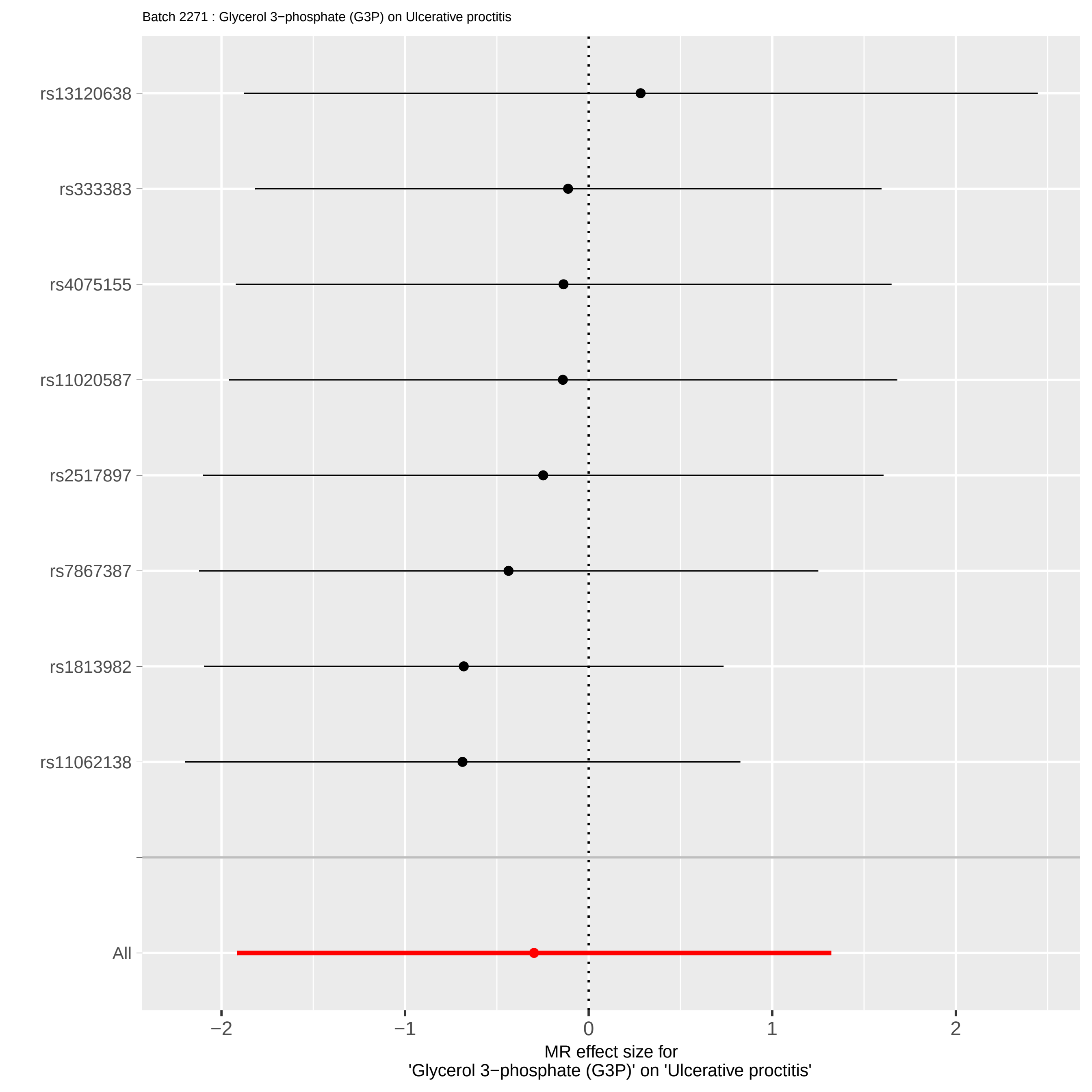

Batch 2271 : Glycerol 3−phosphate (G3P) on Ulcerative proctitis
rs13120638
rs333383
rs4075155
rs11020587
rs2517897
rs7867387
rs1813982
rs11062138
All
−2
−1
0
1
2
MR effect size for
'Glycerol 3−phosphate (G3P)' on 'Ulcerative proctitis'

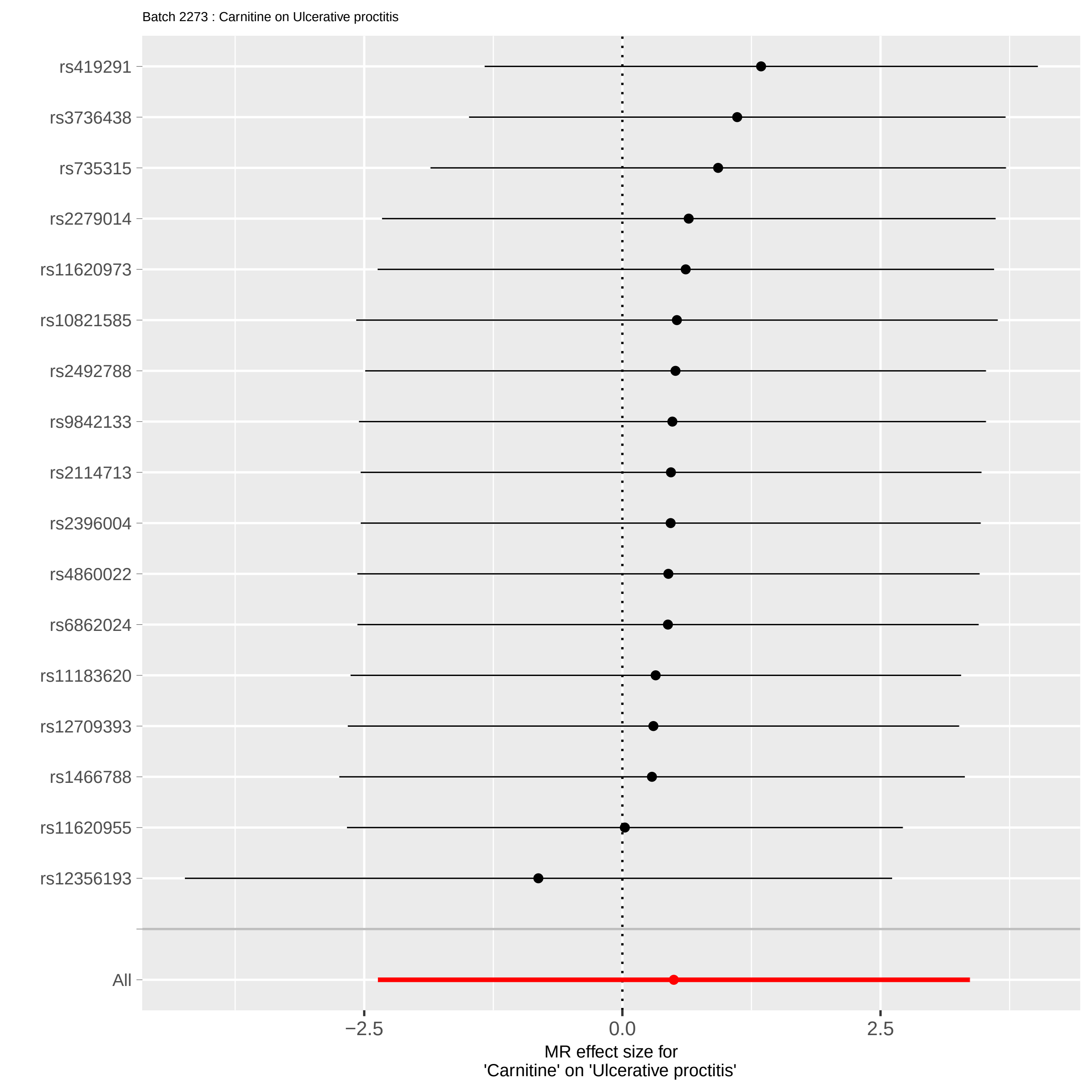
Batch 2273 : Carnitine on Ulcerative proctitis
rs419291
rs3736438
rs735315
rs2279014
rs11620973
rs10821585
rs2492788
rs9842133
rs2114713
rs2396004
rs4860022
rs6862024
rs11183620
rs12709393
rs1466788
rs11620955
rs12356193
All
−2.5
0.0
2.5
MR effect size for
'Carnitine' on 'Ulcerative proctitis'

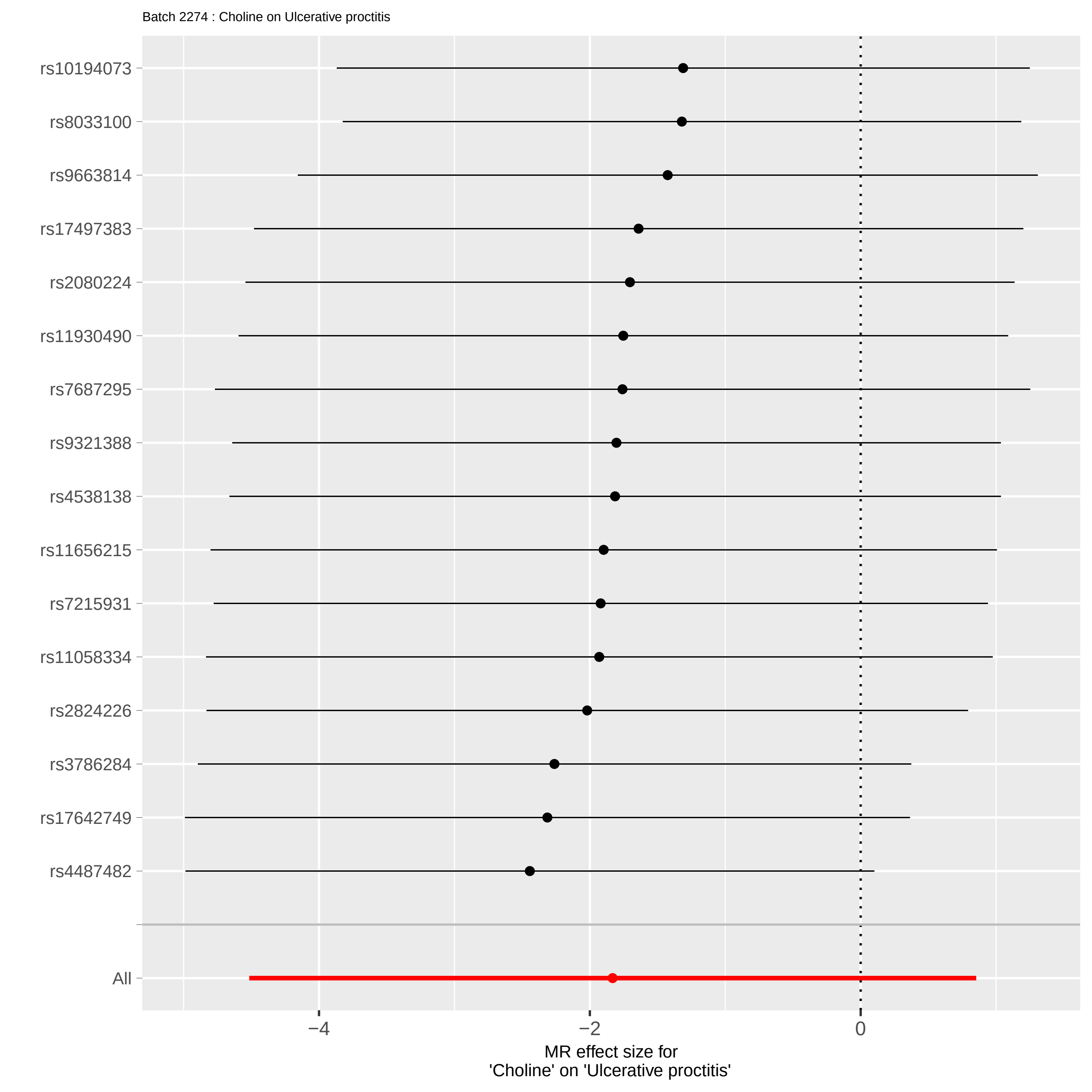
Batch 2274 : Choline on Ulcerative proctitis
rs10194073
rs8033100
rs9663814
rs17497383
rs2080224
rs11930490
rs7687295
rs9321388
rs4538138
rs11656215
rs7215931
rs11058334
rs2824226
rs3786284
rs17642749
rs4487482
All
−4
−2
0
MR effect size for
'Choline' on 'Ulcerative proctitis'

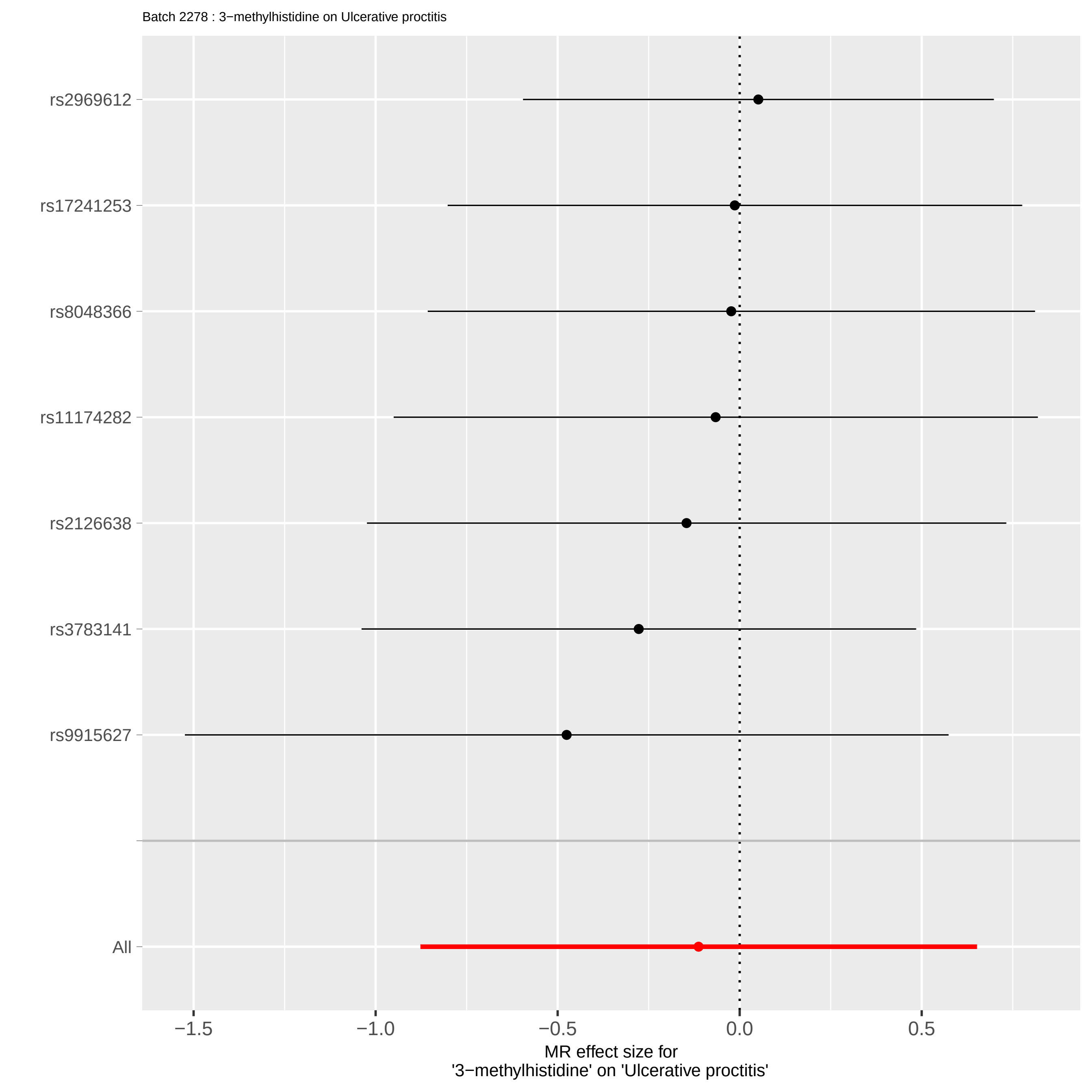

Batch 2278 : 3−methylhistidine on Ulcerative proctitis
rs2969612
rs17241253
rs8048366
rs11174282
rs2126638
rs3783141
rs9915627
All
−1.5
−1.0
−0.5
0.0
0.5
MR effect size for
'3−methylhistidine' on 'Ulcerative proctitis'

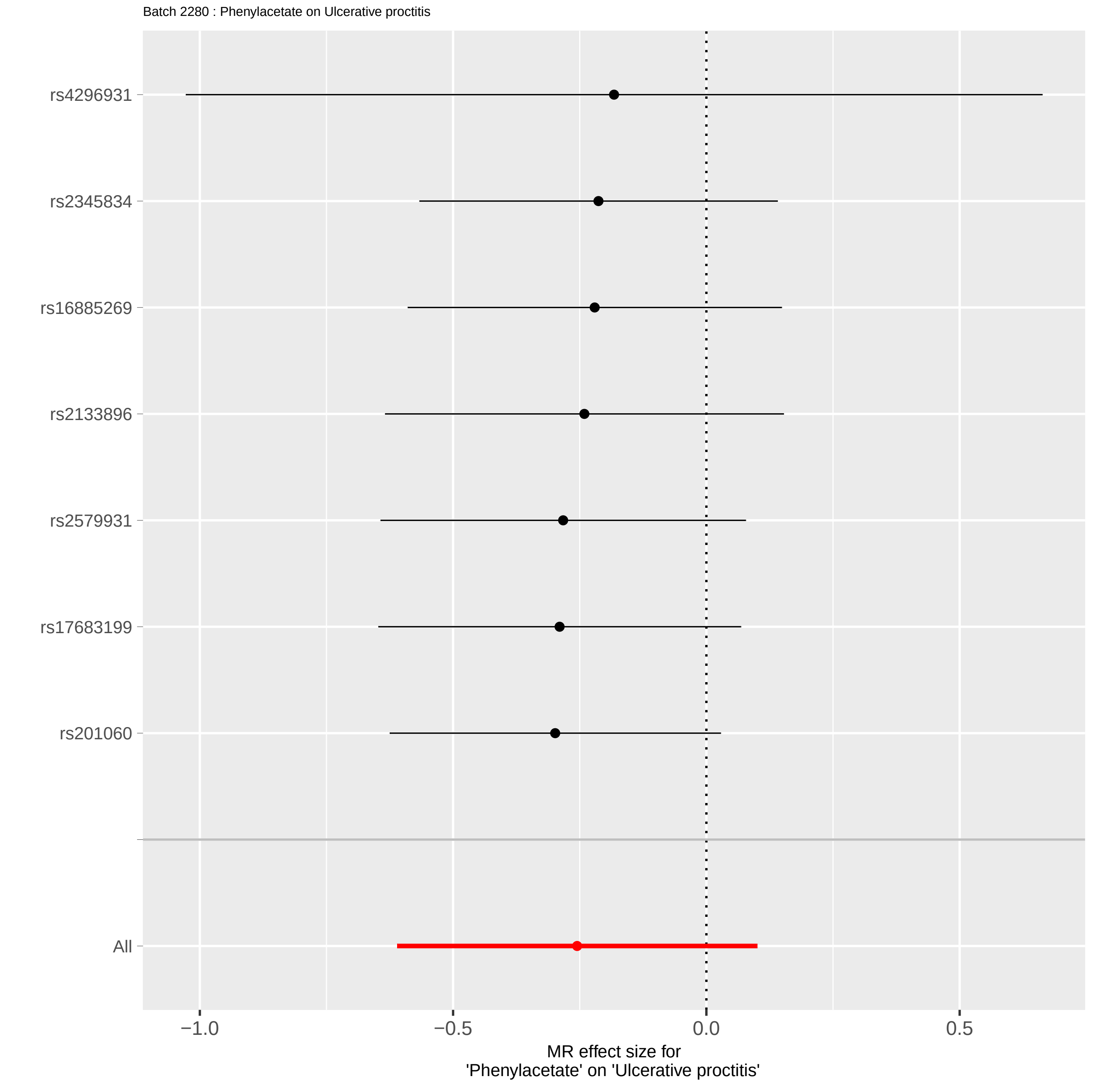

Batch 2280 : Phenylacetate on Ulcerative proctitis
rs4296931
rs2345834
rs16885269
rs2133896
rs2579931
rs17683199
rs201060
All
−1.0
−0.5
0.0
0.5
MR effect size for
'Phenylacetate' on 'Ulcerative proctitis'

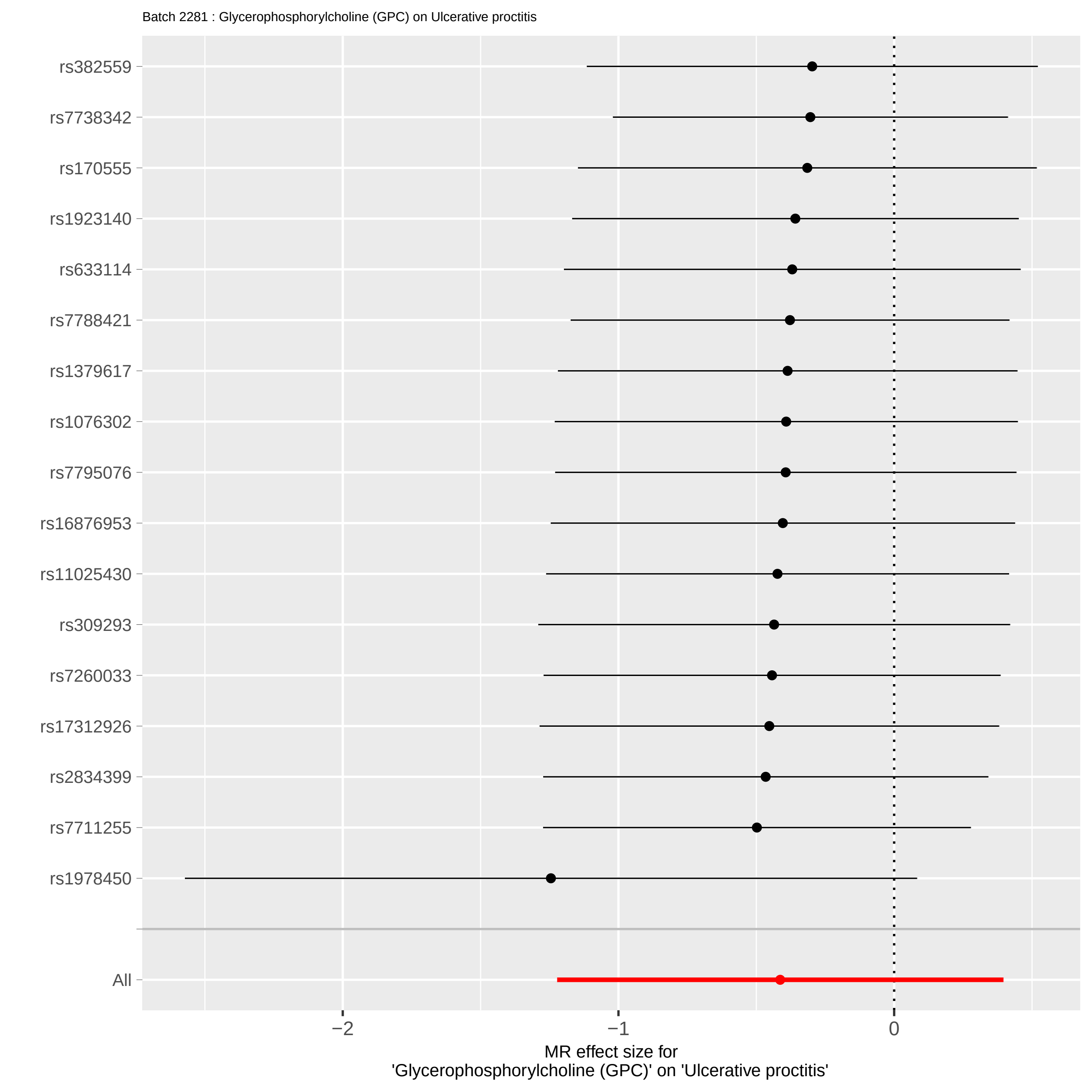
Batch 2281 : Glycerophosphorylcholine (GPC) on Ulcerative proctitis
rs382559
rs7738342
rs170555
rs1923140
rs633114
rs7788421
rs1379617
rs1076302
rs7795076
rs16876953
rs11025430
rs309293
rs7260033
rs17312926
rs2834399
rs7711255
rs1978450
All
−2
−1
0
MR effect size for
'Glycerophosphorylcholine (GPC)' on 'Ulcerative proctitis'

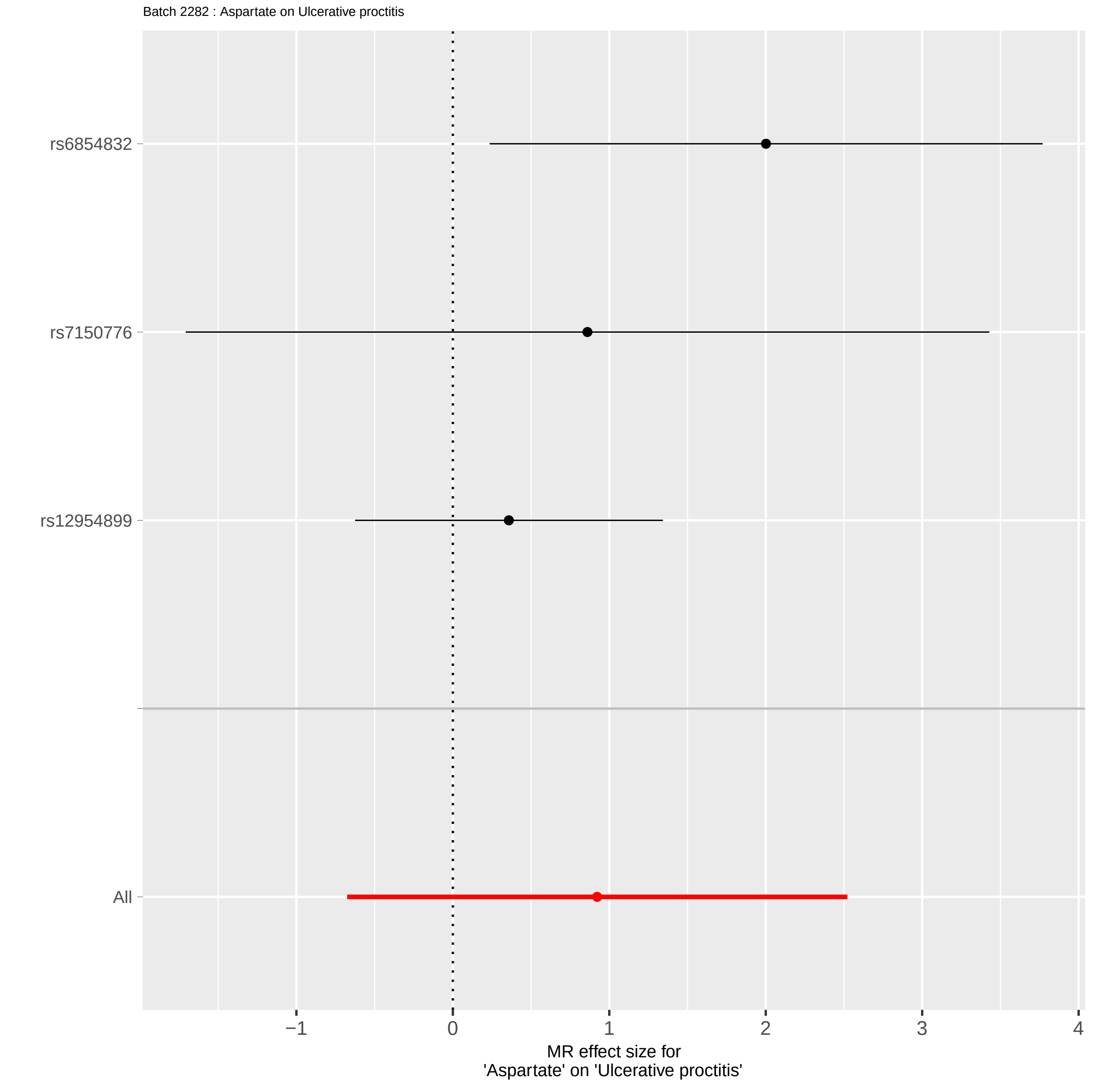
Batch 2282 : Aspartate on Ulcerative proctitis
rs6854832
rs7150776
rs12954899
All
−1
0
1
2
3
4
MR effect size for
'Aspartate' on 'Ulcerative proctitis'

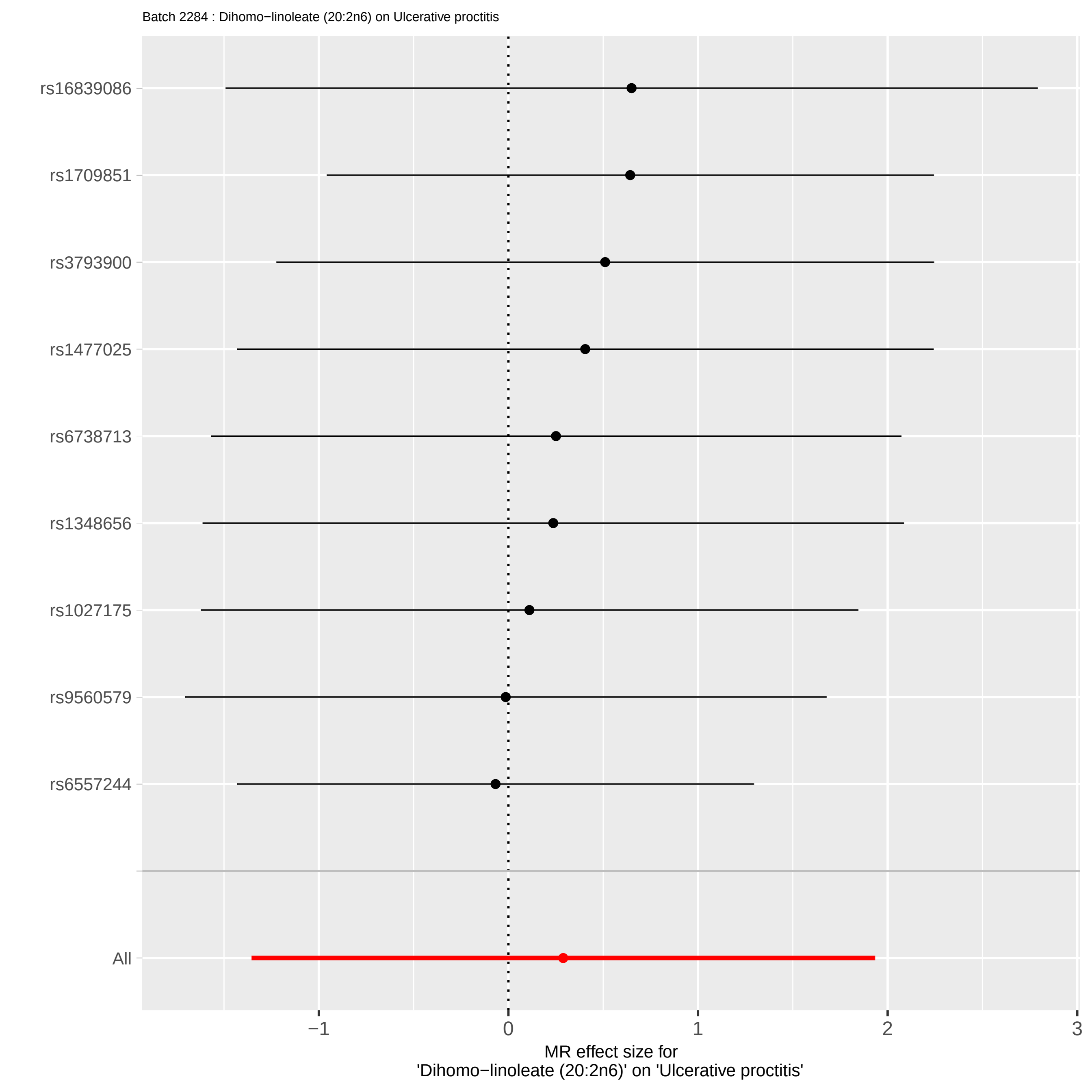

Batch 2284 : Dihomo−linoleate (20:2n6) on Ulcerative proctitis
rs16839086
rs1709851
rs3793900
rs1477025
rs6738713
rs1348656
rs1027175
rs9560579
rs6557244
All
−1
0
1
2
3
MR effect size for
'Dihomo−linoleate (20:2n6)' on 'Ulcerative proctitis'

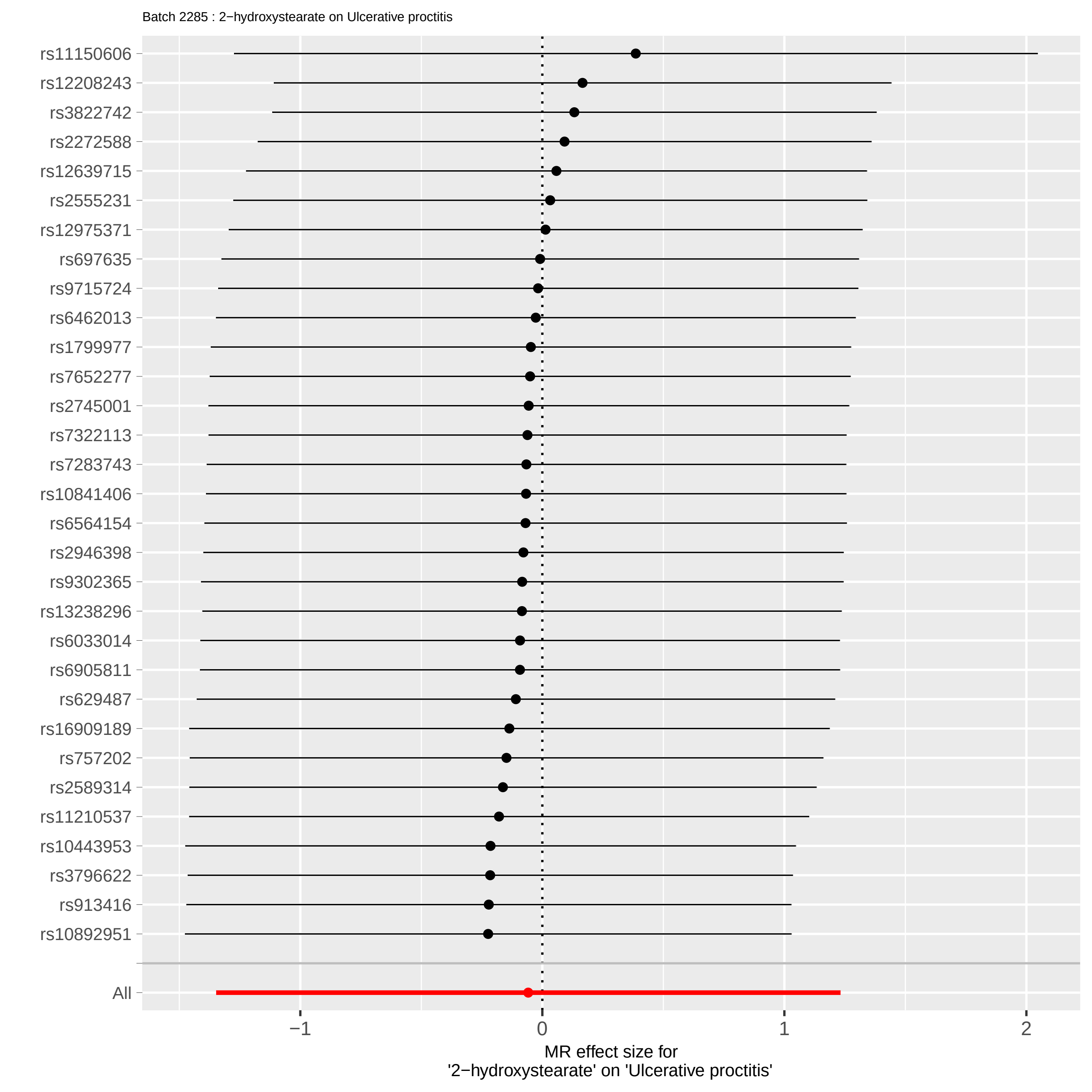
Batch 2285 : 2−hydroxystearate on Ulcerative proctitis
rs11150606
rs12208243
rs3822742
rs2272588
rs12639715
rs2555231
rs12975371
rs697635
rs9715724
rs6462013
rs1799977
rs7652277
rs2745001
rs7322113
rs7283743
rs10841406
rs6564154
rs2946398
rs9302365
rs13238296
rs6033014
rs6905811
rs629487
rs16909189
rs757202
rs2589314
rs11210537
rs10443953
rs3796622
rs913416
rs10892951
All
−1
0
1
2
MR effect size for
'2−hydroxystearate' on 'Ulcerative proctitis'

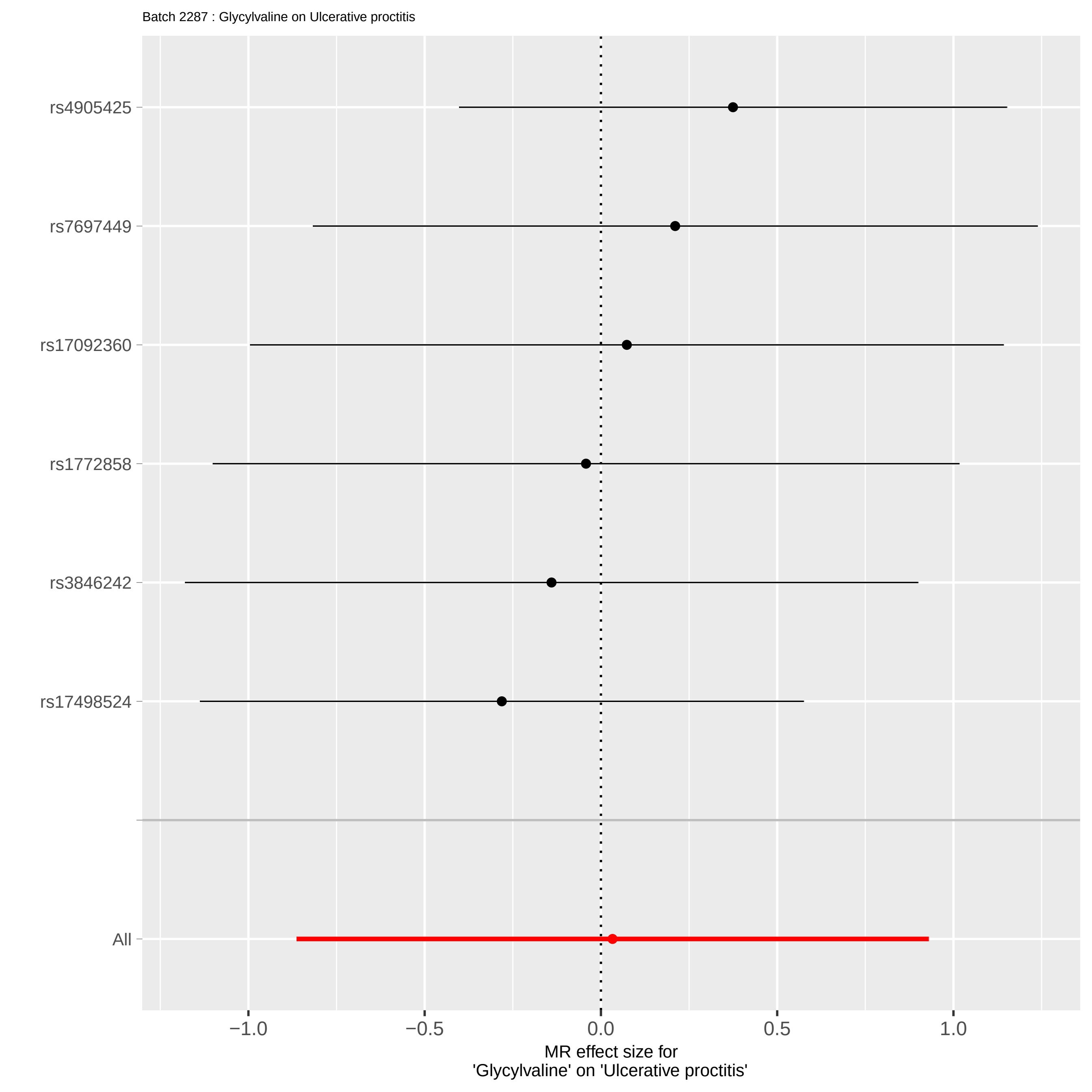
Batch 2287 : Glycylvaline on Ulcerative proctitis
rs4905425
rs7697449
rs17092360
rs1772858
rs3846242
rs17498524
All
−1.0
−0.5
0.0
0.5
1.0
MR effect size for
'Glycylvaline' on 'Ulcerative proctitis'

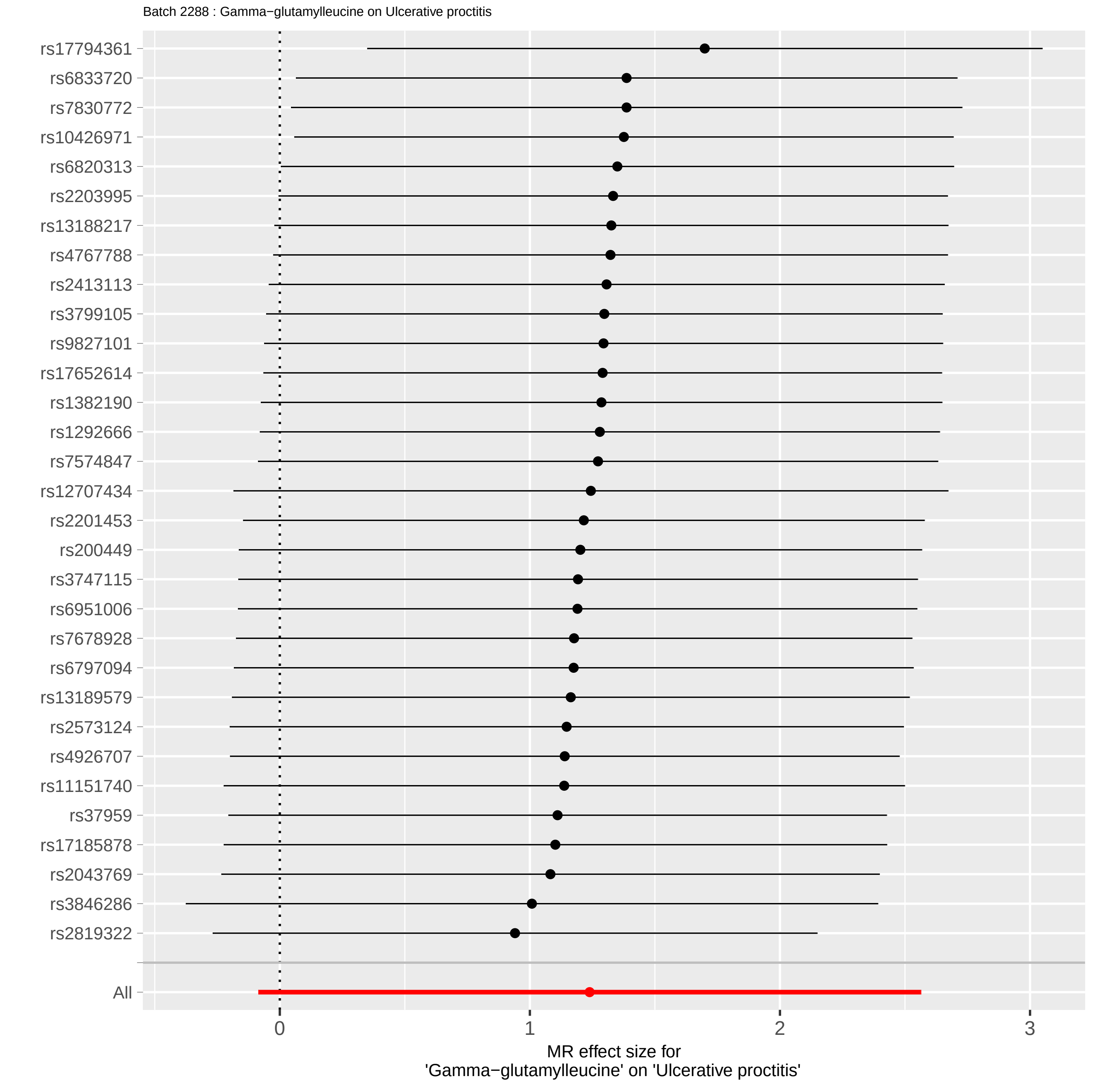
Batch 2288 : Gamma−glutamylleucine on Ulcerative proctitis
rs17794361
rs6833720
rs7830772
rs10426971
rs6820313
rs2203995
rs13188217
rs4767788
rs2413113
rs3799105
rs9827101
rs17652614
rs1382190
rs1292666
rs7574847
rs12707434
rs2201453
rs200449
rs3747115
rs6951006
rs7678928
rs6797094
rs13189579
rs2573124
rs4926707
rs11151740
rs37959
rs17185878
rs2043769
rs3846286
rs2819322
All
0
1
2
3
MR effect size for
'Gamma−glutamylleucine' on 'Ulcerative proctitis'

Batch 2291 : Glycocholate on Ulcerative proctitis

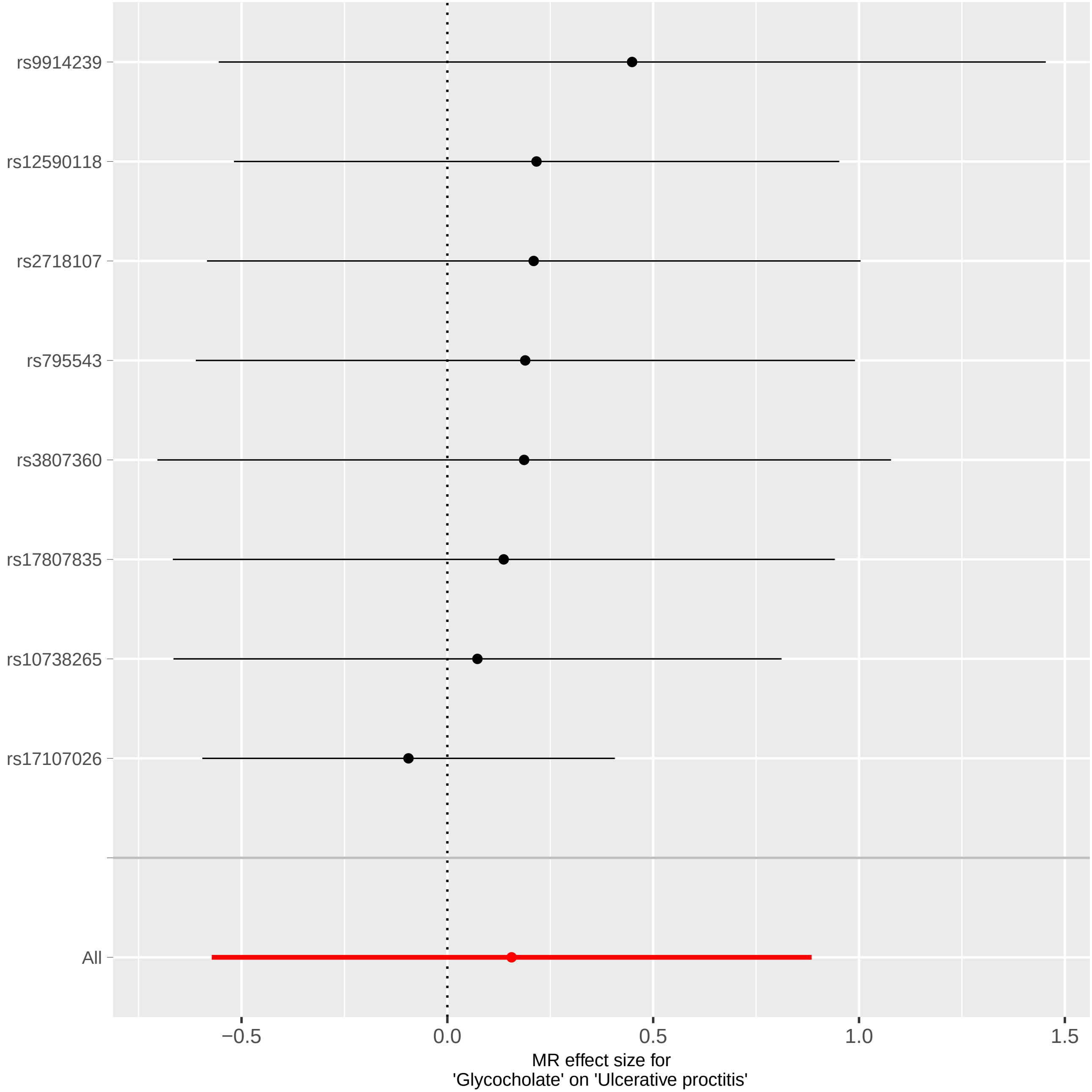

Batch 2292 : Glycodeoxycholate on Ulcerative proctitis

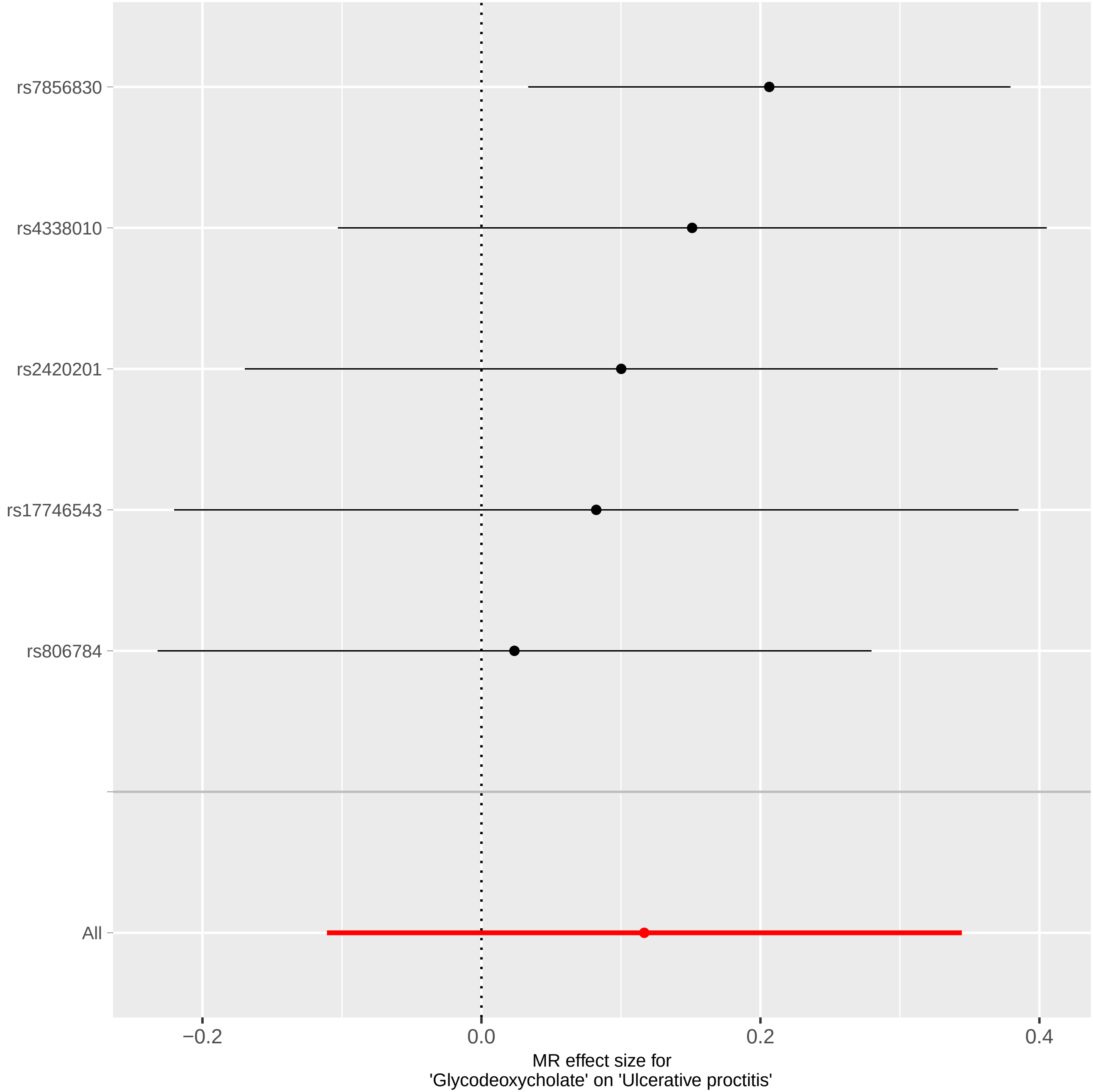

Batch 2293 : Taurochenodeoxycholate on Ulcerative proctitis

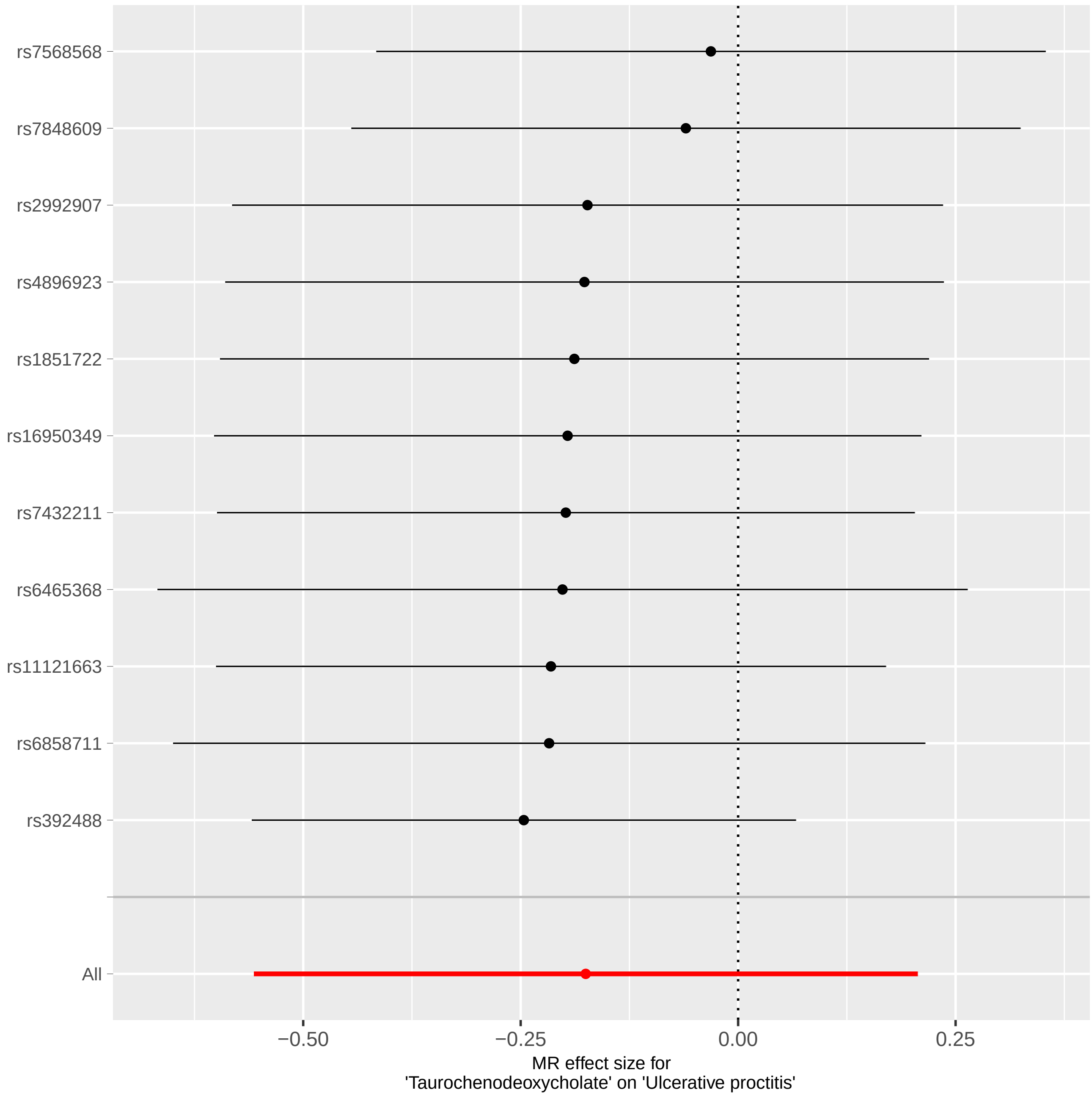

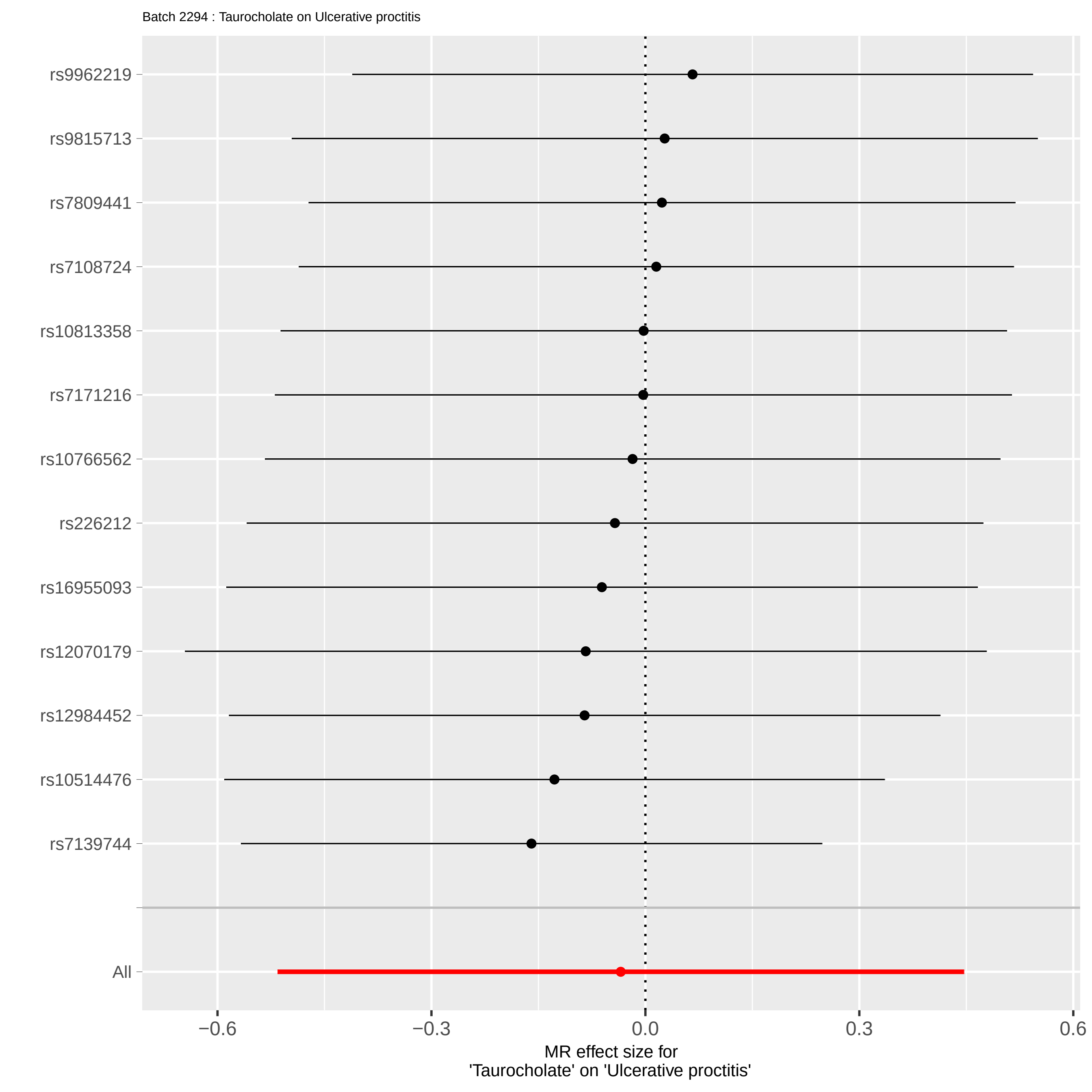

Batch 2294 : Taurocholate on Ulcerative proctitis
rs9962219
rs9815713
rs7809441
rs7108724
rs10813358
rs7171216
rs10766562
rs226212
rs16955093
rs12070179
rs12984452
rs10514476
rs7139744
All
−0.6
−0.3
0.0
0.3
0.6
MR effect size for
'Taurocholate' on 'Ulcerative proctitis'

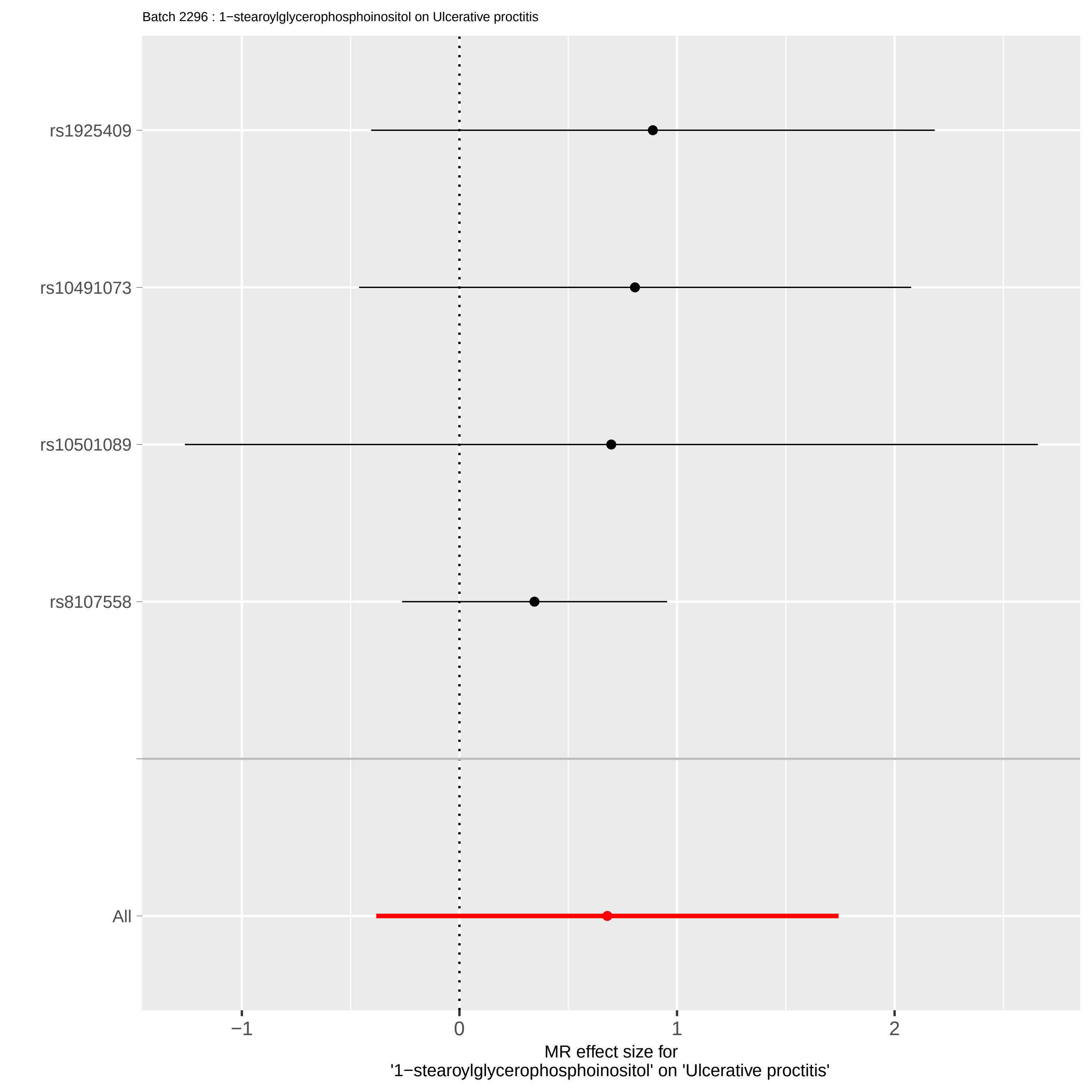
Batch 2296 : 1−stearoylglycerophosphoinositol on Ulcerative proctitis
rs1925409
rs10491073
rs10501089
rs8107558
All
−1
0
1
2
MR effect size for
'1−stearoylglycerophosphoinositol' on 'Ulcerative proctitis'

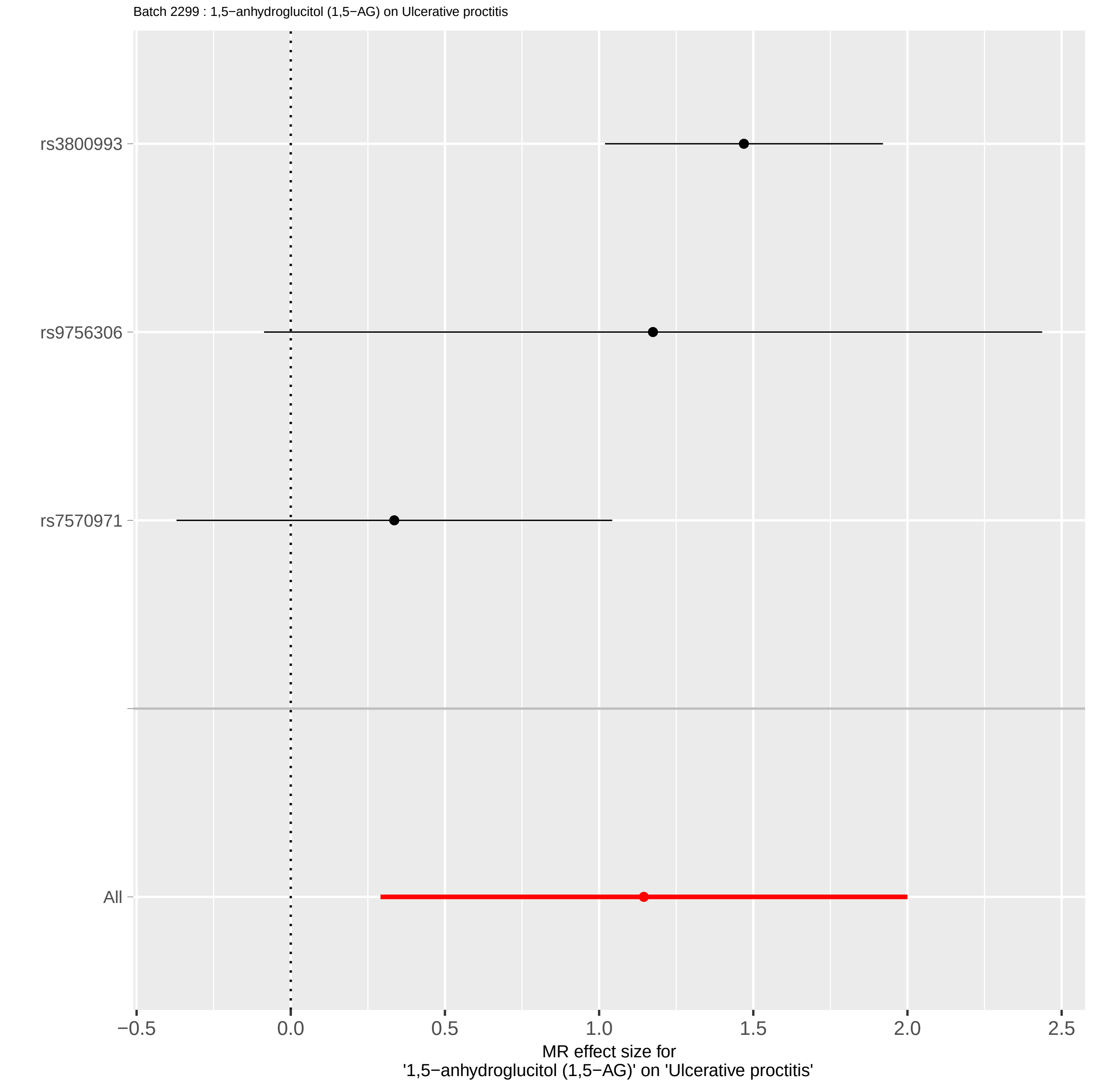
Batch 2299 : 1,5−anhydroglucitol (1,5−AG) on Ulcerative proctitis
rs3800993
rs9756306
rs7570971
All
−0.5
0.0
0.5
1.0
1.5
2.0
2.5
MR effect size for
'1,5−anhydroglucitol (1,5−AG)' on 'Ulcerative proctitis'

Batch 2300 : 2−hydroxybutyrate (AHB) on Ulcerative proctitis

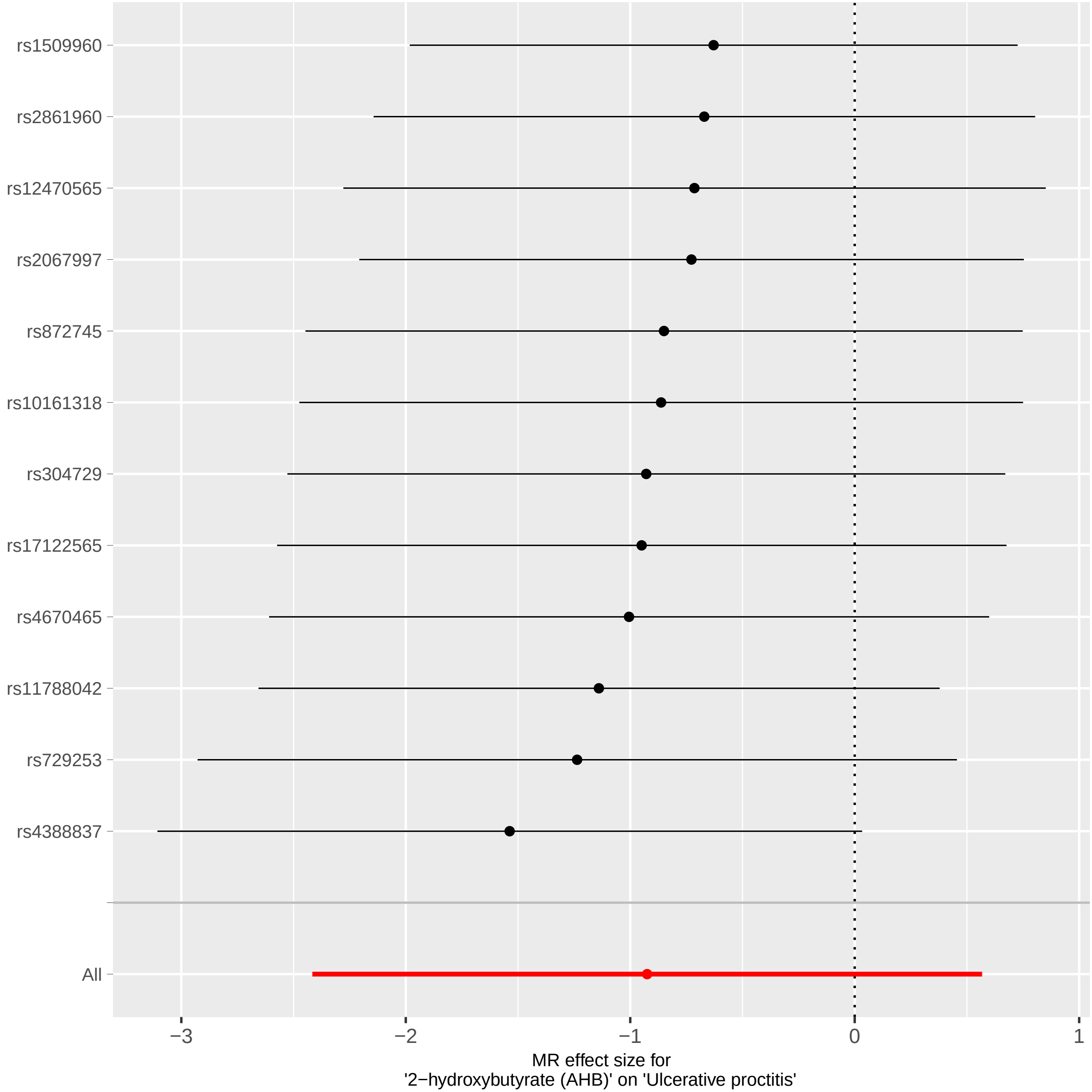

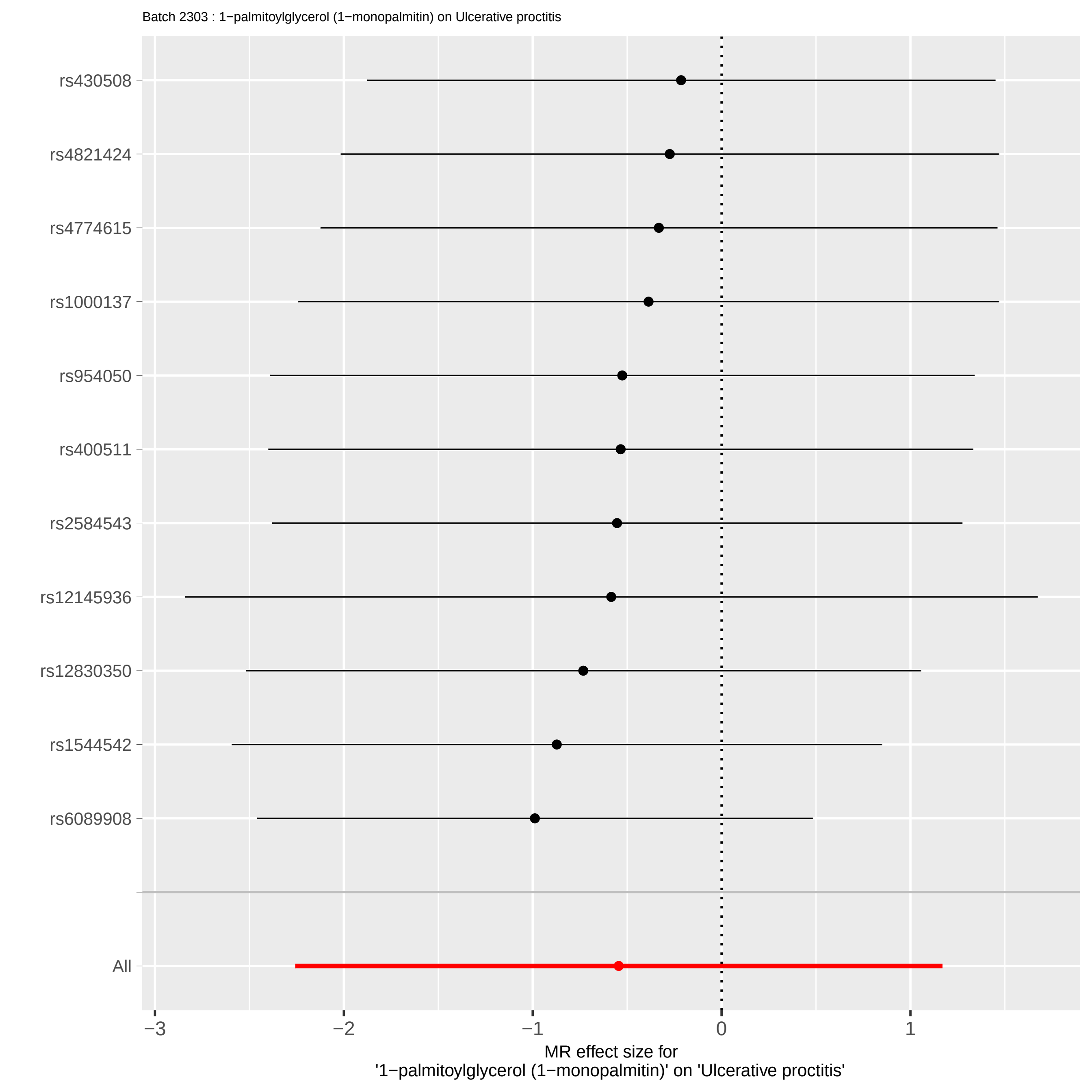

Batch 2303 : 1−palmitoylglycerol (1−monopalmitin) on Ulcerative proctitis
rs430508
rs4821424
rs4774615
rs1000137
rs954050
rs400511
rs2584543
rs12145936
rs12830350
rs1544542
rs6089908
All
−3
−2
−1
0
1
MR effect size for
'1−palmitoylglycerol (1−monopalmitin)' on 'Ulcerative proctitis'

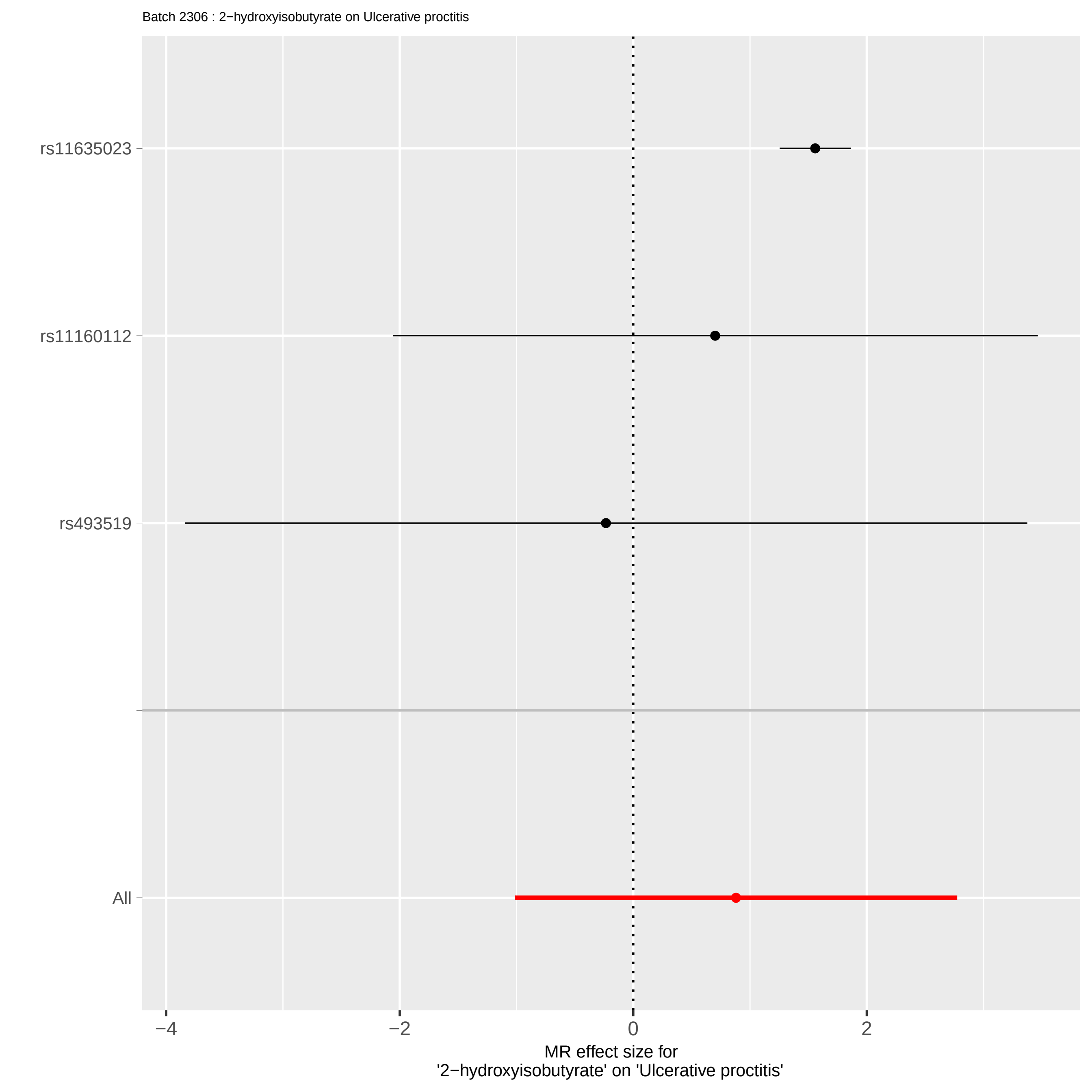
Batch 2306 : 2−hydroxyisobutyrate on Ulcerative proctitis
rs11635023
rs11160112
rs493519
All
−4
−2
0
2
MR effect size for
'2−hydroxyisobutyrate' on 'Ulcerative proctitis'

Batch 2313 : Cholate on Ulcerative proctitis

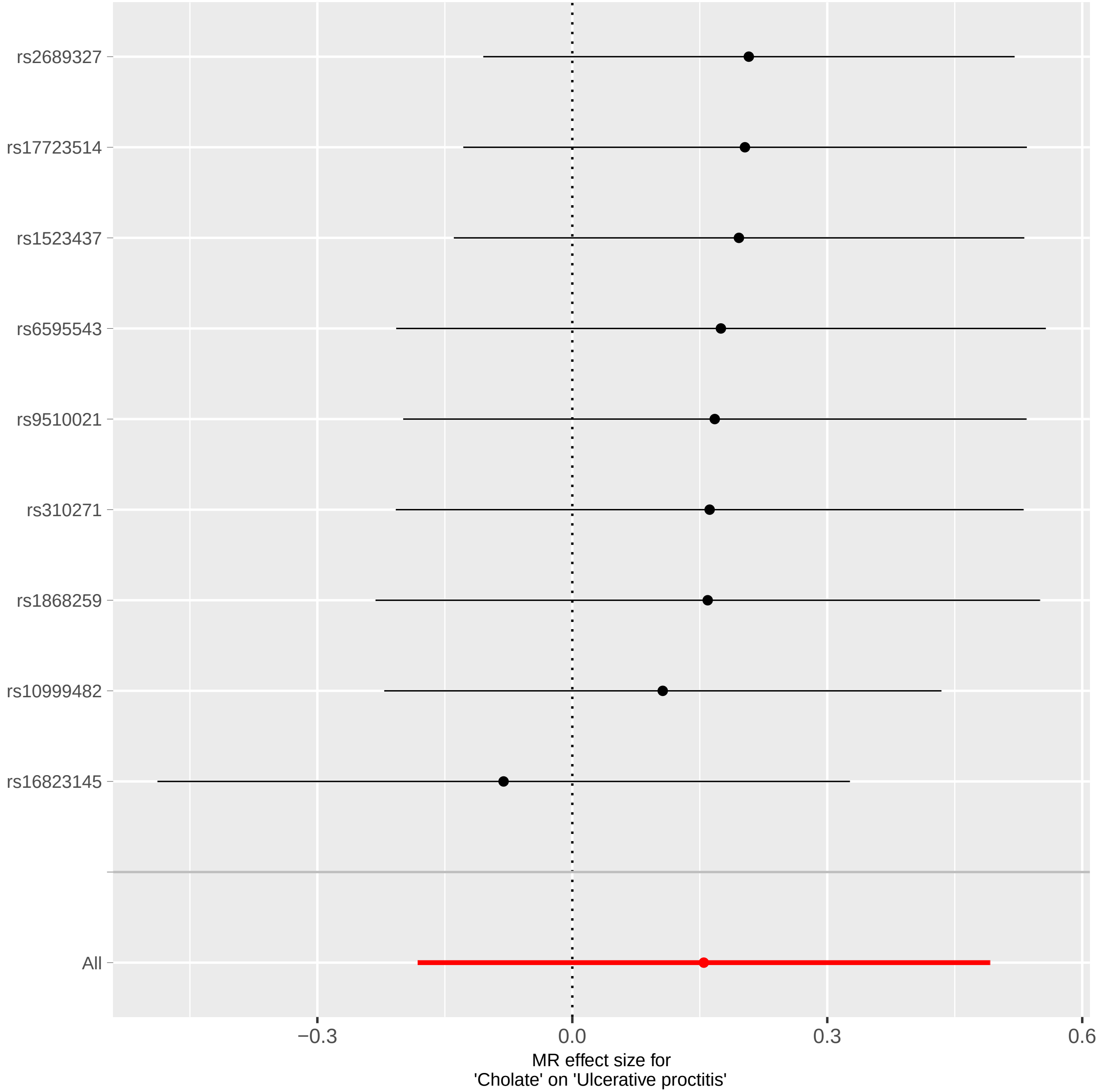

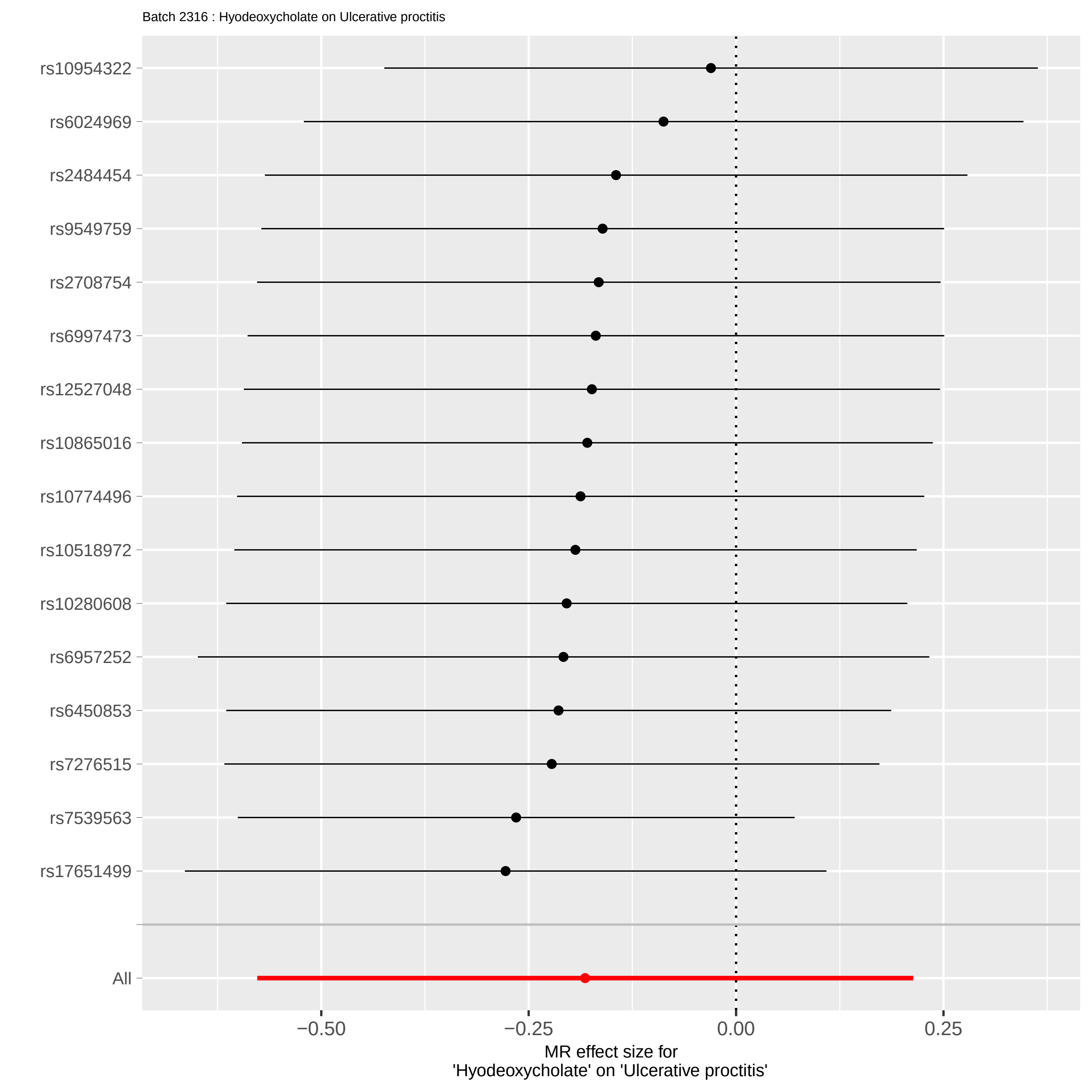

Batch 2316 : Hyodeoxycholate on Ulcerative proctitis
rs10954322
rs6024969
rs2484454
rs9549759
rs2708754
rs6997473
rs12527048
rs10865016
rs10774496
rs10518972
rs10280608
rs6957252
rs6450853
rs7276515
rs7539563
rs17651499
All
−0.50
−0.25
0.00
0.25
MR effect size for
'Hyodeoxycholate' on 'Ulcerative proctitis'

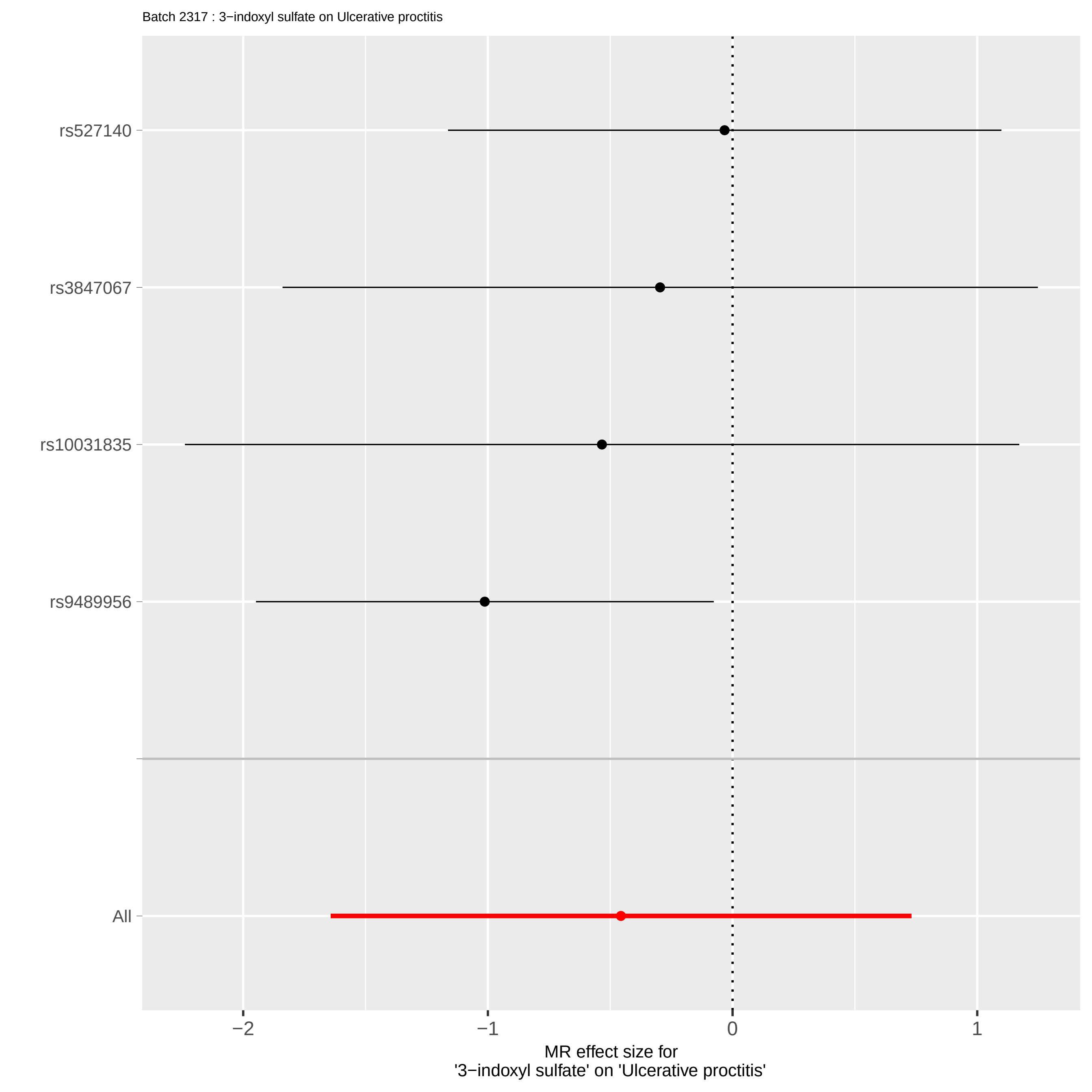
Batch 2317 : 3−indoxyl sulfate on Ulcerative proctitis
rs527140
rs3847067
rs10031835
rs9489956
All
−2
−1
0
1
MR effect size for
'3−indoxyl sulfate' on 'Ulcerative proctitis'

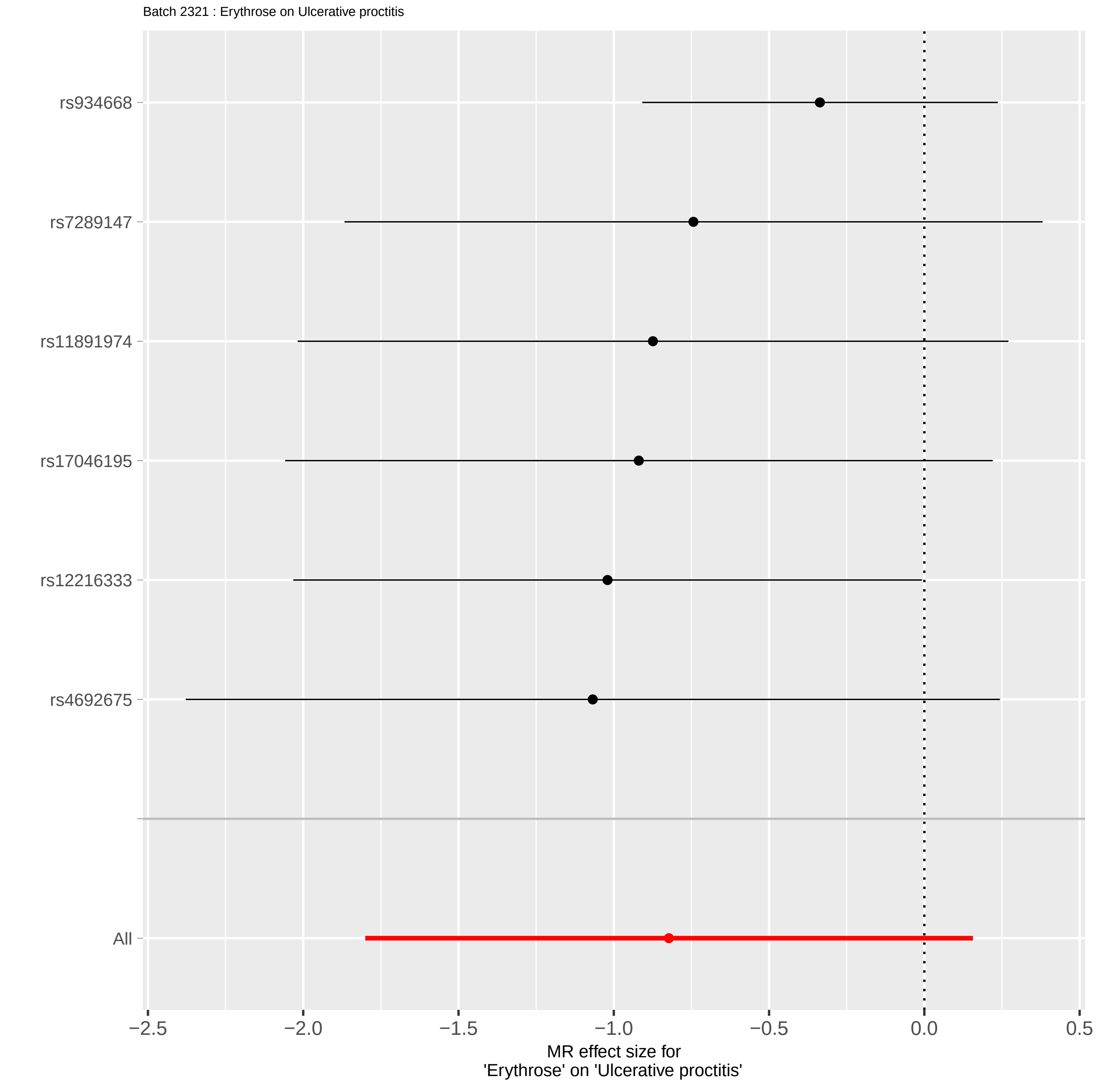

Batch 2321 : Erythrose on Ulcerative proctitis
rs934668
rs7289147
rs11891974
rs17046195
rs12216333
rs4692675
All
−2.5
−2.0
−1.5
−1.0
−0.5
0.0
0.5
MR effect size for
'Erythrose' on 'Ulcerative proctitis'

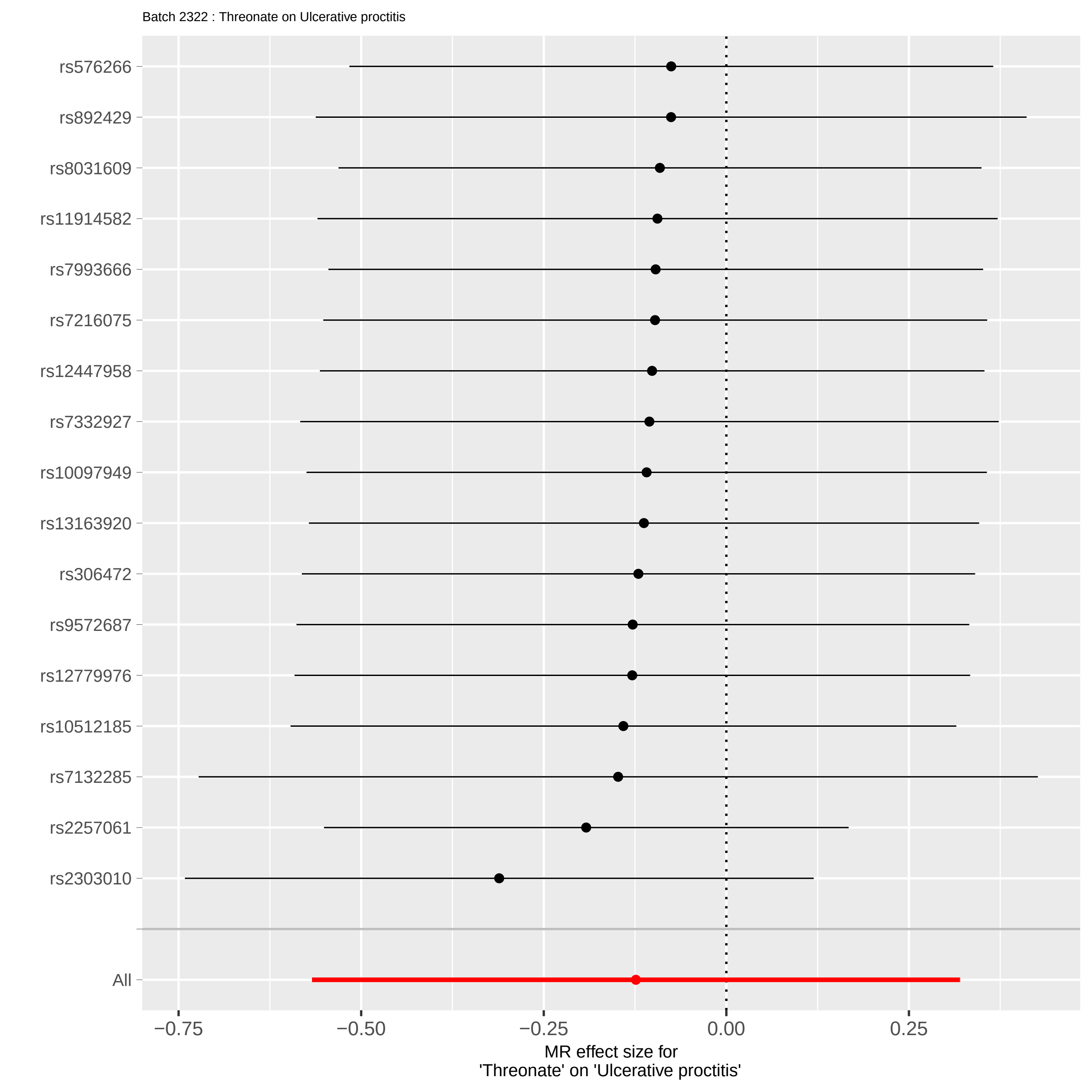

Batch 2322 : Threonate on Ulcerative proctitis
rs576266
rs892429
rs8031609
rs11914582
rs7993666
rs7216075
rs12447958
rs7332927
rs10097949
rs13163920
rs306472
rs9572687
rs12779976
rs10512185
rs7132285
rs2257061
rs2303010
All
−0.75
−0.50
−0.25
0.00
0.25
MR effect size for
'Threonate' on 'Ulcerative proctitis'

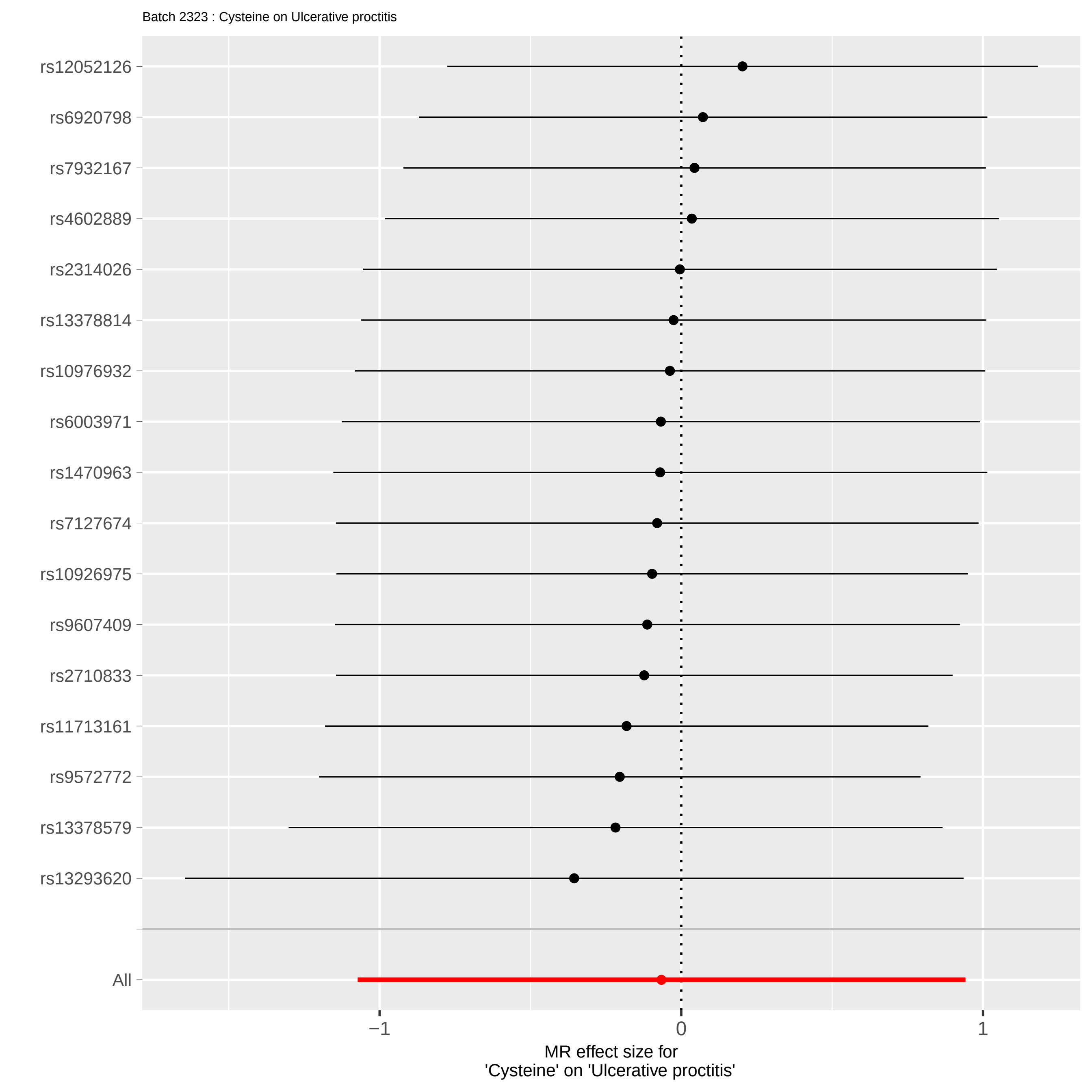

Batch 2323 : Cysteine on Ulcerative proctitis
rs12052126
rs6920798
rs7932167
rs4602889
rs2314026
rs13378814
rs10976932
rs6003971
rs1470963
rs7127674
rs10926975
rs9607409
rs2710833
rs11713161
rs9572772
rs13378579
rs13293620
All
−1
0
1
MR effect size for
'Cysteine' on 'Ulcerative proctitis'

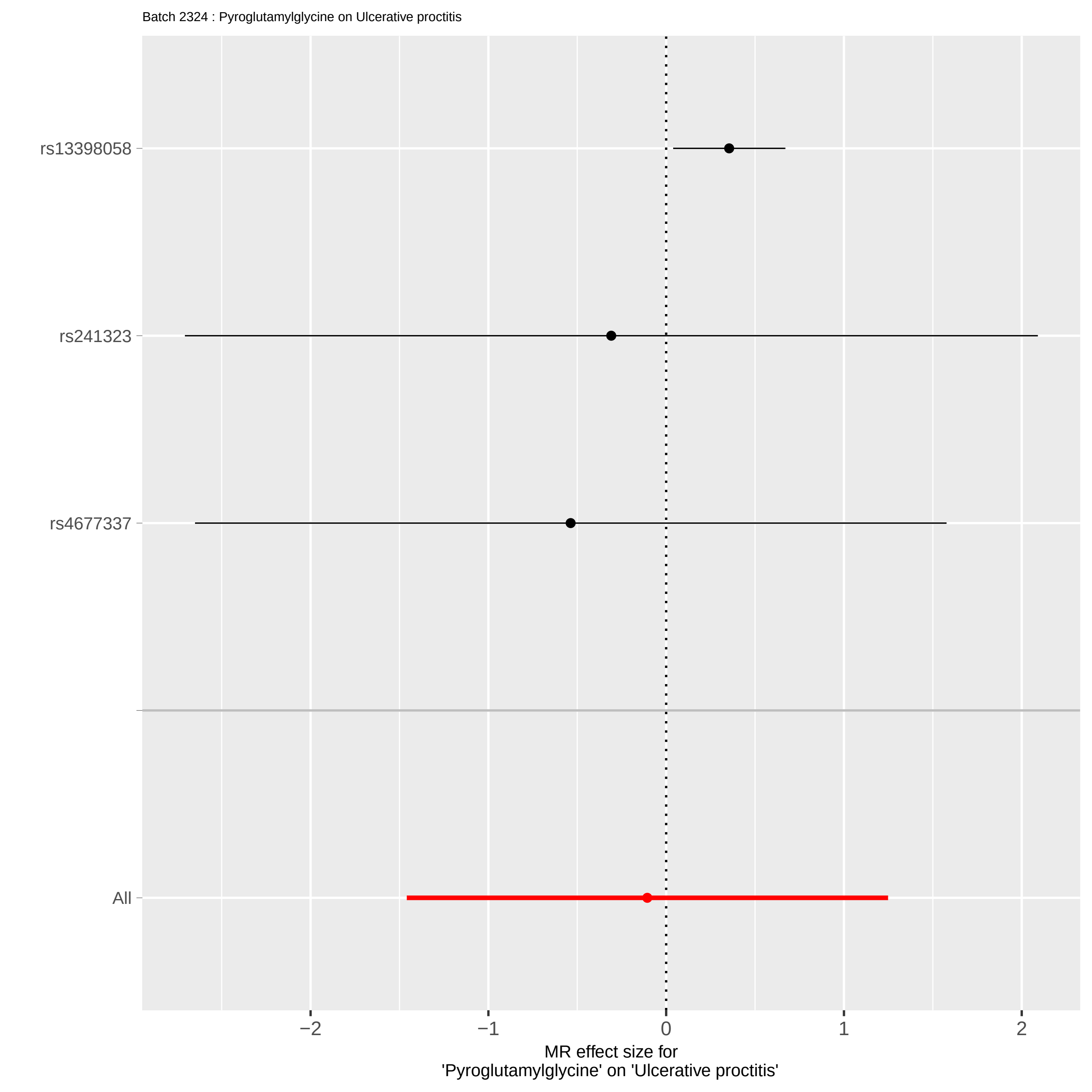
Batch 2324 : Pyroglutamylglycine on Ulcerative proctitis
rs13398058
rs241323
rs4677337
All
−2
−1
0
1
2
MR effect size for
'Pyroglutamylglycine' on 'Ulcerative proctitis'

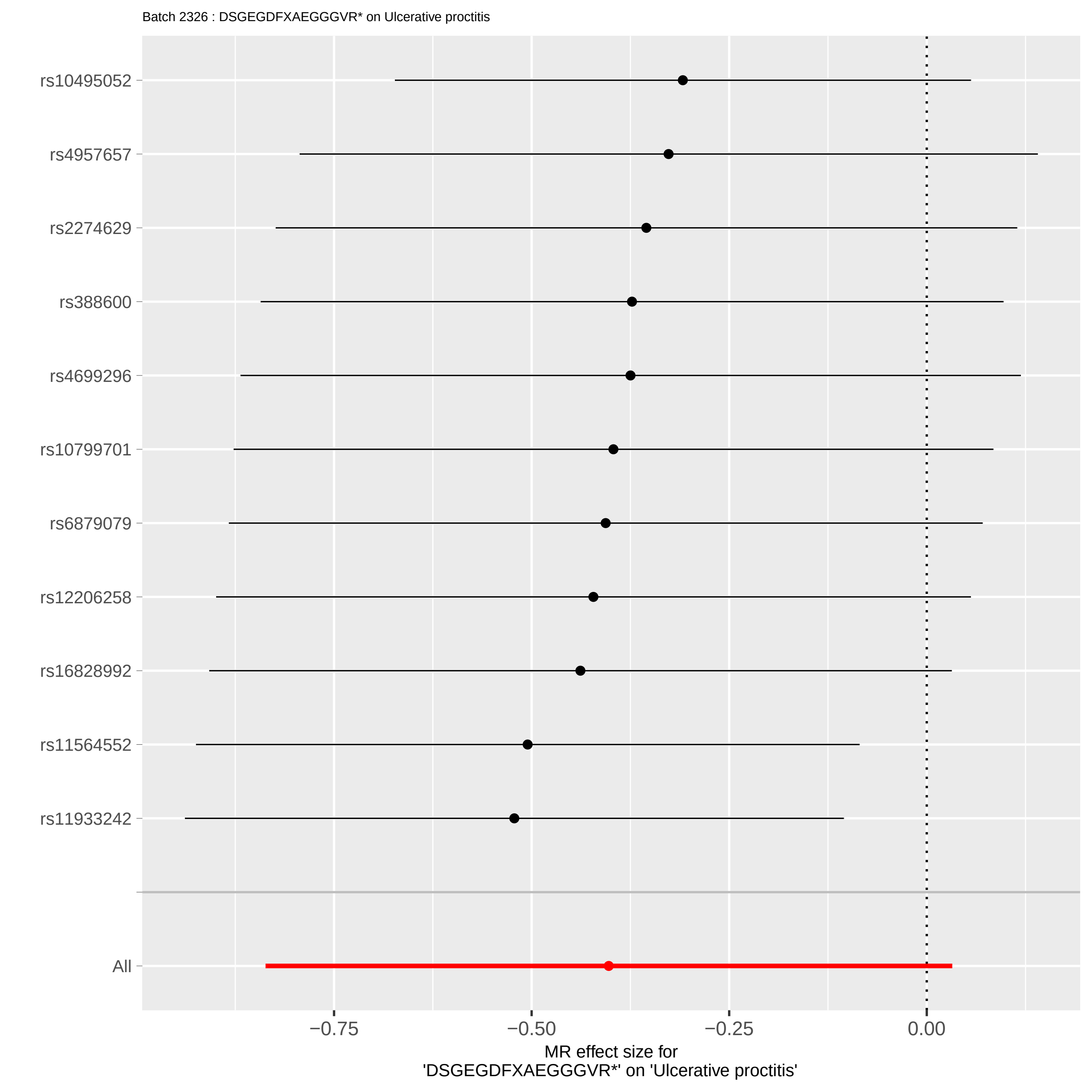

Batch 2326 : DSGEGDFXAEGGGVR* on Ulcerative proctitis
rs10495052
rs4957657
rs2274629
rs388600
rs4699296
rs10799701
rs6879079
rs12206258
rs16828992
rs11564552
rs11933242
All
−0.75
−0.50
−0.25
0.00
MR effect size for
'DSGEGDFXAEGGGVR*' on 'Ulcerative proctitis'

Batch 2327 : Pyridoxate on Ulcerative proctitis

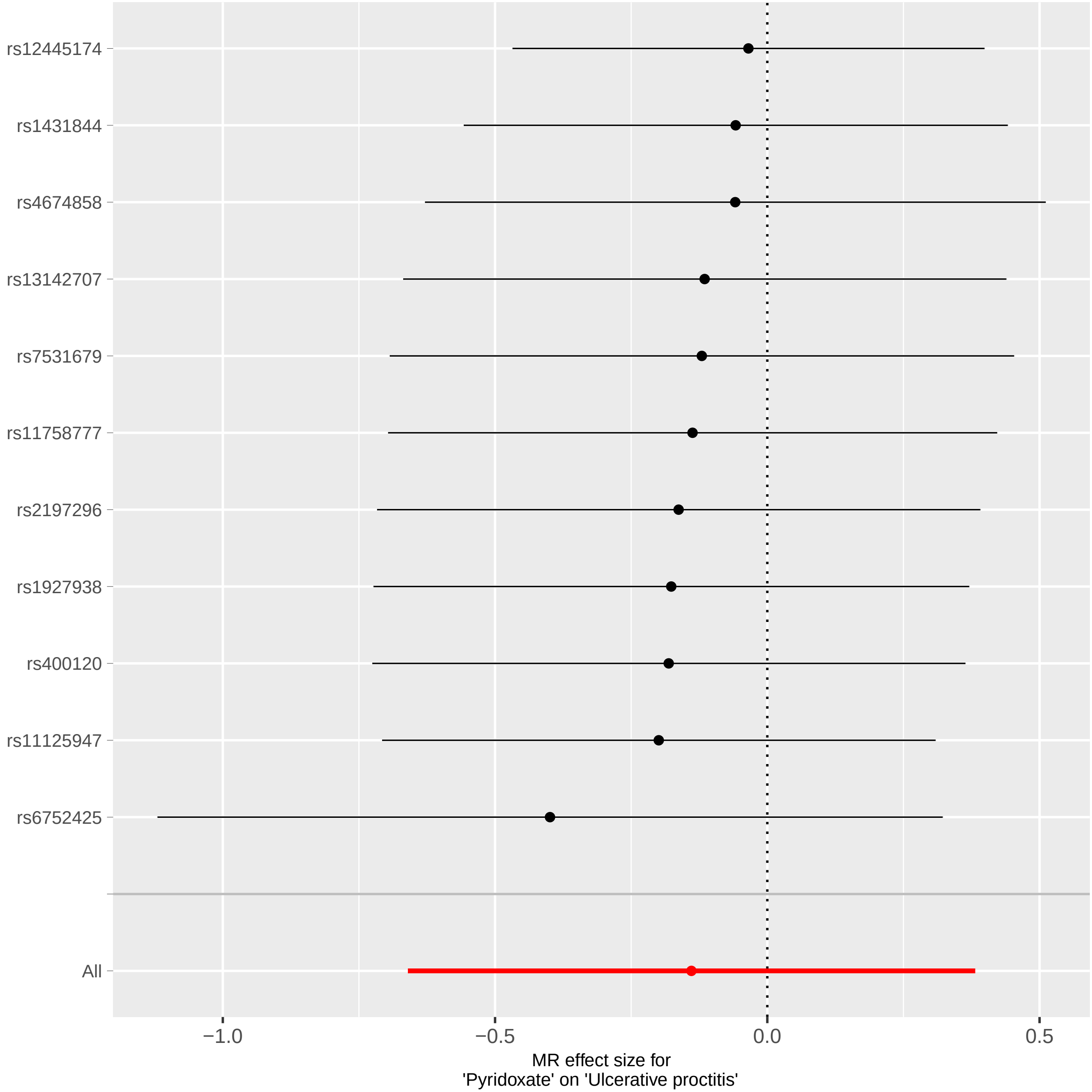

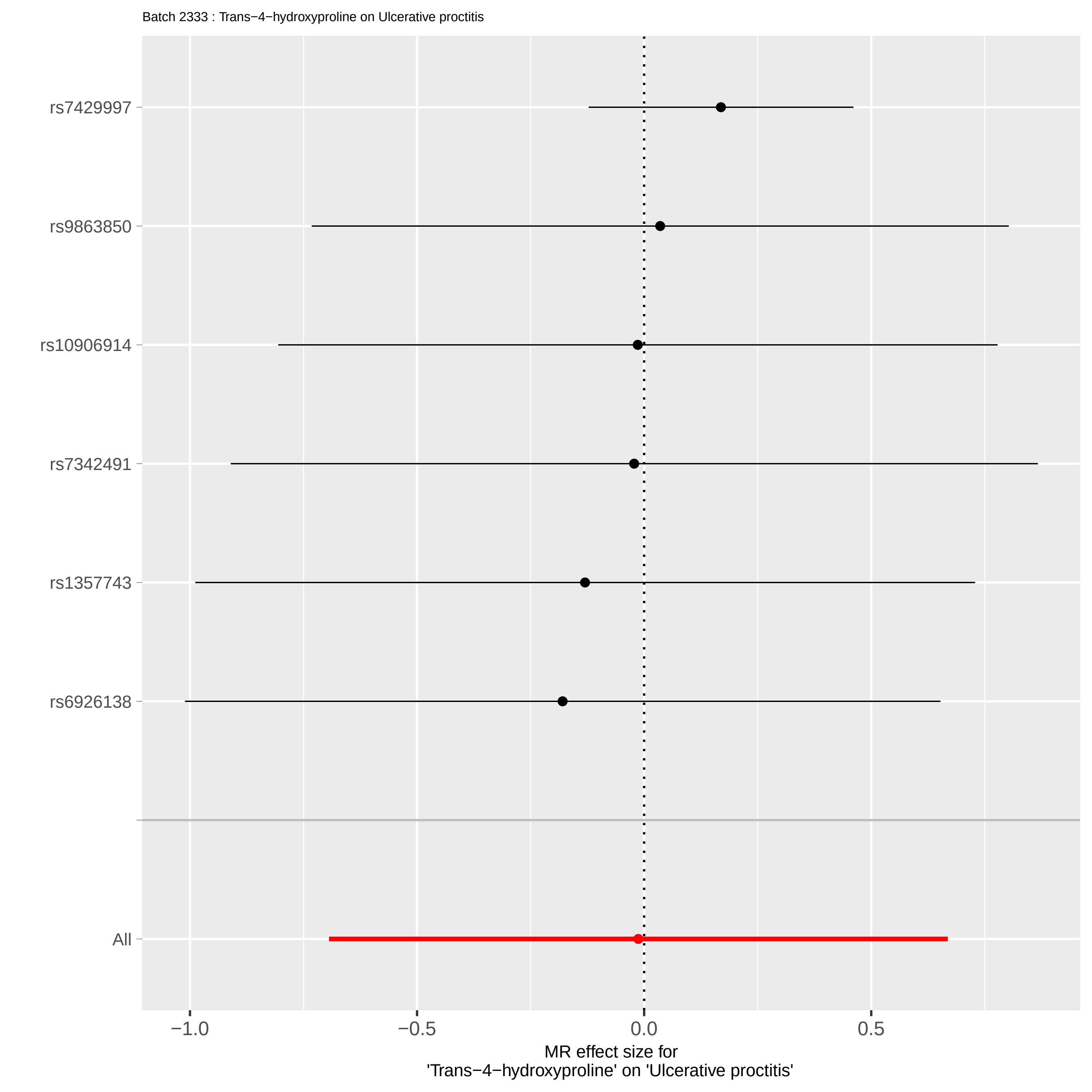

Batch 2333 : Trans−4−hydroxyproline on Ulcerative proctitis
rs7429997
rs9863850
rs10906914
rs7342491
rs1357743
rs6926138
All
−1.0
−0.5
0.0
0.5
MR effect size for
'Trans−4−hydroxyproline' on 'Ulcerative proctitis'

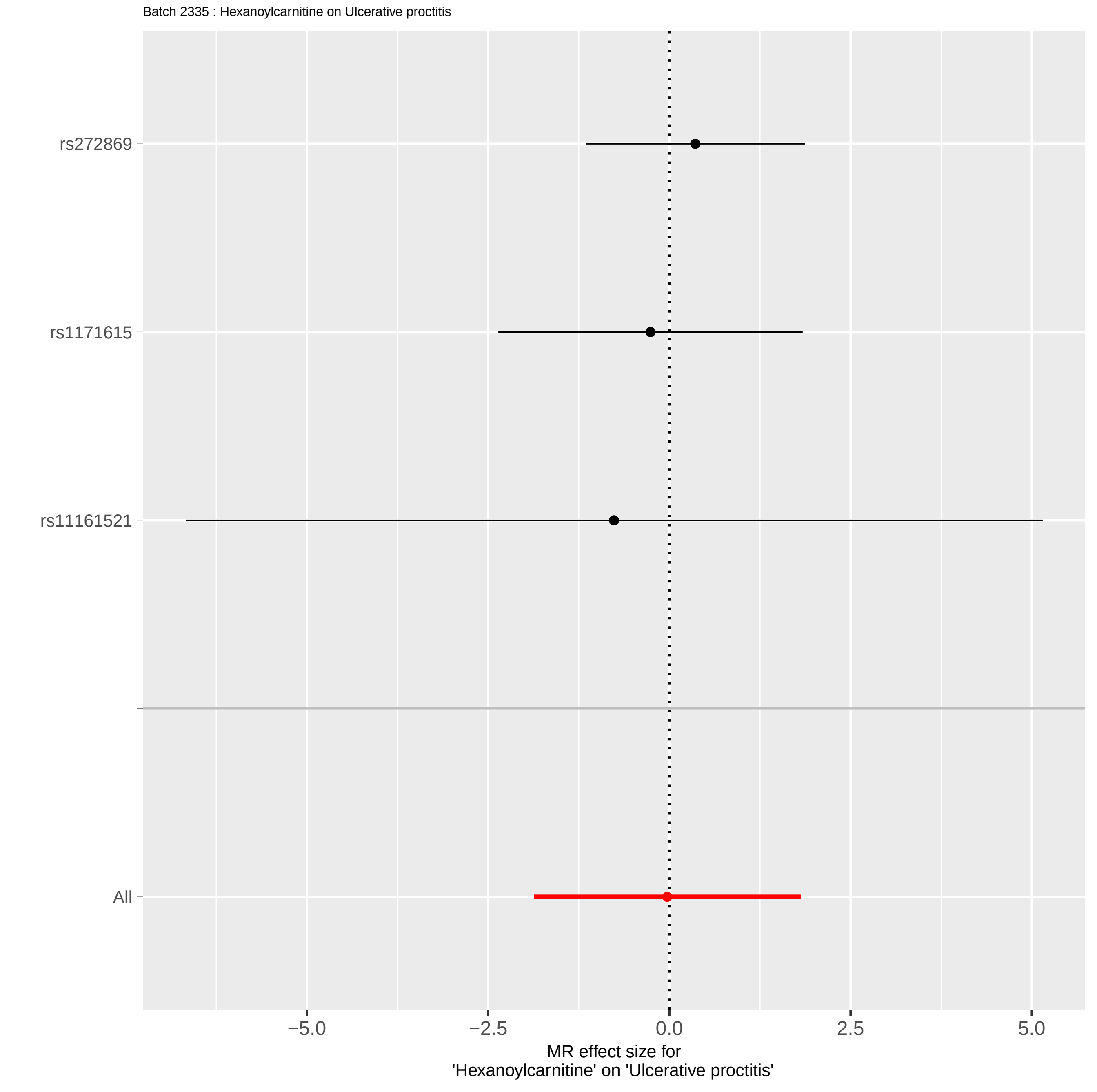

Batch 2335 : Hexanoylcarnitine on Ulcerative proctitis
rs272869
rs1171615
rs11161521
All
−5.0
−2.5
0.0
2.5
5.0
MR effect size for
'Hexanoylcarnitine' on 'Ulcerative proctitis'

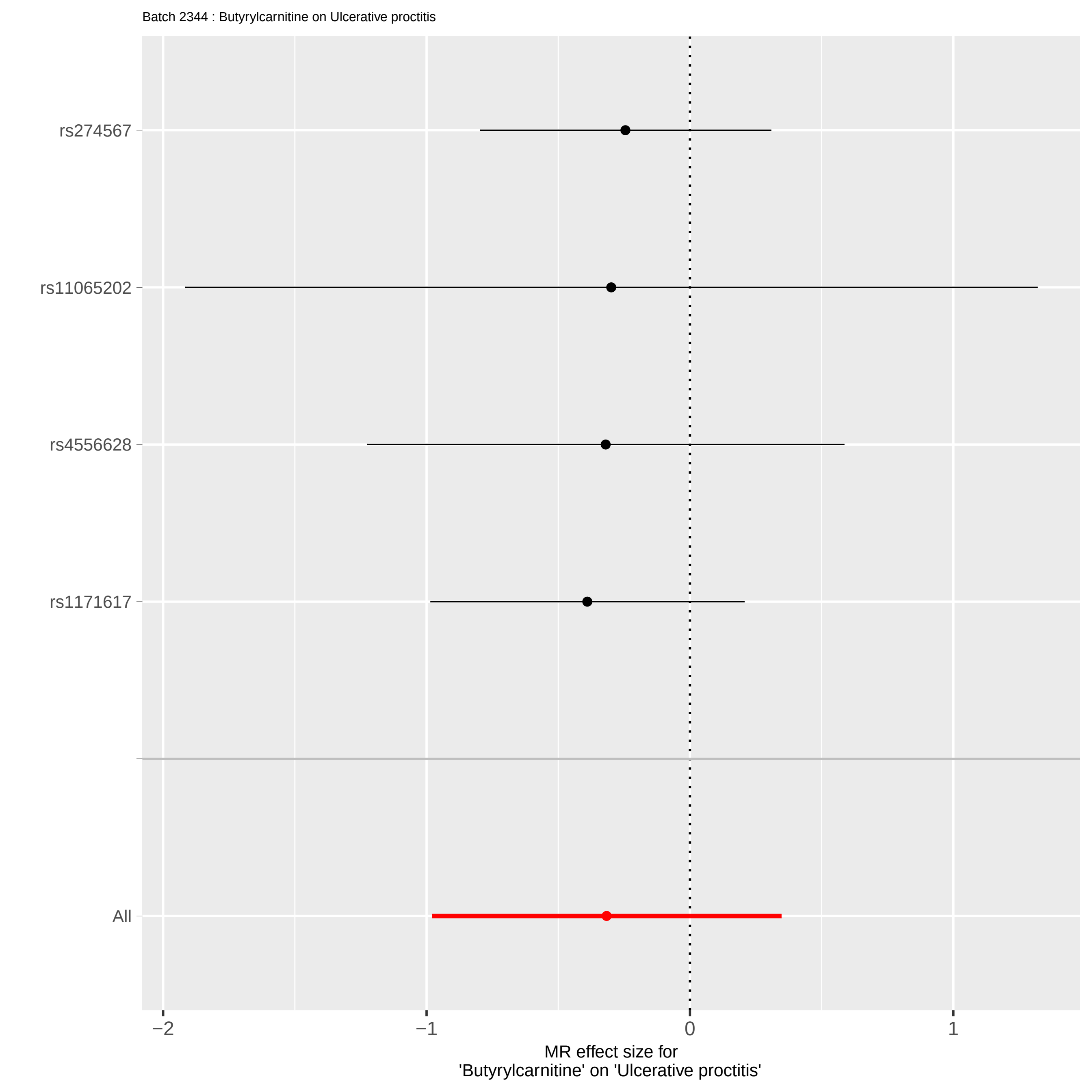

Batch 2344 : Butyrylcarnitine on Ulcerative proctitis
rs274567
rs11065202
rs4556628
rs1171617
All
−2
−1
0
1
MR effect size for
'Butyrylcarnitine' on 'Ulcerative proctitis'

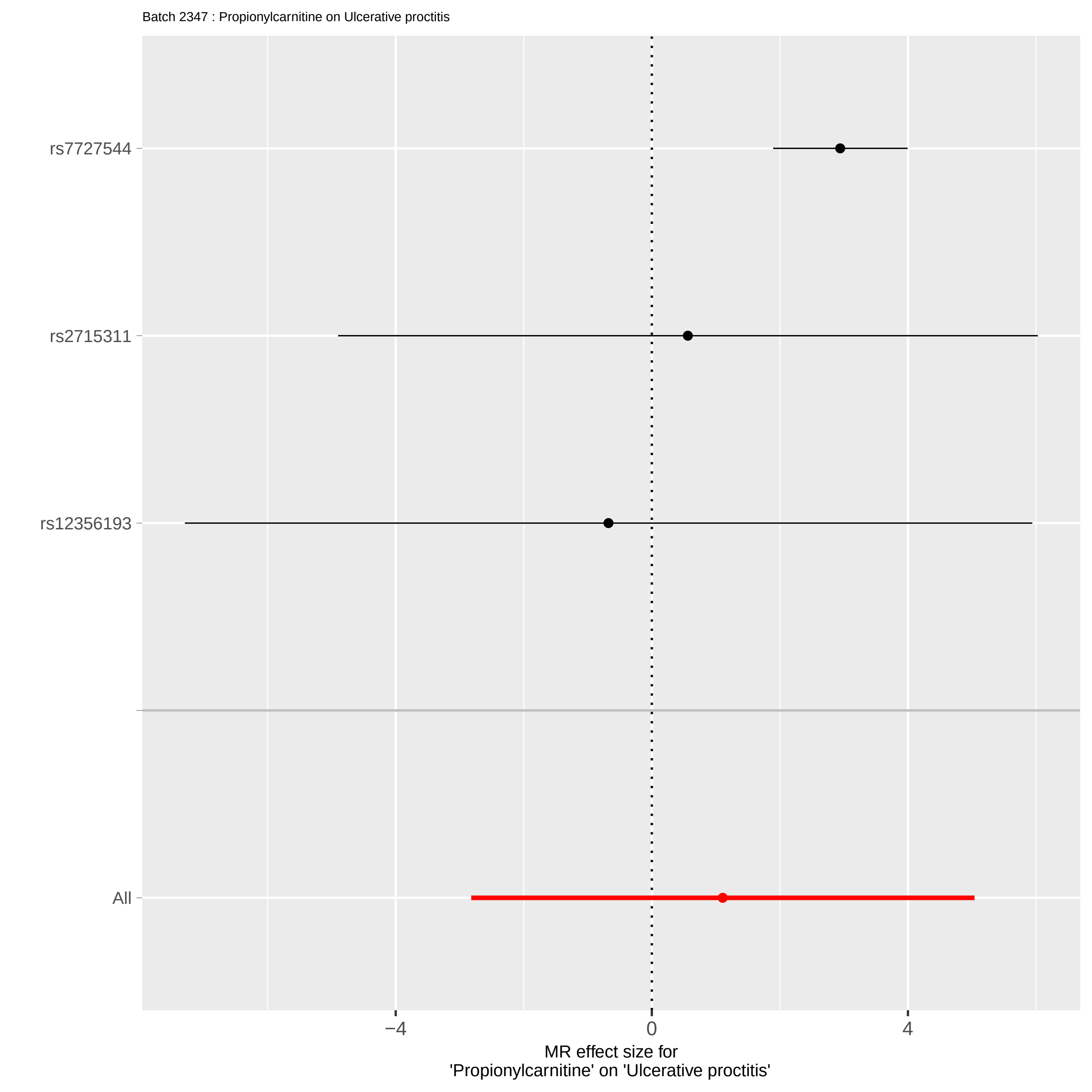

Batch 2347 : Propionylcarnitine on Ulcerative proctitis
rs7727544
rs2715311
rs12356193
All
−4
0
4
MR effect size for
'Propionylcarnitine' on 'Ulcerative proctitis'

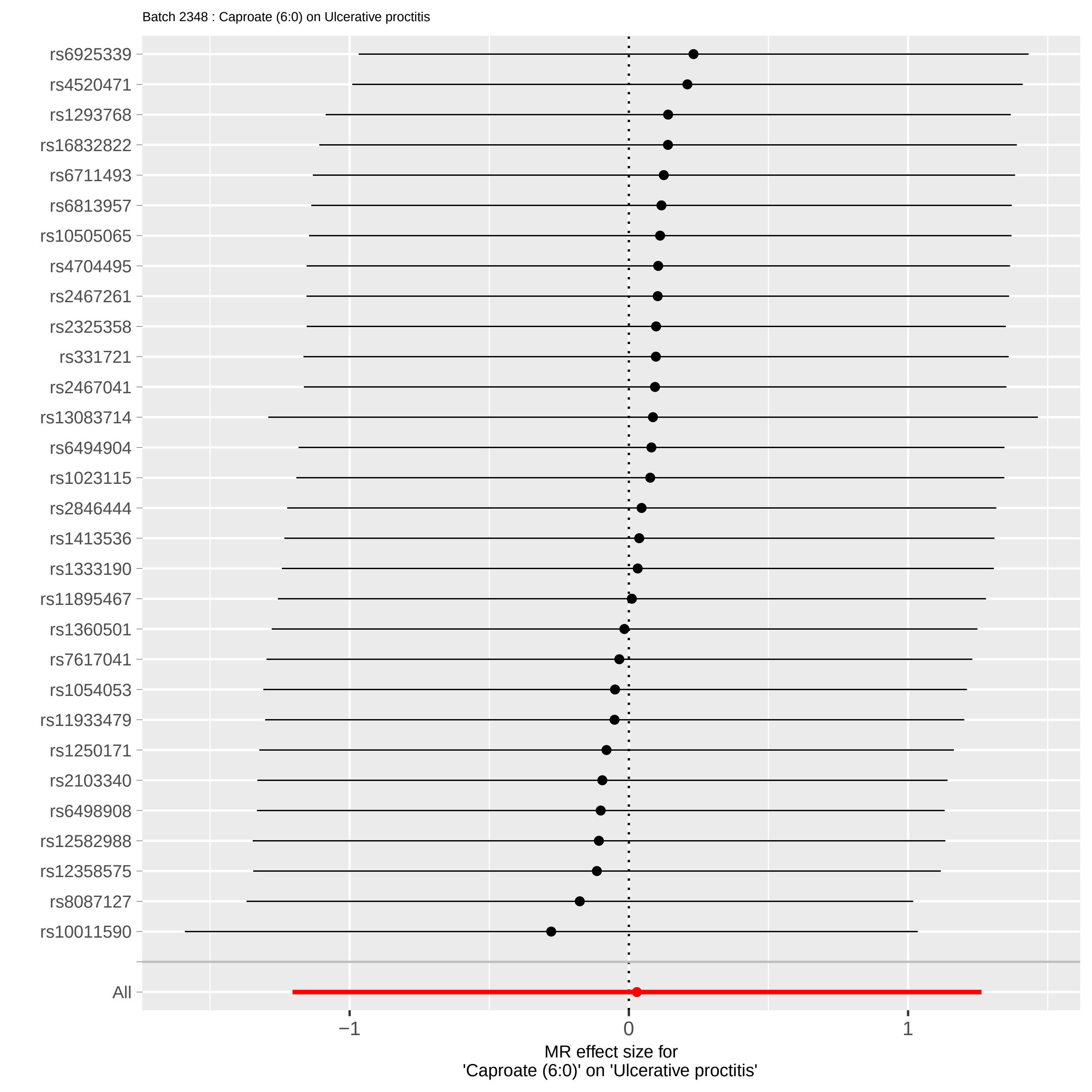

Batch 2348 : Caproate (6:0) on Ulcerative proctitis
rs6925339
rs4520471
rs1293768
rs16832822
rs6711493
rs6813957
rs10505065
rs4704495
rs2467261
rs2325358
rs331721
rs2467041
rs13083714
rs6494904
rs1023115
rs2846444
rs1413536
rs1333190
rs11895467
rs1360501
rs7617041
rs1054053
rs11933479
rs1250171
rs2103340
rs6498908
rs12582988
rs12358575
rs8087127
rs10011590
All
−1
0
1
MR effect size for
'Caproate (6:0)' on 'Ulcerative proctitis'

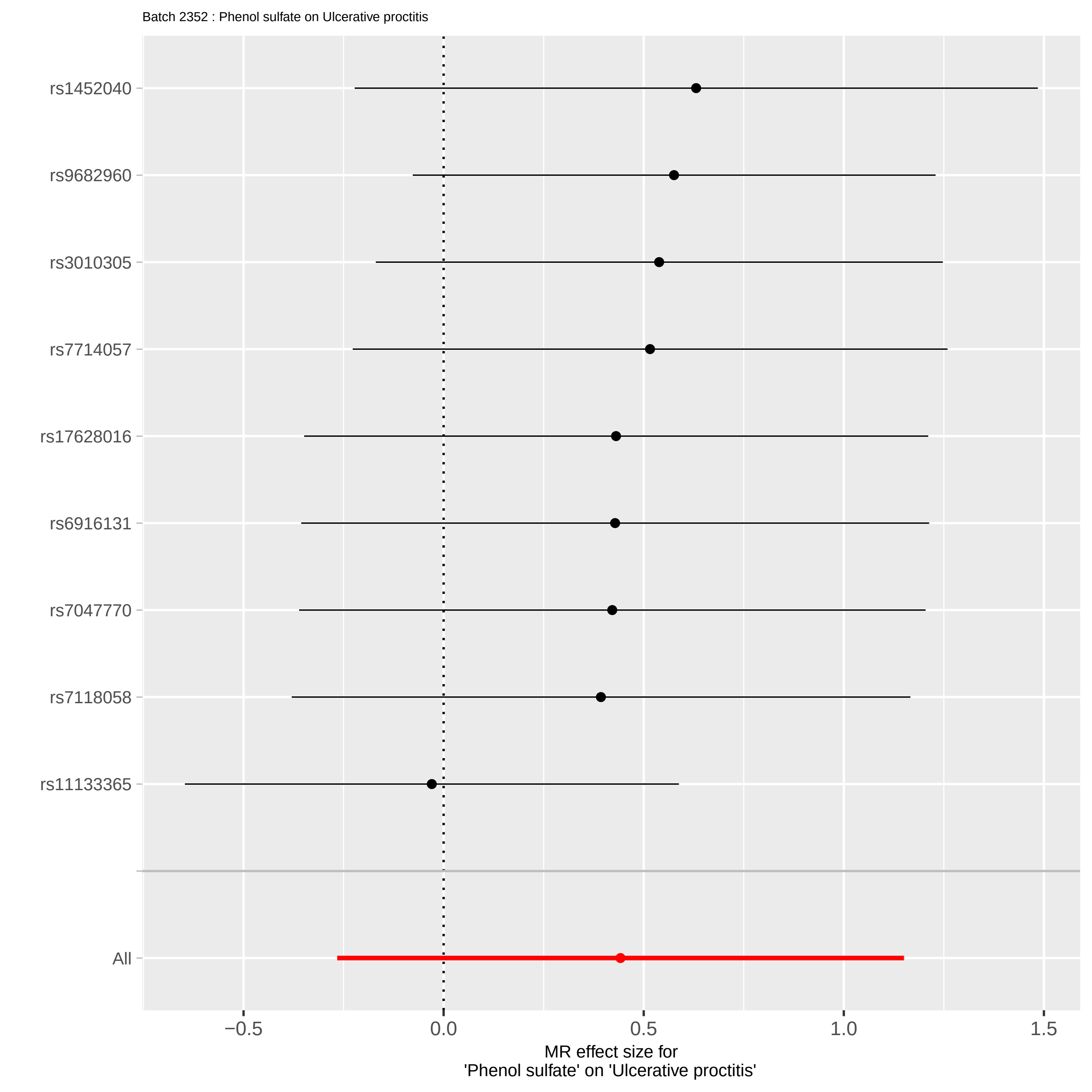

Batch 2352 : Phenol sulfate on Ulcerative proctitis
rs1452040
rs9682960
rs3010305
rs7714057
rs17628016
rs6916131
rs7047770
rs7118058
rs11133365
All
−0.5
0.0
0.5
1.0
1.5
MR effect size for
'Phenol sulfate' on 'Ulcerative proctitis'

Batch 2354 : Heme* on Ulcerative proctitis

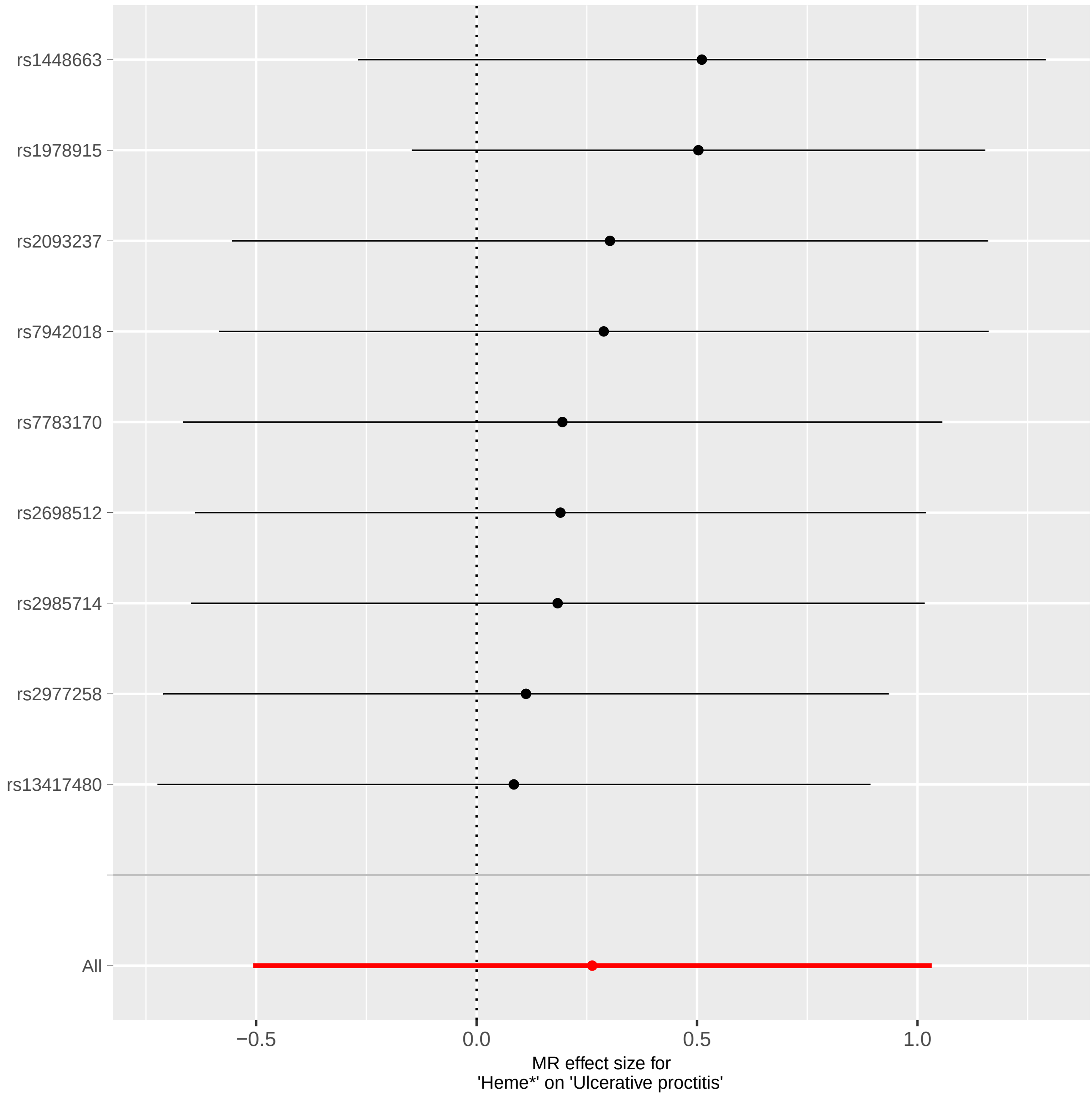

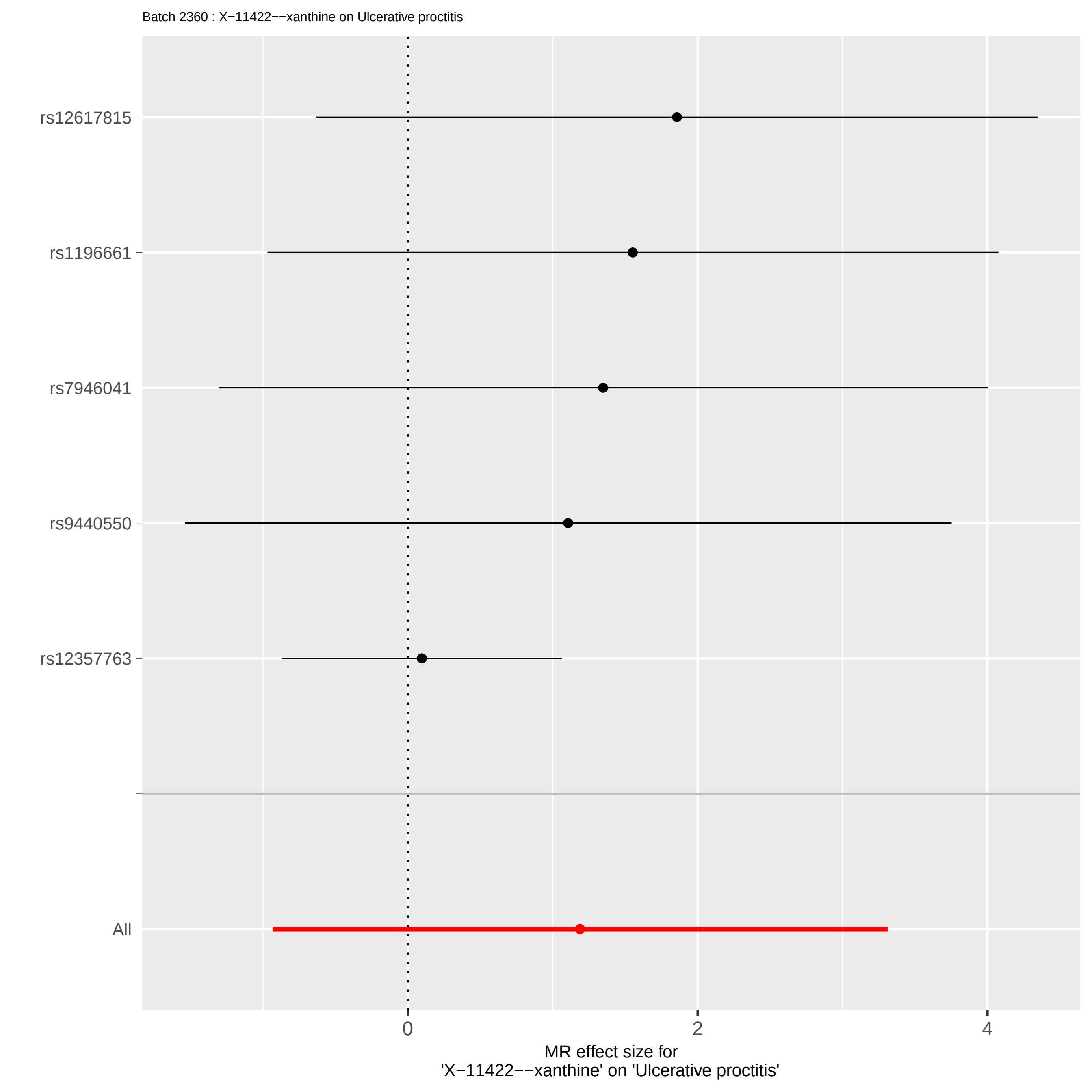

Batch 2360 : X−11422−−xanthine on Ulcerative proctitis
rs12617815
rs1196661
rs7946041
rs9440550
rs12357763
All
0
2
4
MR effect size for
'X−11422−−xanthine' on 'Ulcerative proctitis'

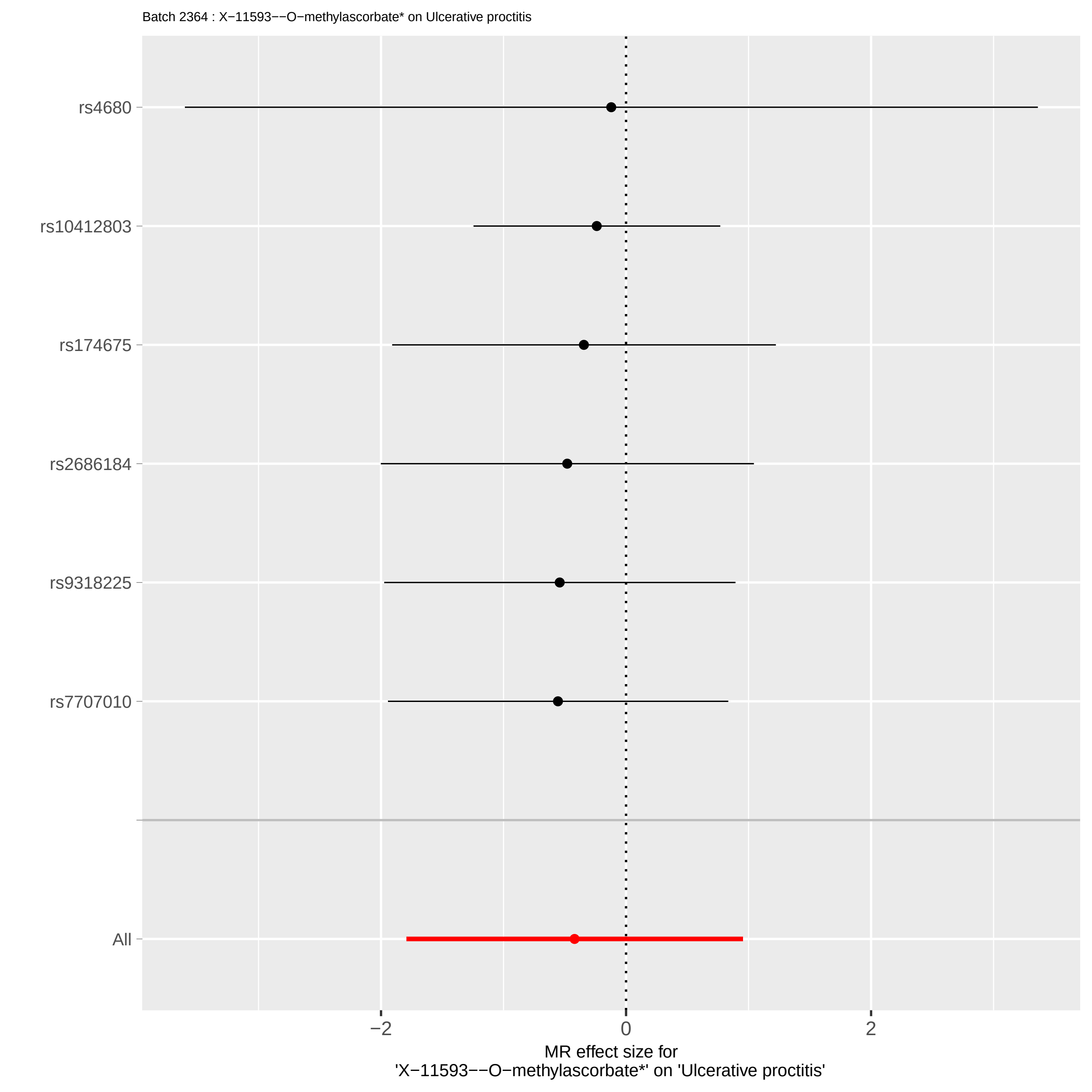
Batch 2364 : X−11593−−O−methylascorbate* on Ulcerative proctitis
rs4680
rs10412803
rs174675
rs2686184
rs9318225
rs7707010
All
−2
0
2
MR effect size for
'X−11593−−O−methylascorbate*' on 'Ulcerative proctitis'

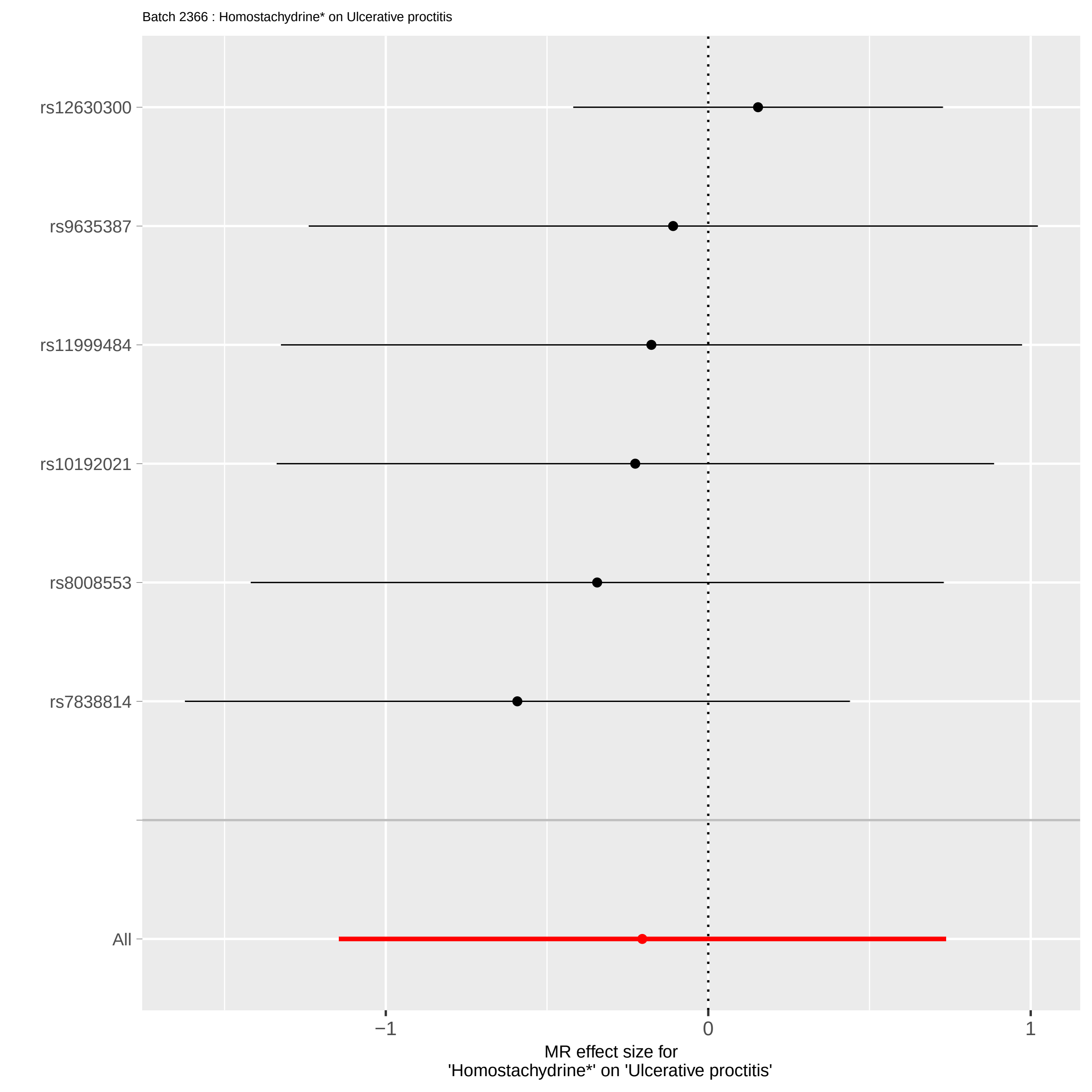

Batch 2366 : Homostachydrine* on Ulcerative proctitis
rs12630300
rs9635387
rs11999484
rs10192021
rs8008553
rs7838814
All
−1
0
1
MR effect size for
'Homostachydrine*' on 'Ulcerative proctitis'

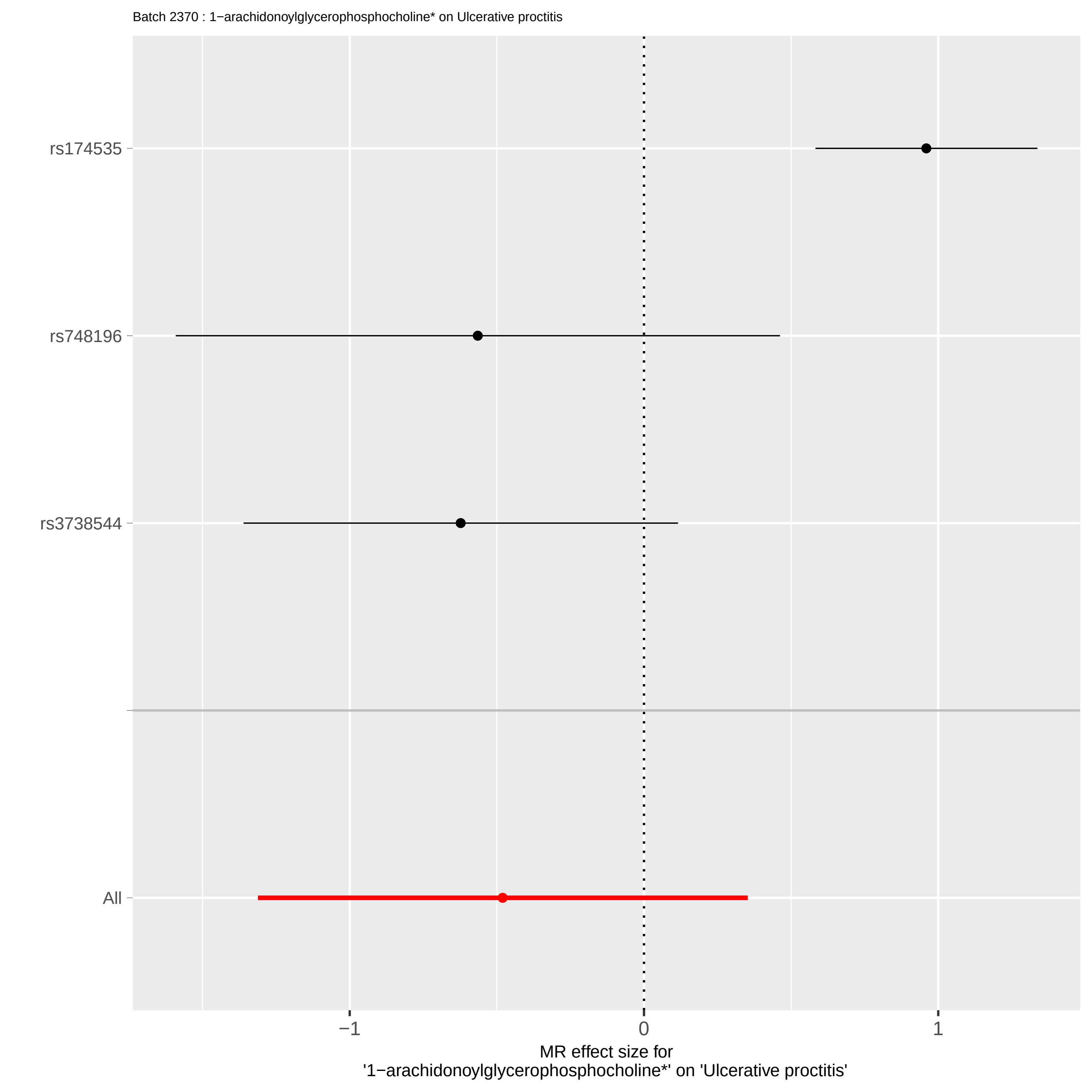
Batch 2370 : 1−arachidonoylglycerophosphocholine* on Ulcerative proctitis
rs174535
rs748196
rs3738544
All
−1
0
1
MR effect size for
'1−arachidonoylglycerophosphocholine*' on 'Ulcerative proctitis'

Batch 2372 : Gamma−glutamylmethionine* on Ulcerative proctitis

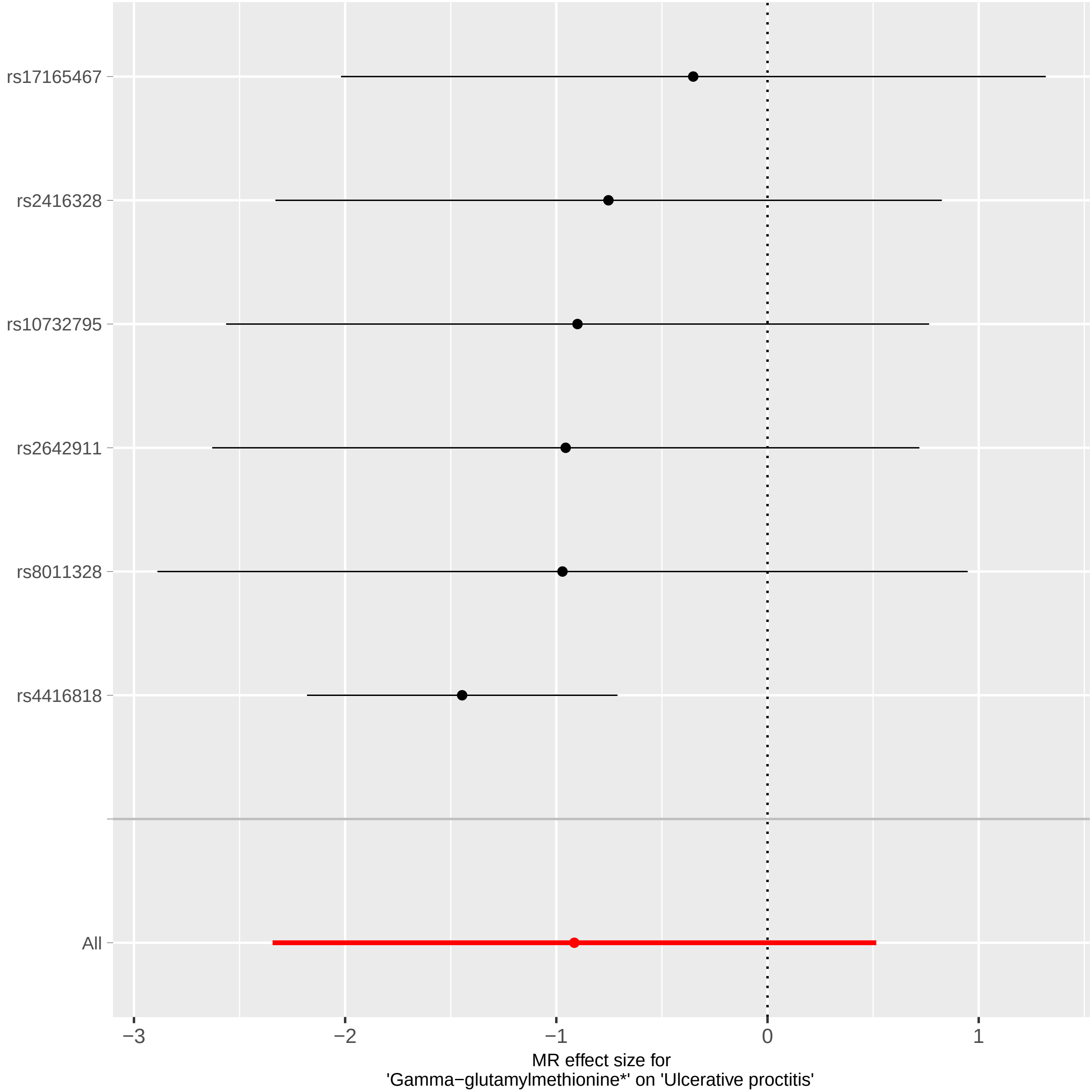

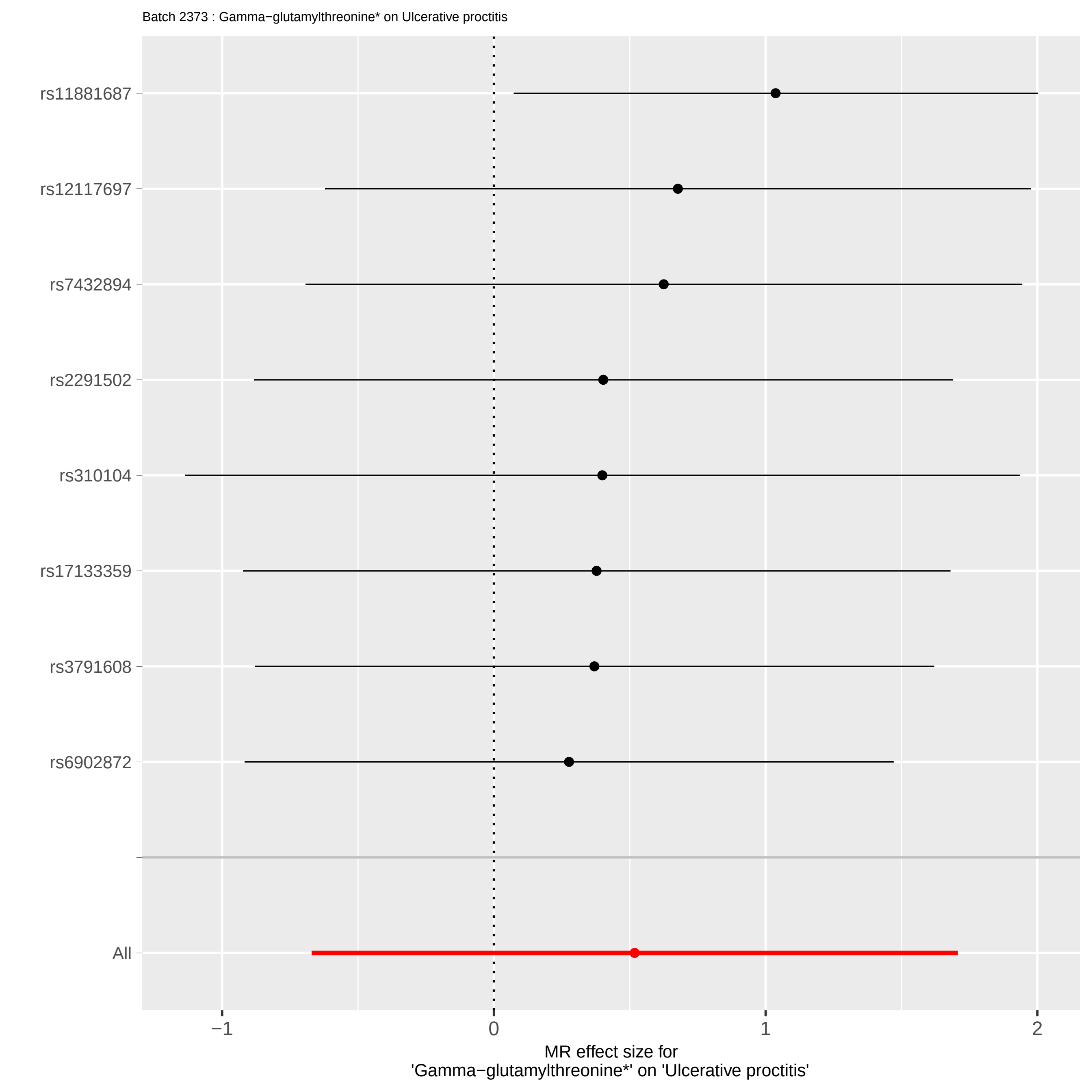

Batch 2373 : Gamma−glutamylthreonine* on Ulcerative proctitis
rs11881687
rs12117697
rs7432894
rs2291502
rs310104
rs17133359
rs3791608
rs6902872
All
−1
0
1
2
MR effect size for
'Gamma−glutamylthreonine*' on 'Ulcerative proctitis'

Batch 2374 : Gamma−tocopherol on Ulcerative proctitis

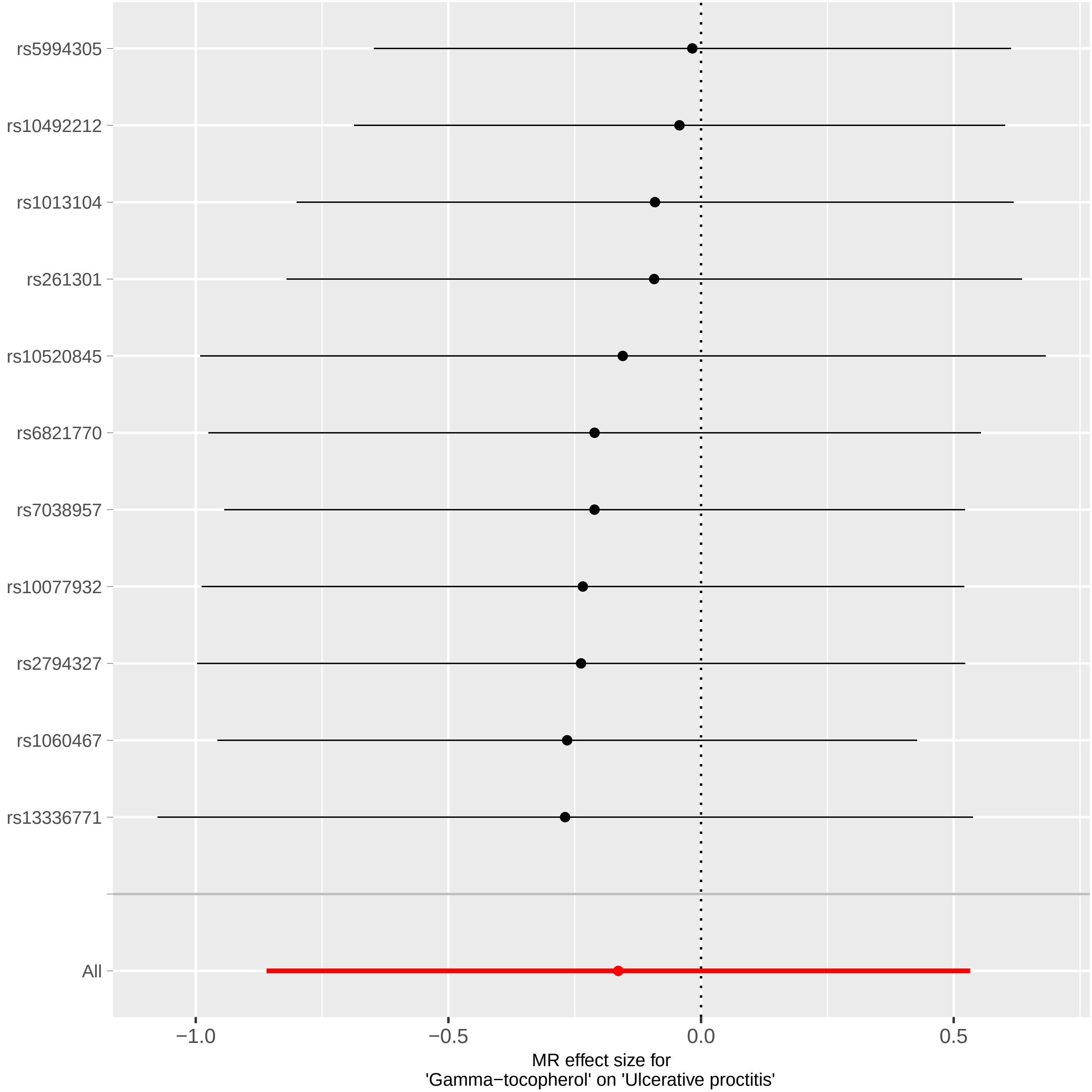

Batch 2377 : Pseudouridine on Ulcerative proctitis

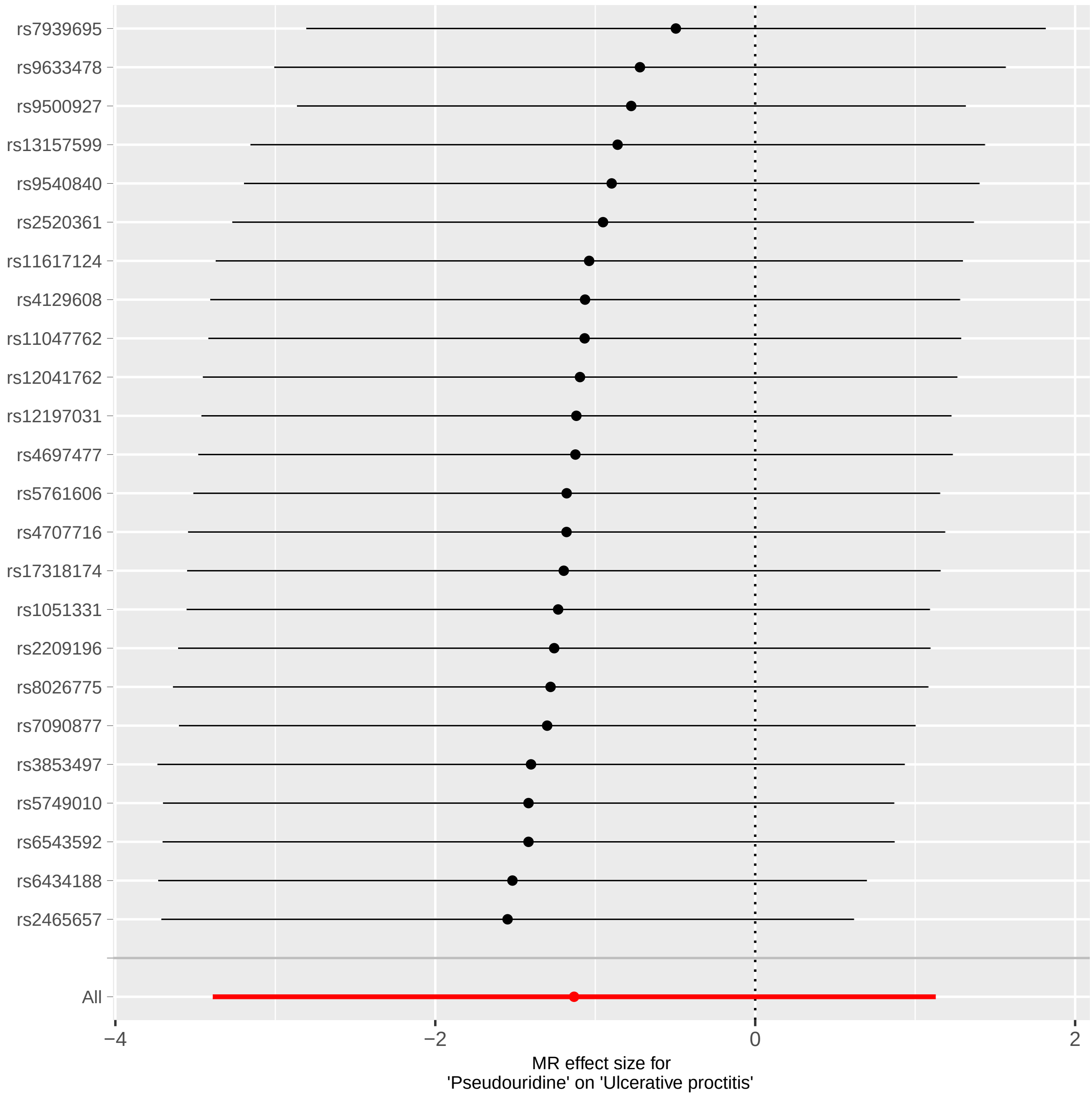

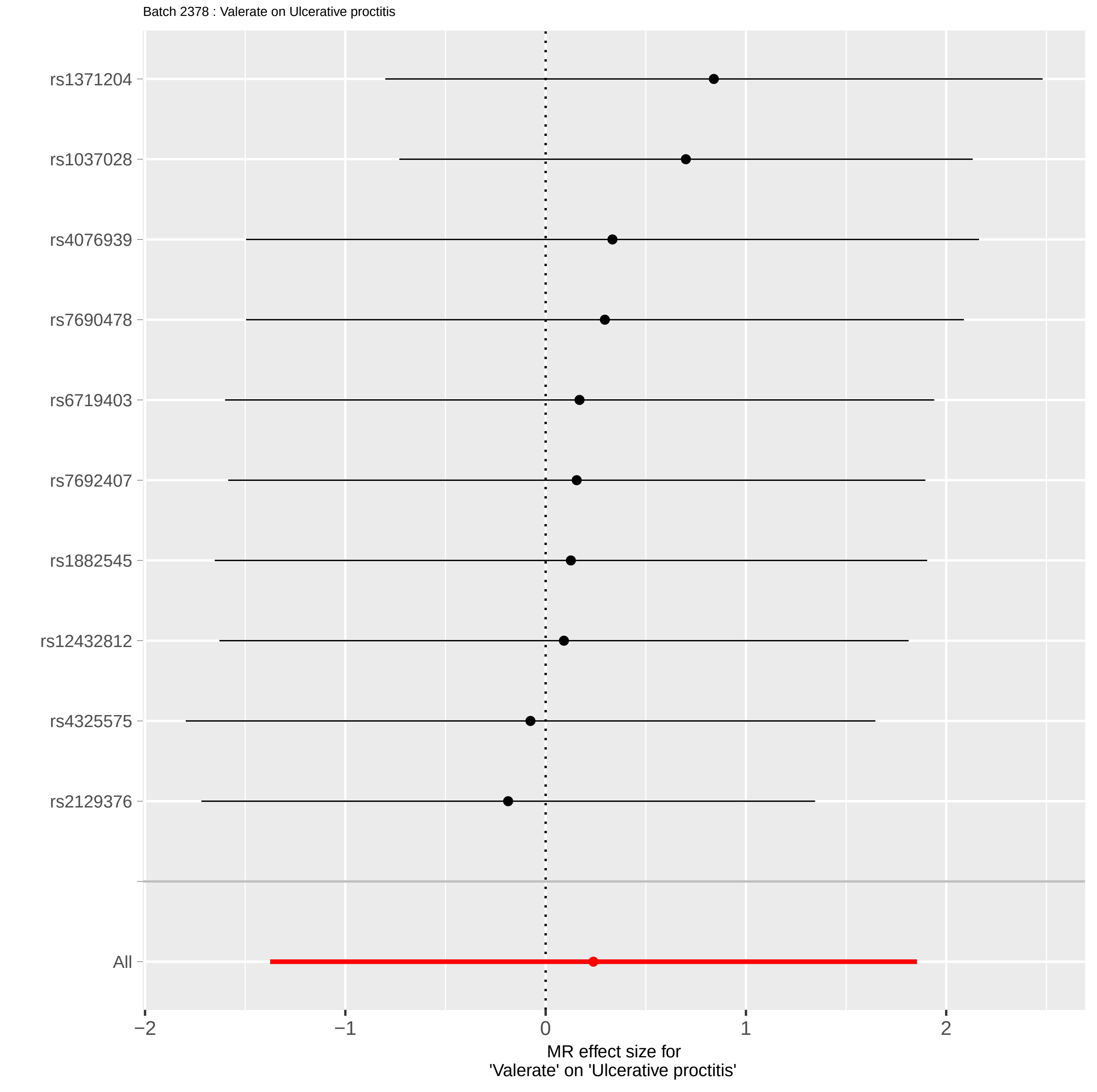
Batch 2378 : Valerate on Ulcerative proctitis
rs1371204
rs1037028
rs4076939
rs7690478
rs6719403
rs7692407
rs1882545
rs12432812
rs4325575
rs2129376
All
−2
−1
0
1
2
MR effect size for
'Valerate' on 'Ulcerative proctitis'

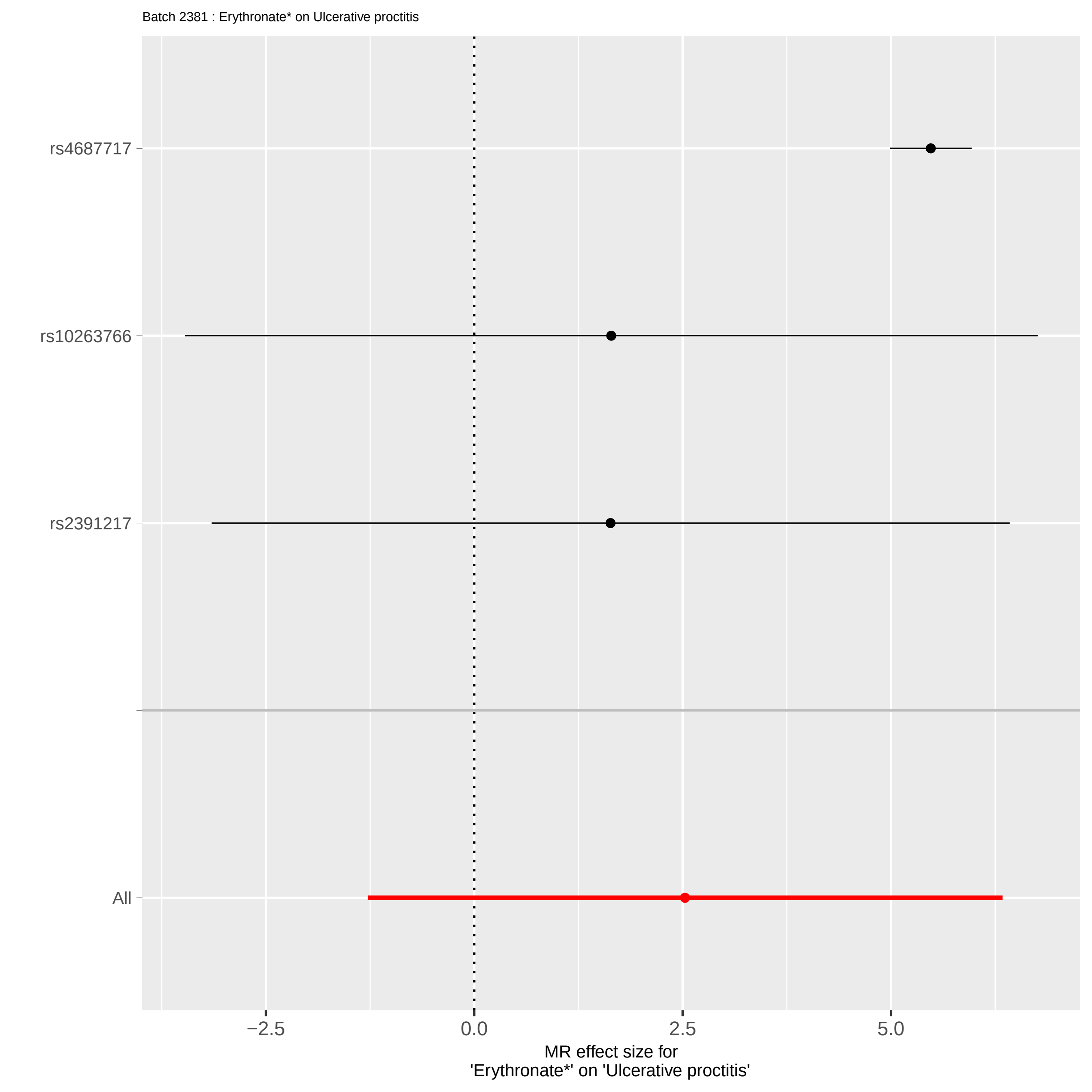

Batch 2381 : Erythronate* on Ulcerative proctitis
rs4687717
rs10263766
rs2391217
All
−2.5
0.0
2.5
5.0
MR effect size for
'Erythronate*' on 'Ulcerative proctitis'

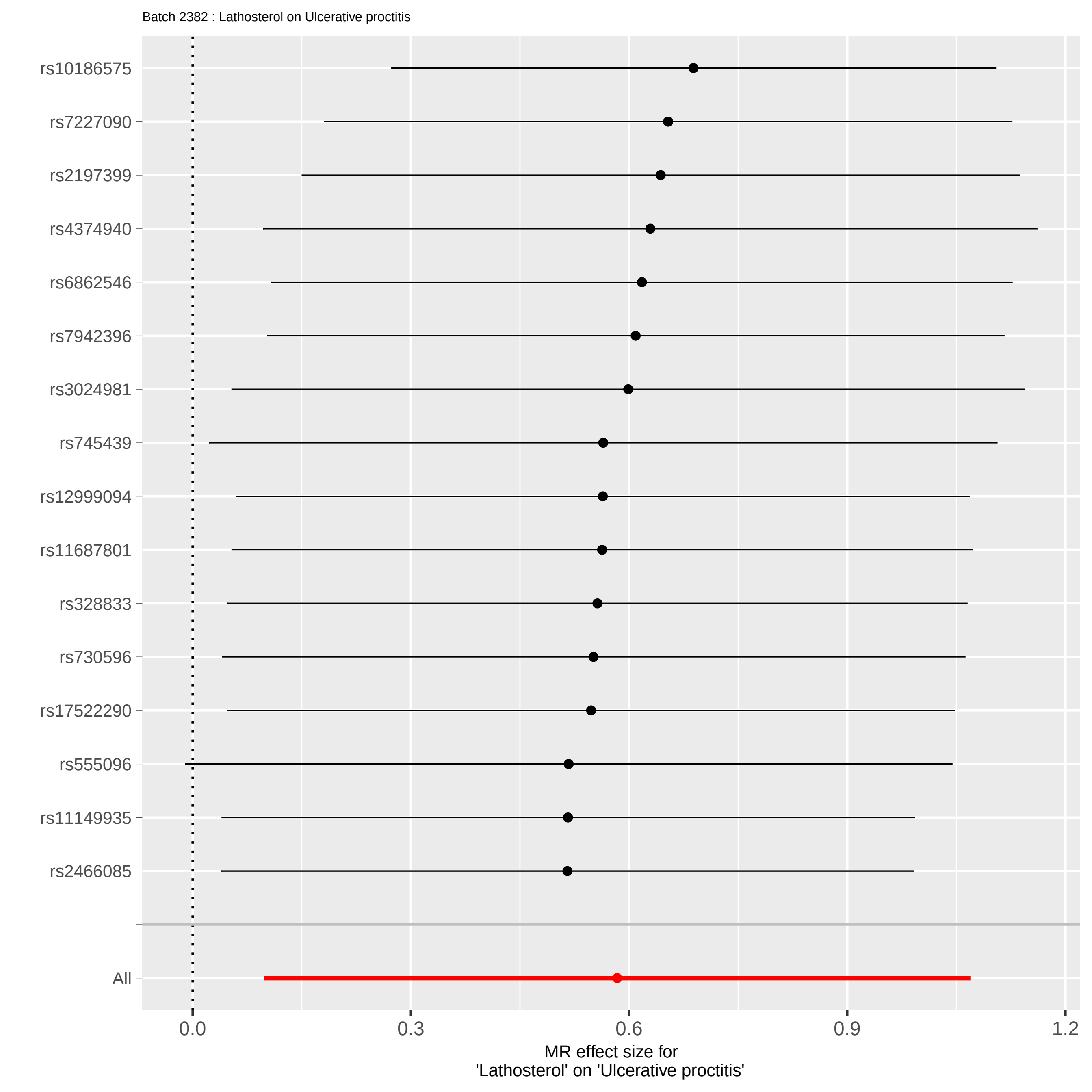
Batch 2382 : Lathosterol on Ulcerative proctitis
rs10186575
rs7227090
rs2197399
rs4374940
rs6862546
rs7942396
rs3024981
rs745439
rs12999094
rs11687801
rs328833
rs730596
rs17522290
rs555096
rs11149935
rs2466085
All
0.0
0.3
0.6
0.9
1.2
MR effect size for
'Lathosterol' on 'Ulcerative proctitis'

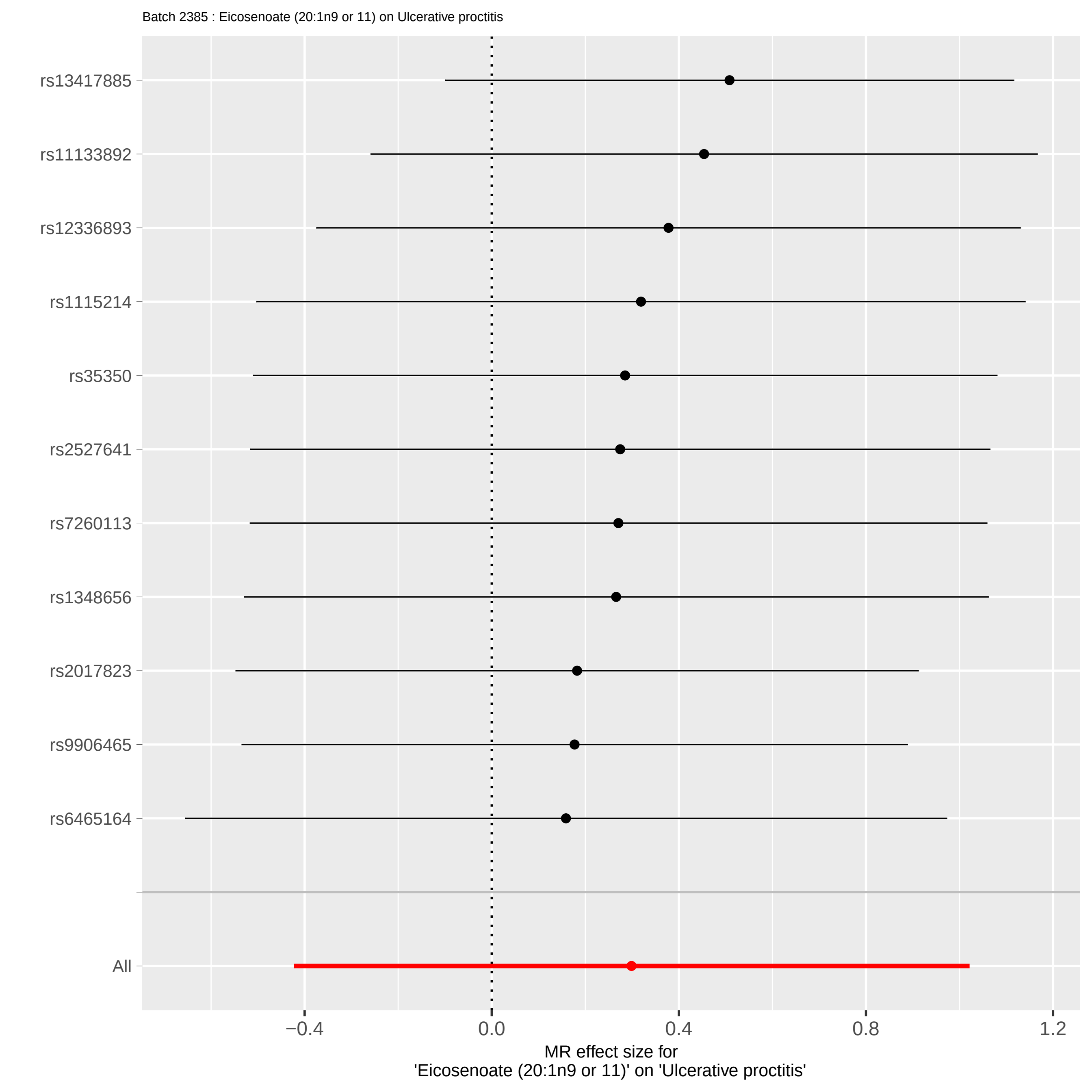

Batch 2385 : Eicosenoate (20:1n9 or 11) on Ulcerative proctitis
rs13417885
rs11133892
rs12336893
rs1115214
rs35350
rs2527641
rs7260113
rs1348656
rs2017823
rs9906465
rs6465164
All
−0.4
0.0
0.4
0.8
1.2
MR effect size for
'Eicosenoate (20:1n9 or 11)' on 'Ulcerative proctitis'

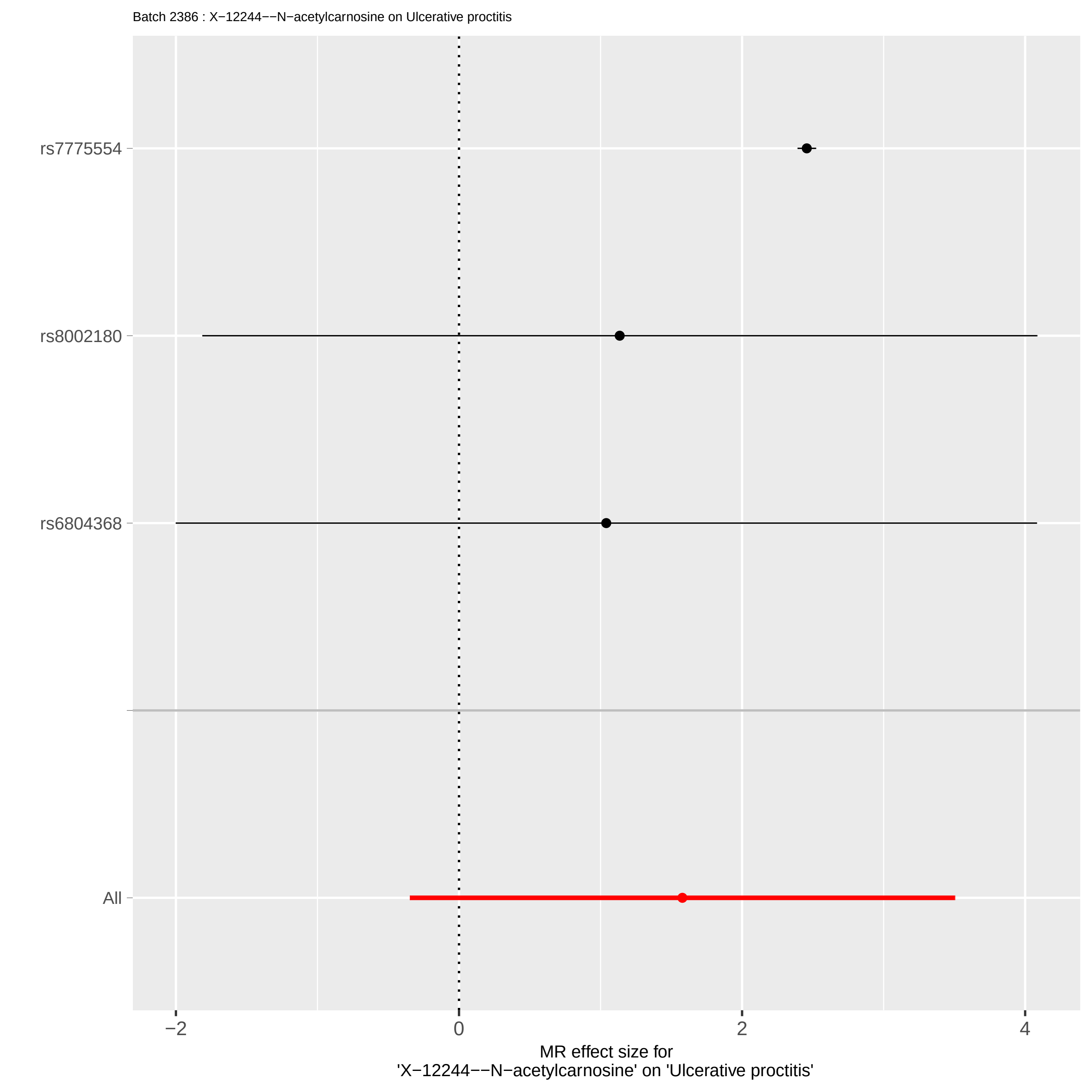

Batch 2386 : X−12244−−N−acetylcarnosine on Ulcerative proctitis
rs7775554
rs8002180
rs6804368
All
−2
0
2
4
MR effect size for
'X−12244−−N−acetylcarnosine' on 'Ulcerative proctitis'

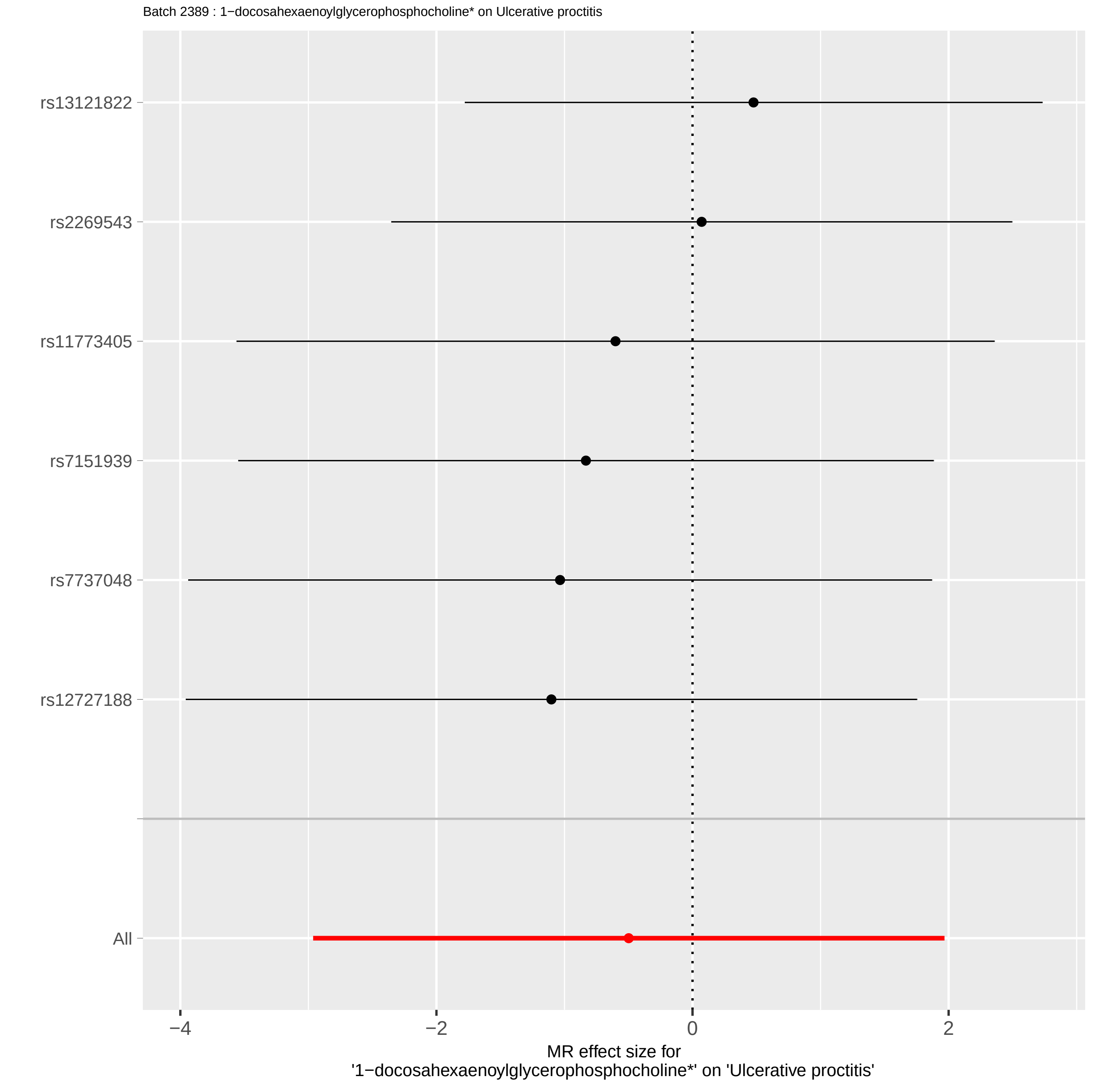

Batch 2389 : 1−docosahexaenoylglycerophosphocholine* on Ulcerative proctitis
rs13121822
rs2269543
rs11773405
rs7151939
rs7737048
rs12727188
All
−4
−2
0
2
MR effect size for
'1−docosahexaenoylglycerophosphocholine*' on 'Ulcerative proctitis'

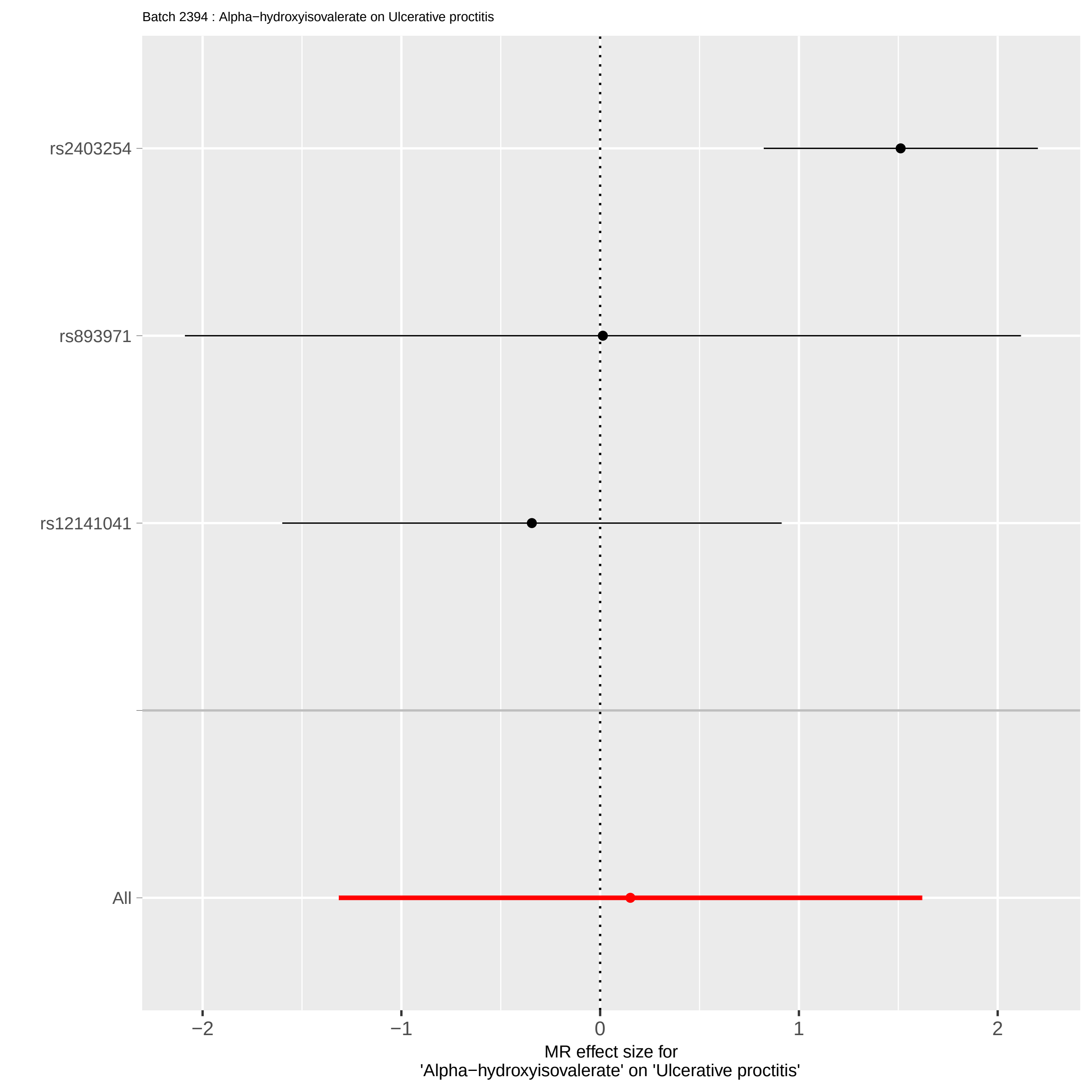

Batch 2394 : Alpha−hydroxyisovalerate on Ulcerative proctitis
rs2403254
rs893971
rs12141041
All
−2
−1
0
1
2
MR effect size for
'Alpha−hydroxyisovalerate' on 'Ulcerative proctitis'

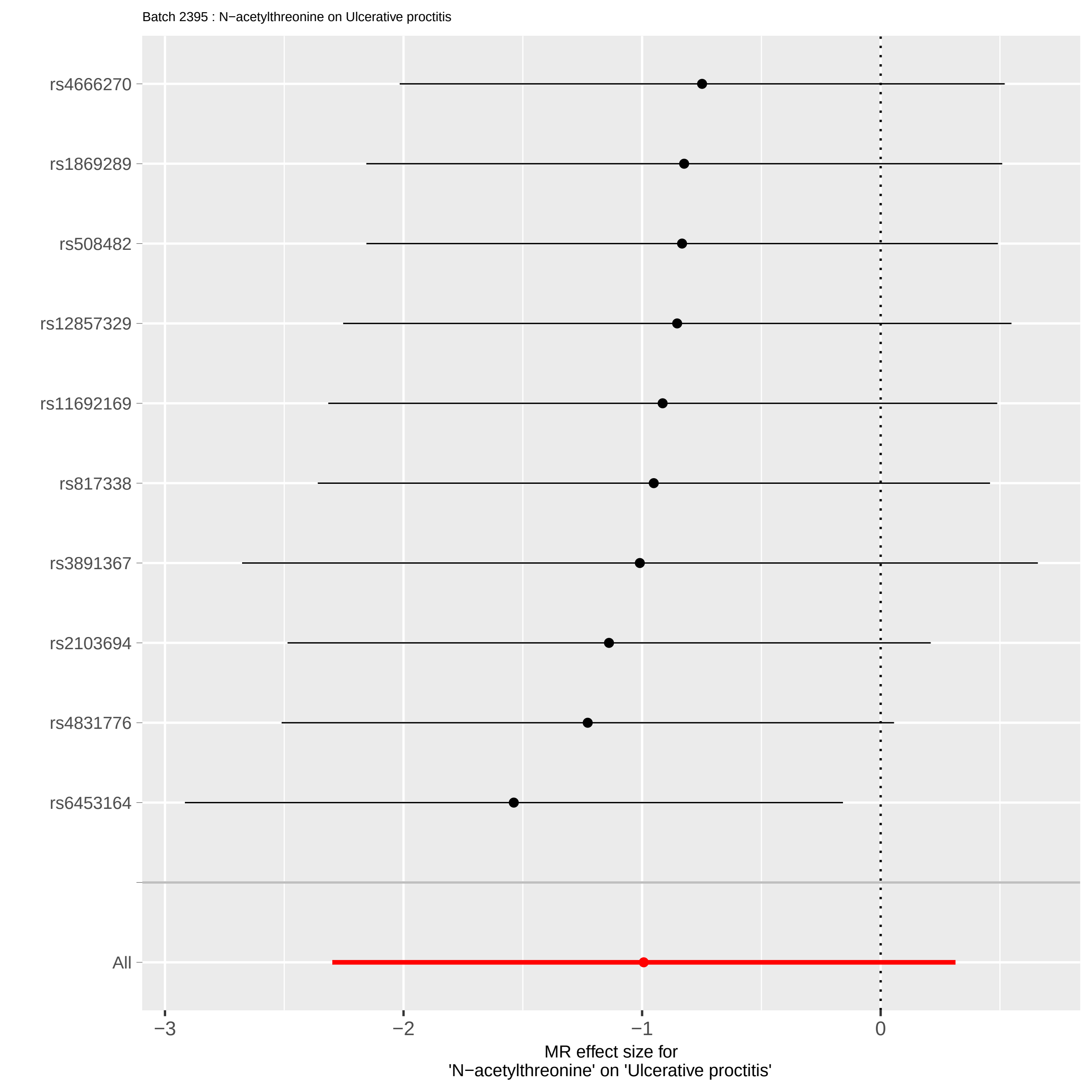

Batch 2395 : N−acetylthreonine on Ulcerative proctitis
rs4666270
rs1869289
rs508482
rs12857329
rs11692169
rs817338
rs3891367
rs2103694
rs4831776
rs6453164
All
−3
−2
−1
0
MR effect size for
'N−acetylthreonine' on 'Ulcerative proctitis'

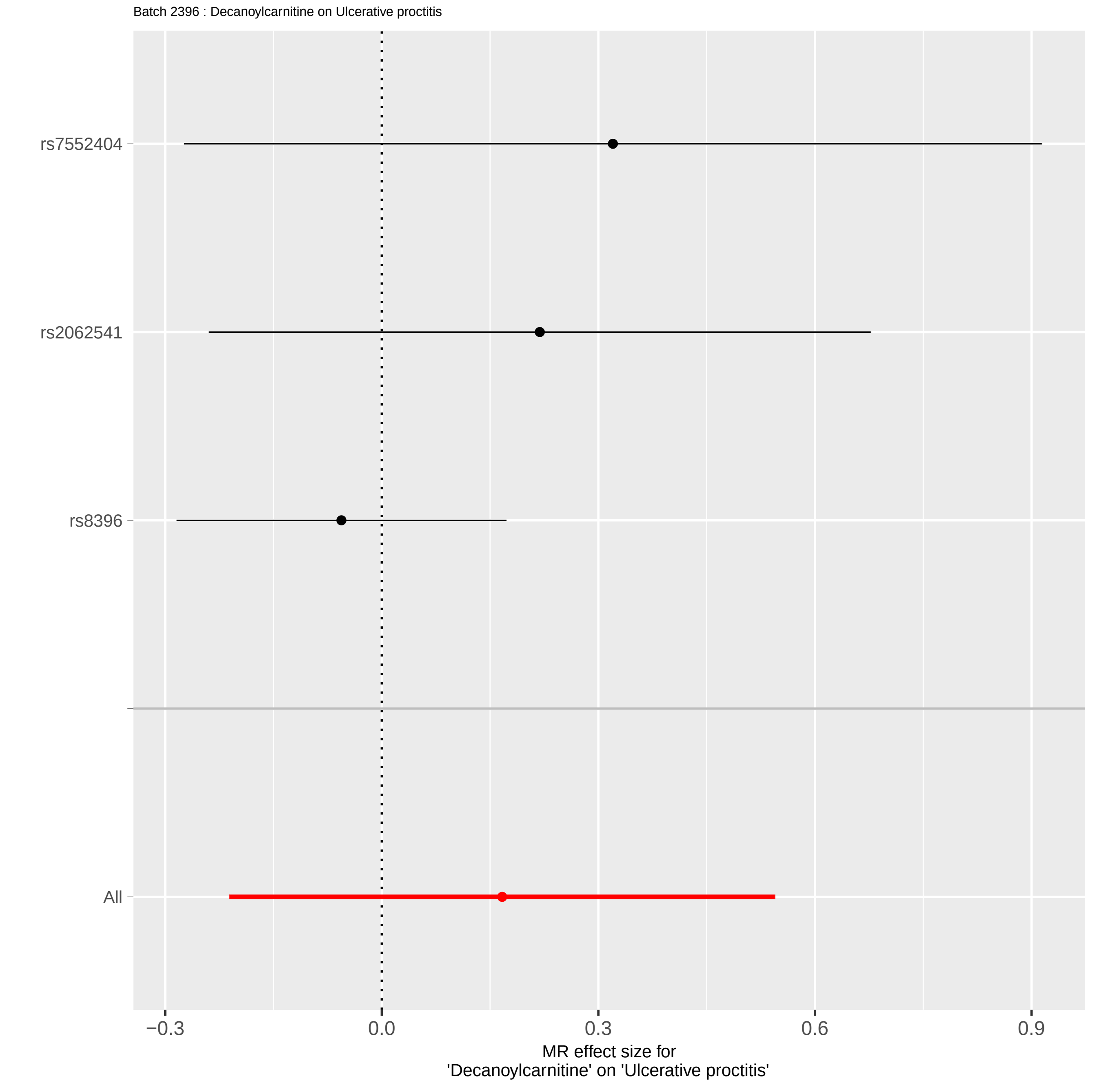

Batch 2396 : Decanoylcarnitine on Ulcerative proctitis
rs7552404
rs2062541
rs8396
All
−0.3
0.0
0.3
0.6
0.9
MR effect size for
'Decanoylcarnitine' on 'Ulcerative proctitis'

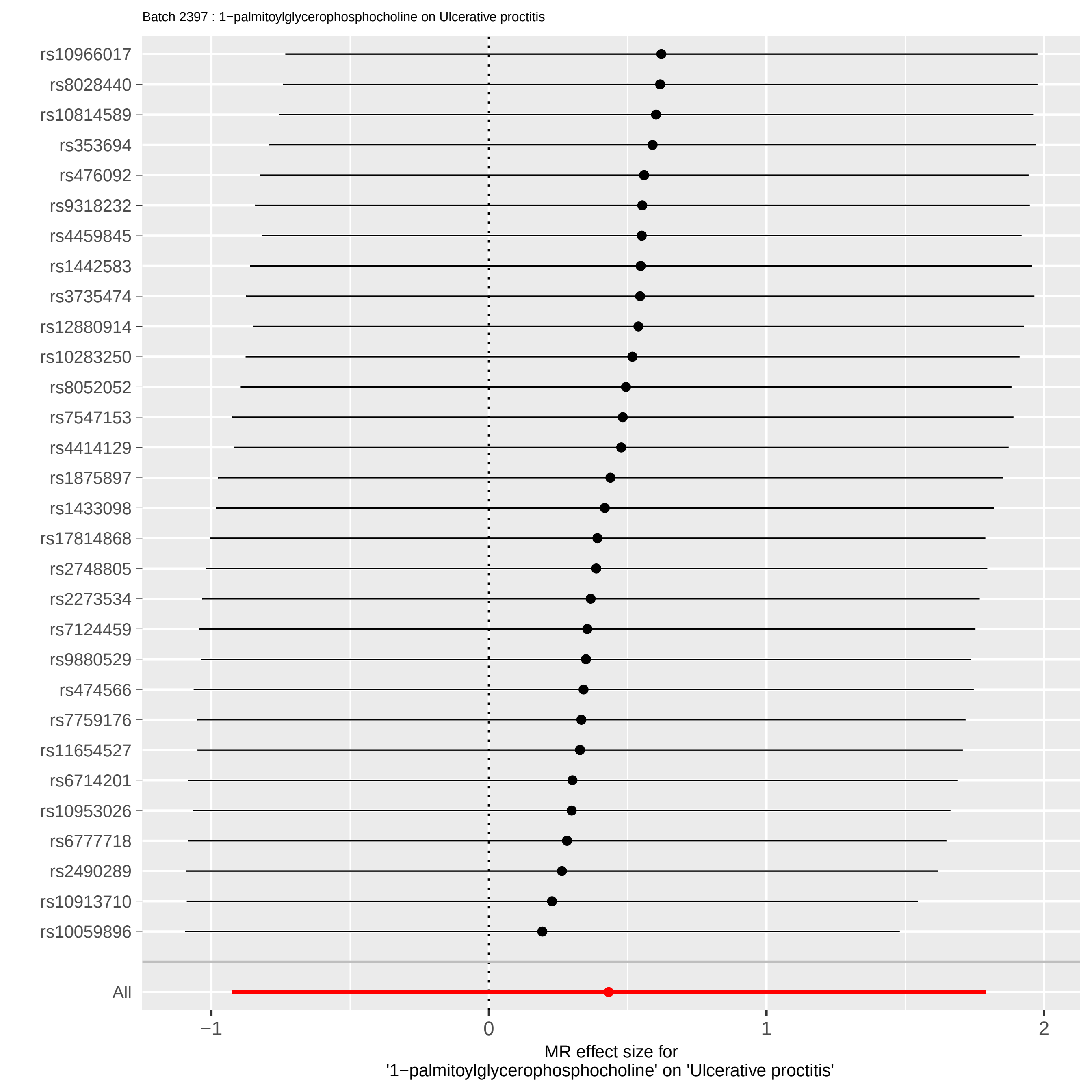

Batch 2397 : 1−palmitoylglycerophosphocholine on Ulcerative proctitis
rs10966017
rs8028440
rs10814589
rs353694
rs476092
rs9318232
rs4459845
rs1442583
rs3735474
rs12880914
rs10283250
rs8052052
rs7547153
rs4414129
rs1875897
rs1433098
rs17814868
rs2748805
rs2273534
rs7124459
rs9880529
rs474566
rs7759176
rs11654527
rs6714201
rs10953026
rs6777718
rs2490289
rs10913710
rs10059896
All
−1
0
1
2
MR effect size for
'1−palmitoylglycerophosphocholine' on 'Ulcerative proctitis'

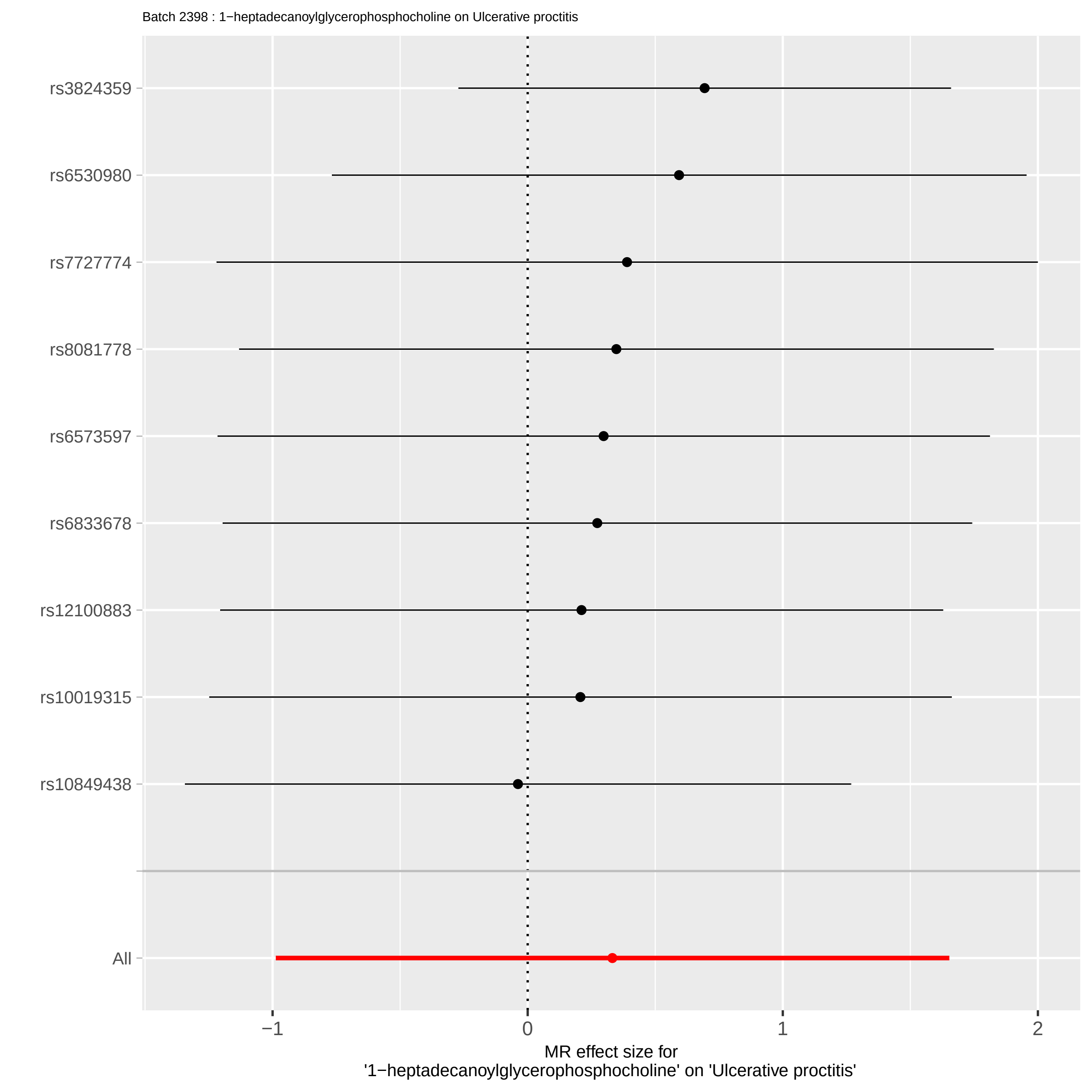

Batch 2398 : 1−heptadecanoylglycerophosphocholine on Ulcerative proctitis
rs3824359
rs6530980
rs7727774
rs8081778
rs6573597
rs6833678
rs12100883
rs10019315
rs10849438
All
−1
0
1
2
MR effect size for
'1−heptadecanoylglycerophosphocholine' on 'Ulcerative proctitis'

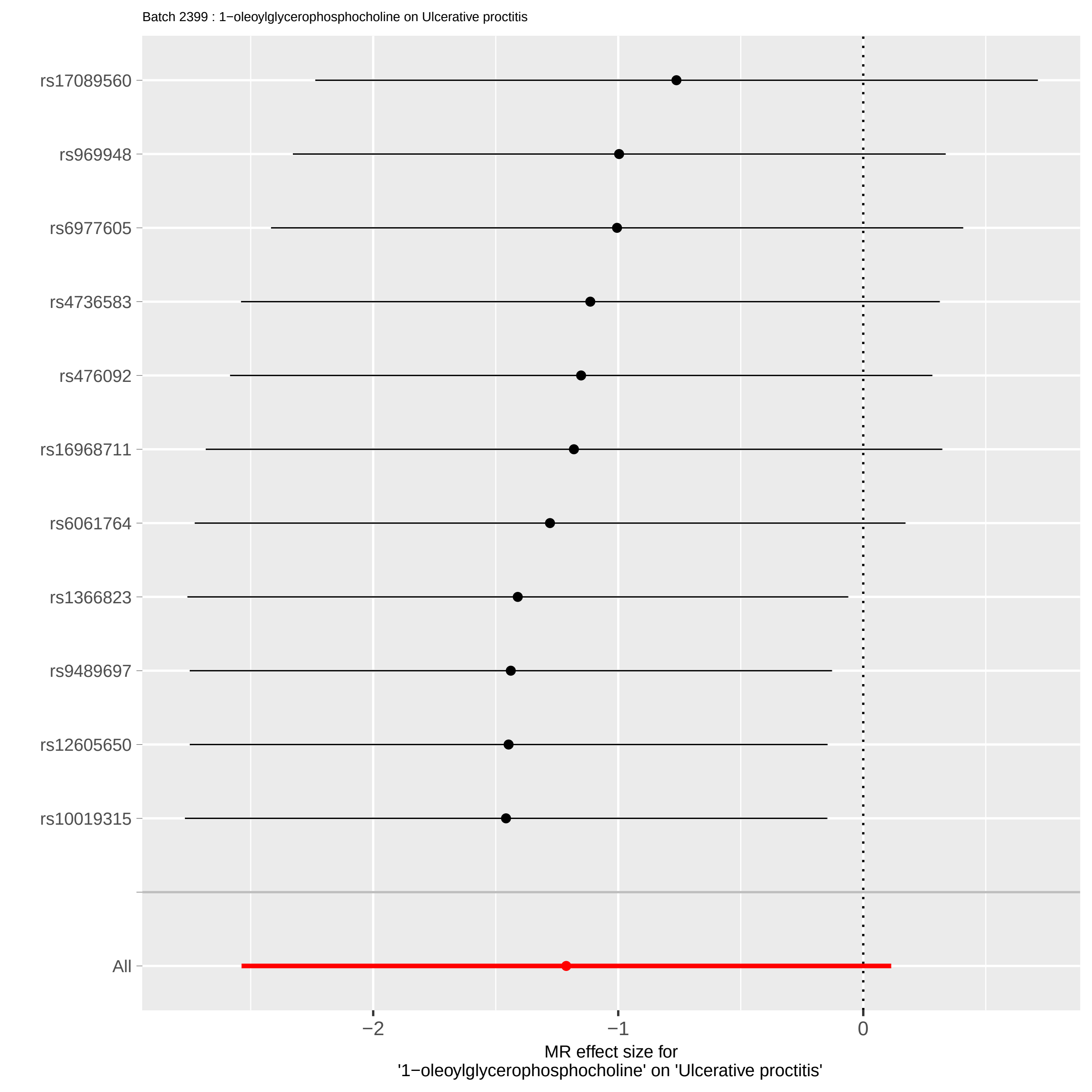
Batch 2399 : 1−oleoylglycerophosphocholine on Ulcerative proctitis
rs17089560
rs969948
rs6977605
rs4736583
rs476092
rs16968711
rs6061764
rs1366823
rs9489697
rs12605650
rs10019315
All
−2
−1
0
MR effect size for
'1−oleoylglycerophosphocholine' on 'Ulcerative proctitis'

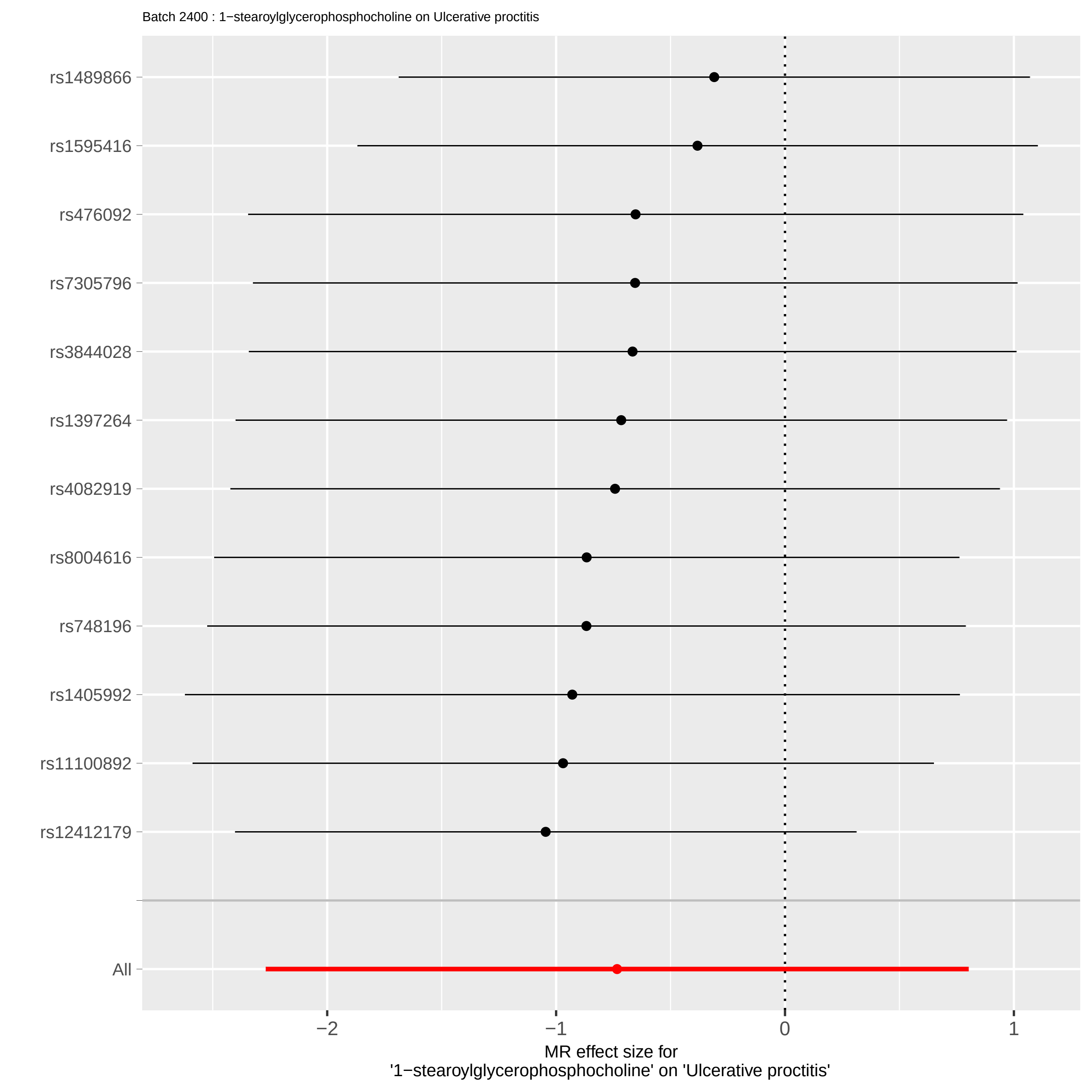

Batch 2400 : 1−stearoylglycerophosphocholine on Ulcerative proctitis
rs1489866
rs1595416
rs476092
rs7305796
rs3844028
rs1397264
rs4082919
rs8004616
rs748196
rs1405992
rs11100892
rs12412179
All
−2
−1
0
1
MR effect size for
'1−stearoylglycerophosphocholine' on 'Ulcerative proctitis'

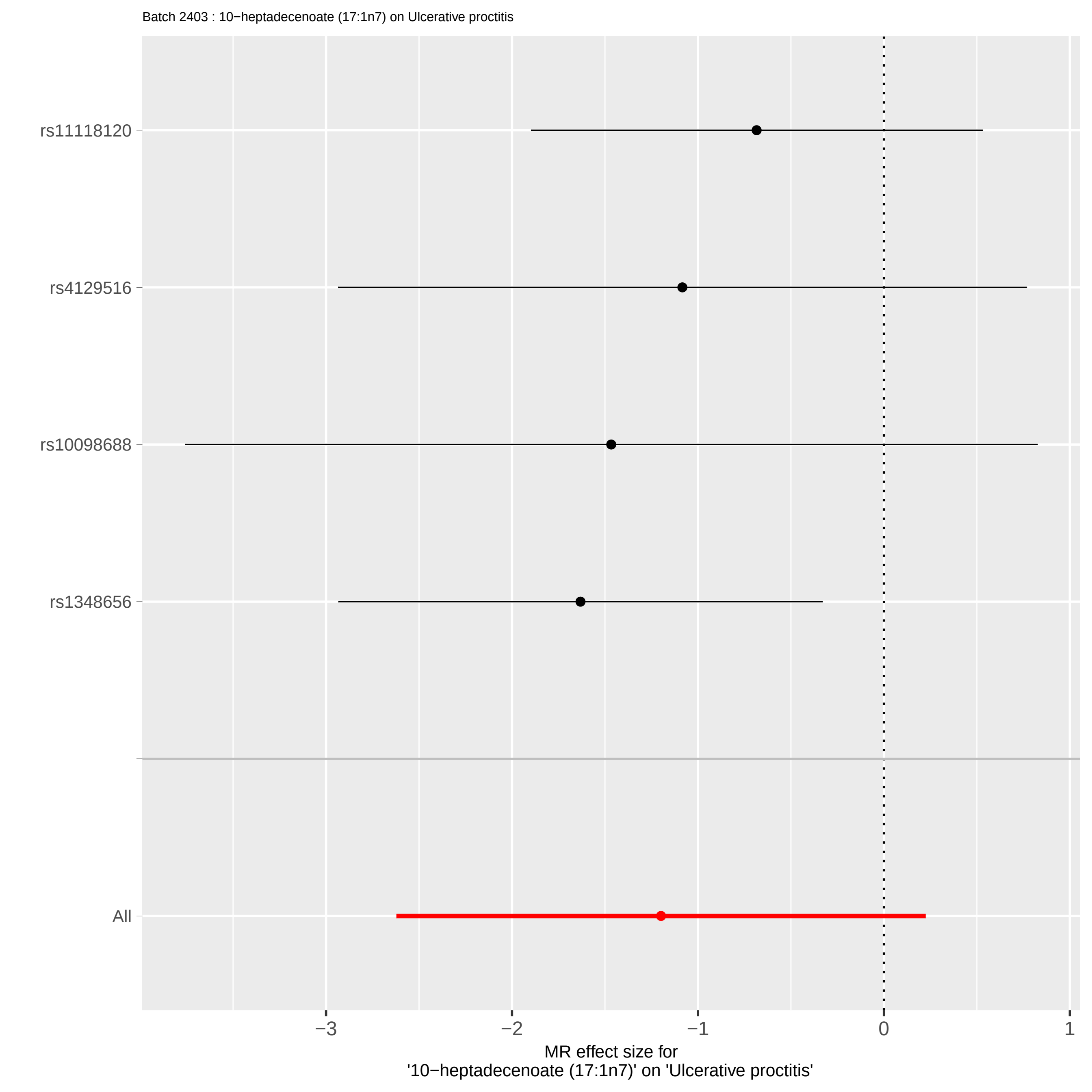
Batch 2403 : 10−heptadecenoate (17:1n7) on Ulcerative proctitis
rs11118120
rs4129516
rs10098688
rs1348656
All
−3
−2
−1
0
1
MR effect size for
'10−heptadecenoate (17:1n7)' on 'Ulcerative proctitis'

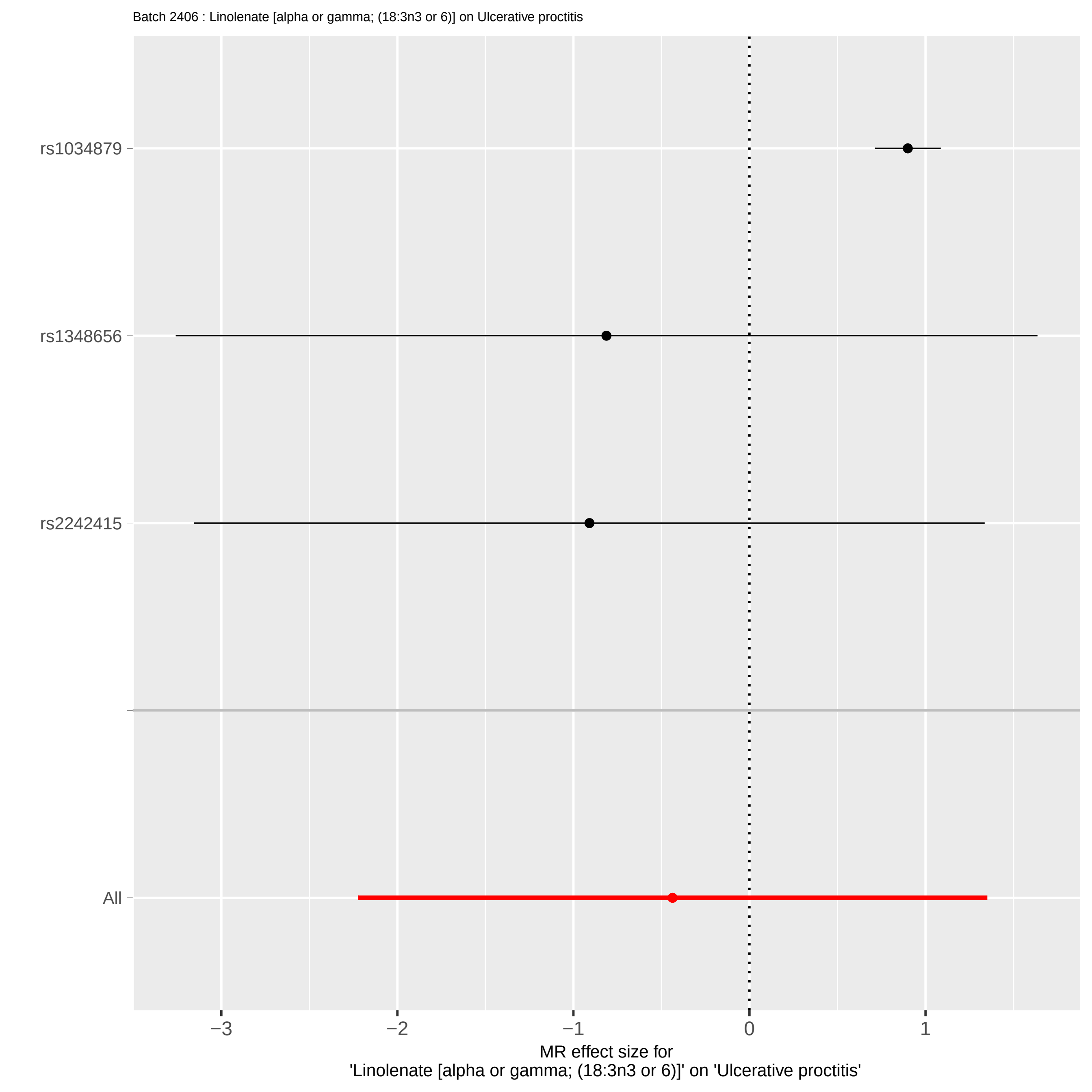
Batch 2406 : Linolenate [alpha or gamma; (18:3n3 or 6)] on Ulcerative proctitis
rs1034879
rs1348656
rs2242415
All
−3
−2
−1
0
1
MR effect size for
'Linolenate [alpha or gamma; (18:3n3 or 6)]' on 'Ulcerative proctitis'

Batch 2407 : X−12510−−2−aminooctanoic acid on Ulcerative proctitis

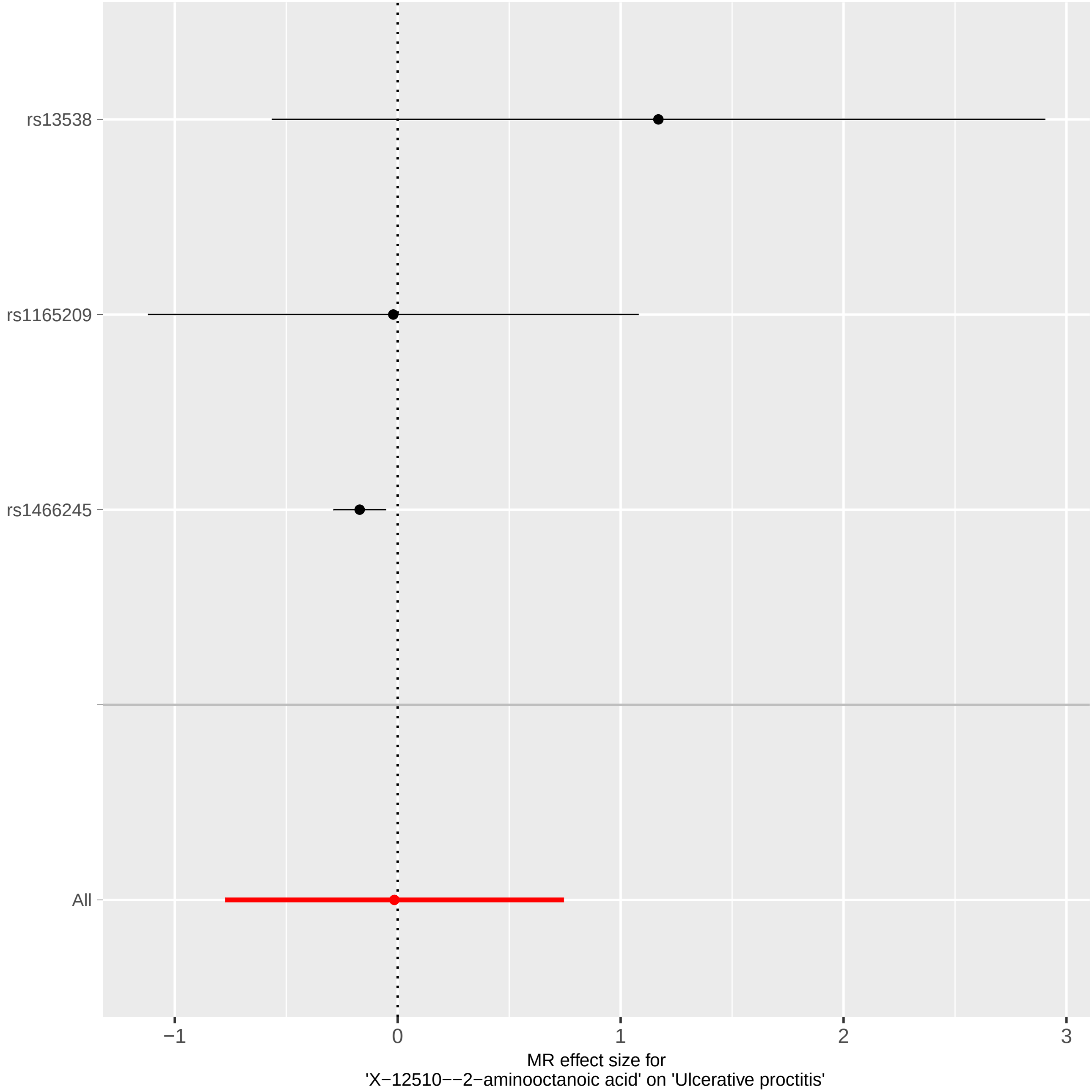

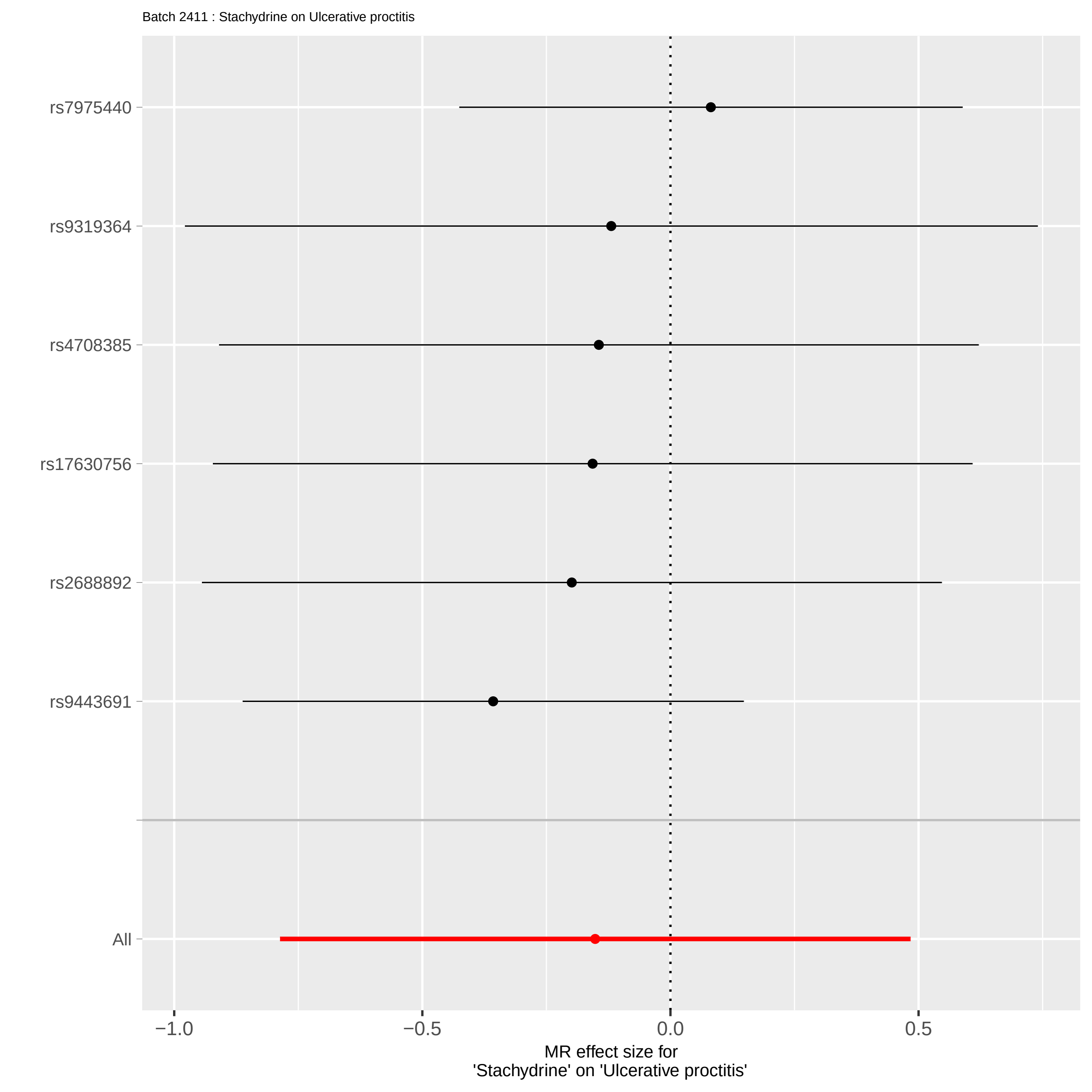
Batch 2411 : Stachydrine on Ulcerative proctitis
rs7975440
rs9319364
rs4708385
rs17630756
rs2688892
rs9443691
All
−1.0
−0.5
0.0
0.5
MR effect size for
'Stachydrine' on 'Ulcerative proctitis'

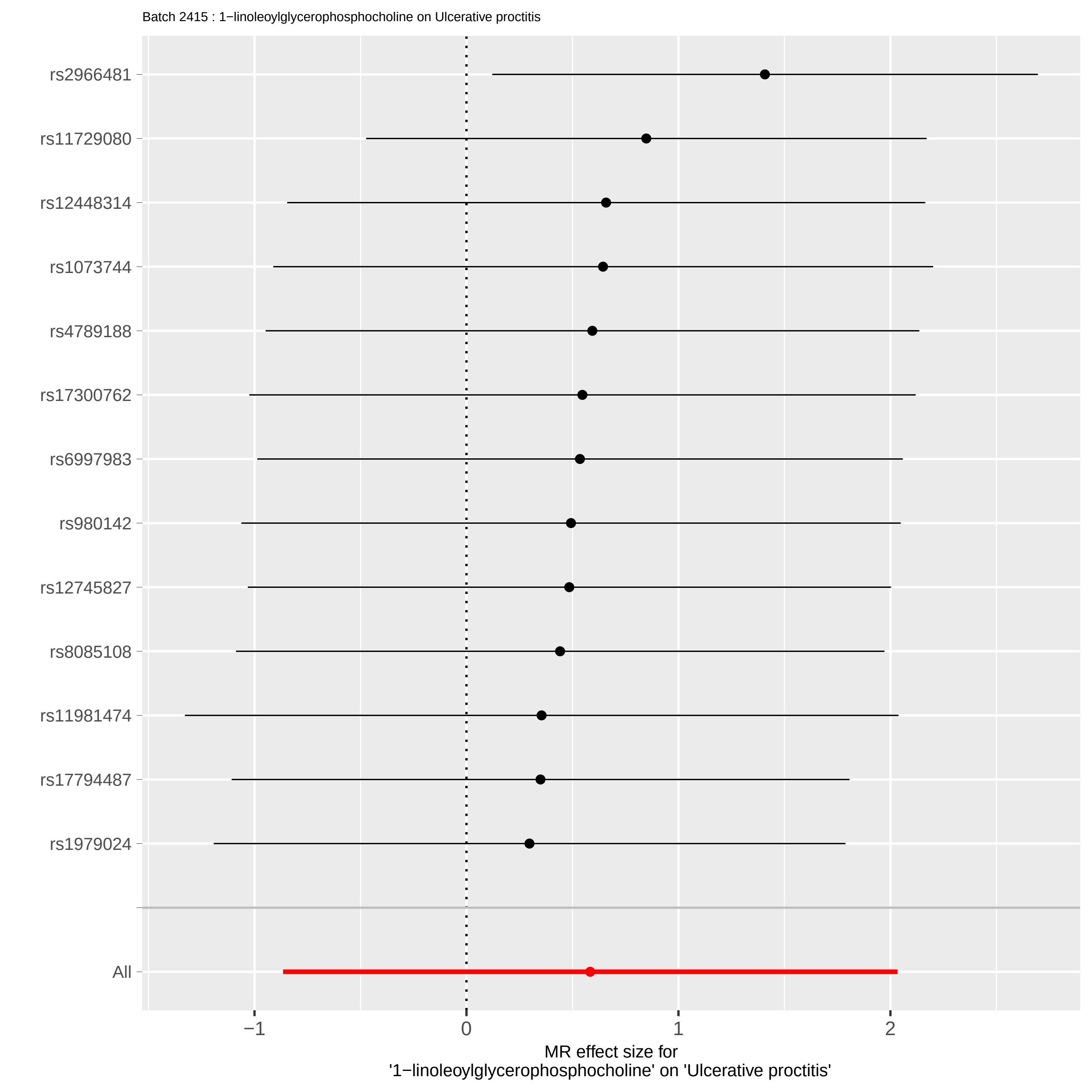

Batch 2415 : 1−linoleoylglycerophosphocholine on Ulcerative proctitis
rs2966481
rs11729080
rs12448314
rs1073744
rs4789188
rs17300762
rs6997983
rs980142
rs12745827
rs8085108
rs11981474
rs17794487
rs1979024
All
−1
0
1
2
MR effect size for
'1−linoleoylglycerophosphocholine' on 'Ulcerative proctitis'

Batch 2416 : Bradykinin, des−arg(9) on Ulcerative proctitis

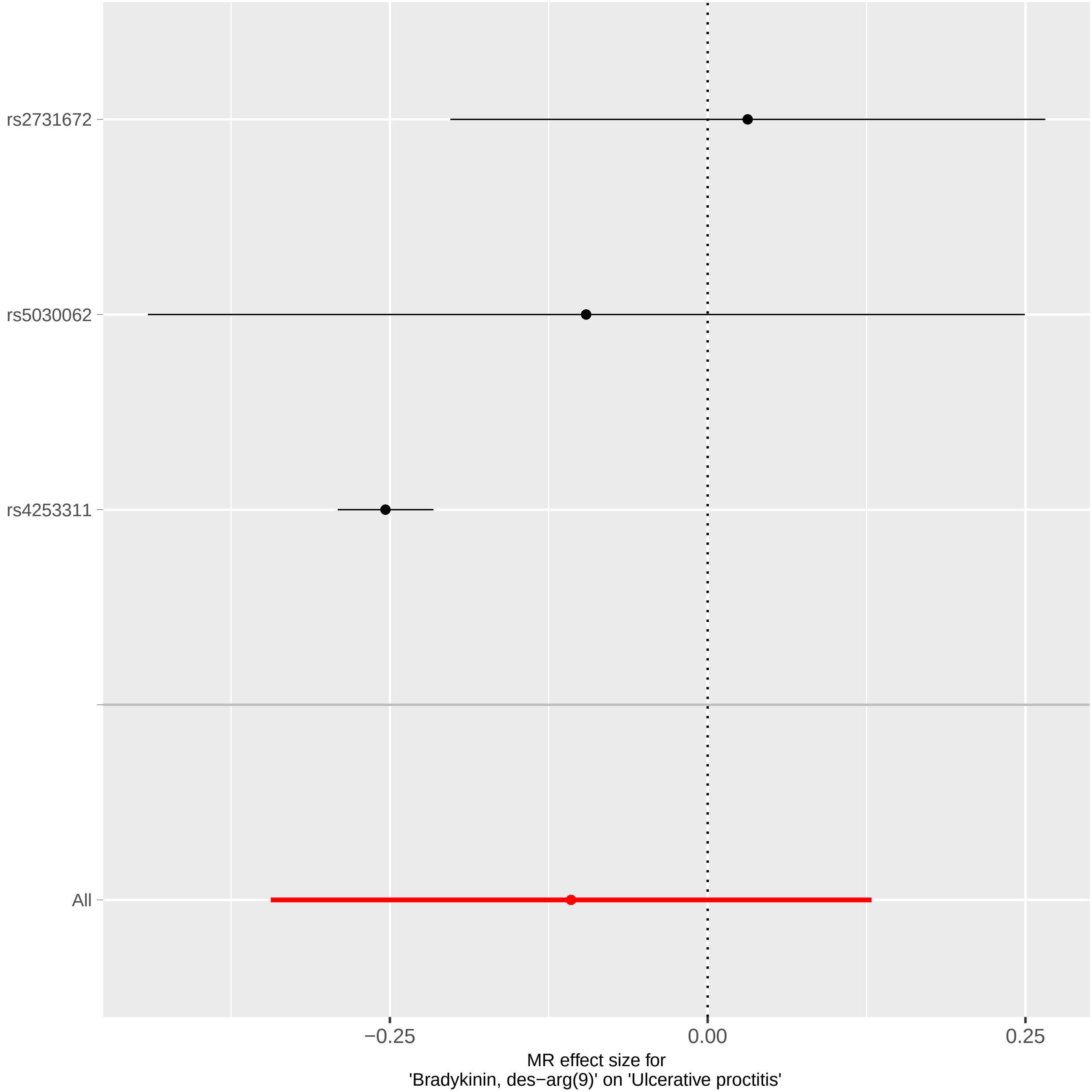

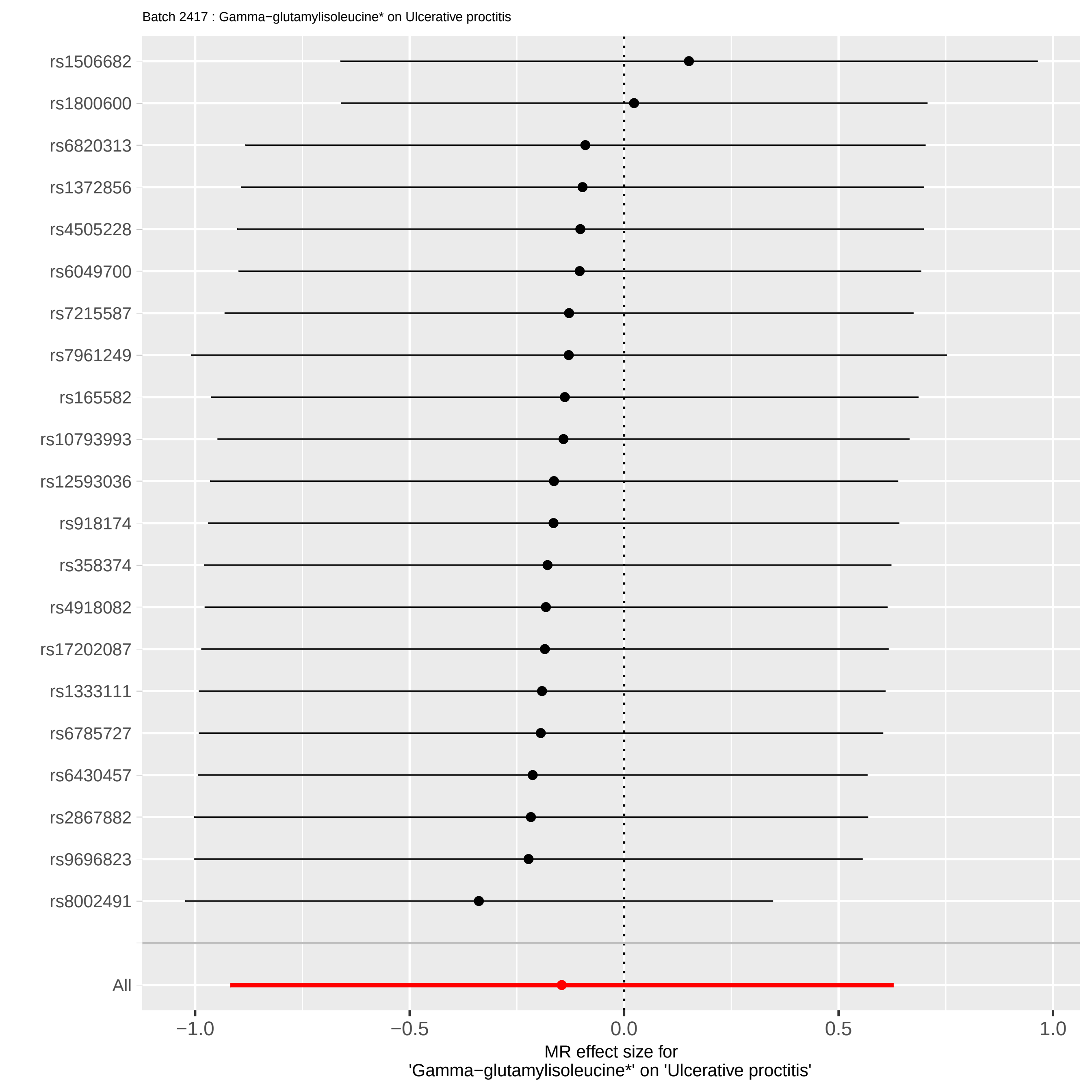

Batch 2417 : Gamma−glutamylisoleucine* on Ulcerative proctitis
rs1506682
rs1800600
rs6820313
rs1372856
rs4505228
rs6049700
rs7215587
rs7961249
rs165582
rs10793993
rs12593036
rs918174
rs358374
rs4918082
rs17202087
rs1333111
rs6785727
rs6430457
rs2867882
rs9696823
rs8002491
All
−1.0
−0.5
0.0
0.5
1.0
MR effect size for
'Gamma−glutamylisoleucine*' on 'Ulcerative proctitis'

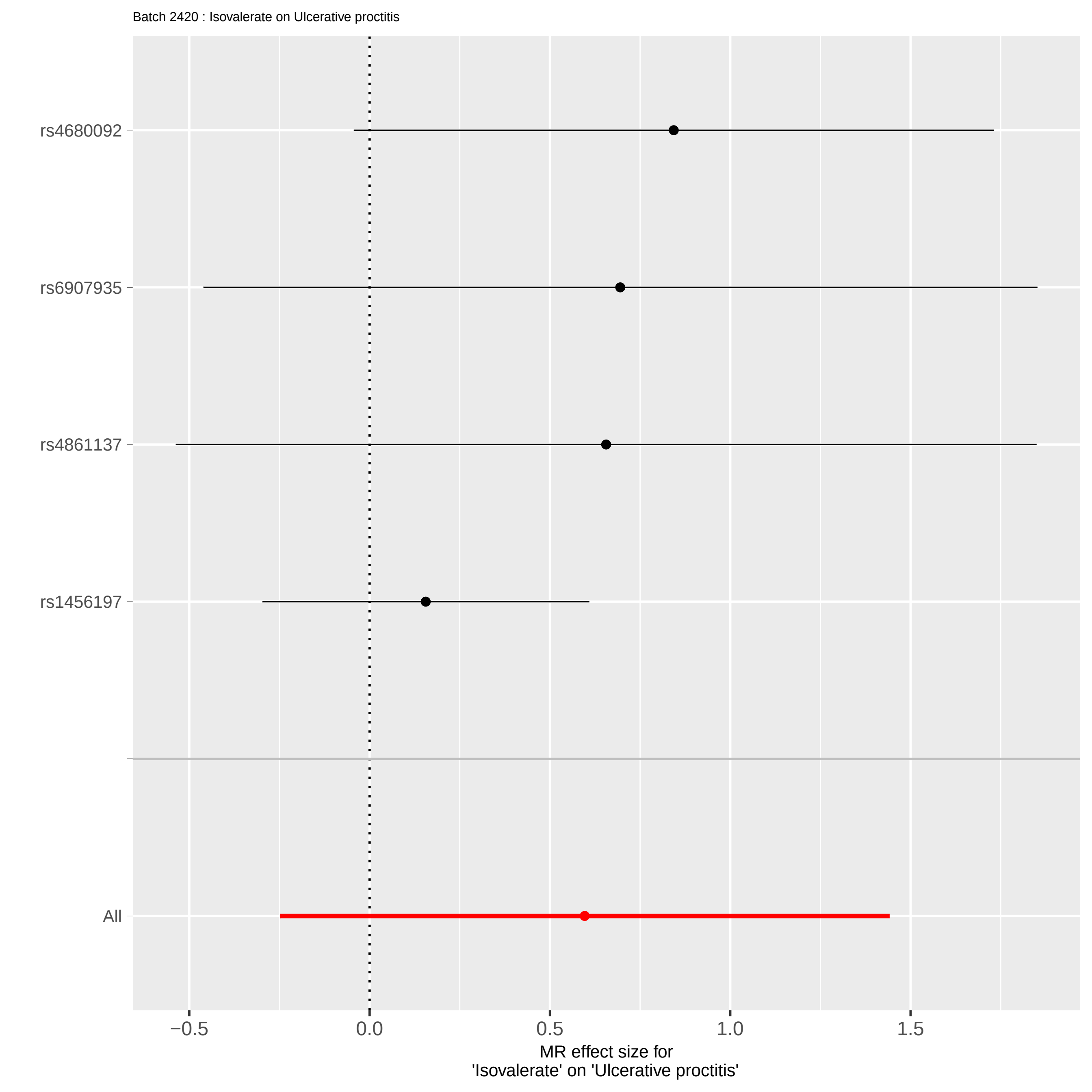

Batch 2420 : Isovalerate on Ulcerative proctitis
rs4680092
rs6907935
rs4861137
rs1456197
All
−0.5
0.0
0.5
1.0
1.5
MR effect size for
'Isovalerate' on 'Ulcerative proctitis'

Batch 2421 : X−13183−−stearamide on Ulcerative proctitis

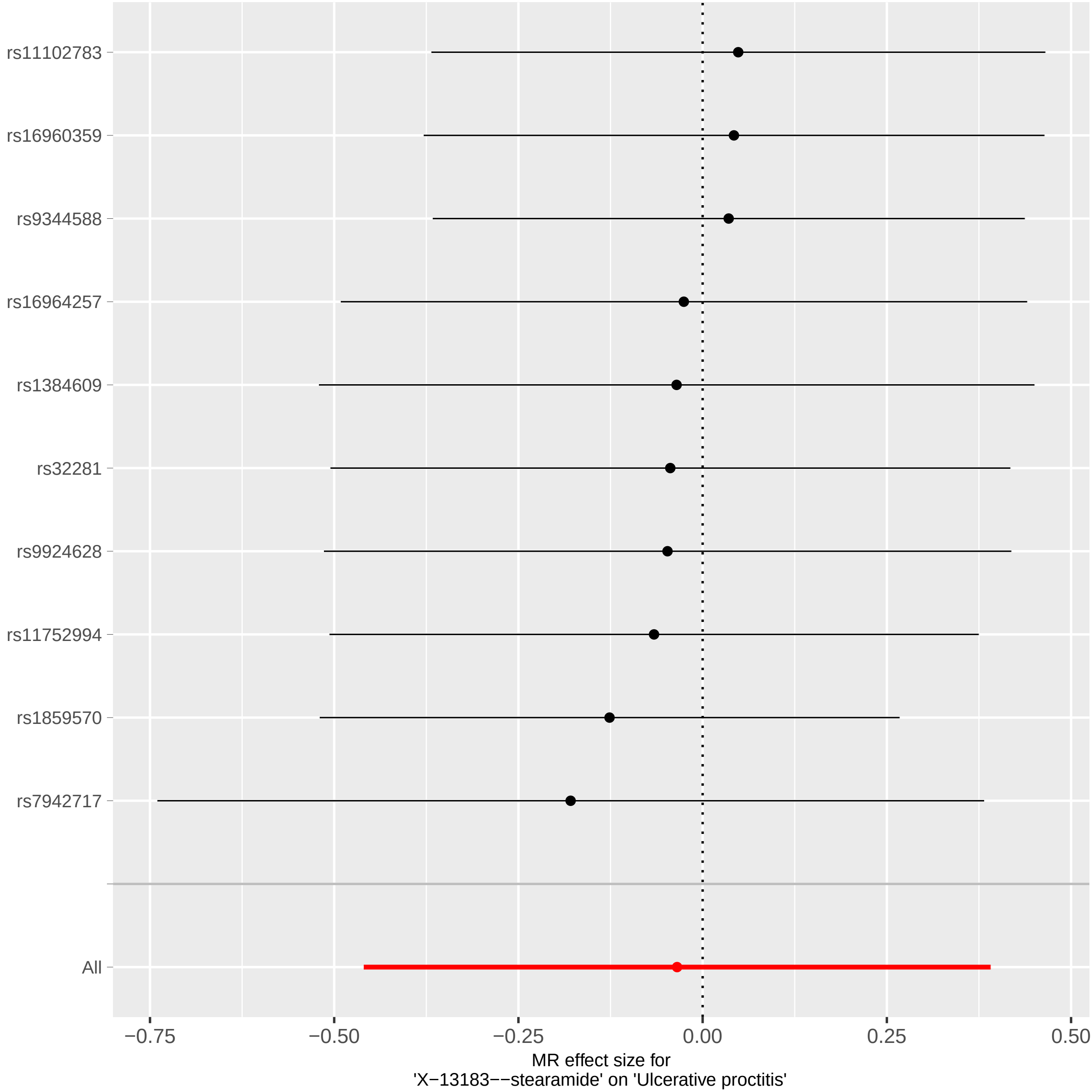

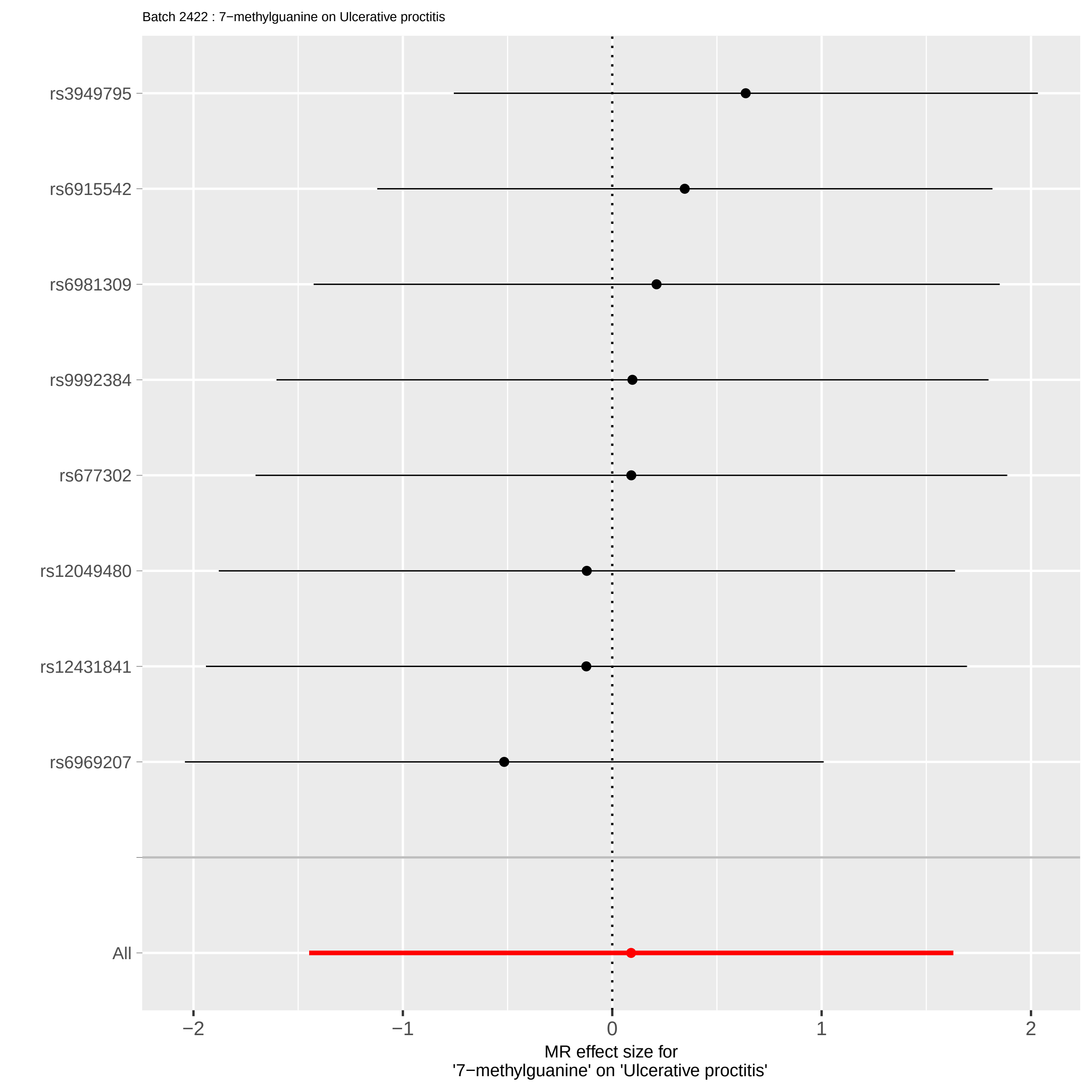

Batch 2422 : 7−methylguanine on Ulcerative proctitis
rs3949795
rs6915542
rs6981309
rs9992384
rs677302
rs12049480
rs12431841
rs6969207
All
−2
−1
0
1
2
MR effect size for
'7−methylguanine' on 'Ulcerative proctitis'

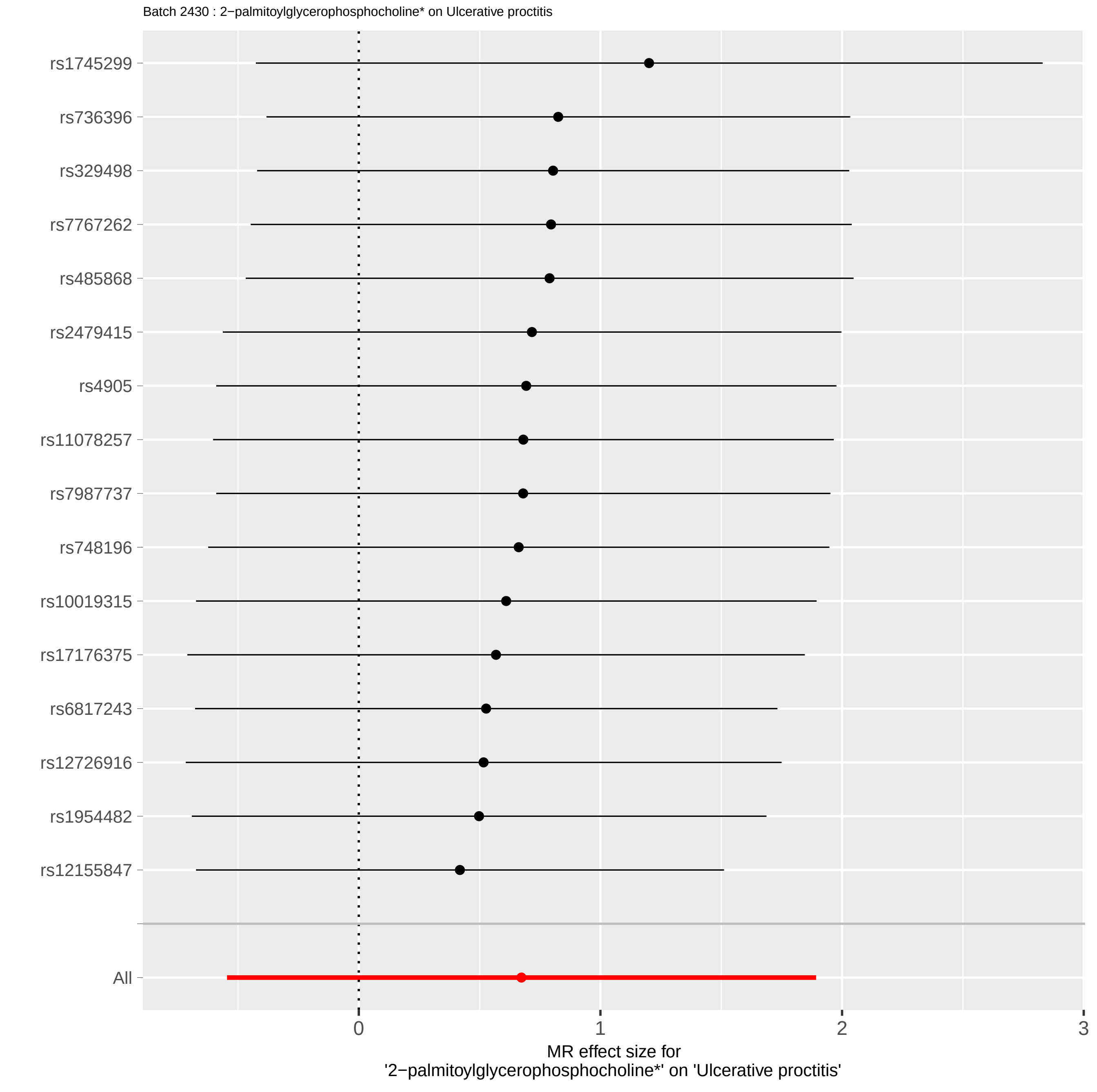
Batch 2430 : 2−palmitoylglycerophosphocholine* on Ulcerative proctitis
rs1745299
rs736396
rs329498
rs7767262
rs485868
rs2479415
rs4905
rs11078257
rs7987737
rs748196
rs10019315
rs17176375
rs6817243
rs12726916
rs1954482
rs12155847
All
0
1
2
3
MR effect size for
'2−palmitoylglycerophosphocholine*' on 'Ulcerative proctitis'

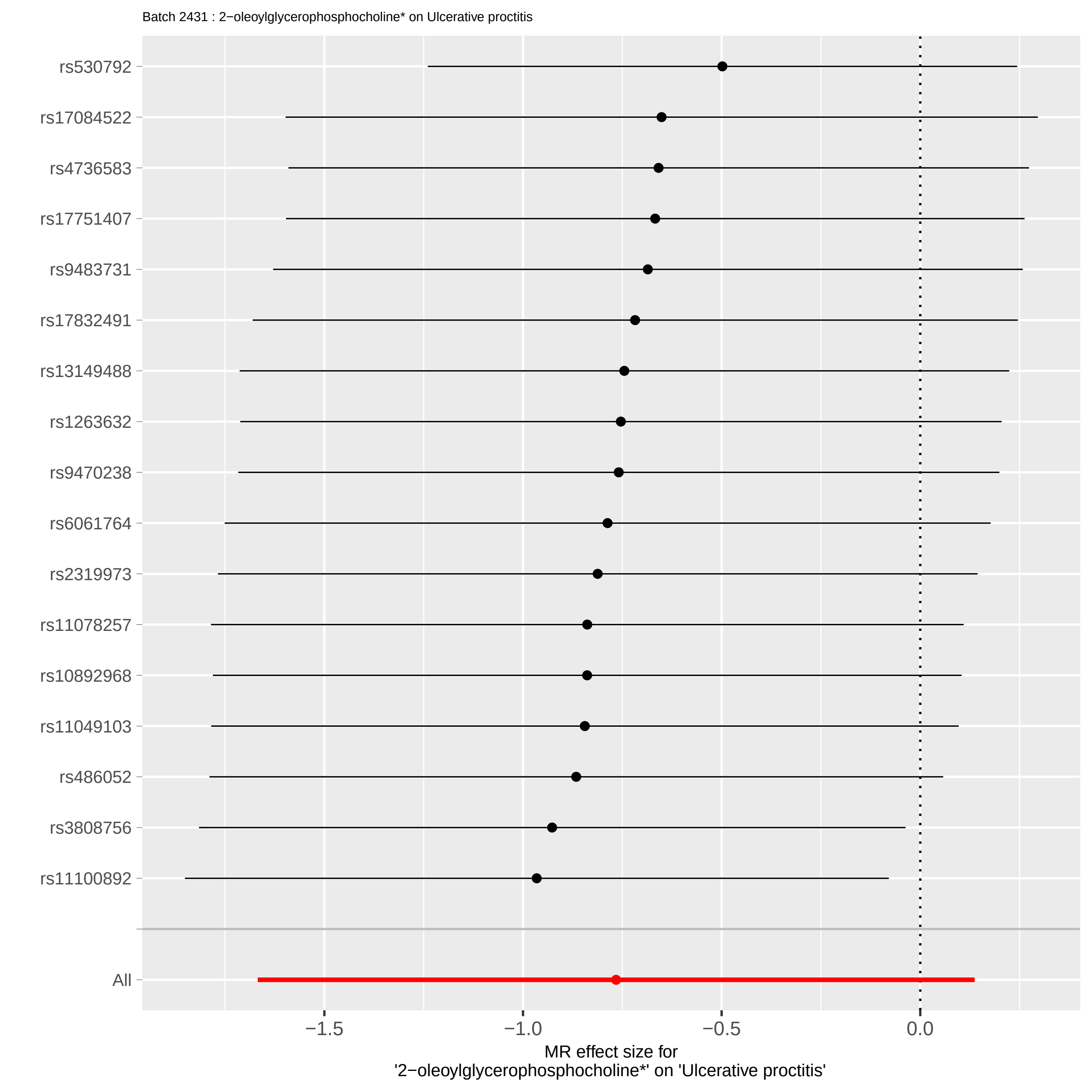

Batch 2431 : 2−oleoylglycerophosphocholine* on Ulcerative proctitis
rs530792
rs17084522
rs4736583
rs17751407
rs9483731
rs17832491
rs13149488
rs1263632
rs9470238
rs6061764
rs2319973
rs11078257
rs10892968
rs11049103
rs486052
rs3808756
rs11100892
All
−1.5
−1.0
−0.5
0.0
MR effect size for
'2−oleoylglycerophosphocholine*' on 'Ulcerative proctitis'

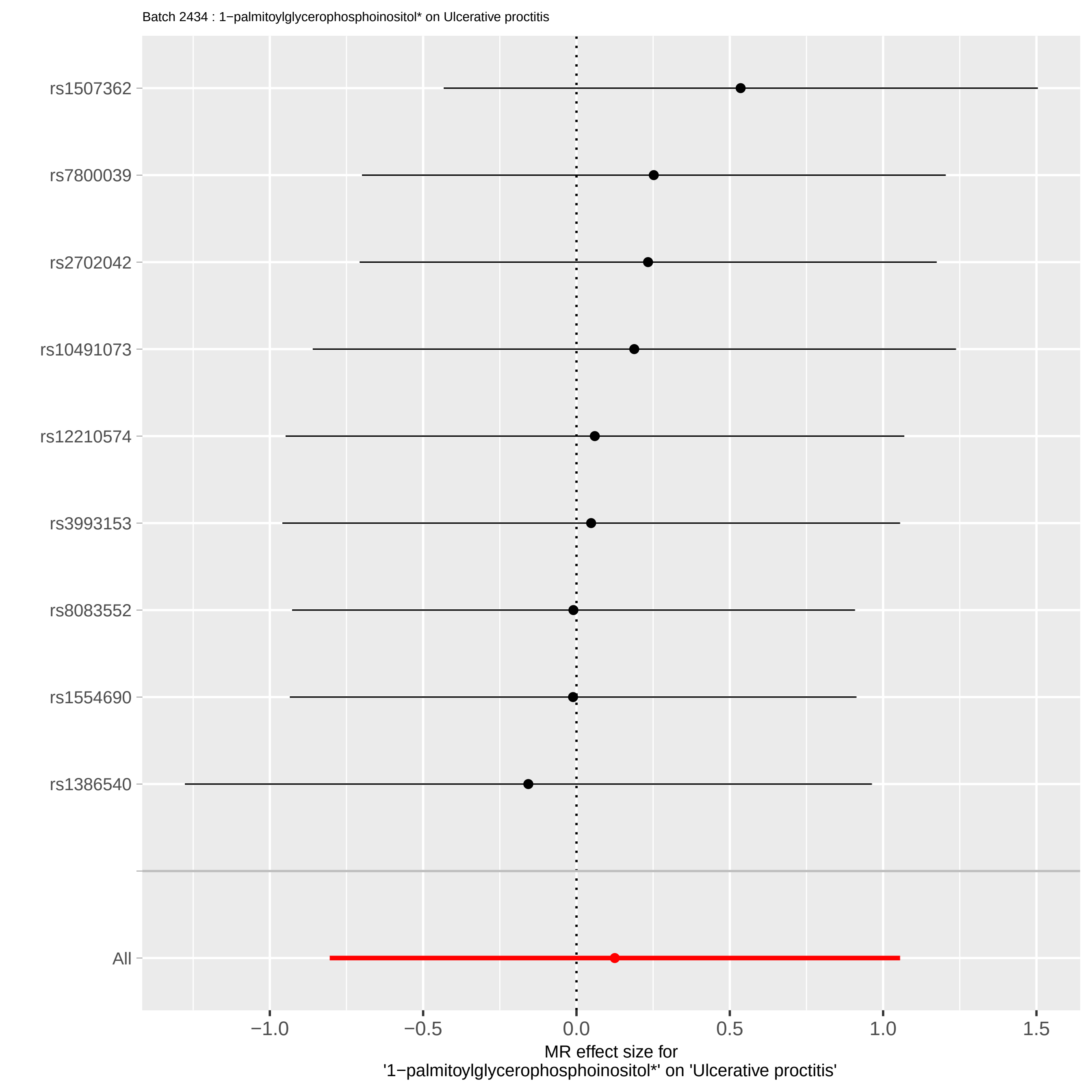
Batch 2434 : 1−palmitoylglycerophosphoinositol* on Ulcerative proctitis
rs1507362
rs7800039
rs2702042
rs10491073
rs12210574
rs3993153
rs8083552
rs1554690
rs1386540
All
−1.0
−0.5
0.0
0.5
1.0
1.5
MR effect size for
'1−palmitoylglycerophosphoinositol*' on 'Ulcerative proctitis'

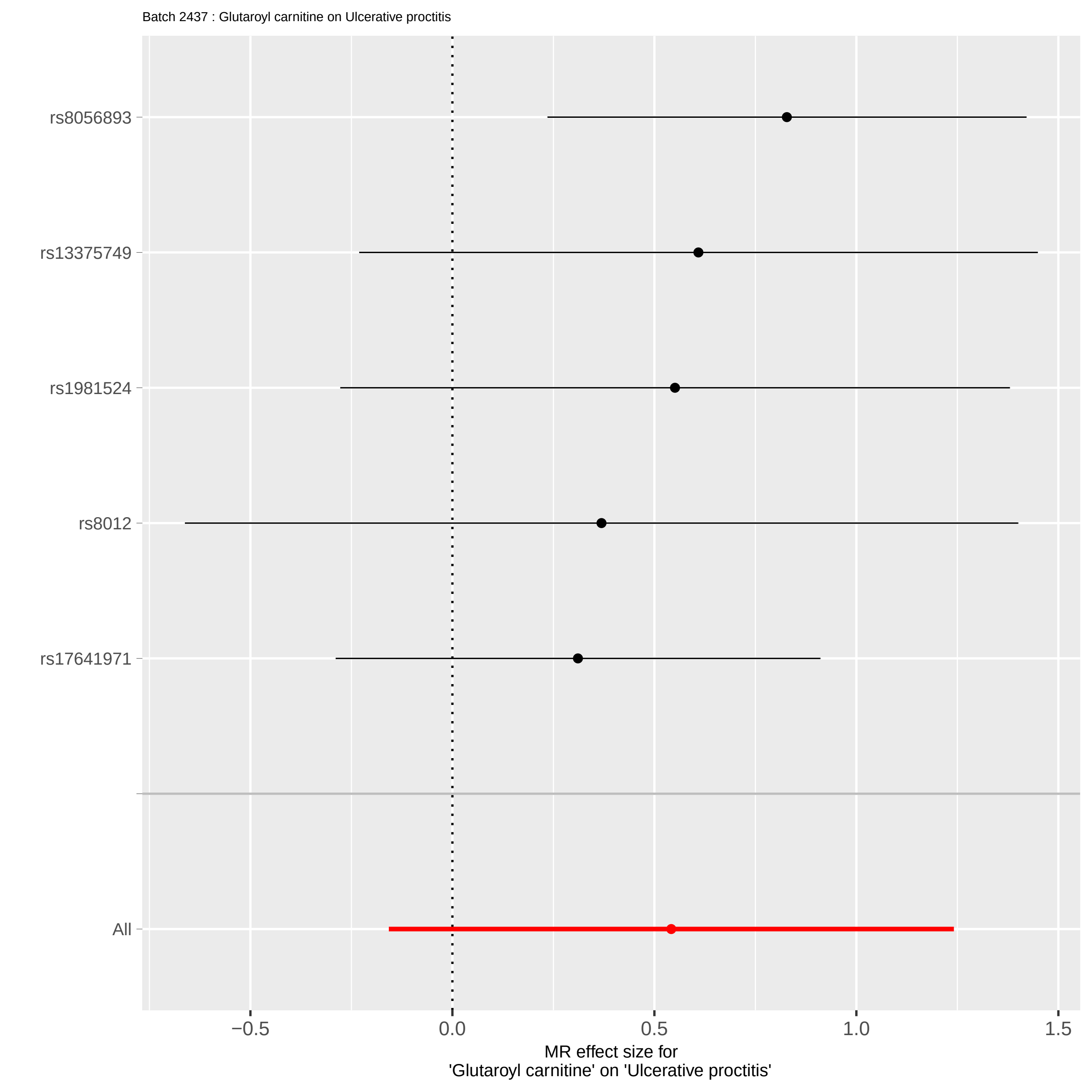

Batch 2437 : Glutaroyl carnitine on Ulcerative proctitis
rs8056893
rs13375749
rs1981524
rs8012
rs17641971
All
−0.5
0.0
0.5
1.0
1.5
MR effect size for
'Glutaroyl carnitine' on 'Ulcerative proctitis'

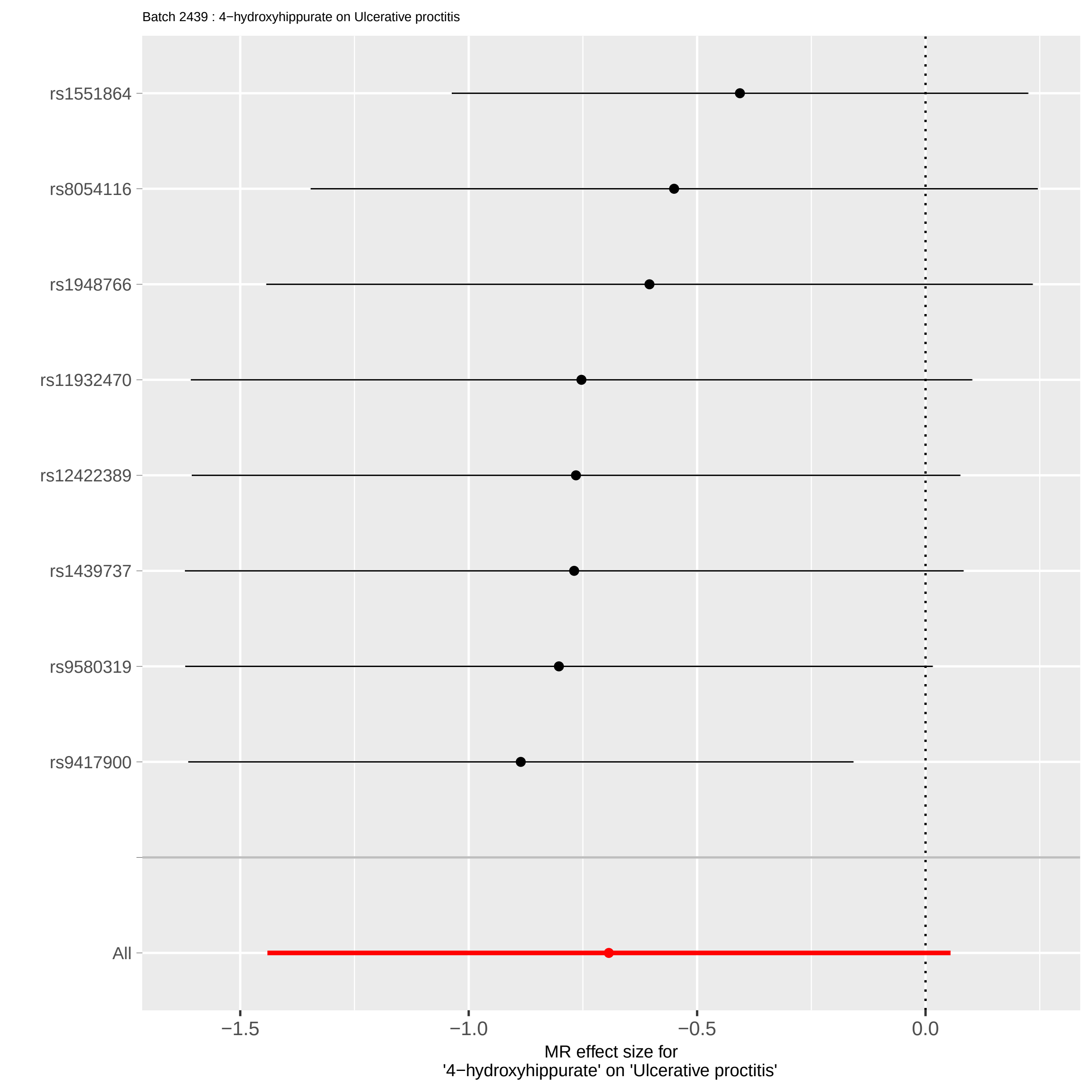
Batch 2439 : 4−hydroxyhippurate on Ulcerative proctitis
rs1551864
rs8054116
rs1948766
rs11932470
rs12422389
rs1439737
rs9580319
rs9417900
All
−1.5
−1.0
−0.5
0.0
MR effect size for
'4−hydroxyhippurate' on 'Ulcerative proctitis'

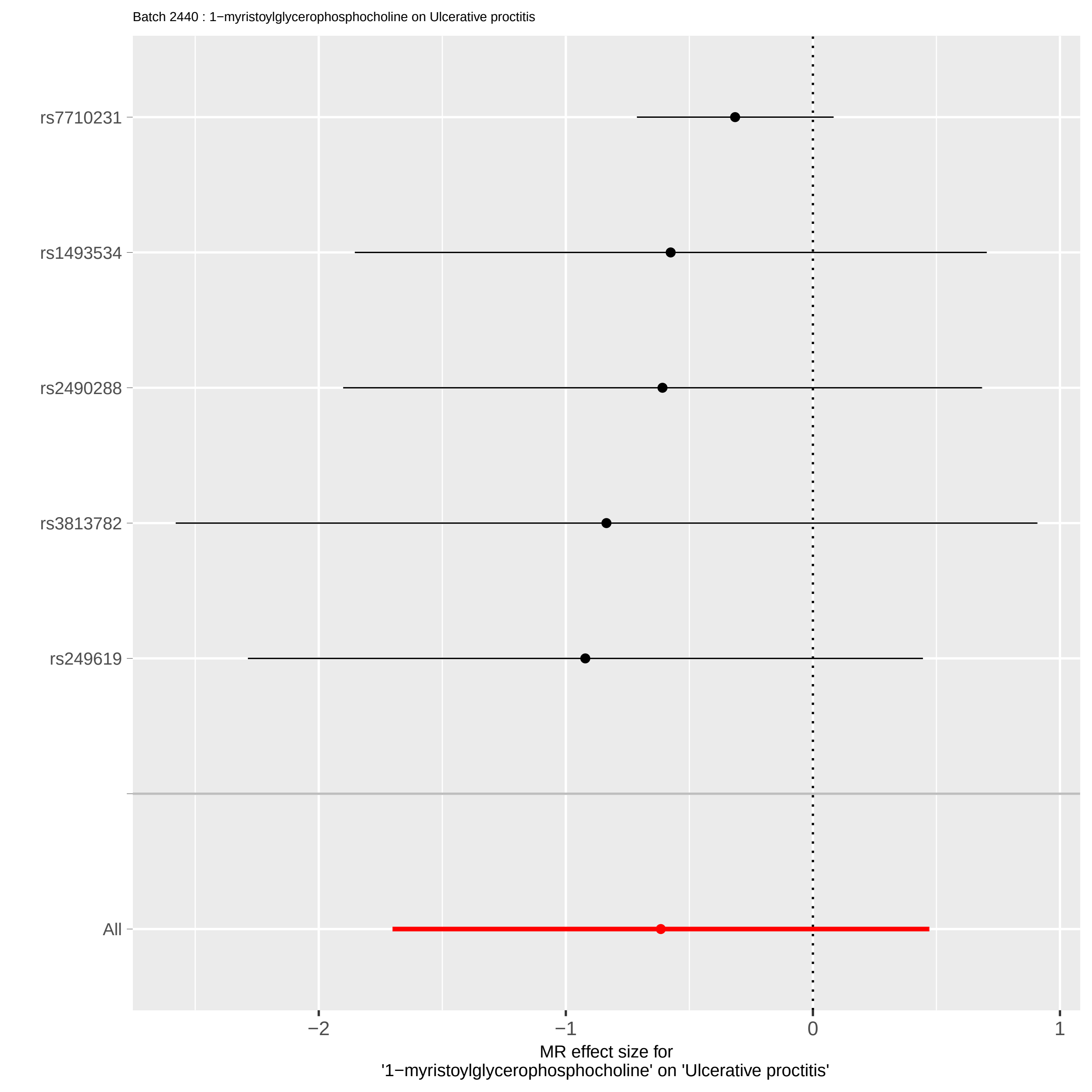
Batch 2440 : 1−myristoylglycerophosphocholine on Ulcerative proctitis
rs7710231
rs1493534
rs2490288
rs3813782
rs249619
All
−2
−1
0
1
MR effect size for
'1−myristoylglycerophosphocholine' on 'Ulcerative proctitis'

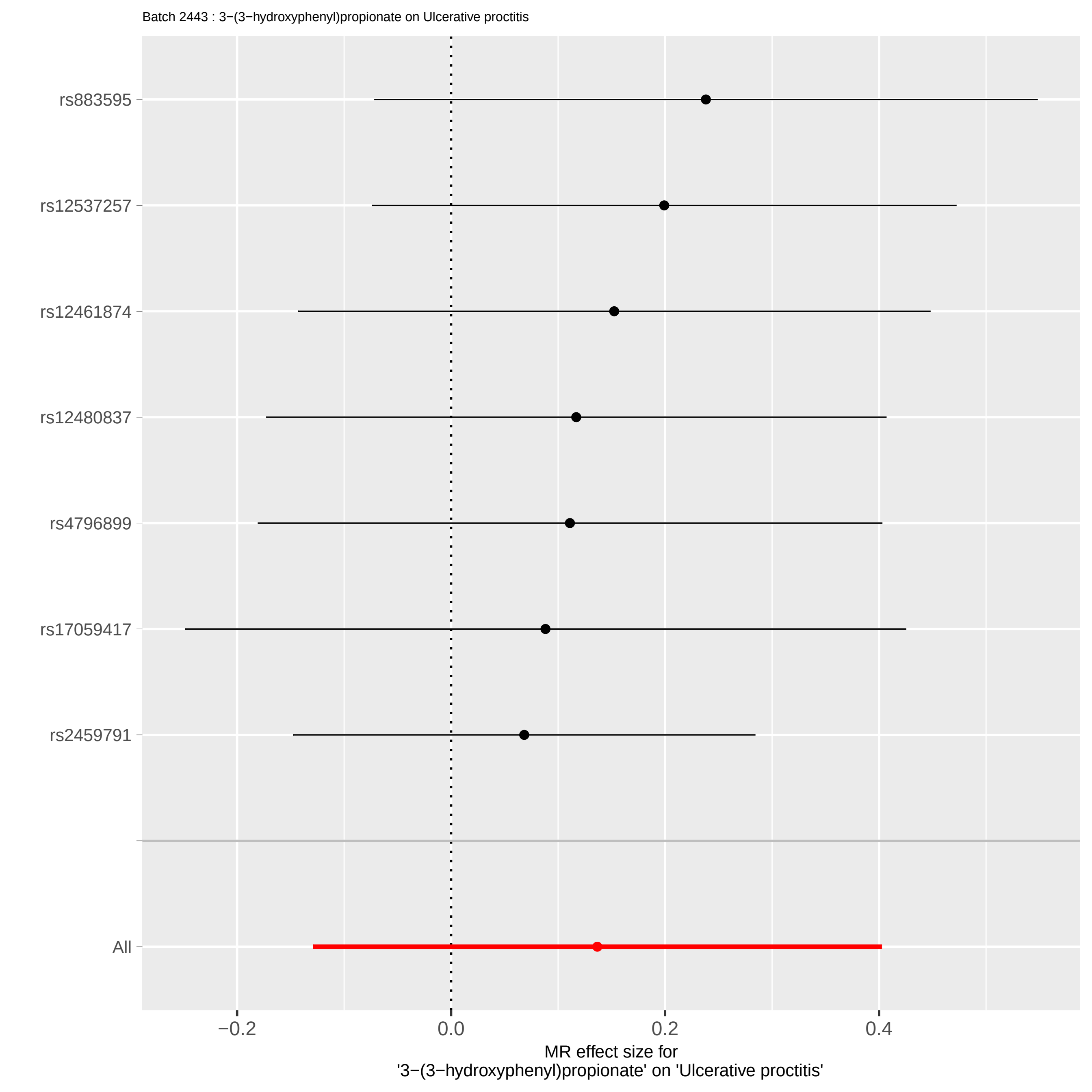

Batch 2443 : 3−(3−hydroxyphenyl)propionate on Ulcerative proctitis
rs883595
rs12537257
rs12461874
rs12480837
rs4796899
rs17059417
rs2459791
All
−0.2
0.0
0.2
0.4
MR effect size for
'3−(3−hydroxyphenyl)propionate' on 'Ulcerative proctitis'

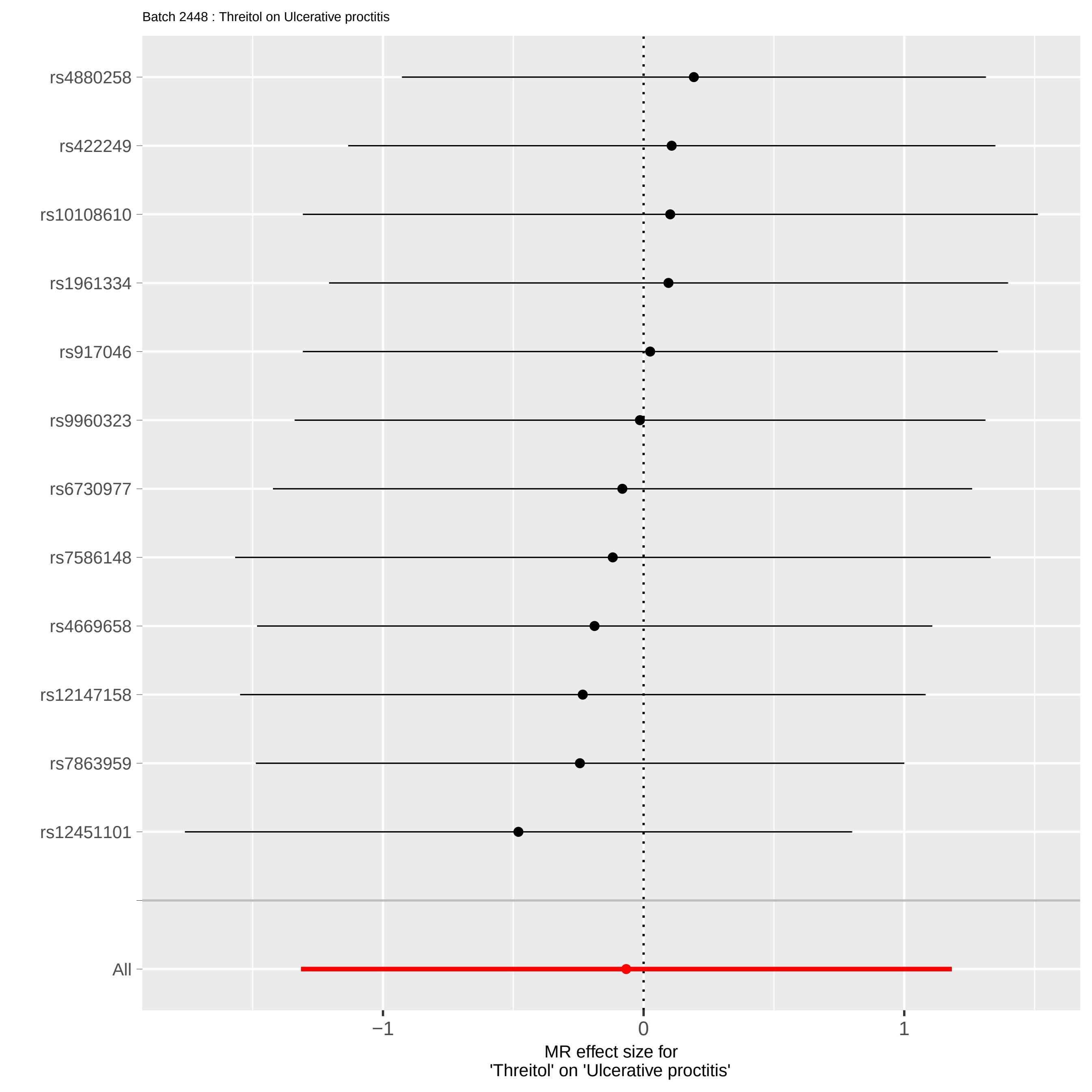

Batch 2448 : Threitol on Ulcerative proctitis
rs4880258
rs422249
rs10108610
rs1961334
rs917046
rs9960323
rs6730977
rs7586148
rs4669658
rs12147158
rs7863959
rs12451101
All
−1
0
1
MR effect size for
'Threitol' on 'Ulcerative proctitis'

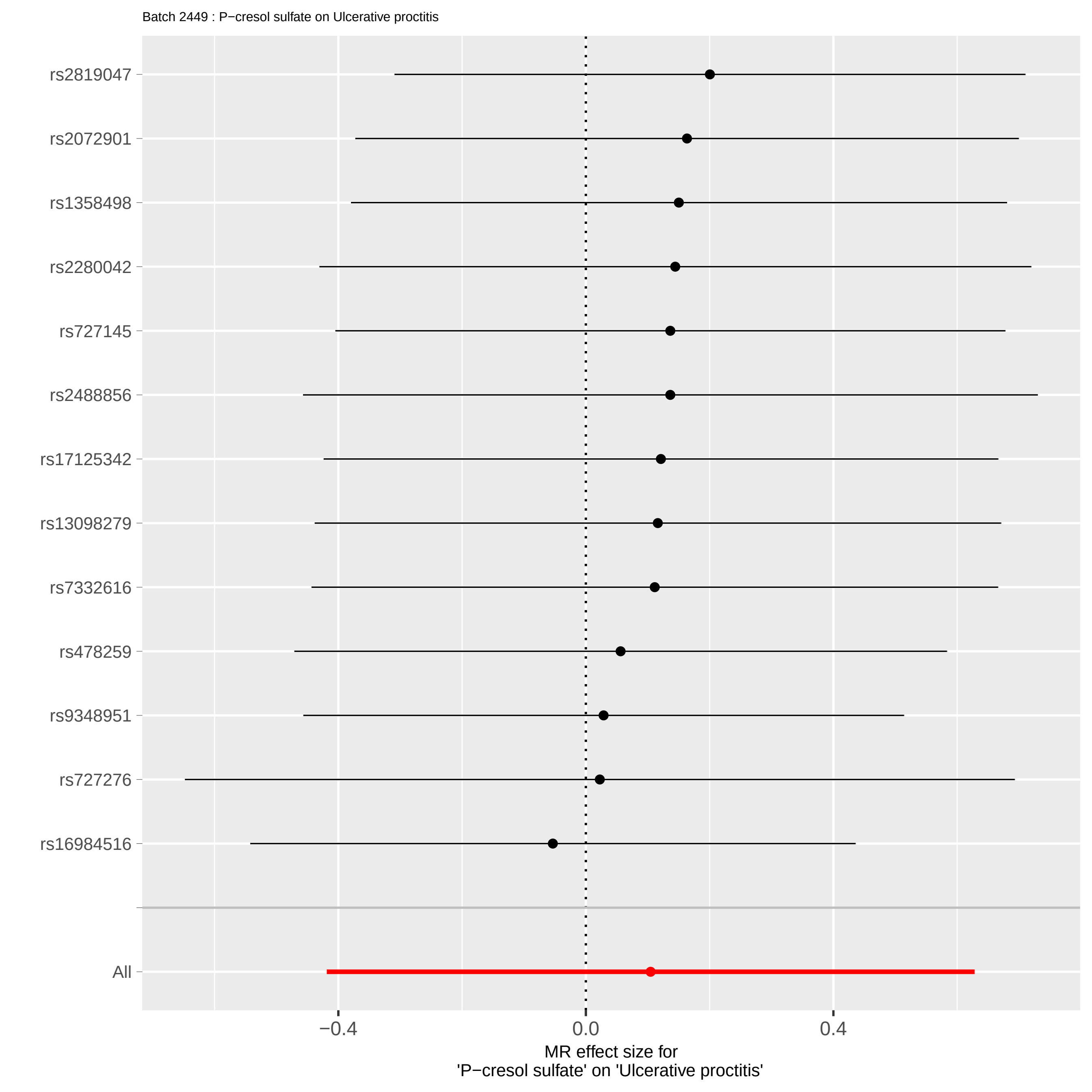
Batch 2449 : P−cresol sulfate on Ulcerative proctitis
rs2819047
rs2072901
rs1358498
rs2280042
rs727145
rs2488856
rs17125342
rs13098279
rs7332616
rs478259
rs9348951
rs727276
rs16984516
All
−0.4
0.0
0.4
MR effect size for
'P−cresol sulfate' on 'Ulcerative proctitis'

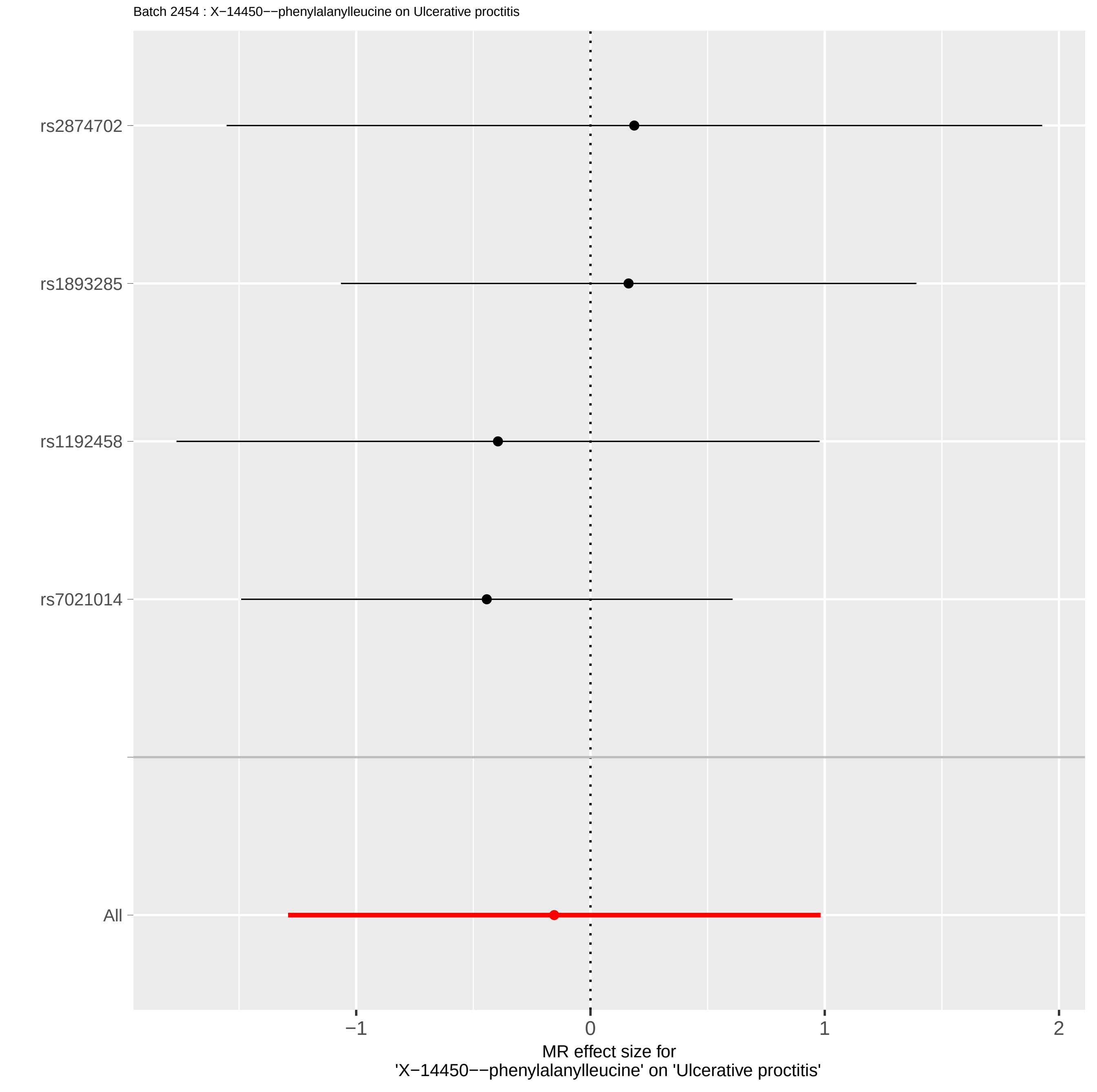
Batch 2454 : X−14450−−phenylalanylleucine on Ulcerative proctitis
rs2874702
rs1893285
rs1192458
rs7021014
All
−1
0
1
2
MR effect size for
'X−14450−−phenylalanylleucine' on 'Ulcerative proctitis'

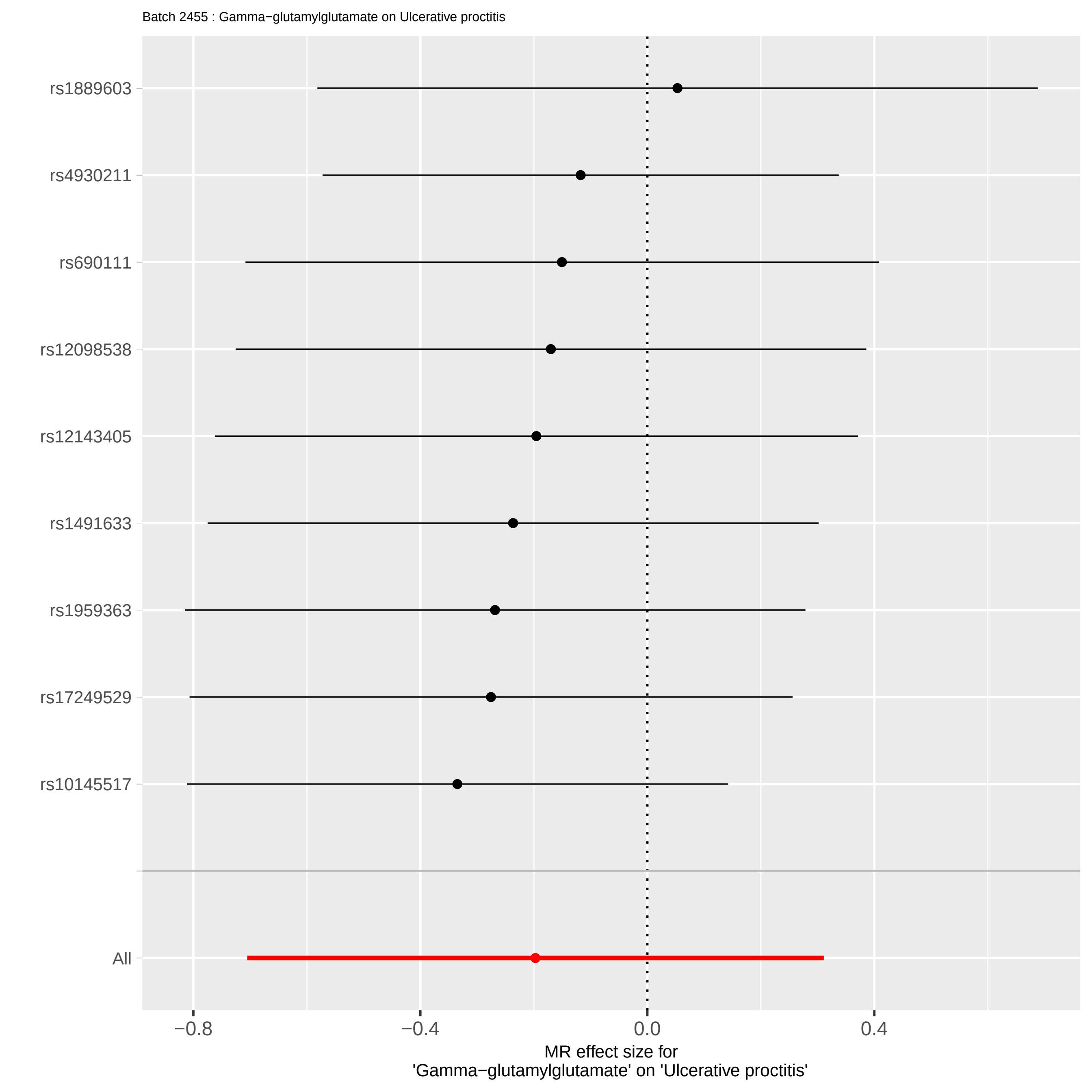

Batch 2455 : Gamma−glutamylglutamate on Ulcerative proctitis
rs1889603
rs4930211
rs690111
rs12098538
rs12143405
rs1491633
rs1959363
rs17249529
rs10145517
All
−0.8
−0.4
0.0
0.4
MR effect size for
'Gamma−glutamylglutamate' on 'Ulcerative proctitis'

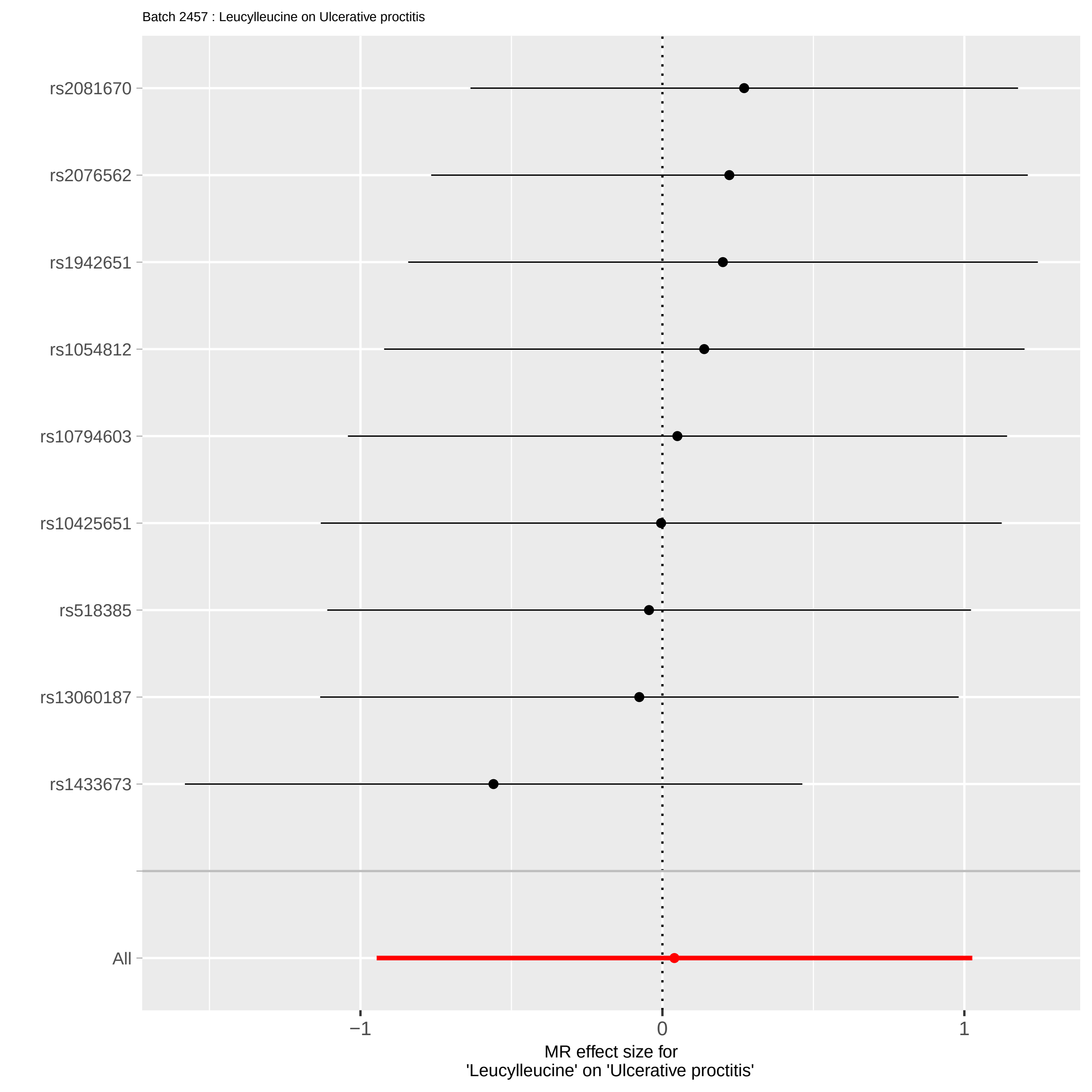

Batch 2457 : Leucylleucine on Ulcerative proctitis
rs2081670
rs2076562
rs1942651
rs1054812
rs10794603
rs10425651
rs518385
rs13060187
rs1433673
All
−1
0
1
MR effect size for
'Leucylleucine' on 'Ulcerative proctitis'

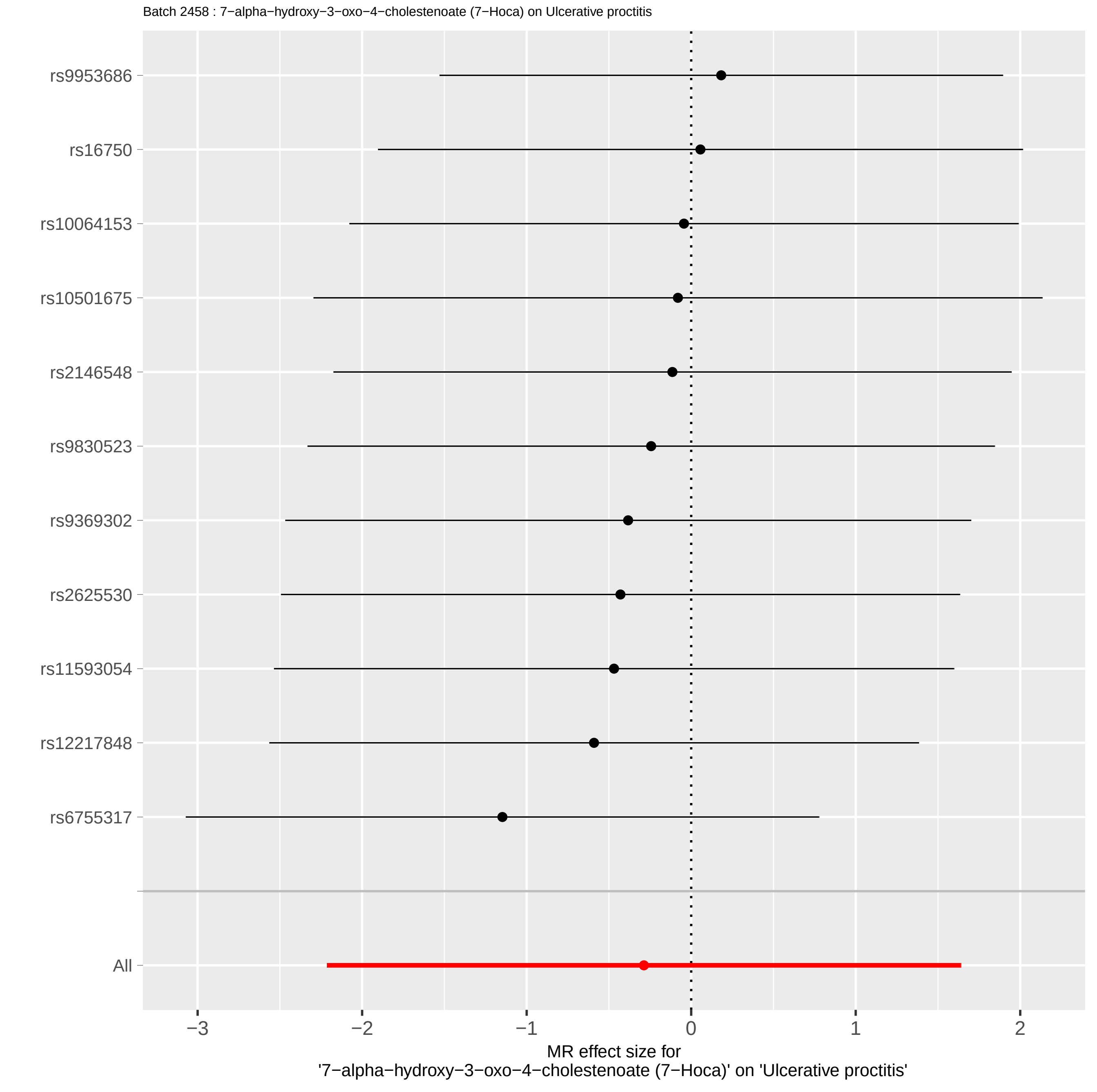
Batch 2458 : 7−alpha−hydroxy−3−oxo−4−cholestenoate (7−Hoca) on Ulcerative proctitis
rs9953686
rs16750
rs10064153
rs10501675
rs2146548
rs9830523
rs9369302
rs2625530
rs11593054
rs12217848
rs6755317
All
−3
−2
−1
0
1
2
MR effect size for
'7−alpha−hydroxy−3−oxo−4−cholestenoate (7−Hoca)' on 'Ulcerative proctitis'

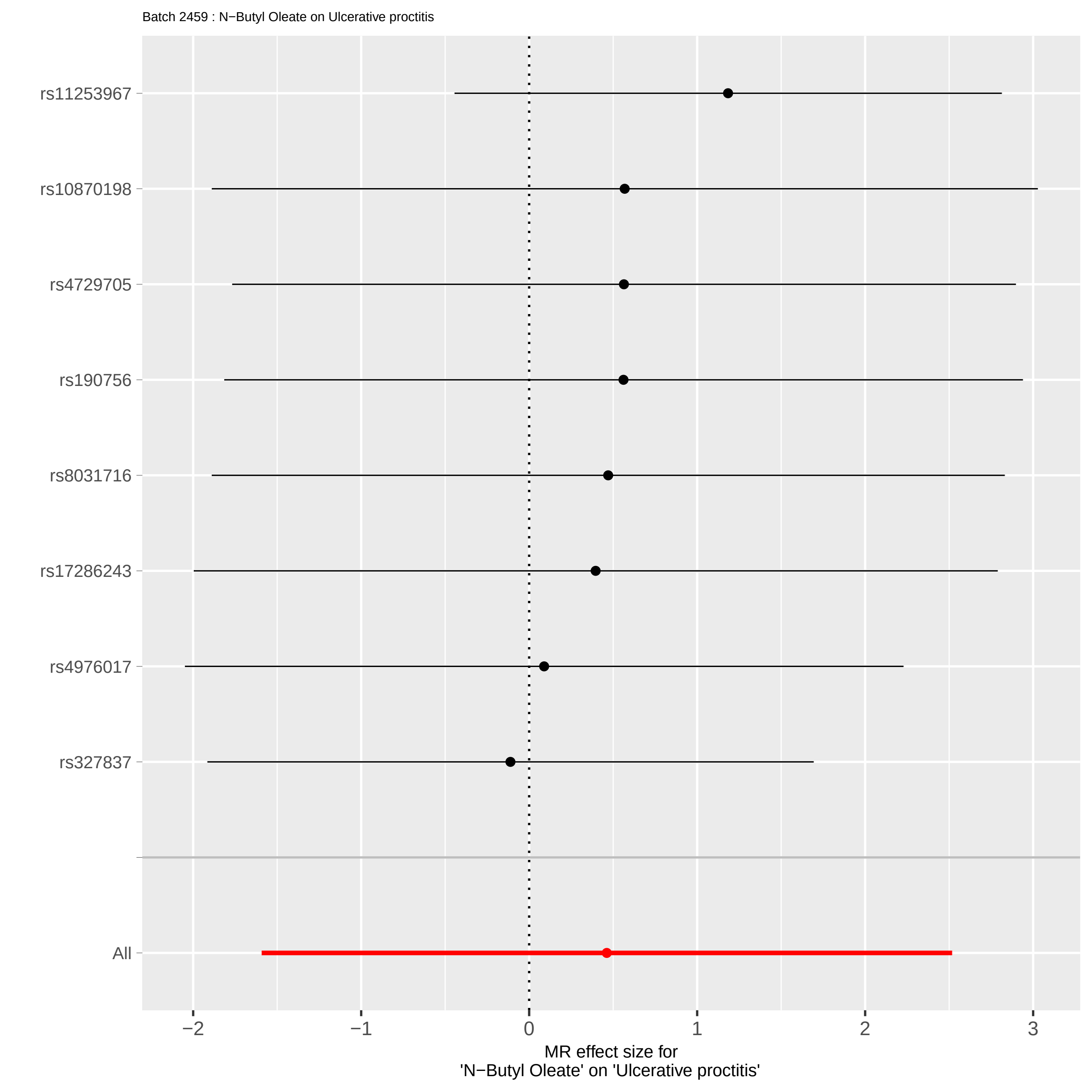
Batch 2459 : N−Butyl Oleate on Ulcerative proctitis
rs11253967
rs10870198
rs4729705
rs190756
rs8031716
rs17286243
rs4976017
rs327837
All
−2
−1
0
1
2
3
MR effect size for
'N−Butyl Oleate' on 'Ulcerative proctitis'

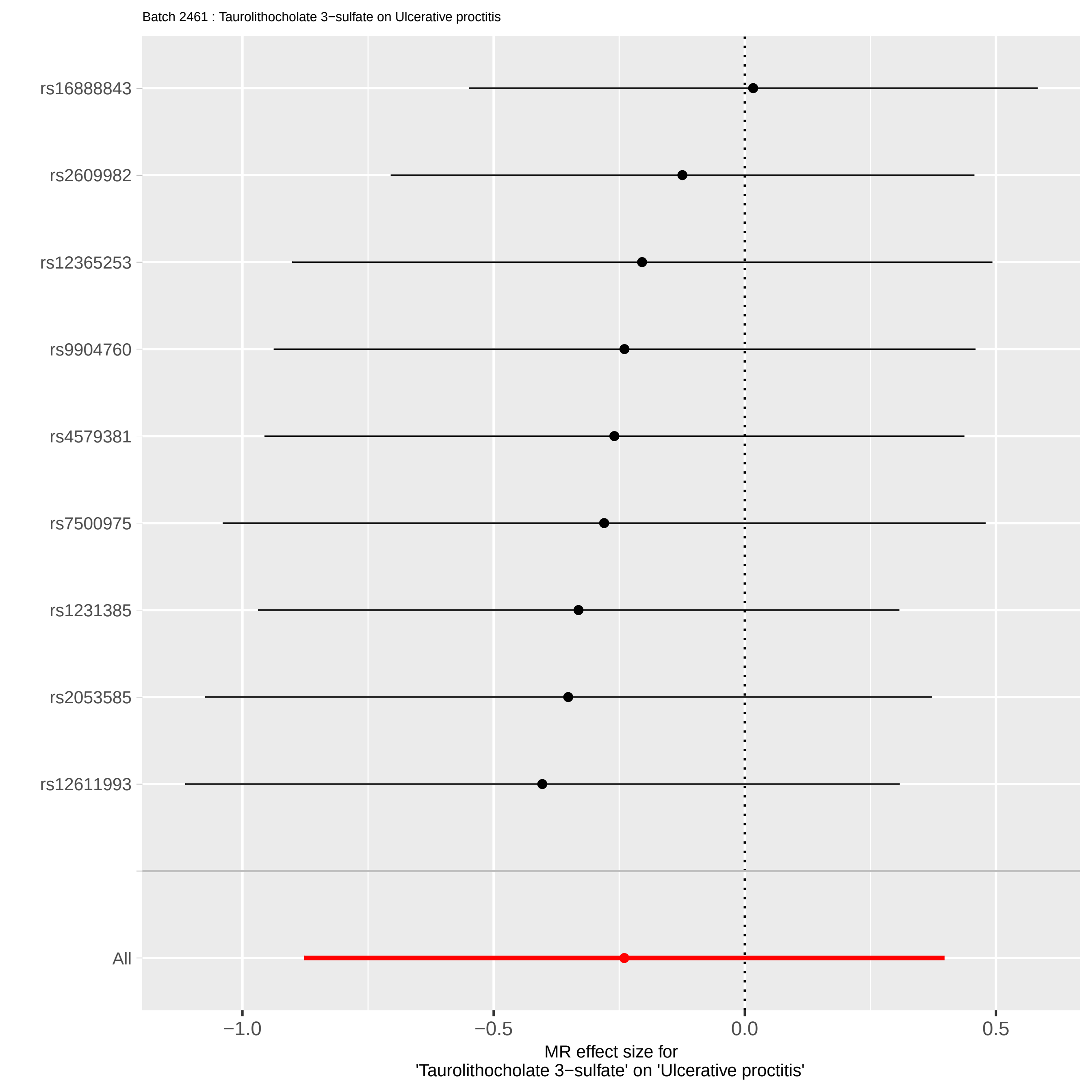

Batch 2461 : Taurolithocholate 3−sulfate on Ulcerative proctitis
rs16888843
rs2609982
rs12365253
rs9904760
rs4579381
rs7500975
rs1231385
rs2053585
rs12611993
All
−1.0
−0.5
0.0
0.5
MR effect size for
'Taurolithocholate 3−sulfate' on 'Ulcerative proctitis'

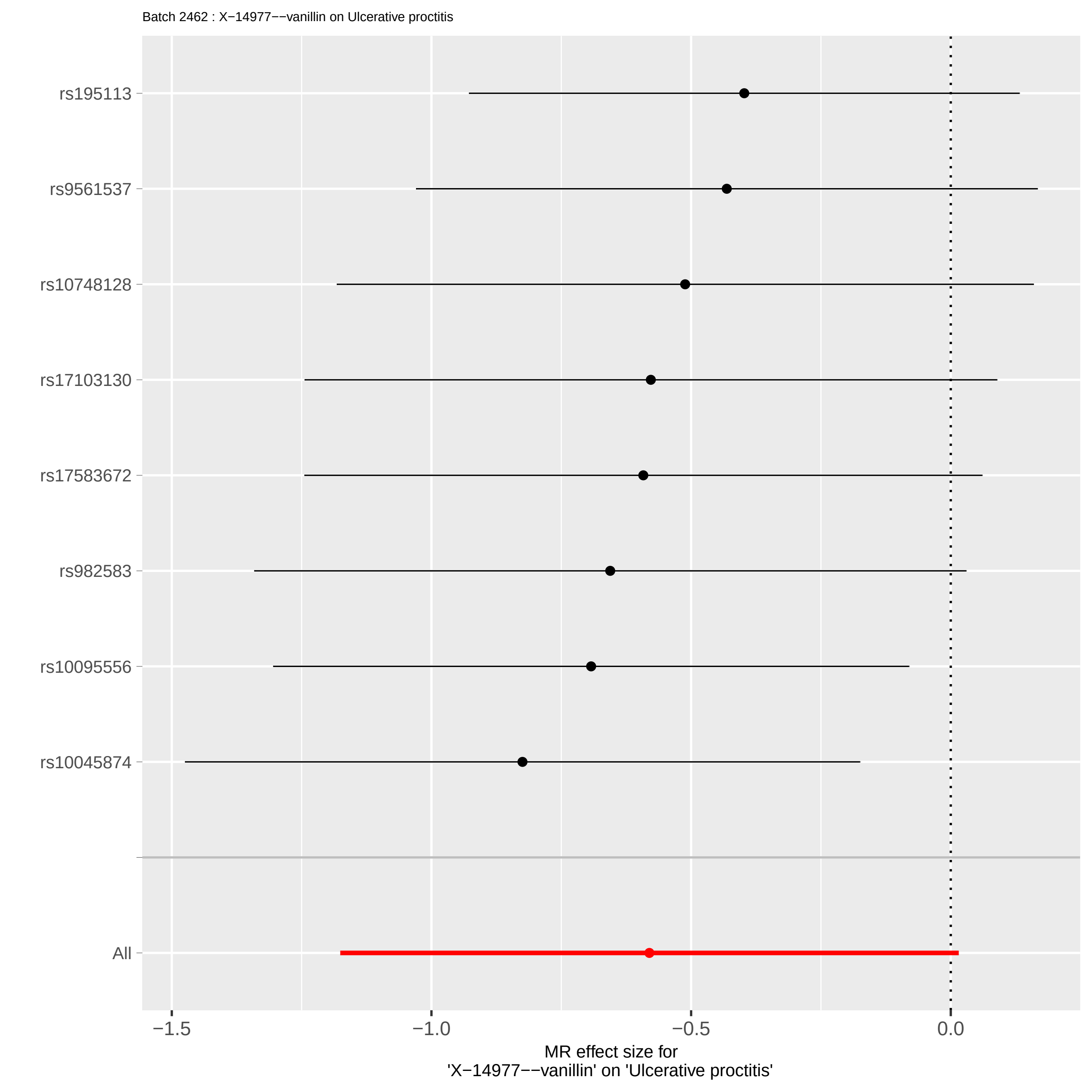
Batch 2462 : X−14977−−vanillin on Ulcerative proctitis
rs195113
rs9561537
rs10748128
rs17103130
rs17583672
rs982583
rs10095556
rs10045874
All
−1.5
−1.0
−0.5
0.0
MR effect size for
'X−14977−−vanillin' on 'Ulcerative proctitis'

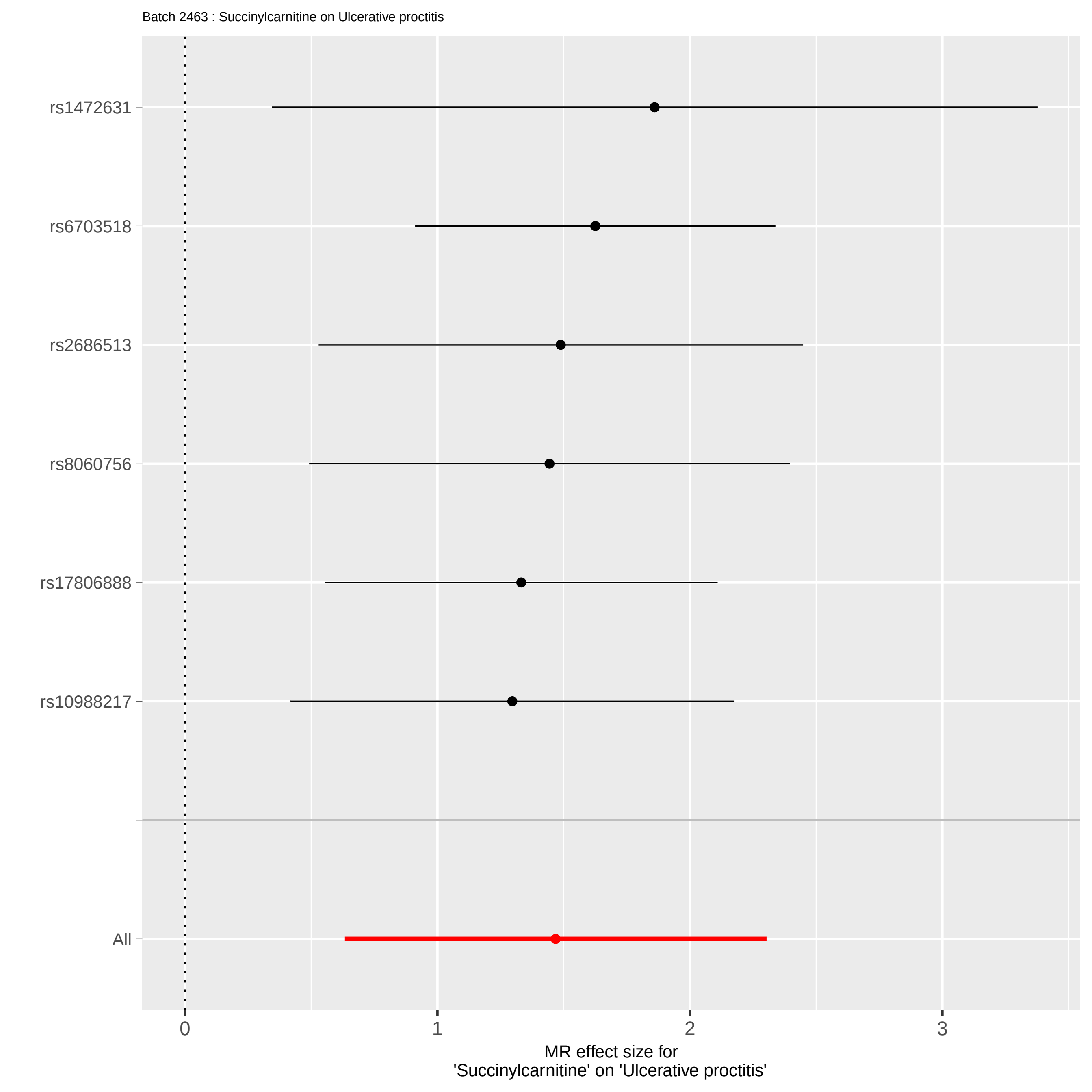
Batch 2463 : Succinylcarnitine on Ulcerative proctitis
rs1472631
rs6703518
rs2686513
rs8060756
rs17806888
rs10988217
All
0
1
2
3
MR effect size for
'Succinylcarnitine' on 'Ulcerative proctitis'

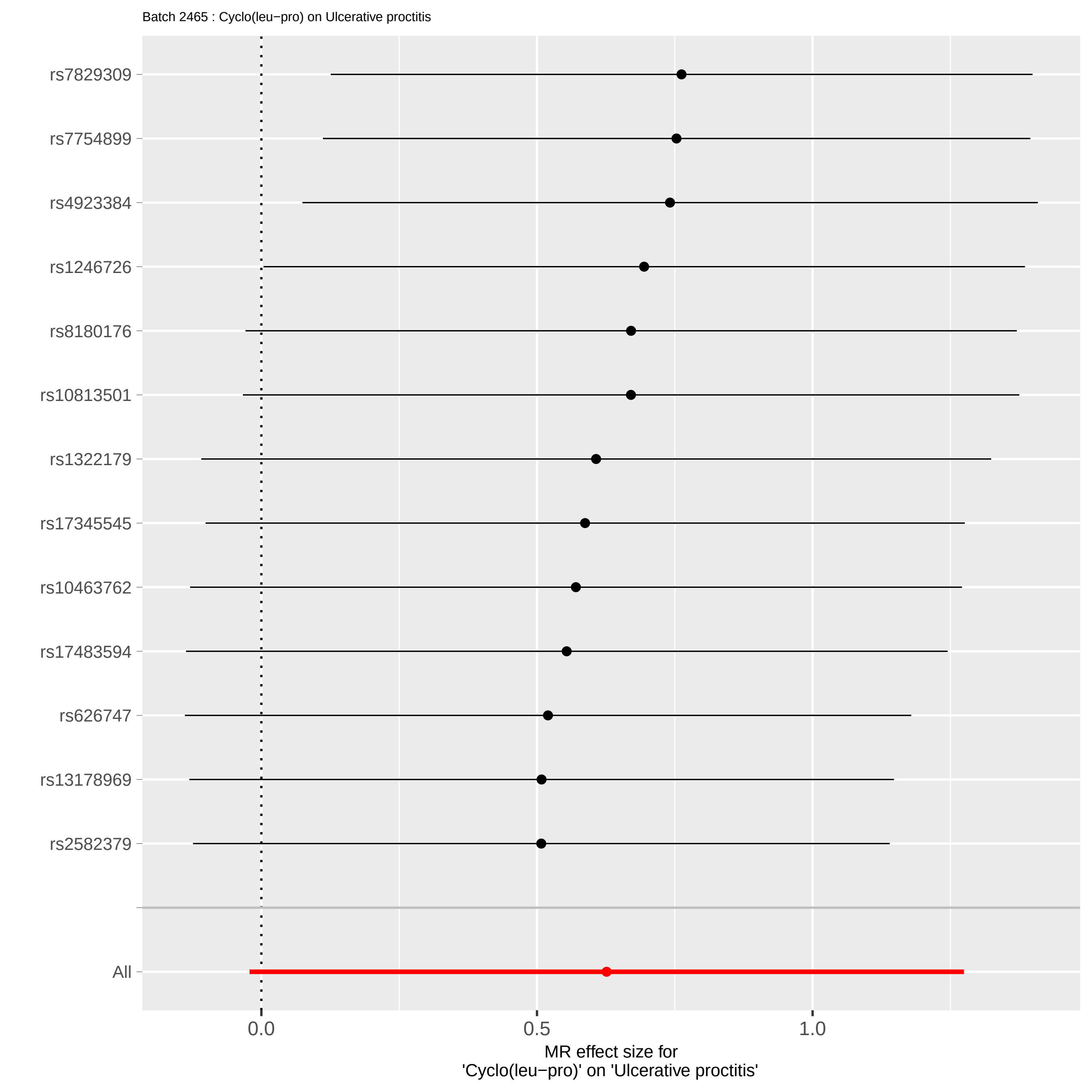
Batch 2465 : Cyclo(leu−pro) on Ulcerative proctitis
rs7829309
rs7754899
rs4923384
rs1246726
rs8180176
rs10813501
rs1322179
rs17345545
rs10463762
rs17483594
rs626747
rs13178969
rs2582379
All
0.0
0.5
1.0
MR effect size for
'Cyclo(leu−pro)' on 'Ulcerative proctitis'

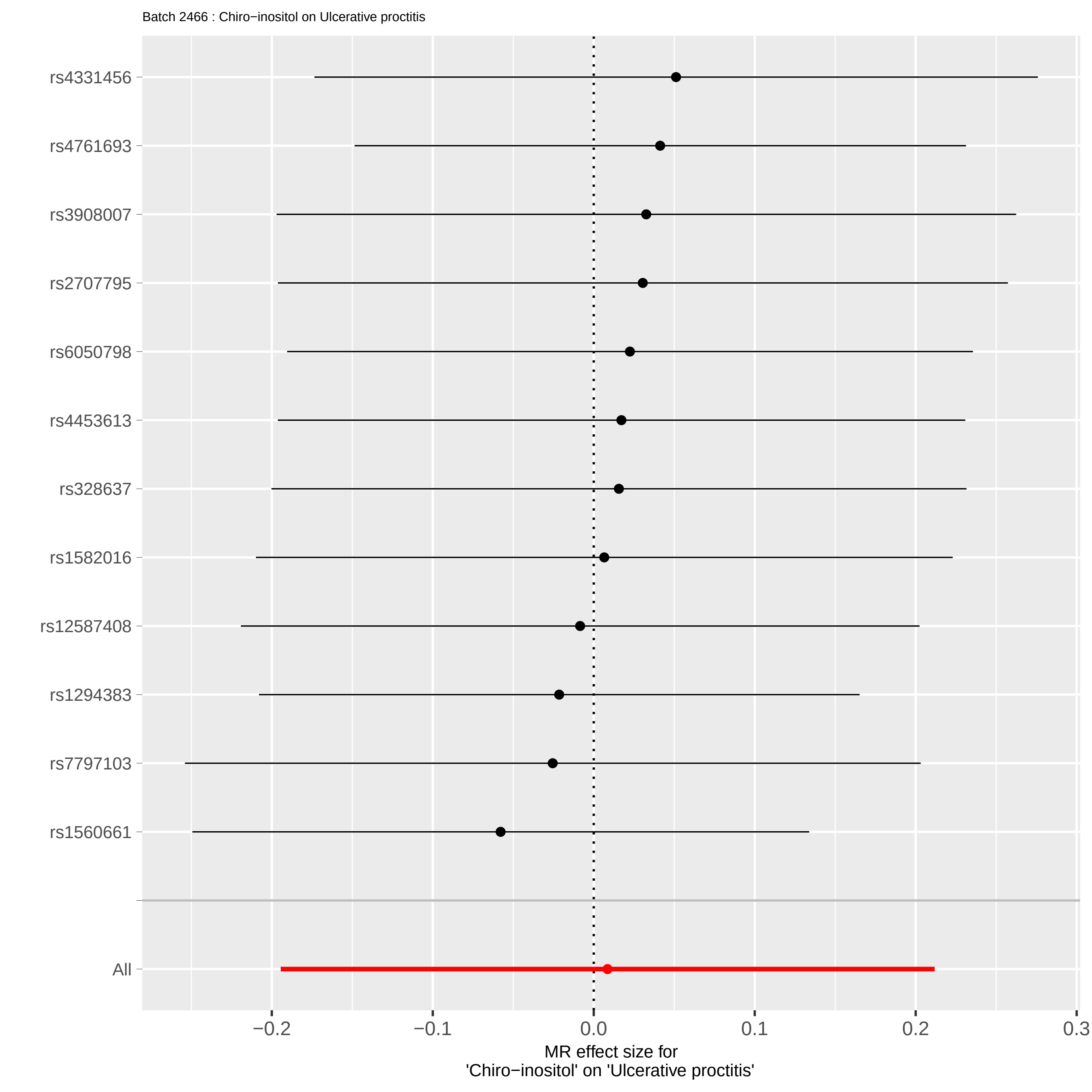

Batch 2466 : Chiro−inositol on Ulcerative proctitis
rs4331456
rs4761693
rs3908007
rs2707795
rs6050798
rs4453613
rs328637
rs1582016
rs12587408
rs1294383
rs7797103
rs1560661
All
−0.2
−0.1
0.0
0.1
0.2
0.3
MR effect size for
'Chiro−inositol' on 'Ulcerative proctitis'

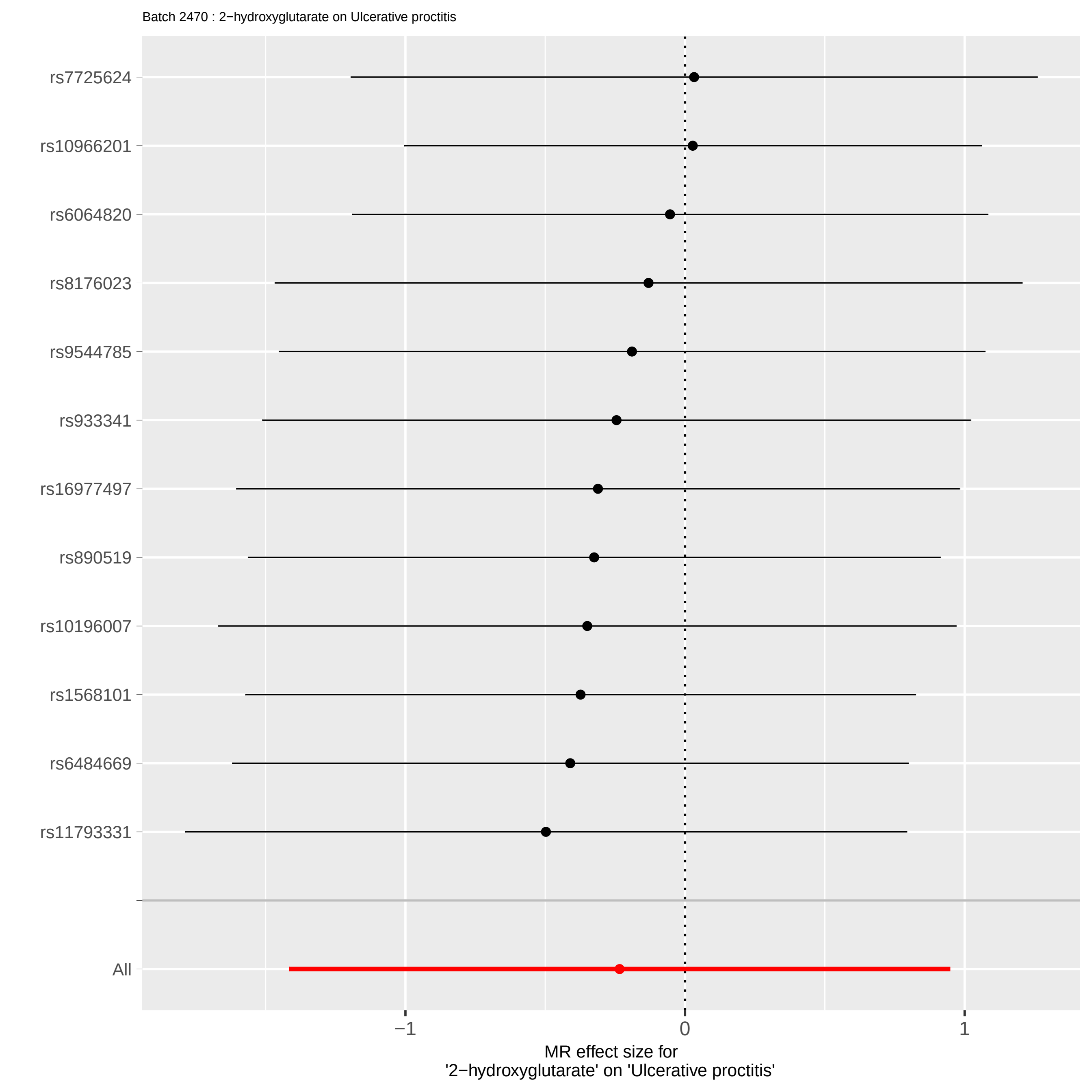
Batch 2470 : 2−hydroxyglutarate on Ulcerative proctitis
rs7725624
rs10966201
rs6064820
rs8176023
rs9544785
rs933341
rs16977497
rs890519
rs10196007
rs1568101
rs6484669
rs11793331
All
−1
0
1
MR effect size for
'2−hydroxyglutarate' on 'Ulcerative proctitis'

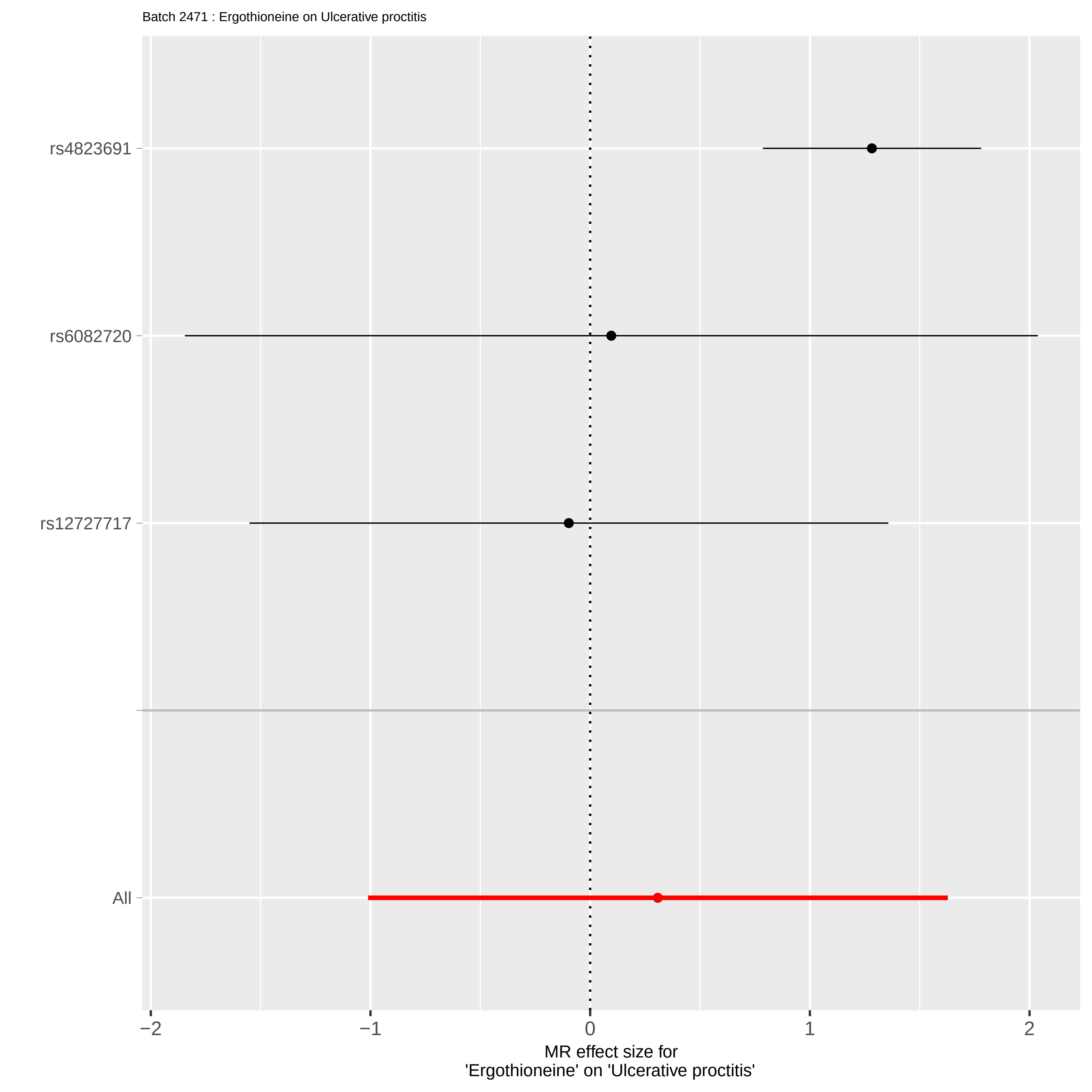
Batch 2471 : Ergothioneine on Ulcerative proctitis
rs4823691
rs6082720
rs12727717
All
−2
−1
0
1
2
MR effect size for
'Ergothioneine' on 'Ulcerative proctitis'

Batch 2473 : Phenylalanylphenylalanine on Ulcerative proctitis

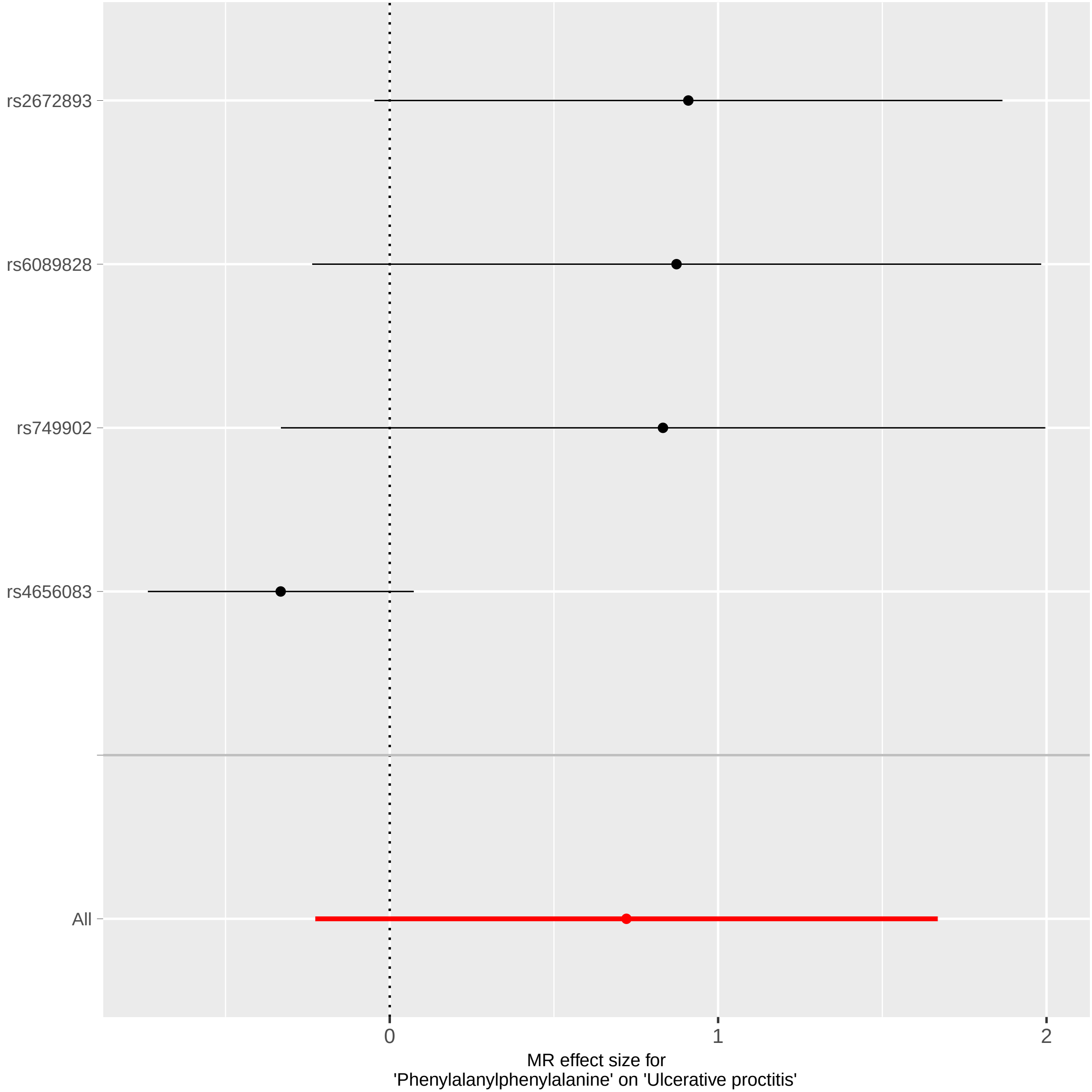

Batch 2475 : 15−methylpalmitate (isobar with 2−methylpalmitate) on Ulcerative proctitis

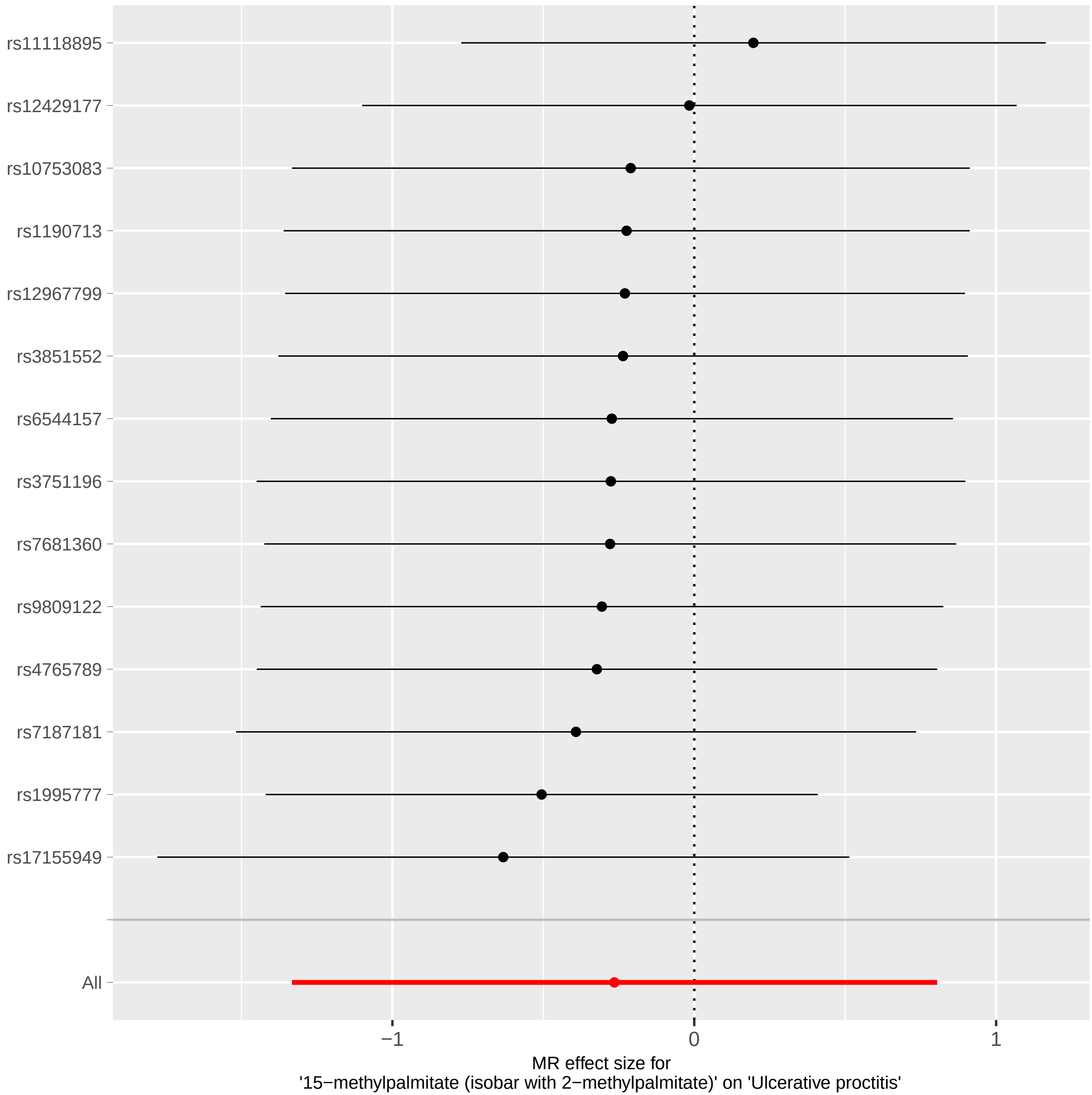

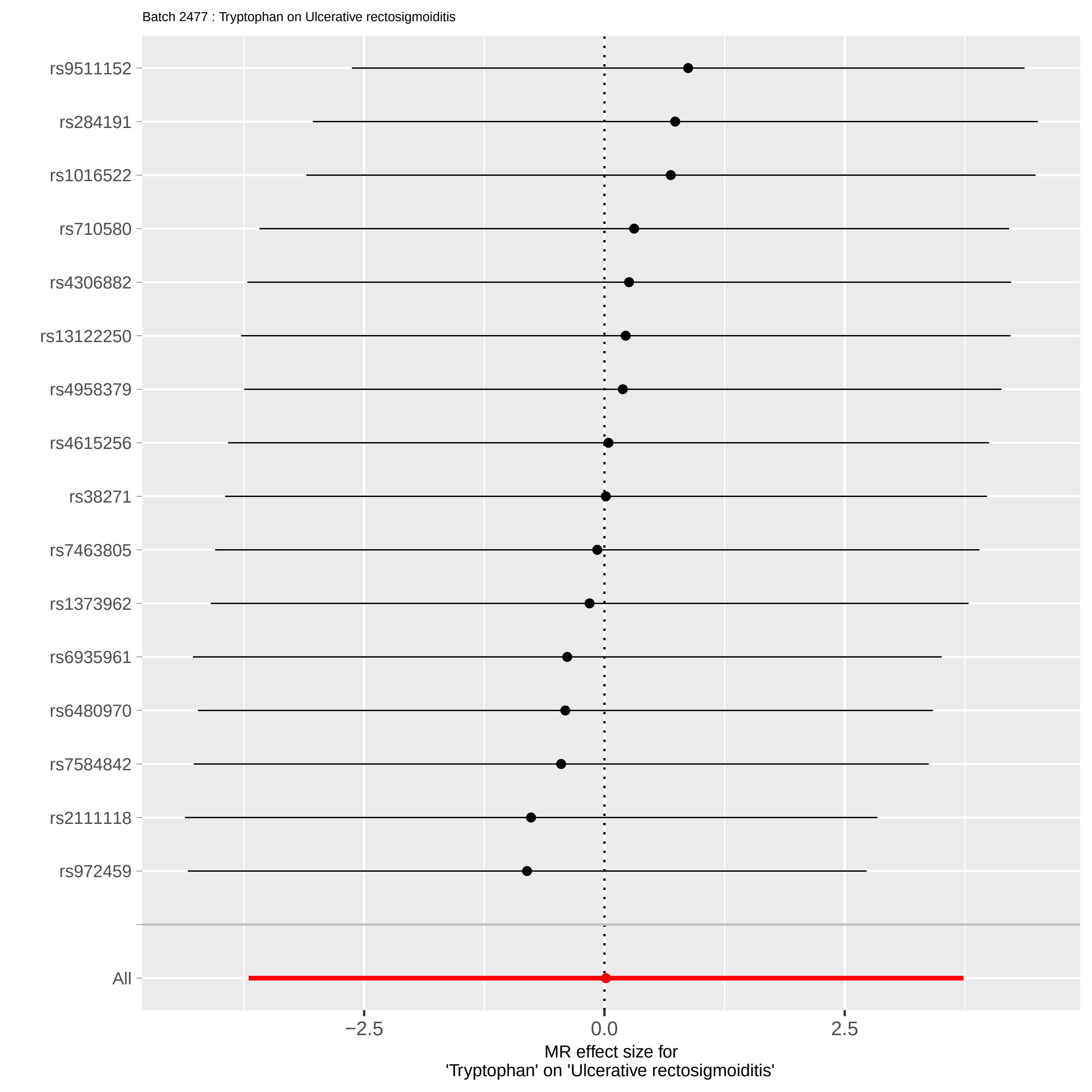
Batch 2477 : Tryptophan on Ulcerative rectosigmoiditis
rs9511152
rs284191
rs1016522
rs710580
rs4306882
rs13122250
rs4958379
rs4615256
rs38271
rs7463805
rs1373962
rs6935961
rs6480970
rs7584842
rs2111118
rs972459
All
−2.5
0.0
2.5
MR effect size for
'Tryptophan' on 'Ulcerative rectosigmoiditis'

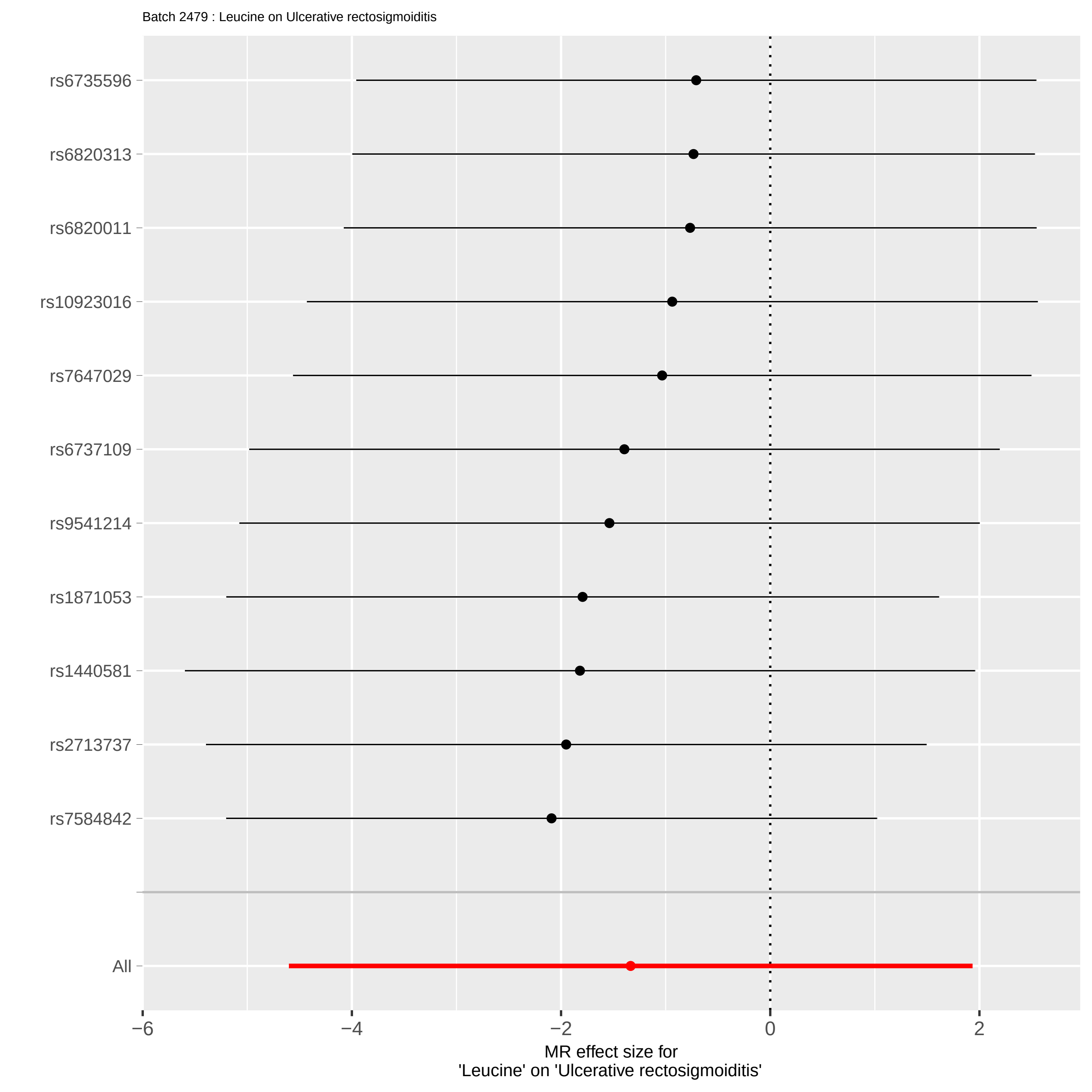
Batch 2479 : Leucine on Ulcerative rectosigmoiditis
rs6735596
rs6820313
rs6820011
rs10923016
rs7647029
rs6737109
rs9541214
rs1871053
rs1440581
rs2713737
rs7584842
All
−6
−4
−2
0
2
MR effect size for
'Leucine' on 'Ulcerative rectosigmoiditis'

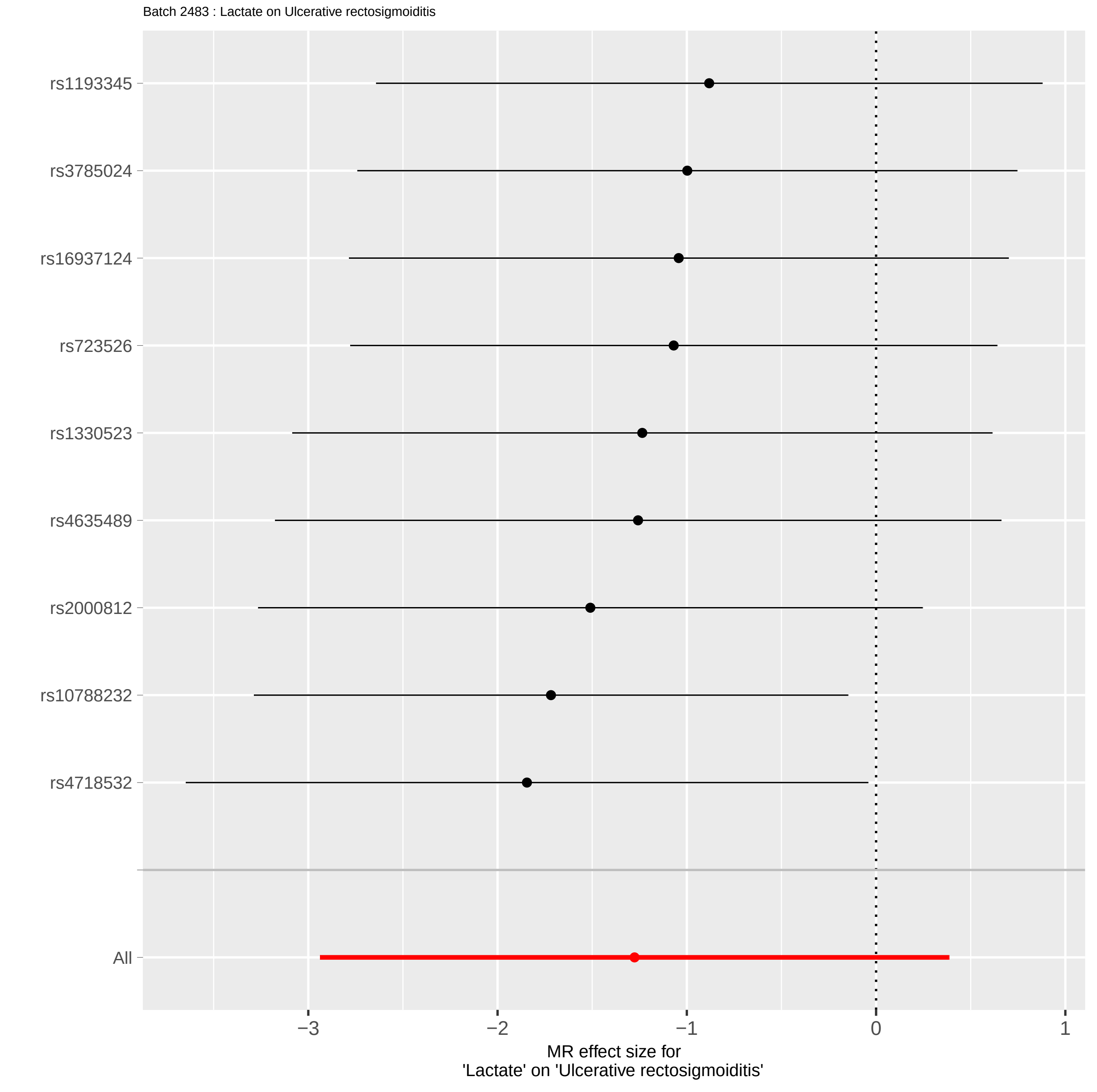

Batch 2483 : Lactate on Ulcerative rectosigmoiditis
rs1193345
rs3785024
rs16937124
rs723526
rs1330523
rs4635489
rs2000812
rs10788232
rs4718532
All
−3
−2
−1
0
1
MR effect size for
'Lactate' on 'Ulcerative rectosigmoiditis'

Batch 2484 : 3−hydroxybutyrate (BHBA) on Ulcerative rectosigmoiditis

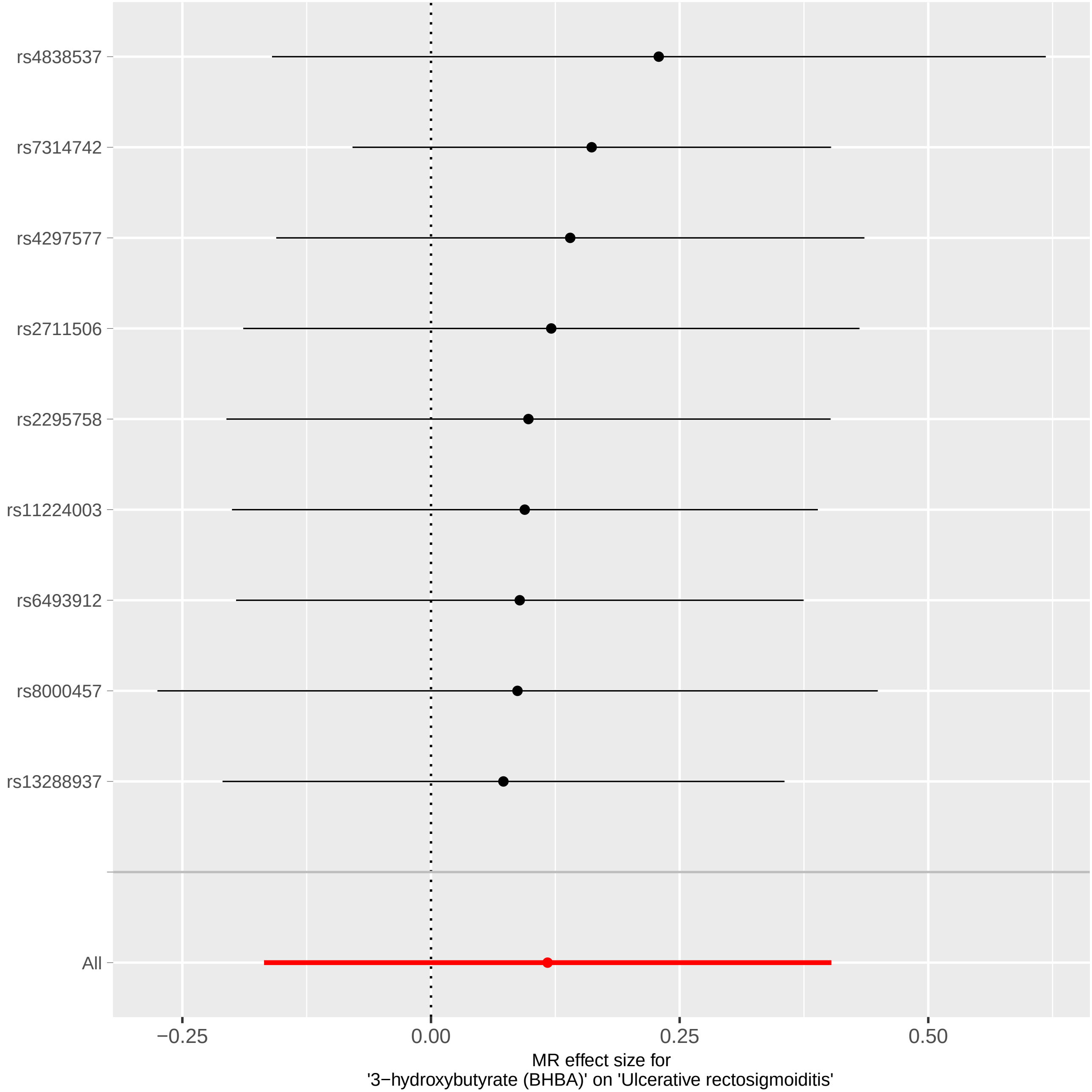

Batch 2485 : Arabinose on Ulcerative rectosigmoiditis

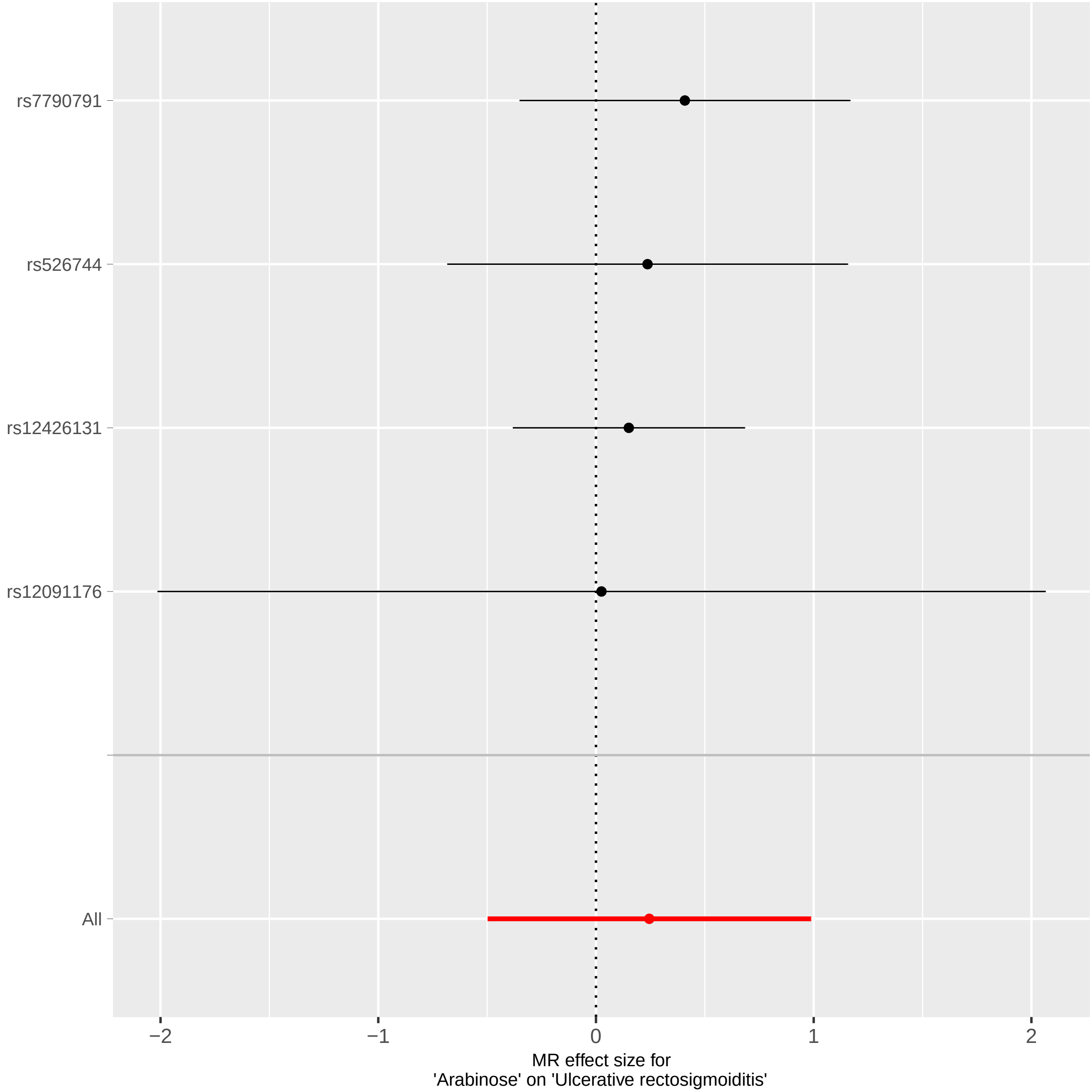

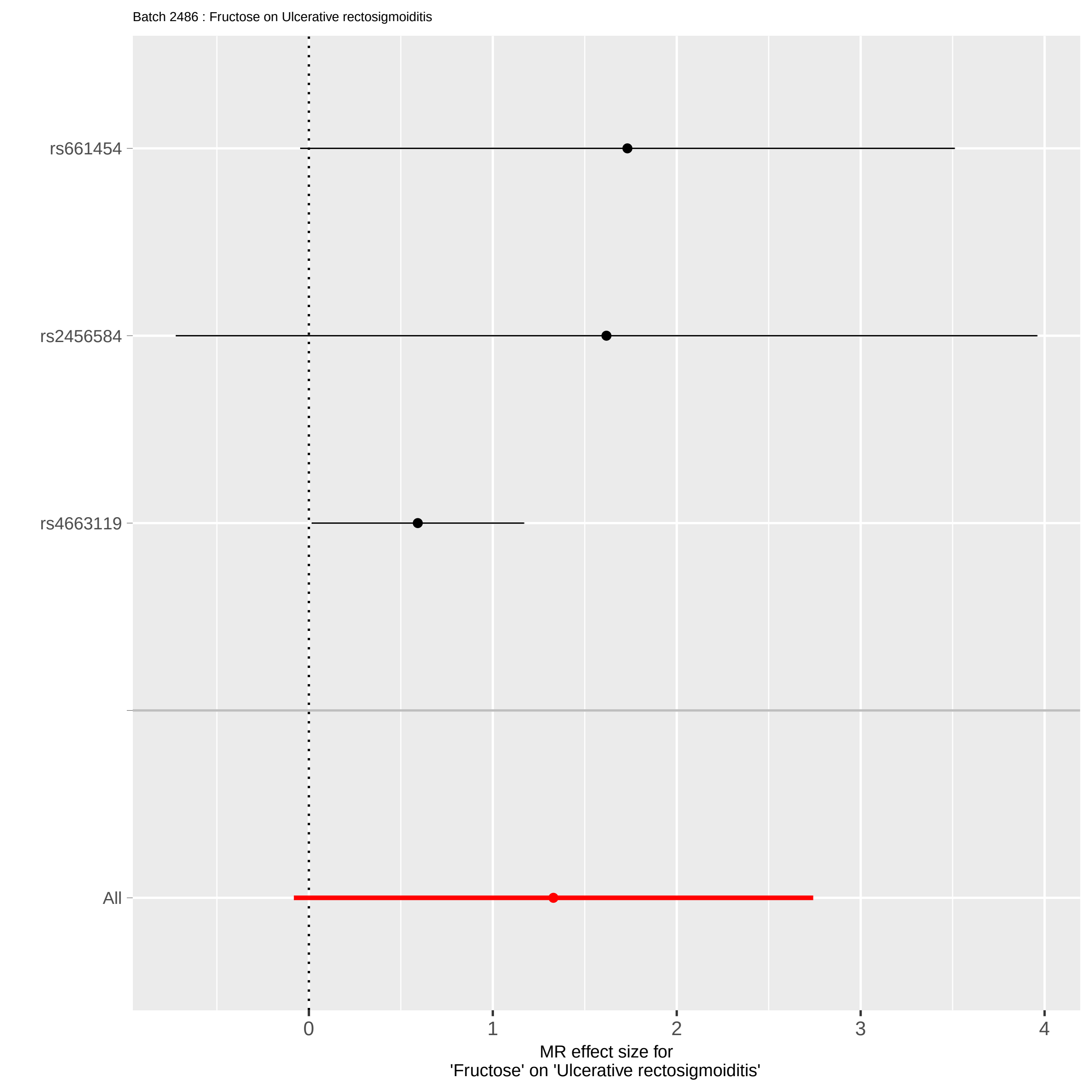
Batch 2486 : Fructose on Ulcerative rectosigmoiditis
rs661454
rs2456584
rs4663119
All
0
1
2
3
4
MR effect size for
'Fructose' on 'Ulcerative rectosigmoiditis'

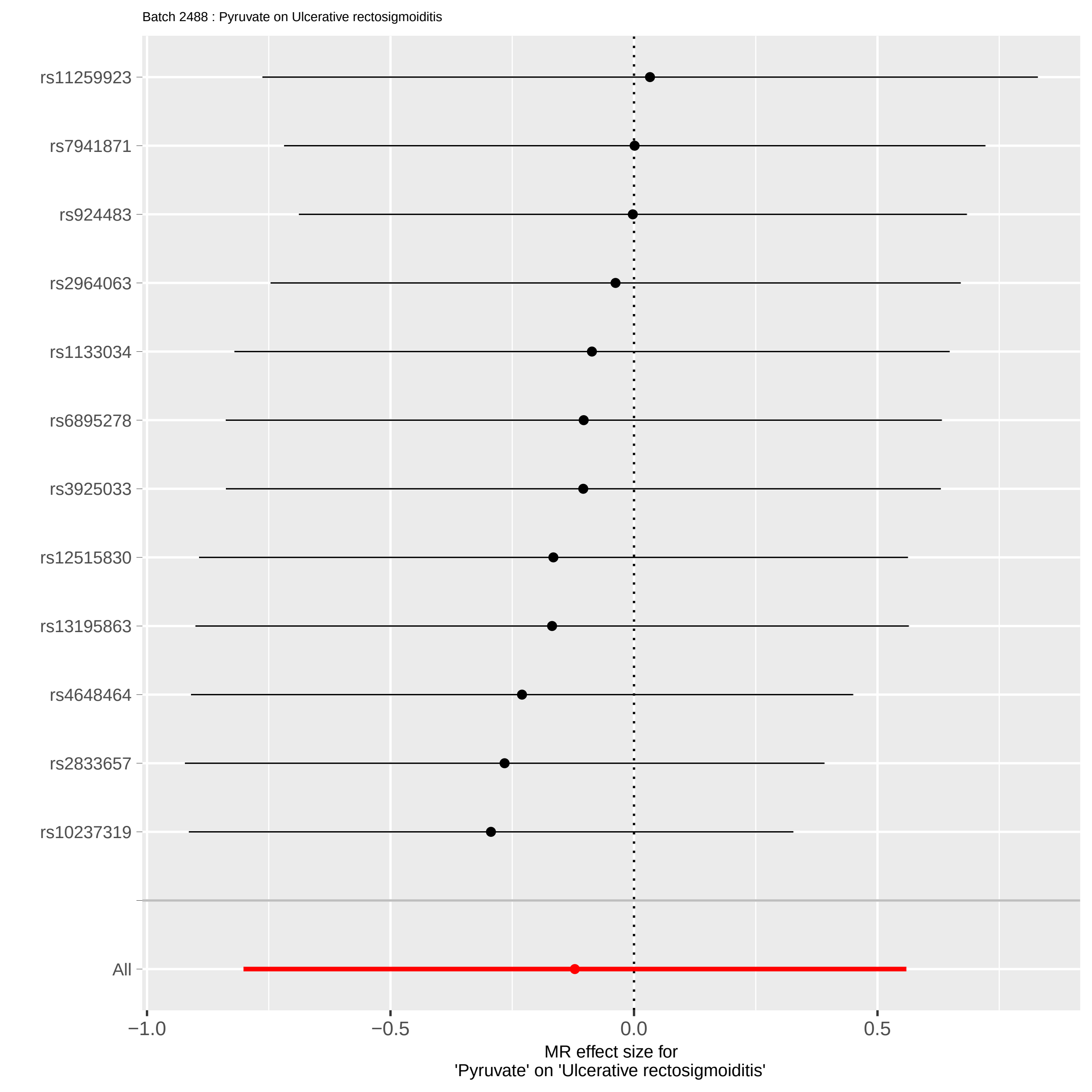
Batch 2488 : Pyruvate on Ulcerative rectosigmoiditis
rs11259923
rs7941871
rs924483
rs2964063
rs1133034
rs6895278
rs3925033
rs12515830
rs13195863
rs4648464
rs2833657
rs10237319
All
−1.0
−0.5
0.0
0.5
MR effect size for
'Pyruvate' on 'Ulcerative rectosigmoiditis'

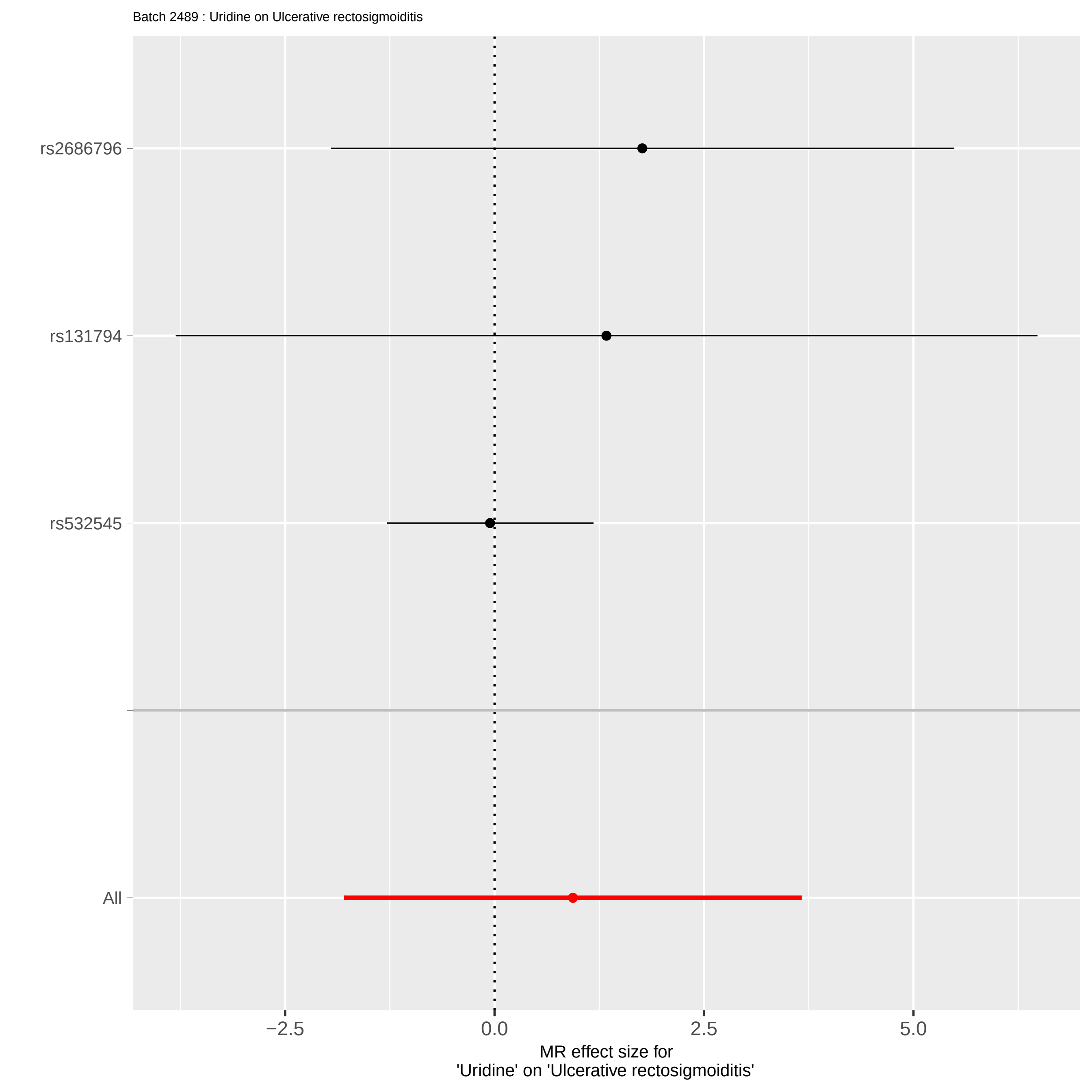

Batch 2489 : Uridine on Ulcerative rectosigmoiditis
rs2686796
rs131794
rs532545
All
−2.5
0.0
2.5
5.0
MR effect size for
'Uridine' on 'Ulcerative rectosigmoiditis'

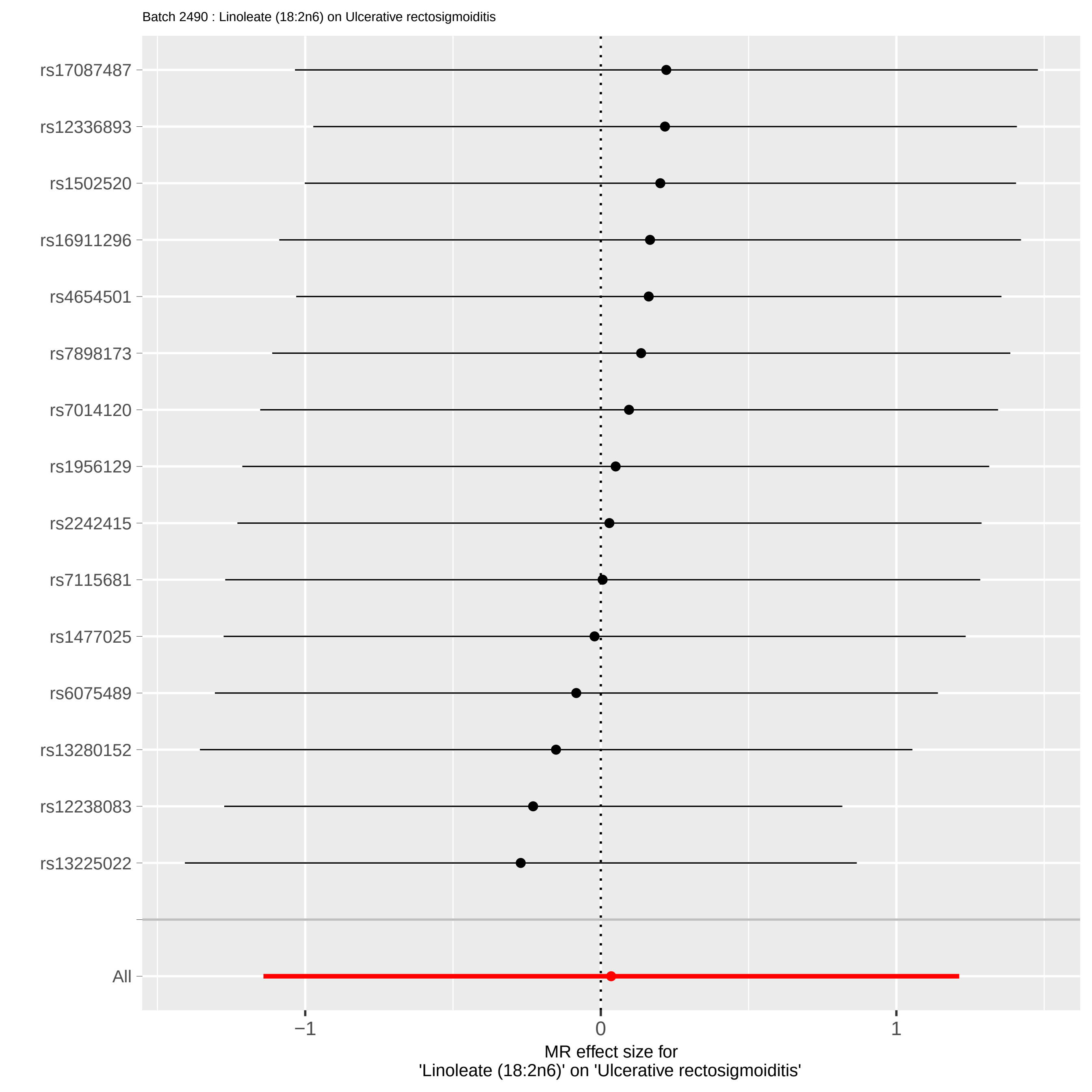
Batch 2490 : Linoleate (18:2n6) on Ulcerative rectosigmoiditis
rs17087487
rs12336893
rs1502520
rs16911296
rs4654501
rs7898173
rs7014120
rs1956129
rs2242415
rs7115681
rs1477025
rs6075489
rs13280152
rs12238083
rs13225022
All
−1
0
1
MR effect size for
'Linoleate (18:2n6)' on 'Ulcerative rectosigmoiditis'

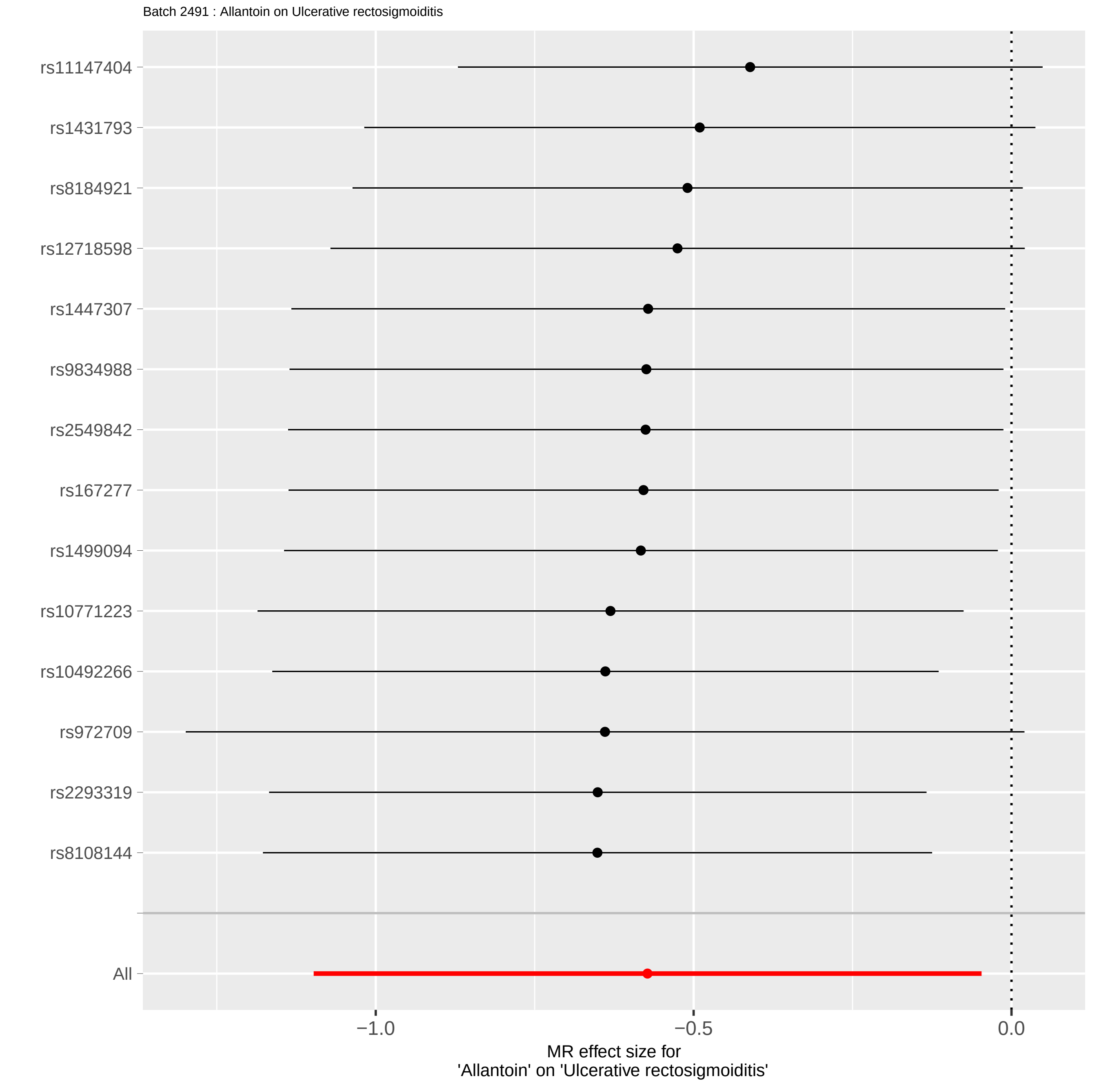

Batch 2491 : Allantoin on Ulcerative rectosigmoiditis
rs11147404
rs1431793
rs8184921
rs12718598
rs1447307
rs9834988
rs2549842
rs167277
rs1499094
rs10771223
rs10492266
rs972709
rs2293319
rs8108144
All
−1.0
−0.5
0.0
MR effect size for
'Allantoin' on 'Ulcerative rectosigmoiditis'

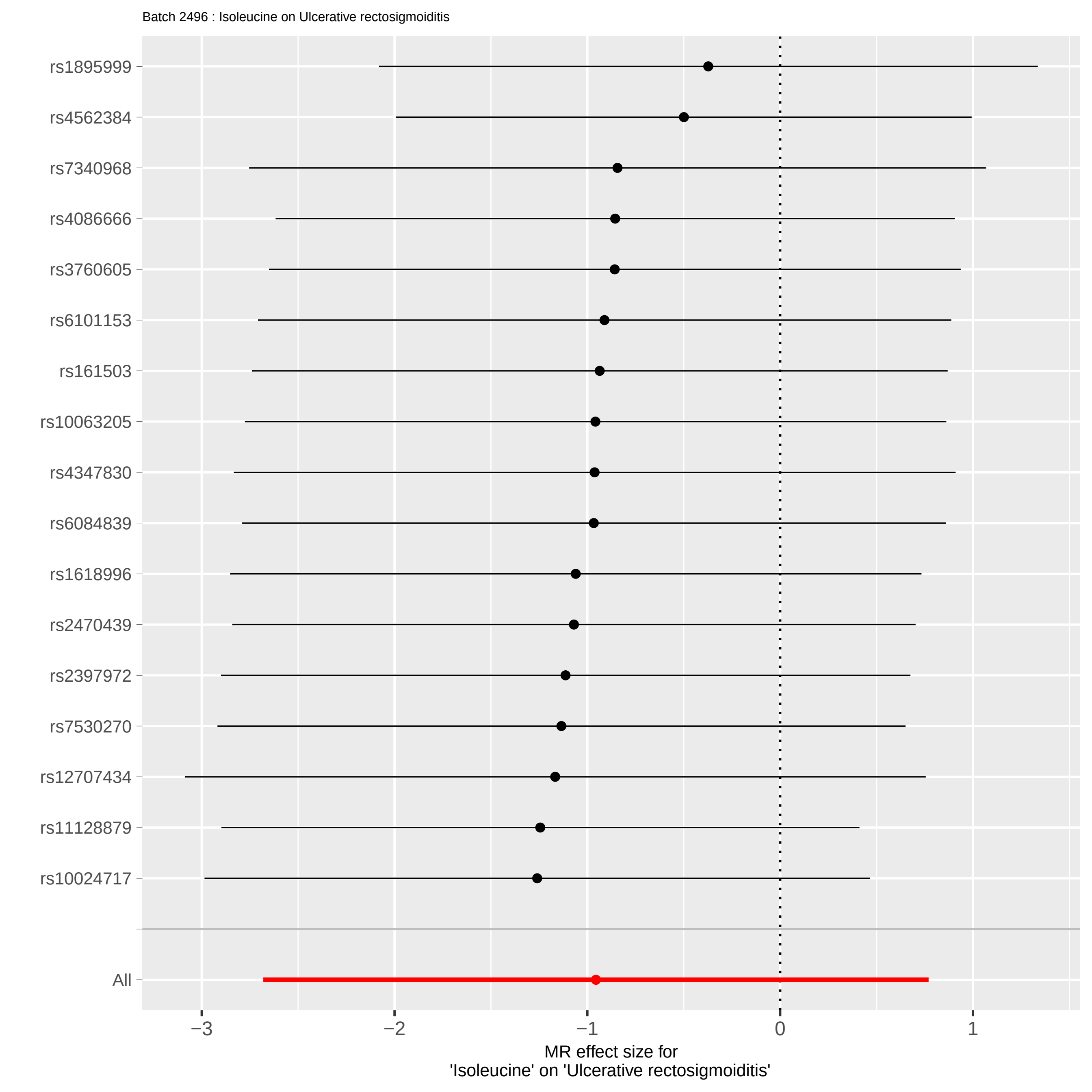

Batch 2496 : Isoleucine on Ulcerative rectosigmoiditis
rs1895999
rs4562384
rs7340968
rs4086666
rs3760605
rs6101153
rs161503
rs10063205
rs4347830
rs6084839
rs1618996
rs2470439
rs2397972
rs7530270
rs12707434
rs11128879
rs10024717
All
−3
−2
−1
0
1
MR effect size for
'Isoleucine' on 'Ulcerative rectosigmoiditis'

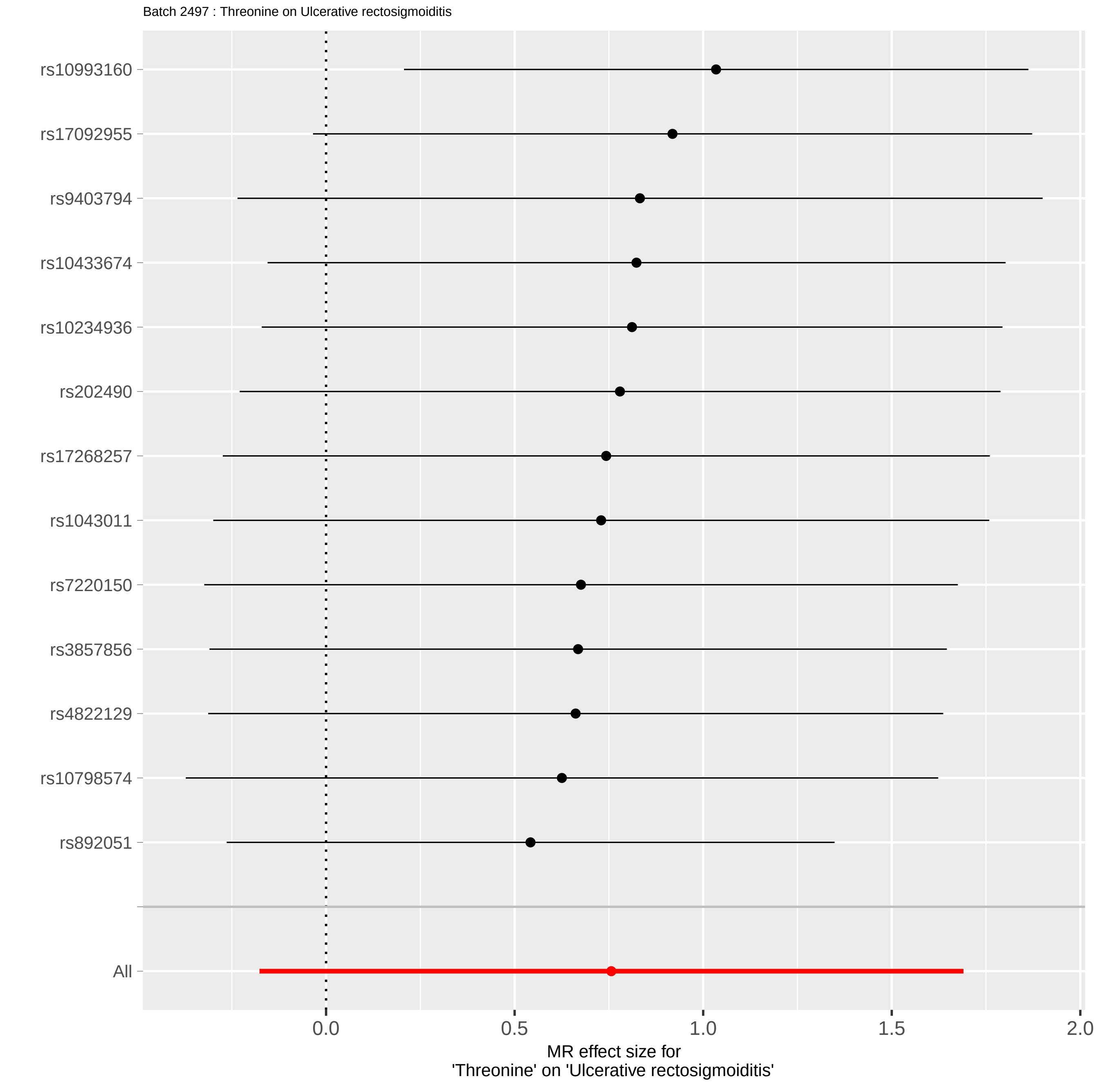
Batch 2497 : Threonine on Ulcerative rectosigmoiditis
rs10993160
rs17092955
rs9403794
rs10433674
rs10234936
rs202490
rs17268257
rs1043011
rs7220150
rs3857856
rs4822129
rs10798574
rs892051
All
0.0
0.5
1.0
1.5
2.0
MR effect size for
'Threonine' on 'Ulcerative rectosigmoiditis'

Batch 2498 : Tyrosine on Ulcerative rectosigmoiditis

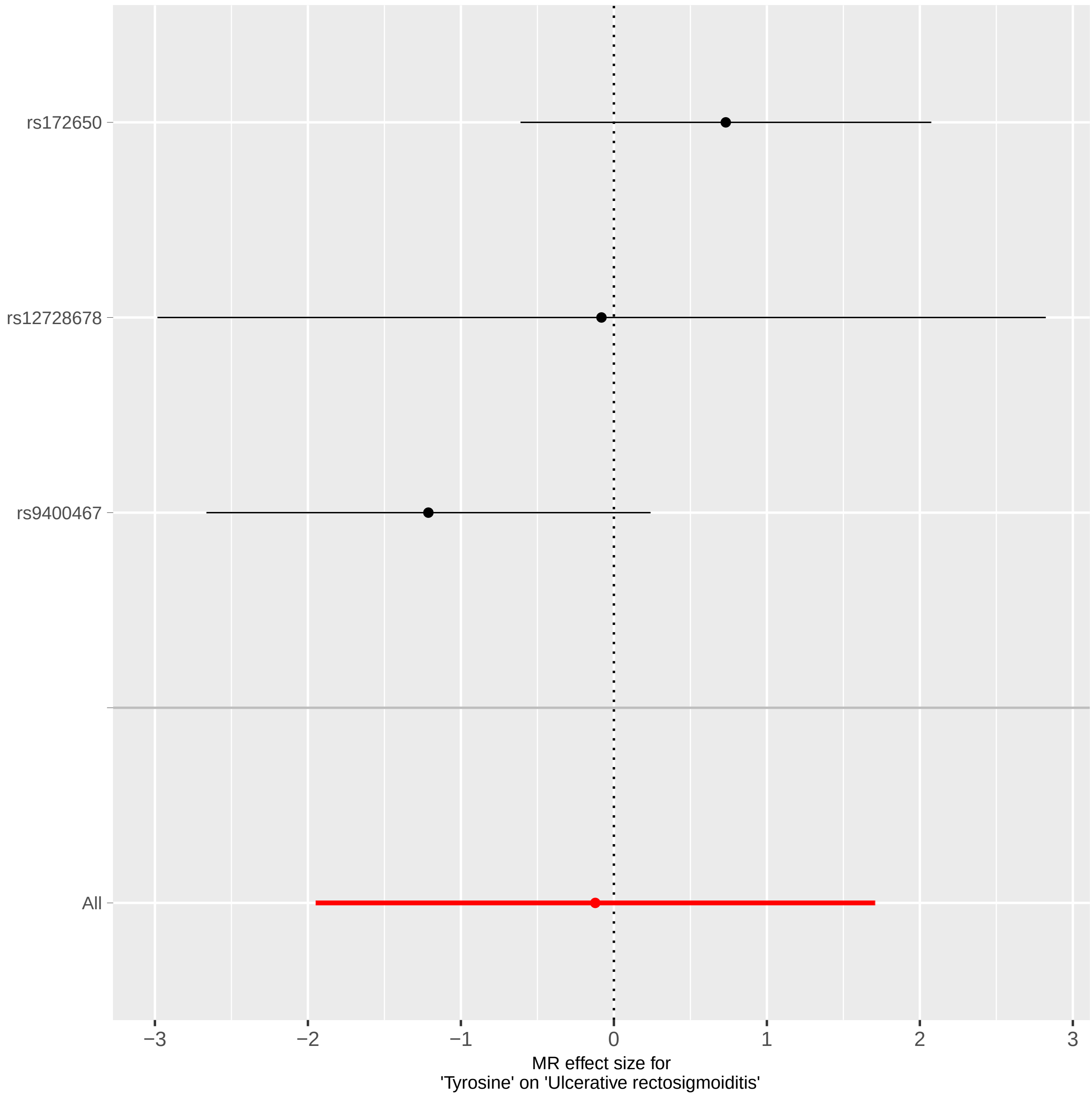

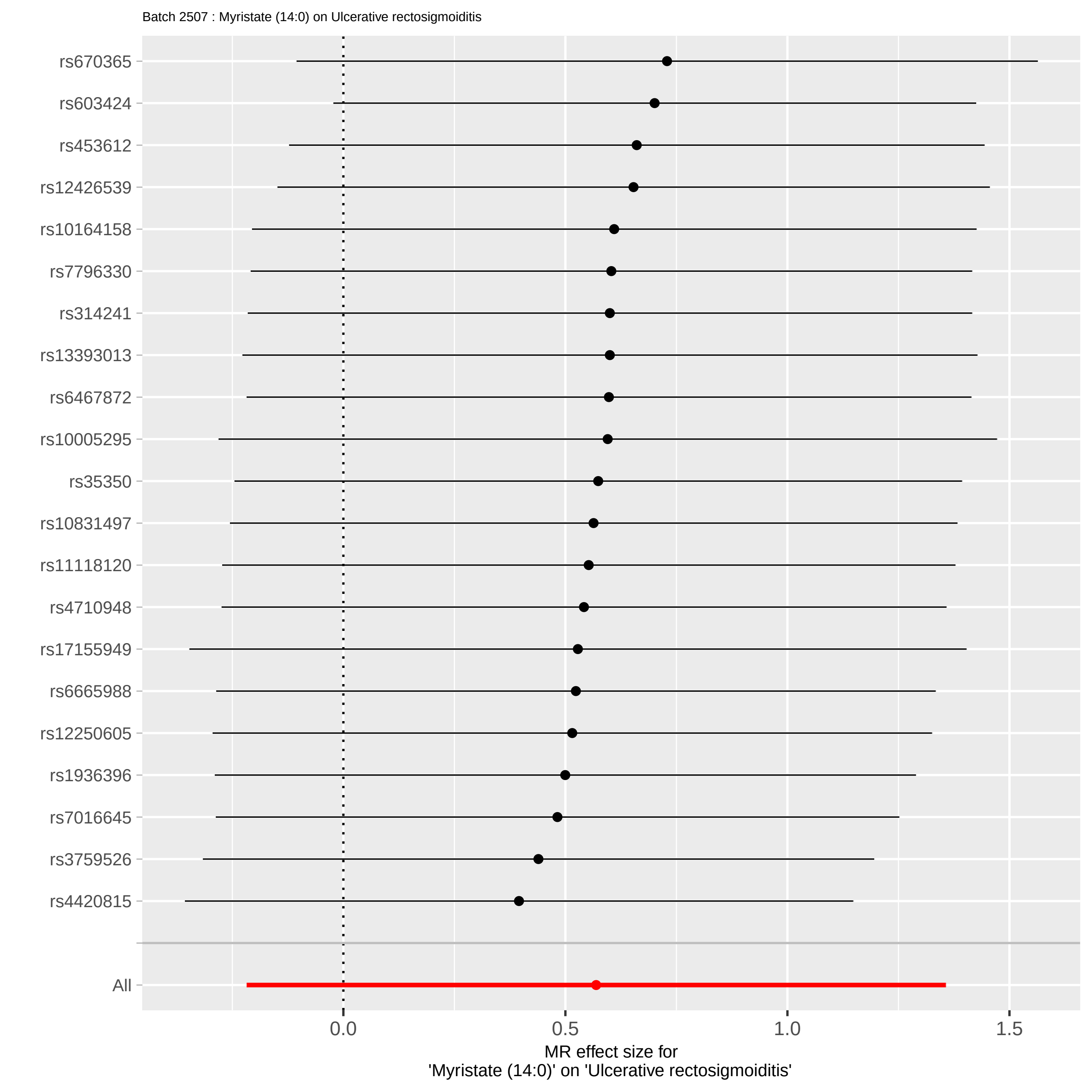
Batch 2507 : Myristate (14:0) on Ulcerative rectosigmoiditis
rs670365
rs603424
rs453612
rs12426539
rs10164158
rs7796330
rs314241
rs13393013
rs6467872
rs10005295
rs35350
rs10831497
rs11118120
rs4710948
rs17155949
rs6665988
rs12250605
rs1936396
rs7016645
rs3759526
rs4420815
All
0.0
0.5
1.0
1.5
MR effect size for
'Myristate (14:0)' on 'Ulcerative rectosigmoiditis'

Batch 2509 : Ornithine on Ulcerative rectosigmoiditis

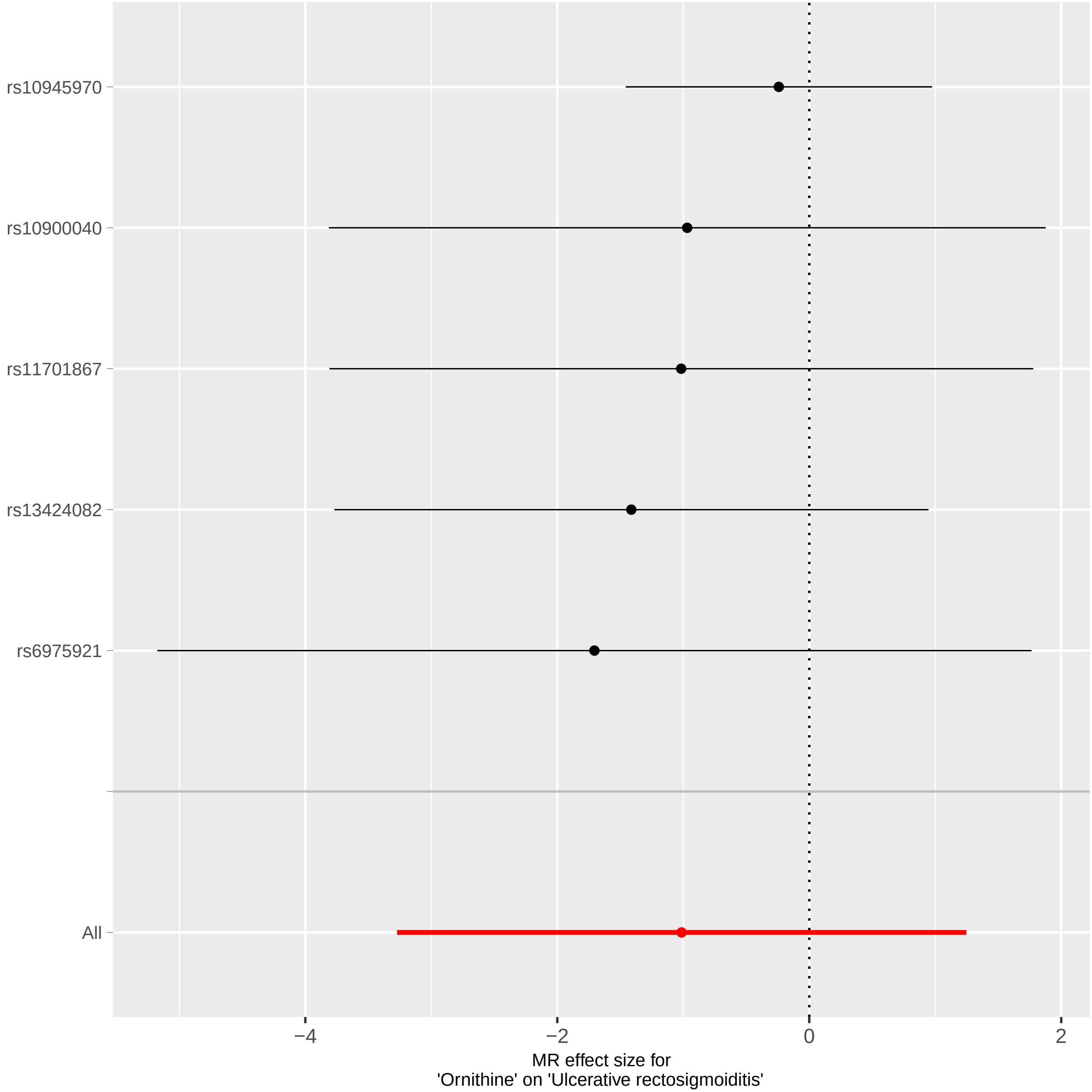

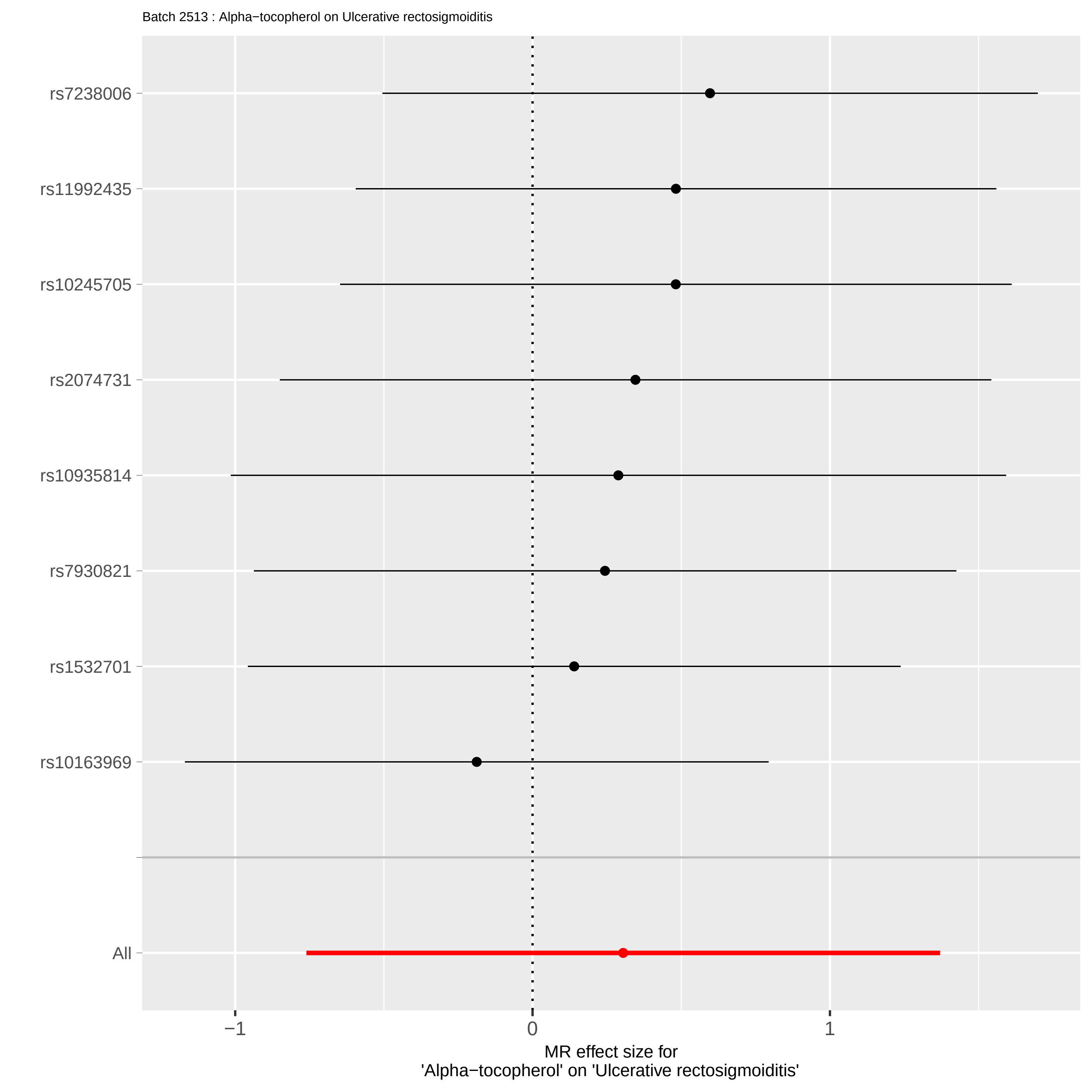
Batch 2513 : Alpha−tocopherol on Ulcerative rectosigmoiditis
rs7238006
rs11992435
rs10245705
rs2074731
rs10935814
rs7930821
rs1532701
rs10163969
All
−1
0
1
MR effect size for
'Alpha−tocopherol' on 'Ulcerative rectosigmoiditis'

Batch 2514 : Citrate on Ulcerative rectosigmoiditis

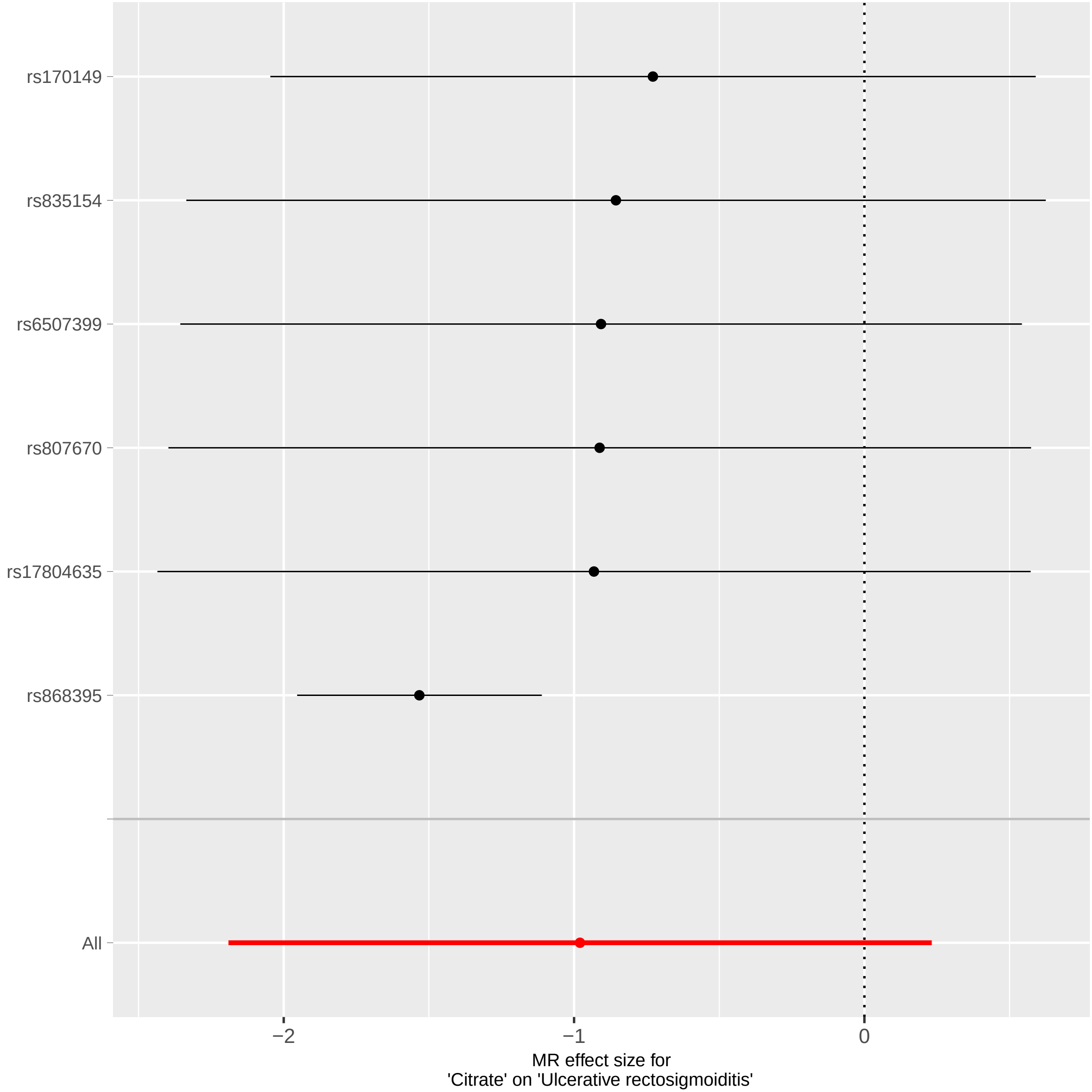

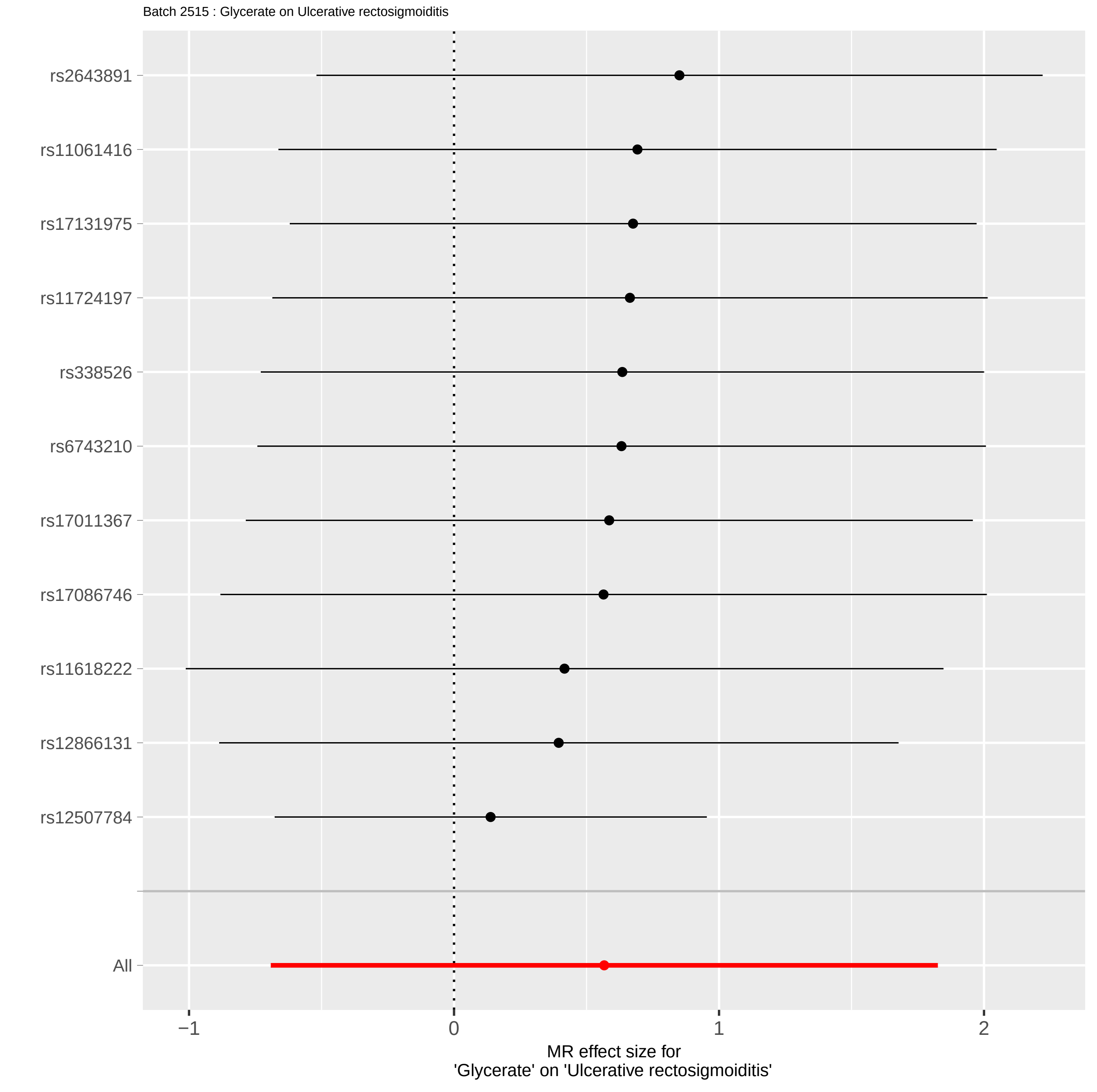

Batch 2515 : Glycerate on Ulcerative rectosigmoiditis
rs2643891
rs11061416
rs17131975
rs11724197
rs338526
rs6743210
rs17011367
rs17086746
rs11618222
rs12866131
rs12507784
All
−1
0
1
2
MR effect size for
'Glycerate' on 'Ulcerative rectosigmoiditis'

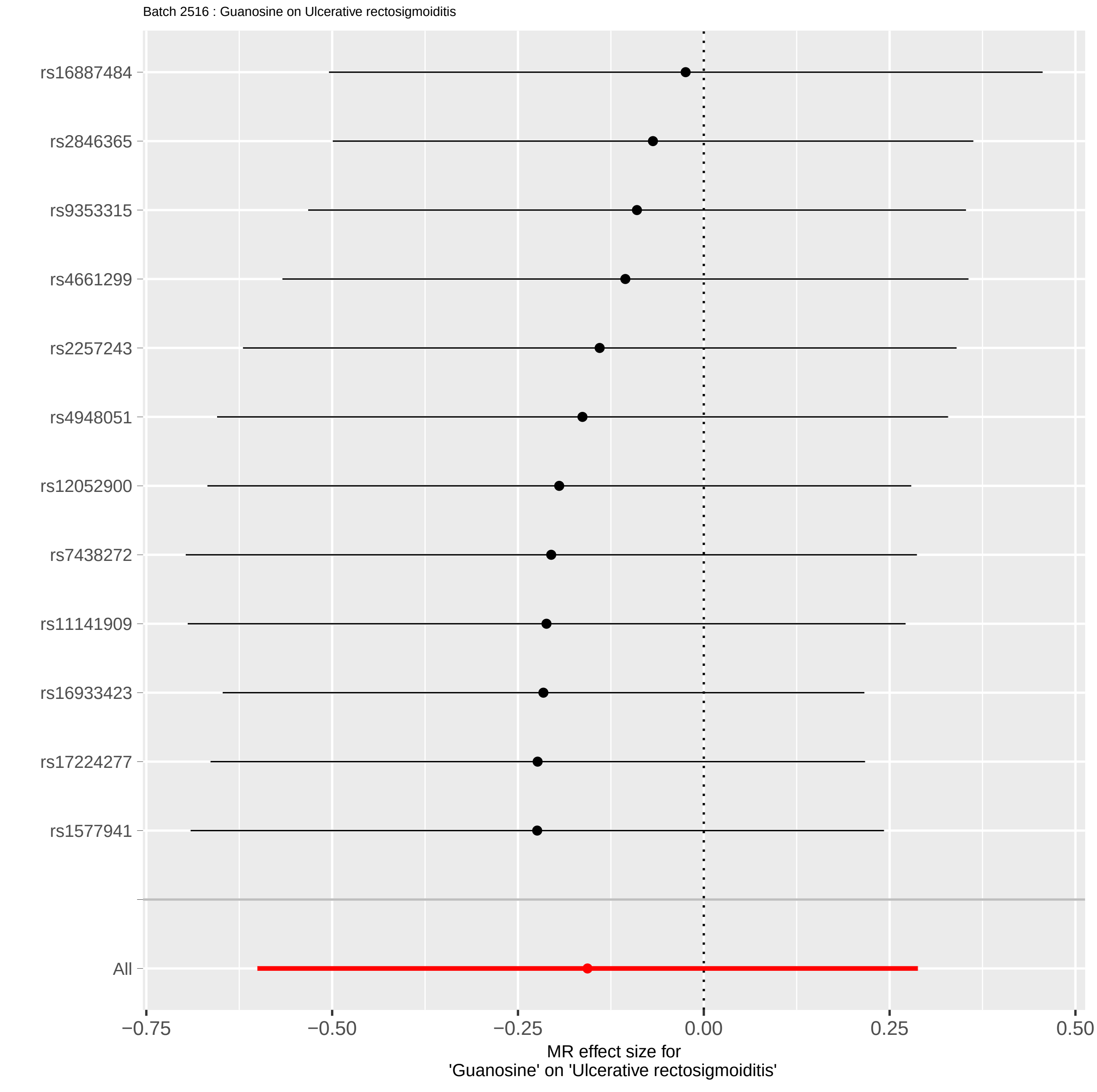

Batch 2516 : Guanosine on Ulcerative rectosigmoiditis
rs16887484
rs2846365
rs9353315
rs4661299
rs2257243
rs4948051
rs12052900
rs7438272
rs11141909
rs16933423
rs17224277
rs1577941
All
−0.75
−0.50
−0.25
0.00
0.25
0.50
MR effect size for
'Guanosine' on 'Ulcerative rectosigmoiditis'

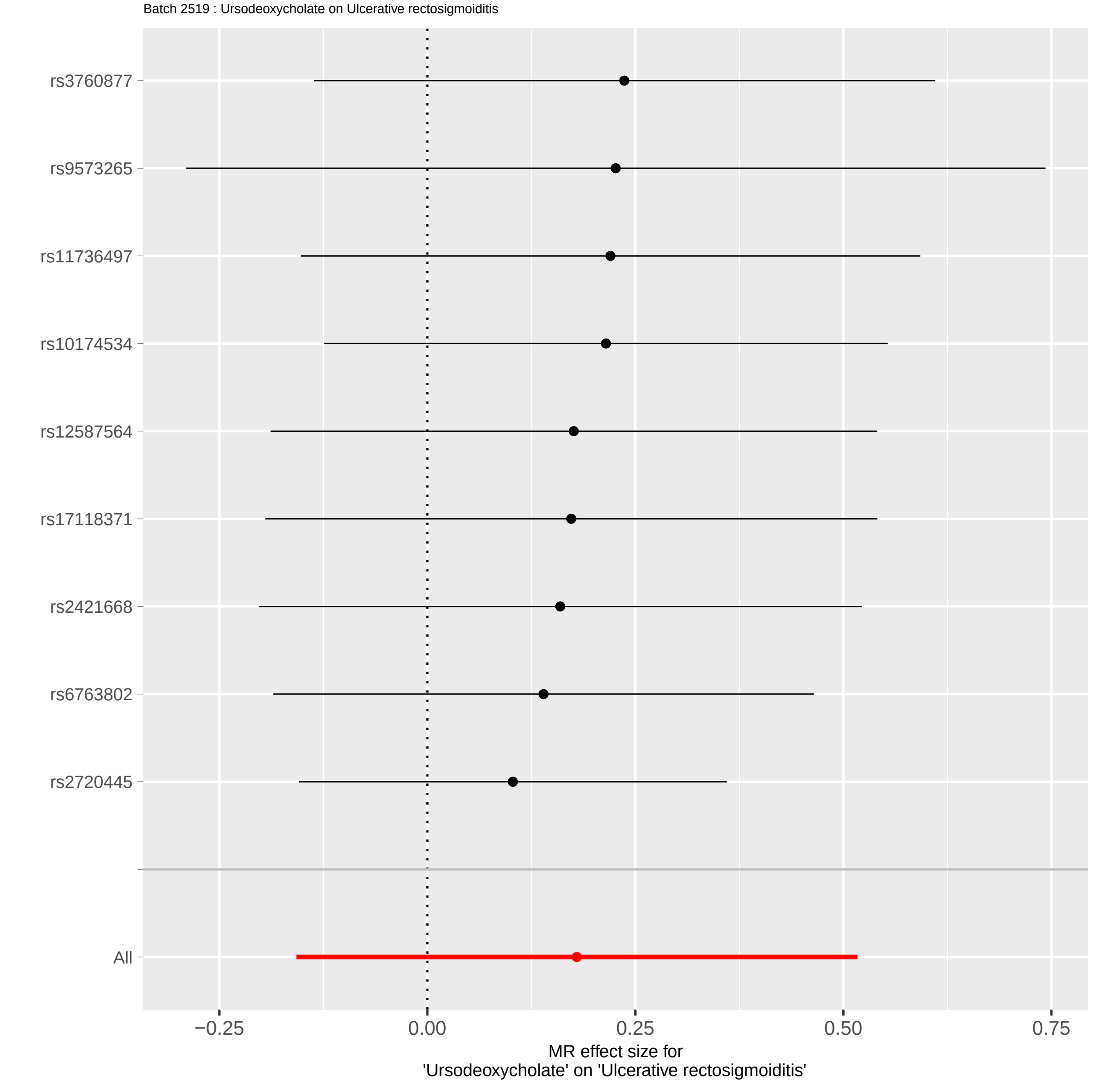

Batch 2519 : Ursodeoxycholate on Ulcerative rectosigmoiditis
rs3760877
rs9573265
rs11736497
rs10174534
rs12587564
rs17118371
rs2421668
rs6763802
rs2720445
All
−0.25
0.00
0.25
0.50
0.75
MR effect size for
'Ursodeoxycholate' on 'Ulcerative rectosigmoiditis'

Batch 2520 : Arginine on Ulcerative rectosigmoiditis

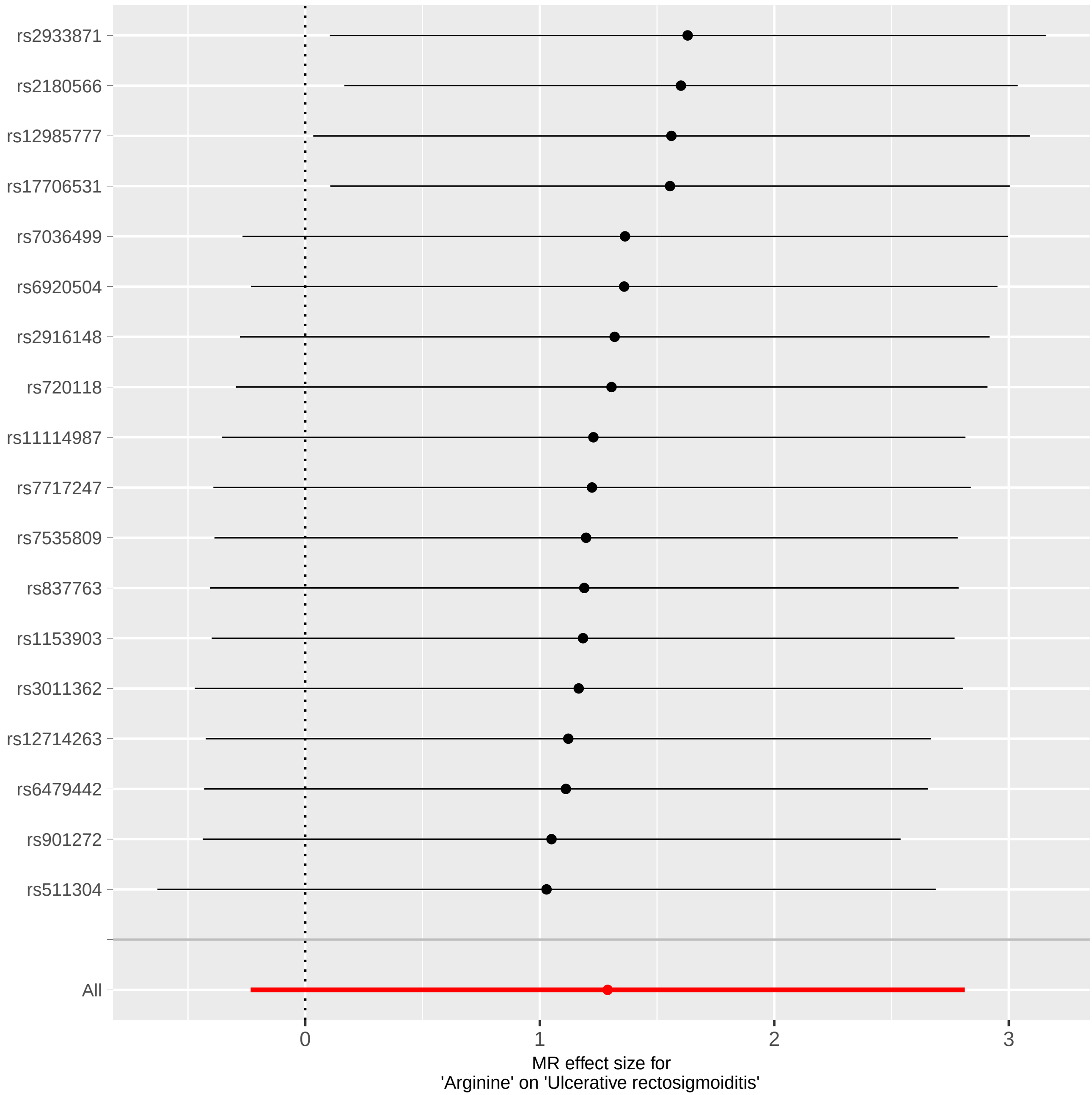

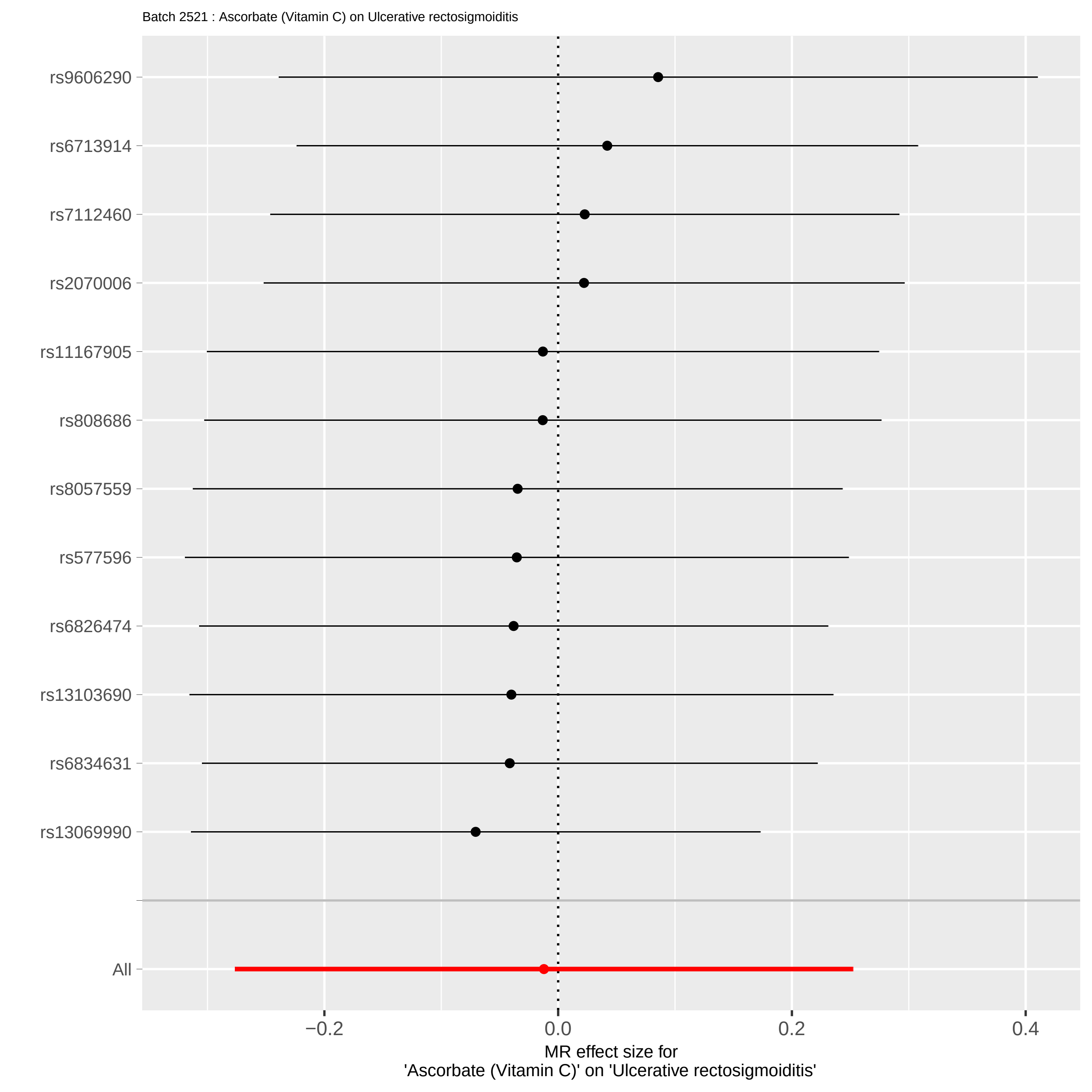

Batch 2521 : Ascorbate (Vitamin C) on Ulcerative rectosigmoiditis
rs9606290
rs6713914
rs7112460
rs2070006
rs11167905
rs808686
rs8057559
rs577596
rs6826474
rs13103690
rs6834631
rs13069990
All
−0.2
0.0
0.2
0.4
MR effect size for
'Ascorbate (Vitamin C)' on 'Ulcerative rectosigmoiditis'

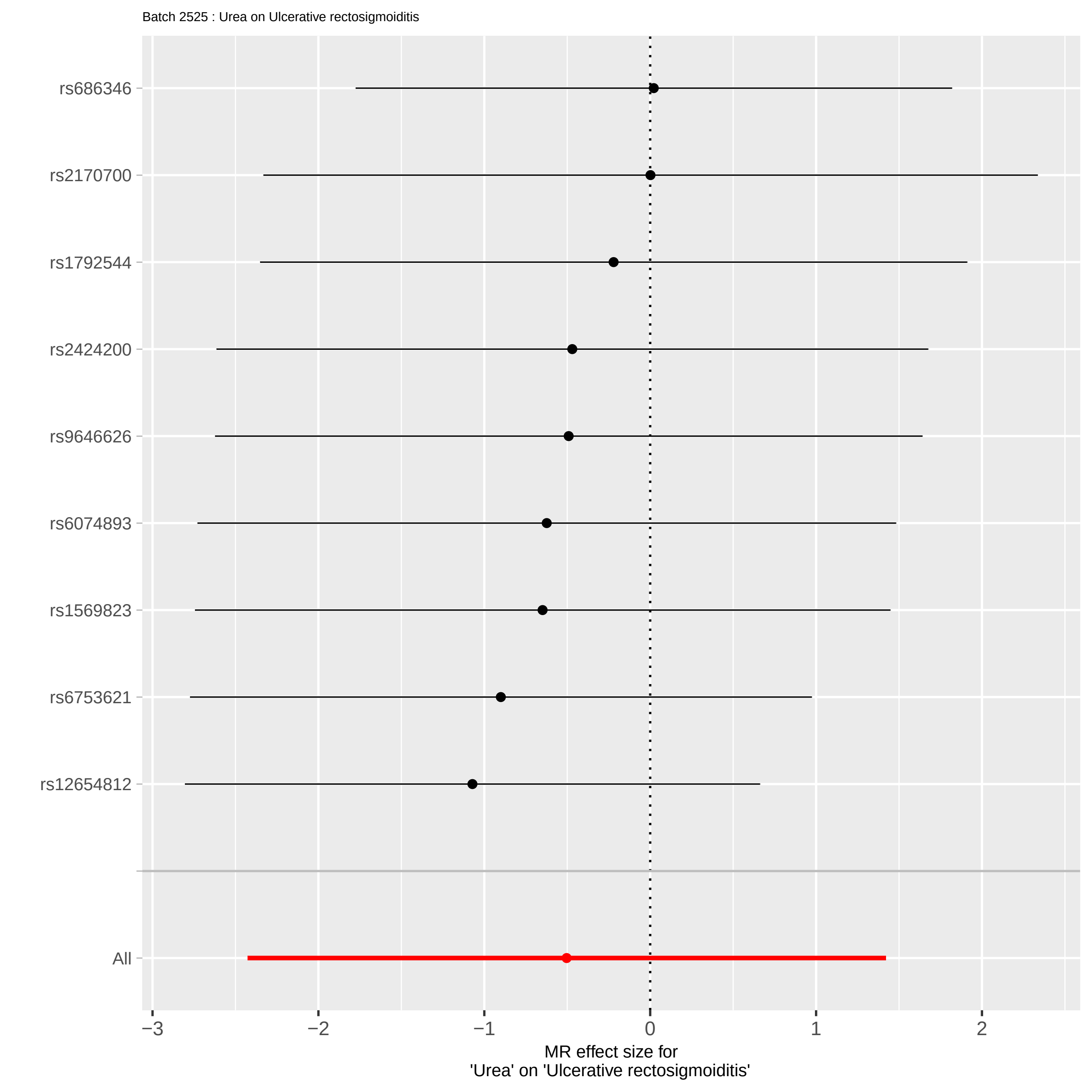
Batch 2525 : Urea on Ulcerative rectosigmoiditis
rs686346
rs2170700
rs1792544
rs2424200
rs9646626
rs6074893
rs1569823
rs6753621
rs12654812
All
−3
−2
−1
0
1
2
MR effect size for
'Urea' on 'Ulcerative rectosigmoiditis'

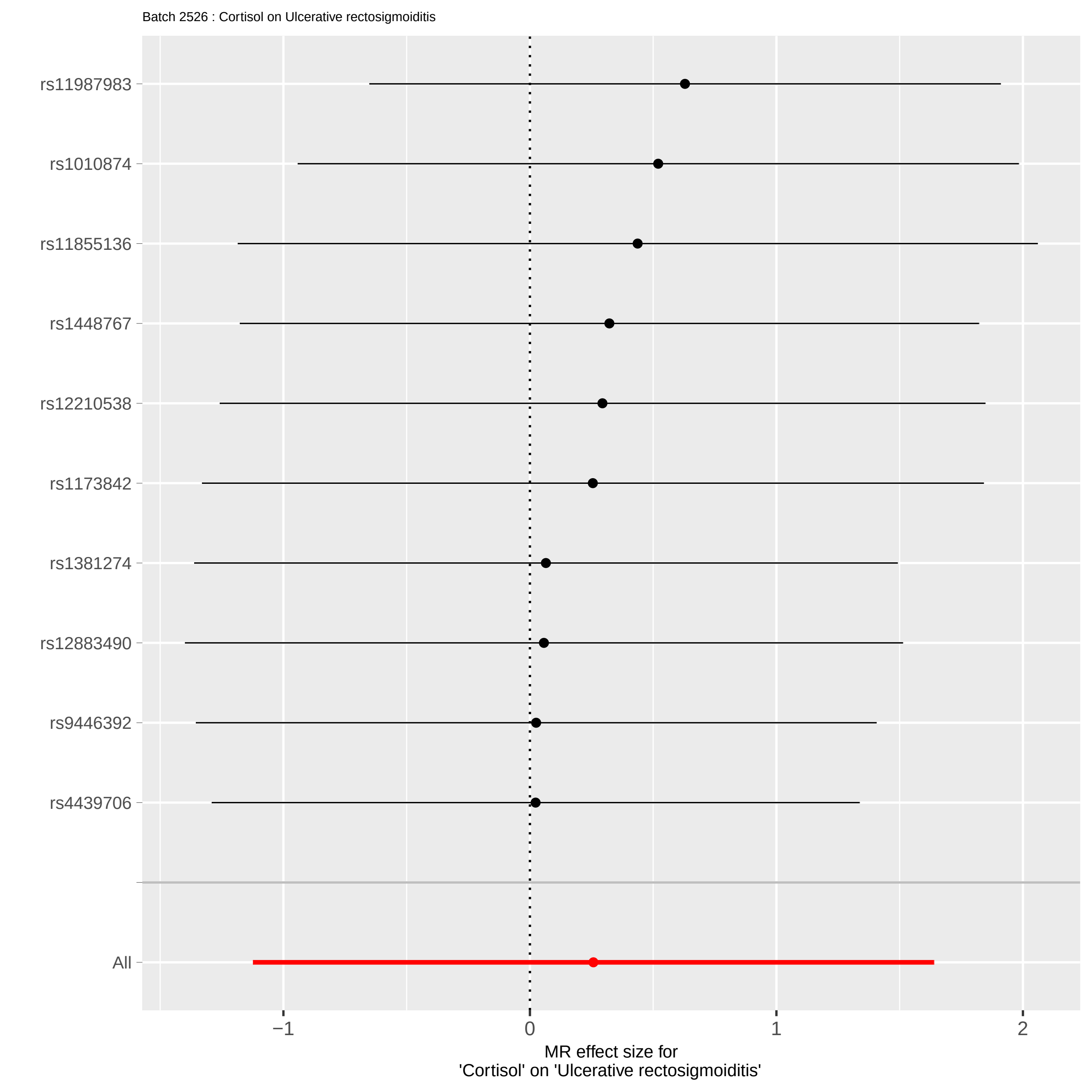
Batch 2526 : Cortisol on Ulcerative rectosigmoiditis
rs11987983
rs1010874
rs11855136
rs1448767
rs12210538
rs1173842
rs1381274
rs12883490
rs9446392
rs4439706
All
−1
0
1
2
MR effect size for
'Cortisol' on 'Ulcerative rectosigmoiditis'

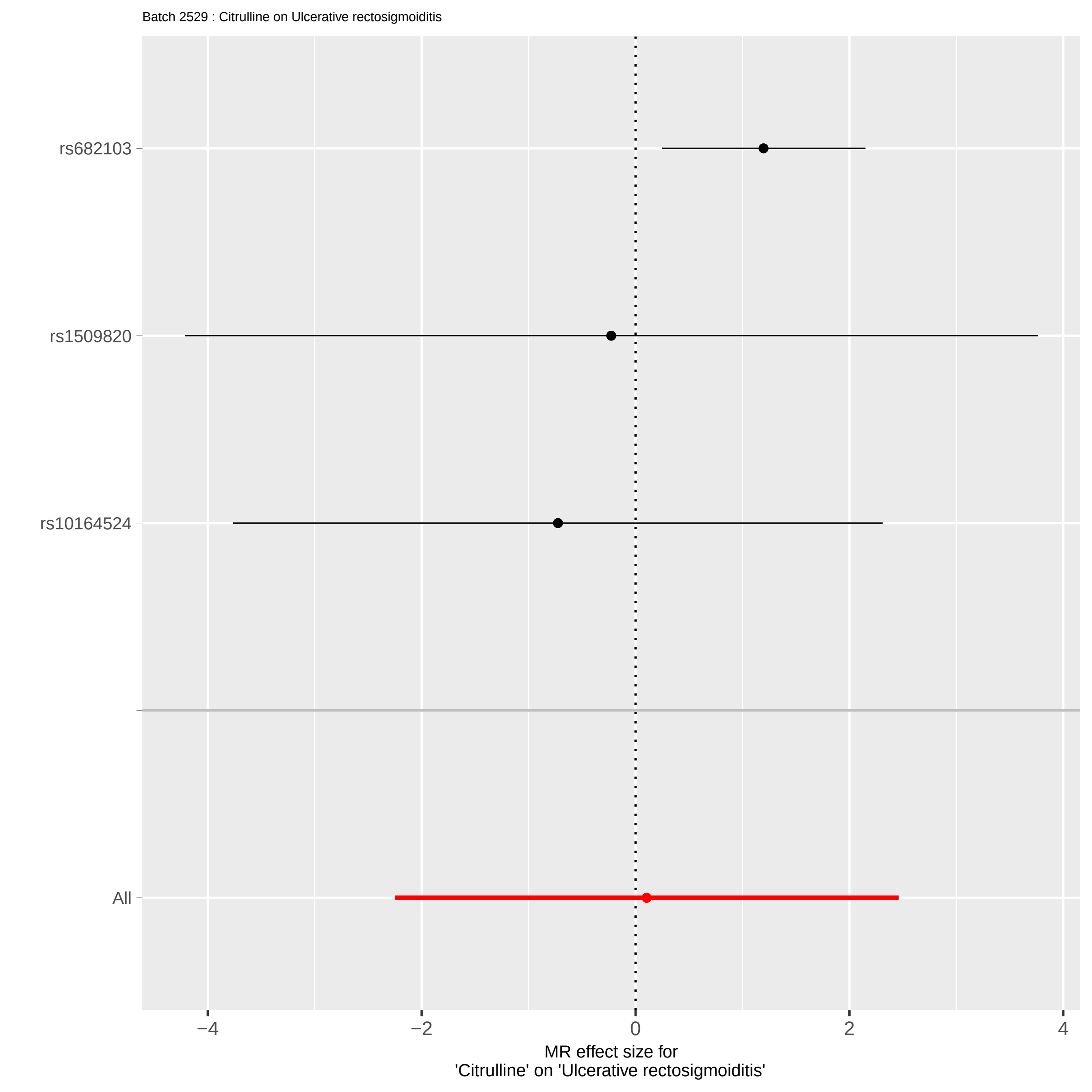
Batch 2529 : Citrulline on Ulcerative rectosigmoiditis
rs682103
rs1509820
rs10164524
All
−4
−2
0
2
4
MR effect size for
'Citrulline' on 'Ulcerative rectosigmoiditis'

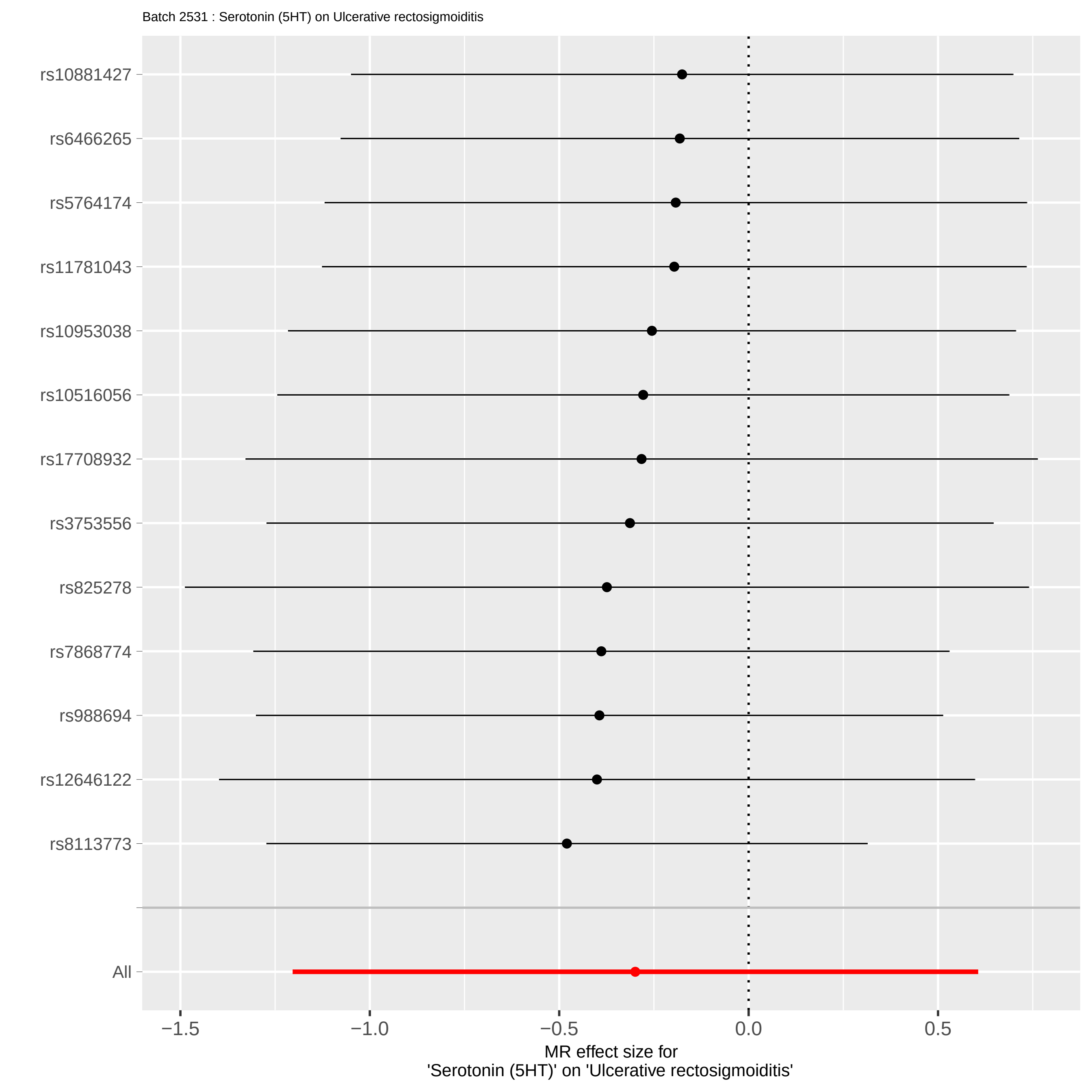

Batch 2531 : Serotonin (5HT) on Ulcerative rectosigmoiditis
rs10881427
rs6466265
rs5764174
rs11781043
rs10953038
rs10516056
rs17708932
rs3753556
rs825278
rs7868774
rs988694
rs12646122
rs8113773
All
−1.5
−1.0
−0.5
0.0
0.5
MR effect size for
'Serotonin (5HT)' on 'Ulcerative rectosigmoiditis'

Batch 2533 : Gamma−glutamyltyrosine on Ulcerative rectosigmoiditis

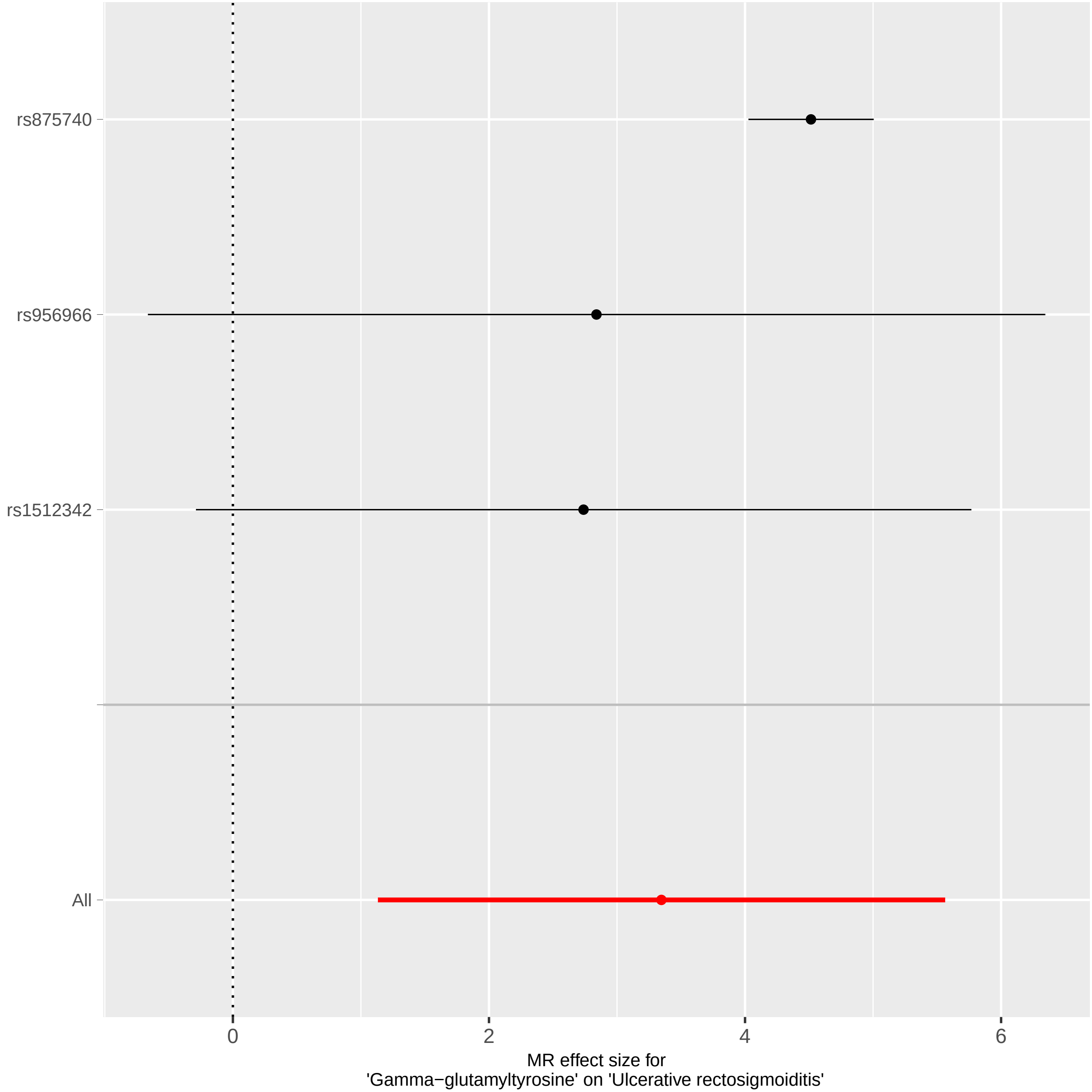

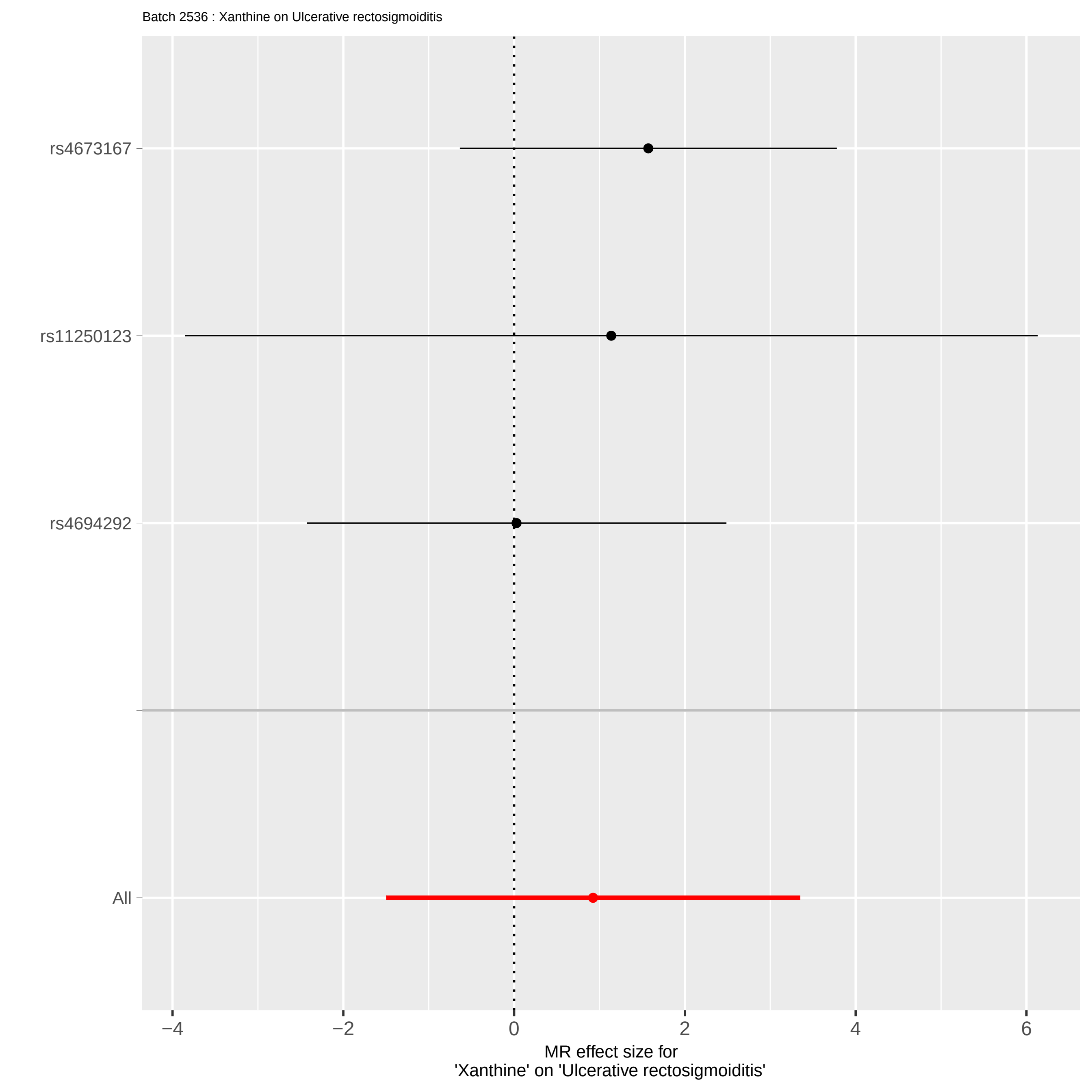

Batch 2536 : Xanthine on Ulcerative rectosigmoiditis
rs4673167
rs11250123
rs4694292
All
−4
−2
0
2
4
6
MR effect size for
'Xanthine' on 'Ulcerative rectosigmoiditis'

Batch 2537 : Phosphate on Ulcerative rectosigmoiditis

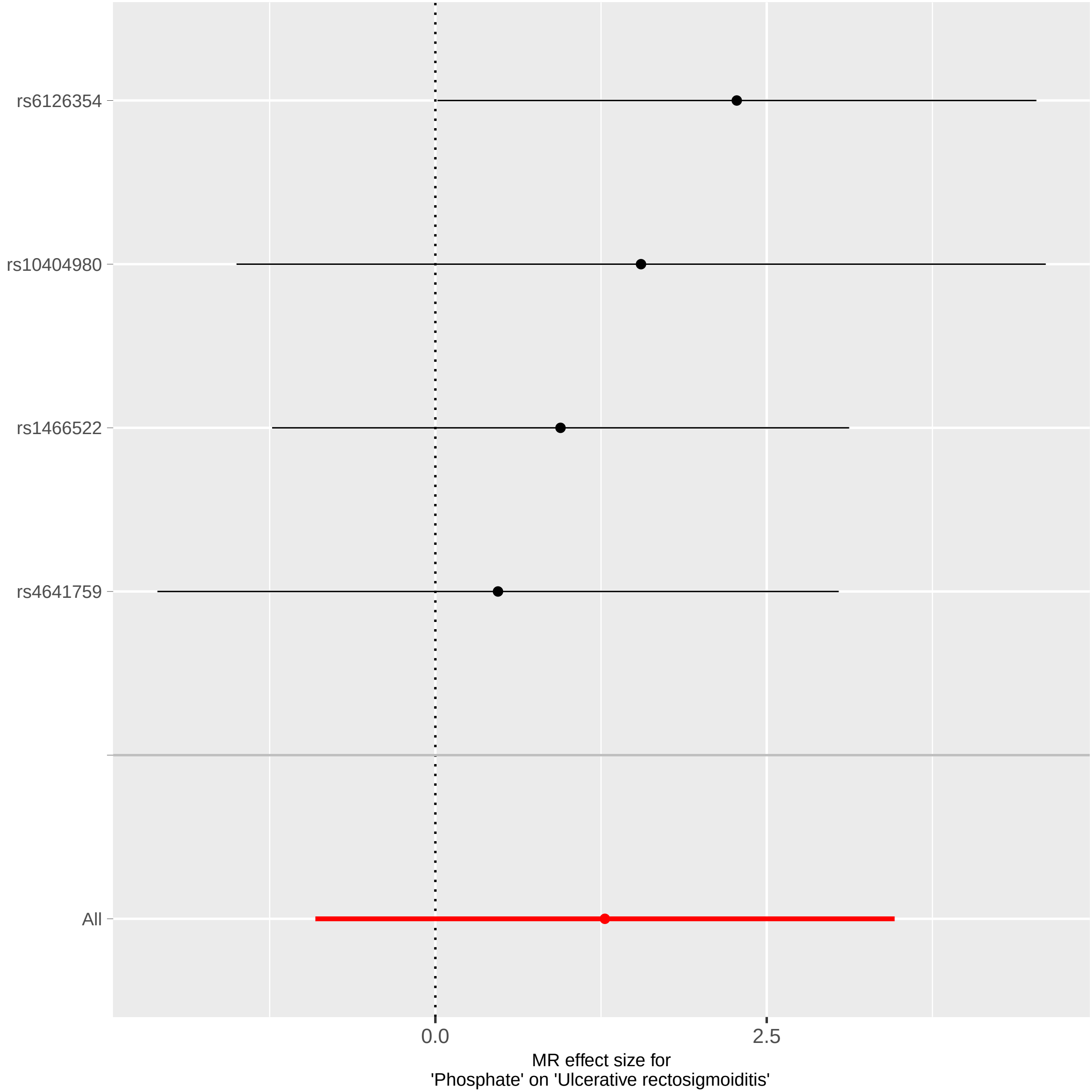

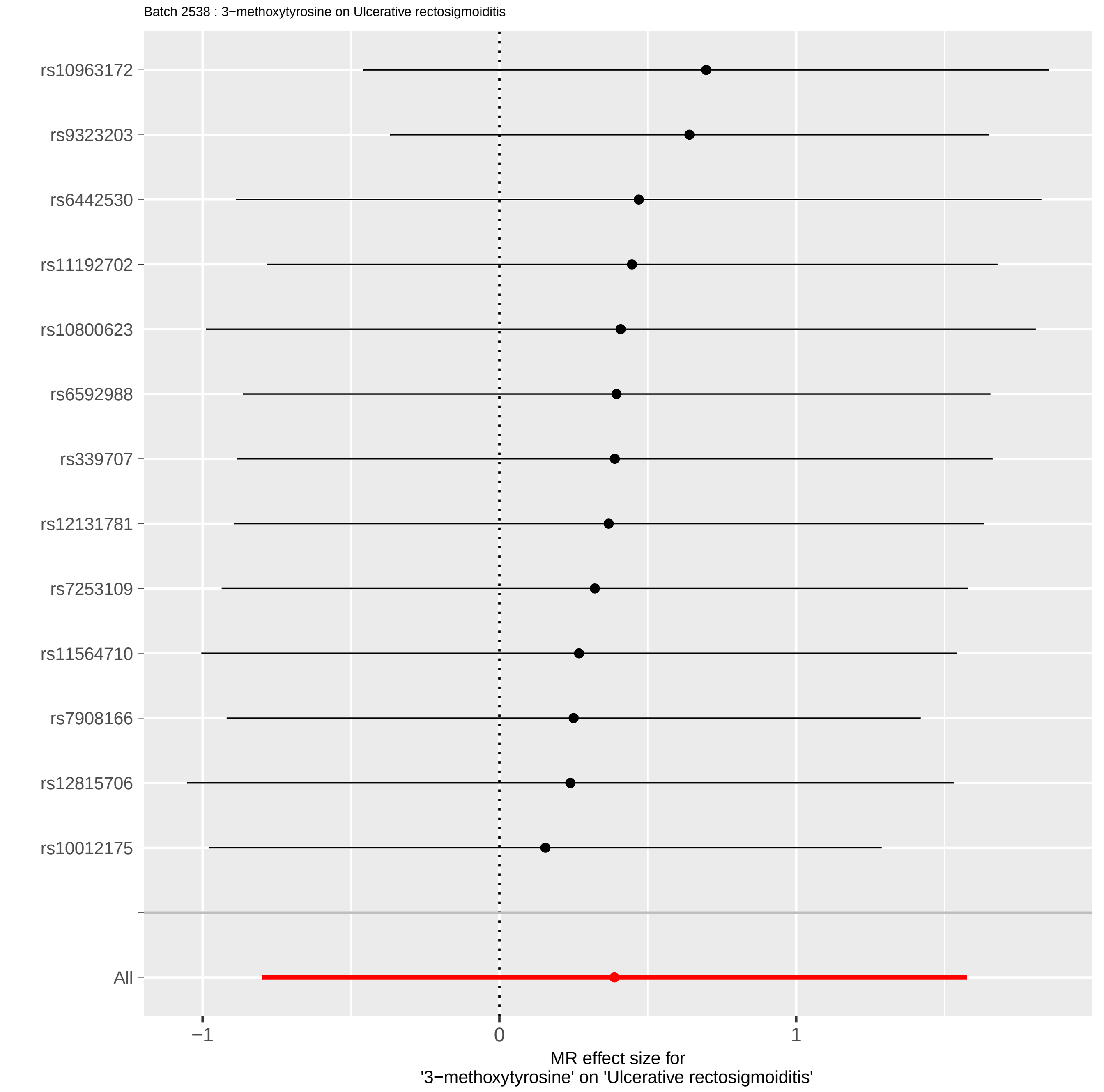

Batch 2538 : 3−methoxytyrosine on Ulcerative rectosigmoiditis
rs10963172
rs9323203
rs6442530
rs11192702
rs10800623
rs6592988
rs339707
rs12131781
rs7253109
rs11564710
rs7908166
rs12815706
rs10012175
All
−1
0
1
MR effect size for
'3−methoxytyrosine' on 'Ulcerative rectosigmoiditis'

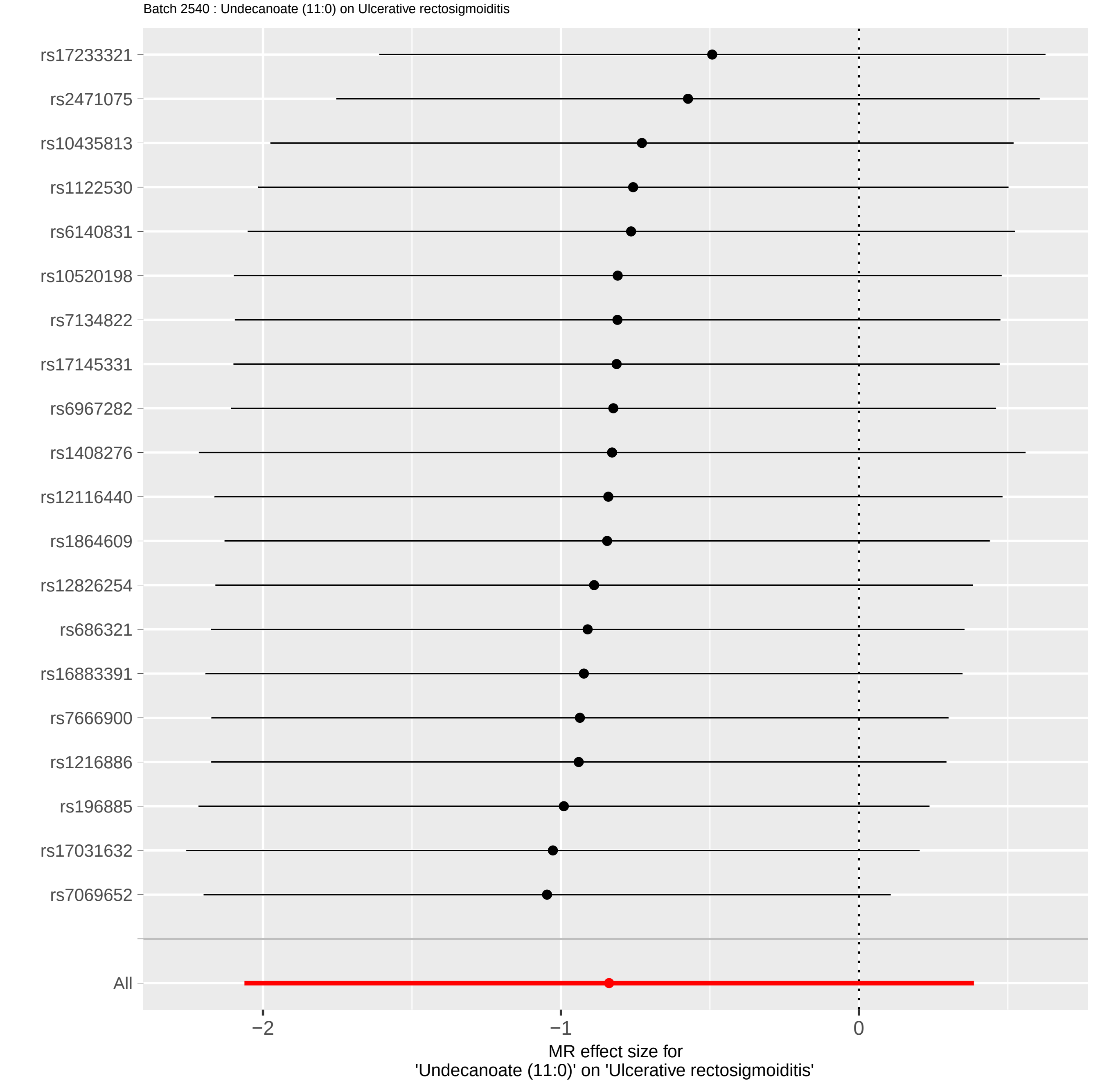

Batch 2540 : Undecanoate (11:0) on Ulcerative rectosigmoiditis
rs17233321
rs2471075
rs10435813
rs1122530
rs6140831
rs10520198
rs7134822
rs17145331
rs6967282
rs1408276
rs12116440
rs1864609
rs12826254
rs686321
rs16883391
rs7666900
rs1216886
rs196885
rs17031632
rs7069652
All
−2
−1
0
MR effect size for
'Undecanoate (11:0)' on 'Ulcerative rectosigmoiditis'

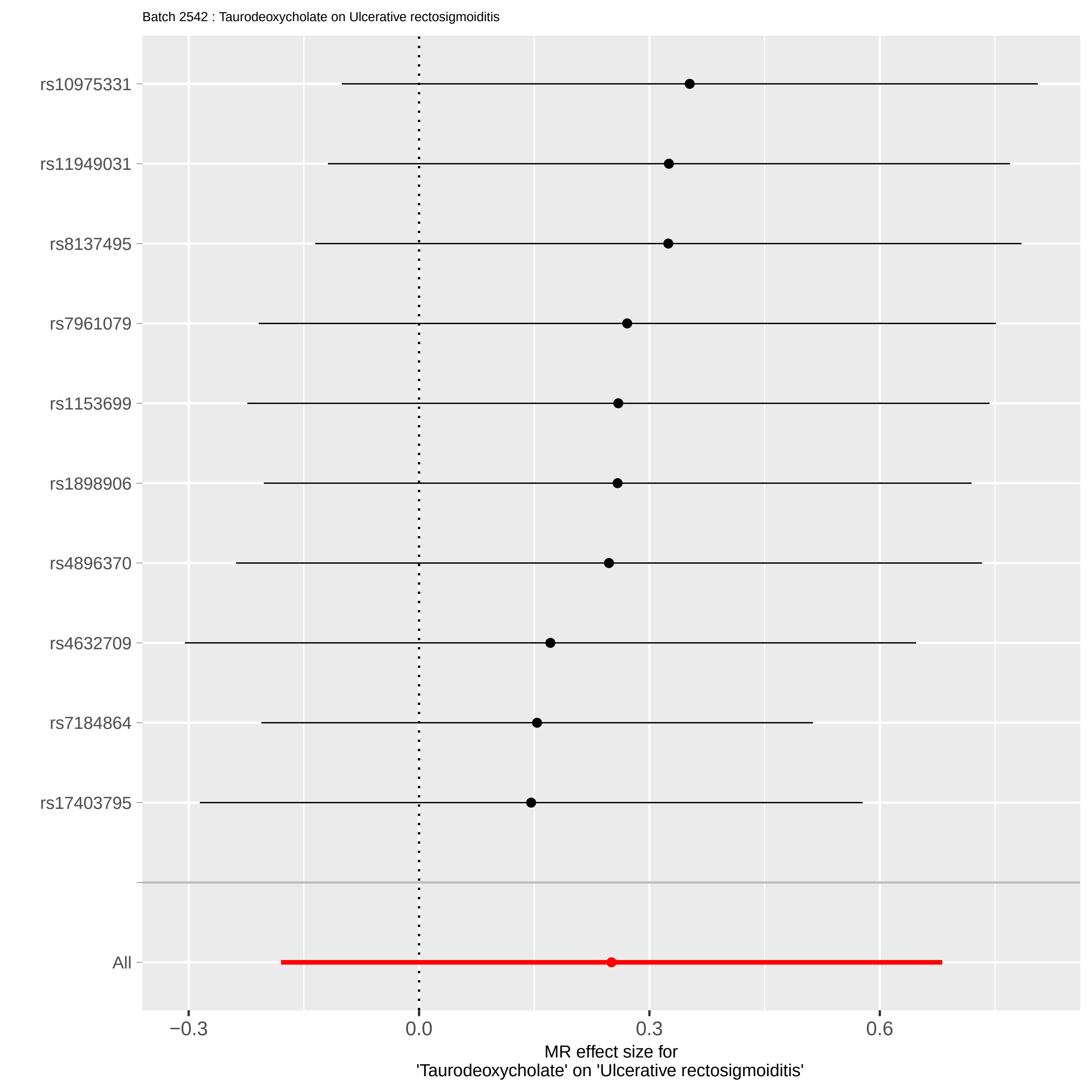

Batch 2542 : Taurodeoxycholate on Ulcerative rectosigmoiditis
rs10975331
rs11949031
rs8137495
rs7961079
rs1153699
rs1898906
rs4896370
rs4632709
rs7184864
rs17403795
All
−0.3
0.0
0.3
0.6
MR effect size for
'Taurodeoxycholate' on 'Ulcerative rectosigmoiditis'

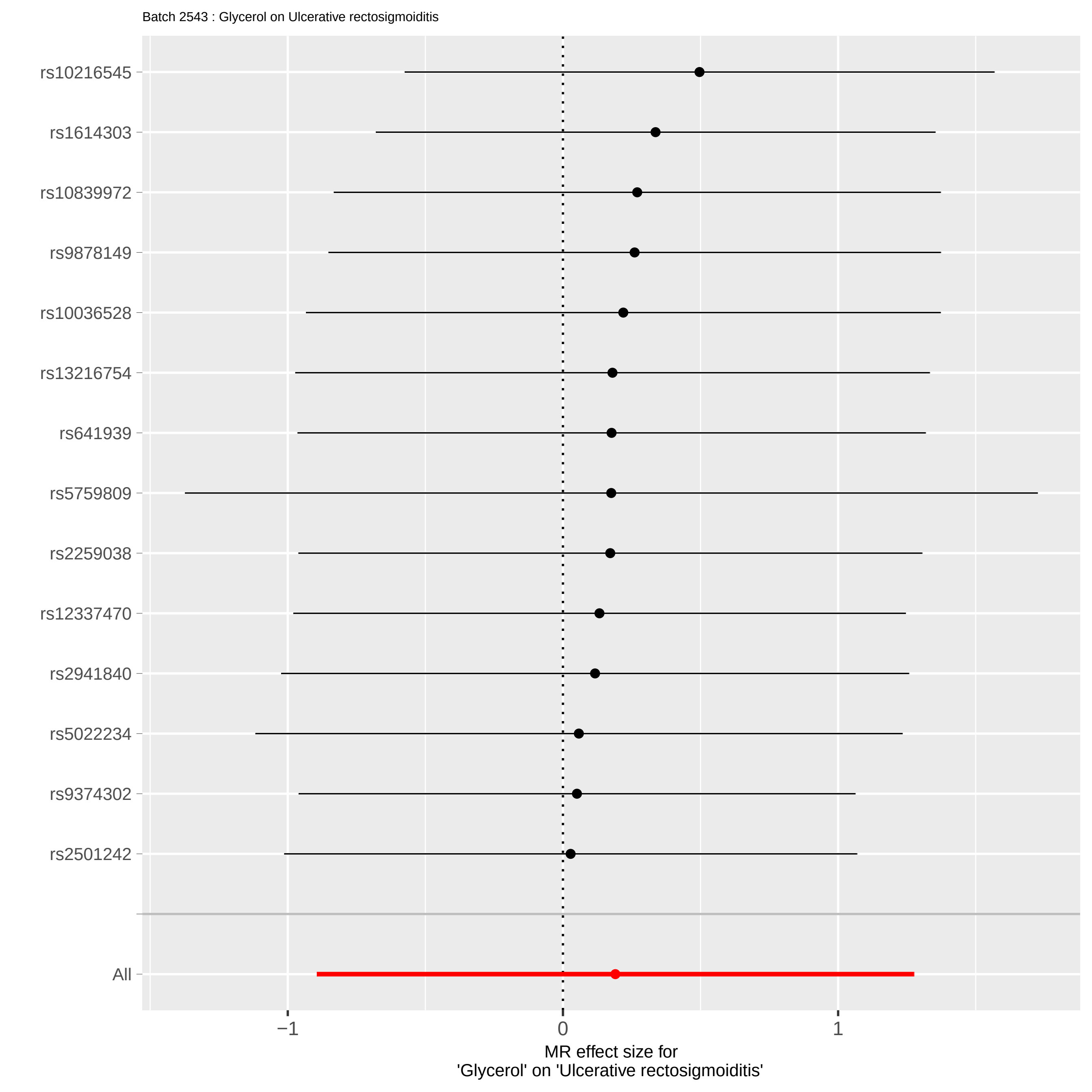

Batch 2543 : Glycerol on Ulcerative rectosigmoiditis
rs10216545
rs1614303
rs10839972
rs9878149
rs10036528
rs13216754
rs641939
rs5759809
rs2259038
rs12337470
rs2941840
rs5022234
rs9374302
rs2501242
All
−1
0
1
MR effect size for
'Glycerol' on 'Ulcerative rectosigmoiditis'

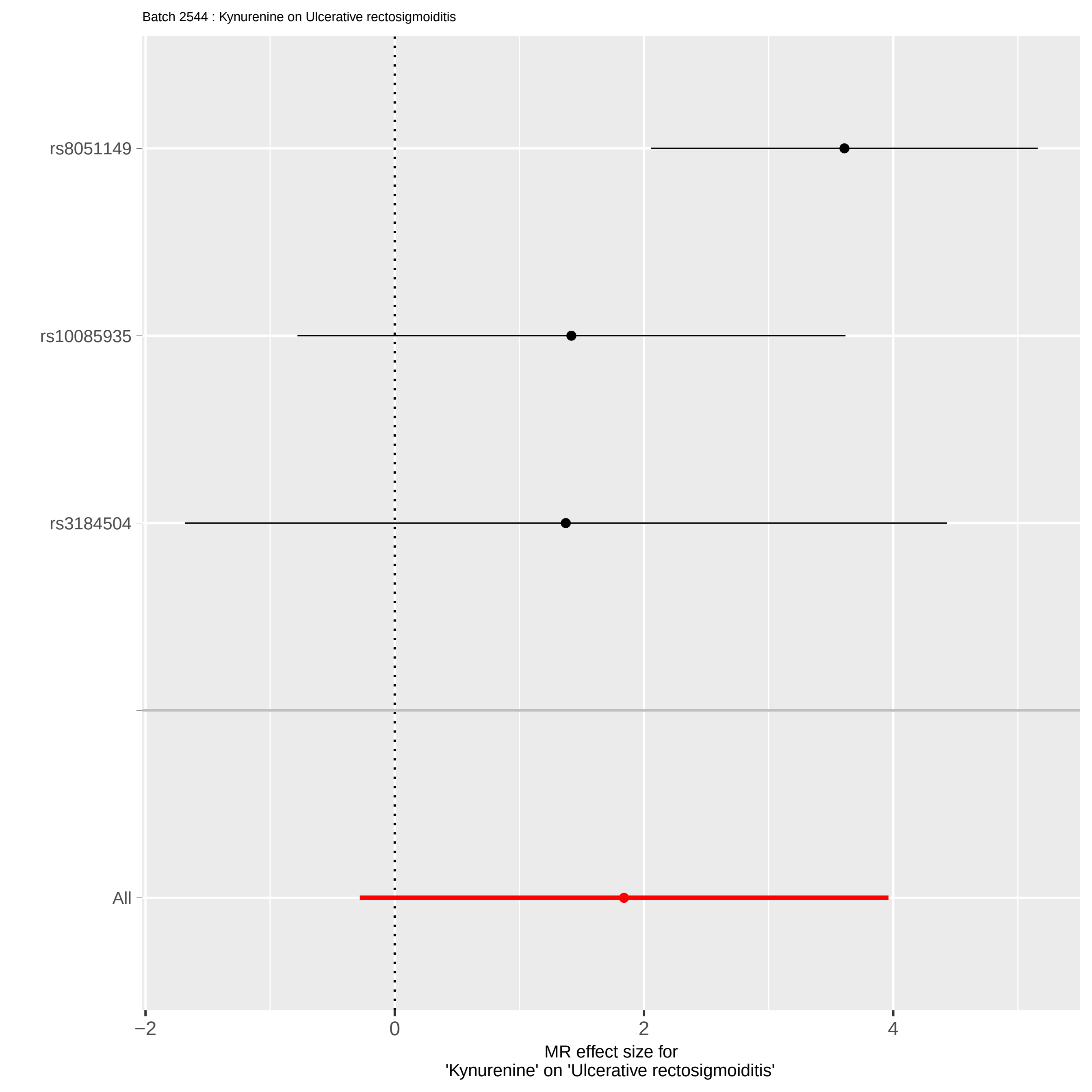

Batch 2544 : Kynurenine on Ulcerative rectosigmoiditis
rs8051149
rs10085935
rs3184504
All
−2
0
2
4
MR effect size for
'Kynurenine' on 'Ulcerative rectosigmoiditis'

Batch 2545 : Mannitol on Ulcerative rectosigmoiditis

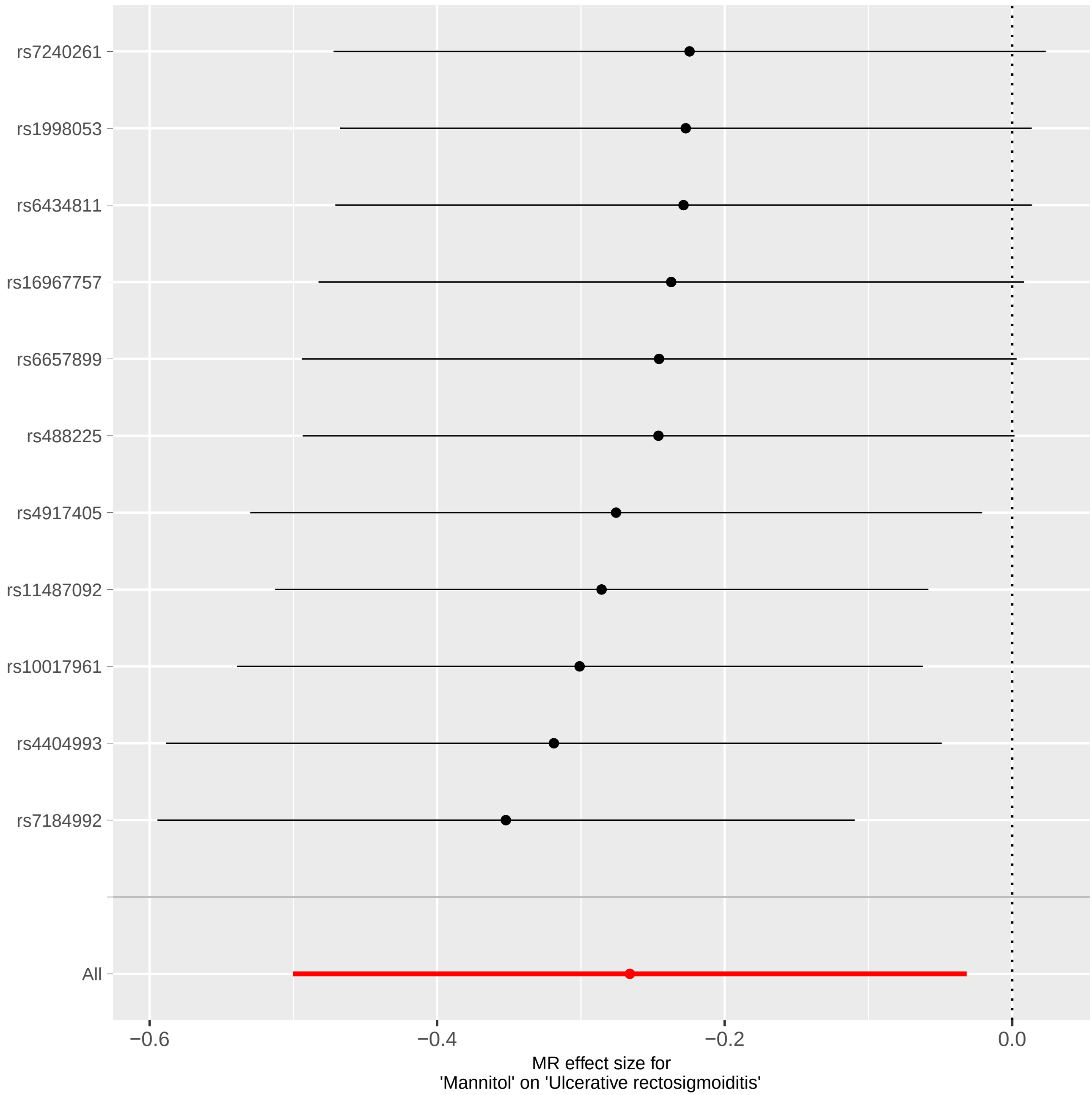

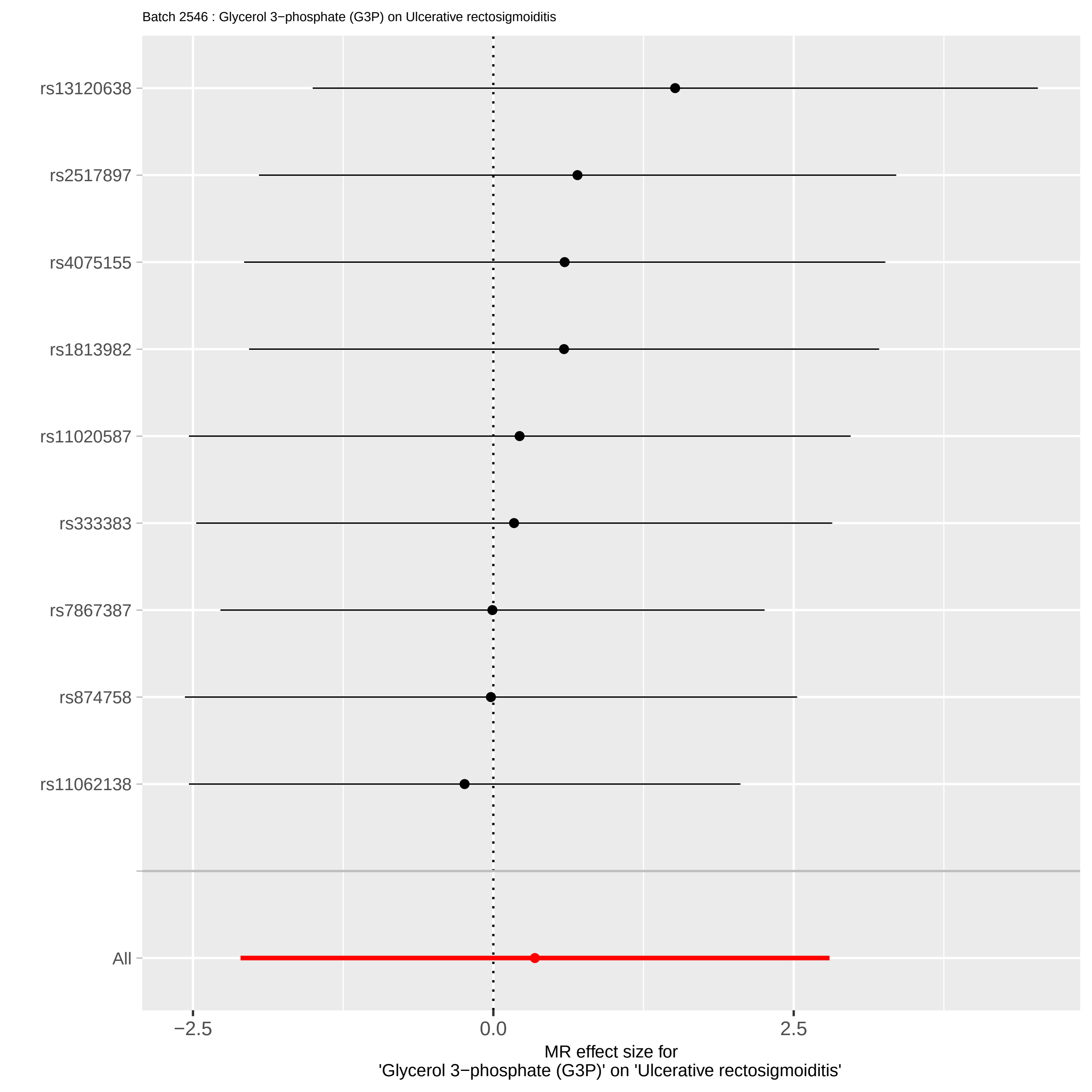
Batch 2546 : Glycerol 3−phosphate (G3P) on Ulcerative rectosigmoiditis
rs13120638
rs2517897
rs4075155
rs1813982
rs11020587
rs333383
rs7867387
rs874758
rs11062138
All
−2.5
0.0
2.5
MR effect size for
'Glycerol 3−phosphate (G3P)' on 'Ulcerative rectosigmoiditis'

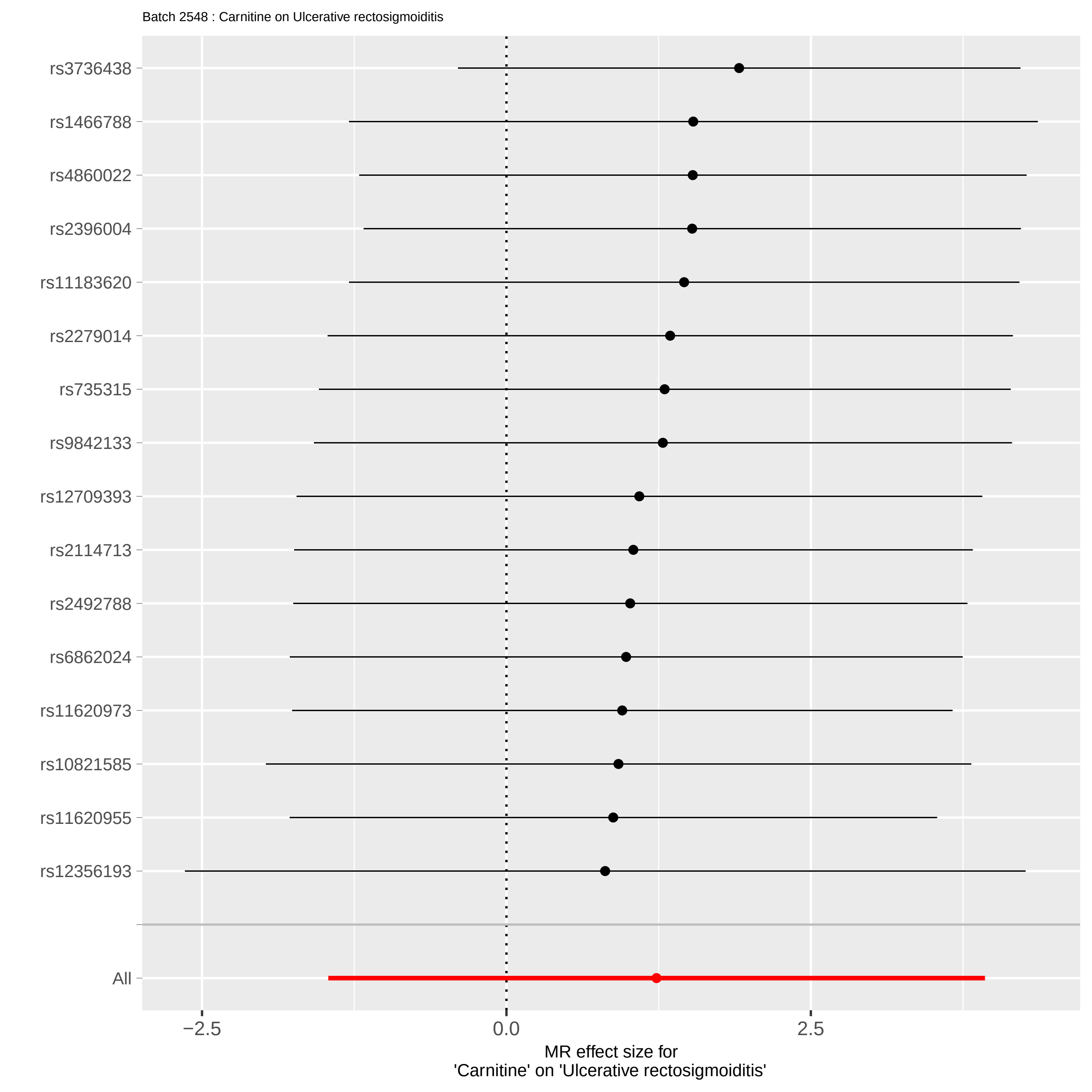
Batch 2548 : Carnitine on Ulcerative rectosigmoiditis
rs3736438
rs1466788
rs4860022
rs2396004
rs11183620
rs2279014
rs735315
rs9842133
rs12709393
rs2114713
rs2492788
rs6862024
rs11620973
rs10821585
rs11620955
rs12356193
All
−2.5
0.0
2.5
MR effect size for
'Carnitine' on 'Ulcerative rectosigmoiditis'

Batch 2549 : Choline on Ulcerative rectosigmoiditis

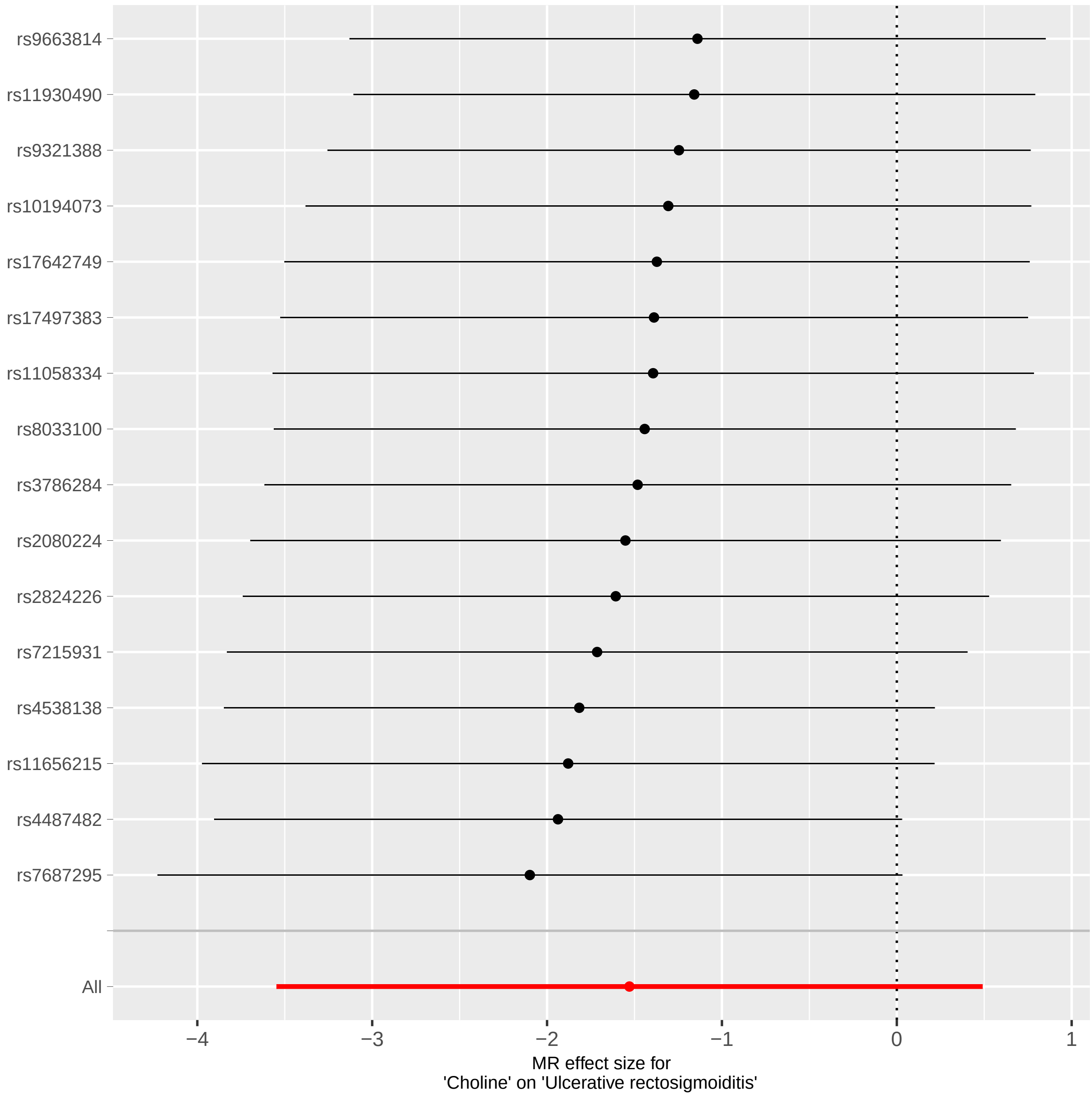

Batch 2553 : 3−methylhistidine on Ulcerative rectosigmoiditis

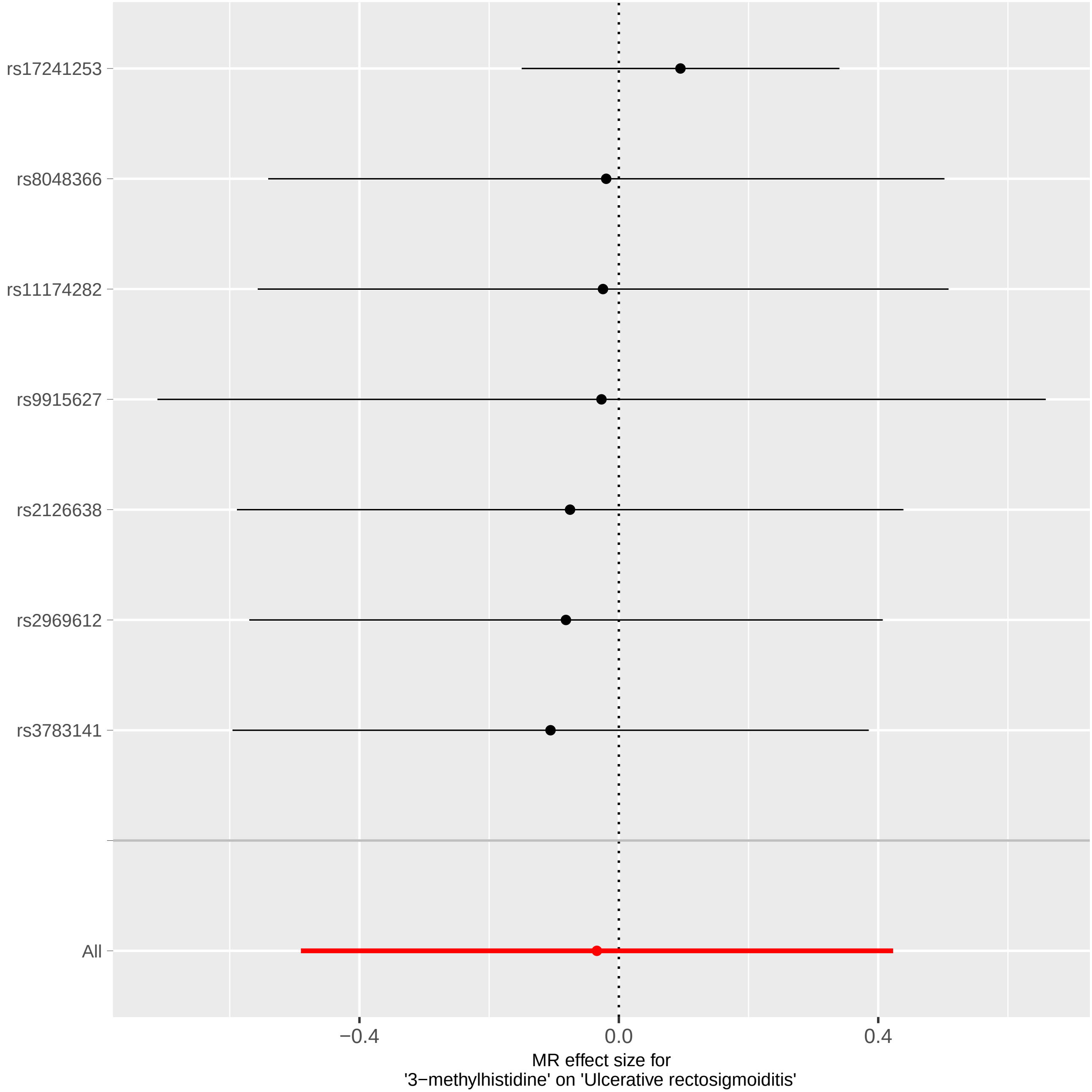

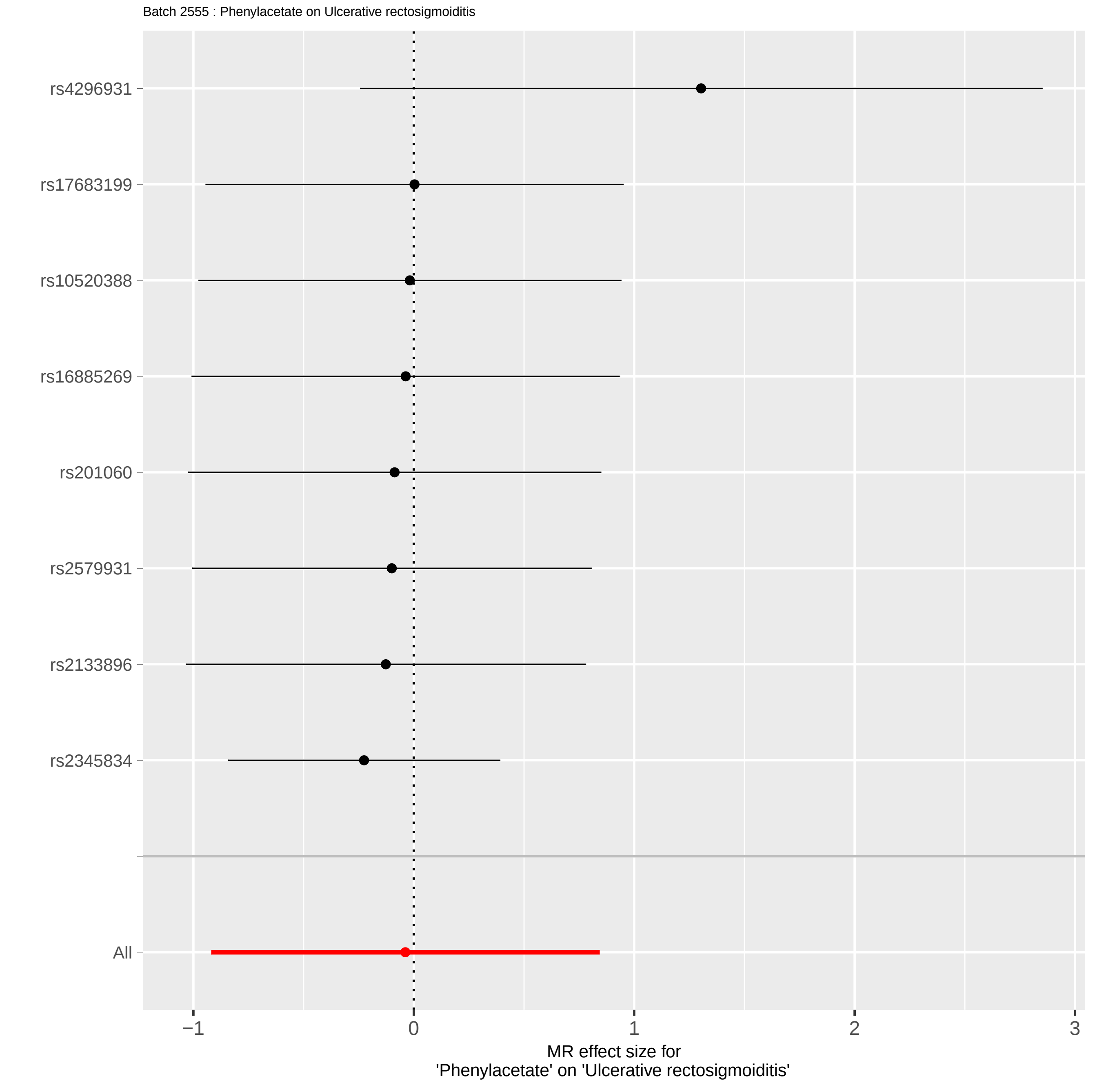
Batch 2555 : Phenylacetate on Ulcerative rectosigmoiditis
rs4296931
rs17683199
rs10520388
rs16885269
rs201060
rs2579931
rs2133896
rs2345834
All
−1
0
1
2
3
MR effect size for
'Phenylacetate' on 'Ulcerative rectosigmoiditis'

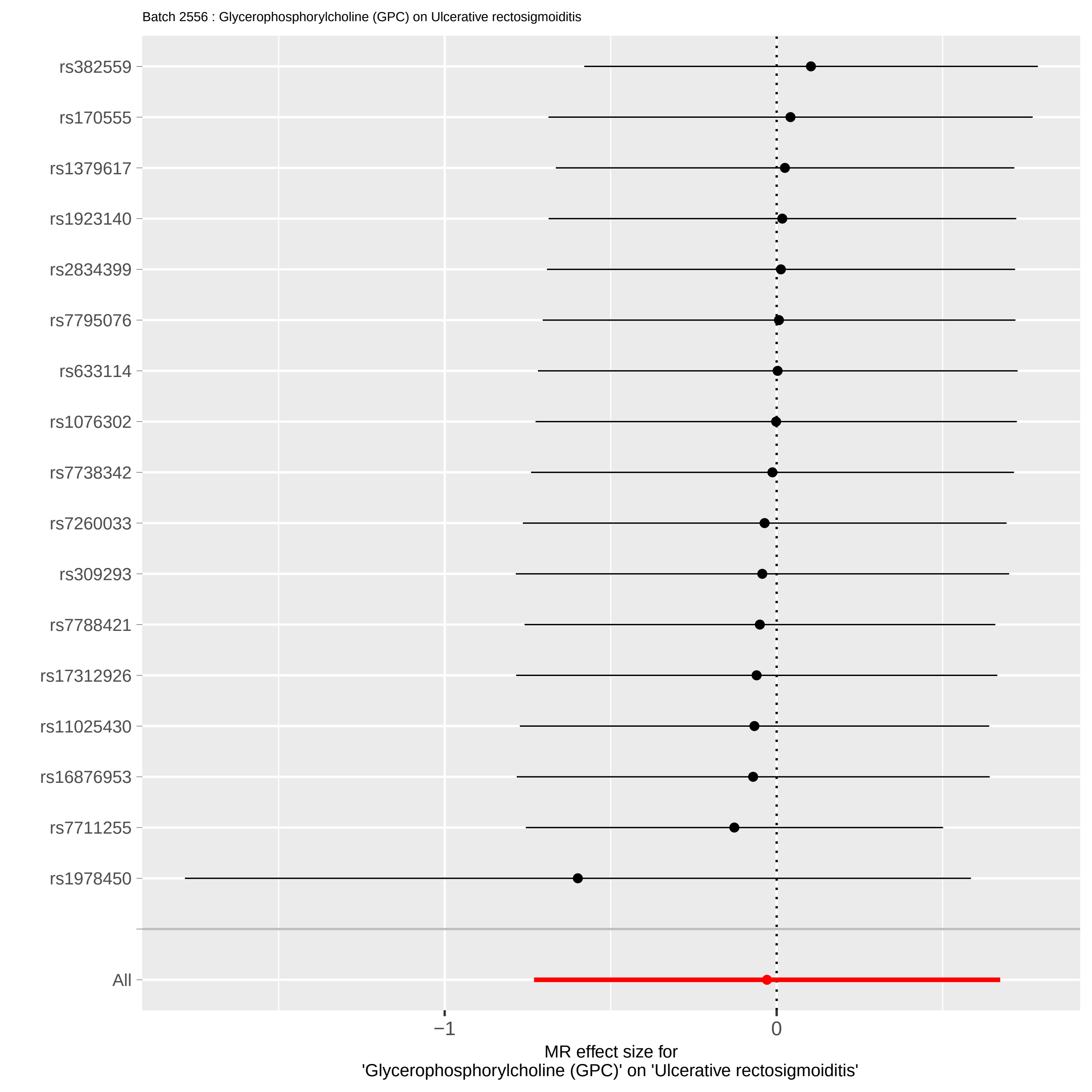

Batch 2556 : Glycerophosphorylcholine (GPC) on Ulcerative rectosigmoiditis
rs382559
rs170555
rs1379617
rs1923140
rs2834399
rs7795076
rs633114
rs1076302
rs7738342
rs7260033
rs309293
rs7788421
rs17312926
rs11025430
rs16876953
rs7711255
rs1978450
All
−1
0
MR effect size for
'Glycerophosphorylcholine (GPC)' on 'Ulcerative rectosigmoiditis'

Batch 2557 : Aspartate on Ulcerative rectosigmoiditis

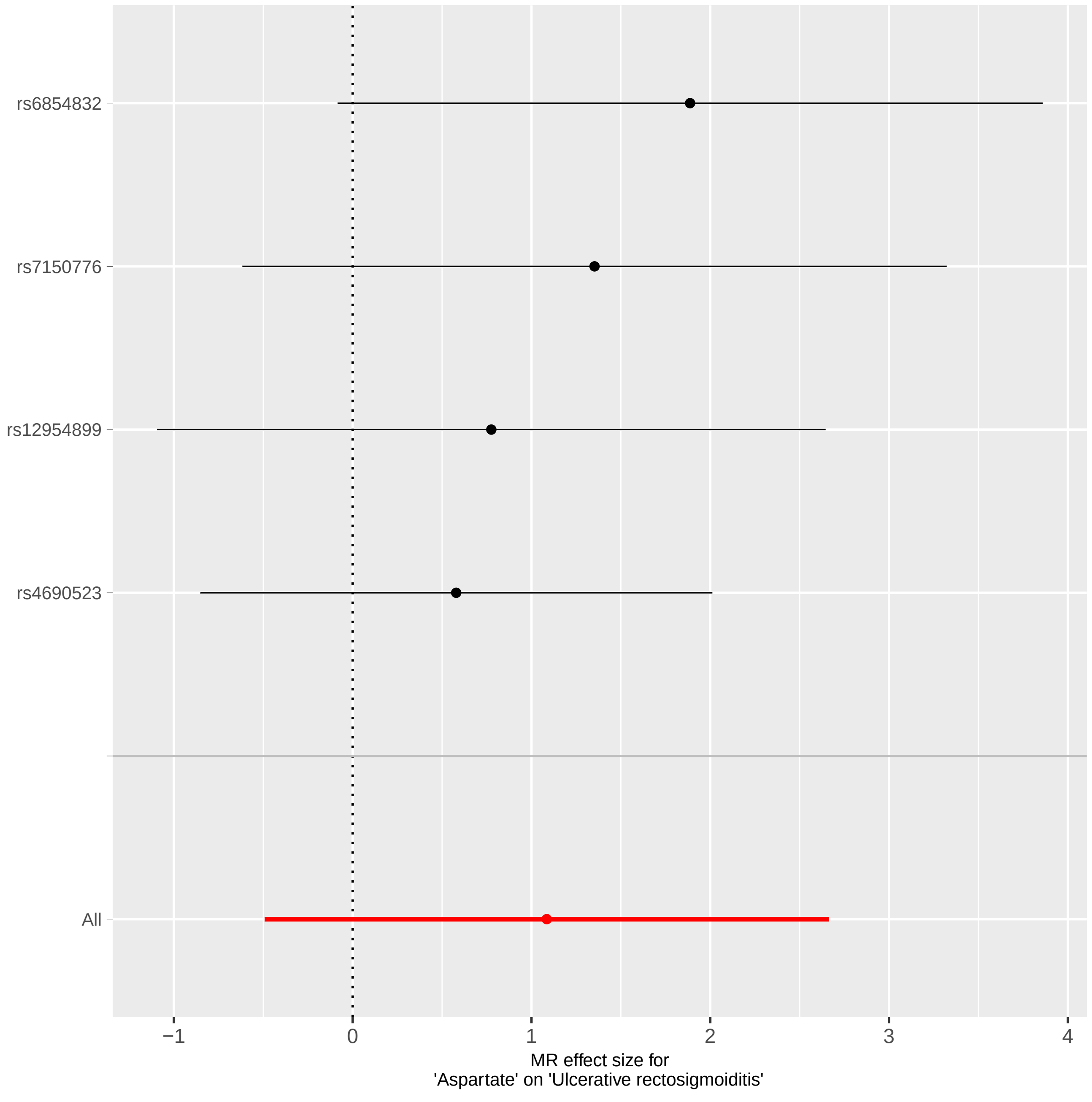

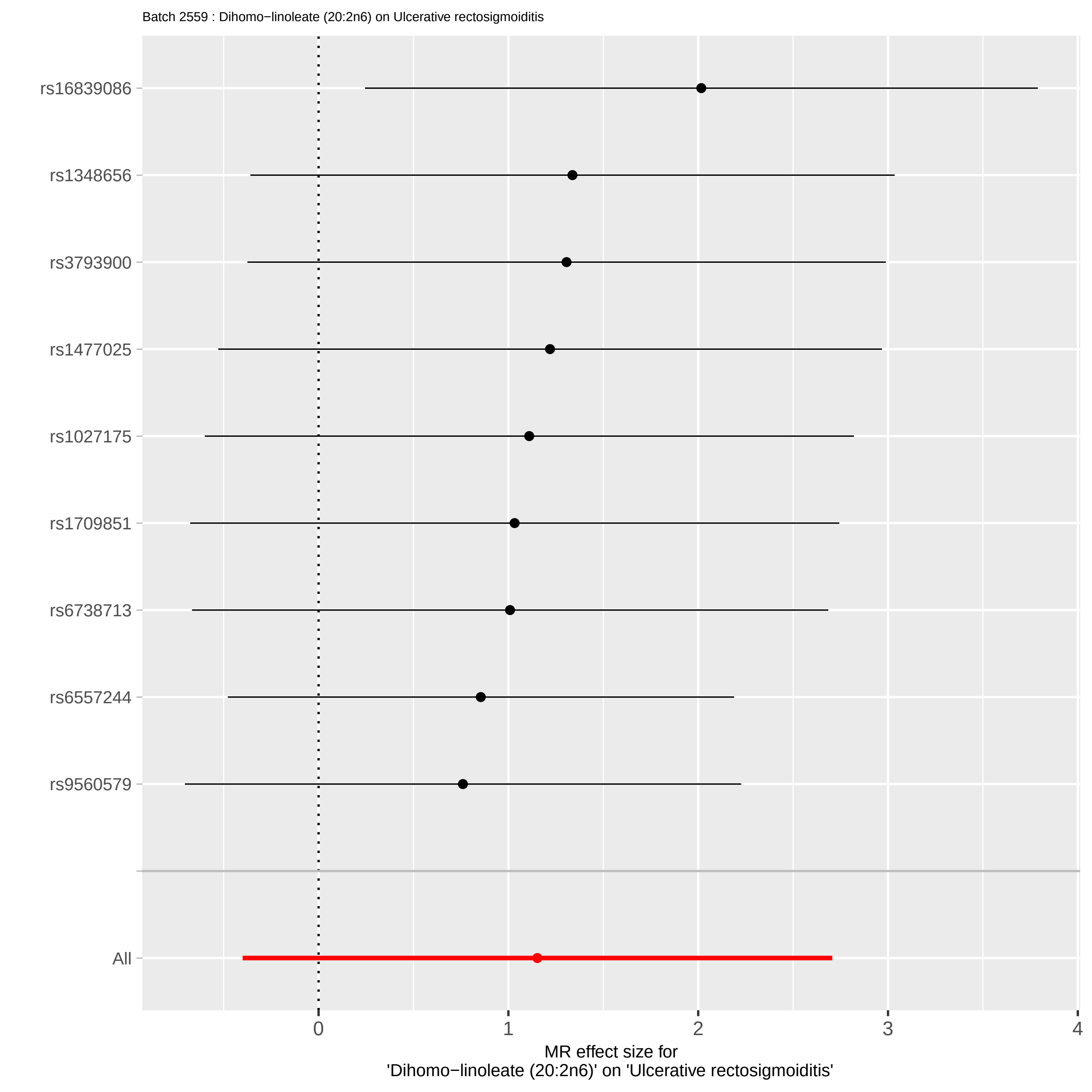
Batch 2559 : Dihomo−linoleate (20:2n6) on Ulcerative rectosigmoiditis
rs16839086
rs1348656
rs3793900
rs1477025
rs1027175
rs1709851
rs6738713
rs6557244
rs9560579
All
0
1
2
3
4
MR effect size for
'Dihomo−linoleate (20:2n6)' on 'Ulcerative rectosigmoiditis'

Batch 2560 : 2−hydroxystearate on Ulcerative rectosigmoiditis

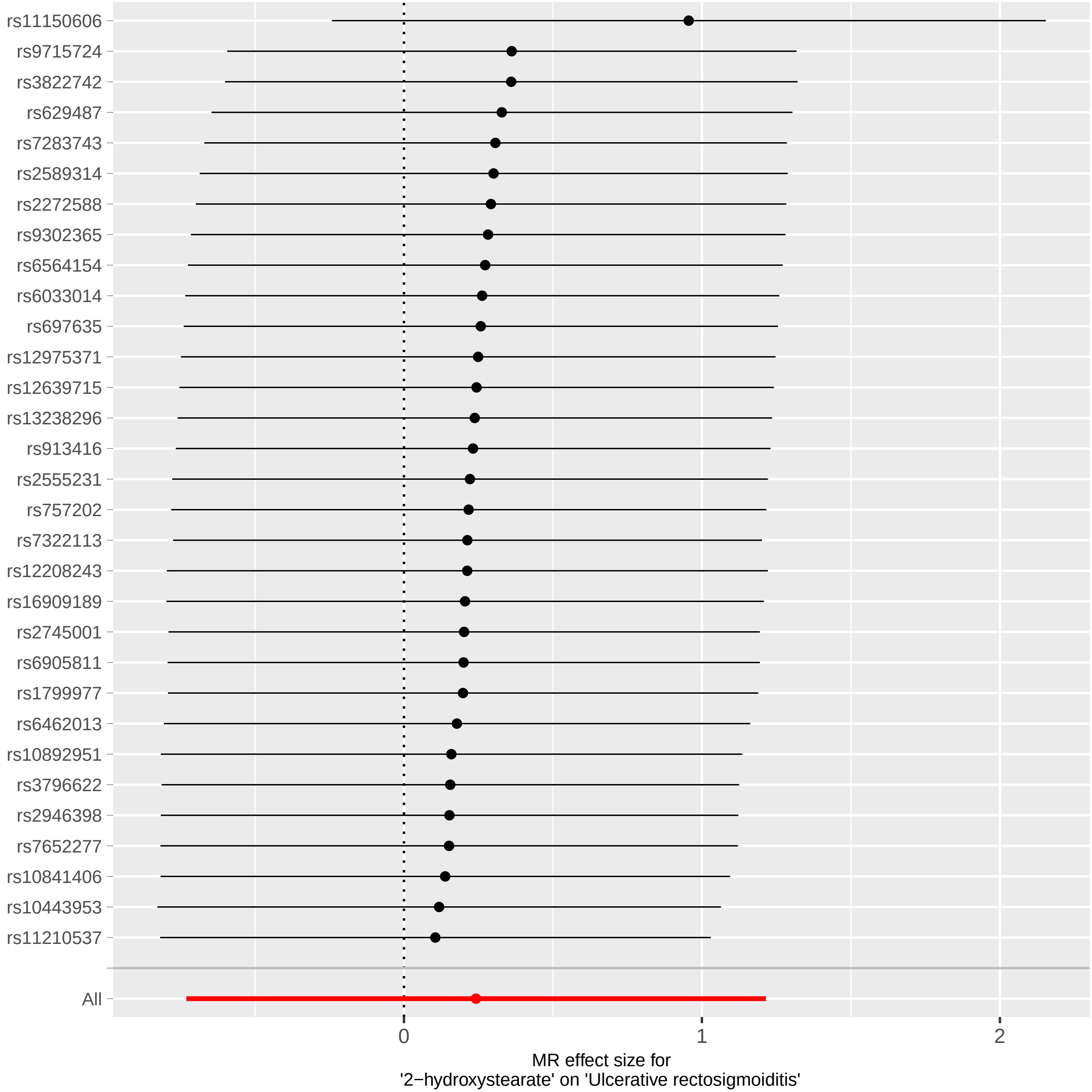

Batch 2562 : Glycylvaline on Ulcerative rectosigmoiditis

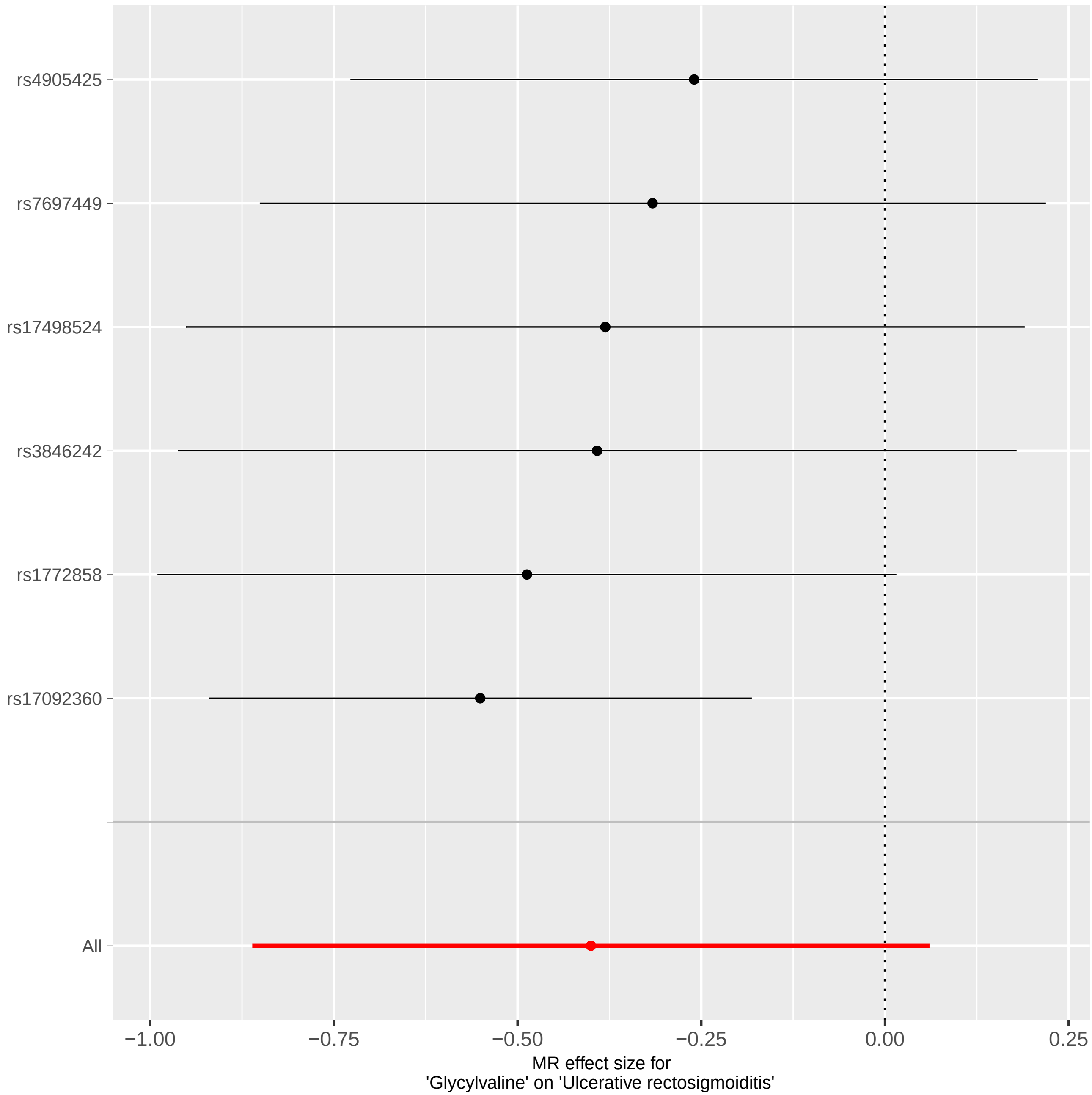

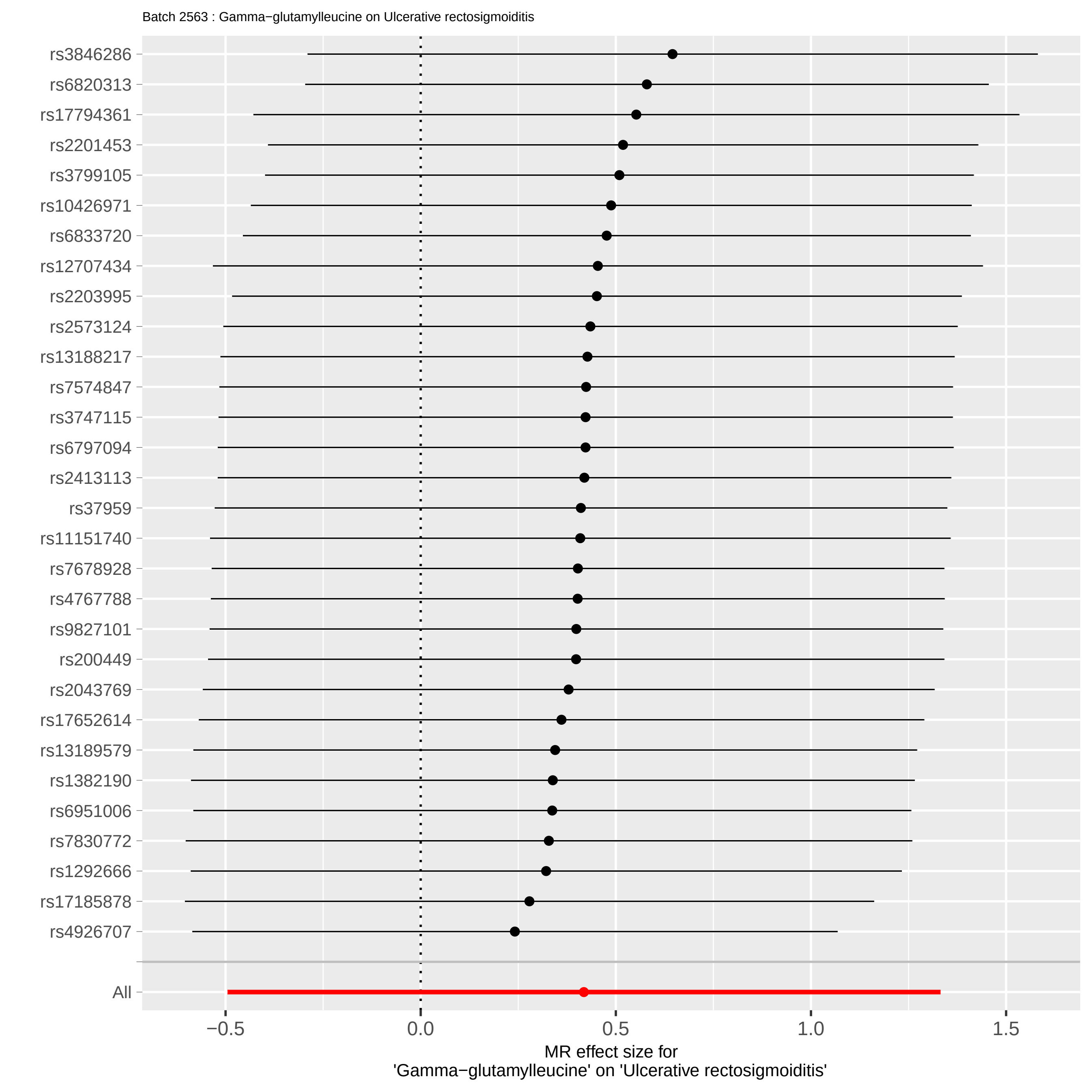

Batch 2563 : Gamma−glutamylleucine on Ulcerative rectosigmoiditis
rs3846286
rs6820313
rs17794361
rs2201453
rs3799105
rs10426971
rs6833720
rs12707434
rs2203995
rs2573124
rs13188217
rs7574847
rs3747115
rs6797094
rs2413113
rs37959
rs11151740
rs7678928
rs4767788
rs9827101
rs200449
rs2043769
rs17652614
rs13189579
rs1382190
rs6951006
rs7830772
rs1292666
rs17185878
rs4926707
All
−0.5
0.0
0.5
1.0
1.5
MR effect size for
'Gamma−glutamylleucine' on 'Ulcerative rectosigmoiditis'

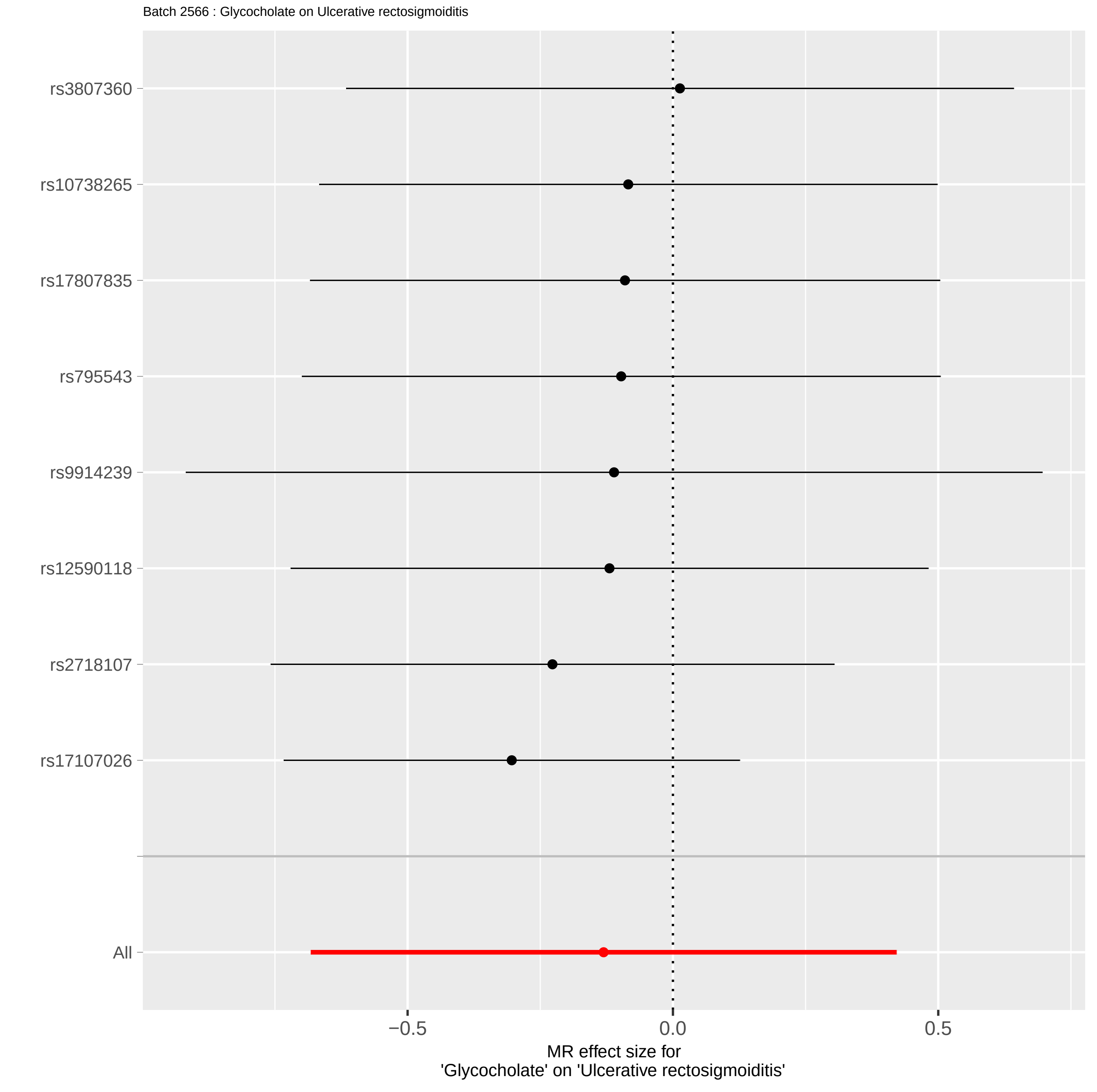
Batch 2566 : Glycocholate on Ulcerative rectosigmoiditis
rs3807360
rs10738265
rs17807835
rs795543
rs9914239
rs12590118
rs2718107
rs17107026
All
−0.5
0.0
0.5
MR effect size for
'Glycocholate' on 'Ulcerative rectosigmoiditis'

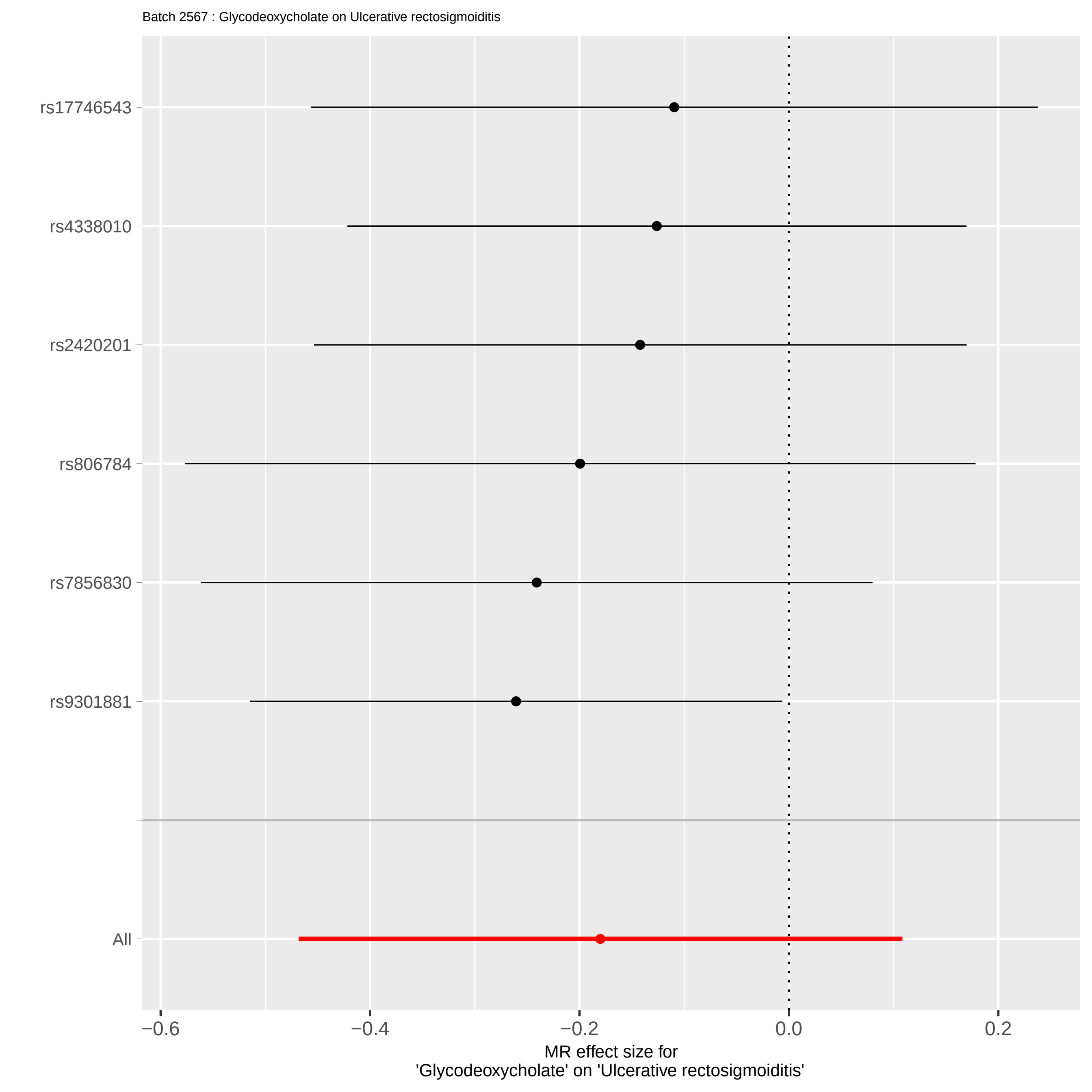
Batch 2567 : Glycodeoxycholate on Ulcerative rectosigmoiditis
rs17746543
rs4338010
rs2420201
rs806784
rs7856830
rs9301881
All
−0.6
−0.4
−0.2
0.0
0.2
MR effect size for
'Glycodeoxycholate' on 'Ulcerative rectosigmoiditis'

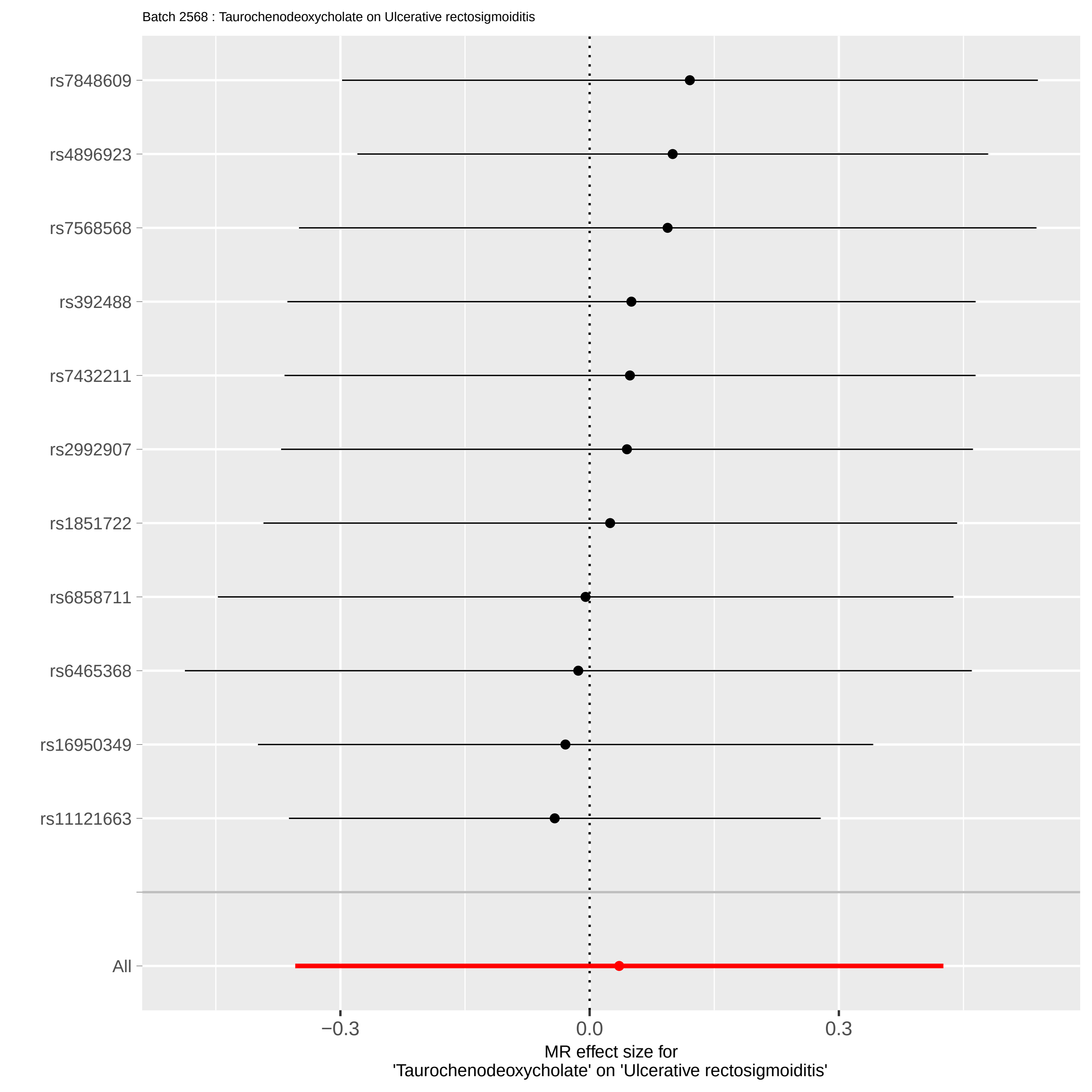

Batch 2568 : Taurochenodeoxycholate on Ulcerative rectosigmoiditis
rs7848609
rs4896923
rs7568568
rs392488
rs7432211
rs2992907
rs1851722
rs6858711
rs6465368
rs16950349
rs11121663
All
−0.3
0.0
0.3
MR effect size for
'Taurochenodeoxycholate' on 'Ulcerative rectosigmoiditis'

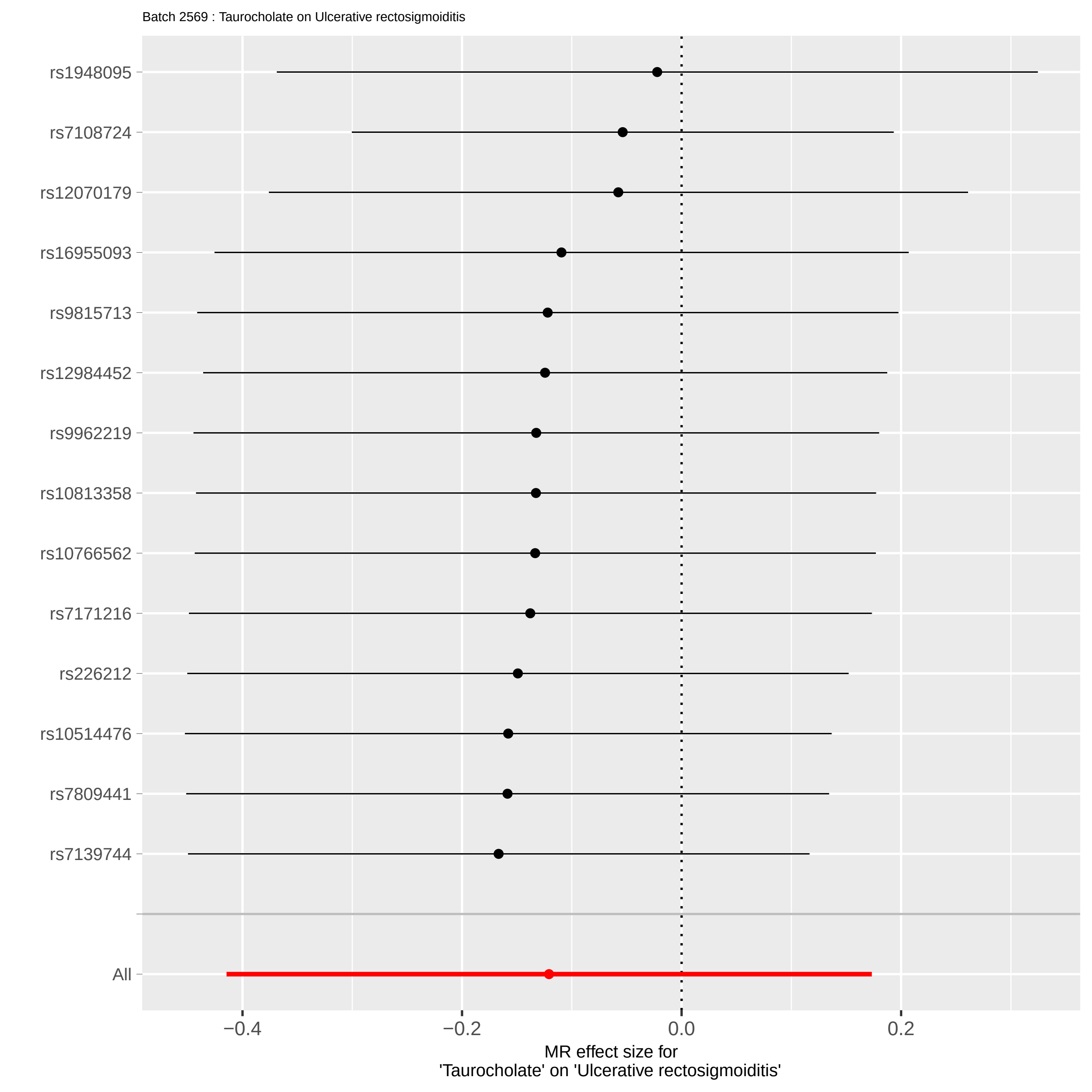
Batch 2569 : Taurocholate on Ulcerative rectosigmoiditis
rs1948095
rs7108724
rs12070179
rs16955093
rs9815713
rs12984452
rs9962219
rs10813358
rs10766562
rs7171216
rs226212
rs10514476
rs7809441
rs7139744
All
−0.4
−0.2
0.0
0.2
MR effect size for
'Taurocholate' on 'Ulcerative rectosigmoiditis'

Batch 2571 : 1−stearoylglycerophosphoinositol on Ulcerative rectosigmoiditis

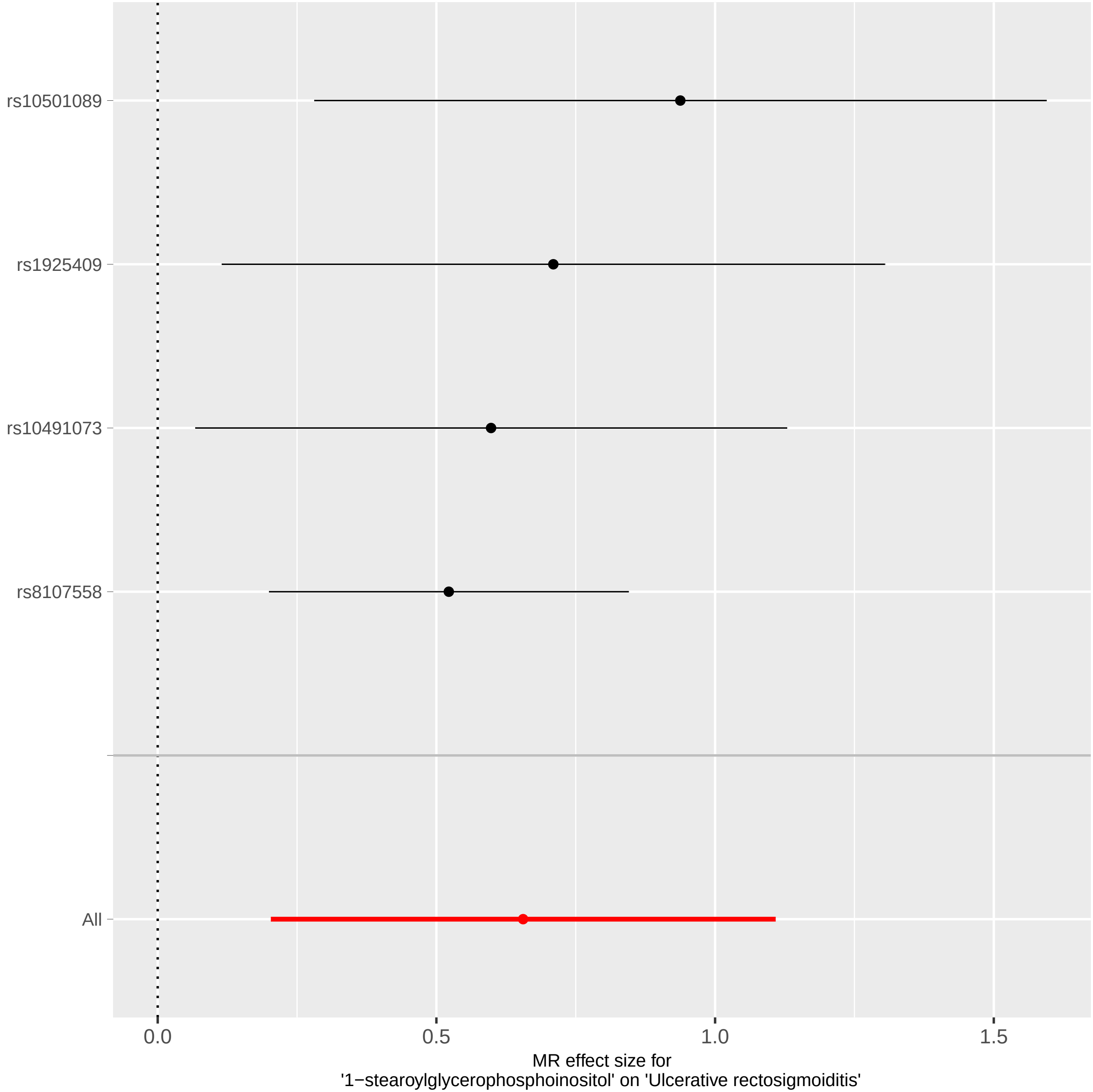

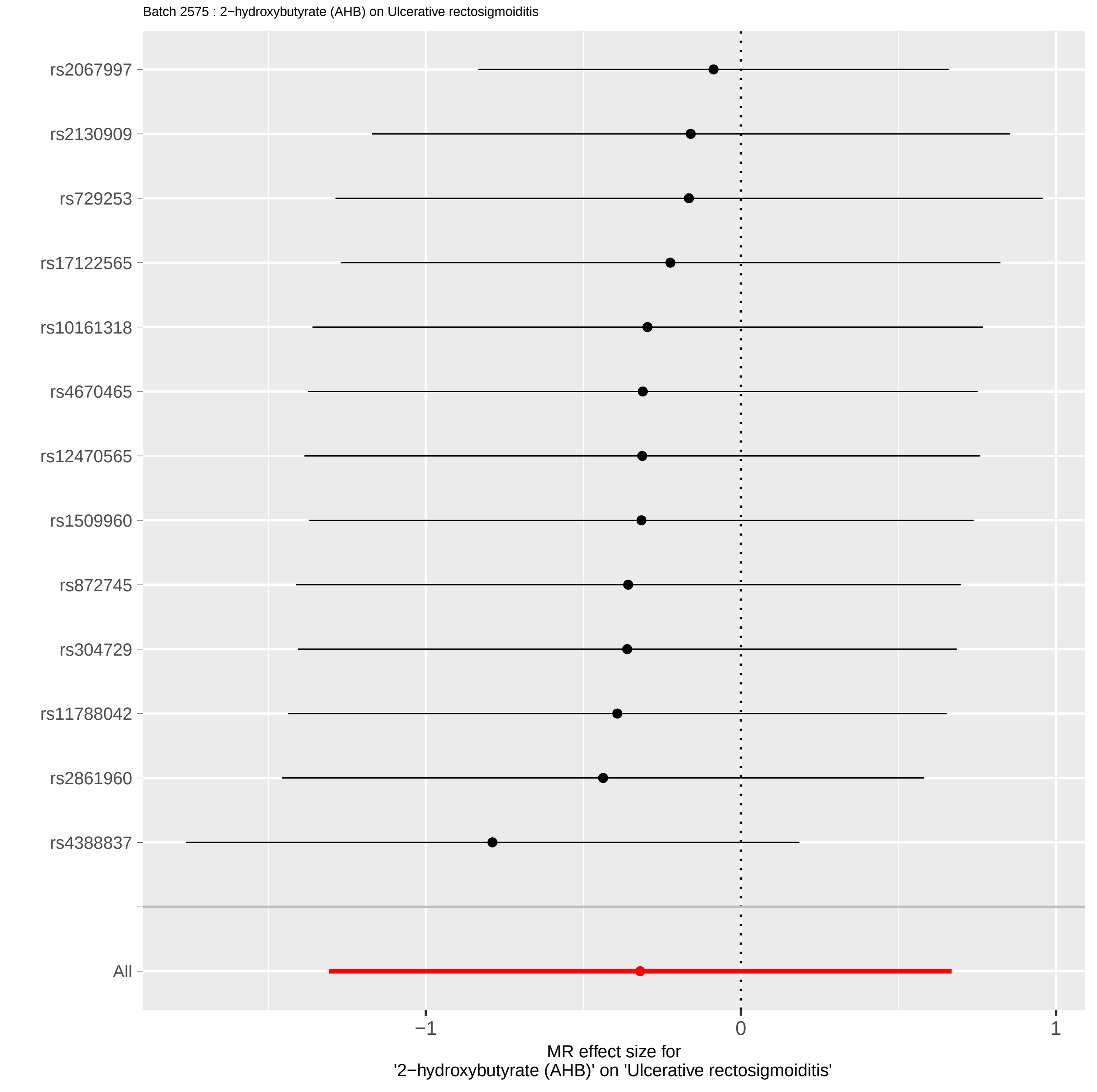

Batch 2575 : 2−hydroxybutyrate (AHB) on Ulcerative rectosigmoiditis
rs2067997
rs2130909
rs729253
rs17122565
rs10161318
rs4670465
rs12470565
rs1509960
rs872745
rs304729
rs11788042
rs2861960
rs4388837
All
−1
0
1
MR effect size for
'2−hydroxybutyrate (AHB)' on 'Ulcerative rectosigmoiditis'

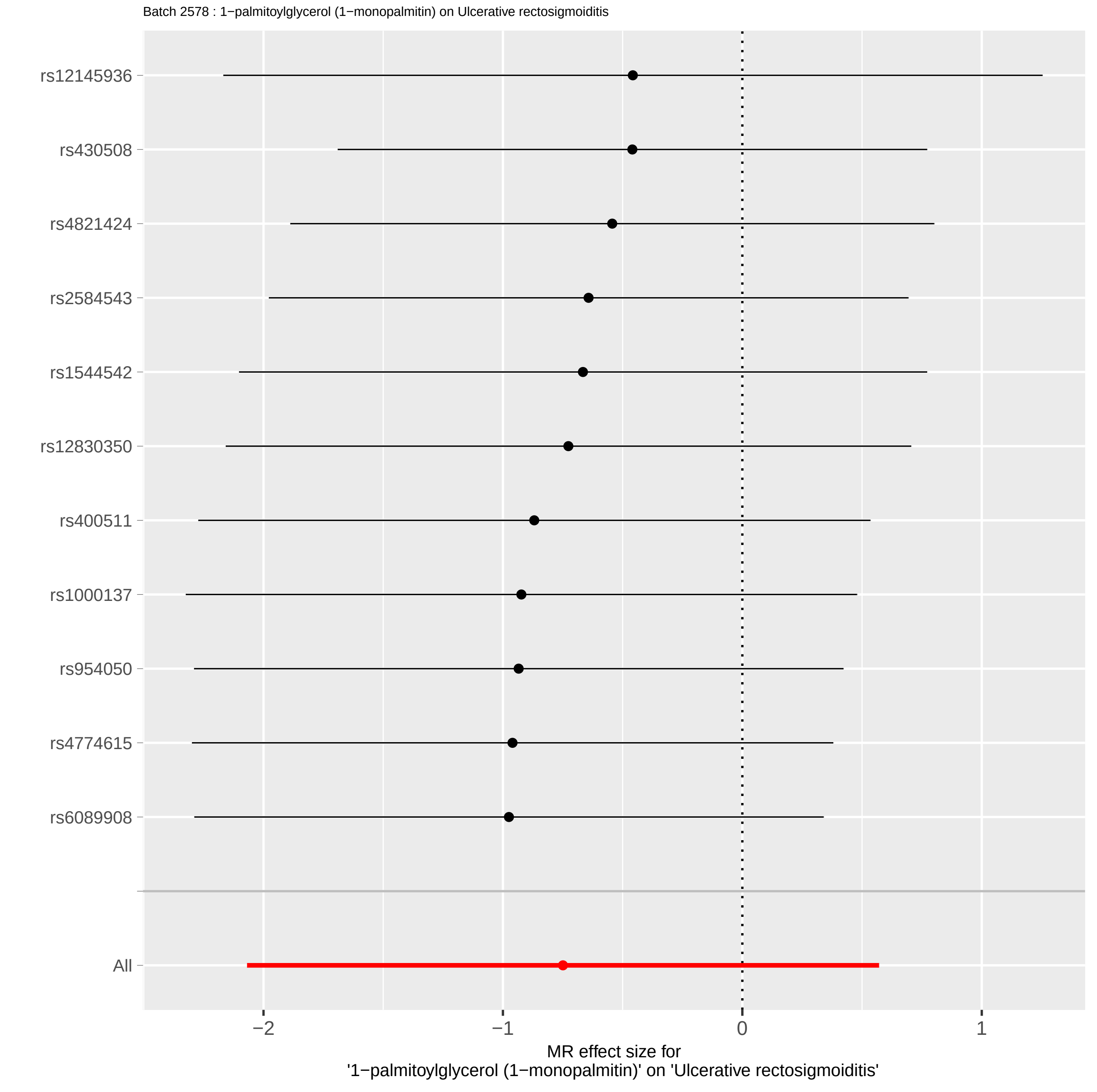

Batch 2578 : 1−palmitoylglycerol (1−monopalmitin) on Ulcerative rectosigmoiditis
rs12145936
rs430508
rs4821424
rs2584543
rs1544542
rs12830350
rs400511
rs1000137
rs954050
rs4774615
rs6089908
All
−2
−1
0
1
MR effect size for
'1−palmitoylglycerol (1−monopalmitin)' on 'Ulcerative rectosigmoiditis'

Batch 2581 : 2−hydroxyisobutyrate on Ulcerative rectosigmoiditis

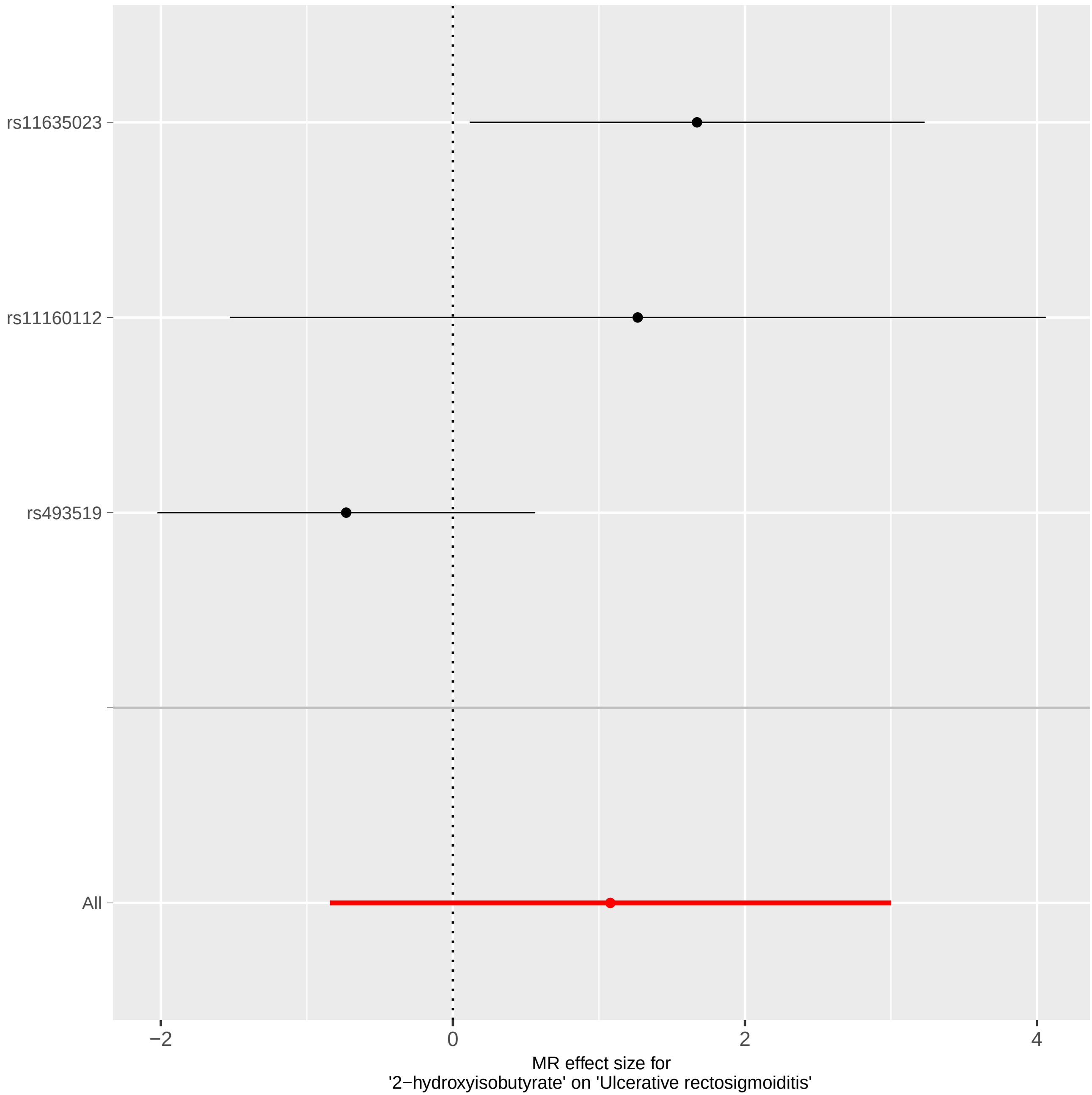

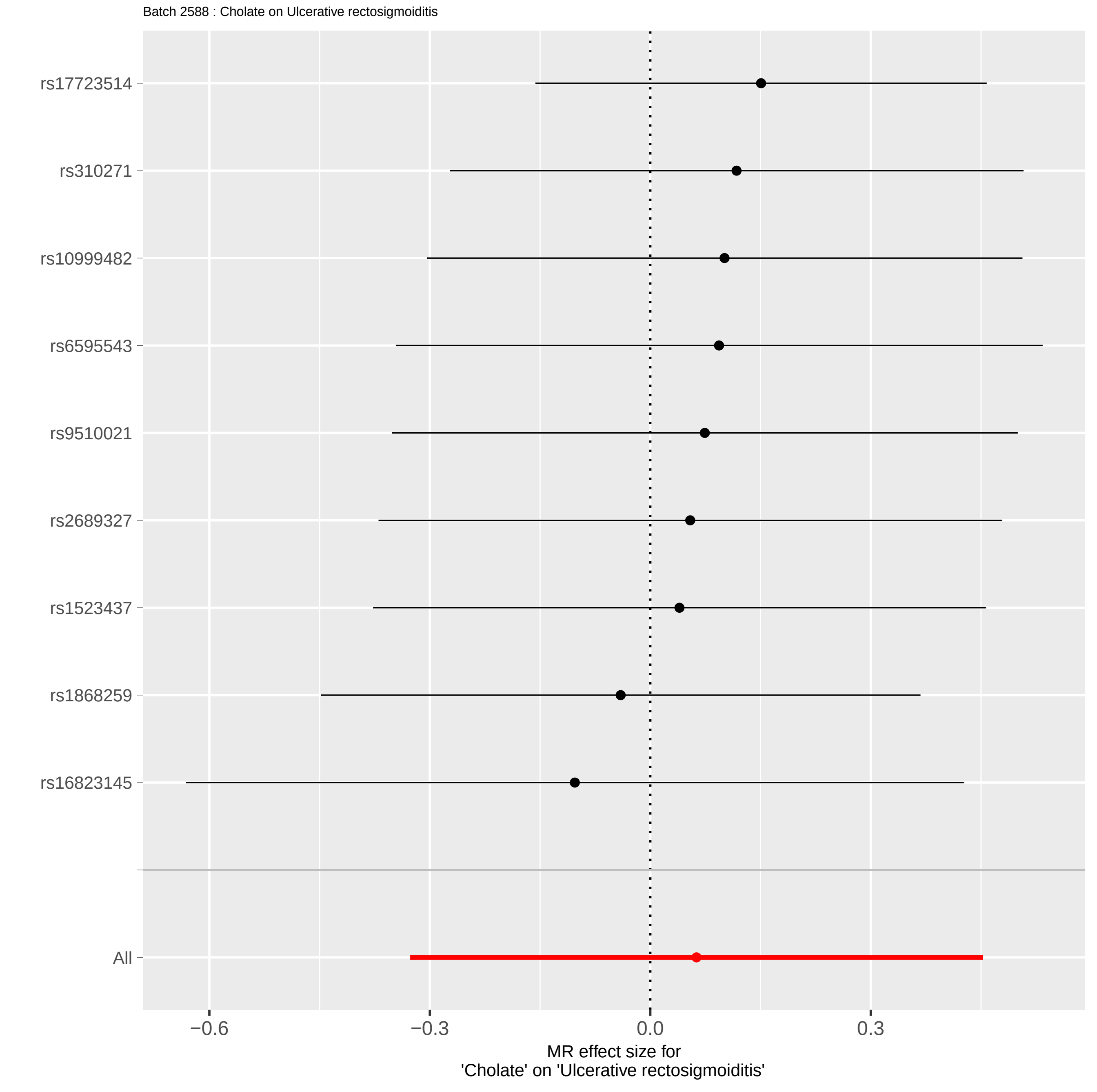

Batch 2588 : Cholate on Ulcerative rectosigmoiditis
rs17723514
rs310271
rs10999482
rs6595543
rs9510021
rs2689327
rs1523437
rs1868259
rs16823145
All
−0.6
−0.3
0.0
0.3
MR effect size for
'Cholate' on 'Ulcerative rectosigmoiditis'

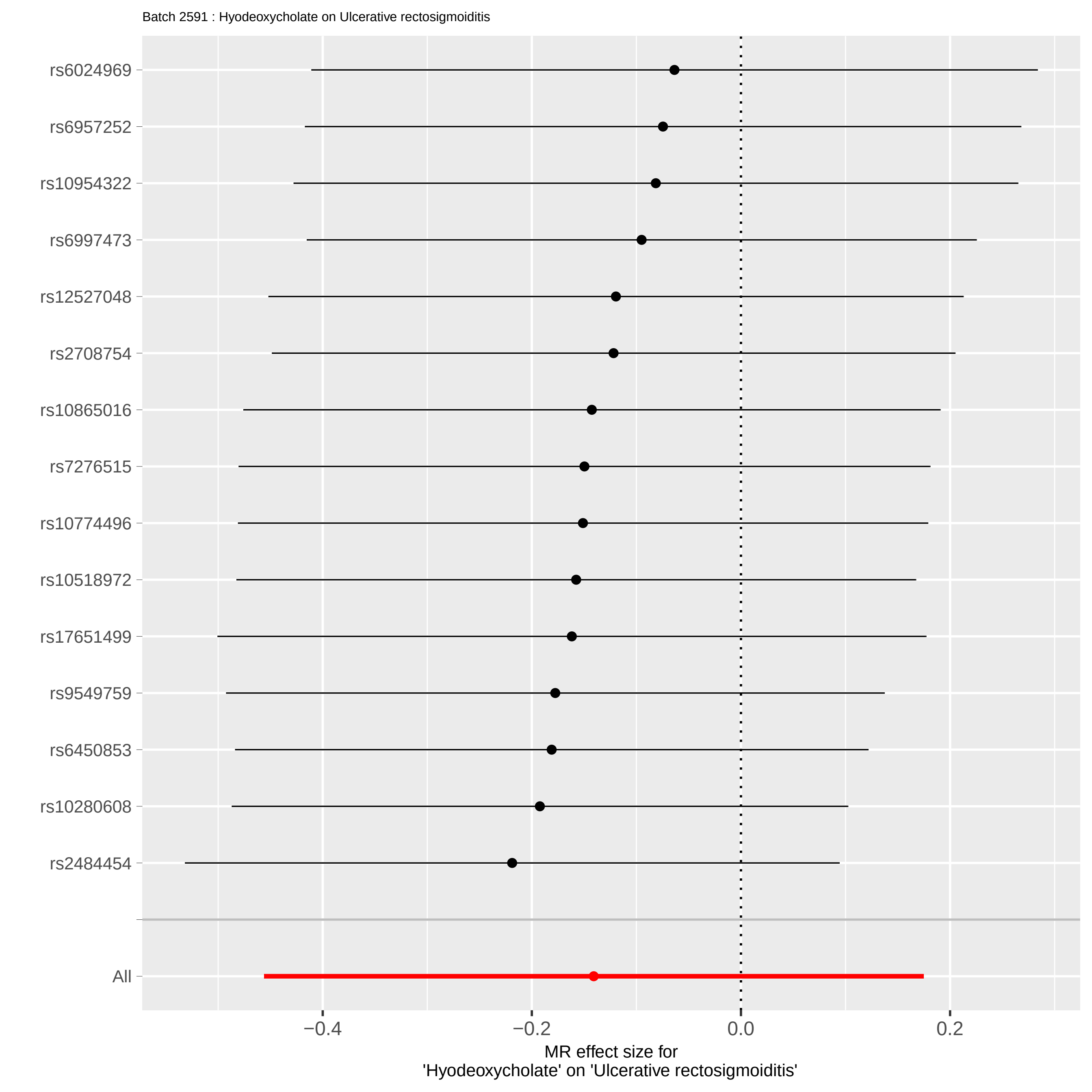

Batch 2591 : Hyodeoxycholate on Ulcerative rectosigmoiditis
rs6024969
rs6957252
rs10954322
rs6997473
rs12527048
rs2708754
rs10865016
rs7276515
rs10774496
rs10518972
rs17651499
rs9549759
rs6450853
rs10280608
rs2484454
All
−0.4
−0.2
0.0
0.2
MR effect size for
'Hyodeoxycholate' on 'Ulcerative rectosigmoiditis'

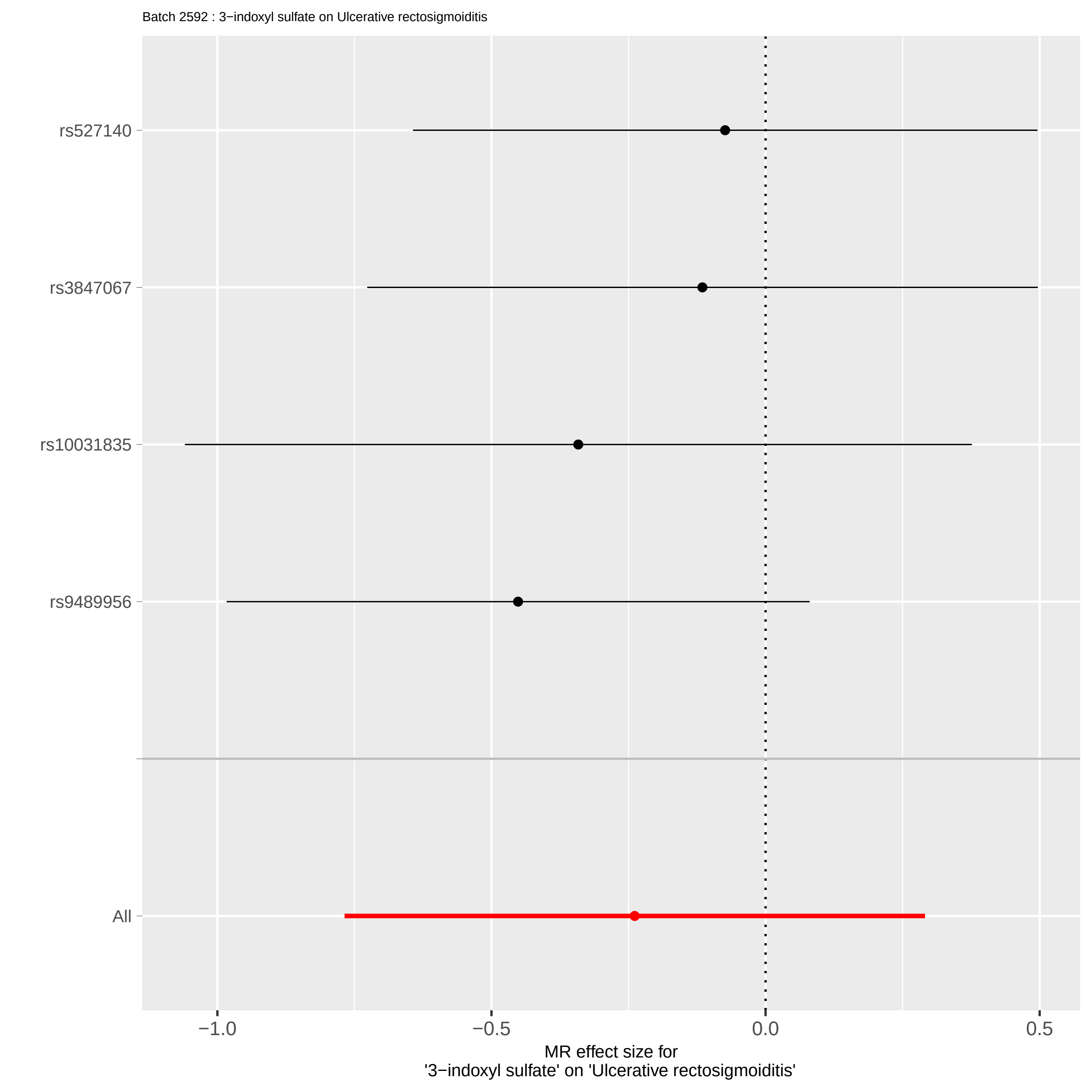
Batch 2592 : 3−indoxyl sulfate on Ulcerative rectosigmoiditis
rs527140
rs3847067
rs10031835
rs9489956
All
−1.0
−0.5
0.0
0.5
MR effect size for
'3−indoxyl sulfate' on 'Ulcerative rectosigmoiditis'

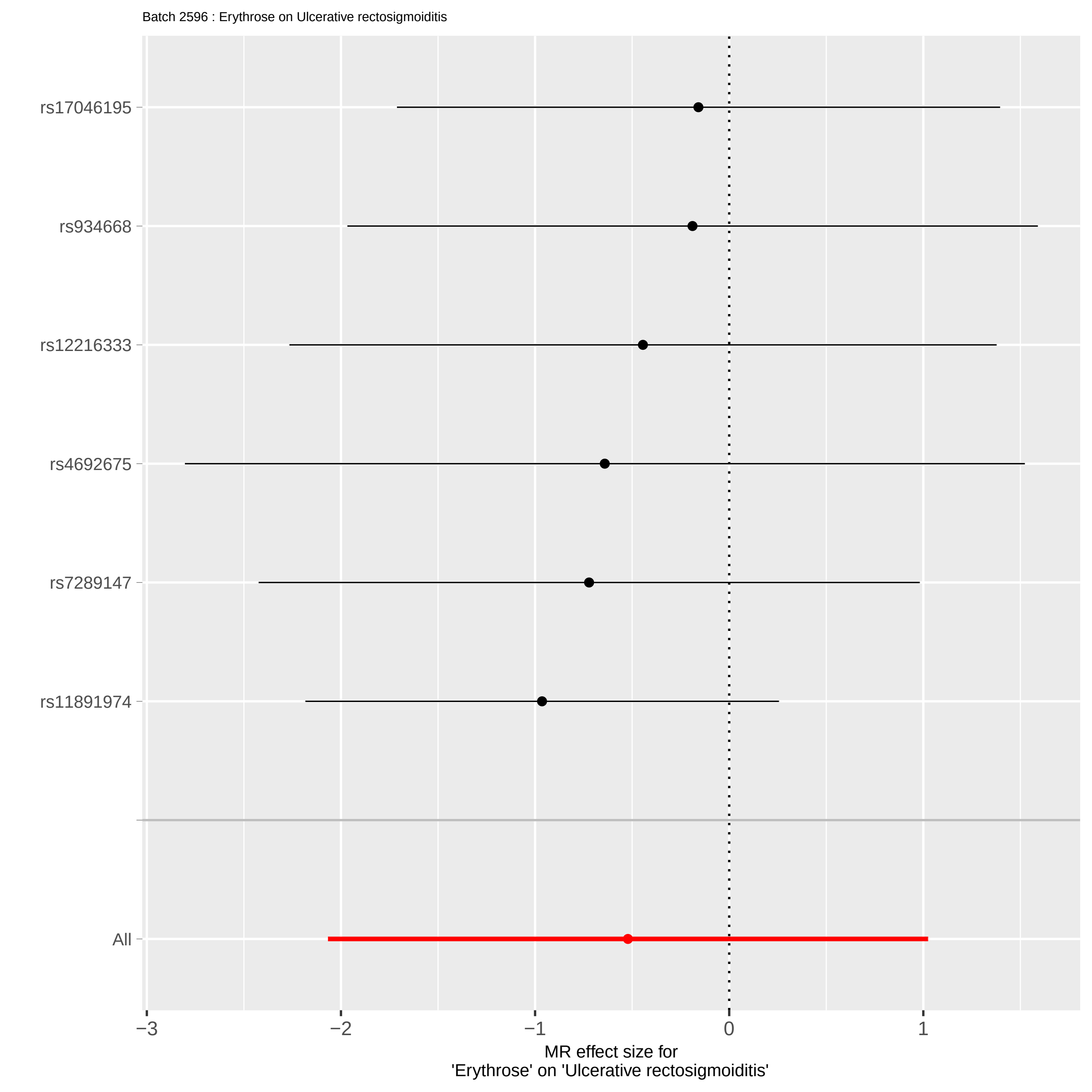

Batch 2596 : Erythrose on Ulcerative rectosigmoiditis
rs17046195
rs934668
rs12216333
rs4692675
rs7289147
rs11891974
All
−3
−2
−1
0
1
MR effect size for
'Erythrose' on 'Ulcerative rectosigmoiditis'

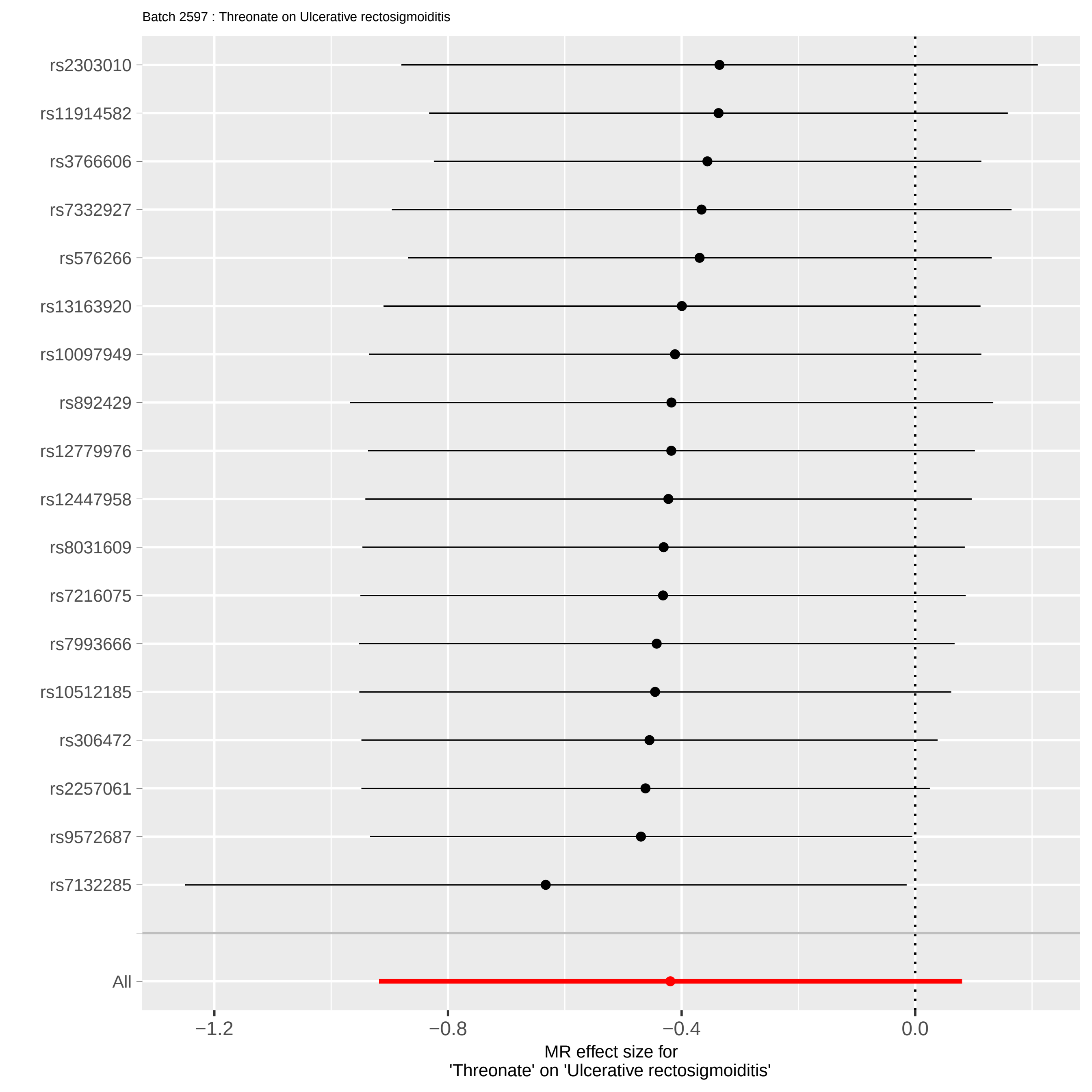

Batch 2597 : Threonate on Ulcerative rectosigmoiditis
rs2303010
rs11914582
rs3766606
rs7332927
rs576266
rs13163920
rs10097949
rs892429
rs12779976
rs12447958
rs8031609
rs7216075
rs7993666
rs10512185
rs306472
rs2257061
rs9572687
rs7132285
All
−1.2
−0.8
−0.4
0.0
MR effect size for
'Threonate' on 'Ulcerative rectosigmoiditis'

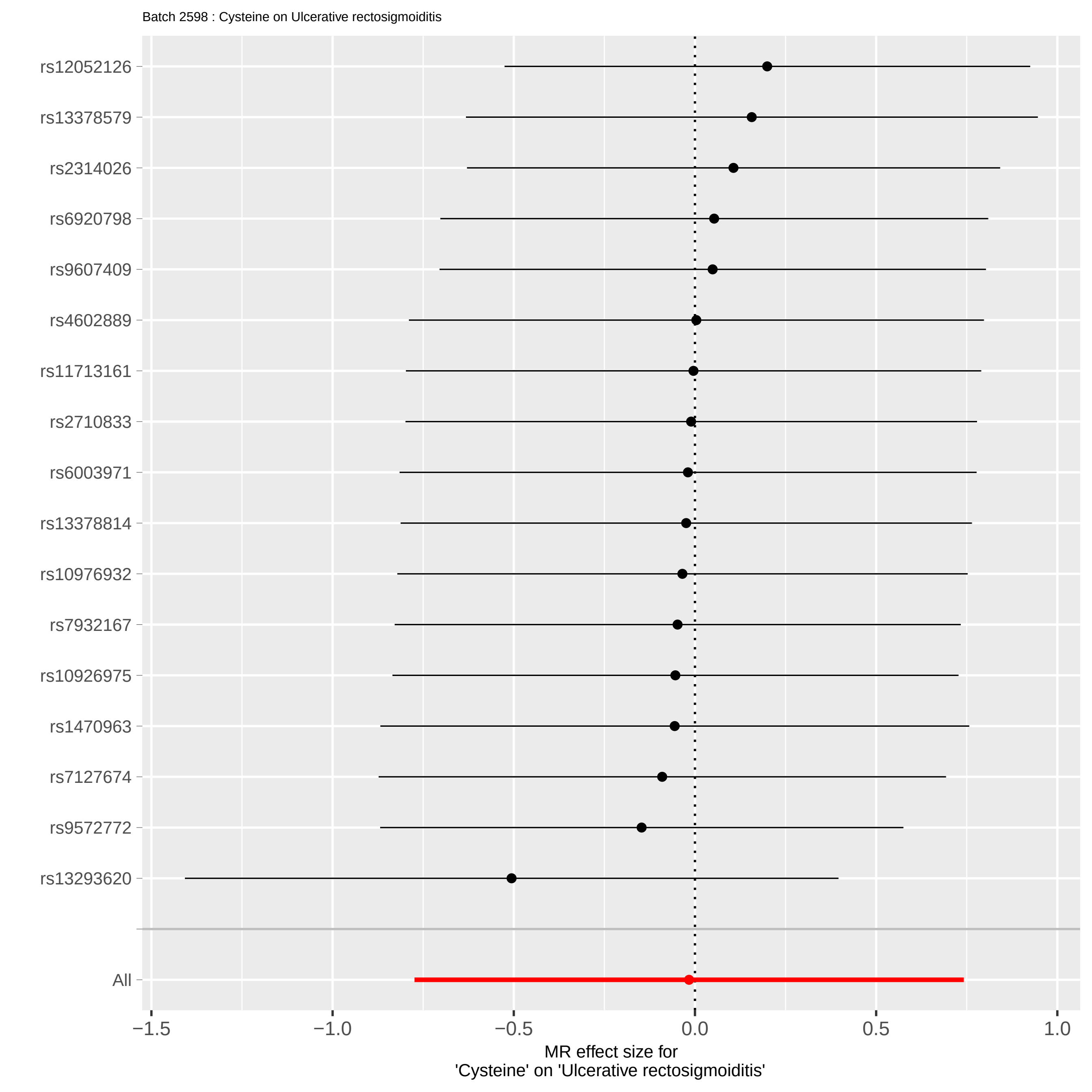
Batch 2598 : Cysteine on Ulcerative rectosigmoiditis
rs12052126
rs13378579
rs2314026
rs6920798
rs9607409
rs4602889
rs11713161
rs2710833
rs6003971
rs13378814
rs10976932
rs7932167
rs10926975
rs1470963
rs7127674
rs9572772
rs13293620
All
−1.5
−1.0
−0.5
0.0
0.5
1.0
MR effect size for
'Cysteine' on 'Ulcerative rectosigmoiditis'

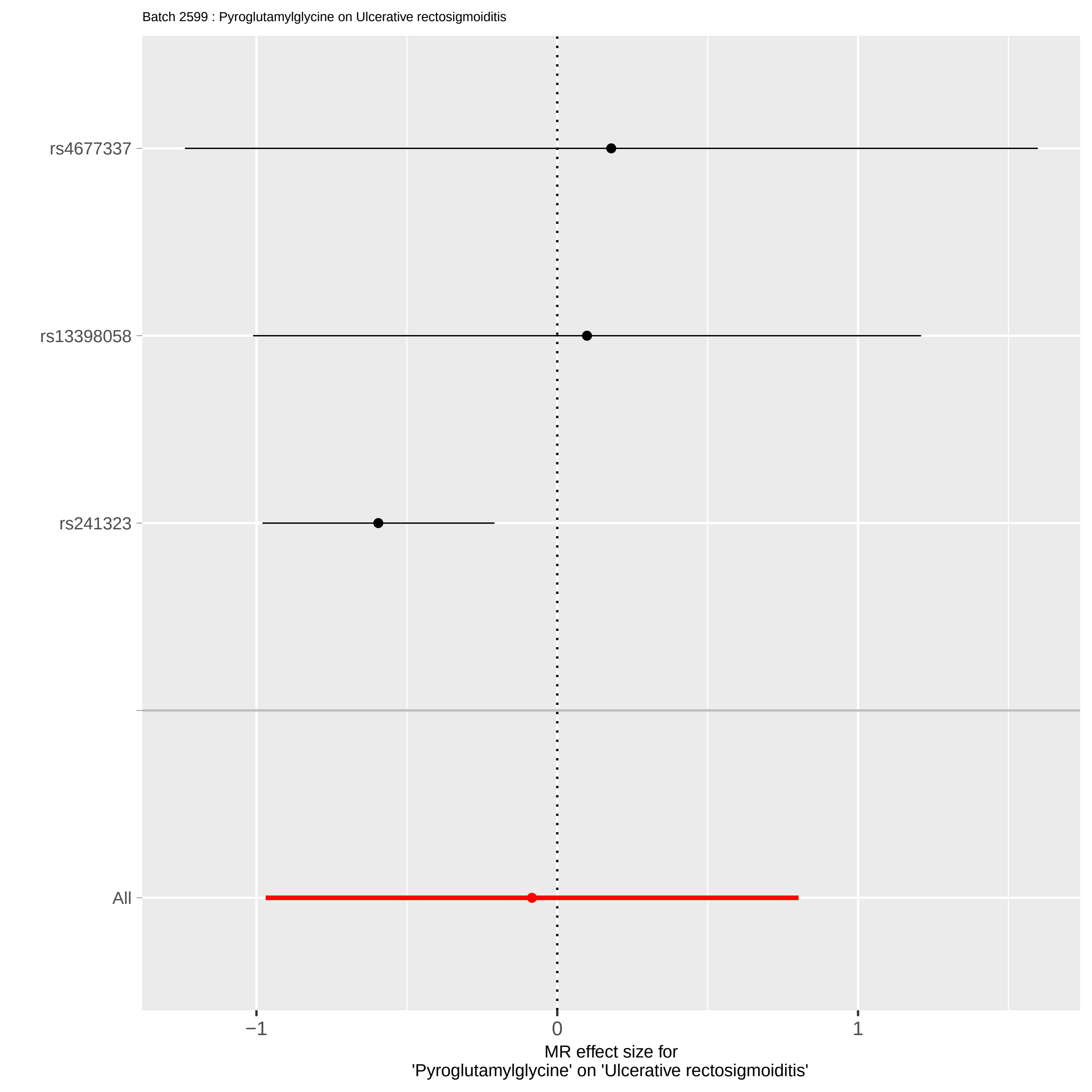
Batch 2599 : Pyroglutamylglycine on Ulcerative rectosigmoiditis
rs4677337
rs13398058
rs241323
All
−1
0
1
MR effect size for
'Pyroglutamylglycine' on 'Ulcerative rectosigmoiditis'

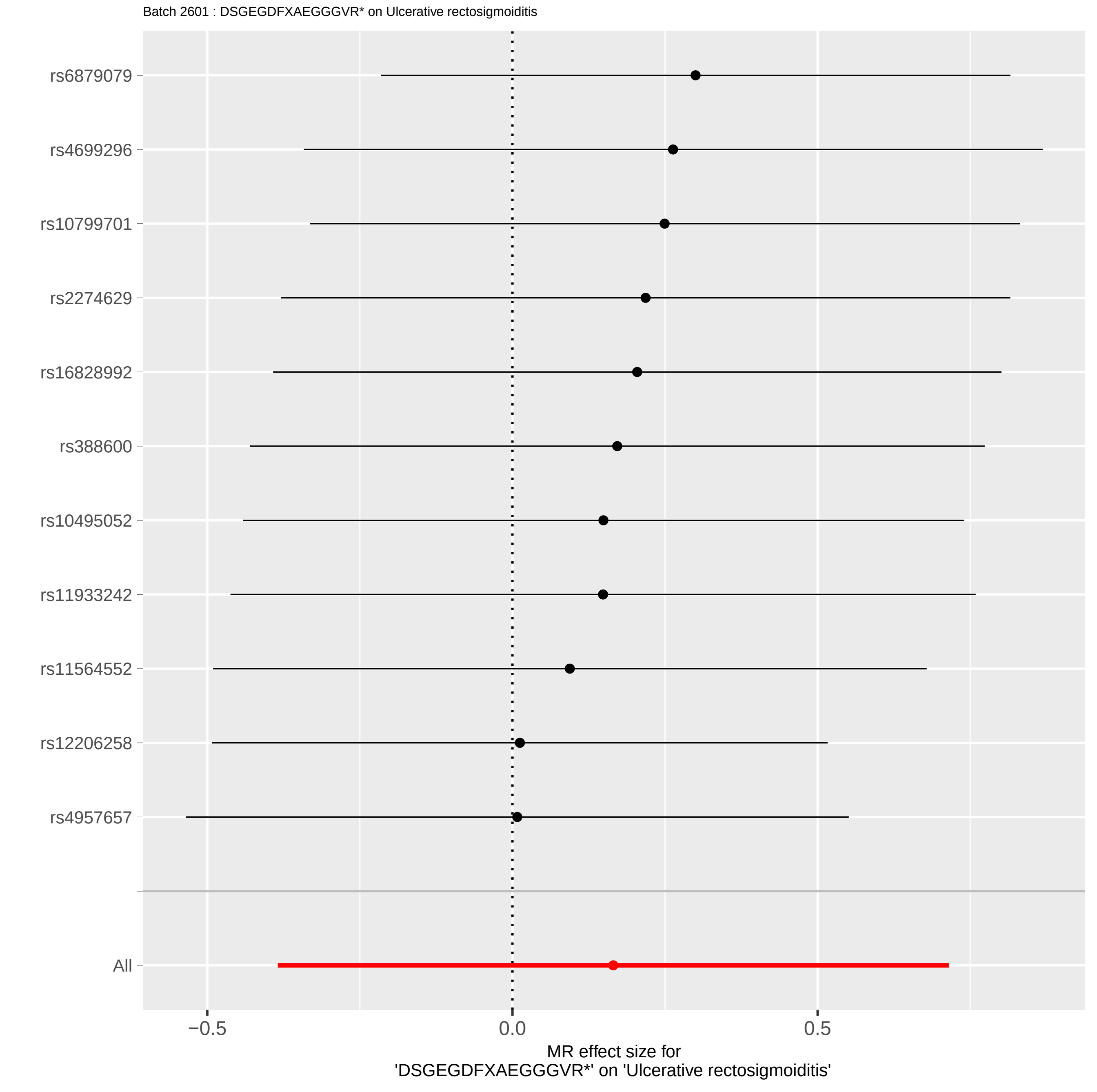
Batch 2601 : DSGEGDFXAEGGGVR* on Ulcerative rectosigmoiditis
rs6879079
rs4699296
rs10799701
rs2274629
rs16828992
rs388600
rs10495052
rs11933242
rs11564552
rs12206258
rs4957657
All
−0.5
0.0
0.5
MR effect size for
'DSGEGDFXAEGGGVR*' on 'Ulcerative rectosigmoiditis'

Batch 2602 : Pyridoxate on Ulcerative rectosigmoiditis

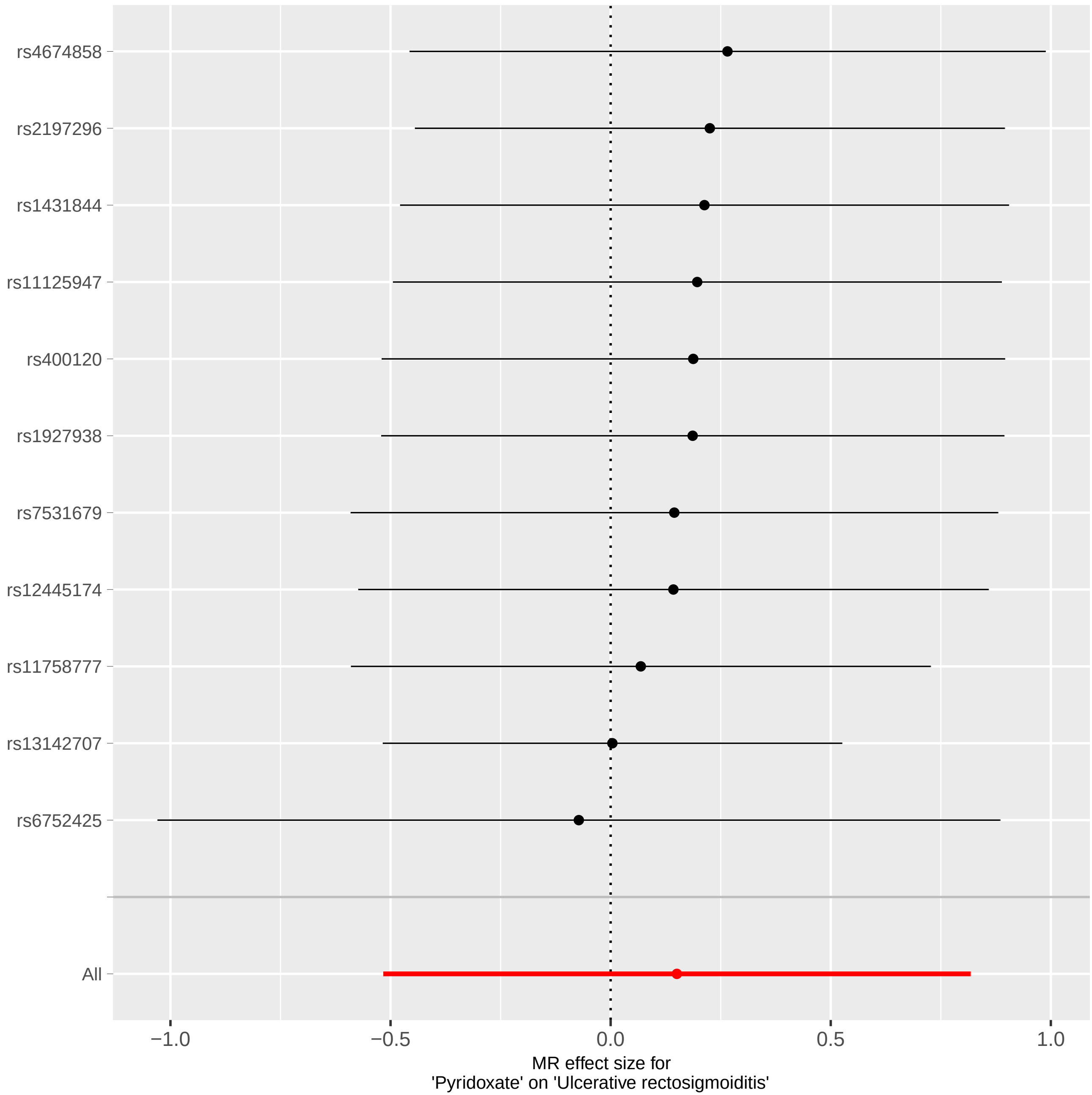

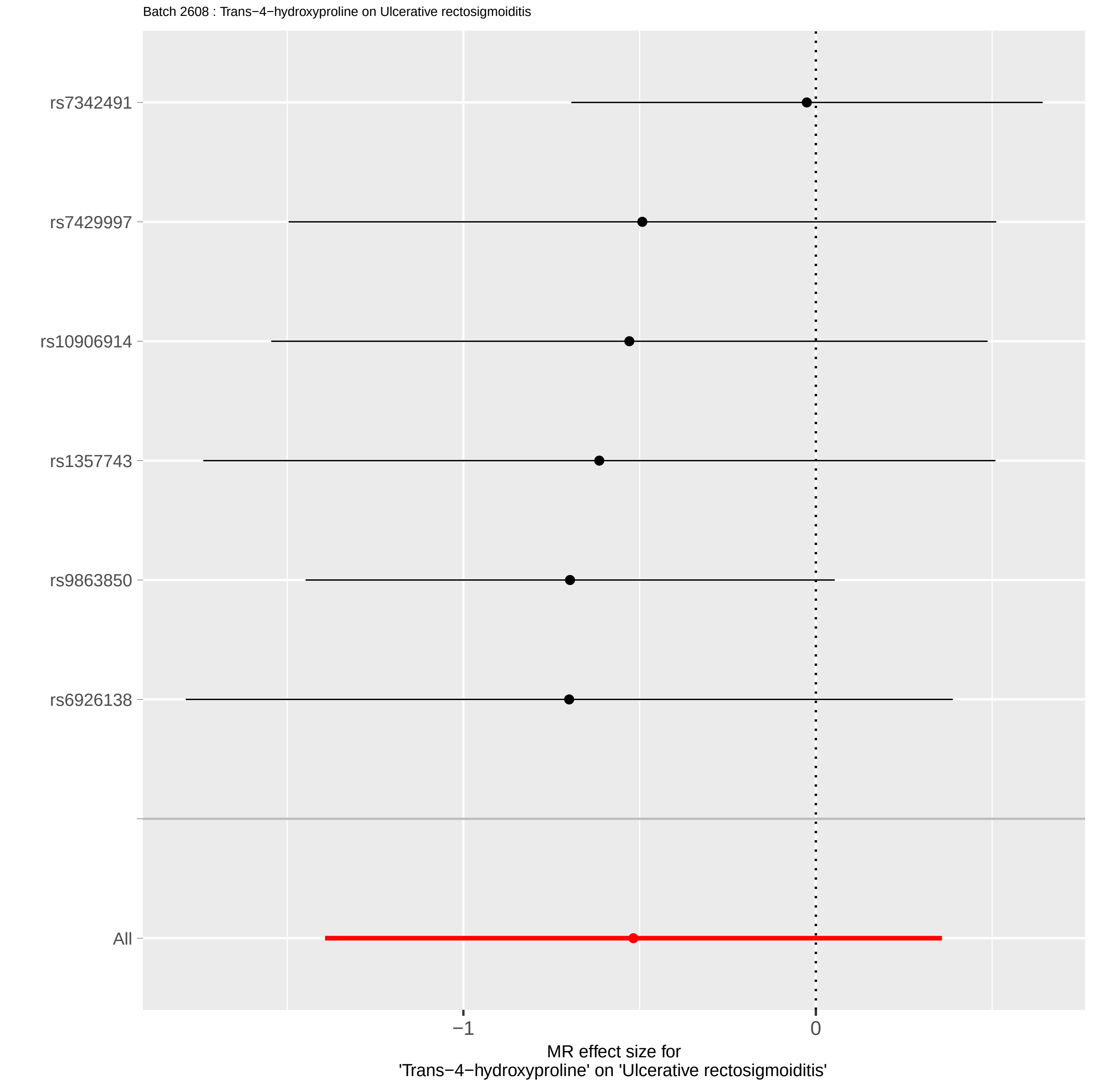
Batch 2608 : Trans−4−hydroxyproline on Ulcerative rectosigmoiditis
rs7342491
rs7429997
rs10906914
rs1357743
rs9863850
rs6926138
All
−1
0
MR effect size for
'Trans−4−hydroxyproline' on 'Ulcerative rectosigmoiditis'

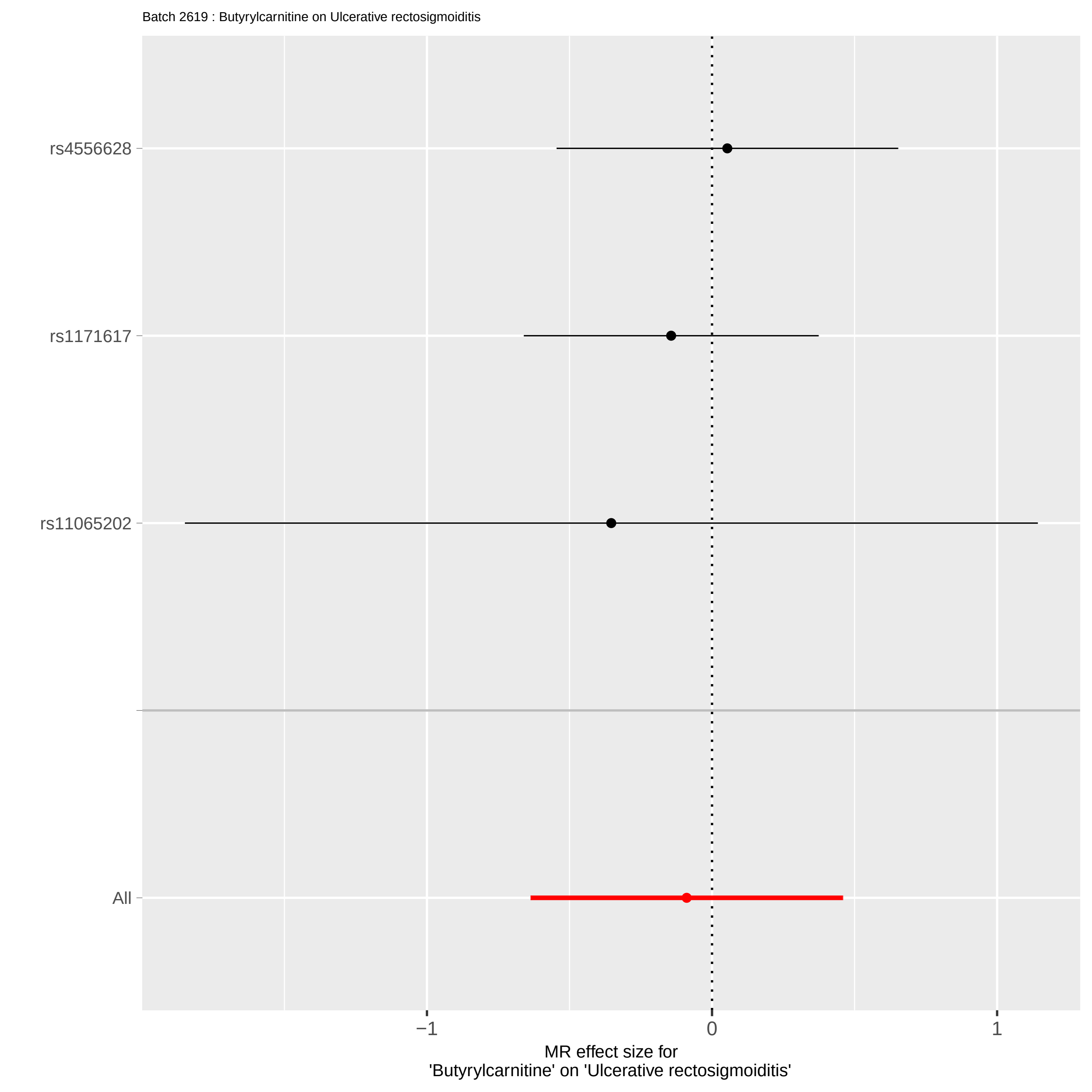

Batch 2619 : Butyrylcarnitine on Ulcerative rectosigmoiditis
rs4556628
rs1171617
rs11065202
All
−1
0
1
MR effect size for
'Butyrylcarnitine' on 'Ulcerative rectosigmoiditis'

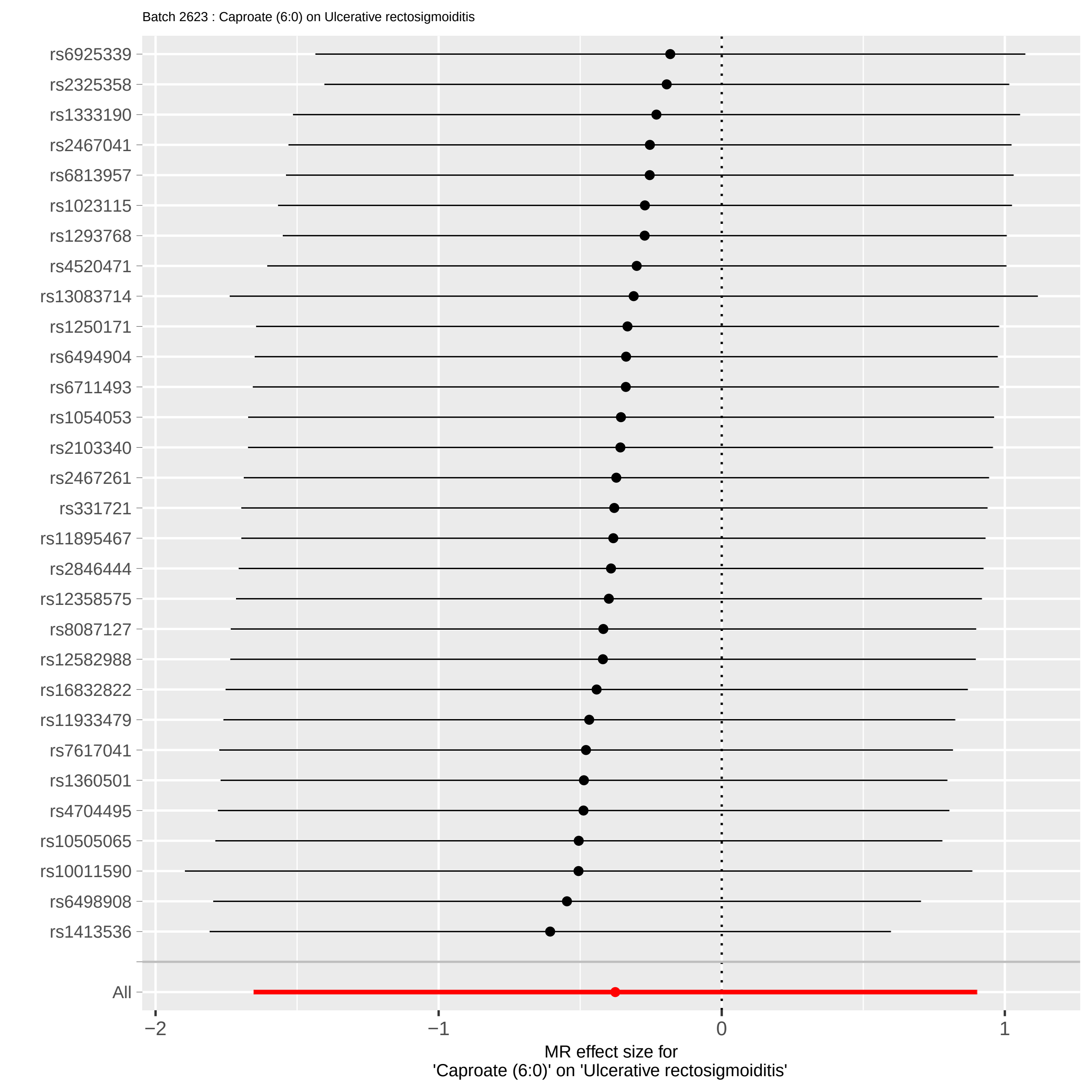
Batch 2623 : Caproate (6:0) on Ulcerative rectosigmoiditis
rs6925339
rs2325358
rs1333190
rs2467041
rs6813957
rs1023115
rs1293768
rs4520471
rs13083714
rs1250171
rs6494904
rs6711493
rs1054053
rs2103340
rs2467261
rs331721
rs11895467
rs2846444
rs12358575
rs8087127
rs12582988
rs16832822
rs11933479
rs7617041
rs1360501
rs4704495
rs10505065
rs10011590
rs6498908
rs1413536
All
−2
−1
0
1
MR effect size for
'Caproate (6:0)' on 'Ulcerative rectosigmoiditis'

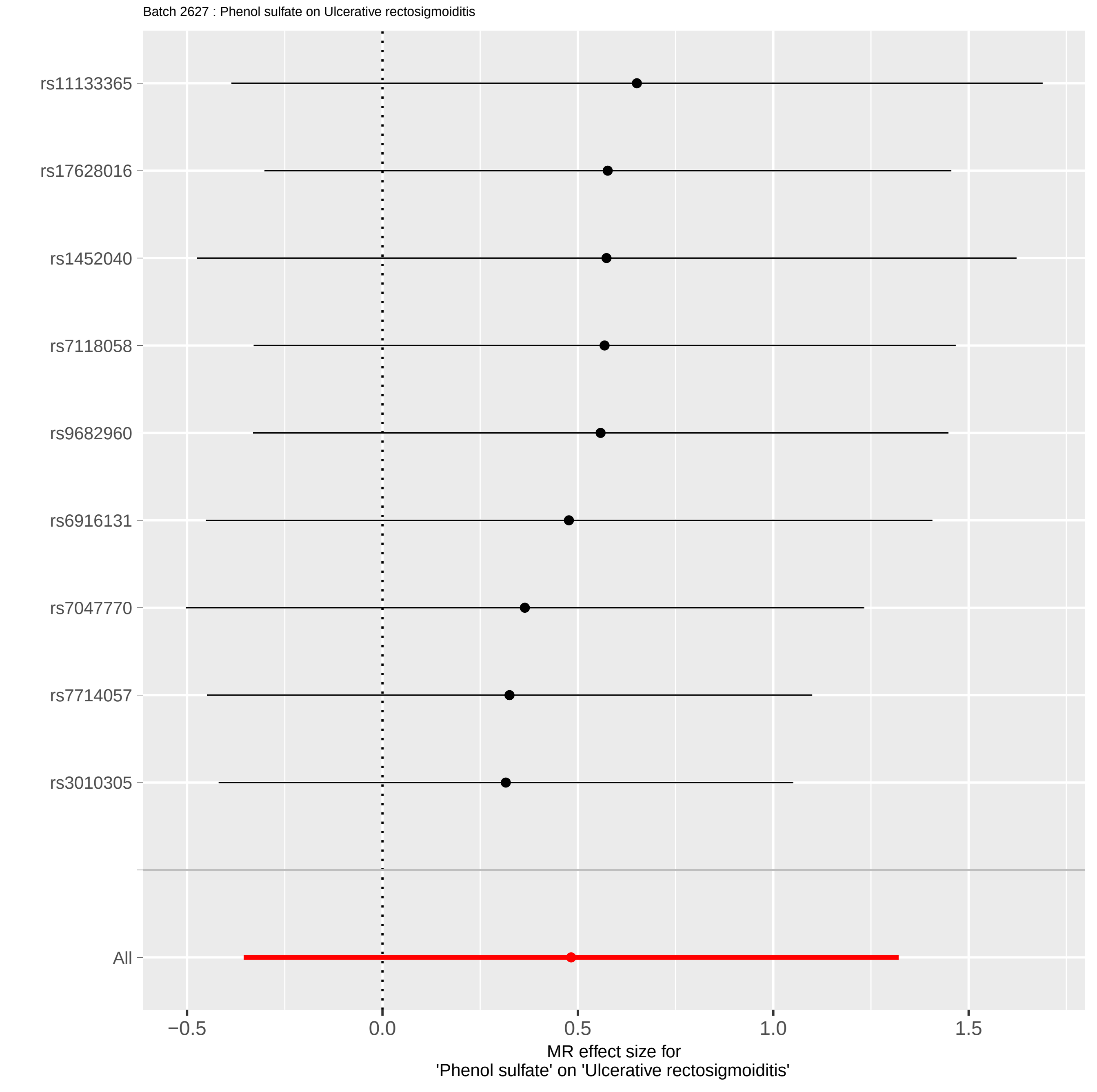

Batch 2627 : Phenol sulfate on Ulcerative rectosigmoiditis
rs11133365
rs17628016
rs1452040
rs7118058
rs9682960
rs6916131
rs7047770
rs7714057
rs3010305
All
−0.5
0.0
0.5
1.0
1.5
MR effect size for
'Phenol sulfate' on 'Ulcerative rectosigmoiditis'

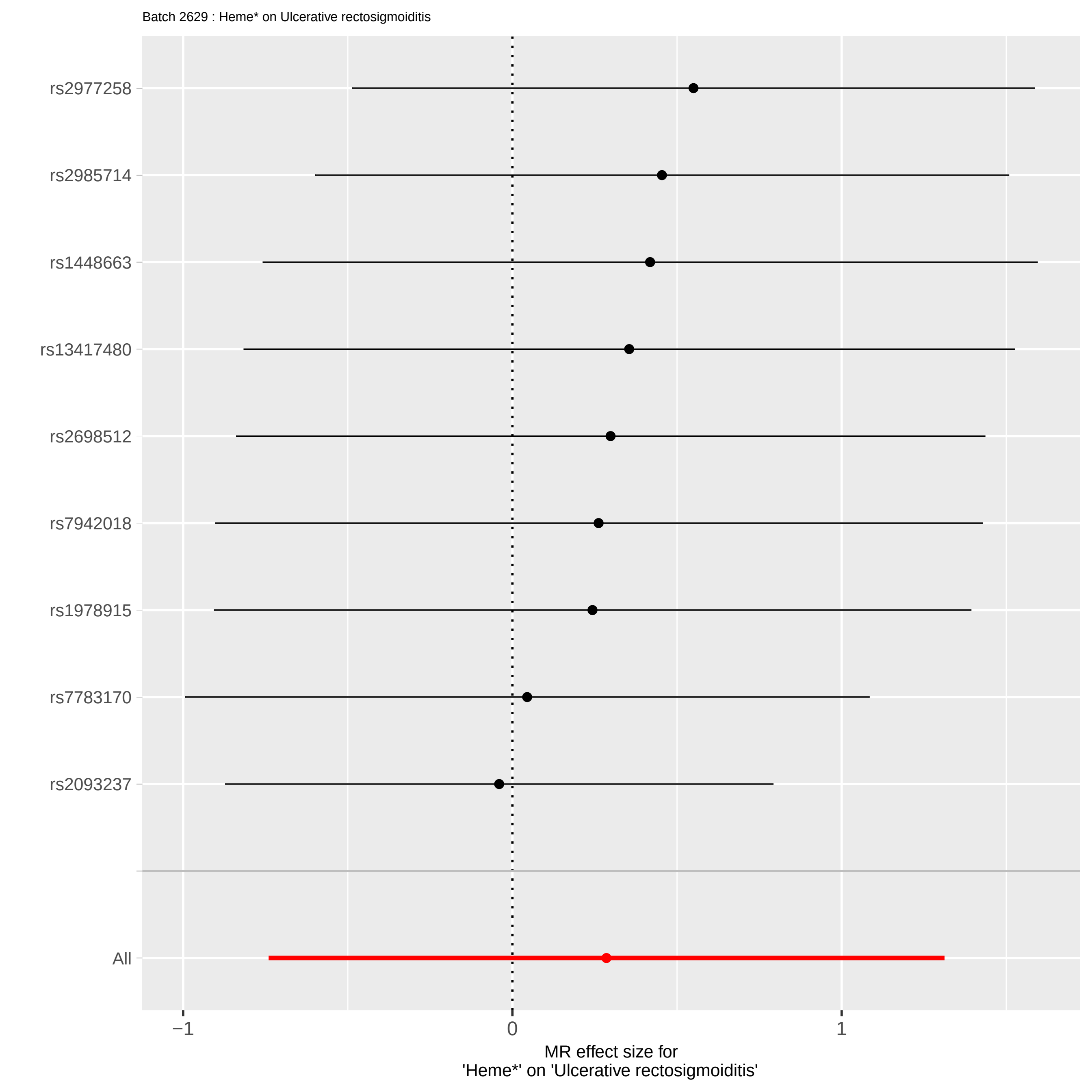

Batch 2629 : Heme* on Ulcerative rectosigmoiditis
rs2977258
rs2985714
rs1448663
rs13417480
rs2698512
rs7942018
rs1978915
rs7783170
rs2093237
All
−1
0
1
MR effect size for
'Heme*' on 'Ulcerative rectosigmoiditis'

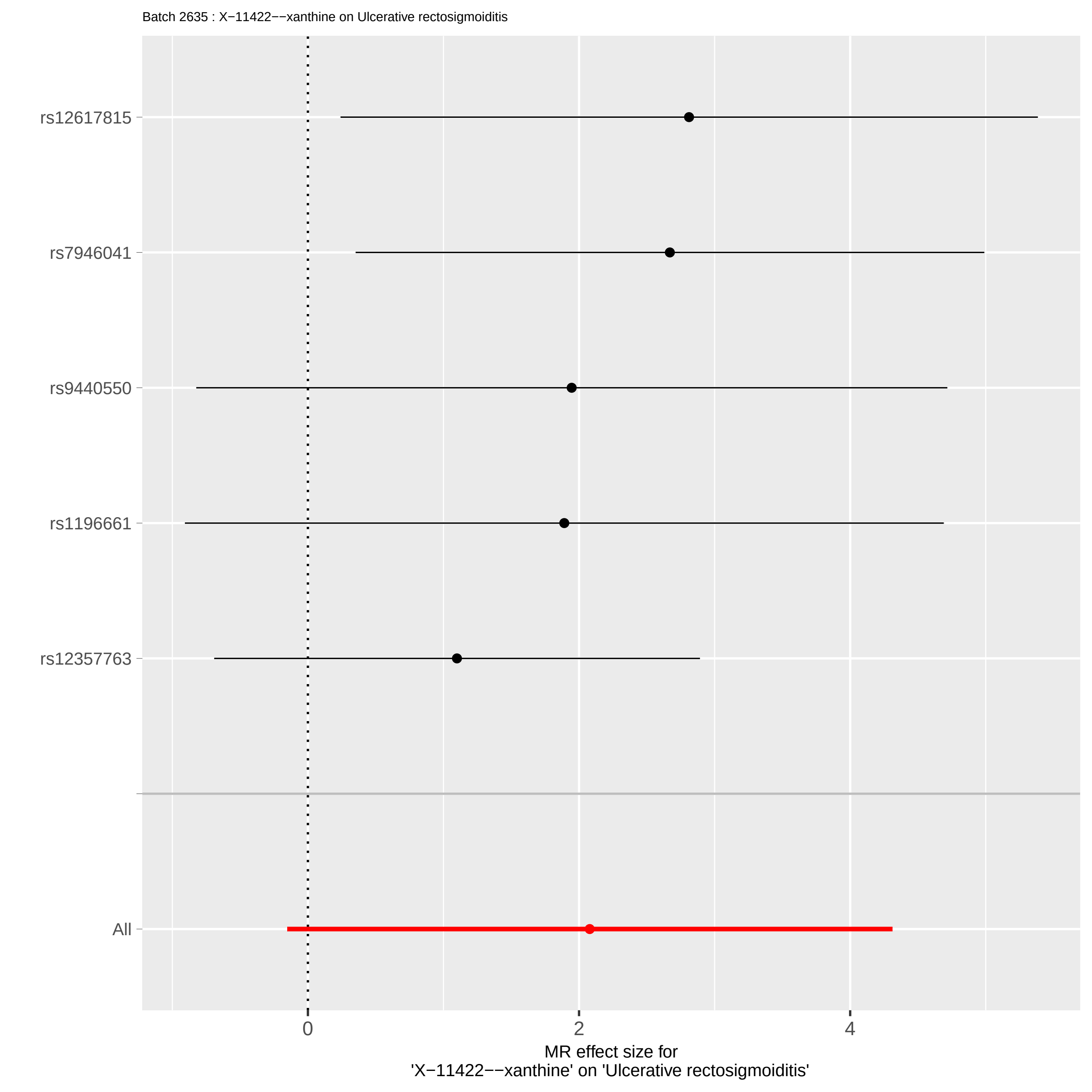

Batch 2635 : X−11422−−xanthine on Ulcerative rectosigmoiditis
rs12617815
rs7946041
rs9440550
rs1196661
rs12357763
All
0
2
4
MR effect size for
'X−11422−−xanthine' on 'Ulcerative rectosigmoiditis'

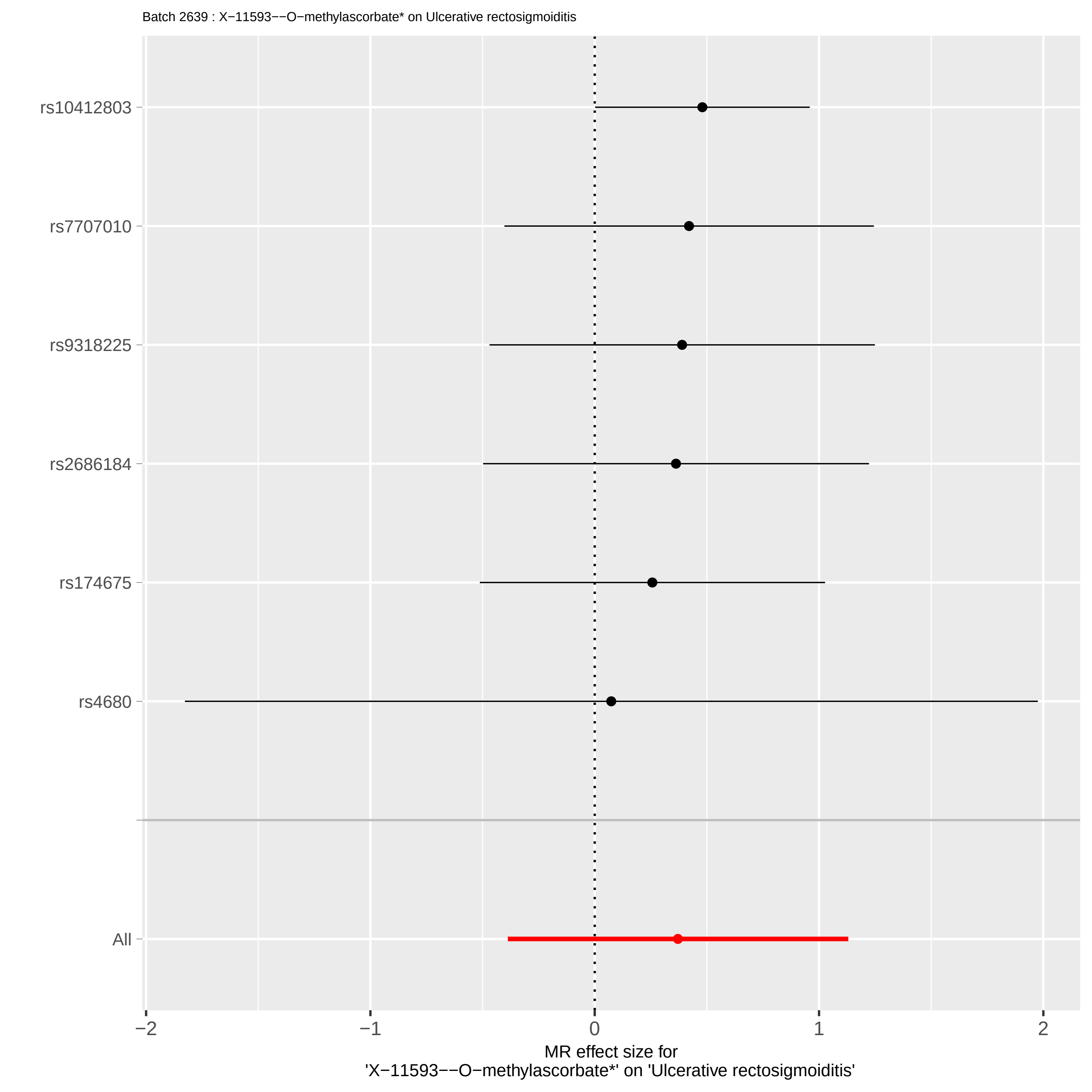
Batch 2639 : X−11593−−O−methylascorbate* on Ulcerative rectosigmoiditis
rs10412803
rs7707010
rs9318225
rs2686184
rs174675
rs4680
All
−2
−1
0
1
2
MR effect size for
'X−11593−−O−methylascorbate*' on 'Ulcerative rectosigmoiditis'

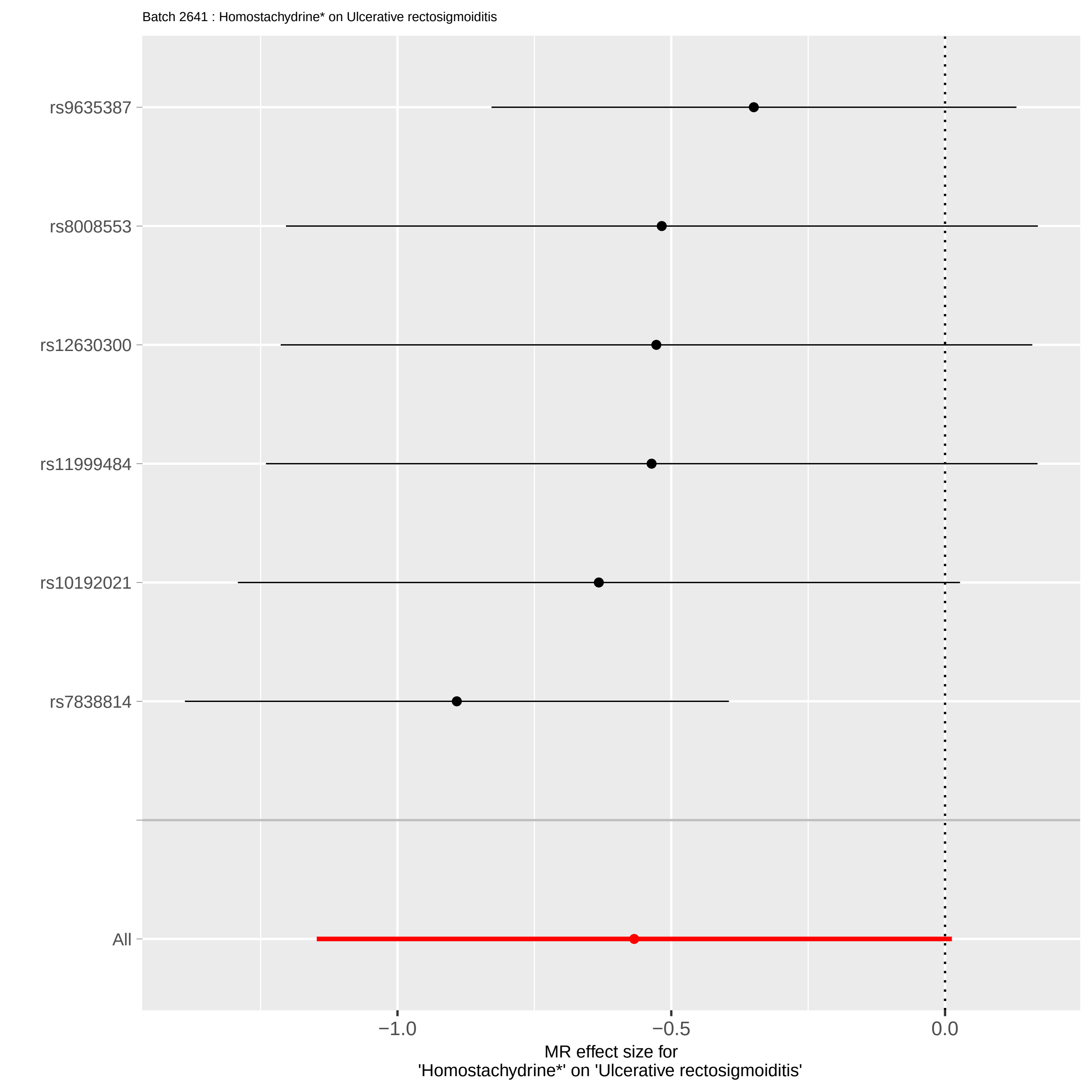

Batch 2641 : Homostachydrine* on Ulcerative rectosigmoiditis
rs9635387
rs8008553
rs12630300
rs11999484
rs10192021
rs7838814
All
−1.0
−0.5
0.0
MR effect size for
'Homostachydrine*' on 'Ulcerative rectosigmoiditis'

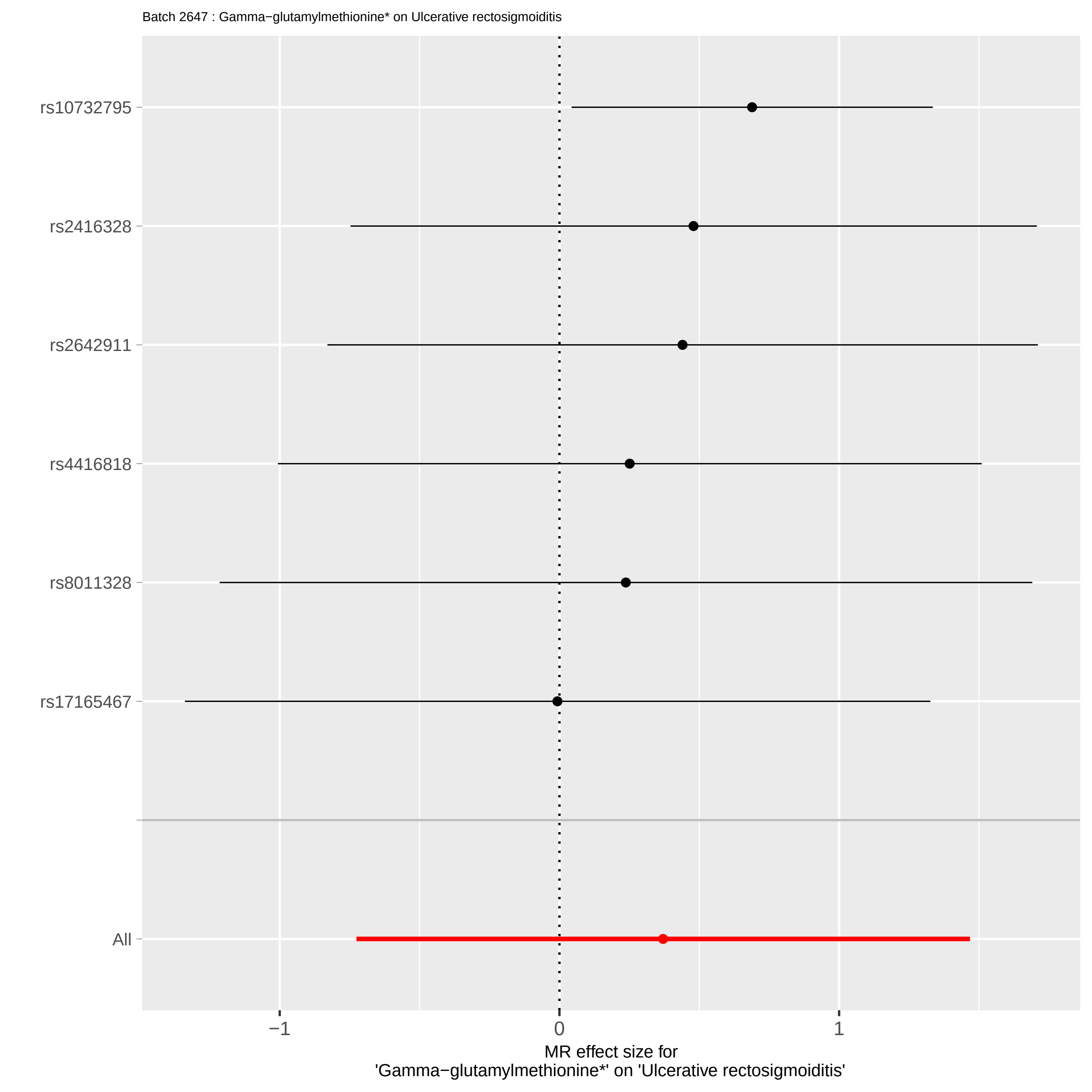
Batch 2647 : Gamma−glutamylmethionine* on Ulcerative rectosigmoiditis
rs10732795
rs2416328
rs2642911
rs4416818
rs8011328
rs17165467
All
−1
0
1
MR effect size for
'Gamma−glutamylmethionine*' on 'Ulcerative rectosigmoiditis'

Batch 2648 : Gamma−glutamylthreonine* on Ulcerative rectosigmoiditis

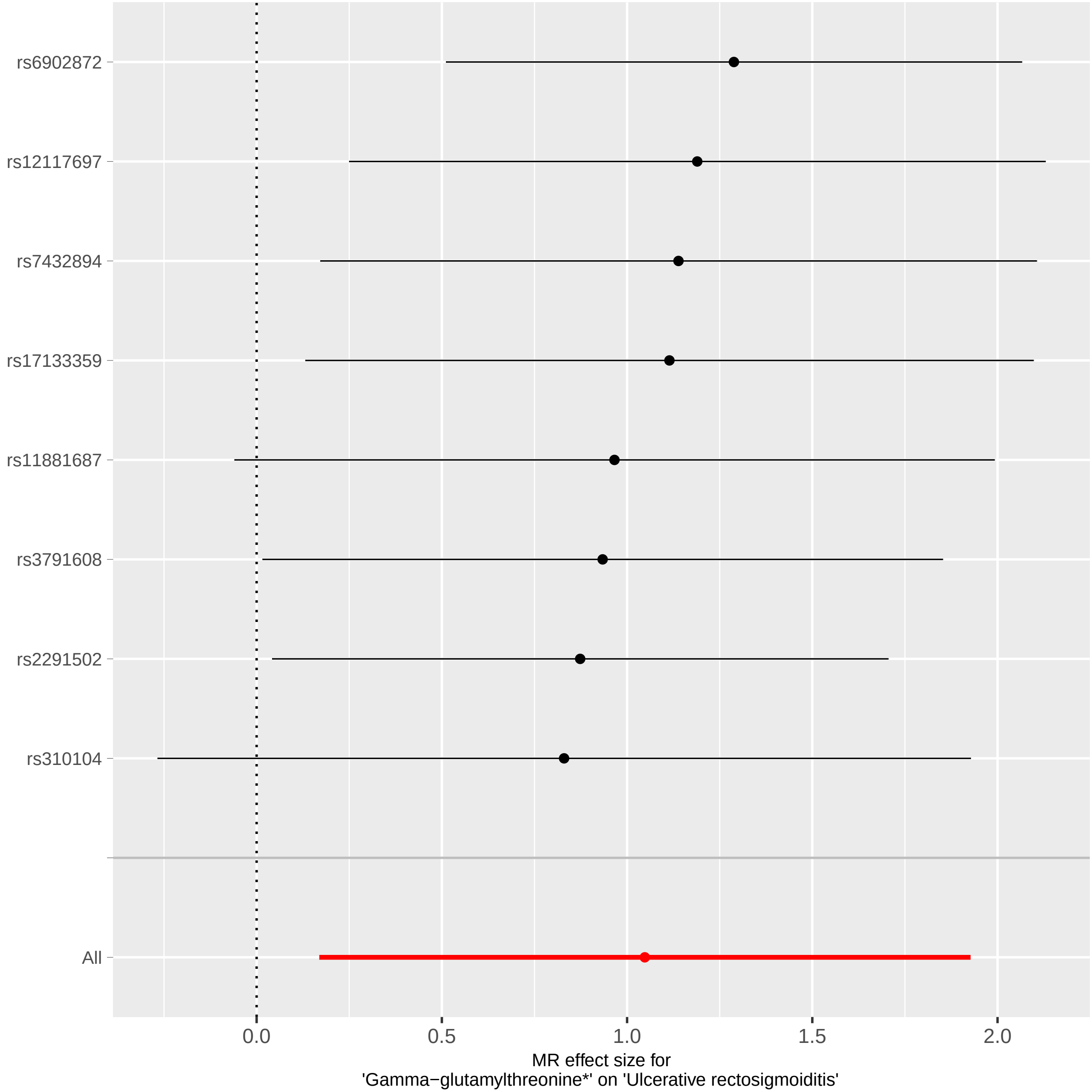

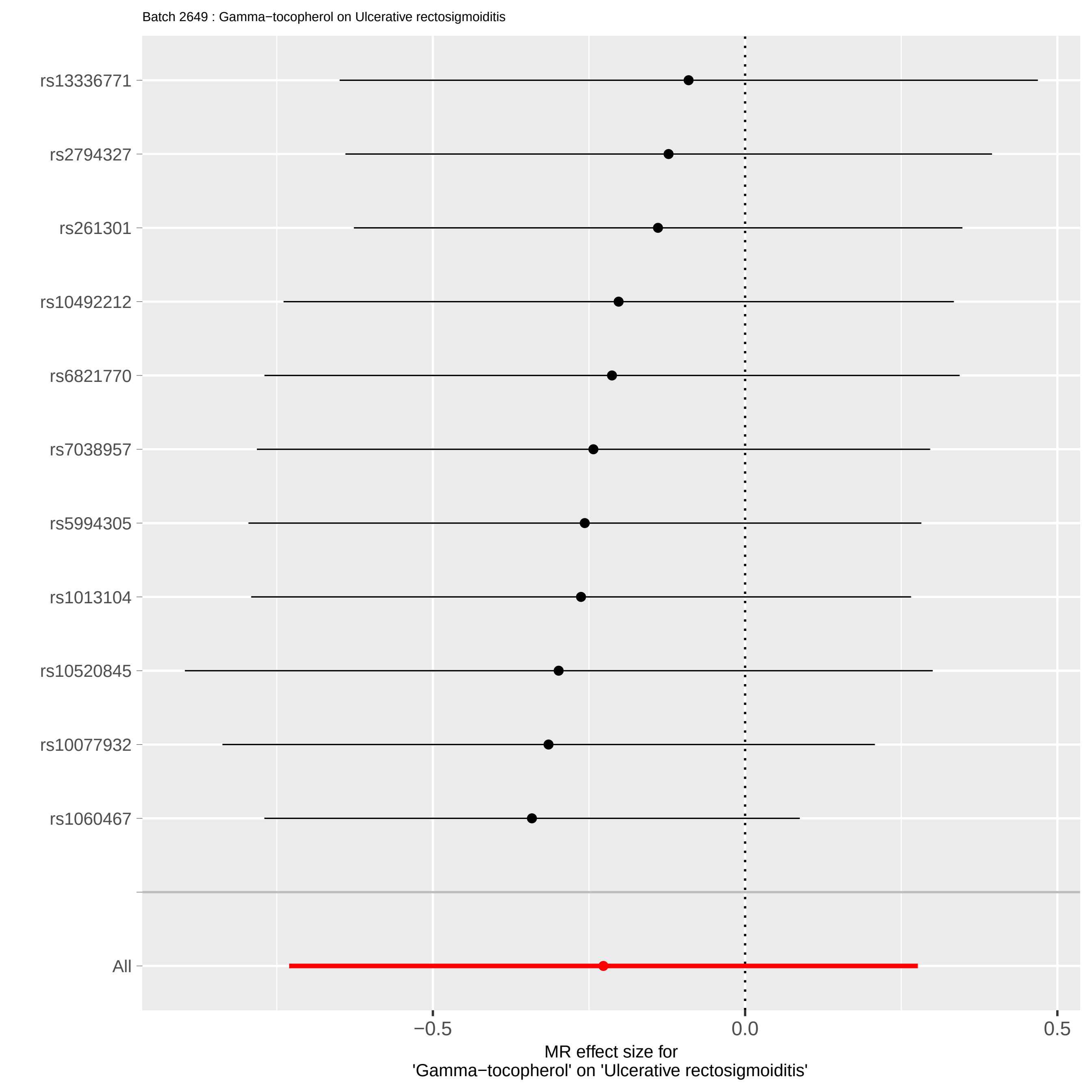

Batch 2649 : Gamma−tocopherol on Ulcerative rectosigmoiditis
rs13336771
rs2794327
rs261301
rs10492212
rs6821770
rs7038957
rs5994305
rs1013104
rs10520845
rs10077932
rs1060467
All
−0.5
0.0
0.5
MR effect size for
'Gamma−tocopherol' on 'Ulcerative rectosigmoiditis'

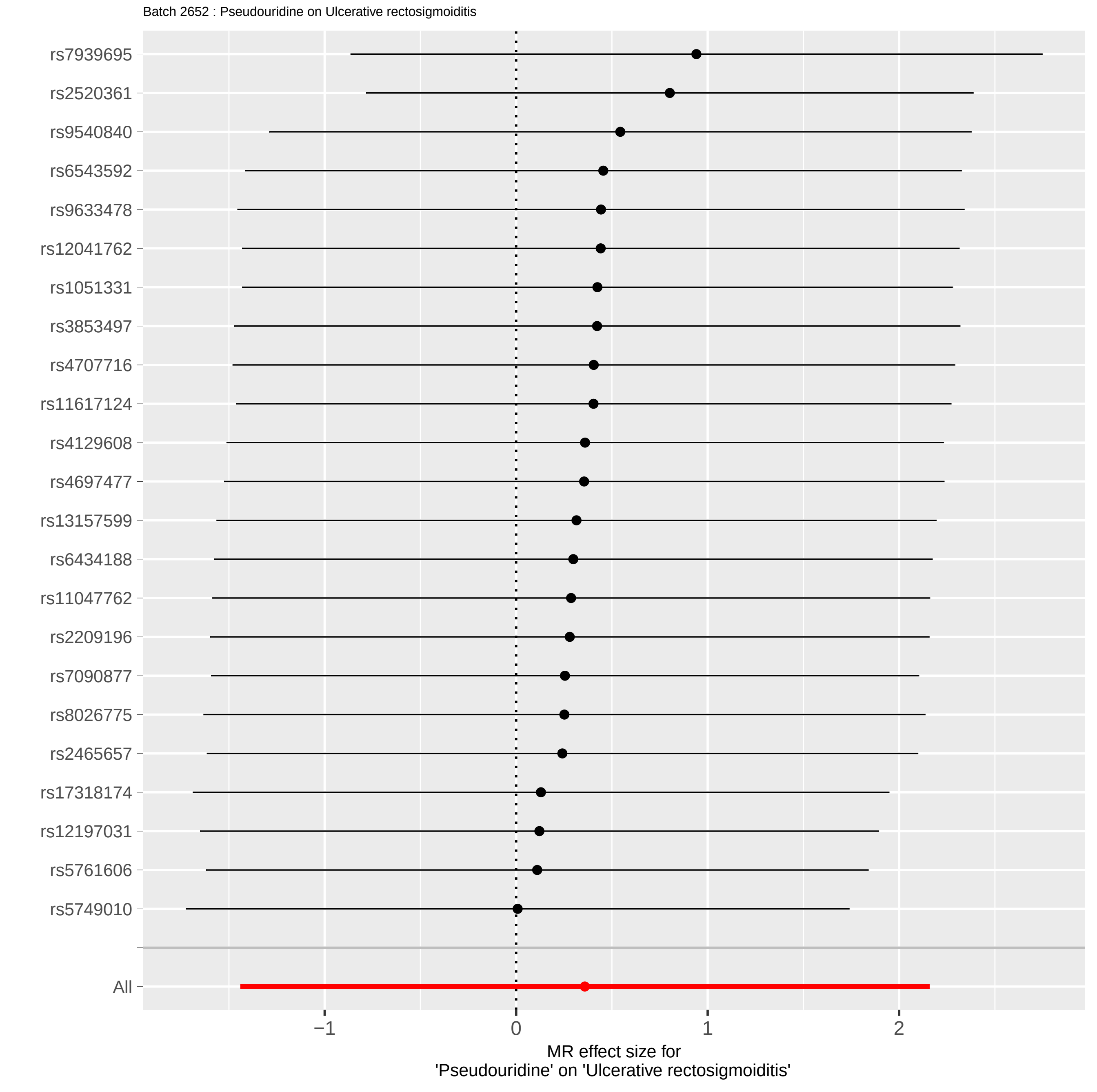

Batch 2652 : Pseudouridine on Ulcerative rectosigmoiditis
rs7939695
rs2520361
rs9540840
rs6543592
rs9633478
rs12041762
rs1051331
rs3853497
rs4707716
rs11617124
rs4129608
rs4697477
rs13157599
rs6434188
rs11047762
rs2209196
rs7090877
rs8026775
rs2465657
rs17318174
rs12197031
rs5761606
rs5749010
All
−1
0
1
2
MR effect size for
'Pseudouridine' on 'Ulcerative rectosigmoiditis'

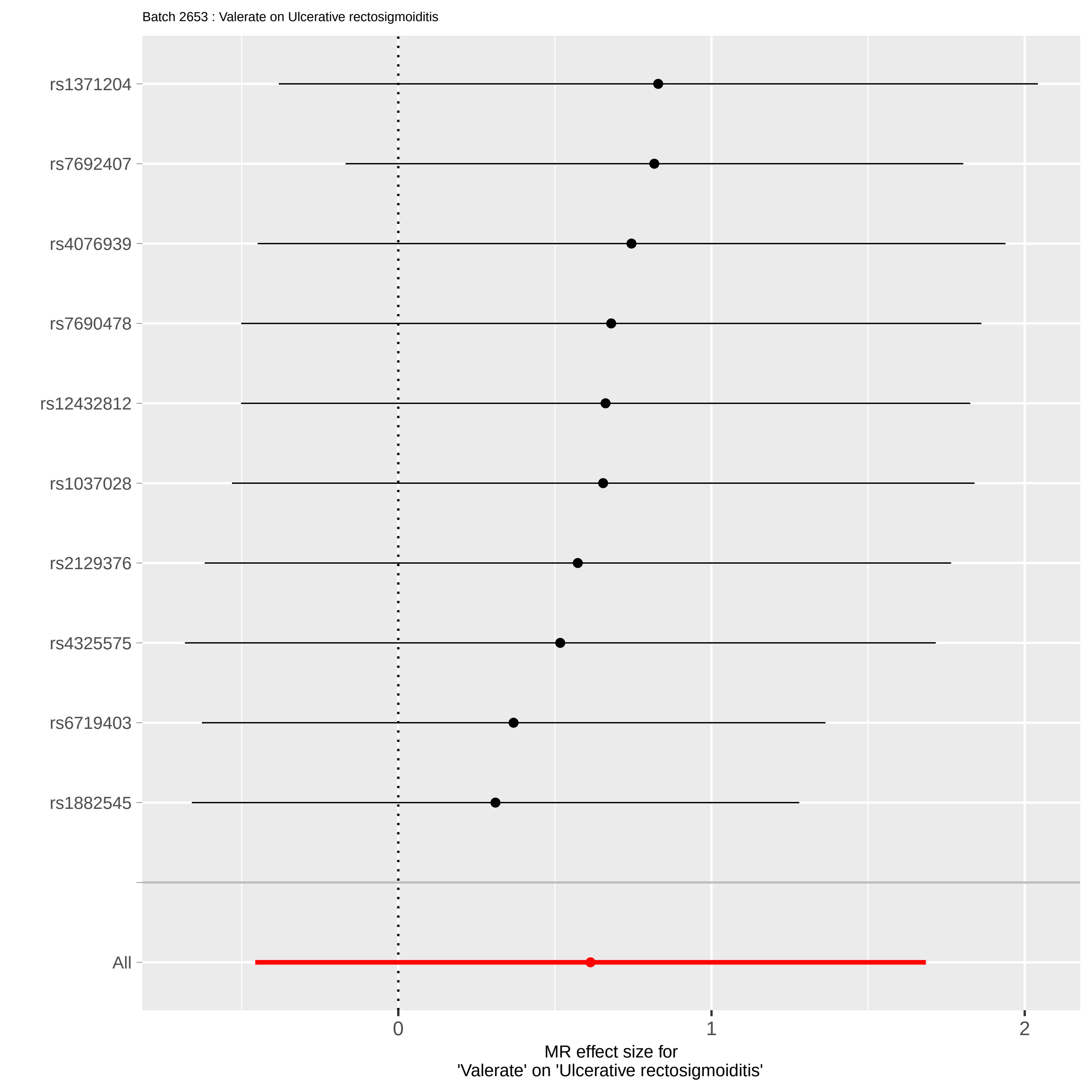

Batch 2653 : Valerate on Ulcerative rectosigmoiditis
rs1371204
rs7692407
rs4076939
rs7690478
rs12432812
rs1037028
rs2129376
rs4325575
rs6719403
rs1882545
All
0
1
2
MR effect size for
'Valerate' on 'Ulcerative rectosigmoiditis'

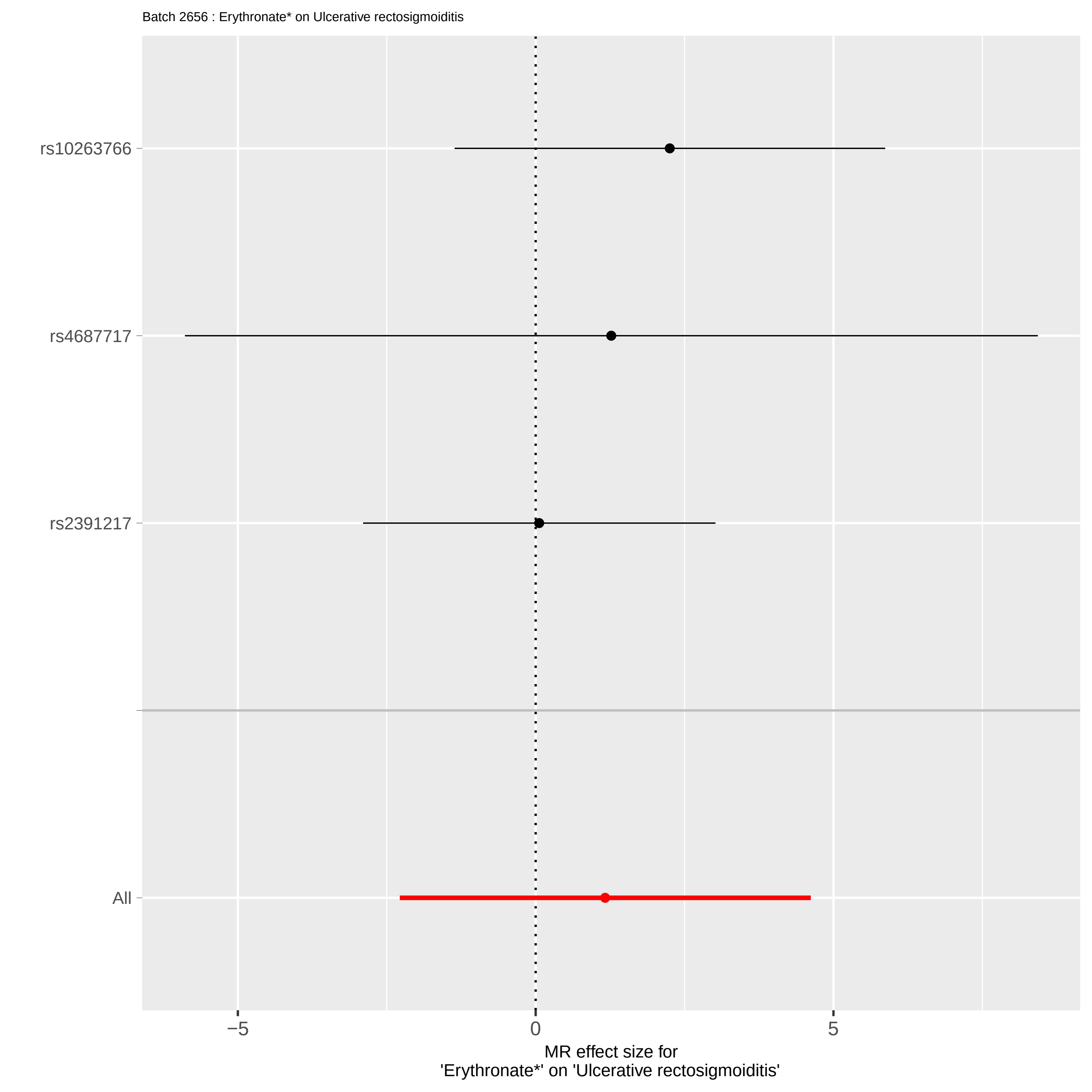
Batch 2656 : Erythronate* on Ulcerative rectosigmoiditis
rs10263766
rs4687717
rs2391217
All
−5
0
5
MR effect size for
'Erythronate*' on 'Ulcerative rectosigmoiditis'

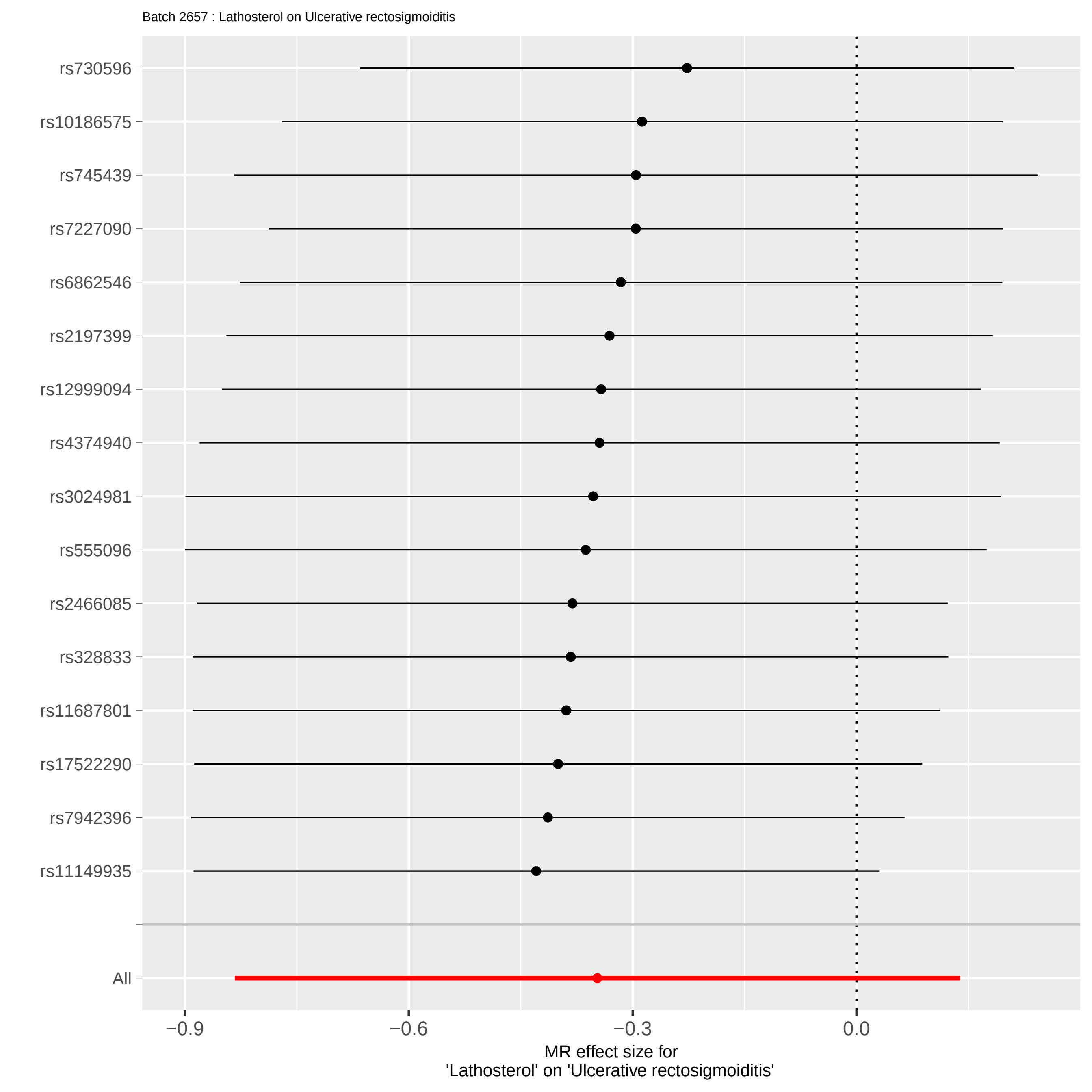
Batch 2657 : Lathosterol on Ulcerative rectosigmoiditis
rs730596
rs10186575
rs745439
rs7227090
rs6862546
rs2197399
rs12999094
rs4374940
rs3024981
rs555096
rs2466085
rs328833
rs11687801
rs17522290
rs7942396
rs11149935
All
−0.9
−0.6
−0.3
0.0
MR effect size for
'Lathosterol' on 'Ulcerative rectosigmoiditis'

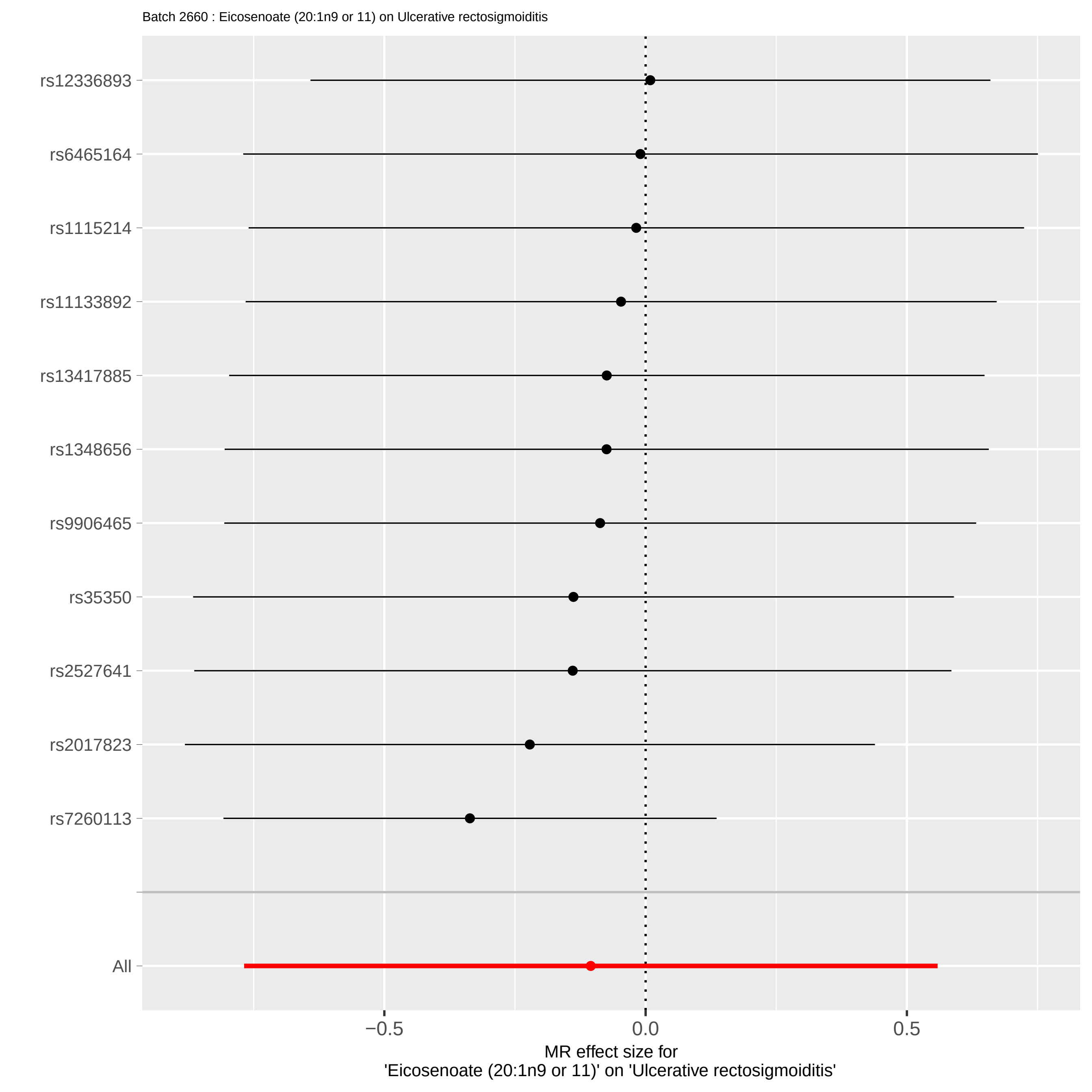

Batch 2660 : Eicosenoate (20:1n9 or 11) on Ulcerative rectosigmoiditis
rs12336893
rs6465164
rs1115214
rs11133892
rs13417885
rs1348656
rs9906465
rs35350
rs2527641
rs2017823
rs7260113
All
−0.5
0.0
0.5
MR effect size for
'Eicosenoate (20:1n9 or 11)' on 'Ulcerative rectosigmoiditis'

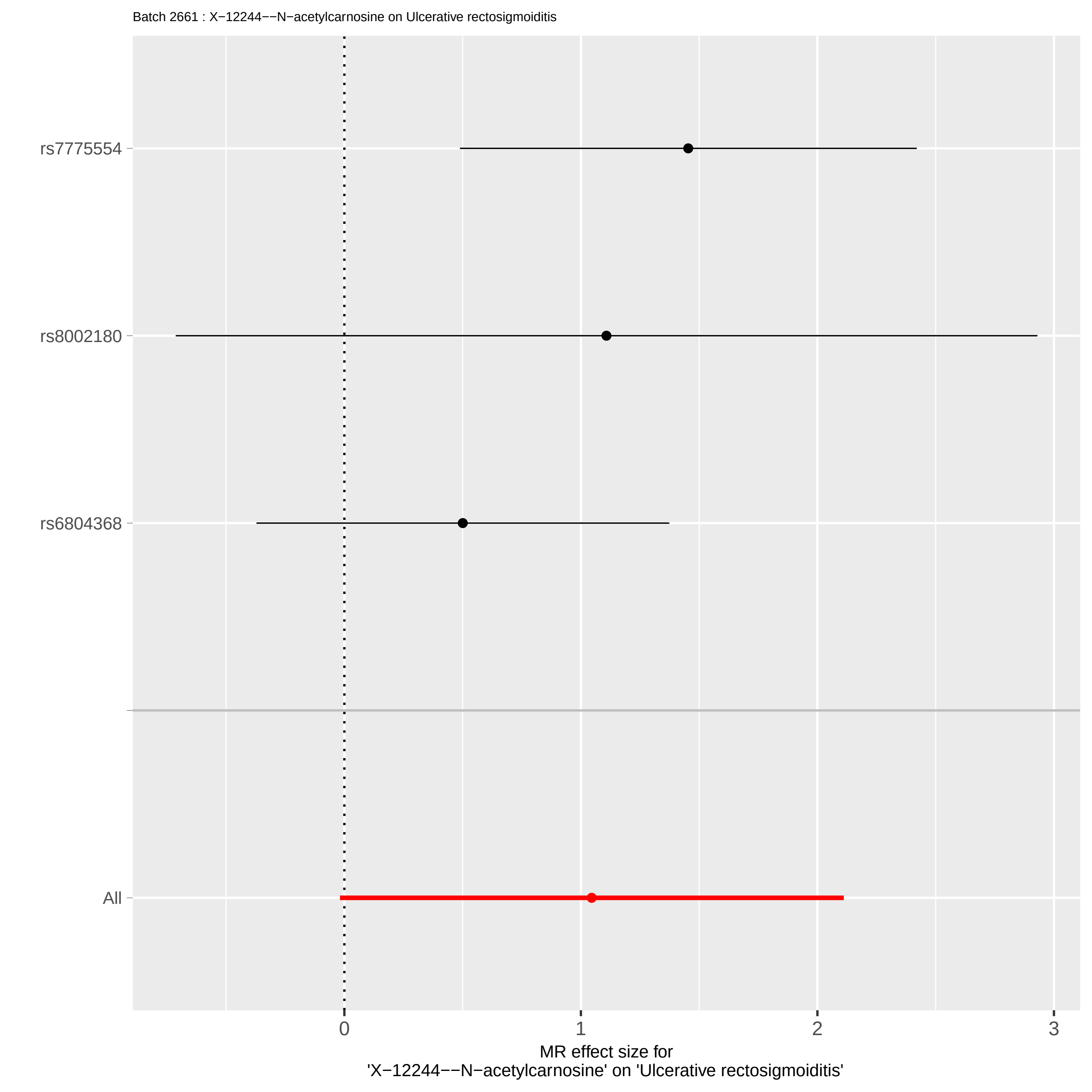

Batch 2661 : X−12244−−N−acetylcarnosine on Ulcerative rectosigmoiditis
rs7775554
rs8002180
rs6804368
All
0
1
2
3
MR effect size for
'X−12244−−N−acetylcarnosine' on 'Ulcerative rectosigmoiditis'

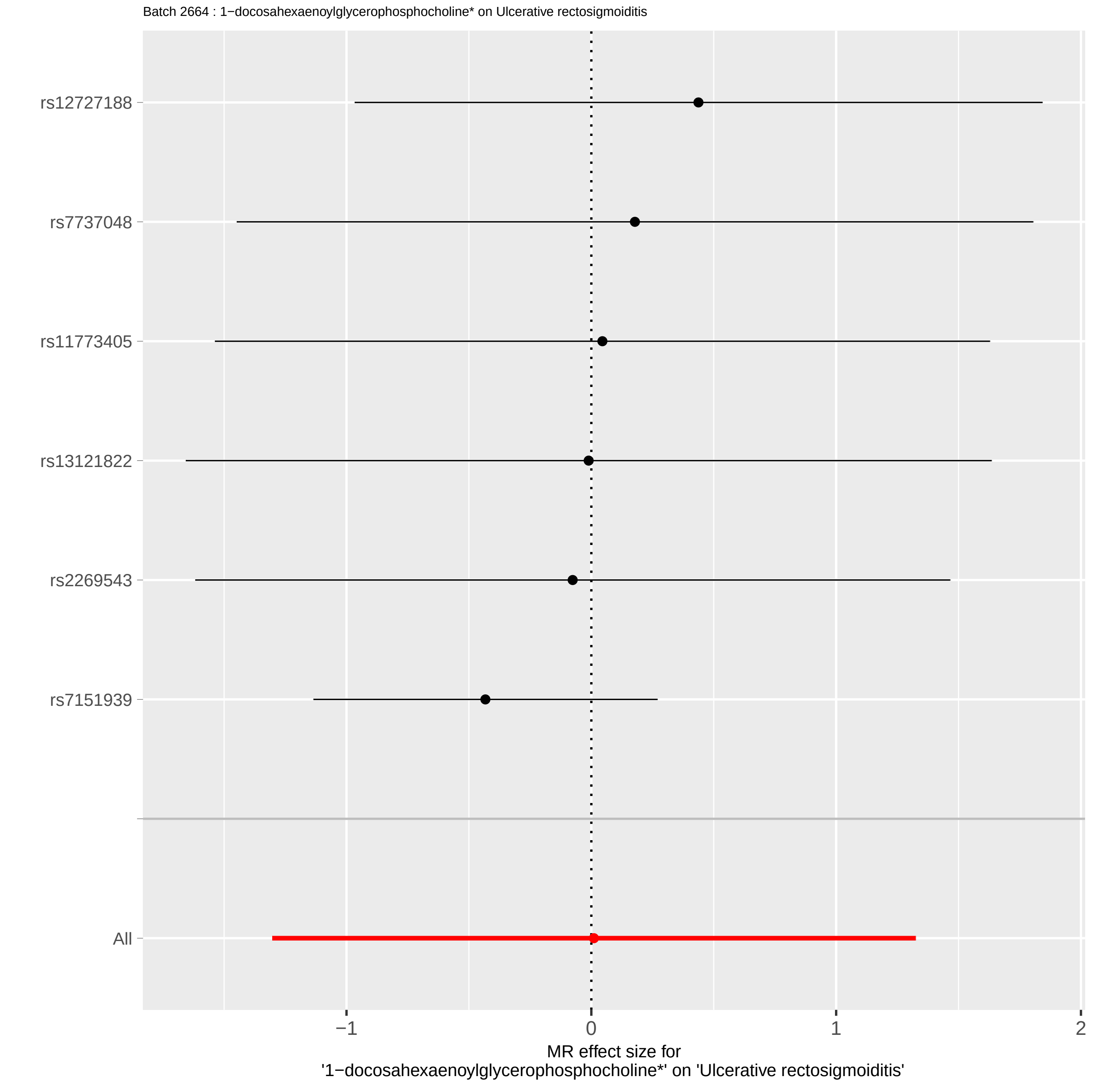

Batch 2664 : 1−docosahexaenoylglycerophosphocholine* on Ulcerative rectosigmoiditis
rs12727188
rs7737048
rs11773405
rs13121822
rs2269543
rs7151939
All
−1
0
1
2
MR effect size for
'1−docosahexaenoylglycerophosphocholine*' on 'Ulcerative rectosigmoiditis'

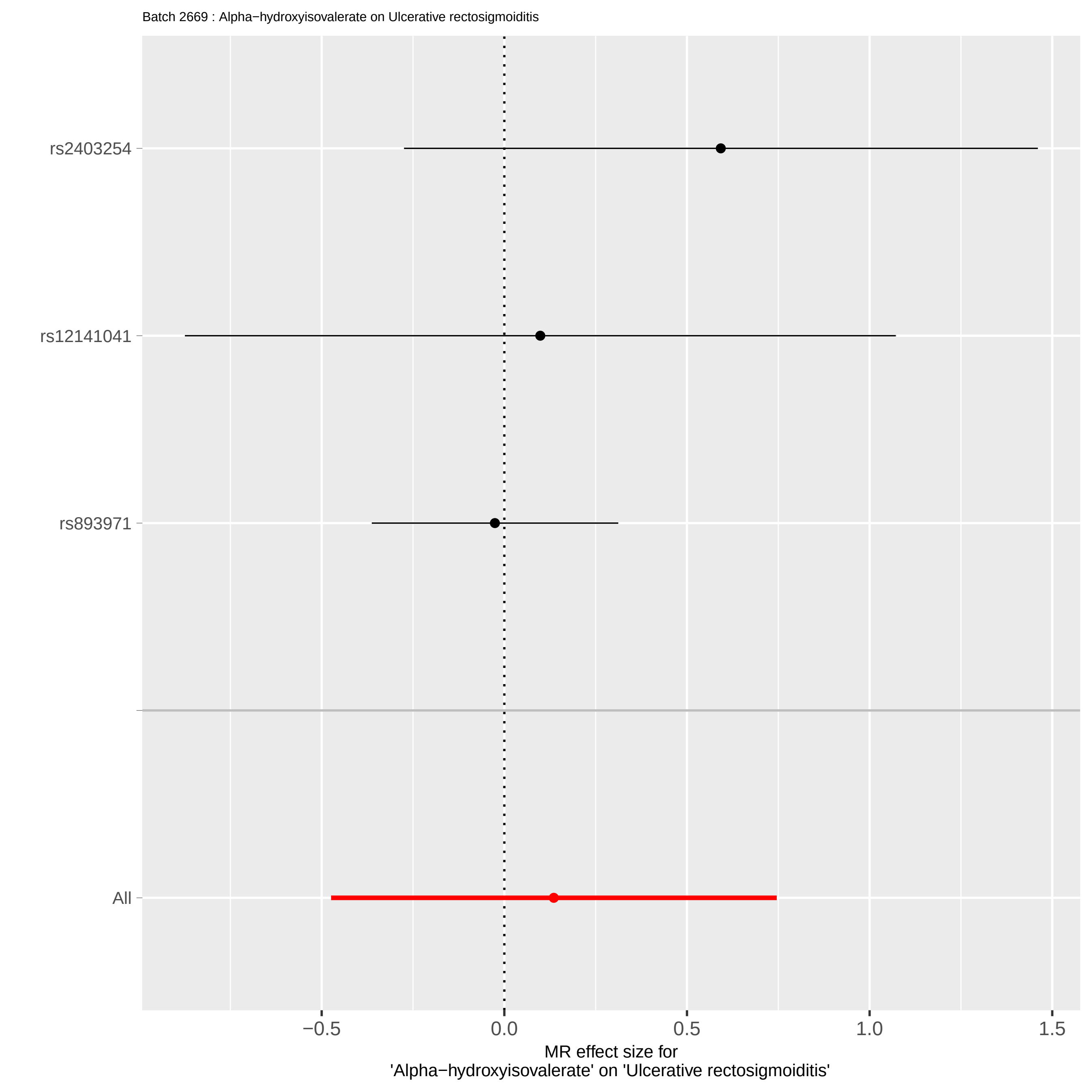
Batch 2669 : Alpha−hydroxyisovalerate on Ulcerative rectosigmoiditis
rs2403254
rs12141041
rs893971
All
−0.5
0.0
0.5
1.0
1.5
MR effect size for
'Alpha−hydroxyisovalerate' on 'Ulcerative rectosigmoiditis'

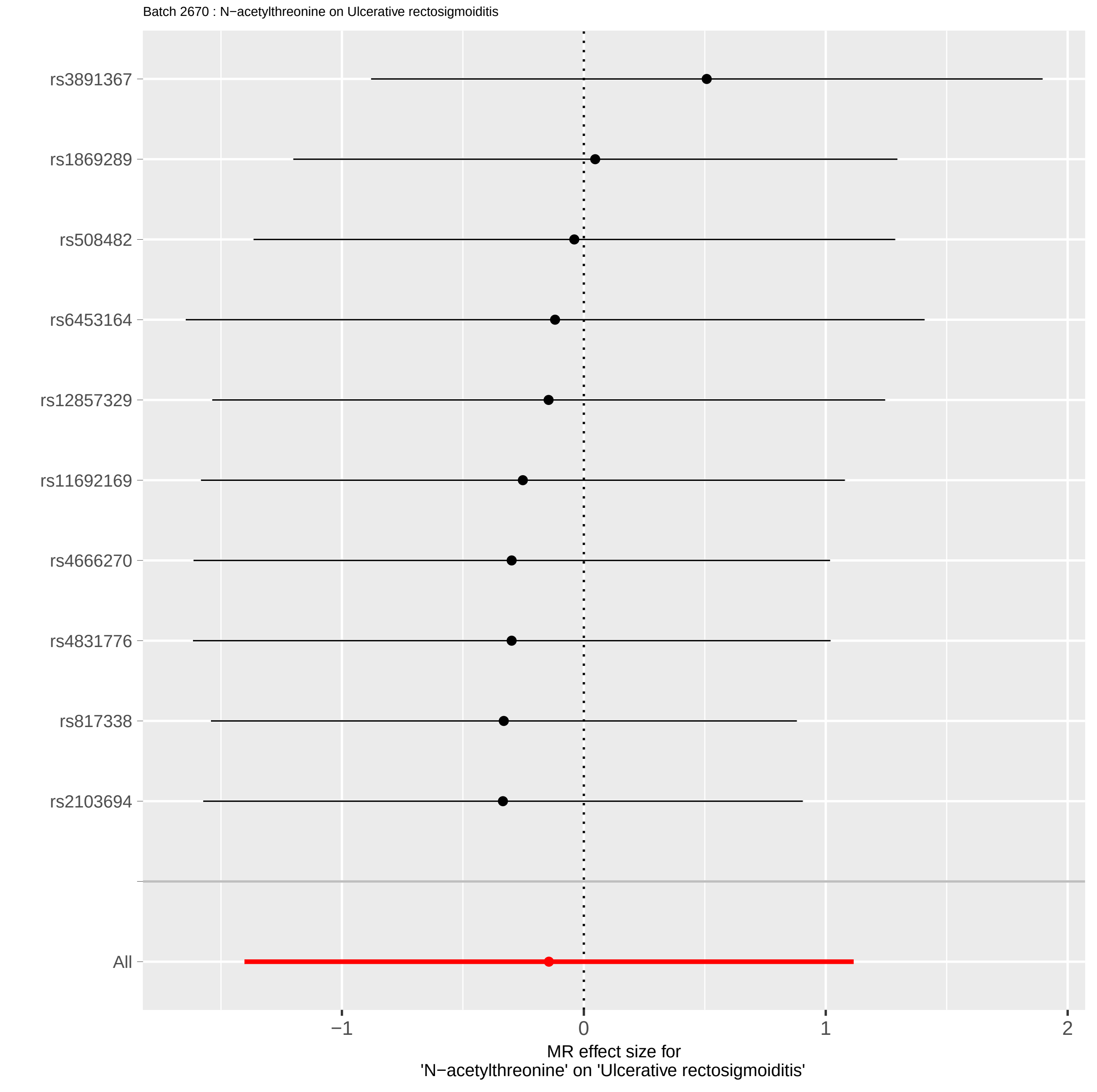

Batch 2670 : N−acetylthreonine on Ulcerative rectosigmoiditis
rs3891367
rs1869289
rs508482
rs6453164
rs12857329
rs11692169
rs4666270
rs4831776
rs817338
rs2103694
All
−1
0
1
2
MR effect size for
'N−acetylthreonine' on 'Ulcerative rectosigmoiditis'

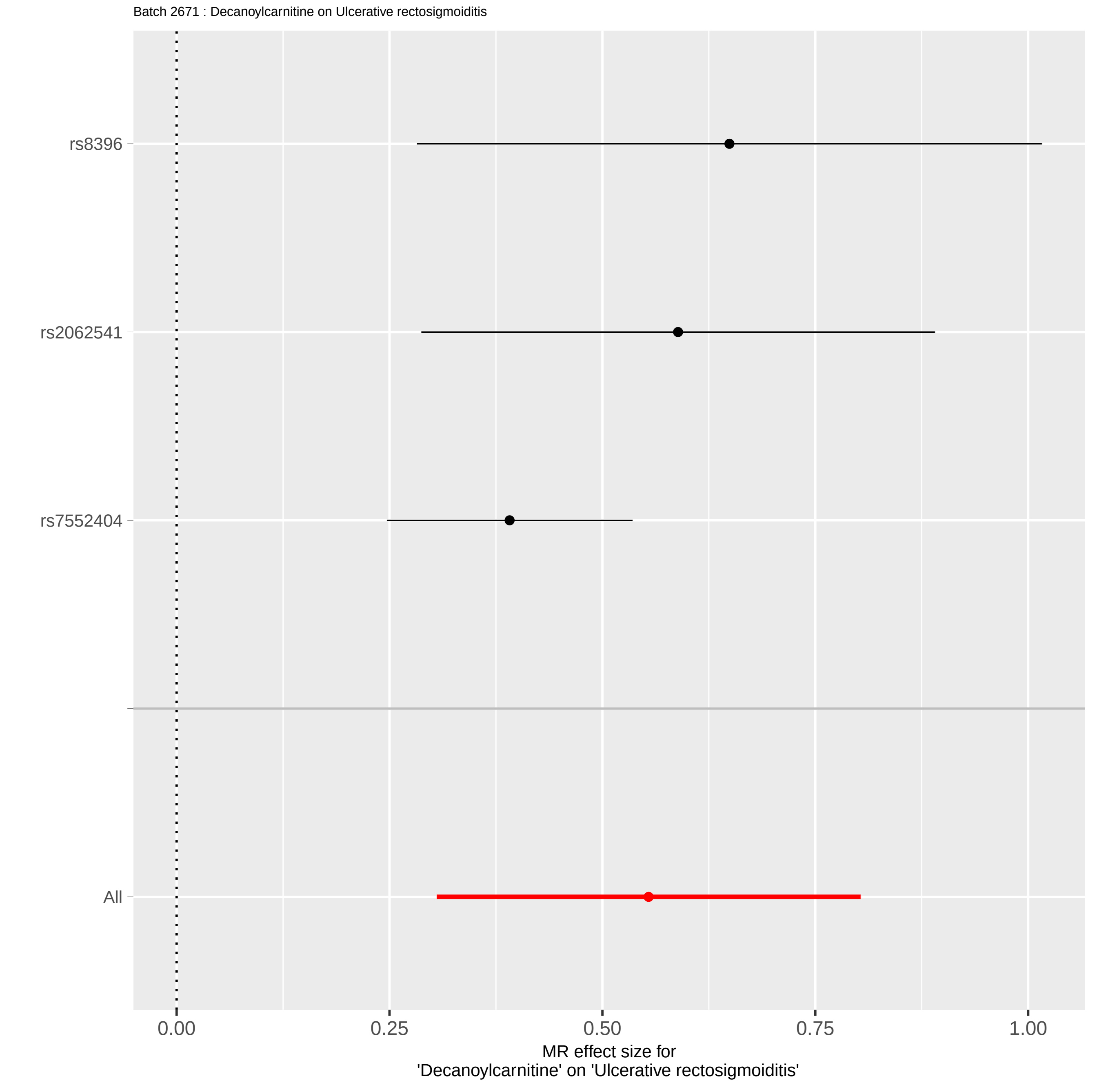

Batch 2671 : Decanoylcarnitine on Ulcerative rectosigmoiditis
rs8396
rs2062541
rs7552404
All
0.00
0.25
0.50
0.75
1.00
MR effect size for
'Decanoylcarnitine' on 'Ulcerative rectosigmoiditis'

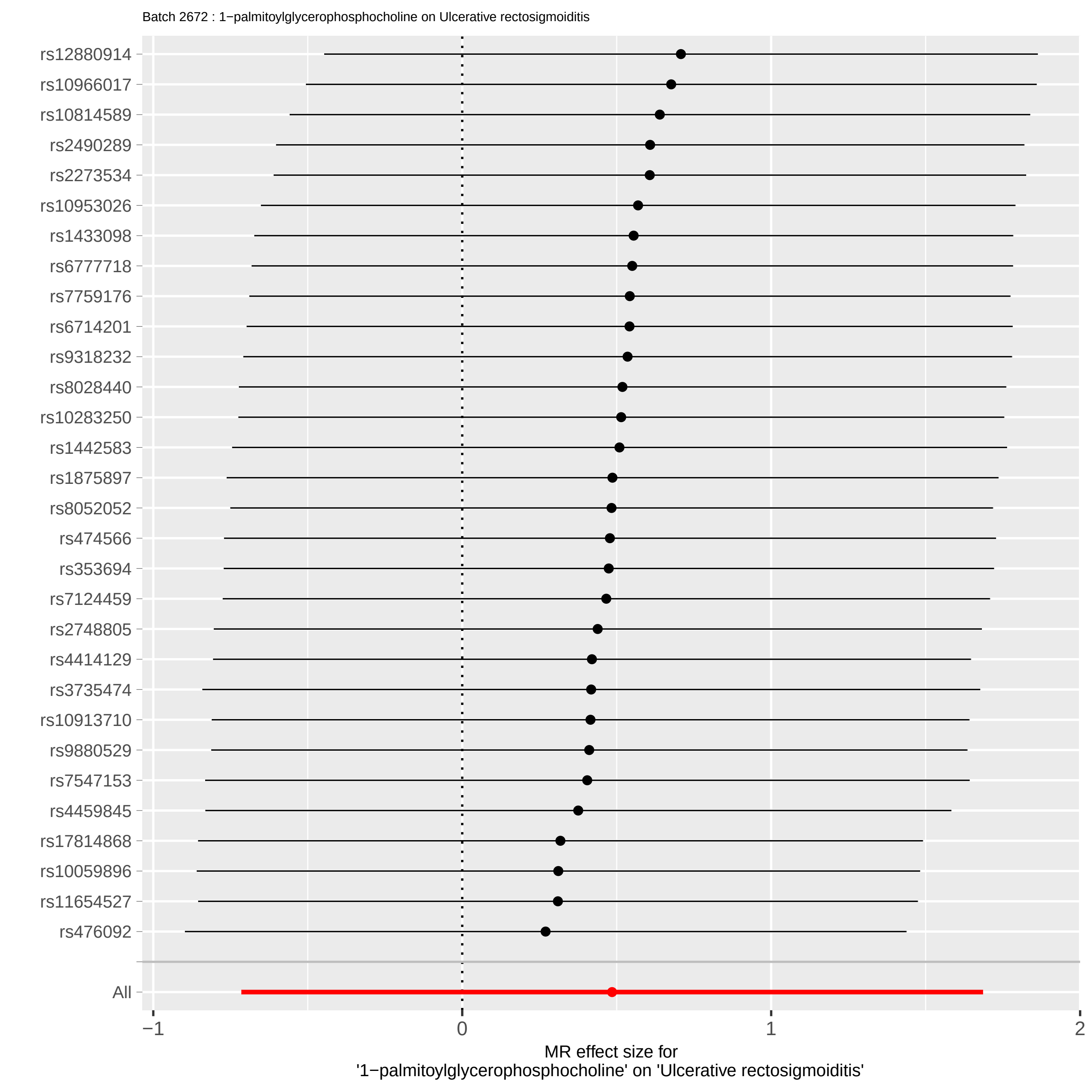

Batch 2672 : 1−palmitoylglycerophosphocholine on Ulcerative rectosigmoiditis
rs12880914
rs10966017
rs10814589
rs2490289
rs2273534
rs10953026
rs1433098
rs6777718
rs7759176
rs6714201
rs9318232
rs8028440
rs10283250
rs1442583
rs1875897
rs8052052
rs474566
rs353694
rs7124459
rs2748805
rs4414129
rs3735474
rs10913710
rs9880529
rs7547153
rs4459845
rs17814868
rs10059896
rs11654527
rs476092
All
−1
0
1
2
MR effect size for
'1−palmitoylglycerophosphocholine' on 'Ulcerative rectosigmoiditis'

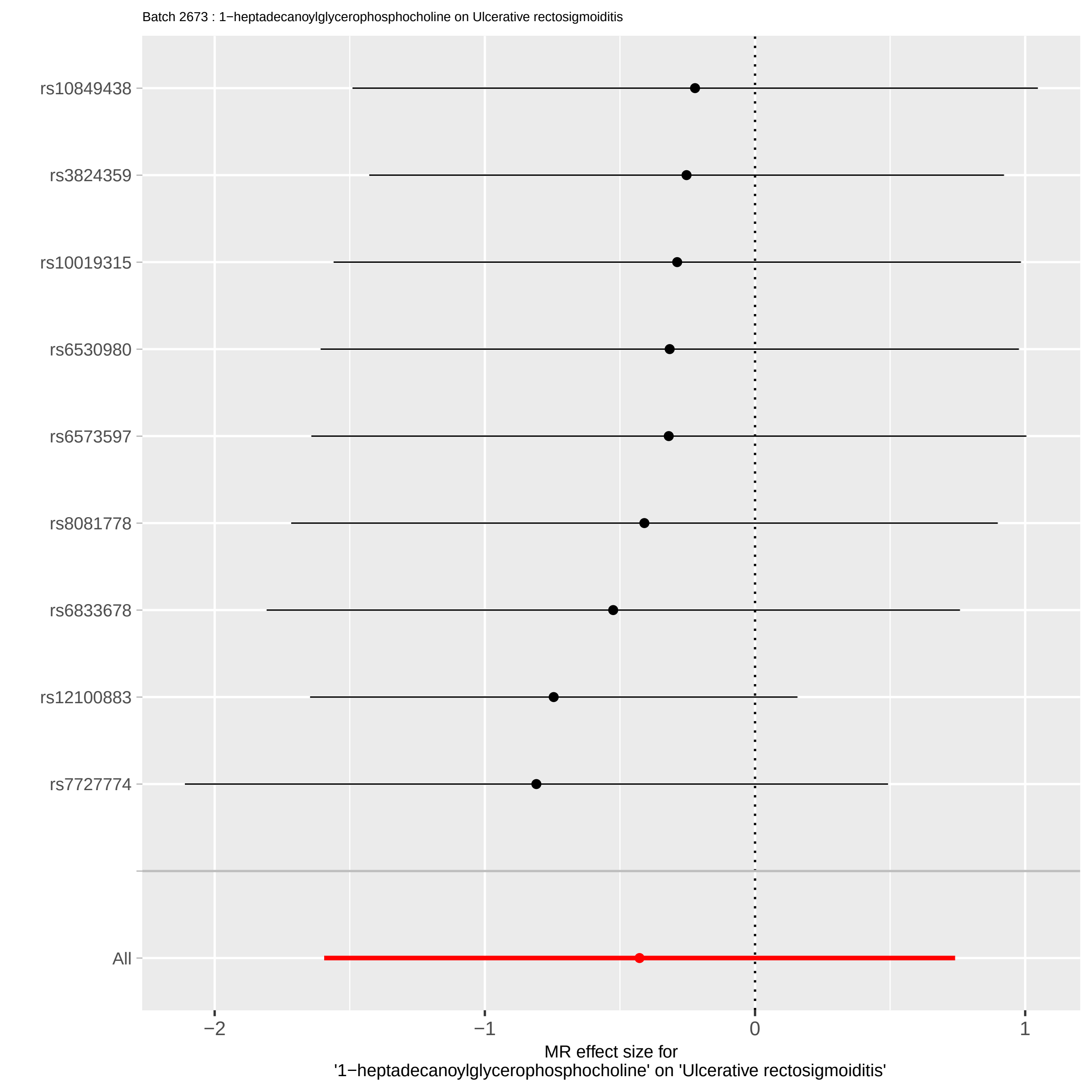

Batch 2673 : 1−heptadecanoylglycerophosphocholine on Ulcerative rectosigmoiditis
rs10849438
rs3824359
rs10019315
rs6530980
rs6573597
rs8081778
rs6833678
rs12100883
rs7727774
All
−2
−1
0
1
MR effect size for
'1−heptadecanoylglycerophosphocholine' on 'Ulcerative rectosigmoiditis'

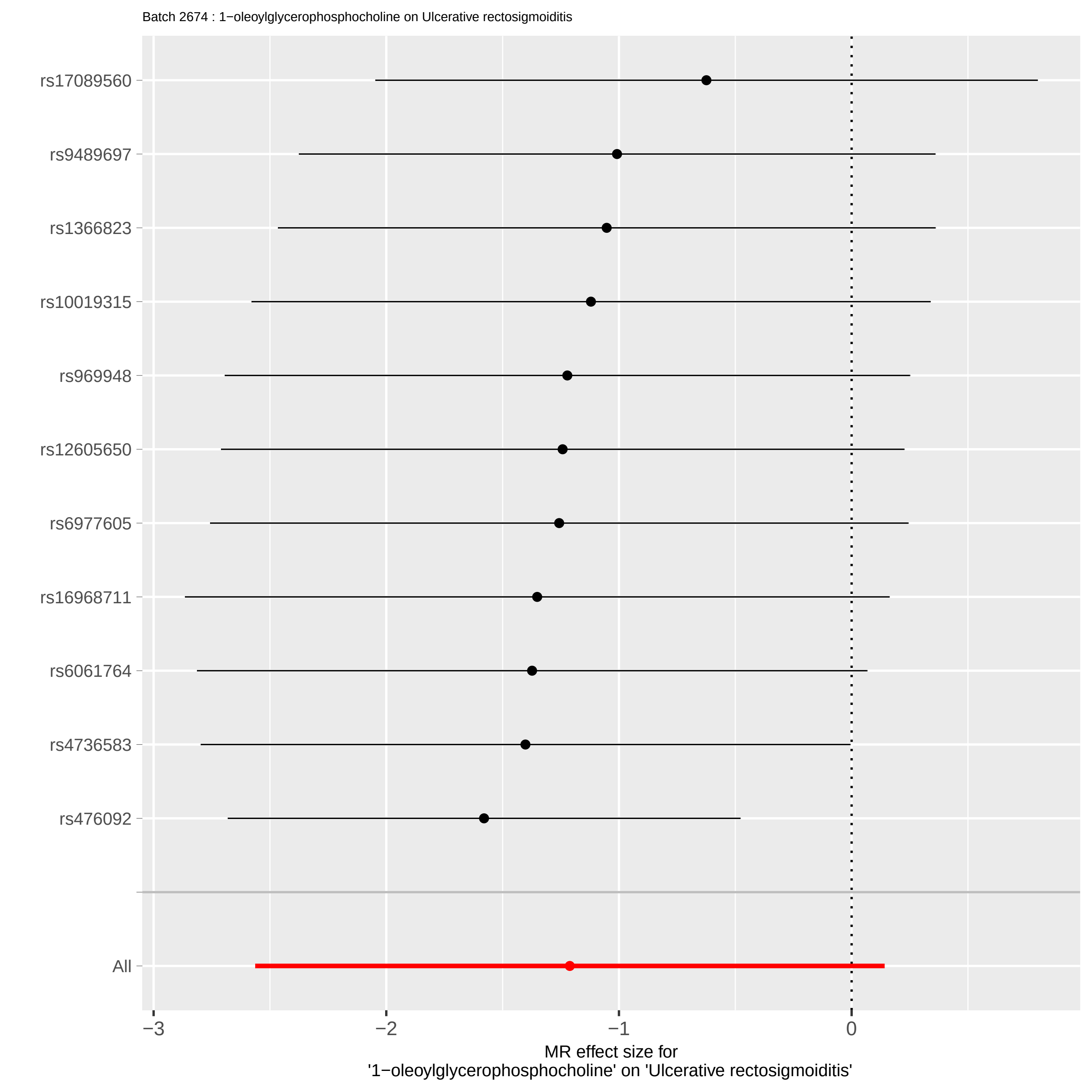

Batch 2674 : 1−oleoylglycerophosphocholine on Ulcerative rectosigmoiditis
rs17089560
rs9489697
rs1366823
rs10019315
rs969948
rs12605650
rs6977605
rs16968711
rs6061764
rs4736583
rs476092
All
−3
−2
−1
0
MR effect size for
'1−oleoylglycerophosphocholine' on 'Ulcerative rectosigmoiditis'

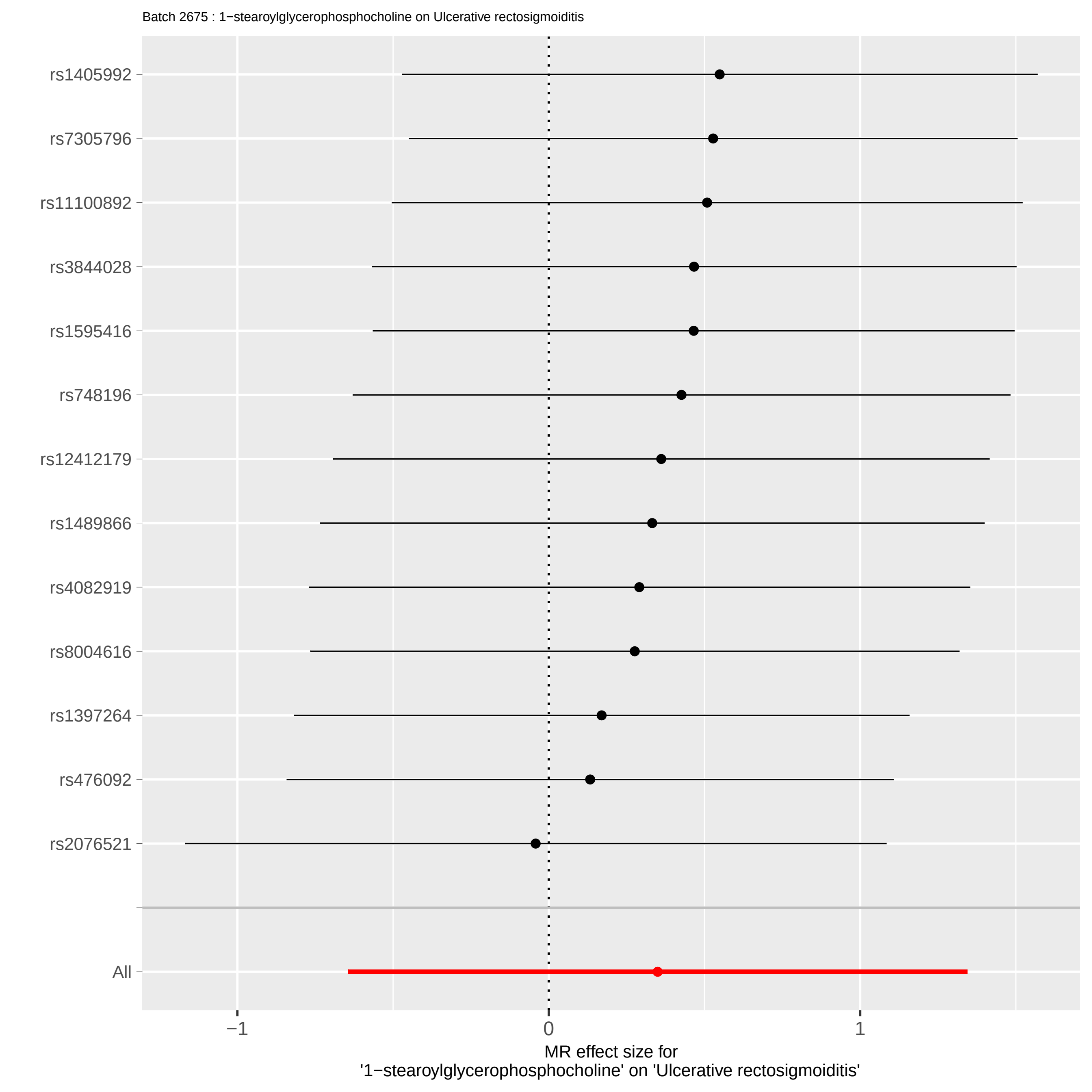

Batch 2675 : 1−stearoylglycerophosphocholine on Ulcerative rectosigmoiditis
rs1405992
rs7305796
rs11100892
rs3844028
rs1595416
rs748196
rs12412179
rs1489866
rs4082919
rs8004616
rs1397264
rs476092
rs2076521
All
−1
0
1
MR effect size for
'1−stearoylglycerophosphocholine' on 'Ulcerative rectosigmoiditis'

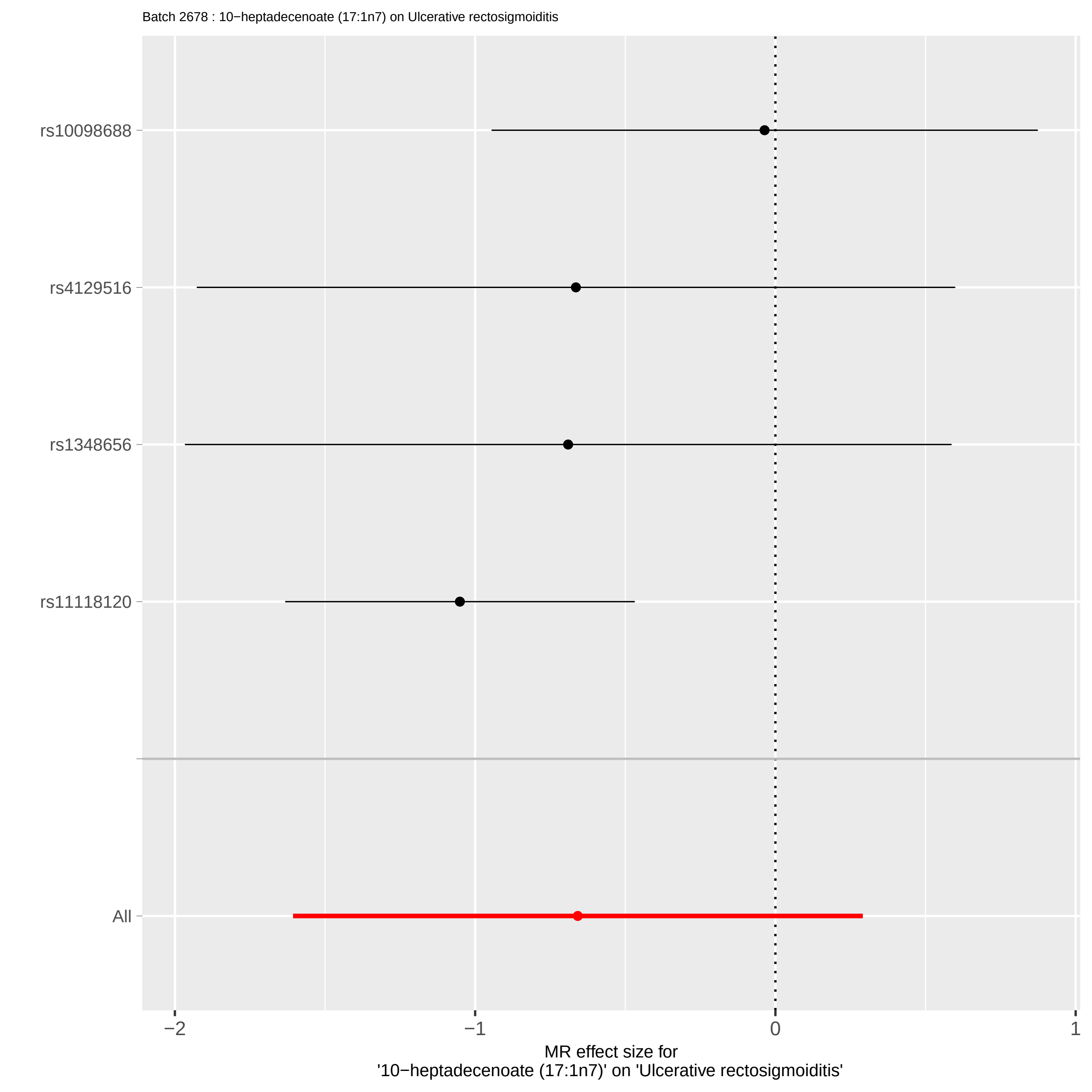
Batch 2678 : 10−heptadecenoate (17:1n7) on Ulcerative rectosigmoiditis
rs10098688
rs4129516
rs1348656
rs11118120
All
−2
−1
0
1
MR effect size for
'10−heptadecenoate (17:1n7)' on 'Ulcerative rectosigmoiditis'

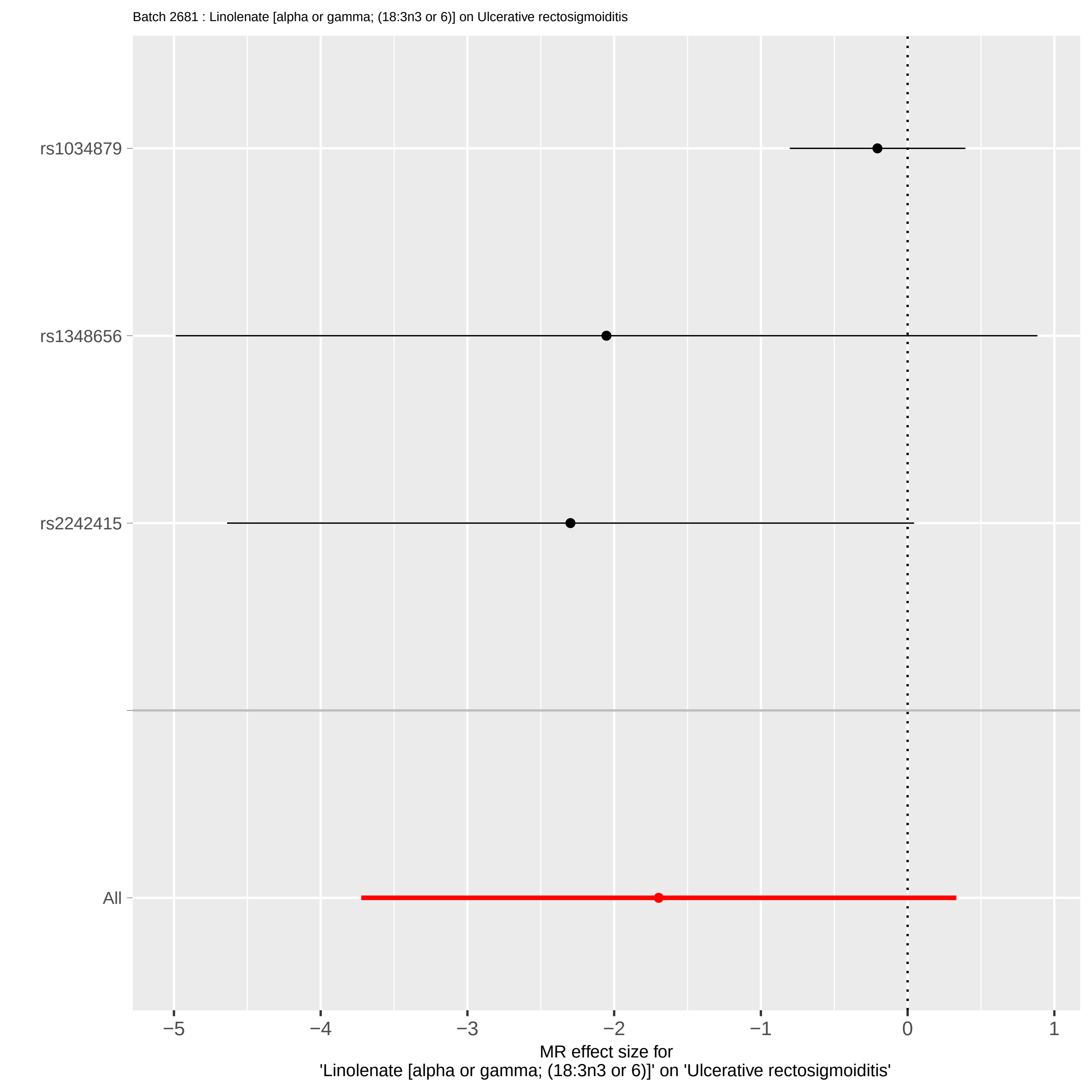
Batch 2681 : Linolenate [alpha or gamma; (18:3n3 or 6)] on Ulcerative rectosigmoiditis
rs1034879
rs1348656
rs2242415
All
−5
−4
−3
−2
−1
0
1
MR effect size for
'Linolenate [alpha or gamma; (18:3n3 or 6)]' on 'Ulcerative rectosigmoiditis'

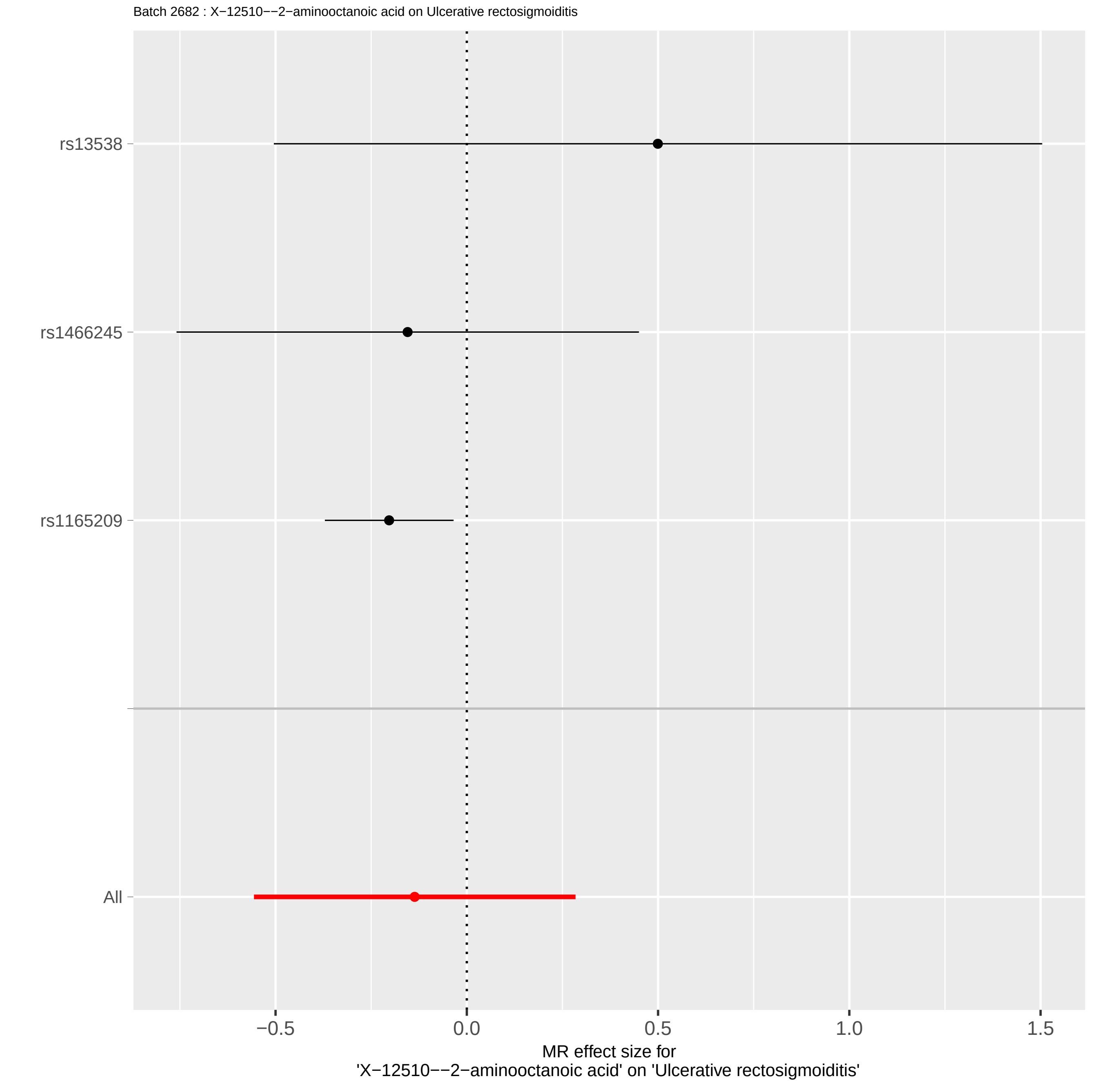
Batch 2682 : X−12510−−2−aminooctanoic acid on Ulcerative rectosigmoiditis
rs13538
rs1466245
rs1165209
All
−0.5
0.0
0.5
1.0
1.5
MR effect size for
'X−12510−−2−aminooctanoic acid' on 'Ulcerative rectosigmoiditis'

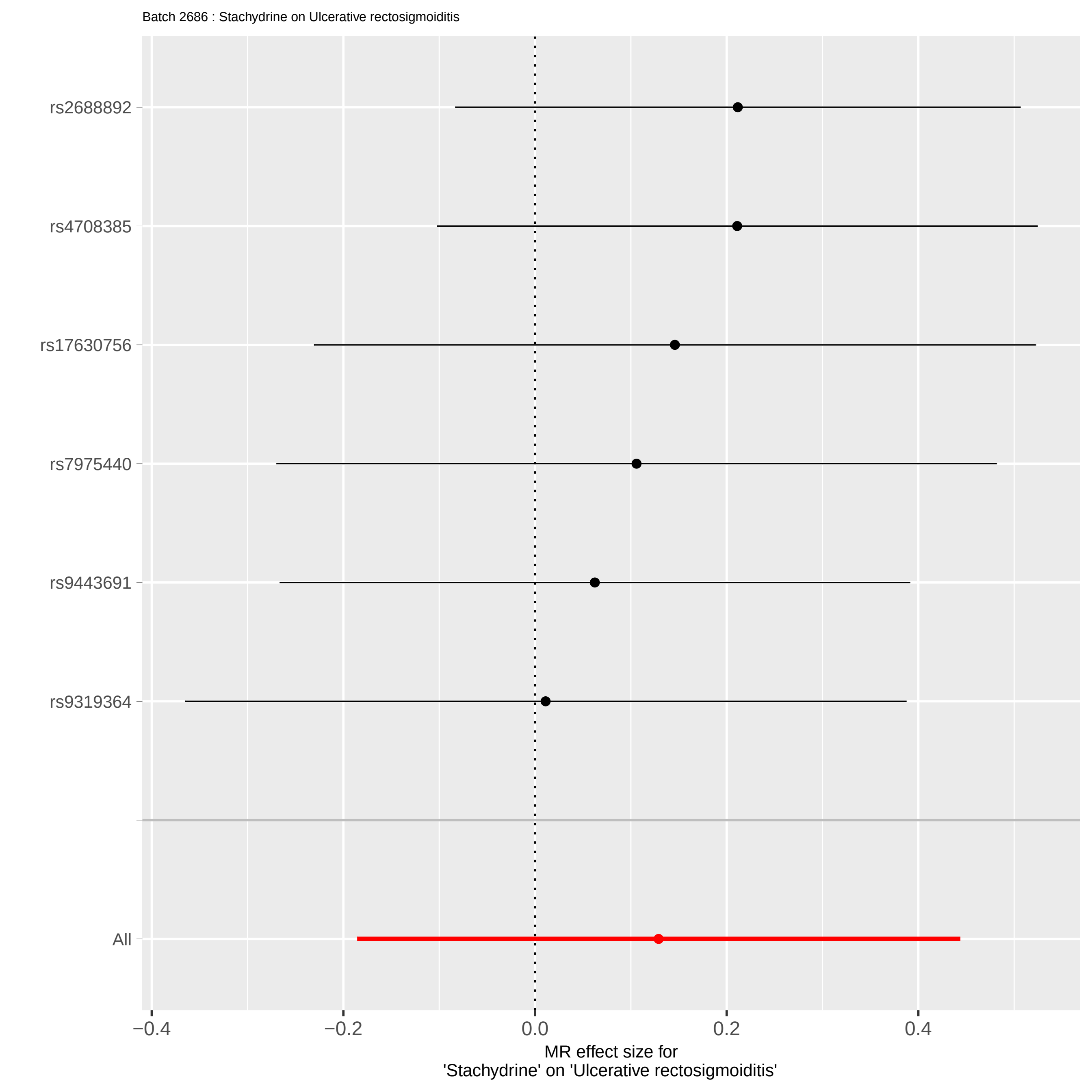
Batch 2686 : Stachydrine on Ulcerative rectosigmoiditis
rs2688892
rs4708385
rs17630756
rs7975440
rs9443691
rs9319364
All
−0.4
−0.2
0.0
0.2
0.4
MR effect size for
'Stachydrine' on 'Ulcerative rectosigmoiditis'

Batch 2690 : 1−linoleoylglycerophosphocholine on Ulcerative rectosigmoiditis

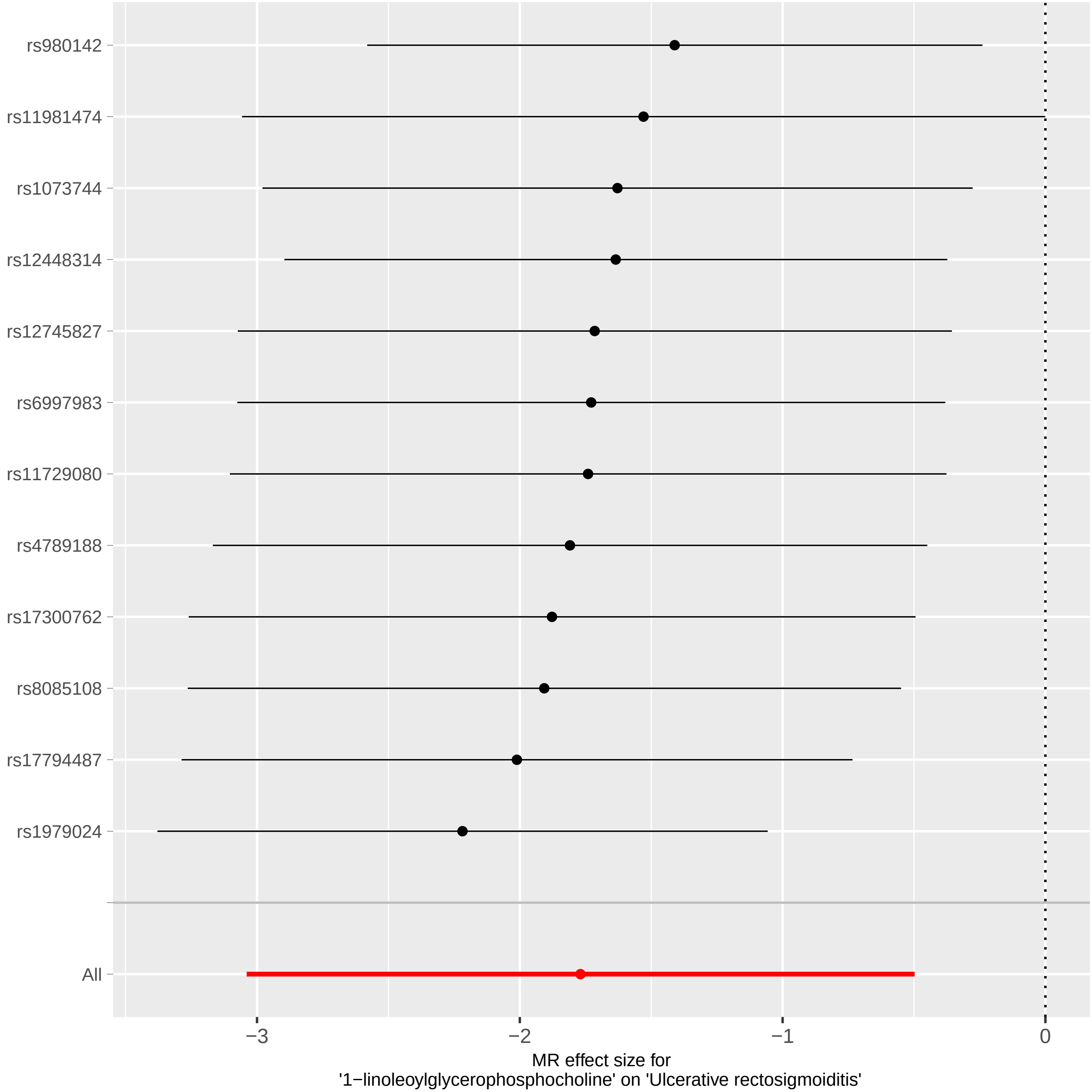

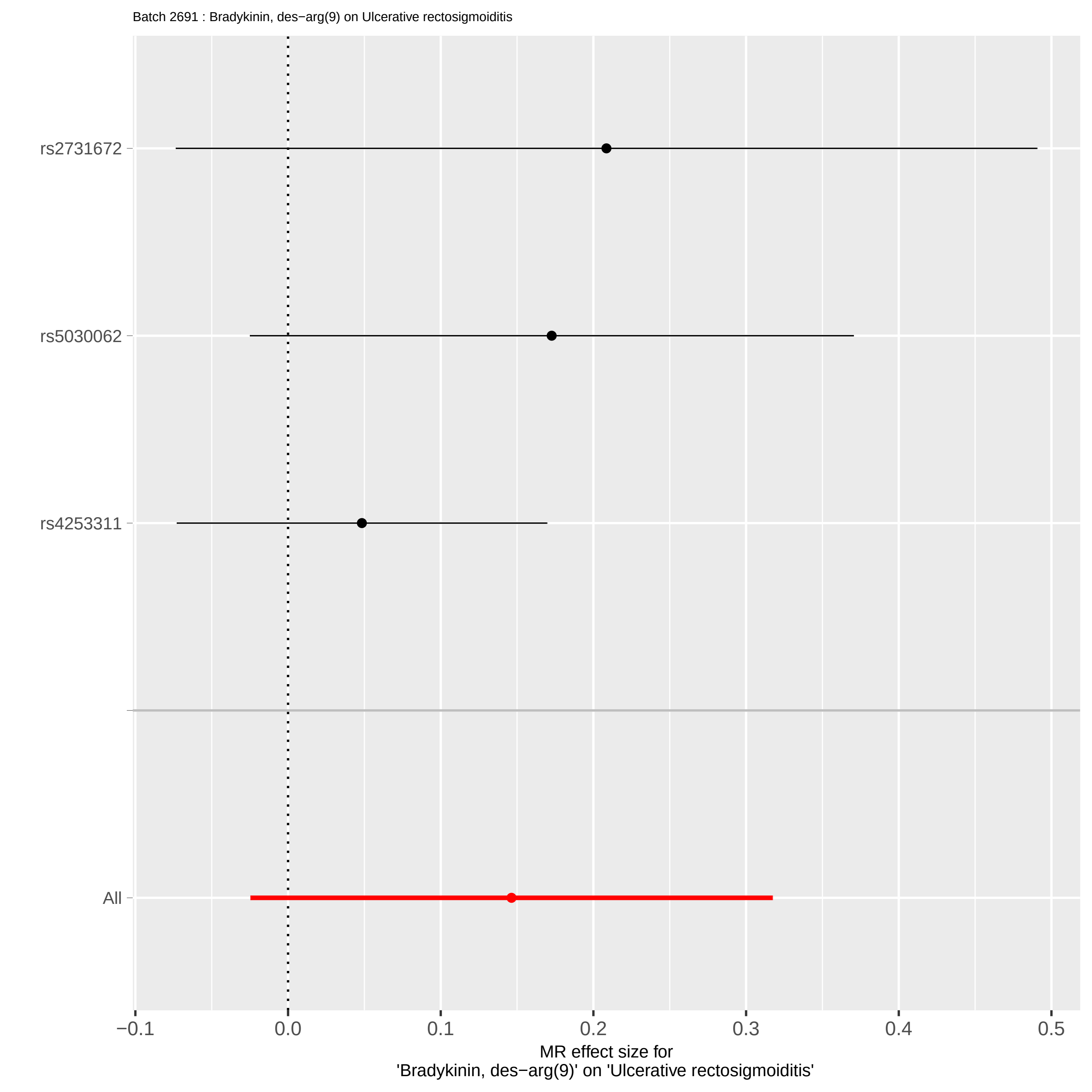
Batch 2691 : Bradykinin, des−arg(9) on Ulcerative rectosigmoiditis
rs2731672
rs5030062
rs4253311
All
−0.1
0.0
0.1
0.2
0.3
0.4
0.5
MR effect size for
'Bradykinin, des−arg(9)' on 'Ulcerative rectosigmoiditis'

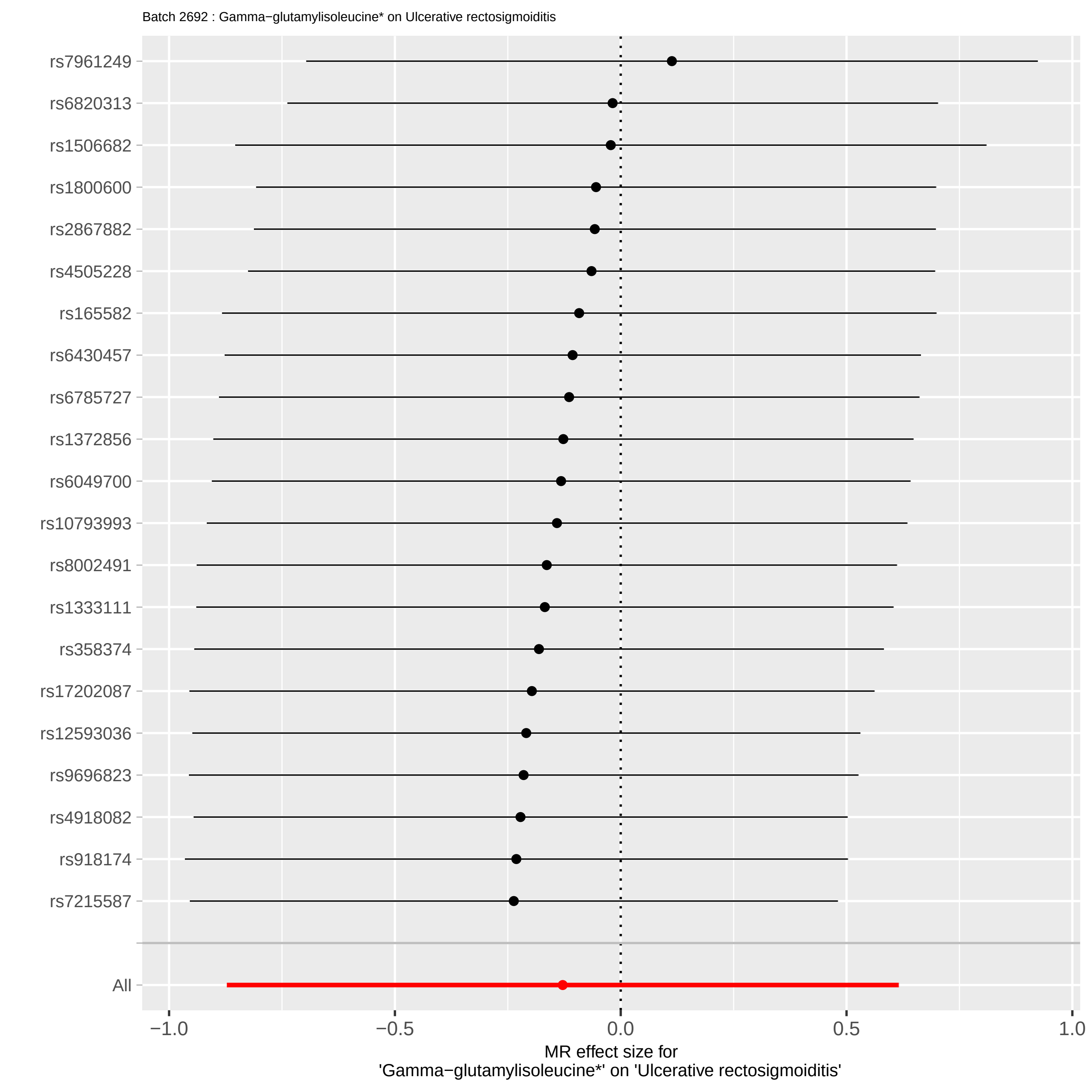

Batch 2692 : Gamma−glutamylisoleucine* on Ulcerative rectosigmoiditis
rs7961249
rs6820313
rs1506682
rs1800600
rs2867882
rs4505228
rs165582
rs6430457
rs6785727
rs1372856
rs6049700
rs10793993
rs8002491
rs1333111
rs358374
rs17202087
rs12593036
rs9696823
rs4918082
rs918174
rs7215587
All
−1.0
−0.5
0.0
0.5
1.0
MR effect size for
'Gamma−glutamylisoleucine*' on 'Ulcerative rectosigmoiditis'

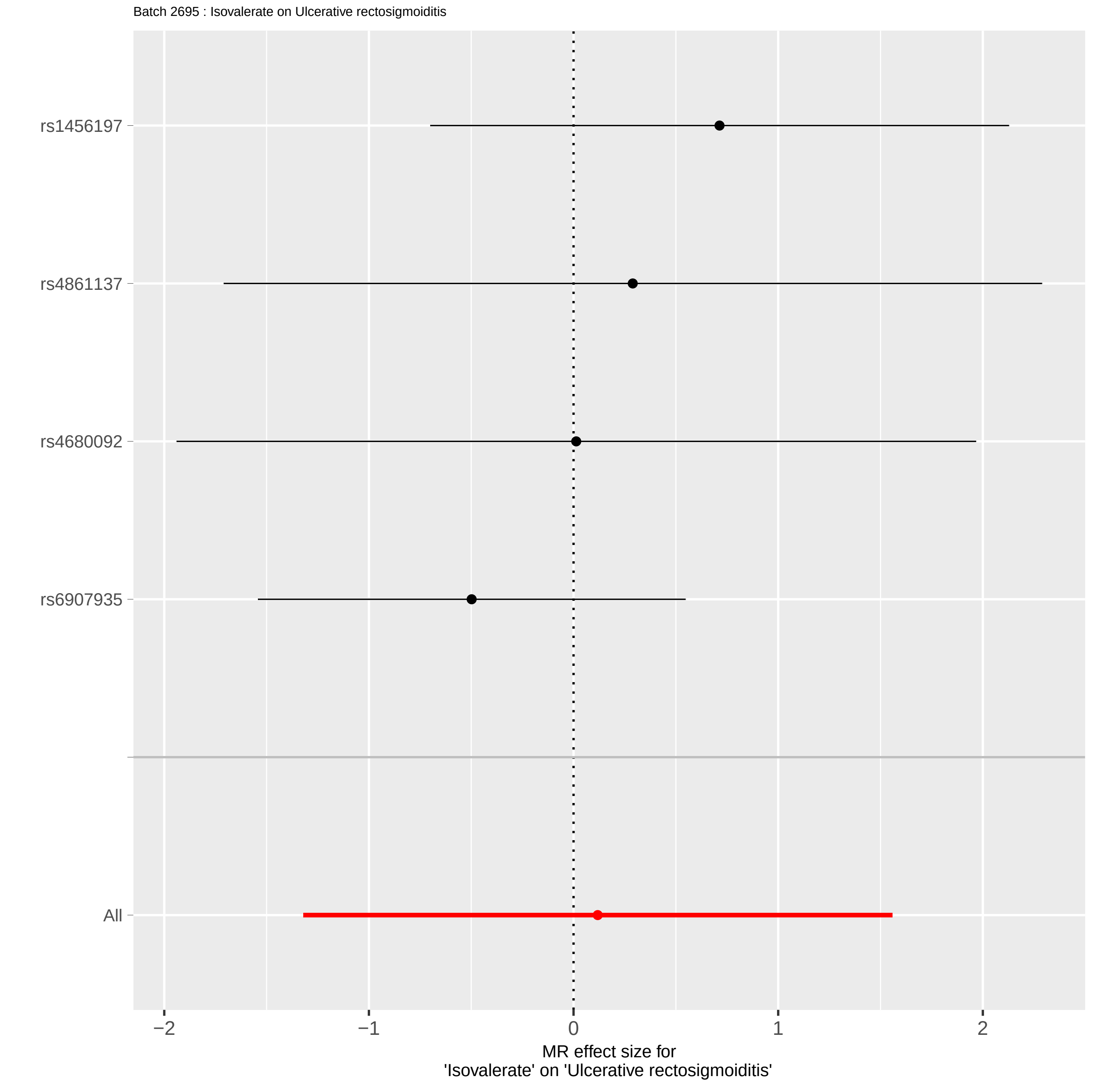
Batch 2695 : Isovalerate on Ulcerative rectosigmoiditis
rs1456197
rs4861137
rs4680092
rs6907935
All
−2
−1
0
1
2
MR effect size for
'Isovalerate' on 'Ulcerative rectosigmoiditis'

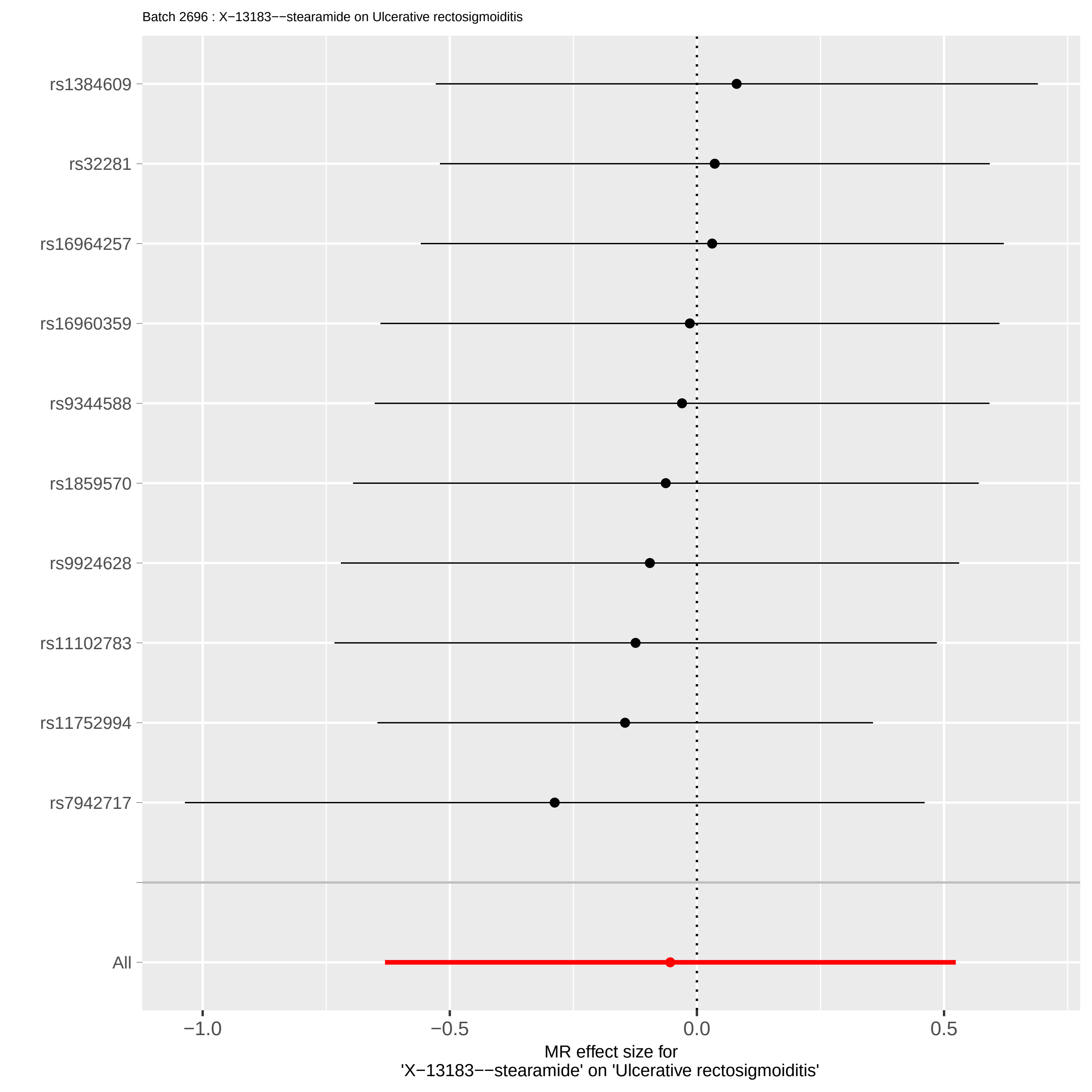

Batch 2696 : X−13183−−stearamide on Ulcerative rectosigmoiditis
rs1384609
rs32281
rs16964257
rs16960359
rs9344588
rs1859570
rs9924628
rs11102783
rs11752994
rs7942717
All
−1.0
−0.5
0.0
0.5
MR effect size for
'X−13183−−stearamide' on 'Ulcerative rectosigmoiditis'

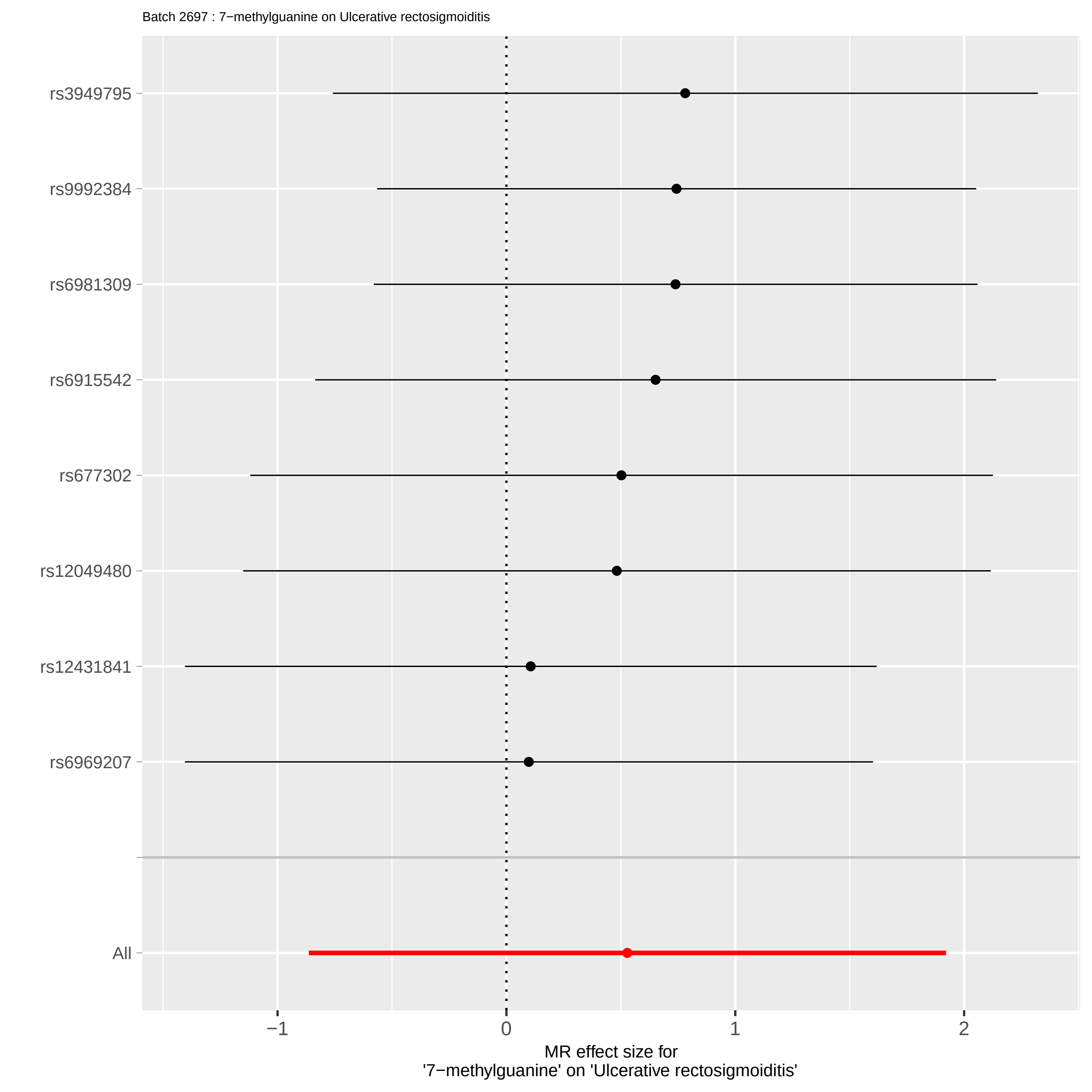
Batch 2697 : 7−methylguanine on Ulcerative rectosigmoiditis
rs3949795
rs9992384
rs6981309
rs6915542
rs677302
rs12049480
rs12431841
rs6969207
All
−1
0
1
2
MR effect size for
'7−methylguanine' on 'Ulcerative rectosigmoiditis'

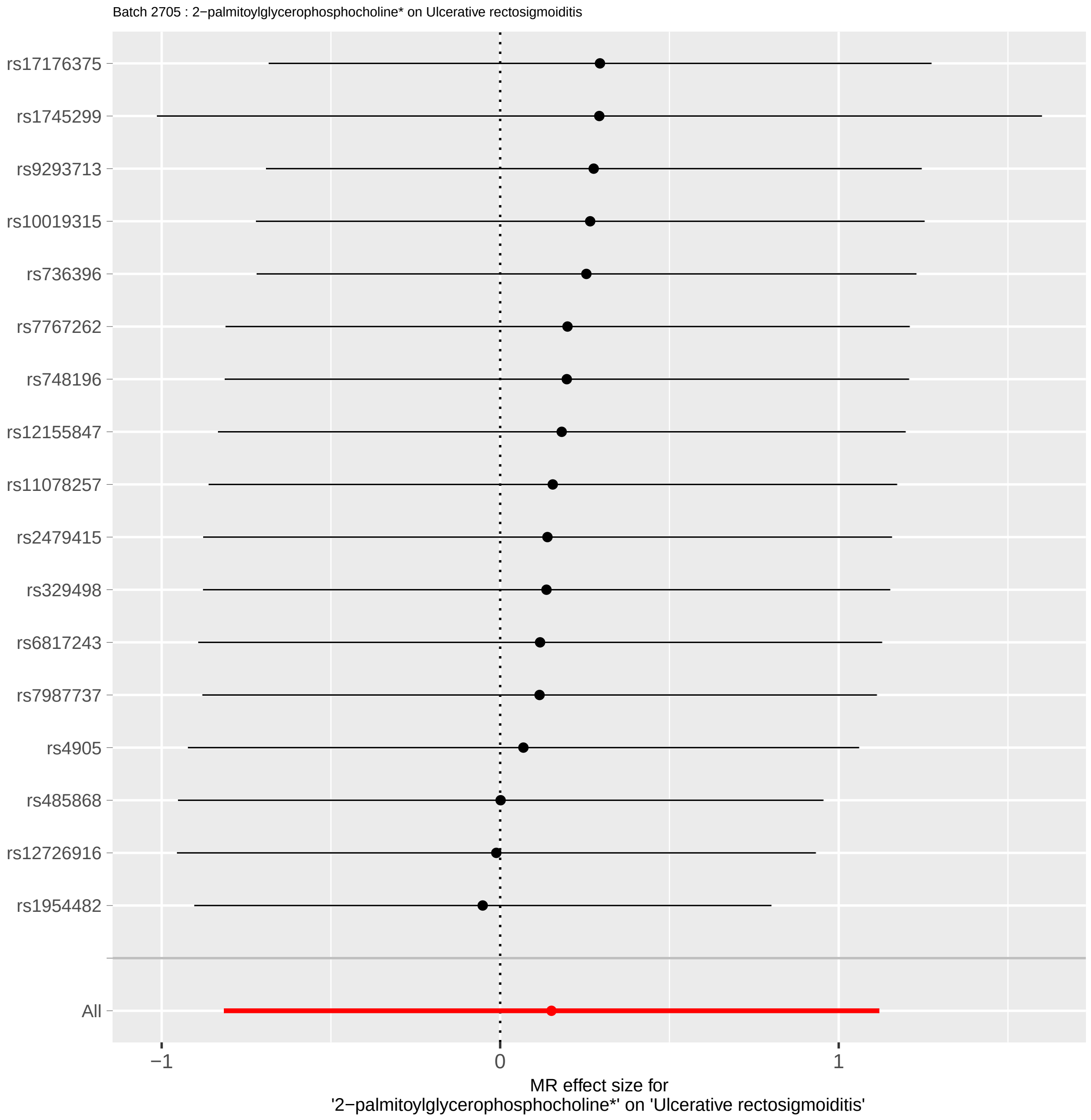

Batch 2705 : 2−palmitoylglycerophosphocholine* on Ulcerative rectosigmoiditis
rs17176375
rs1745299
rs9293713
rs10019315
rs736396
rs7767262
rs748196
rs12155847
rs11078257
rs2479415
rs329498
rs6817243
rs7987737
rs4905
rs485868
rs12726916
rs1954482
All
−1
0
1
MR effect size for
'2−palmitoylglycerophosphocholine*' on 'Ulcerative rectosigmoiditis'

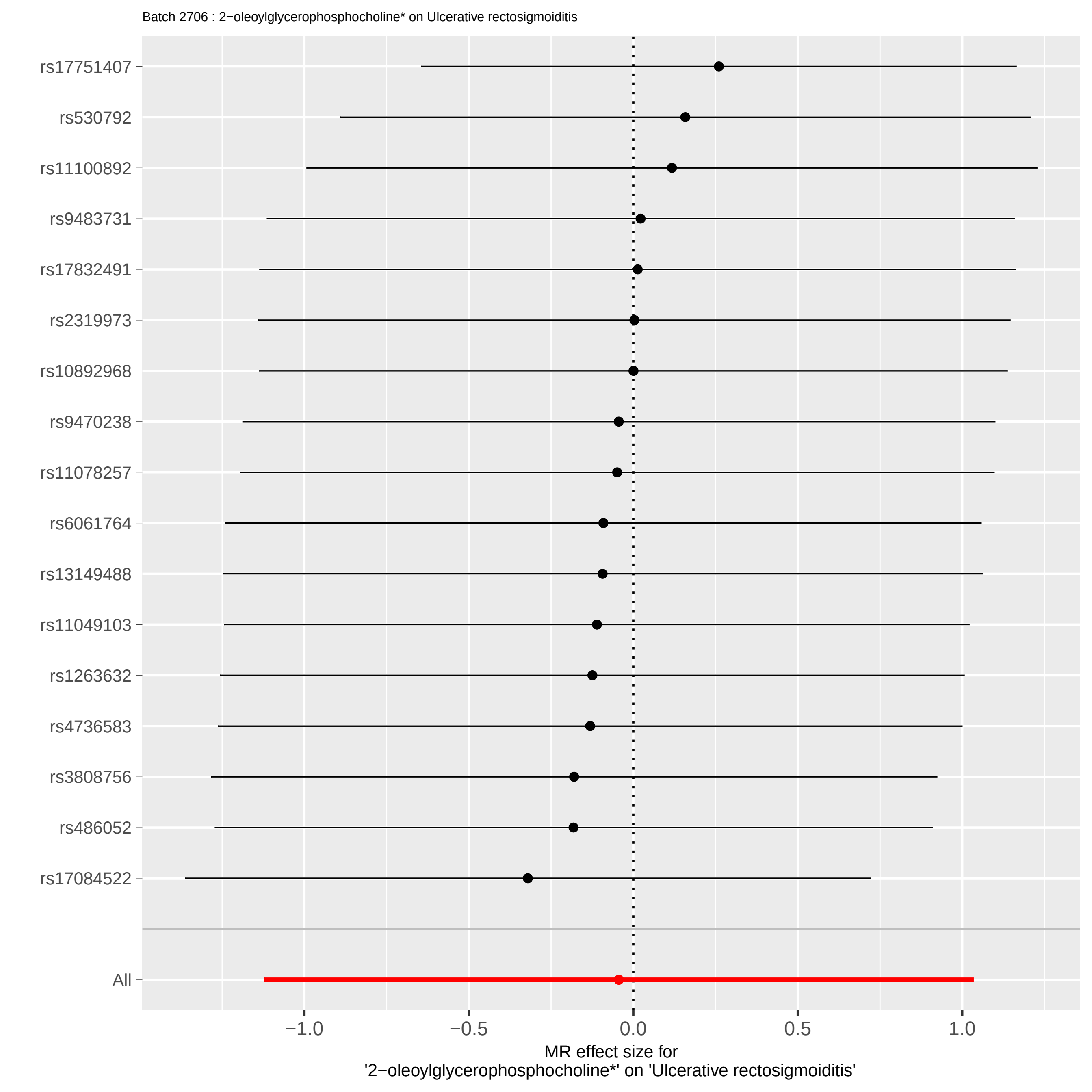

Batch 2706 : 2−oleoylglycerophosphocholine* on Ulcerative rectosigmoiditis
rs17751407
rs530792
rs11100892
rs9483731
rs17832491
rs2319973
rs10892968
rs9470238
rs11078257
rs6061764
rs13149488
rs11049103
rs1263632
rs4736583
rs3808756
rs486052
rs17084522
All
−1.0
−0.5
0.0
0.5
1.0
MR effect size for
'2−oleoylglycerophosphocholine*' on 'Ulcerative rectosigmoiditis'

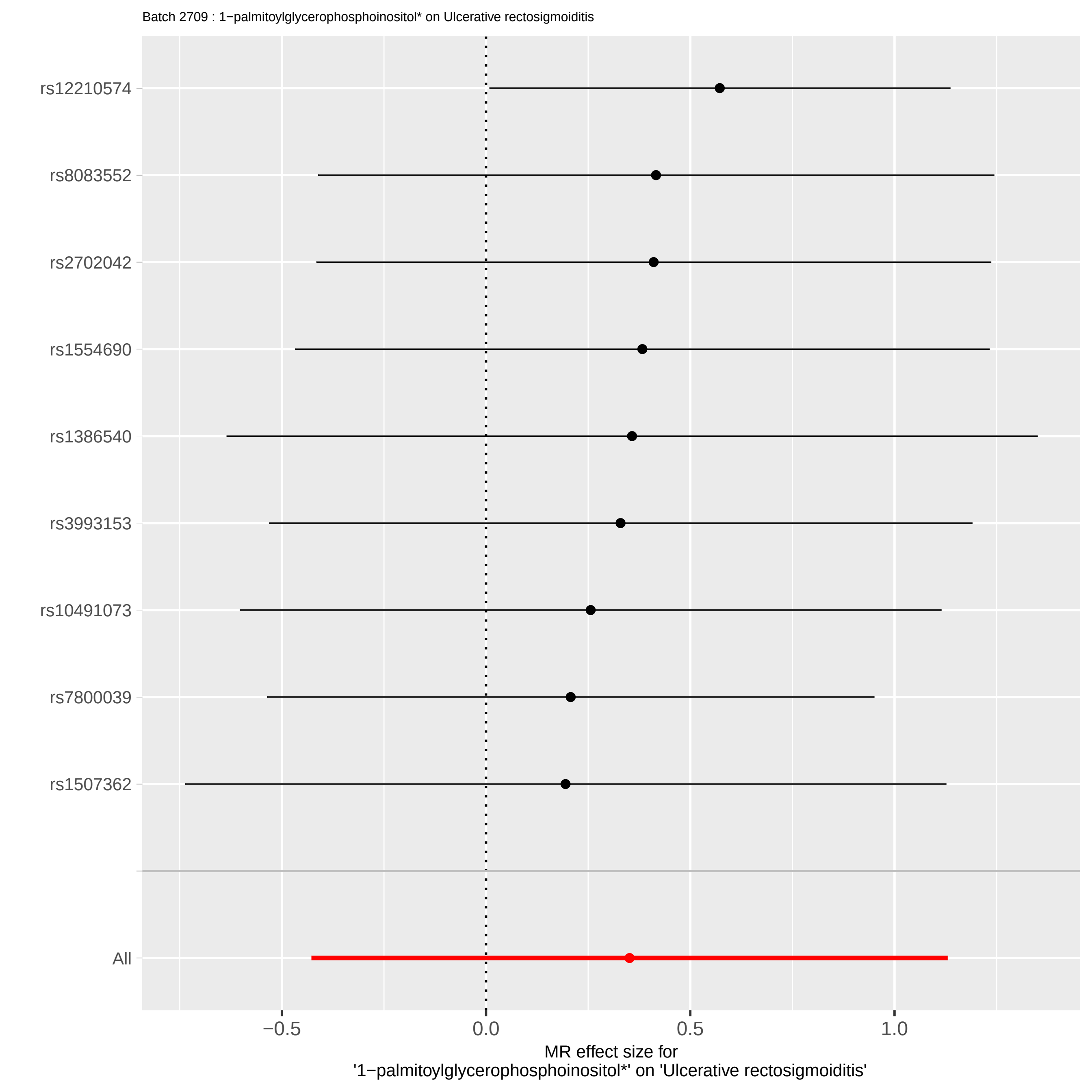

Batch 2709 : 1−palmitoylglycerophosphoinositol* on Ulcerative rectosigmoiditis
rs12210574
rs8083552
rs2702042
rs1554690
rs1386540
rs3993153
rs10491073
rs7800039
rs1507362
All
−0.5
0.0
0.5
1.0
MR effect size for
'1−palmitoylglycerophosphoinositol*' on 'Ulcerative rectosigmoiditis'

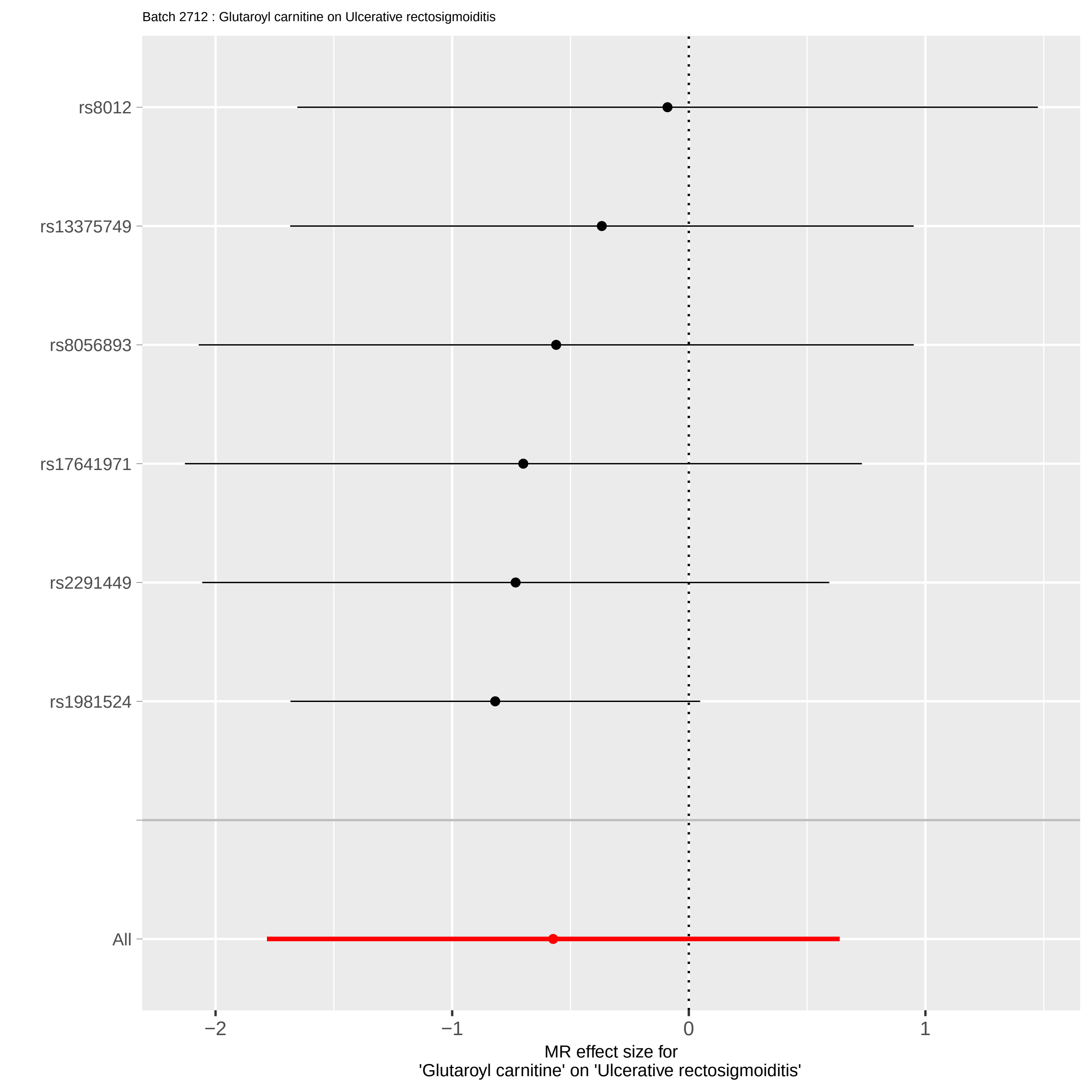

Batch 2712 : Glutaroyl carnitine on Ulcerative rectosigmoiditis
rs8012
rs13375749
rs8056893
rs17641971
rs2291449
rs1981524
All
−2
−1
0
1
MR effect size for
'Glutaroyl carnitine' on 'Ulcerative rectosigmoiditis'

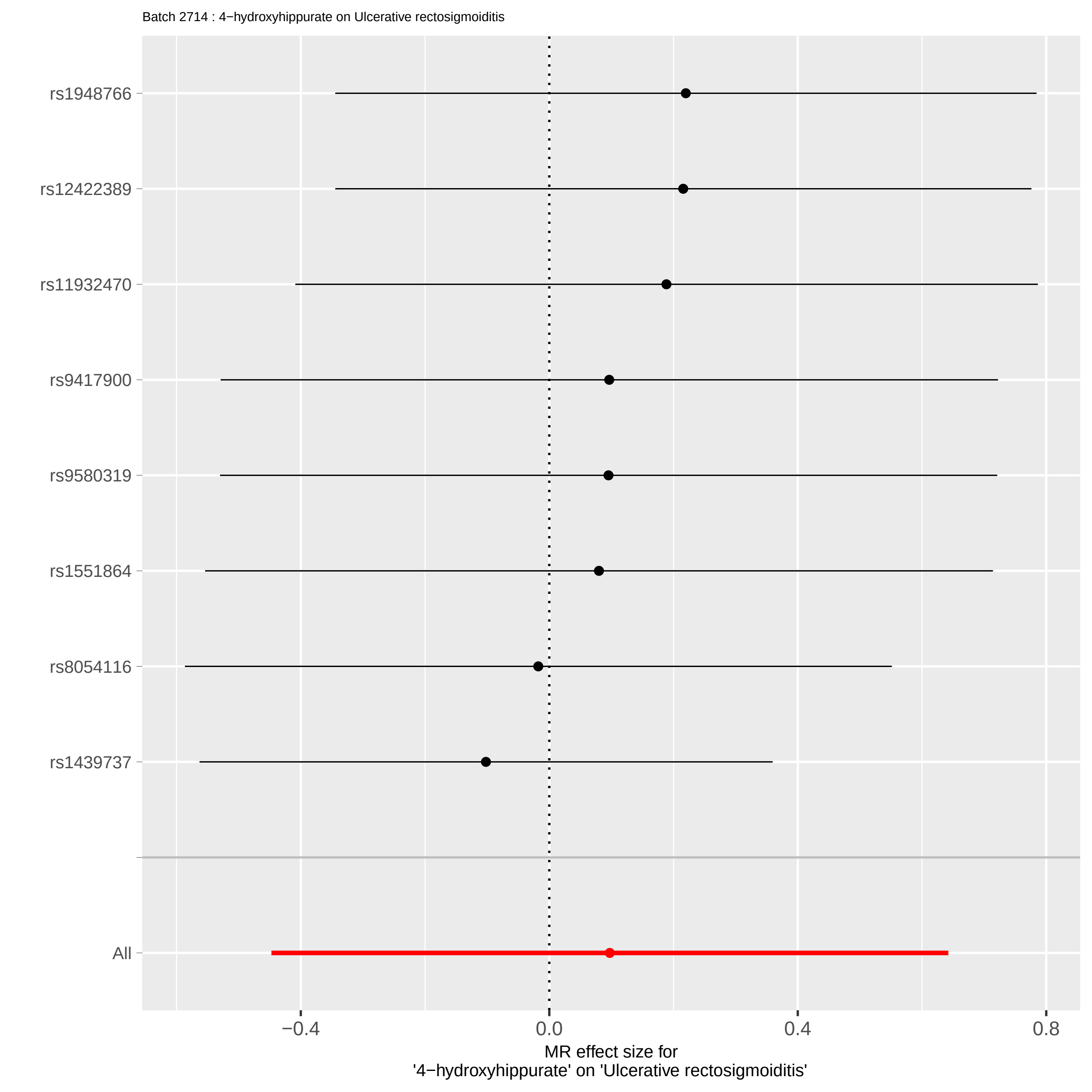

Batch 2714 : 4−hydroxyhippurate on Ulcerative rectosigmoiditis
rs1948766
rs12422389
rs11932470
rs9417900
rs9580319
rs1551864
rs8054116
rs1439737
All
−0.4
0.0
0.4
0.8
MR effect size for
'4−hydroxyhippurate' on 'Ulcerative rectosigmoiditis'

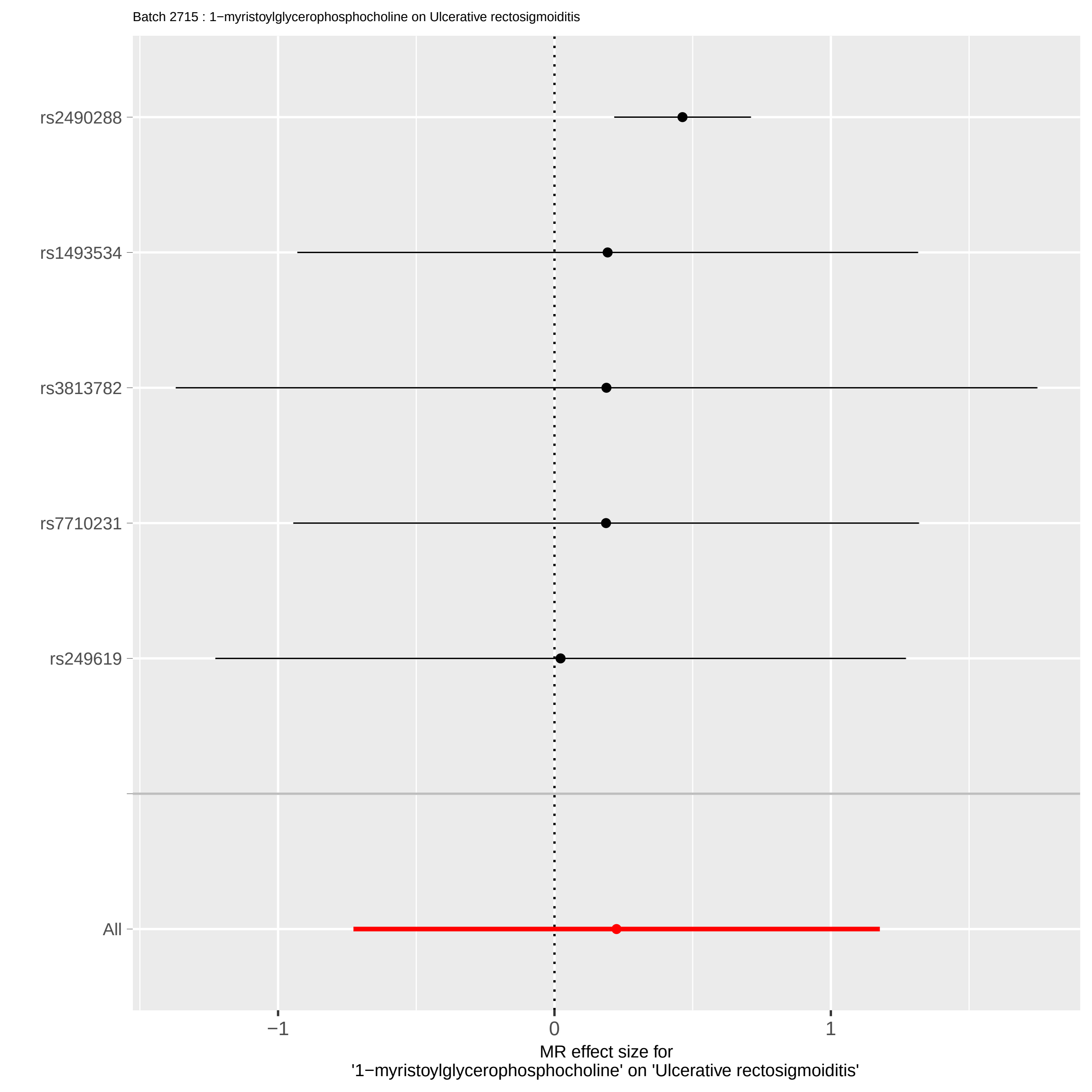
Batch 2715 : 1−myristoylglycerophosphocholine on Ulcerative rectosigmoiditis
rs2490288
rs1493534
rs3813782
rs7710231
rs249619
All
−1
0
1
MR effect size for
'1−myristoylglycerophosphocholine' on 'Ulcerative rectosigmoiditis'

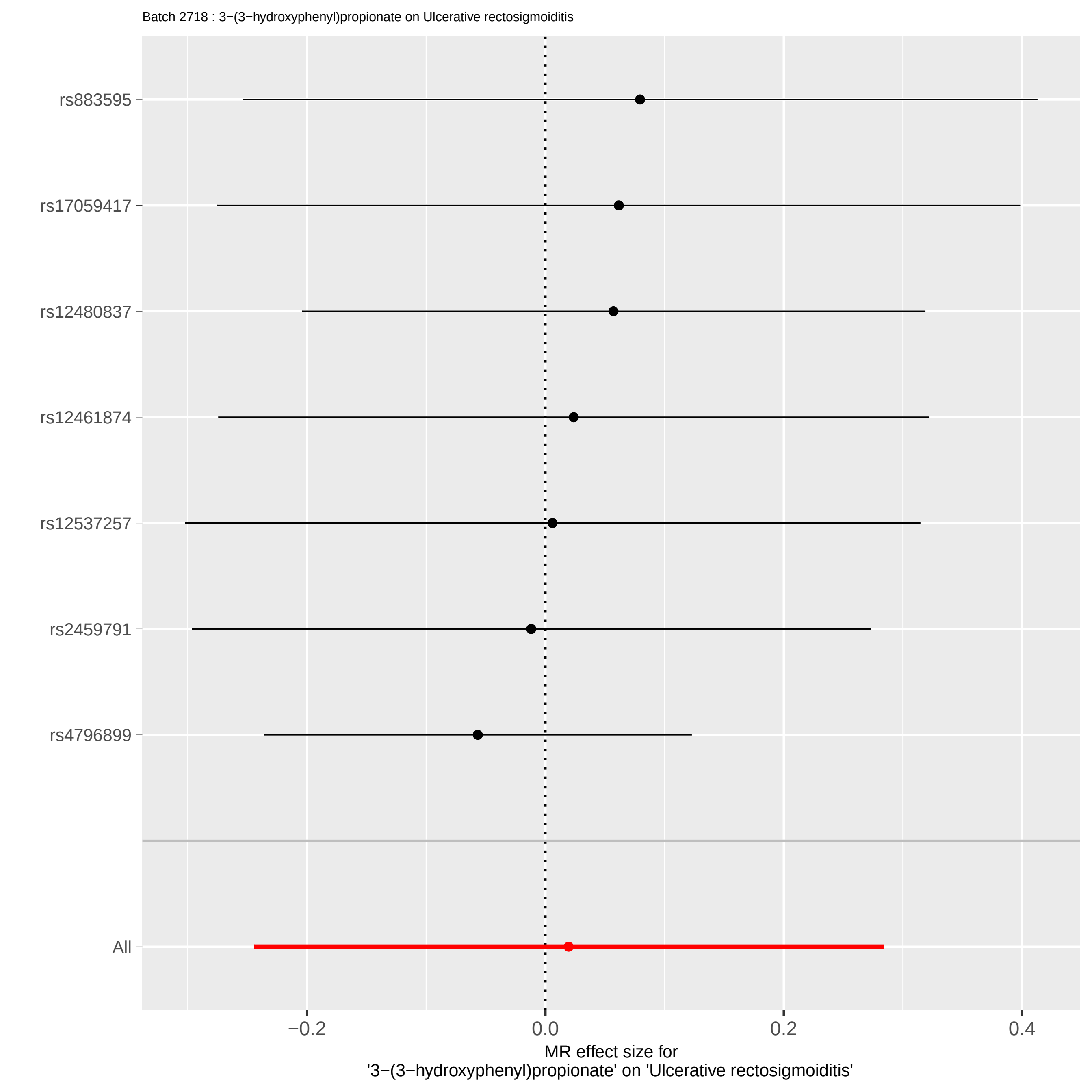

Batch 2718 : 3−(3−hydroxyphenyl)propionate on Ulcerative rectosigmoiditis
rs883595
rs17059417
rs12480837
rs12461874
rs12537257
rs2459791
rs4796899
All
−0.2
0.0
0.2
0.4
MR effect size for
'3−(3−hydroxyphenyl)propionate' on 'Ulcerative rectosigmoiditis'

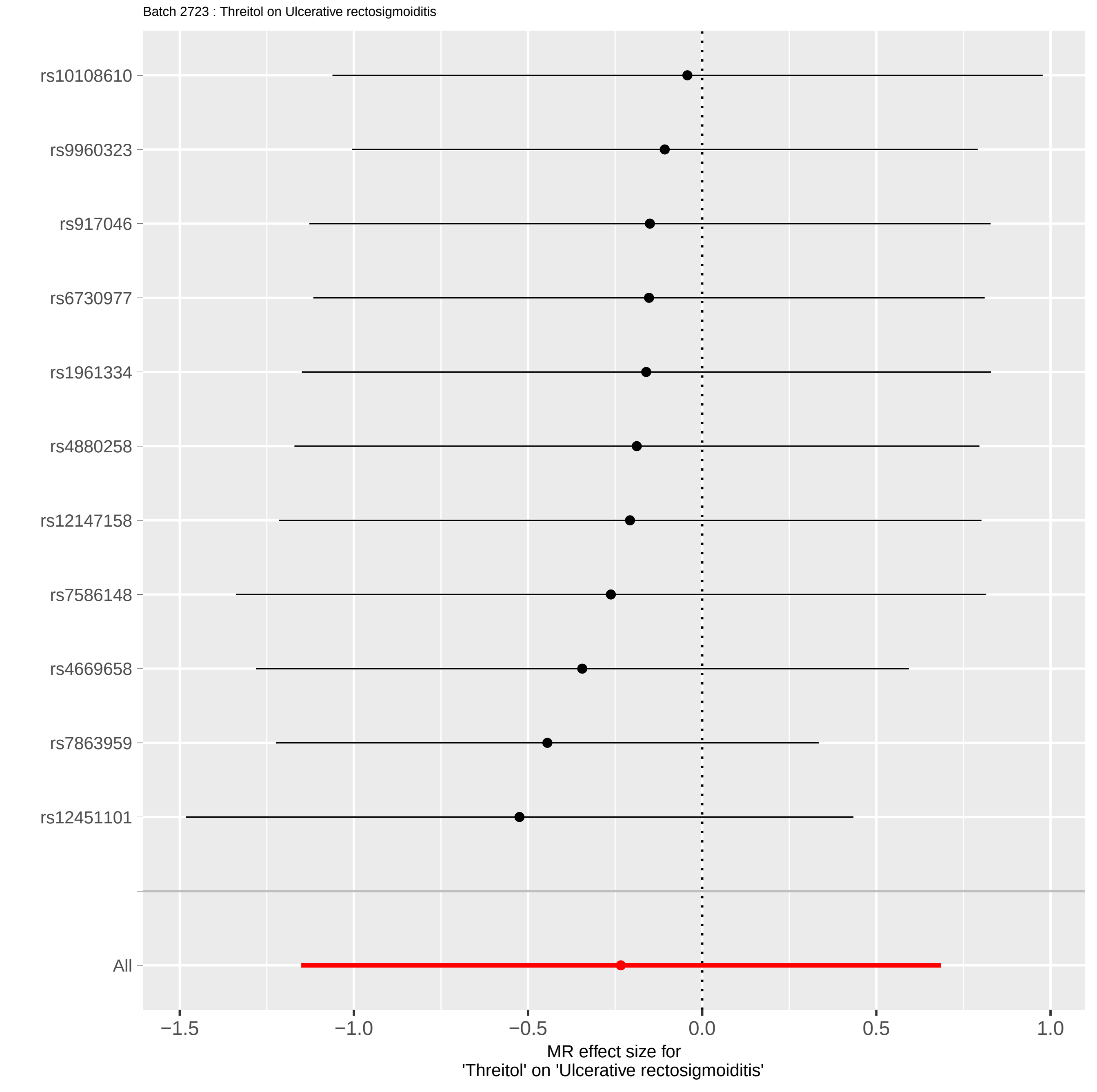

Batch 2723 : Threitol on Ulcerative rectosigmoiditis
rs10108610
rs9960323
rs917046
rs6730977
rs1961334
rs4880258
rs12147158
rs7586148
rs4669658
rs7863959
rs12451101
All
−1.5
−1.0
−0.5
0.0
0.5
1.0
MR effect size for
'Threitol' on 'Ulcerative rectosigmoiditis'

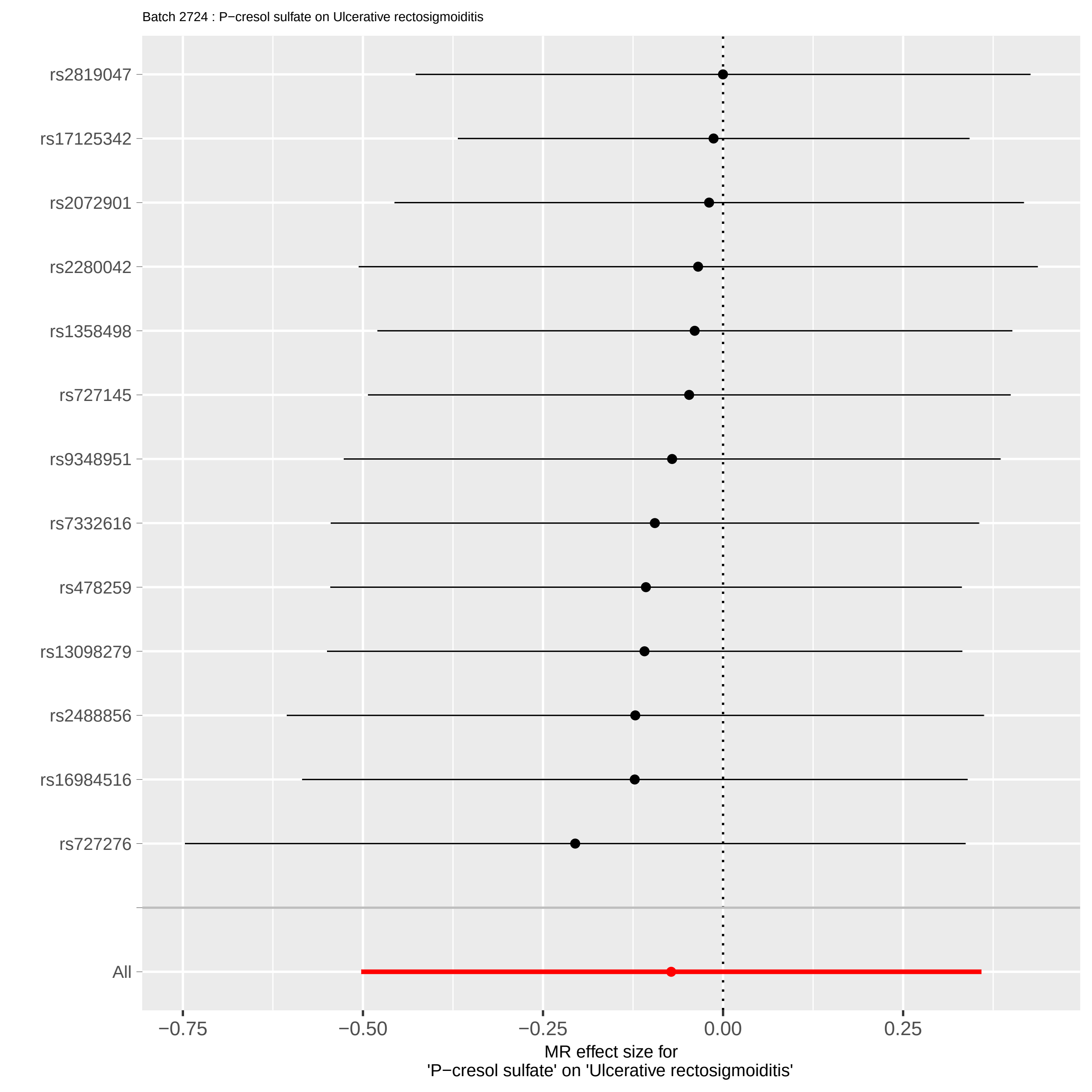

Batch 2724 : P−cresol sulfate on Ulcerative rectosigmoiditis
rs2819047
rs17125342
rs2072901
rs2280042
rs1358498
rs727145
rs9348951
rs7332616
rs478259
rs13098279
rs2488856
rs16984516
rs727276
All
−0.75
−0.50
−0.25
0.00
0.25
MR effect size for
'P−cresol sulfate' on 'Ulcerative rectosigmoiditis'

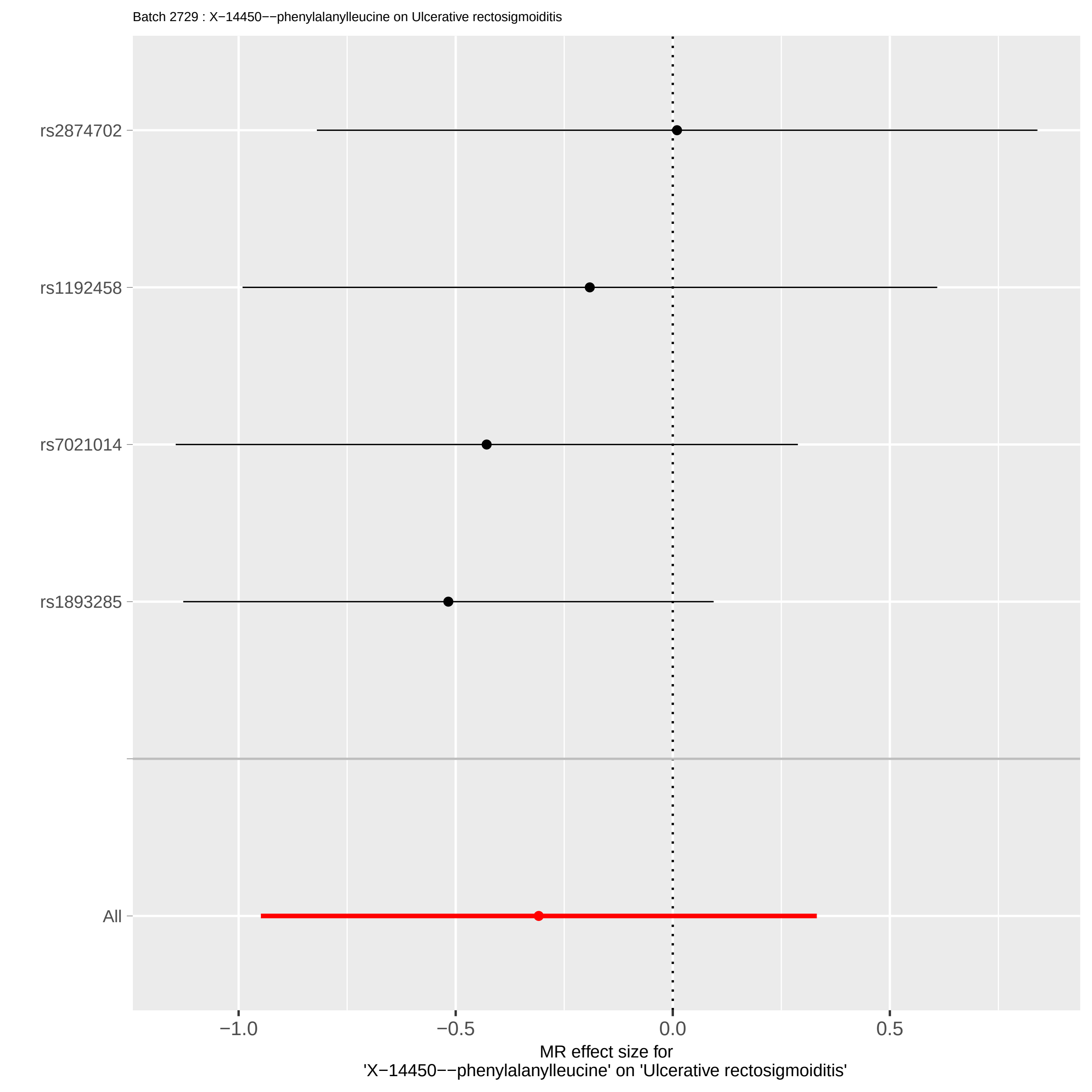
Batch 2729 : X−14450−−phenylalanylleucine on Ulcerative rectosigmoiditis
rs2874702
rs1192458
rs7021014
rs1893285
All
−1.0
−0.5
0.0
0.5
MR effect size for
'X−14450−−phenylalanylleucine' on 'Ulcerative rectosigmoiditis'

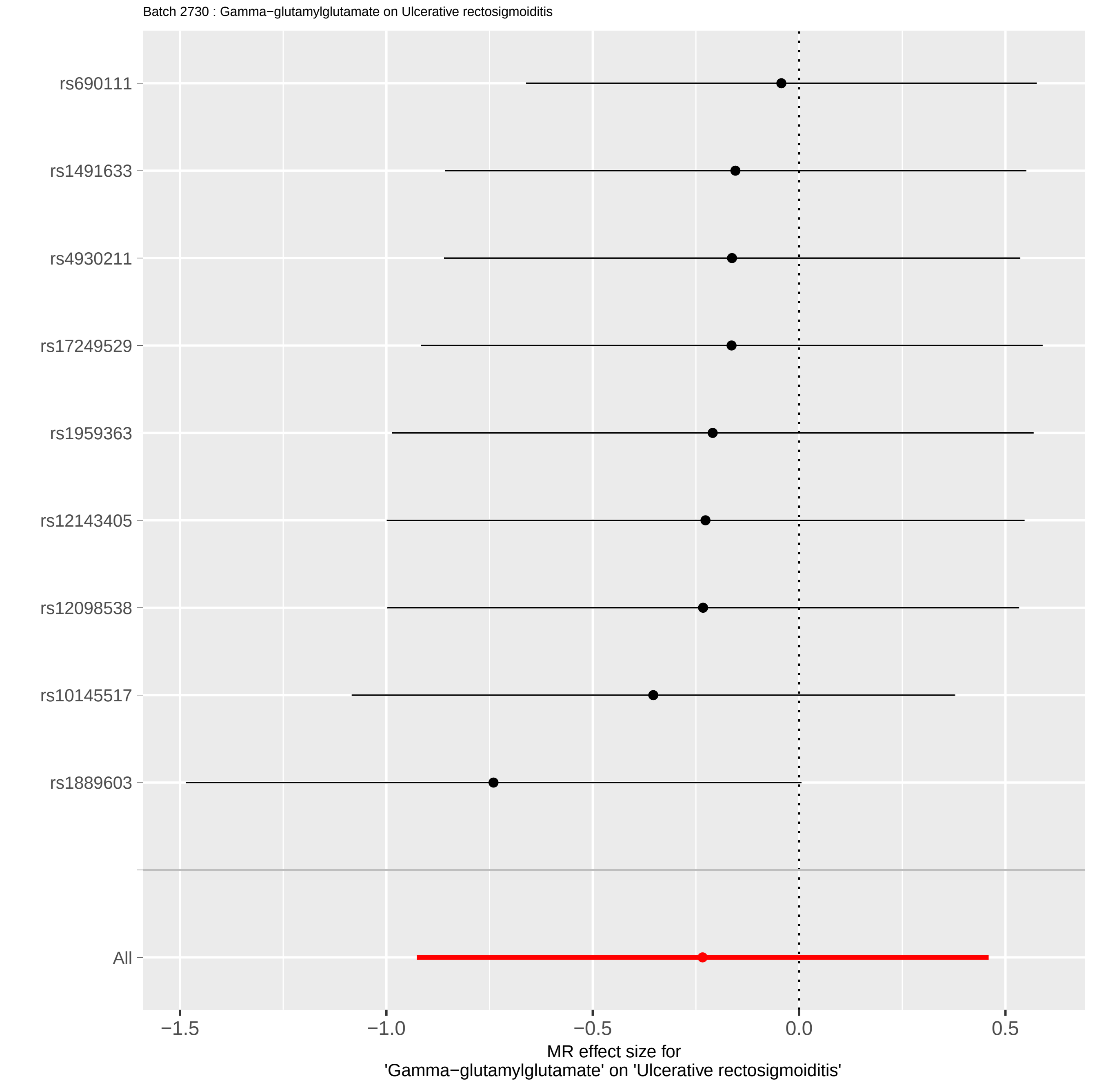

Batch 2730 : Gamma−glutamylglutamate on Ulcerative rectosigmoiditis
rs690111
rs1491633
rs4930211
rs17249529
rs1959363
rs12143405
rs12098538
rs10145517
rs1889603
All
−1.5
−1.0
−0.5
0.0
0.5
MR effect size for
'Gamma−glutamylglutamate' on 'Ulcerative rectosigmoiditis'

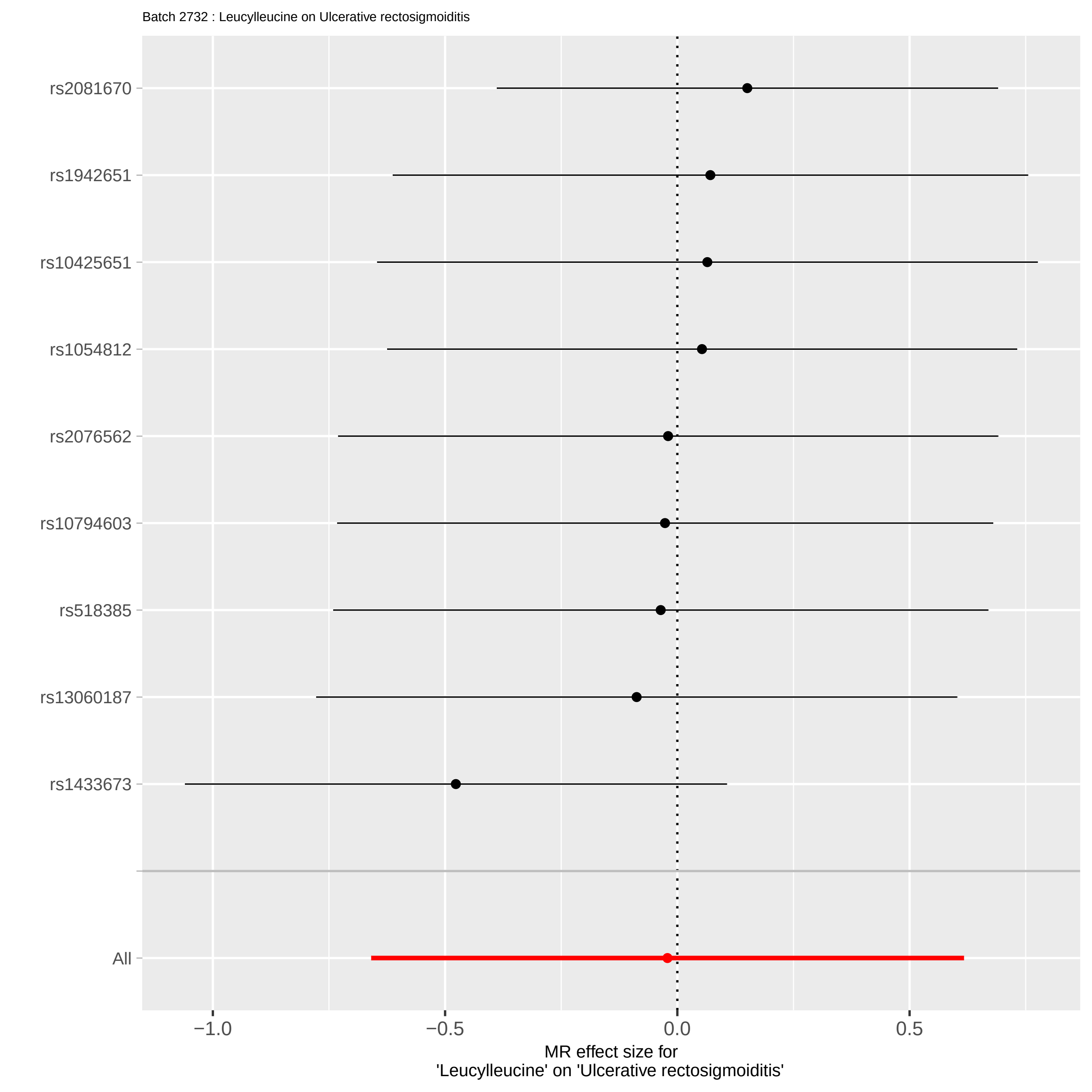

Batch 2732 : Leucylleucine on Ulcerative rectosigmoiditis
rs2081670
rs1942651
rs10425651
rs1054812
rs2076562
rs10794603
rs518385
rs13060187
rs1433673
All
−1.0
−0.5
0.0
0.5
MR effect size for
'Leucylleucine' on 'Ulcerative rectosigmoiditis'

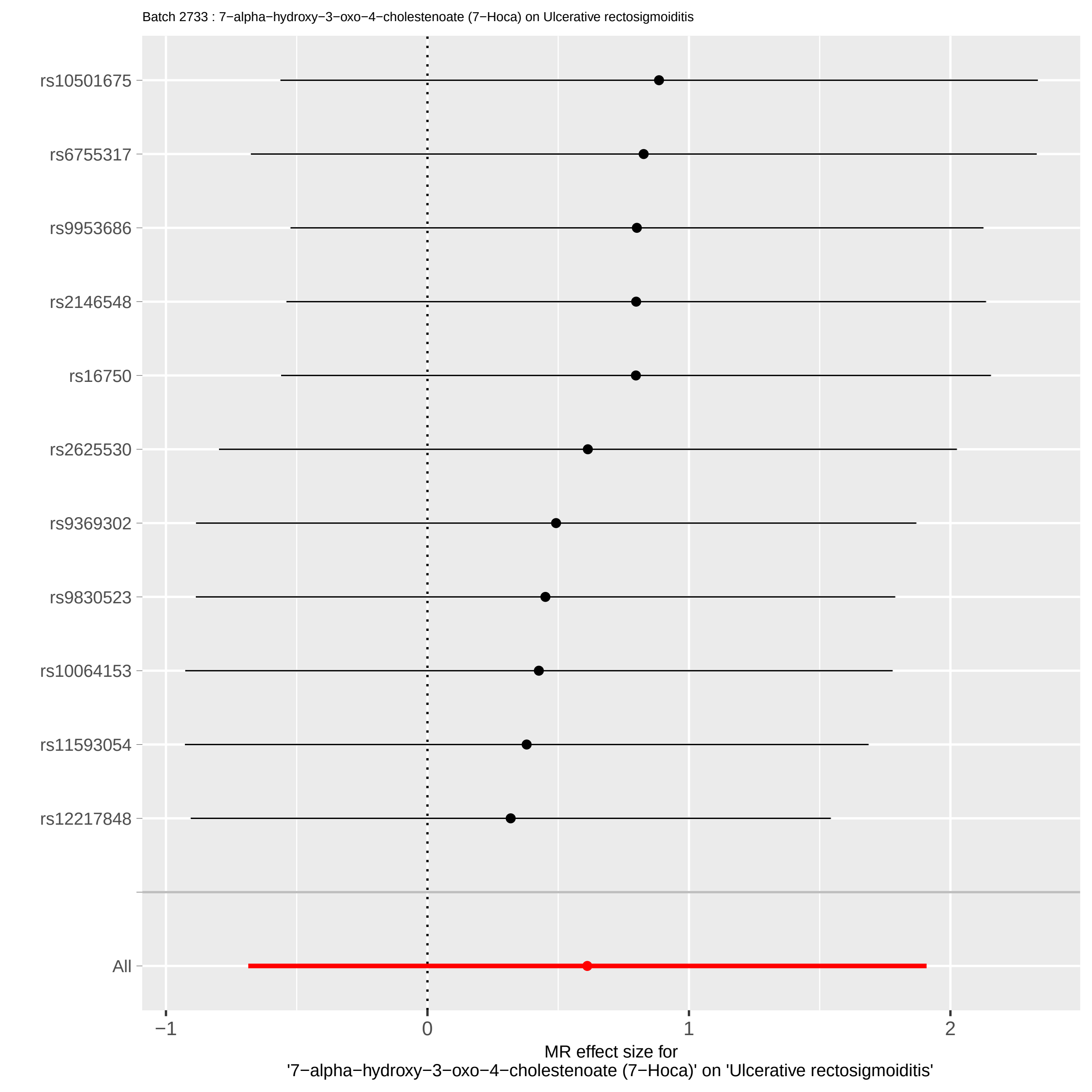

Batch 2733 : 7−alpha−hydroxy−3−oxo−4−cholestenoate (7−Hoca) on Ulcerative rectosigmoiditis
rs10501675
rs6755317
rs9953686
rs2146548
rs16750
rs2625530
rs9369302
rs9830523
rs10064153
rs11593054
rs12217848
All
−1
0
1
2
MR effect size for
'7−alpha−hydroxy−3−oxo−4−cholestenoate (7−Hoca)' on 'Ulcerative rectosigmoiditis'

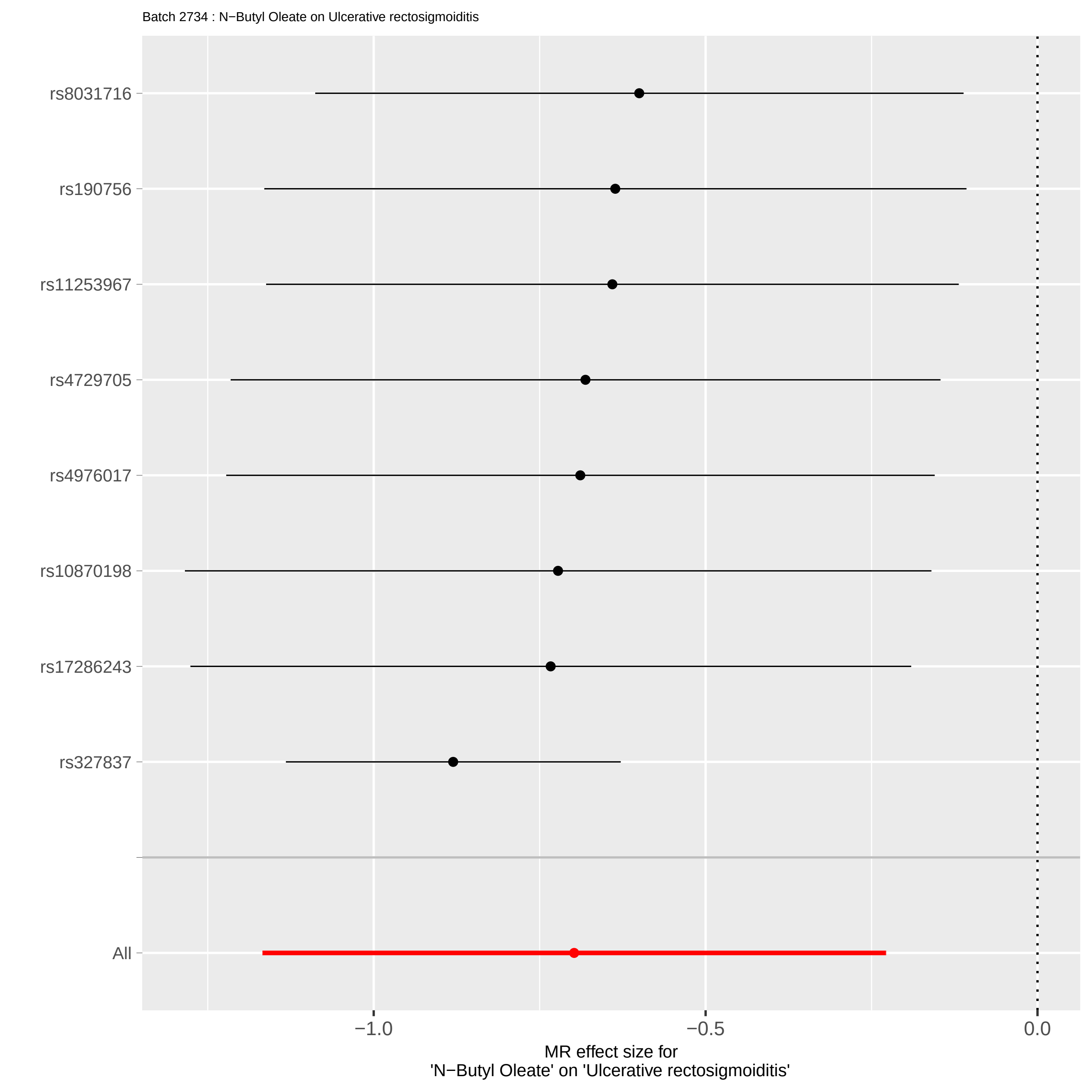
Batch 2734 : N−Butyl Oleate on Ulcerative rectosigmoiditis
rs8031716
rs190756
rs11253967
rs4729705
rs4976017
rs10870198
rs17286243
rs327837
All
−1.0
−0.5
0.0
MR effect size for
'N−Butyl Oleate' on 'Ulcerative rectosigmoiditis'

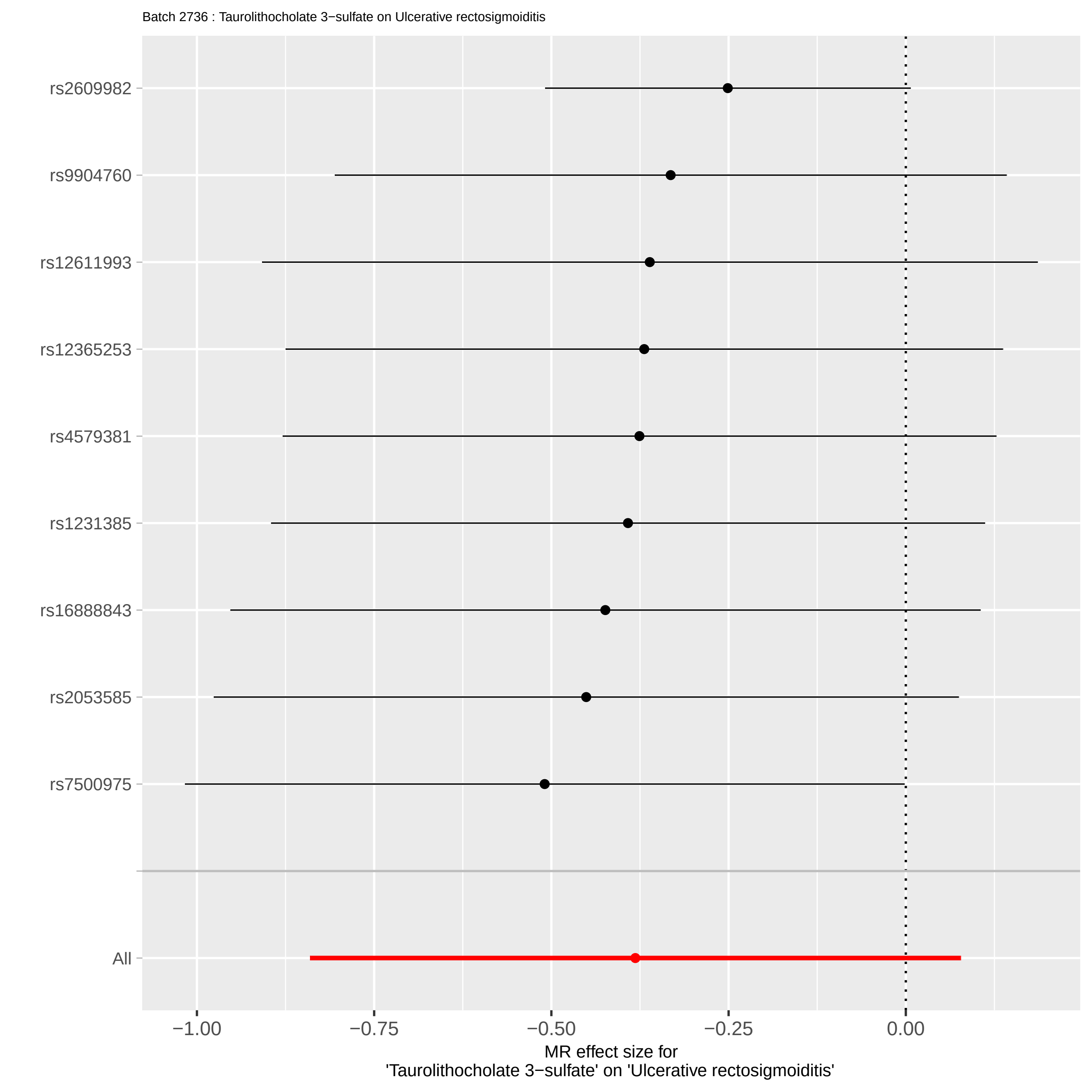
Batch 2736 : Taurolithocholate 3−sulfate on Ulcerative rectosigmoiditis
rs2609982
rs9904760
rs12611993
rs12365253
rs4579381
rs1231385
rs16888843
rs2053585
rs7500975
All
−1.00
−0.75
−0.50
−0.25
0.00
MR effect size for
'Taurolithocholate 3−sulfate' on 'Ulcerative rectosigmoiditis'

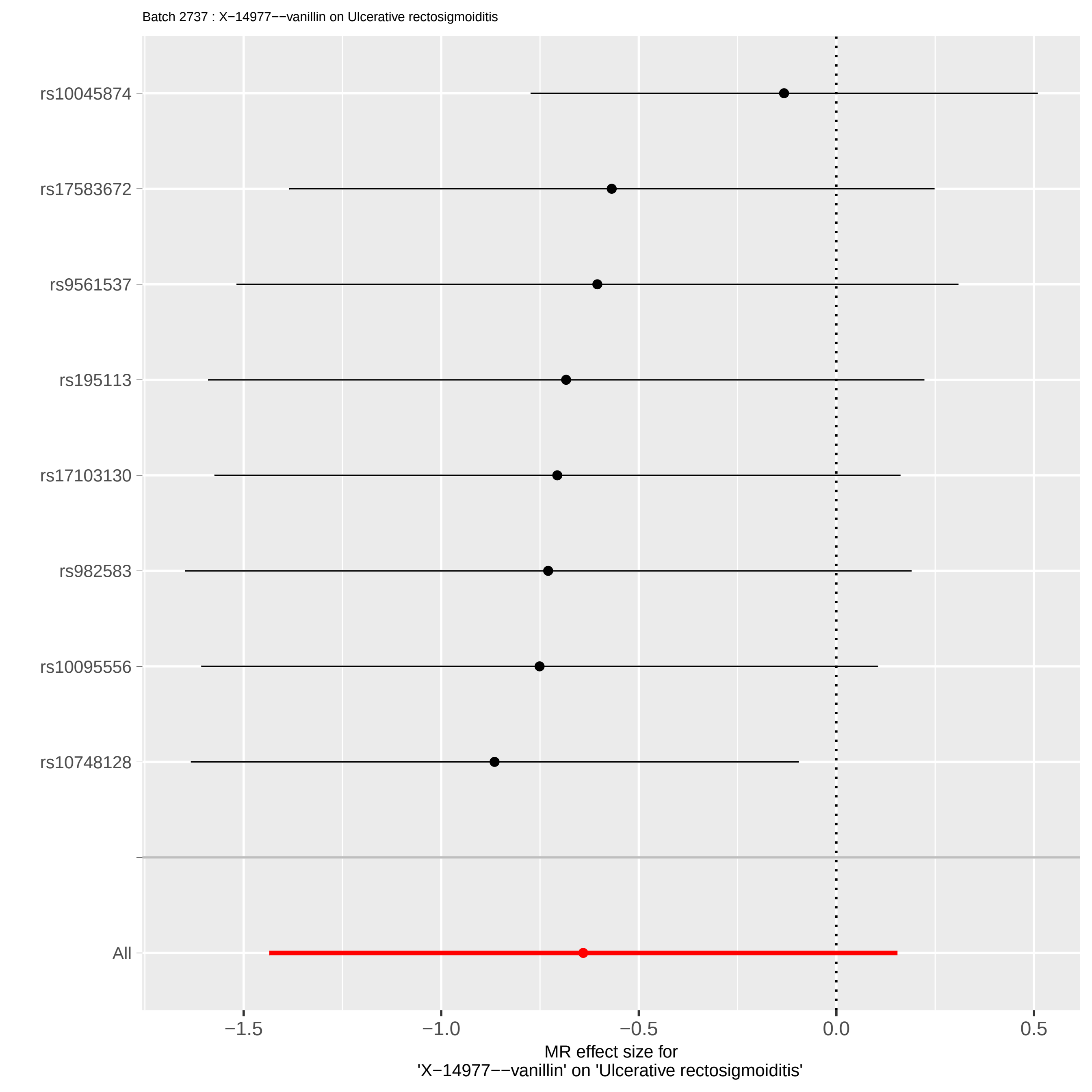
Batch 2737 : X−14977−−vanillin on Ulcerative rectosigmoiditis
rs10045874
rs17583672
rs9561537
rs195113
rs17103130
rs982583
rs10095556
rs10748128
All
−1.5
−1.0
−0.5
0.0
0.5
MR effect size for
'X−14977−−vanillin' on 'Ulcerative rectosigmoiditis'

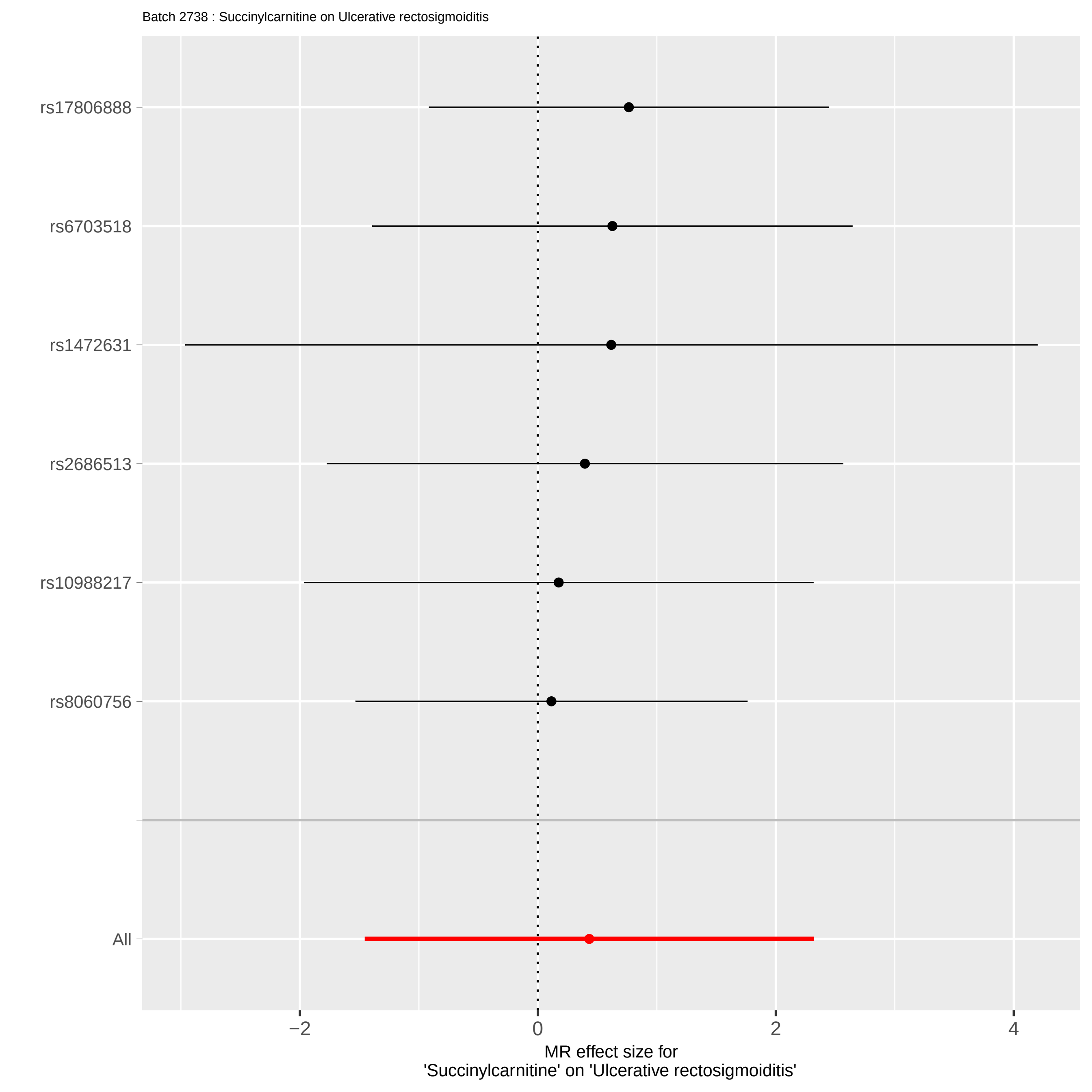

Batch 2738 : Succinylcarnitine on Ulcerative rectosigmoiditis
rs17806888
rs6703518
rs1472631
rs2686513
rs10988217
rs8060756
All
−2
0
2
4
MR effect size for
'Succinylcarnitine' on 'Ulcerative rectosigmoiditis'

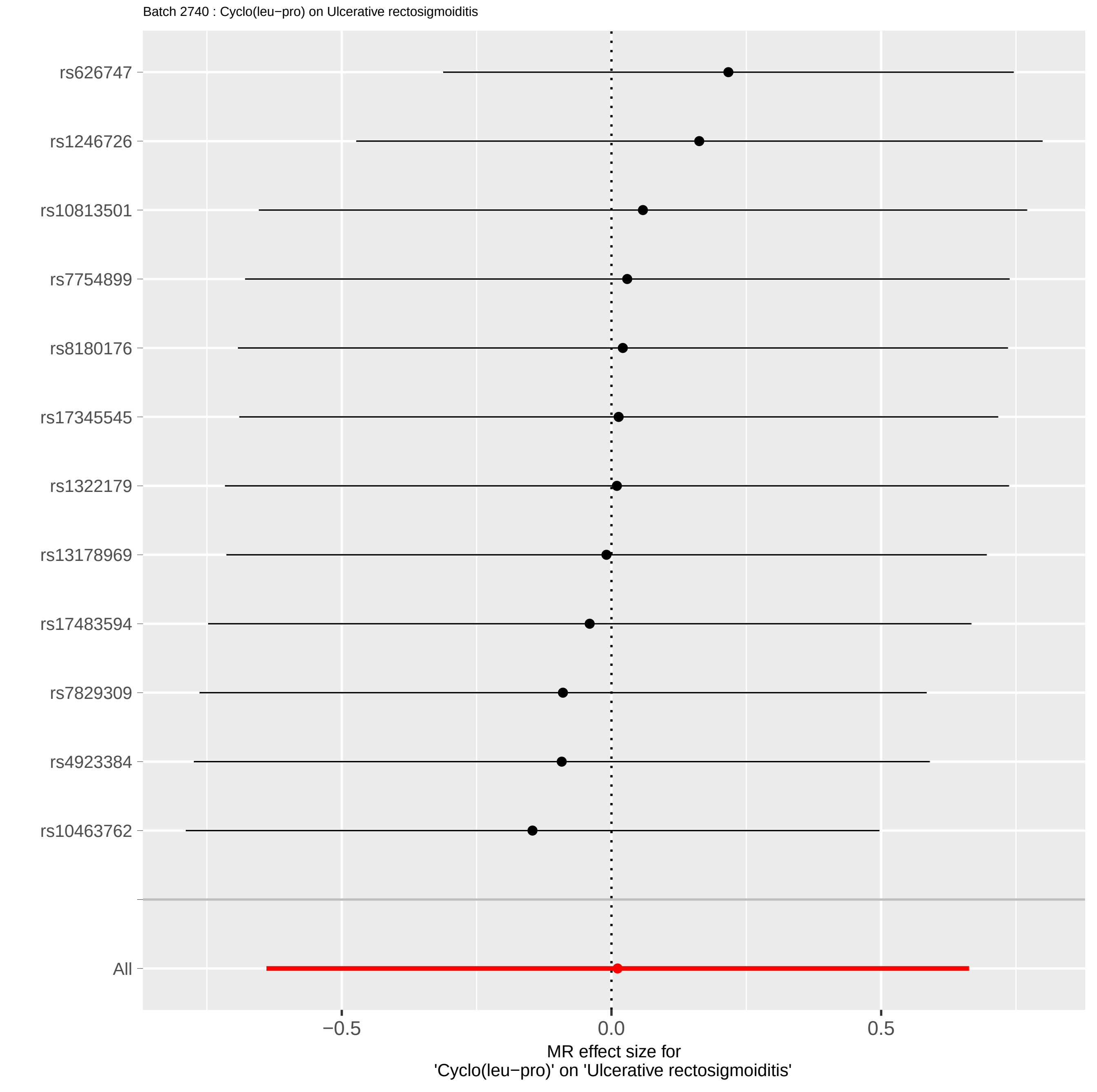

Batch 2740 : Cyclo(leu−pro) on Ulcerative rectosigmoiditis
rs626747
rs1246726
rs10813501
rs7754899
rs8180176
rs17345545
rs1322179
rs13178969
rs17483594
rs7829309
rs4923384
rs10463762
All
−0.5
0.0
0.5
MR effect size for
'Cyclo(leu−pro)' on 'Ulcerative rectosigmoiditis'

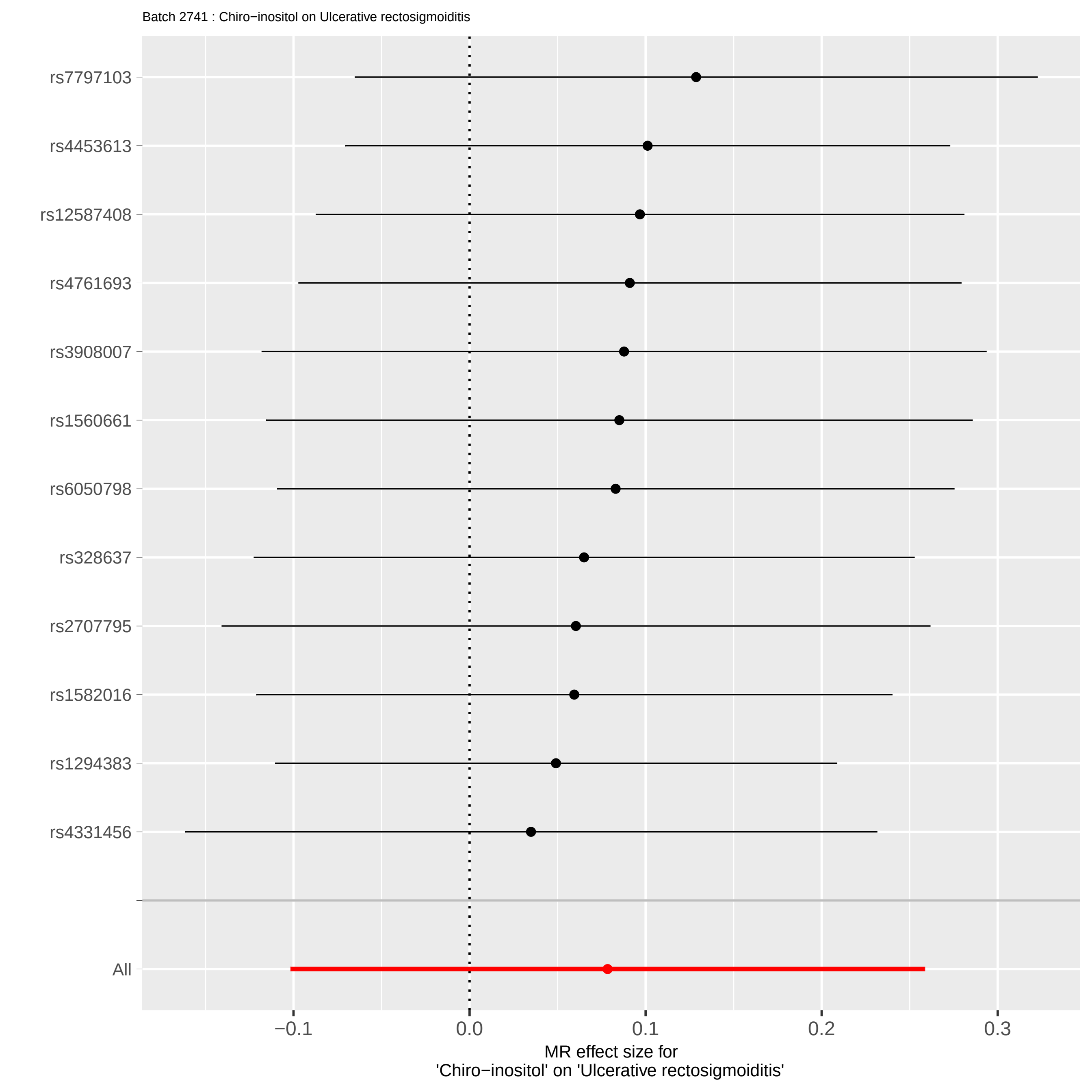
Batch 2741 : Chiro−inositol on Ulcerative rectosigmoiditis
rs7797103
rs4453613
rs12587408
rs4761693
rs3908007
rs1560661
rs6050798
rs328637
rs2707795
rs1582016
rs1294383
rs4331456
All
−0.1
0.0
0.1
0.2
0.3
MR effect size for
'Chiro−inositol' on 'Ulcerative rectosigmoiditis'

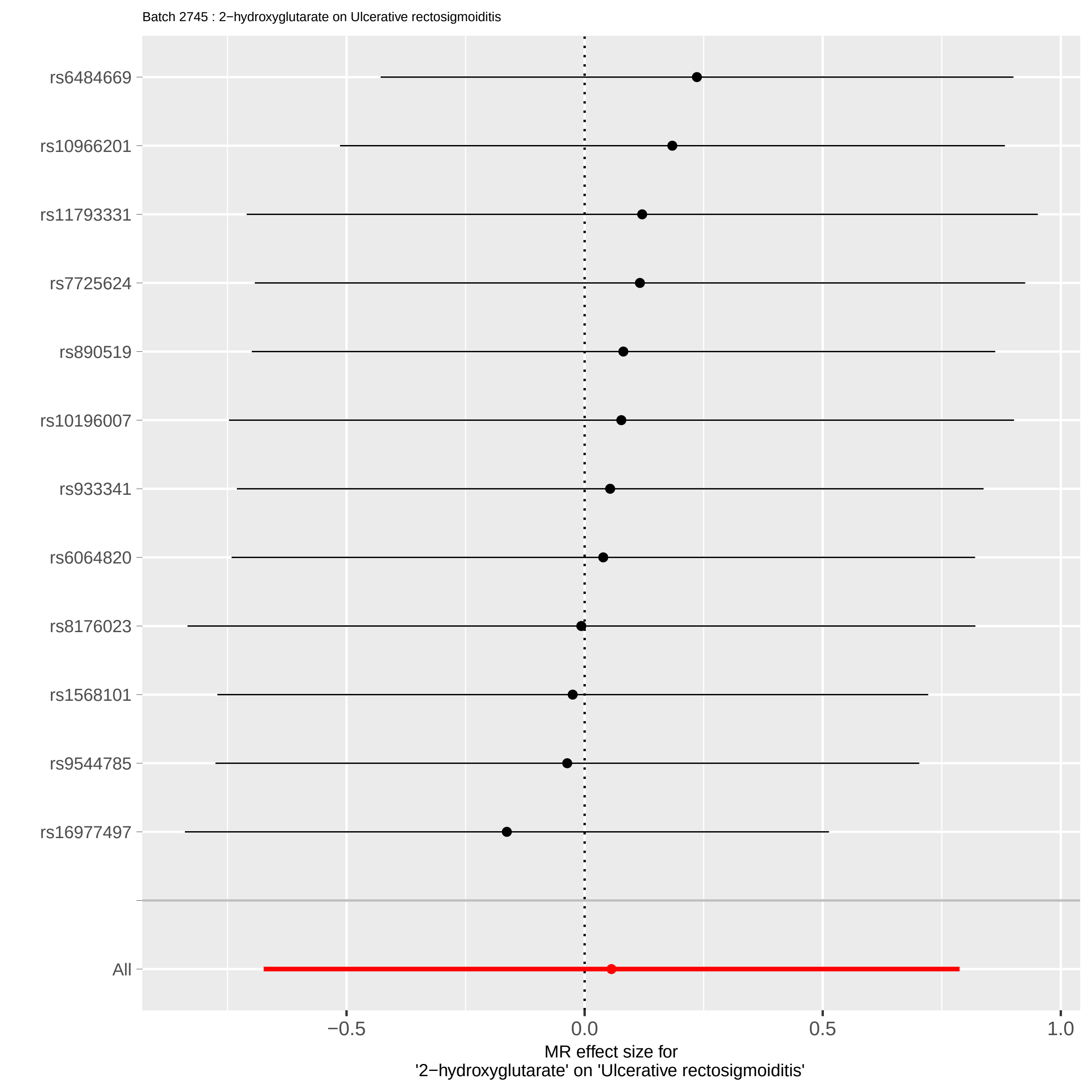
Batch 2745 : 2−hydroxyglutarate on Ulcerative rectosigmoiditis
rs6484669
rs10966201
rs11793331
rs7725624
rs890519
rs10196007
rs933341
rs6064820
rs8176023
rs1568101
rs9544785
rs16977497
All
−0.5
0.0
0.5
1.0
MR effect size for
'2−hydroxyglutarate' on 'Ulcerative rectosigmoiditis'

Batch 2746 : Ergothioneine on Ulcerative rectosigmoiditis

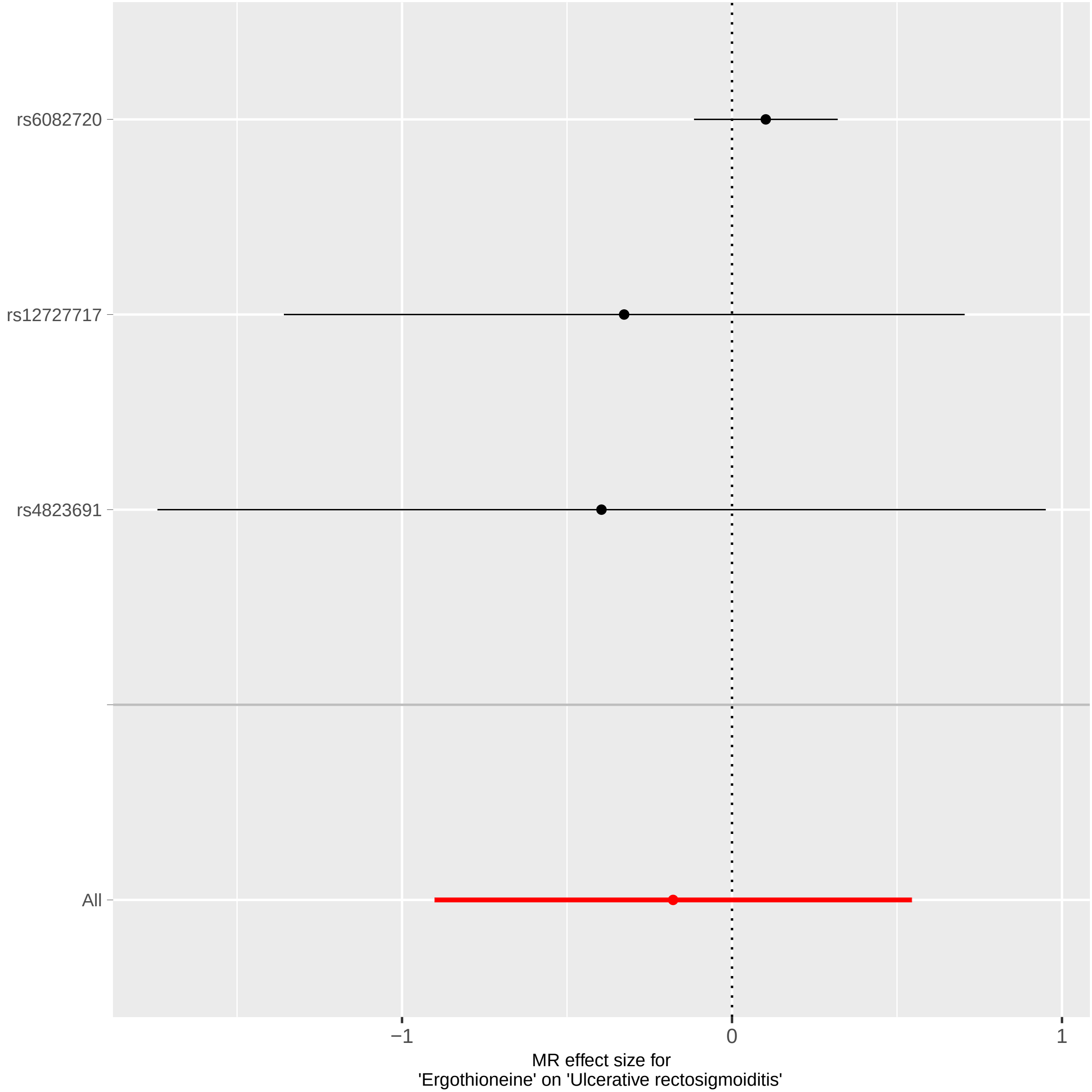

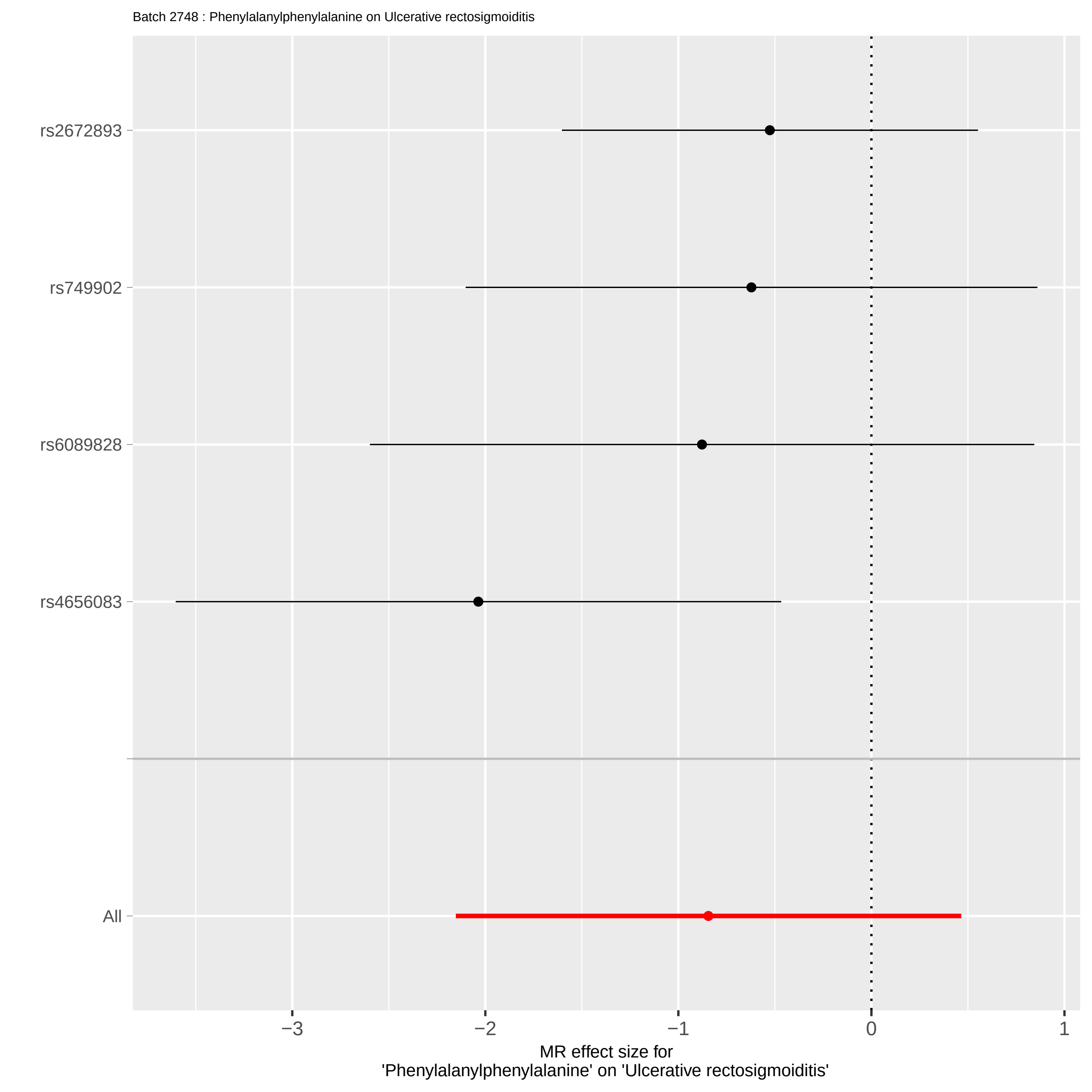
Batch 2748 : Phenylalanylphenylalanine on Ulcerative rectosigmoiditis
rs2672893
rs749902
rs6089828
rs4656083
All
−3
−2
−1
0
1
MR effect size for
'Phenylalanylphenylalanine' on 'Ulcerative rectosigmoiditis'

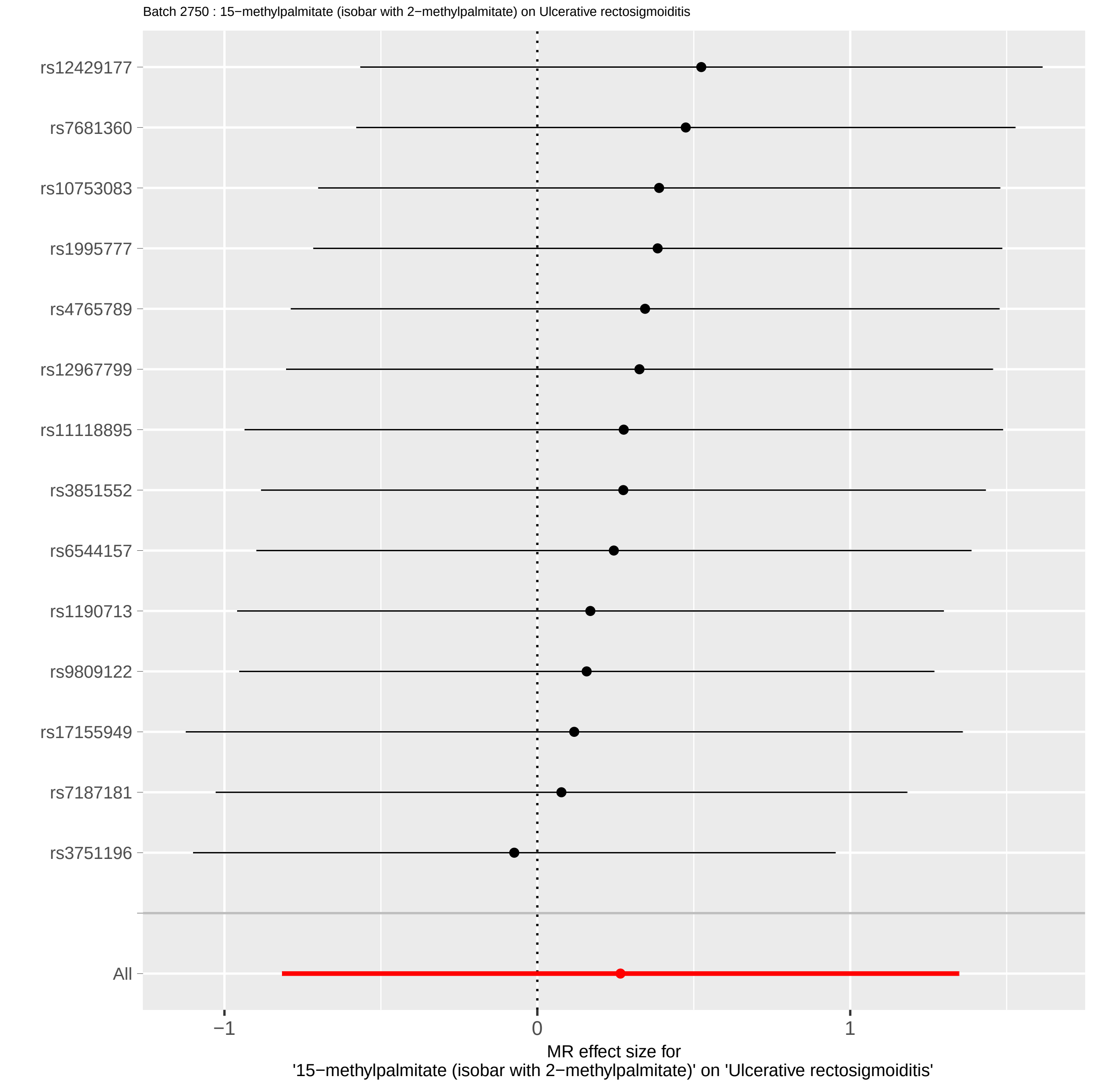

Batch 2750 : 15−methylpalmitate (isobar with 2−methylpalmitate) on Ulcerative rectosigmoiditis
rs12429177
rs7681360
rs10753083
rs1995777
rs4765789
rs12967799
rs11118895
rs3851552
rs6544157
rs1190713
rs9809122
rs17155949
rs7187181
rs3751196
All
−1
0
1
MR effect size for
'15−methylpalmitate (isobar with 2−methylpalmitate)' on 'Ulcerative rectosigmoiditis'
