# Supplementary material for: From serum metabolites to the gut: revealing metabolic clues to susceptibility to subtypes of Crohn’s disease and ulcerative colitis
Source: Front Endocrinol (Lausanne). 2024 Aug 8;15:1375896. doi: 10.3389/fendo.2024.1375896 (PMC11338916; doi:10.3389/fendo.2024.1375896)

Batch 2 : Tryptophan on Crohn's disease of colon

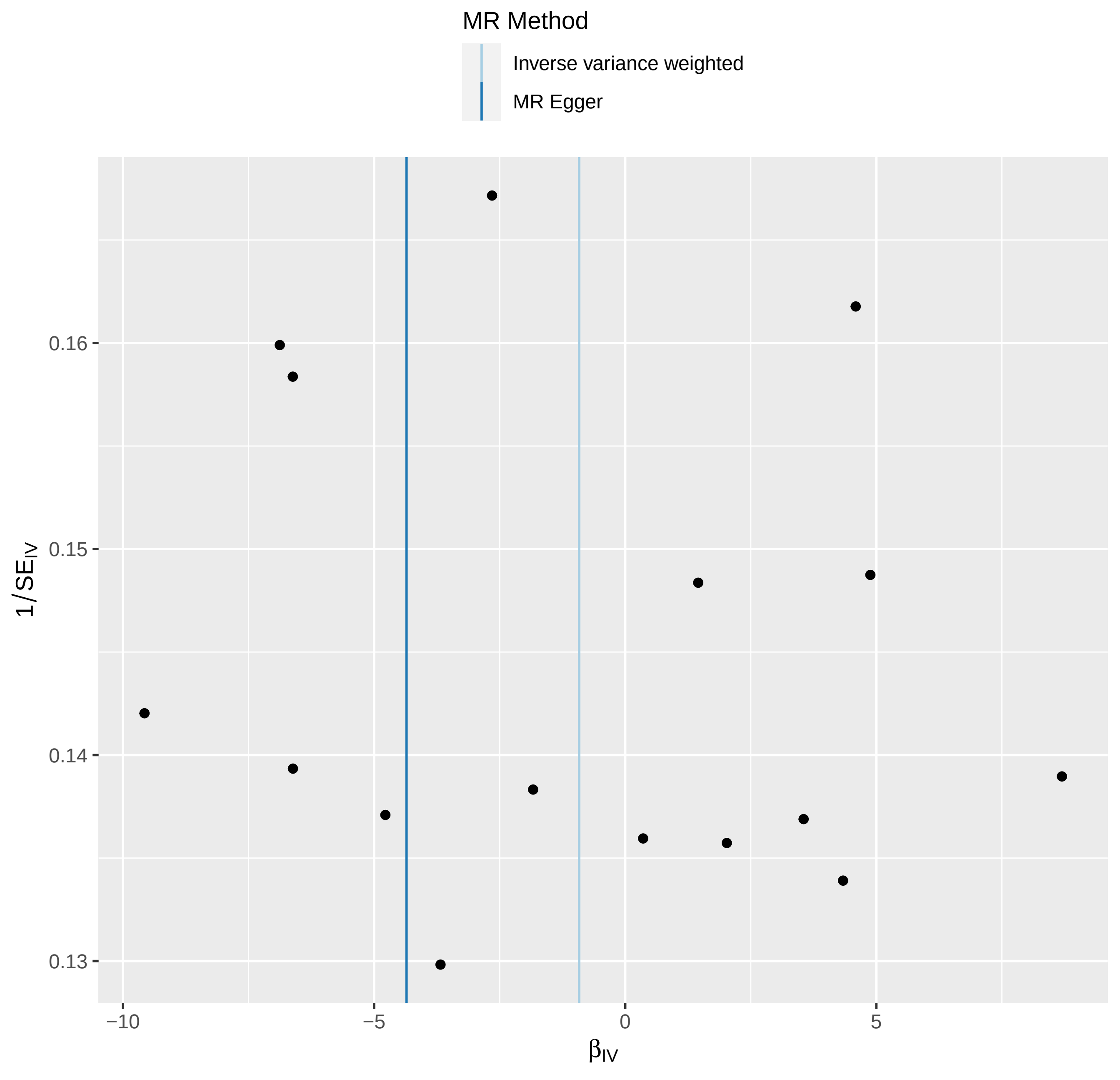

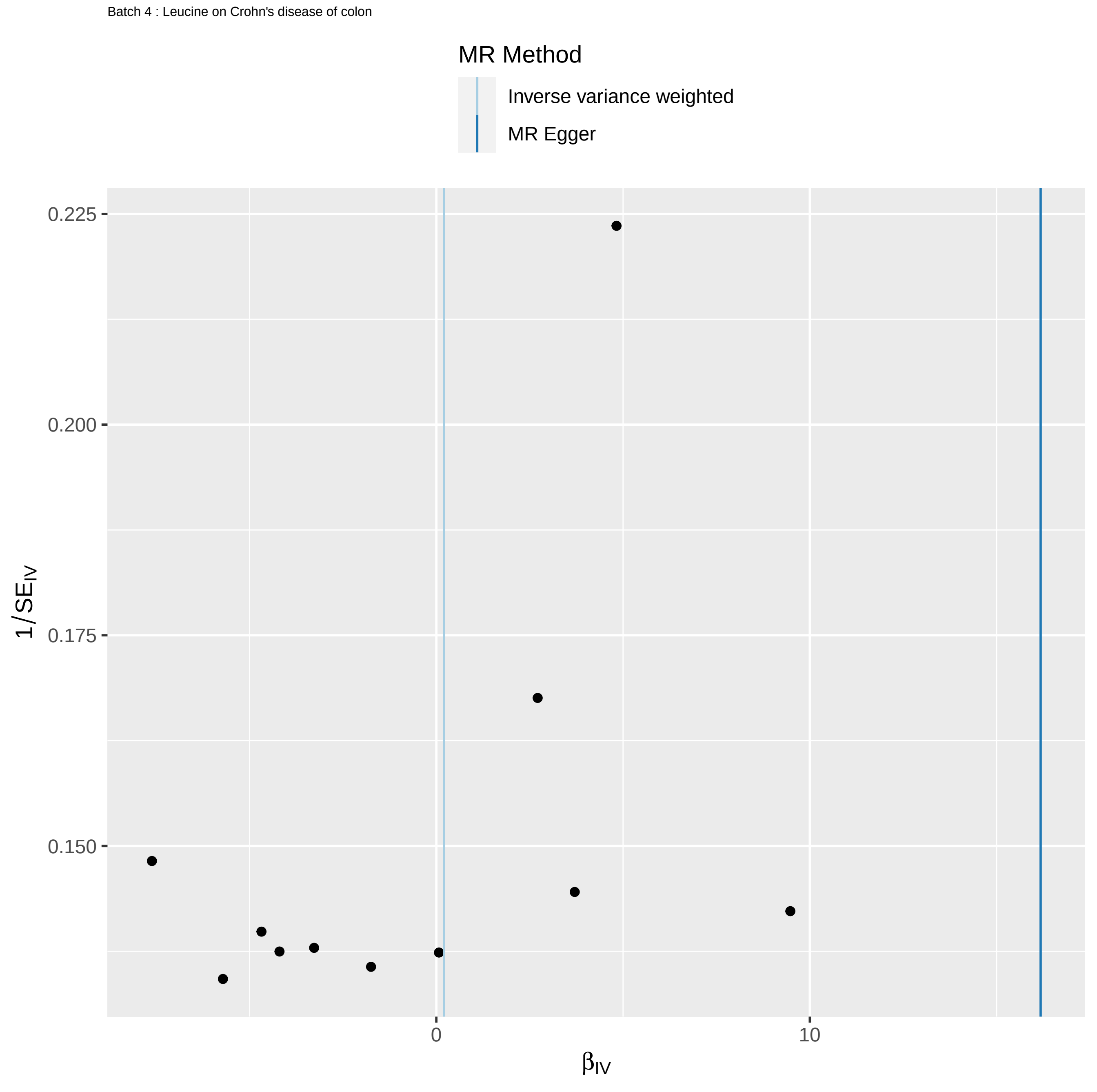
Batch 4 : Leucine on Crohn's disease of colon
MR Method
Inverse variance weighted
MR Egger
0.225
0.200
0.175
0.150
1/SE$_{IV}$
0
10
β$_{IV}$

Batch 8 : Lactate on Crohn's disease of colon

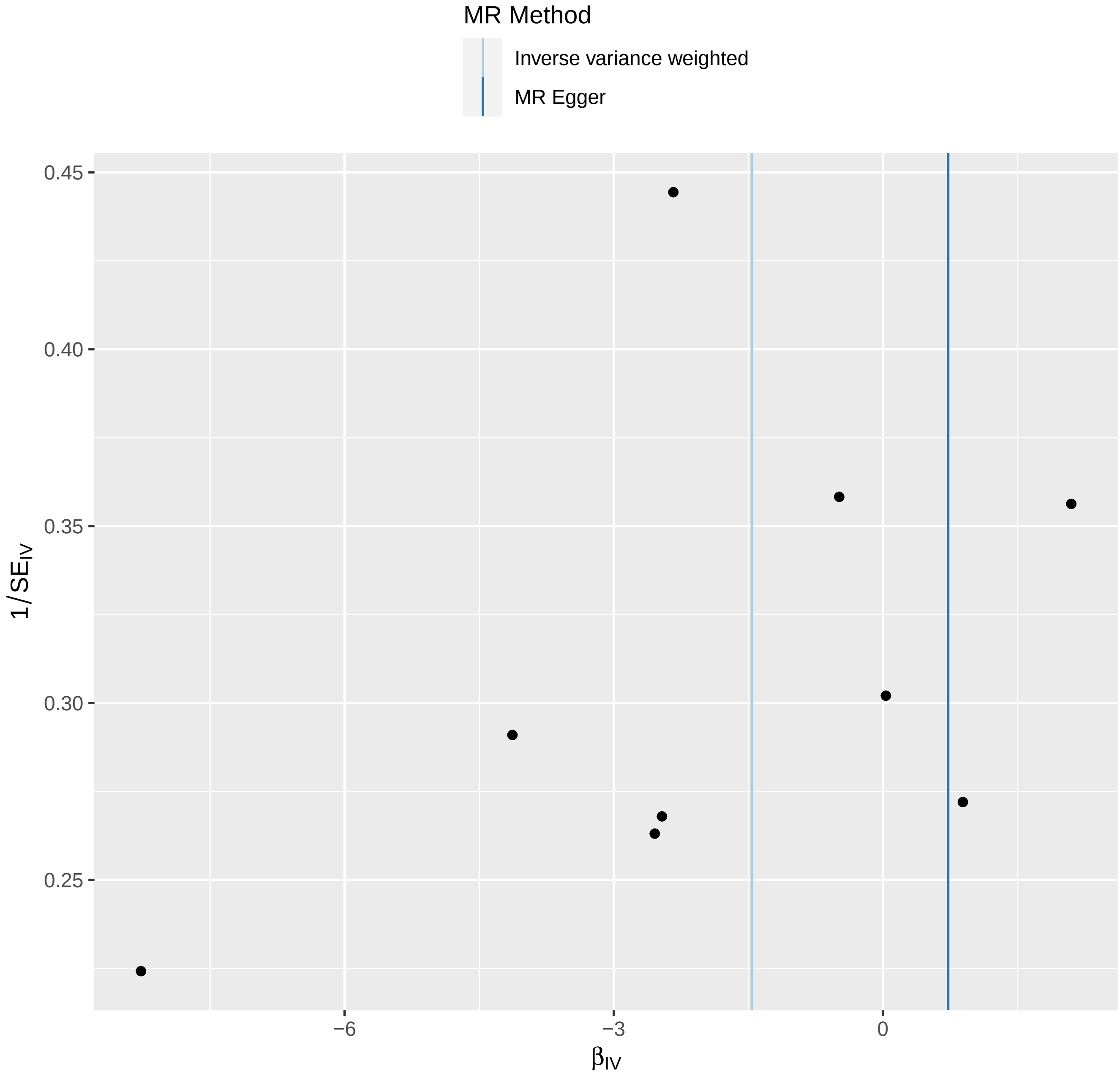

Batch 9 : 3−hydroxybutyrate (BHBA) on Crohn's disease of colon

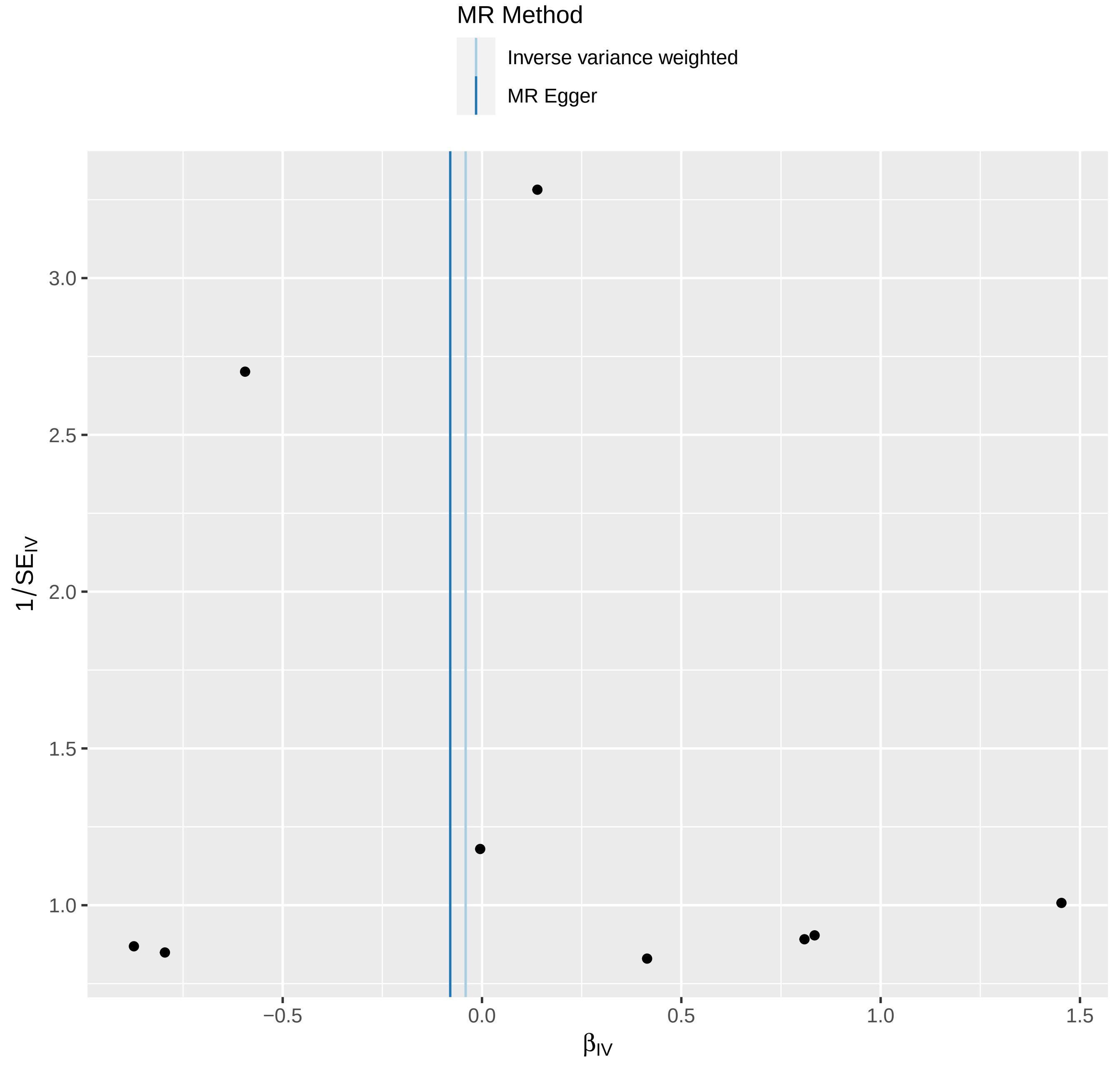

Batch 10 : Arabinose on Crohn's disease of colon

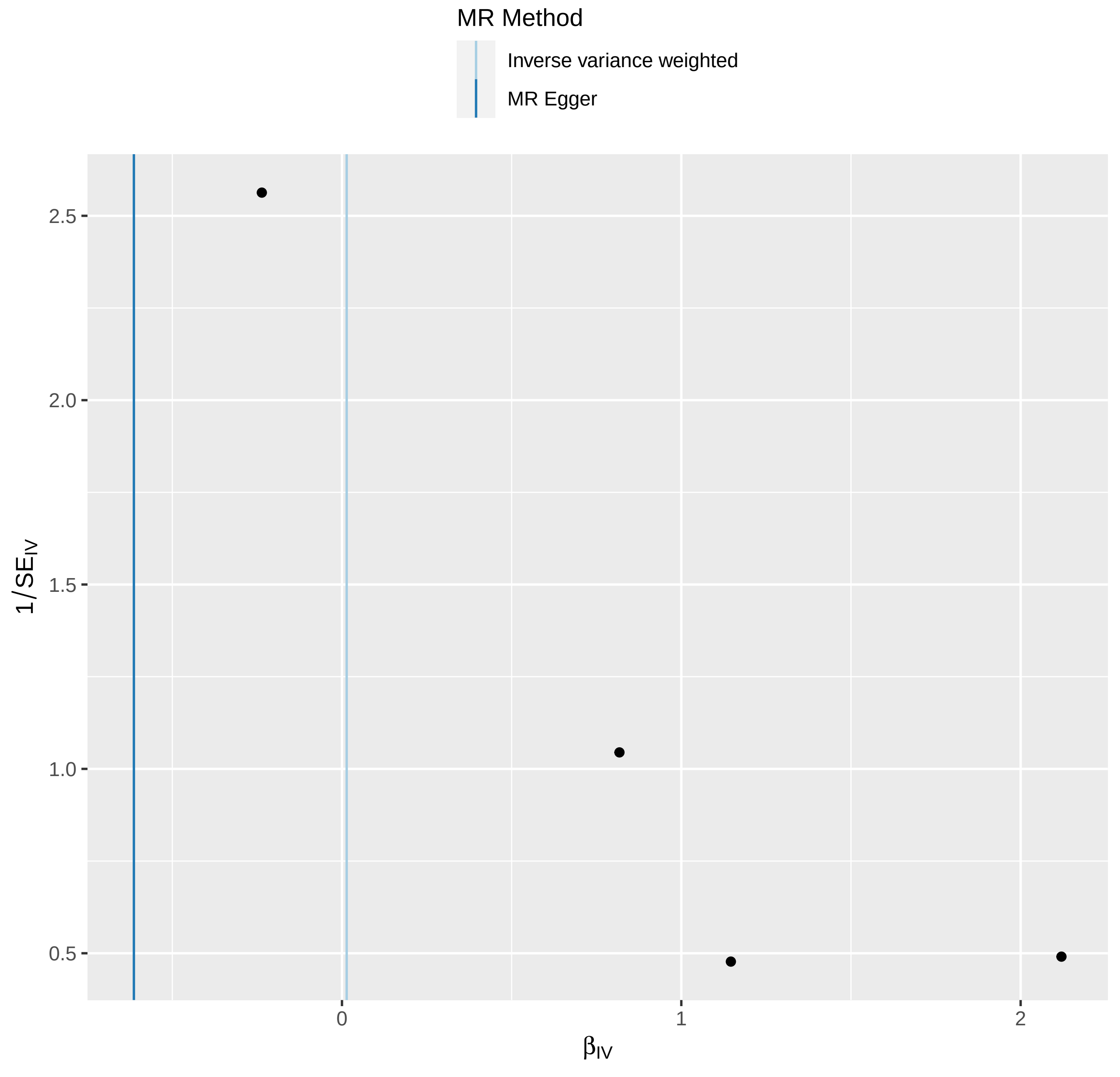

Batch 11 : Fructose on Crohn's disease of colon

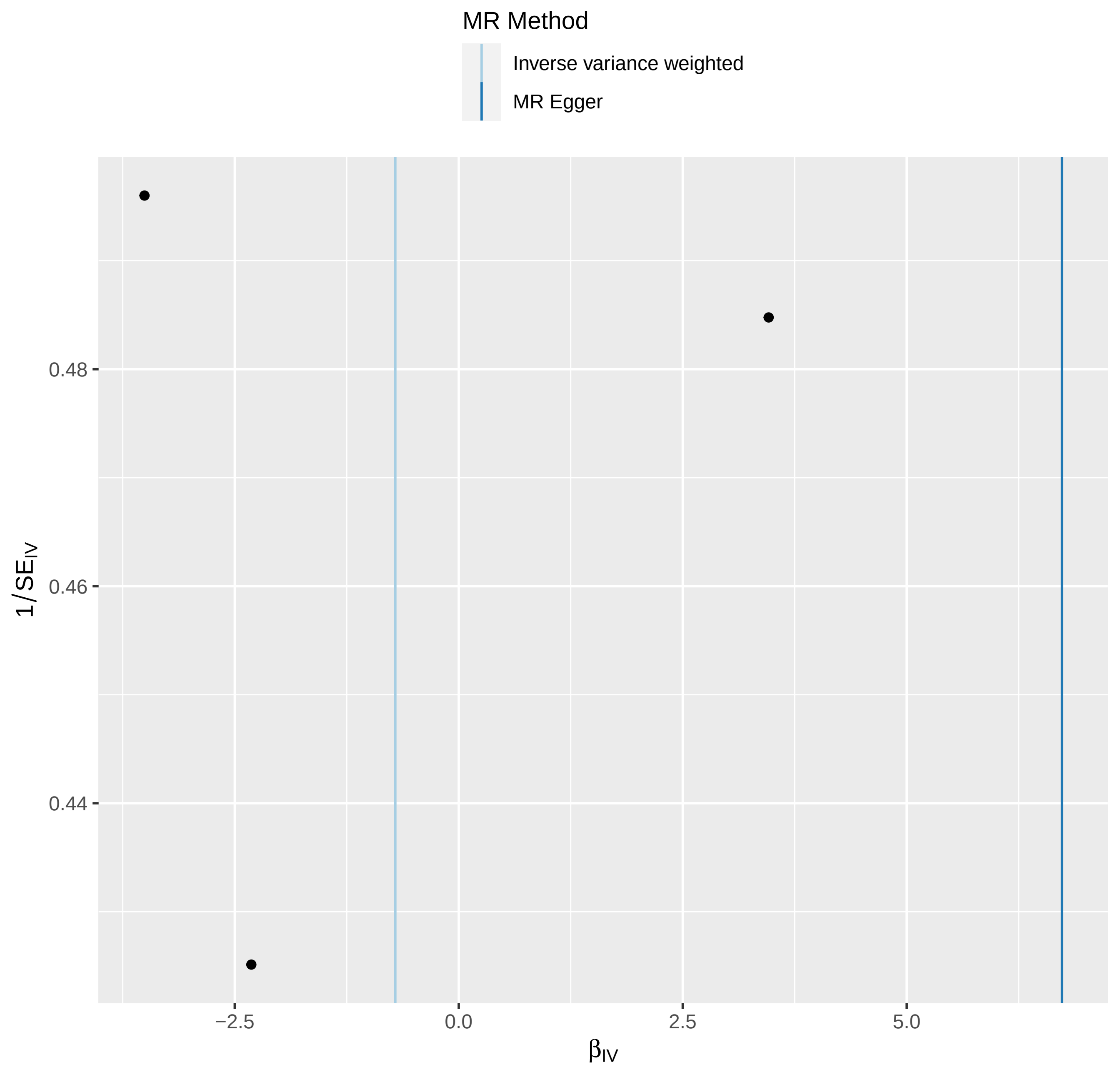

Batch 13 : Pyruvate on Crohn's disease of colon

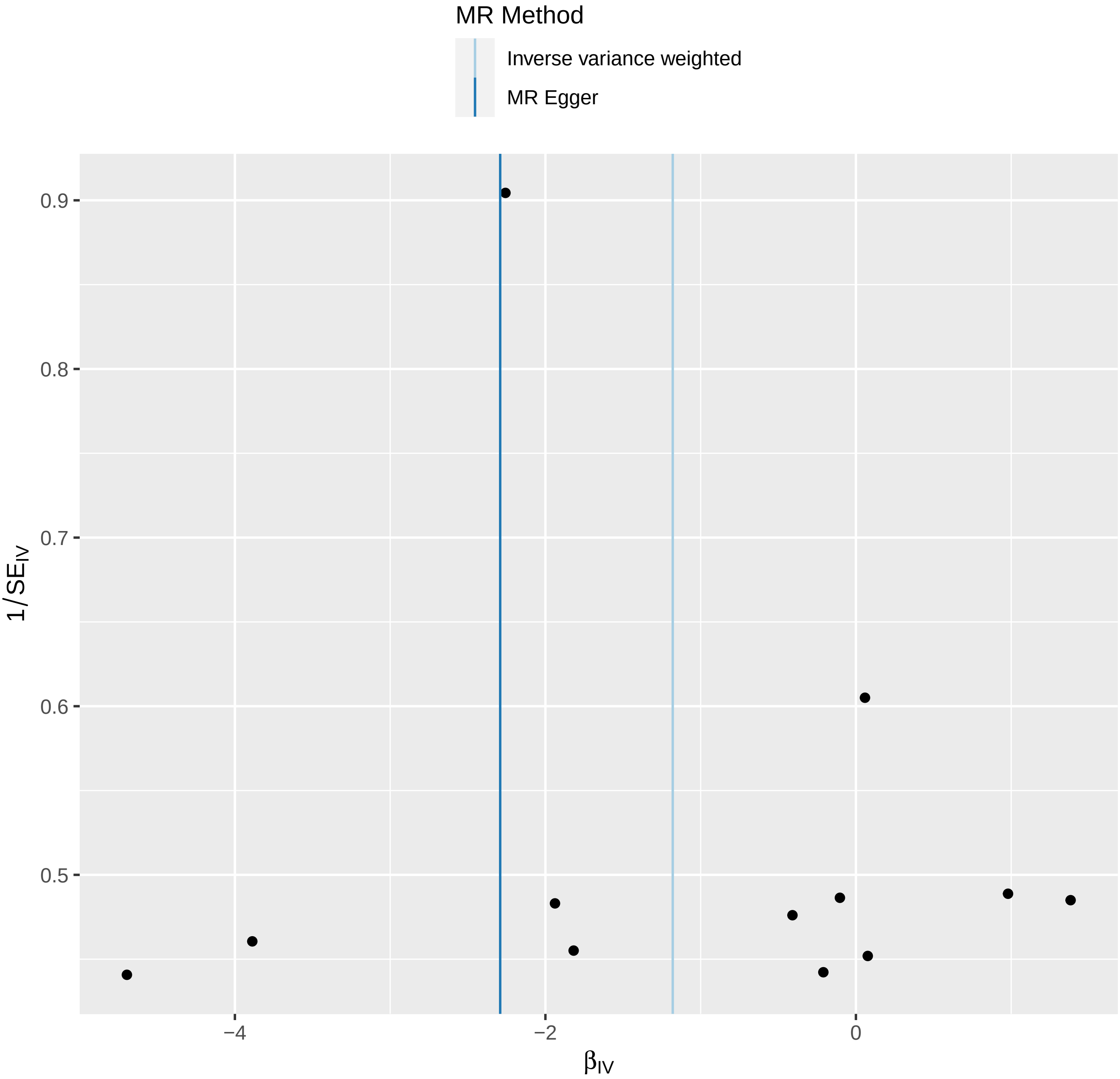

Batch 14 : Uridine on Crohn's disease of colon

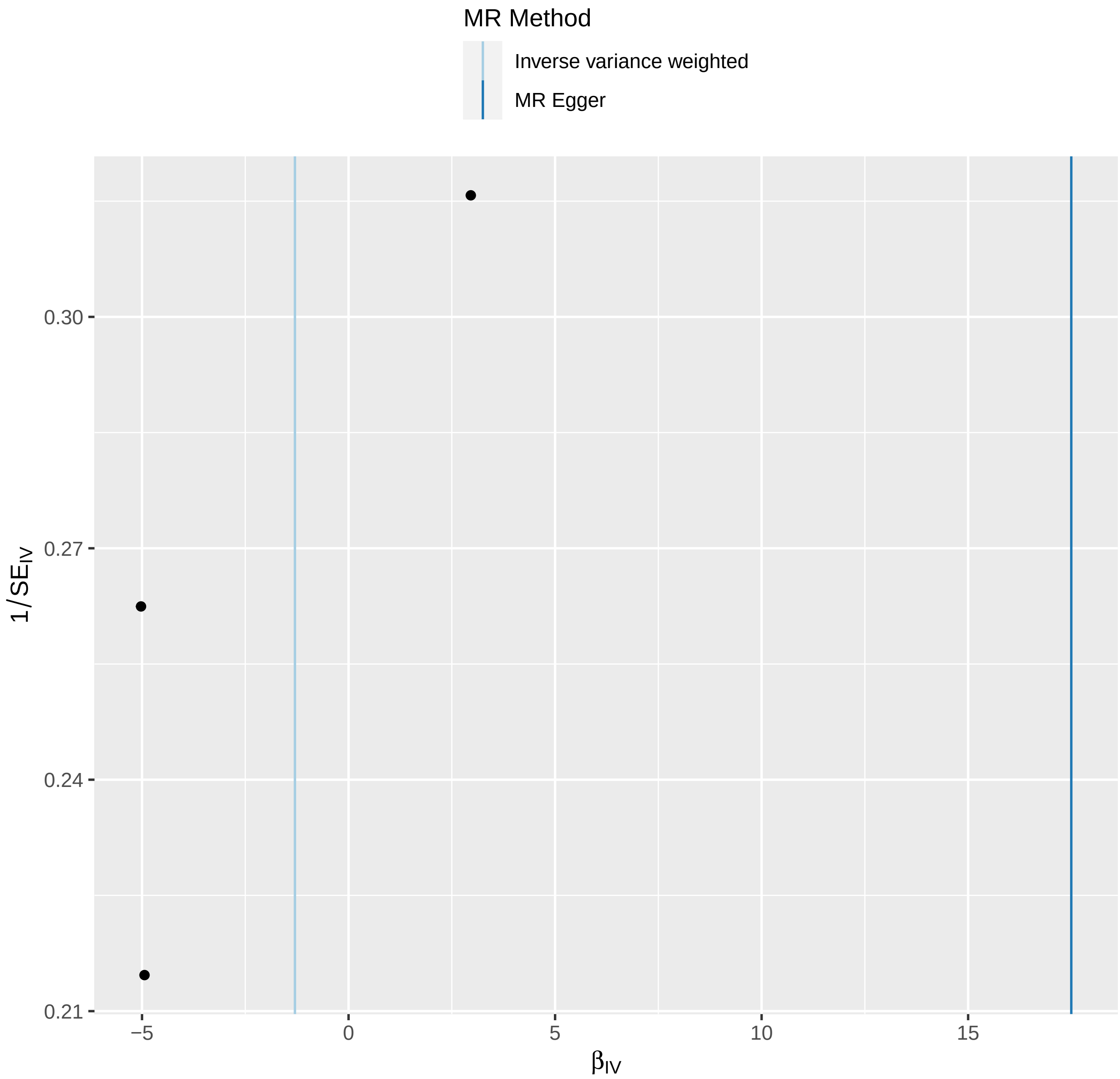

Batch 15 : Linoleate (18:2n6) on Crohn's disease of colon

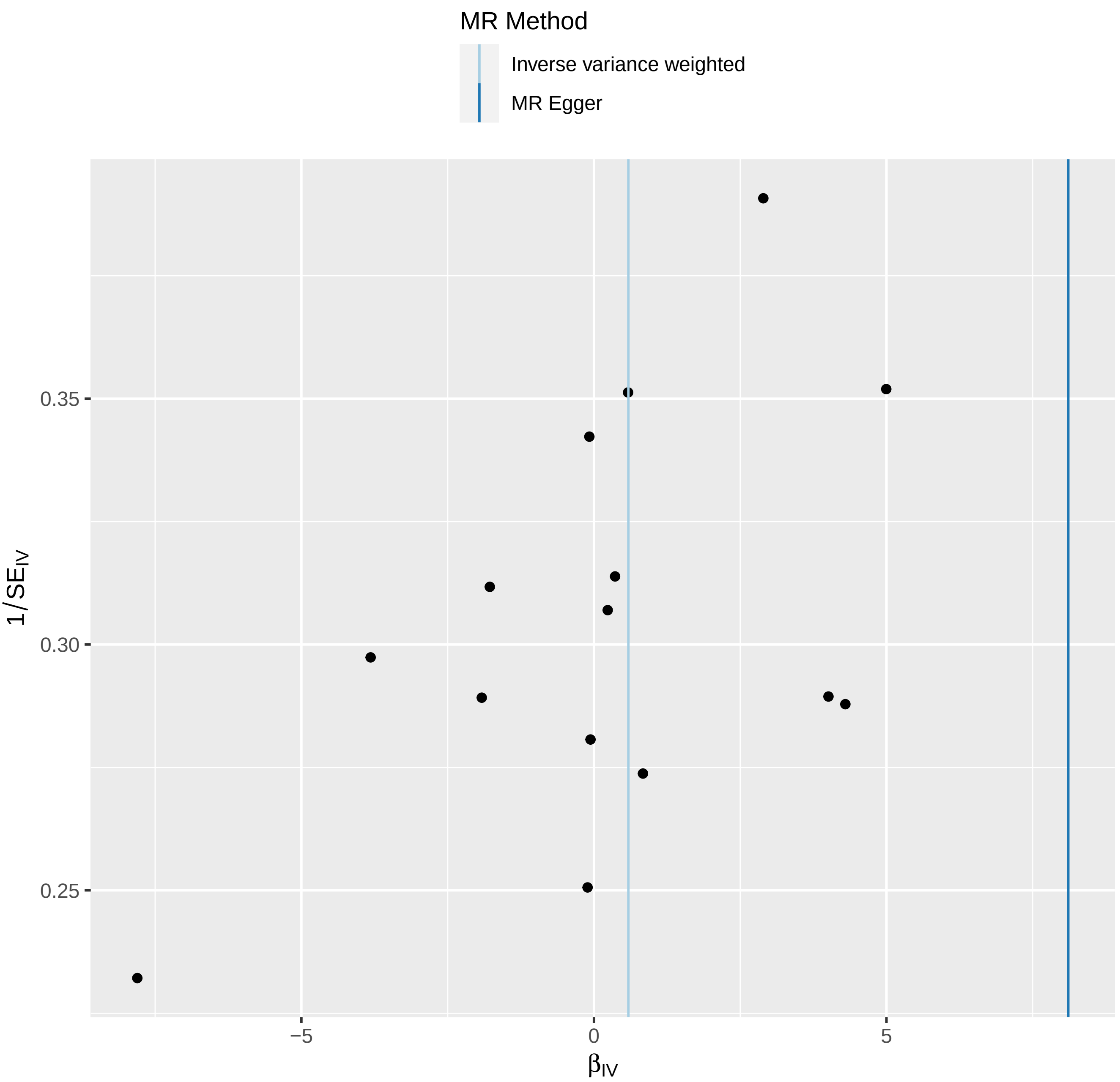

Batch 16 : Allantoin on Crohn's disease of colon

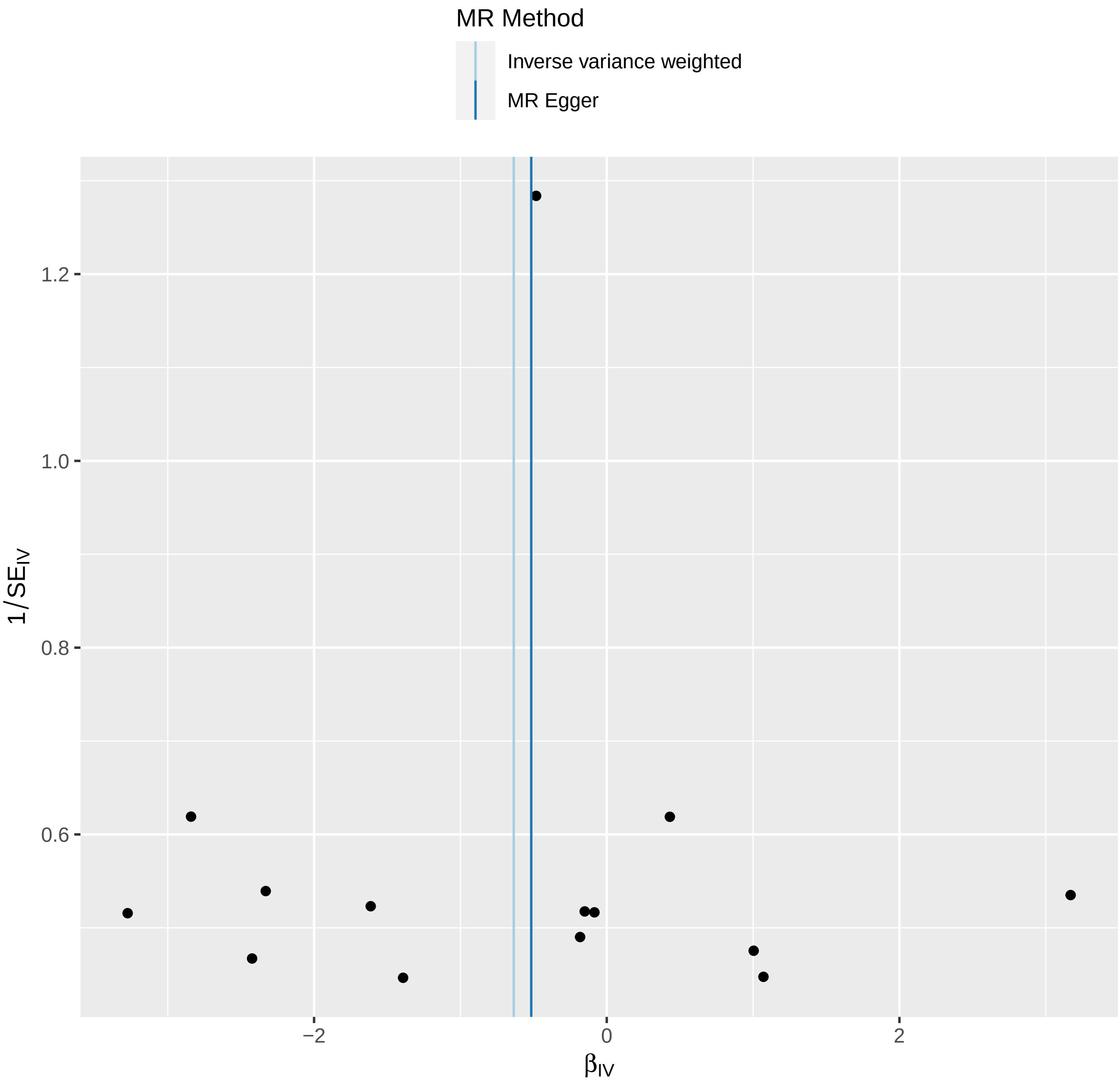

Batch 21 : Isoleucine on Crohn's disease of colon

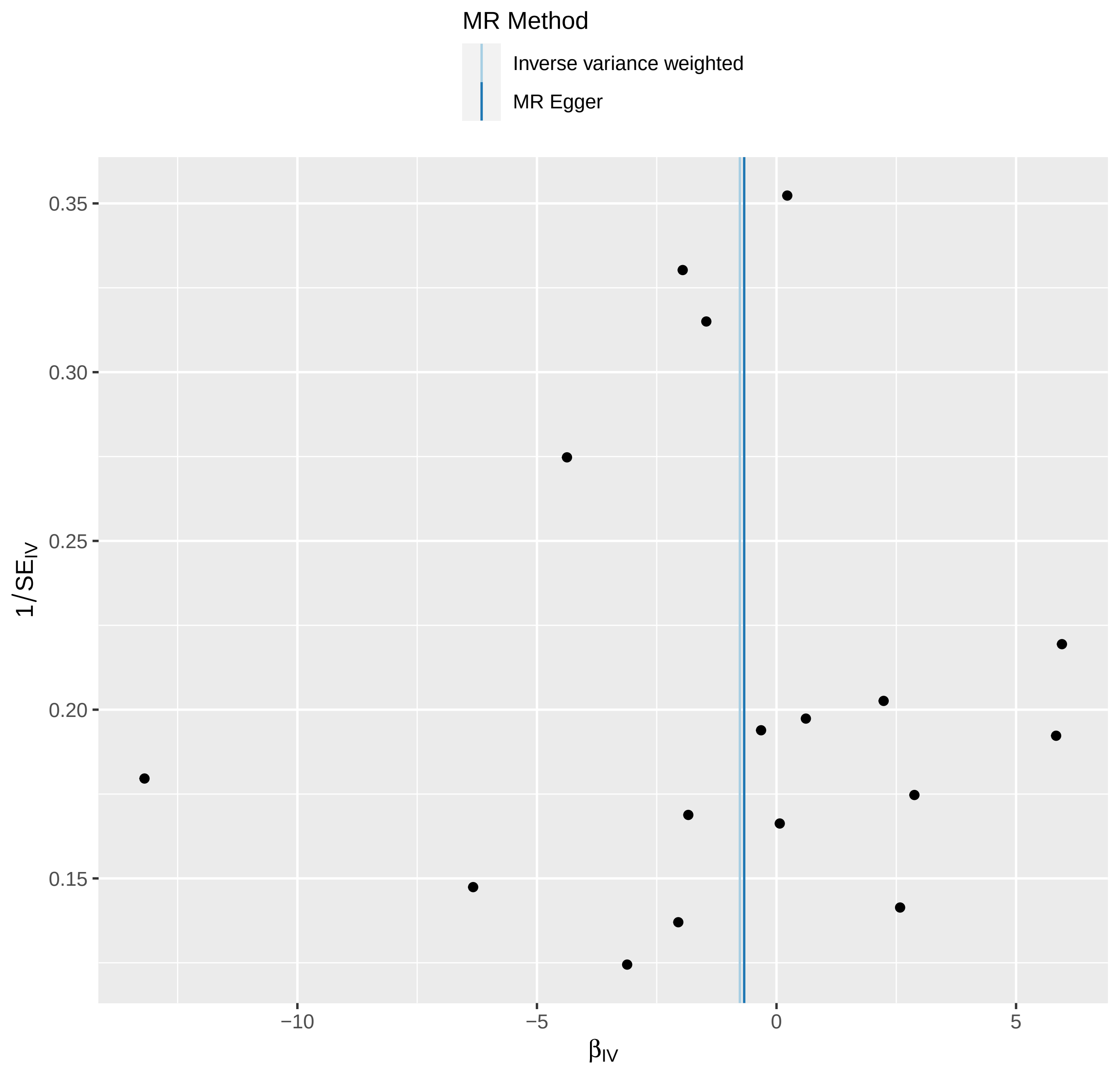

Batch 22 : Threonine on Crohn's disease of colon

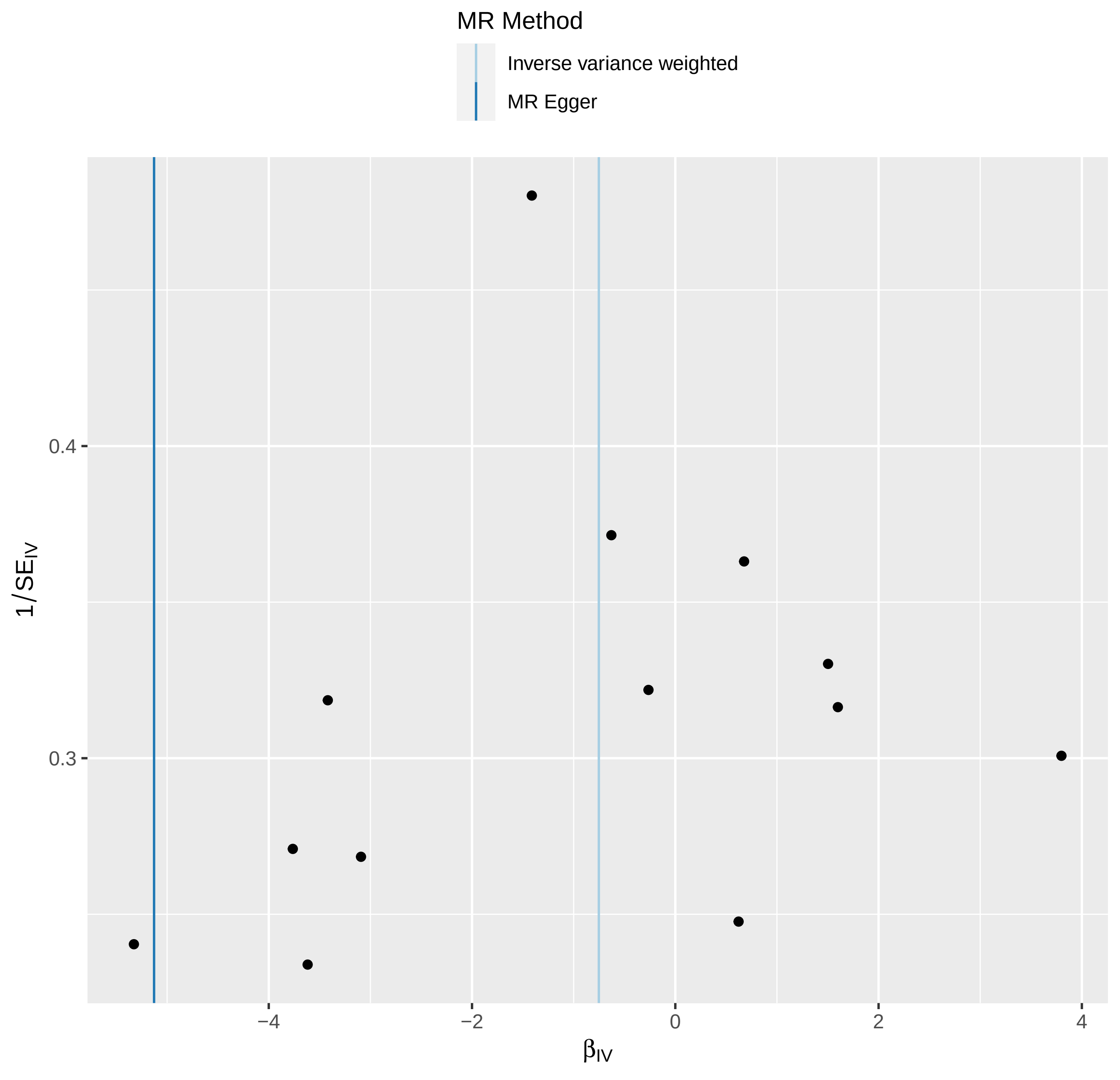

Batch 23 : Tyrosine on Crohn's disease of colon

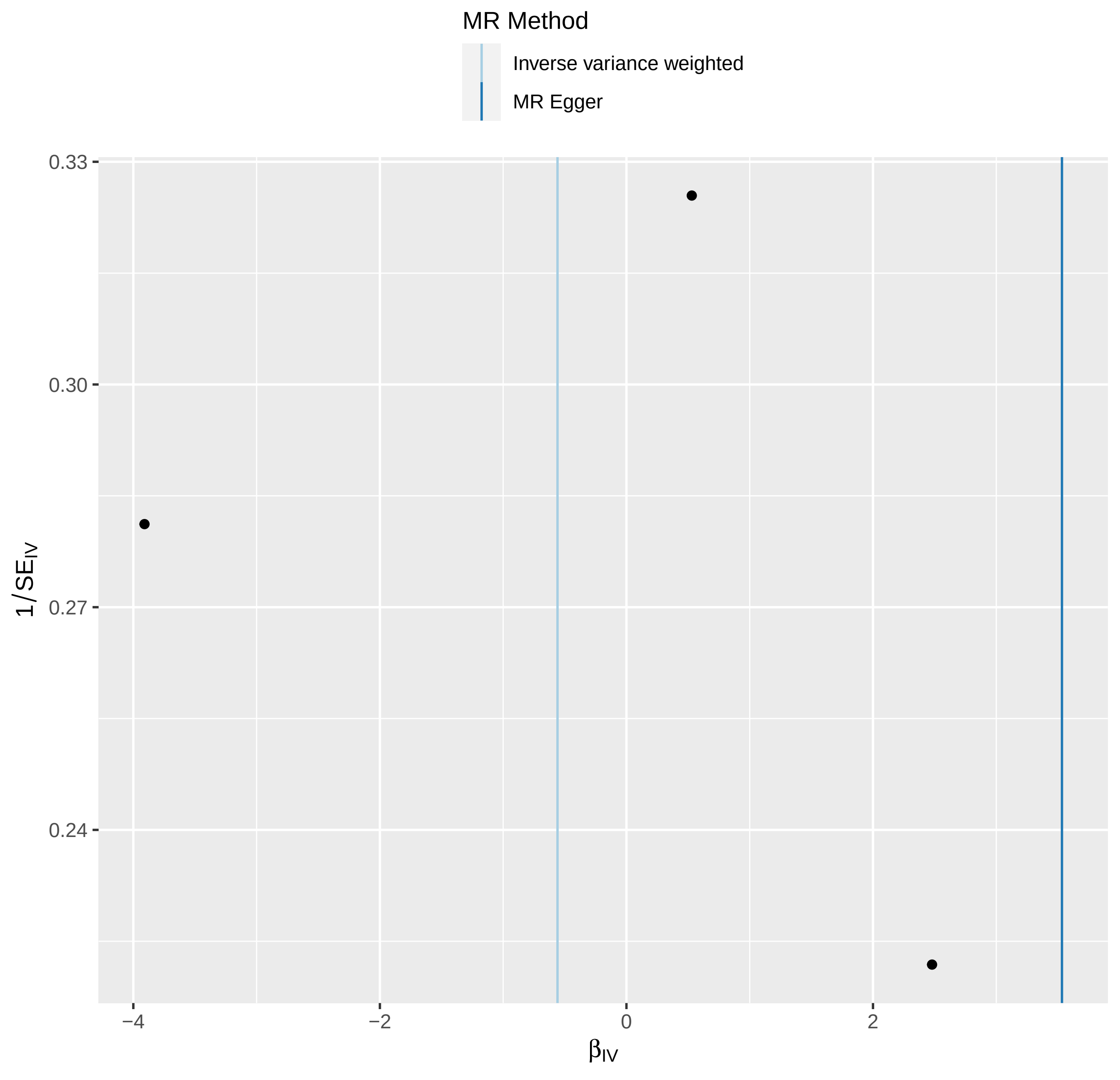

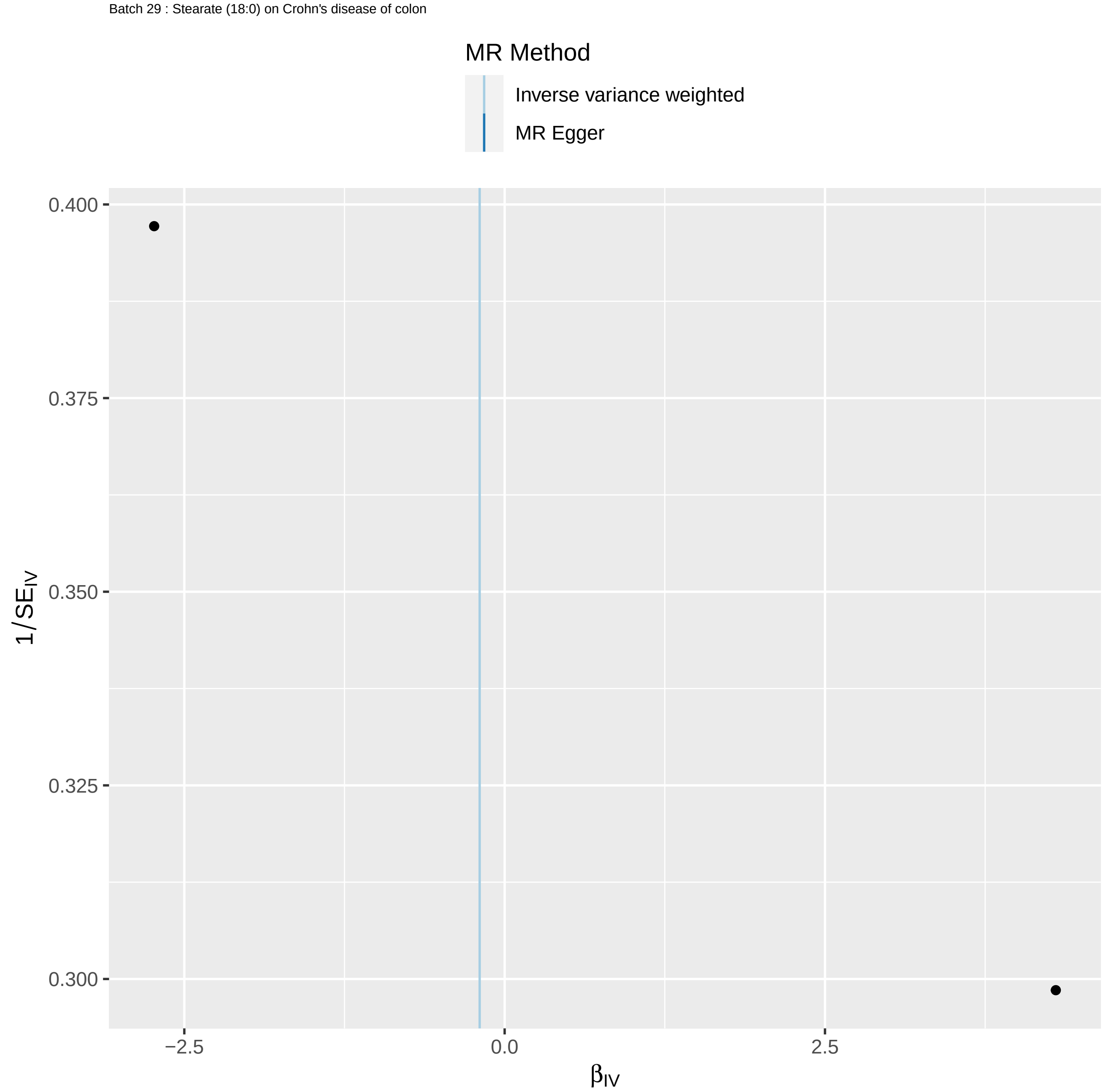
Batch 29 : Stearate (18:0) on Crohn's disease of colon
MR Method
Inverse variance weighted
MR Egger
0.400
0.375
0.350
0.325
0.300
1/SE_IV
−2.5
0.0
2.5
β_IV

Batch 32 : Myristate (14:0) on Crohn's disease of colon

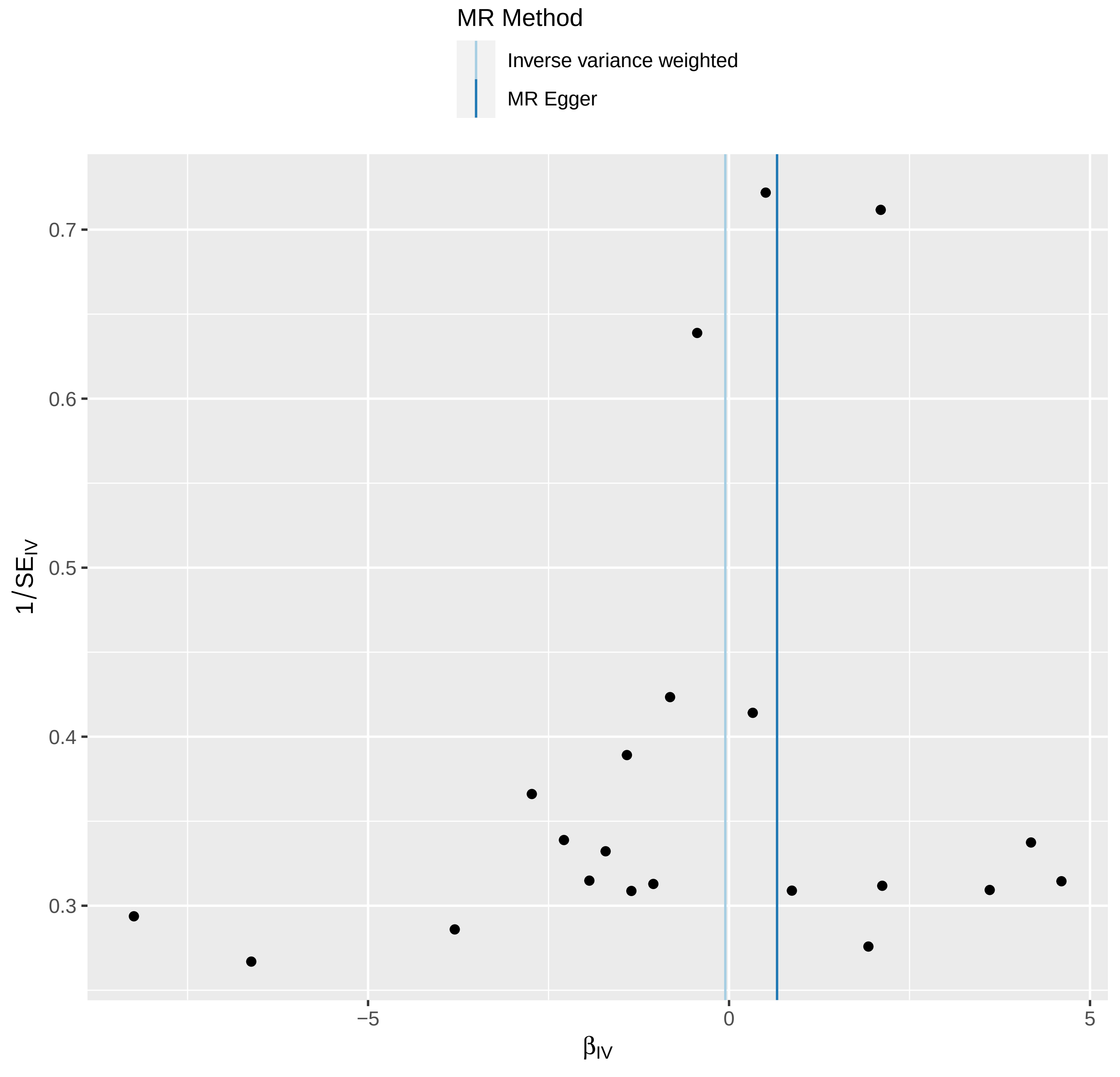

Batch 34 : Ornithine on Crohn's disease of colon

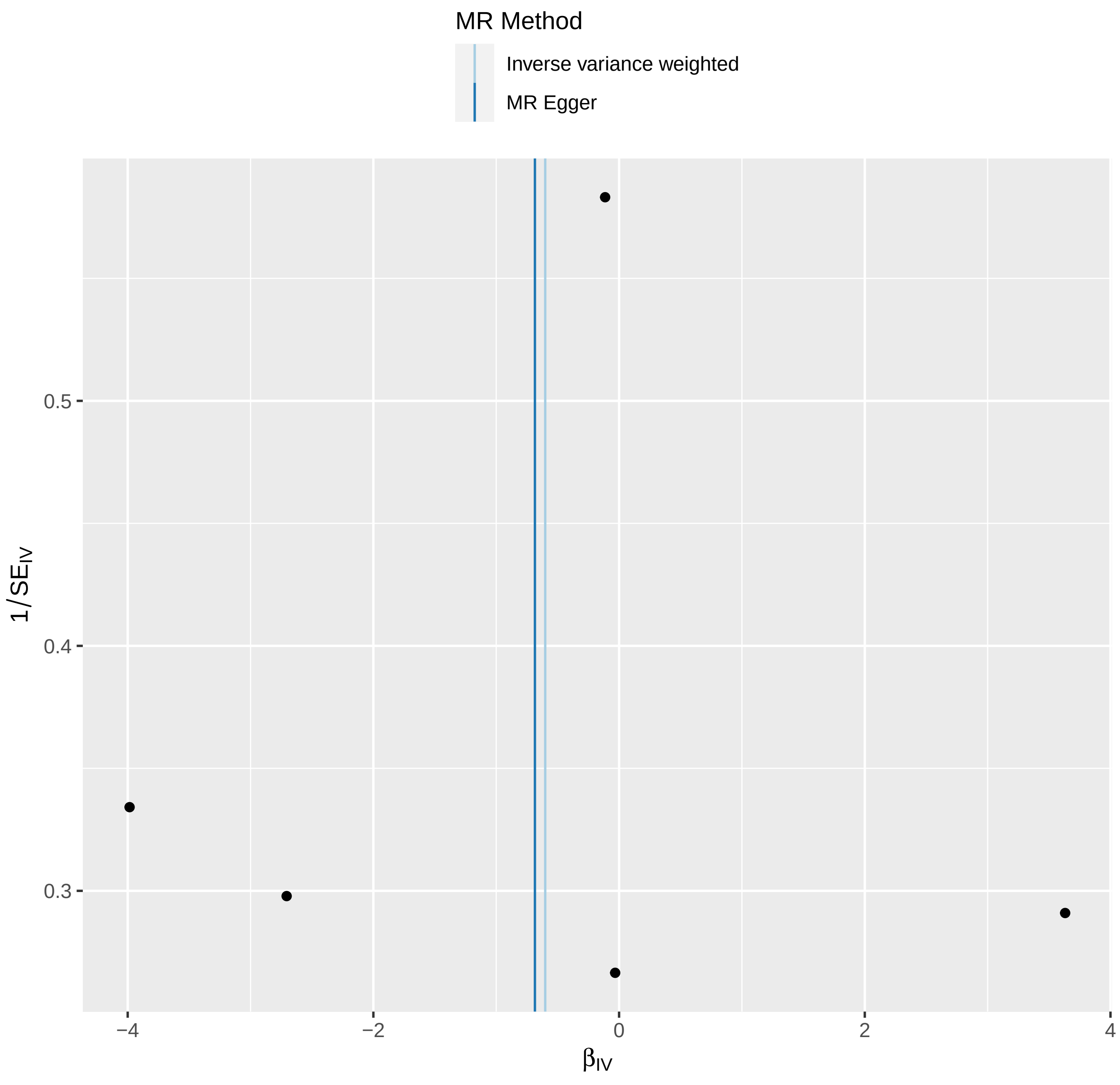

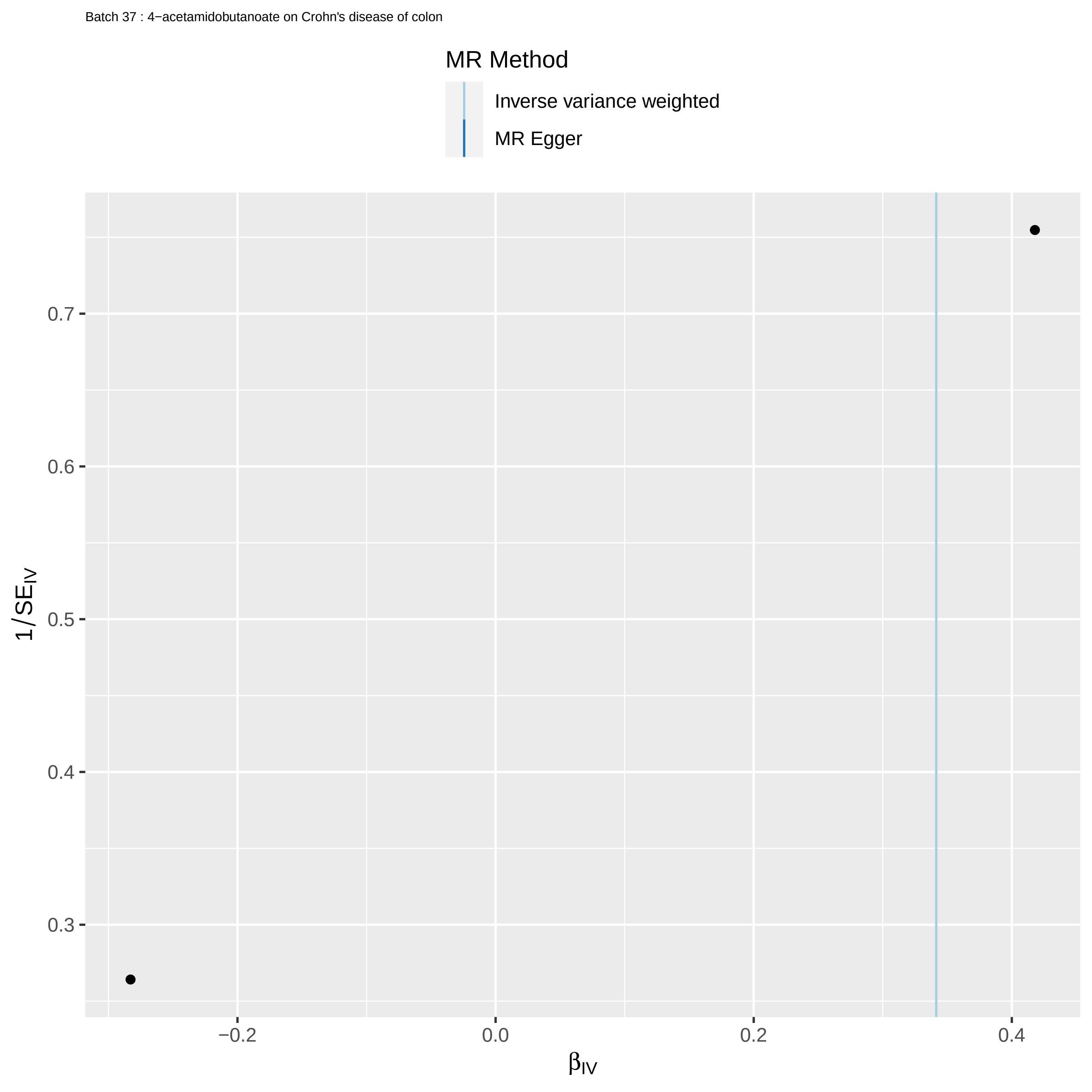
Batch 37 : 4−acetamidobutanoate on Crohn's disease of colon
MR Method
Inverse variance weighted
MR Egger
0.7
0.6
0.5
0.4
0.3
1/SE_IV
−0.2
0.0
0.2
0.4
β_IV

Batch 38 : Alpha−tocopherol on Crohn's disease of colon

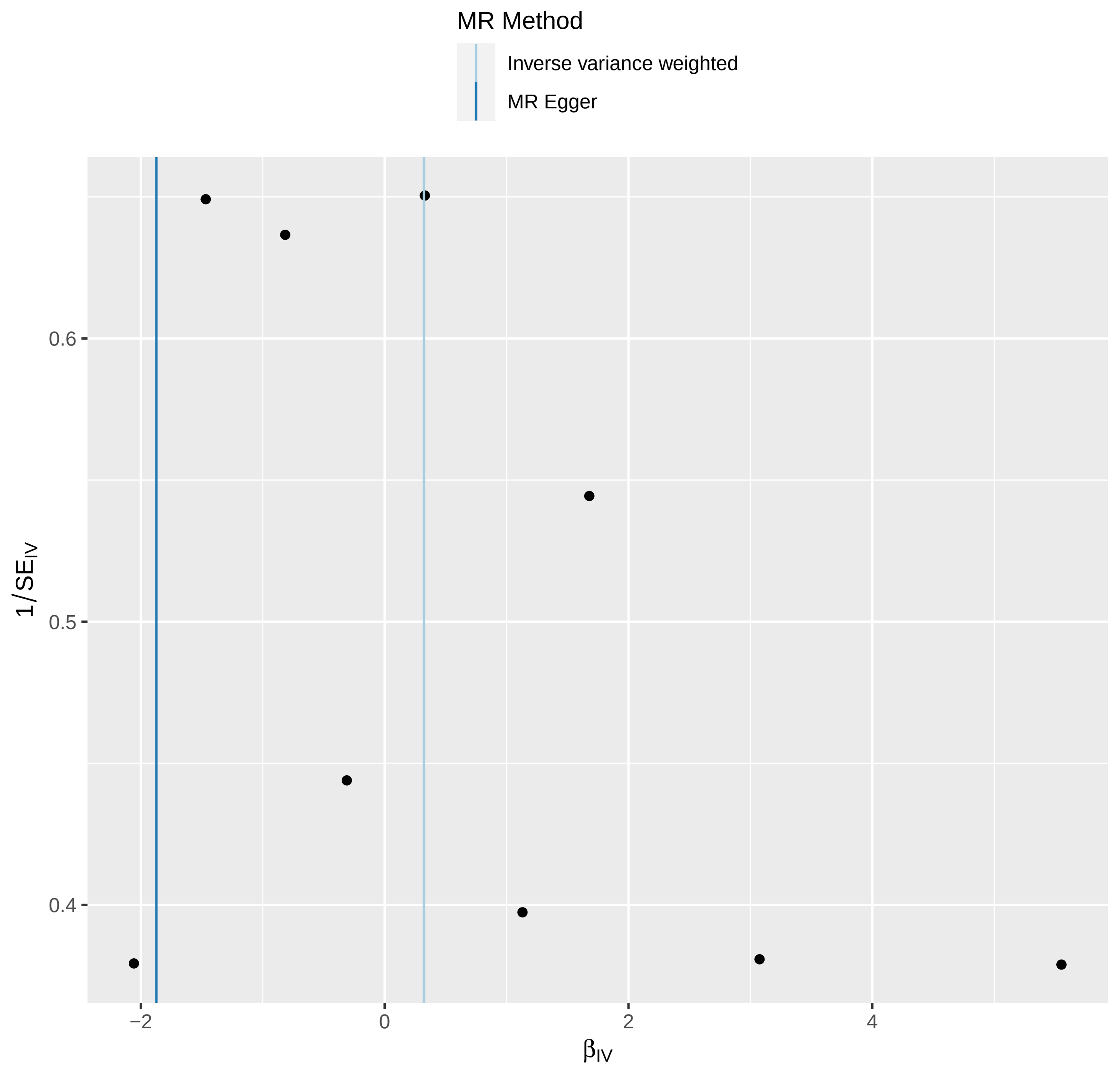

Batch 39 : Citrate on Crohn's disease of colon

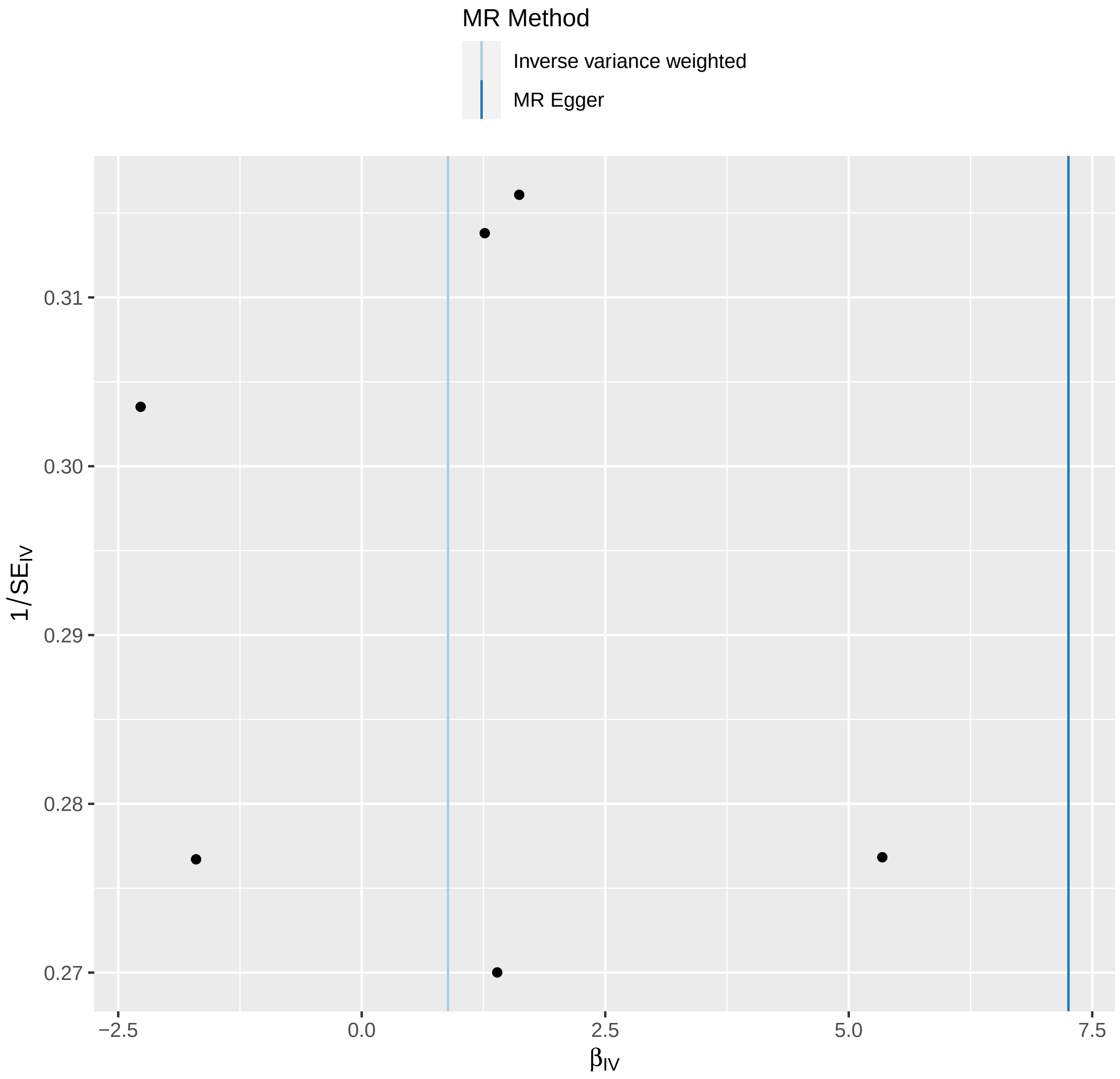

Batch 40 : Glycerate on Crohn's disease of colon

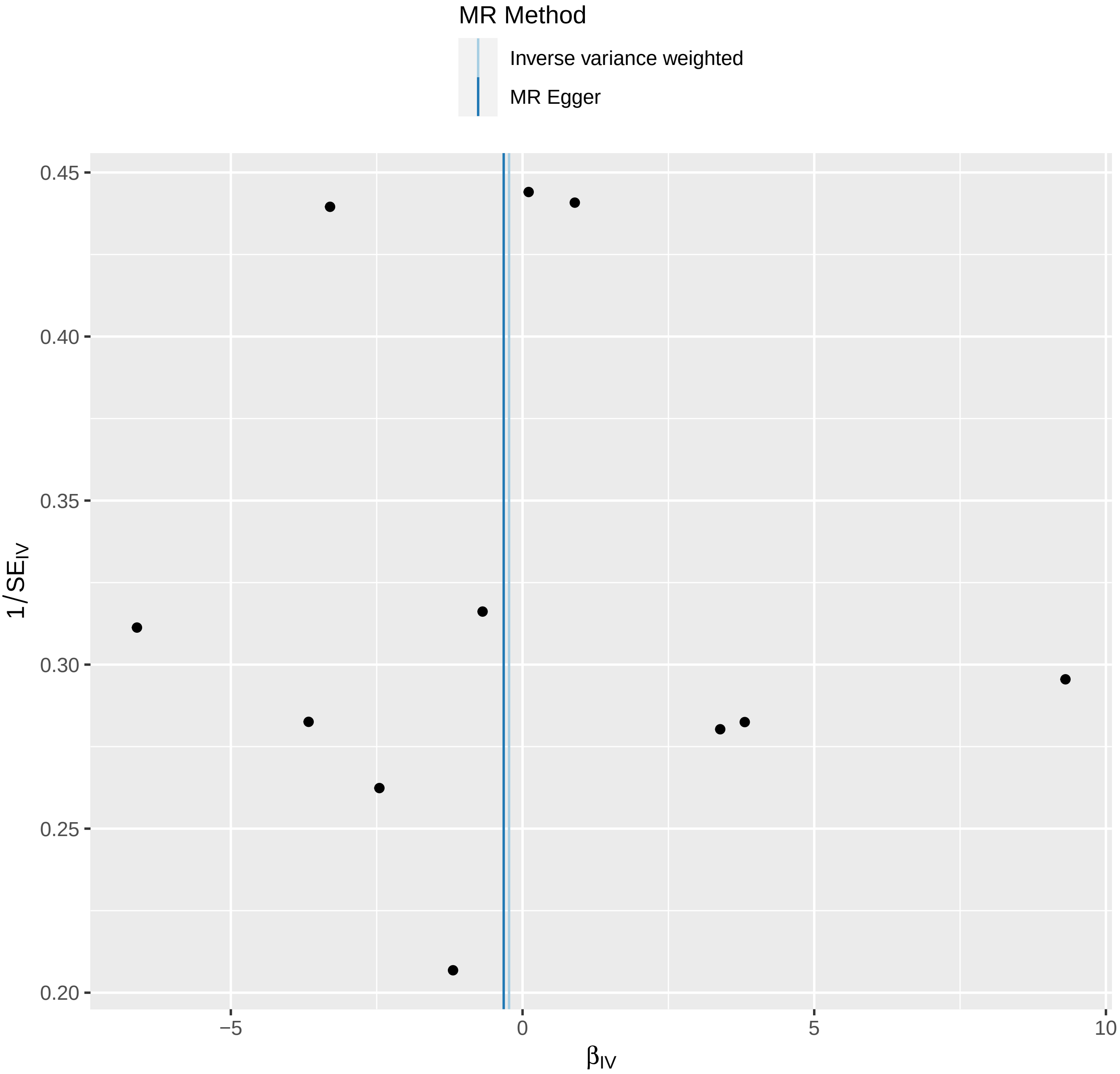

Batch 41 : Guanosine on Crohn's disease of colon

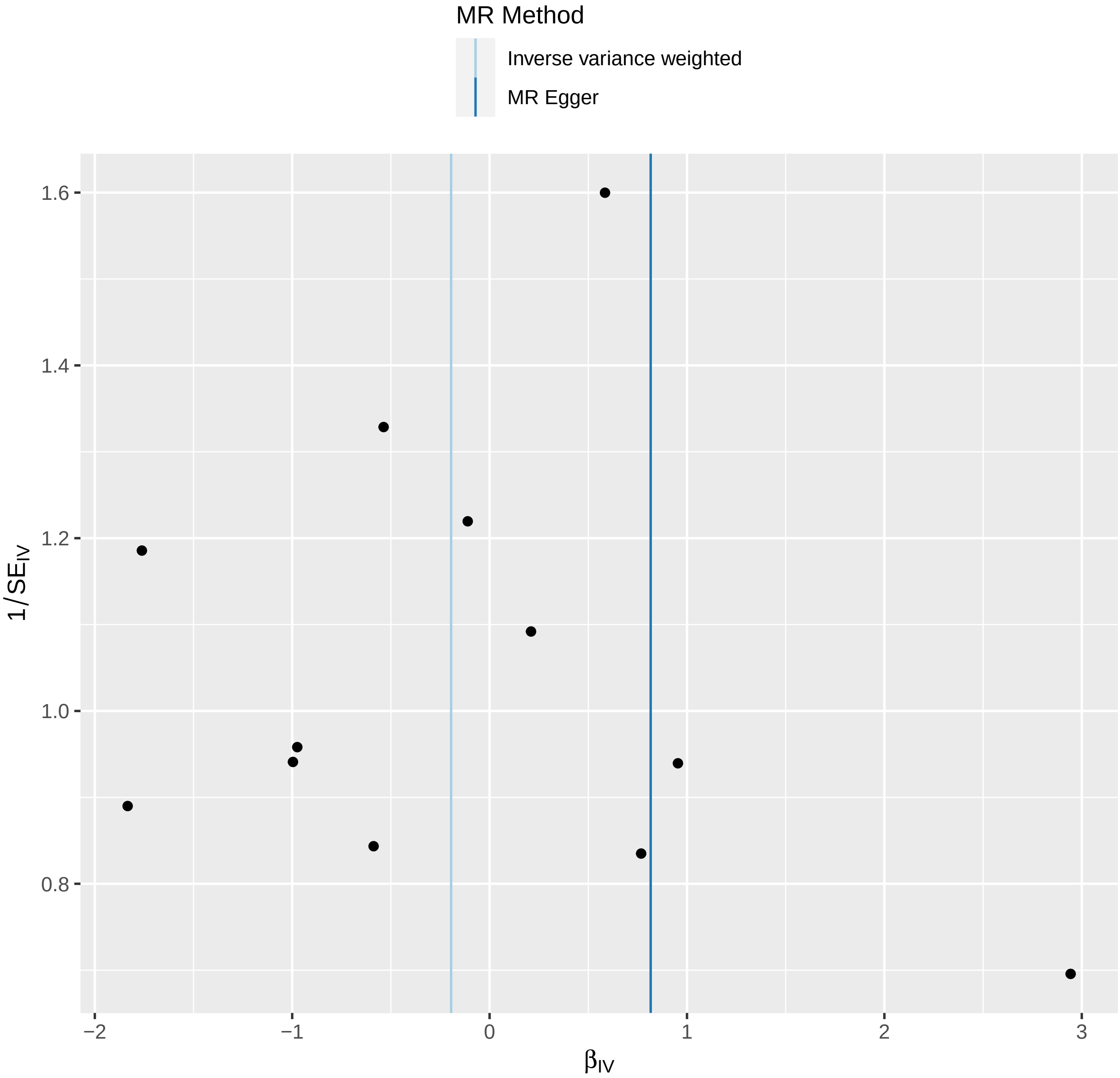

Batch 44 : Ursodeoxycholate on Crohn's disease of colon

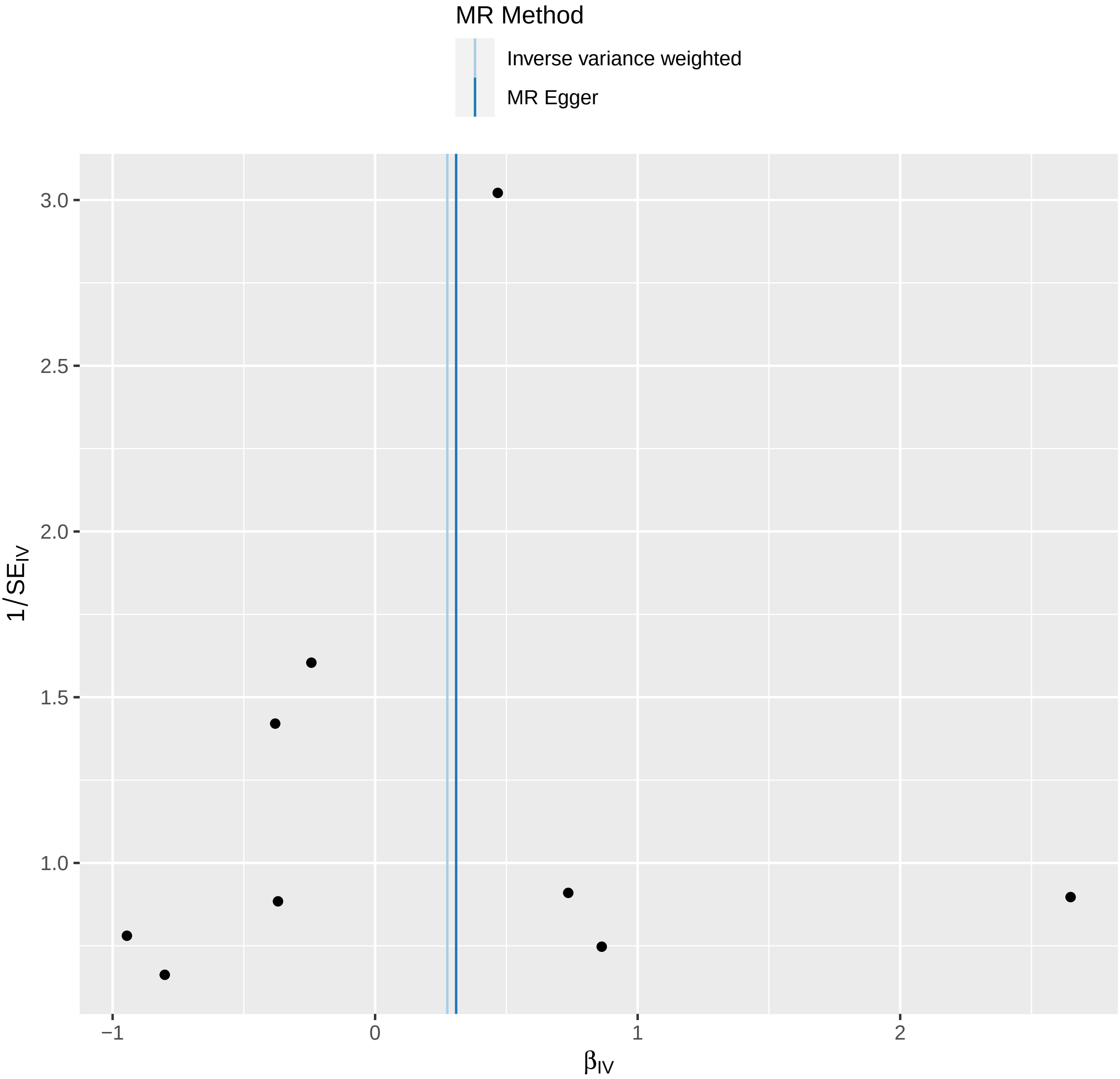

Batch 45 : Arginine on Crohn's disease of colon

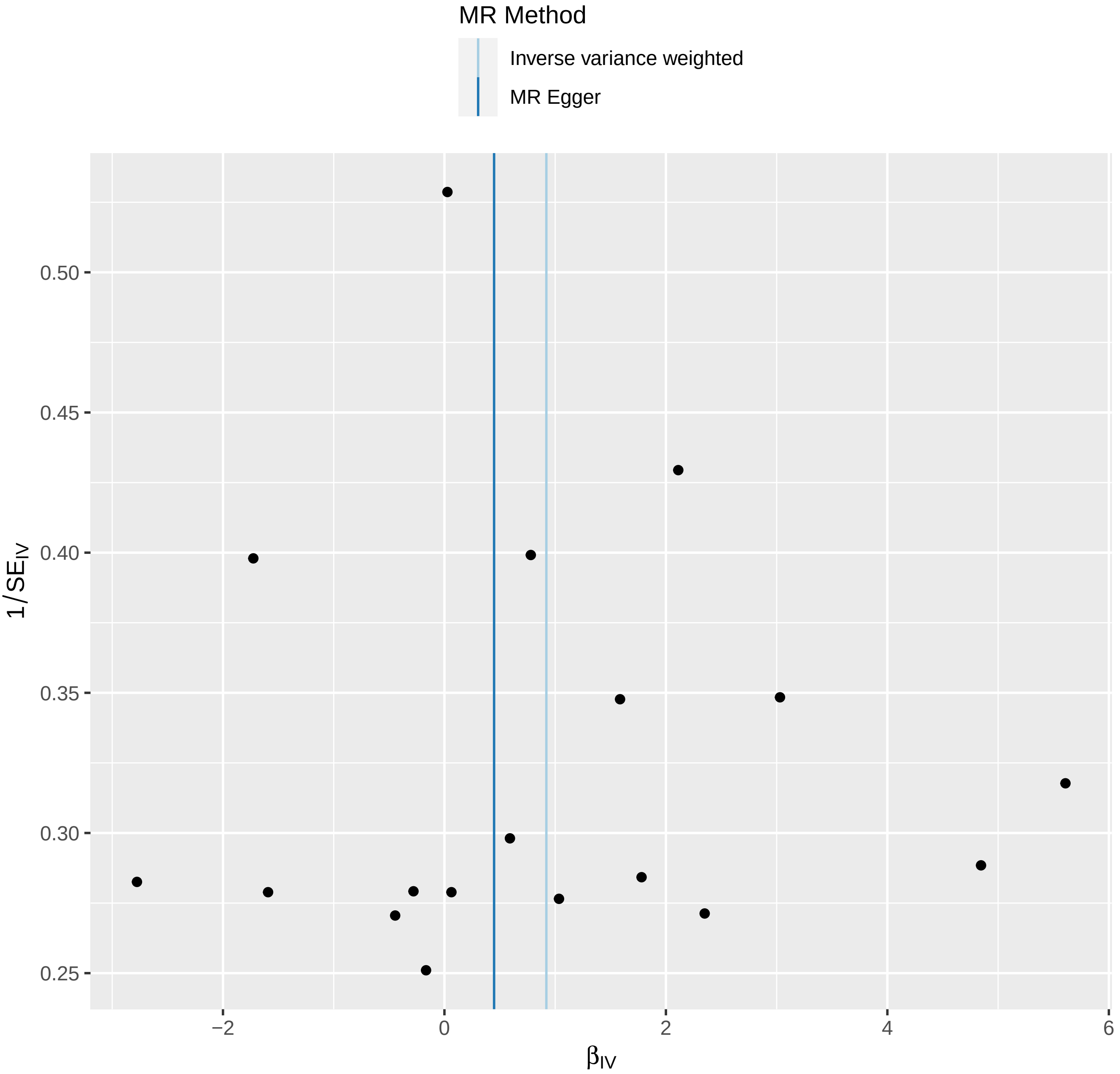

Batch 46 : Ascorbate (Vitamin C) on Crohn's disease of colon

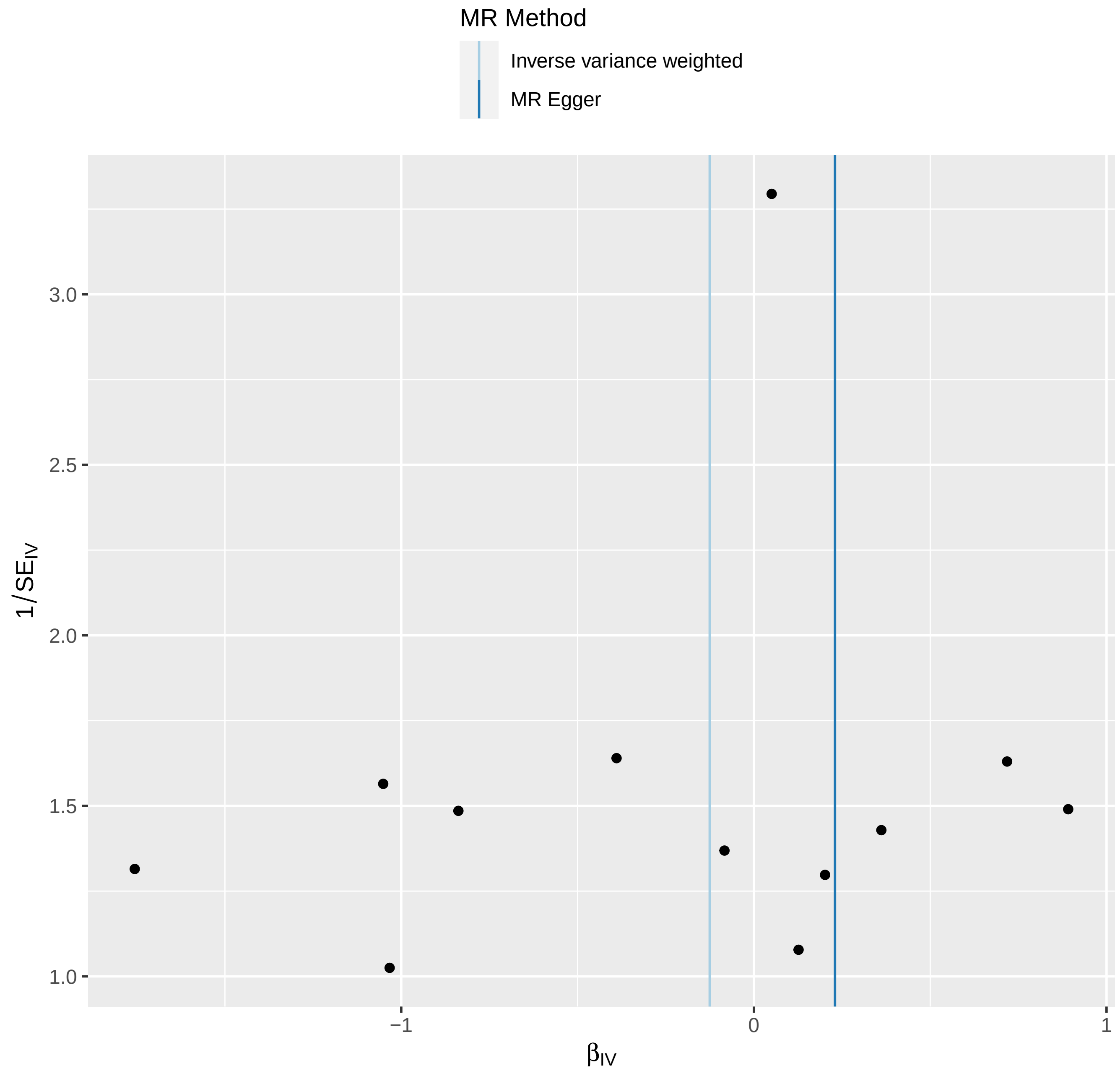

Batch 47 : Heptanoate (7:0) on Crohn's disease of colon

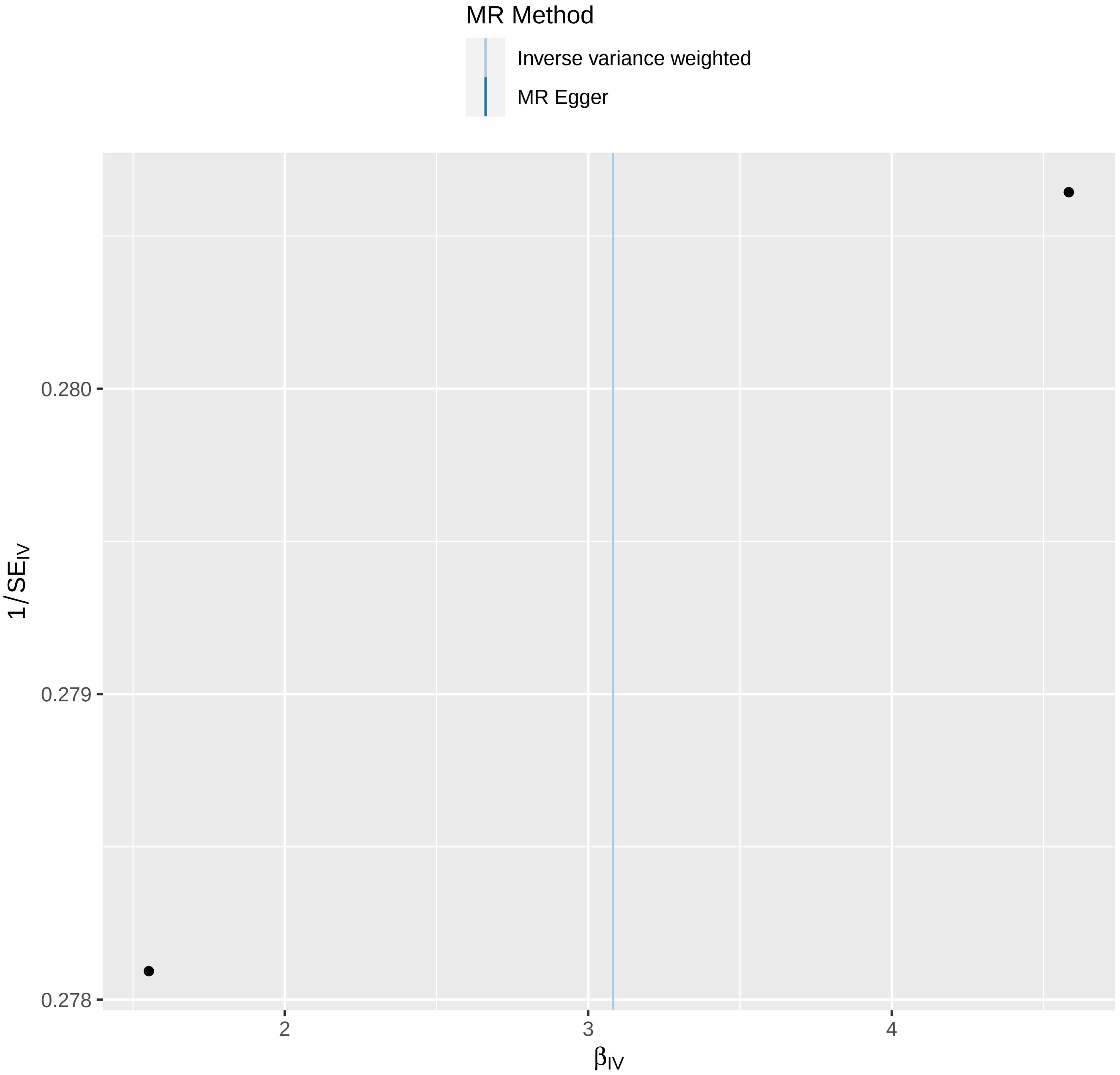

Batch 48 : Laurate (12:0) on Crohn's disease of colon

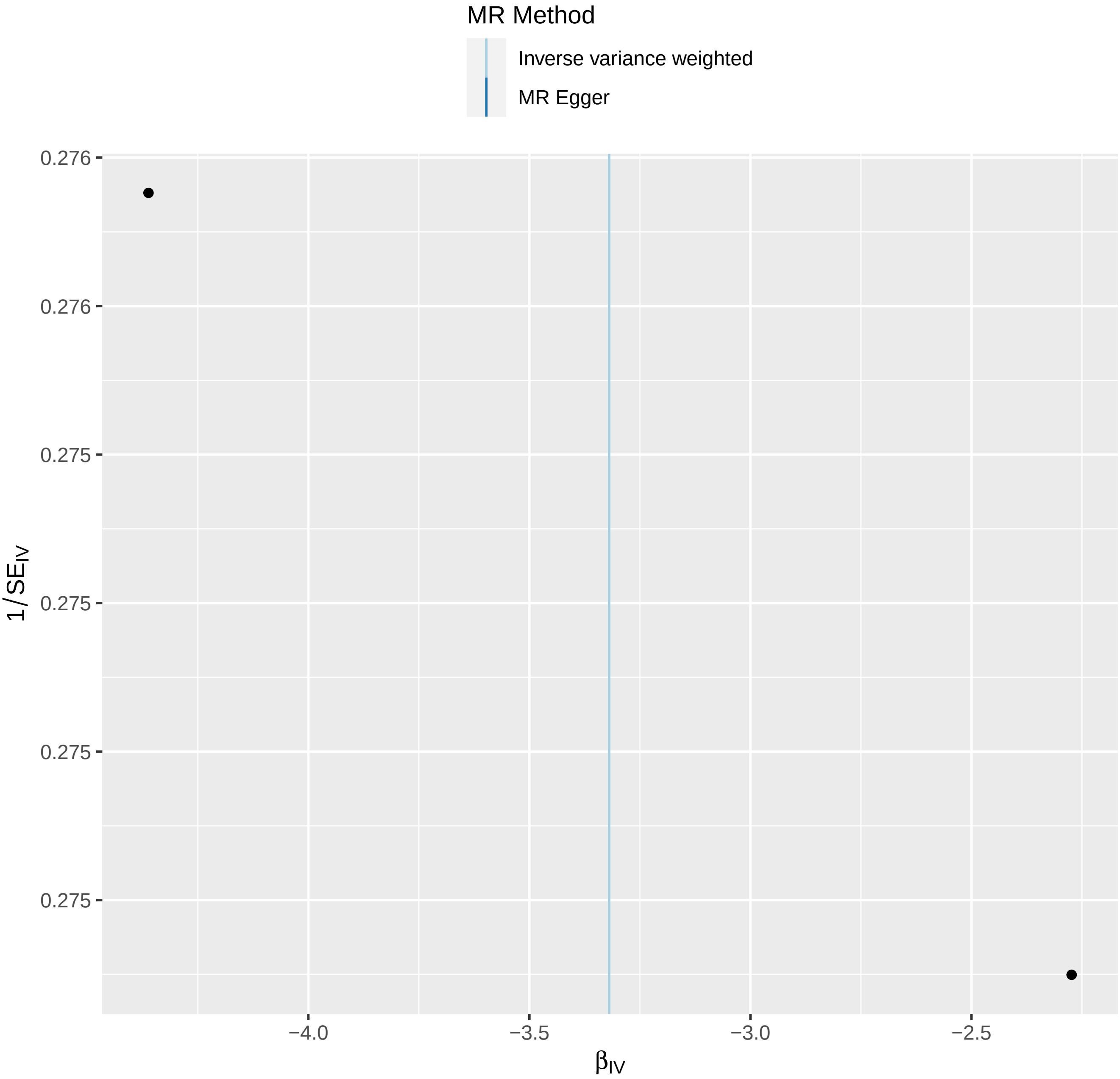

Batch 50 : Urea on Crohn's disease of colon

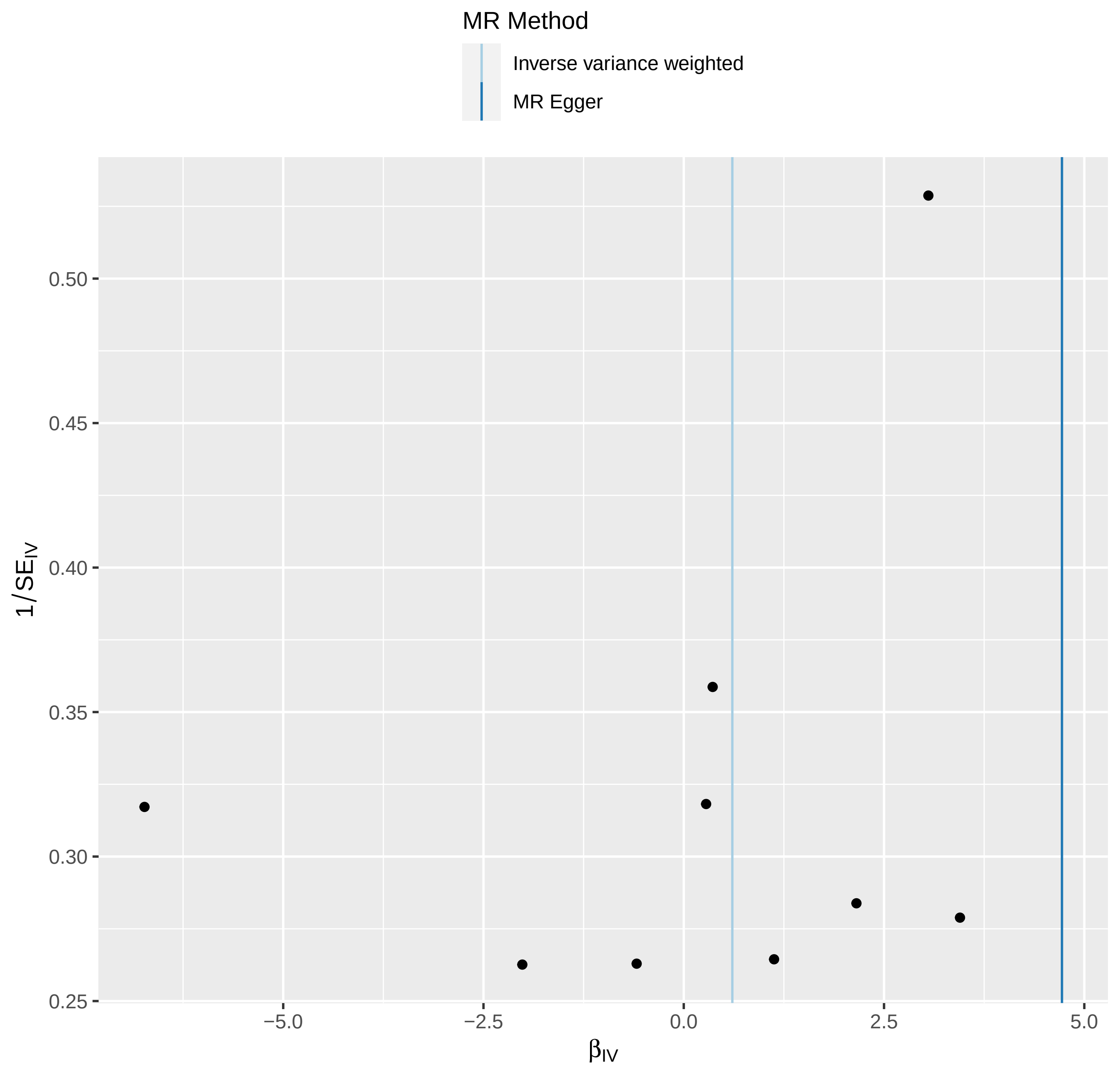

Batch 51 : Cortisol on Crohn's disease of colon

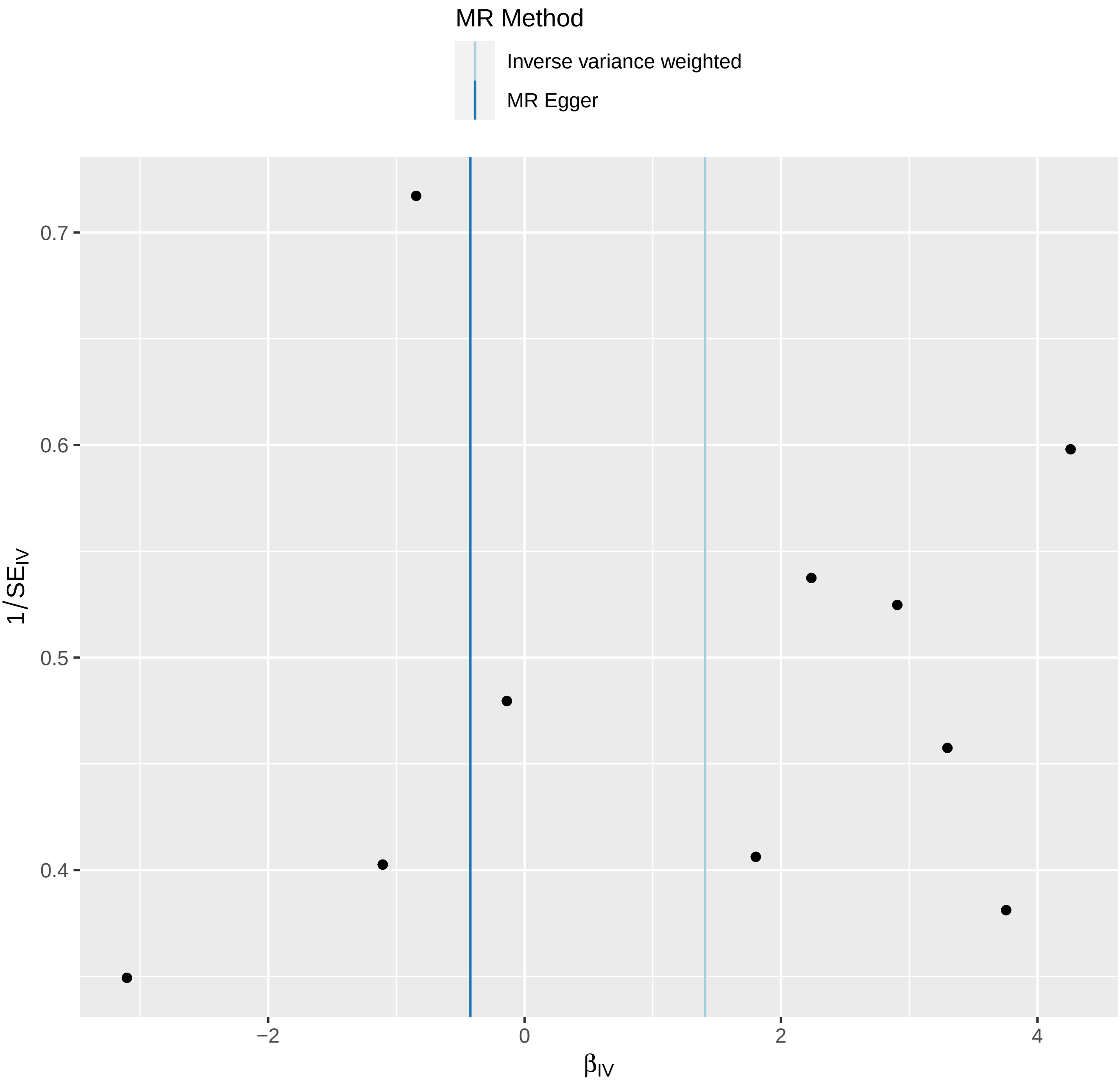

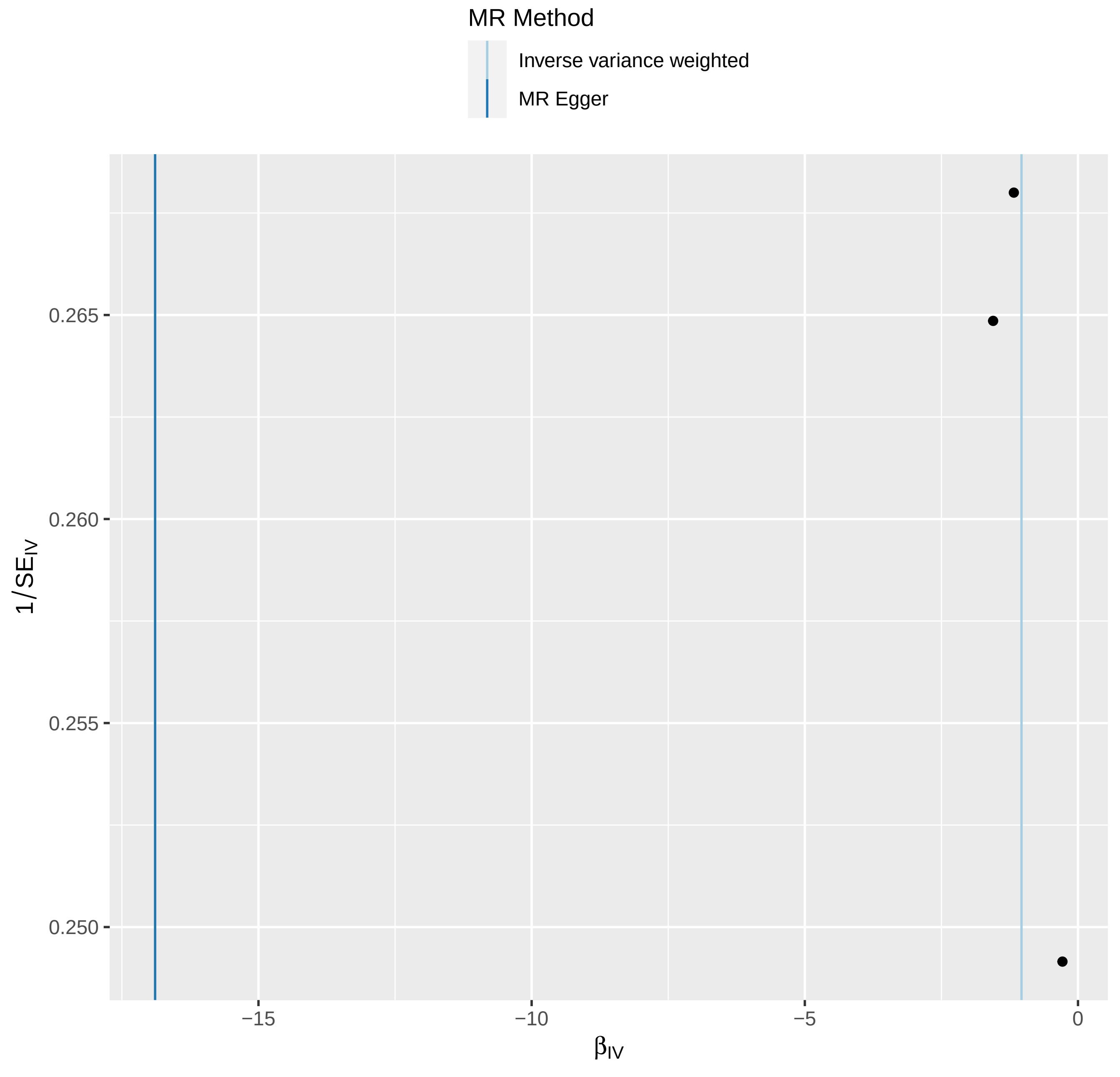
MR Method
Inverse variance weighted
MR Egger
0.265
0.260
0.255
0.250
1/SE_IV
−15
−10
−5
0
β_IV

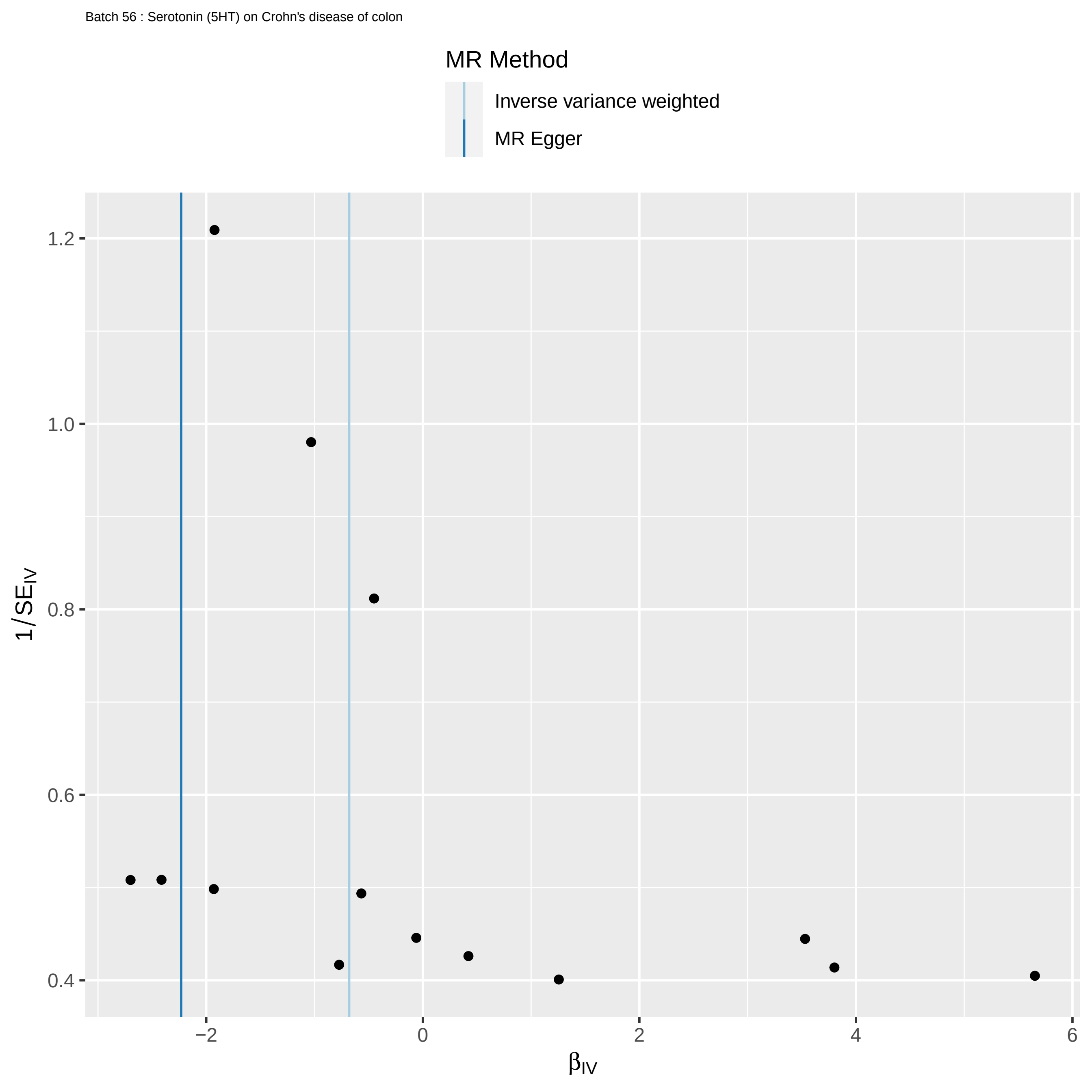
Batch 56 : Serotonin (5HT) on Crohn's disease of colon
MR Method
Inverse variance weighted
MR Egger
1.2
1.0
0.8
0.6
0.4
1/SE_IV
−2
0
2
4
6
β_IV

Batch 57 : Gamma−glutamylglutamine on Crohn's disease of colon

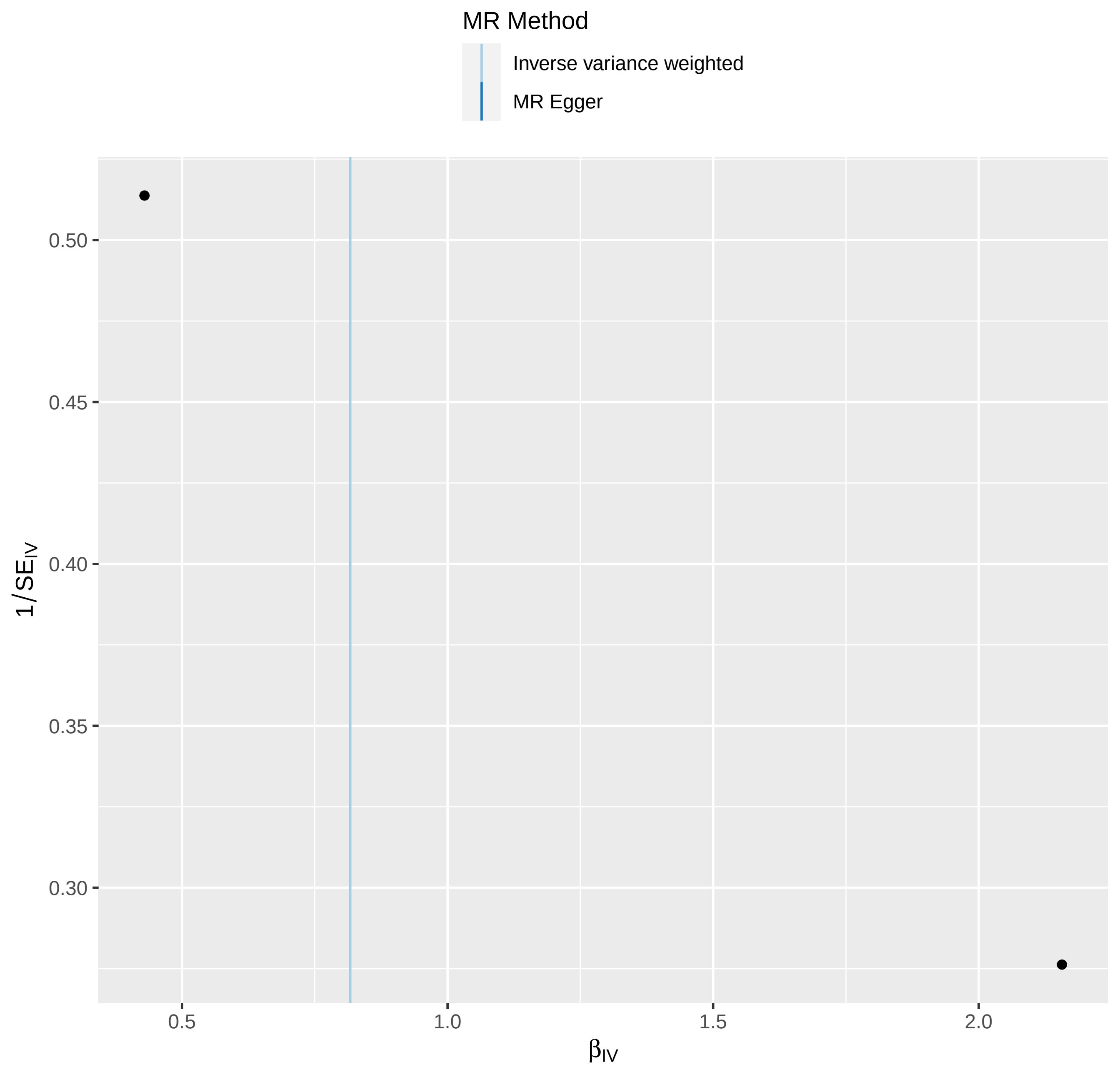

Batch 58 : Gamma−glutamyltyrosine on Crohn's disease of colon

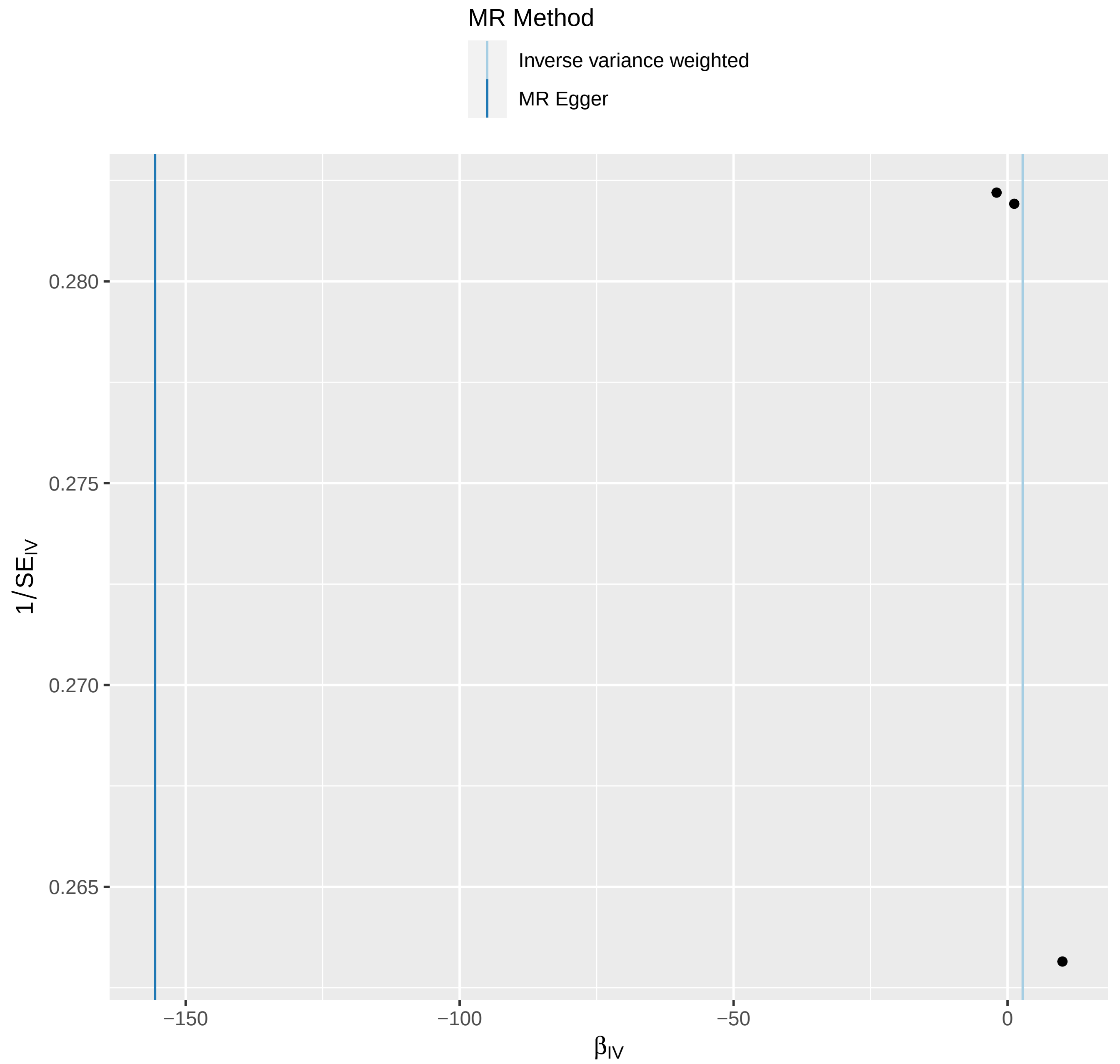

Batch 60 : Betaine on Crohn's disease of colon

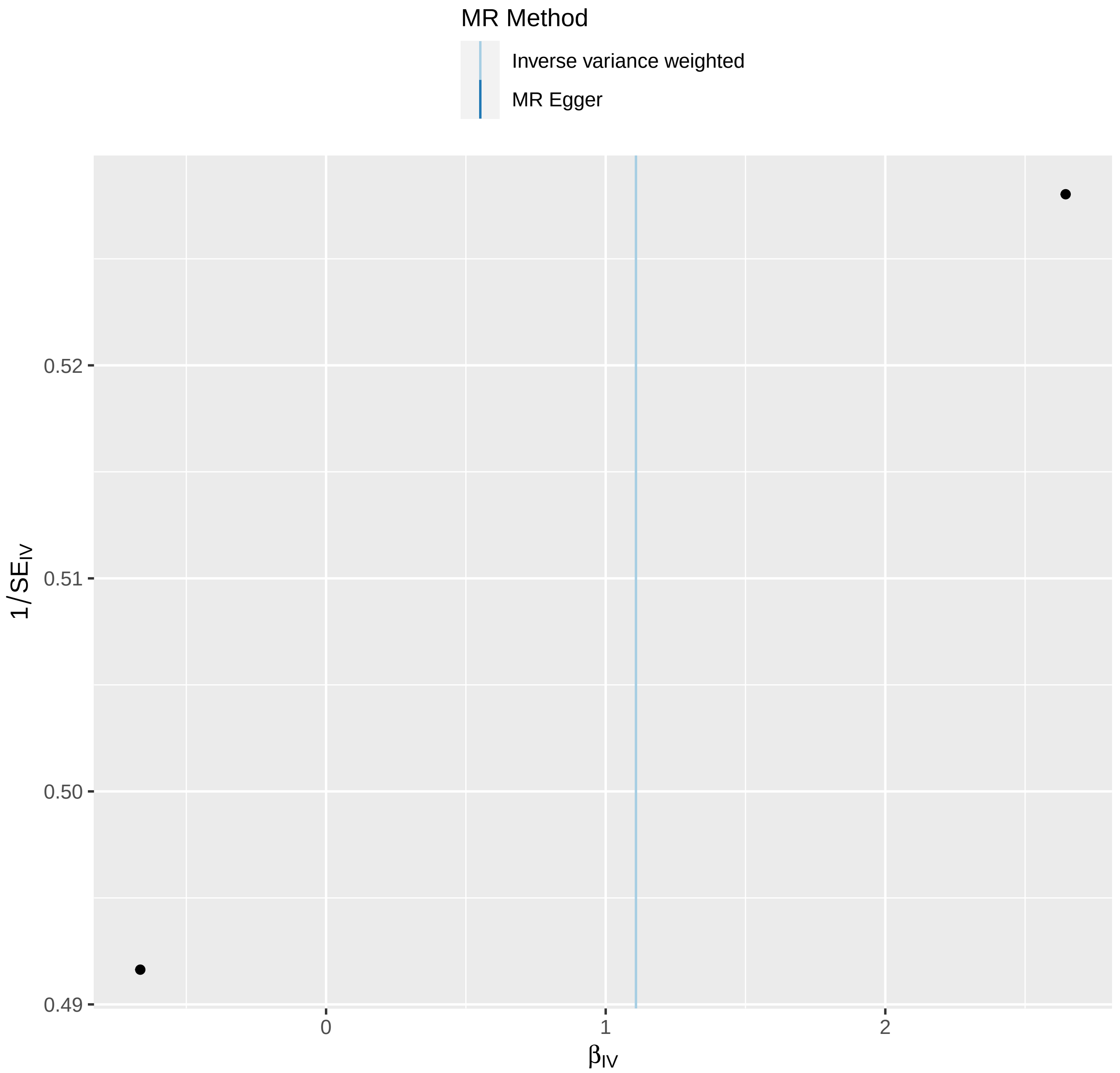

Batch 61 : Xanthine on Crohn's disease of colon

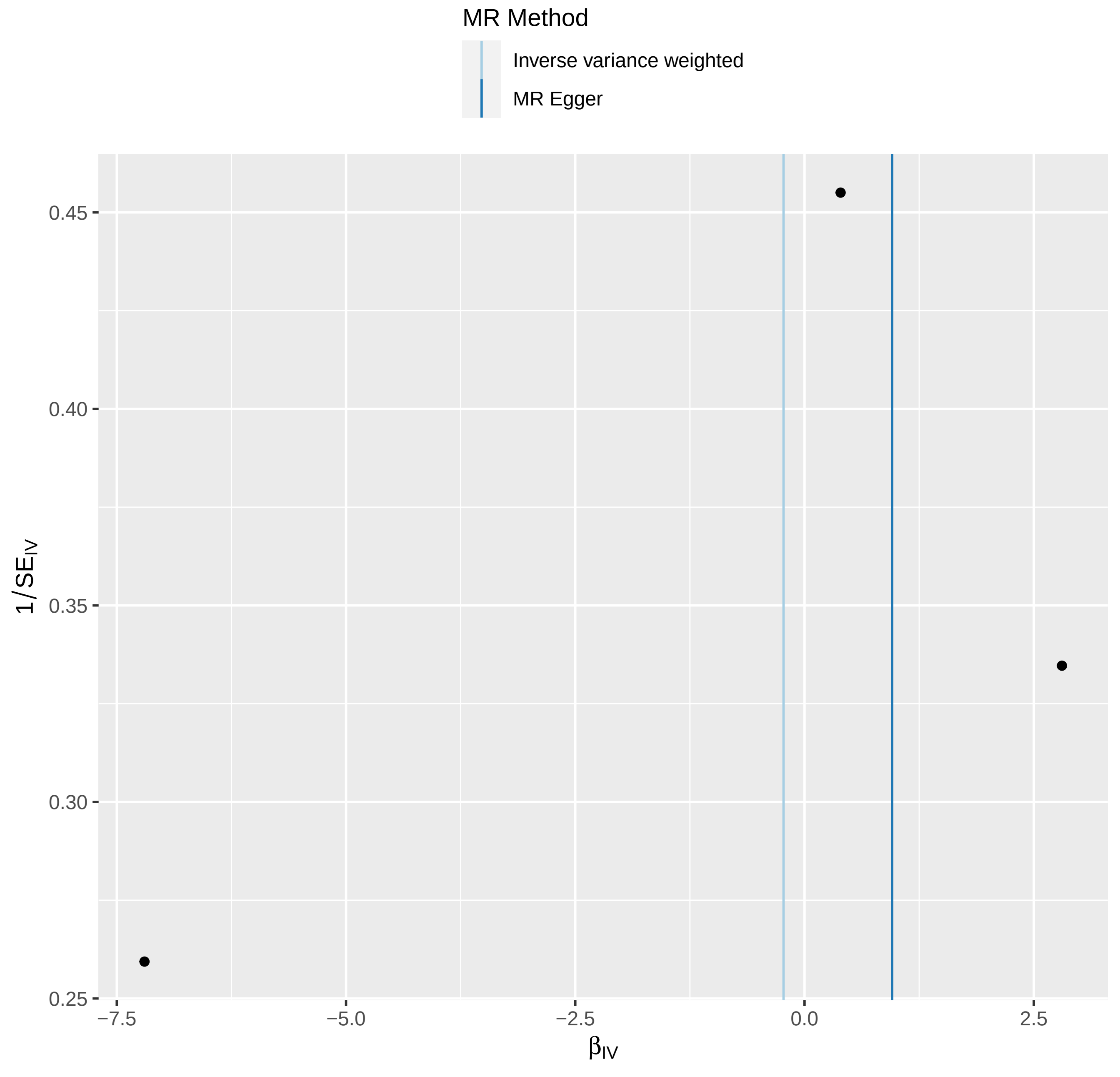

Batch 62 : Phosphate on Crohn's disease of colon

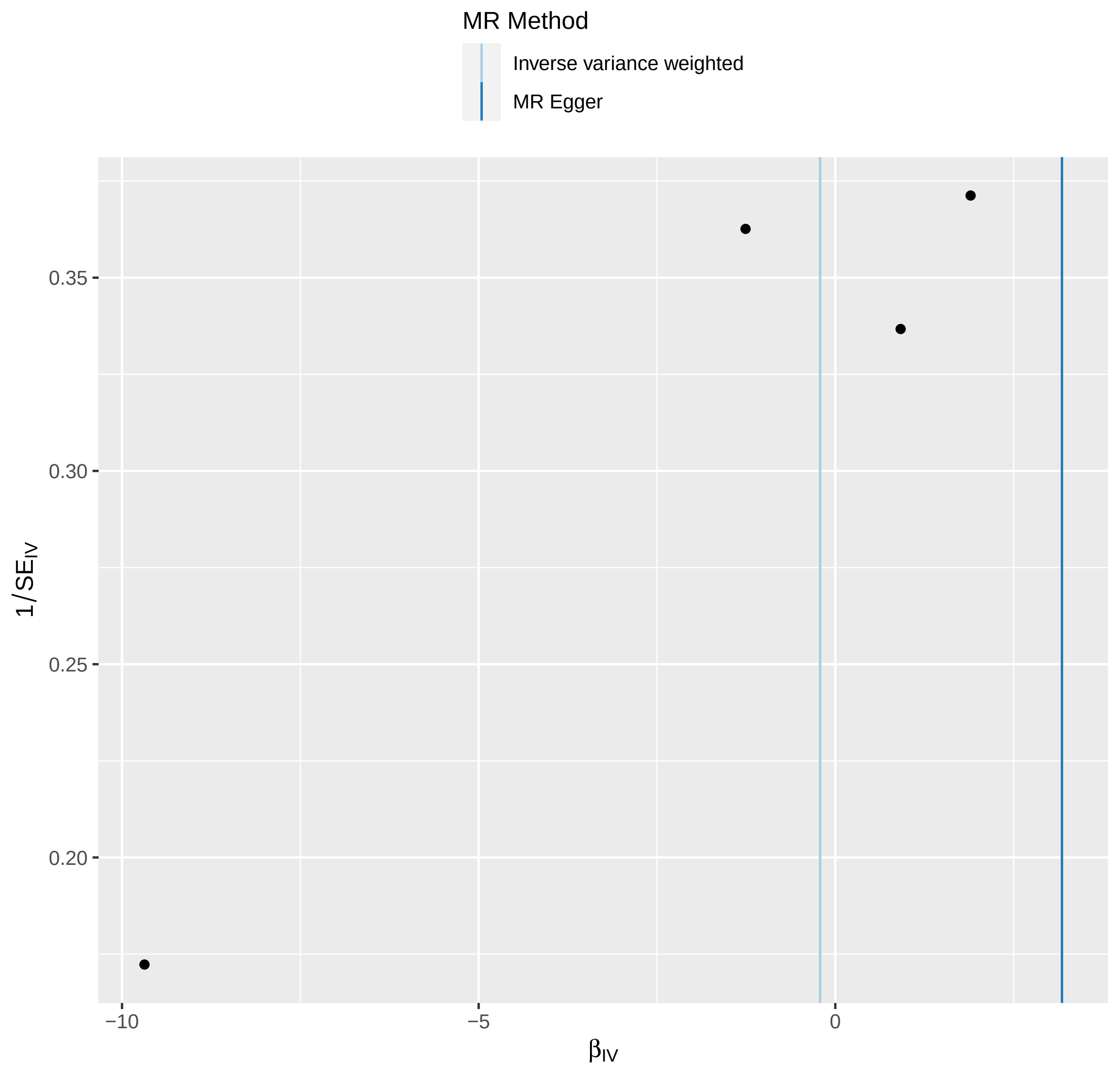

Batch 63 : 3−methoxytyrosine on Crohn's disease of colon

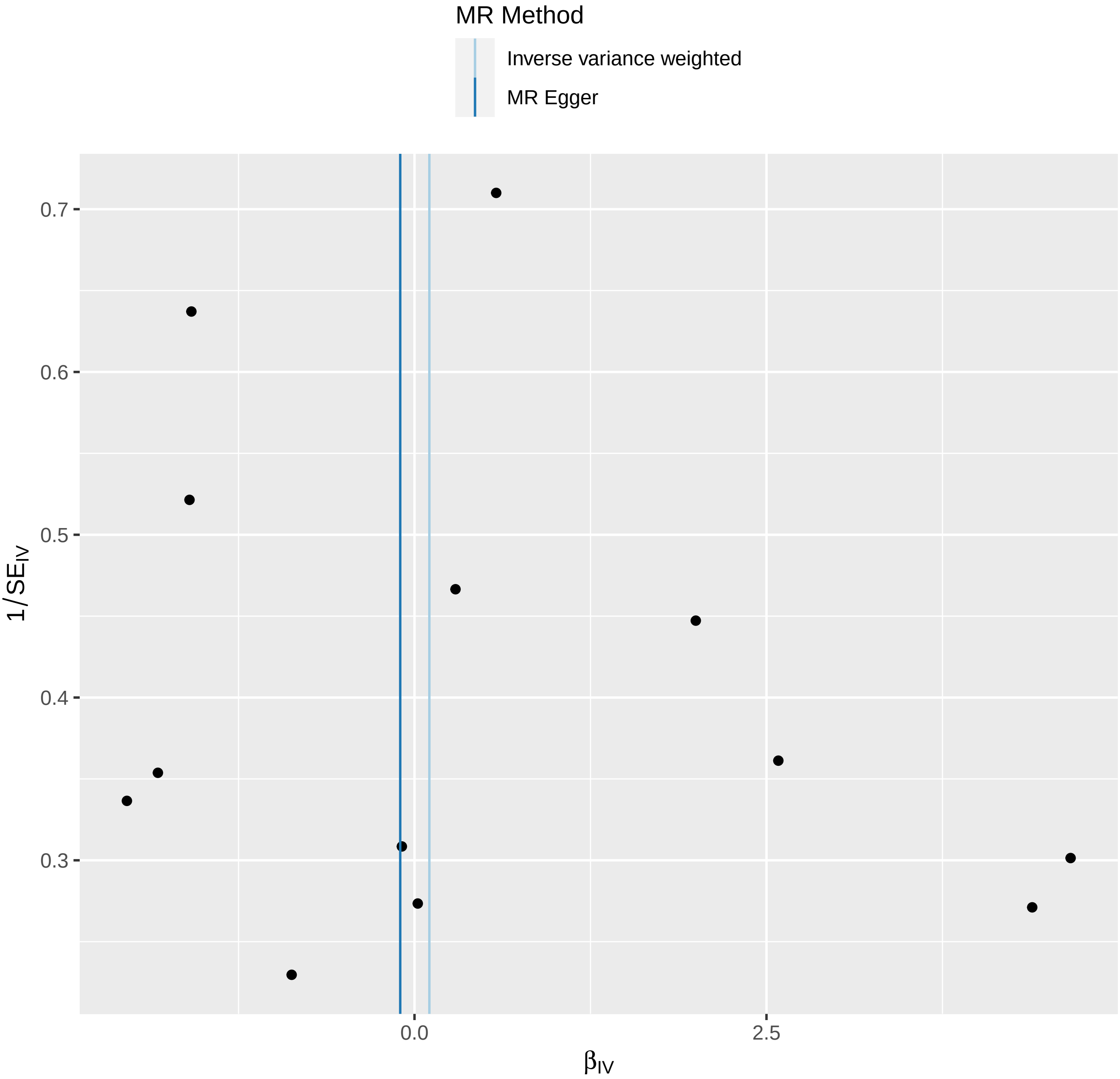

Batch 65 : Undecanoate (11:0) on Crohn's disease of colon

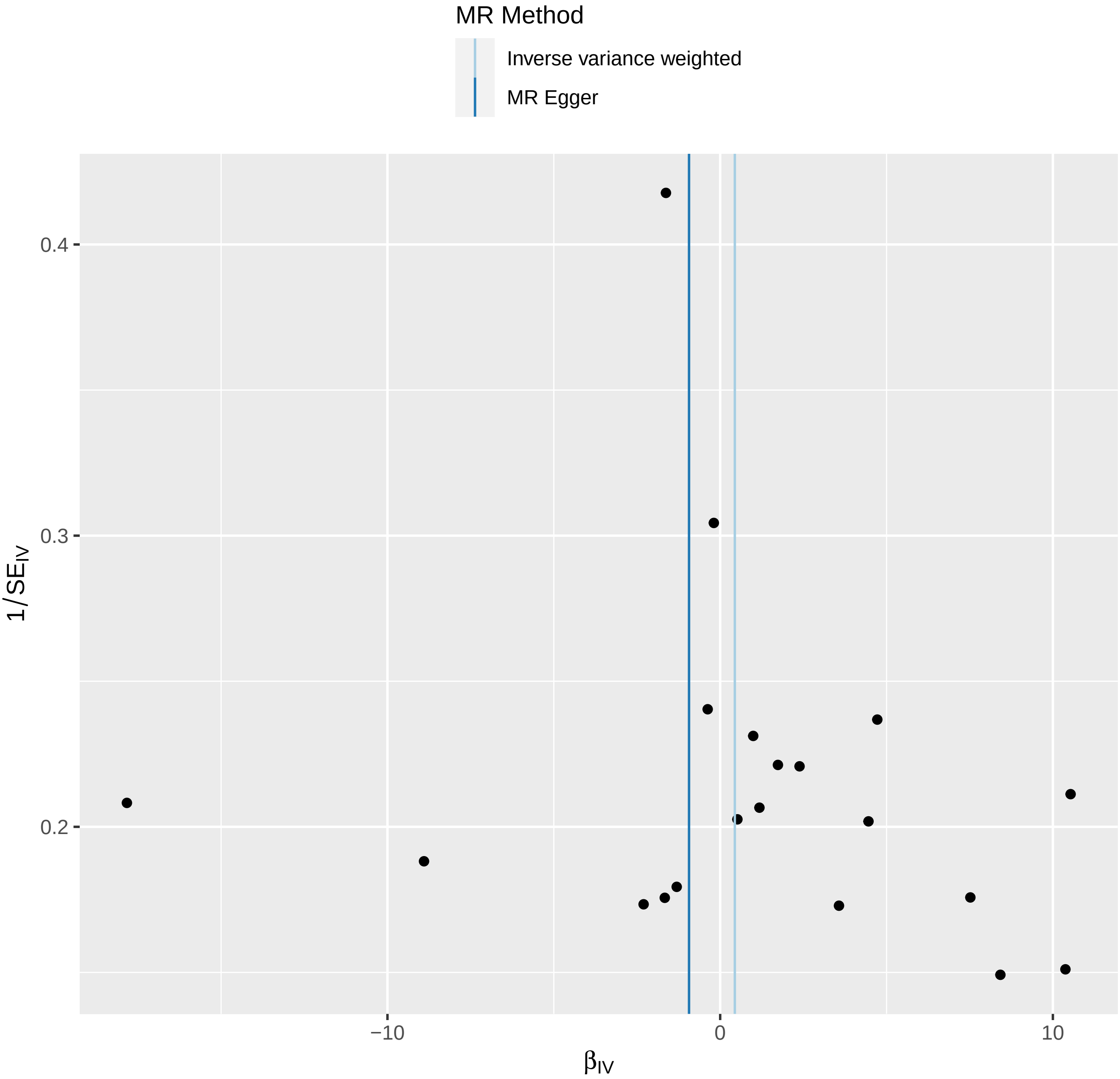

Batch 67 : Taurodeoxycholate on Crohn's disease of colon

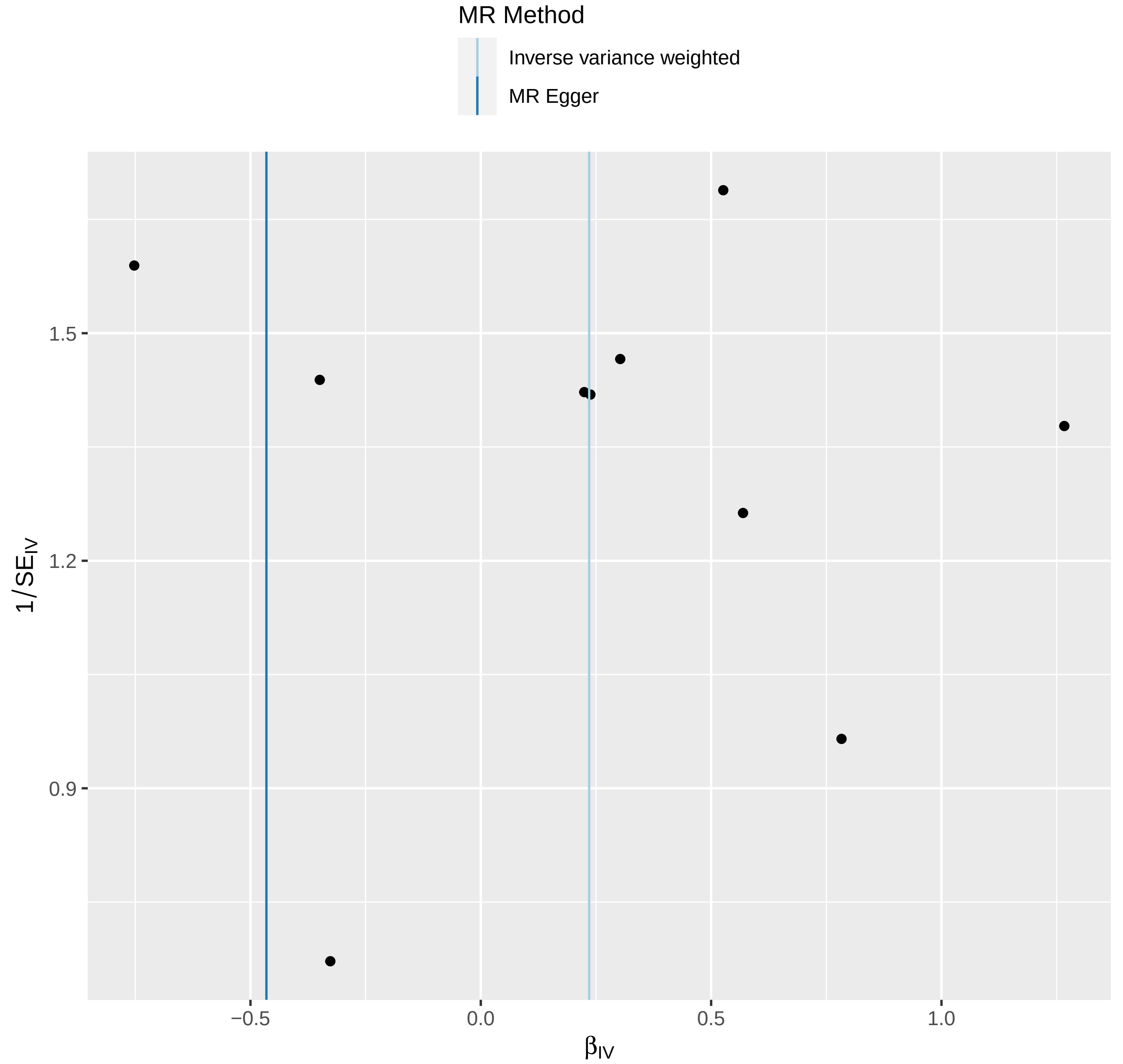

Batch 68 : Glycerol on Crohn's disease of colon

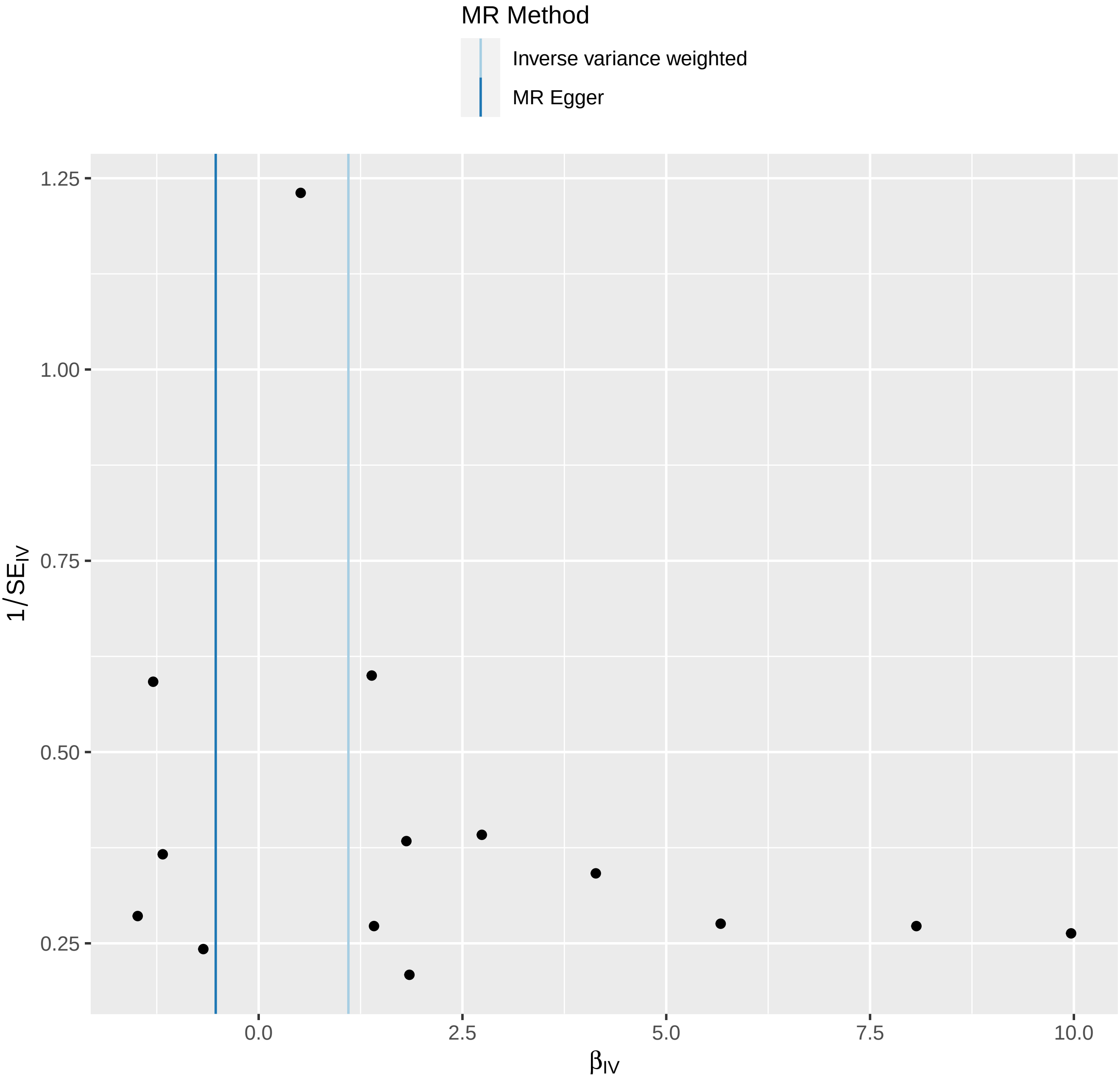

Batch 69 : Kynurenine on Crohn's disease of colon

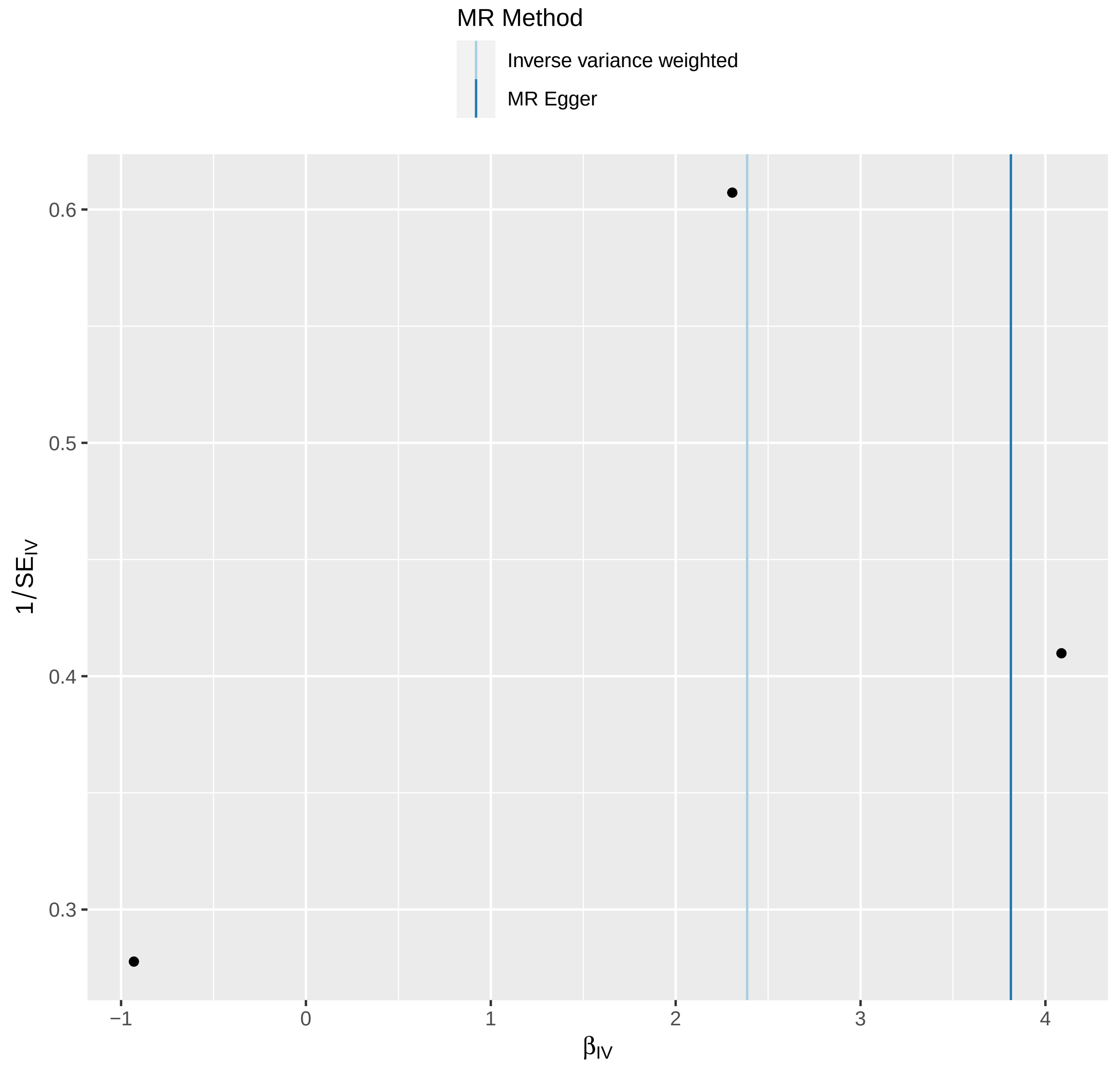

Batch 70 : Mannitol on Crohn's disease of colon

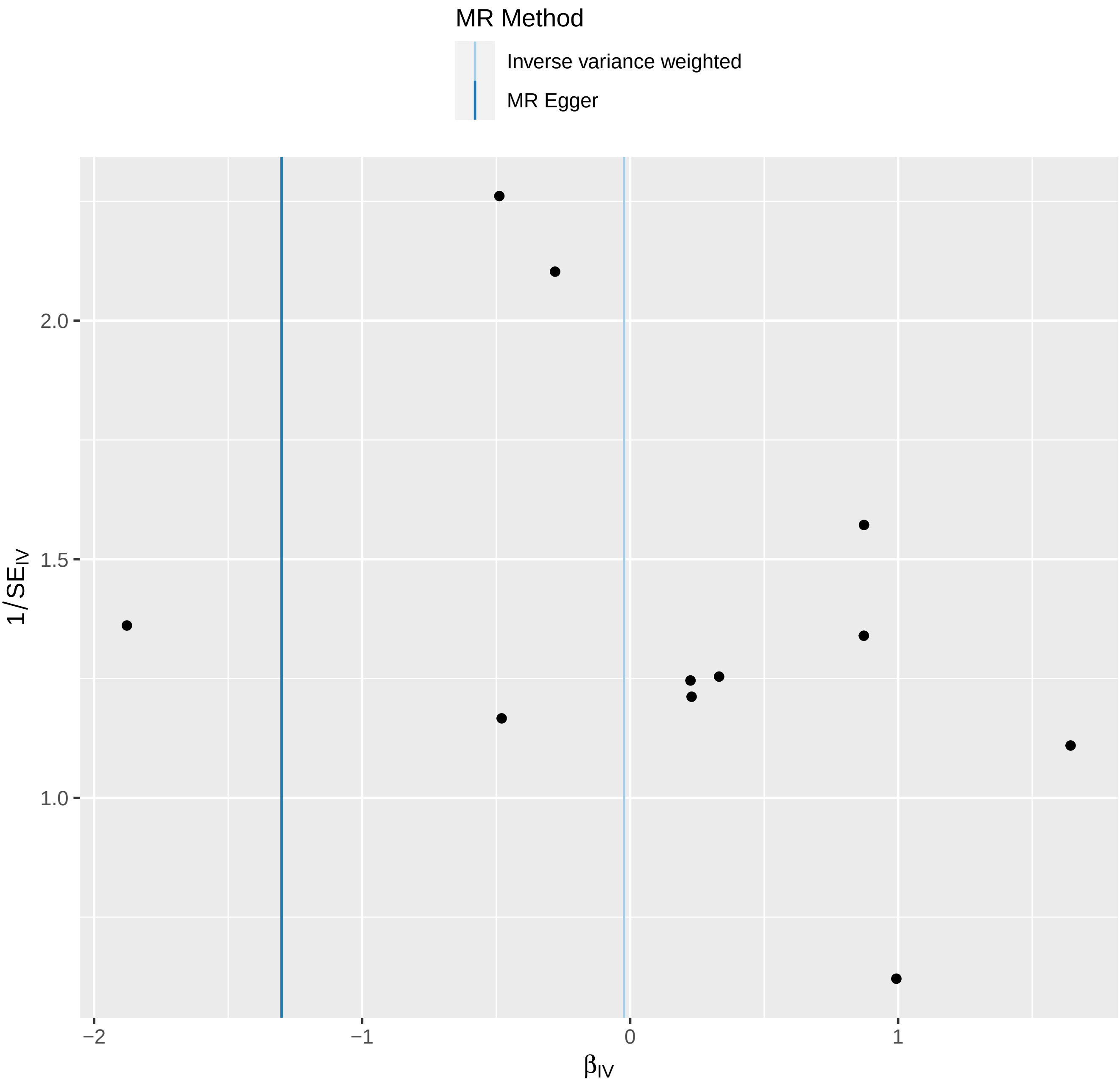

Batch 71 : Glycerol 3−phosphate (G3P) on Crohn's disease of colon

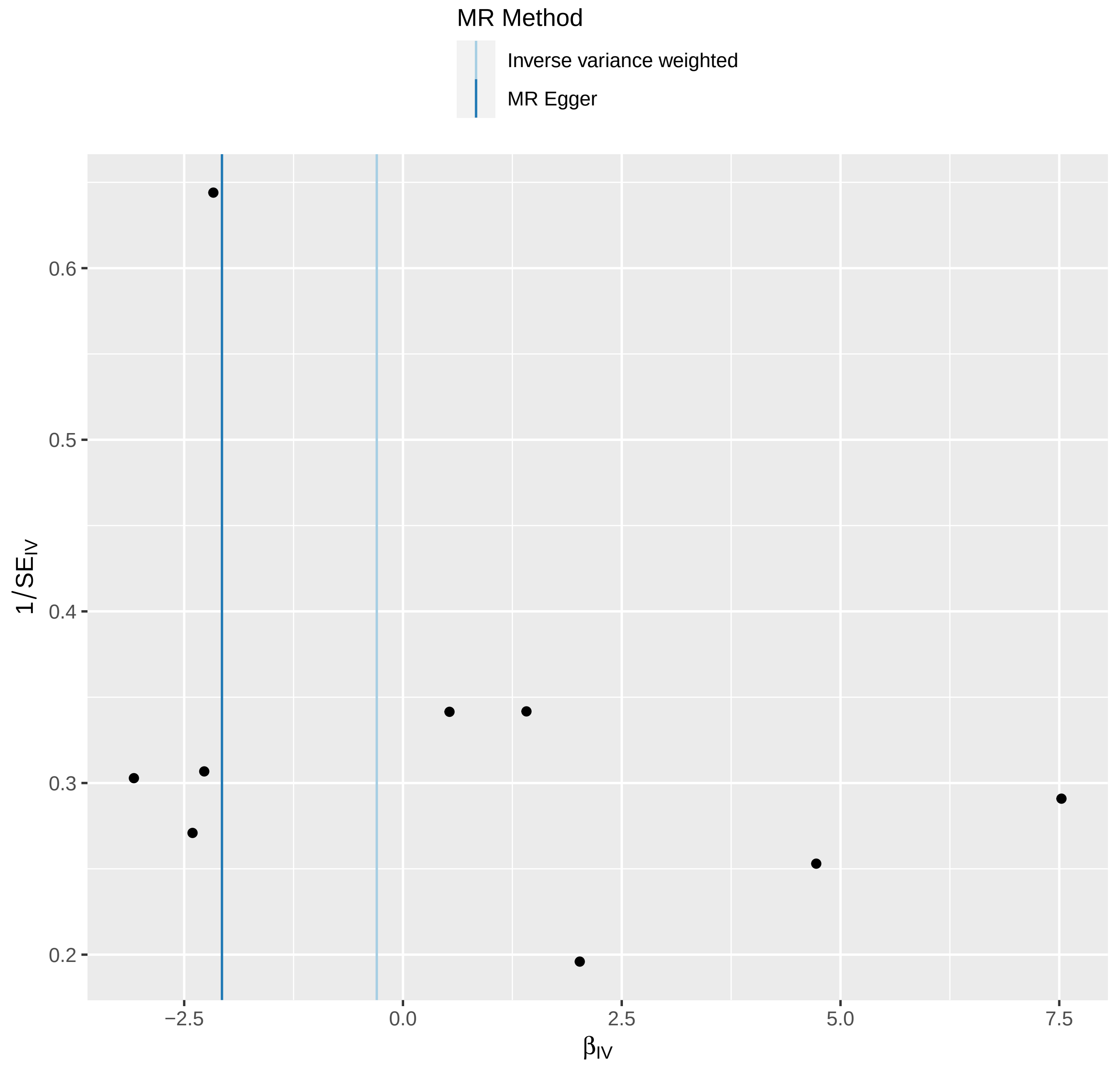

Batch 73 : Carnitine on Crohn's disease of colon

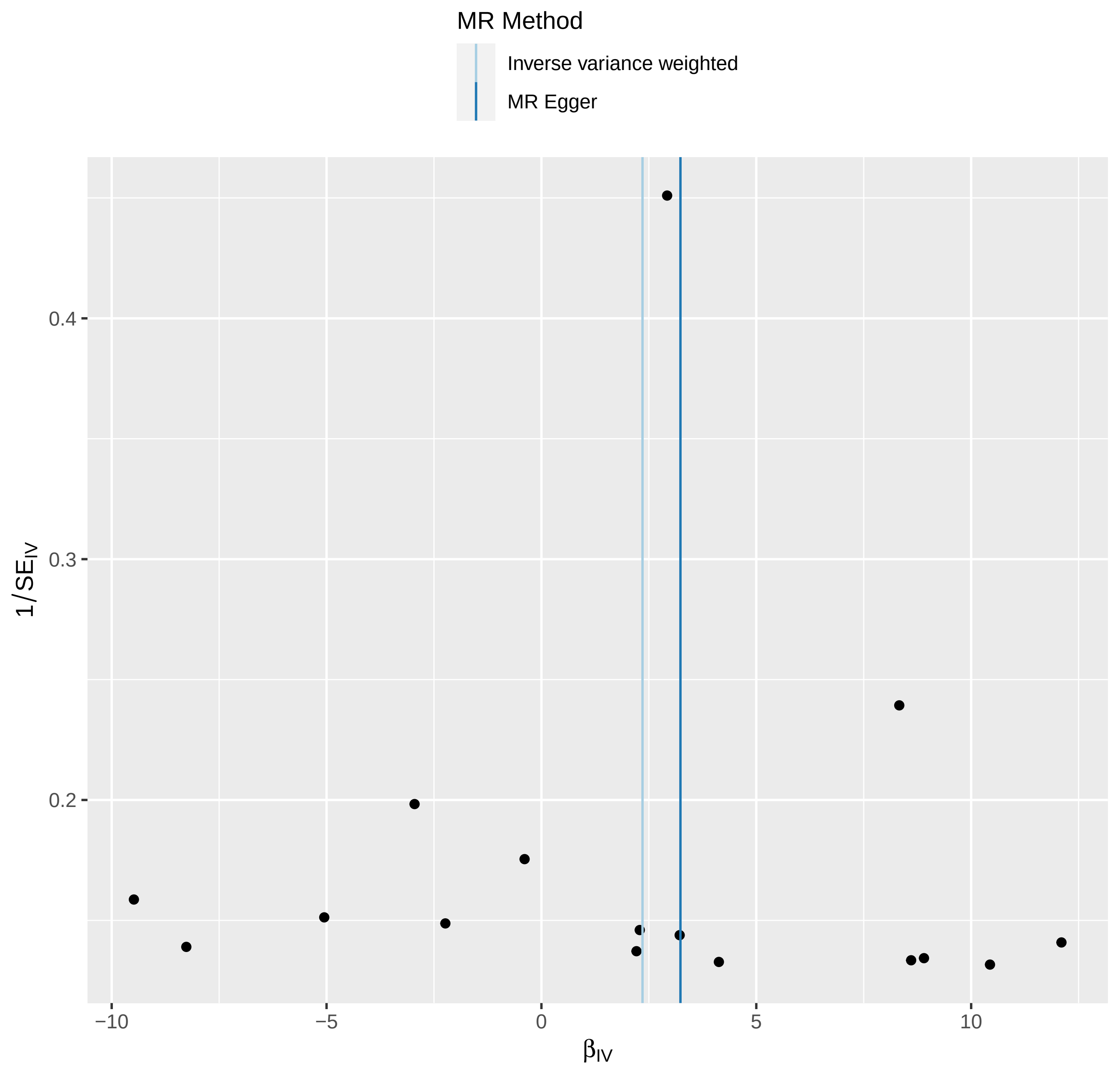

Batch 74 : Choline on Crohn's disease of colon

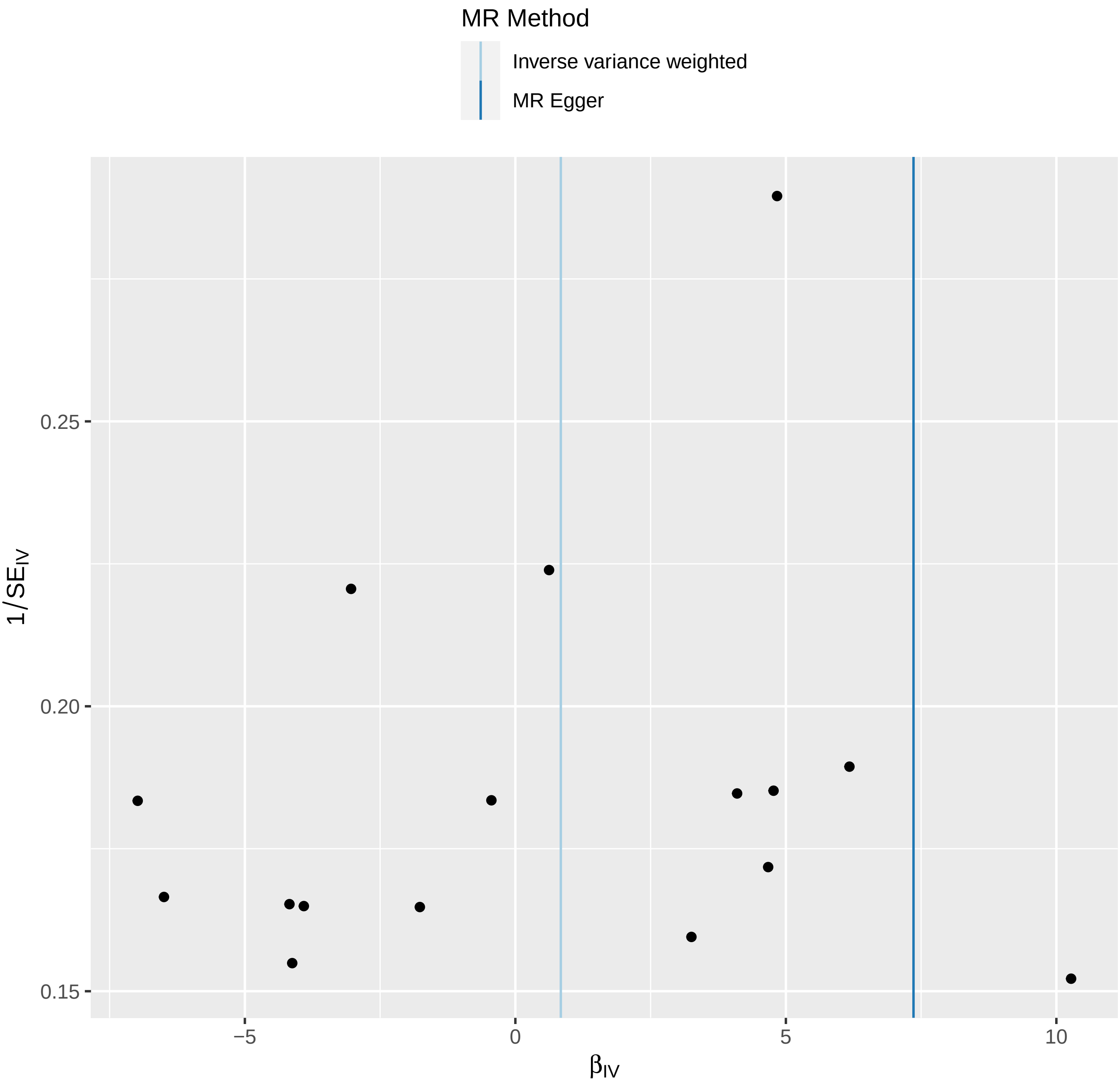

Batch 77 : 3−methyl−2−oxovalerate on Crohn's disease of colon

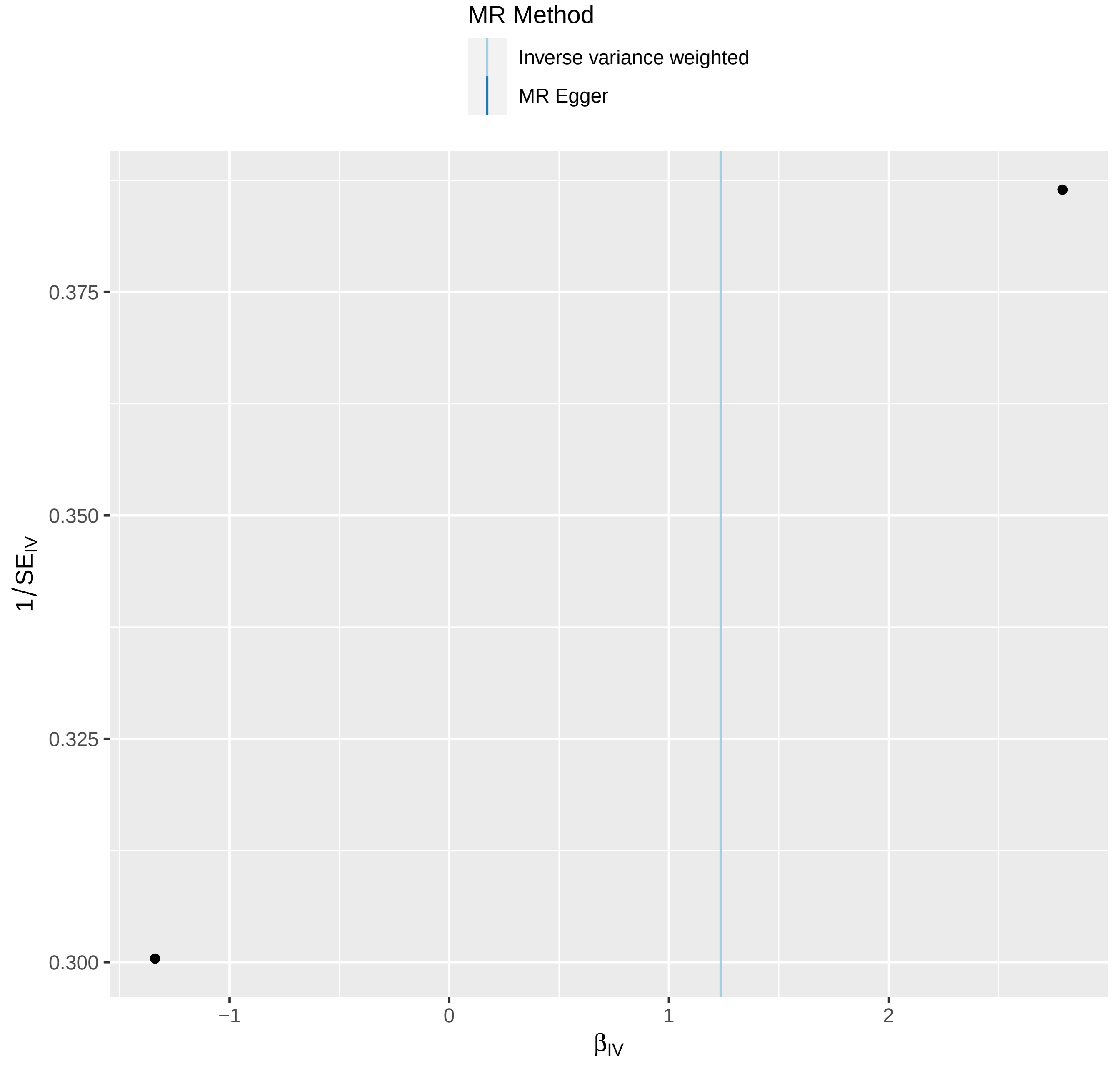

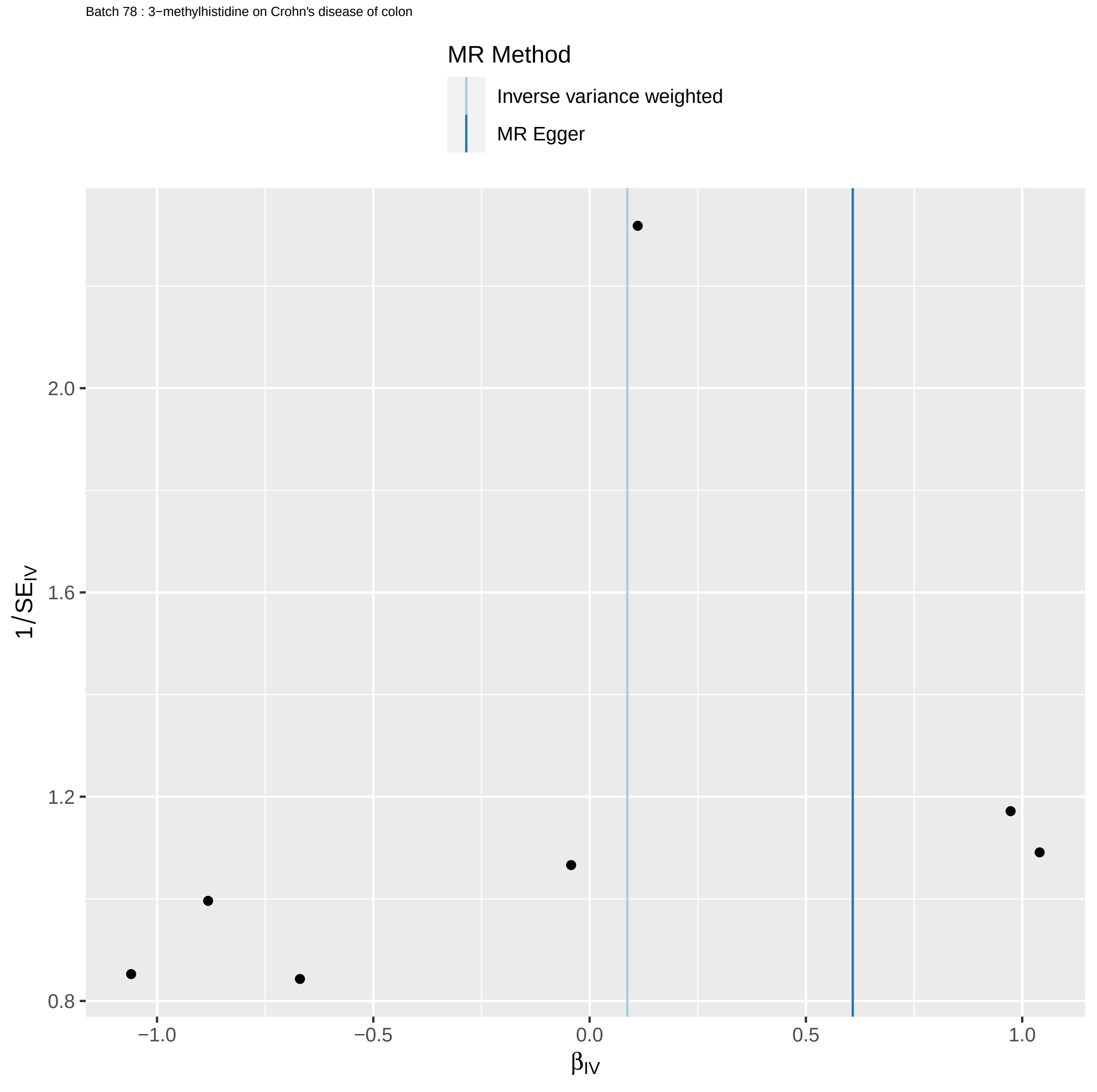

Batch 78 : 3−methylhistidine on Crohn's disease of colon
MR Method
Inverse variance weighted
MR Egger
2.0
1.6
1.2
0.8
1/SE_IV
−1.0
−0.5
0.0
0.5
1.0
β_IV

Batch 80 : Phenylacetate on Crohn's disease of colon

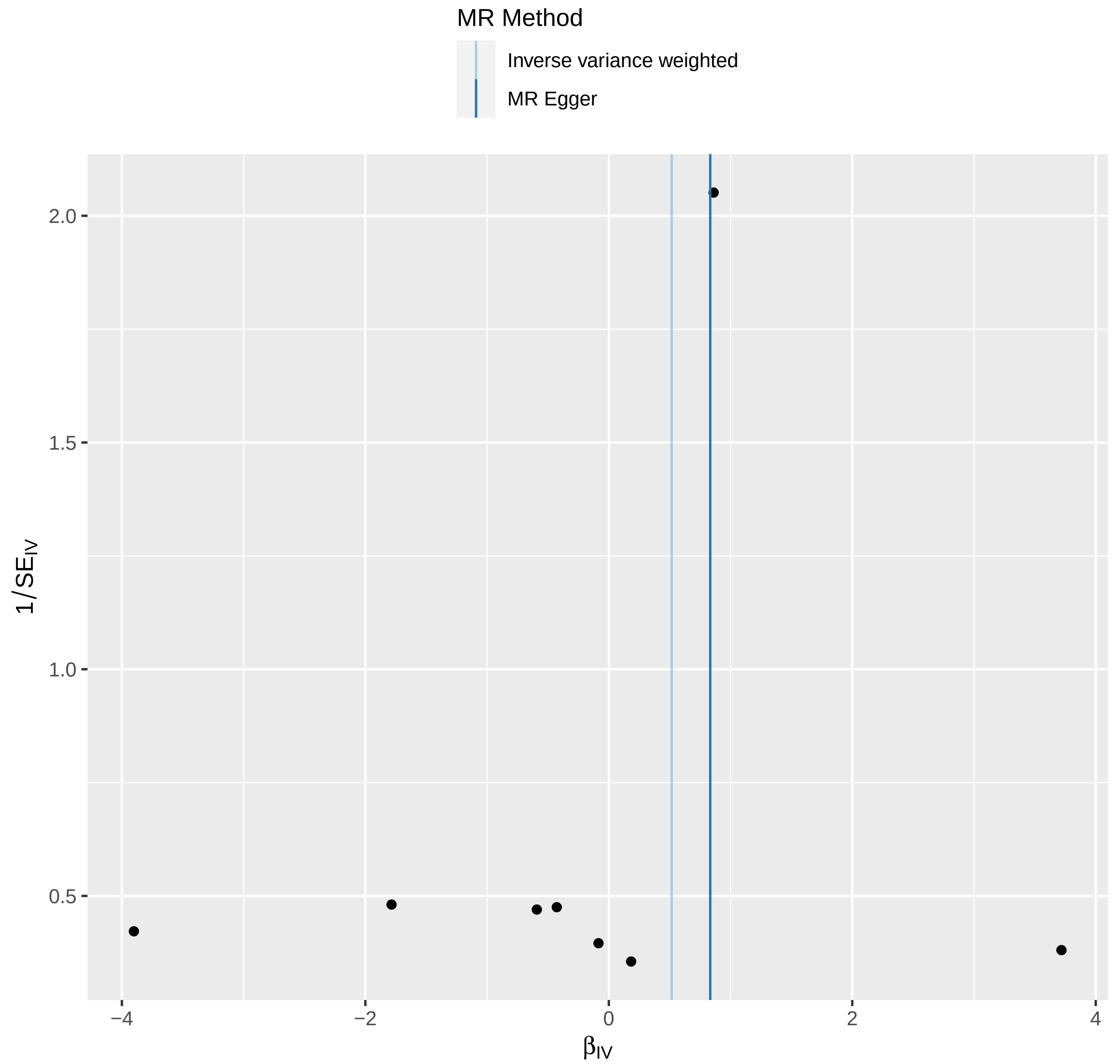

Batch 81 : Glycerophosphorylcholine (GPC) on Crohn's disease of colon

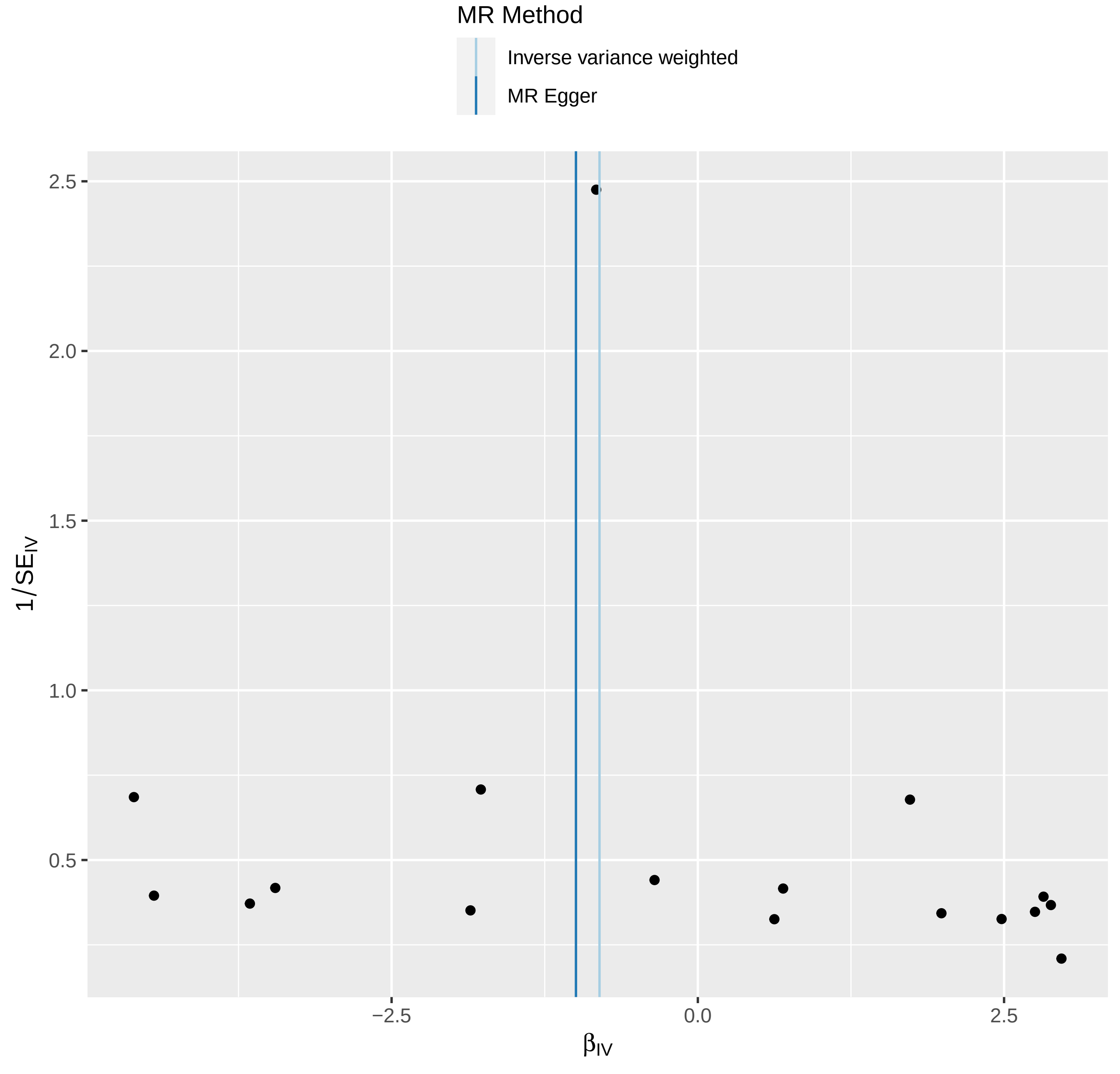

Batch 82 : Aspartate on Crohn's disease of colon

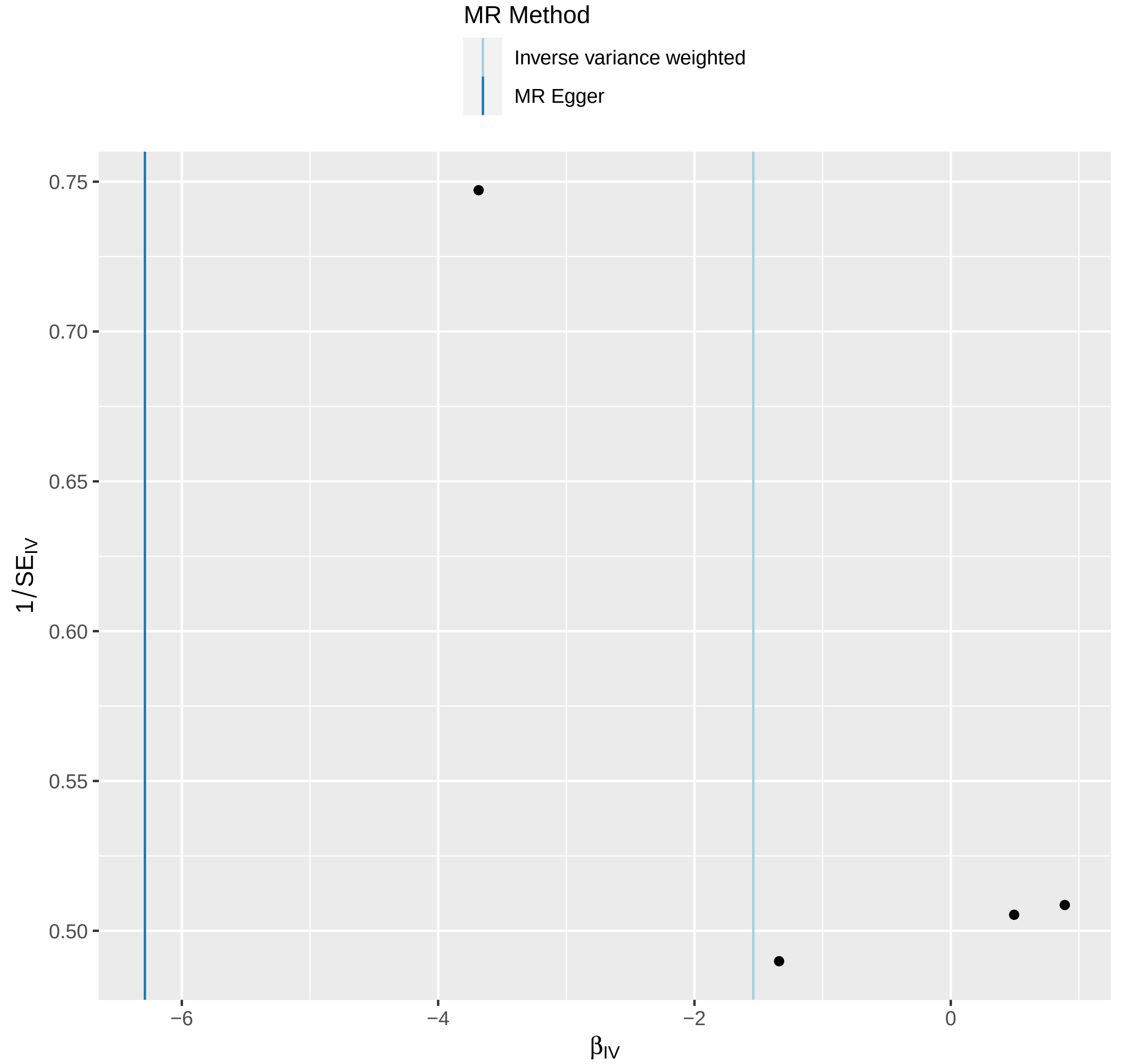

Batch 84 : Dihomo−linoleate (20:2n6) on Crohn's disease of colon

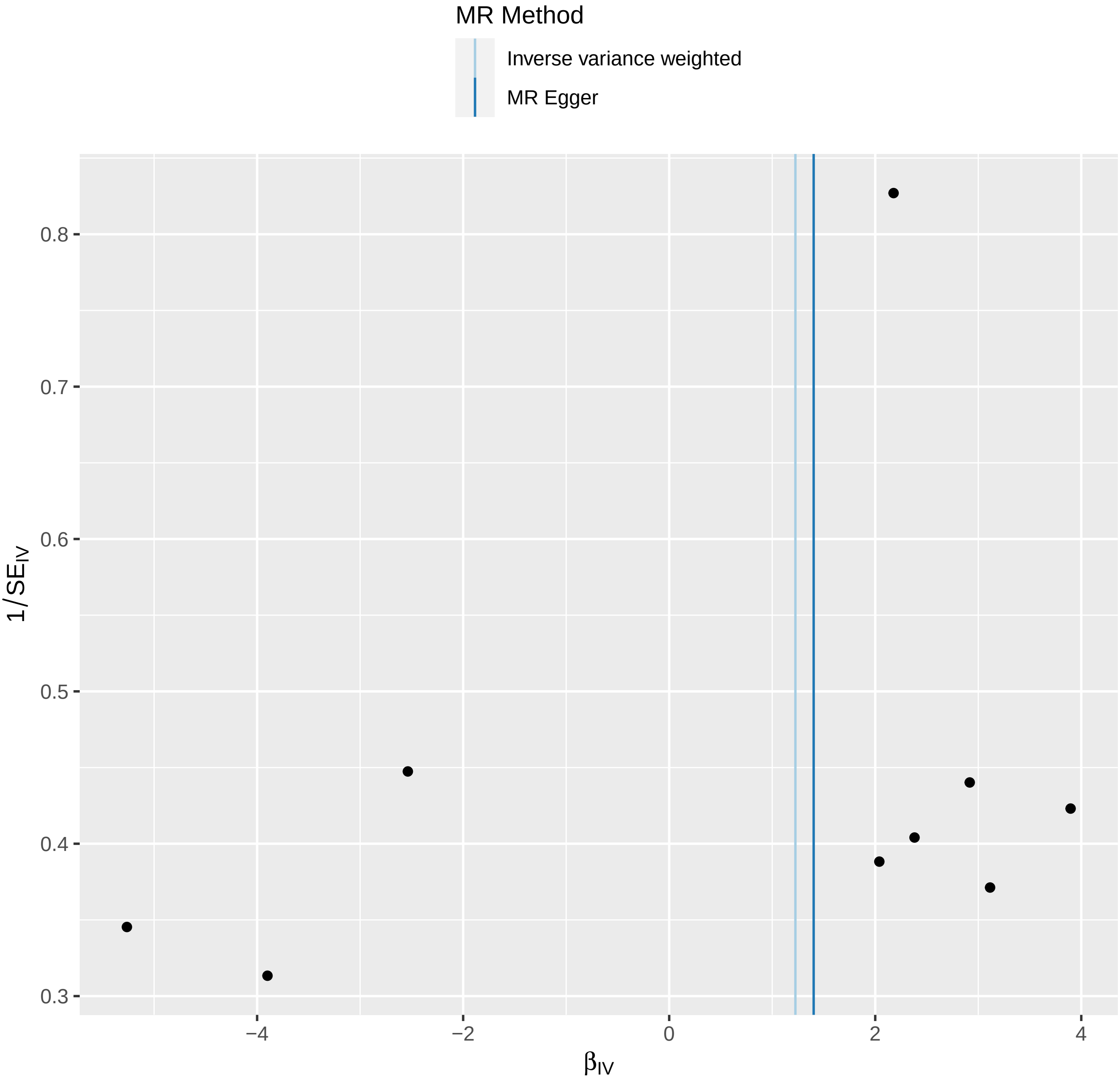

Batch 85 : 2−hydroxystearate on Crohn's disease of colon

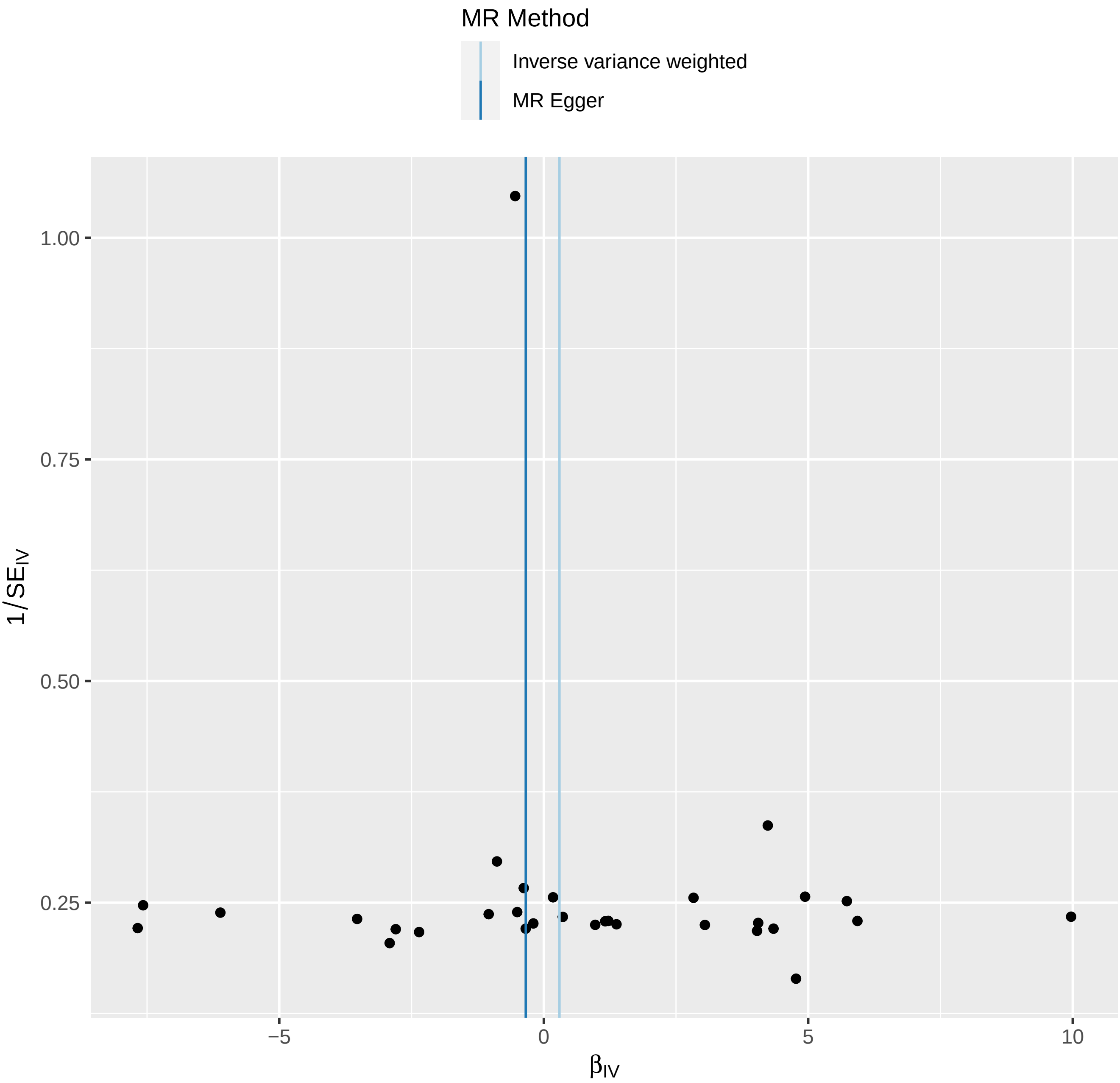

Batch 87 : Glycylvaline on Crohn's disease of colon

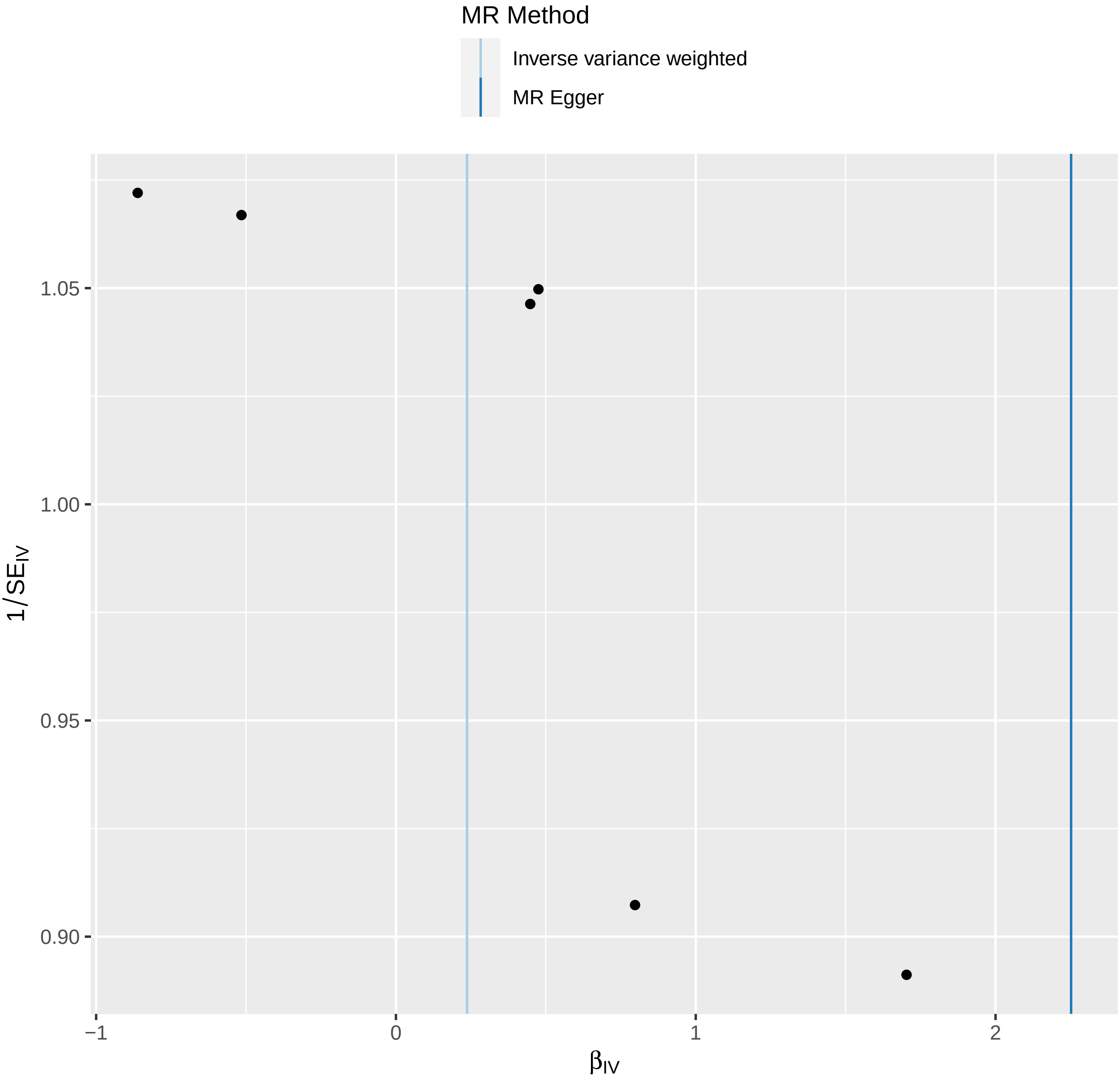

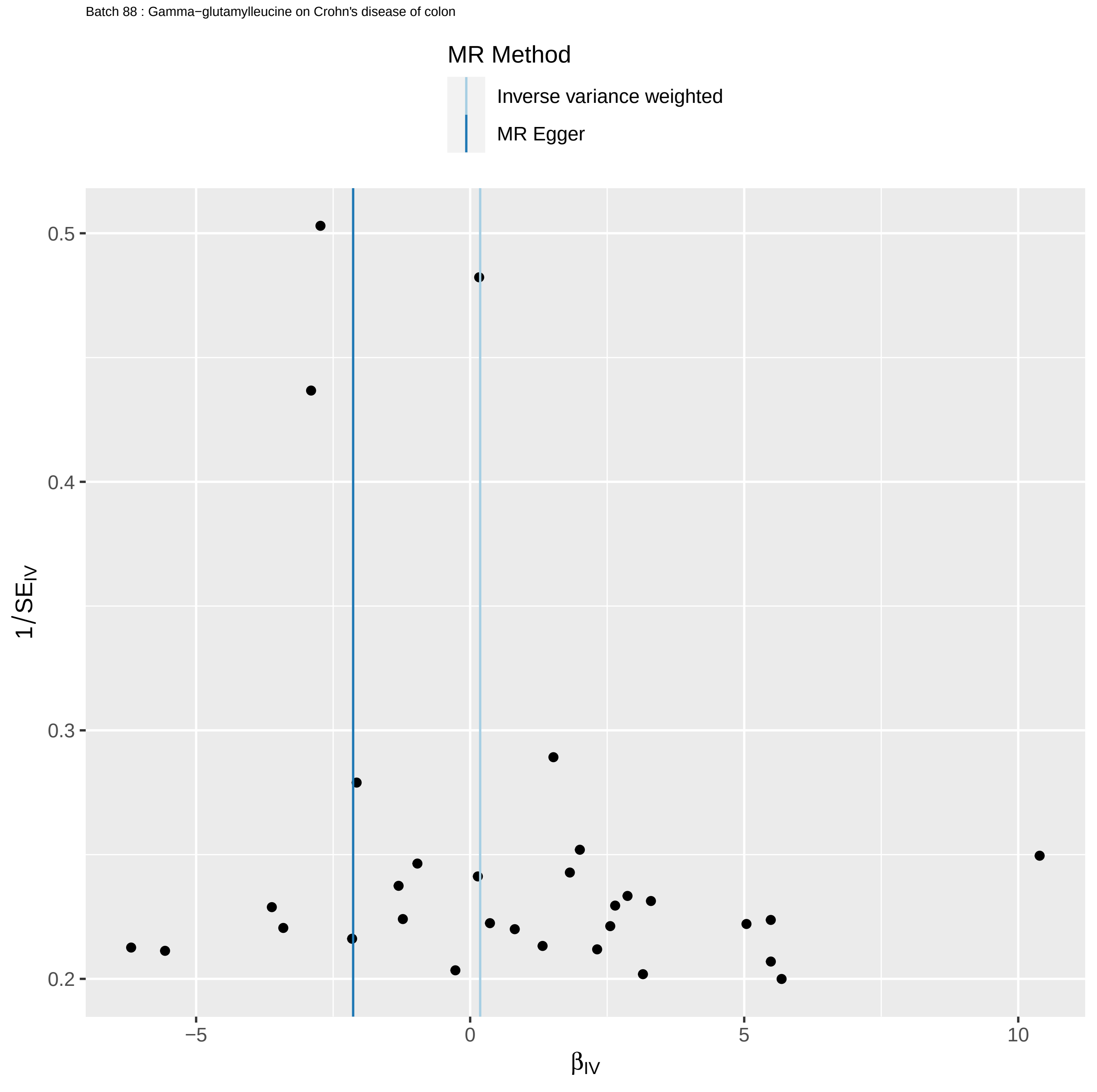

Batch 88 : Gamma−glutamylleucine on Crohn's disease of colon
MR Method
Inverse variance weighted
MR Egger
0.5
0.4
0.3
0.2
1/SE_IV
−5
0
5
10
β_IV

Batch 91 : Glycocholate on Crohn's disease of colon

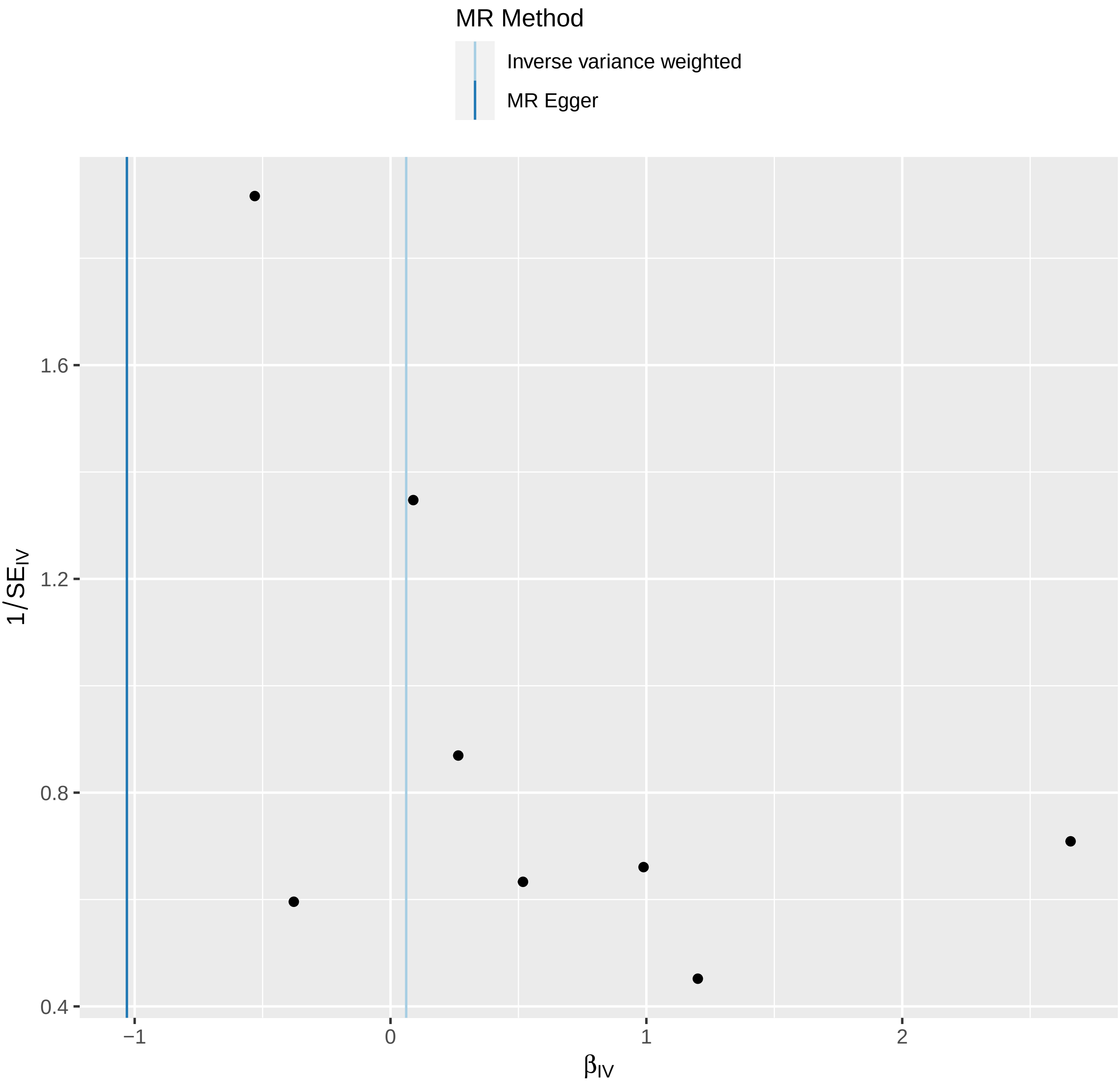

Batch 92 : Glycodeoxycholate on Crohn's disease of colon

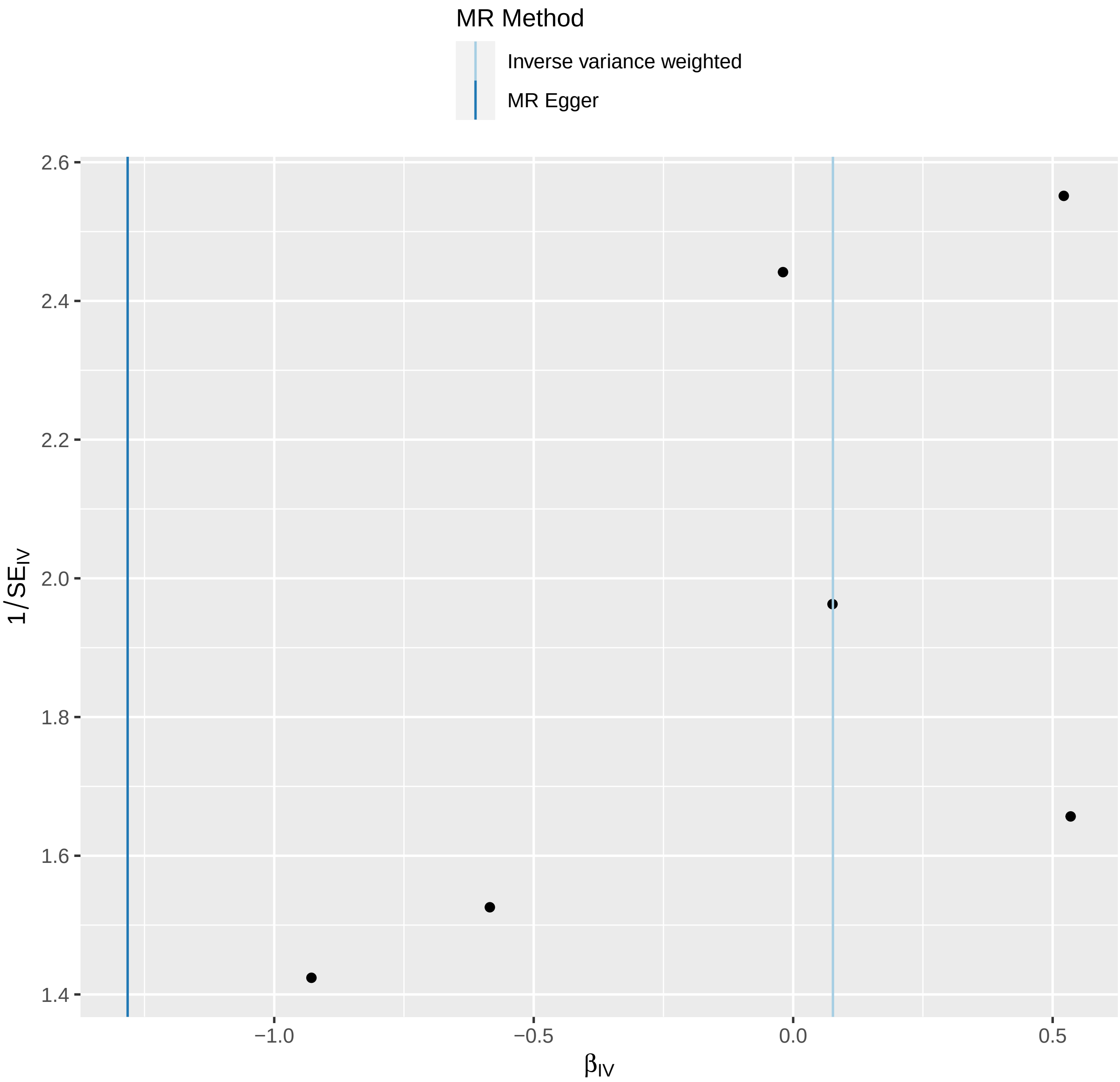

Batch 93 : Taurochenodeoxycholate on Crohn's disease of colon

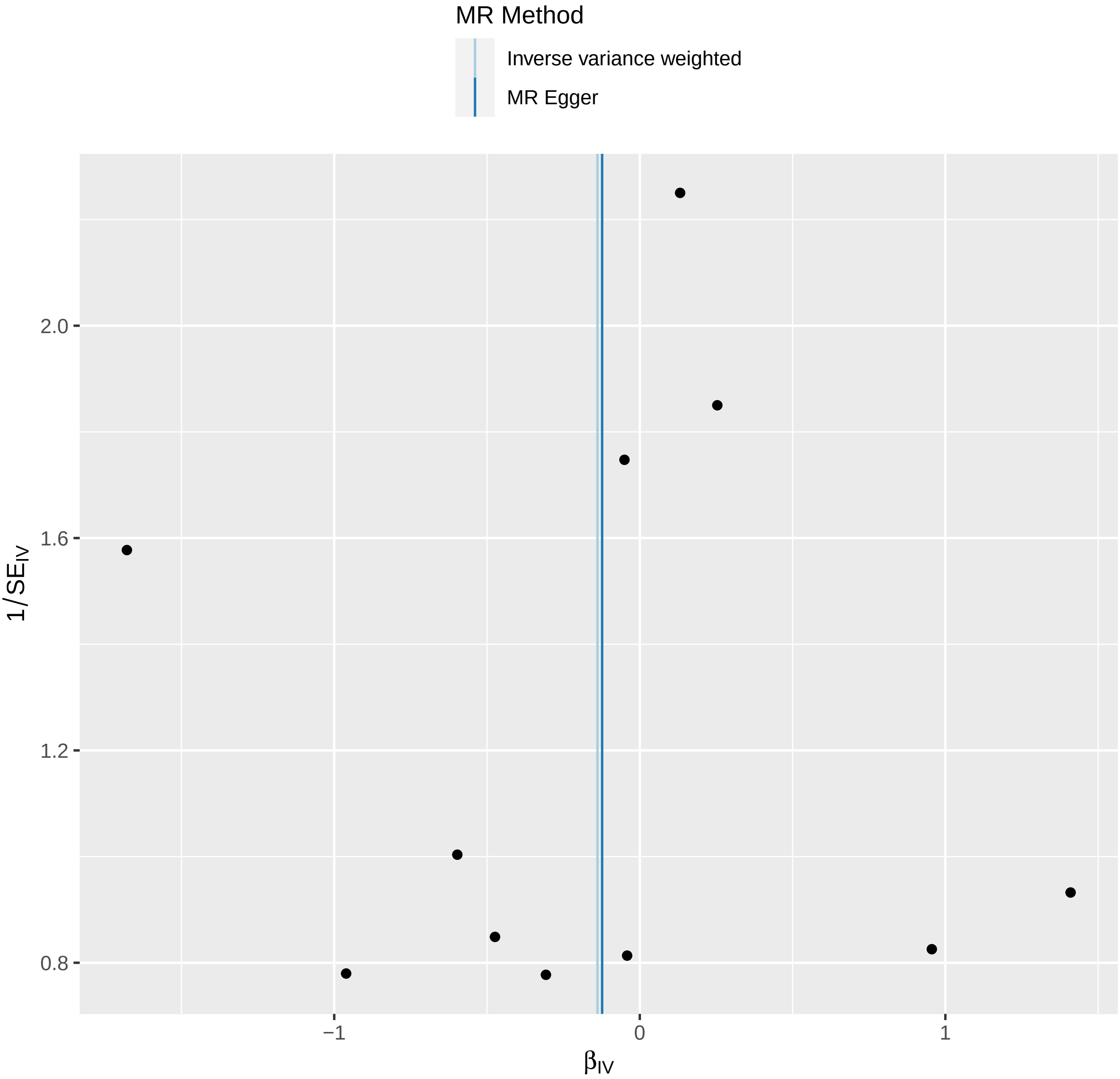

Batch 94 : Taurocholate on Crohn's disease of colon

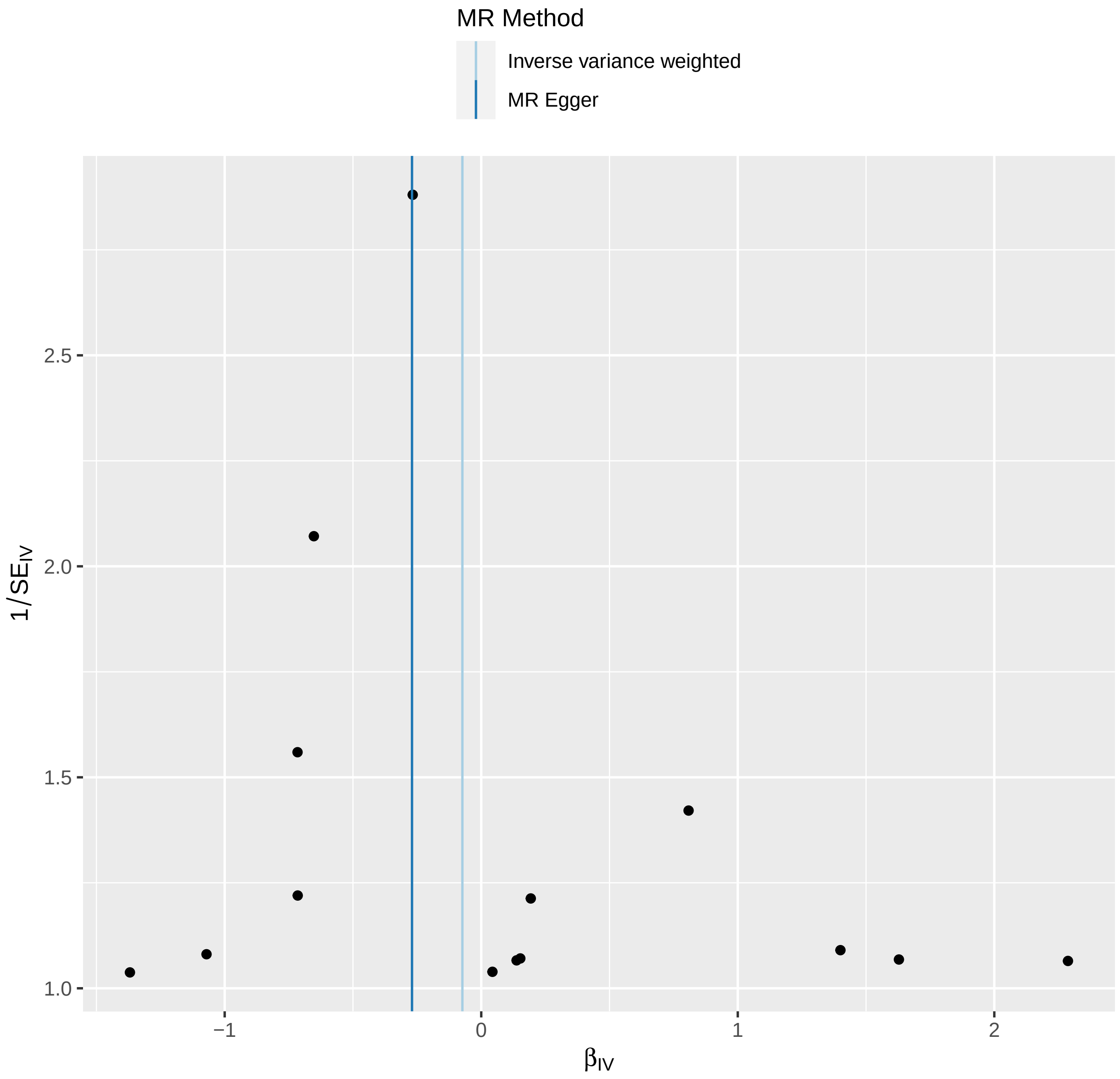

Batch 96 : 1−stearoylglycerophosphoinositol on Crohn's disease of colon

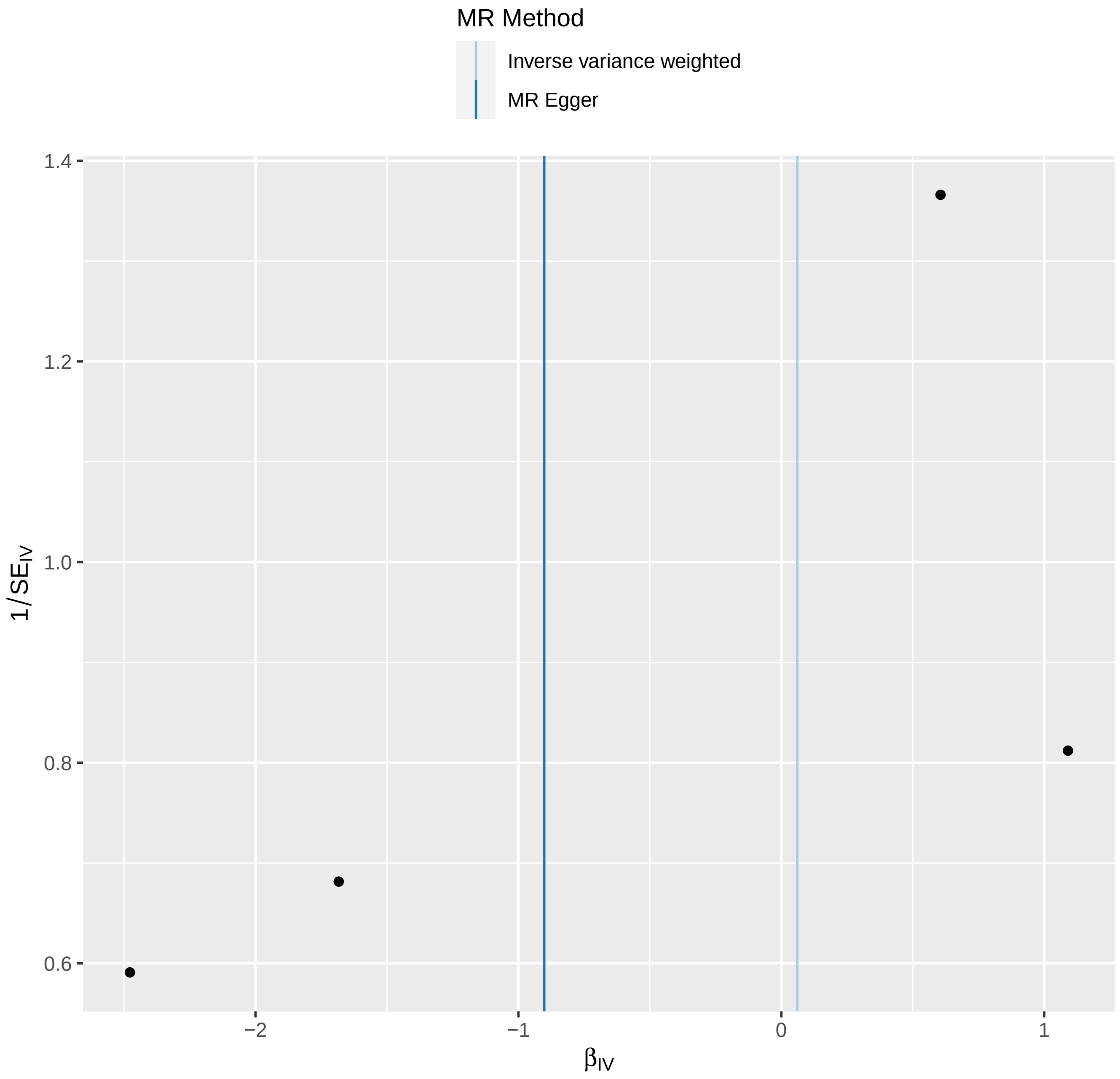

Batch 97 : Myo−inositol on Crohn's disease of colon

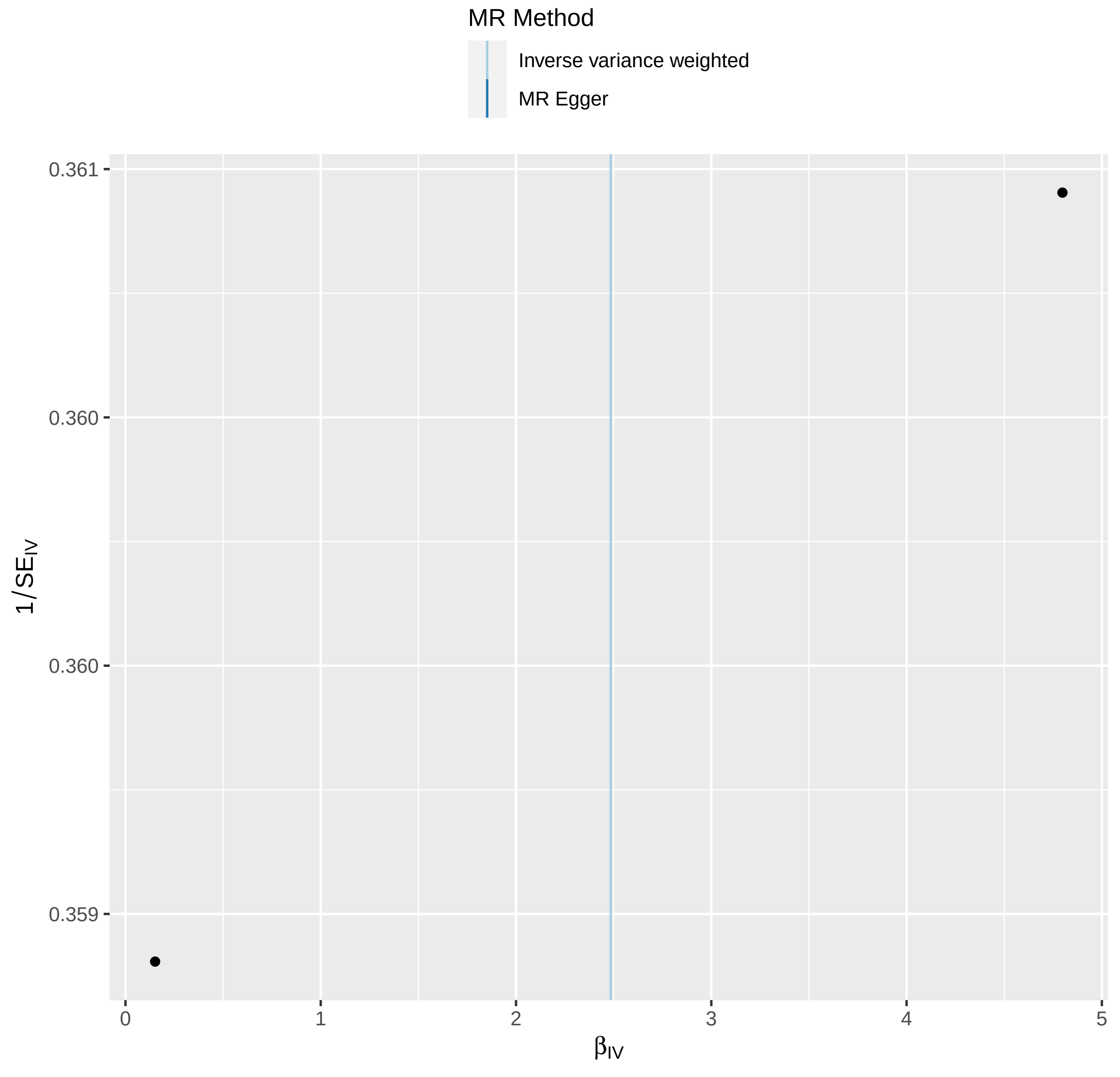

Batch 99 : 1,5−anhydroglucitol (1,5−AG) on Crohn's disease of colon

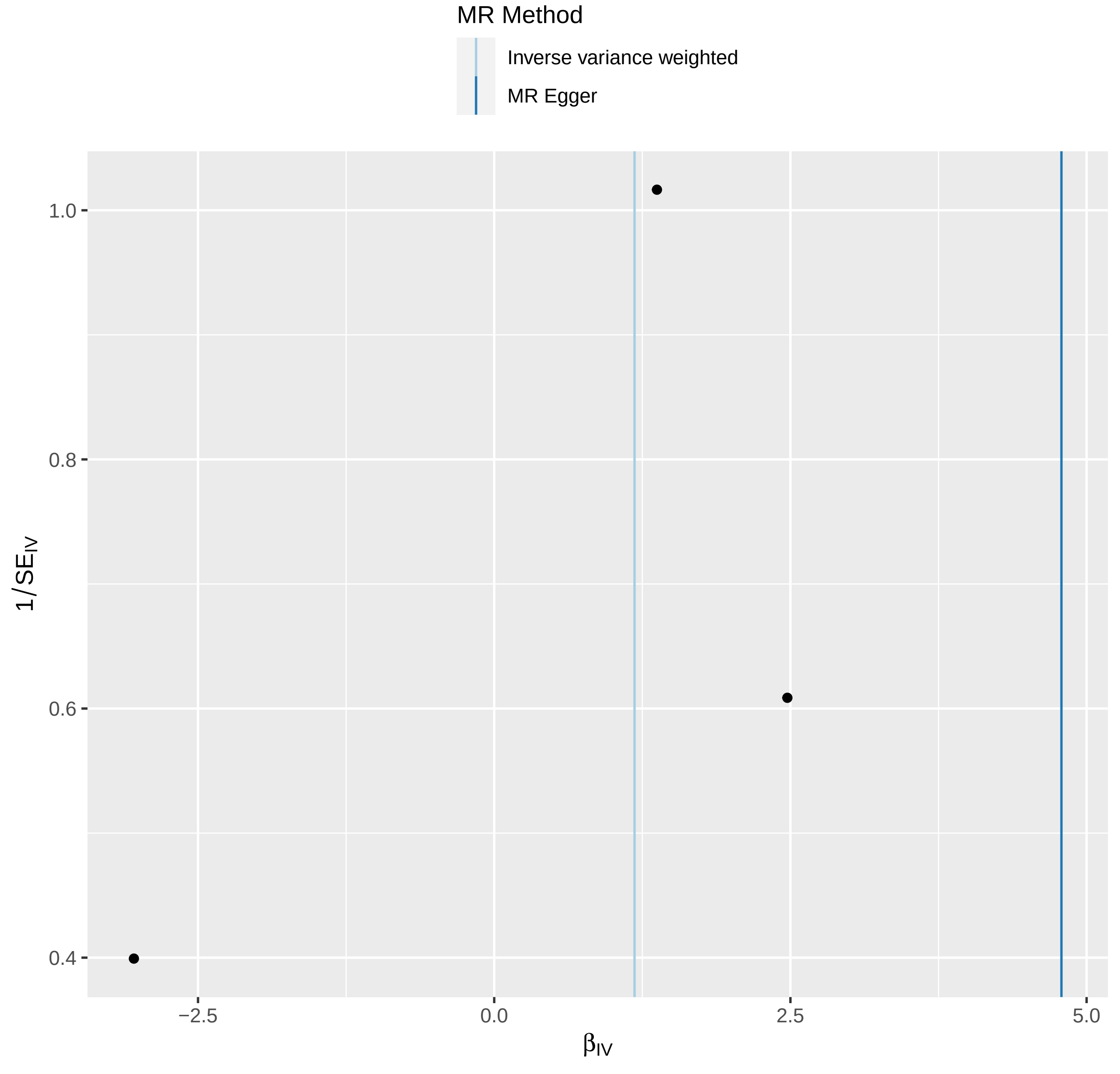

Batch 100 : 2−hydroxybutyrate (AHB) on Crohn's disease of colon

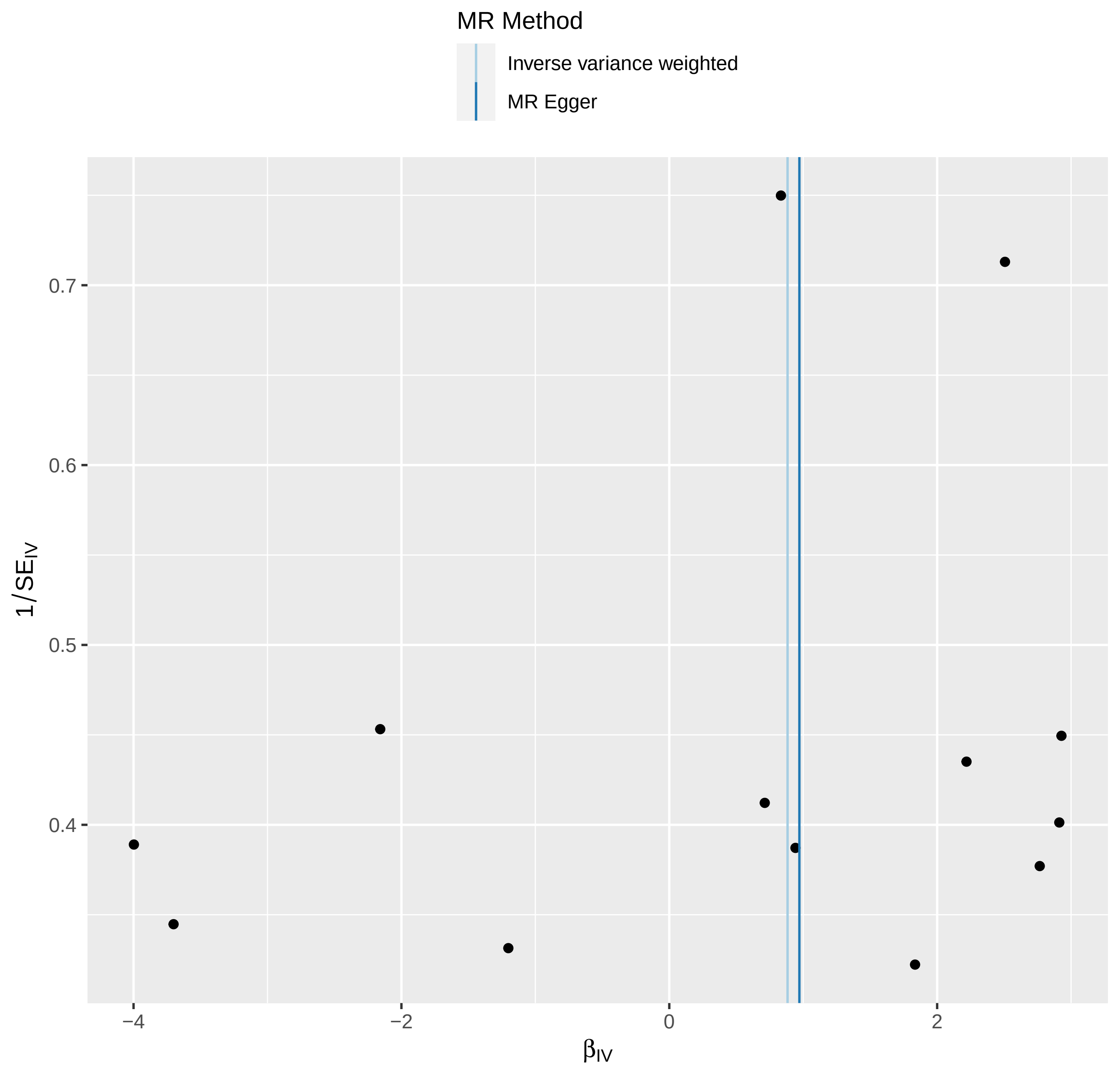

Batch 103 : 1−palmitoylglycerol (1−monopalmitin) on Crohn's disease of colon

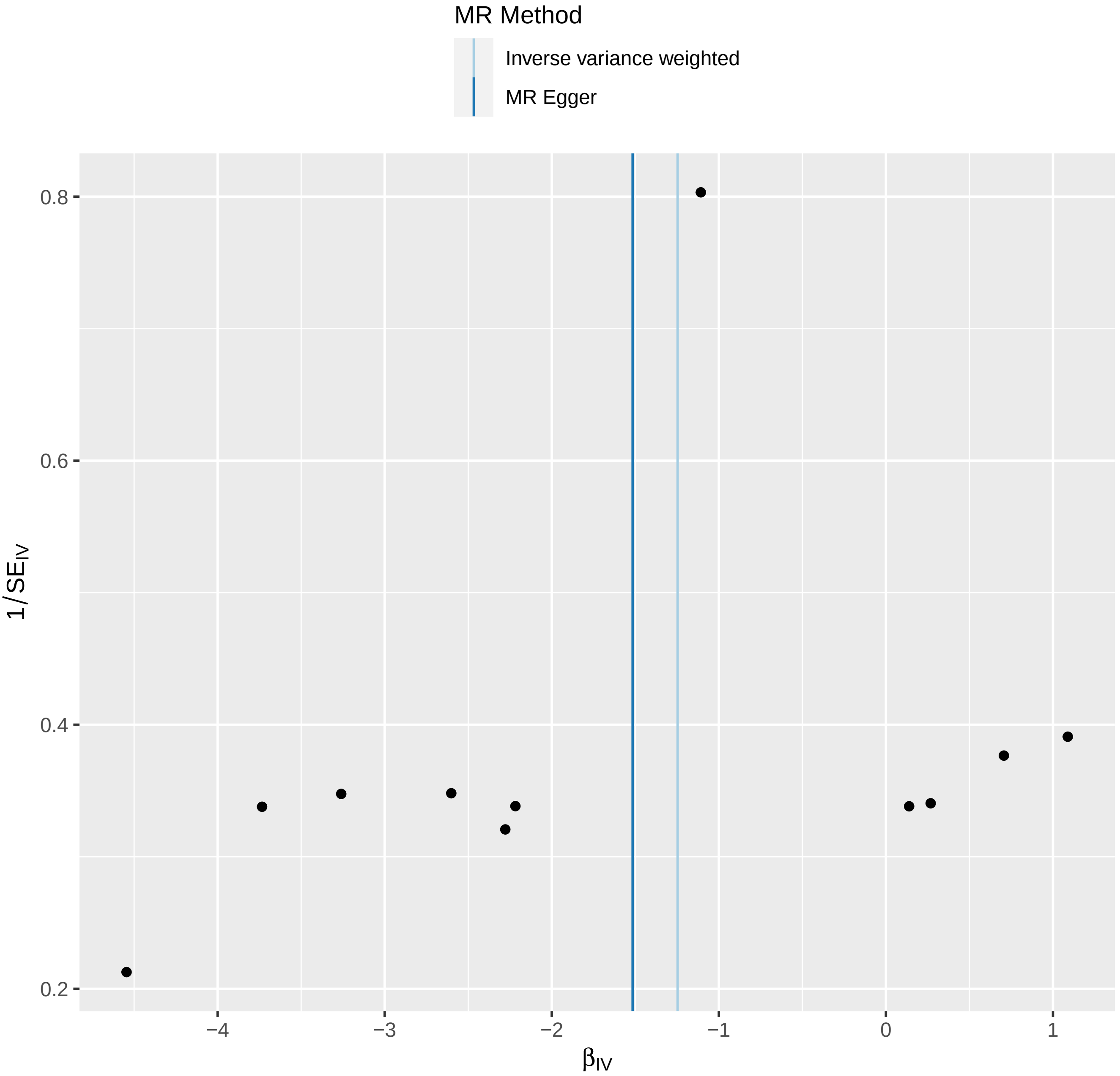

Batch 106 : 2−hydroxyisobutyrate on Crohn's disease of colon

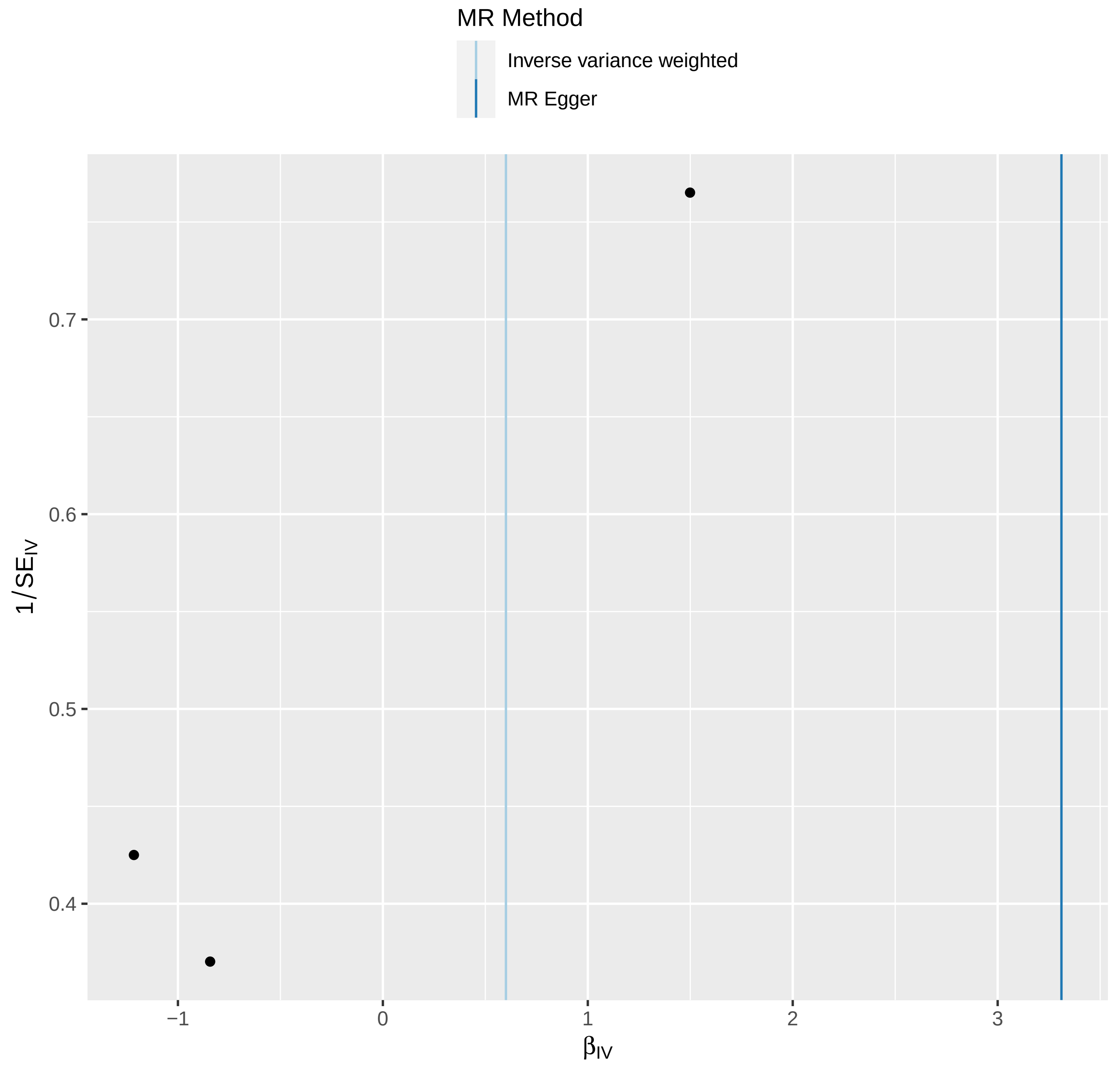

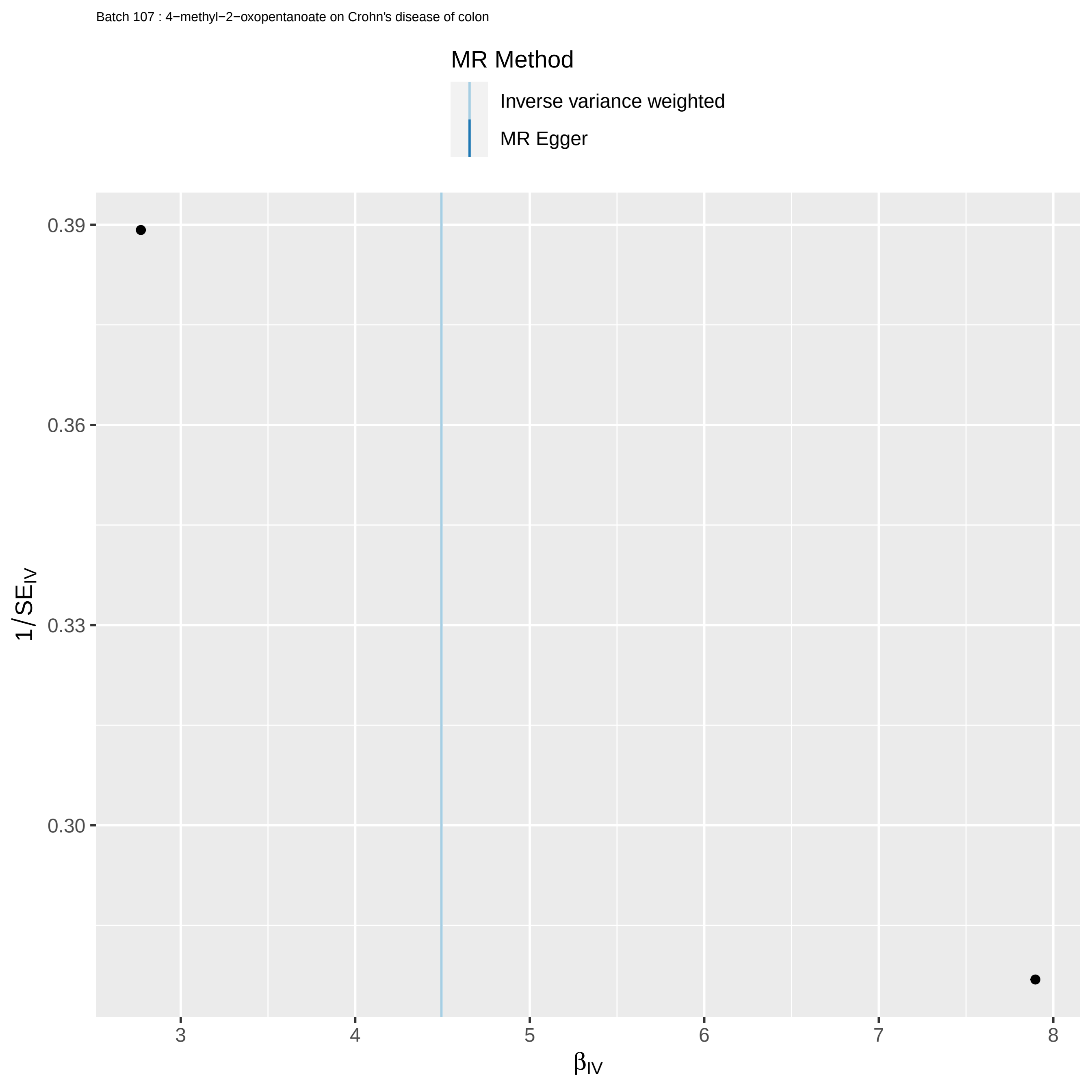
Batch 107 : 4−methyl−2−oxopentanoate on Crohn's disease of colon
MR Method
Inverse variance weighted
MR Egger
0.39
0.36
0.33
0.30
1/SE_IV
3
4
5
6
7
8
β_IV

Batch 111 : Levulinate (4−oxovalerate) on Crohn's disease of colon

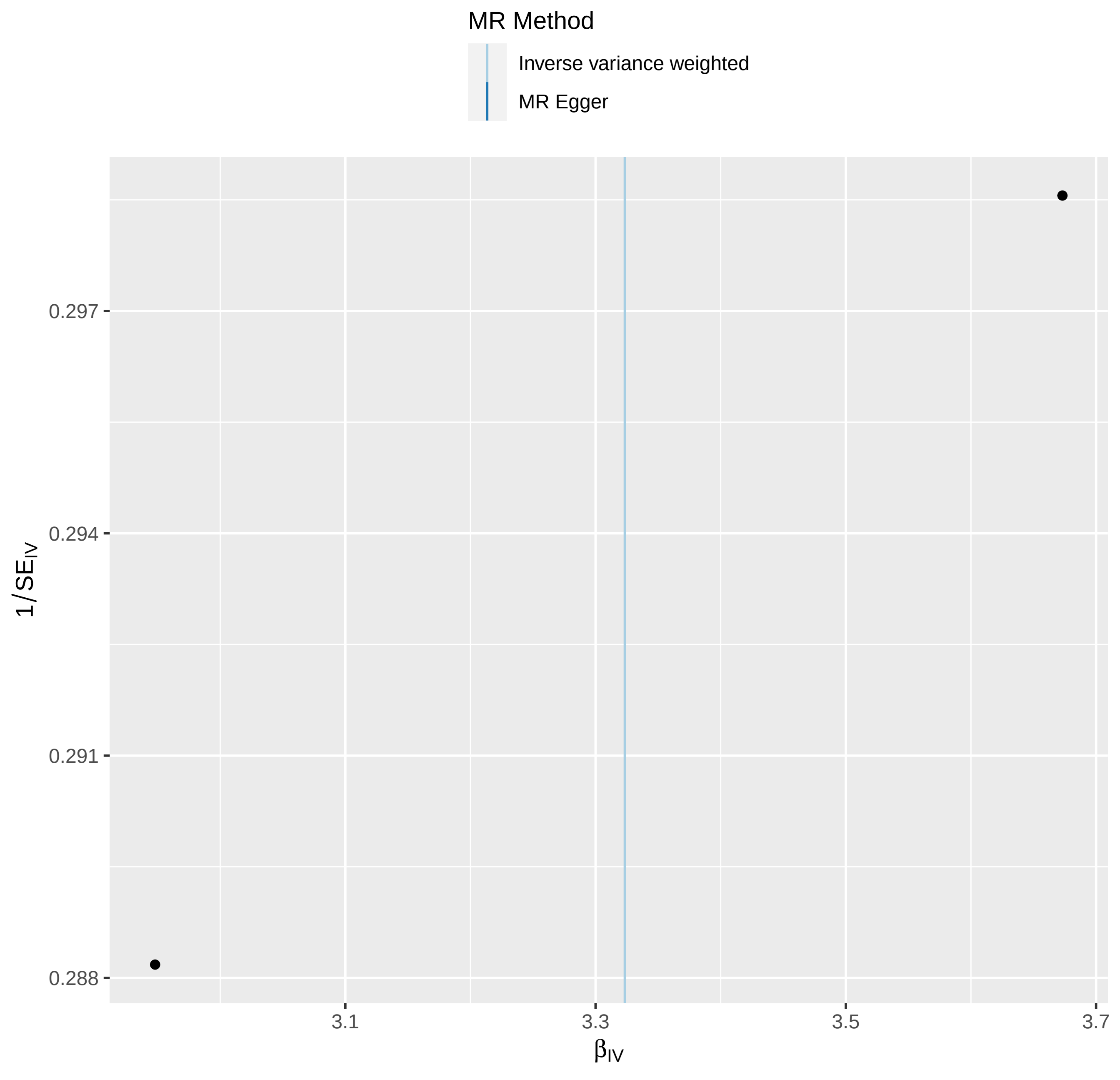

Batch 113 : Cholate on Crohn's disease of colon

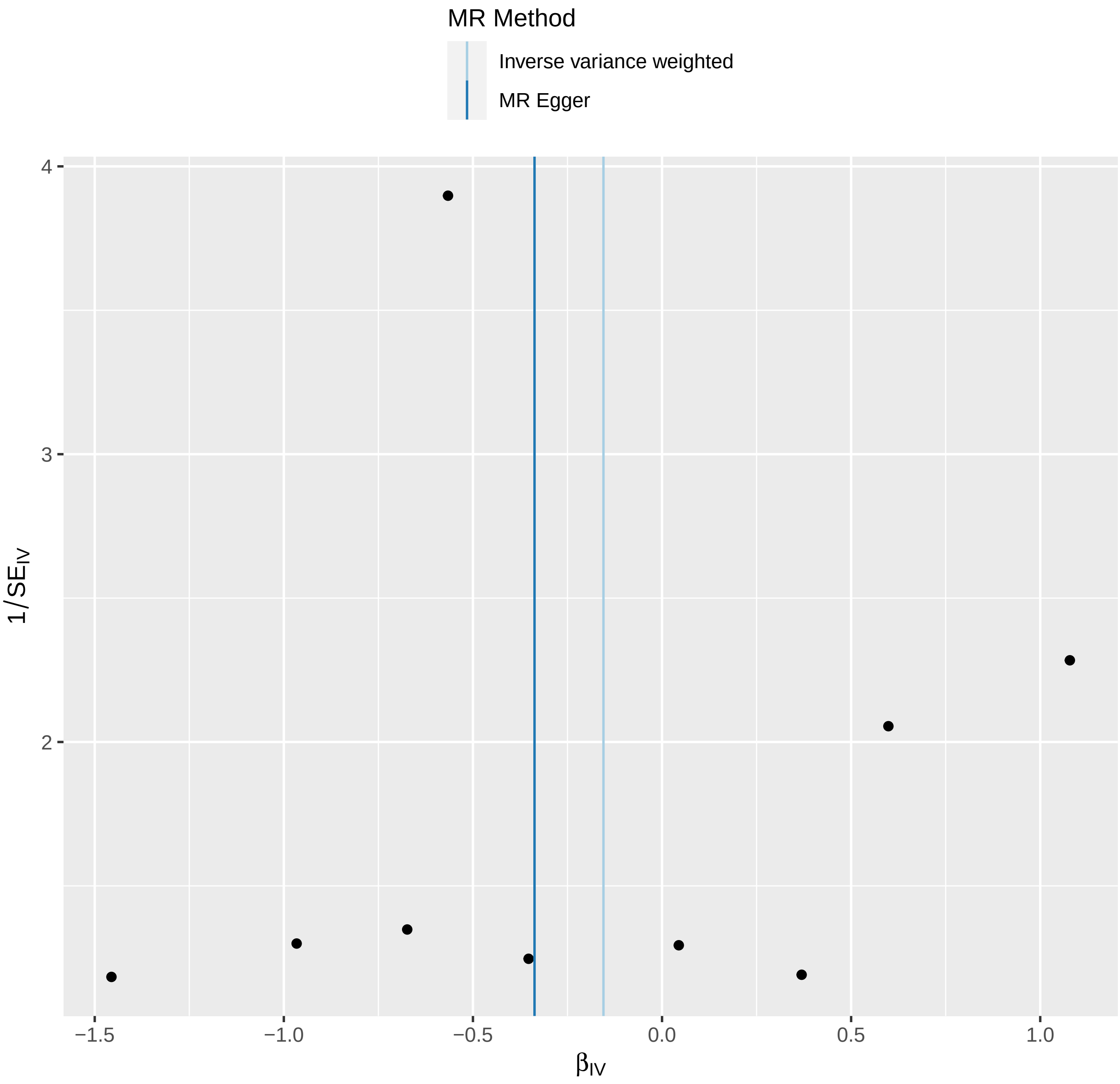

Batch 116 : Hyodeoxycholate on Crohn's disease of colon

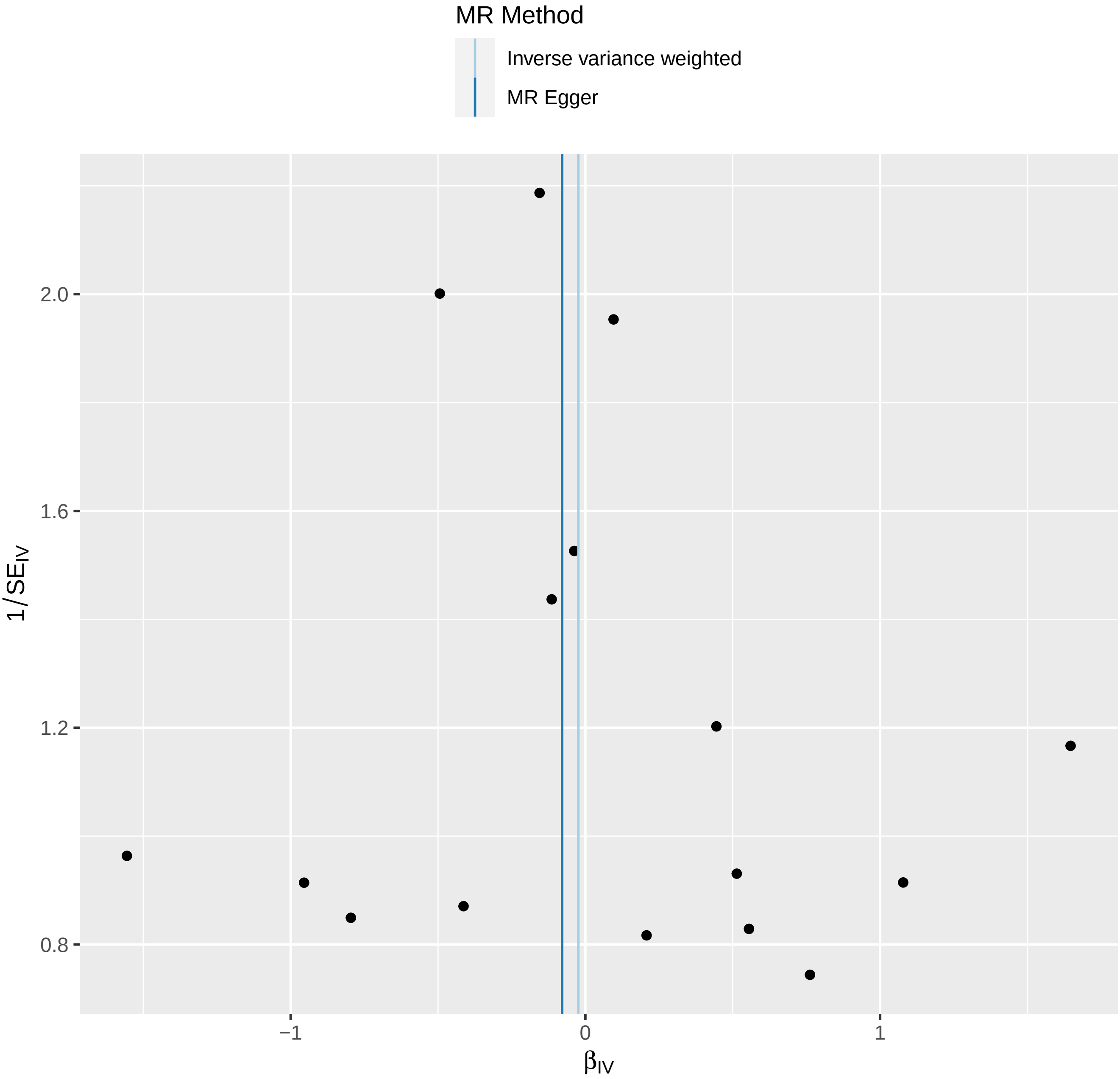

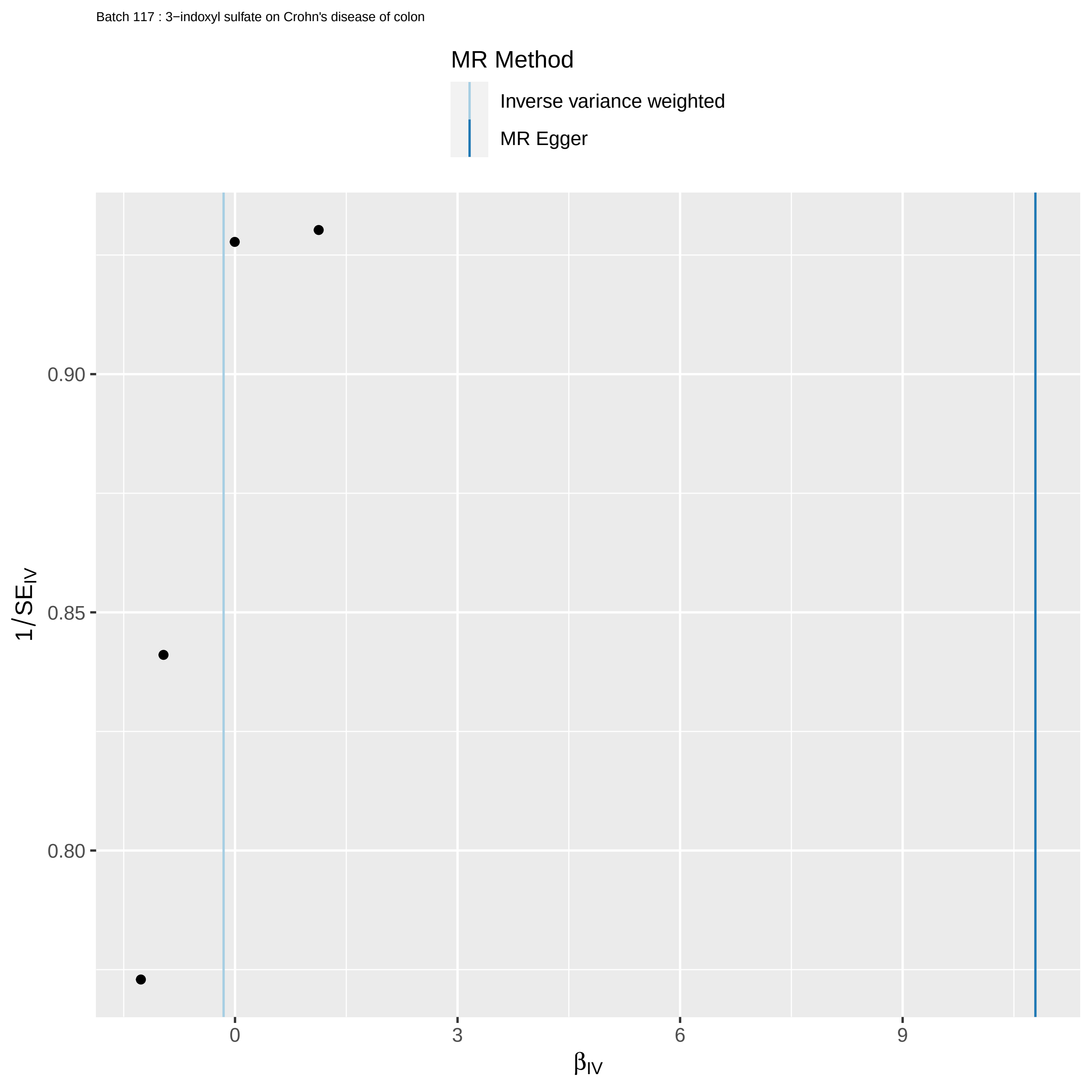

Batch 117 : 3−indoxyl sulfate on Crohn's disease of colon
MR Method
Inverse variance weighted
MR Egger
0.90
0.85
0.80
1/SE_IV
0
3
6
9
β_IV

Batch 121 : Erythrose on Crohn's disease of colon

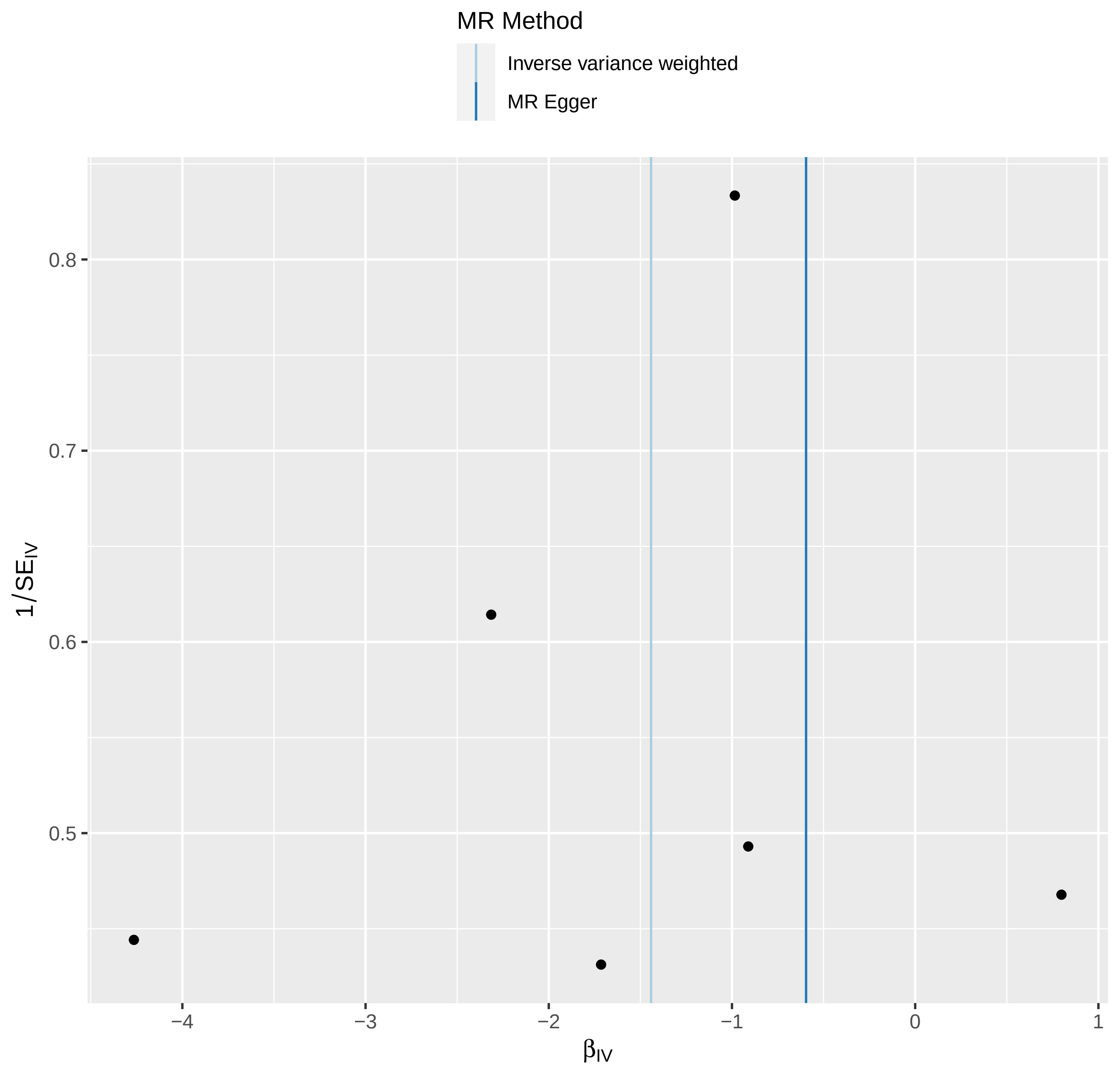

Batch 122 : Threonate on Crohn's disease of colon

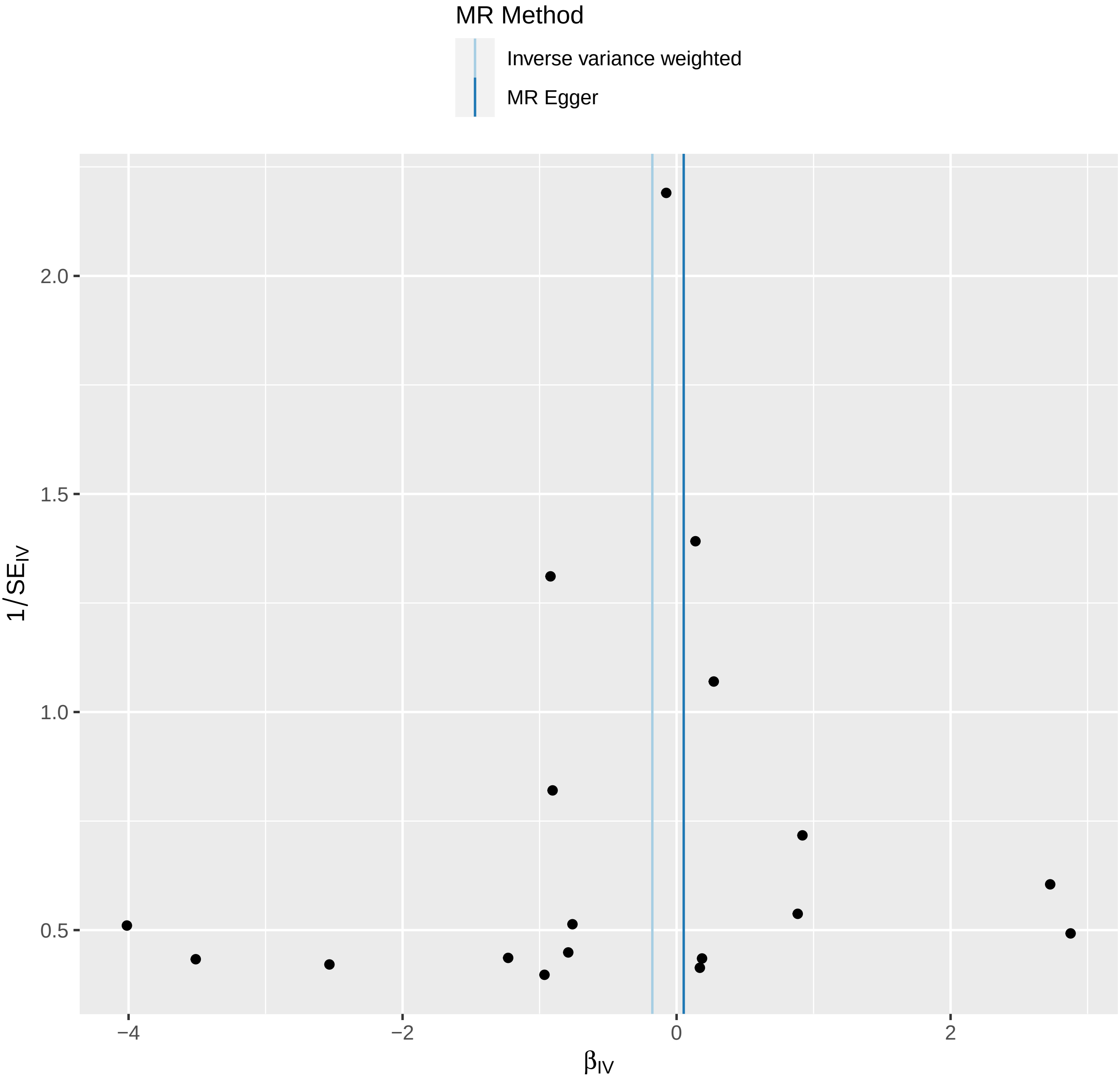

Batch 123 : Cysteine on Crohn's disease of colon

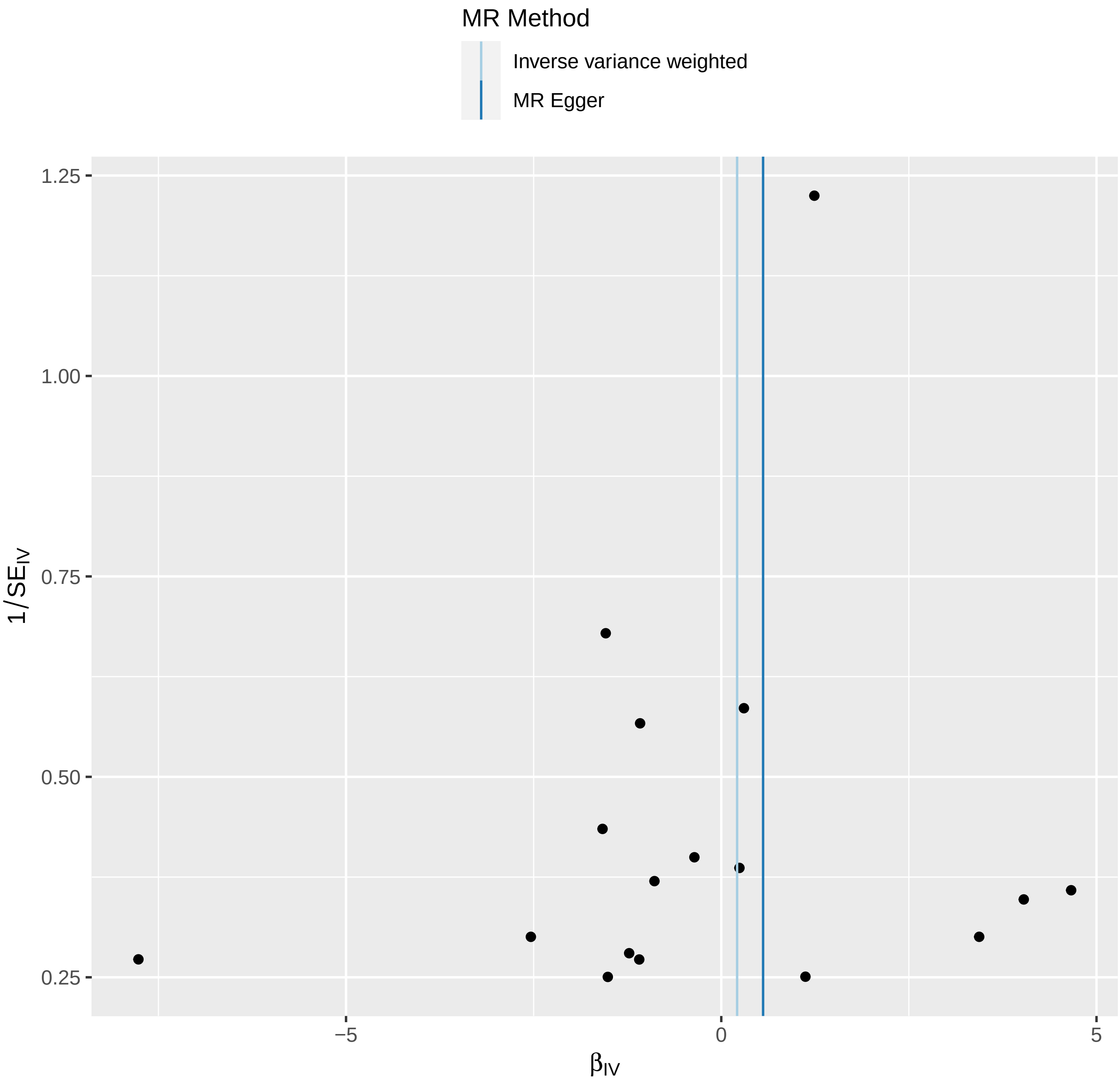

Batch 124 : Pyroglutamylglycine on Crohn's disease of colon

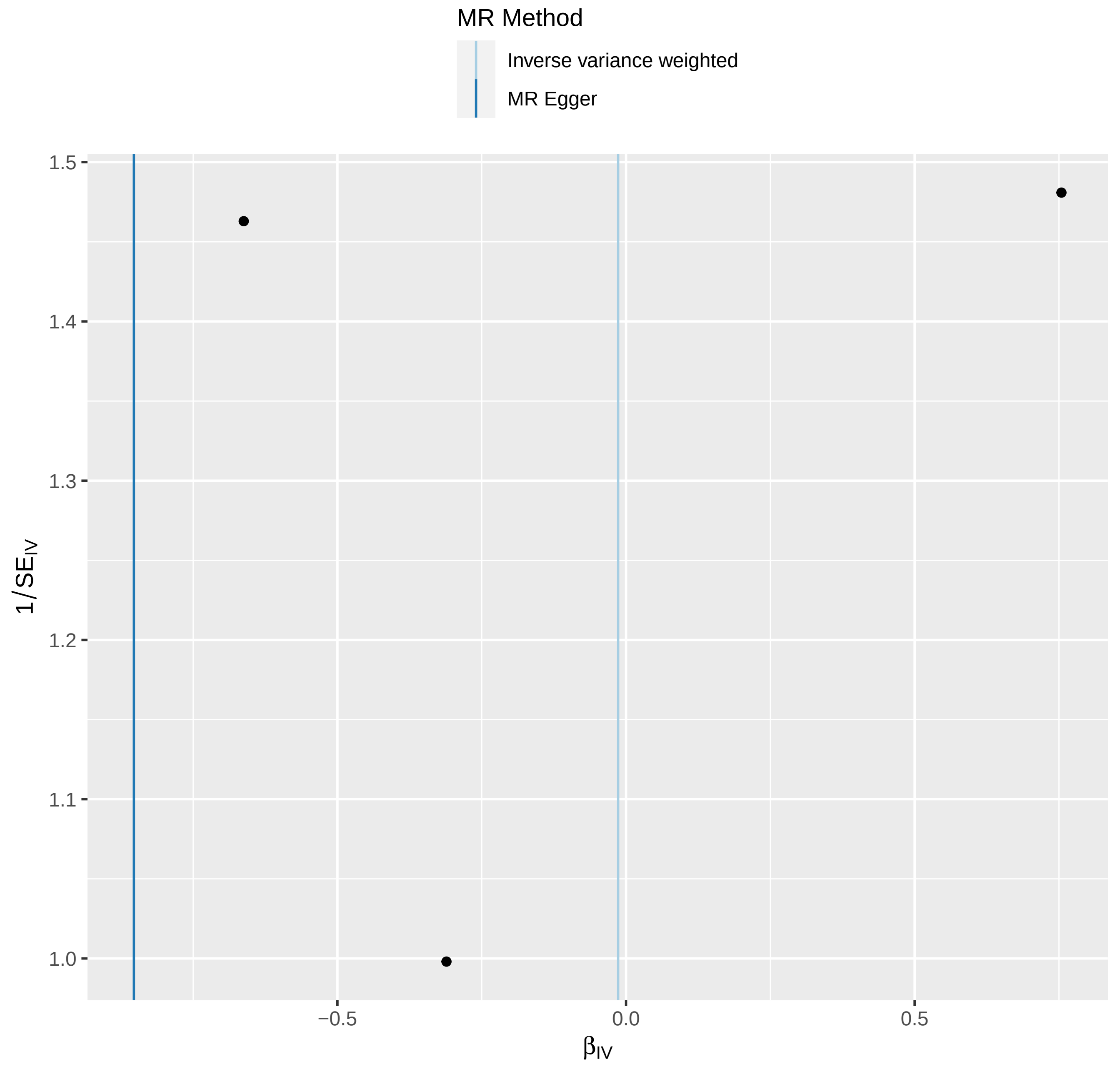

Batch 126 : DSGEGDFXAEGGGVR* on Crohn's disease of colon

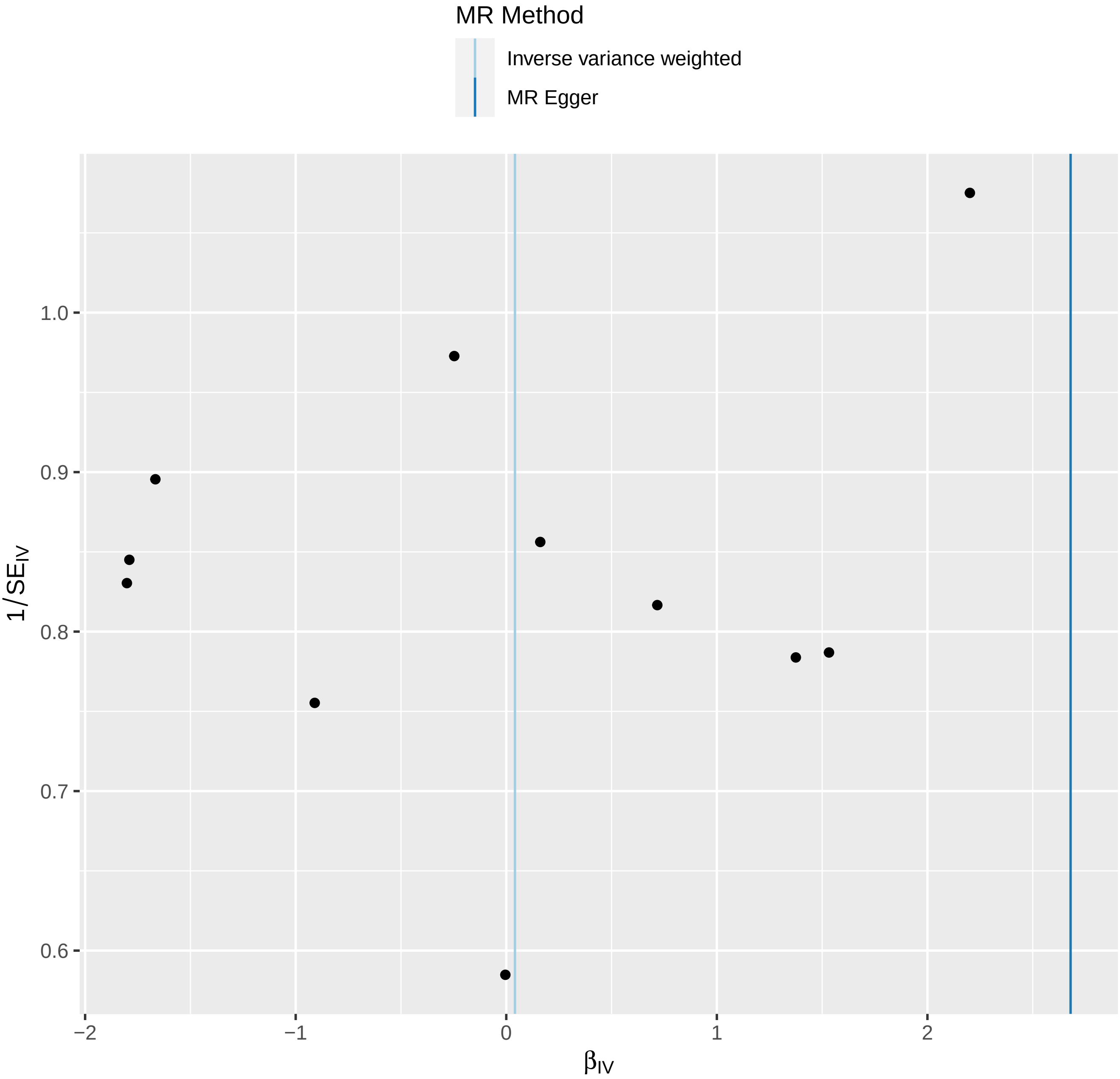

Batch 127 : Pyridoxate on Crohn's disease of colon

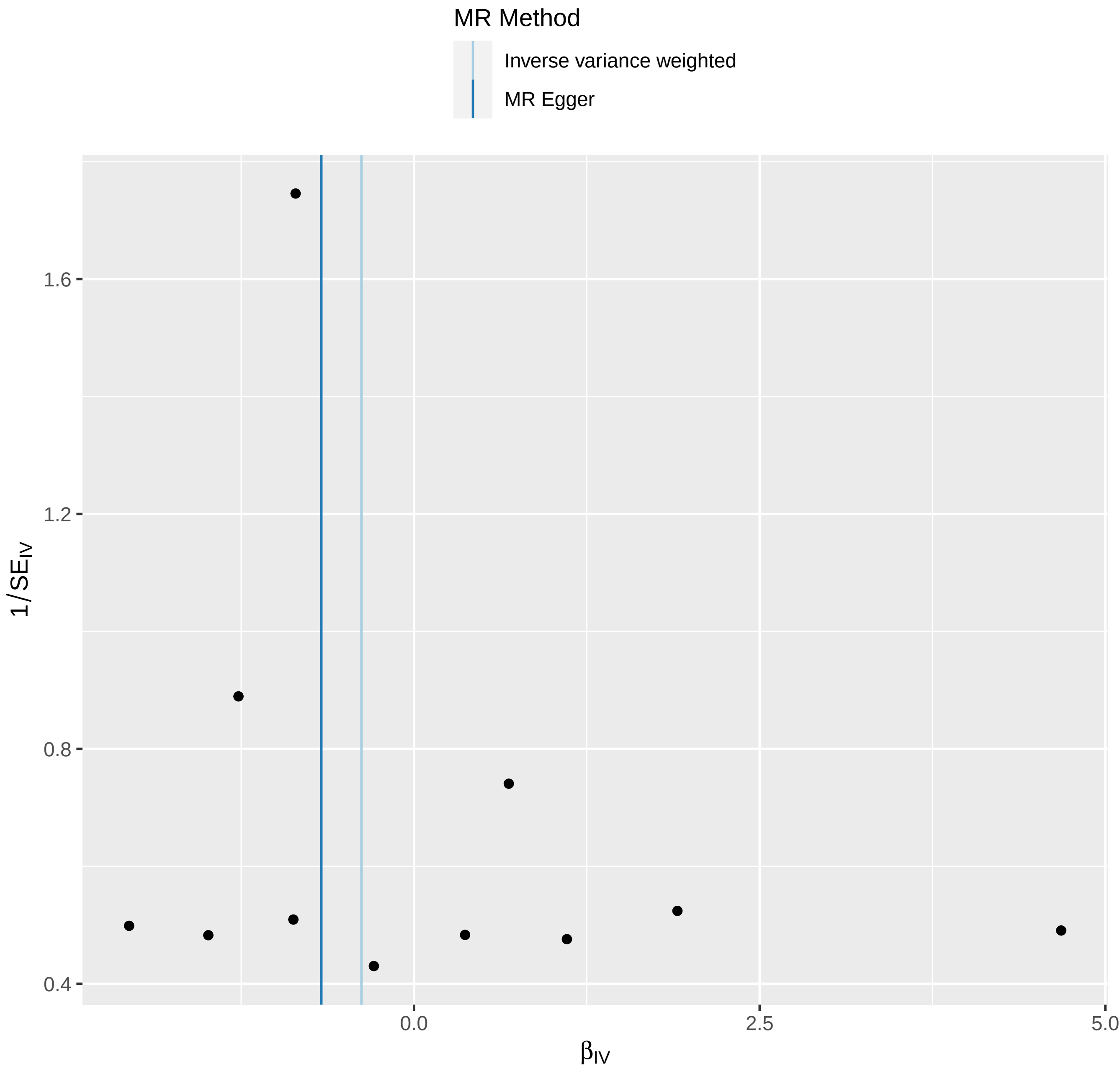

Batch 128 : Androsterone sulfate on Crohn's disease of colon

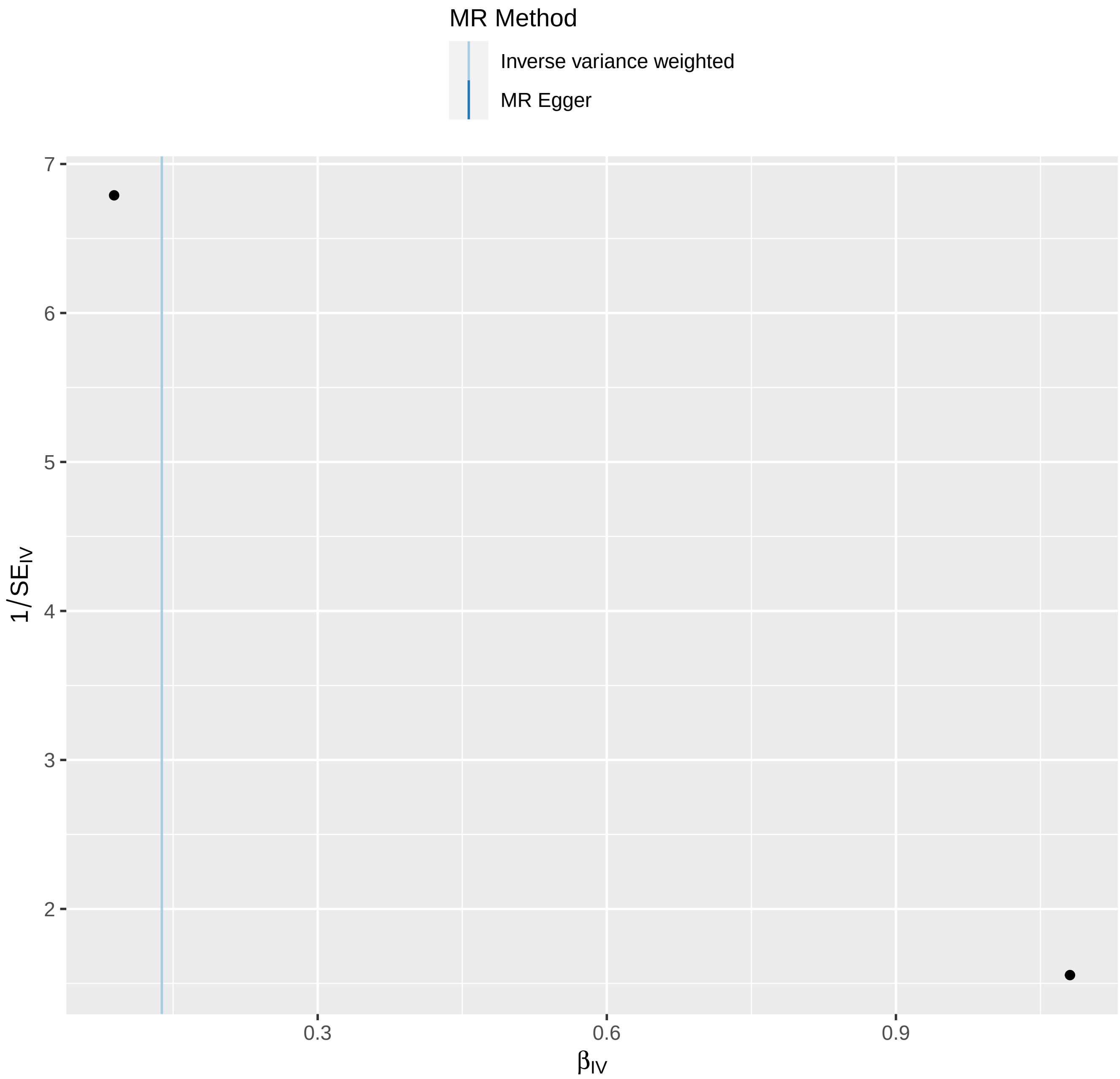

Batch 132 : Serine on Crohn's disease of colon

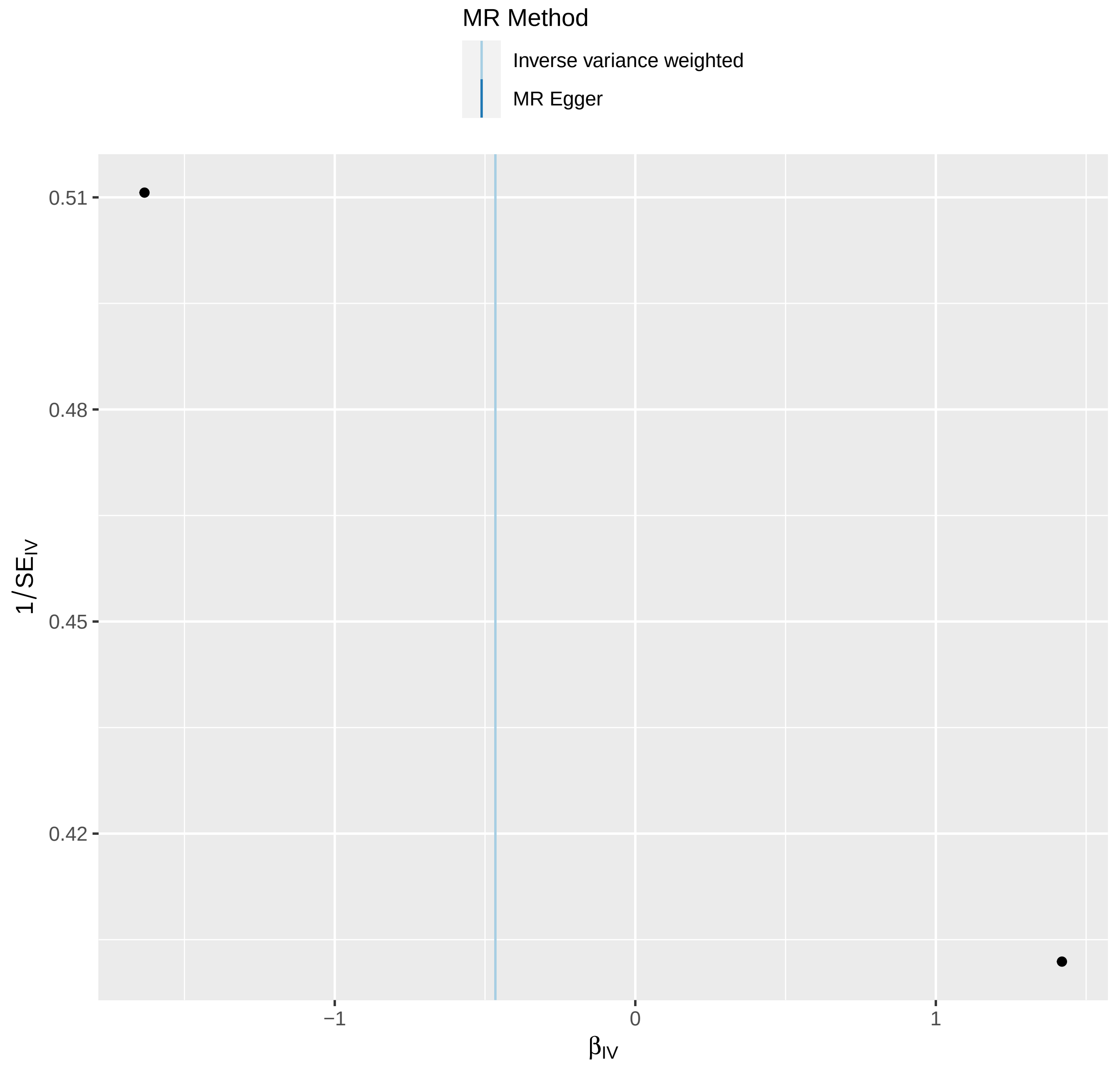

Batch 133 : Trans−4−hydroxyproline on Crohn's disease of colon

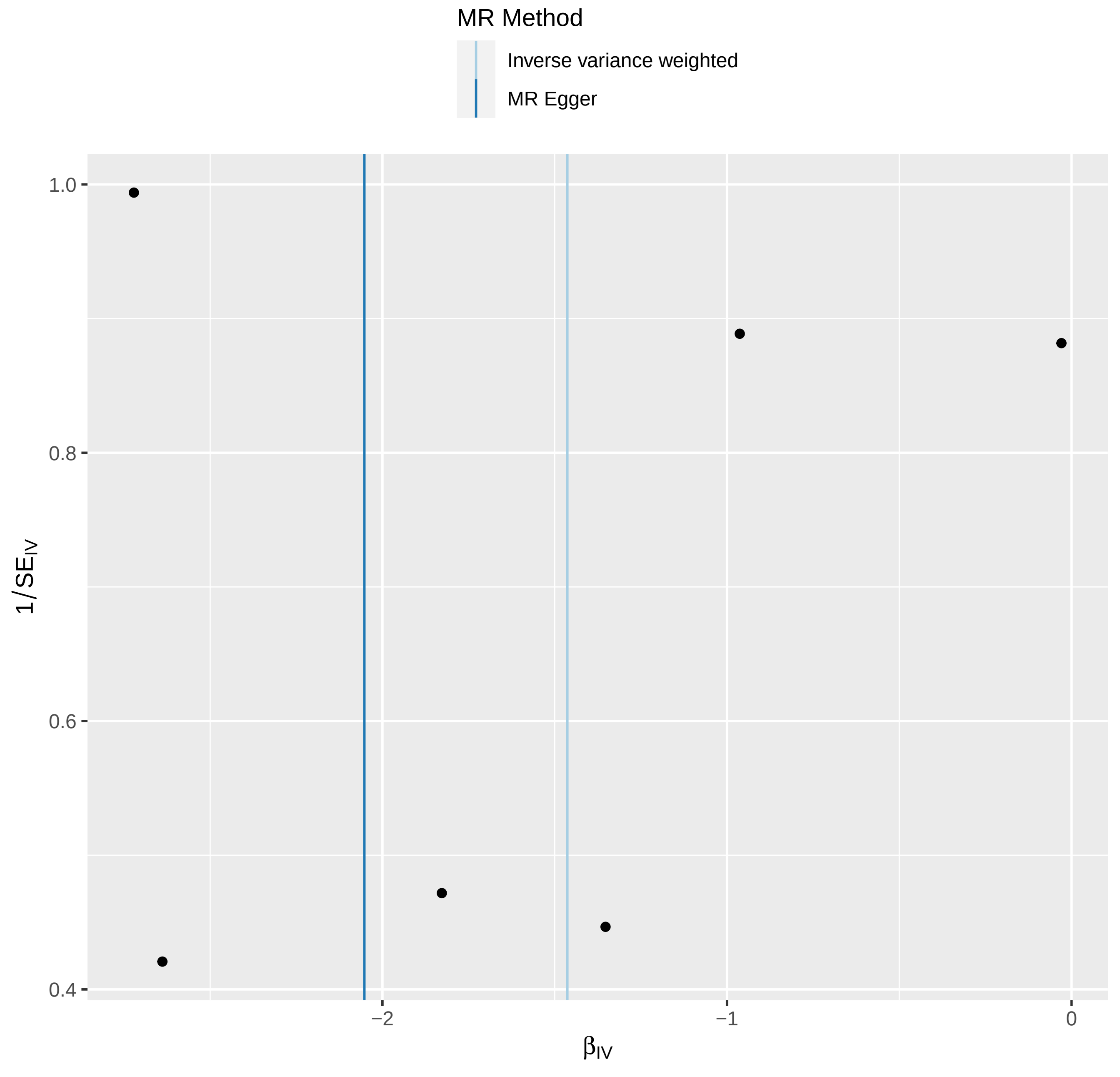

Batch 134 : Glutamate on Crohn's disease of colon

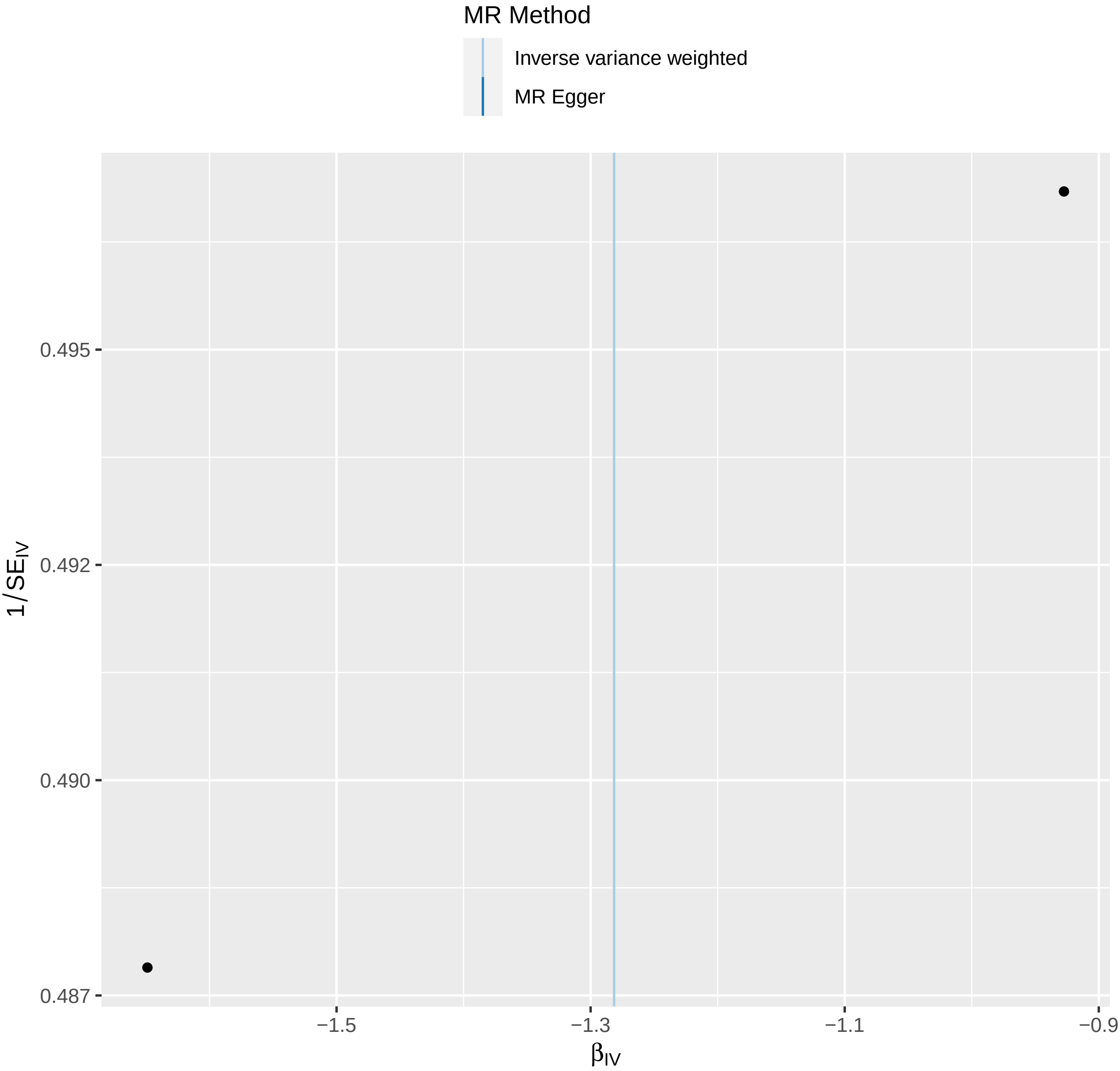

Batch 135 : Hexanoylcarnitine on Crohn's disease of colon

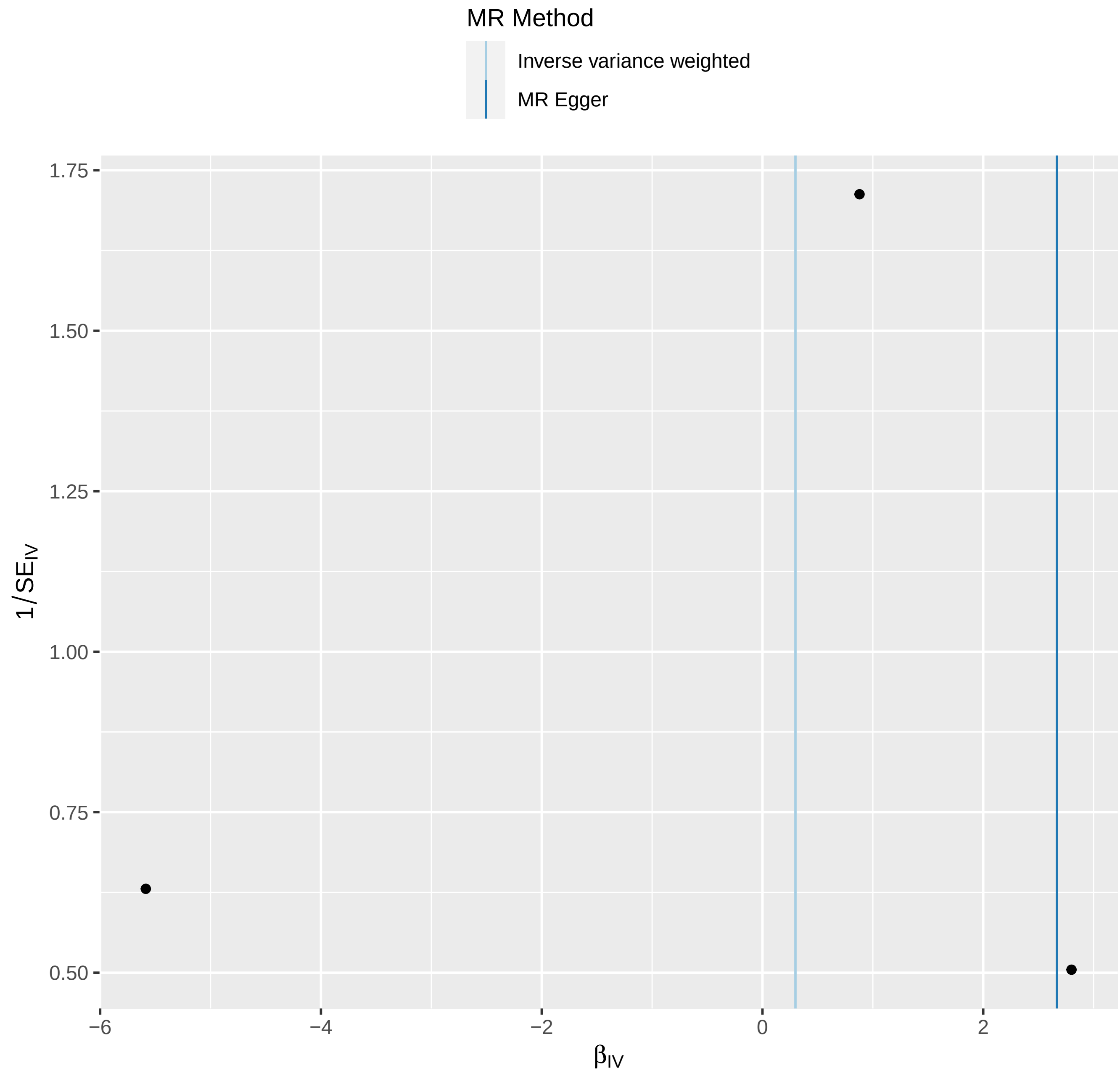

Batch 139 : 2−aminobutyrate on Crohn's disease of colon

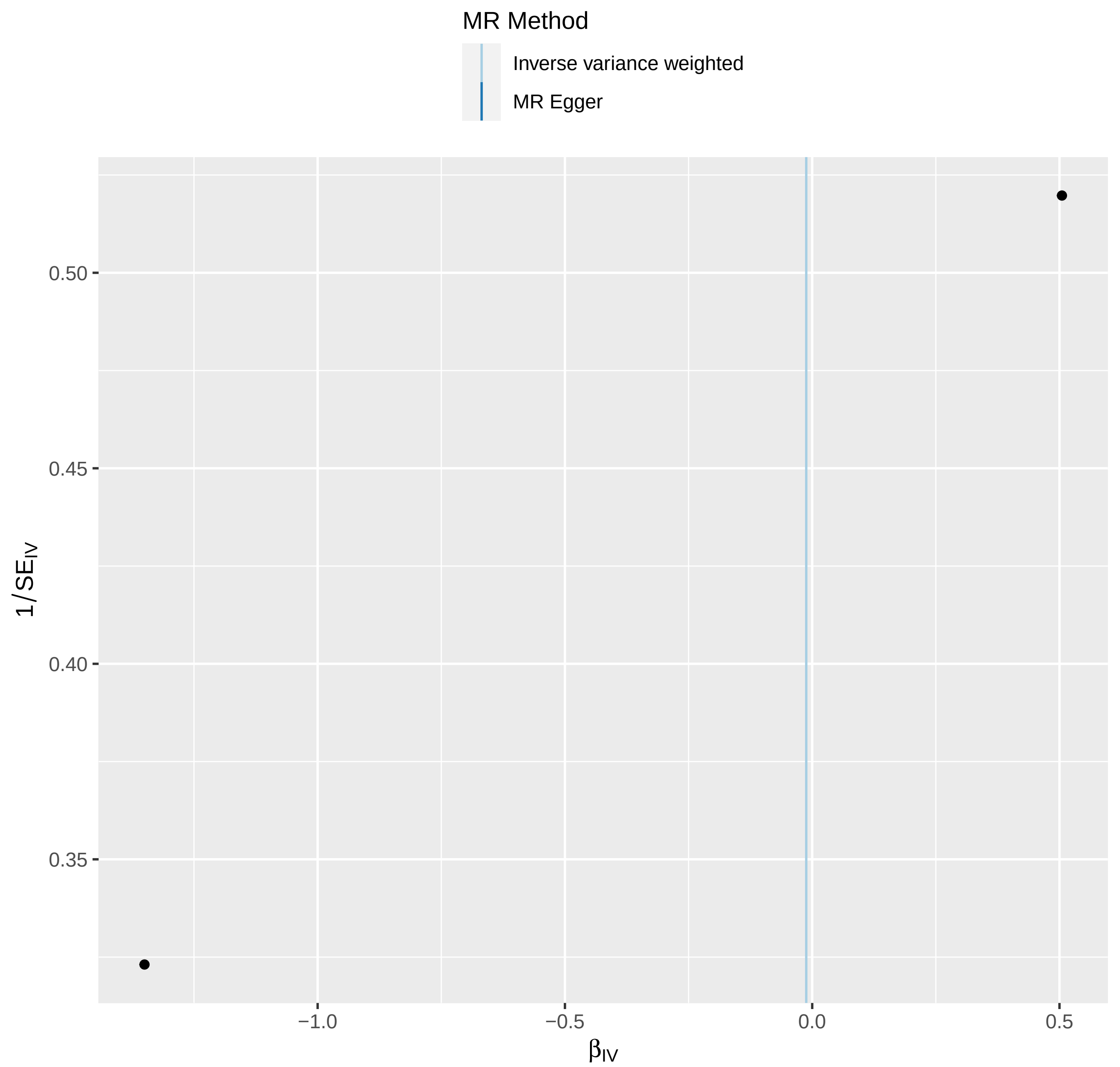

Batch 144 : Butyrylcarnitine on Crohn's disease of colon

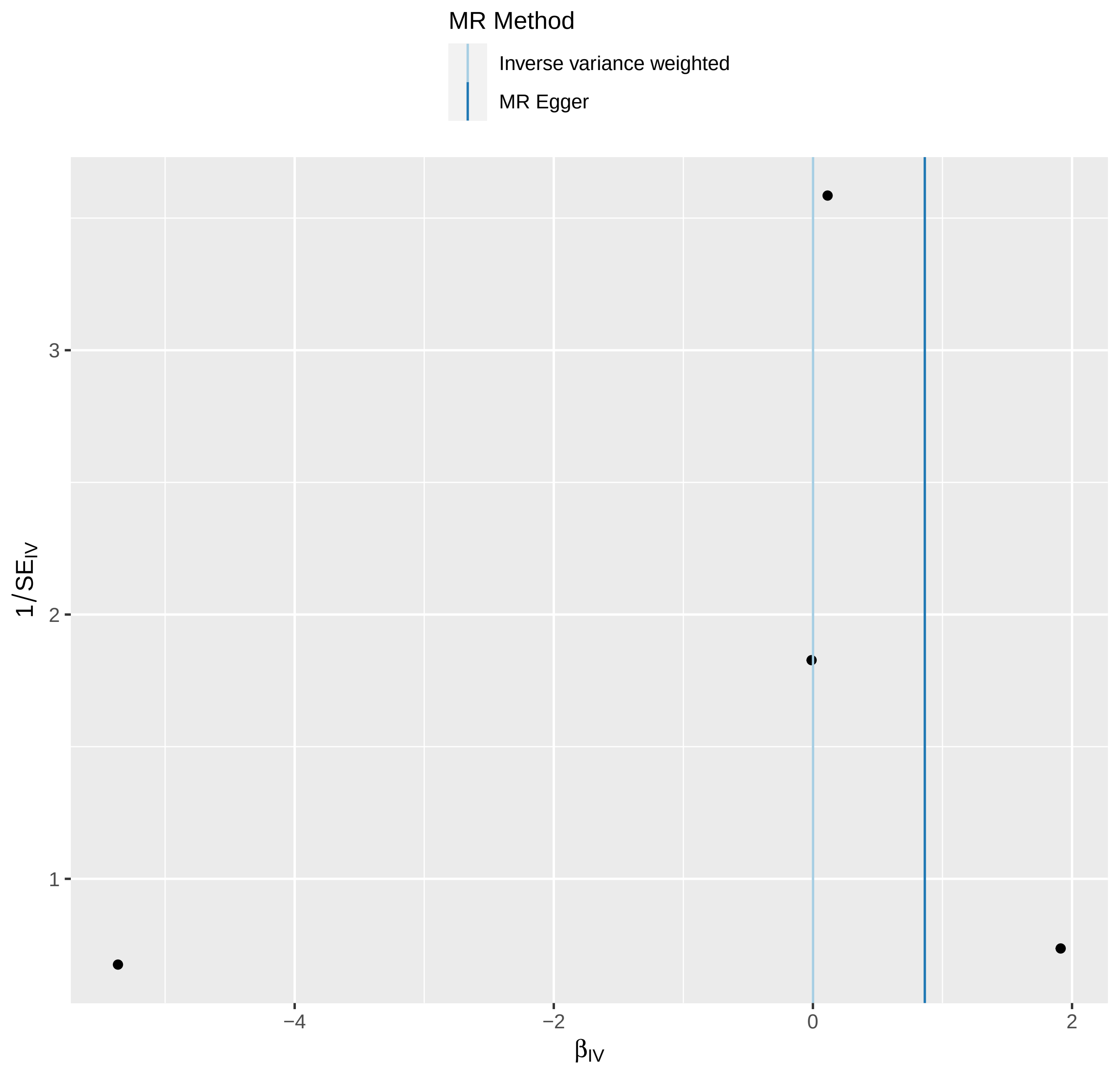

Batch 147 : Propionylcarnitine on Crohn's disease of colon

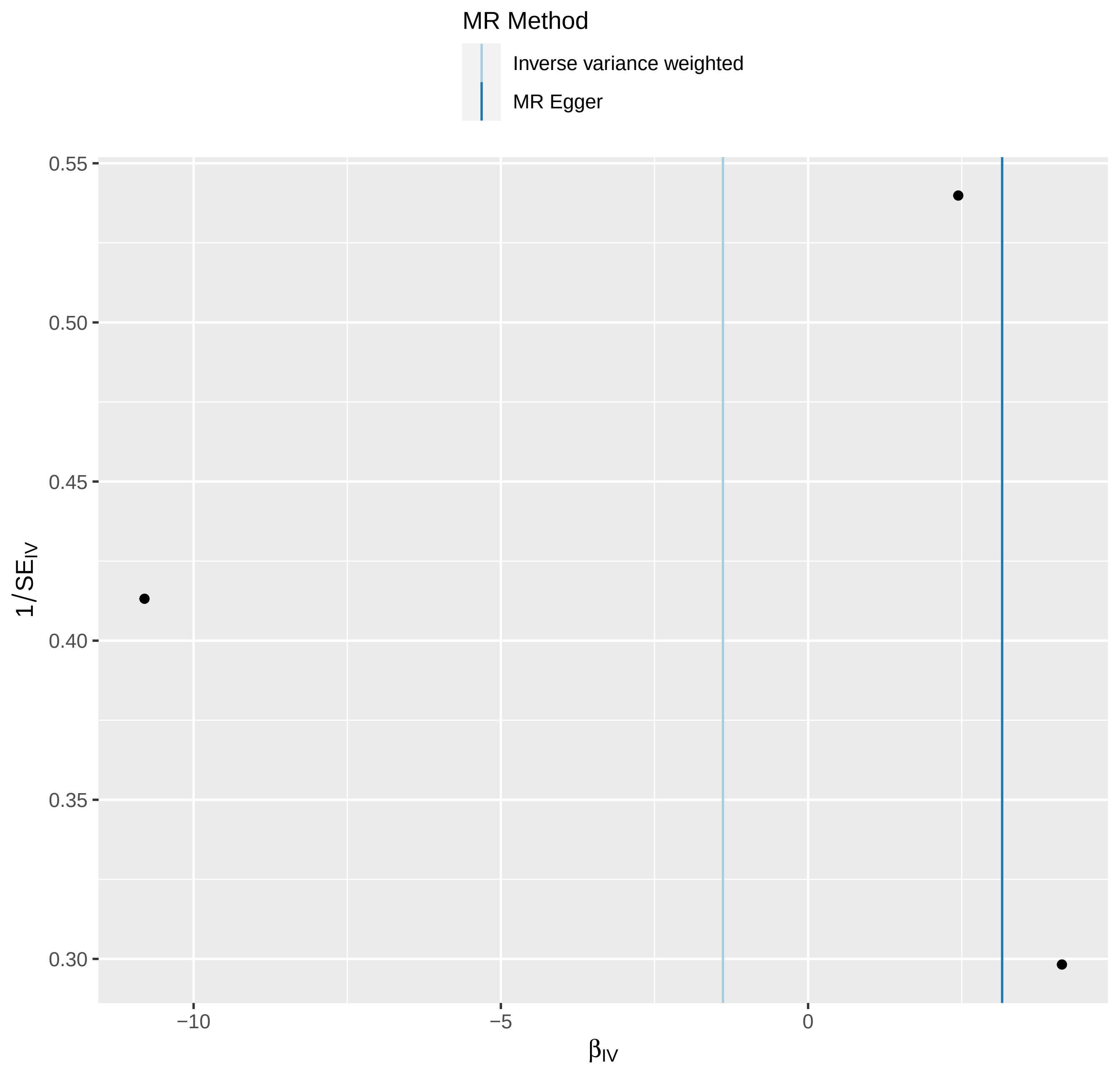

Batch 148 : Caproate (6:0) on Crohn's disease of colon

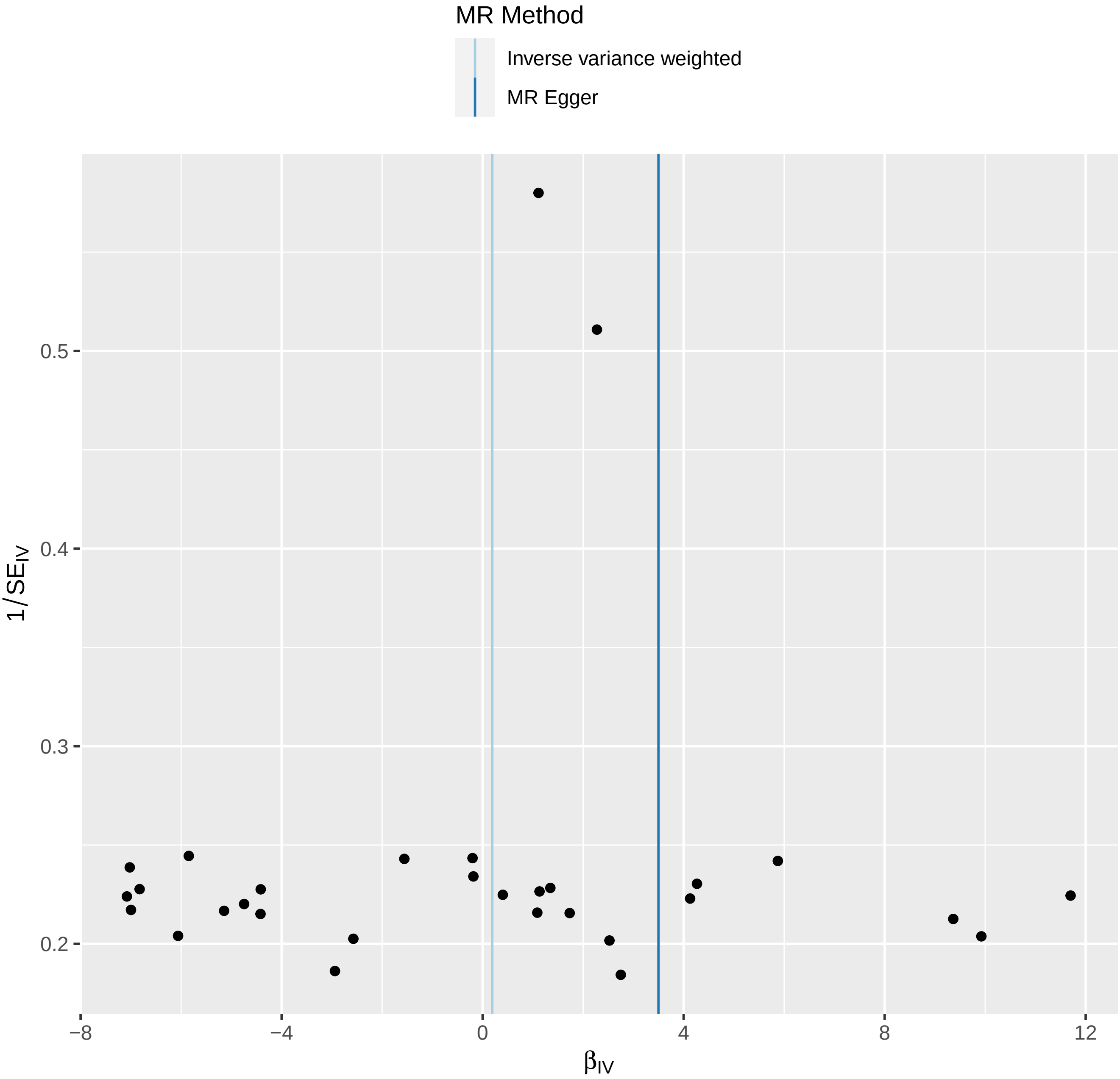

Batch 149 : Caprylate (8:0) on Crohn's disease of colon

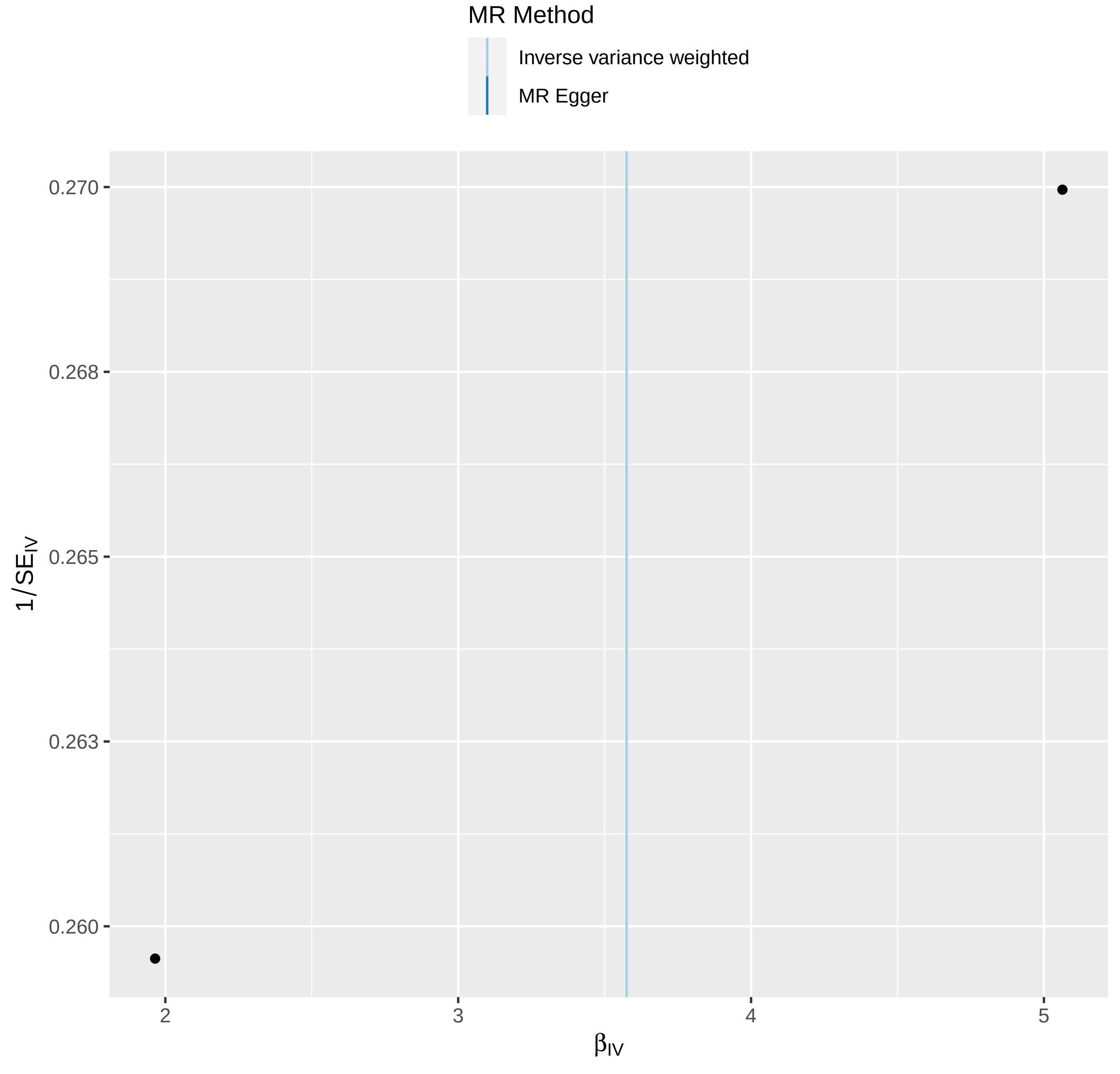

Batch 150 : 10−undecenoate (11:1n1) on Crohn's disease of colon

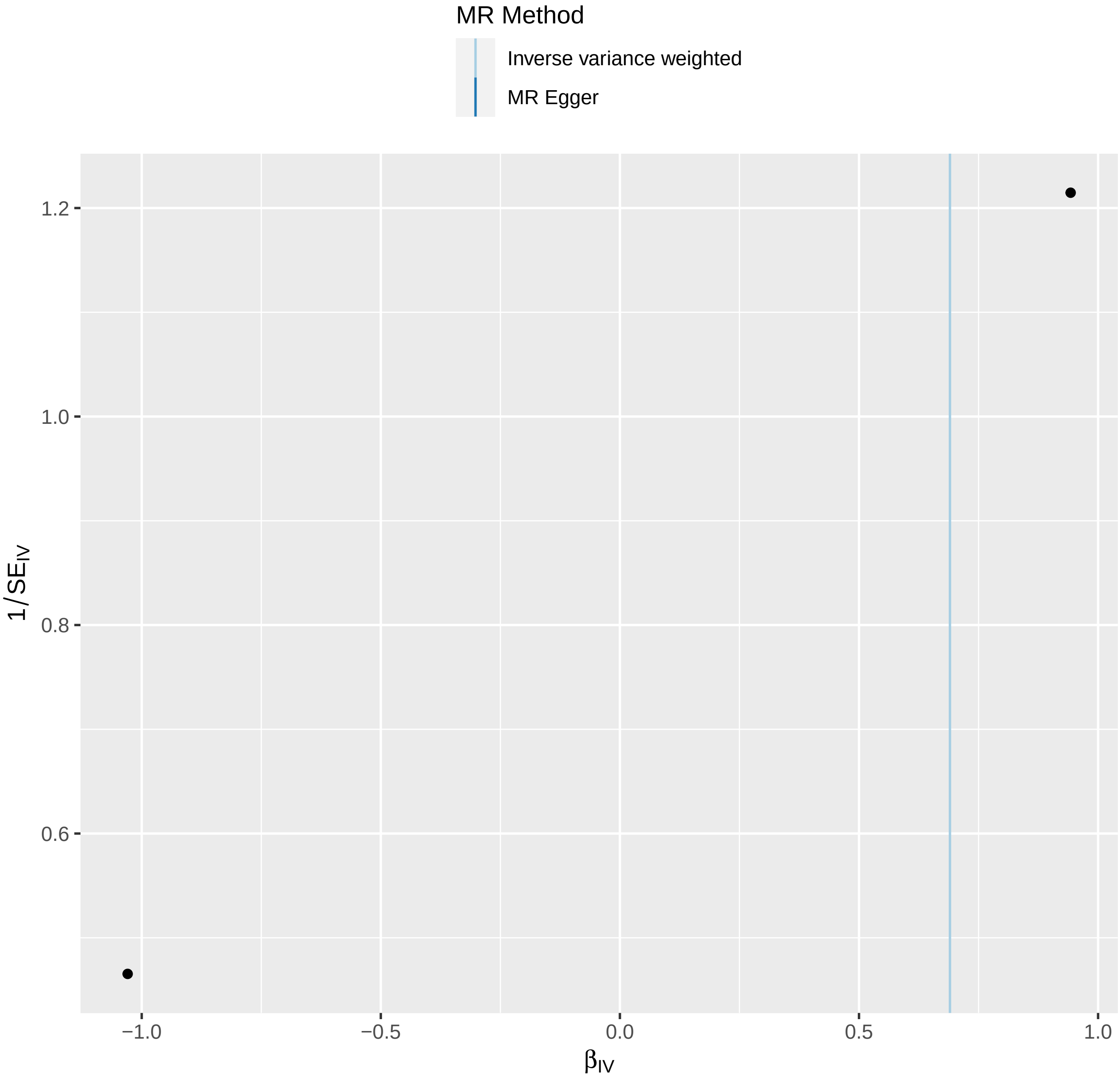

Batch 152 : Phenol sulfate on Crohn's disease of colon

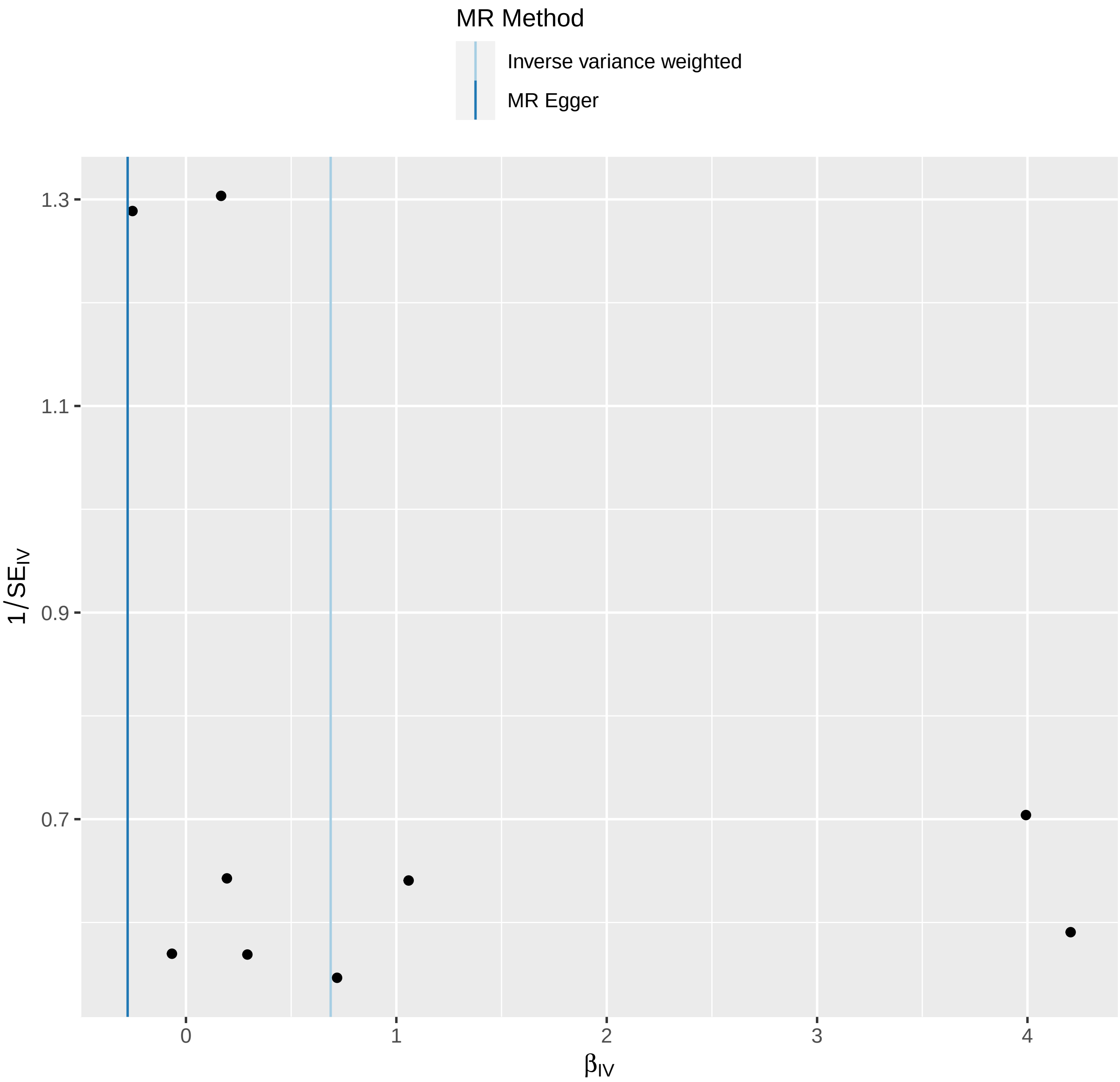

Batch 154 : Heme* on Crohn's disease of colon

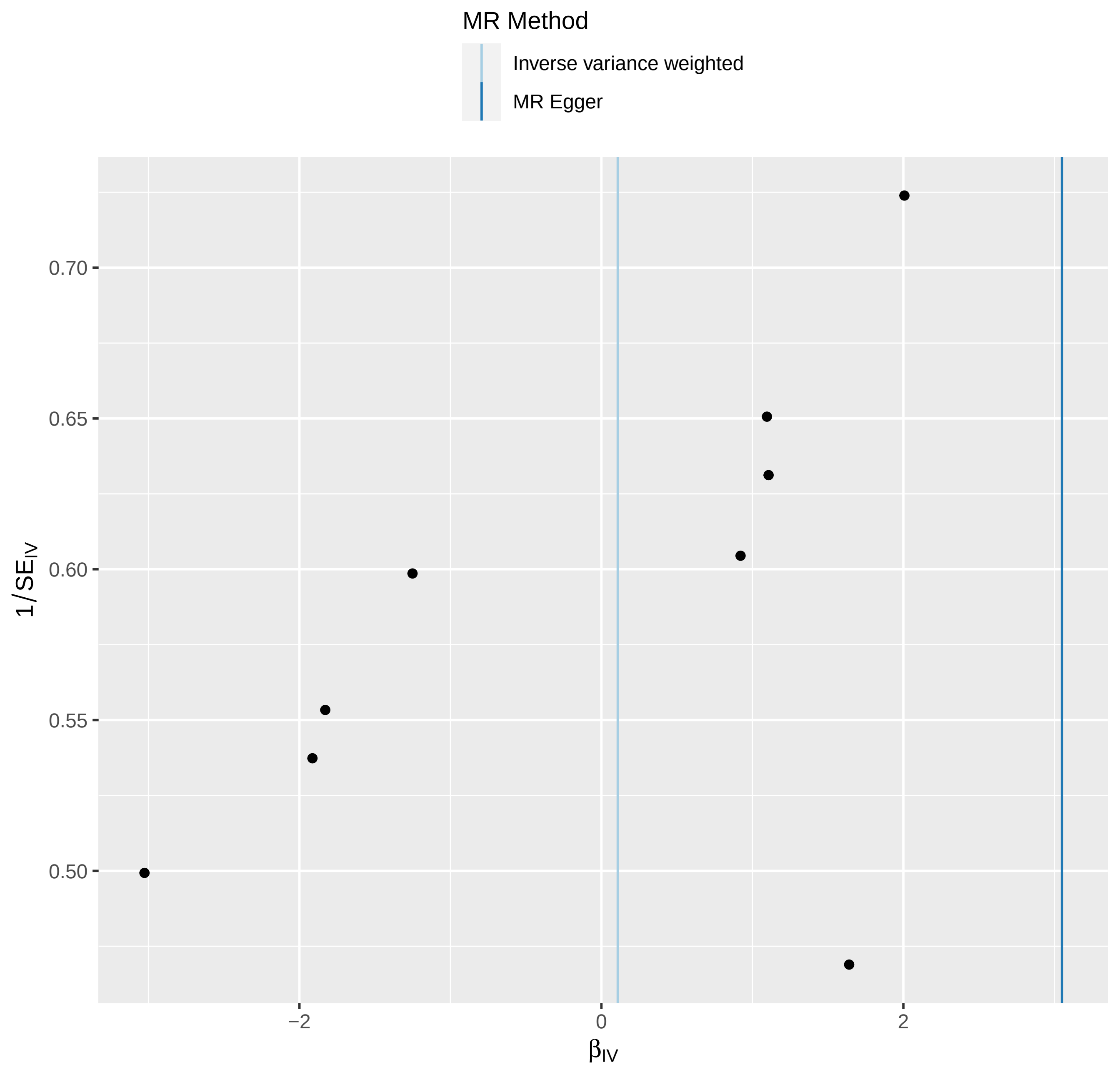

Batch 157 : Pyroglutamine* on Crohn's disease of colon

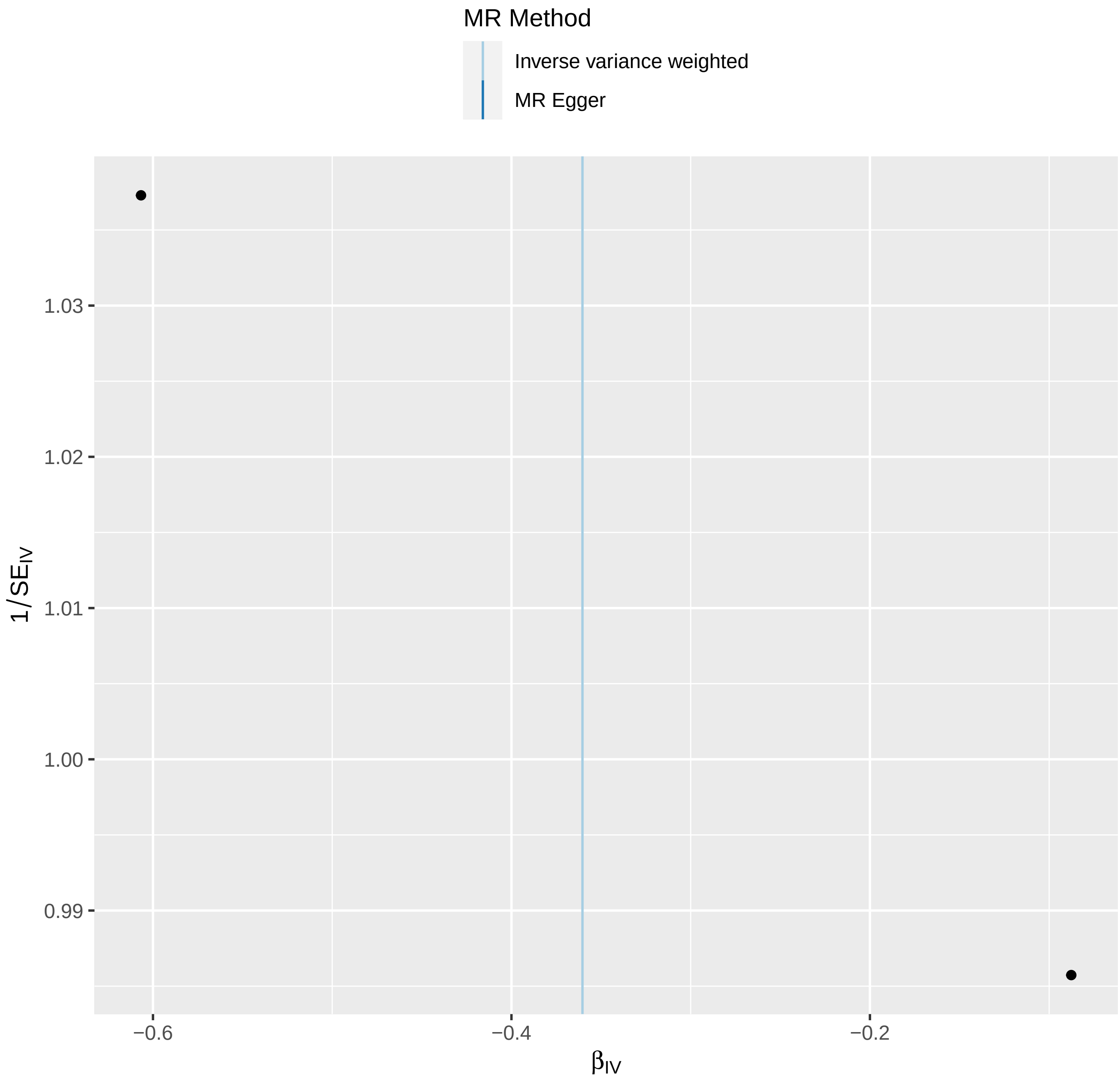

Batch 160 : X−11422−−xanthine on Crohn's disease of colon

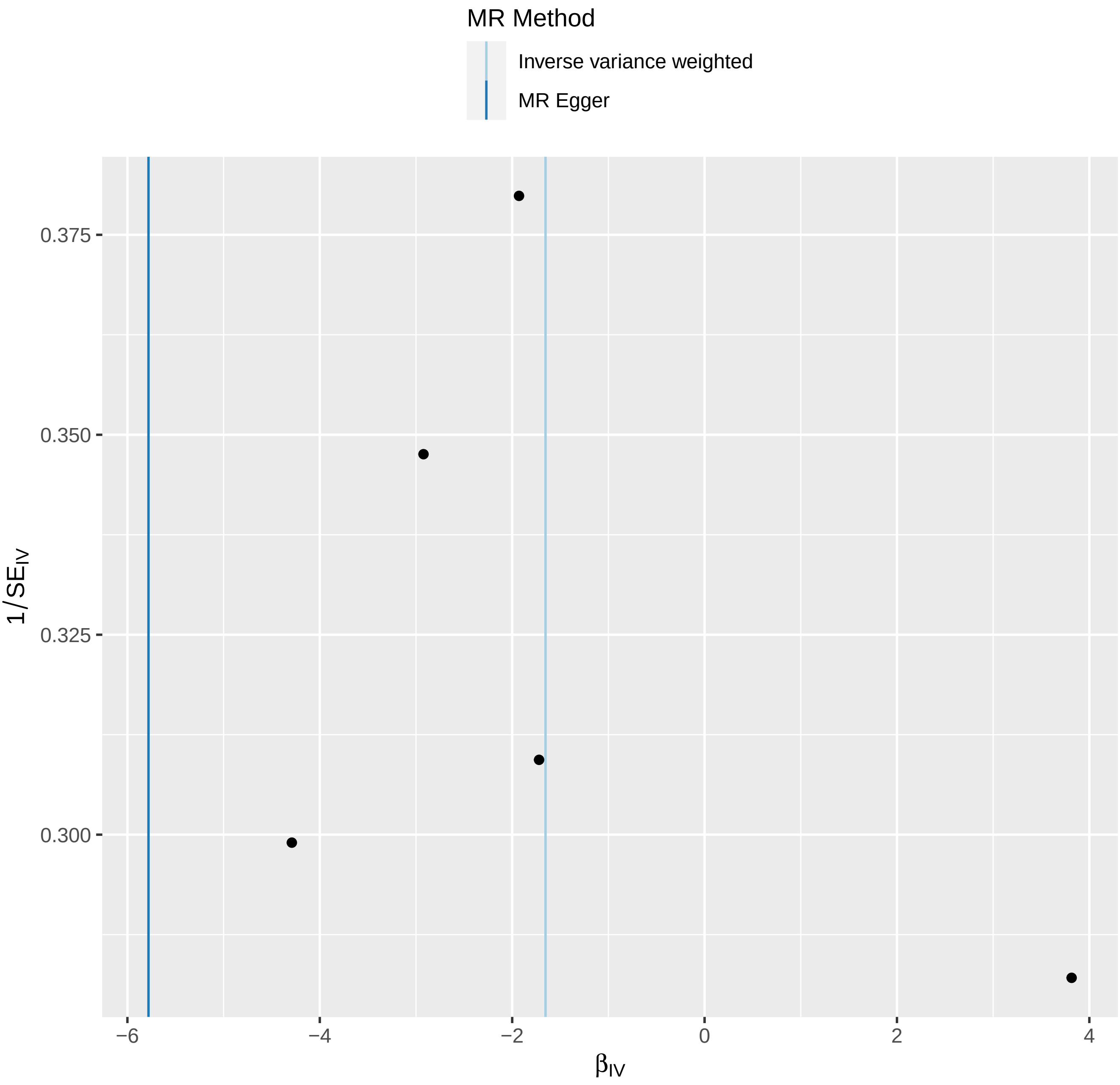

Batch 163 : HWESASXX* on Crohn's disease of colon

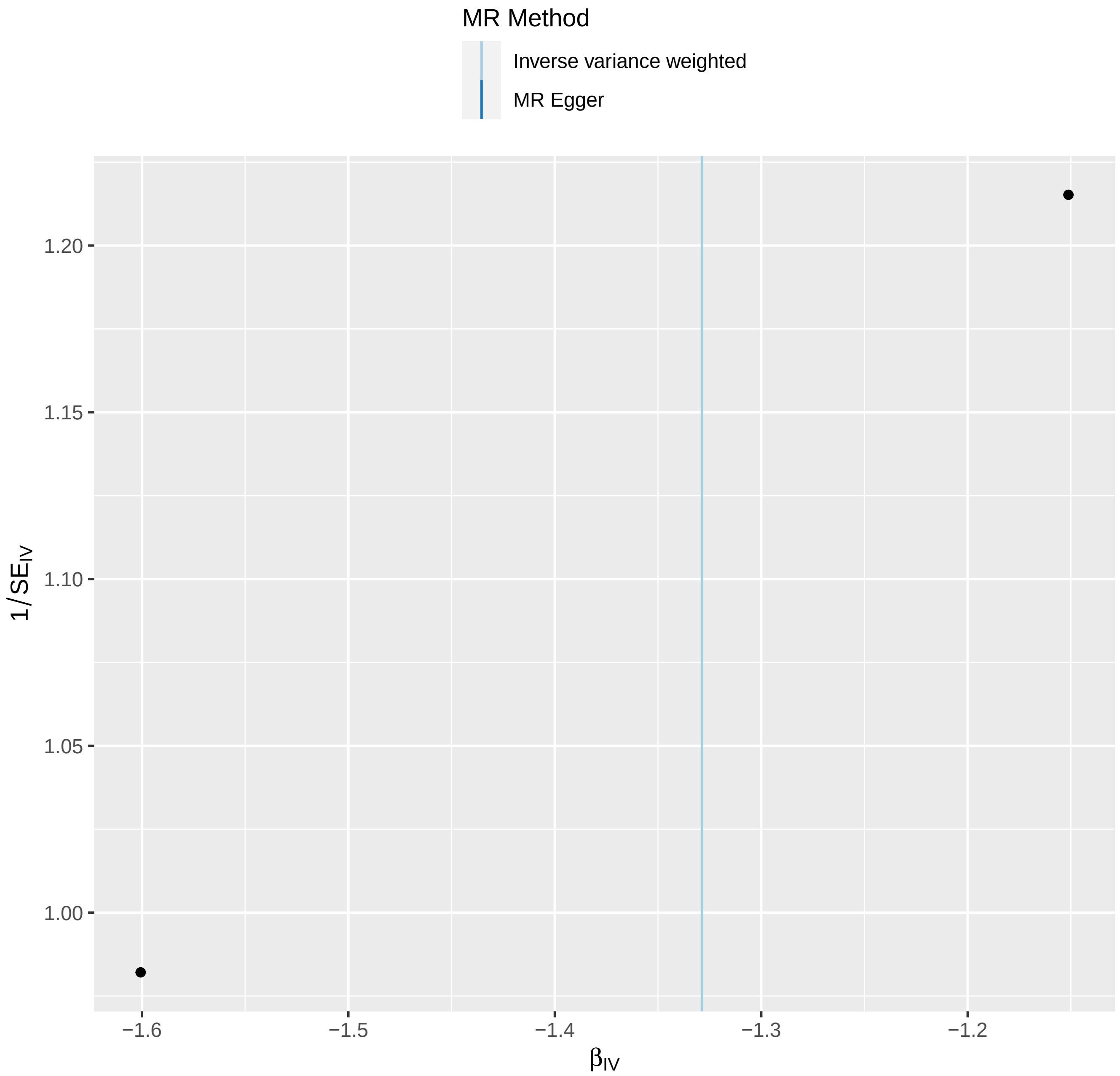

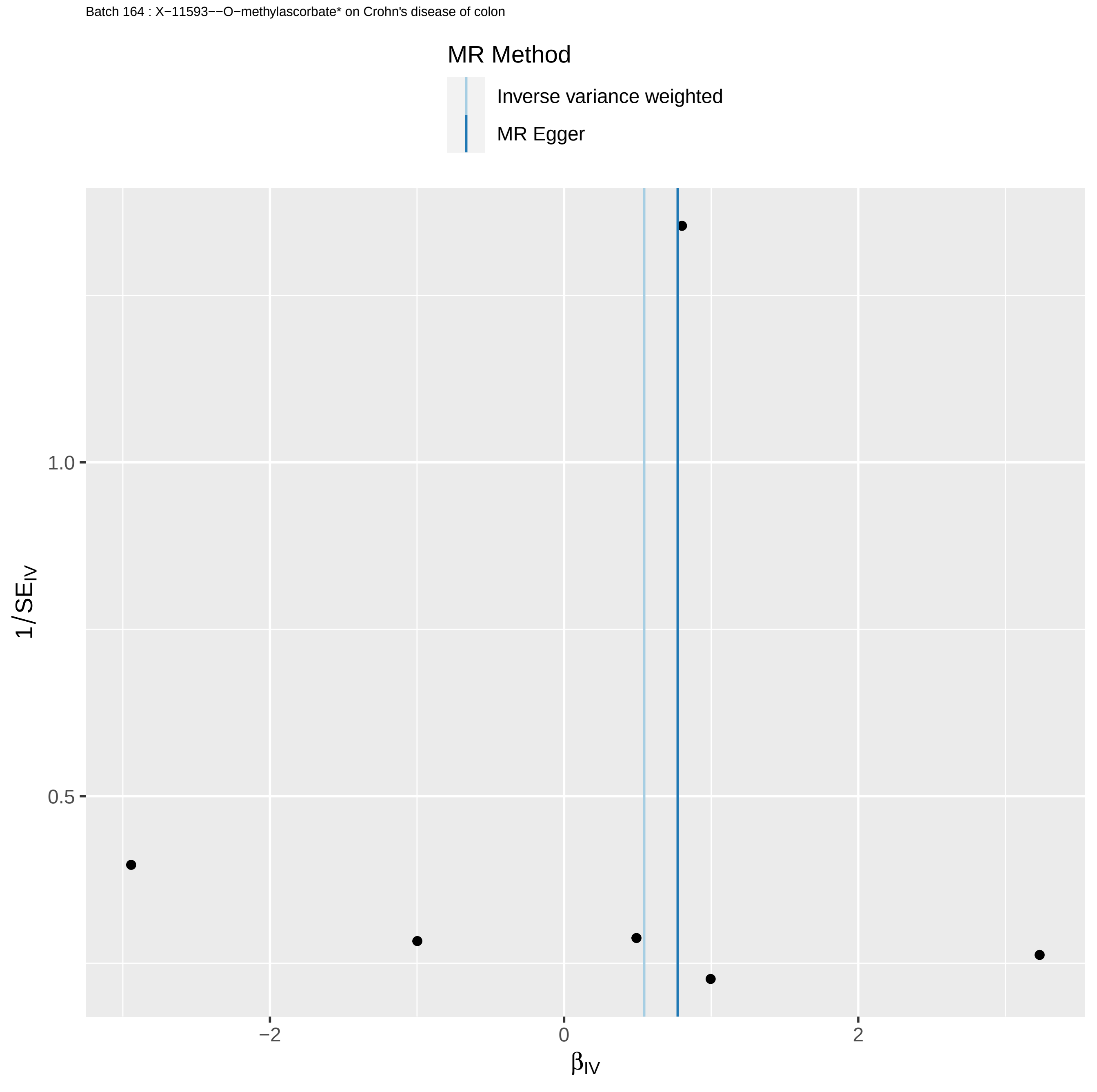

Batch 164 : X−11593−−O−methylascorbate* on Crohn's disease of colon
MR Method
Inverse variance weighted
MR Egger
1.0
0.5
$1/SE_{IV}$
−2
0
2
β_{IV}

Batch 166 : Homostachydrine* on Crohn's disease of colon

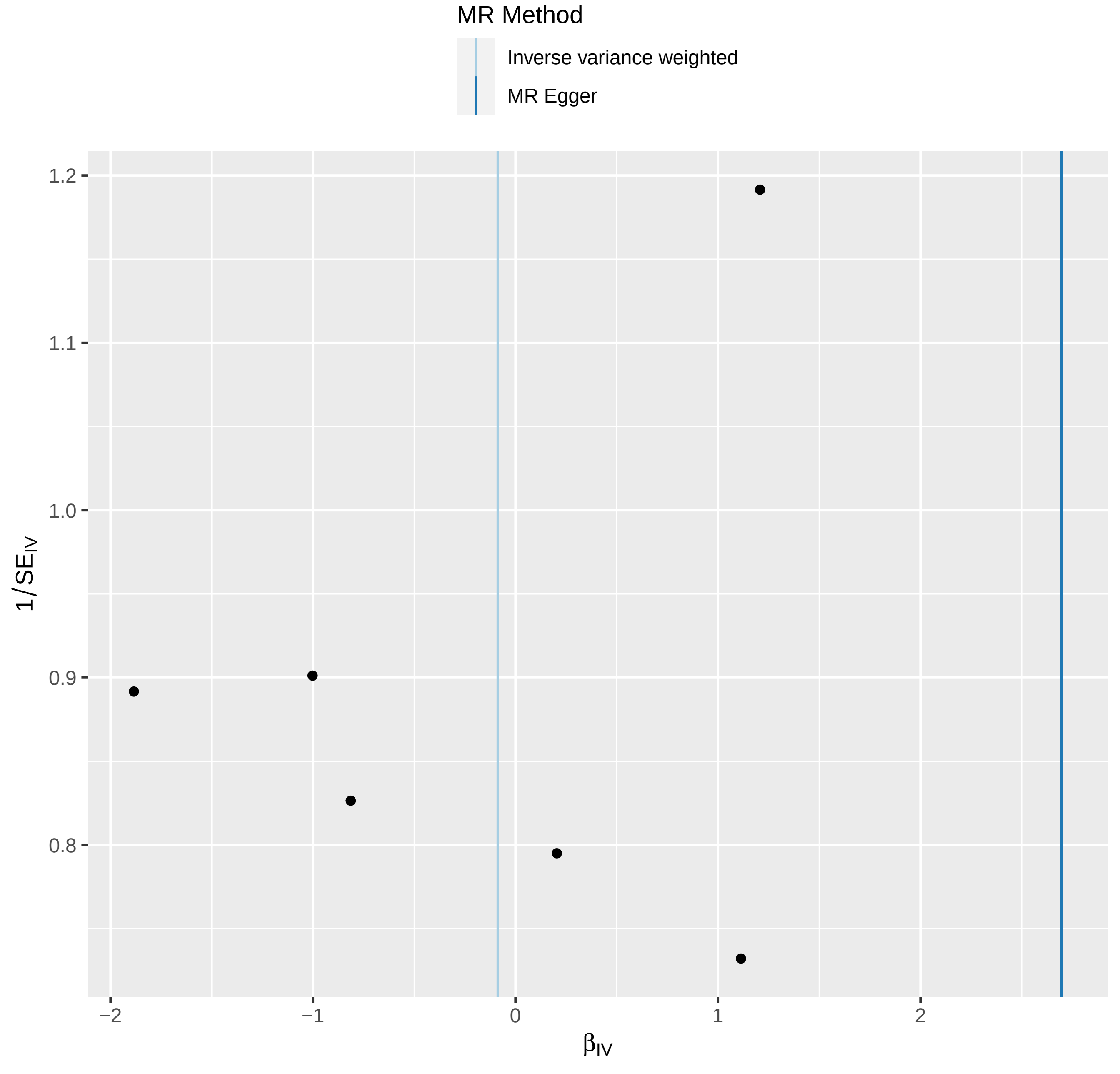

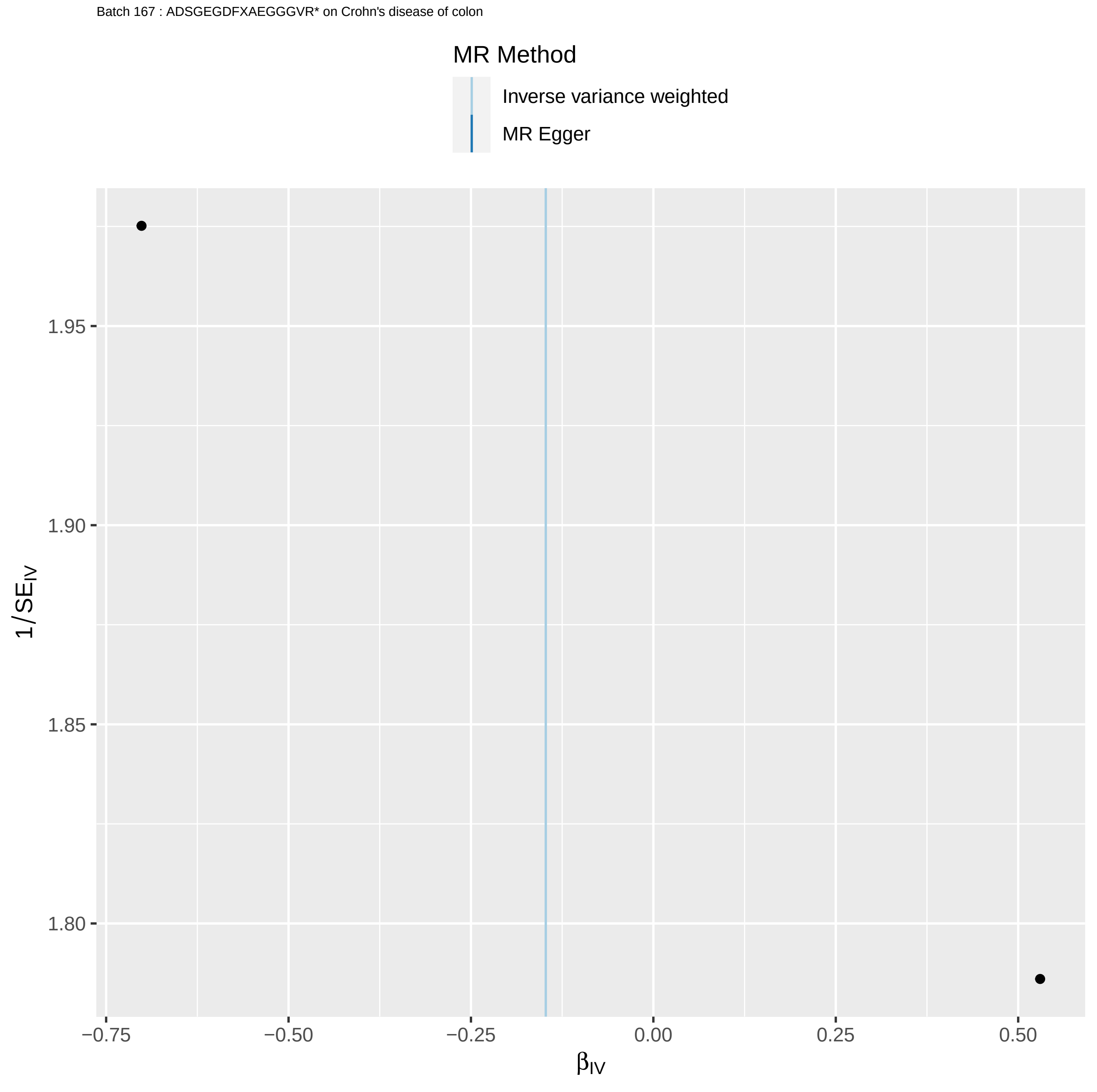
Batch 167 : ADSGEGDFXAEGGGVR* on Crohn's disease of colon
MR Method
Inverse variance weighted
MR Egger
1.95
1.90
1.85
1.80
1/SE_IV
−0.75
−0.50
−0.25
0.00
0.25
0.50
β_IV

Batch 169 : X−11793−−oxidized bilirubin* on Crohn's disease of colon

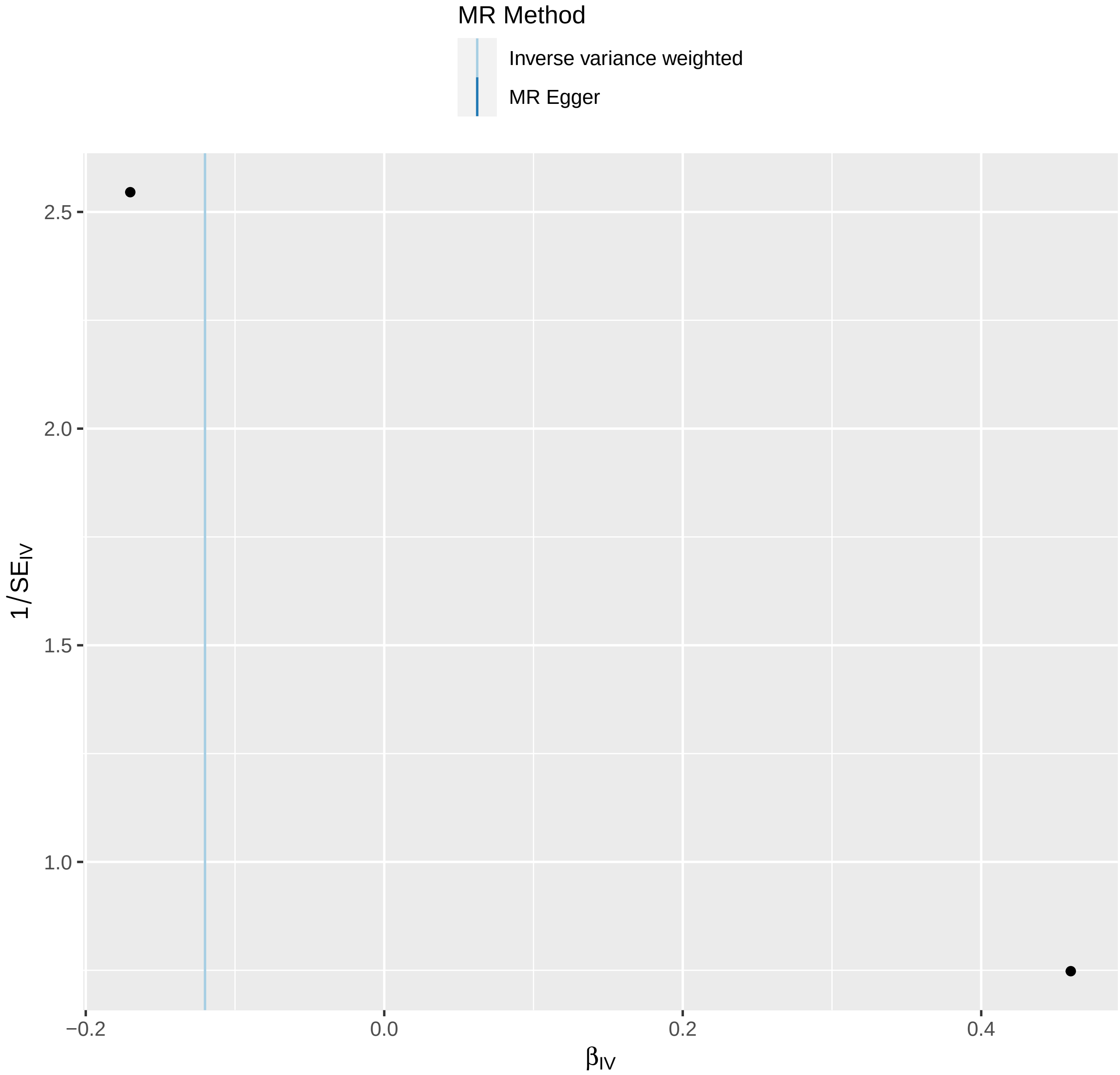

Batch 170 : 1−arachidonoylglycerophosphocholine* on Crohn's disease of colon

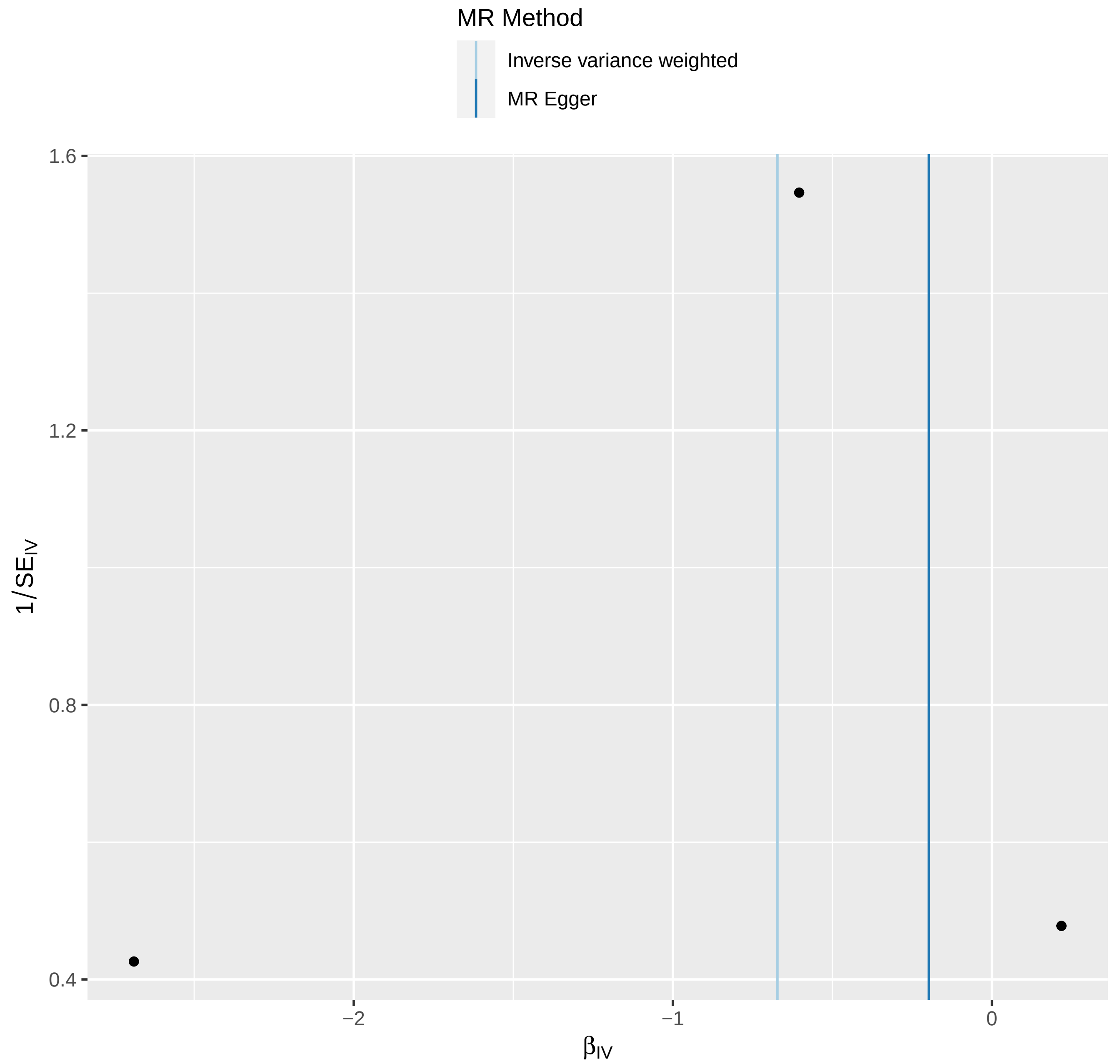

Batch 172 : Gamma−glutamylmethionine* on Crohn's disease of colon

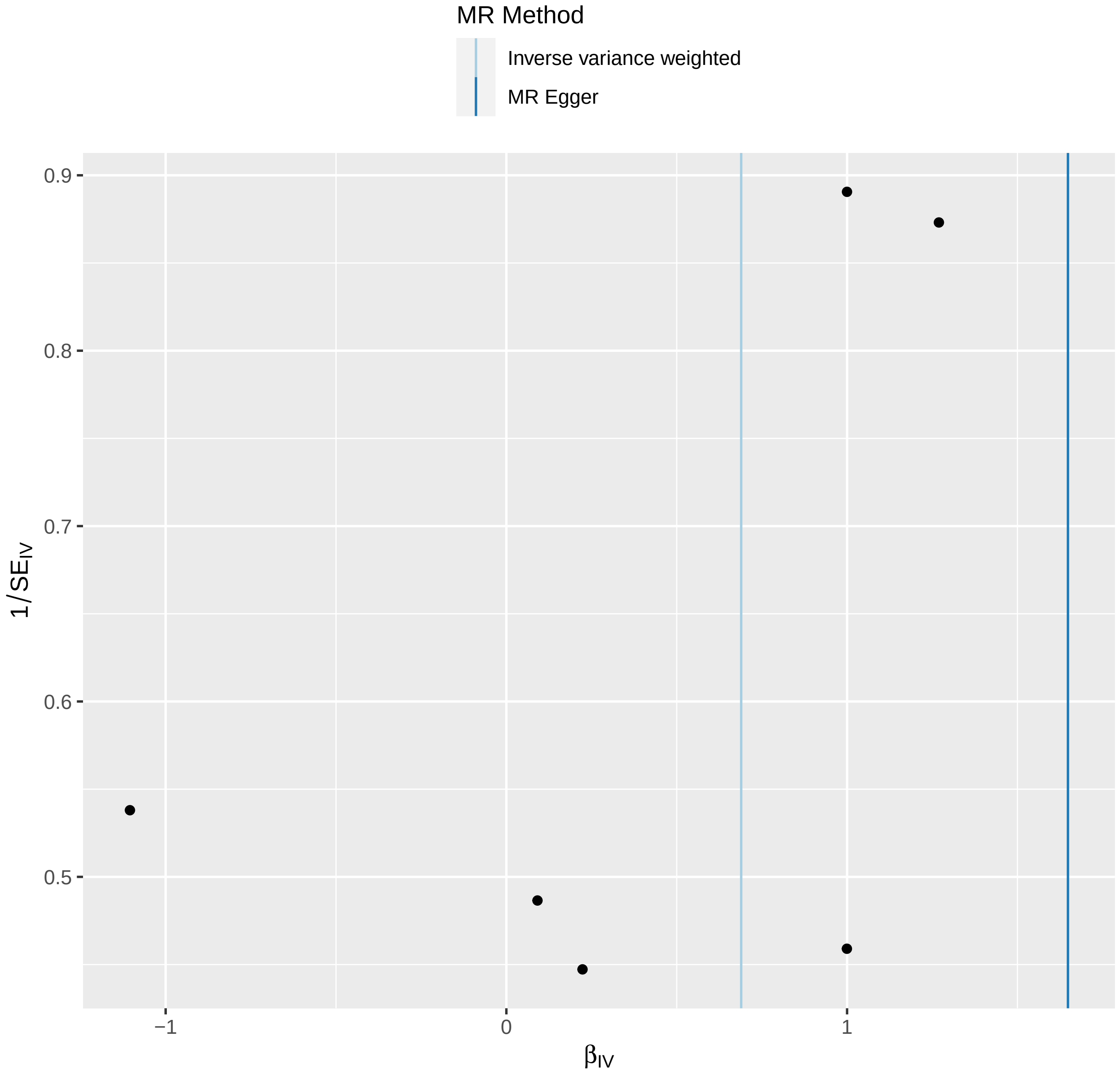

Batch 173 : Gamma−glutamylthreonine* on Crohn's disease of colon

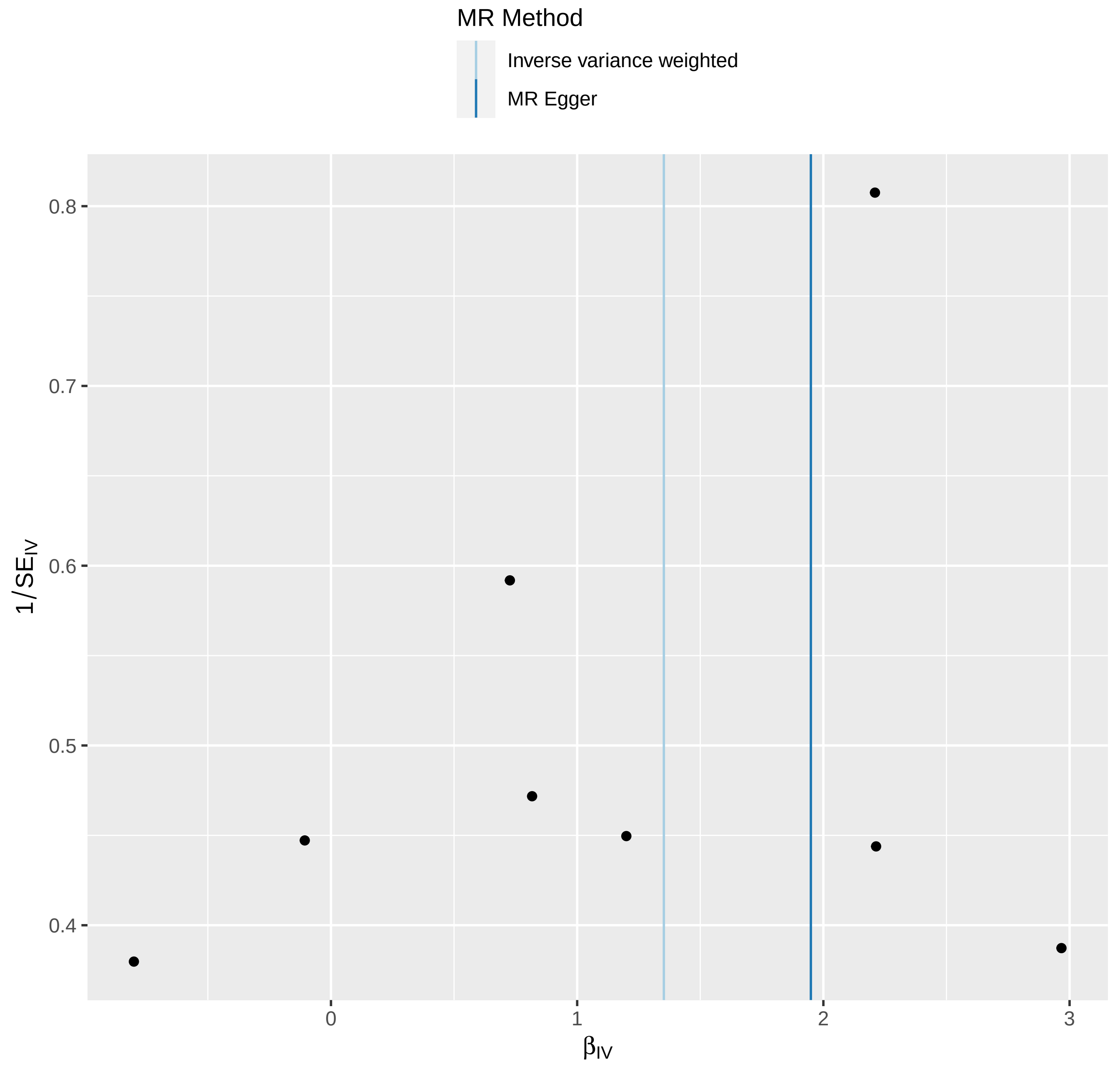

Batch 174 : Gamma−tocopherol on Crohn's disease of colon

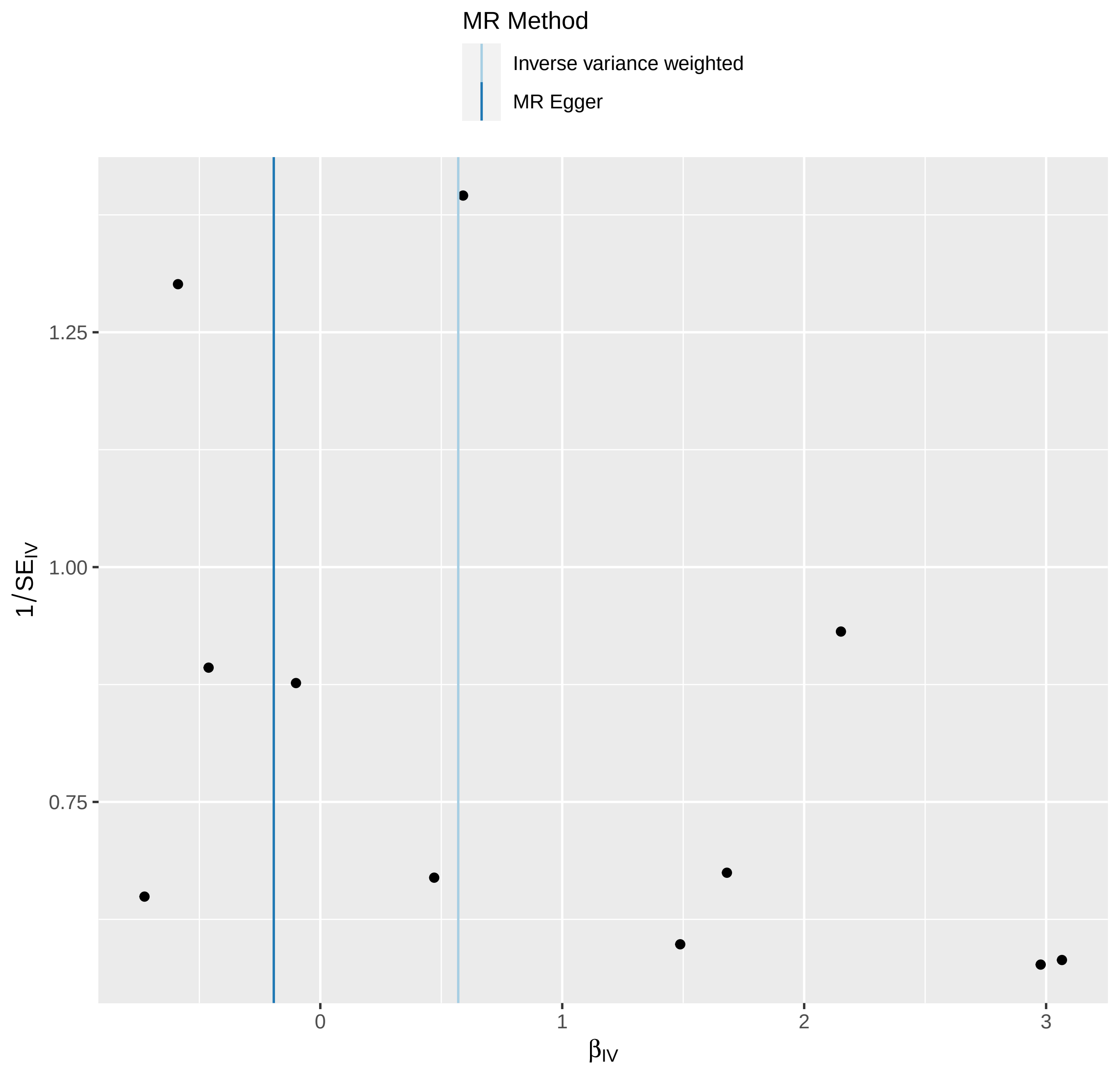

Batch 175 : Gamma−glutamylphenylalanine on Crohn's disease of colon

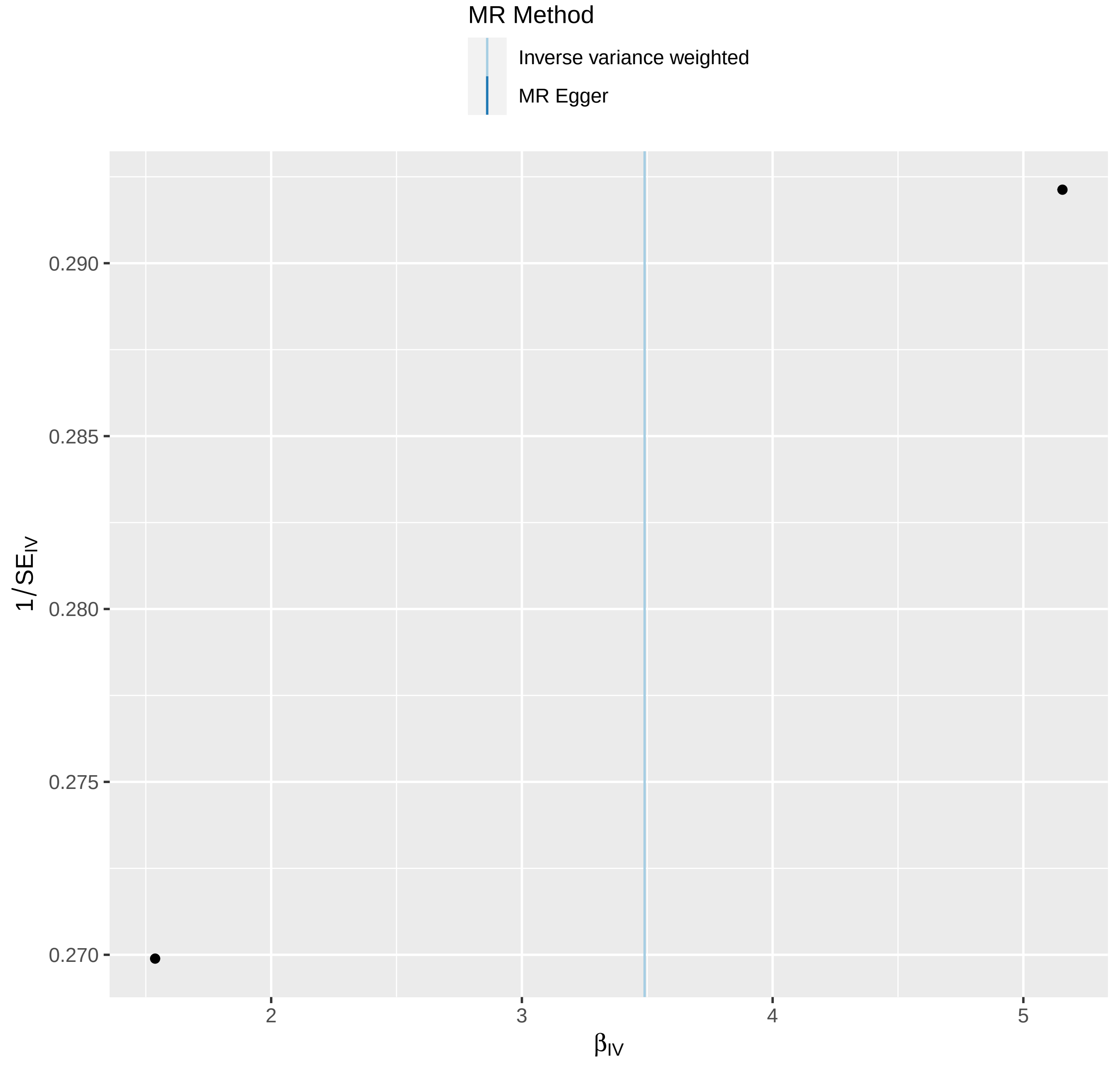

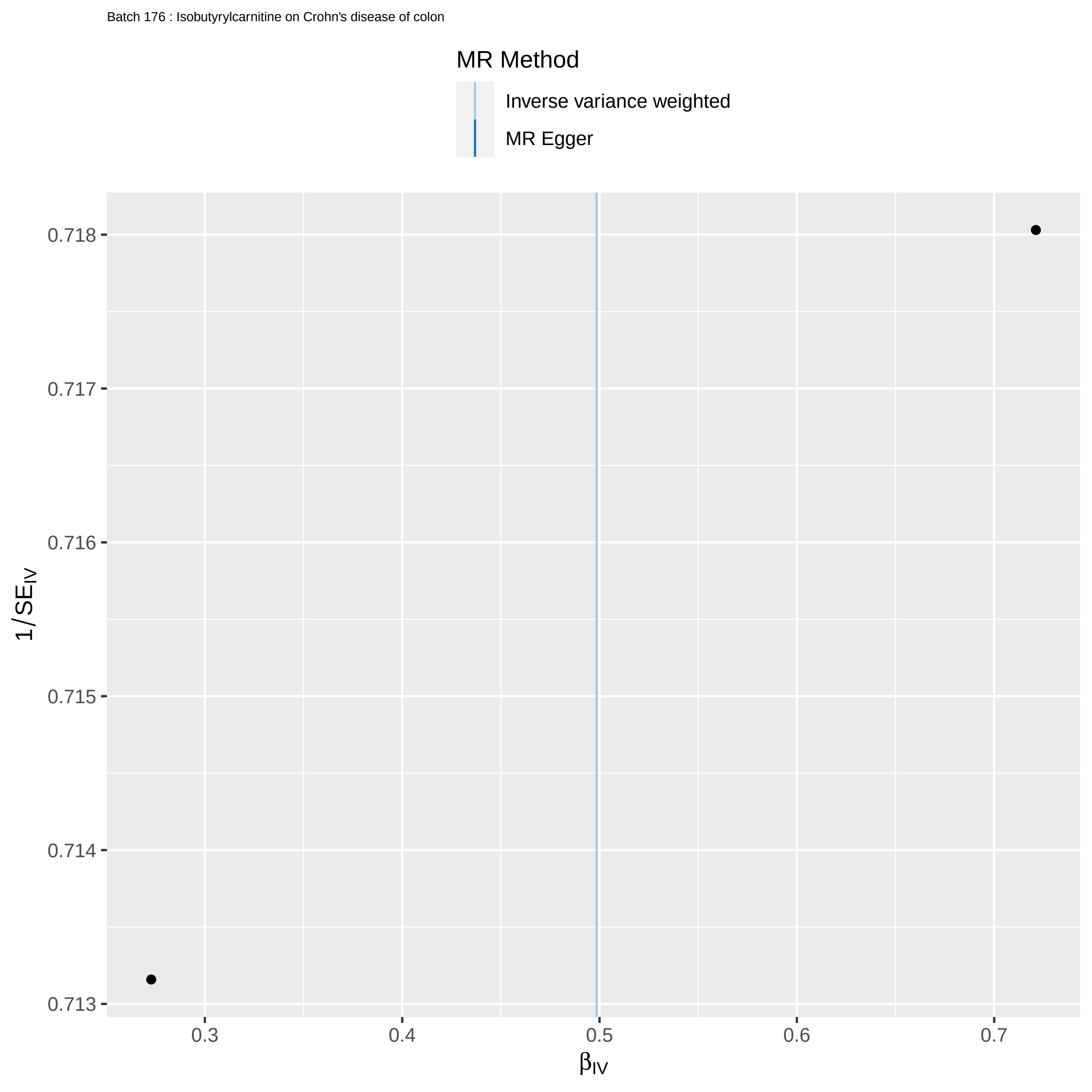
Batch 176 : Isobutyrylcarnitine on Crohn's disease of colon
MR Method
Inverse variance weighted
MR Egger
0.718
0.717
0.716
0.715
0.714
0.713
0.3
0.4
0.5
0.6
0.7
1/SE_IV
β_IV

Batch 177 : Pseudouridine on Crohn's disease of colon

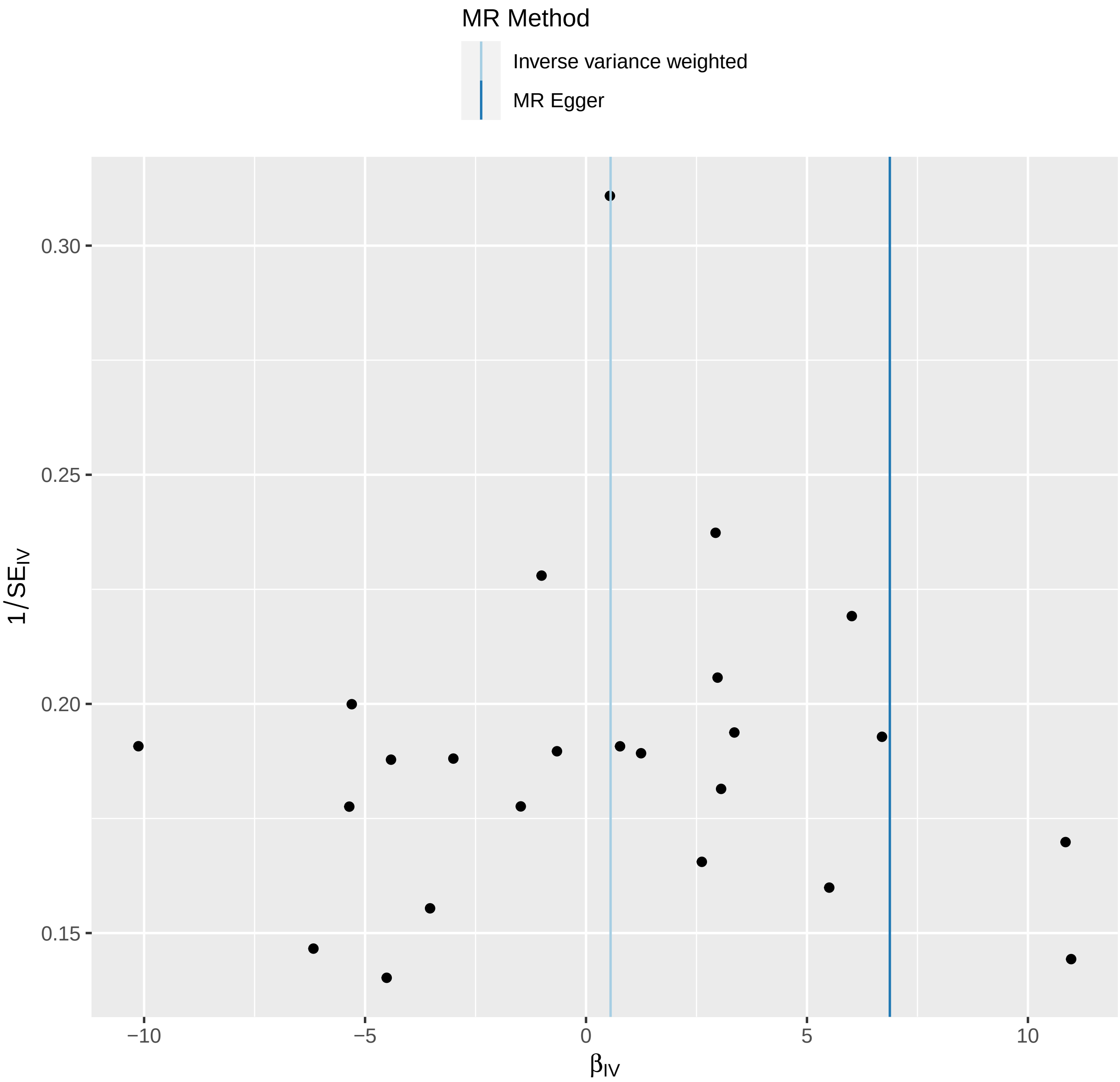

Batch 178 : Valerate on Crohn's disease of colon

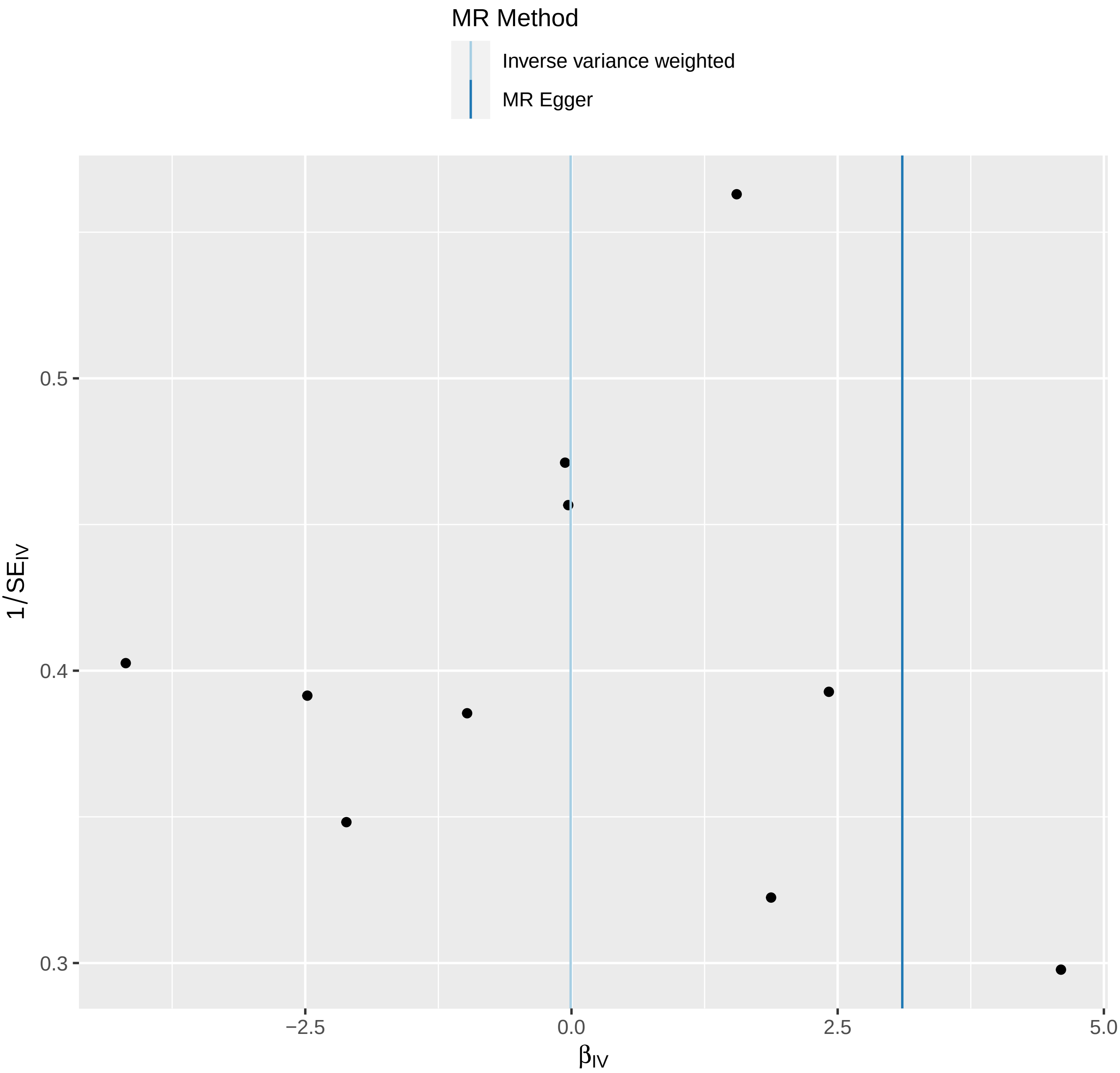

Batch 181 : Erythronate* on Crohn's disease of colon

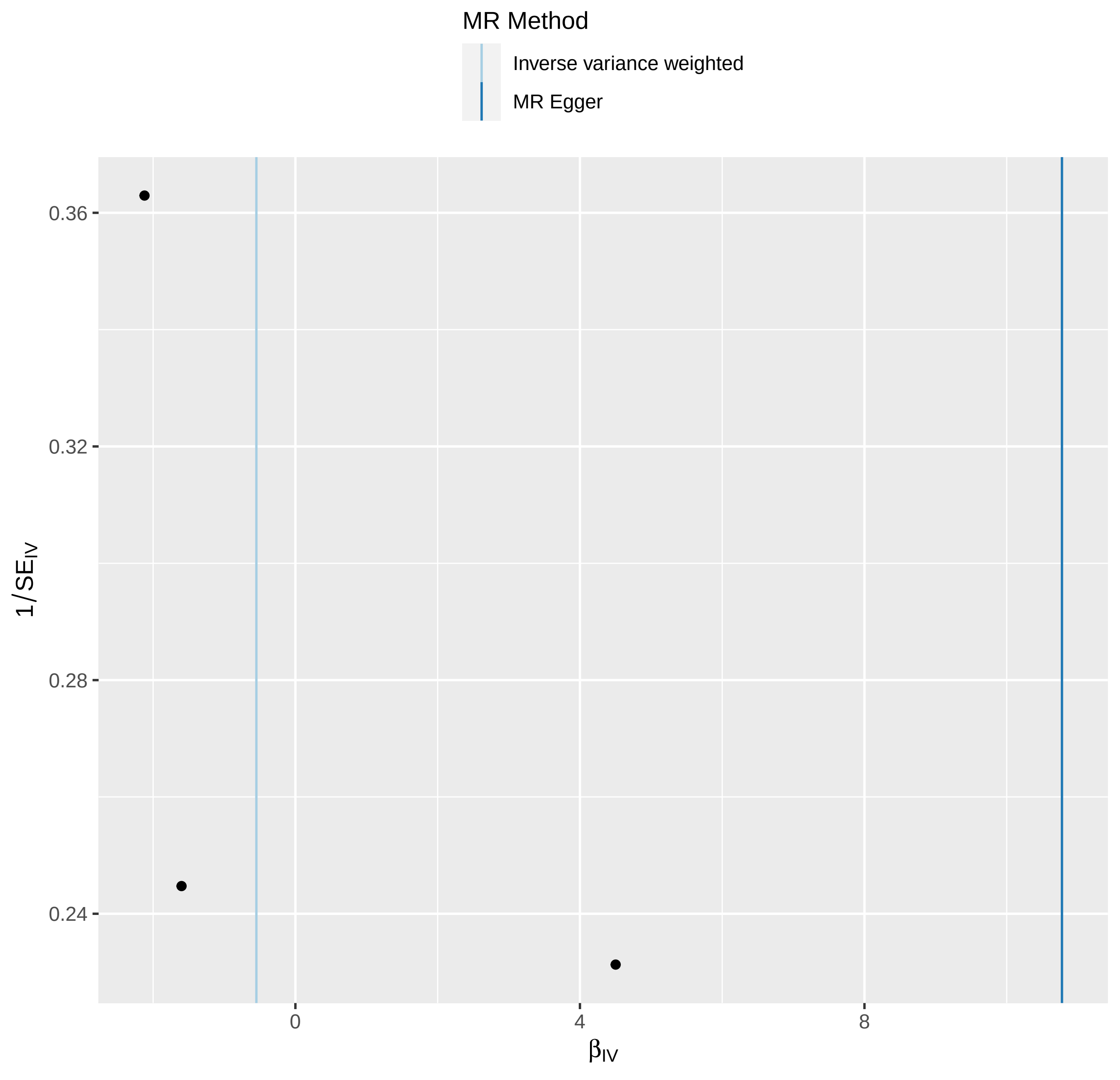

Batch 182 : Lathosterol on Crohn's disease of colon

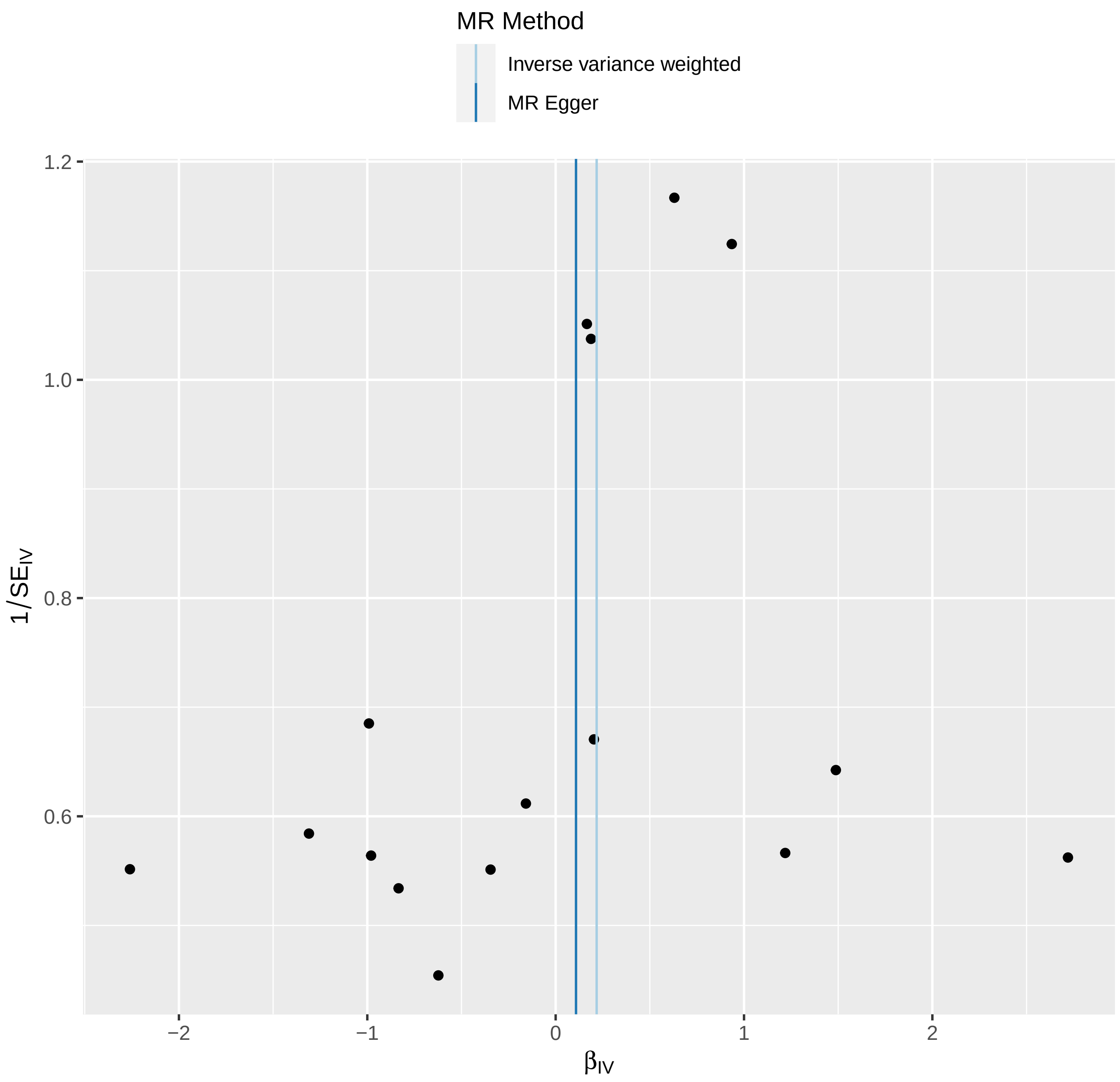

Batch 185 : Eicosenoate (20:1n9 or 11) on Crohn's disease of colon

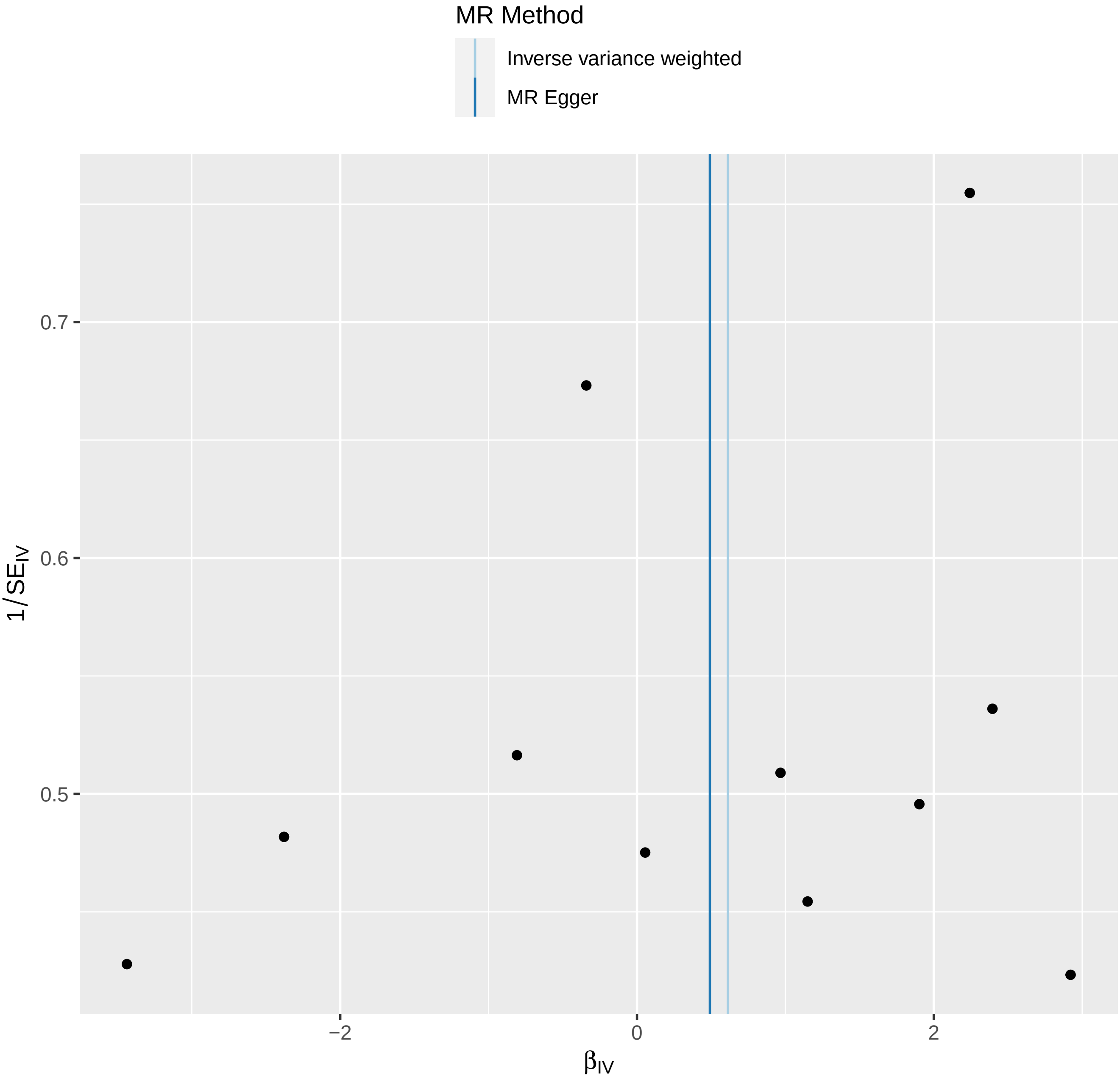

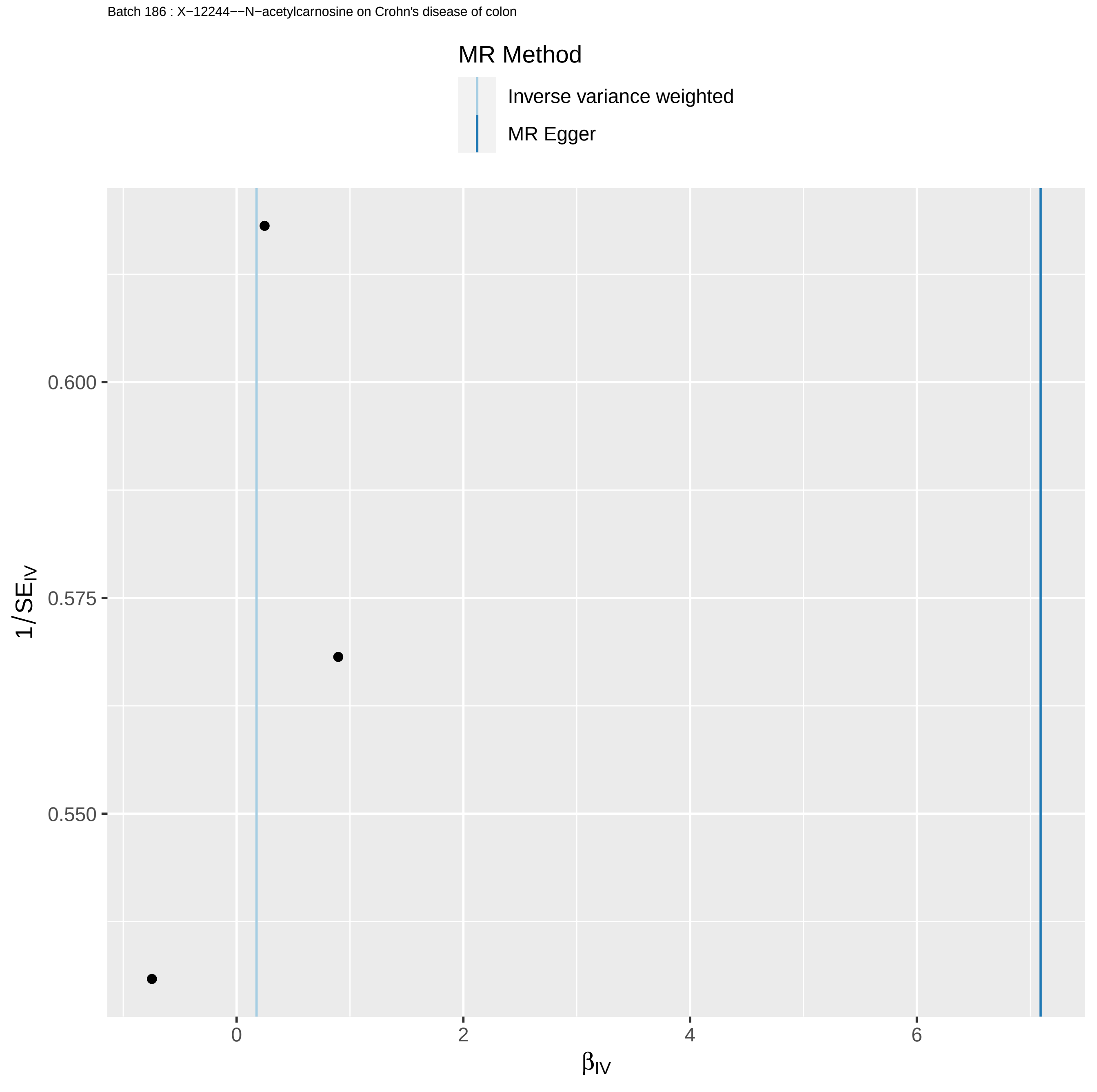
Batch 186 : X−12244−−N−acetylcarnosine on Crohn's disease of colon
MR Method
Inverse variance weighted
MR Egger
0.600
0.575
0.550
1/SE$_{IV}$
0
2
4
6
β$_{IV}$

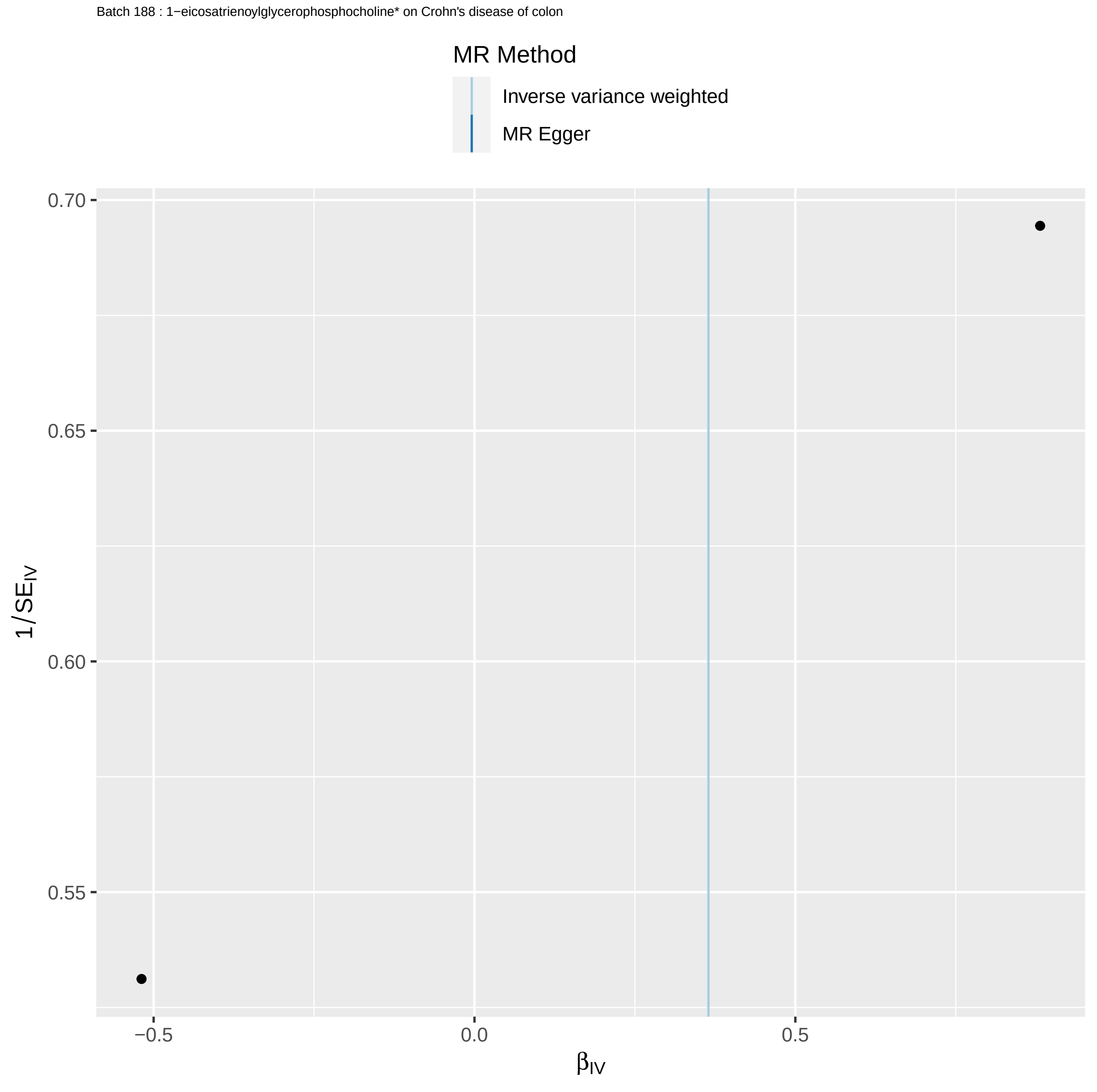

Batch 188 : 1−eicosatrienoylglycerophosphocholine* on Crohn's disease of colon
MR Method
Inverse variance weighted
MR Egger
0.70
0.65
0.60
0.55
1/SE_IV
−0.5
0.0
0.5
β_IV

Batch 189 : 1−docosahexaenoylglycerophosphocholine* on Crohn's disease of colon

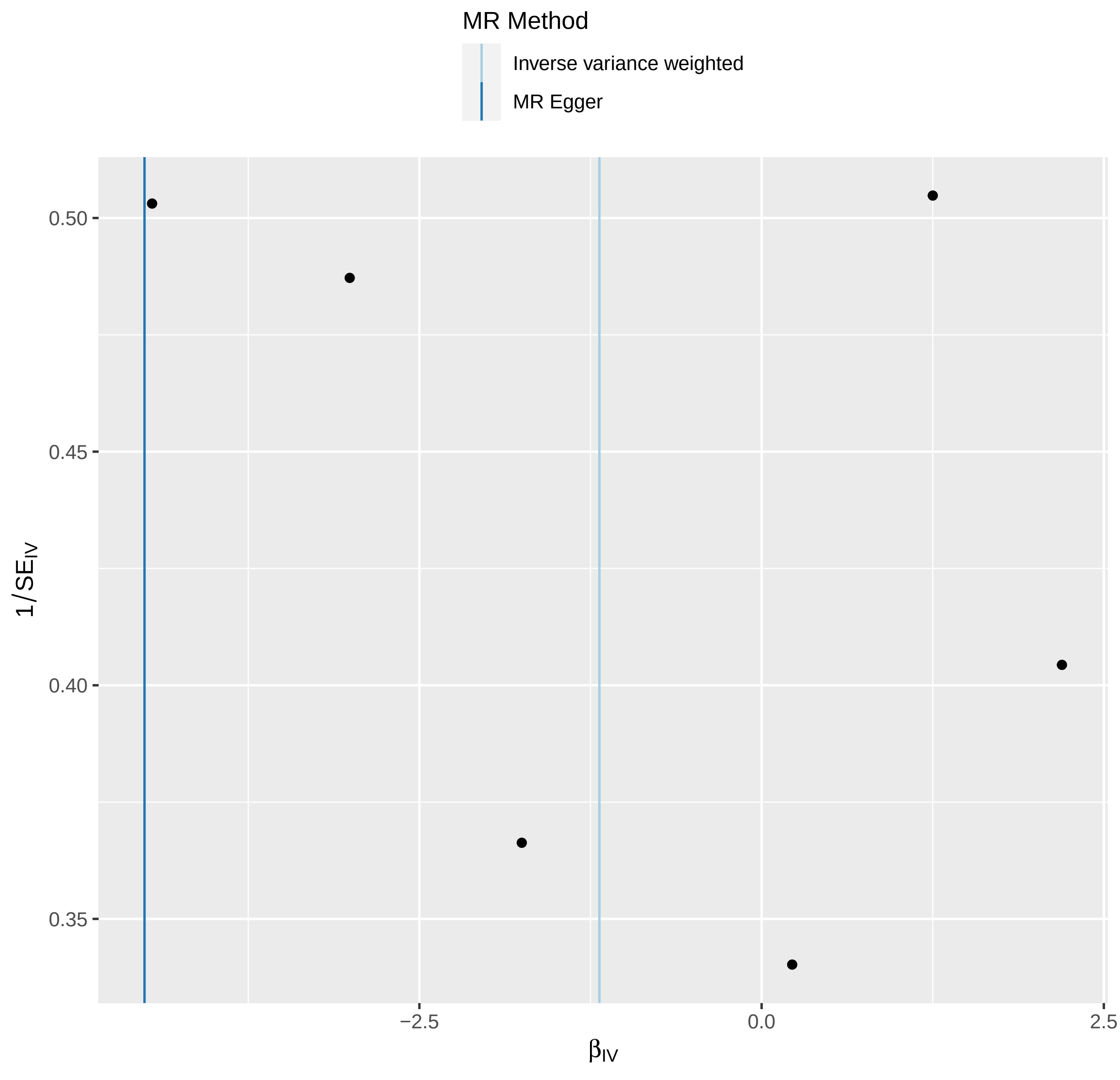

Batch 193 : Octanoylcarnitine on Crohn's disease of colon

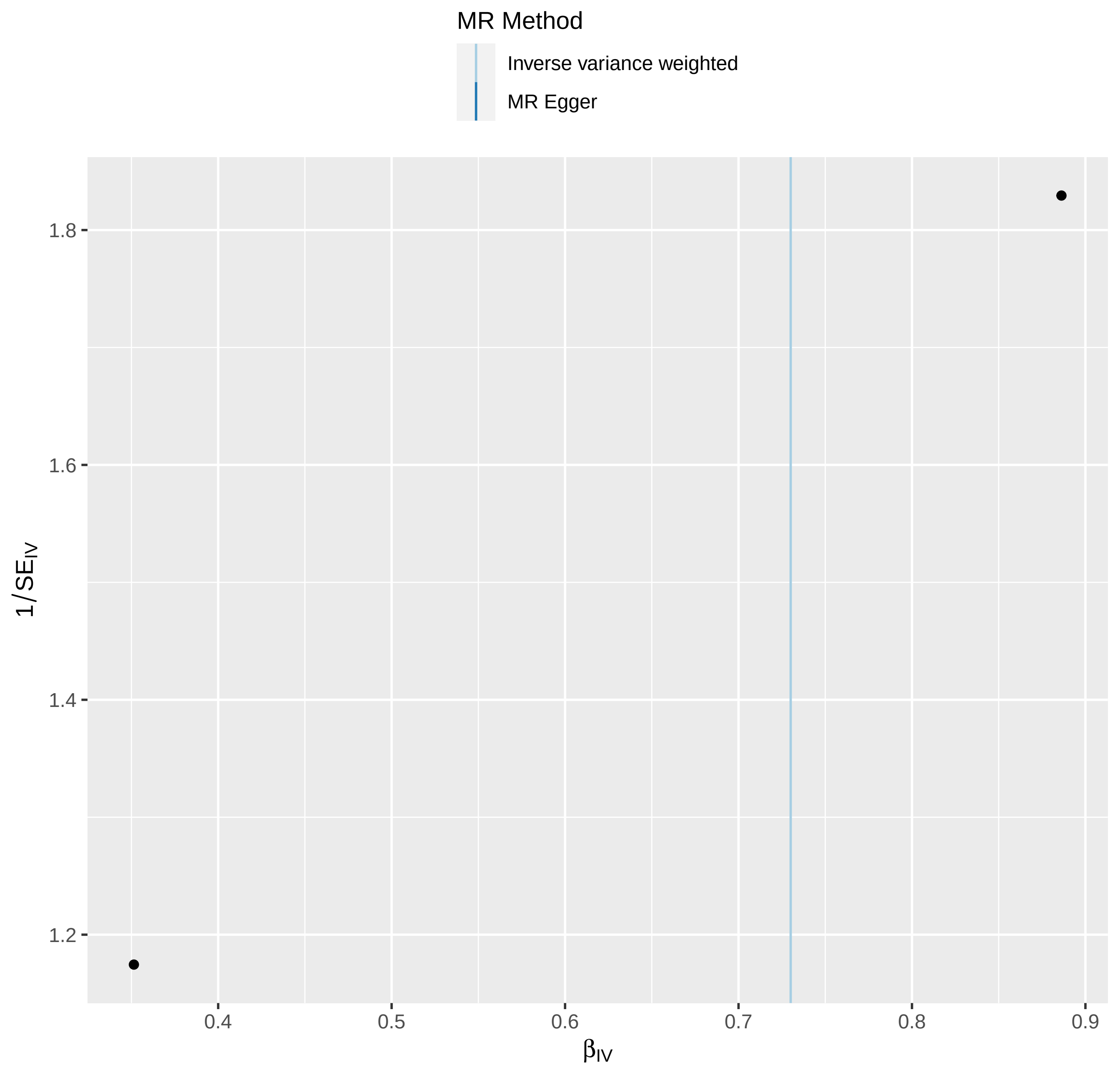

Batch 194 : Alpha−hydroxyisovalerate on Crohn's disease of colon

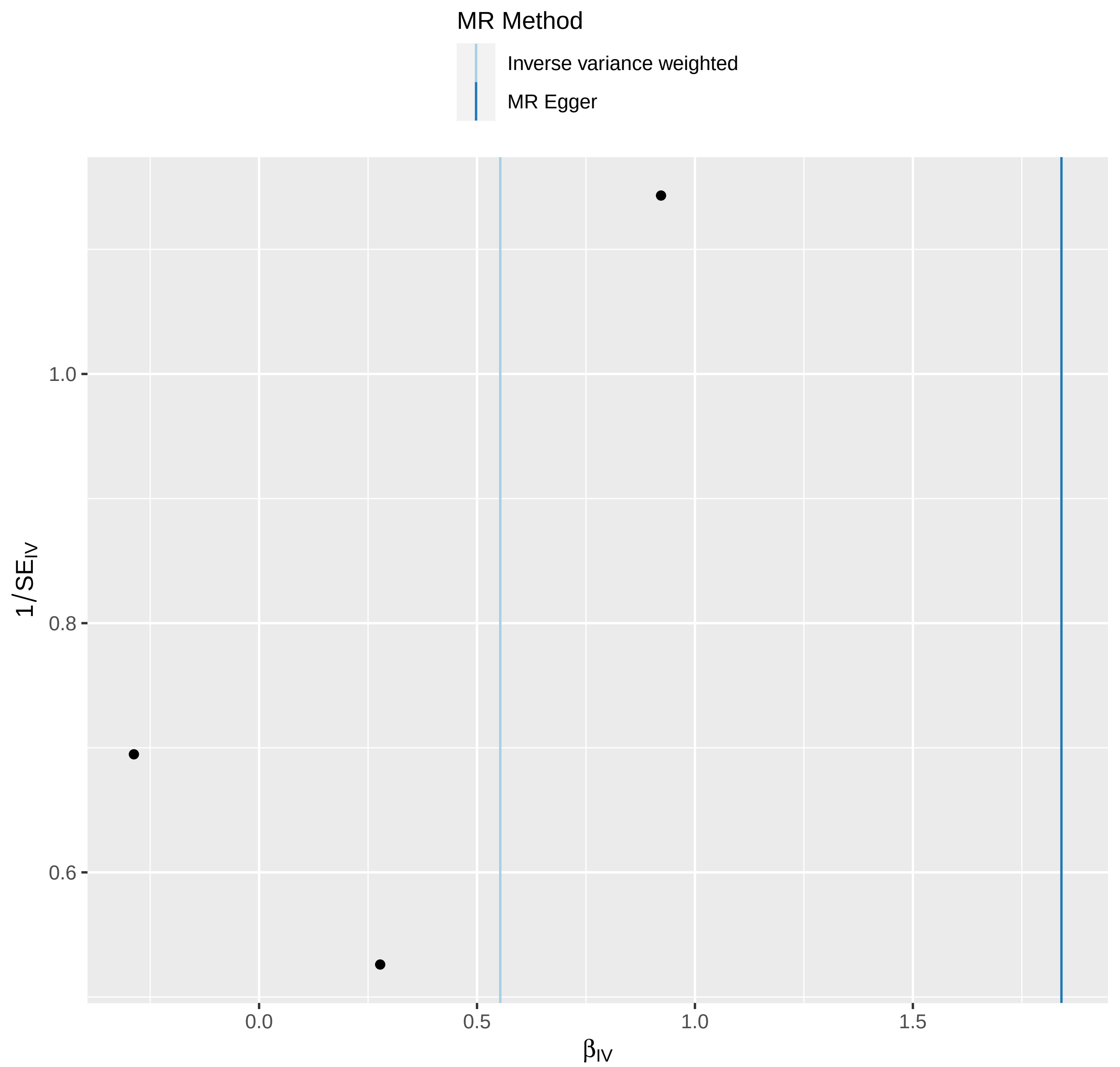

Batch 195 : N−acetylthreonine on Crohn's disease of colon

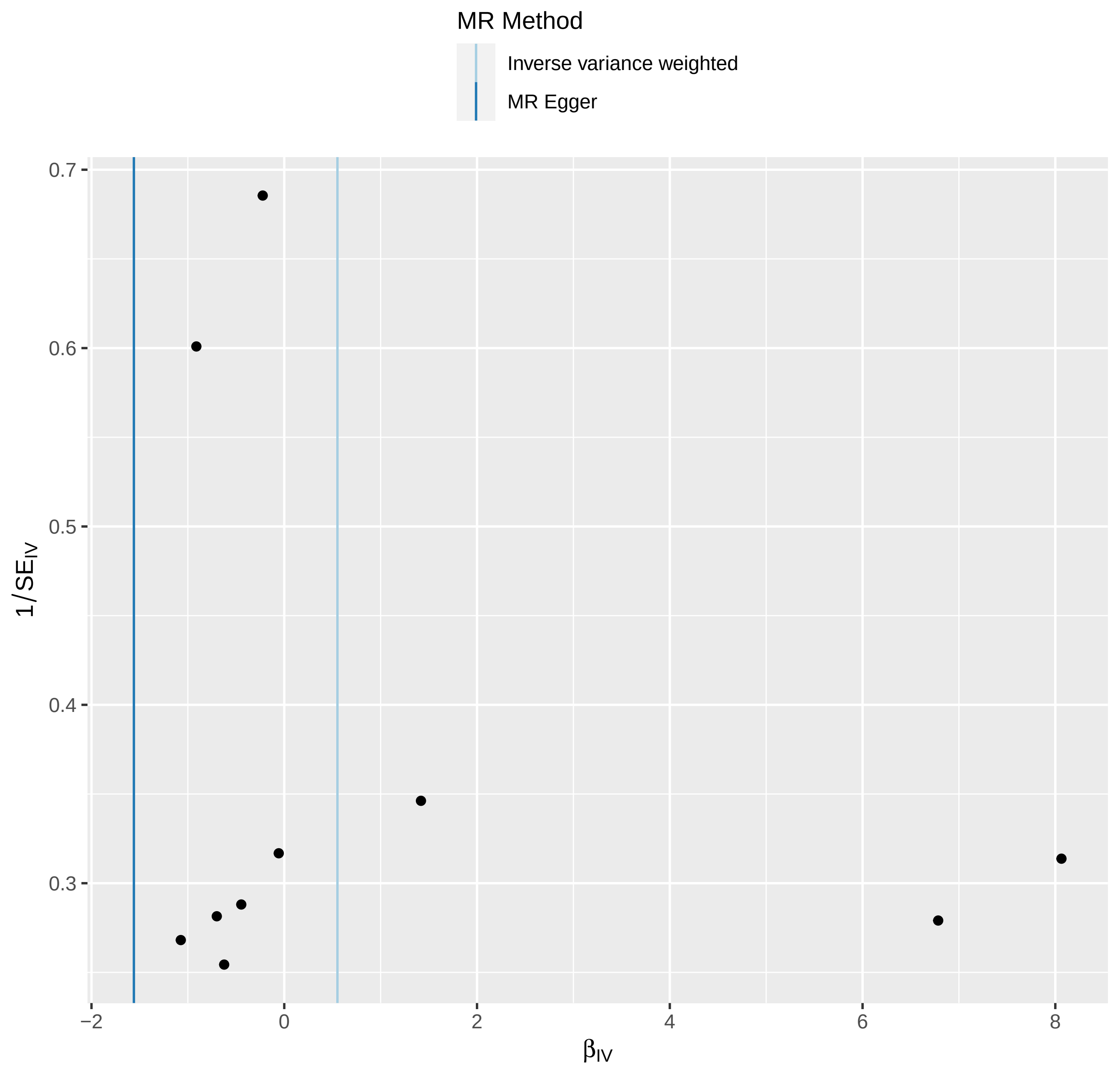

Batch 196 : Decanoylcarnitine on Crohn's disease of colon

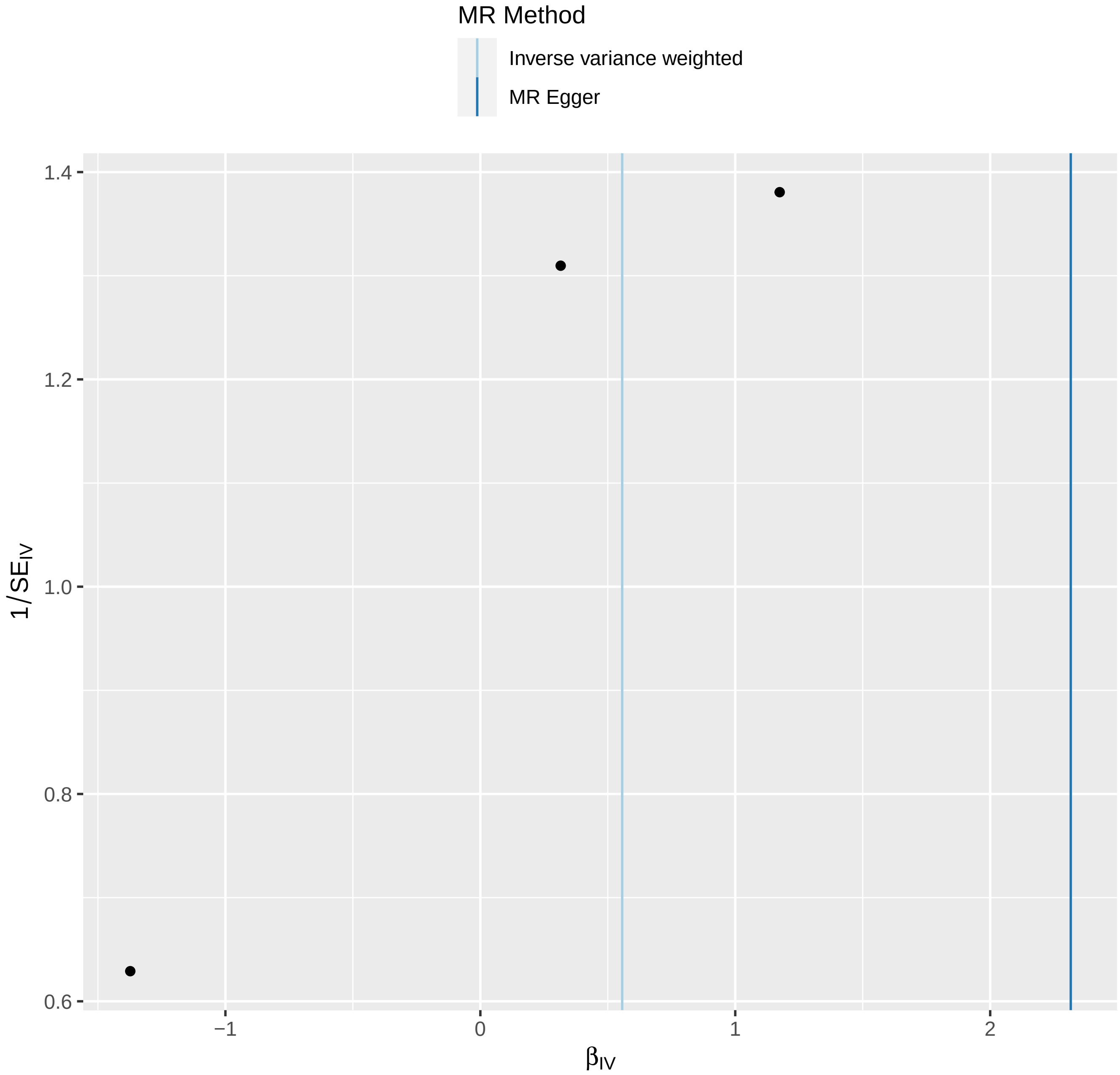

Batch 197 : 1−palmitoylglycerophosphocholine on Crohn's disease of colon

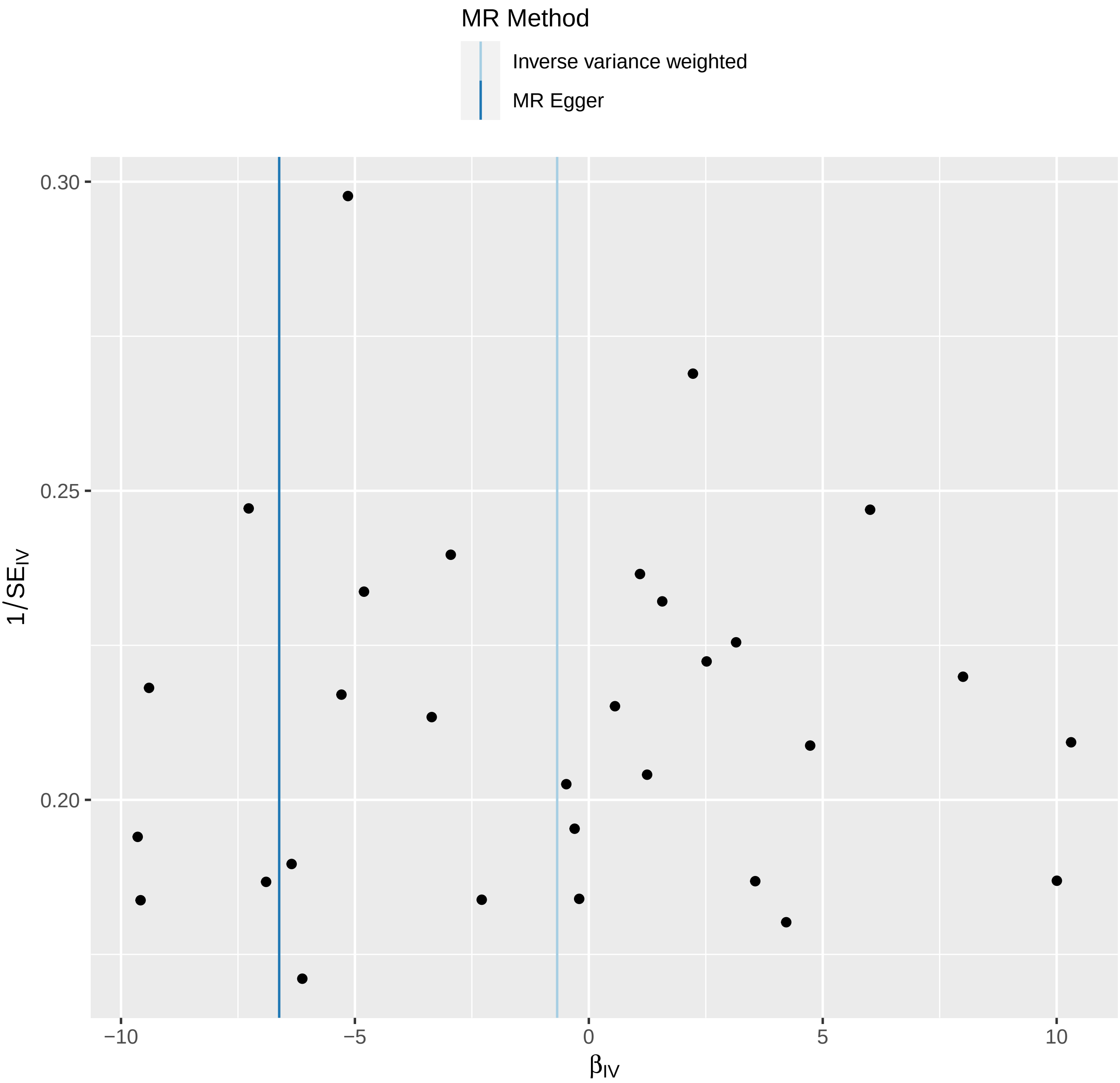

Batch 198 : 1−heptadecanoylglycerophosphocholine on Crohn's disease of colon

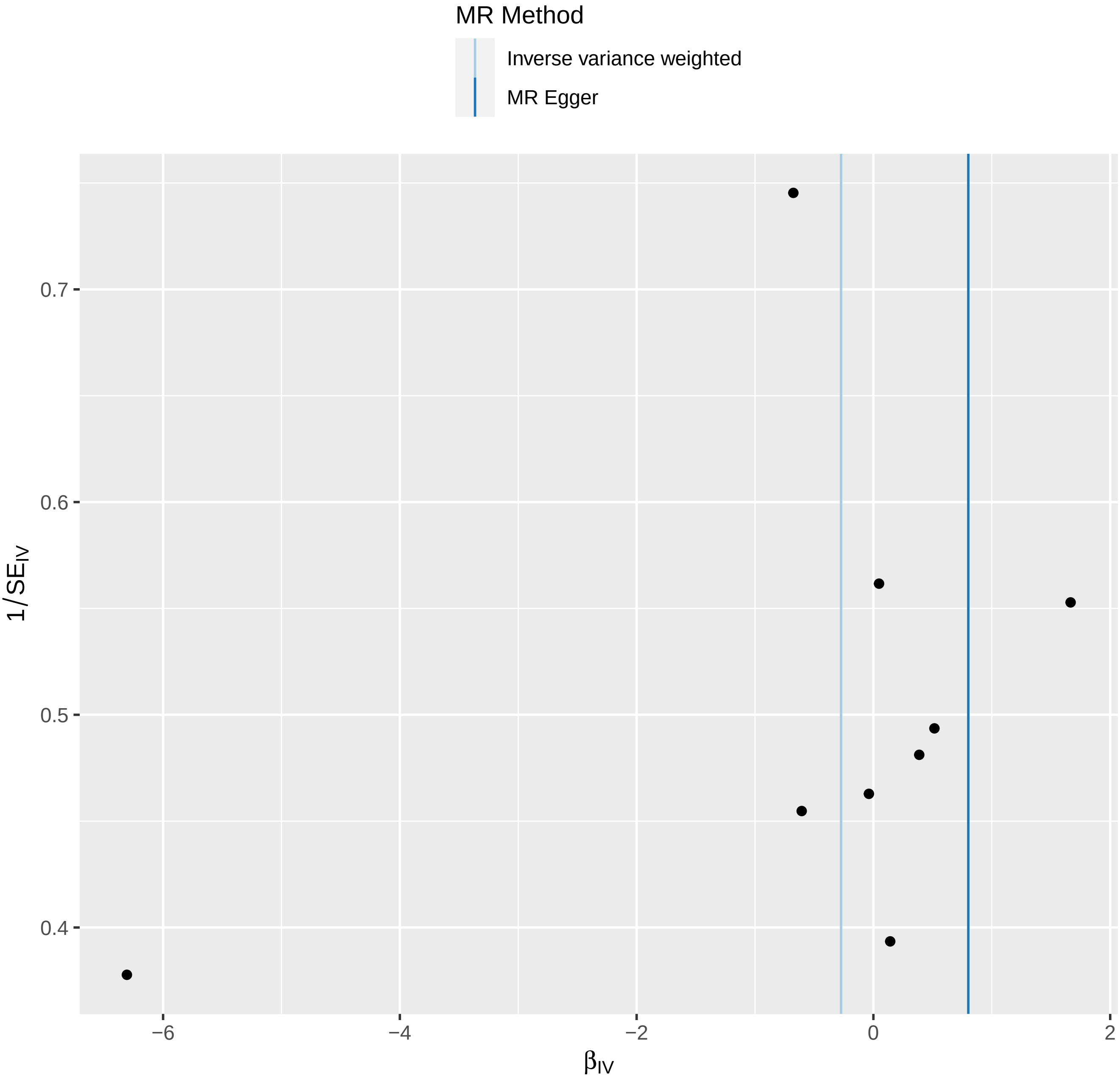

Batch 199 : 1−oleoylglycerophosphocholine on Crohn's disease of colon

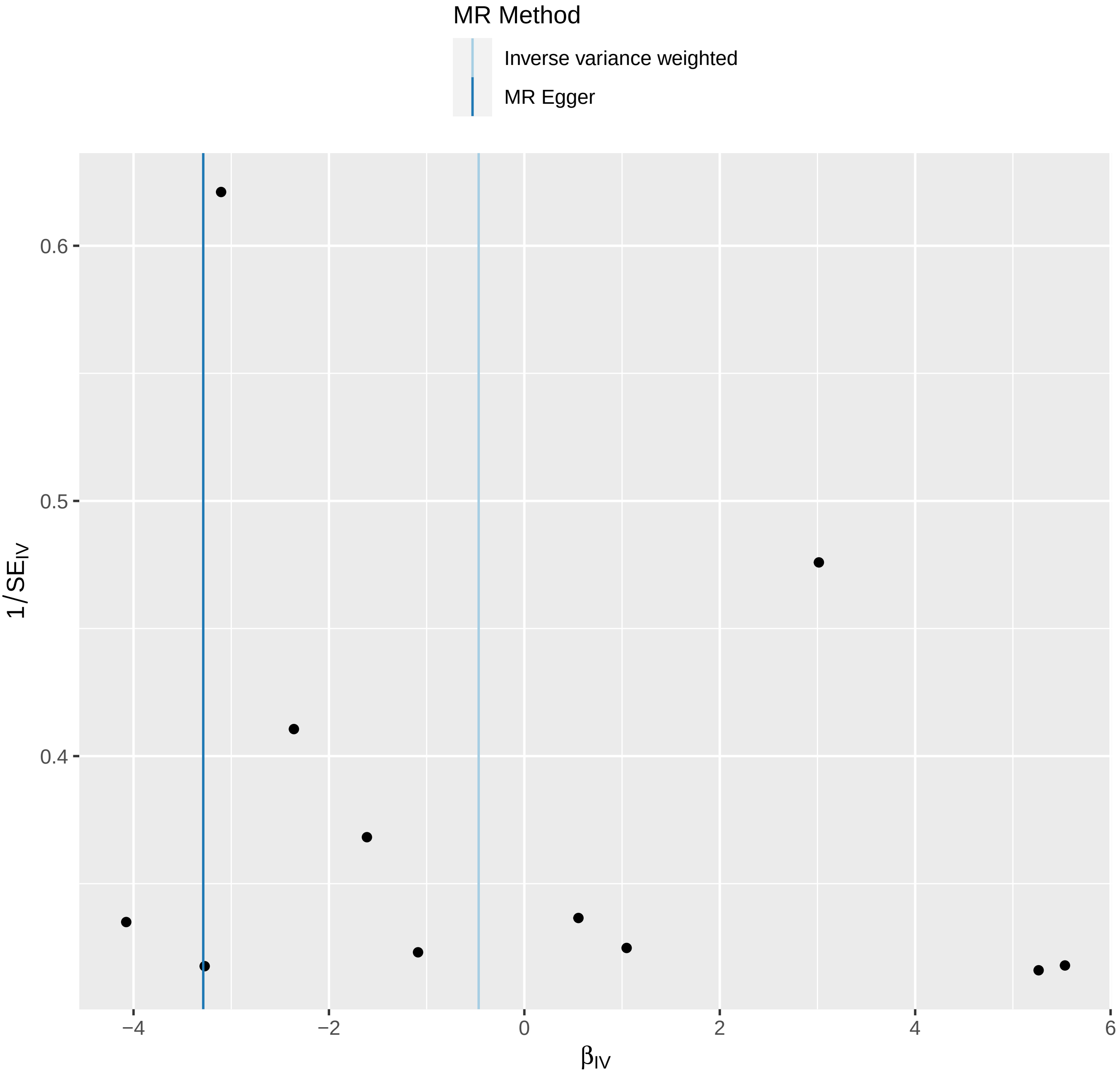

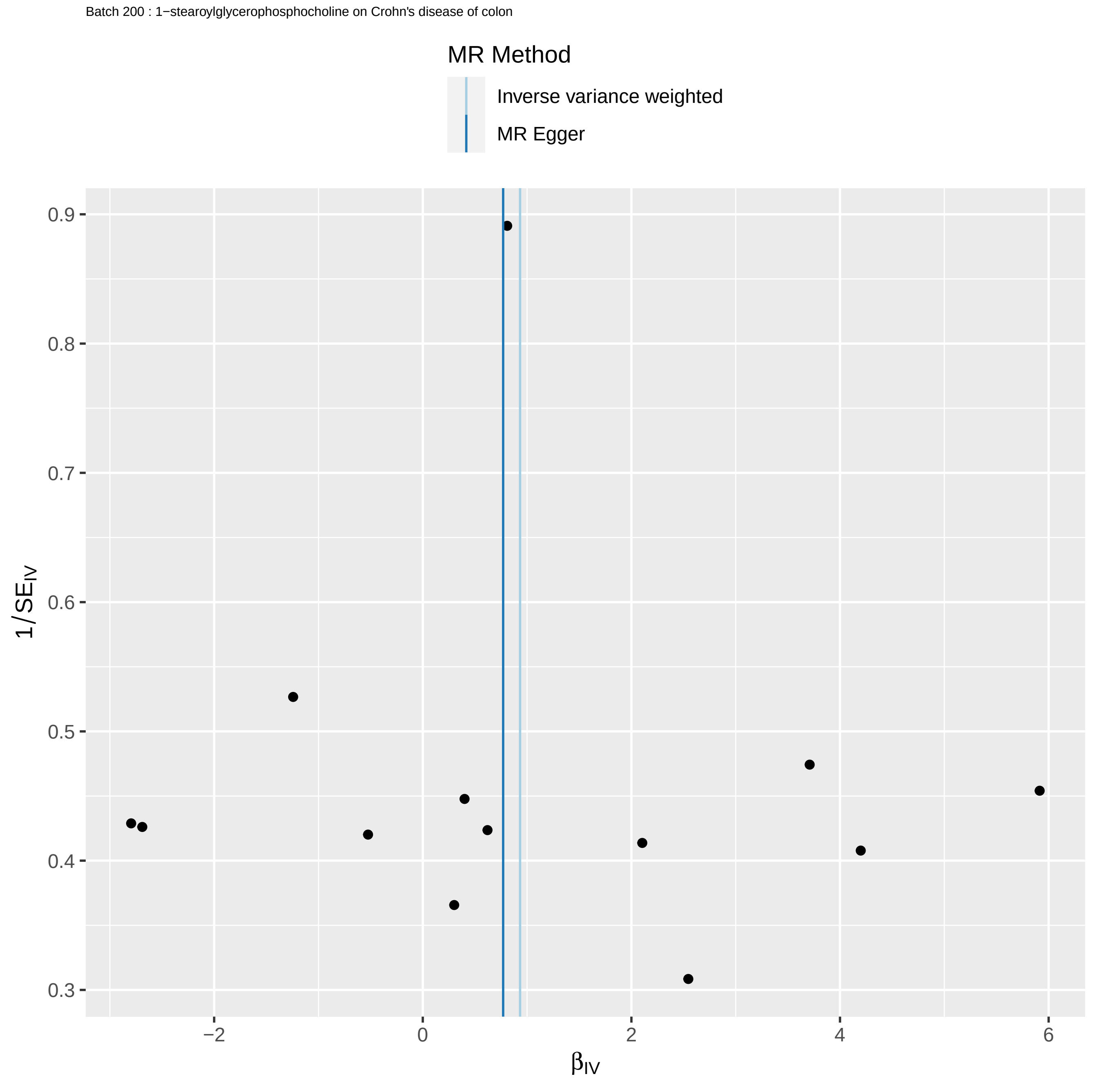
Batch 200 : 1−stearoylglycerophosphocholine on Crohn's disease of colon
MR Method
Inverse variance weighted
MR Egger
0.9
0.8
0.7
0.6
0.5
0.4
0.3
−2
0
2
4
6
1/SE_IV
β_IV

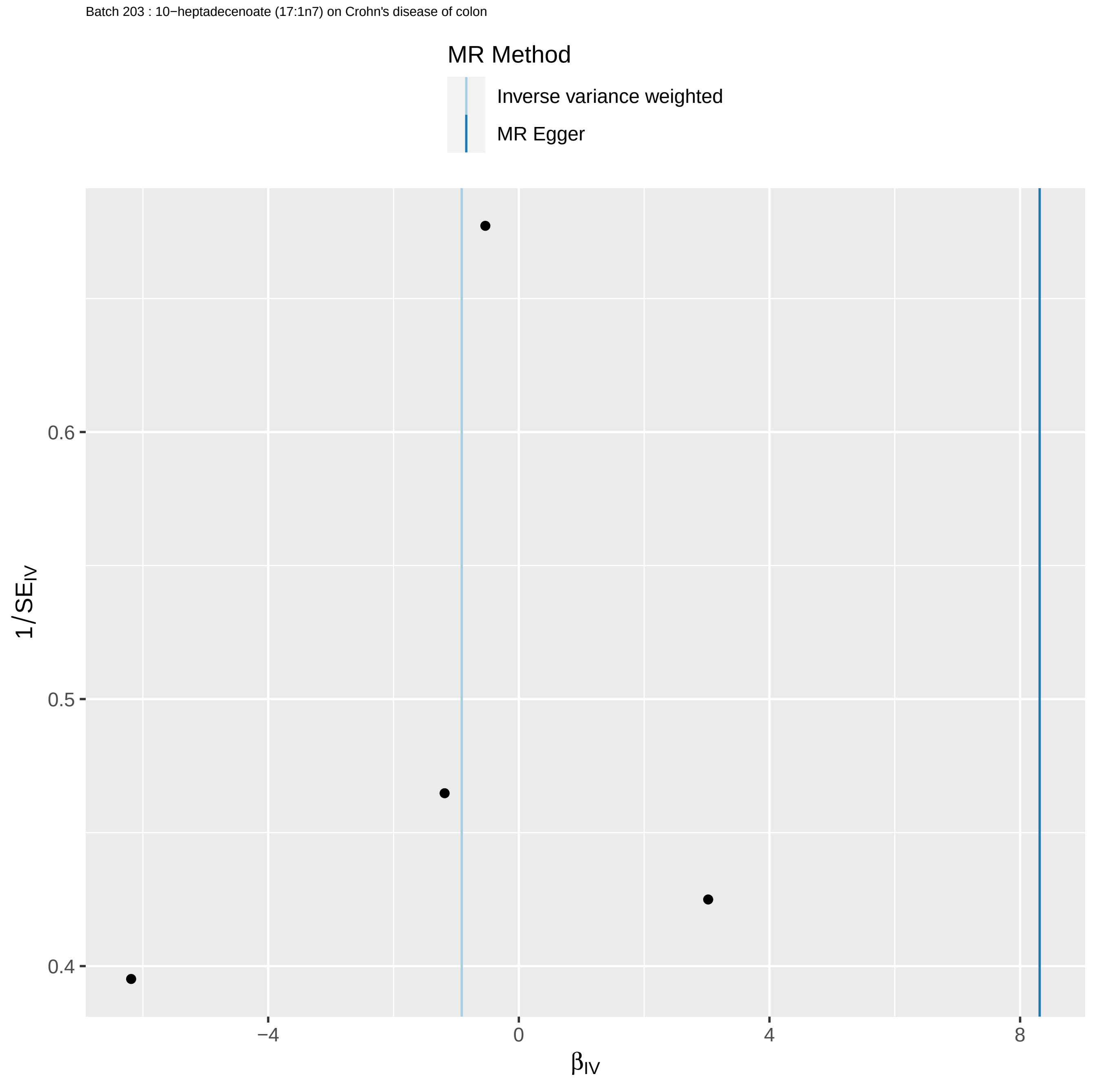

Batch 203 : 10−heptadecenoate (17:1n7) on Crohn's disease of colon
MR Method
Inverse variance weighted
MR Egger
0.6
0.5
0.4
1/SE_IV
−4
0
4
8
β_IV

Batch 205 : Epiandrosterone sulfate on Crohn's disease of colon

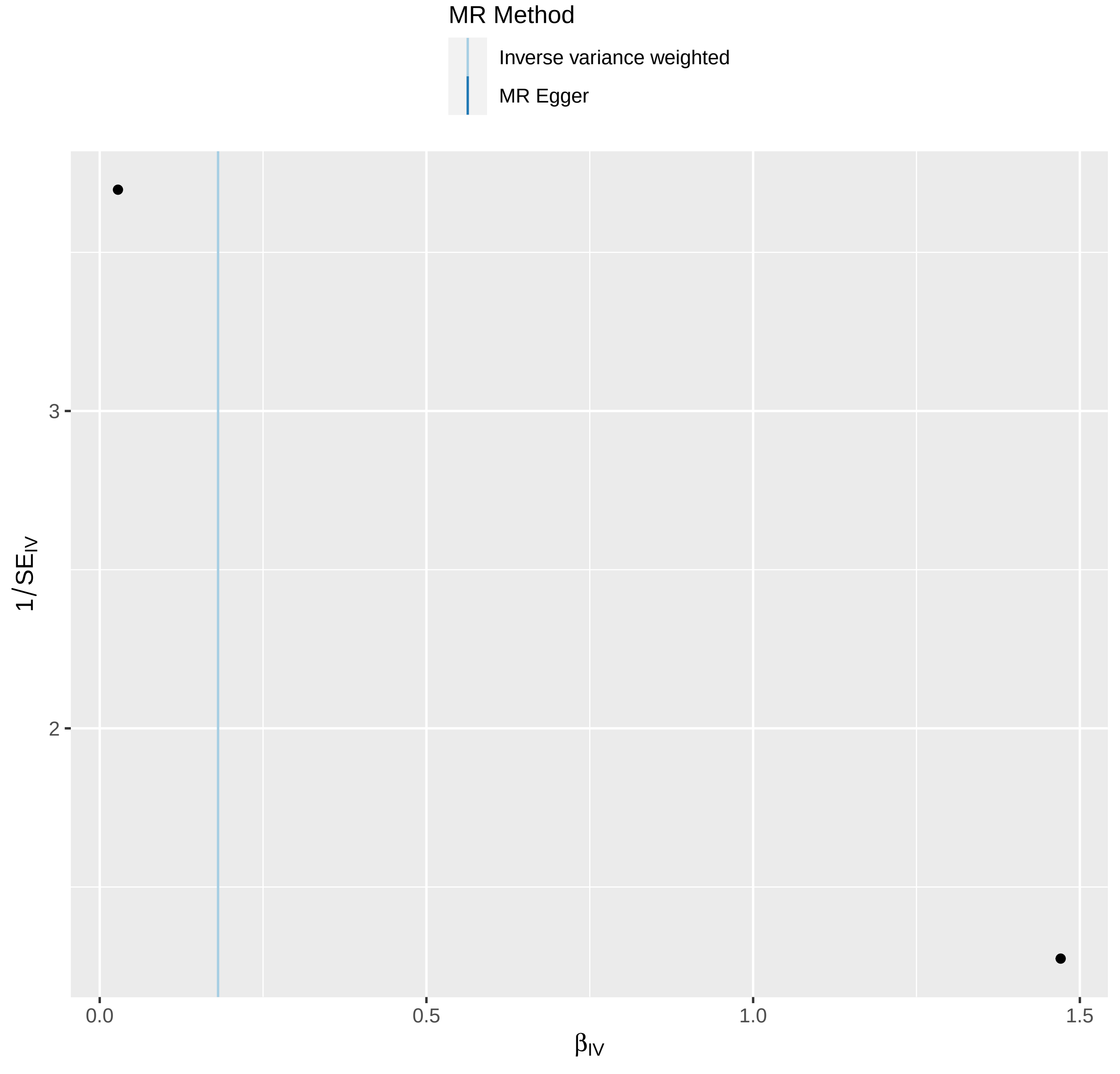

Batch 206 : Linolenate [alpha or gamma; (18:3n3 or 6)] on Crohn's disease of colon

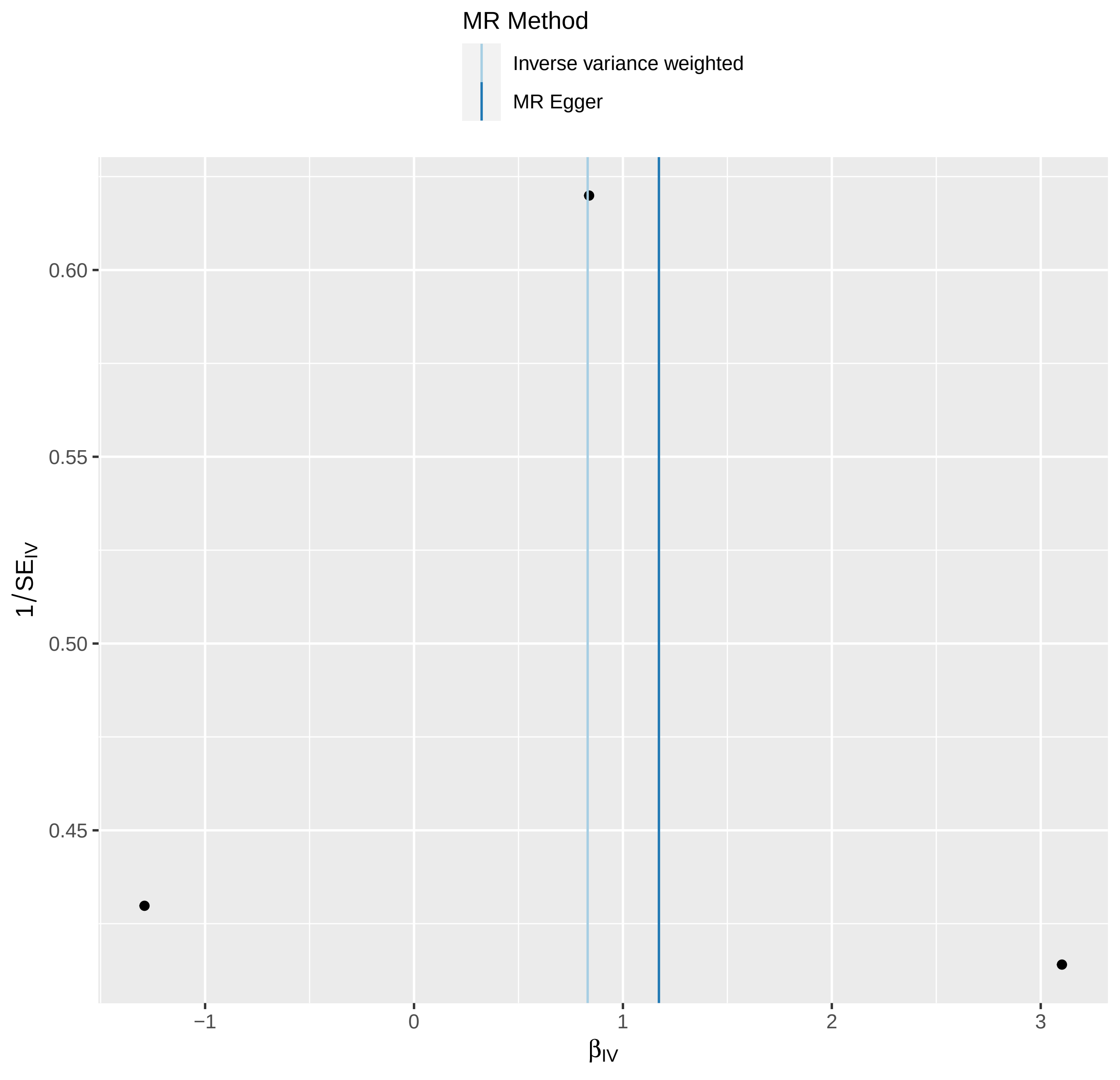

Batch 207 : X−12510−−2−aminooctanoic acid on Crohn's disease of colon

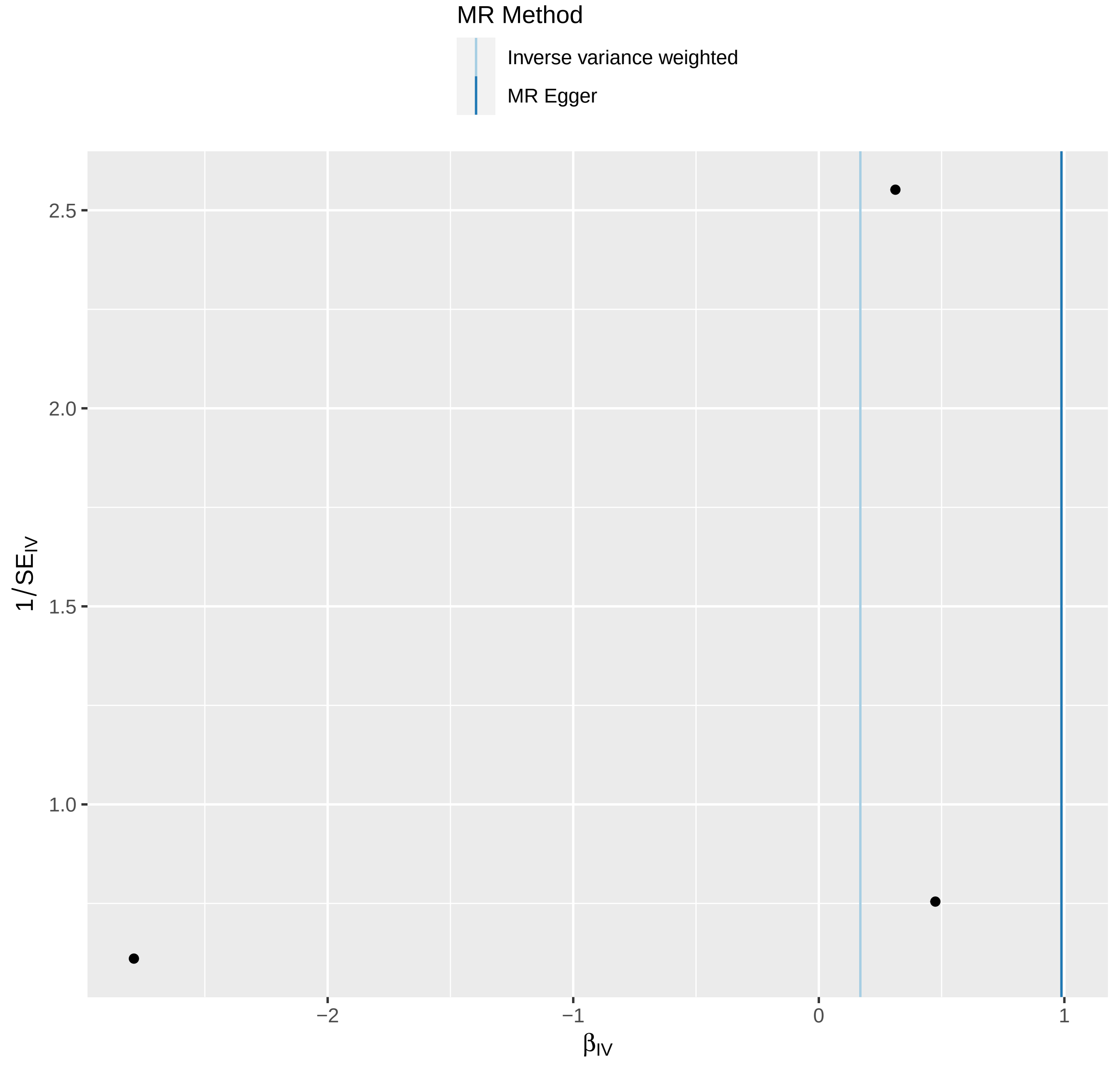

Batch 210 : Asparagine on Crohn's disease of colon

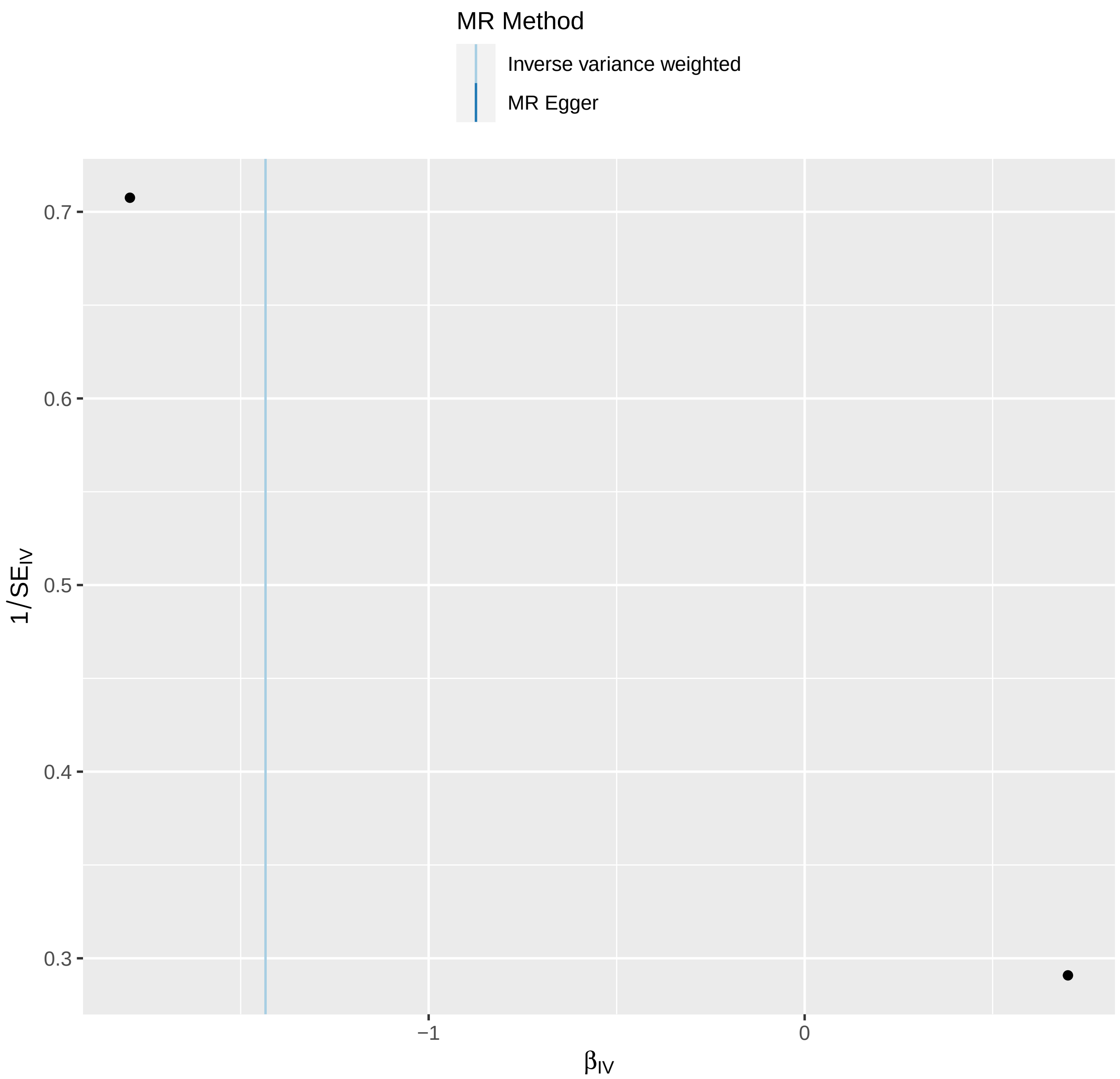

Batch 211 : Stachydrine on Crohn's disease of colon

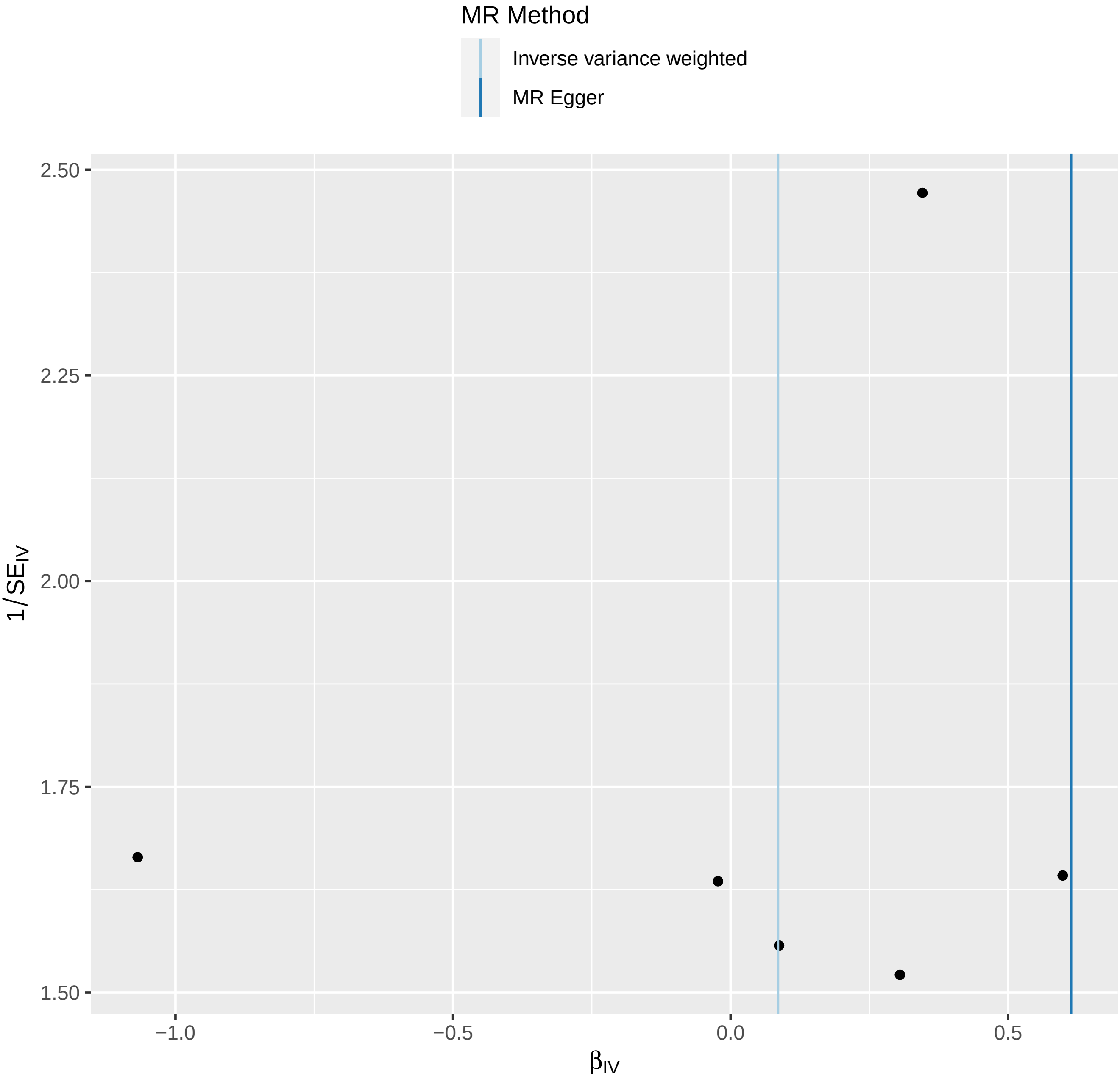

Batch 212 : Isovalerylcarnitine on Crohn's disease of colon

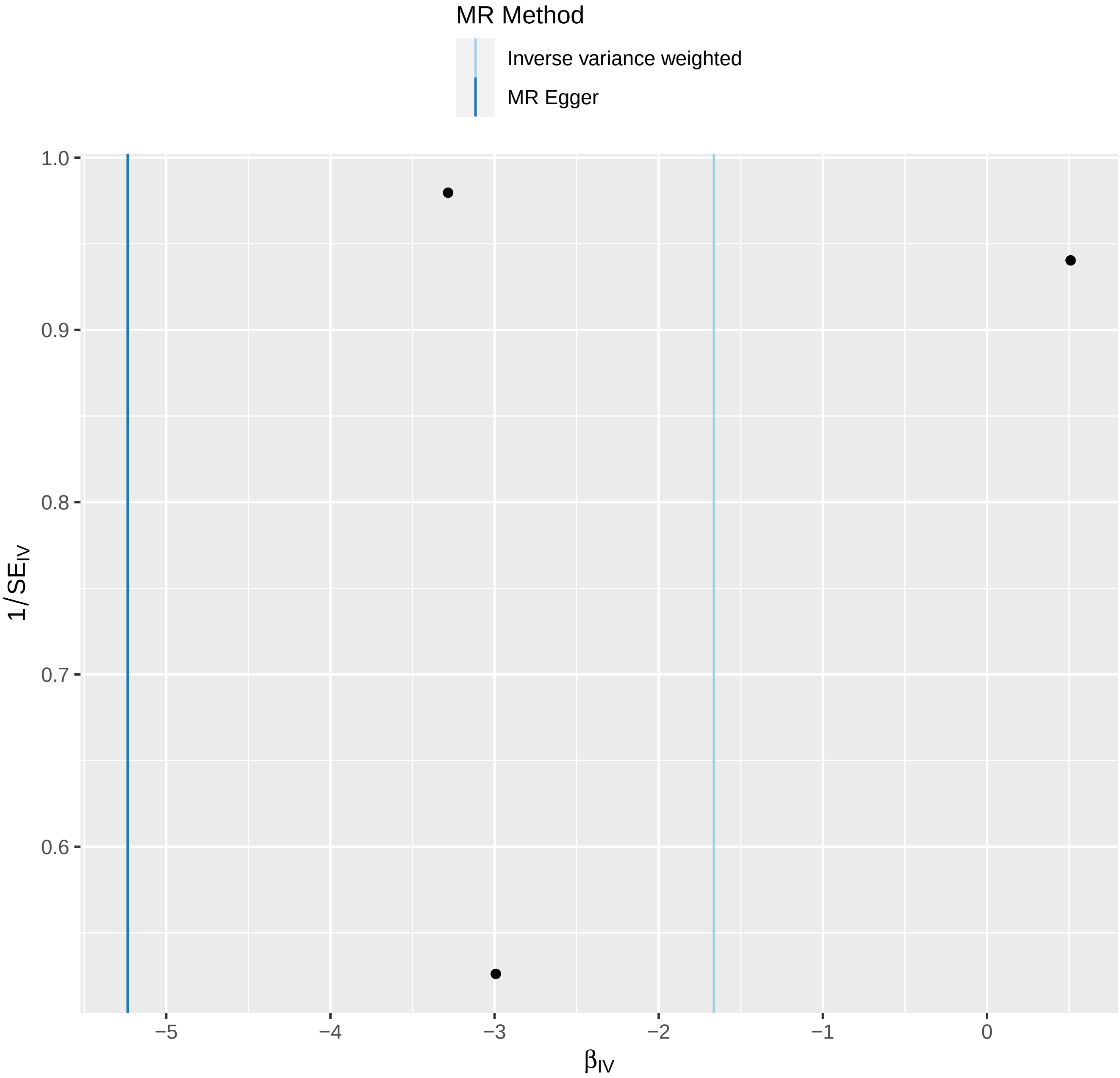

Batch 215 : 1−linoleoylglycerophosphocholine on Crohn's disease of colon

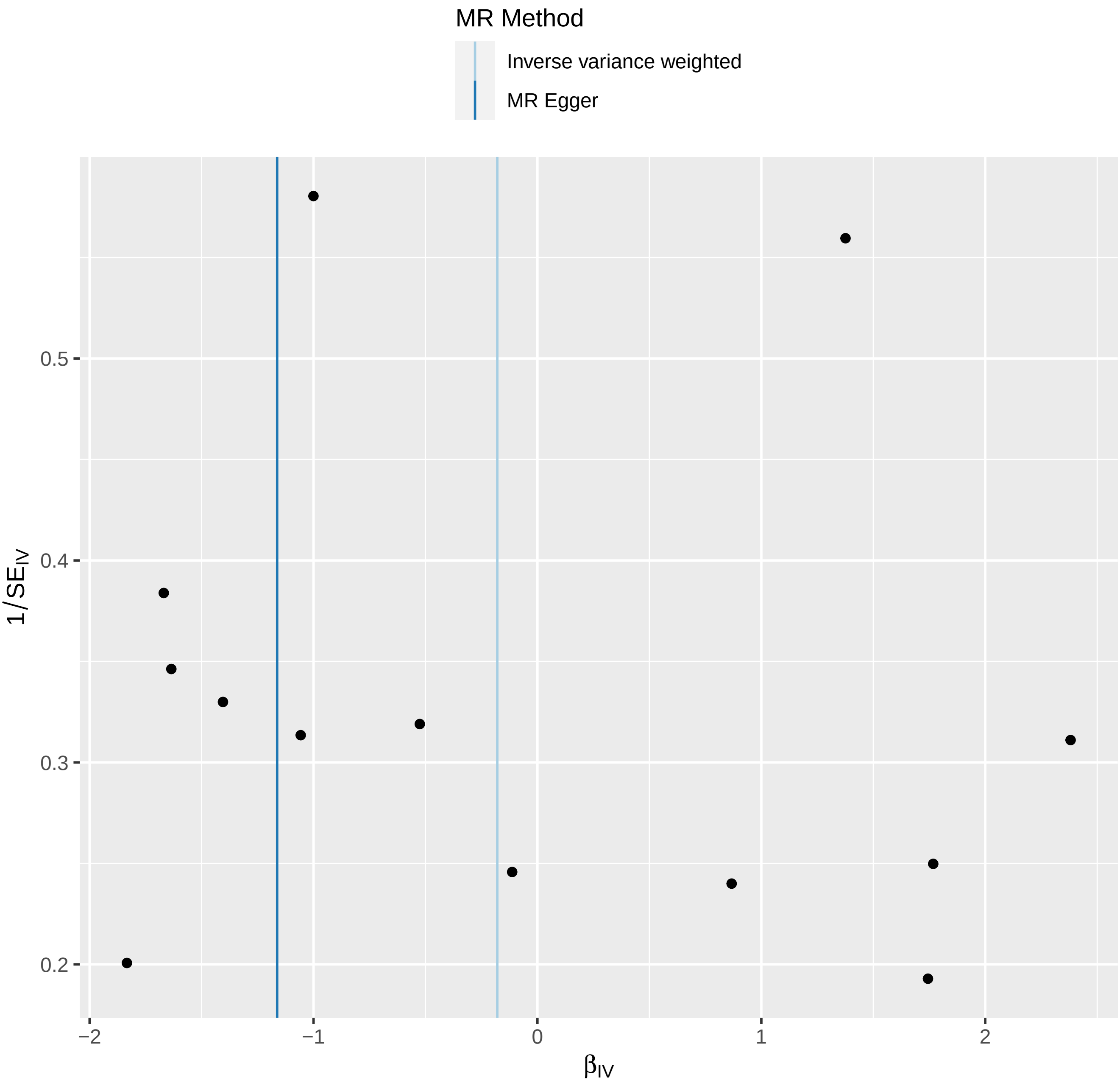

Batch 216 : Bradykinin, des−arg(9) on Crohn's disease of colon

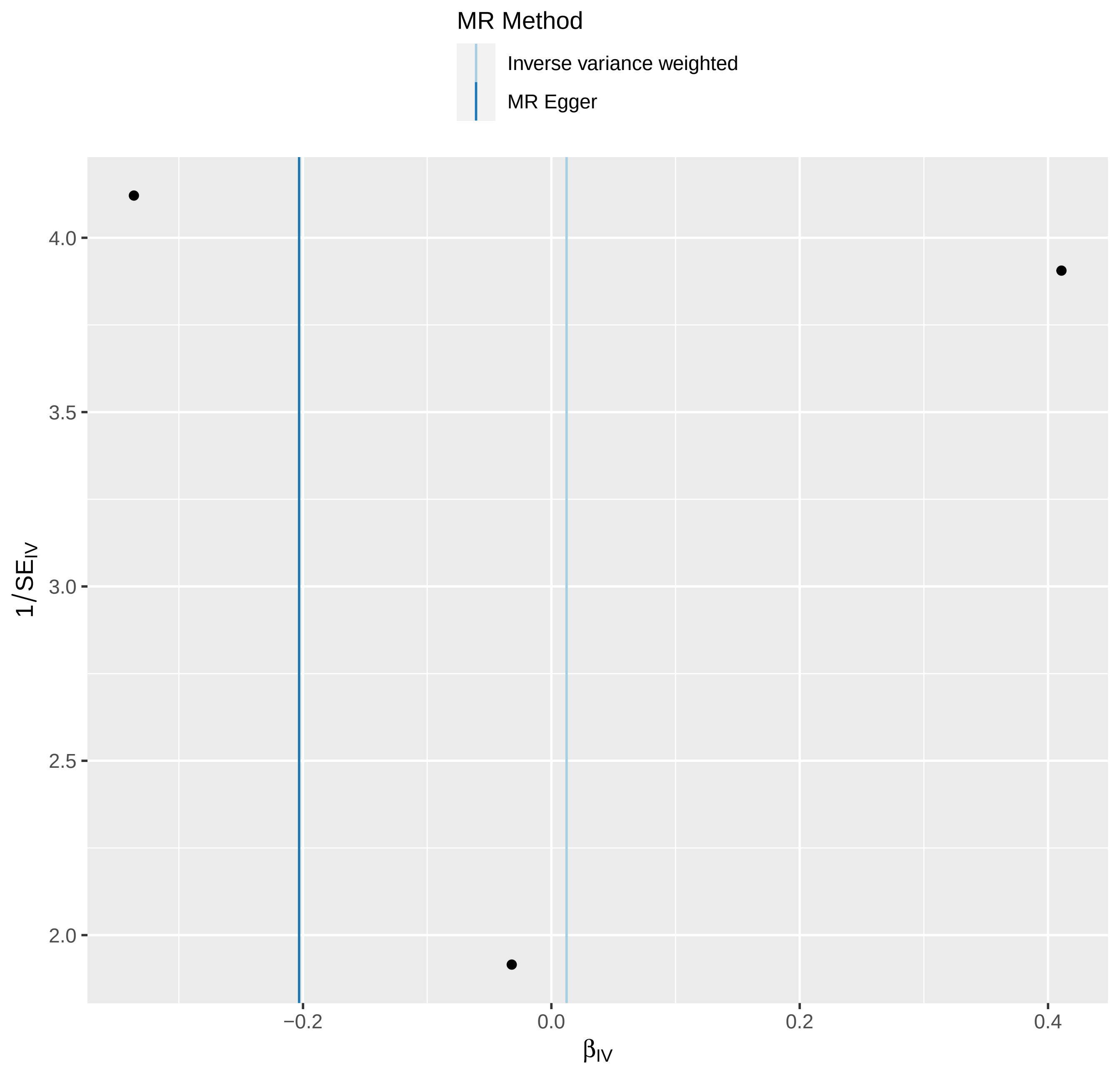

Batch 217 : Gamma−glutamylisoleucine* on Crohn's disease of colon

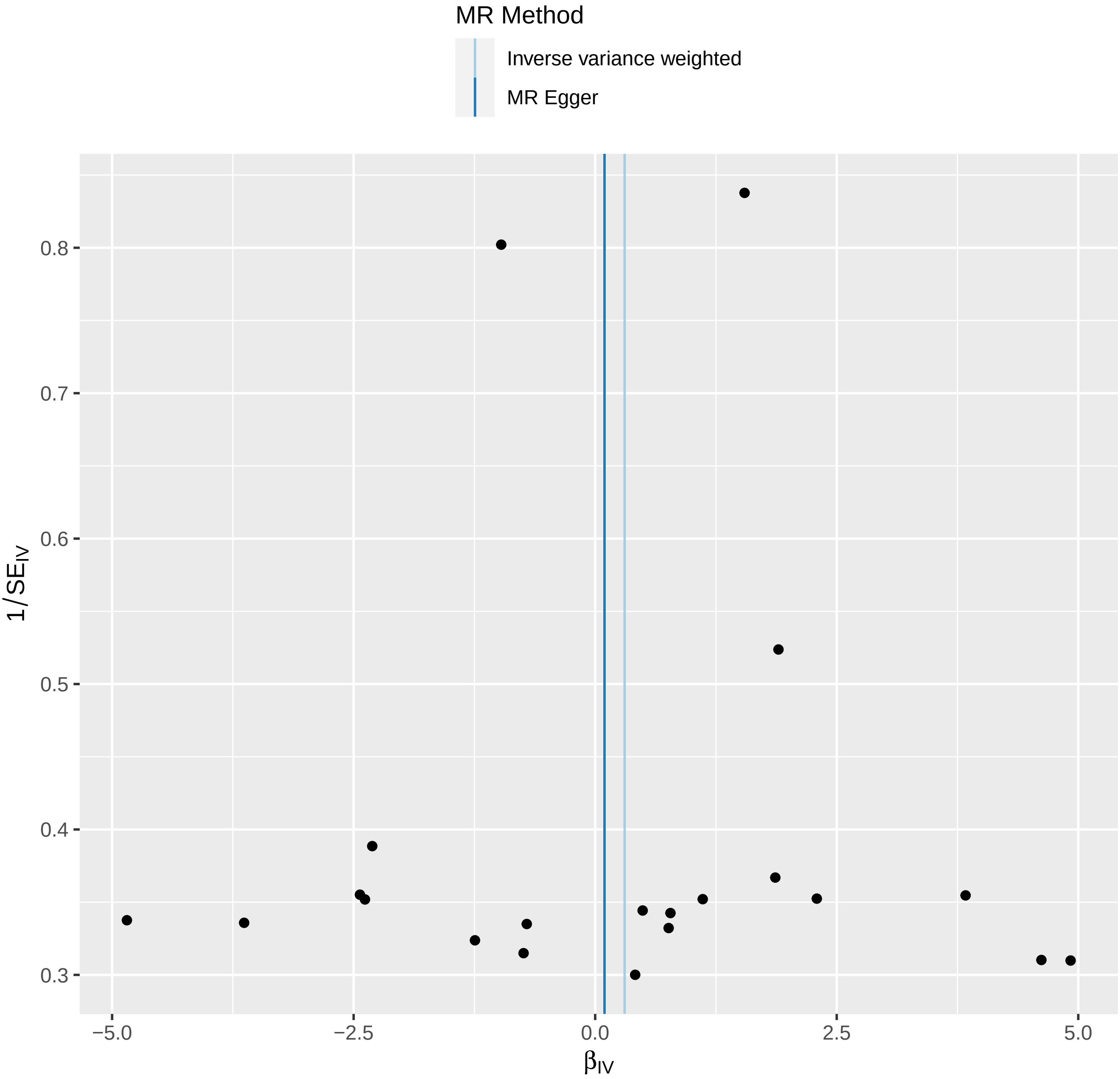

Batch 220 : Isovalerate on Crohn's disease of colon

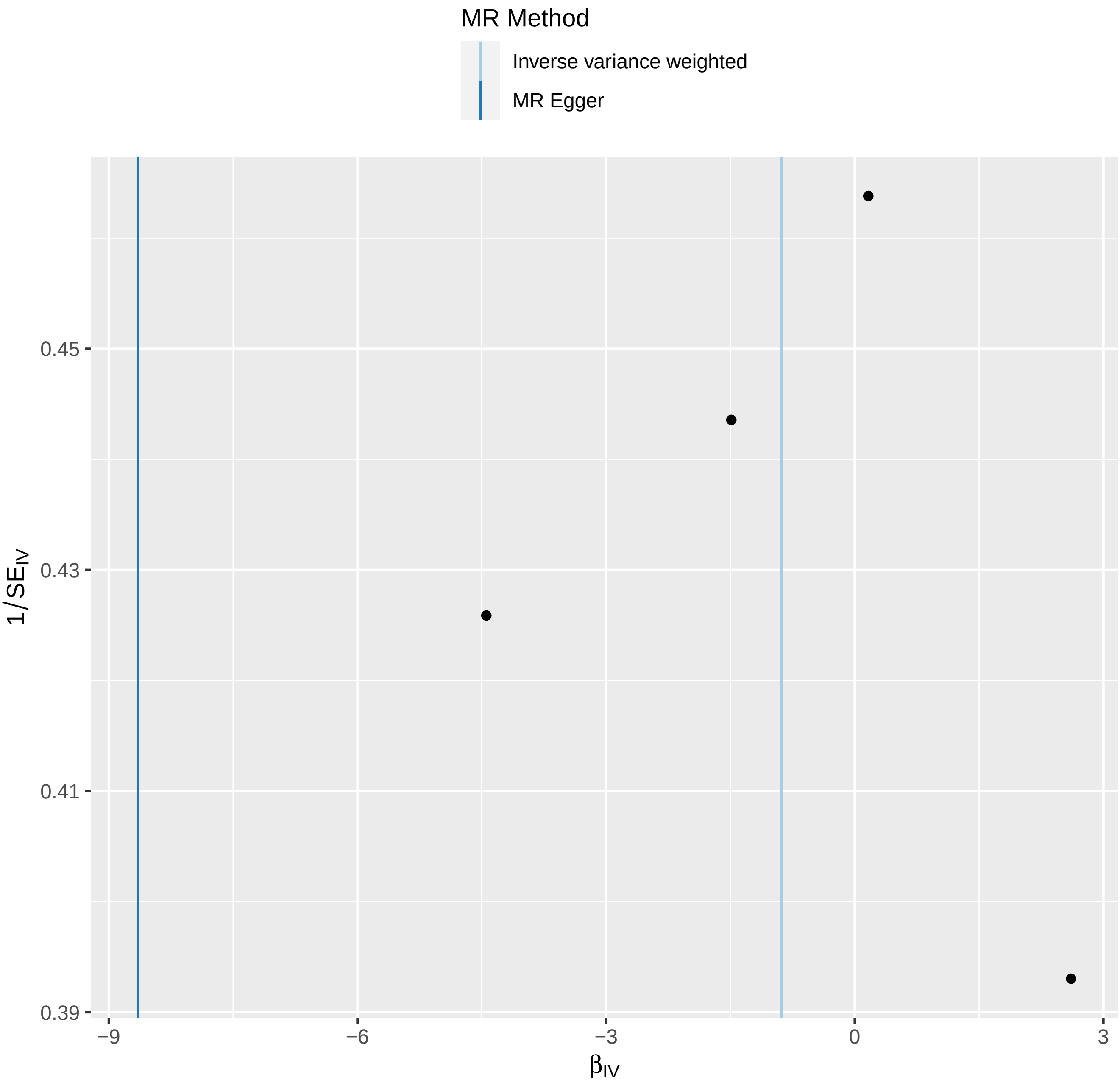

Batch 221 : X−13183−−stearamide on Crohn's disease of colon

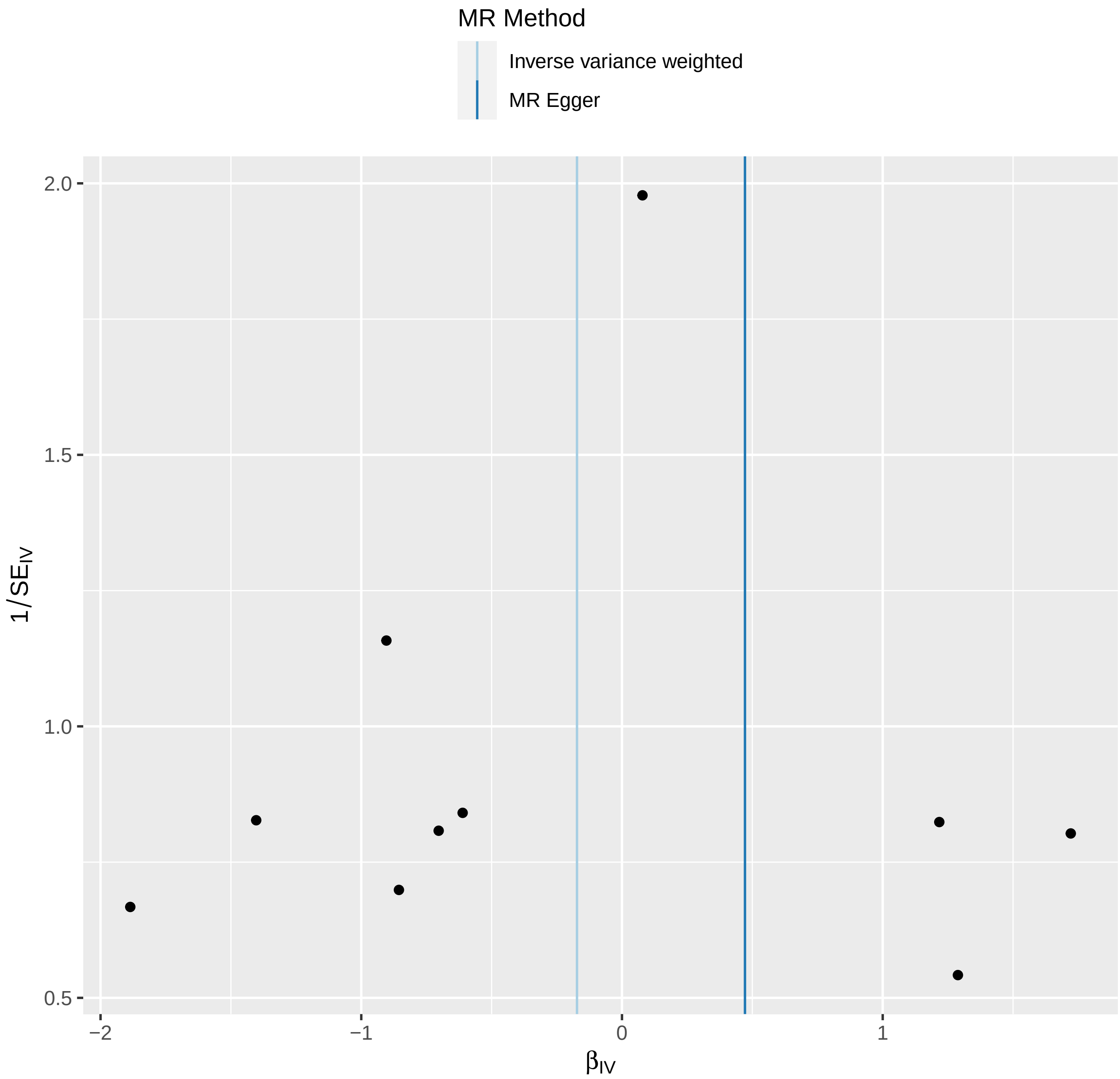

Batch 222 : 7−methylguanine on Crohn's disease of colon

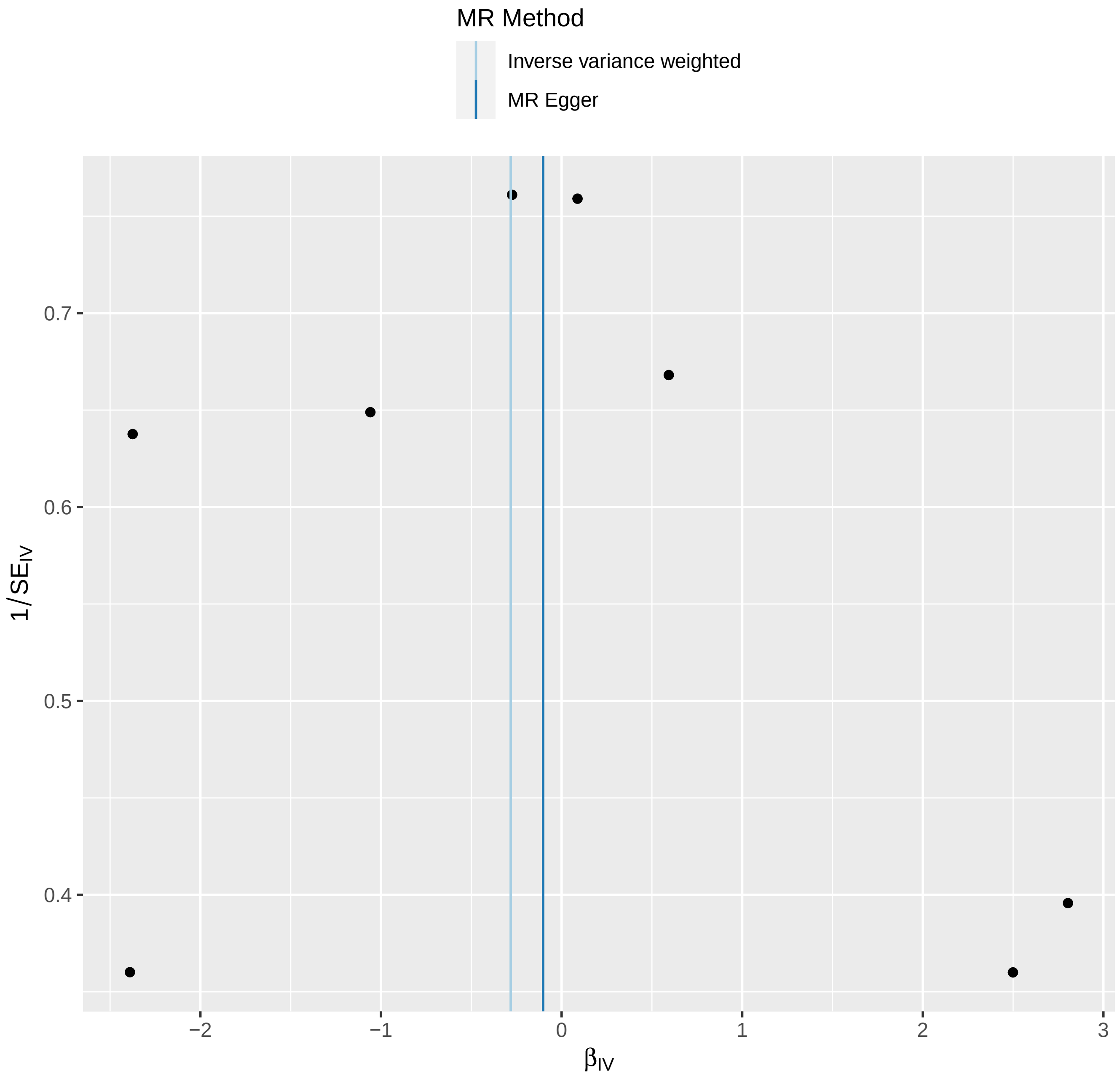

Batch 229 : X−13431−−nonanoylcarnitine* on Crohn's disease of colon

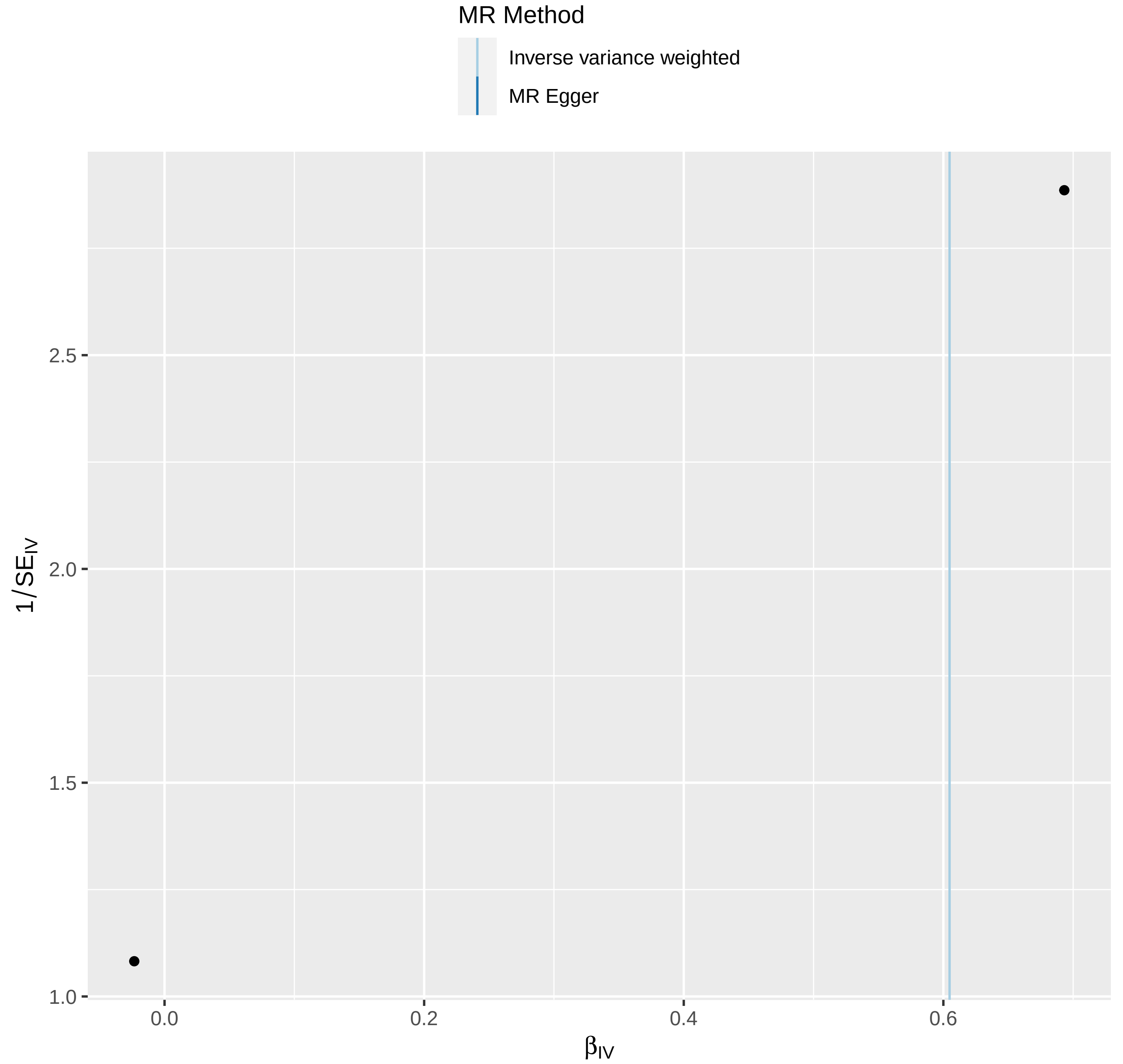

Batch 230 : 2−palmitoylglycerophosphocholine* on Crohn's disease of colon

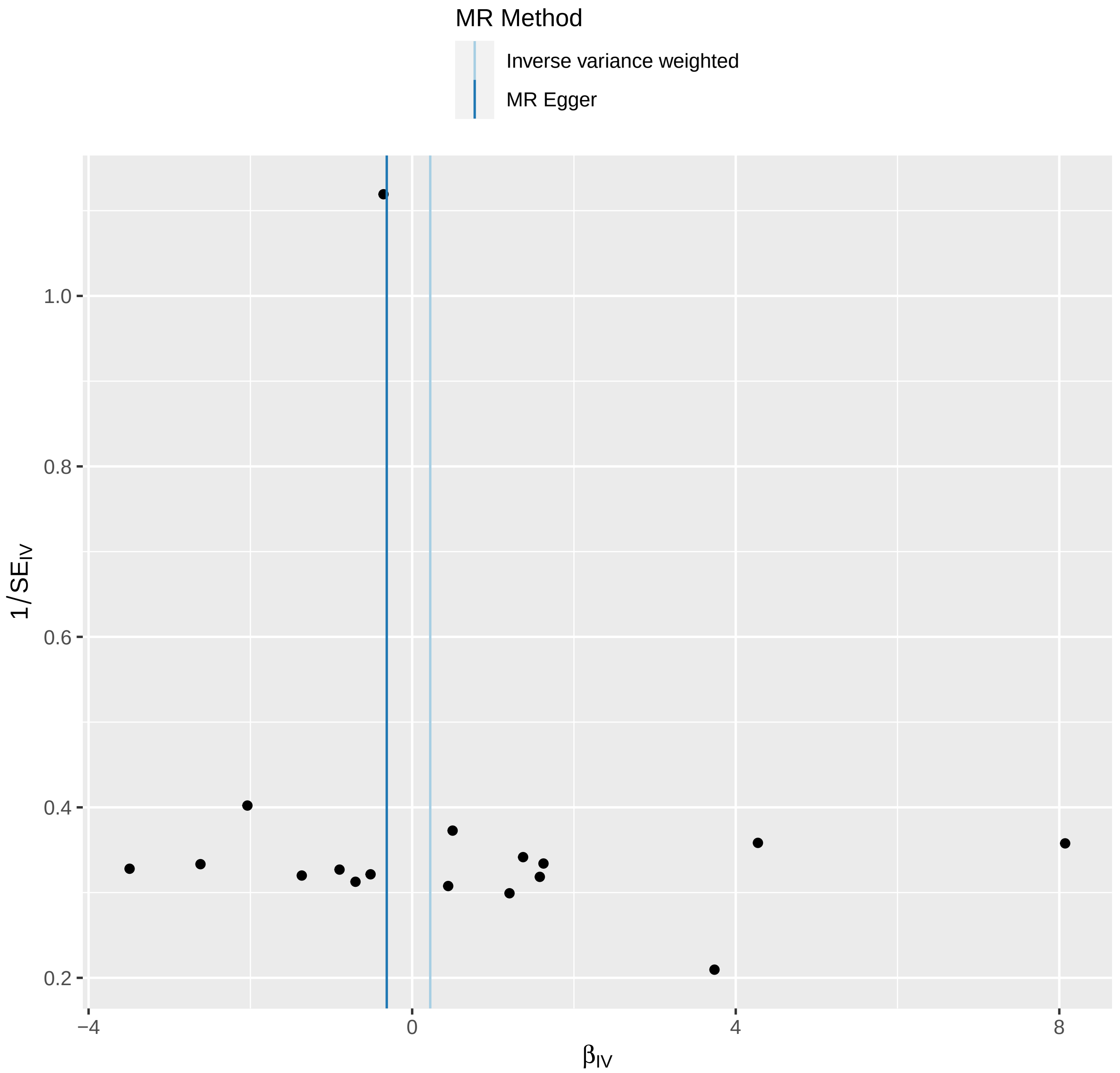

Batch 231 : 2−oleoylglycerophosphocholine* on Crohn's disease of colon

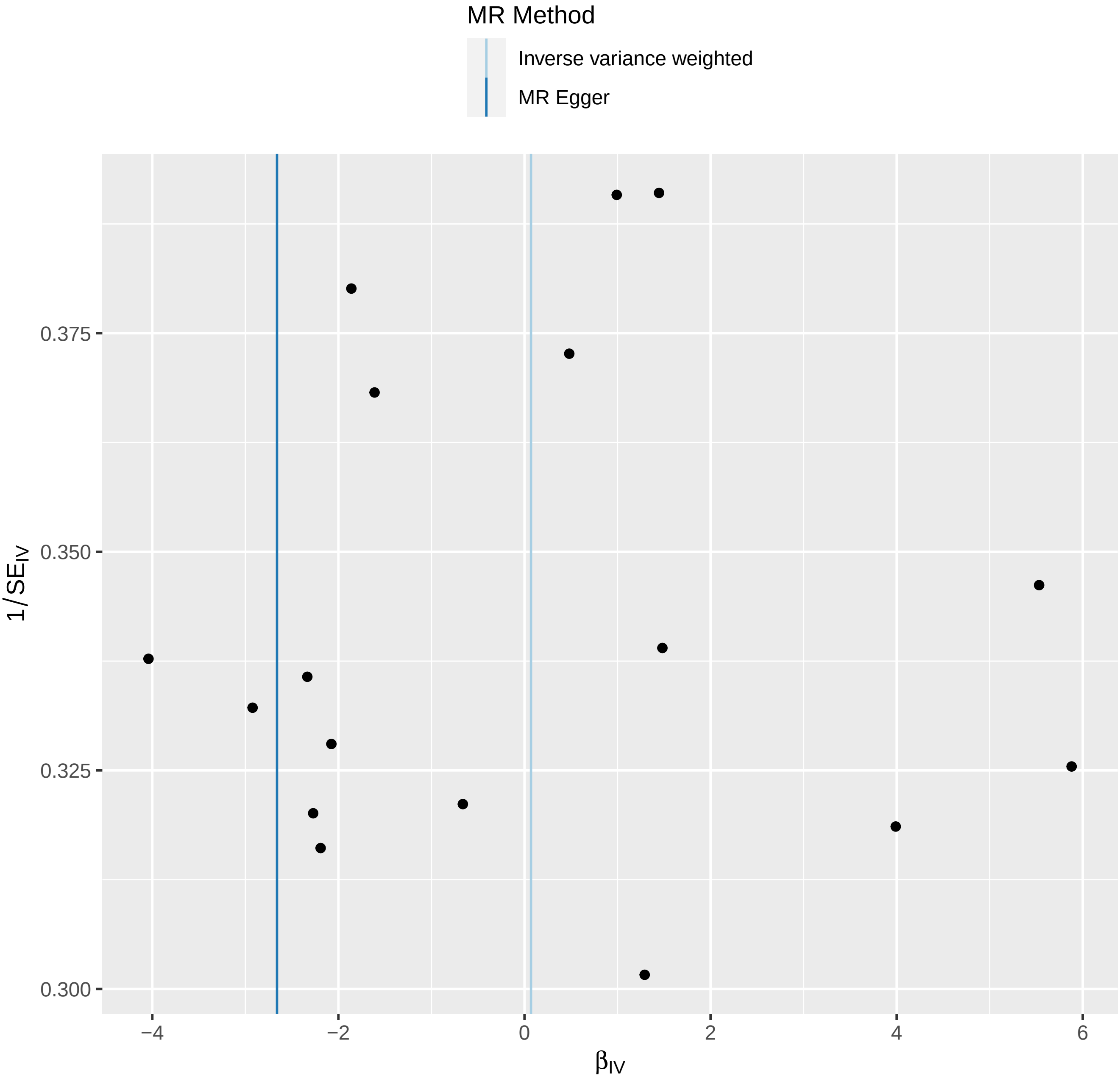

Batch 234 : 1−palmitoylglycerophosphoinositol* on Crohn's disease of colon

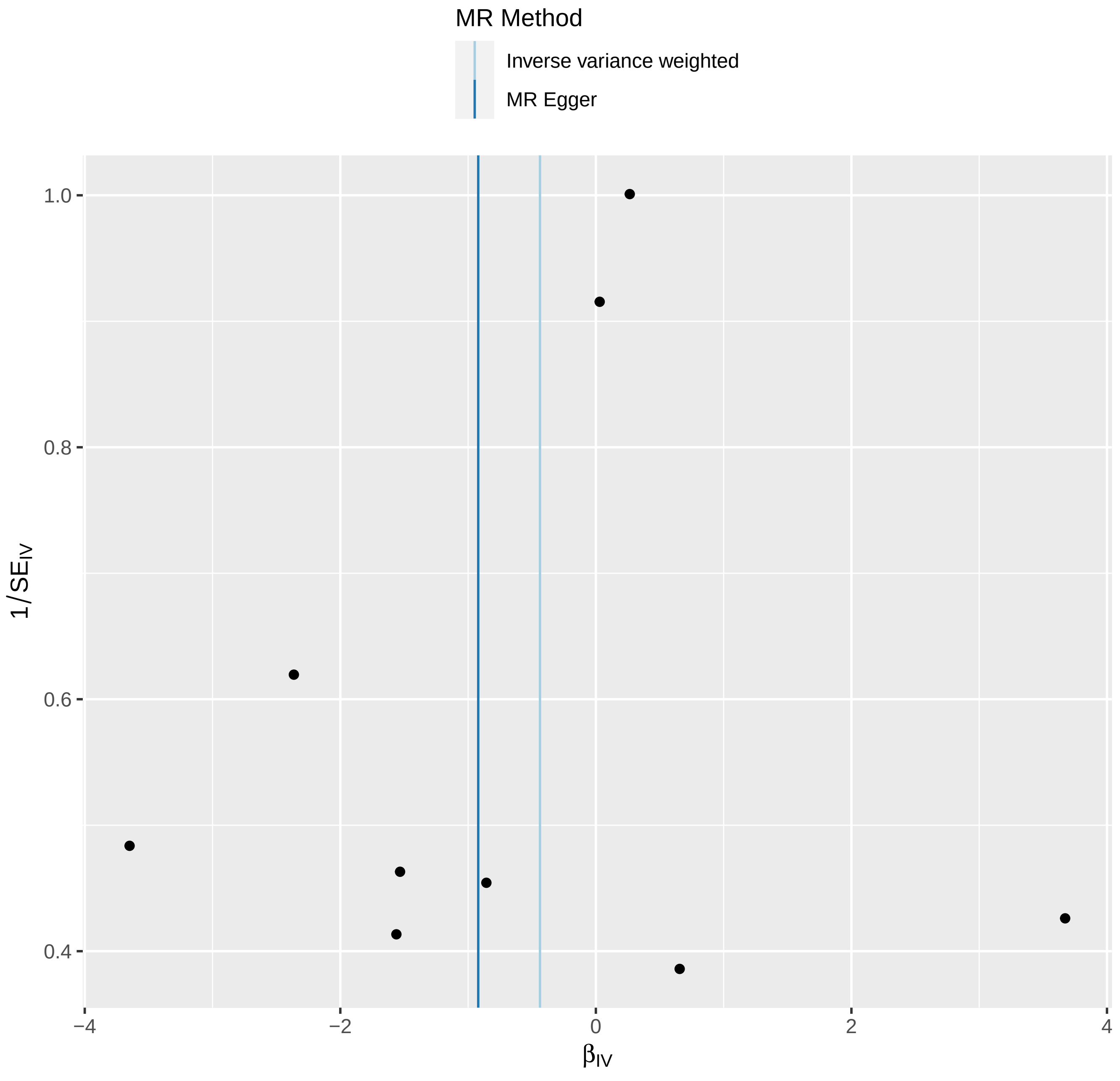

Batch 237 : Glutaroyl carnitine on Crohn's disease of colon

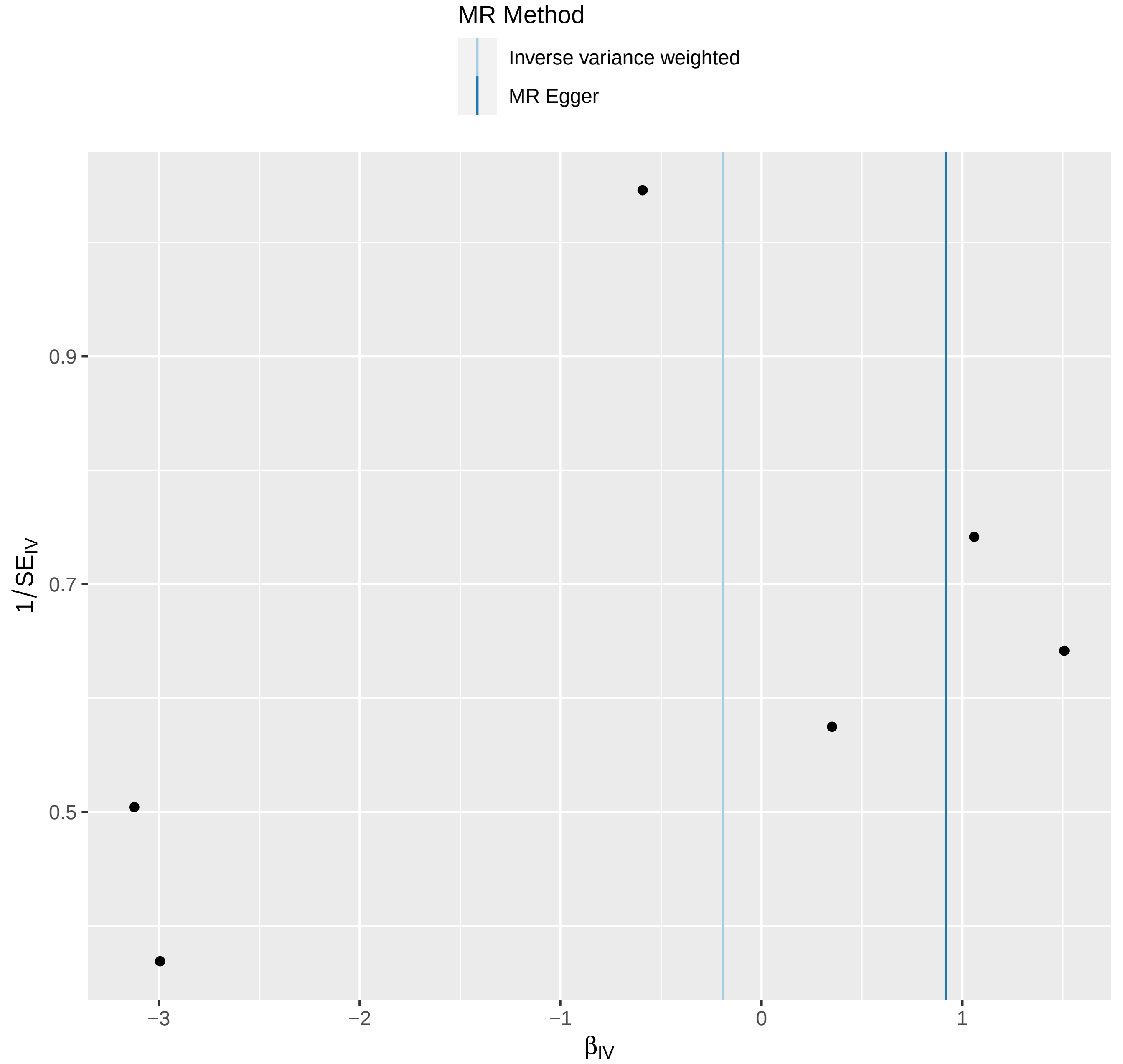

Batch 239 : 4−hydroxyhippurate on Crohn's disease of colon

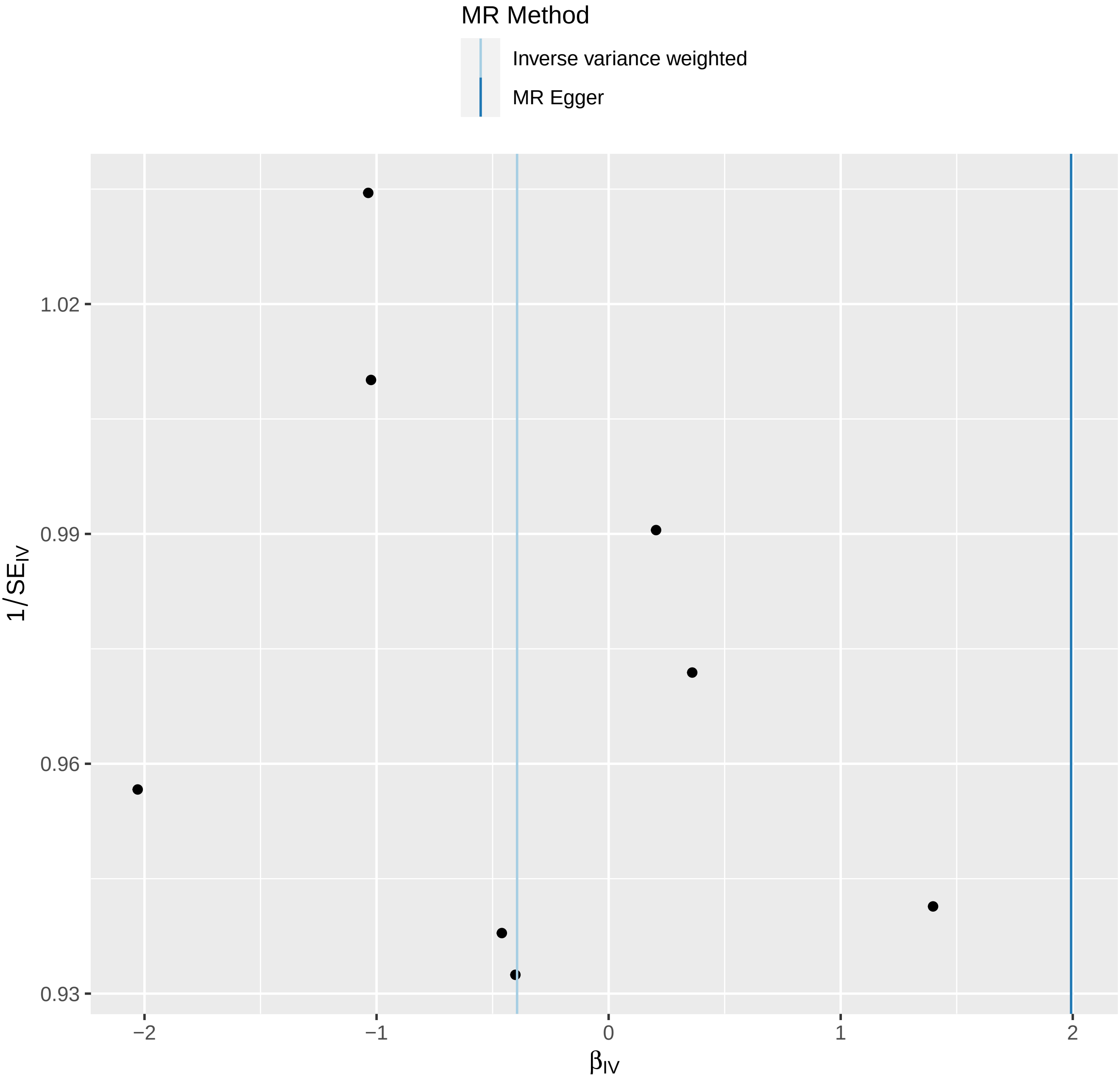

Batch 240 : 1−myristoylglycerophosphocholine on Crohn's disease of colon

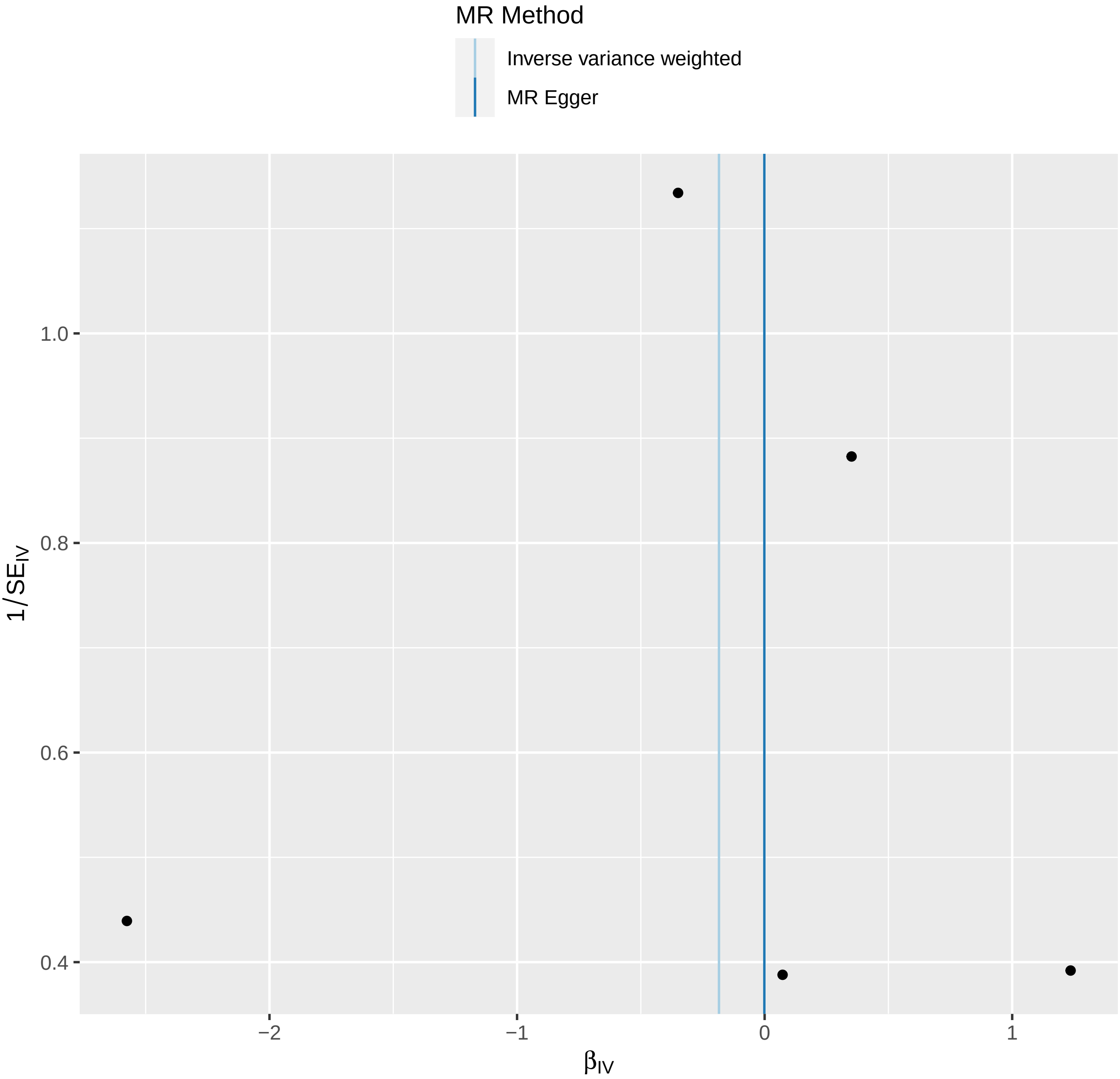

Batch 243 : 3−(3−hydroxyphenyl)propionate on Crohn's disease of colon

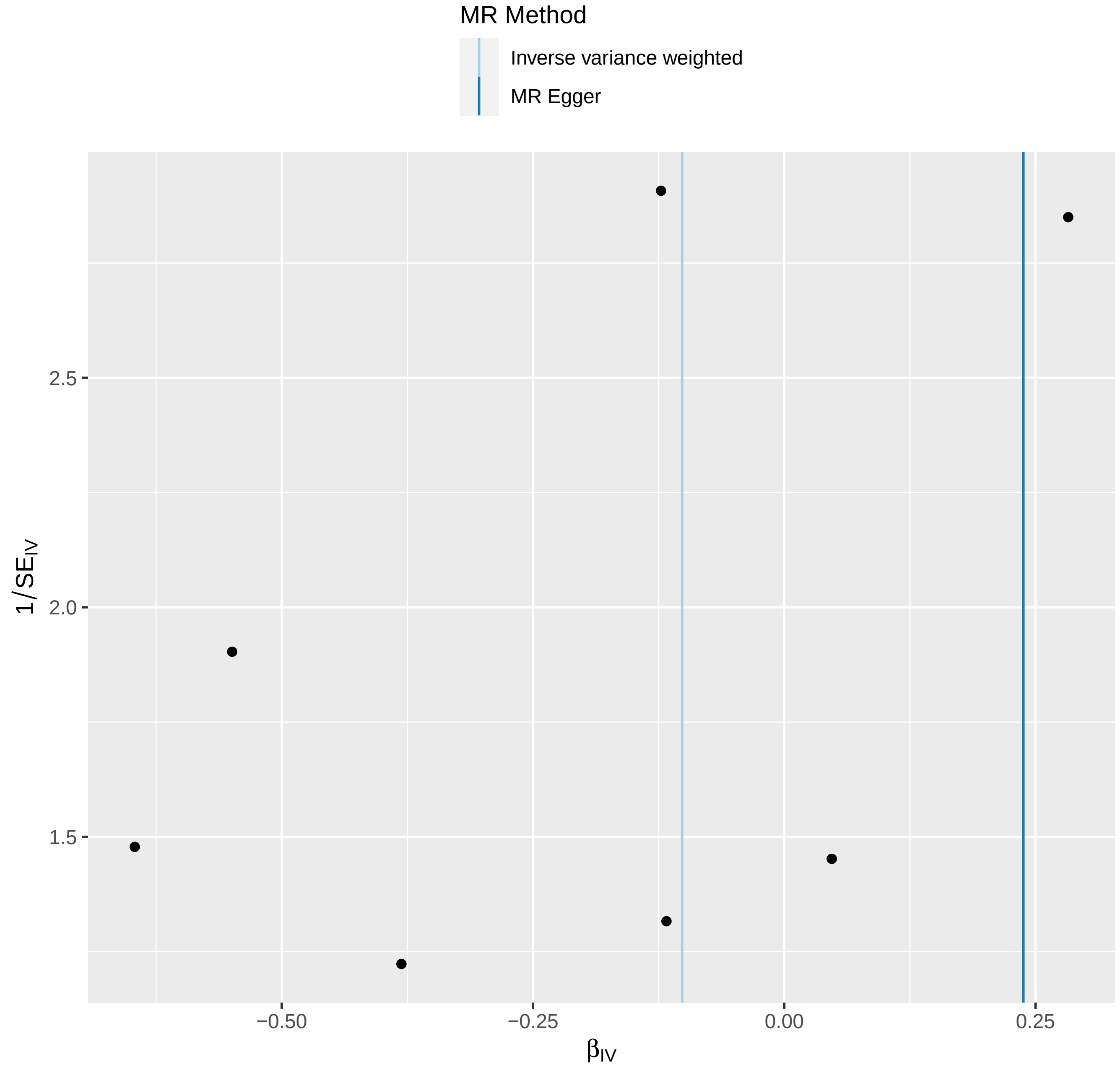

Batch 247 : Dihomo−linolenate (20:3n3 or n6) on Crohn's disease of colon

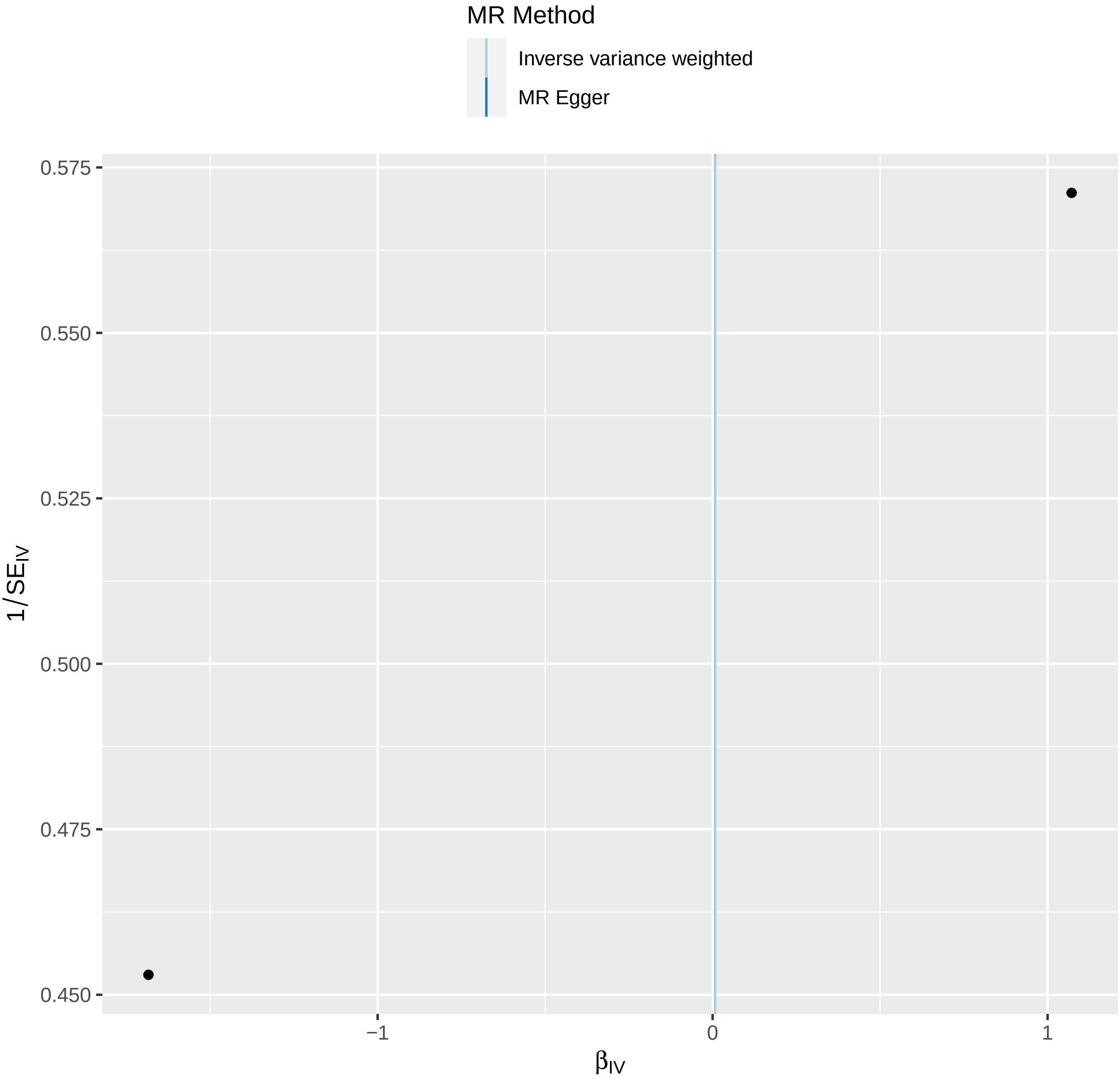

Batch 248 : Threitol on Crohn's disease of colon

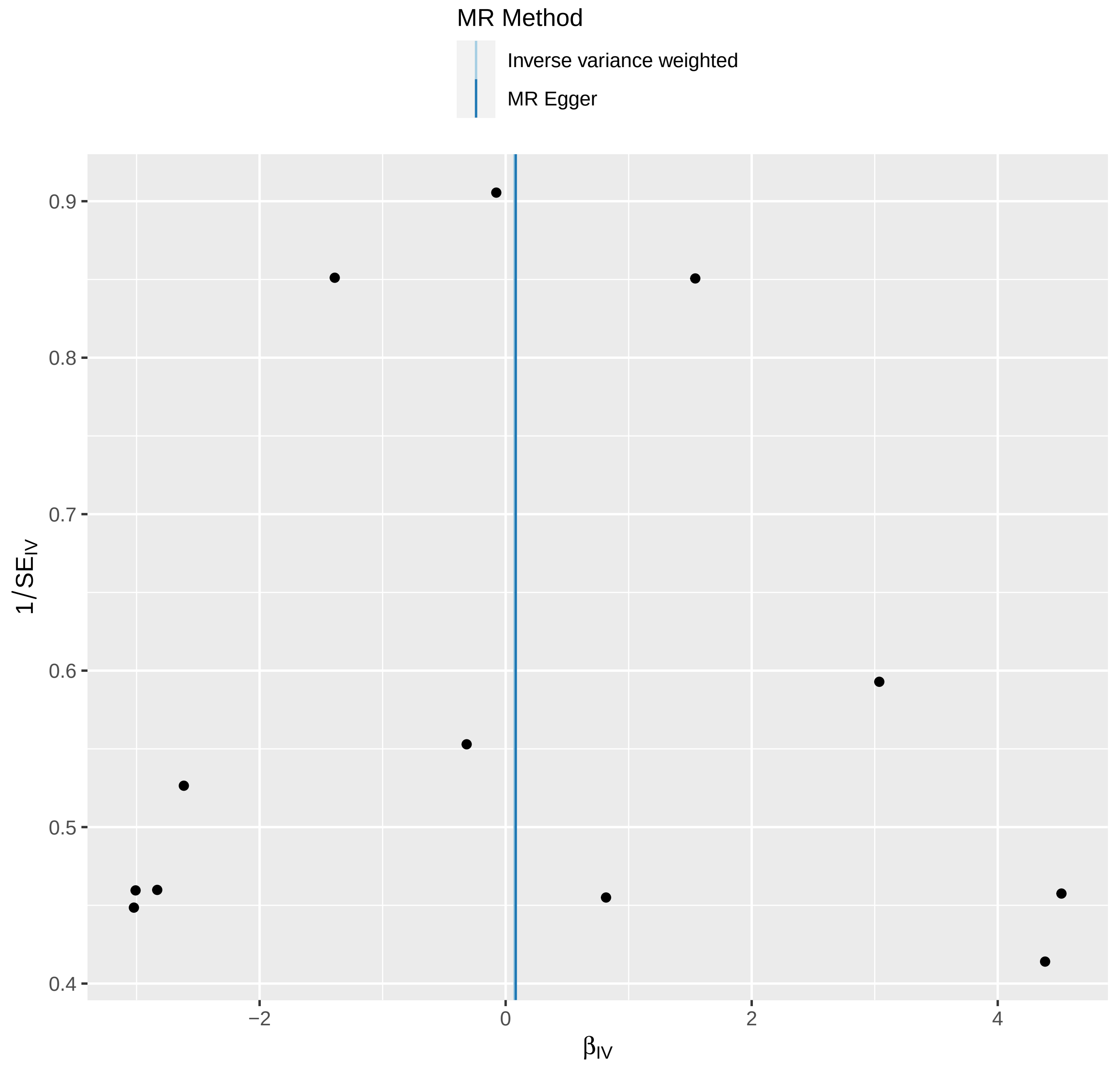

Batch 249 : P−cresol sulfate on Crohn's disease of colon

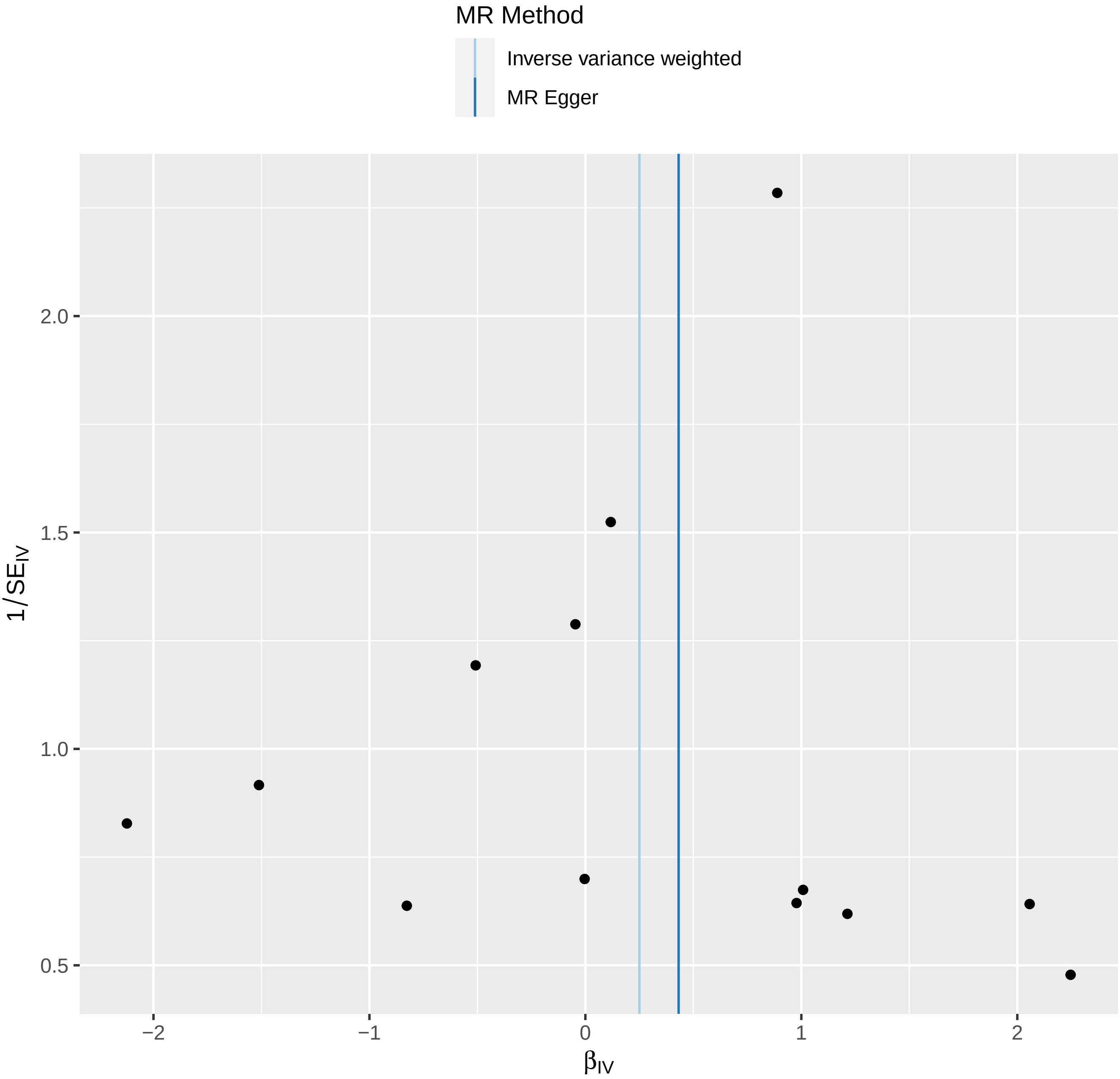

Batch 252 : X−14208−−phenylalanylserine on Crohn's disease of colon

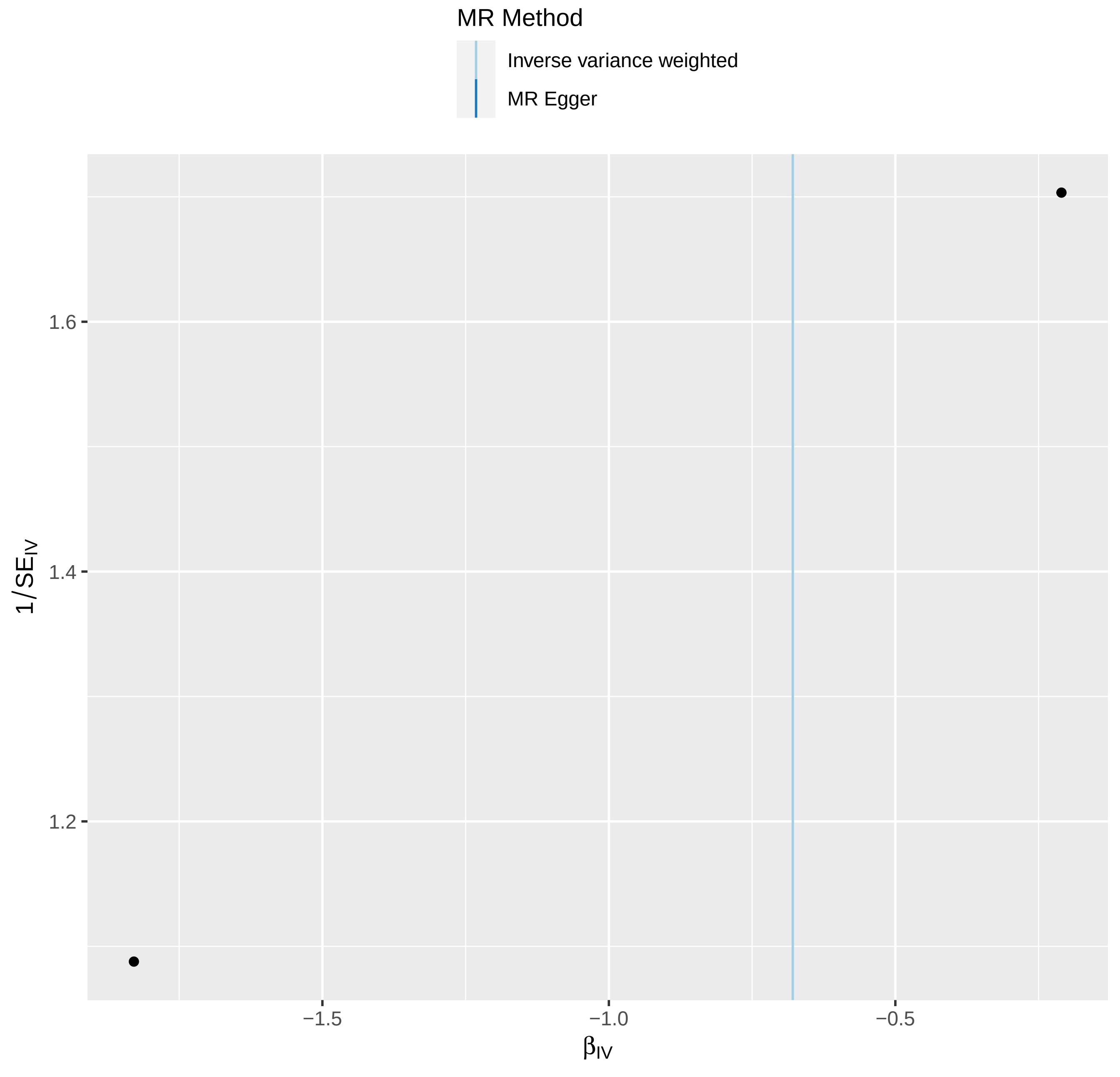

Batch 254 : X−14450−−phenylalanylleucine on Crohn's disease of colon

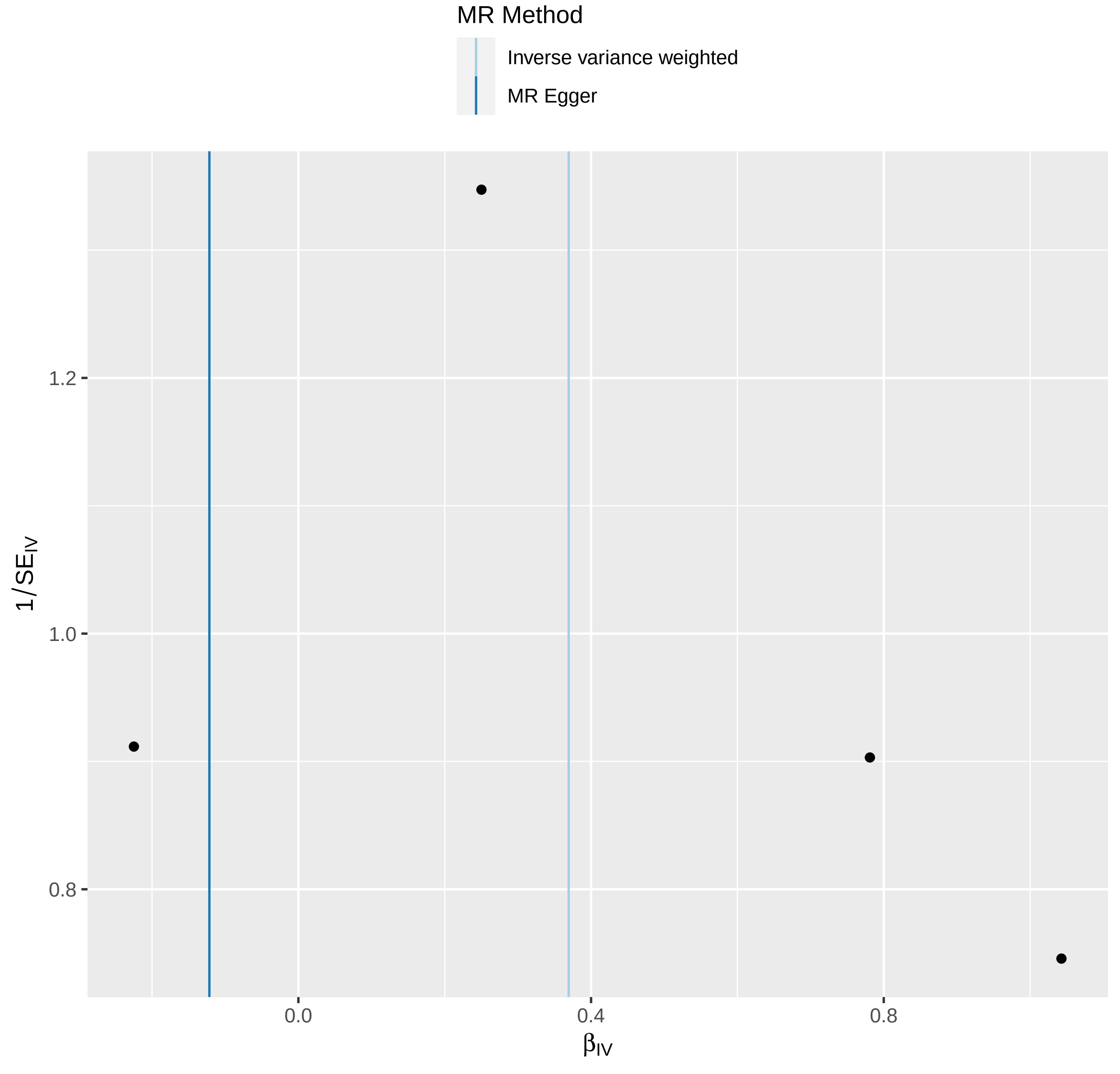

Batch 255 : Gamma−glutamylglutamate on Crohn's disease of colon

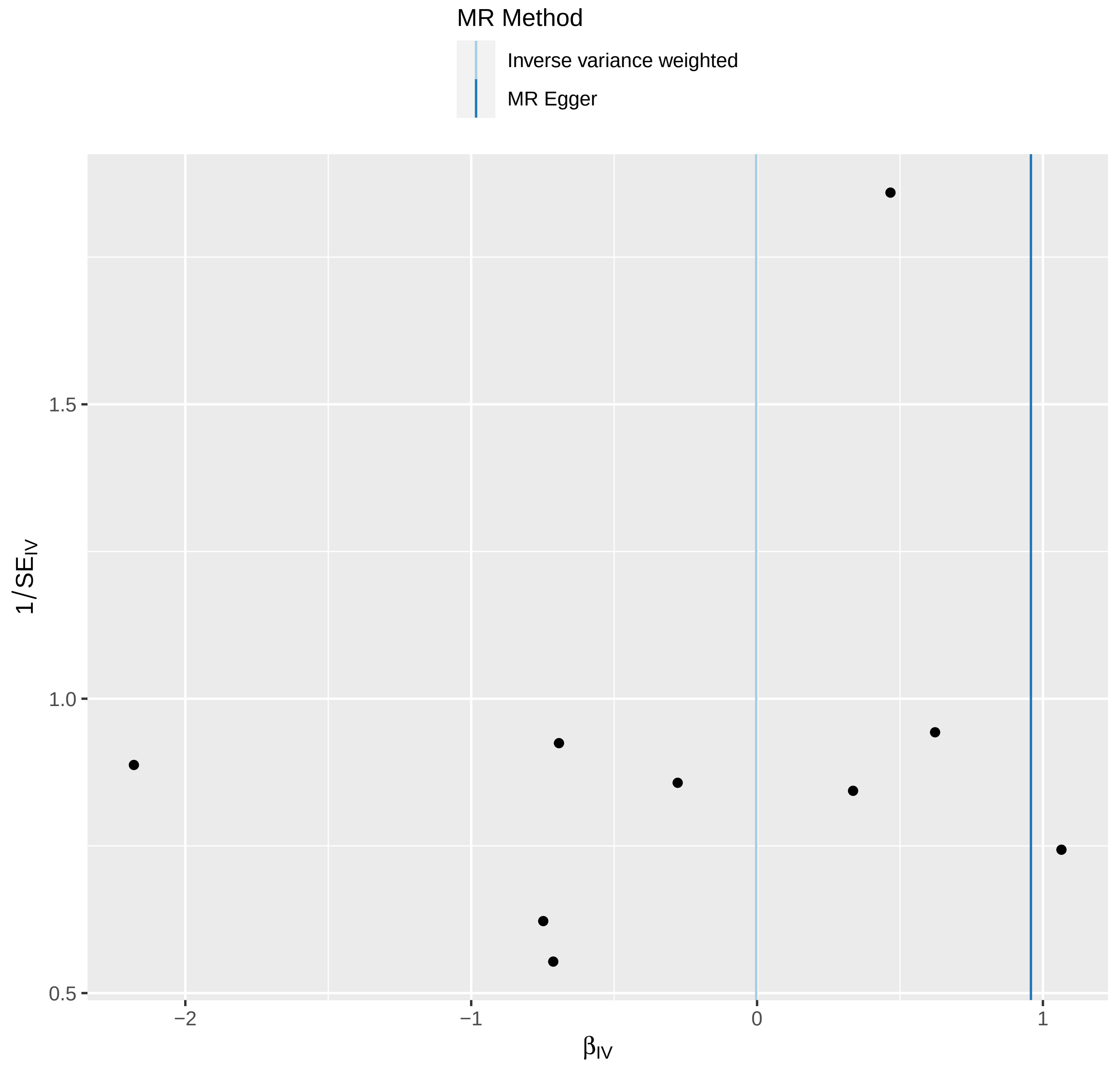

Batch 256 : Octadecanedioate on Crohn's disease of colon

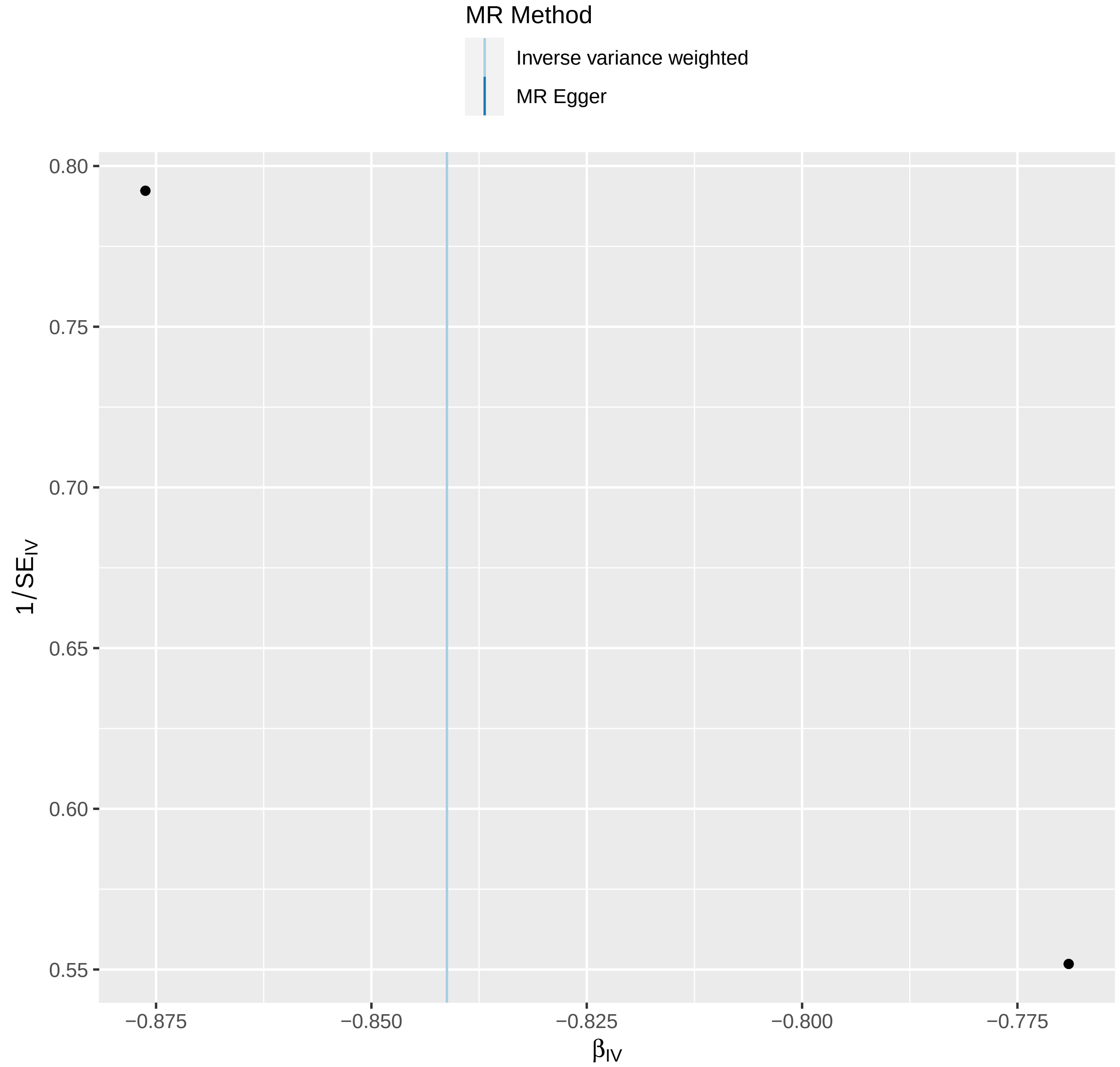

Batch 257 : Leucylleucine on Crohn's disease of colon

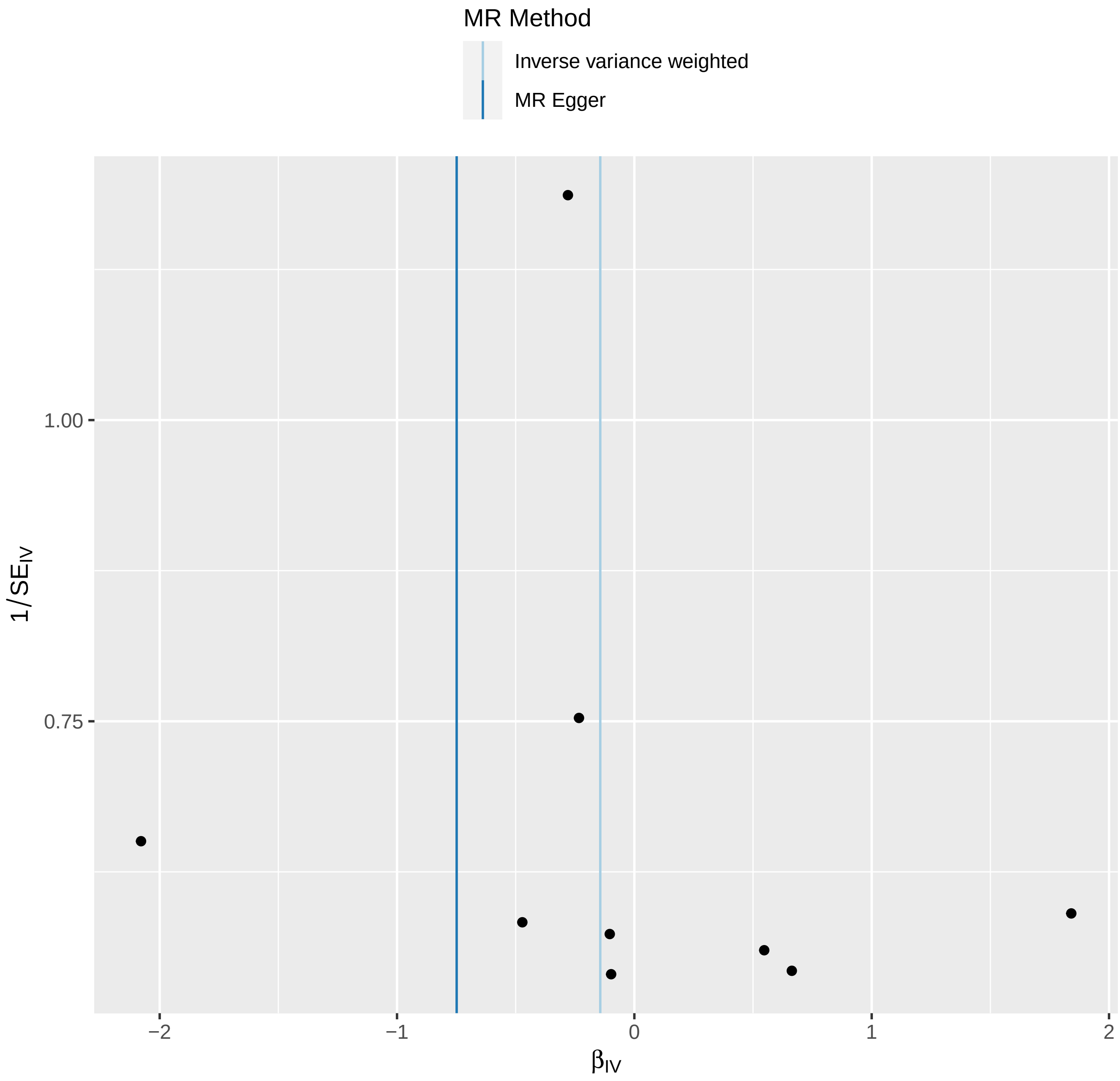

Batch 258 : 7−alpha−hydroxy−3−oxo−4−cholestenoate (7−Hoca) on Crohn's disease of colon

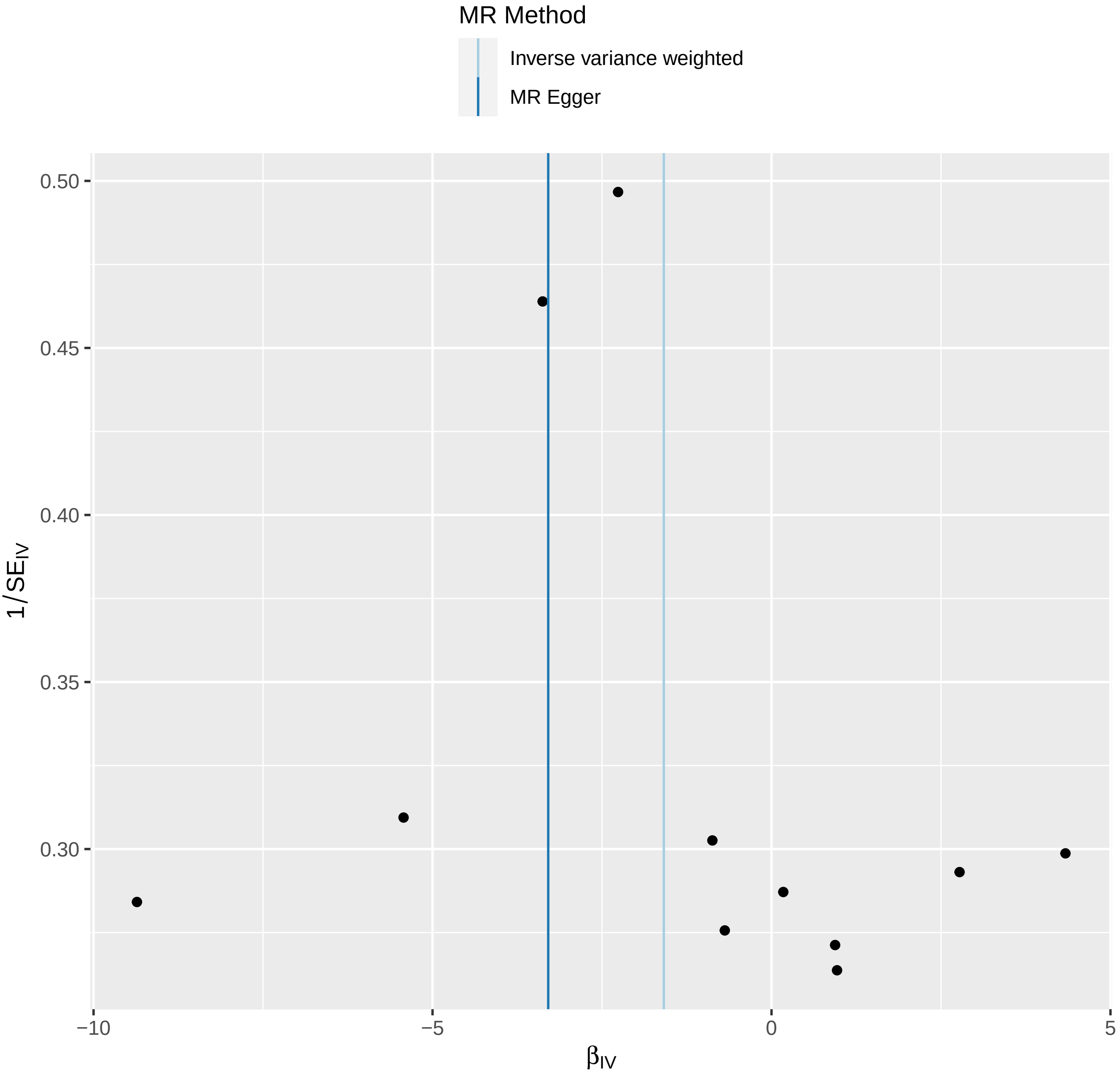

Batch 259 : N−Butyl Oleate on Crohn's disease of colon

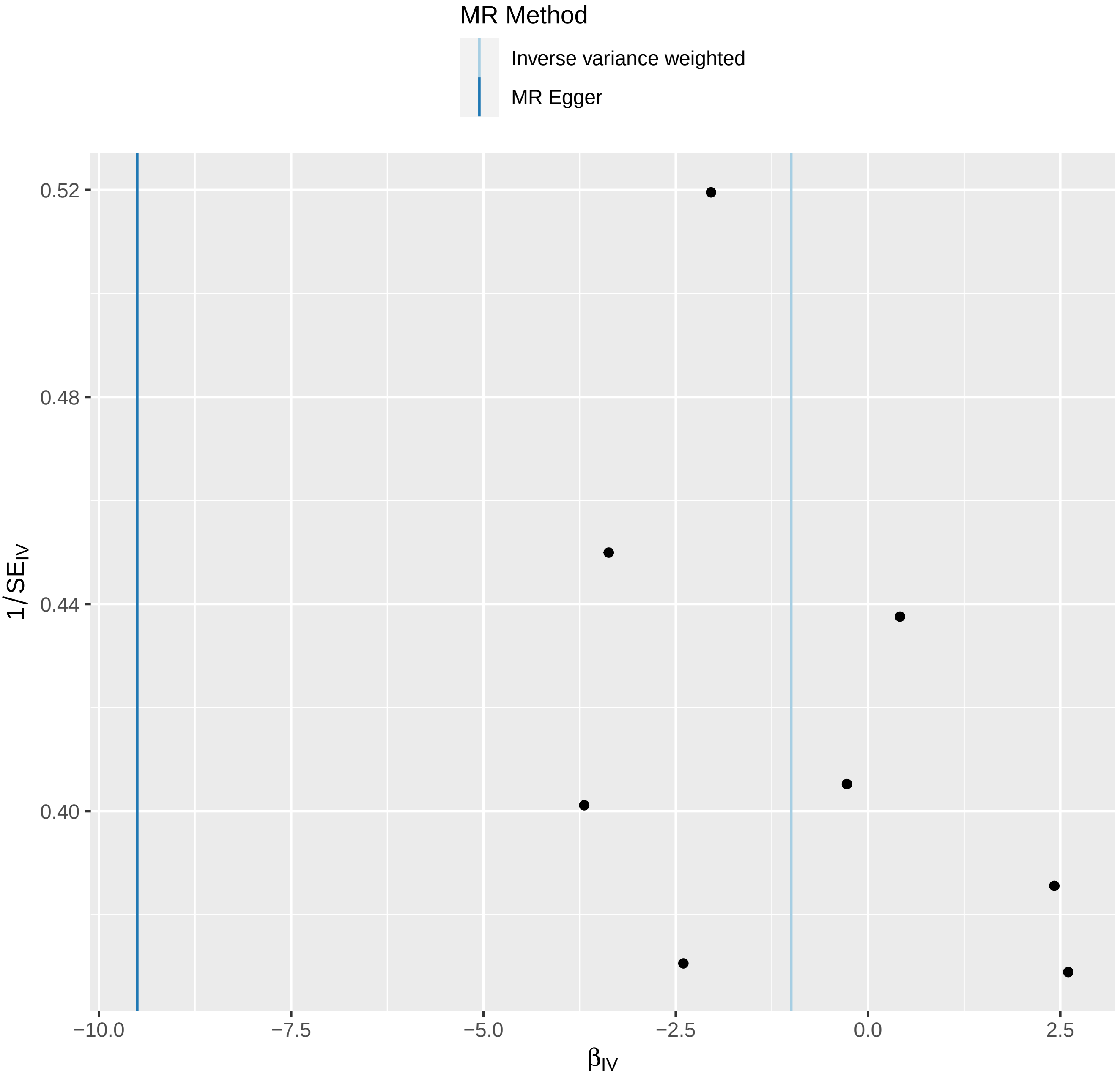

Batch 261 : Taurolithocholate 3−sulfate on Crohn's disease of colon

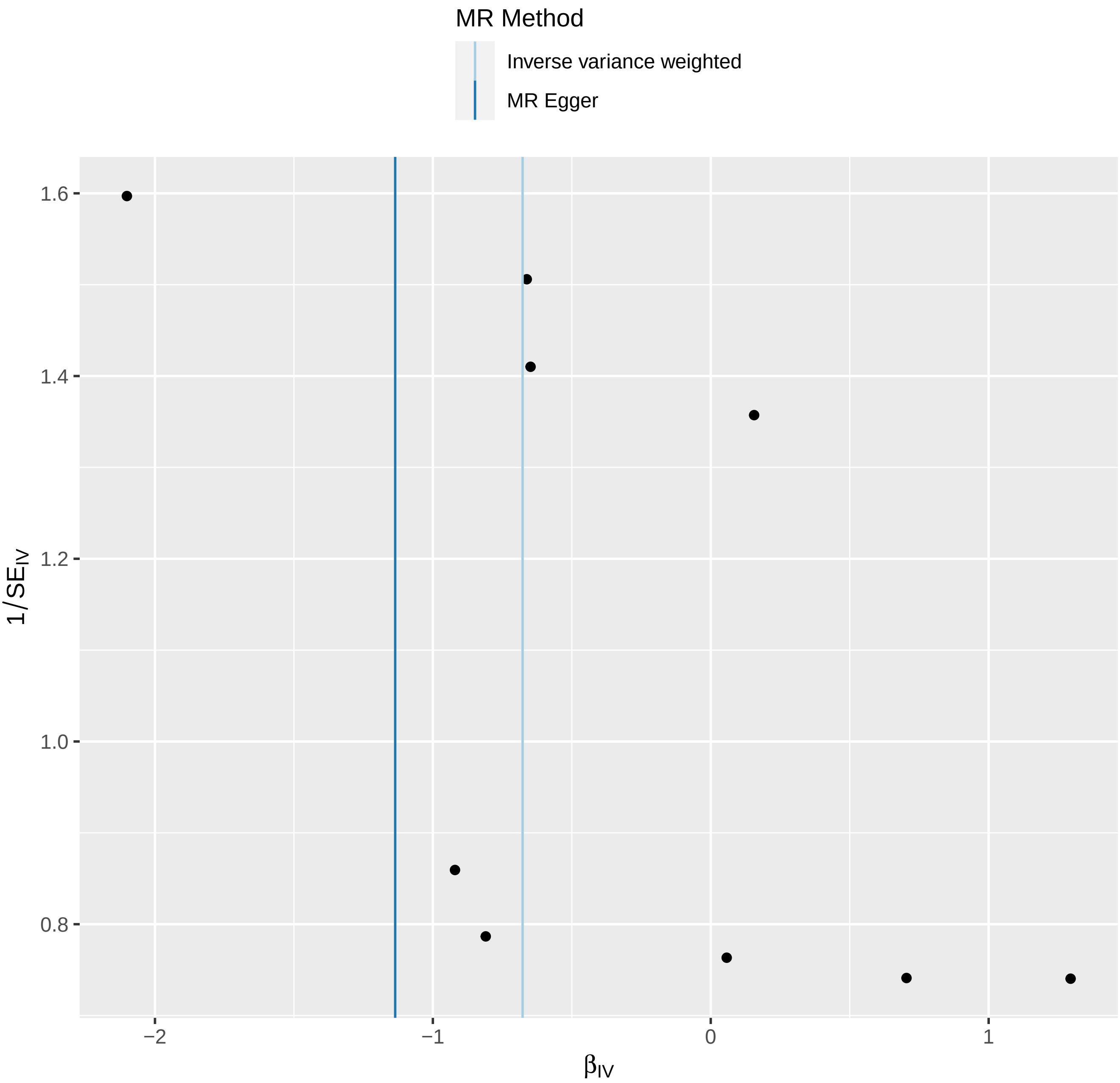

Batch 262 : X−14977−−vanillin on Crohn's disease of colon

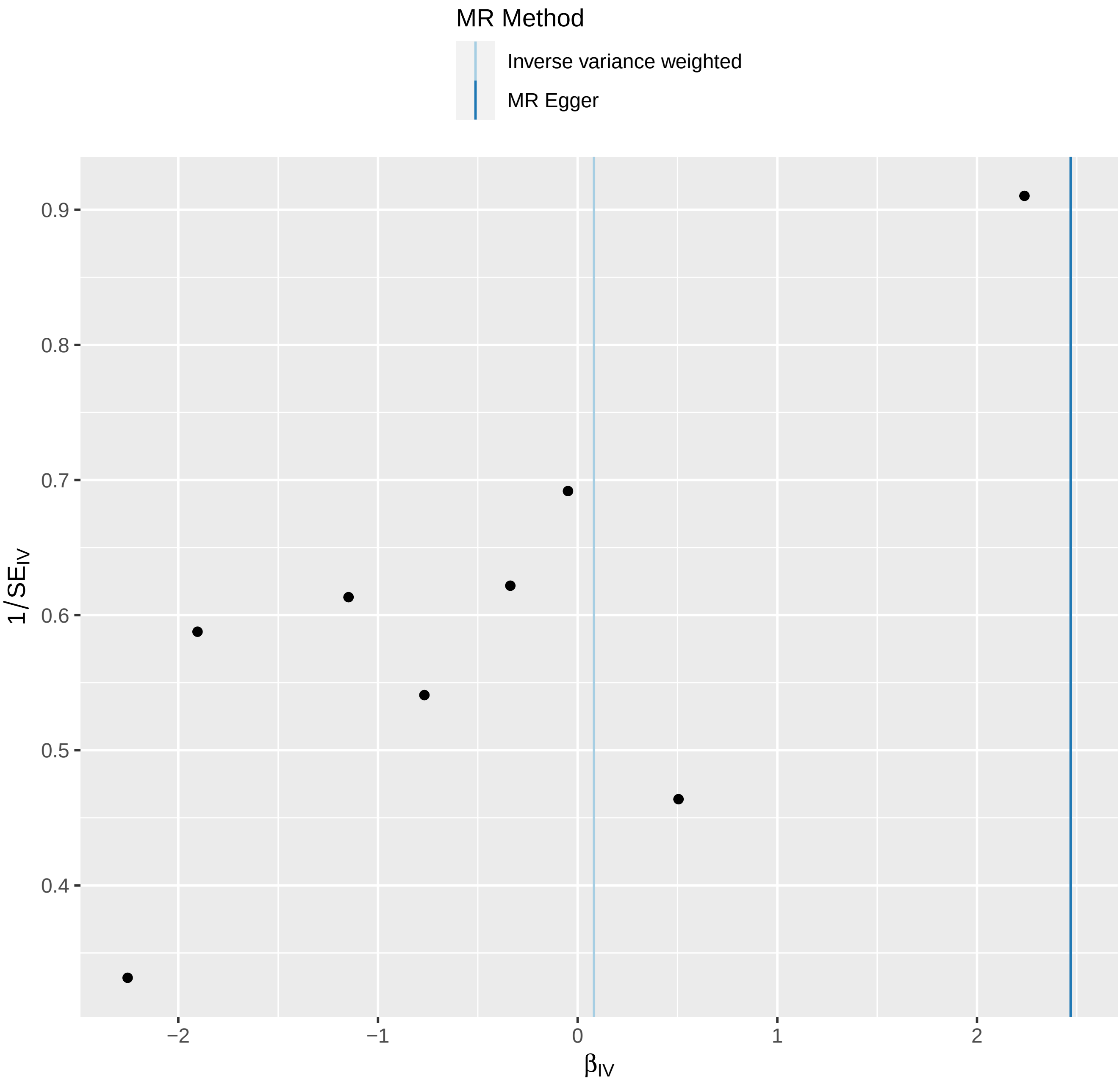

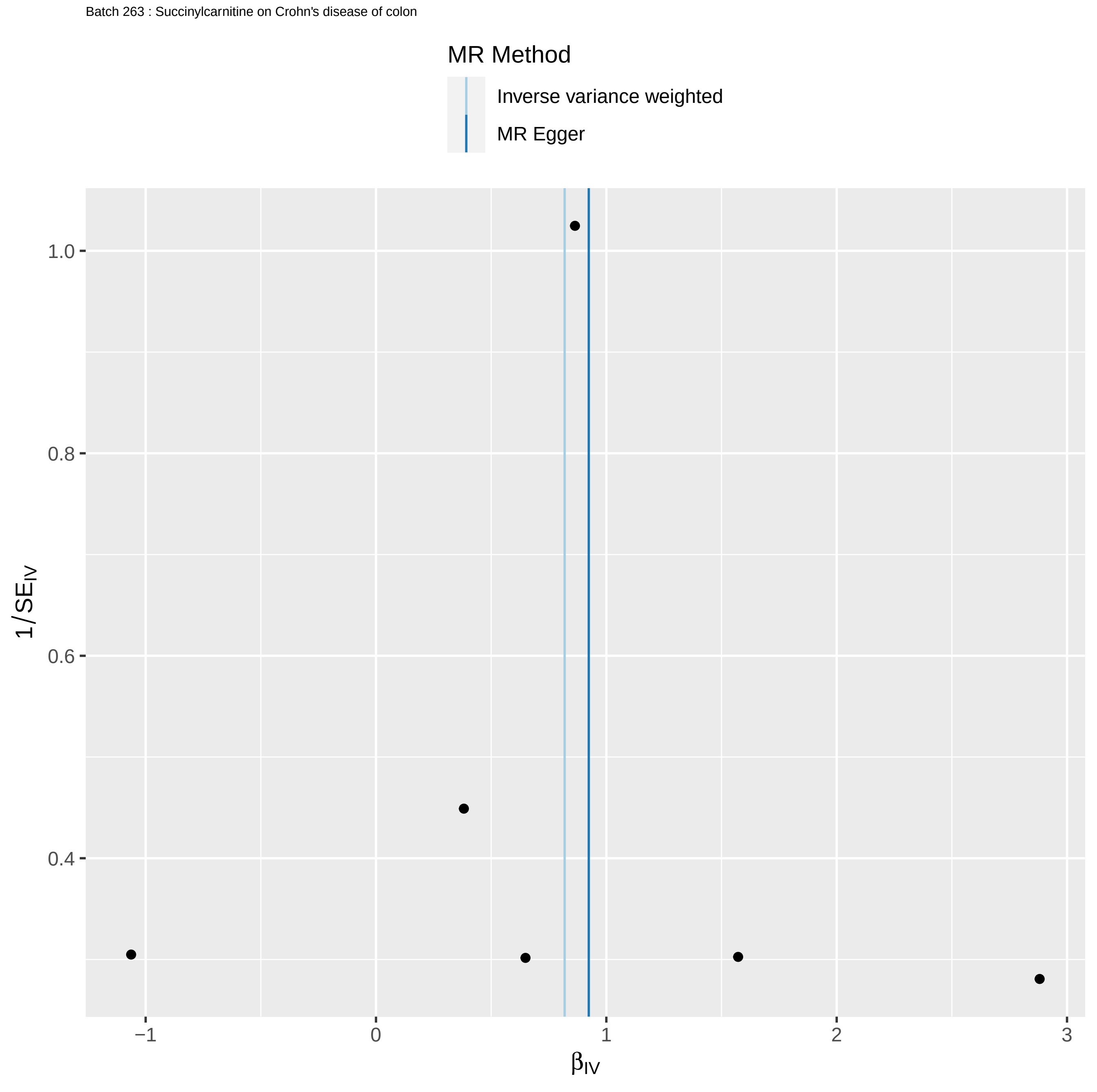

Batch 263 : Succinylcarnitine on Crohn's disease of colon
MR Method
Inverse variance weighted
MR Egger
1.0
0.8
0.6
0.4
1/SE_IV
−1
0
1
2
3
β_IV

Batch 265 : Cyclo(leu−pro) on Crohn's disease of colon

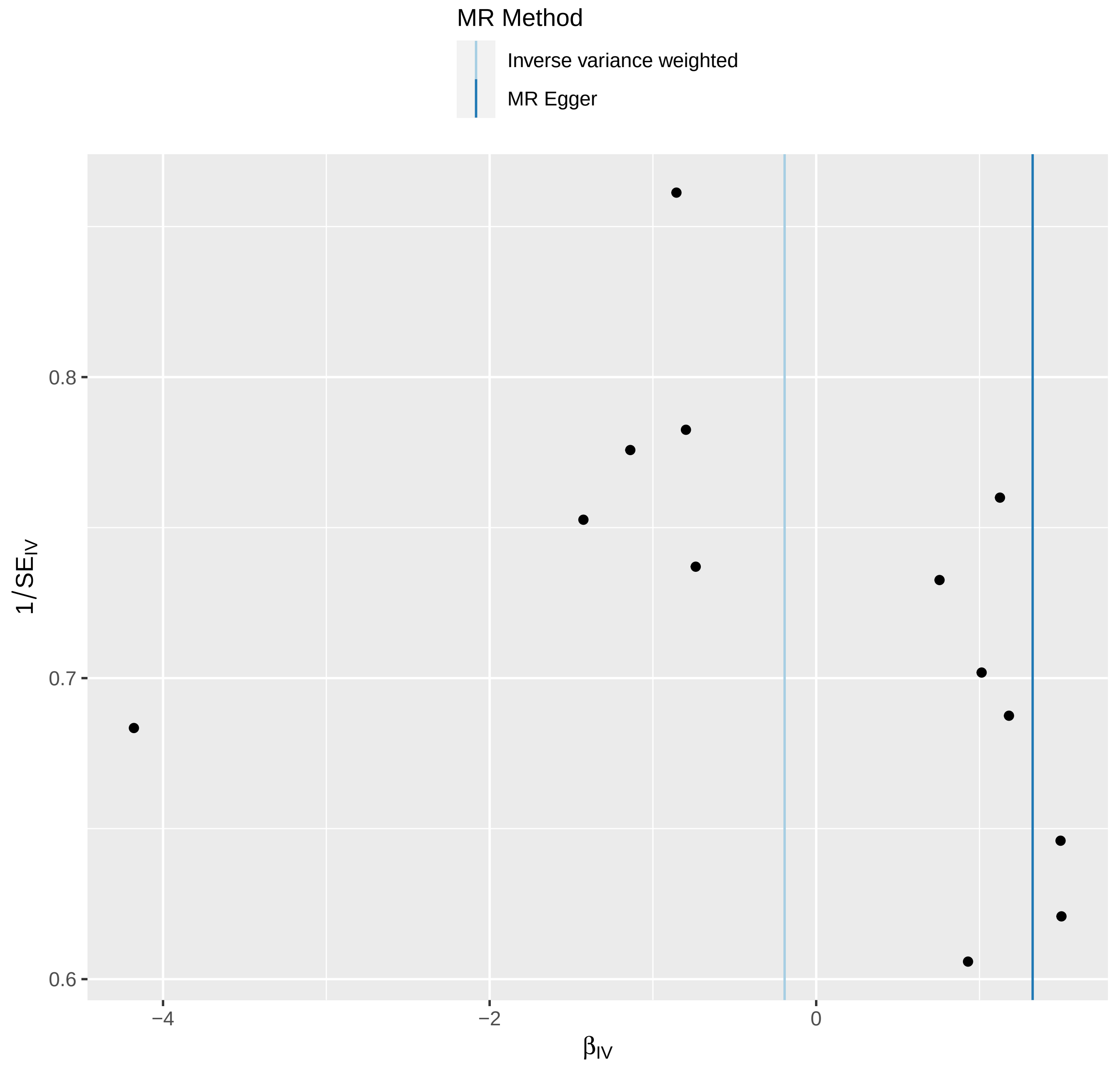

Batch 266 : Chiro−inositol on Crohn's disease of colon

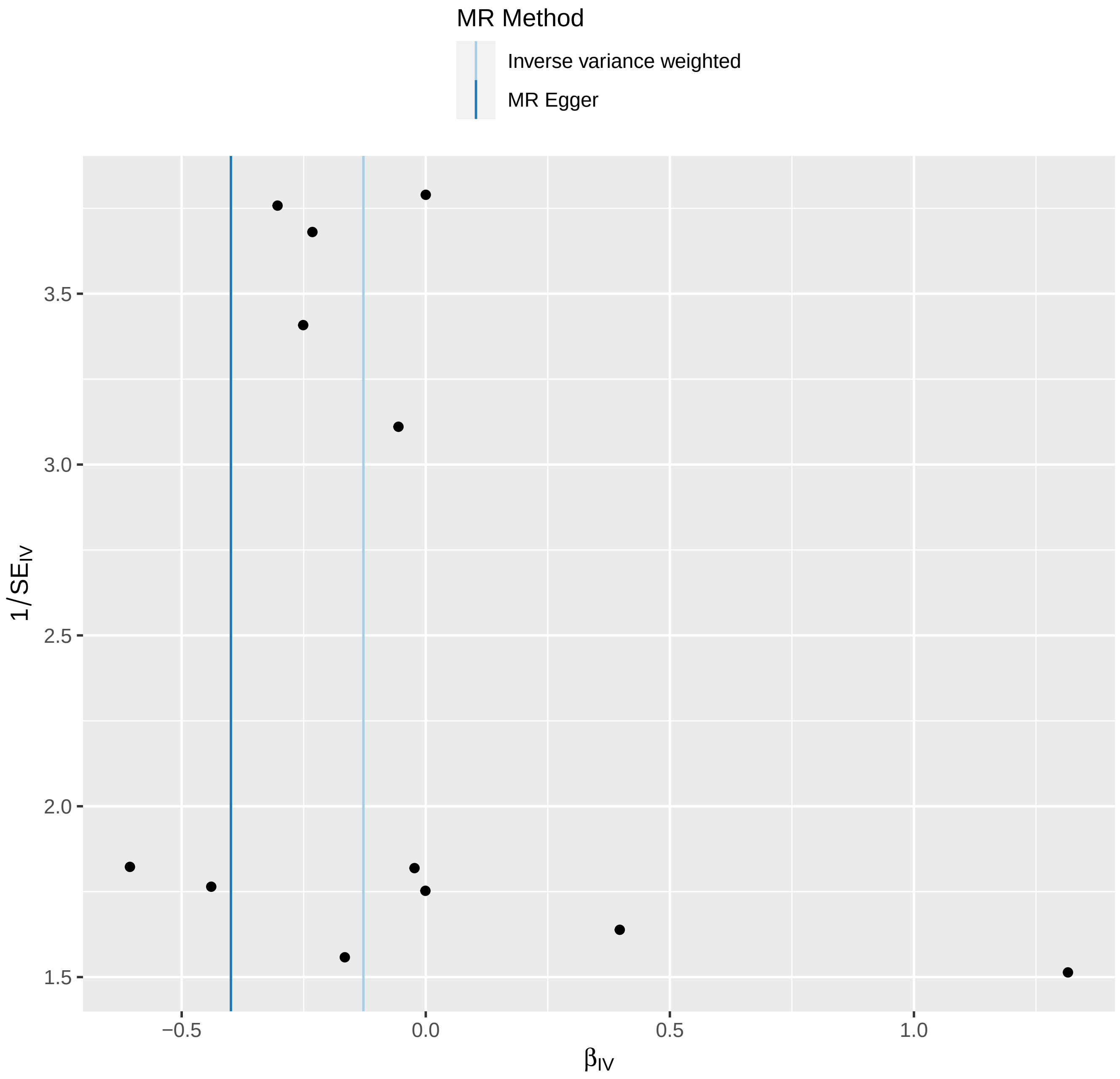

Batch 270 : 2−hydroxyglutarate on Crohn's disease of colon

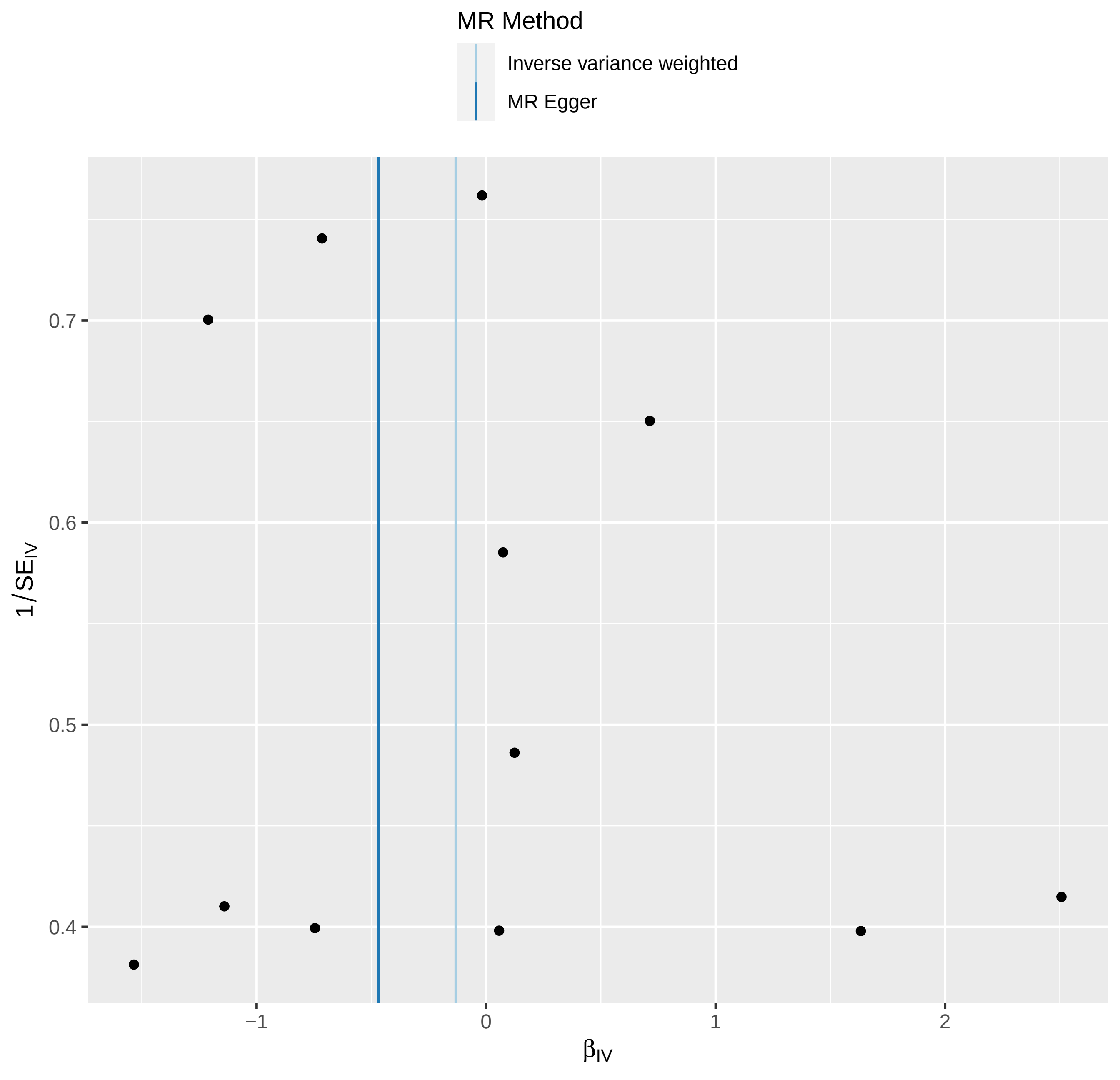

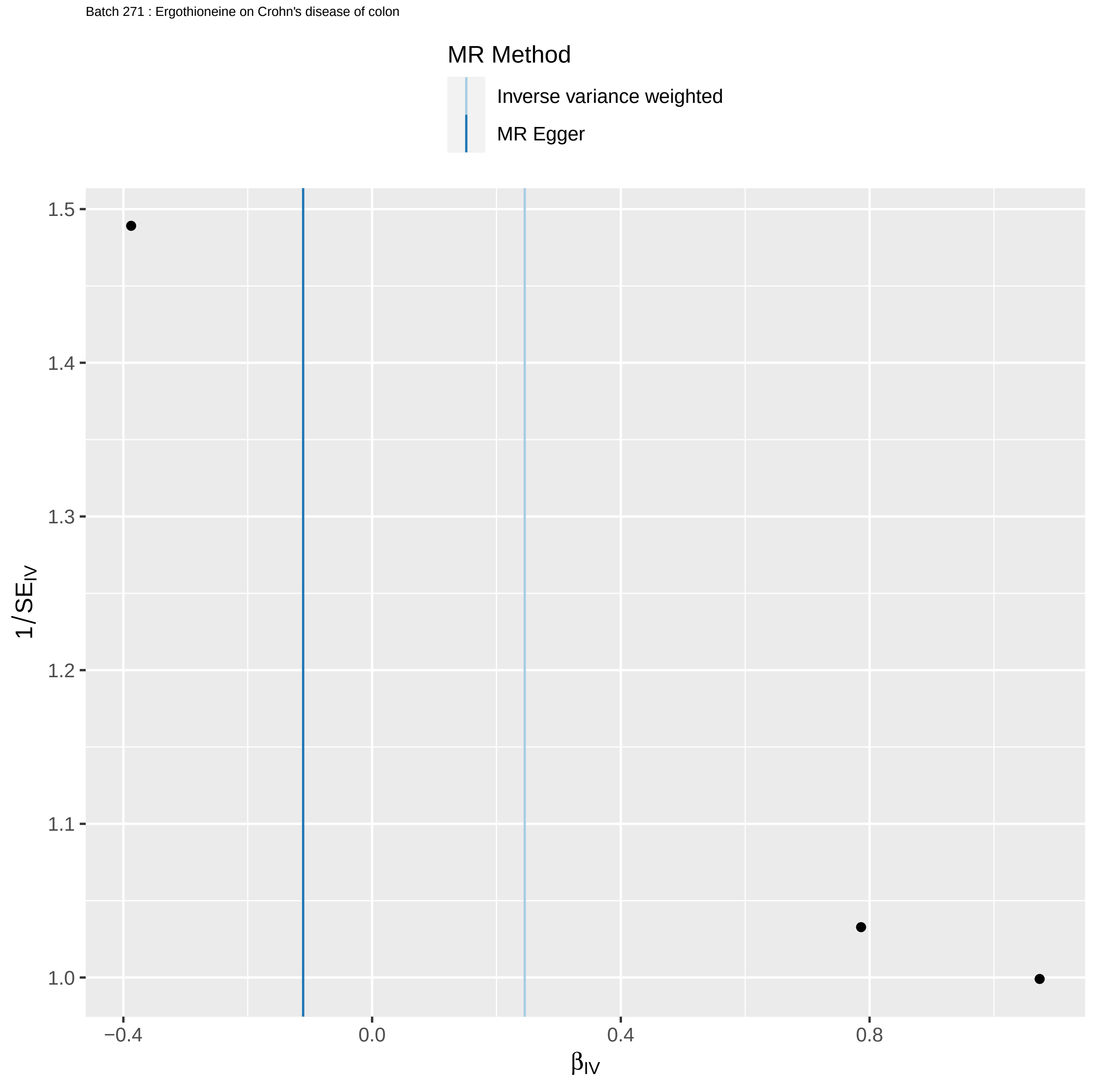
Batch 271 : Ergothioneine on Crohn's disease of colon
MR Method
Inverse variance weighted
MR Egger
1.5
1.4
1.3
1.2
1.1
1.0
1/SE_IV
−0.4
0.0
0.4
0.8
β_IV

Batch 273 : Phenylalanylphenylalanine on Crohn's disease of colon

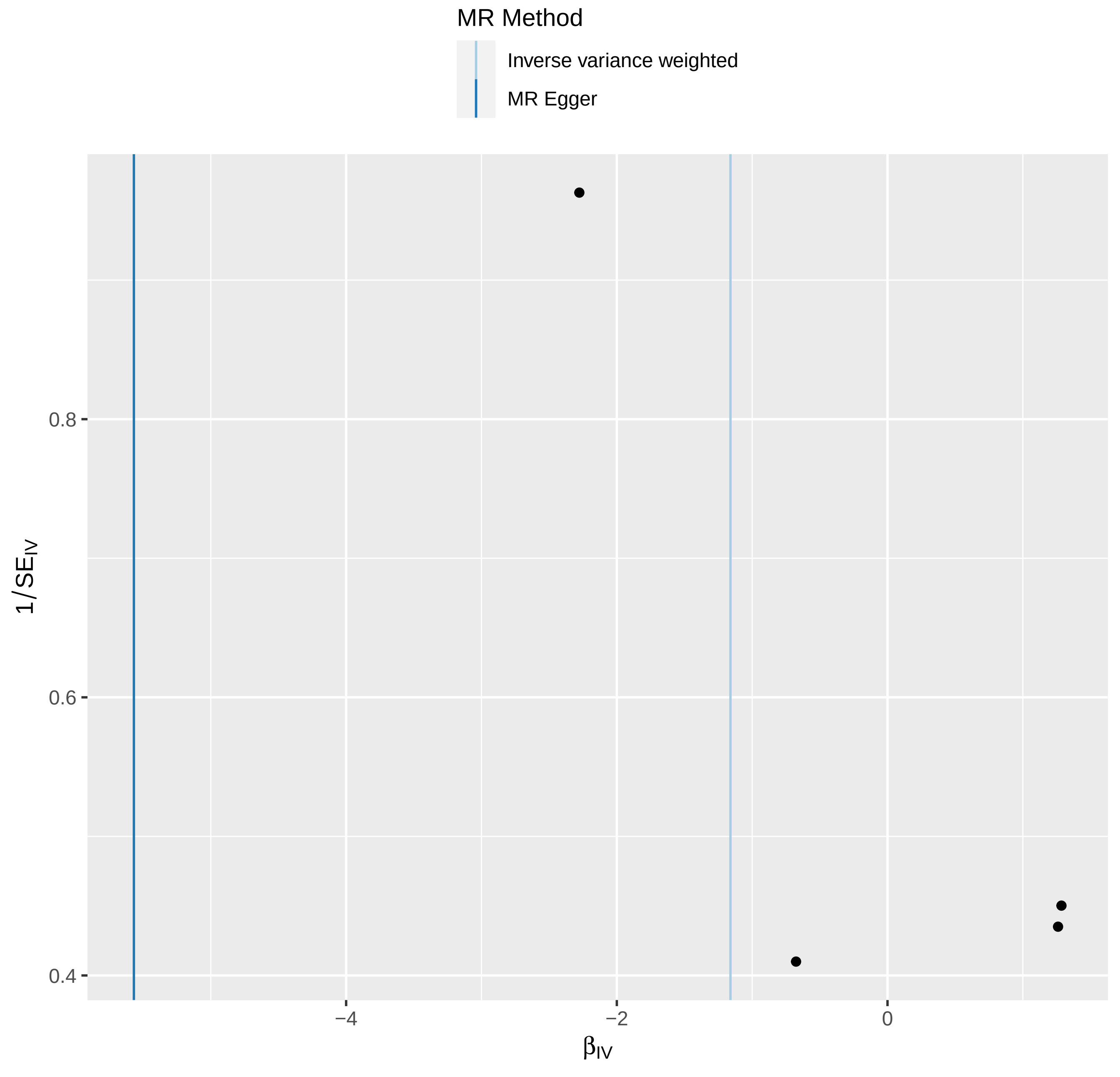

Batch 274 : Cis−4−decenoyl carnitine on Crohn's disease of colon

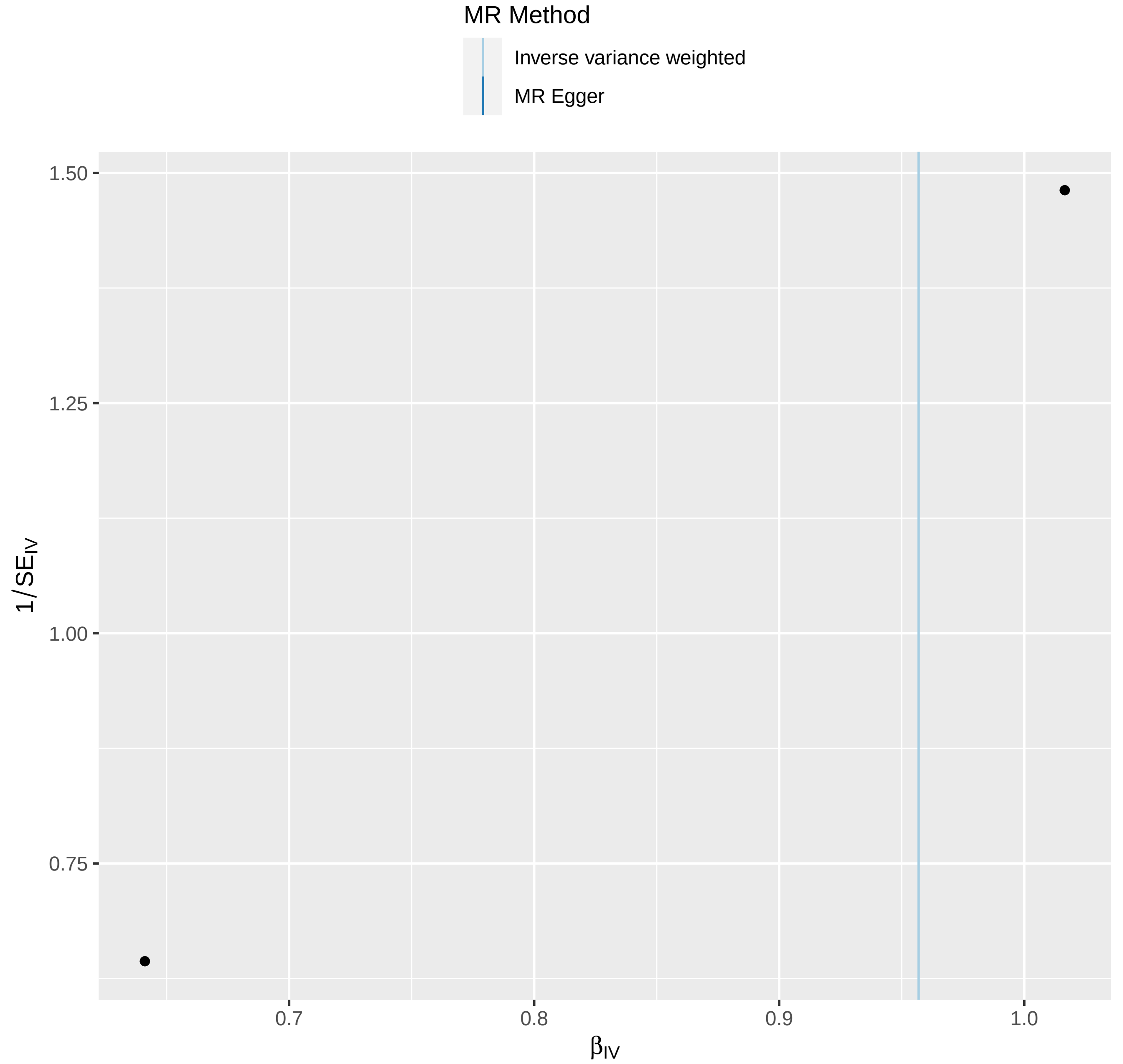

Batch 275 : 15−methylpalmitate (isobar with 2−methylpalmitate) on Crohn's disease of colon

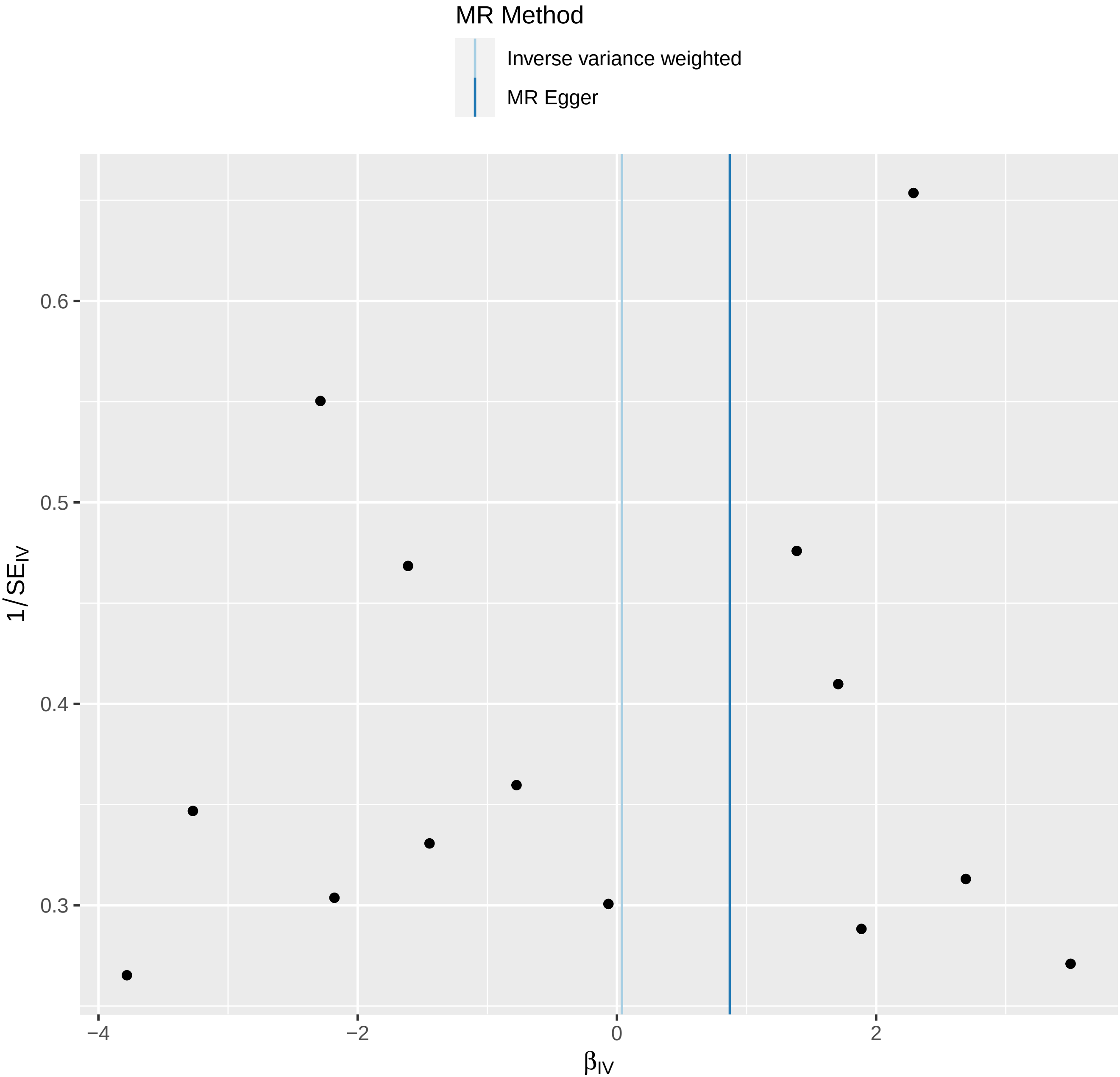

Batch 277 : Tryptophan on Crohn's disease of ileocolon

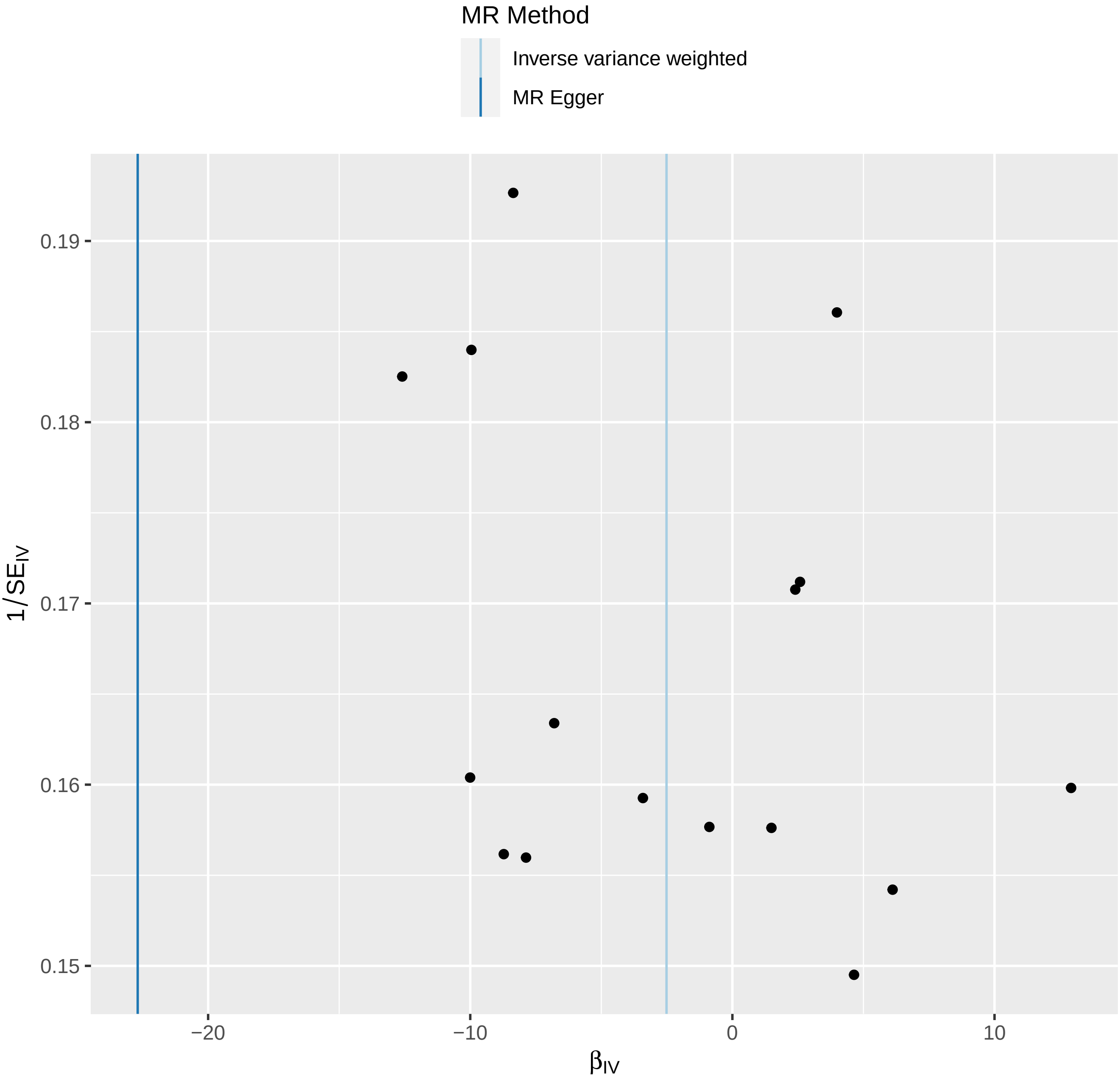

Batch 279 : Leucine on Crohn's disease of ileocolon

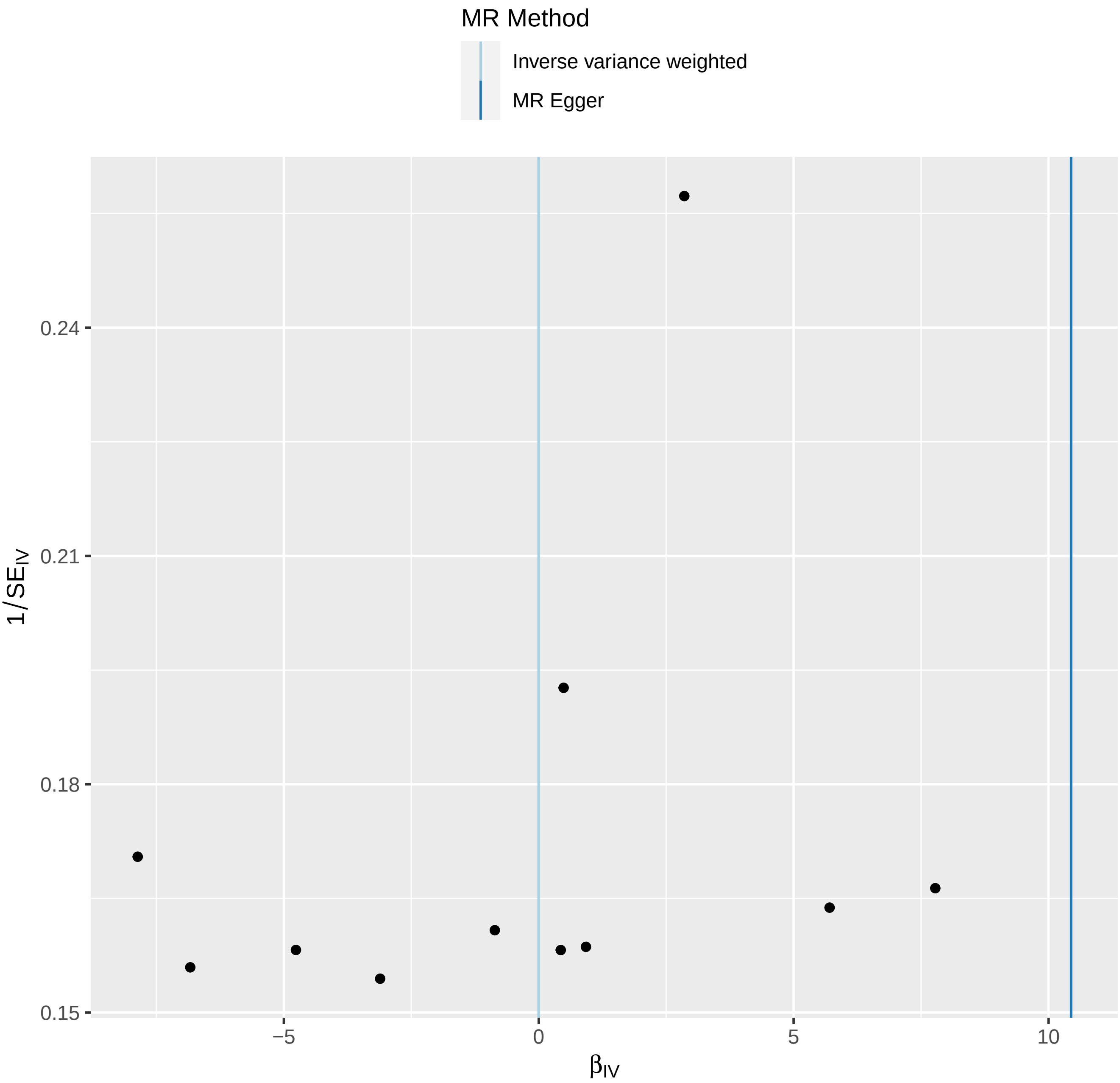

Batch 283 : Lactate on Crohn's disease of ileocolon

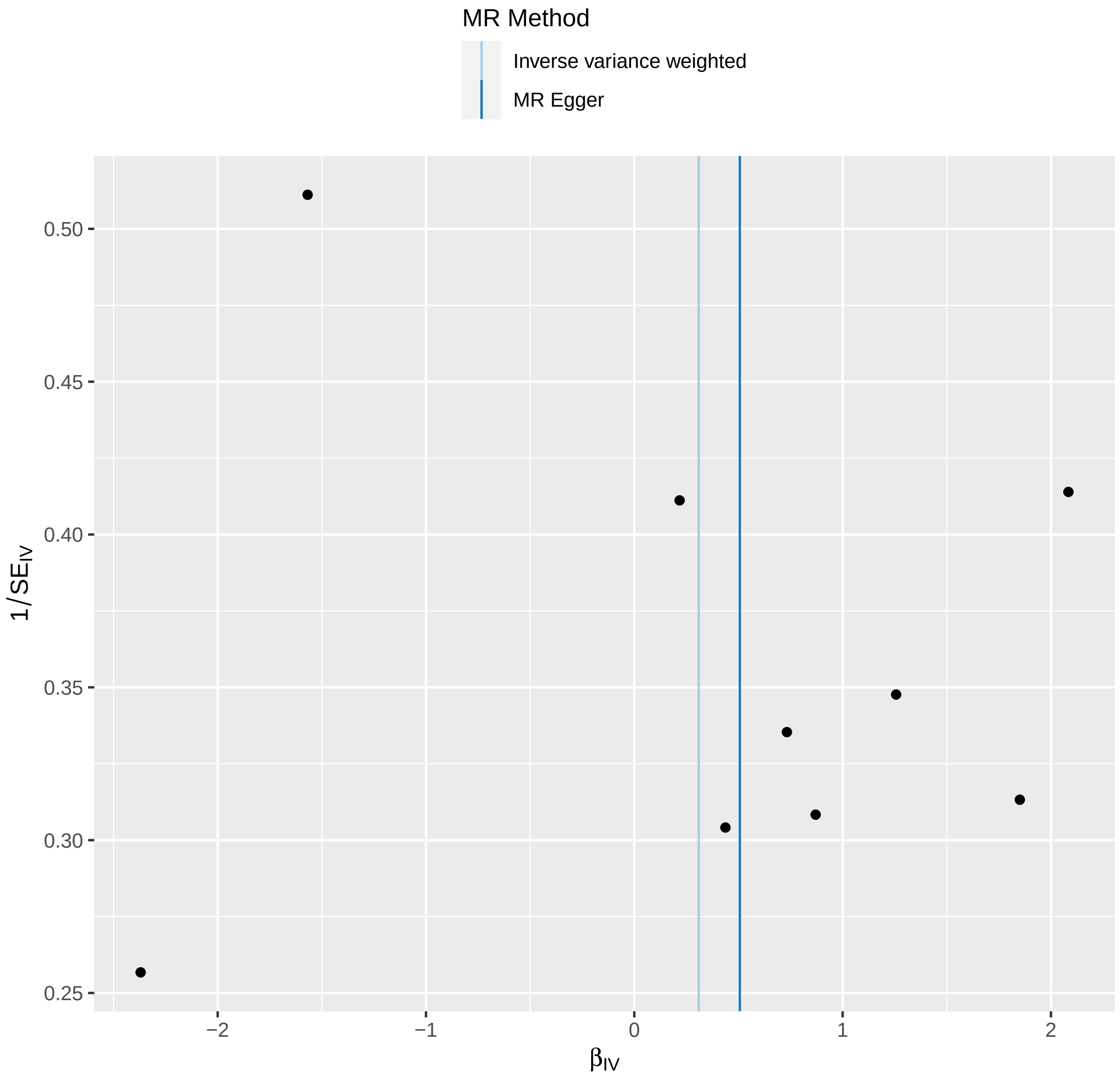

Batch 284 : 3−hydroxybutyrate (BHBA) on Crohn's disease of ileocolon

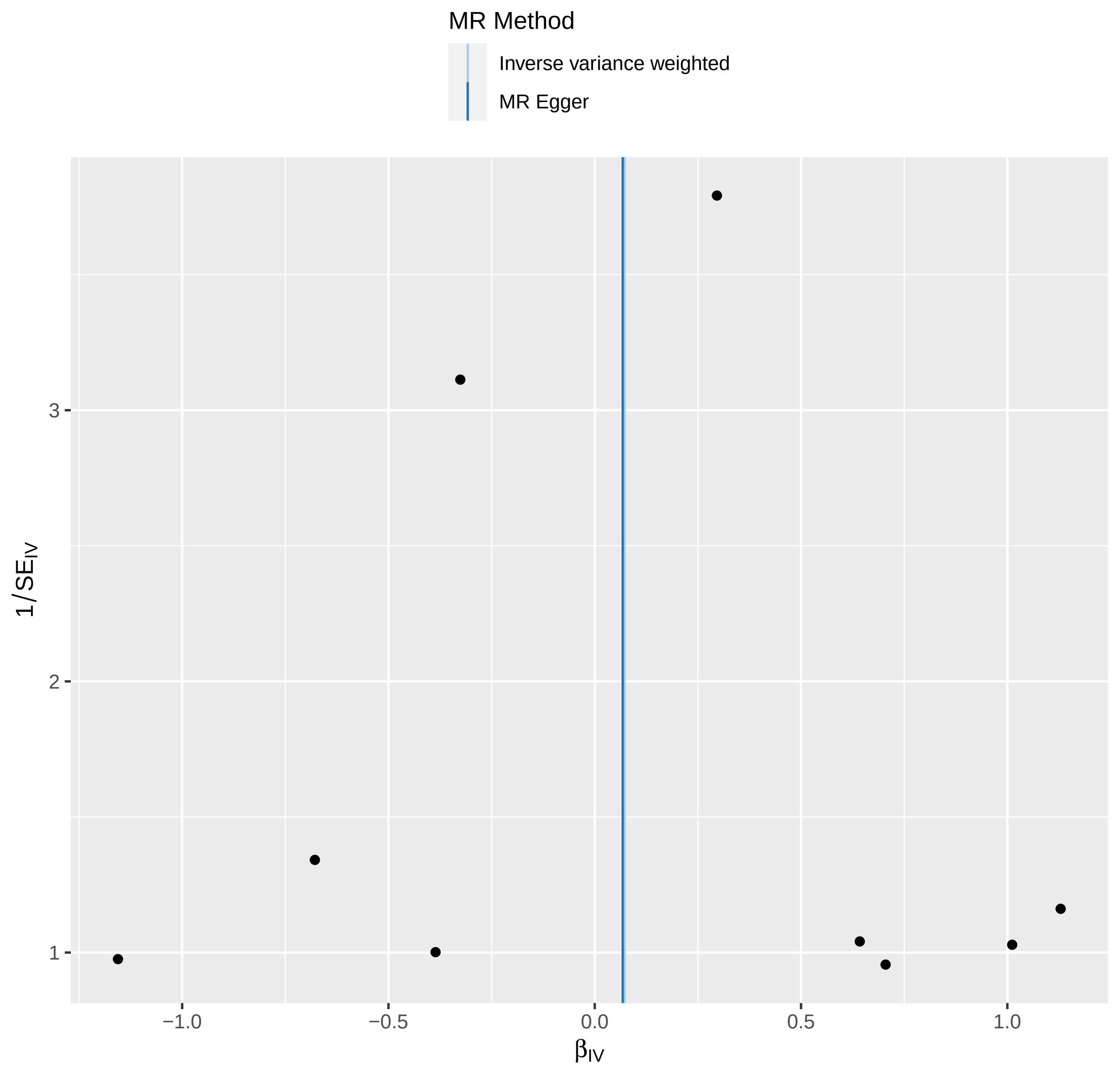

Batch 285 : Arabinose on Crohn's disease of ileocolon

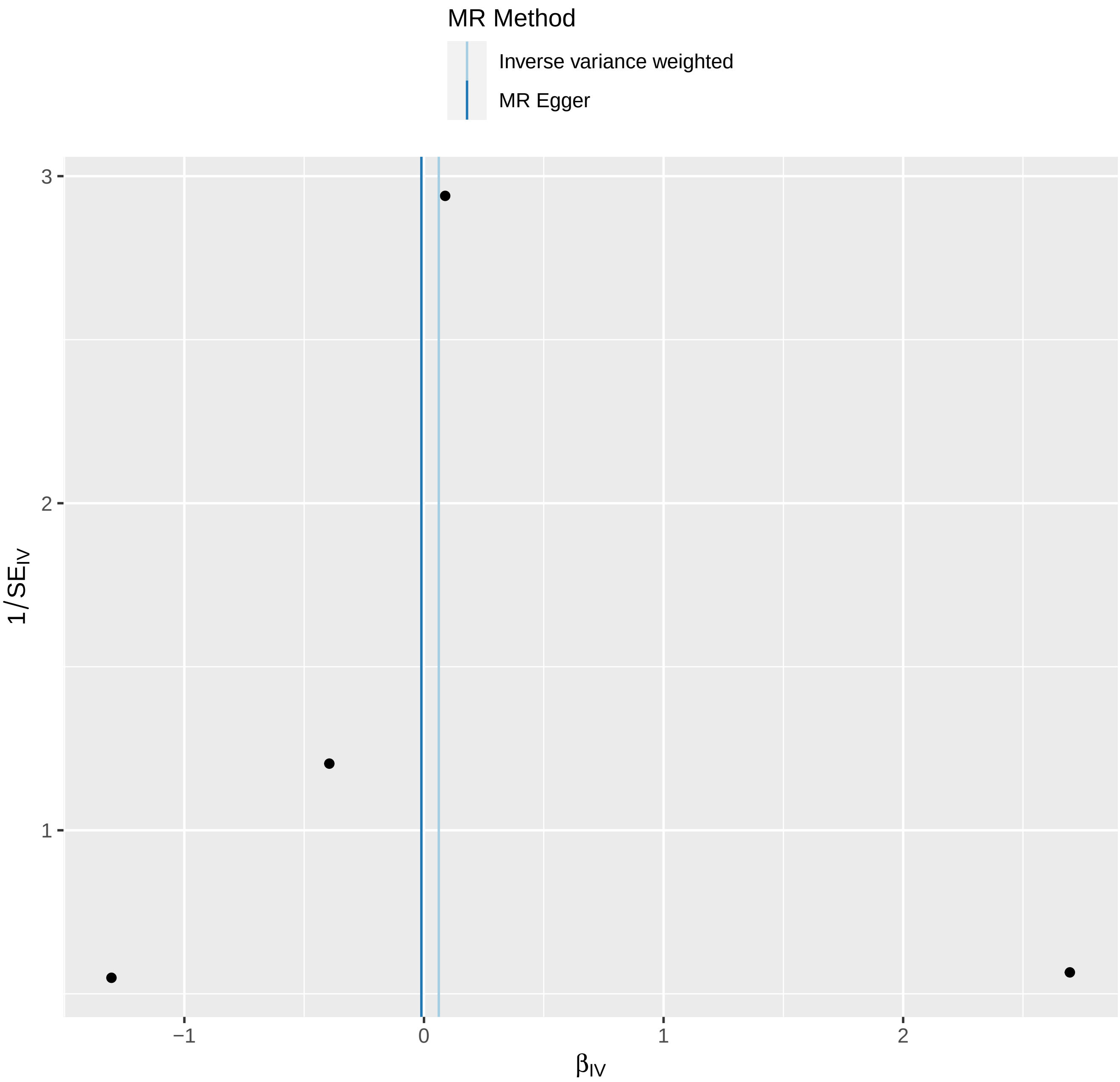

Batch 286 : Fructose on Crohn's disease of ileocolon

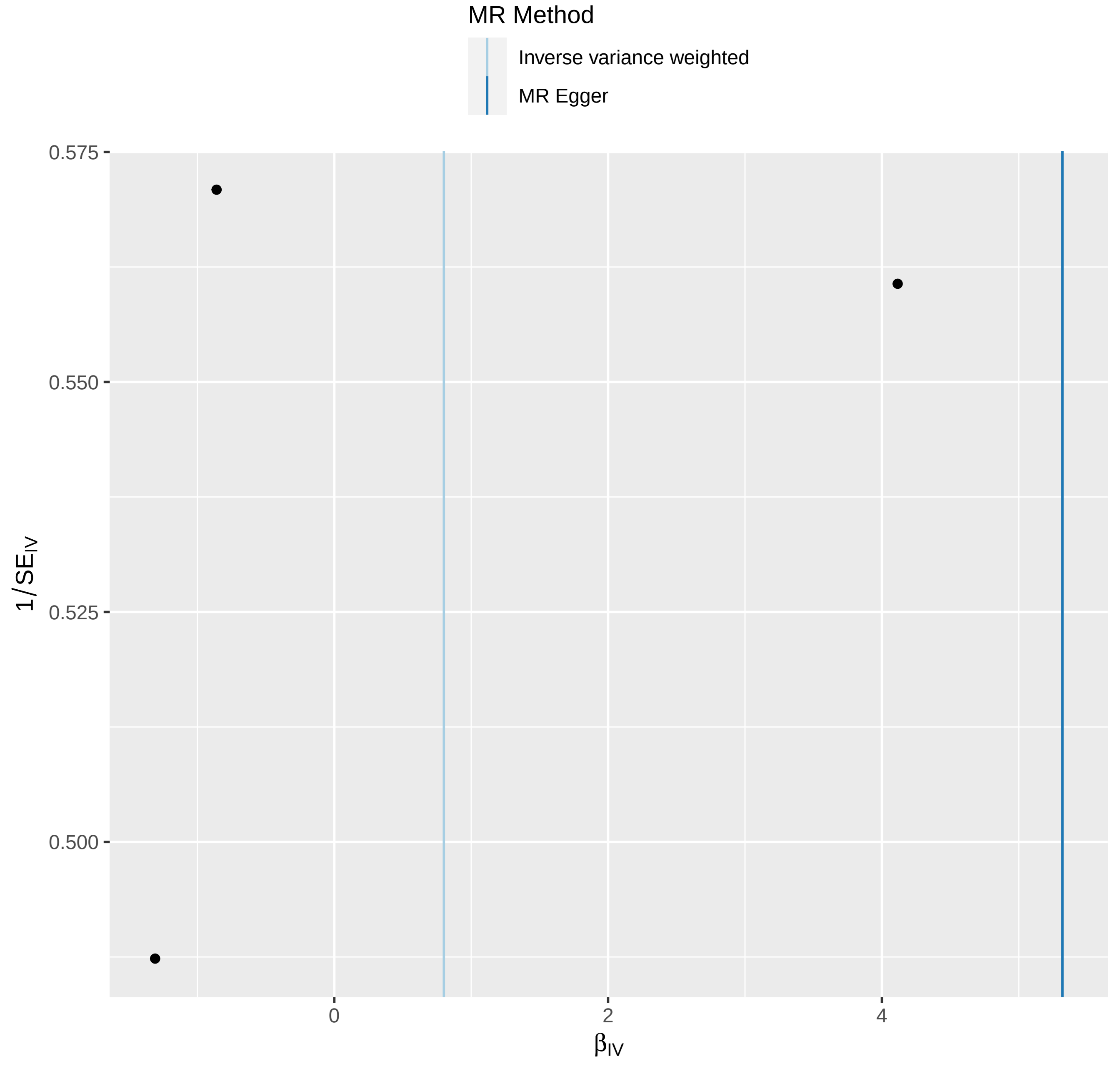

Batch 288 : Pyruvate on Crohn's disease of ileocolon

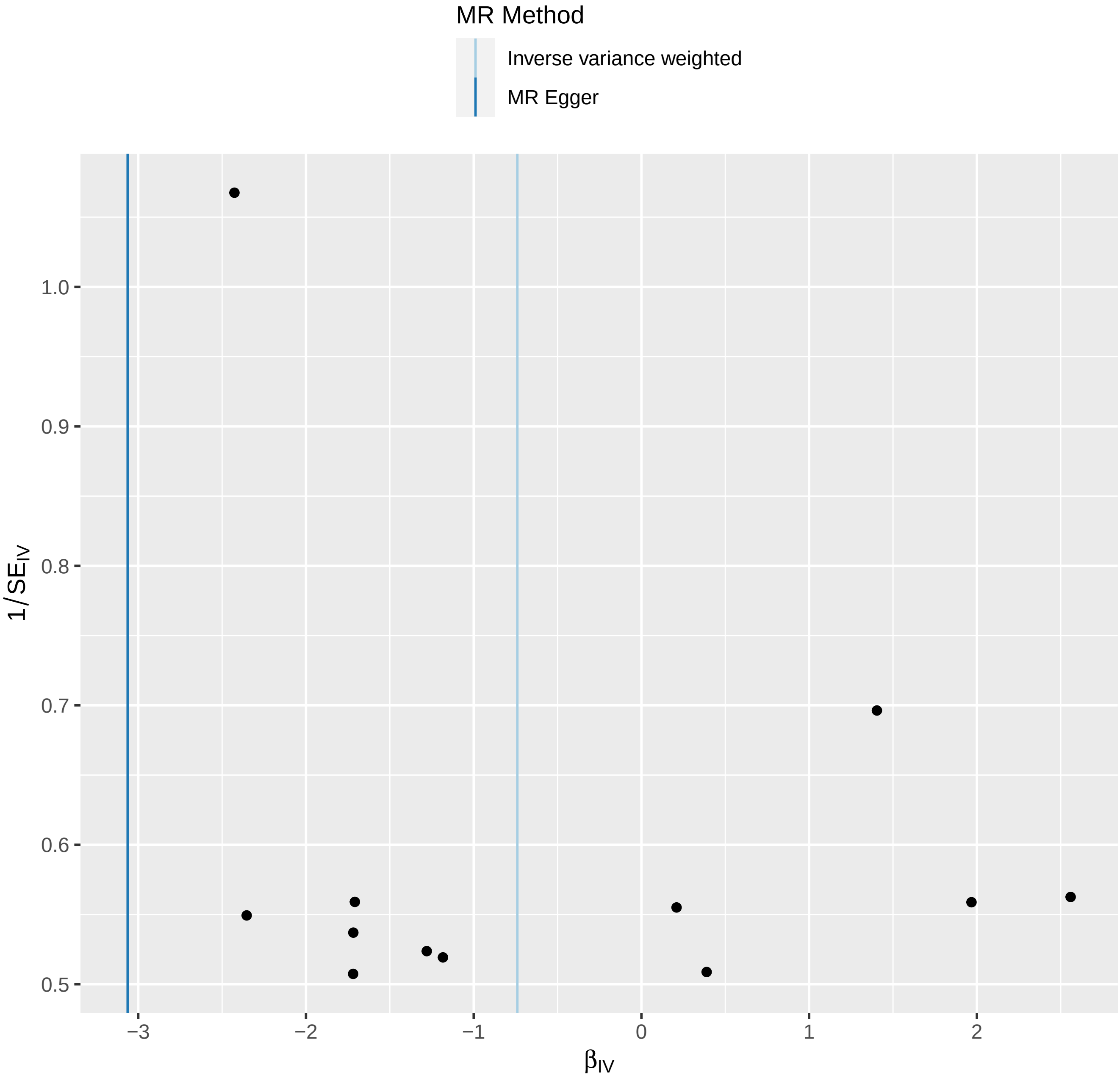

Batch 289 : Uridine on Crohn's disease of ileocolon

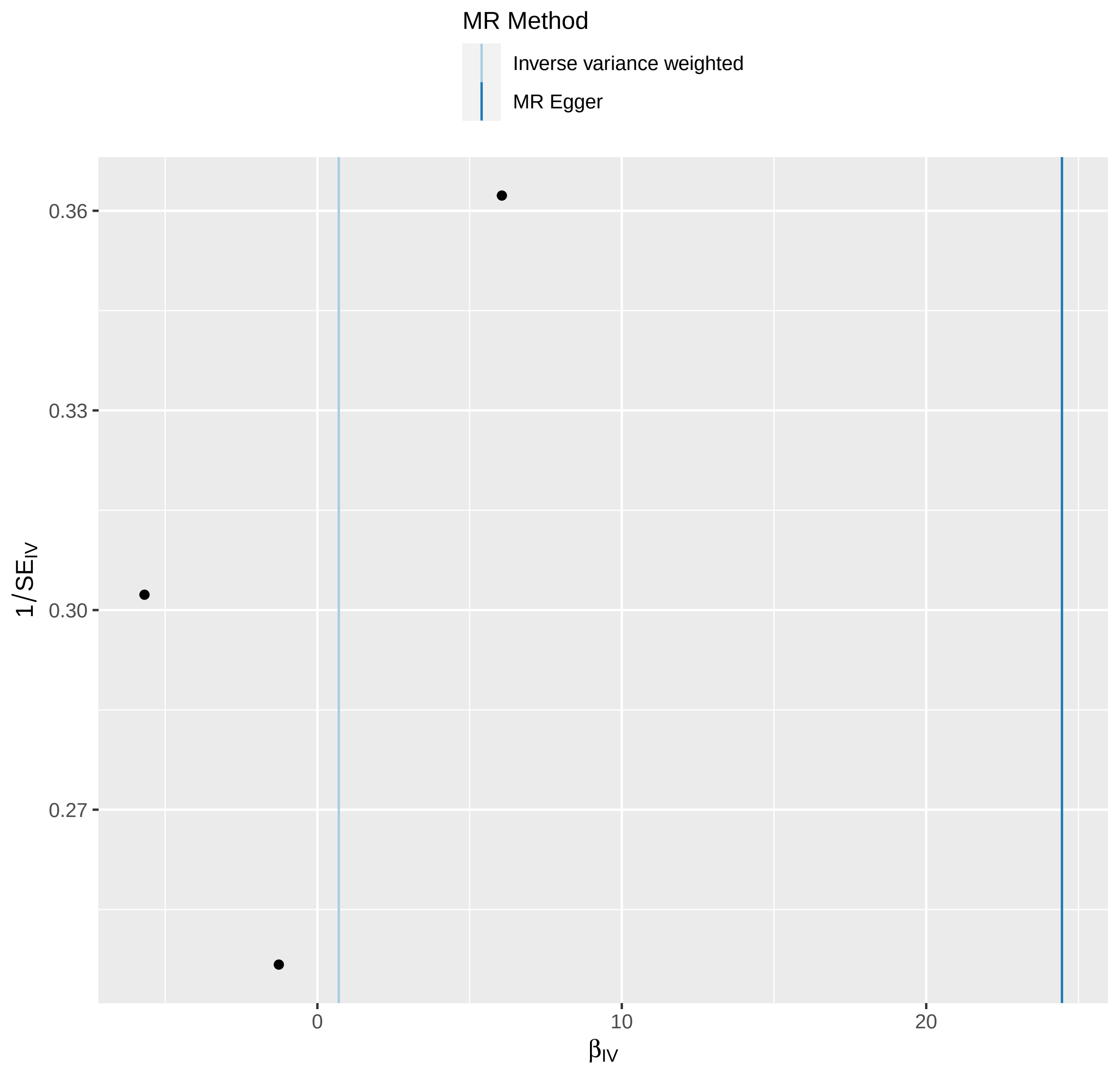

Batch 290 : Linoleate (18:2n6) on Crohn's disease of ileocolon

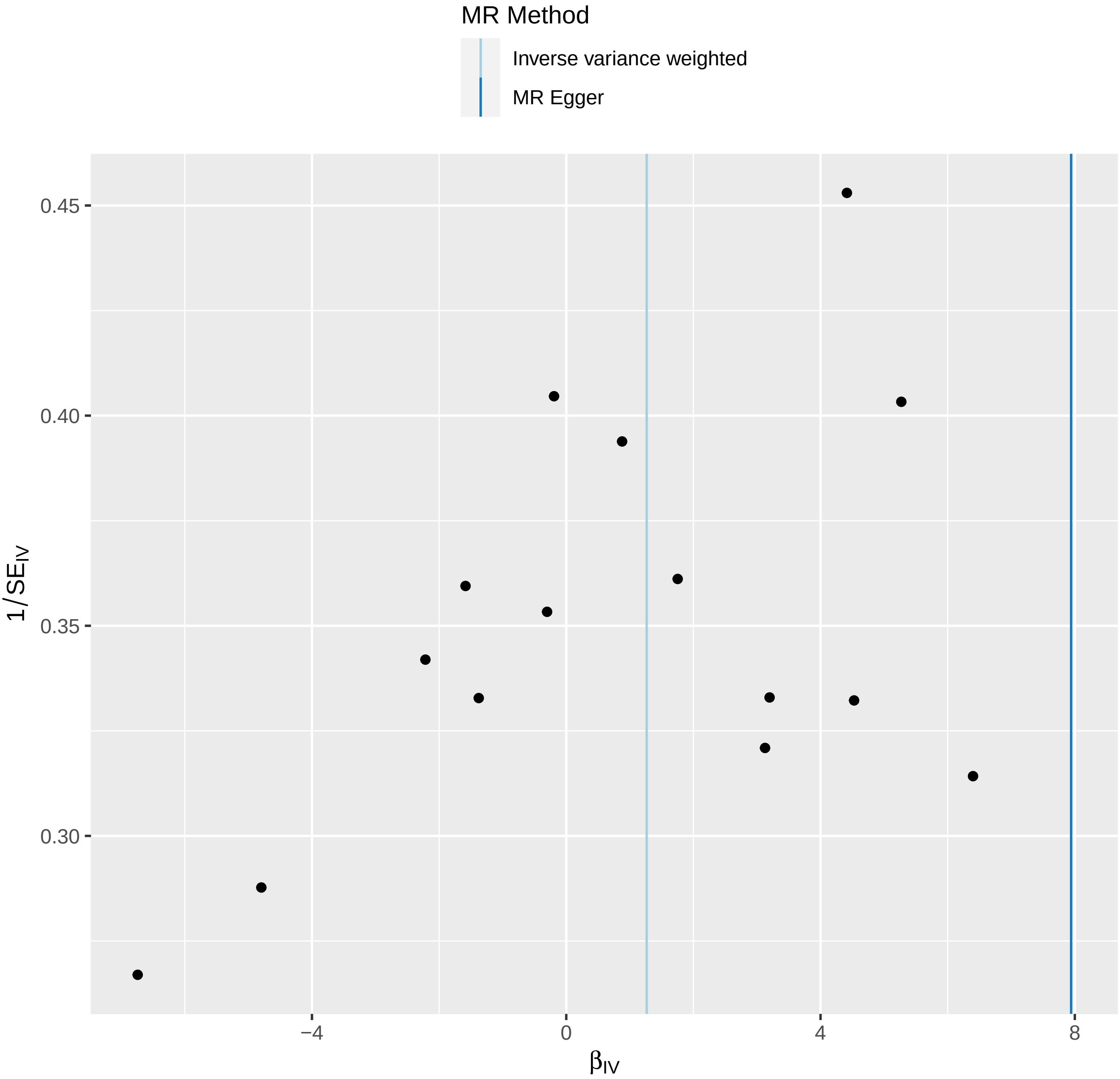

Batch 291 : Allantoin on Crohn's disease of ileocolon

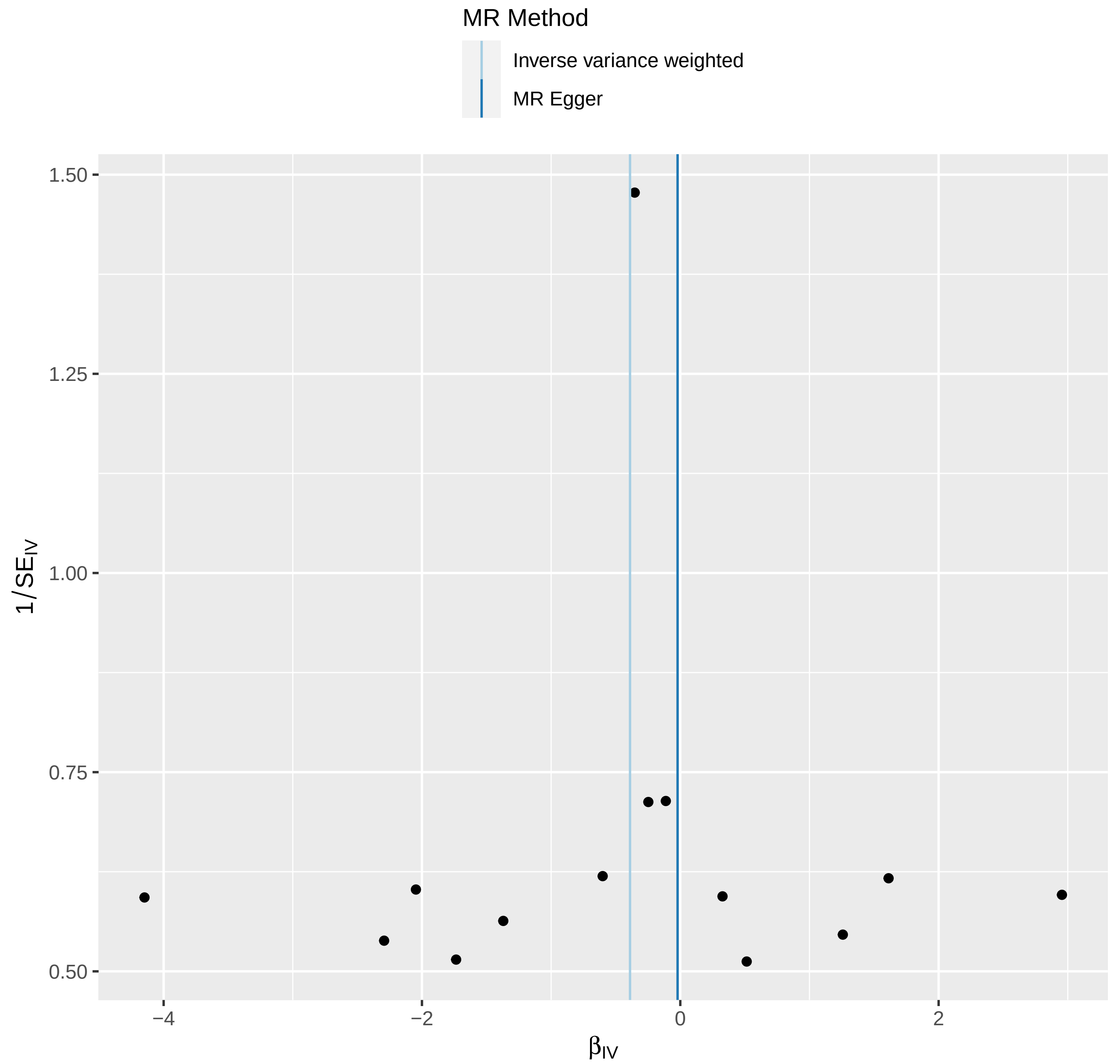

Batch 296 : Isoleucine on Crohn's disease of ileocolon

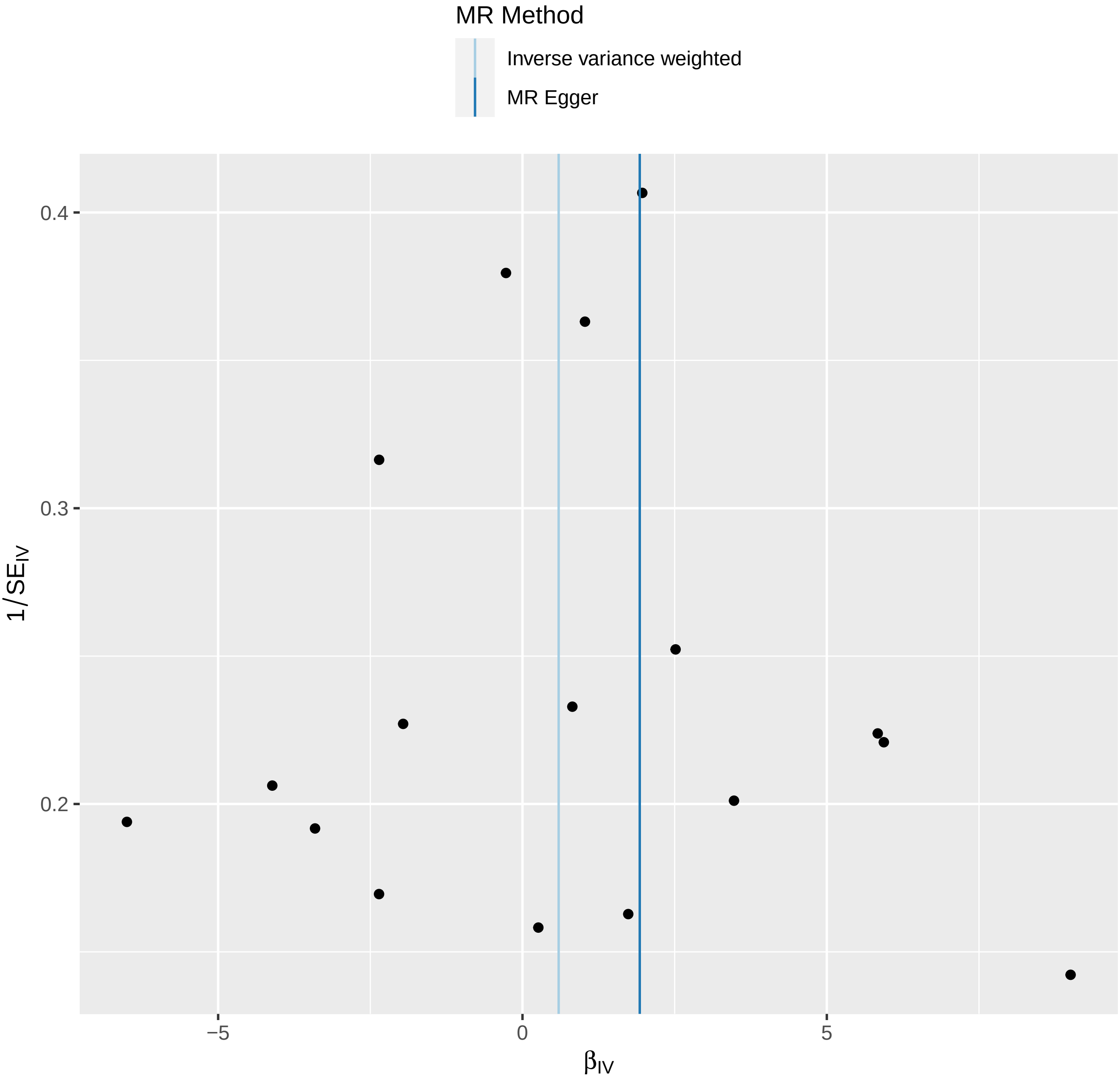

Batch 297 : Threonine on Crohn's disease of ileocolon

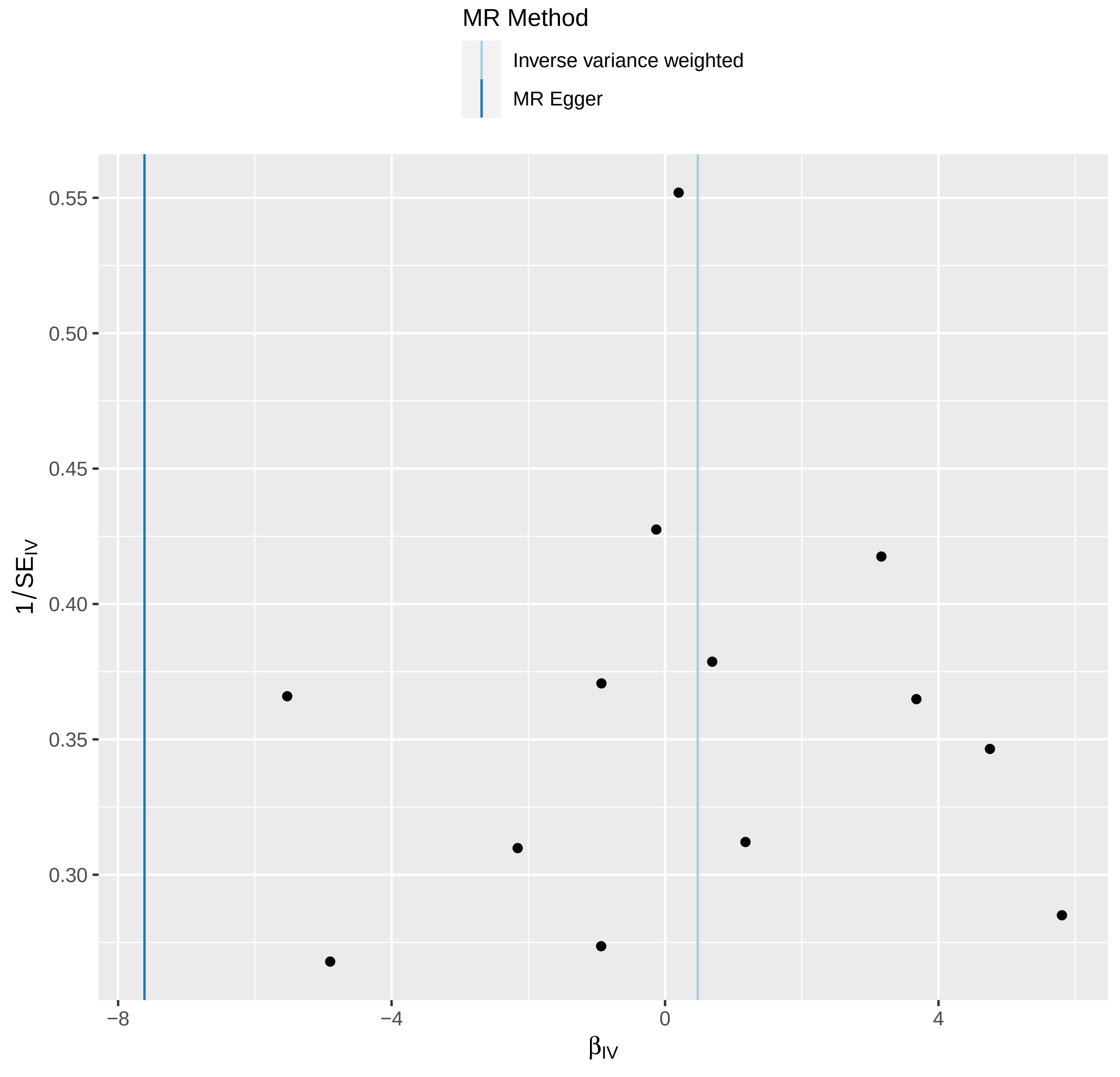

Batch 298 : Tyrosine on Crohn's disease of ileocolon

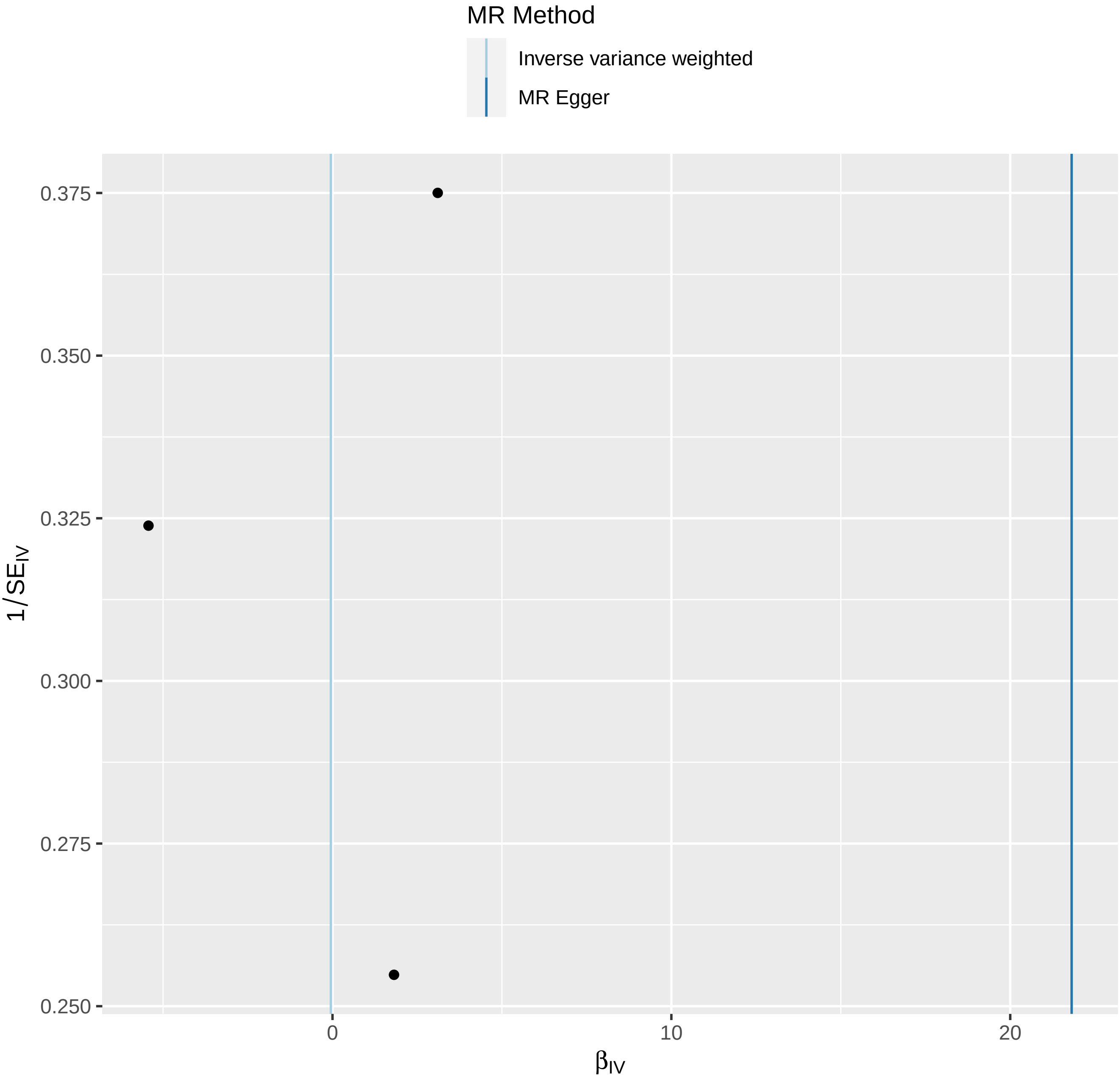

Batch 304 : Stearate (18:0) on Crohn's disease of ileocolon

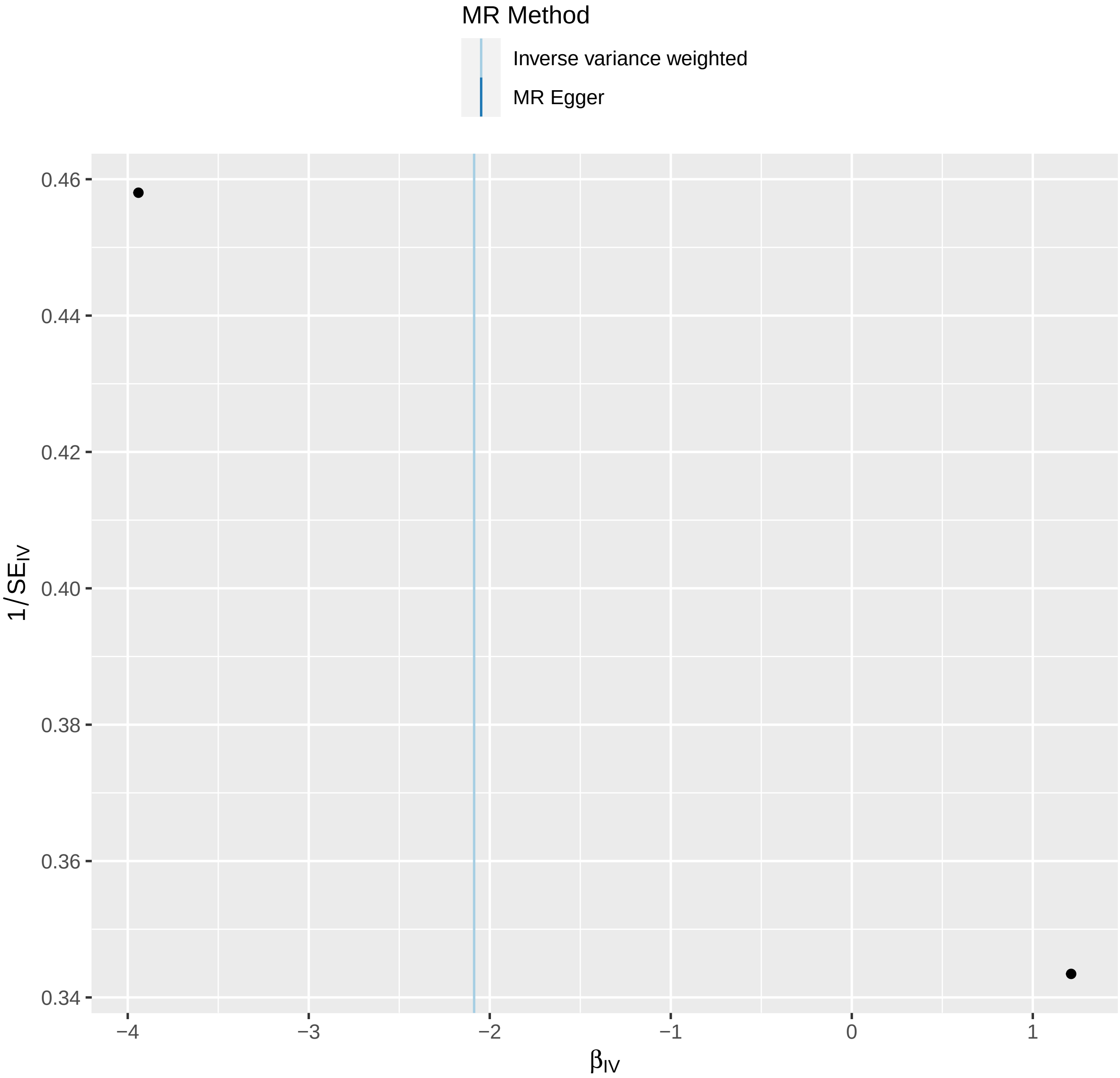

Batch 307 : Myristate (14:0) on Crohn's disease of ileocolon

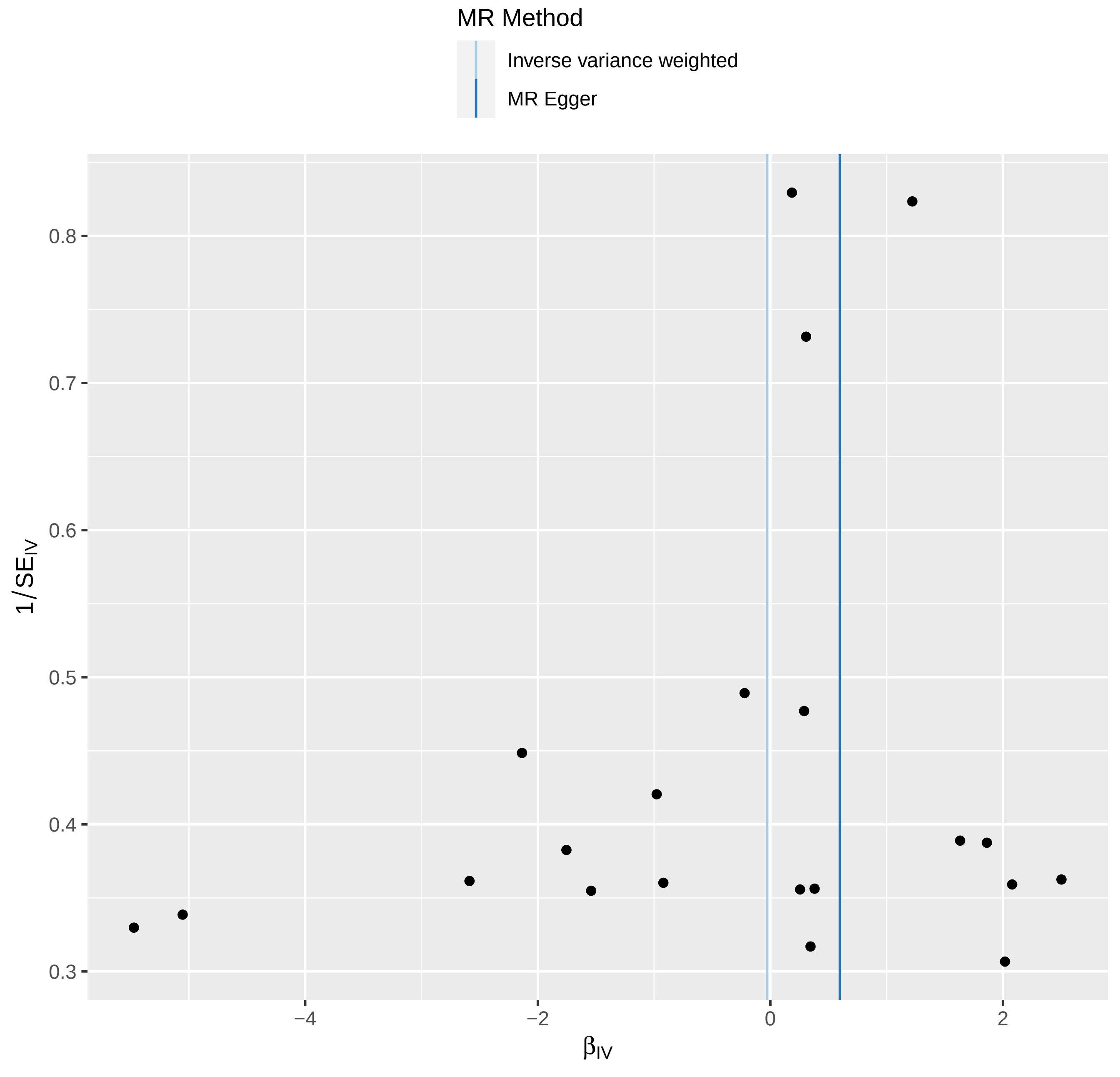

Batch 309 : Ornithine on Crohn's disease of ileocolon

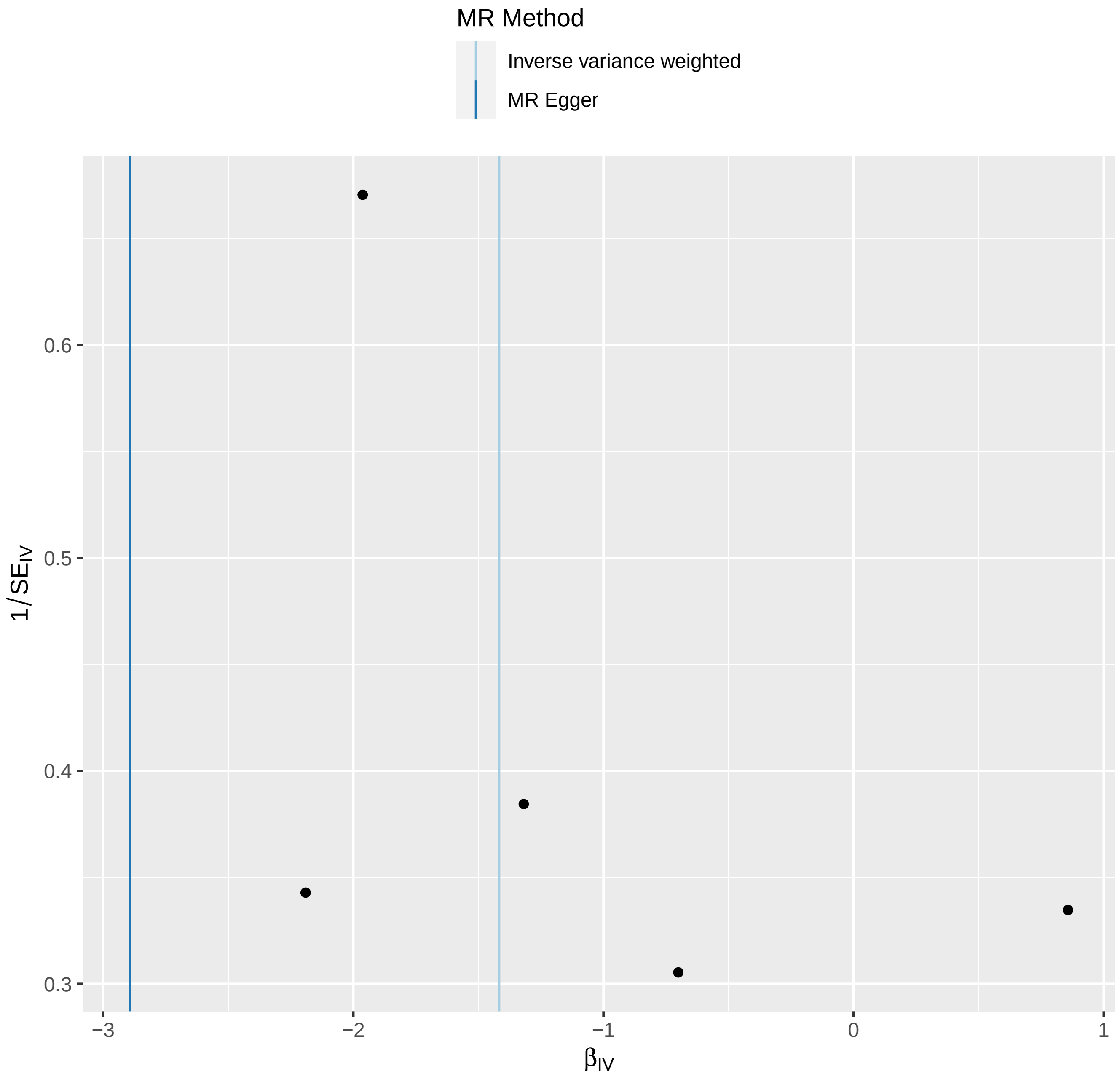

Batch 312 : 4−acetamidobutanoate on Crohn's disease of ileocolon

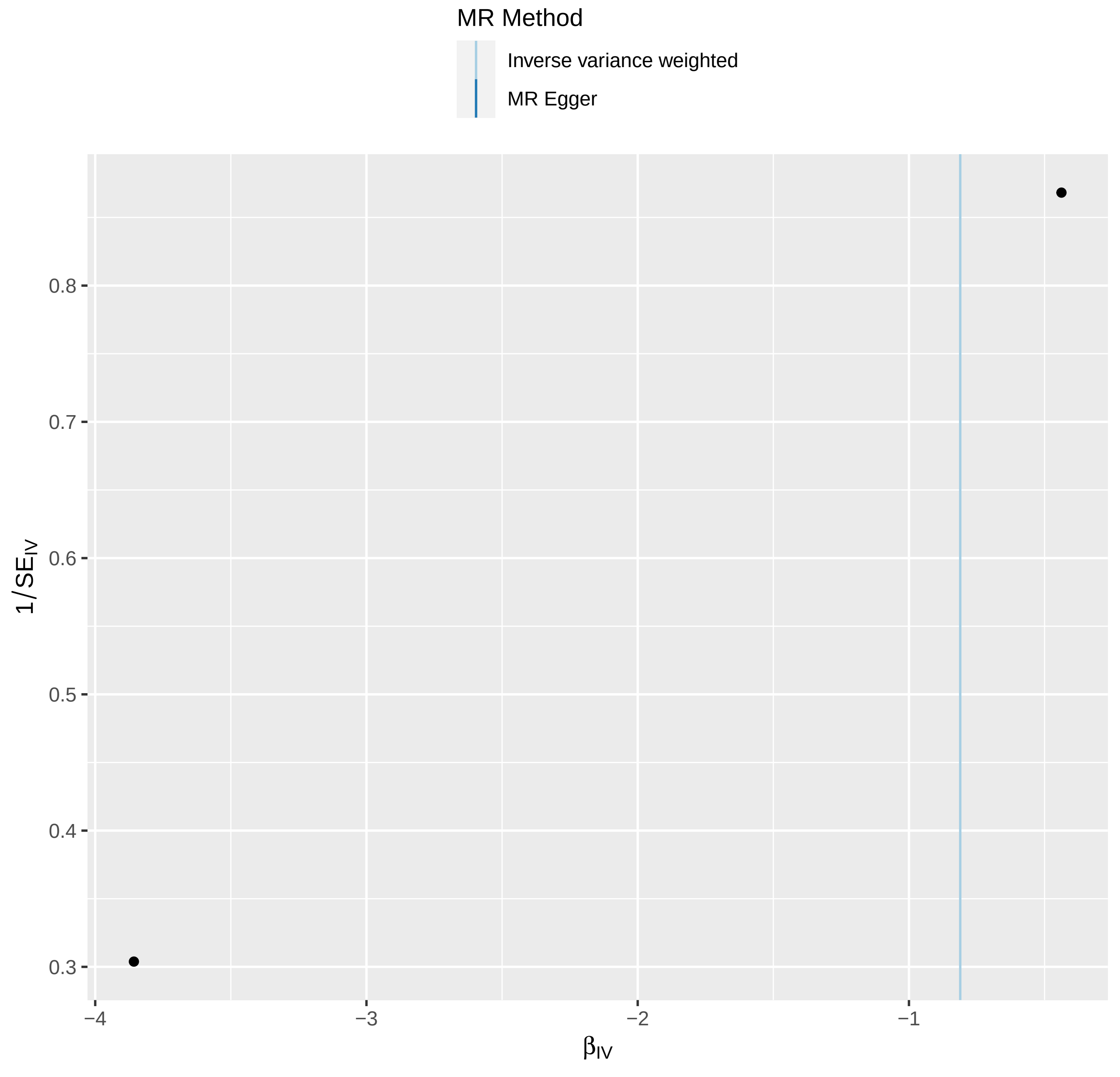

Batch 313 : Alpha−tocopherol on Crohn's disease of ileocolon

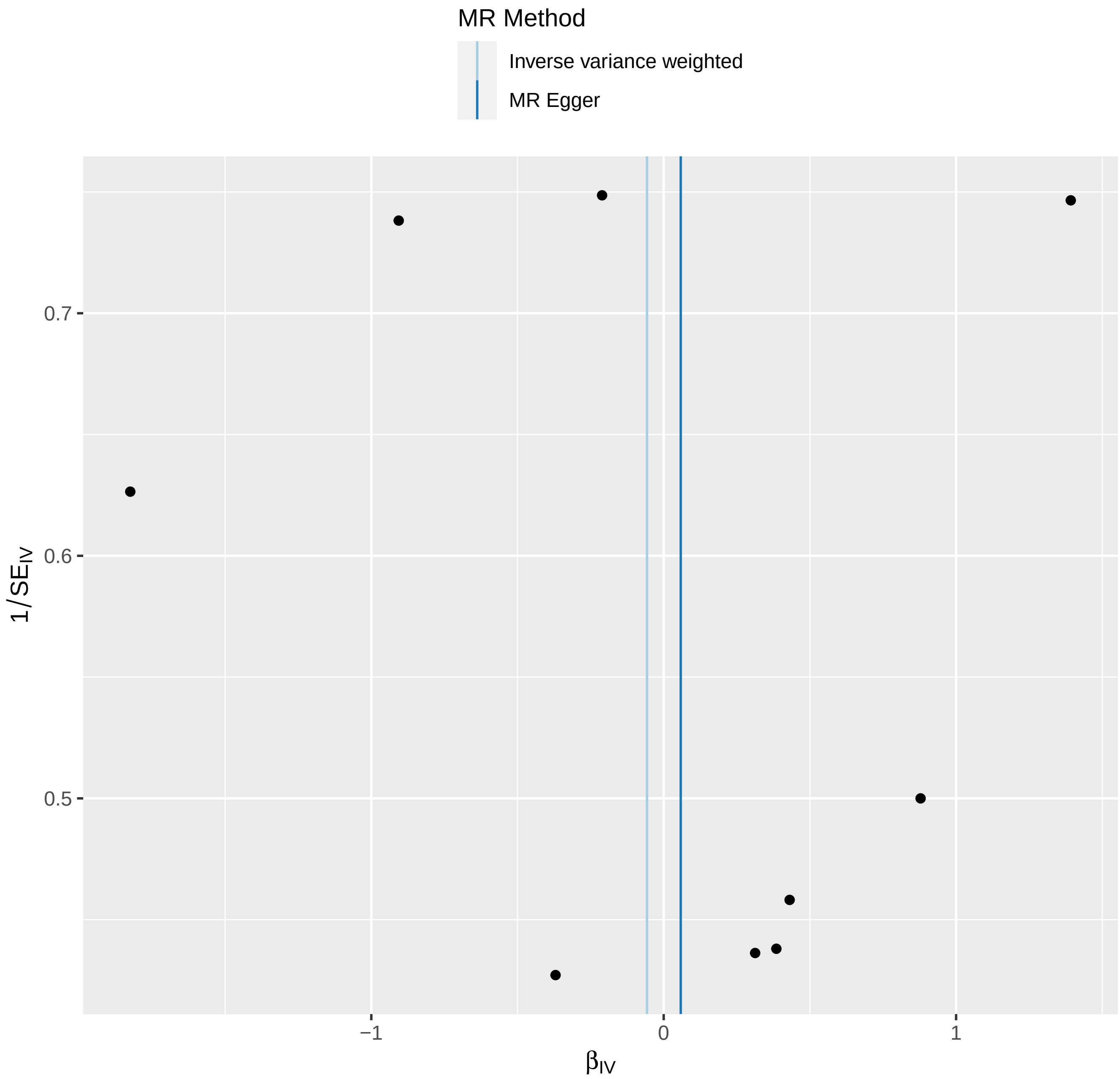

Batch 314 : Citrate on Crohn's disease of ileocolon

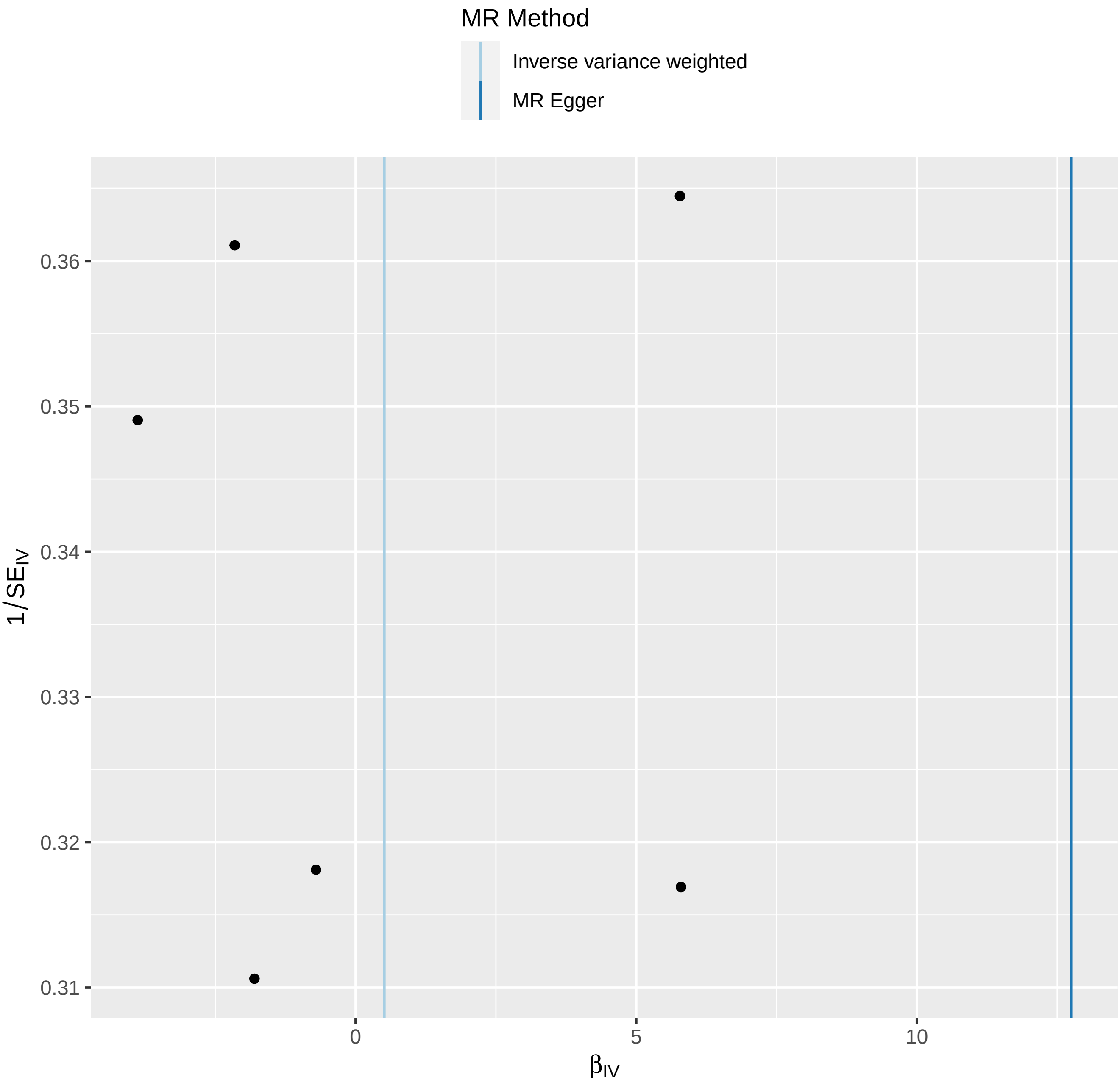

Batch 315 : Glycerate on Crohn's disease of ileocolon

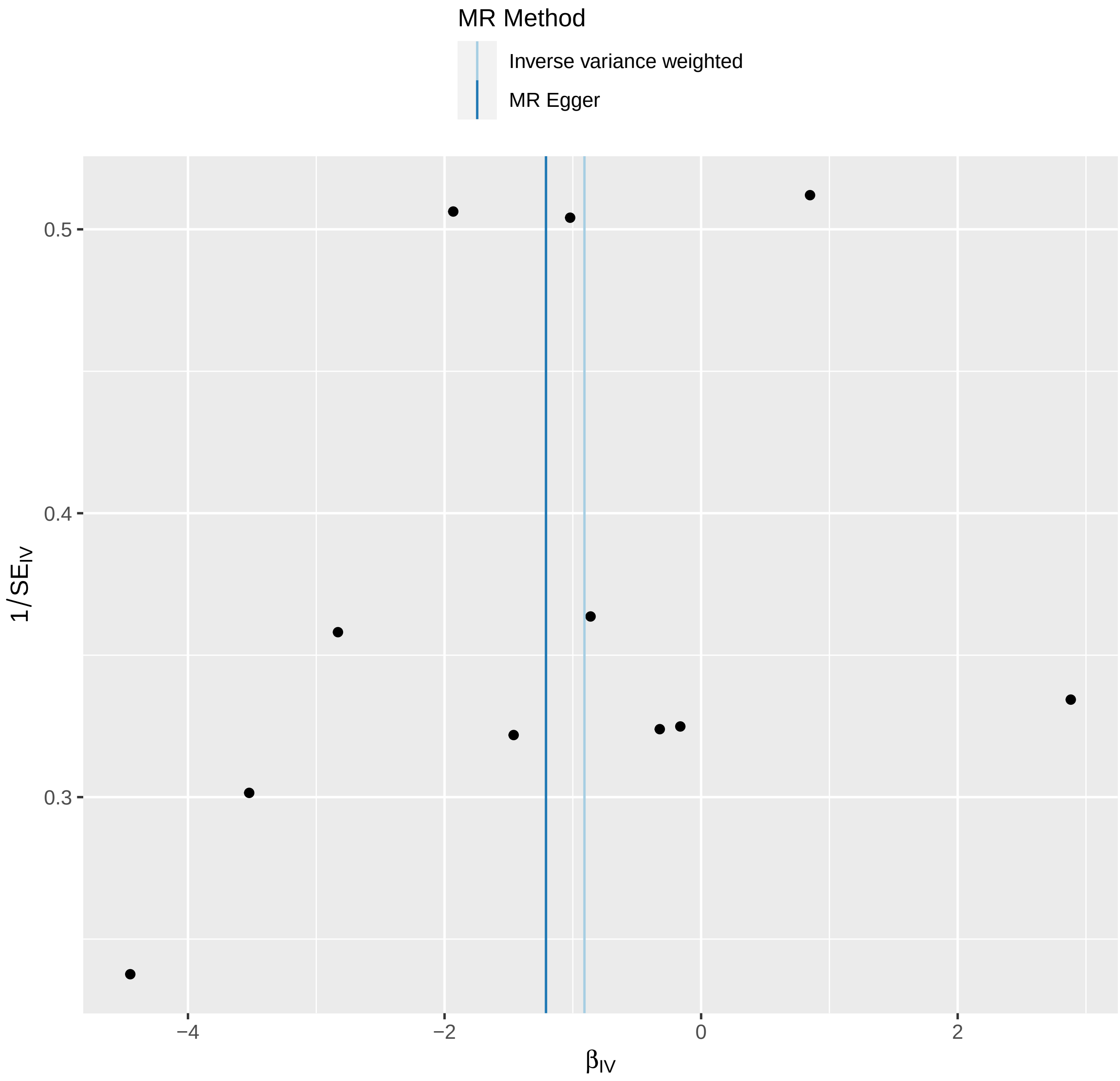

Batch 316 : Guanosine on Crohn's disease of ileocolon

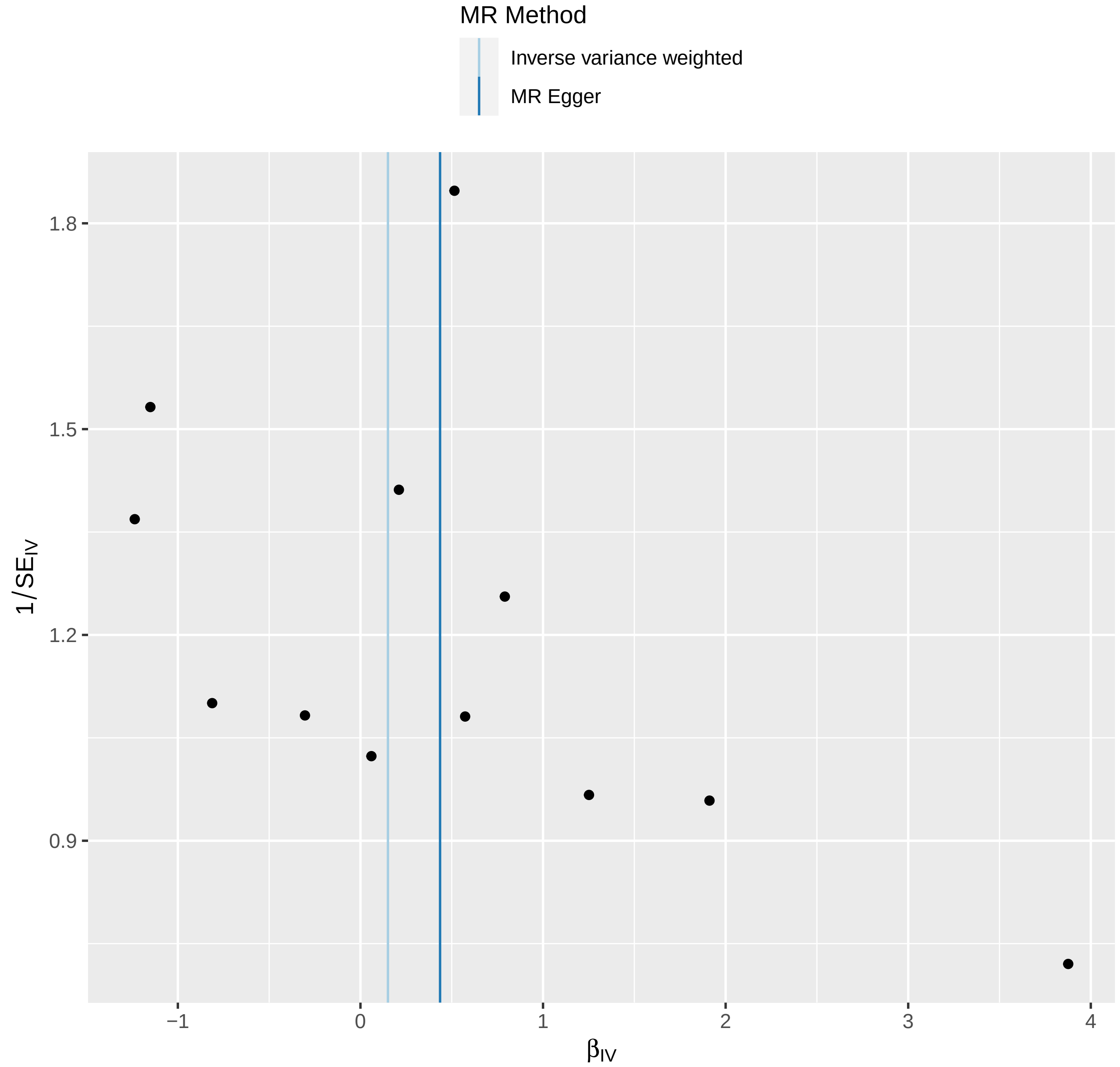

Batch 319 : Ursodeoxycholate on Crohn's disease of ileocolon

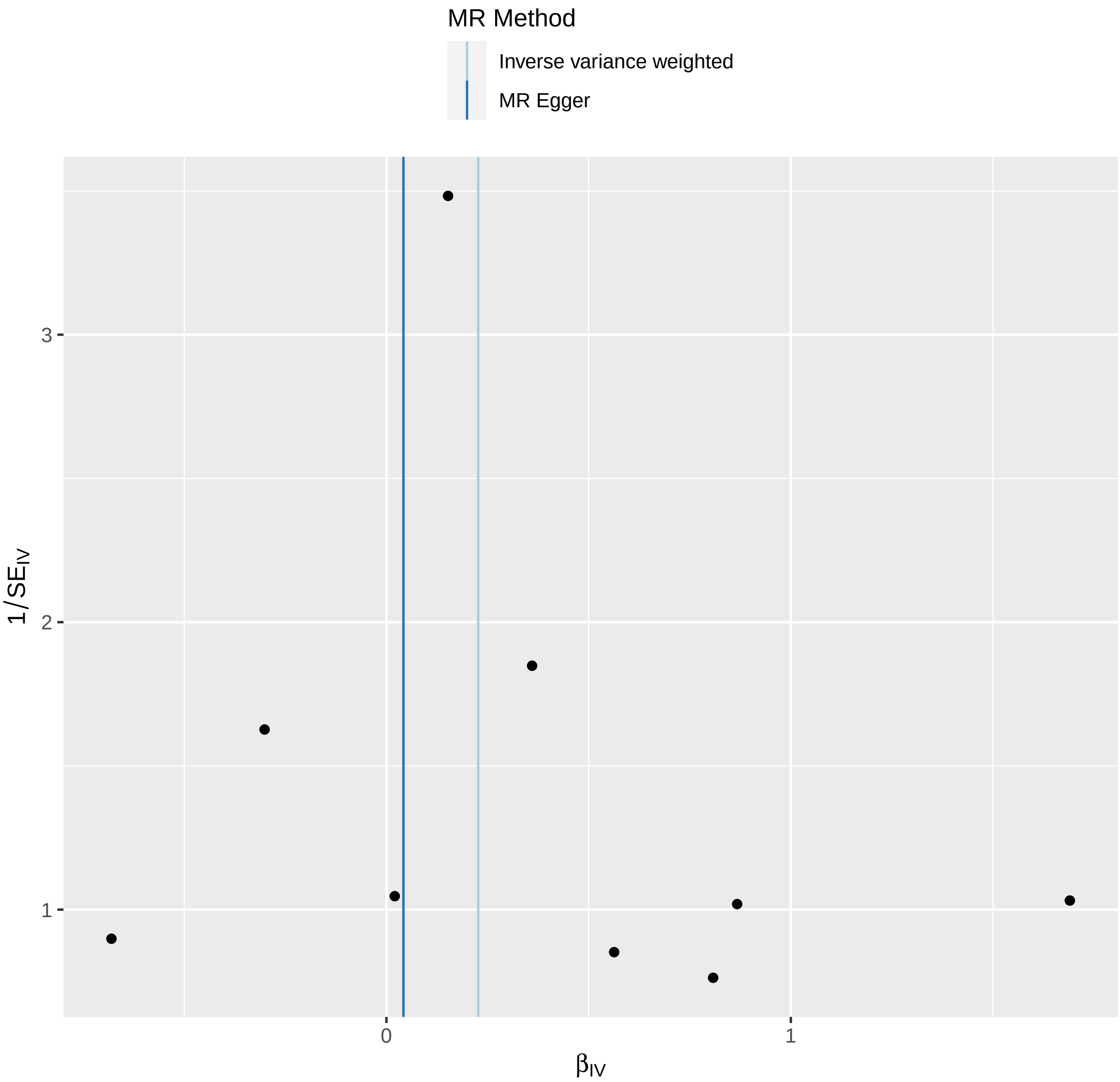

Batch 320 : Arginine on Crohn's disease of ileocolon

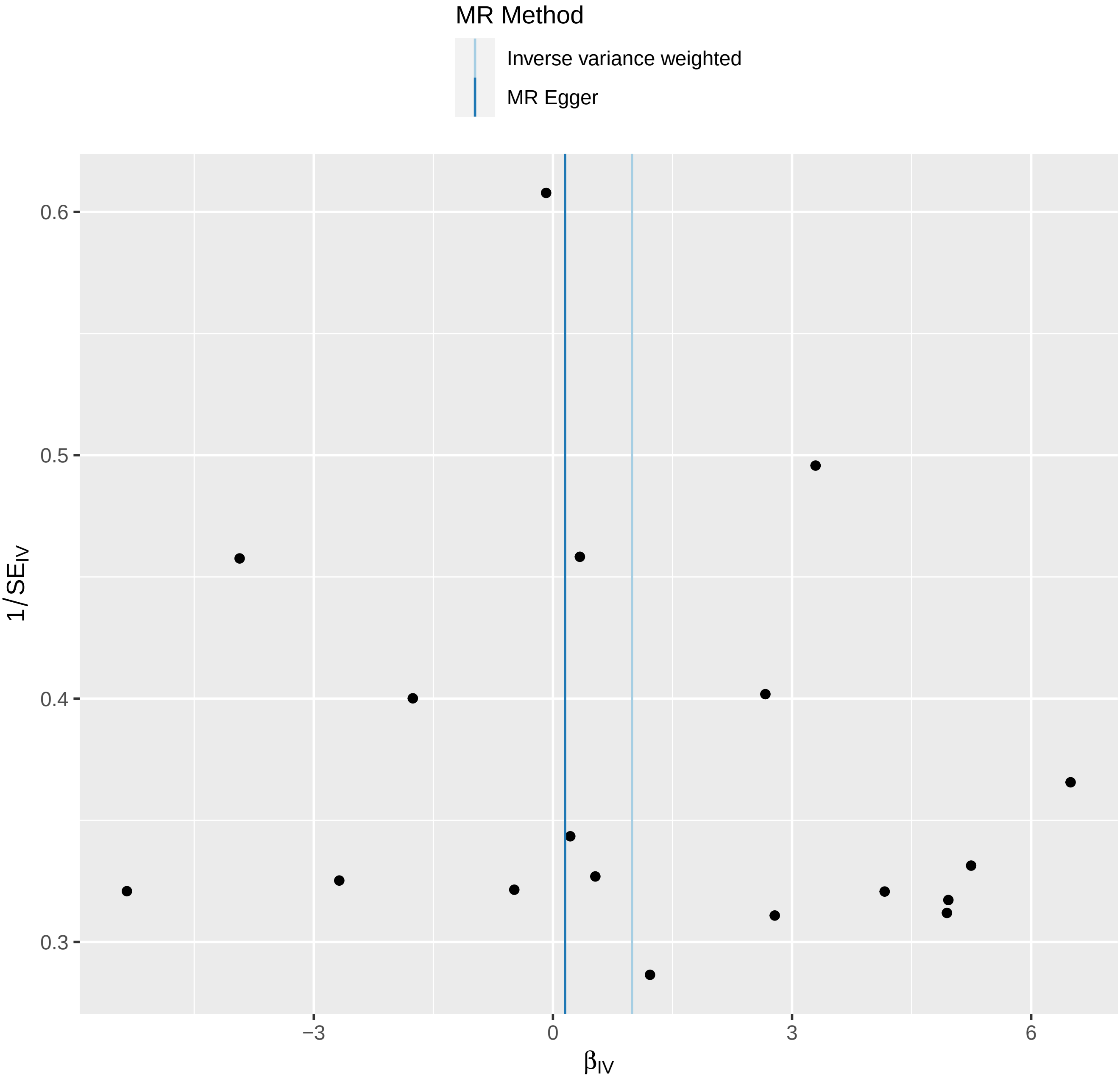

Batch 321 : Ascorbate (Vitamin C) on Crohn's disease of ileocolon

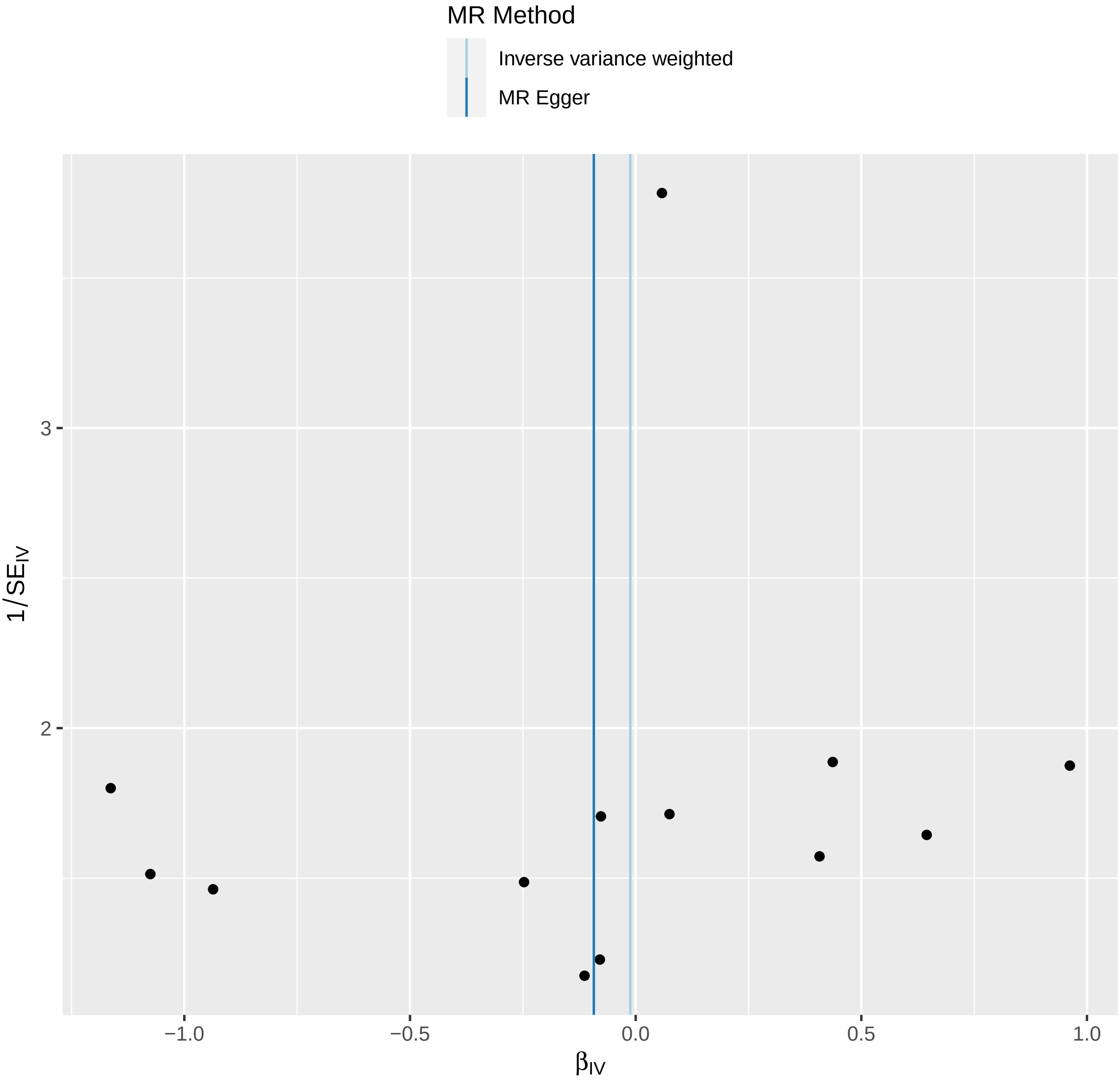

Batch 322 : Heptanoate (7:0) on Crohn's disease of ileocolon

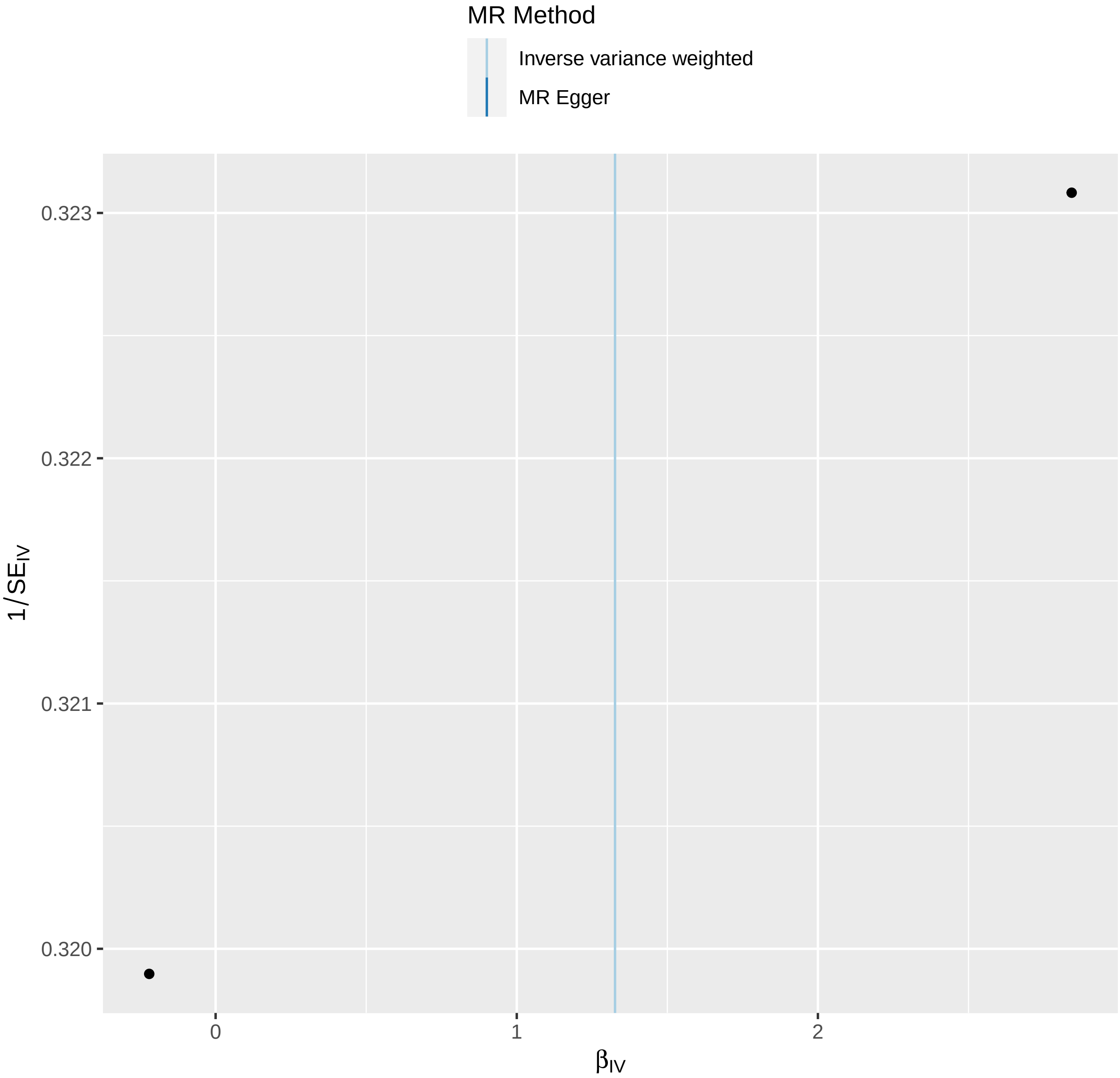

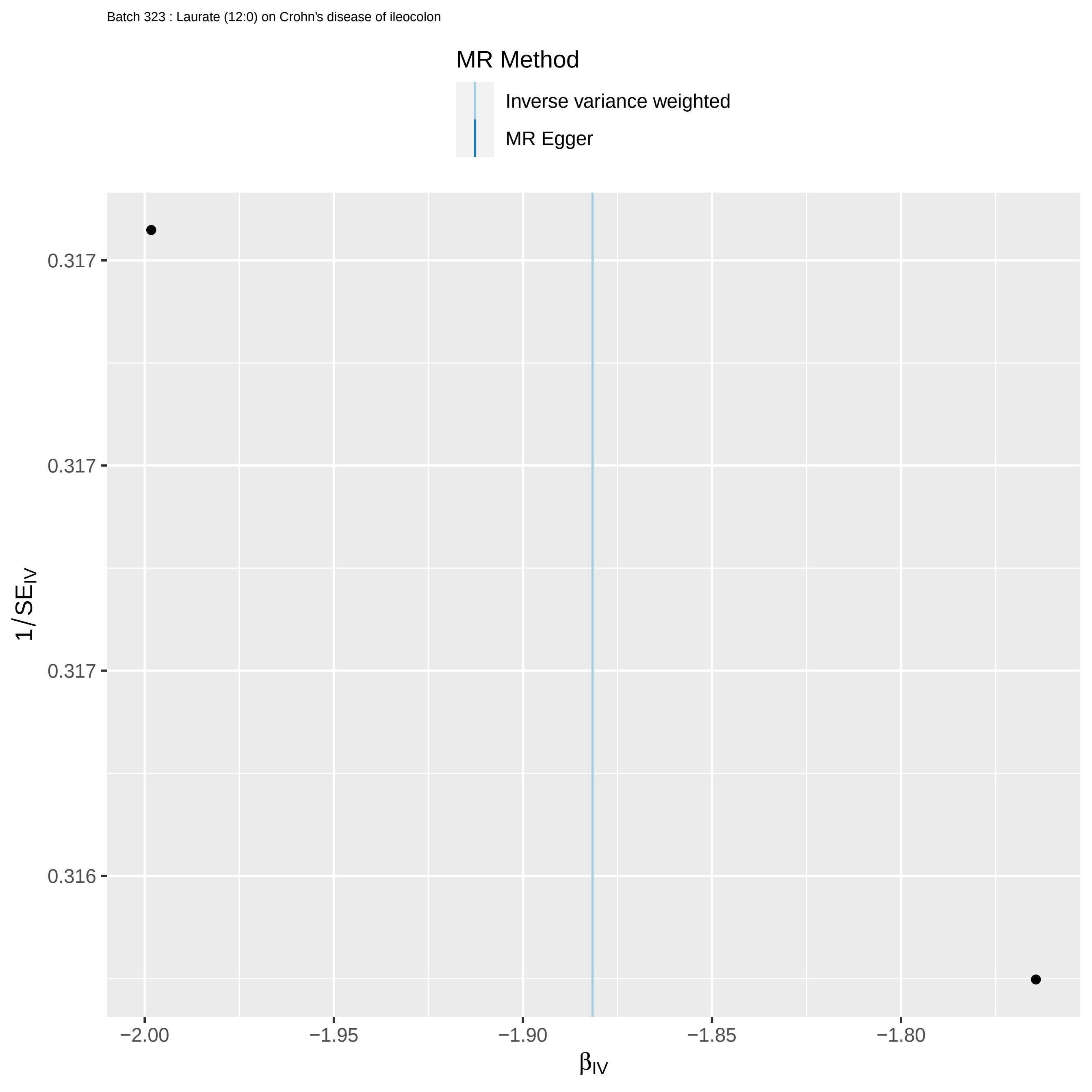
Batch 323 : Laurate (12:0) on Crohn's disease of ileocolon
MR Method
Inverse variance weighted
MR Egger
0.317
0.317
0.317
0.316
1/SE_IV
−2.00
−1.95
−1.90
−1.85
−1.80
β_IV

Batch 325 : Urea on Crohn's disease of ileocolon

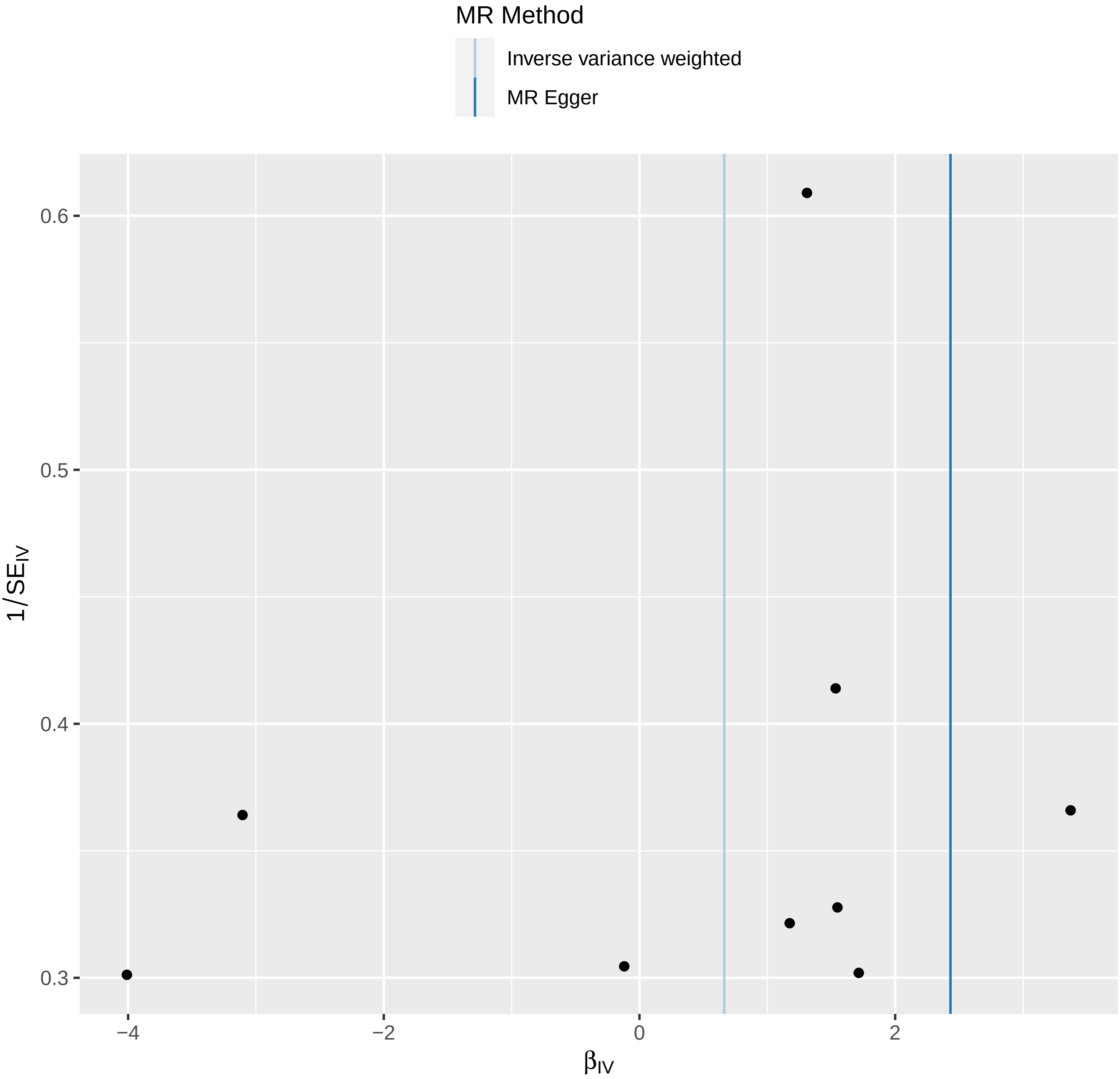

Batch 326 : Cortisol on Crohn's disease of ileocolon

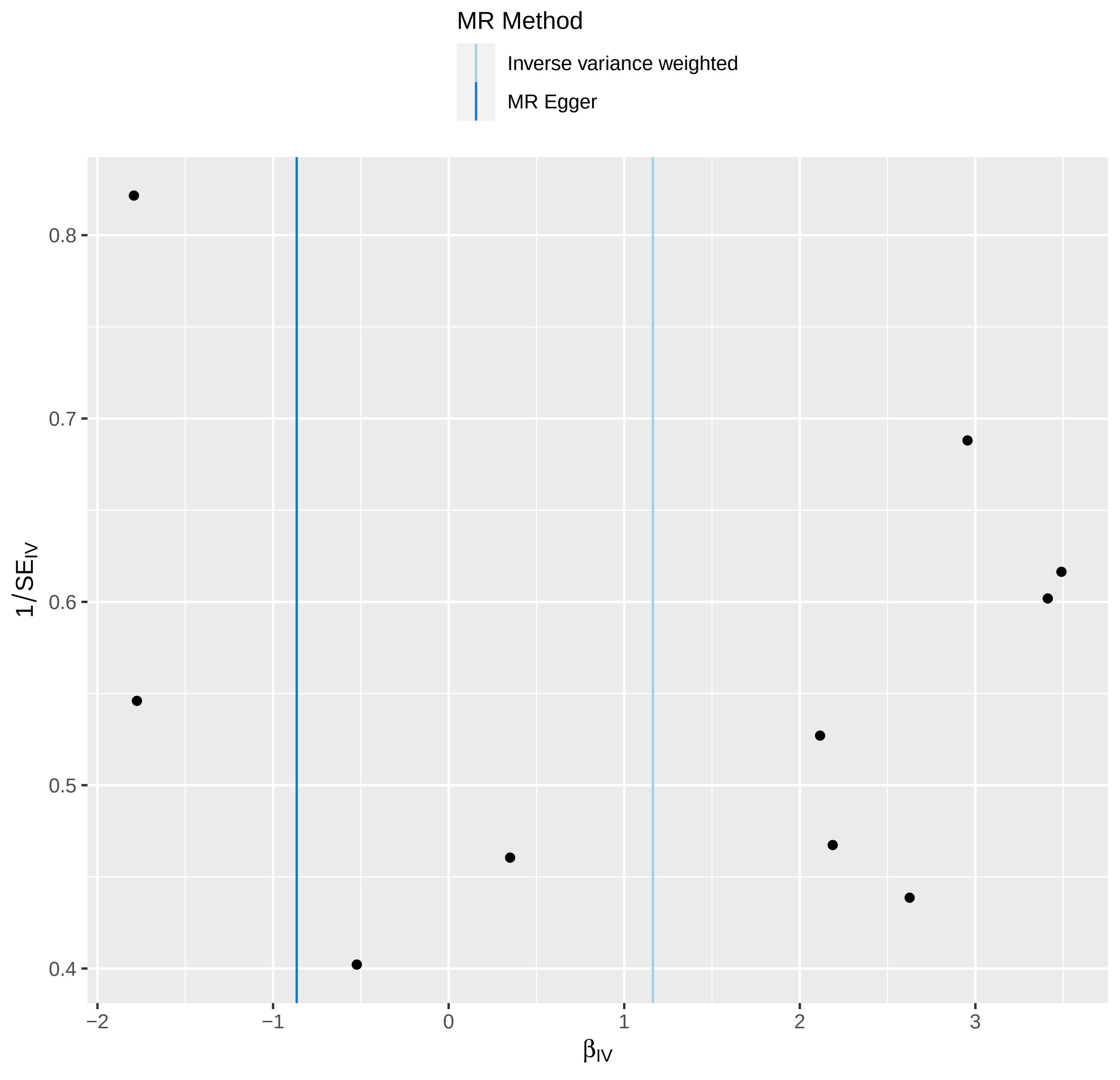

Batch 329 : Citrulline on Crohn's disease of ileocolon

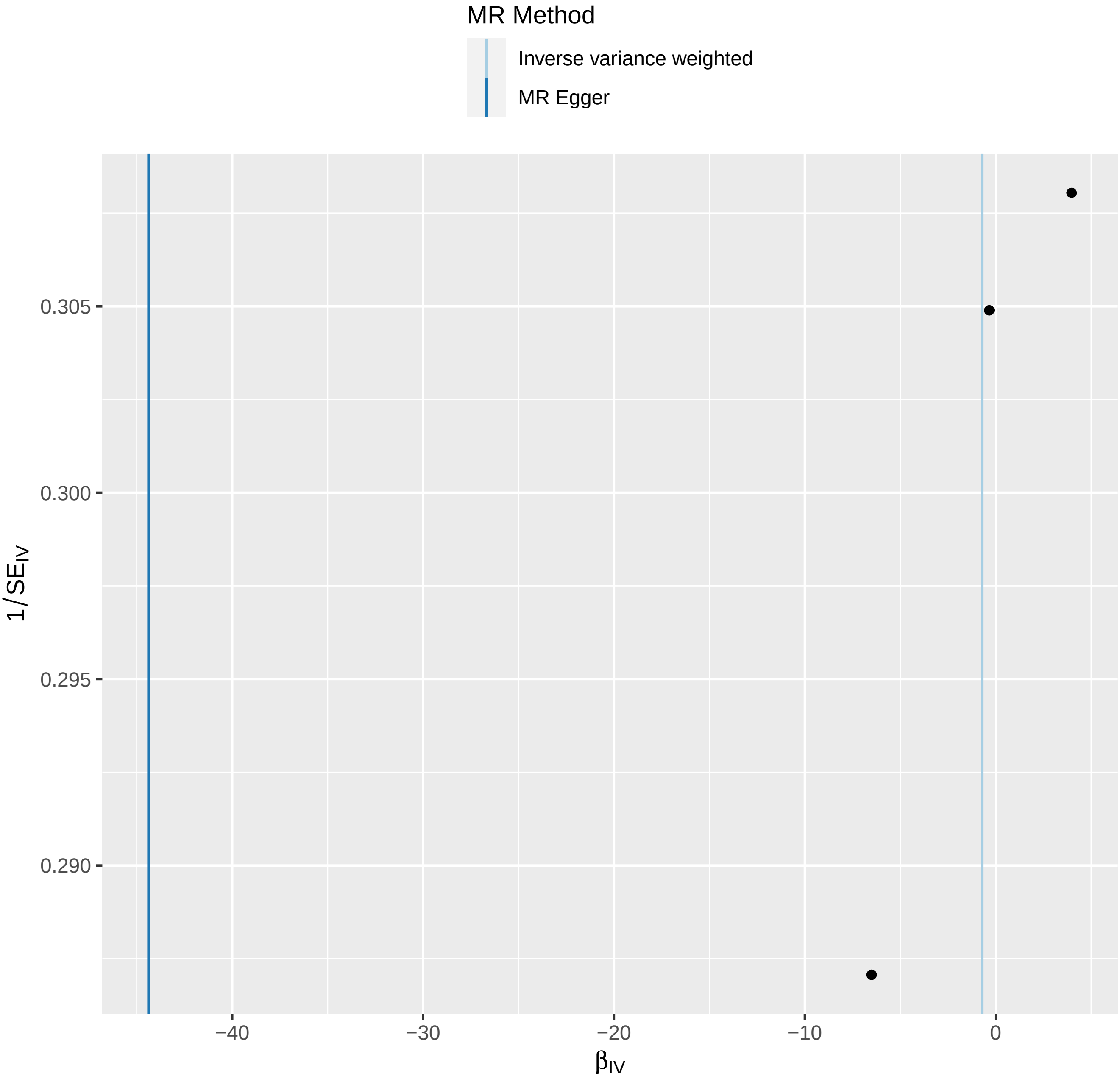

Batch 331 : Serotonin (5HT) on Crohn's disease of ileocolon

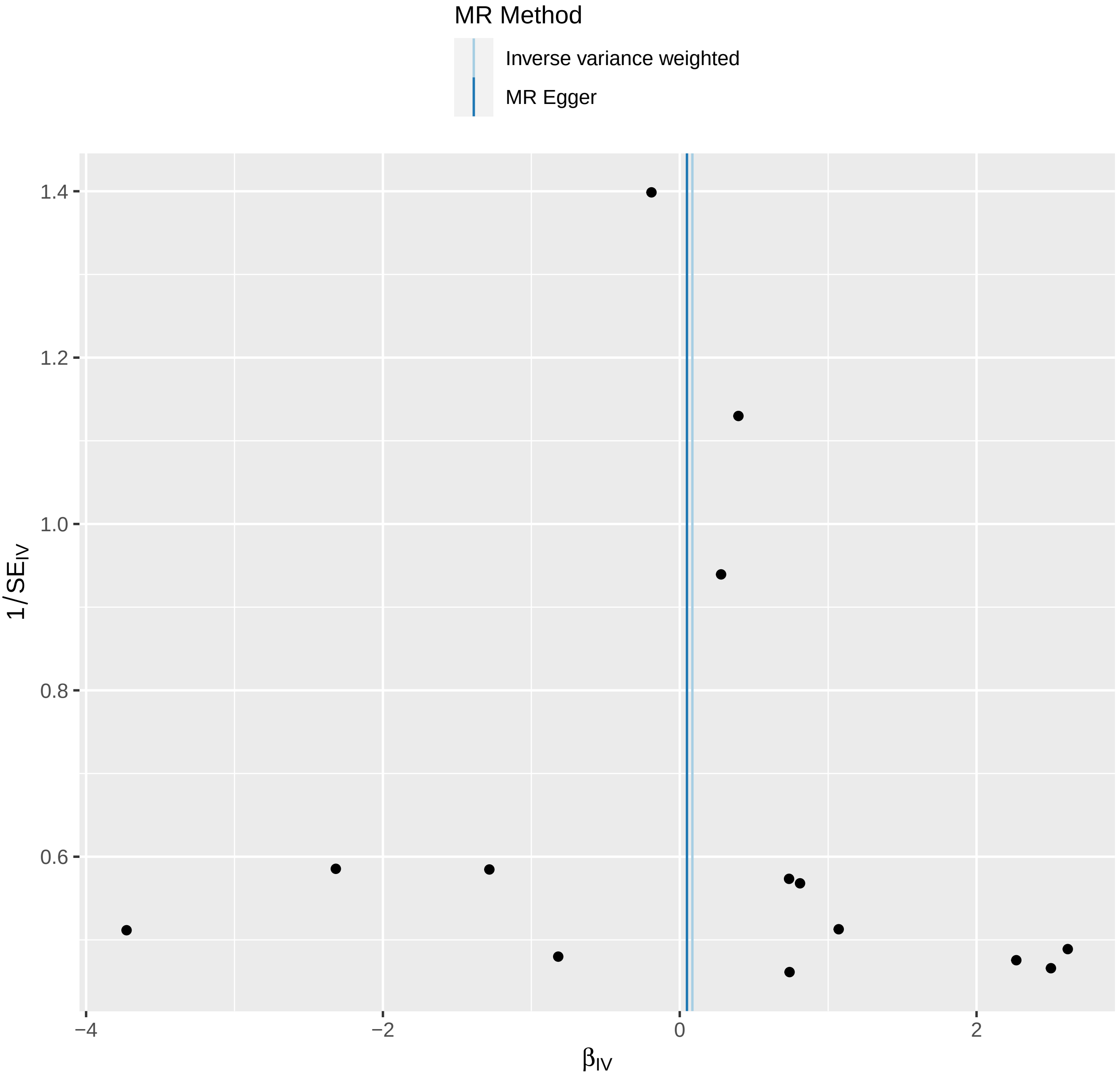

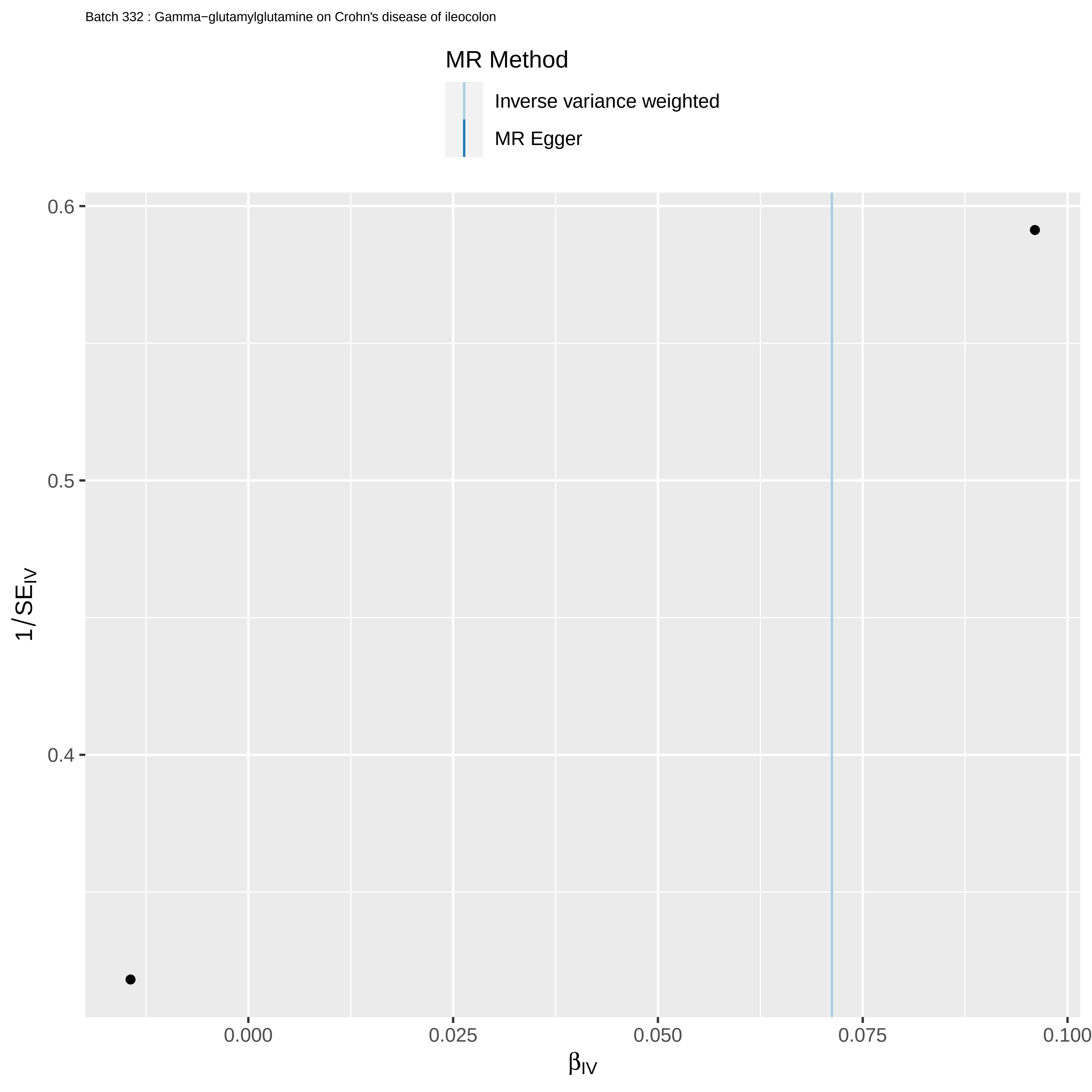

Batch 332 : Gamma−glutamylglutamine on Crohn's disease of ileocolon
MR Method
Inverse variance weighted
MR Egger
0.6
0.5
0.4
1/SEIV
0.000
0.025
0.050
0.075
0.100
βIV

Batch 333 : Gamma−glutamyltyrosine on Crohn's disease of ileocolon

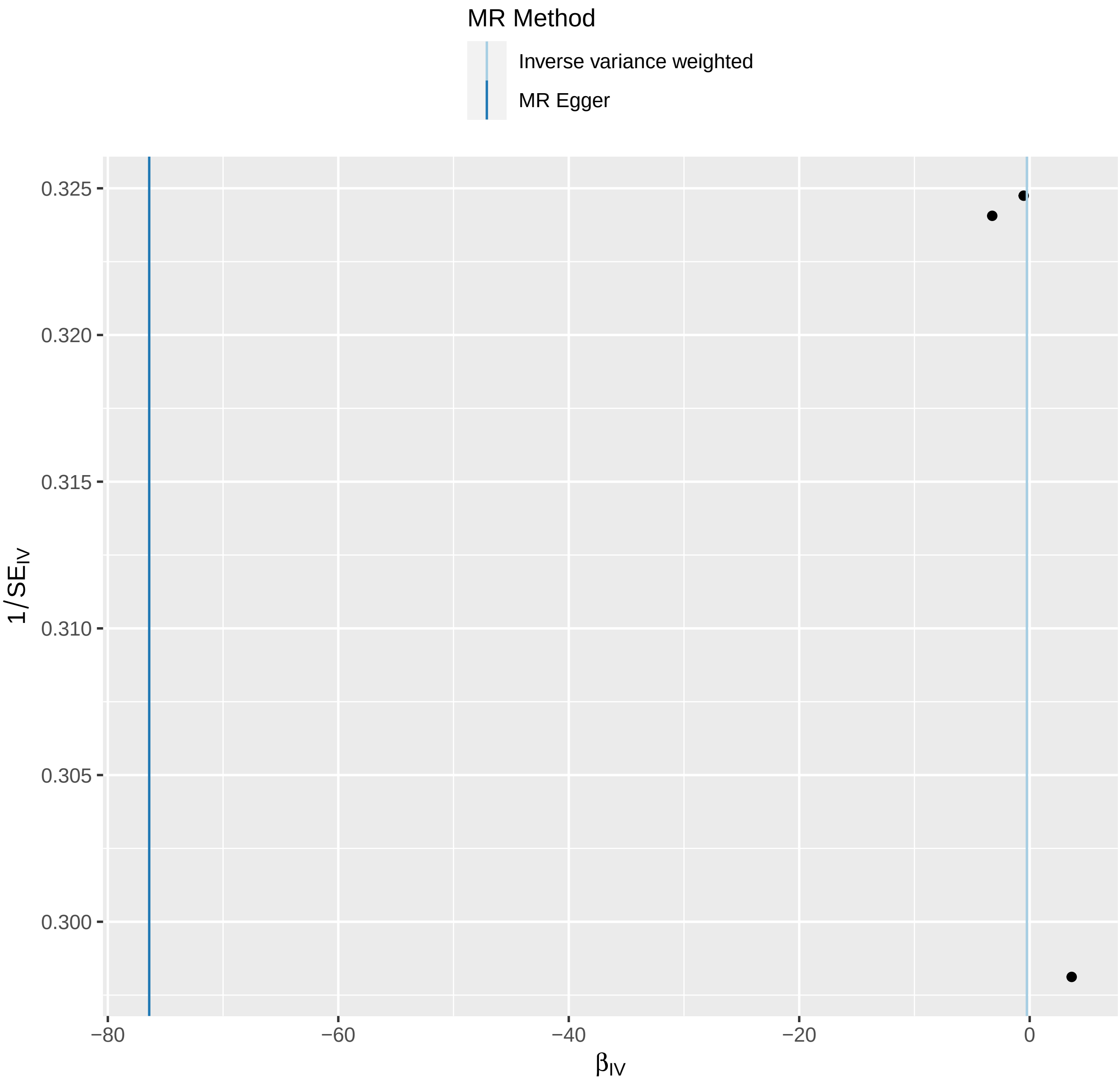

Batch 335 : Betaine on Crohn's disease of ileocolon

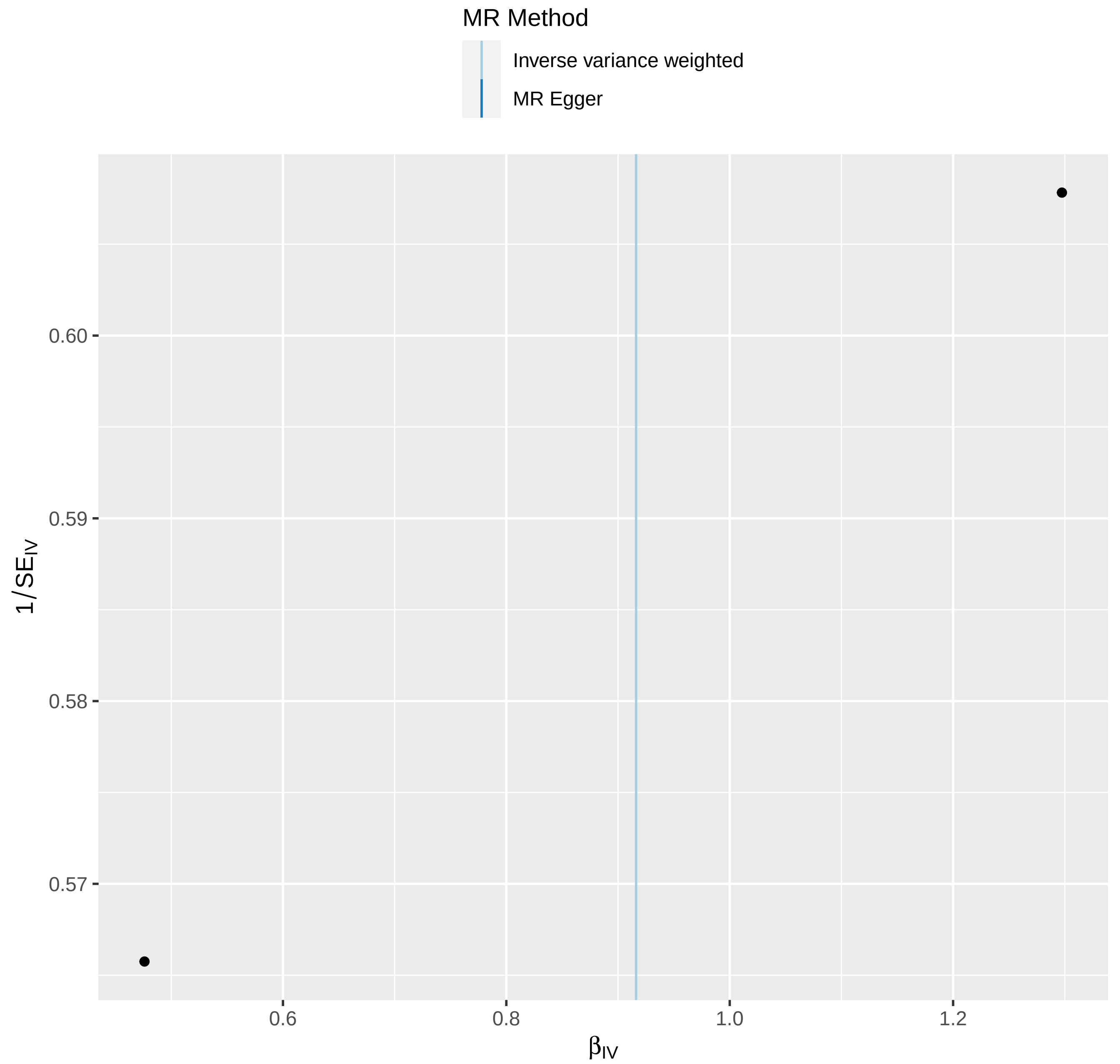

Batch 336 : Xanthine on Crohn's disease of ileocolon

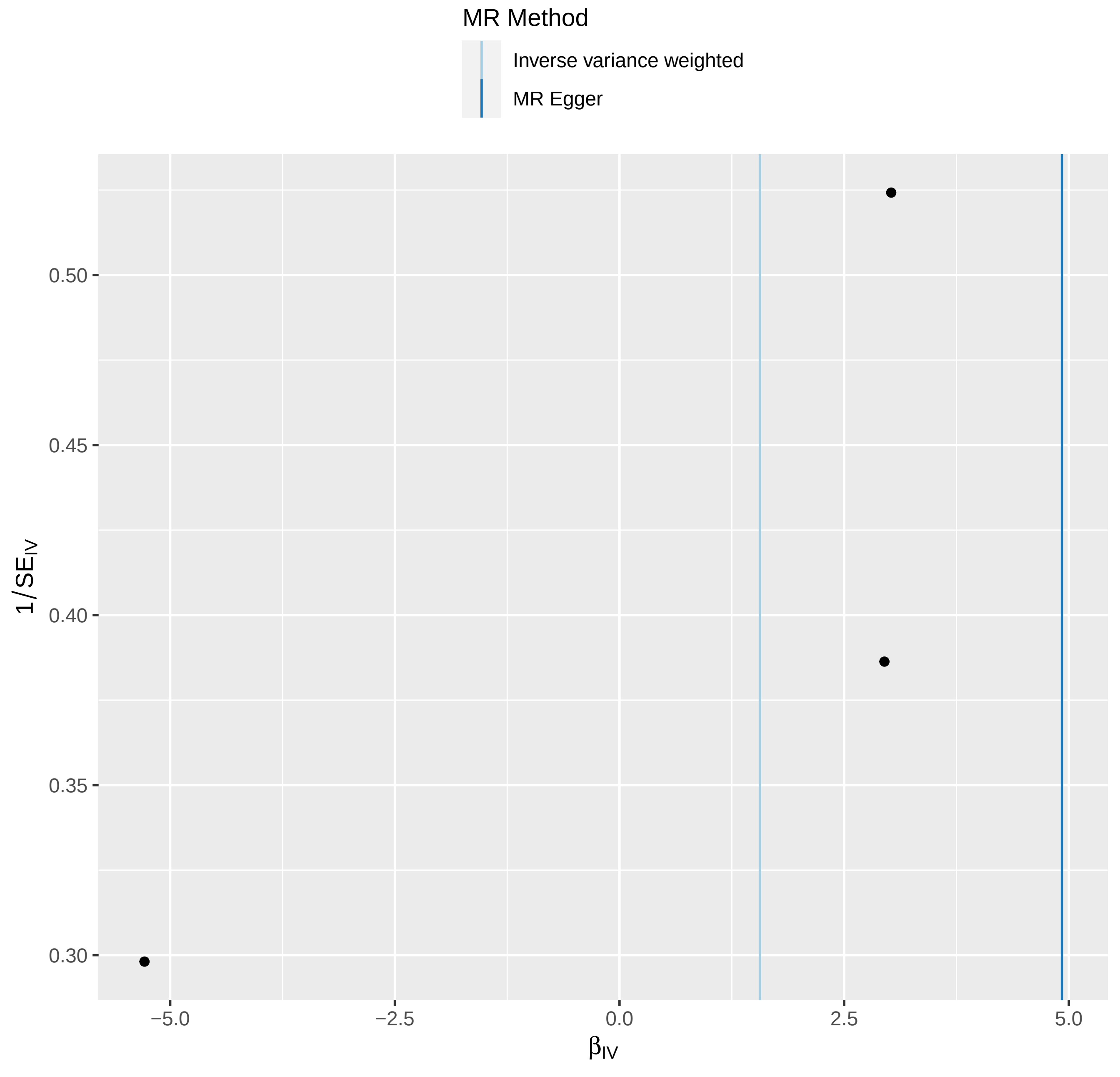

Batch 337 : Phosphate on Crohn's disease of ileocolon

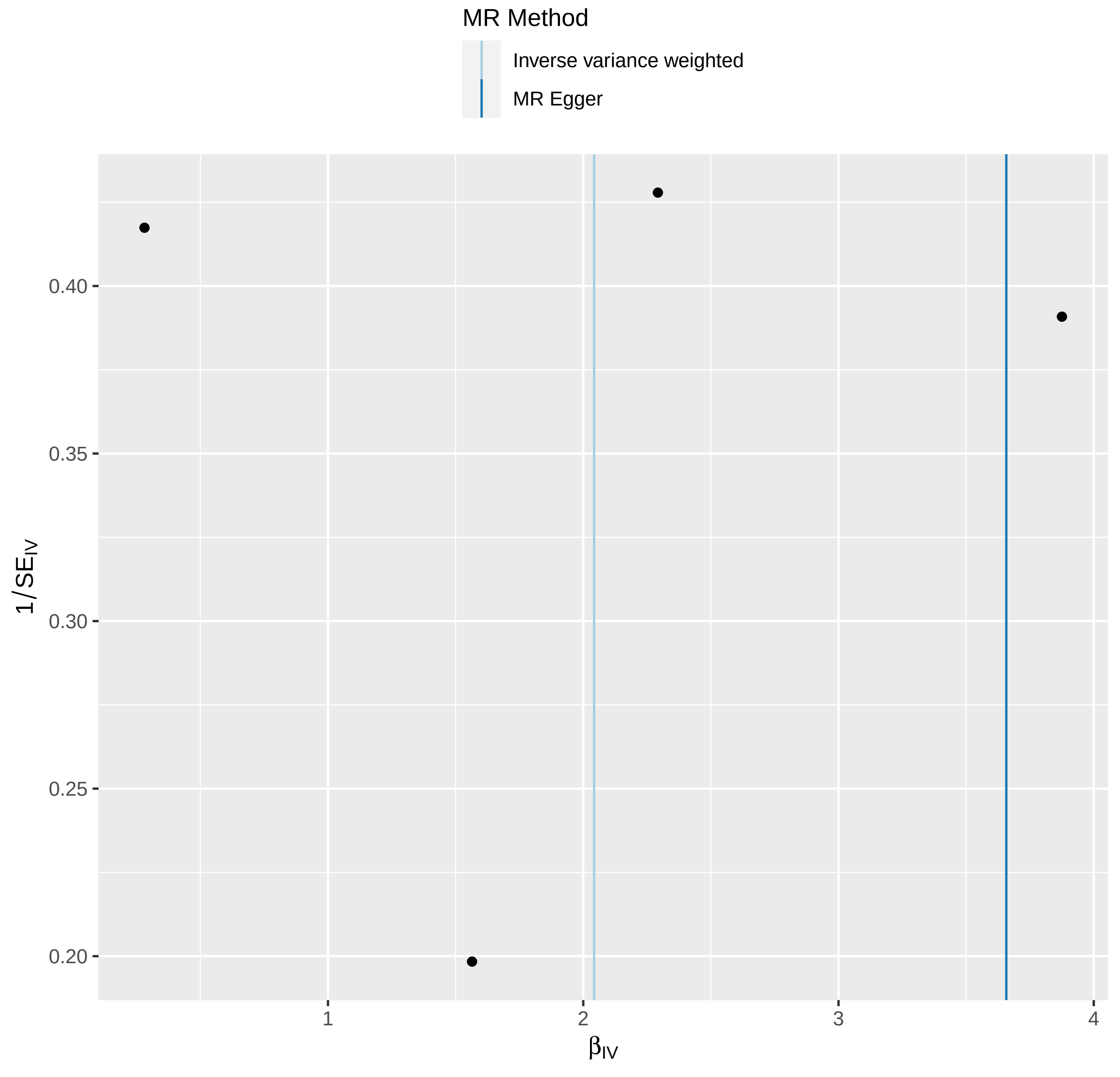

Batch 338 : 3−methoxytyrosine on Crohn's disease of ileocolon

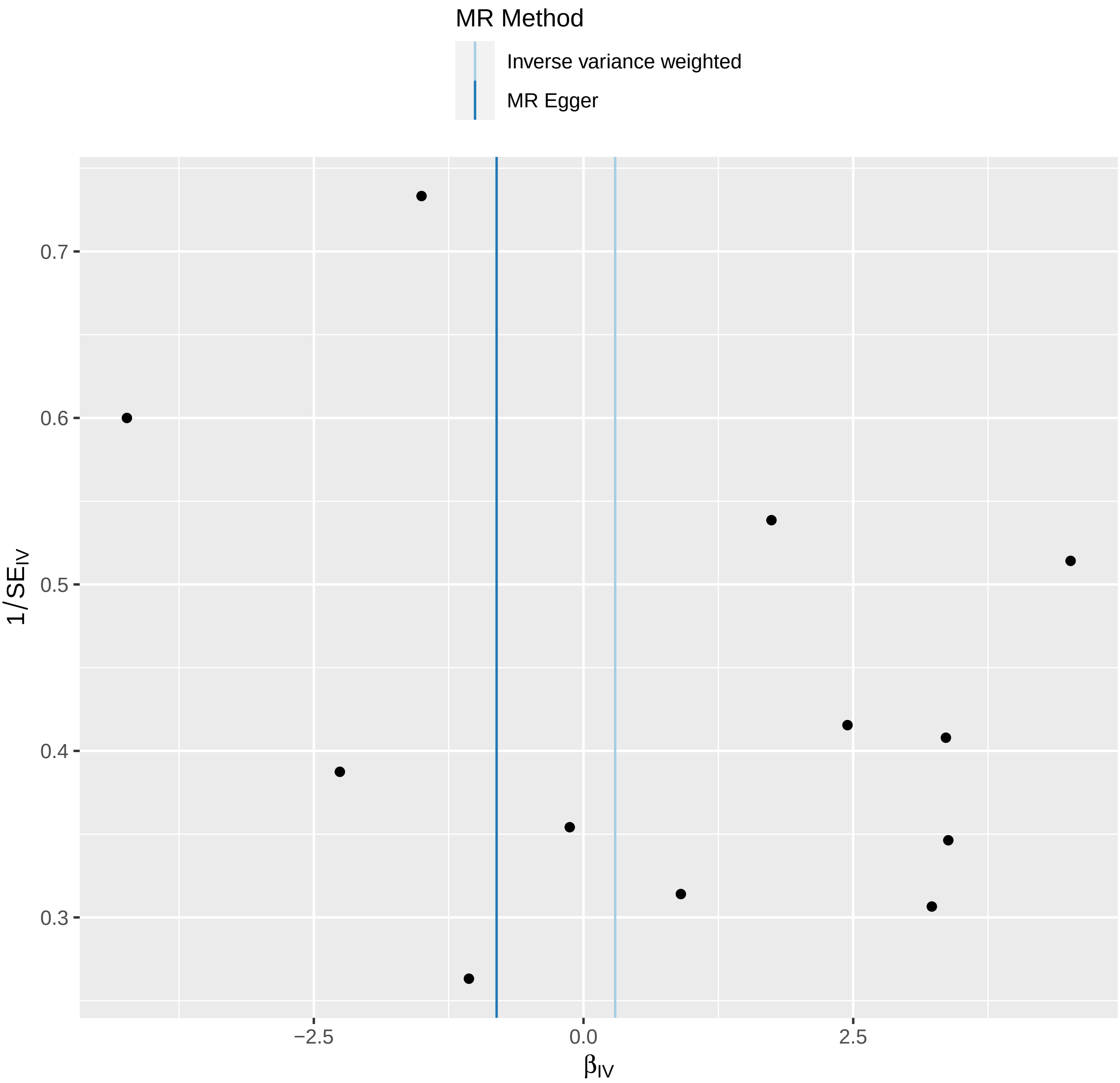

Batch 340 : Undecanoate (11:0) on Crohn's disease of ileocolon

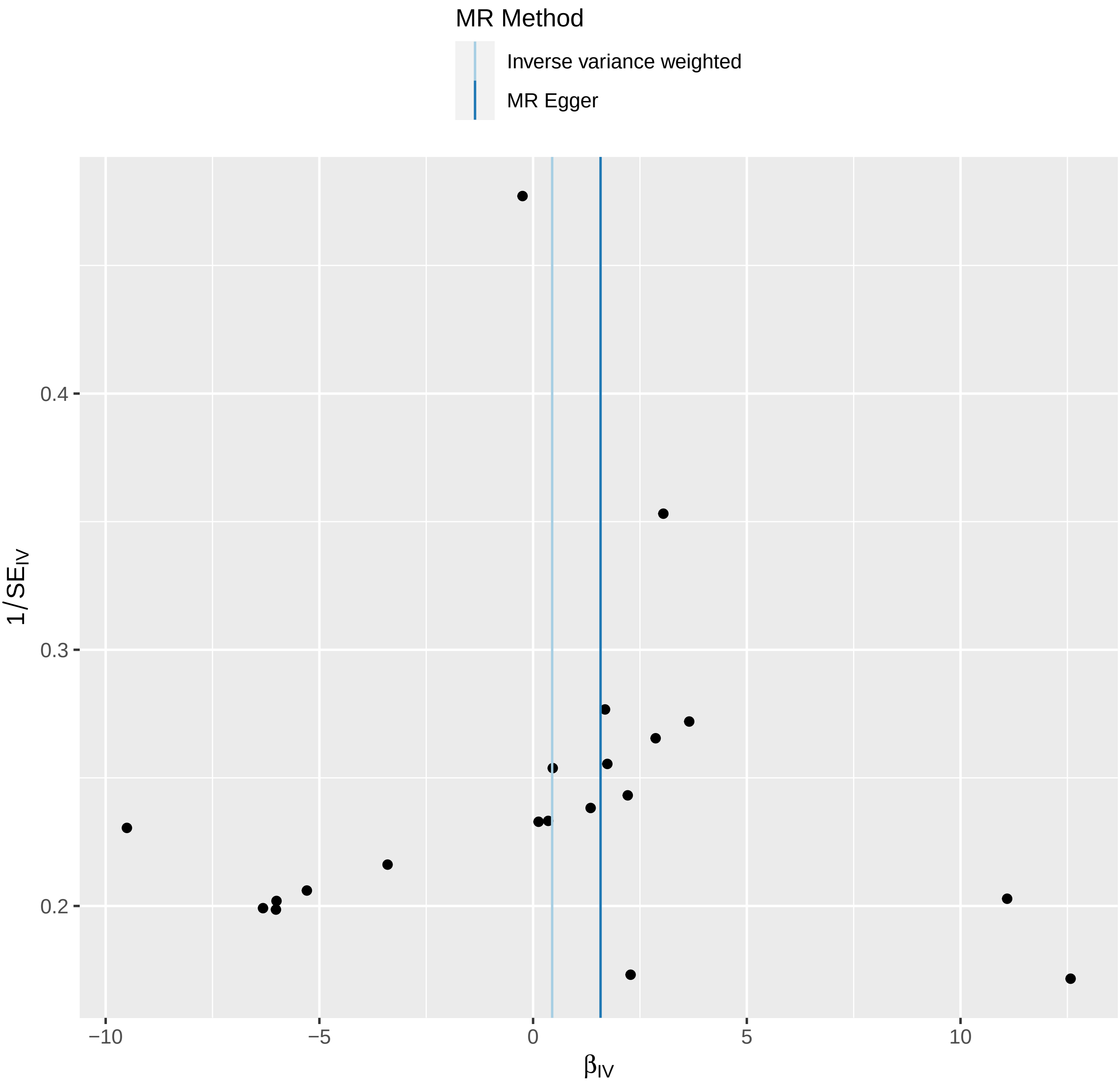

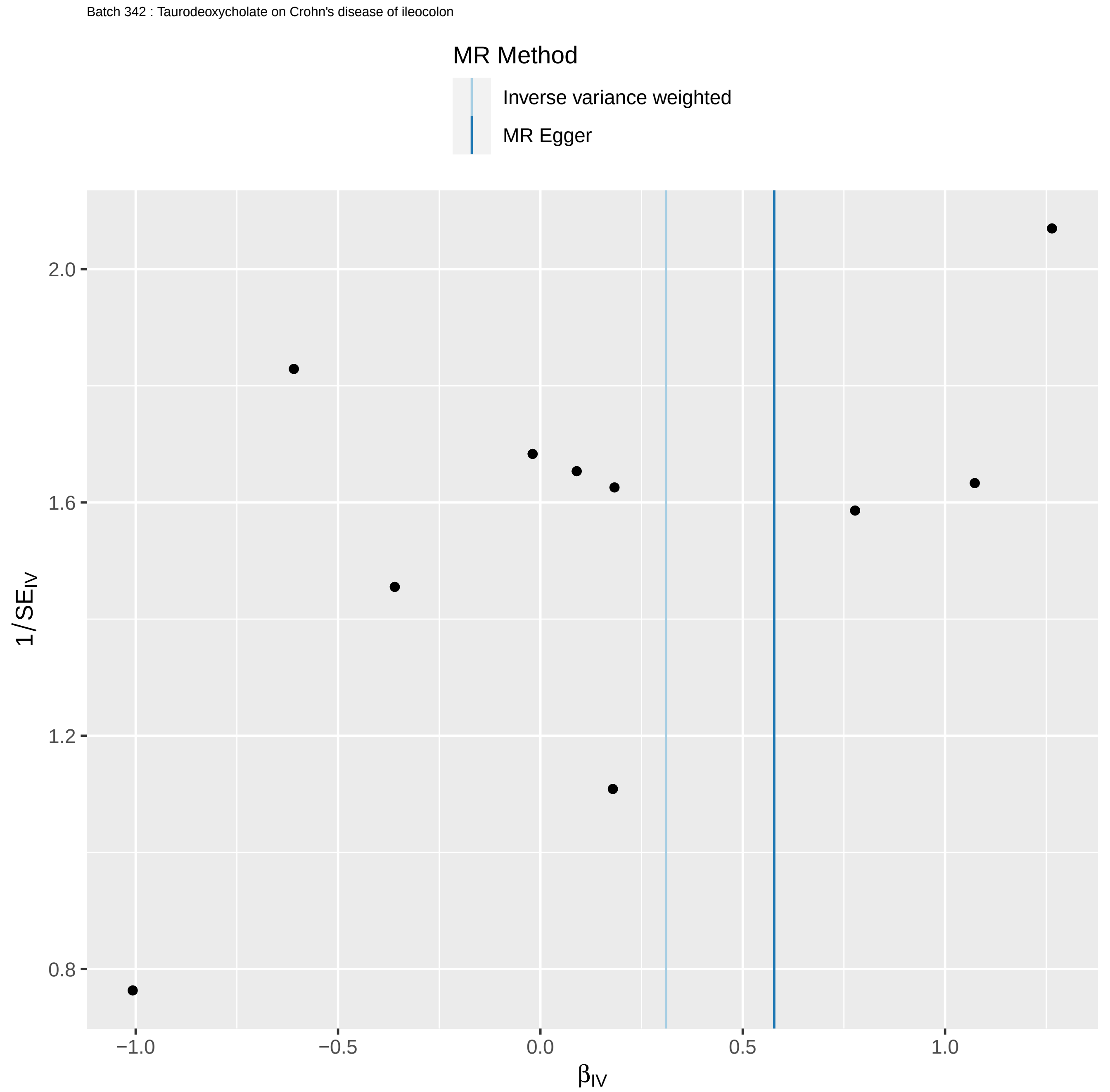
Batch 342 : Taurodeoxycholate on Crohn's disease of ileocolon
MR Method
Inverse variance weighted
MR Egger
2.0
1.6
1.2
0.8
1/SE_IV
−1.0
−0.5
0.0
0.5
1.0
β_IV

Batch 343 : Glycerol on Crohn's disease of ileocolon

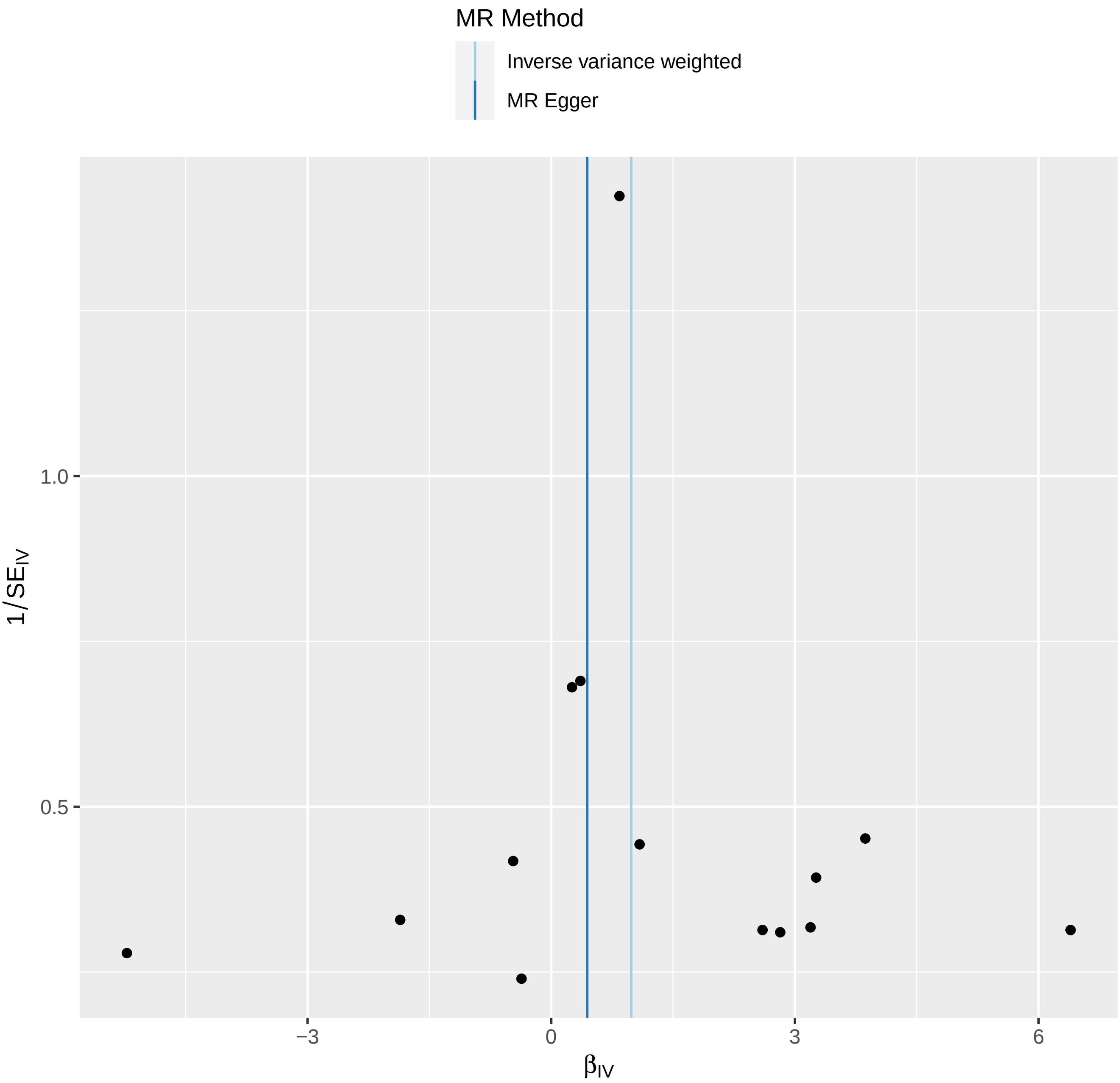

Batch 344 : Kynurenine on Crohn's disease of ileocolon

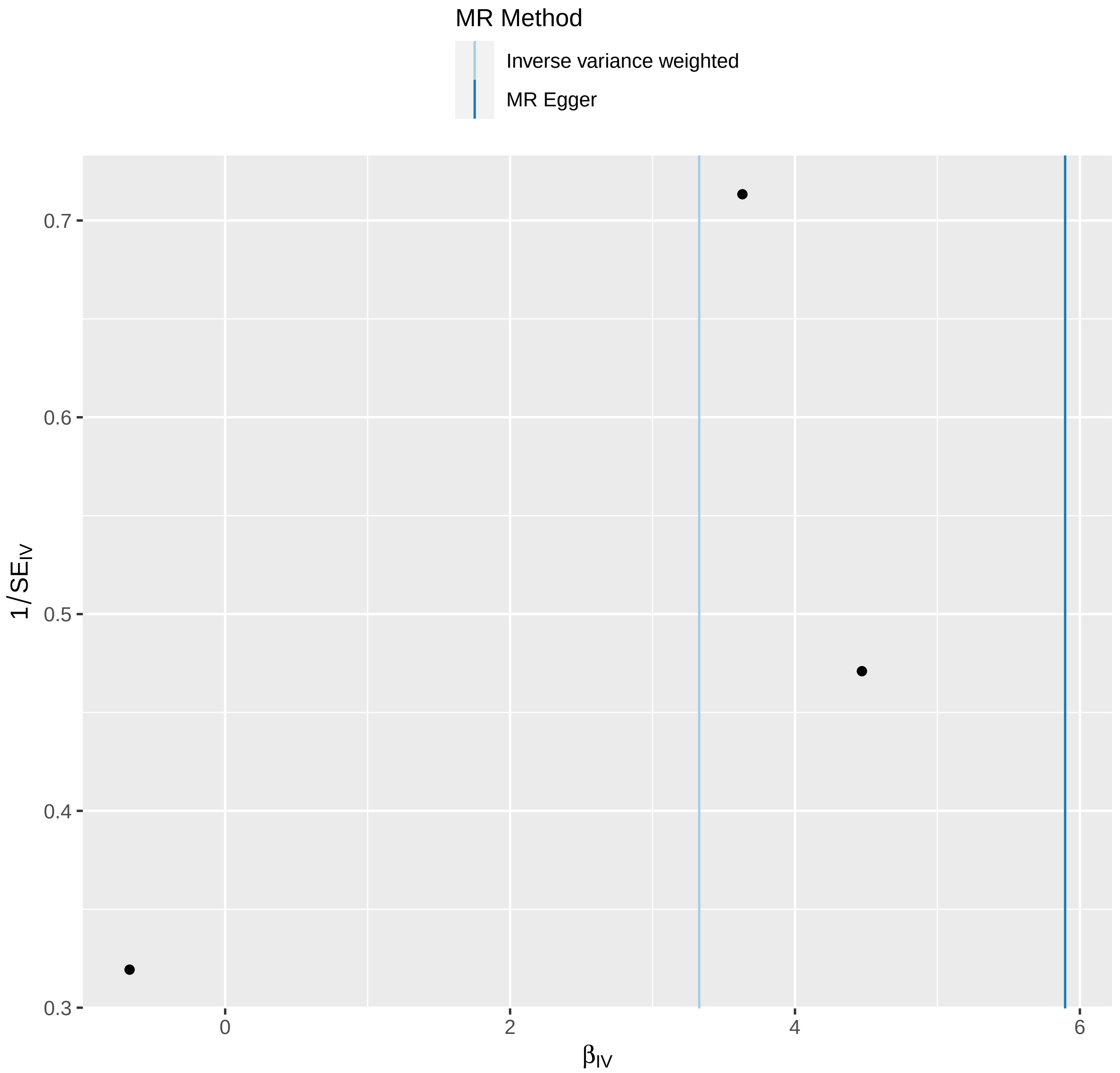

Batch 345 : Mannitol on Crohn's disease of ileocolon

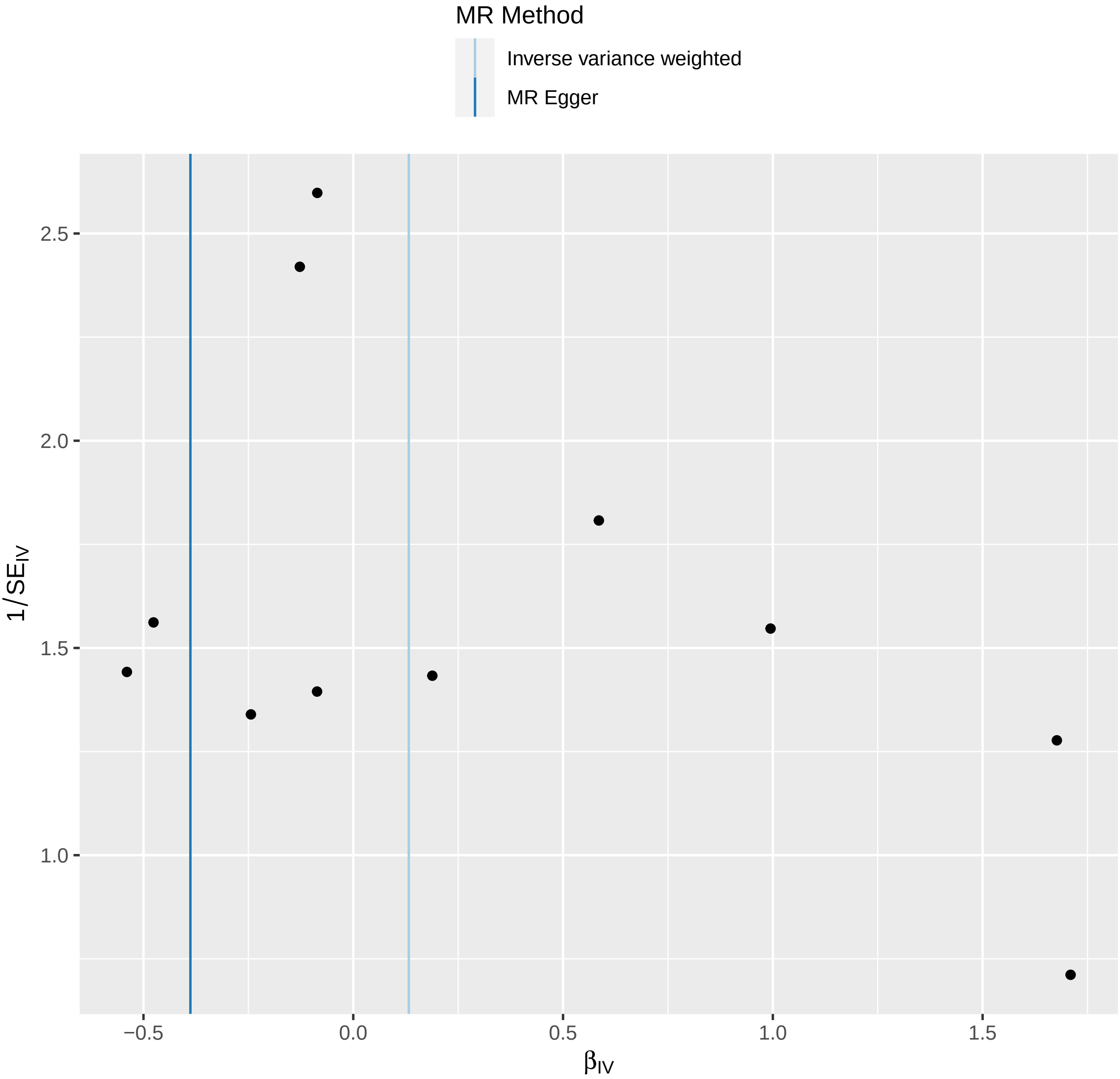

Batch 346 : Glycerol 3−phosphate (G3P) on Crohn's disease of ileocolon

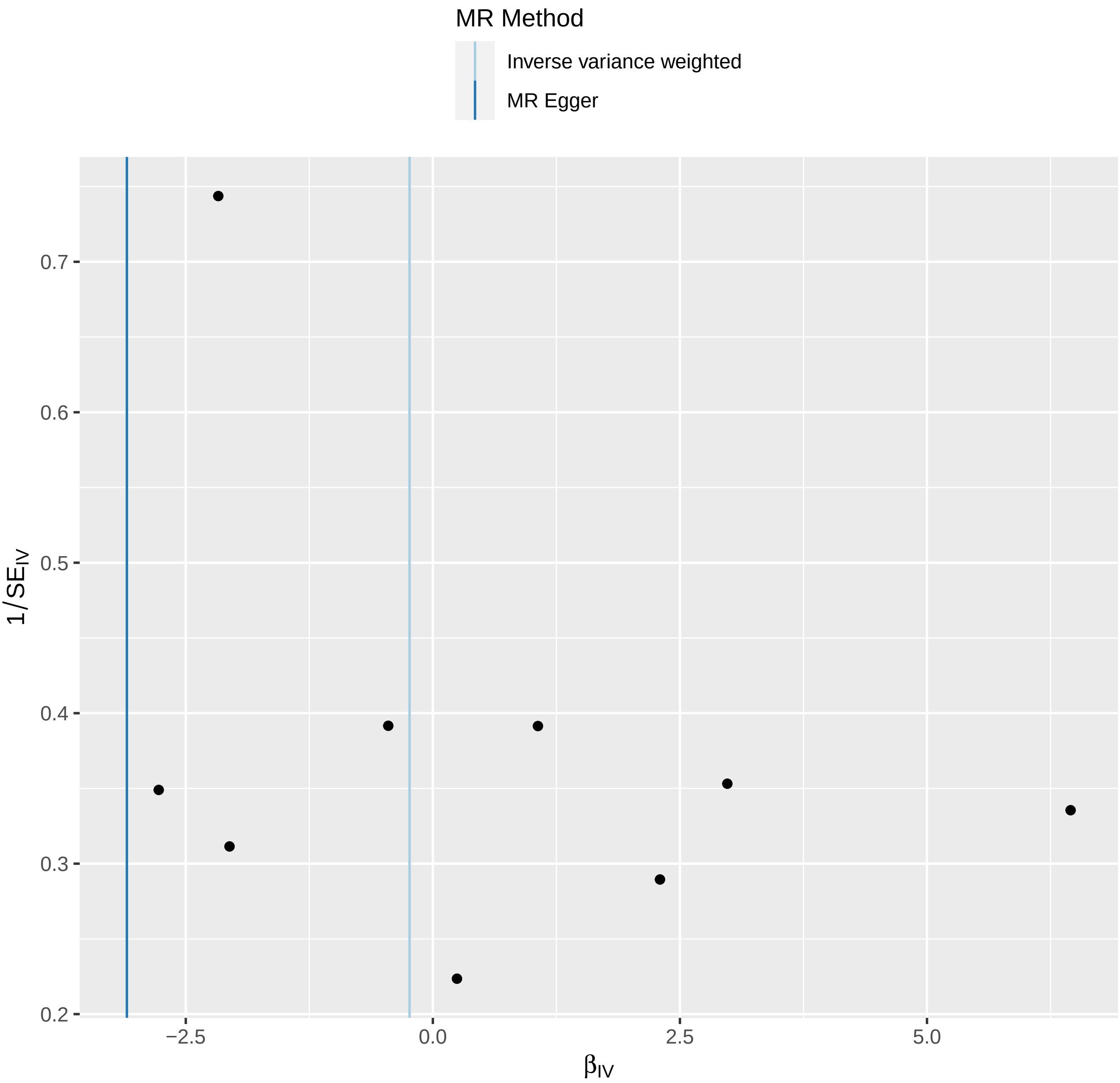

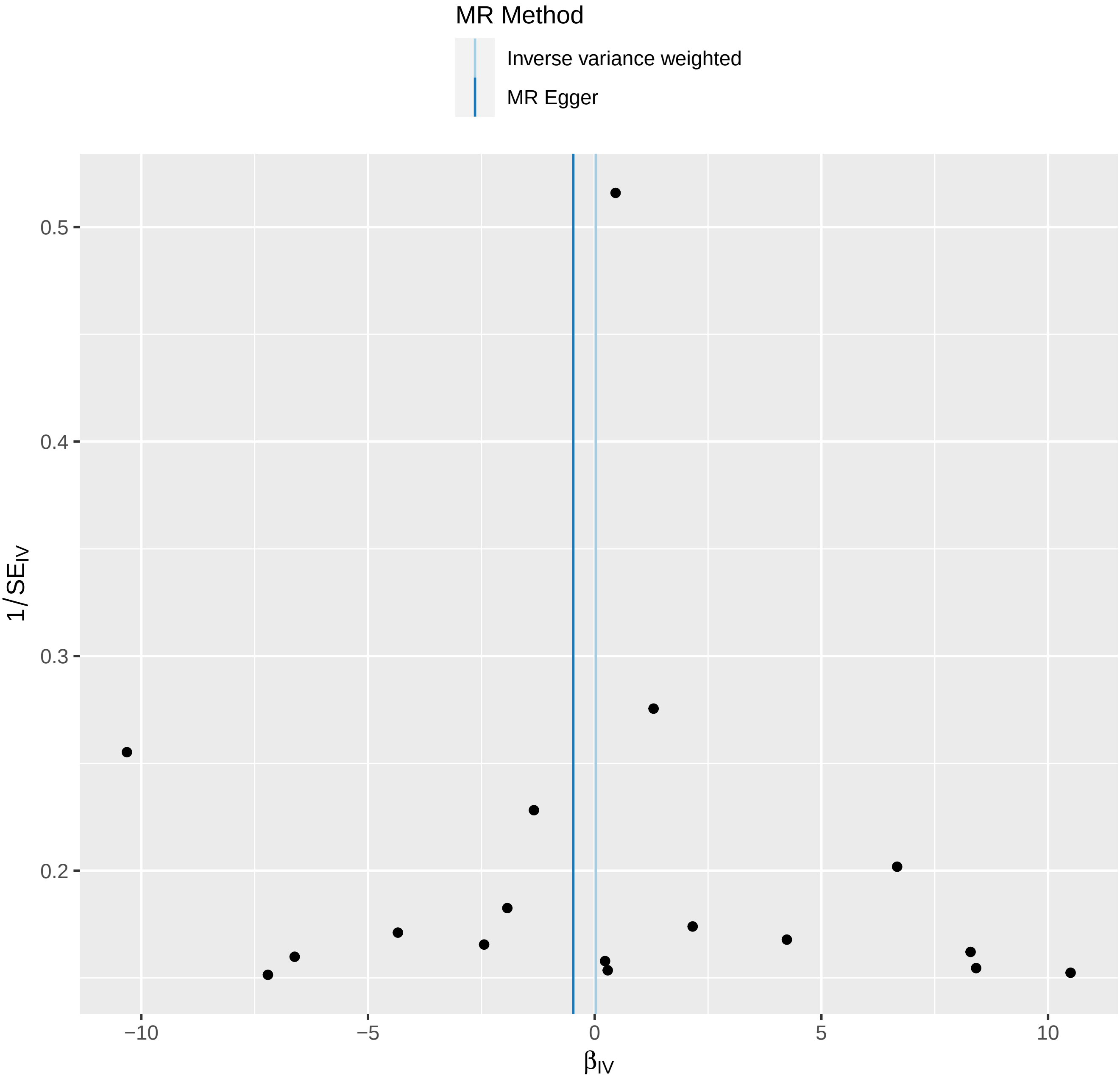
MR Method
Inverse variance weighted
MR Egger
0.5
0.4
0.3
0.2
1/SE$_{IV}$
−10
−5
0
5
10
β$_{IV}$

Batch 349 : Choline on Crohn's disease of ileocolon

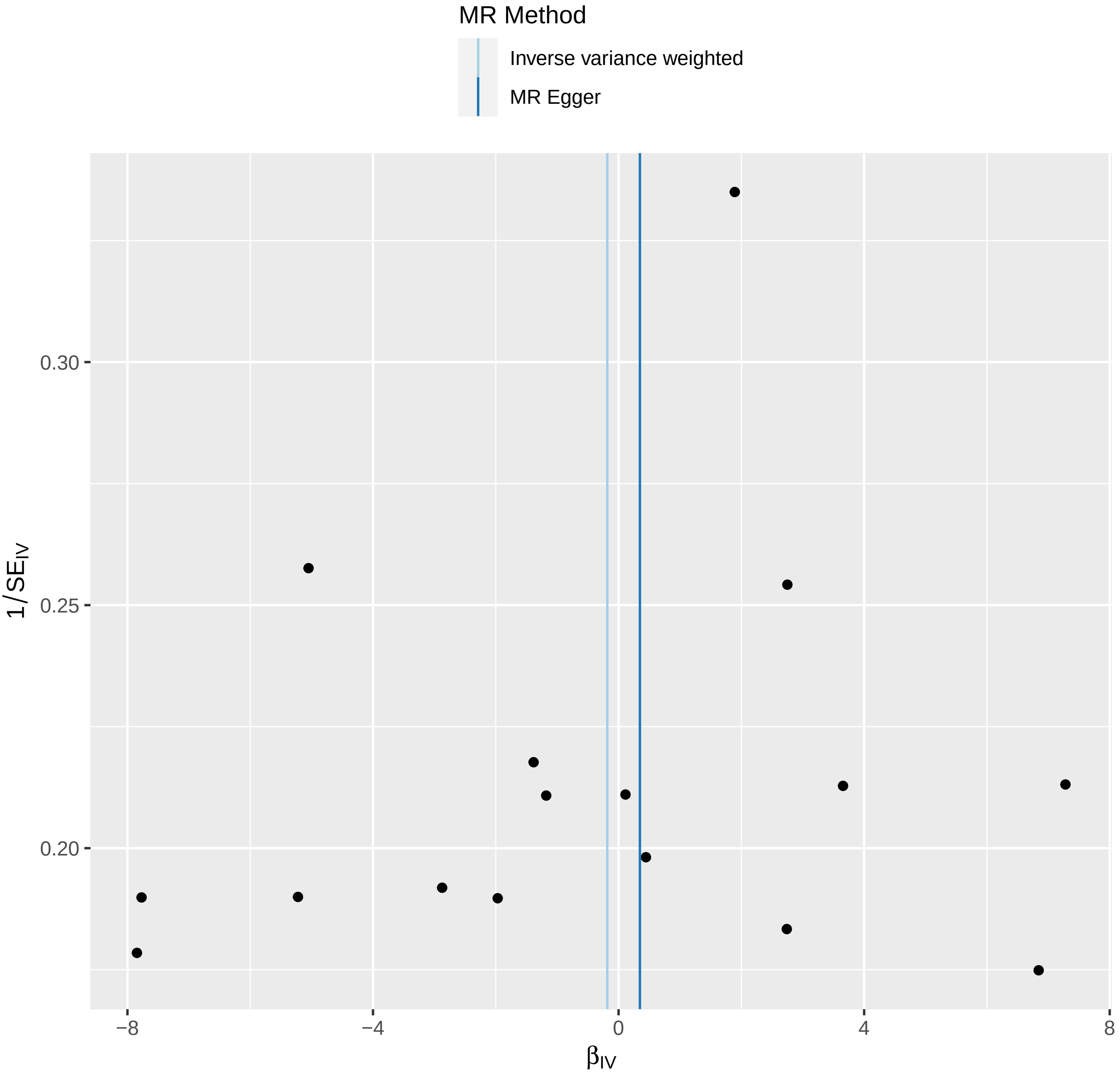

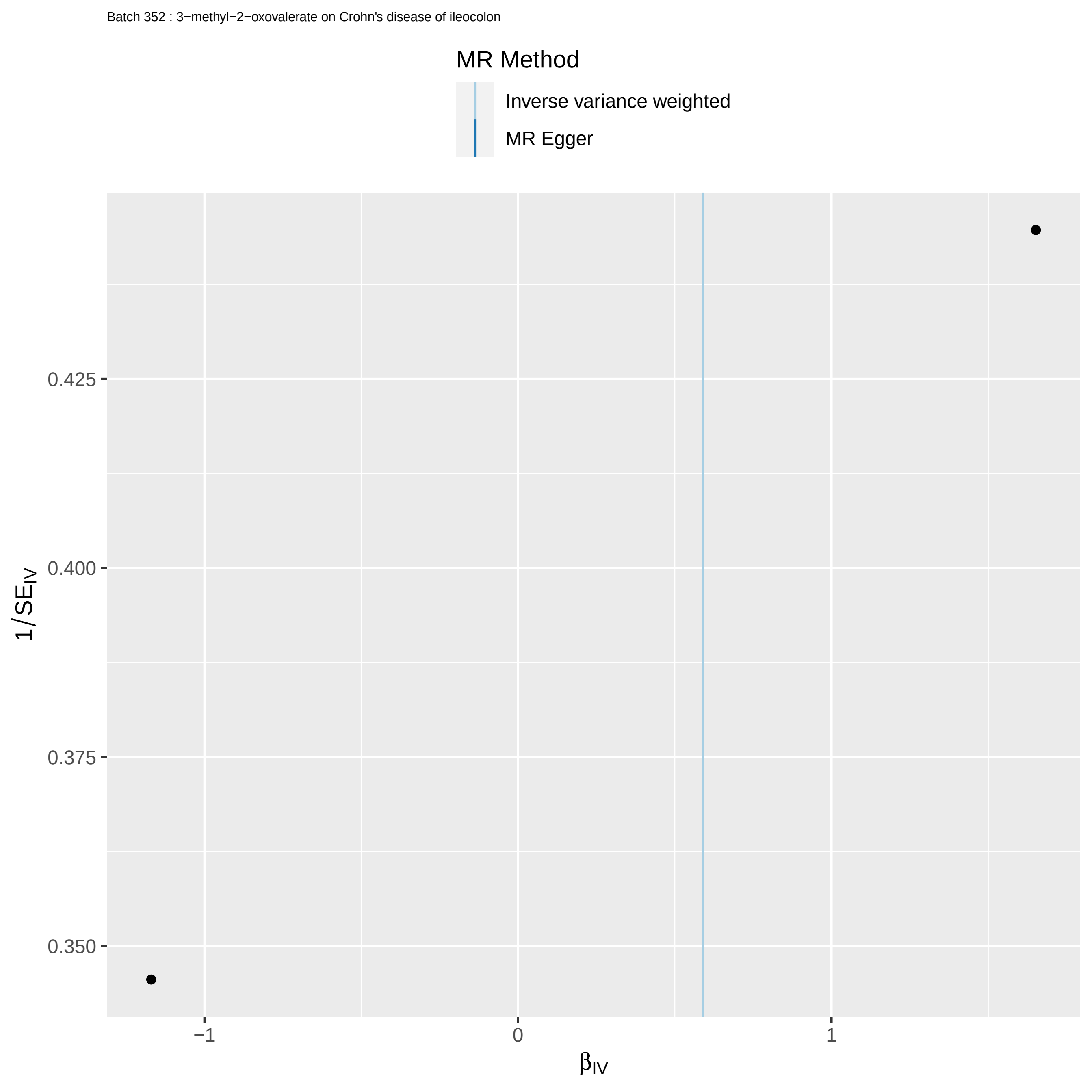

Batch 352 : 3−methyl−2−oxovalerate on Crohn's disease of ileocolon
MR Method
Inverse variance weighted
MR Egger
0.425
0.400
0.375
0.350
1/SE$_{IV}$
−1
0
1
β$_{IV}$

Batch 353 : 3−methylhistidine on Crohn's disease of ileocolon

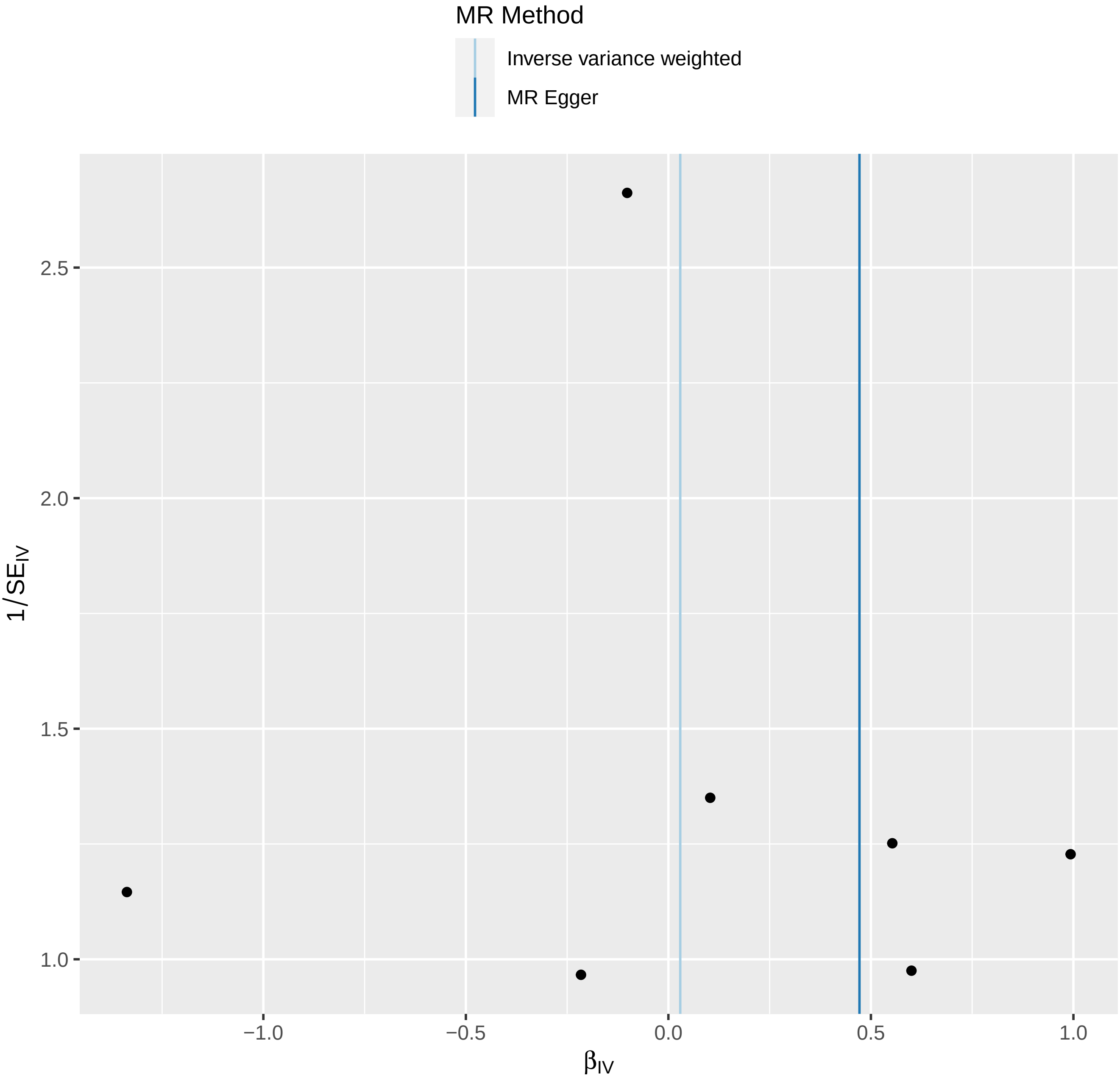

Batch 355 : Phenylacetate on Crohn's disease of ileocolon

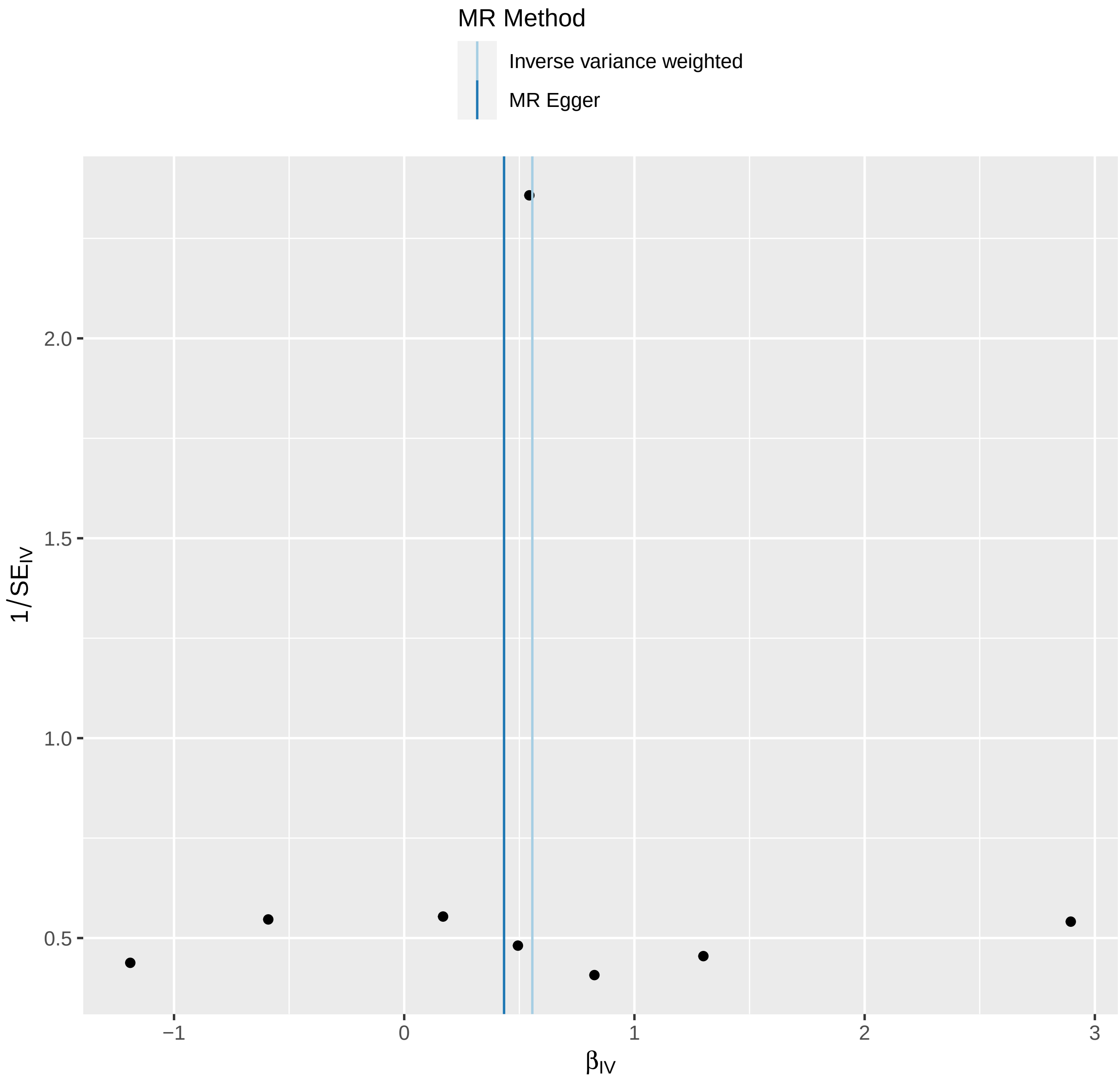

Batch 356 : Glycerophosphorylcholine (GPC) on Crohn's disease of ileocolon

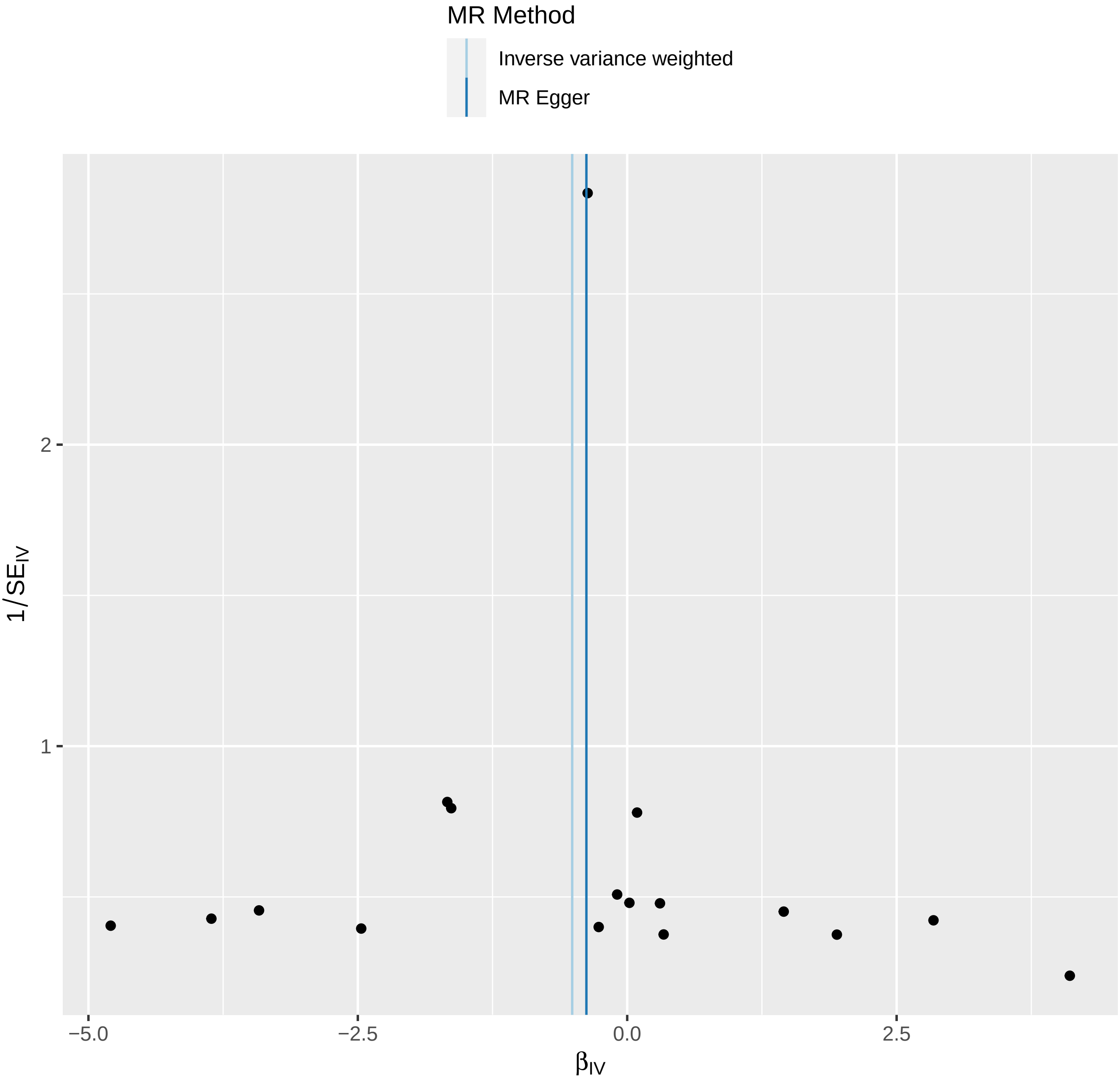

Batch 357 : Aspartate on Crohn's disease of ileocolon

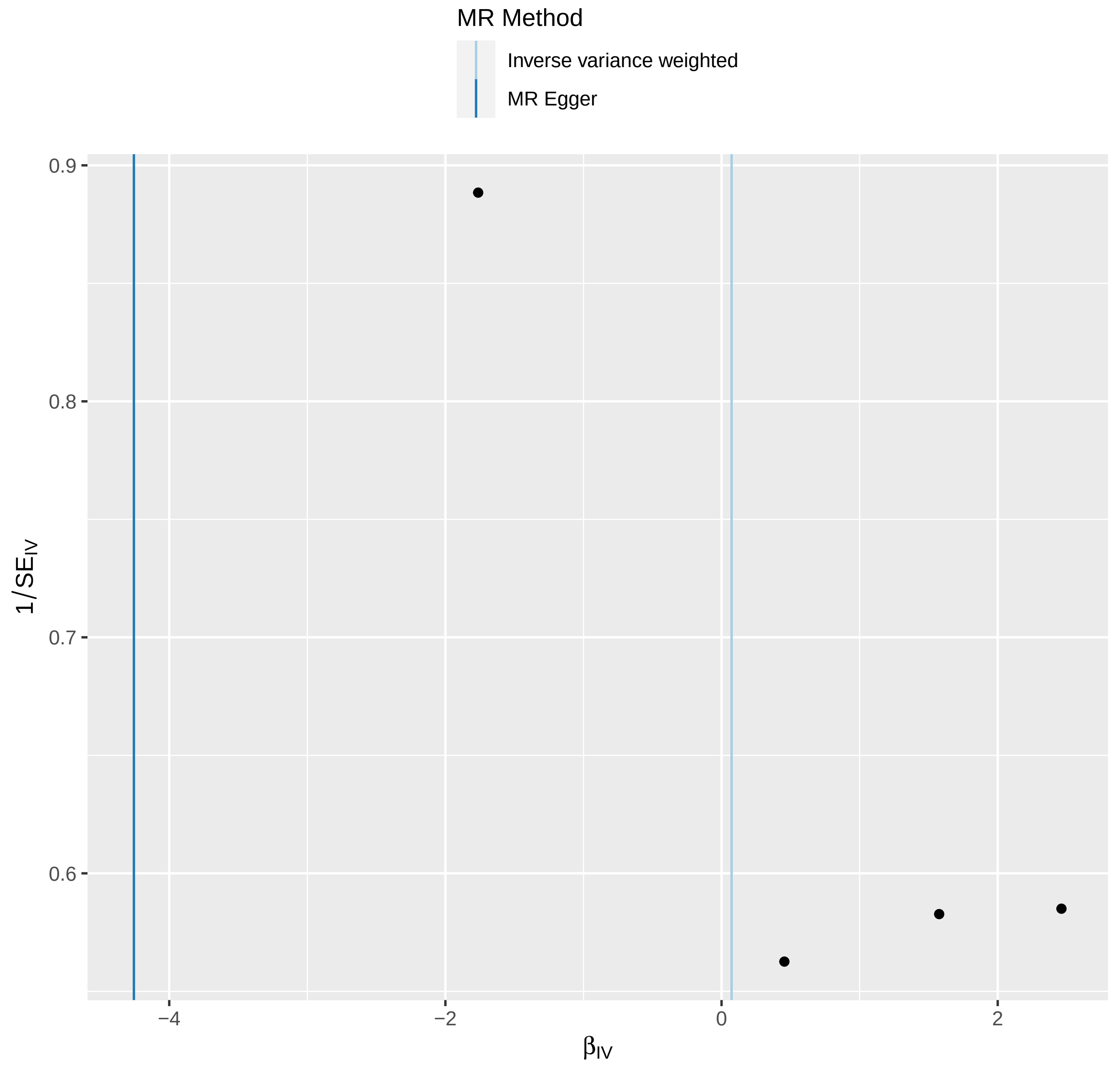

Batch 359 : Dihomo−linoleate (20:2n6) on Crohn's disease of ileocolon

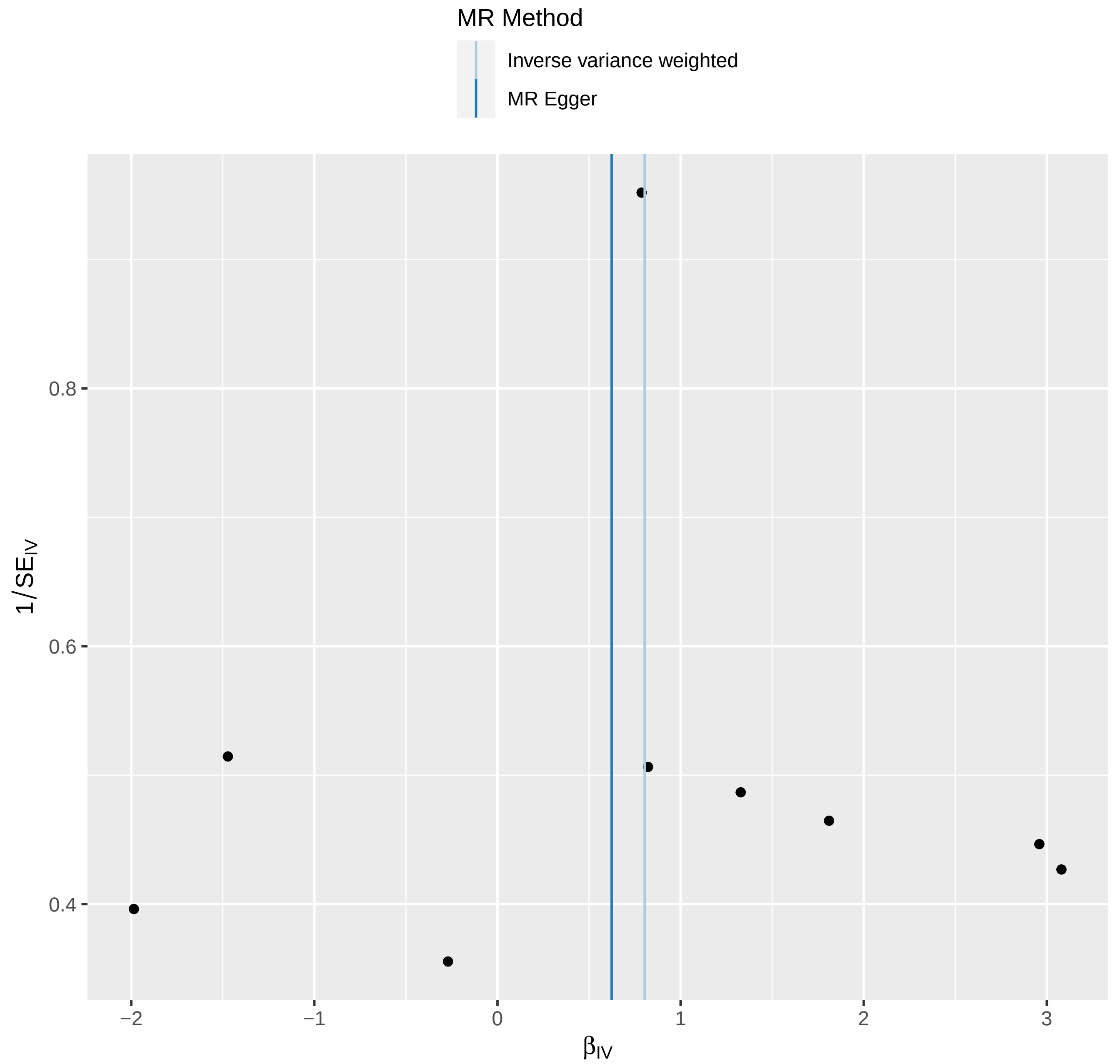

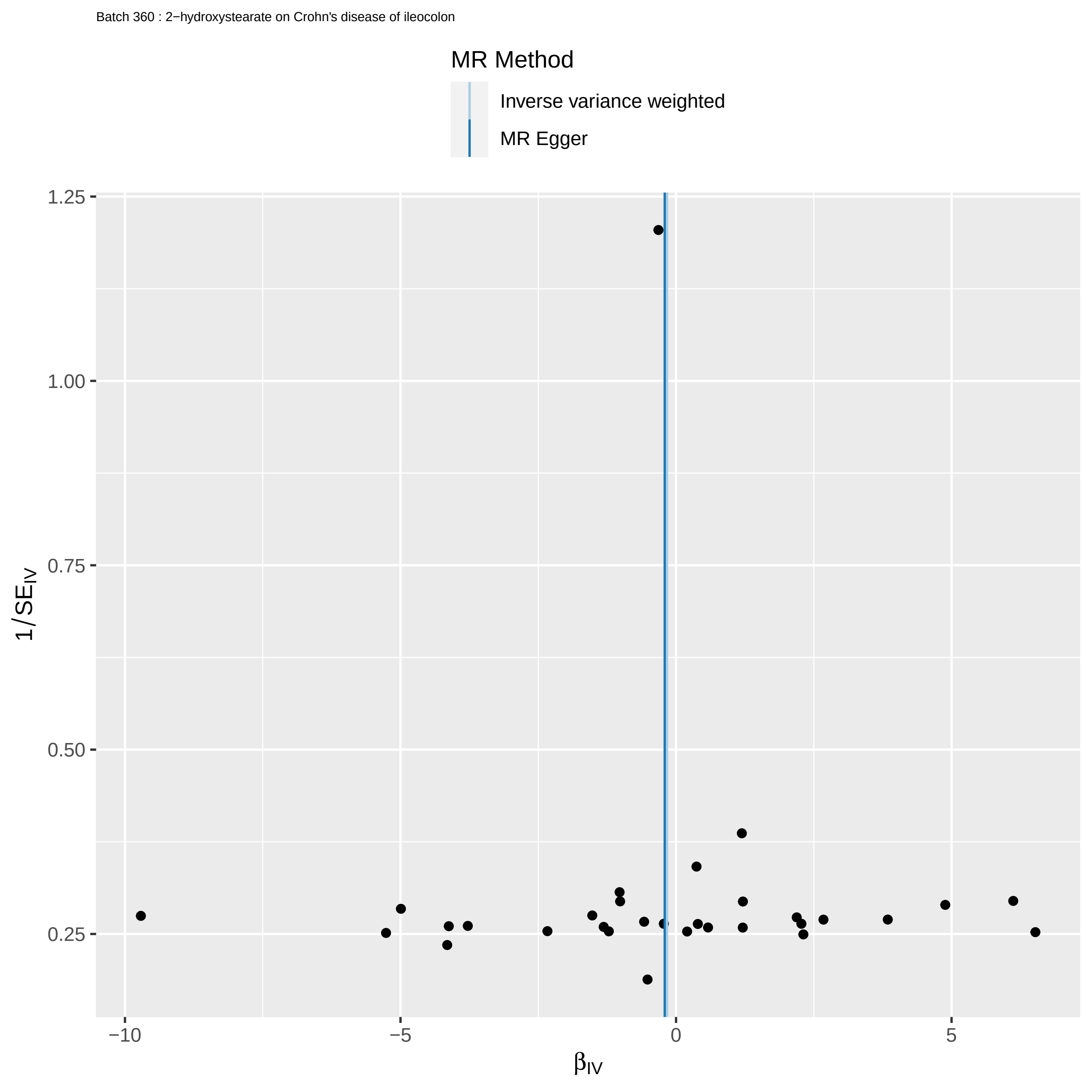

Batch 360 : 2−hydroxystearate on Crohn's disease of ileocolon
MR Method
Inverse variance weighted
MR Egger
1.25
1.00
0.75
0.50
0.25
1/SE$_{IV}$
−10
−5
0
5
β$_{IV}$

Batch 362 : Glycylvaline on Crohn's disease of ileocolon

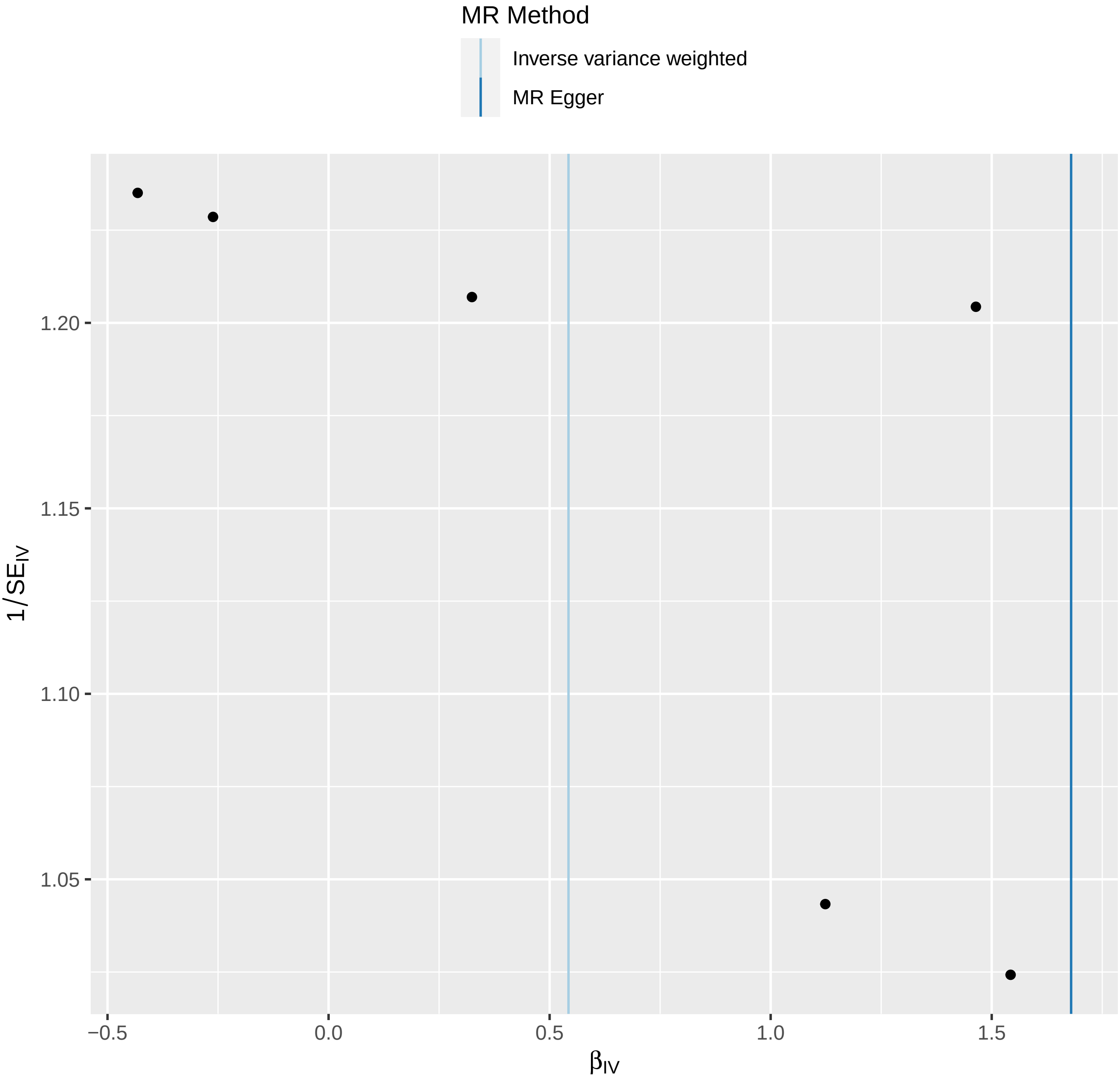

Batch 363 : Gamma−glutamylleucine on Crohn's disease of ileocolon

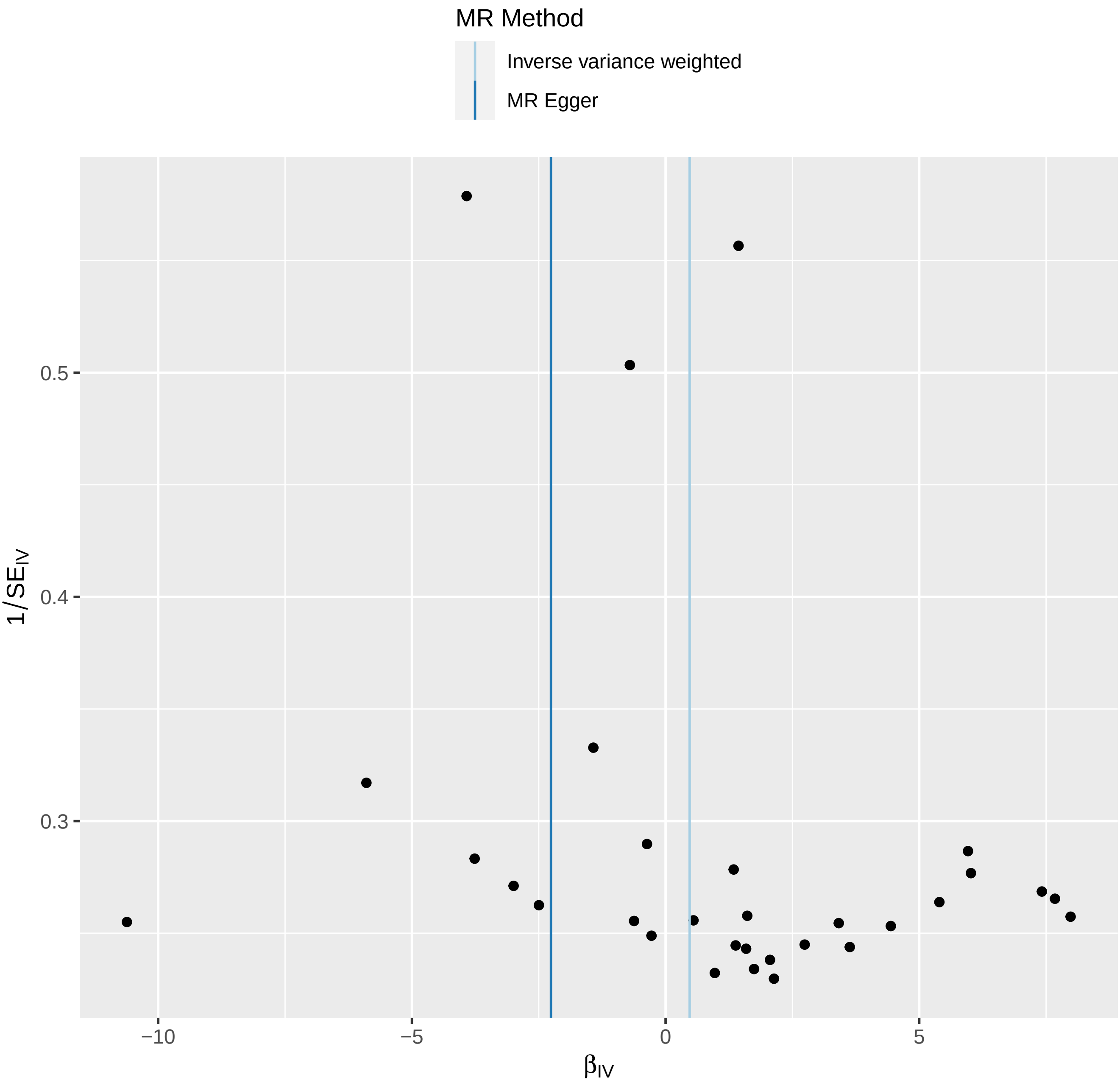

Batch 366 : Glycocholate on Crohn's disease of ileocolon

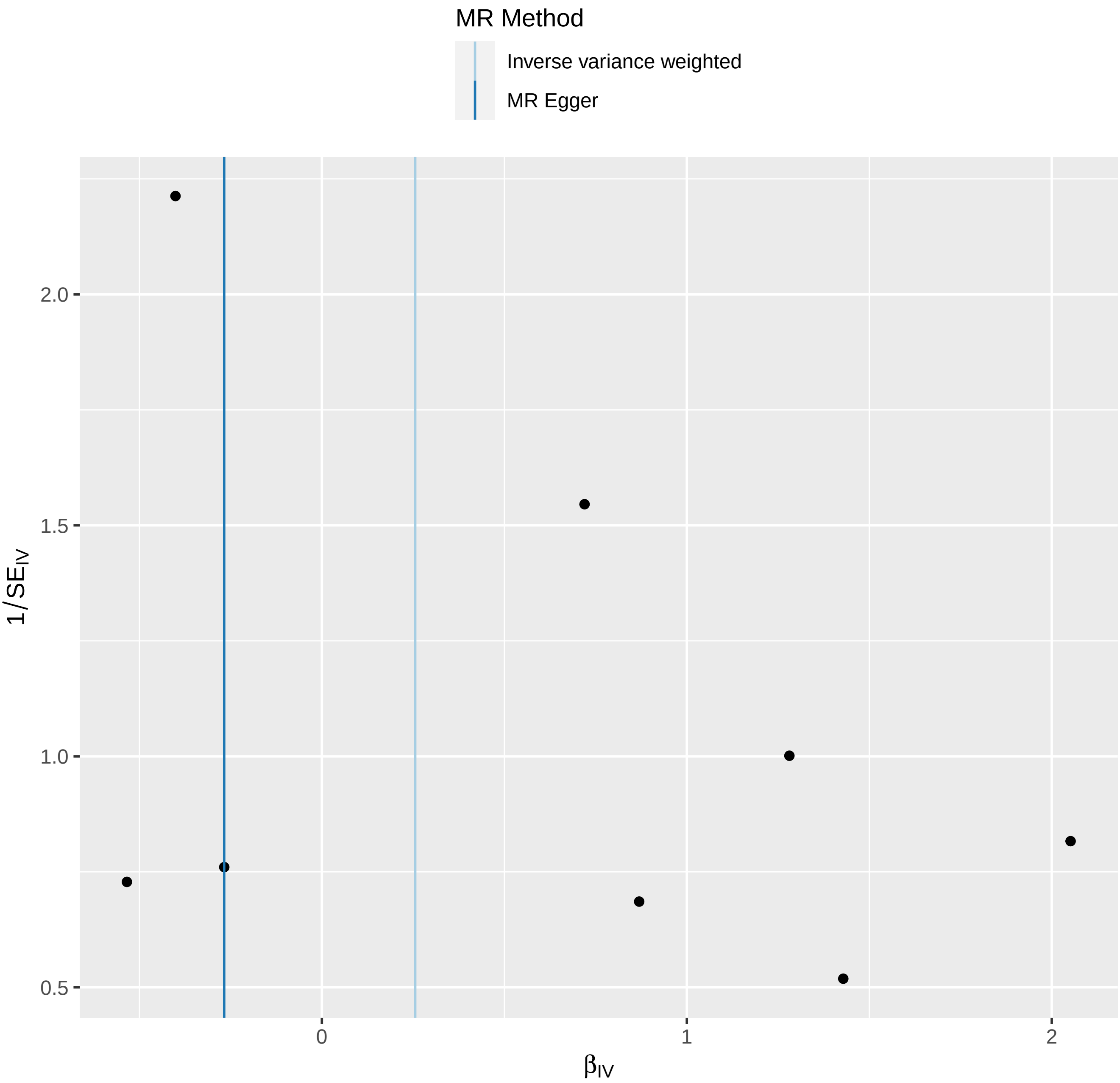

Batch 367 : Glycodeoxycholate on Crohn's disease of ileocolon

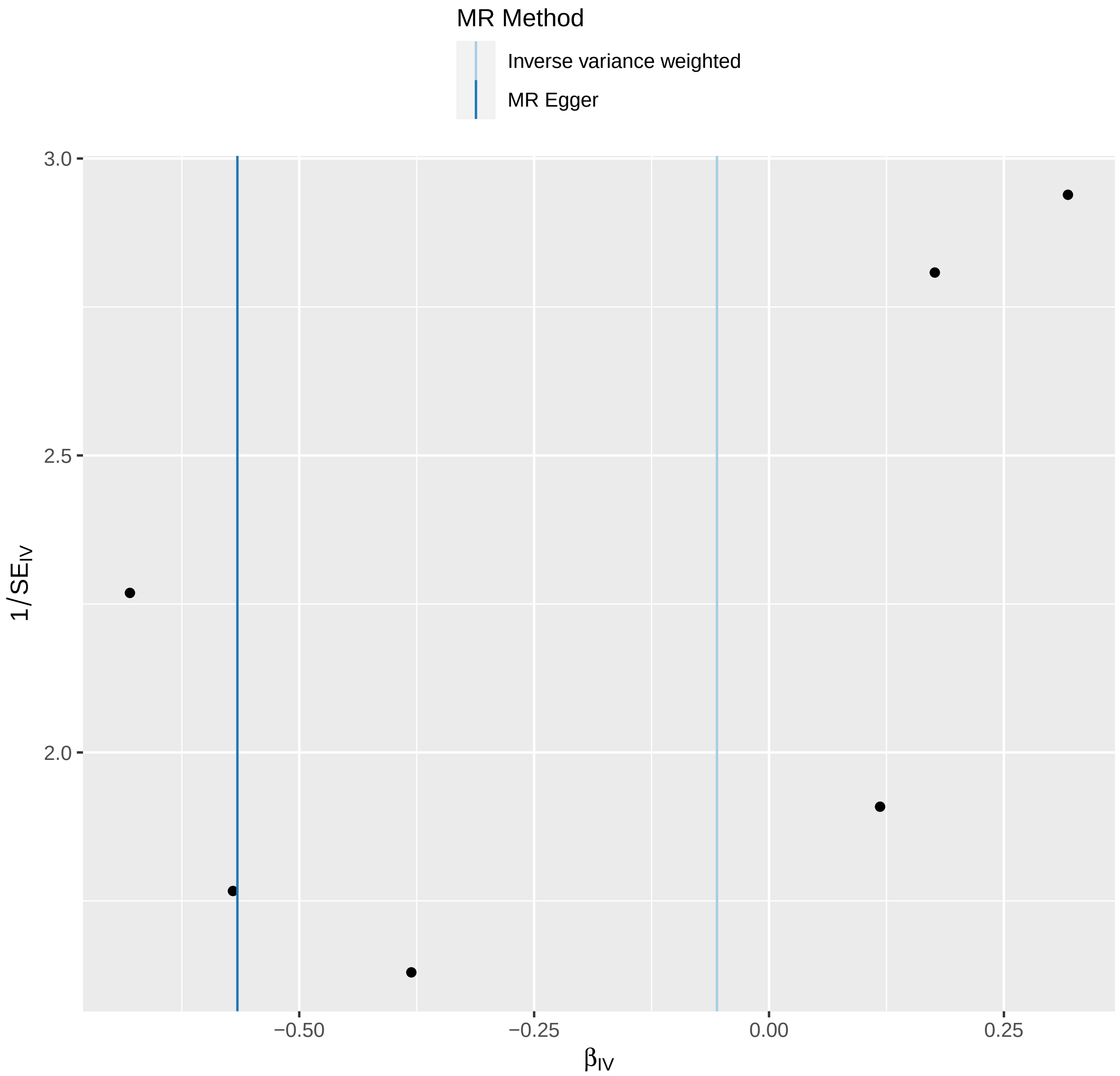

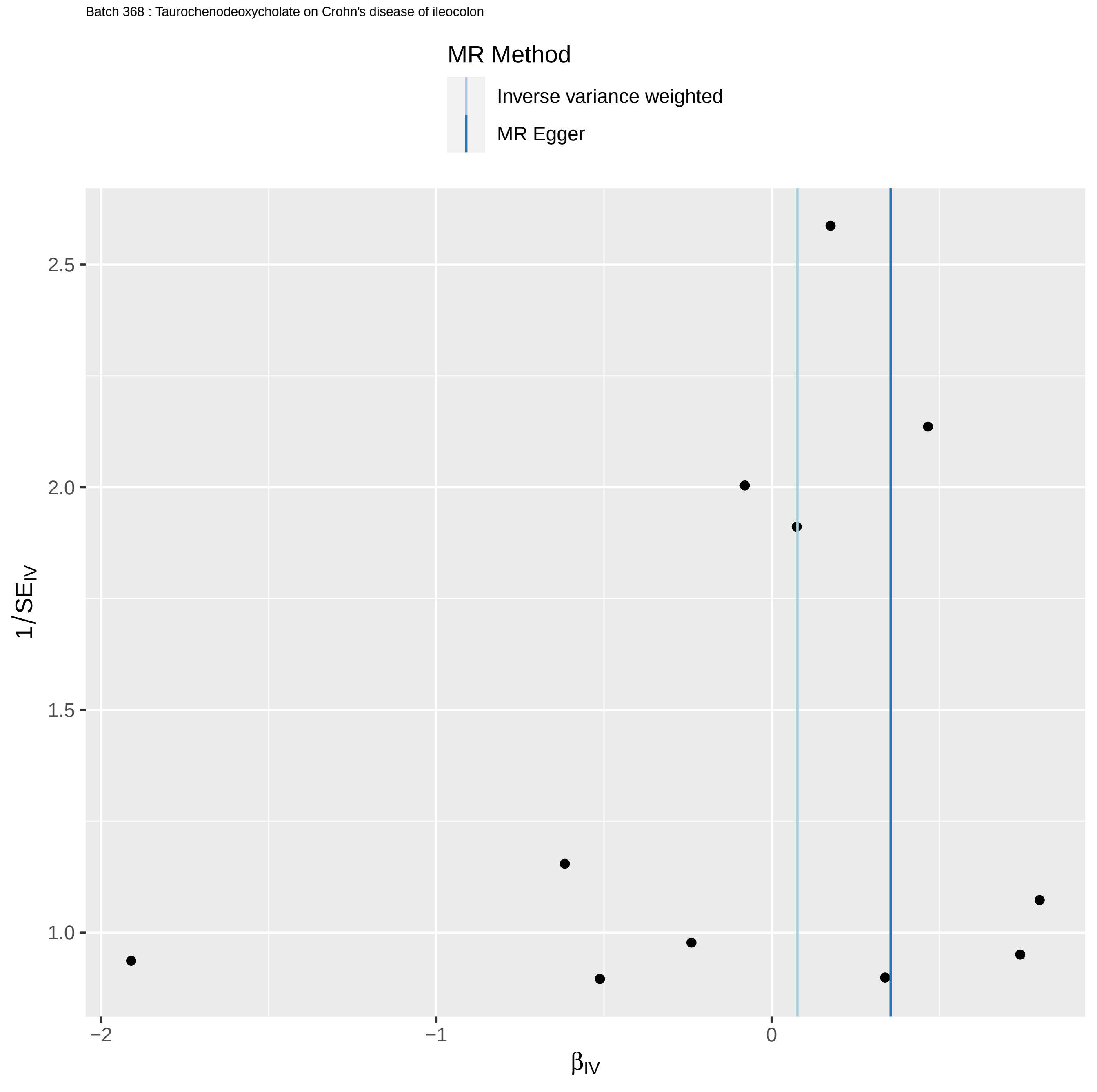

Batch 368 : Taurochenodeoxycholate on Crohn's disease of ileocolon
MR Method
Inverse variance weighted
MR Egger
2.5
2.0
1.5
1.0
1/SE_IV
−2
−1
0
β_IV

Batch 369 : Taurocholate on Crohn's disease of ileocolon

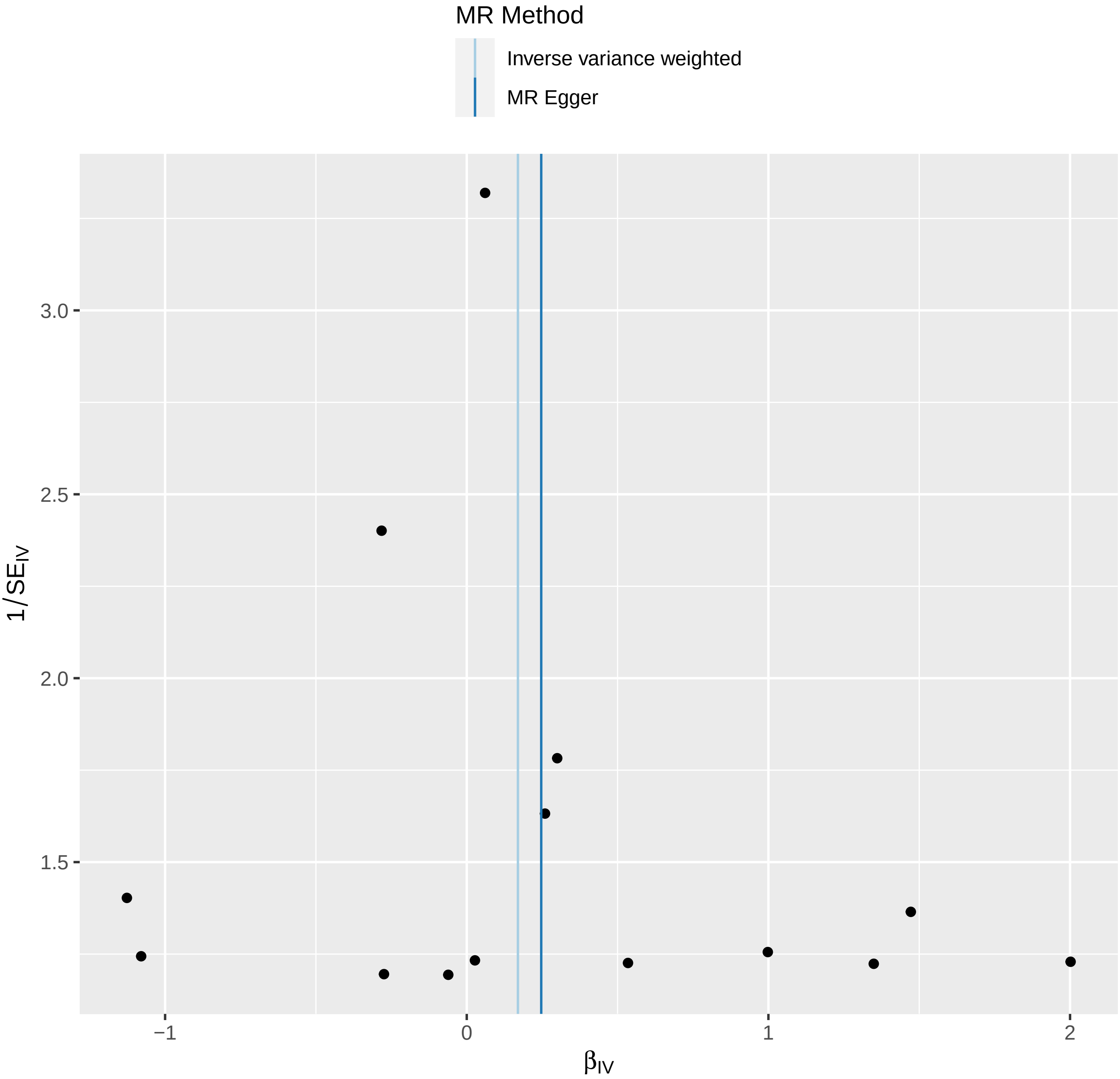

Batch 371 : 1−stearoylglycerophosphoinositol on Crohn's disease of ileocolon

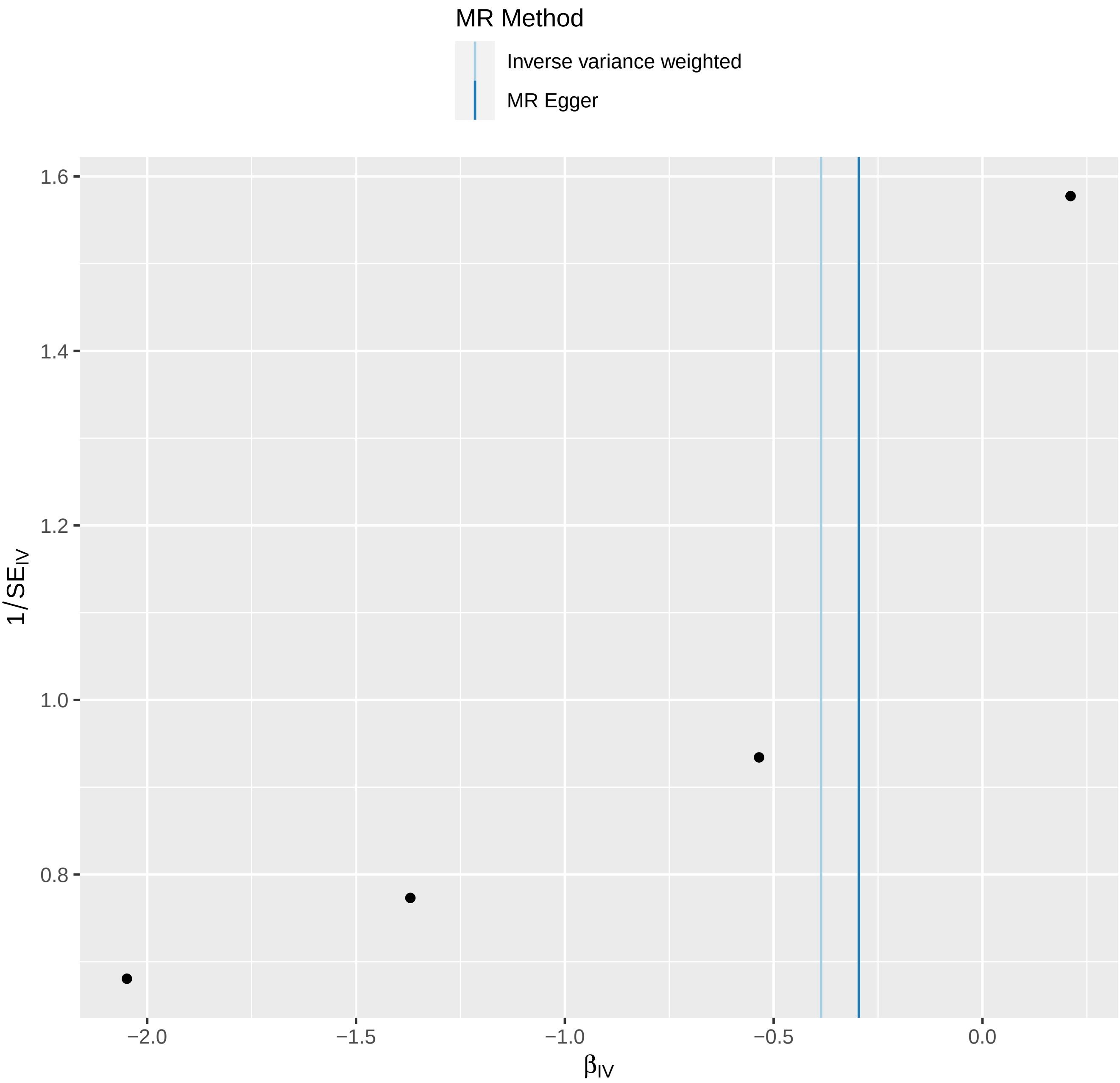

Batch 372 : Myo−inositol on Crohn's disease of ileocolon

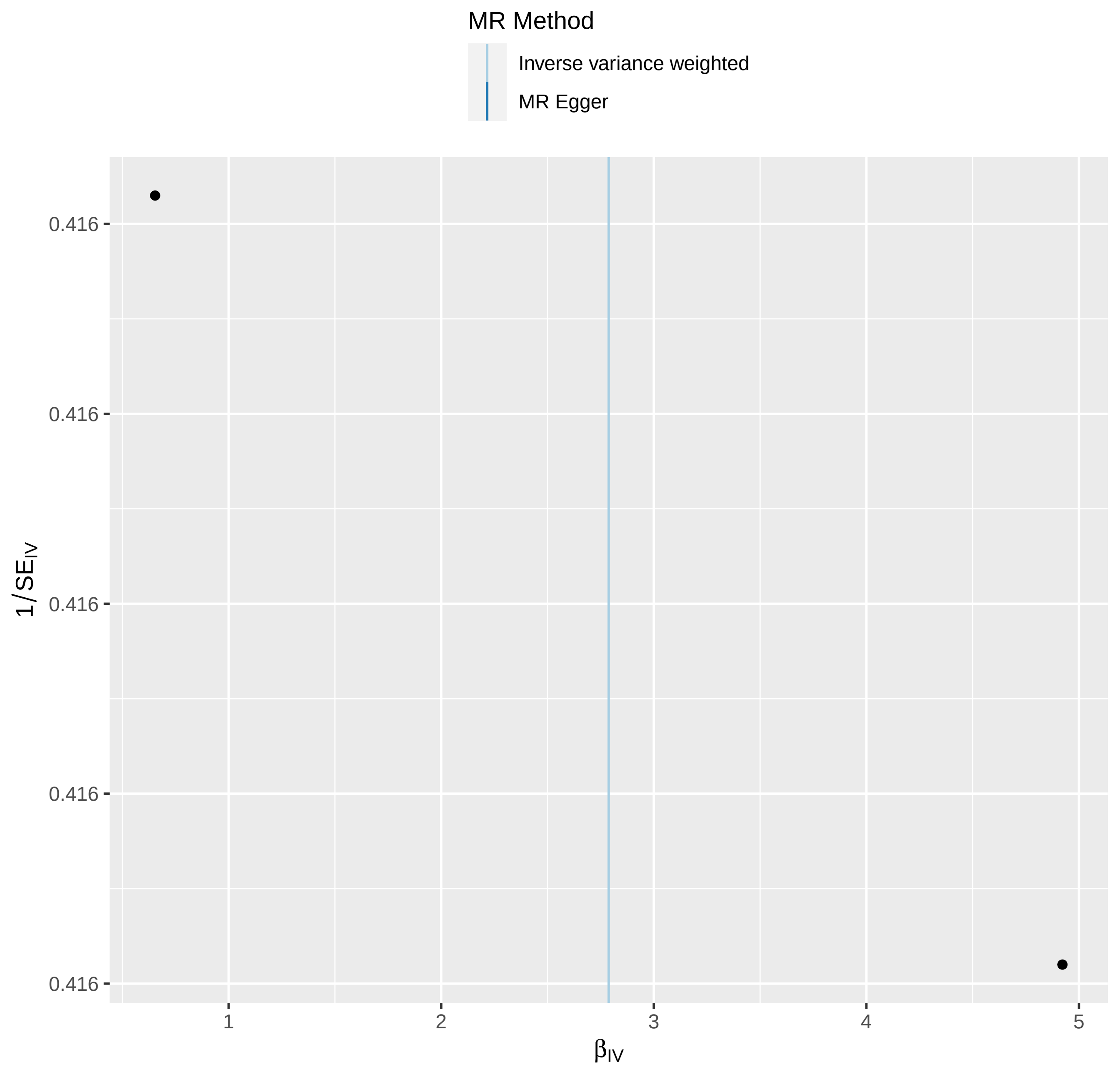

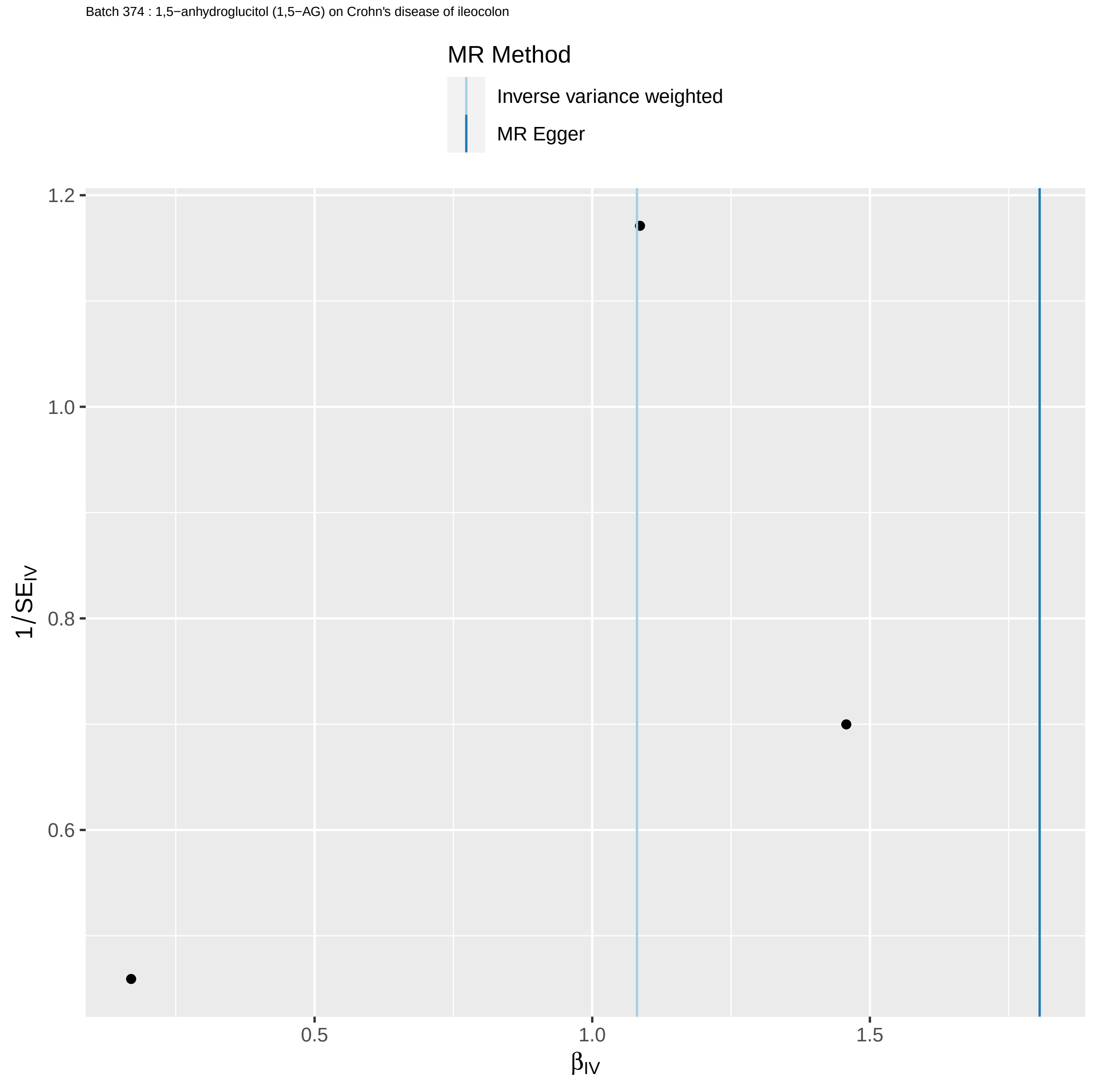

Batch 374 : 1,5−anhydroglucitol (1,5−AG) on Crohn's disease of ileocolon
MR Method
Inverse variance weighted
MR Egger
1.2
1.0
0.8
0.6
1/SE_IV
0.5
1.0
1.5
β_IV

Batch 375 : 2−hydroxybutyrate (AHB) on Crohn's disease of ileocolon

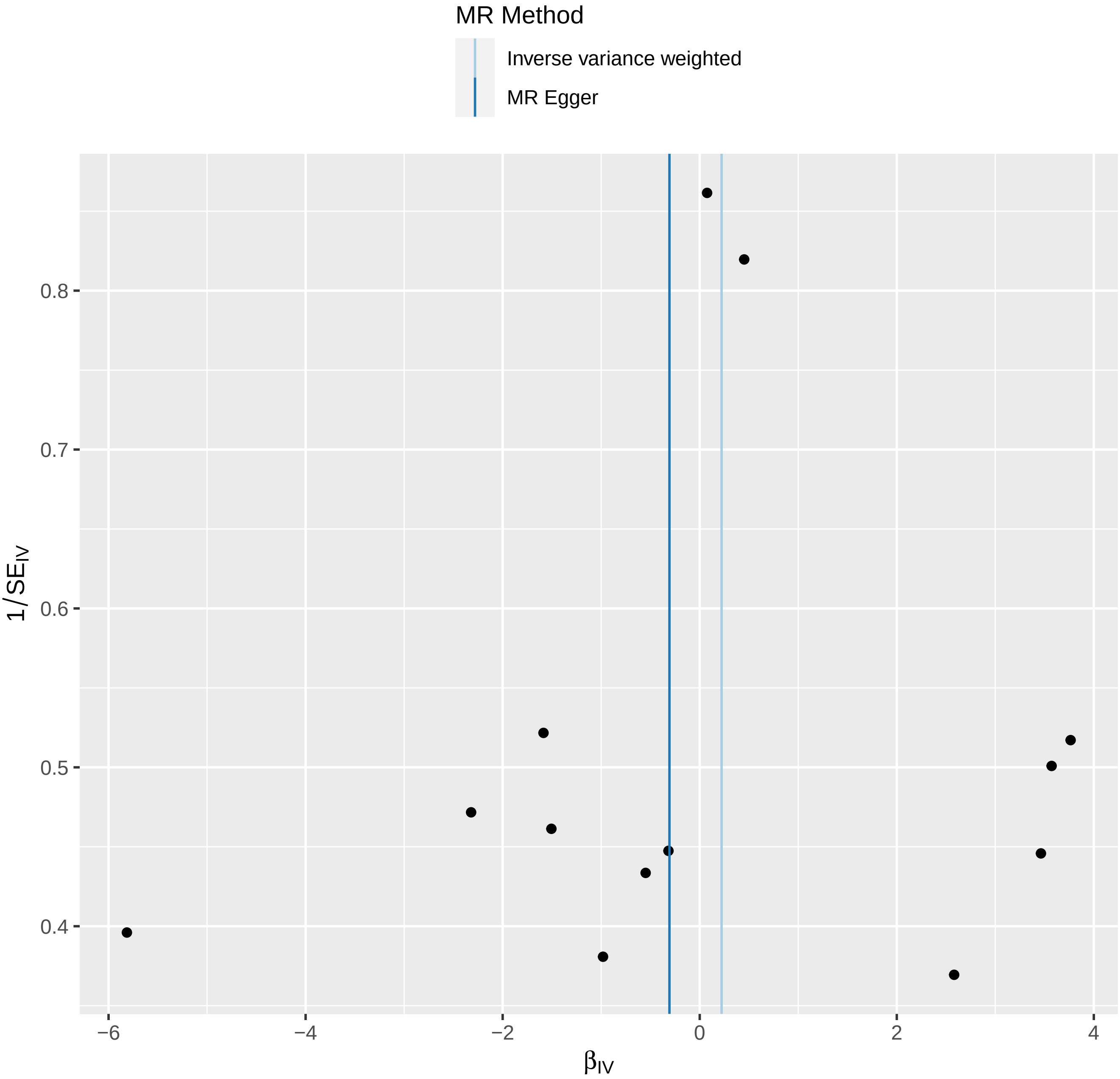

Batch 378 : 1−palmitoylglycerol (1−monopalmitin) on Crohn's disease of ileocolon

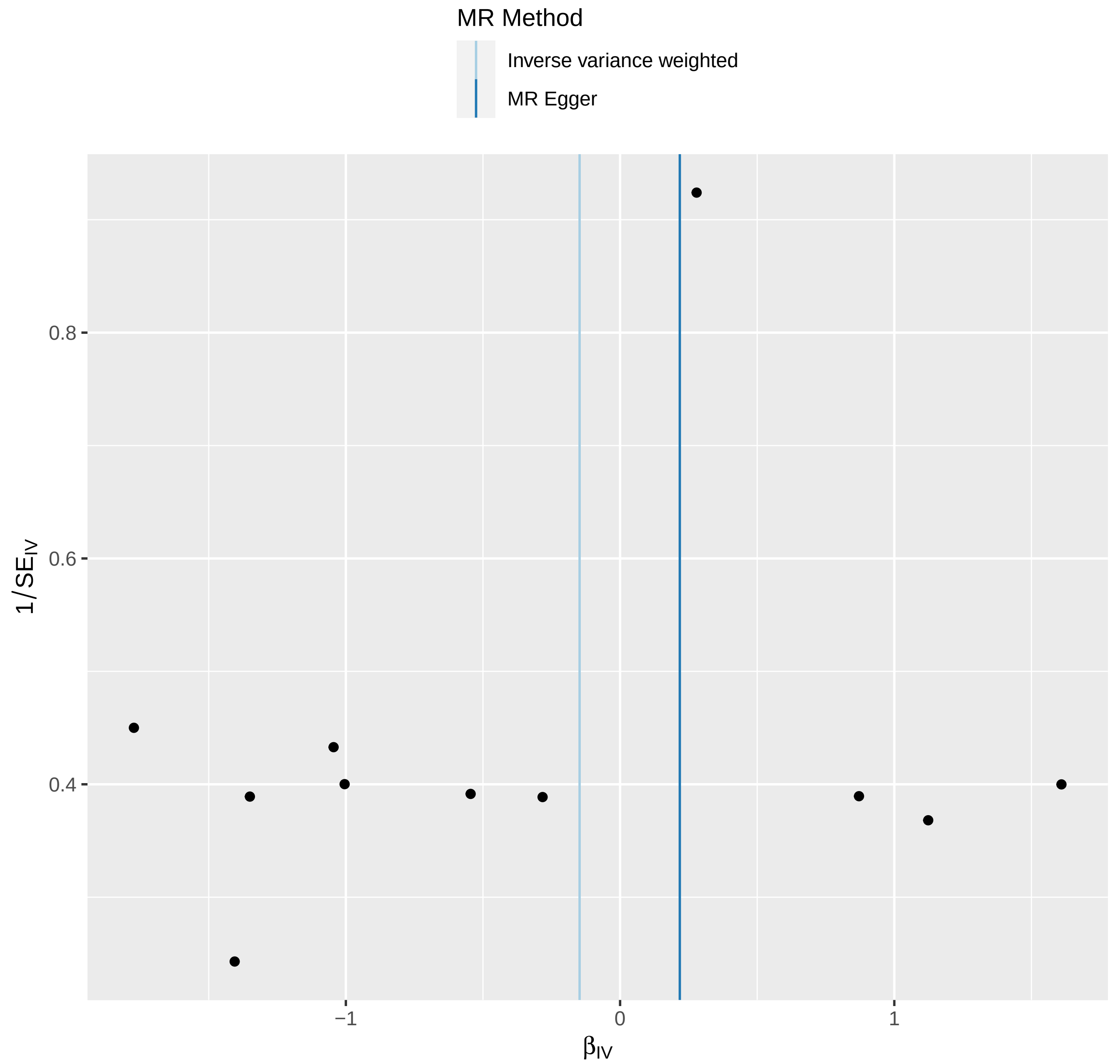

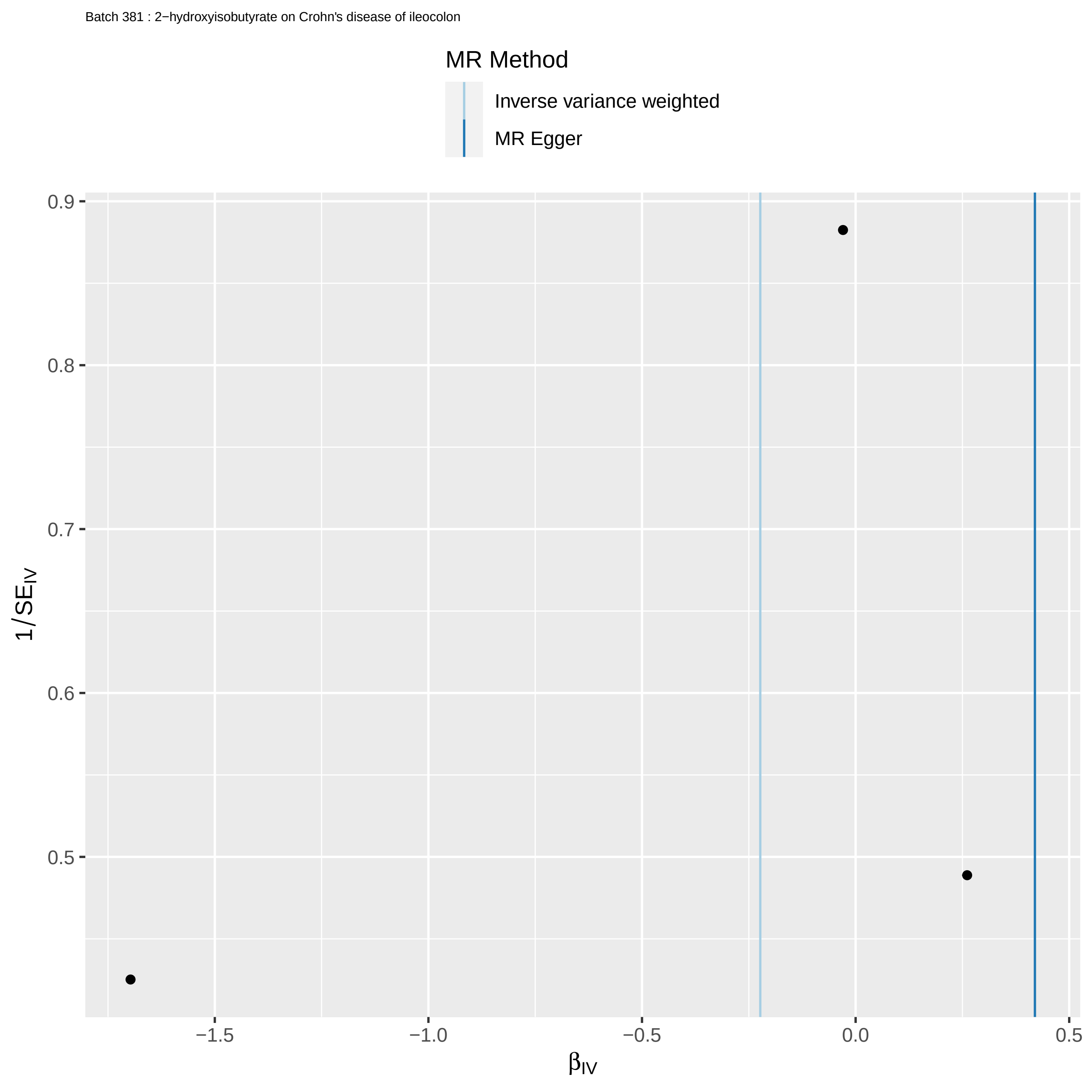

Batch 381 : 2−hydroxyisobutyrate on Crohn's disease of ileocolon
MR Method
Inverse variance weighted
MR Egger
0.9
0.8
0.7
0.6
0.5
1/SE_IV
−1.5
−1.0
−0.5
0.0
0.5
β_IV

Batch 382 : 4−methyl−2−oxopentanoate on Crohn's disease of ileocolon

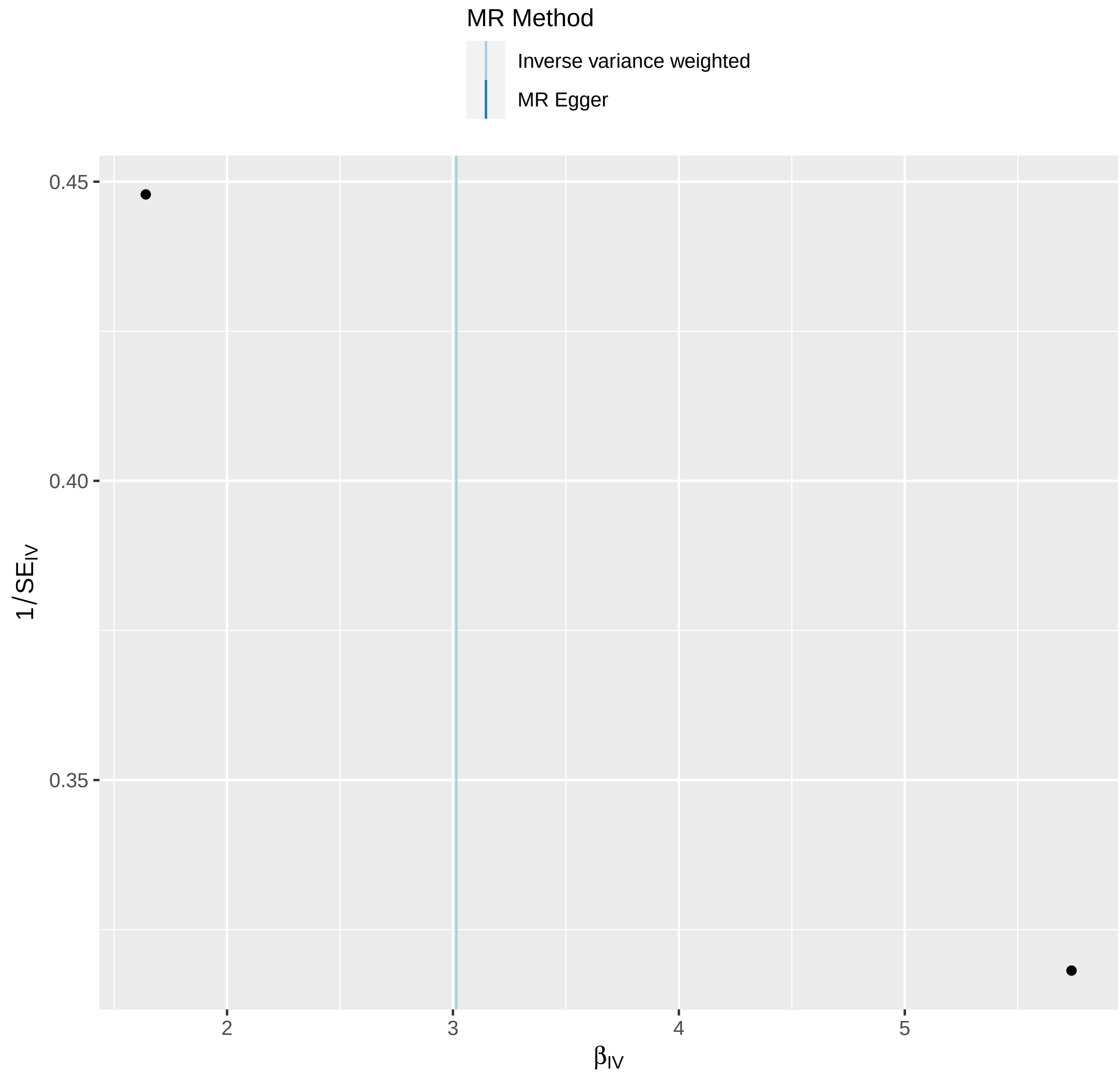

Batch 386 : Levulinate (4−oxovalerate) on Crohn's disease of ileocolon

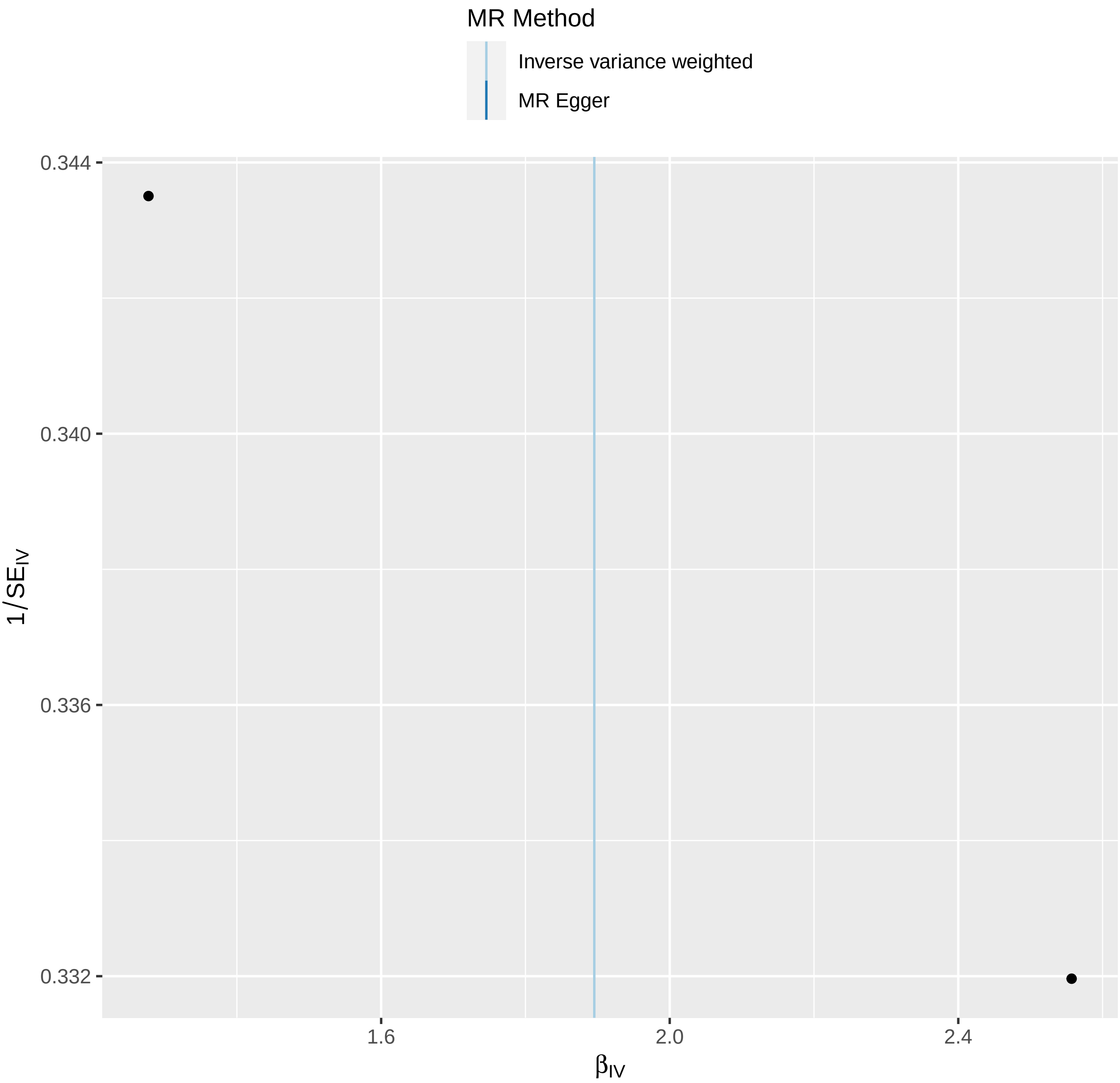

Batch 388 : Cholate on Crohn's disease of ileocolon

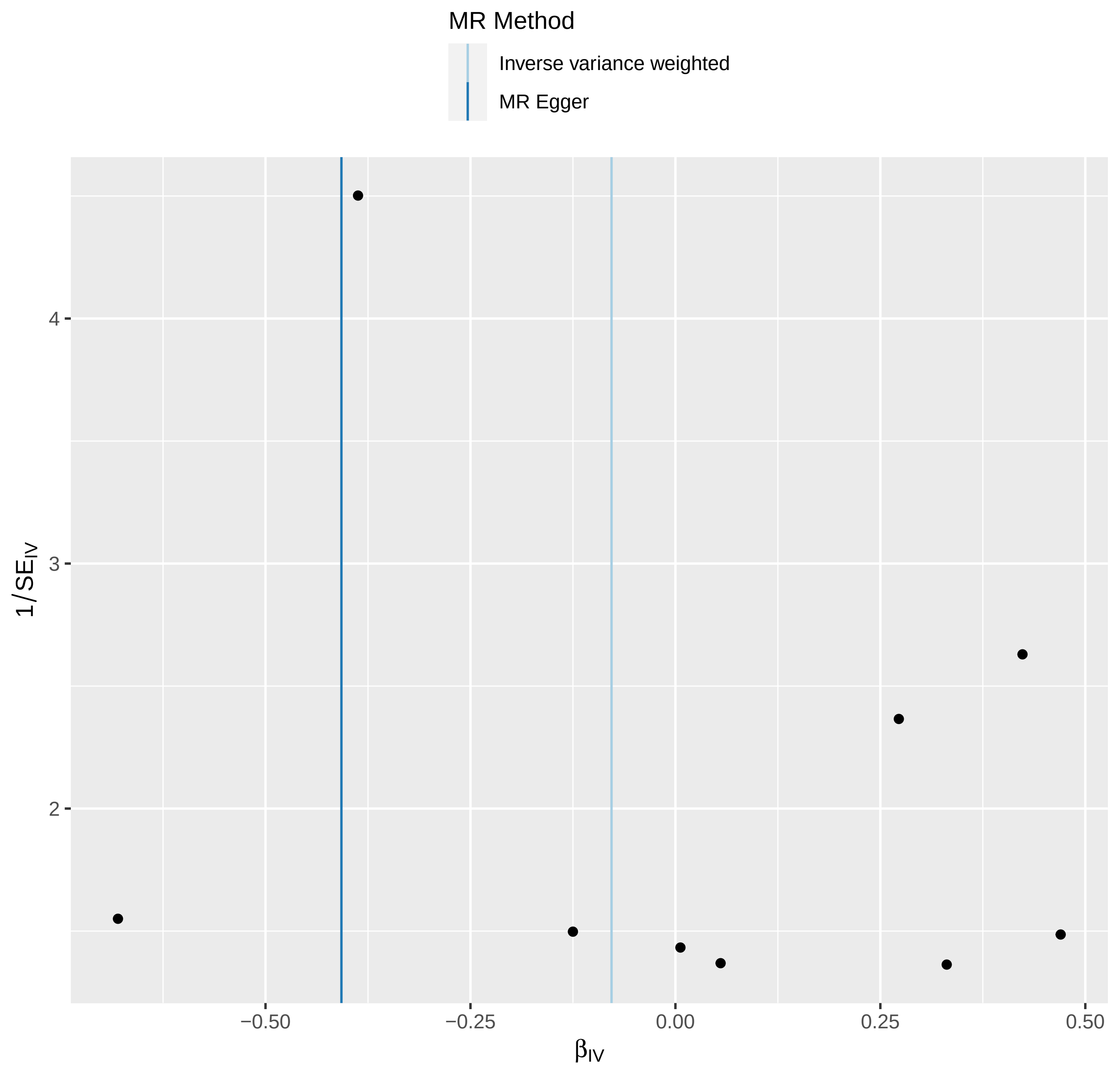

Batch 391 : Hyodeoxycholate on Crohn's disease of ileocolon

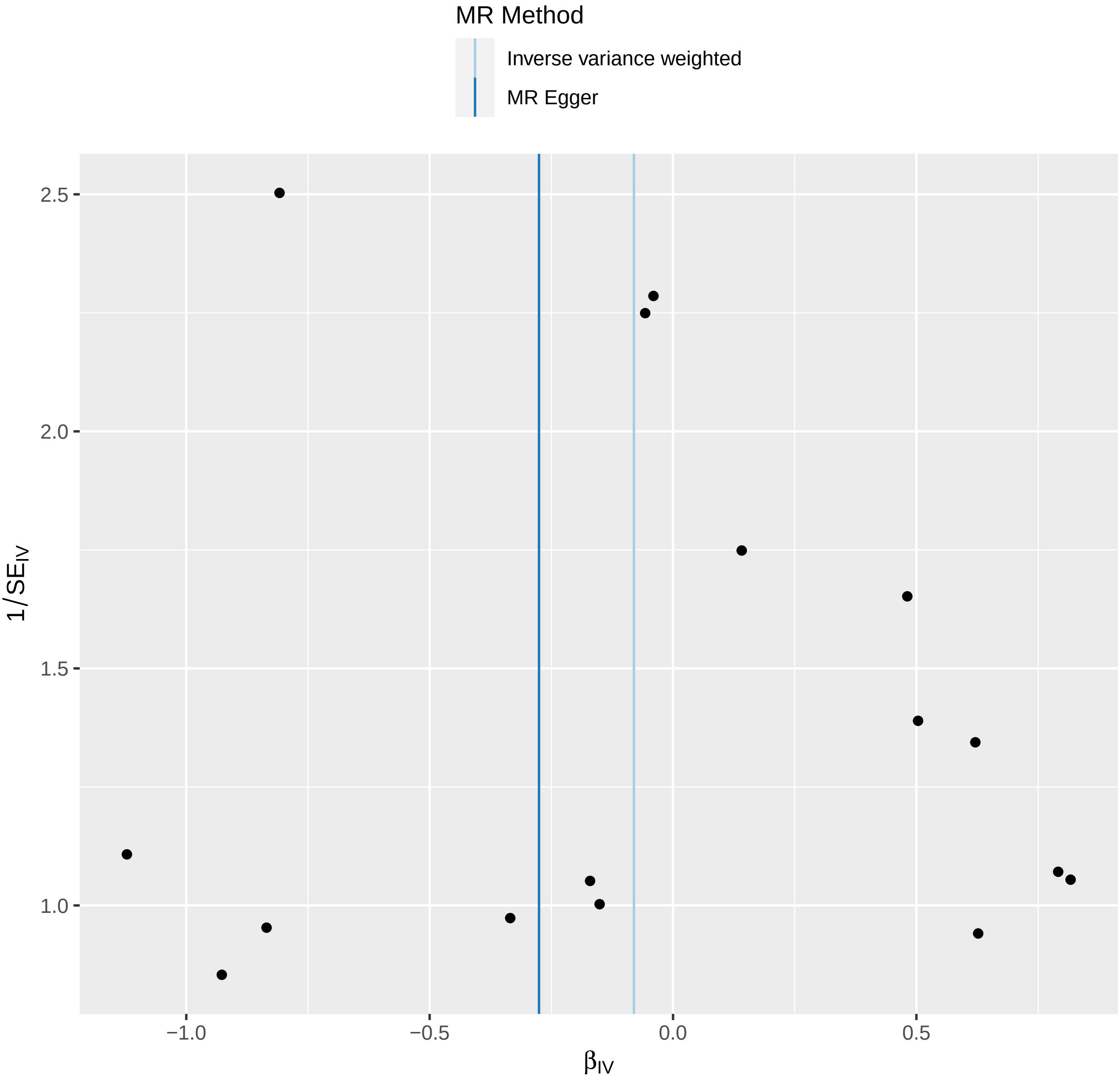

Batch 392 : 3−indoxyl sulfate on Crohn's disease of ileocolon

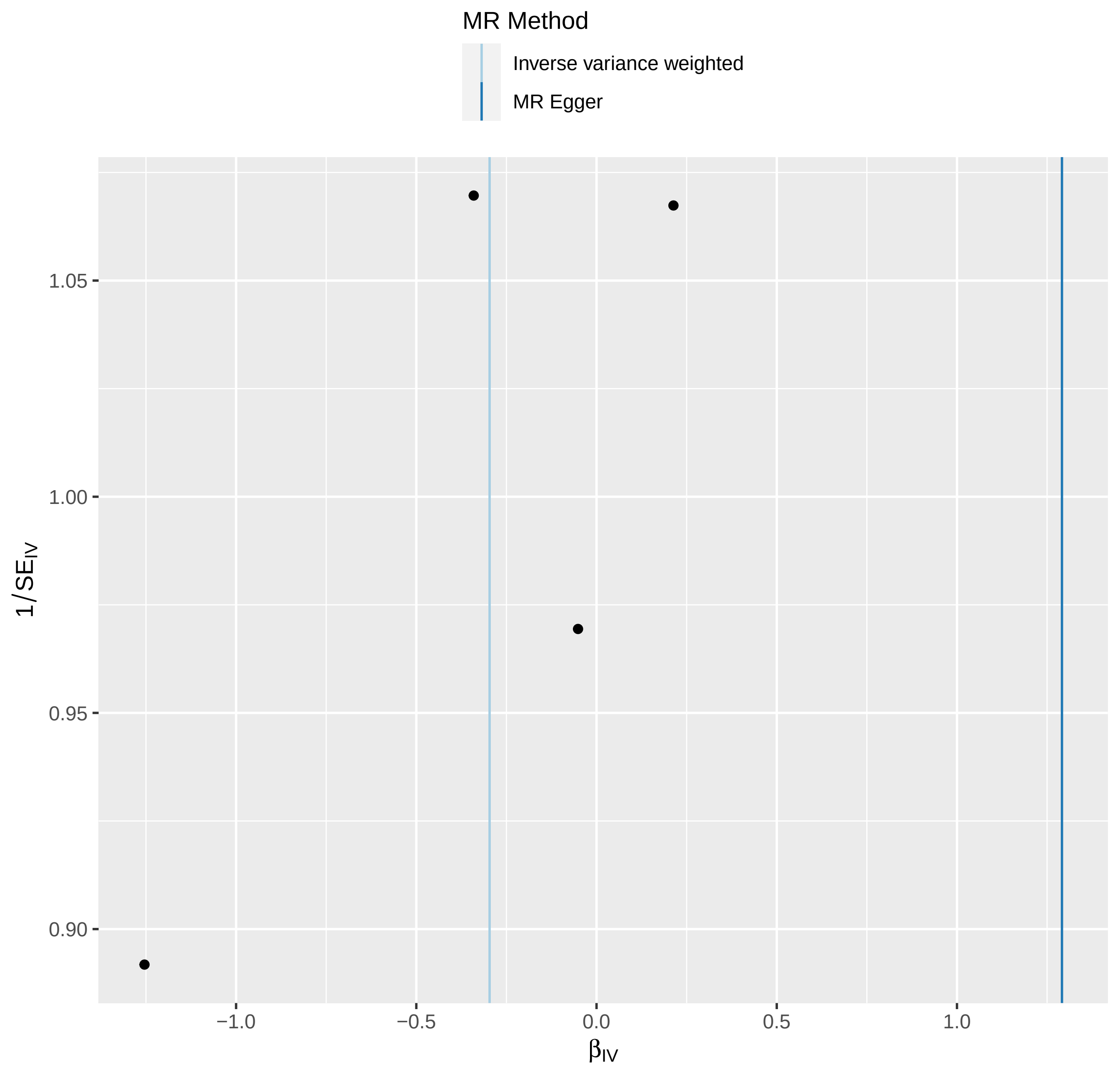

Batch 396 : Erythrose on Crohn's disease of ileocolon

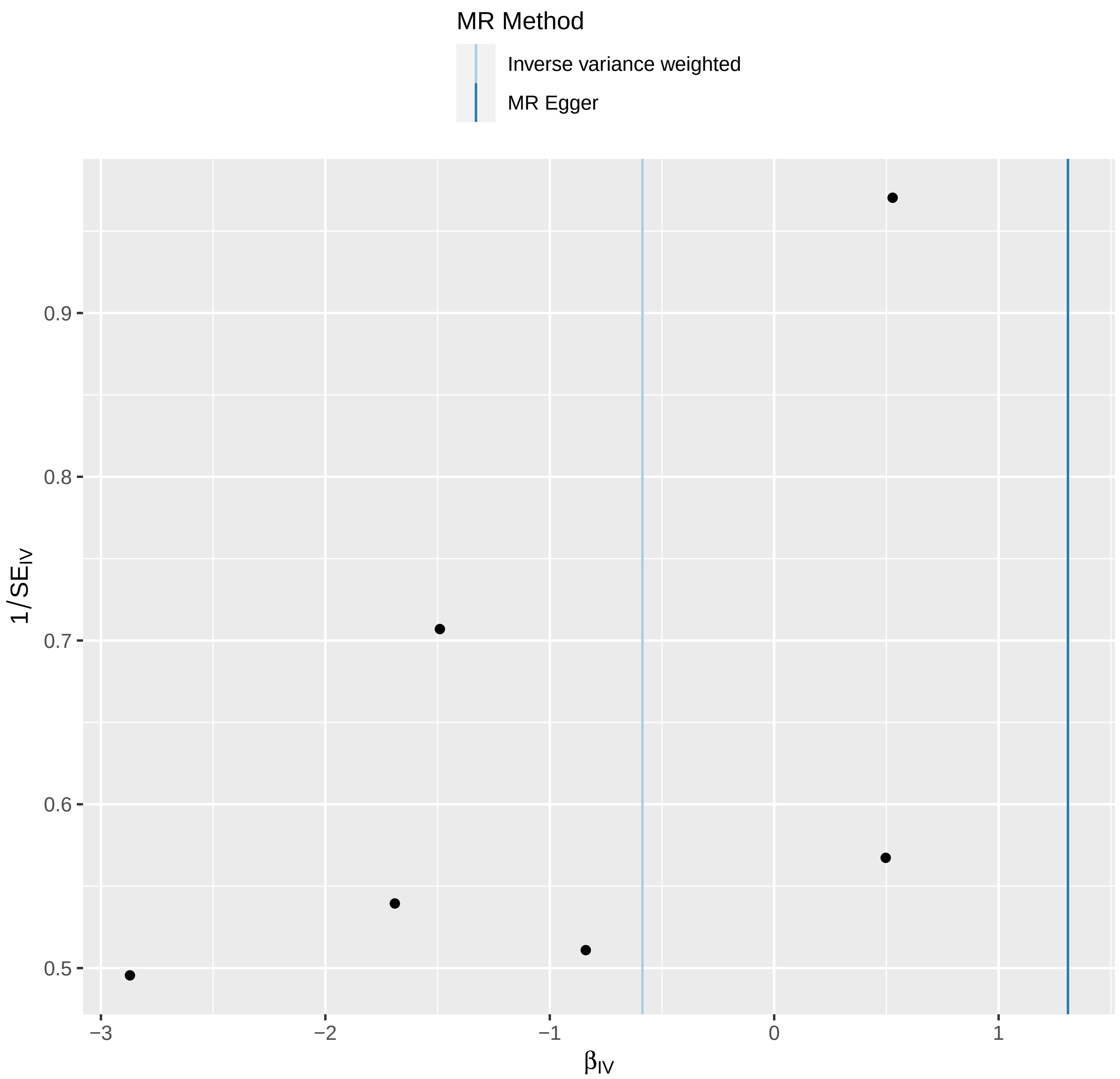

Batch 397 : Threonate on Crohn's disease of ileocolon

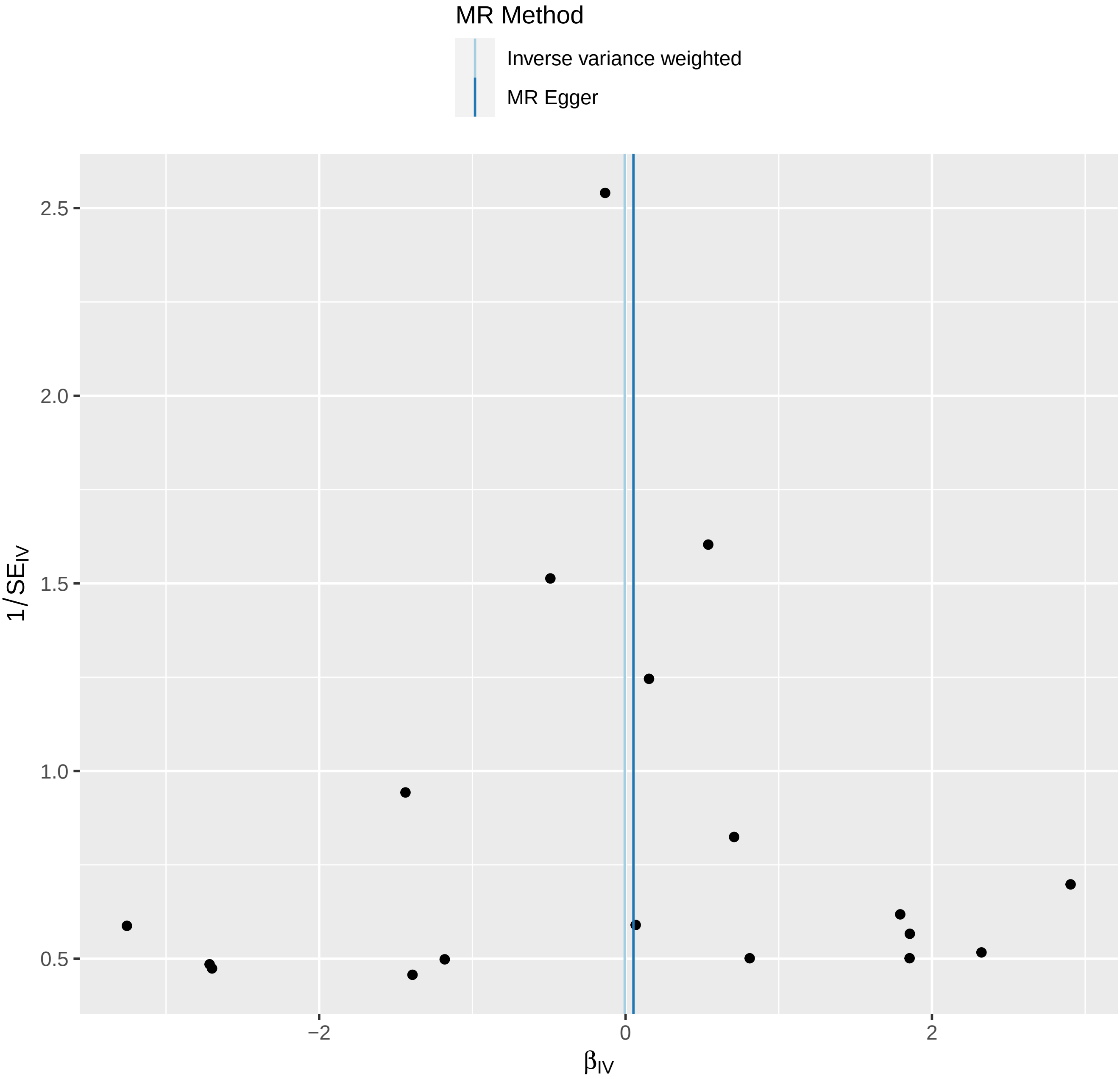

Batch 398 : Cysteine on Crohn's disease of ileocolon

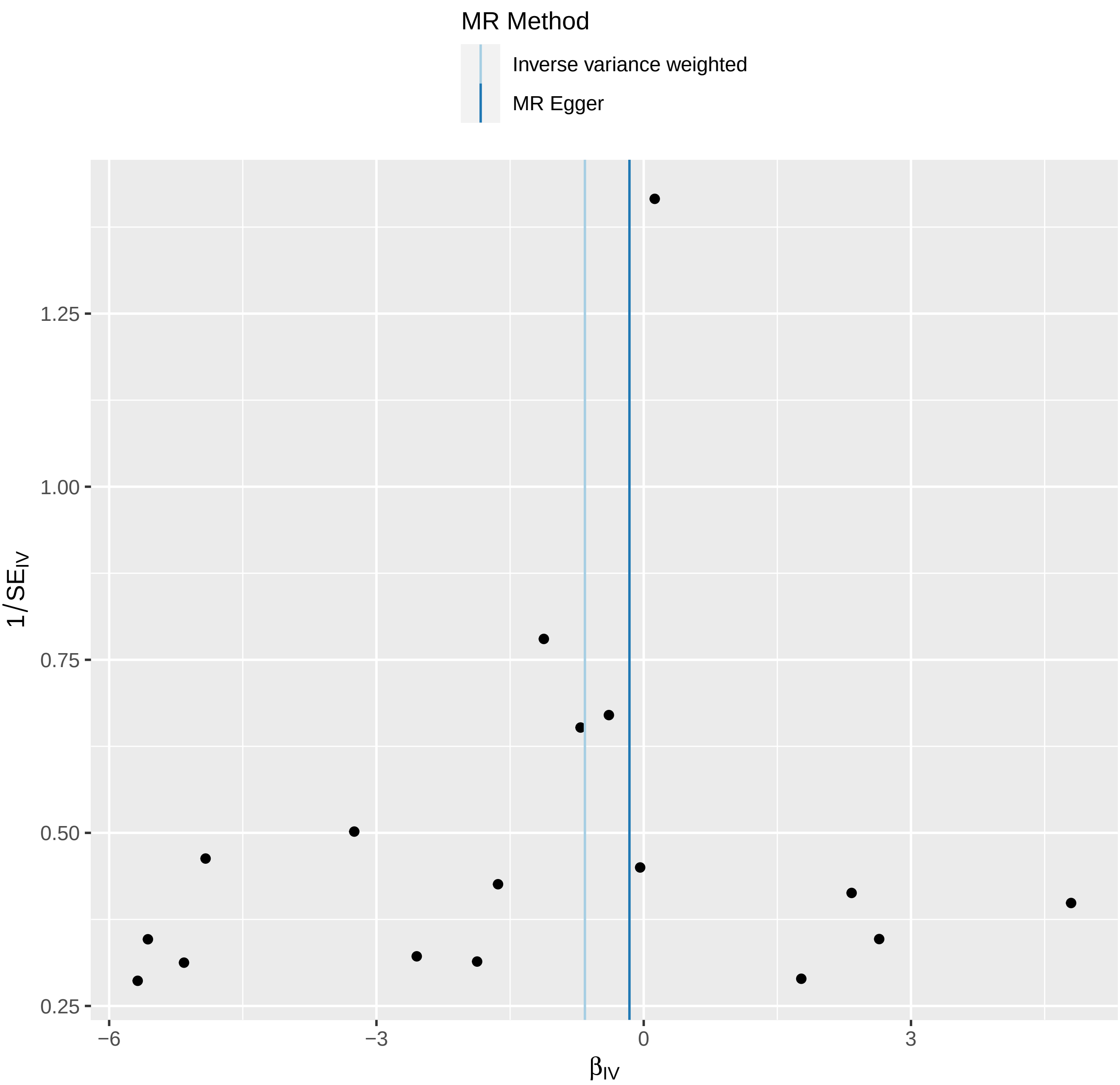

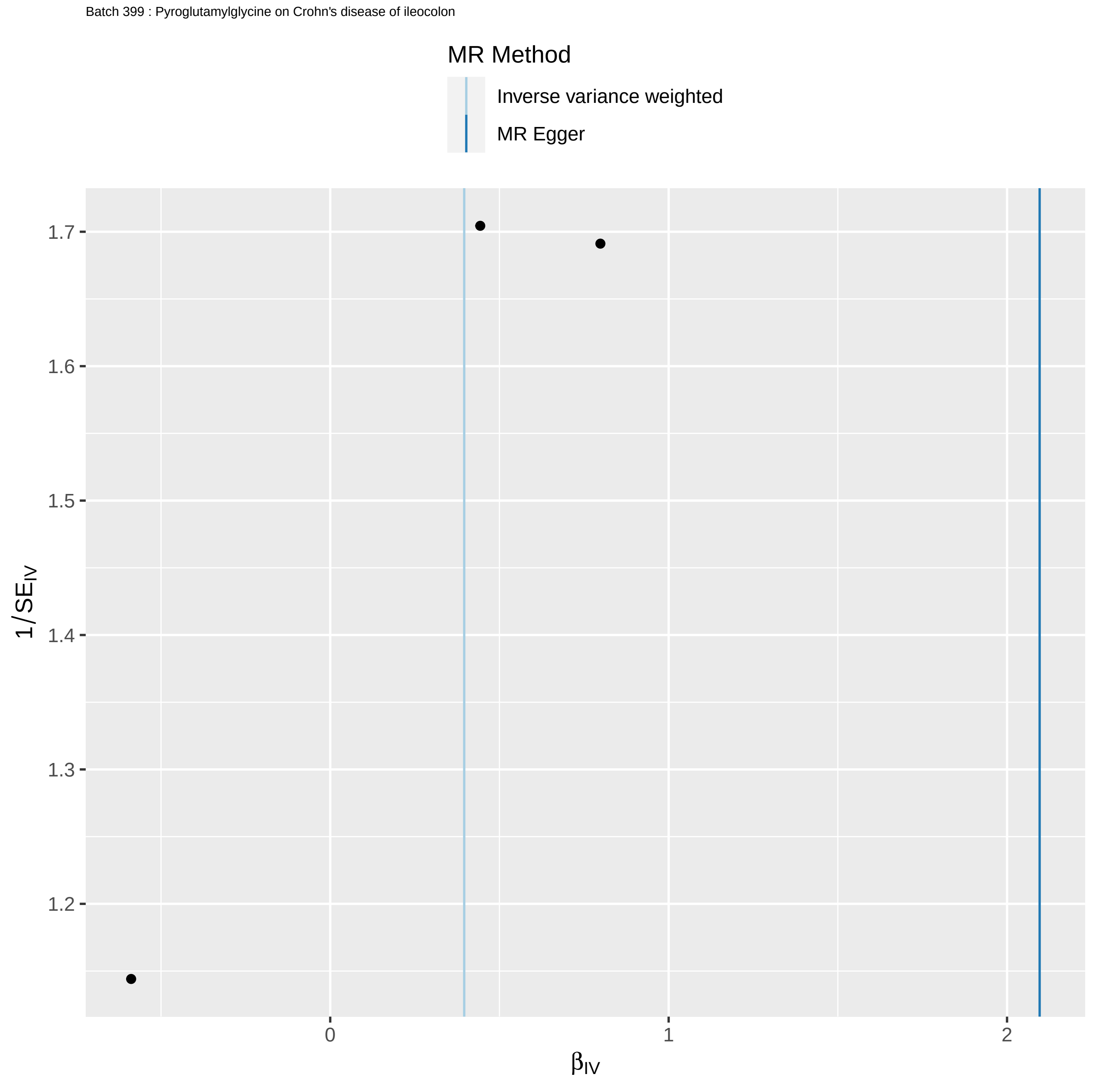
Batch 399 : Pyroglutamylglycine on Crohn's disease of ileocolon
MR Method
Inverse variance weighted
MR Egger
1.7
1.6
1.5
1.4
1.3
1.2
0
1
2
$1/SE_{IV}$
β_{IV}

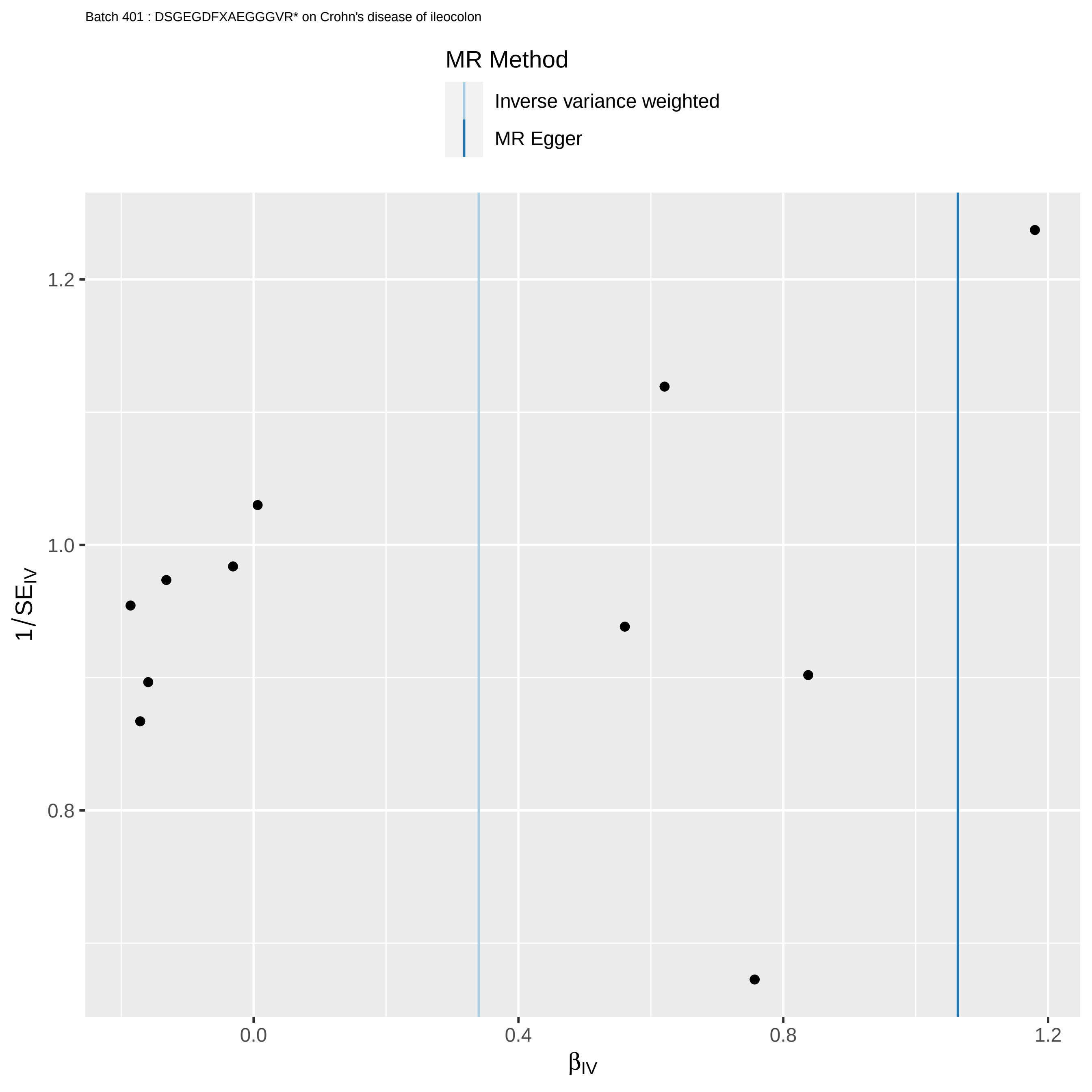

Batch 401 : DSGEGDFXAEGGGVR* on Crohn's disease of ileocolon
MR Method
Inverse variance weighted
MR Egger
1.2
1.0
0.8
1/SE$_{IV}$
0.0
0.4
0.8
1.2
β$_{IV}$

Batch 402 : Pyridoxate on Crohn's disease of ileocolon

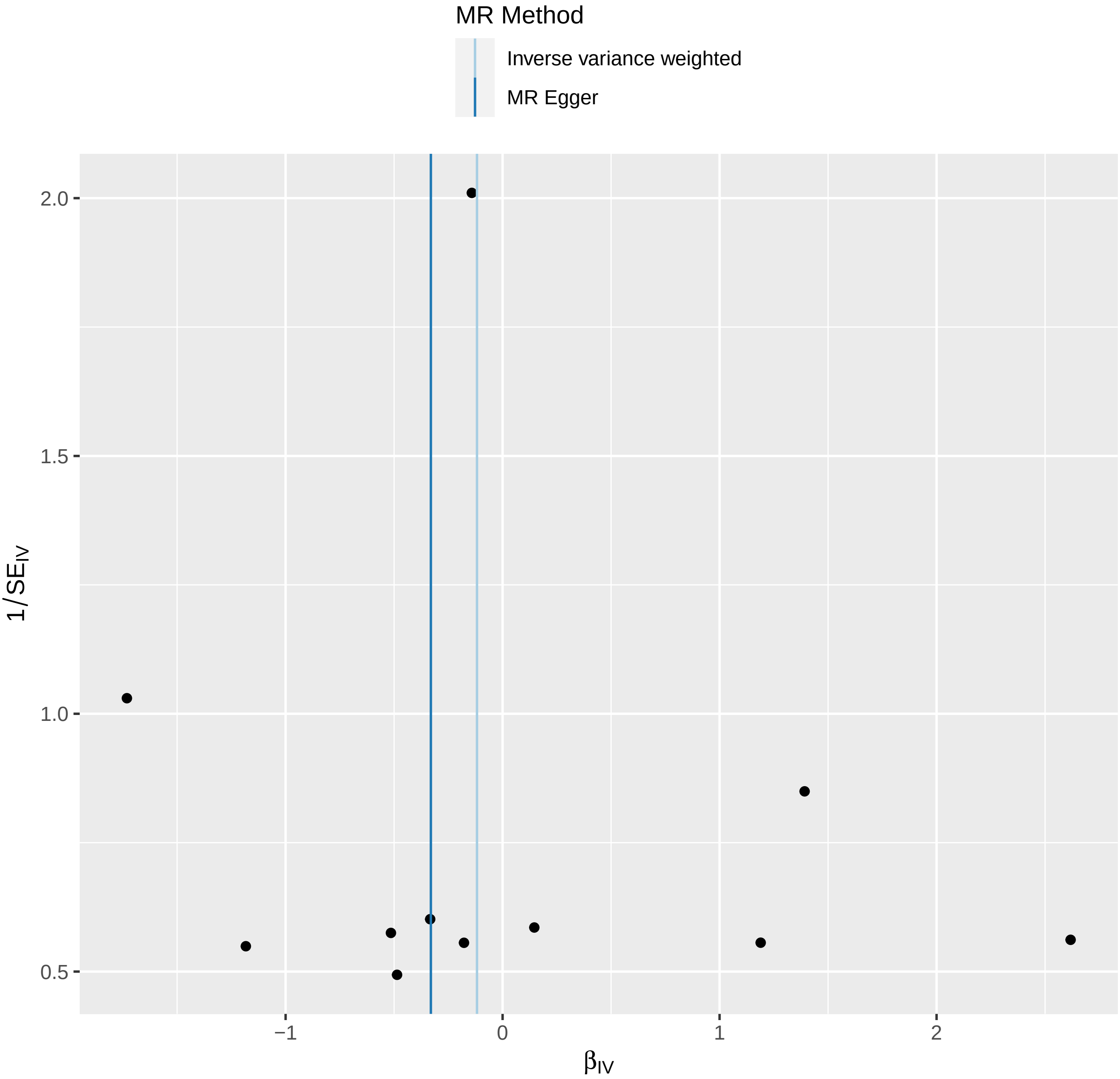

Batch 403 : Androsterone sulfate on Crohn's disease of ileocolon

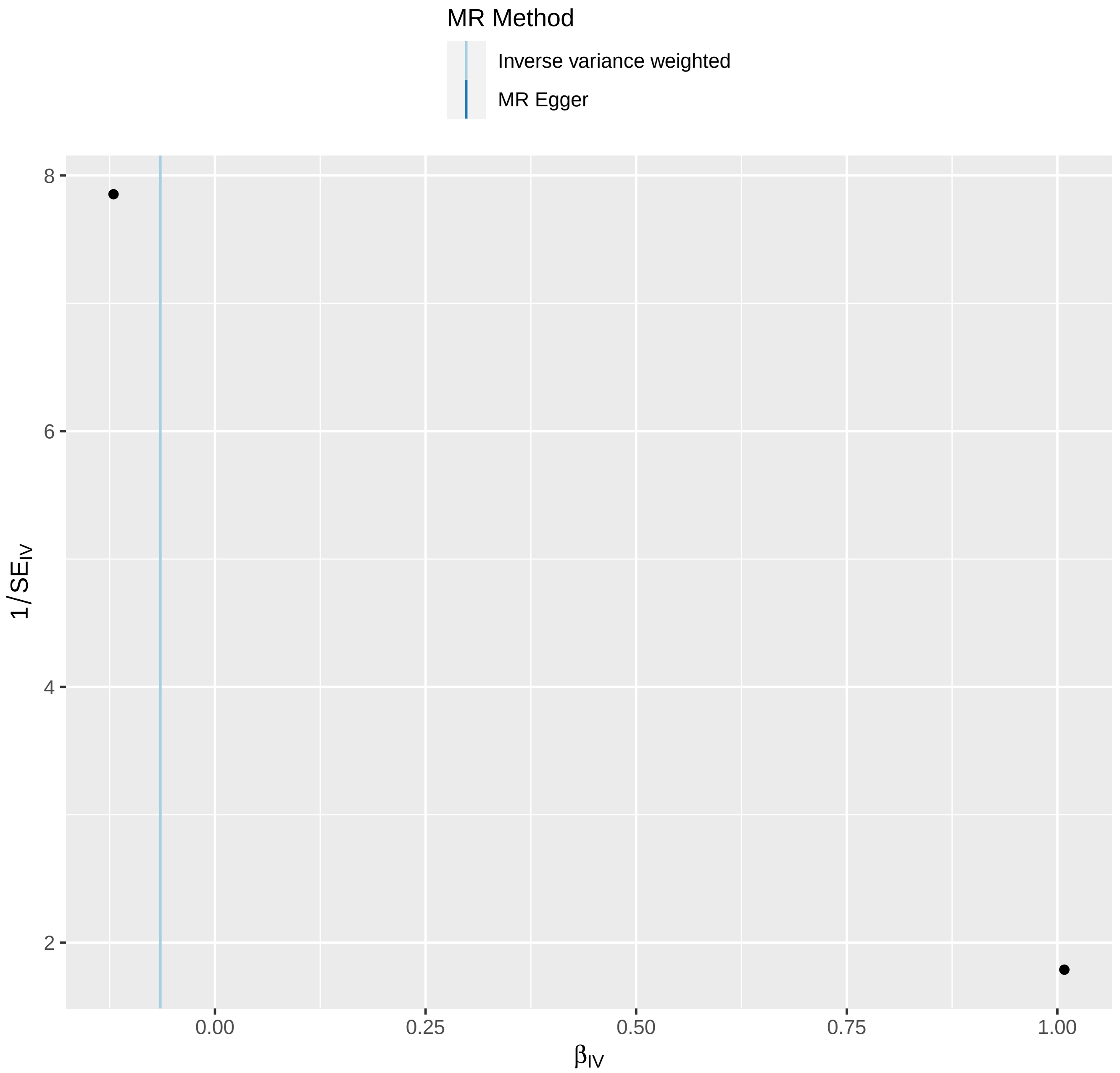

Batch 408 : Trans−4−hydroxyproline on Crohn's disease of ileocolon

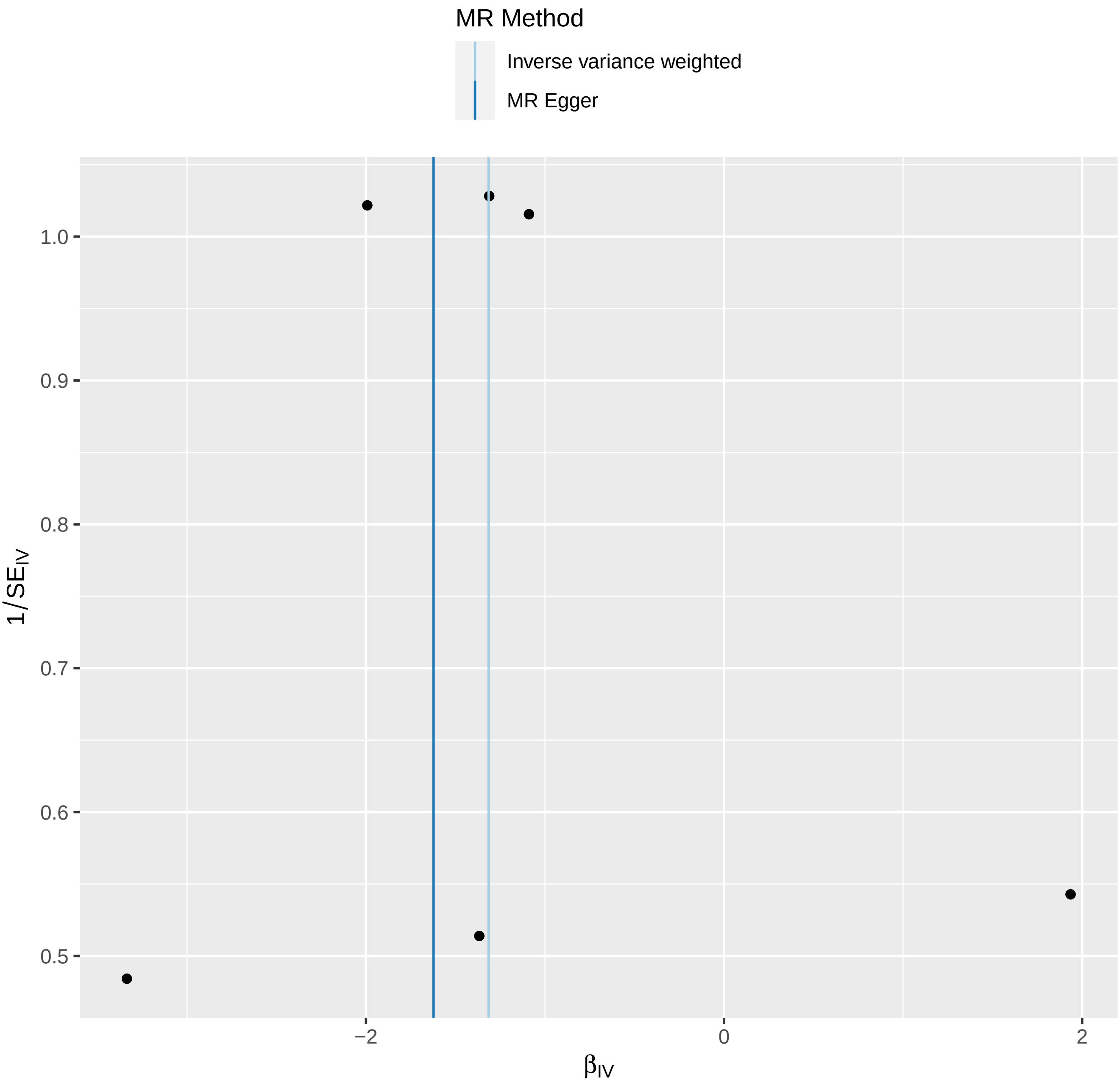

Batch 410 : Hexanoylcarnitine on Crohn's disease of ileocolon

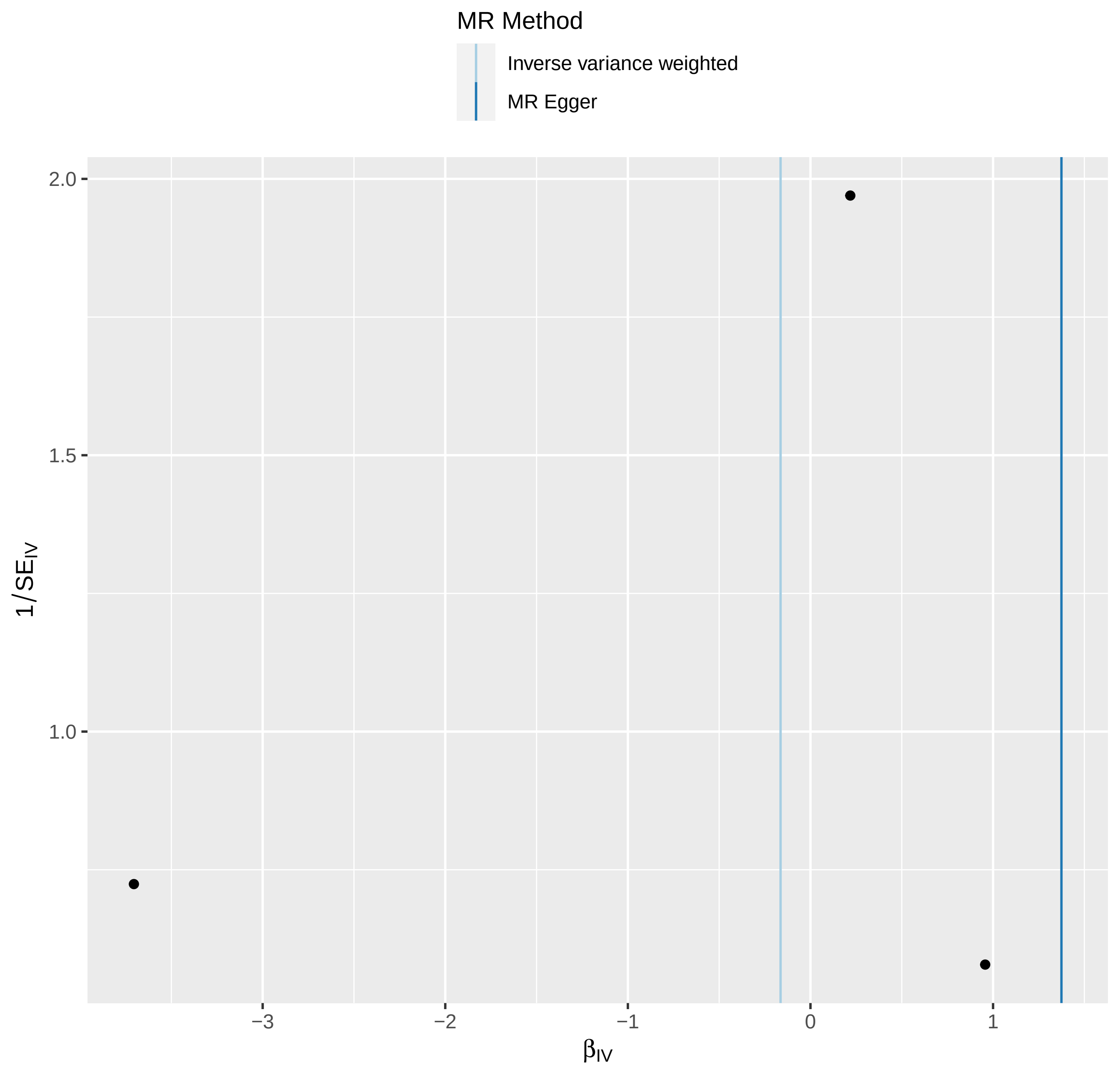

Batch 414 : 2−aminobutyrate on Crohn's disease of ileocolon

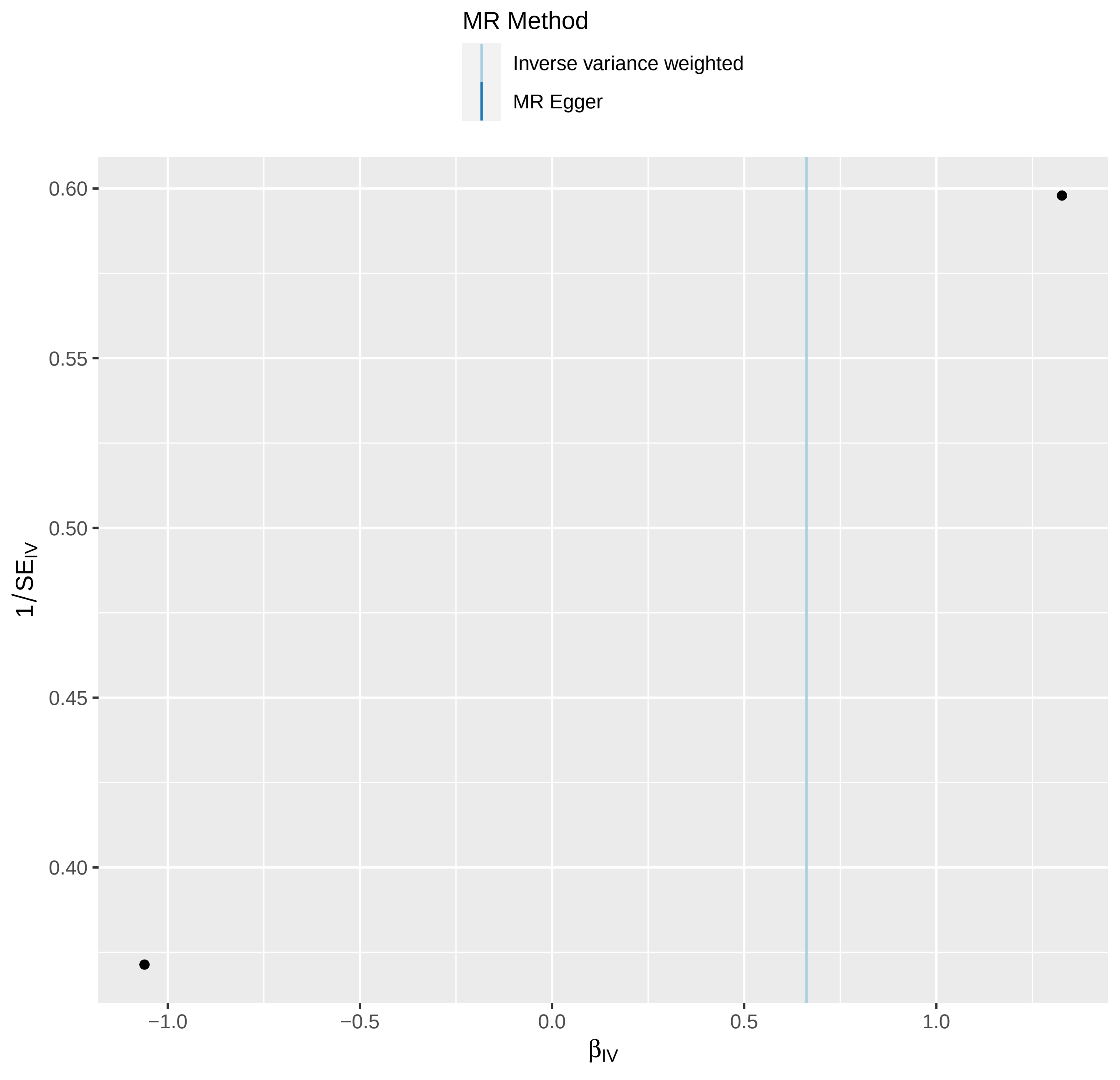

Batch 419 : Butyrylcarnitine on Crohn's disease of ileocolon

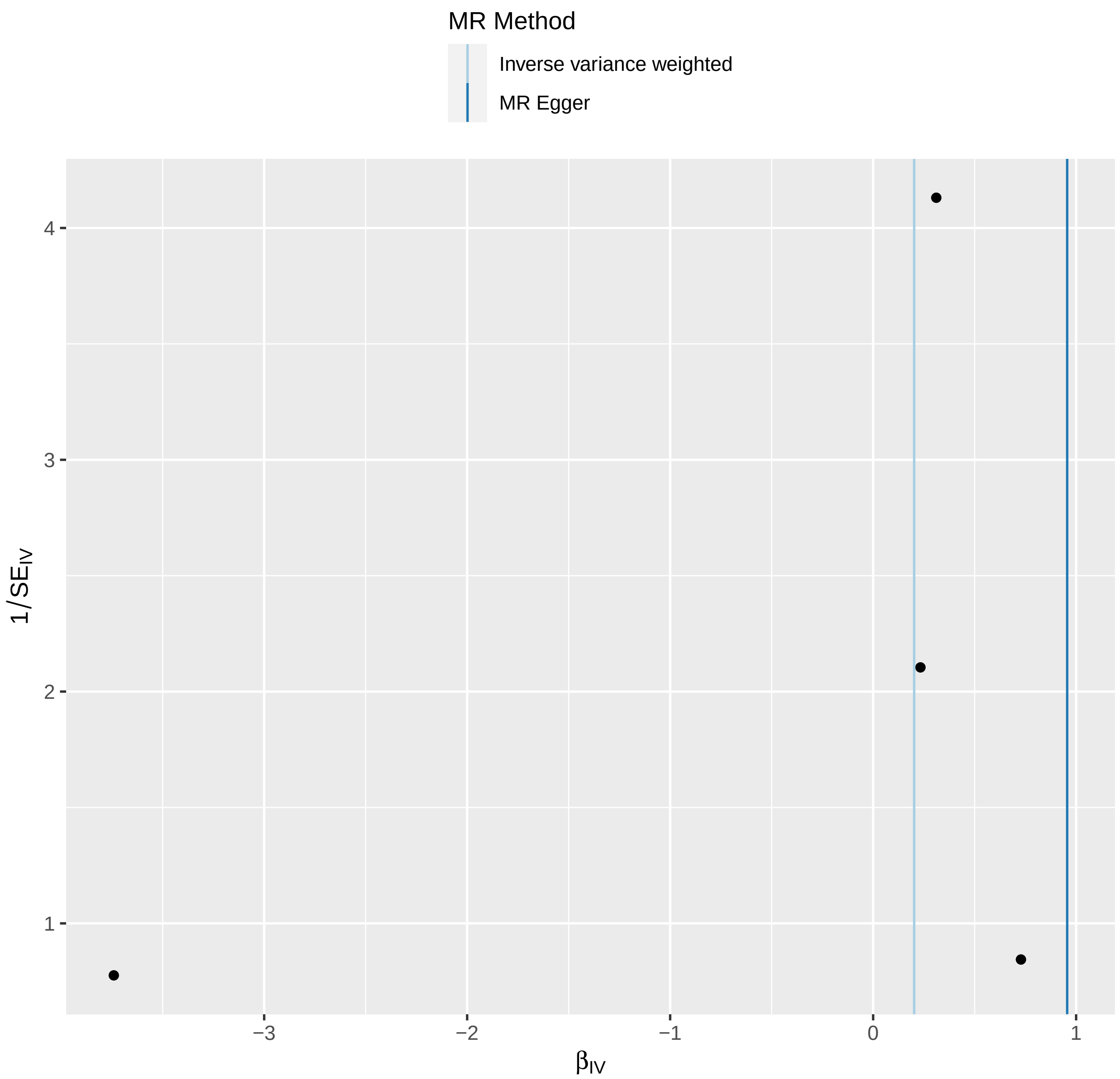

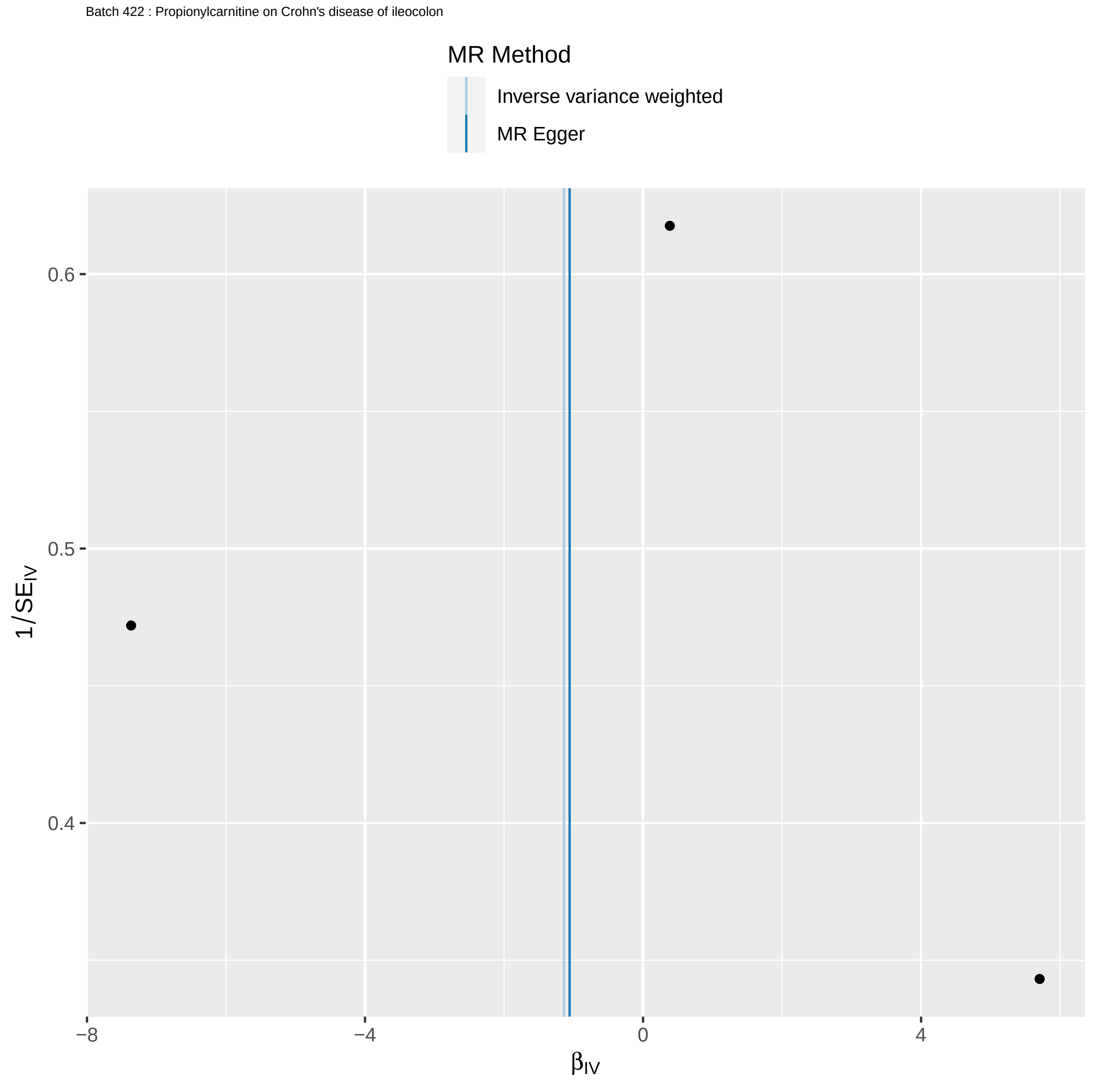

Batch 422 : Propionylcarnitine on Crohn's disease of ileocolon
MR Method
Inverse variance weighted
MR Egger
0.6
0.5
0.4
1/SE_IV
−8
−4
0
4
β_IV

Batch 423 : Caproate (6:0) on Crohn's disease of ileocolon

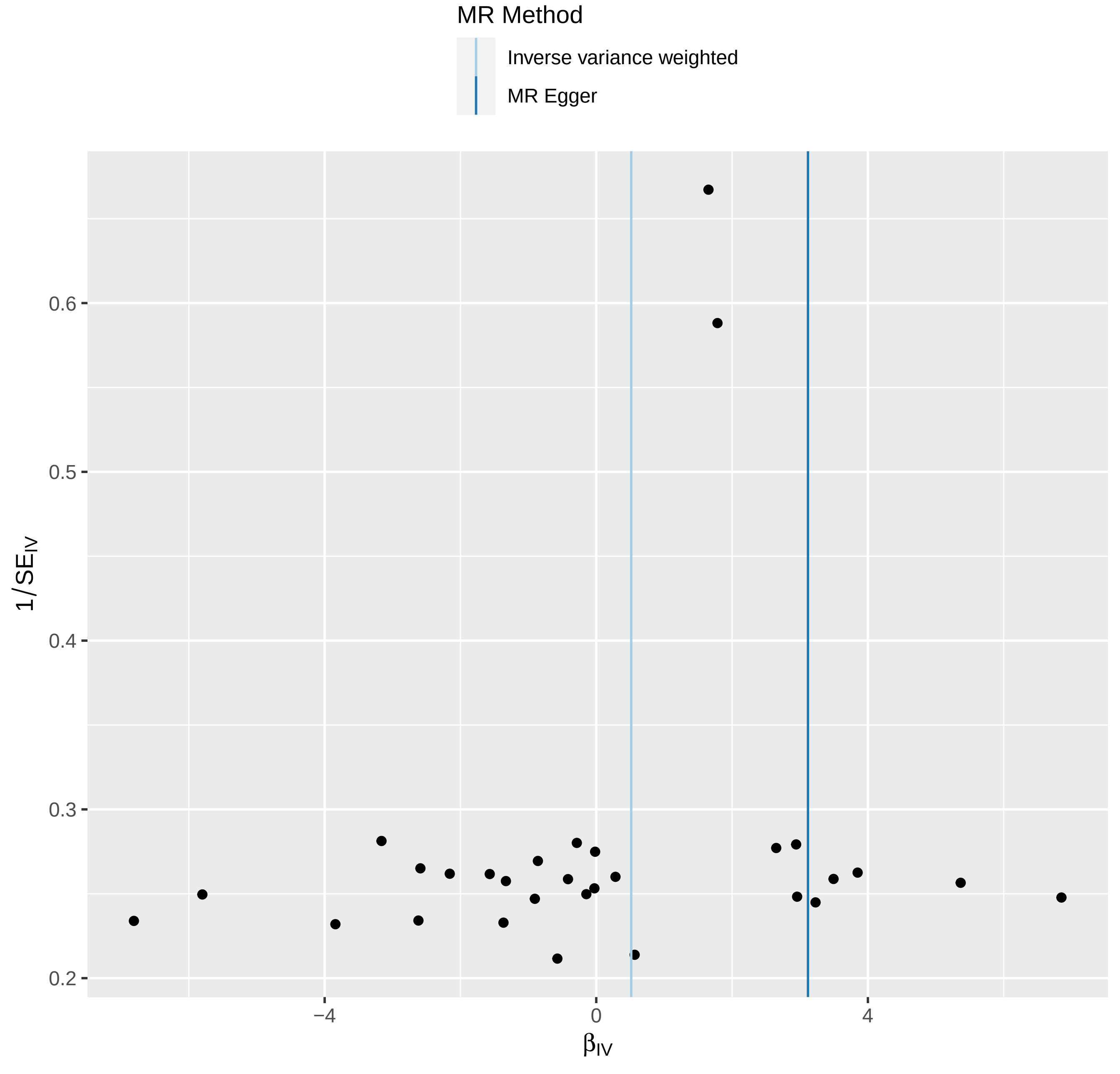

Batch 424 : Caprylate (8:0) on Crohn's disease of ileocolon

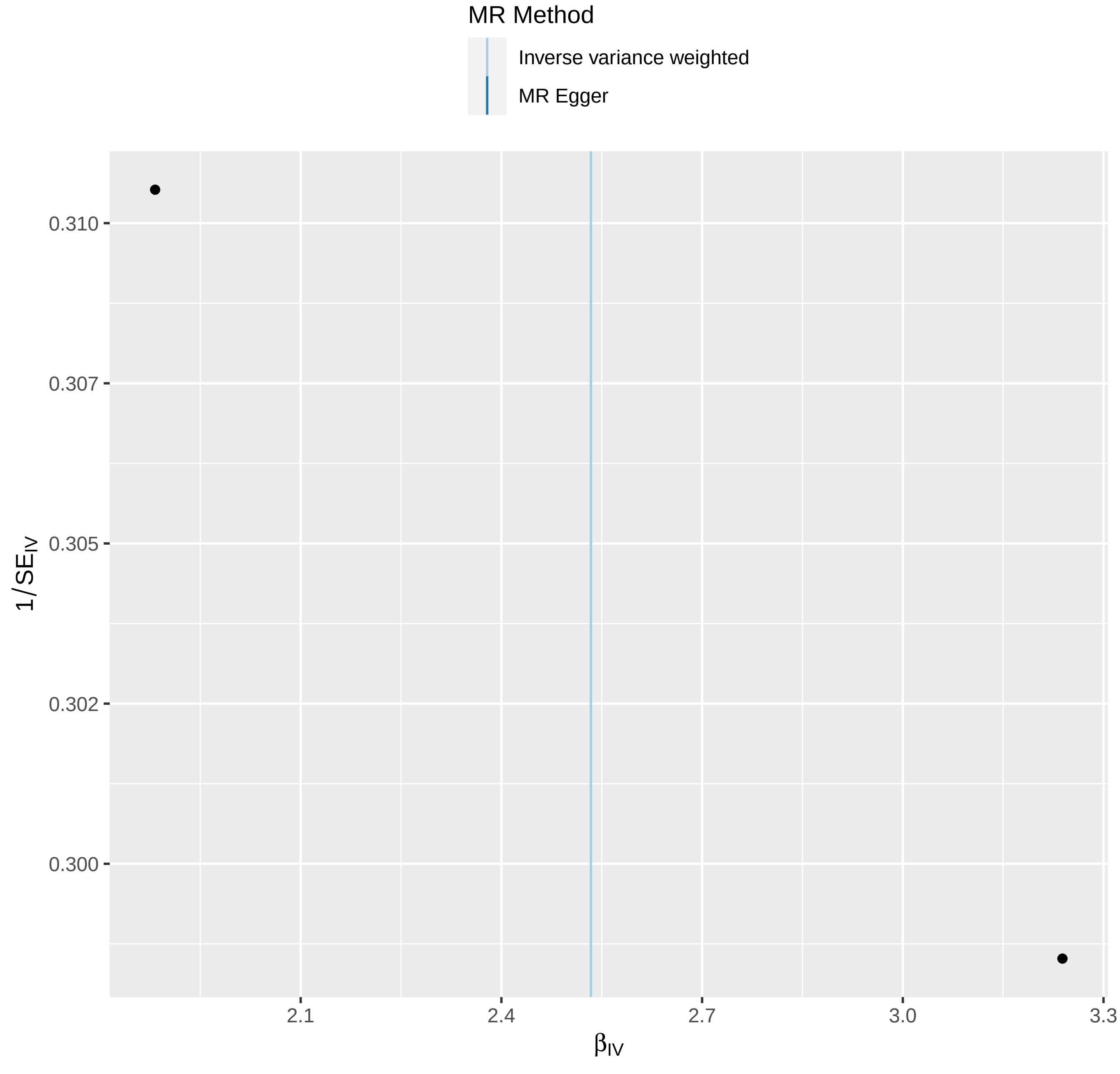

Batch 425 : 10−undecenoate (11:1n1) on Crohn's disease of ileocolon

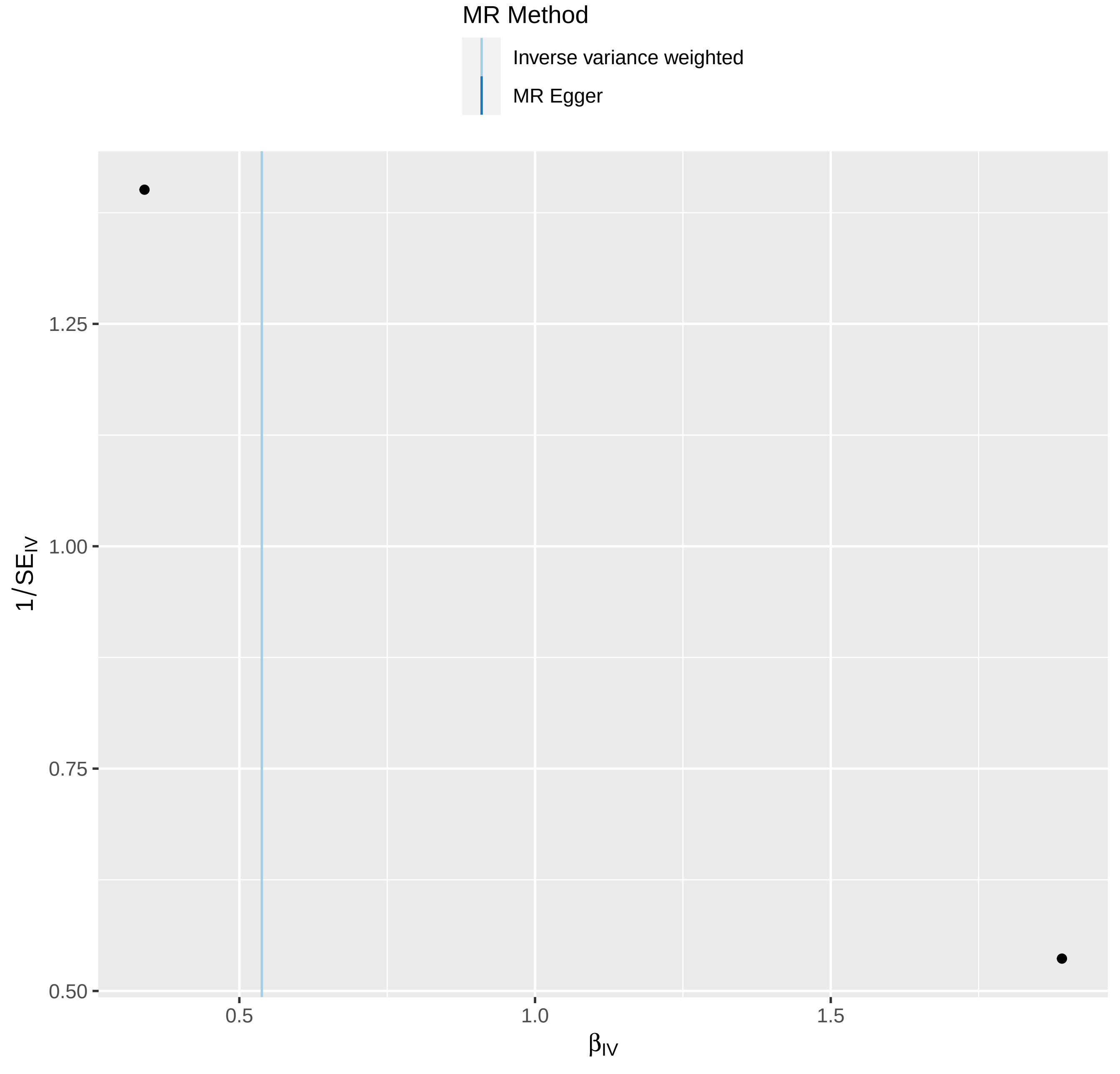

Batch 427 : Phenol sulfate on Crohn's disease of ileocolon

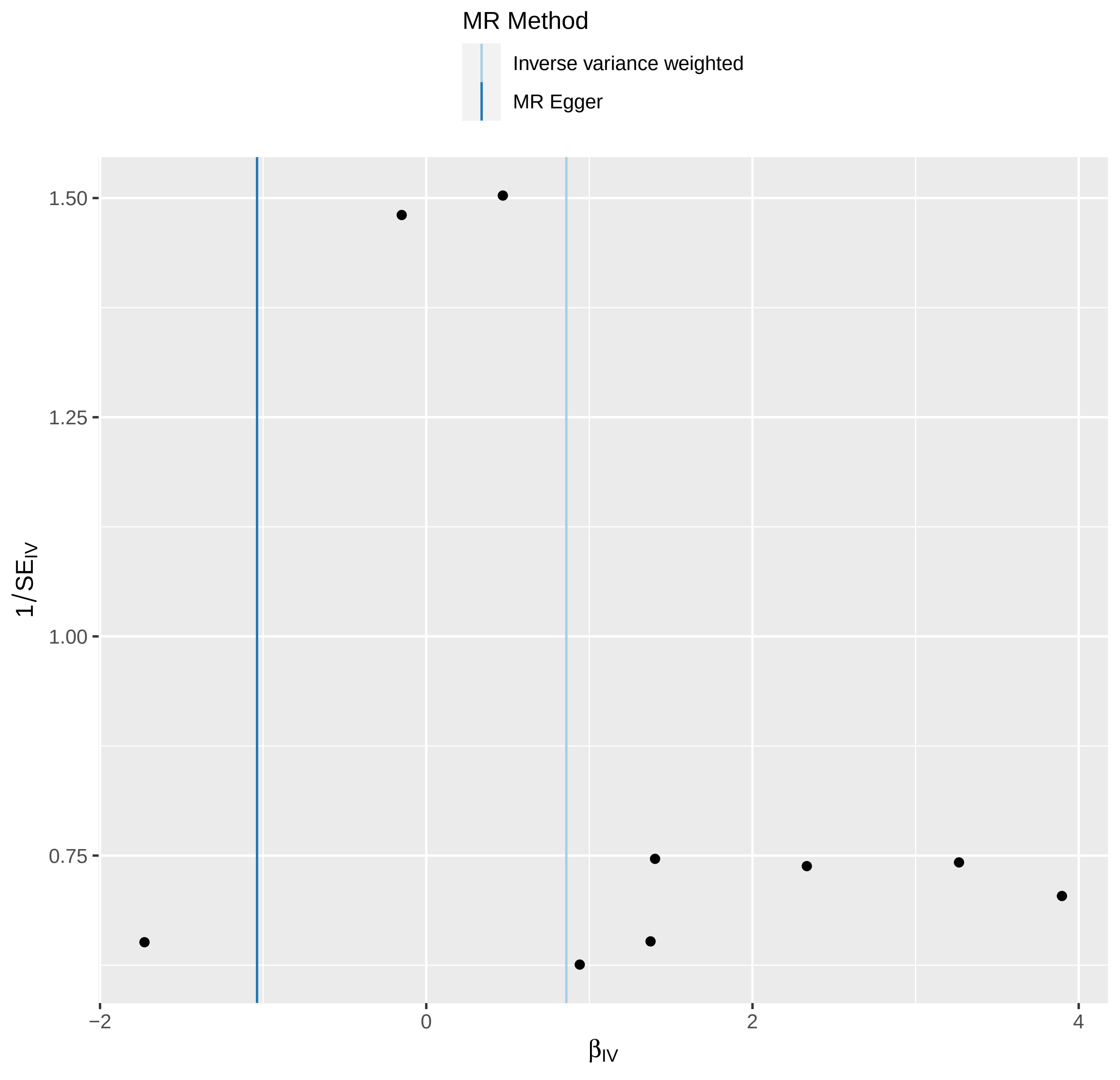

Batch 429 : Heme* on Crohn's disease of ileocolon

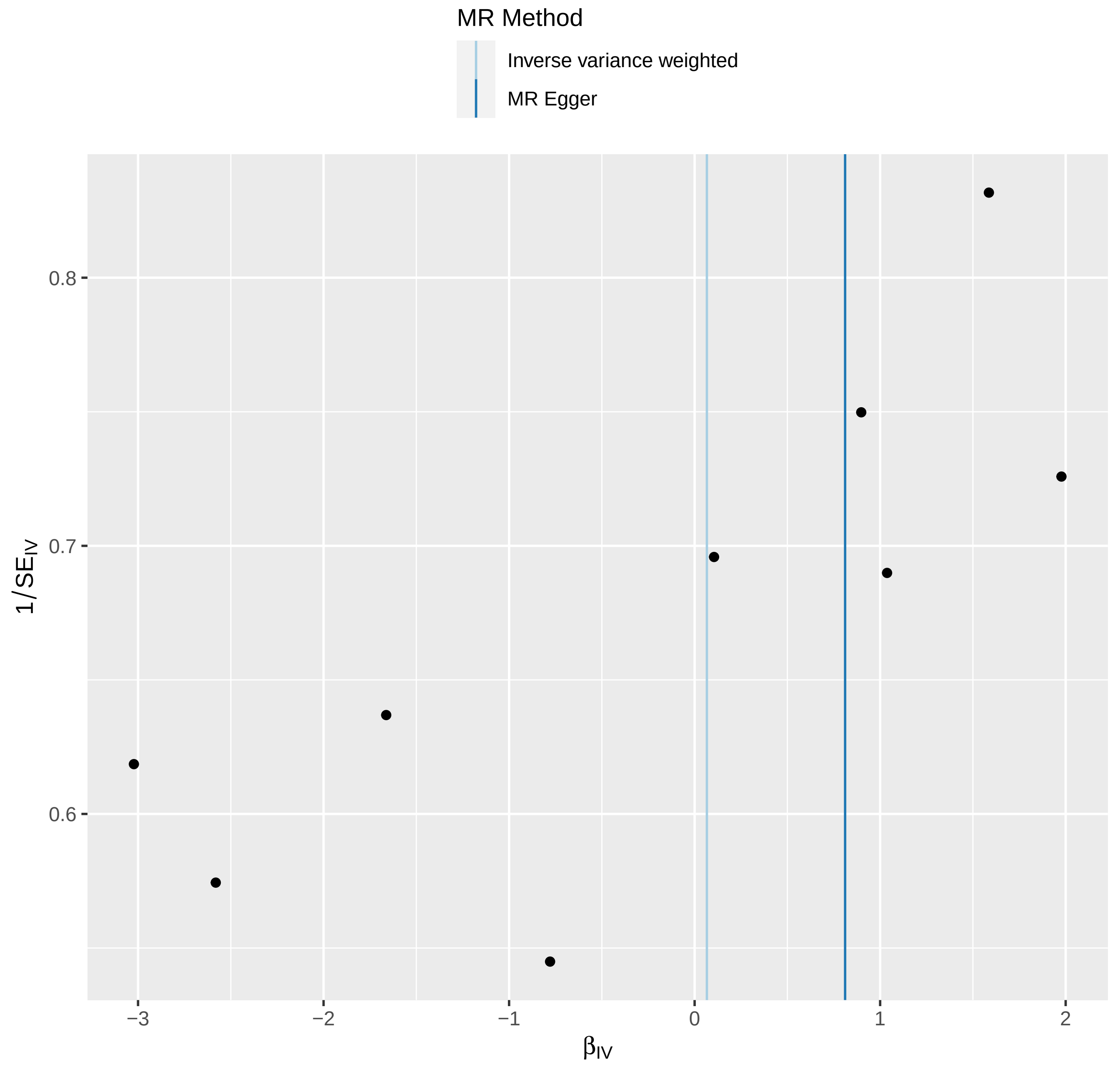

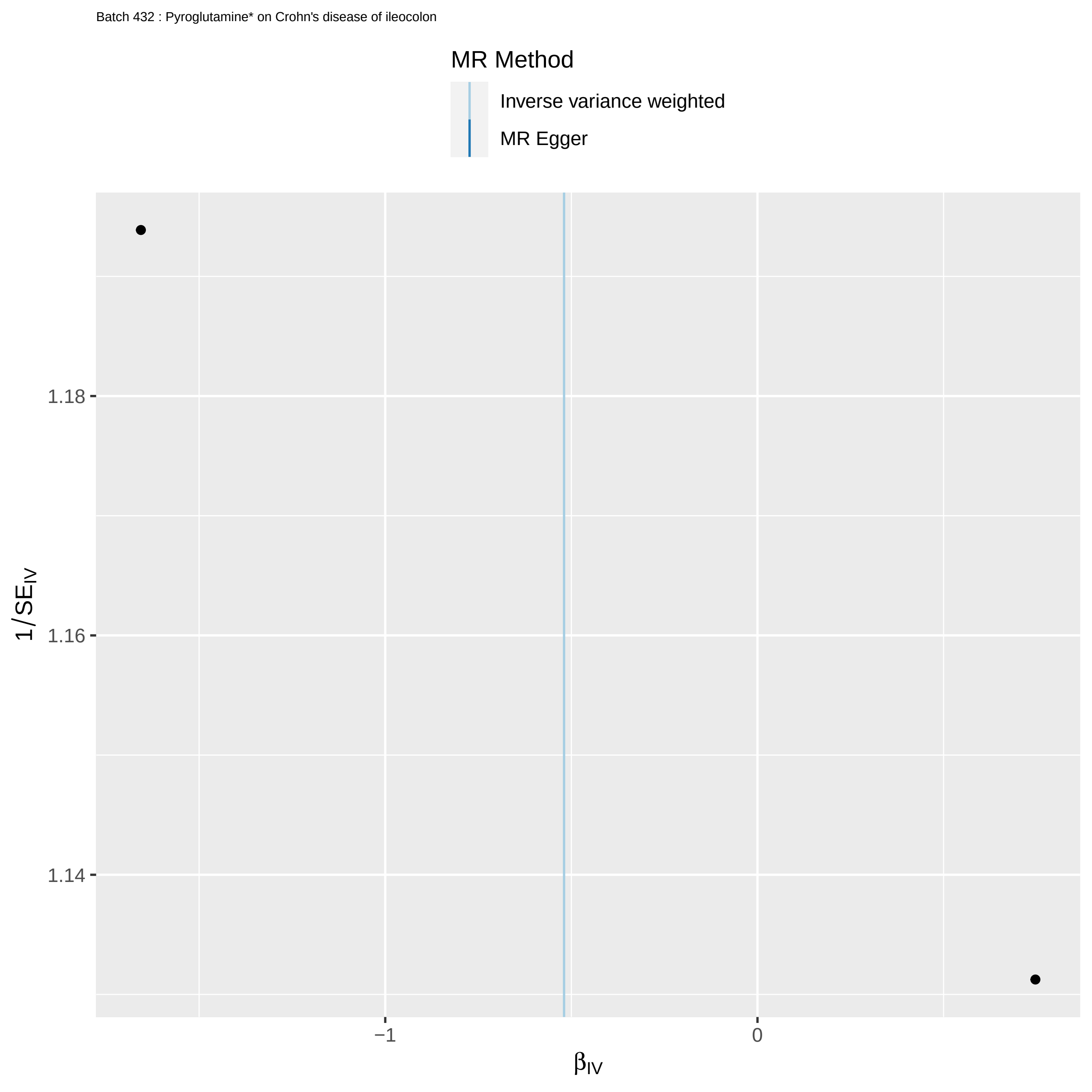
Batch 432 : Pyroglutamine* on Crohn's disease of ileocolon
MR Method
Inverse variance weighted
MR Egger
1.18
1.16
1.14
1/SE$_{IV}$
−1
0
β$_{IV}$

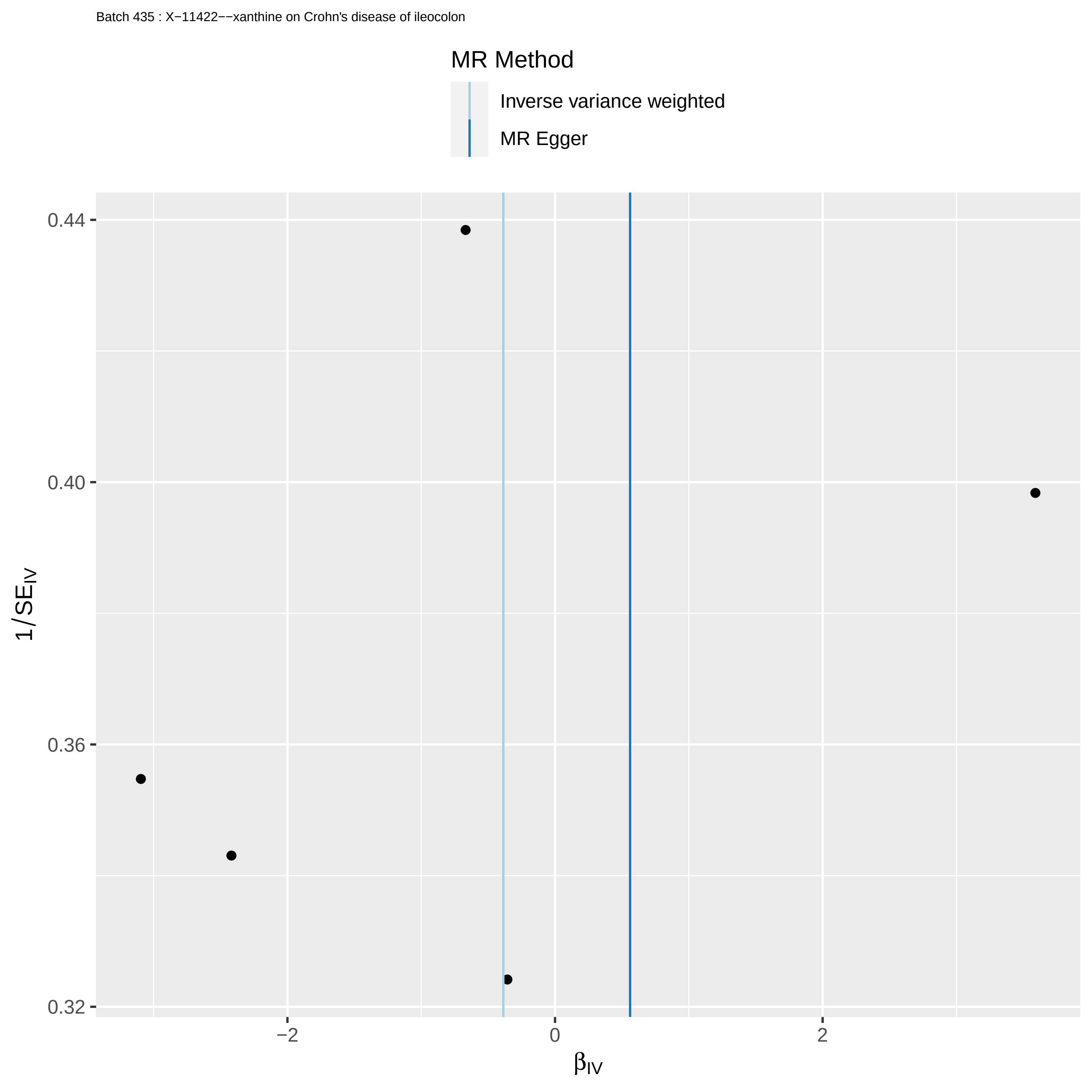

Batch 435 : X−11422−−xanthine on Crohn's disease of ileocolon
MR Method
Inverse variance weighted
MR Egger
0.44
0.40
0.36
0.32
1/SE_IV
−2
0
2
β_IV

Batch 438 : HWESASXX* on Crohn's disease of ileocolon

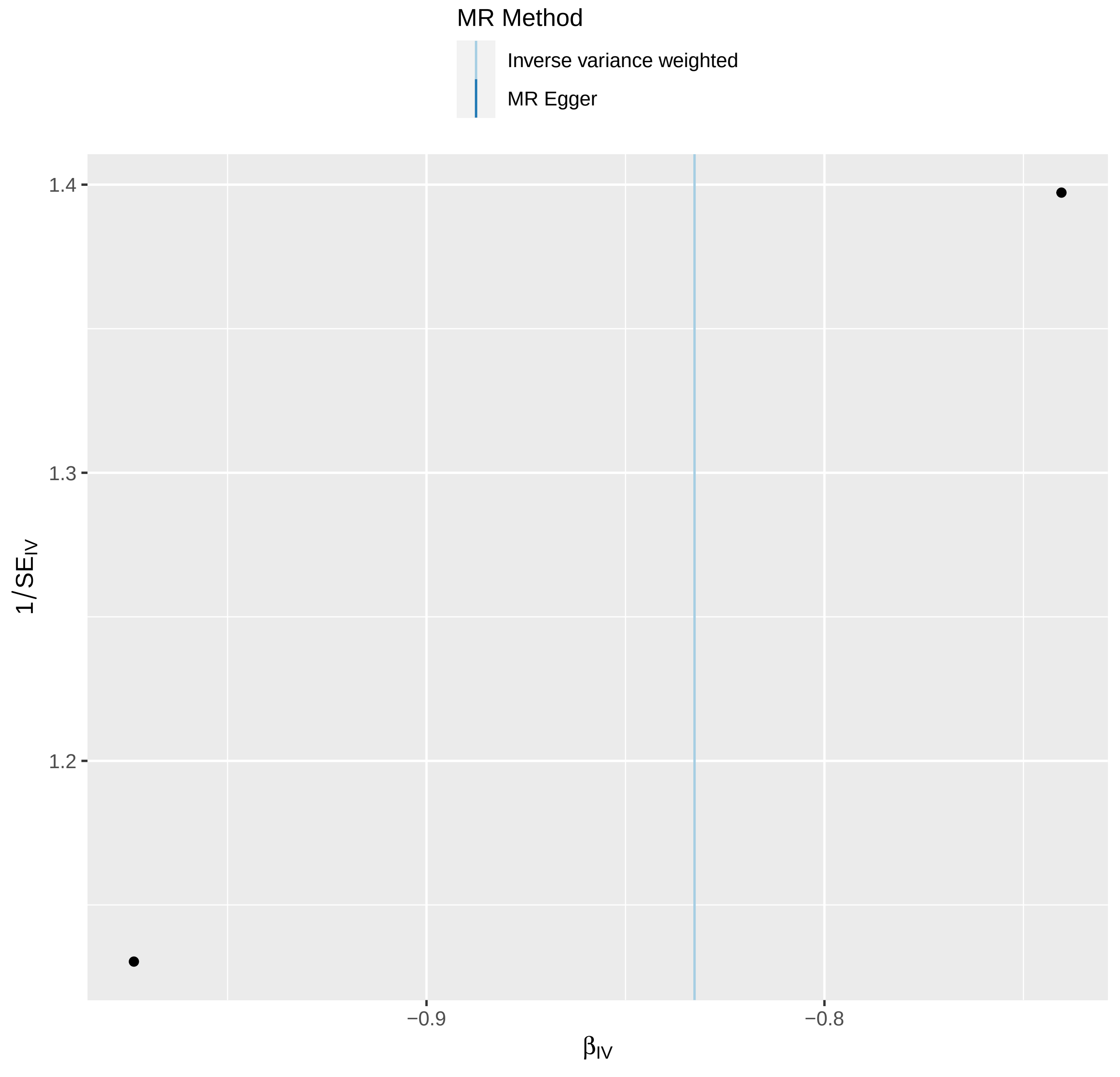

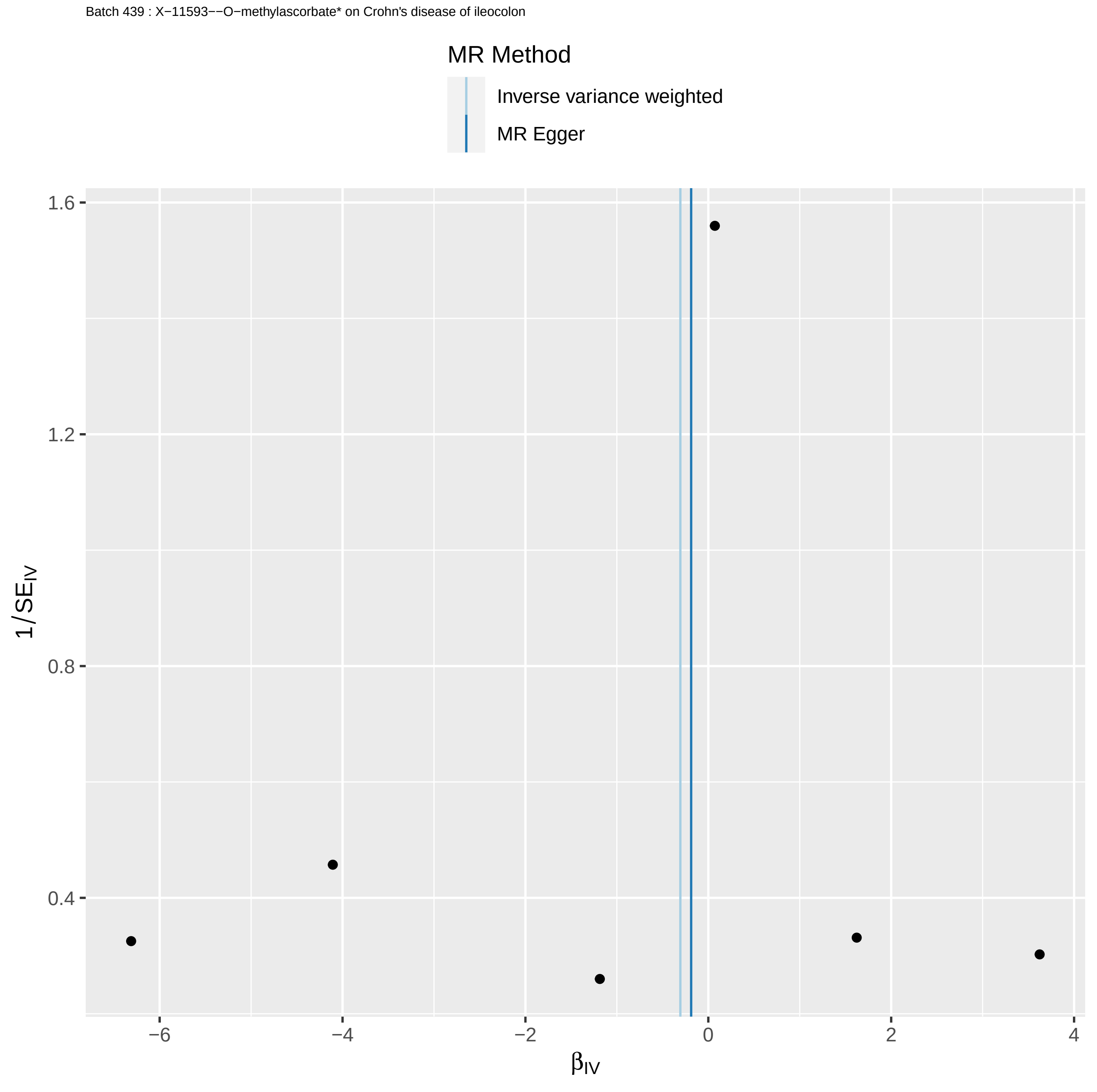

Batch 439 : X−11593−−O−methylascorbate* on Crohn's disease of ileocolon
MR Method
Inverse variance weighted
MR Egger
1.6
1.2
0.8
0.4
1/SE_IV
−6
−4
−2
0
2
4
β_IV

Batch 441 : Homostachydrine* on Crohn's disease of ileocolon

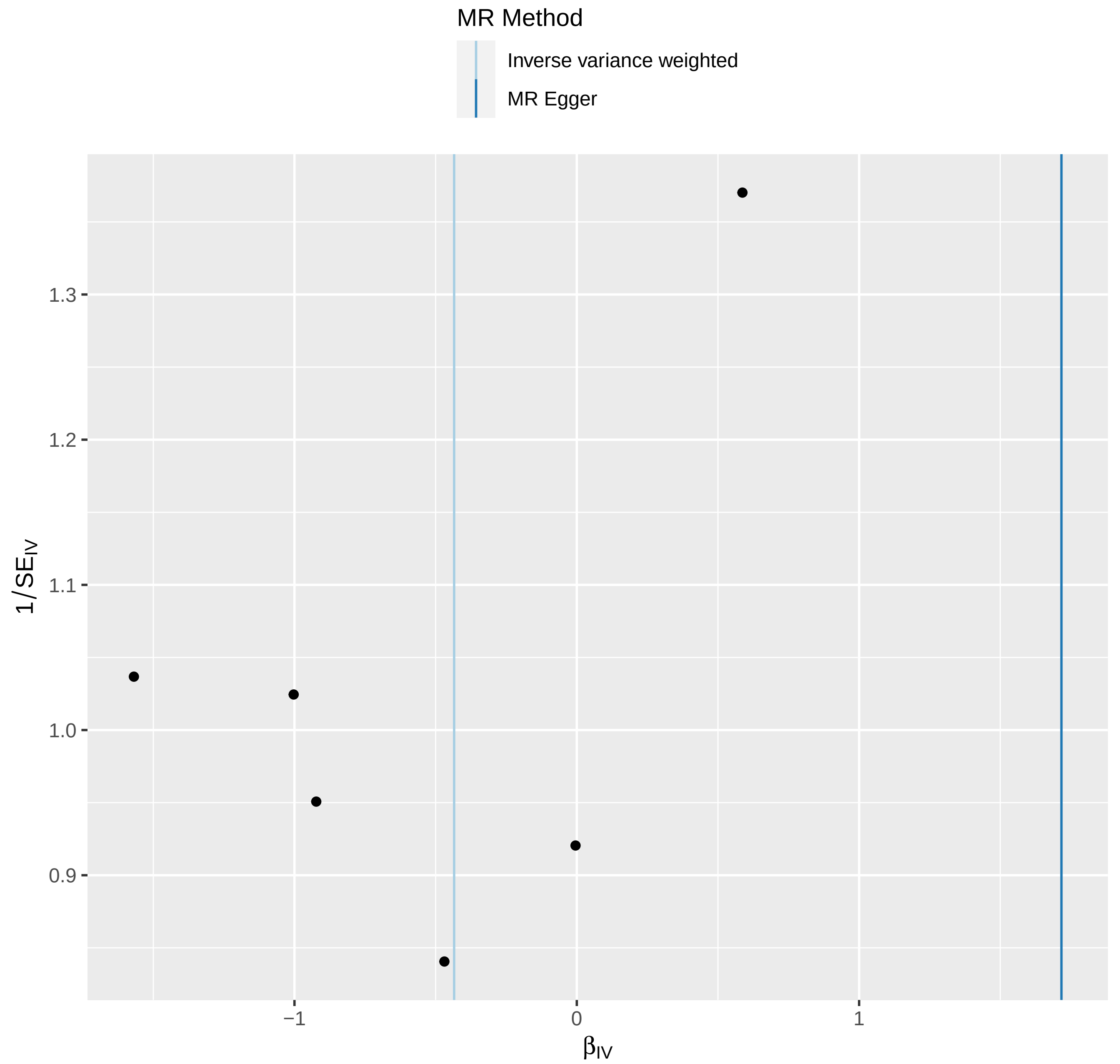

Batch 442 : ADSGEGDFXAEGGGVR* on Crohn's disease of ileocolon

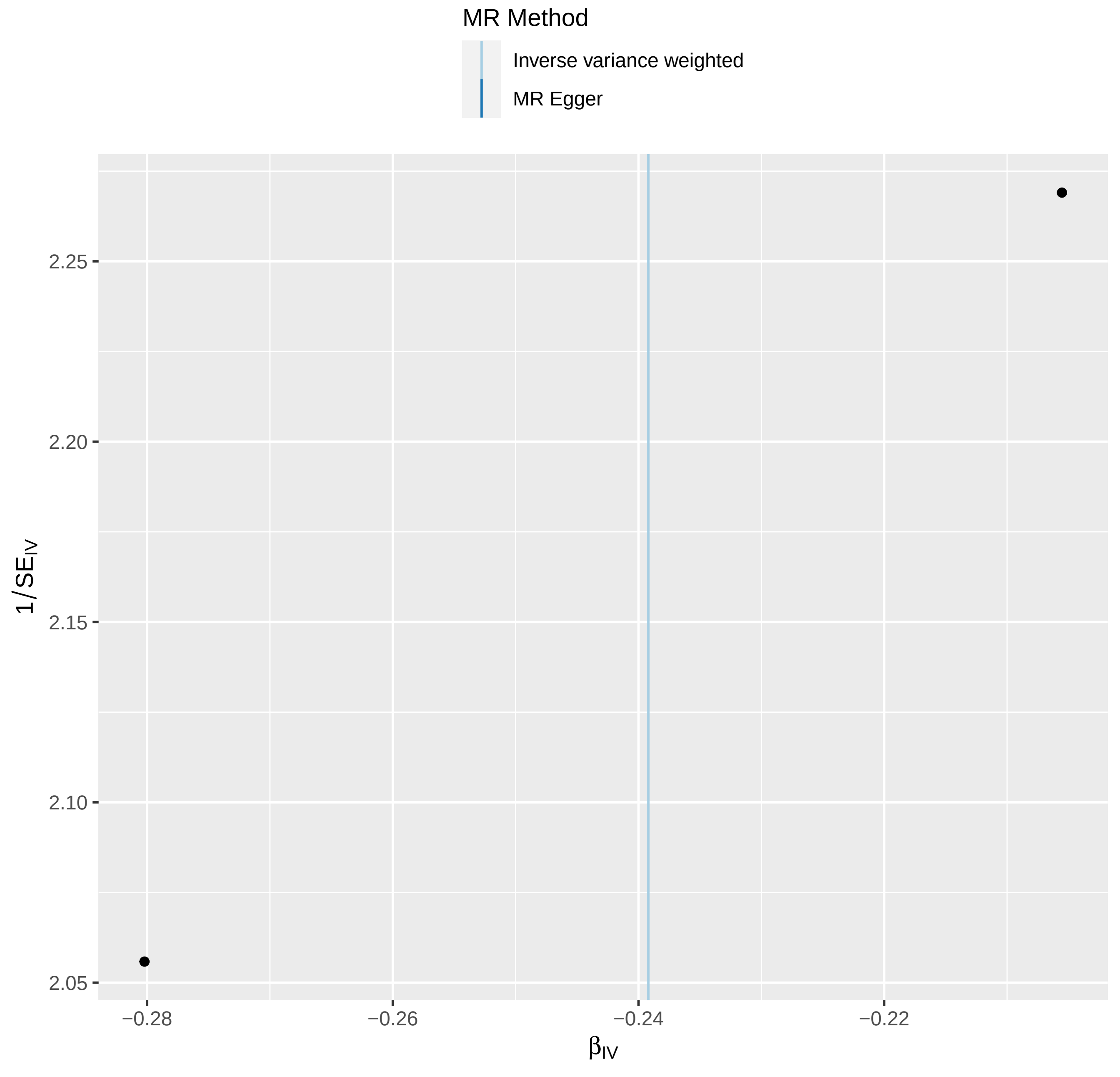

Batch 444 : X−11793−−oxidized bilirubin* on Crohn's disease of ileocolon

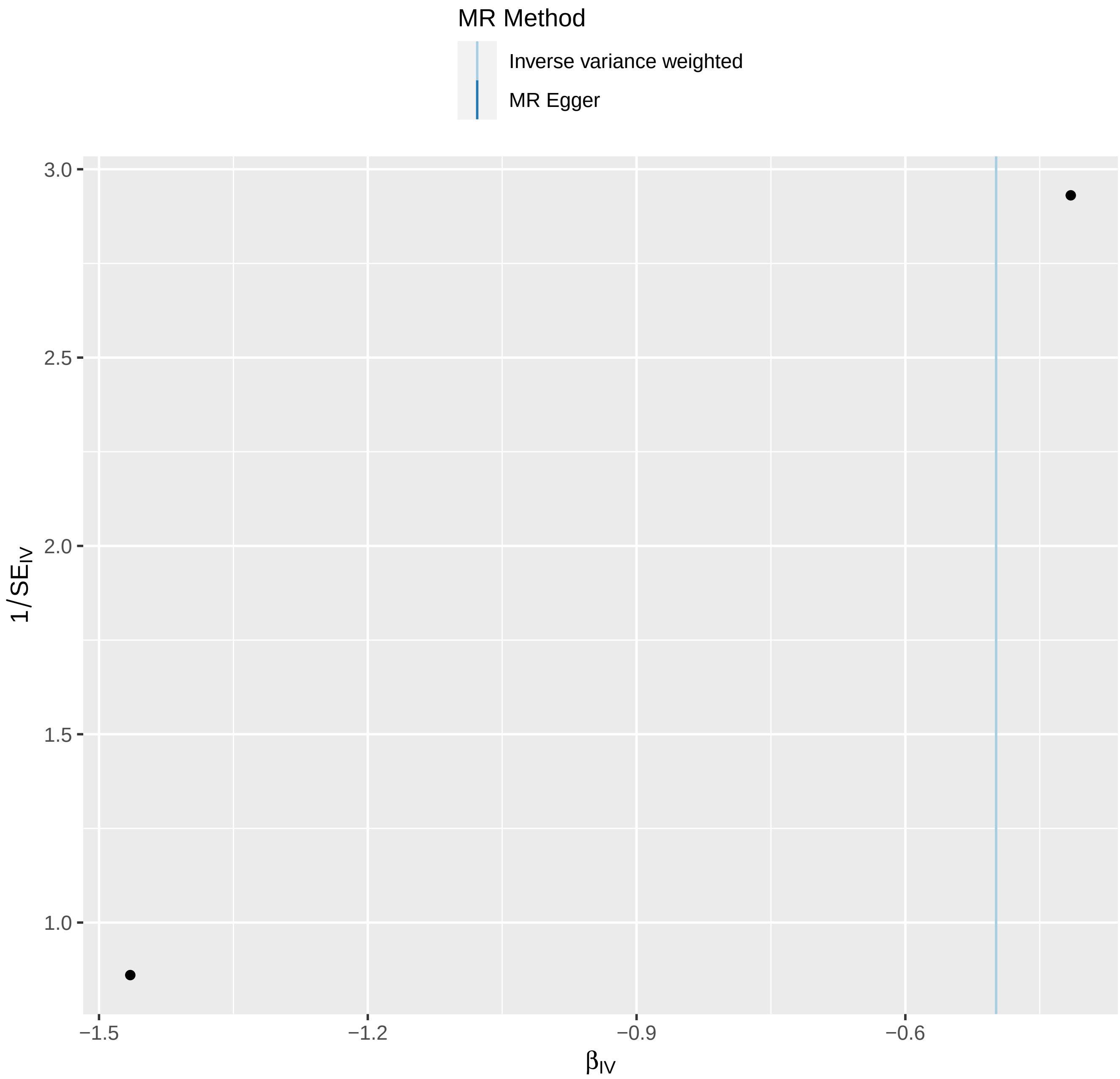

Batch 445 : 1−arachidonoylglycerophosphocholine* on Crohn's disease of ileocolon

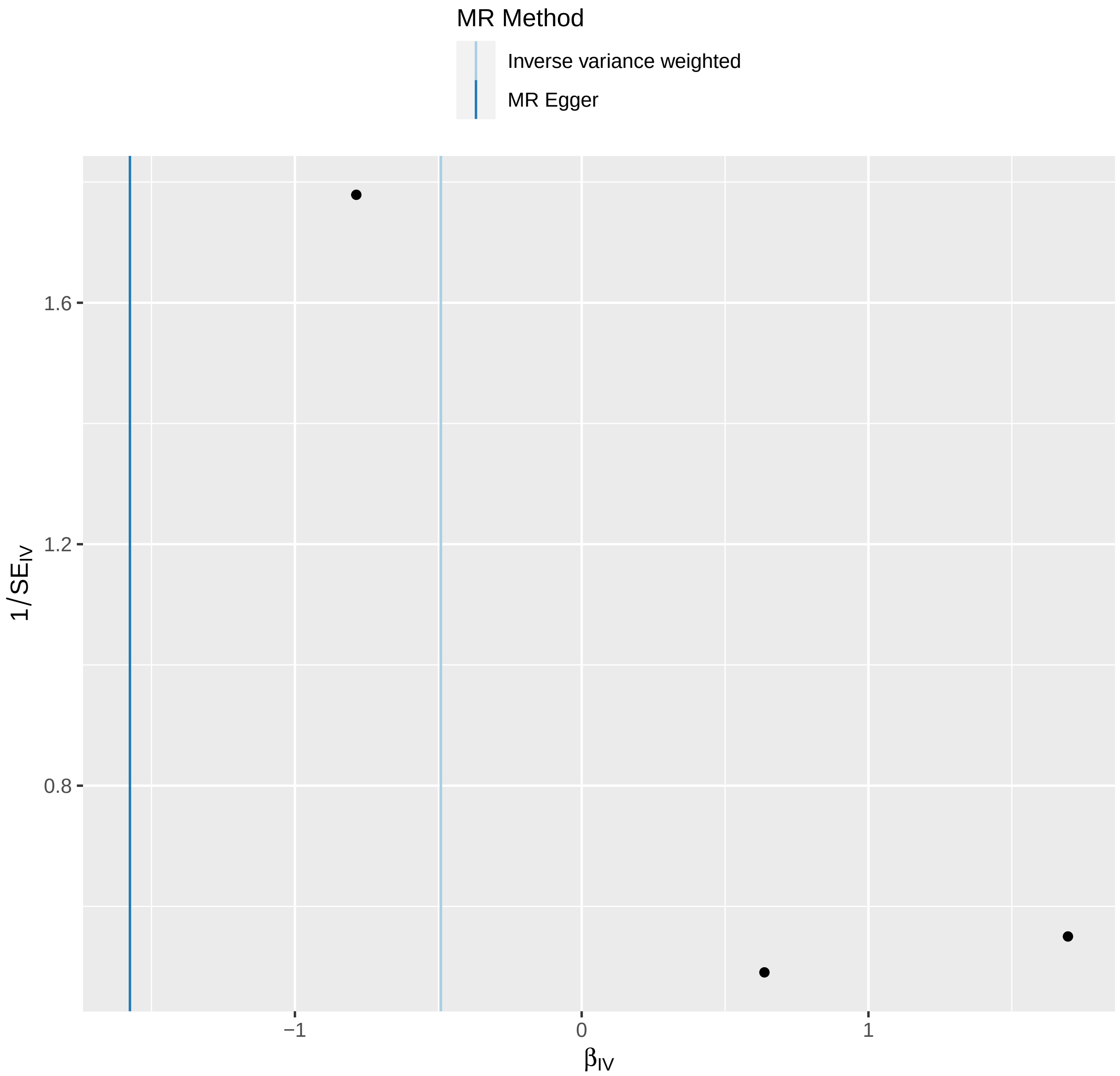

Batch 447 : Gamma−glutamylmethionine* on Crohn's disease of ileocolon

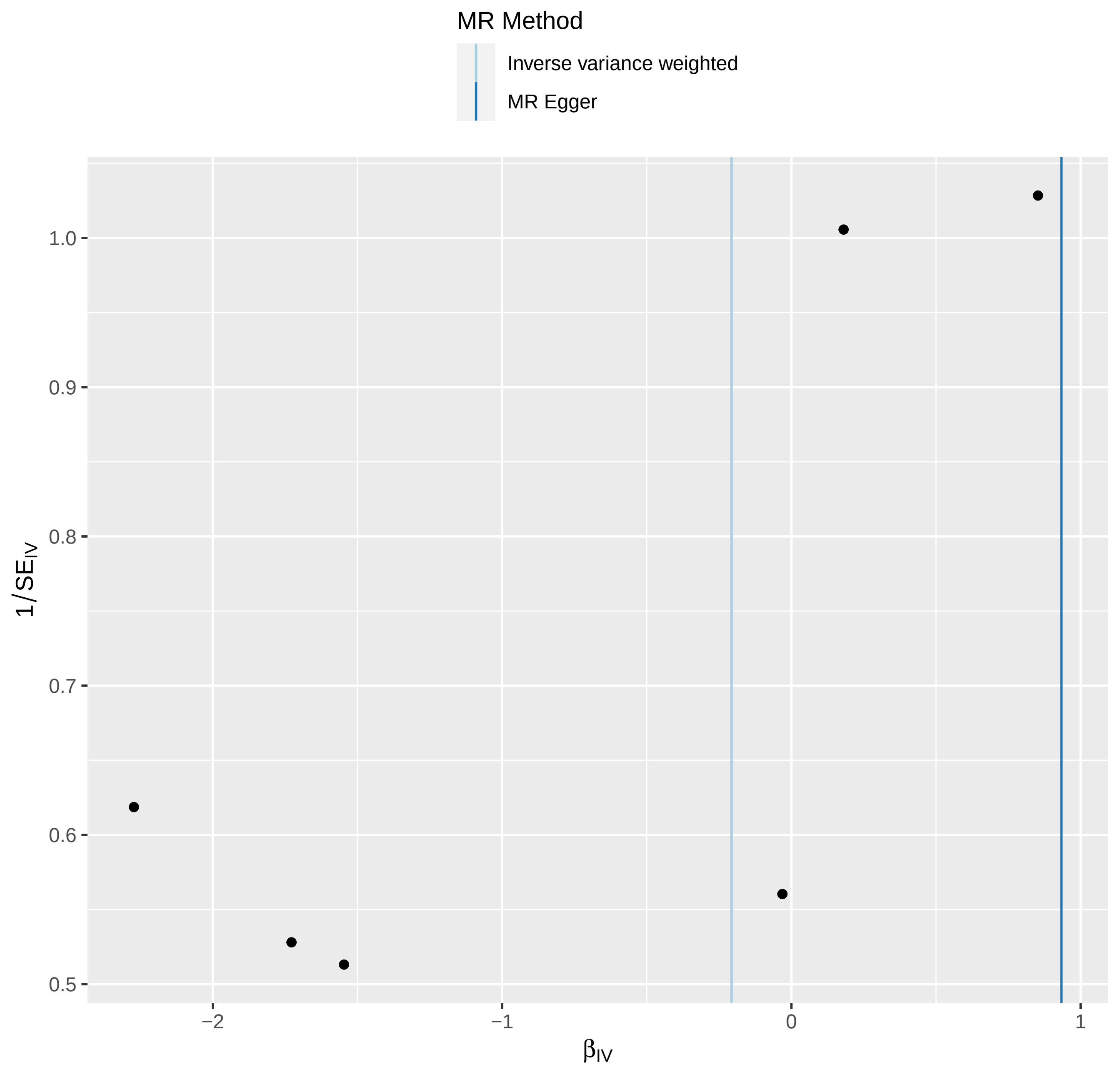

Batch 448 : Gamma−glutamylthreonine* on Crohn's disease of ileocolon

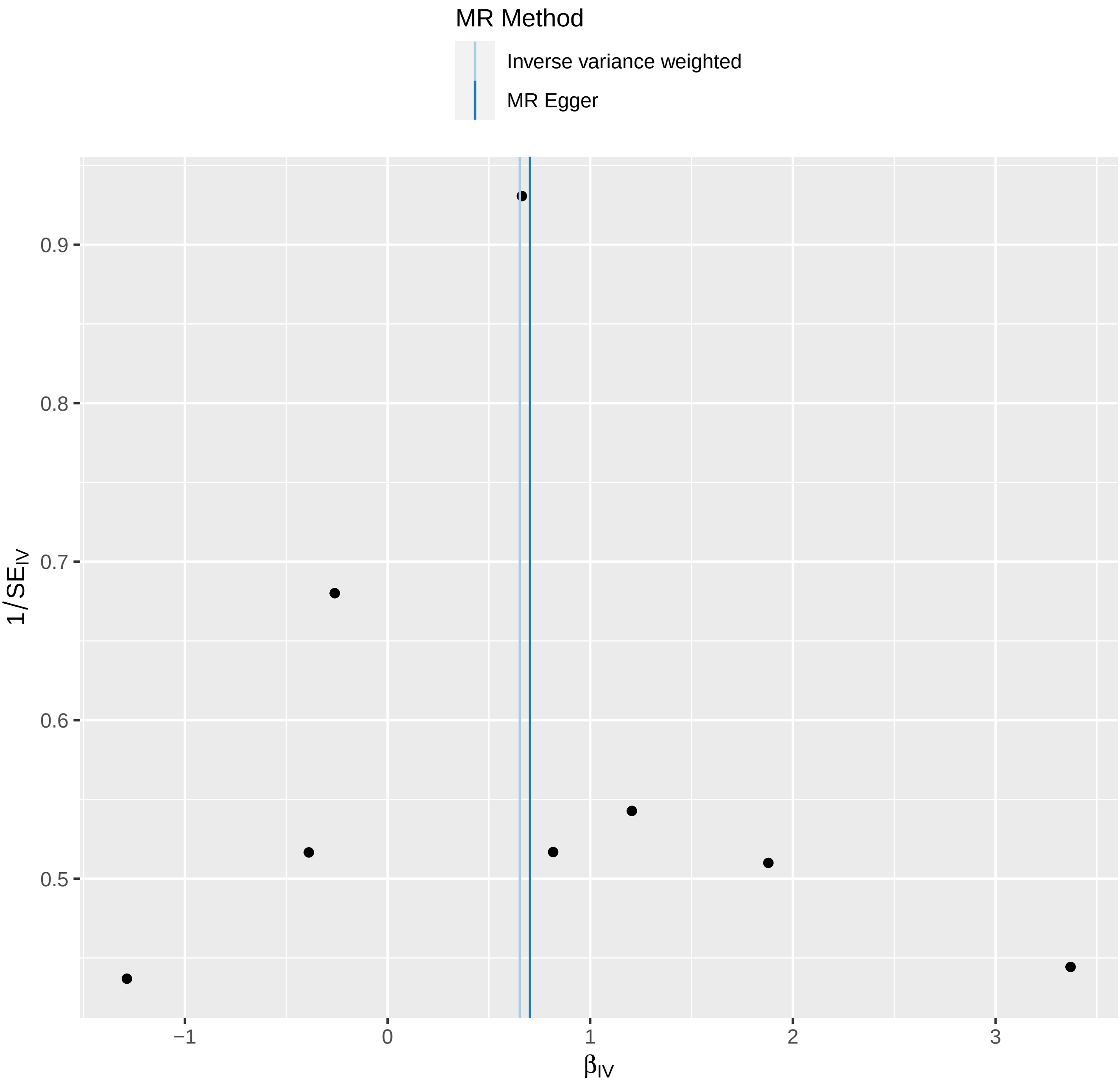

Batch 449 : Gamma−tocopherol on Crohn's disease of ileocolon

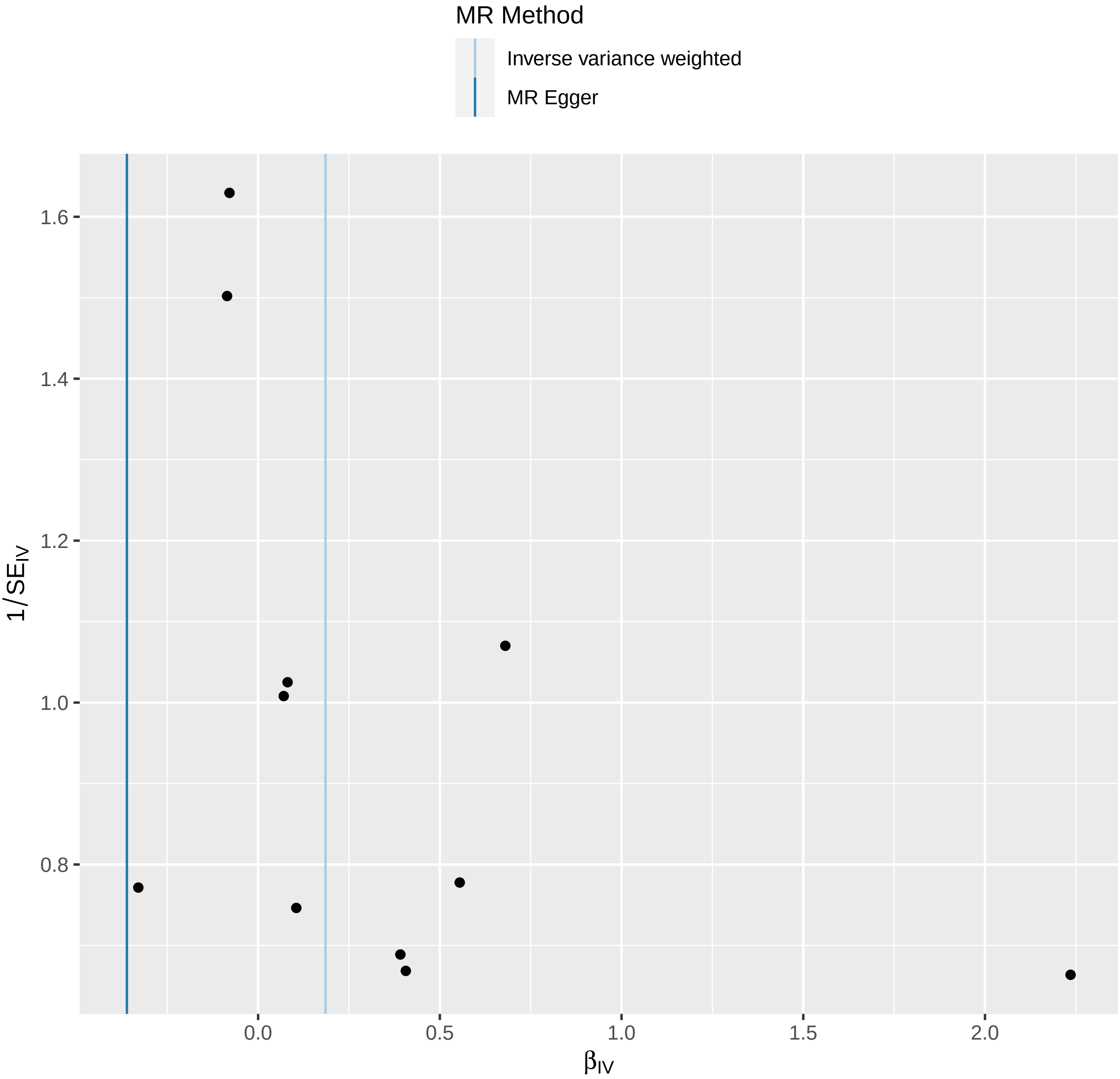

Batch 450 : Gamma−glutamylphenylalanine on Crohn's disease of ileocolon

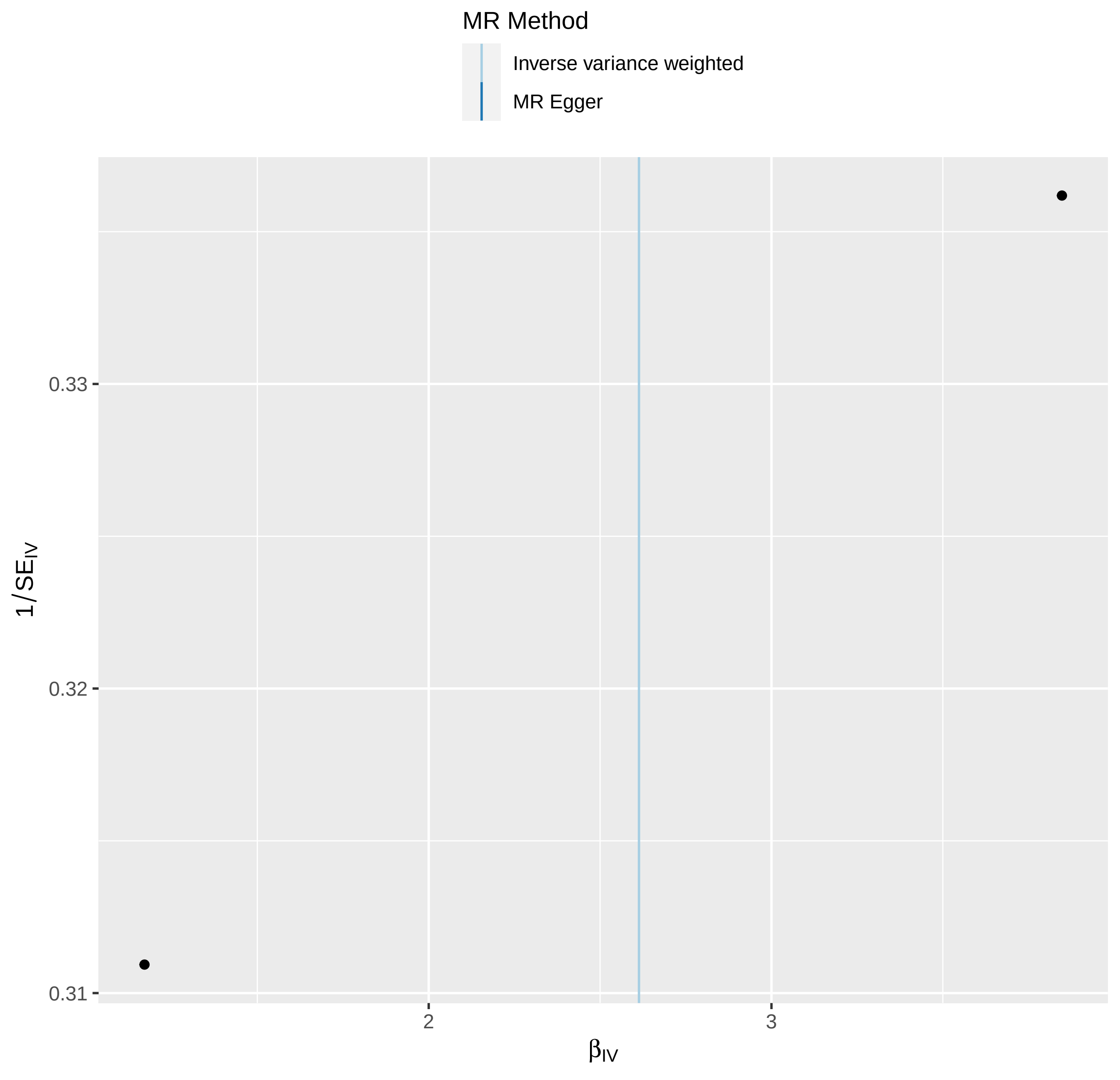

Batch 451 : Isobutyrylcarnitine on Crohn's disease of ileocolon

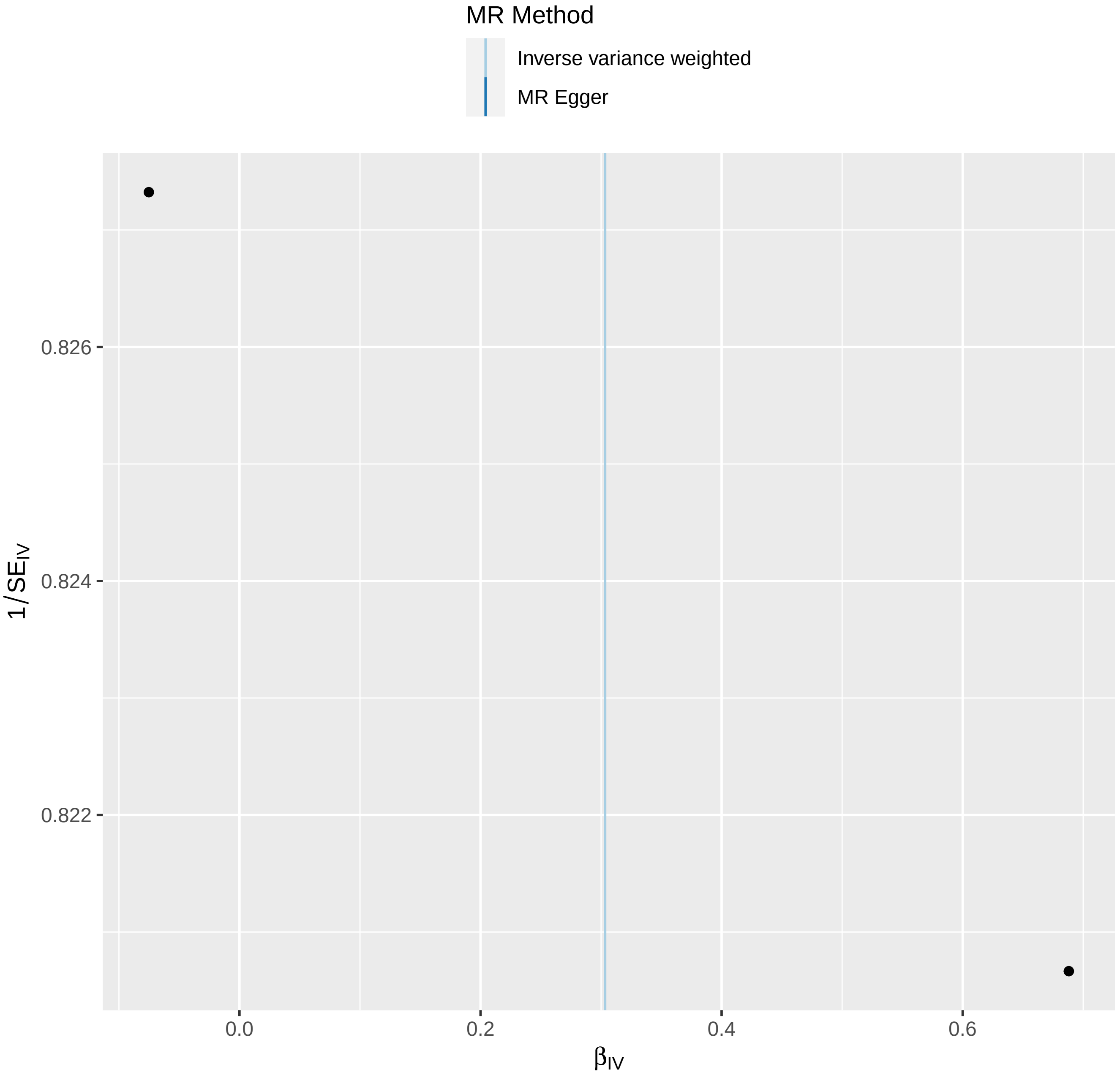

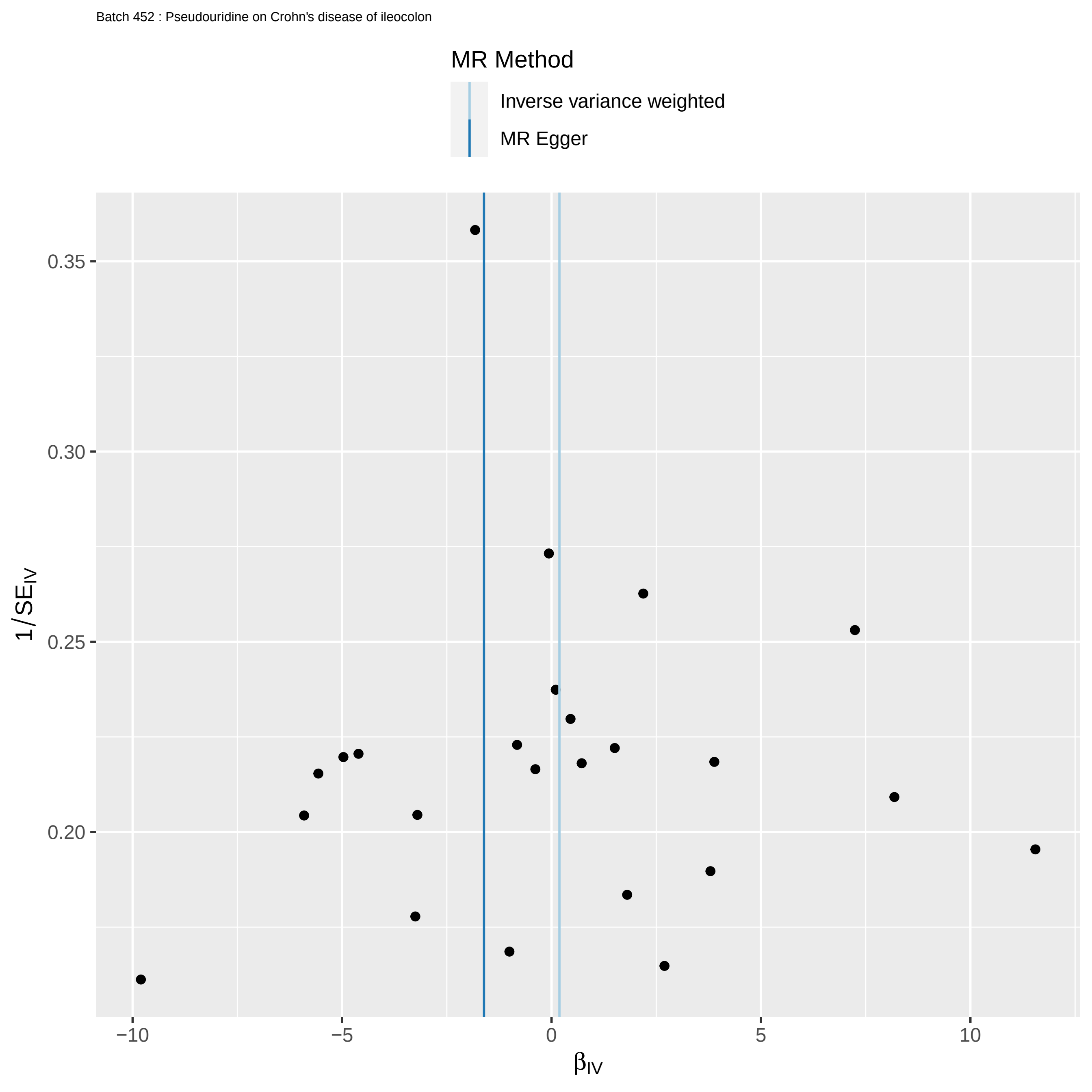

Batch 452 : Pseudouridine on Crohn's disease of ileocolon
MR Method
Inverse variance weighted
MR Egger
0.35
0.30
0.25
0.20
1/SE_IV
−10
−5
0
5
10
β_IV

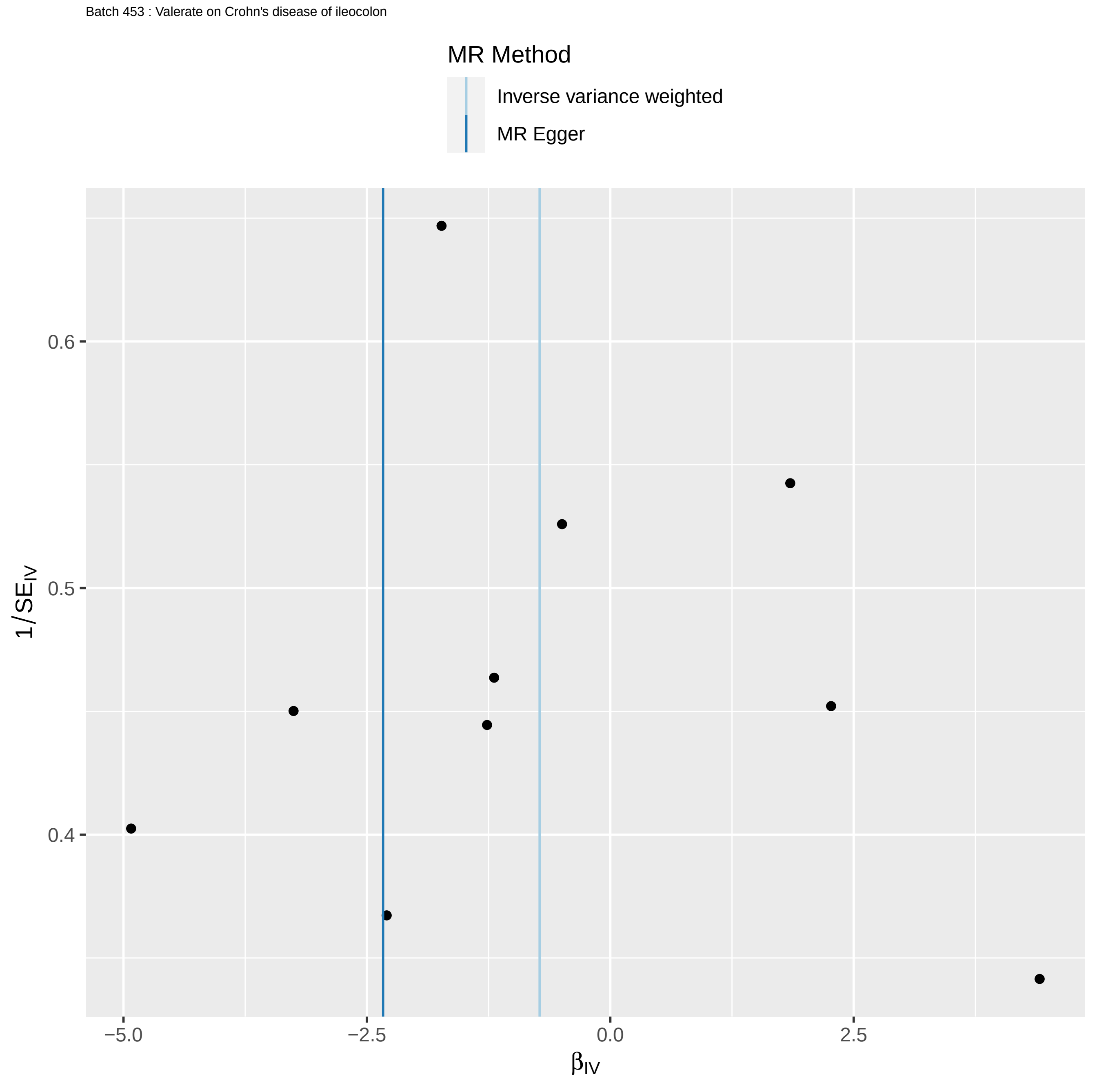
Batch 453 : Valerate on Crohn's disease of ileocolon
MR Method
Inverse variance weighted
MR Egger
0.6
0.5
0.4
1/SE_IV
−5.0
−2.5
0.0
2.5
β_IV

Batch 456 : Erythronate* on Crohn's disease of ileocolon

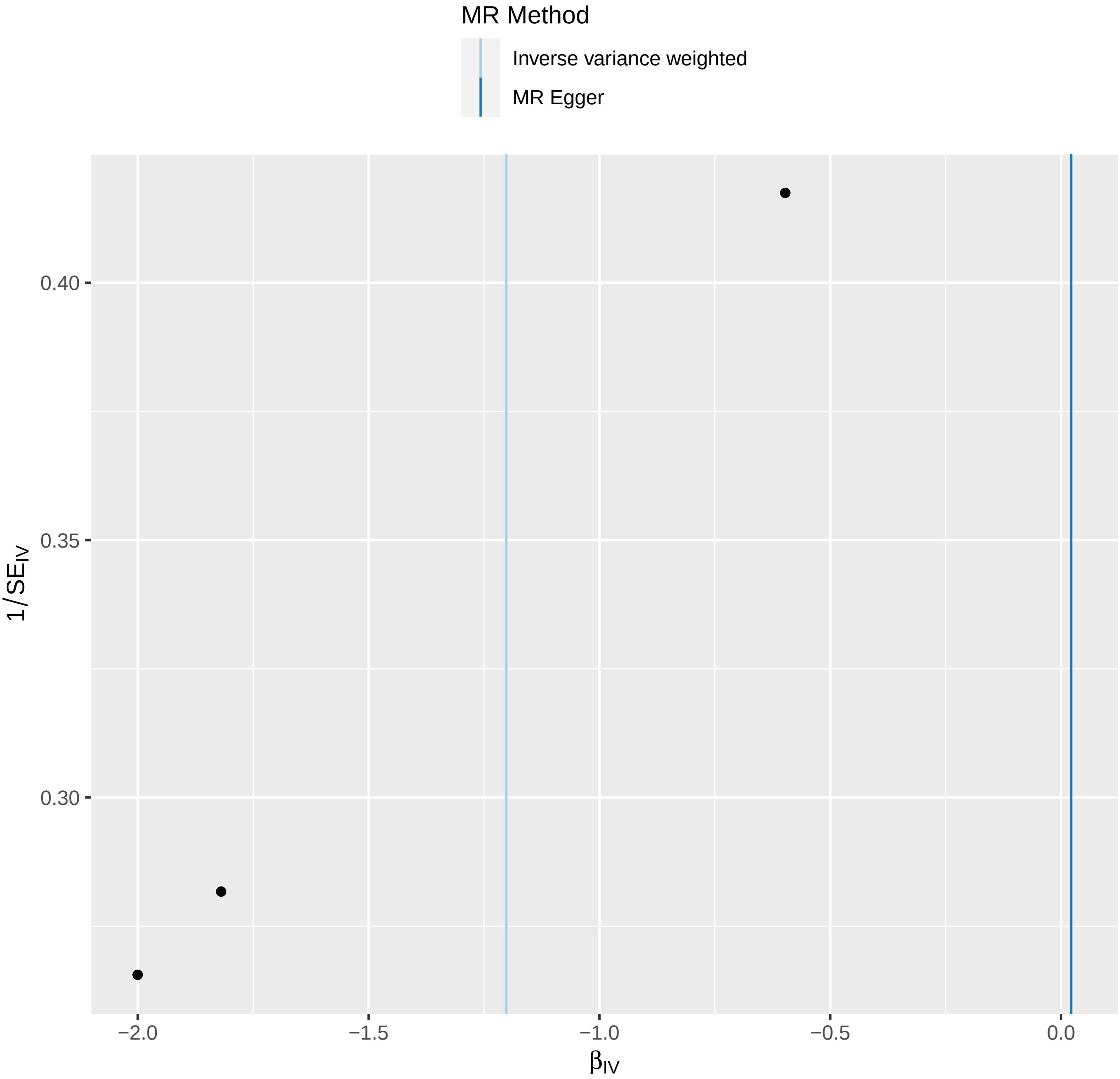

Batch 457 : Lathosterol on Crohn's disease of ileocolon

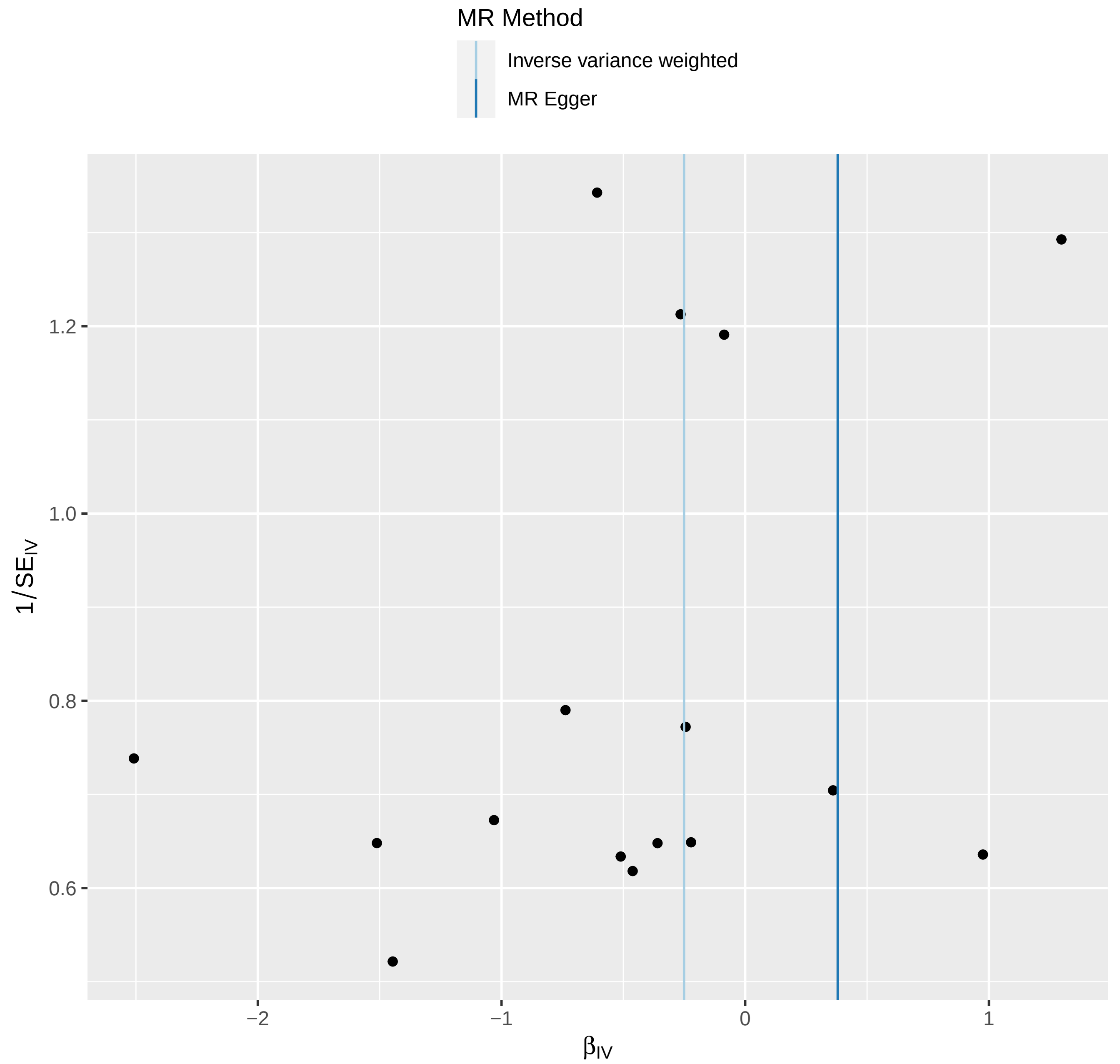

Batch 460 : Eicosenoate (20:1n9 or 11) on Crohn's disease of ileocolon

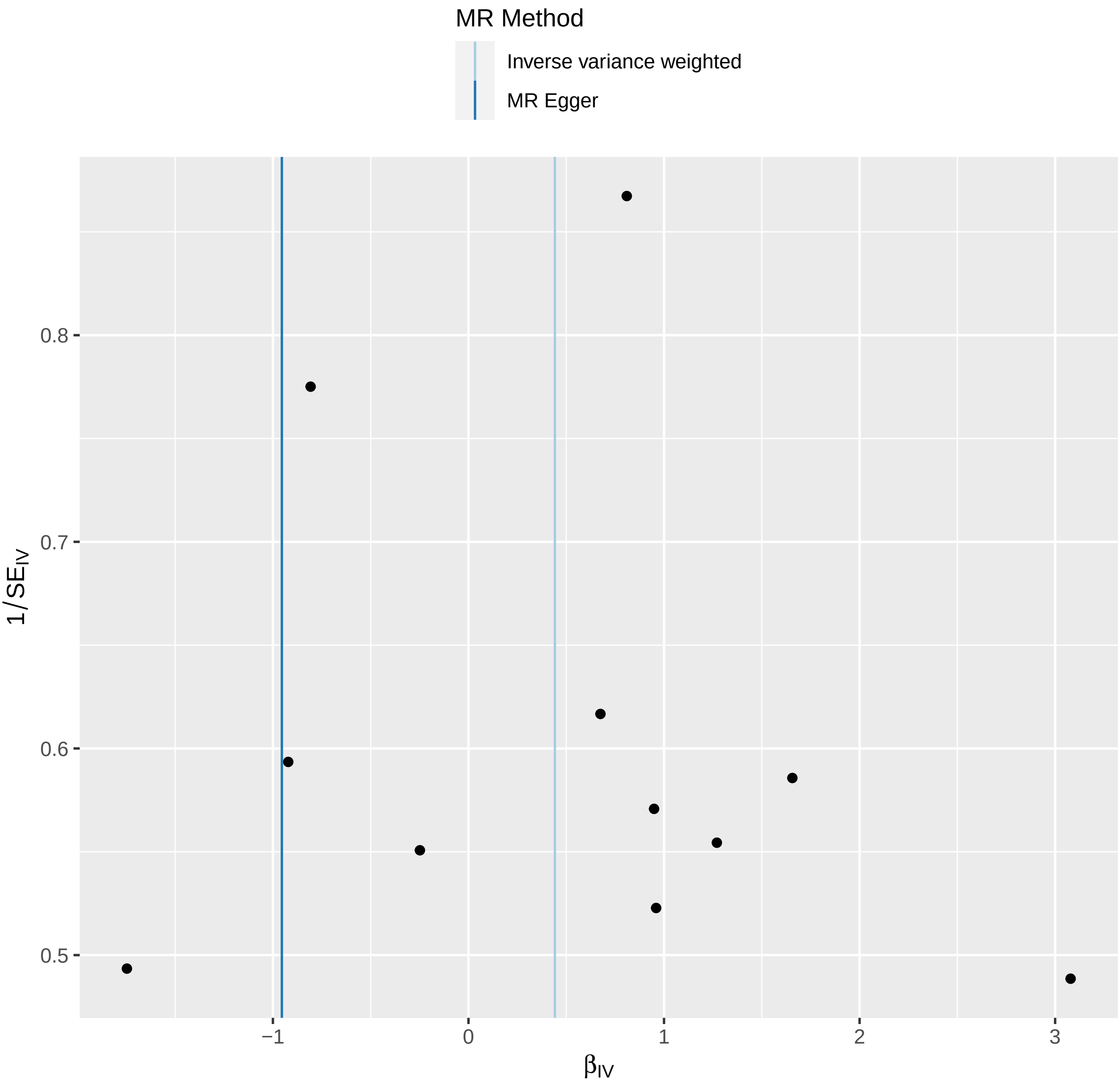

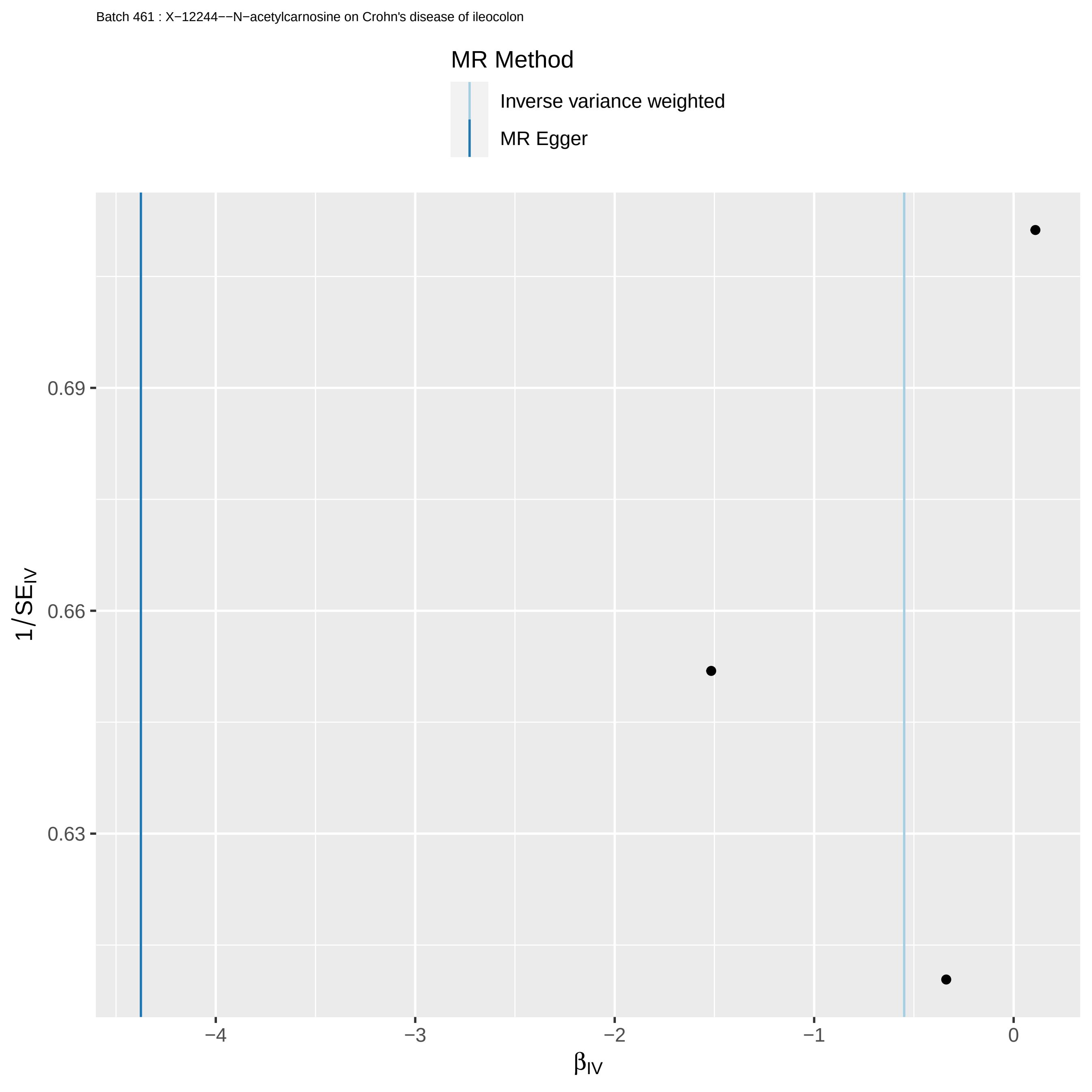

Batch 461 : X−12244−−N−acetylcarnosine on Crohn's disease of ileocolon
MR Method
Inverse variance weighted
MR Egger
0.69
0.66
0.63
1/SE_IV
−4
−3
−2
−1
0
β_IV

Batch 463 : 1−eicosatrienoylglycerophosphocholine* on Crohn's disease of ileocolon

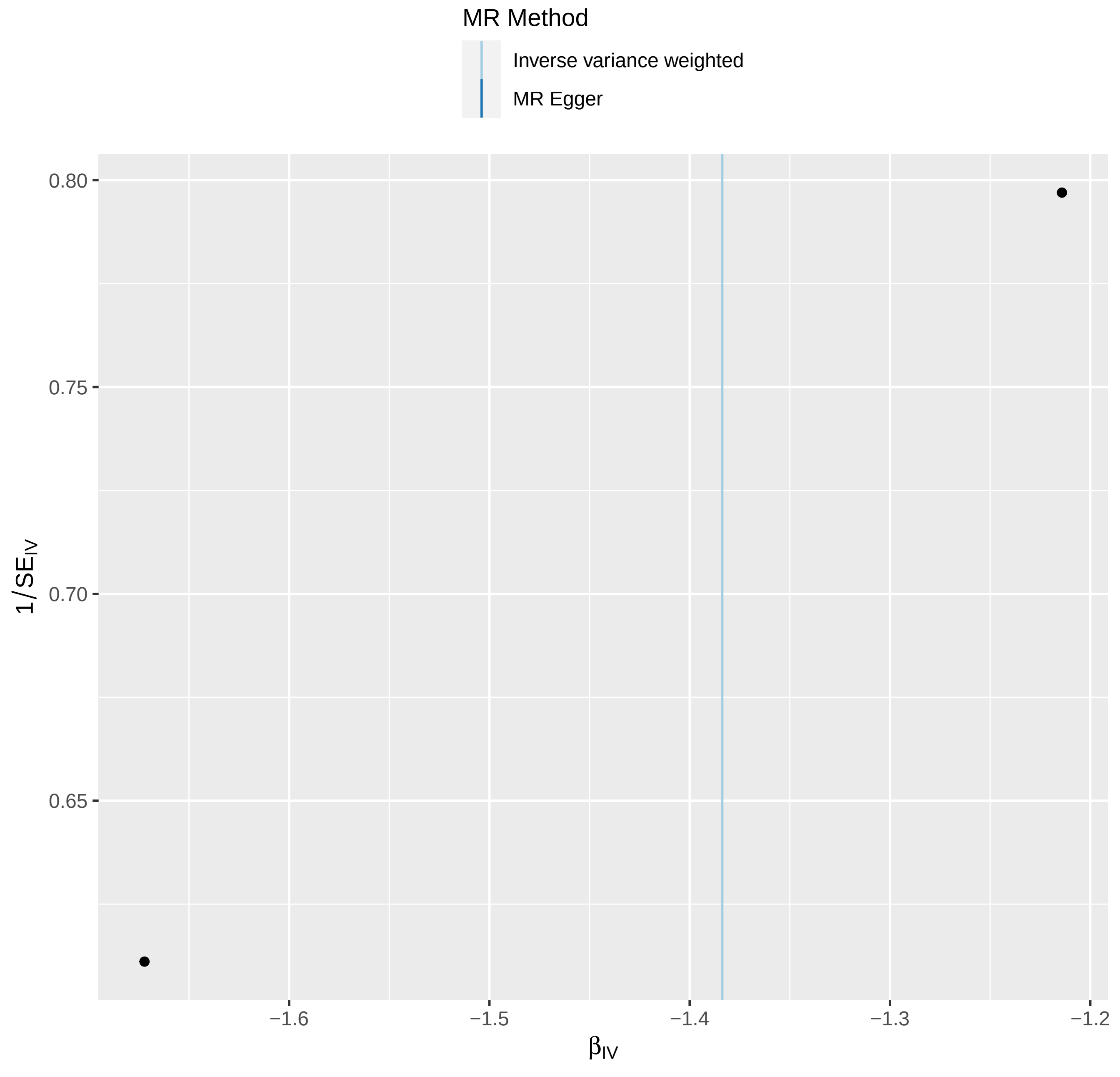

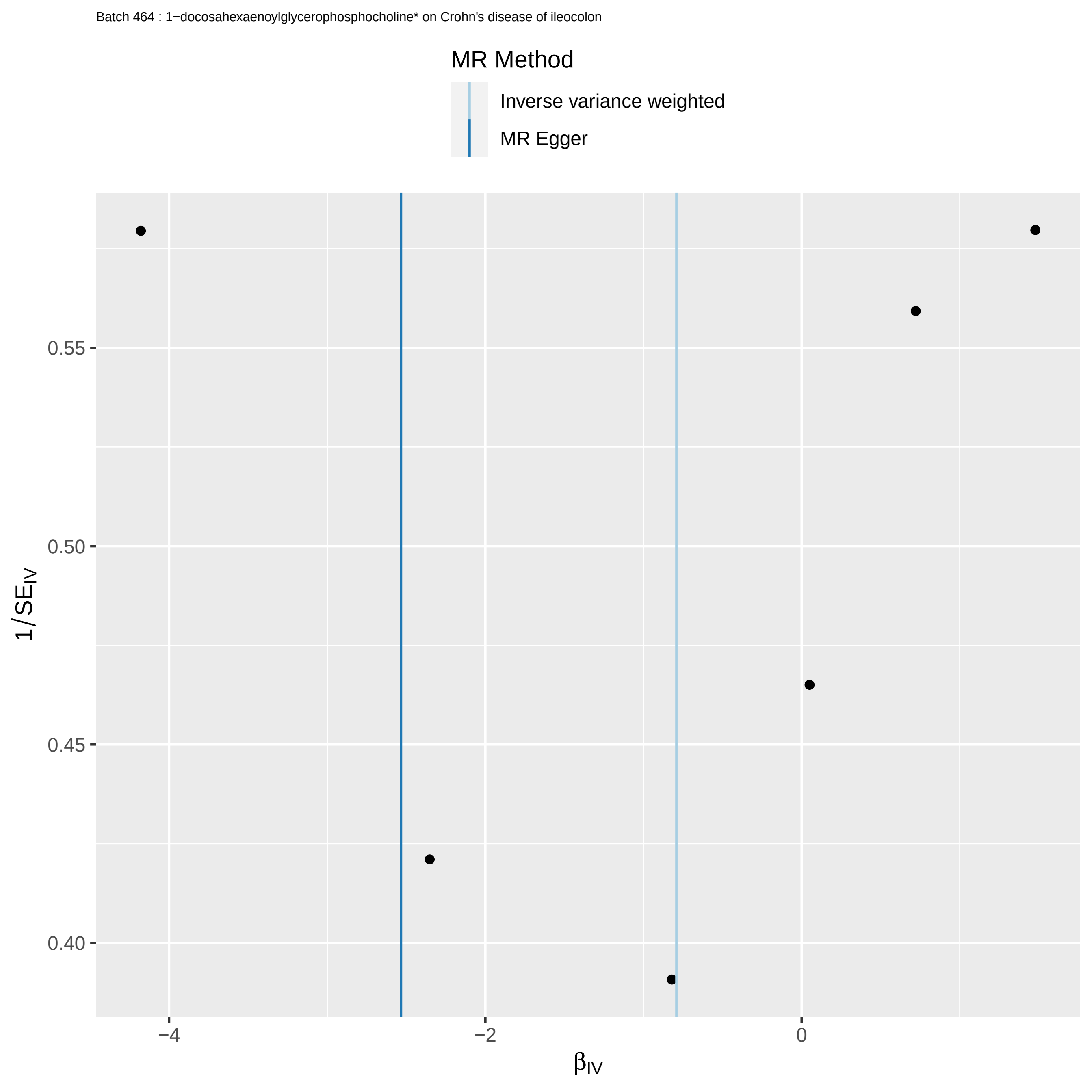

Batch 464 : 1−docosahexaenoylglycerophosphocholine* on Crohn's disease of ileocolon
MR Method
Inverse variance weighted
MR Egger
0.55
0.50
0.45
0.40
1/SE$_{IV}$
−4
−2
0
β$_{IV}$

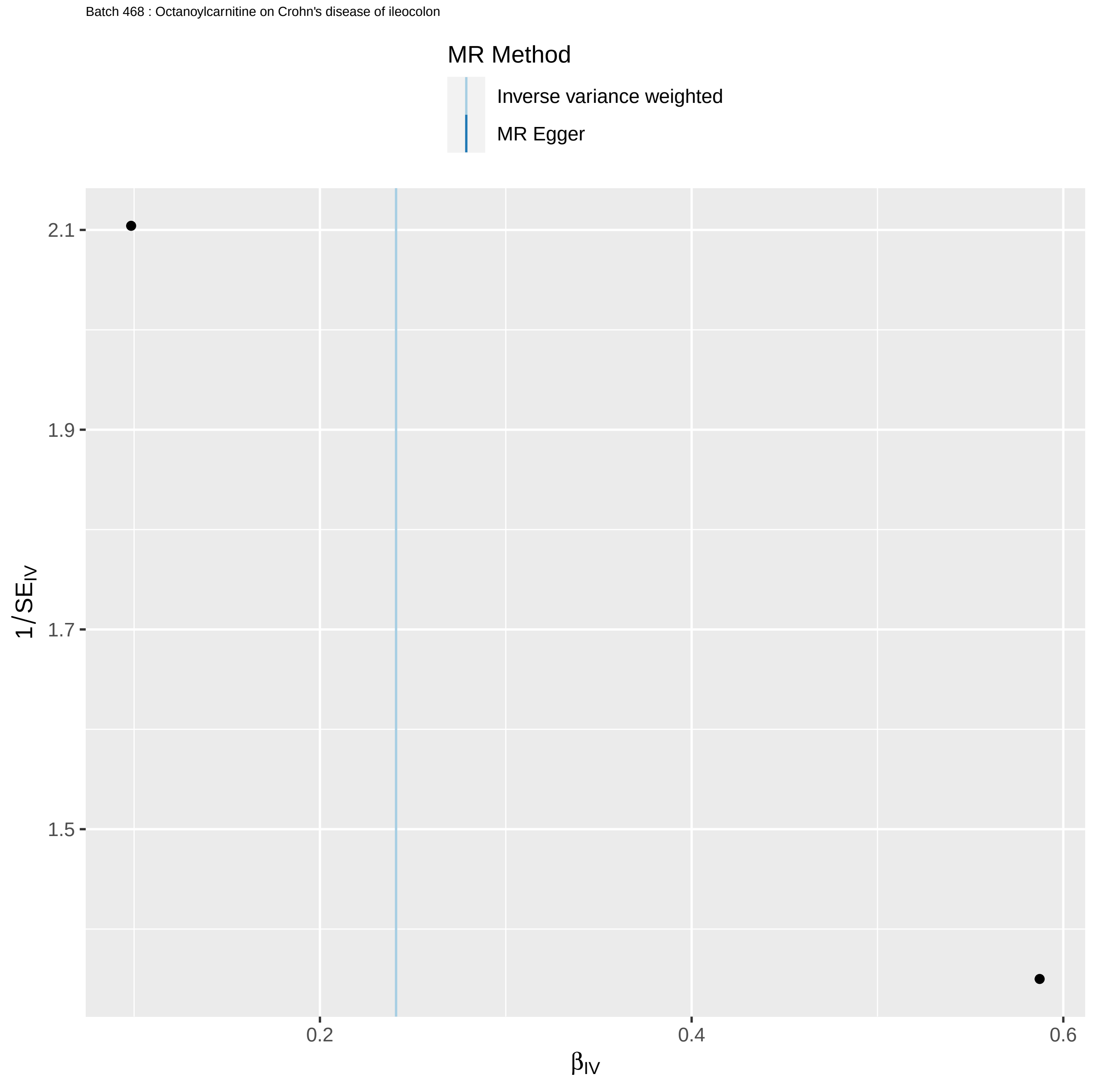

Batch 468 : Octanoylcarnitine on Crohn's disease of ileocolon
MR Method
Inverse variance weighted
MR Egger
2.1
1.9
1.7
1.5
$1/SE_{IV}$
0.2
0.4
0.6
β_{IV}

Batch 469 : Alpha−hydroxyisovalerate on Crohn's disease of ileocolon

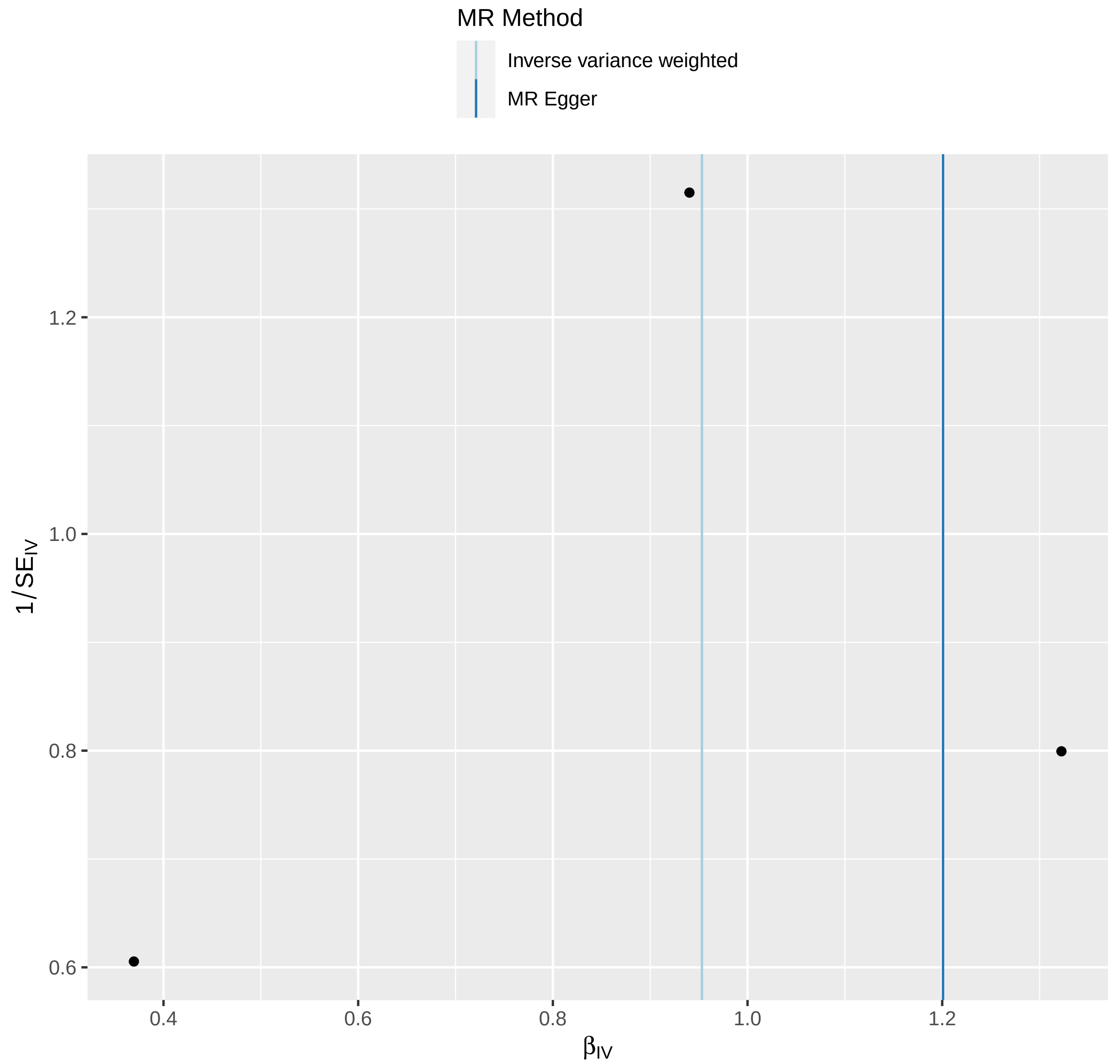

Batch 470 : N−acetylthreonine on Crohn's disease of ileocolon

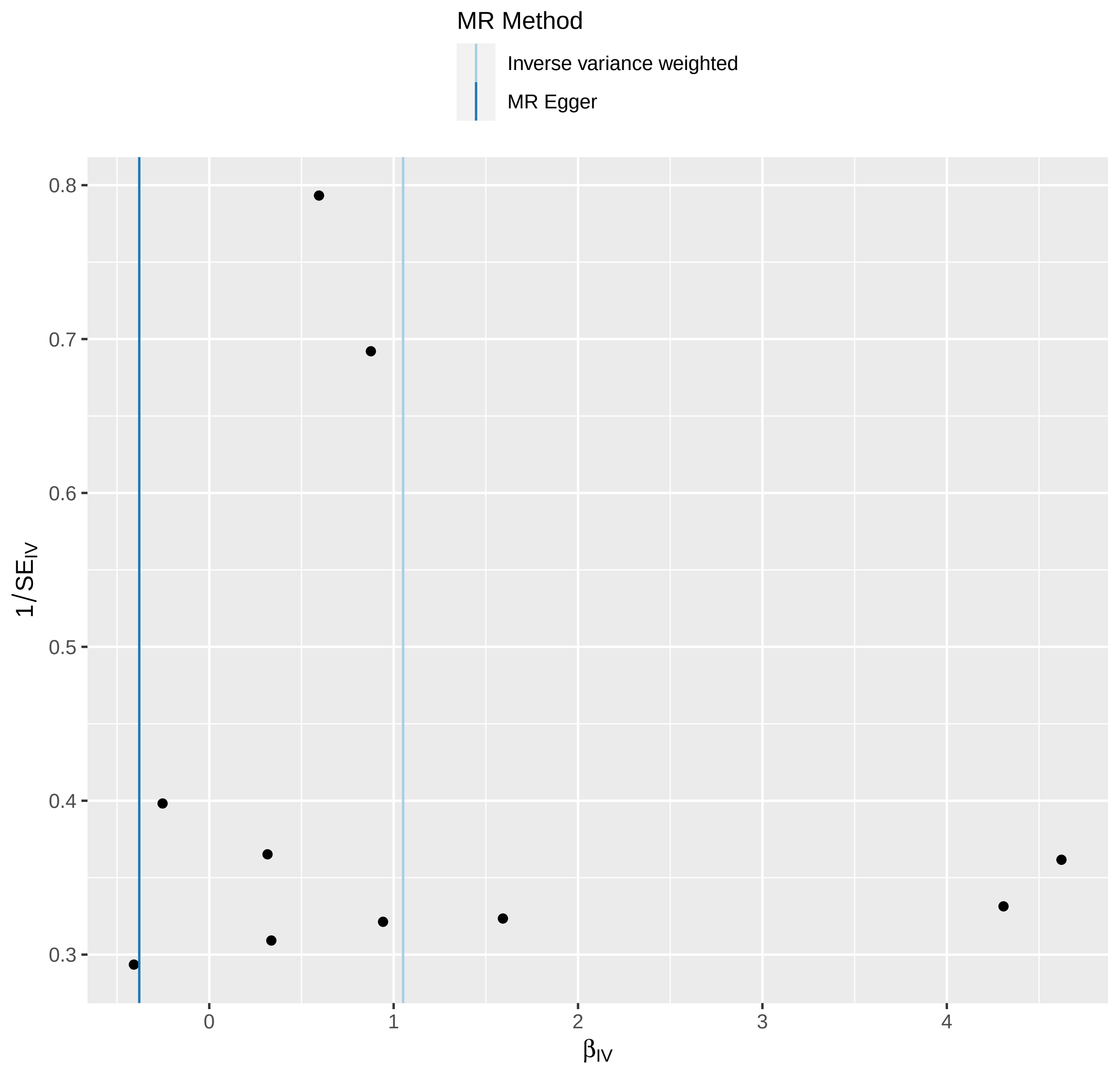

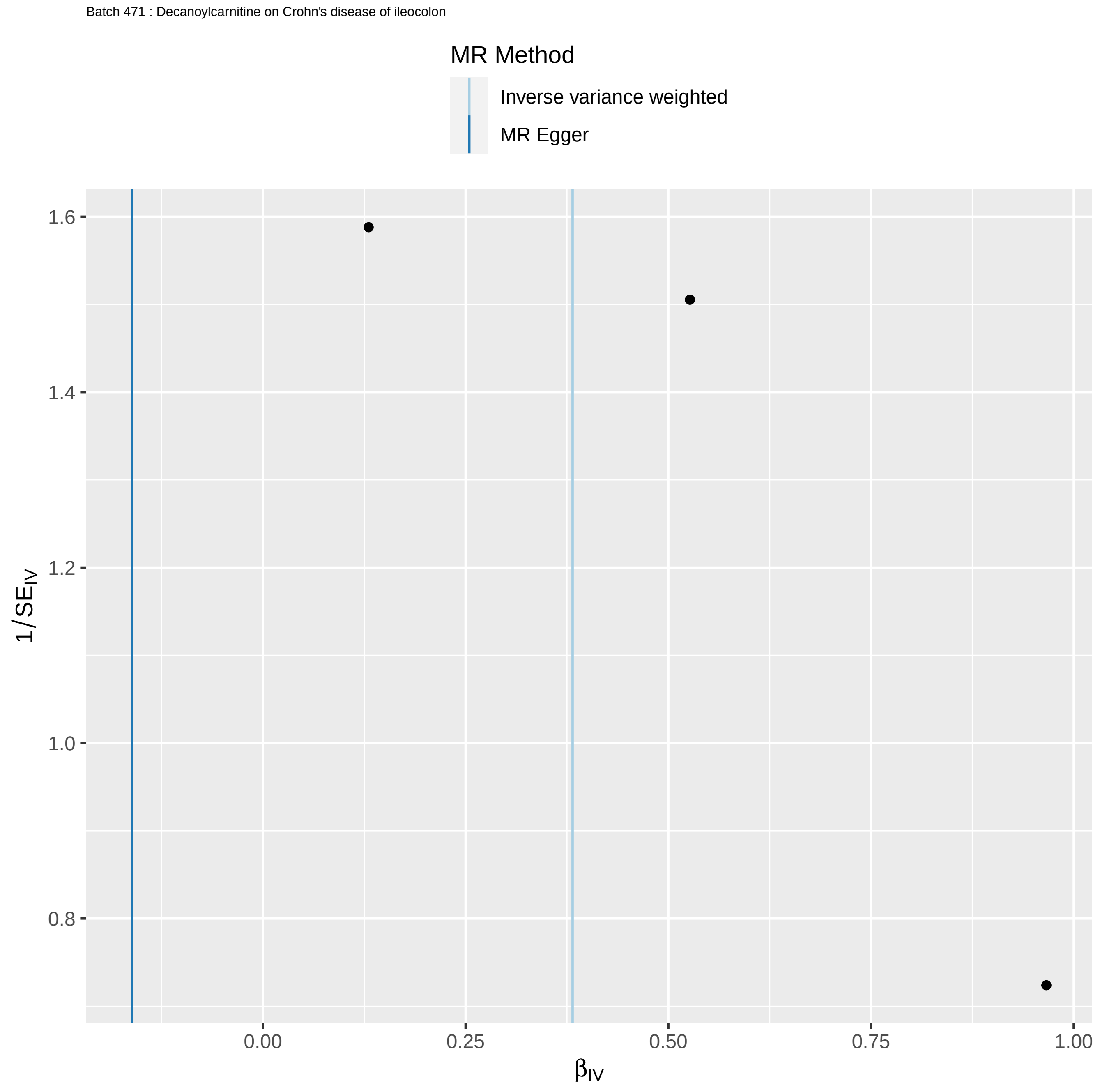
Batch 471 : Decanoylcarnitine on Crohn's disease of ileocolon
MR Method
Inverse variance weighted
MR Egger
1.6
1.4
1.2
1.0
0.8
$1/SE_{IV}$
0.00
0.25
0.50
0.75
1.00
β_{IV}

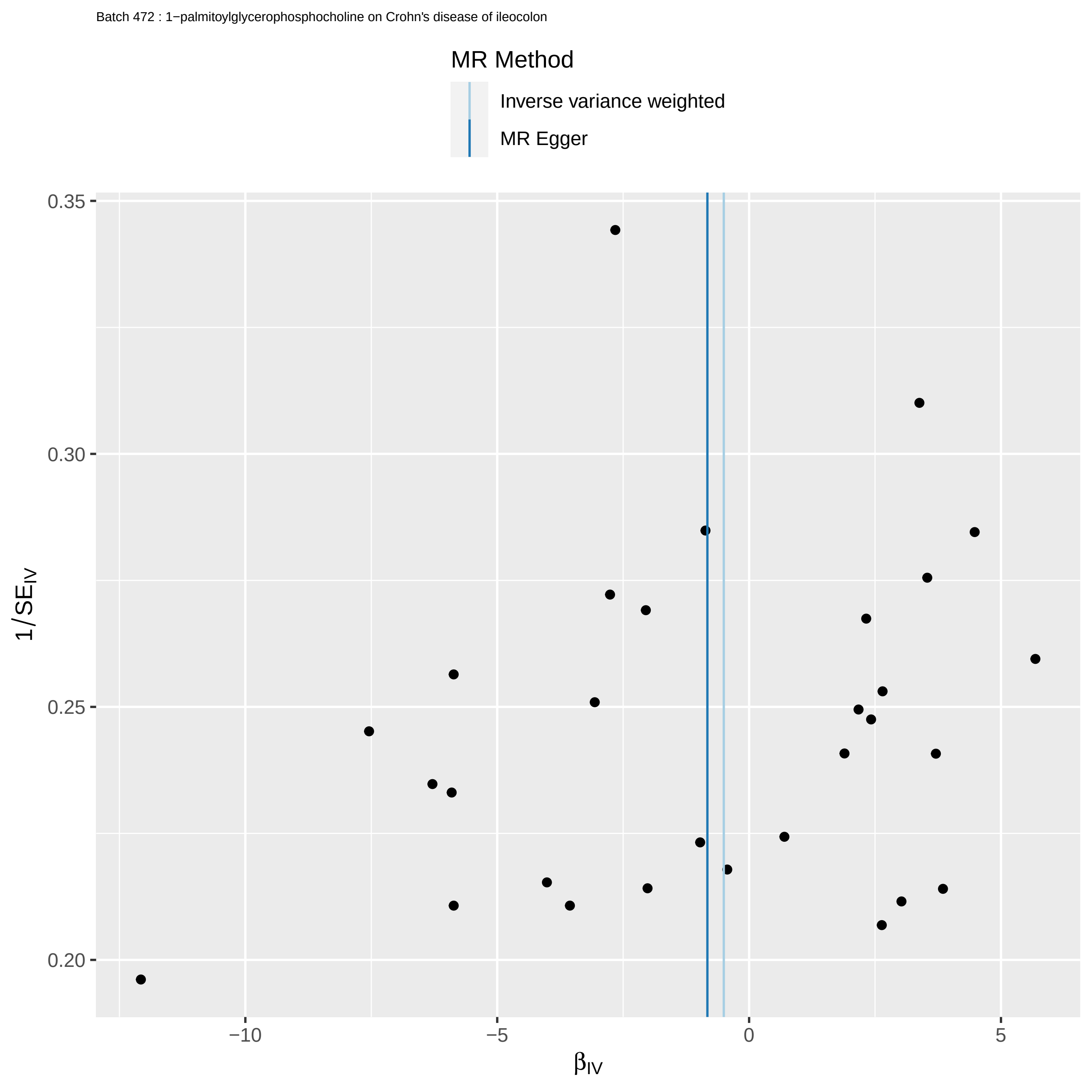

Batch 472 : 1−palmitoylglycerophosphocholine on Crohn's disease of ileocolon
MR Method
Inverse variance weighted
MR Egger
0.35
0.30
0.25
0.20
1/SE_IV
−10
−5
0
5
β_IV

Batch 473 : 1−heptadecanoylglycerophosphocholine on Crohn's disease of ileocolon

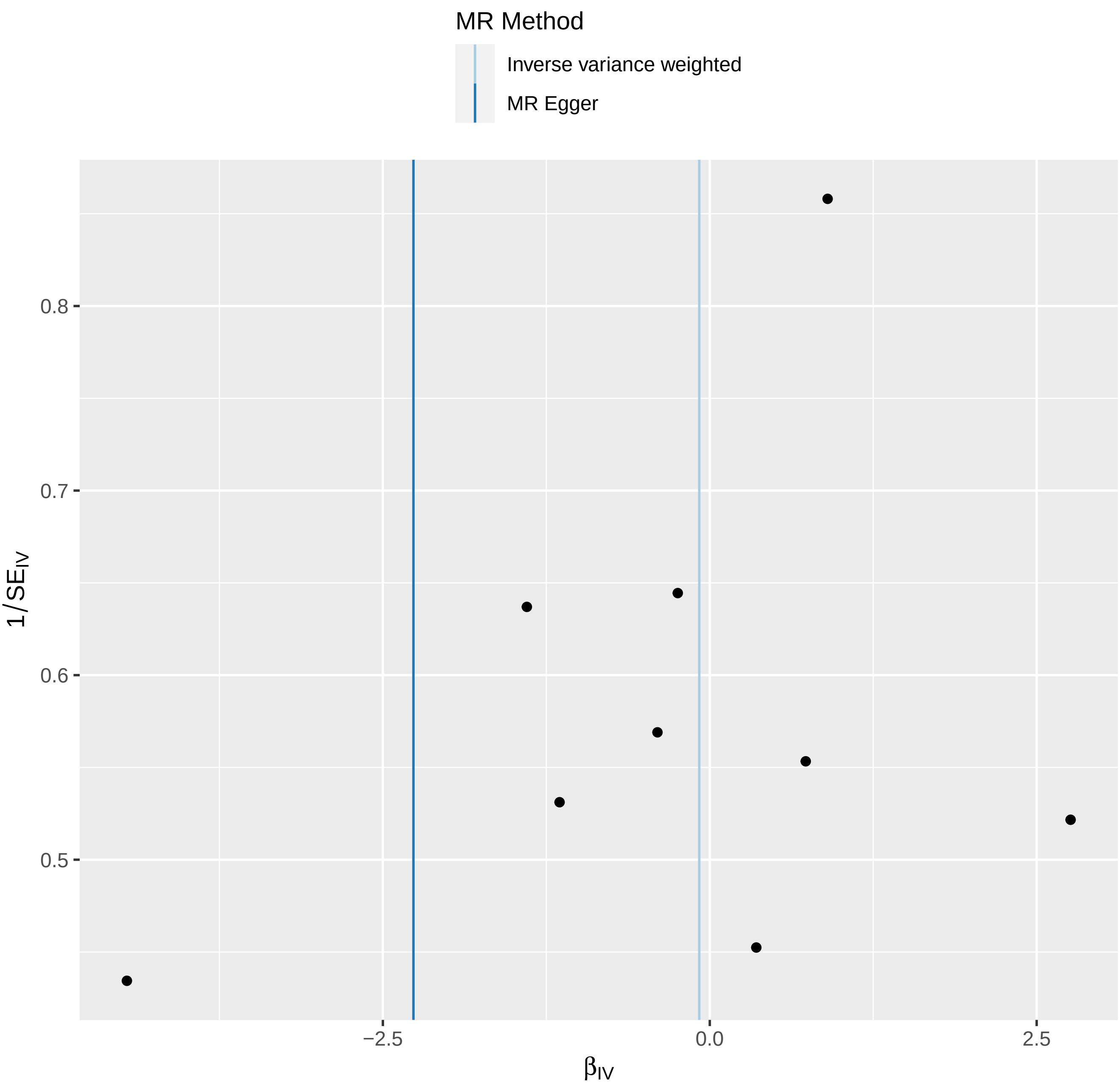

Batch 474 : 1−oleoylglycerophosphocholine on Crohn's disease of ileocolon

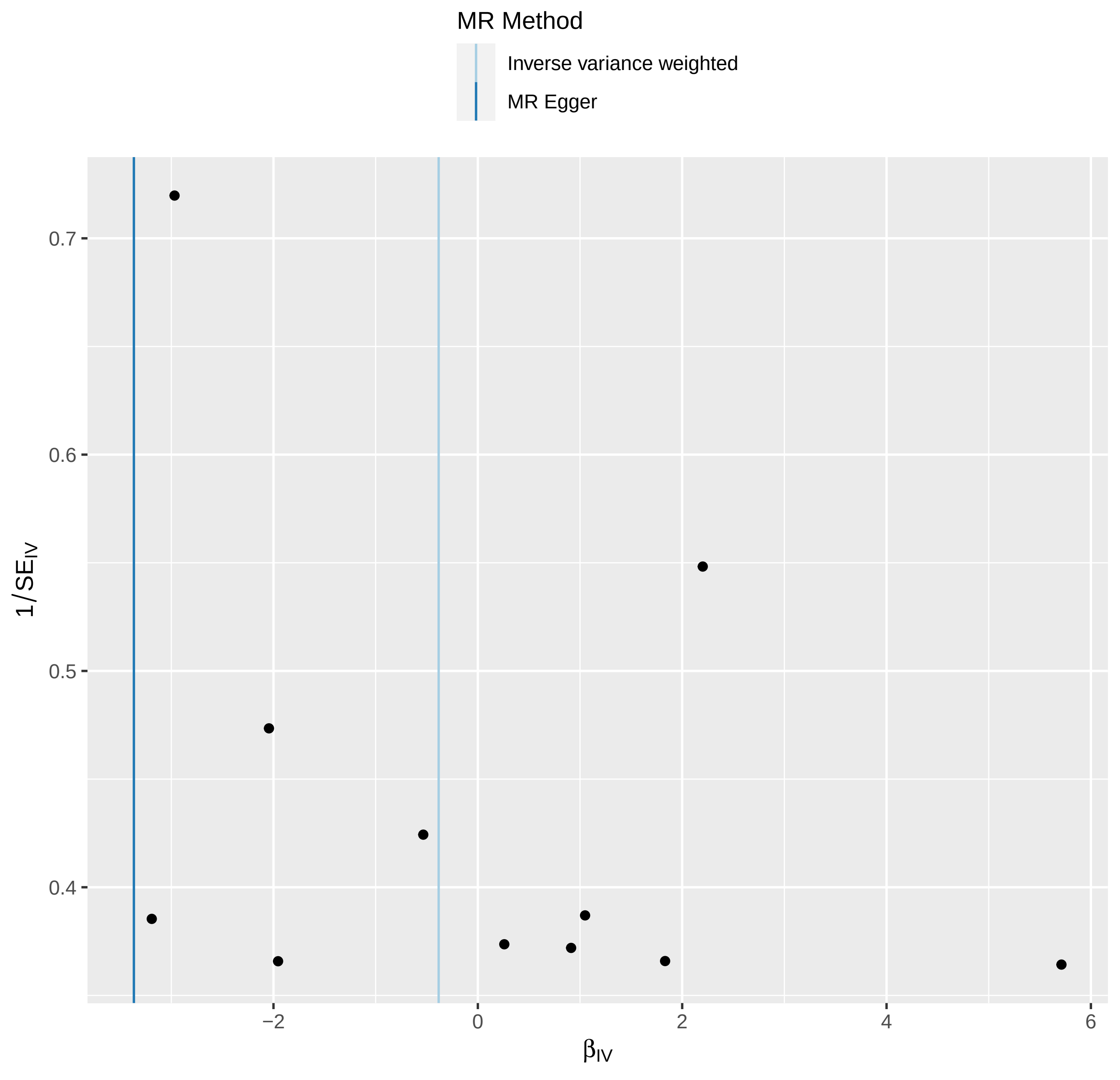

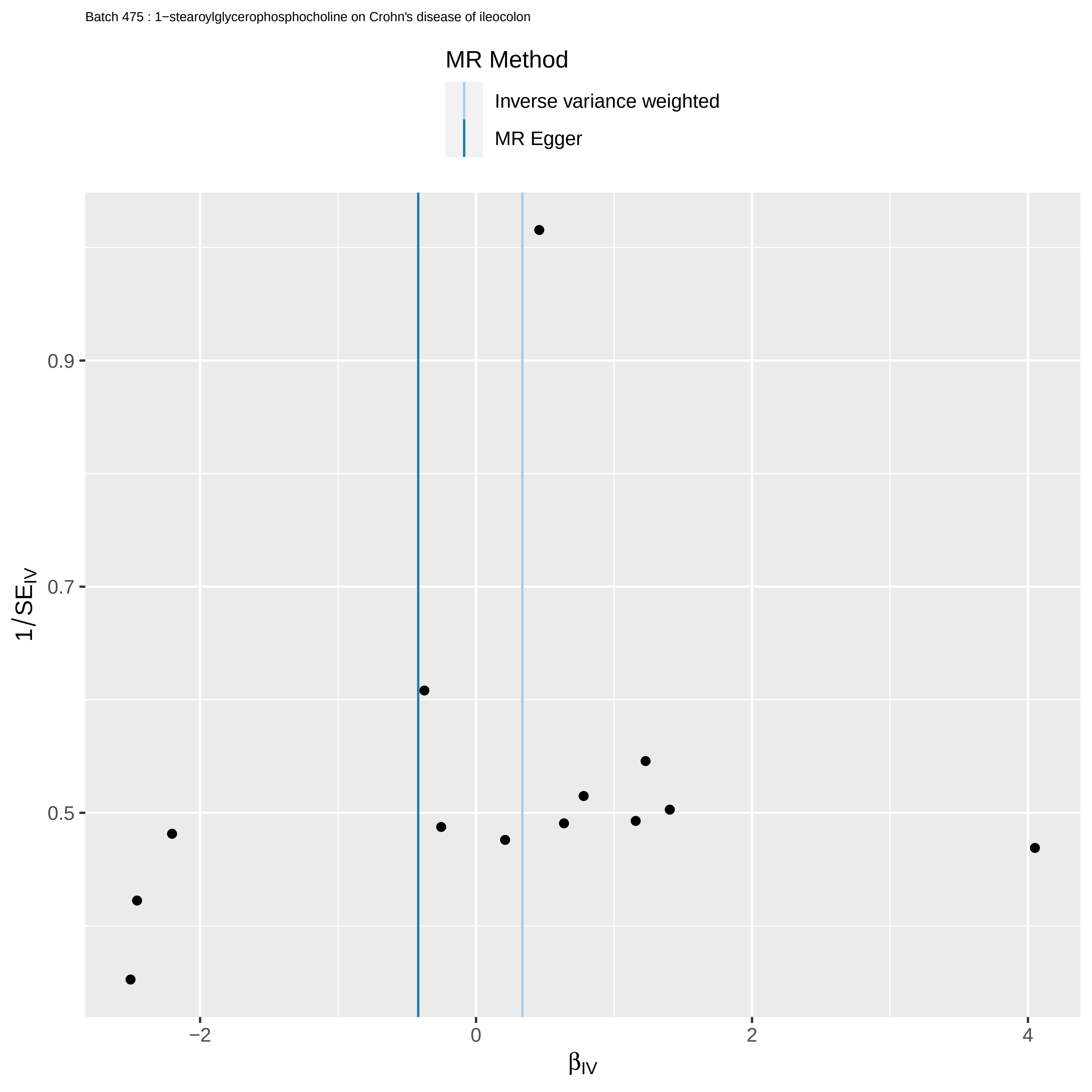
Batch 475 : 1−stearoylglycerophosphocholine on Crohn's disease of ileocolon
MR Method
Inverse variance weighted
MR Egger
0.9
0.7
0.5
1/SE$_{IV}$
−2
0
2
4
β$_{IV}$

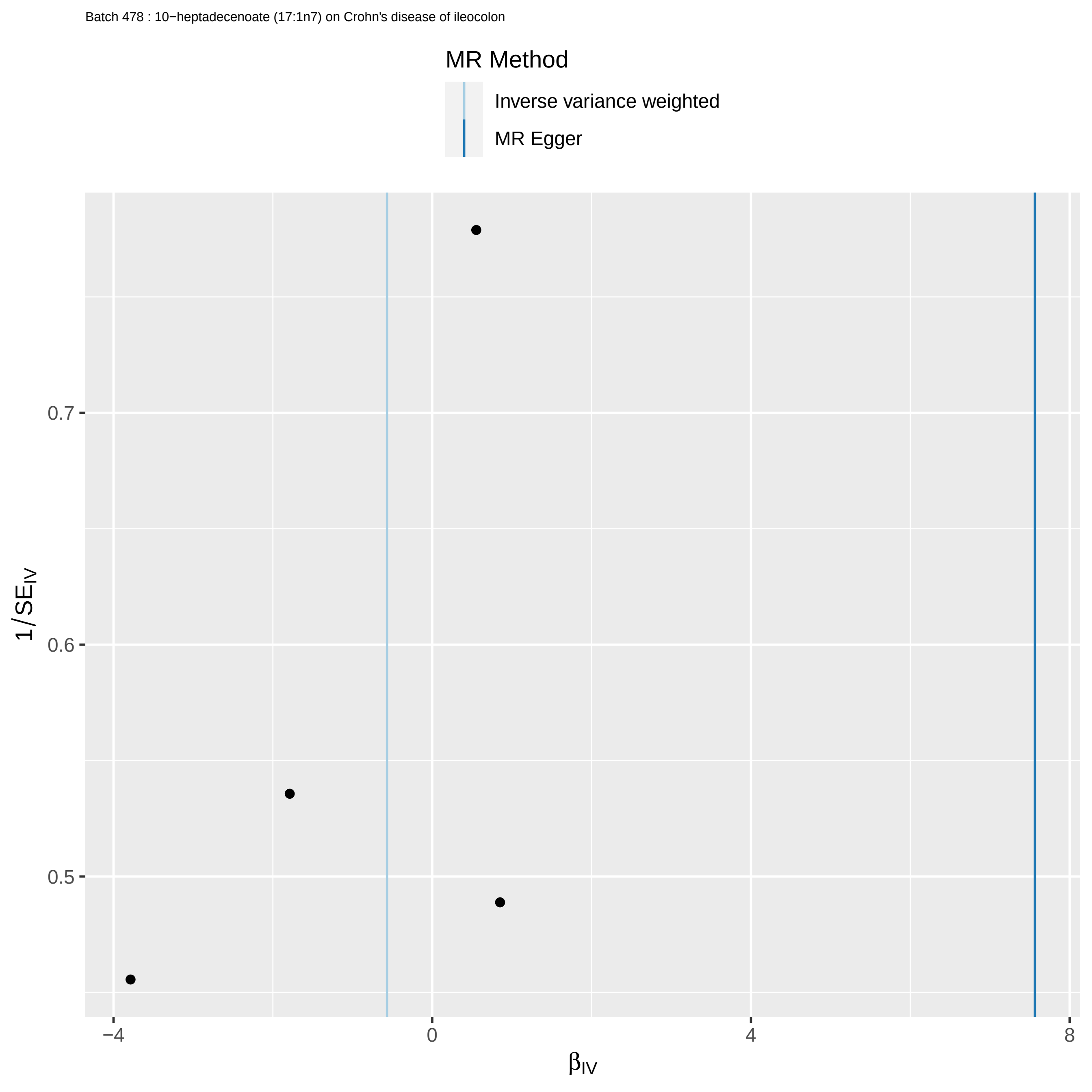
Batch 478 : 10−heptadecenoate (17:1n7) on Crohn's disease of ileocolon
MR Method
Inverse variance weighted
MR Egger
1/SE_IV
0.7
0.6
0.5
β_IV
−4
0
4
8

Batch 480 : Epiandrosterone sulfate on Crohn's disease of ileocolon

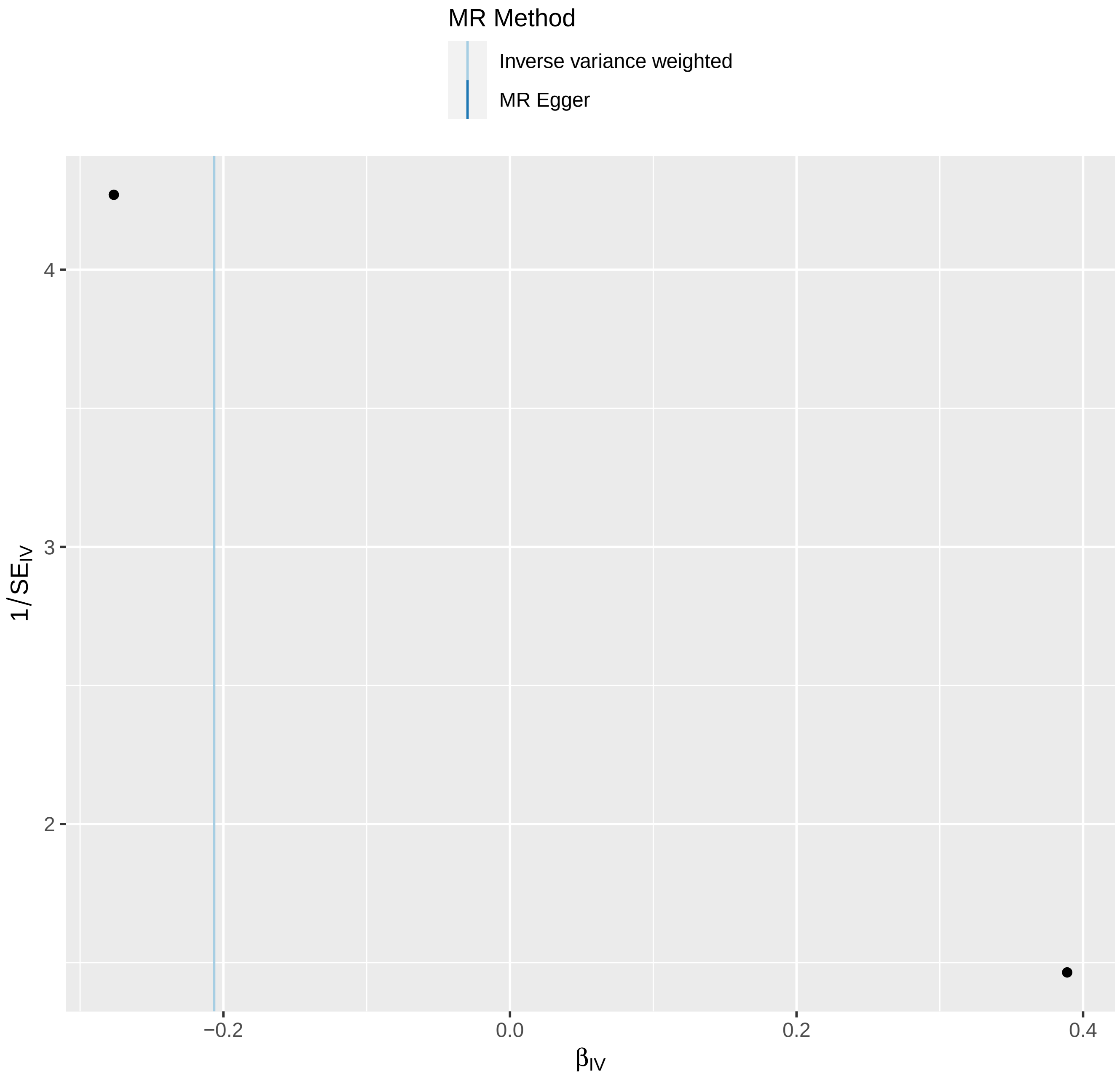

Batch 481 : Linolenate [alpha or gamma; (18:3n3 or 6)] on Crohn's disease of ileocolon

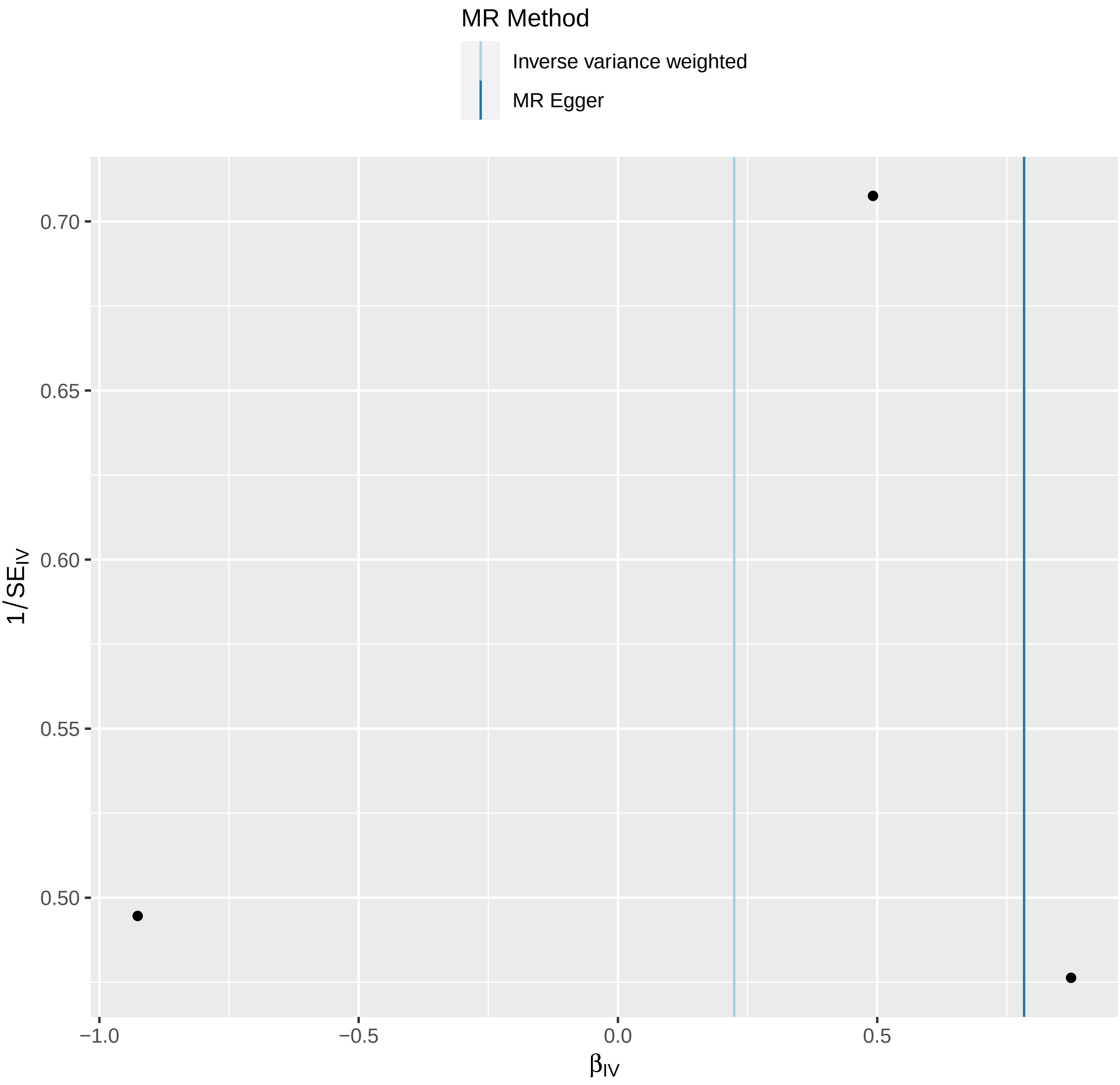

Batch 482 : X−12510−−2−aminooctanoic acid on Crohn's disease of ileocolon

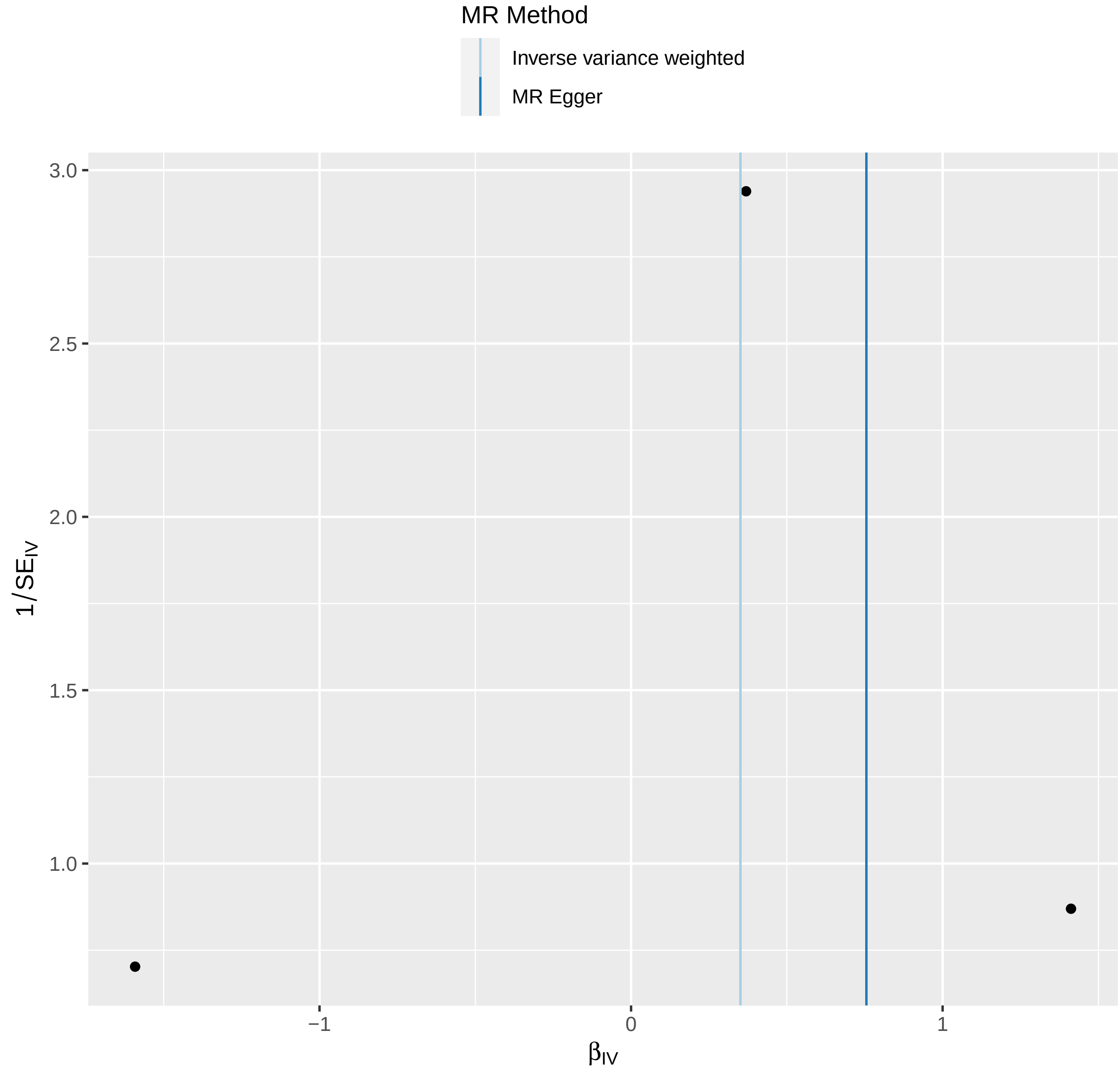

Batch 485 : Asparagine on Crohn's disease of ileocolon

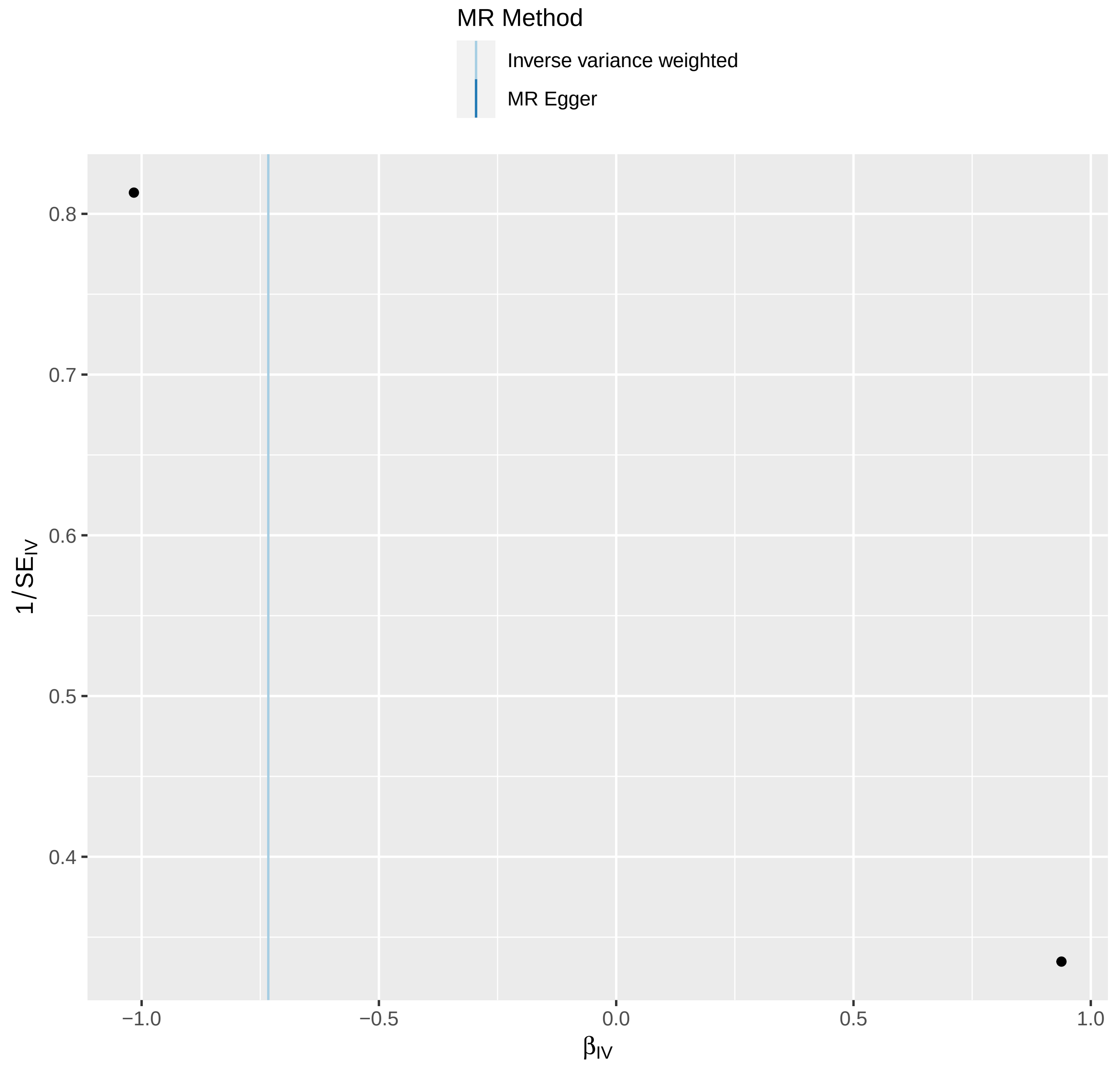

Batch 486 : Stachydrine on Crohn's disease of ileocolon

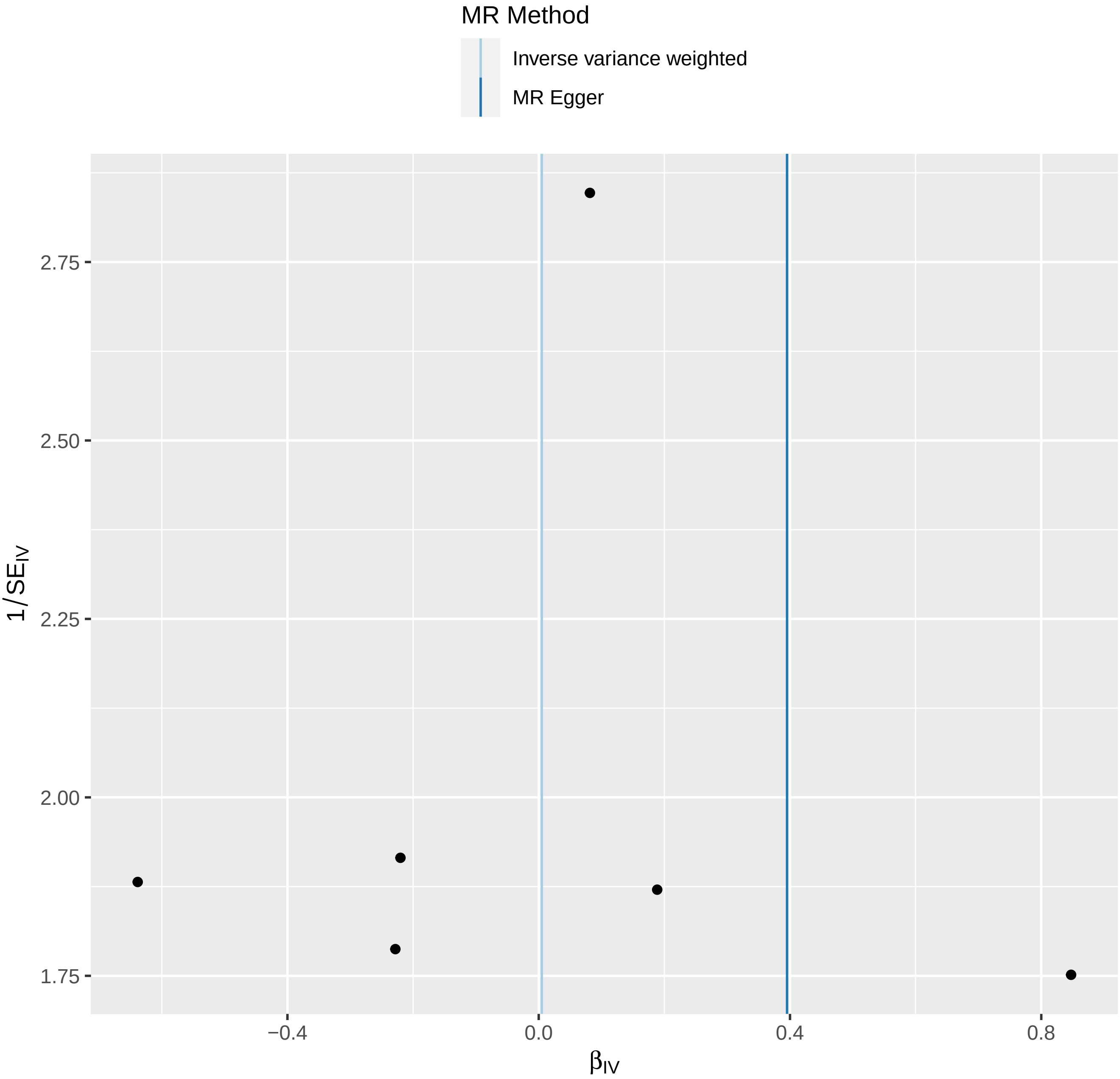

Batch 487 : Isovalerylcarnitine on Crohn's disease of ileocolon

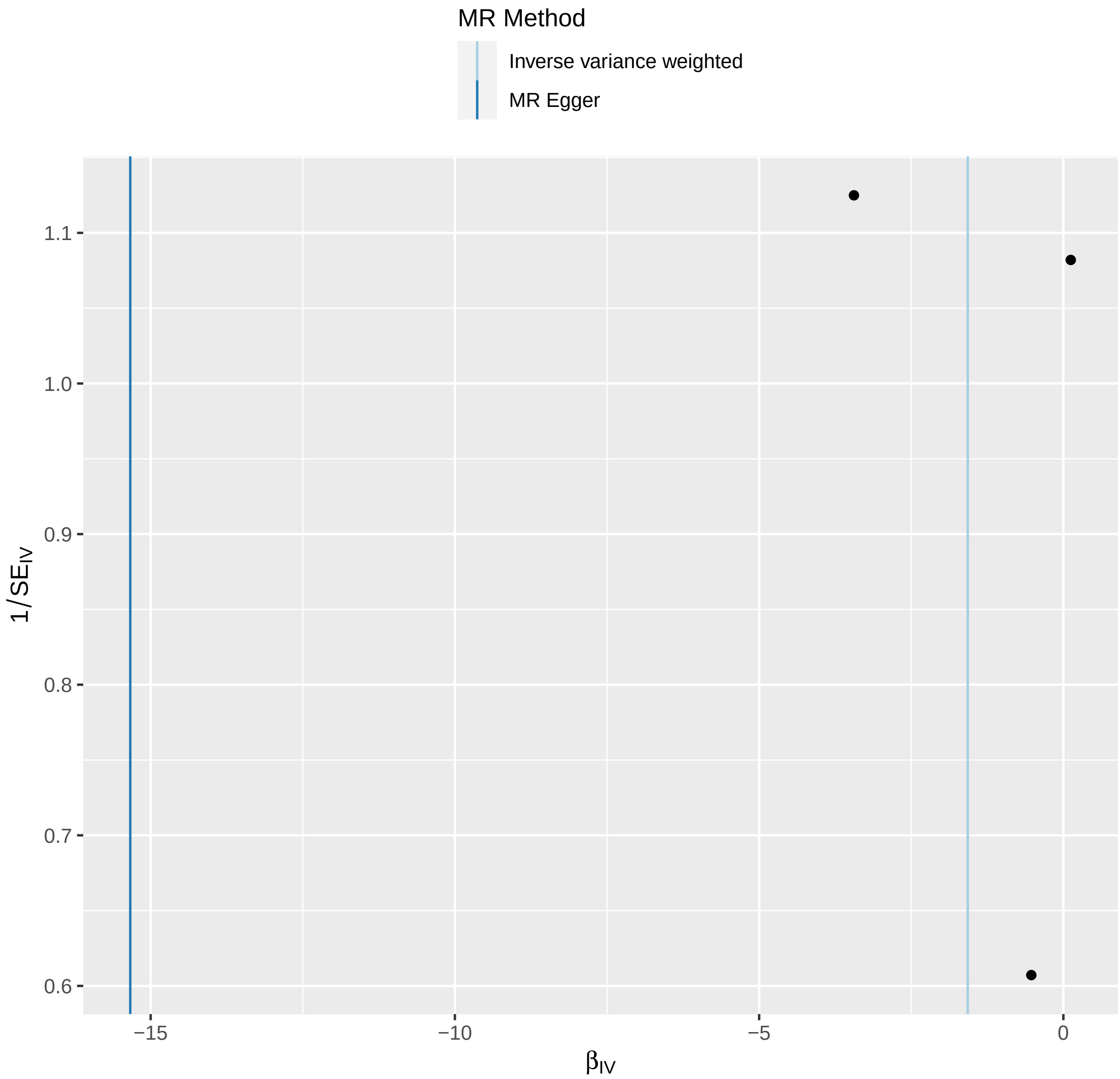

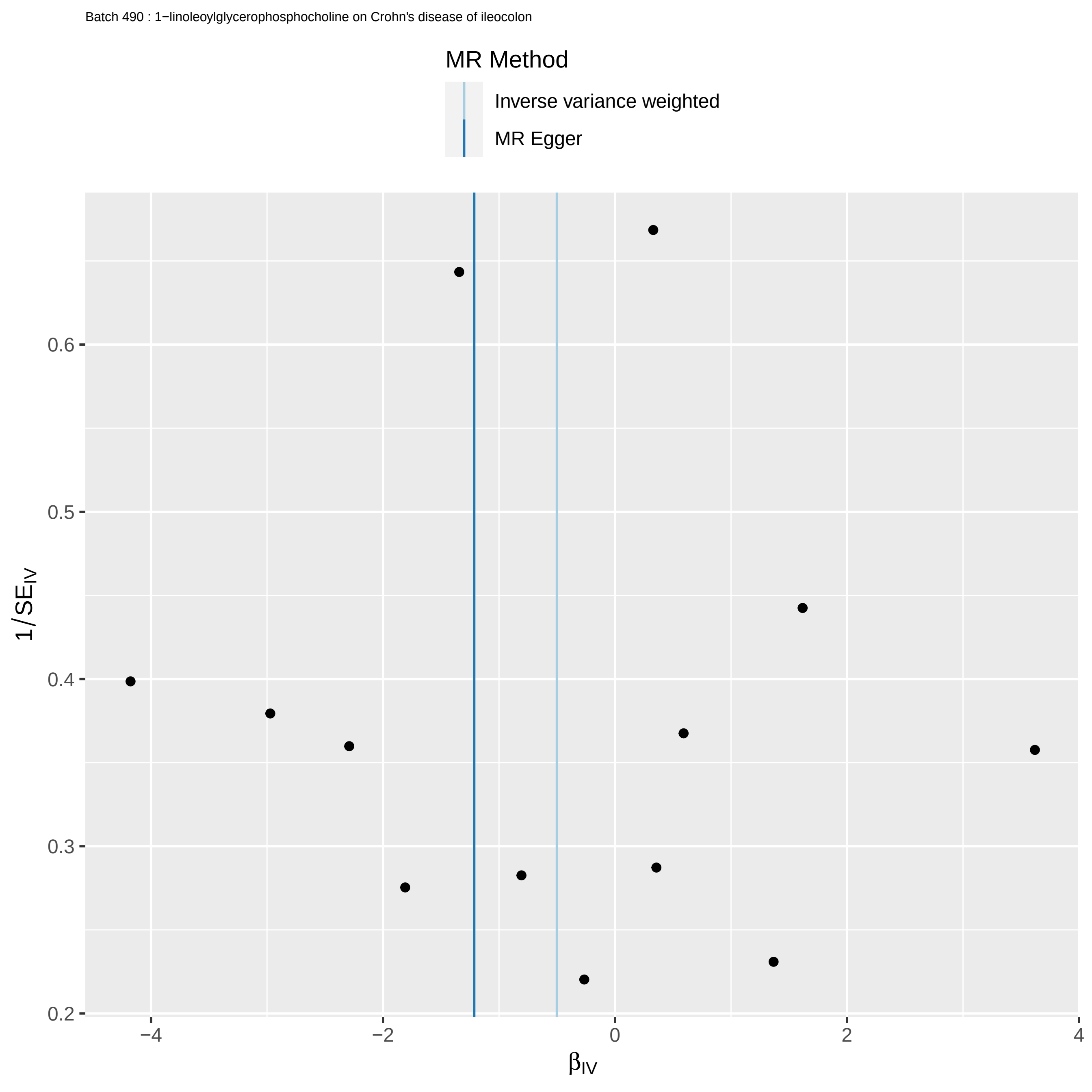

Batch 490 : 1−linoleoylglycerophosphocholine on Crohn's disease of ileocolon
MR Method
Inverse variance weighted
MR Egger
1/SE$_{IV}$
0.6
0.5
0.4
0.3
0.2
−4
−2
0
2
4
β$_{IV}$

Batch 491 : Bradykinin, des−arg(9) on Crohn's disease of ileocolon

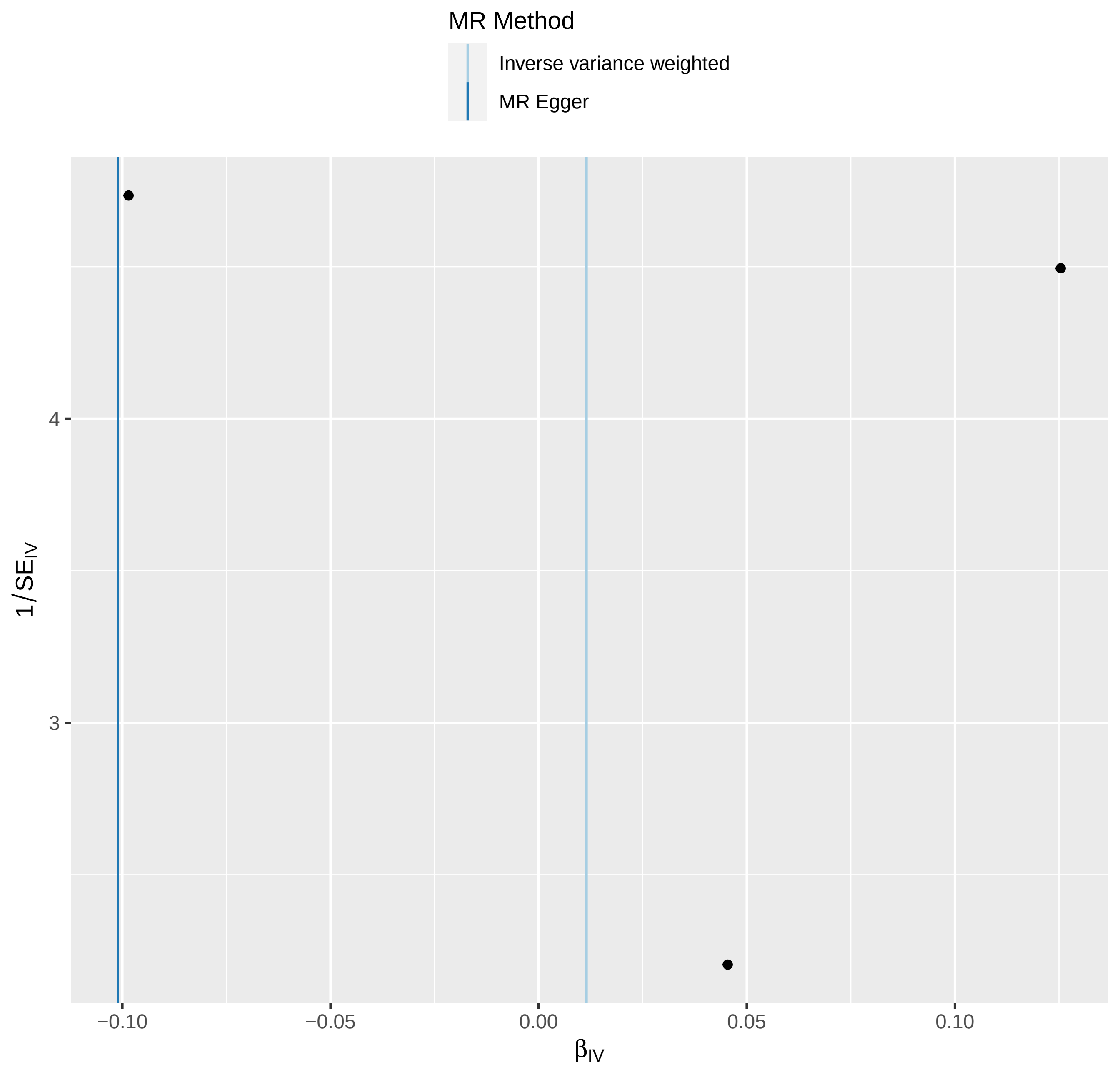

Batch 492 : Gamma−glutamylisoleucine* on Crohn's disease of ileocolon

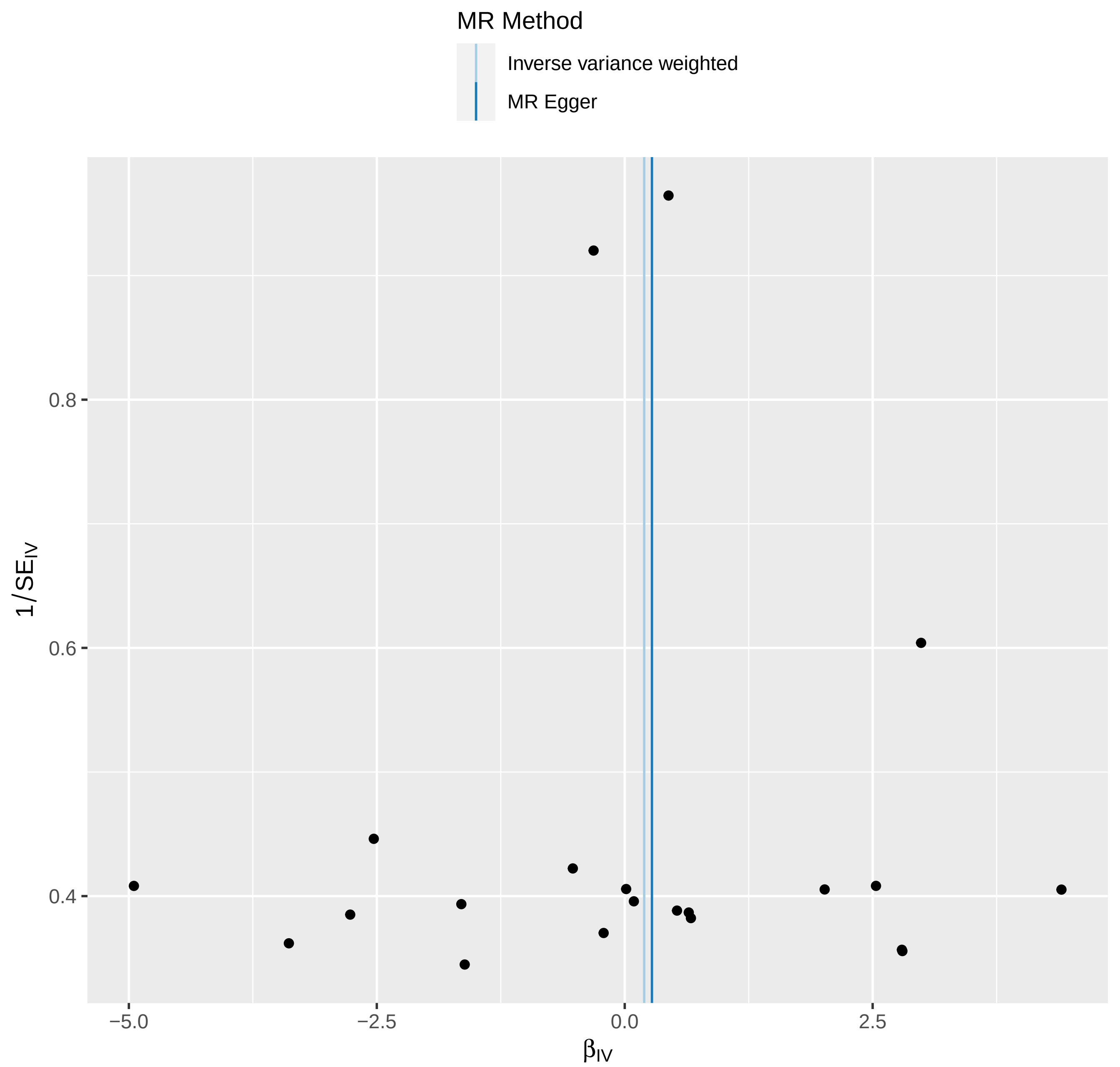

Batch 495 : Isovalerate on Crohn's disease of ileocolon

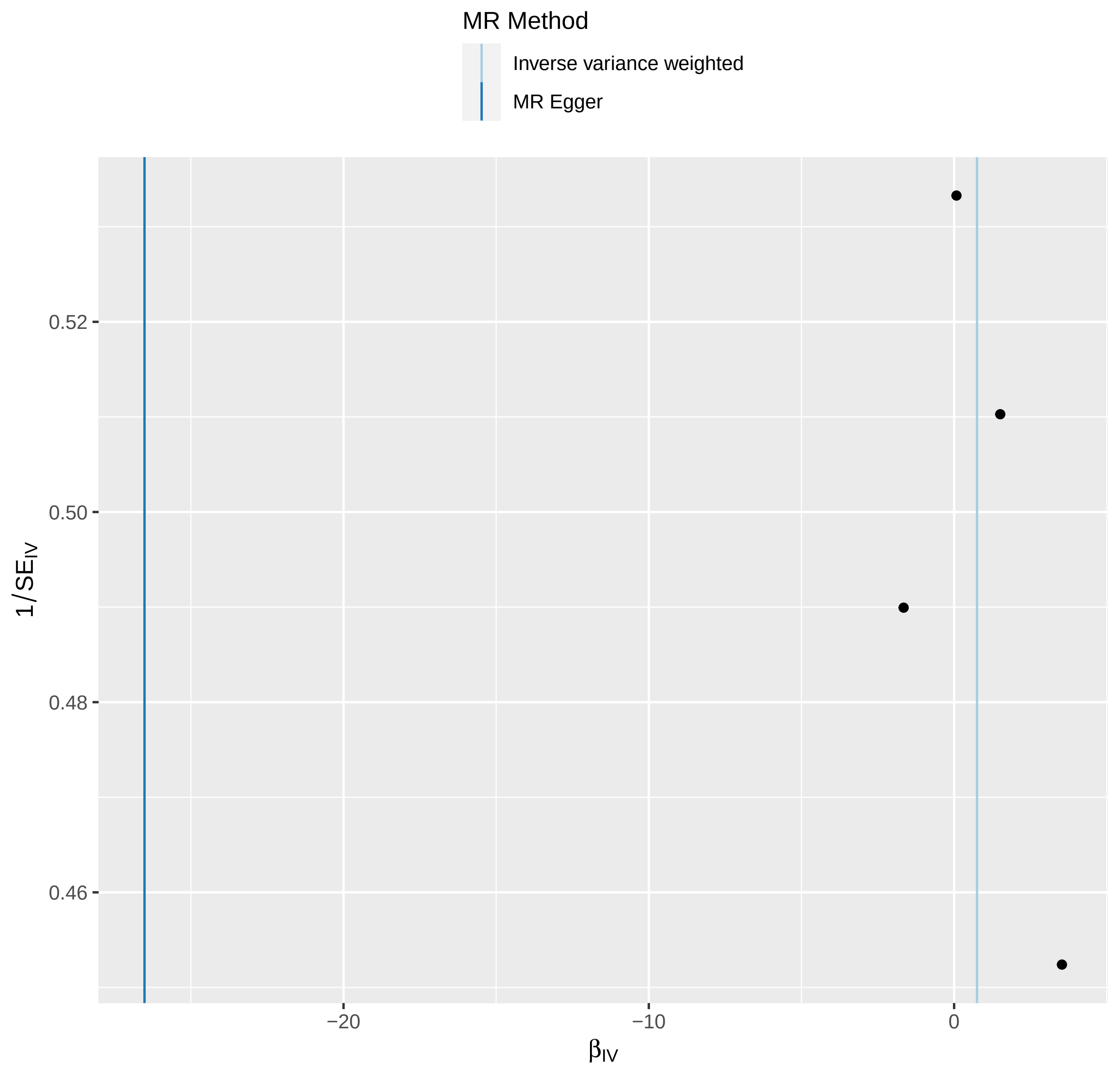

Batch 496 : X−13183−−stearamide on Crohn's disease of ileocolon

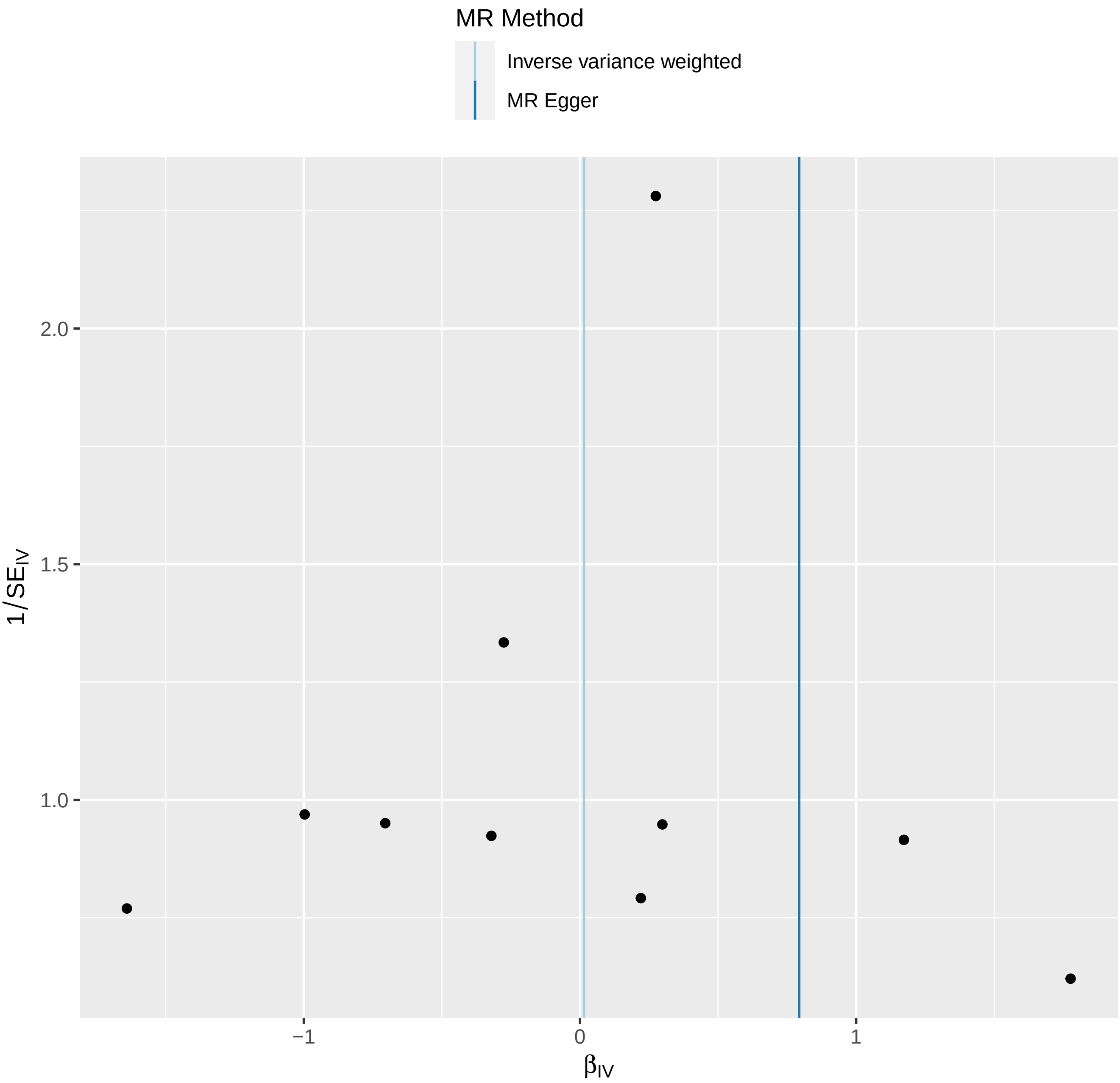

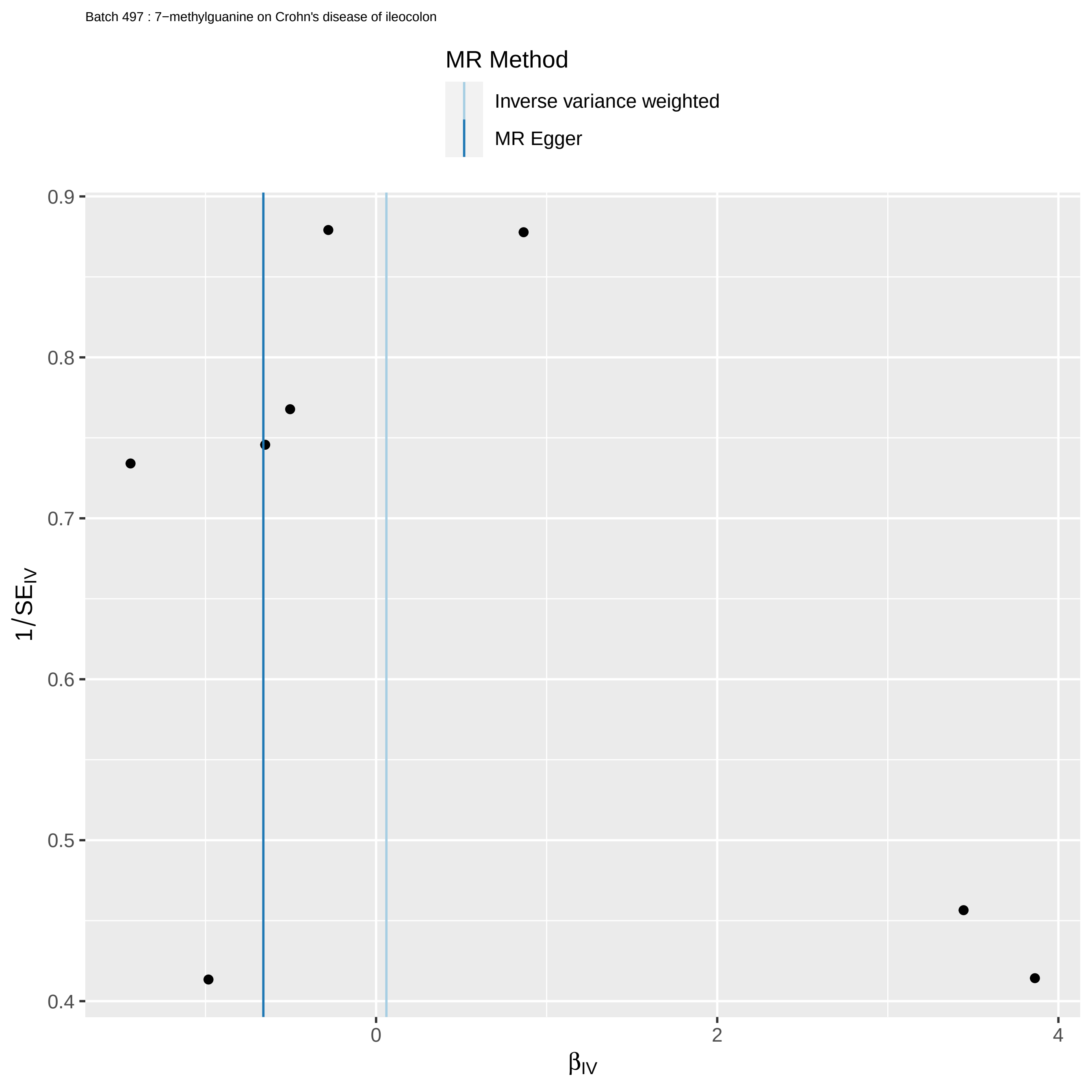
Batch 497 : 7−methylguanine on Crohn's disease of ileocolon
MR Method
Inverse variance weighted
MR Egger
0.9
0.8
0.7
0.6
0.5
0.4
1/SE_IV
0
2
4
β_IV

Batch 505 : 2−palmitoylglycerophosphocholine* on Crohn's disease of ileocolon

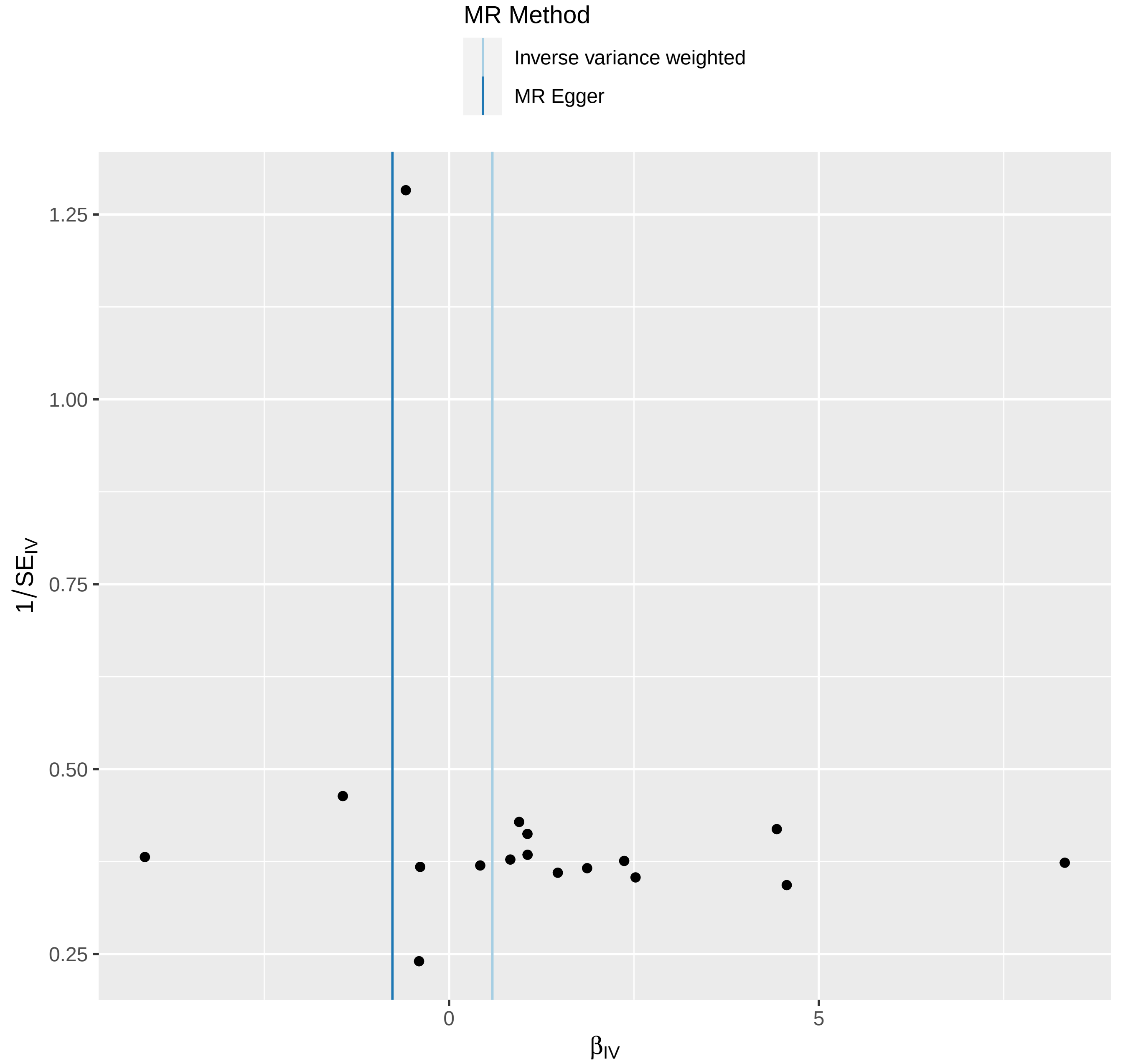

Batch 506 : 2−oleoylglycerophosphocholine* on Crohn's disease of ileocolon

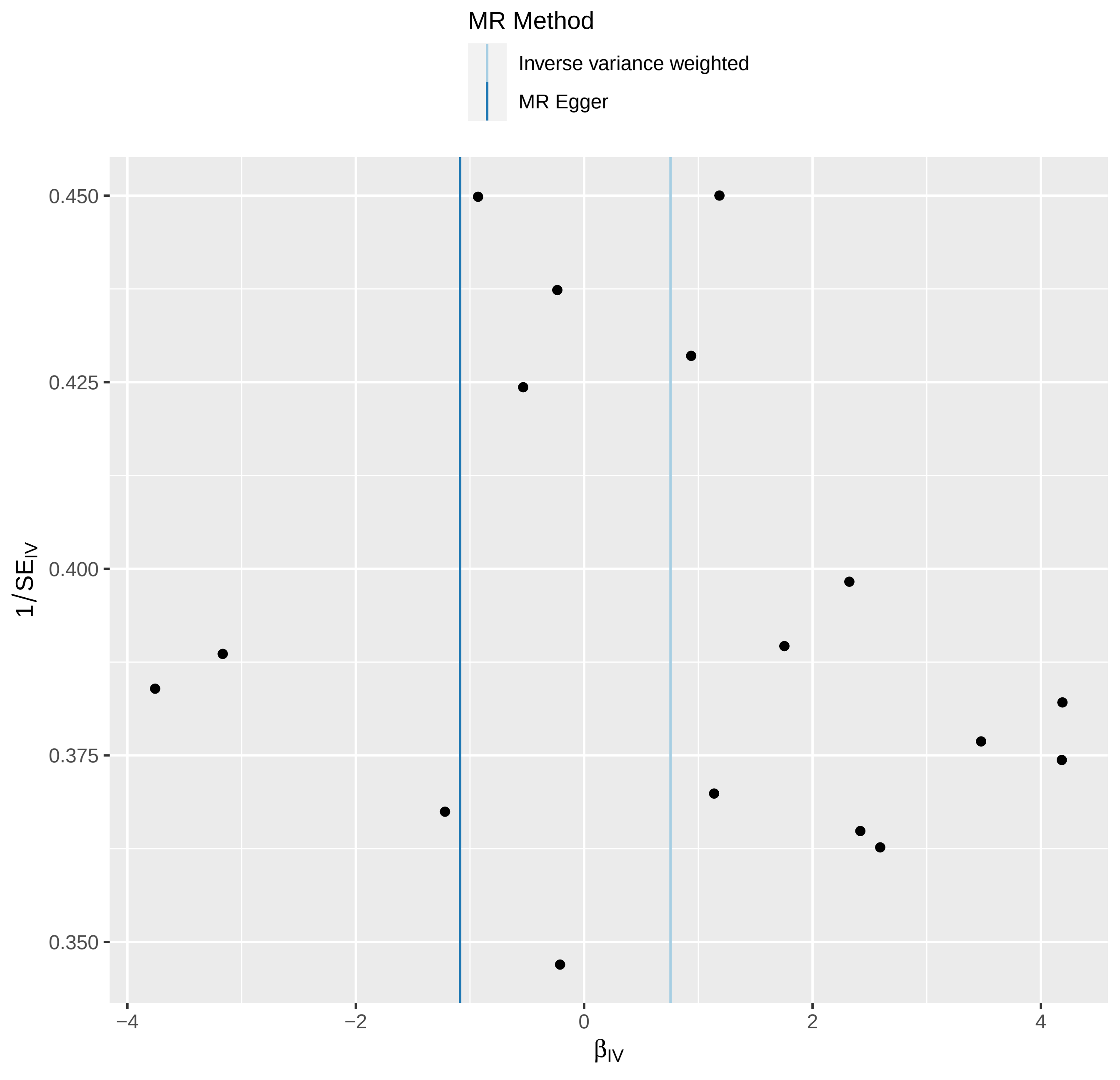

Batch 509 : 1−palmitoylglycerophosphoinositol* on Crohn's disease of ileocolon

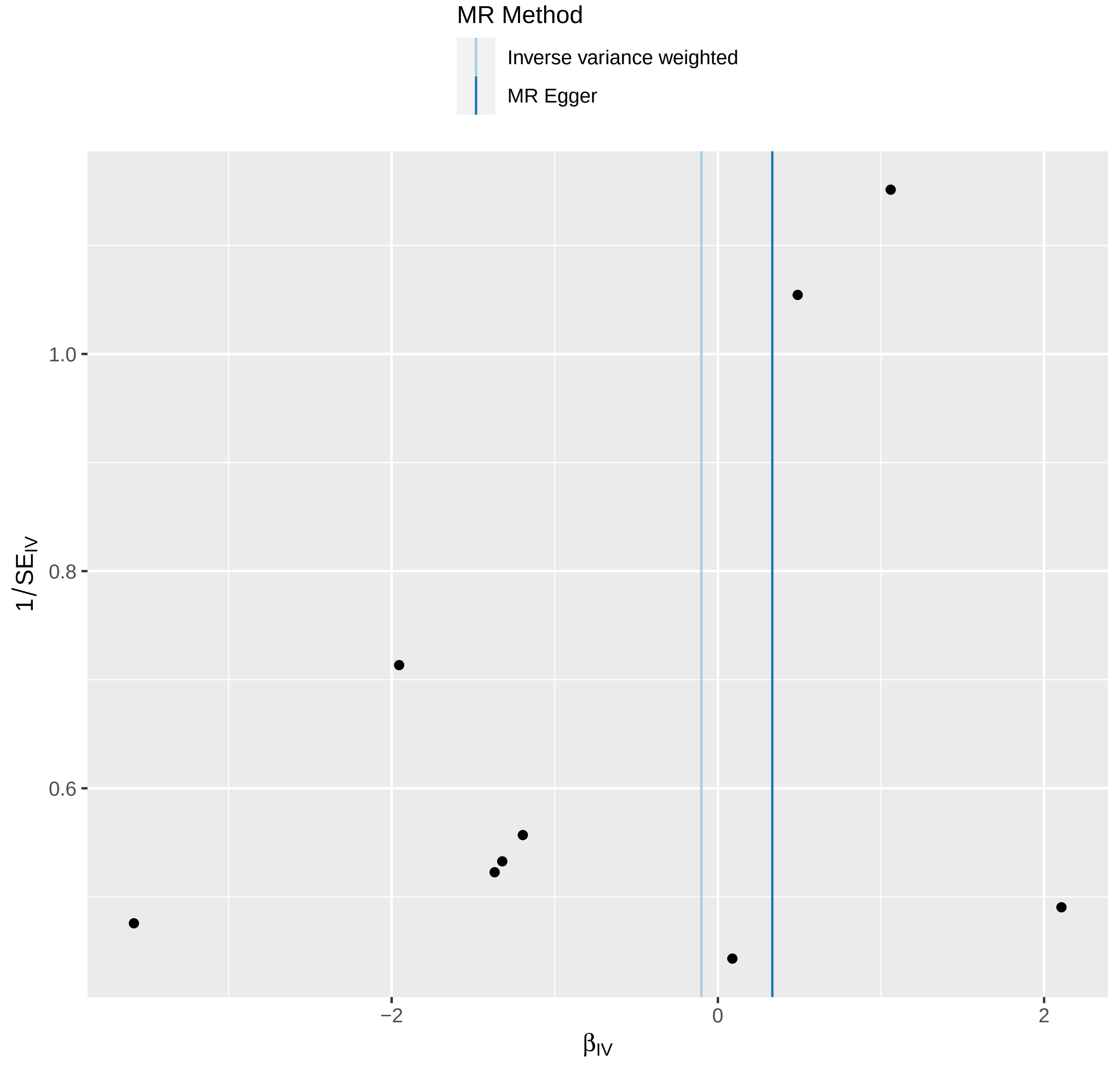

Batch 511 : Hydroxyisovaleroyl carnitine on Crohn's disease of ileocolon

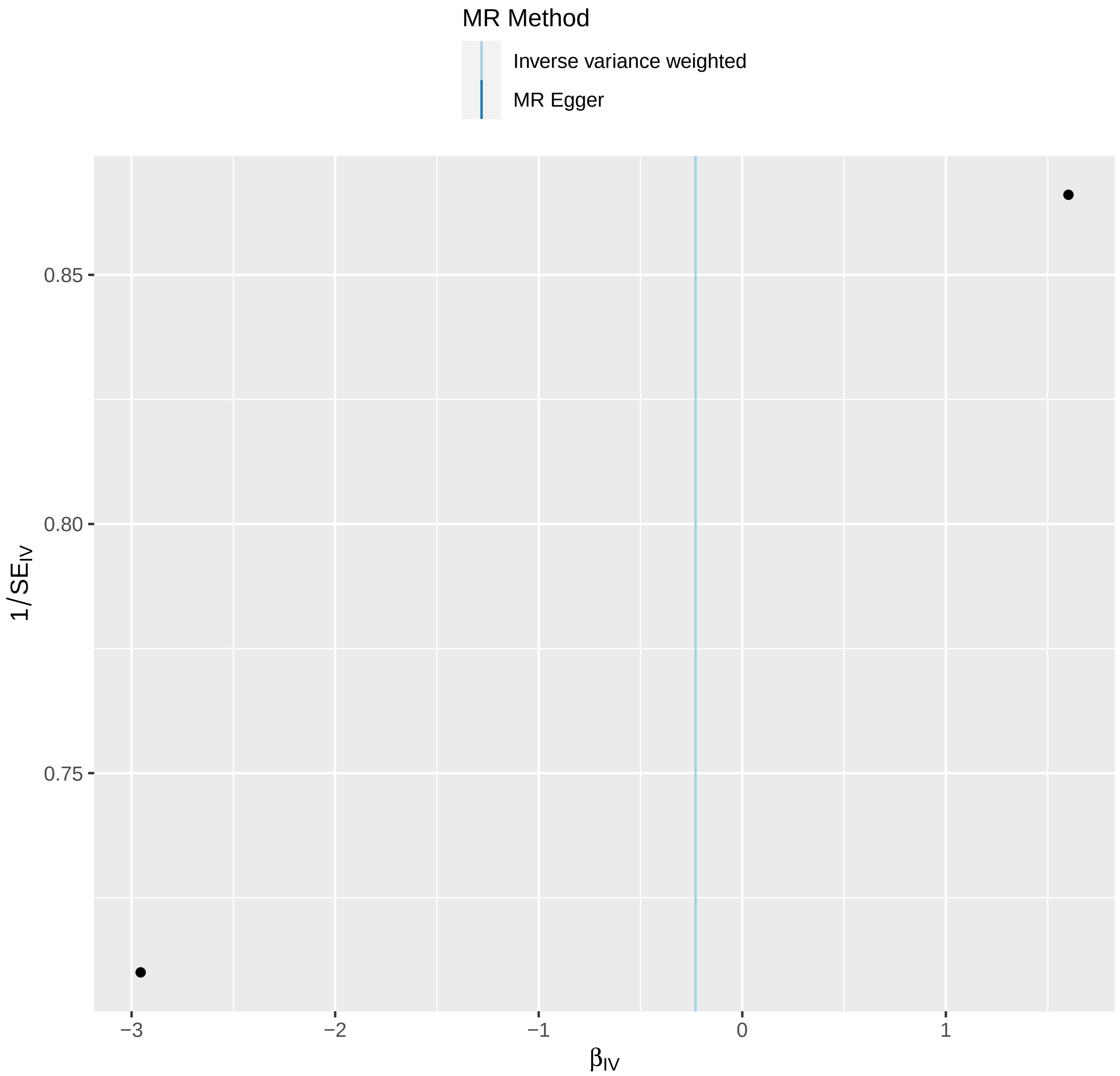

Batch 512 : Glutaroyl carnitine on Crohn's disease of ileocolon

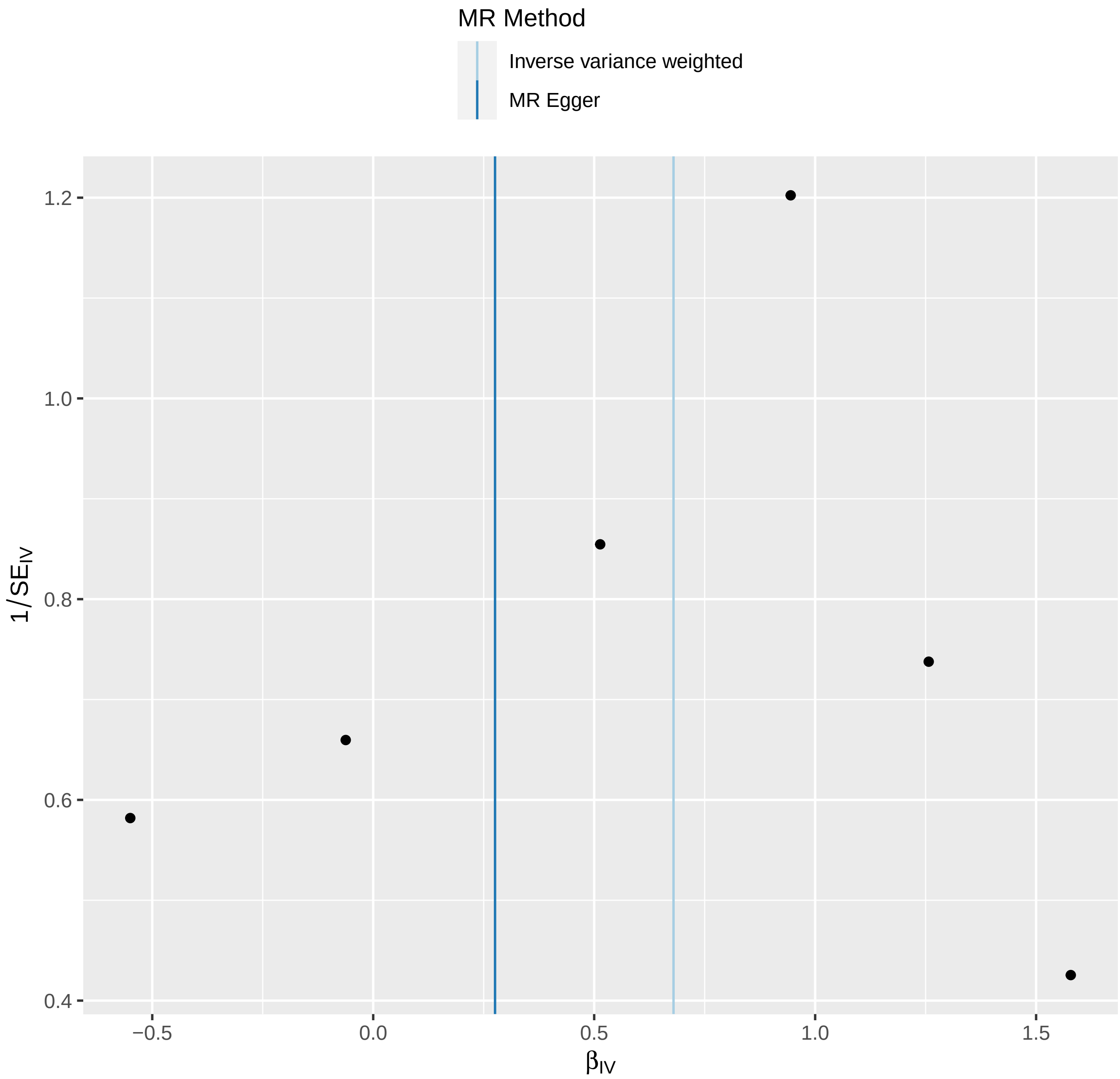

Batch 514 : 4−hydroxyhippurate on Crohn's disease of ileocolon

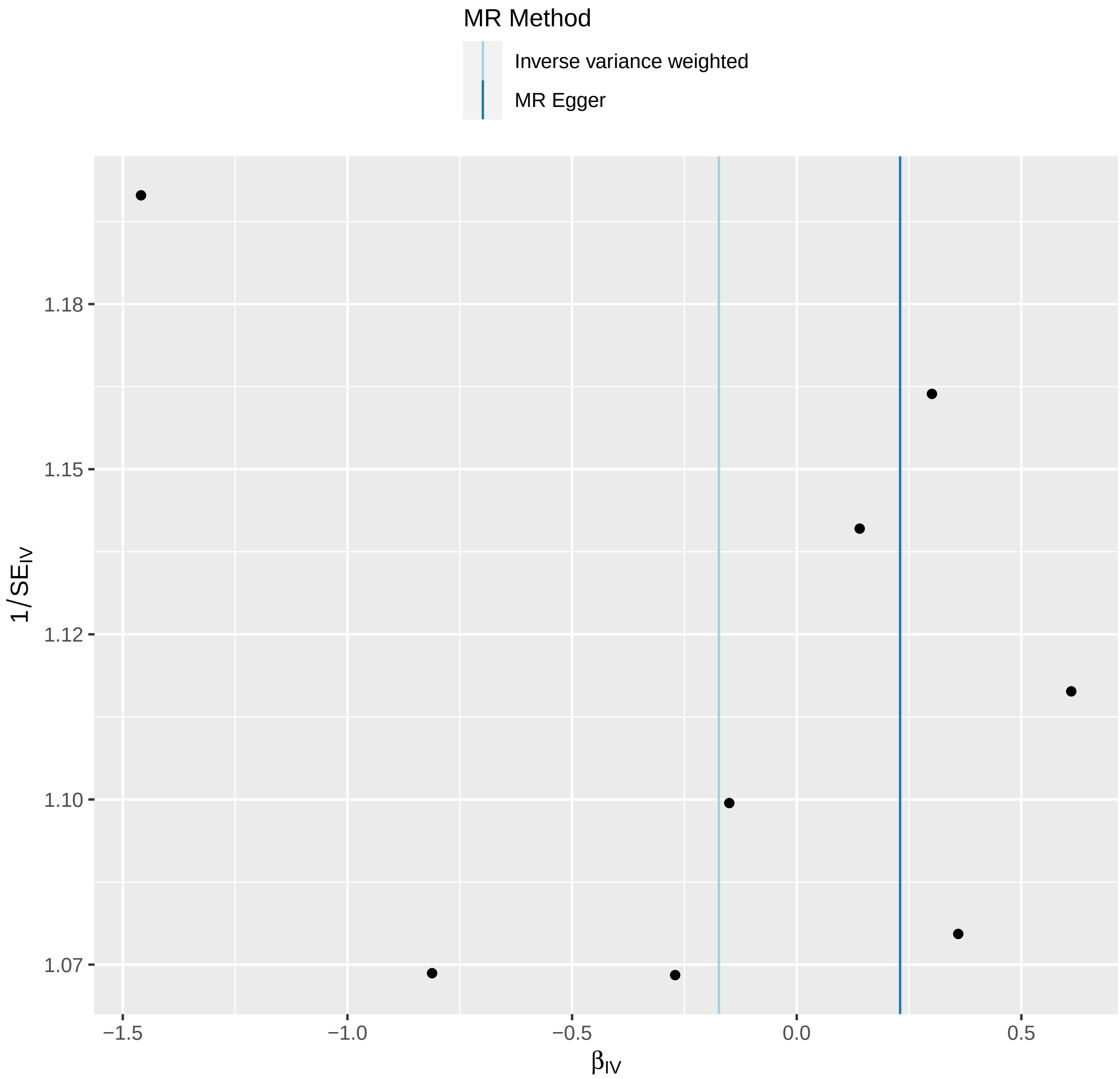

Batch 515 : 1−myristoylglycerophosphocholine on Crohn's disease of ileocolon

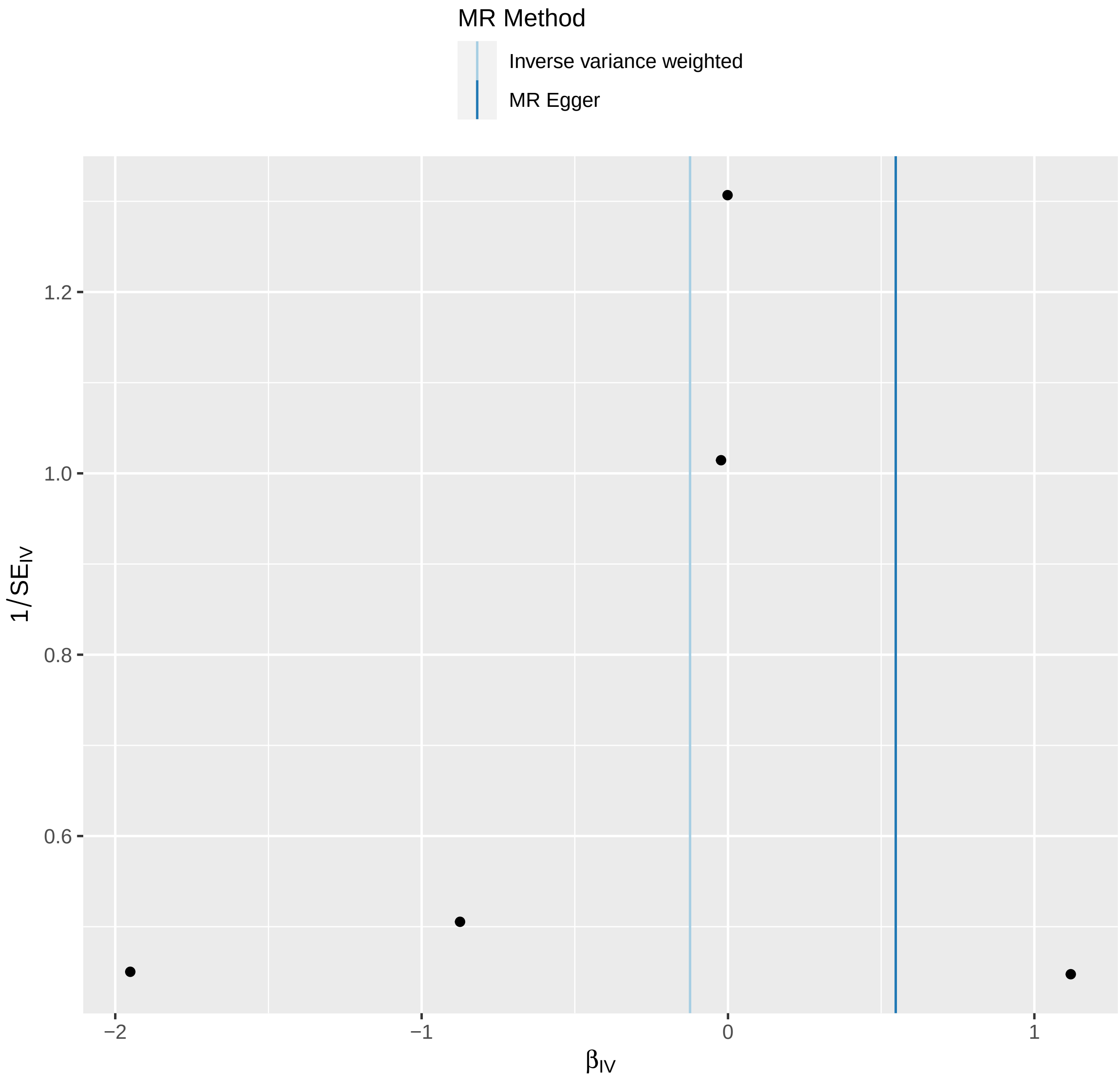

Batch 518 : 3−(3−hydroxyphenyl)propionate on Crohn's disease of ileocolon

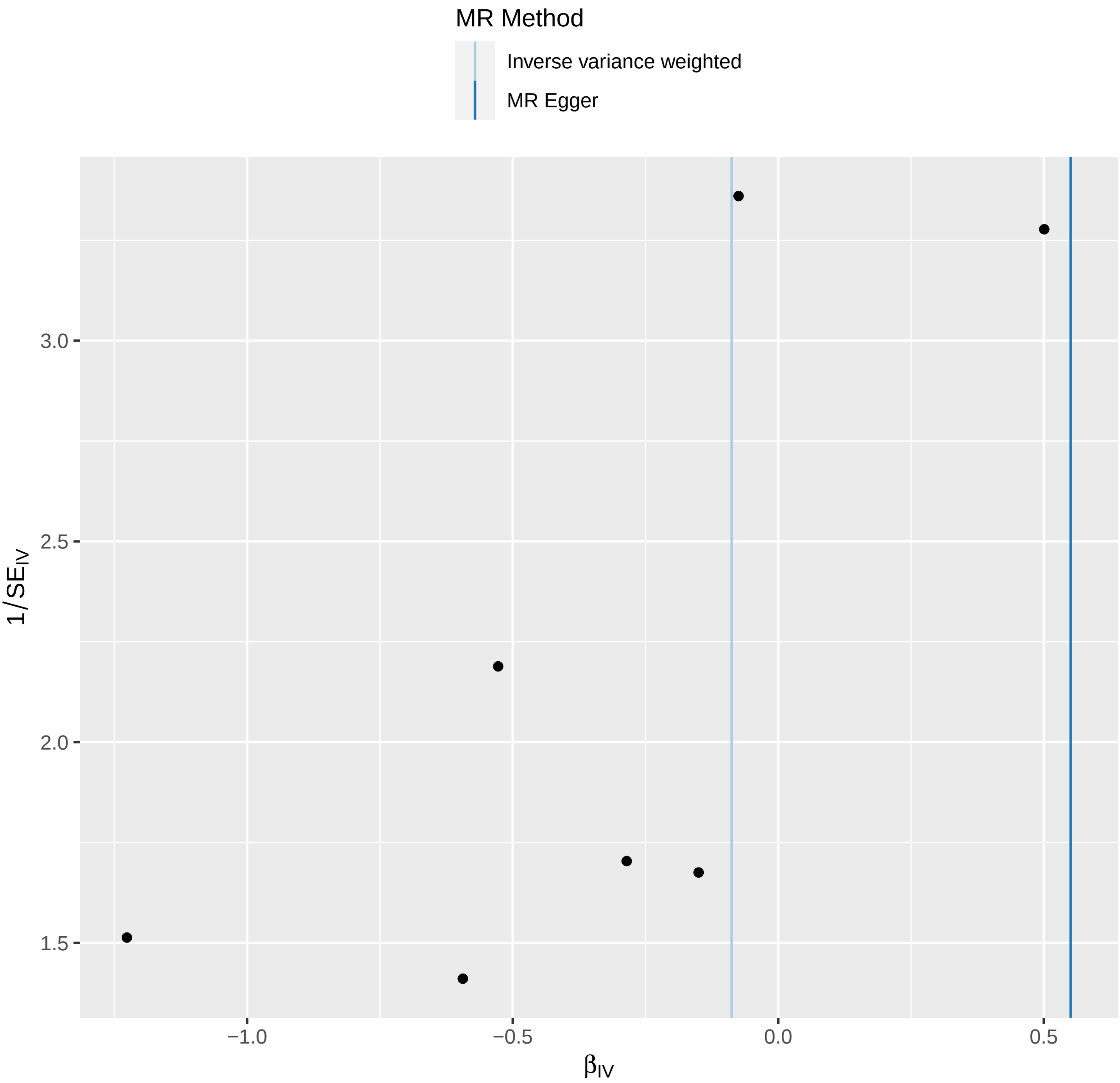

Batch 522 : Dihomo−linolenate (20:3n3 or n6) on Crohn's disease of ileocolon

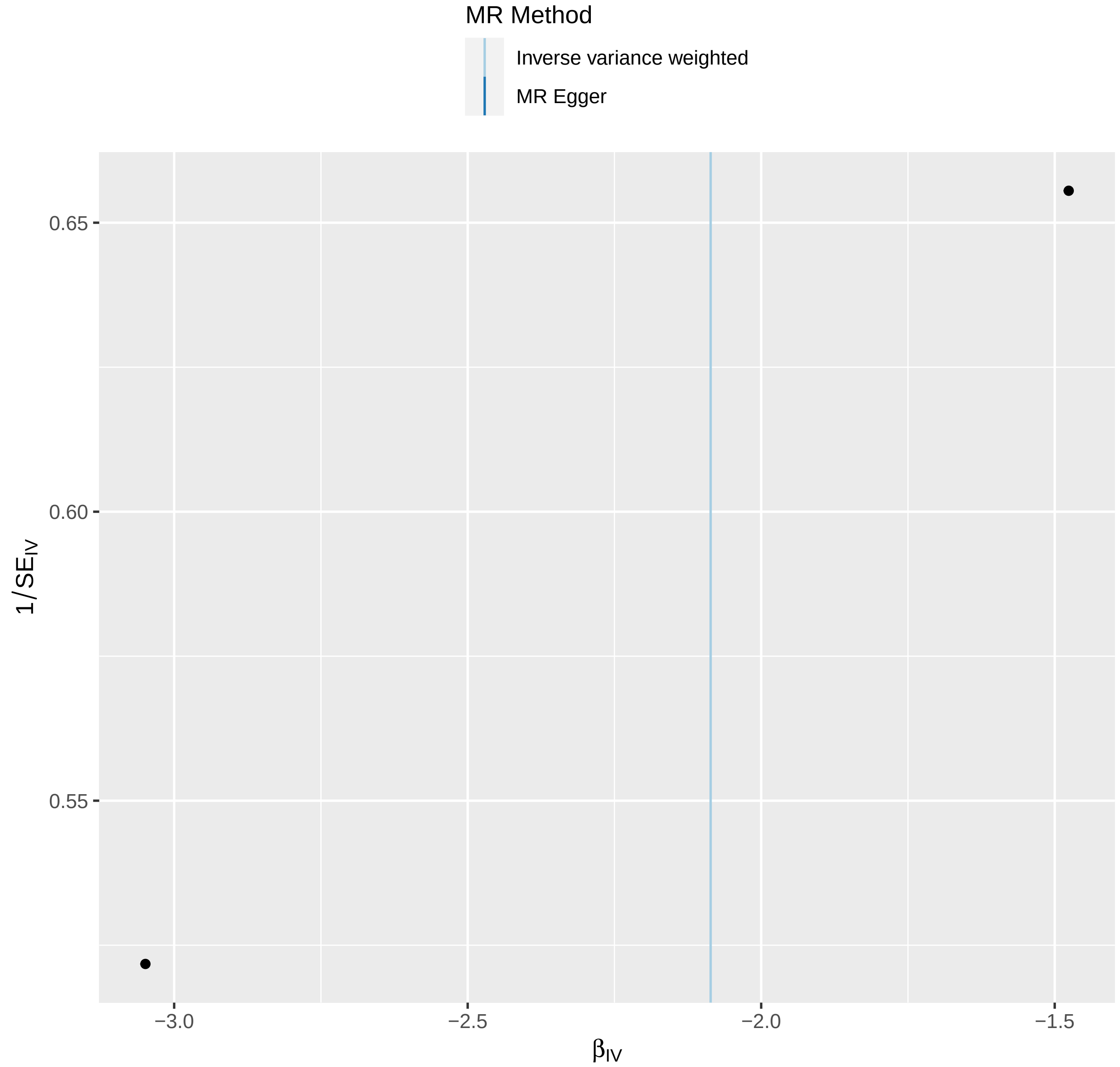

Batch 523 : Threitol on Crohn's disease of ileocolon

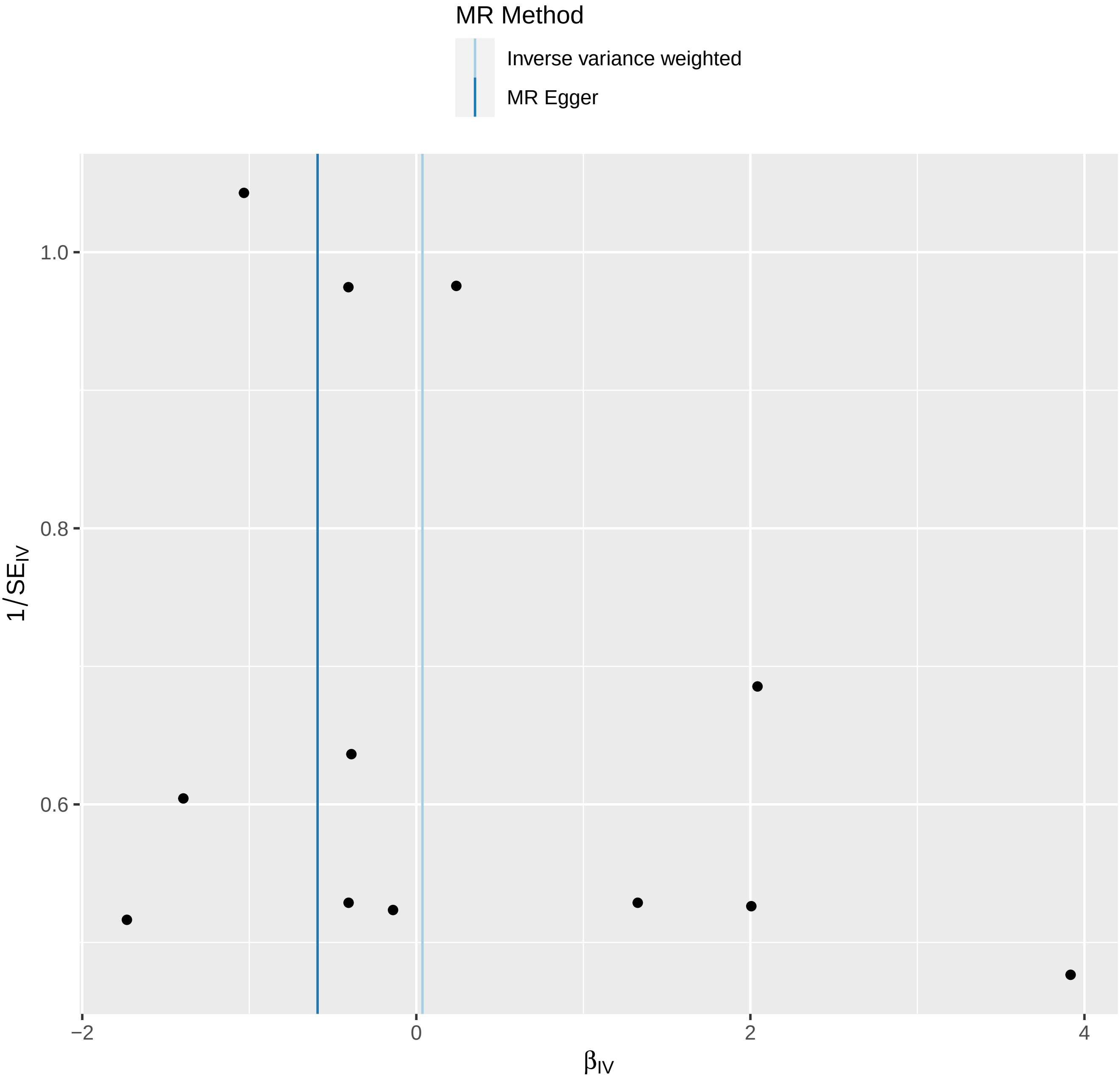

Batch 524 : P−cresol sulfate on Crohn's disease of ileocolon

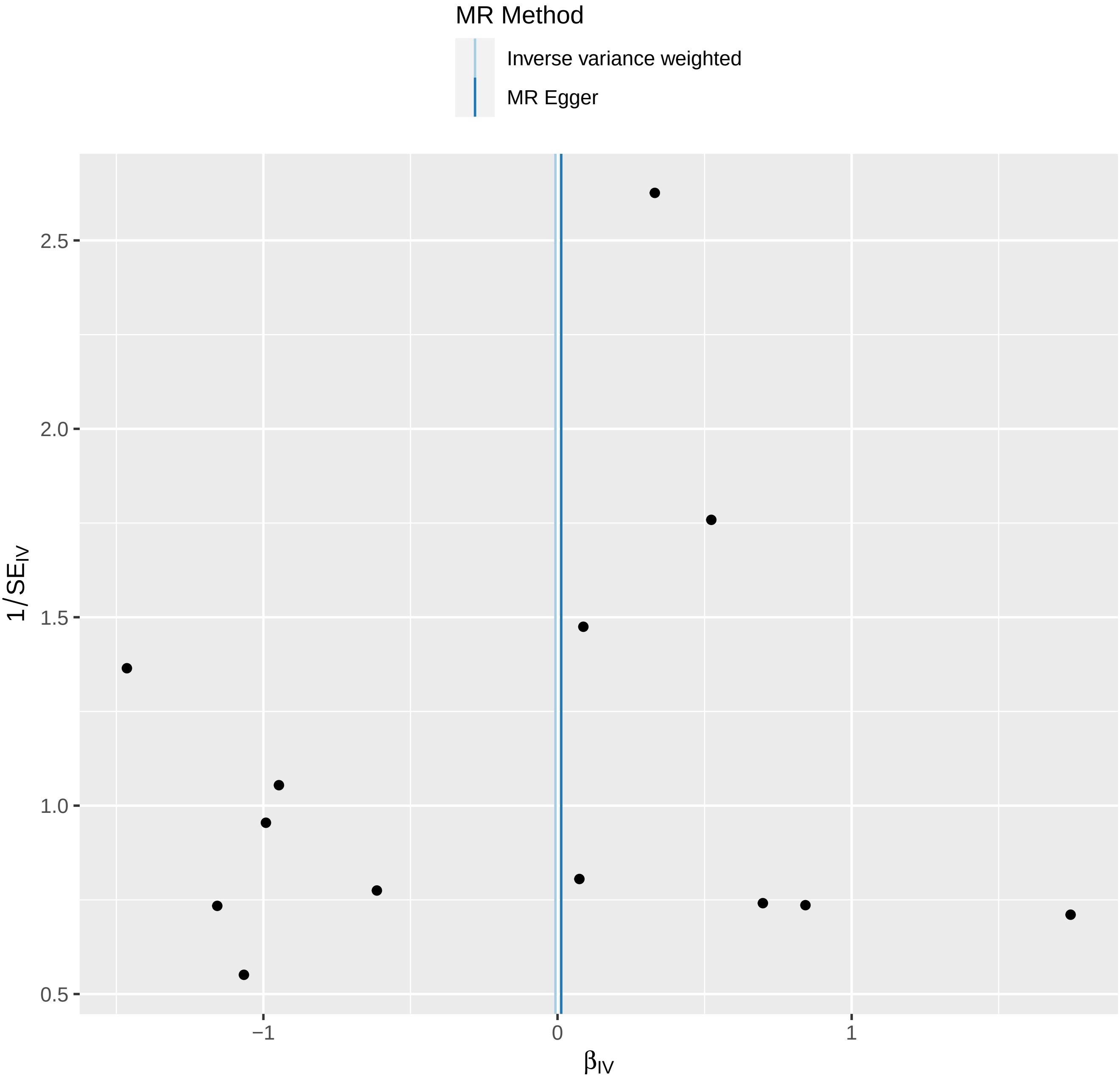

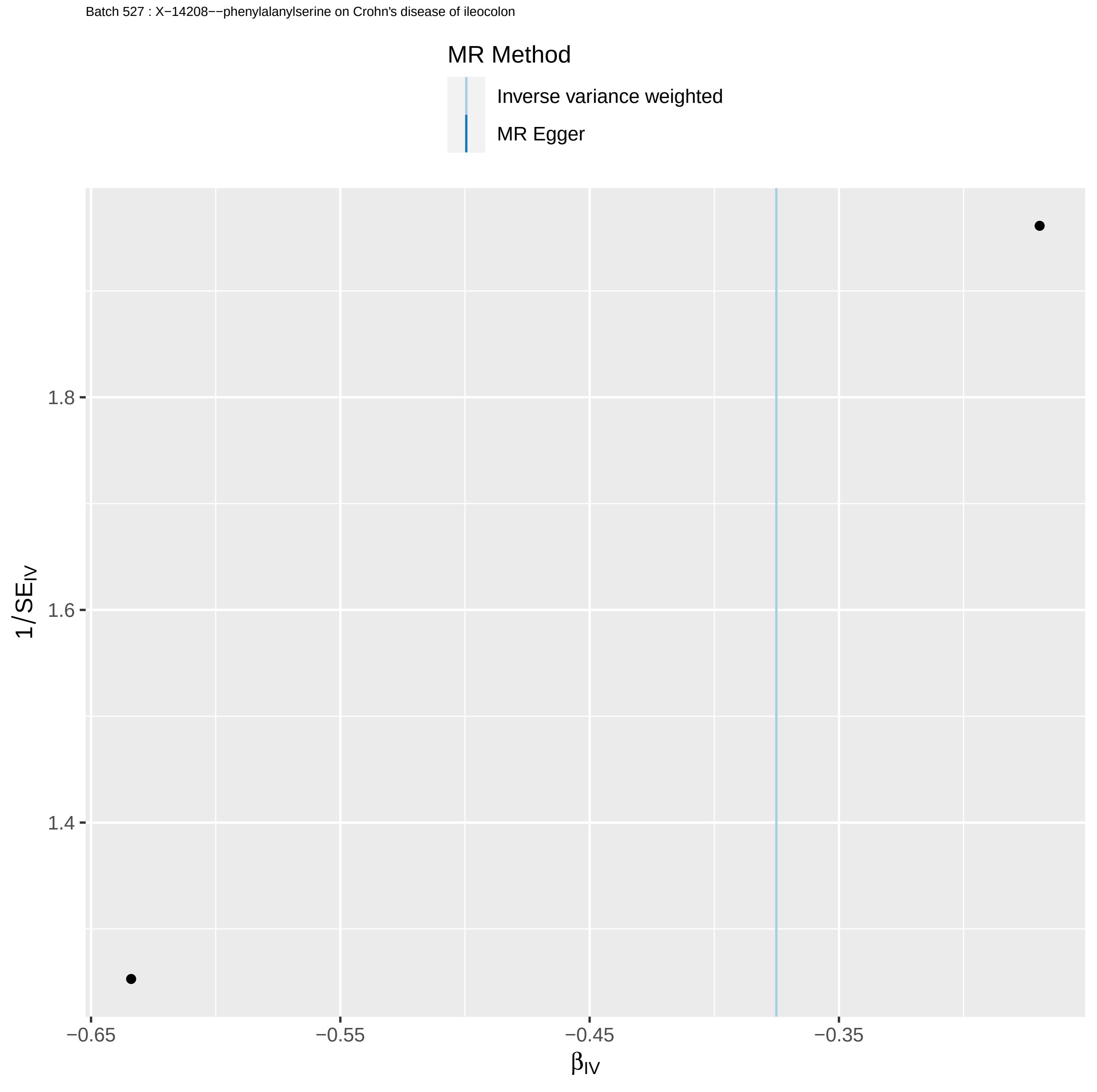
Batch 527 : X−14208−−phenylalanylserine on Crohn's disease of ileocolon
MR Method
Inverse variance weighted
MR Egger
1.8
1.6
1.4
1/SE$_{IV}$
−0.65
−0.55
−0.45
−0.35
β_{IV}

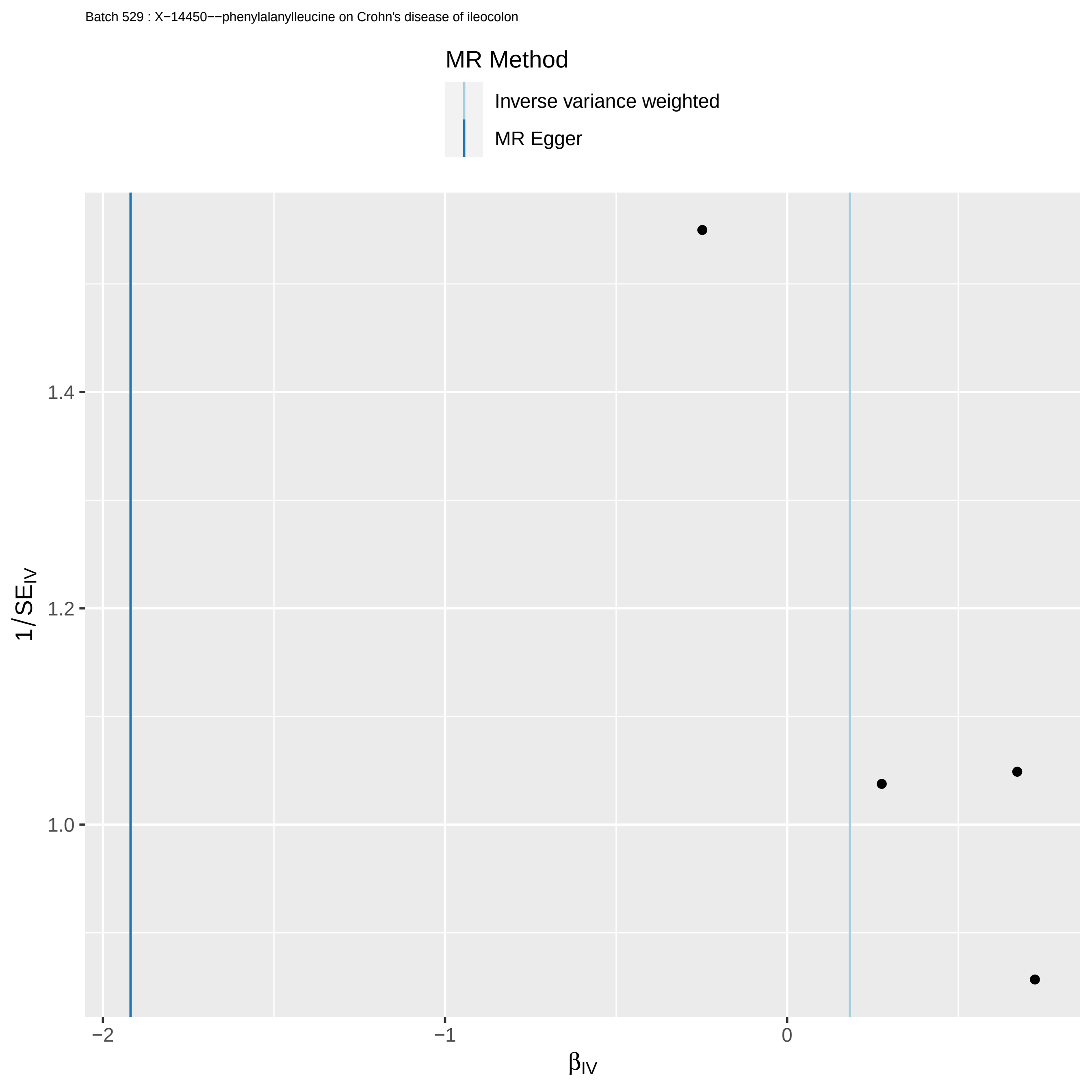

Batch 529 : X−14450−−phenylalanylleucine on Crohn's disease of ileocolon
MR Method
Inverse variance weighted
MR Egger
1.4
1.2
1.0
1/SE_IV
−2
−1
0
β_IV

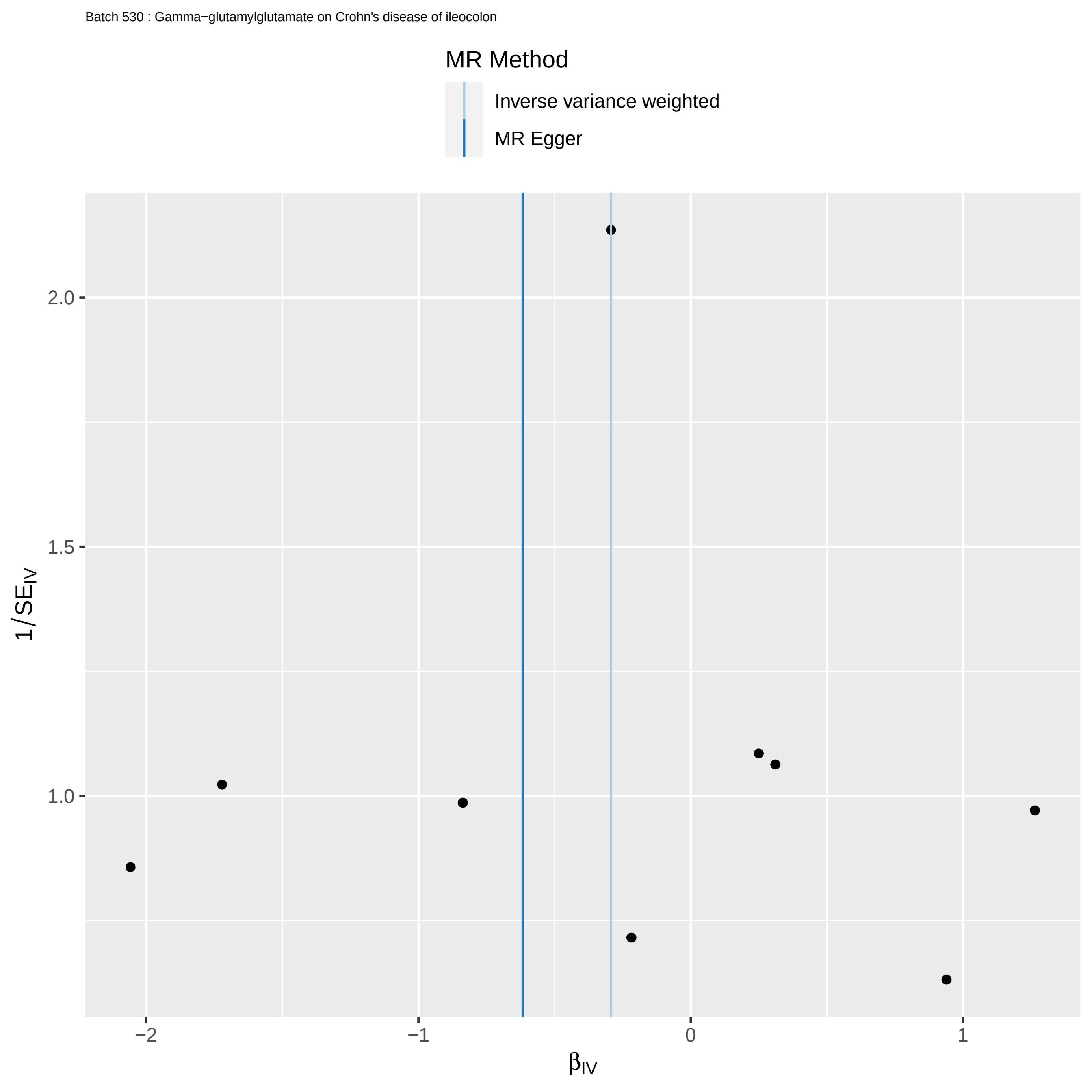

Batch 530 : Gamma−glutamylglutamate on Crohn's disease of ileocolon
MR Method
Inverse variance weighted
MR Egger
2.0
1.5
1.0
1/SE$_{IV}$
−2
−1
0
1
β_{IV}

Batch 531 : Octadecanedioate on Crohn's disease of ileocolon

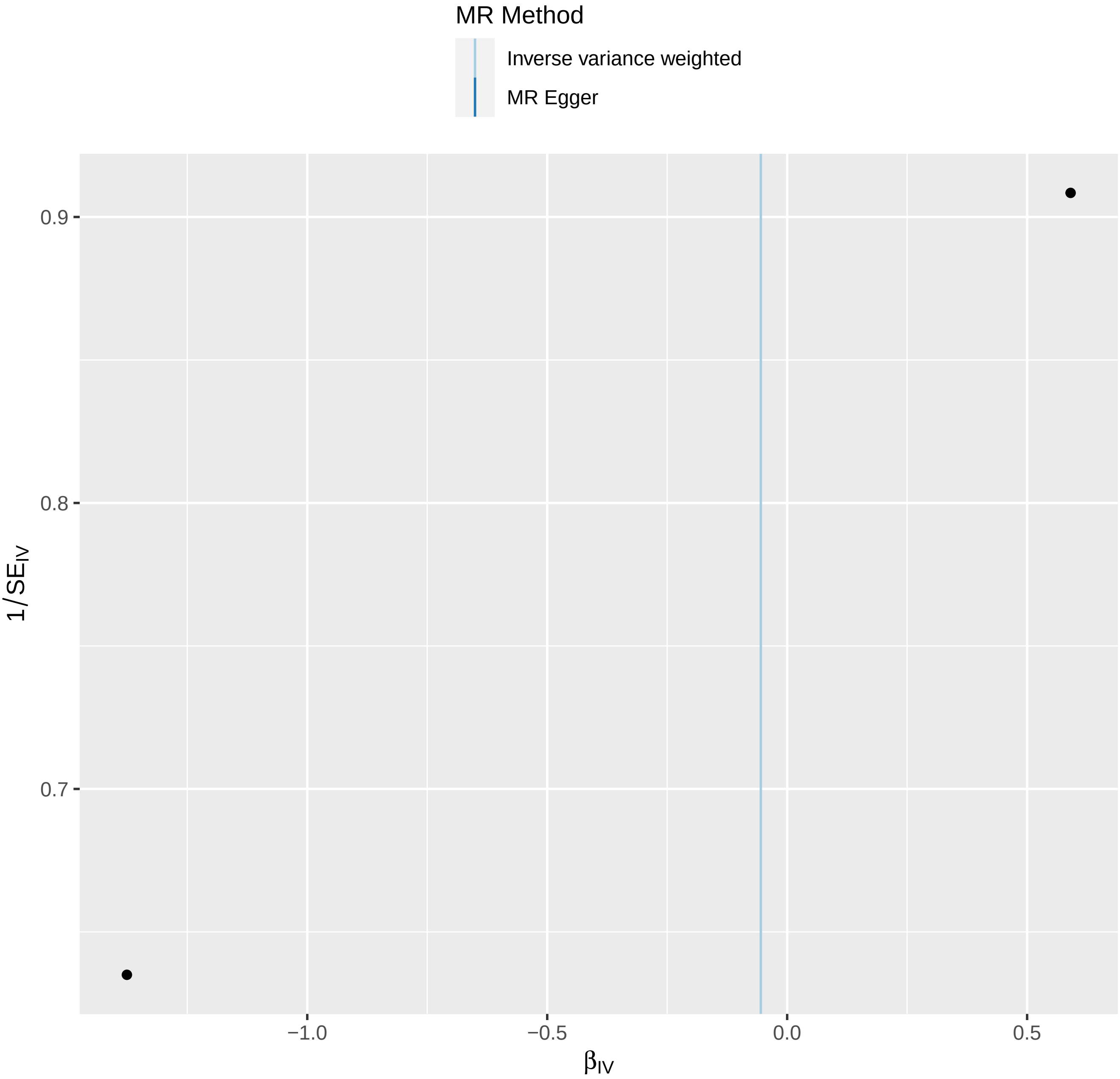

Batch 532 : Leucylleucine on Crohn's disease of ileocolon

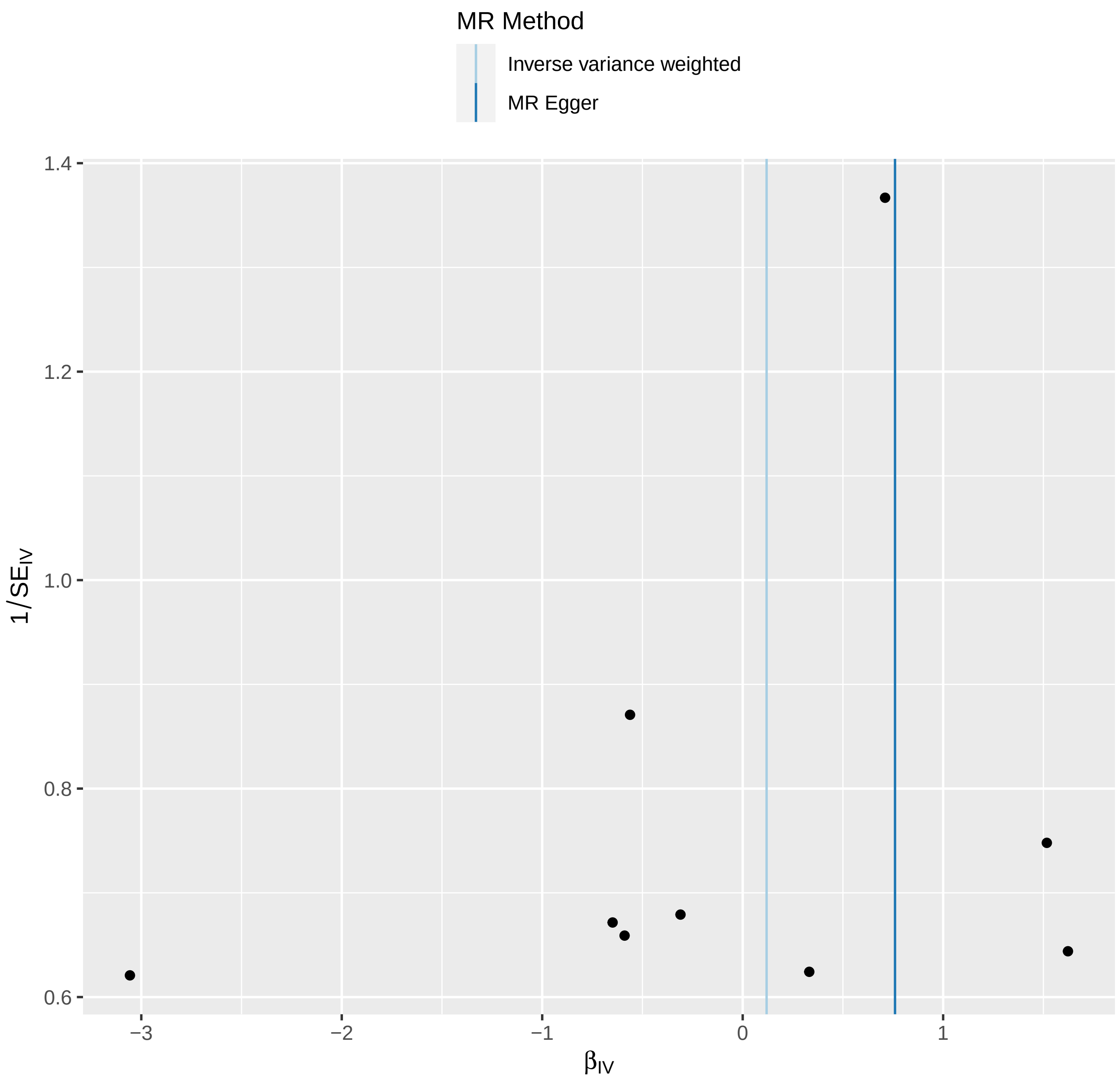

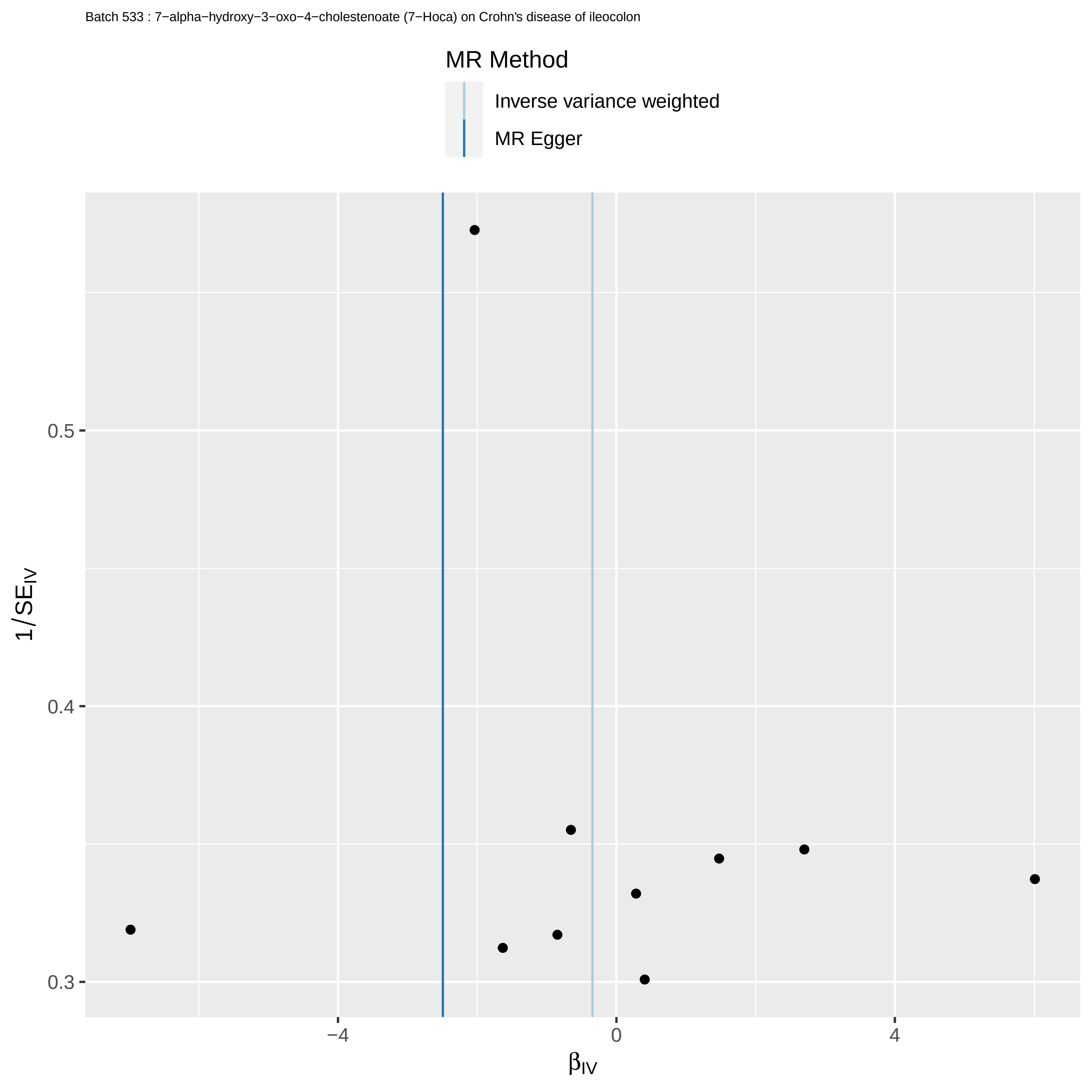
Batch 533 : 7−alpha−hydroxy−3−oxo−4−cholestenoate (7−Hoca) on Crohn's disease of ileocolon
MR Method
Inverse variance weighted
MR Egger
0.5
0.4
0.3
1/SE_IV
−4
0
4
β_IV

Batch 534 : N−Butyl Oleate on Crohn's disease of ileocolon

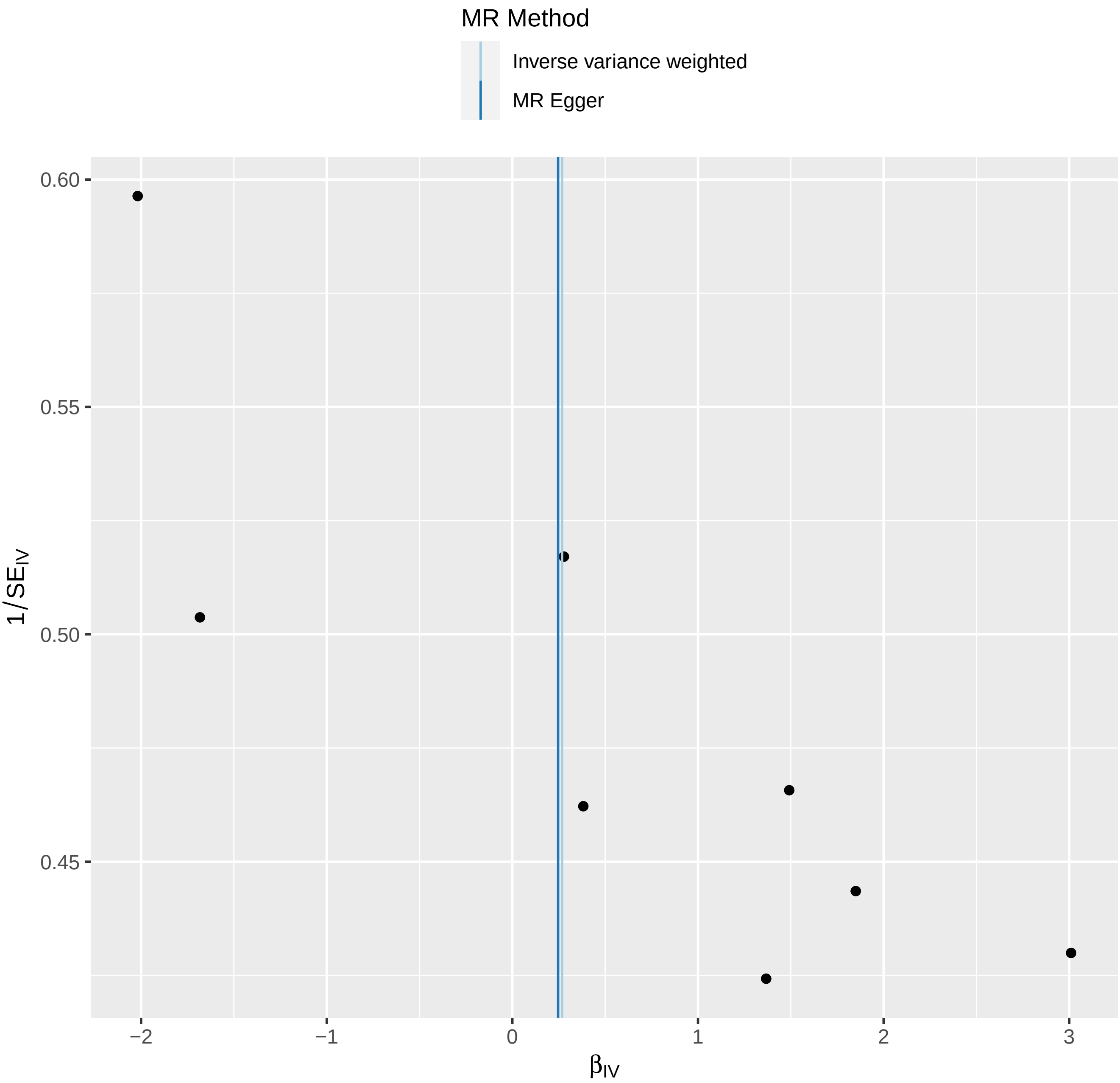

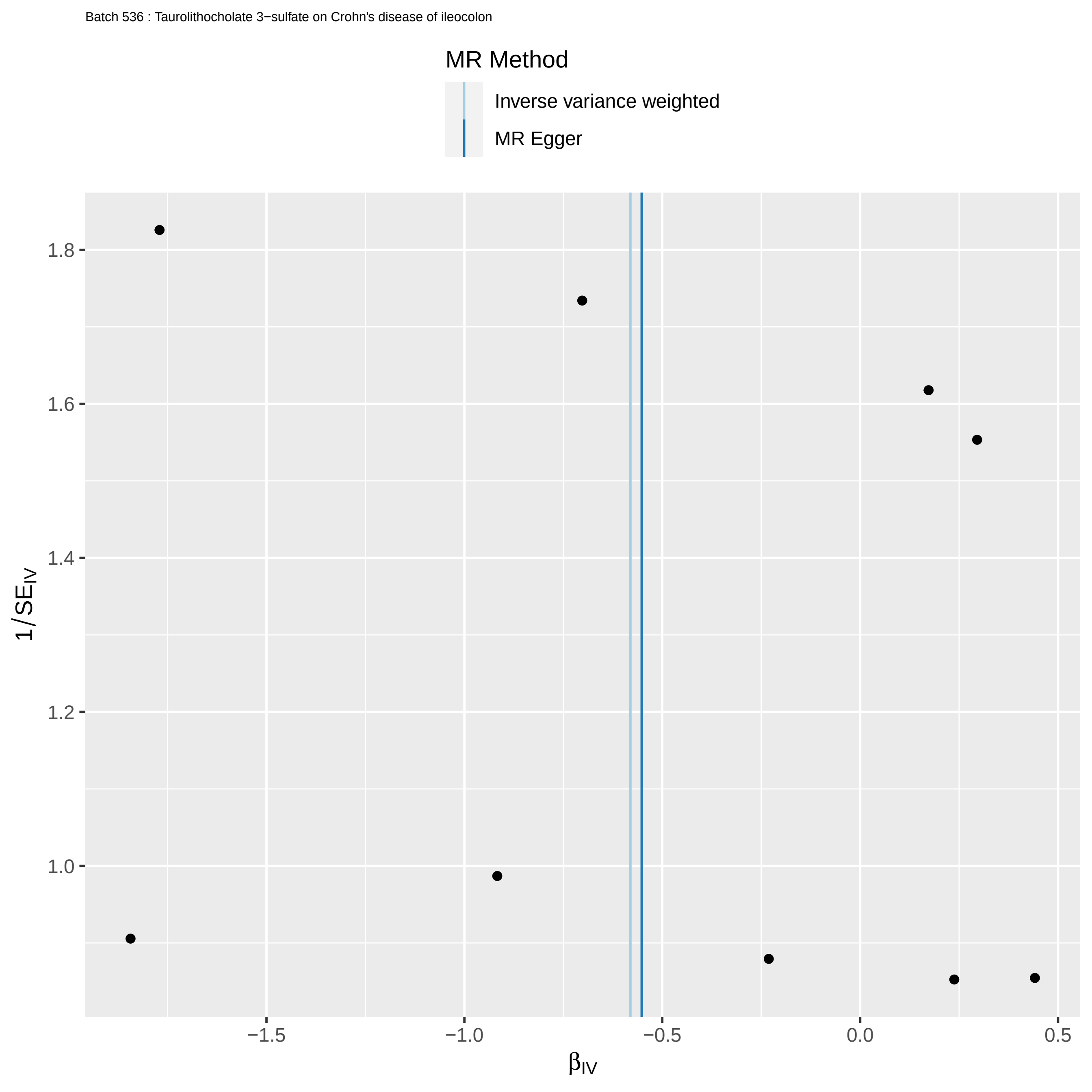

Batch 536 : Taurolithocholate 3−sulfate on Crohn's disease of ileocolon
MR Method
Inverse variance weighted
MR Egger
1/SE_IV
β_IV
1.8
1.6
1.4
1.2
1.0
−1.5
−1.0
−0.5
0.0
0.5

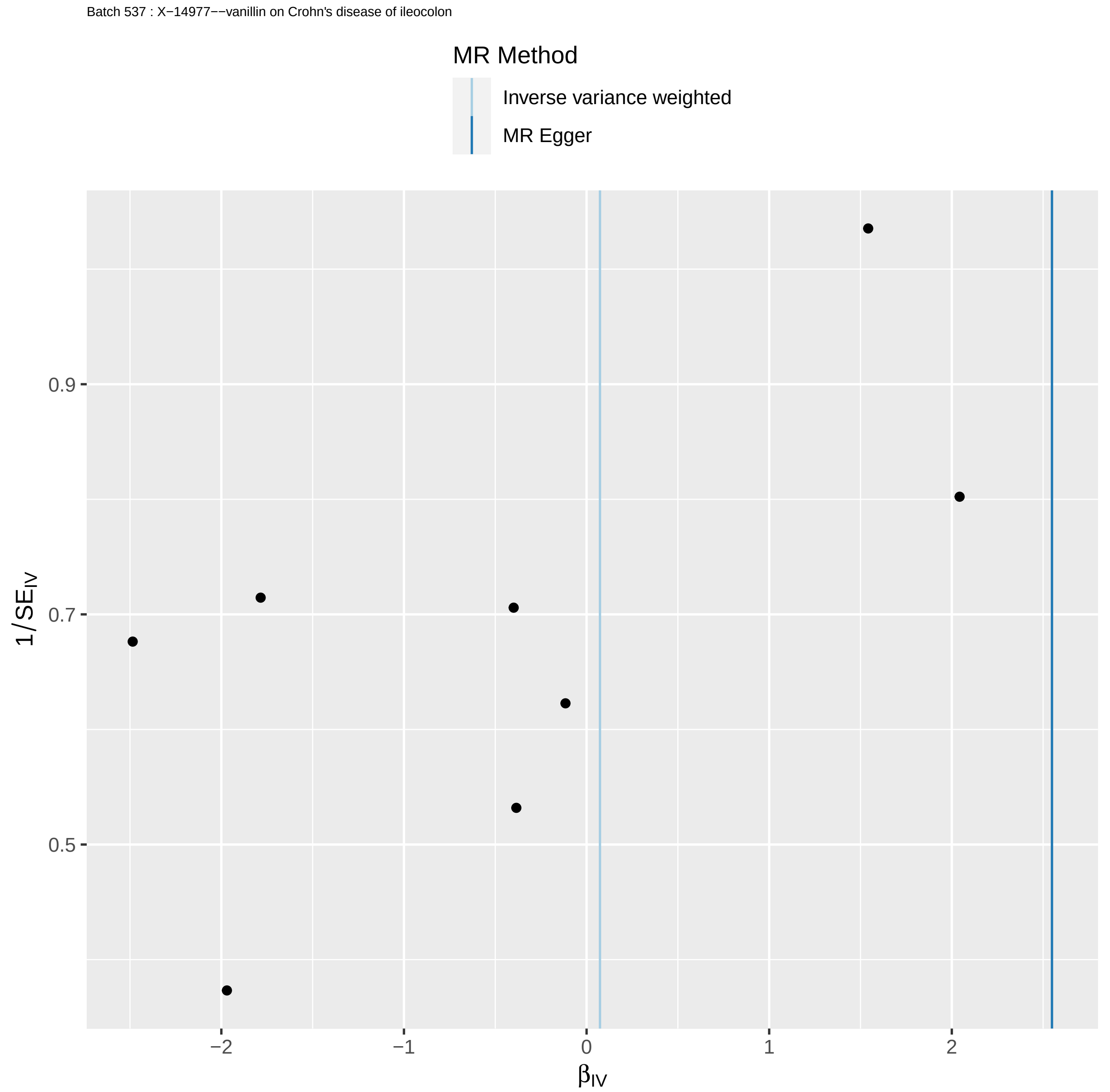
Batch 537 : X−14977−−vanillin on Crohn's disease of ileocolon
MR Method
Inverse variance weighted
MR Egger
0.9
0.7
0.5
1/SE$_{IV}$
−2
−1
0
1
2
β$_{IV}$

Batch 538 : Succinylcarnitine on Crohn's disease of ileocolon

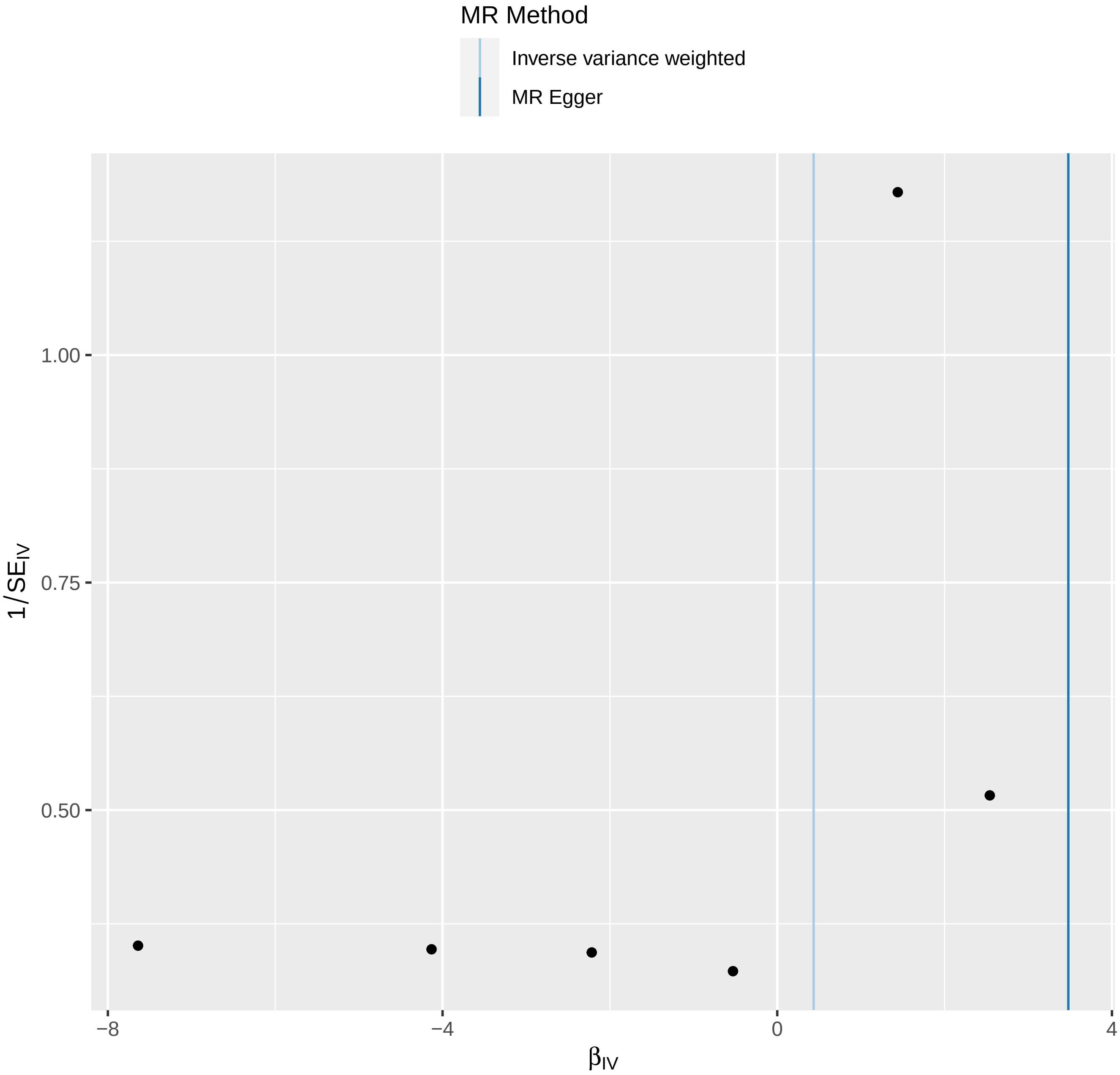

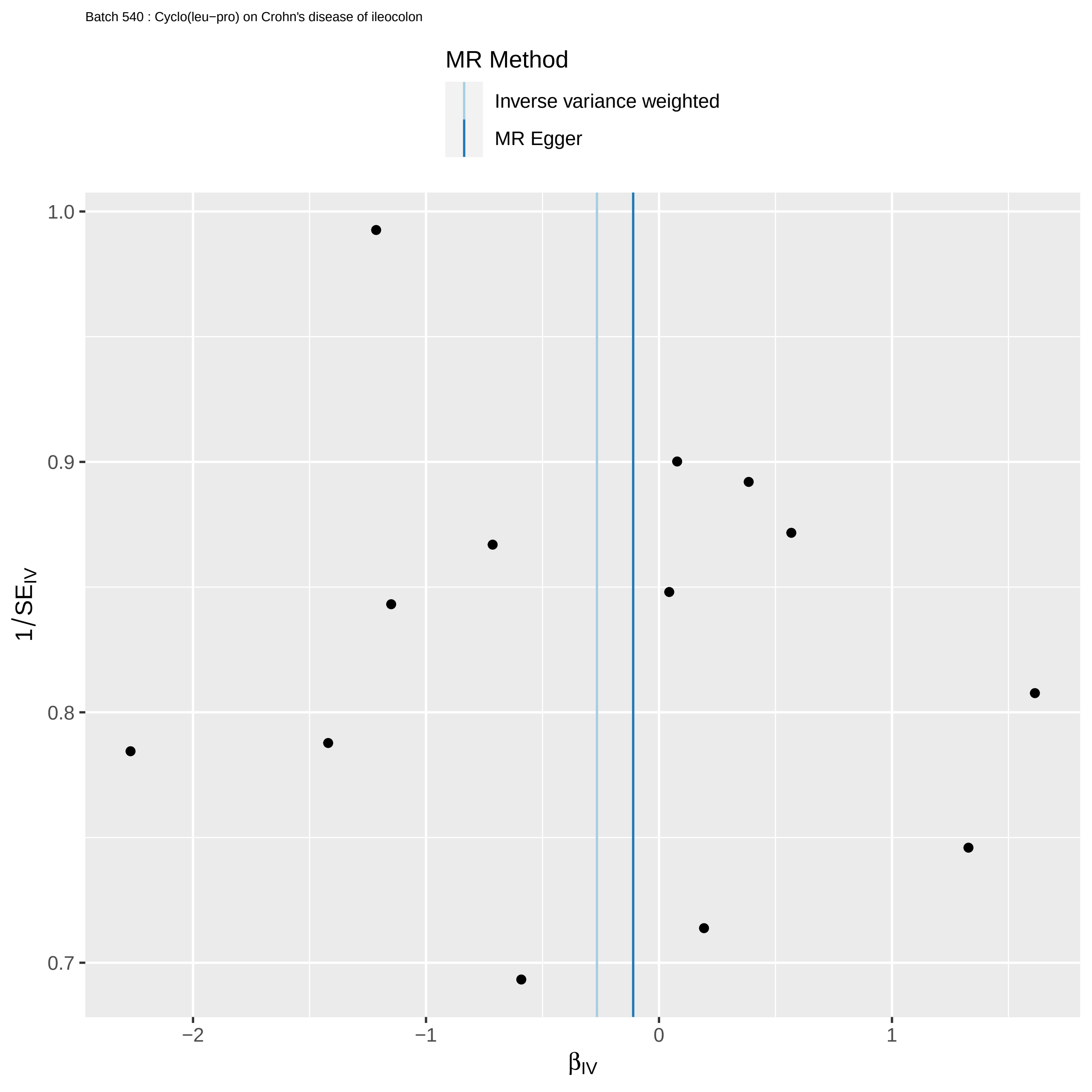

Batch 540 : Cyclo(leu−pro) on Crohn's disease of ileocolon
MR Method
Inverse variance weighted
MR Egger
1.0
0.9
0.8
0.7
1/SE_IV
−2
−1
0
1
β_IV

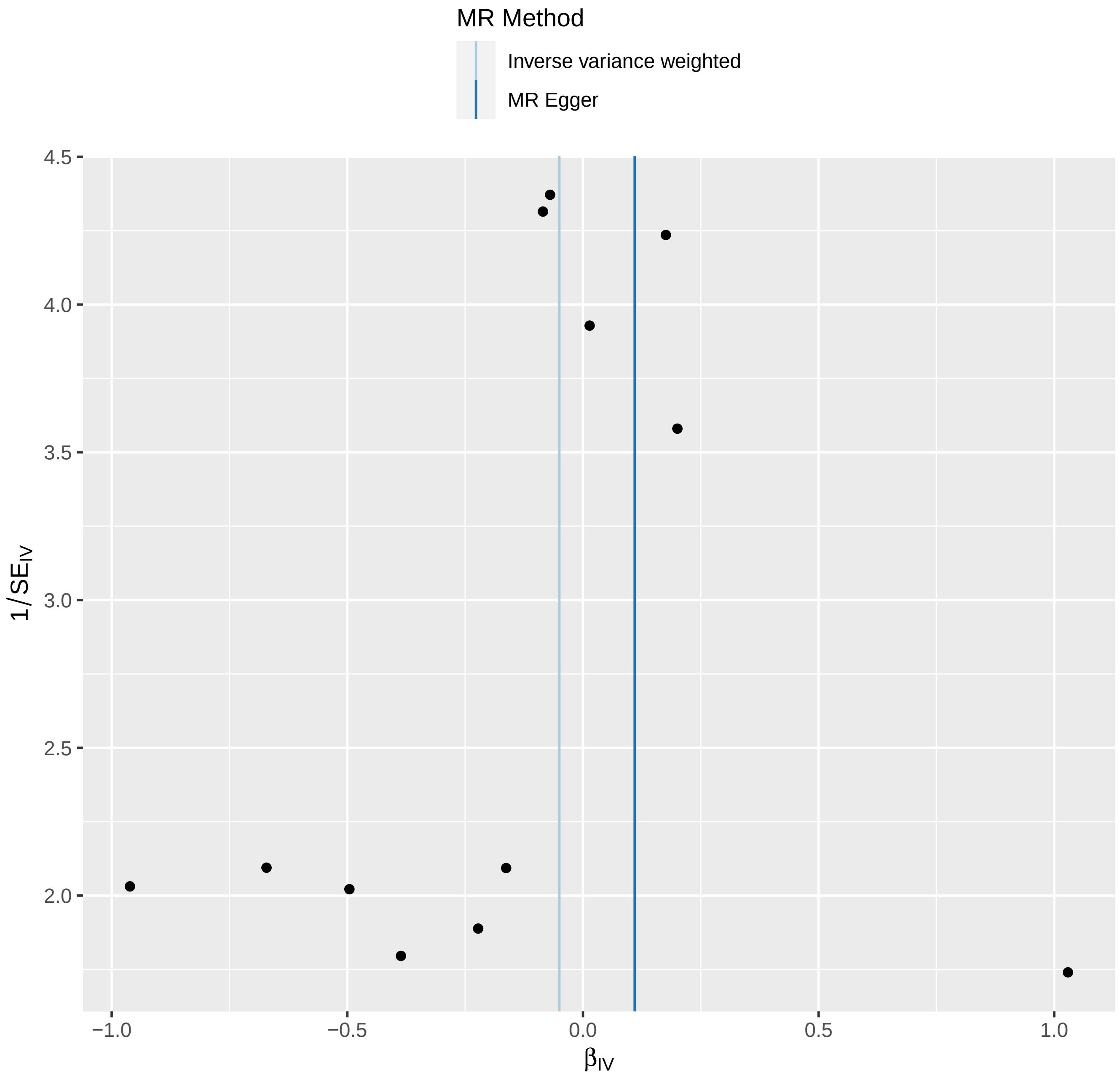
MR Method
Inverse variance weighted
MR Egger
4.5
4.0
3.5
3.0
2.5
2.0
$1/SE_{IV}$
−1.0
−0.5
0.0
0.5
1.0
β_{IV}

Batch 545 : 2−hydroxyglutarate on Crohn's disease of ileocolon

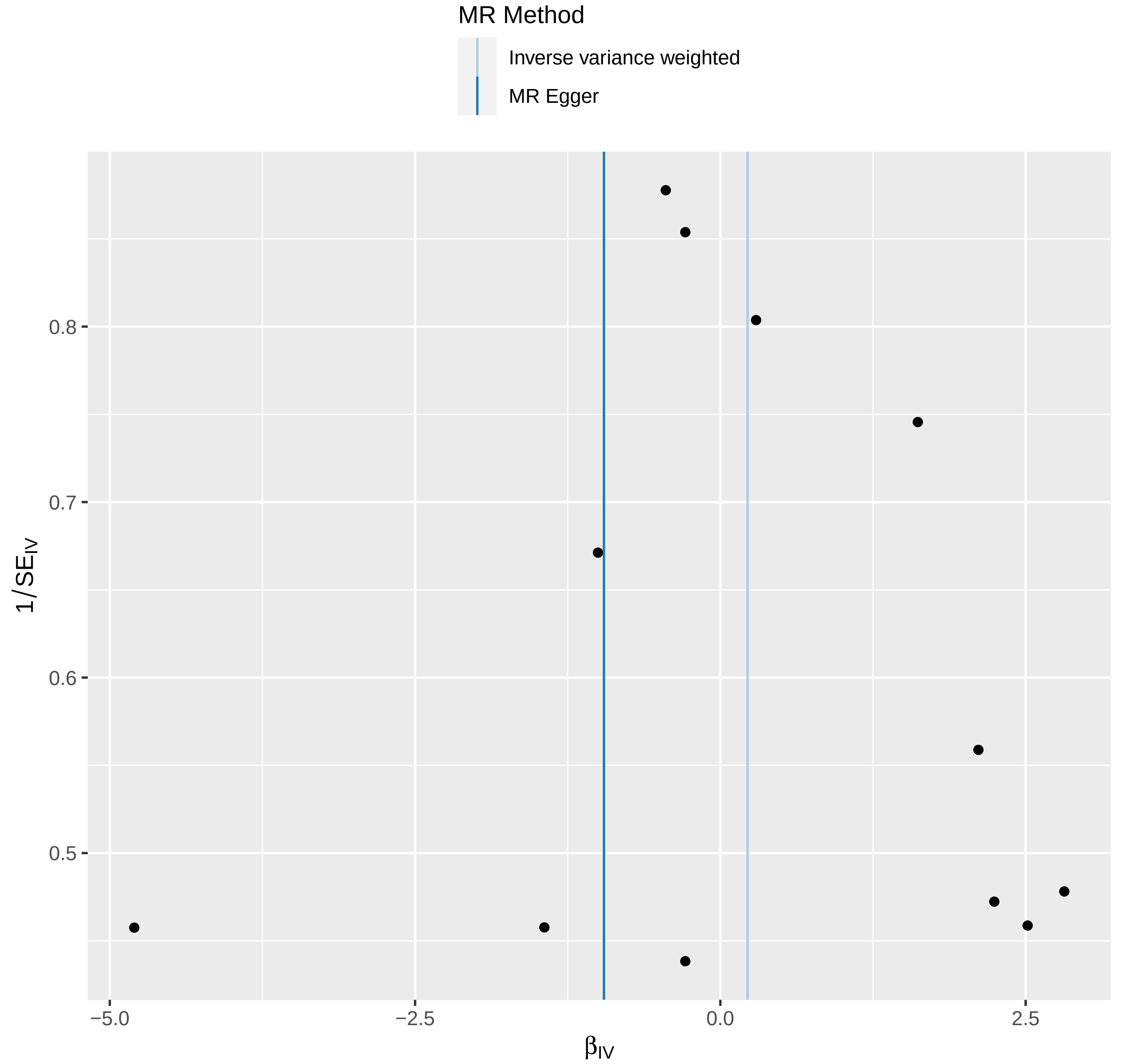

Batch 546 : Ergothioneine on Crohn's disease of ileocolon

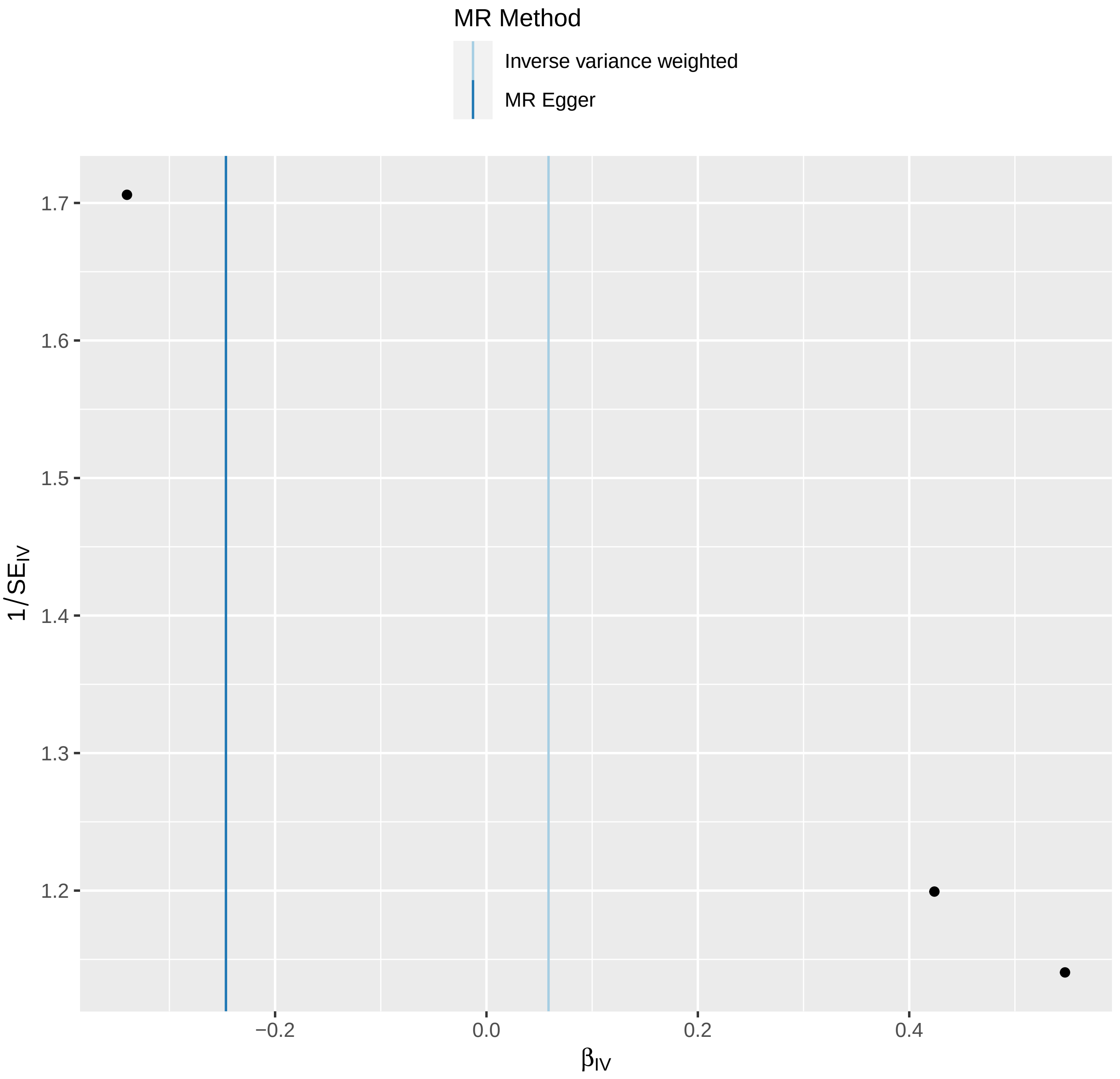

Batch 548 : Phenylalanylphenylalanine on Crohn's disease of ileocolon

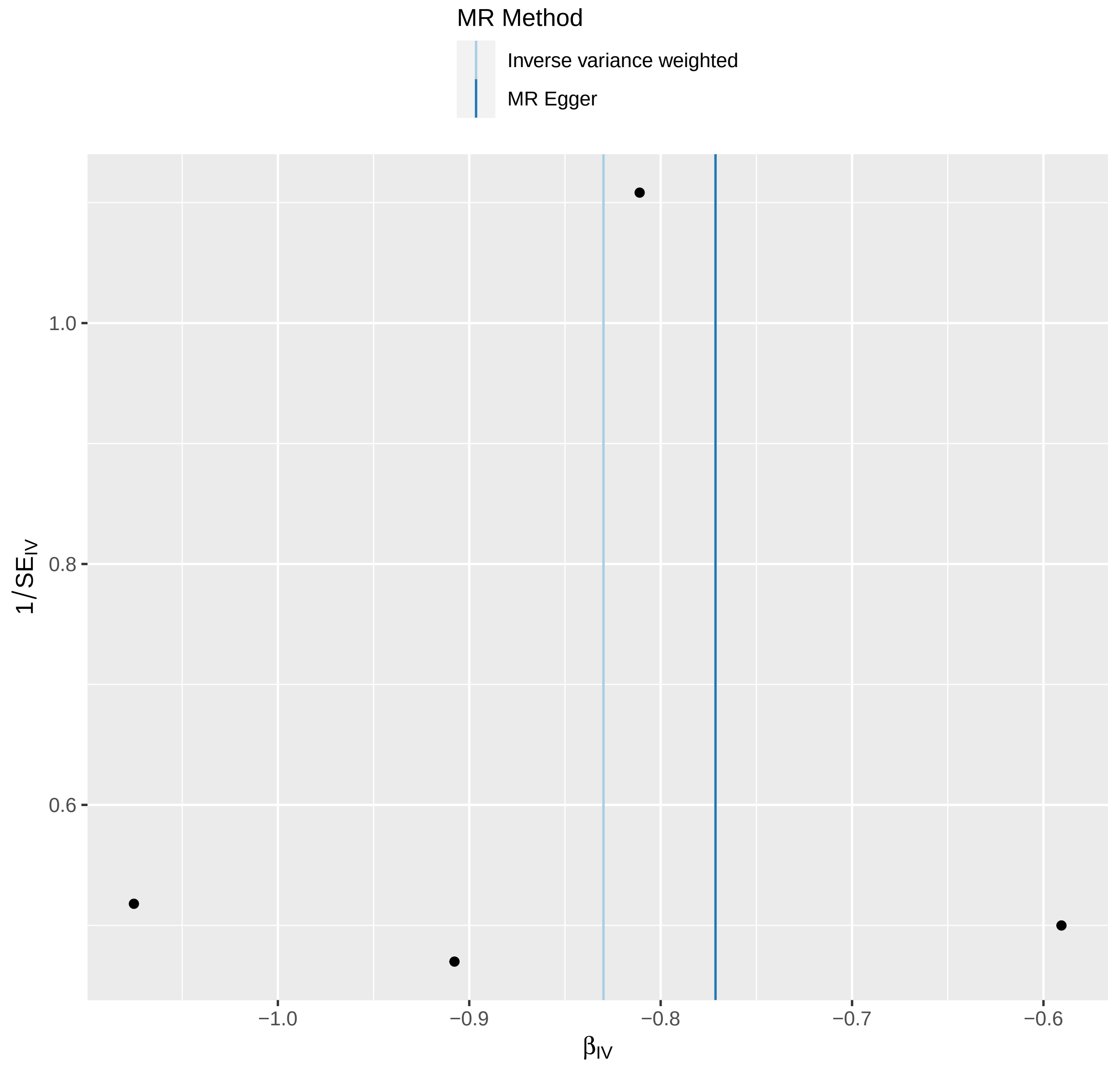

Batch 549 : Cis−4−decenoyl carnitine on Crohn's disease of ileocolon

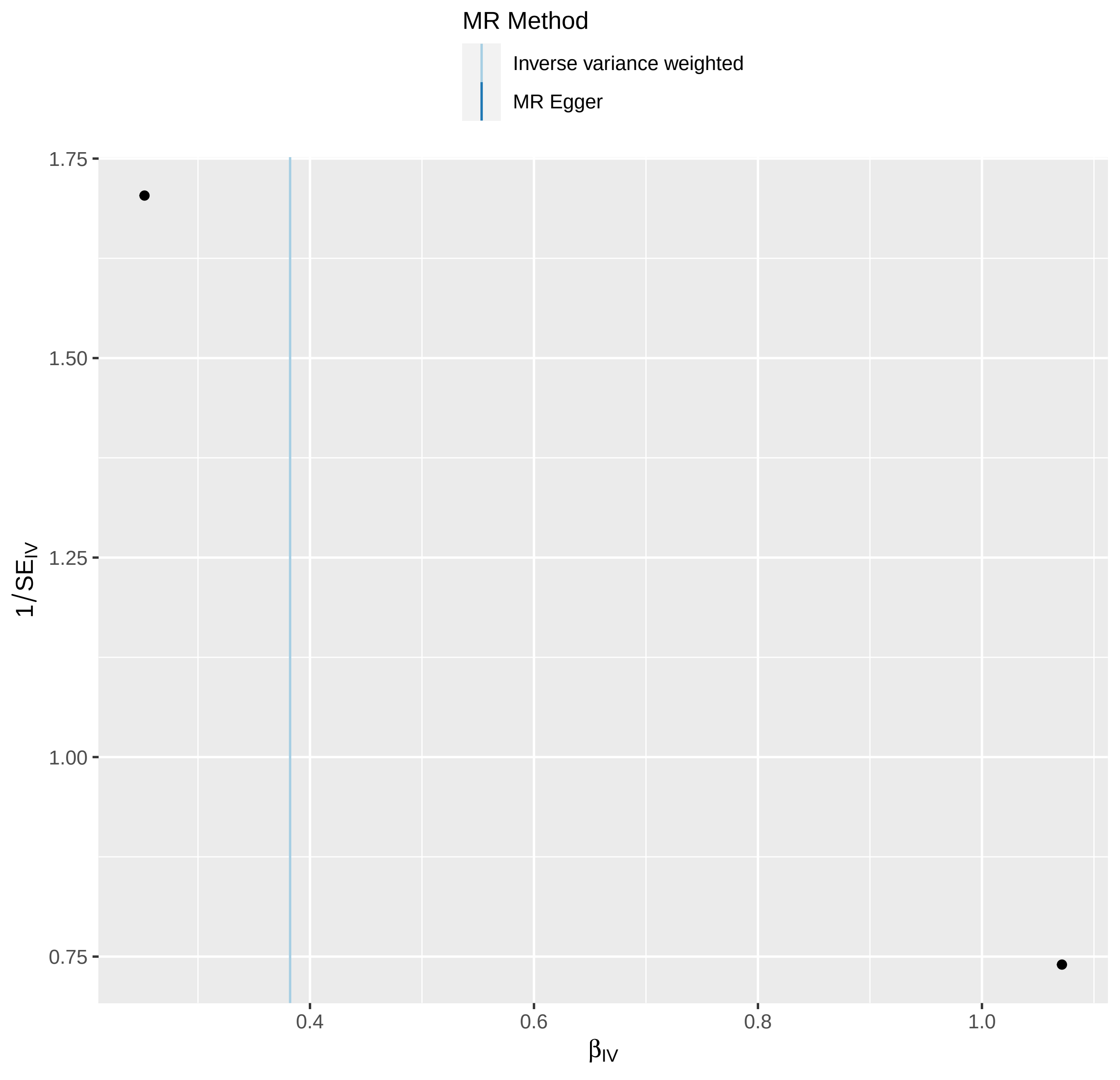

Batch 550 : 15−methylpalmitate (isobar with 2−methylpalmitate) on Crohn's disease of ileocolon

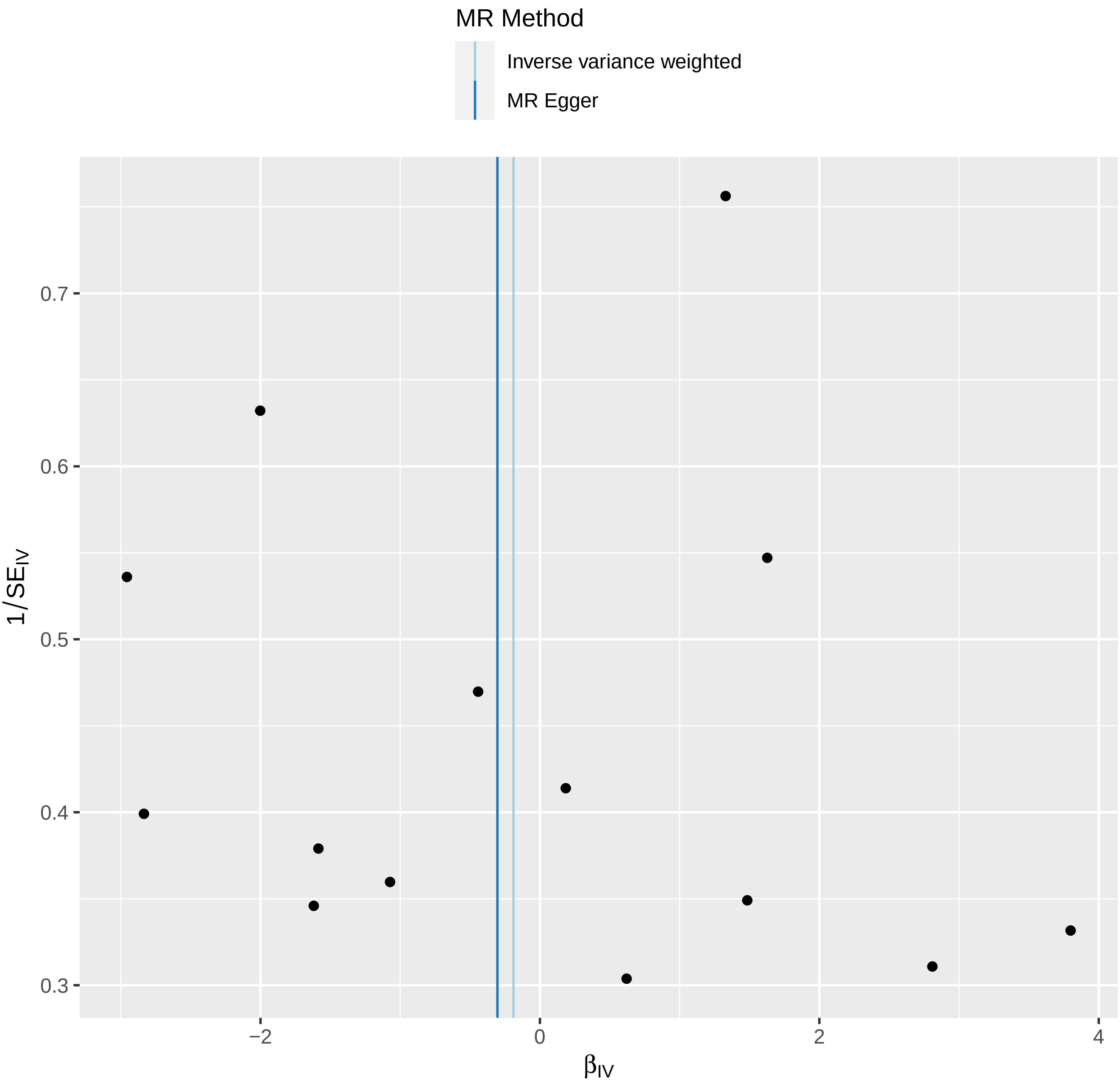

Batch 552 : Tryptophan on Crohn's disease of small intestine

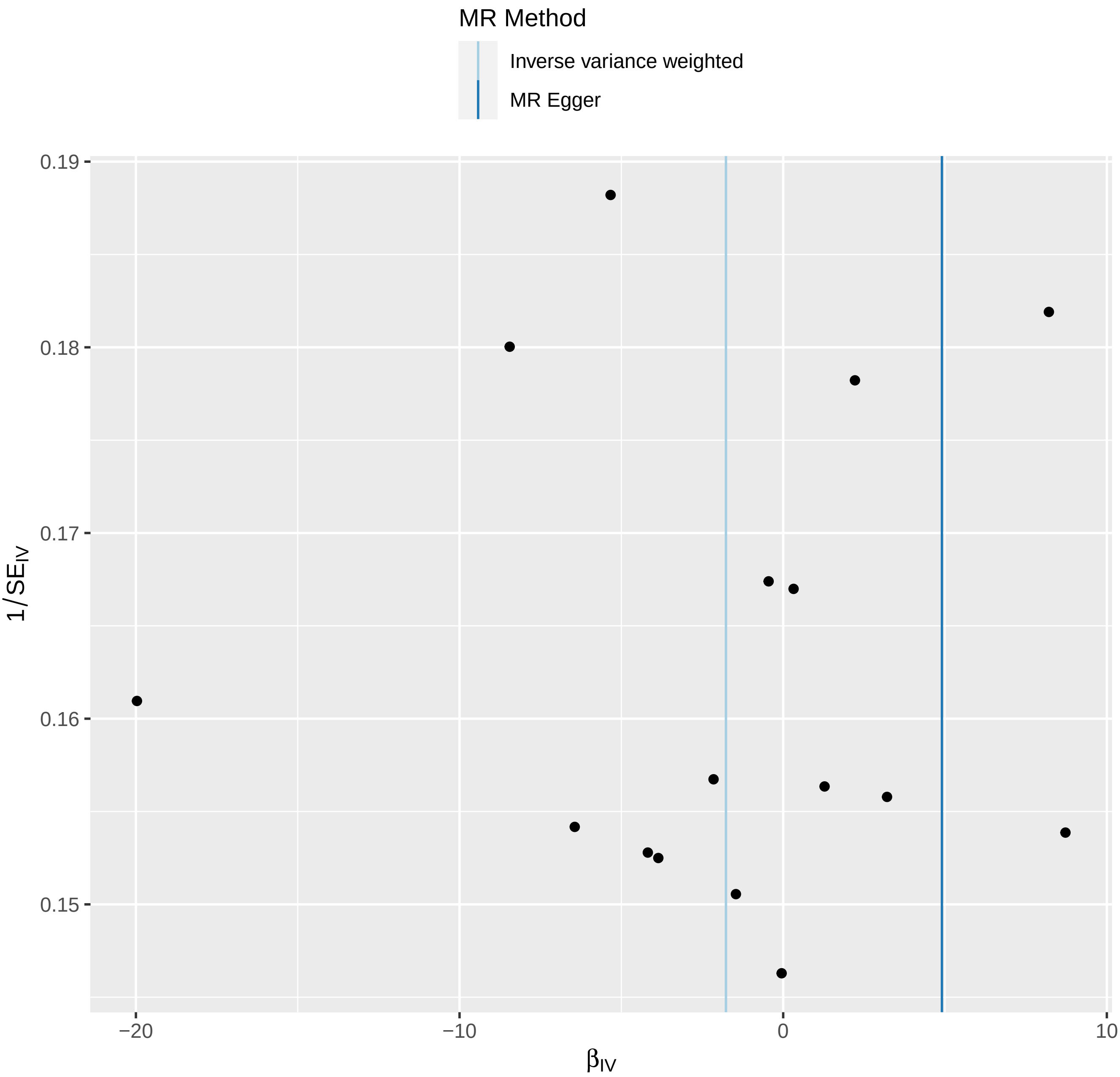

Batch 554 : Leucine on Crohn's disease of small intestine

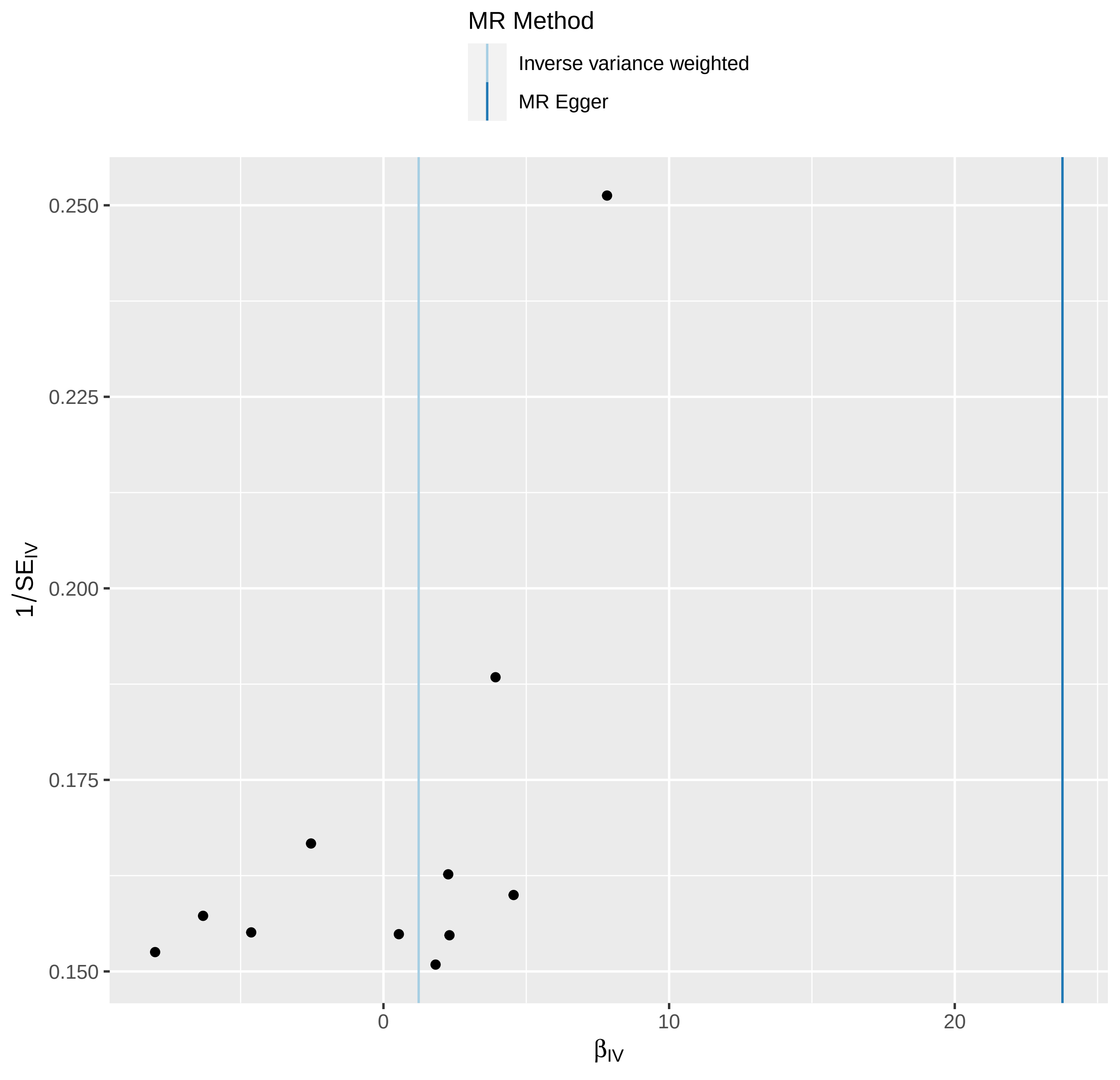

Batch 558 : Lactate on Crohn's disease of small intestine

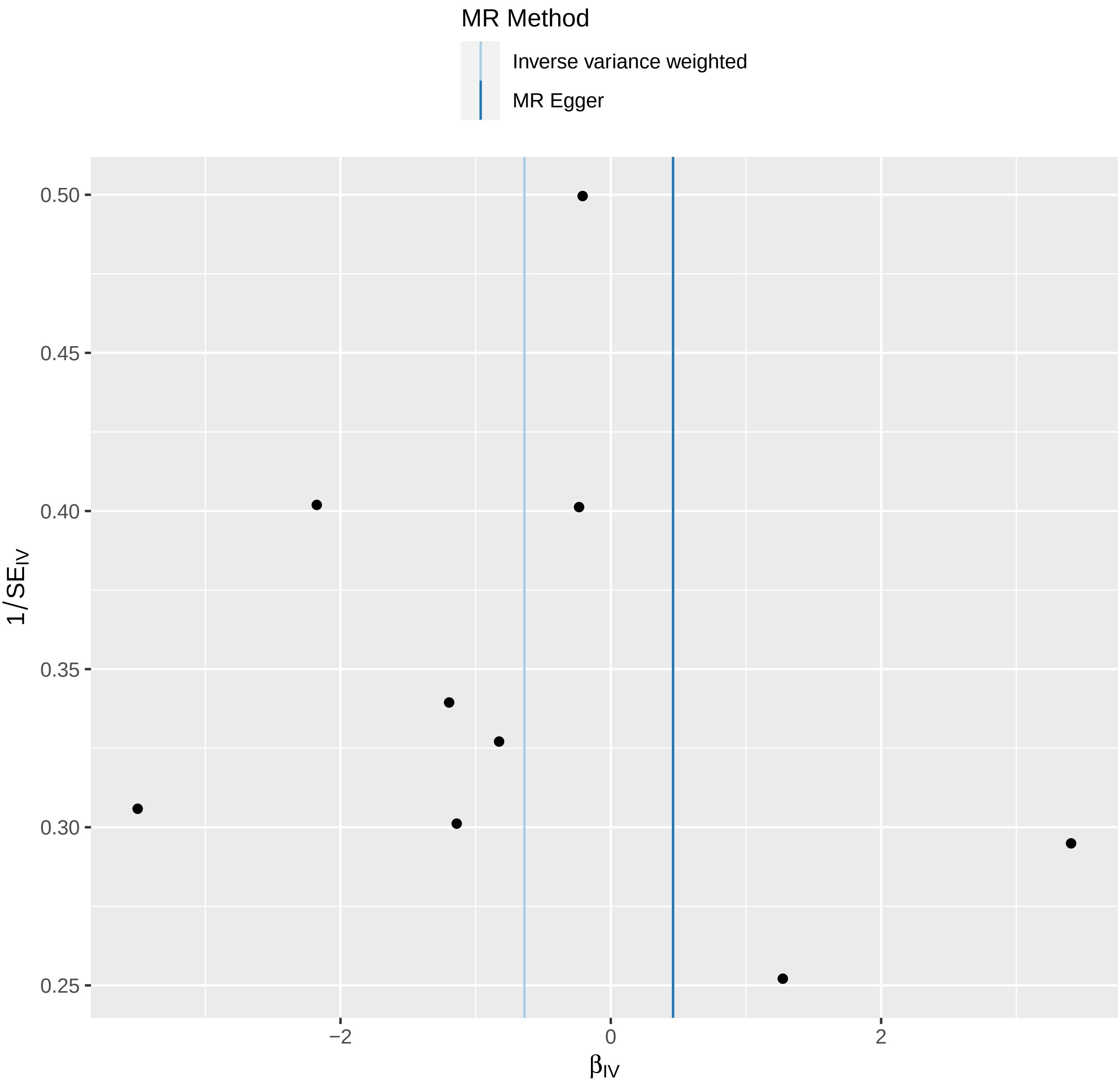

Batch 559 : 3−hydroxybutyrate (BHBA) on Crohn's disease of small intestine

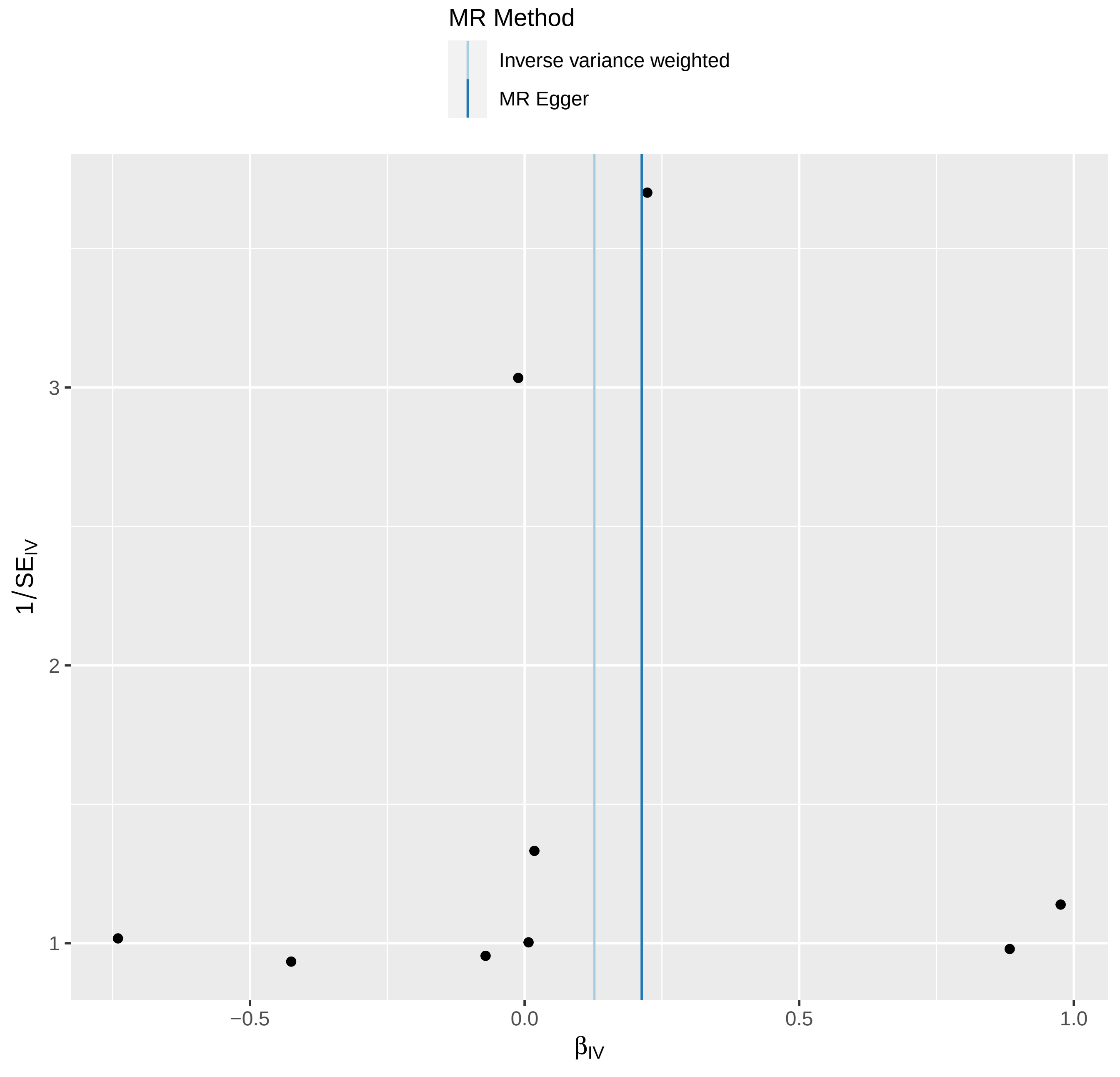

Batch 560 : Arabinose on Crohn's disease of small intestine

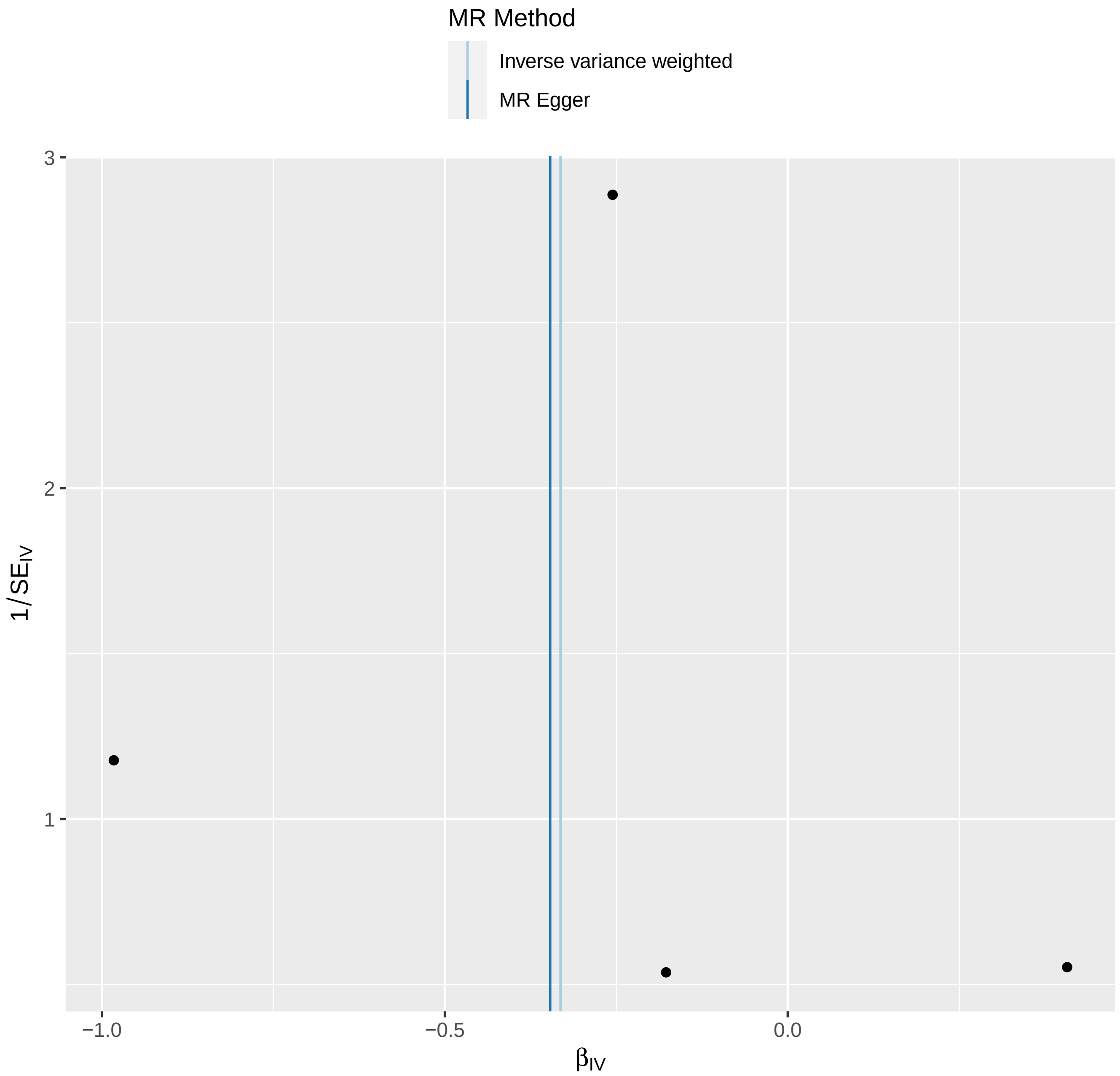

Batch 561 : Fructose on Crohn's disease of small intestine

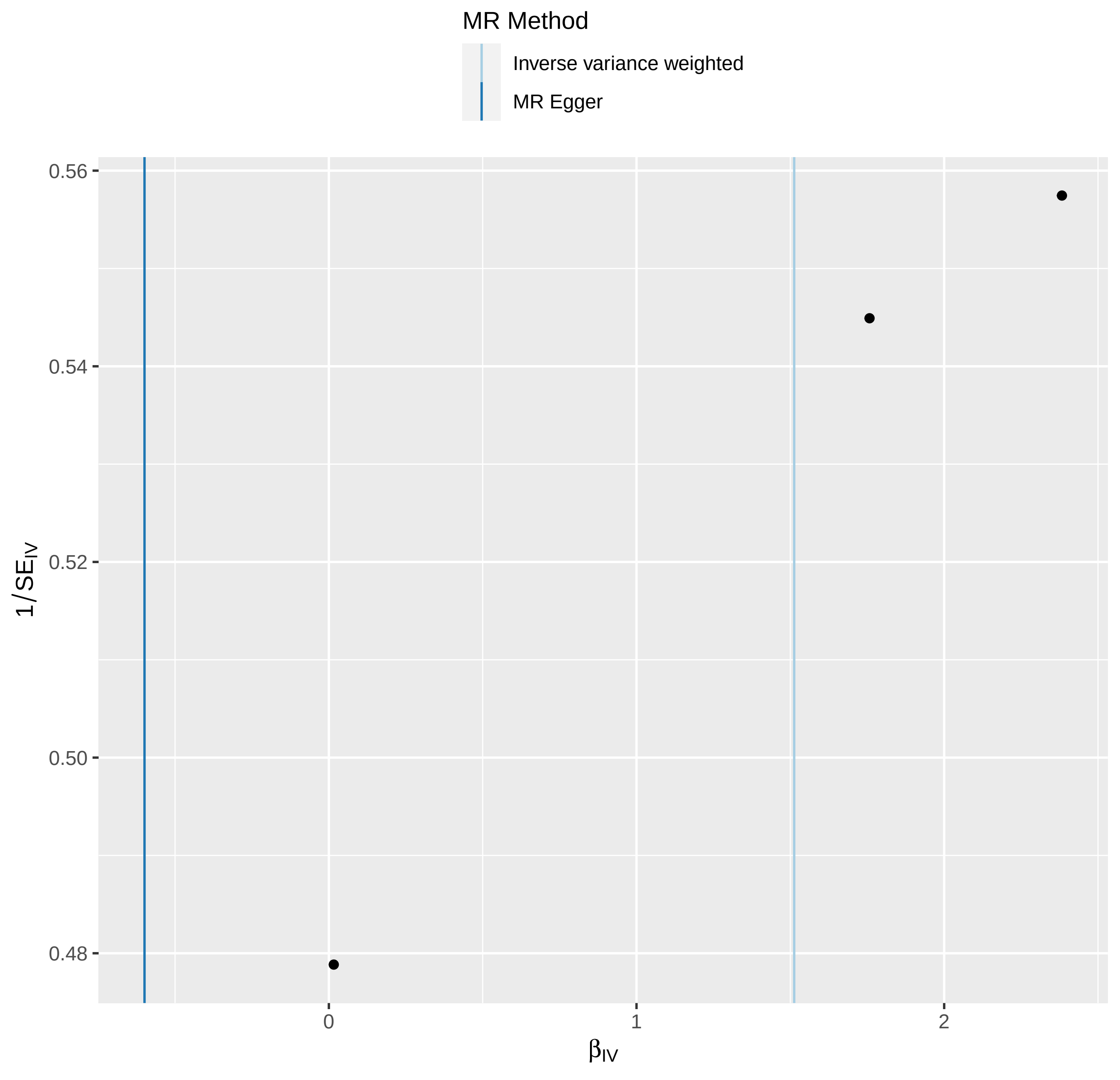

Batch 563 : Pyruvate on Crohn's disease of small intestine

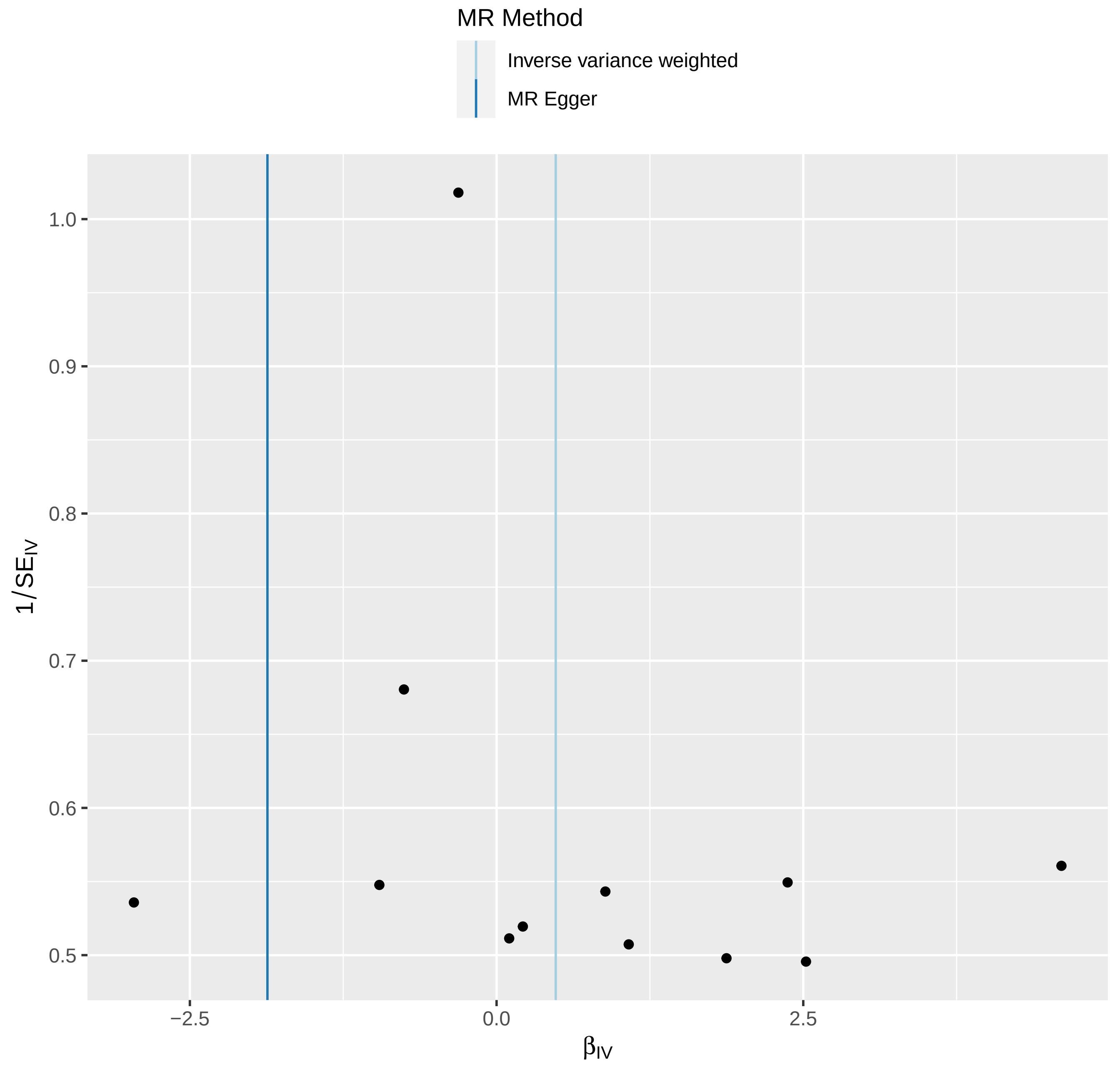

Batch 565 : Linoleate (18:2n6) on Crohn's disease of small intestine

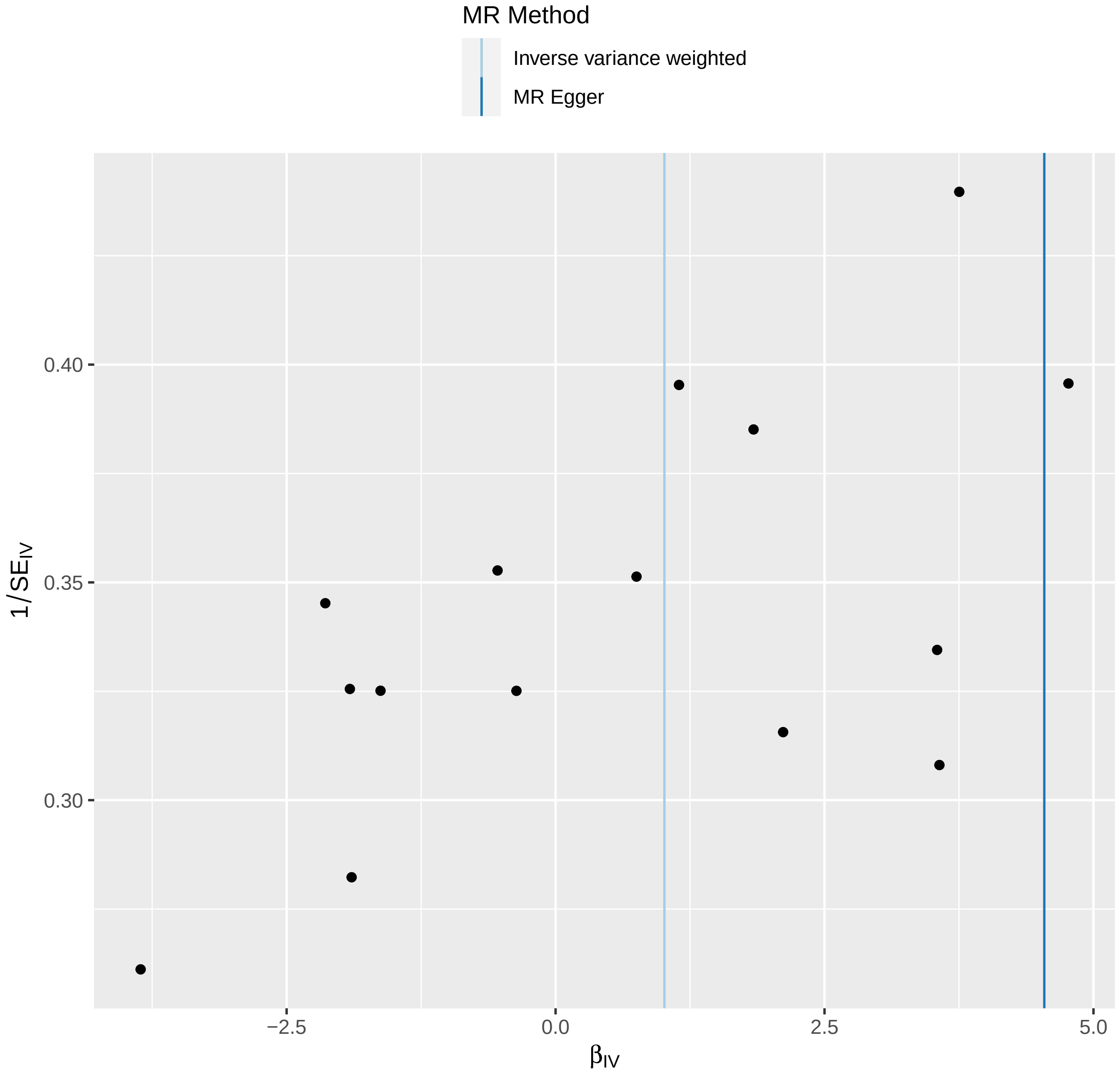

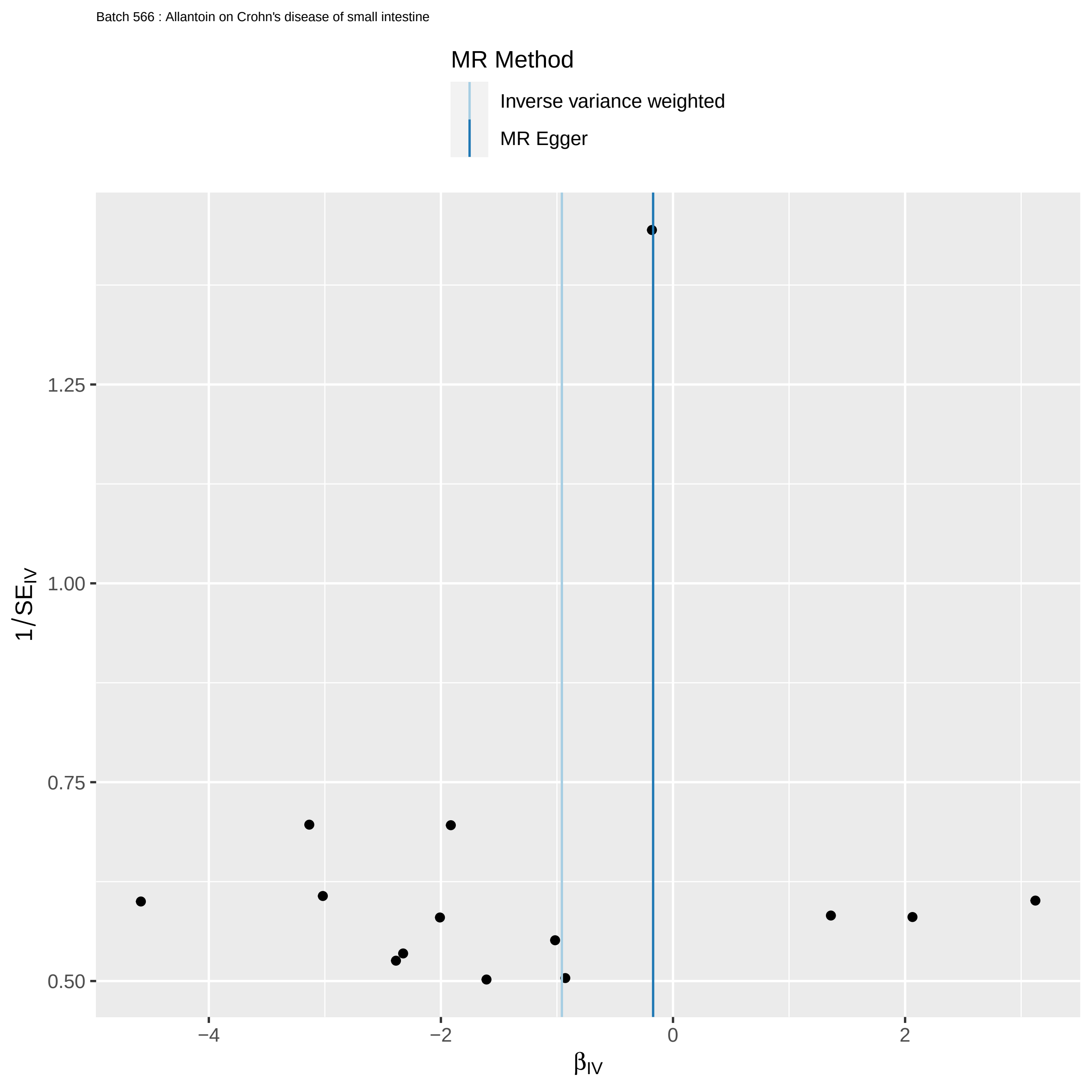
Batch 566 : Allantoin on Crohn's disease of small intestine
MR Method
Inverse variance weighted
MR Egger
1.25
1.00
0.75
0.50
1/SE_IV
−4
−2
0
2
β_IV

Batch 571 : Isoleucine on Crohn's disease of small intestine

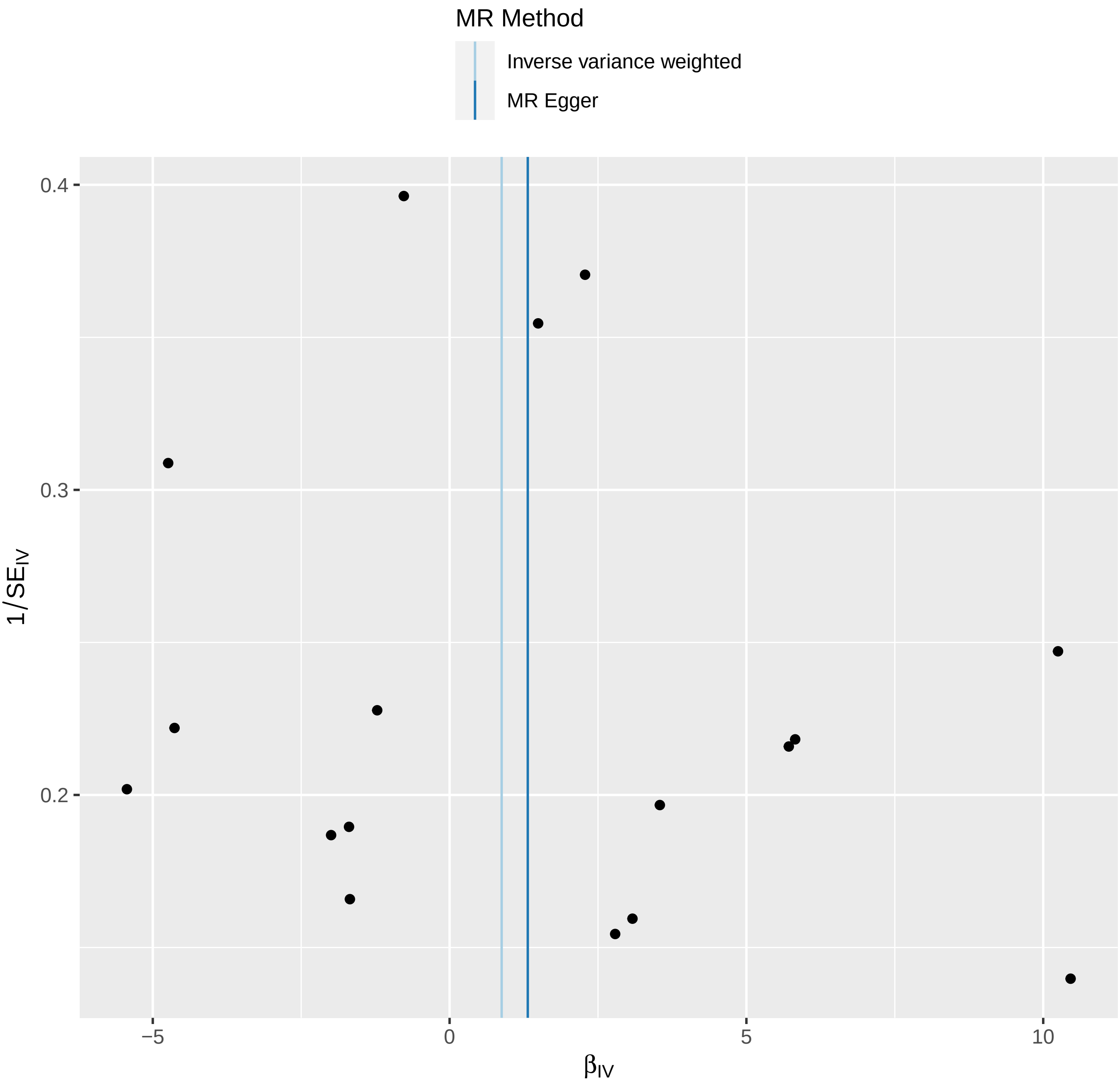

Batch 572 : Threonine on Crohn's disease of small intestine

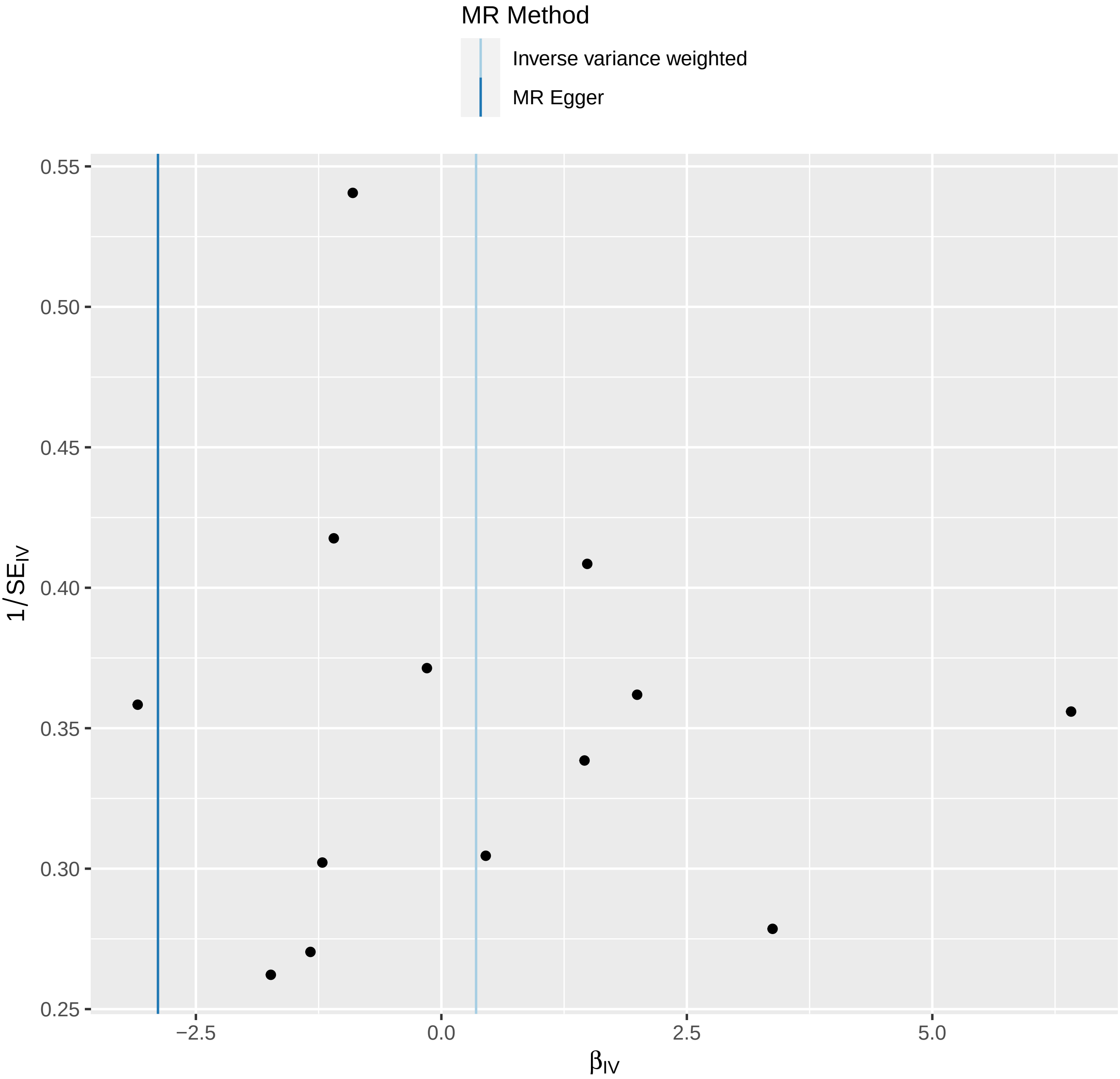

Batch 573 : Tyrosine on Crohn's disease of small intestine

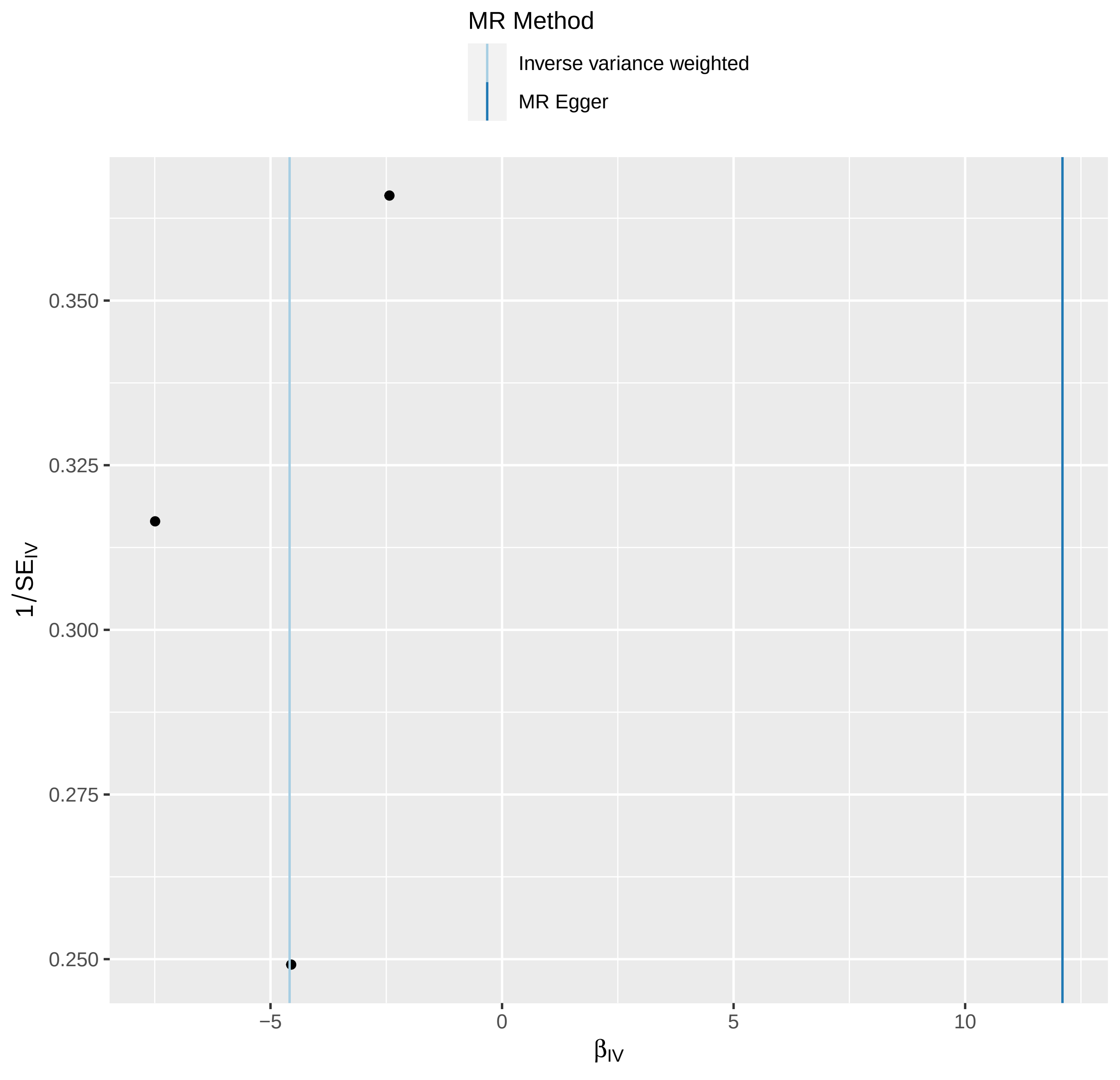

Batch 579 : Stearate (18:0) on Crohn's disease of small intestine

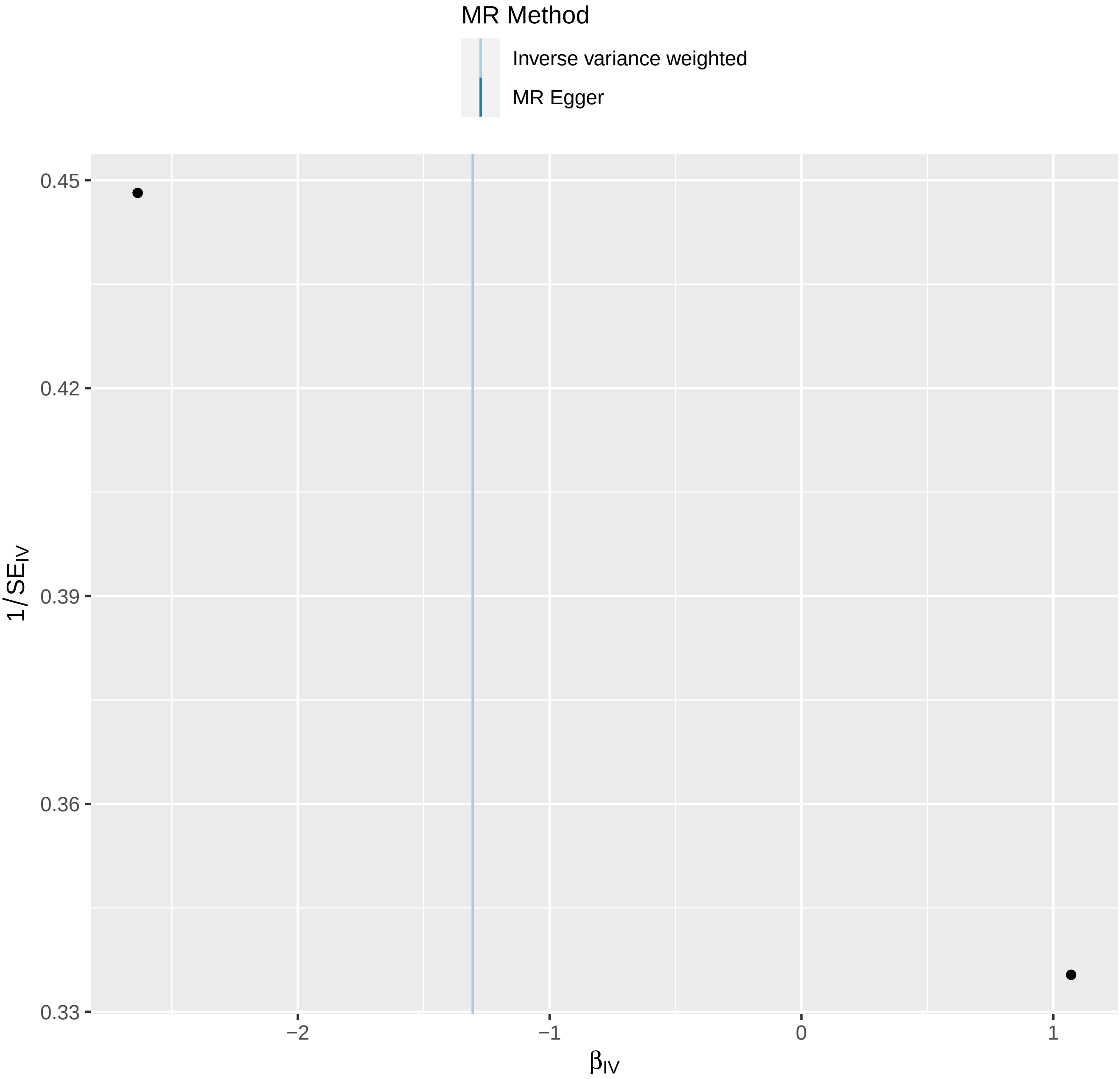

Batch 582 : Myristate (14:0) on Crohn's disease of small intestine

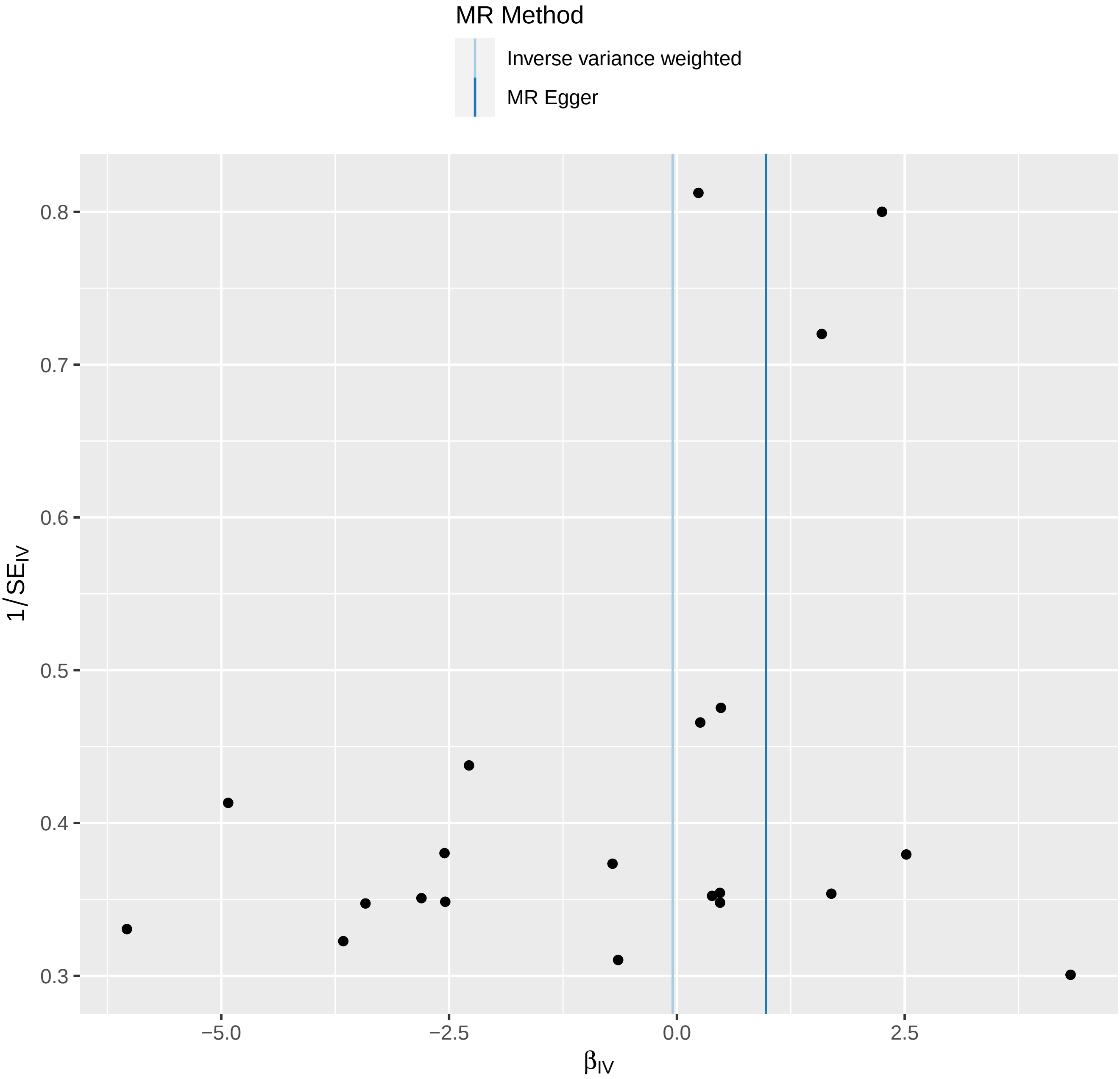

Batch 584 : Ornithine on Crohn's disease of small intestine

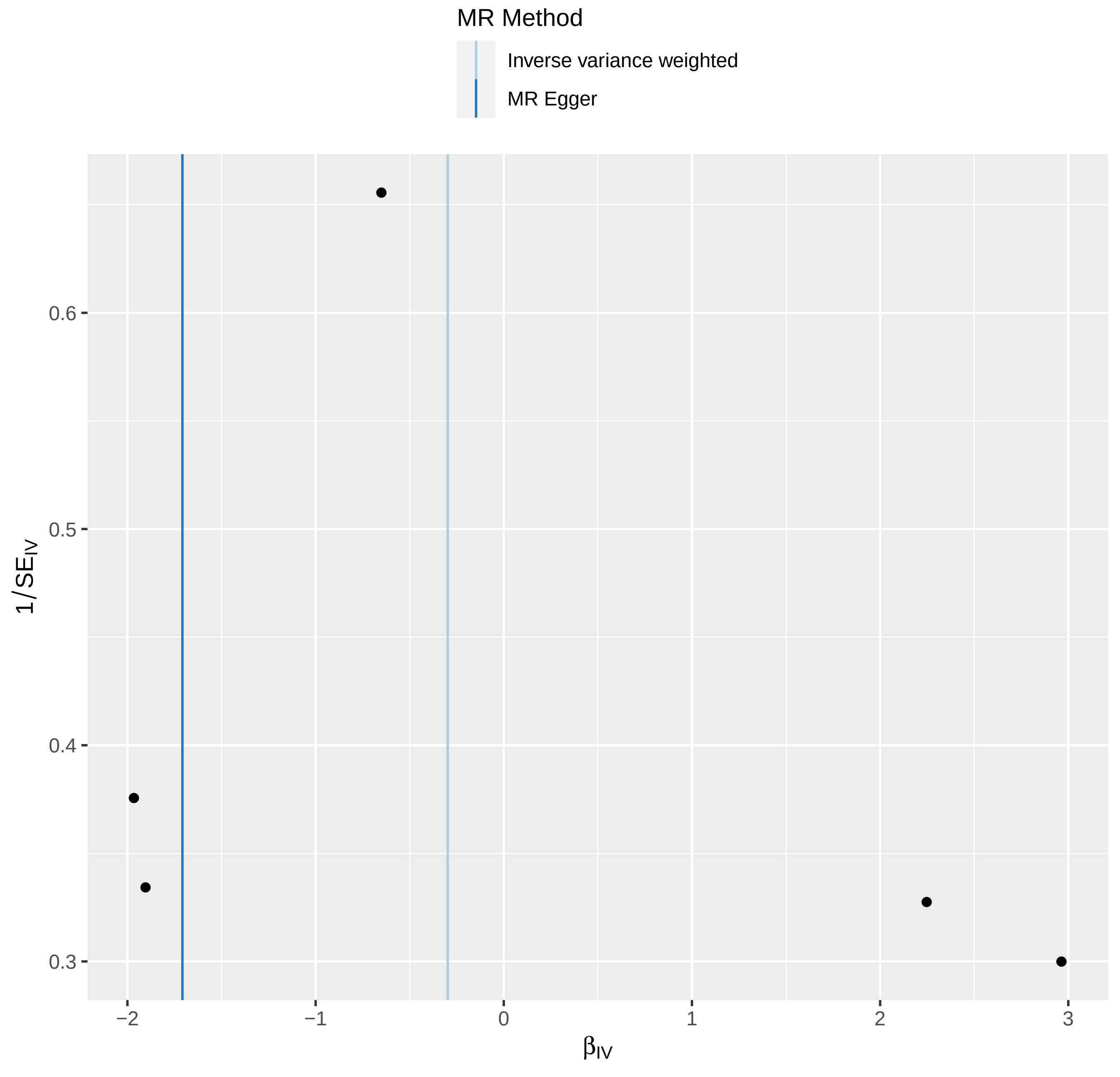

Batch 587 : 4−acetamidobutanoate on Crohn's disease of small intestine

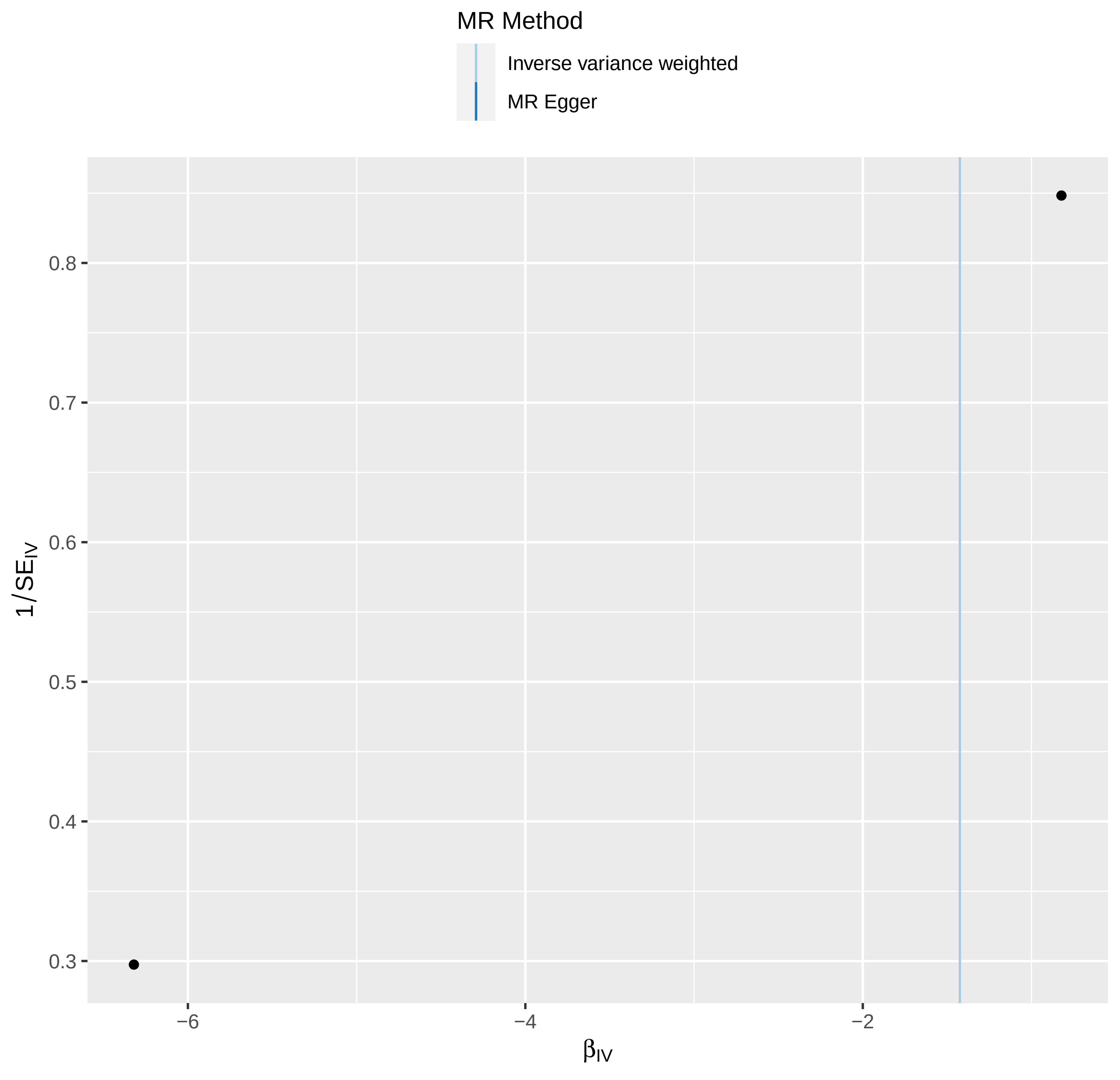

Batch 588 : Alpha−tocopherol on Crohn's disease of small intestine

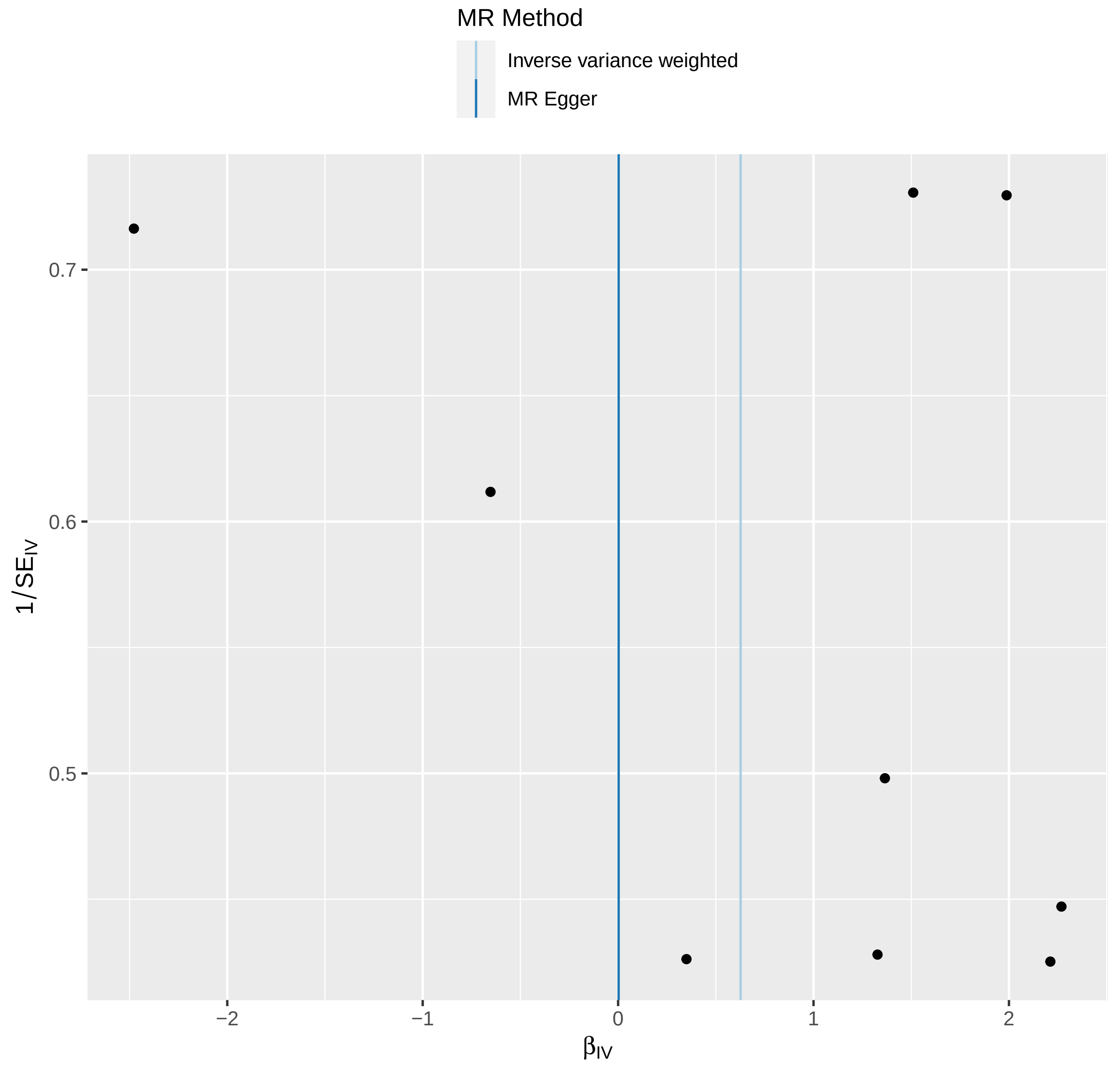

Batch 589 : Citrate on Crohn's disease of small intestine

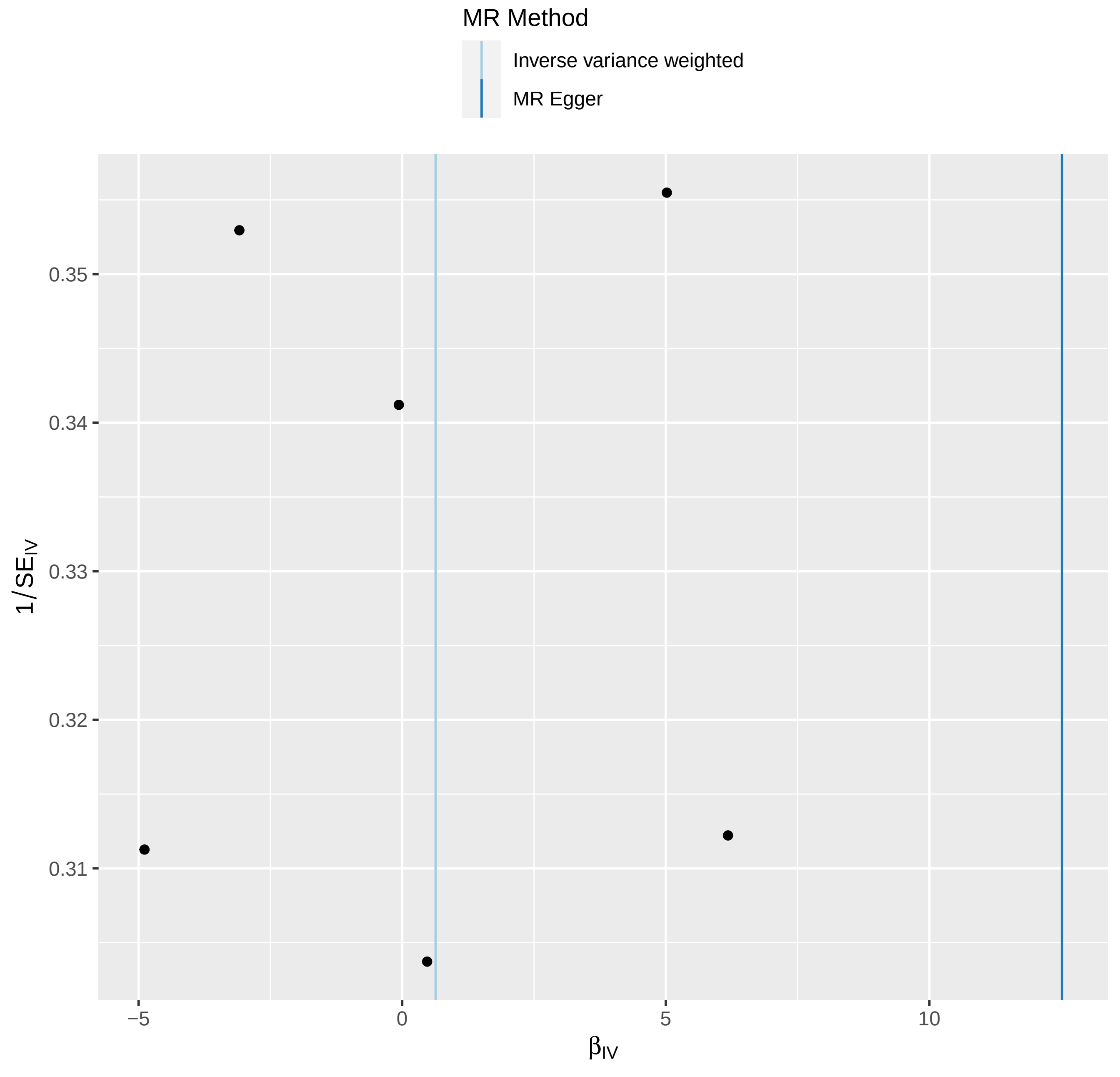

Batch 590 : Glycerate on Crohn's disease of small intestine

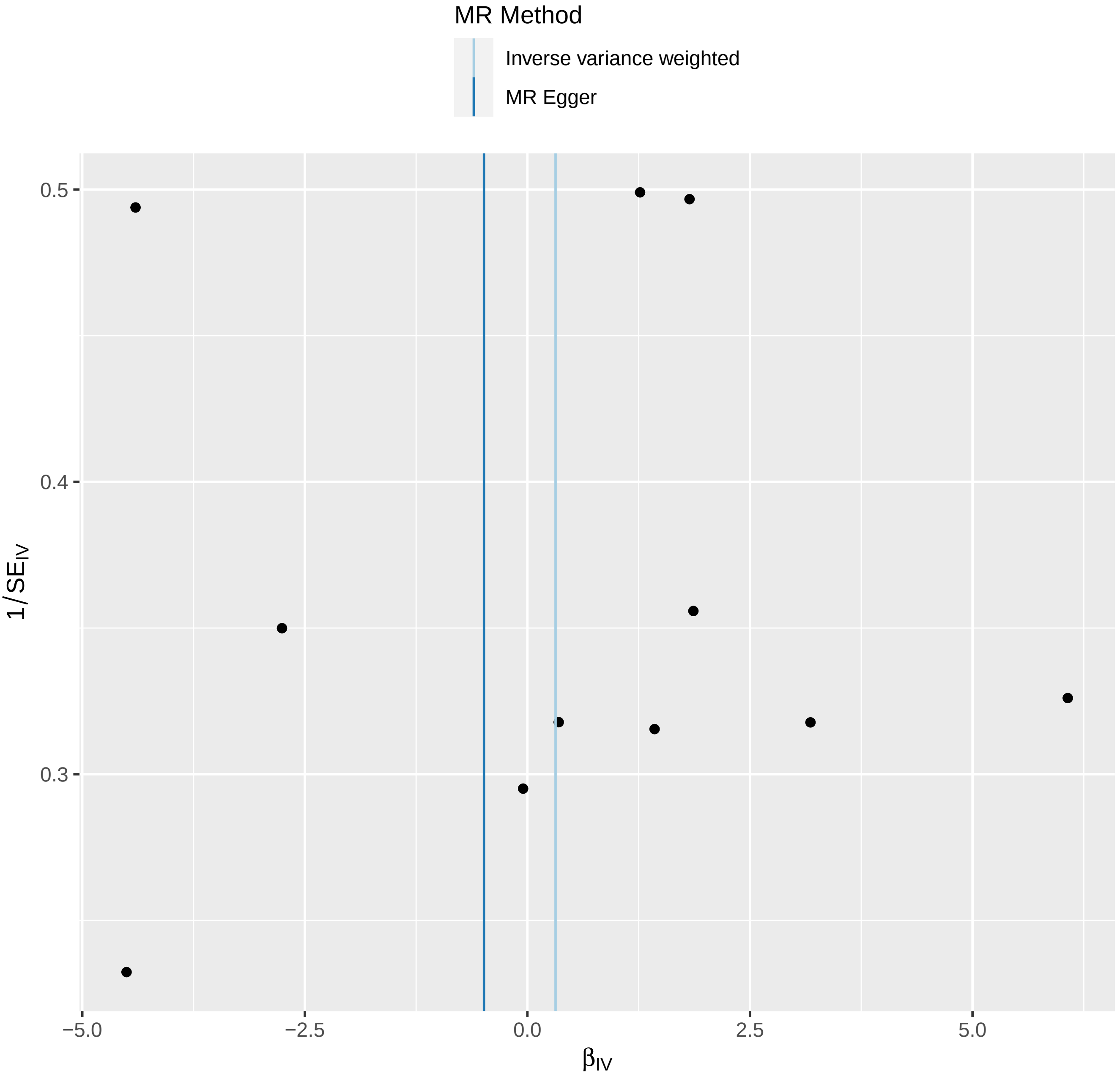

Batch 591 : Guanosine on Crohn's disease of small intestine

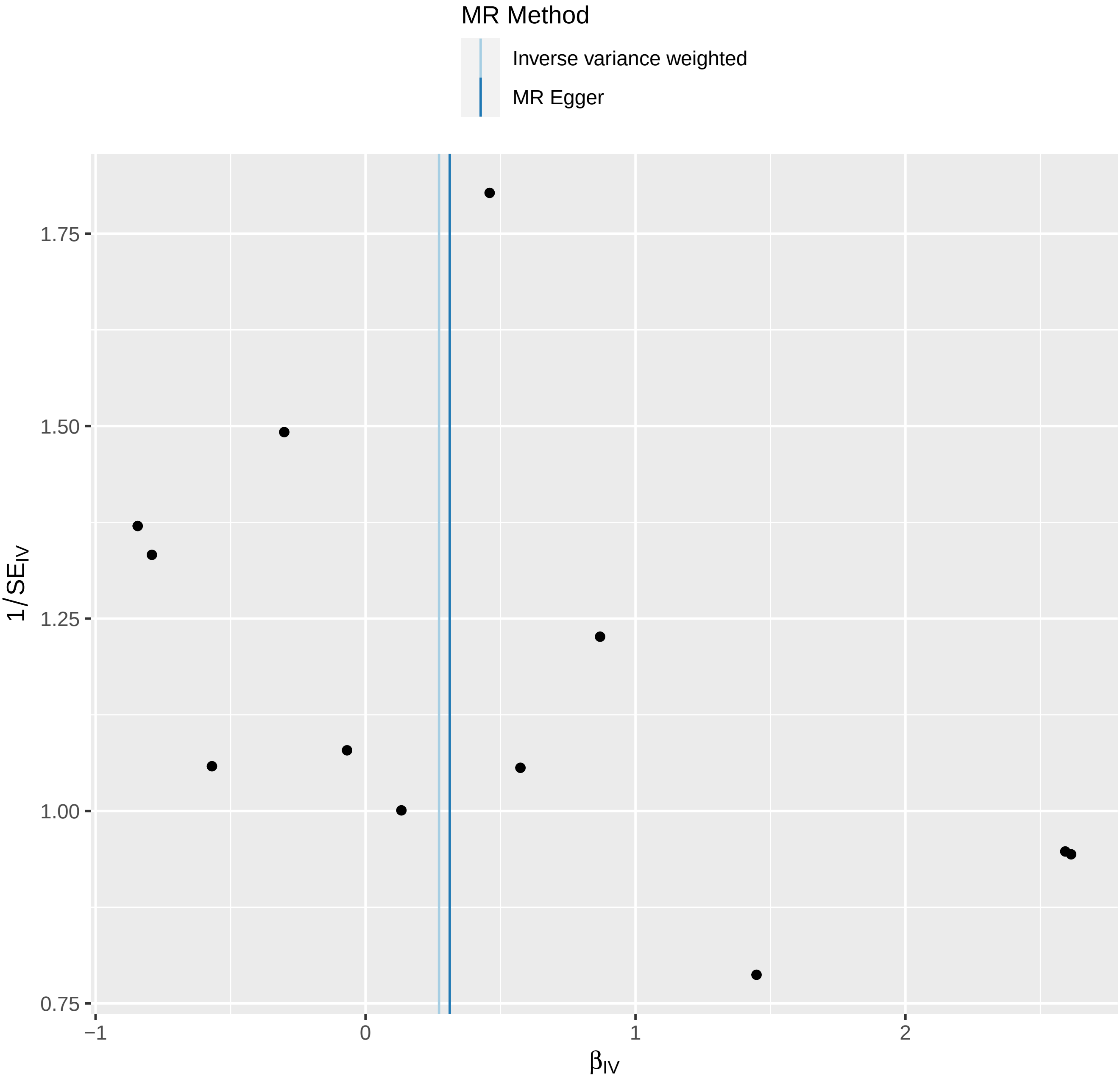

Batch 594 : Ursodeoxycholate on Crohn's disease of small intestine

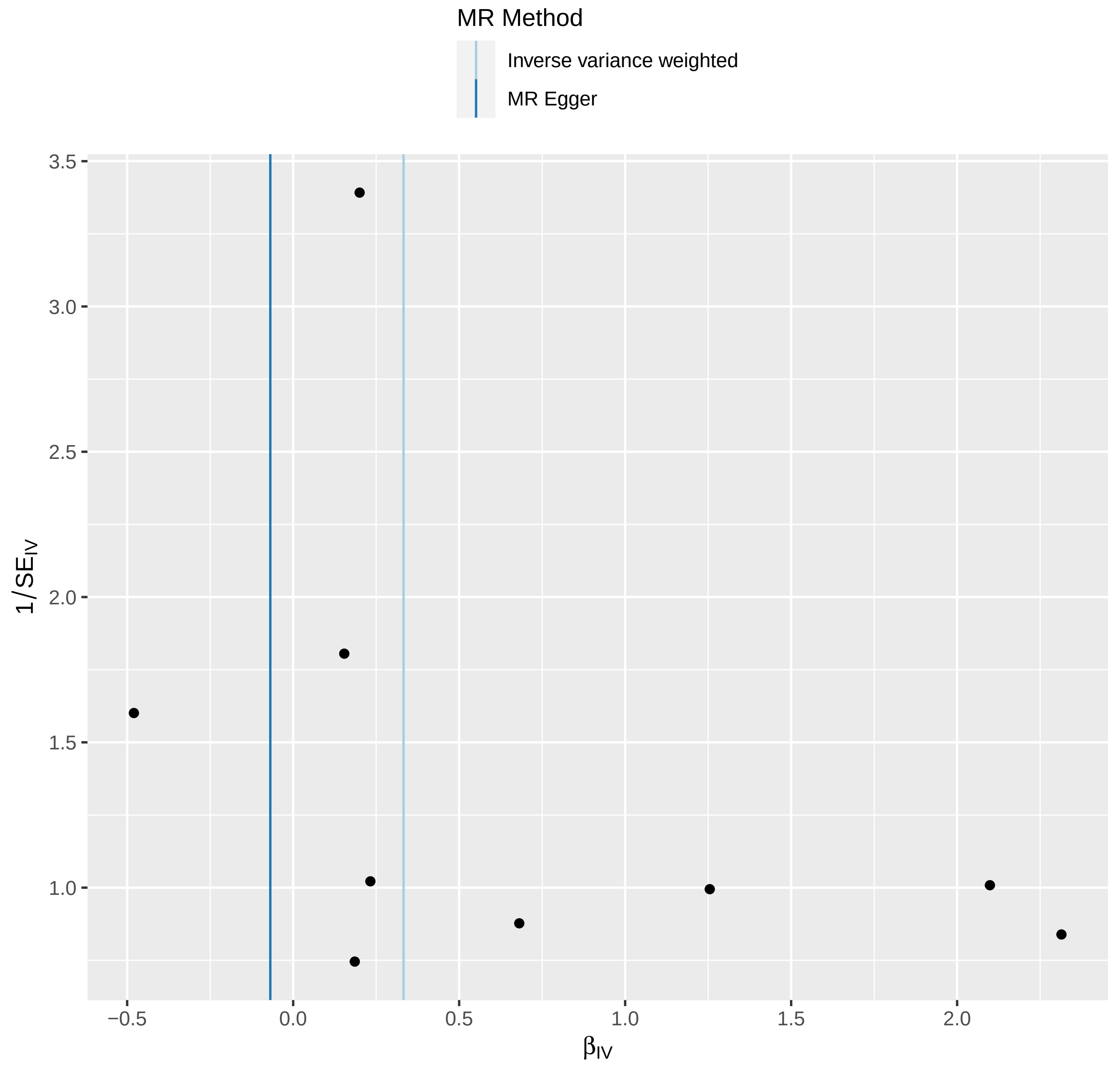

Batch 595 : Arginine on Crohn's disease of small intestine

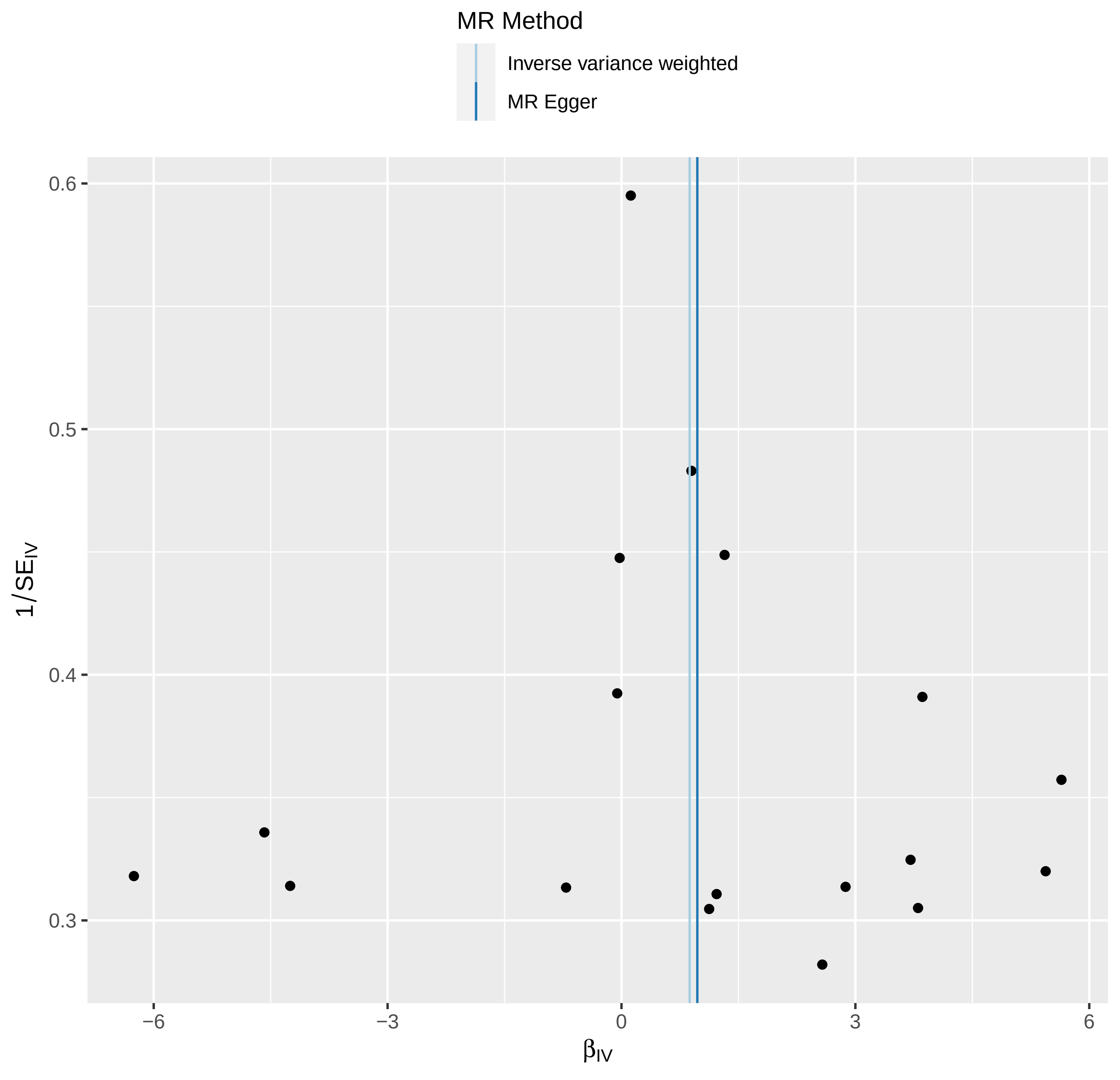

Batch 596 : Ascorbate (Vitamin C) on Crohn's disease of small intestine

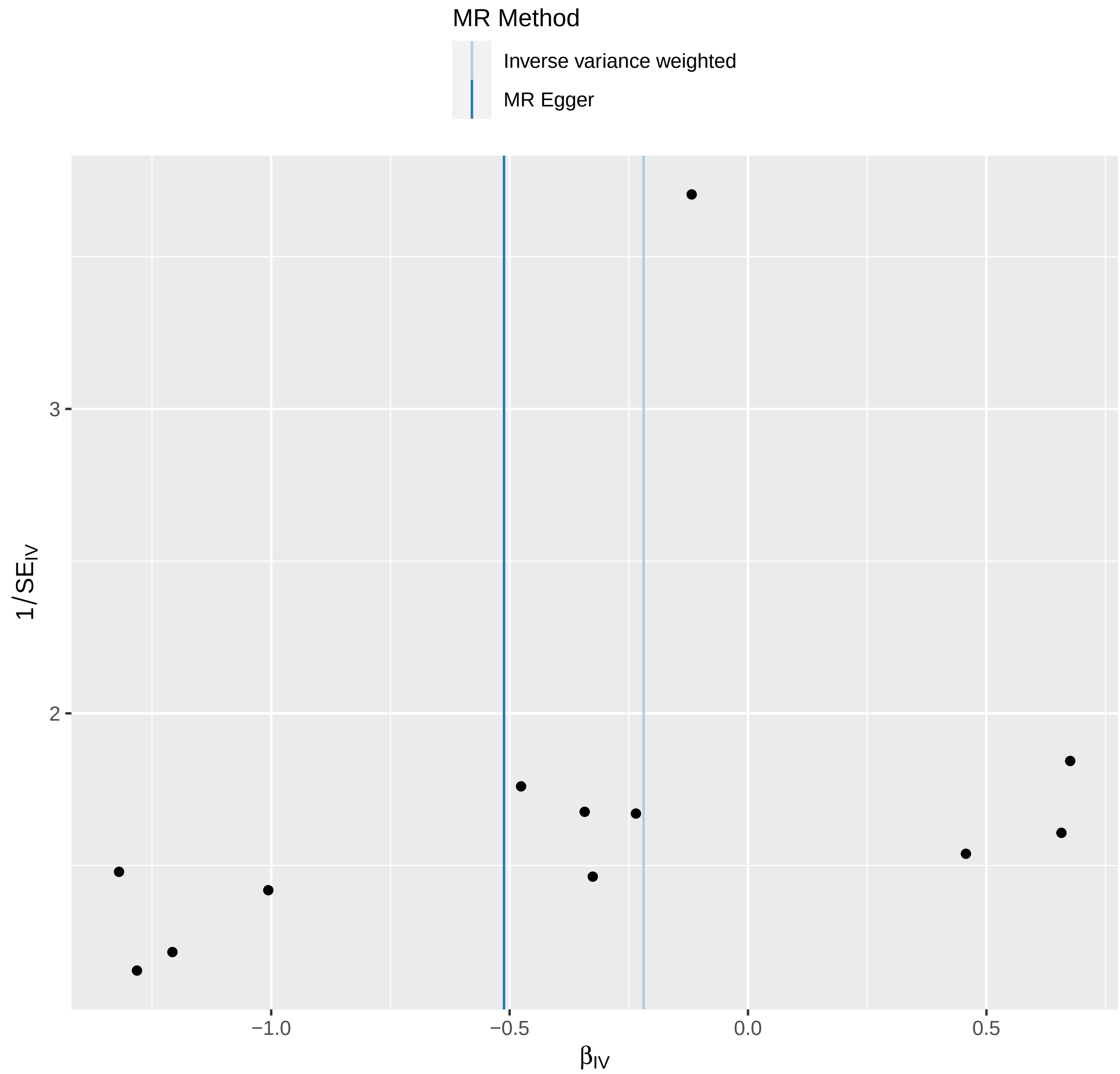

Batch 597 : Heptanoate (7:0) on Crohn's disease of small intestine

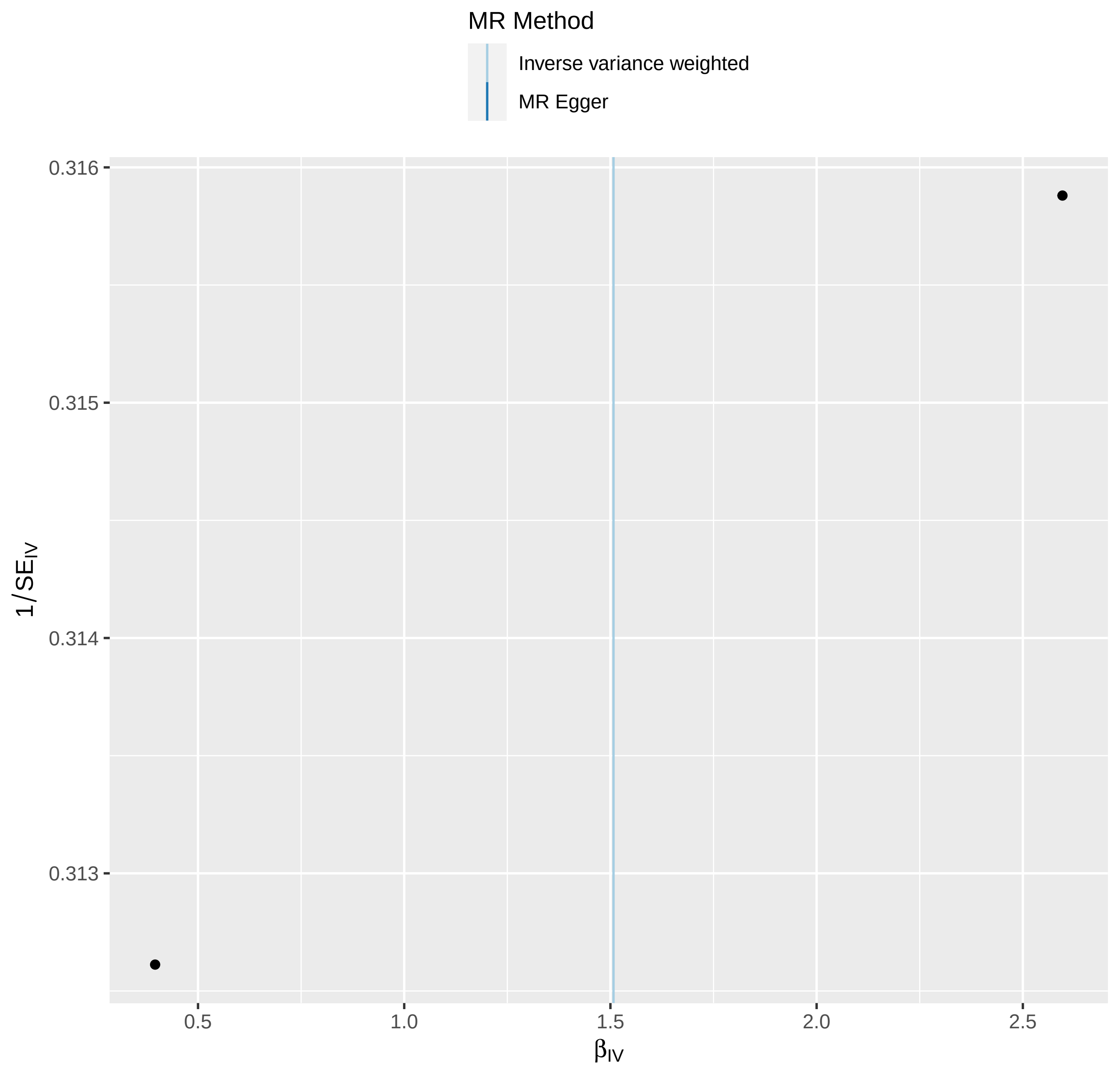

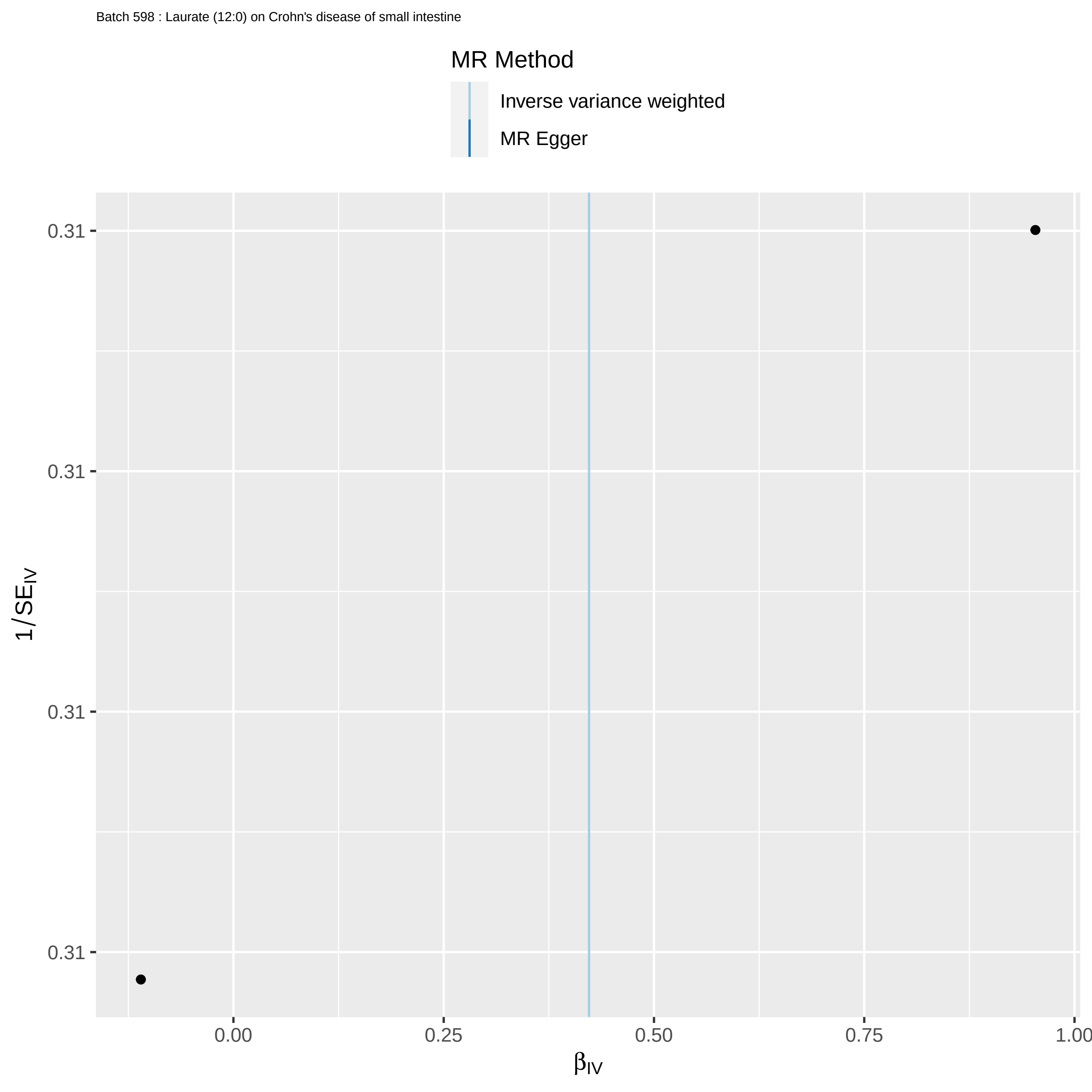
Batch 598 : Laurate (12:0) on Crohn's disease of small intestine
MR Method
Inverse variance weighted
MR Egger
0.31
0.31
0.31
0.31
1/SE_IV
0.00
0.25
0.50
0.75
1.00
β_IV

Batch 600 : Urea on Crohn's disease of small intestine

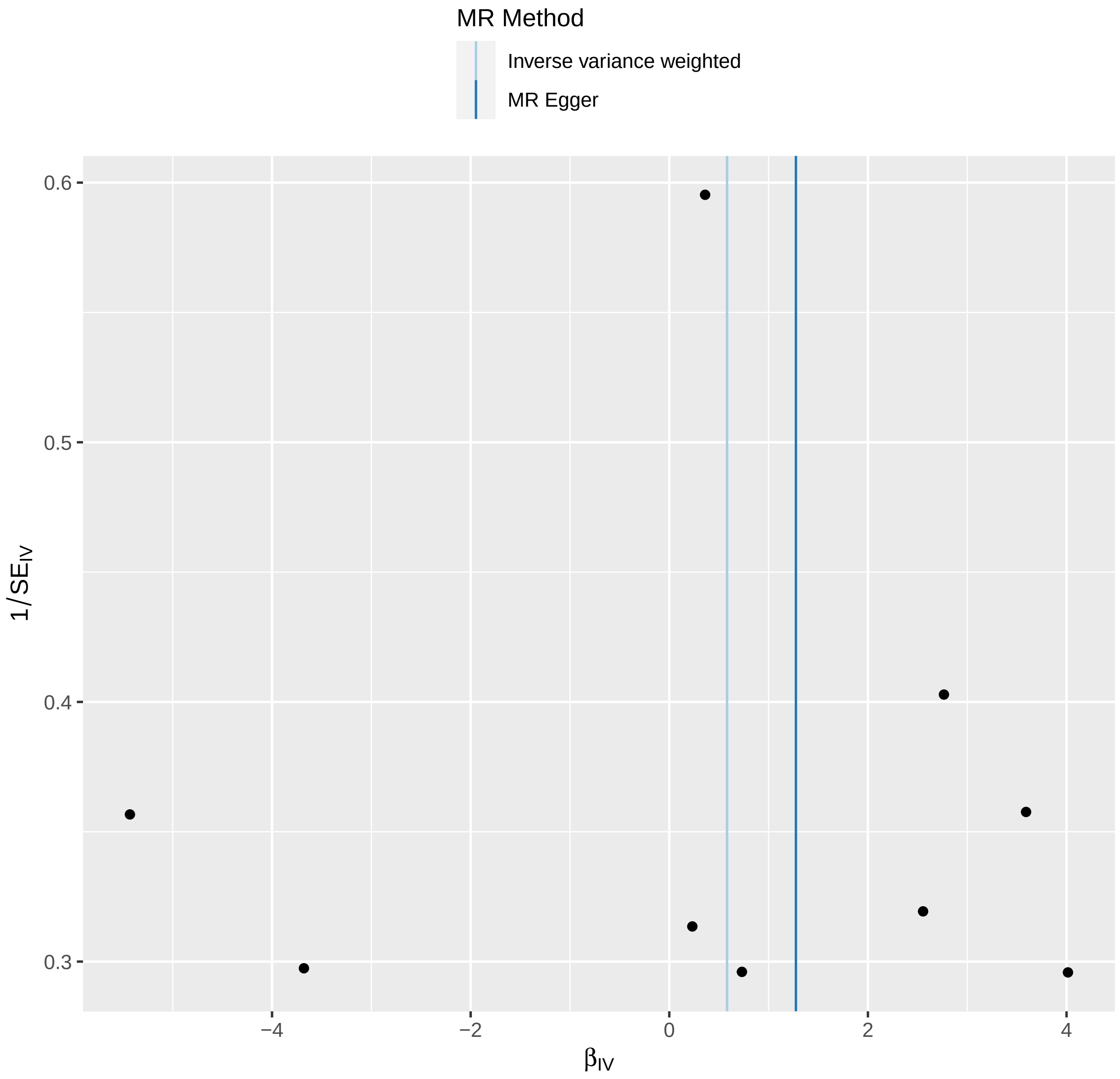

Batch 601 : Cortisol on Crohn's disease of small intestine

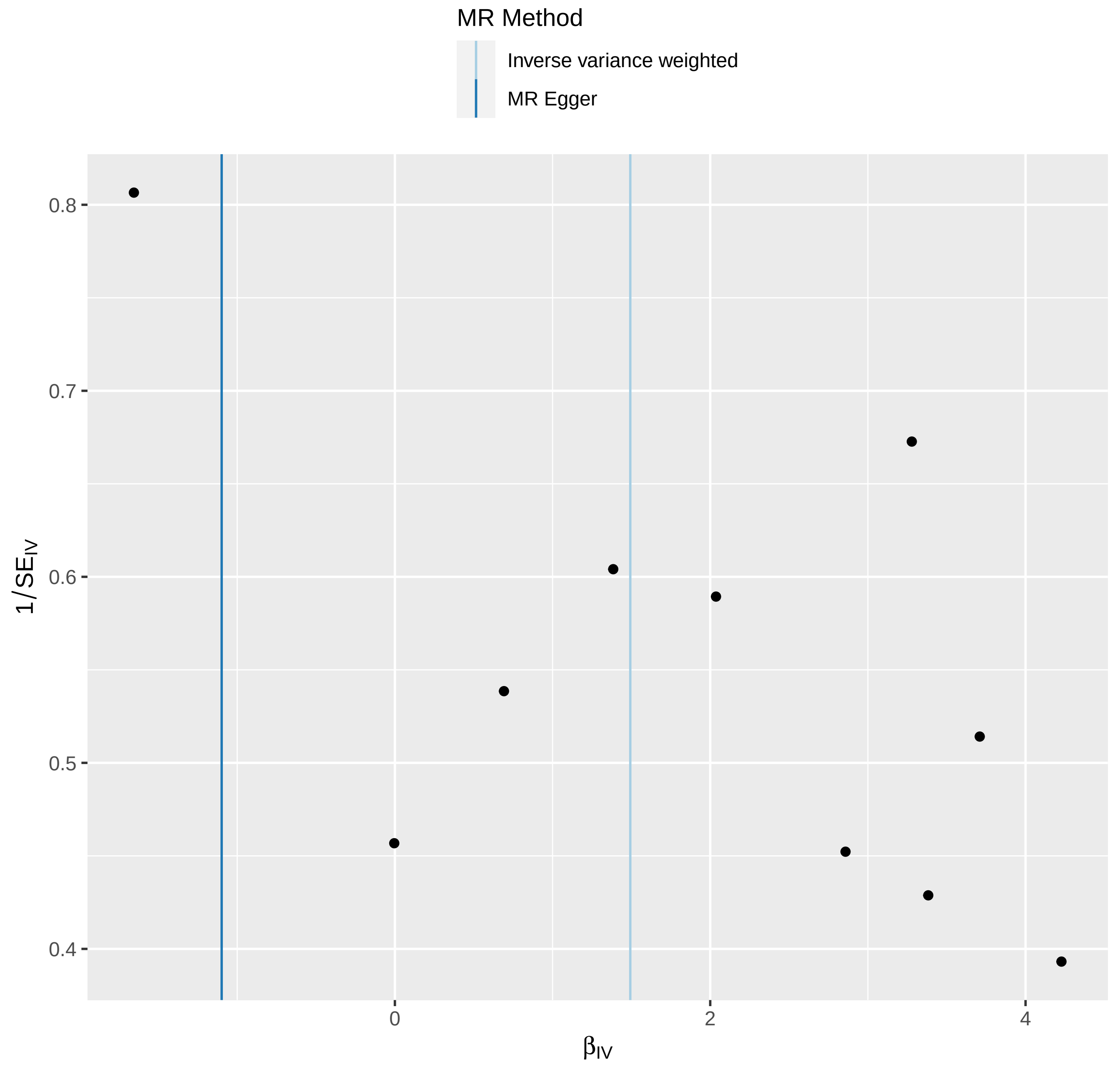

Batch 604 : Citrulline on Crohn's disease of small intestine

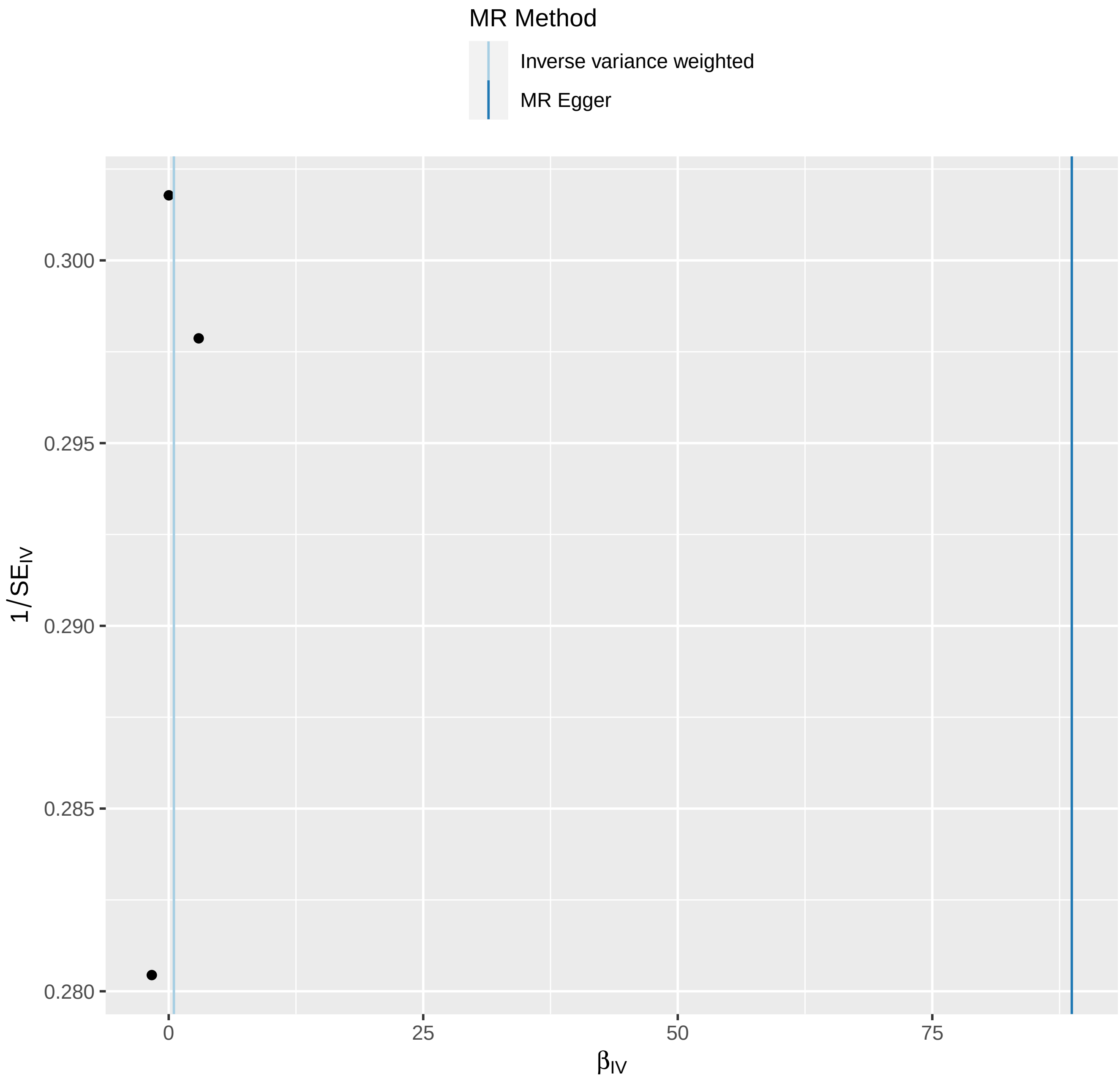

Batch 606 : Serotonin (5HT) on Crohn's disease of small intestine

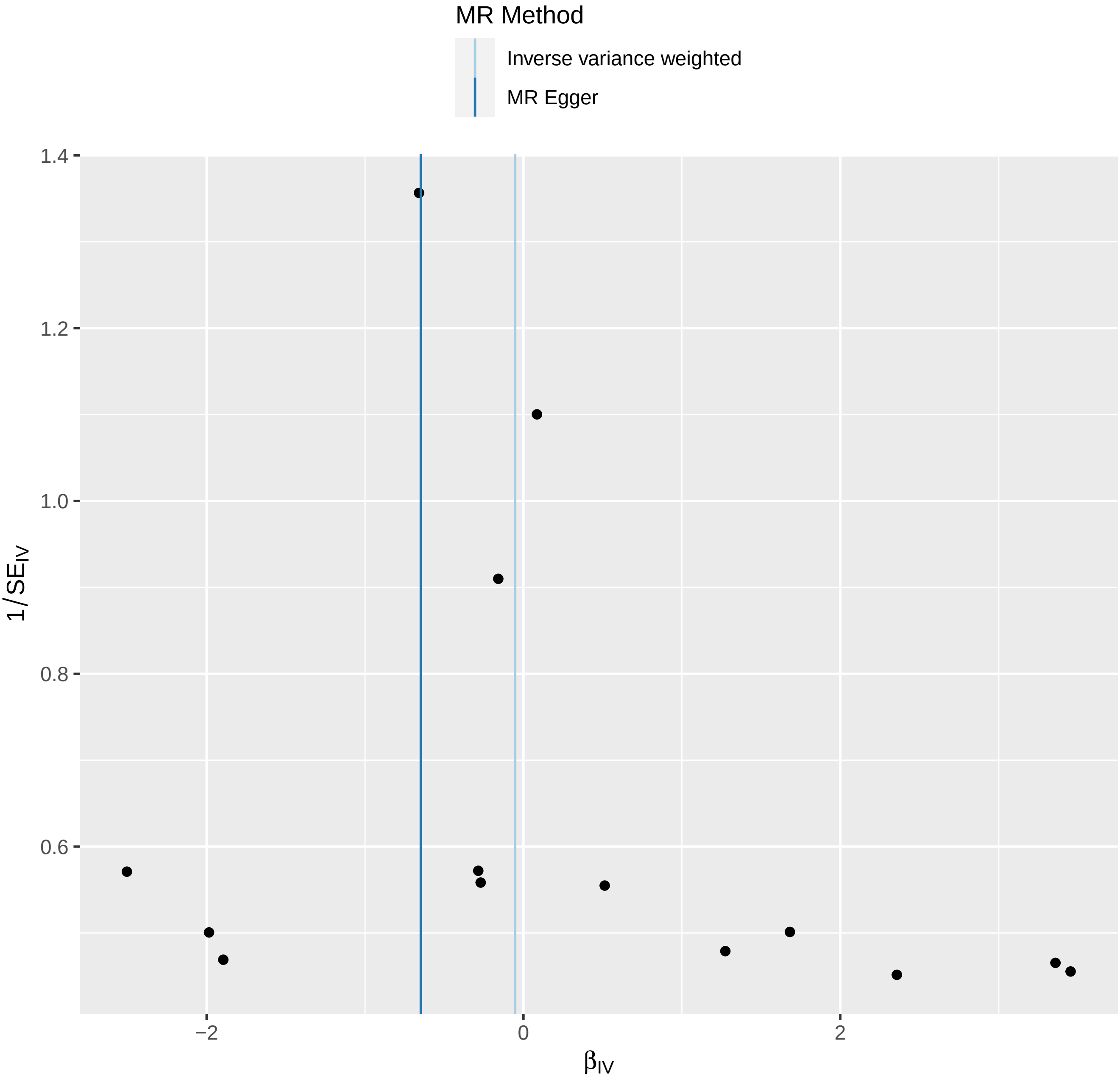

Batch 607 : Gamma−glutamylglutamine on Crohn's disease of small intestine

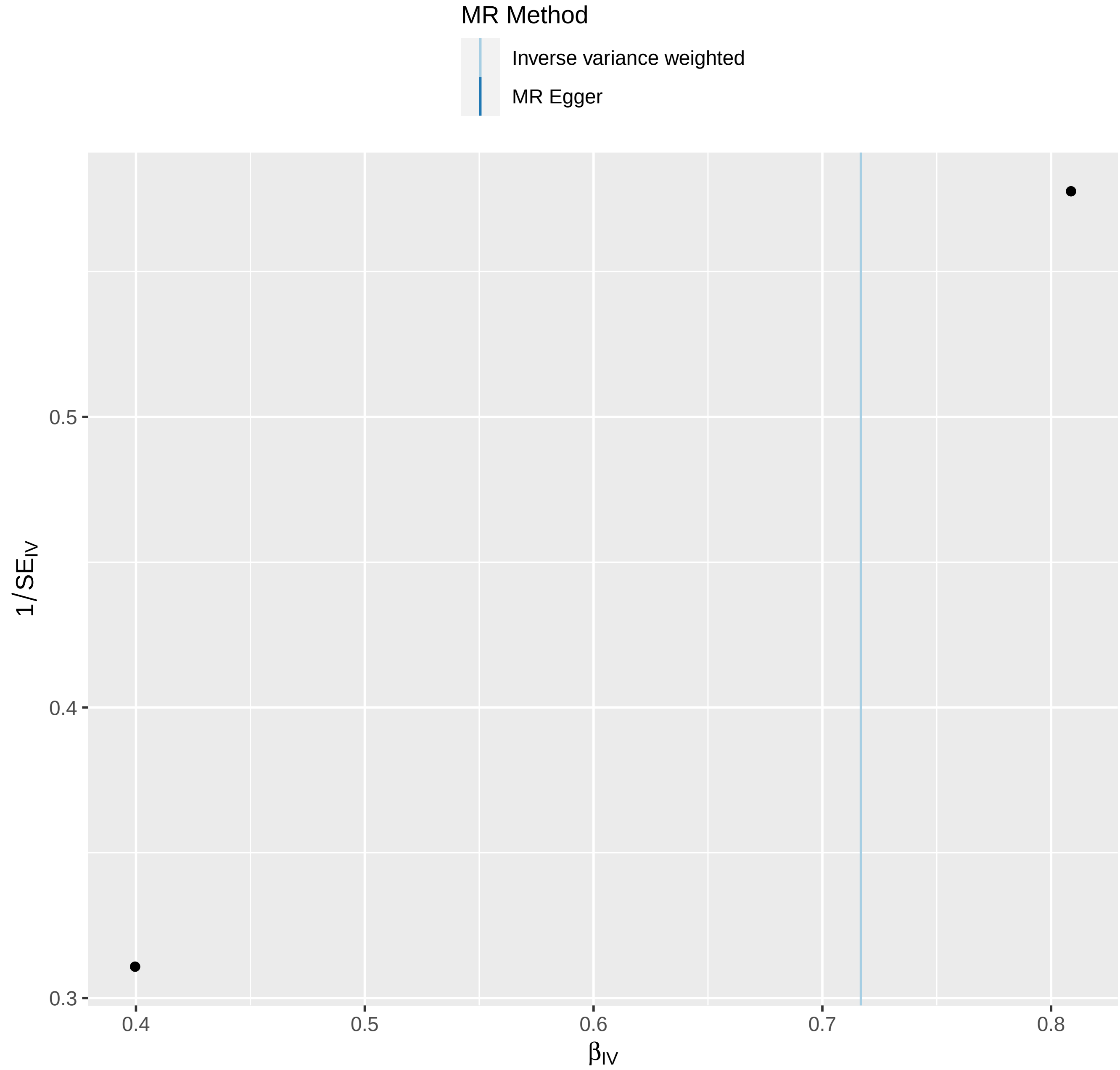

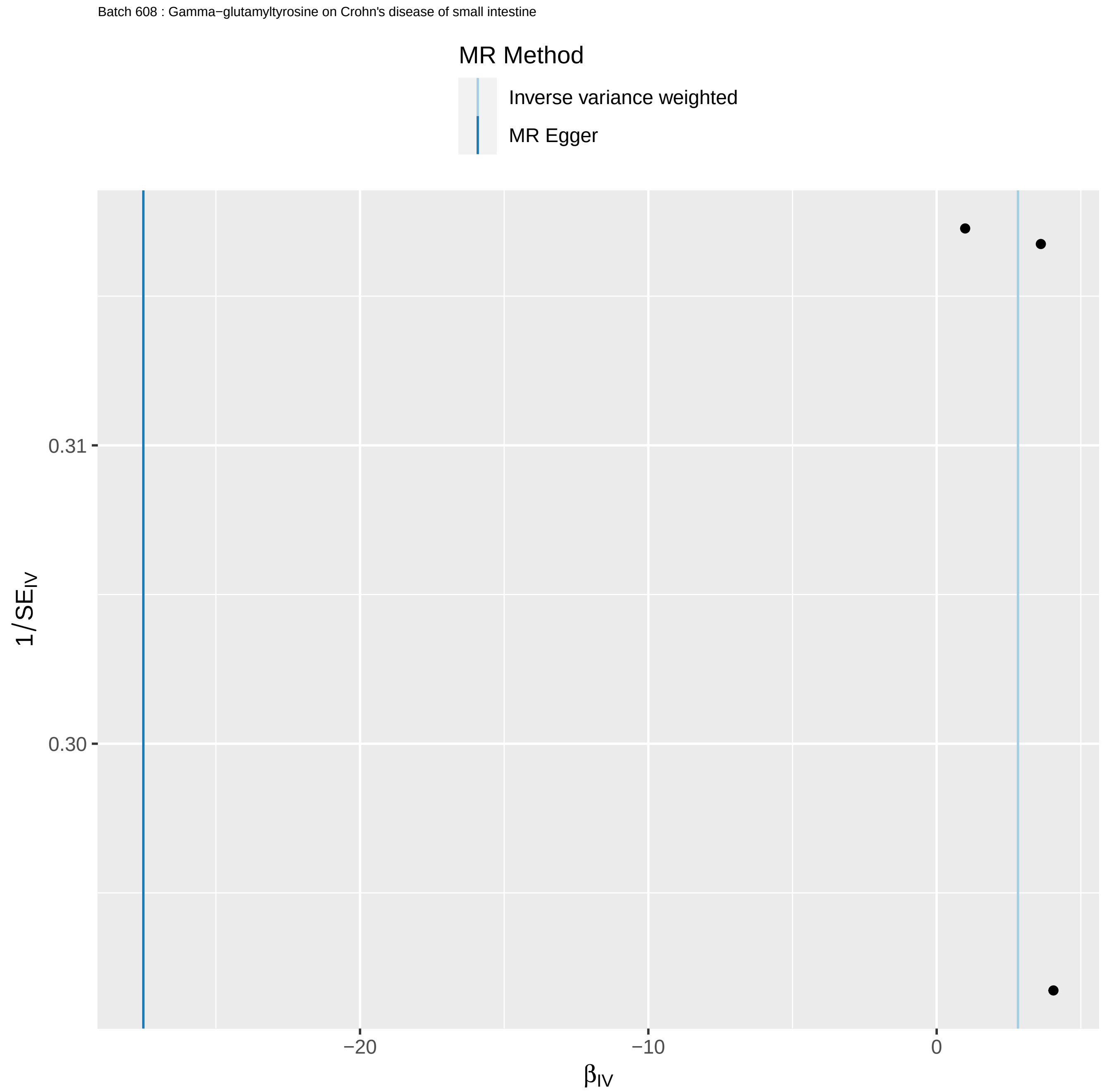
Batch 608 : Gamma−glutamyltyrosine on Crohn's disease of small intestine
MR Method
Inverse variance weighted
MR Egger
0.31
0.30
1/SE_IV
−20
−10
0
β_IV

Batch 610 : Betaine on Crohn's disease of small intestine

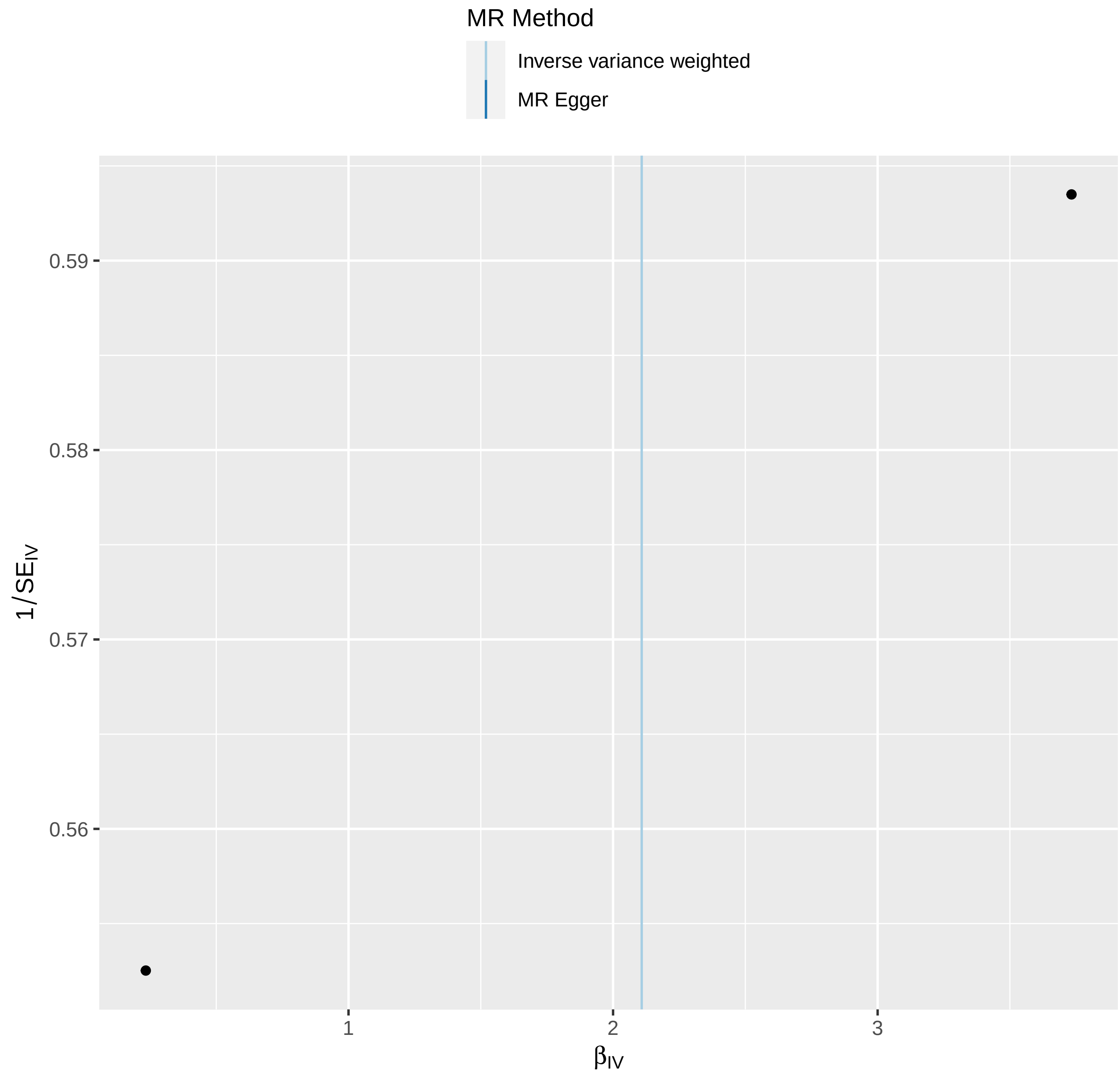

Batch 611 : Xanthine on Crohn's disease of small intestine

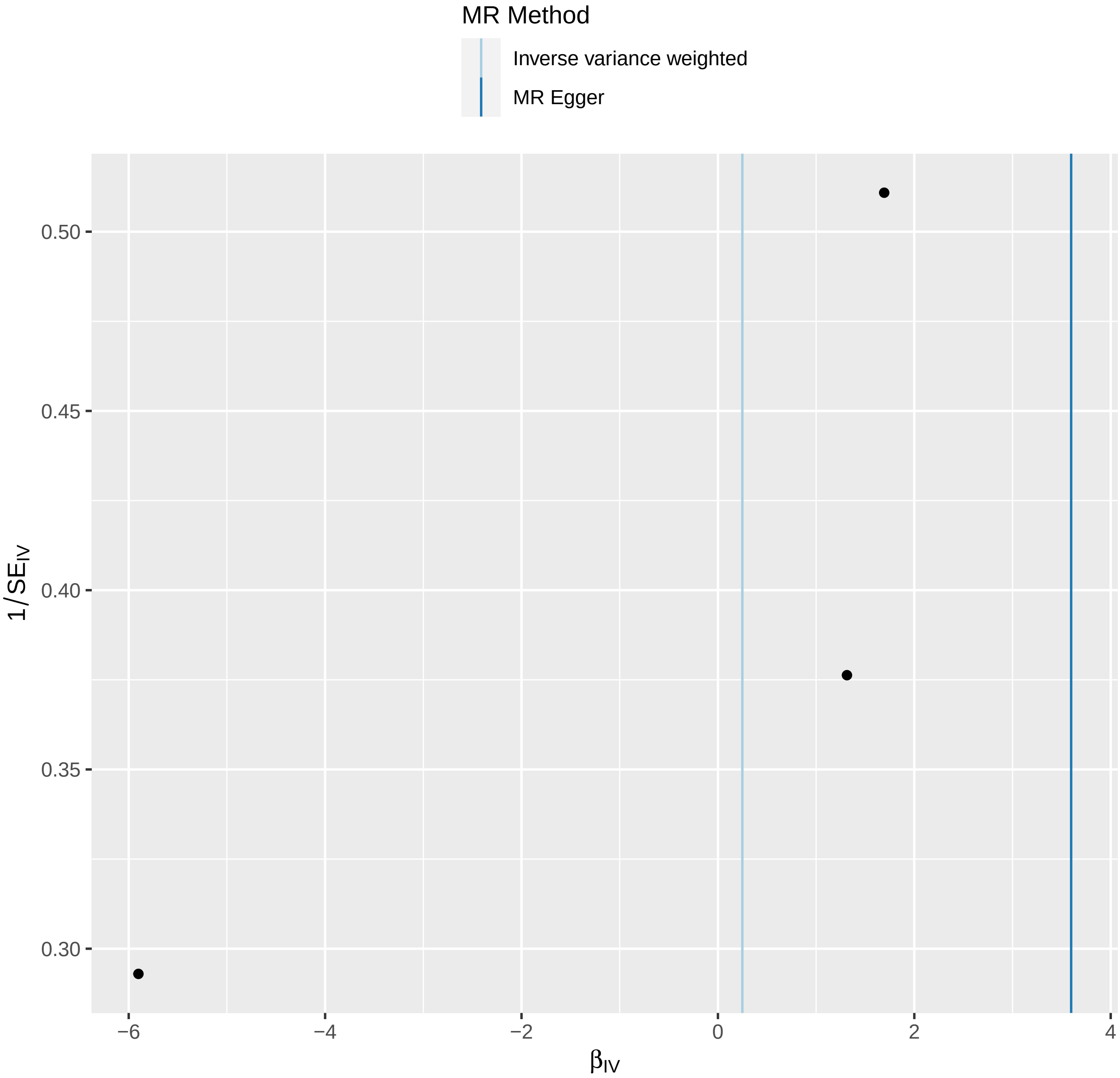

Batch 612 : Phosphate on Crohn's disease of small intestine

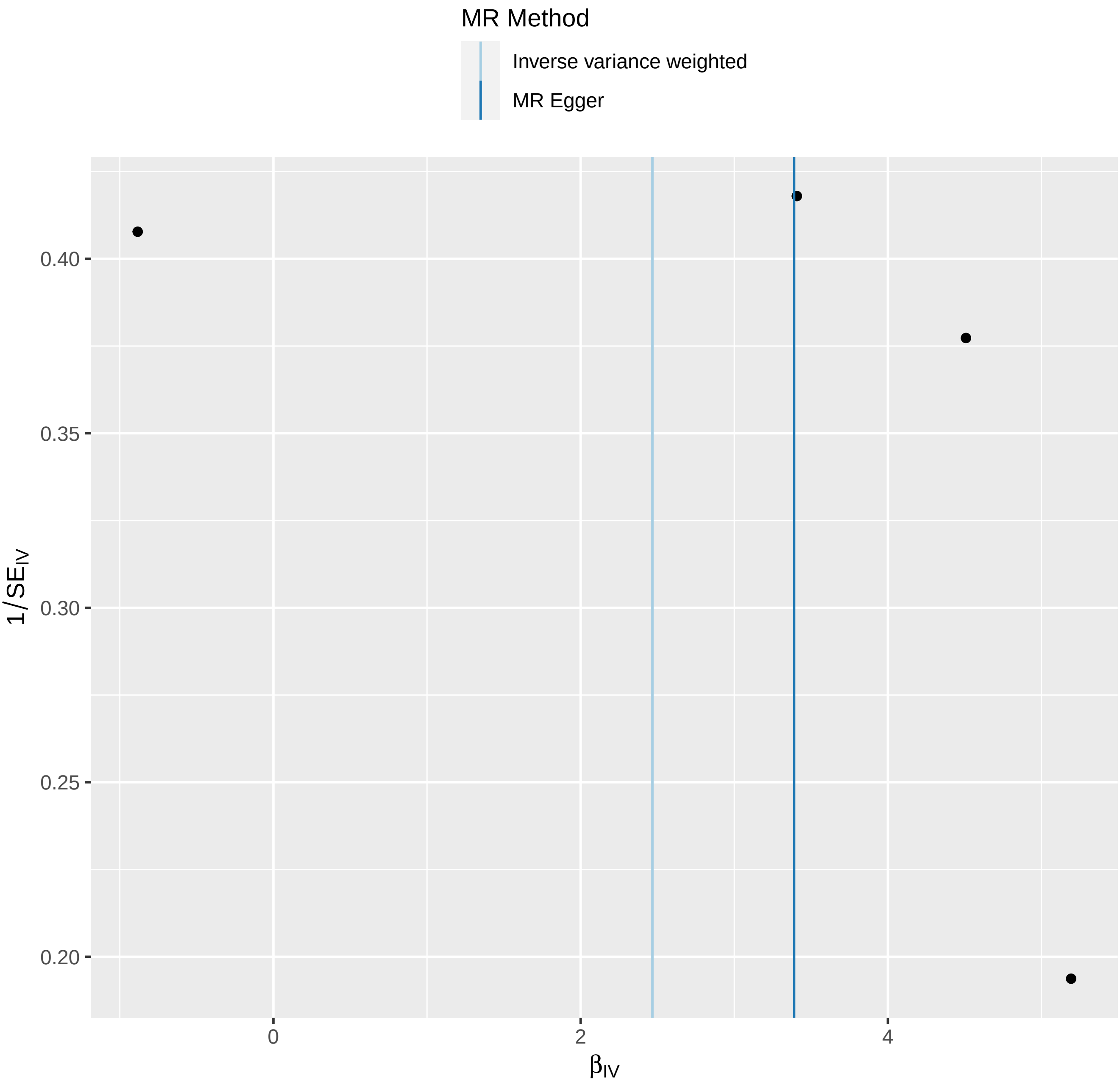

Batch 613 : 3−methoxytyrosine on Crohn's disease of small intestine

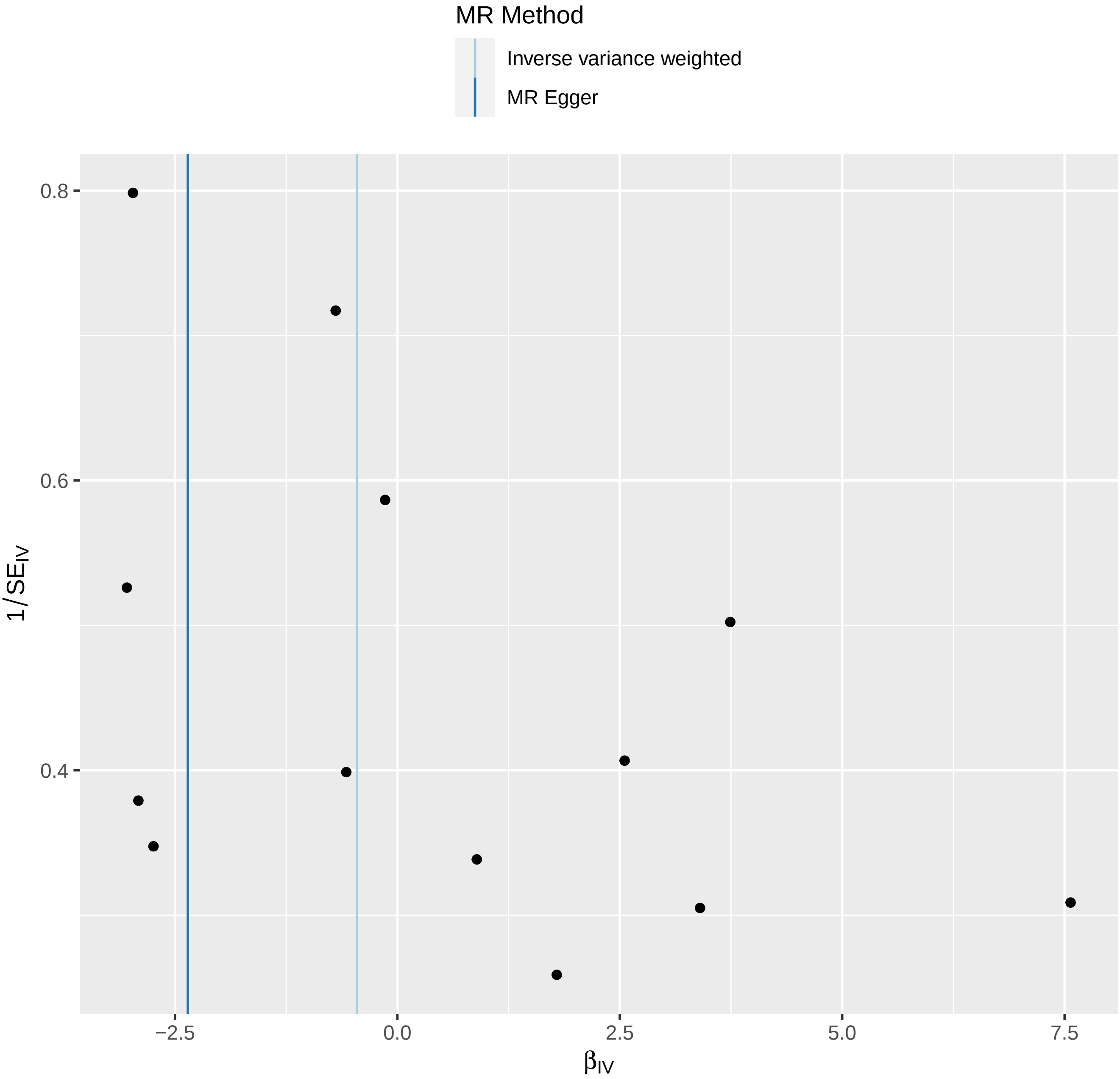

Batch 615 : Undecanoate (11:0) on Crohn's disease of small intestine

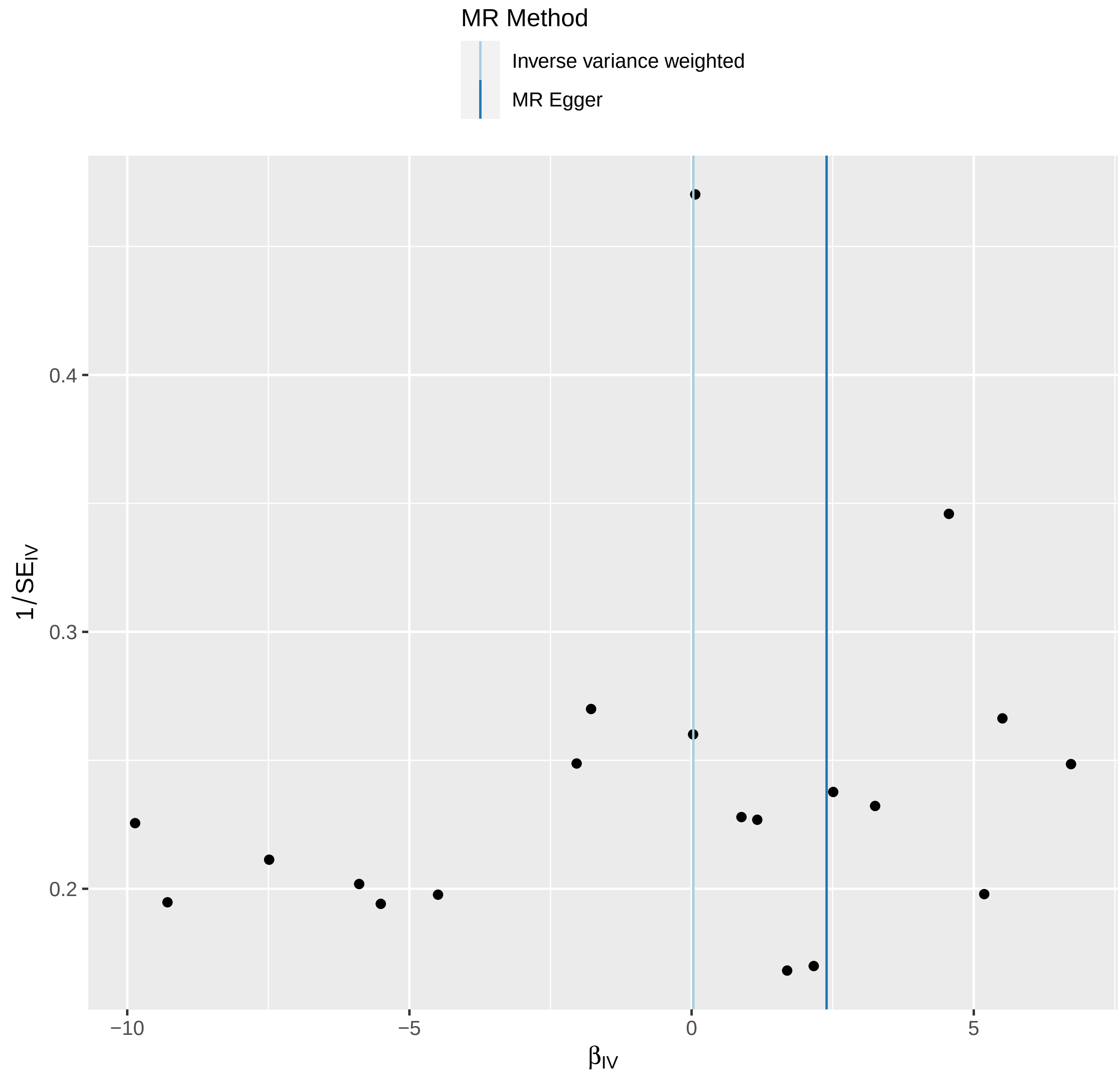

Batch 617 : Taurodeoxycholate on Crohn's disease of small intestine

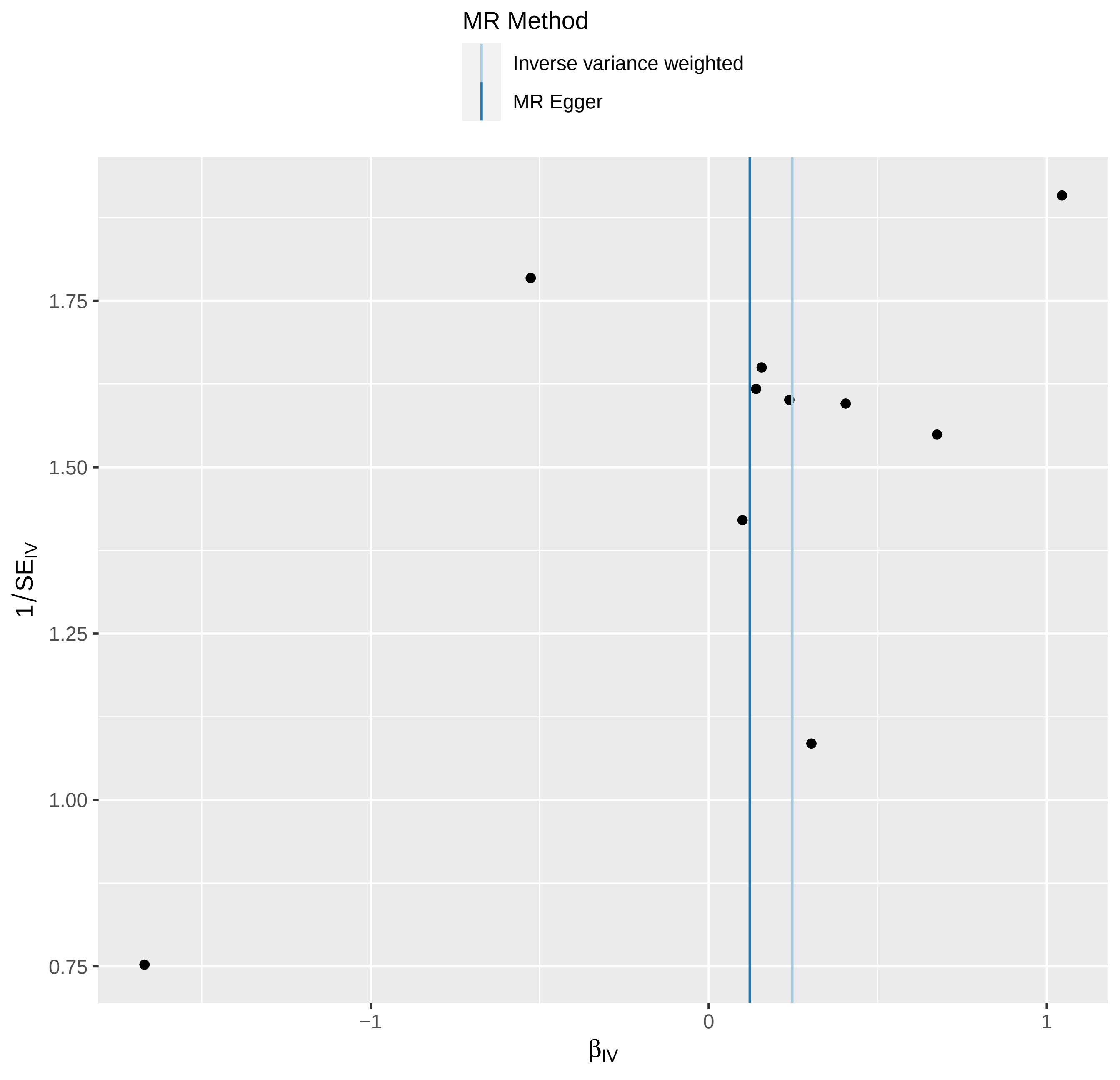

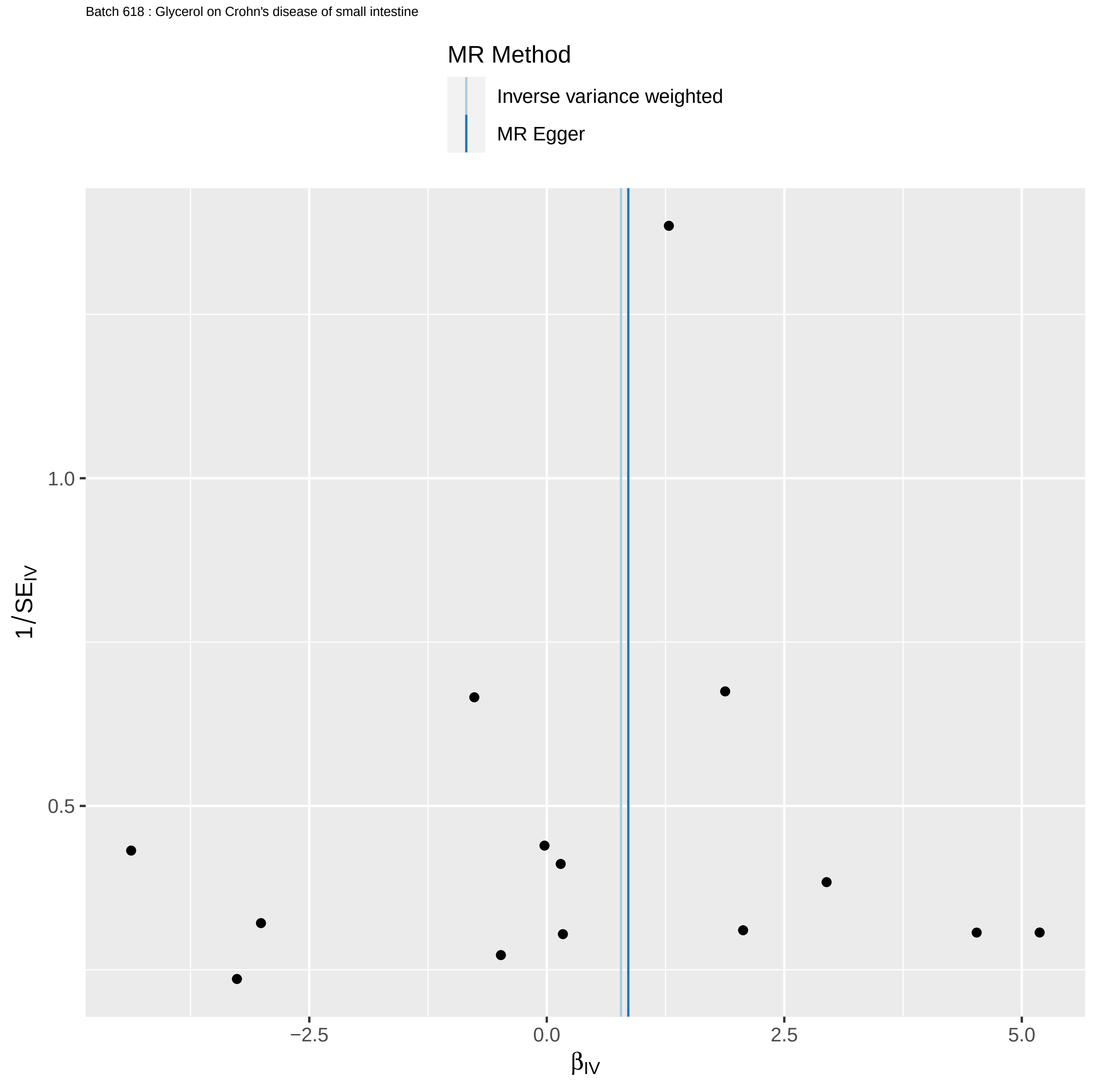

Batch 618 : Glycerol on Crohn's disease of small intestine
MR Method
Inverse variance weighted
MR Egger
1.0
0.5
1/SE_IV
−2.5
0.0
2.5
5.0
β_IV

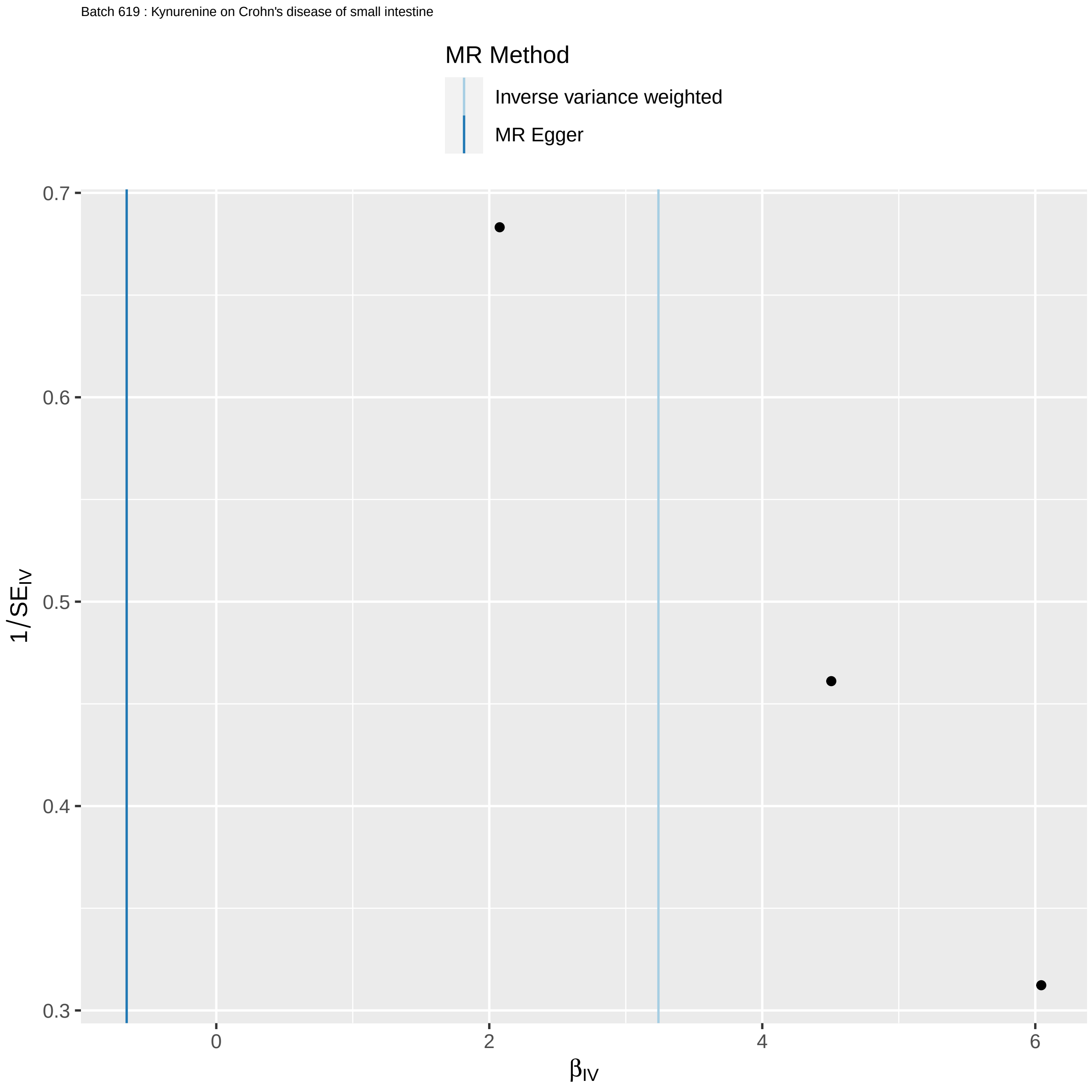
Batch 619 : Kynurenine on Crohn's disease of small intestine
MR Method
Inverse variance weighted
MR Egger
0.7
0.6
0.5
0.4
0.3
1/SE_IV
0
2
4
6
β_IV

Batch 620 : Mannitol on Crohn's disease of small intestine

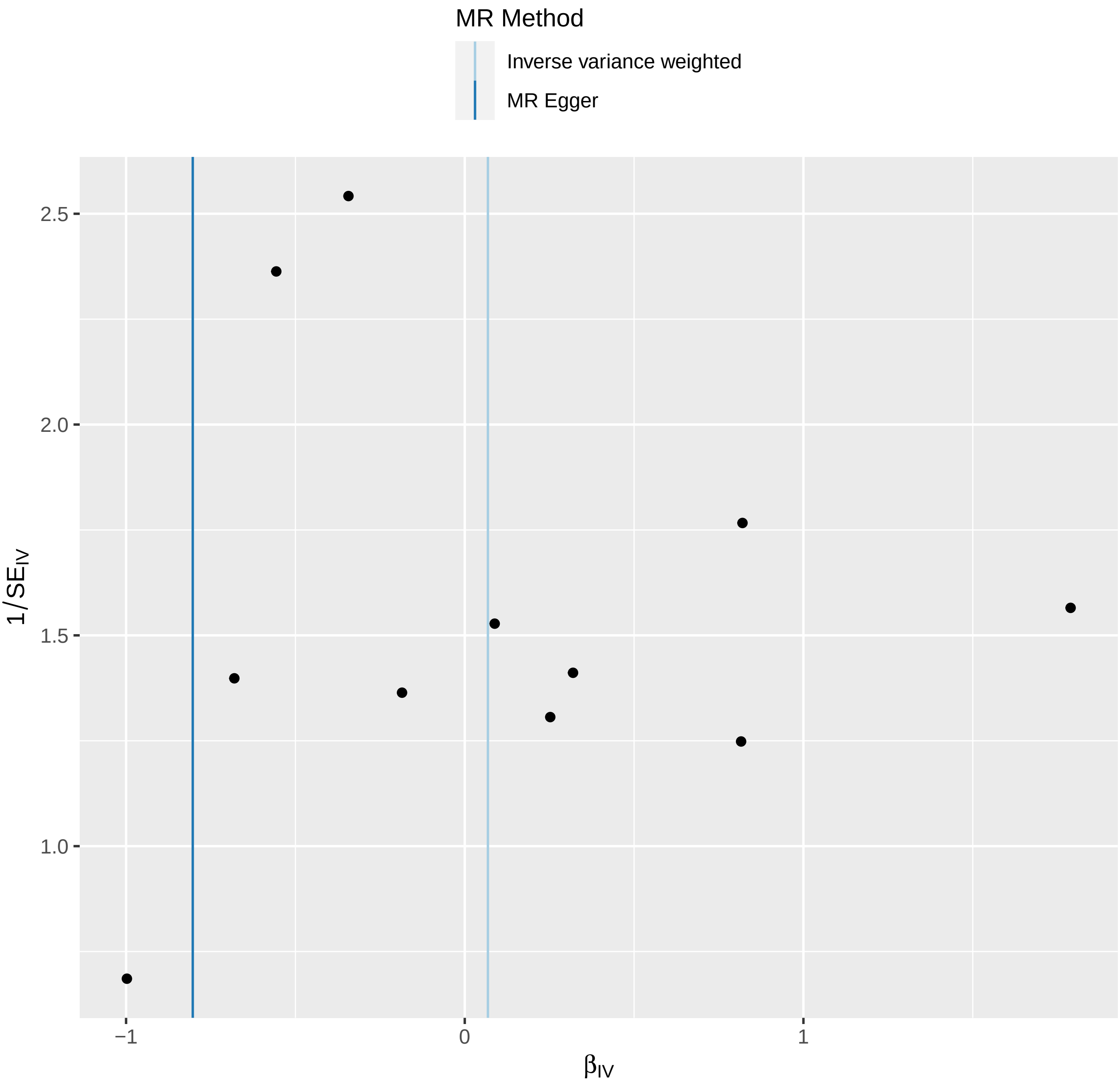

Batch 621 : Glycerol 3−phosphate (G3P) on Crohn's disease of small intestine

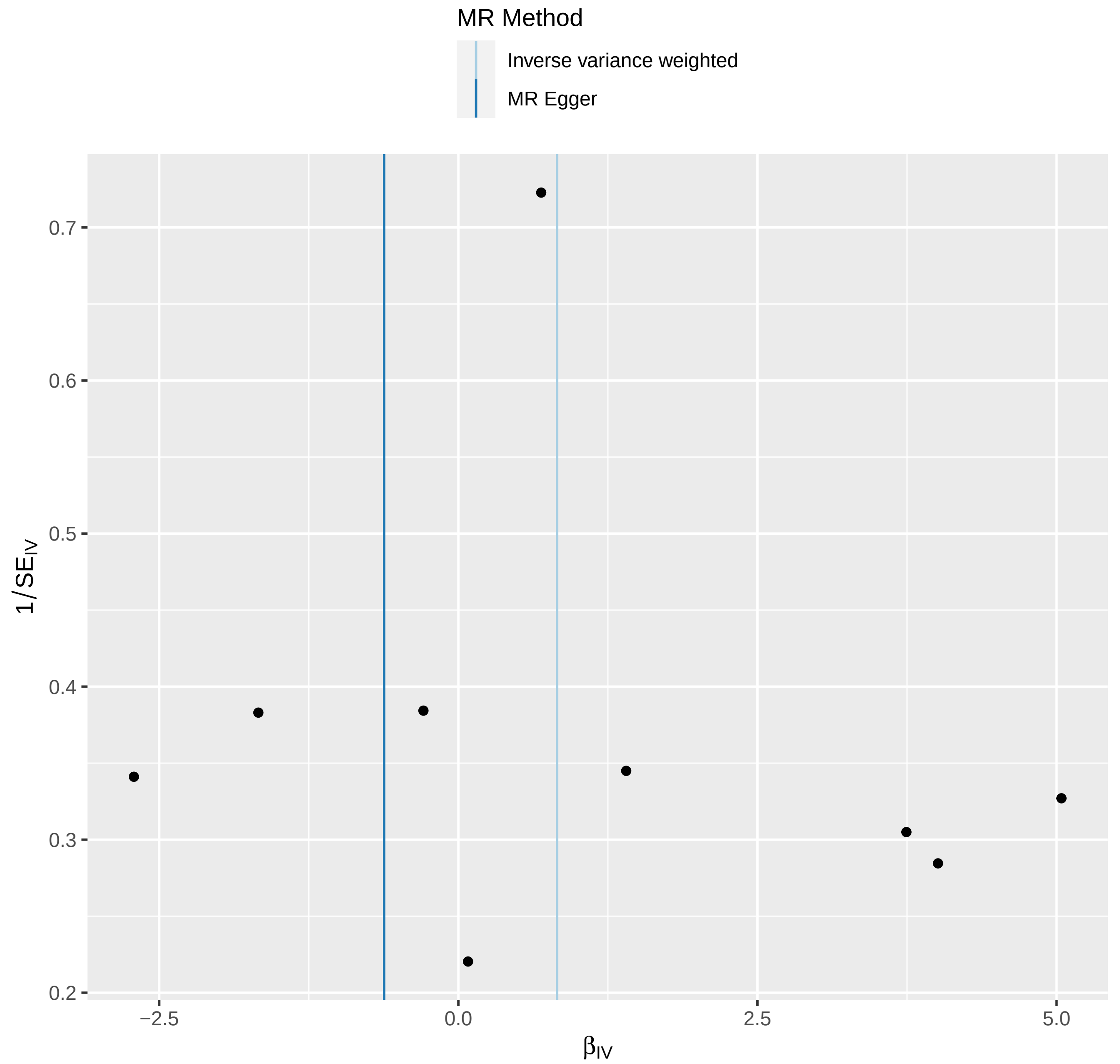

Batch 623 : Carnitine on Crohn's disease of small intestine

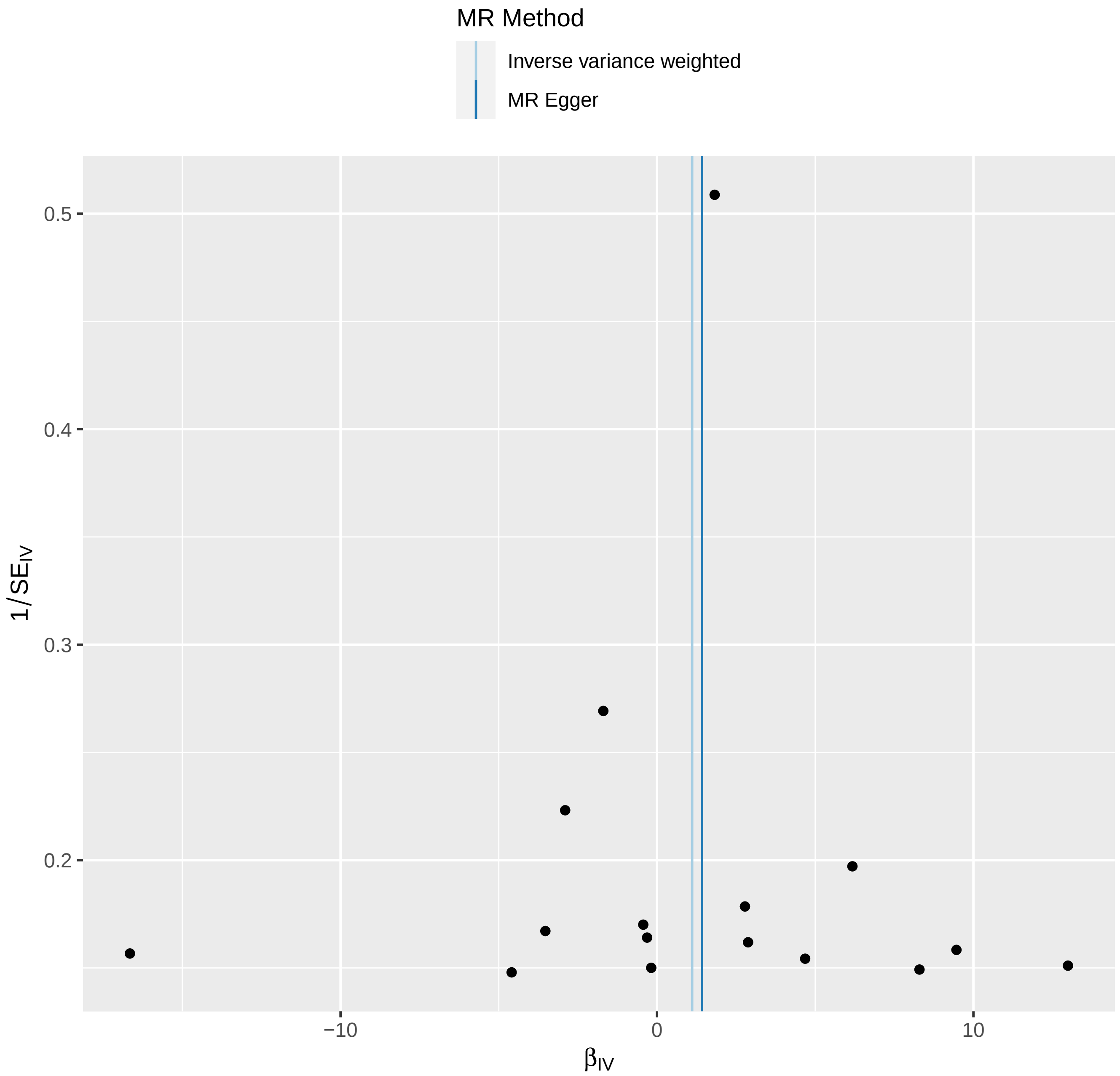

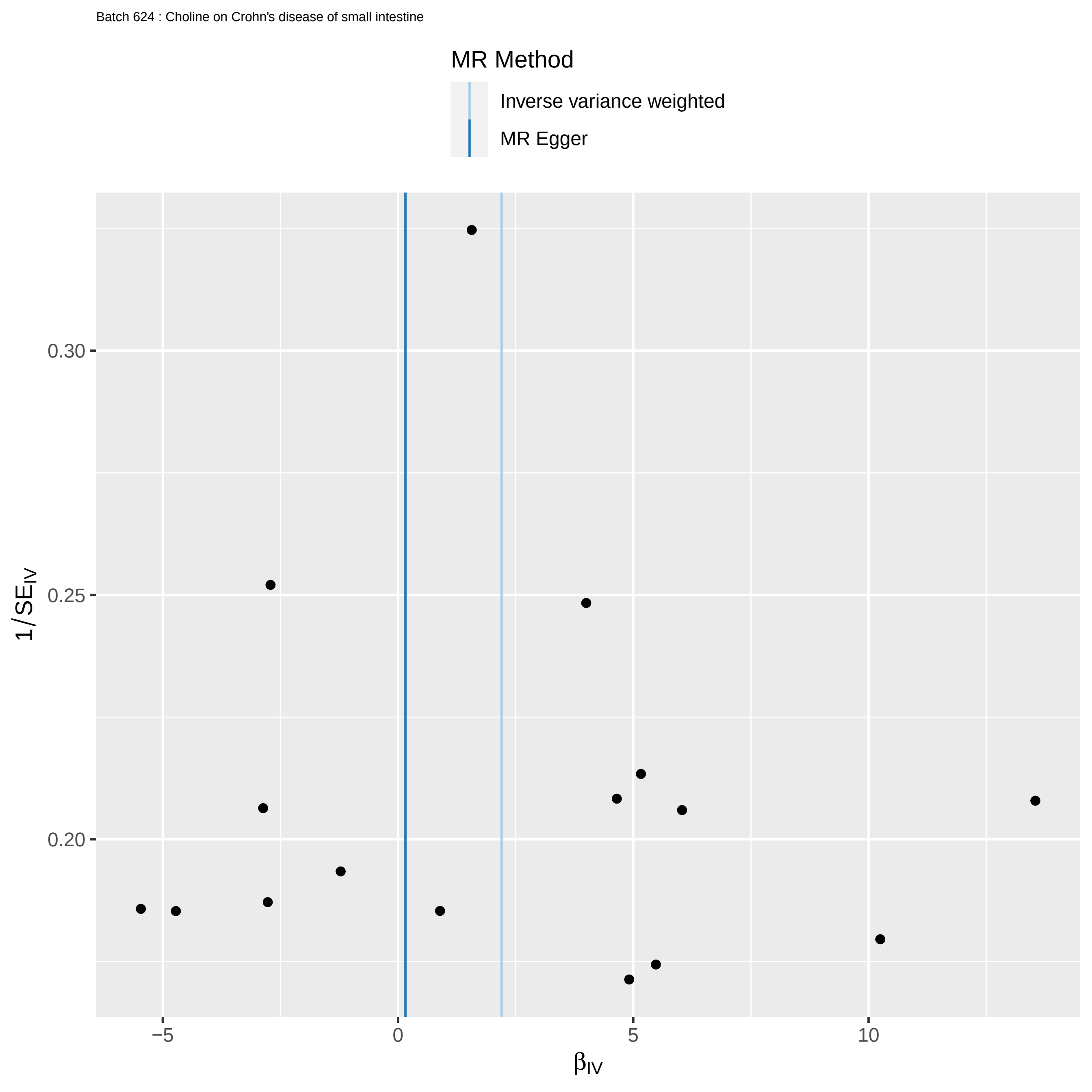

Batch 624 : Choline on Crohn's disease of small intestine
MR Method
Inverse variance weighted
MR Egger
0.30
0.25
0.20
1/SE_IV
−5
0
5
10
β_IV

Batch 627 : 3−methyl−2−oxovalerate on Crohn's disease of small intestine

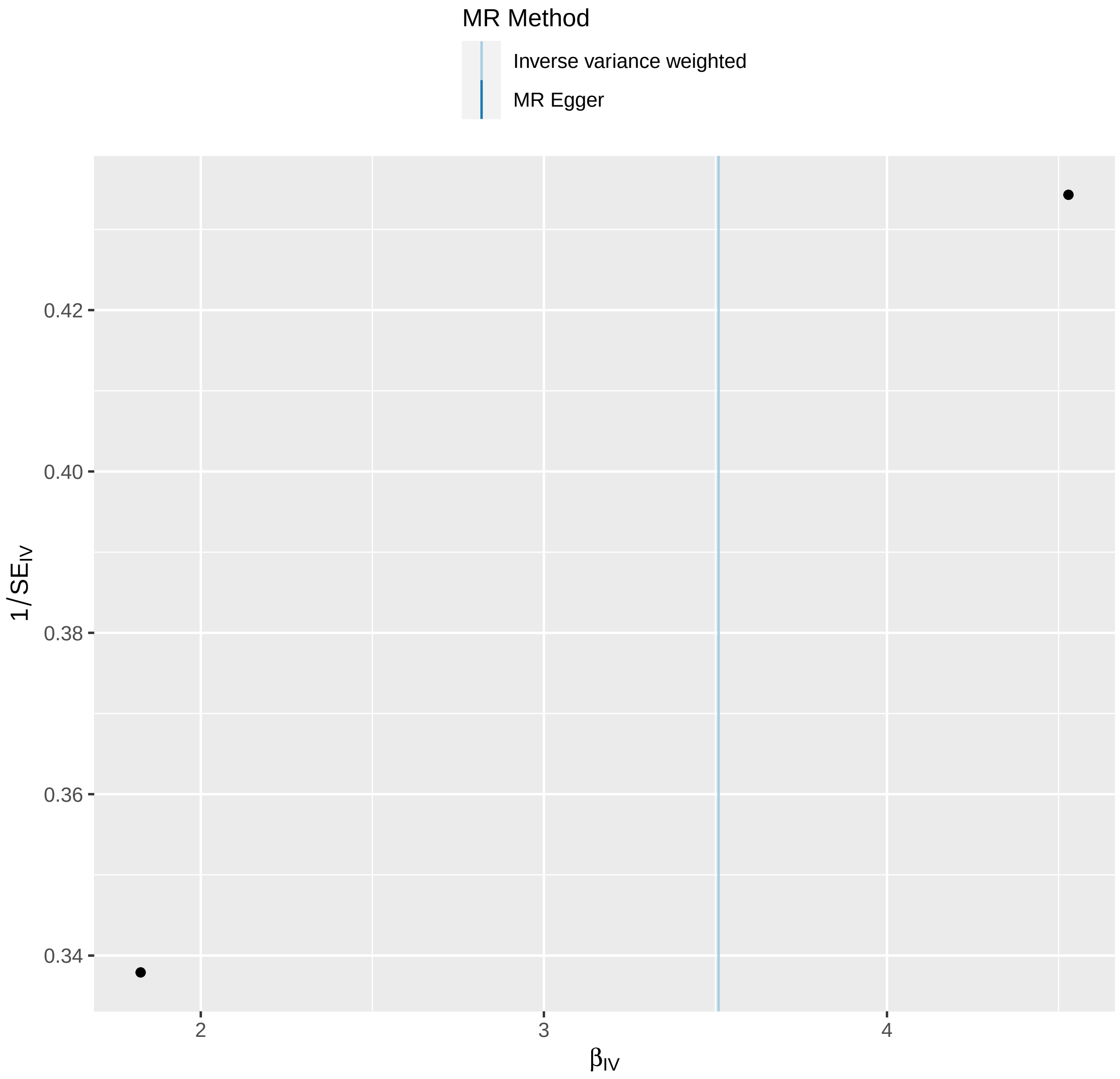

Batch 628 : 3−methylhistidine on Crohn's disease of small intestine

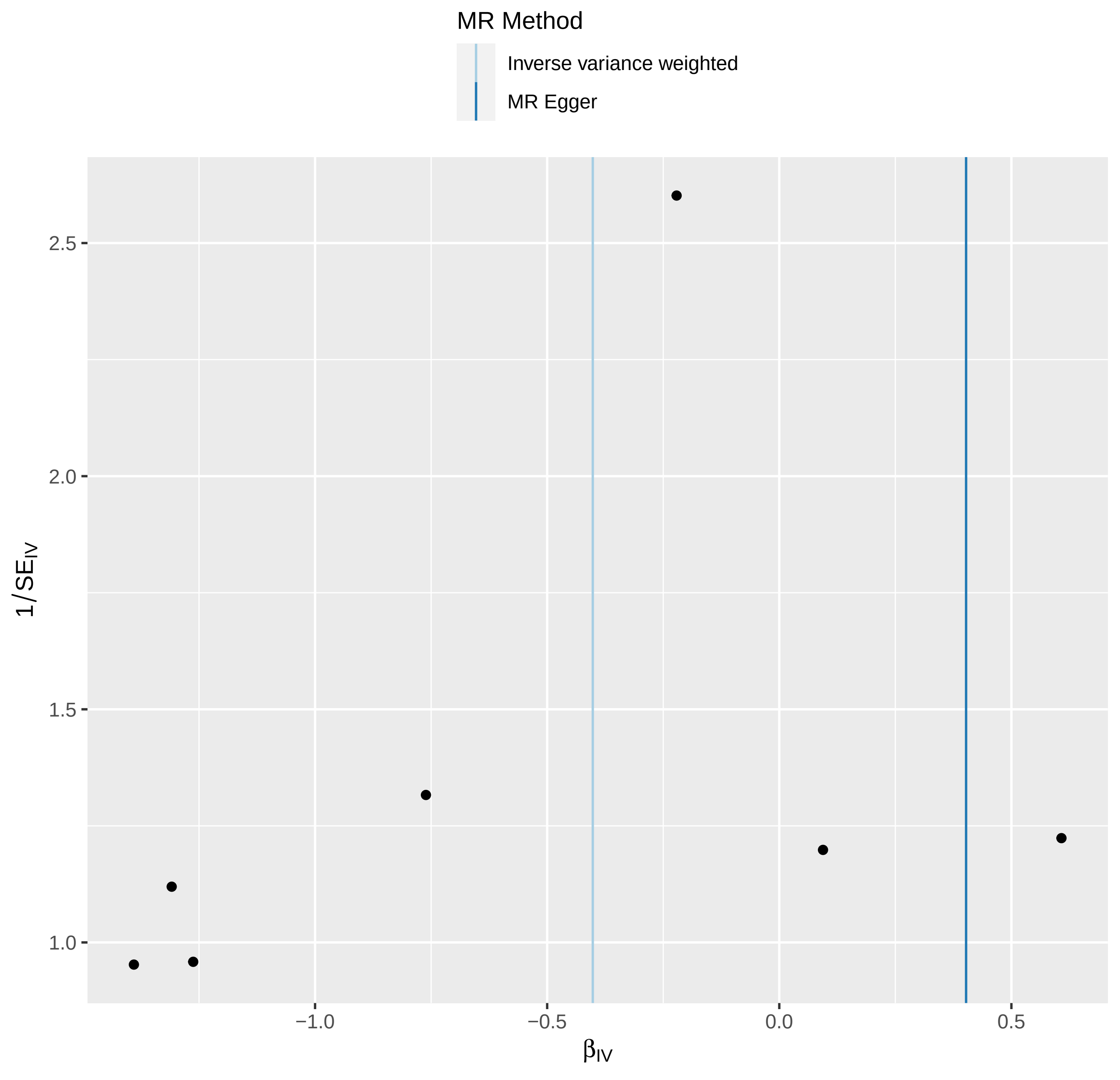

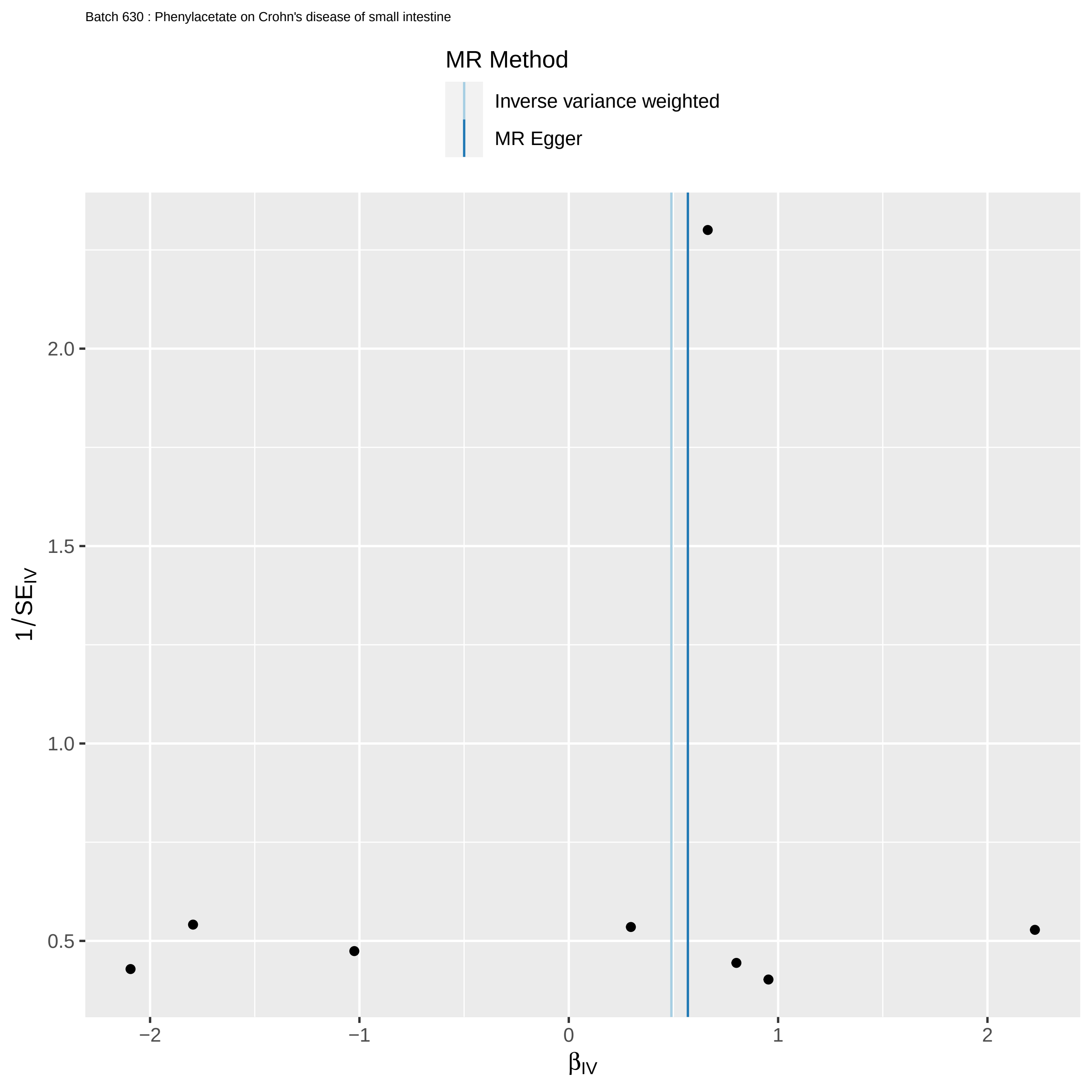
Batch 630 : Phenylacetate on Crohn's disease of small intestine
MR Method
Inverse variance weighted
MR Egger
2.0
1.5
1.0
0.5
1/SE_IV
−2
−1
0
1
2
β_IV

Batch 631 : Glycerophosphorylcholine (GPC) on Crohn's disease of small intestine

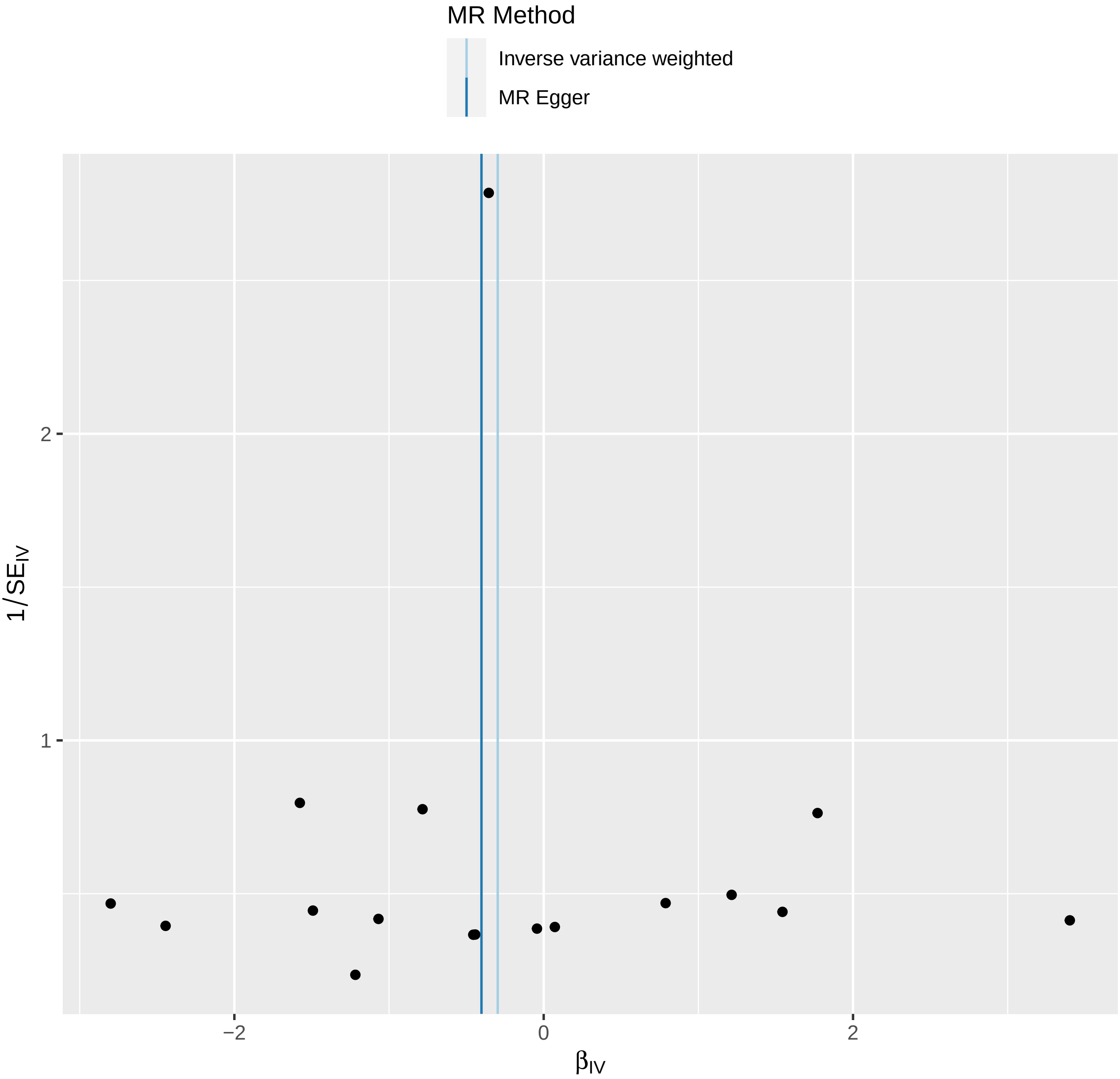

Batch 632 : Aspartate on Crohn's disease of small intestine

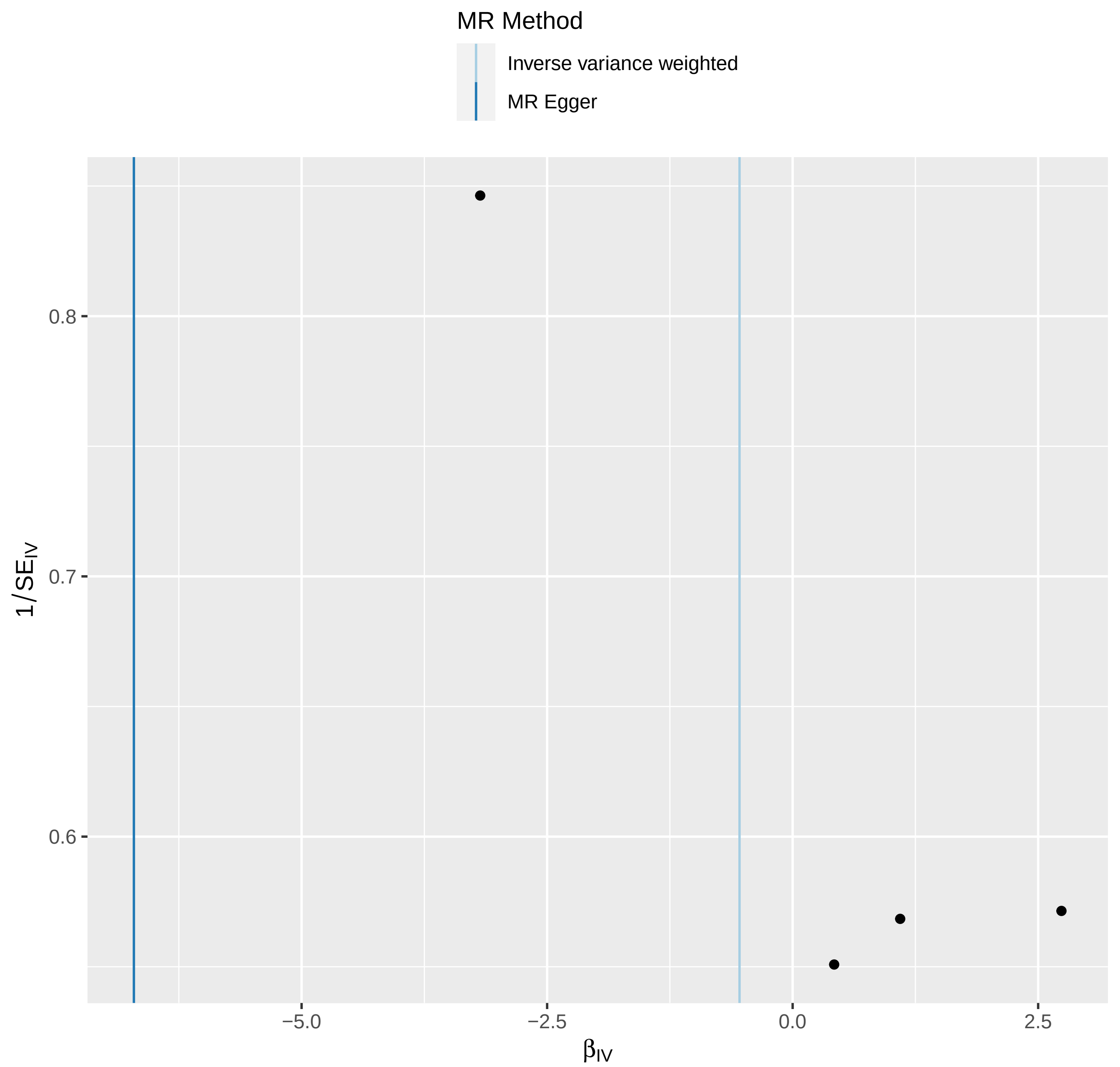

Batch 634 : Dihomo−linoleate (20:2n6) on Crohn's disease of small intestine

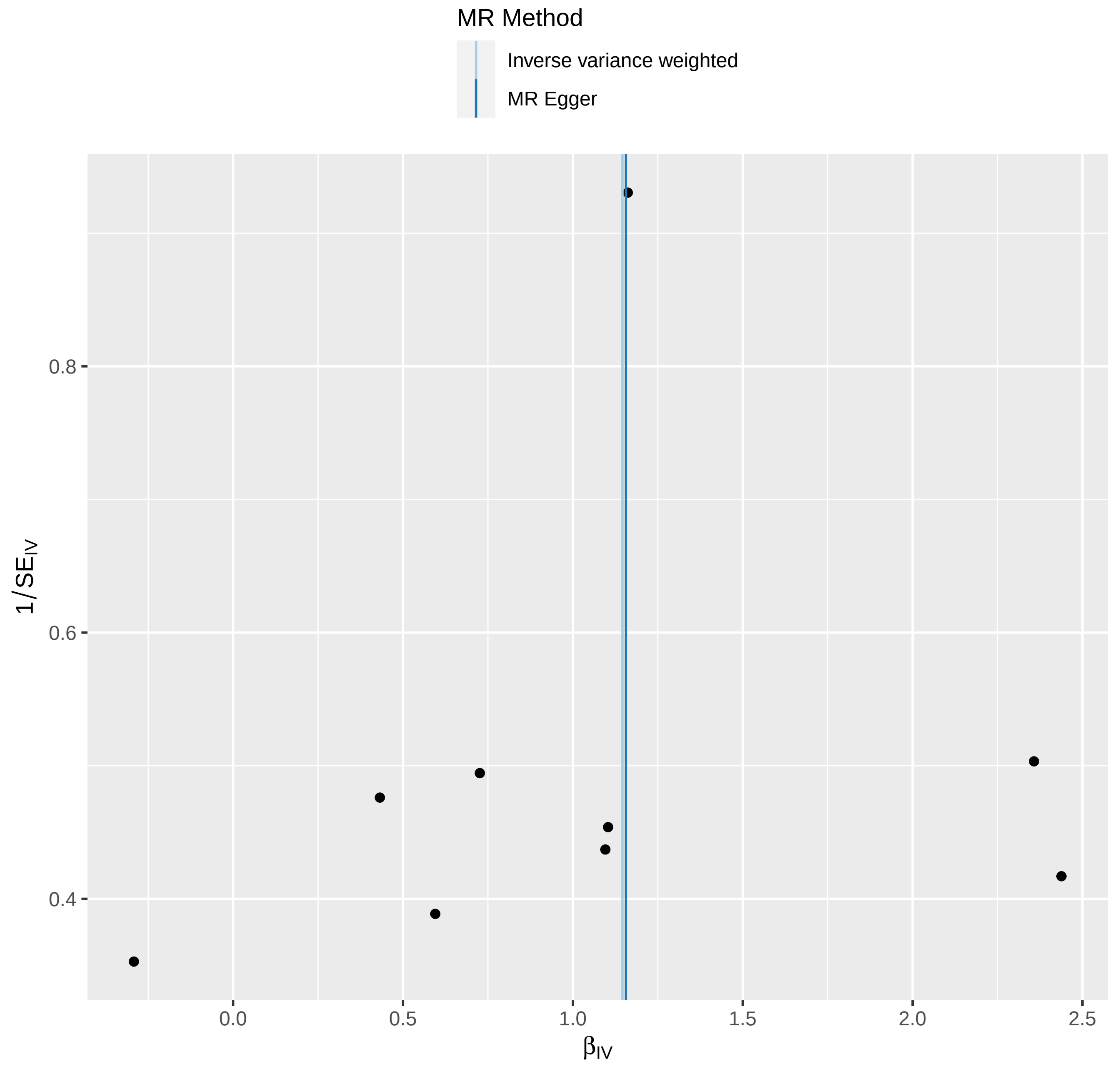

Batch 635 : 2−hydroxystearate on Crohn's disease of small intestine

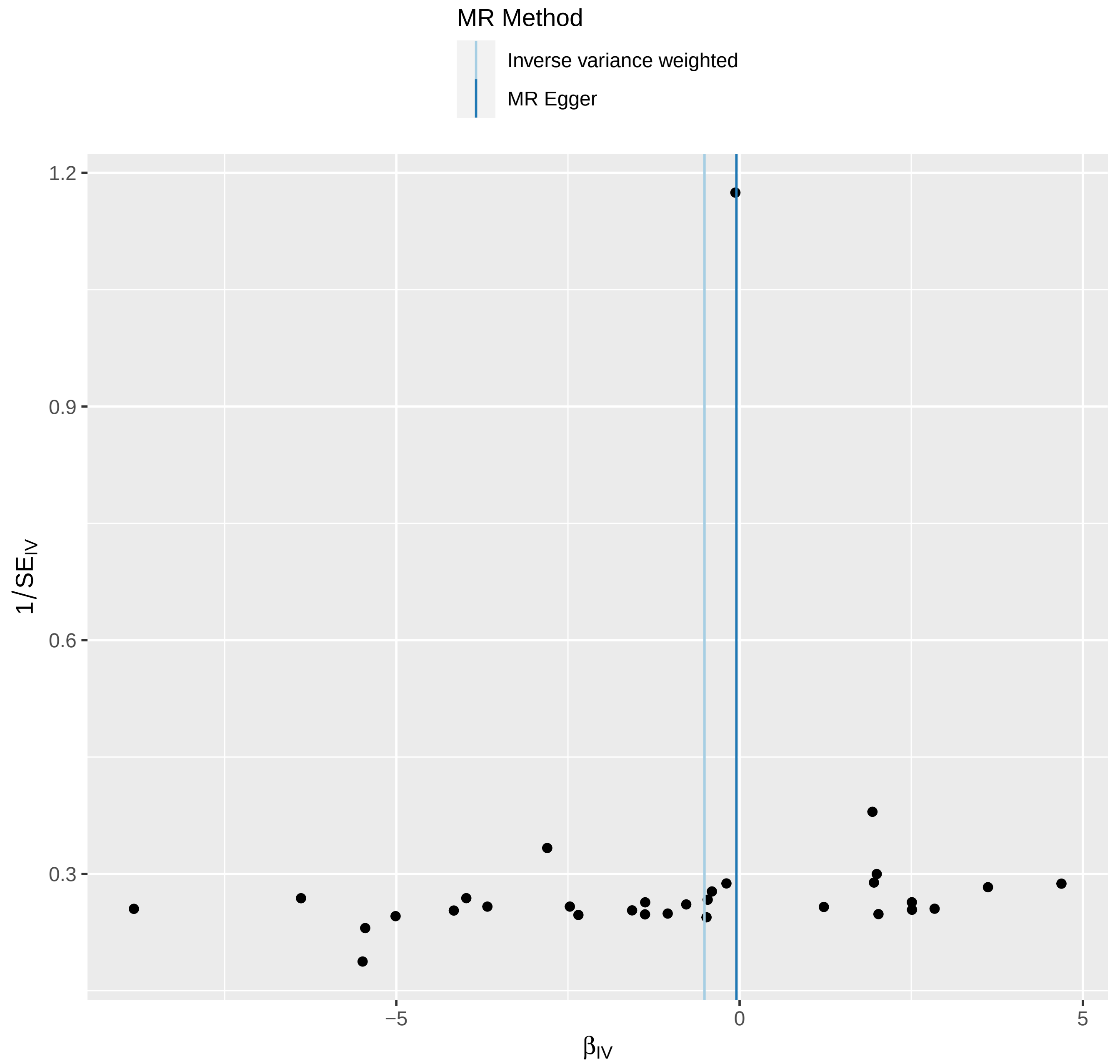

Batch 637 : Glycylvaline on Crohn's disease of small intestine

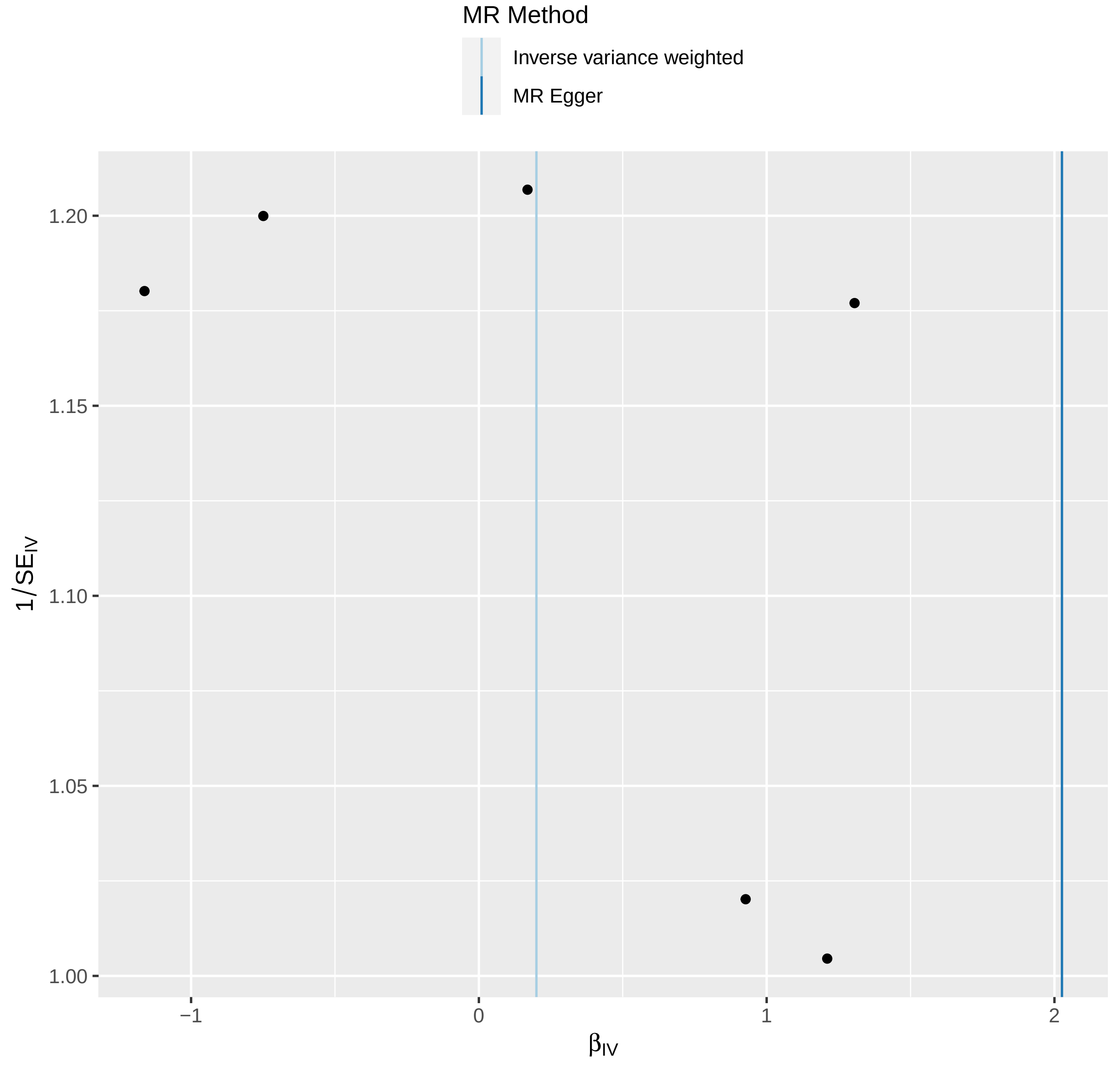

Batch 638 : Gamma−glutamylleucine on Crohn's disease of small intestine

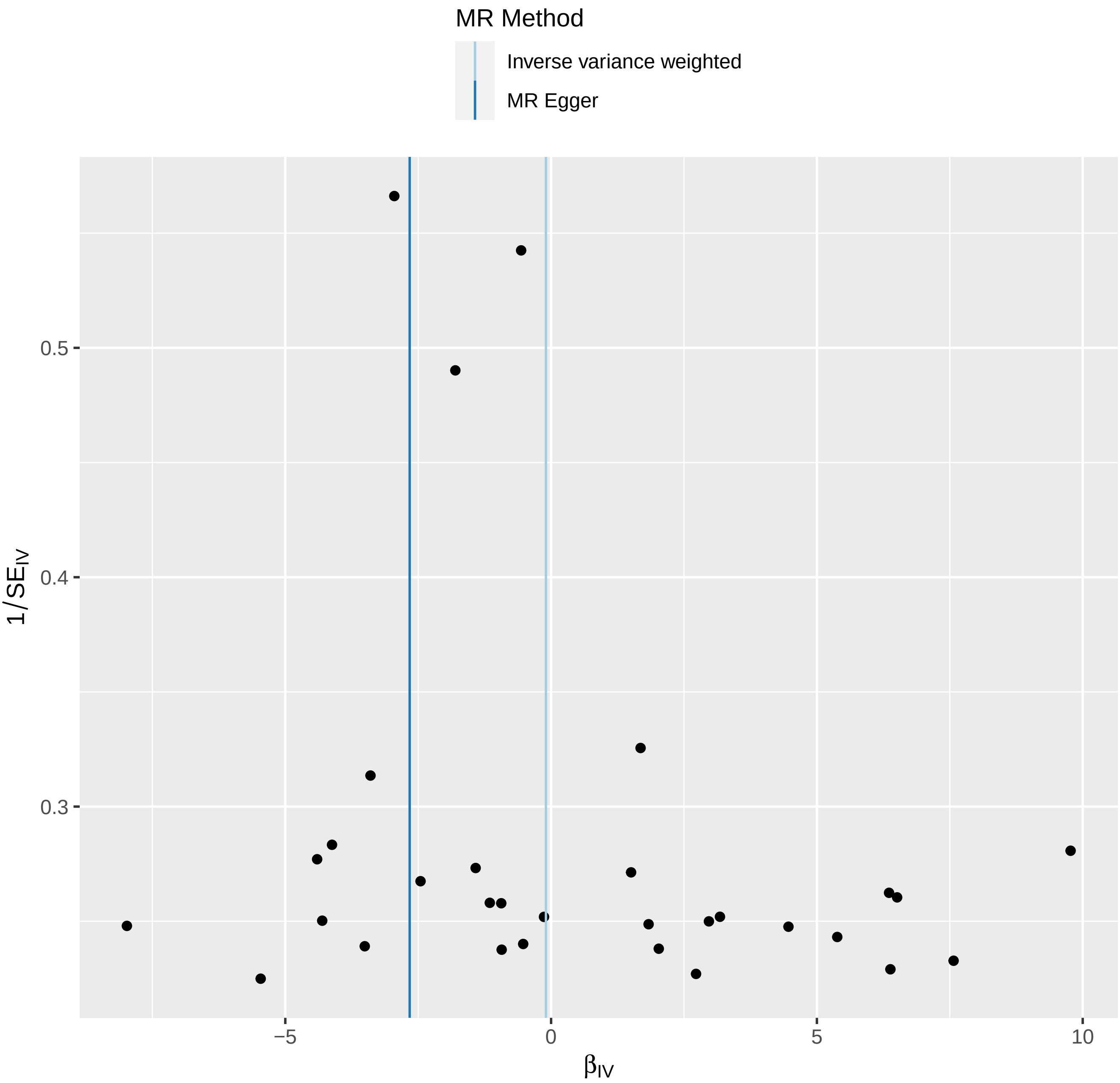

Batch 641 : Glycocholate on Crohn's disease of small intestine

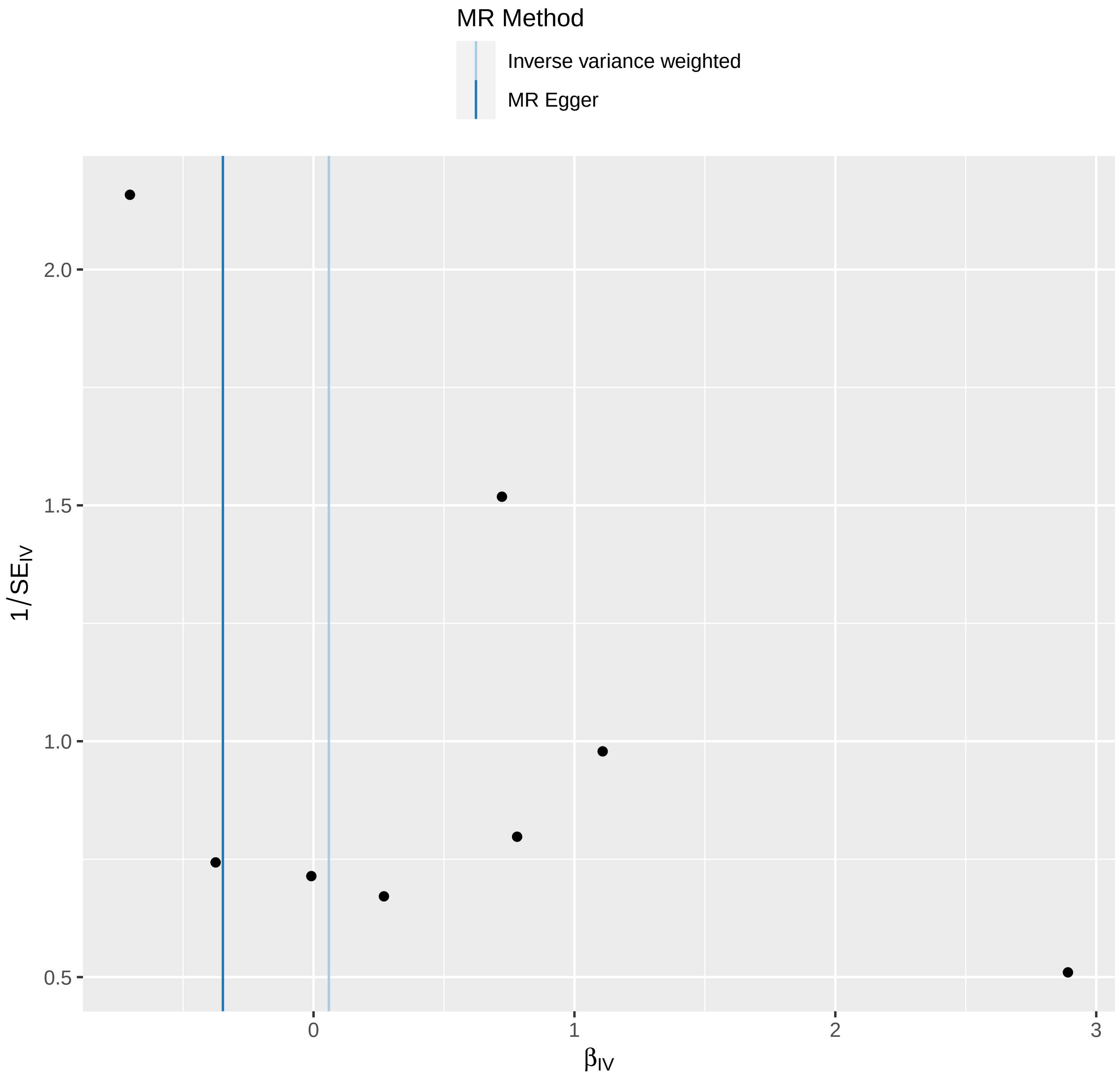

Batch 642 : Glycodeoxycholate on Crohn's disease of small intestine

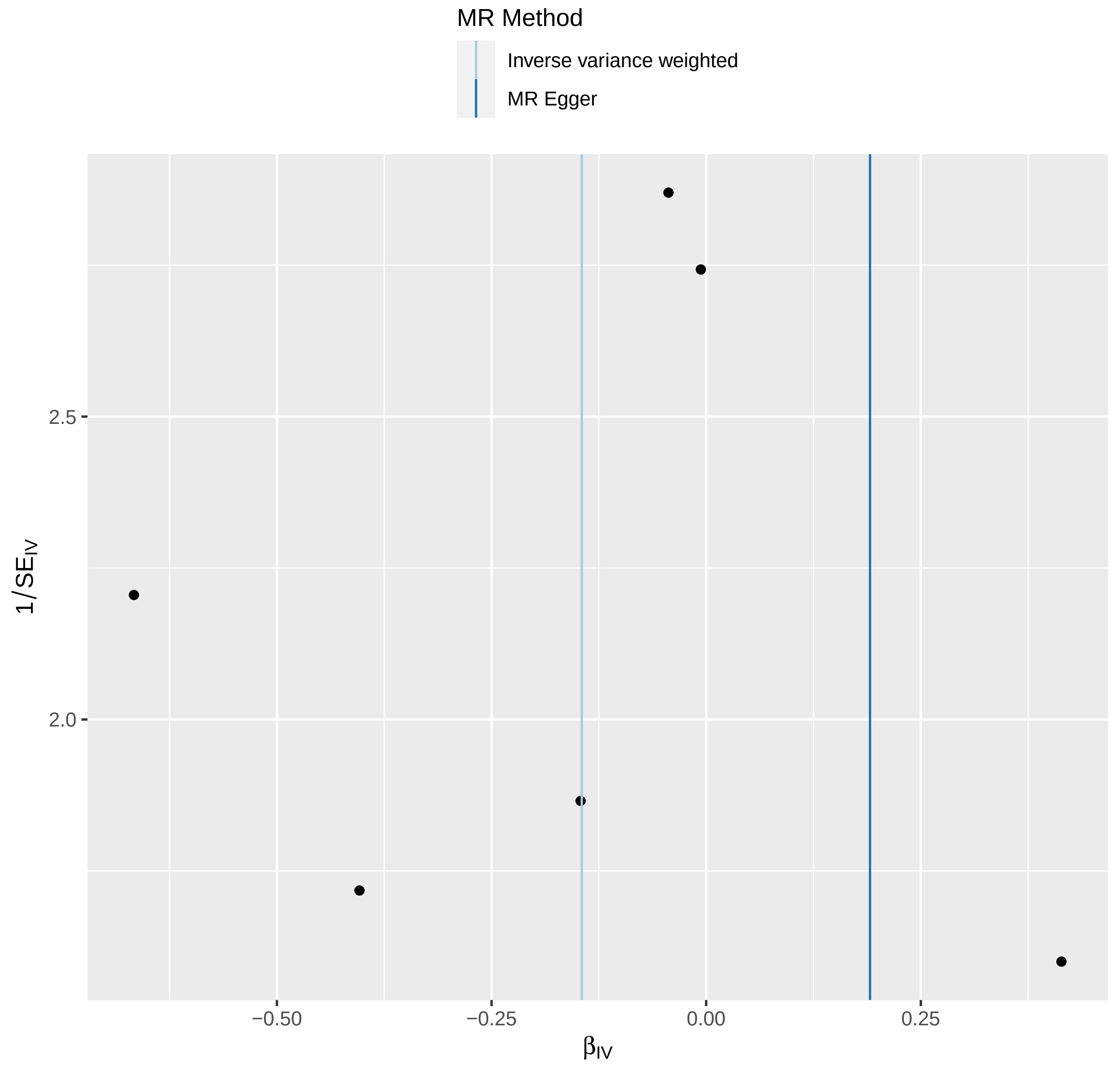

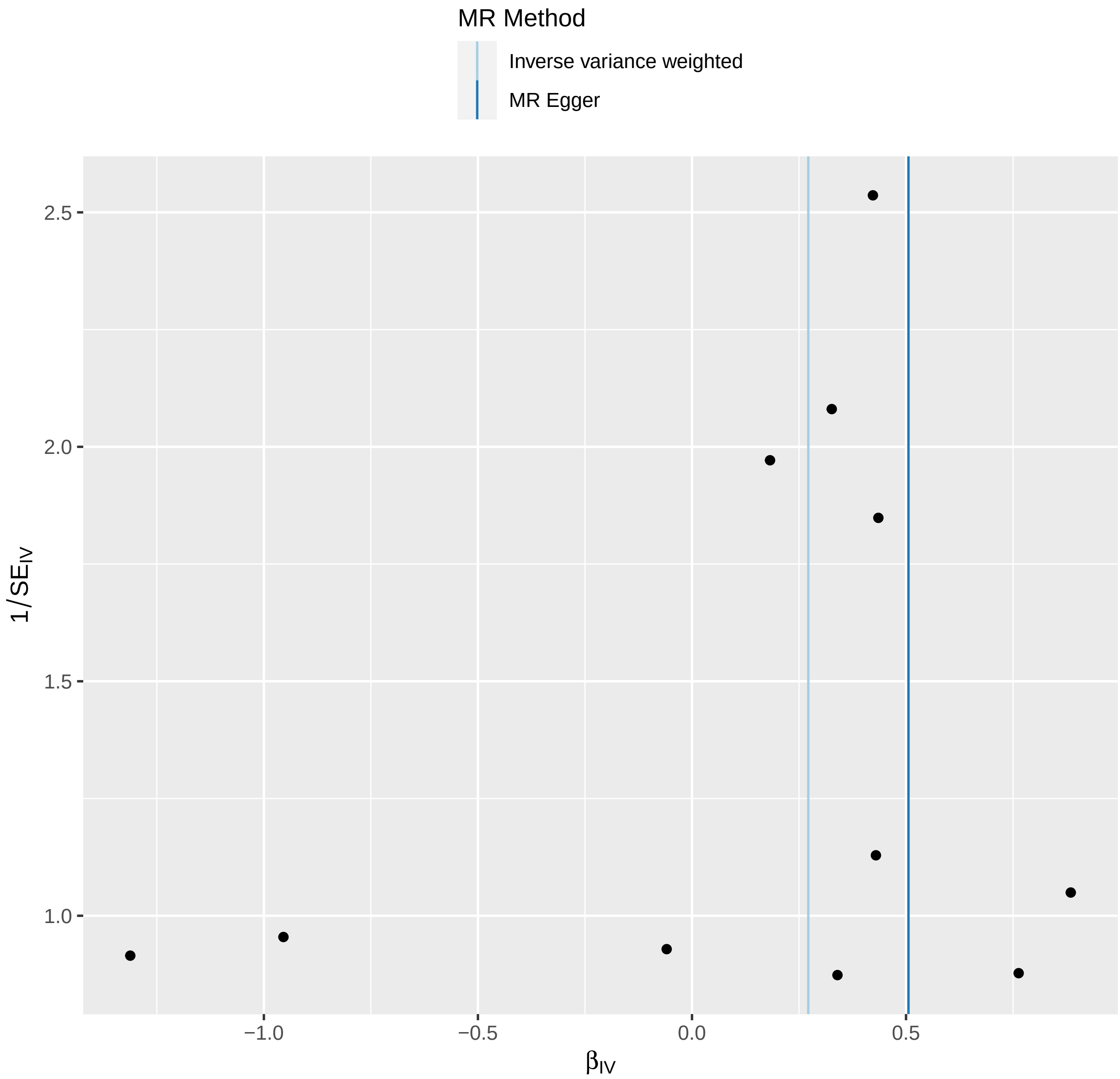
MR Method
Inverse variance weighted
MR Egger
2.5
2.0
1.5
1.0
1/SE_IV
−1.0
−0.5
0.0
0.5
β_IV

Batch 644 : Taurocholate on Crohn's disease of small intestine

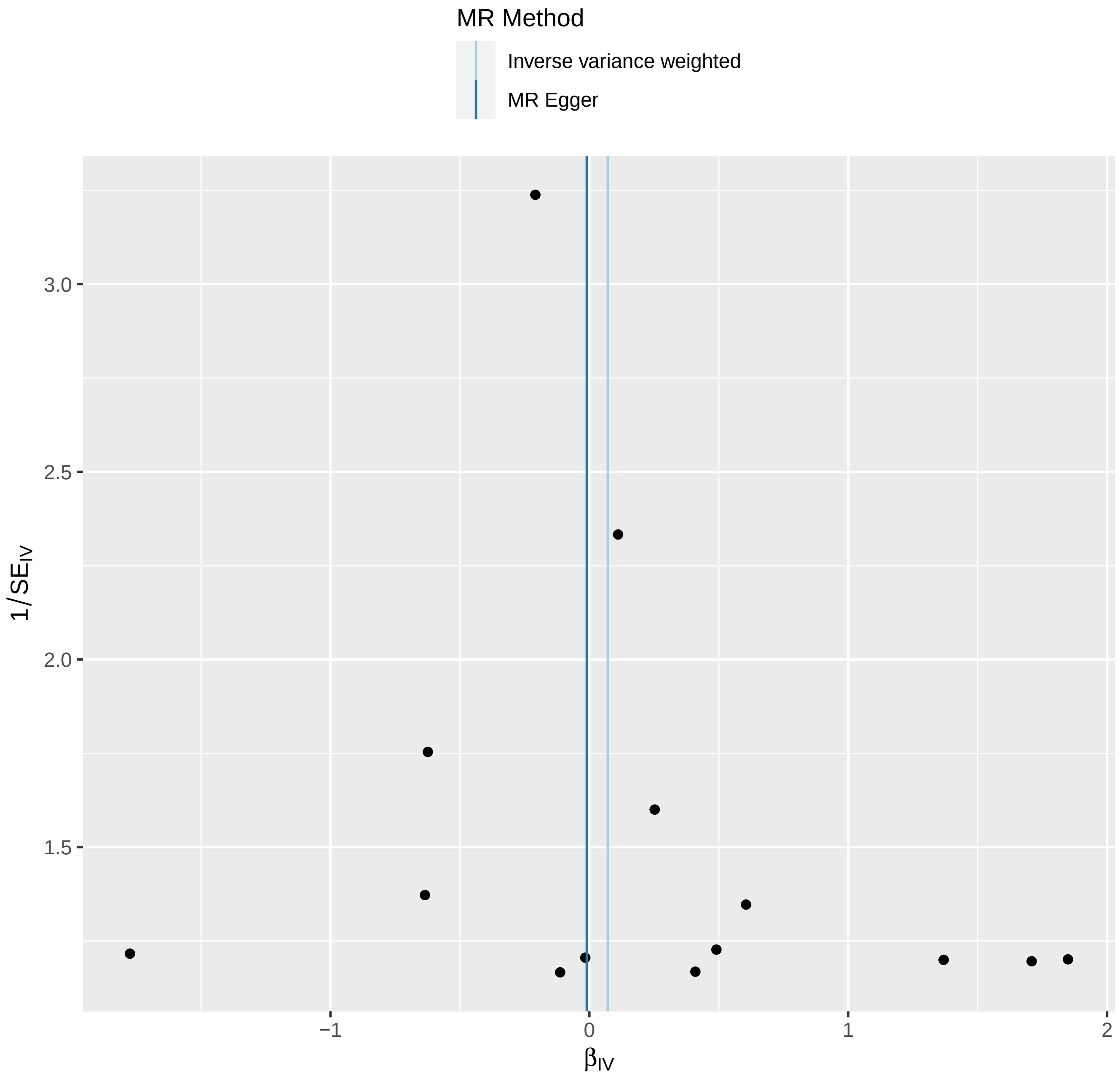

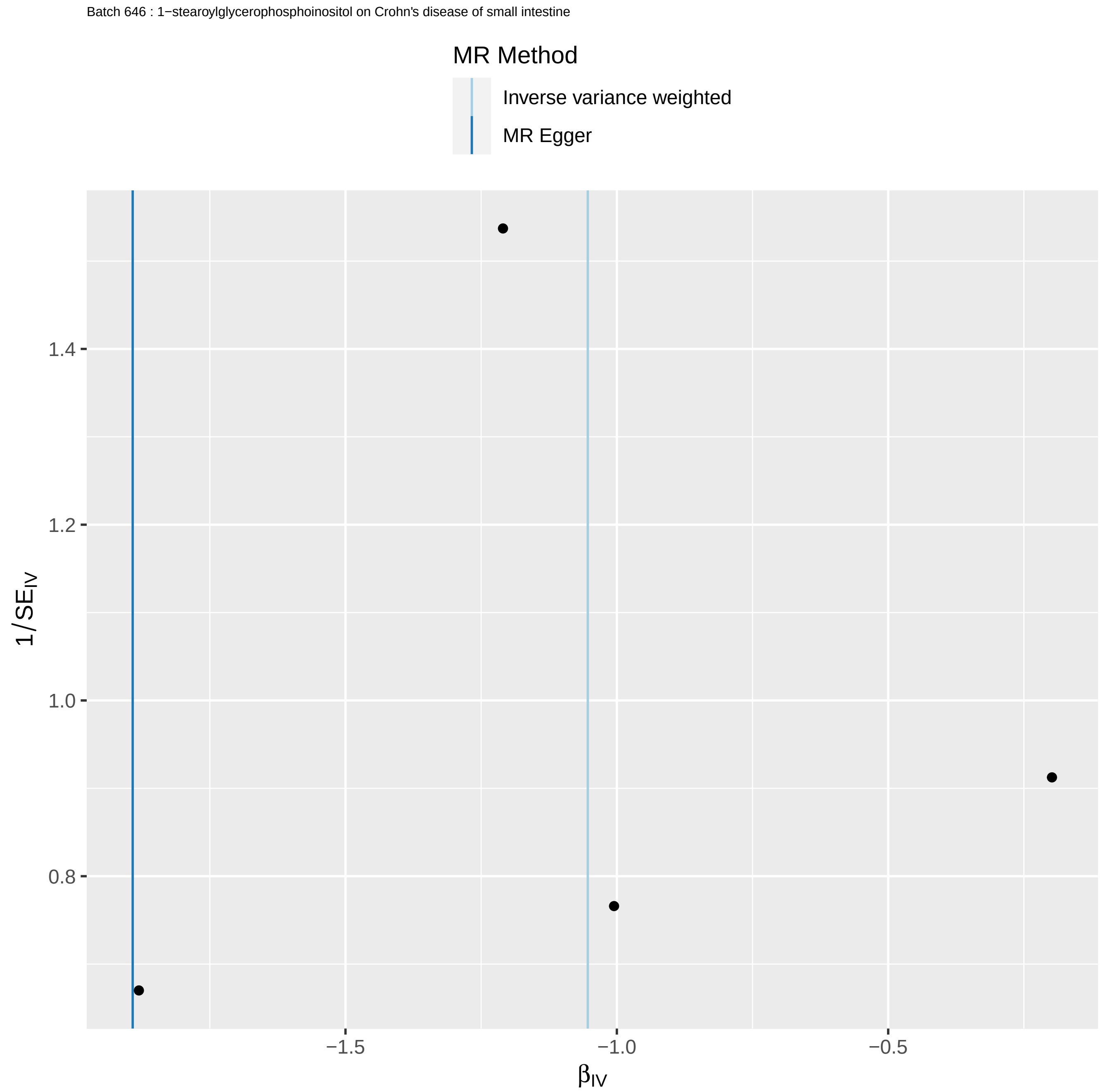
Batch 646 : 1−stearoylglycerophosphoinositol on Crohn's disease of small intestine
MR Method
Inverse variance weighted
MR Egger
1.4
1.2
1.0
0.8
$1/SE_{IV}$
−1.5
−1.0
−0.5
β_{IV}

Batch 647 : Myo−inositol on Crohn's disease of small intestine

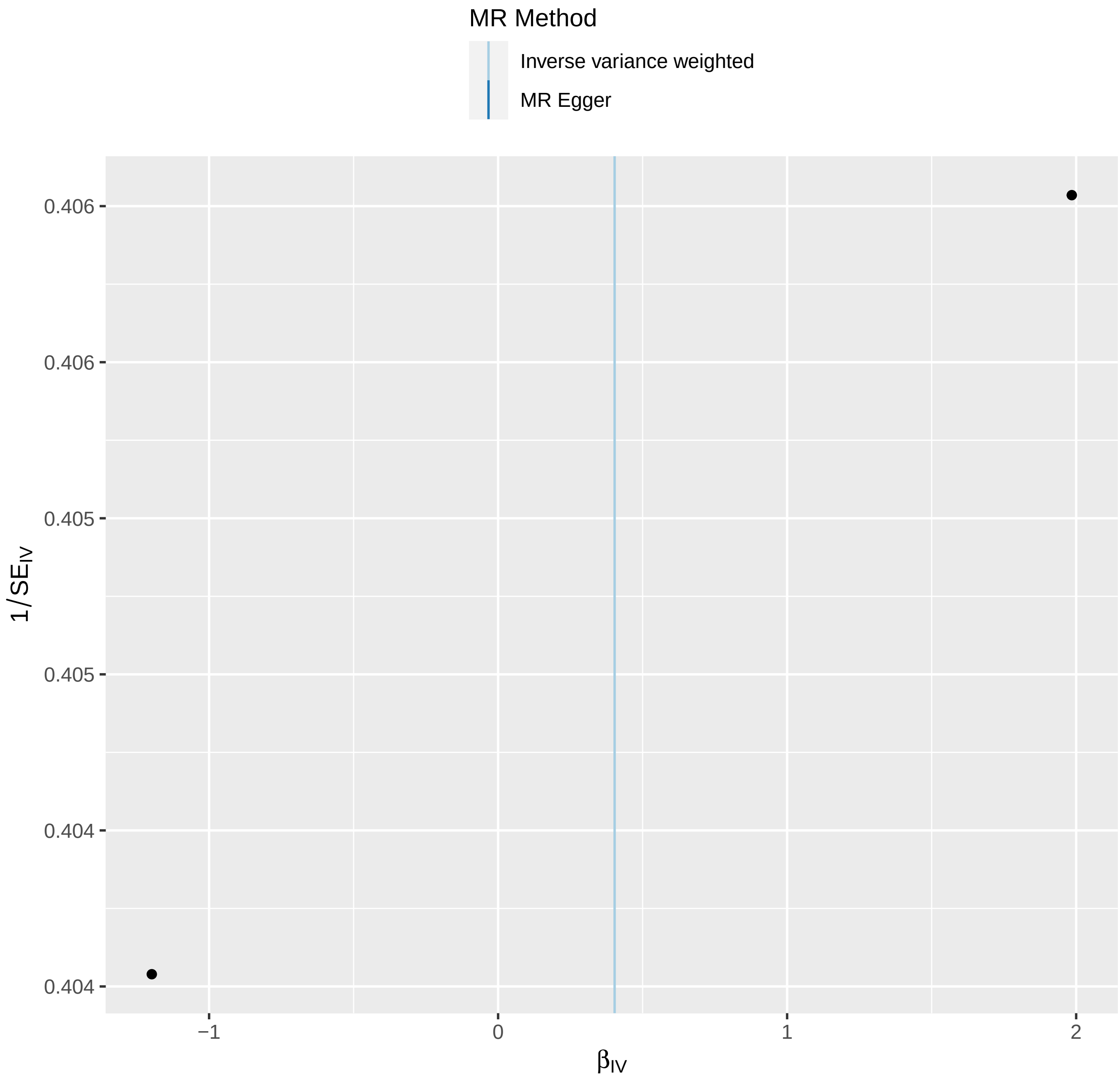

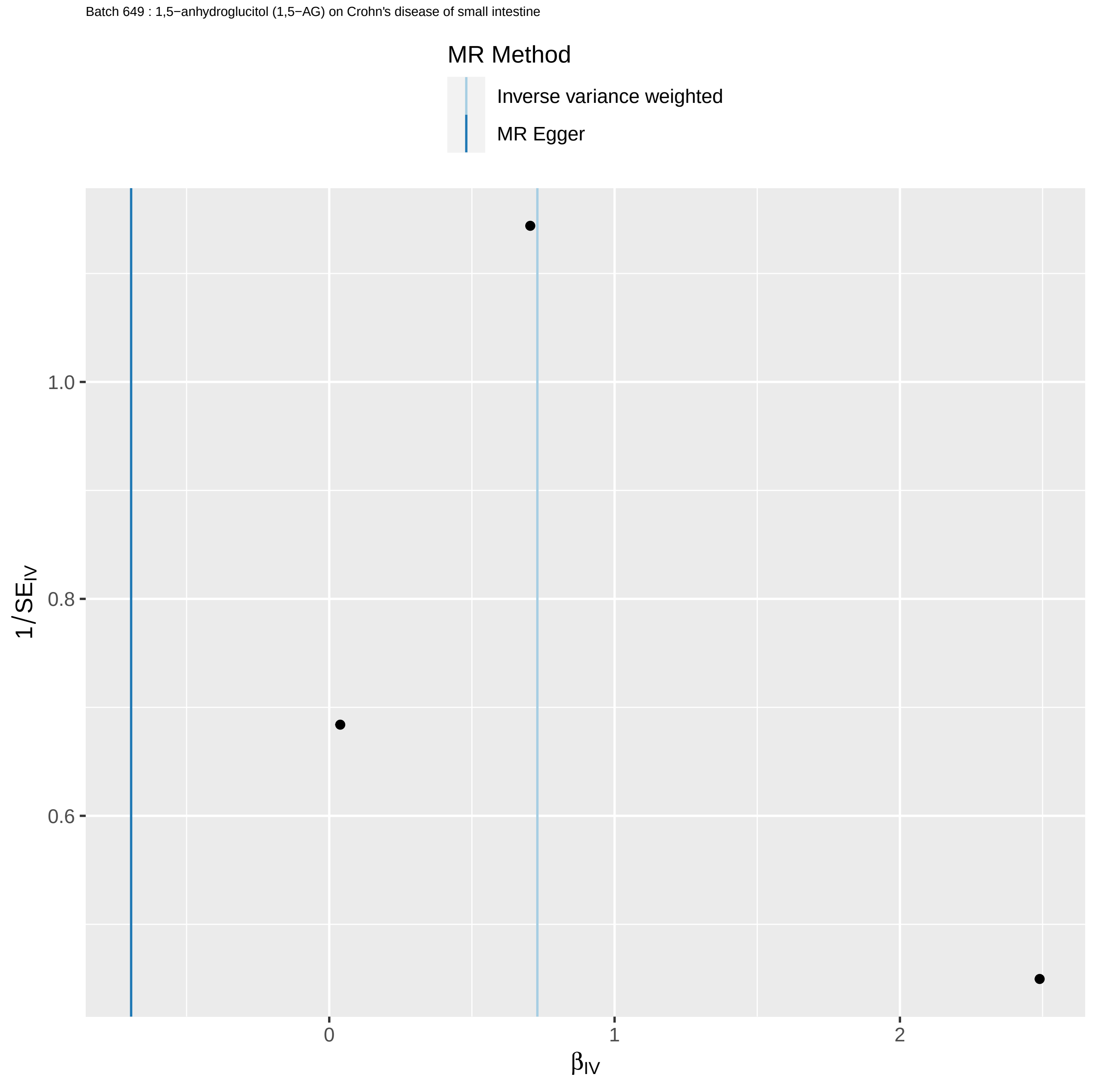

Batch 649 : 1,5−anhydroglucitol (1,5−AG) on Crohn's disease of small intestine
MR Method
Inverse variance weighted
MR Egger
1.0
0.8
0.6
1/SE_IV
0
1
2
β_IV

Batch 650 : 2−hydroxybutyrate (AHB) on Crohn's disease of small intestine

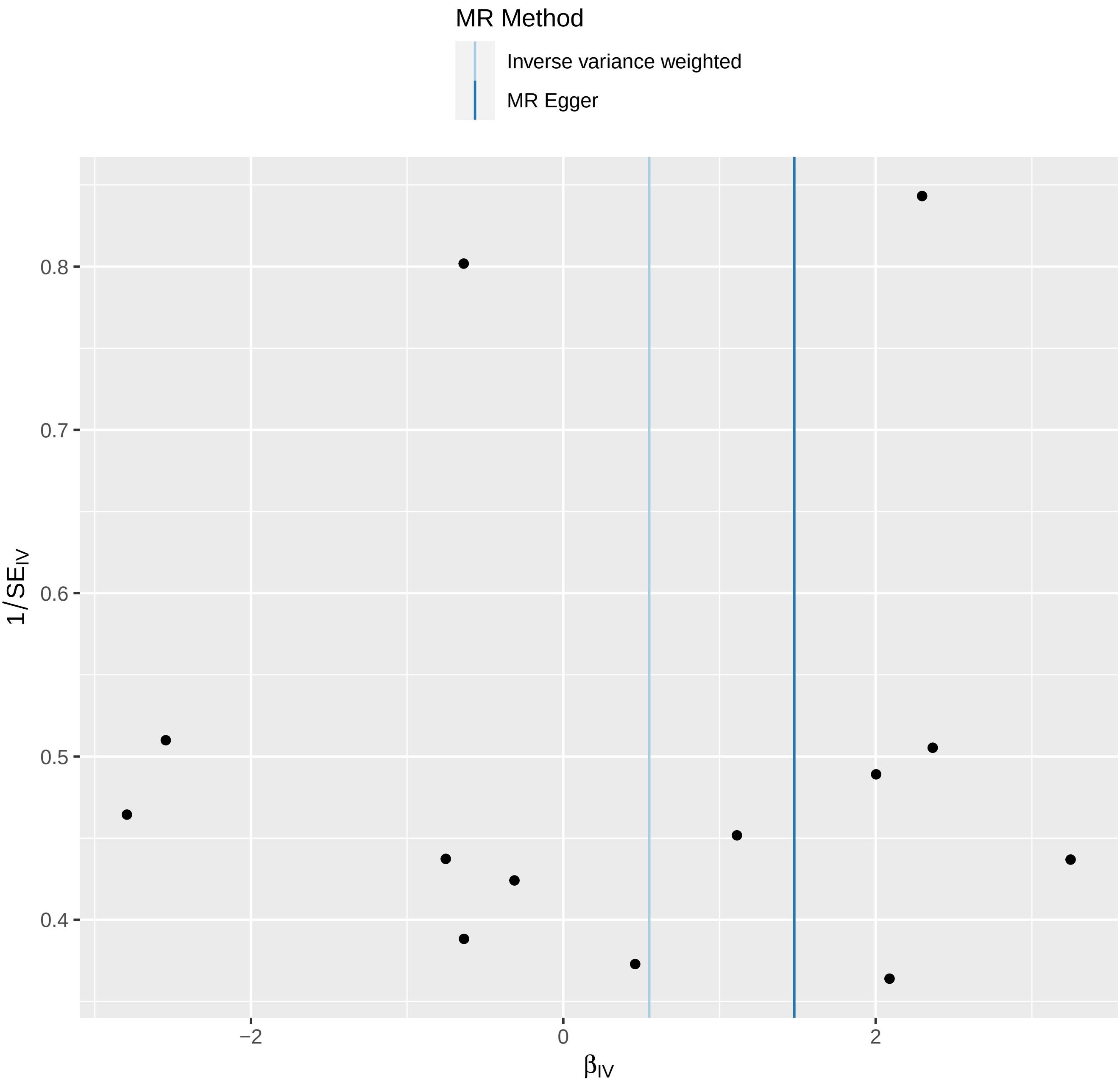

Batch 653 : 1−palmitoylglycerol (1−monopalmitin) on Crohn's disease of small intestine

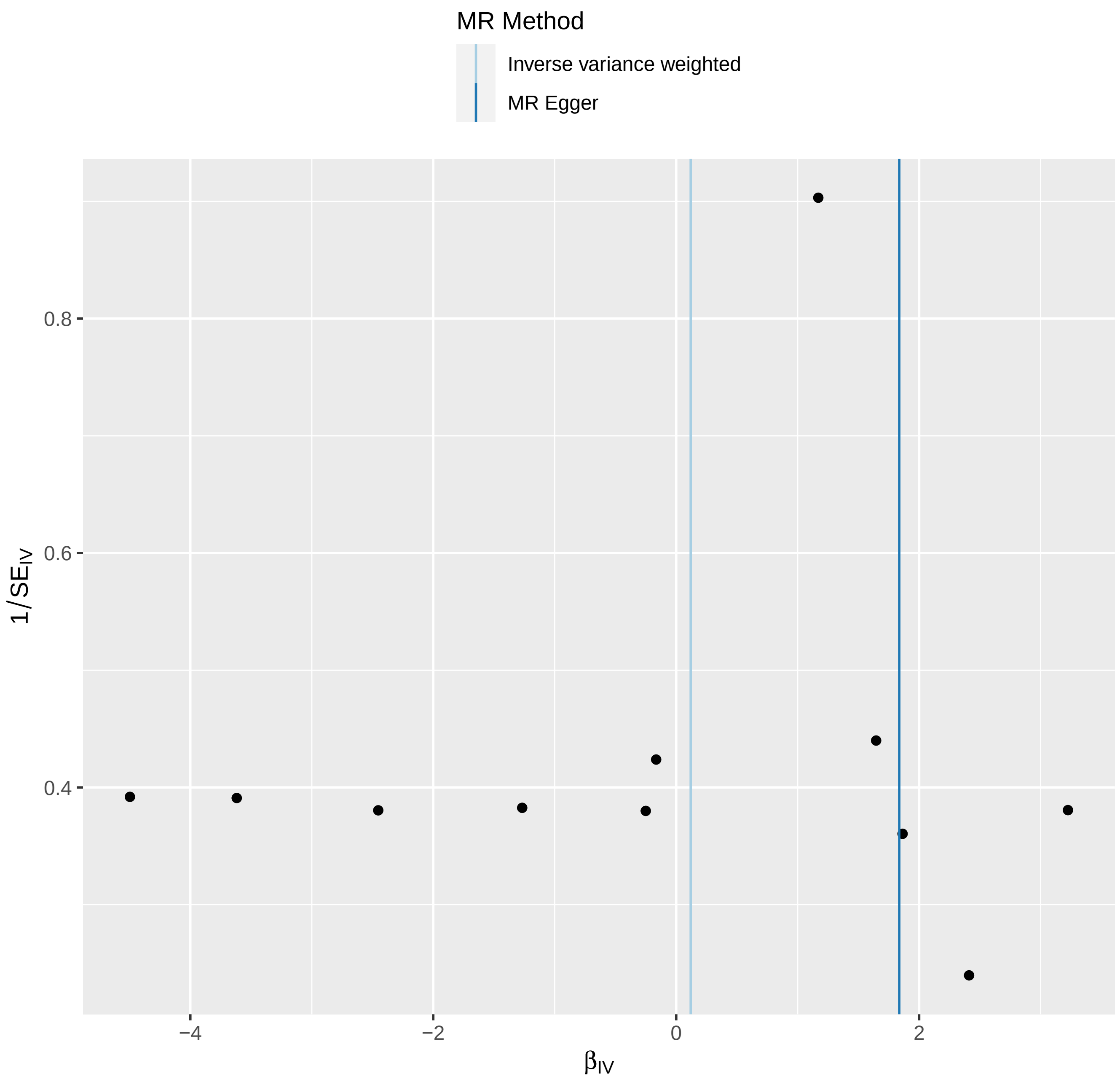

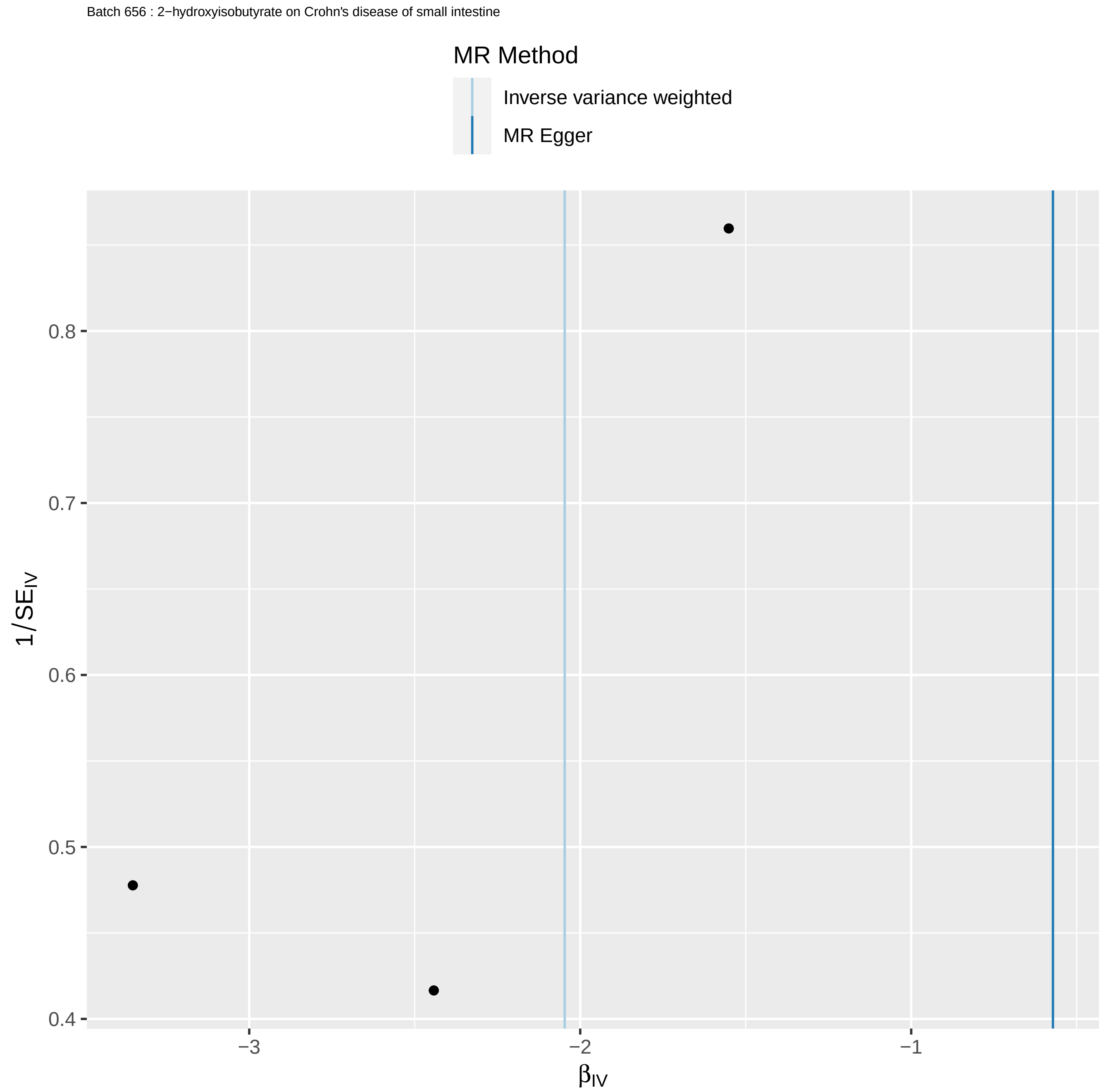

Batch 656 : 2−hydroxyisobutyrate on Crohn's disease of small intestine
MR Method
Inverse variance weighted
MR Egger
0.8
0.7
0.6
0.5
0.4
$1/SE_{IV}$
−3
−2
−1
β_{IV}

Batch 657 : 4−methyl−2−oxopentanoate on Crohn's disease of small intestine

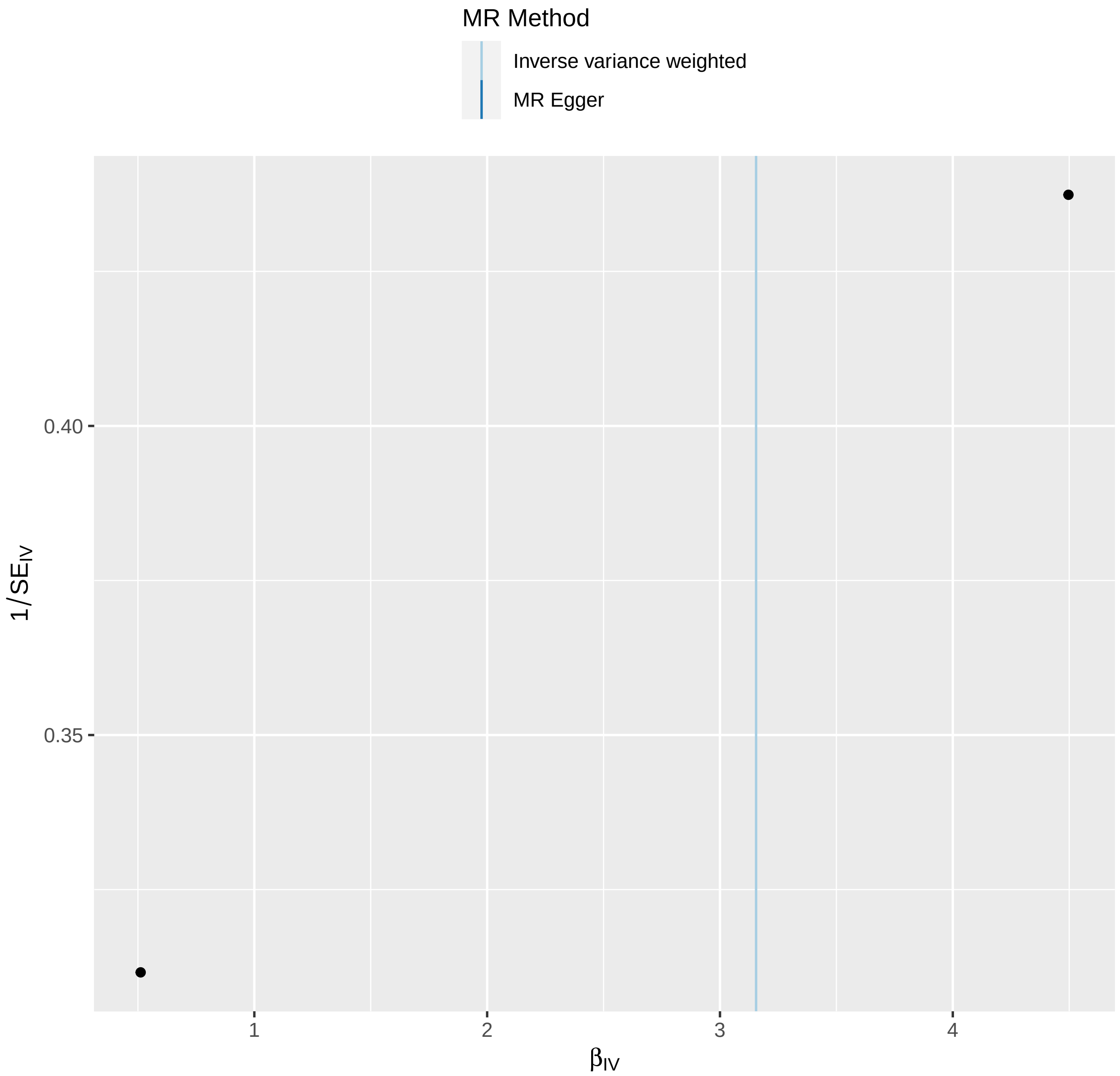

Batch 661 : Levulinate (4−oxovalerate) on Crohn's disease of small intestine

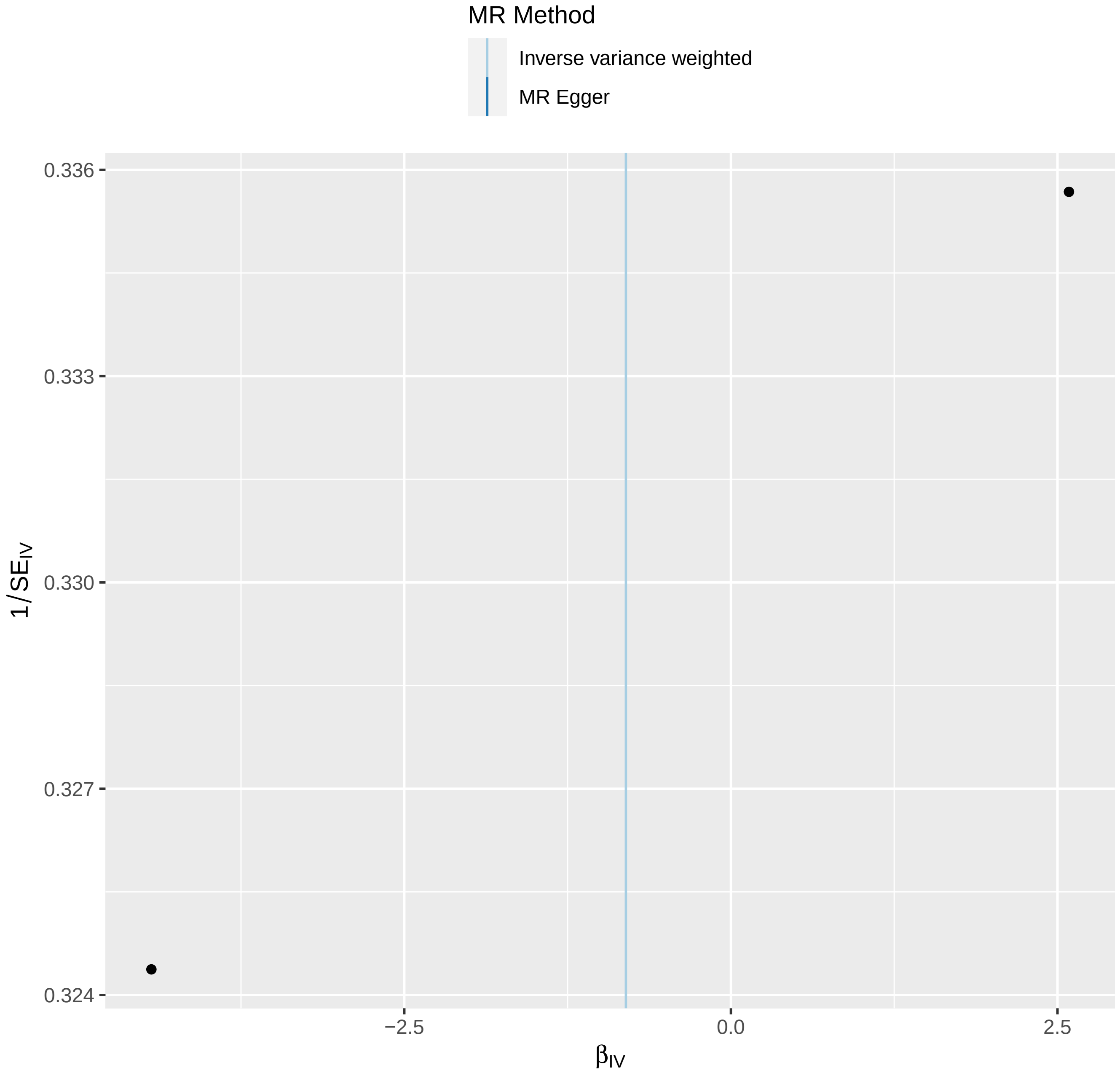

Batch 663 : Cholate on Crohn's disease of small intestine

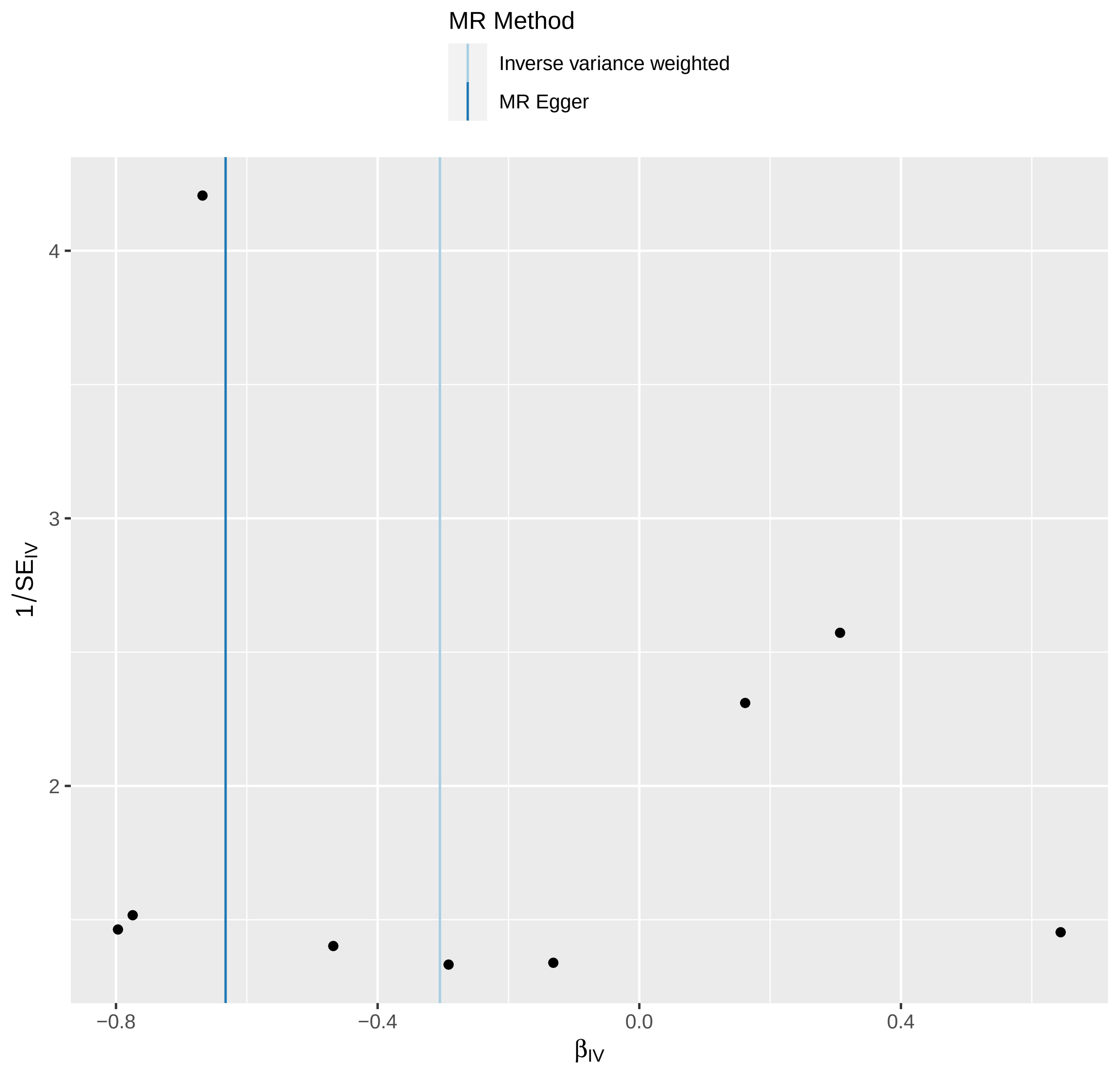

Batch 666 : Hyodeoxycholate on Crohn's disease of small intestine

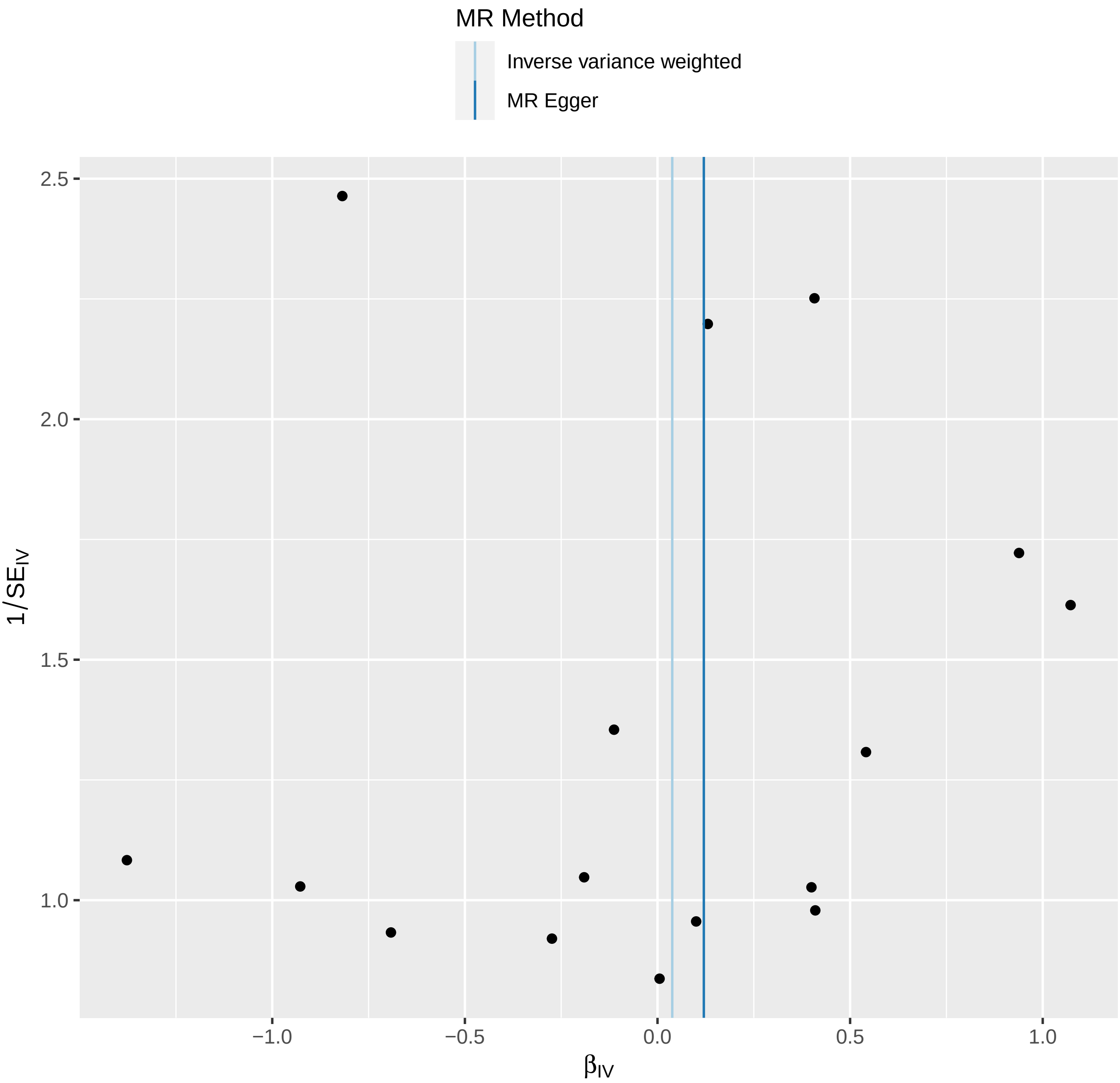

Batch 667 : 3−indoxyl sulfate on Crohn's disease of small intestine

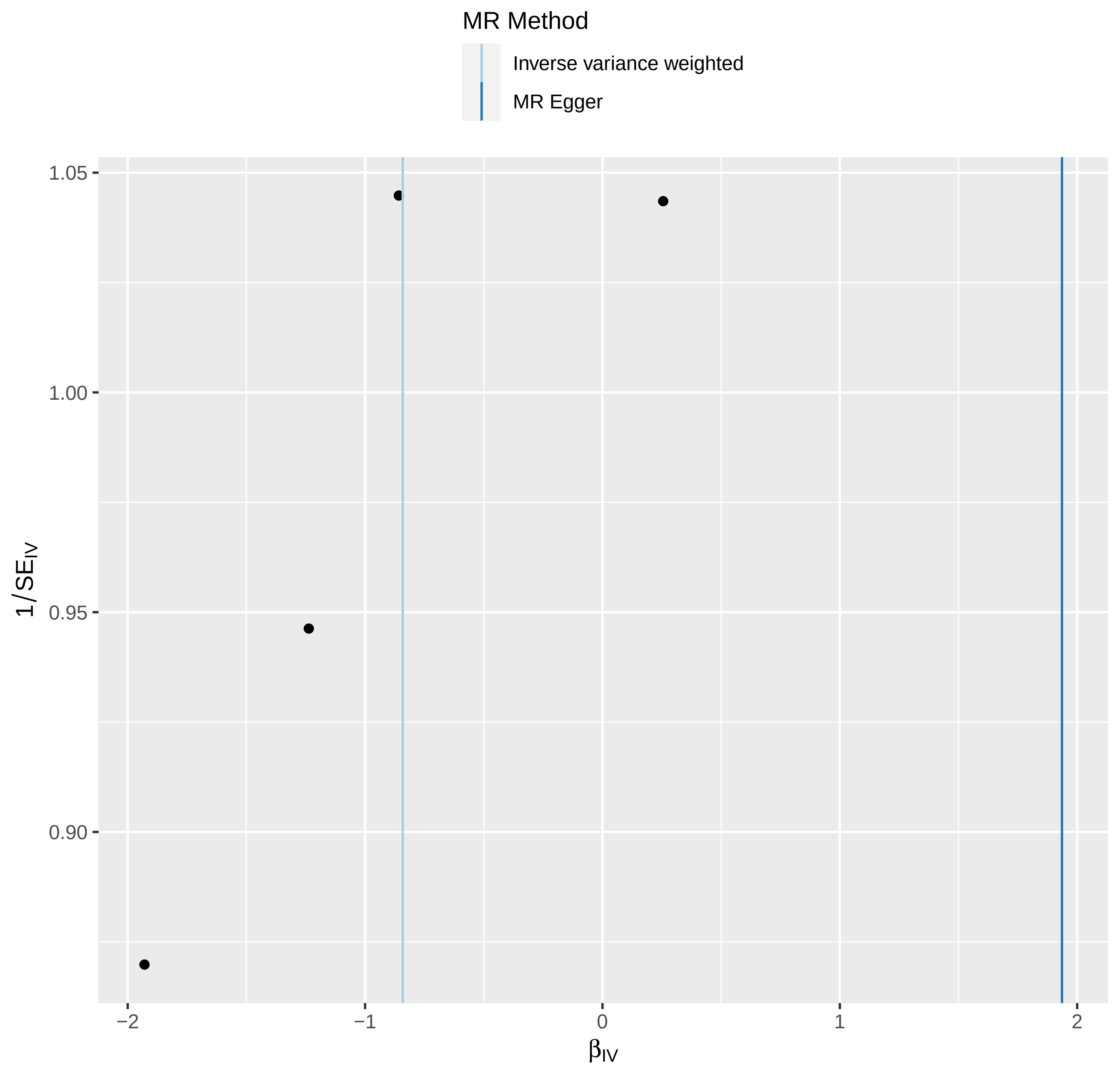

Batch 671 : Erythrose on Crohn's disease of small intestine

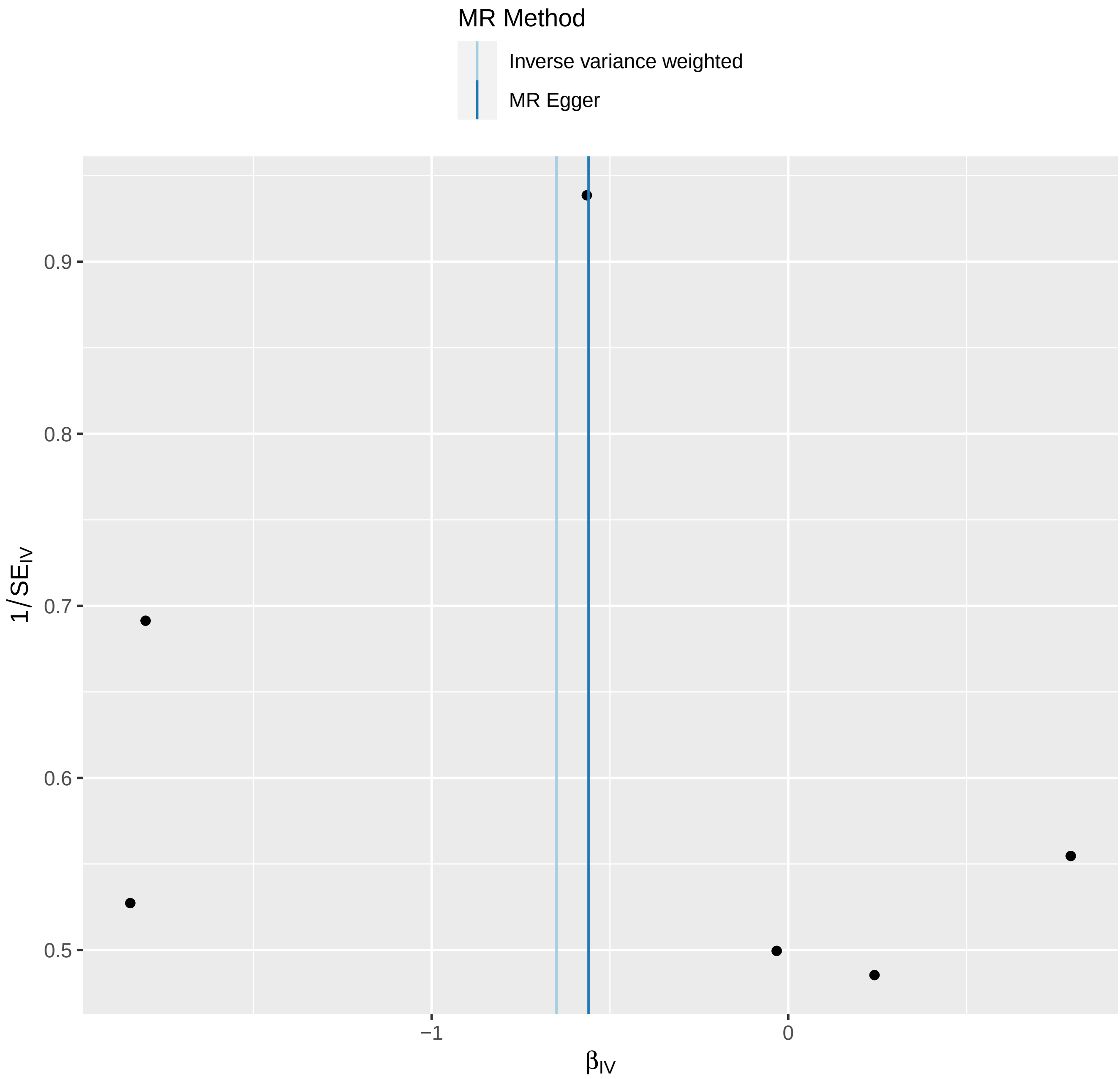

Batch 672 : Threonate on Crohn's disease of small intestine

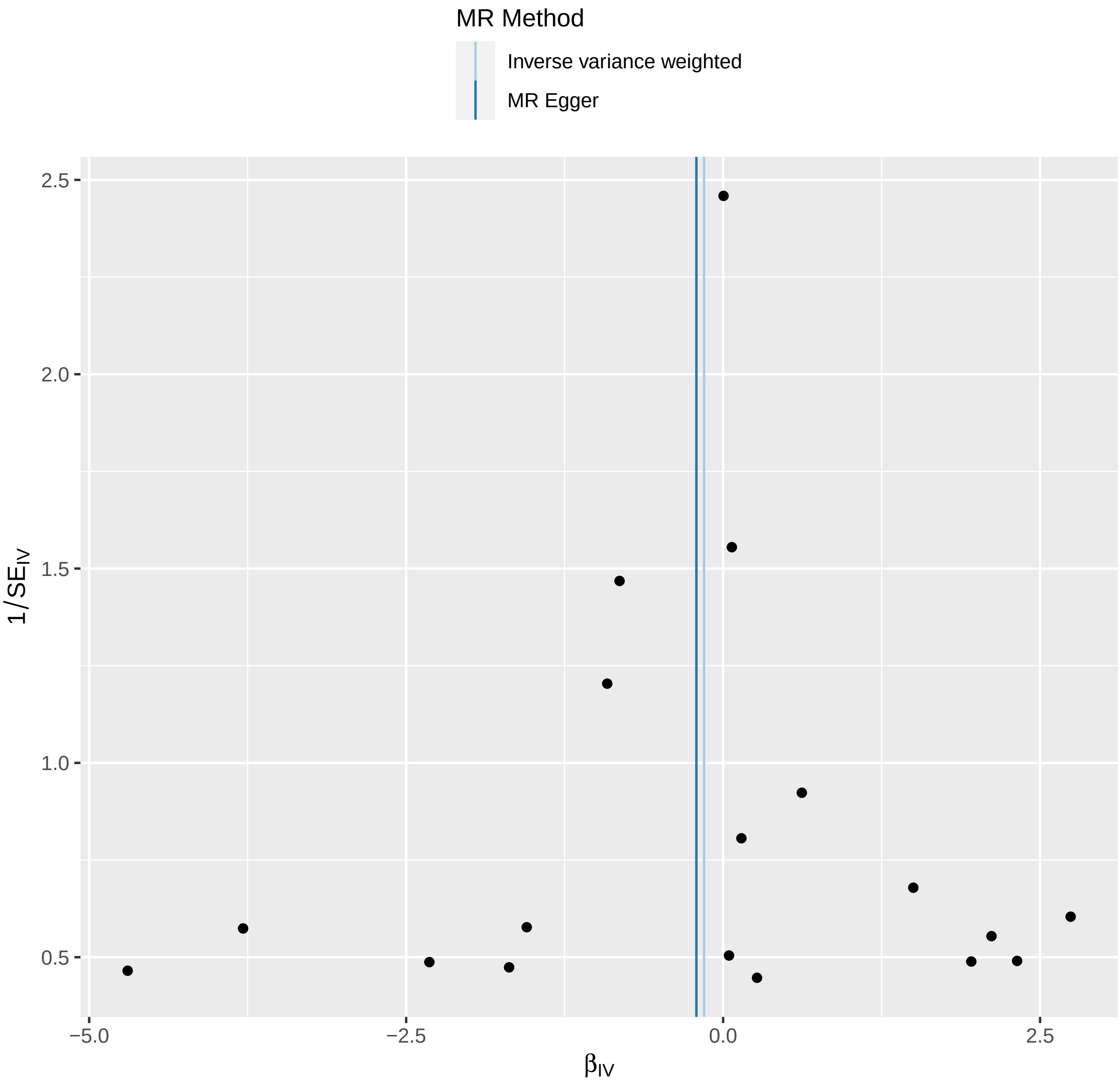

Batch 673 : Cysteine on Crohn's disease of small intestine

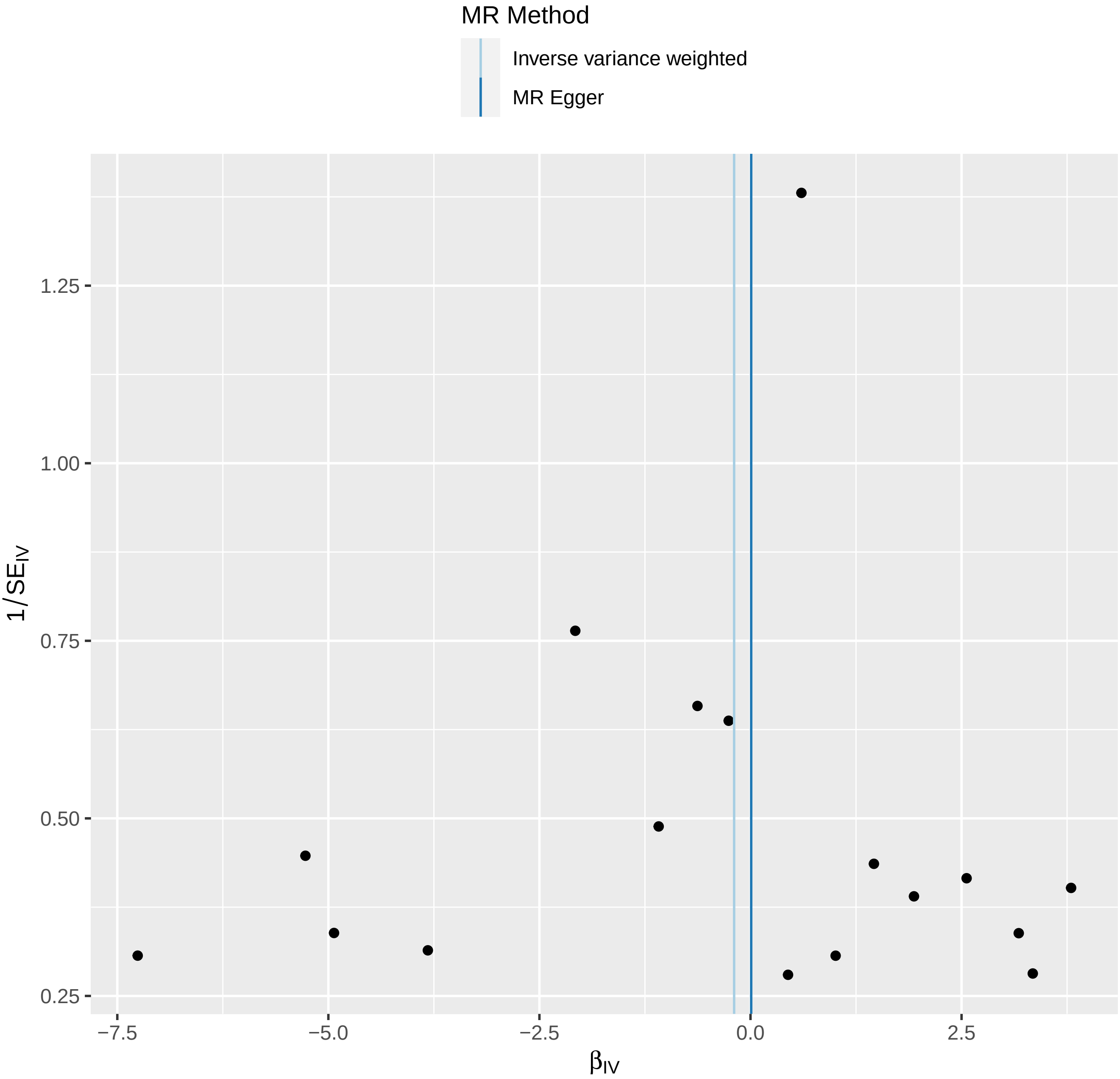

Batch 674 : Pyroglutamylglycine on Crohn's disease of small intestine

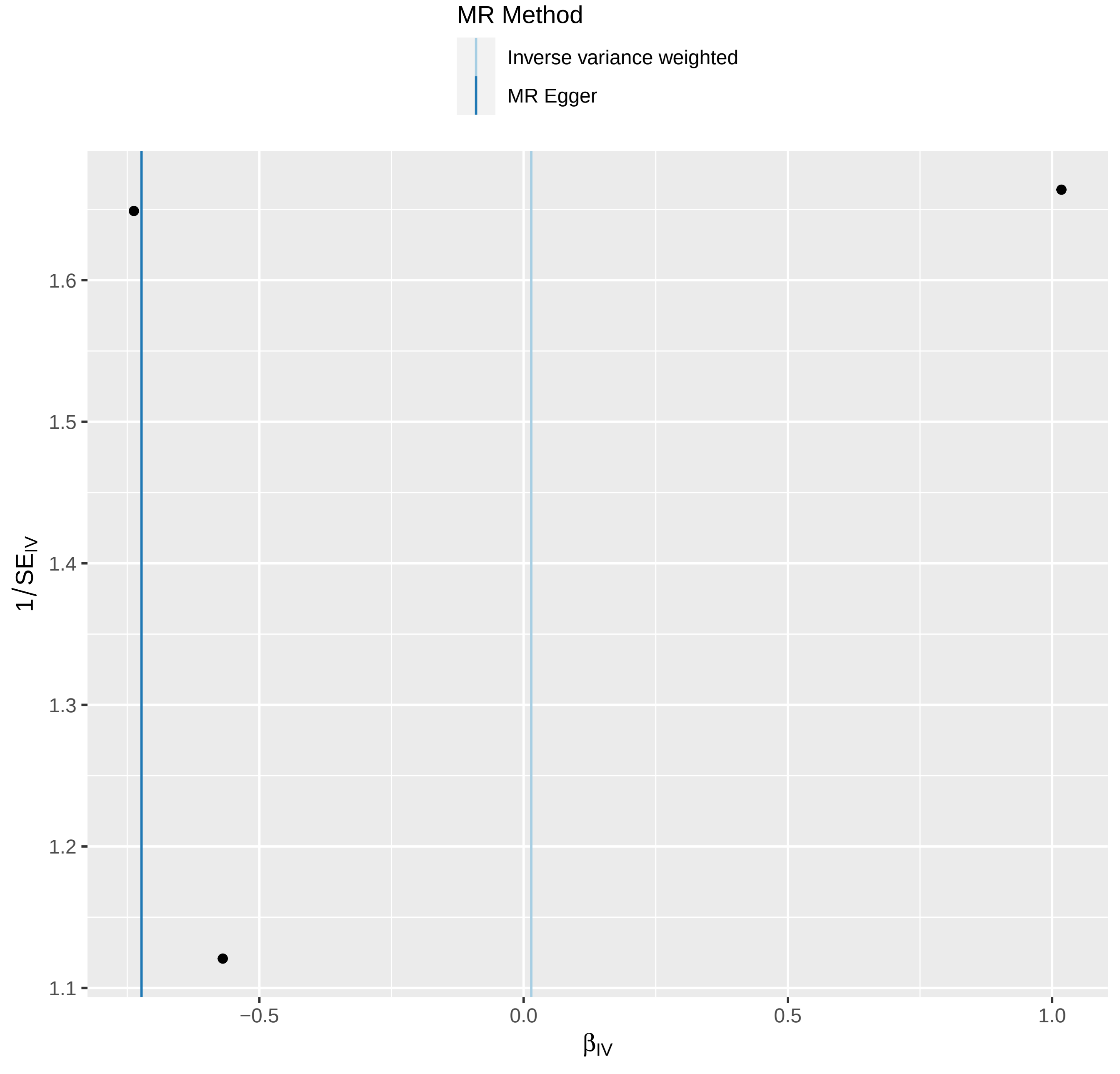

Batch 676 : DSGEGDFXAEGGGVR* on Crohn's disease of small intestine

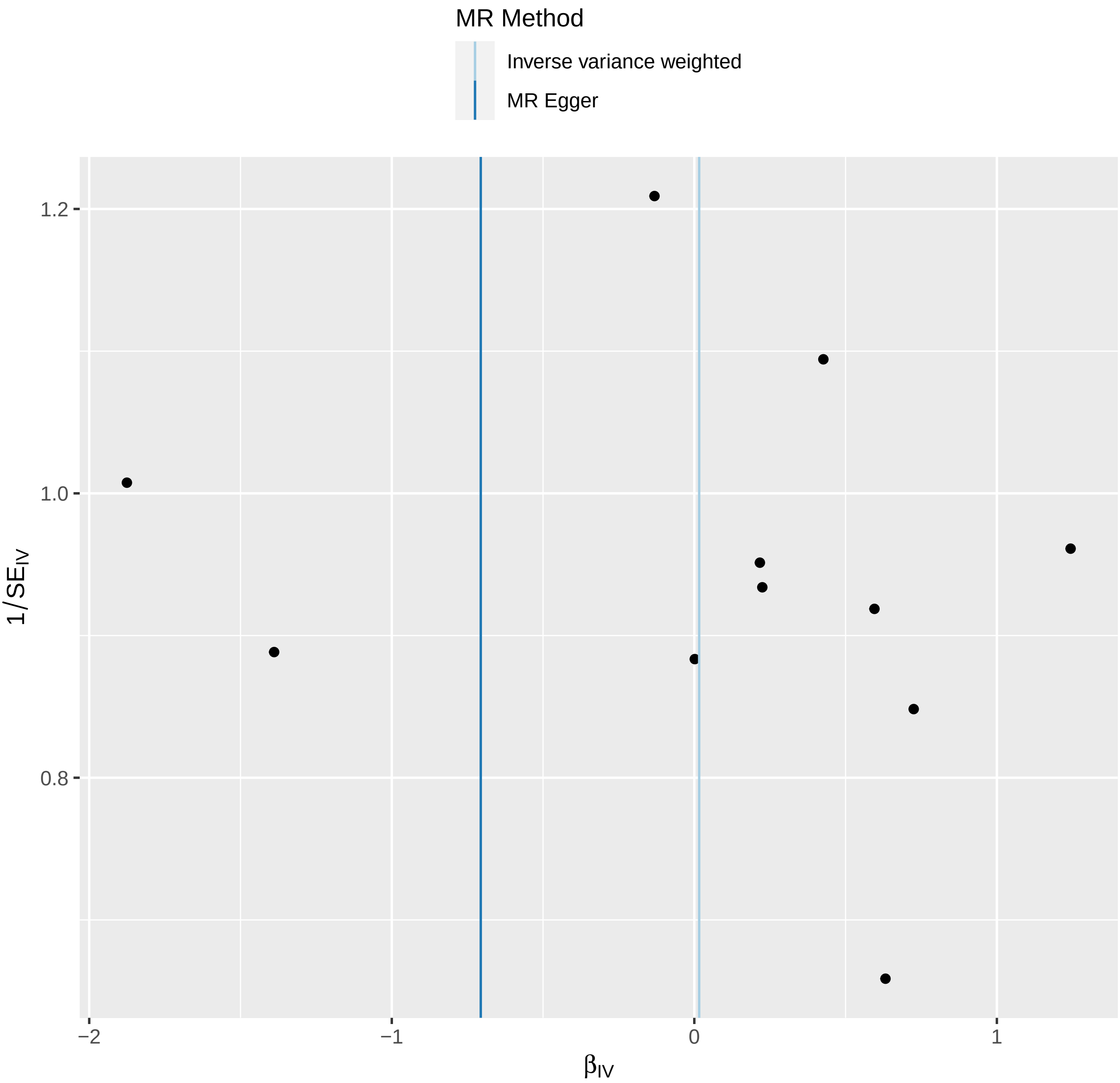

Batch 677 : Pyridoxate on Crohn's disease of small intestine

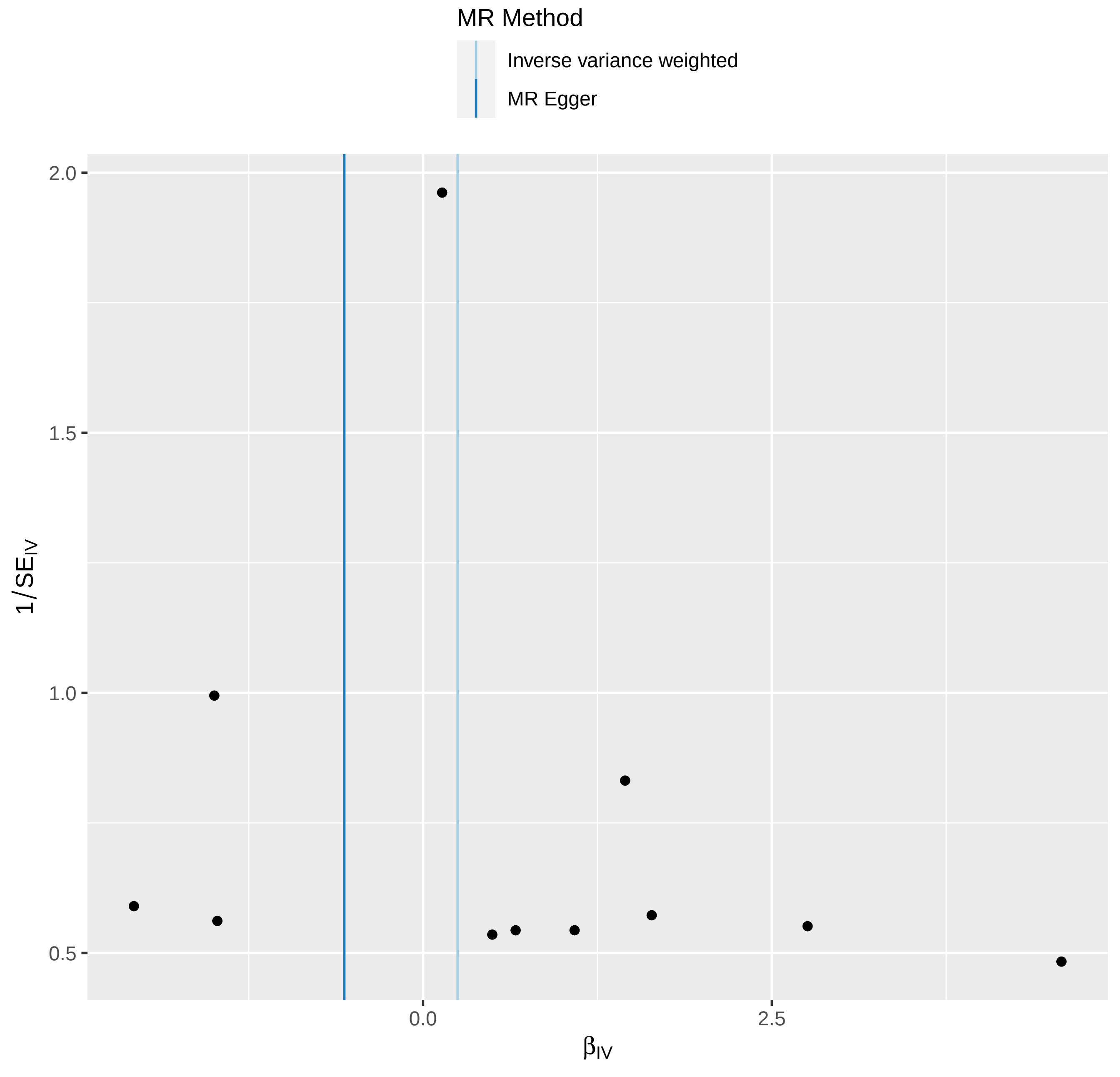

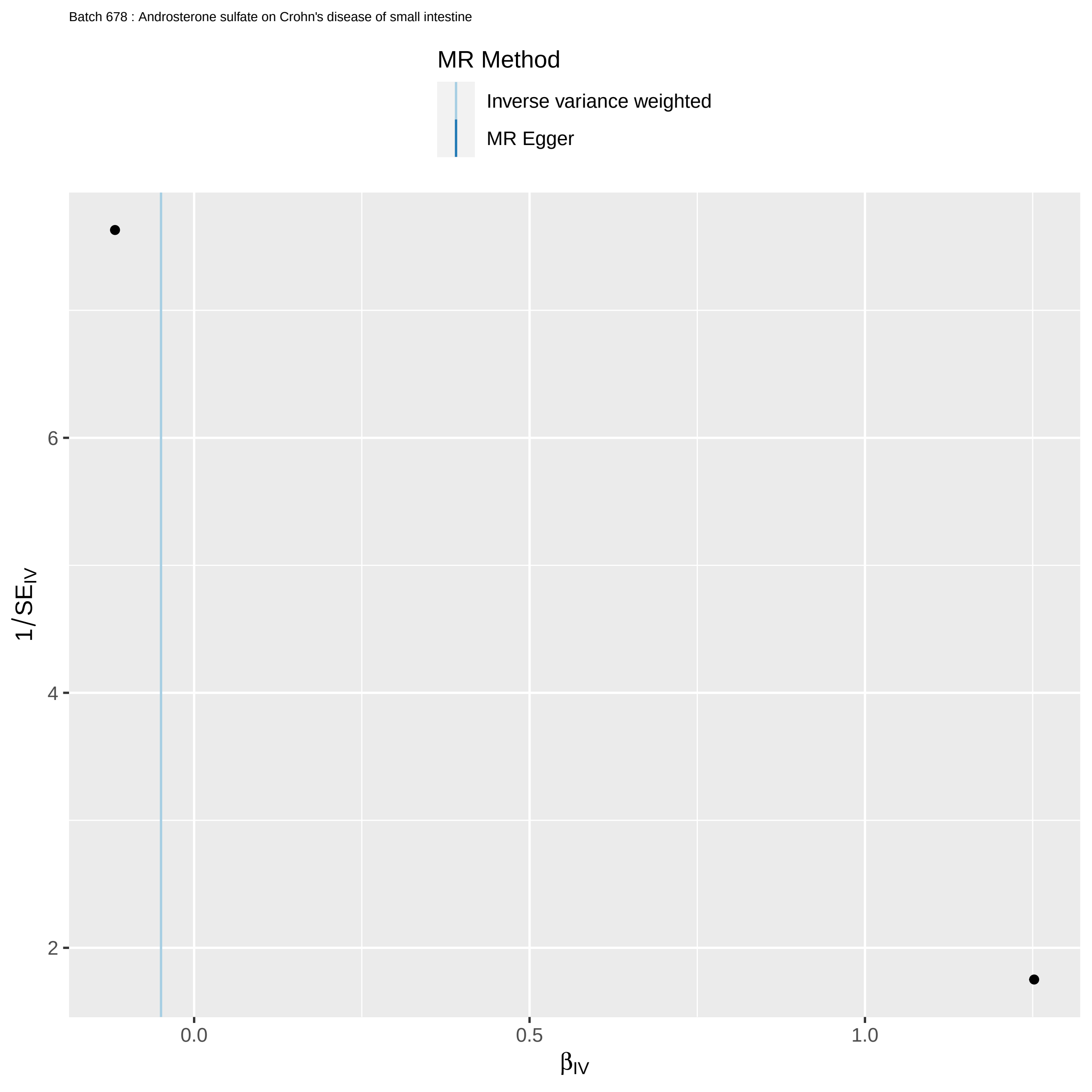

Batch 678 : Androsterone sulfate on Crohn's disease of small intestine
MR Method
Inverse variance weighted
MR Egger
6
4
2
1/SE$_{IV}$
0.0
0.5
1.0
β_{IV}

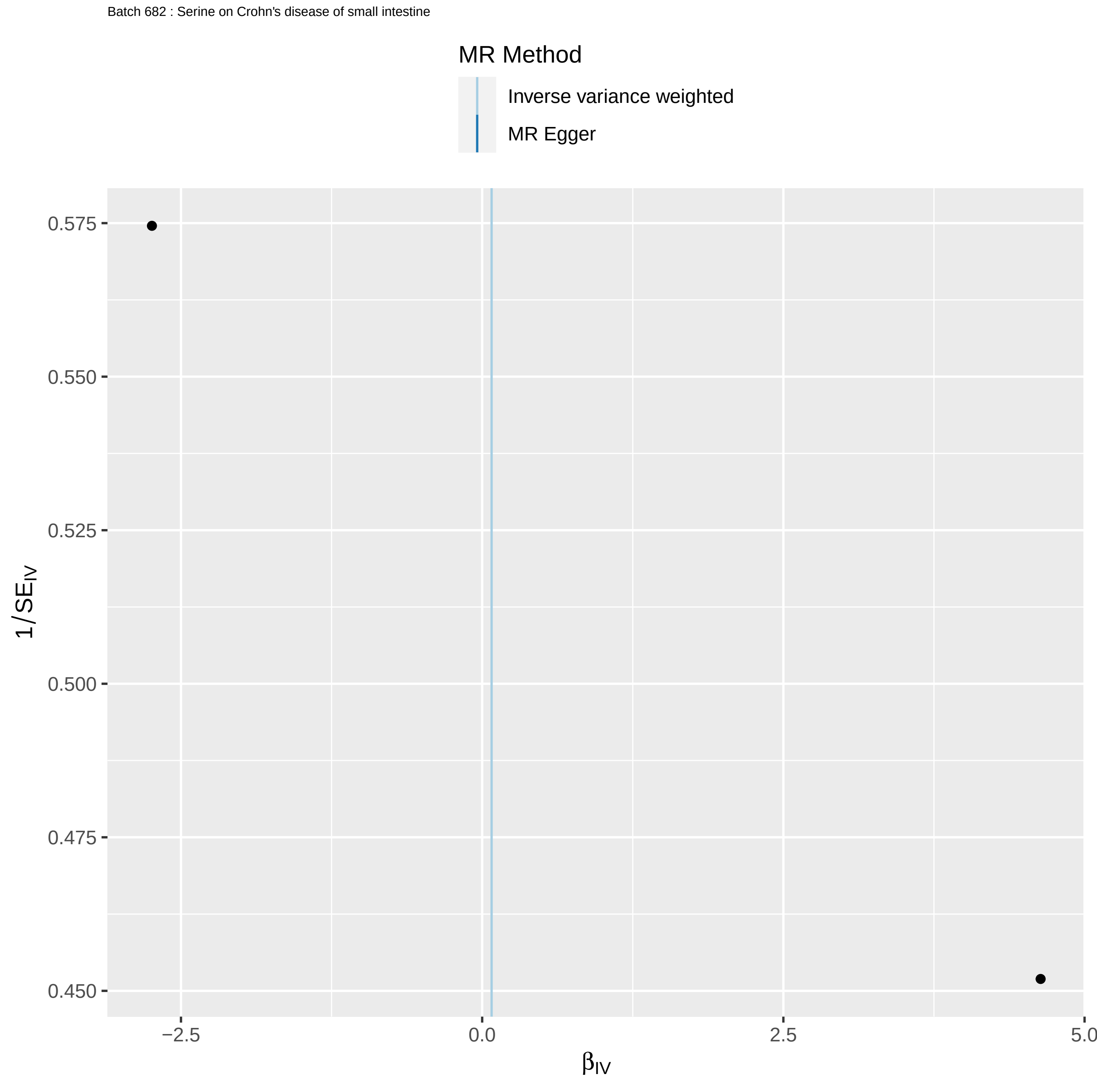

Batch 682 : Serine on Crohn's disease of small intestine
MR Method
Inverse variance weighted
MR Egger
0.575
0.550
0.525
0.500
0.475
0.450
1/SE_IV
−2.5
0.0
2.5
5.0
β_IV

Batch 683 : Trans−4−hydroxyproline on Crohn's disease of small intestine

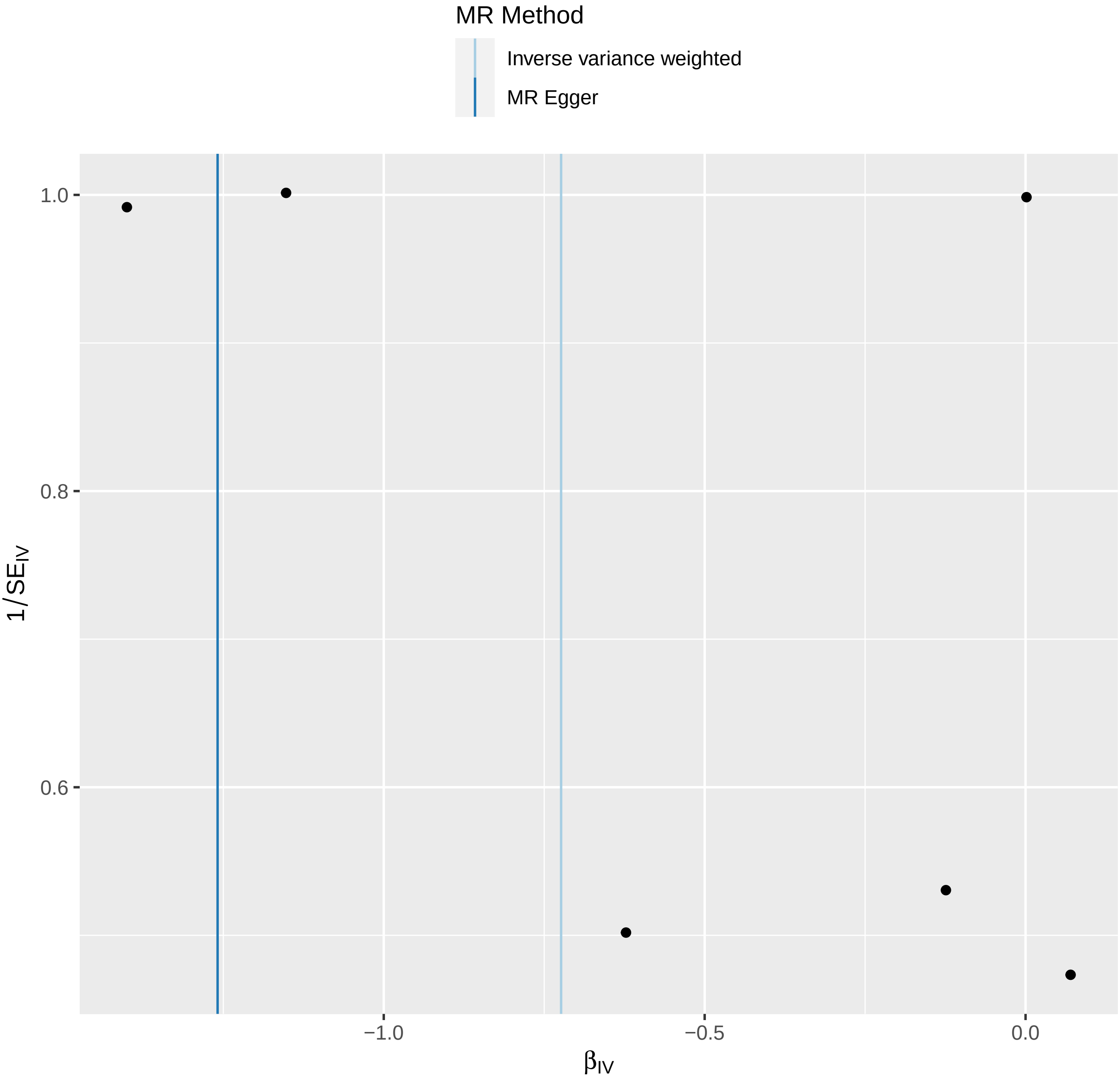

Batch 685 : Hexanoylcarnitine on Crohn's disease of small intestine

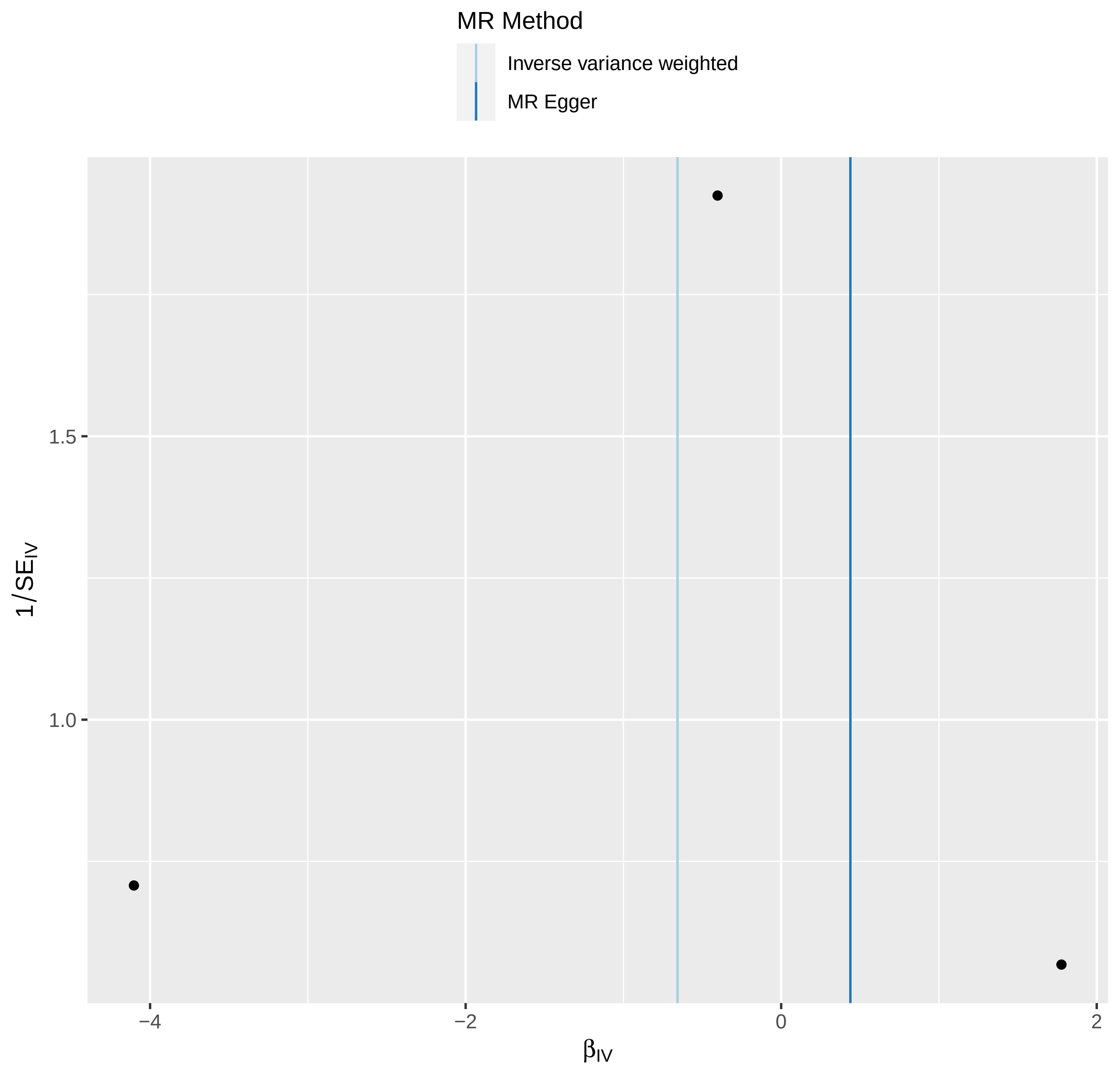

Batch 689 : 2−aminobutyrate on Crohn's disease of small intestine

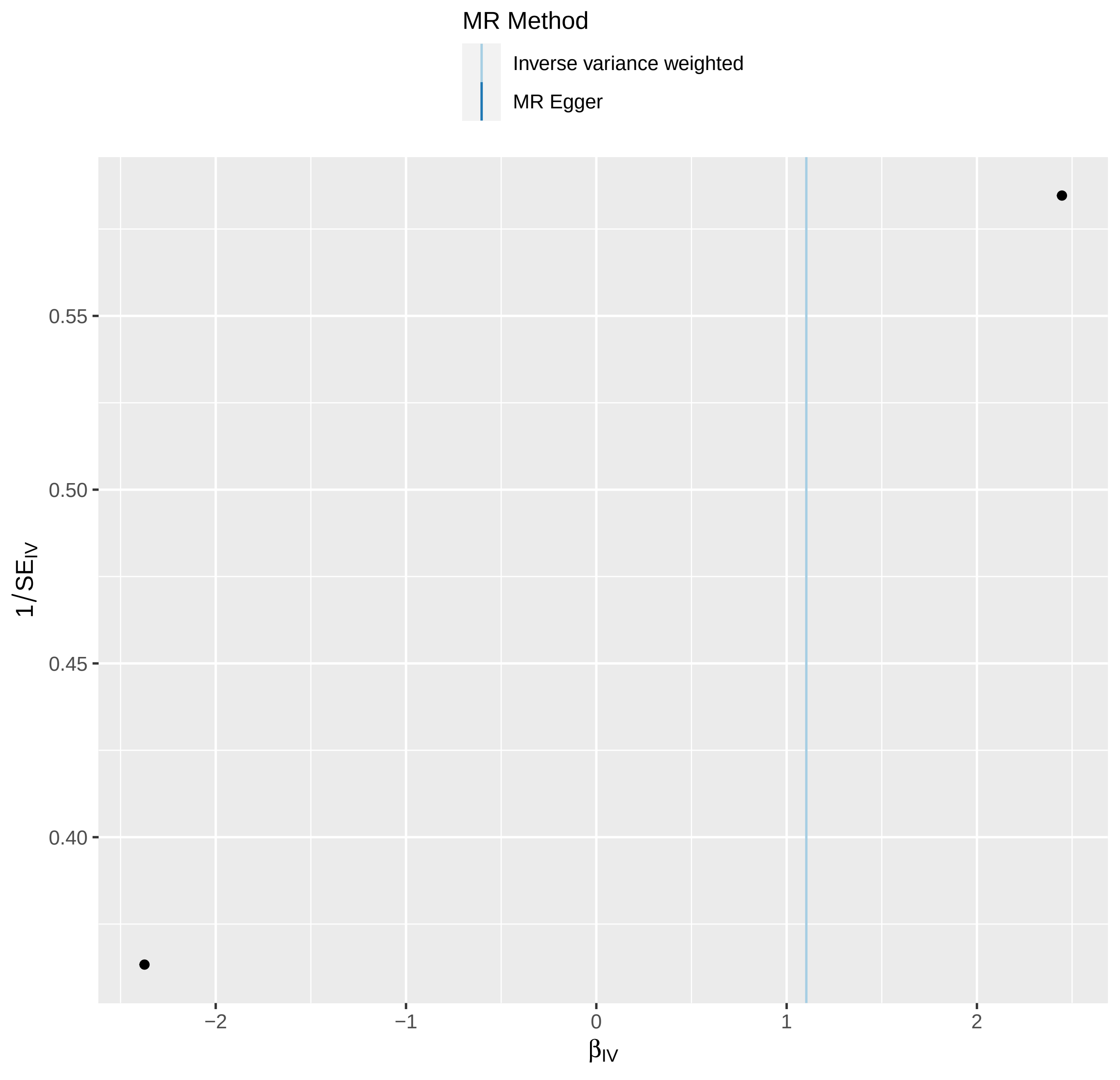

Batch 694 : Butyrylcarnitine on Crohn's disease of small intestine

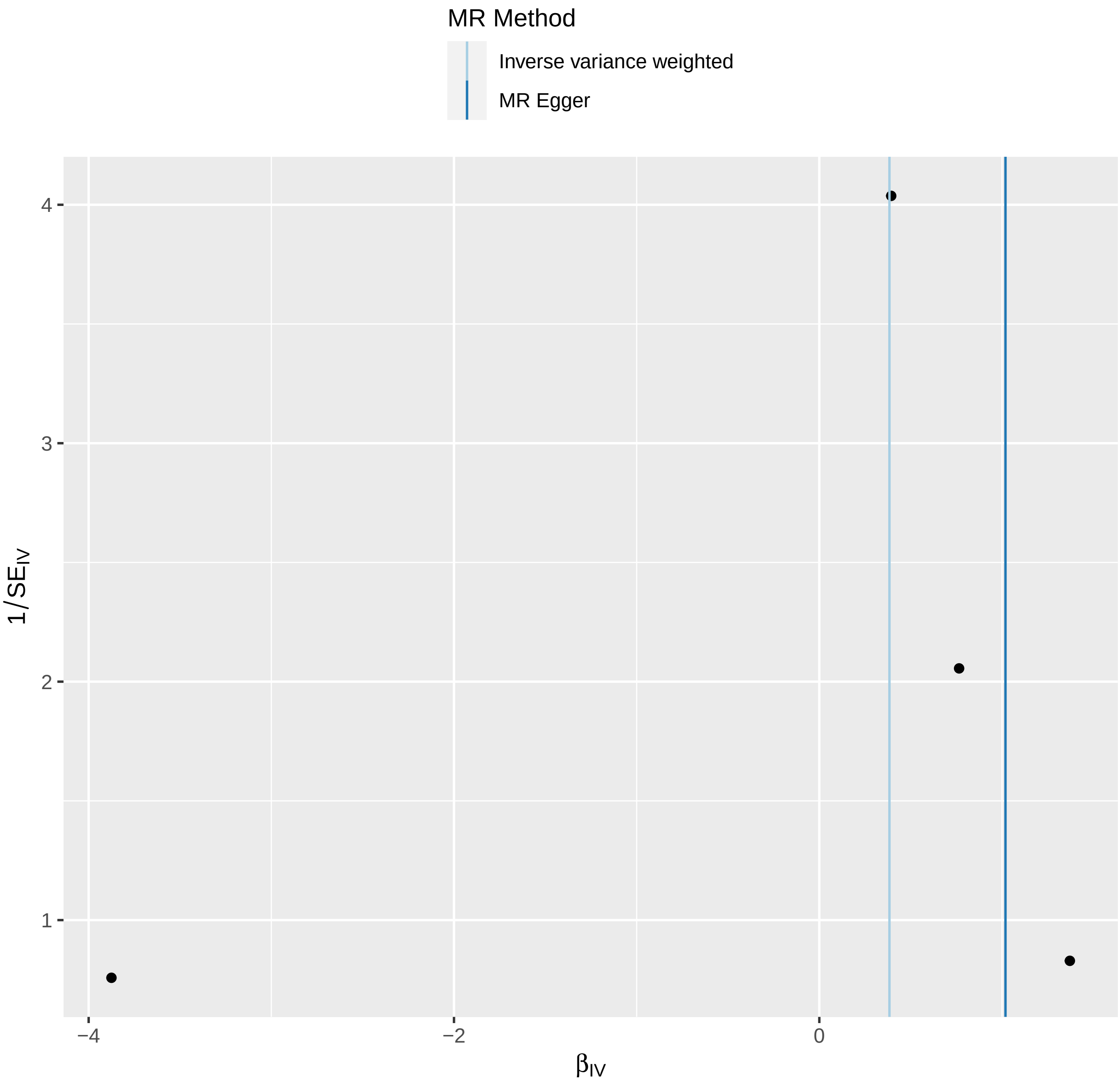

Batch 697 : Propionylcarnitine on Crohn's disease of small intestine

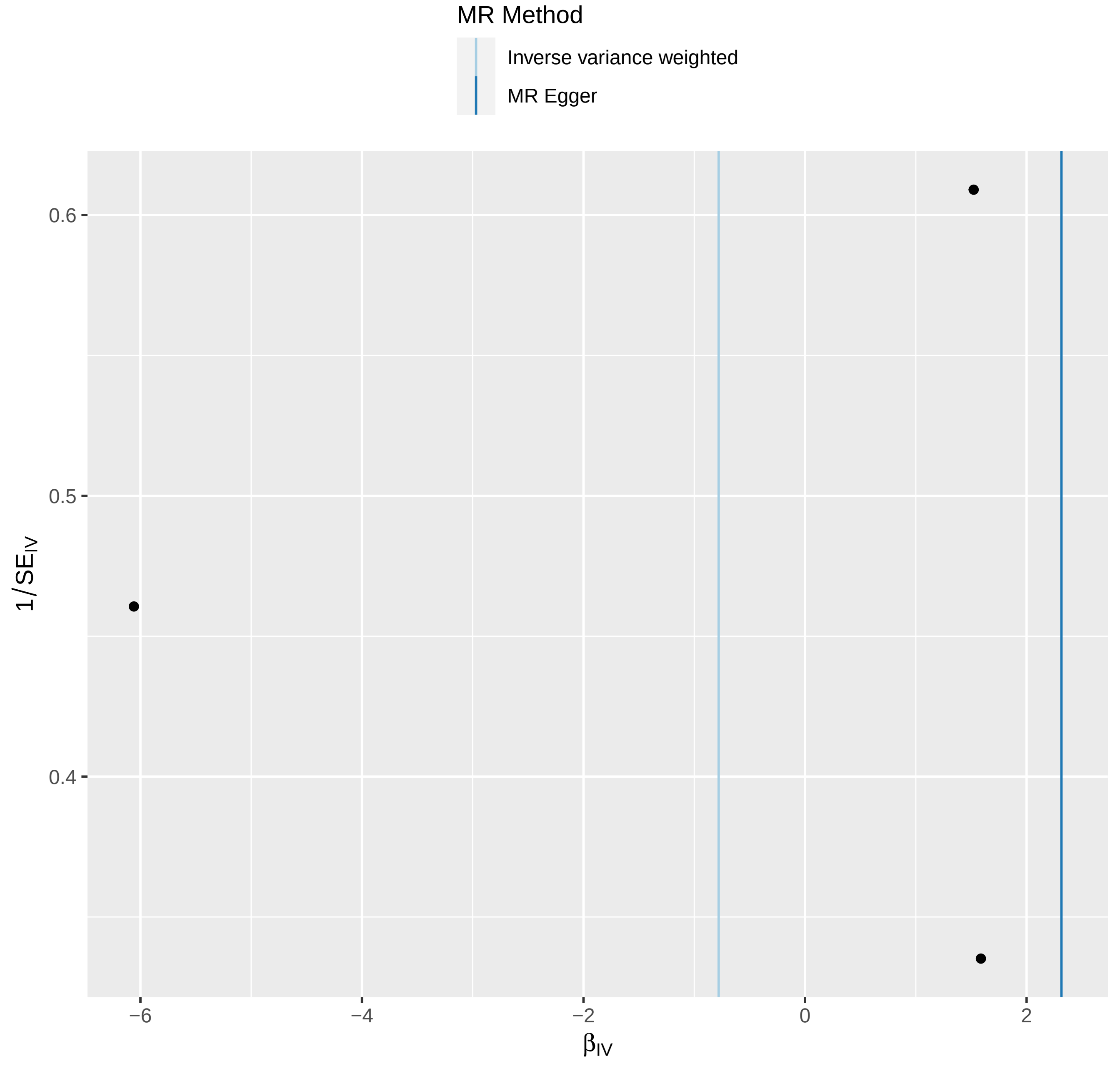

Batch 698 : Caproate (6:0) on Crohn's disease of small intestine

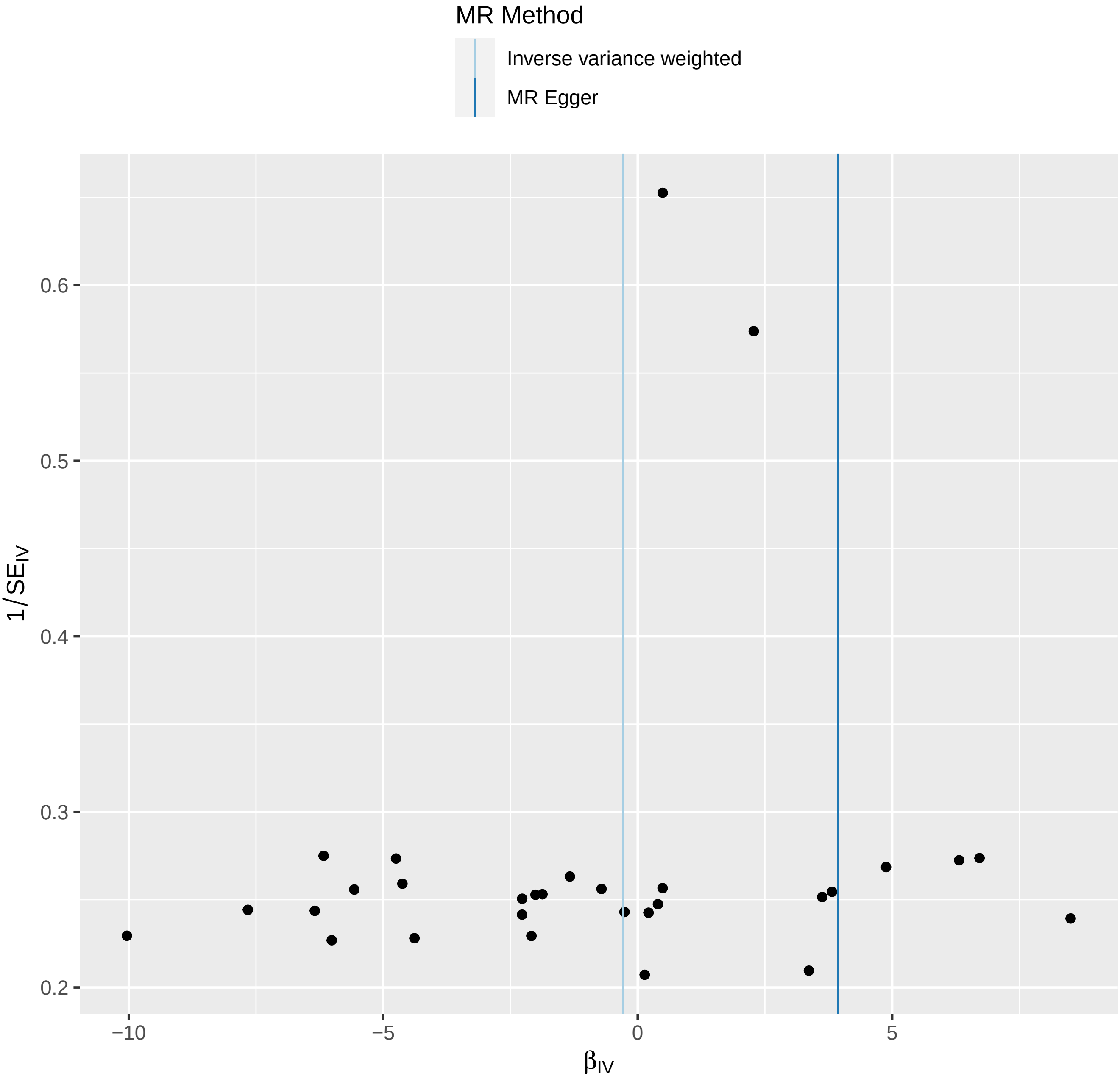

Batch 699 : Caprylate (8:0) on Crohn's disease of small intestine

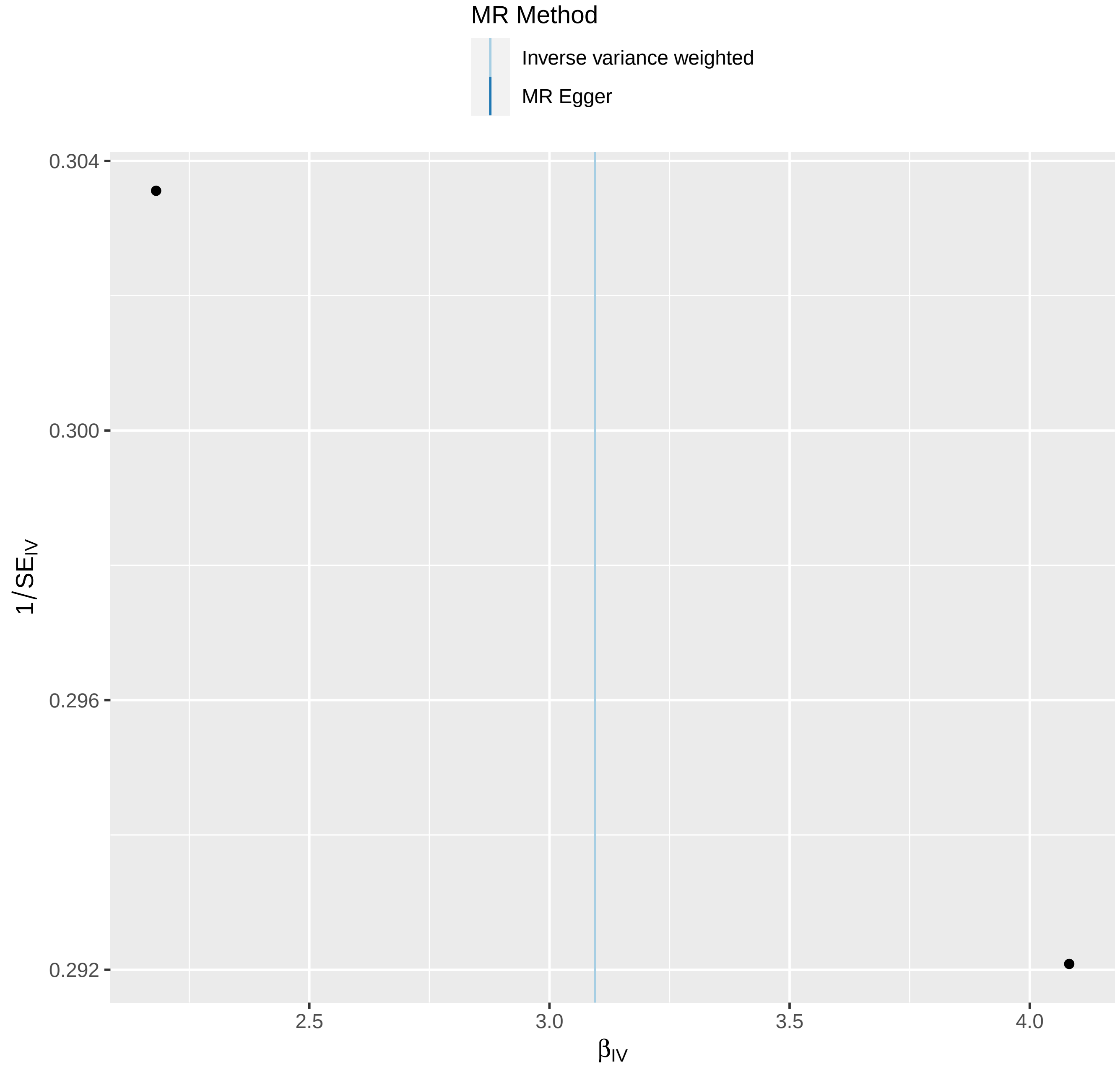

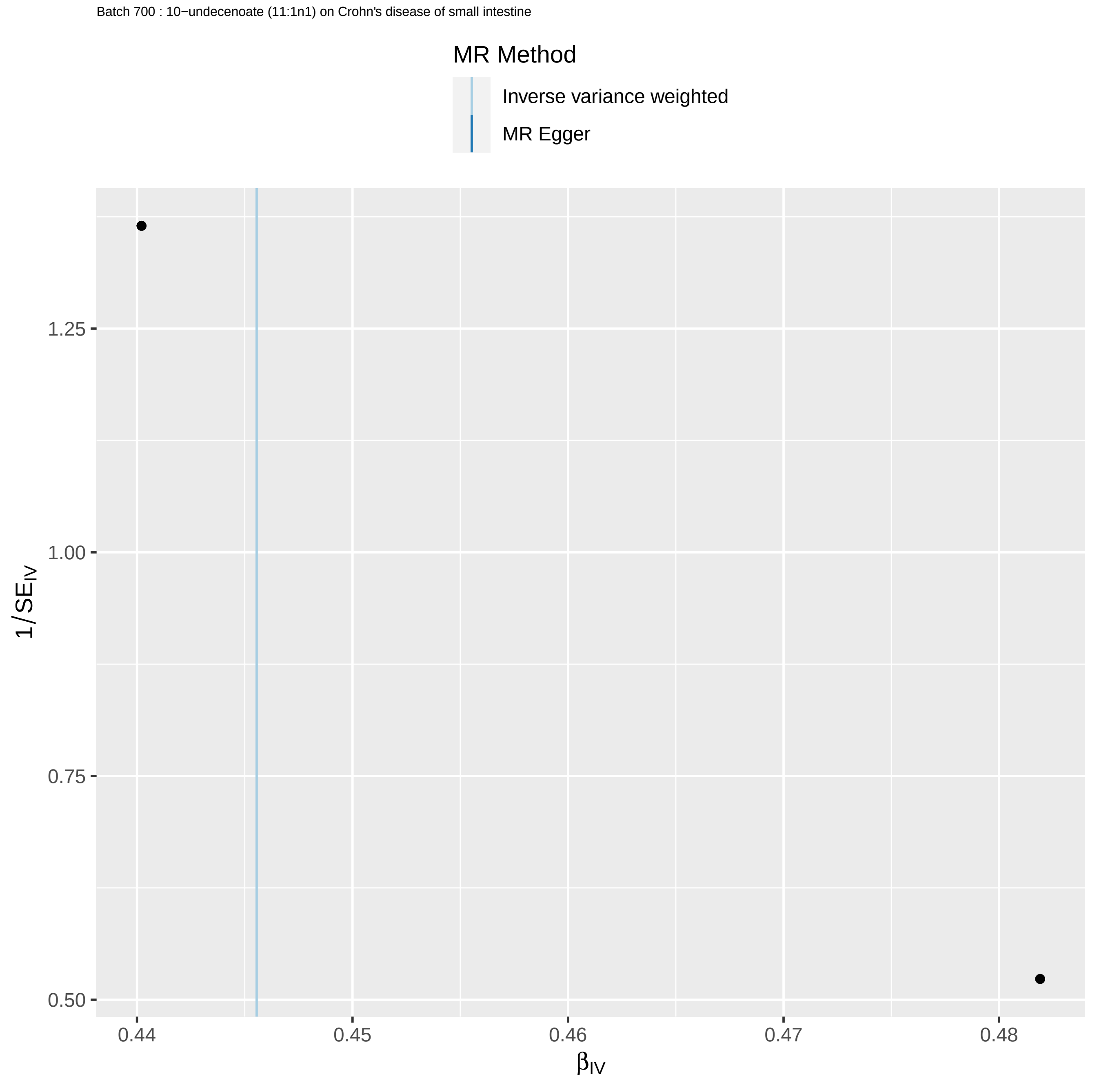

Batch 700 : 10−undecenoate (11:1n1) on Crohn's disease of small intestine
MR Method
Inverse variance weighted
MR Egger
1.25
1.00
0.75
0.50
$1/SE_{IV}$
0.44
0.45
0.46
0.47
0.48
β_{IV}

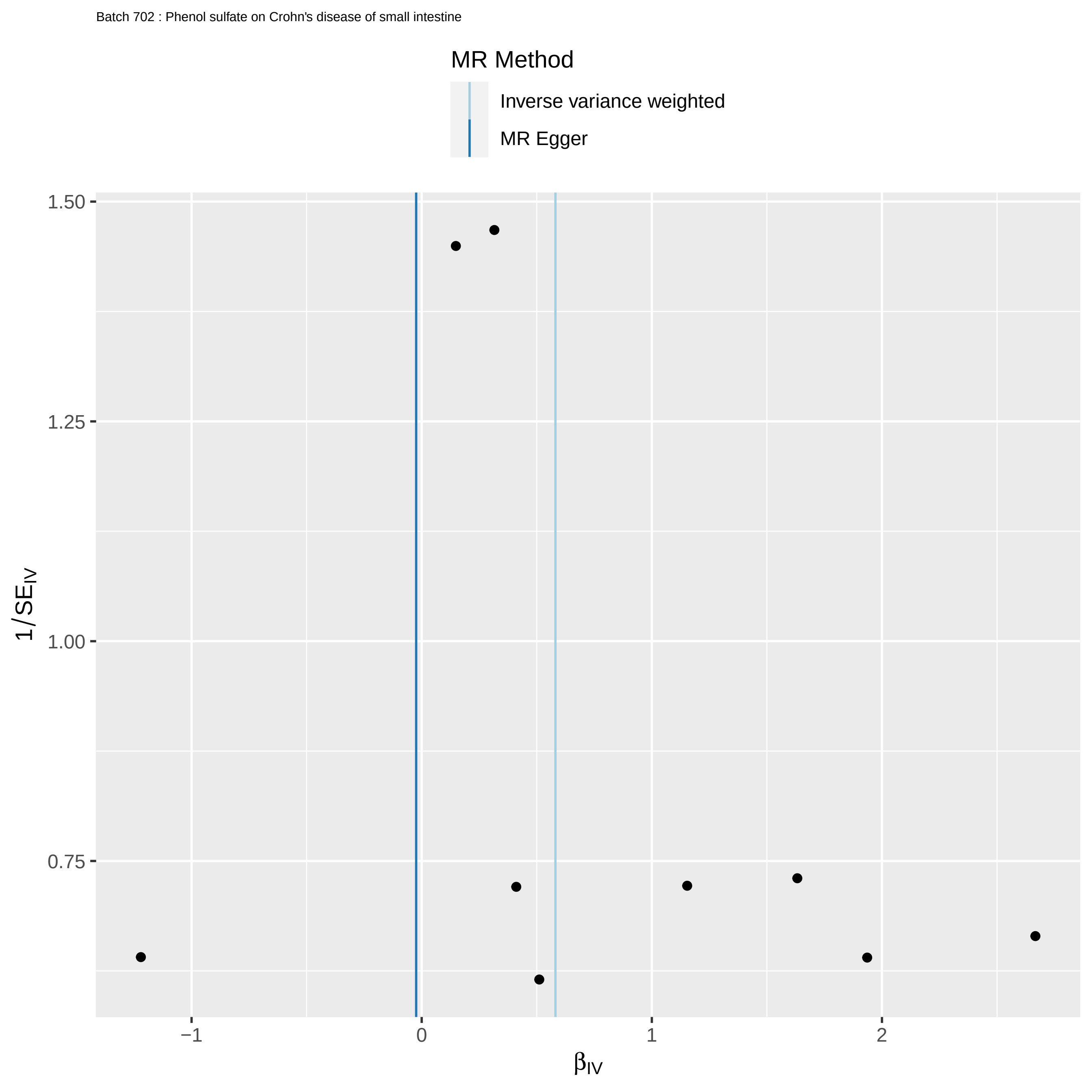
Batch 702 : Phenol sulfate on Crohn's disease of small intestine
MR Method
Inverse variance weighted
MR Egger
1.50
1.25
1.00
0.75
1/SE$_{IV}$
−1
0
1
2
β$_{IV}$

Batch 704 : Heme* on Crohn's disease of small intestine

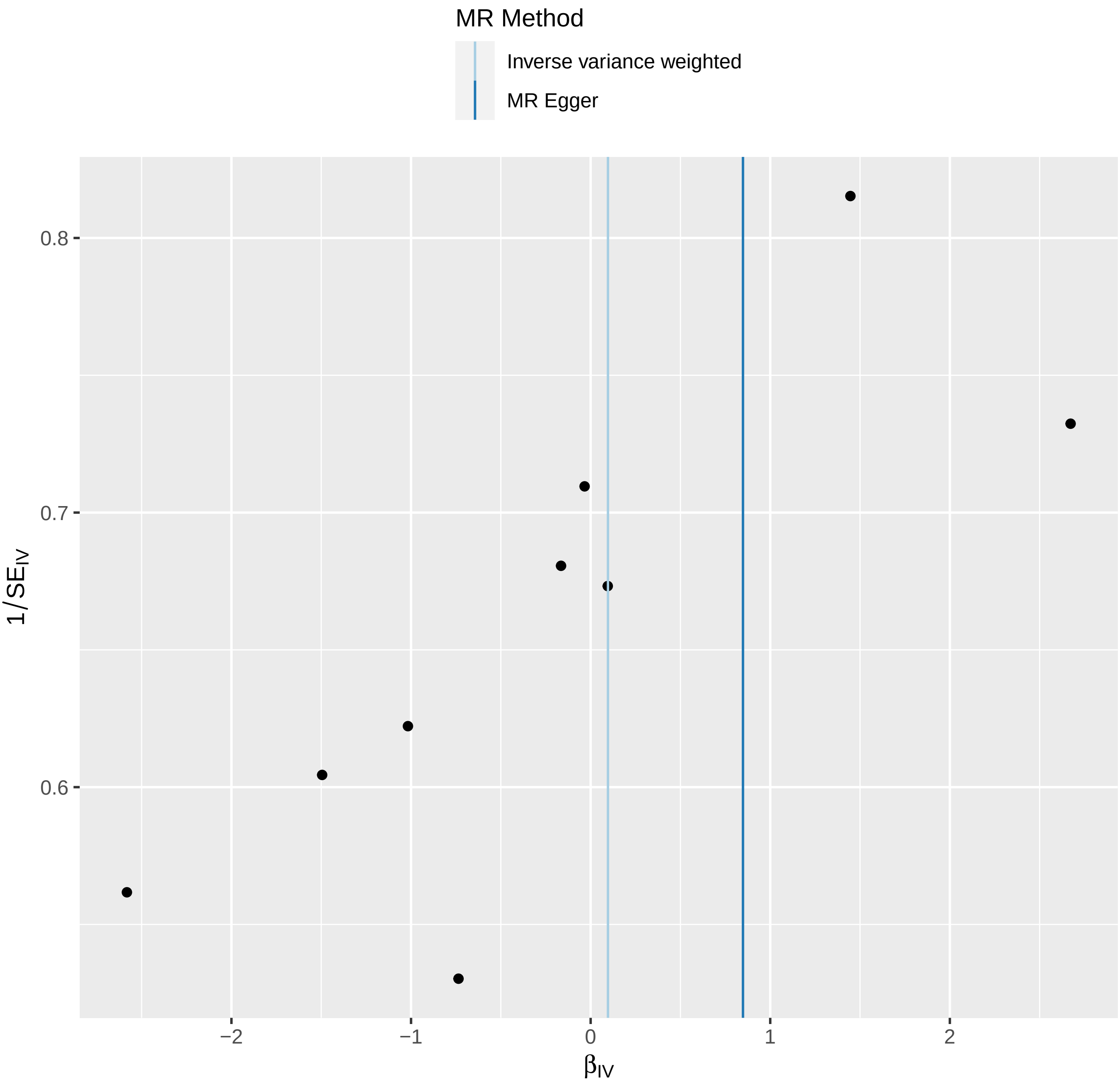

Batch 707 : Pyroglutamine* on Crohn's disease of small intestine

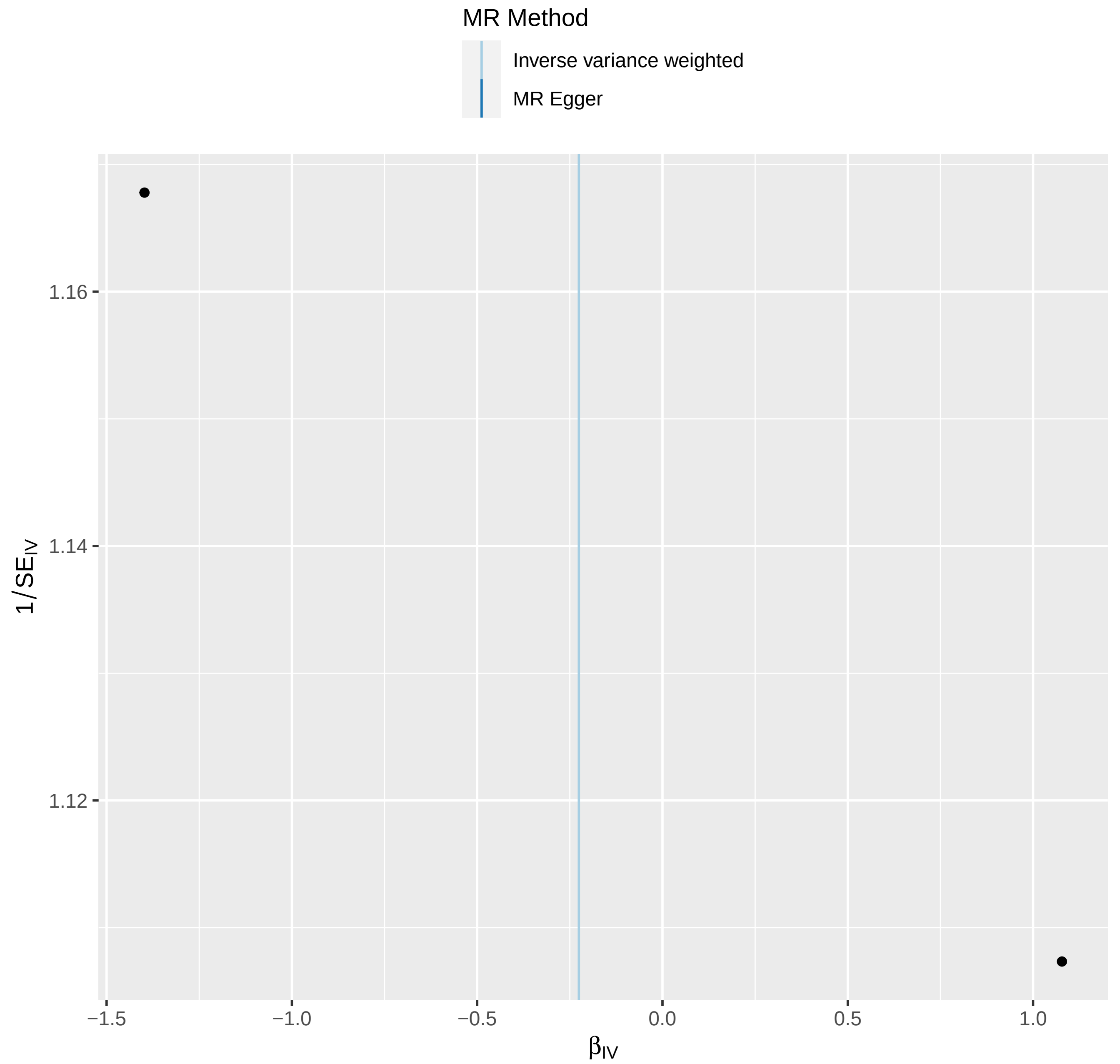

Batch 710 : X−11422−−xanthine on Crohn's disease of small intestine

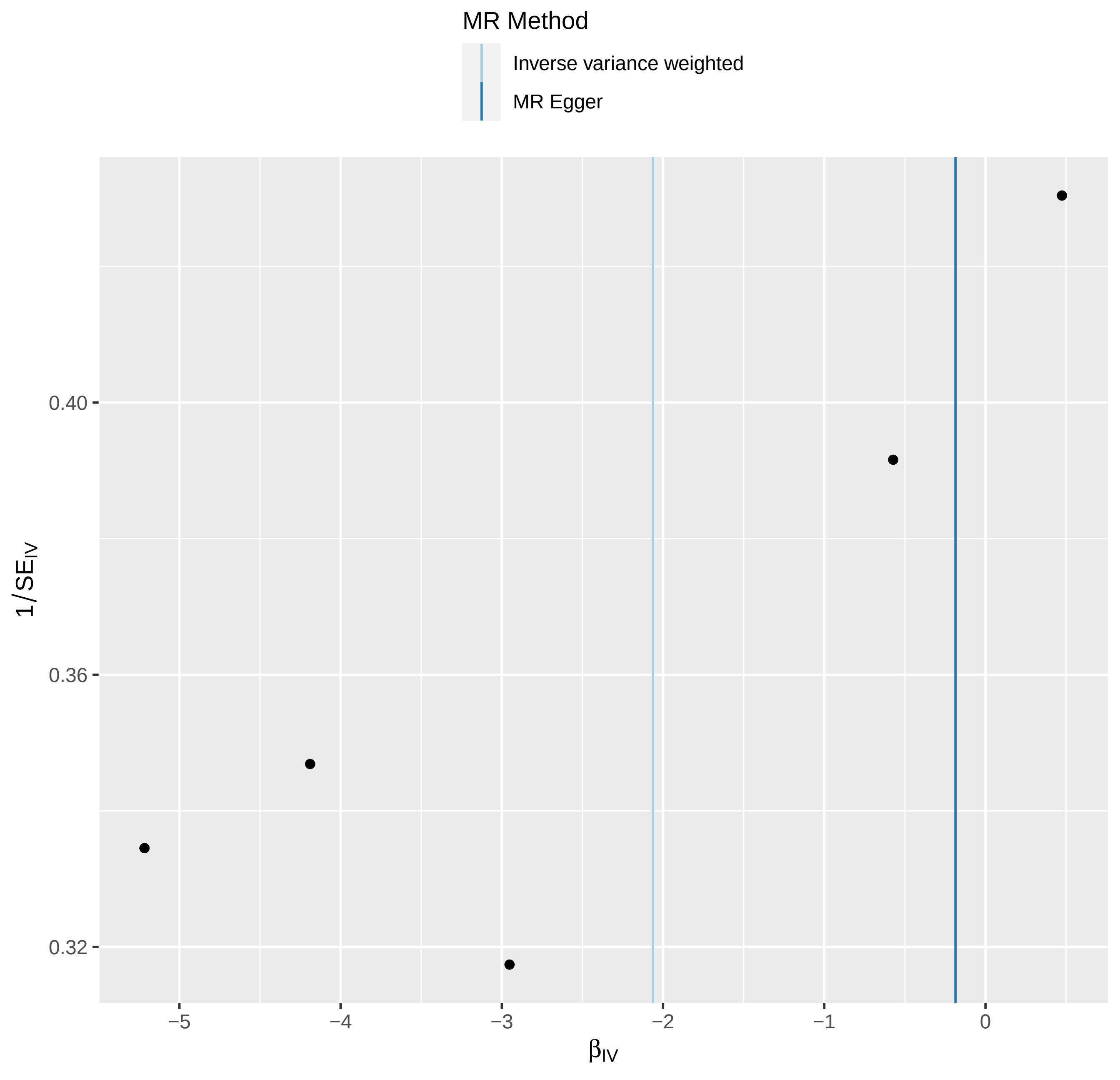

Batch 713 : HWESASXX* on Crohn's disease of small intestine

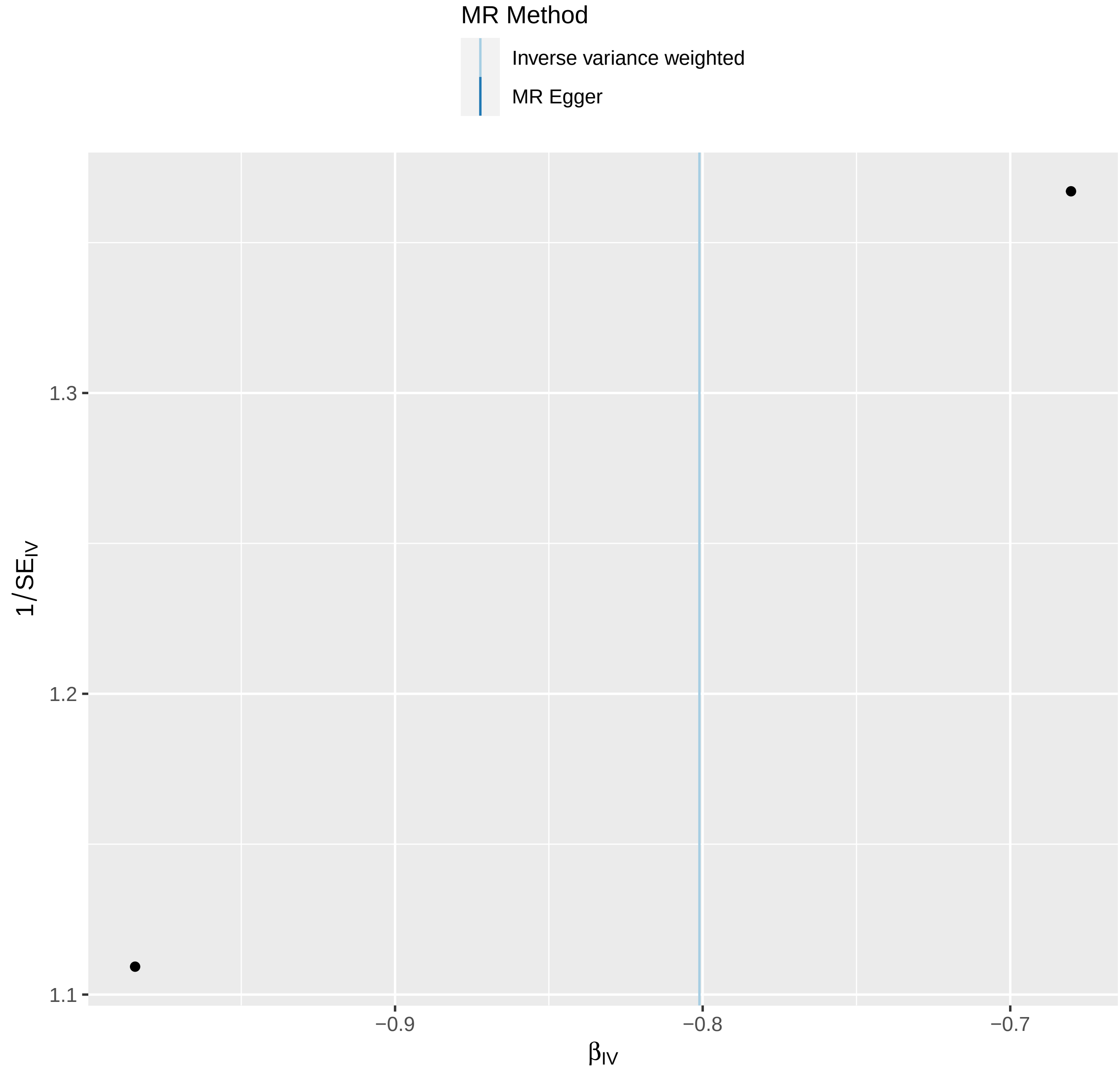

Batch 714 : X−11593−−O−methylascorbate* on Crohn's disease of small intestine

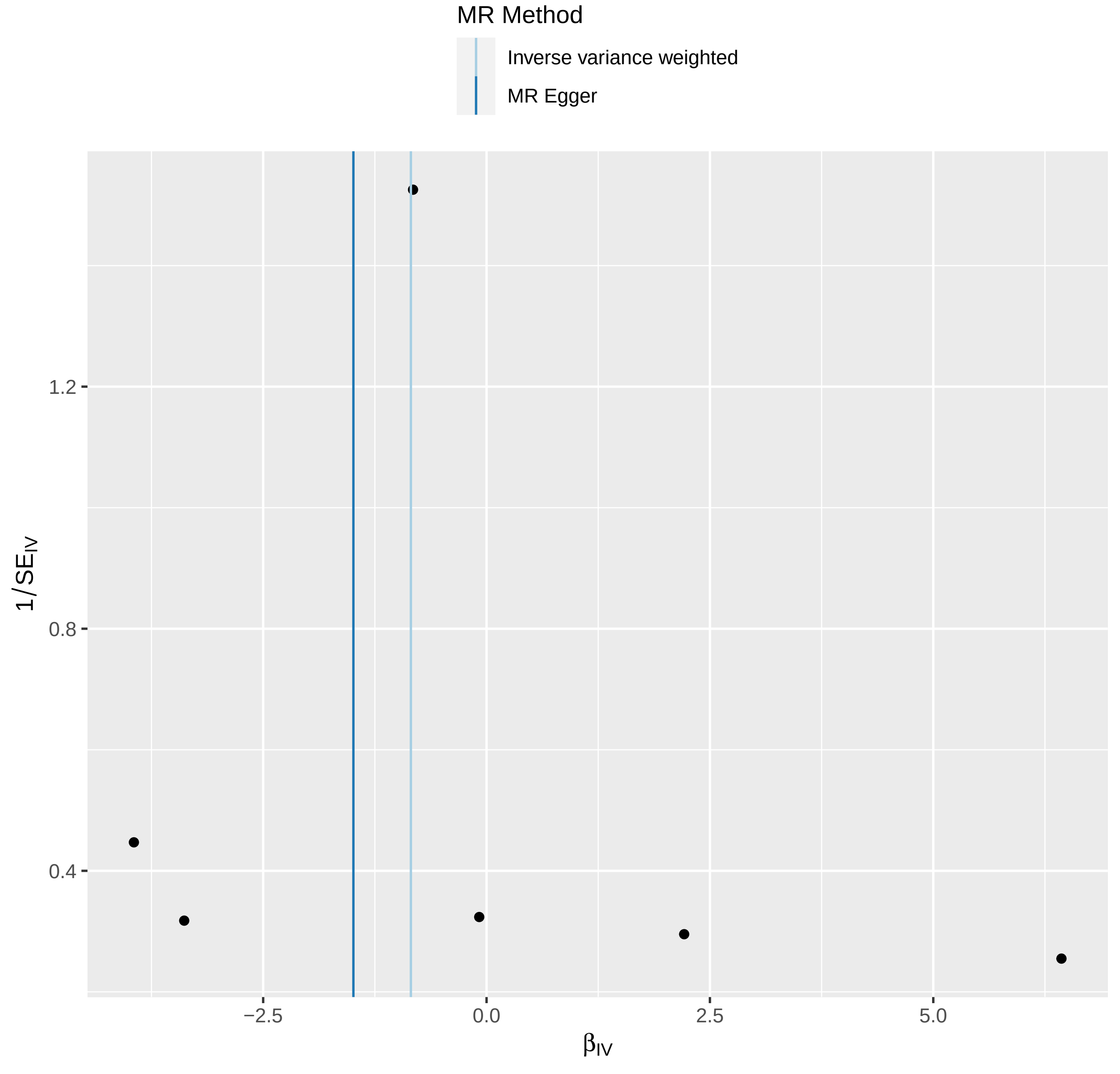

Batch 716 : Homostachydrine* on Crohn's disease of small intestine

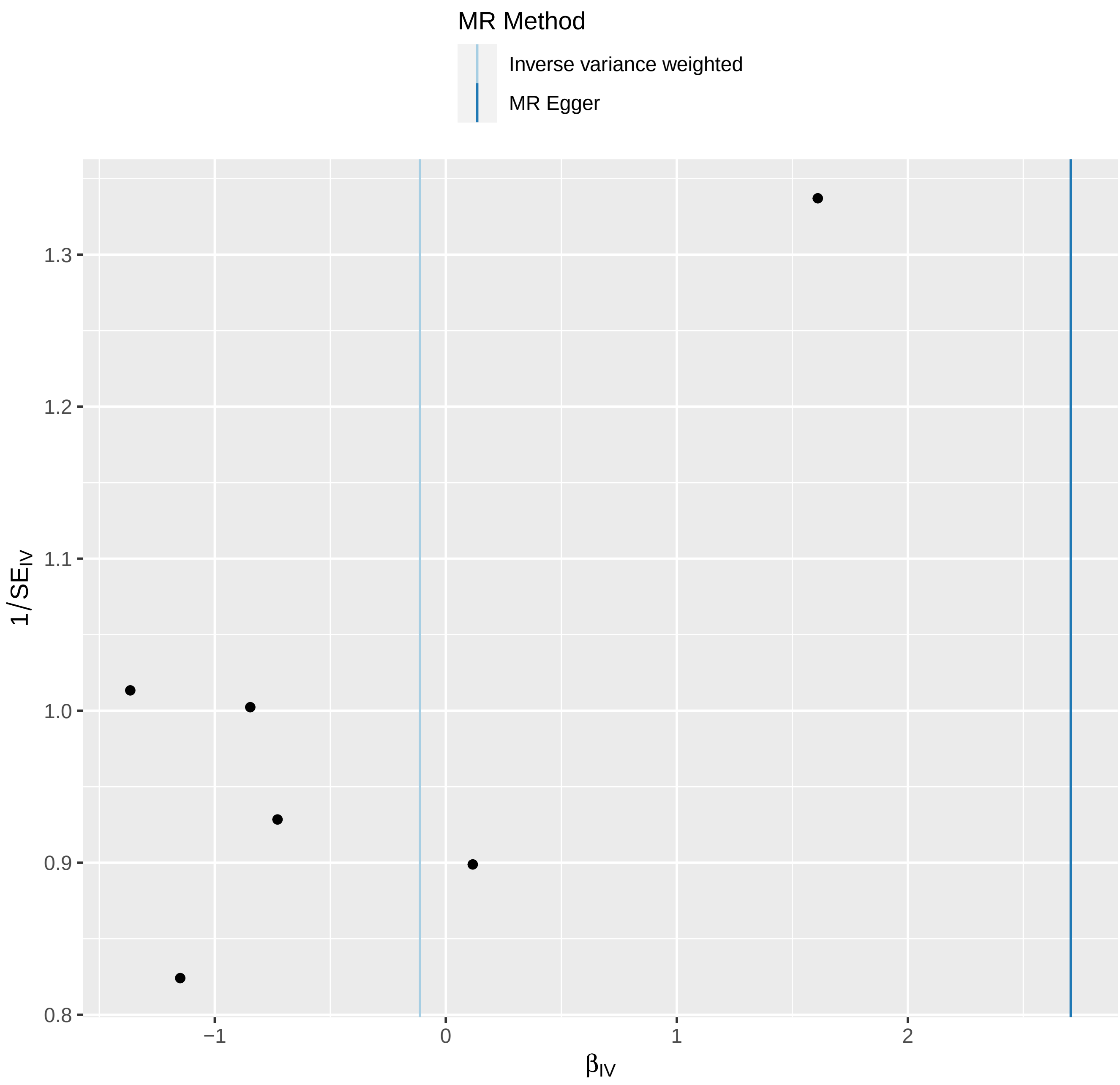

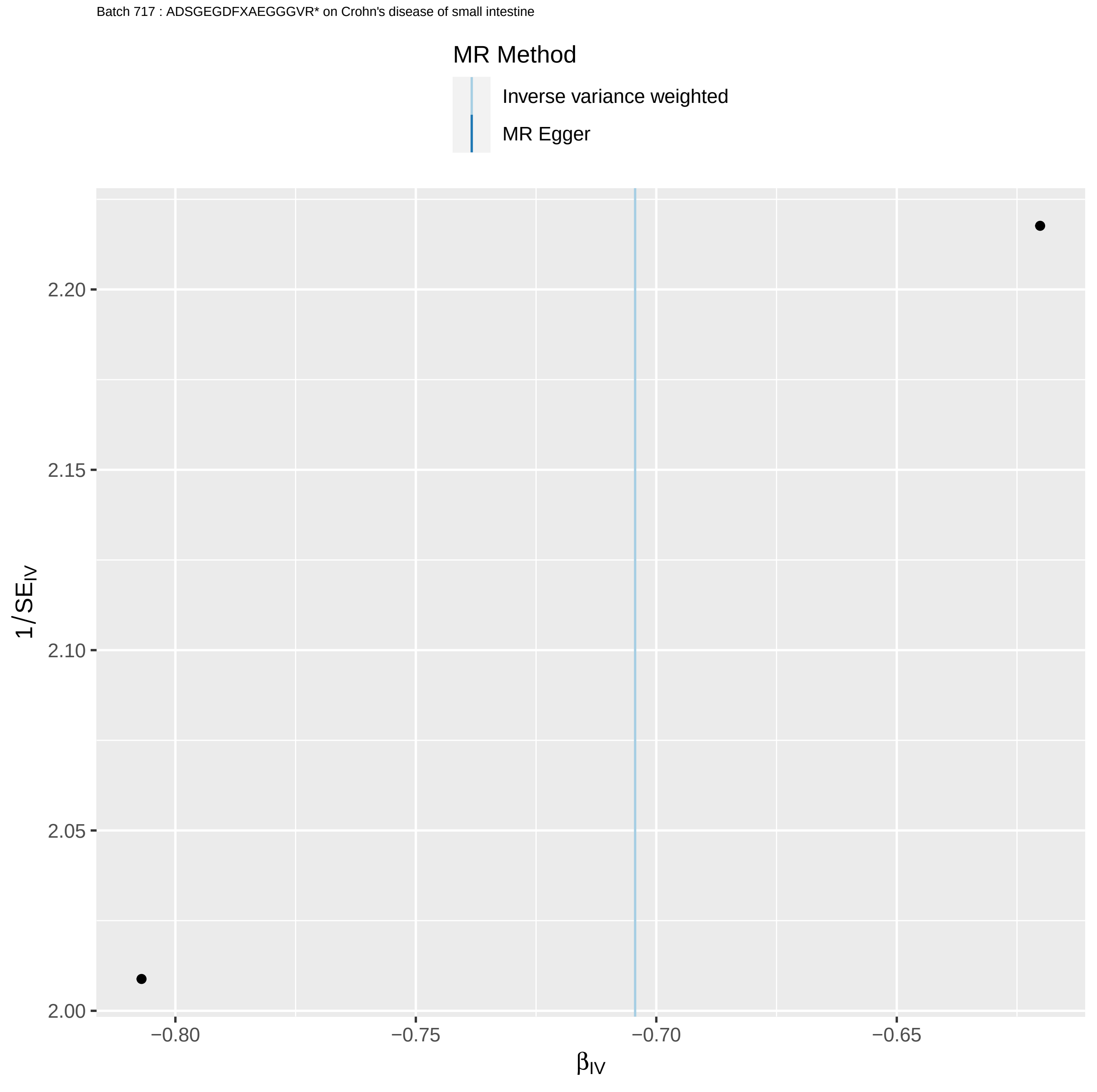
Batch 717 : ADSGEGDFXAEGGGVR* on Crohn's disease of small intestine
MR Method
Inverse variance weighted
MR Egger
2.20
2.15
2.10
2.05
2.00
1/SE$_{IV}$
−0.80
−0.75
−0.70
−0.65
β_{IV}

Batch 719 : X−11793−−oxidized bilirubin* on Crohn's disease of small intestine

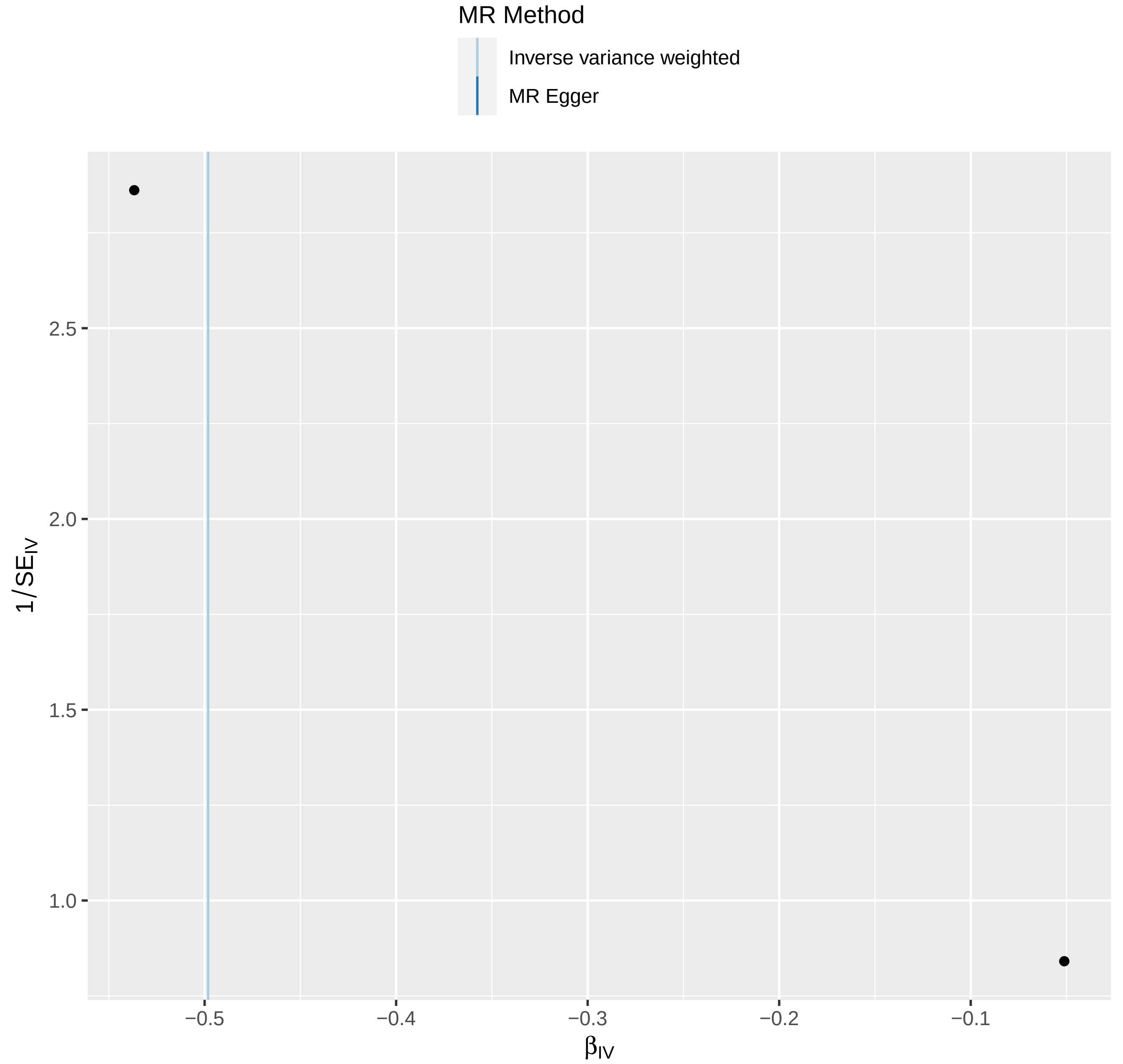

Batch 720 : 1−arachidonoylglycerophosphocholine* on Crohn's disease of small intestine

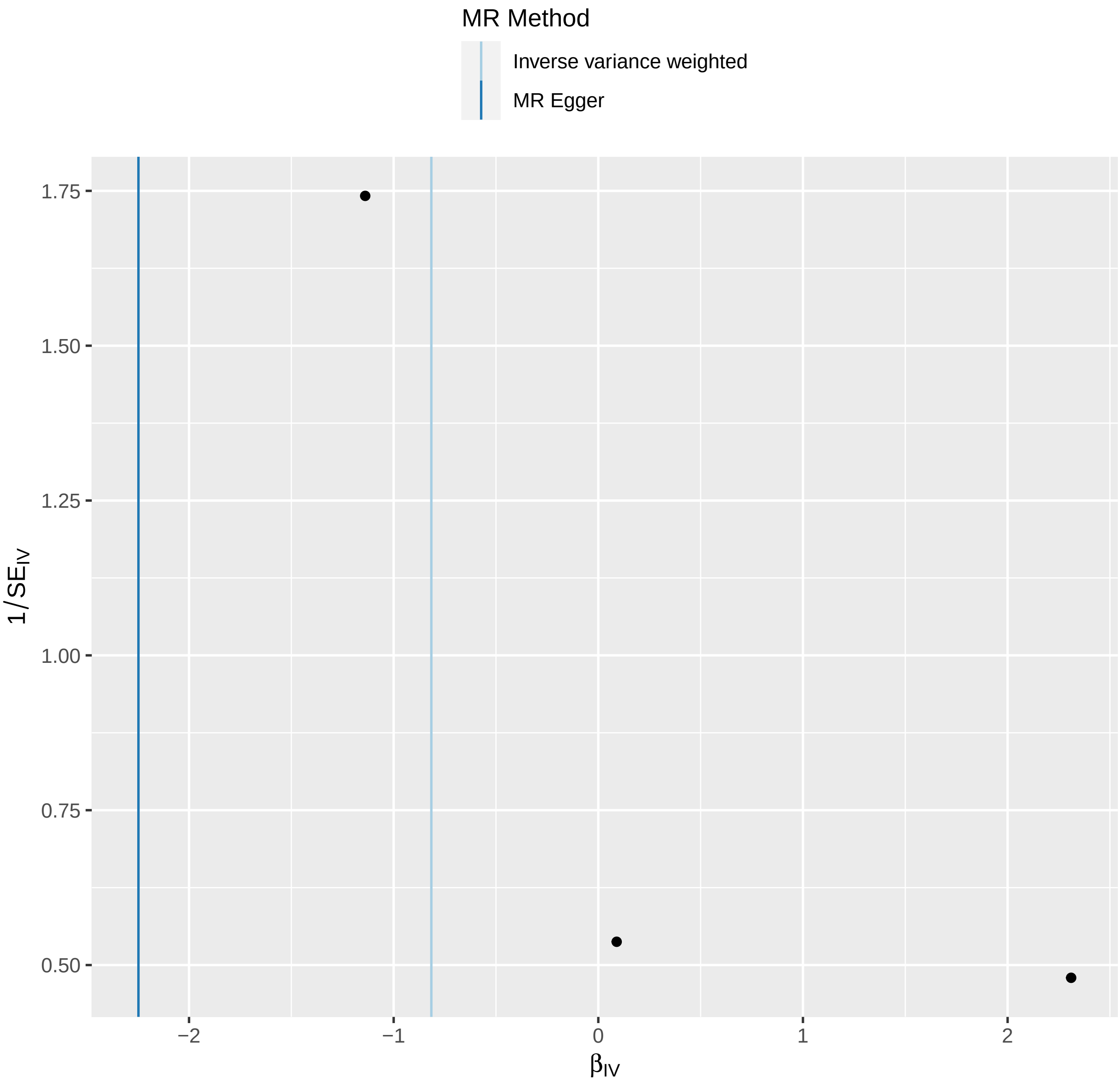

Batch 722 : Gamma−glutamylmethionine* on Crohn's disease of small intestine

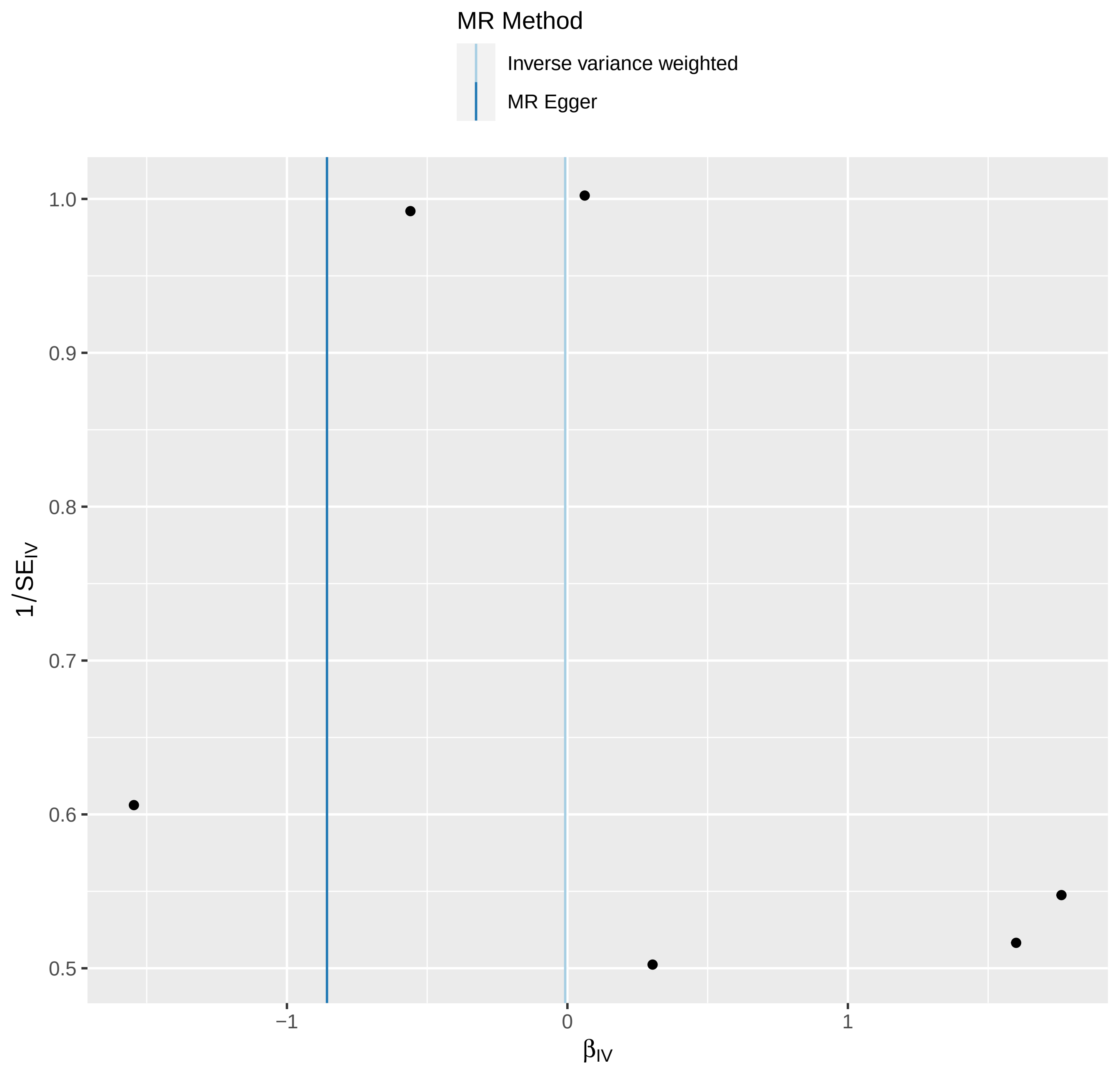

Batch 723 : Gamma−glutamylthreonine* on Crohn's disease of small intestine

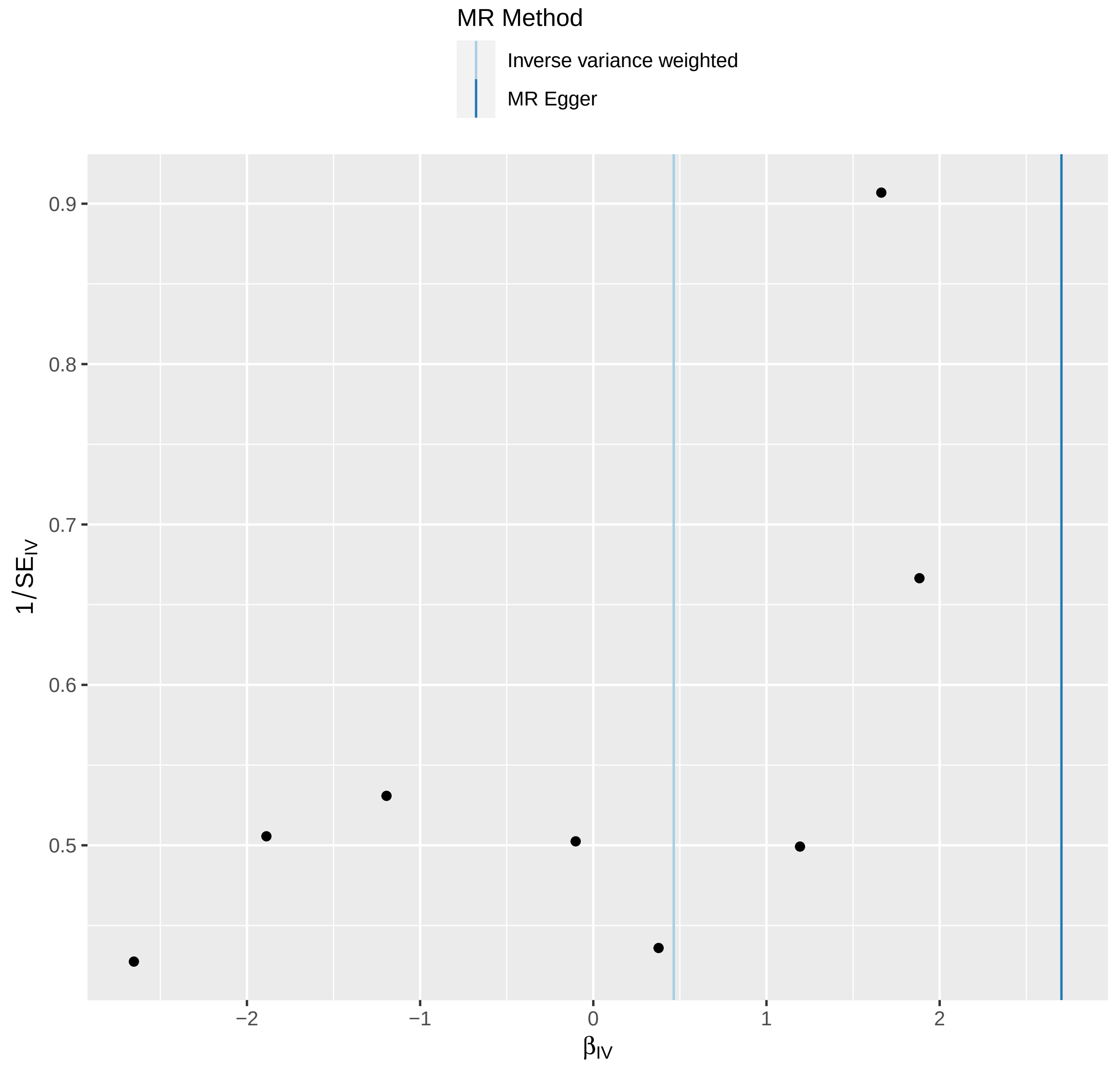

Batch 724 : Gamma−tocopherol on Crohn's disease of small intestine

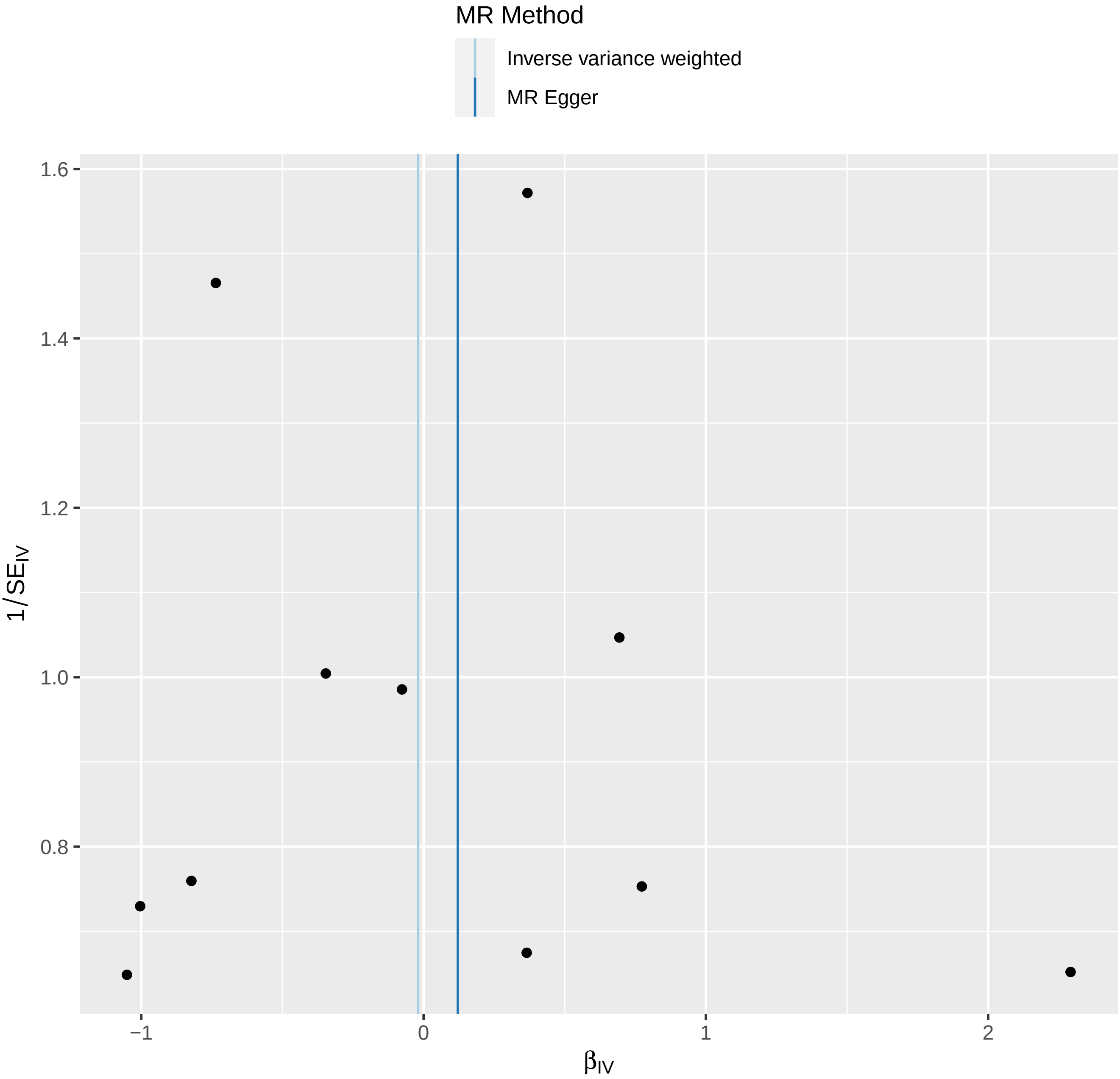

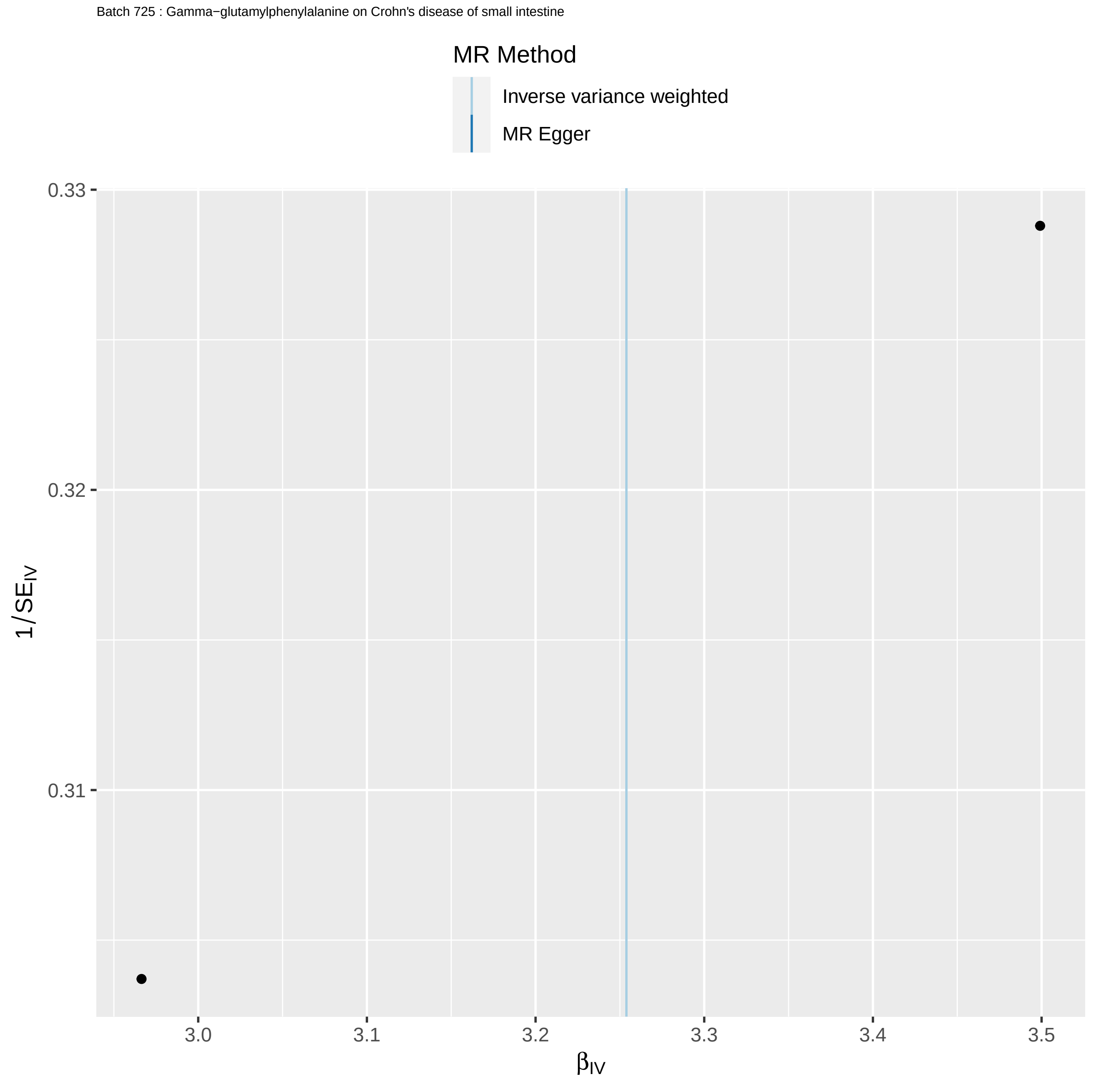
Batch 725 : Gamma−glutamylphenylalanine on Crohn's disease of small intestine
MR Method
Inverse variance weighted
MR Egger
0.33
0.32
0.31
1/SE_IV
3.0
3.1
3.2
3.3
3.4
3.5
β_IV

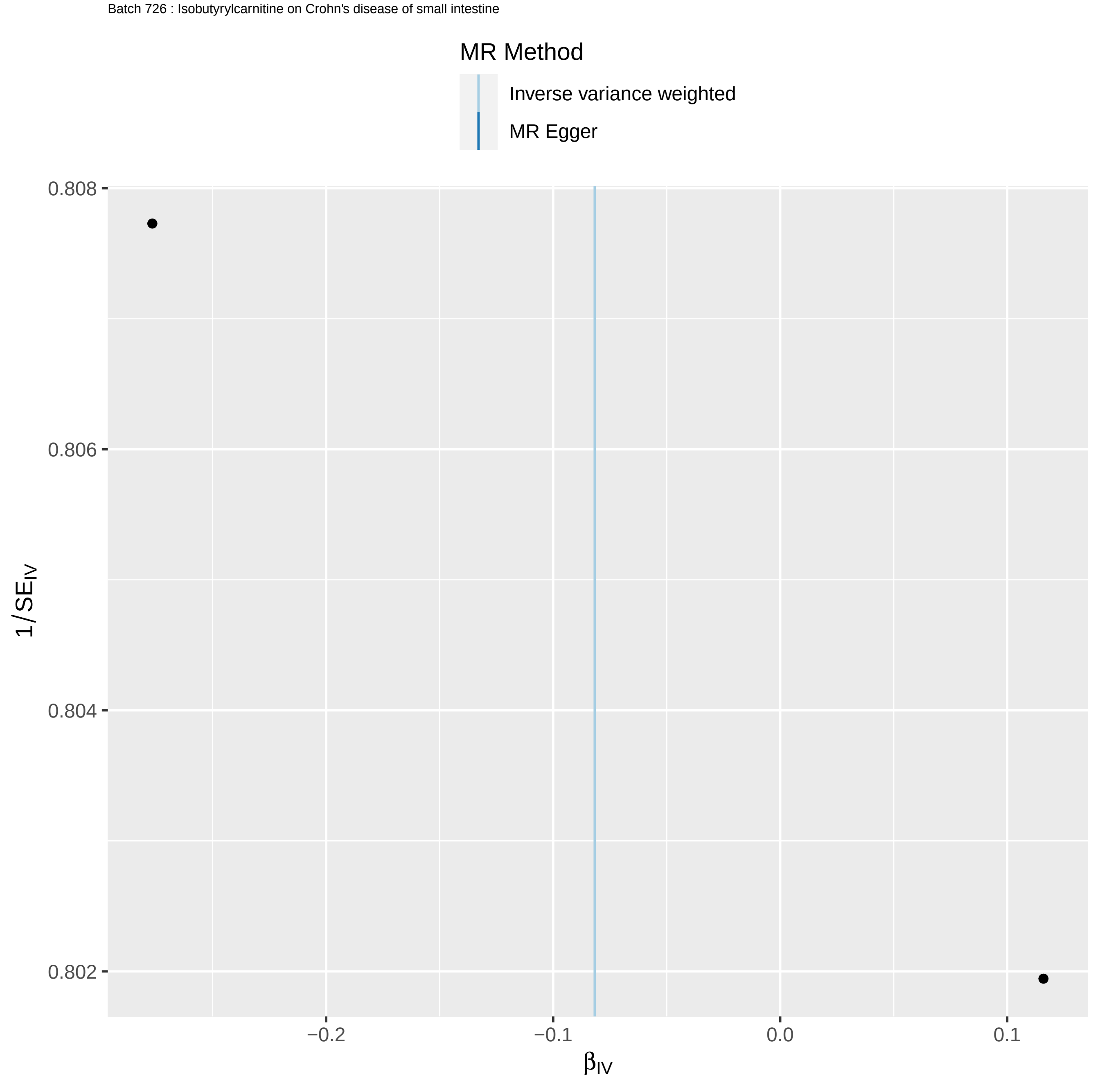

Batch 726 : Isobutyrylcarnitine on Crohn's disease of small intestine
MR Method
Inverse variance weighted
MR Egger
0.808
0.806
0.804
0.802
1/SE_IV
−0.2
−0.1
0.0
0.1
β_IV

Batch 727 : Pseudouridine on Crohn's disease of small intestine

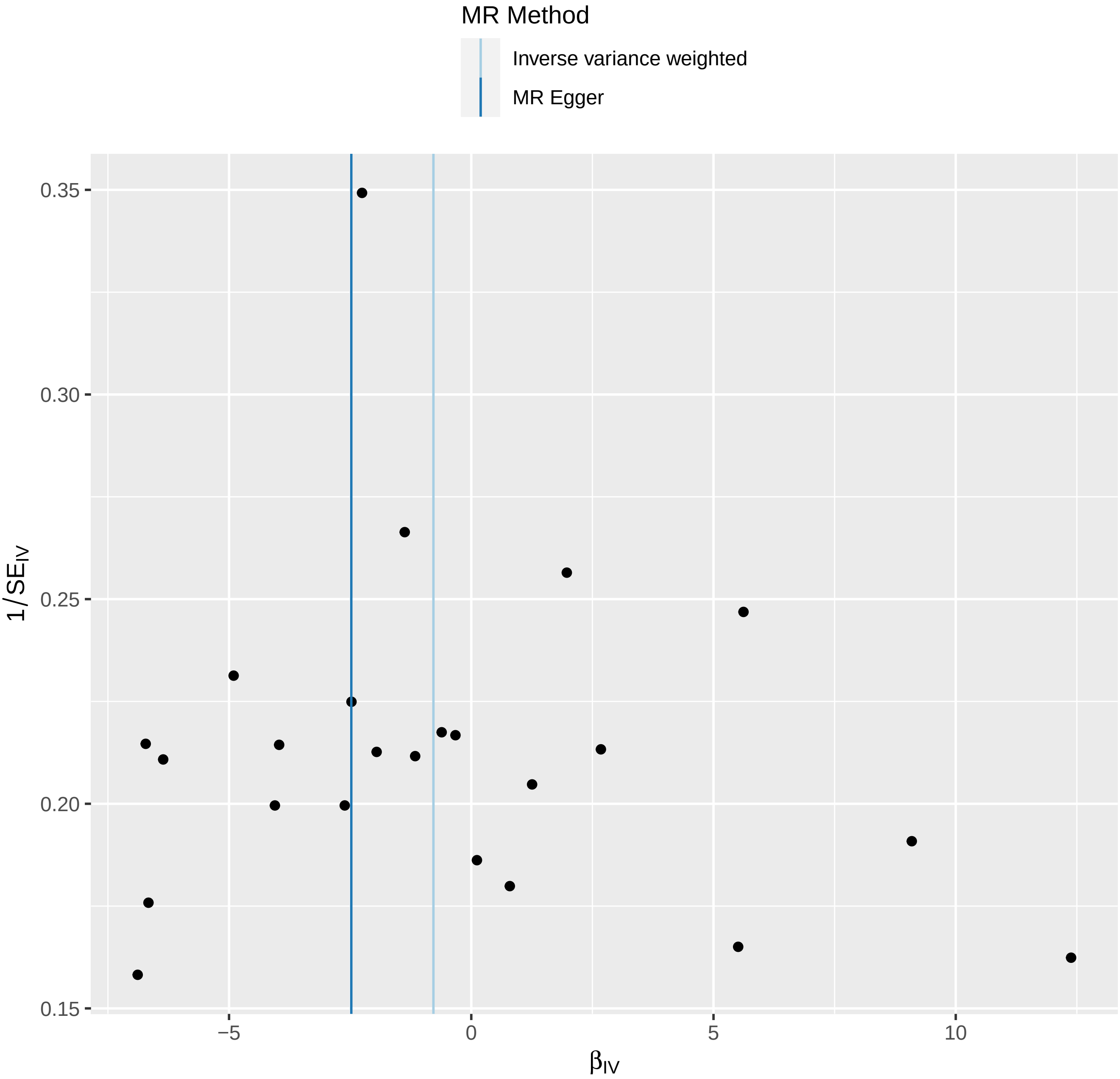

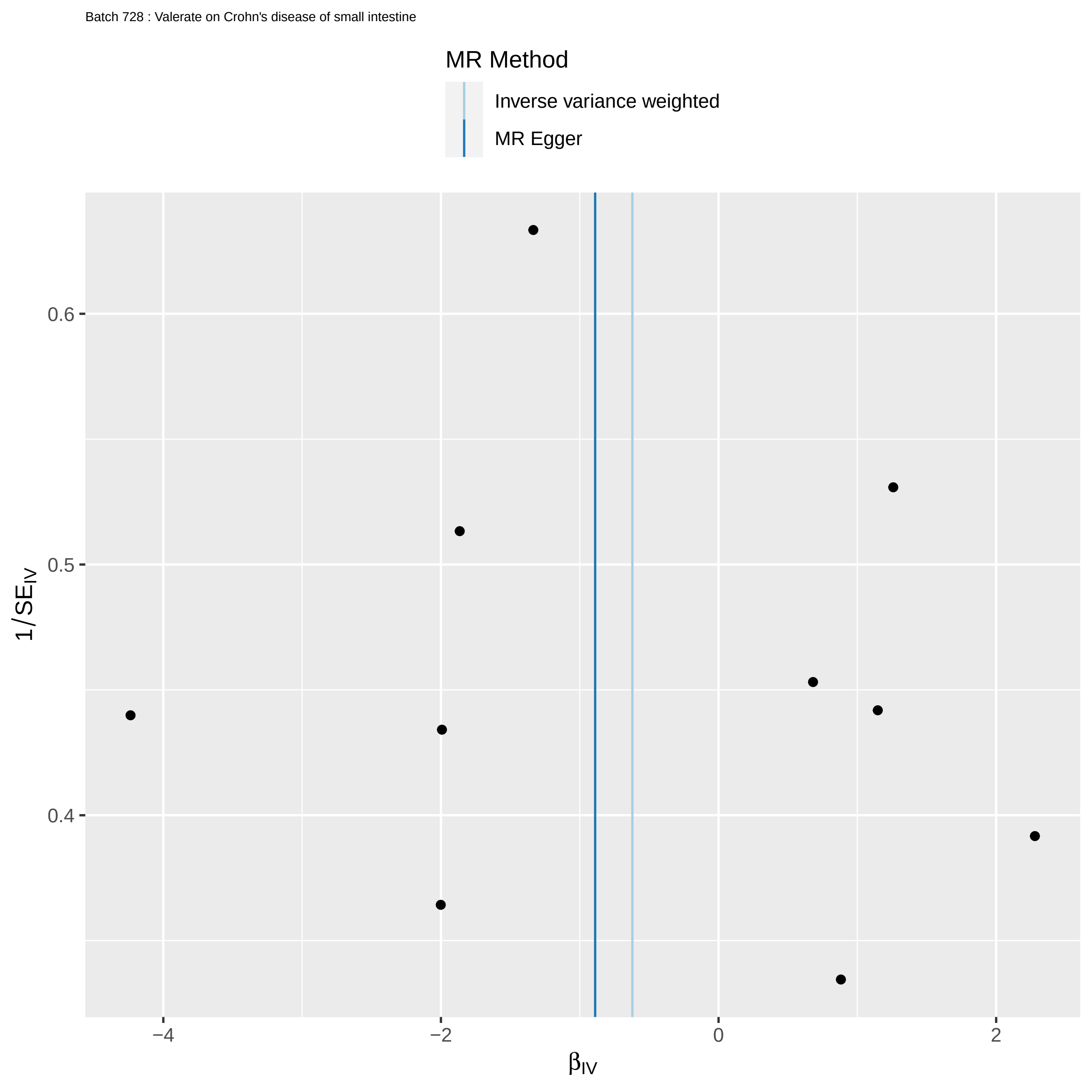
Batch 728 : Valerate on Crohn's disease of small intestine
MR Method
Inverse variance weighted
MR Egger
0.6
0.5
0.4
1/SE$_{IV}$
−4
−2
0
2
β_{IV}

Batch 731 : Erythronate* on Crohn's disease of small intestine

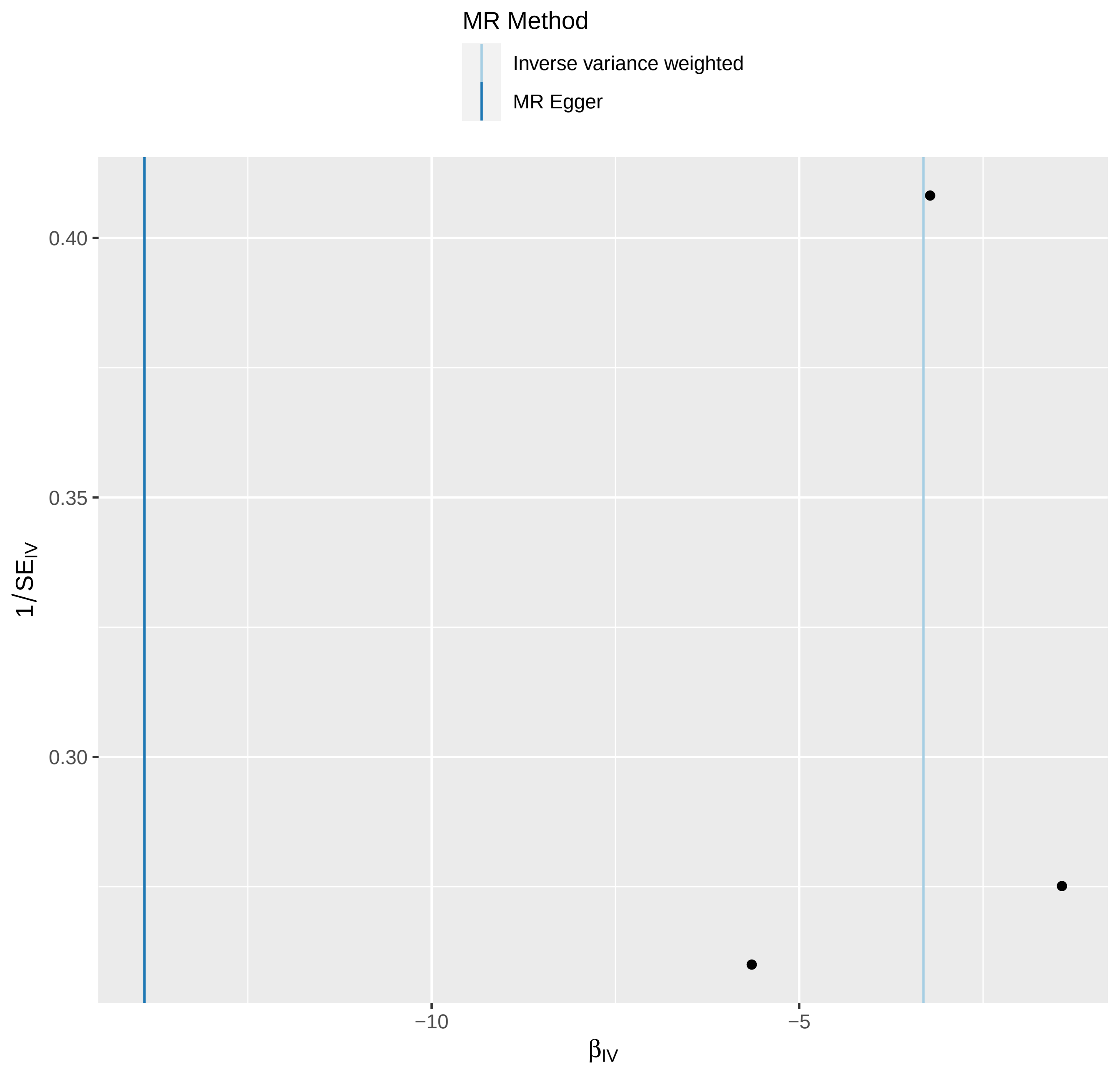

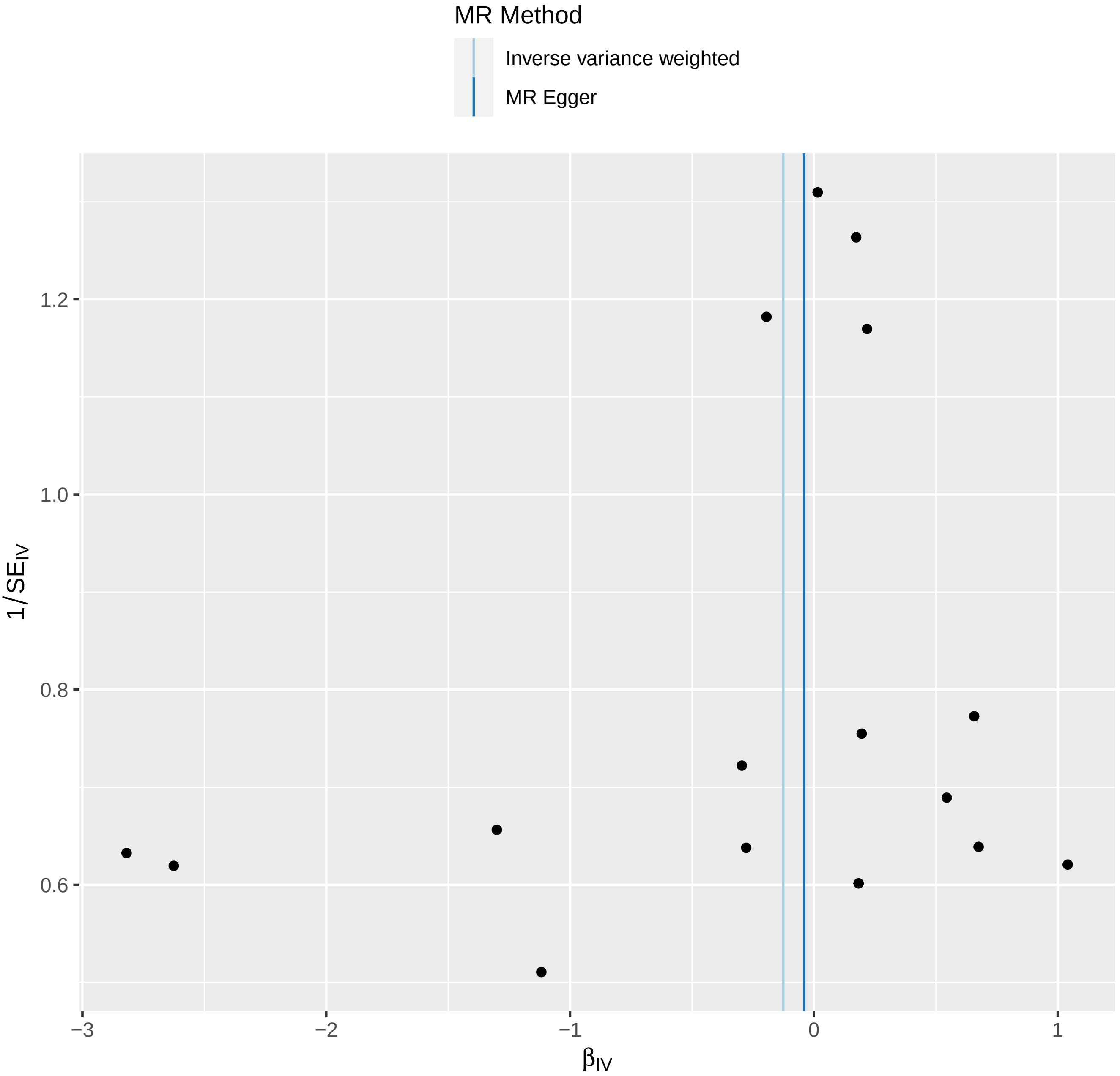
MR Method
Inverse variance weighted
MR Egger
1.2
1.0
0.8
0.6
1/SE_IV
−3
−2
−1
0
1
β_IV

Batch 735 : Eicosenoate (20:1n9 or 11) on Crohn's disease of small intestine

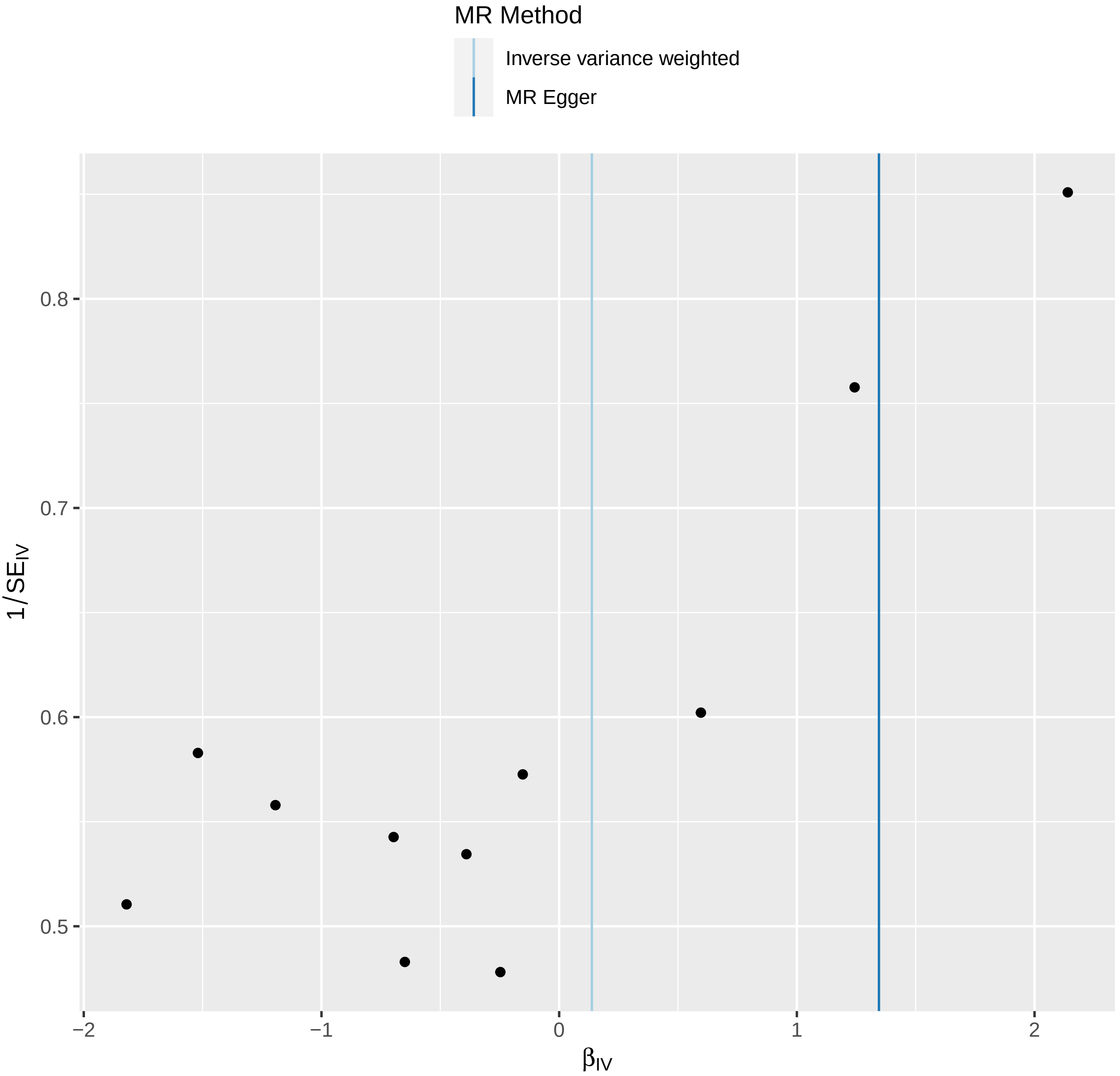

Batch 736 : X−12244−−N−acetylcarnosine on Crohn's disease of small intestine

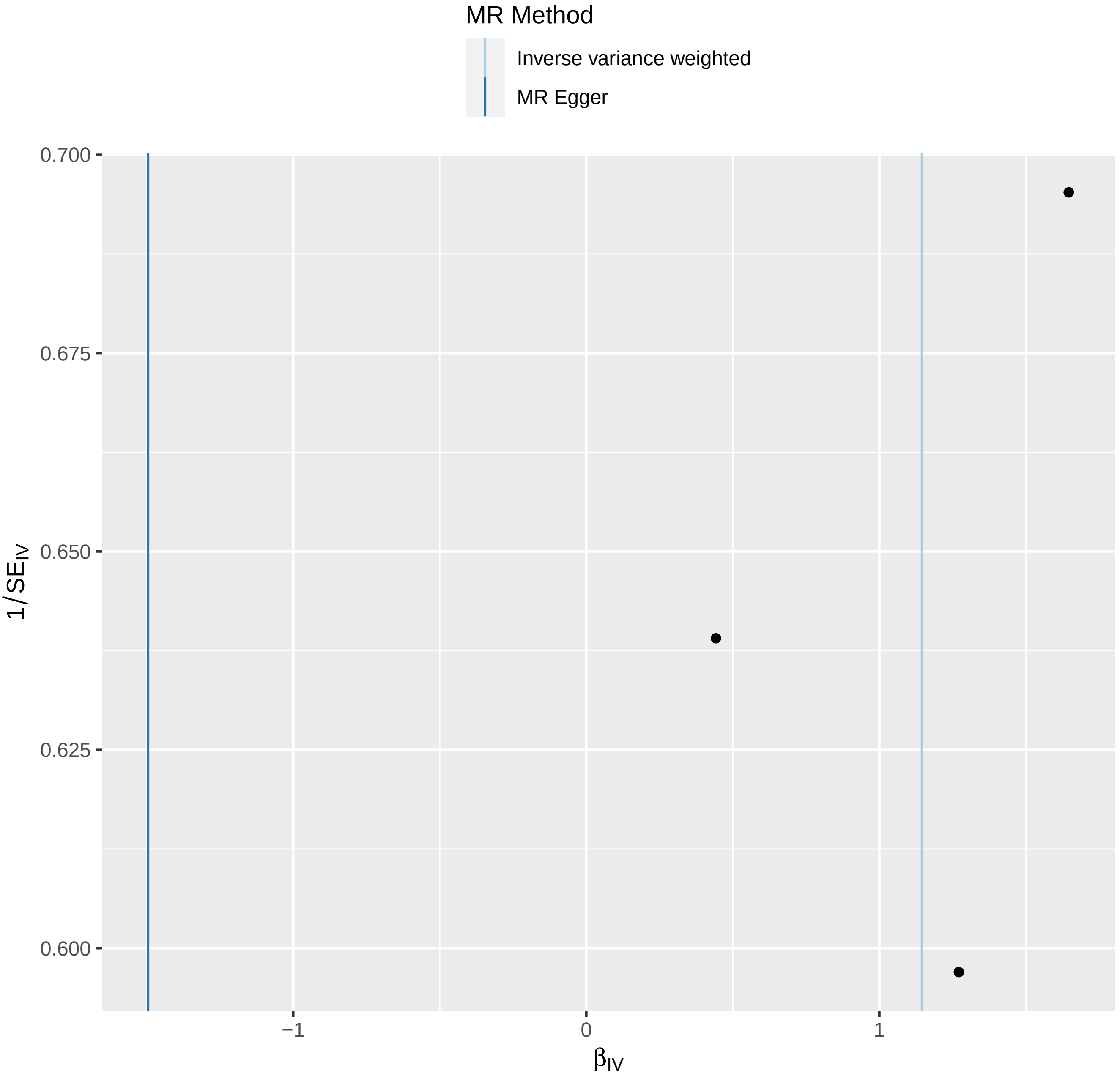

Batch 738 : 1−eicosatrienoylglycerophosphocholine* on Crohn's disease of small intestine

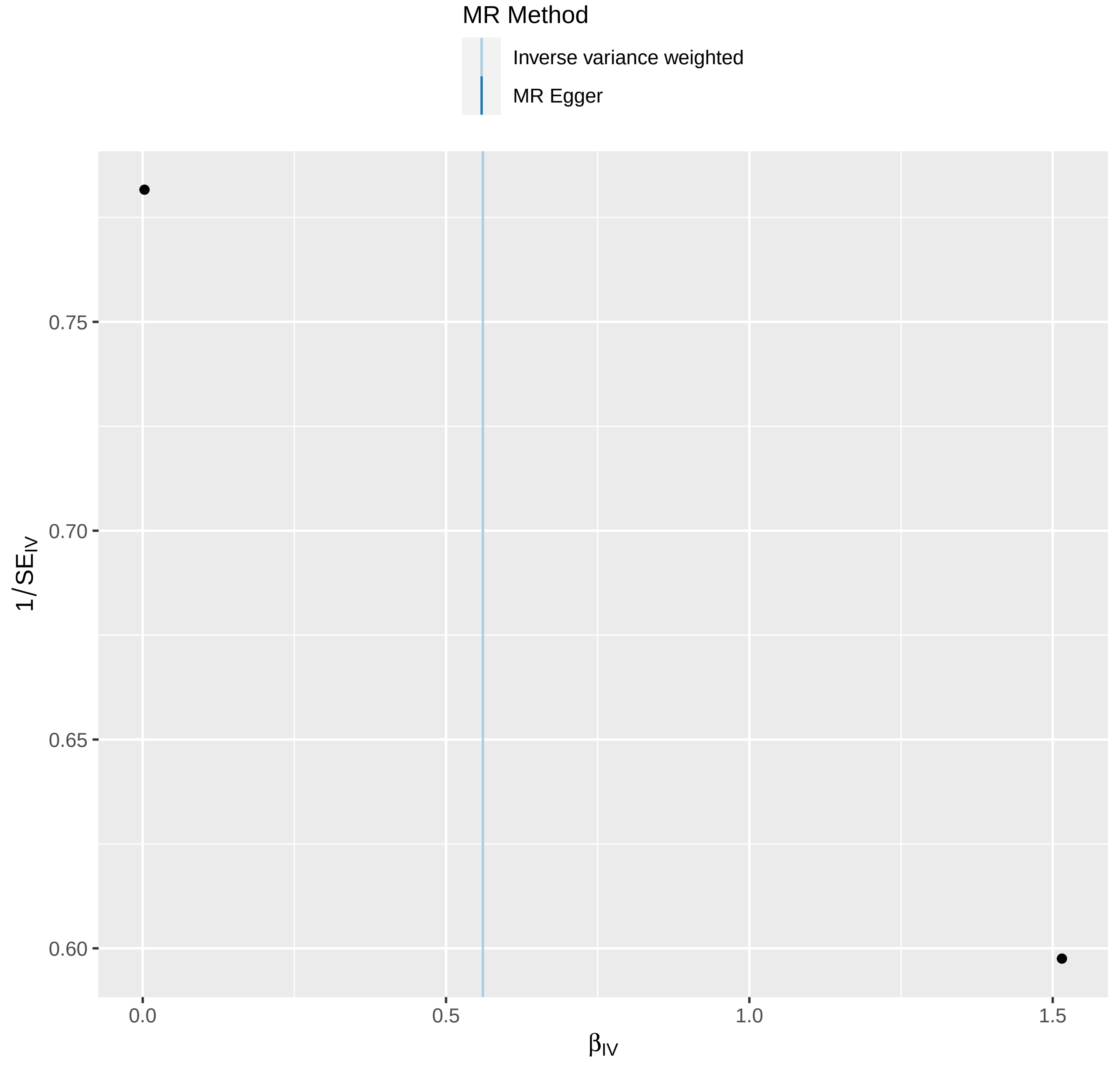

Batch 739 : 1−docosahexaenoylglycerophosphocholine* on Crohn's disease of small intestine

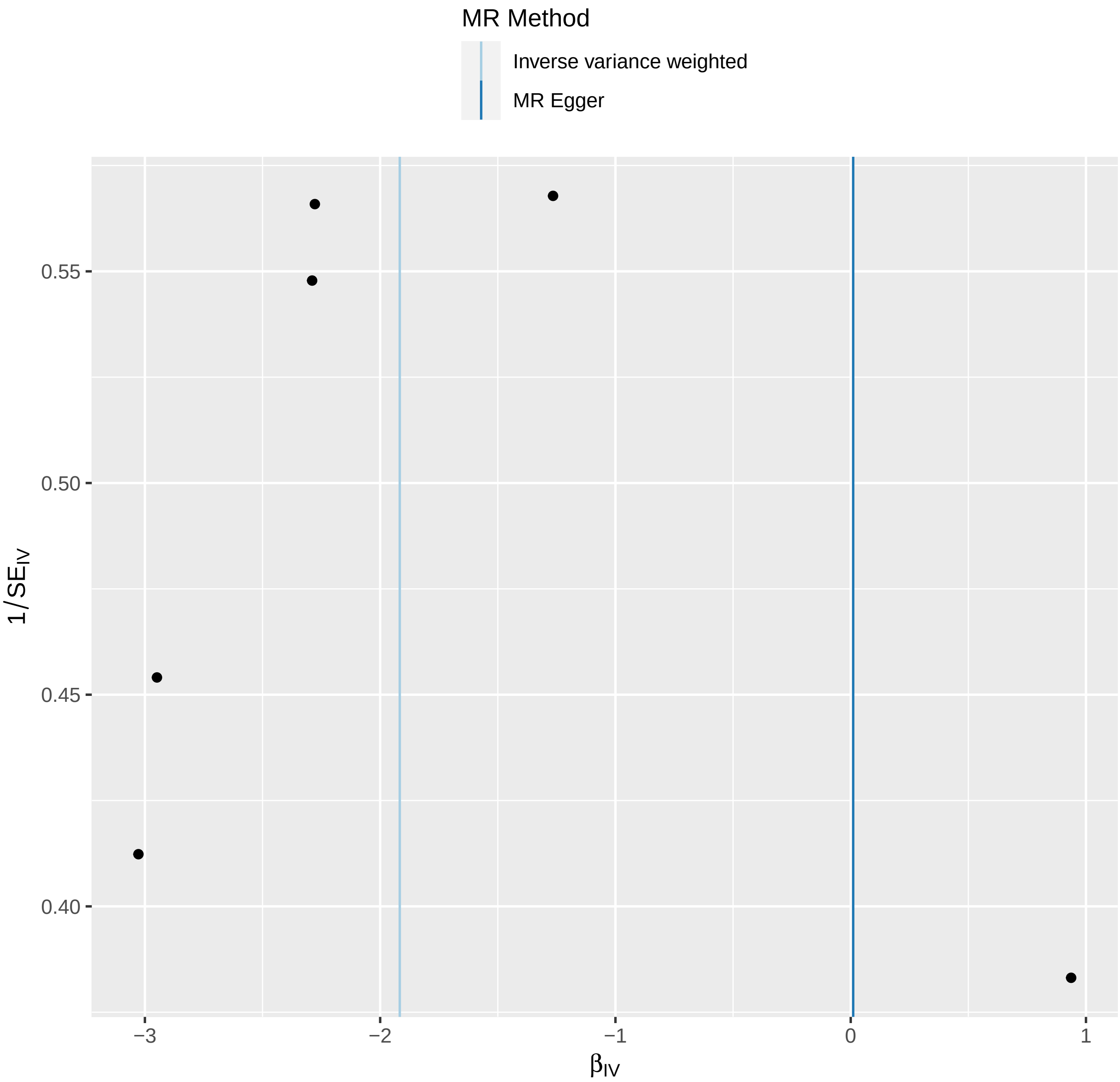

Batch 743 : Octanoylcarnitine on Crohn's disease of small intestine

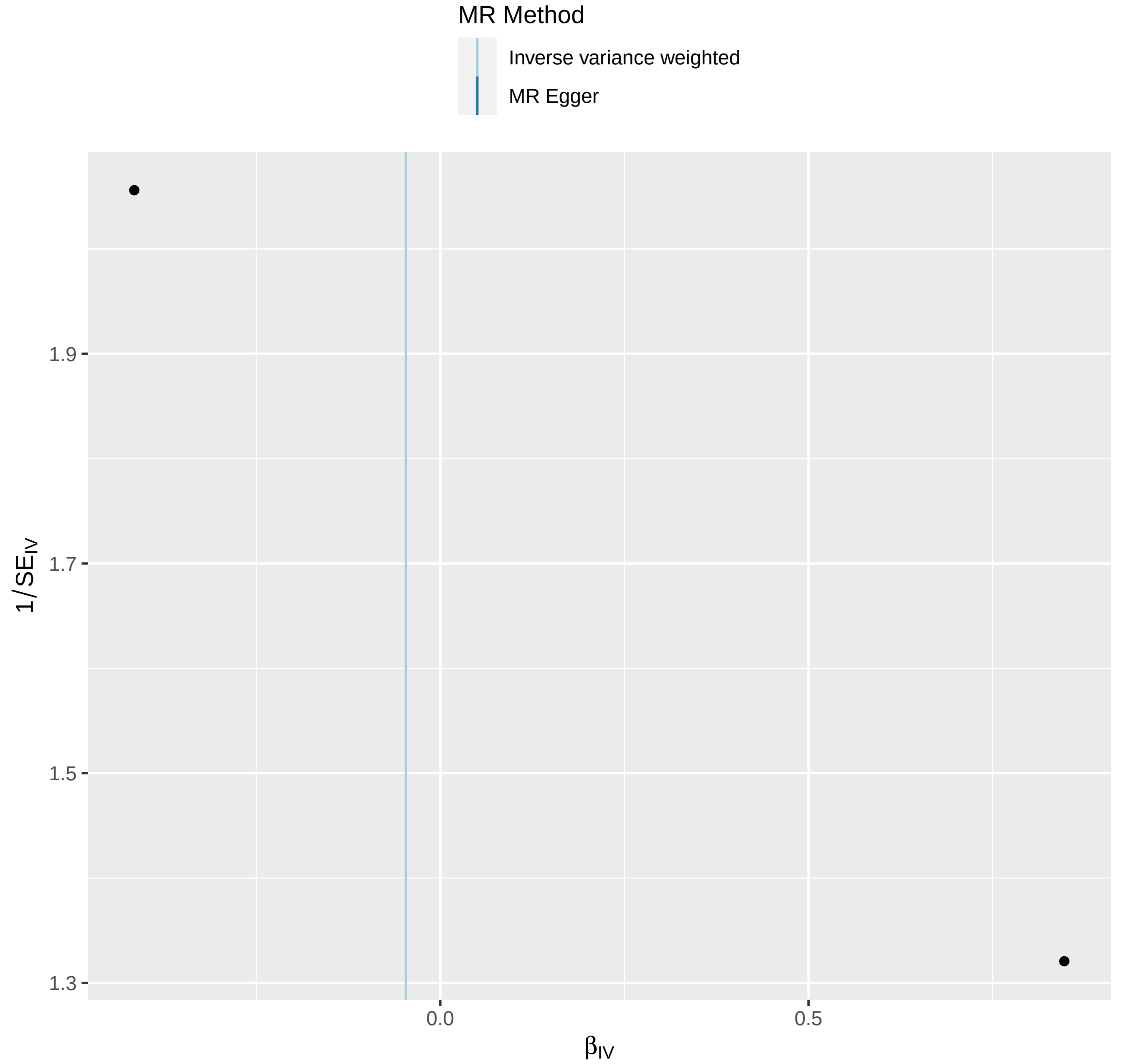

Batch 744 : Alpha−hydroxyisovalerate on Crohn's disease of small intestine

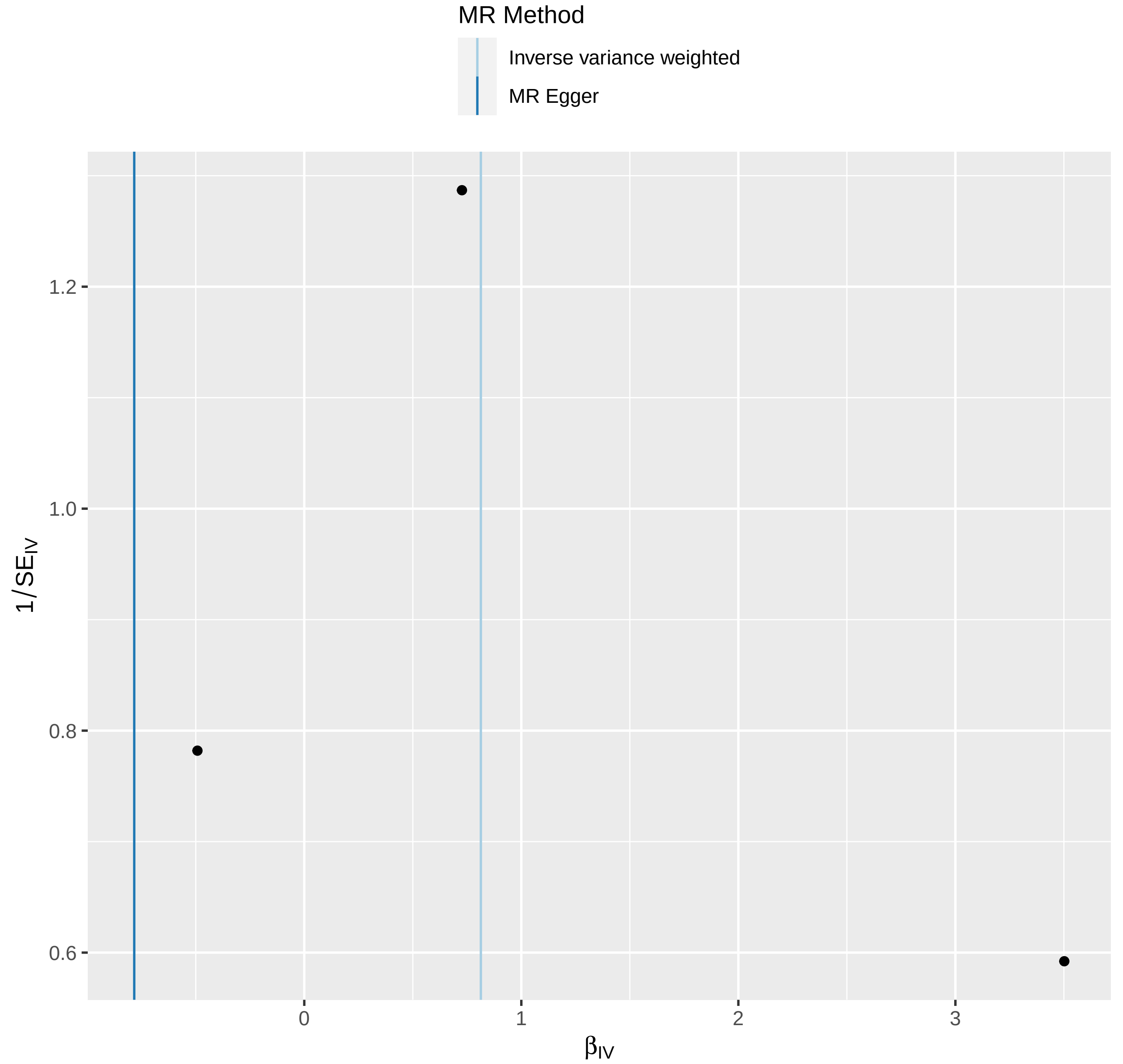

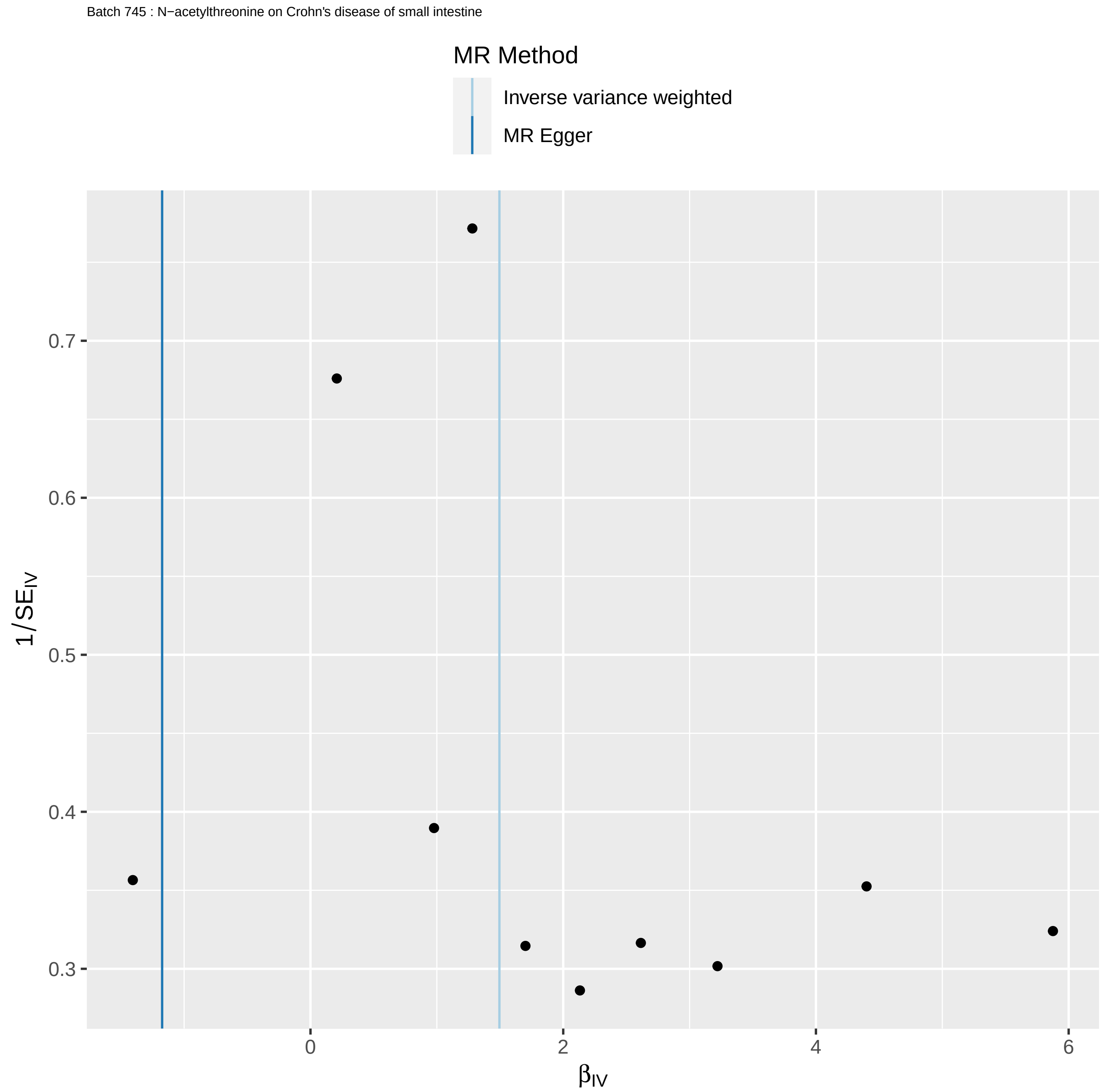
Batch 745 : N−acetylthreonine on Crohn's disease of small intestine
MR Method
Inverse variance weighted
MR Egger
0.7
0.6
0.5
0.4
0.3
1/SE_IV
0
2
4
6
β_IV

Batch 746 : Decanoylcarnitine on Crohn's disease of small intestine

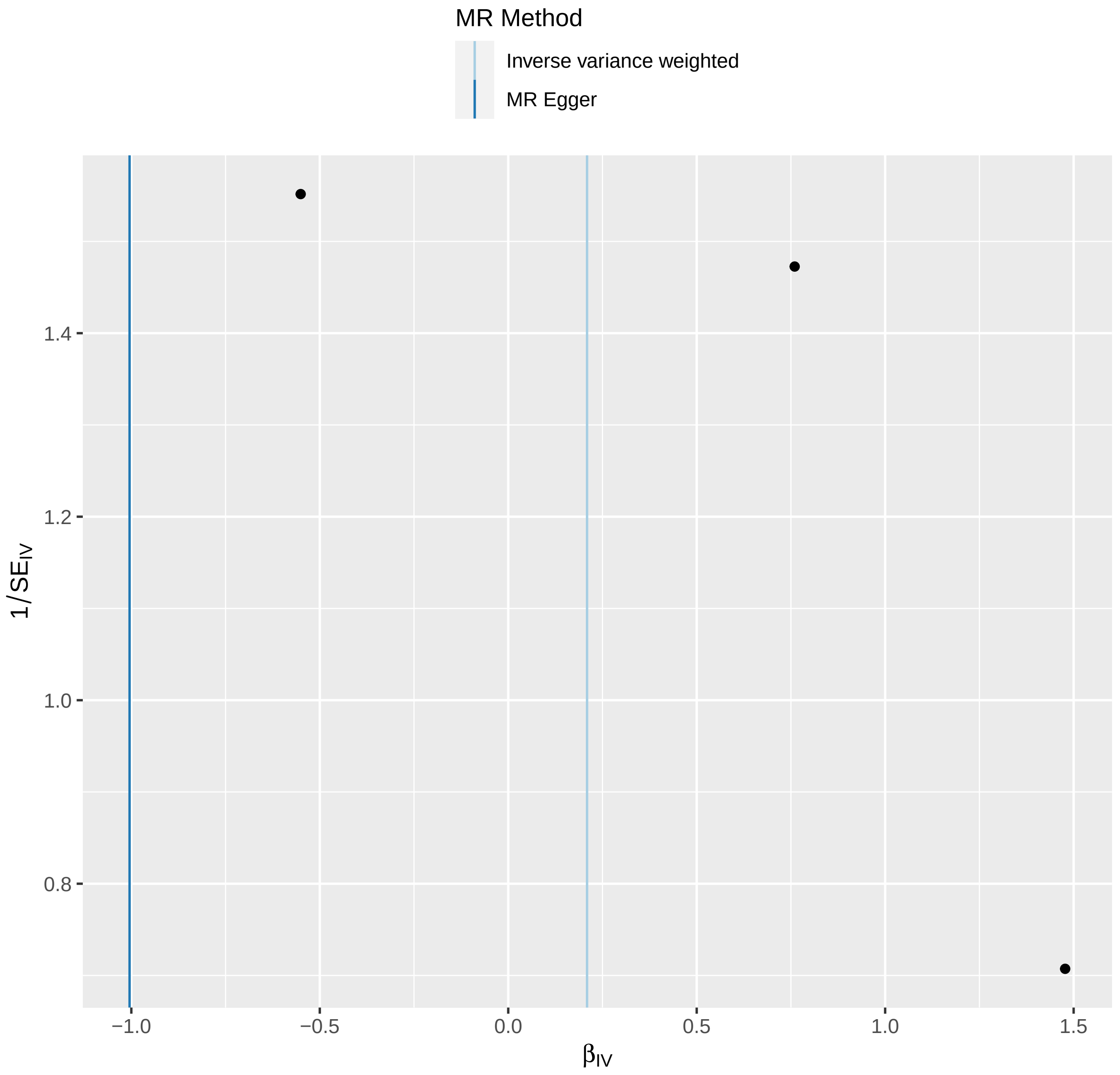

Batch 747 : 1−palmitoylglycerophosphocholine on Crohn's disease of small intestine

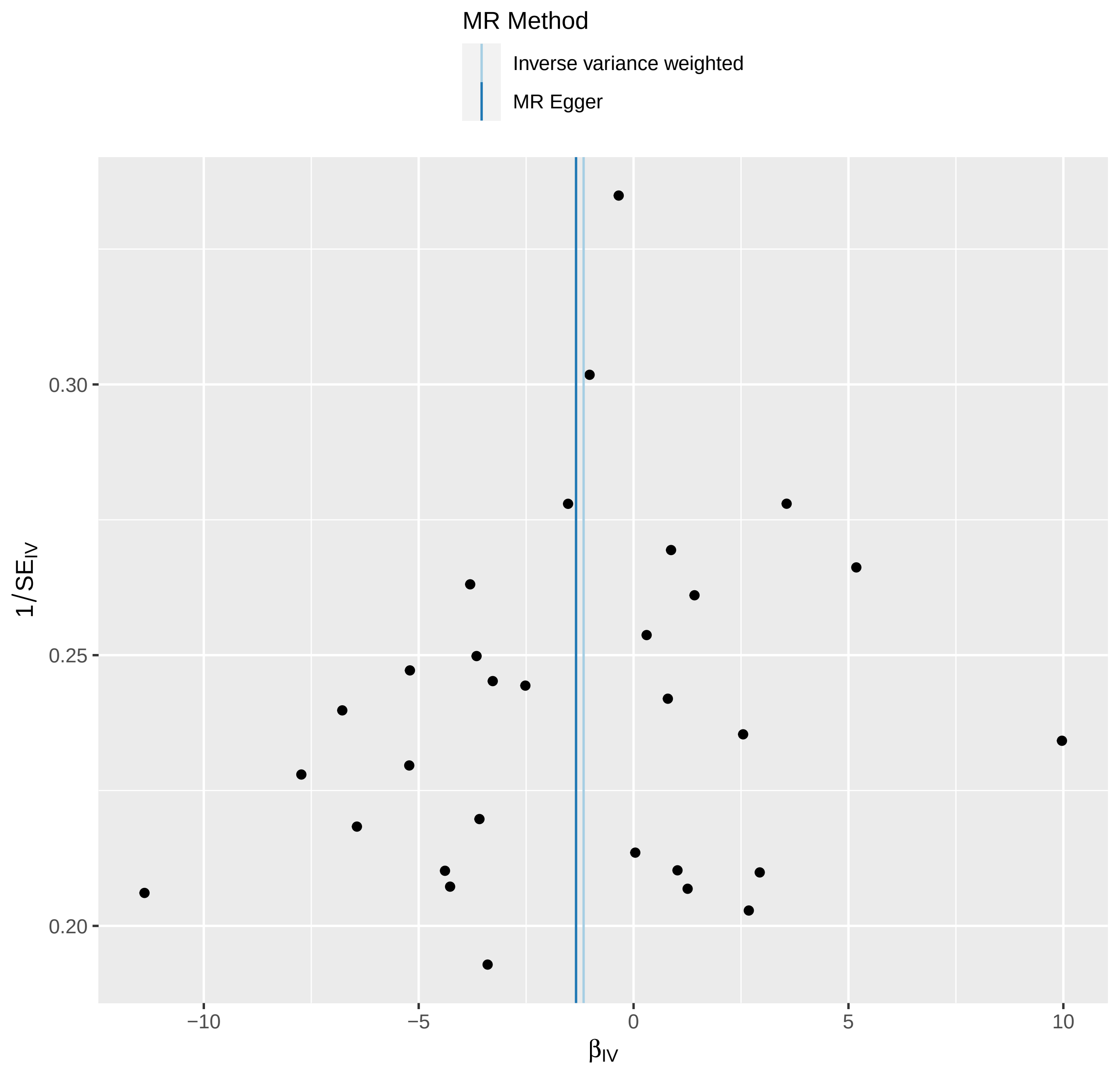

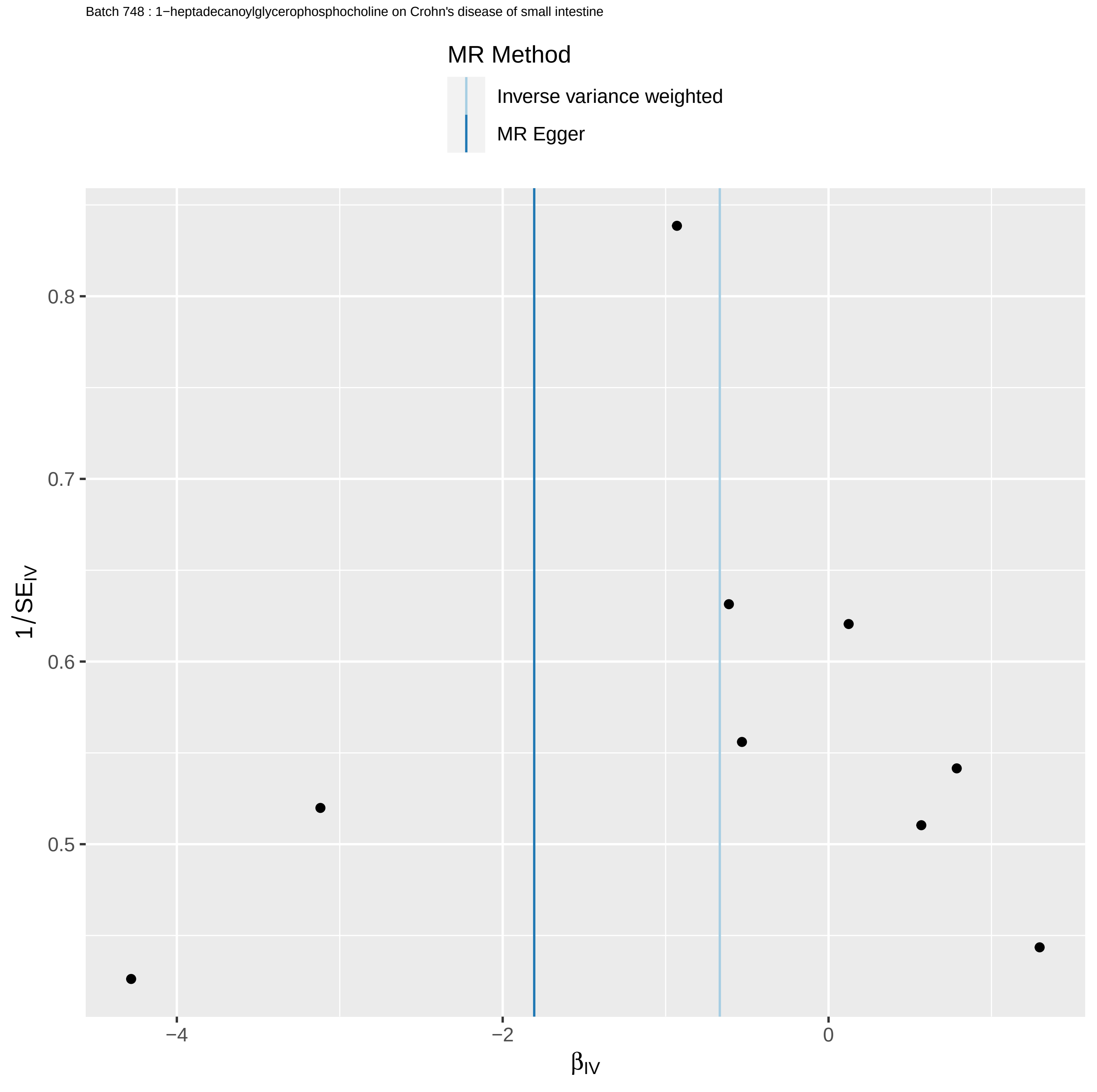

Batch 748 : 1−heptadecanoylglycerophosphocholine on Crohn's disease of small intestine
MR Method
Inverse variance weighted
MR Egger
0.8
0.7
0.6
0.5
1/SE_IV
−4
−2
0
β_IV

Batch 749 : 1−oleoylglycerophosphocholine on Crohn's disease of small intestine

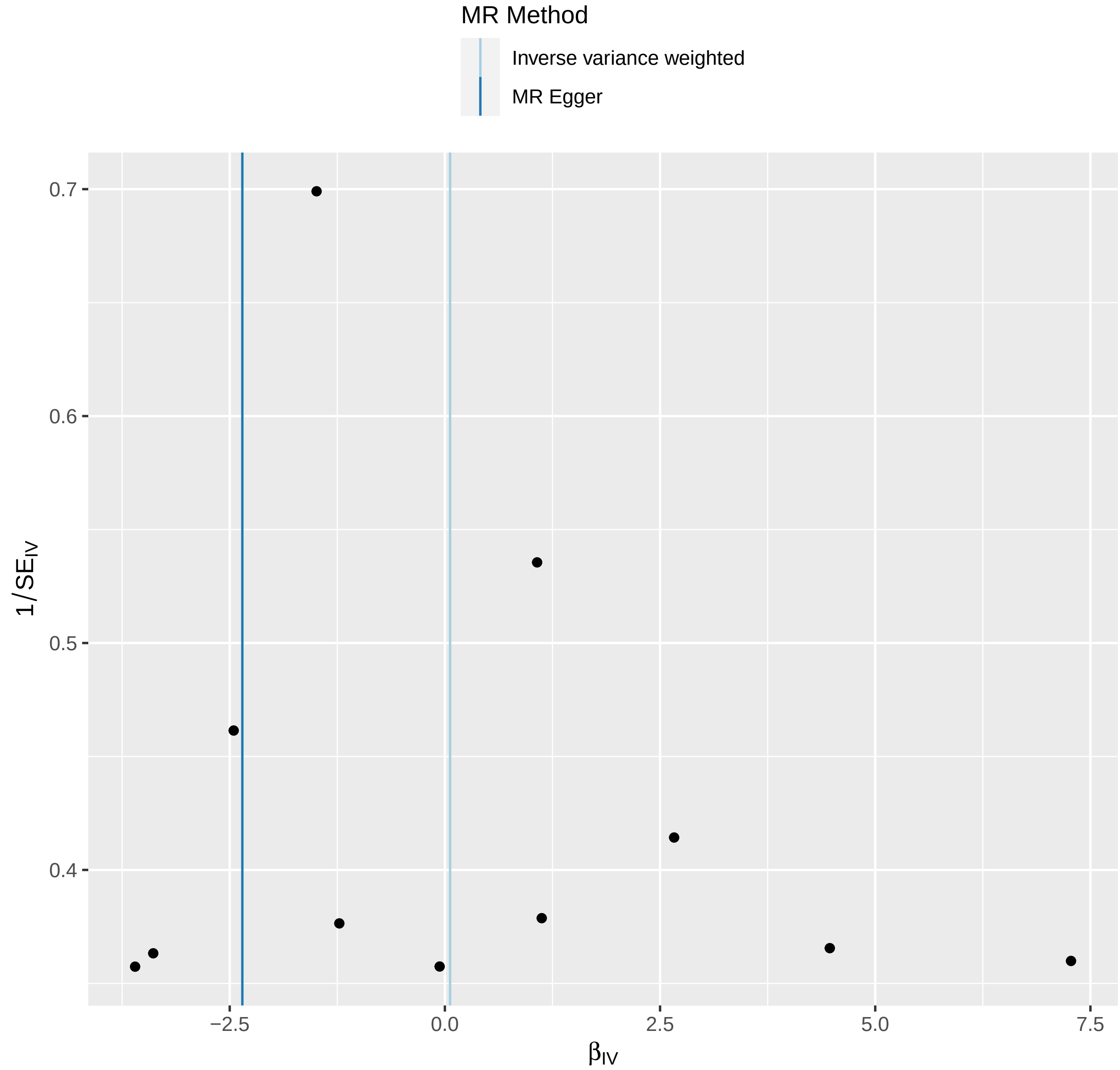

Batch 750 : 1−stearoylglycerophosphocholine on Crohn's disease of small intestine

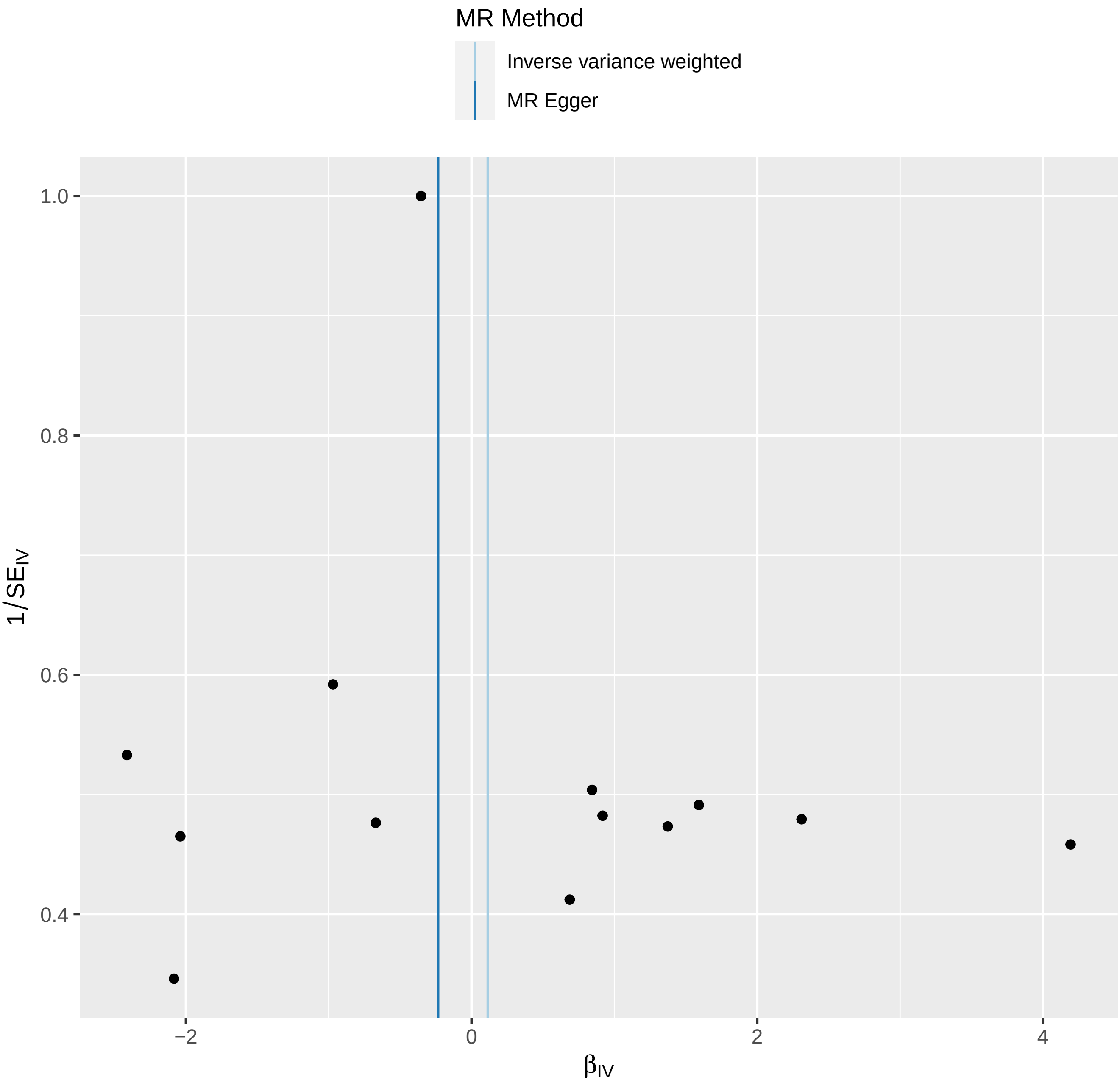

Batch 753 : 10−heptadecenoate (17:1n7) on Crohn's disease of small intestine

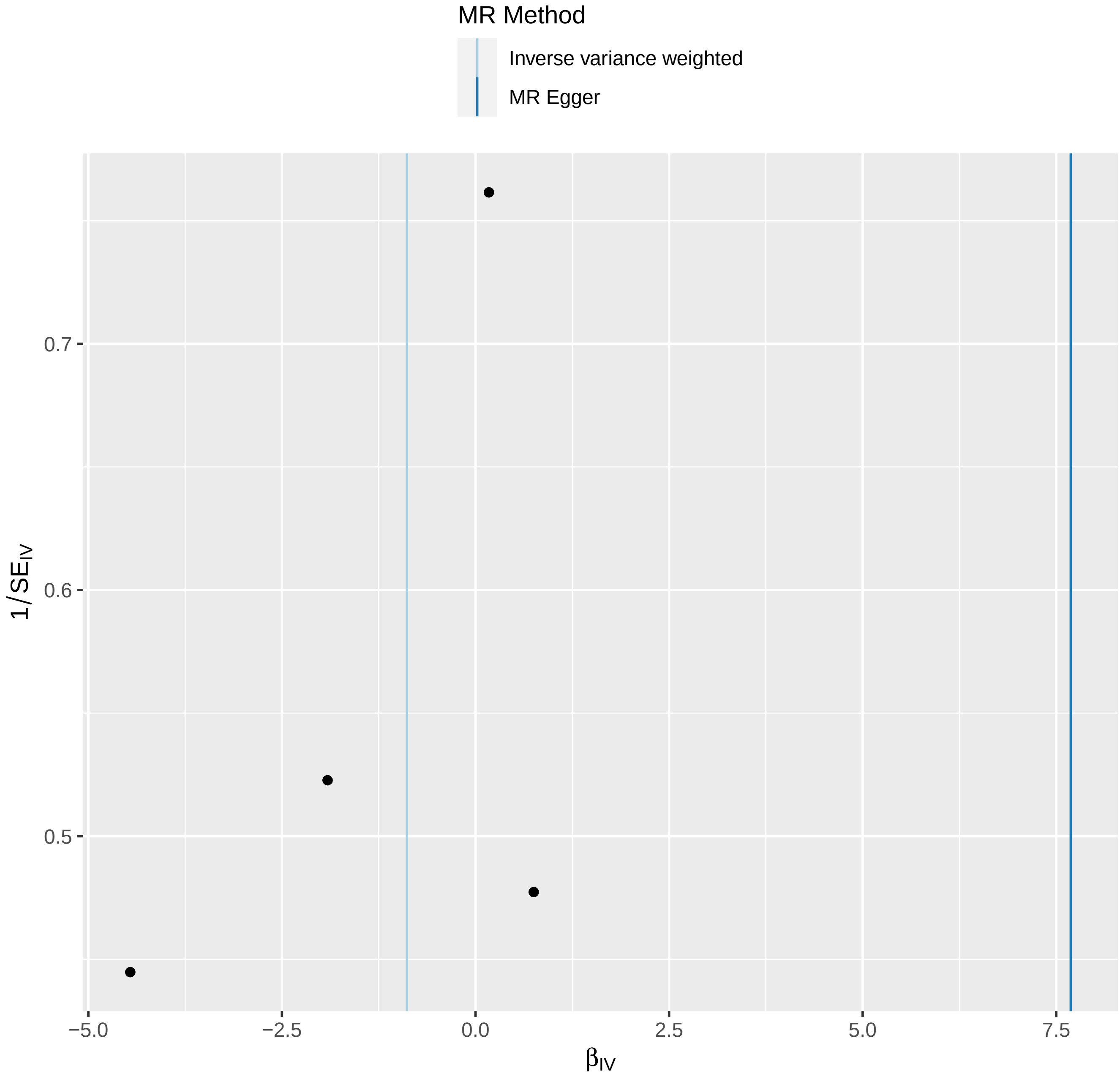

Batch 755 : Epiandrosterone sulfate on Crohn's disease of small intestine

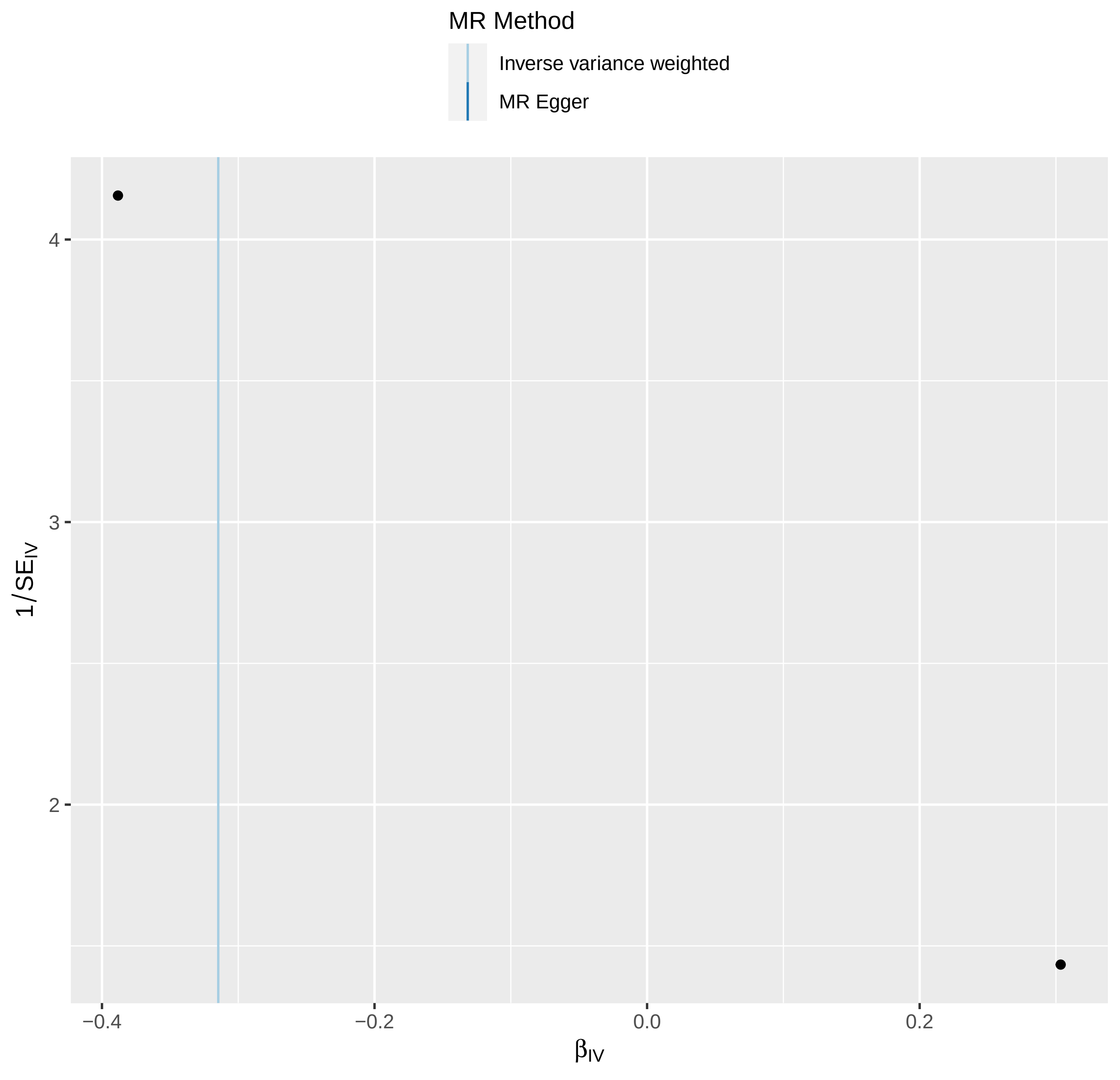

Batch 756 : Linolenate [alpha or gamma; (18:3n3 or 6)] on Crohn's disease of small intestine

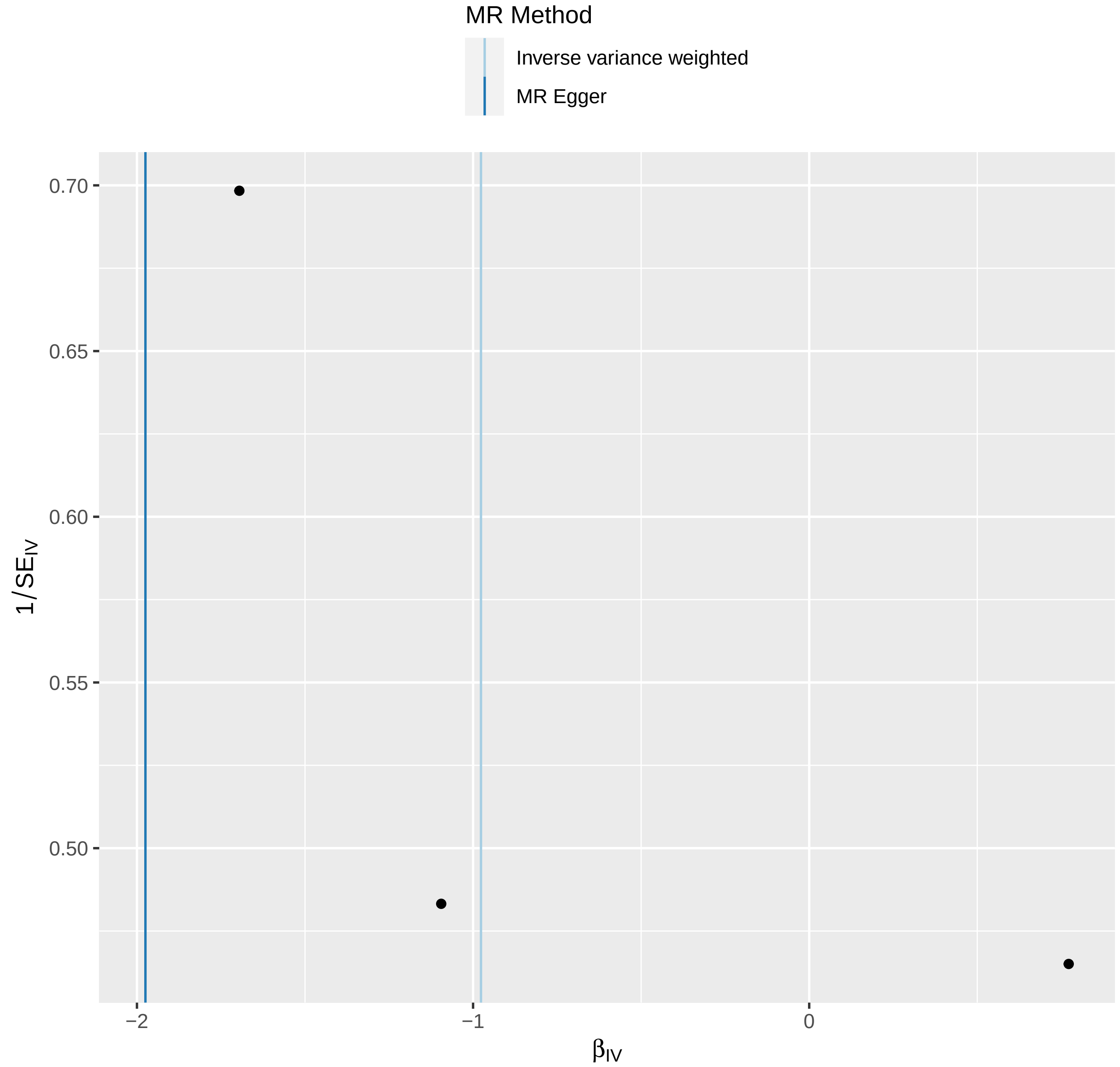

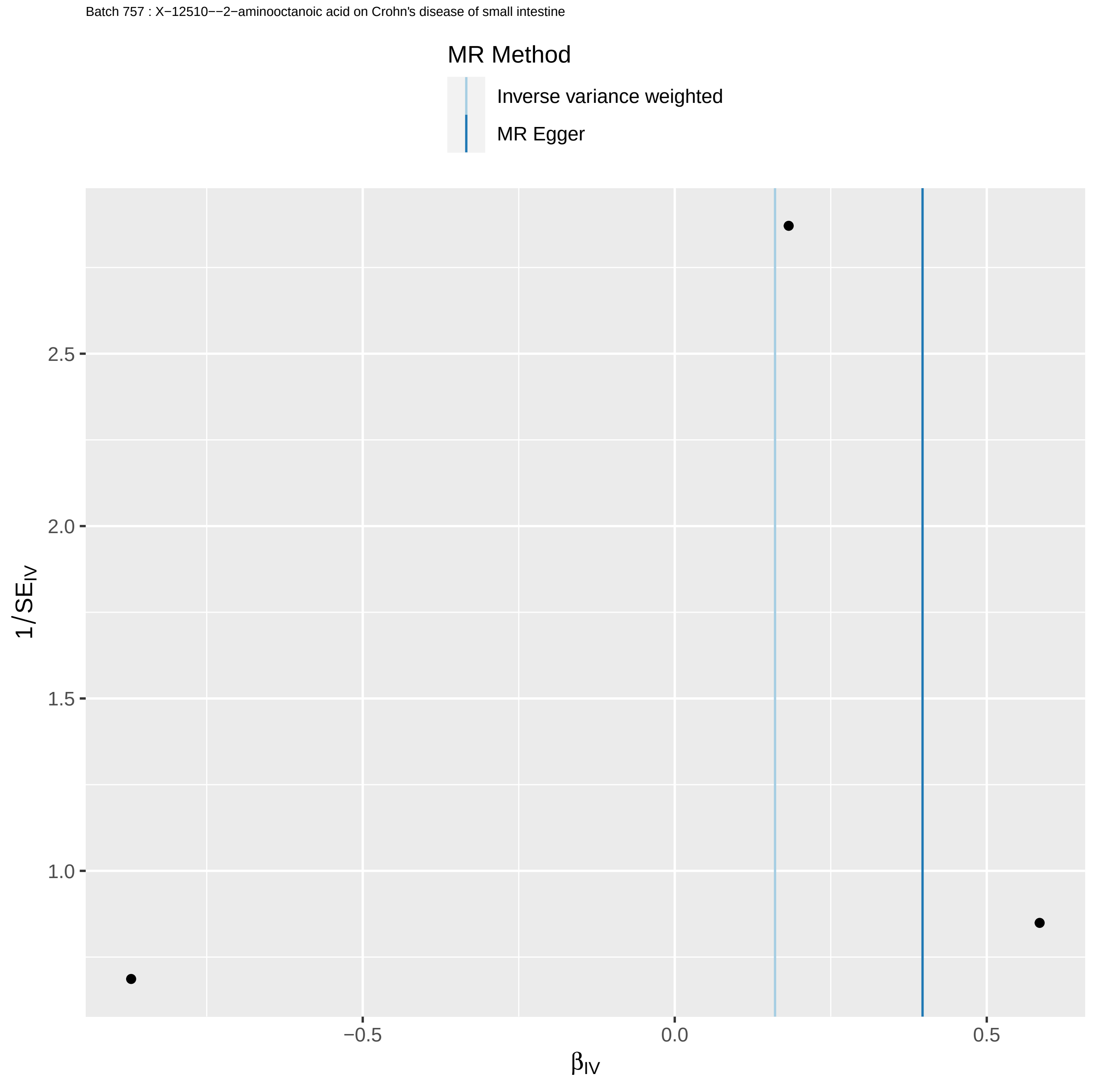
Batch 757 : X−12510−−2−aminooctanoic acid on Crohn's disease of small intestine
MR Method
Inverse variance weighted
MR Egger
2.5
2.0
1.5
1.0
1/SE_IV
−0.5
0.0
0.5
β_IV

Batch 760 : Asparagine on Crohn's disease of small intestine

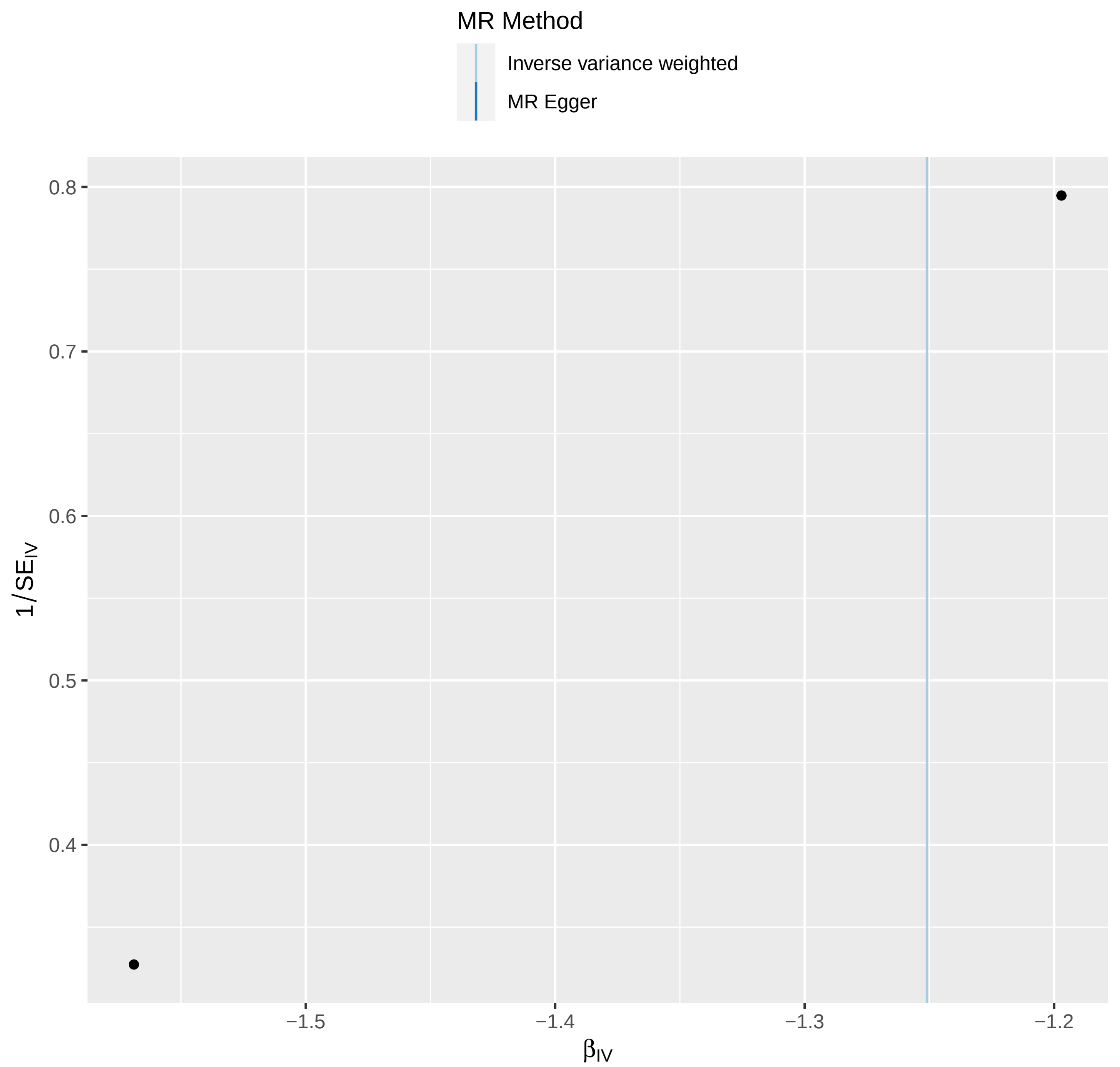

Batch 761 : Stachydrine on Crohn's disease of small intestine

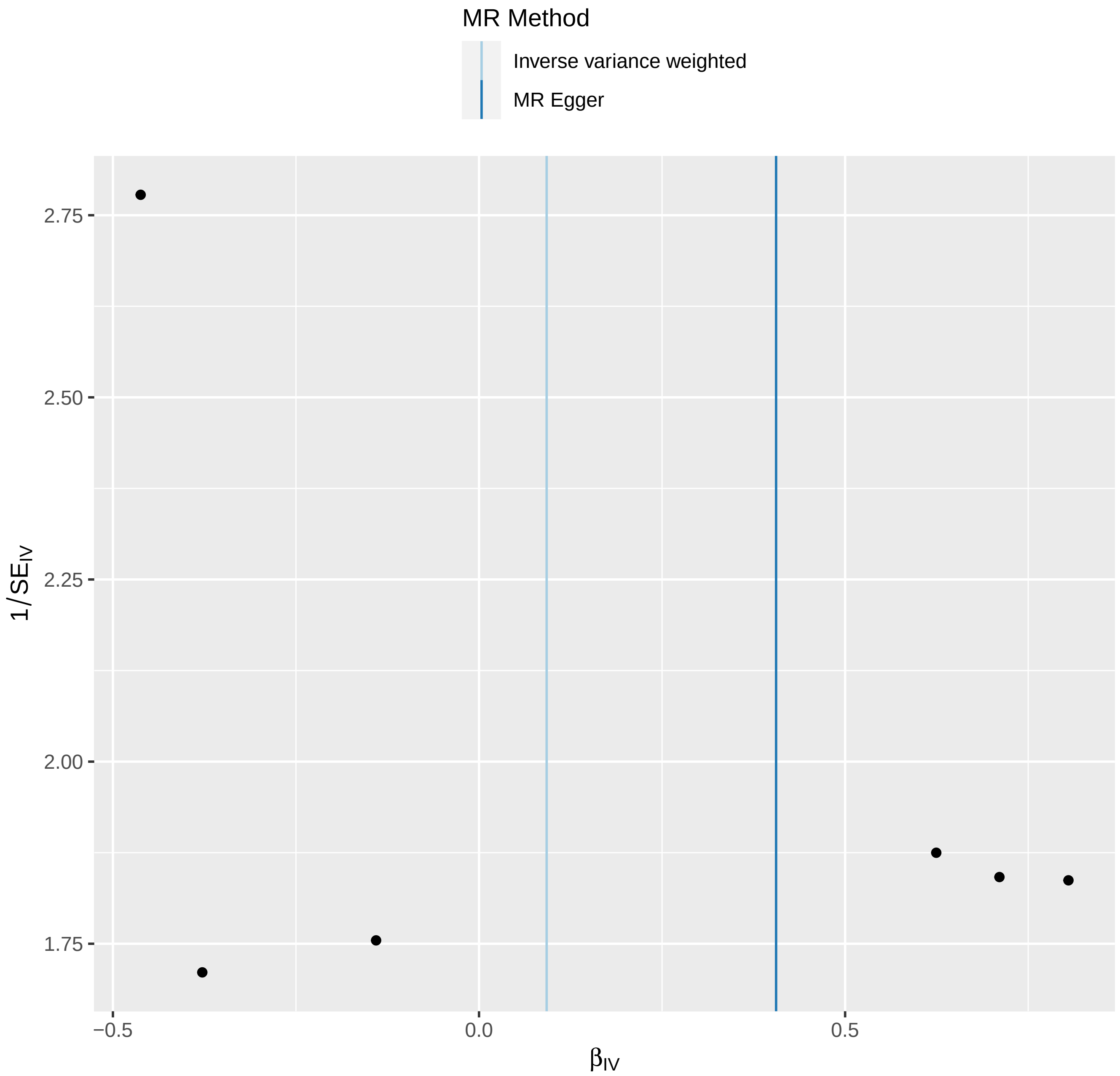

Batch 762 : Isovalerylcarnitine on Crohn's disease of small intestine

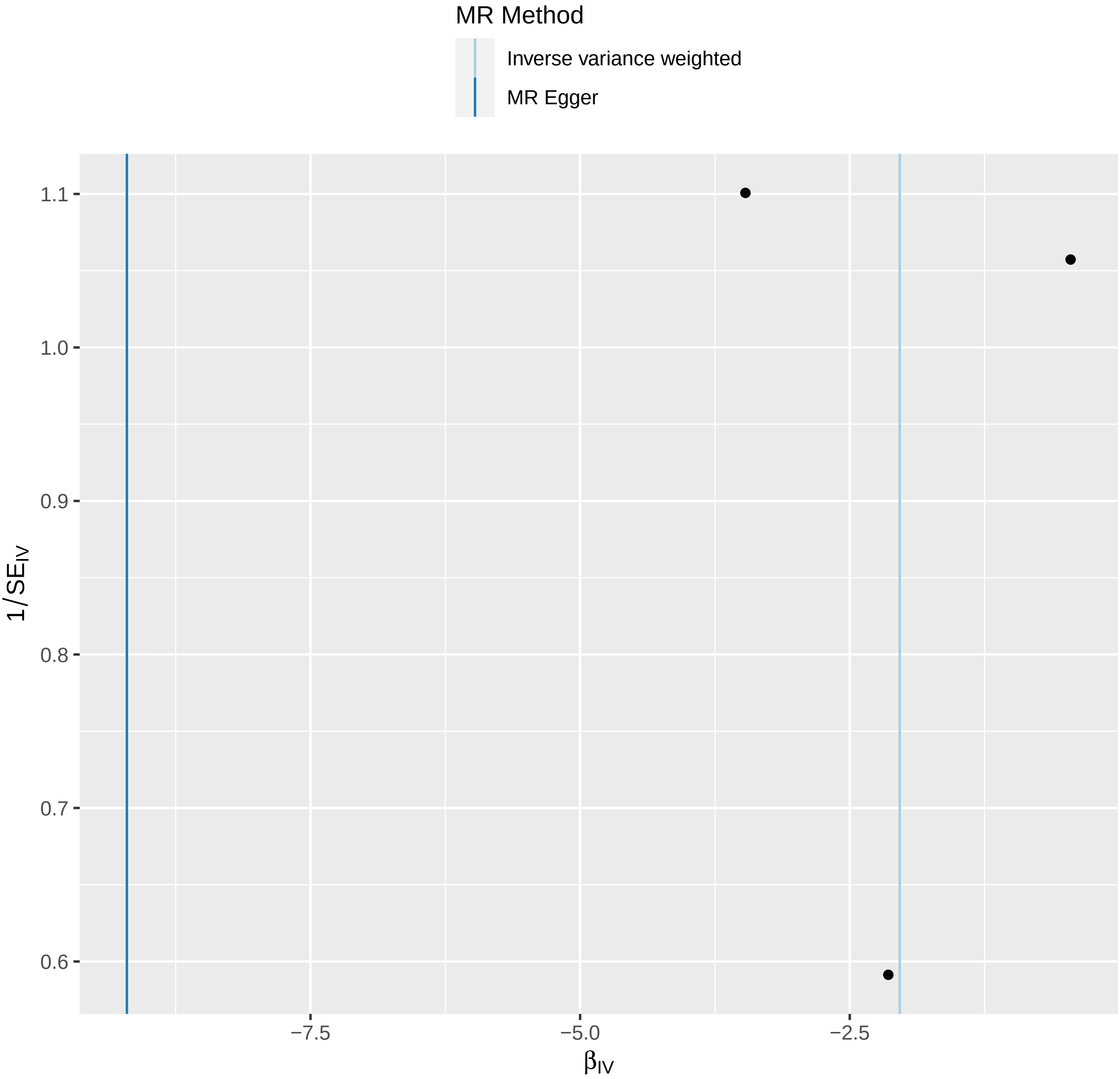

Batch 765 : 1−linoleoylglycerophosphocholine on Crohn's disease of small intestine

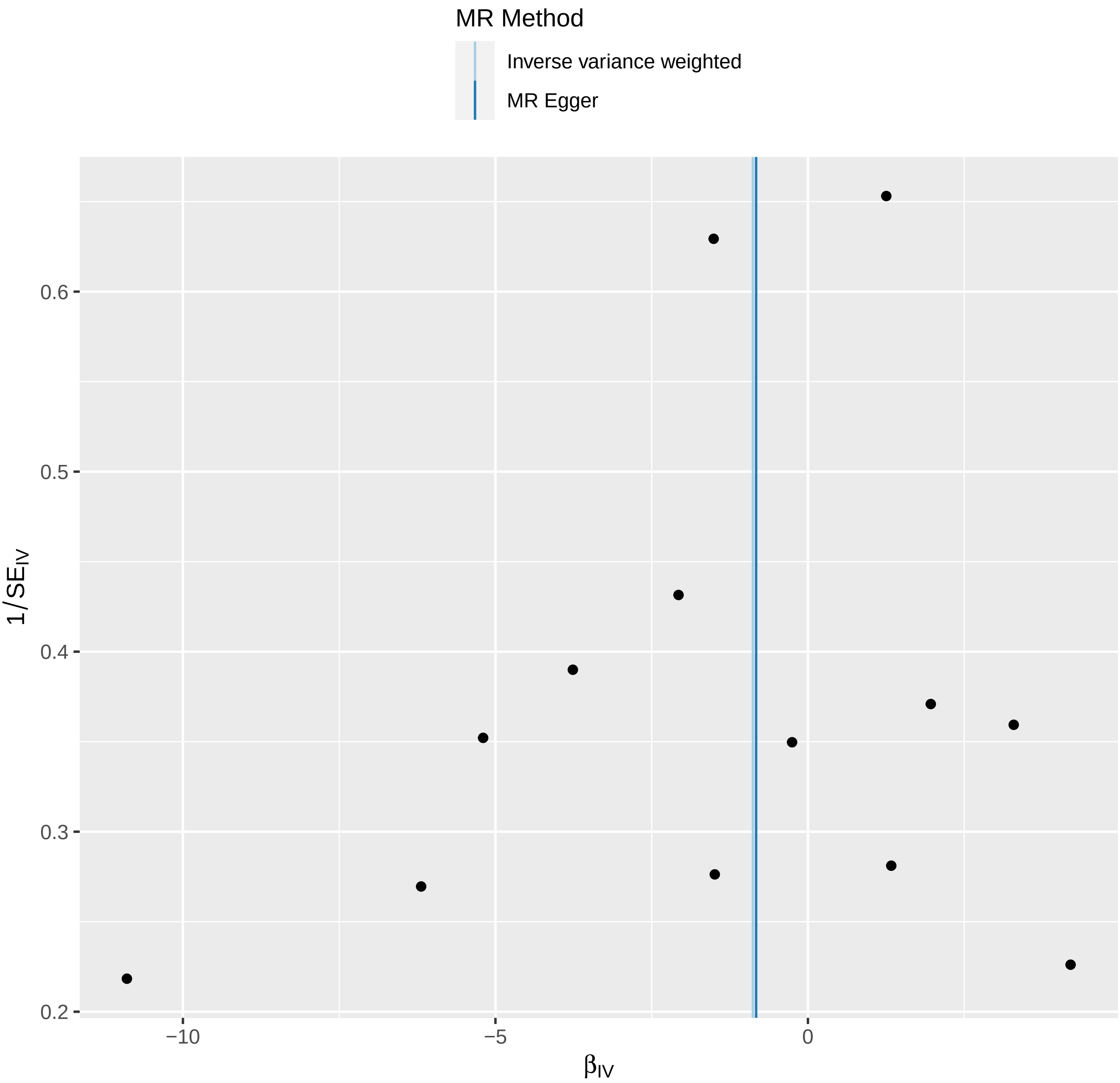

Batch 766 : Bradykinin, des−arg(9) on Crohn's disease of small intestine

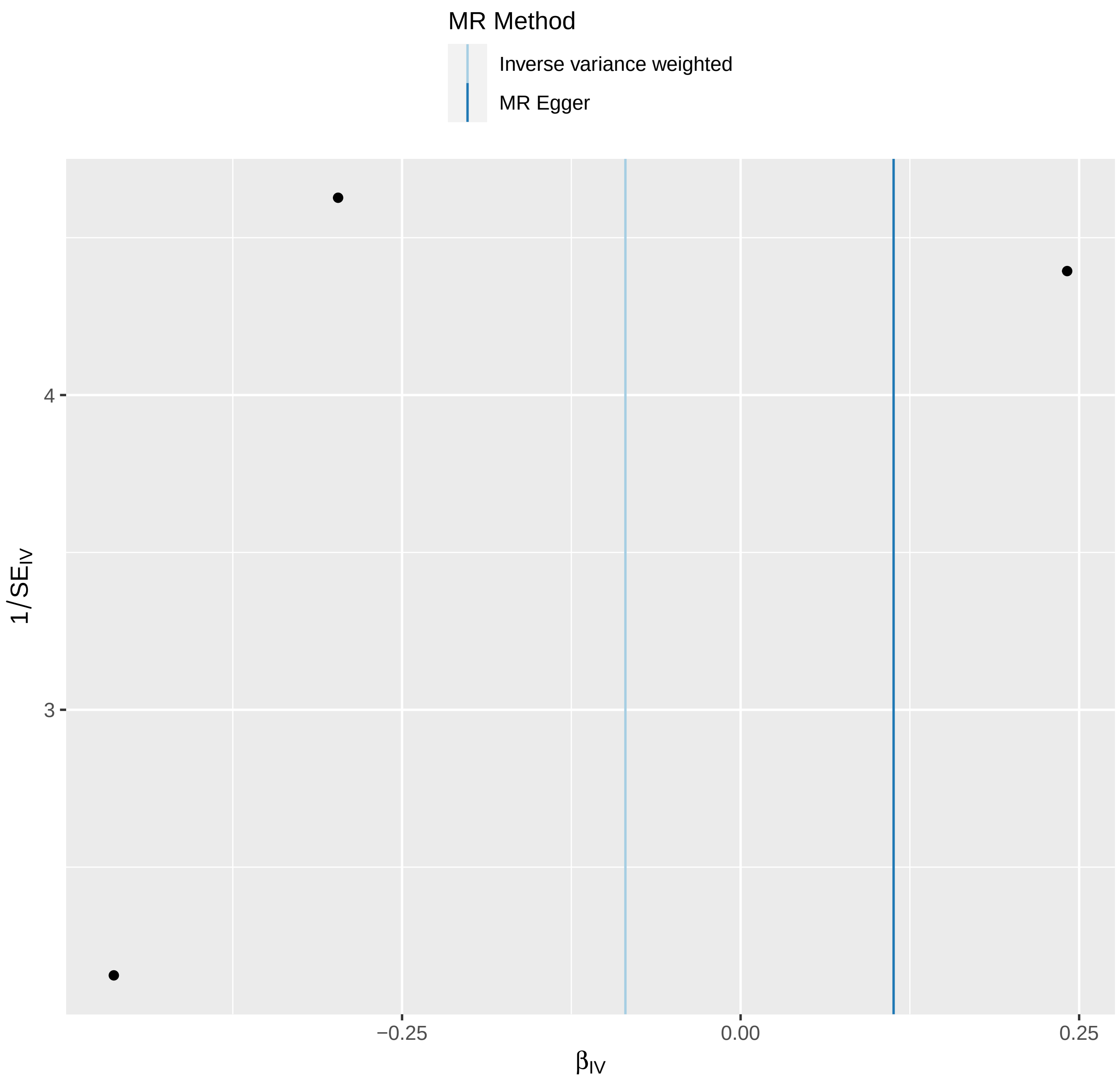

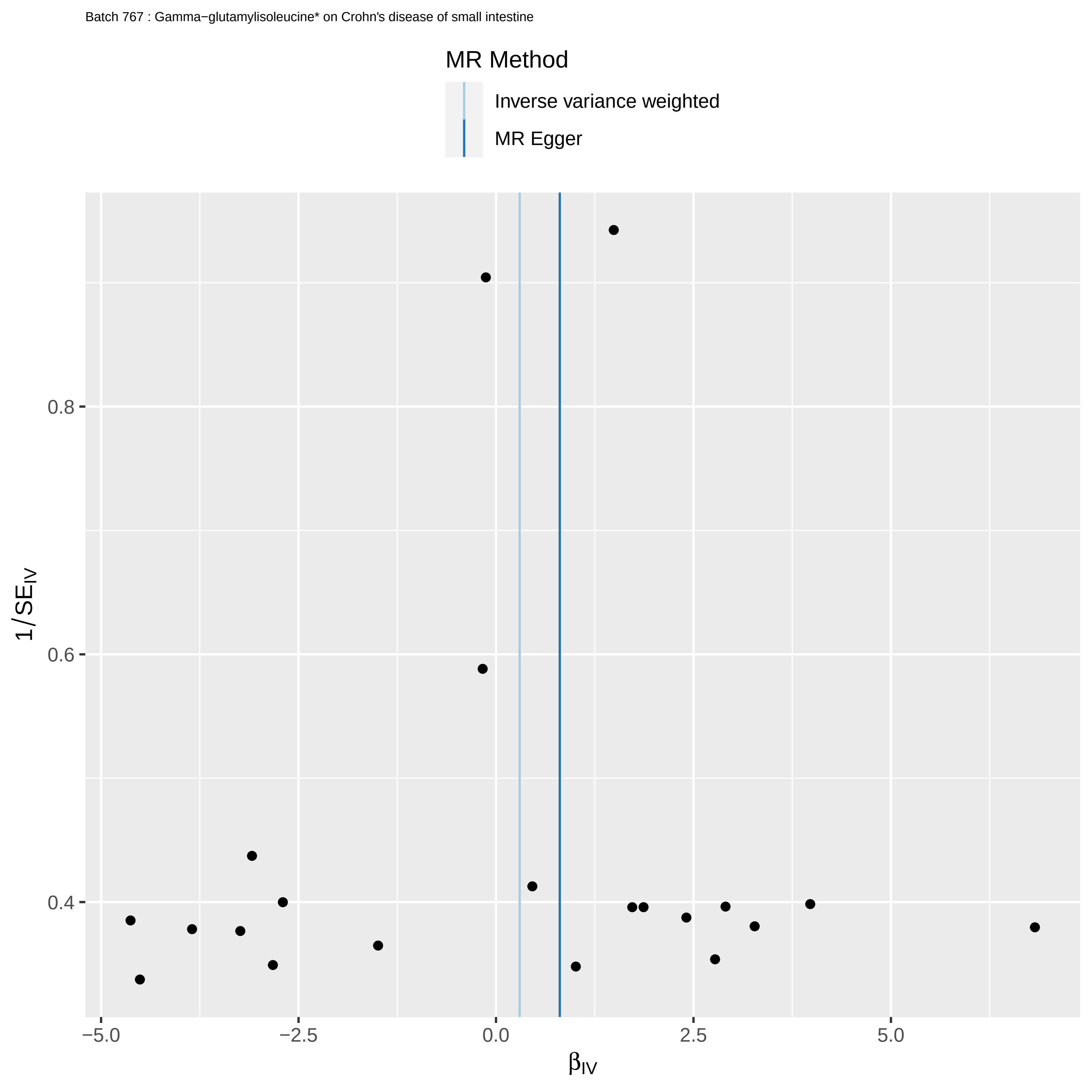

Batch 767 : Gamma−glutamylisoleucine* on Crohn's disease of small intestine
MR Method
Inverse variance weighted
MR Egger
0.8
0.6
0.4
1/SE$_{IV}$
−5.0
−2.5
0.0
2.5
5.0
β$_{IV}$

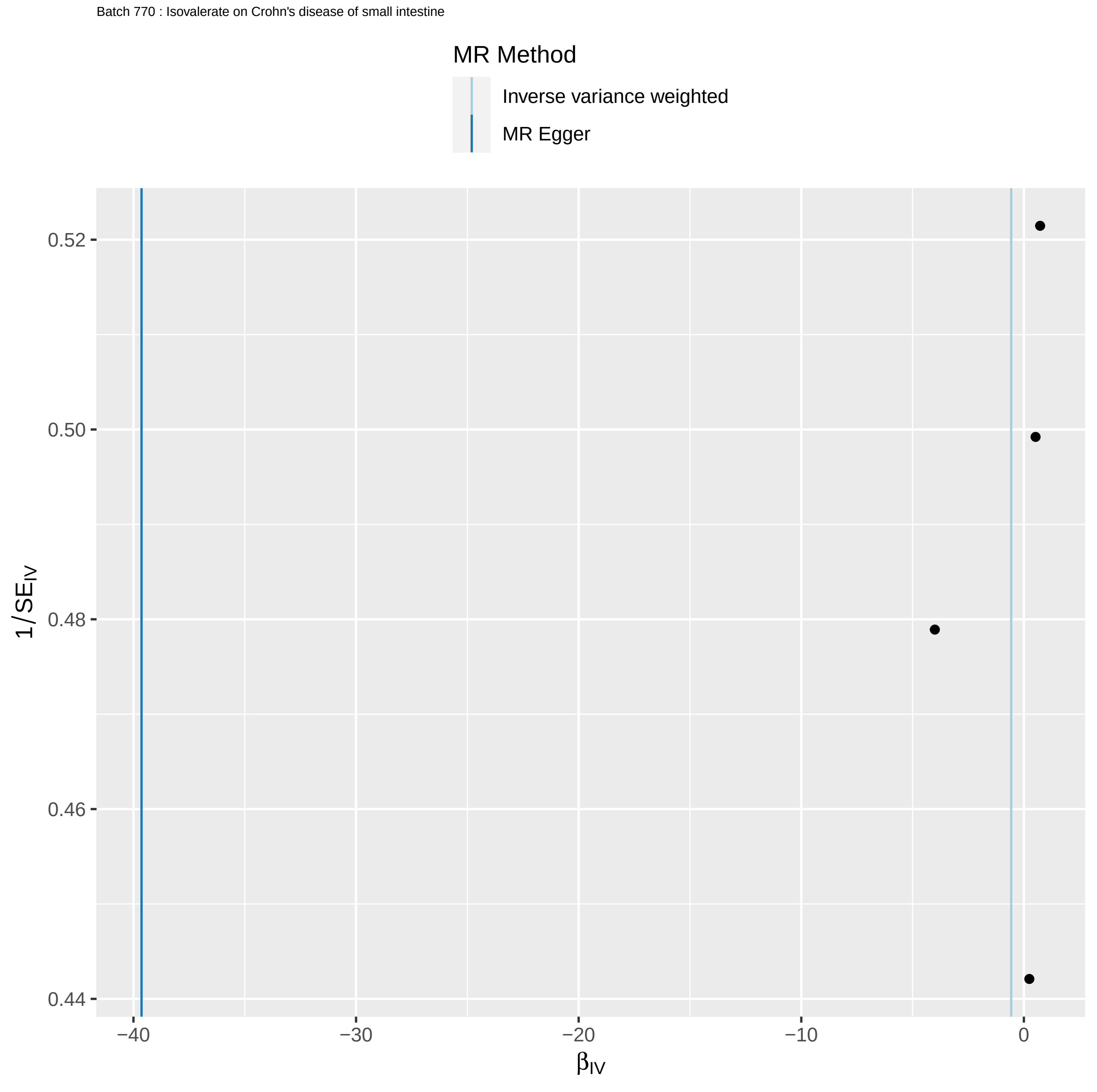

Batch 770 : Isovalerate on Crohn's disease of small intestine
MR Method
Inverse variance weighted
MR Egger
0.52
0.50
0.48
0.46
0.44
1/SE_IV
−40
−30
−20
−10
0
β_IV

Batch 771 : X−13183−−stearamide on Crohn's disease of small intestine

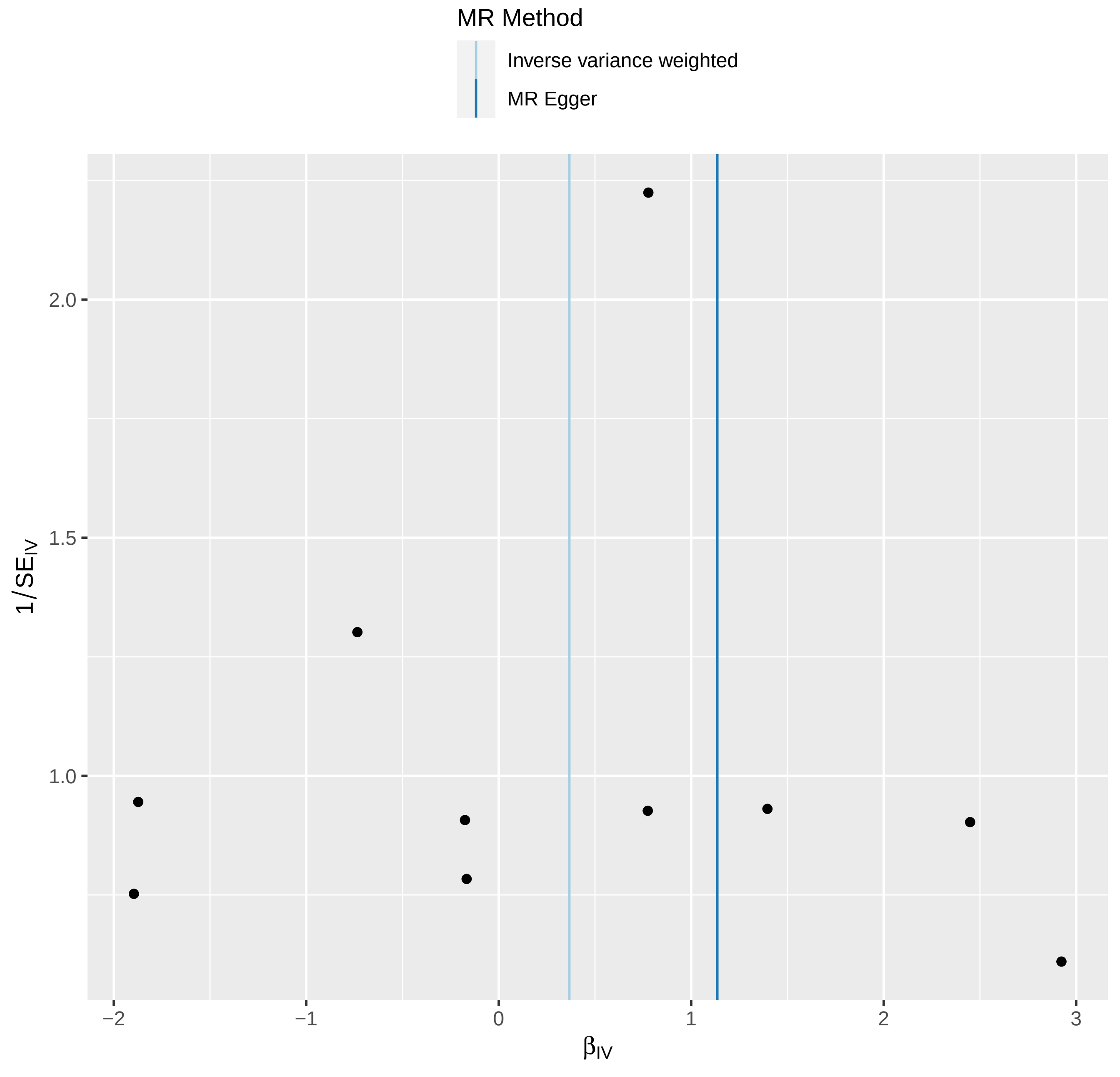

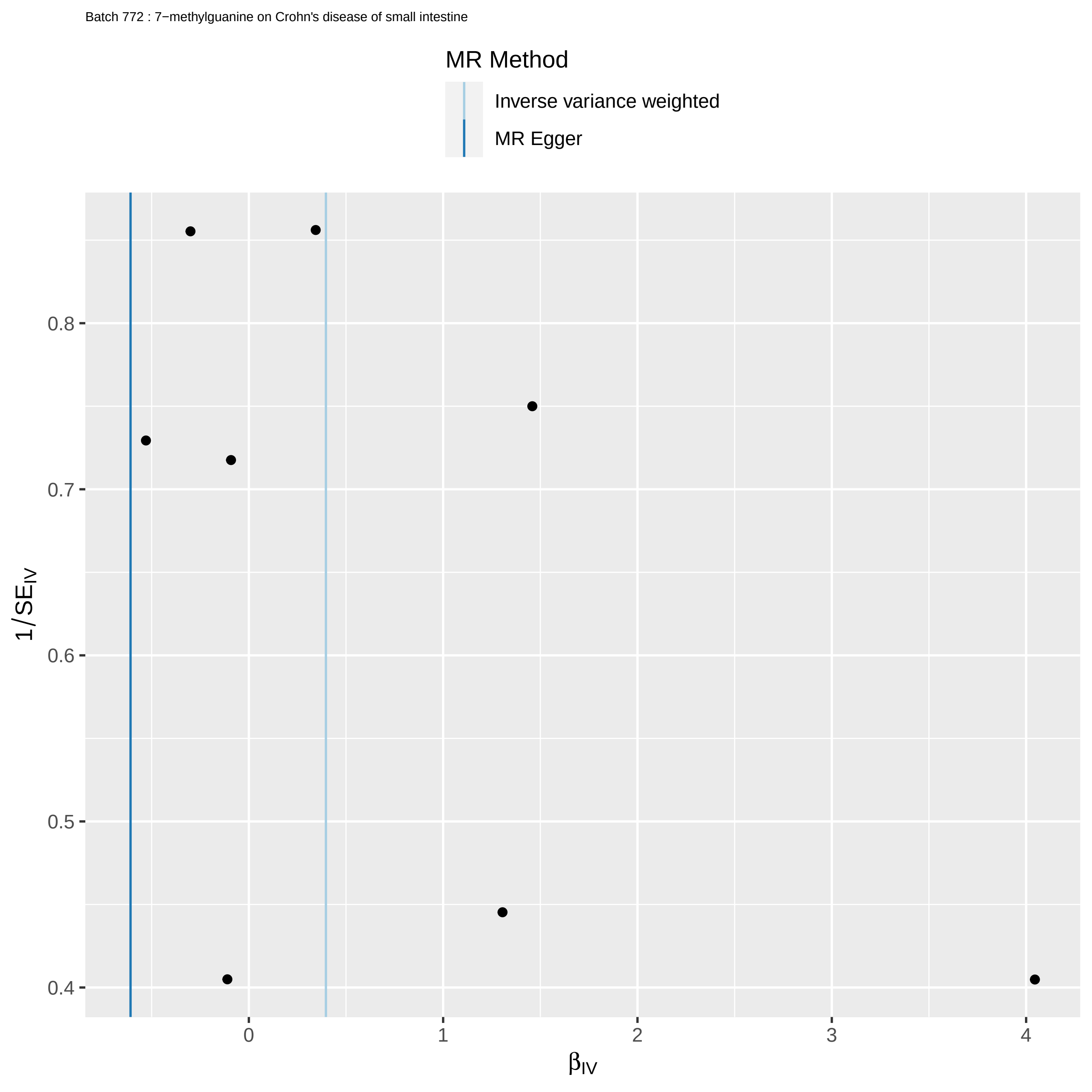

Batch 772 : 7−methylguanine on Crohn's disease of small intestine
MR Method
Inverse variance weighted
MR Egger
1/SE_IV
0.4
0.5
0.6
0.7
0.8
0
1
2
3
4
β_IV

Batch 779 : X−13431−−nonanoylcarnitine* on Crohn's disease of small intestine

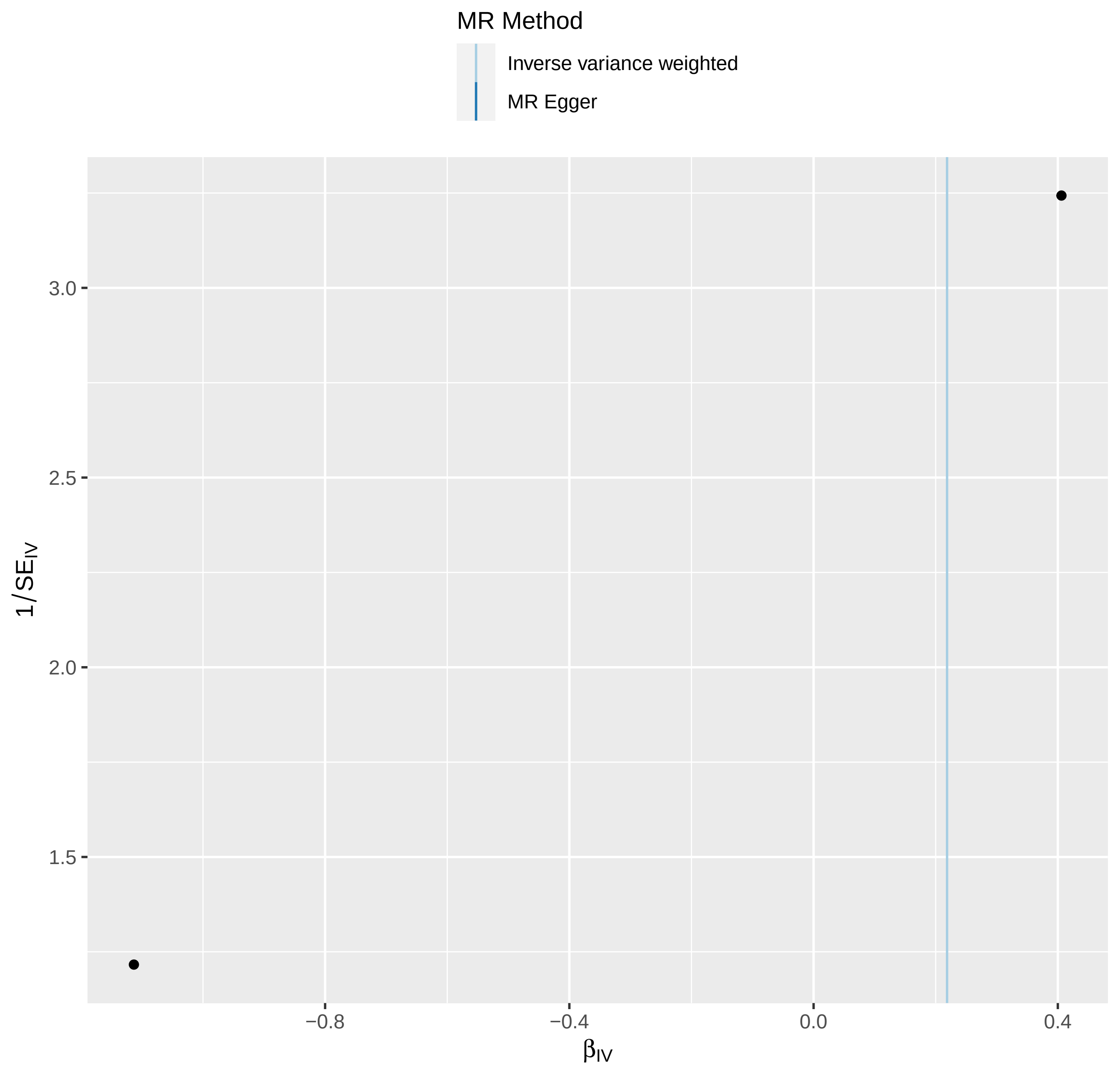

Batch 780 : 2−palmitoylglycerophosphocholine* on Crohn's disease of small intestine

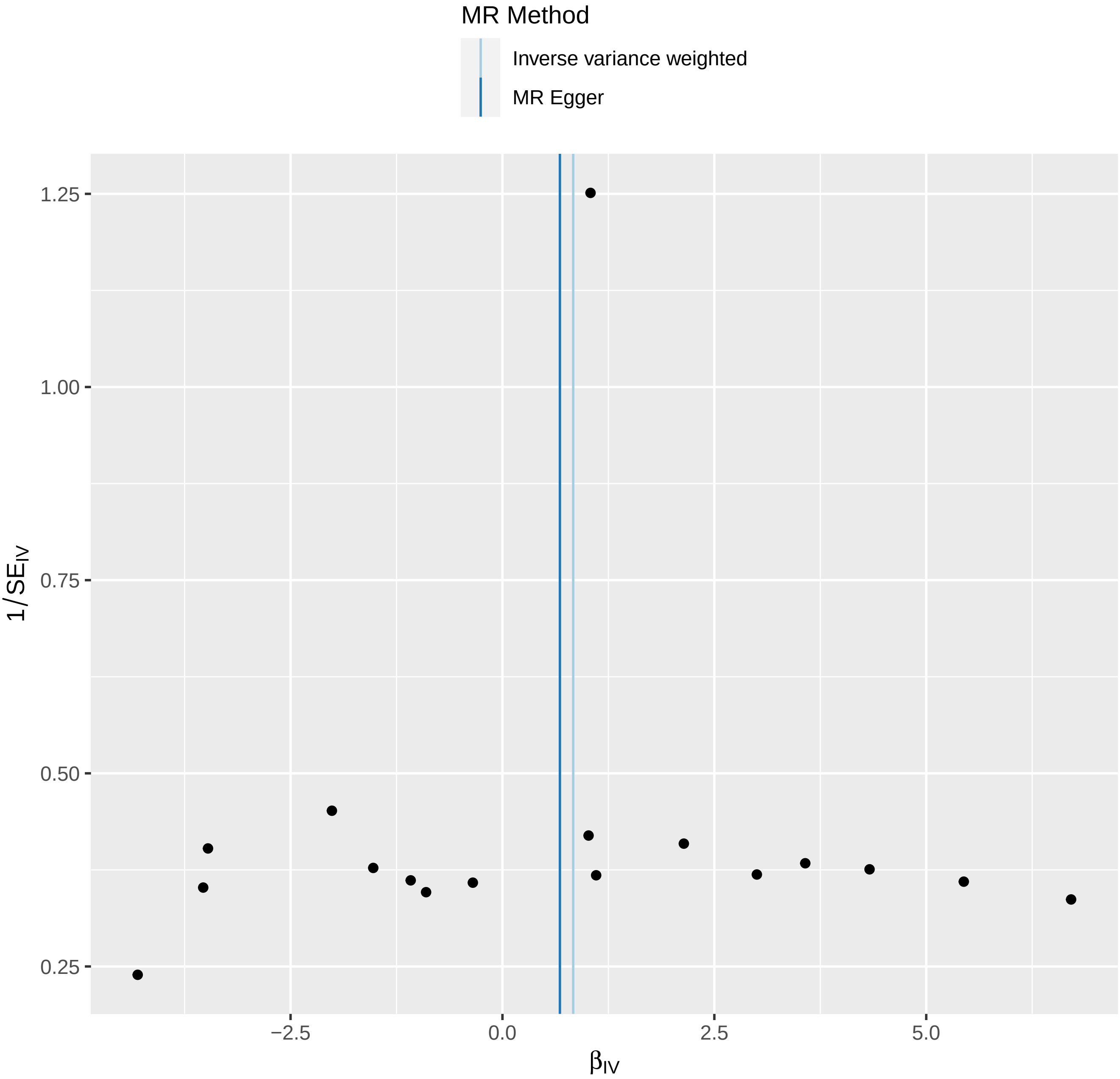

Batch 781 : 2−oleoylglycerophosphocholine* on Crohn's disease of small intestine

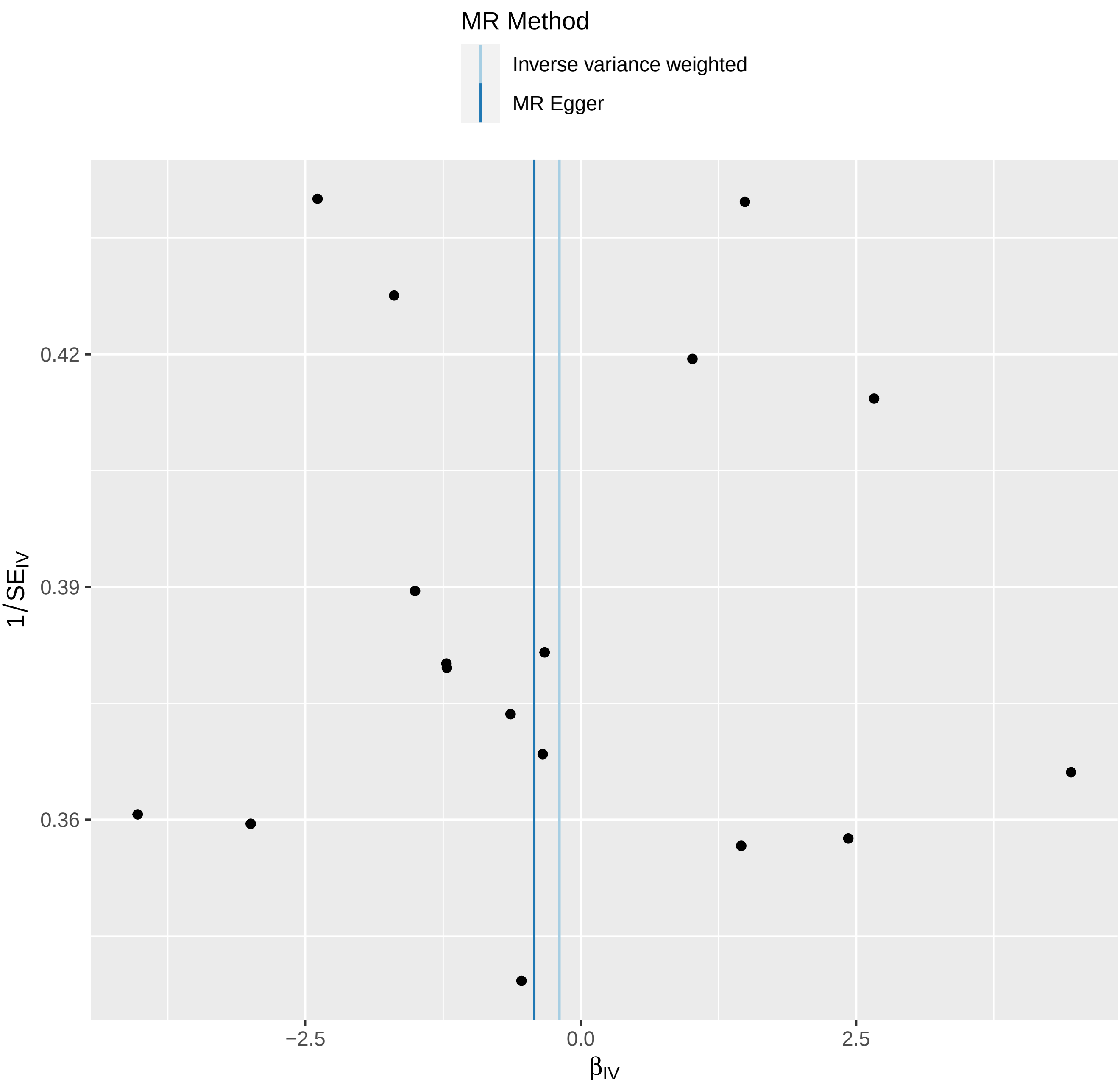

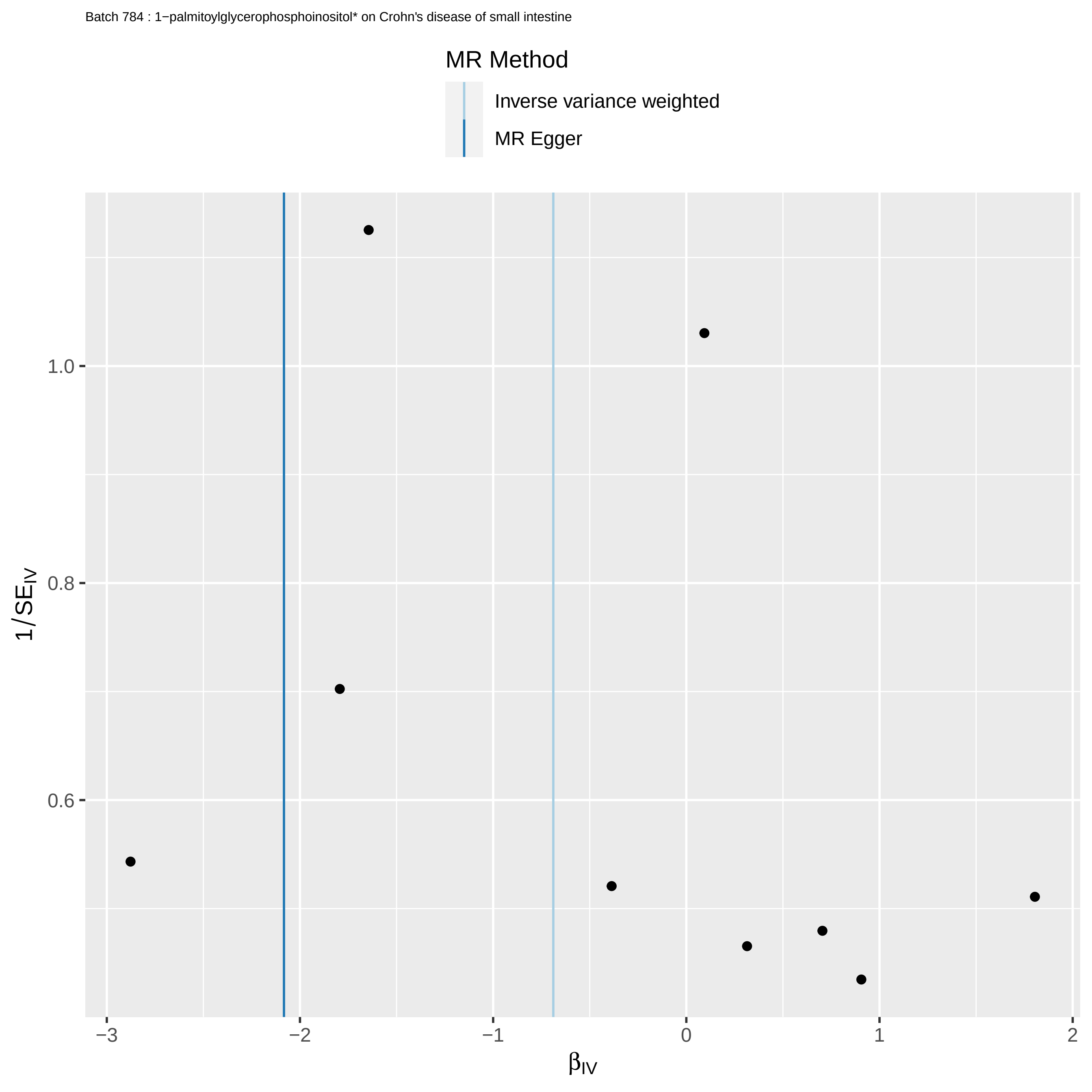

Batch 784 : 1−palmitoylglycerophosphoinositol* on Crohn's disease of small intestine
MR Method
Inverse variance weighted
MR Egger
1.0
0.8
0.6
1/SE$_{IV}$
−3
−2
−1
0
1
2
β$_{IV}$

Batch 787 : Glutaroyl carnitine on Crohn's disease of small intestine

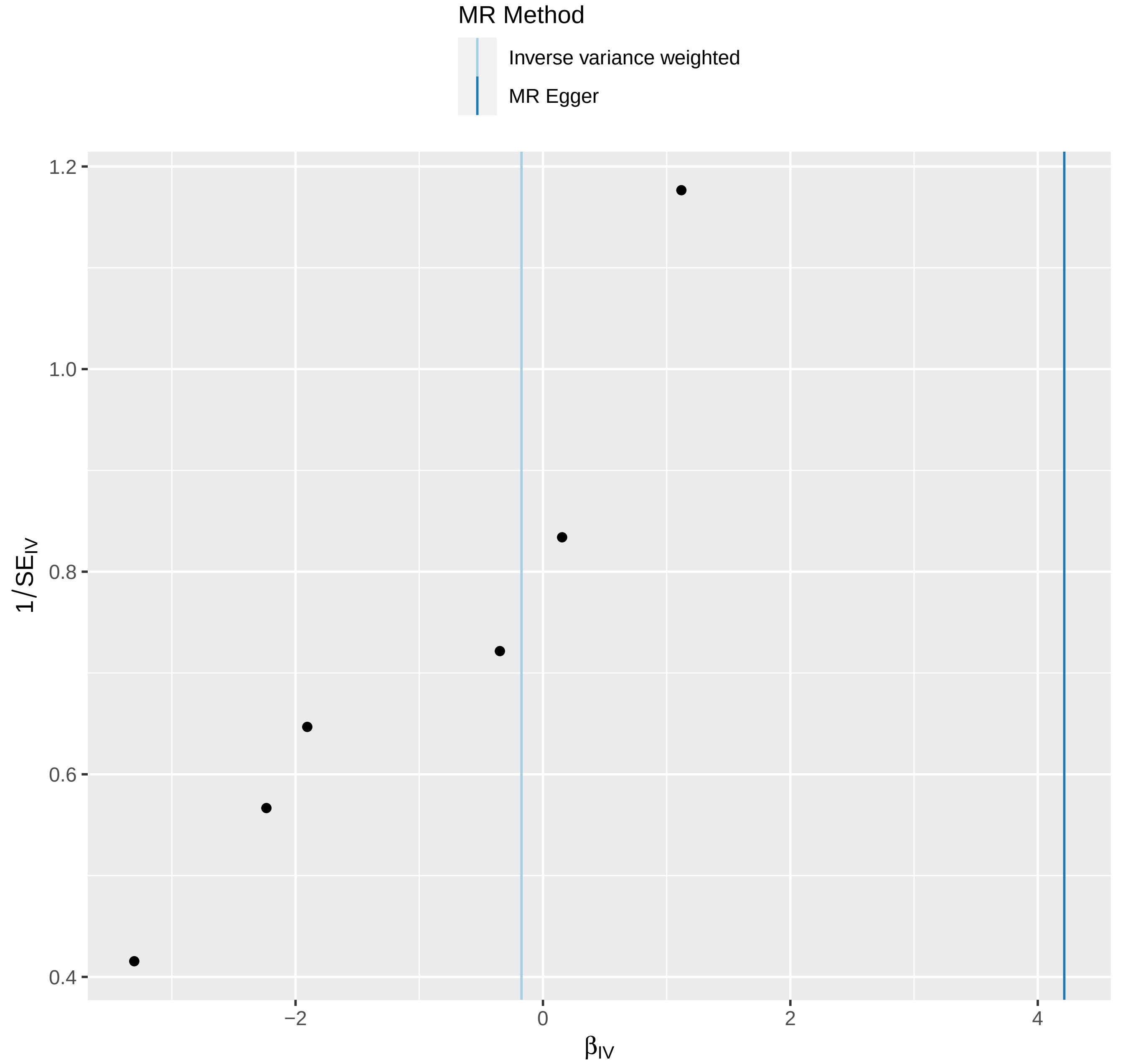

Batch 789 : 4−hydroxyhippurate on Crohn's disease of small intestine

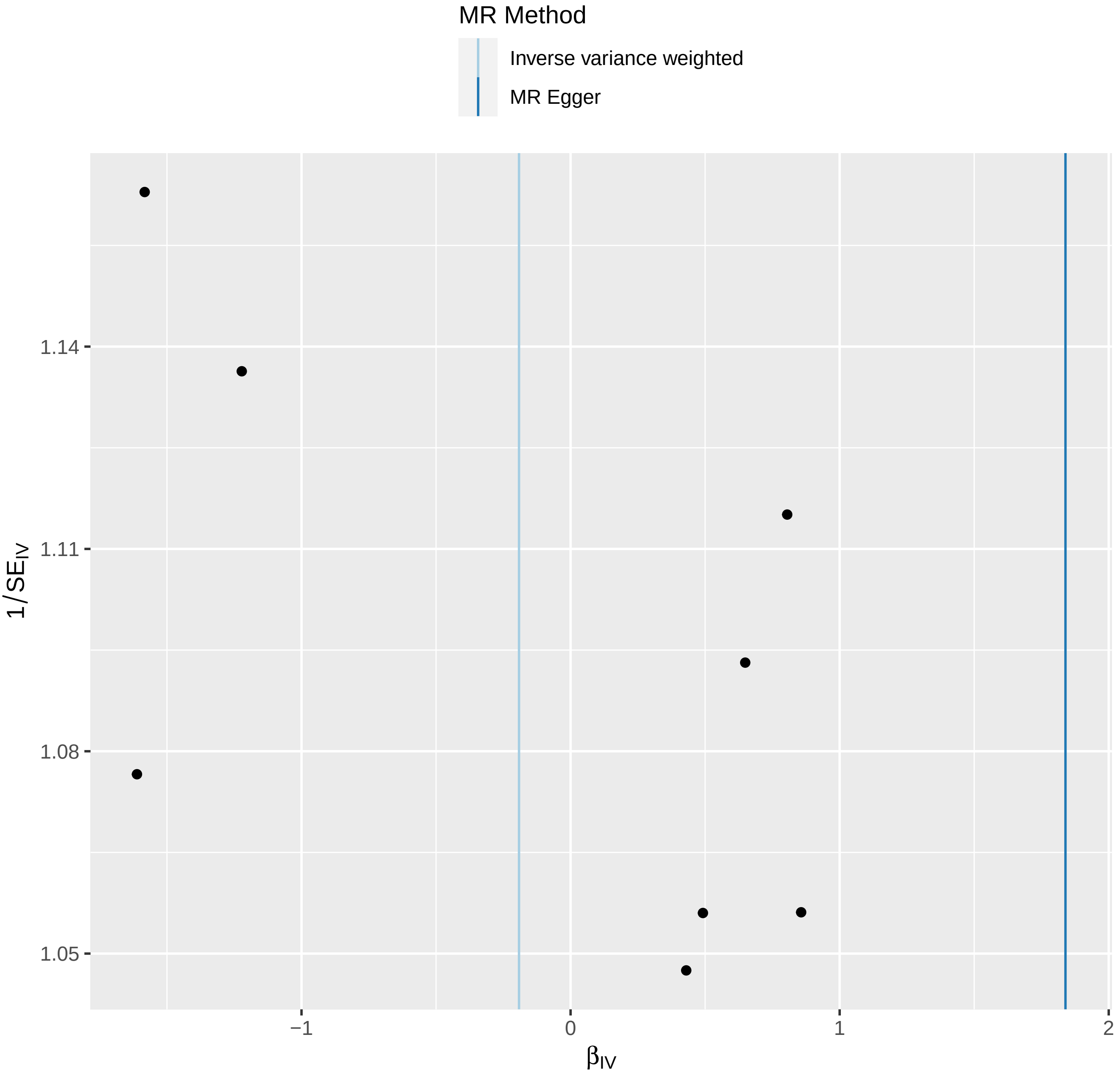

Batch 790 : 1−myristoylglycerophosphocholine on Crohn's disease of small intestine

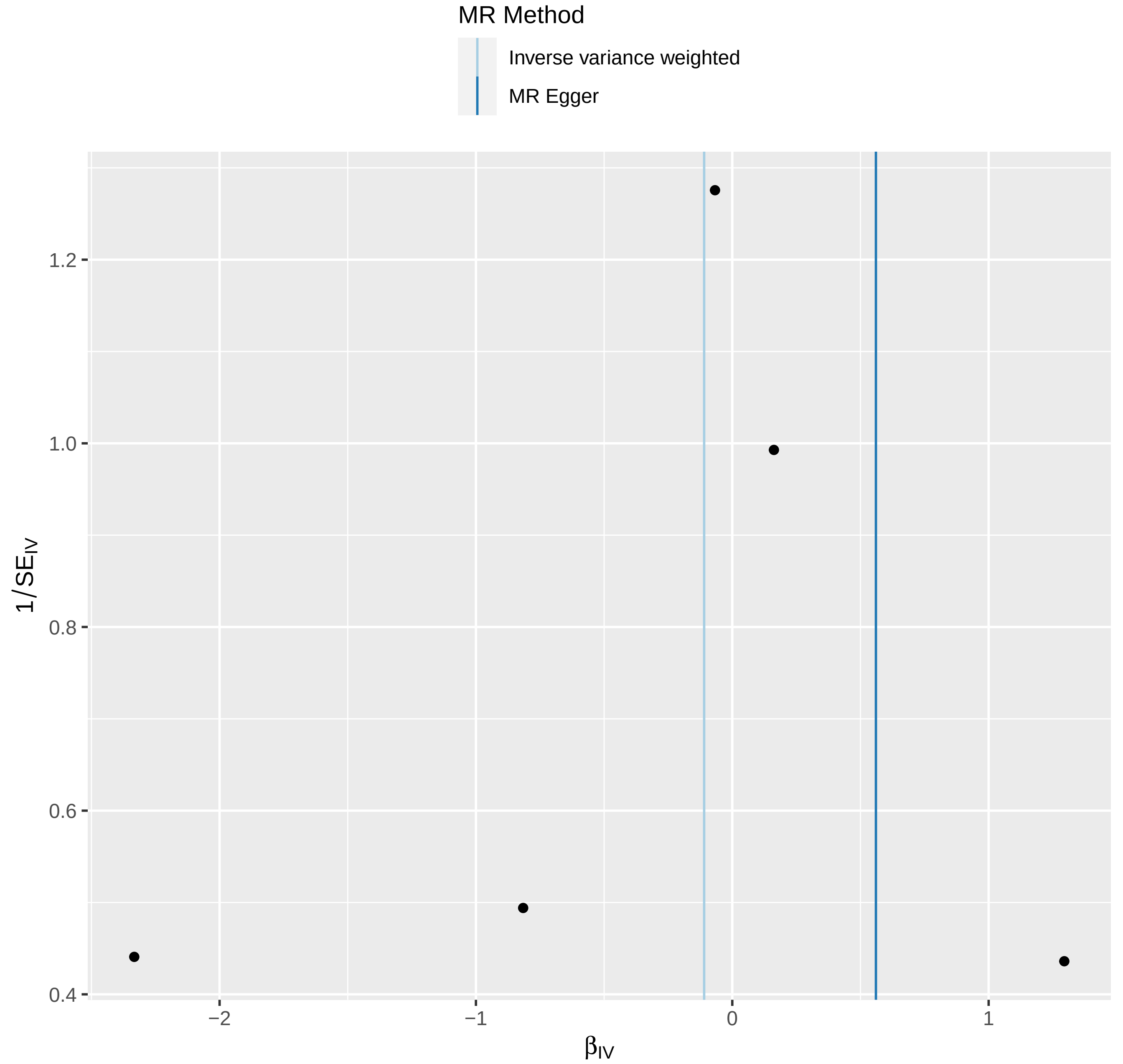

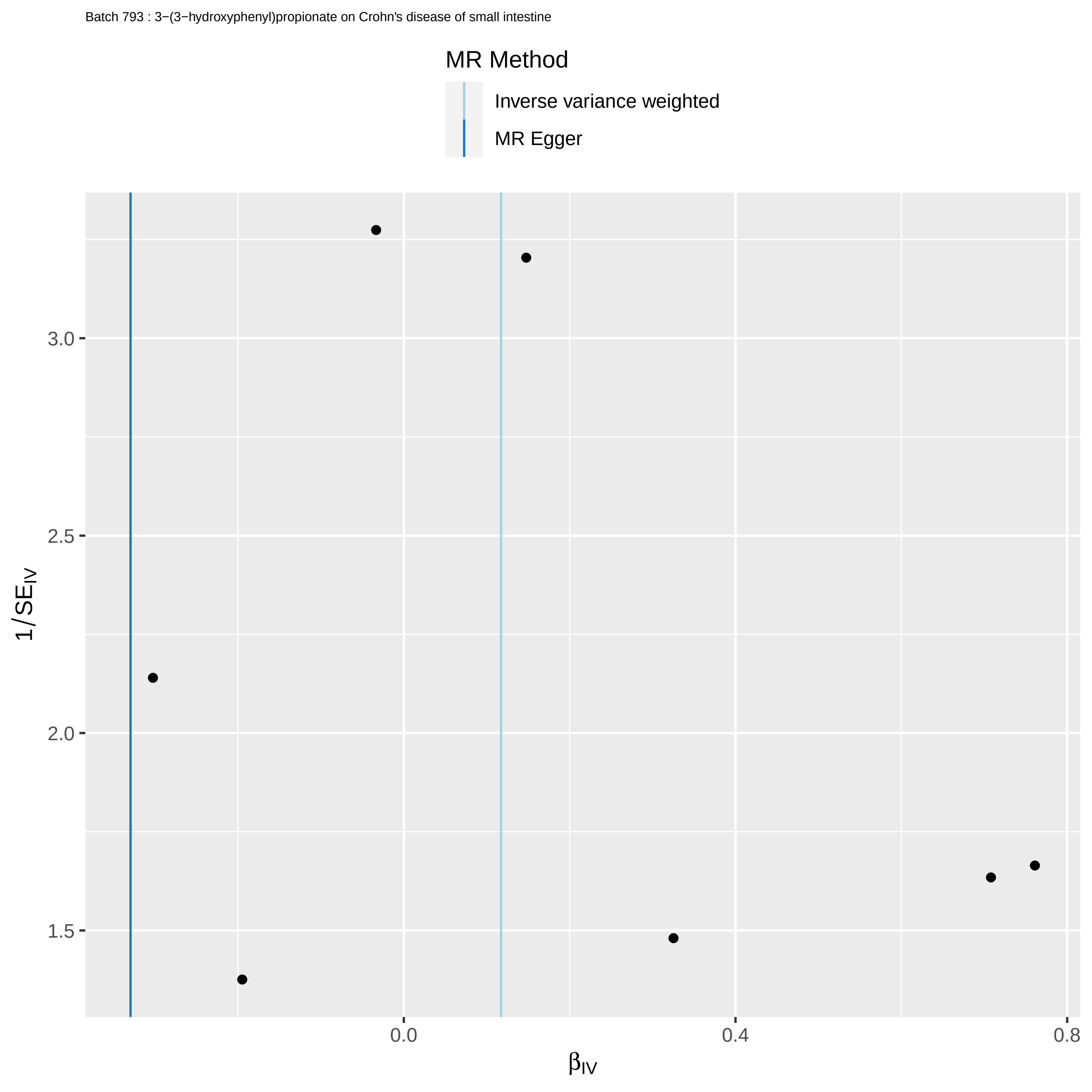

Batch 793 : 3−(3−hydroxyphenyl)propionate on Crohn's disease of small intestine
MR Method
Inverse variance weighted
MR Egger
3.0
2.5
2.0
1.5
1/SE_IV
0.0
0.4
0.8
β_IV

Batch 797 : Dihomo−linolenate (20:3n3 or n6) on Crohn's disease of small intestine

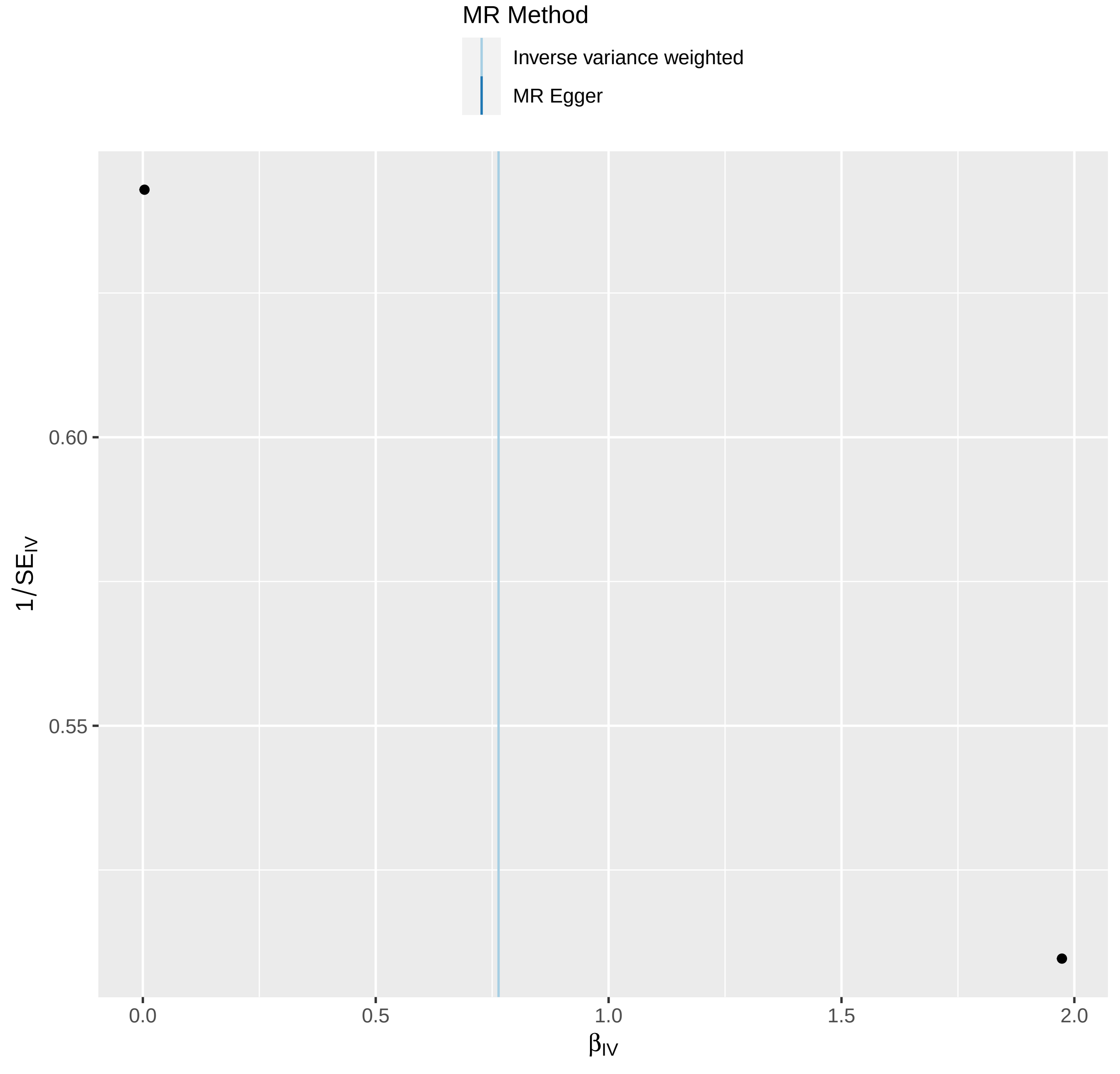

Batch 798 : Threitol on Crohn's disease of small intestine

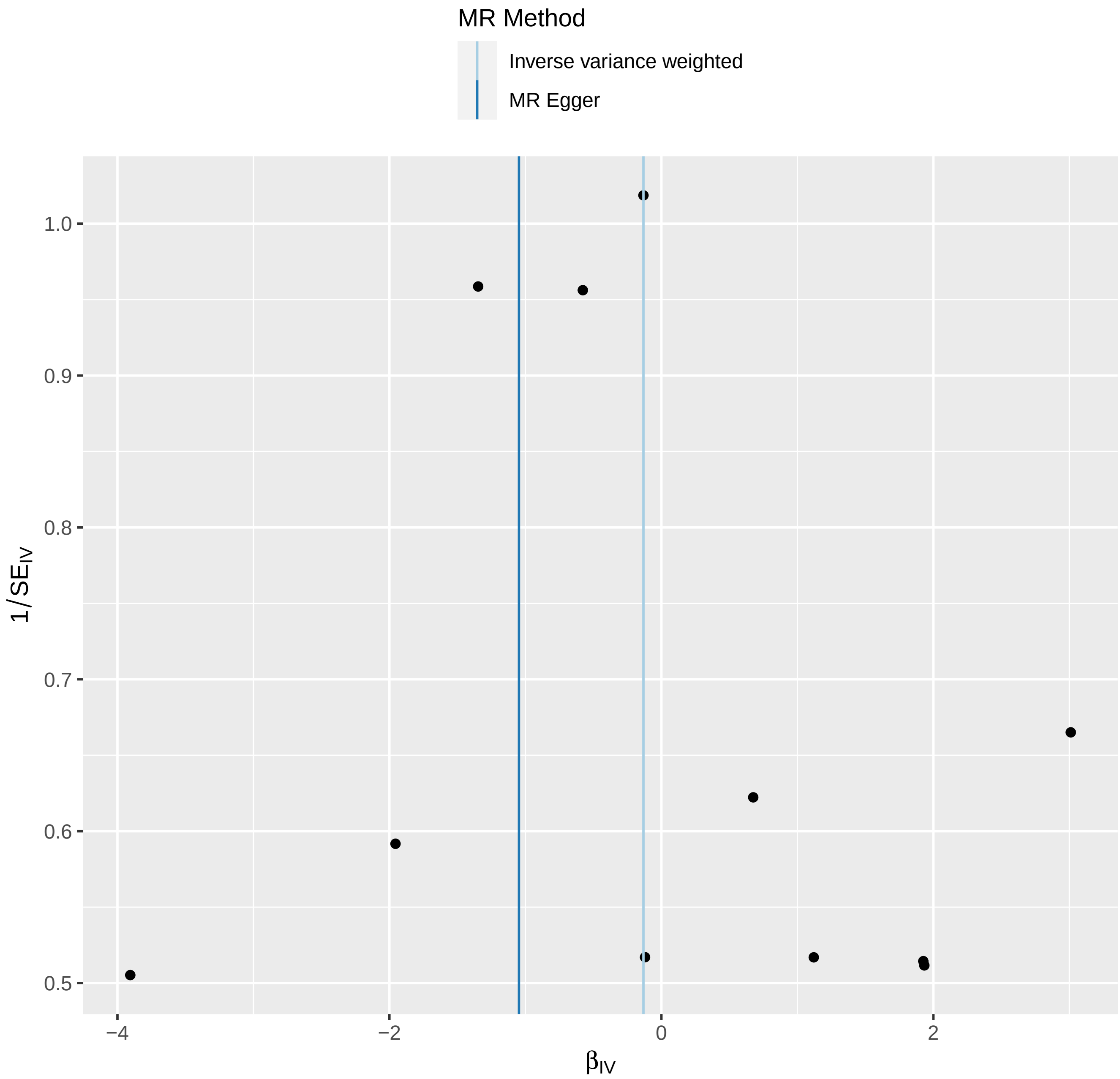

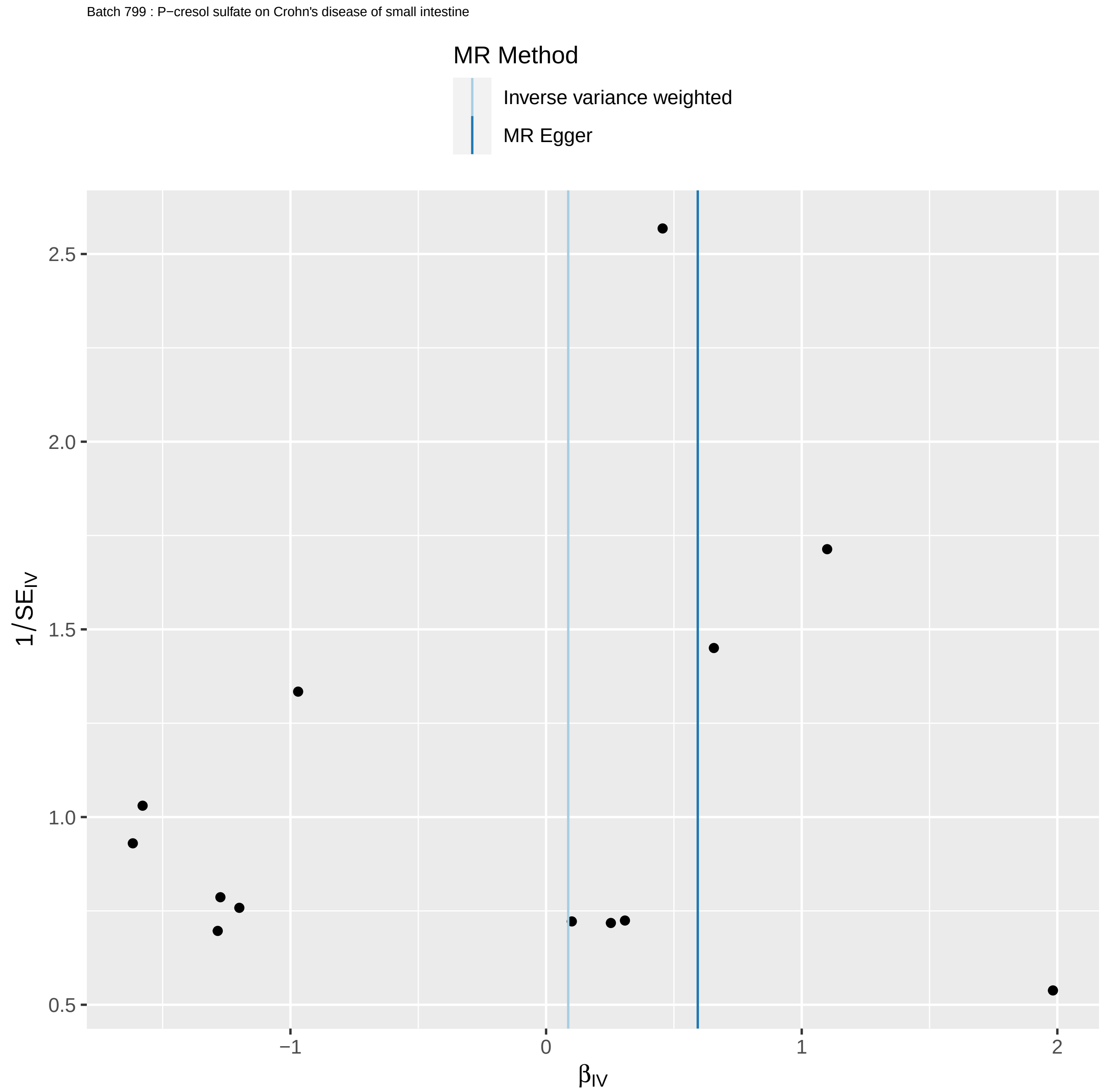

Batch 799 : P−cresol sulfate on Crohn's disease of small intestine
MR Method
Inverse variance weighted
MR Egger
2.5
2.0
1.5
1.0
0.5
1/SE_IV
−1
0
1
2
β_IV

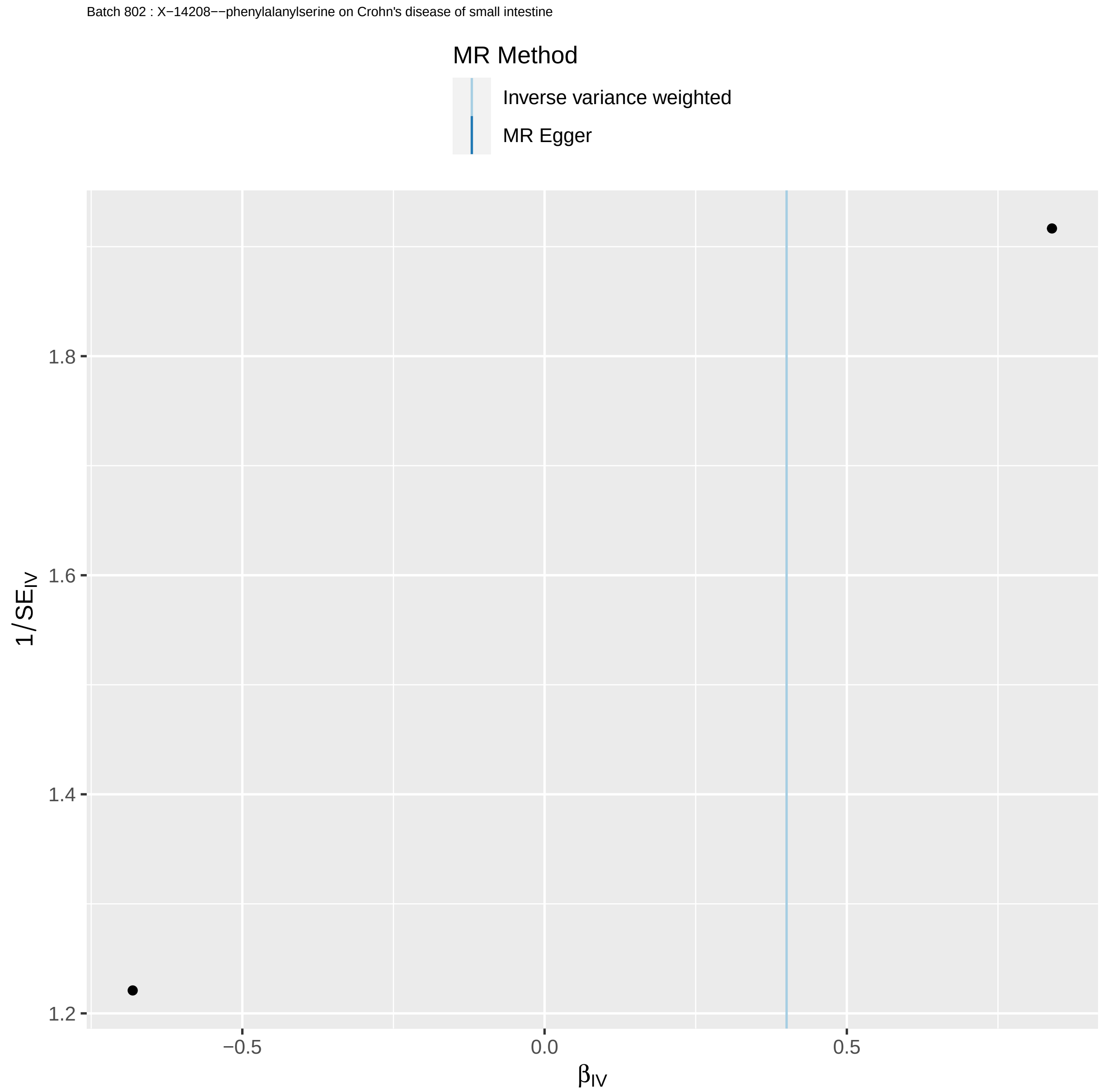

Batch 802 : X−14208−−phenylalanylserine on Crohn's disease of small intestine
MR Method
Inverse variance weighted
MR Egger
1.8
1.6
1.4
1.2
1/SE_IV
−0.5
0.0
0.5
β_IV

Batch 804 : X−14450−−phenylalanylleucine on Crohn's disease of small intestine

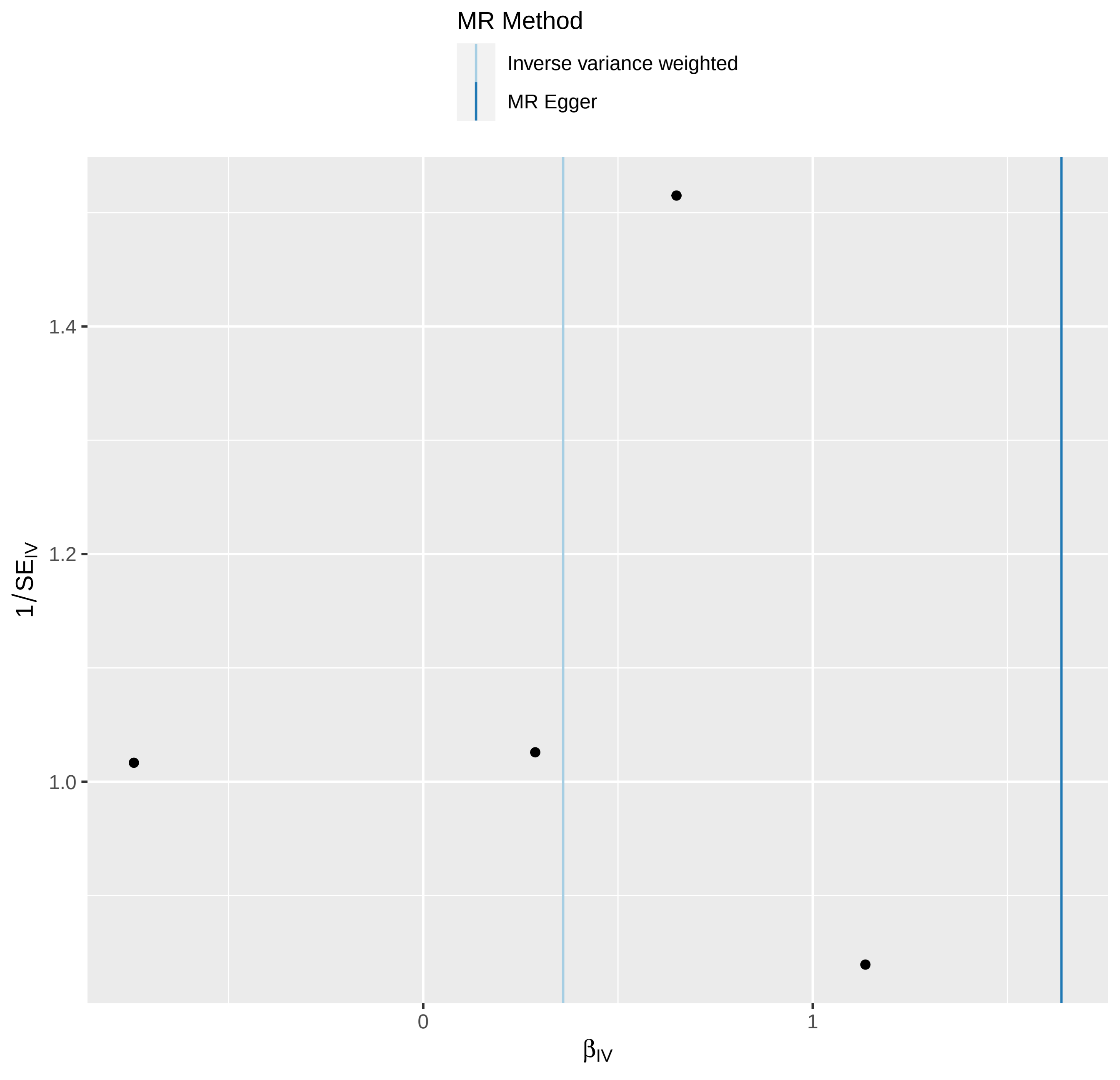

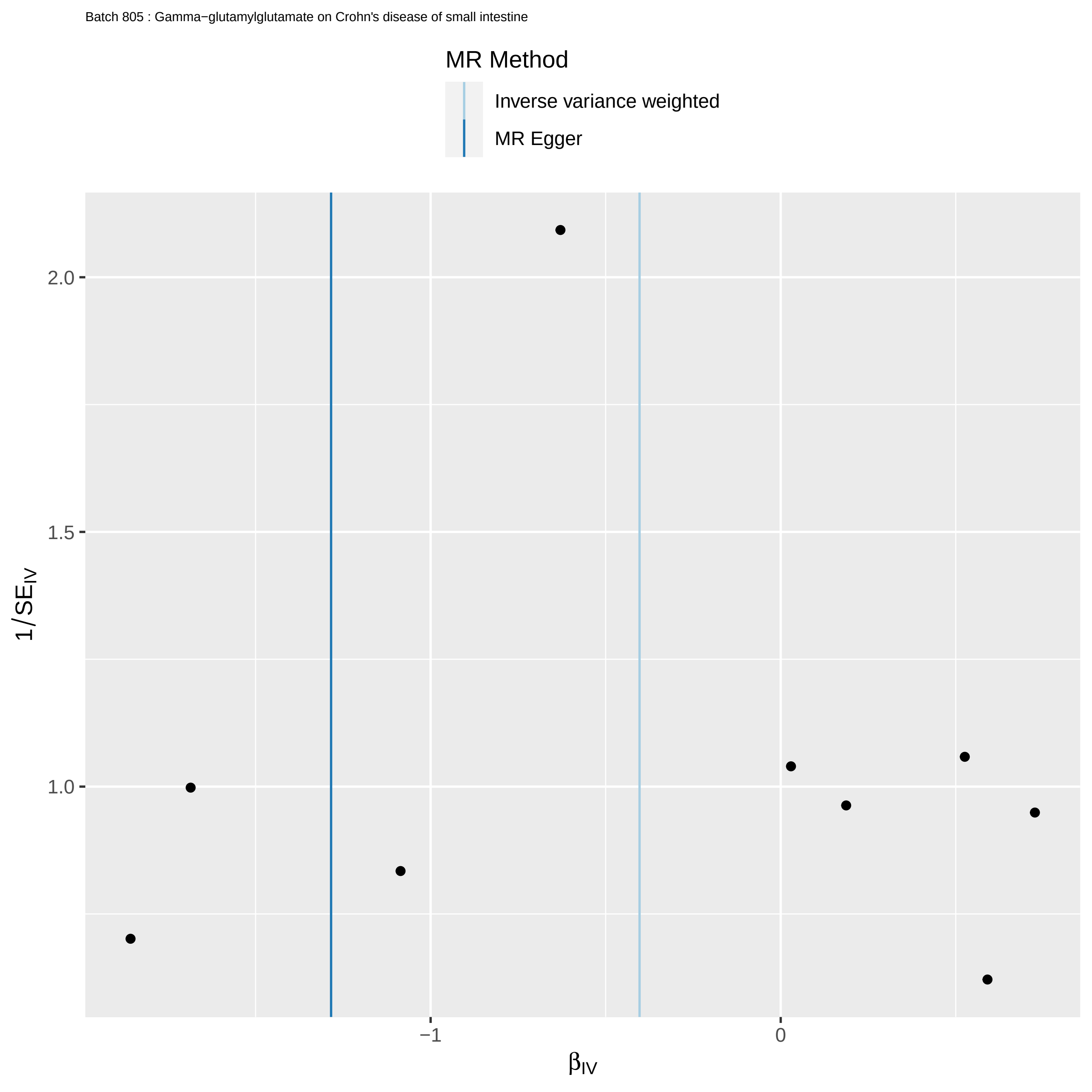
Batch 805 : Gamma−glutamylglutamate on Crohn's disease of small intestine
MR Method
Inverse variance weighted
MR Egger
2.0
1.5
1.0
1/SE_IV
−1
0
β_IV

Batch 806 : Octadecanedioate on Crohn's disease of small intestine

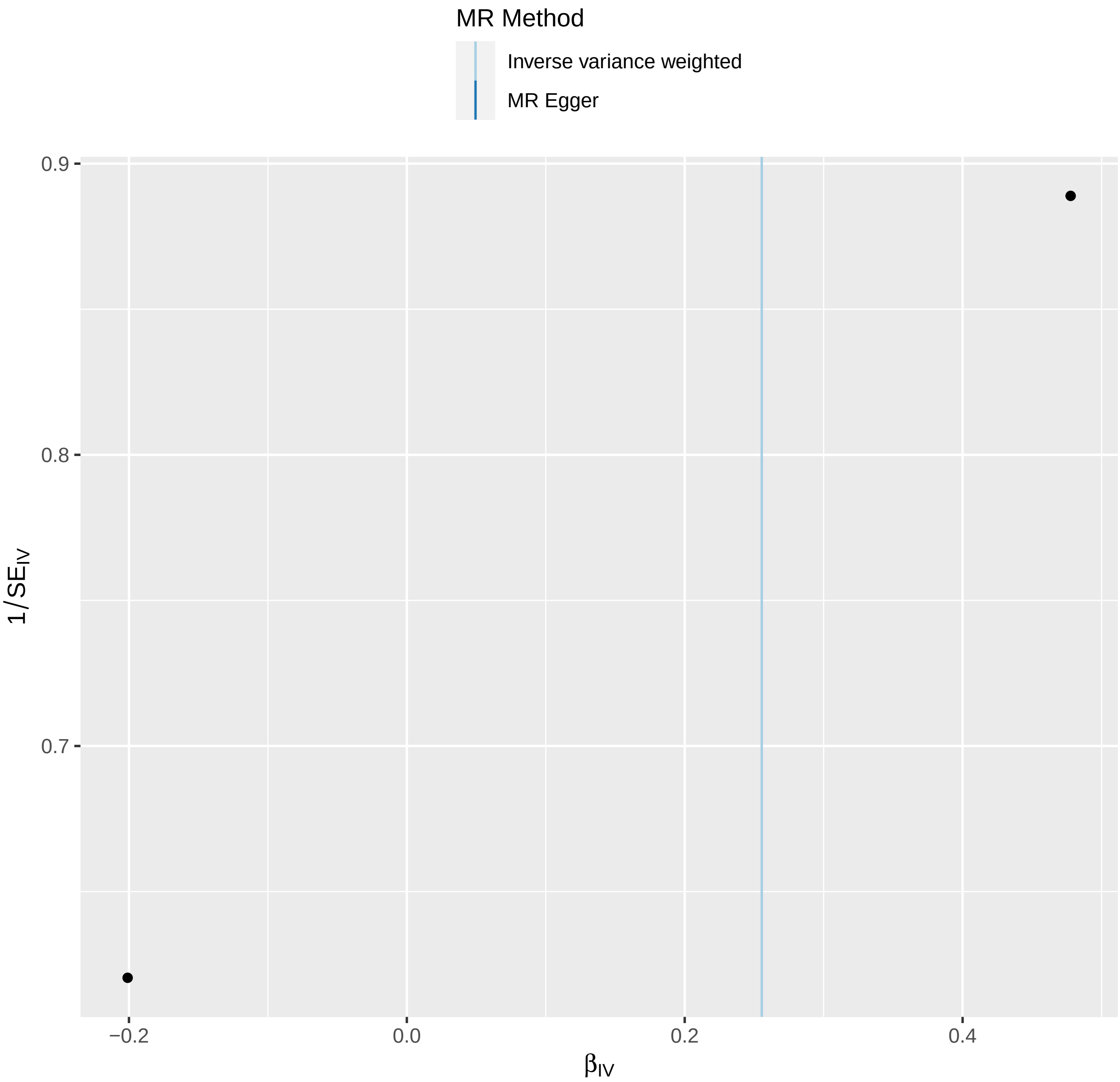

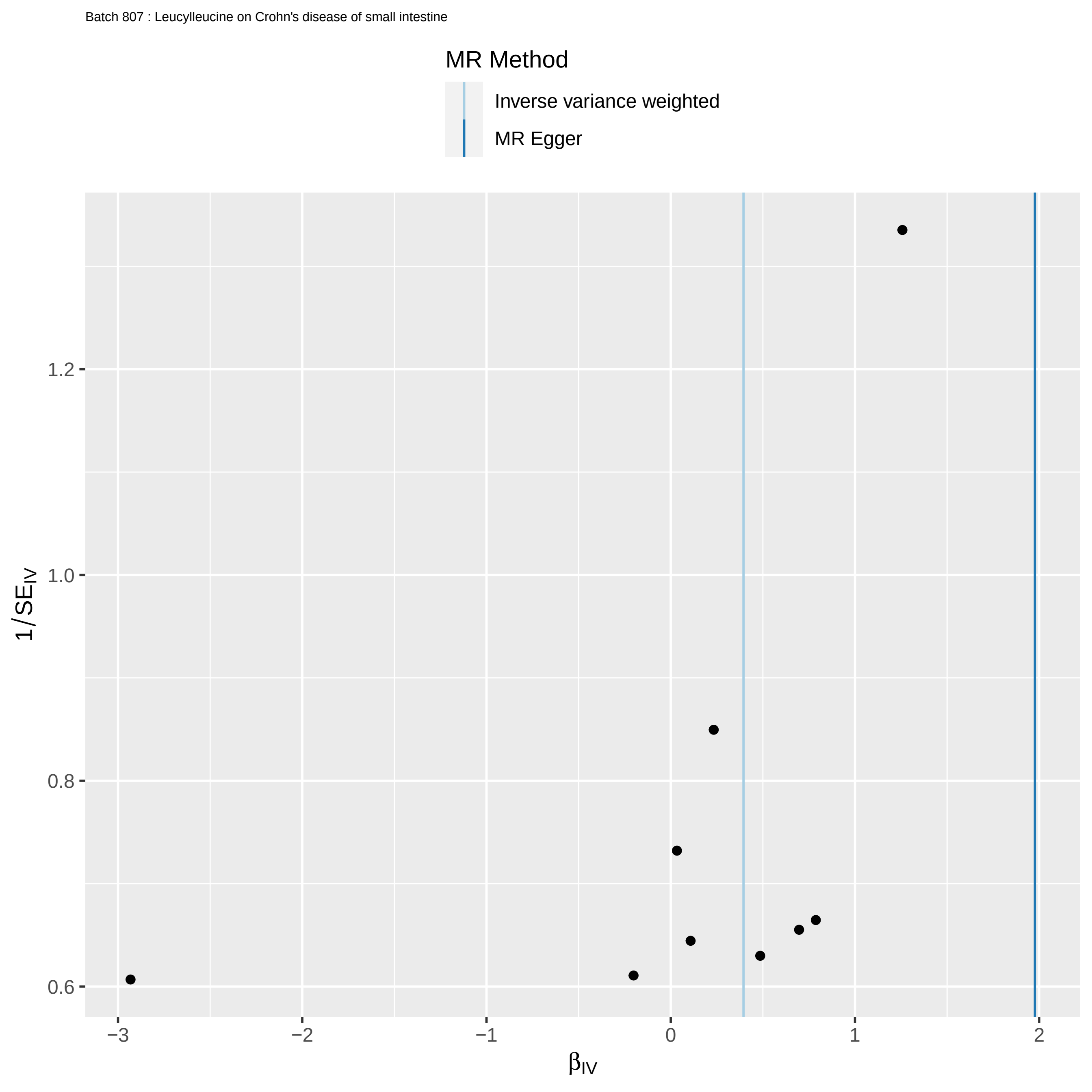

Batch 807 : Leucylleucine on Crohn's disease of small intestine
MR Method
Inverse variance weighted
MR Egger
1.2
1.0
0.8
0.6
$1/SE_{IV}$
−3
−2
−1
0
1
2
β_{IV}

Batch 808 : 7−alpha−hydroxy−3−oxo−4−cholestenoate (7−Hoca) on Crohn's disease of small intestine

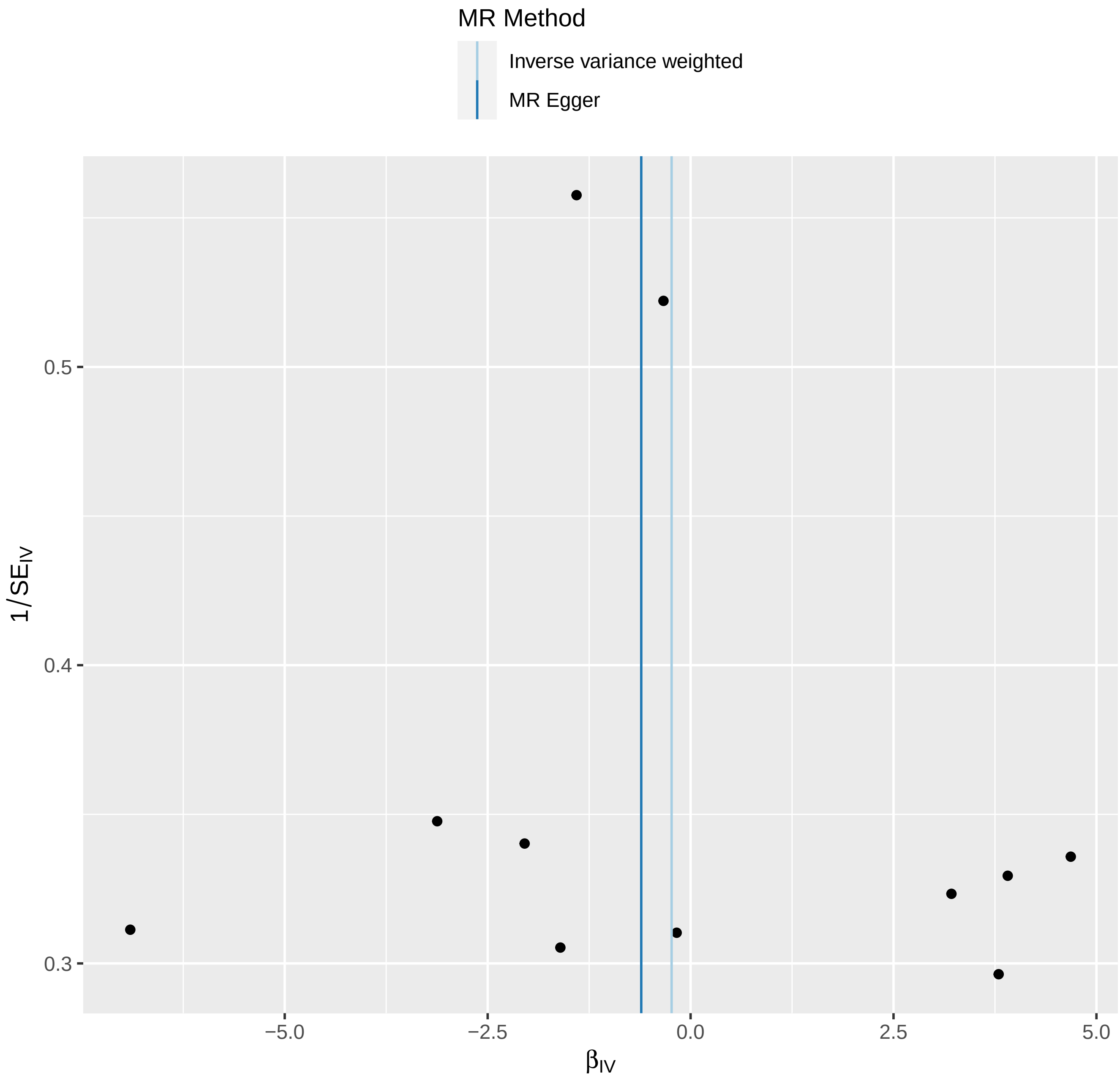

Batch 809 : N−Butyl Oleate on Crohn's disease of small intestine

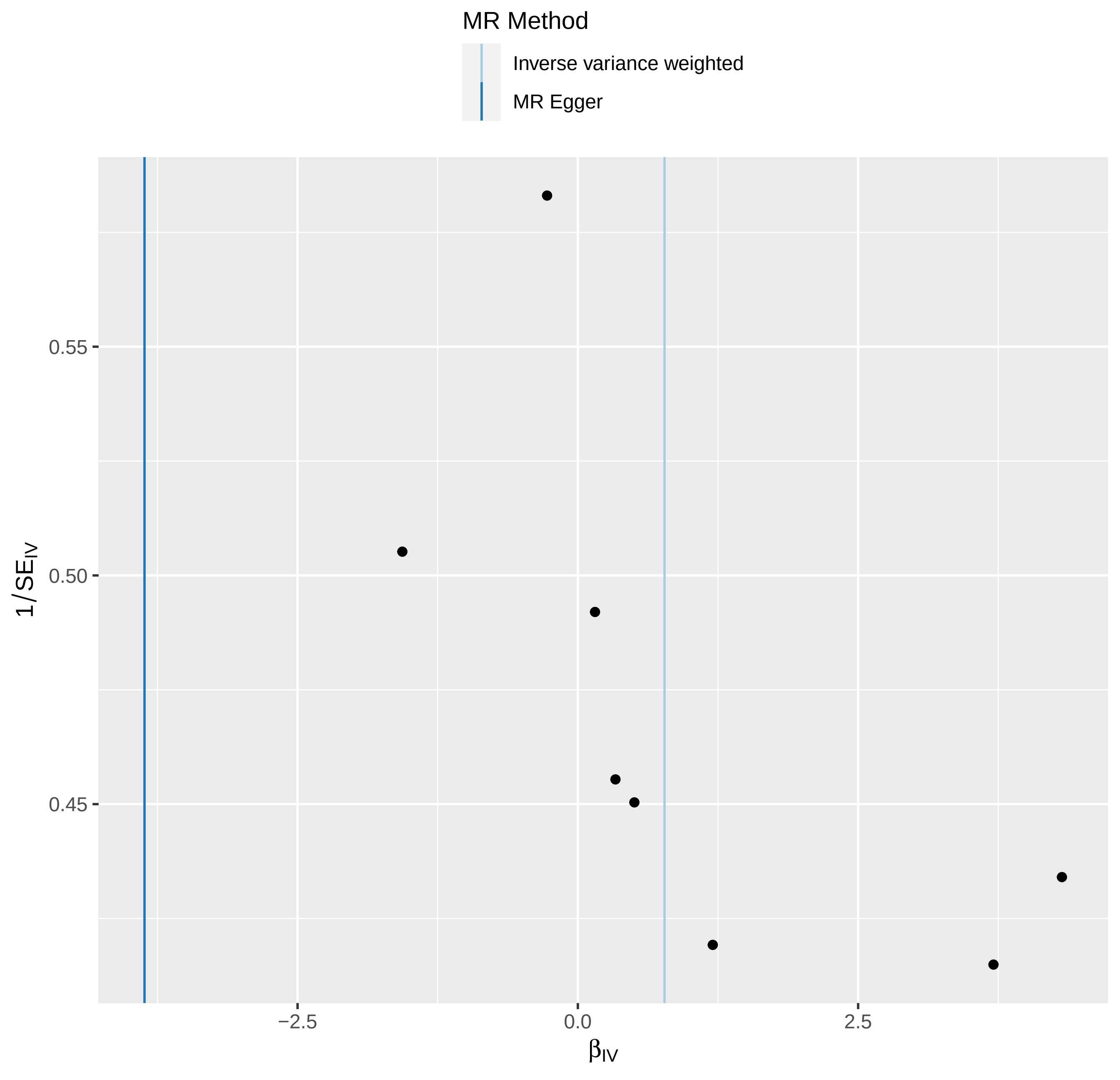

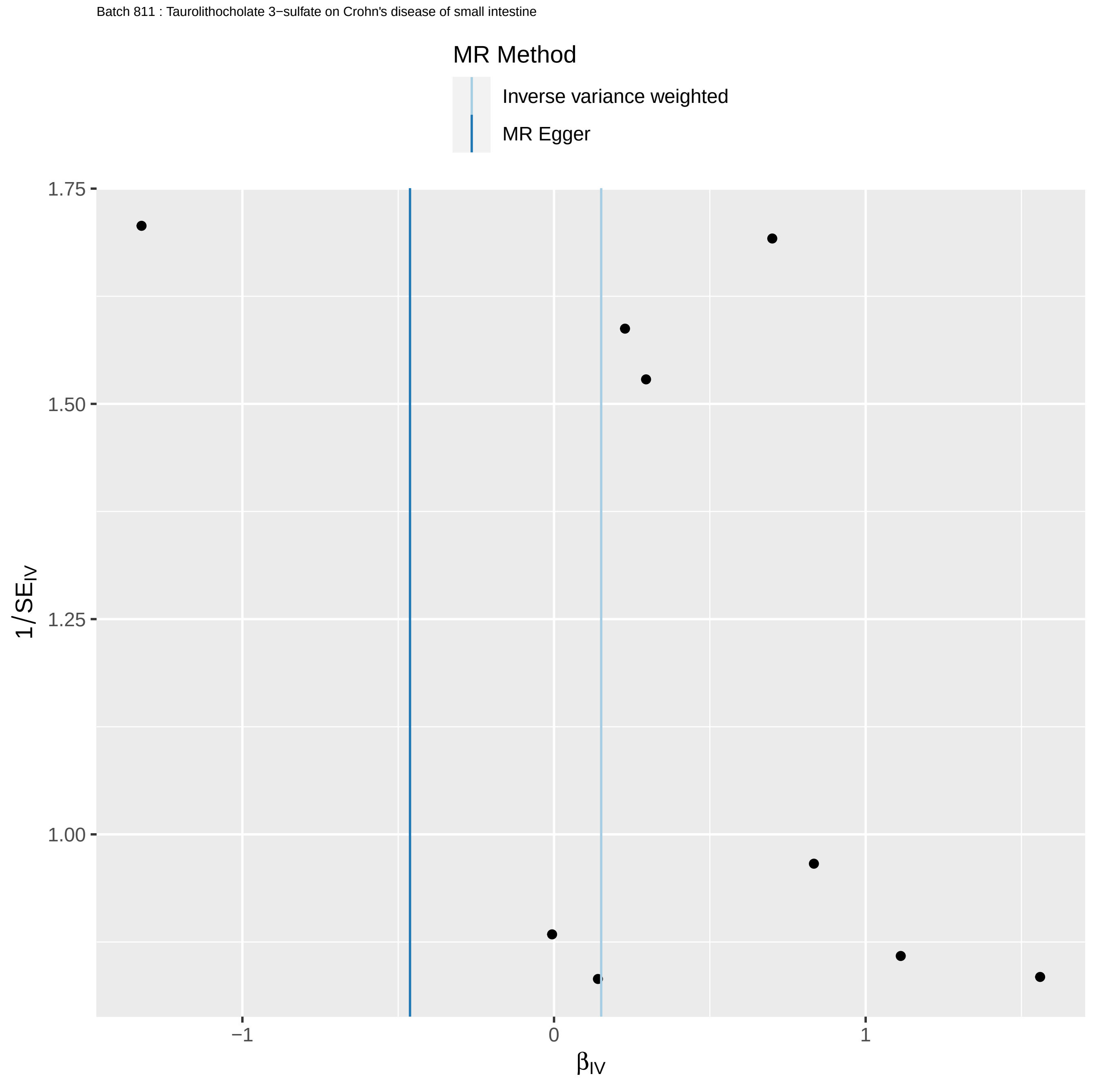

Batch 811 : Taurolithocholate 3−sulfate on Crohn's disease of small intestine
MR Method
Inverse variance weighted
MR Egger
1.75
1.50
1.25
1.00
1/SE_IV
−1
0
1
β_IV

Batch 812 : X−14977−−vanillin on Crohn's disease of small intestine

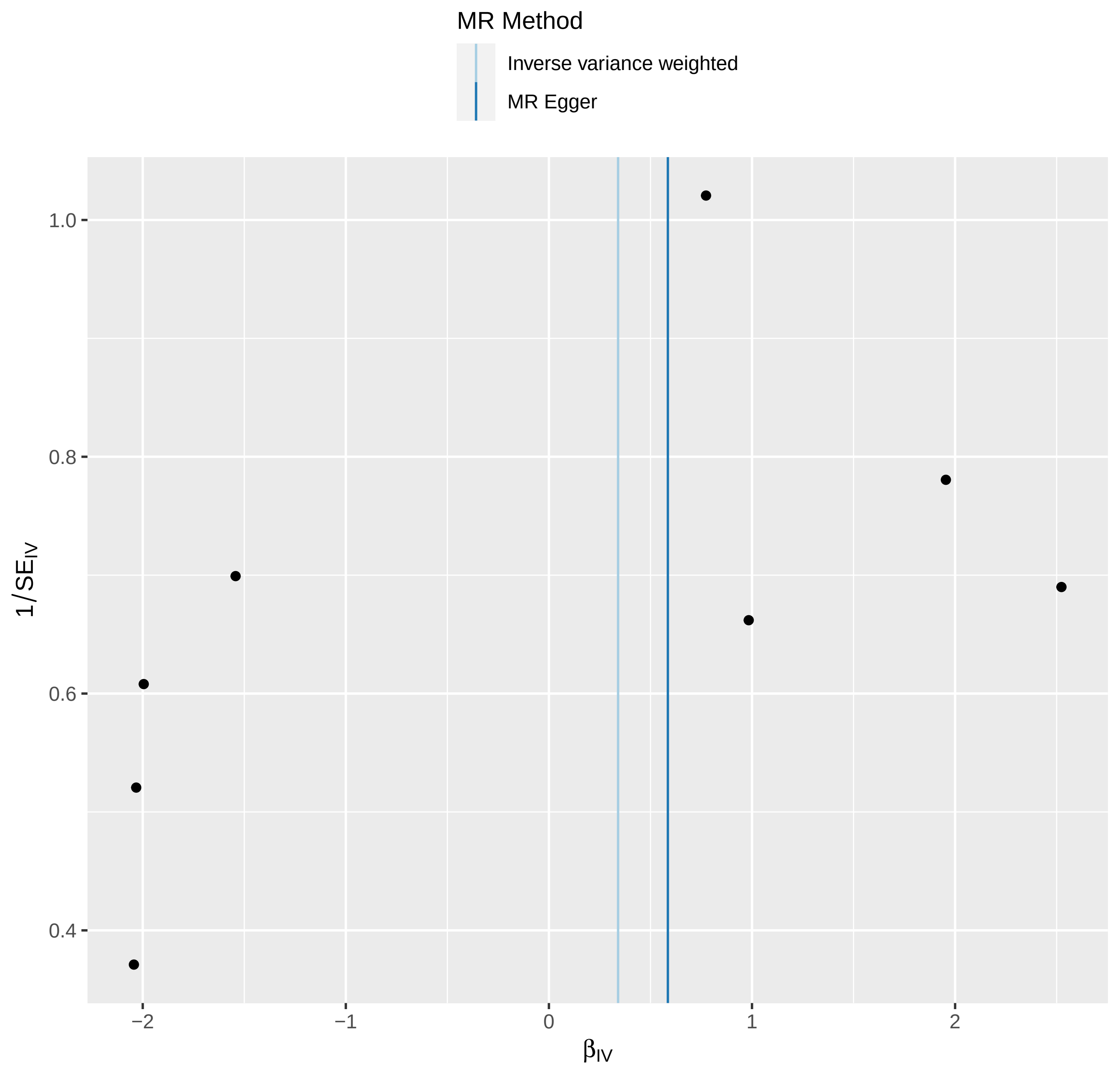

Batch 813 : Succinylcarnitine on Crohn's disease of small intestine

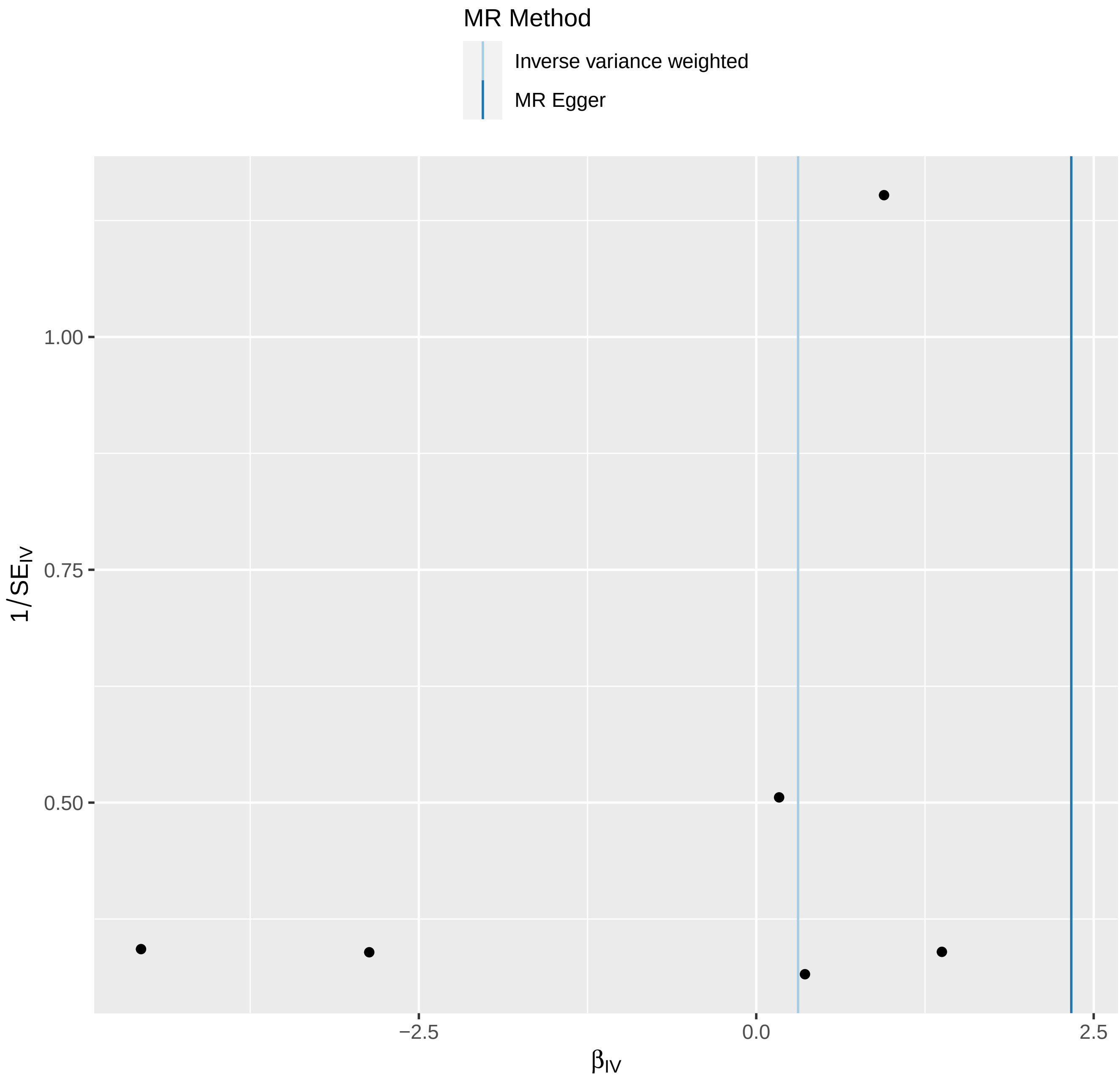

Batch 815 : Cyclo(leu−pro) on Crohn's disease of small intestine

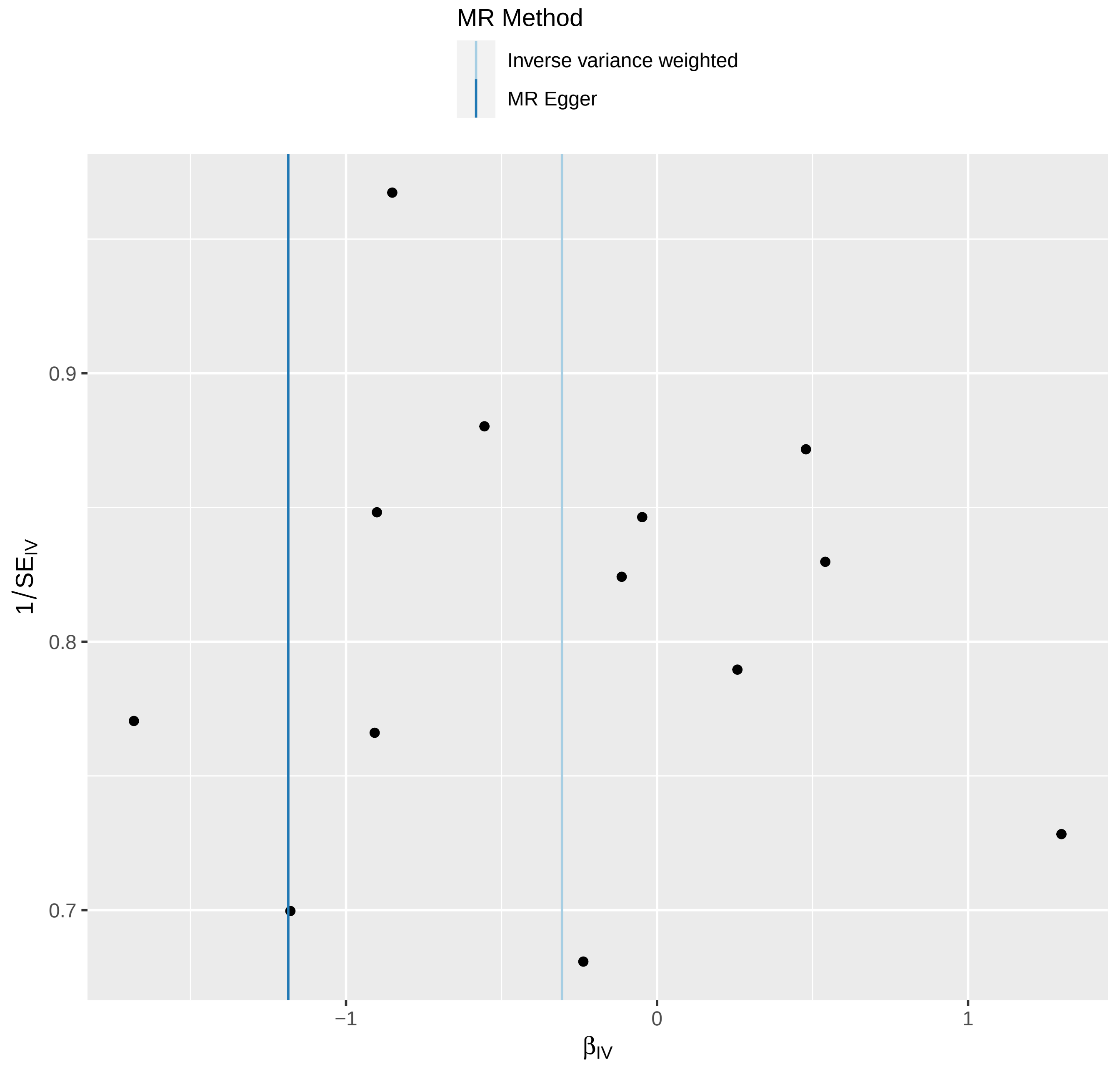

Batch 816 : Chiro−inositol on Crohn's disease of small intestine

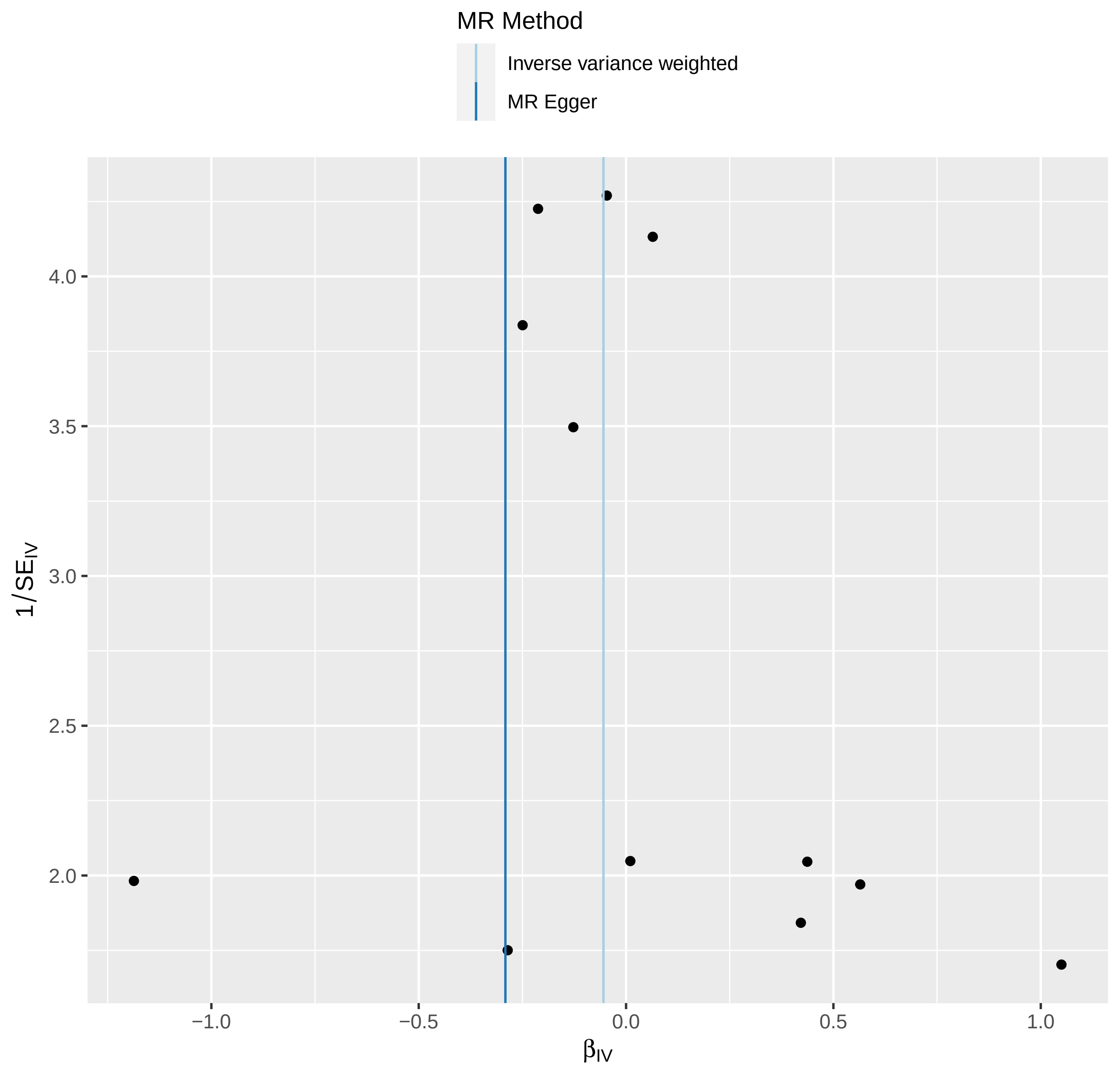

Batch 820 : 2−hydroxyglutarate on Crohn's disease of small intestine

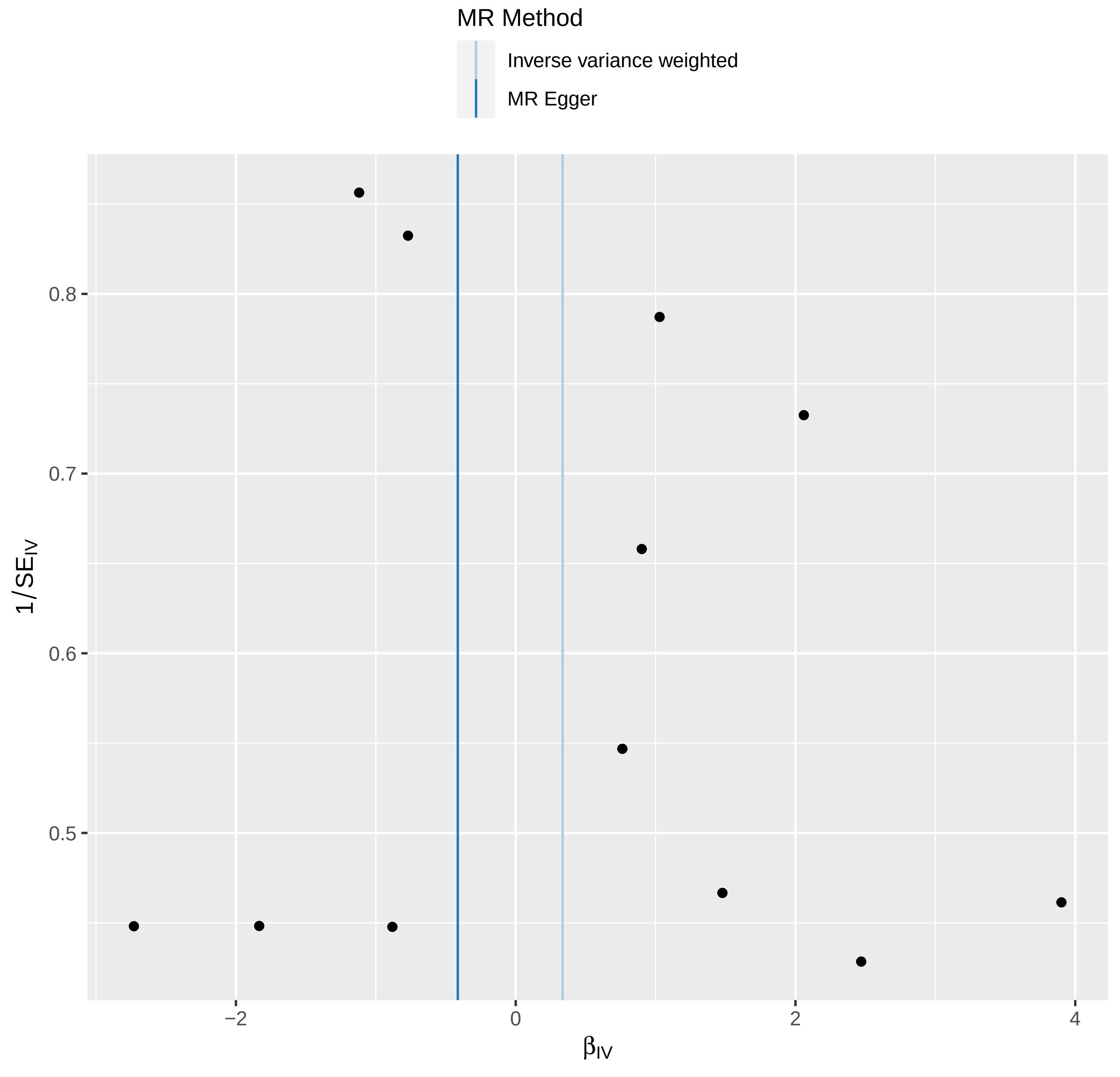

Batch 821 : Ergothioneine on Crohn's disease of small intestine

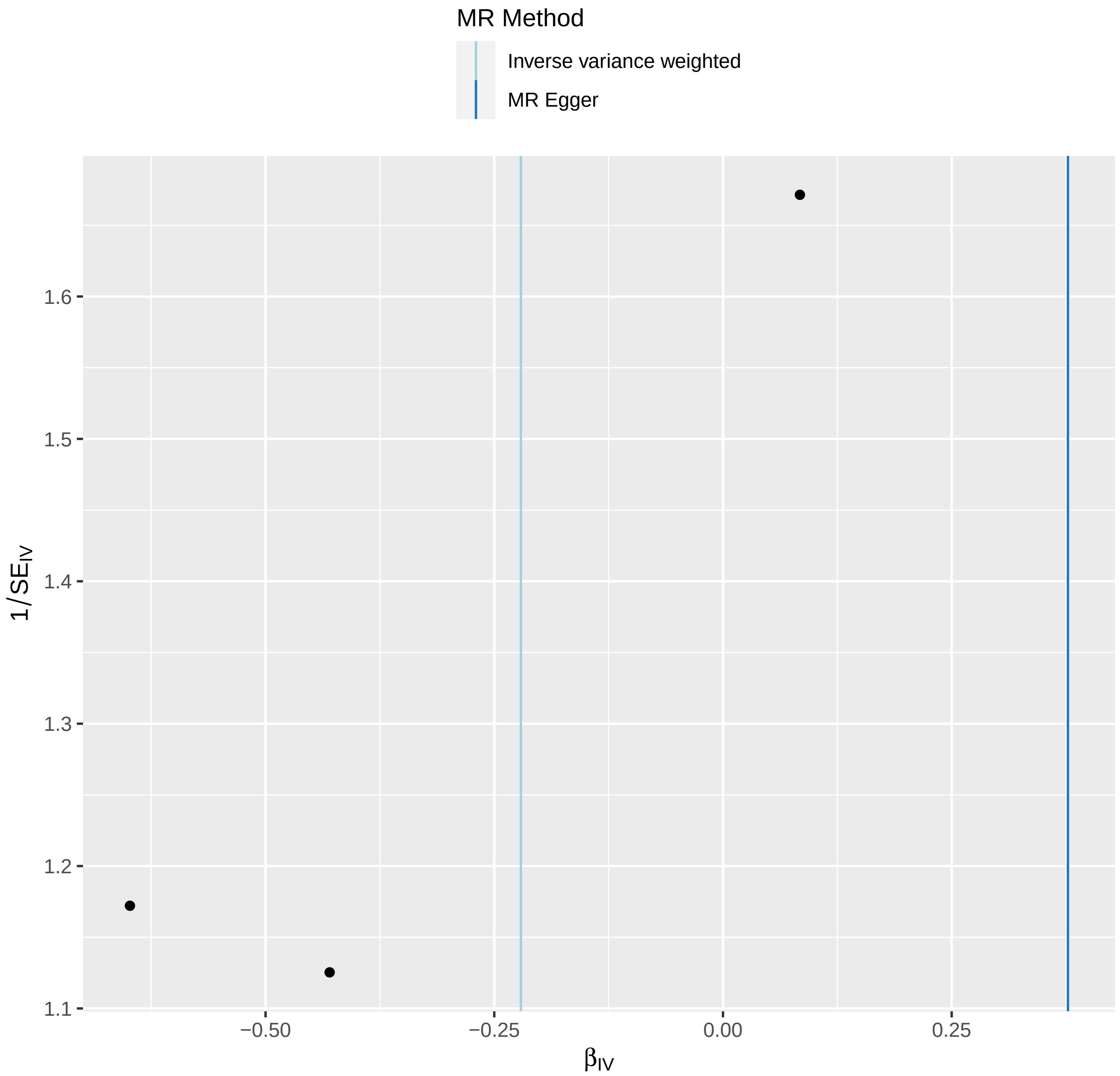

Batch 823 : Phenylalanylphenylalanine on Crohn's disease of small intestine

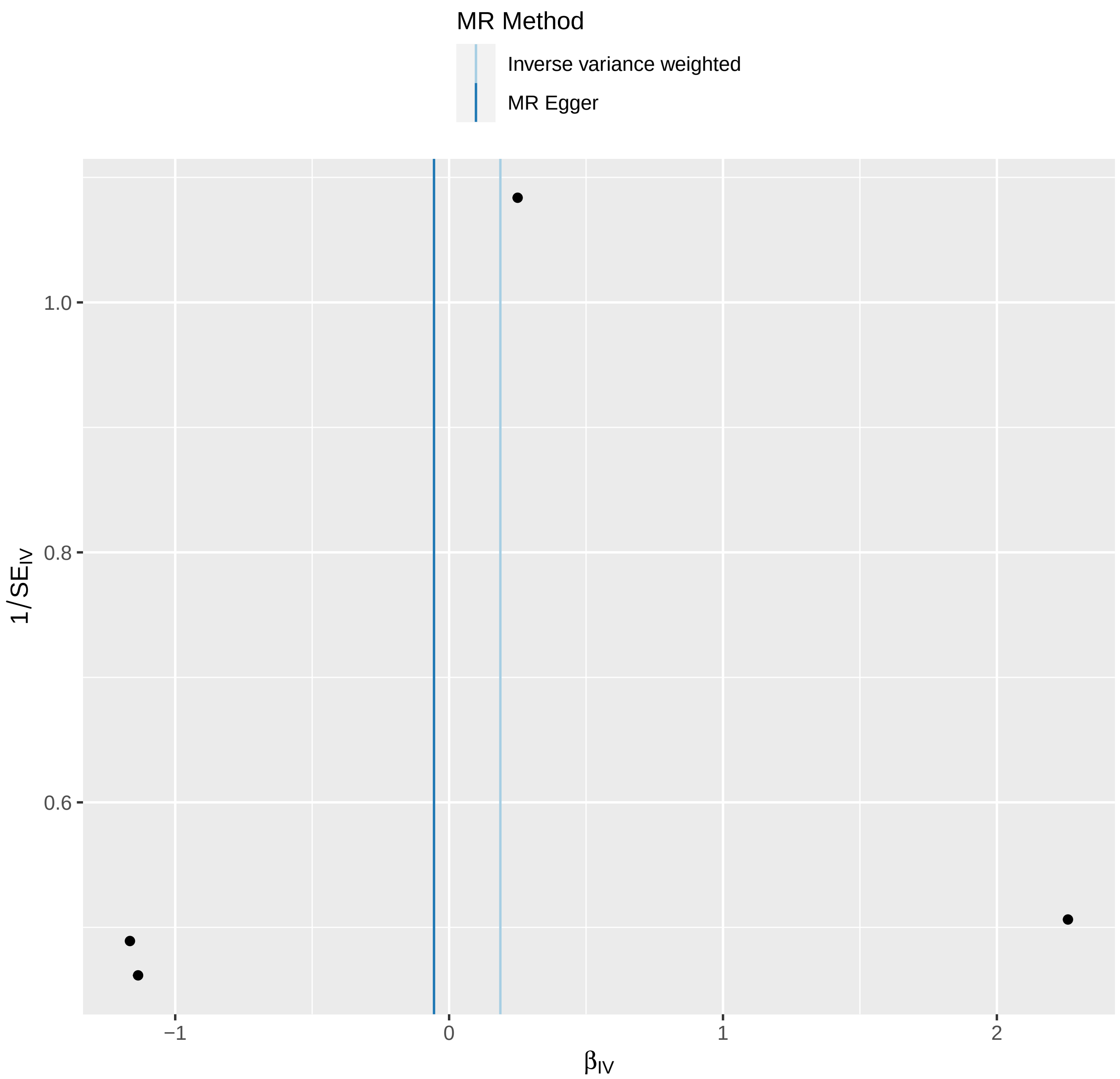

Batch 824 : Cis−4−decenoyl carnitine on Crohn's disease of small intestine

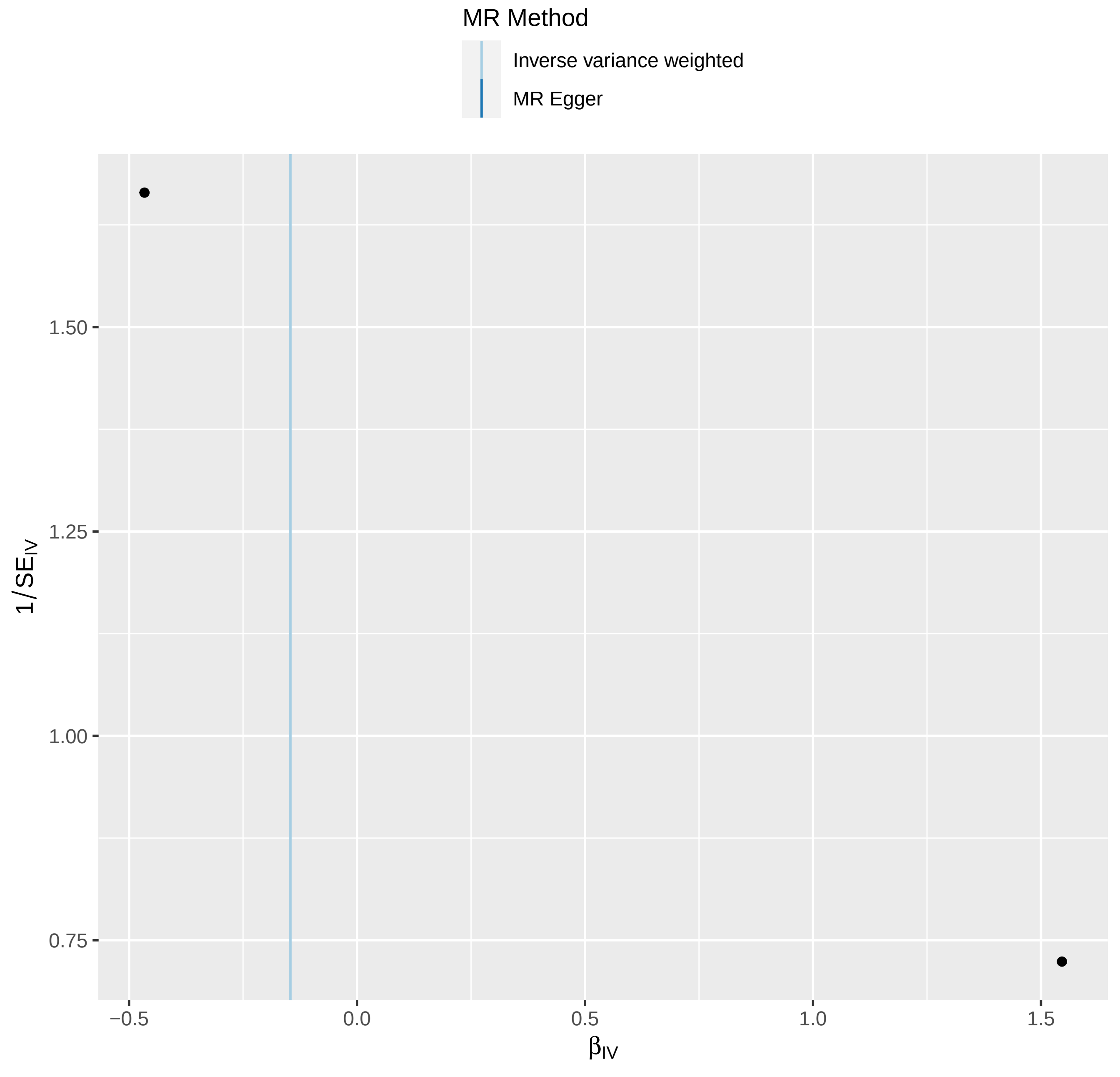

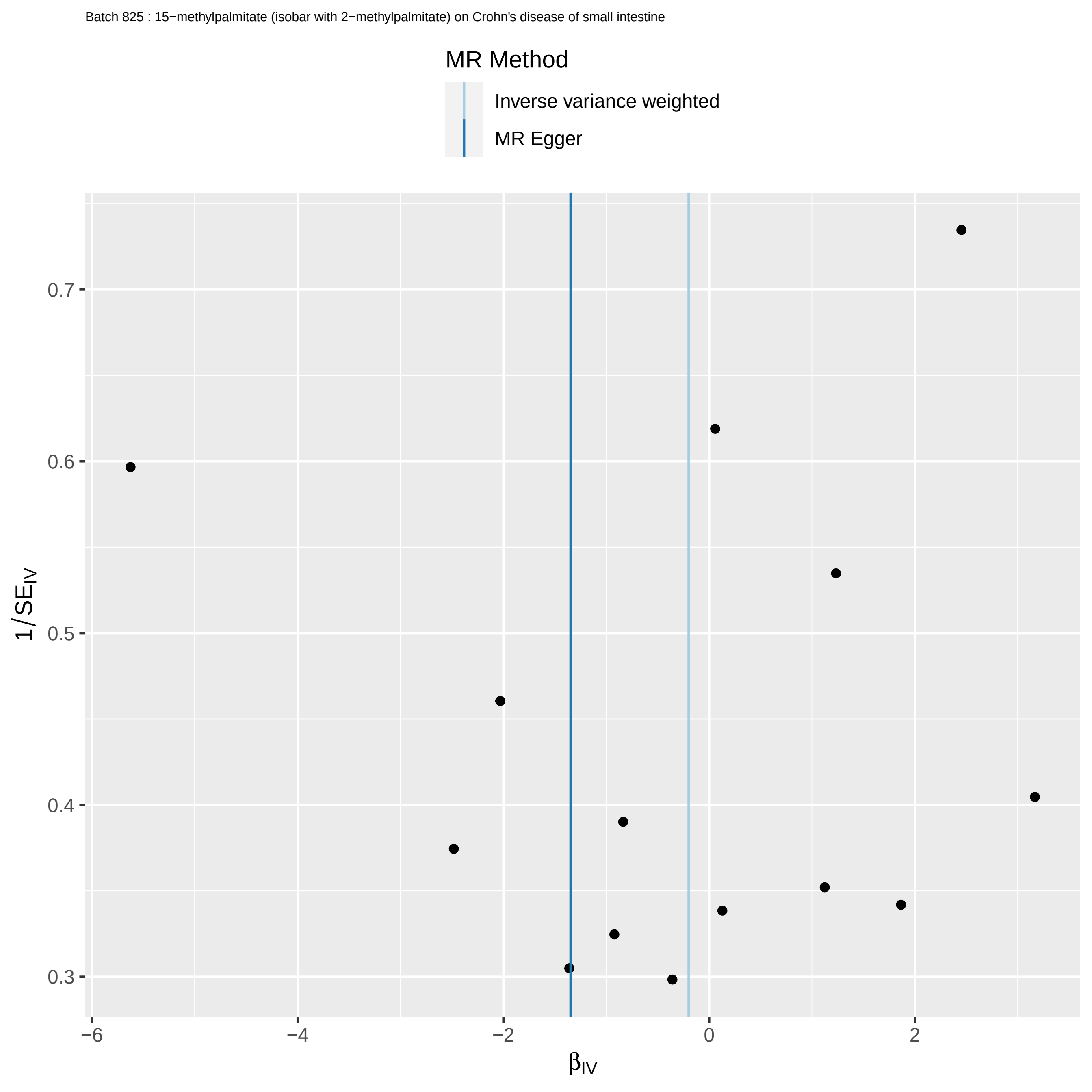

Batch 825 : 15−methylpalmitate (isobar with 2−methylpalmitate) on Crohn's disease of small intestine
MR Method
Inverse variance weighted
MR Egger
0.7
0.6
0.5
0.4
0.3
1/SE_IV
−6
−4
−2
0
2
β_IV

Batch 827 : Tryptophan on Arthropathy in Crohn disease

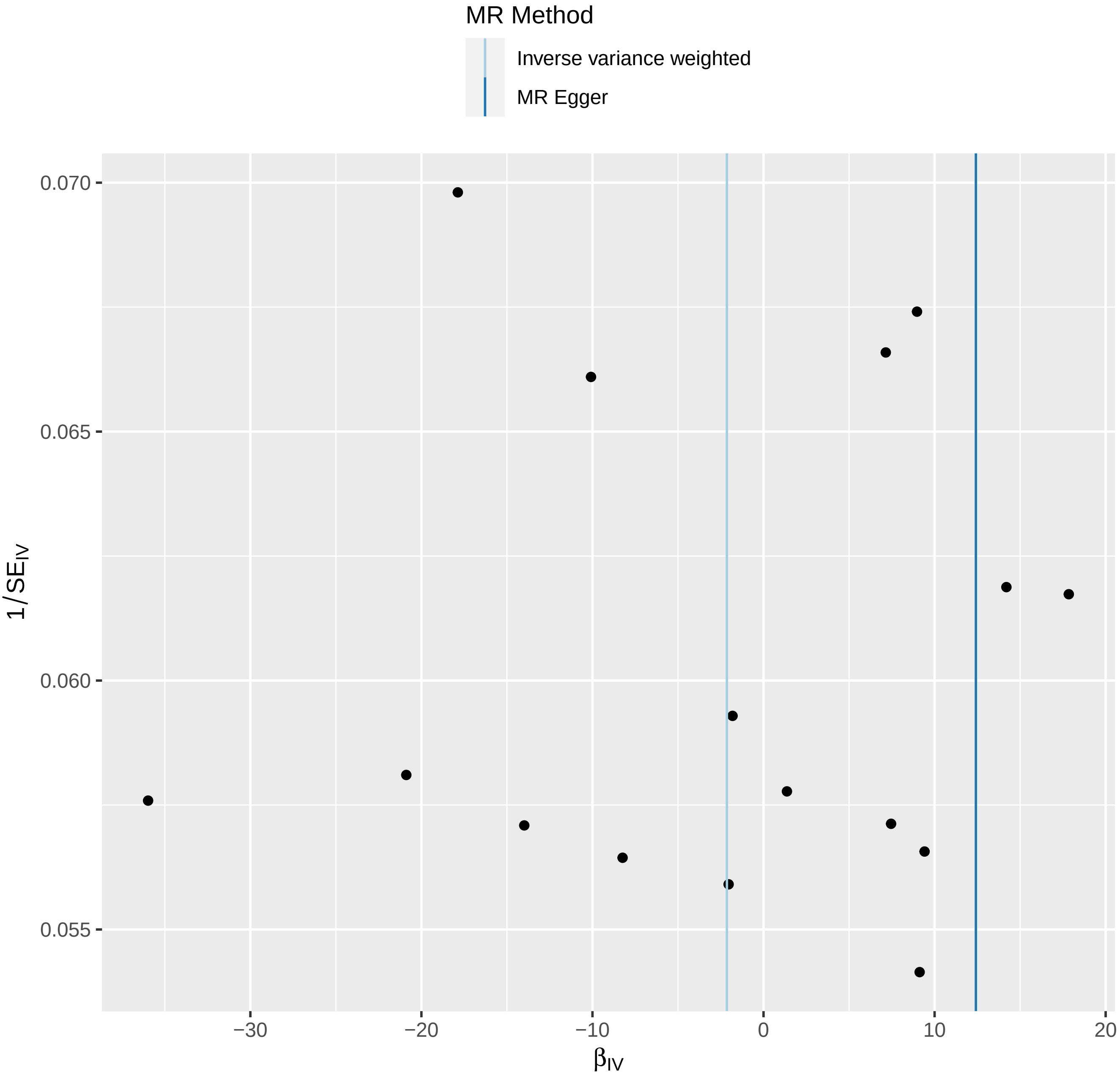

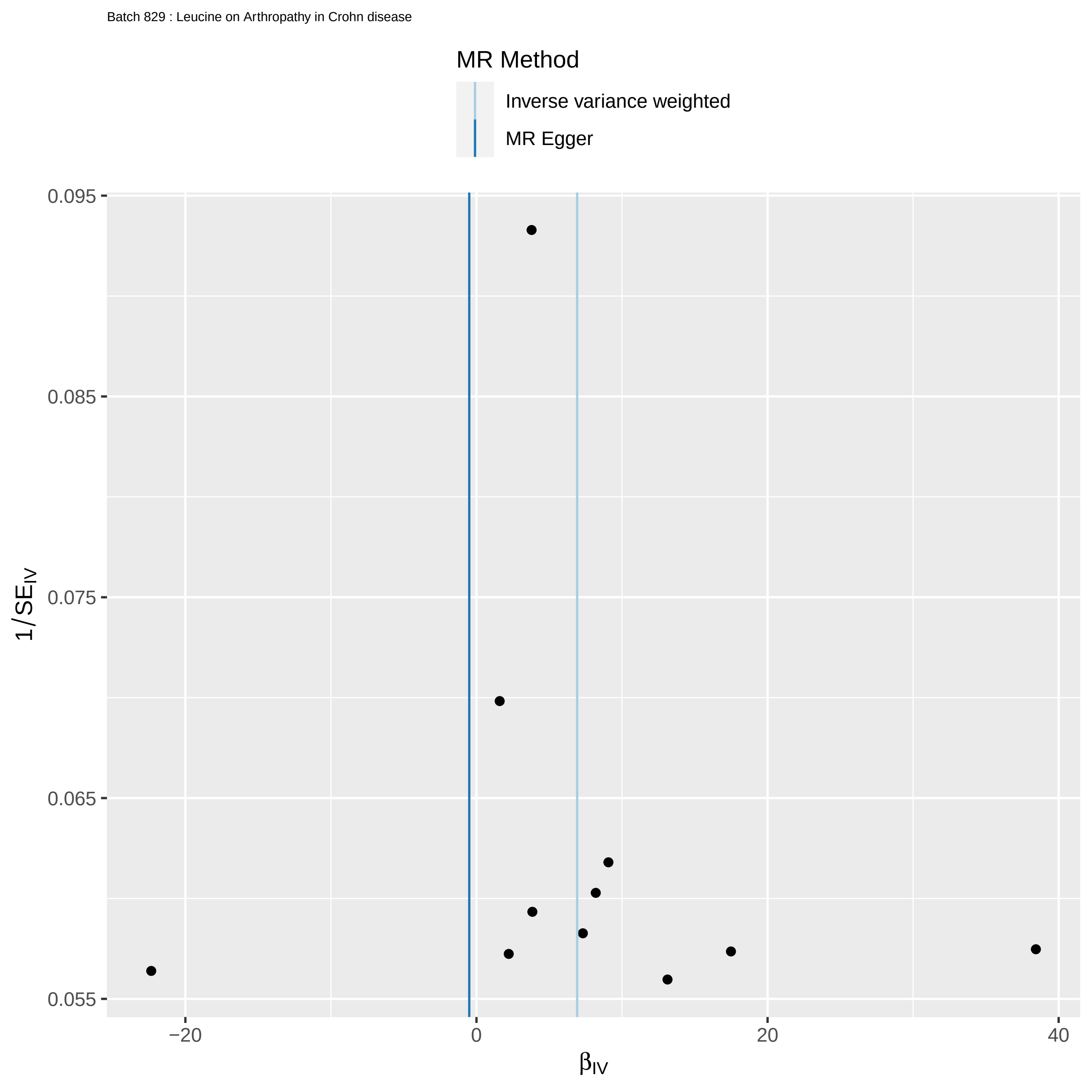
Batch 829 : Leucine on Arthropathy in Crohn disease
MR Method
Inverse variance weighted
MR Egger
0.095
0.085
0.075
0.065
0.055
1/SE_IV
−20
0
20
40
β_IV

Batch 833 : Lactate on Arthropathy in Crohn disease

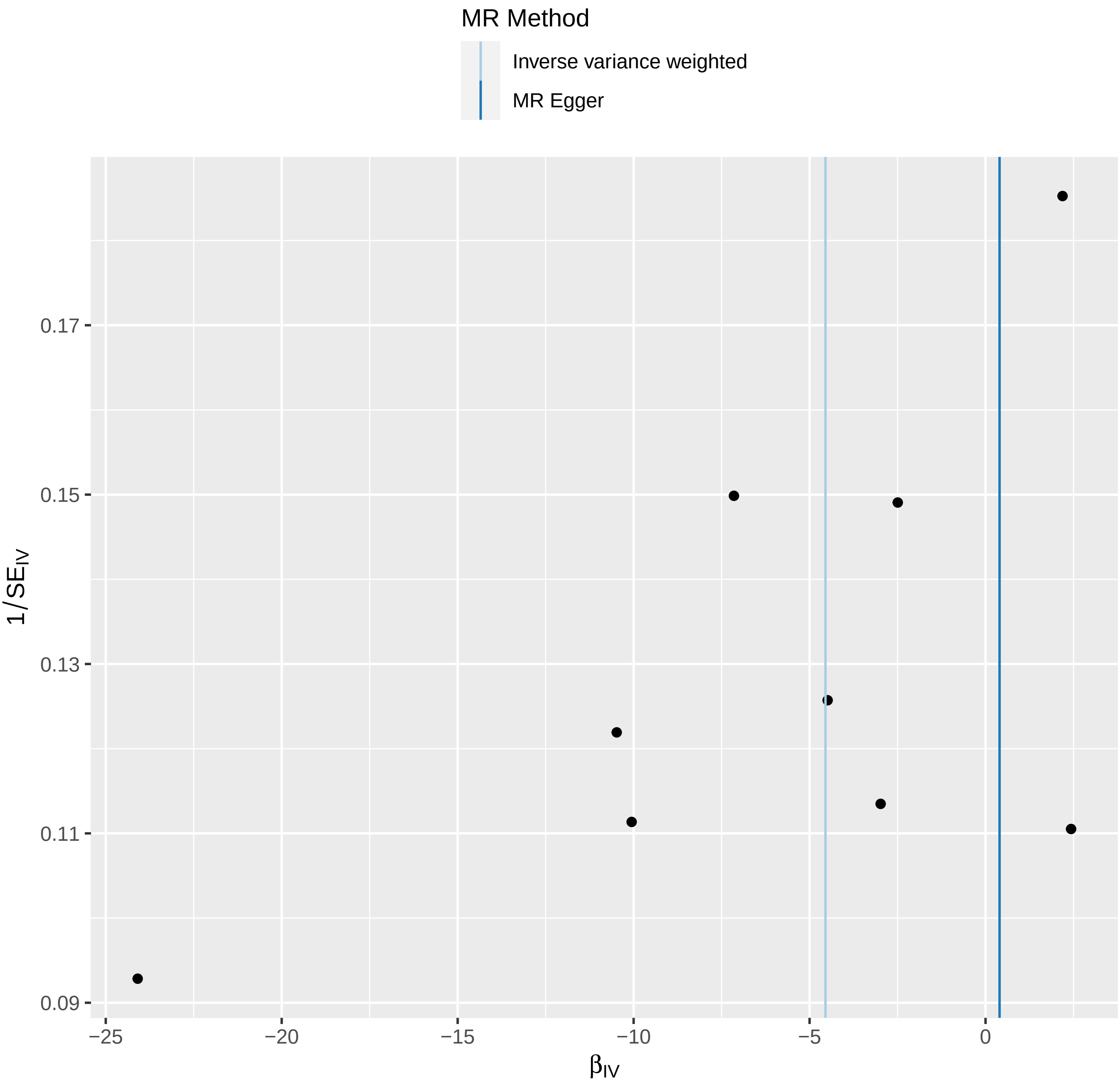

Batch 834 : 3−hydroxybutyrate (BHBA) on Arthropathy in Crohn disease

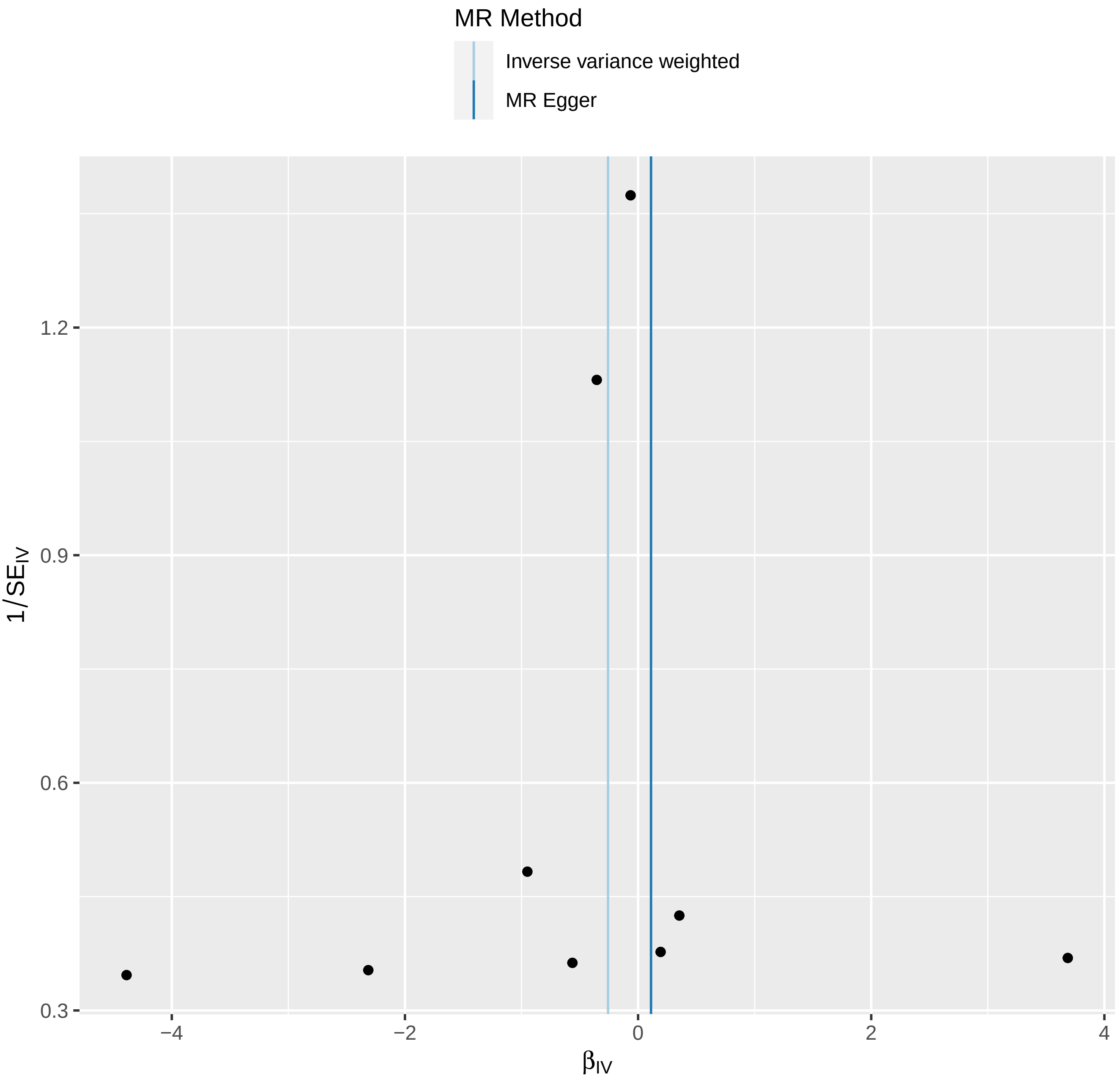

Batch 835 : Arabinose on Arthropathy in Crohn disease

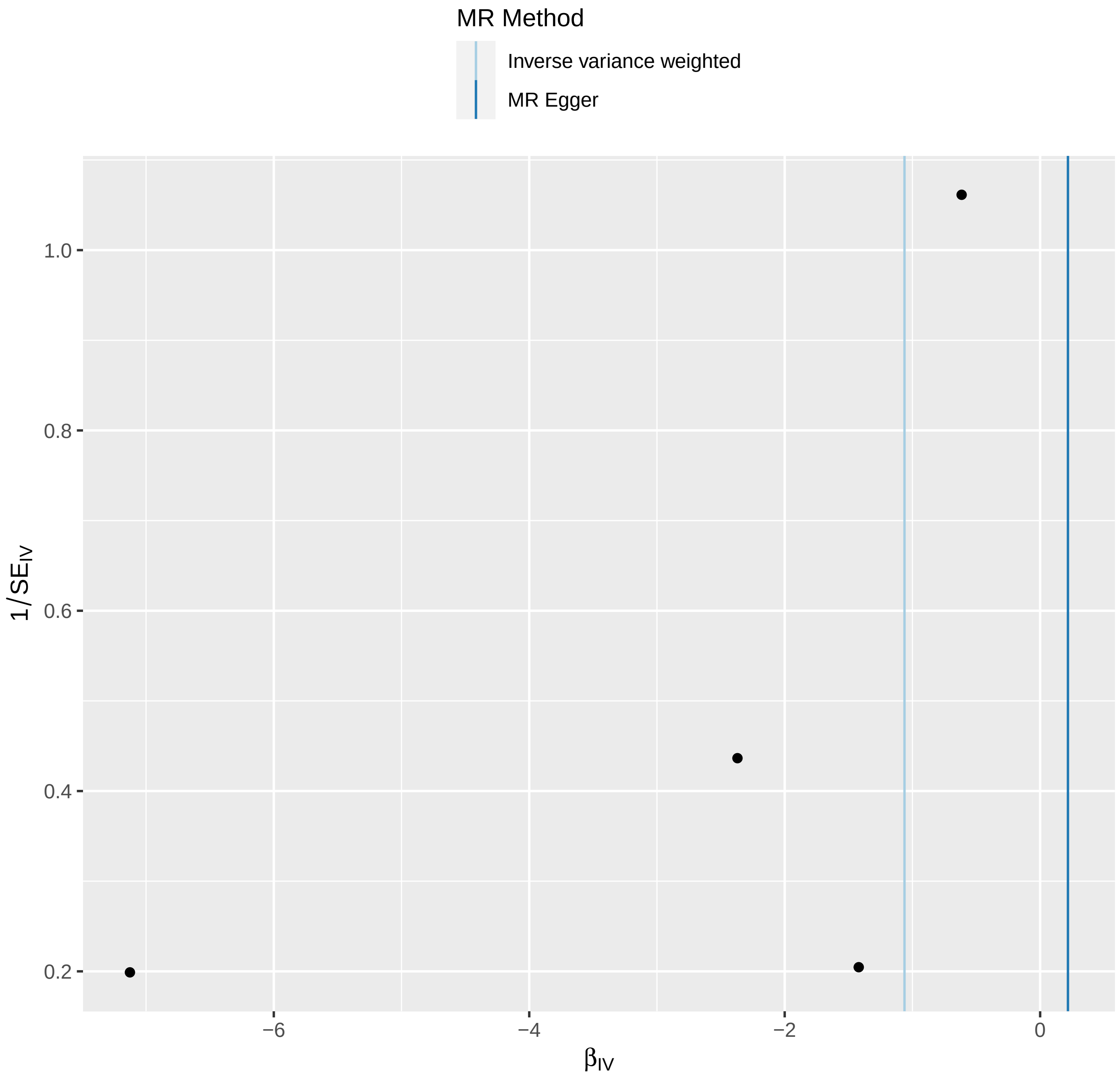

Batch 836 : Fructose on Arthropathy in Crohn disease

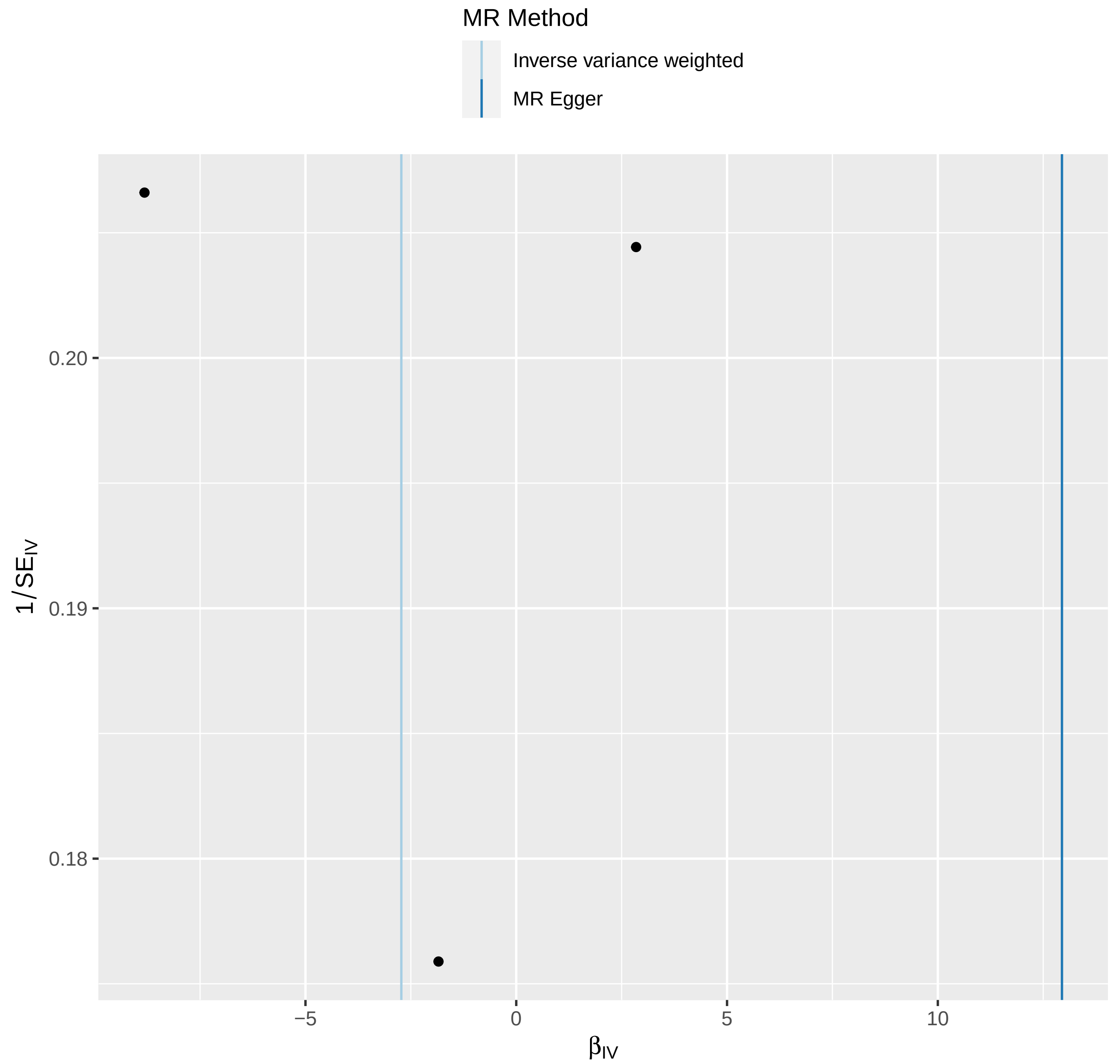

Batch 838 : Pyruvate on Arthropathy in Crohn disease

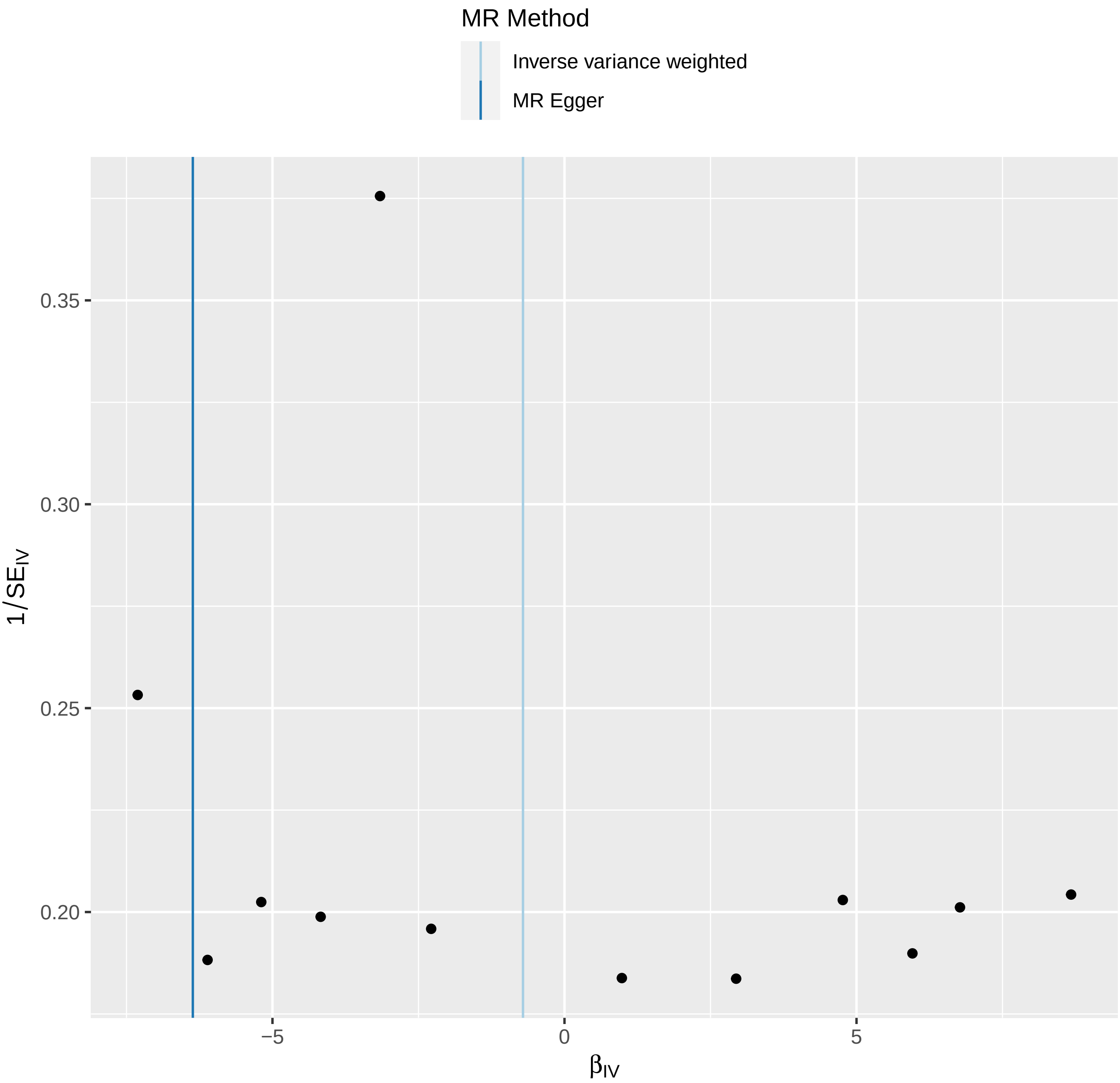

Batch 839 : Uridine on Arthropathy in Crohn disease

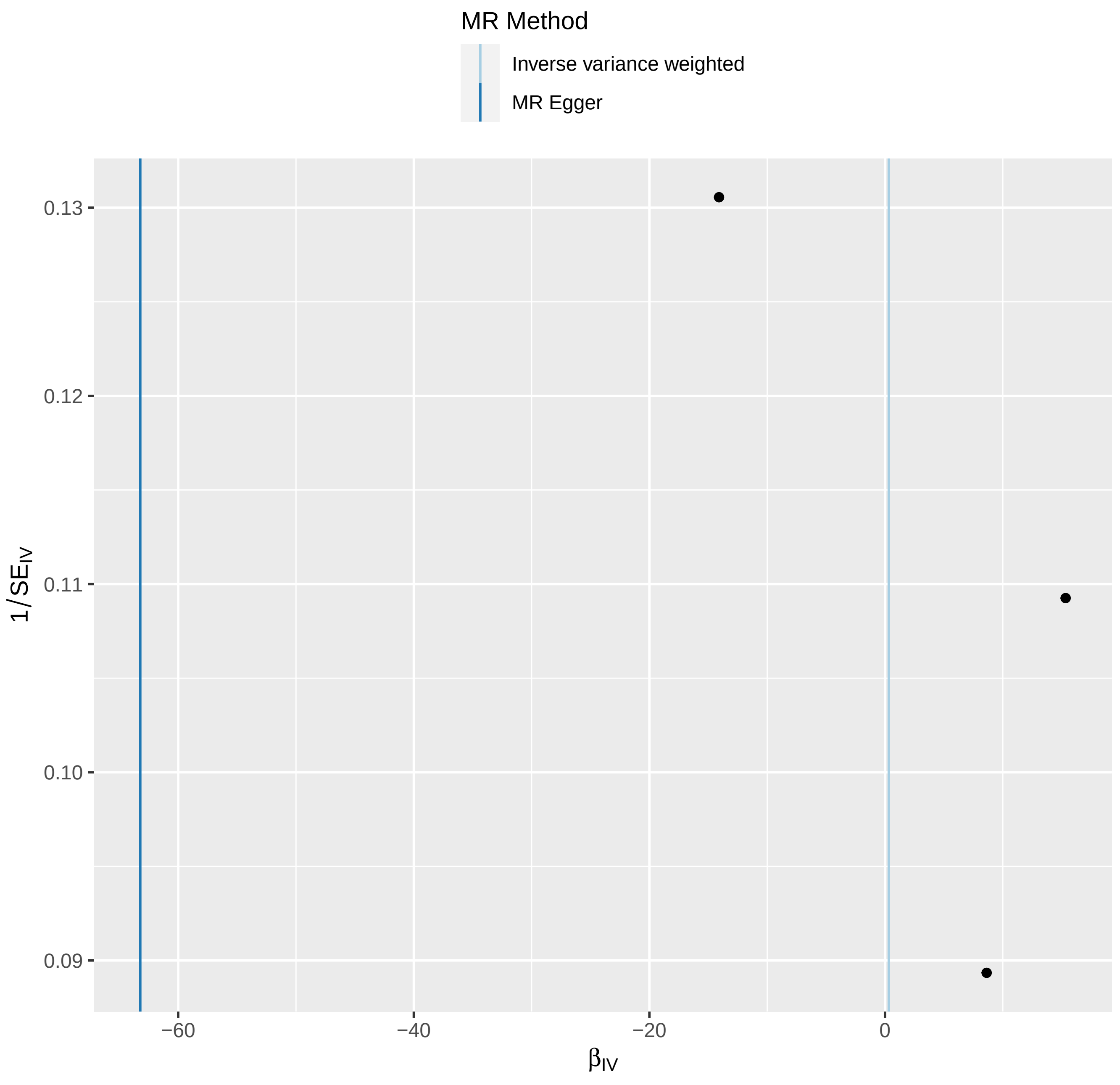

Batch 840 : Linoleate (18:2n6) on Arthropathy in Crohn disease

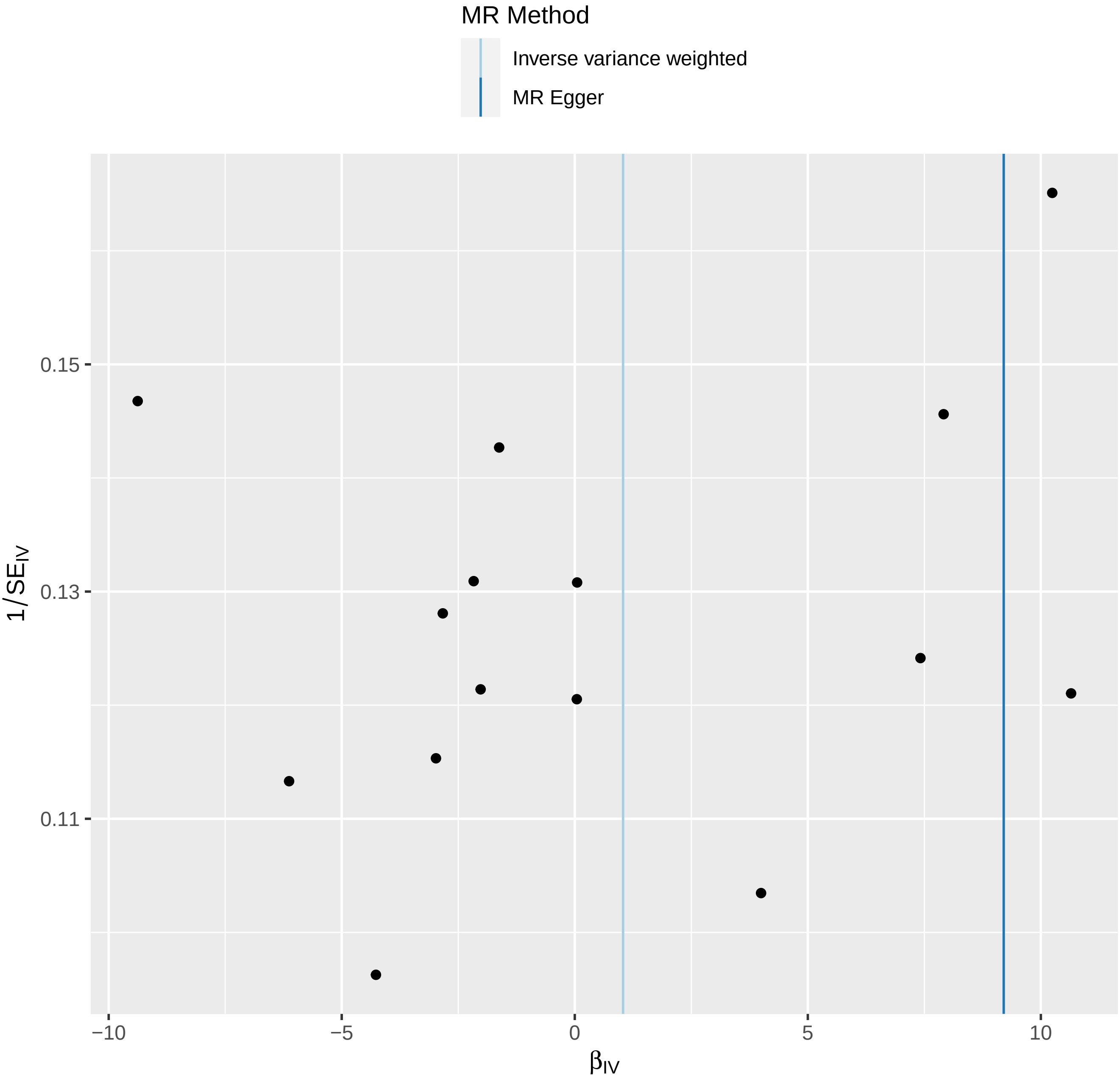

Batch 841 : Allantoin on Arthropathy in Crohn disease

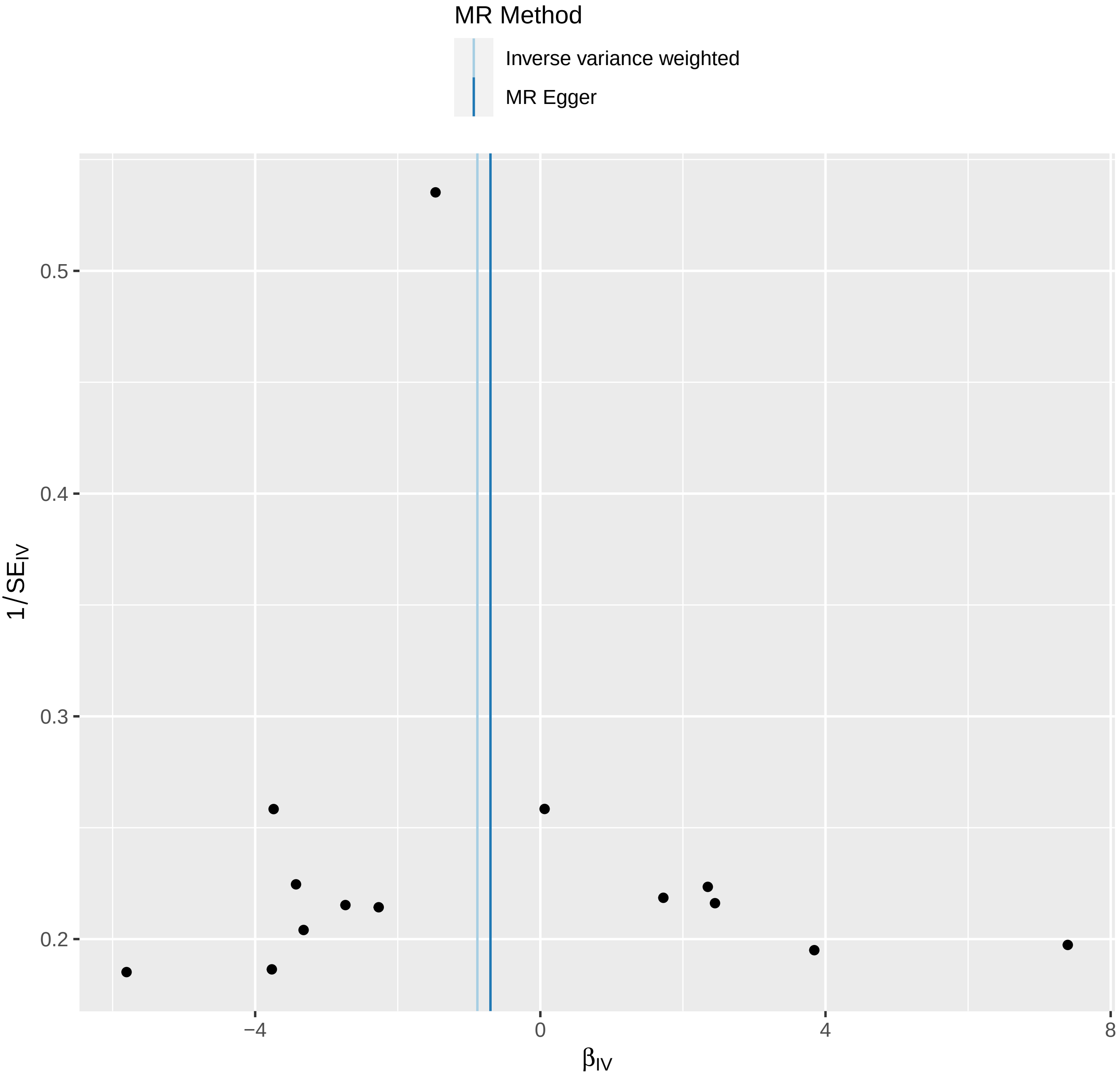

Batch 846 : Isoleucine on Arthropathy in Crohn disease

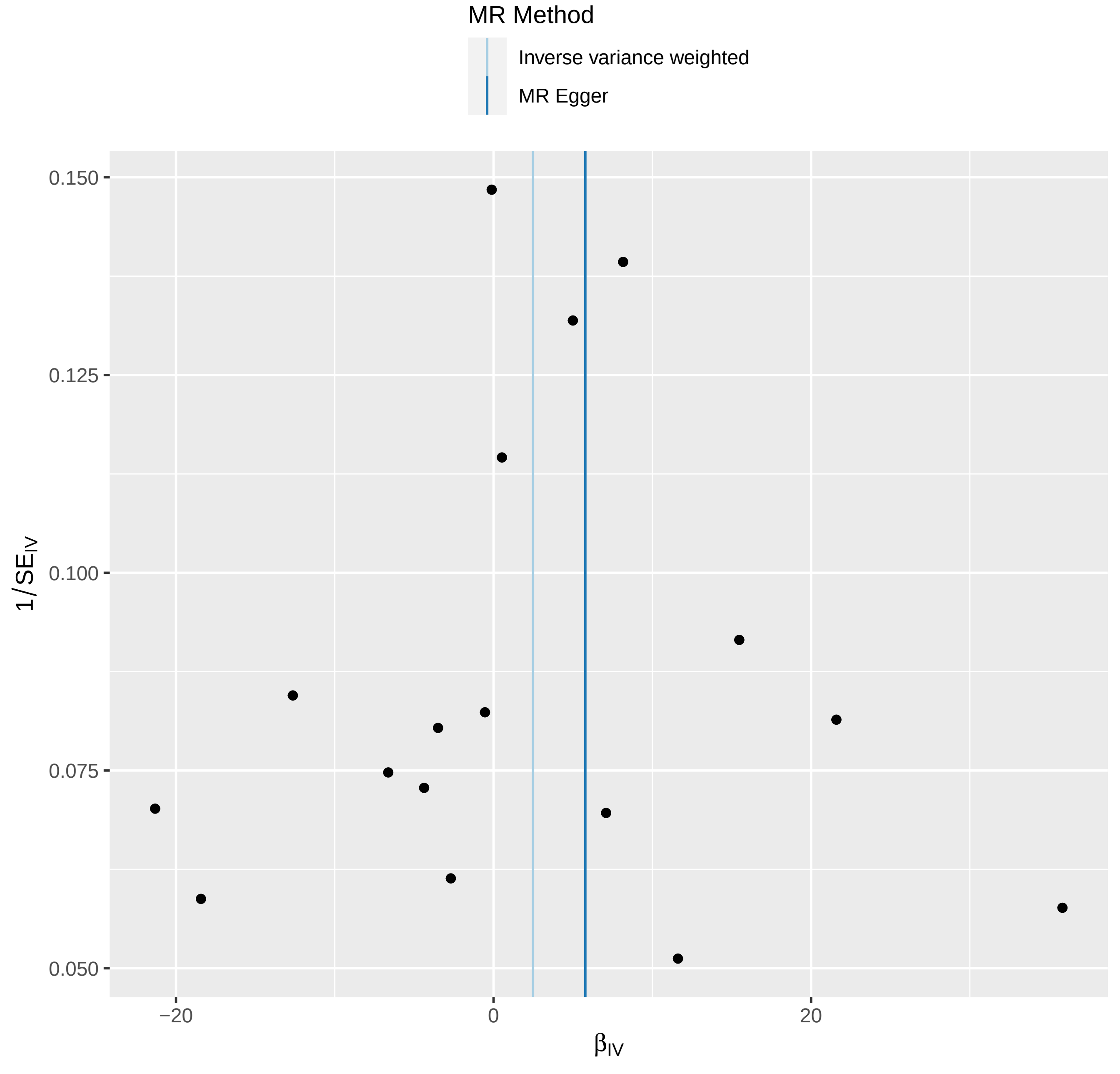

Batch 847 : Threonine on Arthropathy in Crohn disease

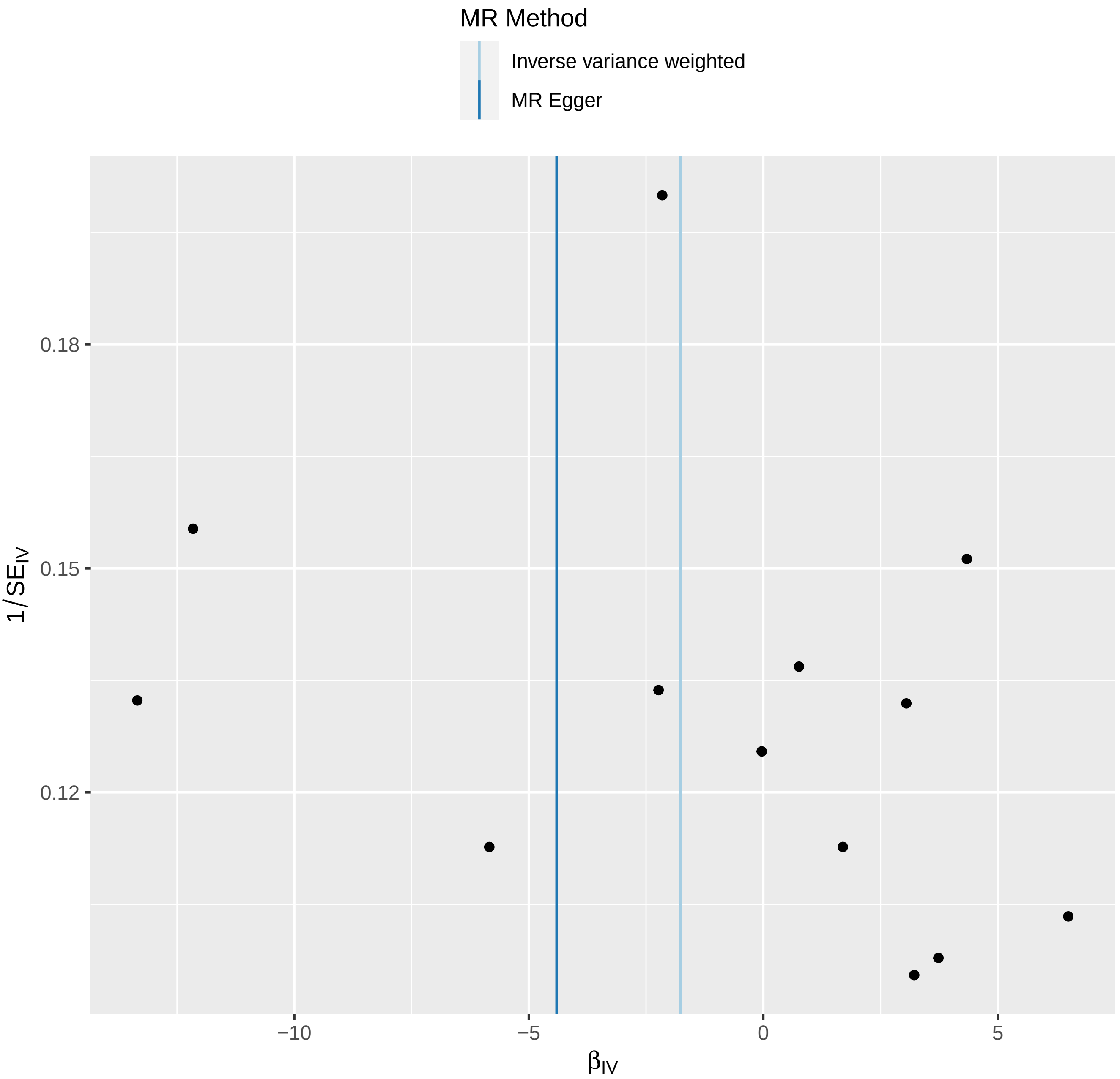

Batch 848 : Tyrosine on Arthropathy in Crohn disease

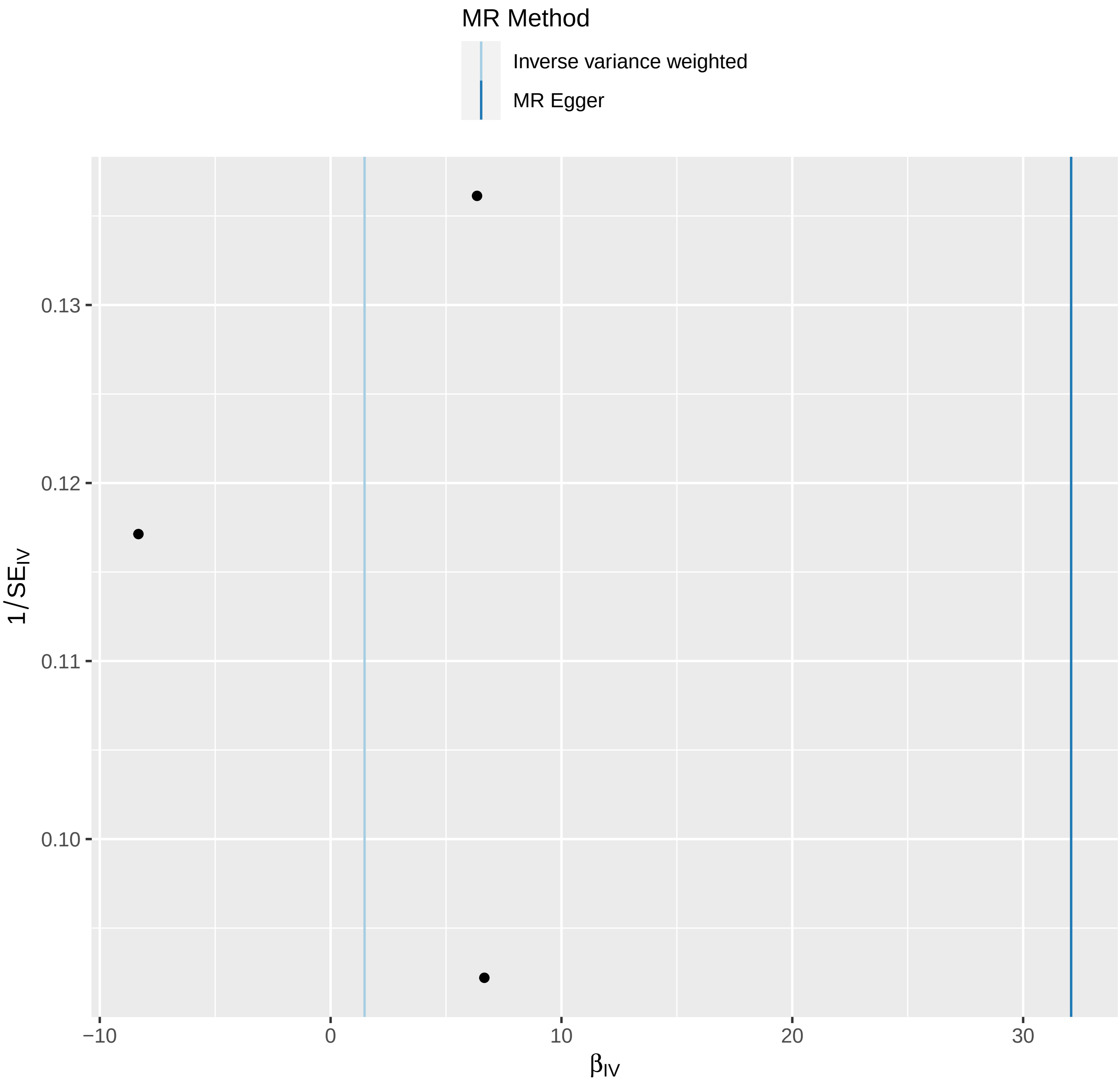

Batch 854 : Stearate (18:0) on Arthropathy in Crohn disease

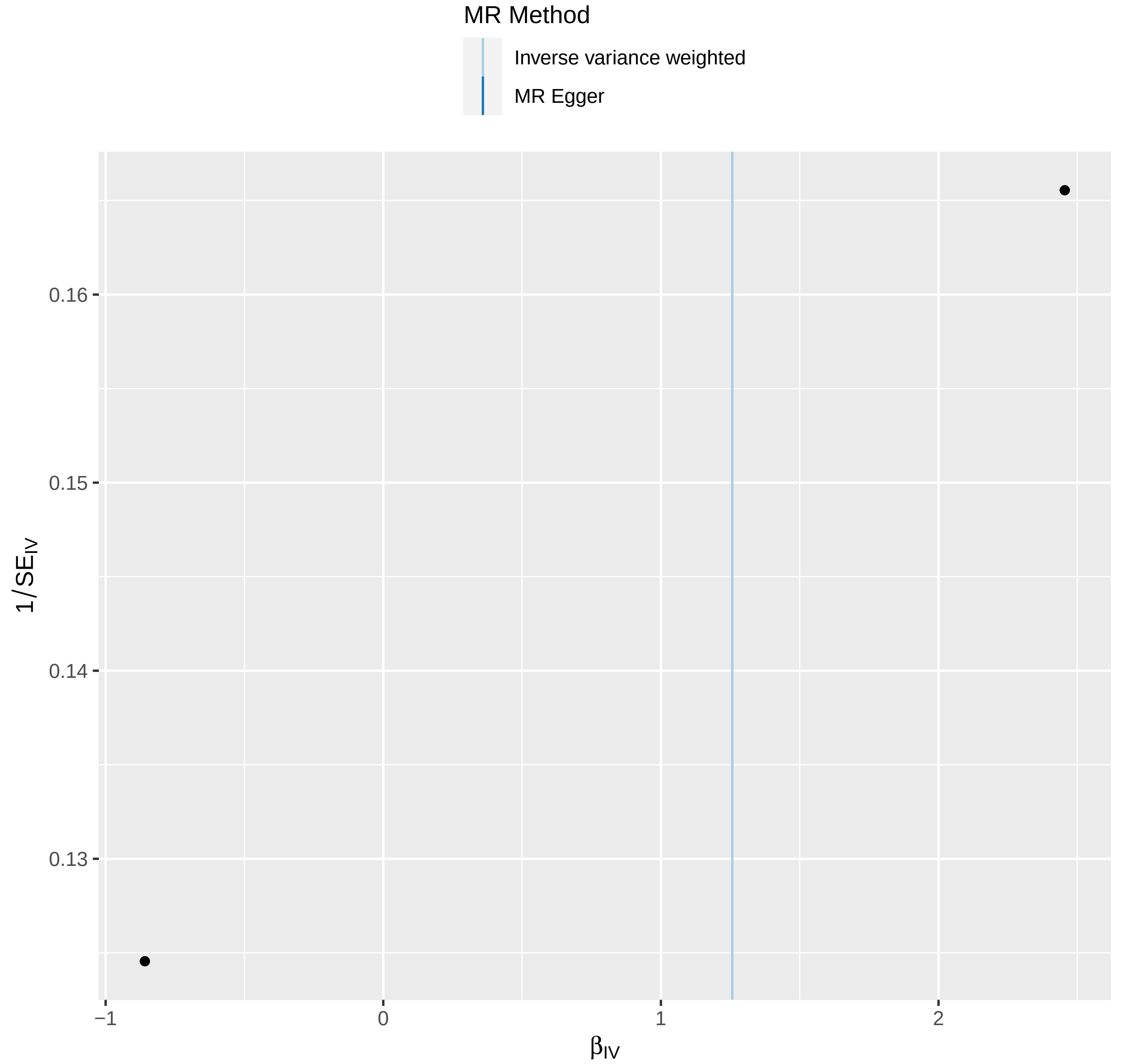

Batch 857 : Myristate (14:0) on Arthropathy in Crohn disease

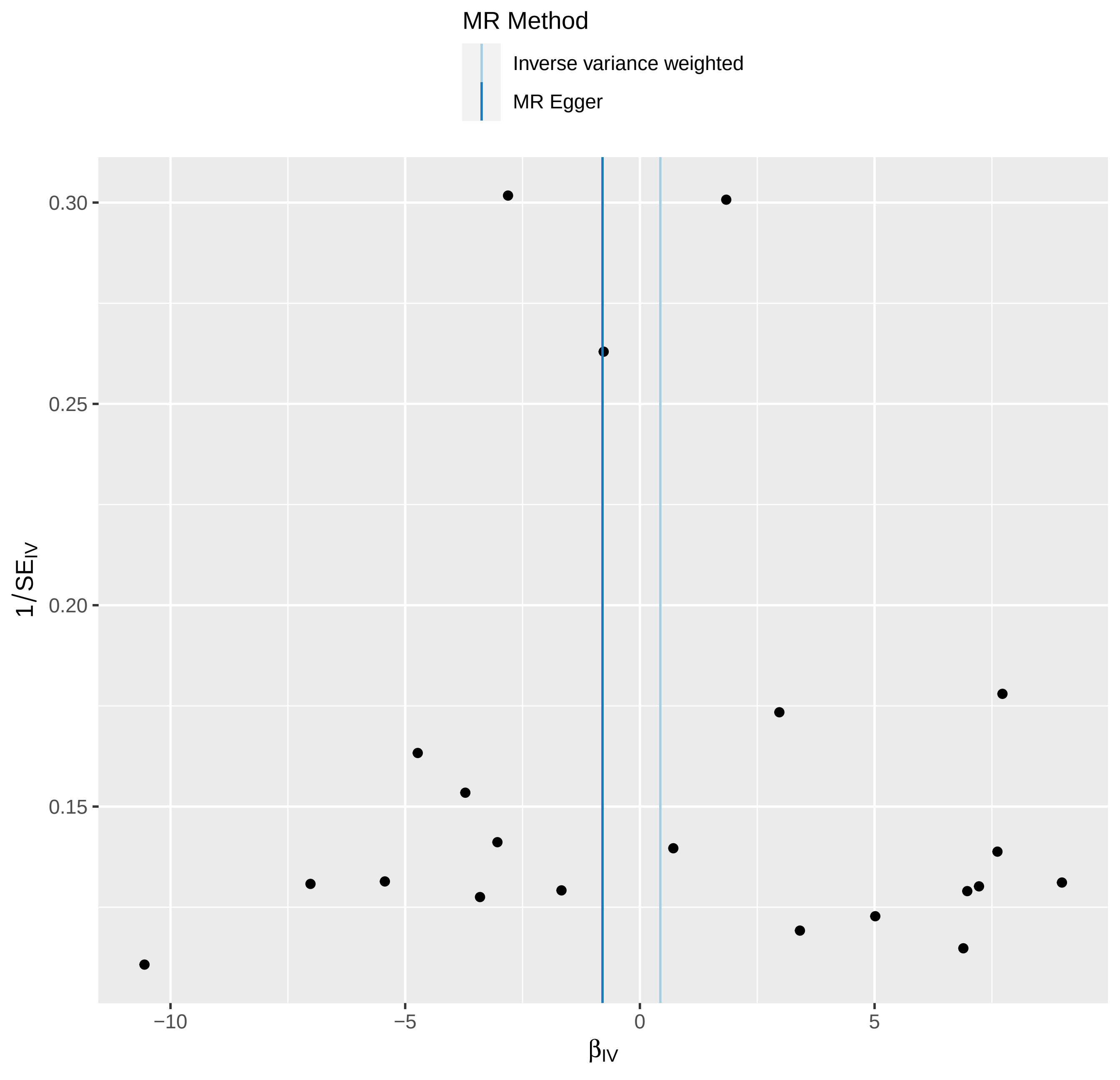

Batch 859 : Ornithine on Arthropathy in Crohn disease

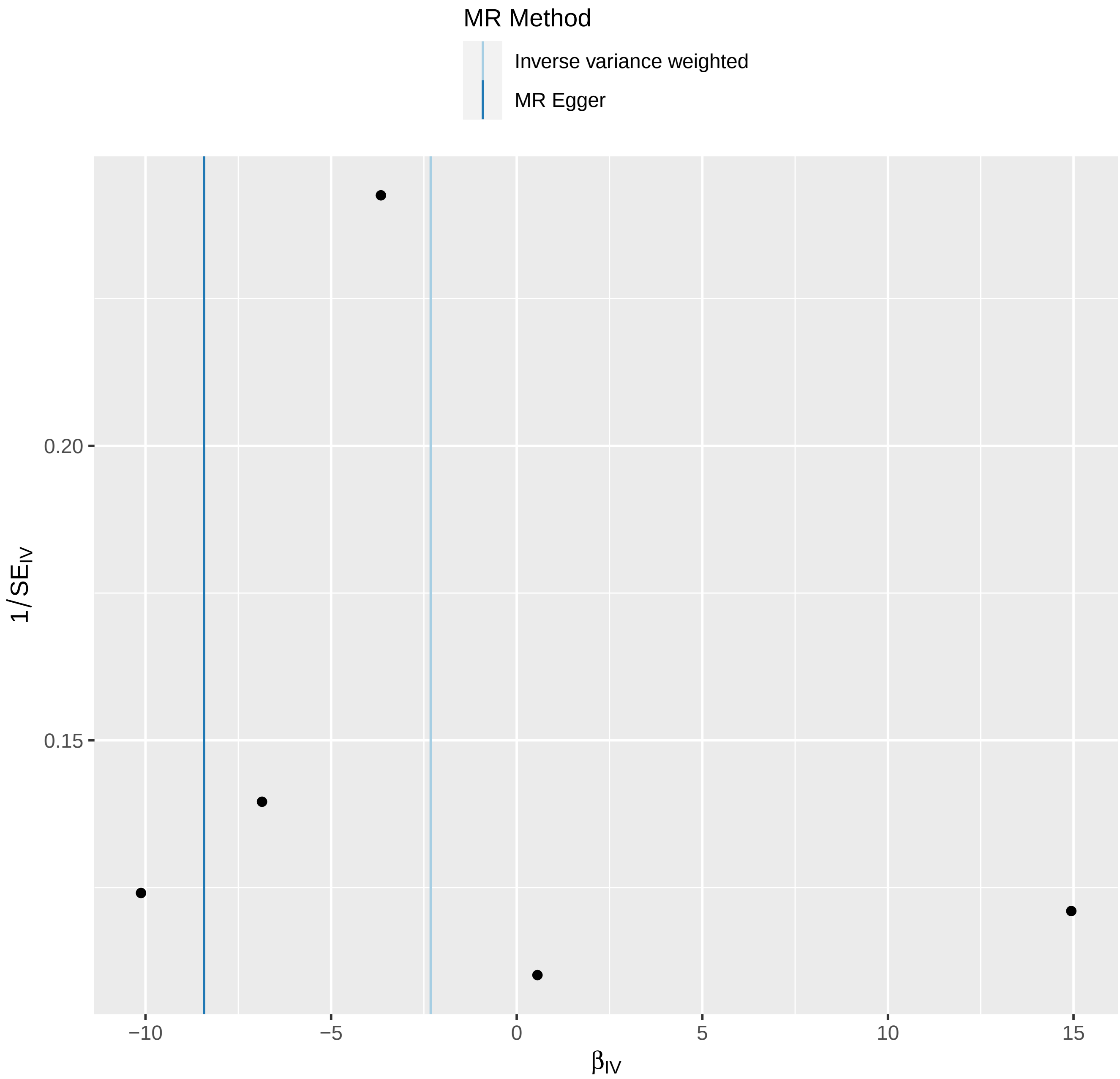

Batch 862 : 4−acetamidobutanoate on Arthropathy in Crohn disease

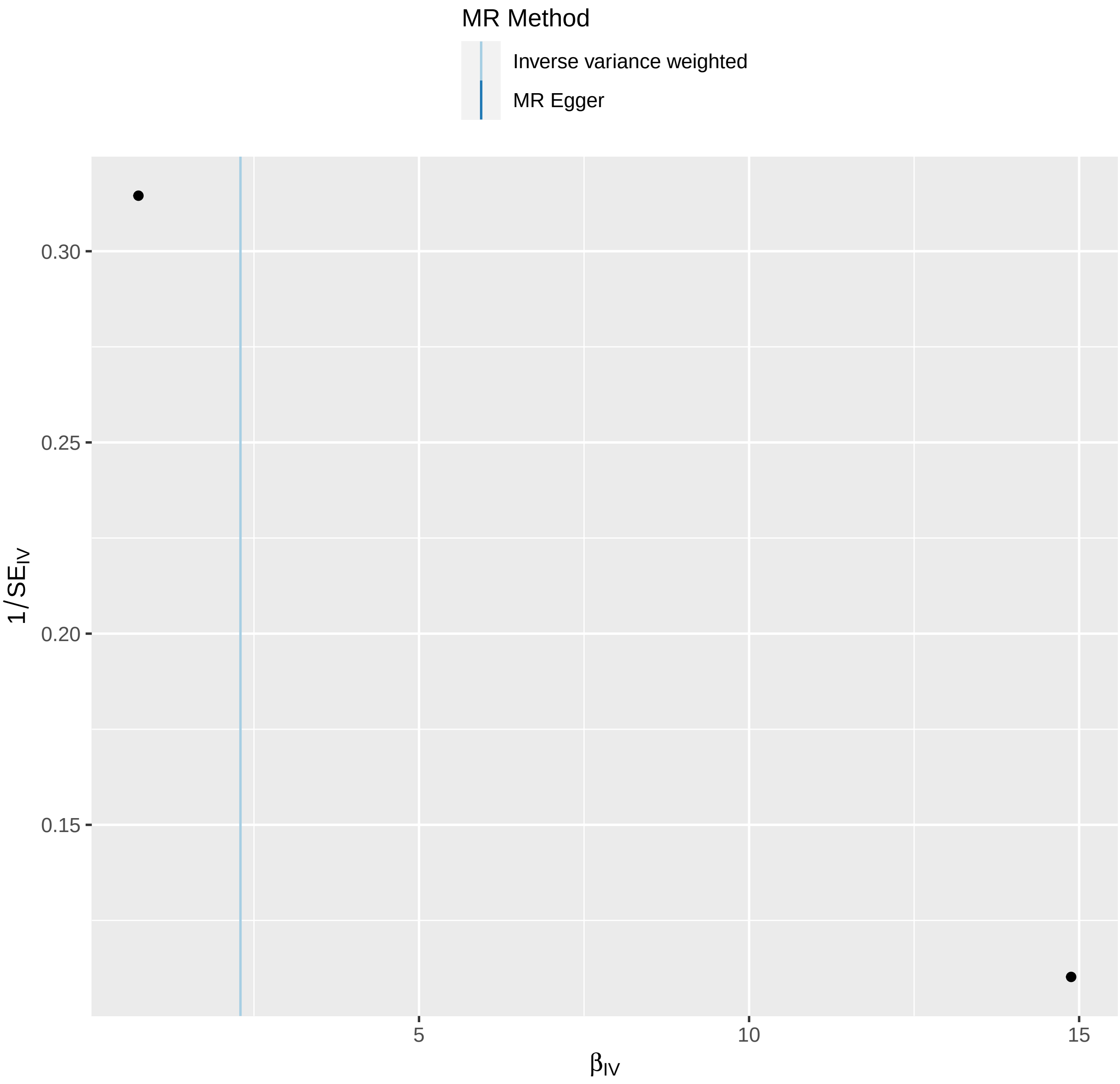

Batch 863 : Alpha−tocopherol on Arthropathy in Crohn disease

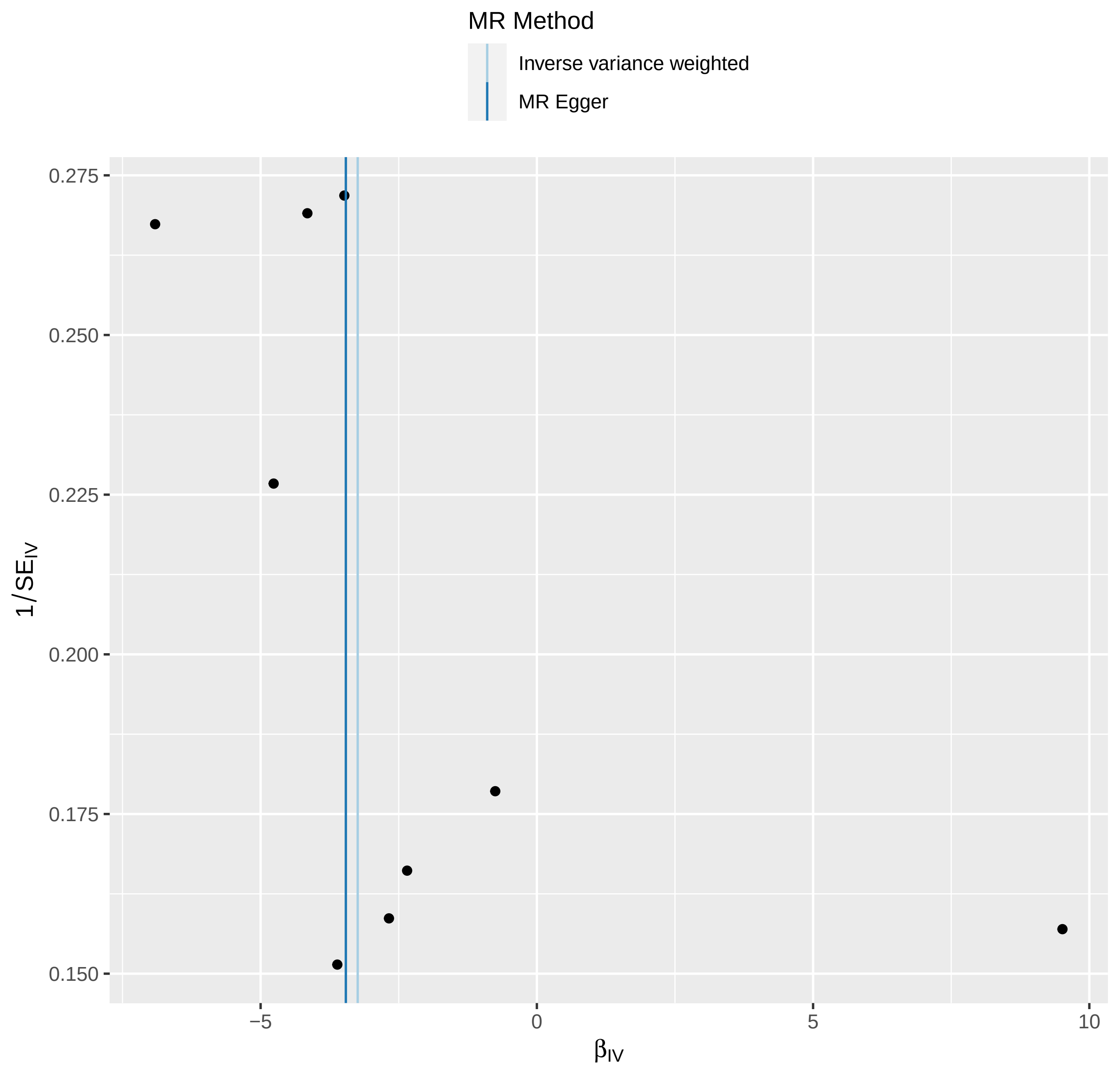

Batch 864 : Citrate on Arthropathy in Crohn disease

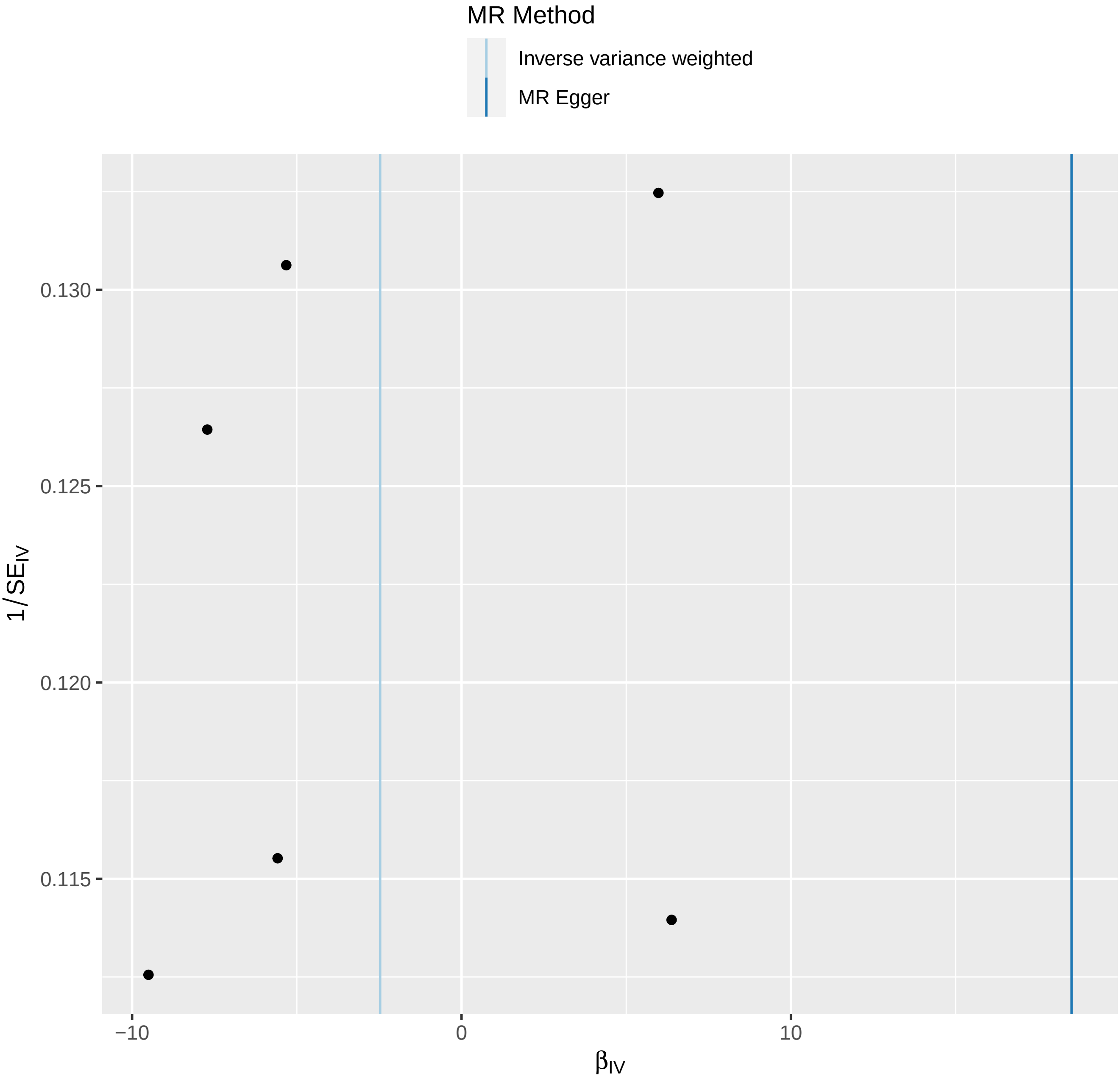

Batch 865 : Glycerate on Arthropathy in Crohn disease

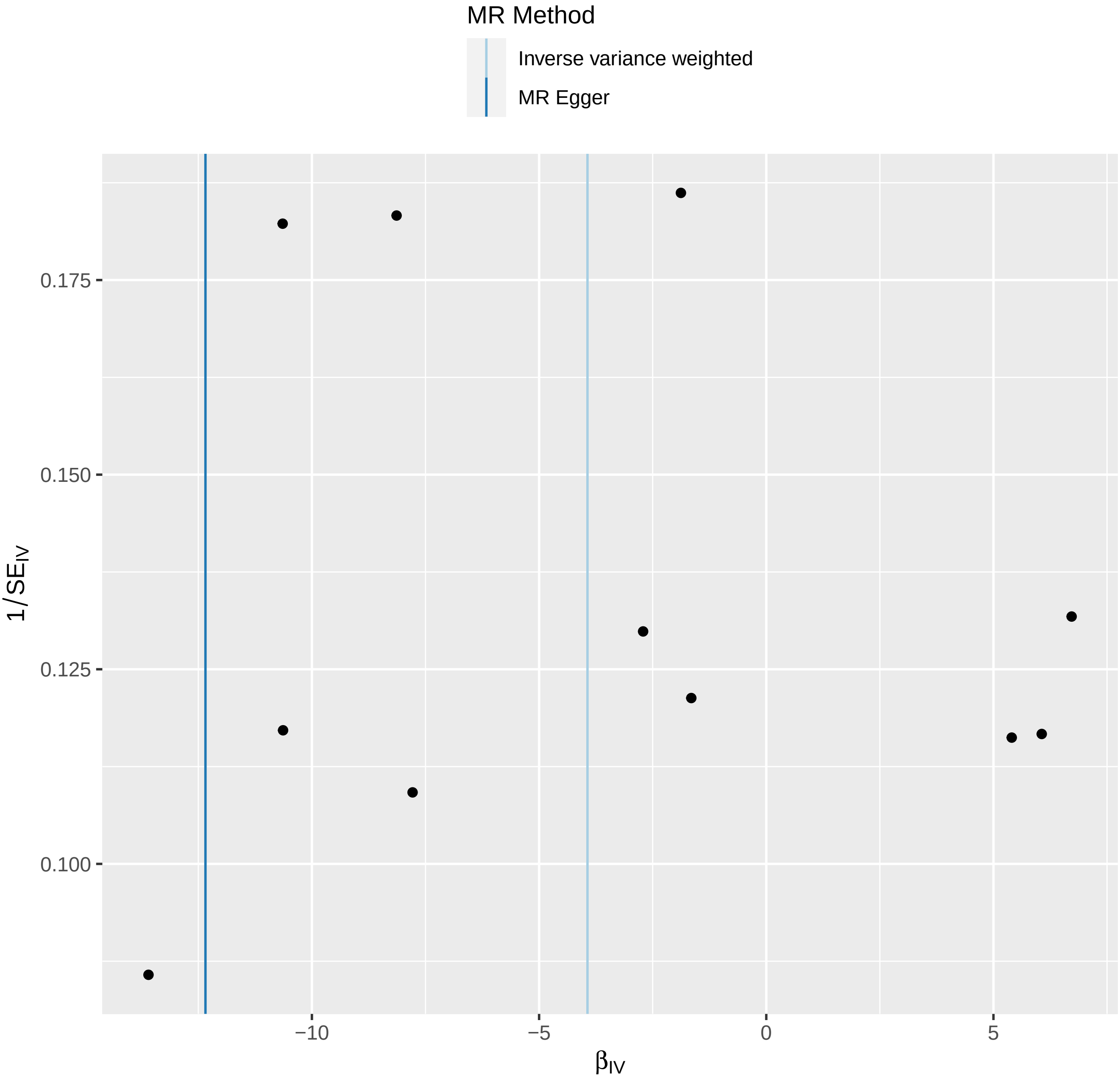

Batch 866 : Guanosine on Arthropathy in Crohn disease

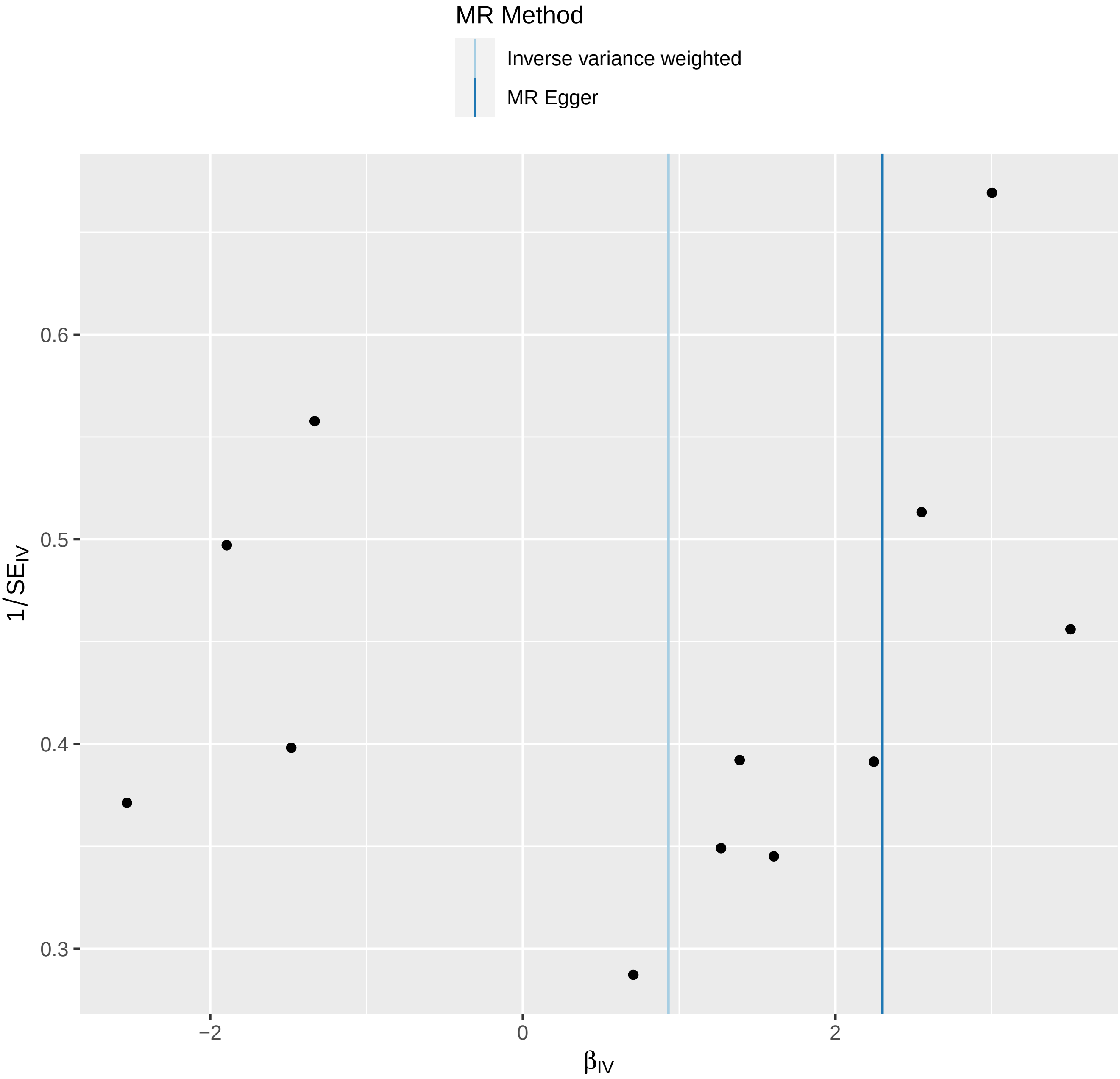

Batch 869 : Ursodeoxycholate on Arthropathy in Crohn disease

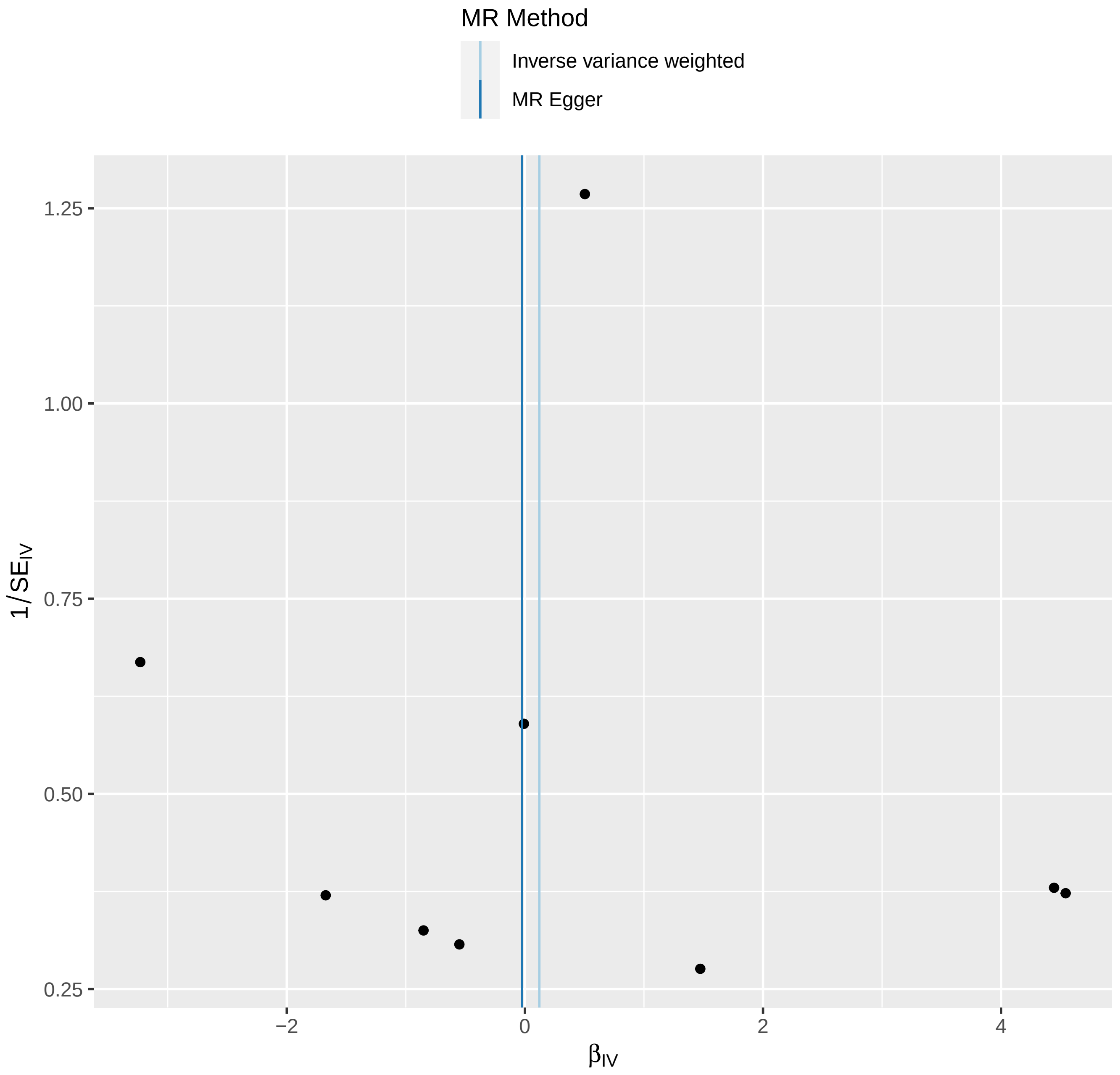

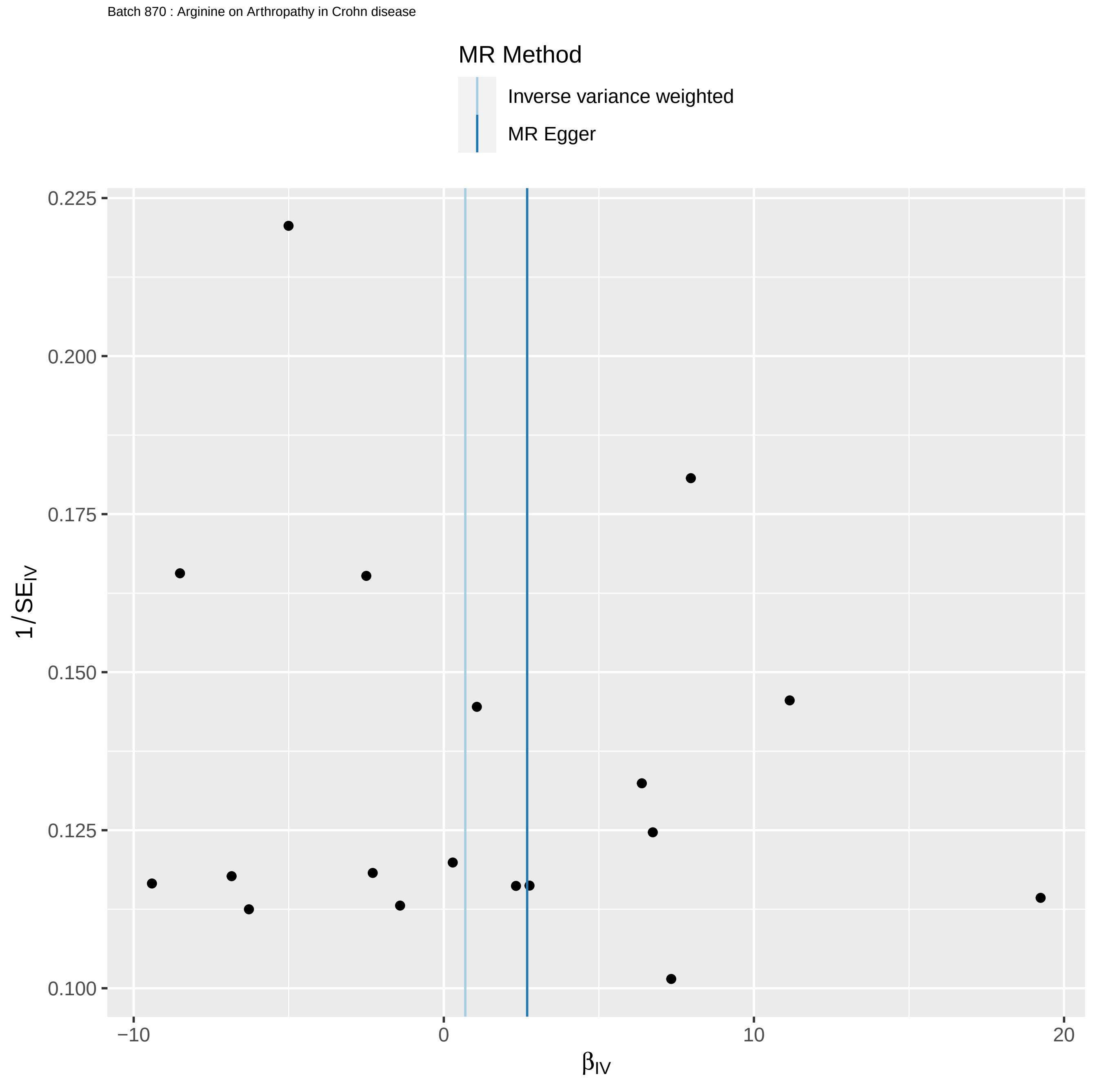
Batch 870 : Arginine on Arthropathy in Crohn disease
MR Method
Inverse variance weighted
MR Egger
0.225
0.200
0.175
0.150
0.125
0.100
$1/SE_{IV}$
−10
0
10
20
β_{IV}

Batch 871 : Ascorbate (Vitamin C) on Arthropathy in Crohn disease

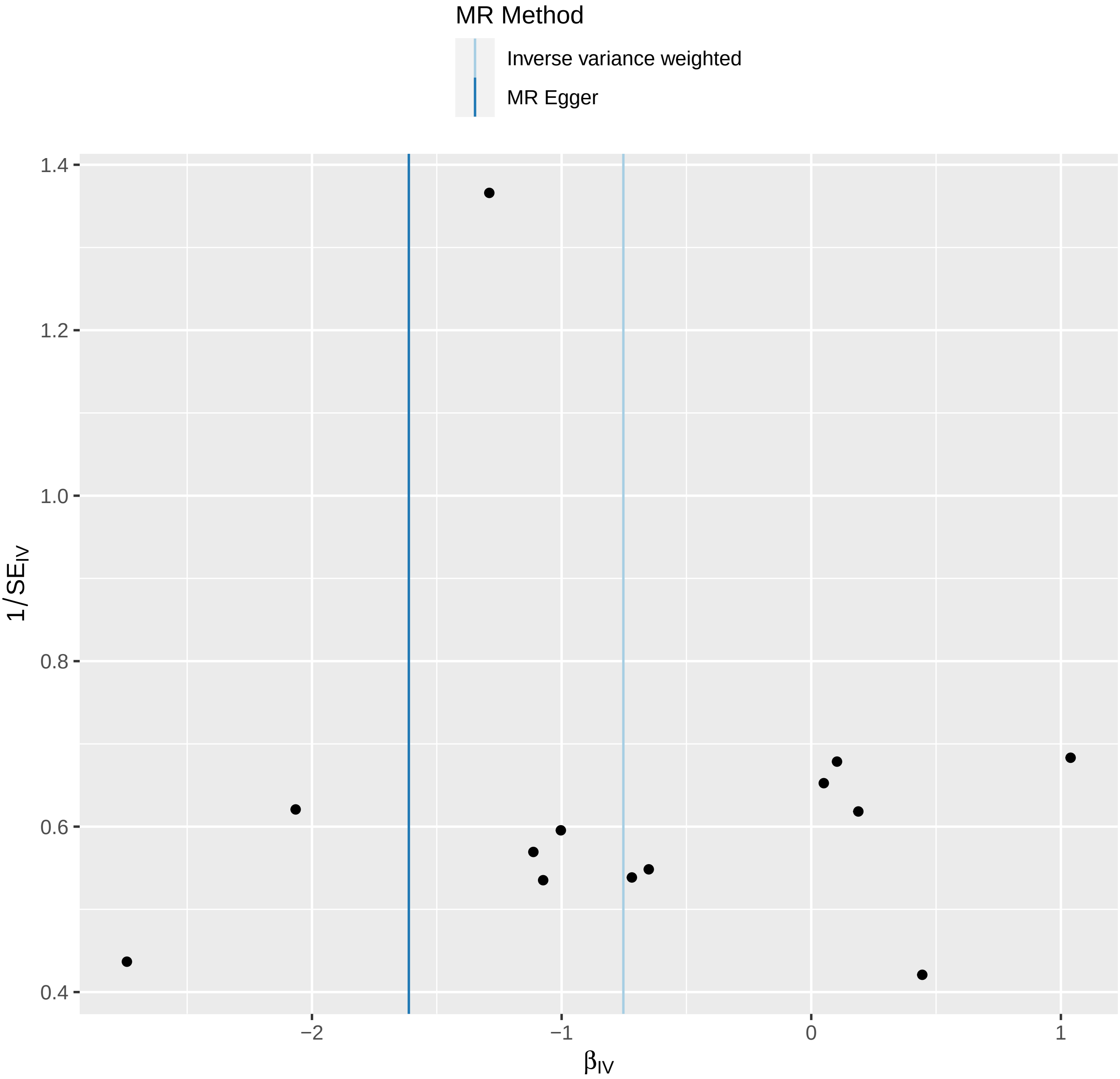

Batch 872 : Heptanoate (7:0) on Arthropathy in Crohn disease

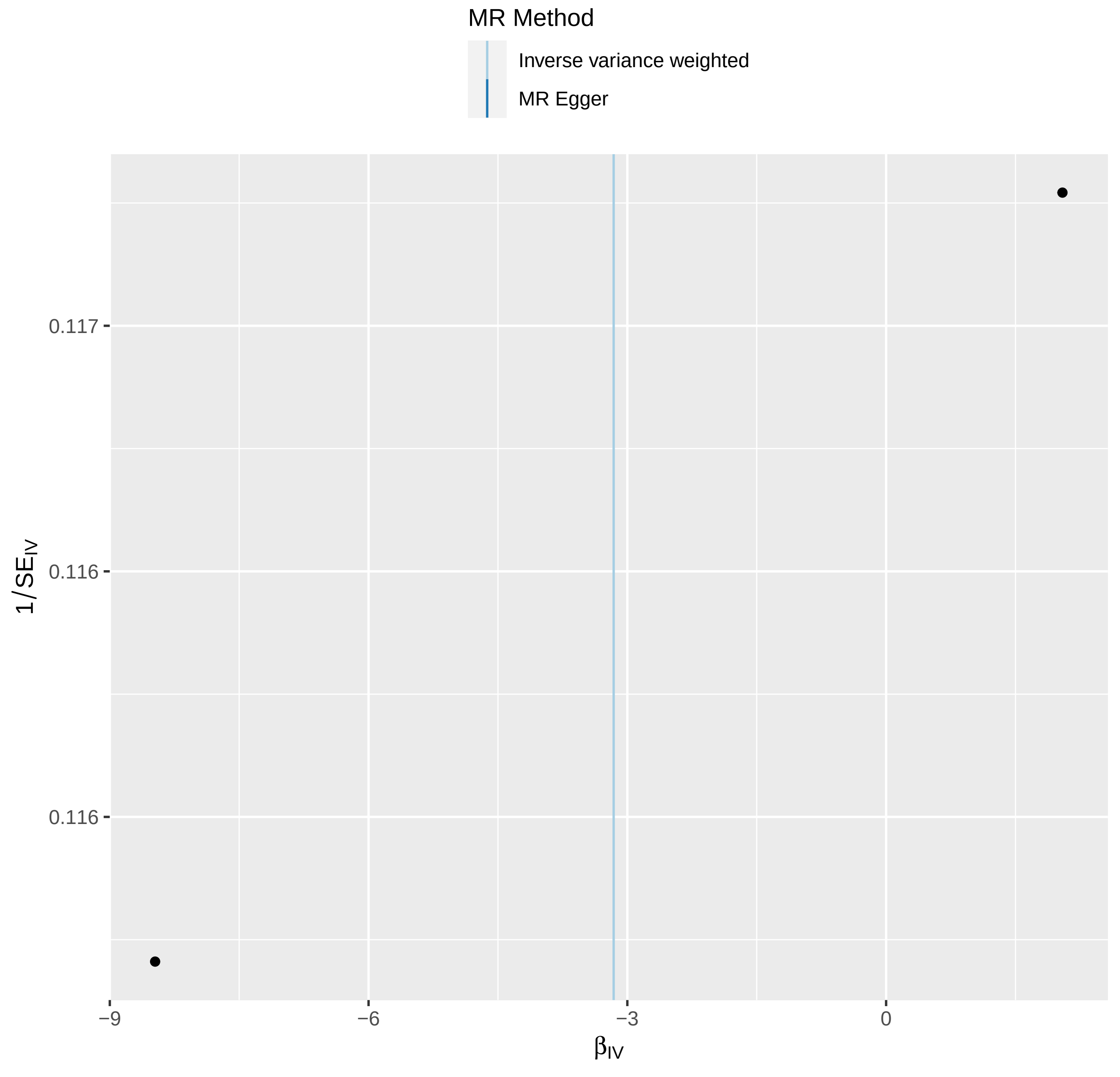

Batch 875 : Urea on Arthropathy in Crohn disease

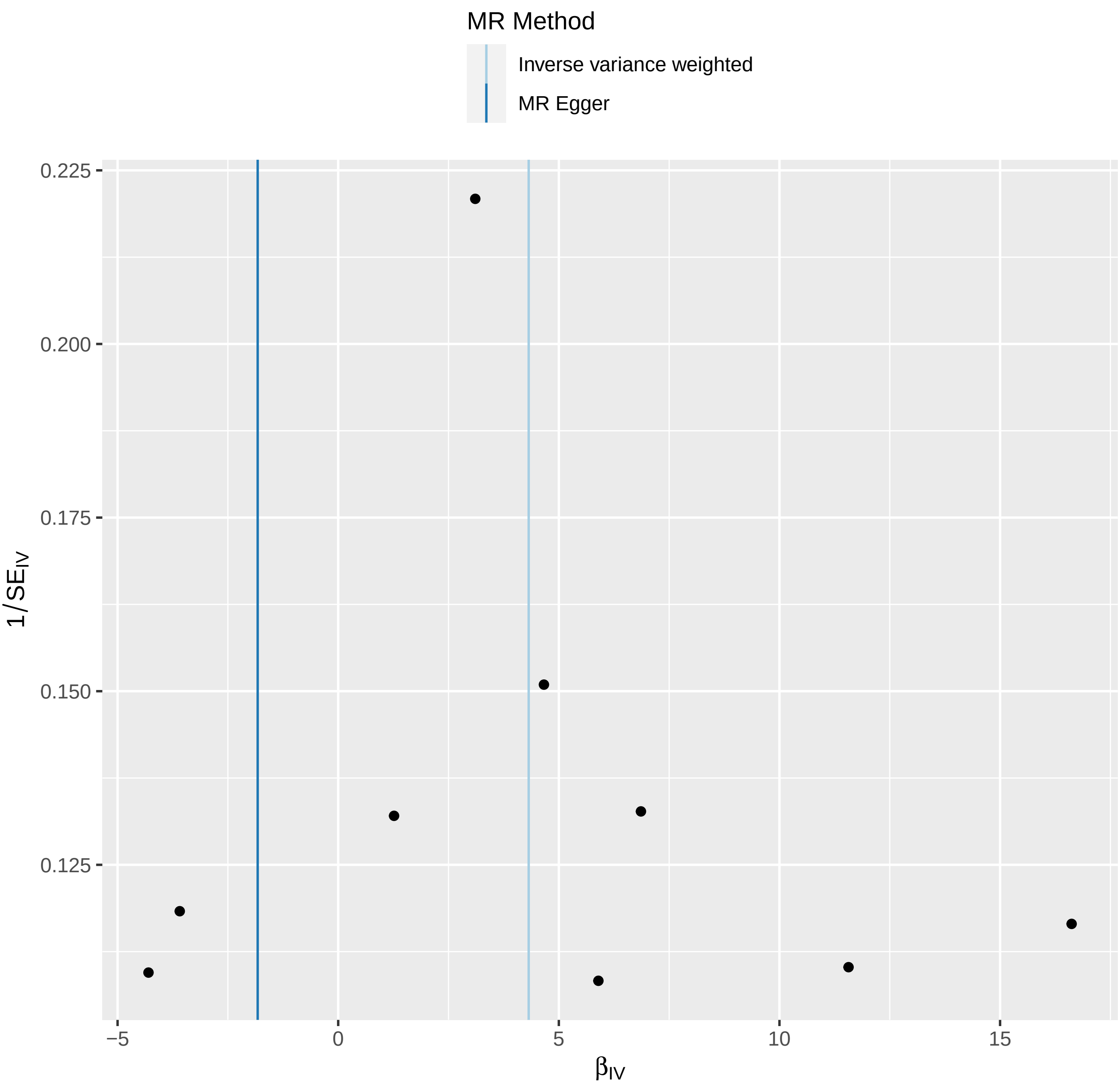

Batch 876 : Cortisol on Arthropathy in Crohn disease

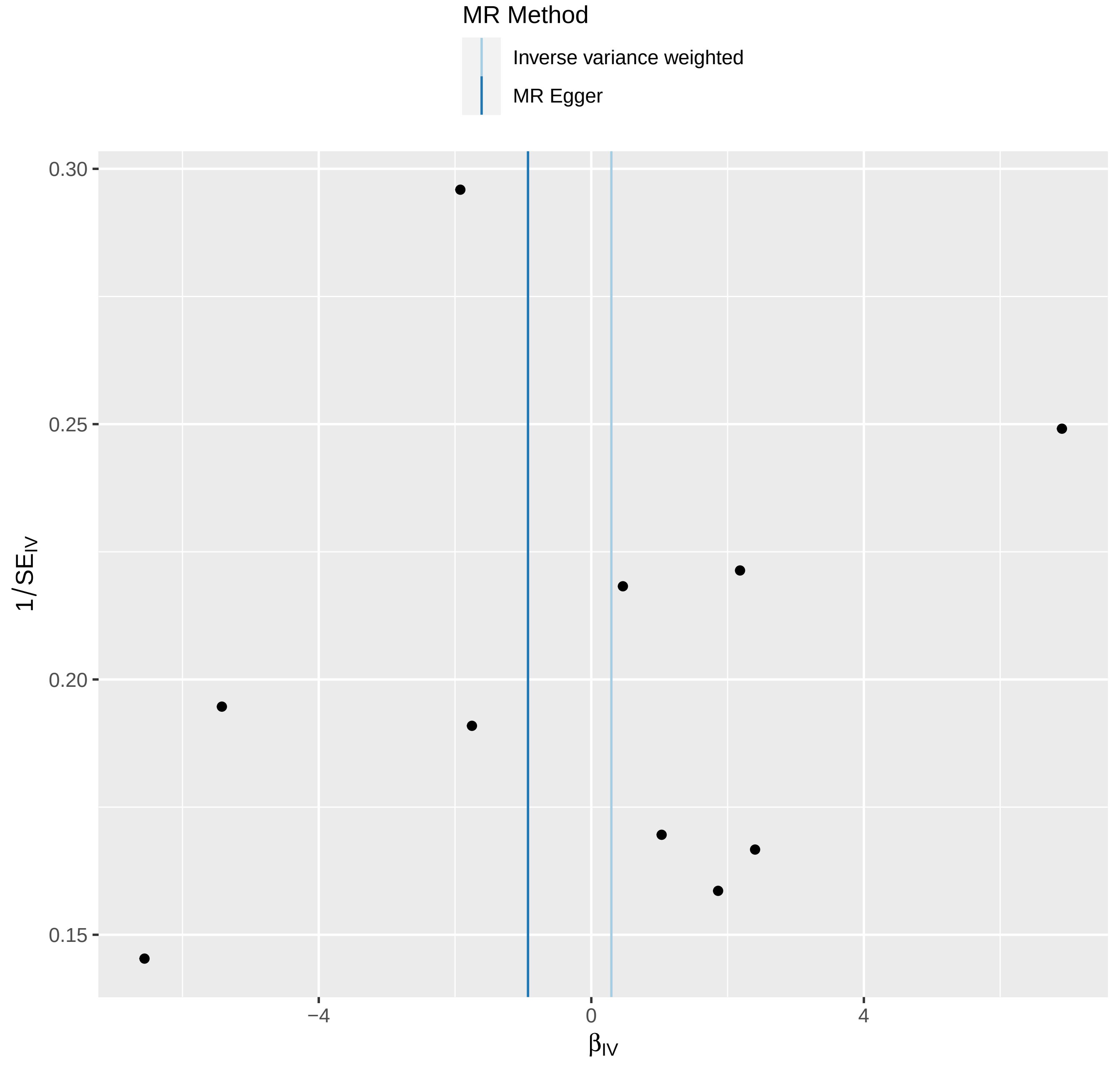

Batch 879 : Citrulline on Arthropathy in Crohn disease

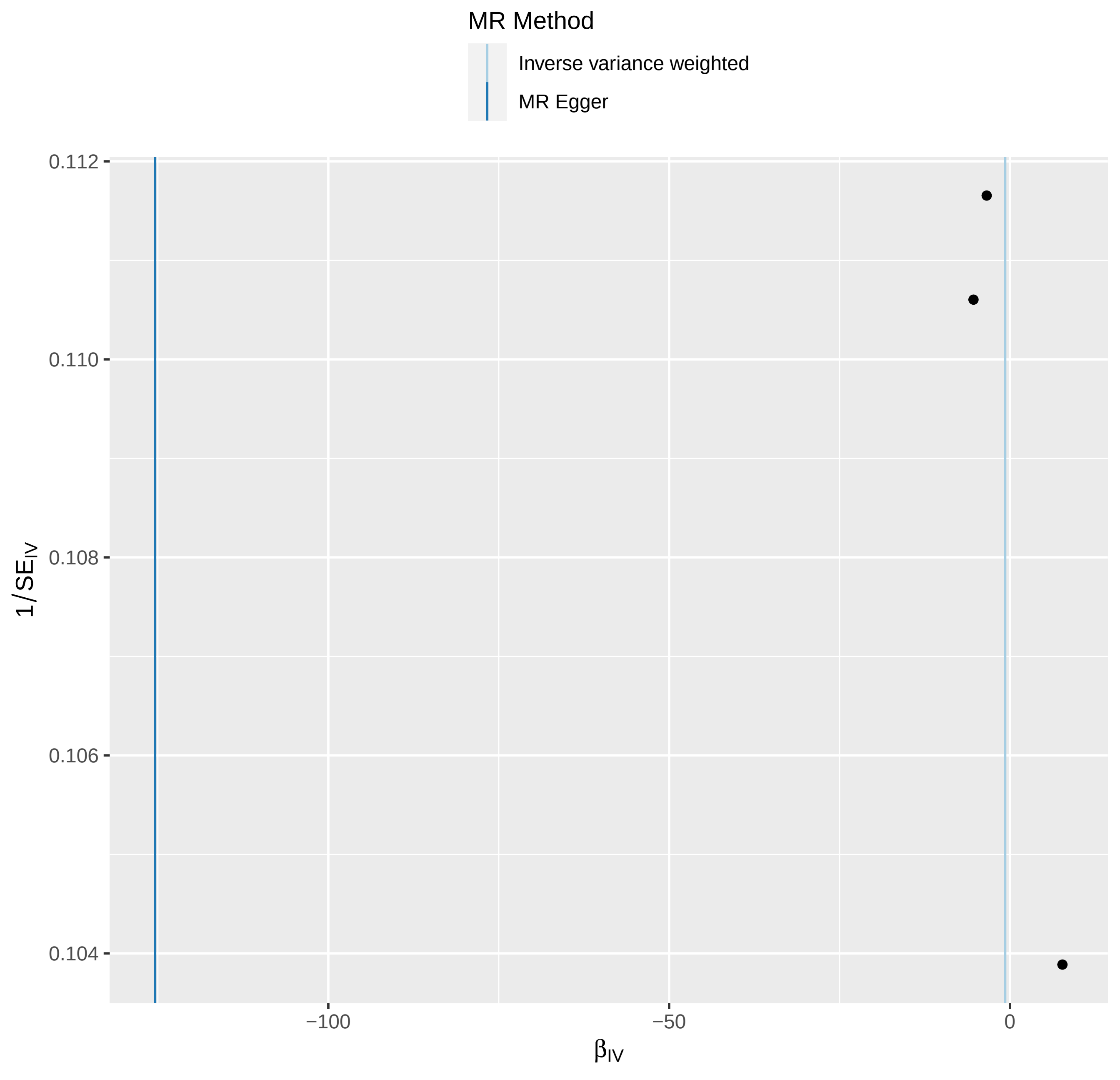

Batch 881 : Serotonin (5HT) on Arthropathy in Crohn disease

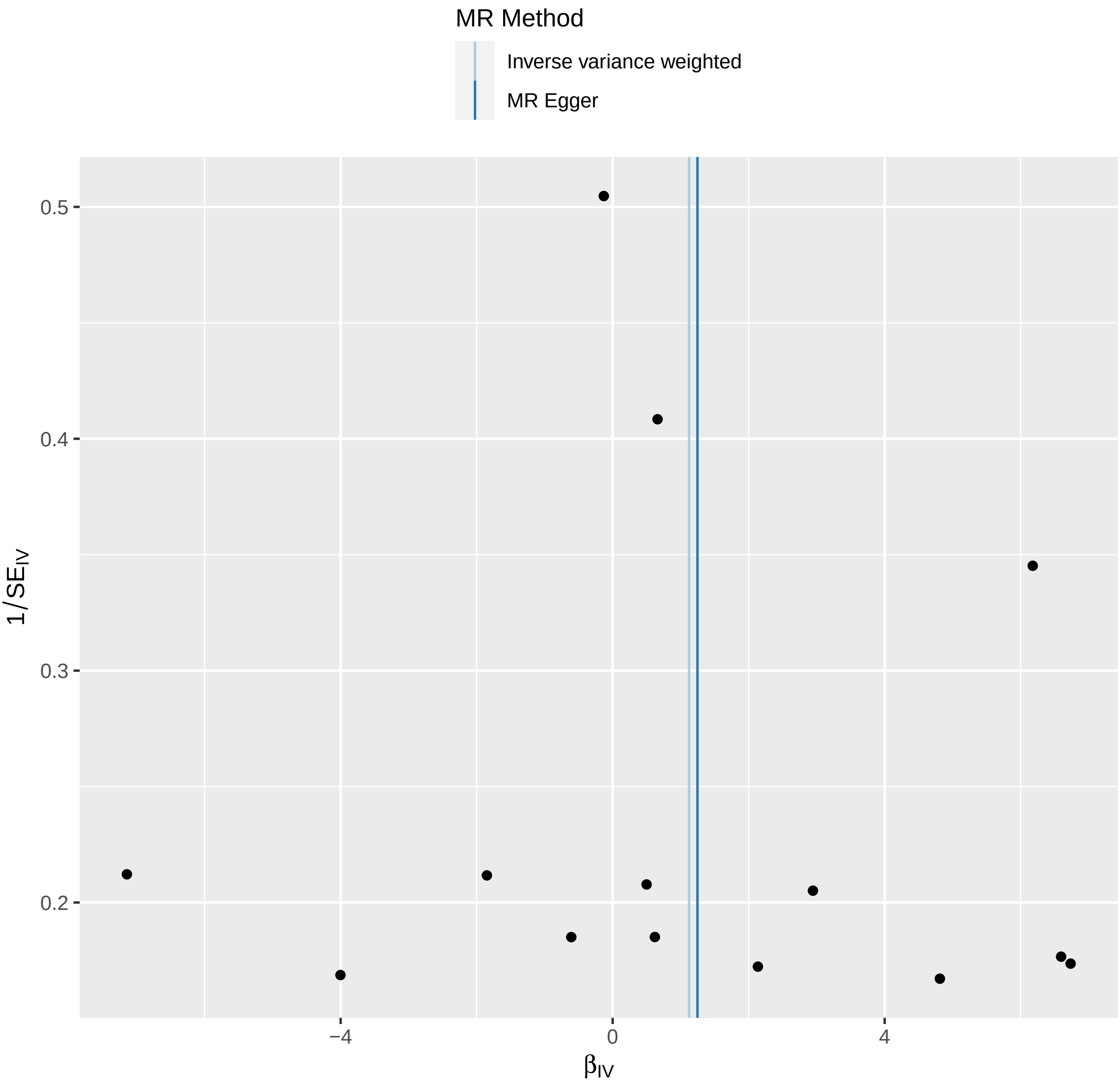

Batch 882 : Gamma−glutamylglutamine on Arthropathy in Crohn disease

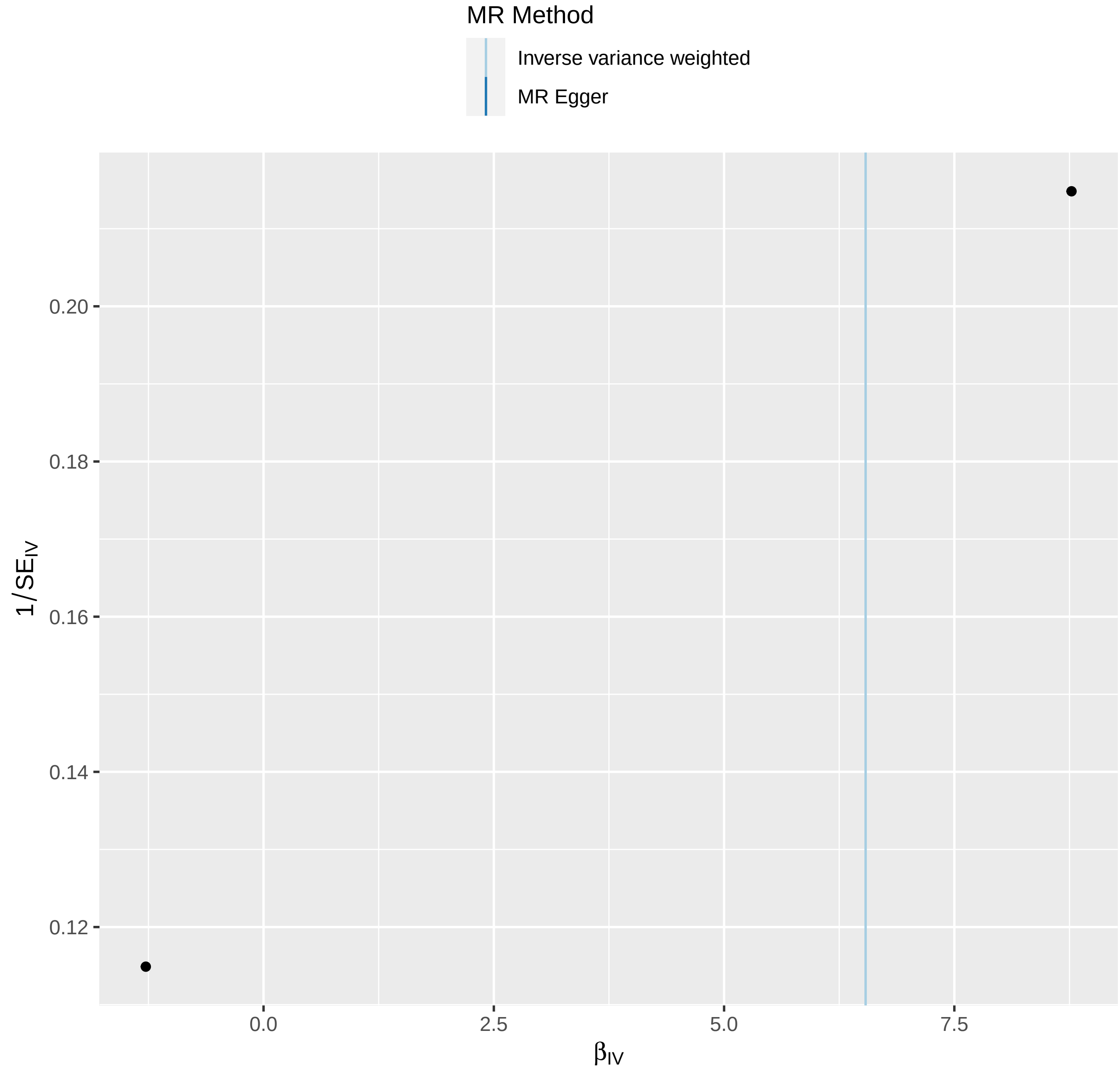

Batch 883 : Gamma−glutamyltyrosine on Arthropathy in Crohn disease

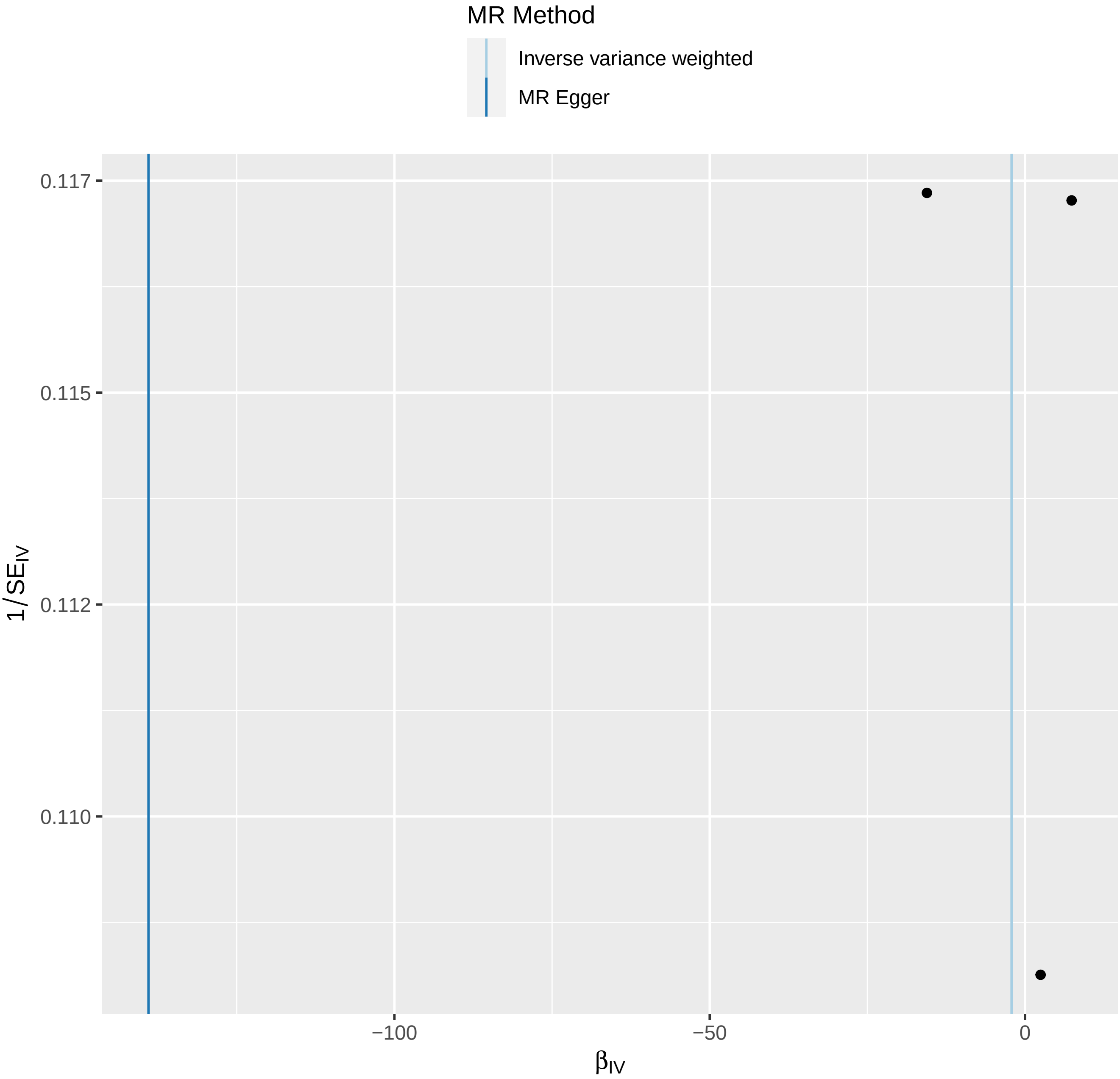

Batch 885 : Betaine on Arthropathy in Crohn disease

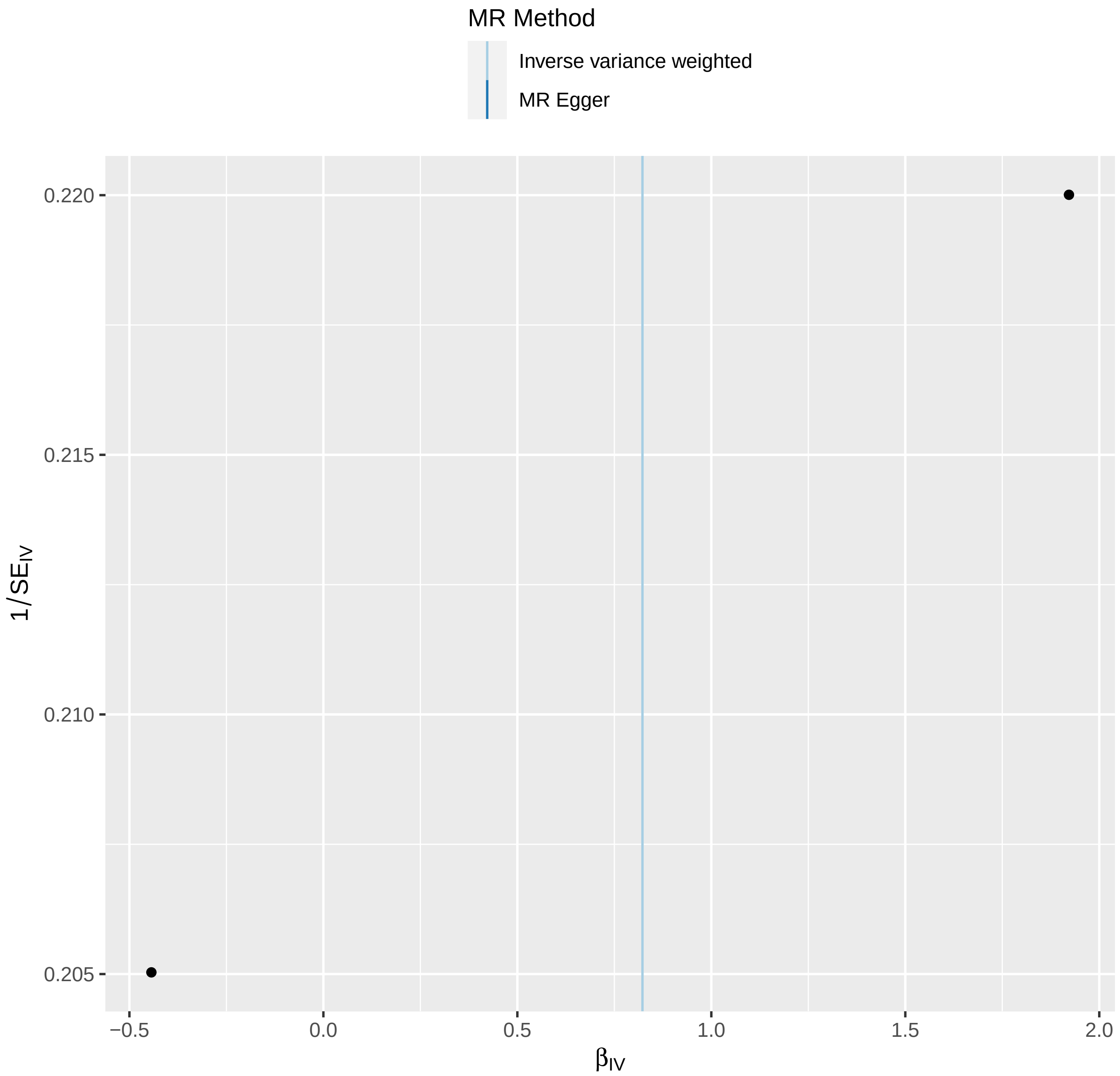

Batch 886 : Xanthine on Arthropathy in Crohn disease

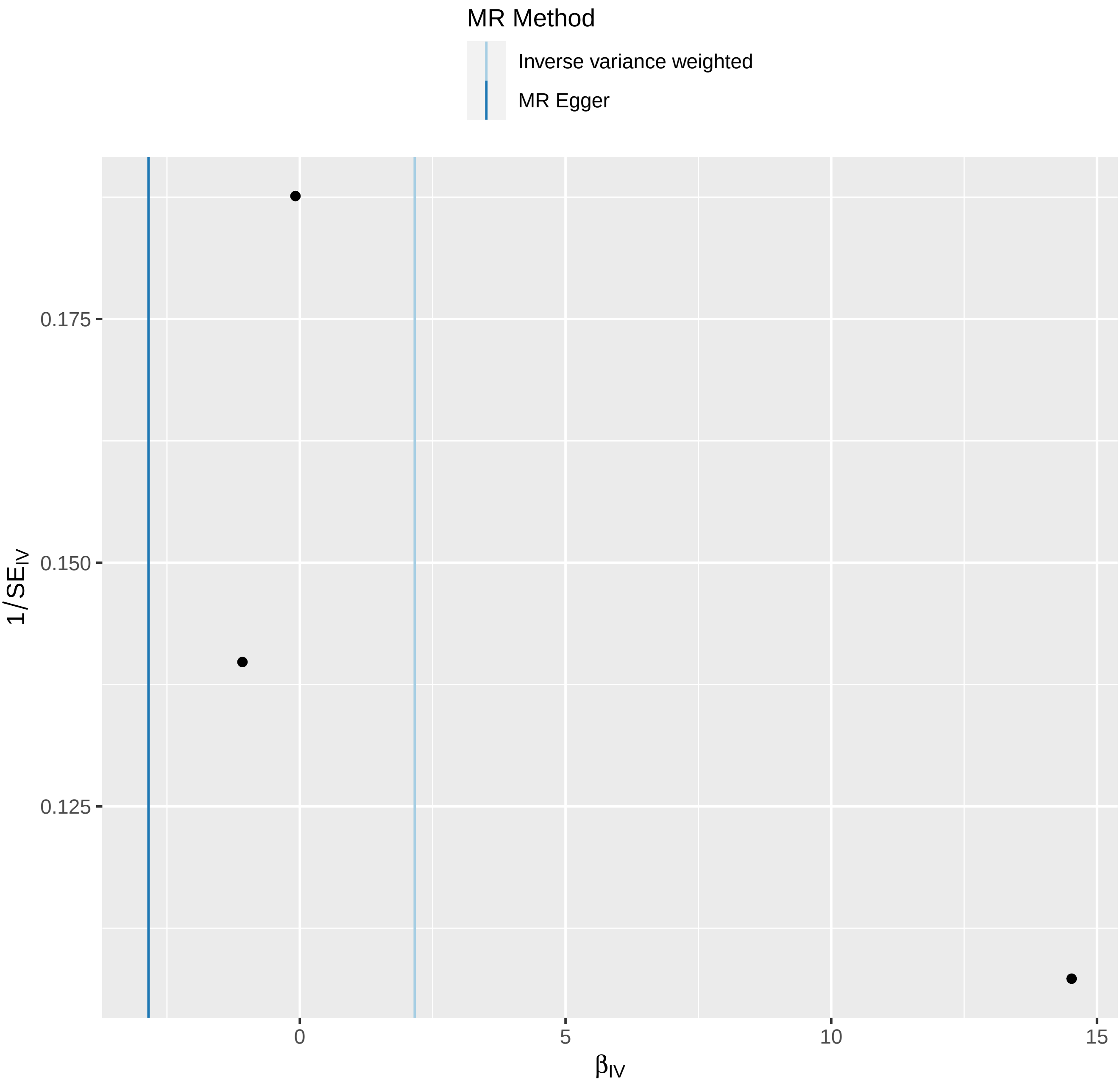

Batch 887 : Phosphate on Arthropathy in Crohn disease

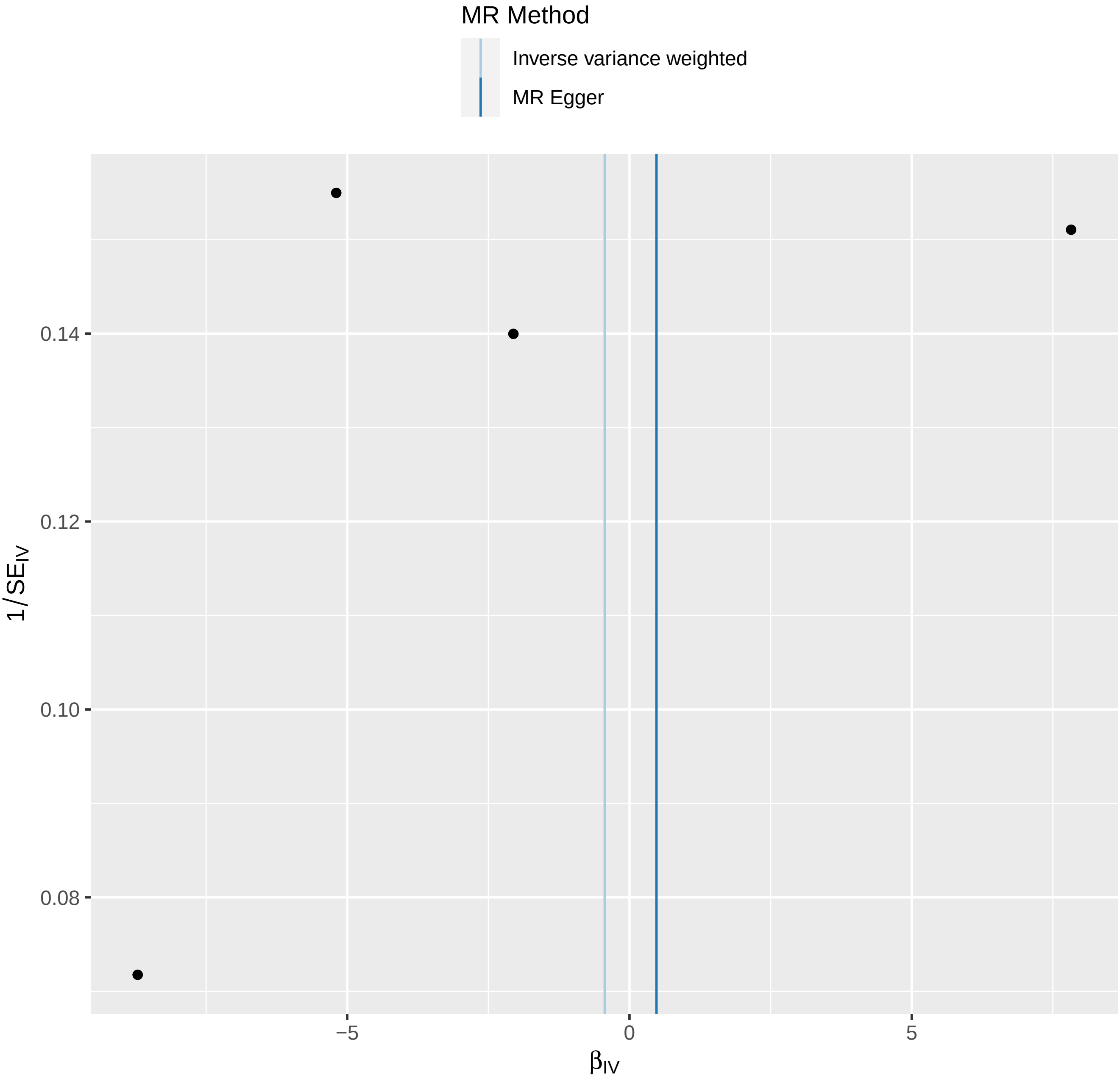

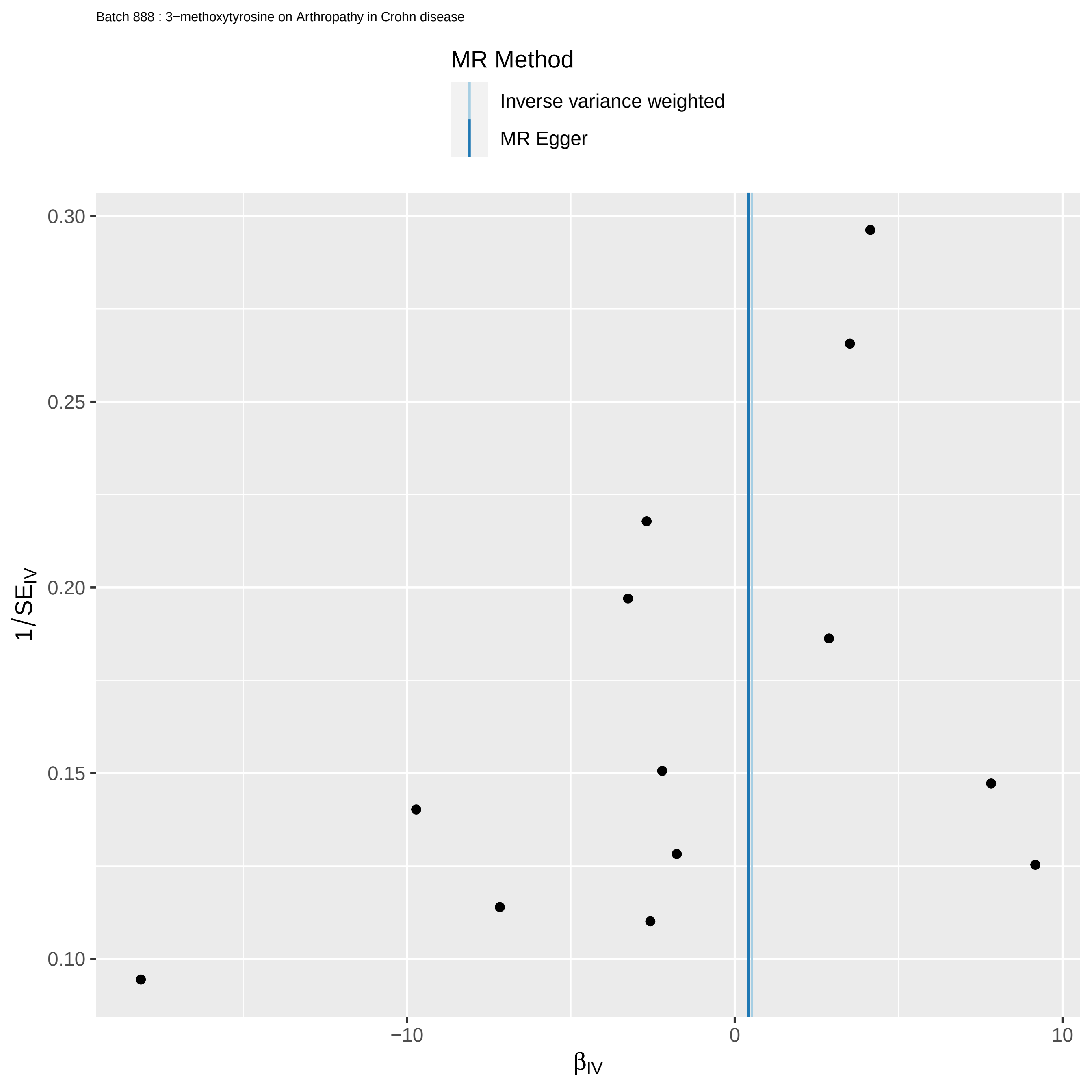

Batch 888 : 3−methoxytyrosine on Arthropathy in Crohn disease
MR Method
Inverse variance weighted
MR Egger
0.30
0.25
0.20
0.15
0.10
1/SE_IV
−10
0
10
β_IV

Batch 890 : Undecanoate (11:0) on Arthropathy in Crohn disease

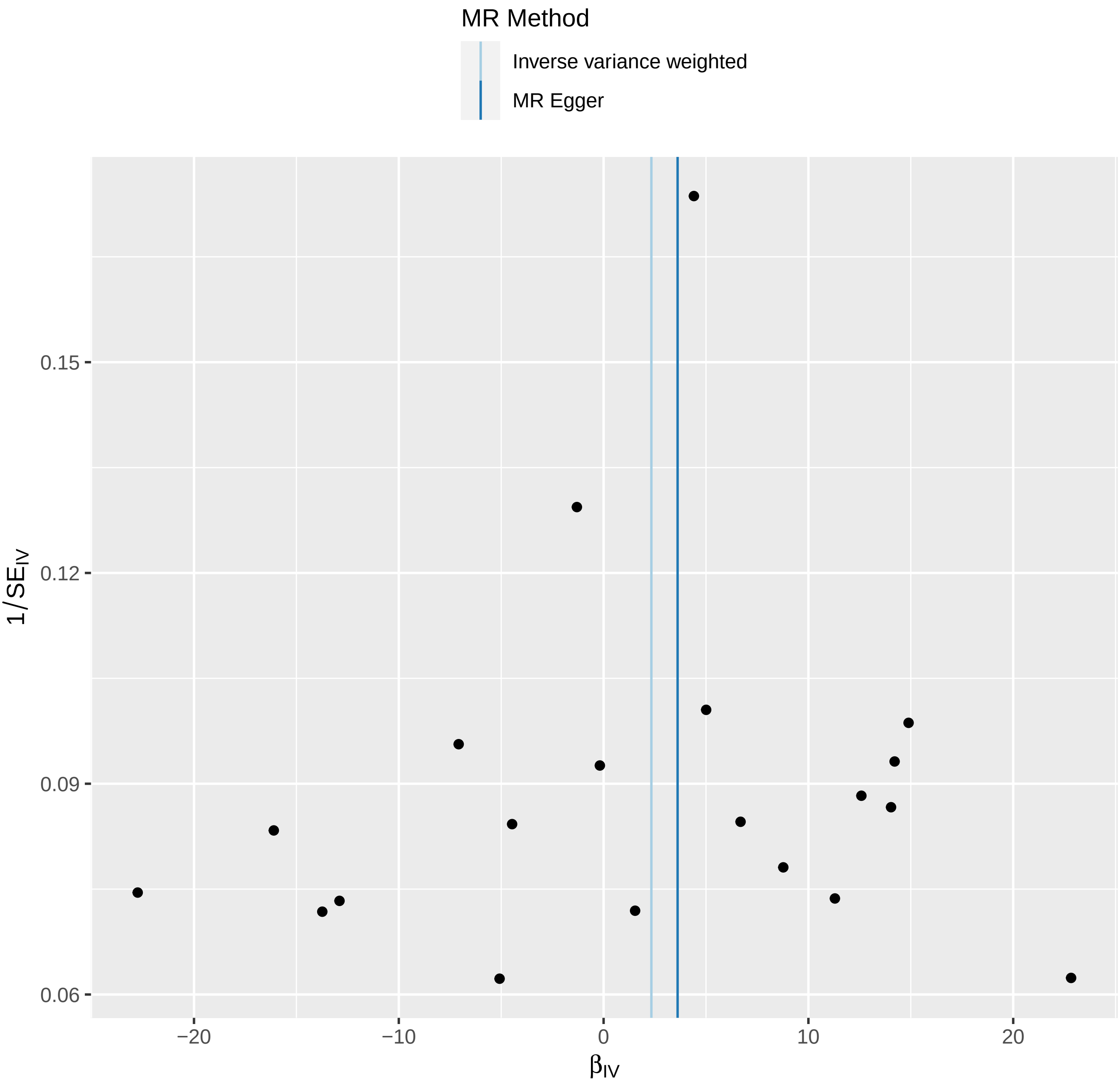

Batch 892 : Taurodeoxycholate on Arthropathy in Crohn disease

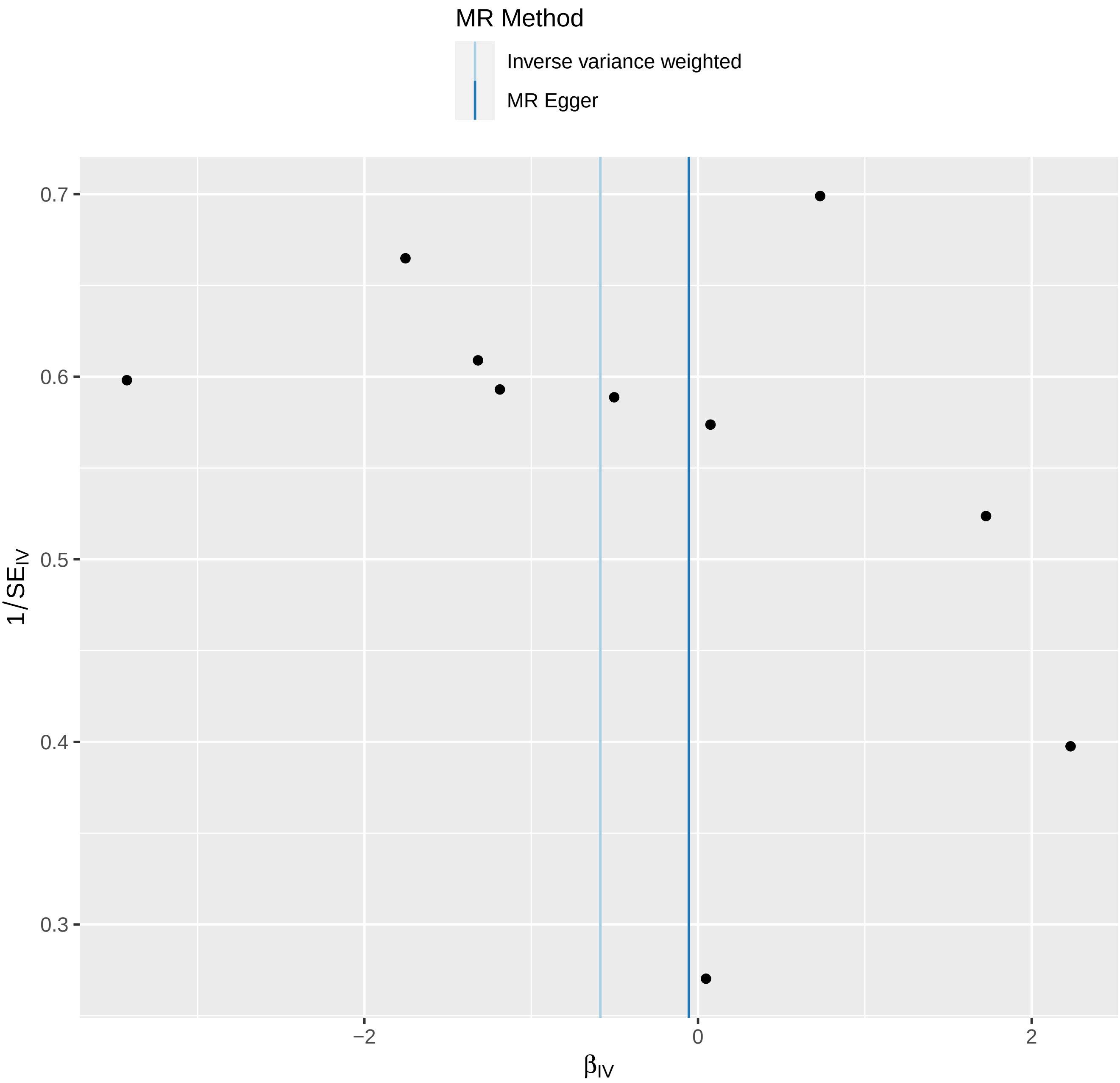

Batch 893 : Glycerol on Arthropathy in Crohn disease

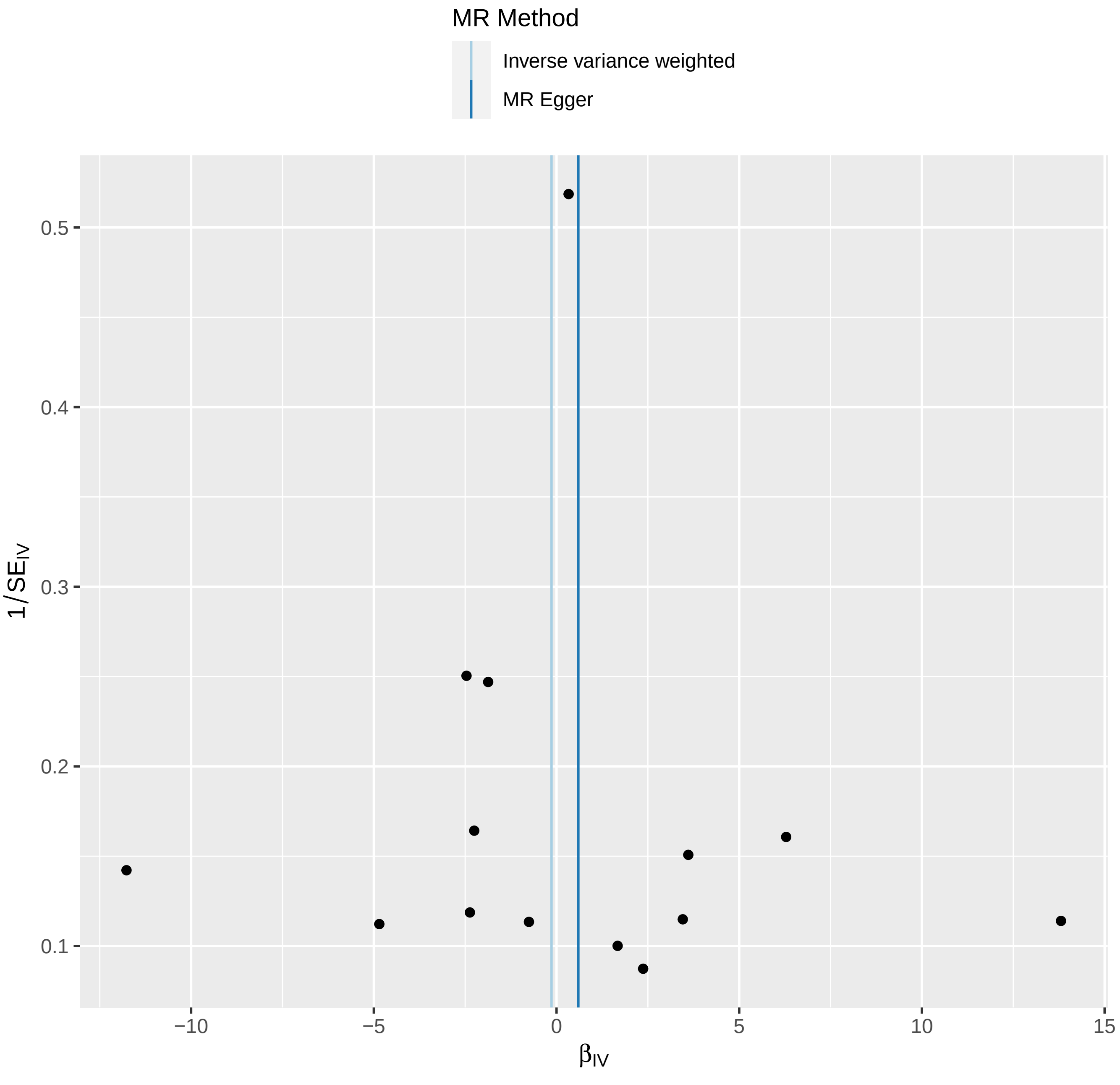

Batch 894 : Kynurenine on Arthropathy in Crohn disease

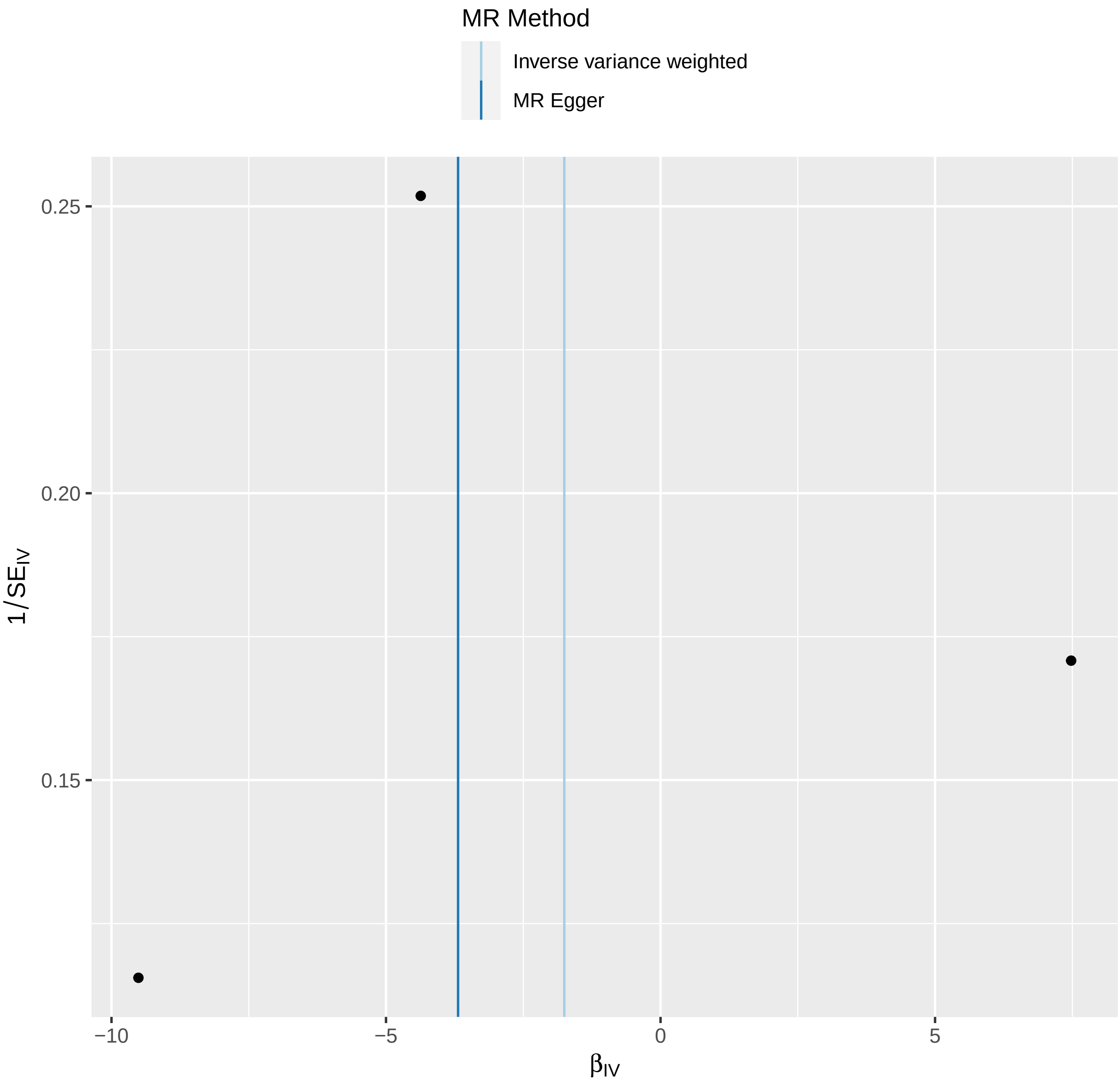

Batch 895 : Mannitol on Arthropathy in Crohn disease

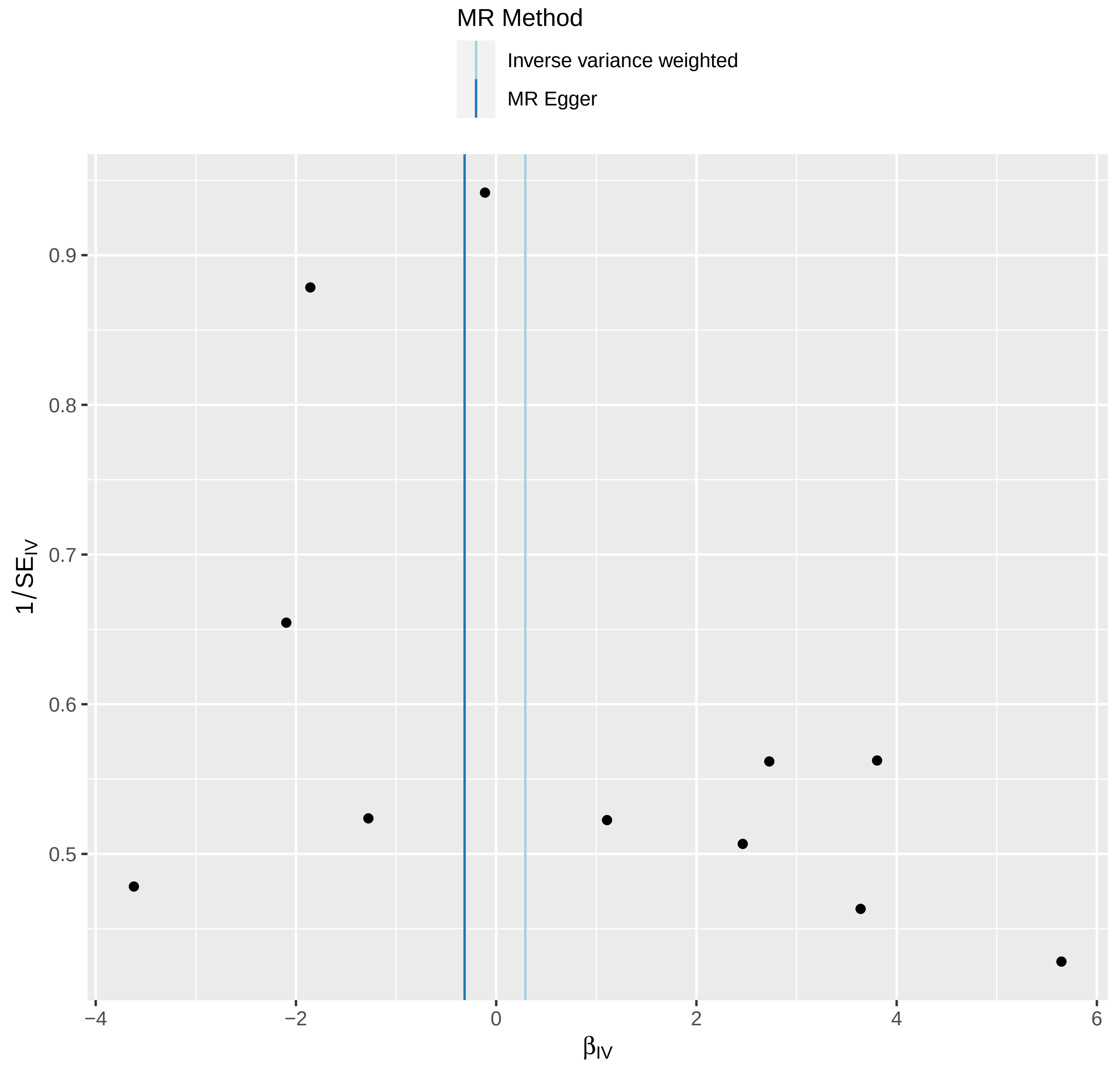

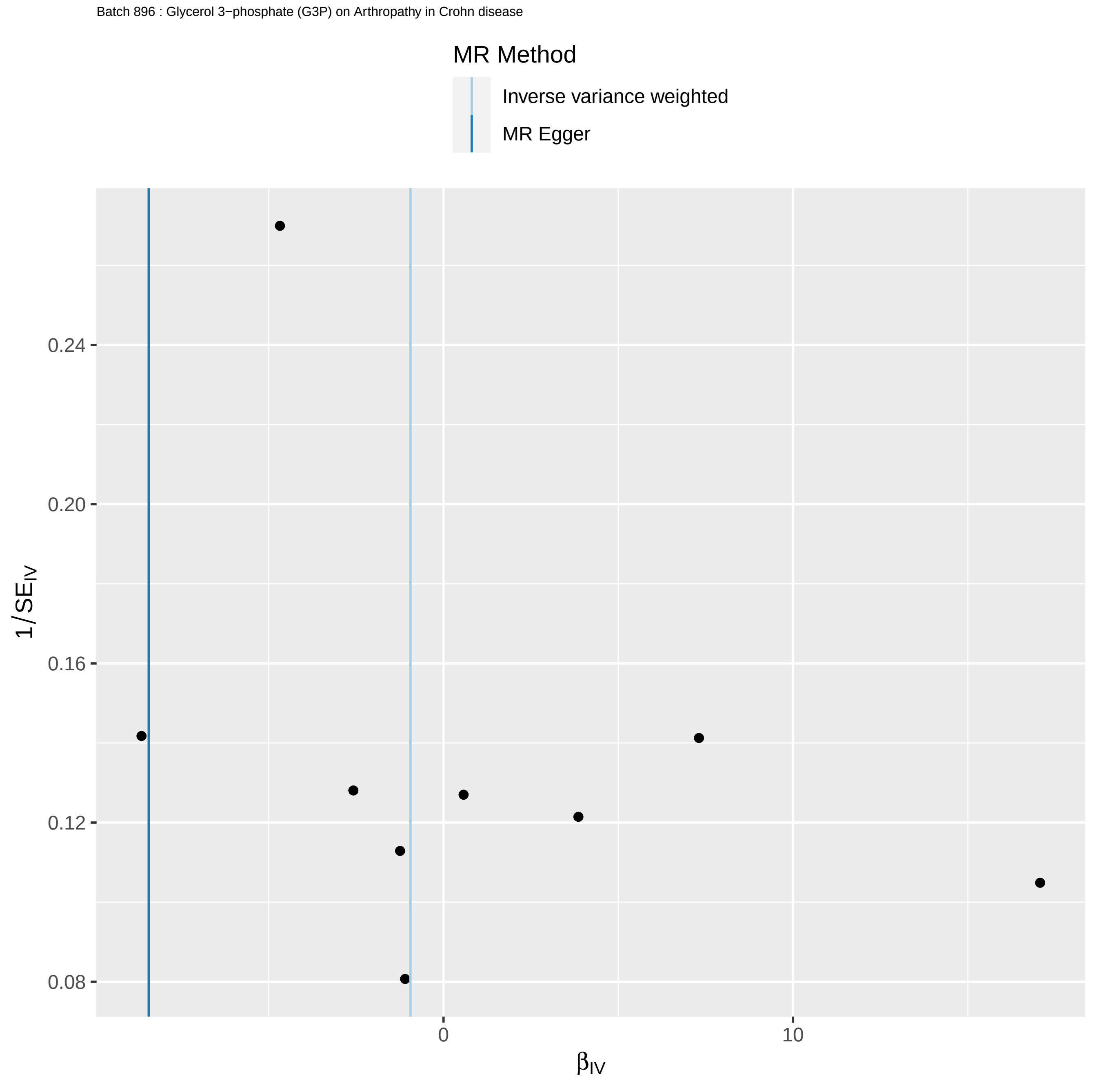

Batch 896 : Glycerol 3−phosphate (G3P) on Arthropathy in Crohn disease
MR Method
Inverse variance weighted
MR Egger
0.24
0.20
0.16
0.12
0.08
1/SE_IV
0
10
β_IV

Batch 898 : Carnitine on Arthropathy in Crohn disease

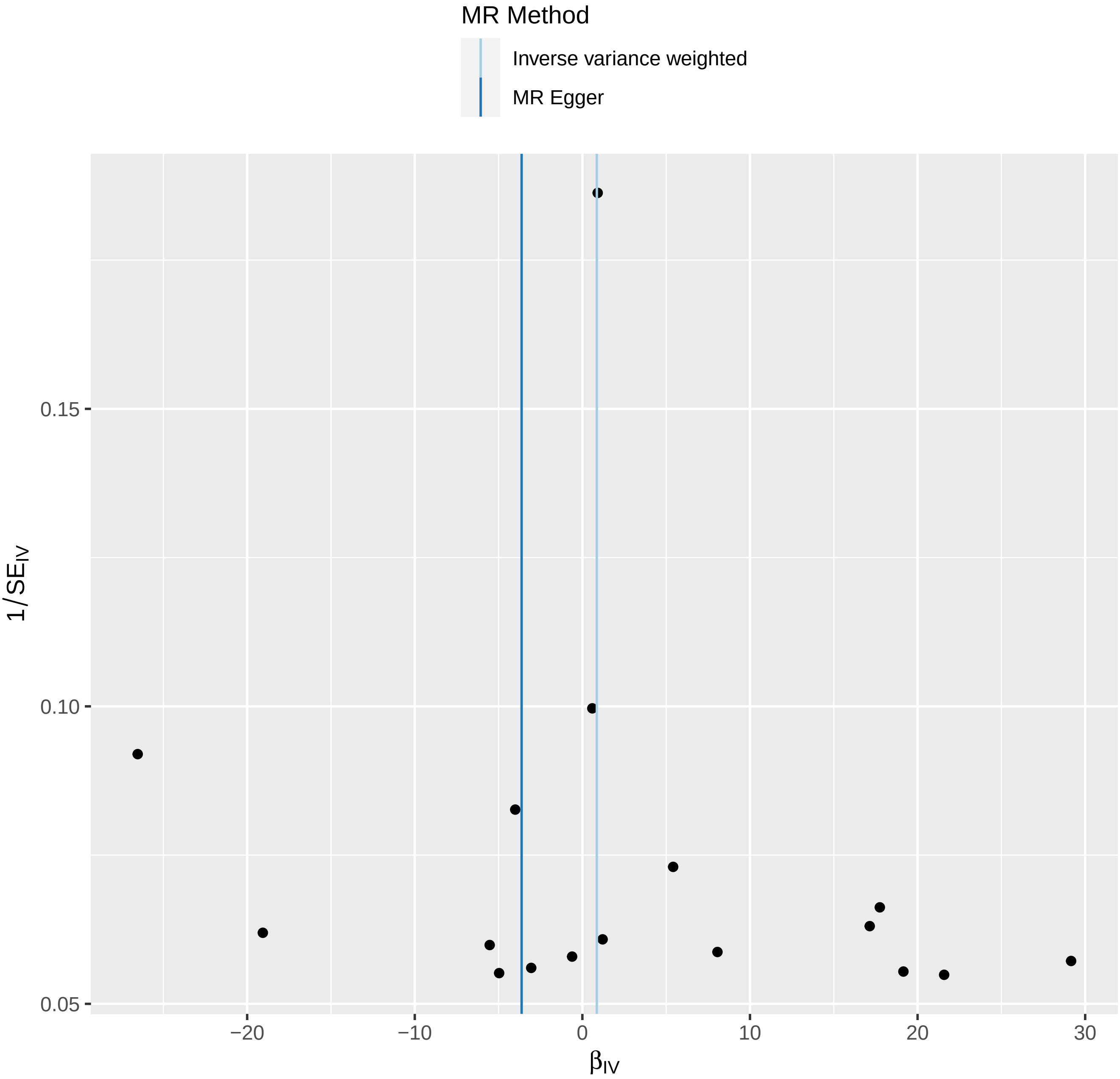

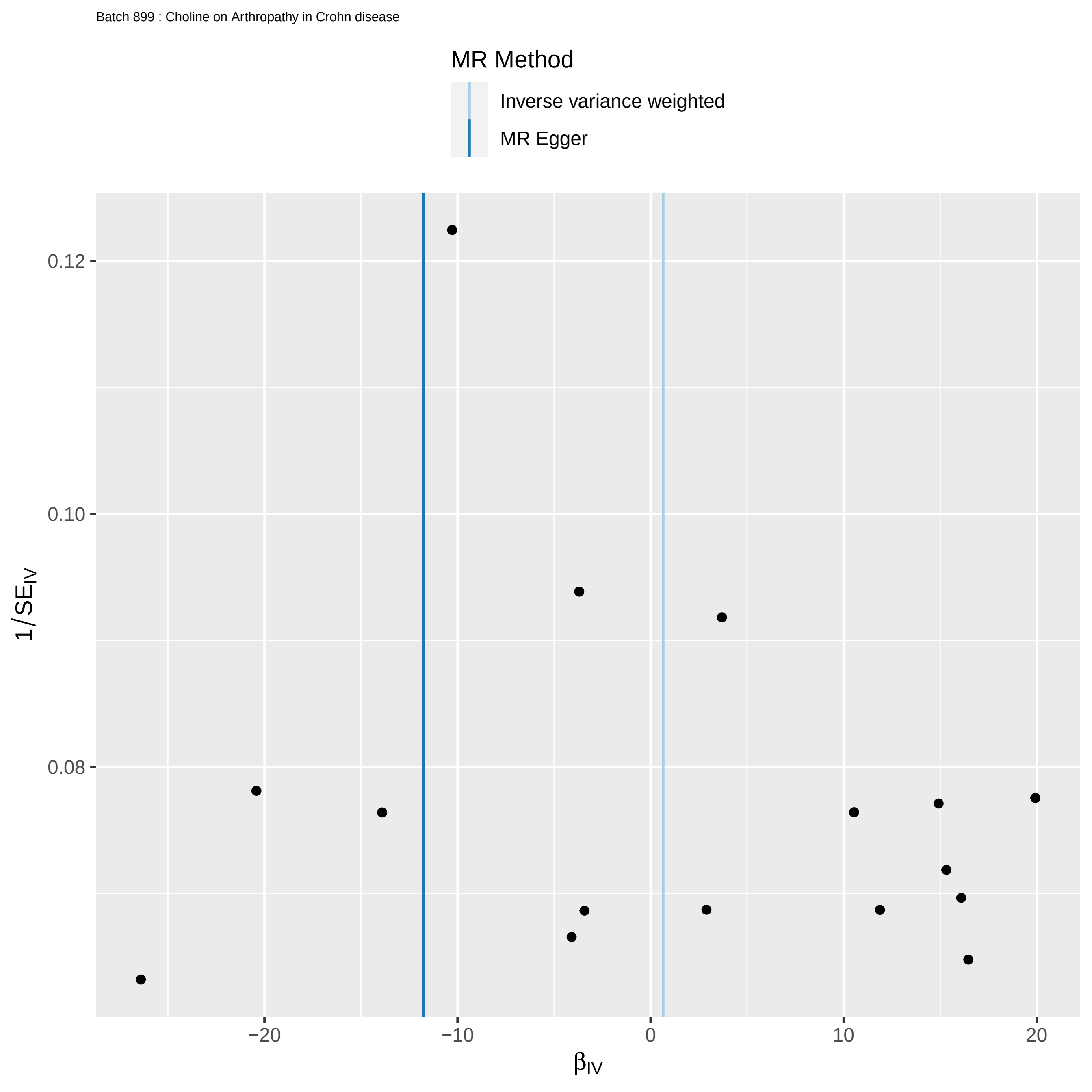

Batch 899 : Choline on Arthropathy in Crohn disease
MR Method
Inverse variance weighted
MR Egger
0.12
0.10
0.08
1/SE$_{IV}$
−20
−10
0
10
20
β$_{IV}$

Batch 902 : 3−methyl−2−oxovalerate on Arthropathy in Crohn disease

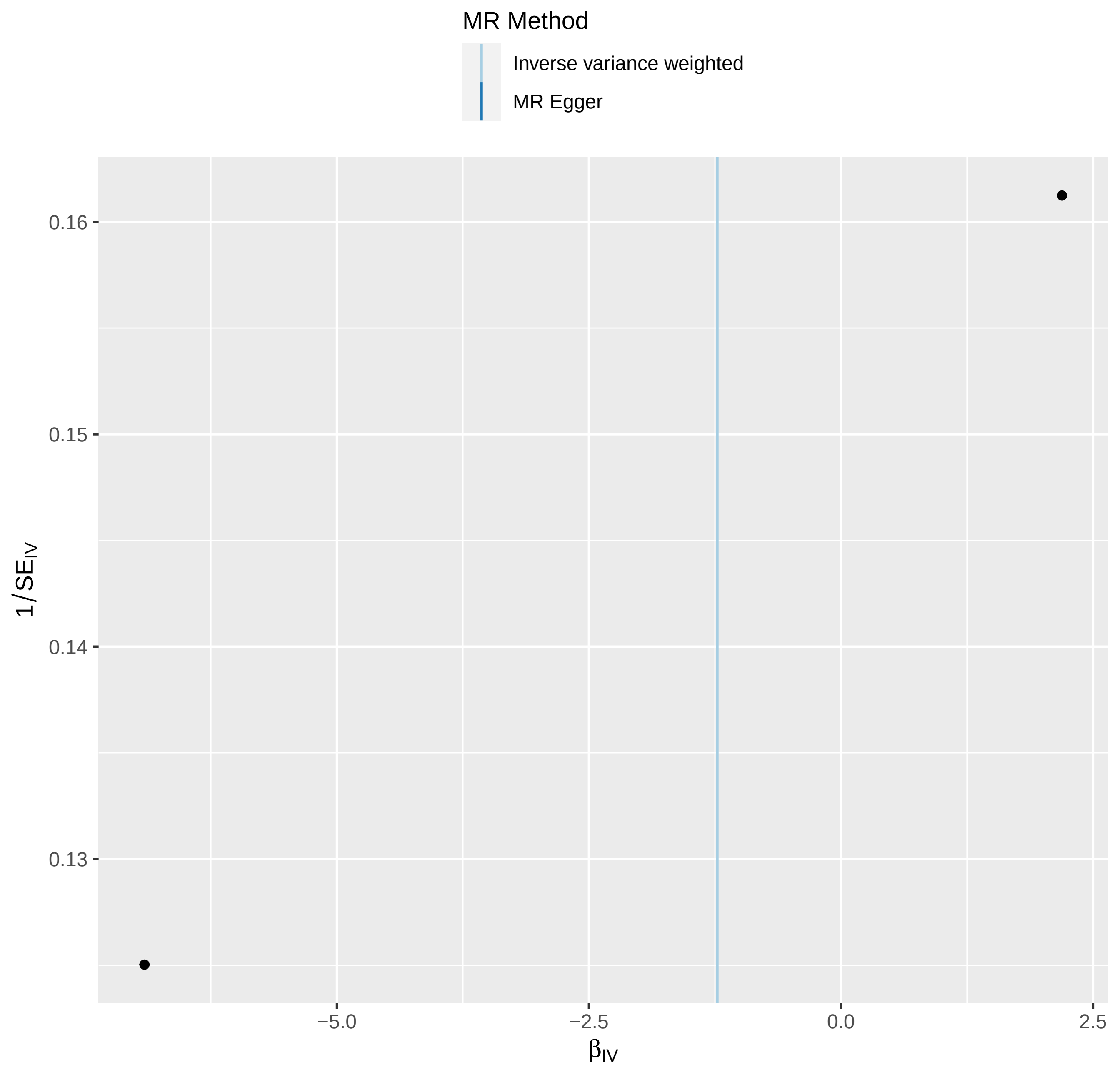

Batch 903 : 3−methylhistidine on Arthropathy in Crohn disease

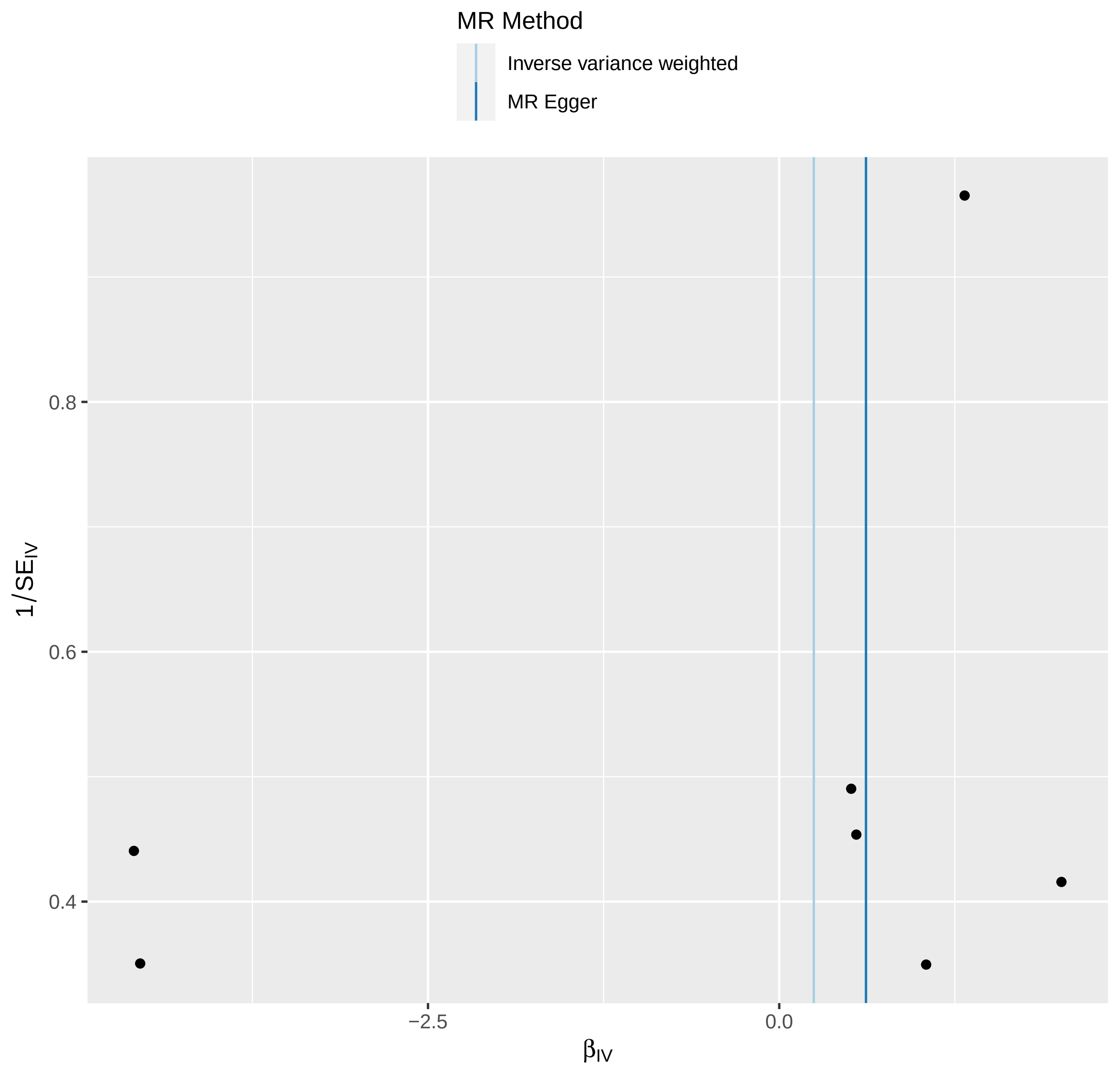

Batch 905 : Phenylacetate on Arthropathy in Crohn disease

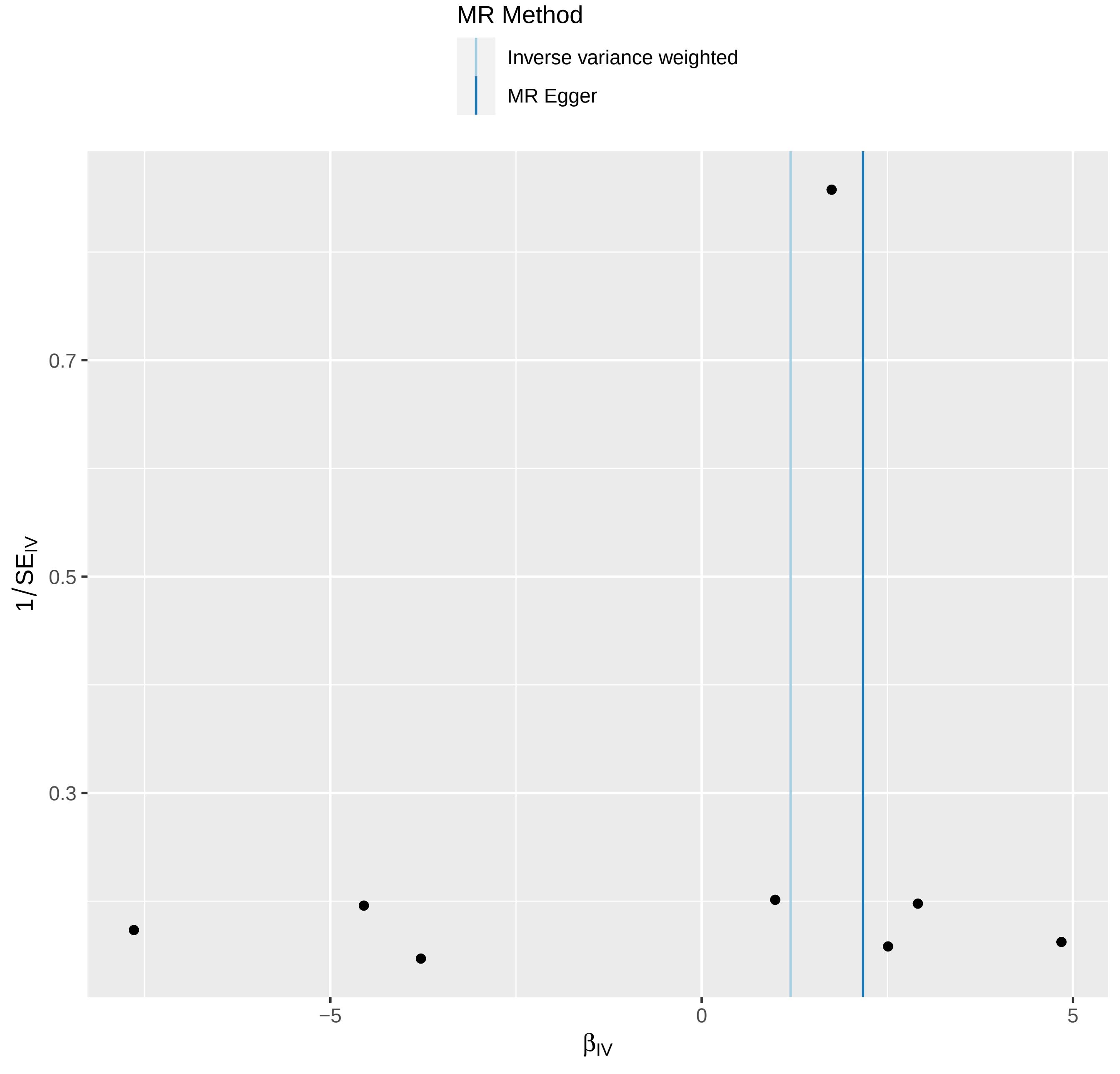

Batch 906 : Glycerophosphorylcholine (GPC) on Arthropathy in Crohn disease

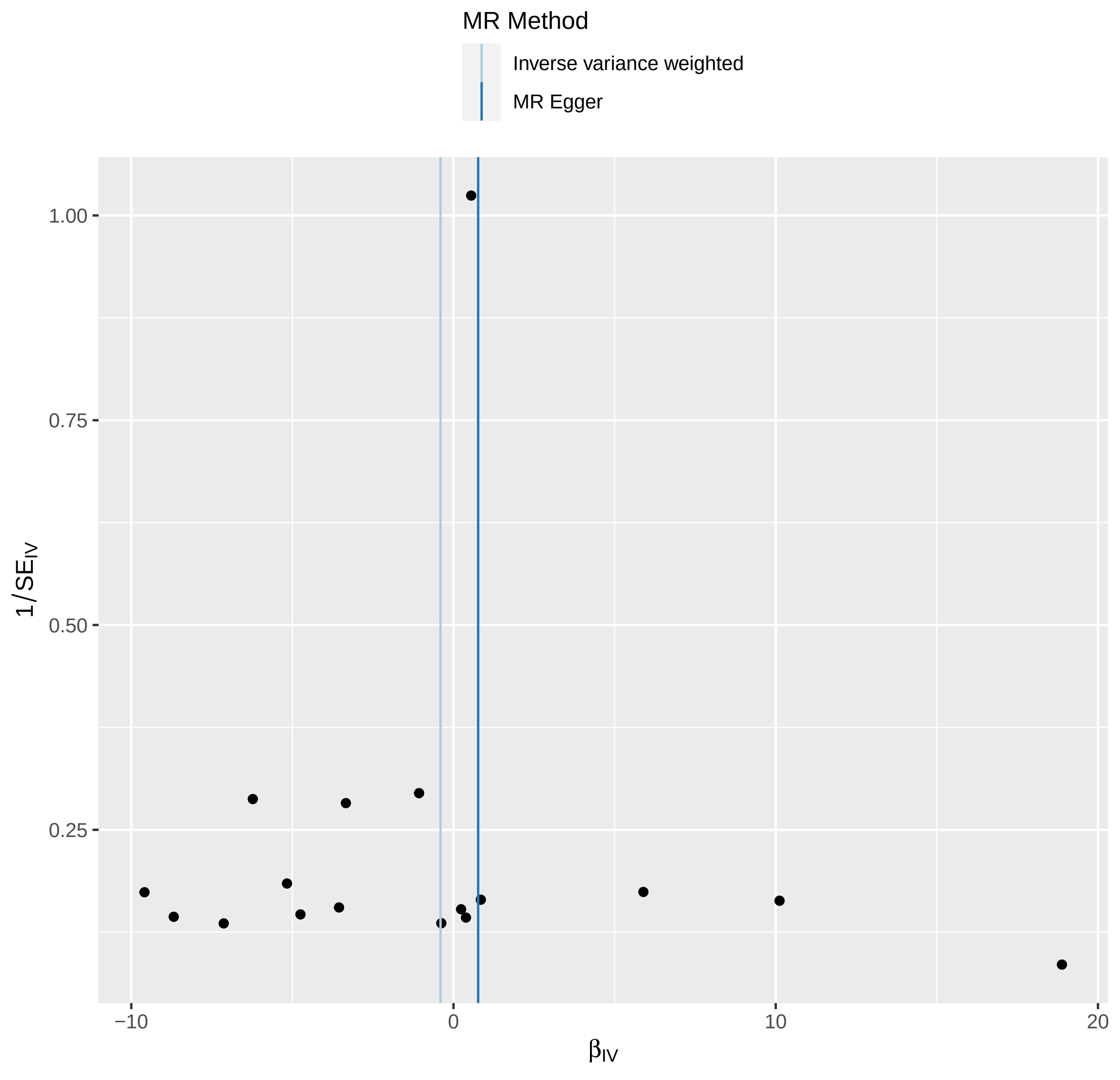

Batch 907 : Aspartate on Arthropathy in Crohn disease

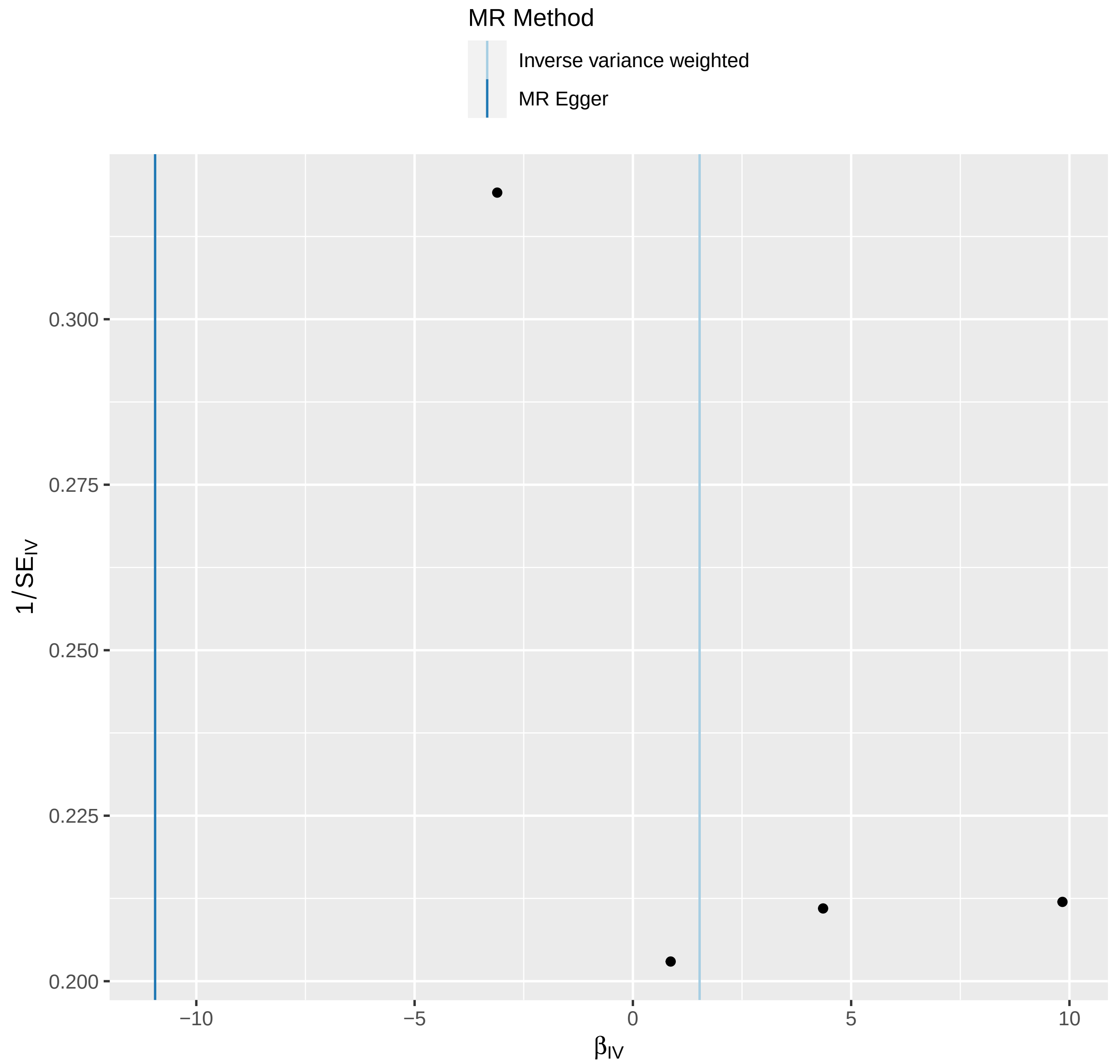

Batch 909 : Dihomo−linoleate (20:2n6) on Arthropathy in Crohn disease

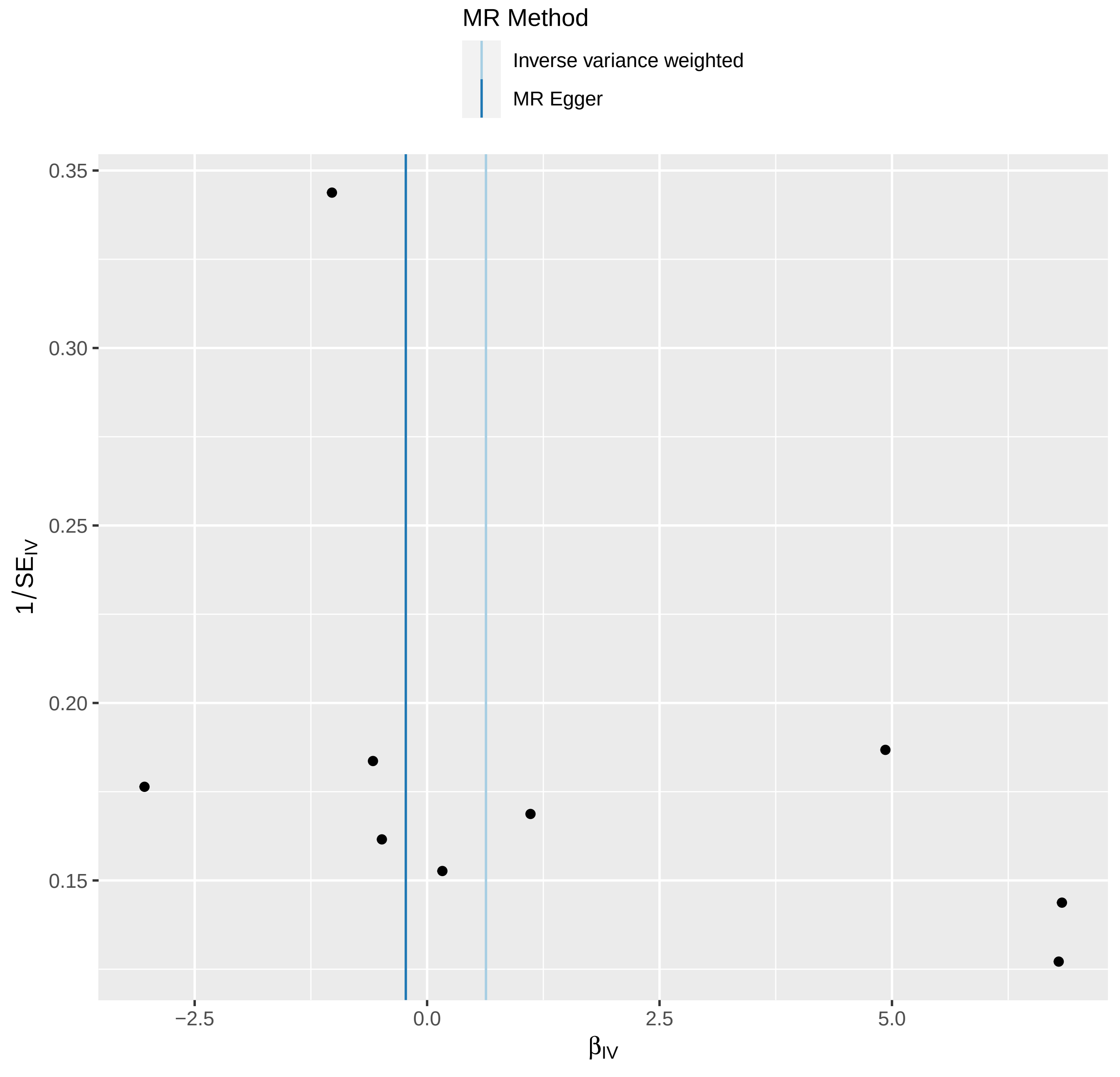

Batch 910 : 2−hydroxystearate on Arthropathy in Crohn disease

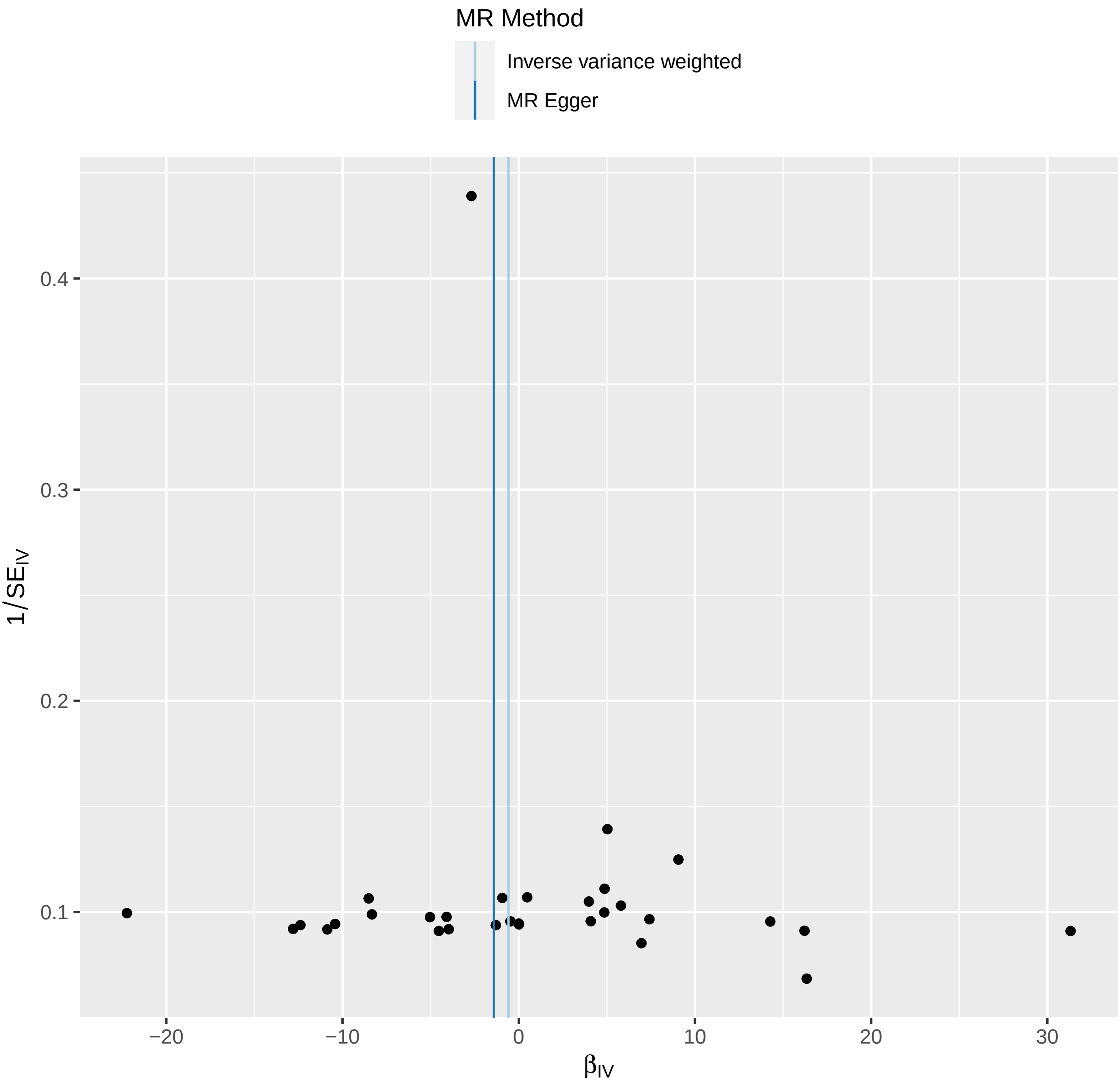

Batch 912 : Glycylvaline on Arthropathy in Crohn disease

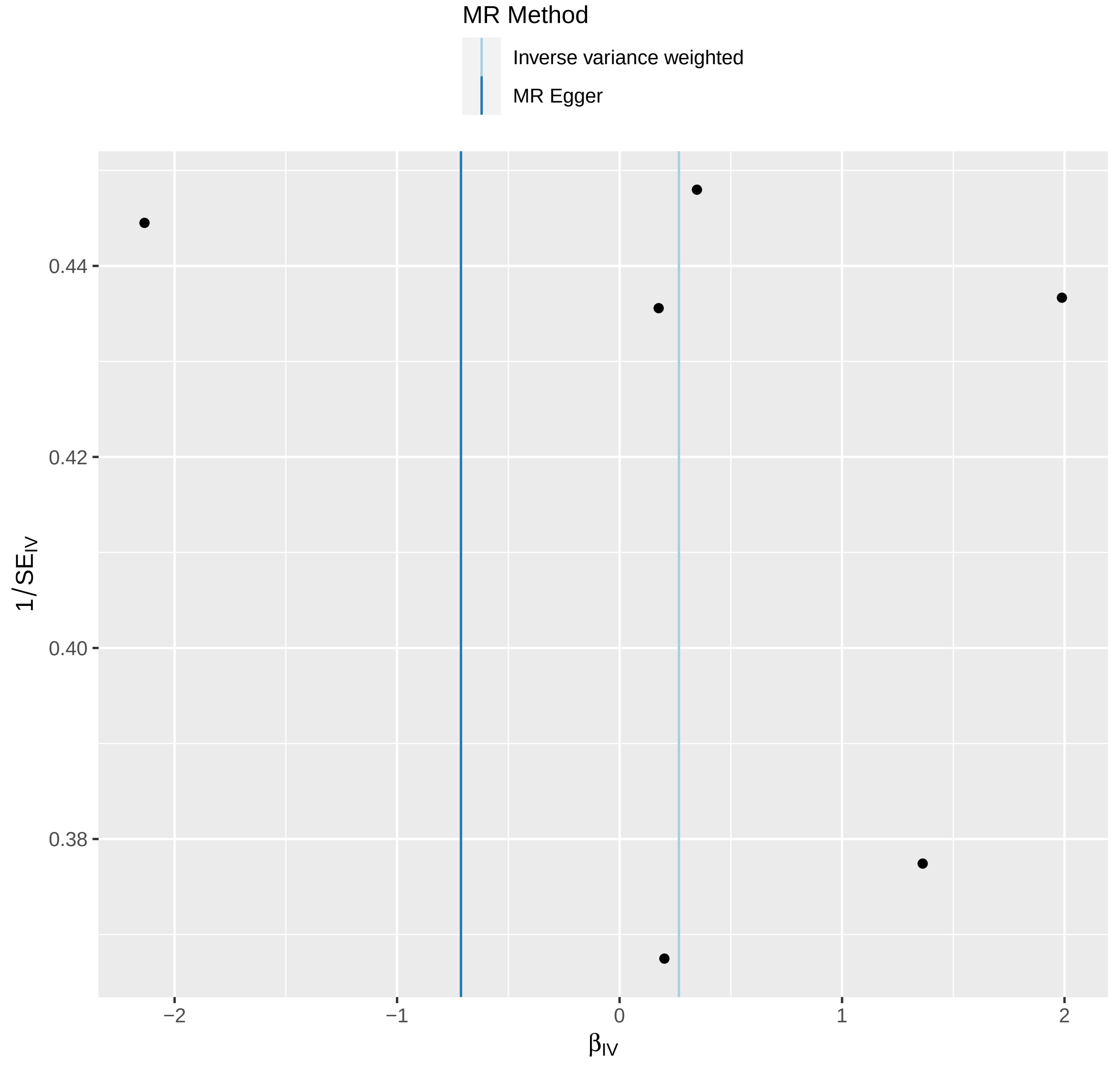

Batch 913 : Gamma−glutamylleucine on Arthropathy in Crohn disease

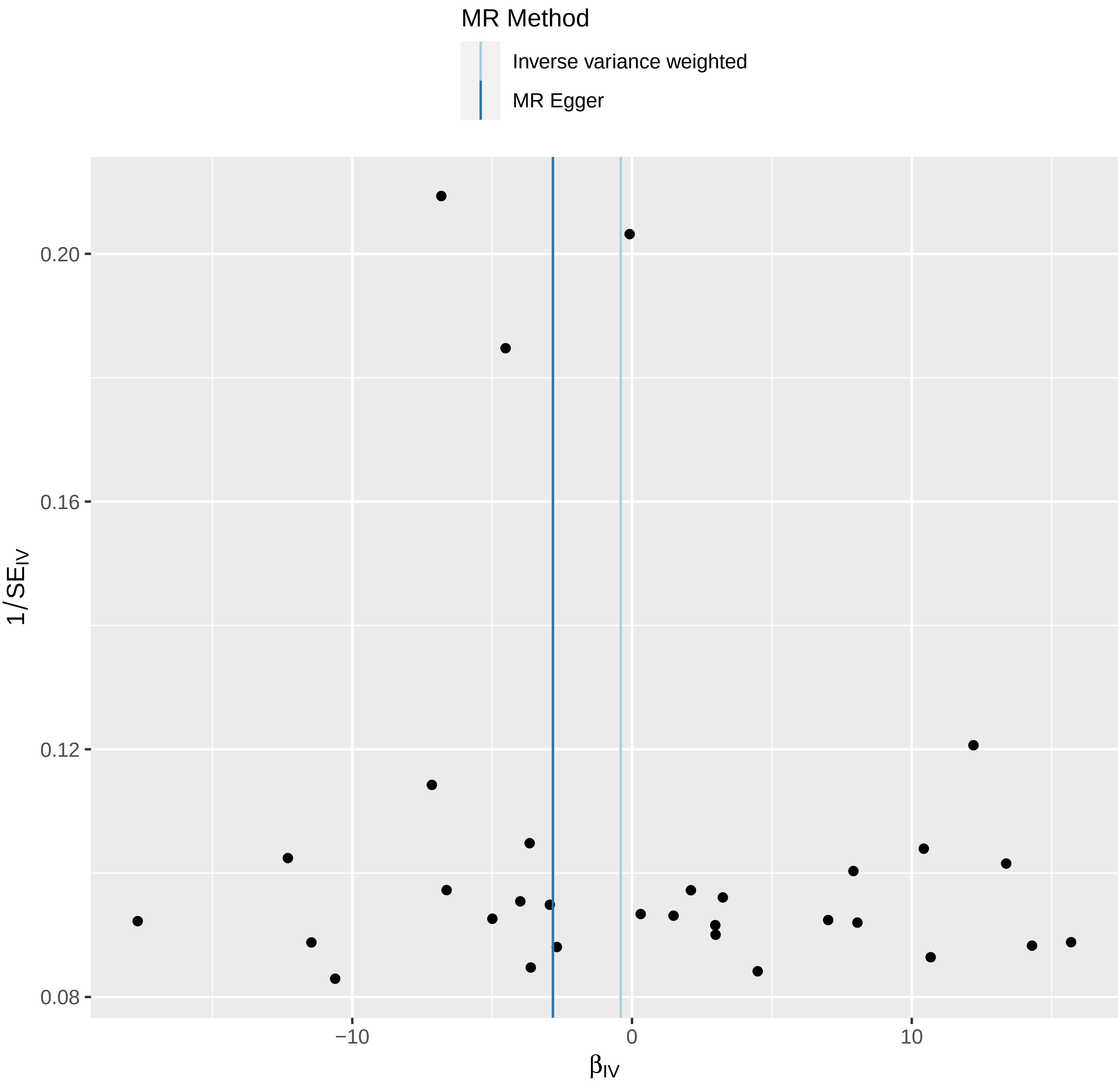

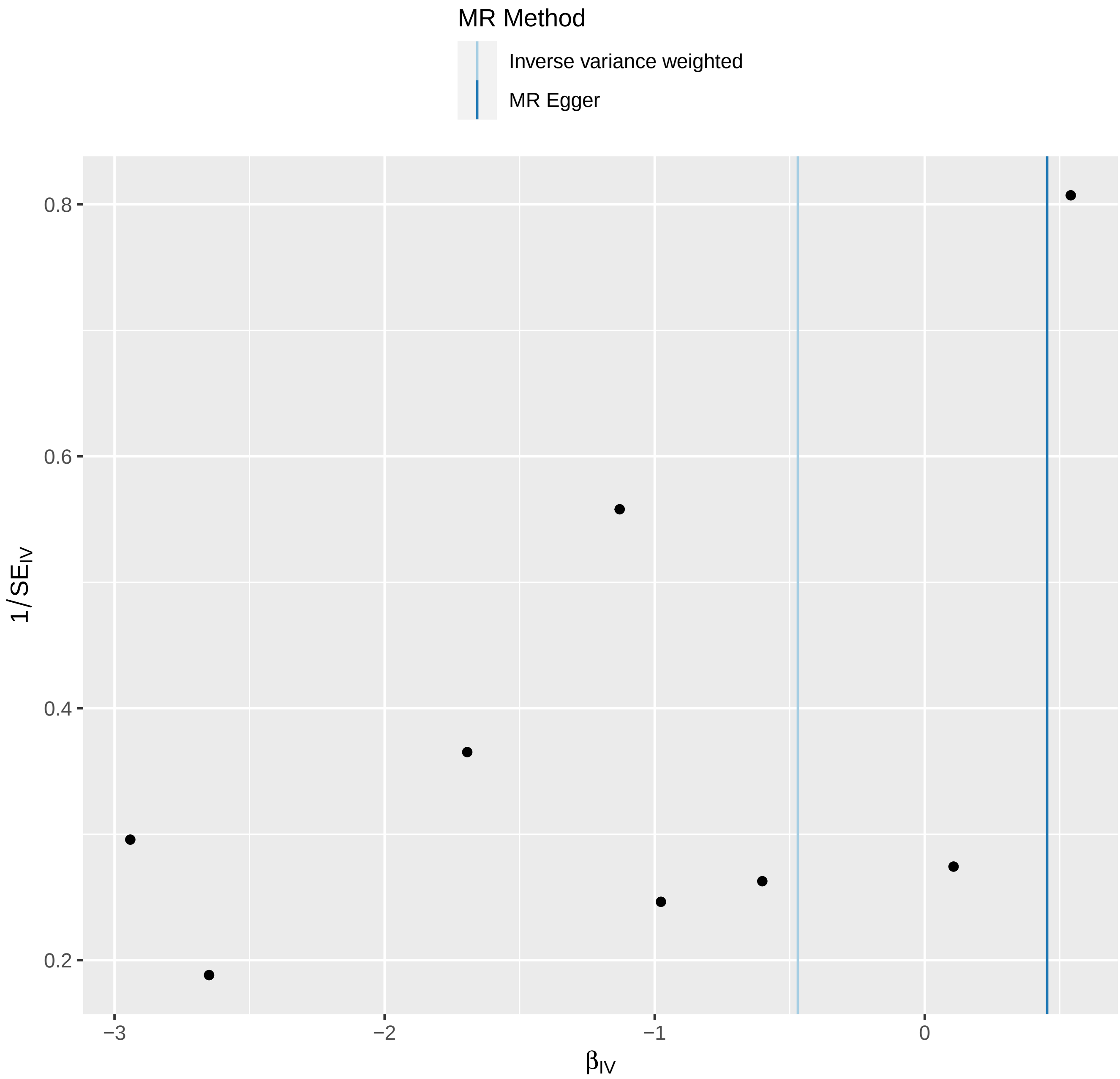

MR Method
Inverse variance weighted
MR Egger
1/SE$_{IV}$
0.8
0.6
0.4
0.2
−3
−2
−1
0
β_{IV}

Batch 917 : Glycodeoxycholate on Arthropathy in Crohn disease

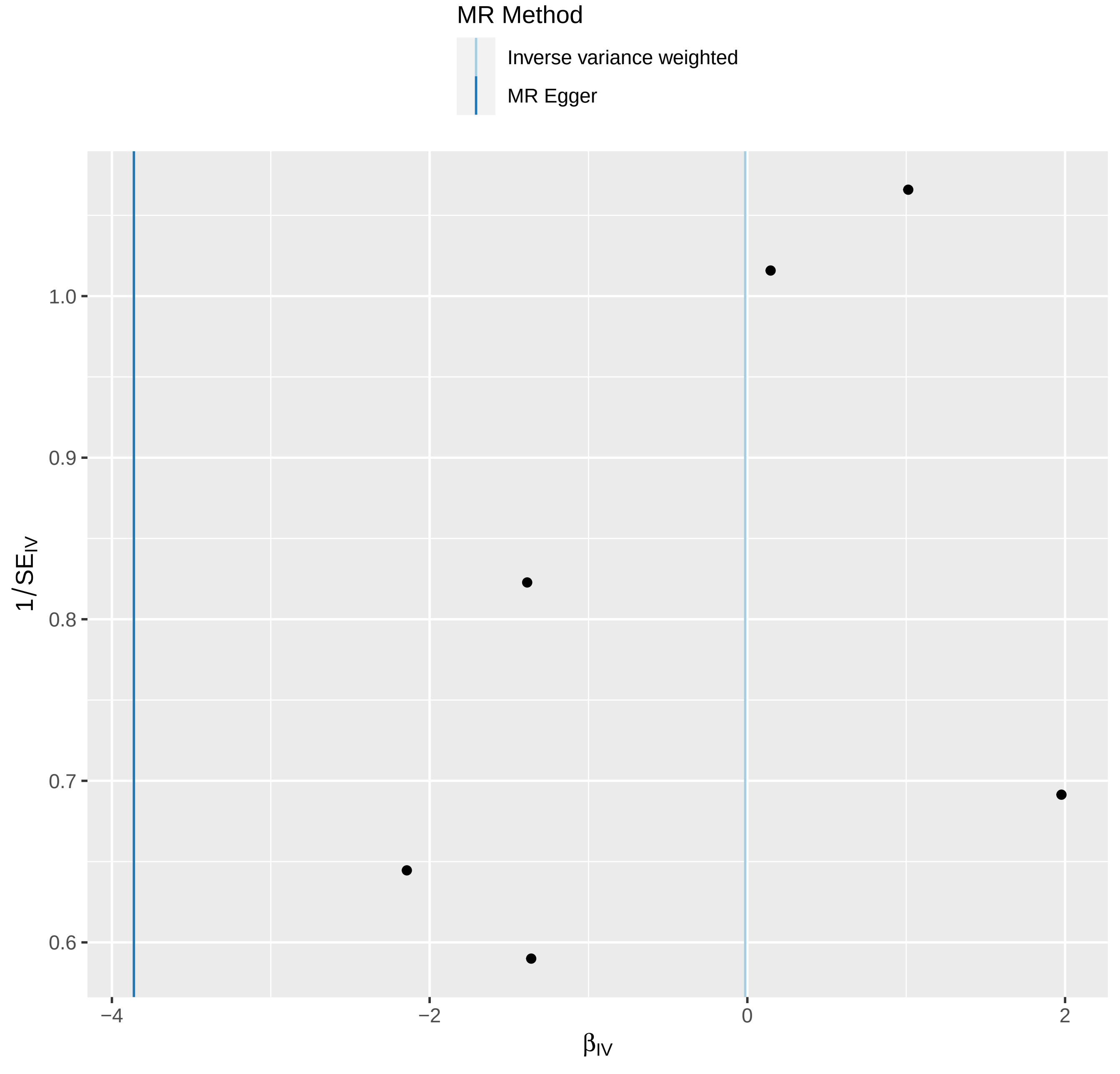

Batch 918 : Taurochenodeoxycholate on Arthropathy in Crohn disease

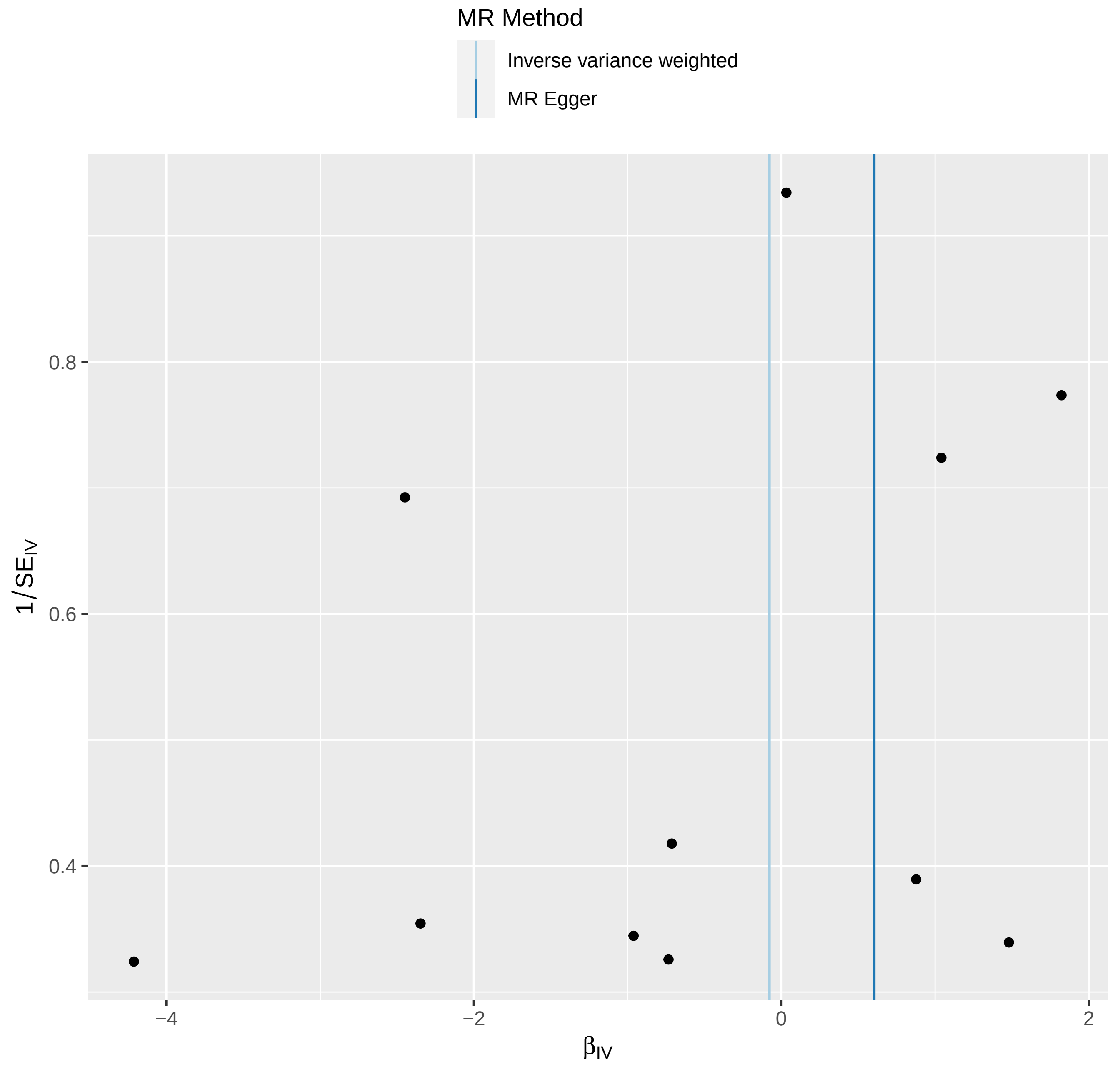

Batch 919 : Taurocholate on Arthropathy in Crohn disease

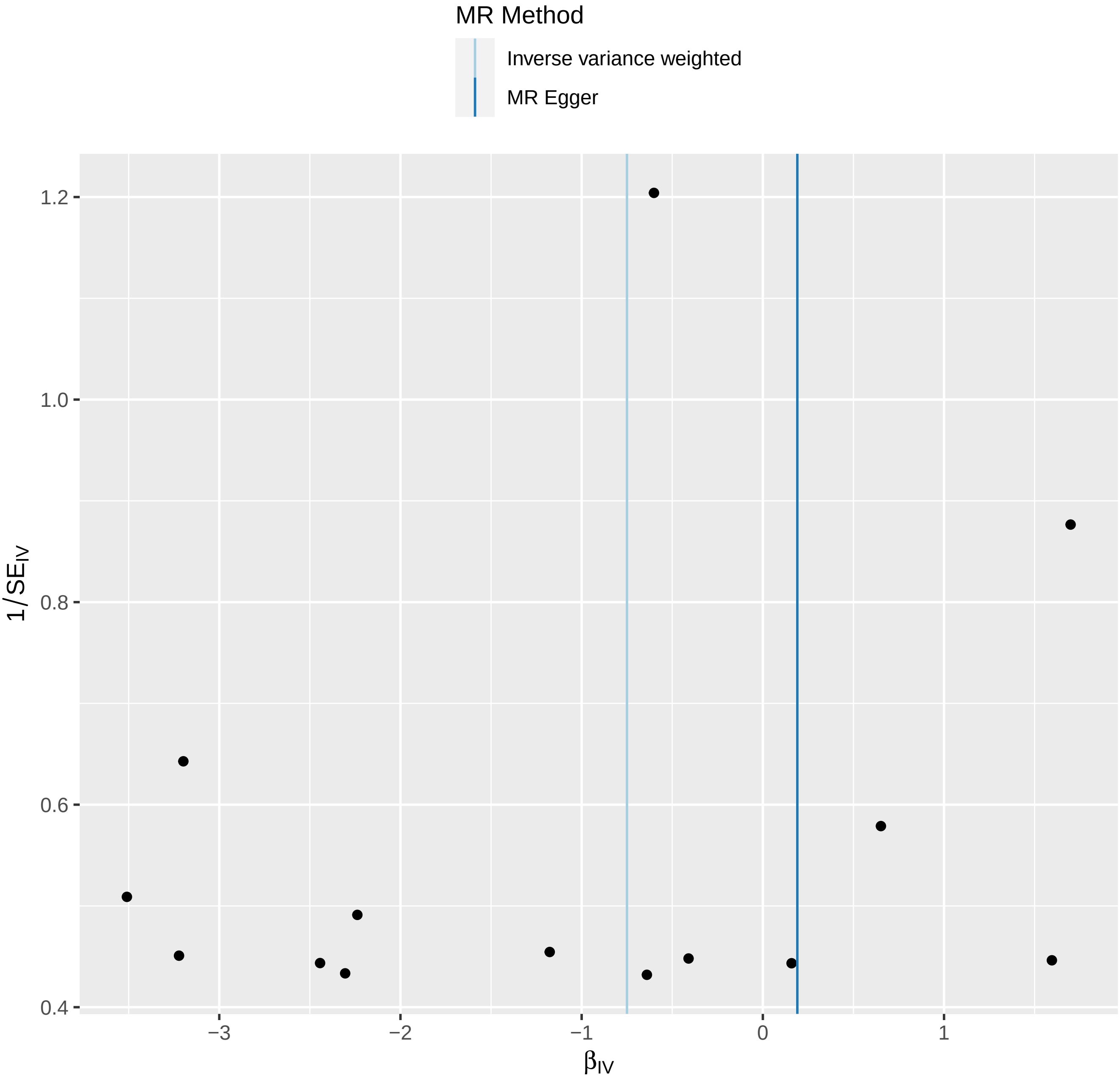

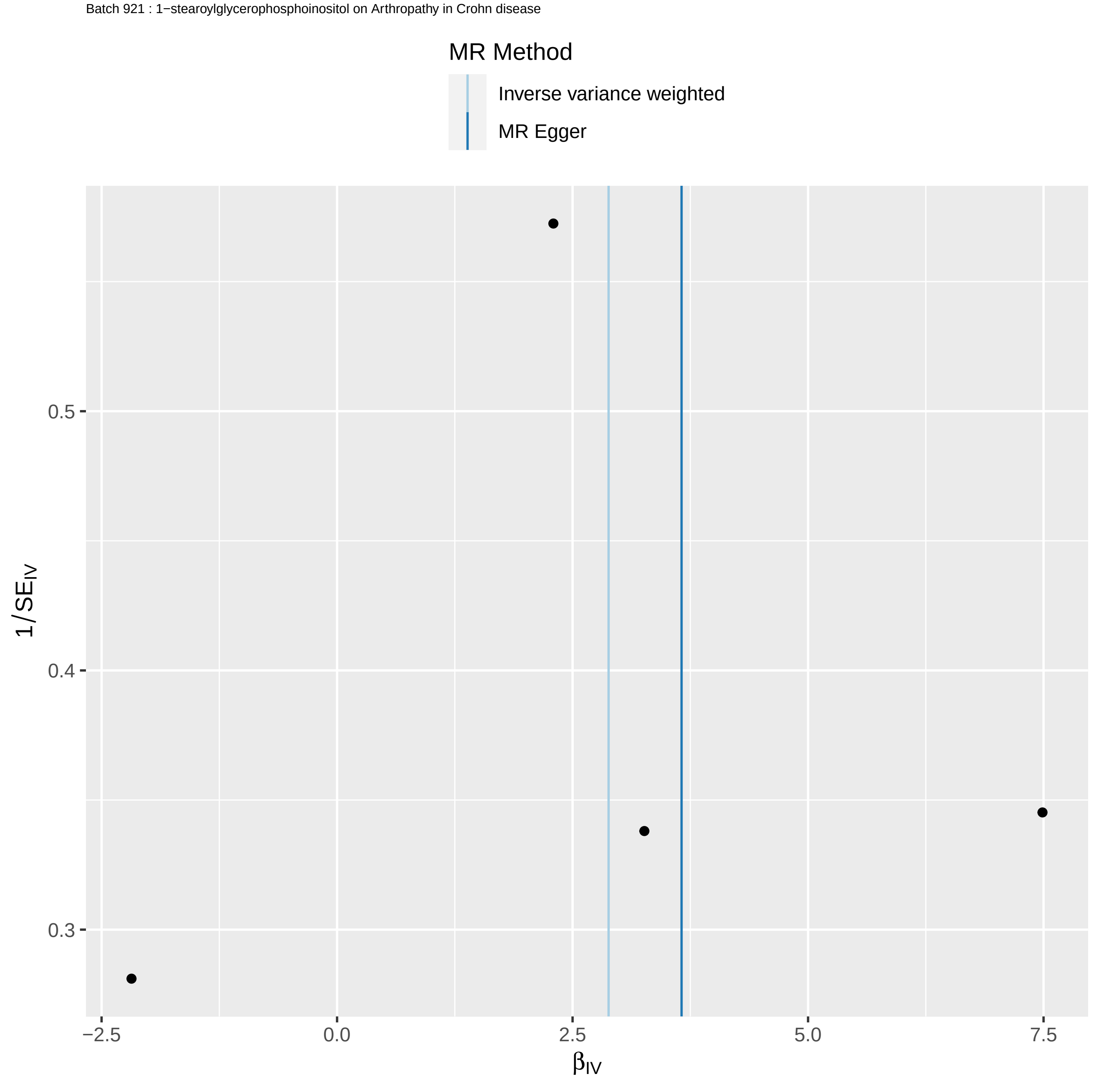

Batch 921 : 1−stearoylglycerophosphoinositol on Arthropathy in Crohn disease
MR Method
Inverse variance weighted
MR Egger
0.5
0.4
0.3
1/SE_IV
−2.5
0.0
2.5
5.0
7.5
β_IV

Batch 922 : Myo−inositol on Arthropathy in Crohn disease

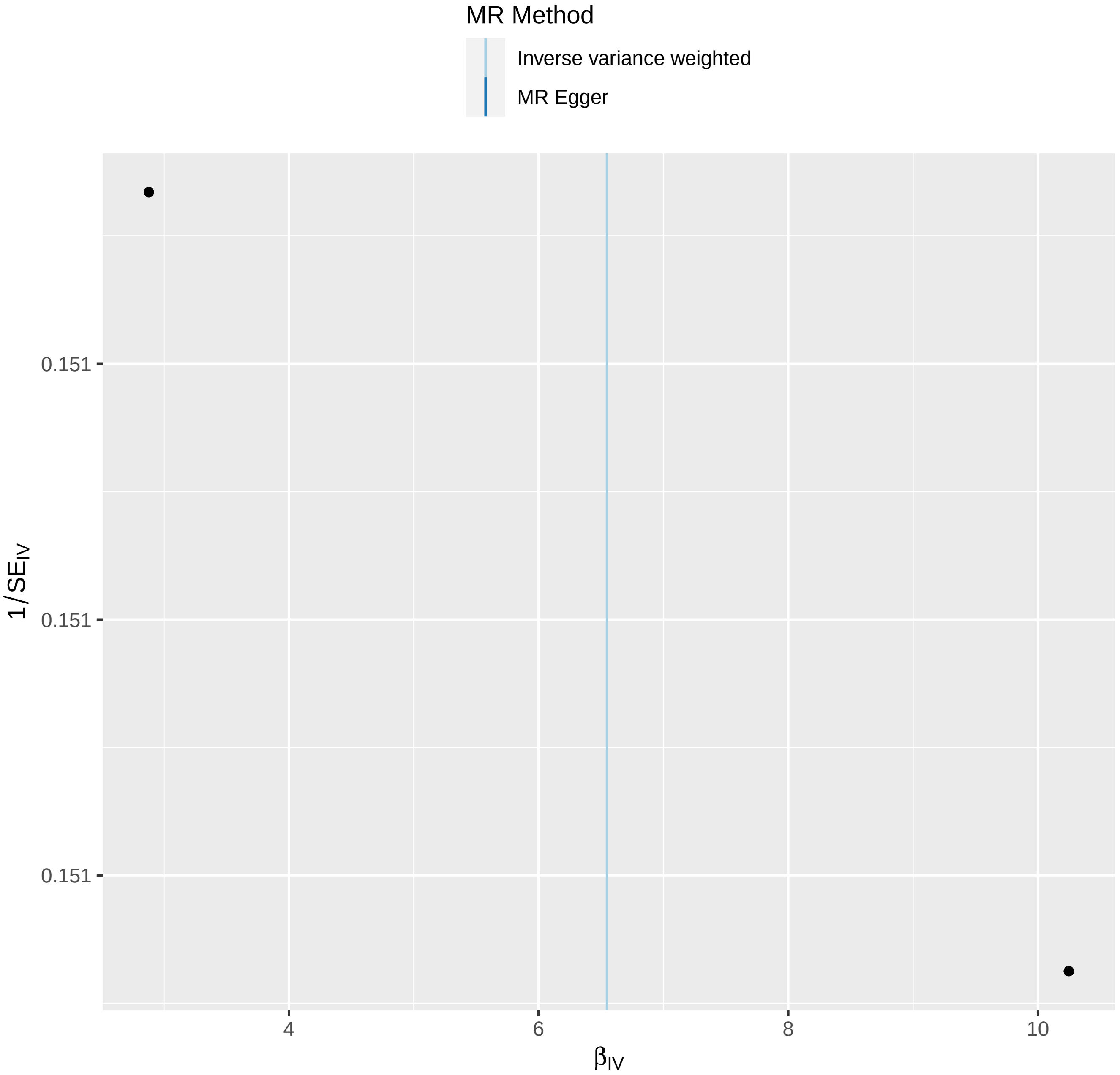

Batch 924 : 1,5−anhydroglucitol (1,5−AG) on Arthropathy in Crohn disease

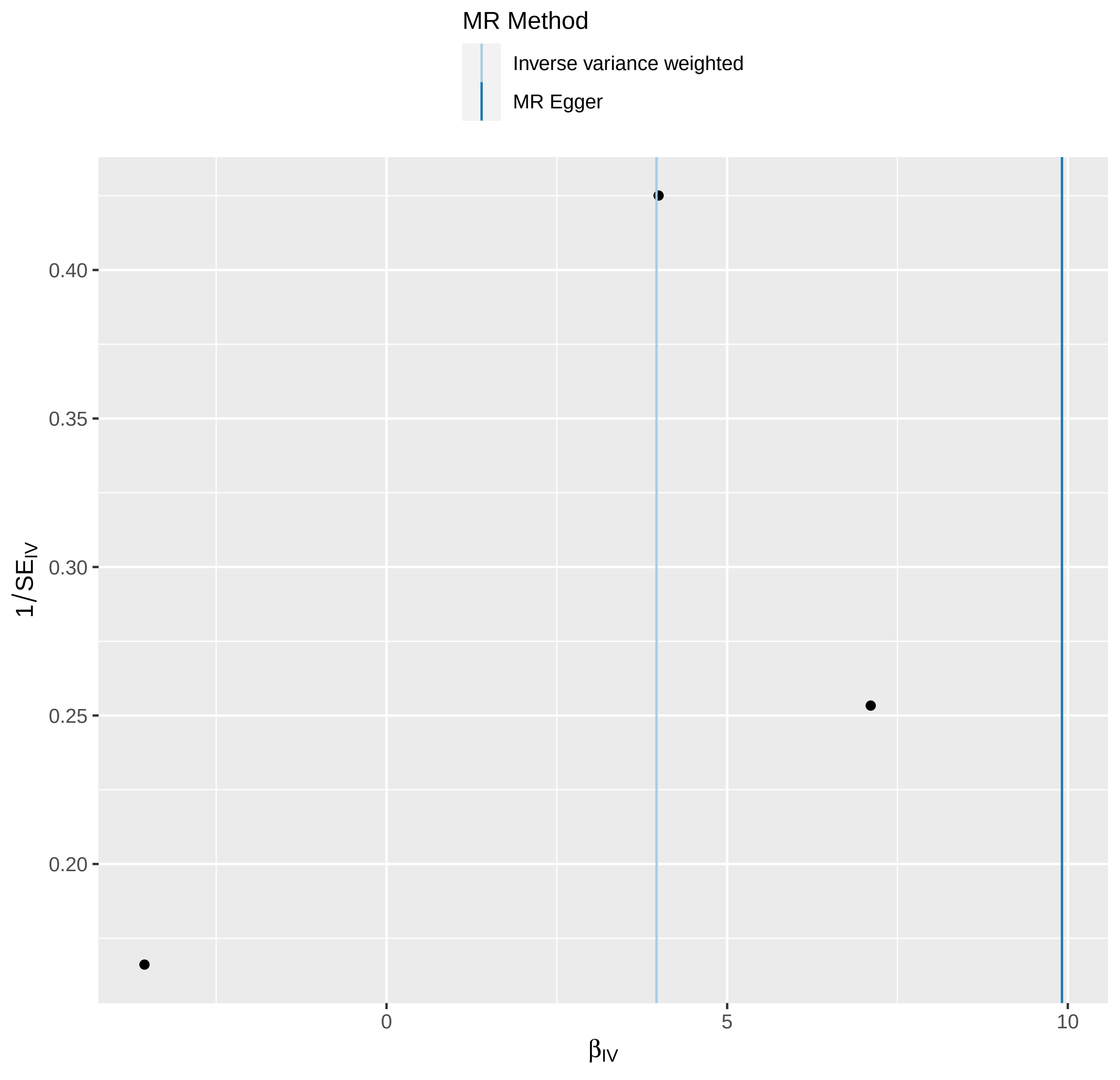

Batch 925 : 2−hydroxybutyrate (AHB) on Arthropathy in Crohn disease

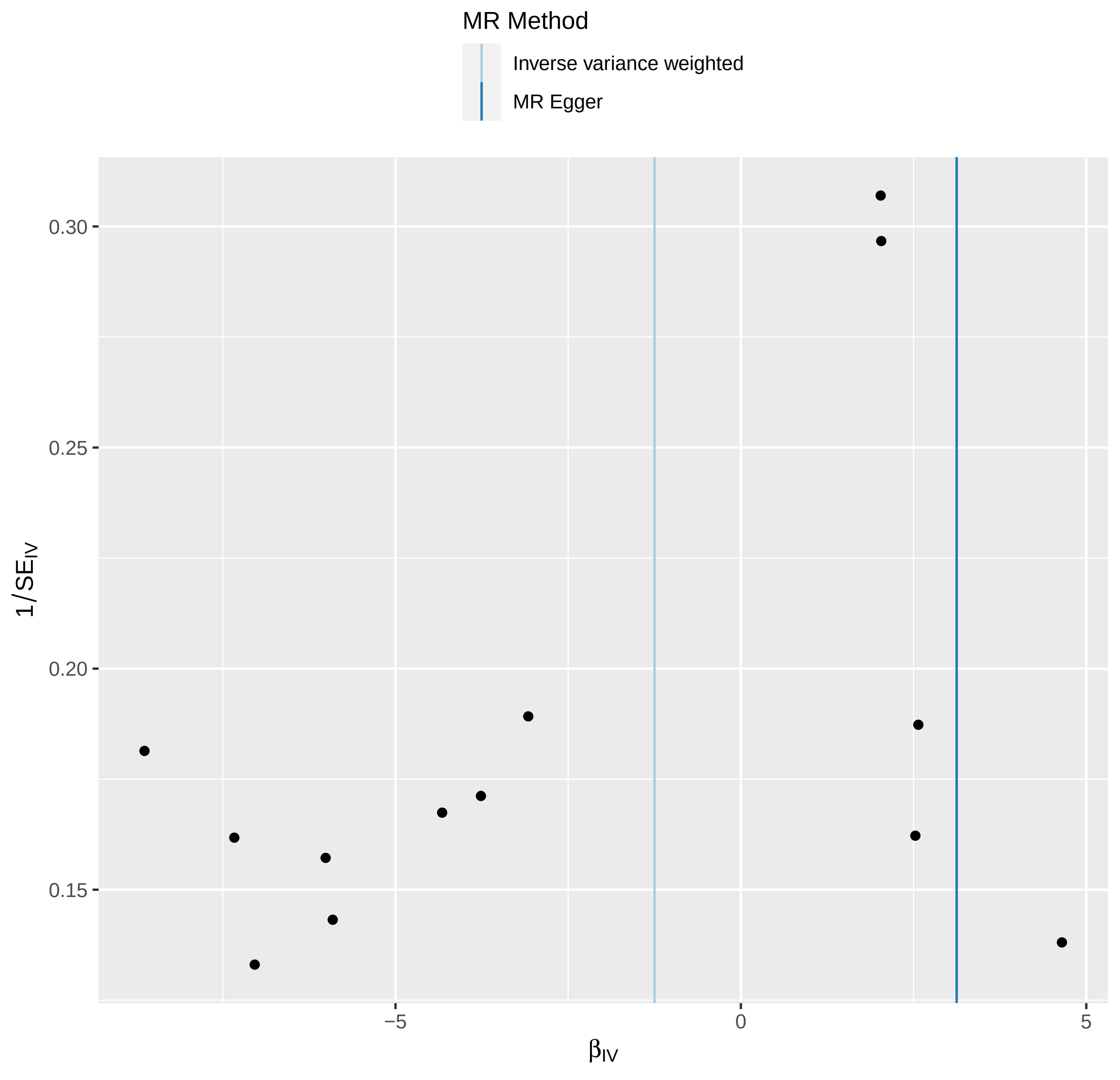

Batch 928 : 1−palmitoylglycerol (1−monopalmitin) on Arthropathy in Crohn disease

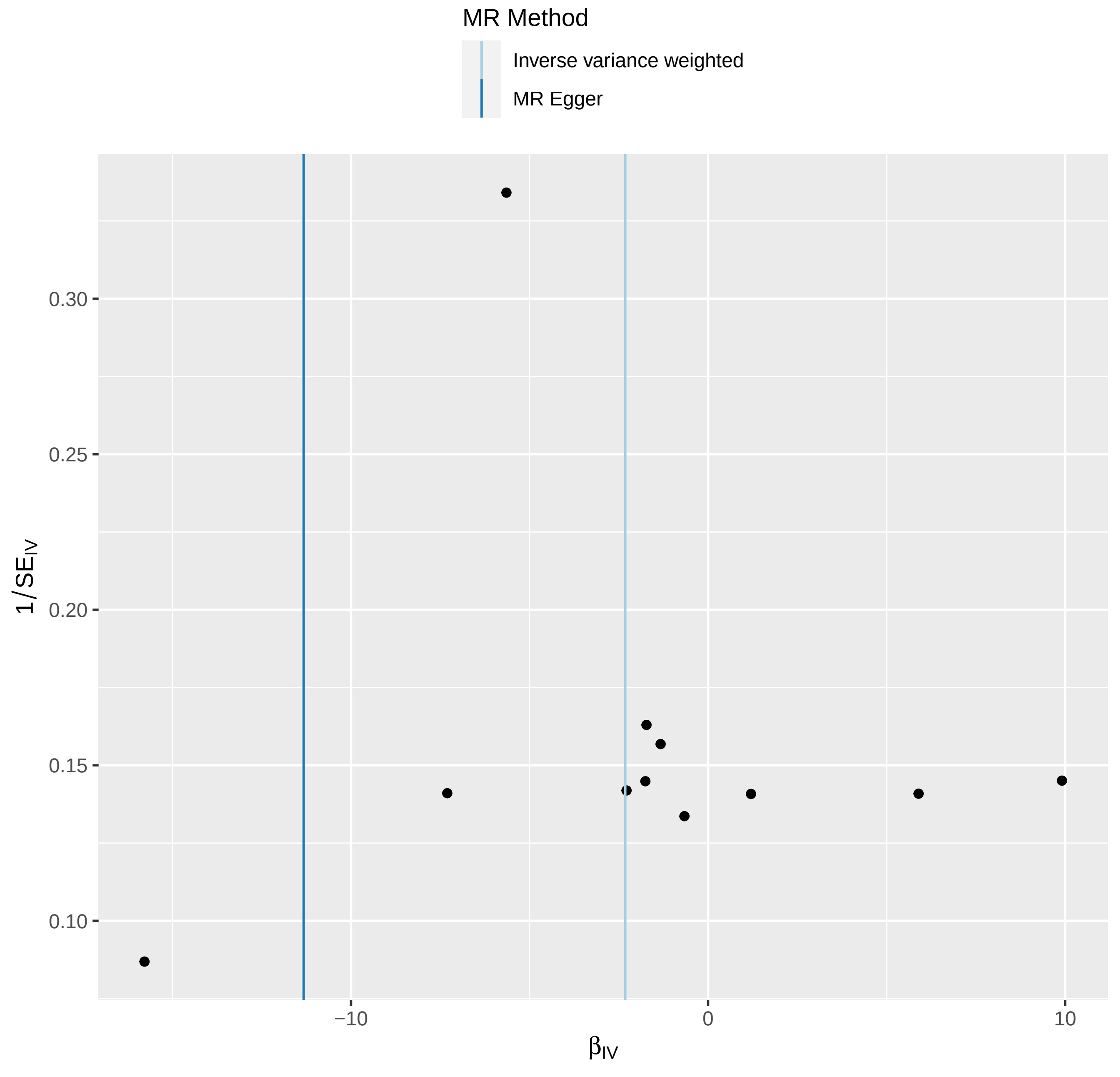

Batch 931 : 2−hydroxyisobutyrate on Arthropathy in Crohn disease

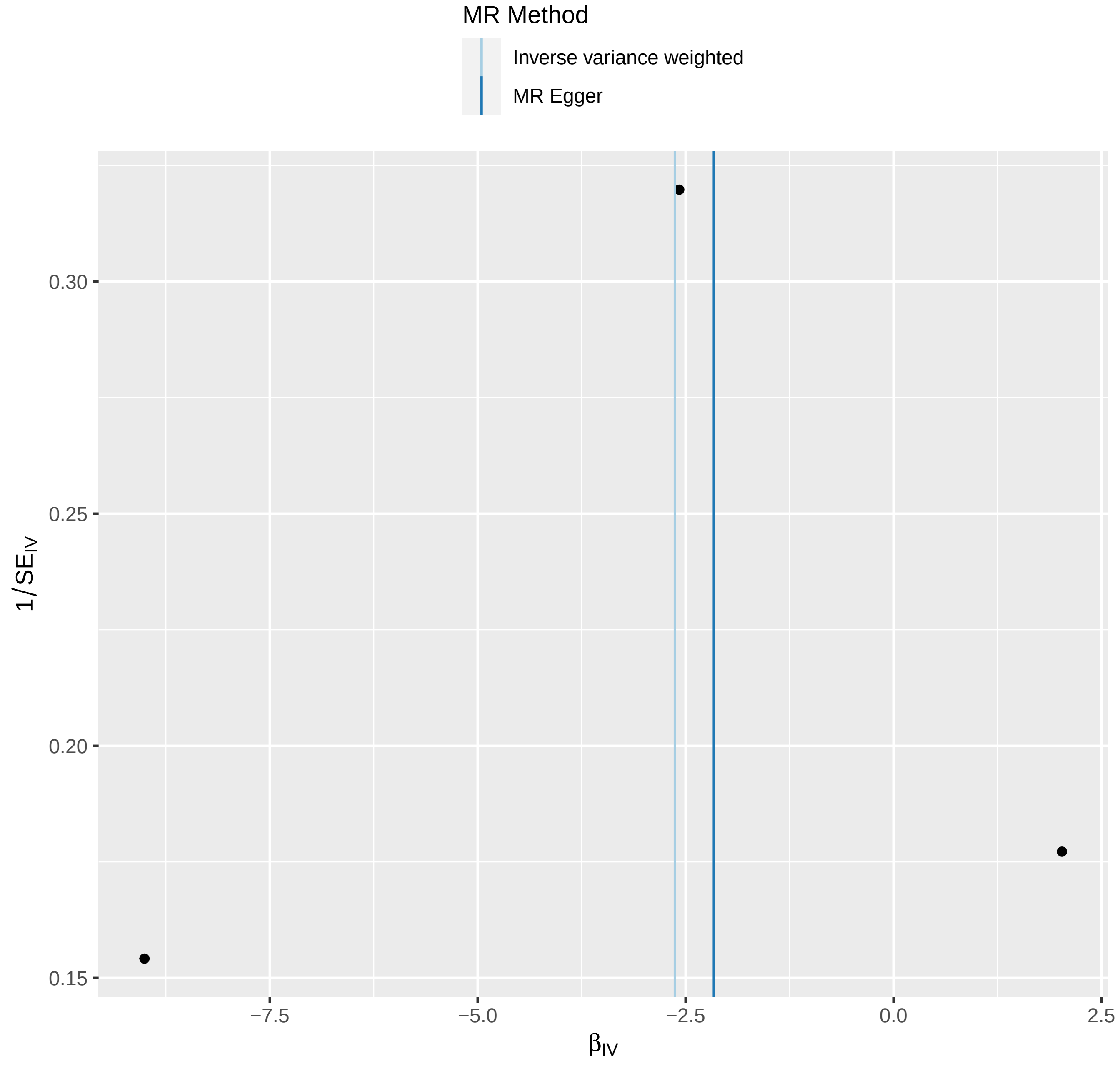

Batch 932 : 4−methyl−2−oxopentanoate on Arthropathy in Crohn disease

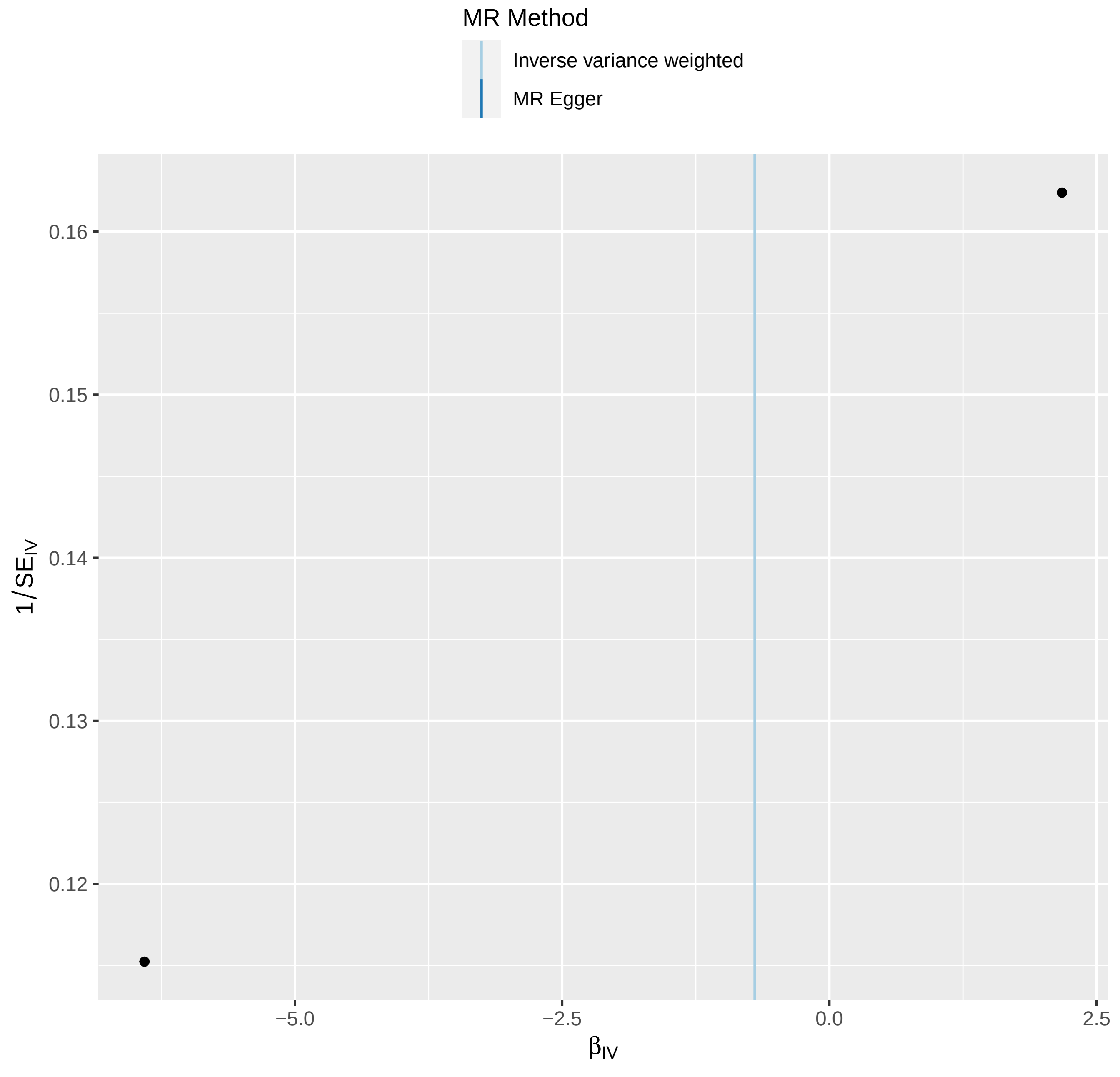

Batch 936 : Levulinate (4−oxovalerate) on Arthropathy in Crohn disease

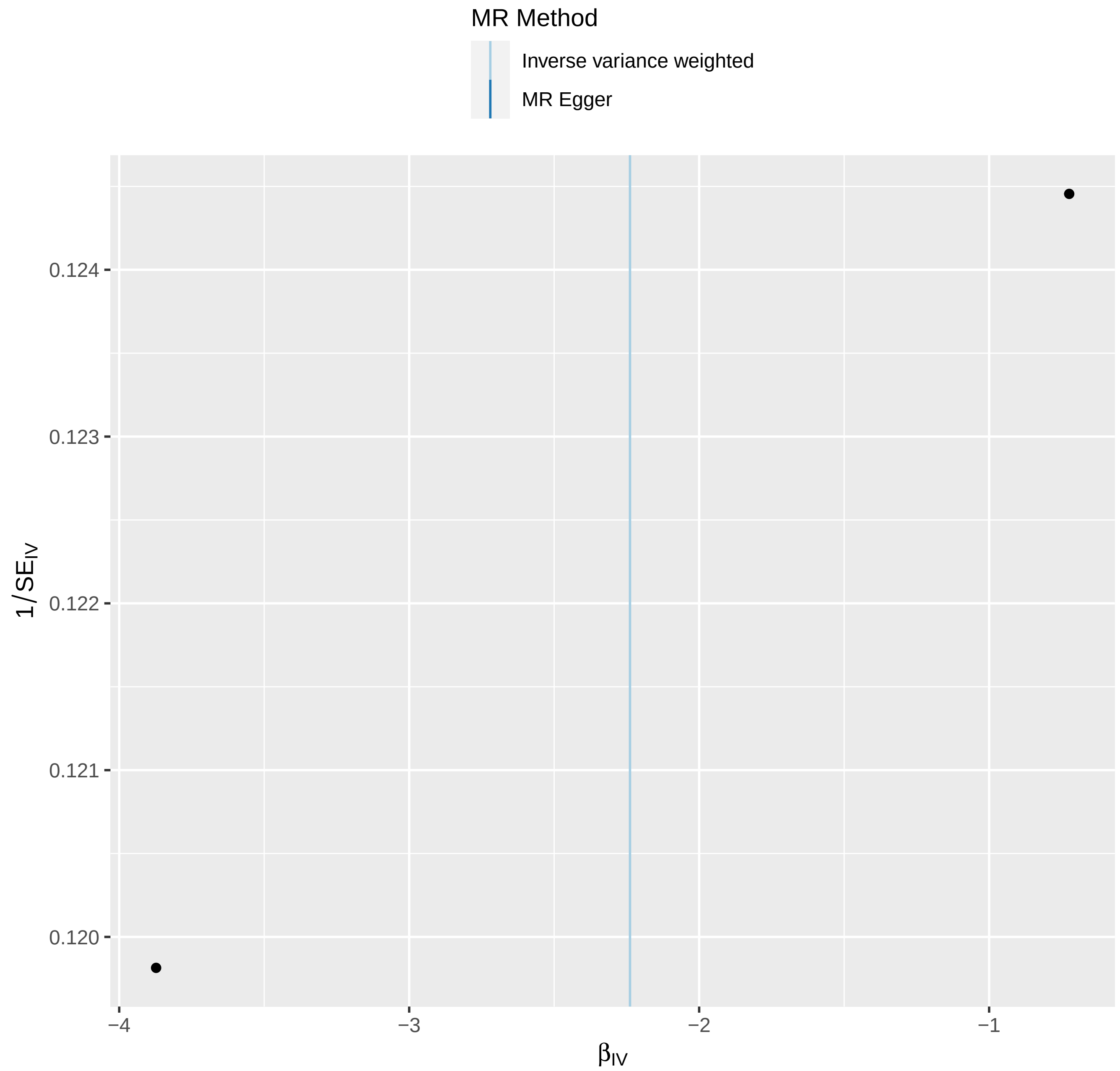

Batch 938 : Cholate on Arthropathy in Crohn disease

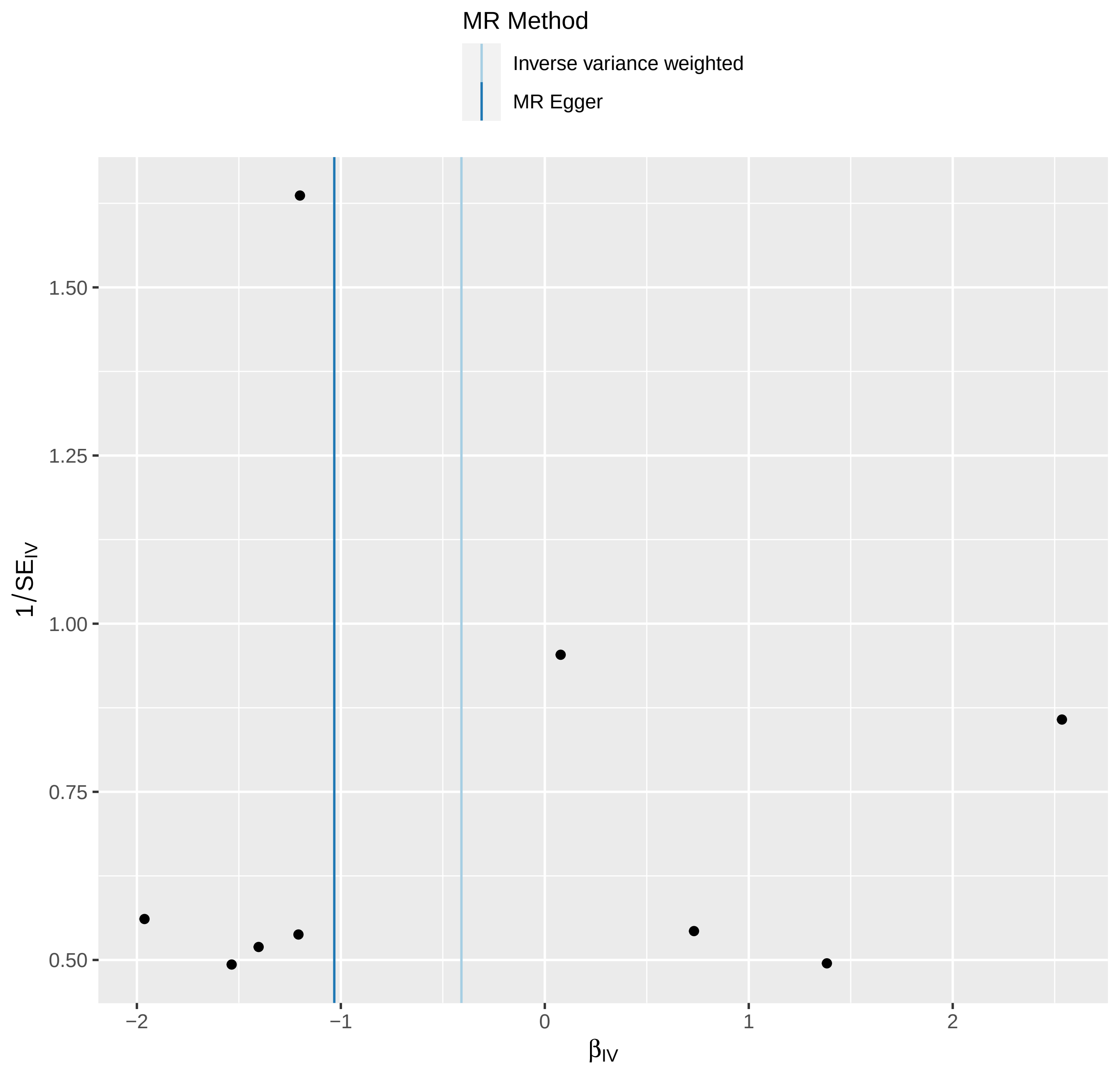

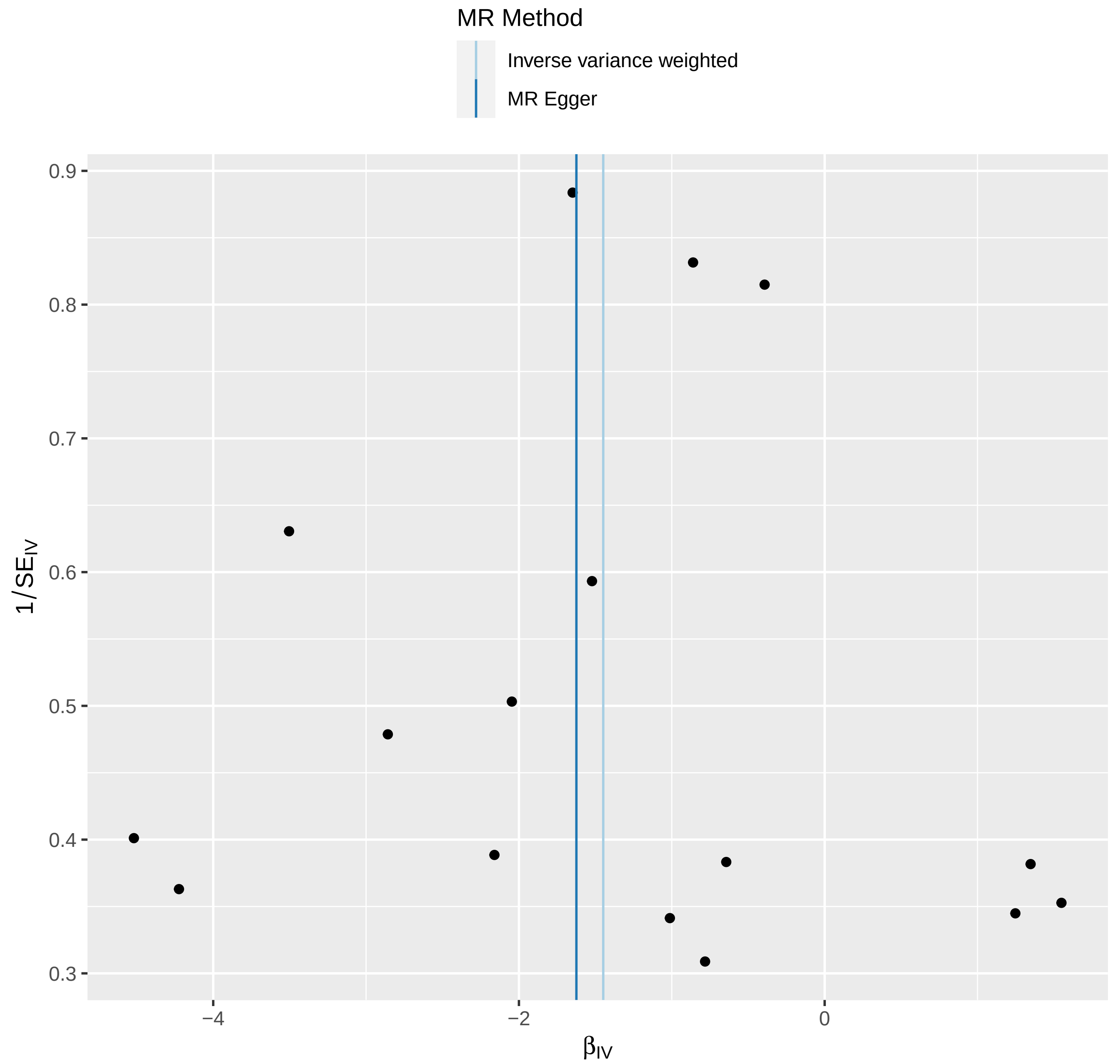
MR Method
Inverse variance weighted
MR Egger
0.9
0.8
0.7
0.6
0.5
0.4
0.3
1/SE_IV
−4
−2
0
β_IV

Batch 942 : 3−indoxyl sulfate on Arthropathy in Crohn disease

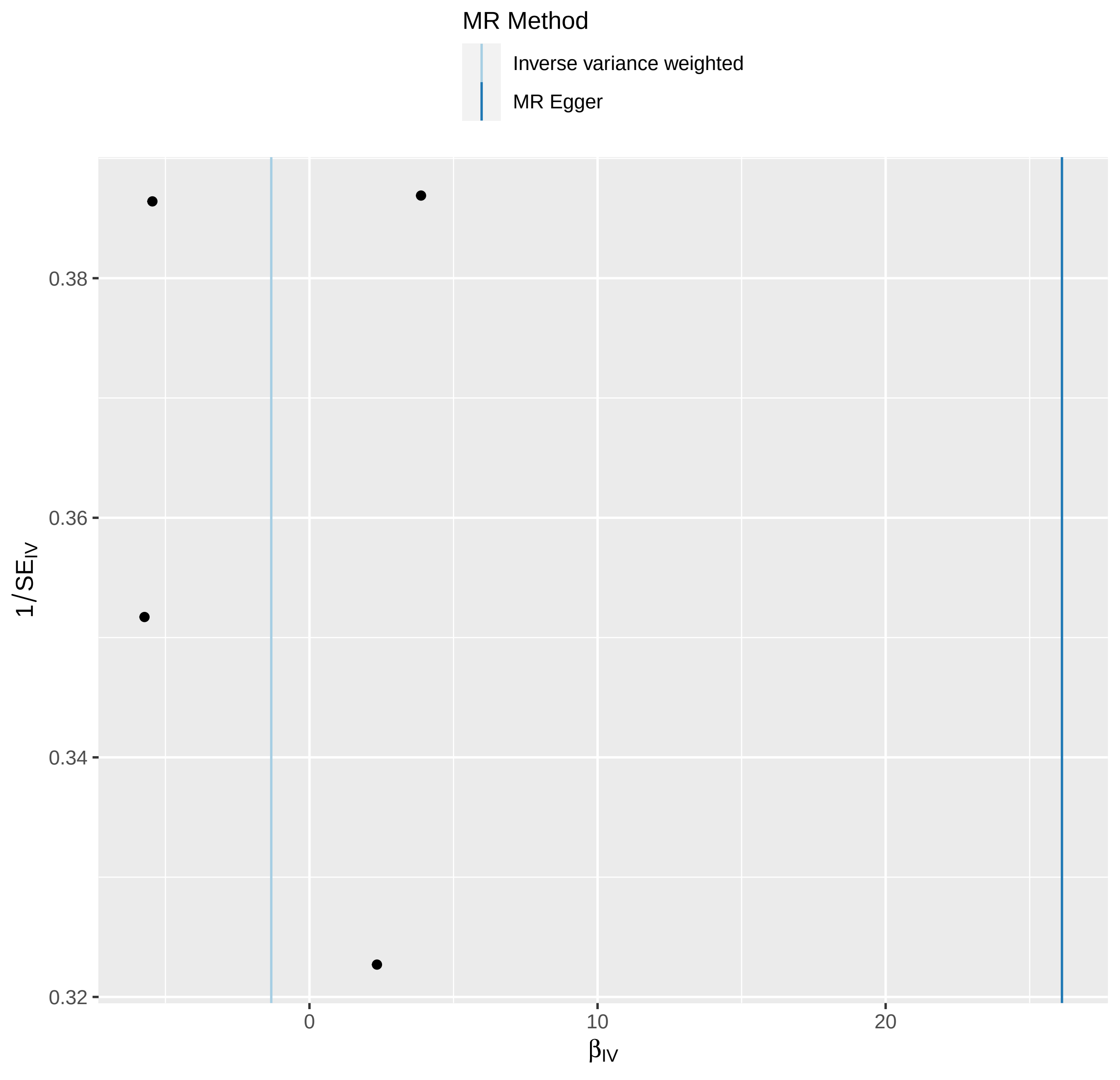

Batch 946 : Erythrose on Arthropathy in Crohn disease

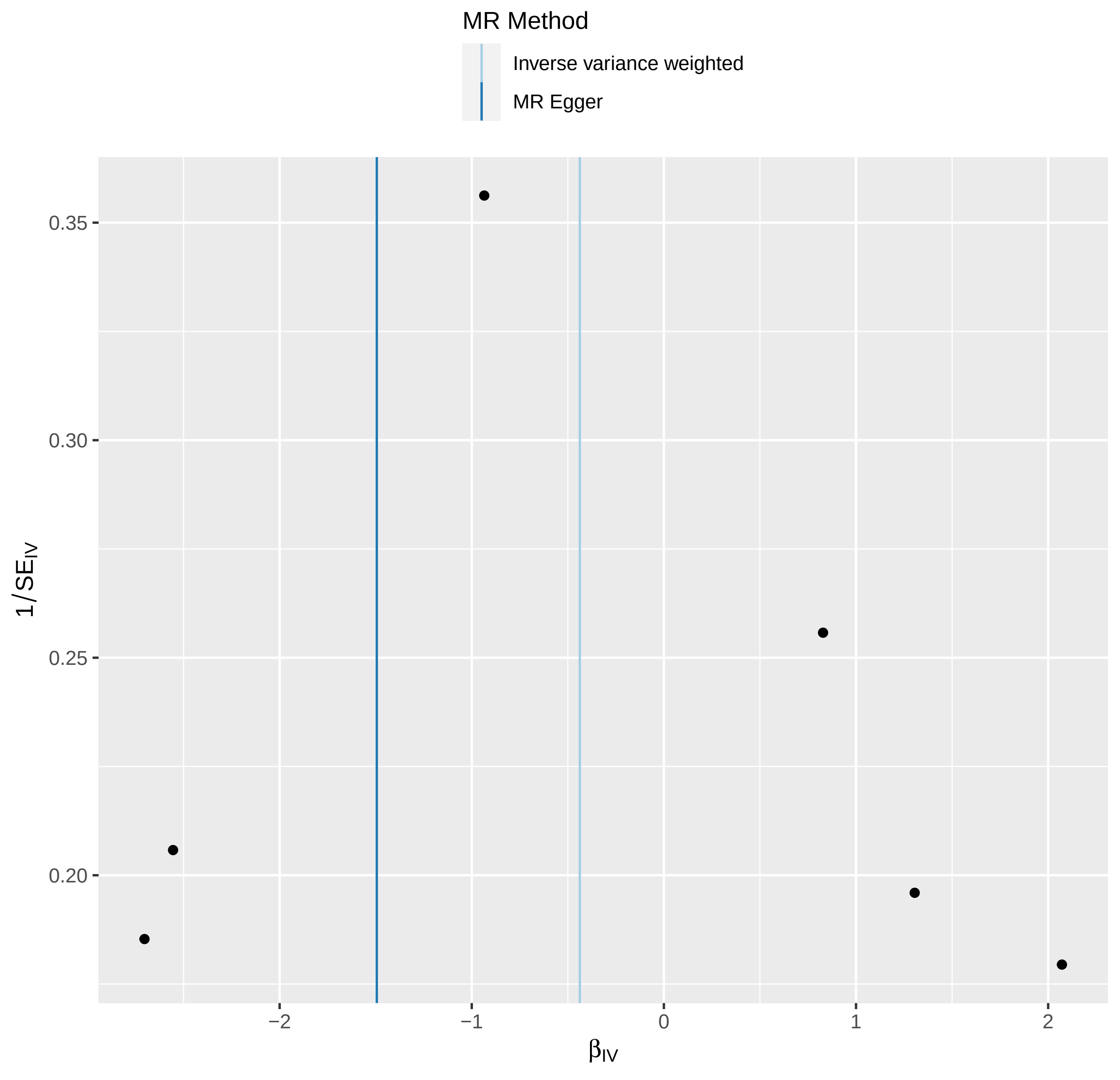

Batch 947 : Threonate on Arthropathy in Crohn disease

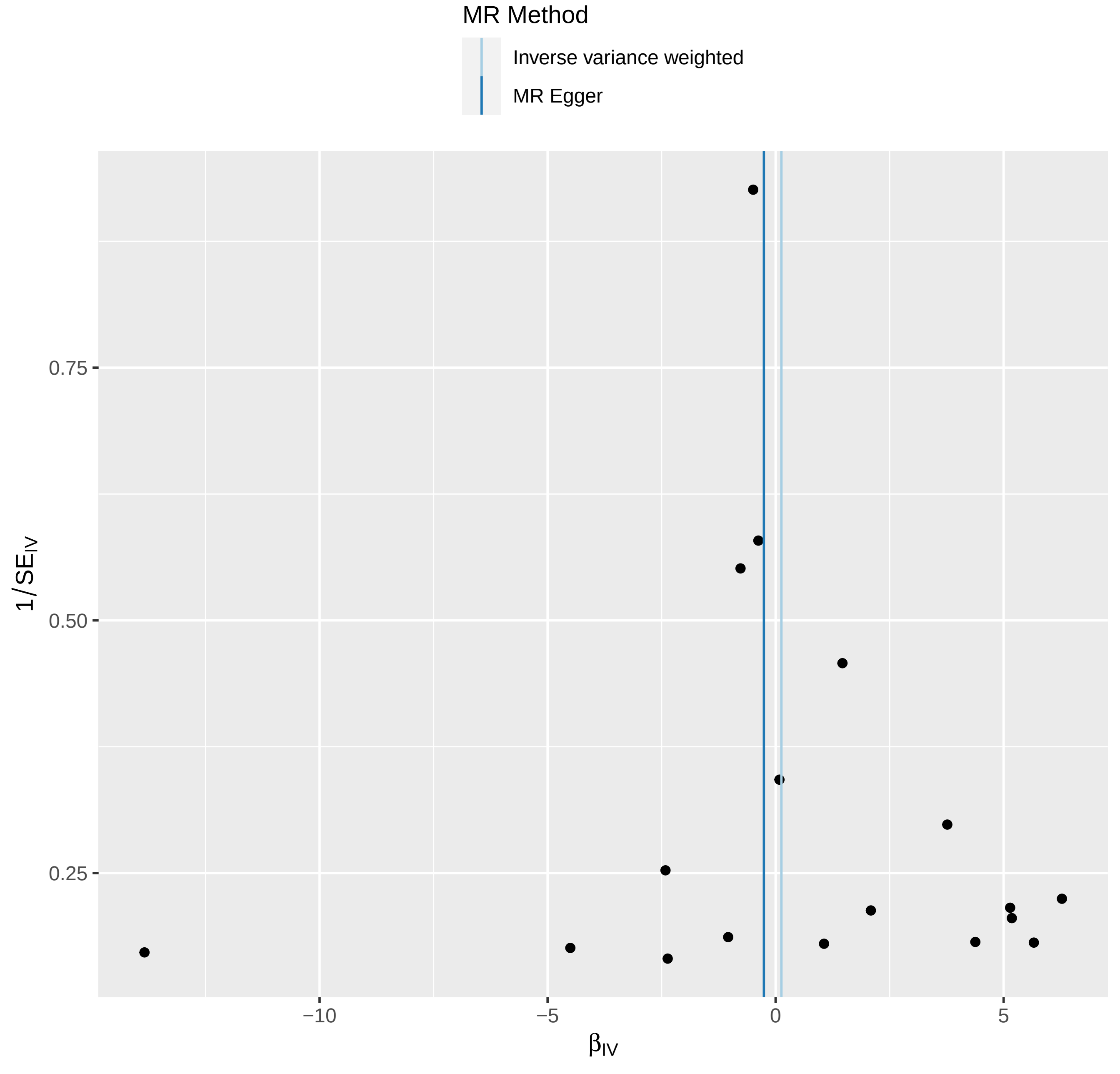

Batch 948 : Cysteine on Arthropathy in Crohn disease

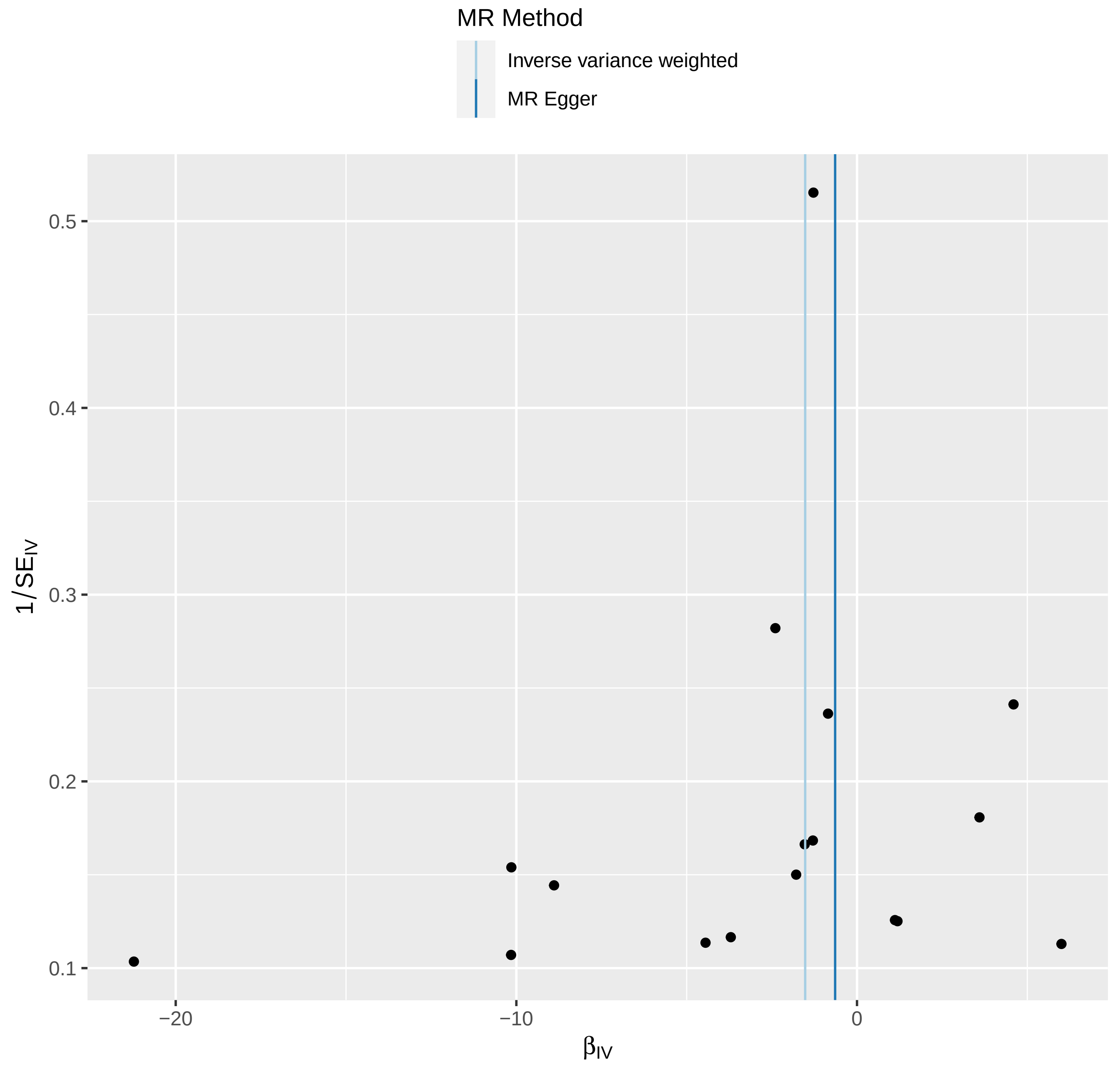

Batch 949 : Pyroglutamylglycine on Arthropathy in Crohn disease

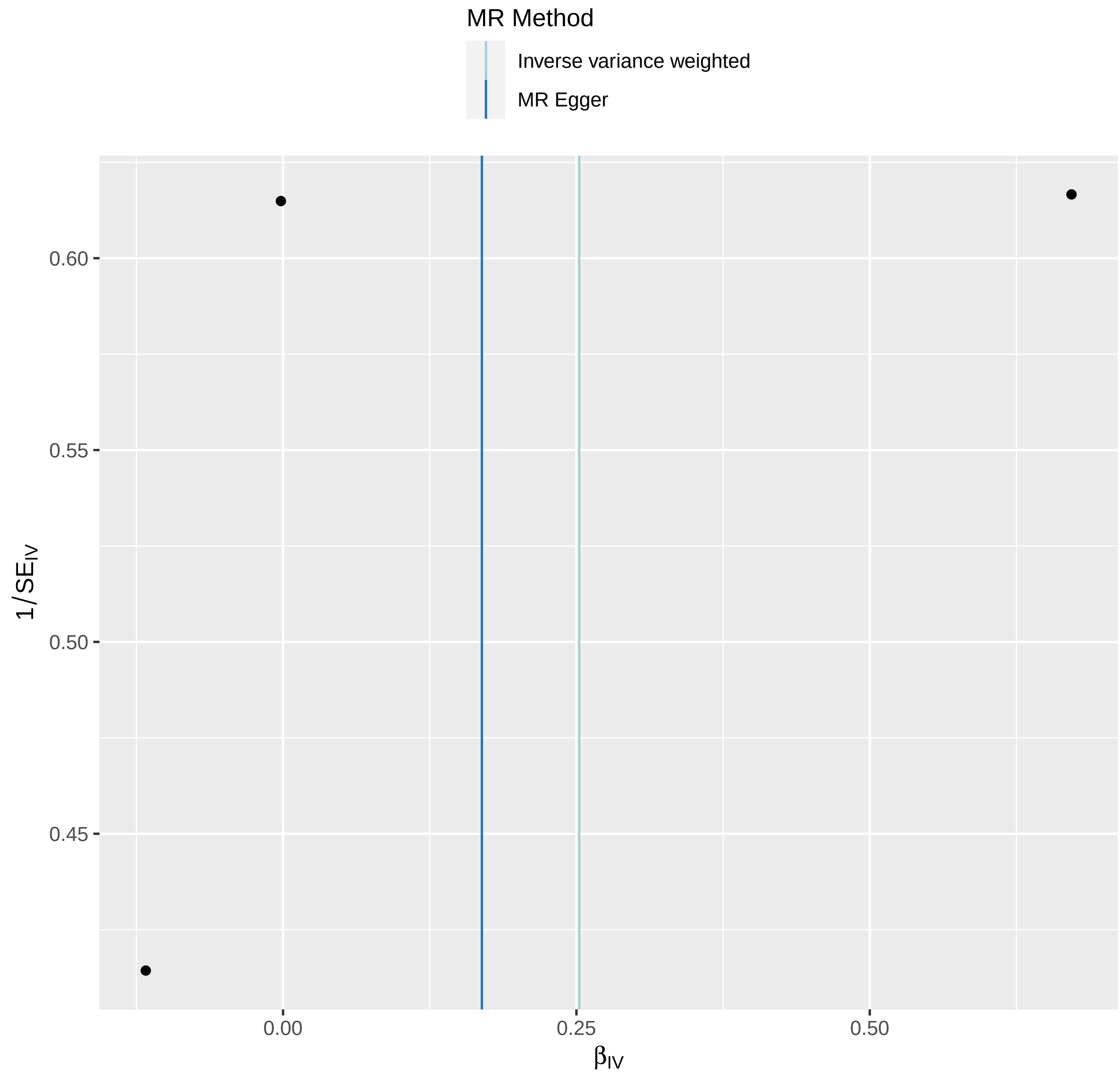

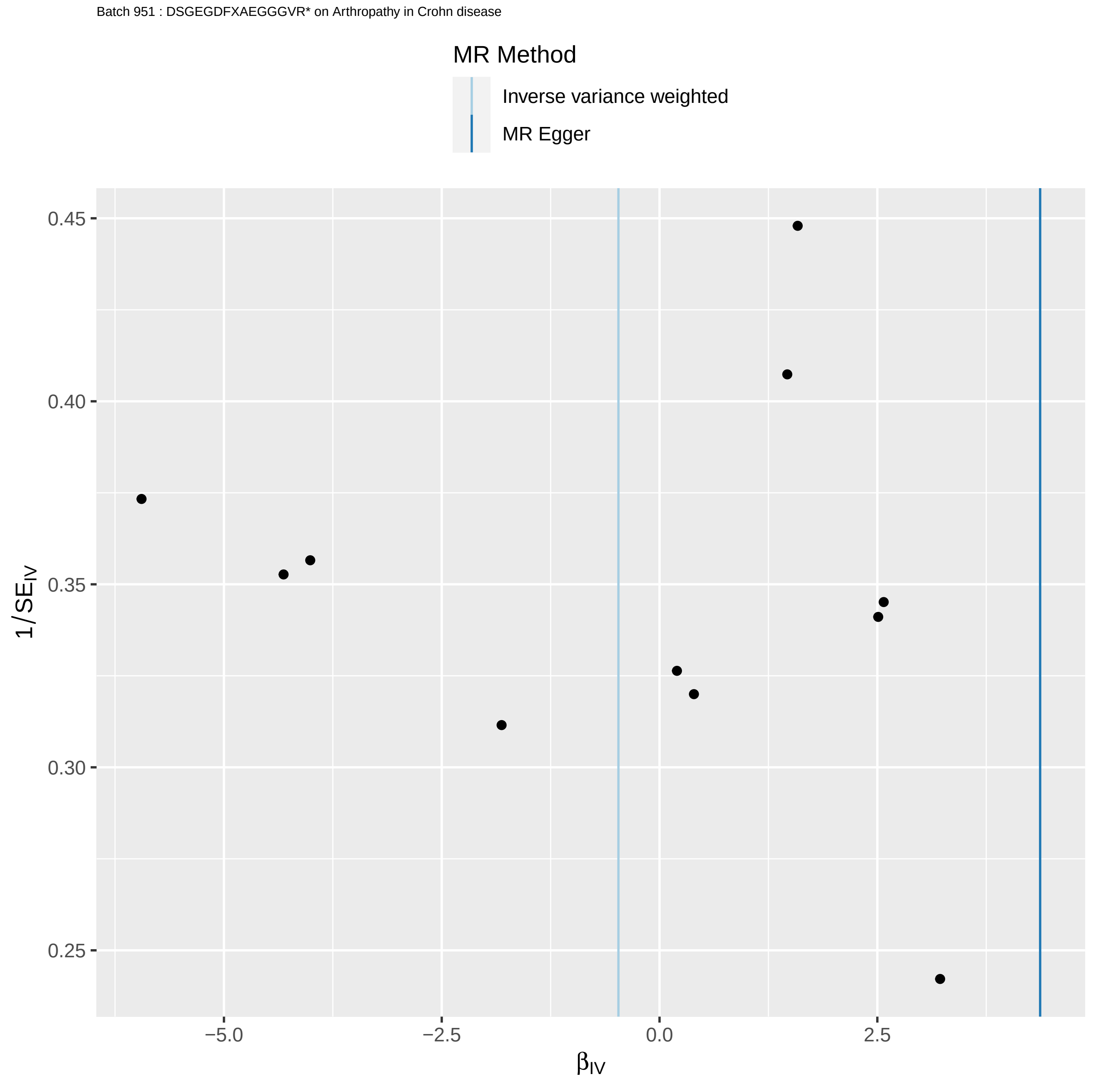

Batch 951 : DSGEGDFXAEGGGVR* on Arthropathy in Crohn disease
MR Method
Inverse variance weighted
MR Egger
0.45
0.40
0.35
0.30
0.25
1/SE_IV
−5.0
−2.5
0.0
2.5
β_IV

Batch 952 : Pyridoxate on Arthropathy in Crohn disease

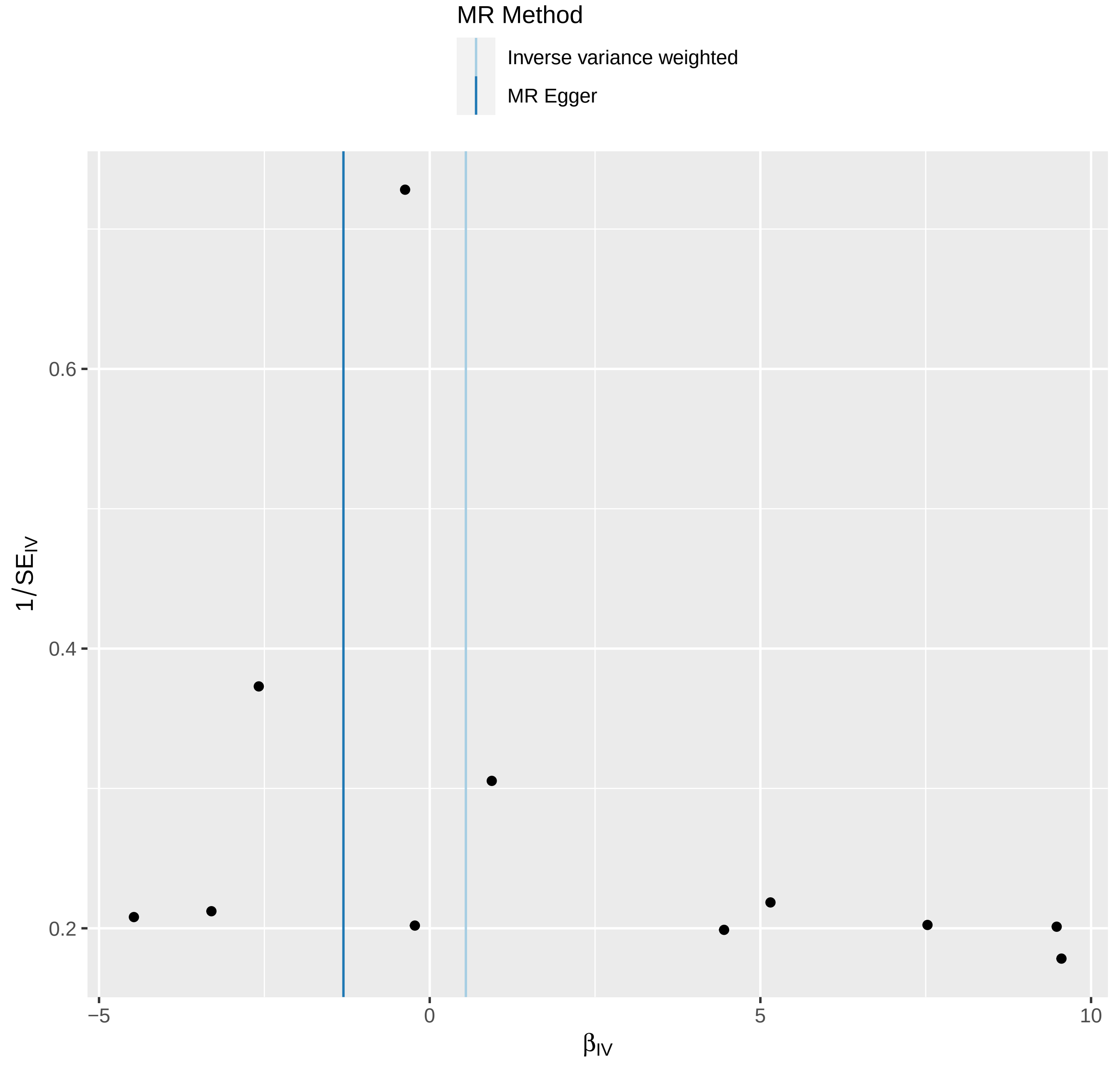

Batch 953 : Androsterone sulfate on Arthropathy in Crohn disease

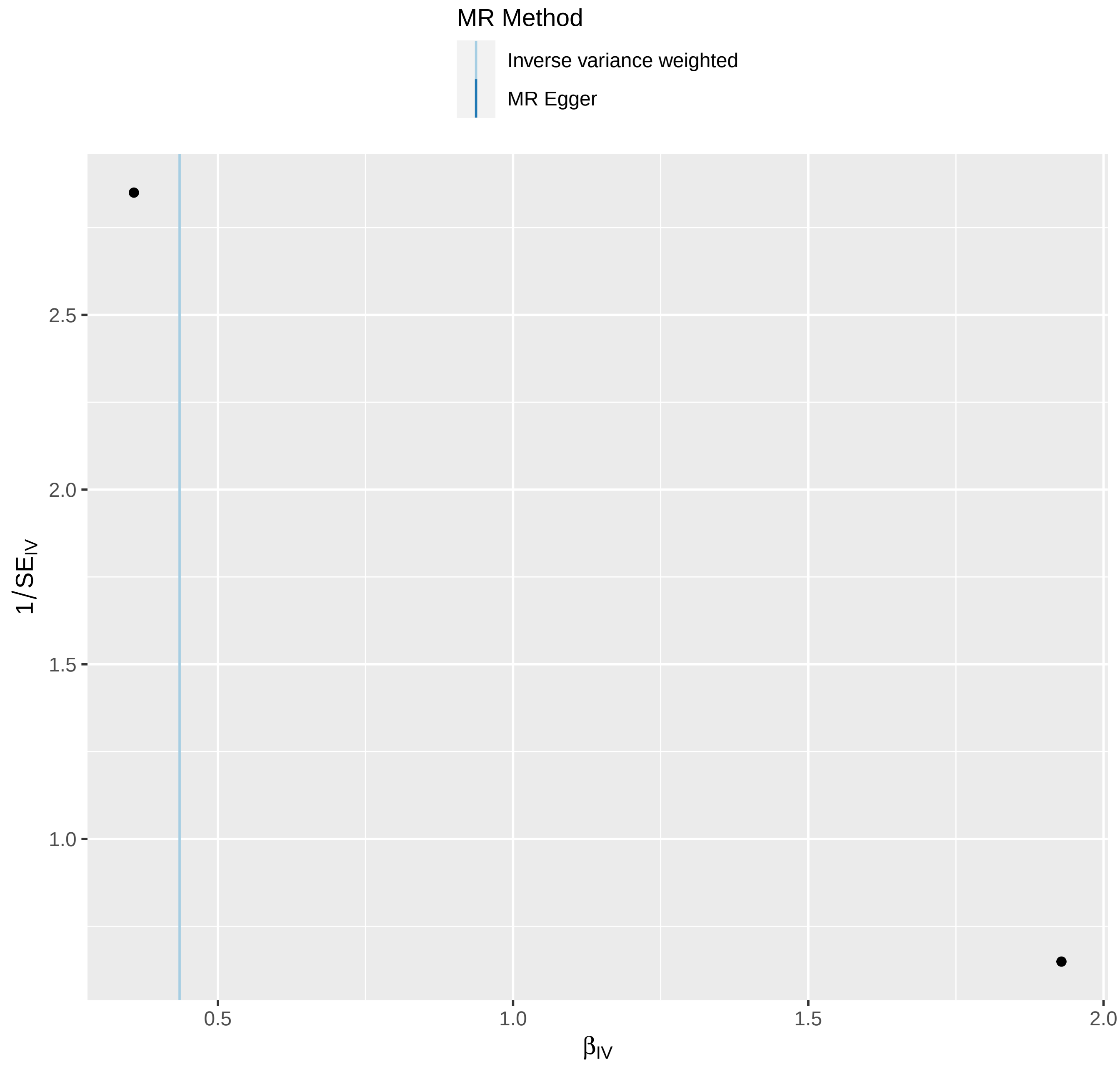

Batch 957 : Serine on Arthropathy in Crohn disease

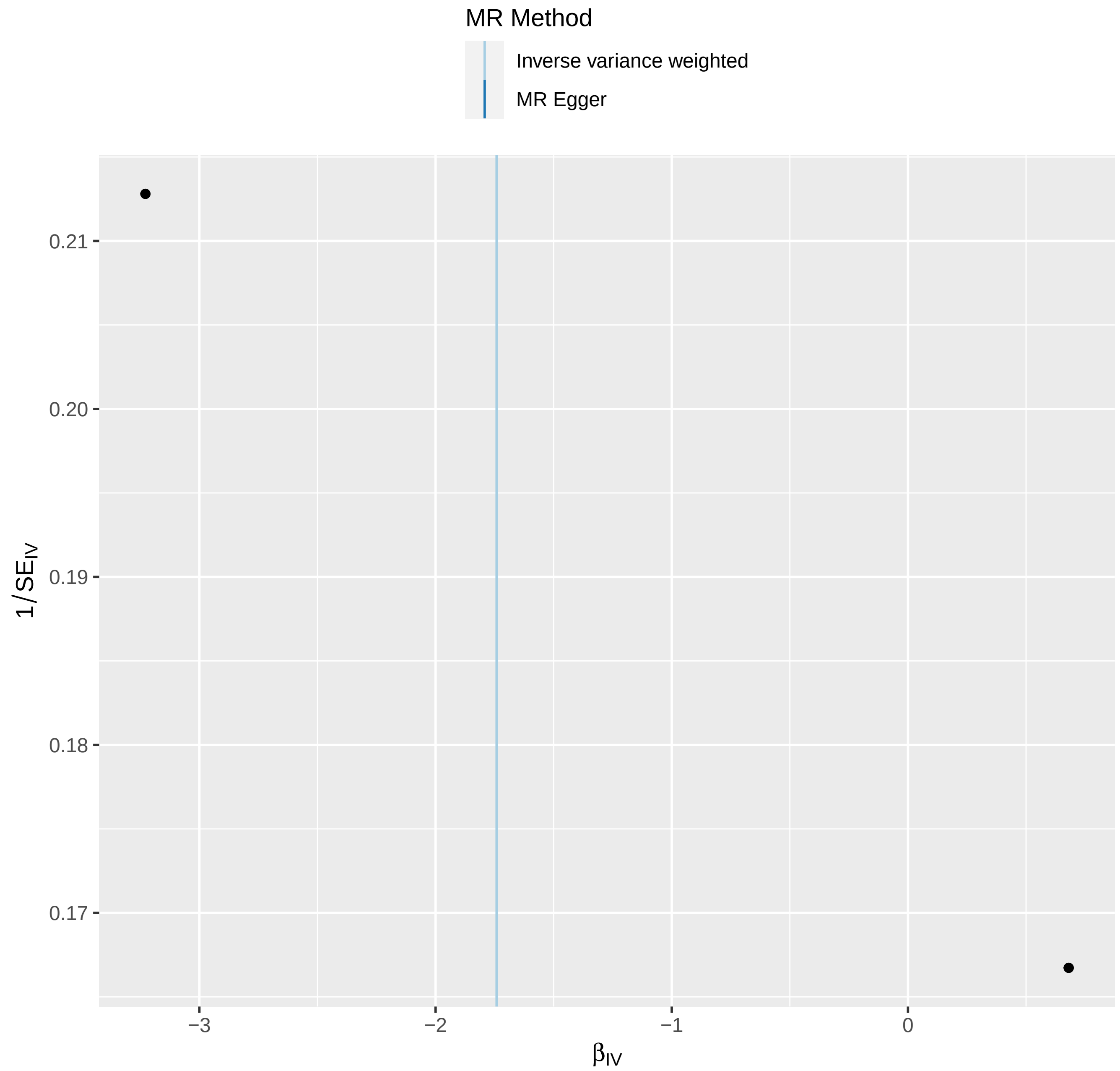

Batch 958 : Trans−4−hydroxyproline on Arthropathy in Crohn disease

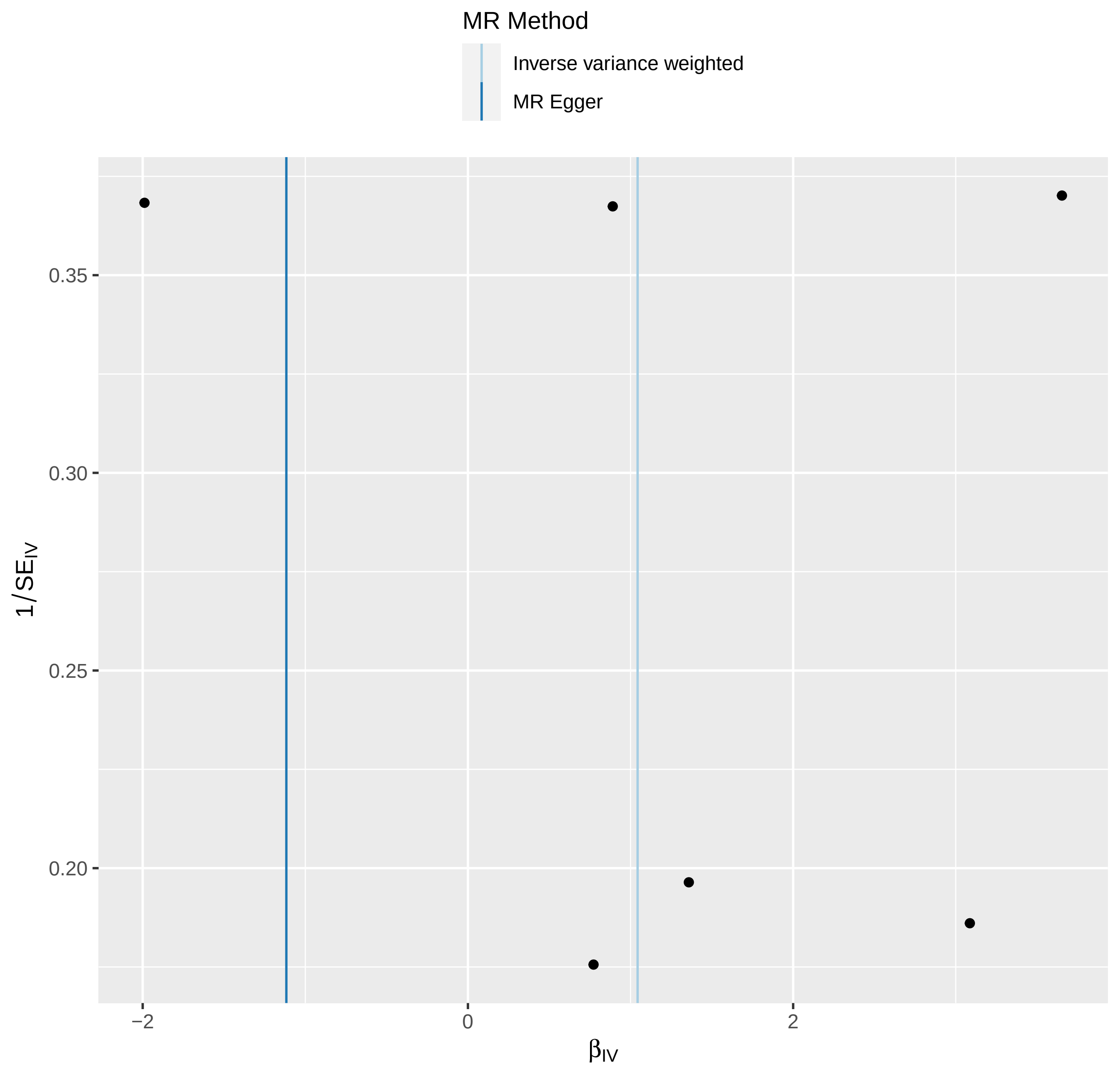

Batch 960 : Hexanoylcarnitine on Arthropathy in Crohn disease

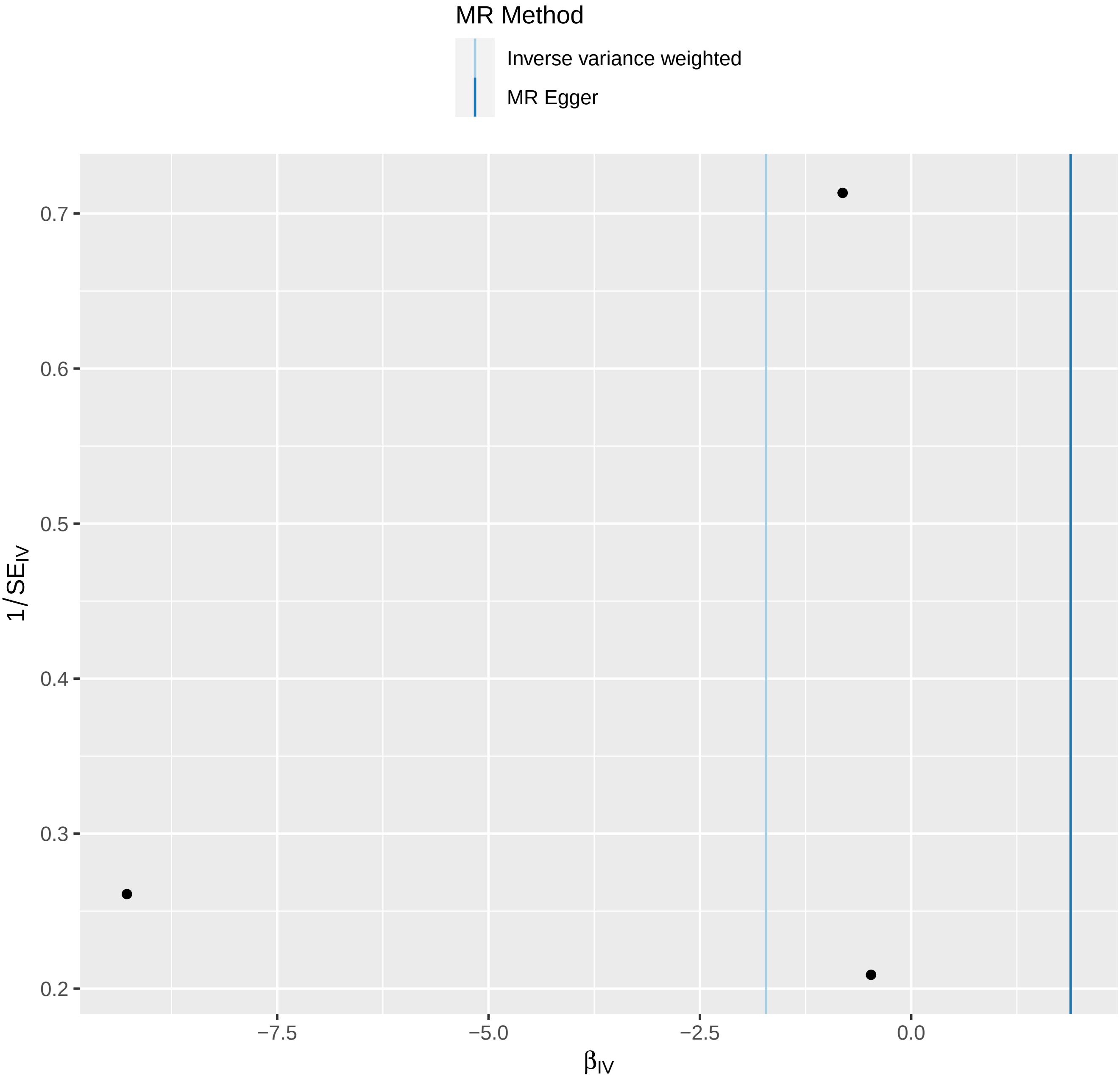

Batch 964 : 2−aminobutyrate on Arthropathy in Crohn disease

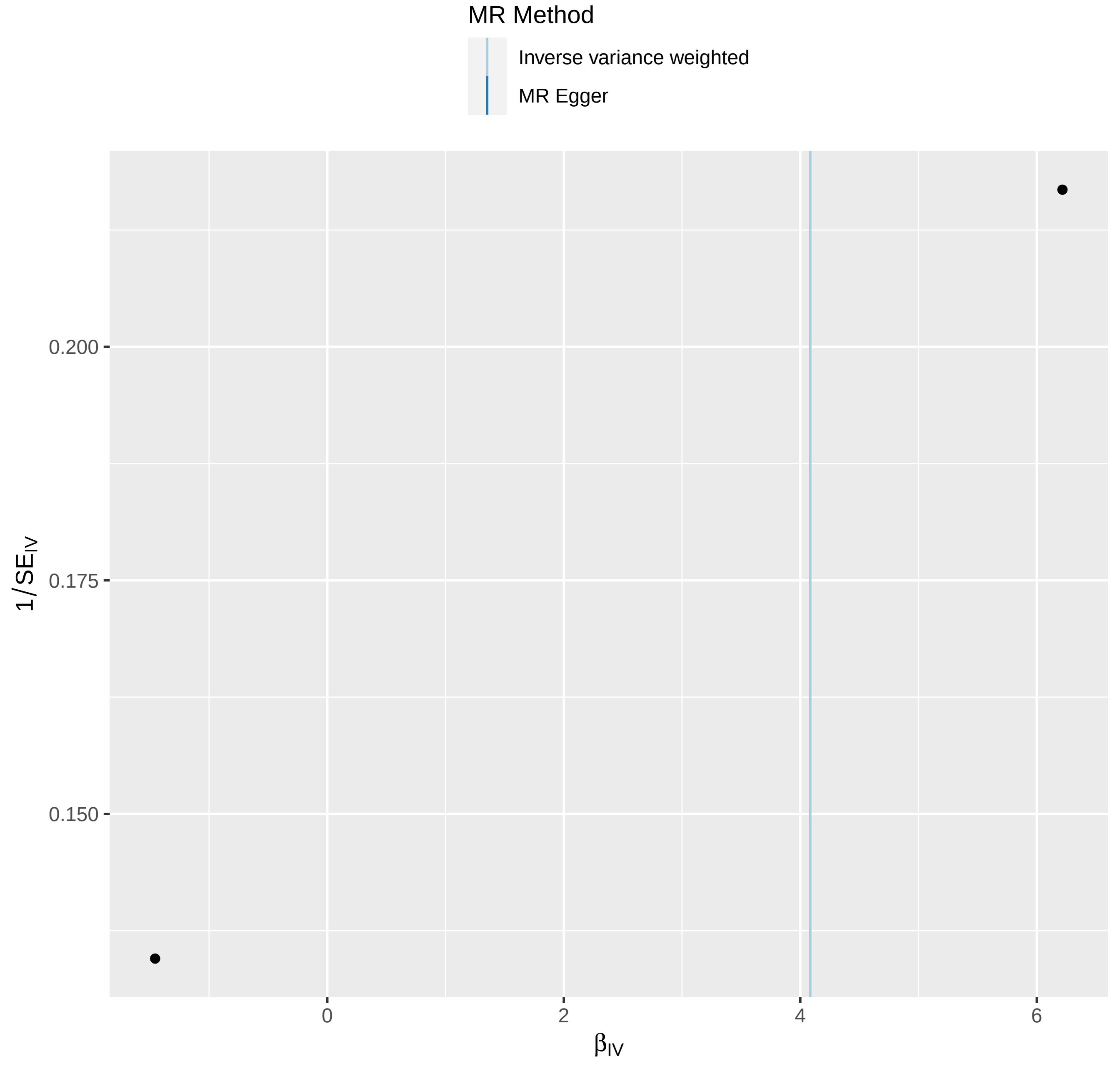

Batch 969 : Butyrylcarnitine on Arthropathy in Crohn disease

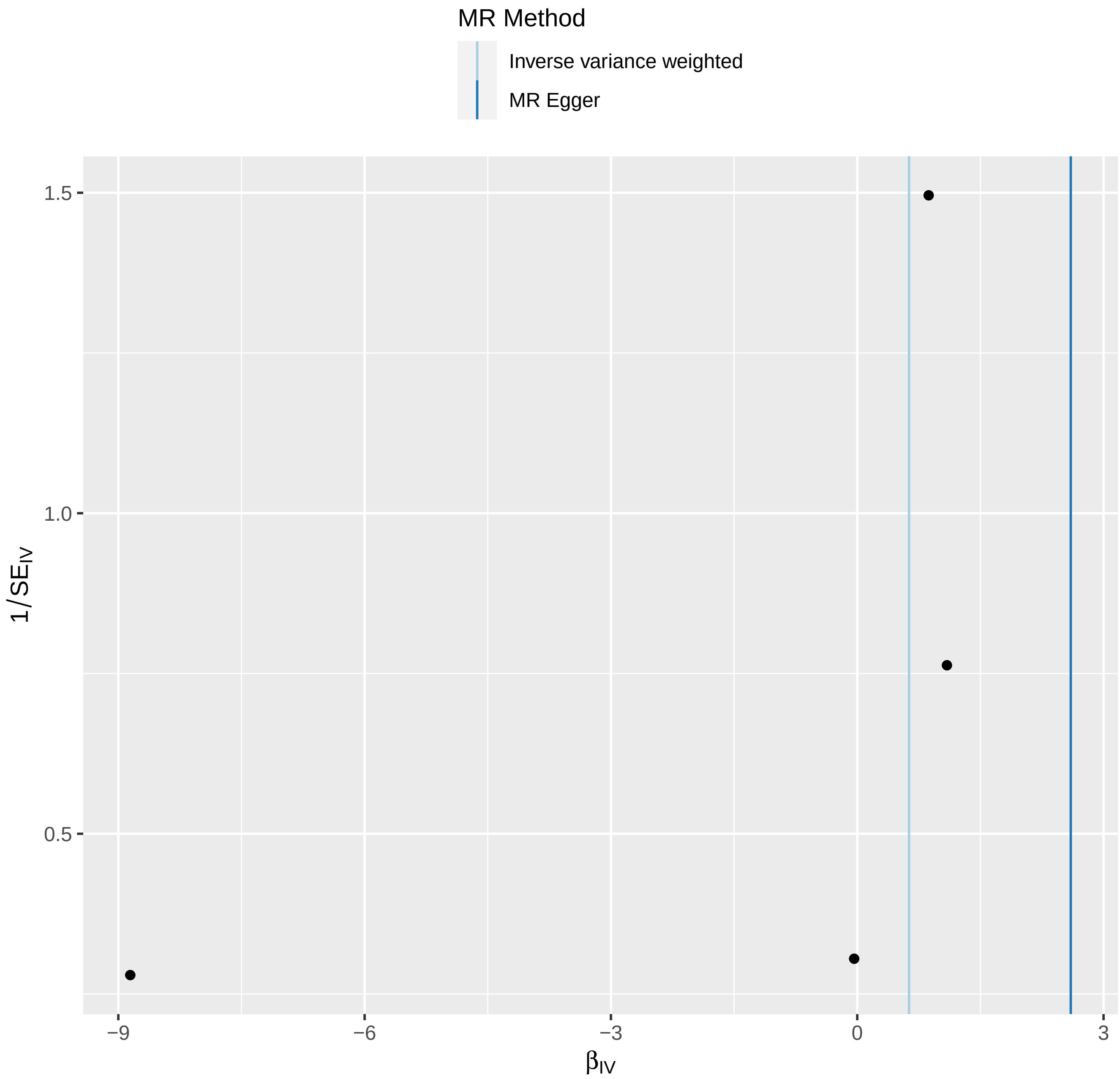

Batch 972 : Propionylcarnitine on Arthropathy in Crohn disease

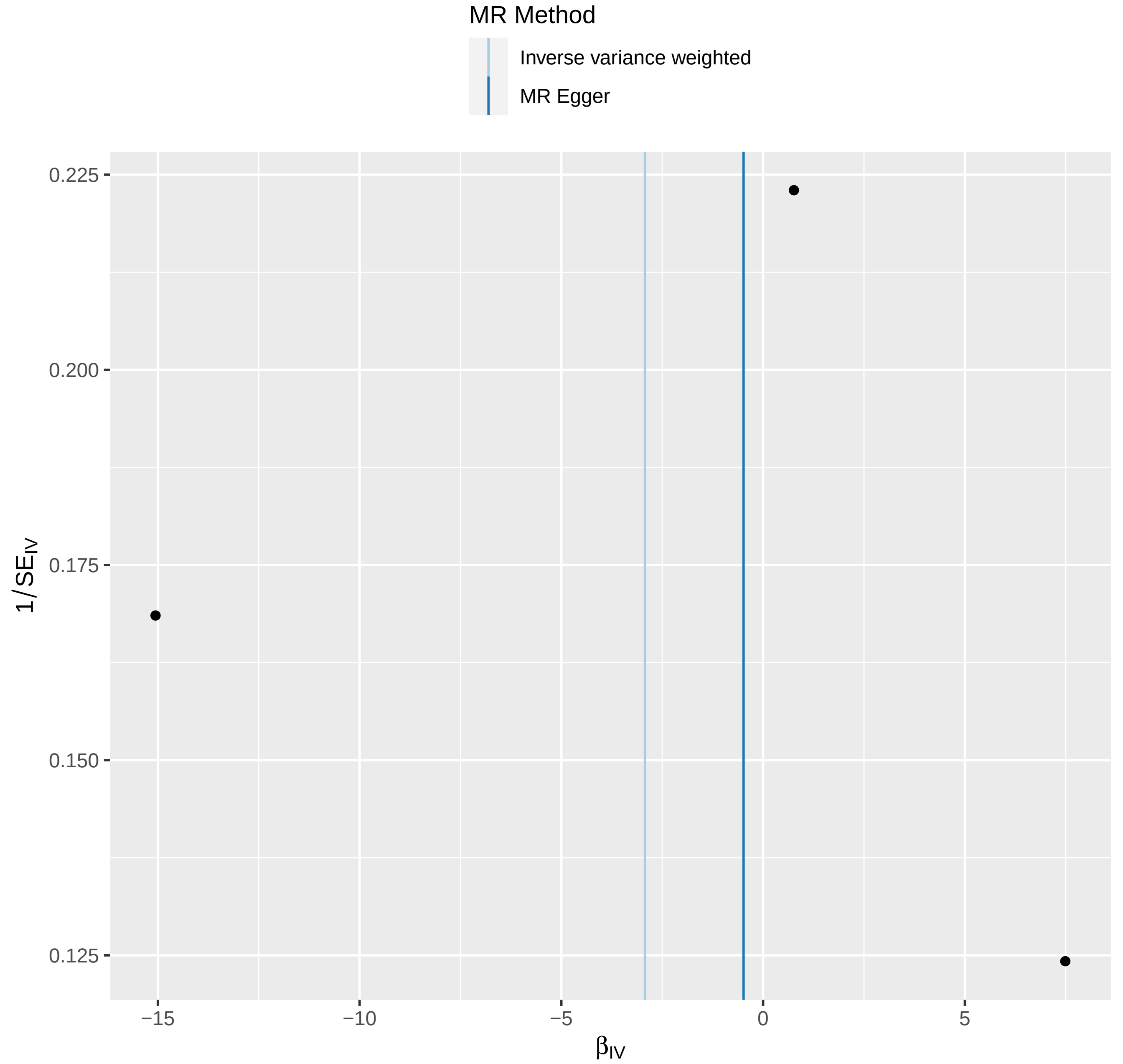

Batch 973 : Caproate (6:0) on Arthropathy in Crohn disease

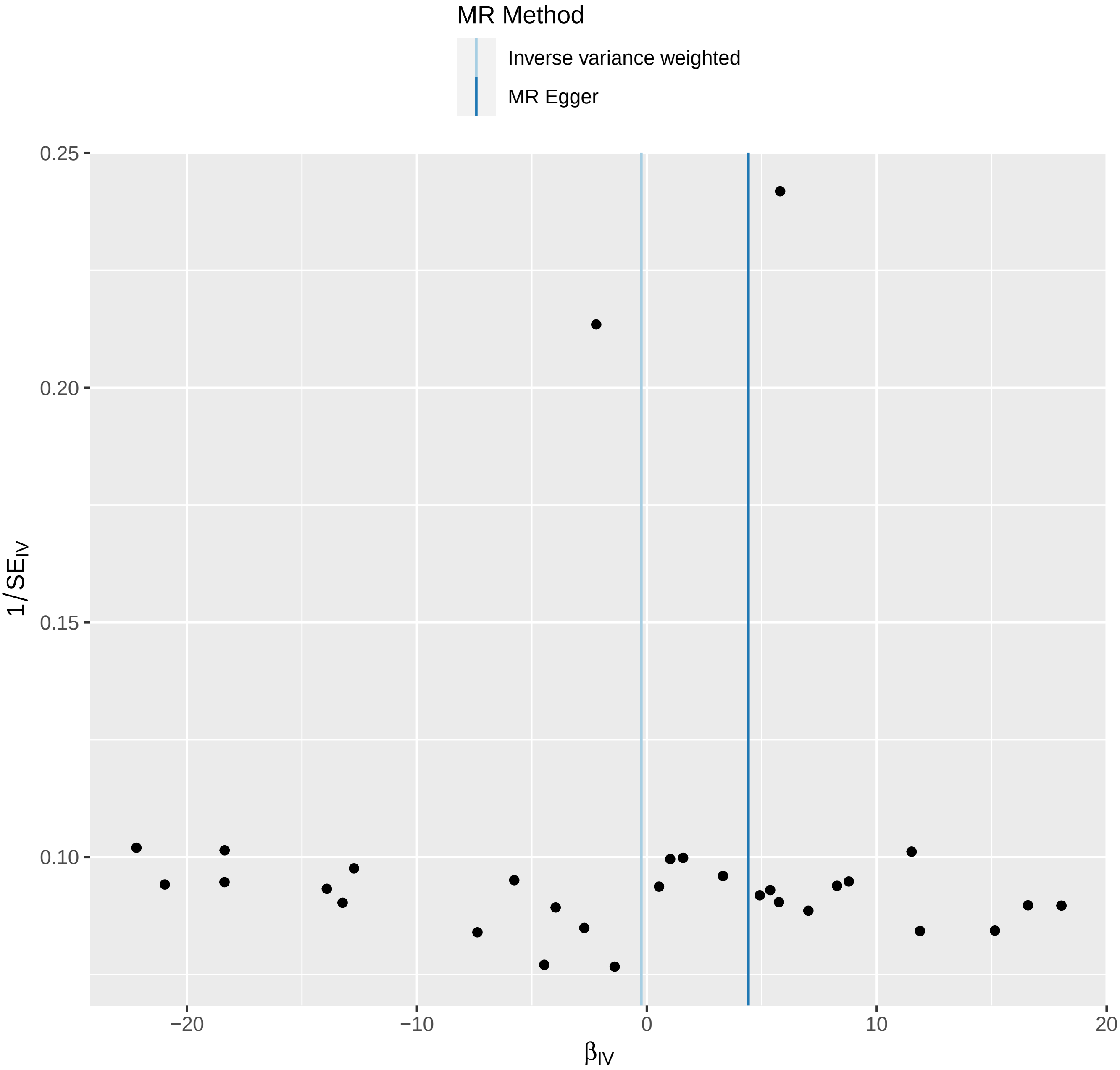

Batch 974 : Caprylate (8:0) on Arthropathy in Crohn disease

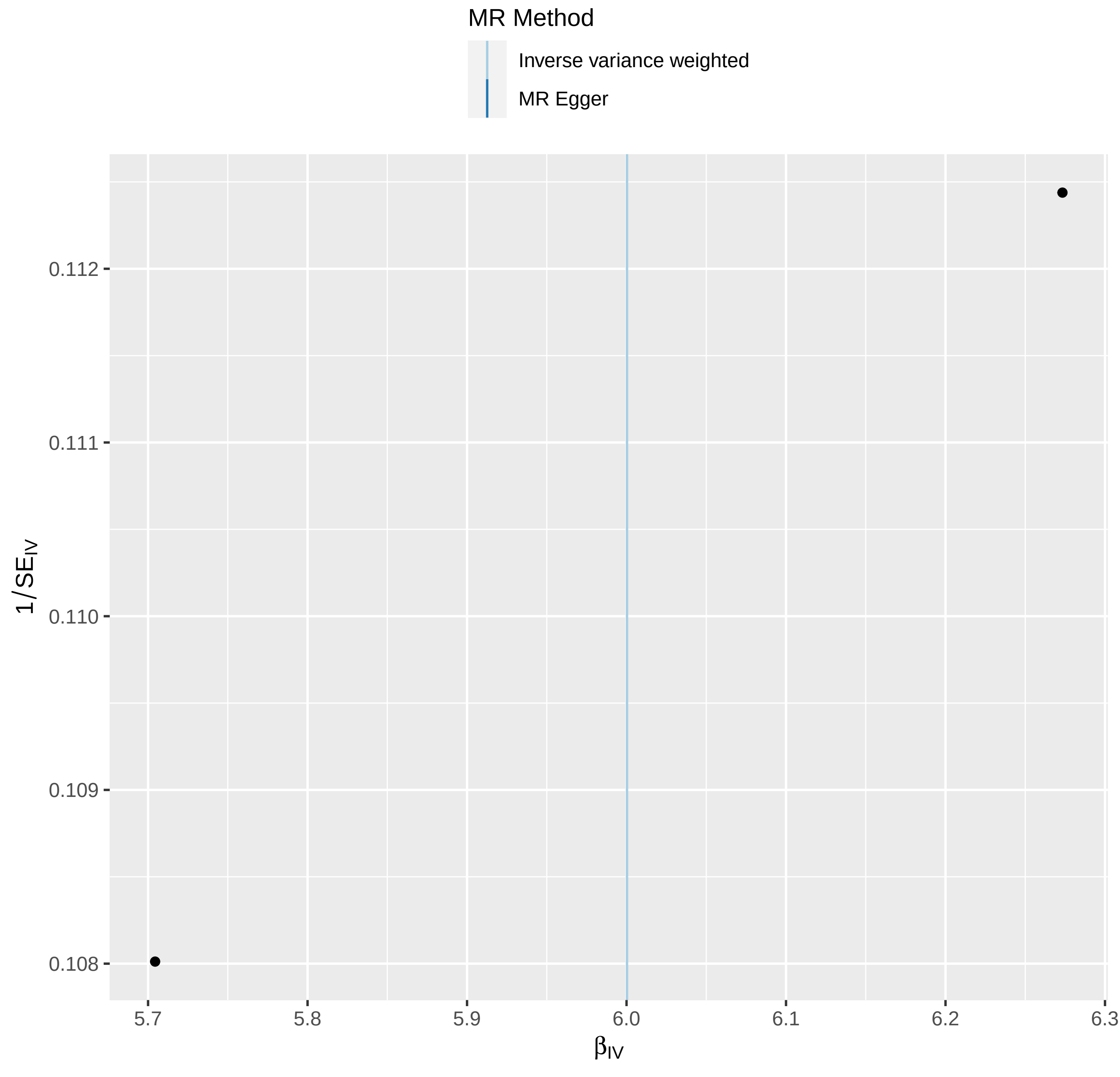

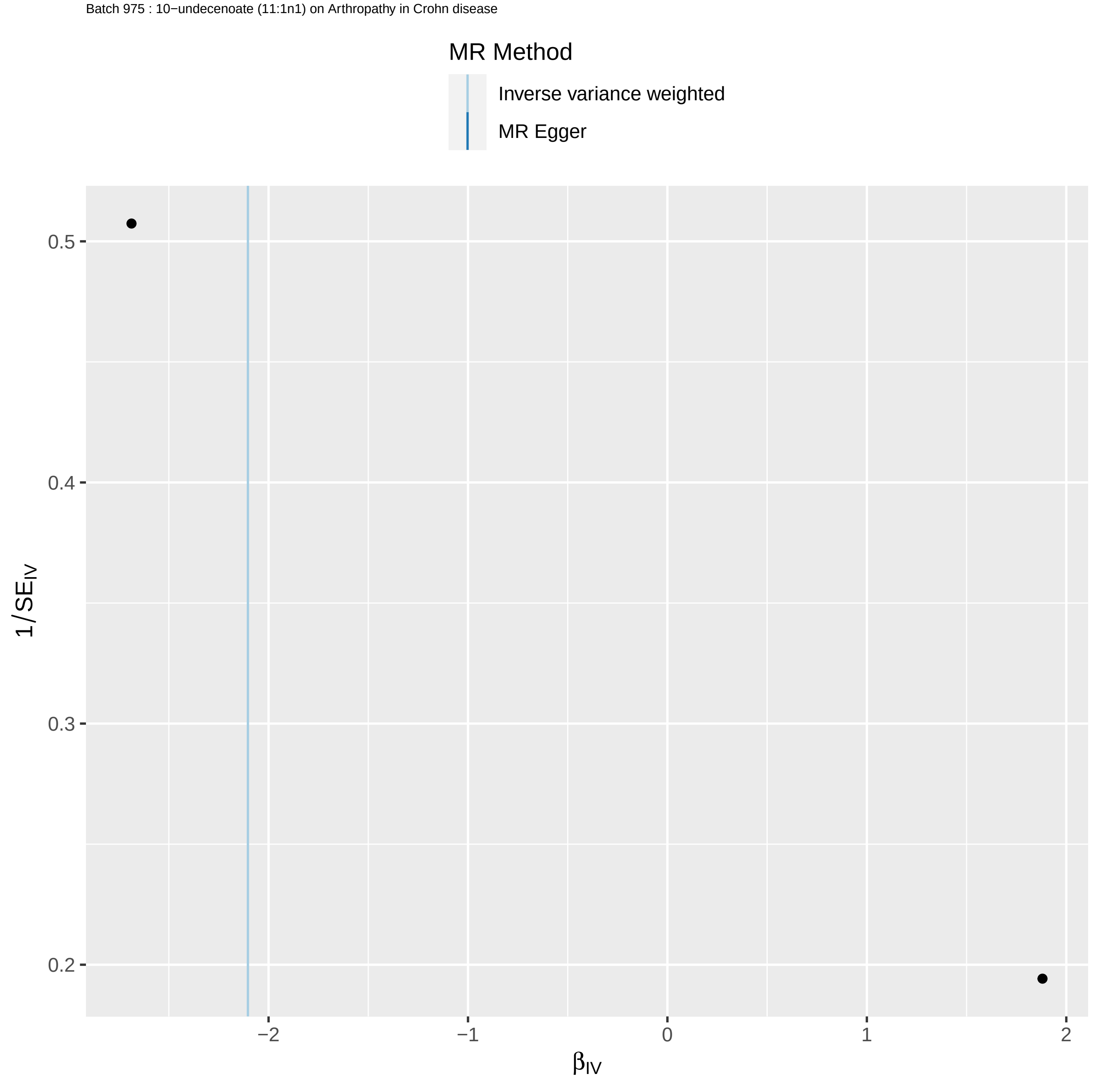
Batch 975 : 10−undecenoate (11:1n1) on Arthropathy in Crohn disease
MR Method
Inverse variance weighted
MR Egger
0.5
0.4
0.3
0.2
1/SE$_{IV}$
−2
−1
0
1
2
β_{IV}

Batch 977 : Phenol sulfate on Arthropathy in Crohn disease

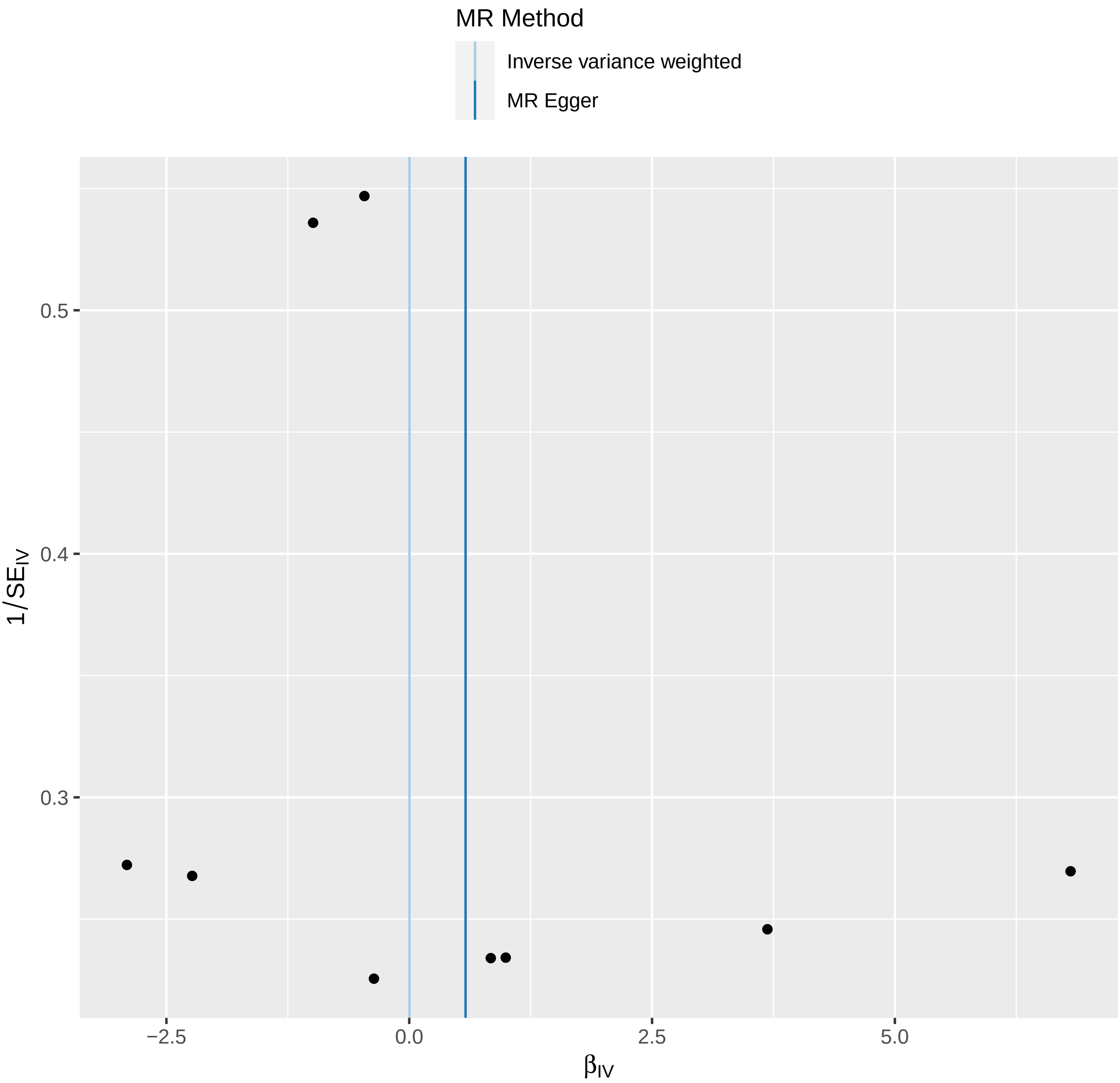

Batch 979 : Heme* on Arthropathy in Crohn disease

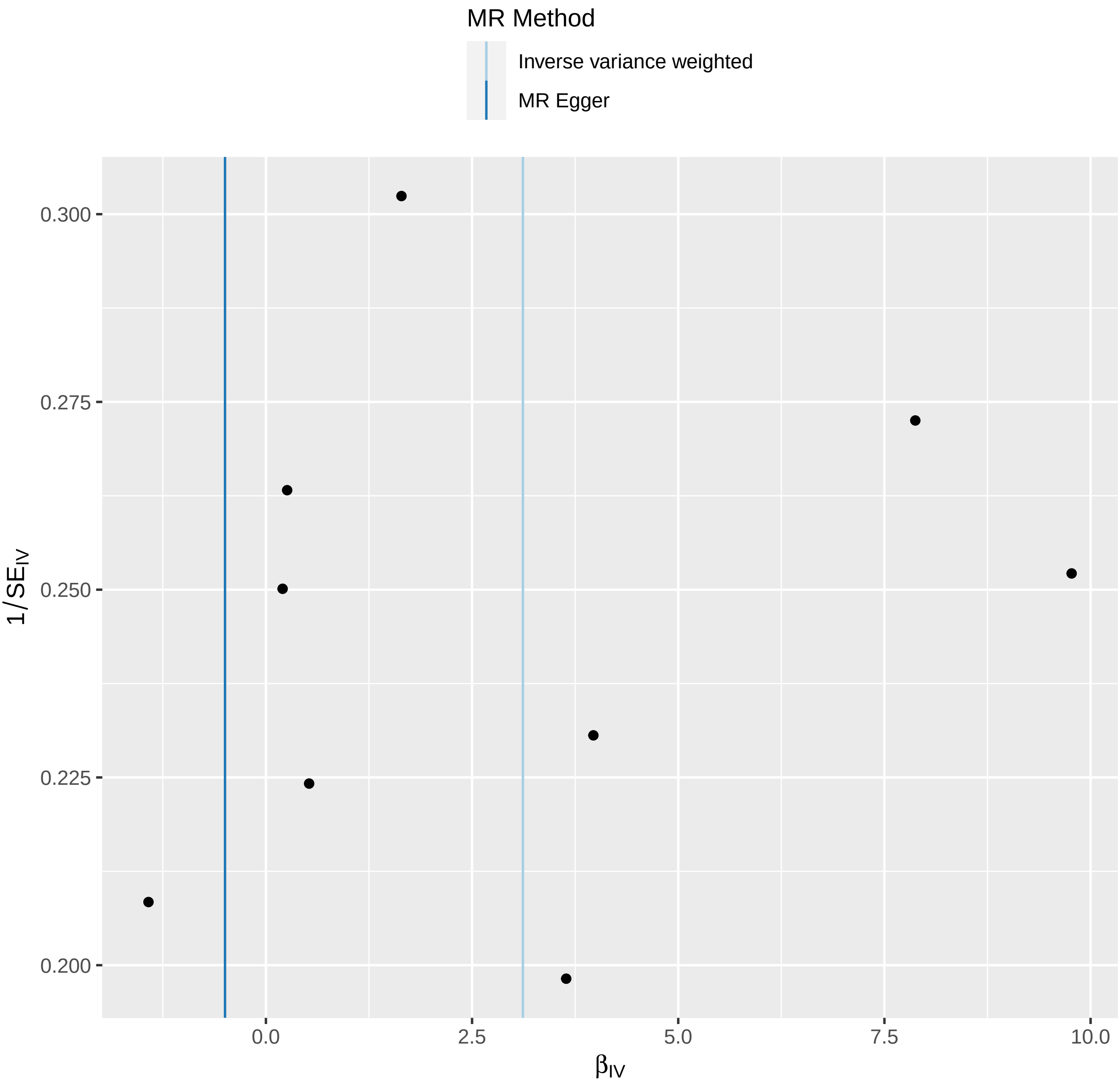

Batch 982 : Pyroglutamine* on Arthropathy in Crohn disease

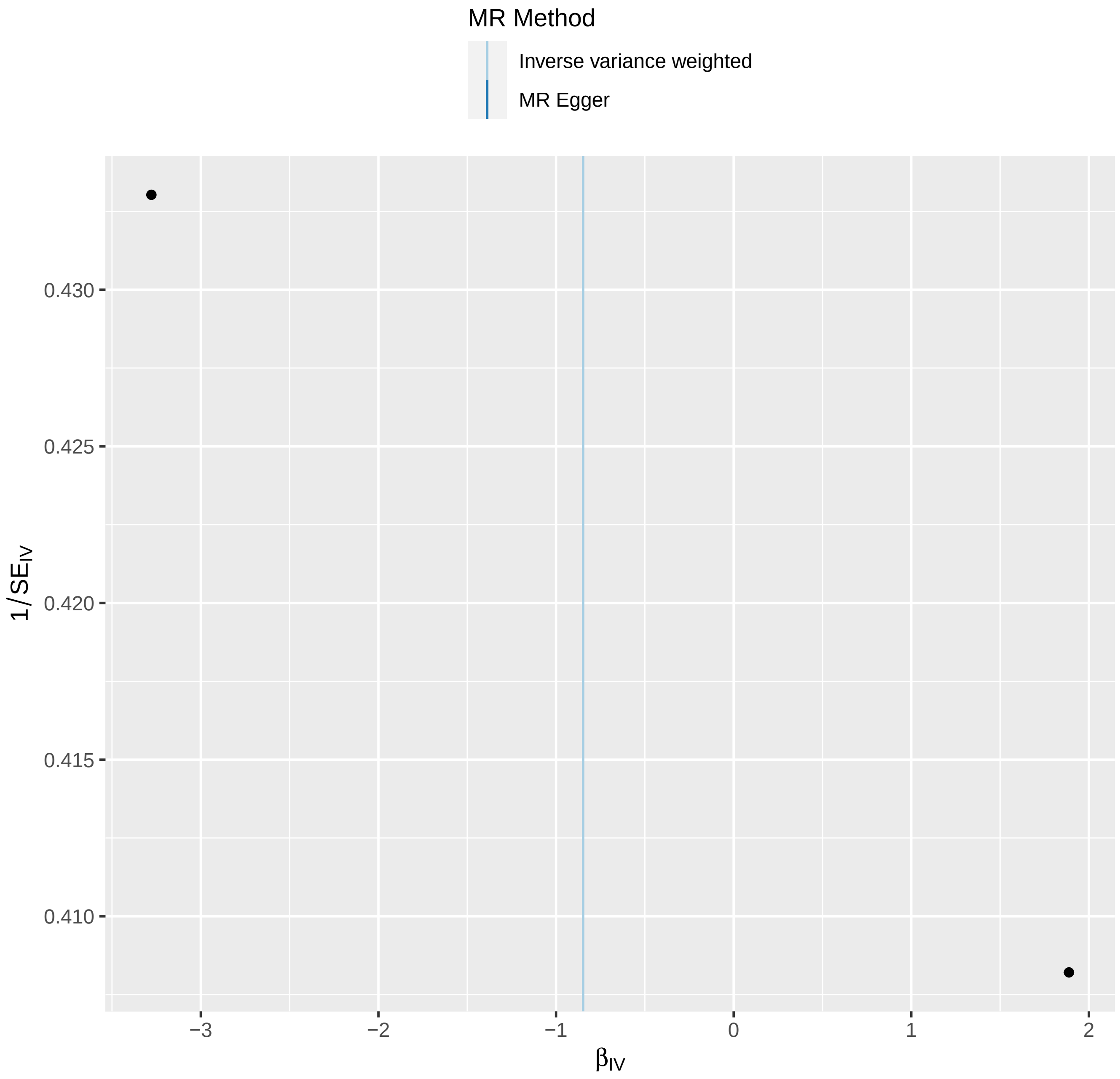

Batch 985 : X−11422−−xanthine on Arthropathy in Crohn disease

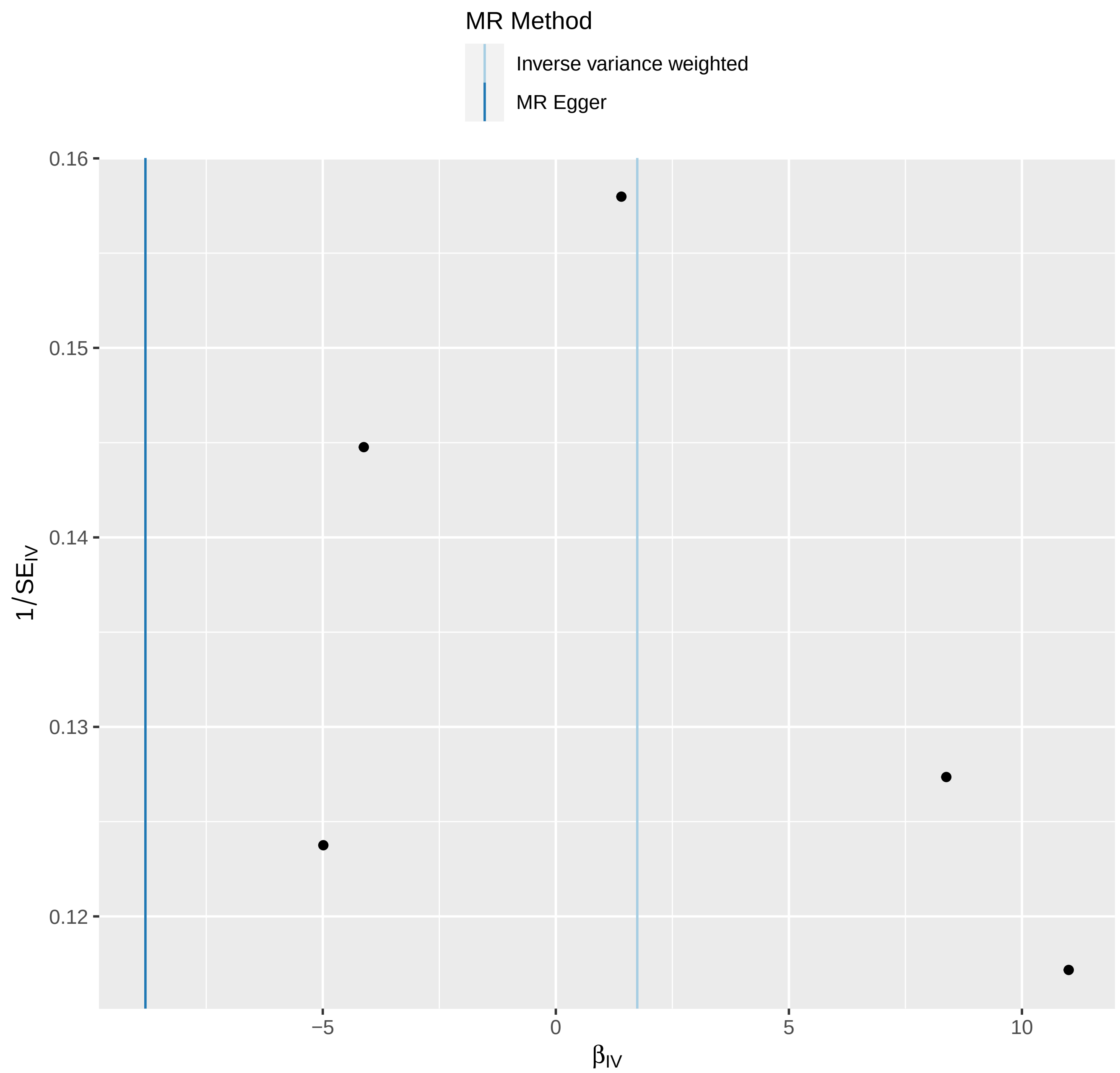

Batch 988 : HWESASXX* on Arthropathy in Crohn disease

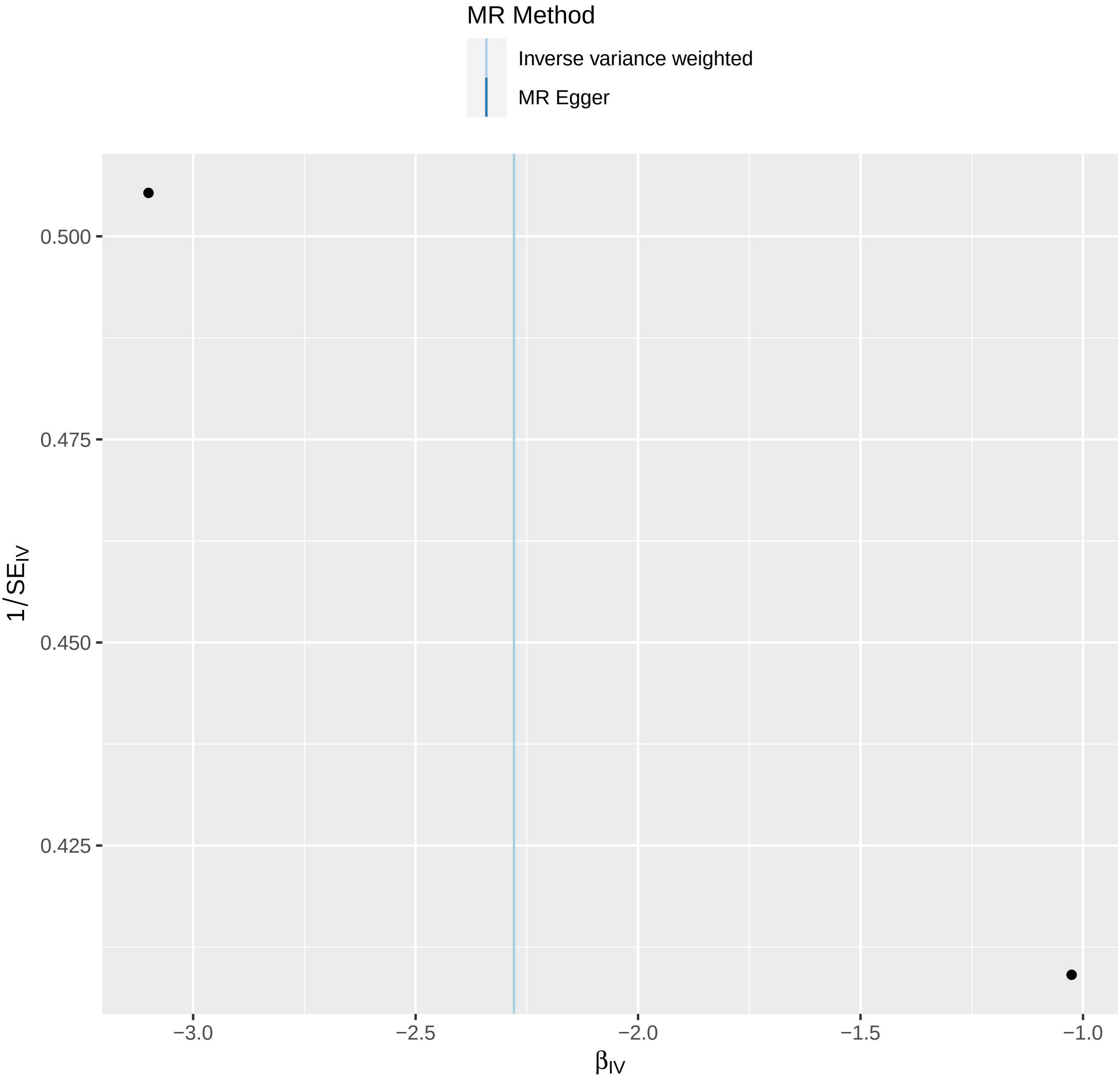

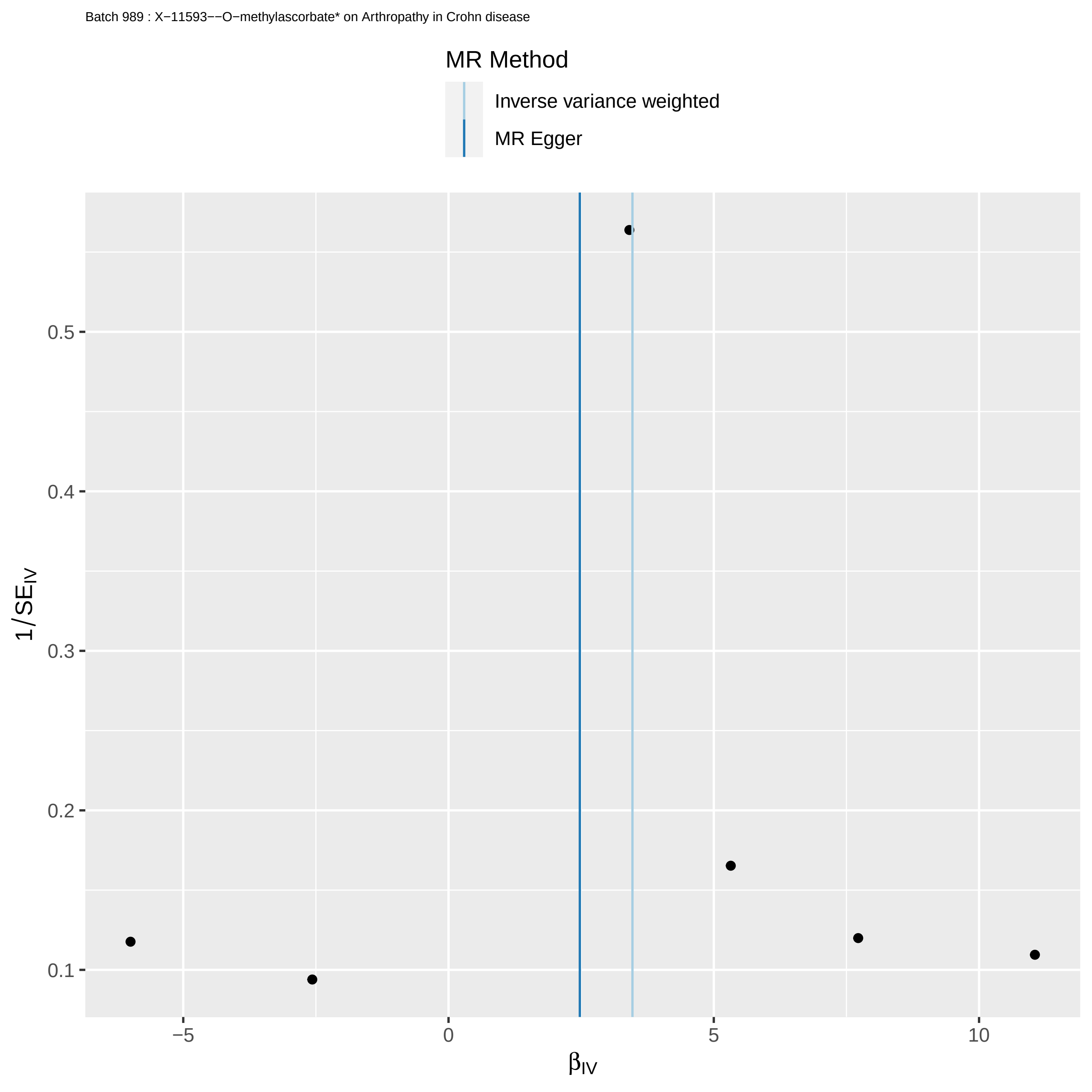

Batch 989 : X−11593−−O−methylascorbate* on Arthropathy in Crohn disease
MR Method
Inverse variance weighted
MR Egger
0.5
0.4
0.3
0.2
0.1
1/SE_IV
−5
0
5
10
β_IV

Batch 991 : Homostachydrine* on Arthropathy in Crohn disease

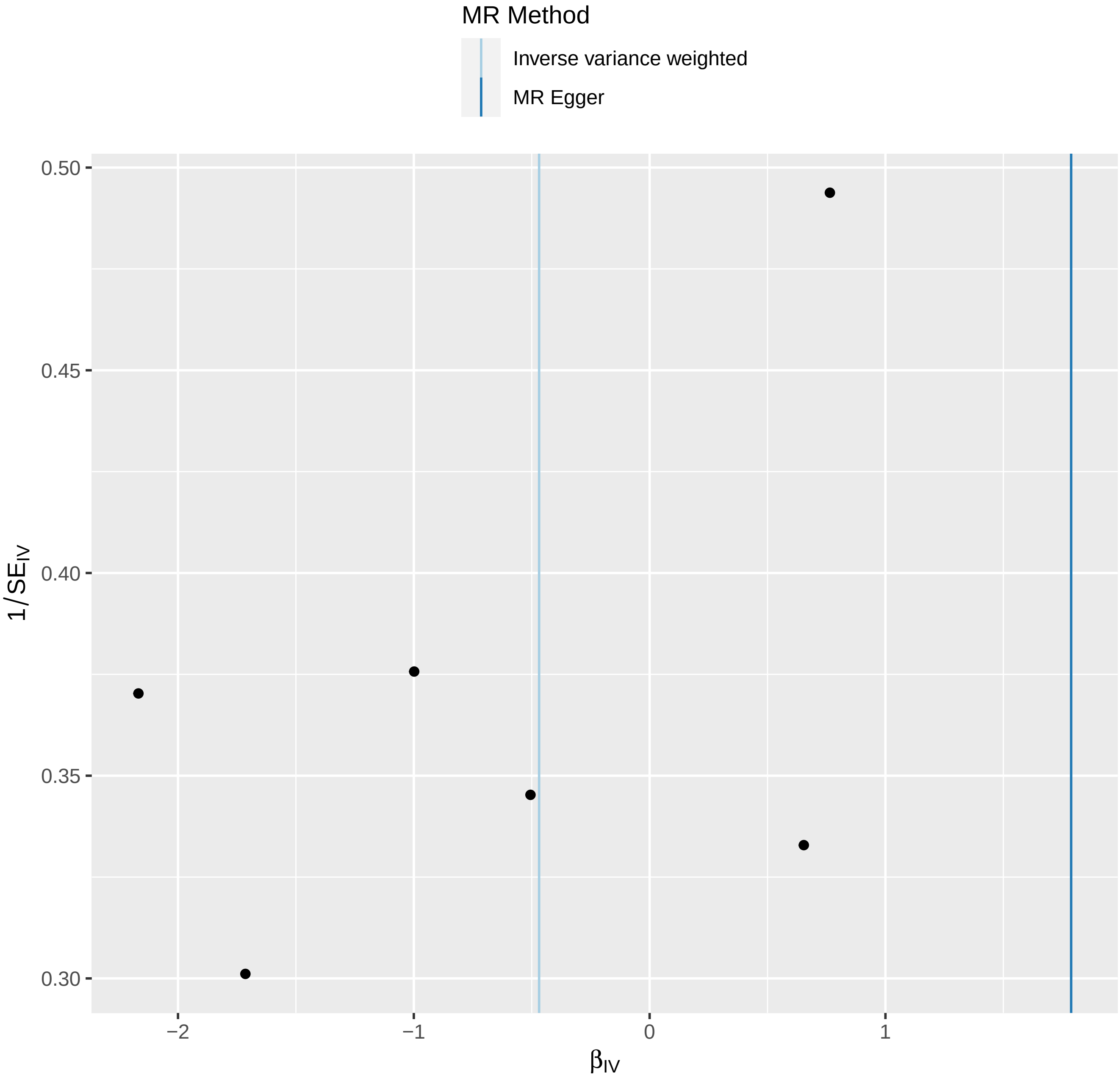

Batch 992 : ADSGEGDFXAEGGGVR* on Arthropathy in Crohn disease

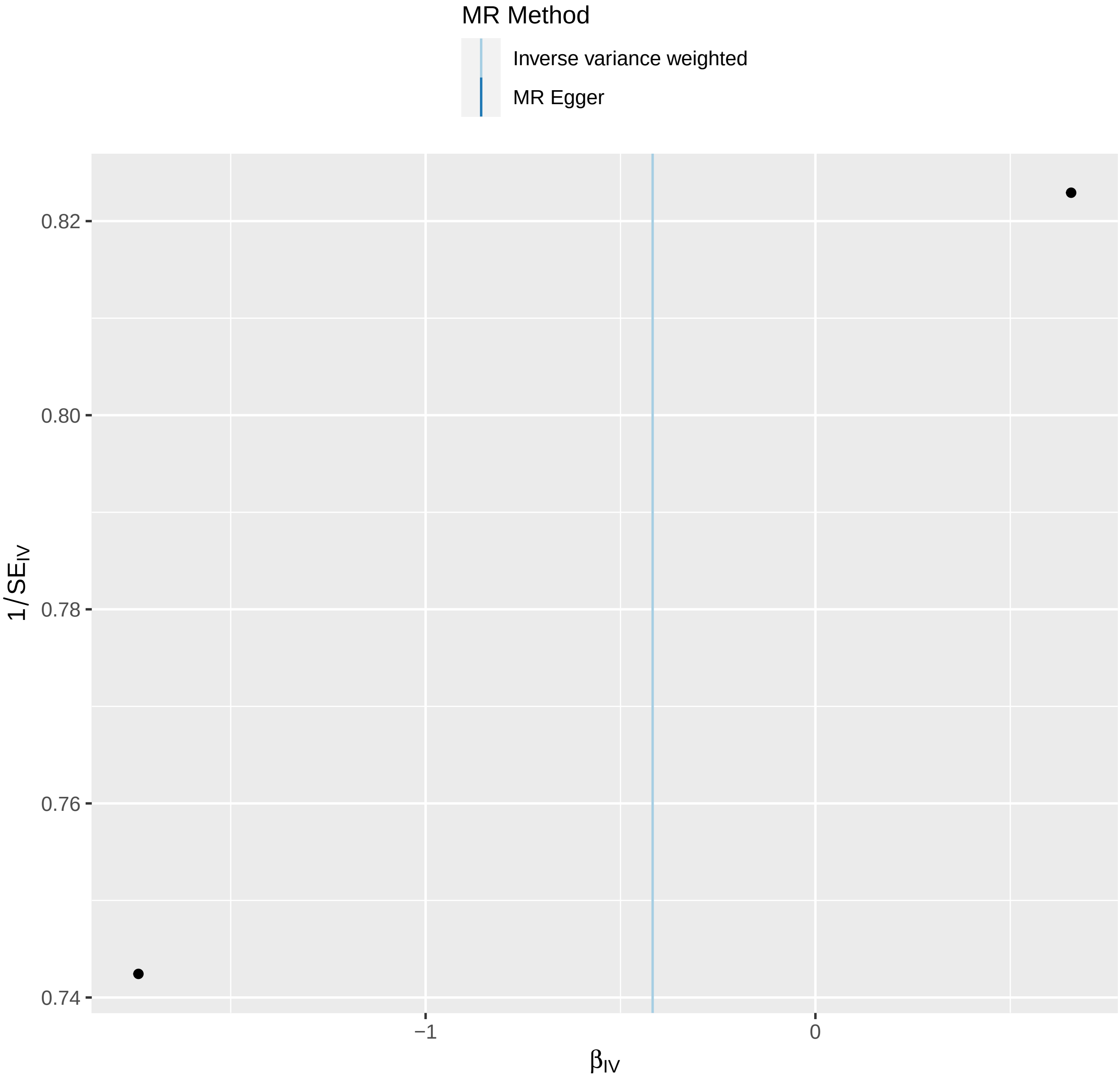

Batch 994 : X−11793−−oxidized bilirubin* on Arthropathy in Crohn disease

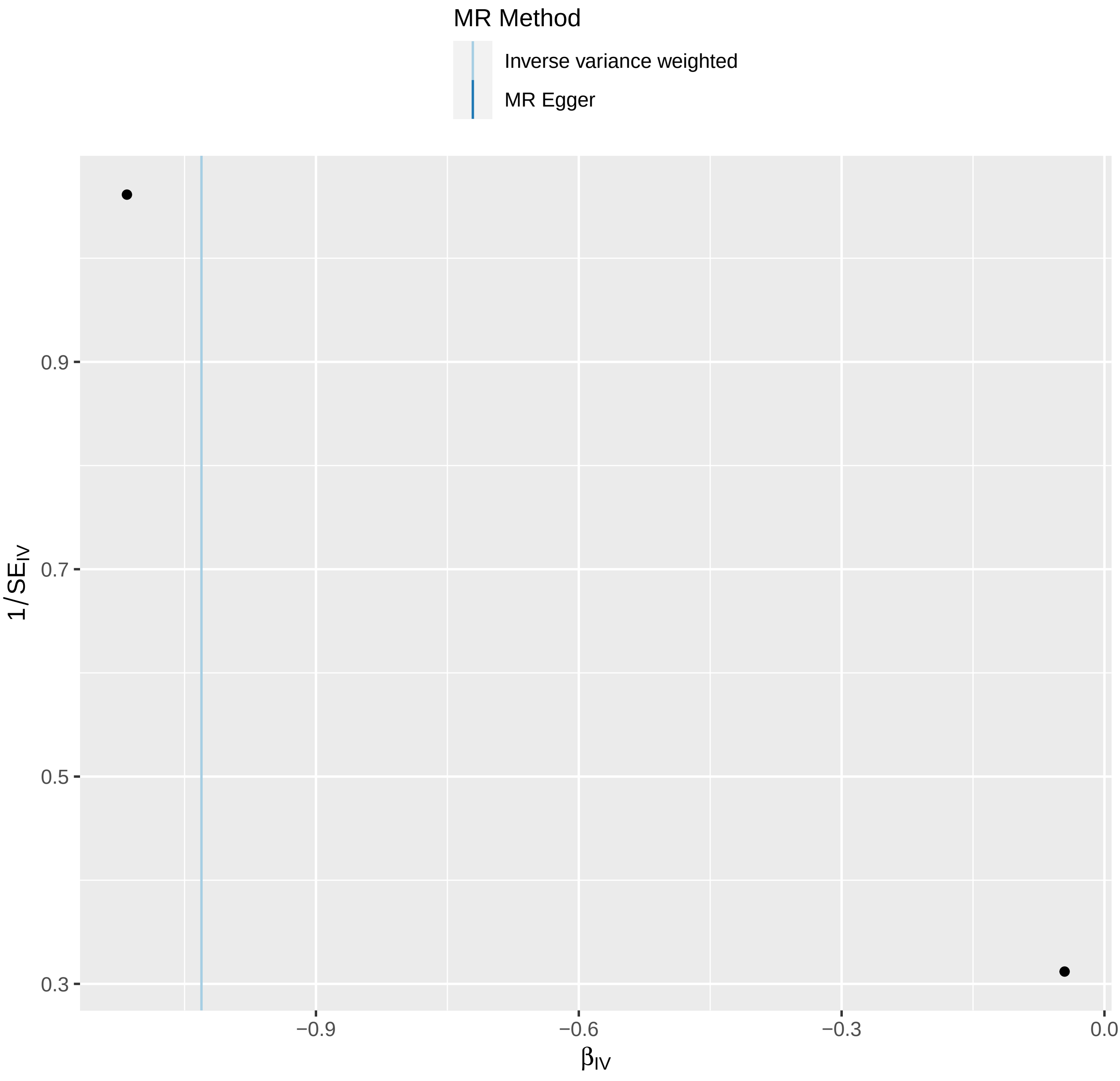

Batch 995 : 1−arachidonoylglycerophosphocholine* on Arthropathy in Crohn disease

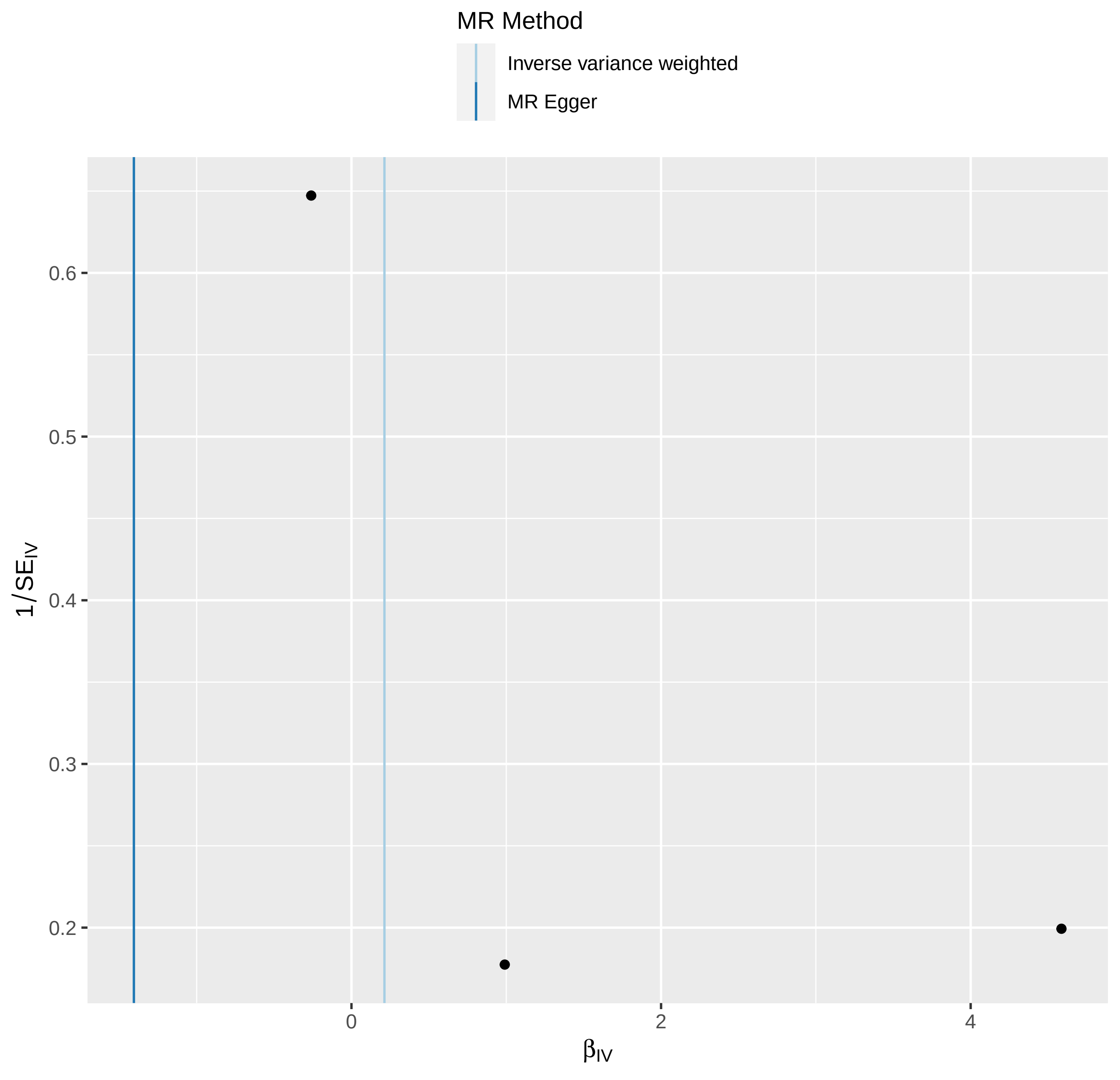

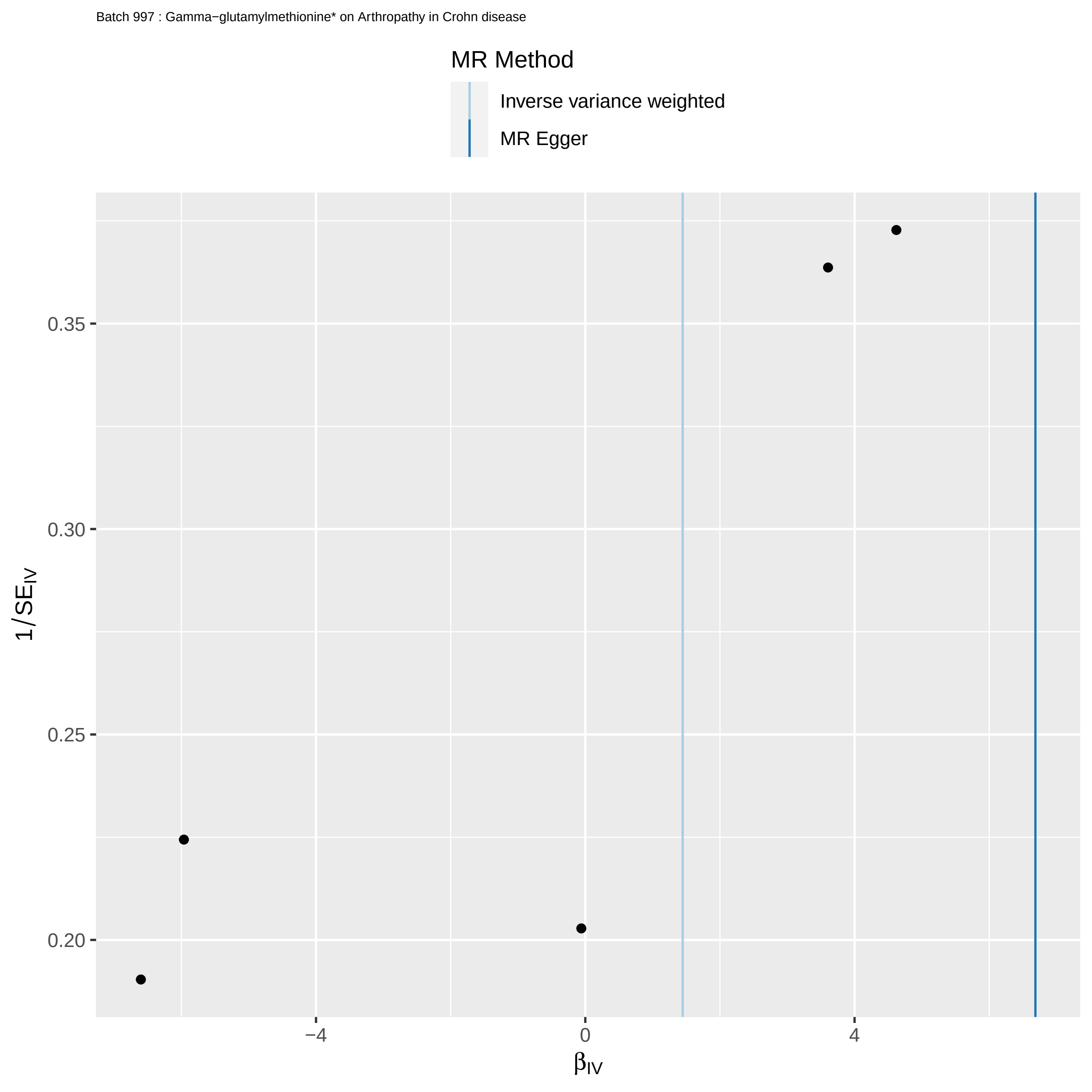
Batch 997 : Gamma−glutamylmethionine* on Arthropathy in Crohn disease
MR Method
Inverse variance weighted
MR Egger
0.35
0.30
0.25
0.20
1/SE_IV
−4
0
4
β_IV

Batch 998 : Gamma−glutamylthreonine* on Arthropathy in Crohn disease

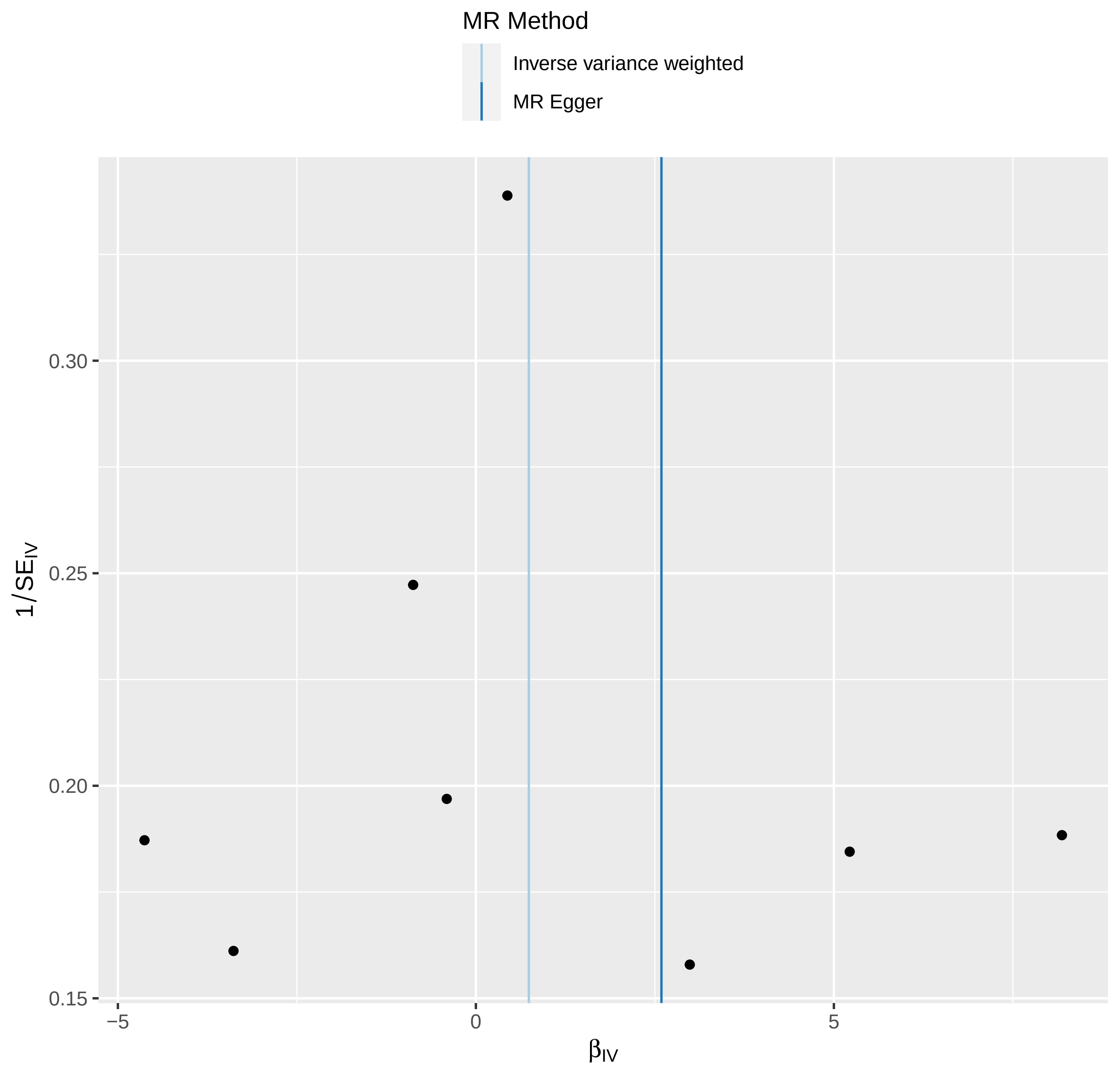

Batch 999 : Gamma−tocopherol on Arthropathy in Crohn disease

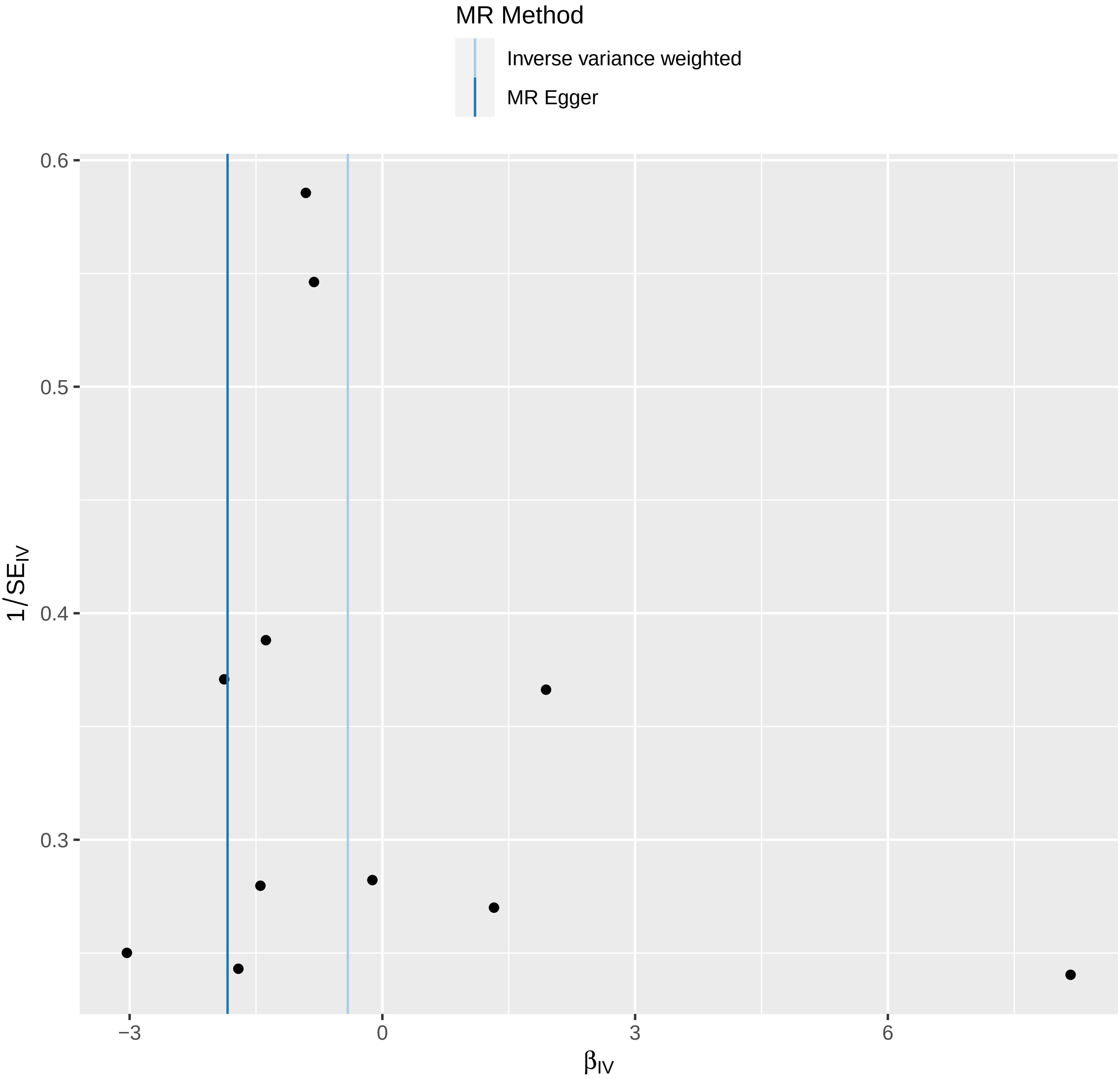

Batch 1000 : Gamma−glutamylphenylalanine on Arthropathy in Crohn disease

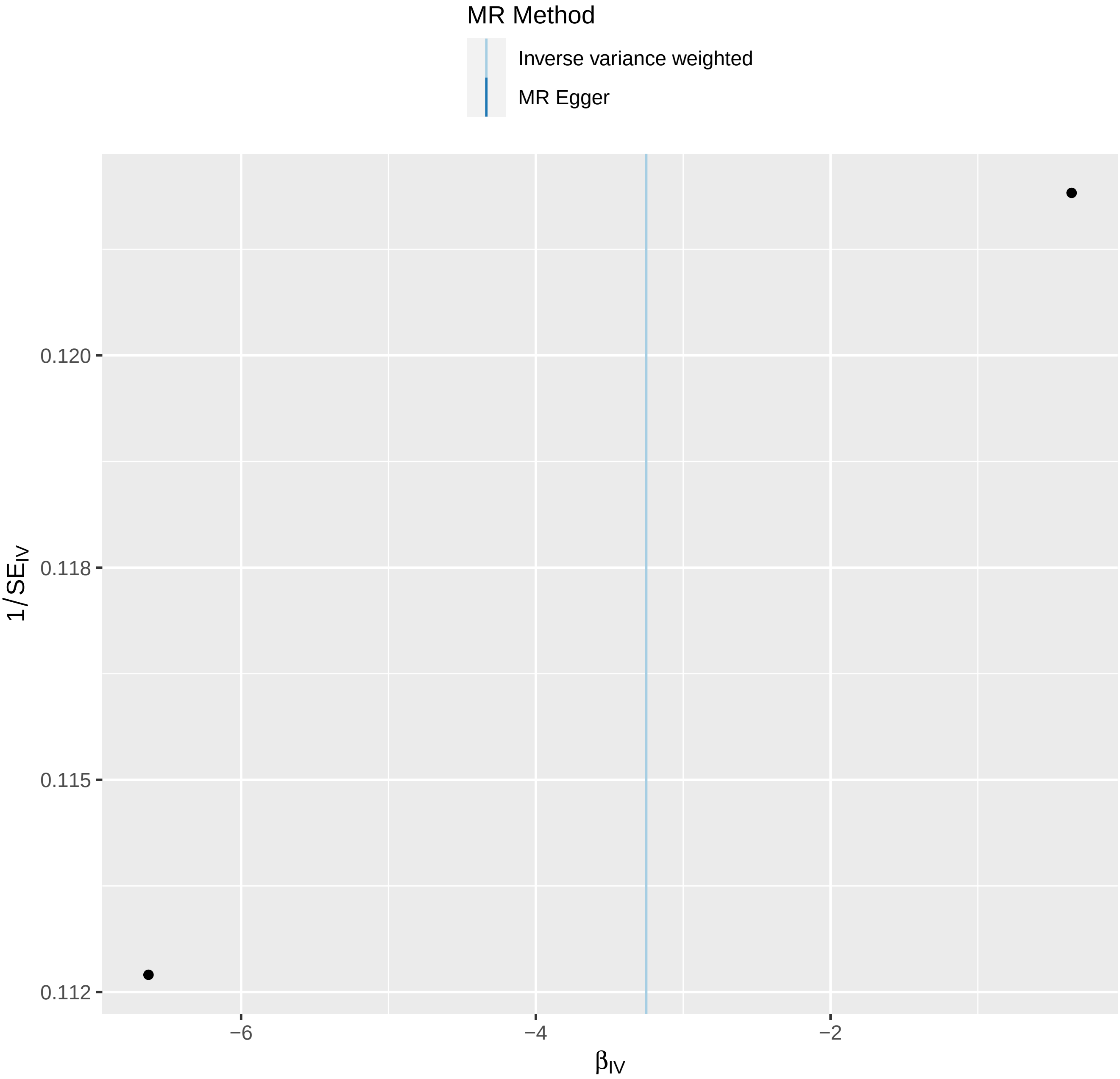

Batch 1002 : Pseudouridine on Arthropathy in Crohn disease

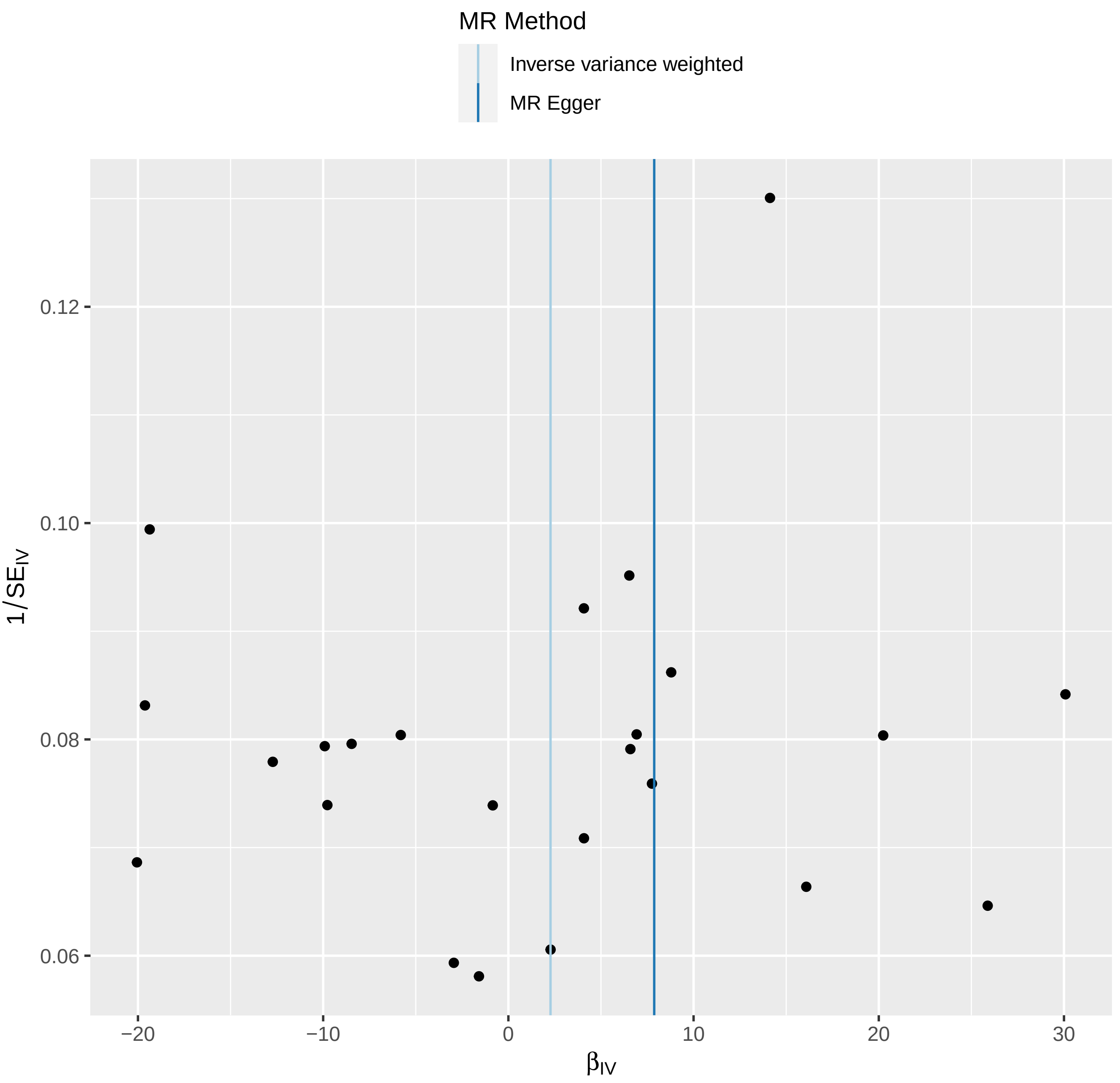

Batch 1003 : Valerate on Arthropathy in Crohn disease

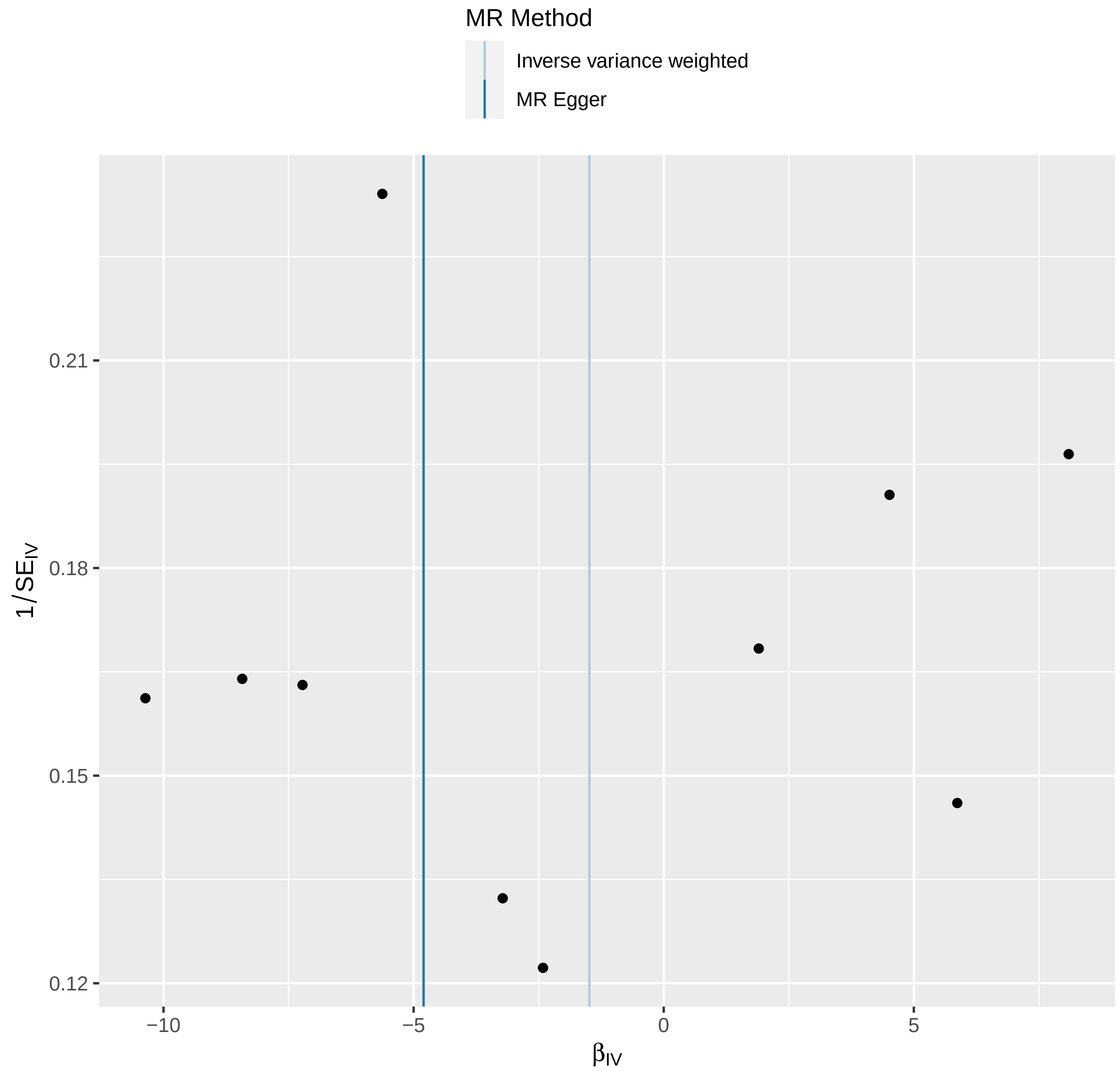

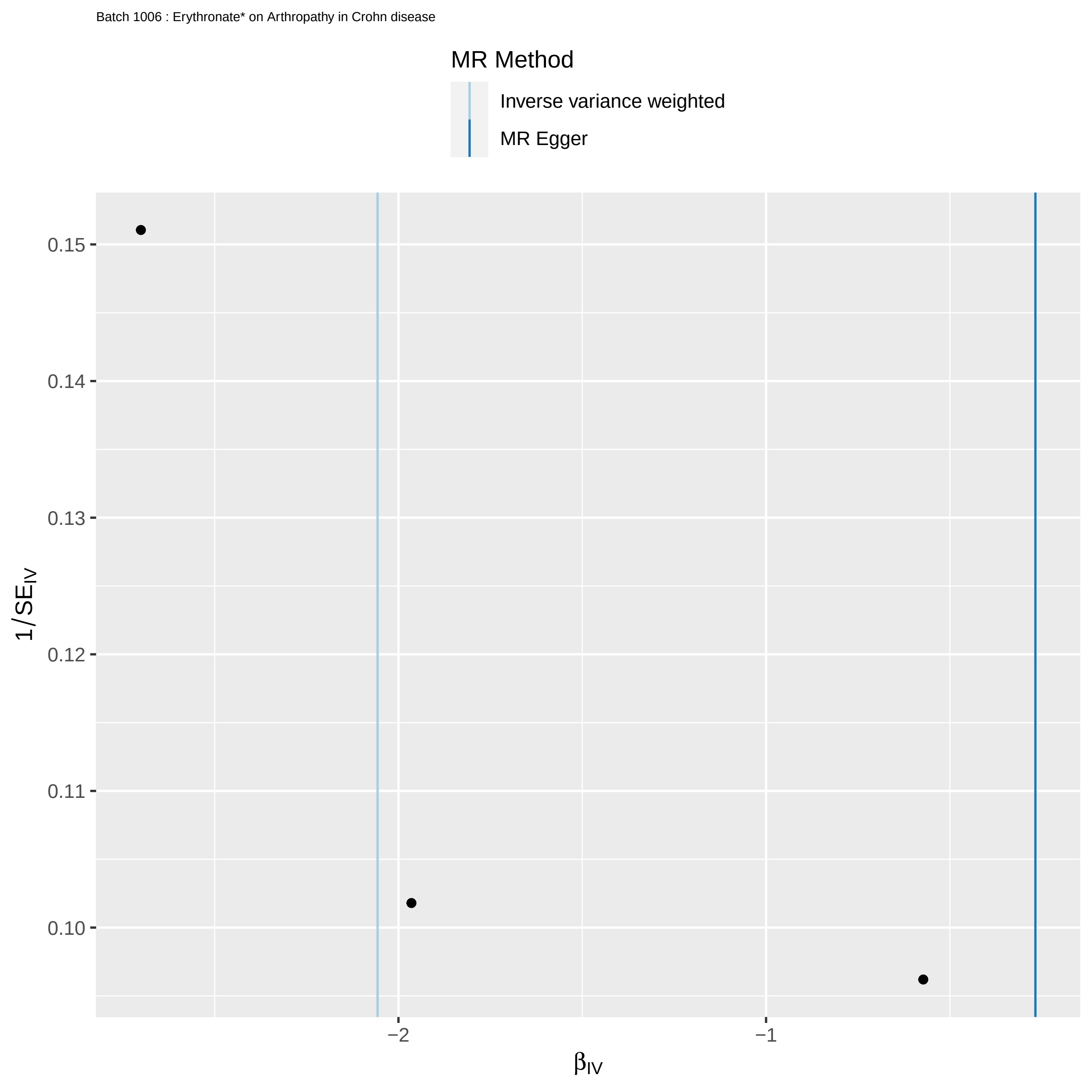
Batch 1006 : Erythronate* on Arthropathy in Crohn disease
MR Method
Inverse variance weighted
MR Egger
1/SE_IV
0.15
0.14
0.13
0.12
0.11
0.10
−2
−1
β_IV

Batch 1007 : Lathosterol on Arthropathy in Crohn disease

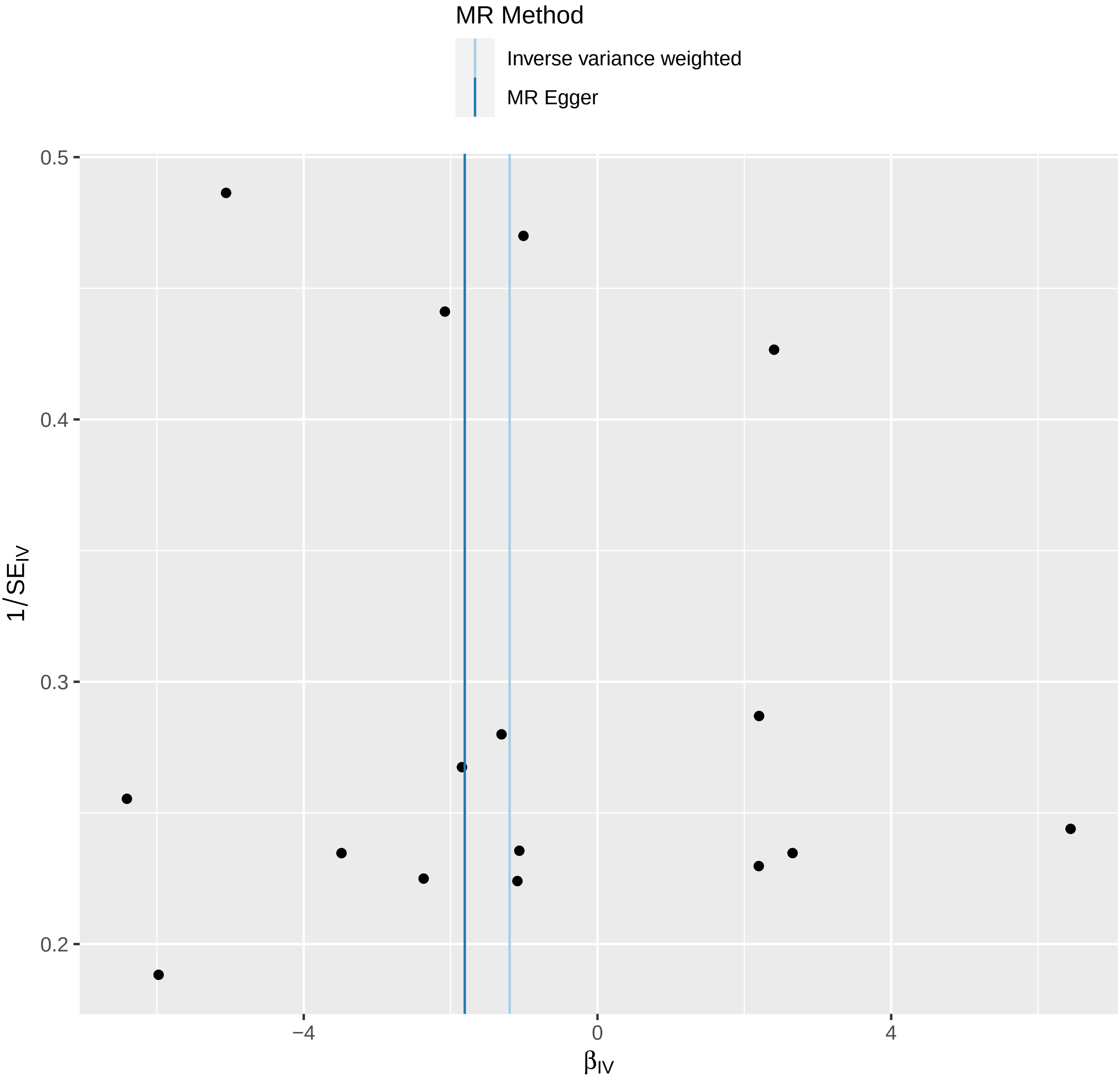

Batch 1010 : Eicosenoate (20:1n9 or 11) on Arthropathy in Crohn disease

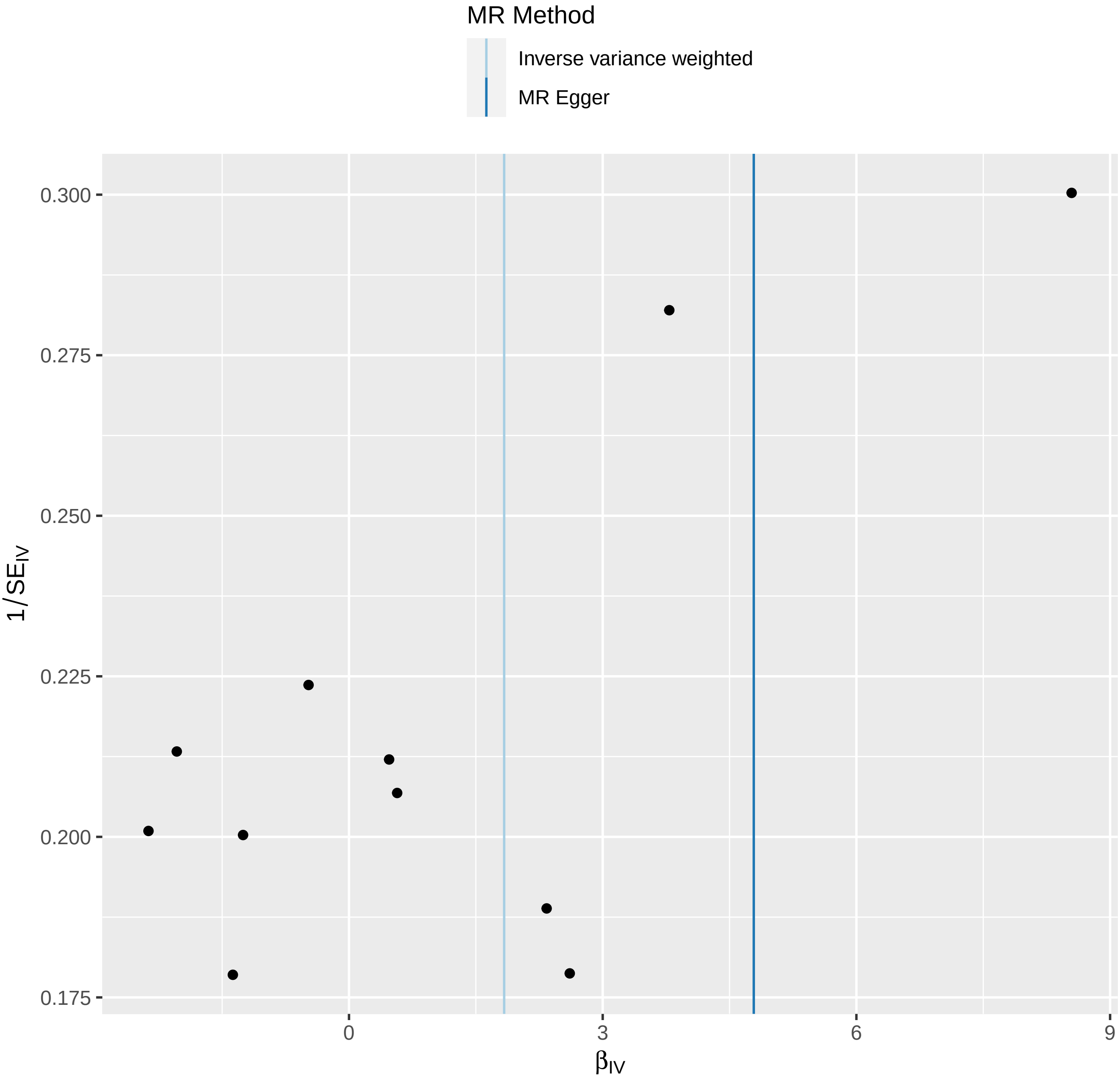

Batch 1011 : X−12244−−N−acetylcarnosine on Arthropathy in Crohn disease

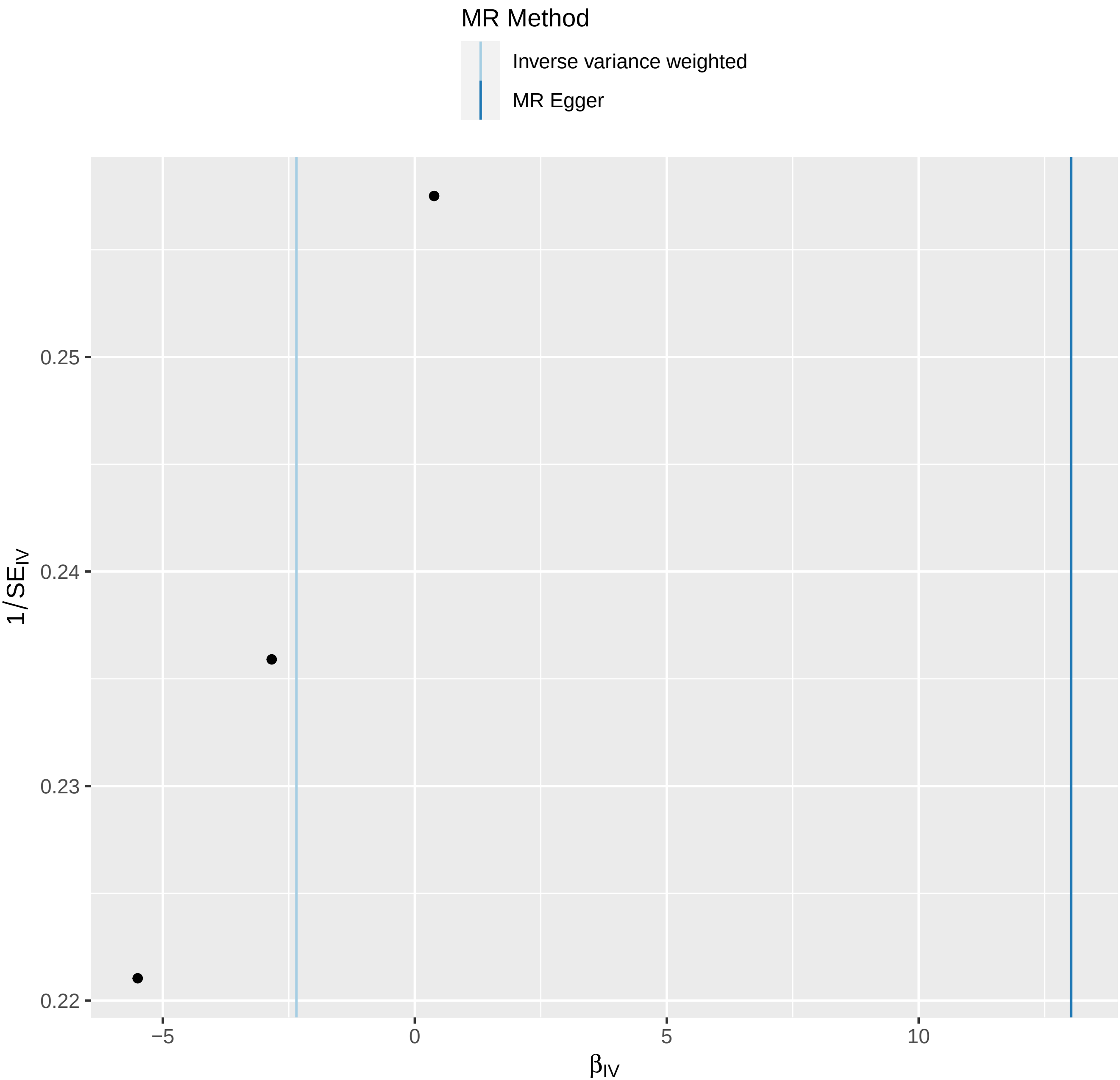

Batch 1013 : 1−eicosatrienoylglycerophosphocholine* on Arthropathy in Crohn disease

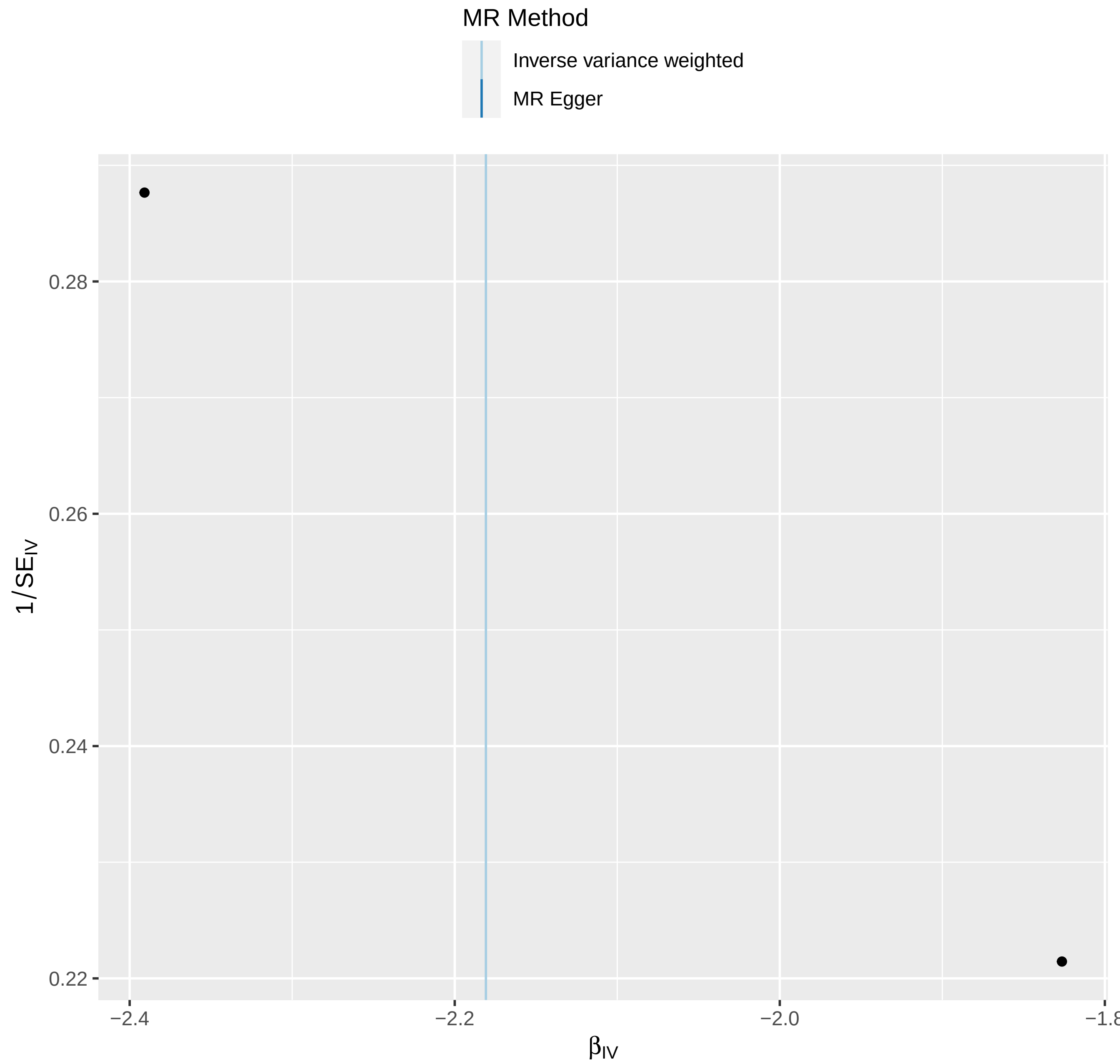

Batch 1014 : 1−docosahexaenoylglycerophosphocholine* on Arthropathy in Crohn disease

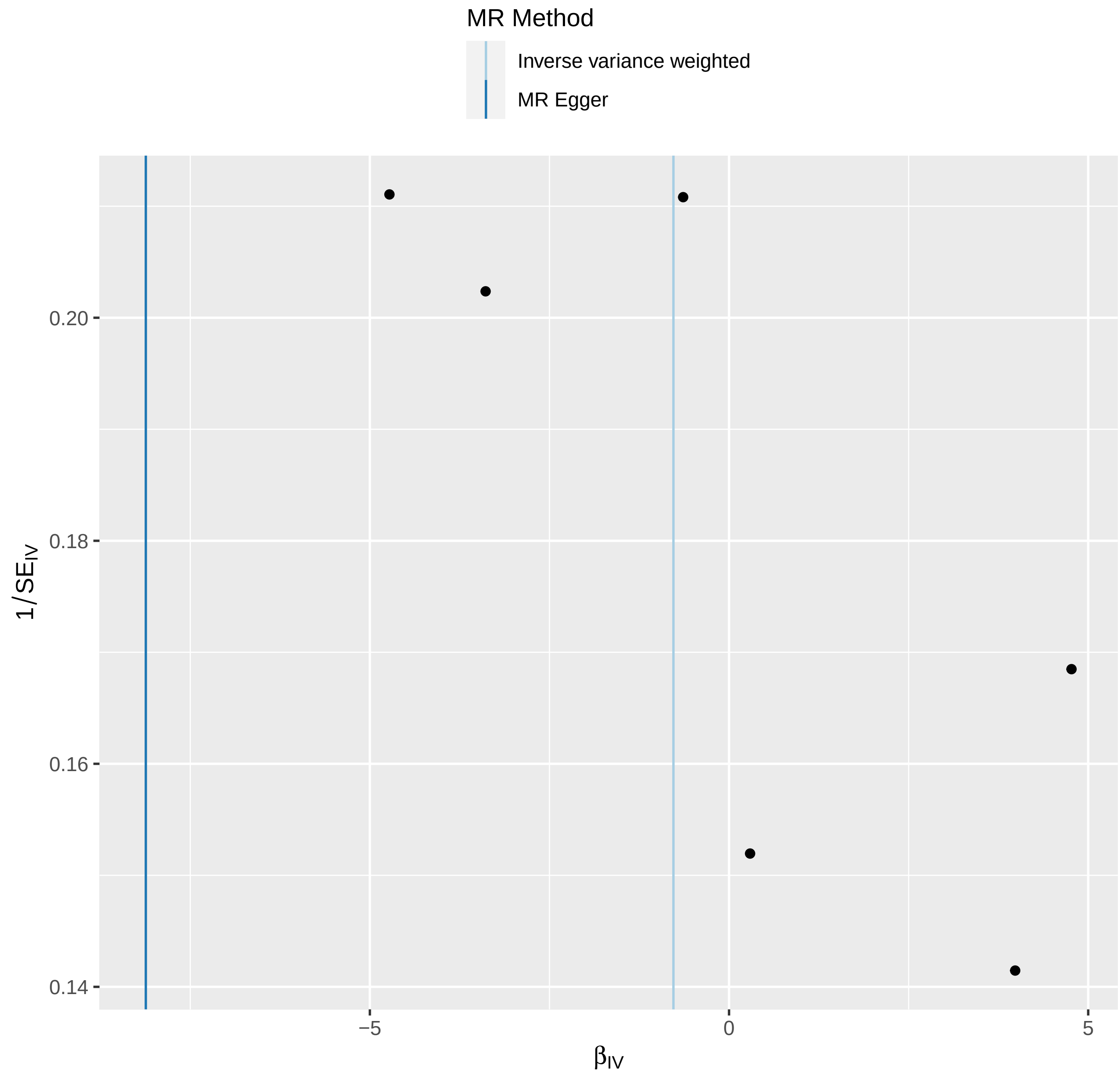

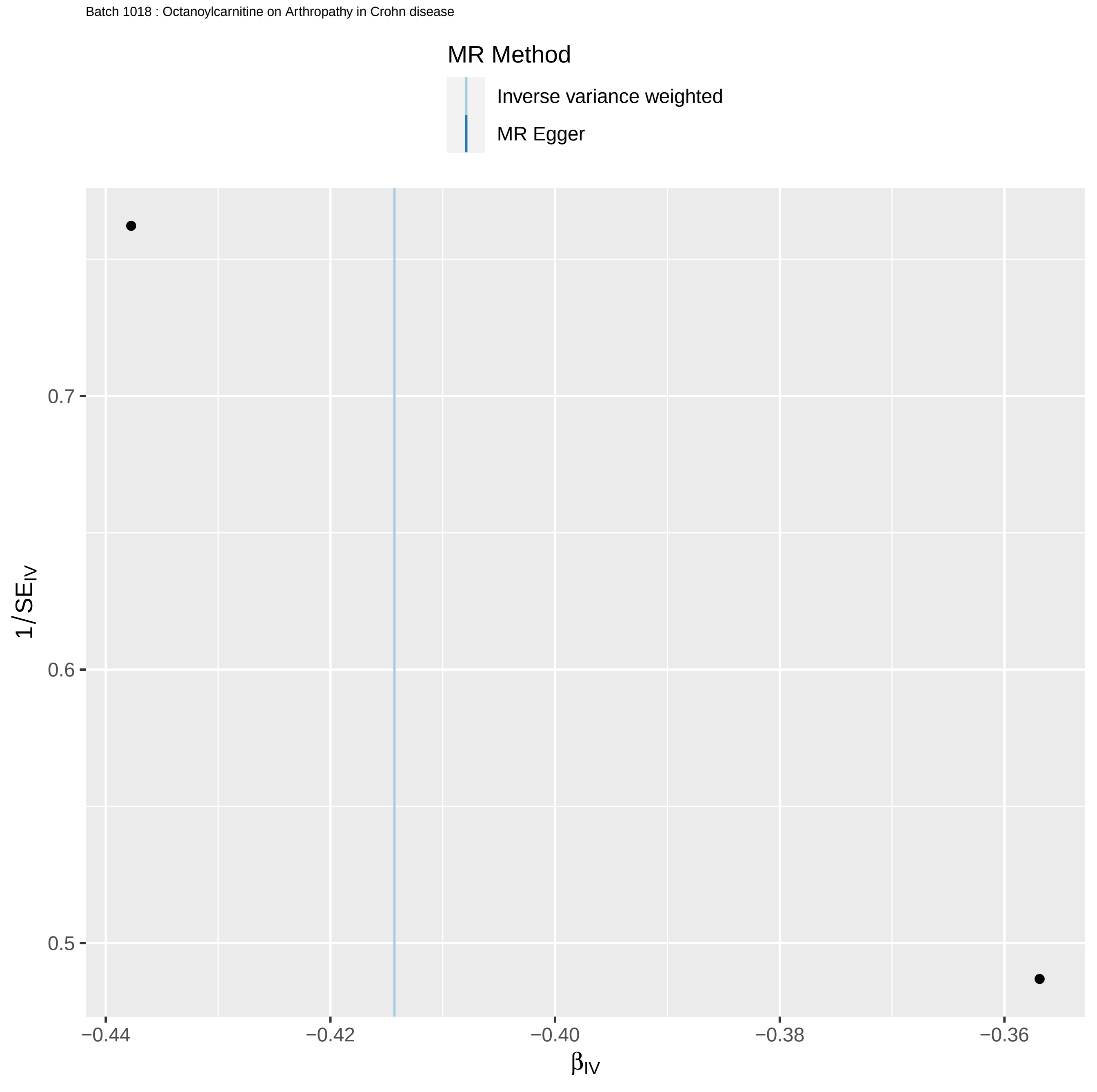

Batch 1018 : Octanoylcarnitine on Arthropathy in Crohn disease
MR Method
Inverse variance weighted
MR Egger
0.7
0.6
0.5
1/SE_IV
−0.44
−0.42
−0.40
−0.38
−0.36
β_IV

Batch 1019 : Alpha−hydroxyisovalerate on Arthropathy in Crohn disease

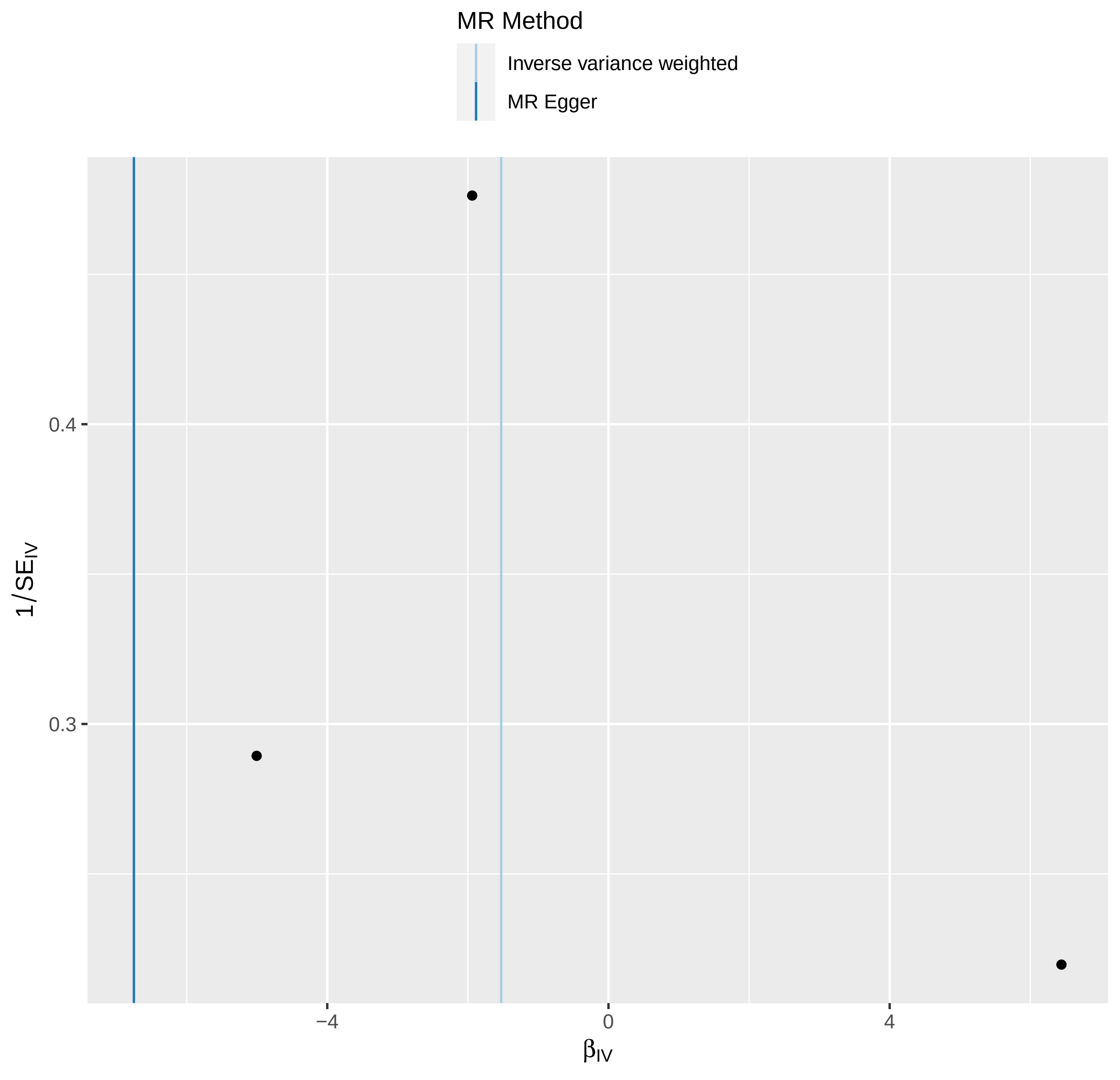

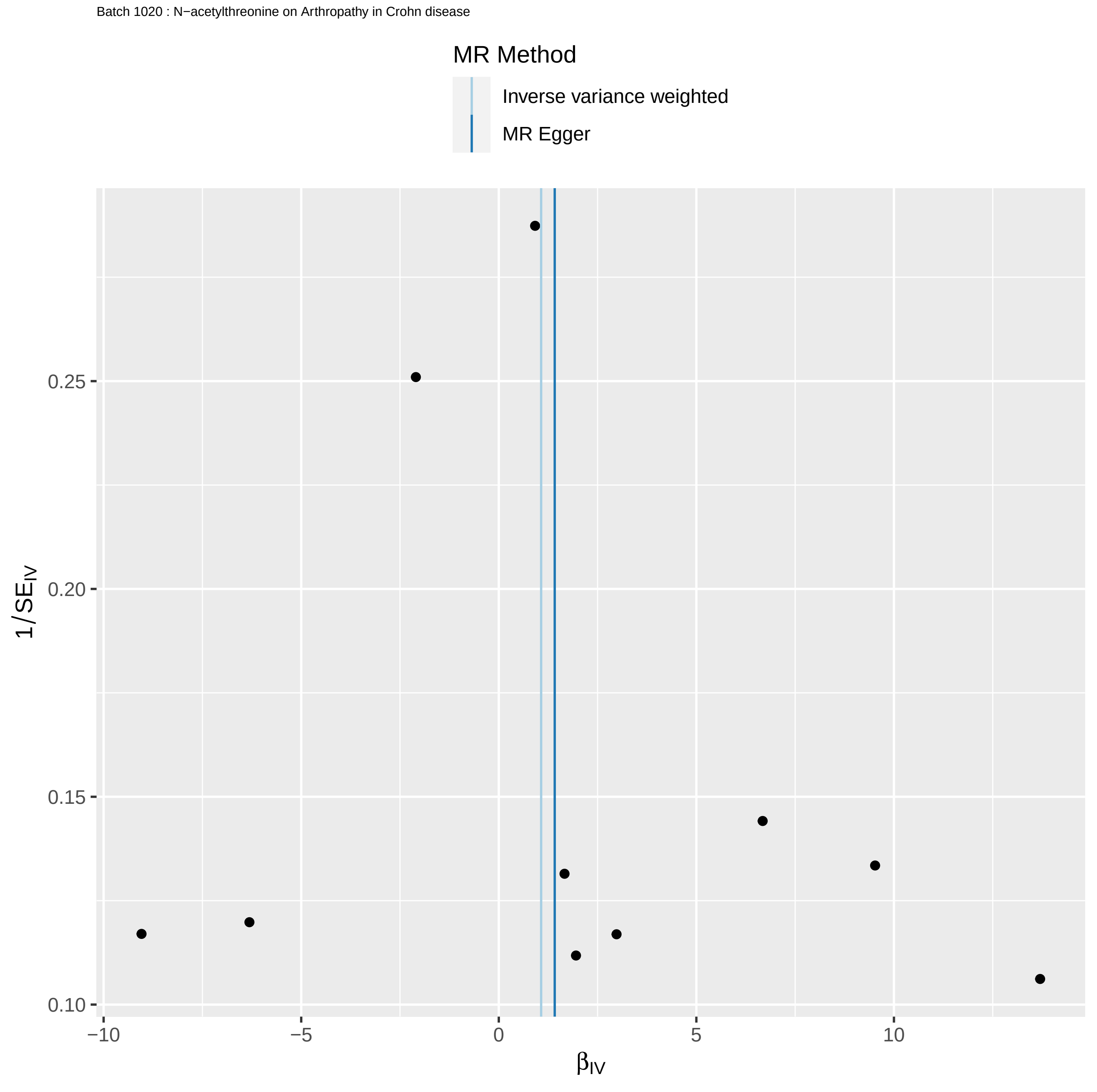

Batch 1020 : N−acetylthreonine on Arthropathy in Crohn disease
MR Method
Inverse variance weighted
MR Egger
0.25
0.20
0.15
0.10
1/SE$_{IV}$
−10
−5
0
5
10
β$_{IV}$

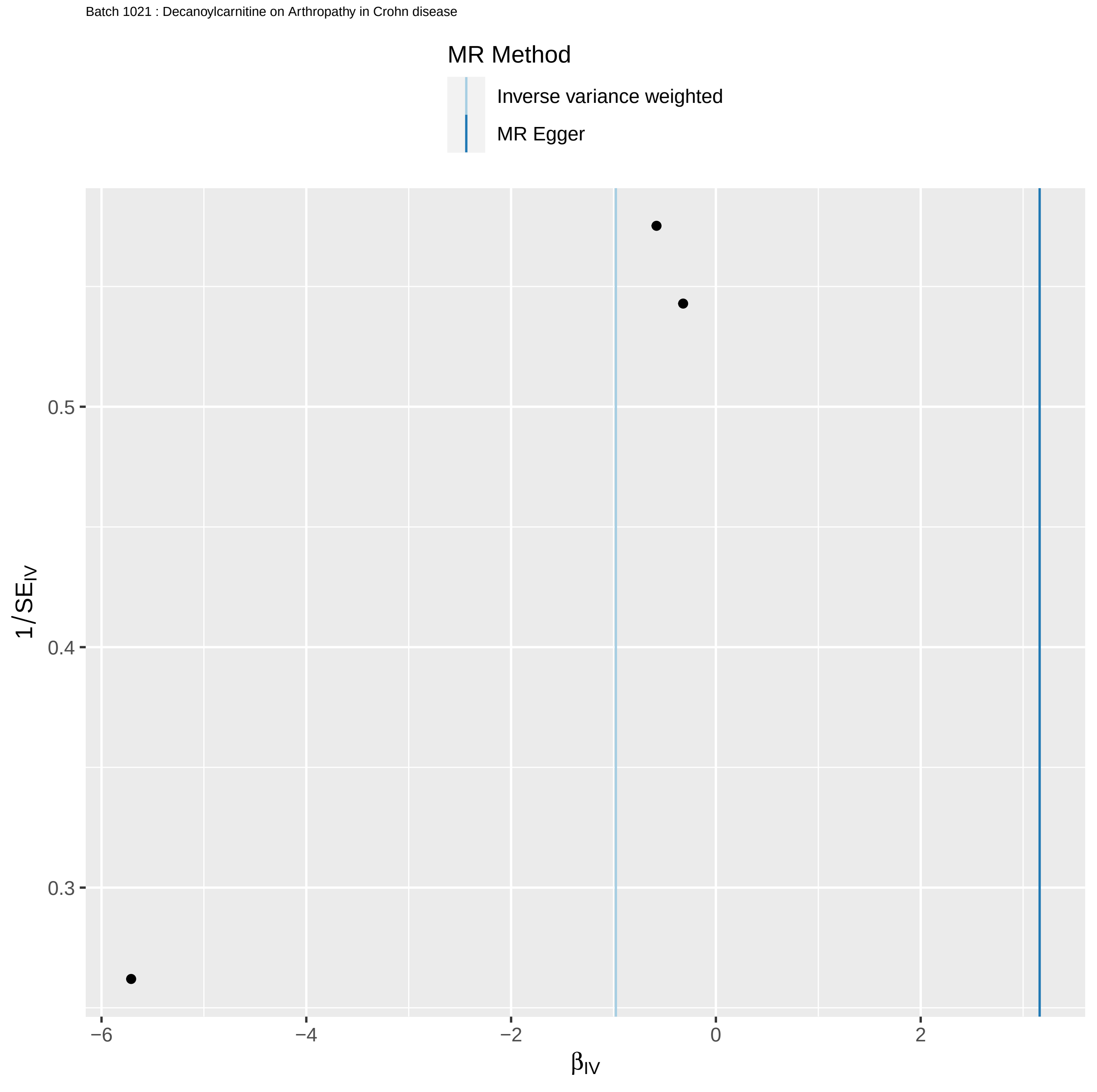
Batch 1021 : Decanoylcarnitine on Arthropathy in Crohn disease
MR Method
Inverse variance weighted
MR Egger
0.5
0.4
0.3
1/SE$_{IV}$
−6
−4
−2
0
2
β$_{IV}$

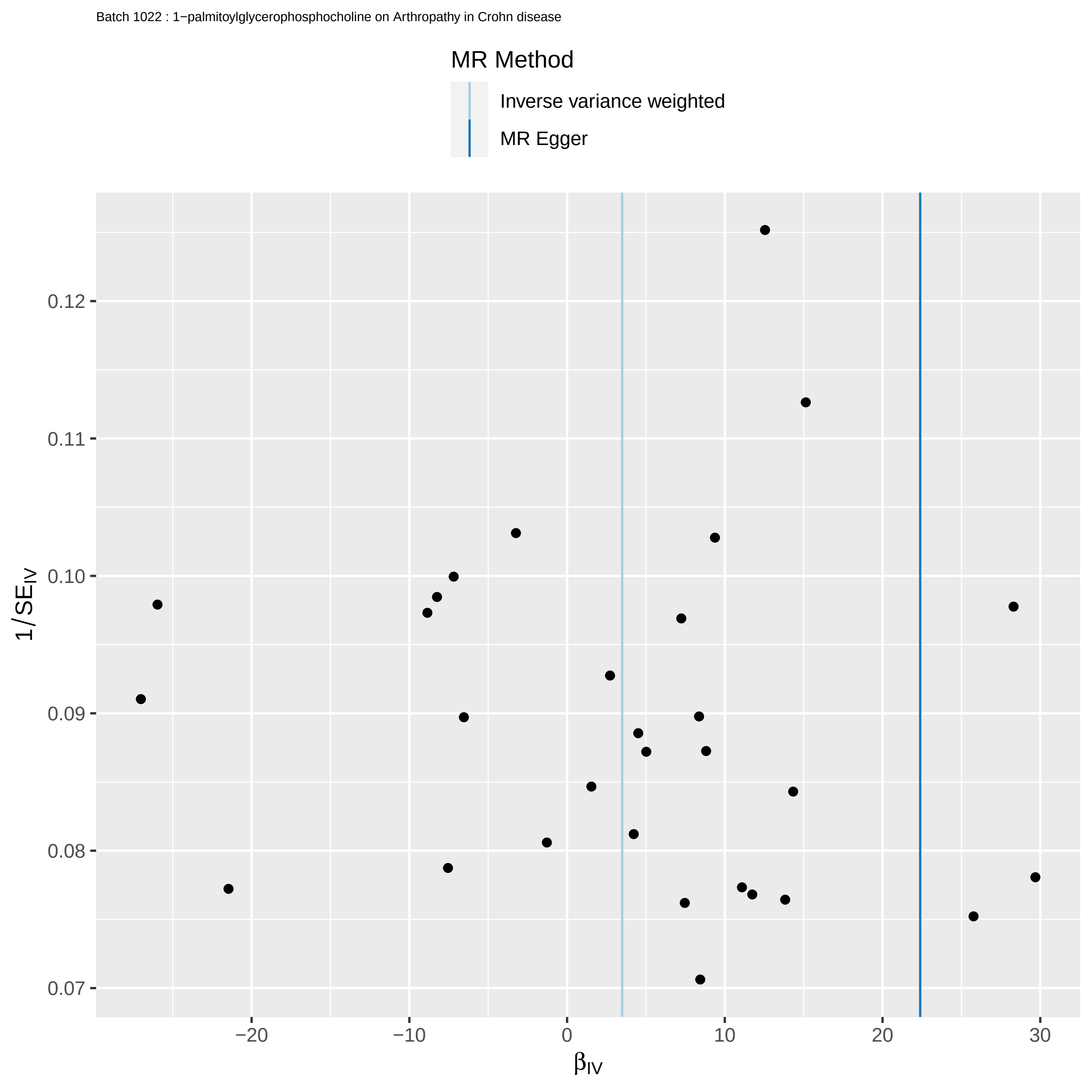

Batch 1022 : 1−palmitoylglycerophosphocholine on Arthropathy in Crohn disease
MR Method
Inverse variance weighted
MR Egger
0.12
0.11
0.10
0.09
0.08
0.07
1/SE$_{IV}$
−20
−10
0
10
20
30
β_{IV}

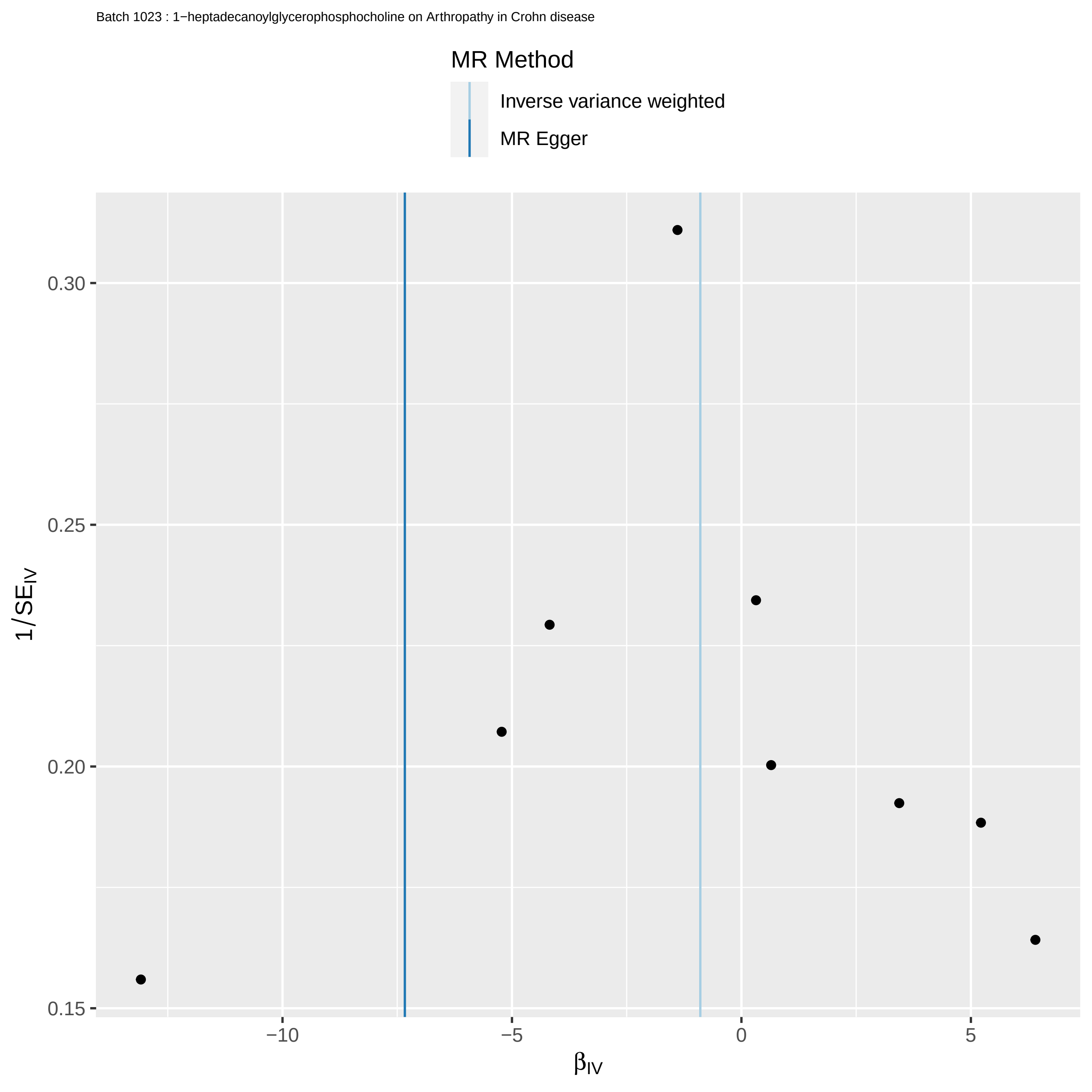
Batch 1023 : 1−heptadecanoylglycerophosphocholine on Arthropathy in Crohn disease
MR Method
Inverse variance weighted
MR Egger
0.30
0.25
0.20
0.15
1/SE_IV
−10
−5
0
5
β_IV

Batch 1024 : 1−oleoylglycerophosphocholine on Arthropathy in Crohn disease

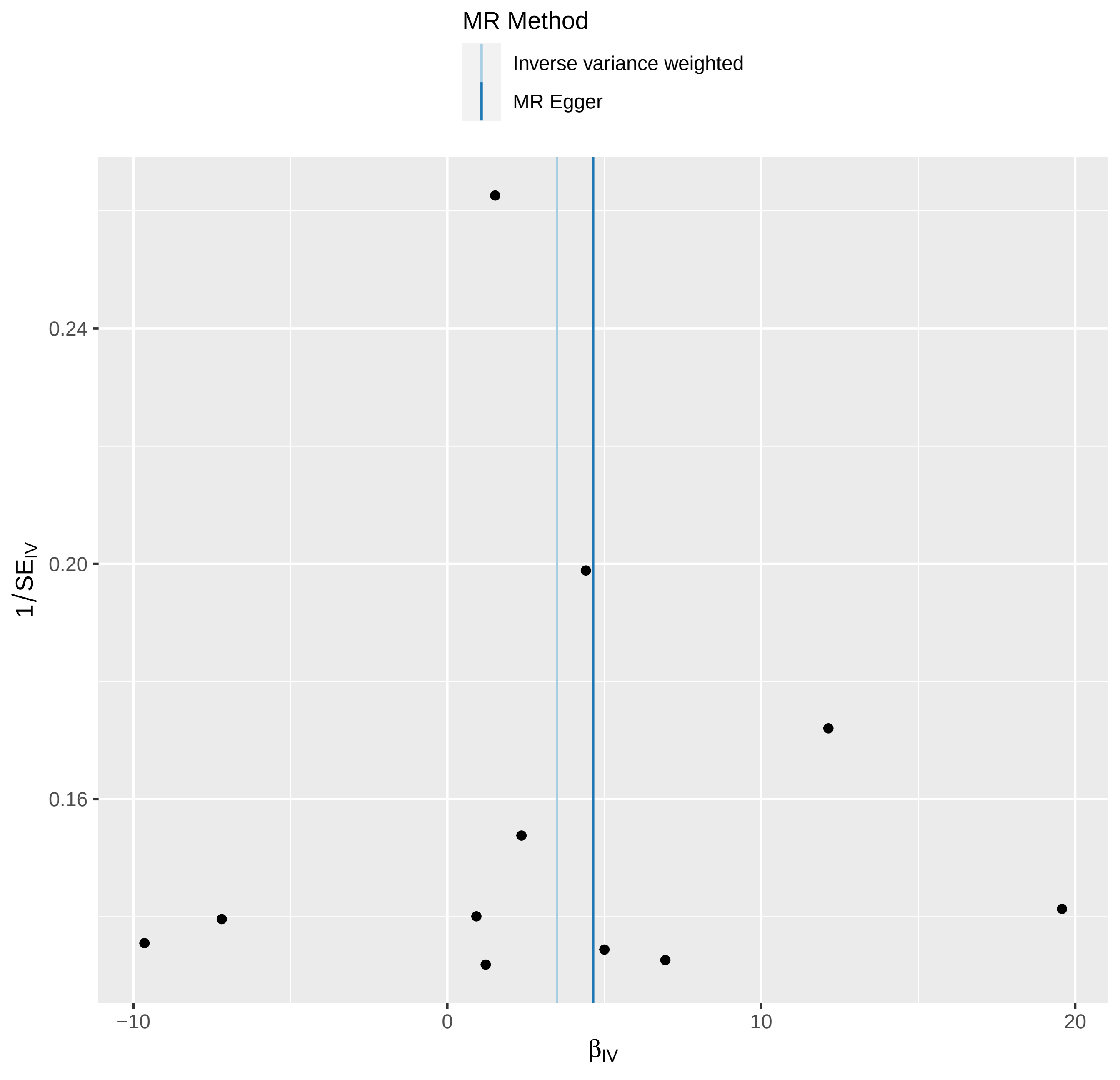

Batch 1025 : 1−stearoylglycerophosphocholine on Arthropathy in Crohn disease

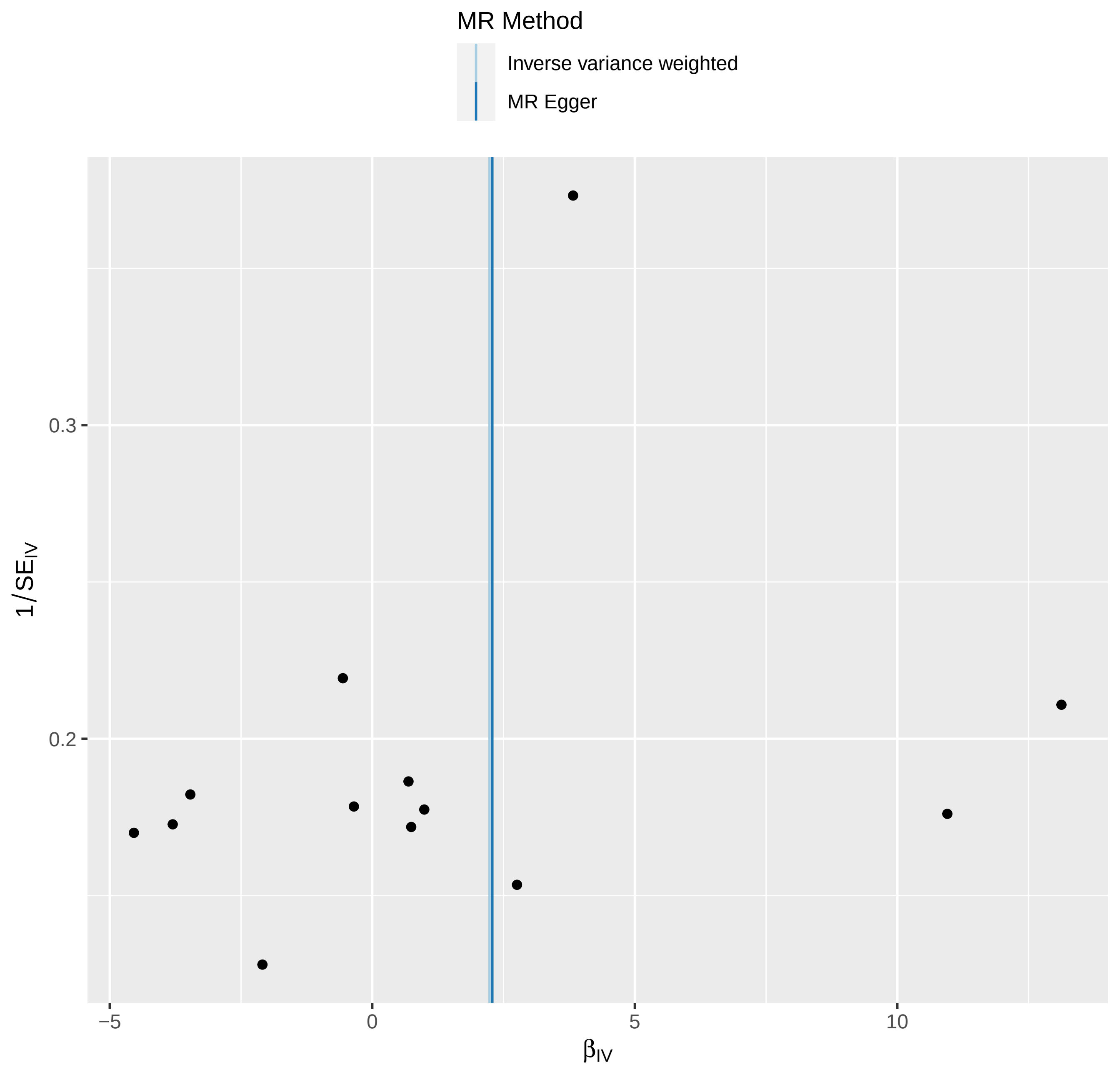

Batch 1028 : 10−heptadecenoate (17:1n7) on Arthropathy in Crohn disease

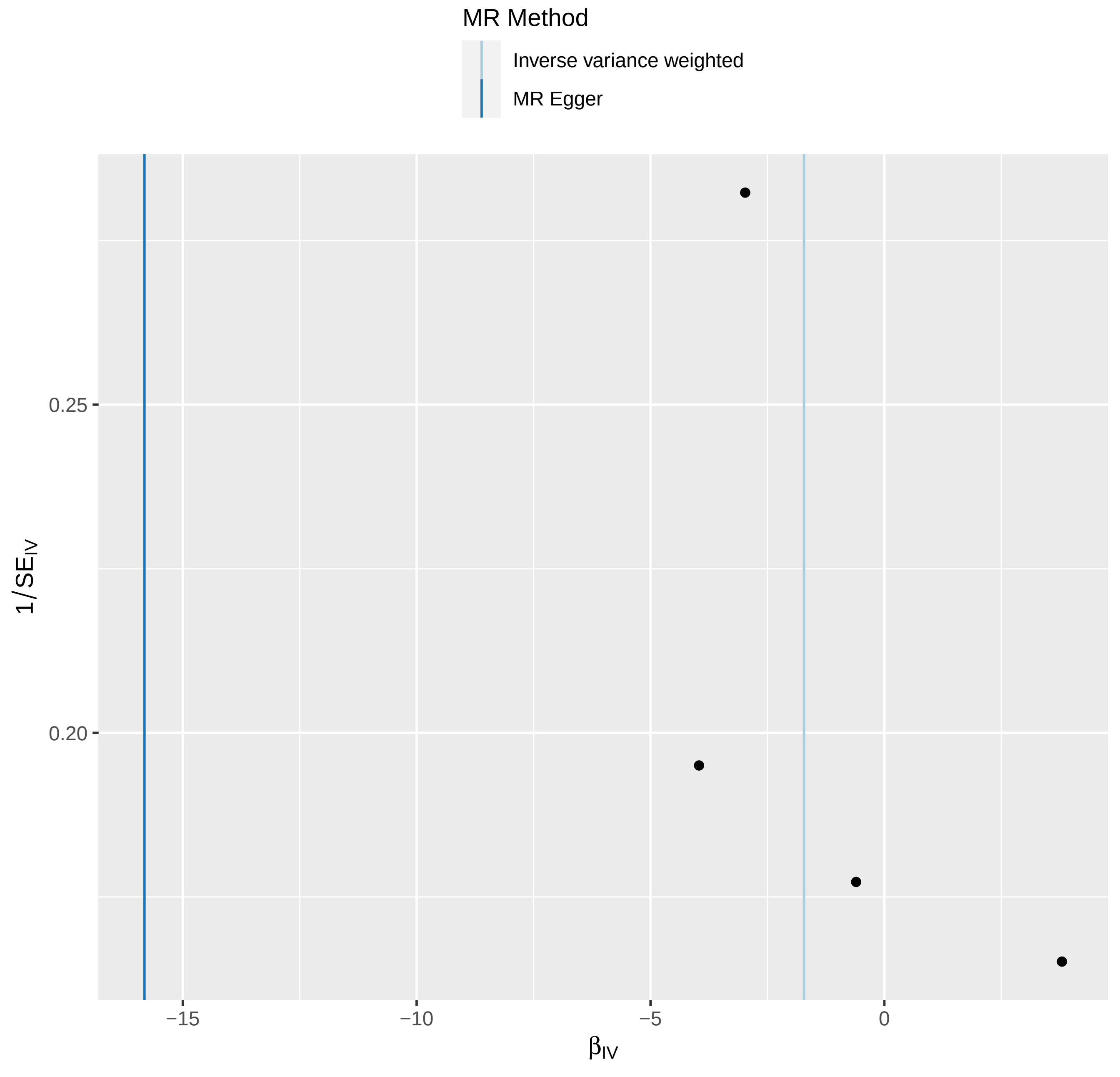

Batch 1030 : Epiandrosterone sulfate on Arthropathy in Crohn disease

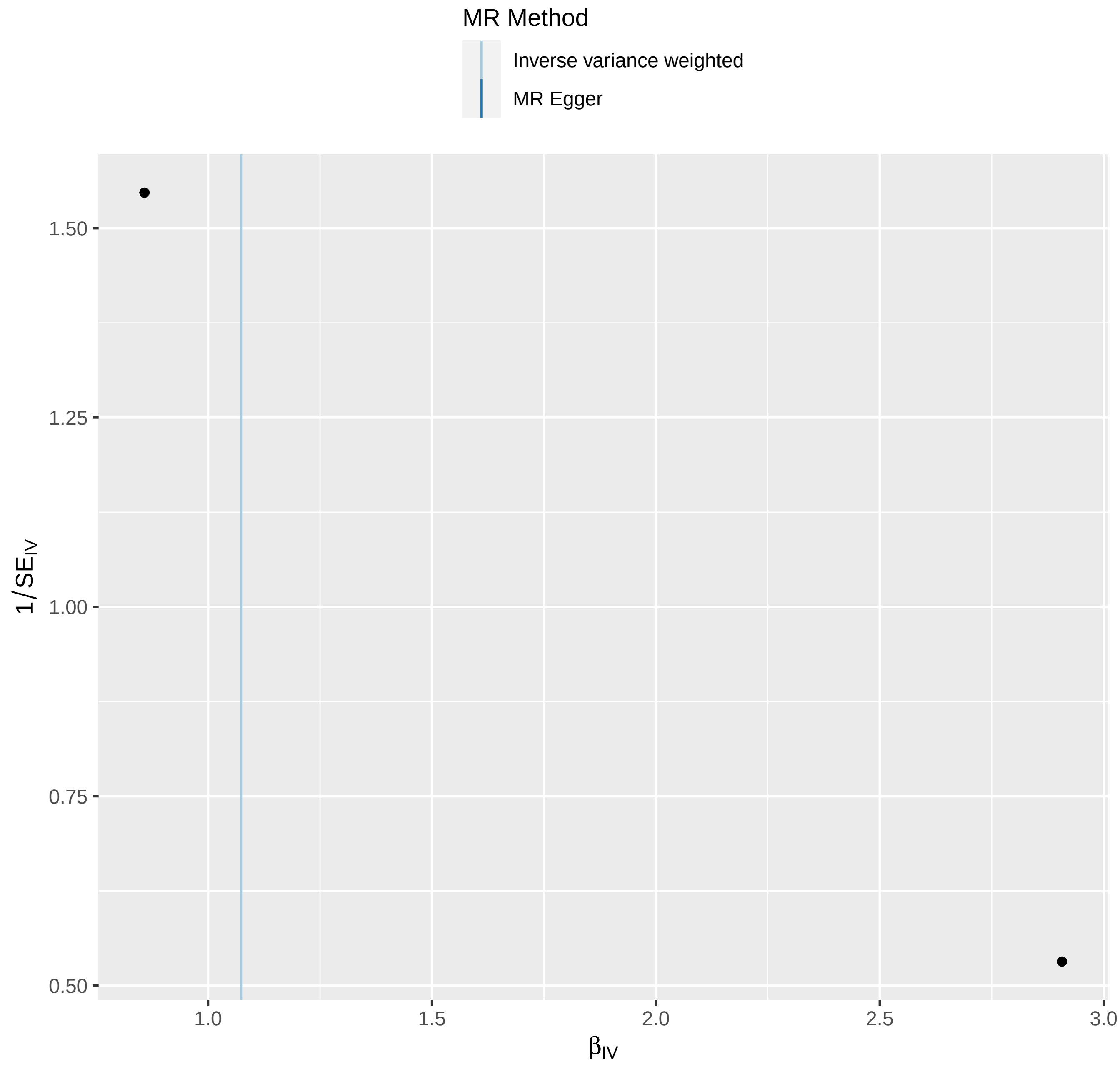

Batch 1031 : Linolenate [alpha or gamma; (18:3n3 or 6)] on Arthropathy in Crohn disease

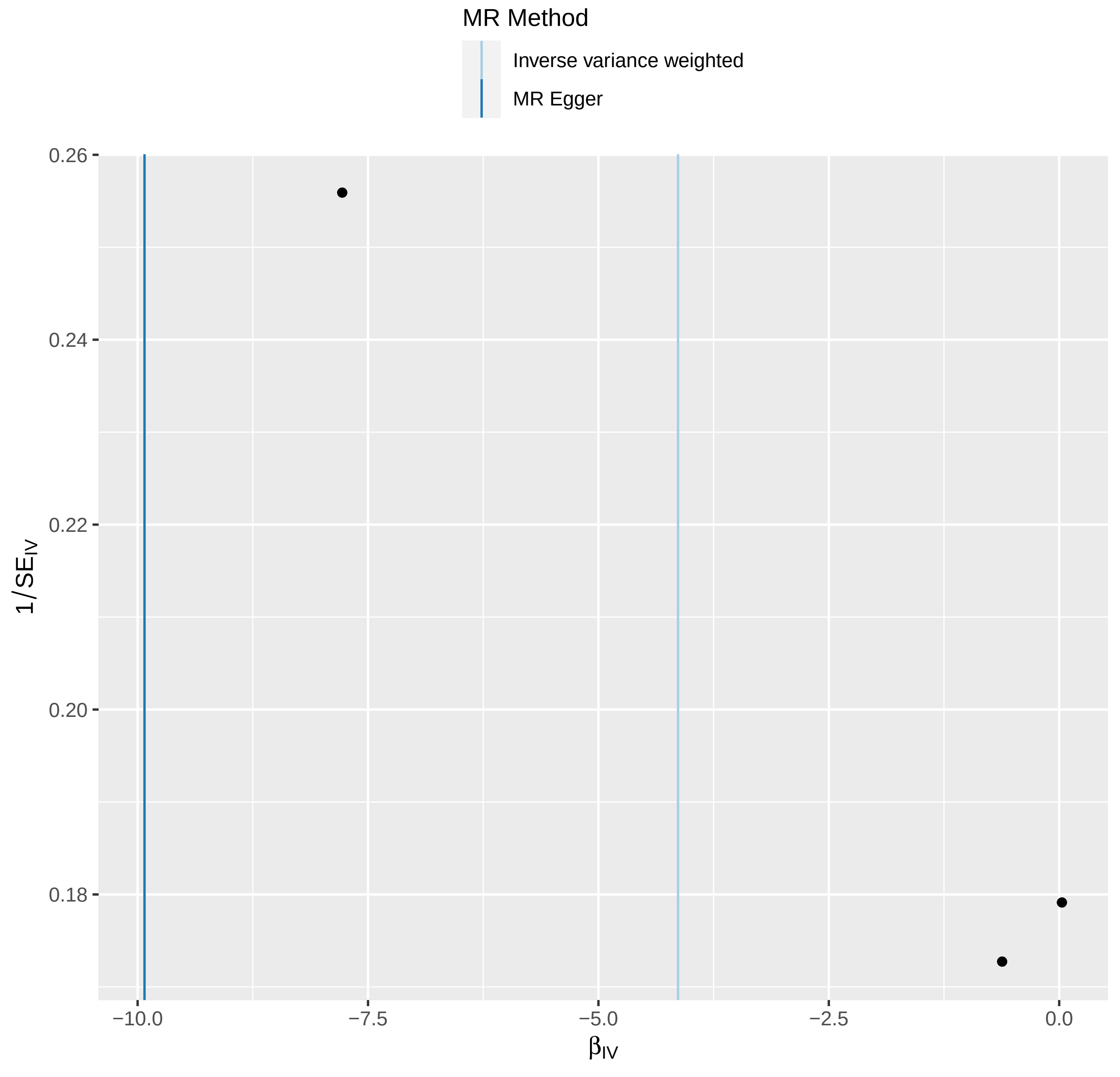

Batch 1032 : X−12510−−2−aminooctanoic acid on Arthropathy in Crohn disease

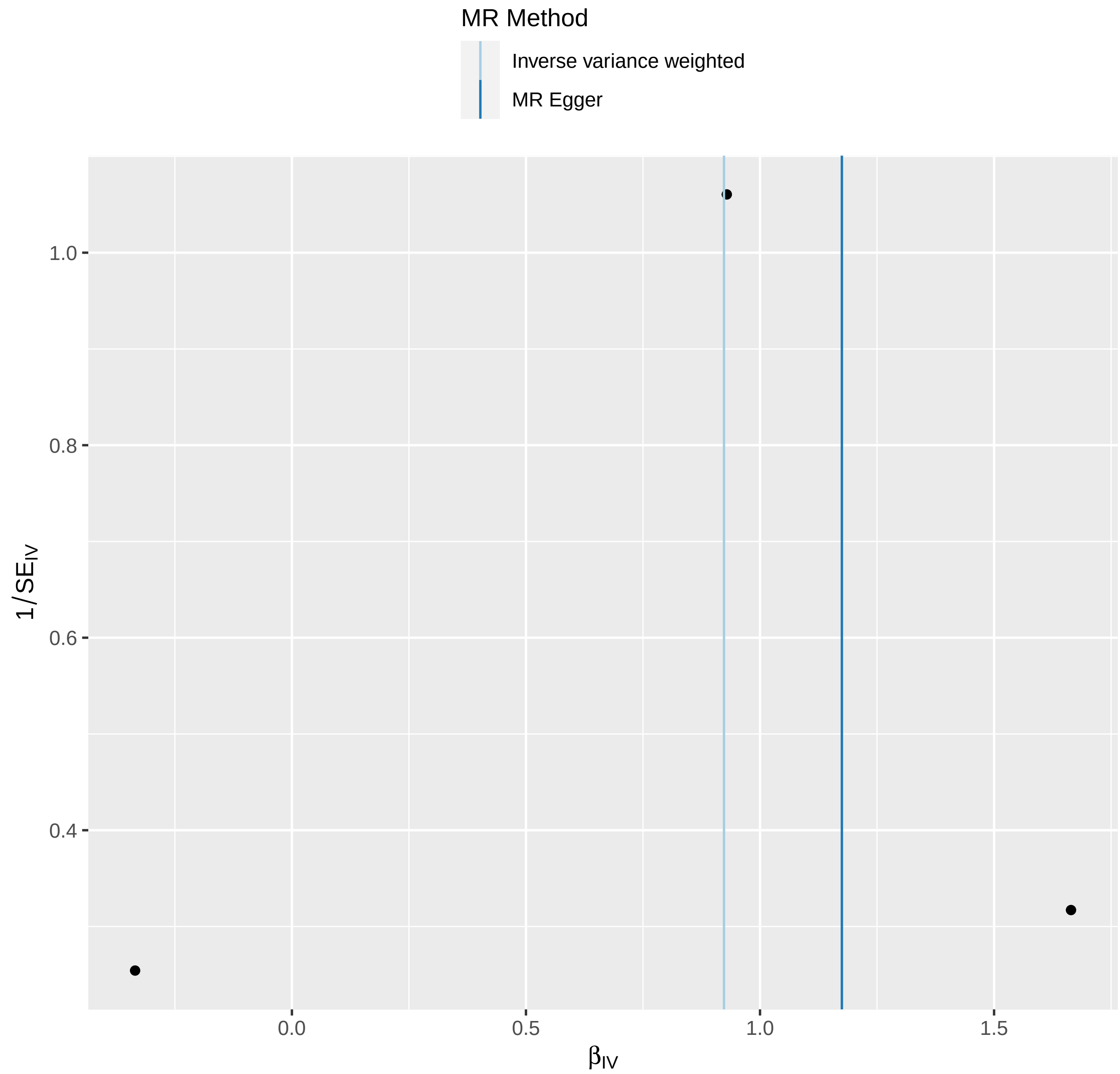

Batch 1035 : Asparagine on Arthropathy in Crohn disease

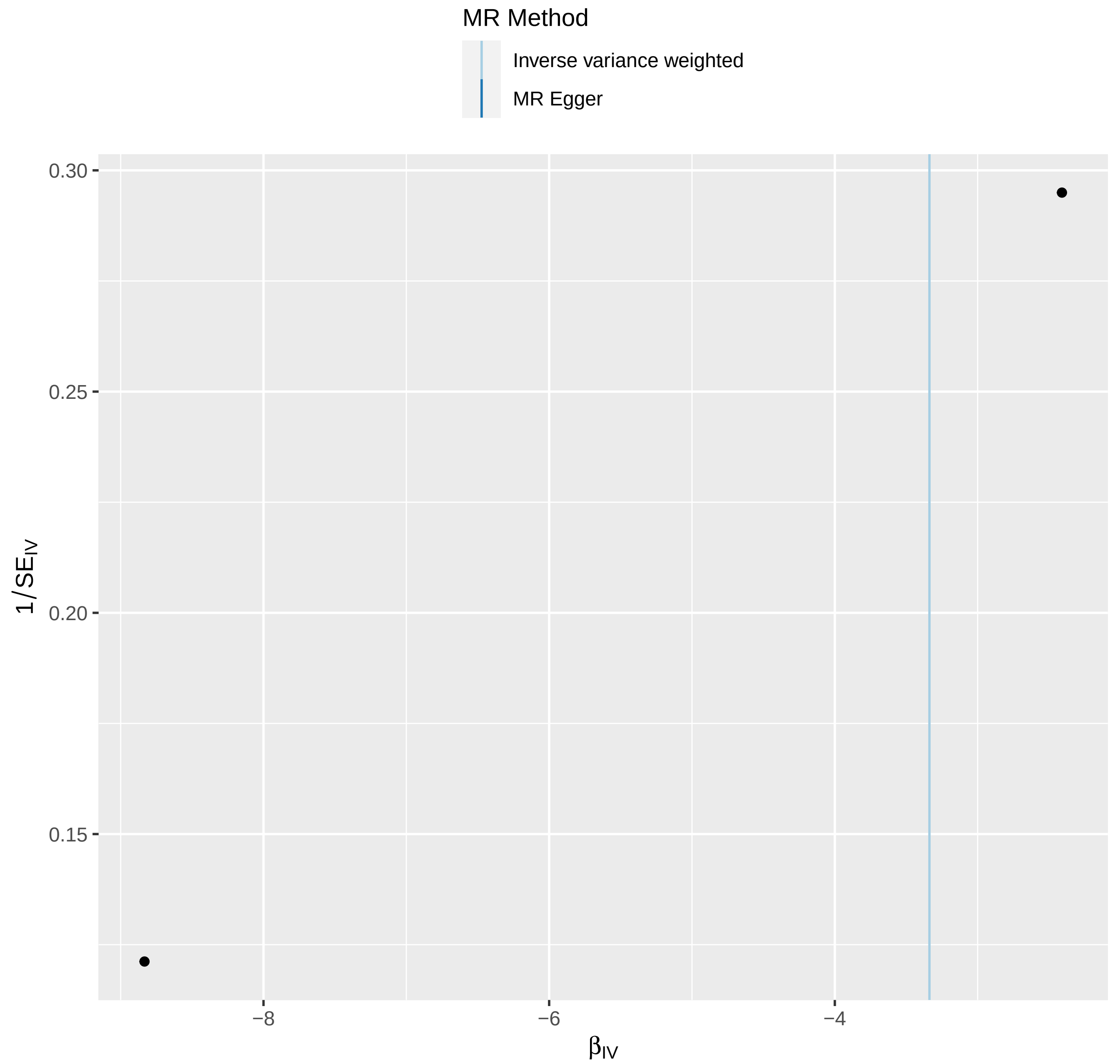

Batch 1036 : Stachydrine on Arthropathy in Crohn disease

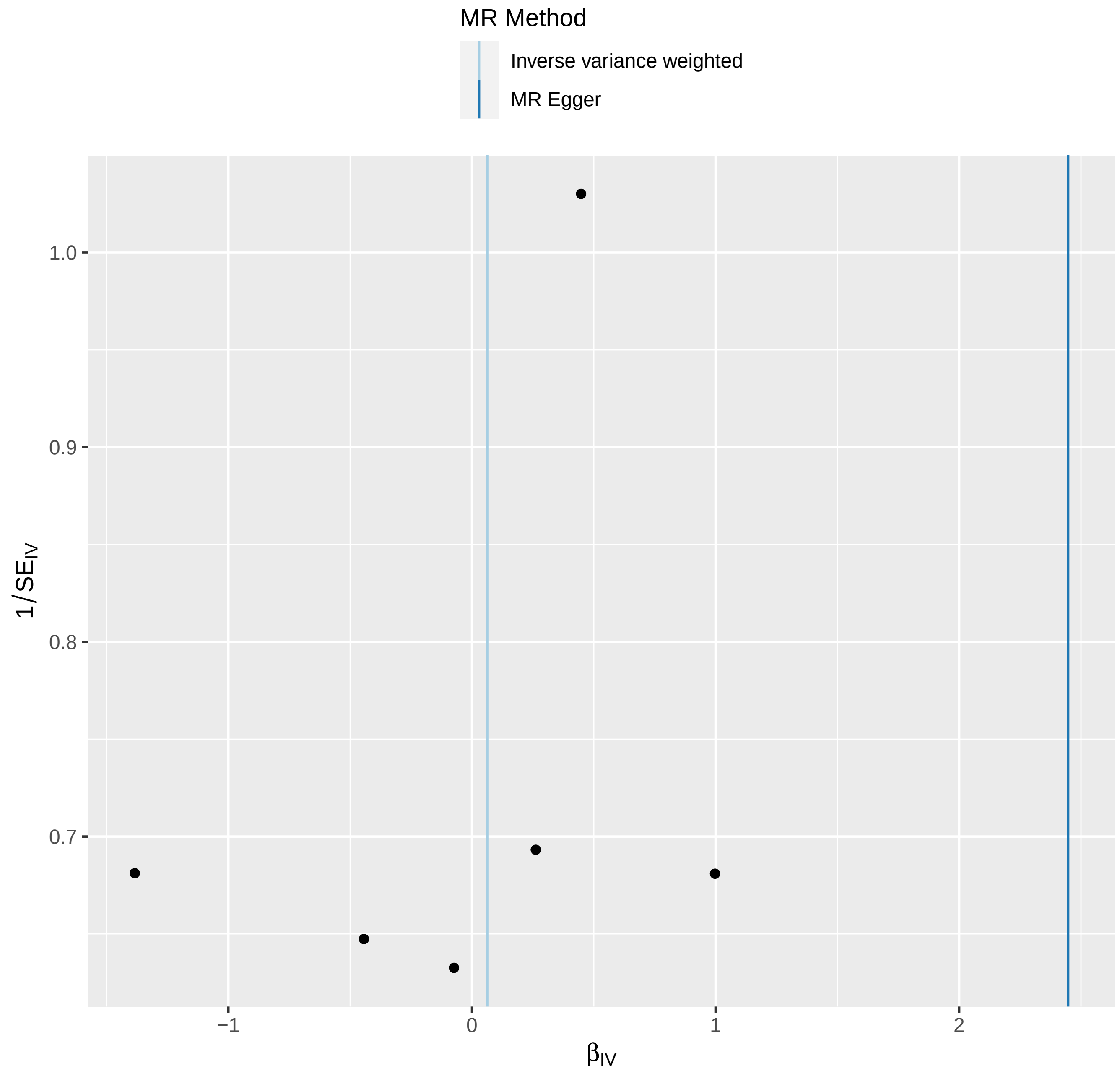

Batch 1037 : Isovalerylcarnitine on Arthropathy in Crohn disease

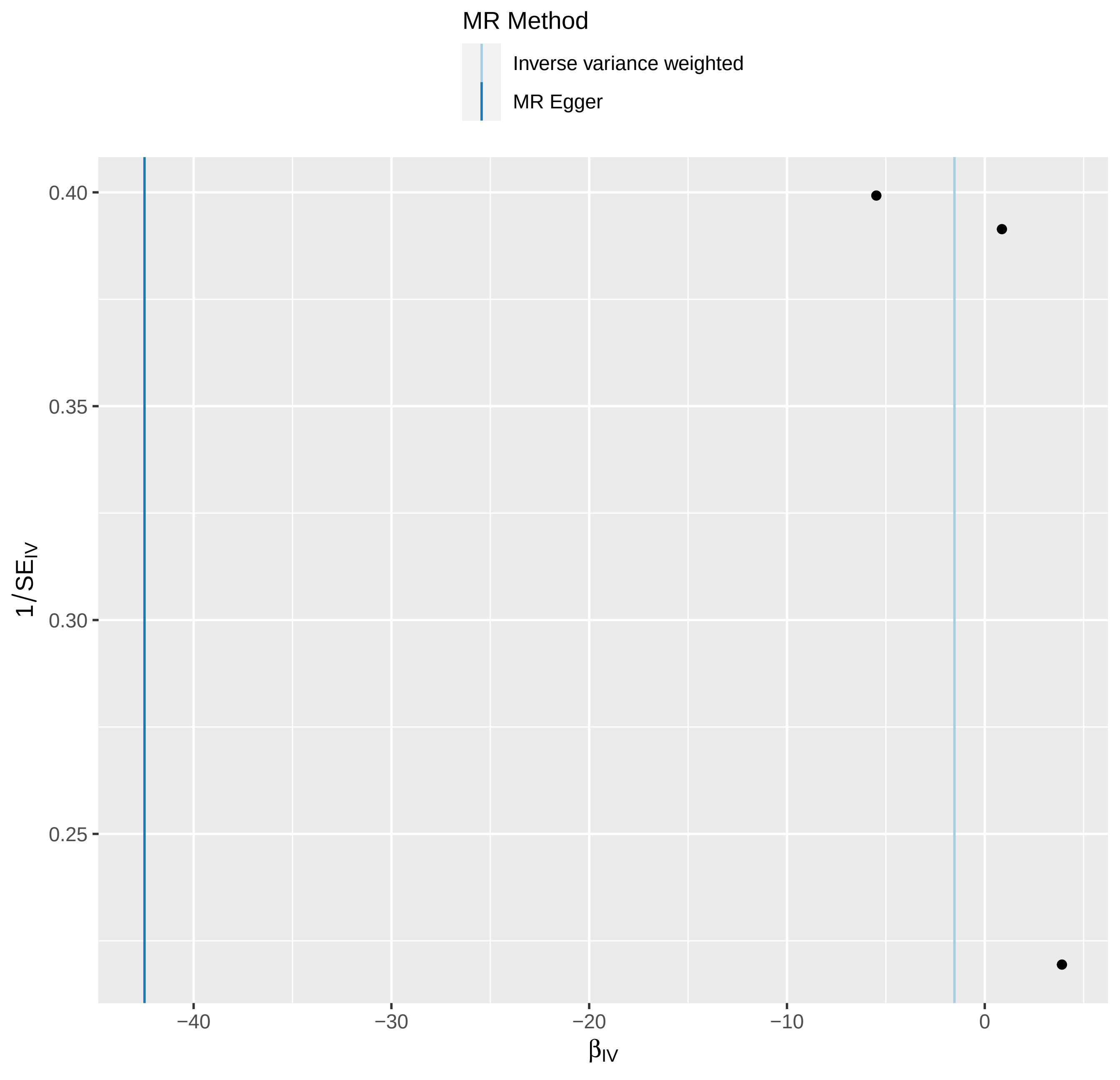

Batch 1040 : 1−linoleoylglycerophosphocholine on Arthropathy in Crohn disease

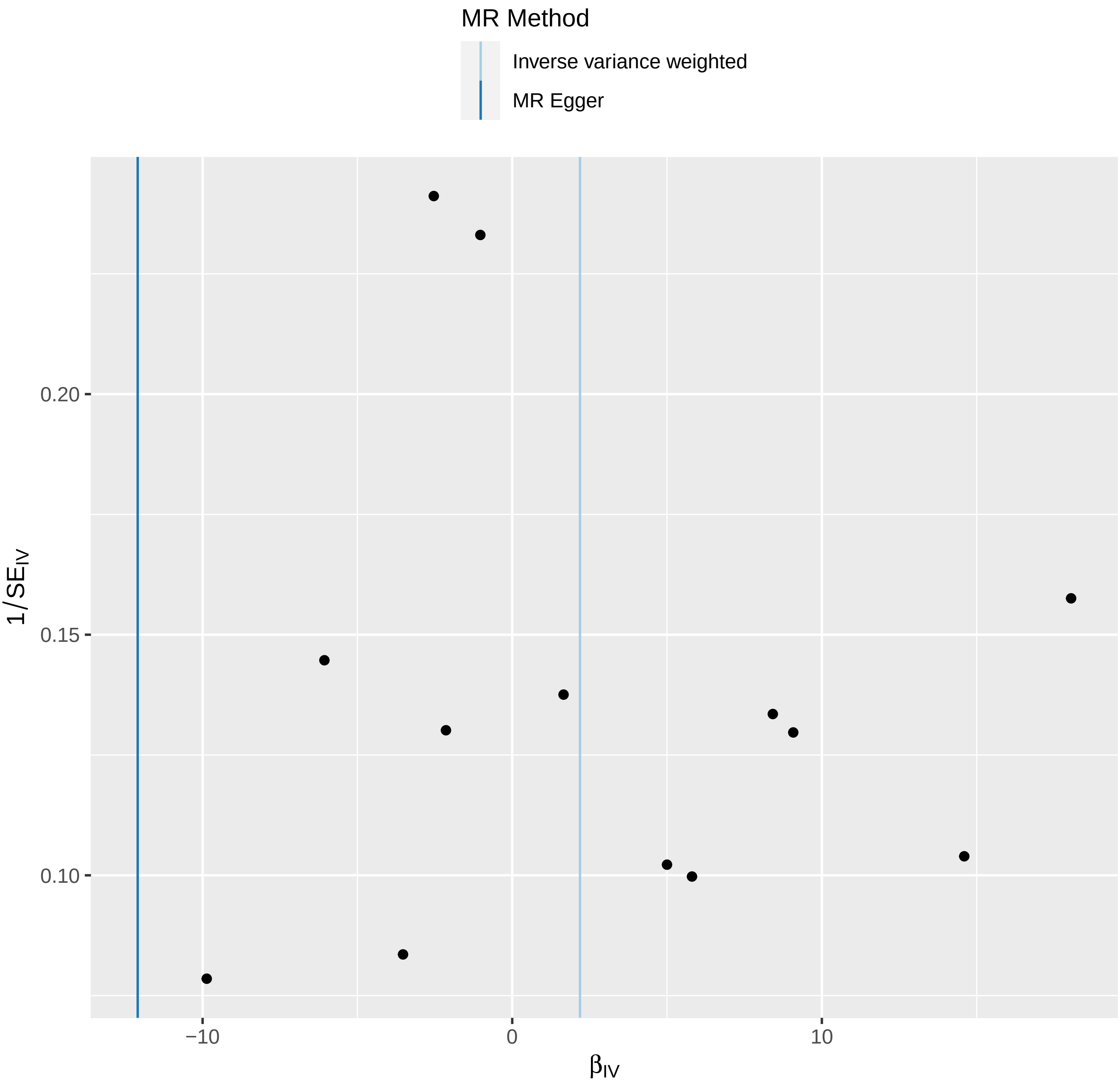

Batch 1041 : Bradykinin, des−arg(9) on Arthropathy in Crohn disease

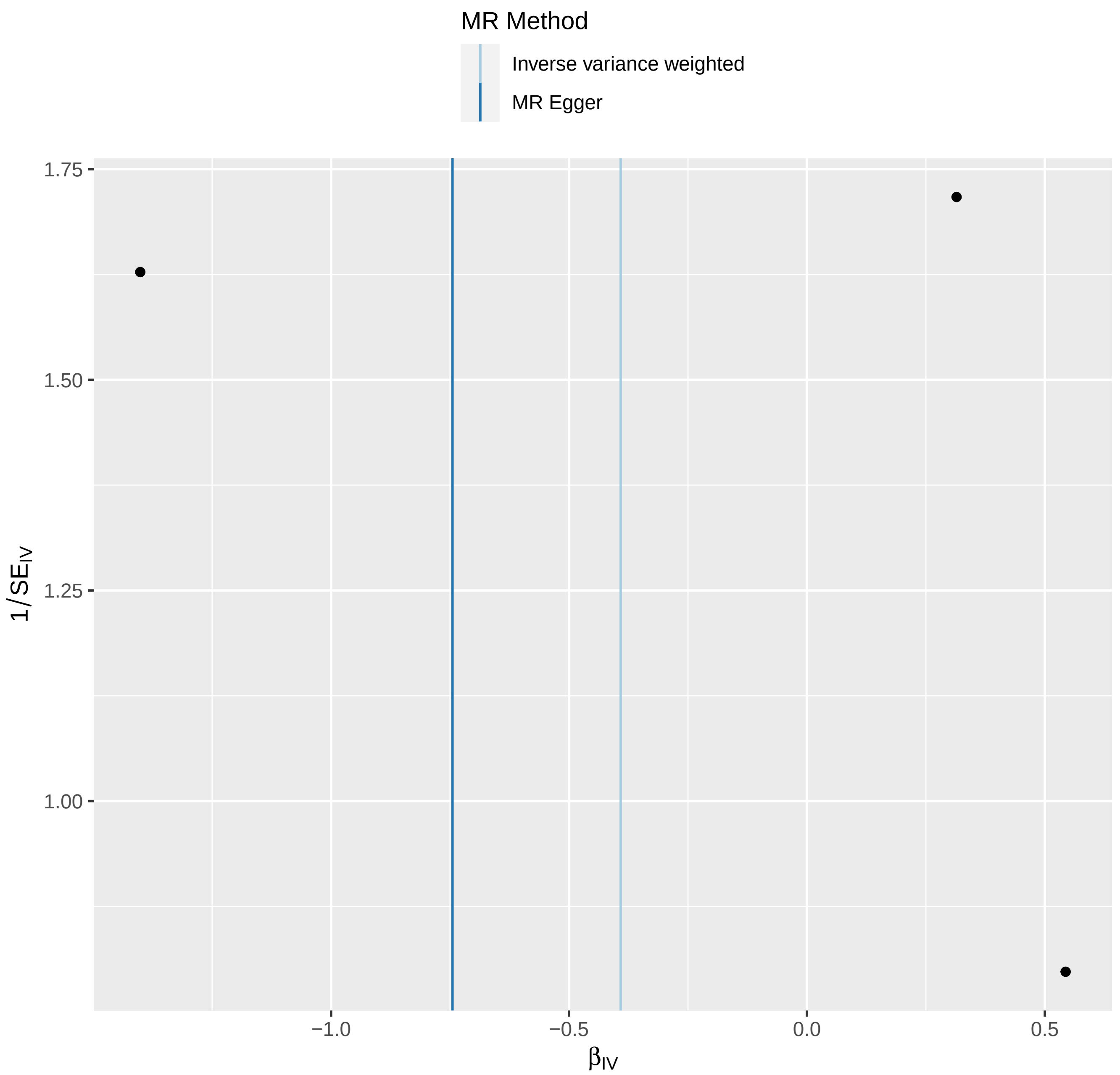

Batch 1042 : Gamma−glutamylisoleucine* on Arthropathy in Crohn disease

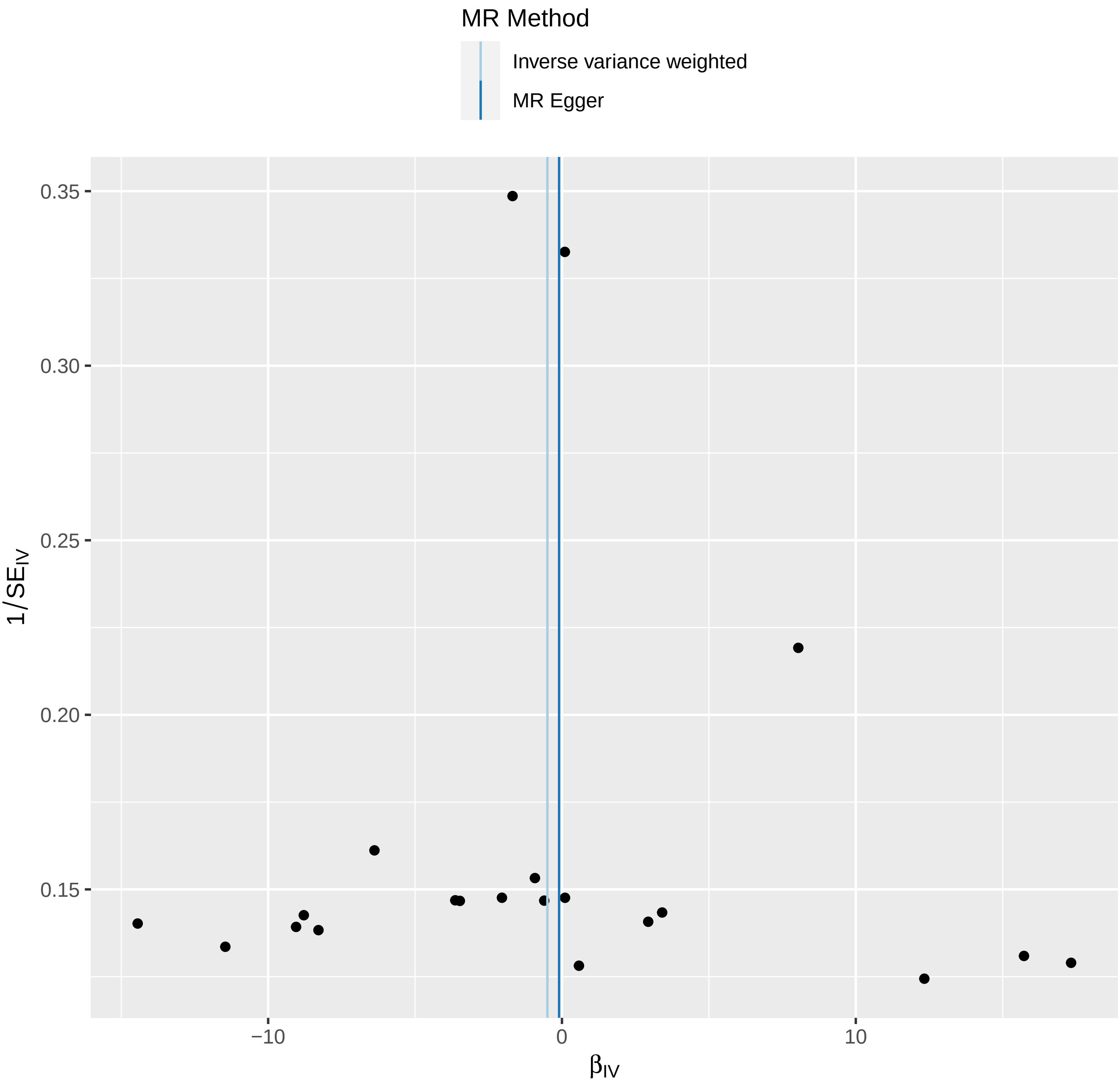

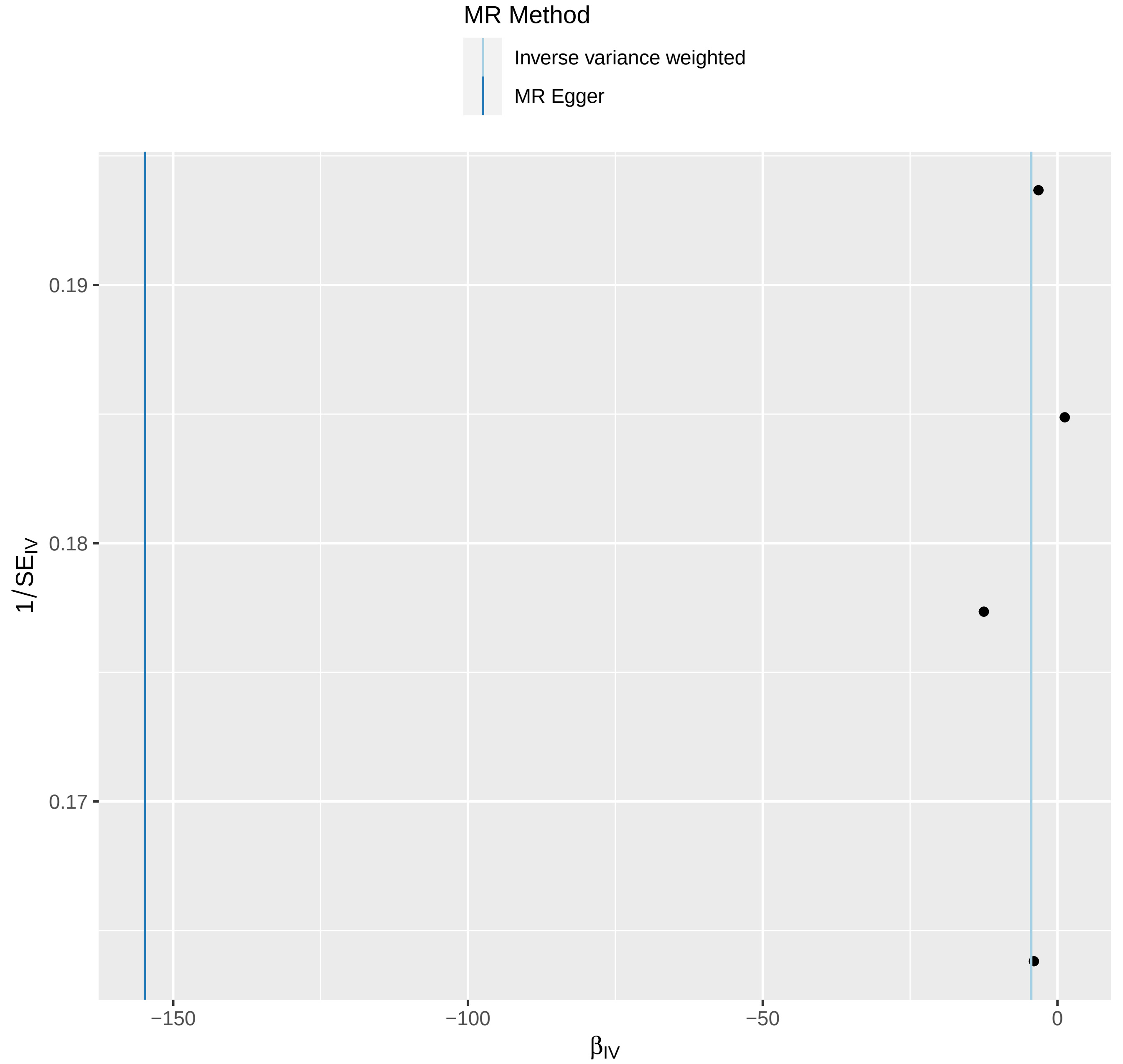
MR Method
Inverse variance weighted
MR Egger
0.19
0.18
0.17
$1/SE_{IV}$
−150
−100
−50
0
β_{IV}

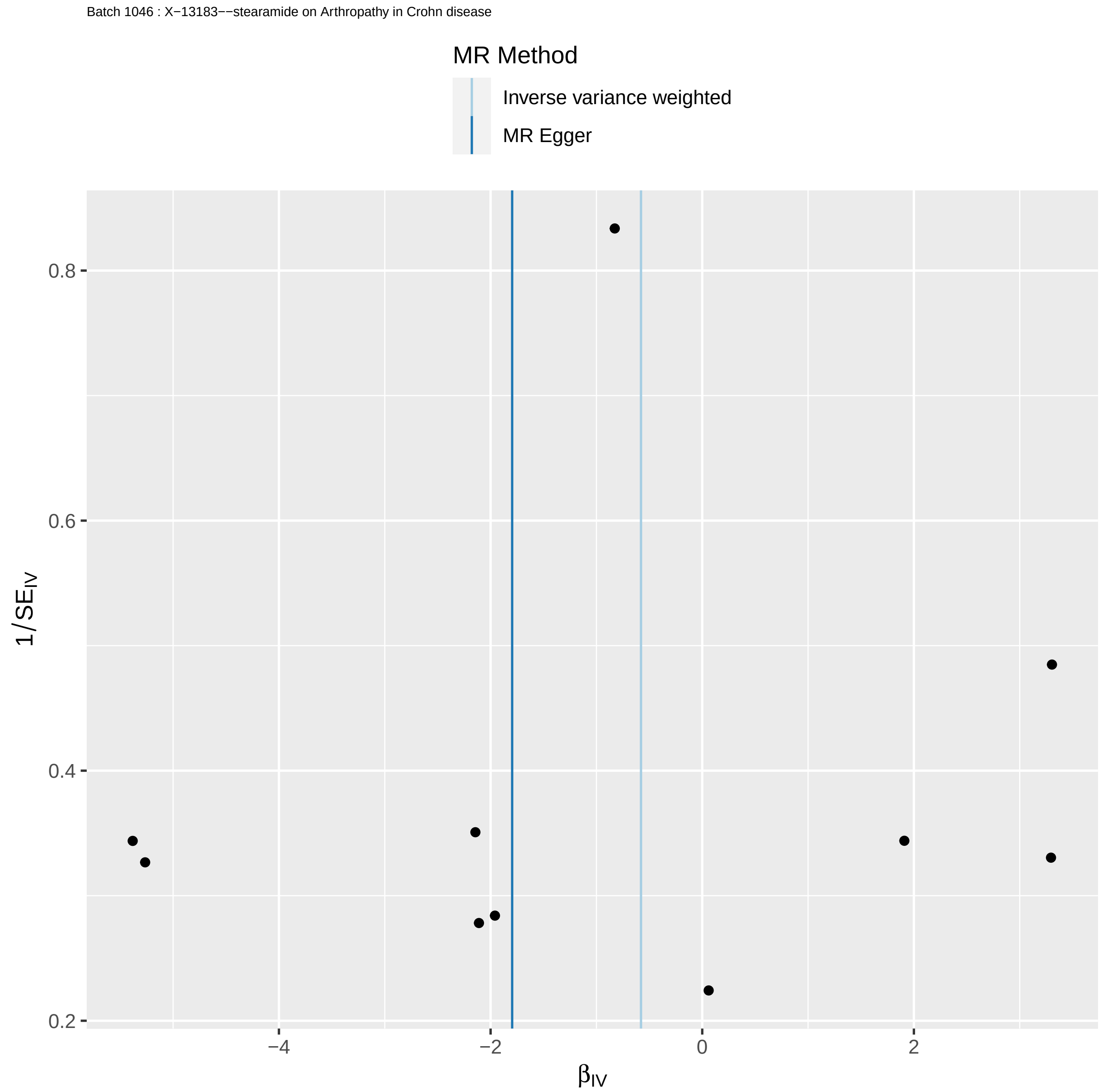

Batch 1046 : X−13183−−stearamide on Arthropathy in Crohn disease
MR Method
Inverse variance weighted
MR Egger
0.8
0.6
0.4
0.2
1/SE$_{IV}$
−4
−2
0
2
β$_{IV}$

Batch 1047 : 7−methylguanine on Arthropathy in Crohn disease

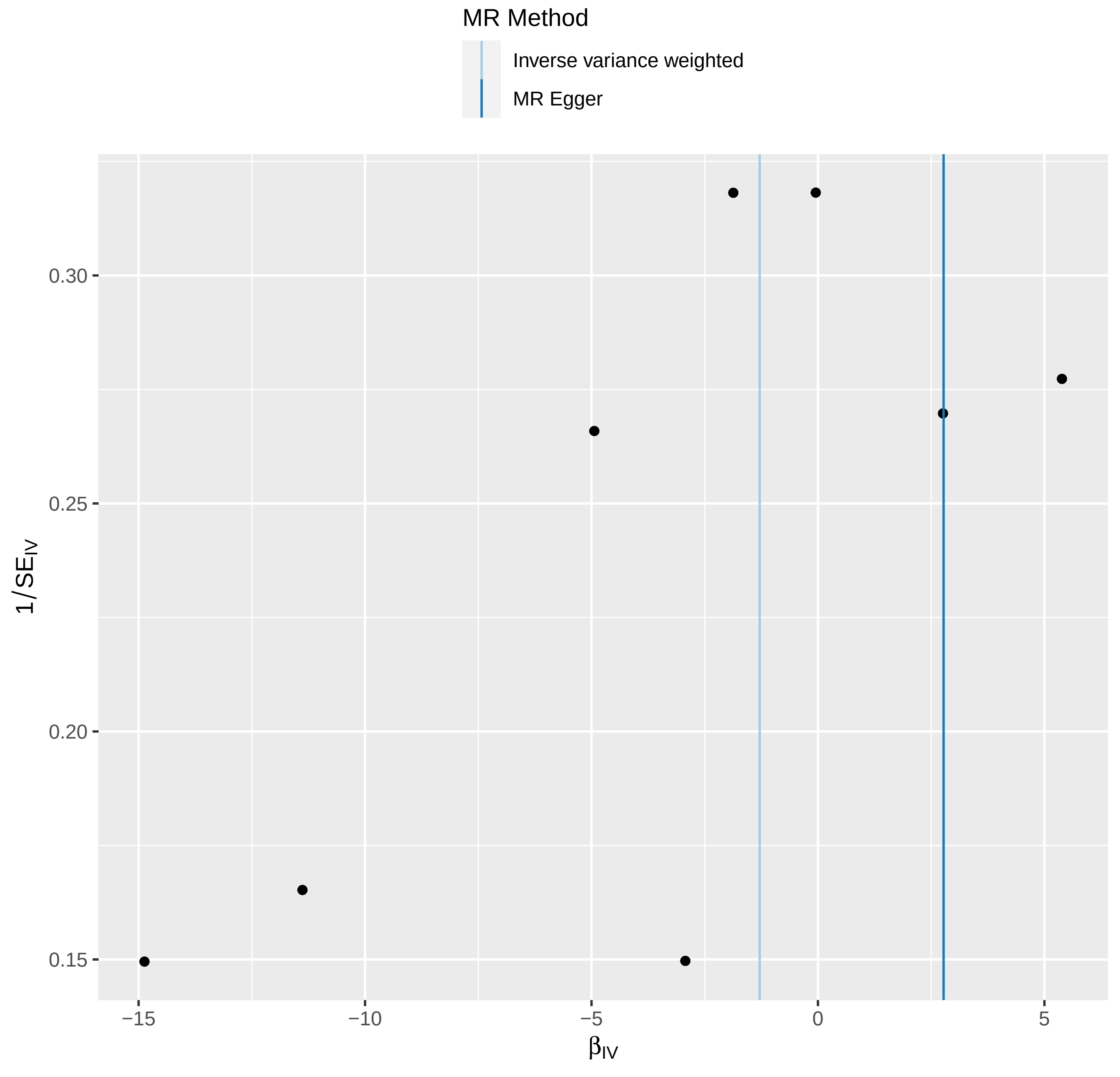

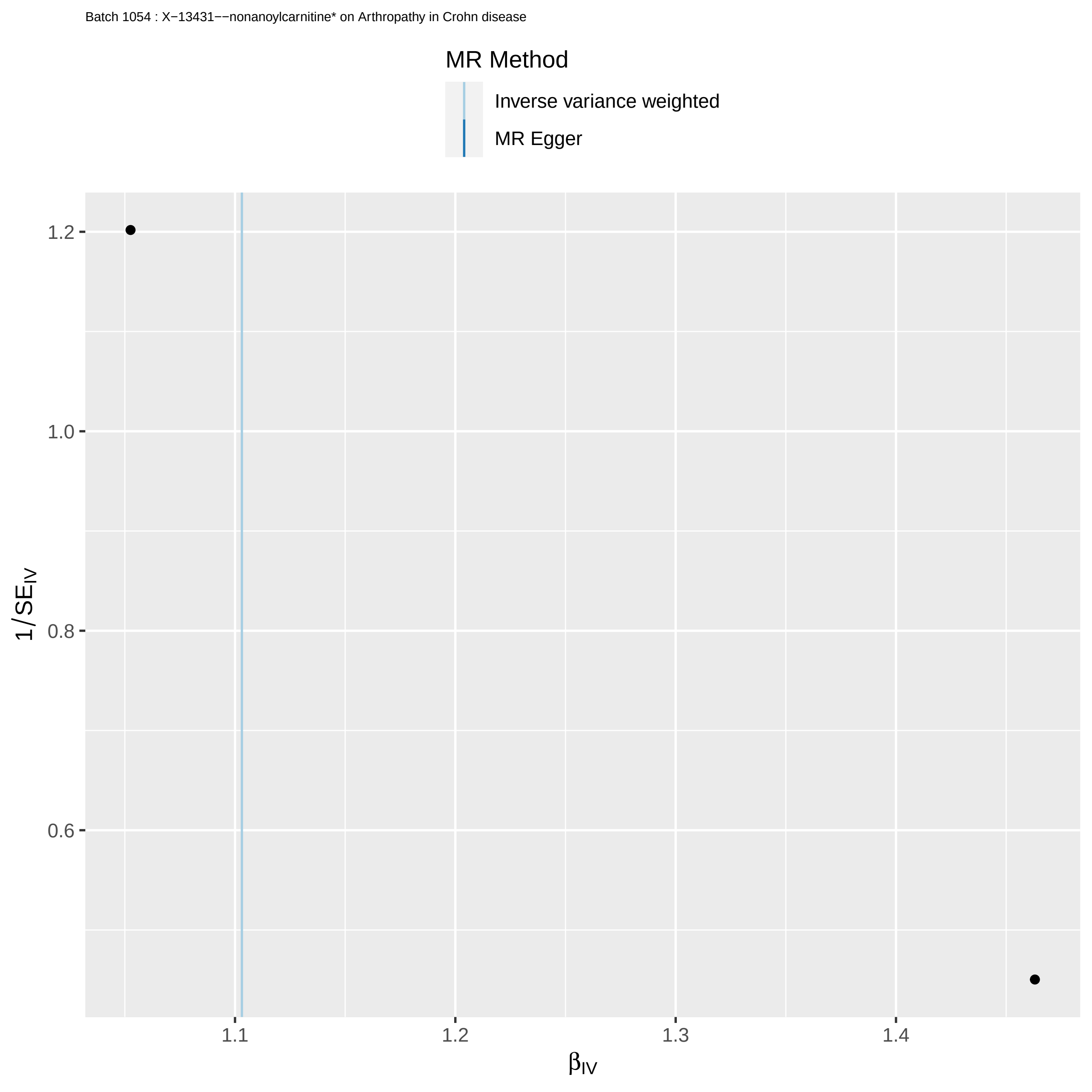
Batch 1054 : X−13431−−nonanoylcarnitine* on Arthropathy in Crohn disease
MR Method
Inverse variance weighted
MR Egger
1.2
1.0
0.8
0.6
1/SE_IV
1.1
1.2
1.3
1.4
β_IV

Batch 1055 : 2−palmitoylglycerophosphocholine* on Arthropathy in Crohn disease

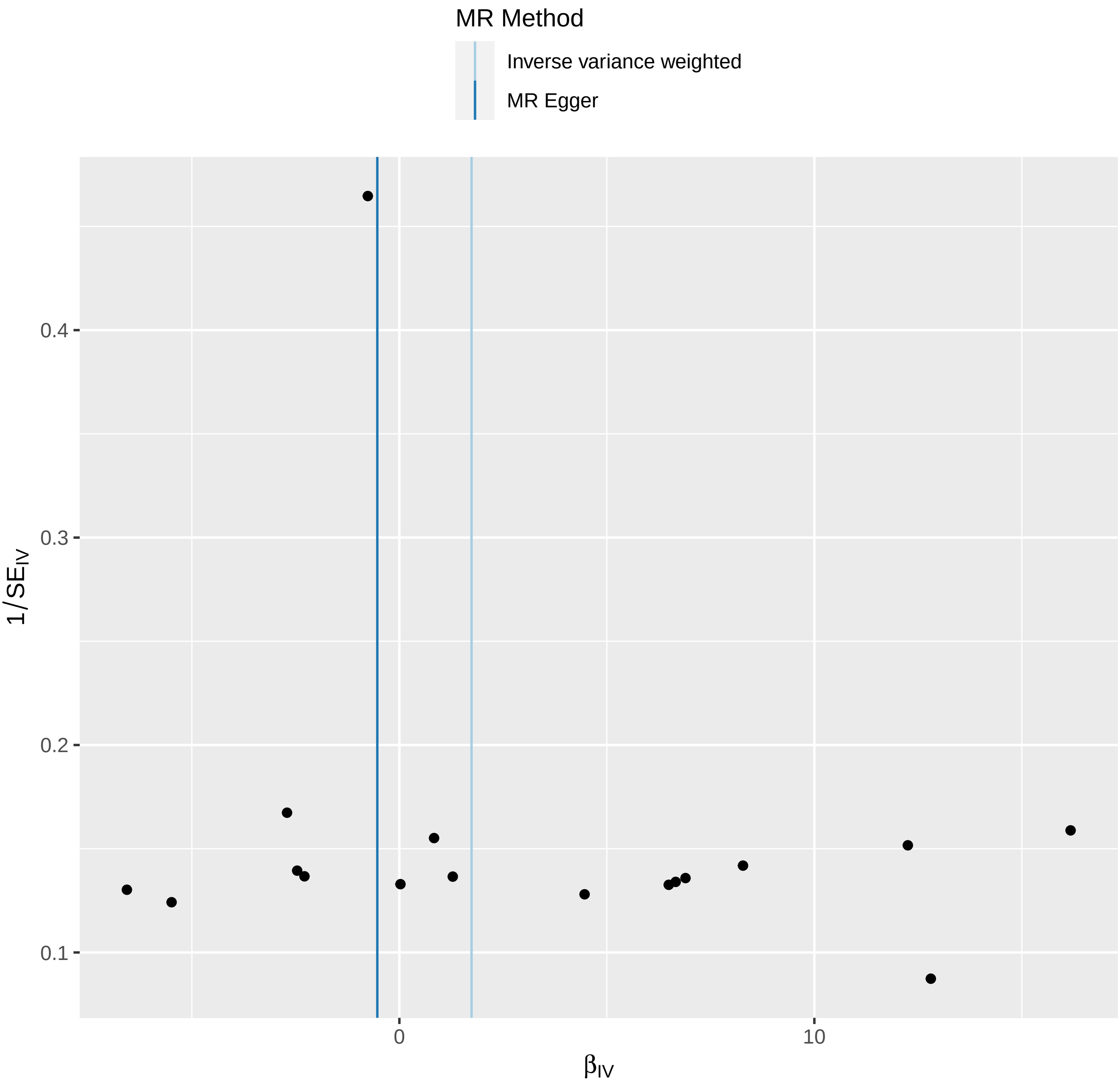

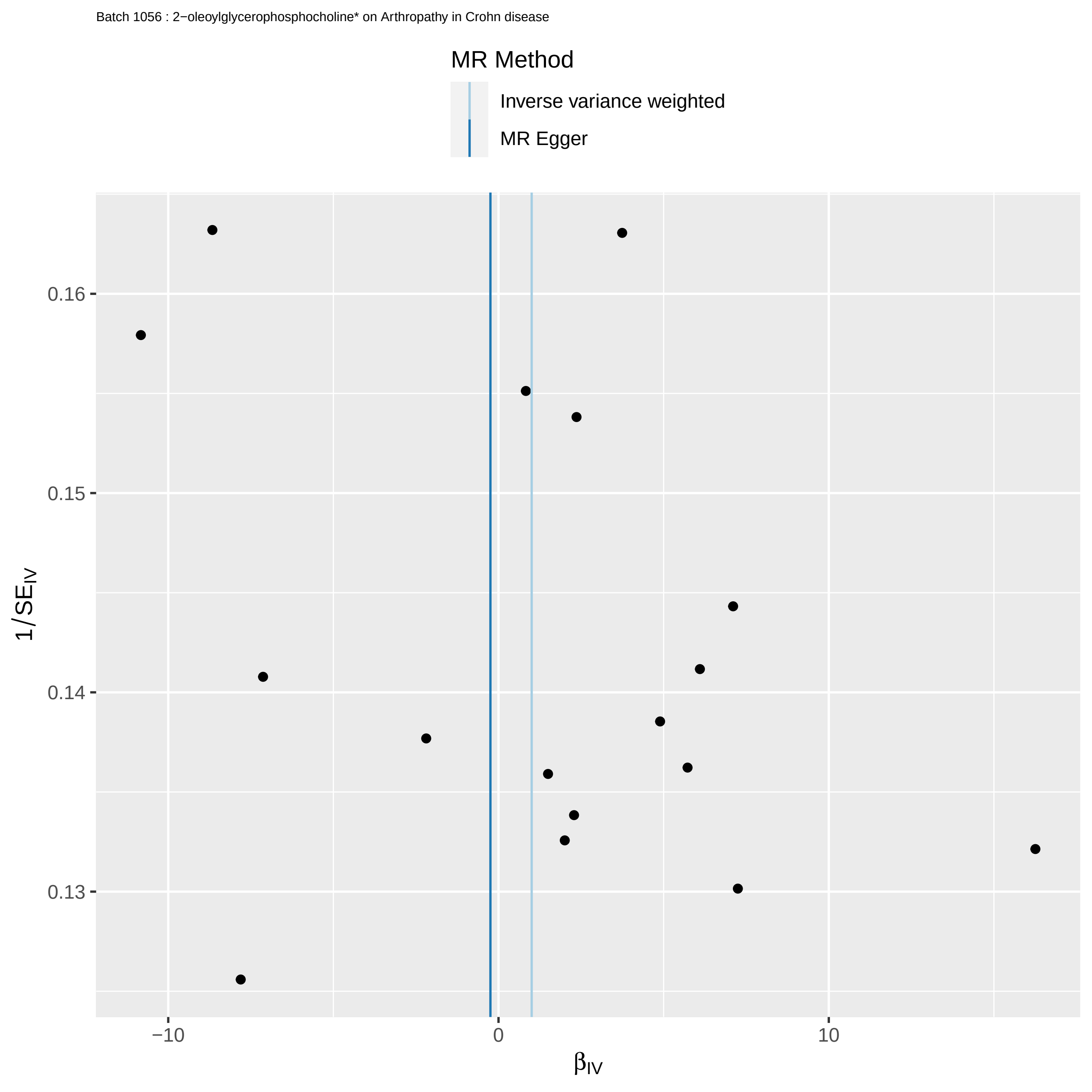

Batch 1056 : 2−oleoylglycerophosphocholine* on Arthropathy in Crohn disease
MR Method
Inverse variance weighted
MR Egger
0.16
0.15
0.14
0.13
1/SE$_{IV}$
−10
0
10
β$_{IV}$

Batch 1059 : 1−palmitoylglycerophosphoinositol* on Arthropathy in Crohn disease

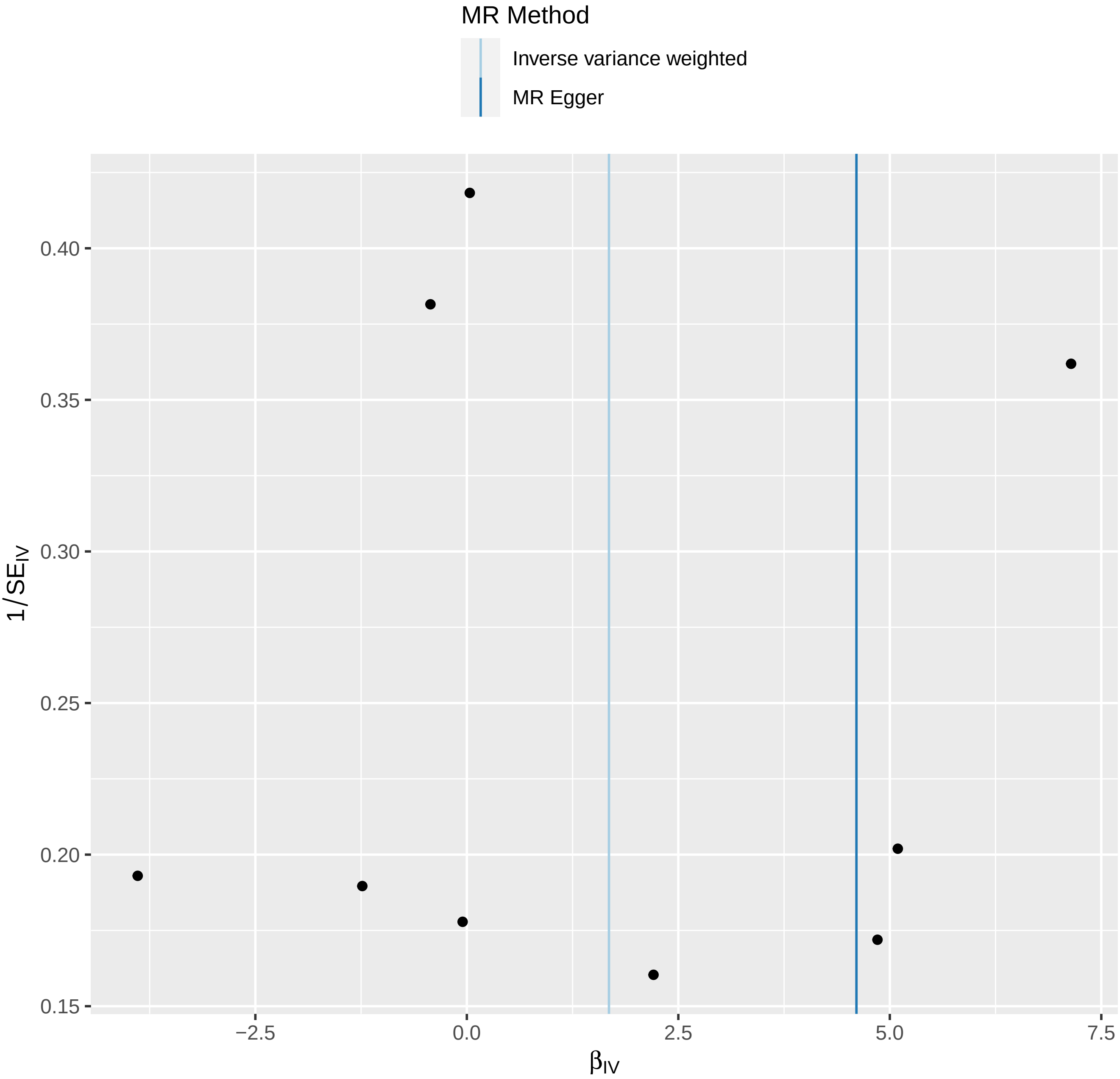

Batch 1061 : Hydroxyisovaleroyl carnitine on Arthropathy in Crohn disease

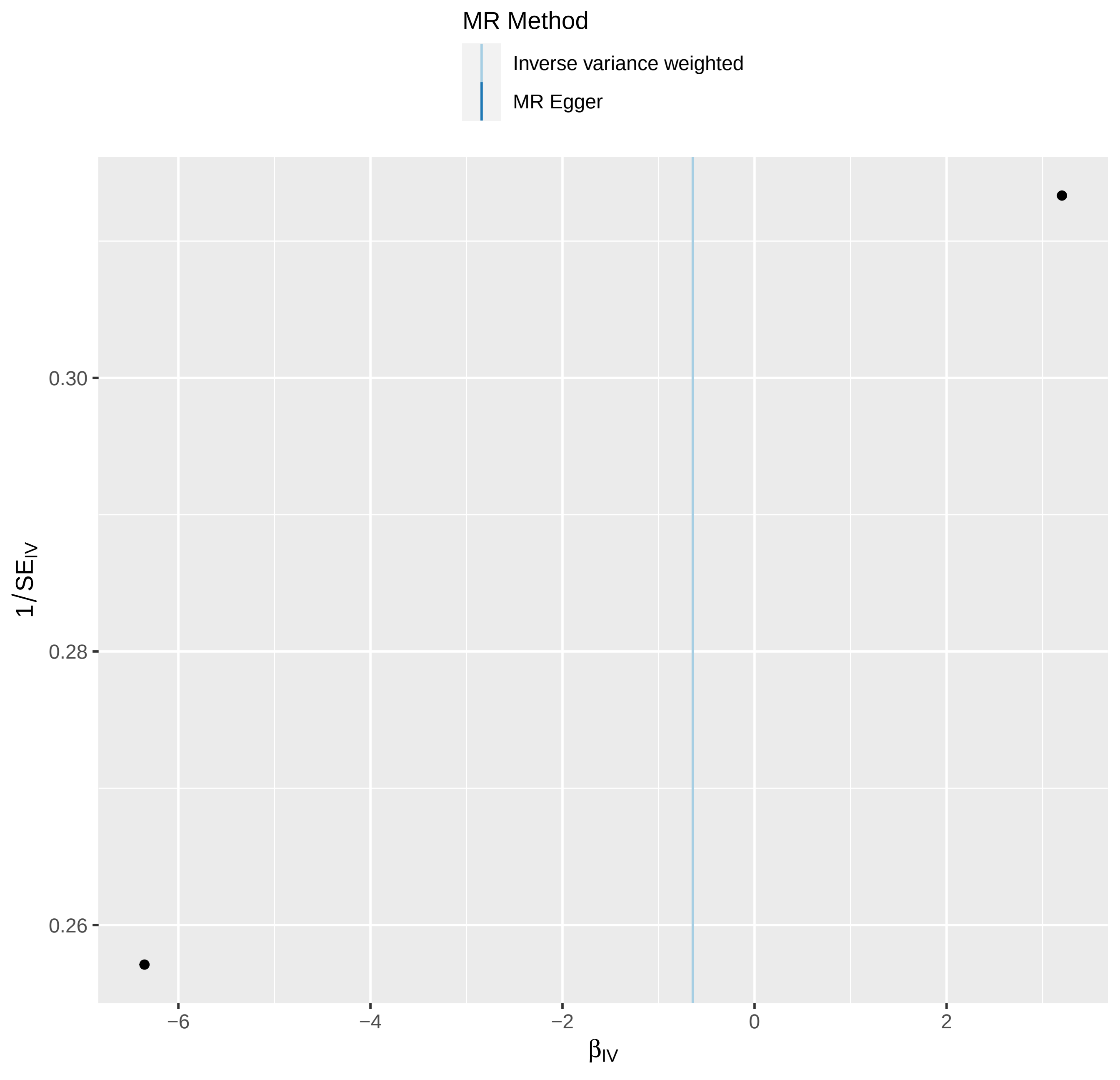

Batch 1062 : Glutaroyl carnitine on Arthropathy in Crohn disease

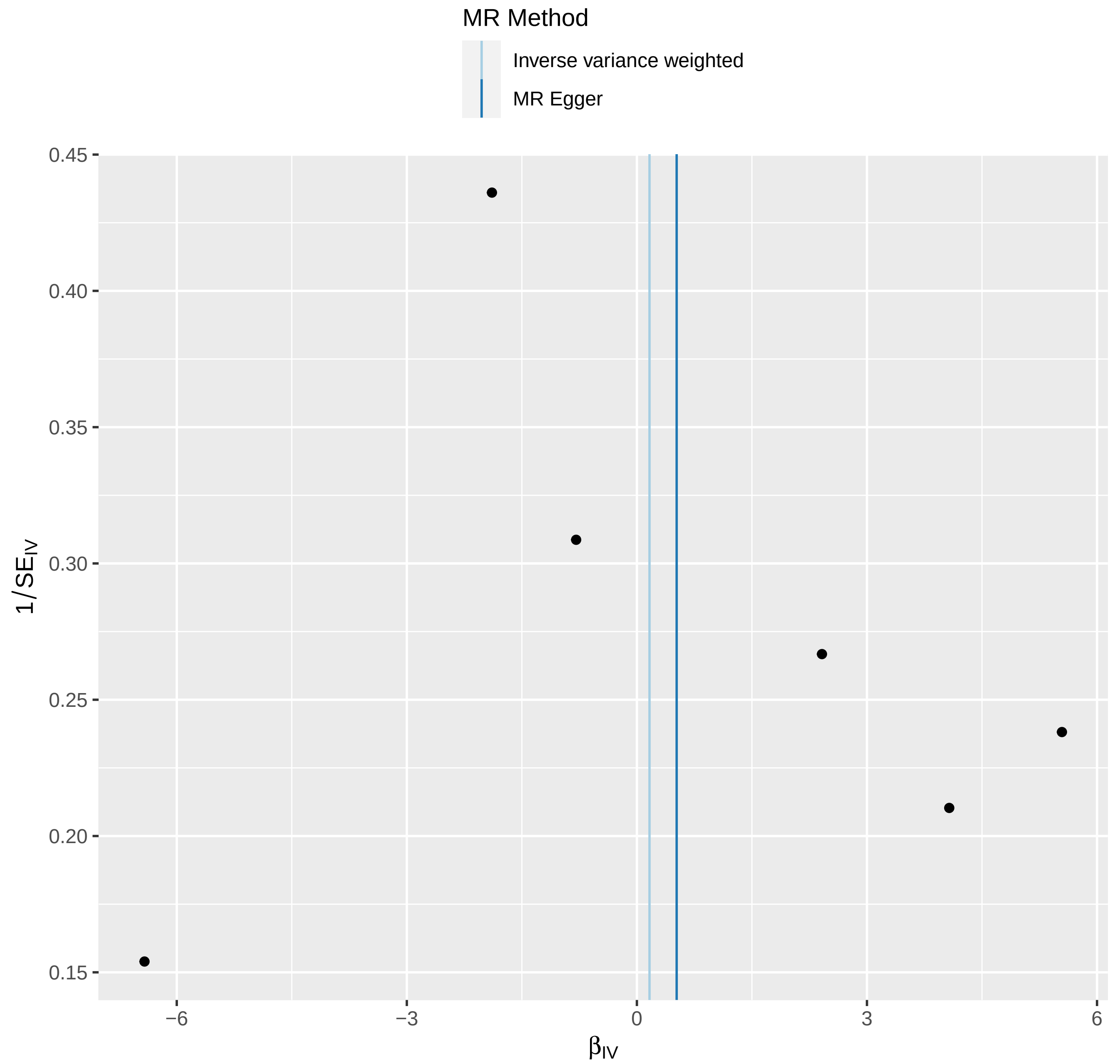

Batch 1064 : 4−hydroxyhippurate on Arthropathy in Crohn disease

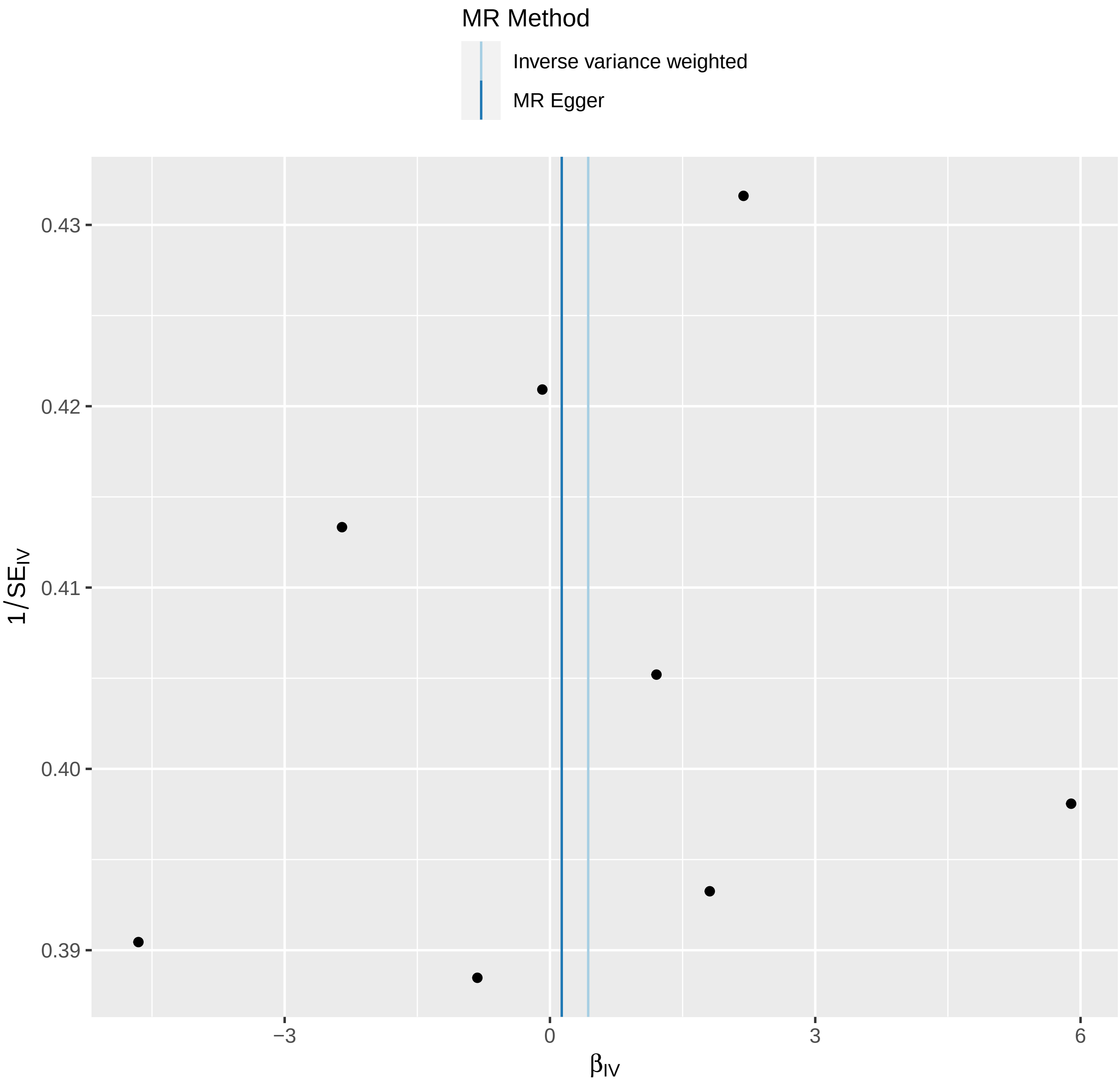

Batch 1065 : 1−myristoylglycerophosphocholine on Arthropathy in Crohn disease

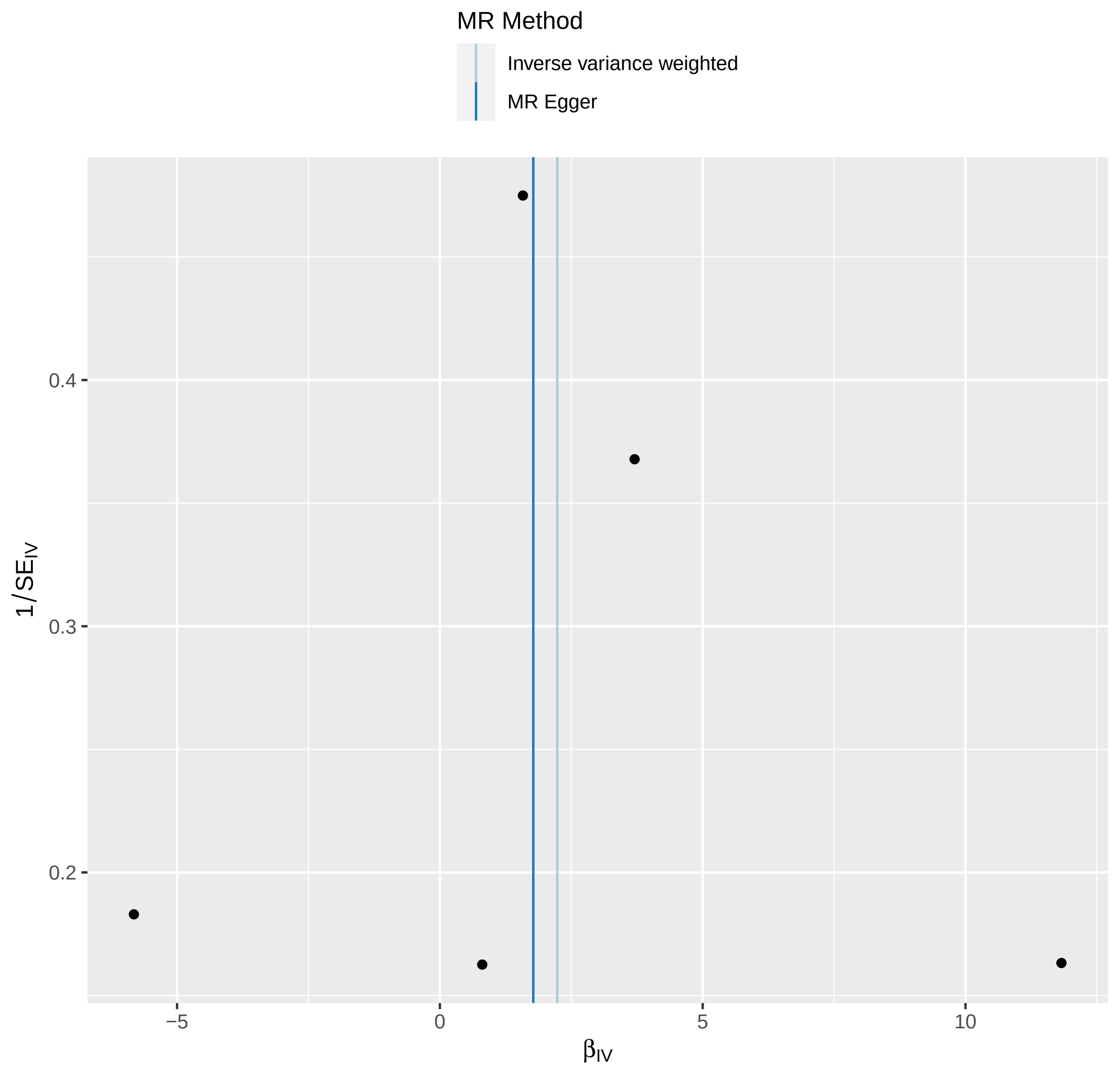

Batch 1068 : 3−(3−hydroxyphenyl)propionate on Arthropathy in Crohn disease

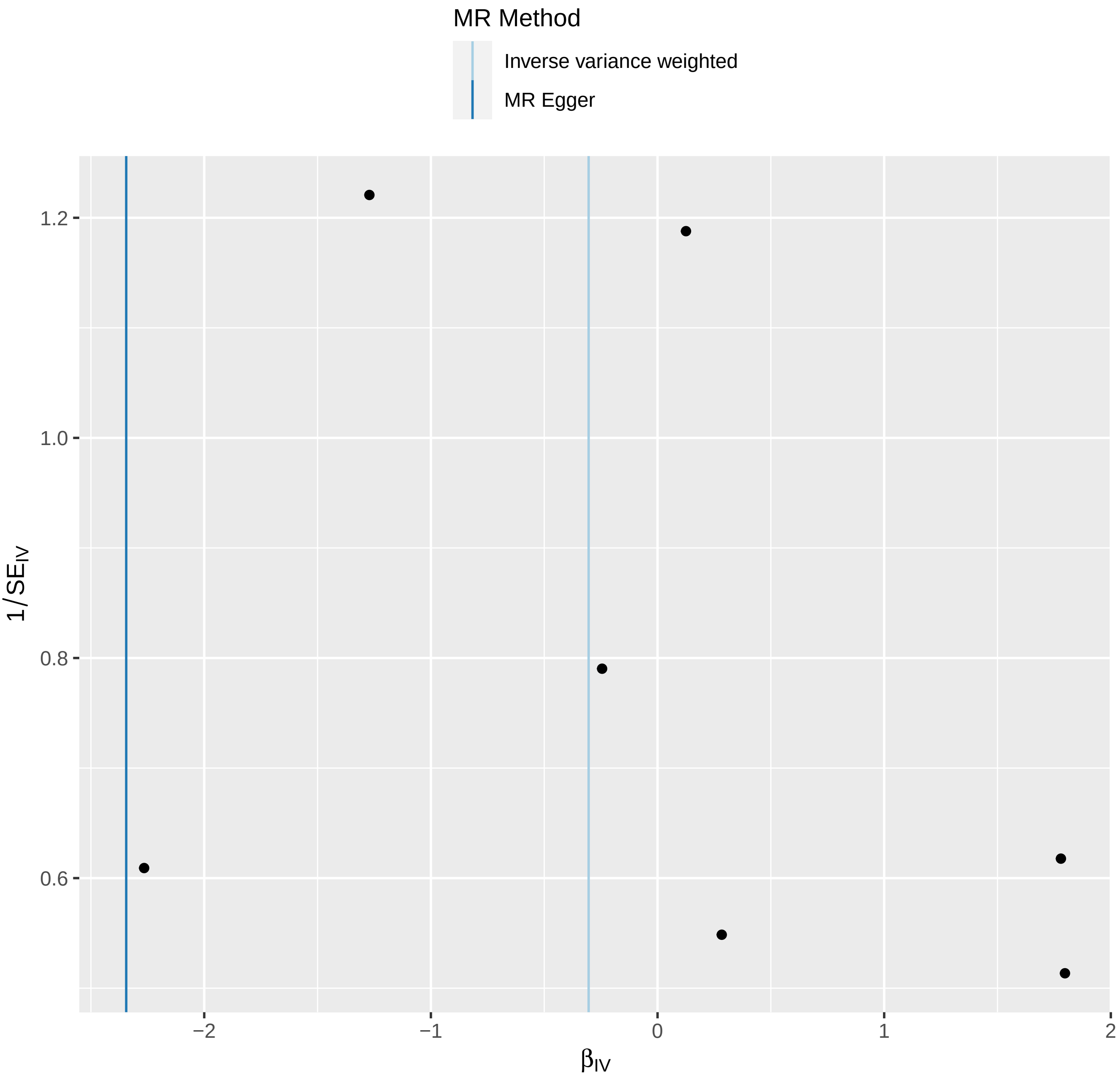

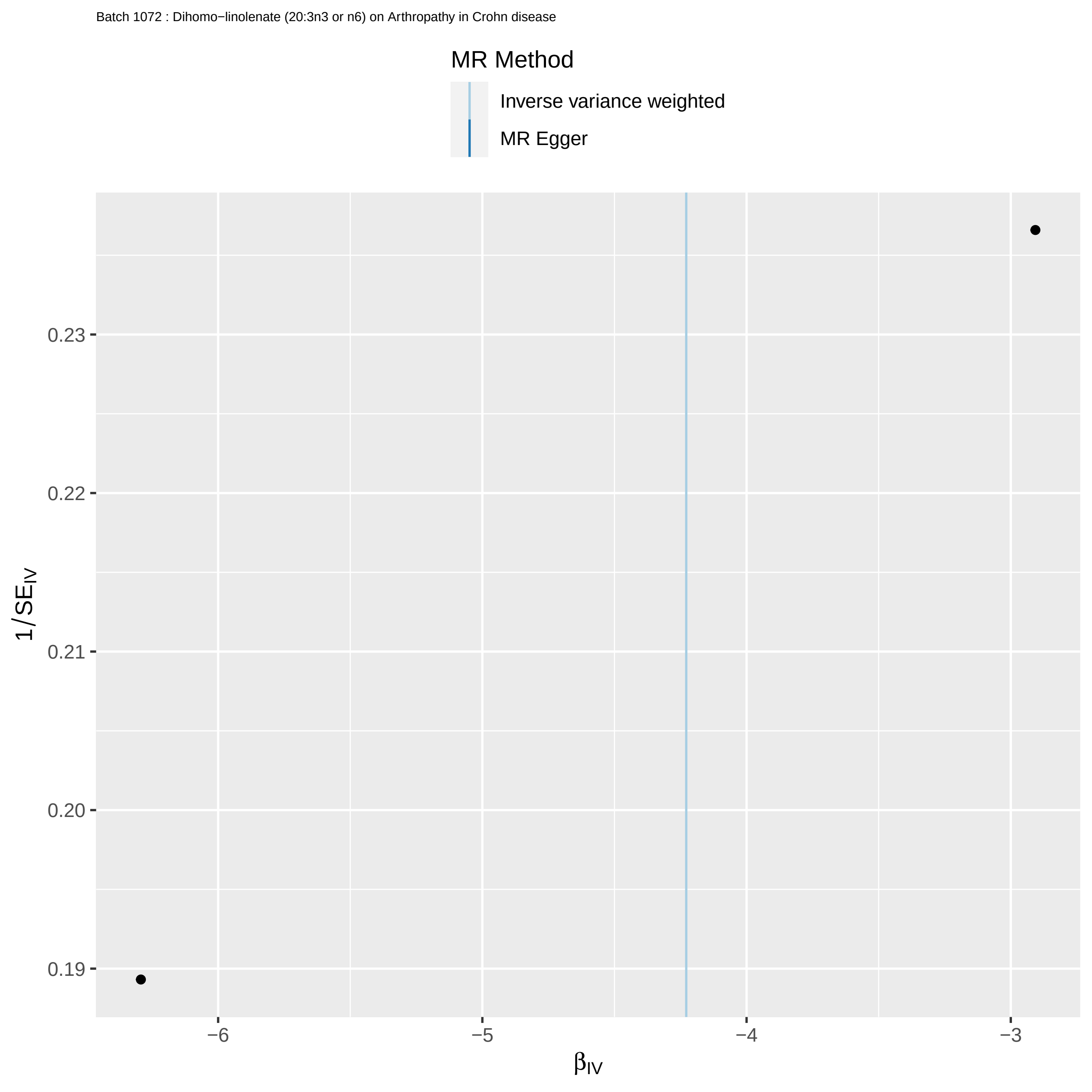

Batch 1072 : Dihomo−linolenate (20:3n3 or n6) on Arthropathy in Crohn disease
MR Method
Inverse variance weighted
MR Egger
0.23
0.22
0.21
0.20
0.19
$1/SE_{IV}$
−6
−5
−4
−3
β_{IV}

Batch 1073 : Threitol on Arthropathy in Crohn disease

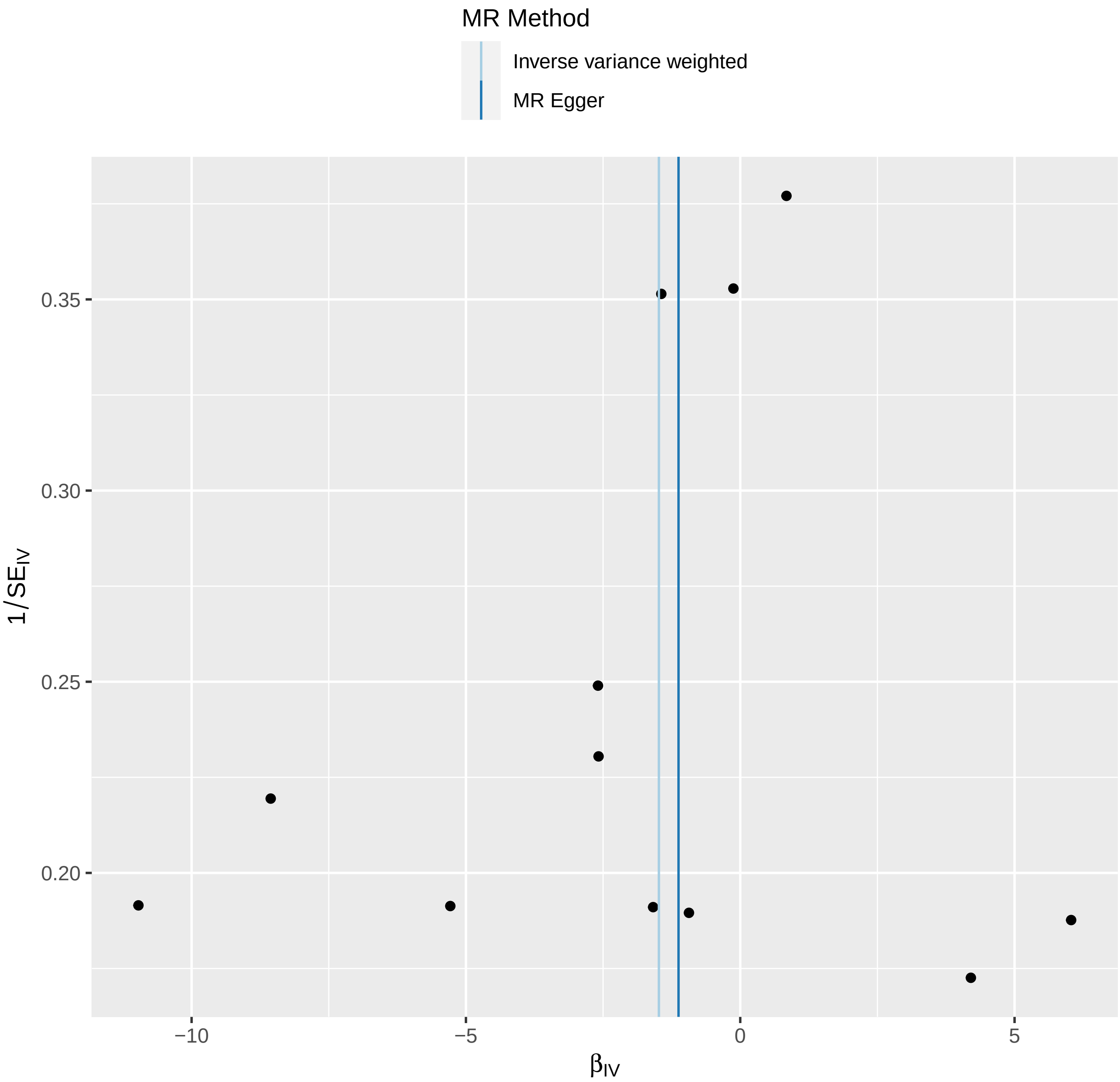

Batch 1074 : P−cresol sulfate on Arthropathy in Crohn disease

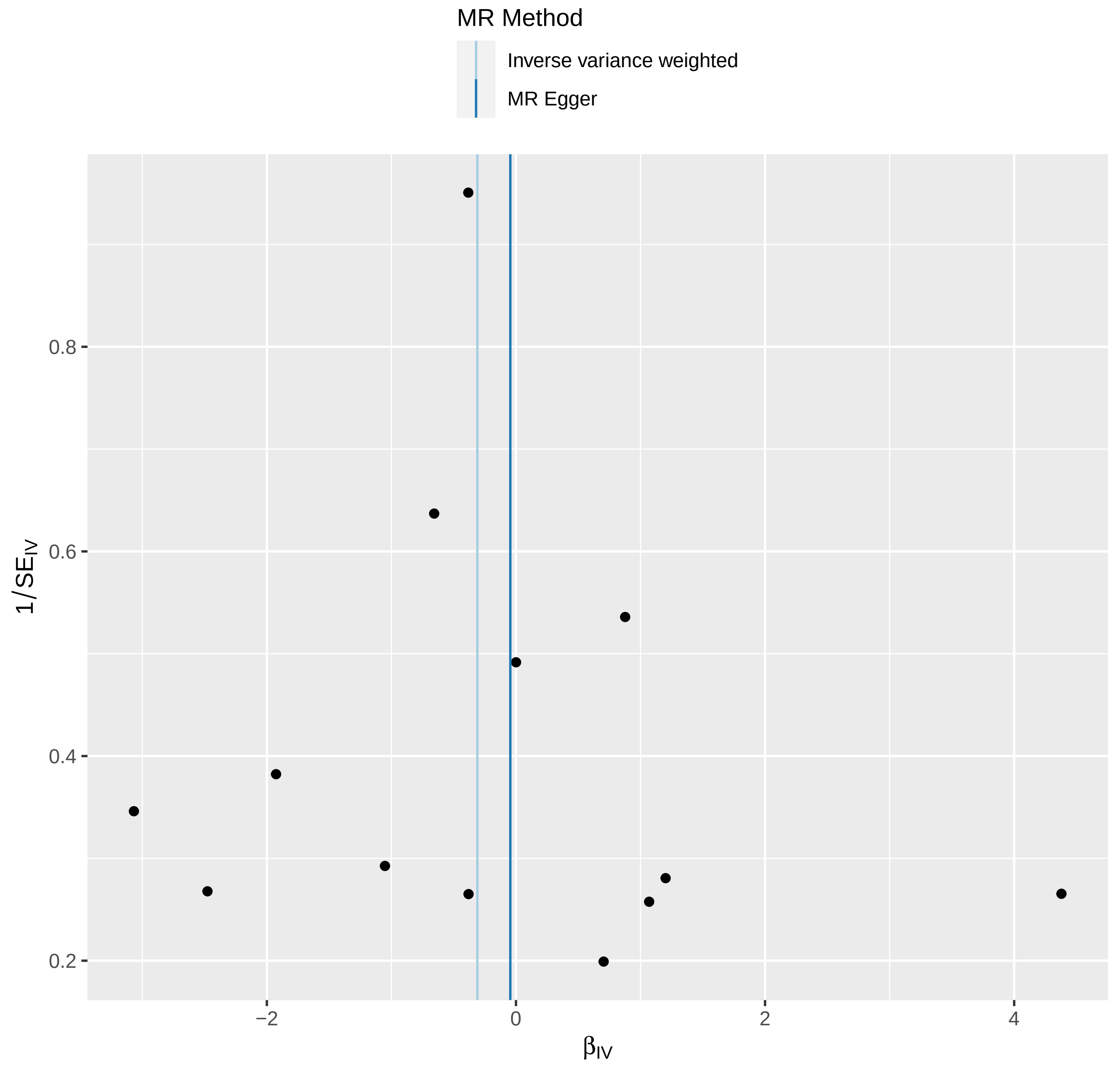

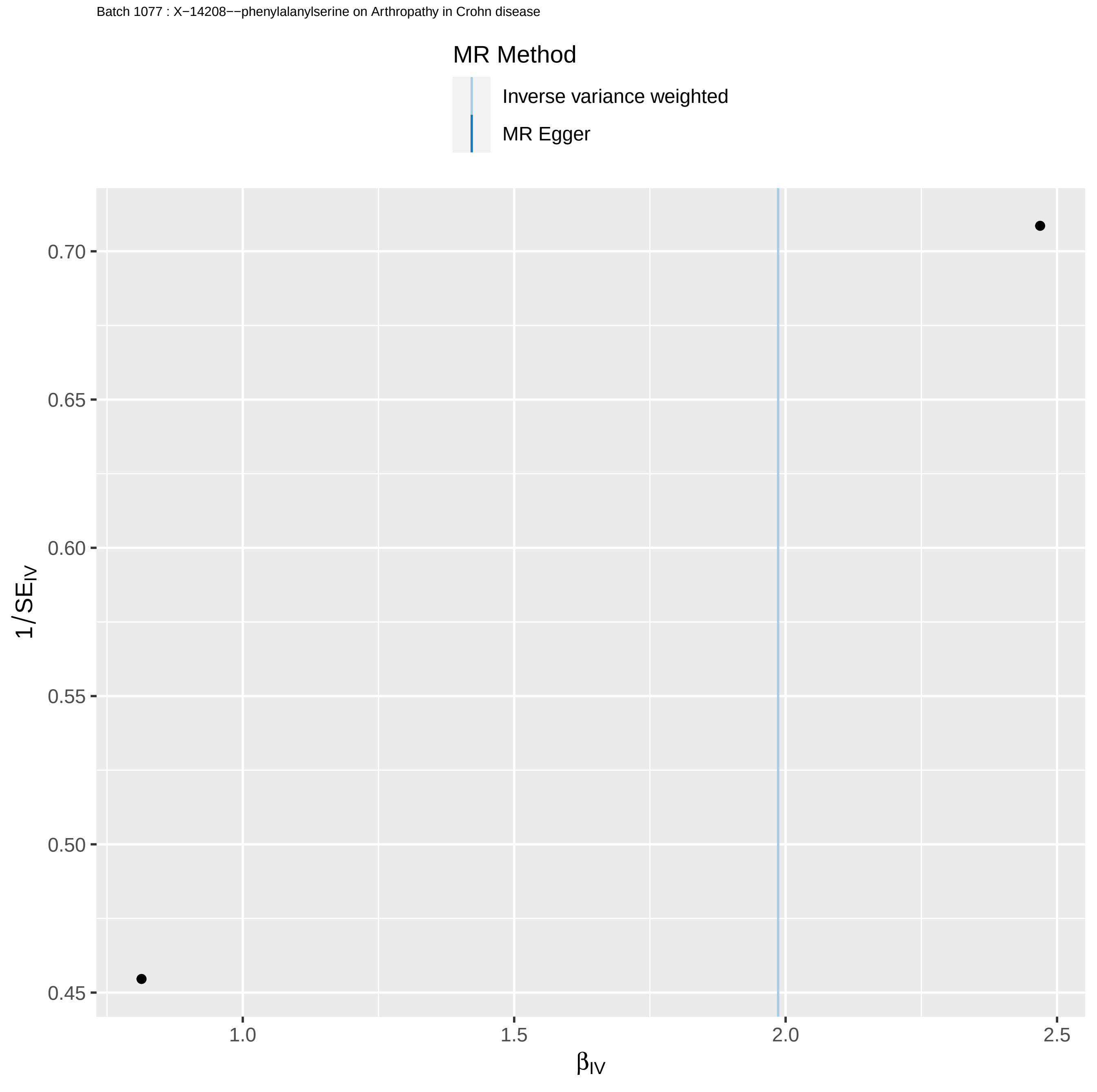

Batch 1077 : X−14208−−phenylalanylserine on Arthropathy in Crohn disease
MR Method
Inverse variance weighted
MR Egger
0.70
0.65
0.60
0.55
0.50
0.45
1/SE$_{IV}$
1.0
1.5
2.0
2.5
β$_{IV}$

Batch 1079 : X−14450−−phenylalanylleucine on Arthropathy in Crohn disease

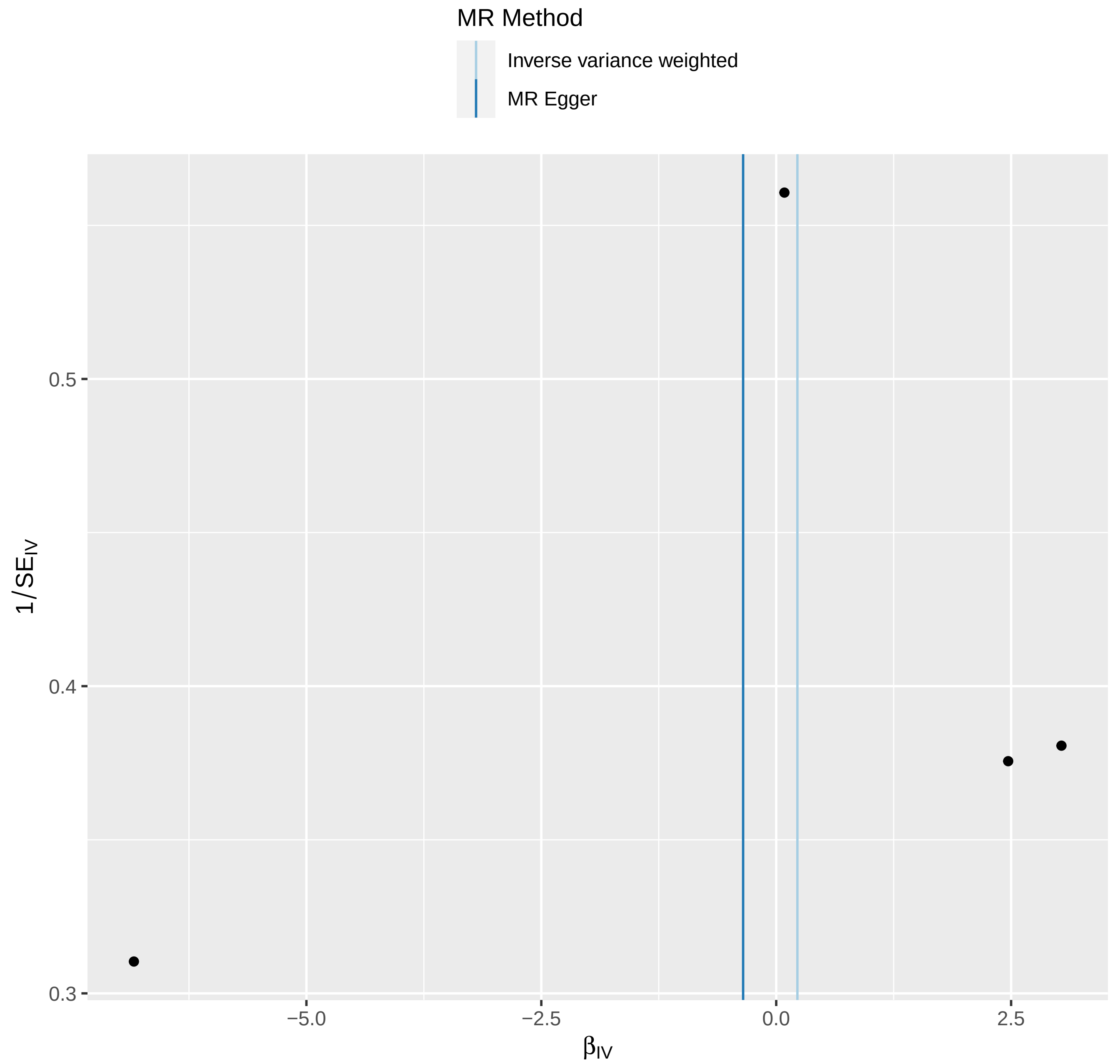

Batch 1080 : Gamma−glutamylglutamate on Arthropathy in Crohn disease

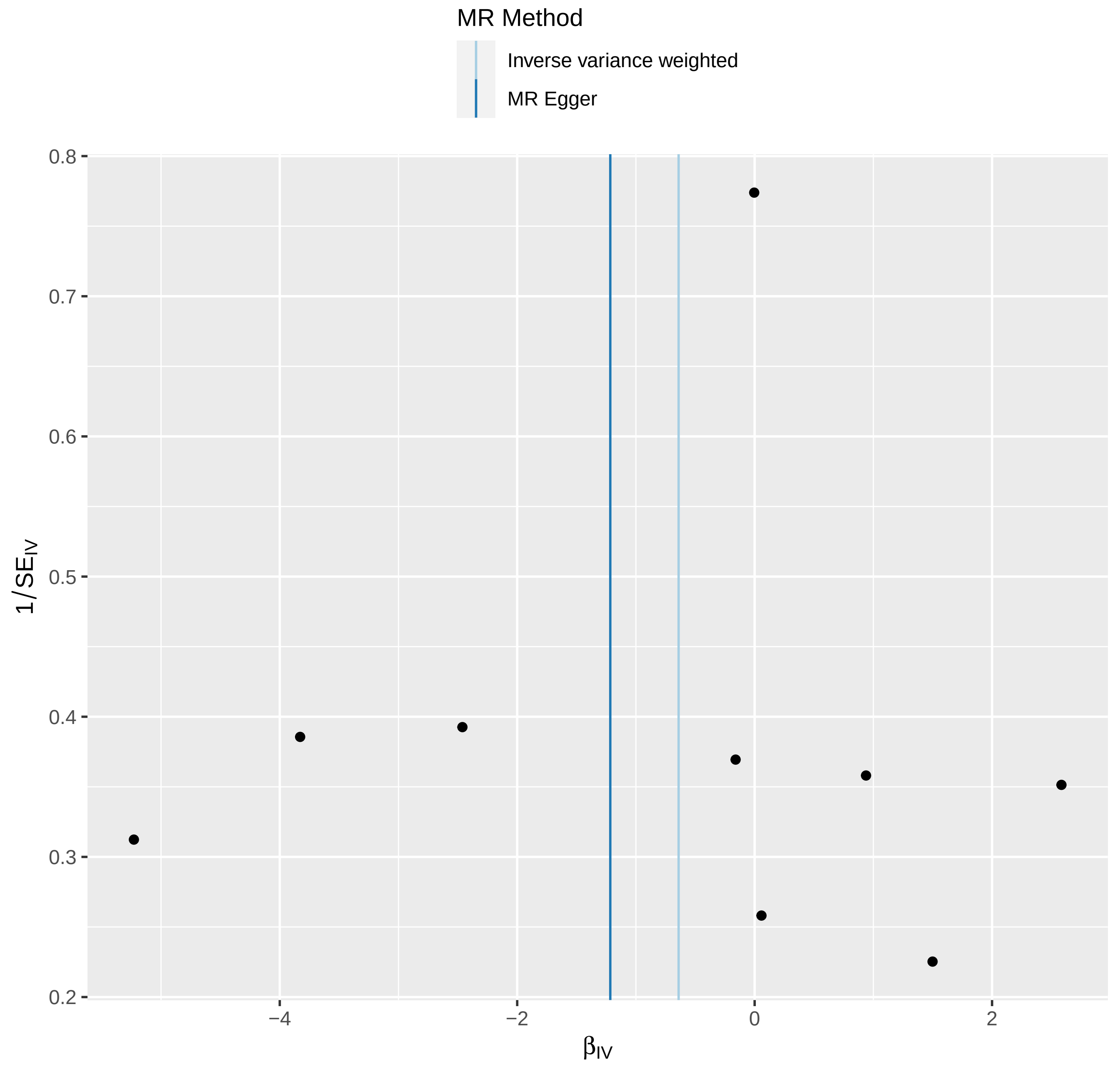

Batch 1081 : Octadecanedioate on Arthropathy in Crohn disease

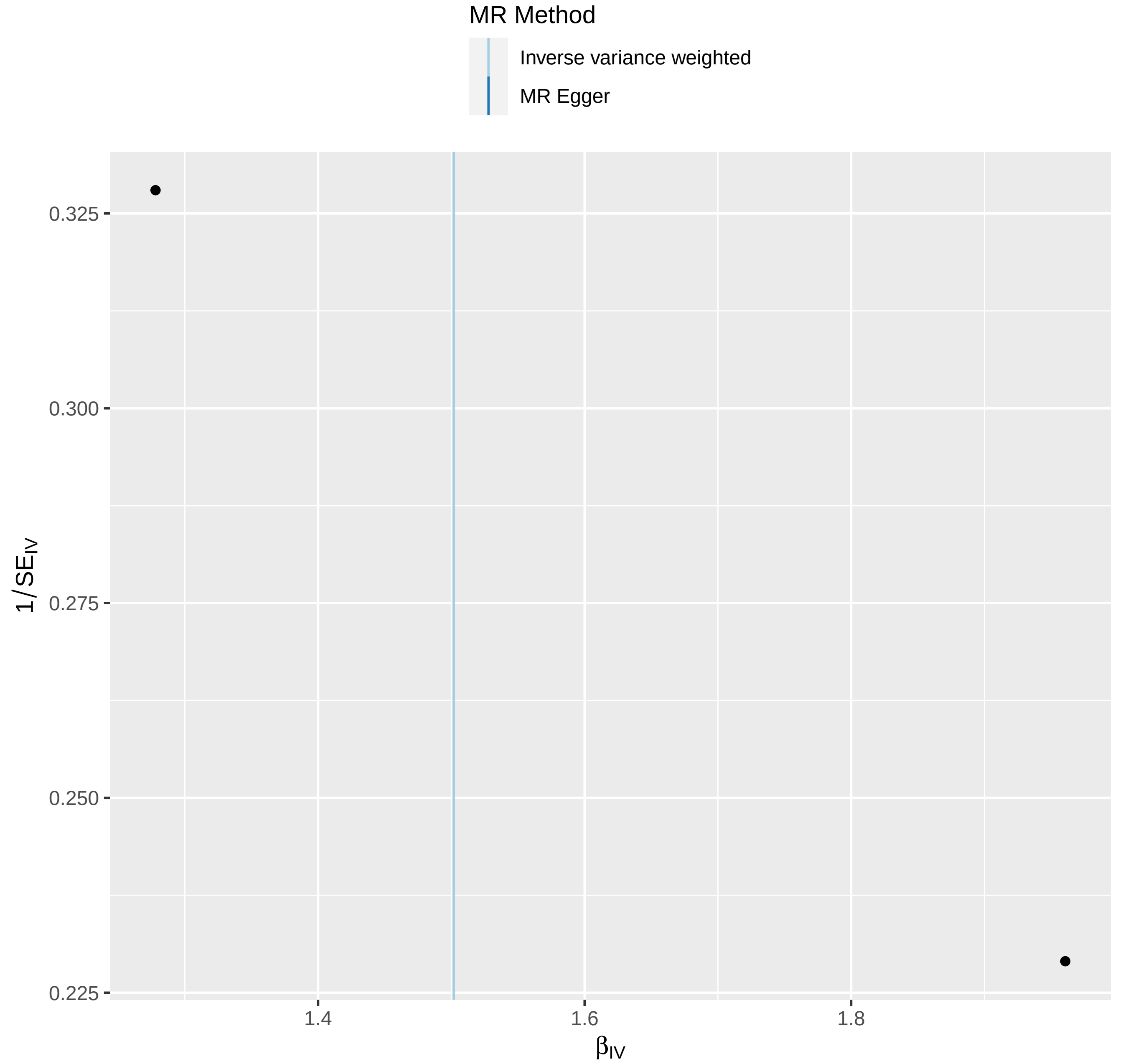

Batch 1082 : Leucylleucine on Arthropathy in Crohn disease

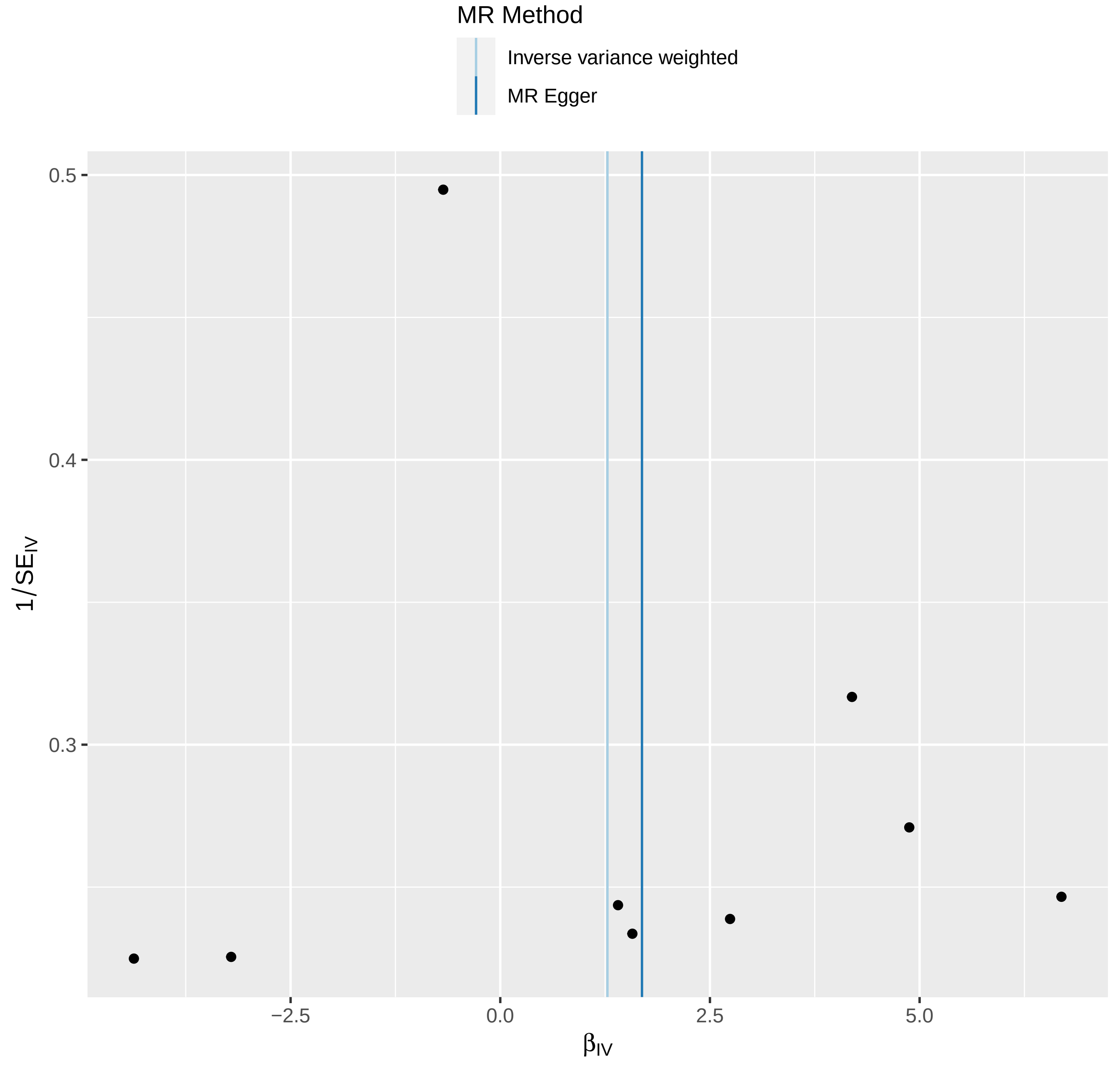

Batch 1083 : 7−alpha−hydroxy−3−oxo−4−cholestenoate (7−Hoca) on Arthropathy in Crohn disease

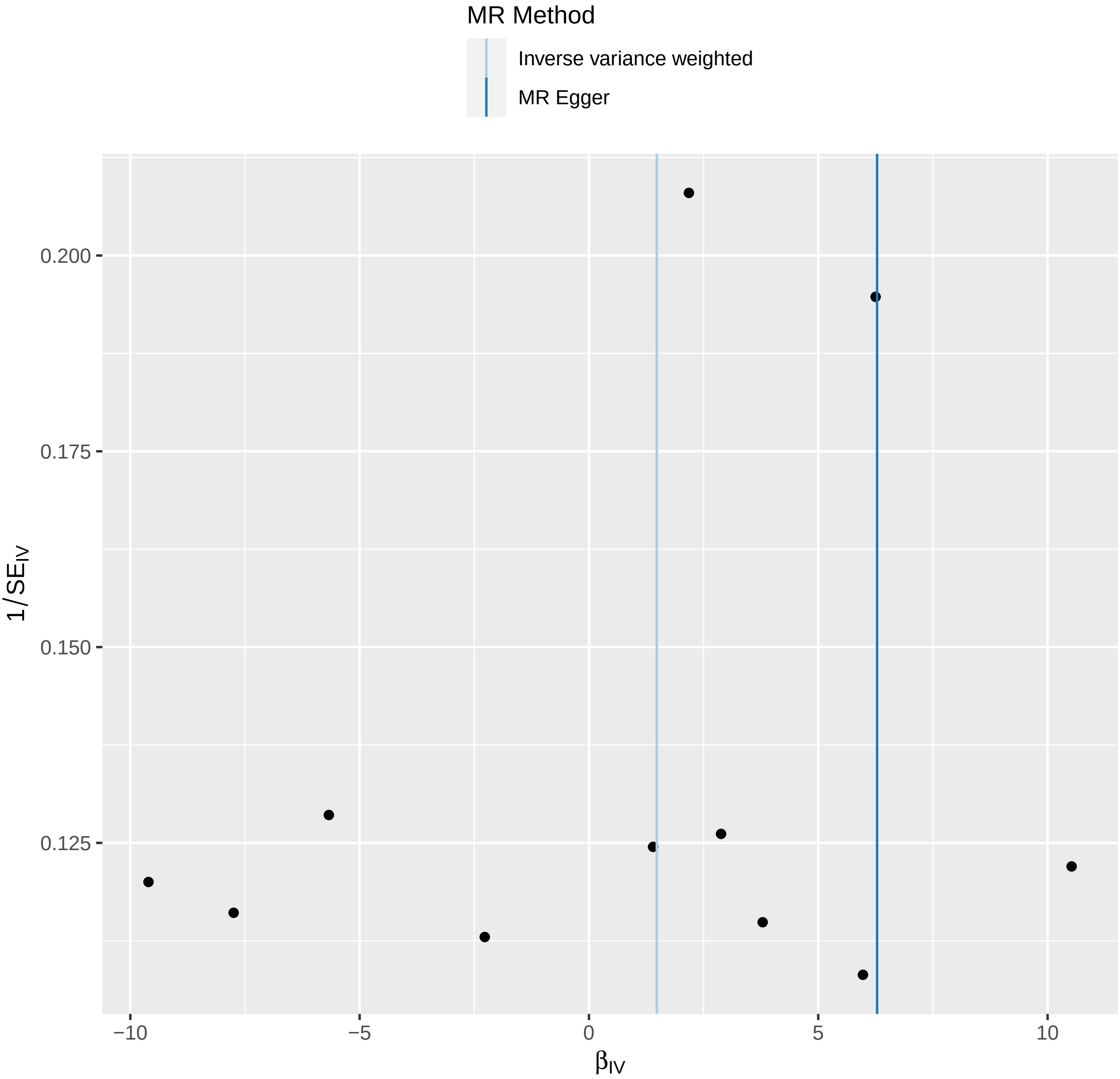

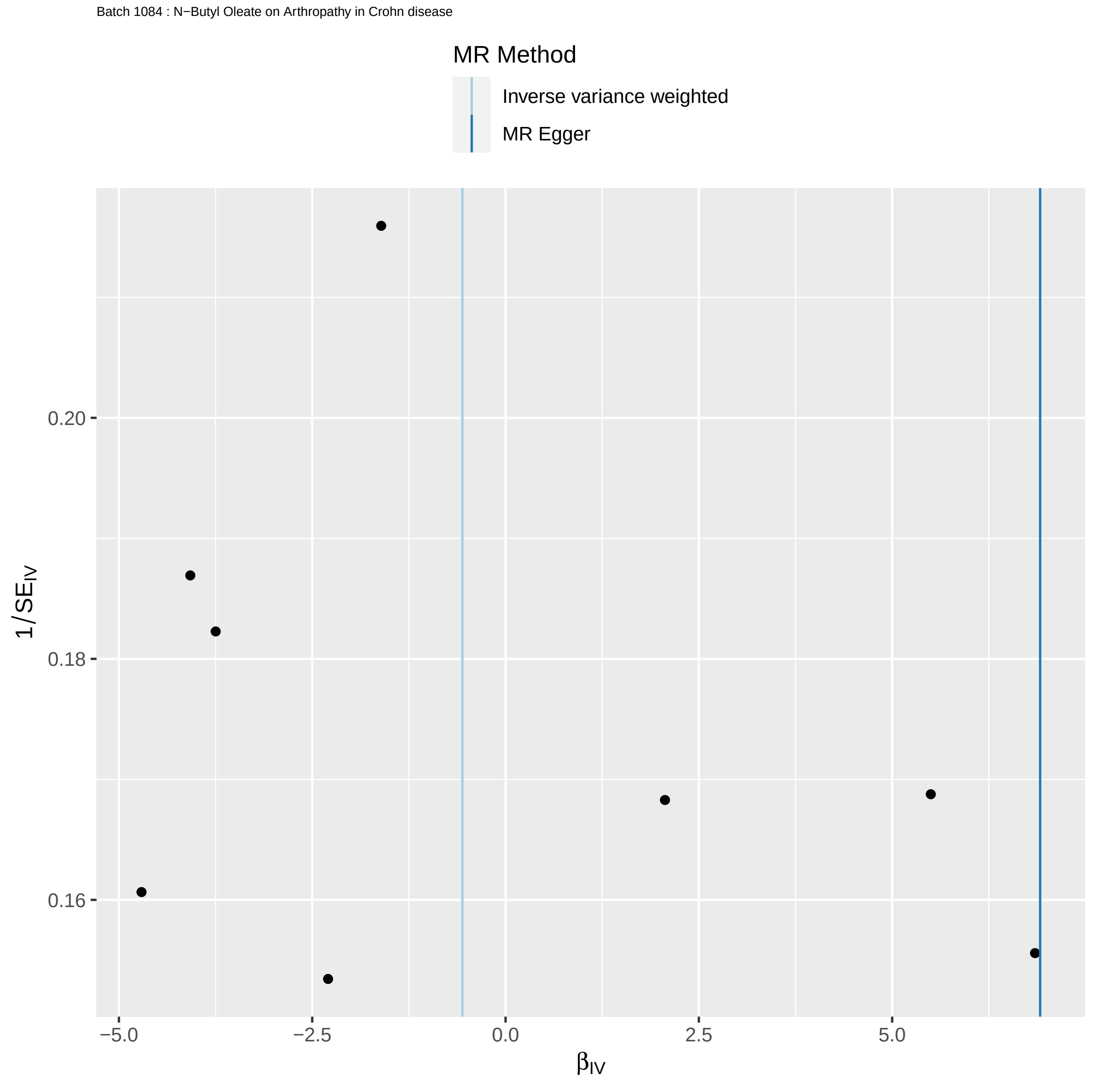

Batch 1084 : N−Butyl Oleate on Arthropathy in Crohn disease
MR Method
Inverse variance weighted
MR Egger
0.20
0.18
0.16
1/SE_IV
−5.0
−2.5
0.0
2.5
5.0
β_IV

Batch 1086 : Taurolithocholate 3−sulfate on Arthropathy in Crohn disease

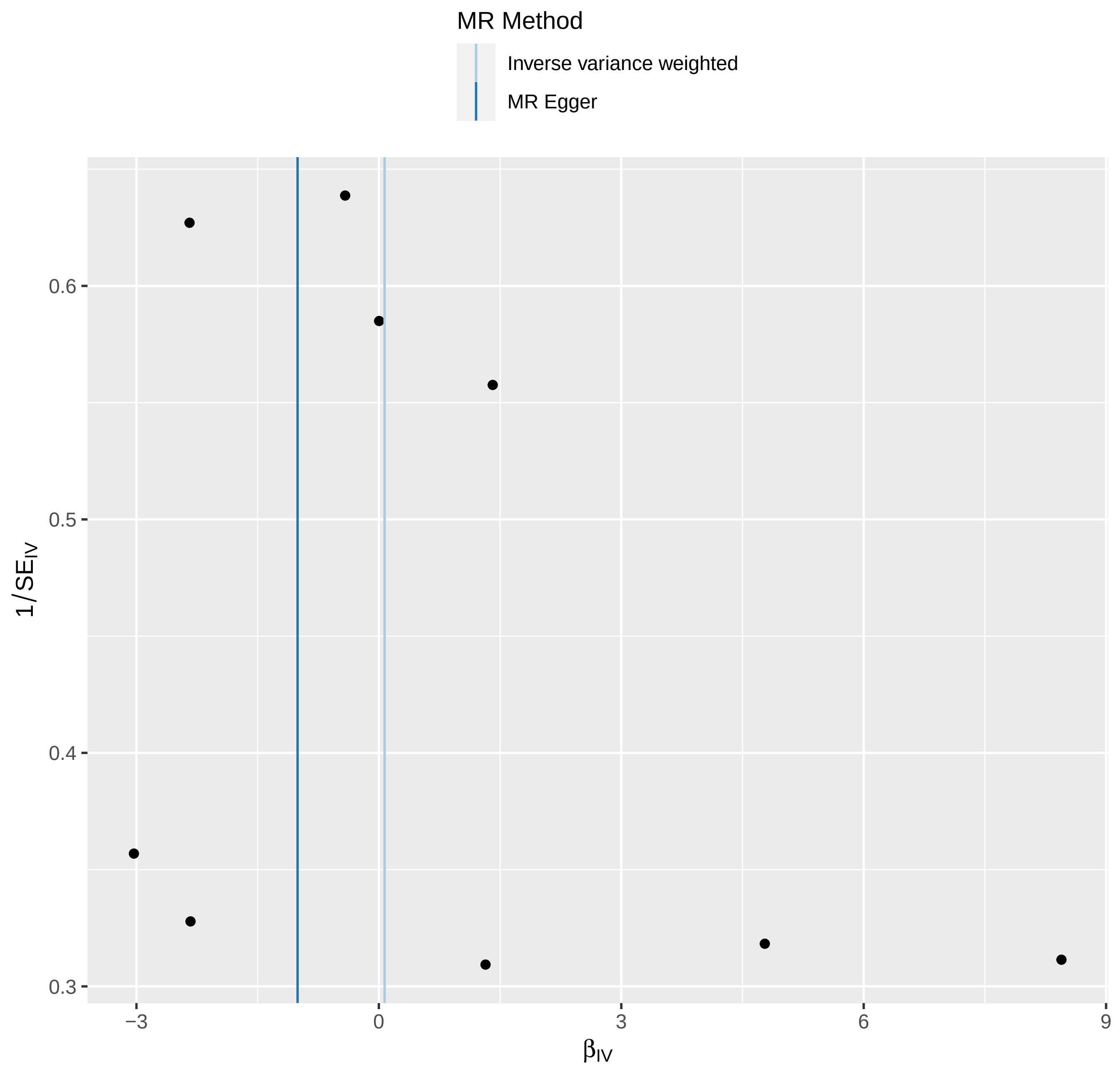

Batch 1087 : X−14977−−vanillin on Arthropathy in Crohn disease

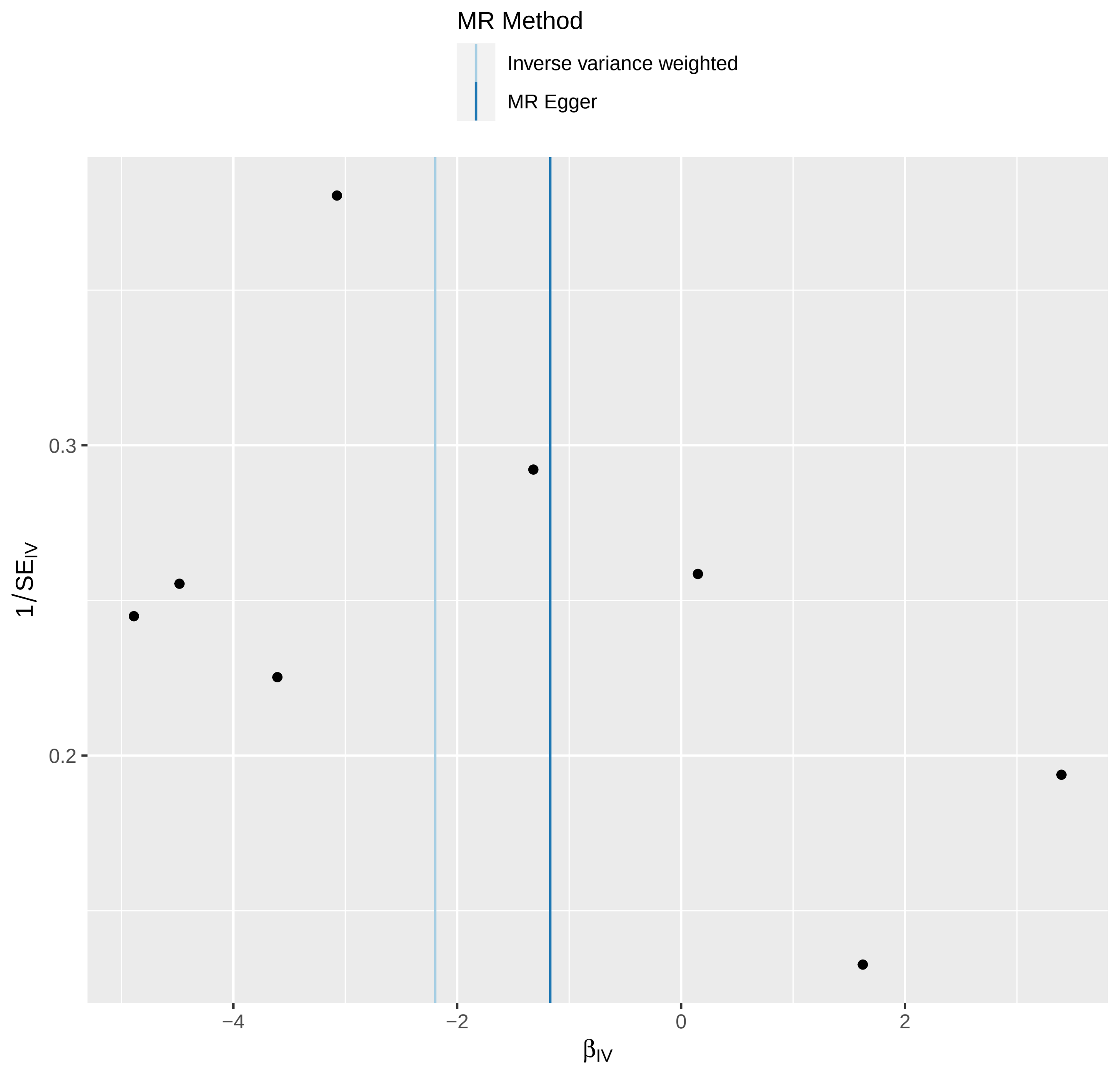

Batch 1088 : Succinylcarnitine on Arthropathy in Crohn disease

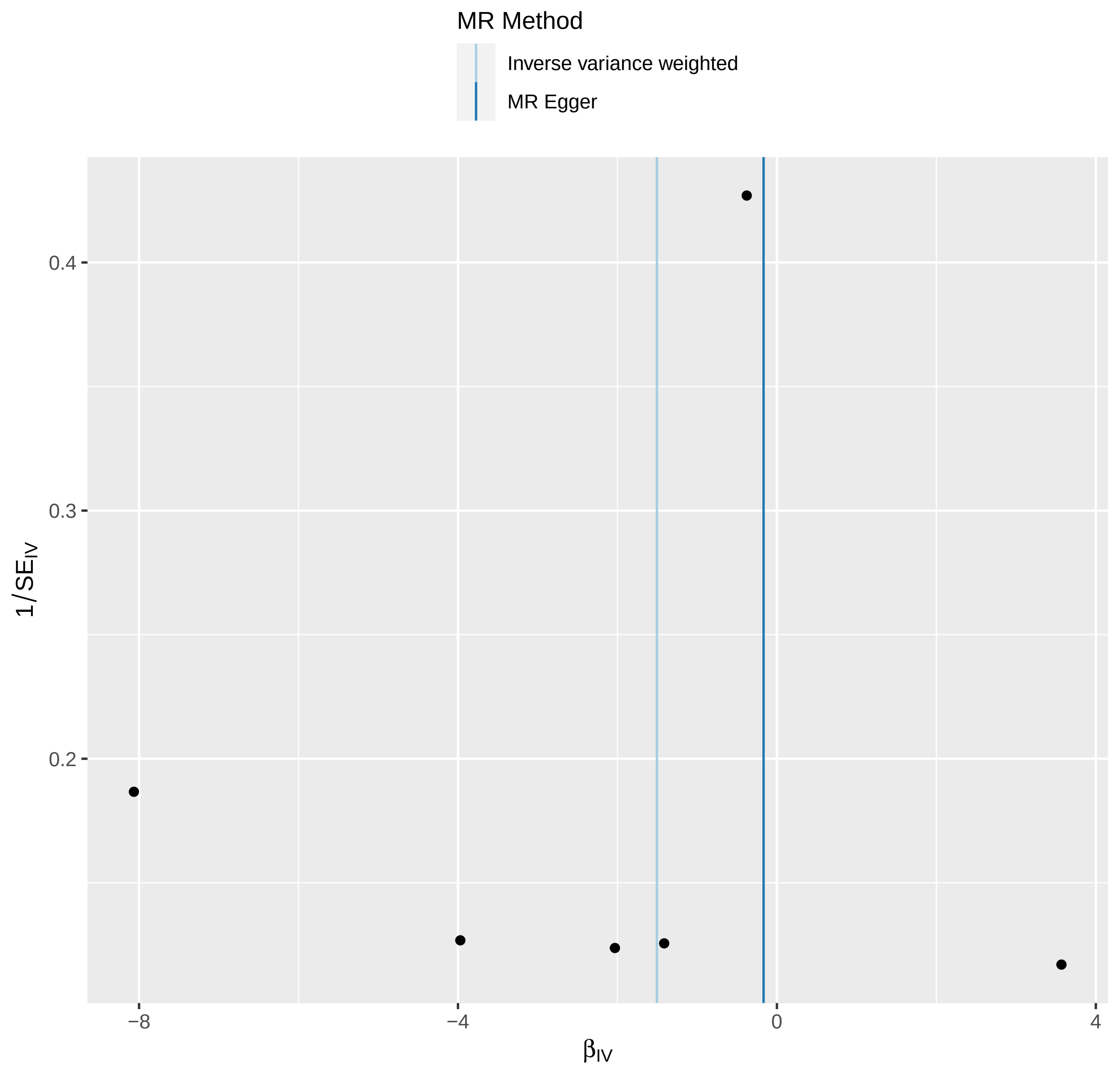

Batch 1090 : Cyclo(leu−pro) on Arthropathy in Crohn disease

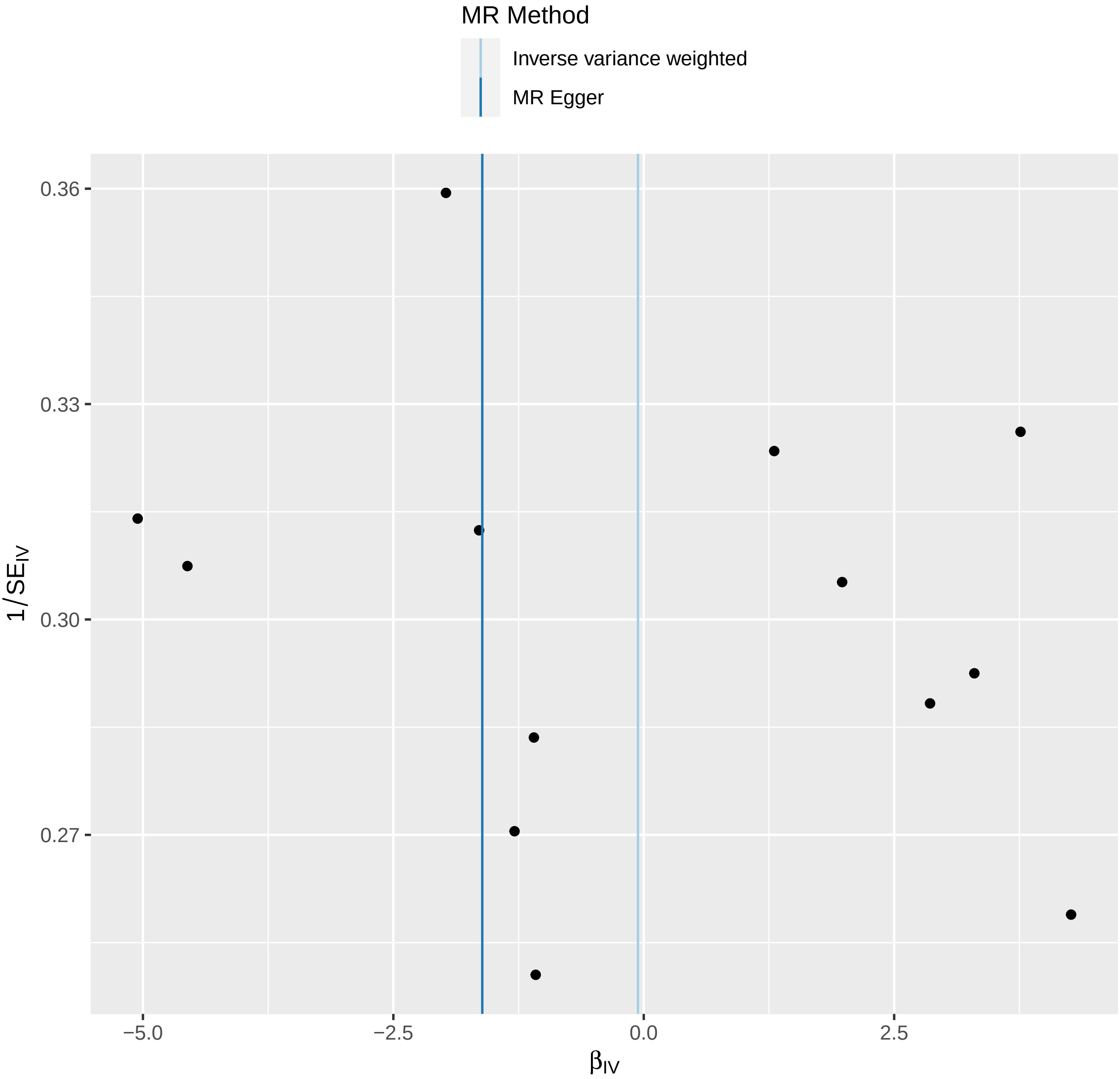

Batch 1091 : Chiro−inositol on Arthropathy in Crohn disease

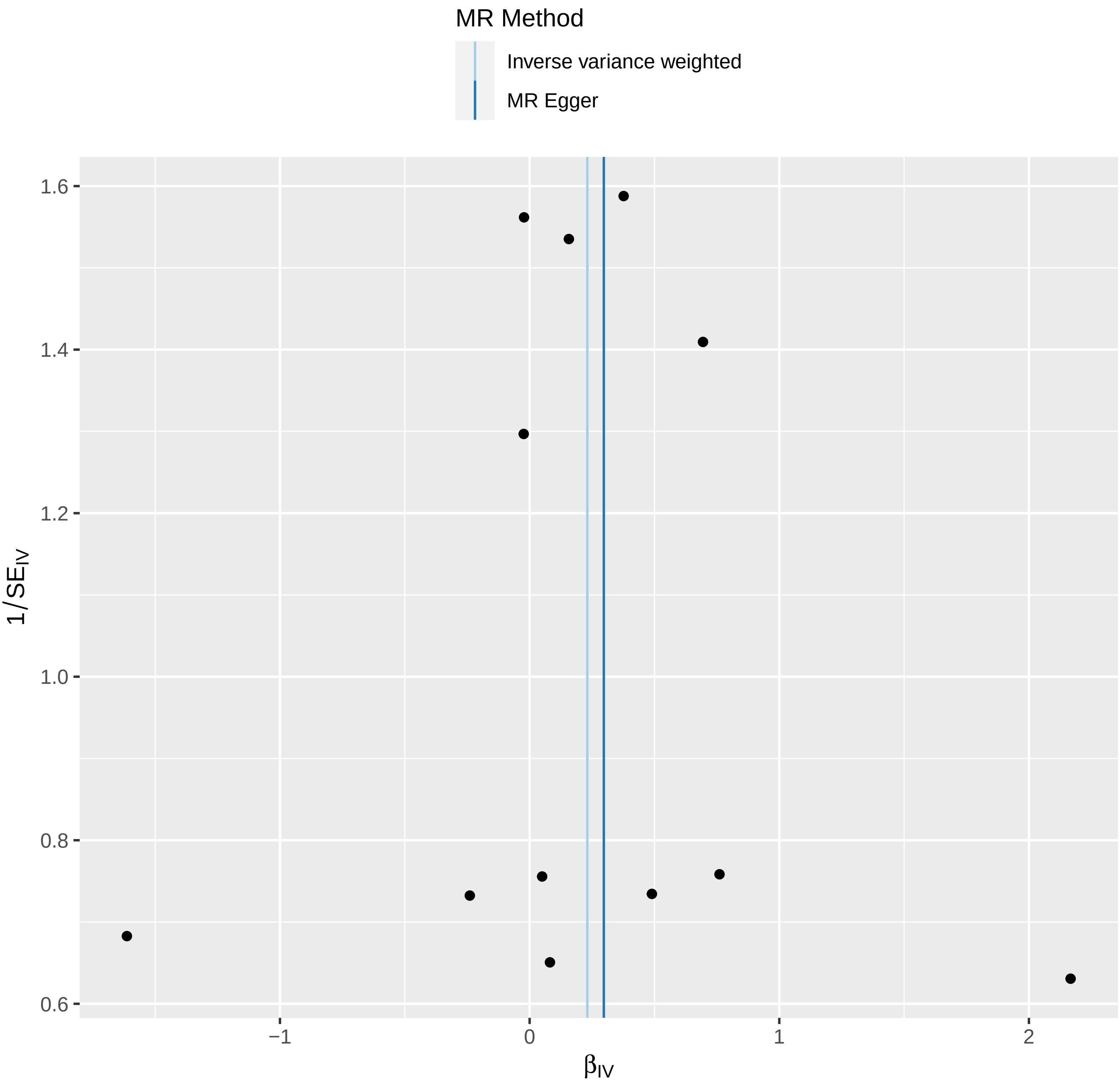

Batch 1095 : 2−hydroxyglutarate on Arthropathy in Crohn disease

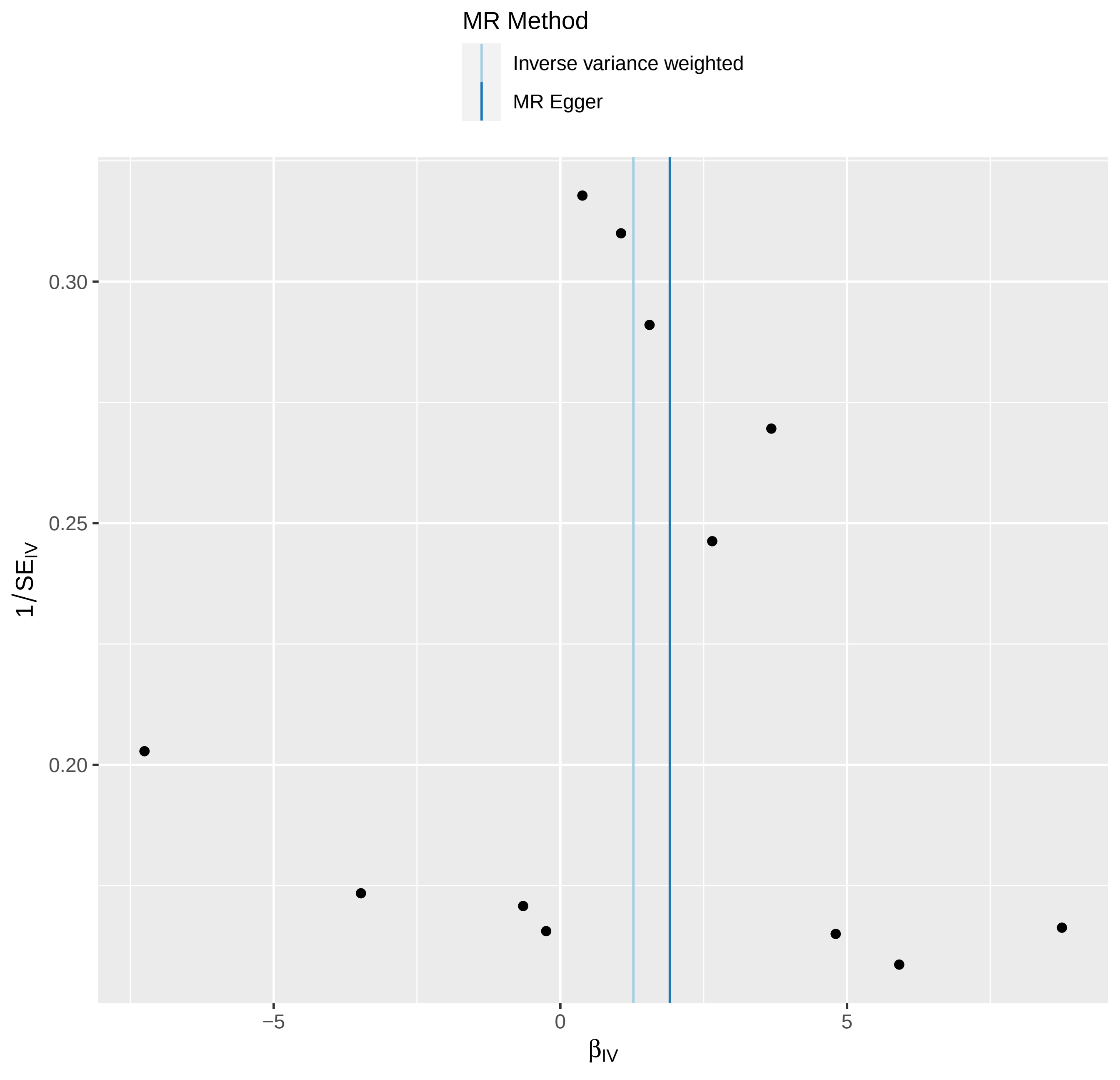

Batch 1096 : Ergothioneine on Arthropathy in Crohn disease

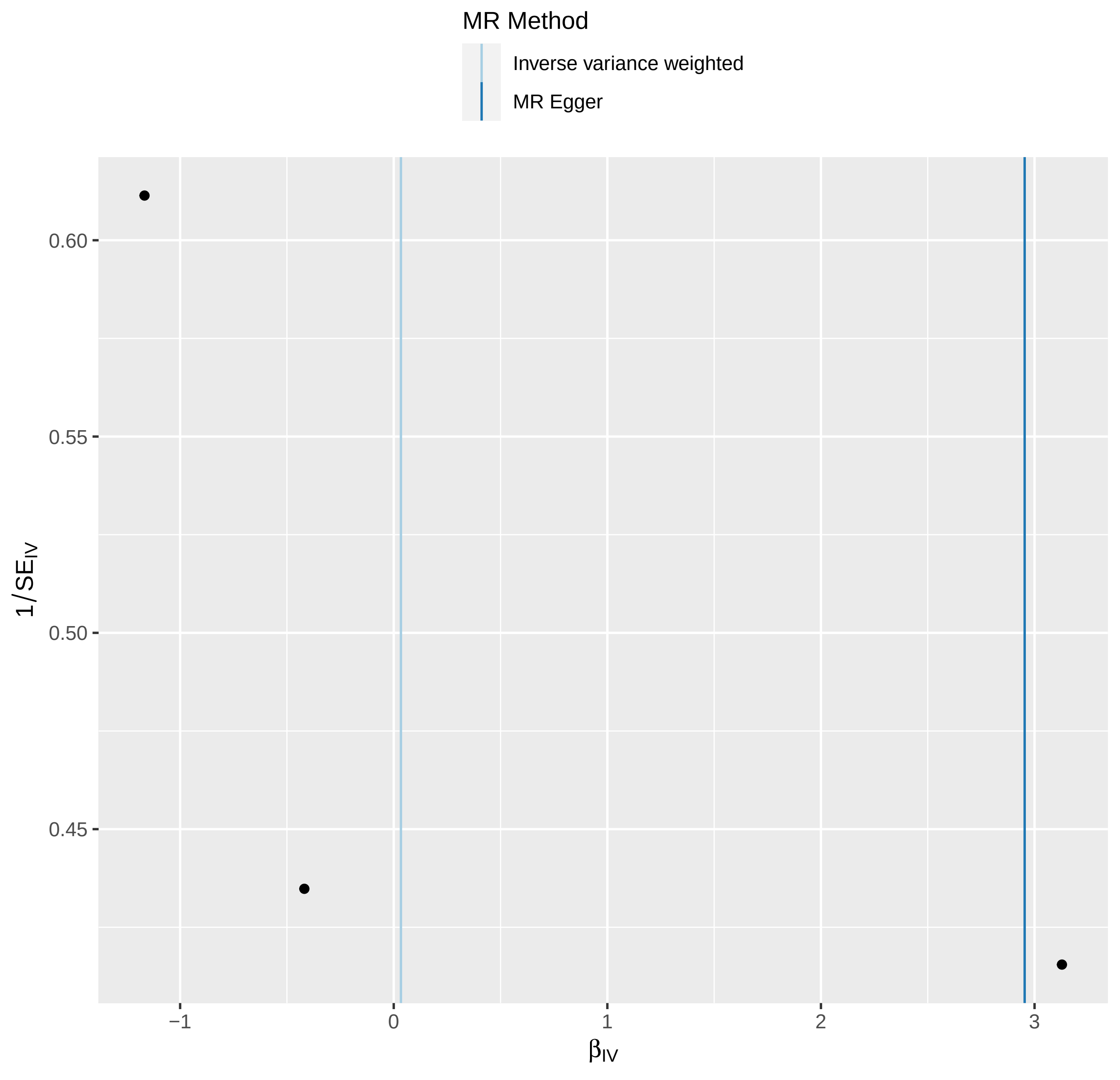

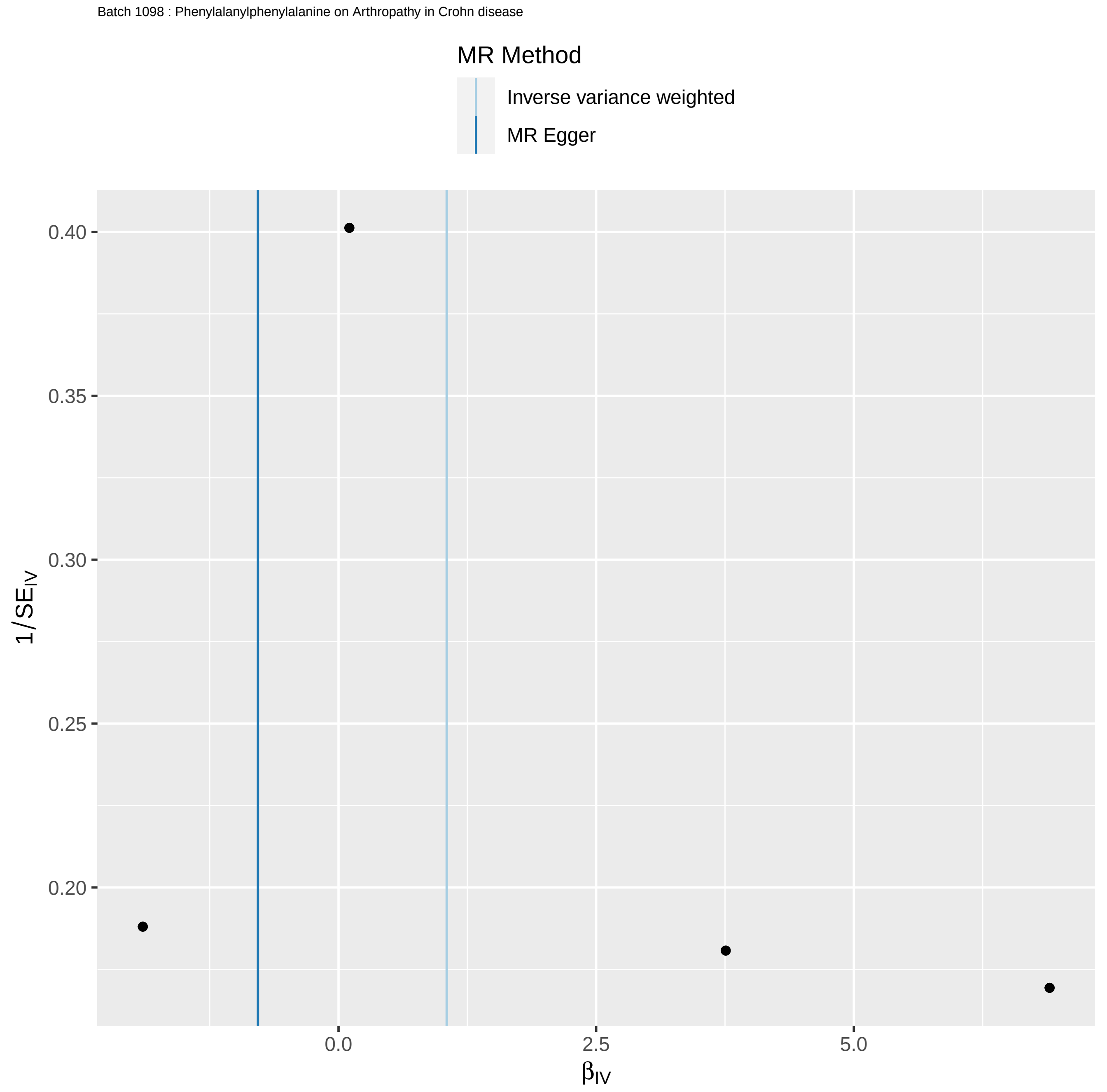
Batch 1098 : Phenylalanylphenylalanine on Arthropathy in Crohn disease
MR Method
Inverse variance weighted
MR Egger
0.40
0.35
0.30
0.25
0.20
$1/SE_{IV}$
0.0
2.5
5.0
β_{IV}

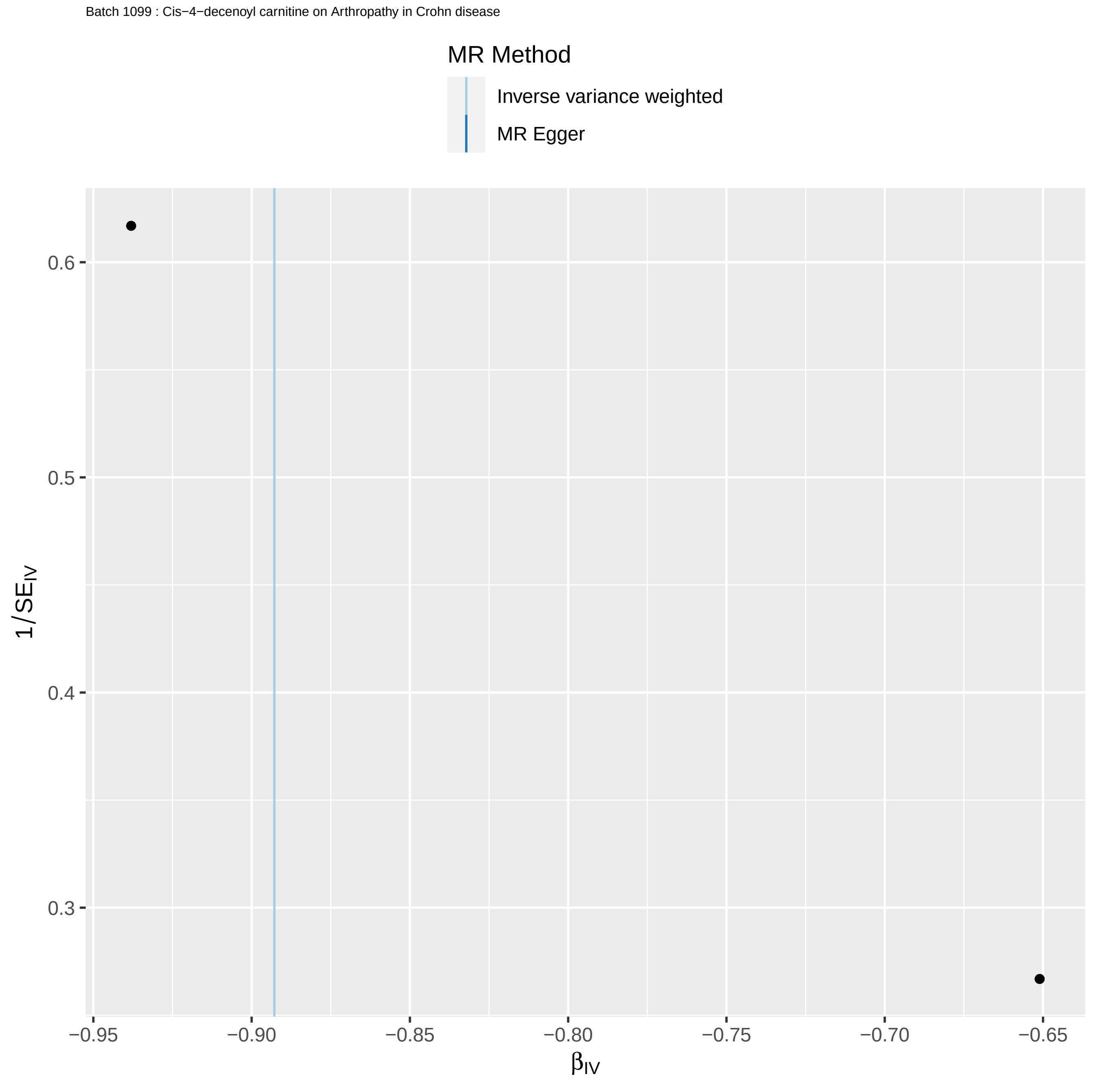

Batch 1099 : Cis−4−decenoyl carnitine on Arthropathy in Crohn disease
MR Method
Inverse variance weighted
MR Egger
0.6
0.5
0.4
0.3
1/SE_IV
−0.95
−0.90
−0.85
−0.80
−0.75
−0.70
−0.65
β_IV

Batch 1100 : 15−methylpalmitate (isobar with 2−methylpalmitate) on Arthropathy in Crohn disease

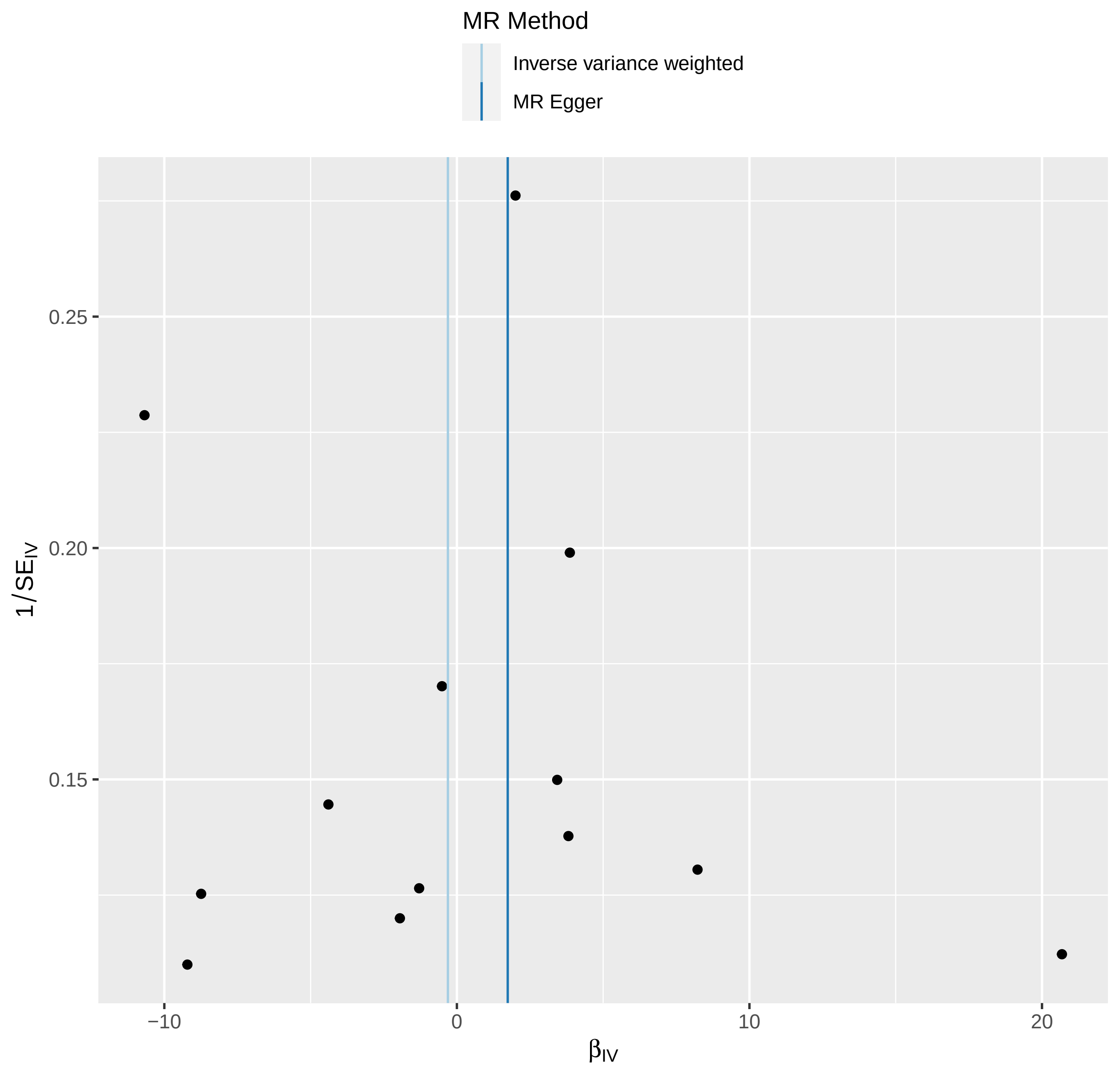

Batch 1102 : Tryptophan on Ulcerative colitis with PSC

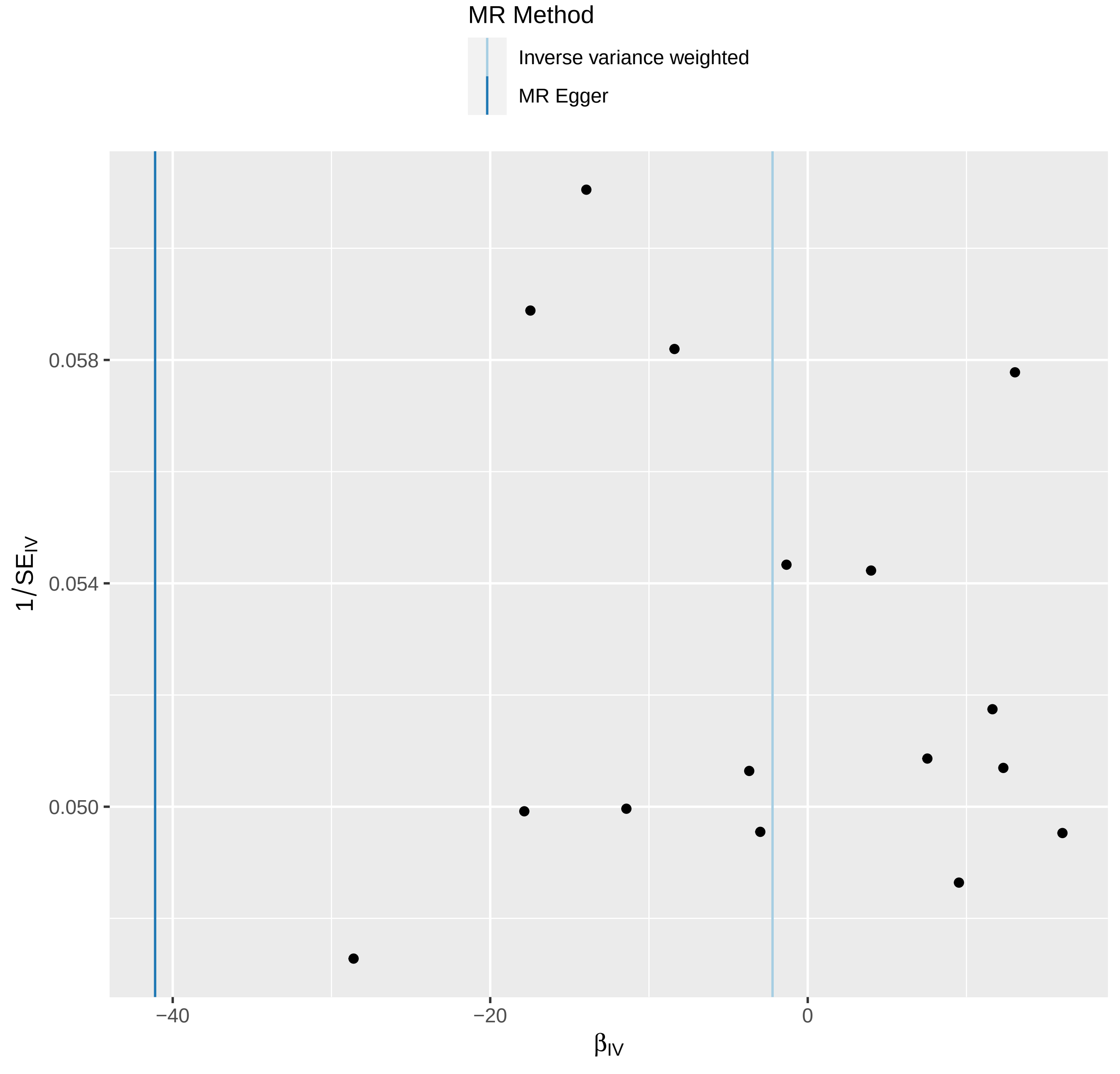

Batch 1104 : Leucine on Ulcerative colitis with PSC

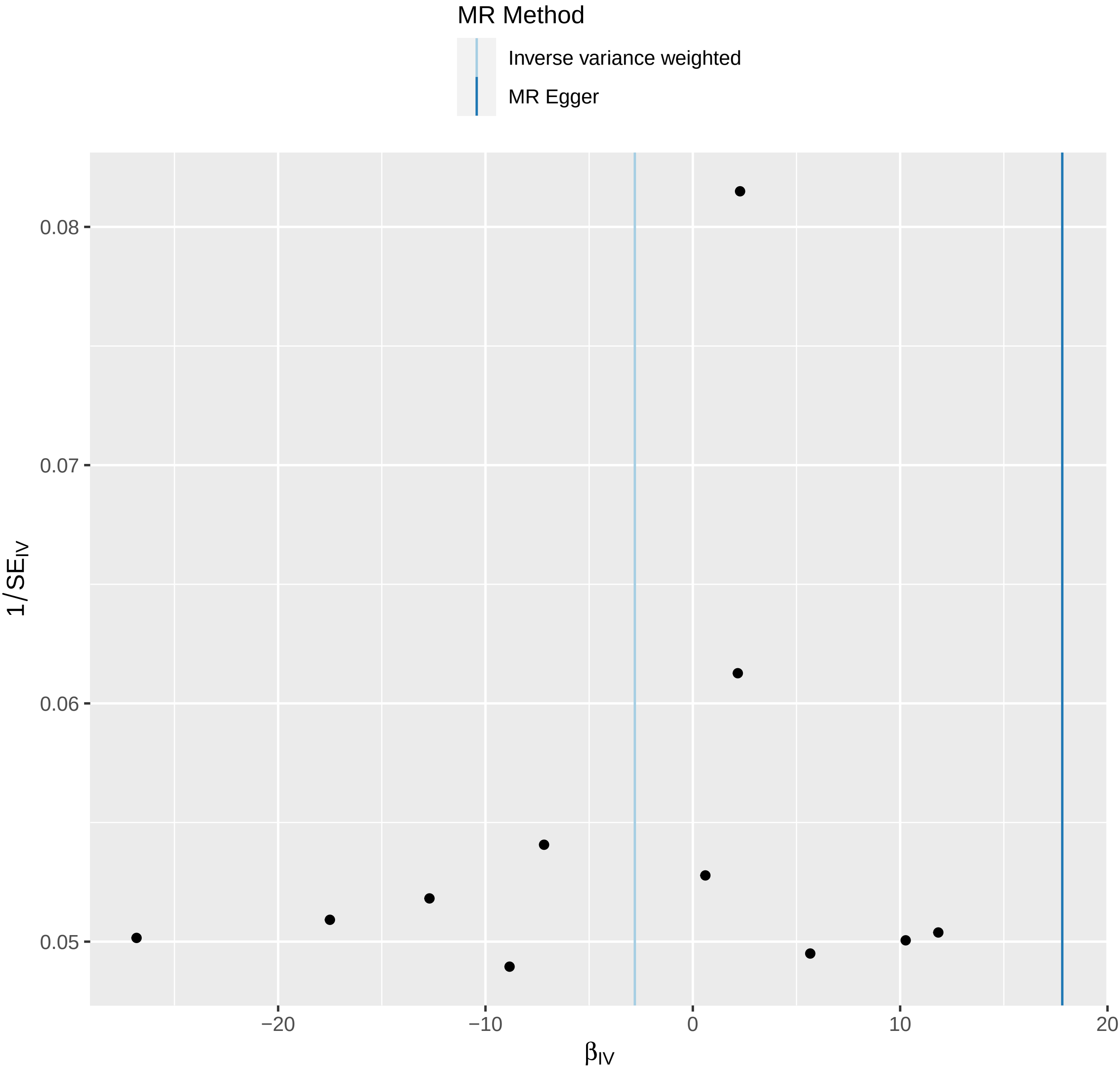

Batch 1108 : Lactate on Ulcerative colitis with PSC

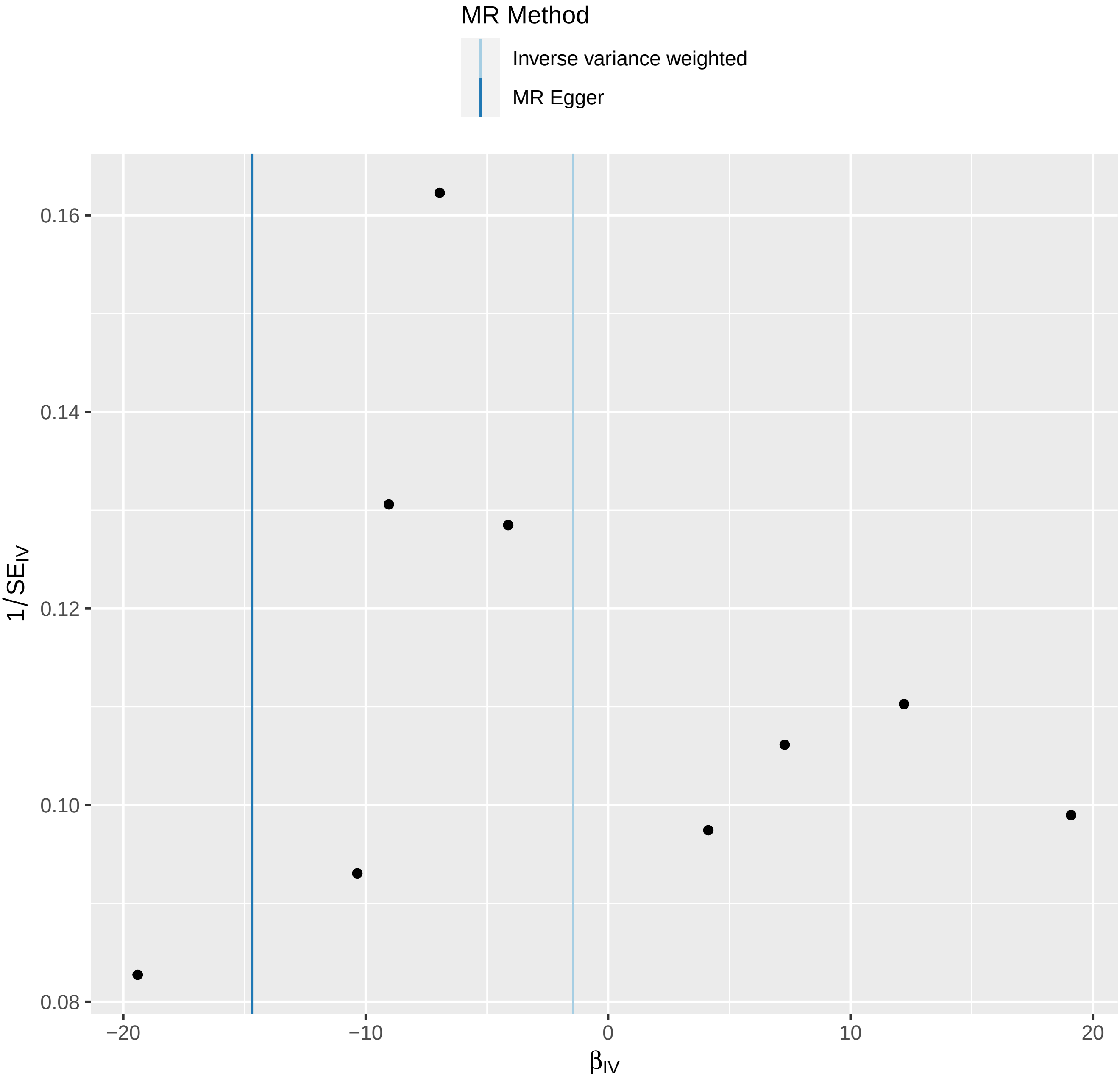

Batch 1109 : 3−hydroxybutyrate (BHBA) on Ulcerative colitis with PSC

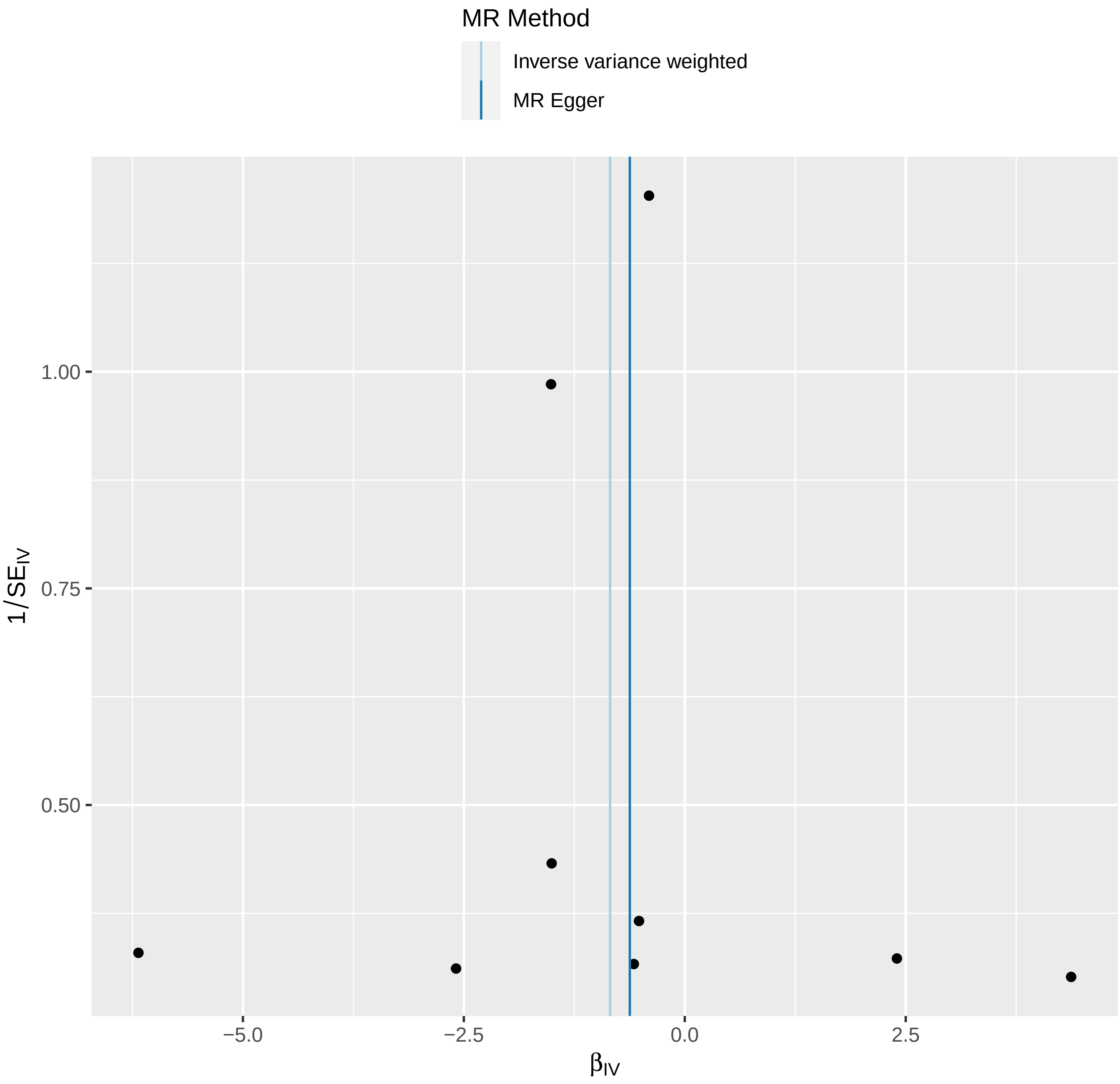

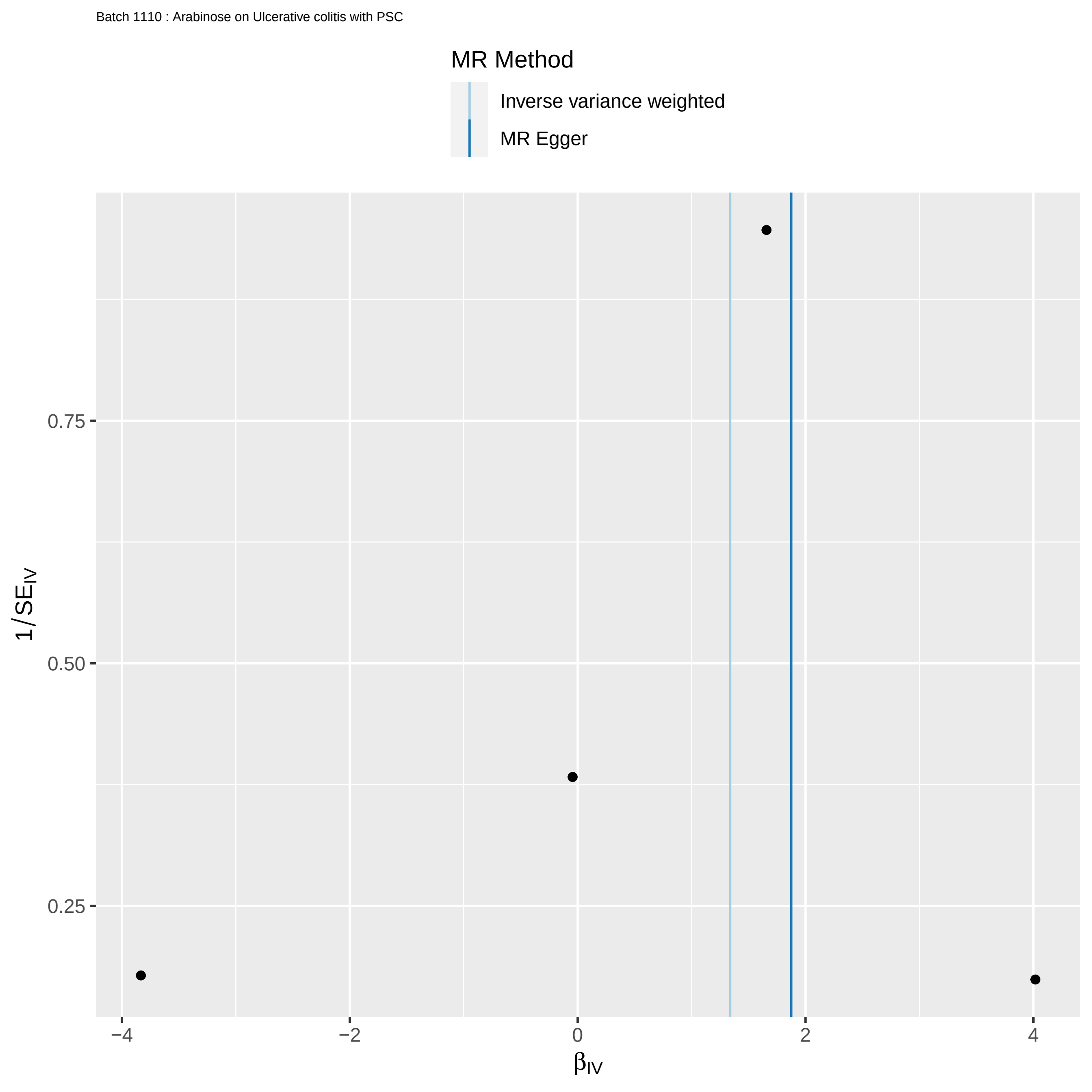
Batch 1110 : Arabinose on Ulcerative colitis with PSC
MR Method
Inverse variance weighted
MR Egger
0.75
0.50
0.25
1/SE$_{IV}$
−4
−2
0
2
4
β_{IV}

Batch 1111 : Fructose on Ulcerative colitis with PSC

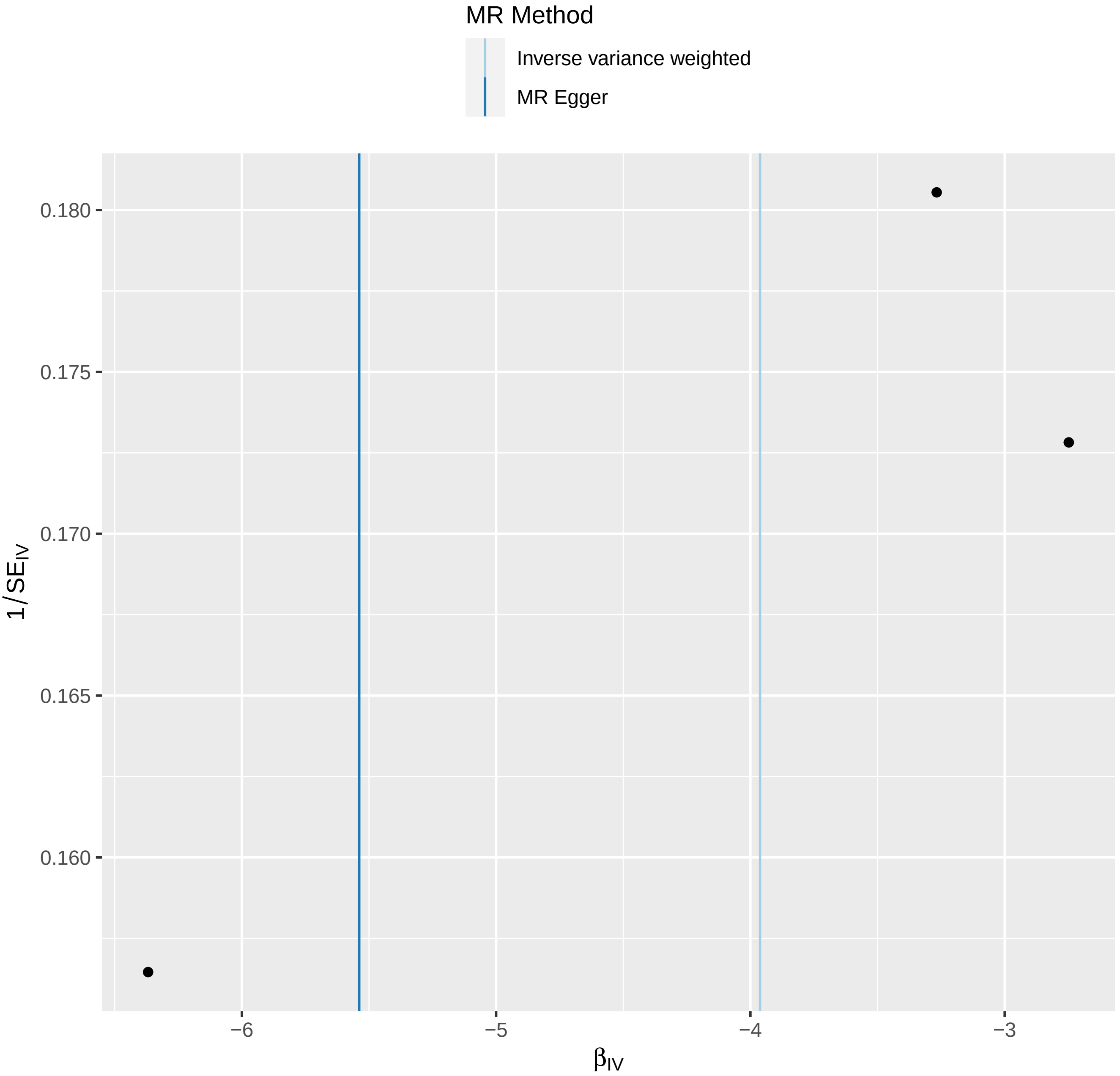

Batch 1113 : Pyruvate on Ulcerative colitis with PSC

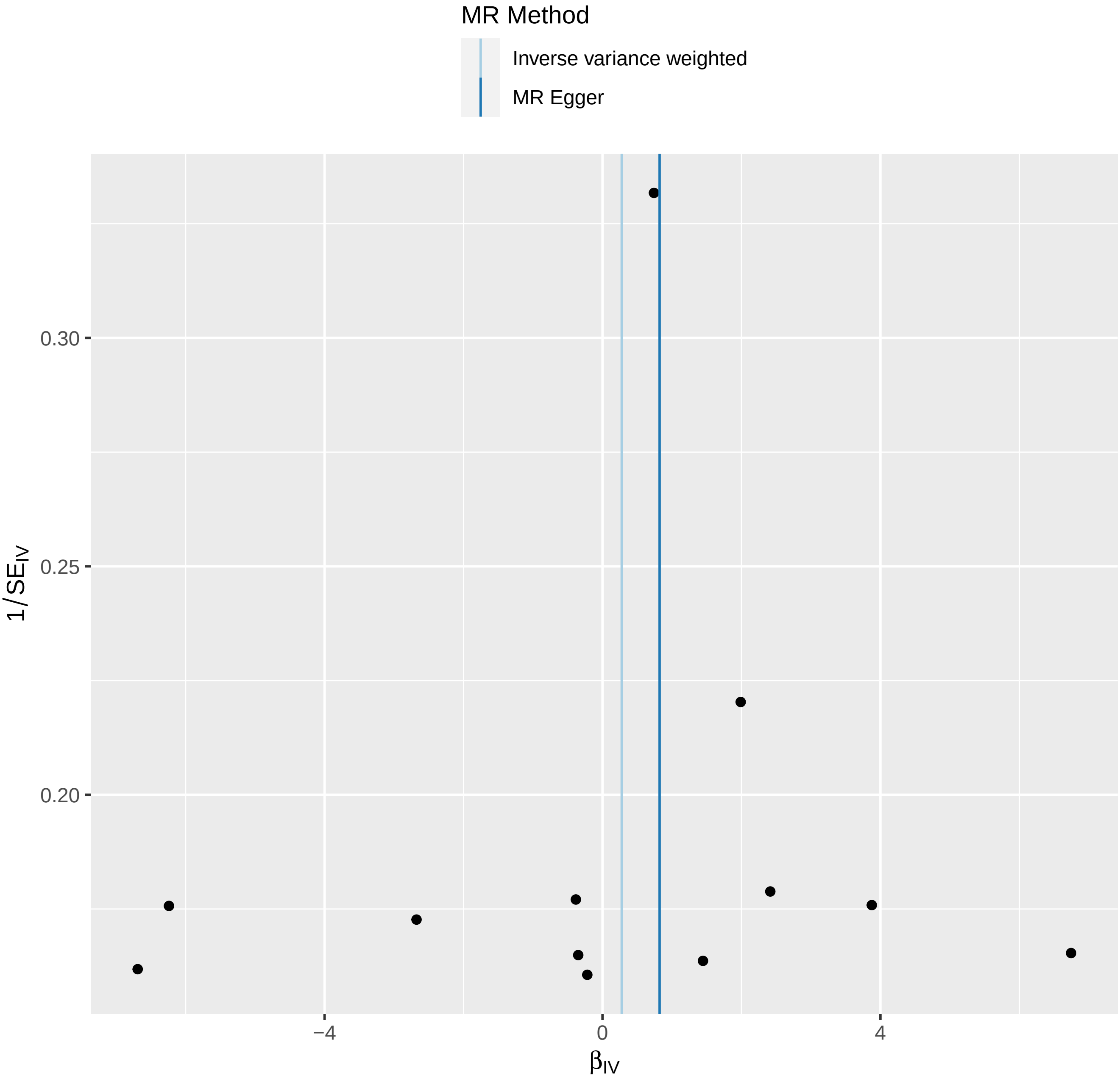

Batch 1114 : Uridine on Ulcerative colitis with PSC

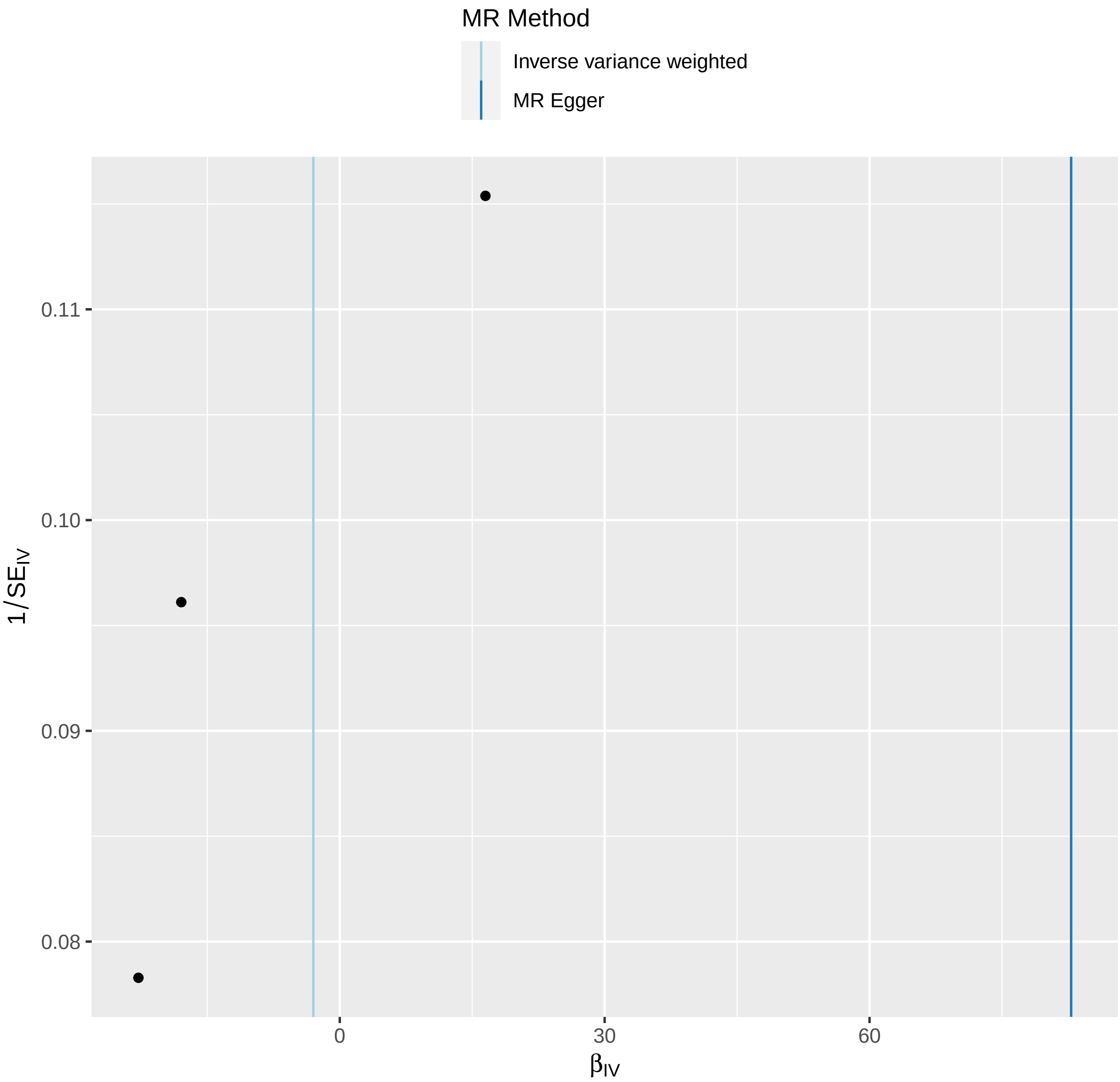

Batch 1115 : Linoleate (18:2n6) on Ulcerative colitis with PSC

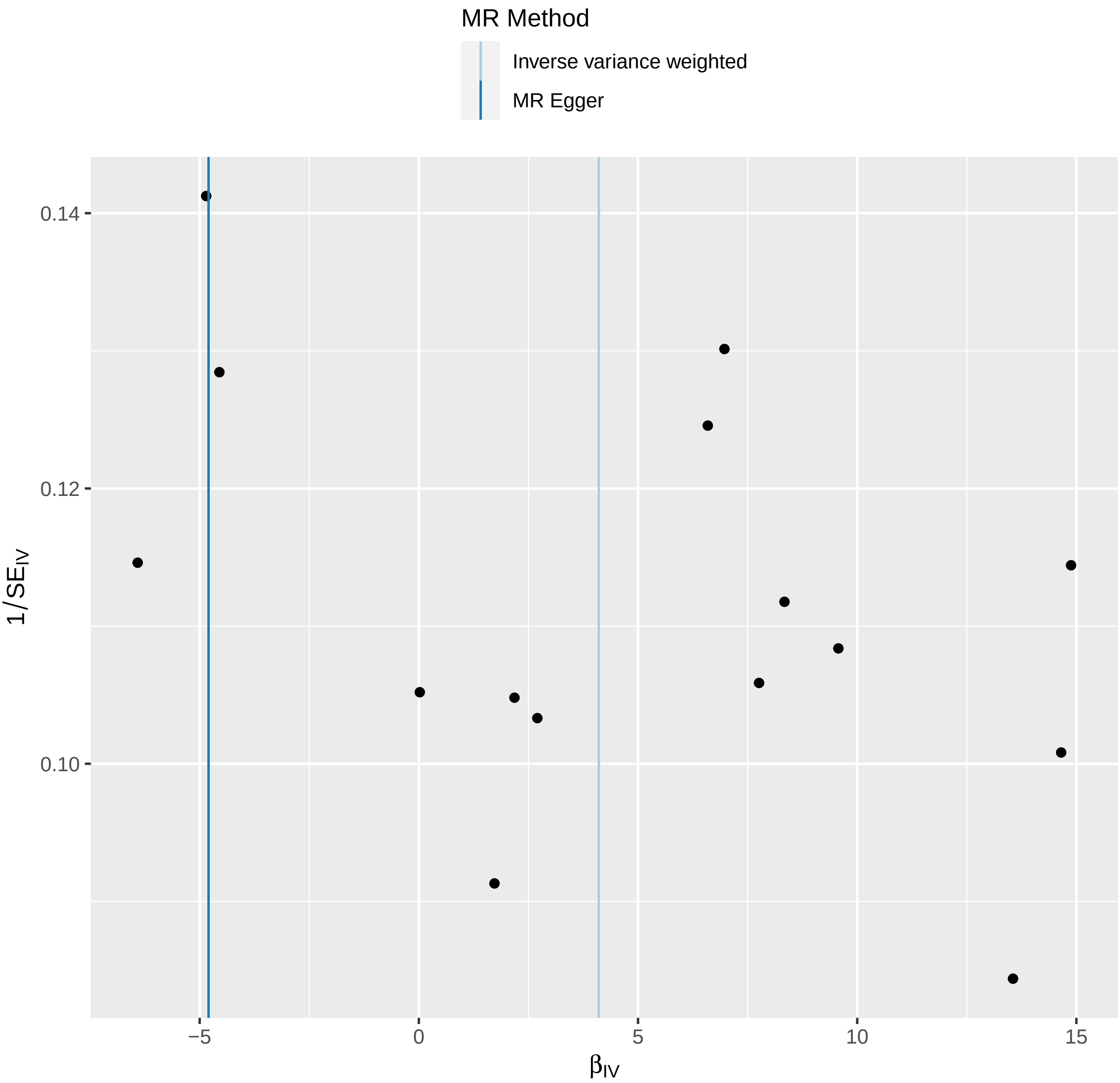

Batch 1116 : Allantoin on Ulcerative colitis with PSC

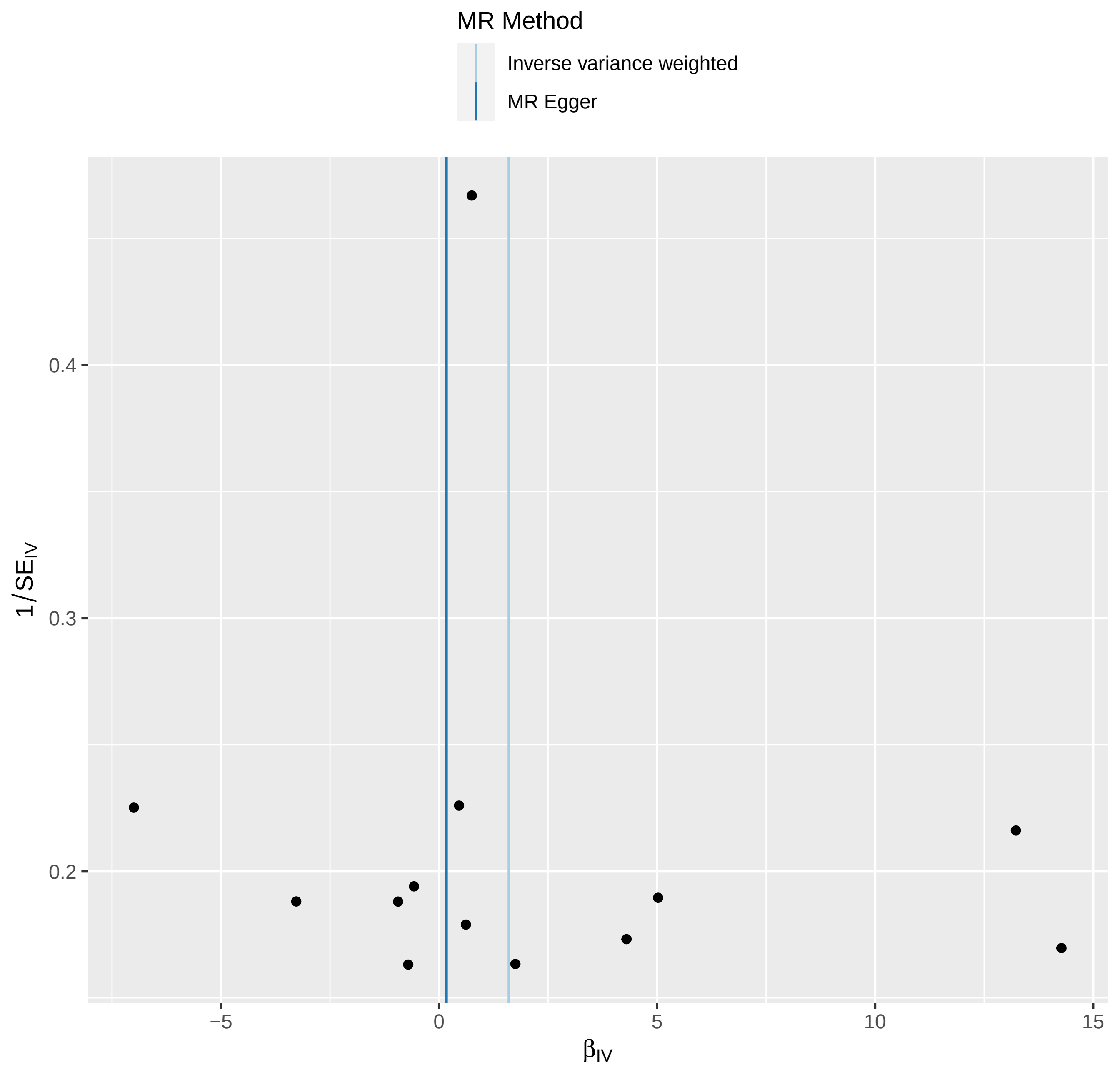

Batch 1121 : Isoleucine on Ulcerative colitis with PSC

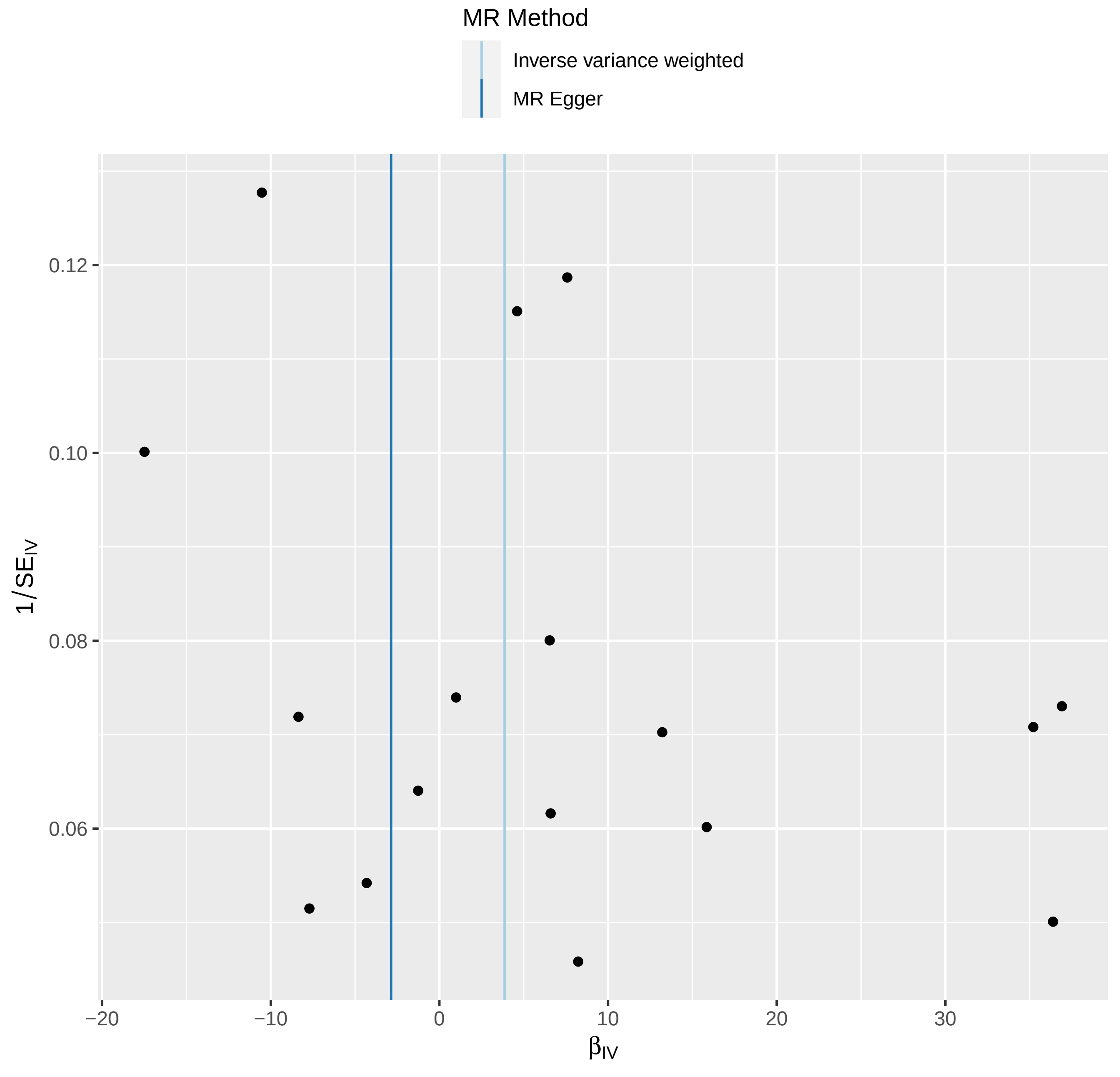

Batch 1122 : Threonine on Ulcerative colitis with PSC

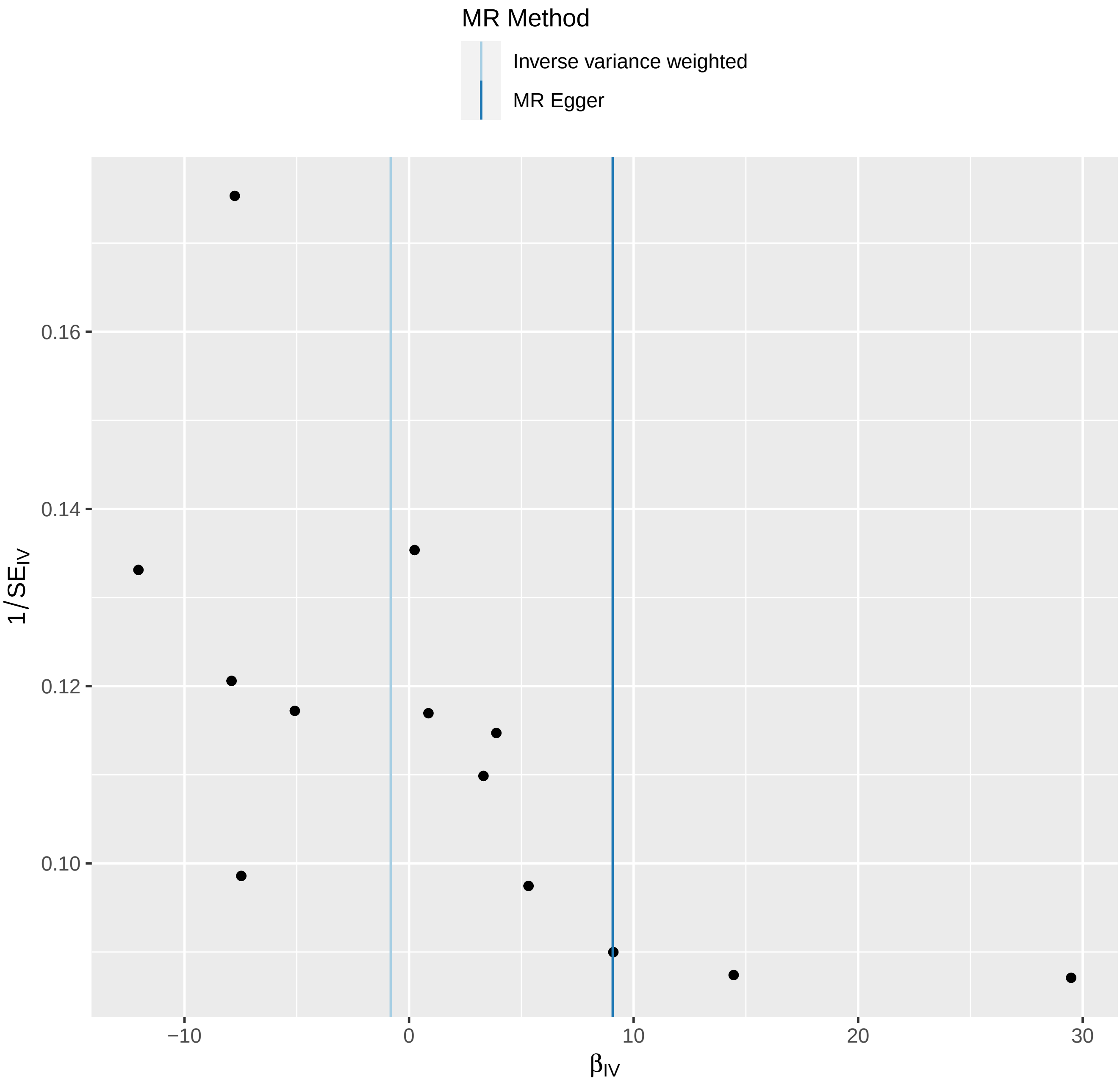

Batch 1123 : Tyrosine on Ulcerative colitis with PSC

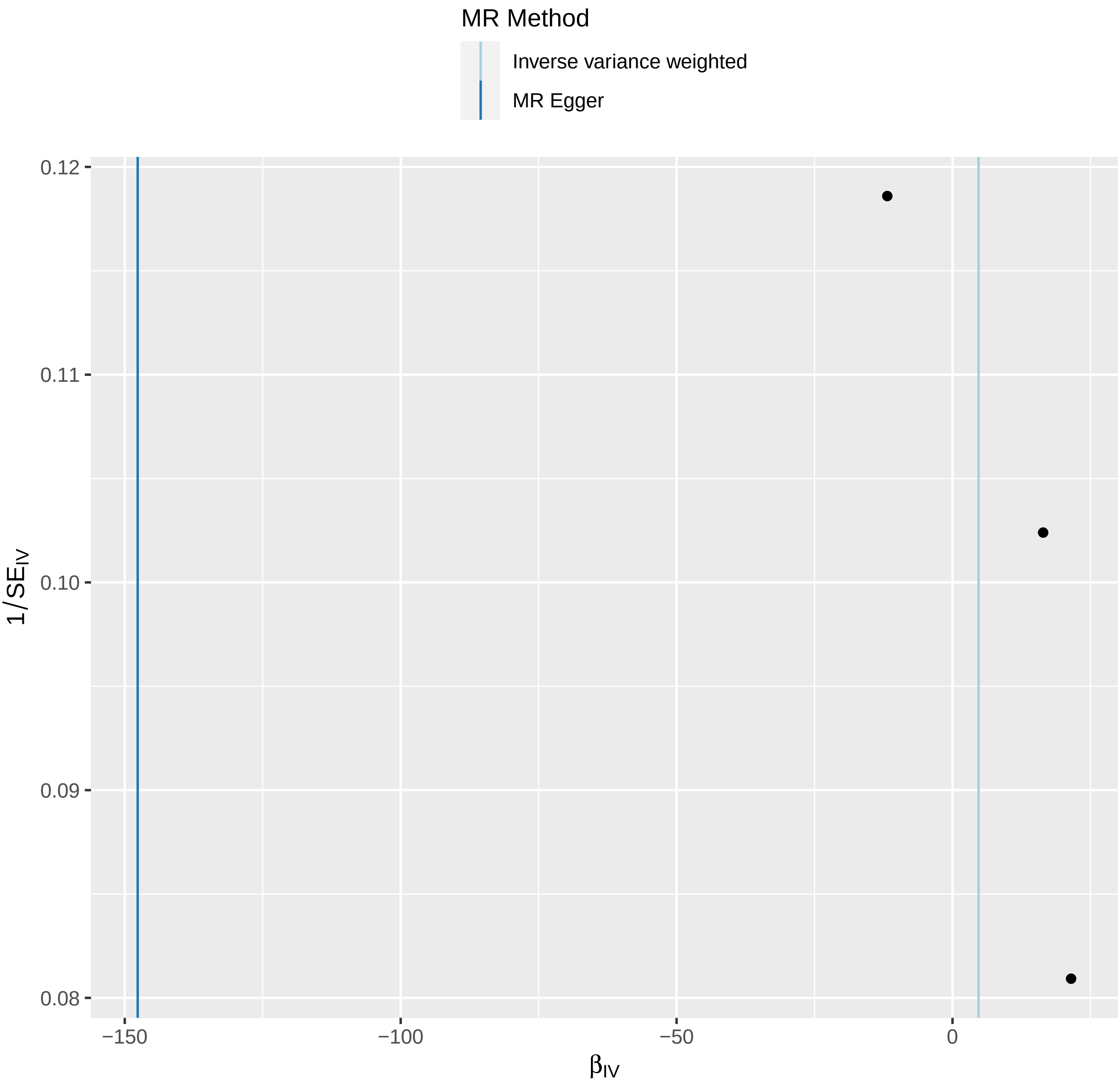

Batch 1129 : Stearate (18:0) on Ulcerative colitis with PSC

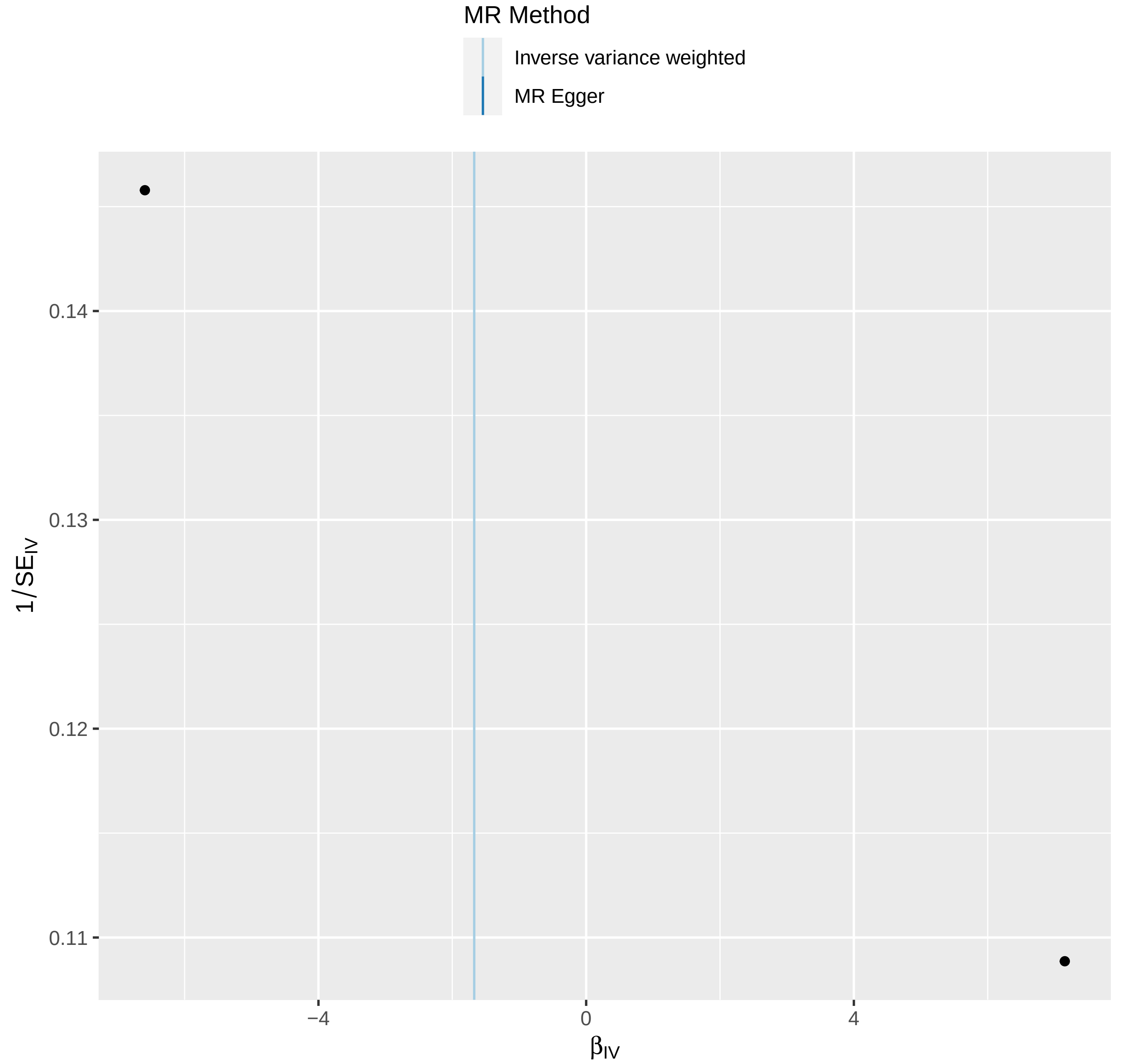

Batch 1132 : Myristate (14:0) on Ulcerative colitis with PSC

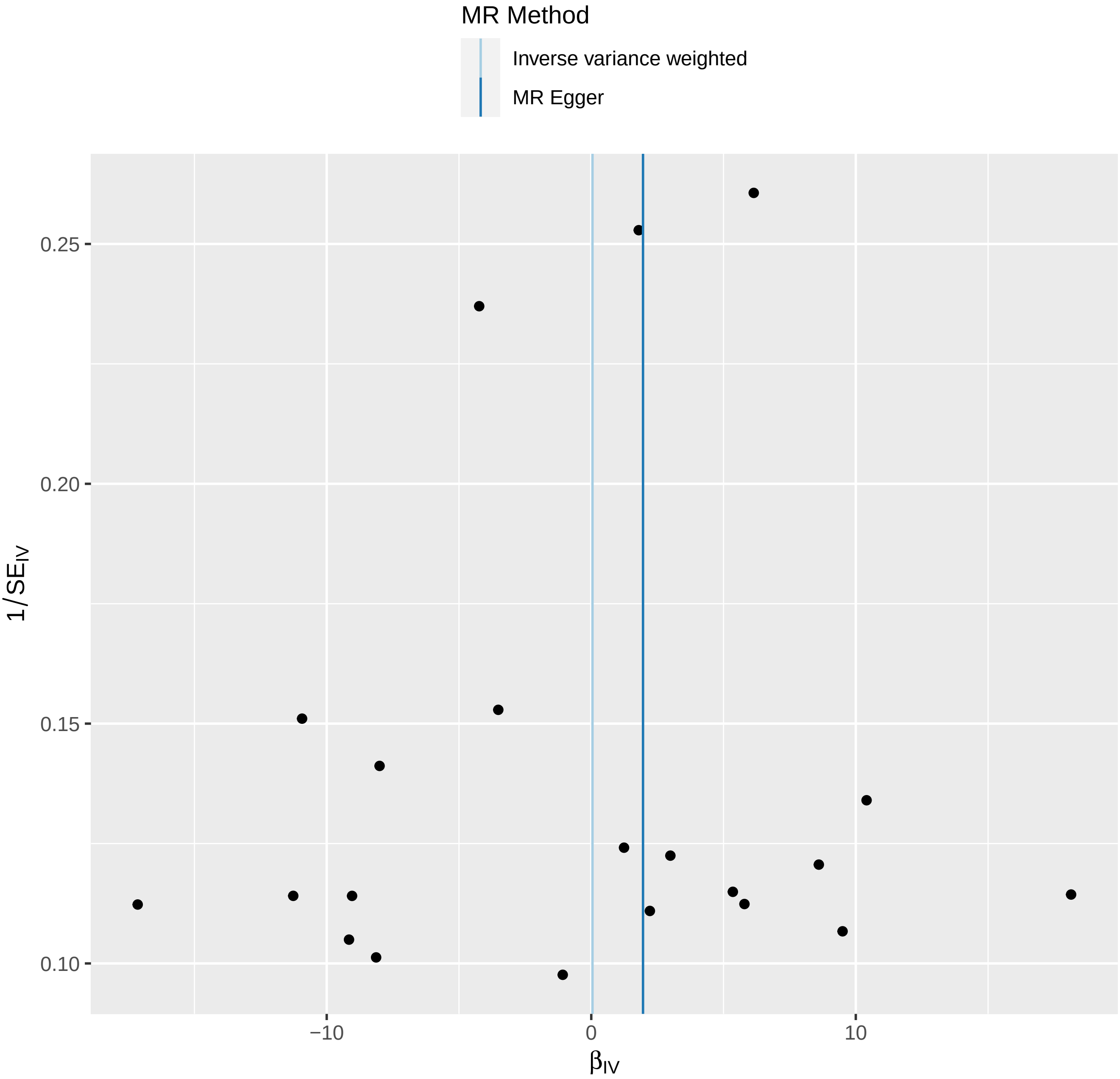

Batch 1134 : Ornithine on Ulcerative colitis with PSC

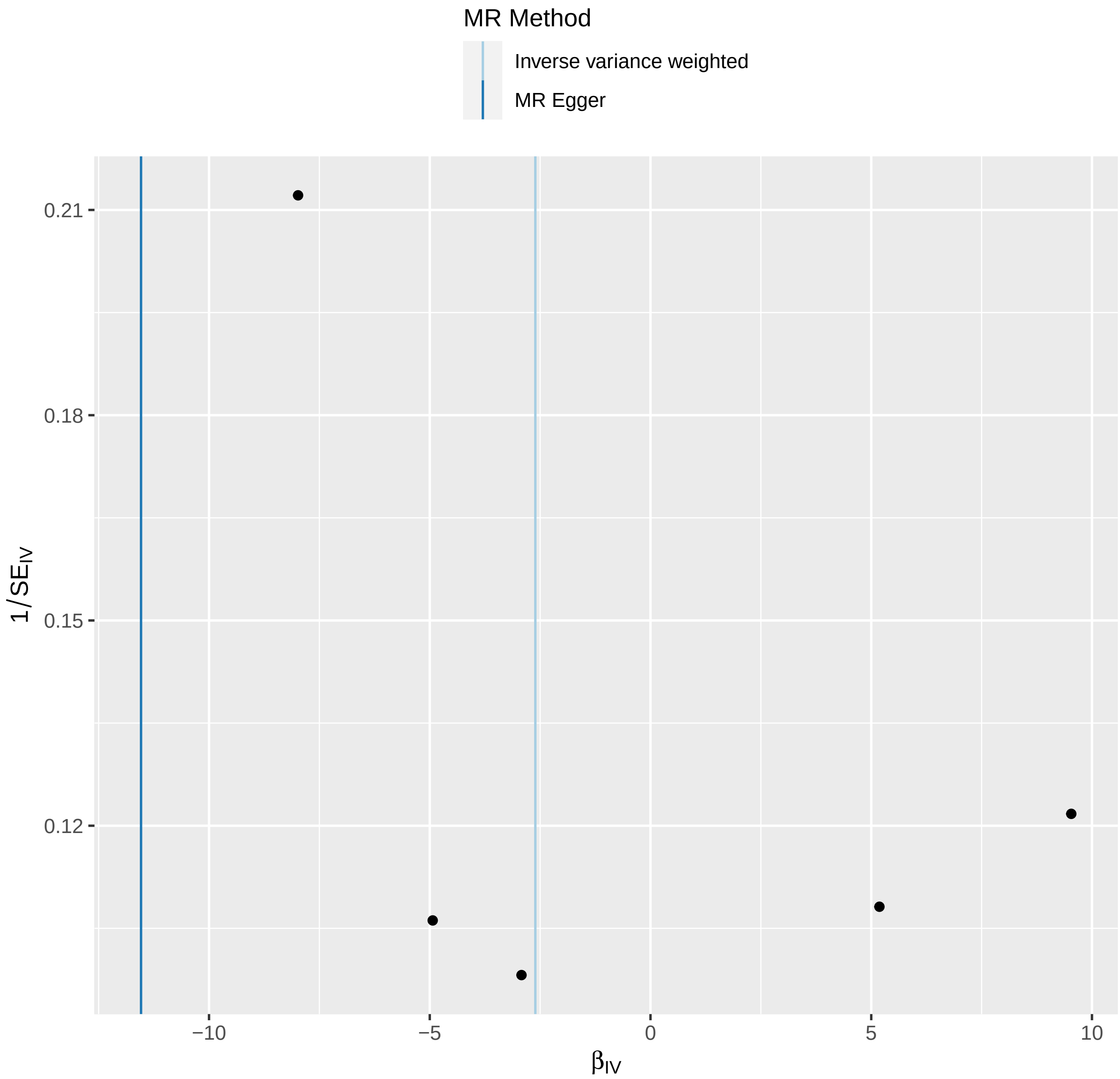

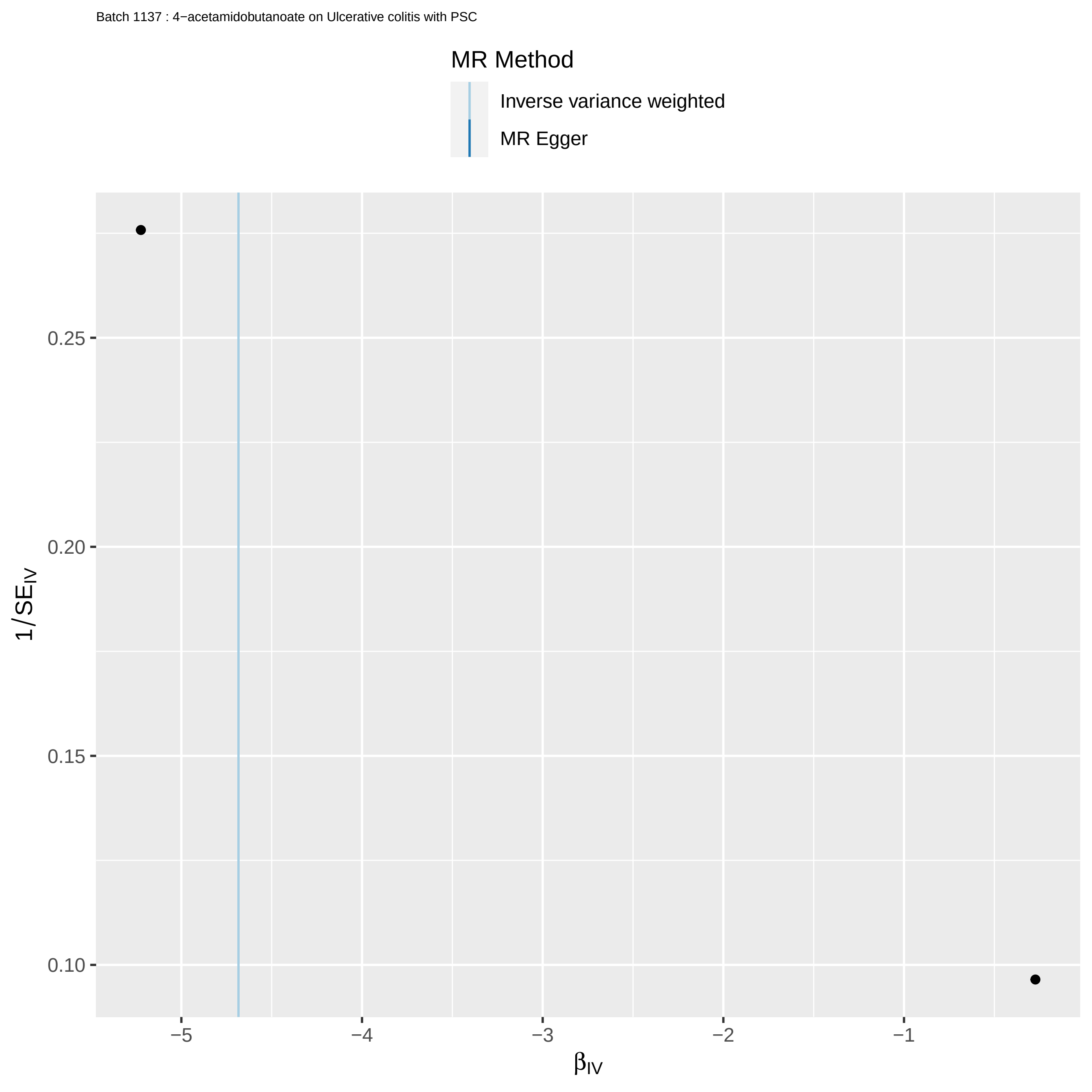
Batch 1137 : 4−acetamidobutanoate on Ulcerative colitis with PSC
MR Method
Inverse variance weighted
MR Egger
0.25
0.20
0.15
0.10
1/SE$_{IV}$
−5
−4
−3
−2
−1
β$_{IV}$

Batch 1138 : Alpha−tocopherol on Ulcerative colitis with PSC

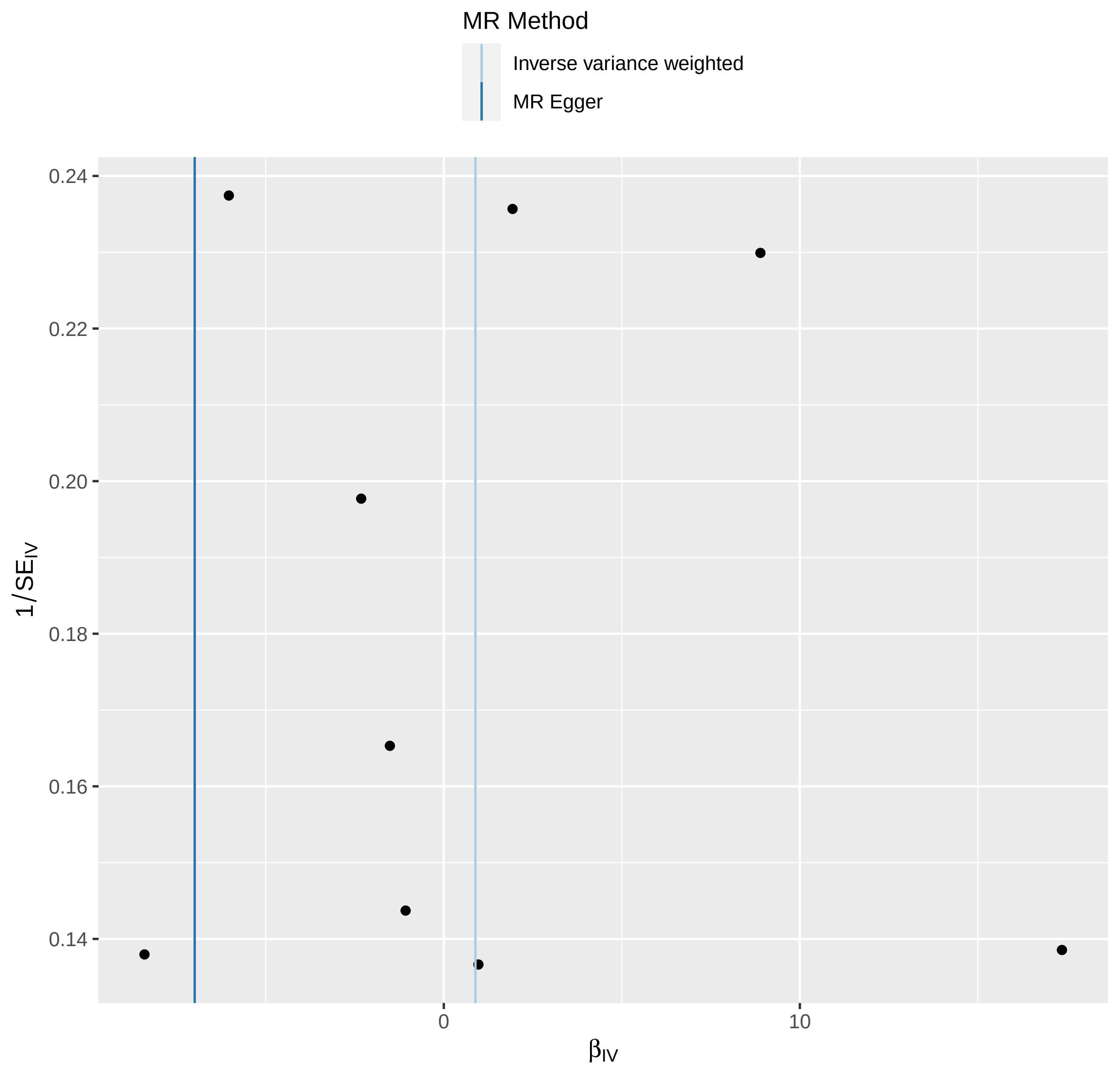

Batch 1139 : Citrate on Ulcerative colitis with PSC

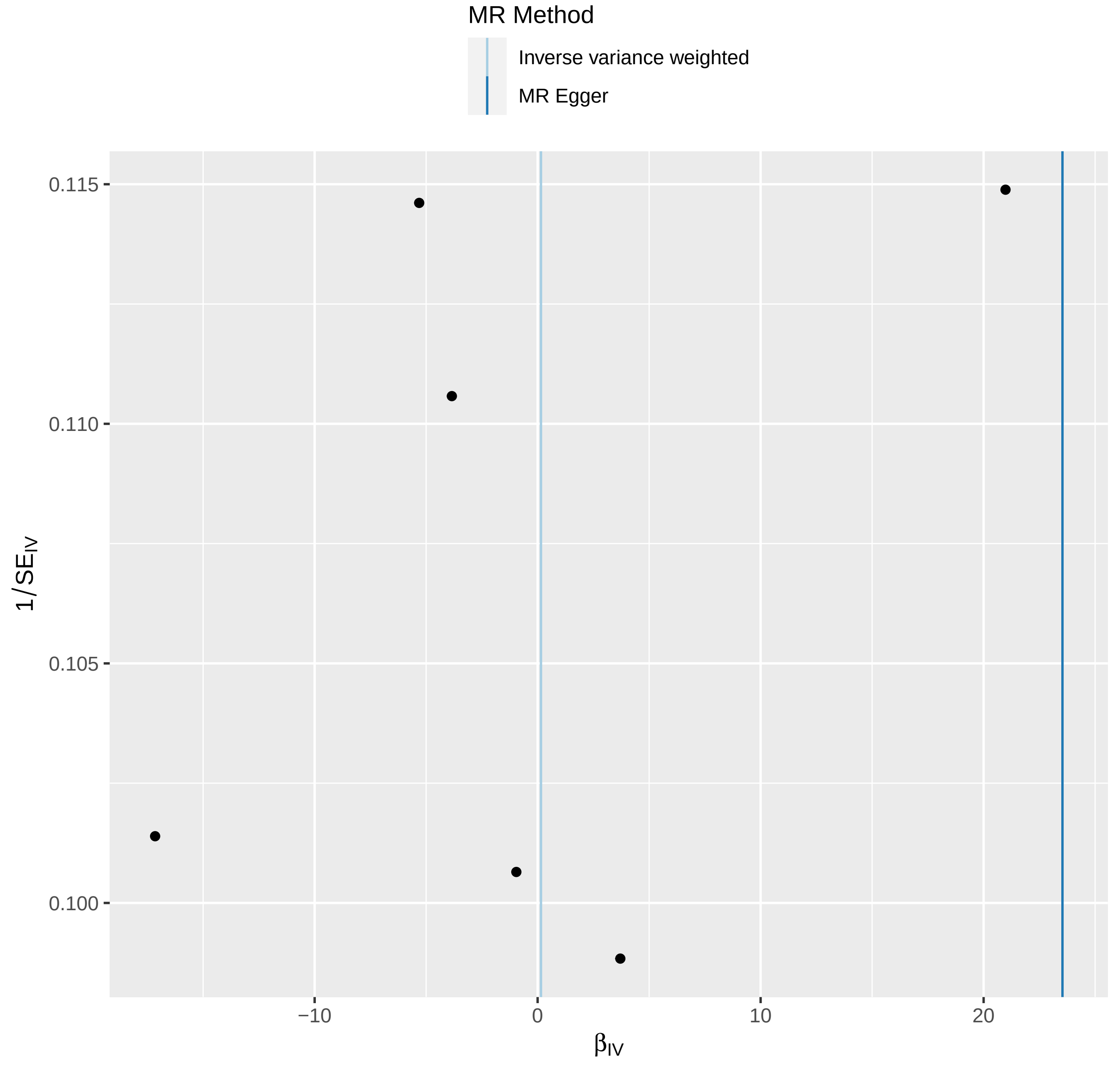

Batch 1140 : Glycerate on Ulcerative colitis with PSC

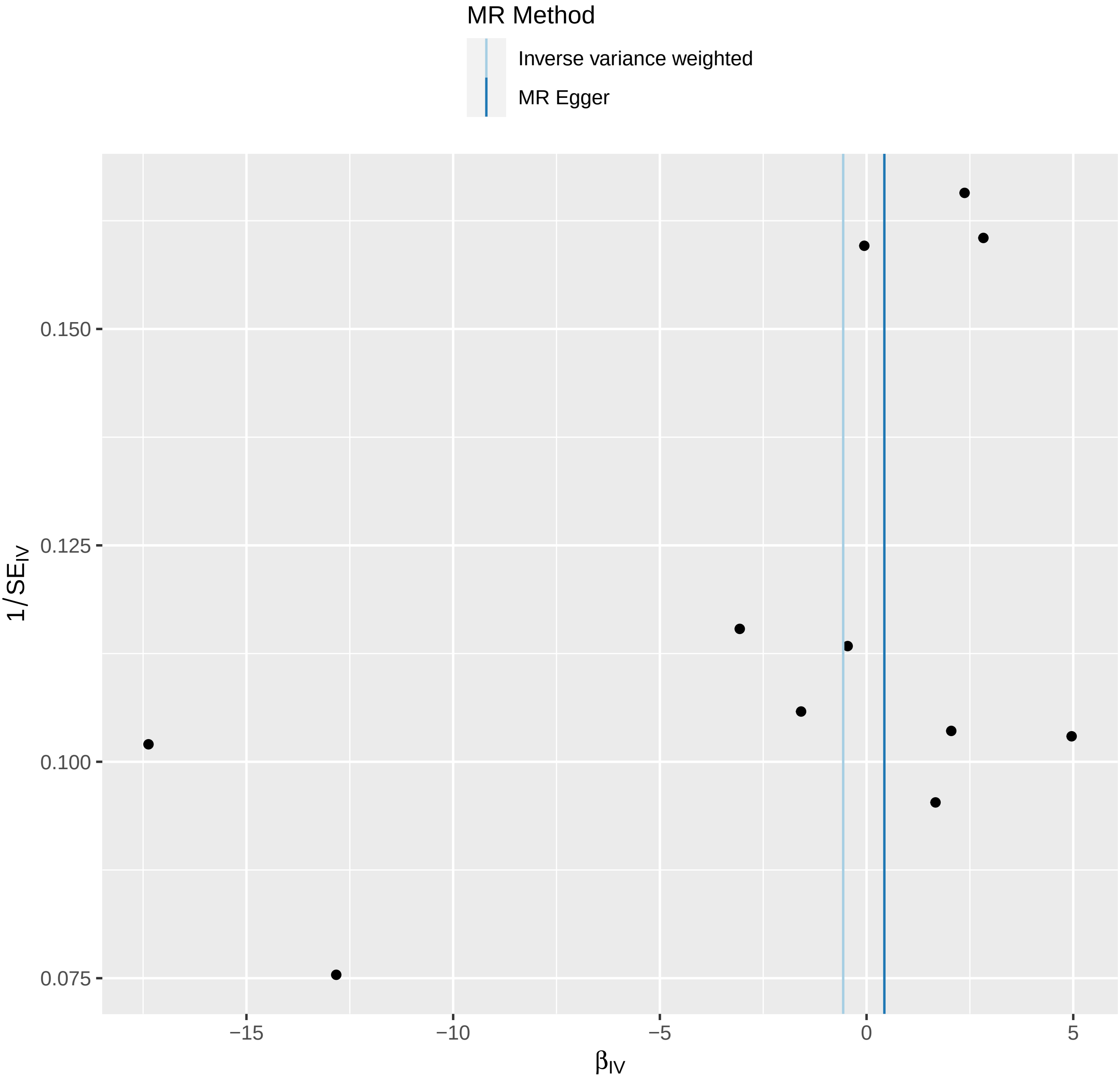

Batch 1141 : Guanosine on Ulcerative colitis with PSC

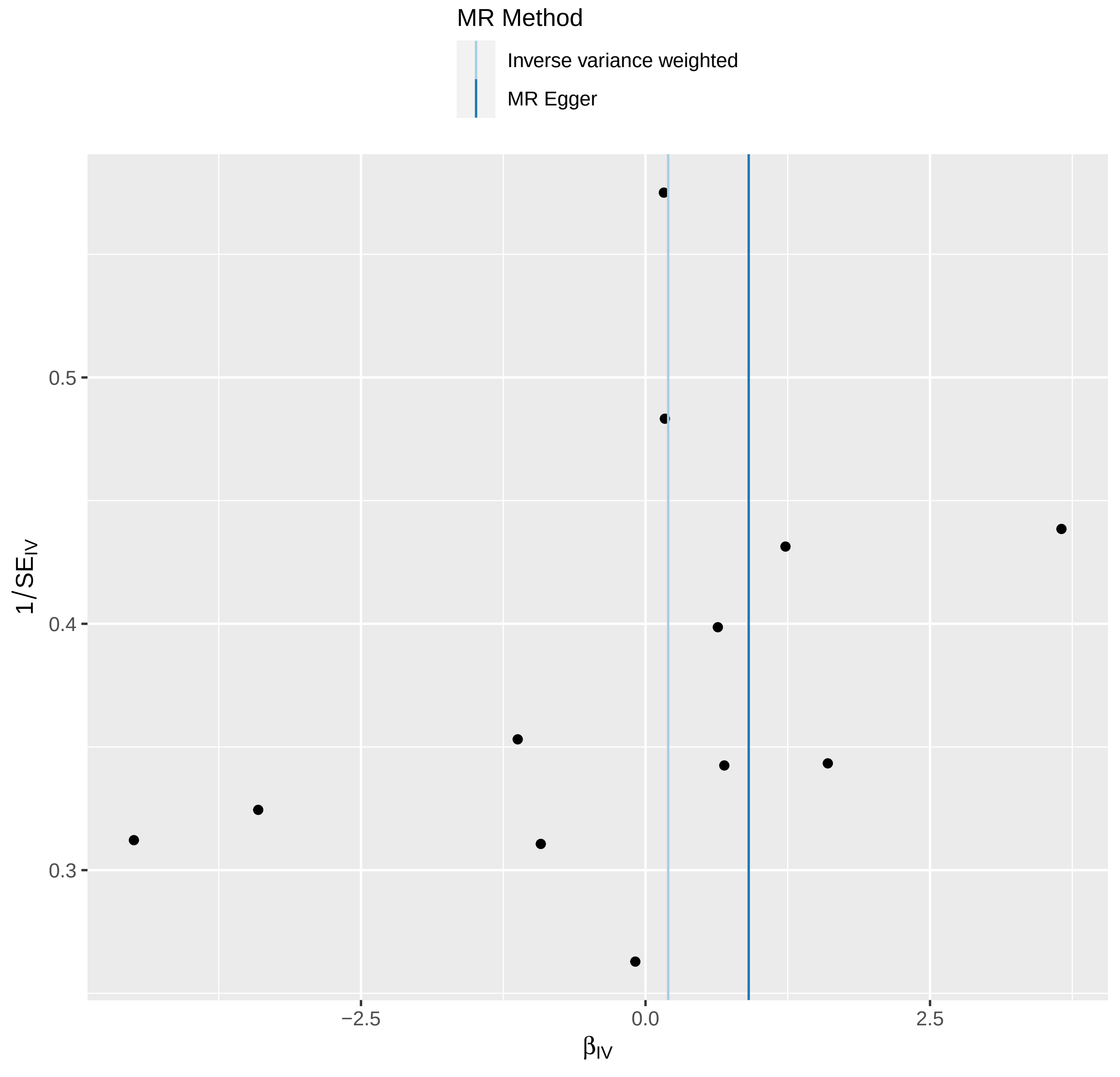

Batch 1144 : Ursodeoxycholate on Ulcerative colitis with PSC

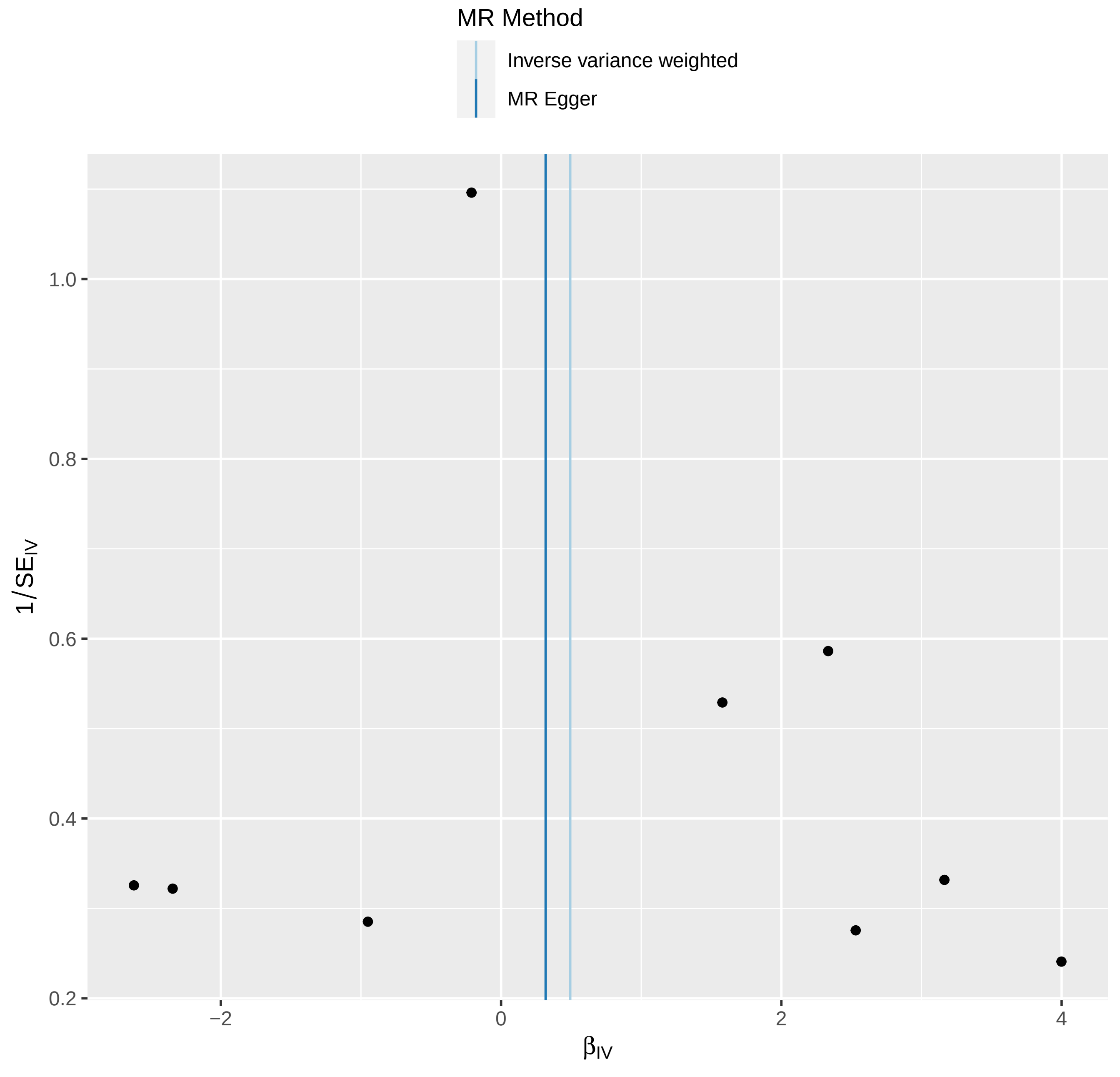

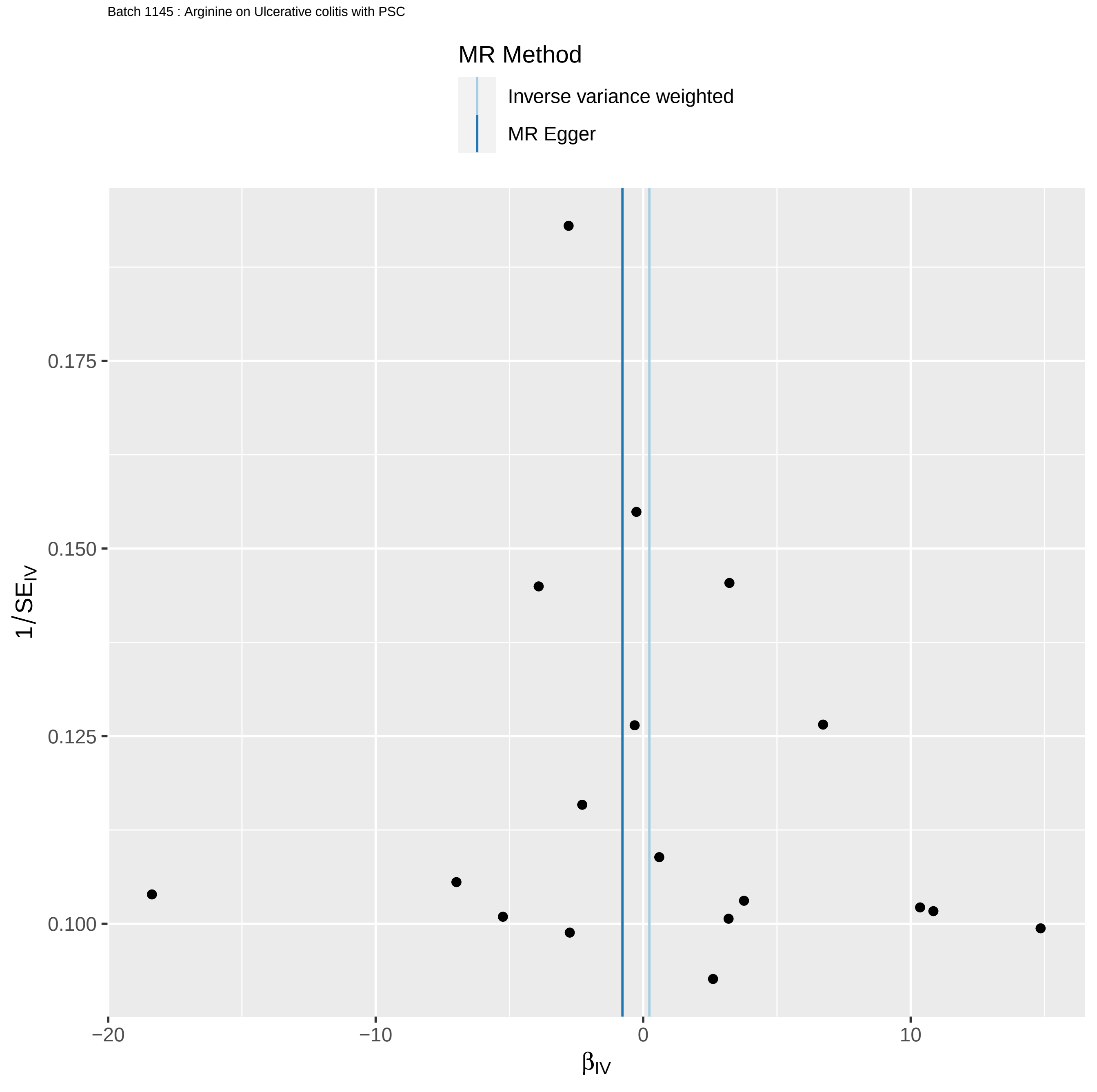

Batch 1145 : Arginine on Ulcerative colitis with PSC
MR Method
Inverse variance weighted
MR Egger
0.175
0.150
0.125
0.100
1/SE$_{IV}$
−20
−10
0
10
β_{IV}

Batch 1146 : Ascorbate (Vitamin C) on Ulcerative colitis with PSC

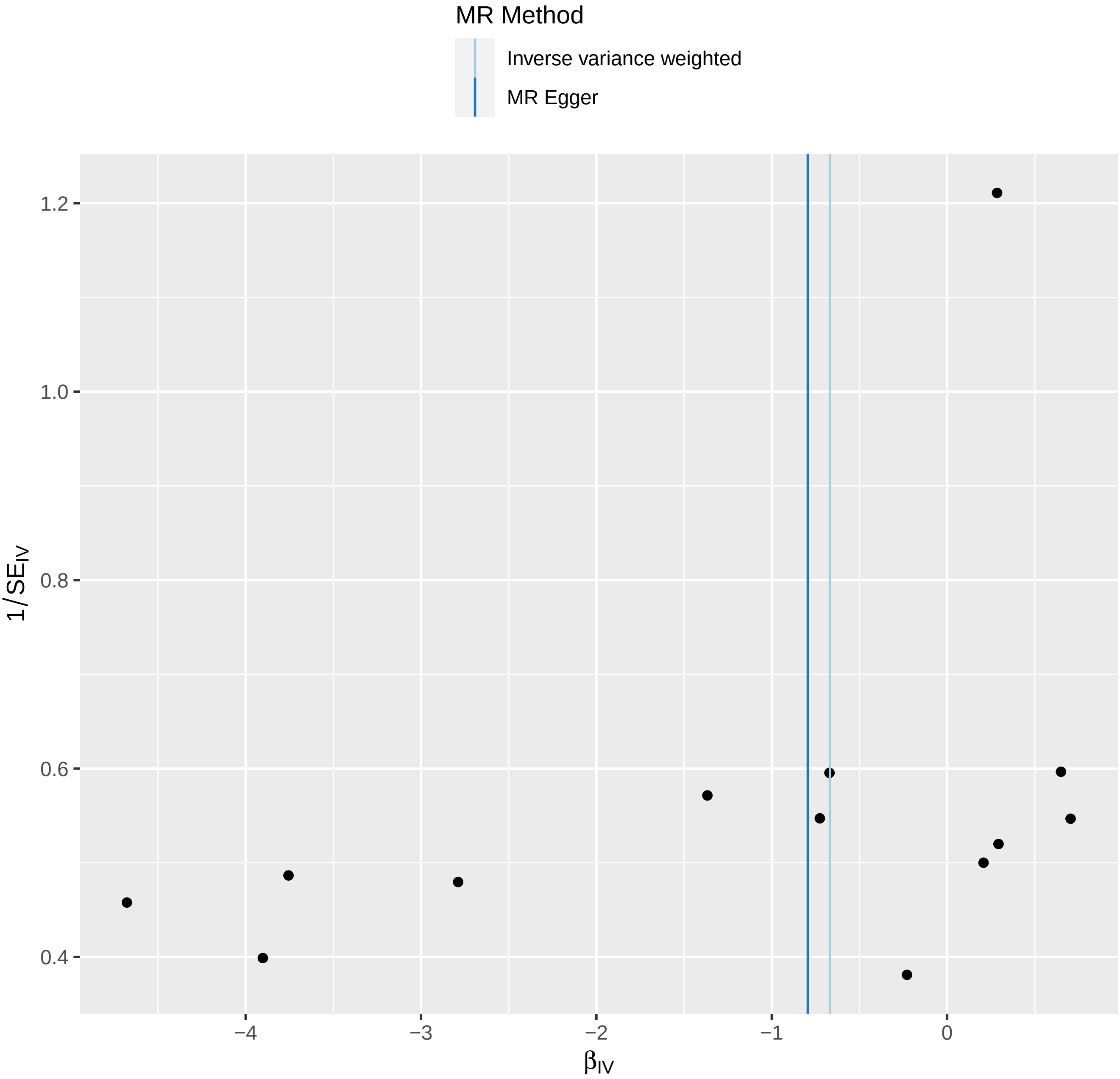

Batch 1147 : Heptanoate (7:0) on Ulcerative colitis with PSC

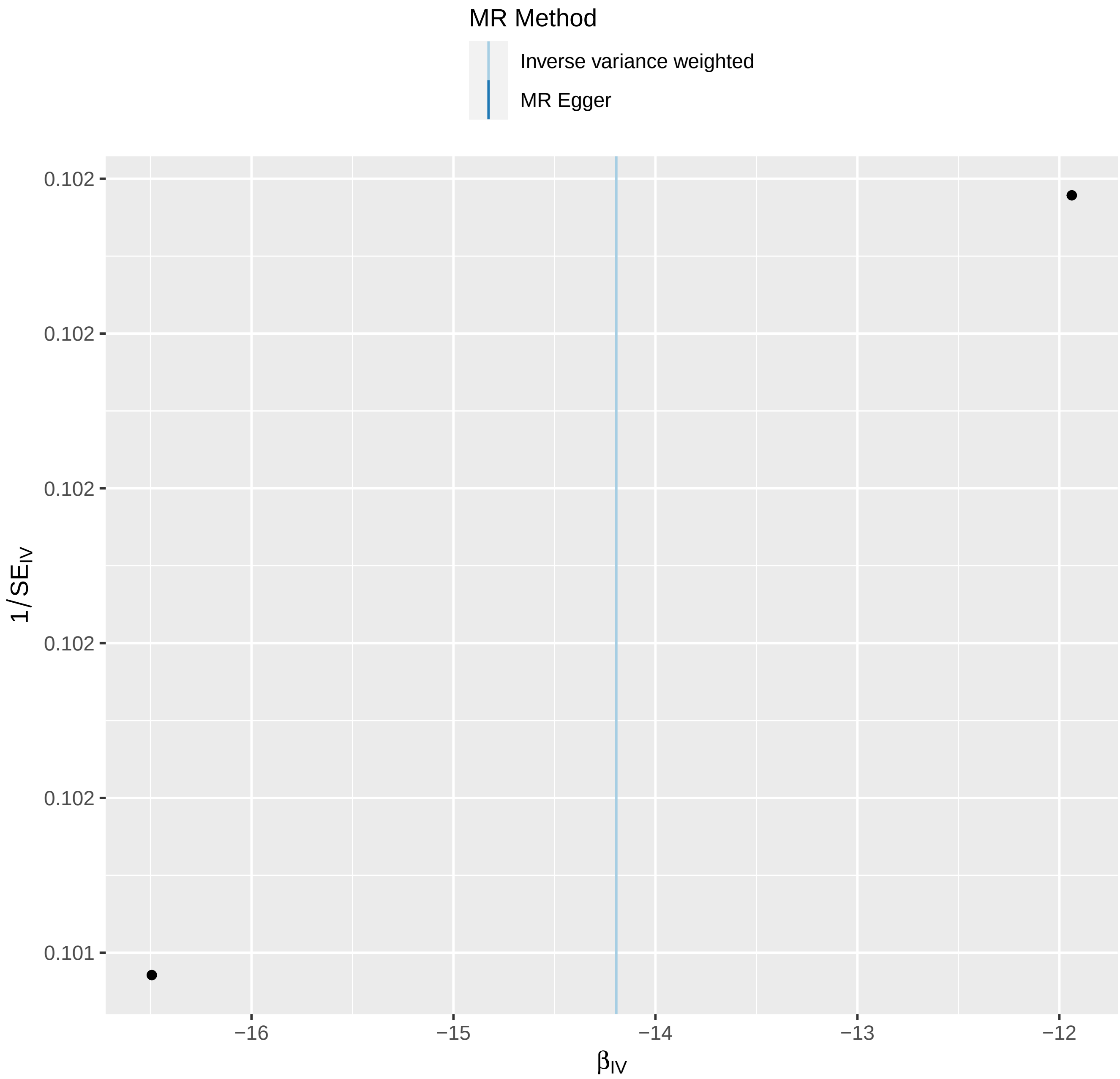

Batch 1148 : Laurate (12:0) on Ulcerative colitis with PSC

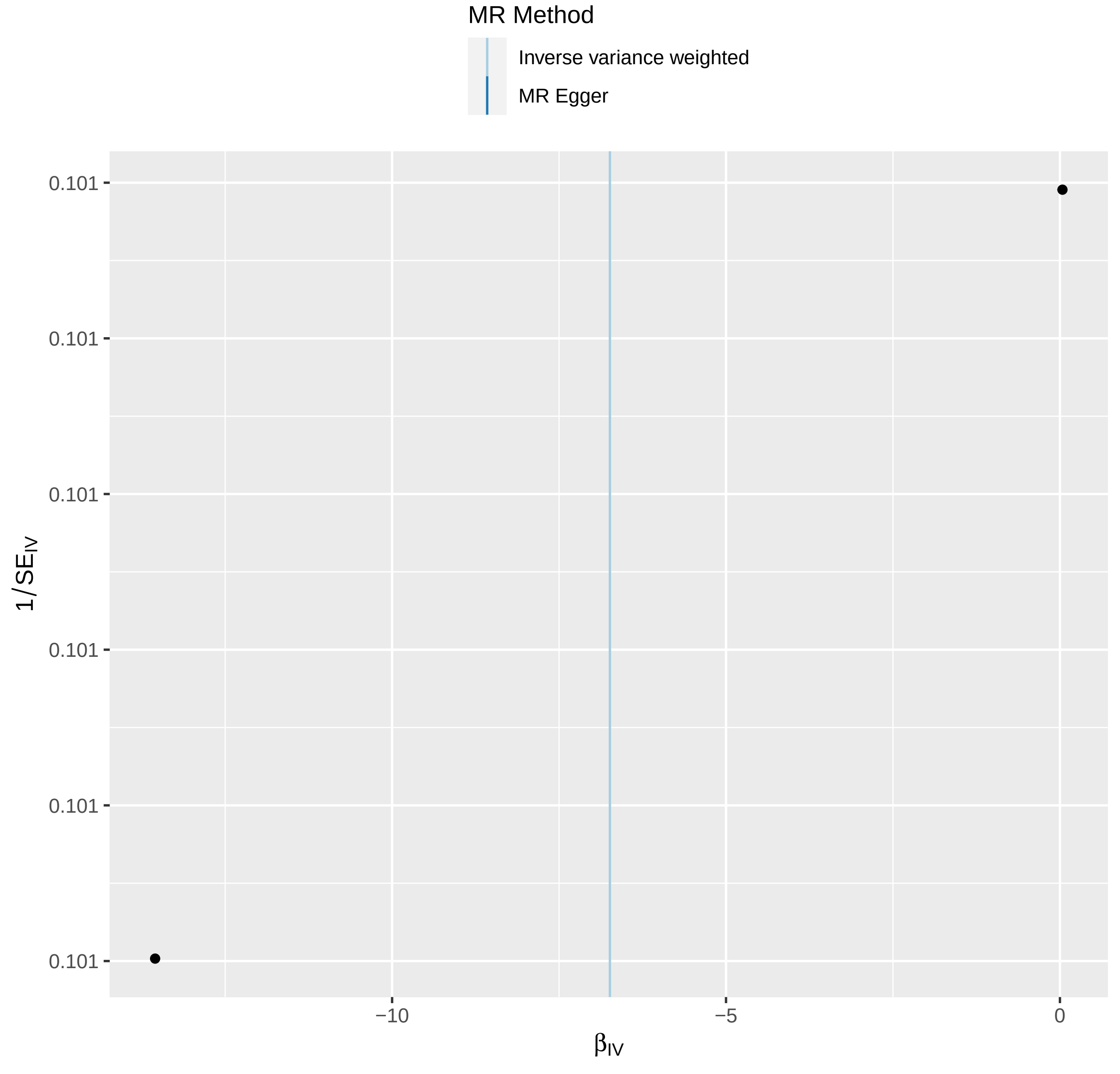

Batch 1150 : Urea on Ulcerative colitis with PSC

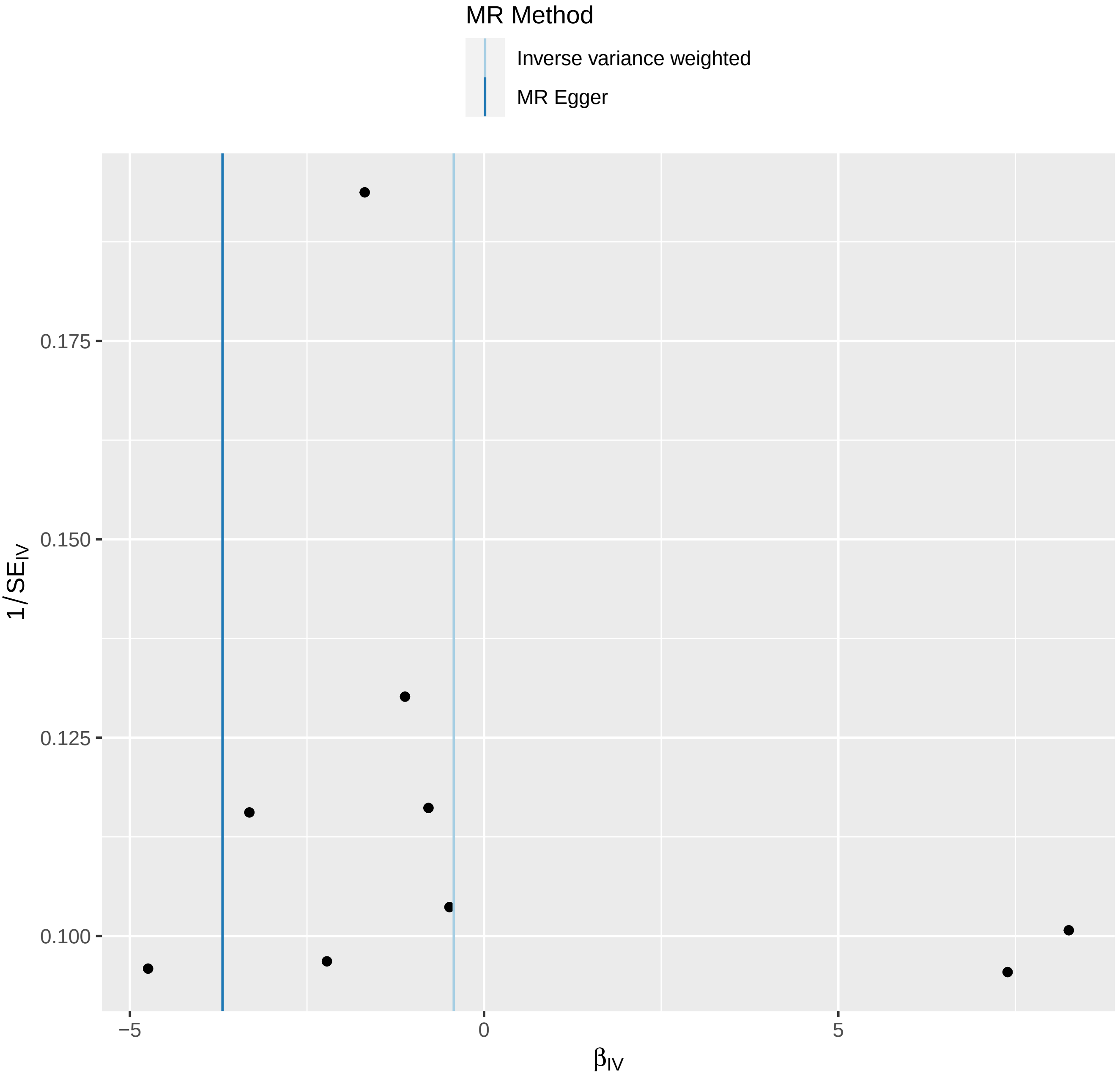

Batch 1151 : Cortisol on Ulcerative colitis with PSC

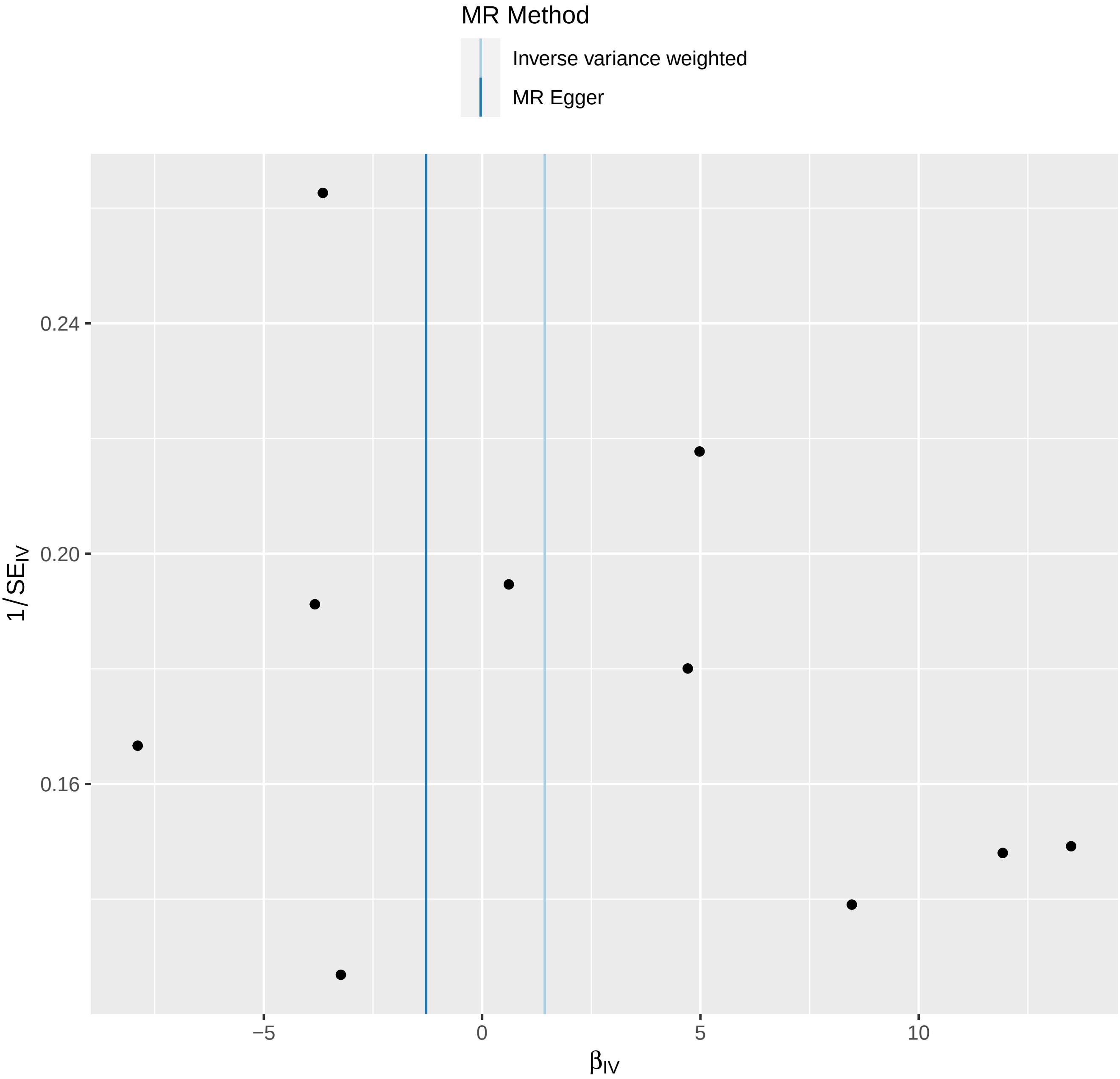

Batch 1154 : Citrulline on Ulcerative colitis with PSC

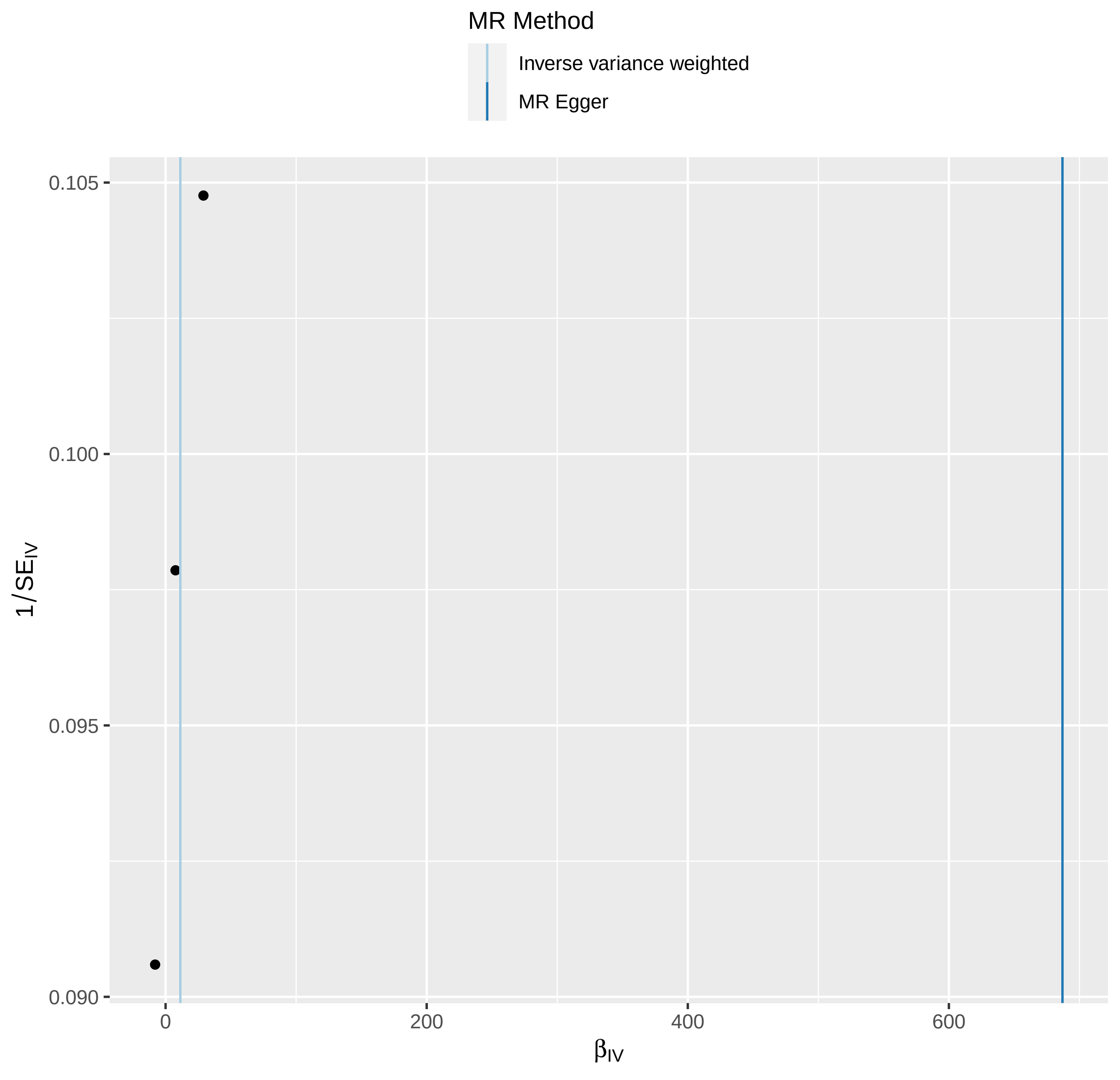

Batch 1156 : Serotonin (5HT) on Ulcerative colitis with PSC

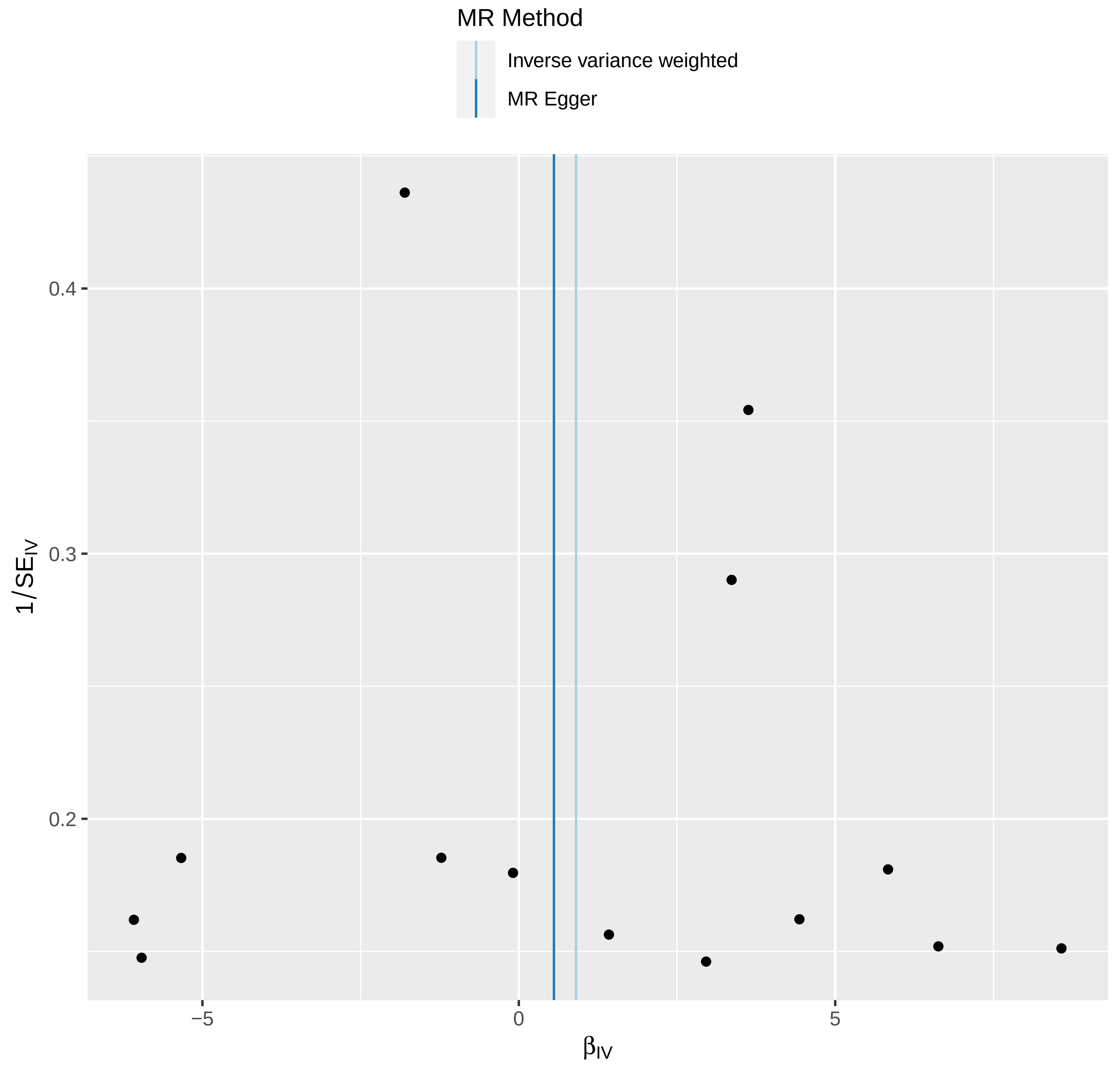

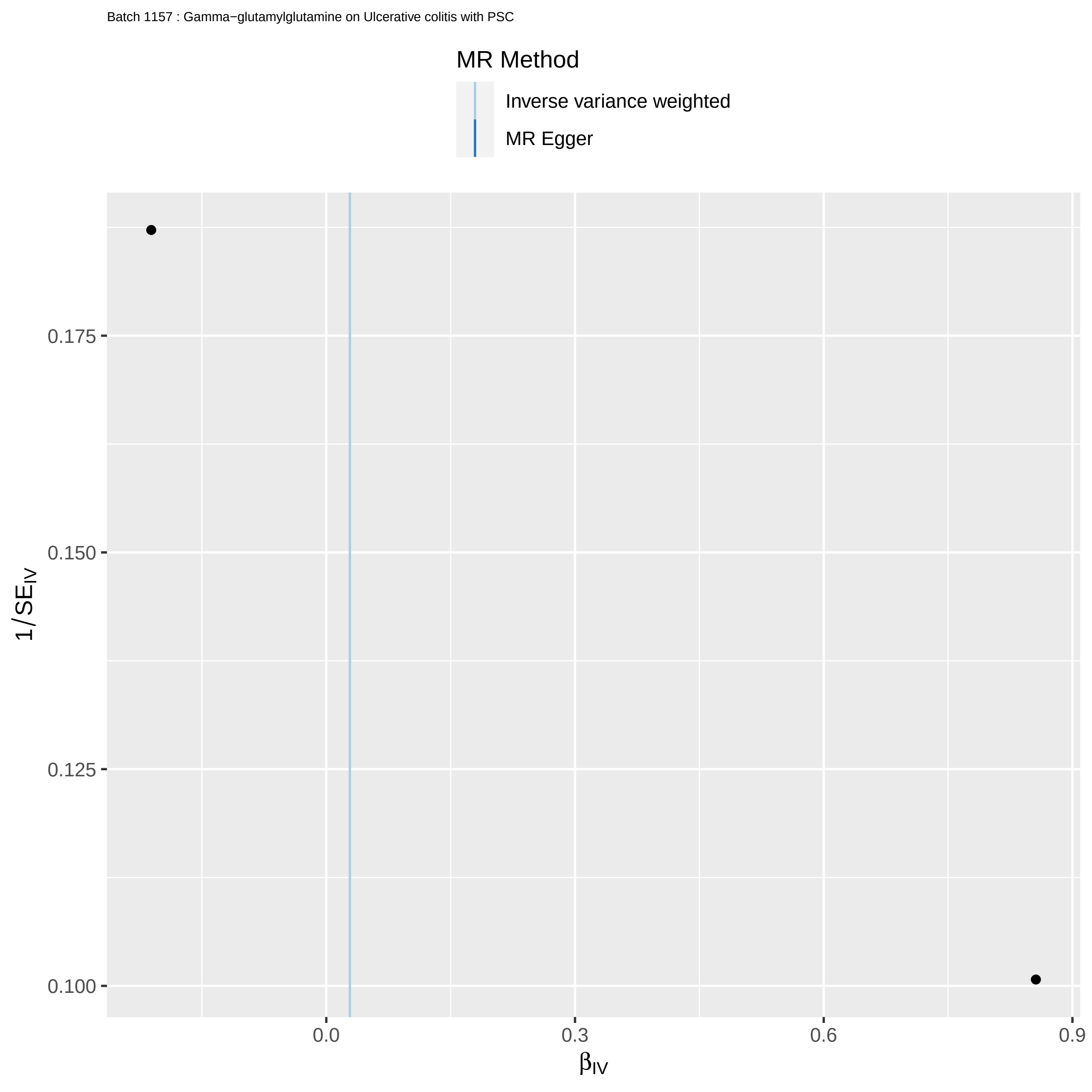
Batch 1157 : Gamma−glutamylglutamine on Ulcerative colitis with PSC
MR Method
Inverse variance weighted
MR Egger
1/SE_IV
0.175
0.150
0.125
0.100
β_IV
0.0
0.3
0.6
0.9

Batch 1158 : Gamma−glutamyltyrosine on Ulcerative colitis with PSC

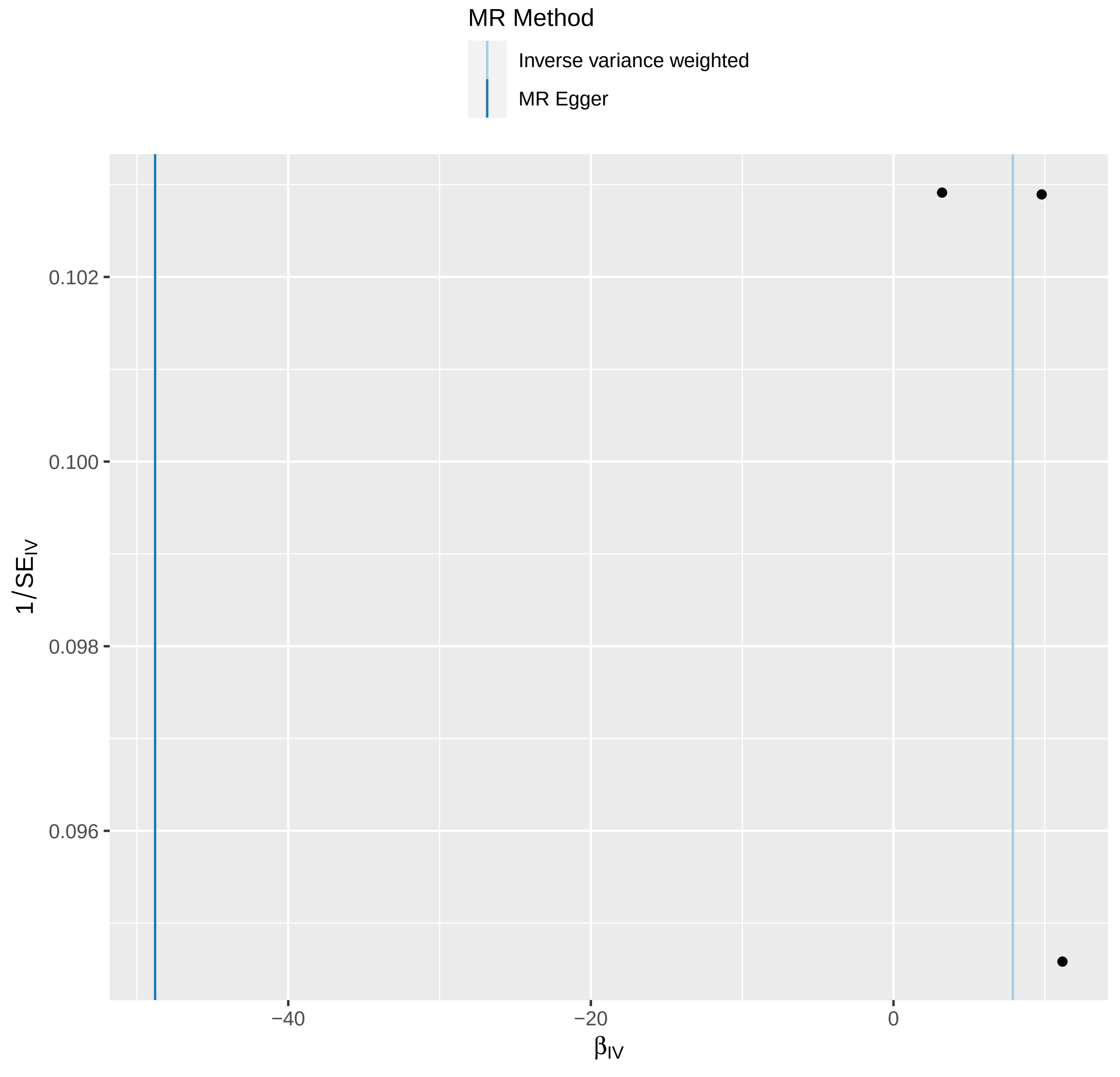

Batch 1160 : Betaine on Ulcerative colitis with PSC

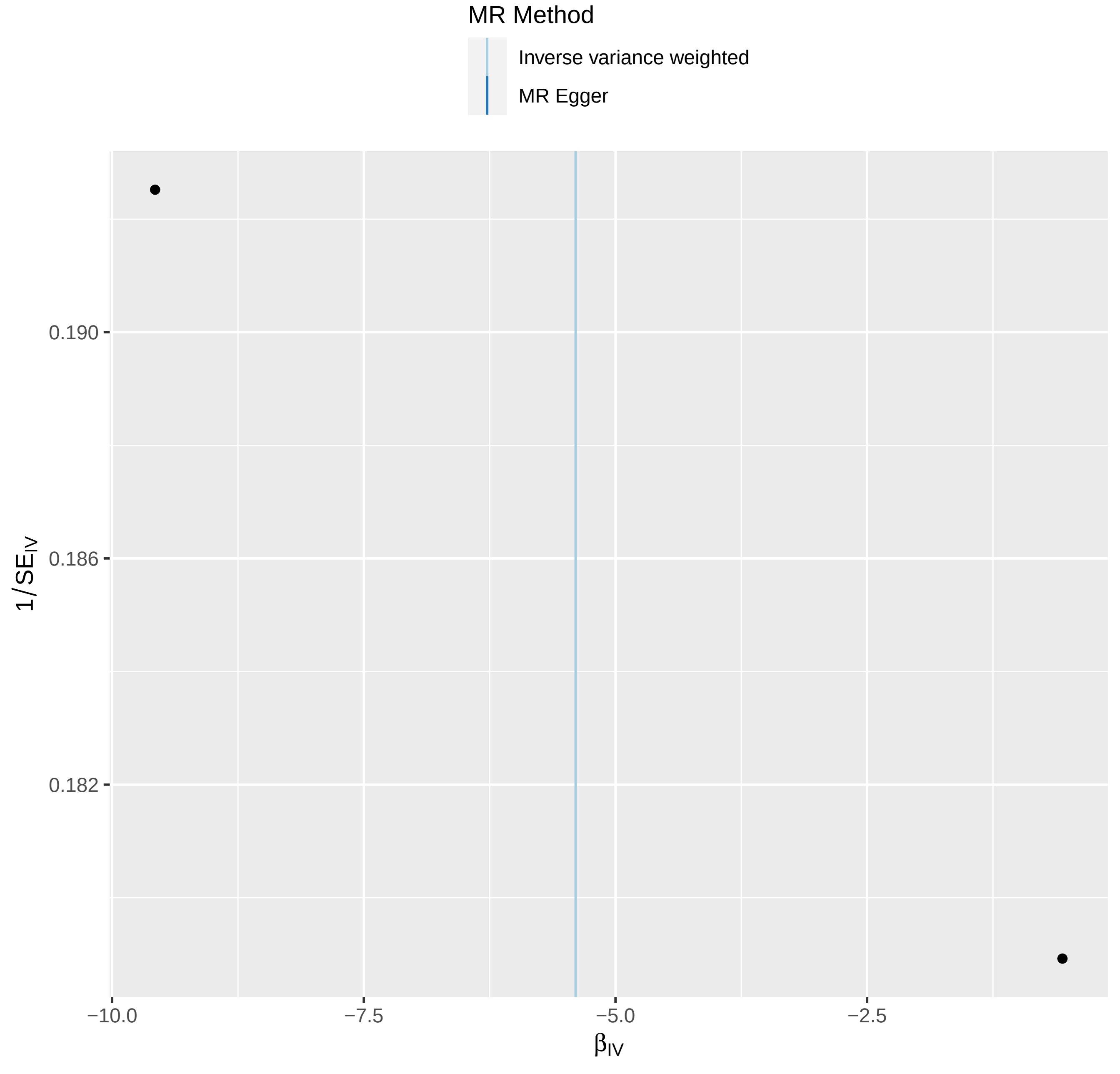

Batch 1161 : Xanthine on Ulcerative colitis with PSC

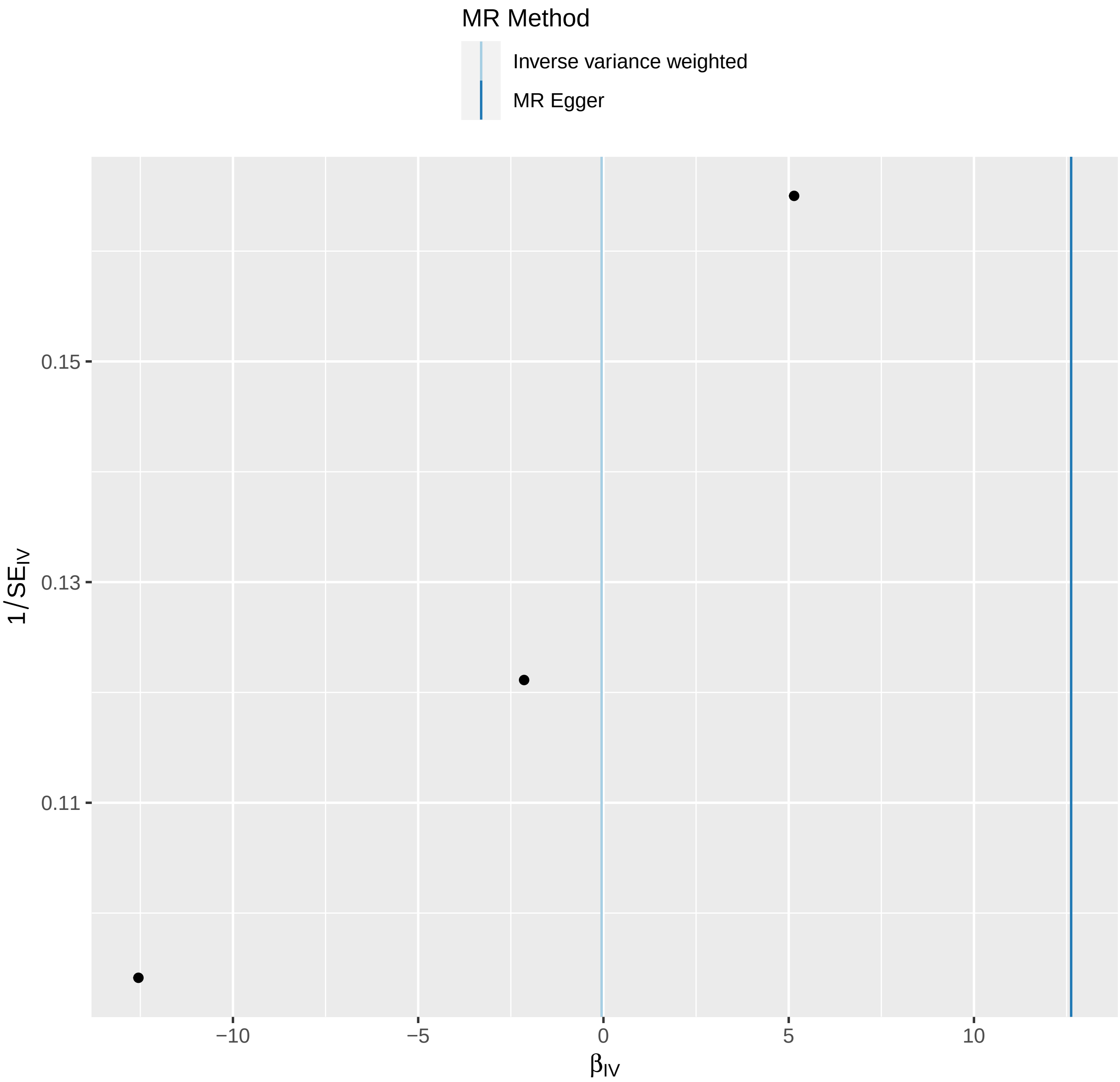

Batch 1162 : Phosphate on Ulcerative colitis with PSC

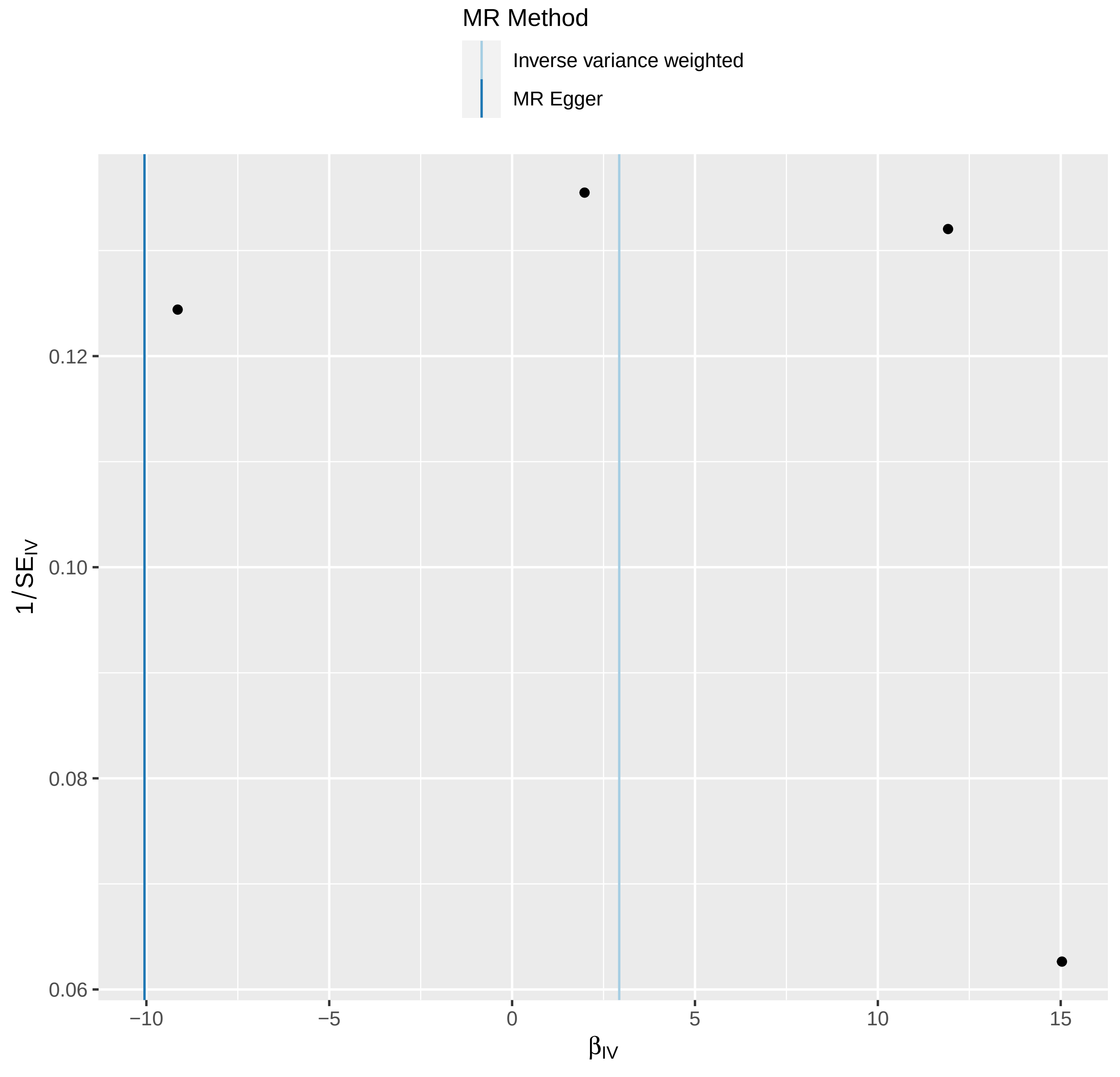

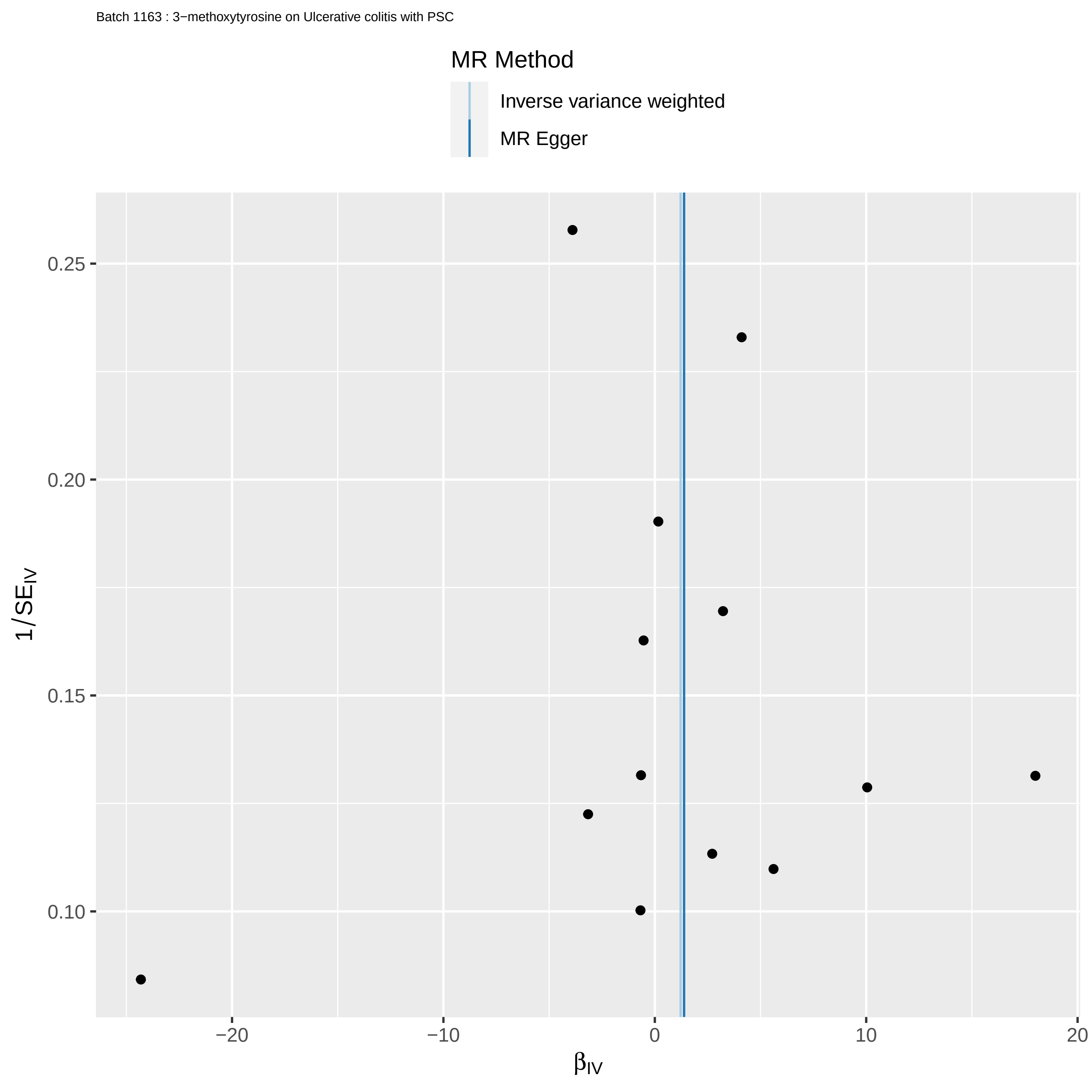

Batch 1163 : 3−methoxytyrosine on Ulcerative colitis with PSC
MR Method
Inverse variance weighted
MR Egger
0.25
0.20
0.15
0.10
1/SE_IV
−20
−10
0
10
20
β_IV

Batch 1165 : Undecanoate (11:0) on Ulcerative colitis with PSC

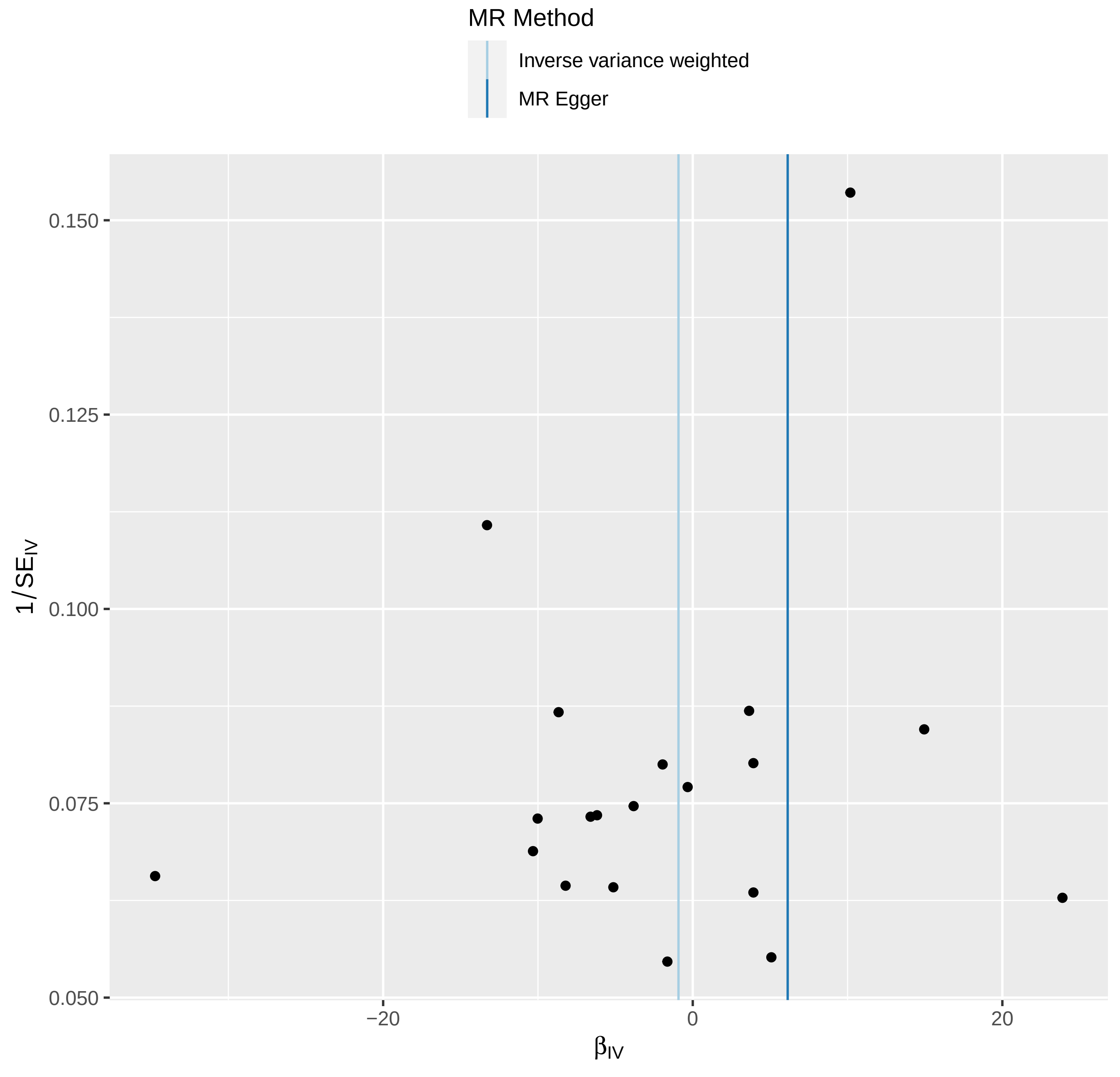

Batch 1167 : Taurodeoxycholate on Ulcerative colitis with PSC

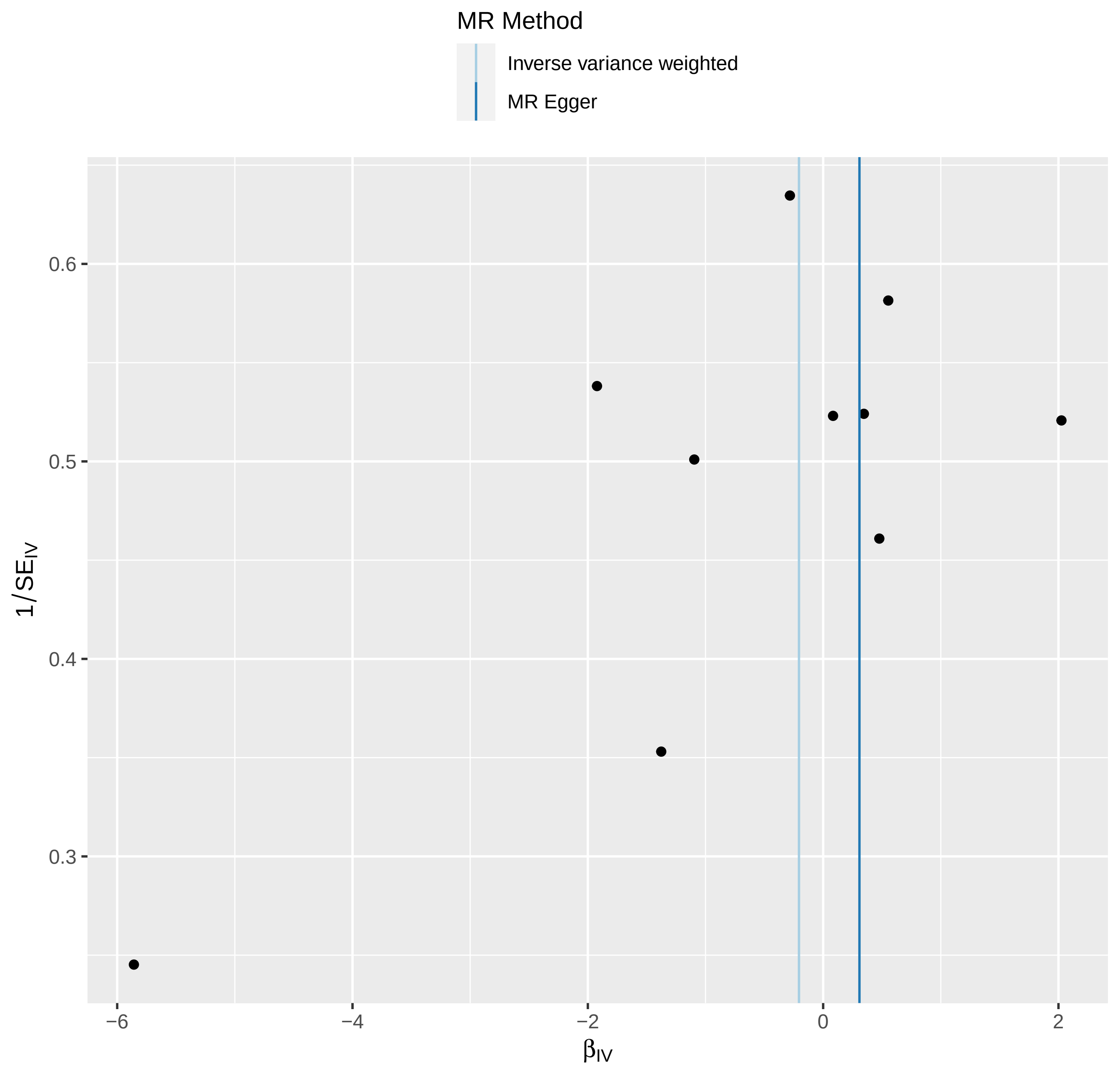

Batch 1168 : Glycerol on Ulcerative colitis with PSC

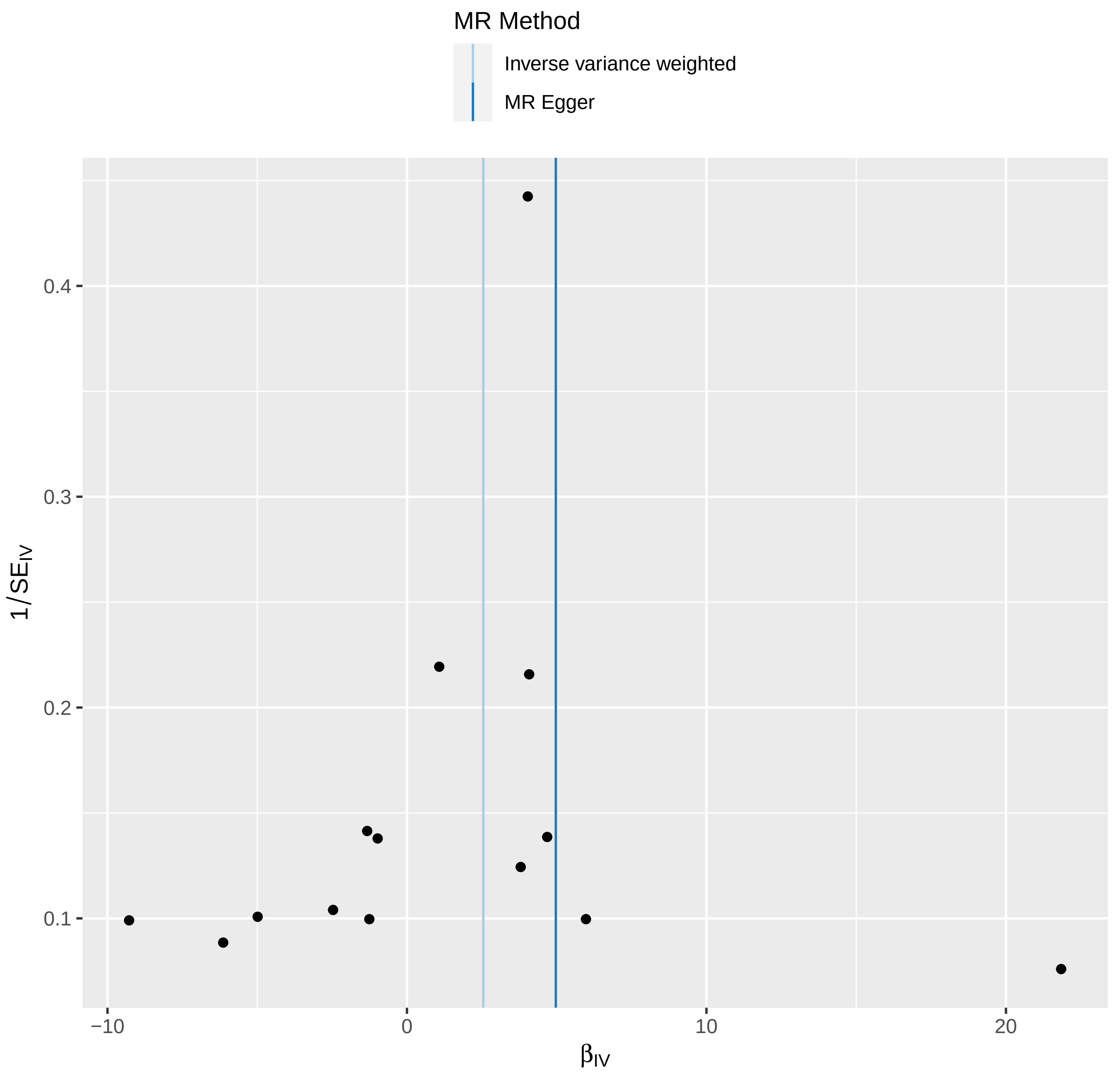

Batch 1169 : Kynurenine on Ulcerative colitis with PSC

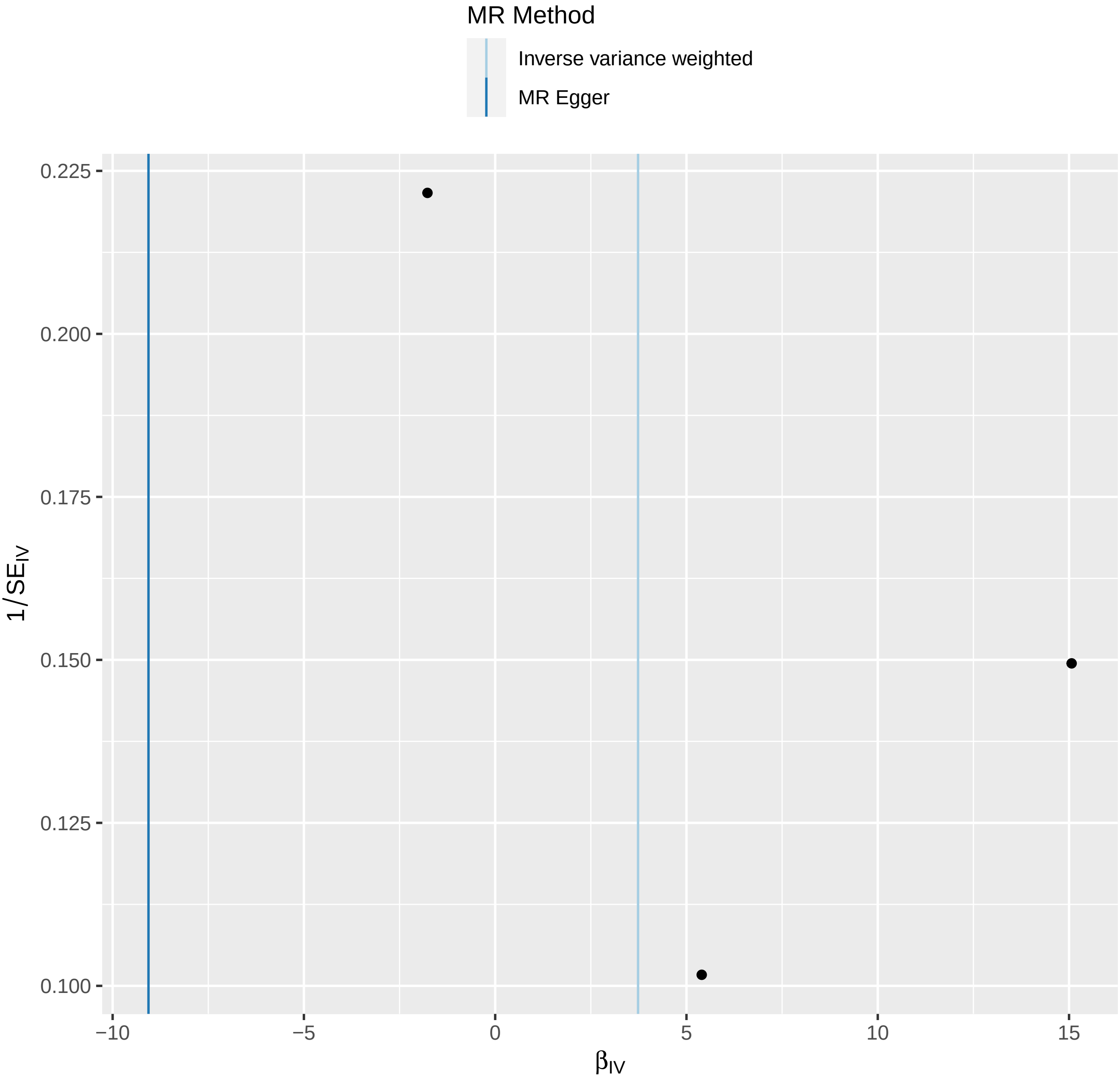

Batch 1170 : Mannitol on Ulcerative colitis with PSC

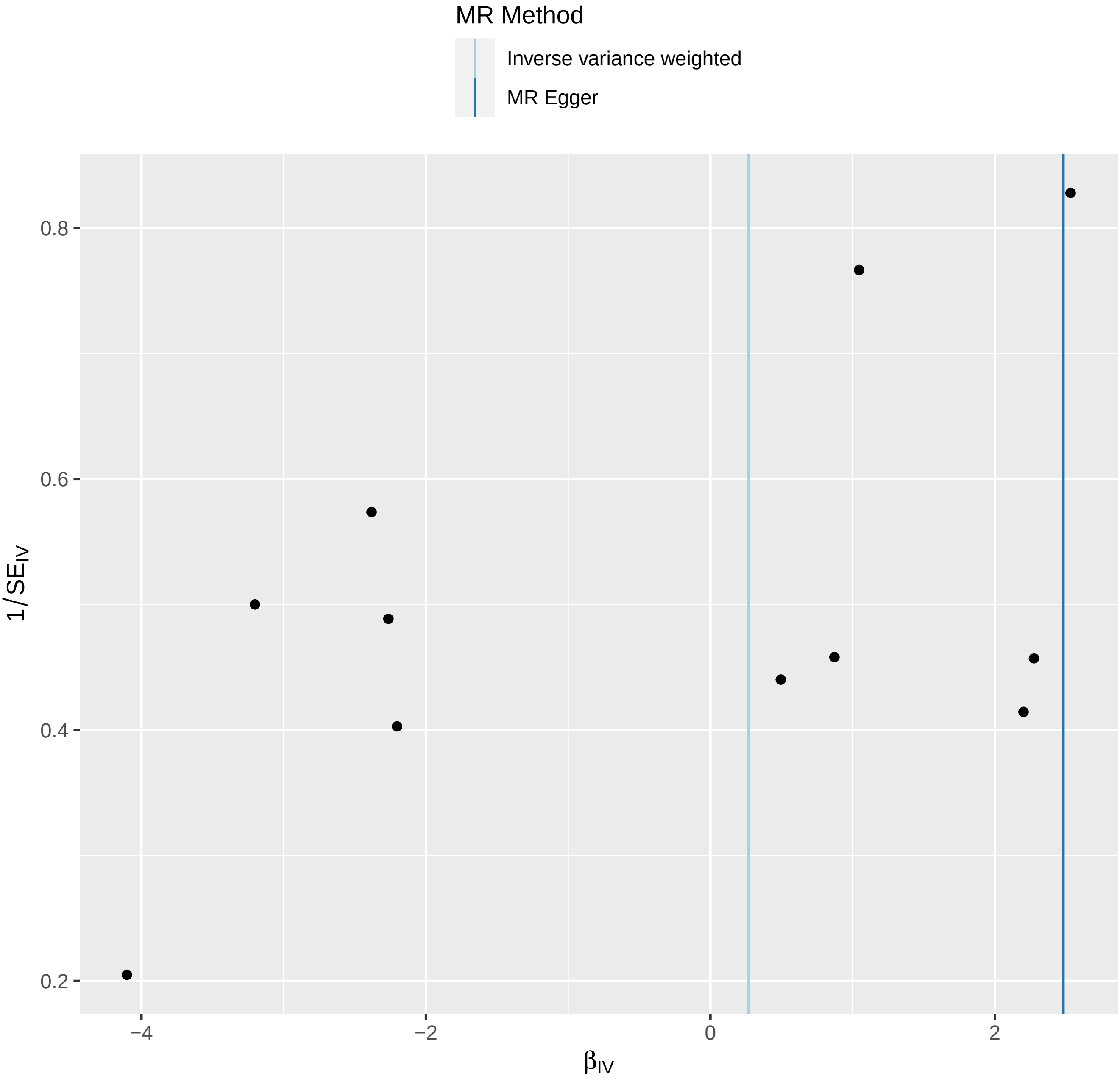

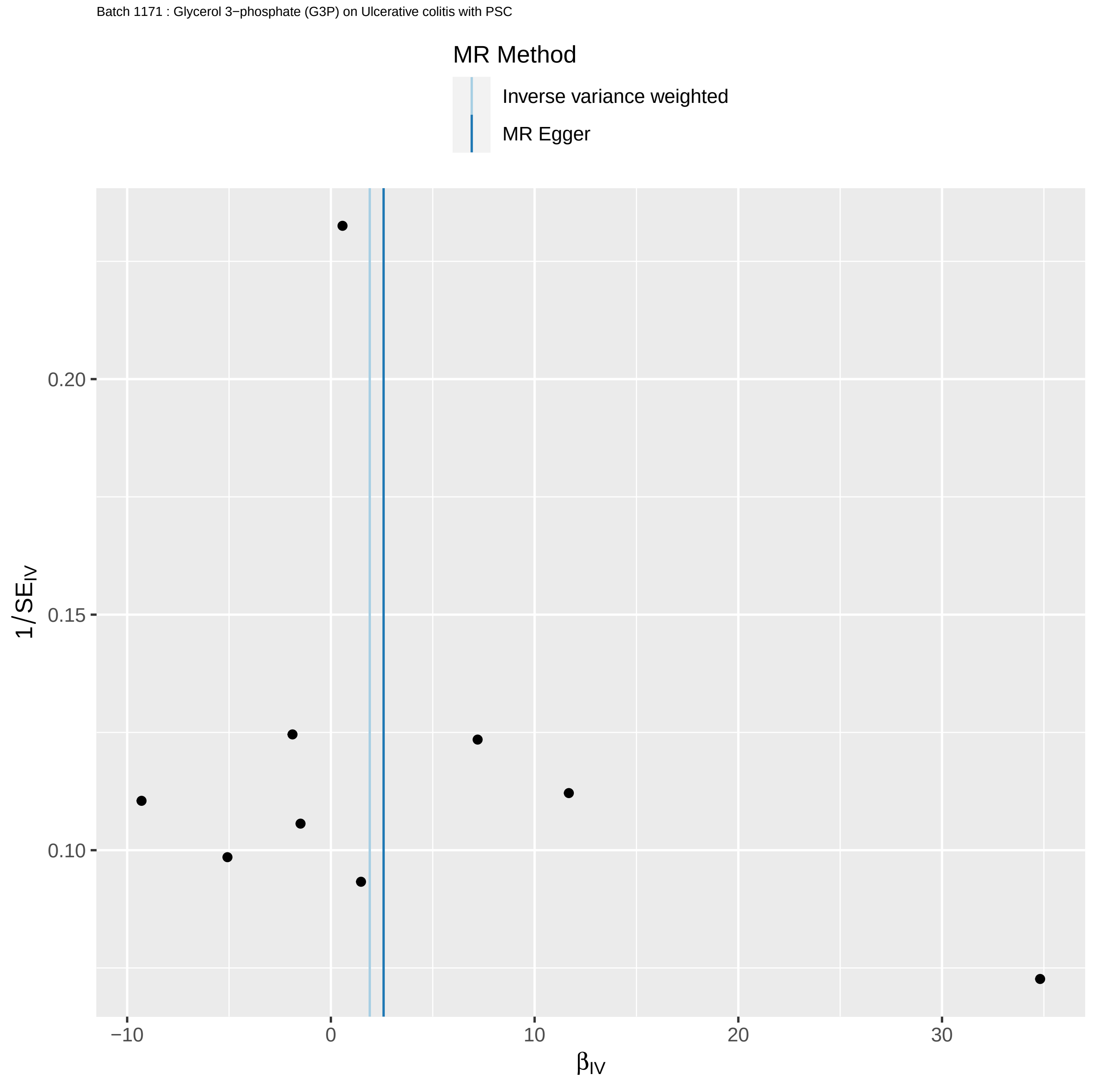

Batch 1171 : Glycerol 3−phosphate (G3P) on Ulcerative colitis with PSC
MR Method
Inverse variance weighted
MR Egger
0.20
0.15
0.10
1/SE_IV
−10
0
10
20
30
β_IV

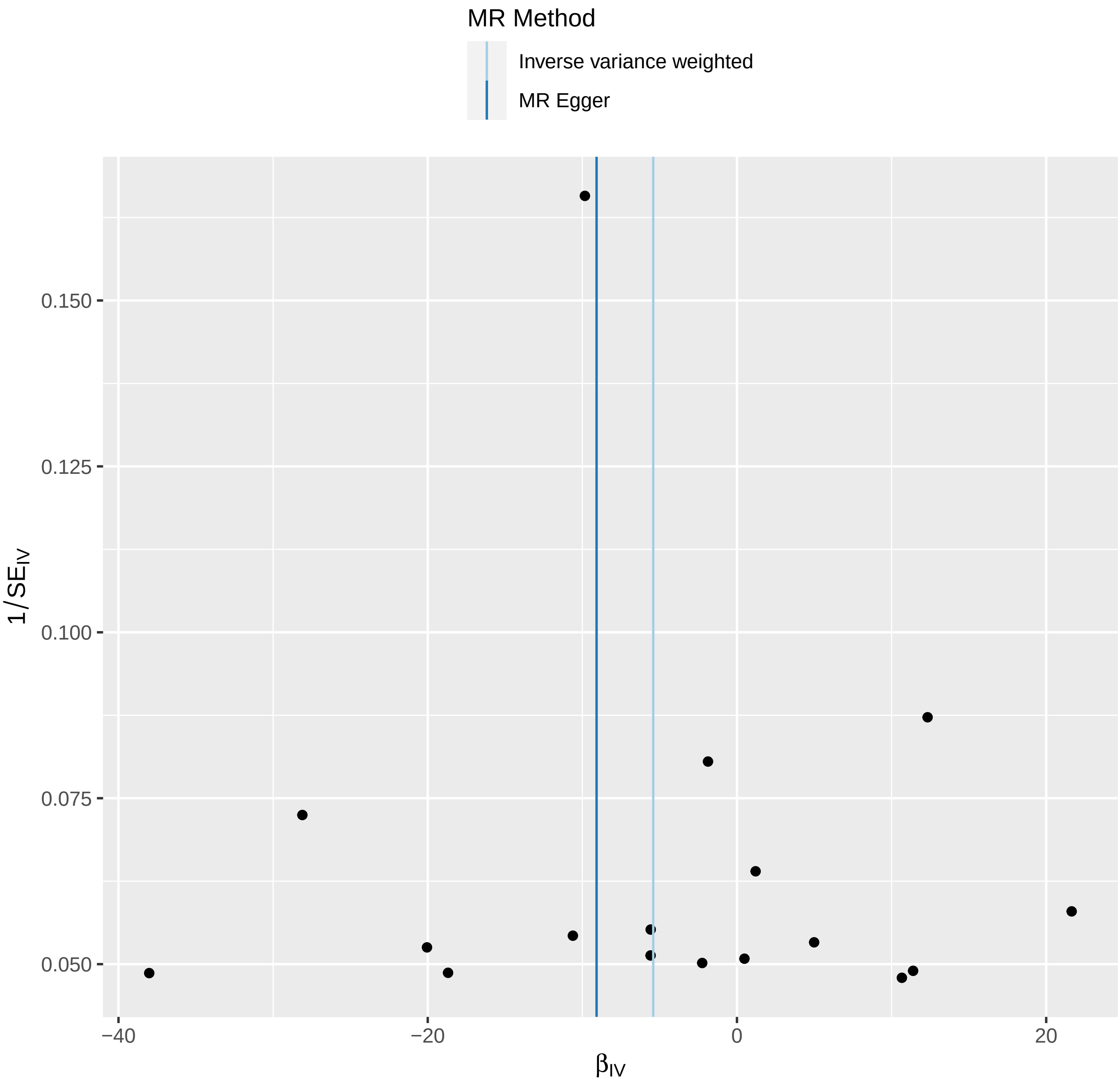
MR Method
Inverse variance weighted
MR Egger
0.150
0.125
0.100
0.075
0.050
−40
−20
0
20
1/SE_IV
β_IV

Batch 1174 : Choline on Ulcerative colitis with PSC

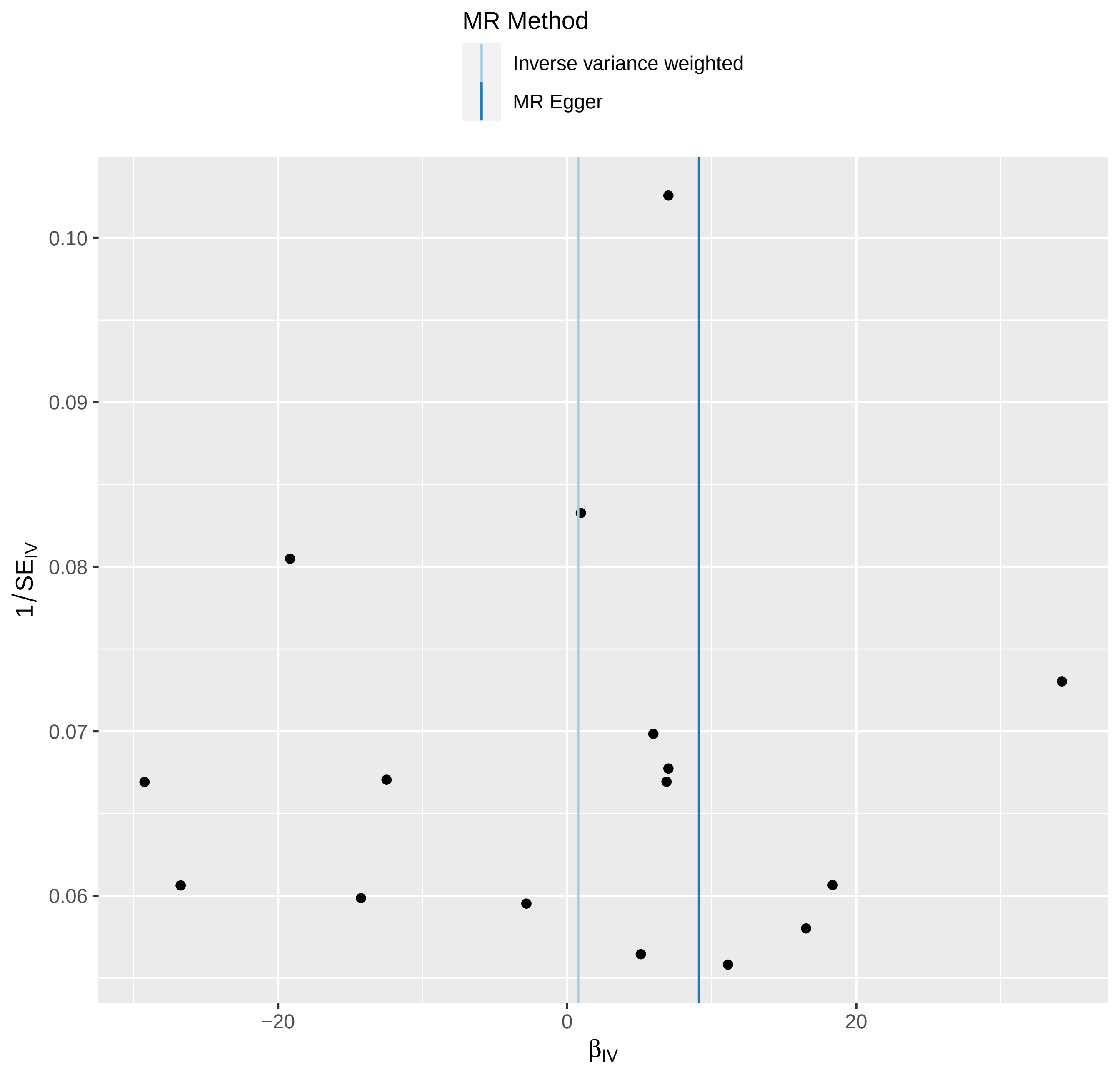

Batch 1177 : 3−methyl−2−oxovalerate on Ulcerative colitis with PSC

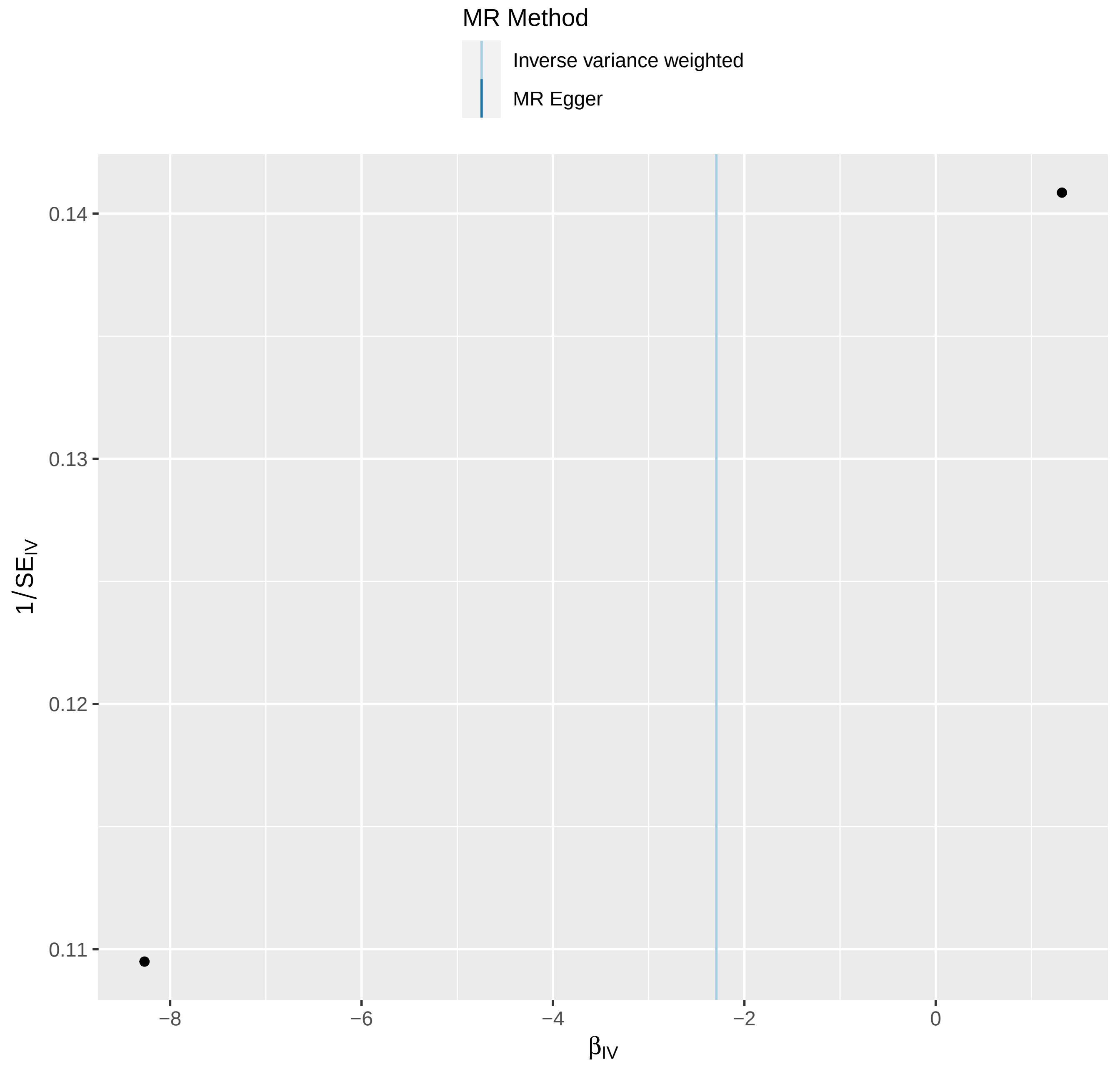

Batch 1178 : 3−methylhistidine on Ulcerative colitis with PSC

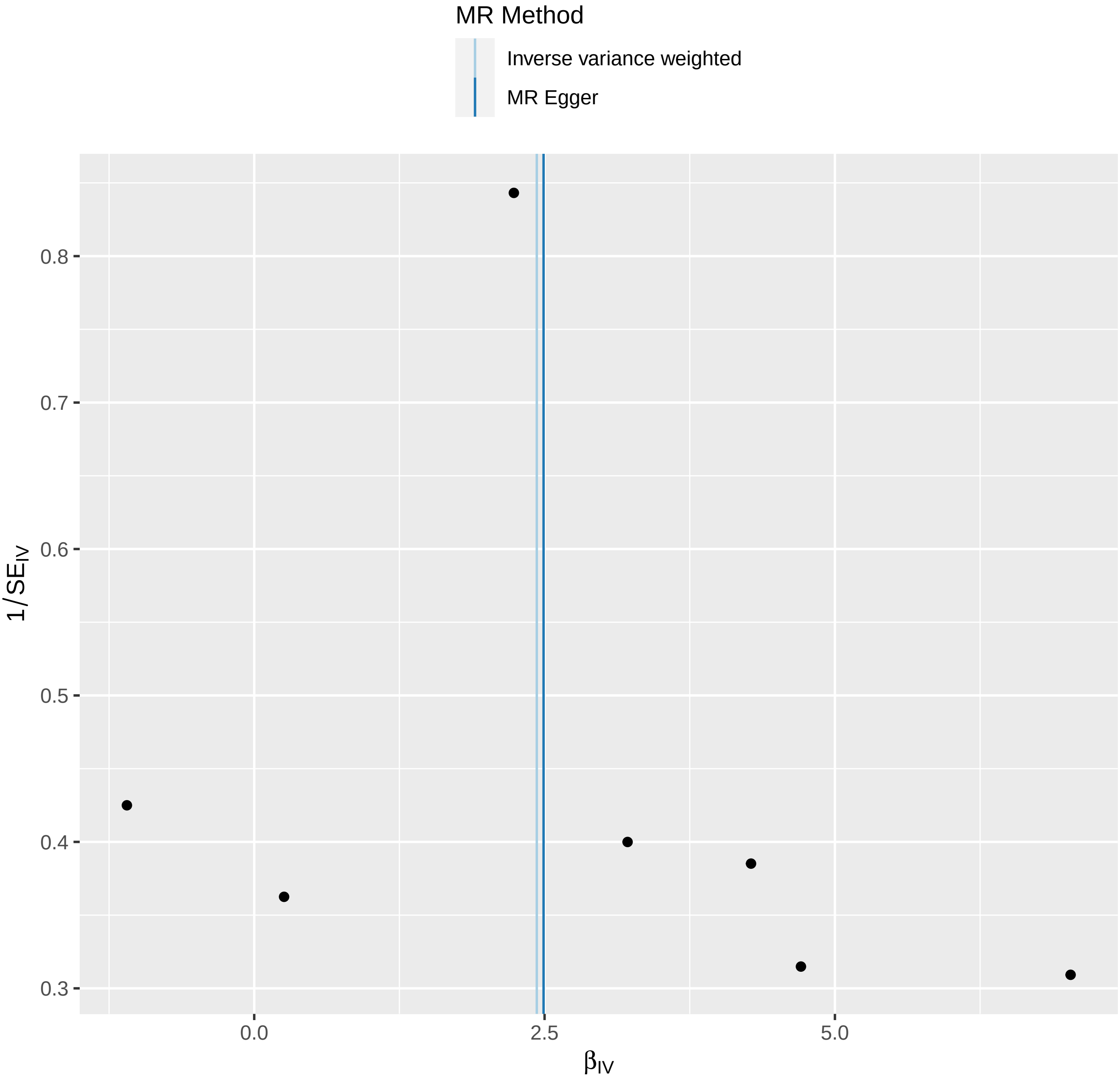

Batch 1180 : Phenylacetate on Ulcerative colitis with PSC

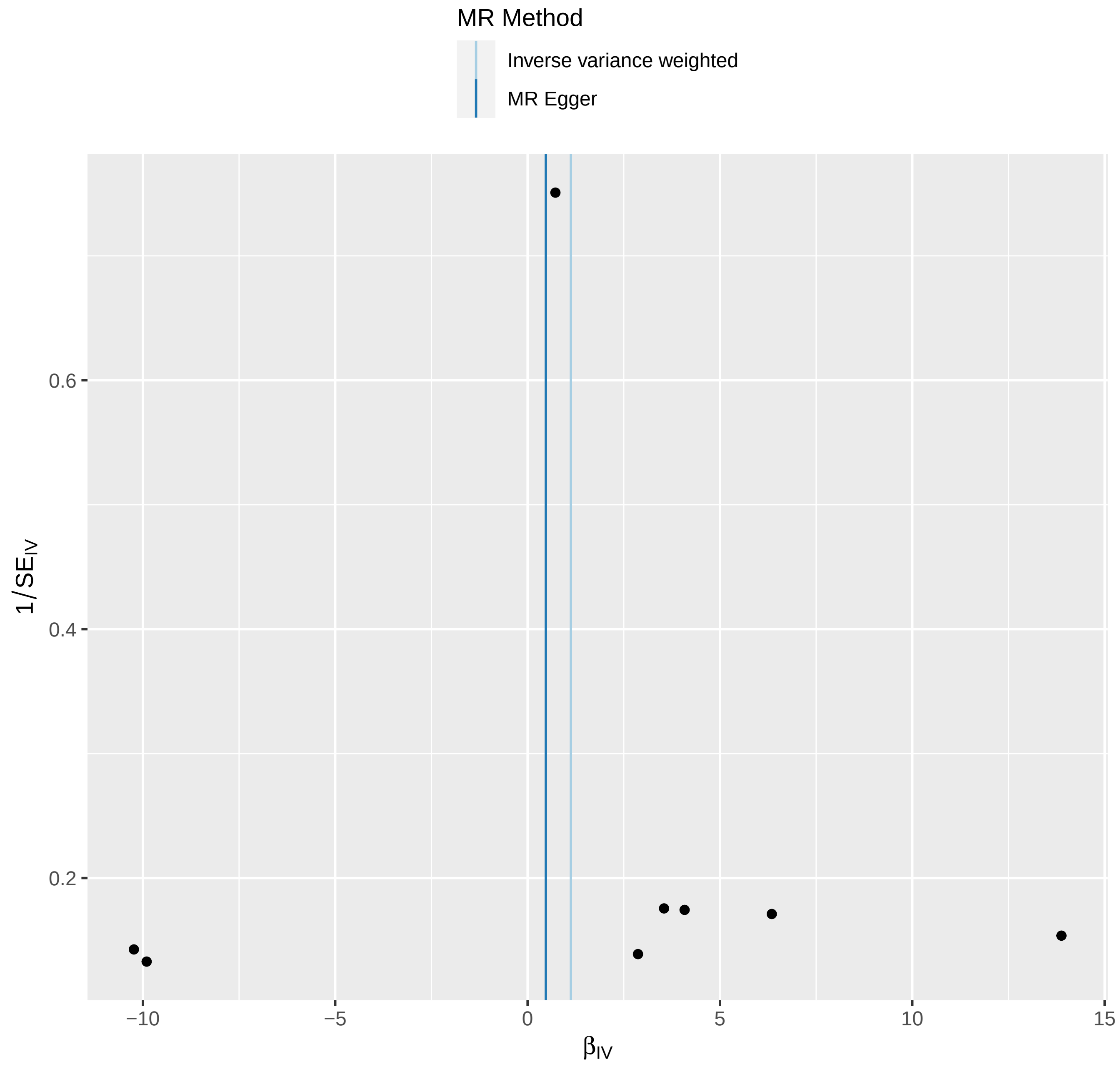

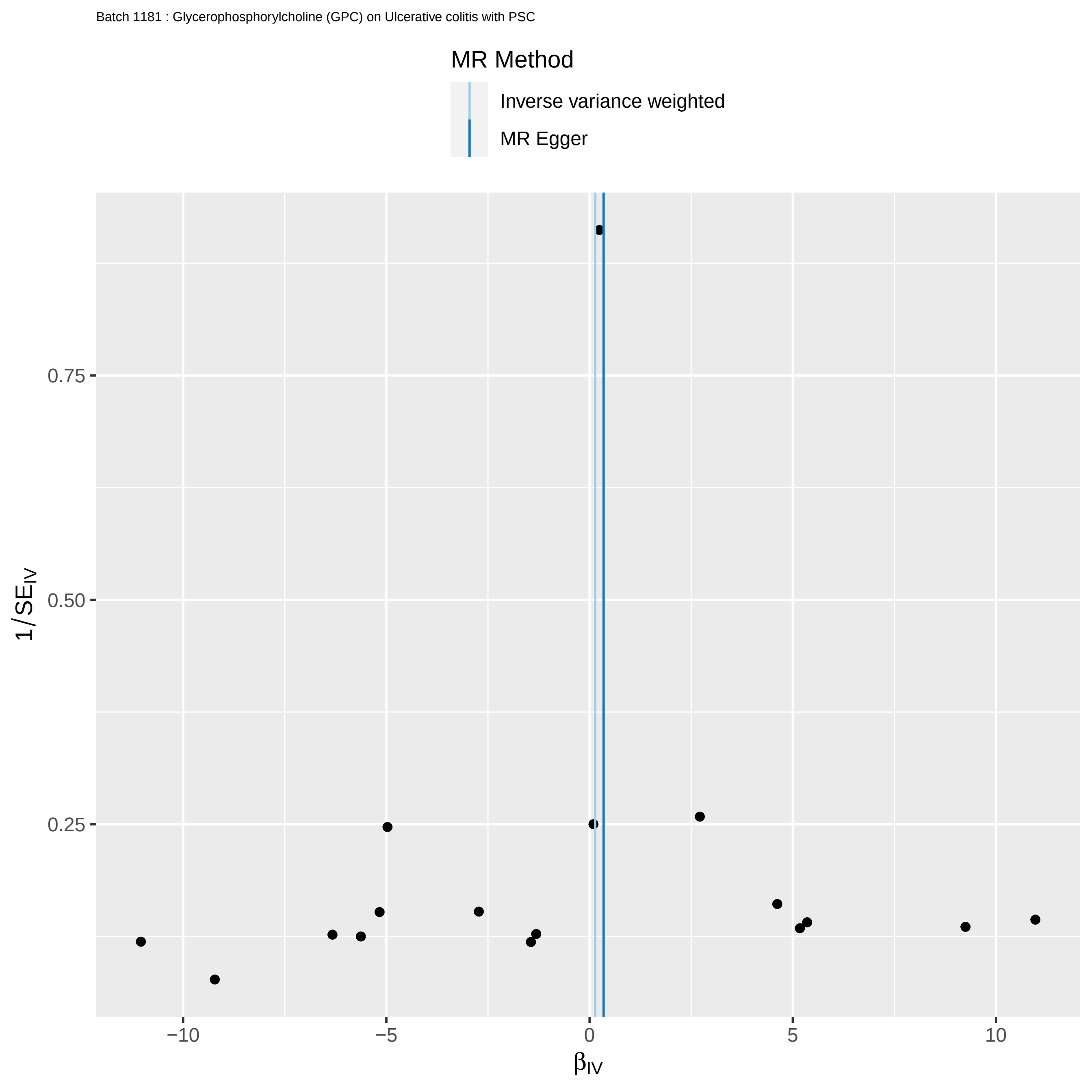

Batch 1181 : Glycerophosphorylcholine (GPC) on Ulcerative colitis with PSC
MR Method
Inverse variance weighted
MR Egger
0.75
0.50
0.25
1/SE_IV
−10
−5
0
5
10
β_IV

Batch 1182 : Aspartate on Ulcerative colitis with PSC

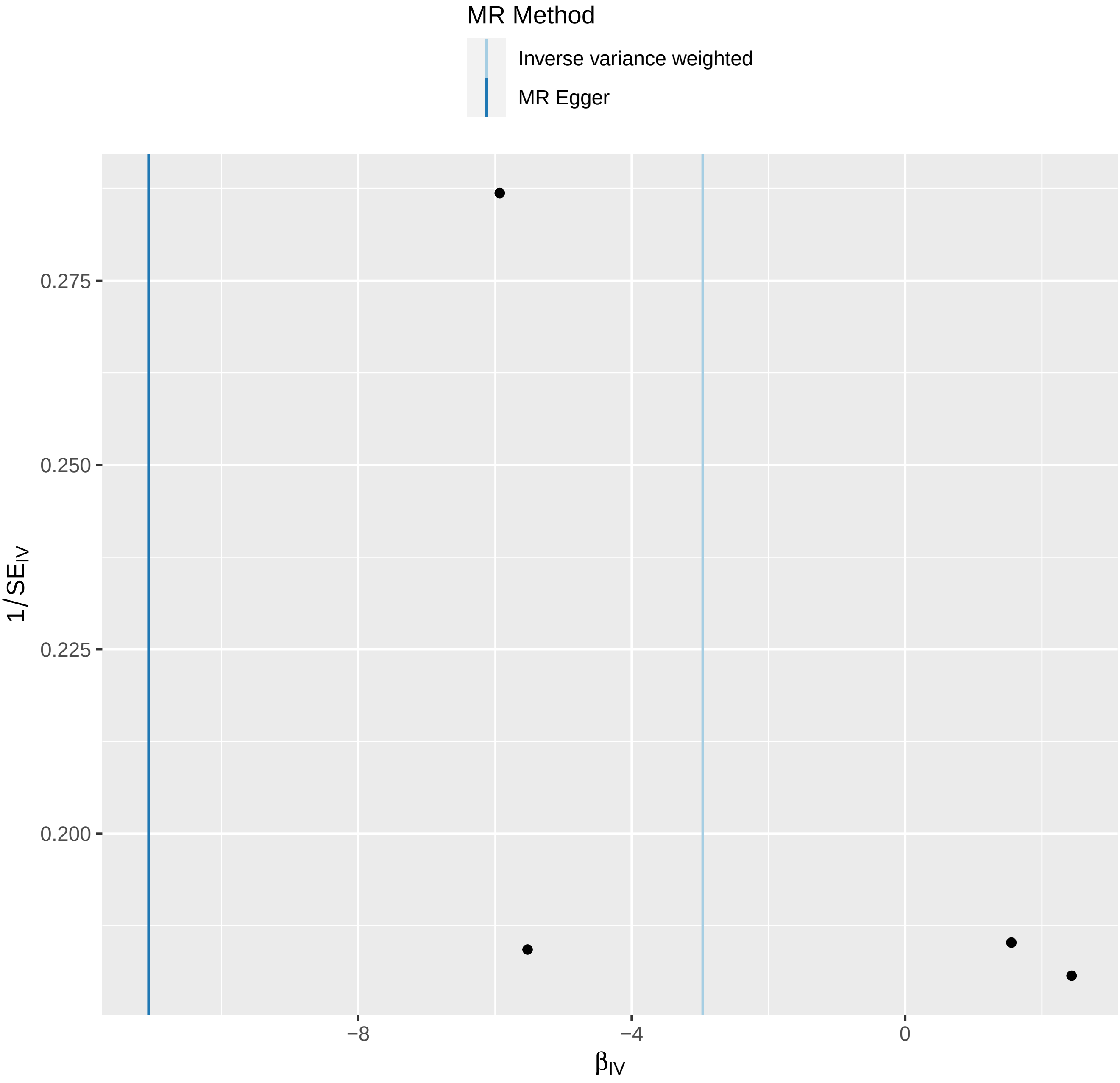

Batch 1184 : Dihomo−linoleate (20:2n6) on Ulcerative colitis with PSC

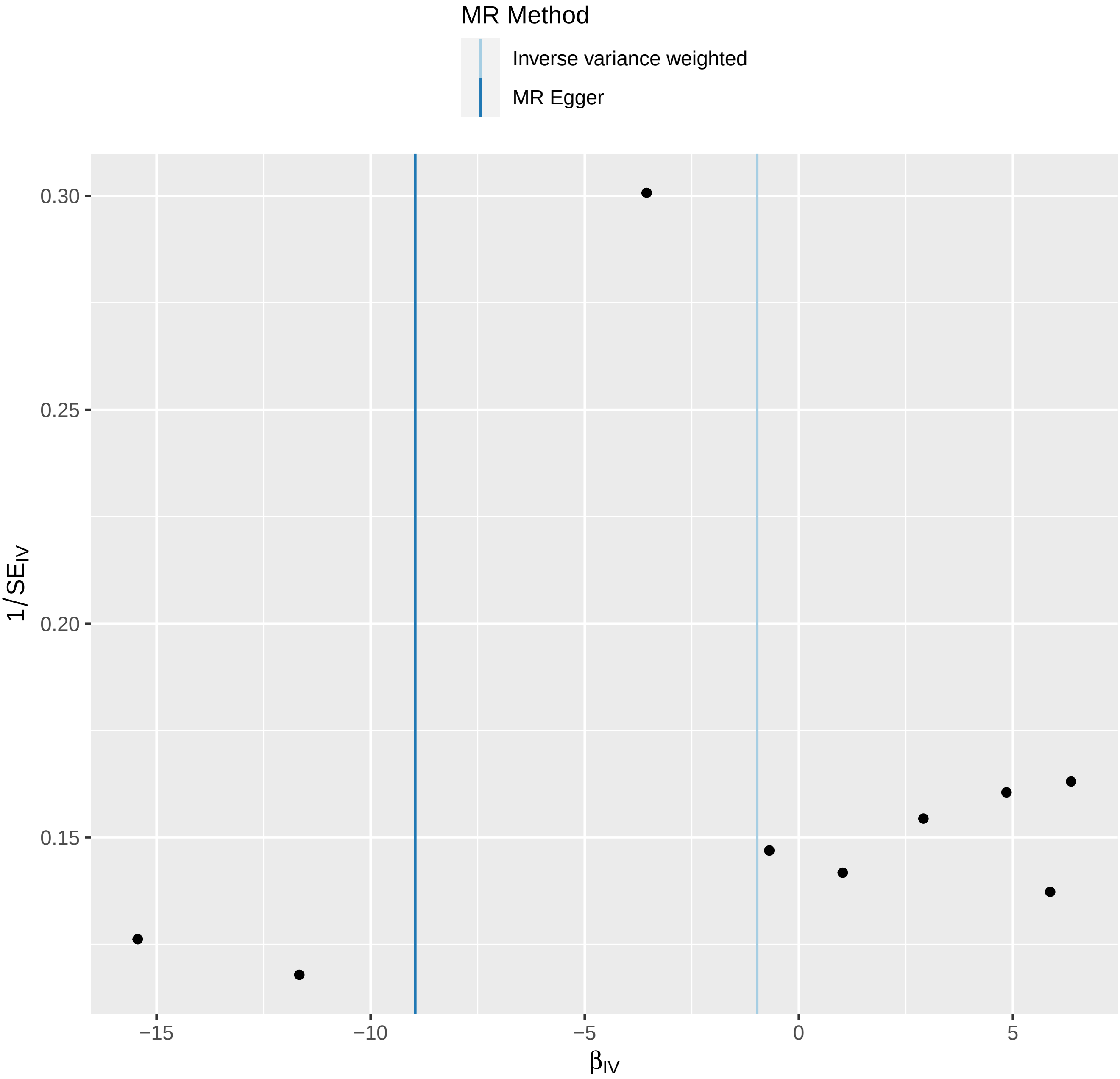

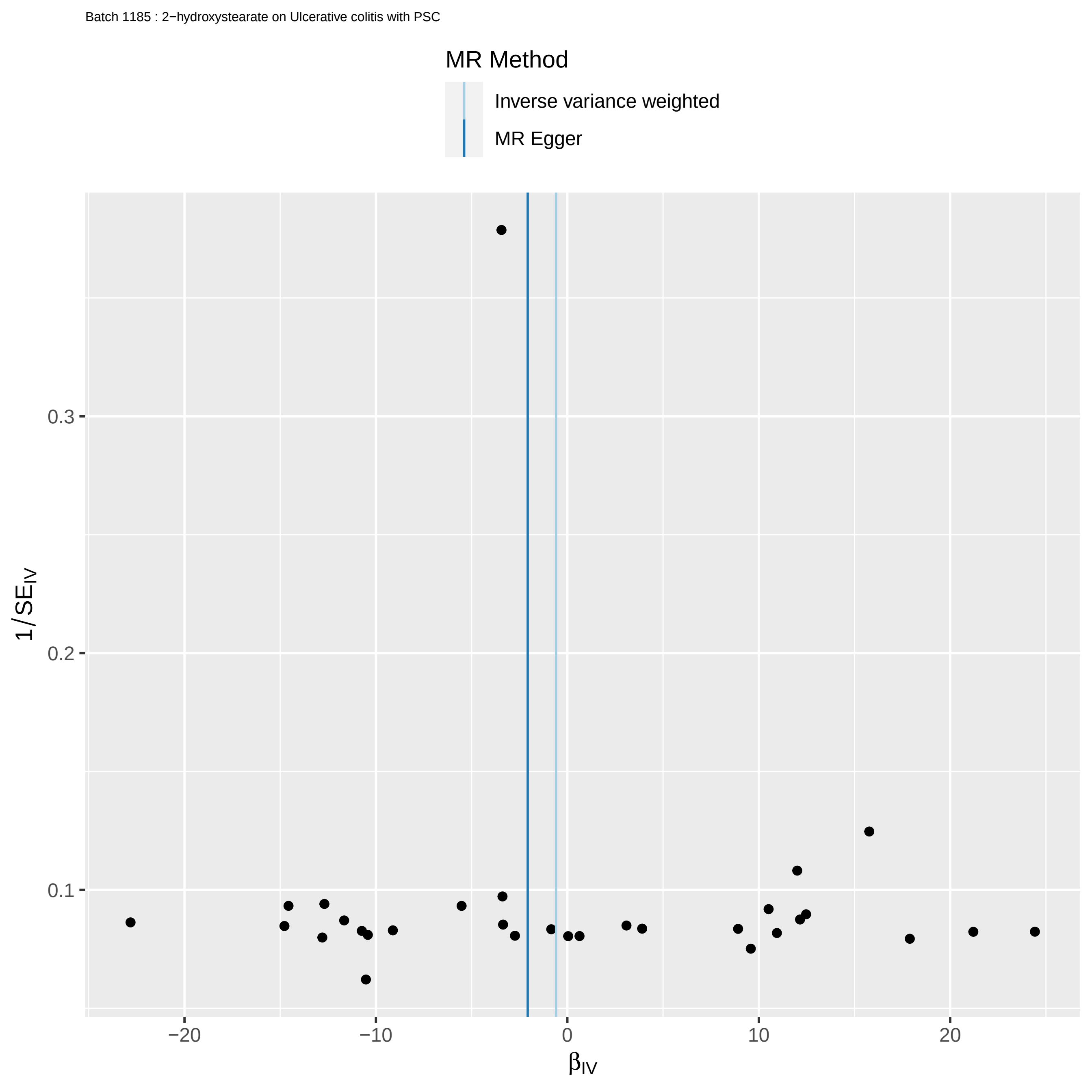

Batch 1185 : 2−hydroxystearate on Ulcerative colitis with PSC
MR Method
Inverse variance weighted
MR Egger
0.3
0.2
0.1
1/SE$_{IV}$
−20
−10
0
10
20
β_{IV}

Batch 1187 : Glycylvaline on Ulcerative colitis with PSC

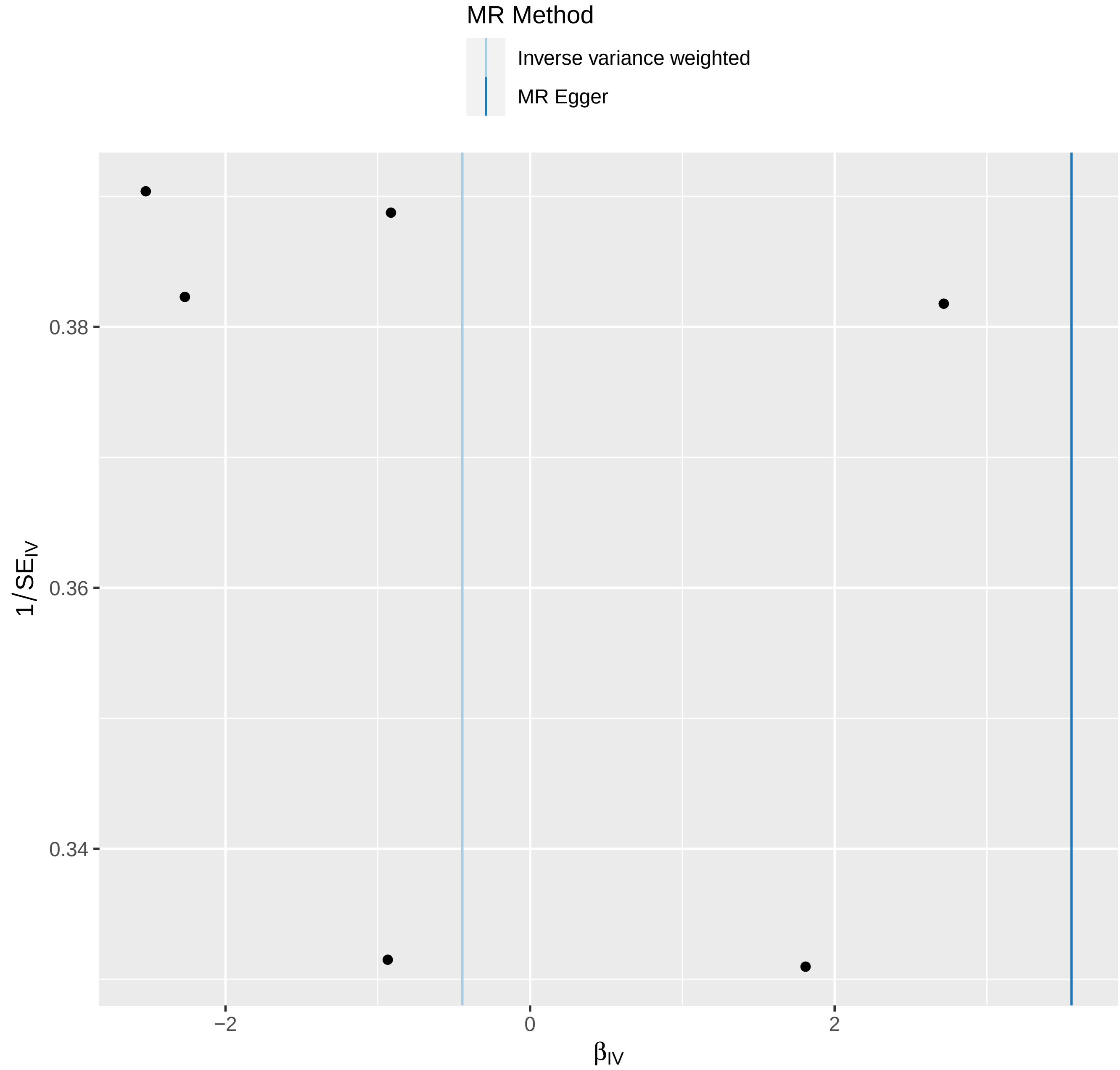

Batch 1188 : Gamma−glutamylleucine on Ulcerative colitis with PSC

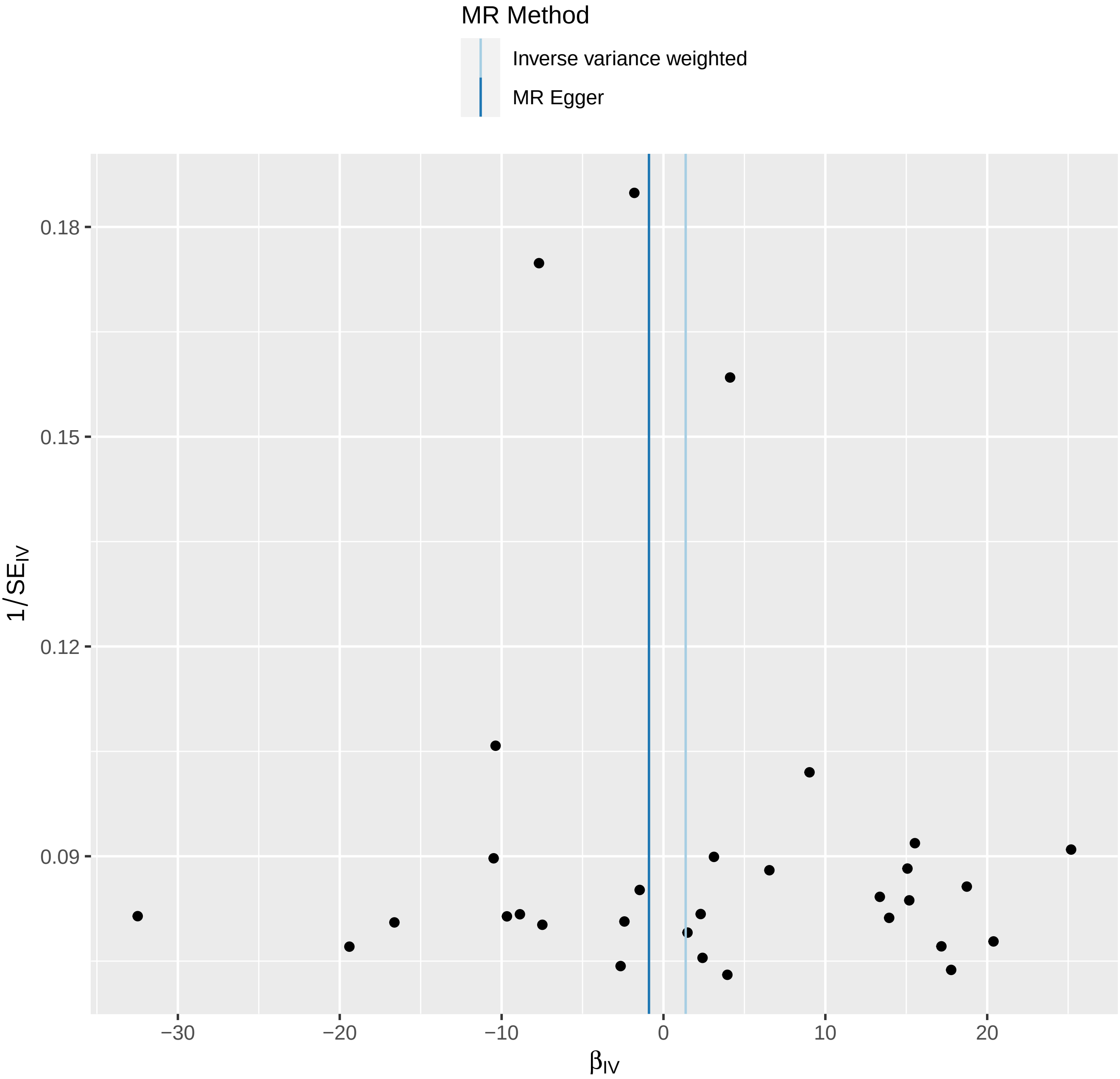

Batch 1191 : Glycocholate on Ulcerative colitis with PSC

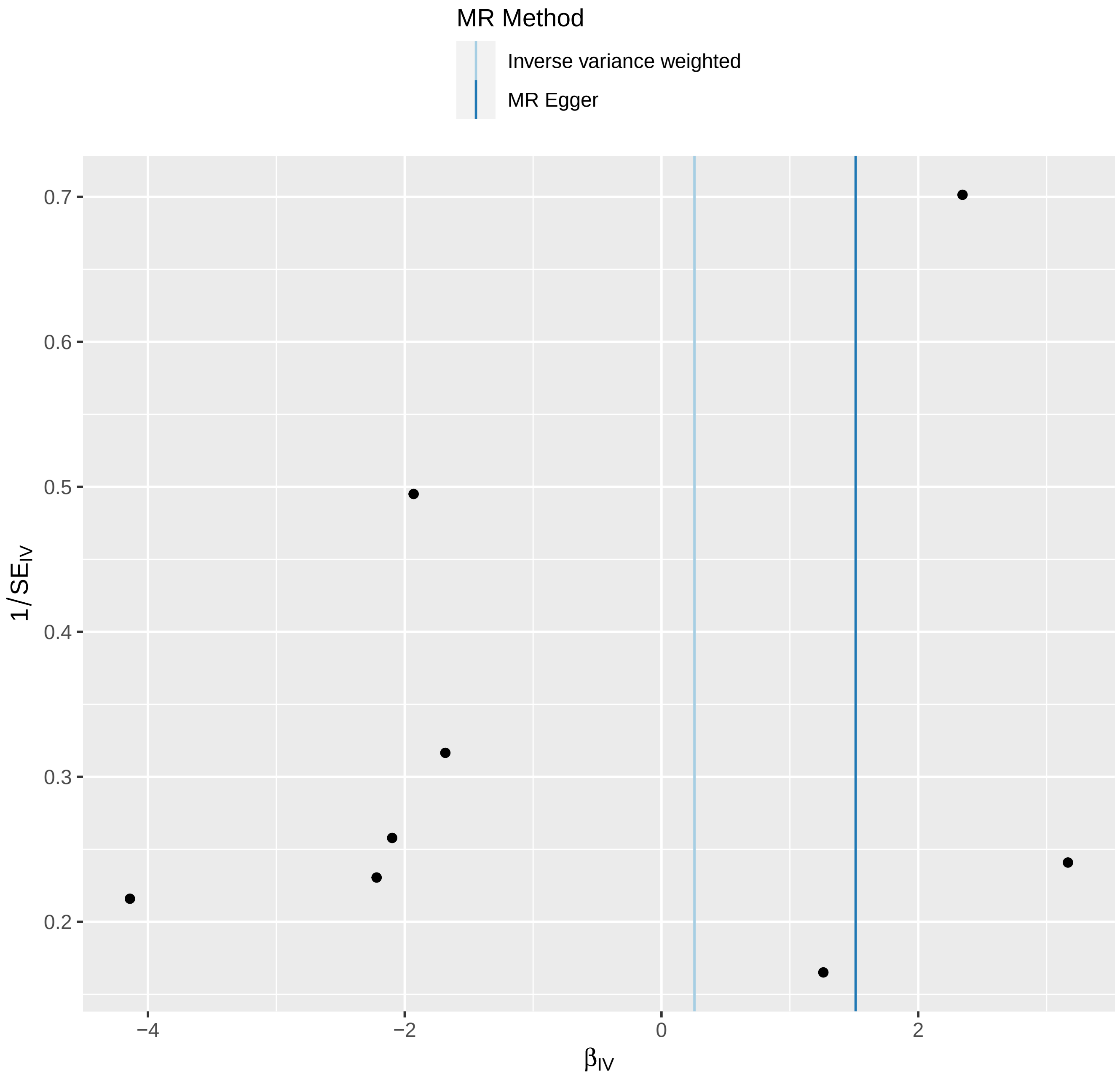

Batch 1192 : Glycodeoxycholate on Ulcerative colitis with PSC

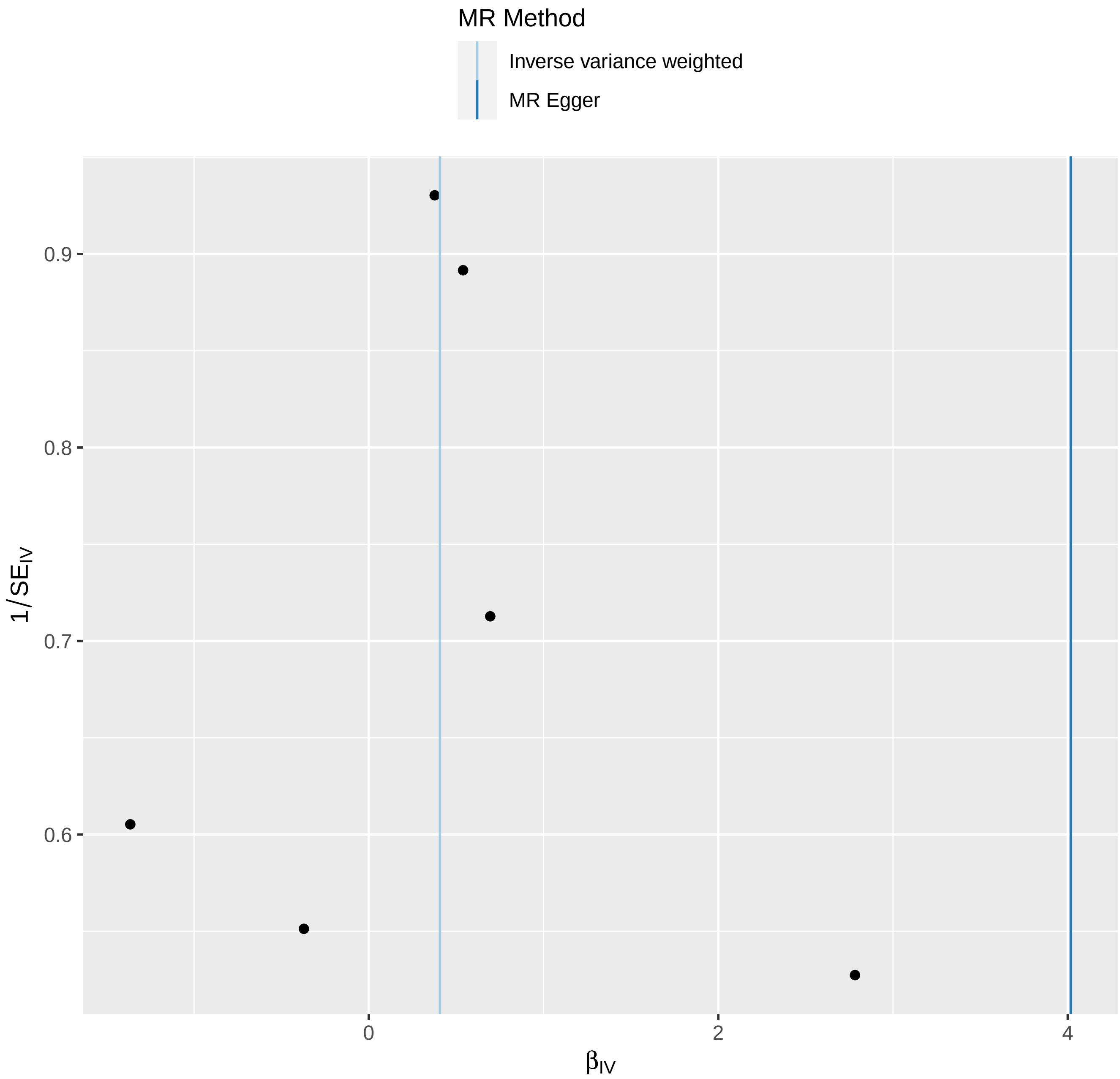

Batch 1193 : Taurochenodeoxycholate on Ulcerative colitis with PSC

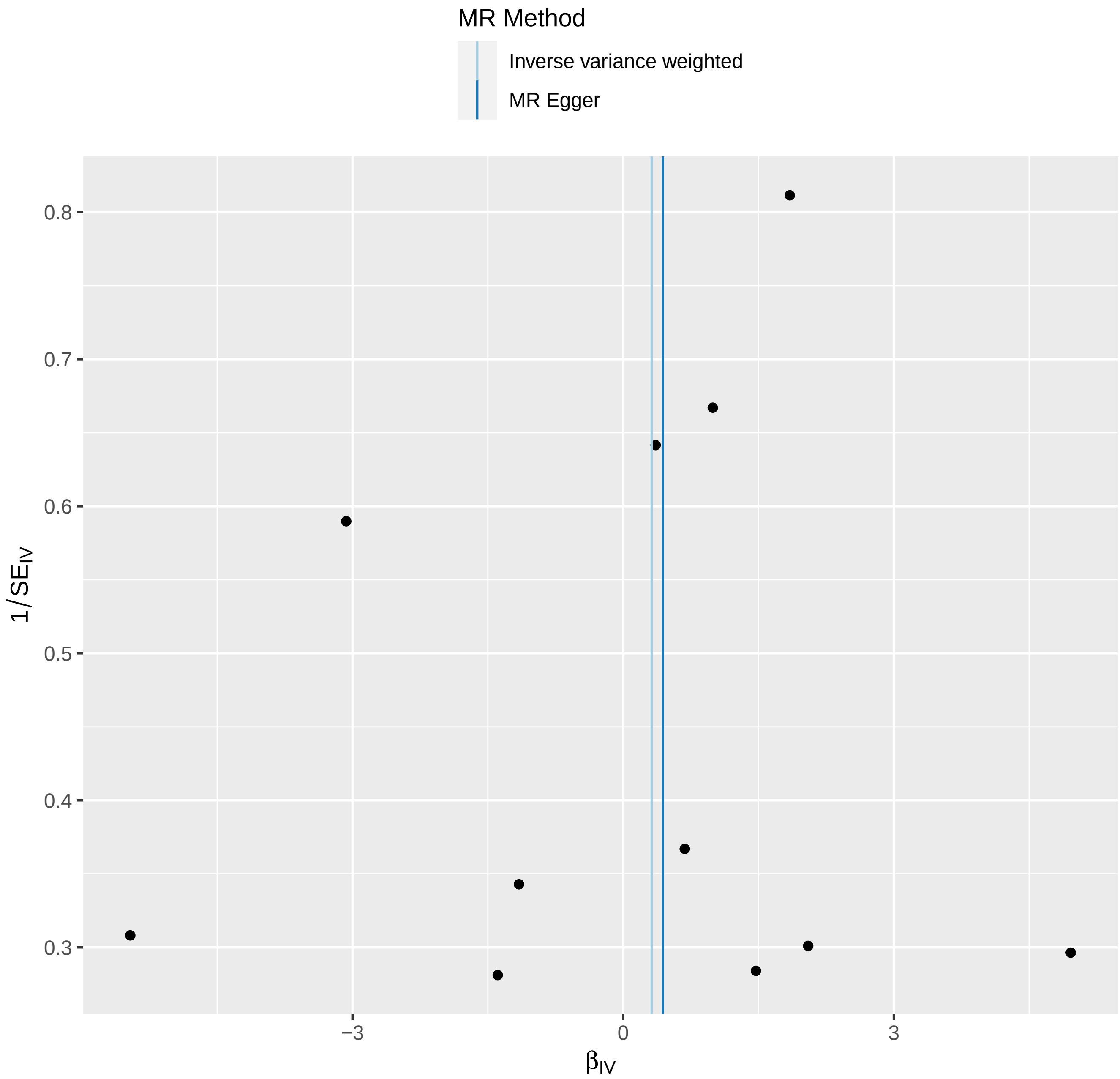

Batch 1194 : Taurocholate on Ulcerative colitis with PSC

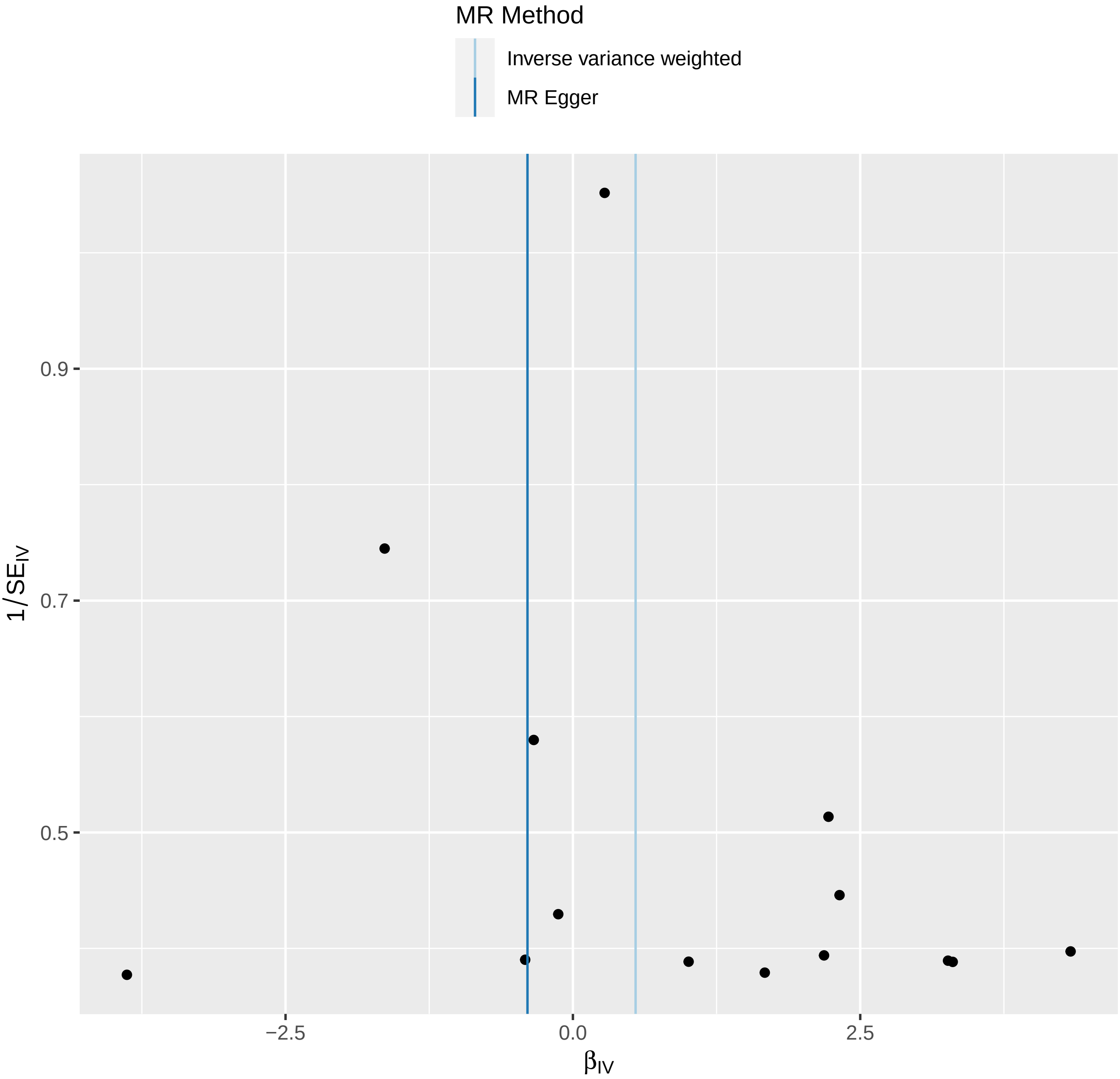

Batch 1196 : 1−stearoylglycerophosphoinositol on Ulcerative colitis with PSC

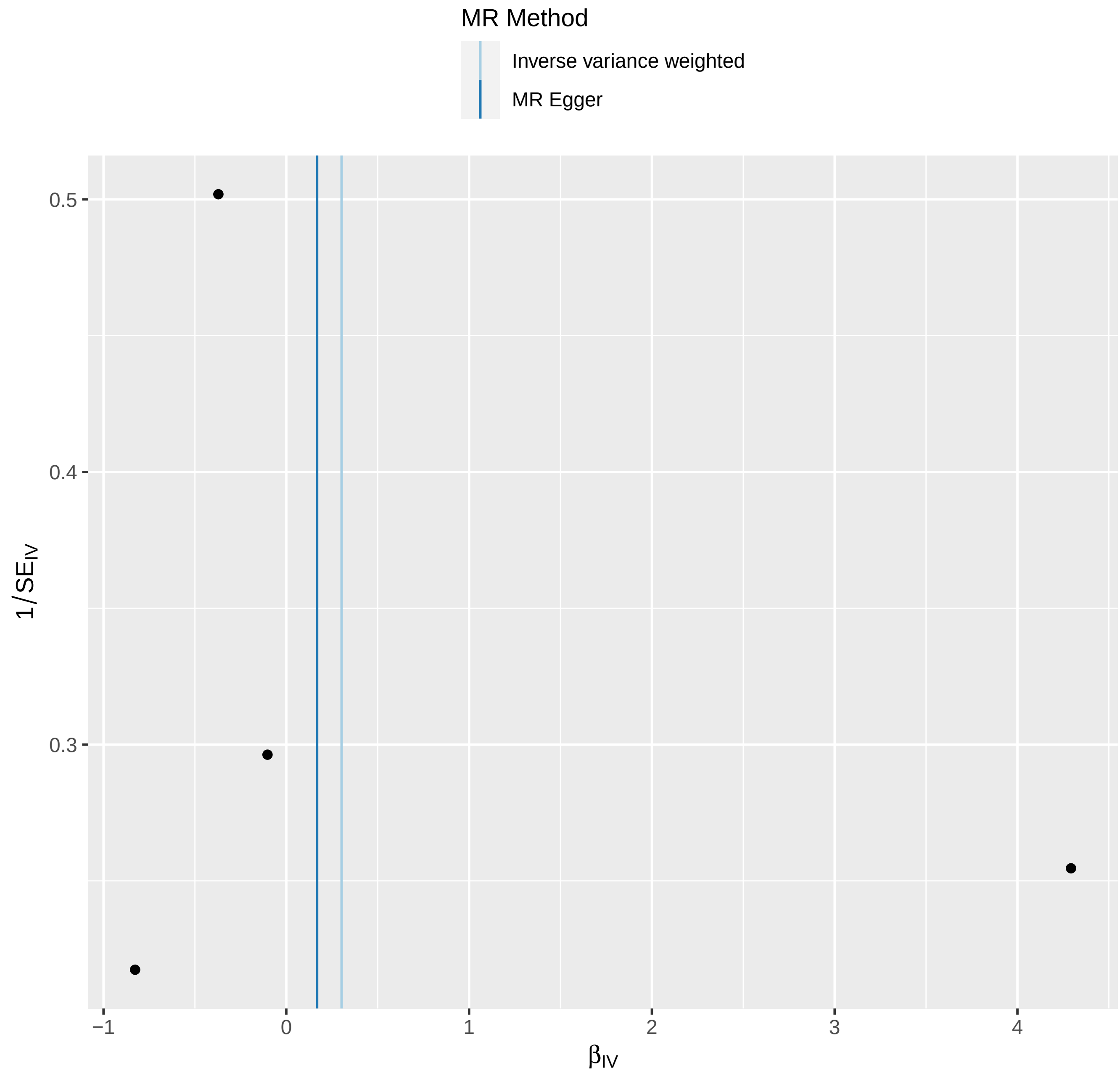

Batch 1197 : Myo−inositol on Ulcerative colitis with PSC

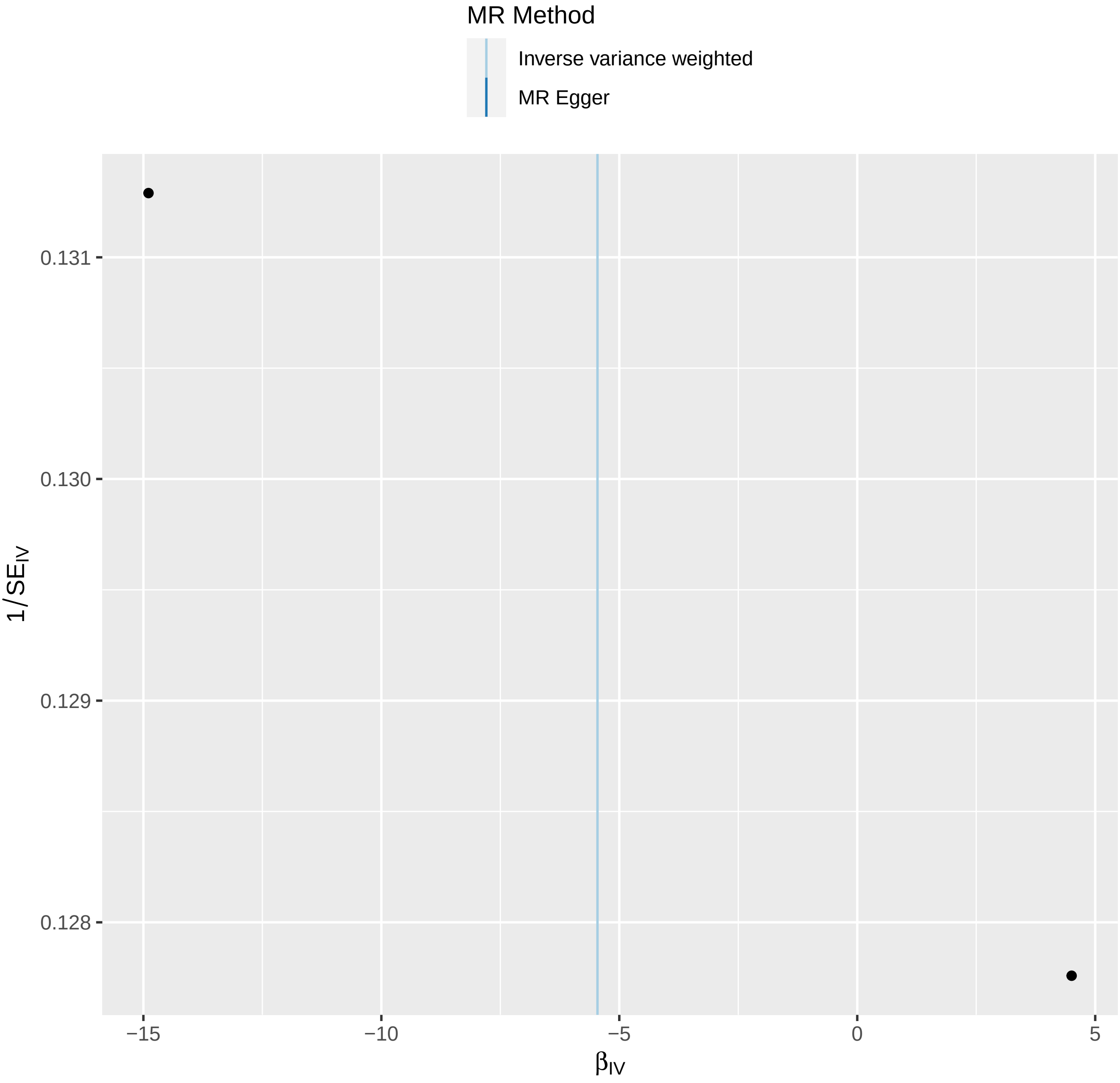

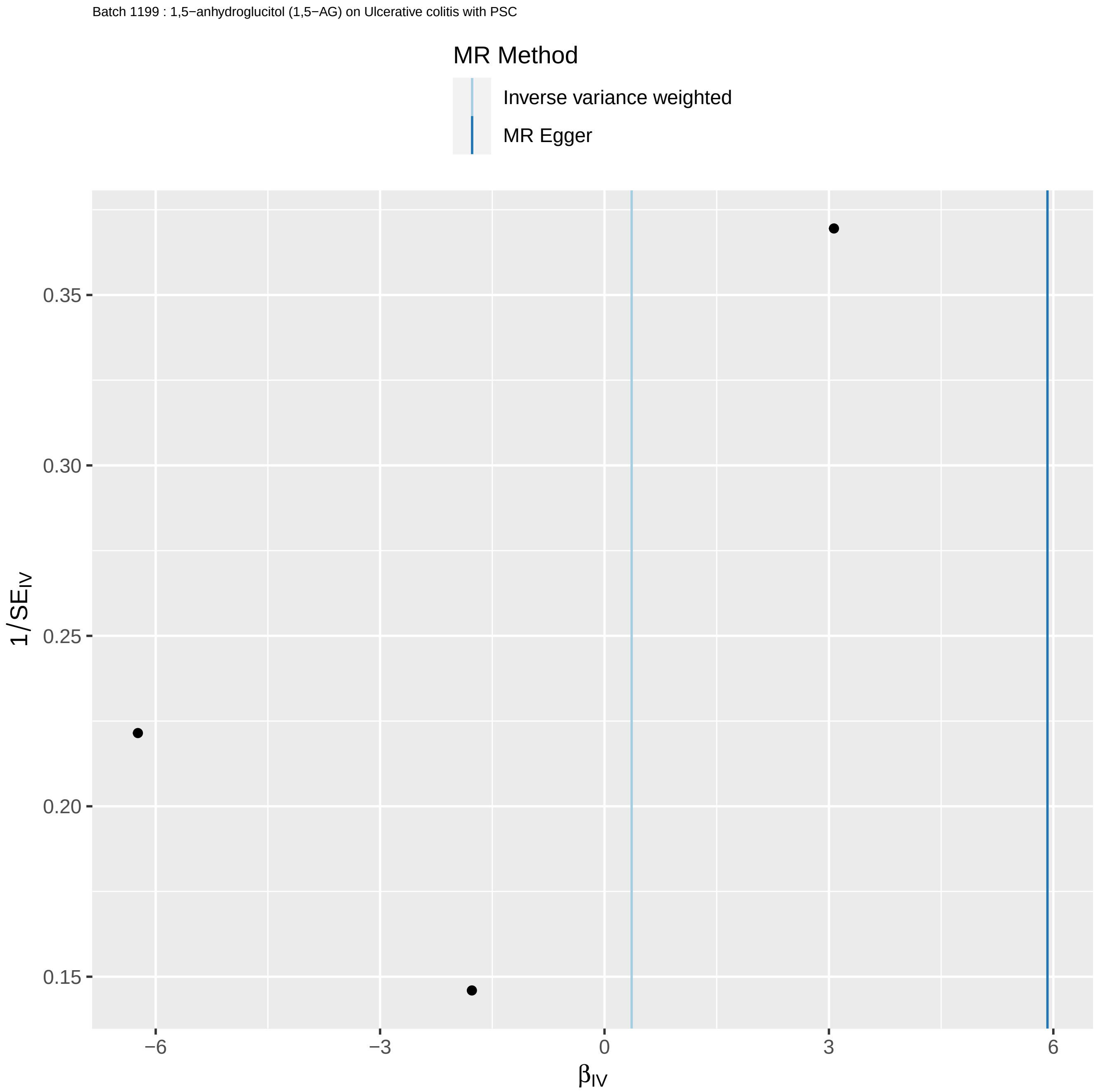

Batch 1199 : 1,5−anhydroglucitol (1,5−AG) on Ulcerative colitis with PSC
MR Method
Inverse variance weighted
MR Egger
0.35
0.30
0.25
0.20
0.15
1/SE$_{IV}$
−6
−3
0
3
6
β$_{IV}$

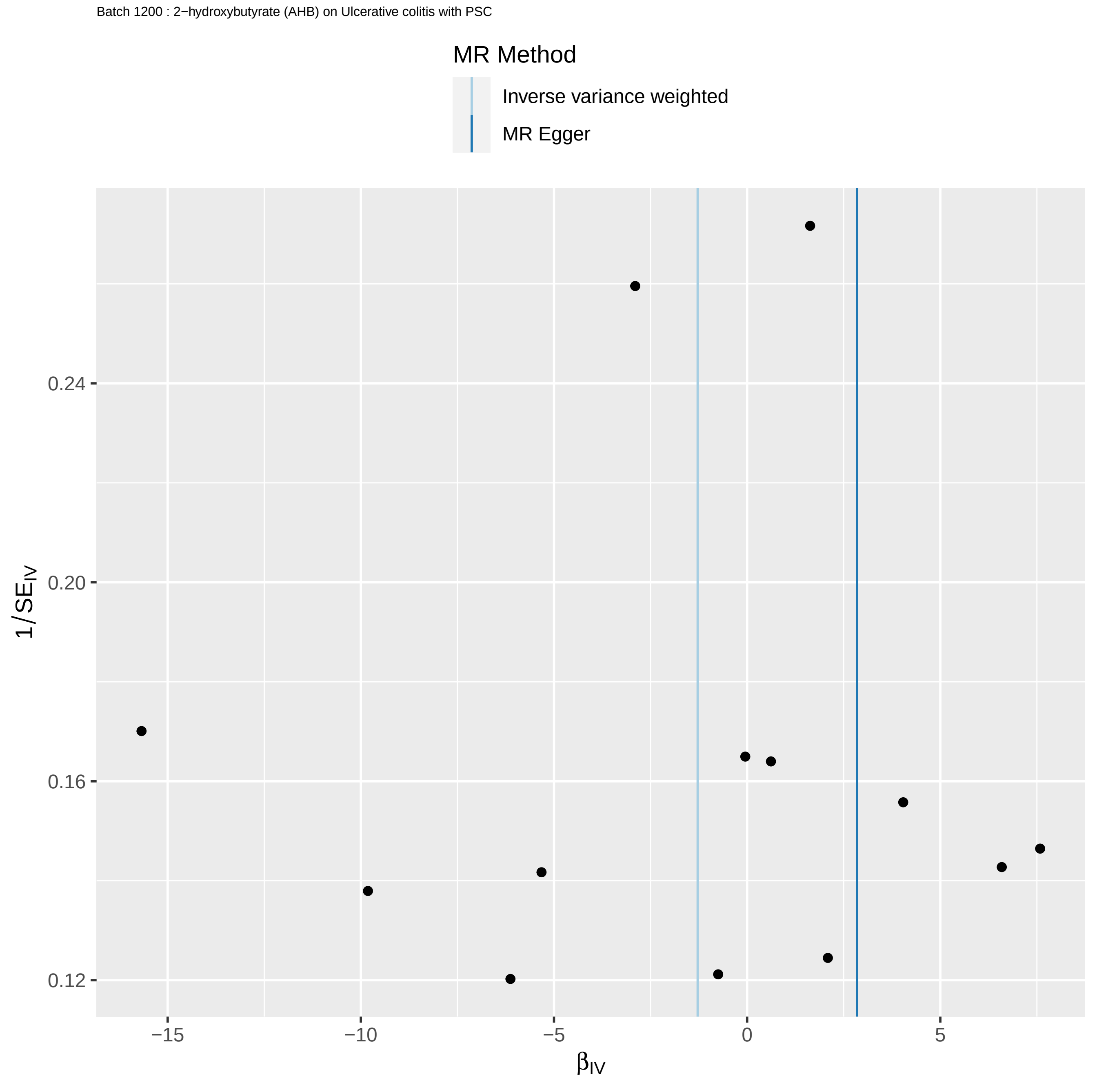

Batch 1200 : 2−hydroxybutyrate (AHB) on Ulcerative colitis with PSC
MR Method
Inverse variance weighted
MR Egger
0.24
0.20
0.16
0.12
1/SE_IV
−15
−10
−5
0
5
β_IV

Batch 1203 : 1−palmitoylglycerol (1−monopalmitin) on Ulcerative colitis with PSC

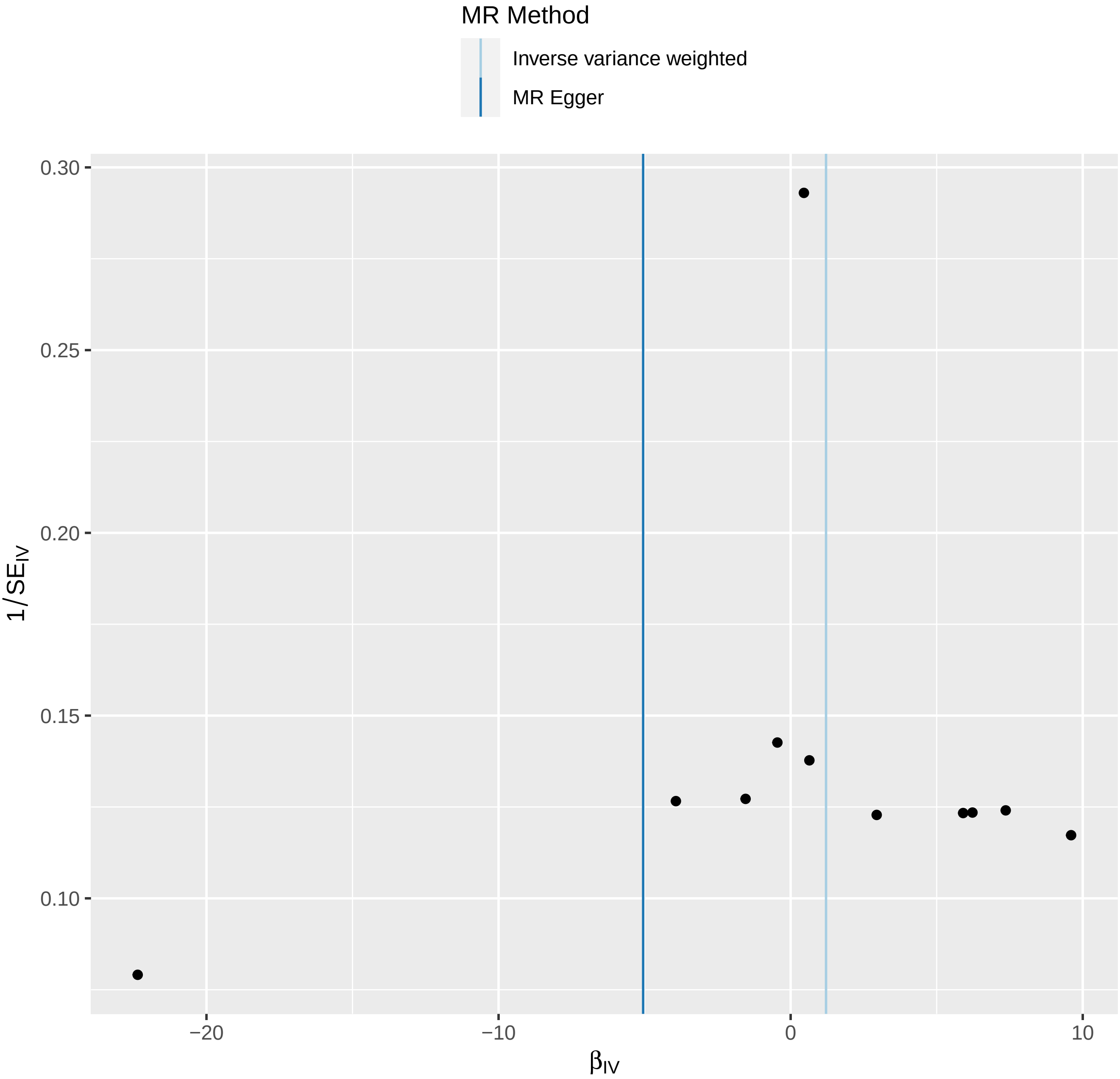

Batch 1206 : 2−hydroxyisobutyrate on Ulcerative colitis with PSC

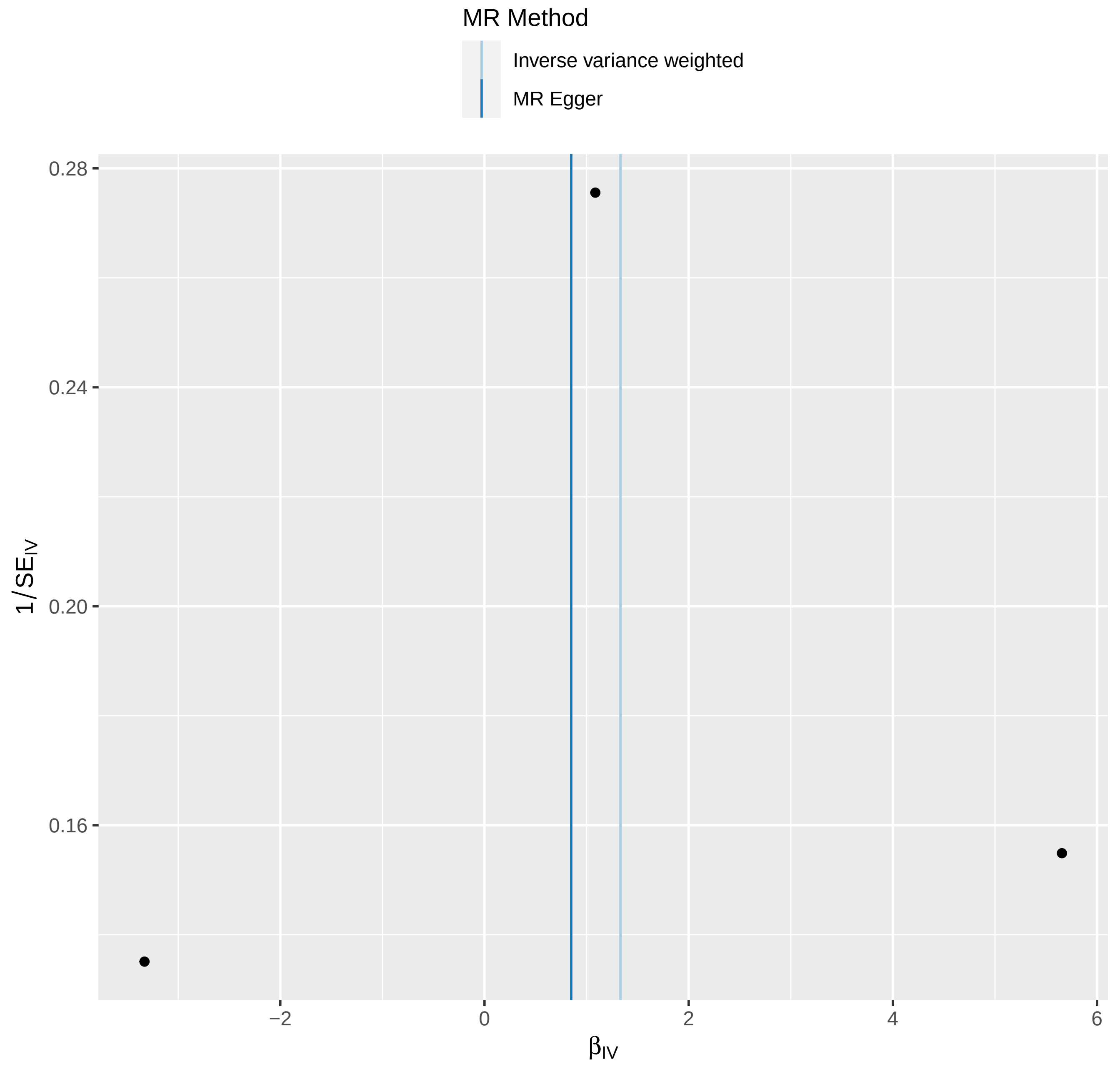

Batch 1207 : 4−methyl−2−oxopentanoate on Ulcerative colitis with PSC

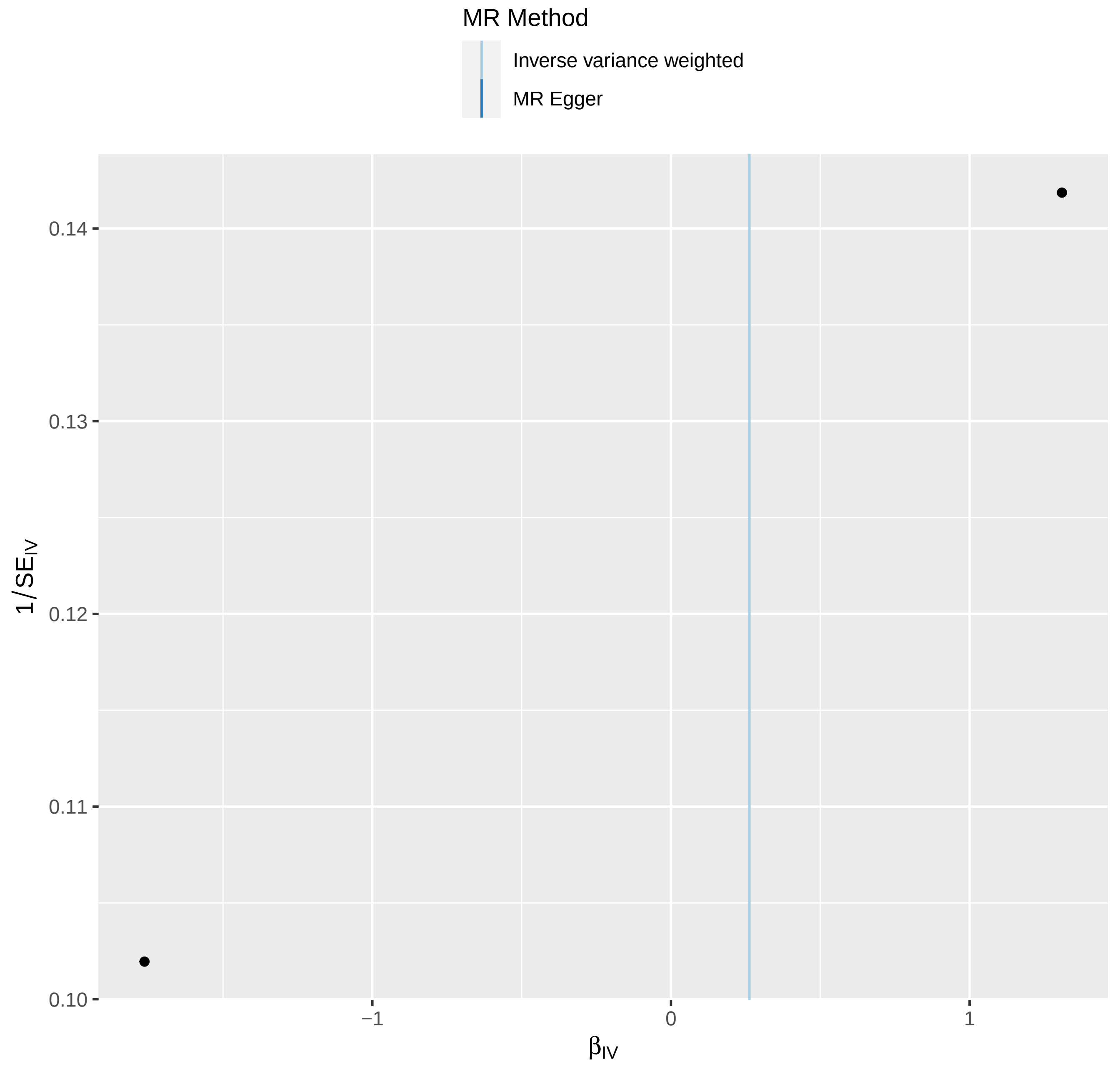

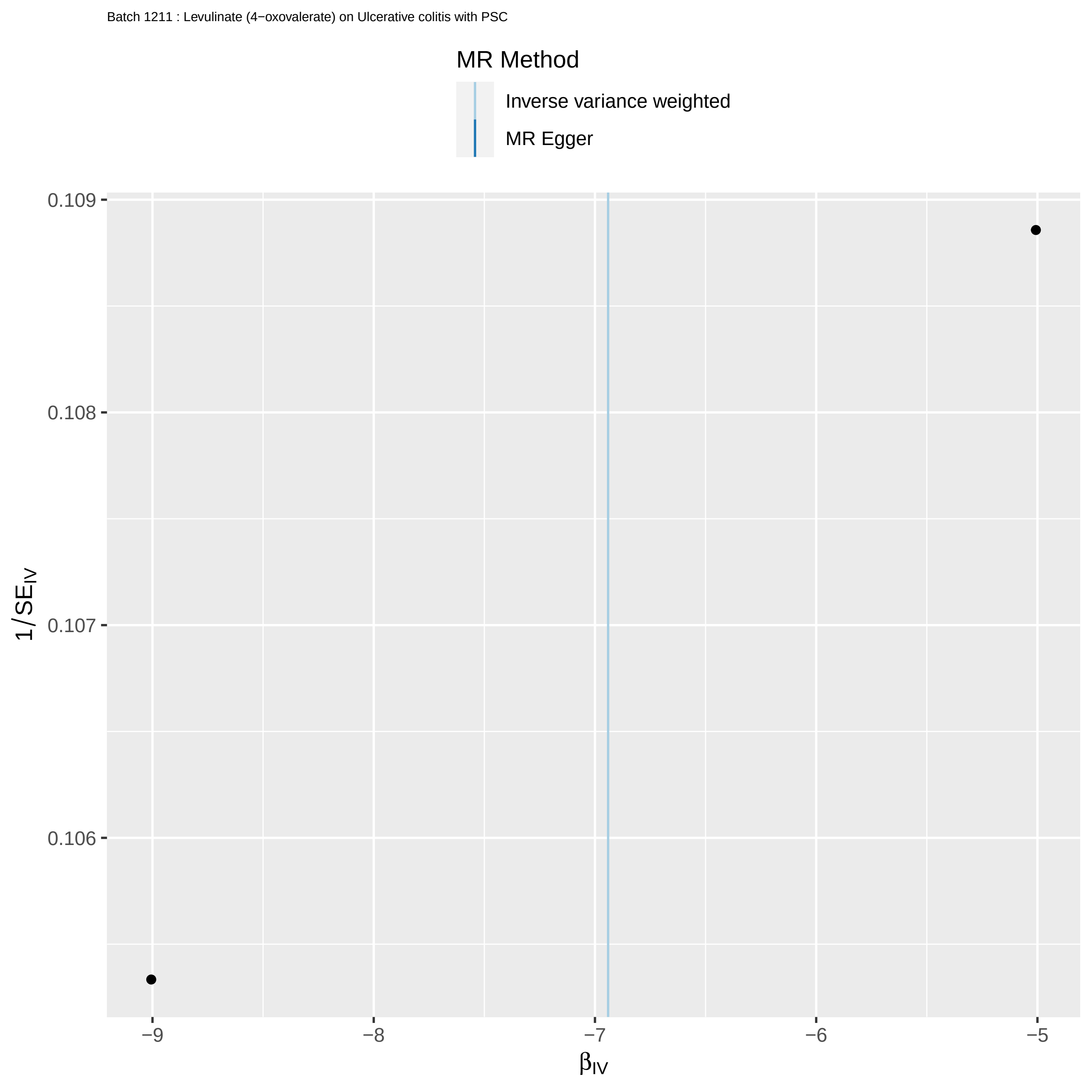

Batch 1211 : Levulinate (4−oxovalerate) on Ulcerative colitis with PSC
MR Method
Inverse variance weighted
MR Egger
0.109
0.108
0.107
0.106
1/SE_IV
−9
−8
−7
−6
−5
β_IV

Batch 1213 : Cholate on Ulcerative colitis with PSC

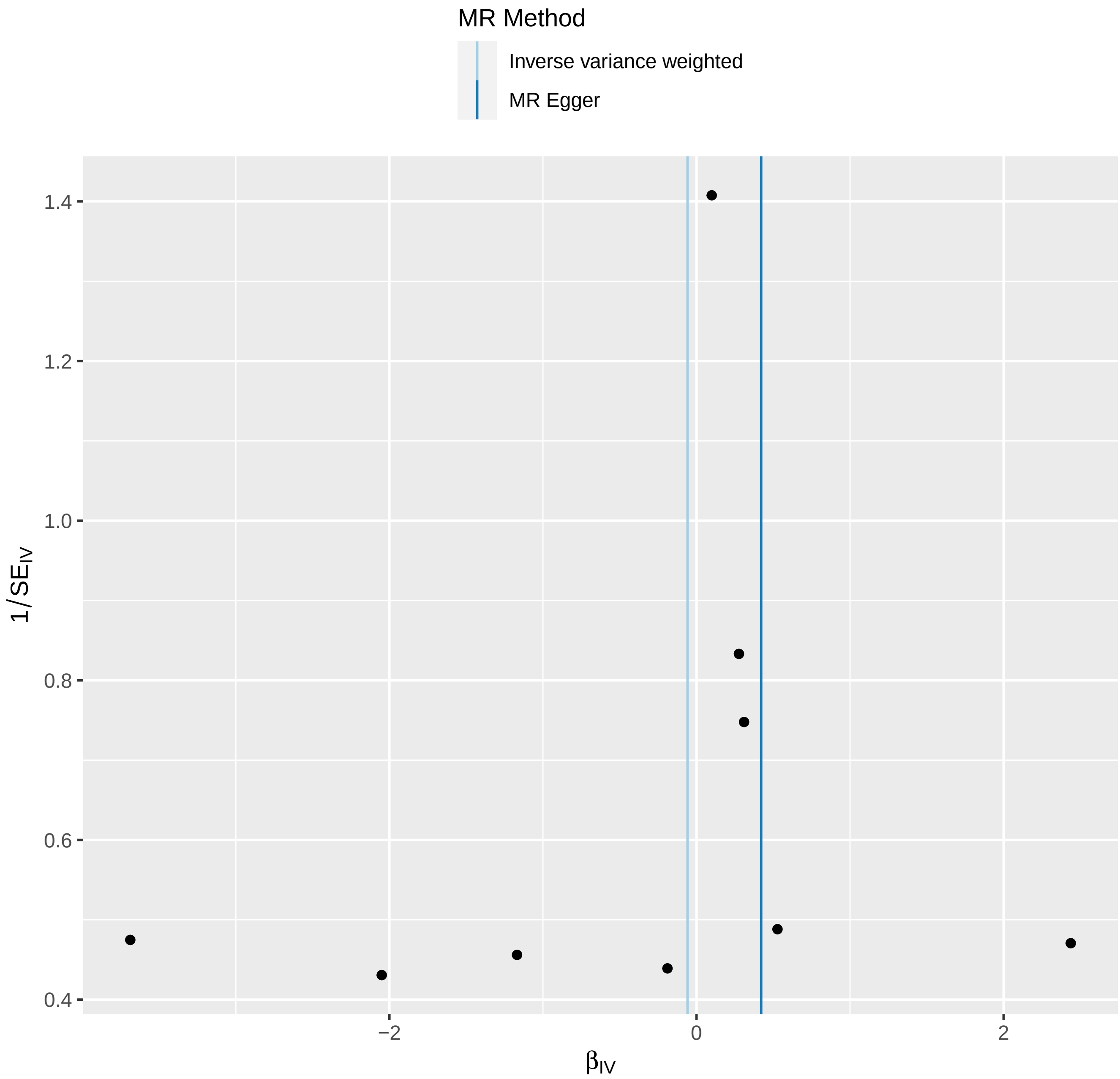

Batch 1216 : Hyodeoxycholate on Ulcerative colitis with PSC

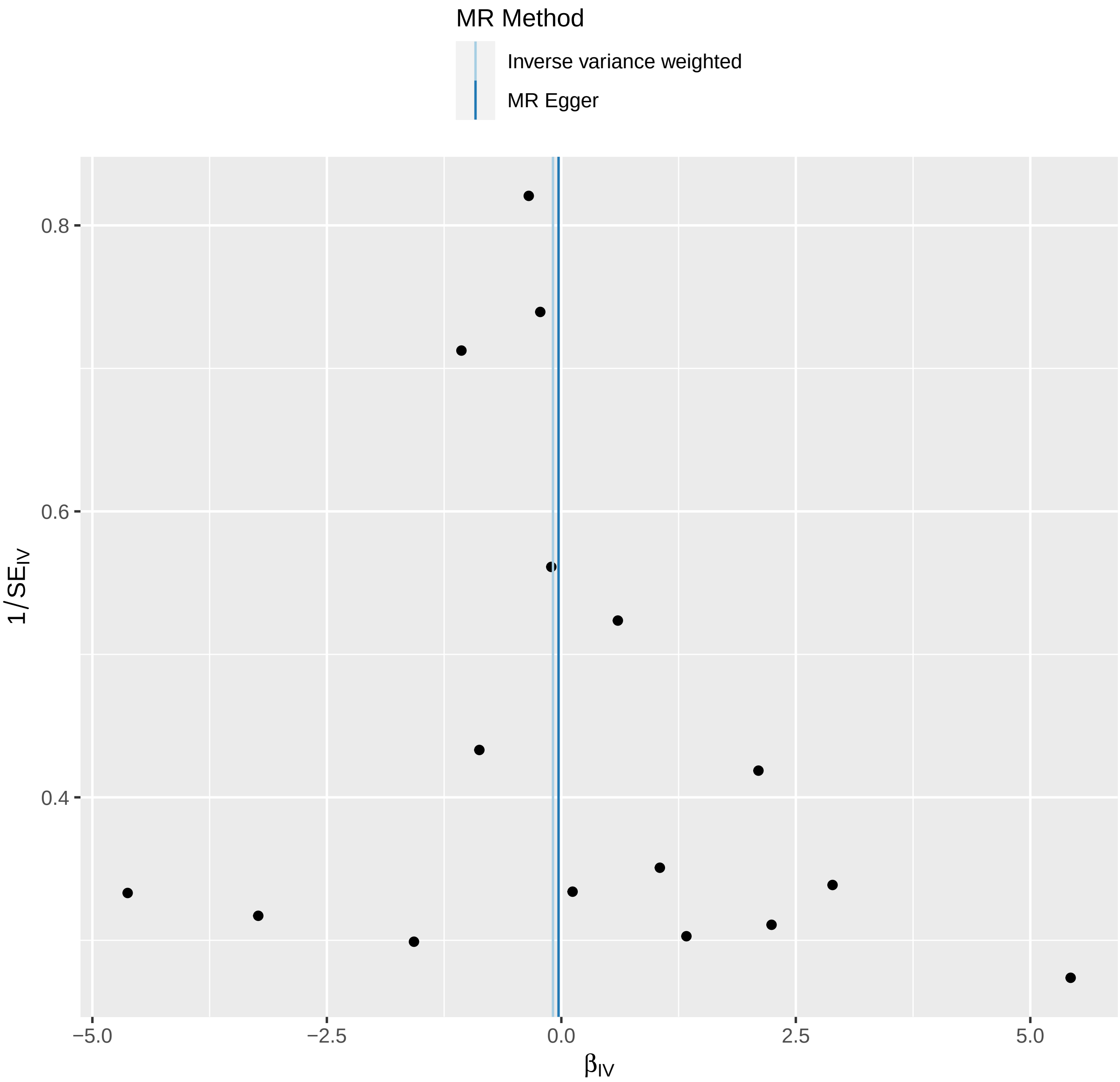

Batch 1217 : 3−indoxyl sulfate on Ulcerative colitis with PSC

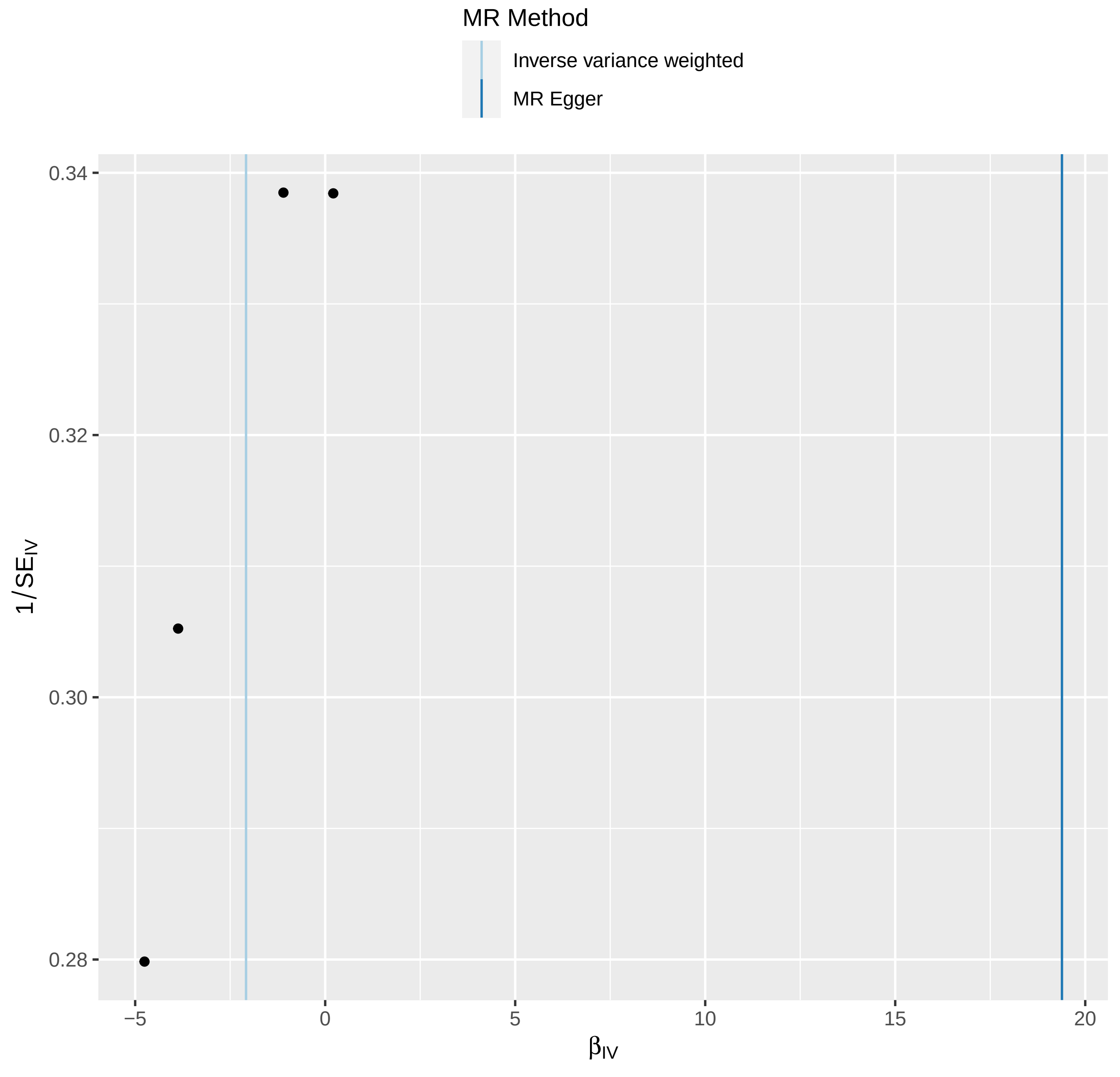

Batch 1221 : Erythrose on Ulcerative colitis with PSC

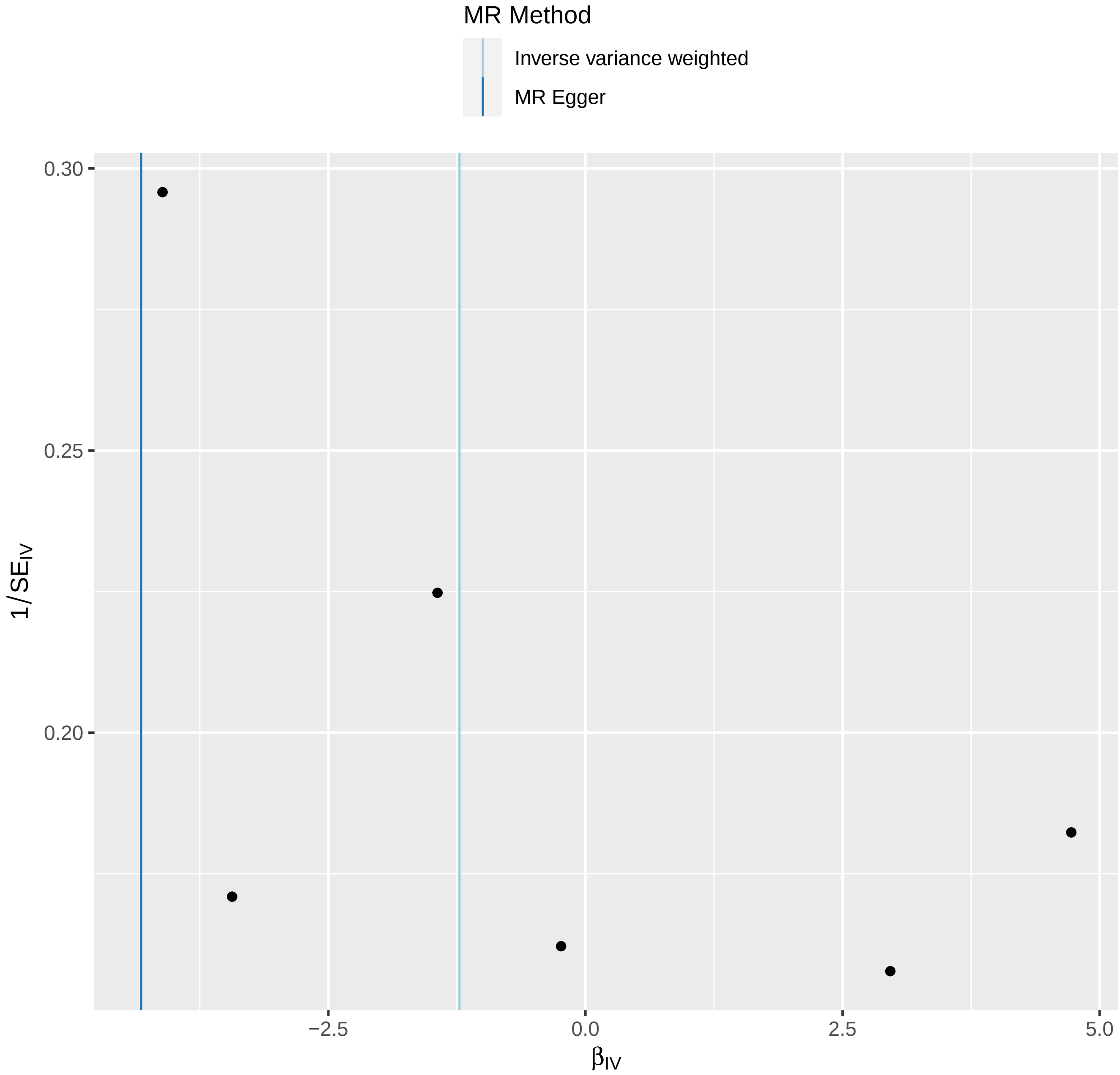

Batch 1222 : Threonate on Ulcerative colitis with PSC

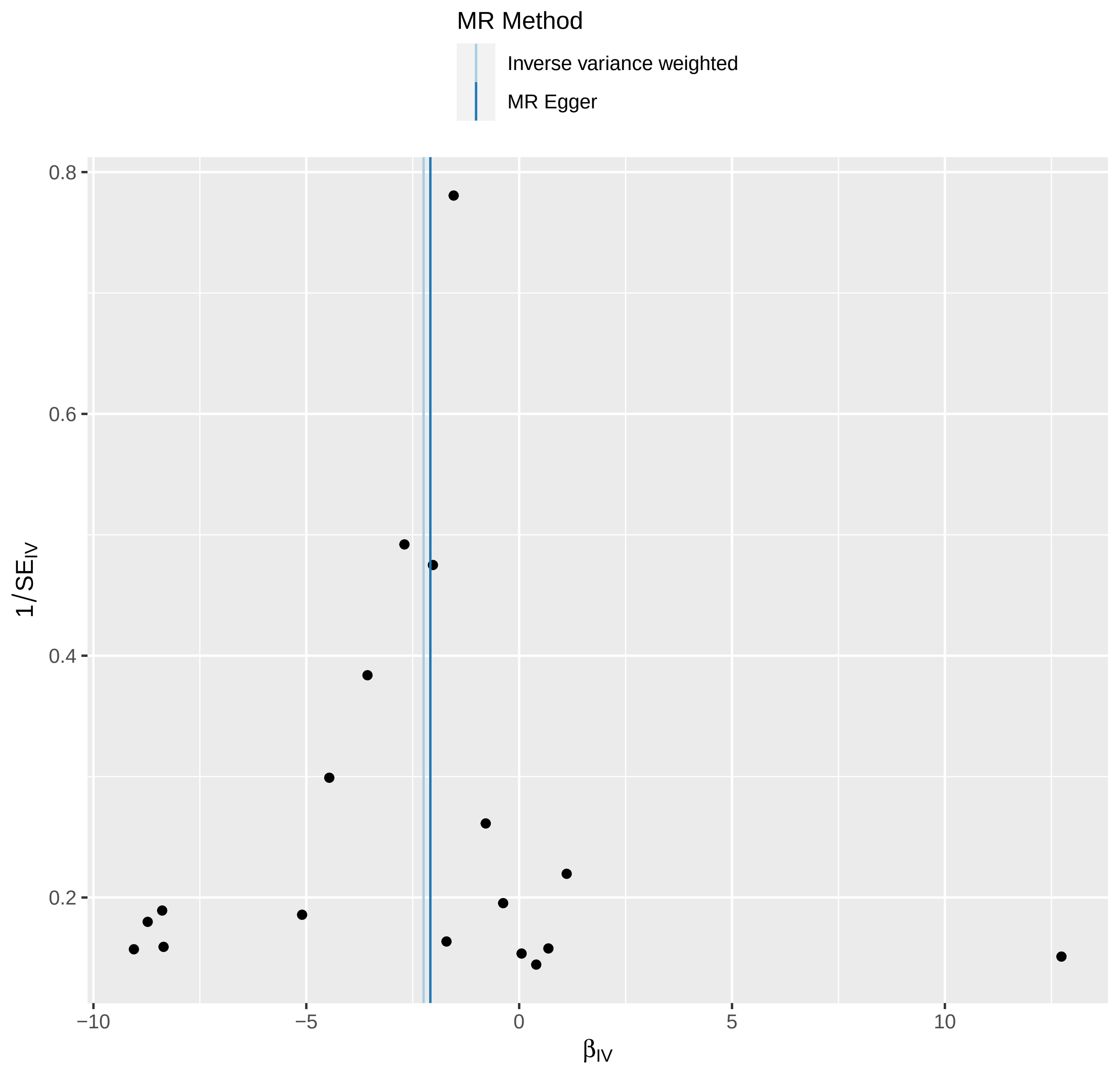

Batch 1223 : Cysteine on Ulcerative colitis with PSC

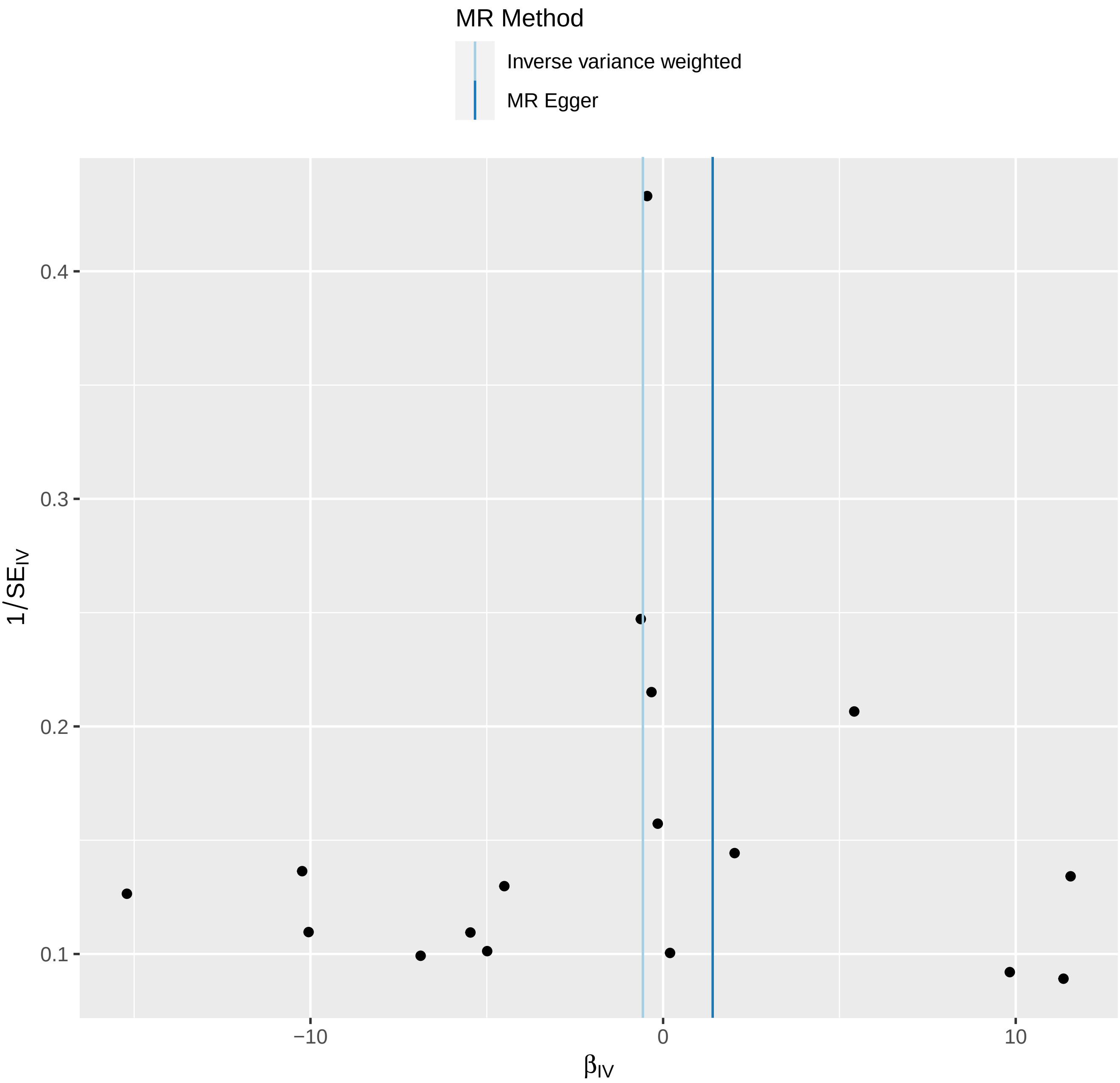

Batch 1224 : Pyroglutamylglycine on Ulcerative colitis with PSC

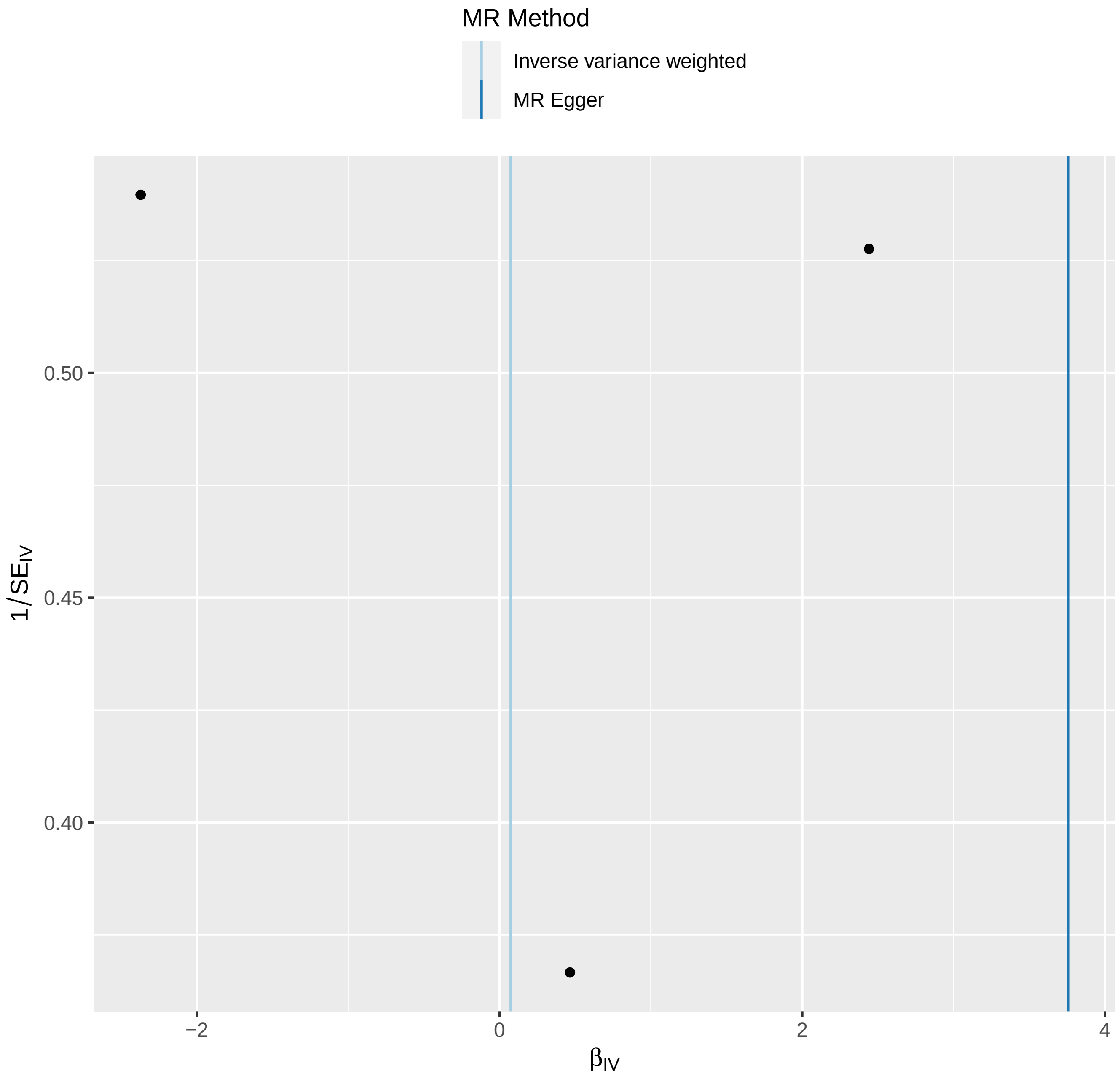

Batch 1226 : DSGEGDFXAEGGGVR* on Ulcerative colitis with PSC

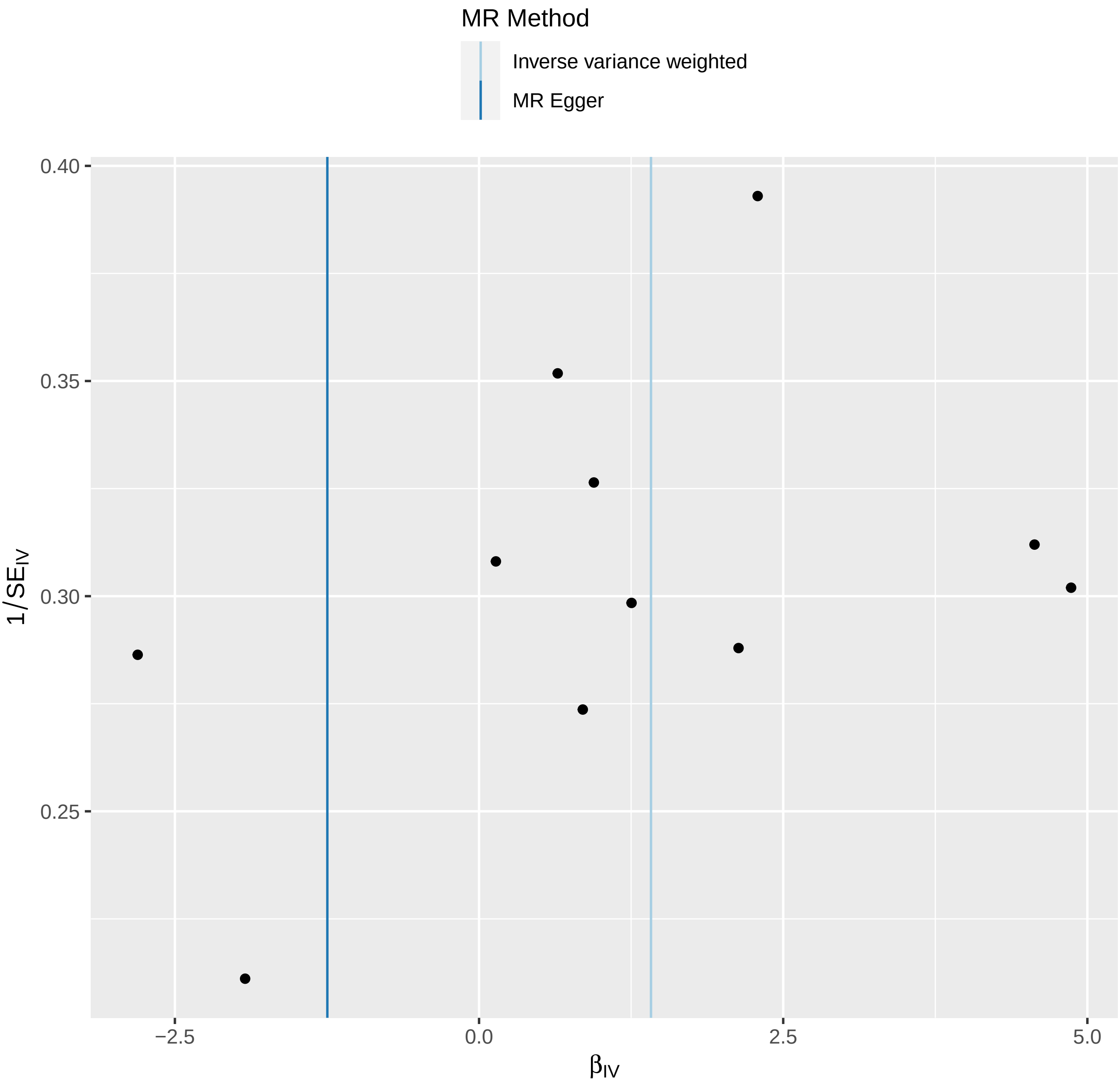

Batch 1227 : Pyridoxate on Ulcerative colitis with PSC

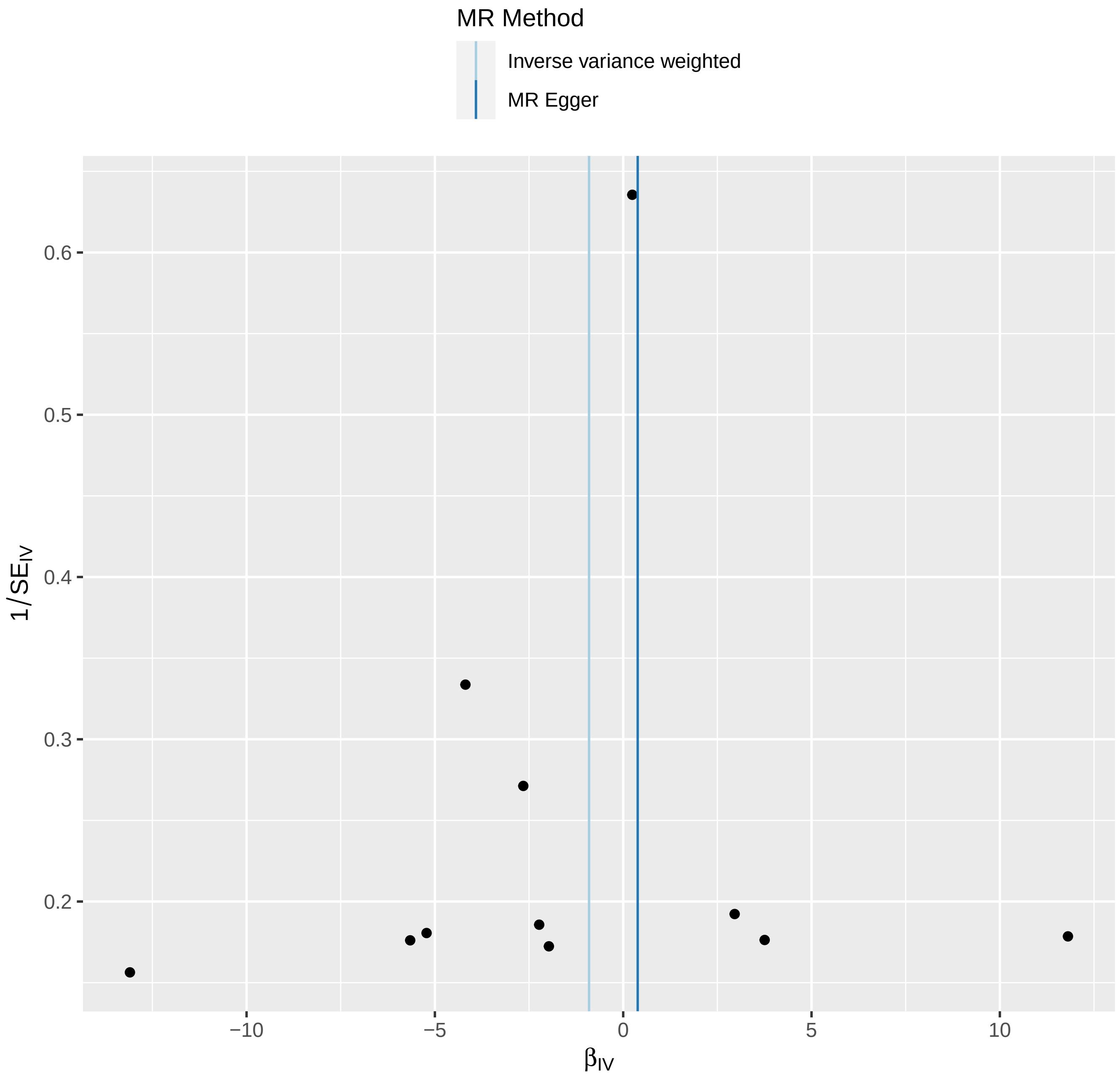

Batch 1228 : Androsterone sulfate on Ulcerative colitis with PSC

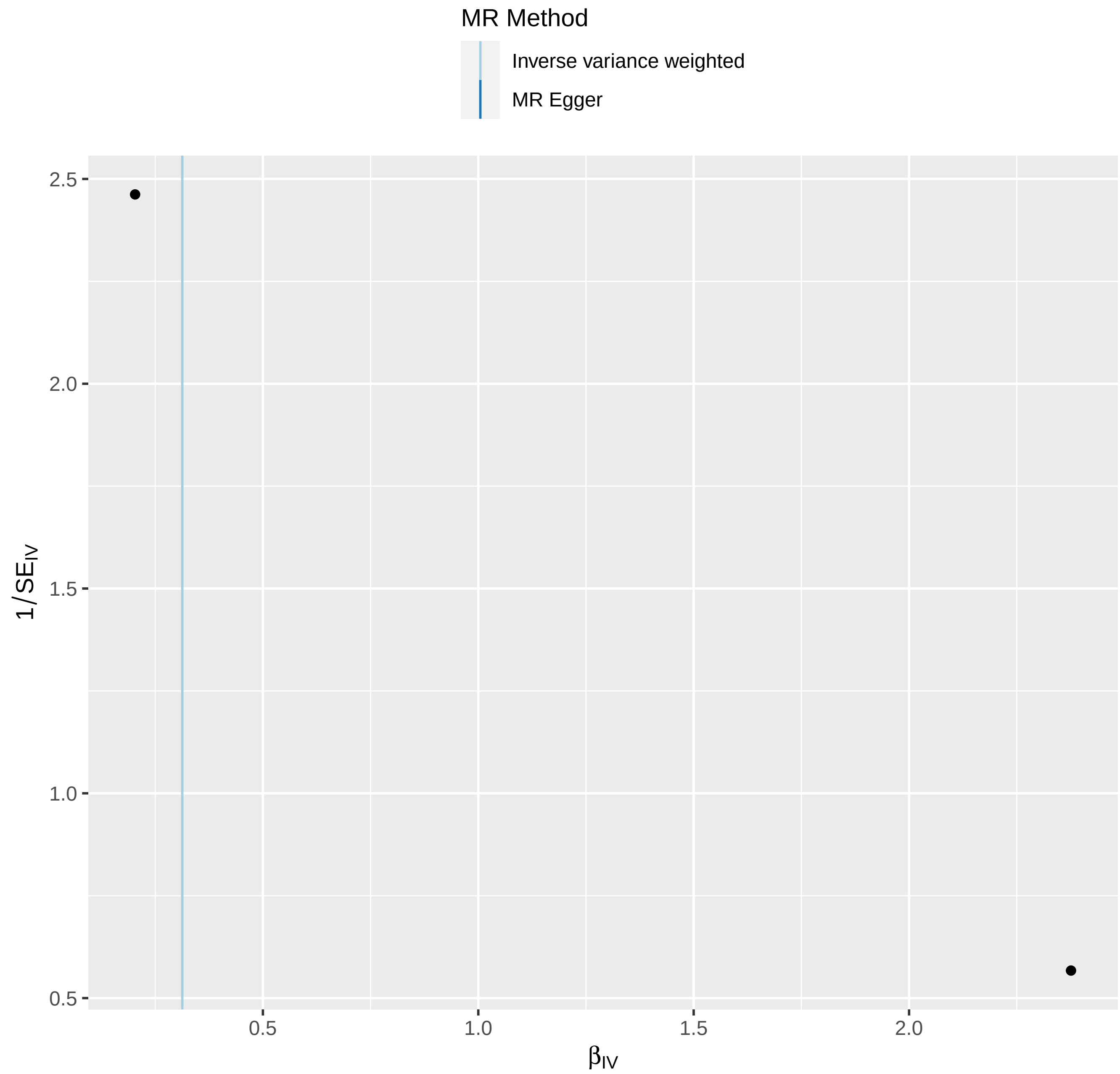

Batch 1231 : Acetylcarnitine on Ulcerative colitis with PSC

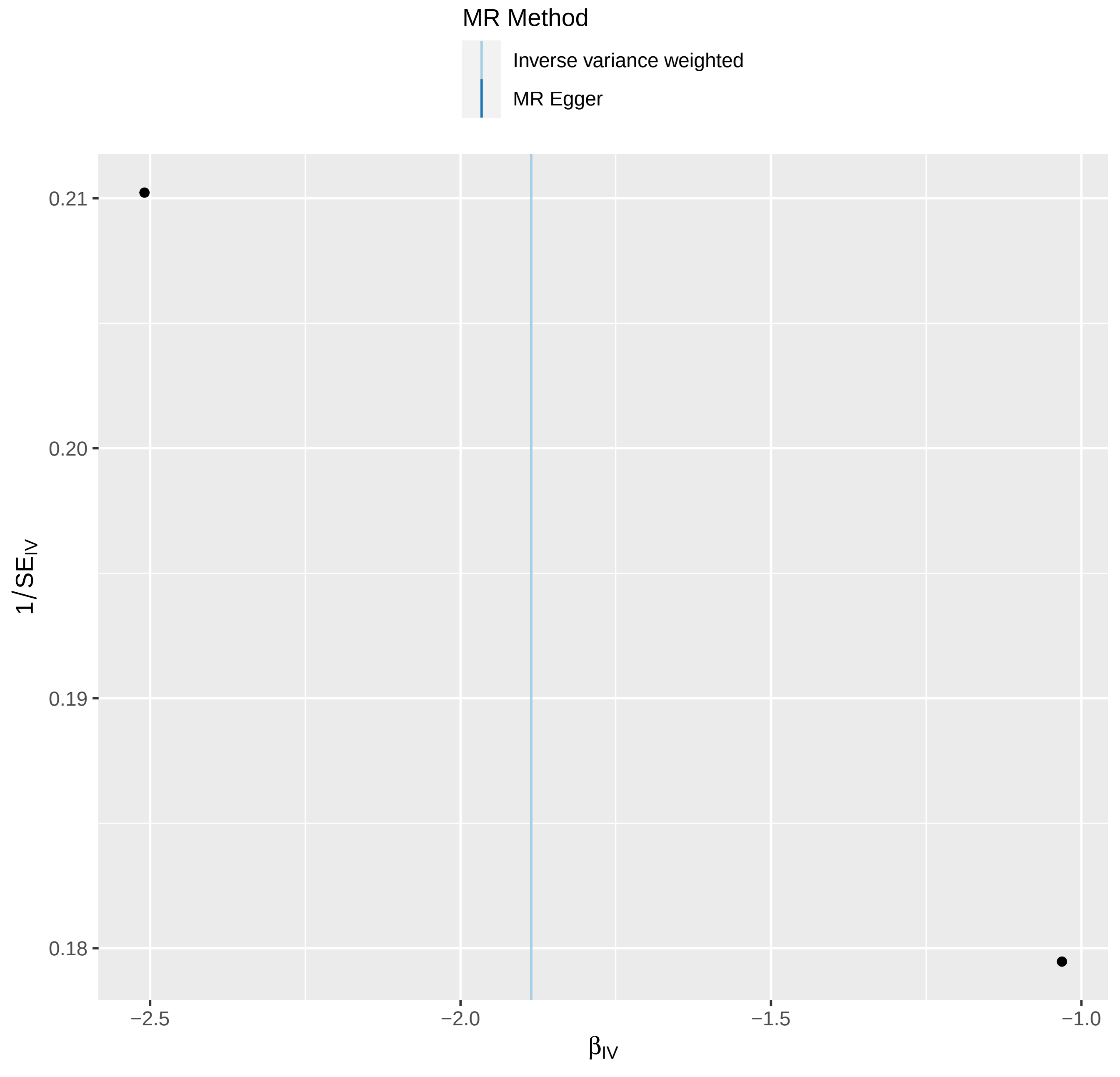

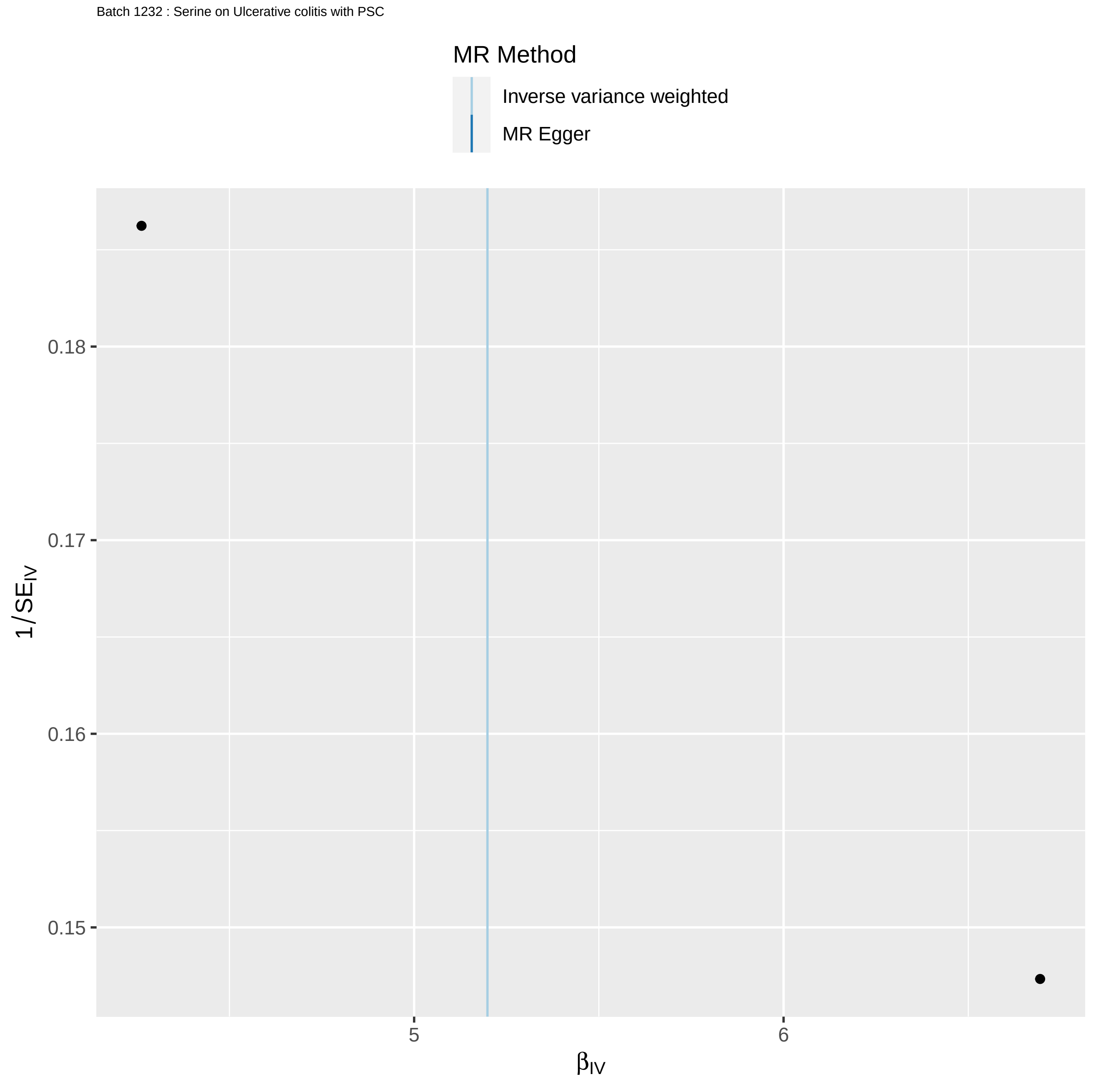

Batch 1232 : Serine on Ulcerative colitis with PSC
MR Method
Inverse variance weighted
MR Egger
0.18
0.17
0.16
0.15
$1/SE_{IV}$
5
6
β_{IV}

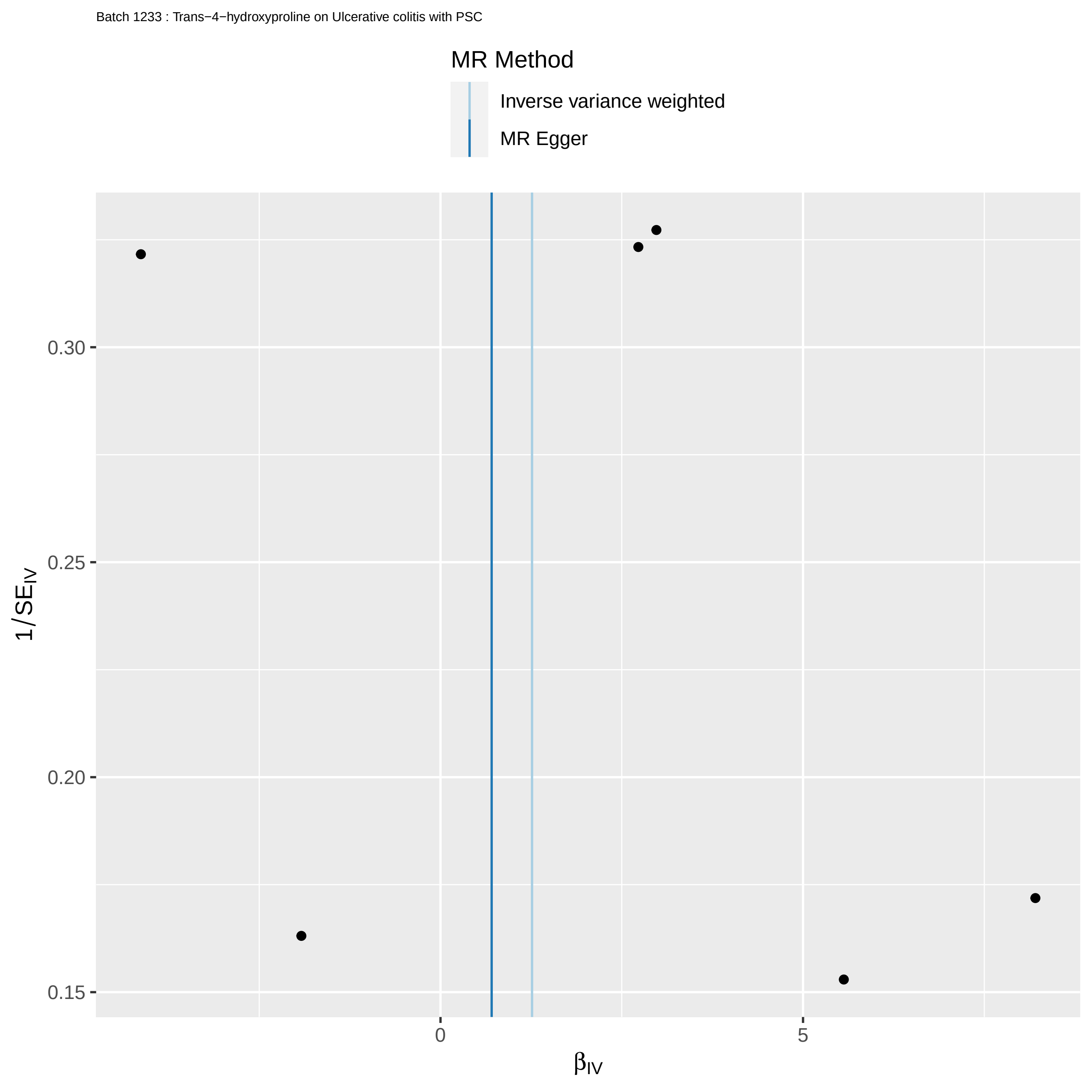

Batch 1233 : Trans−4−hydroxyproline on Ulcerative colitis with PSC
MR Method
Inverse variance weighted
MR Egger
0.30
0.25
0.20
0.15
$1/SE_{IV}$
0
5
β_{IV}

Batch 1234 : Glutamate on Ulcerative colitis with PSC

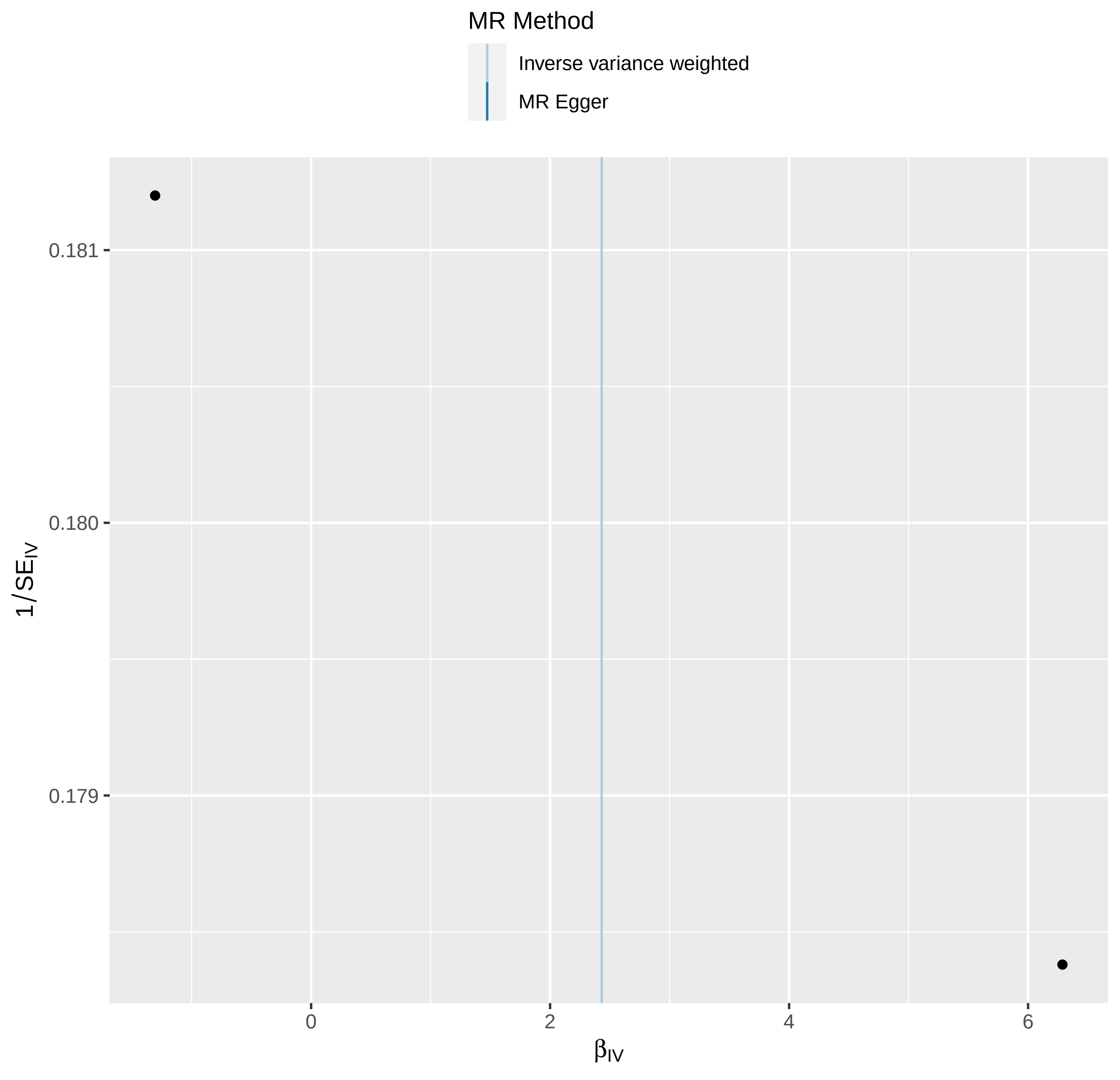

Batch 1235 : Hexanoylcarnitine on Ulcerative colitis with PSC

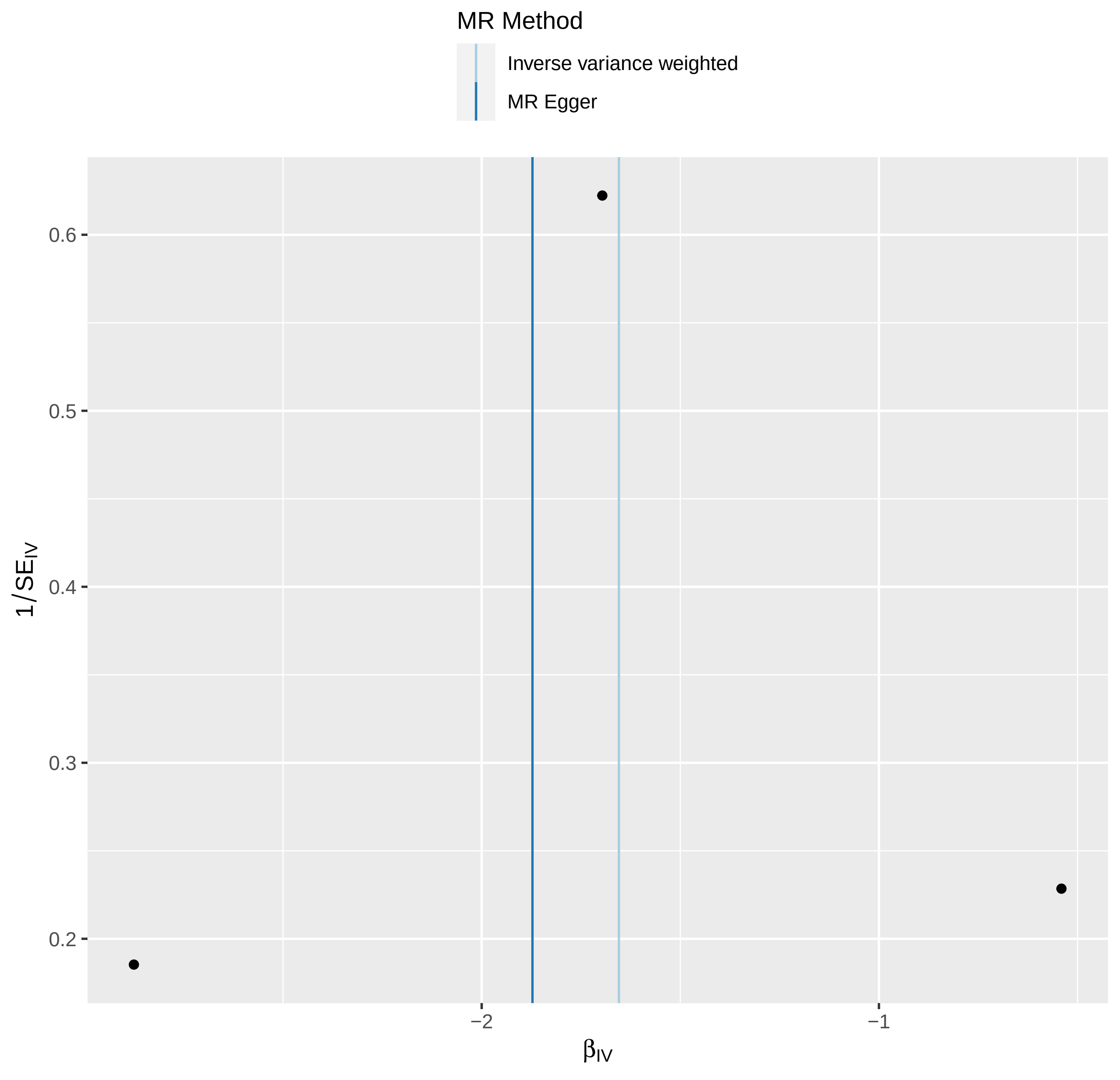

Batch 1239 : 2−aminobutyrate on Ulcerative colitis with PSC

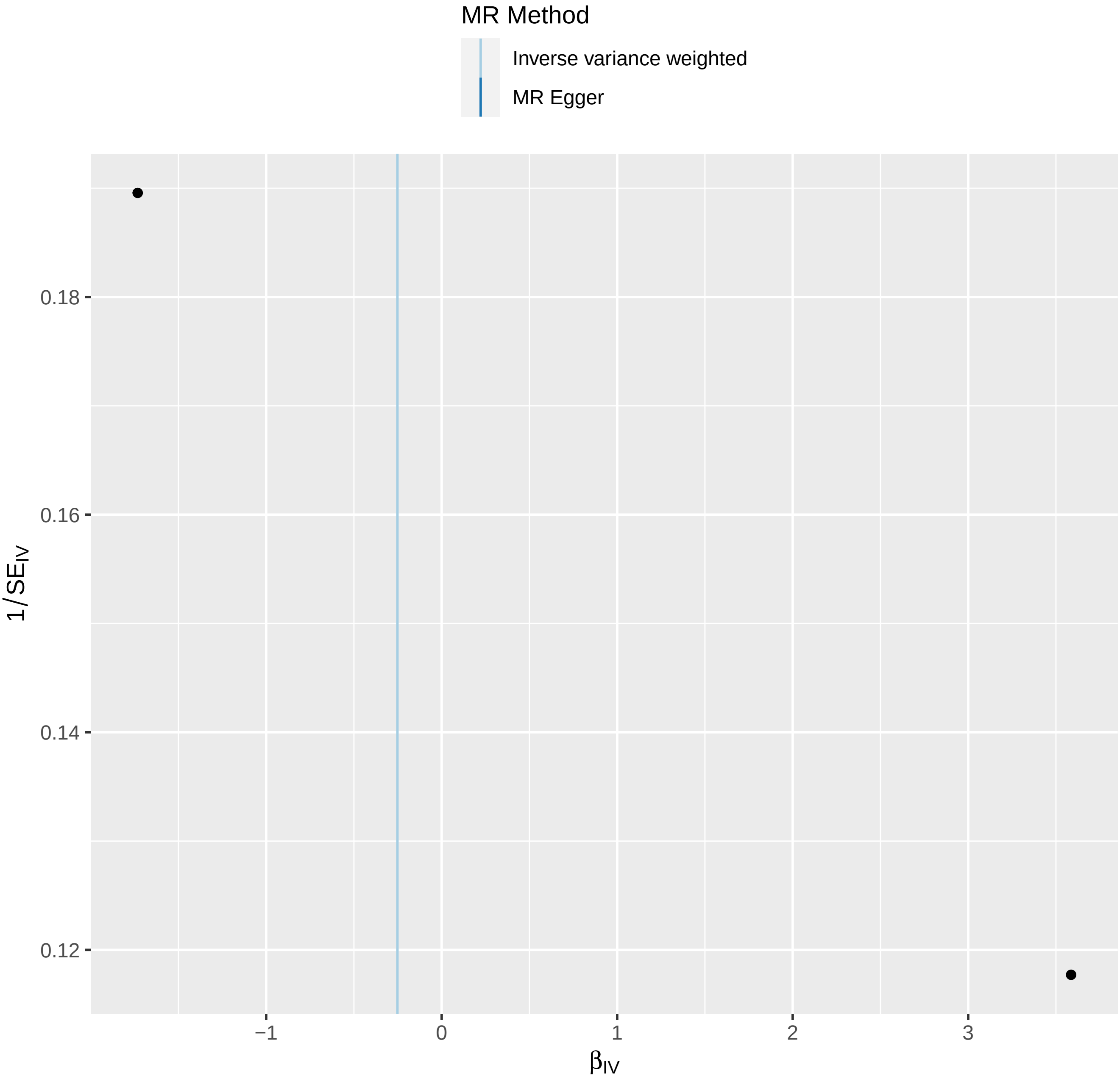

Batch 1244 : Butyrylcarnitine on Ulcerative colitis with PSC

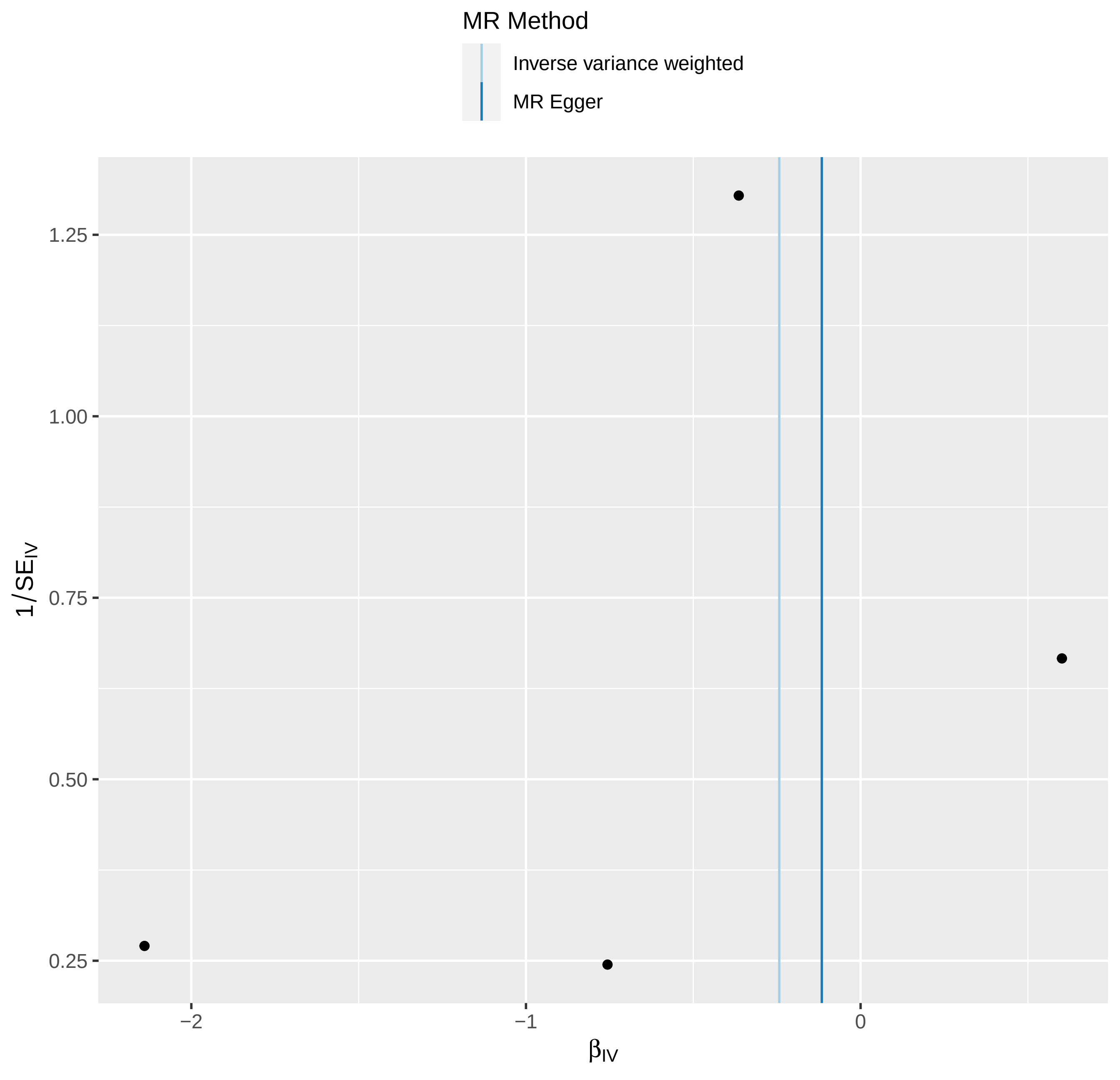

Batch 1247 : Propionylcarnitine on Ulcerative colitis with PSC

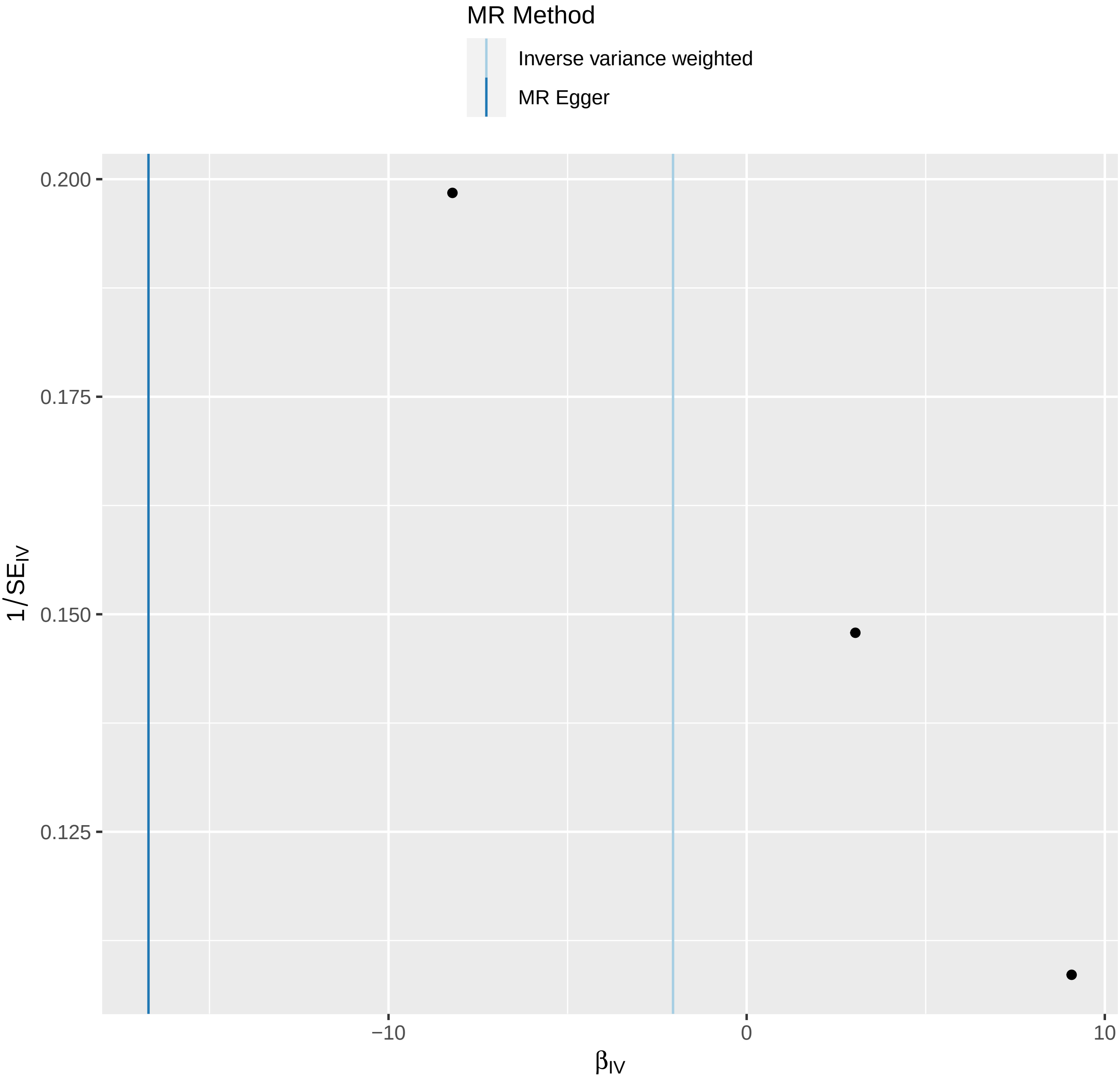

Batch 1248 : Caproate (6:0) on Ulcerative colitis with PSC

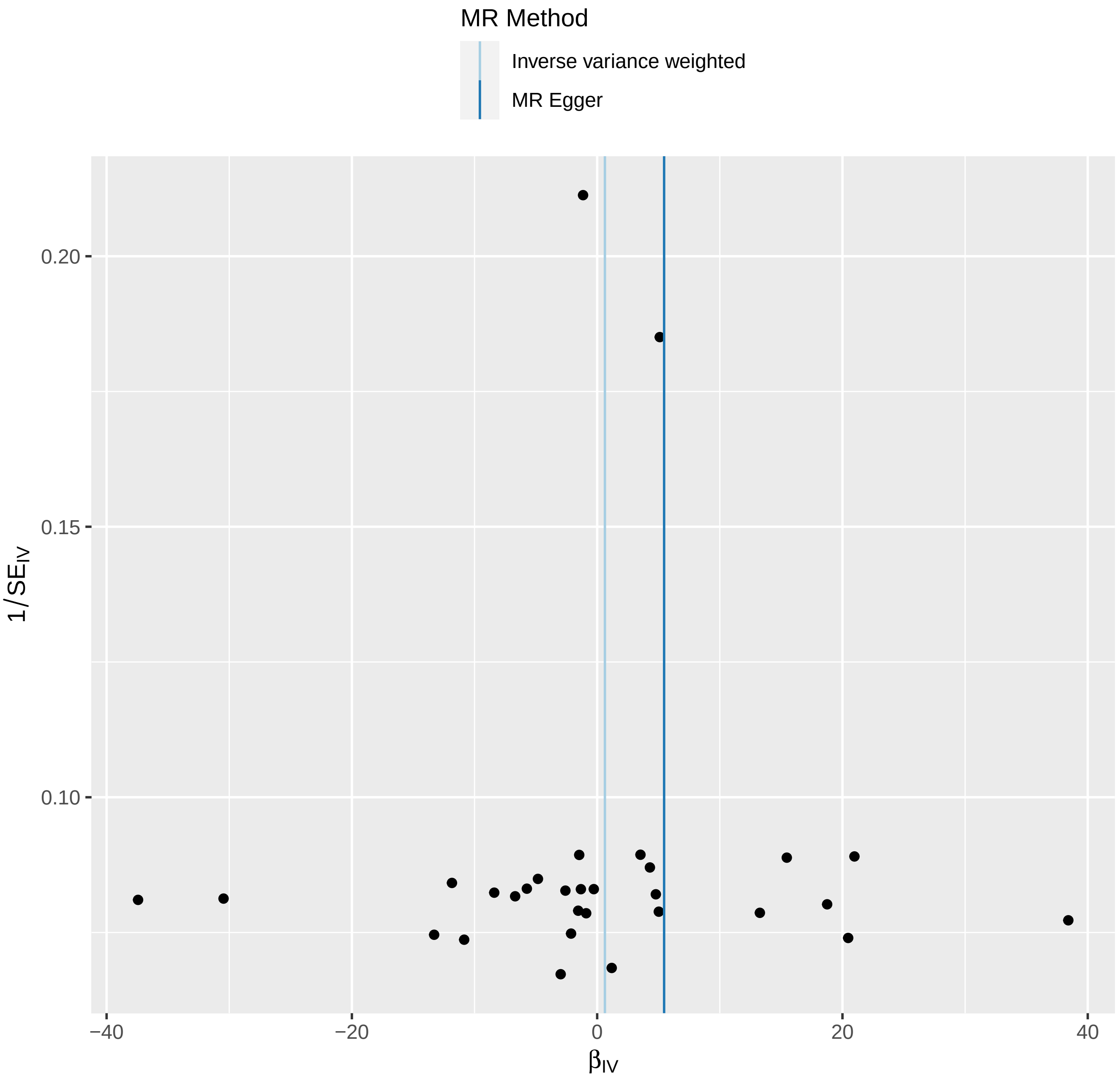

Batch 1249 : Caprylate (8:0) on Ulcerative colitis with PSC

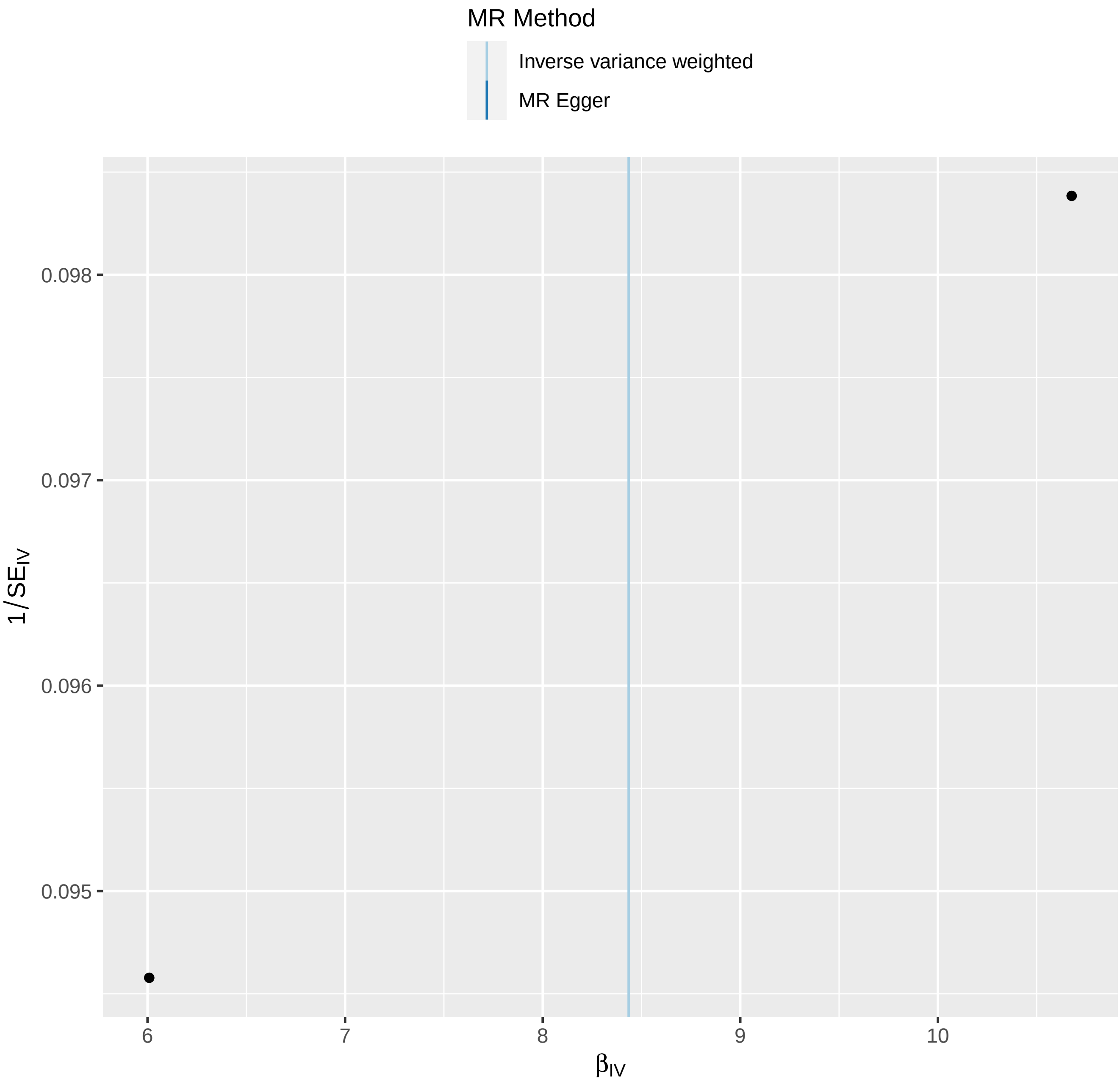

Batch 1250 : 10−undecenoate (11:1n1) on Ulcerative colitis with PSC

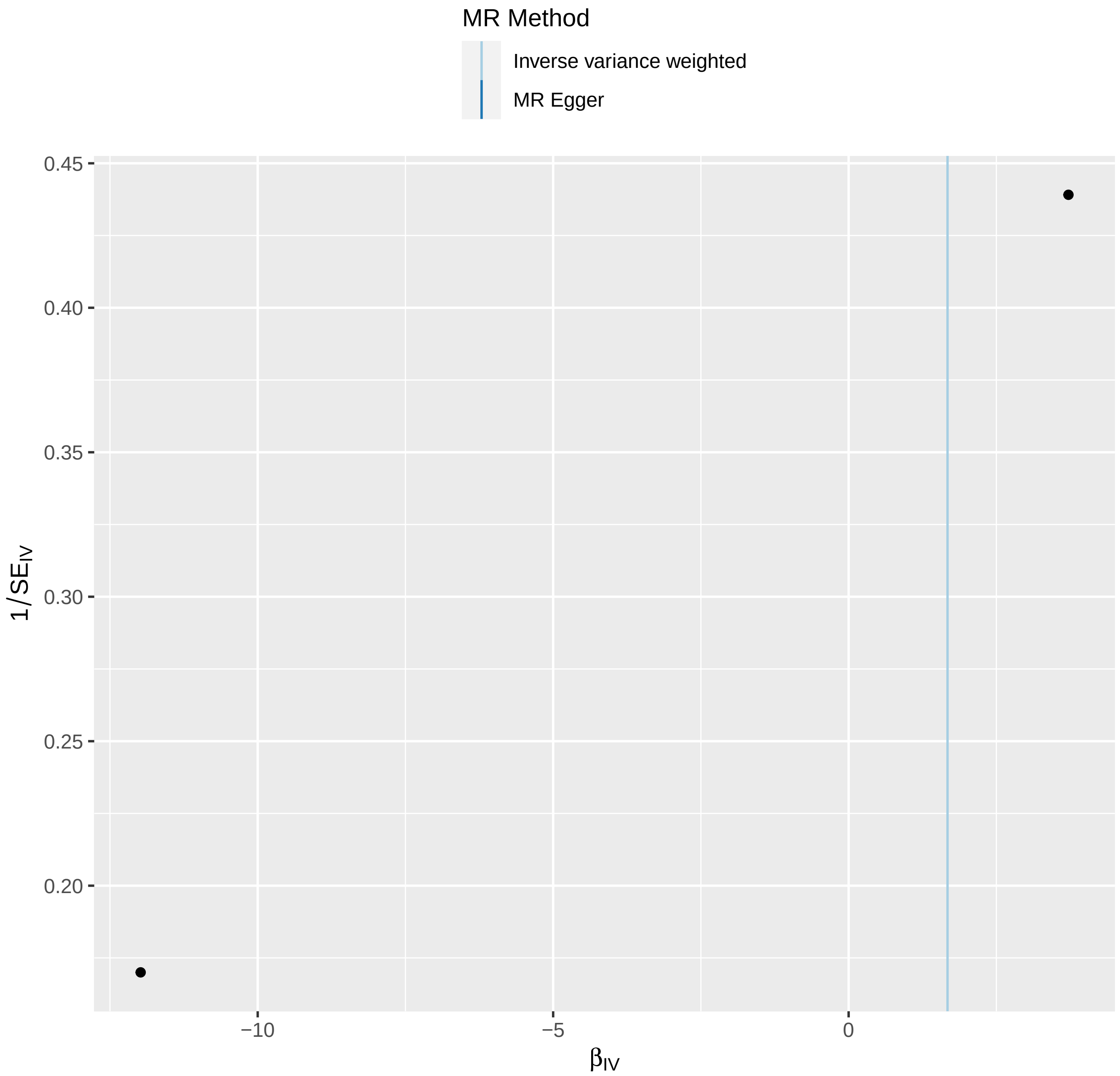

Batch 1252 : Phenol sulfate on Ulcerative colitis with PSC

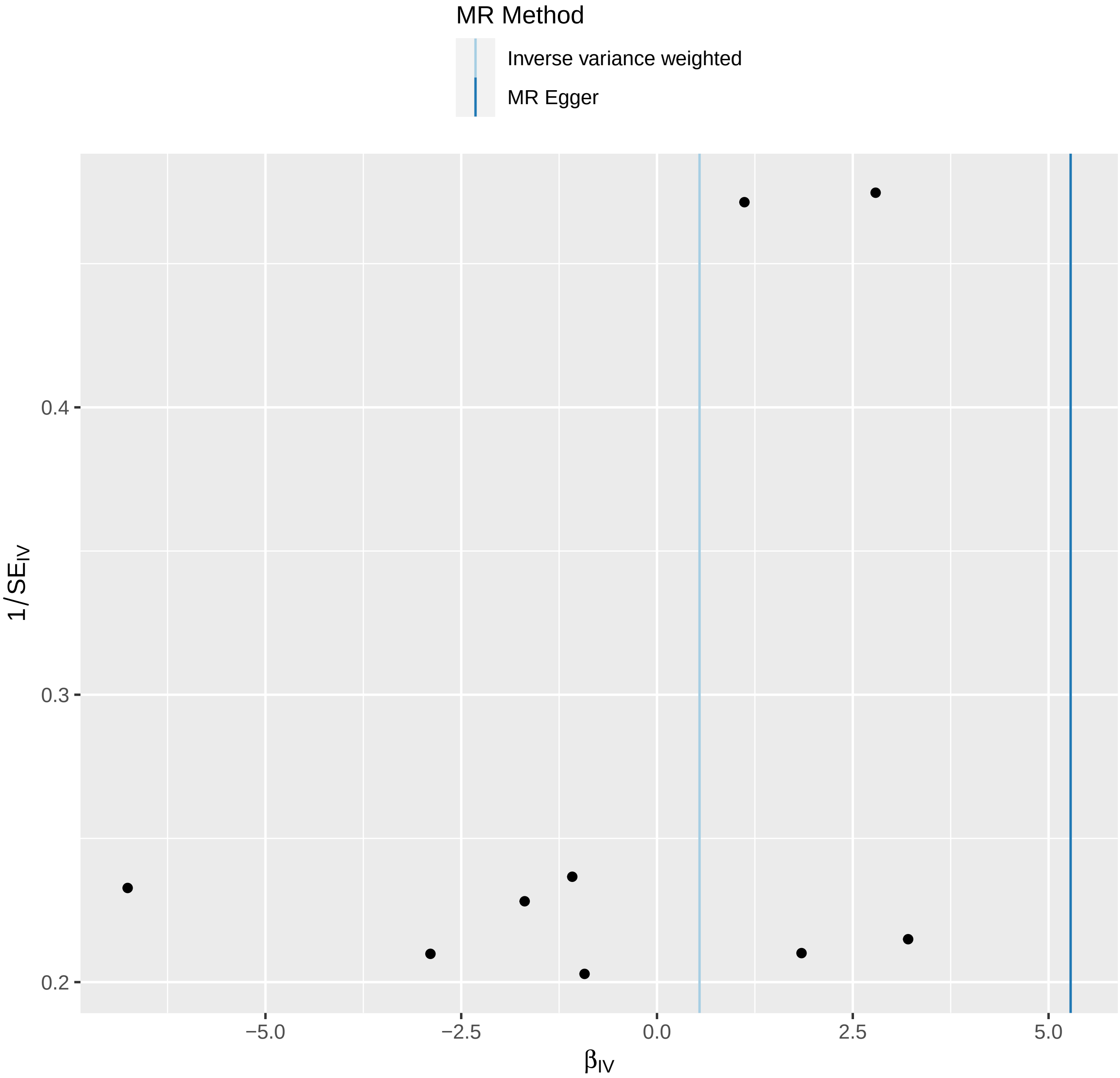

Batch 1254 : Heme* on Ulcerative colitis with PSC

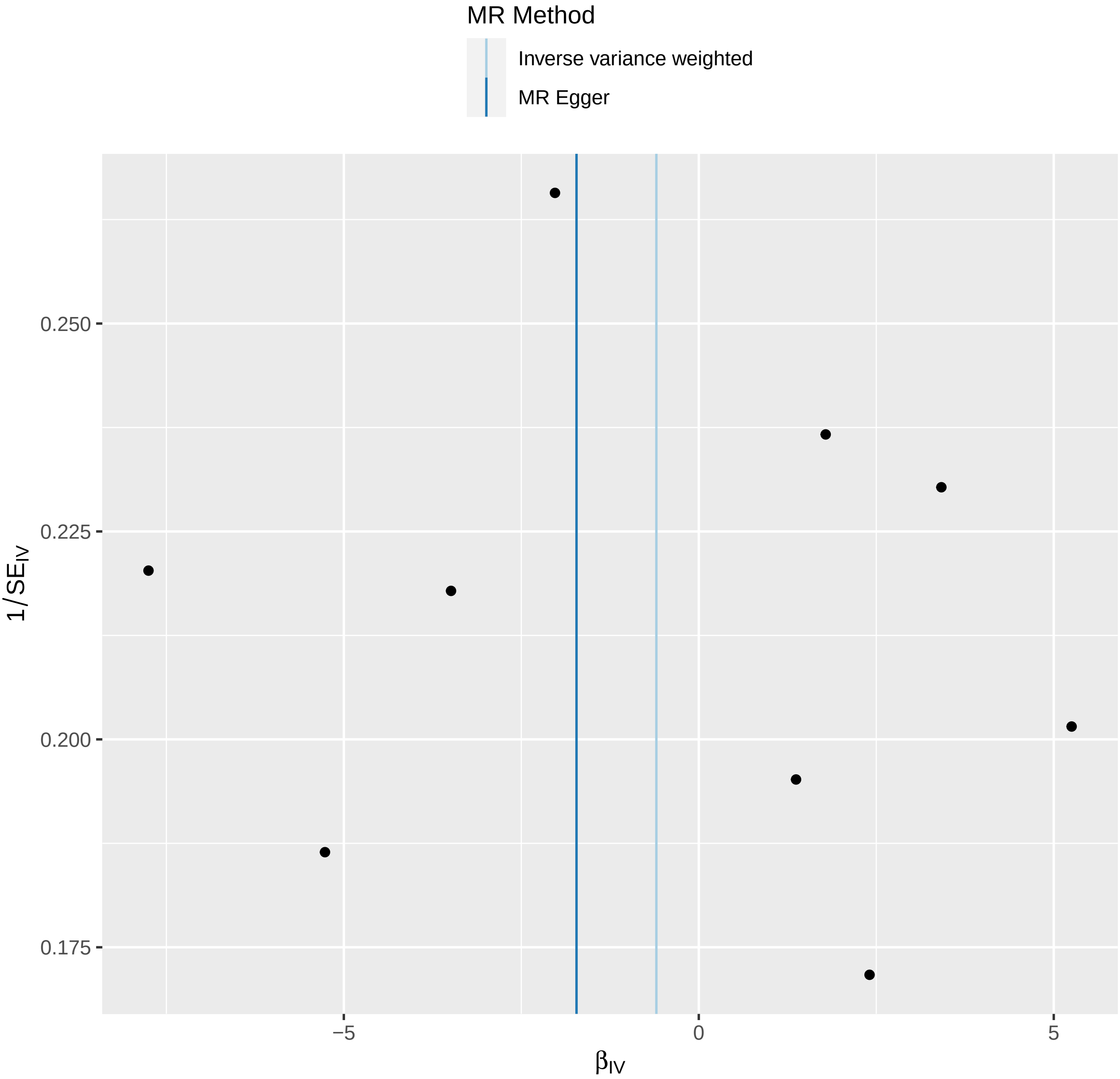

Batch 1256 : 3−dehydrocarnitine* on Ulcerative colitis with PSC

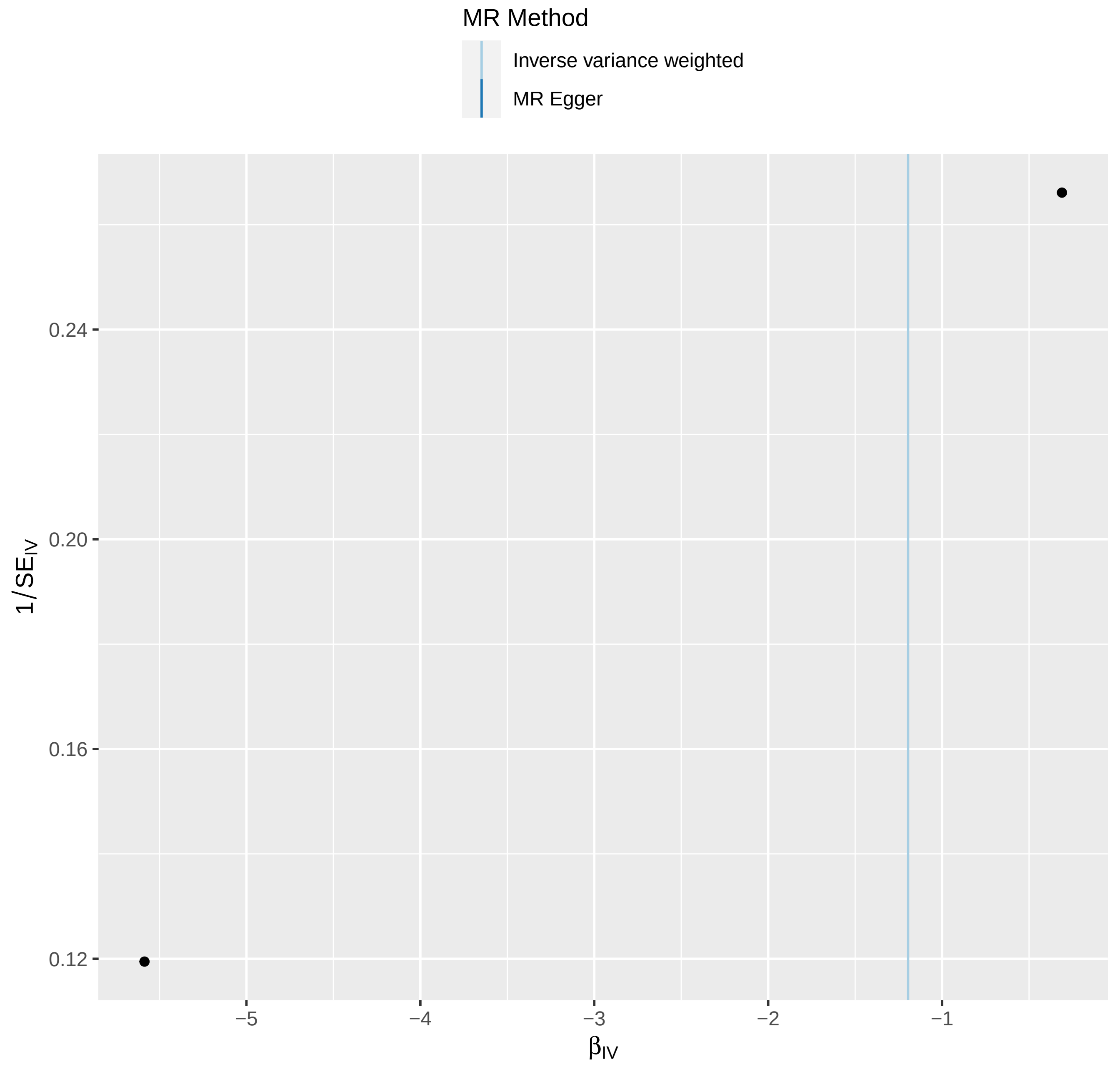

Batch 1257 : Pyroglutamine* on Ulcerative colitis with PSC

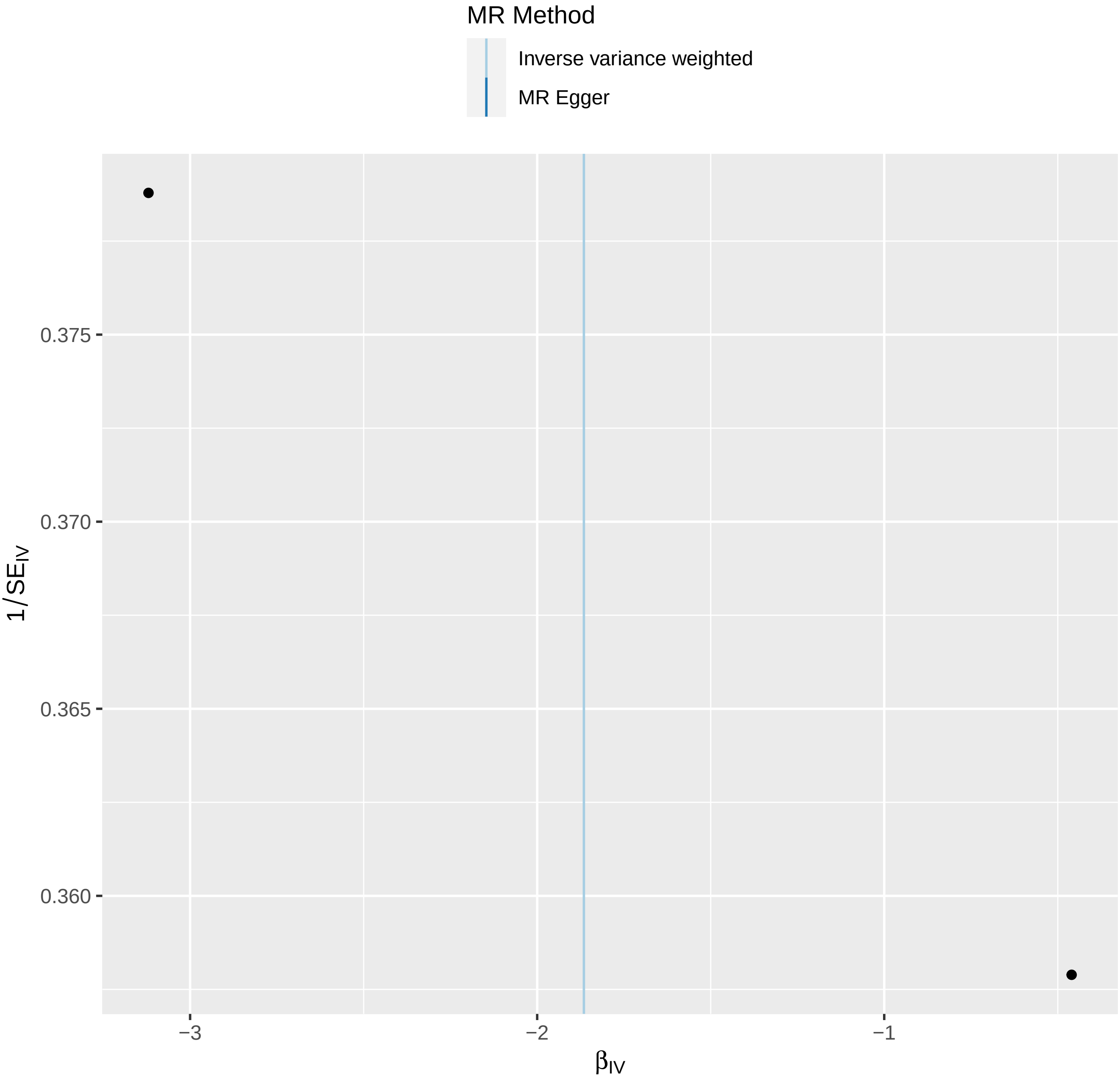

Batch 1260 : X−11422−−xanthine on Ulcerative colitis with PSC

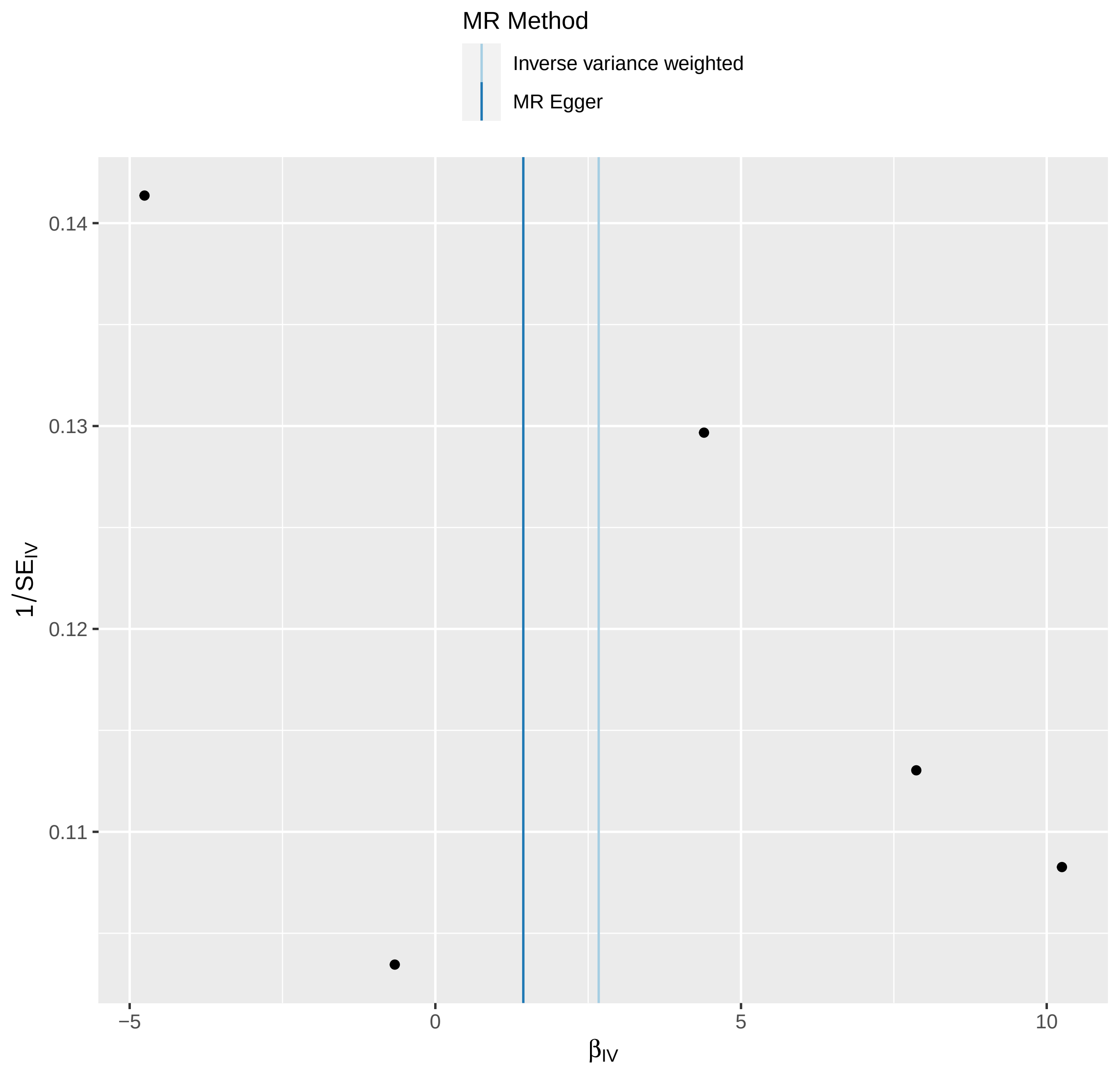

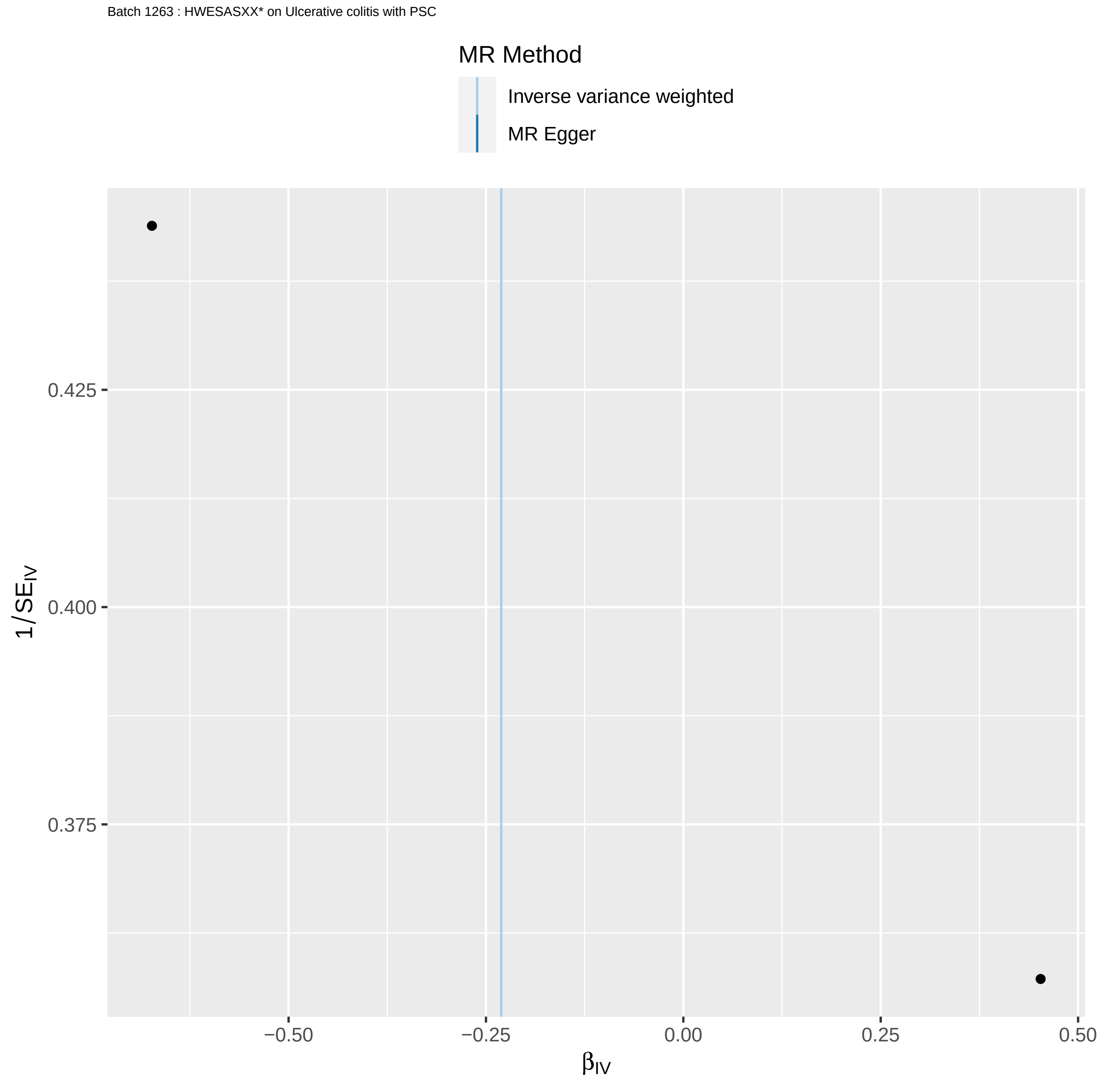

Batch 1263 : HWESASXX* on Ulcerative colitis with PSC
MR Method
Inverse variance weighted
MR Egger
0.425
0.400
0.375
1/SE$_{IV}$
−0.50
−0.25
0.00
0.25
0.50
β$_{IV}$

Batch 1264 : X−11593−−O−methylascorbate* on Ulcerative colitis with PSC

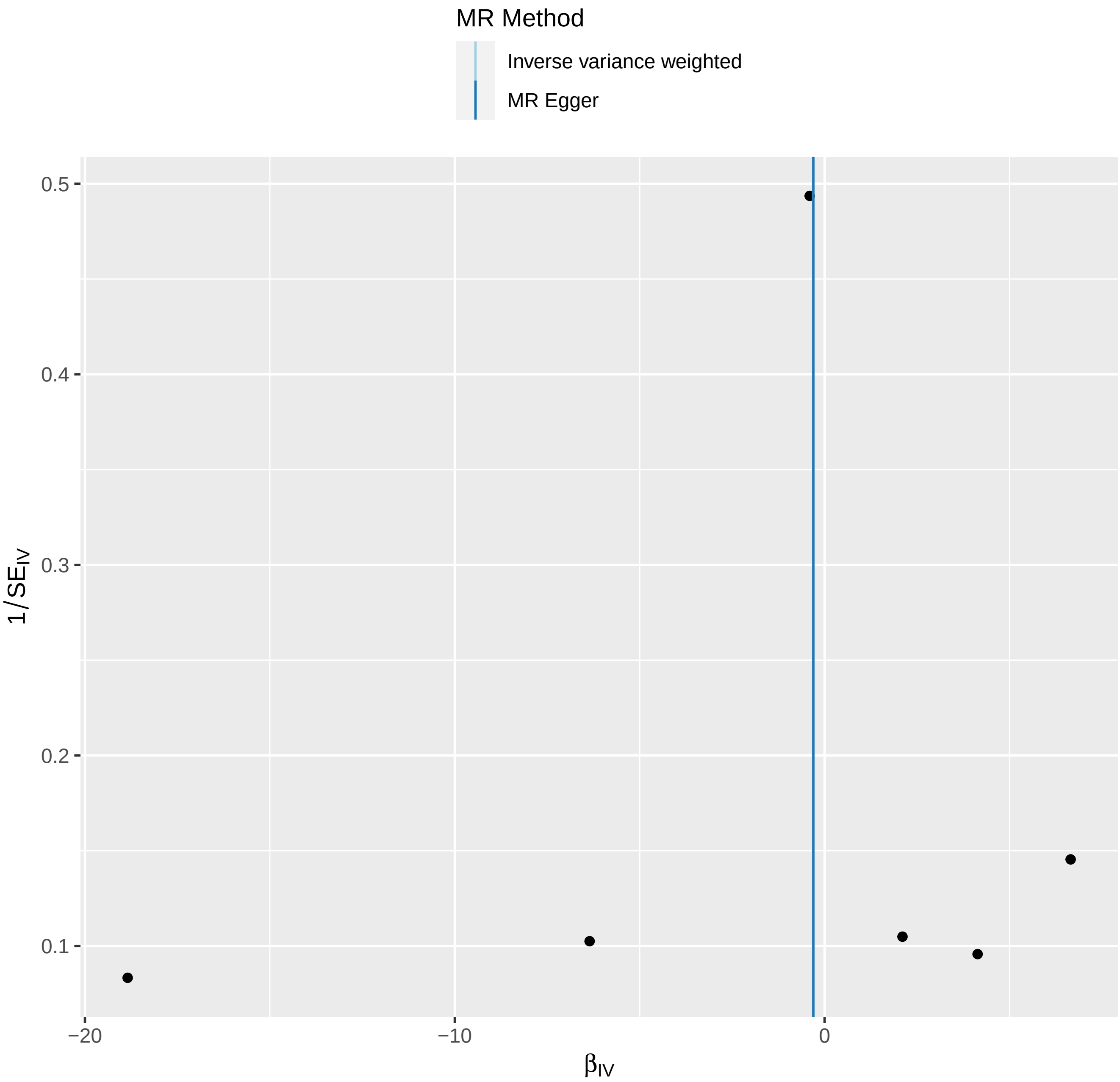

Batch 1266 : Homostachydrine* on Ulcerative colitis with PSC

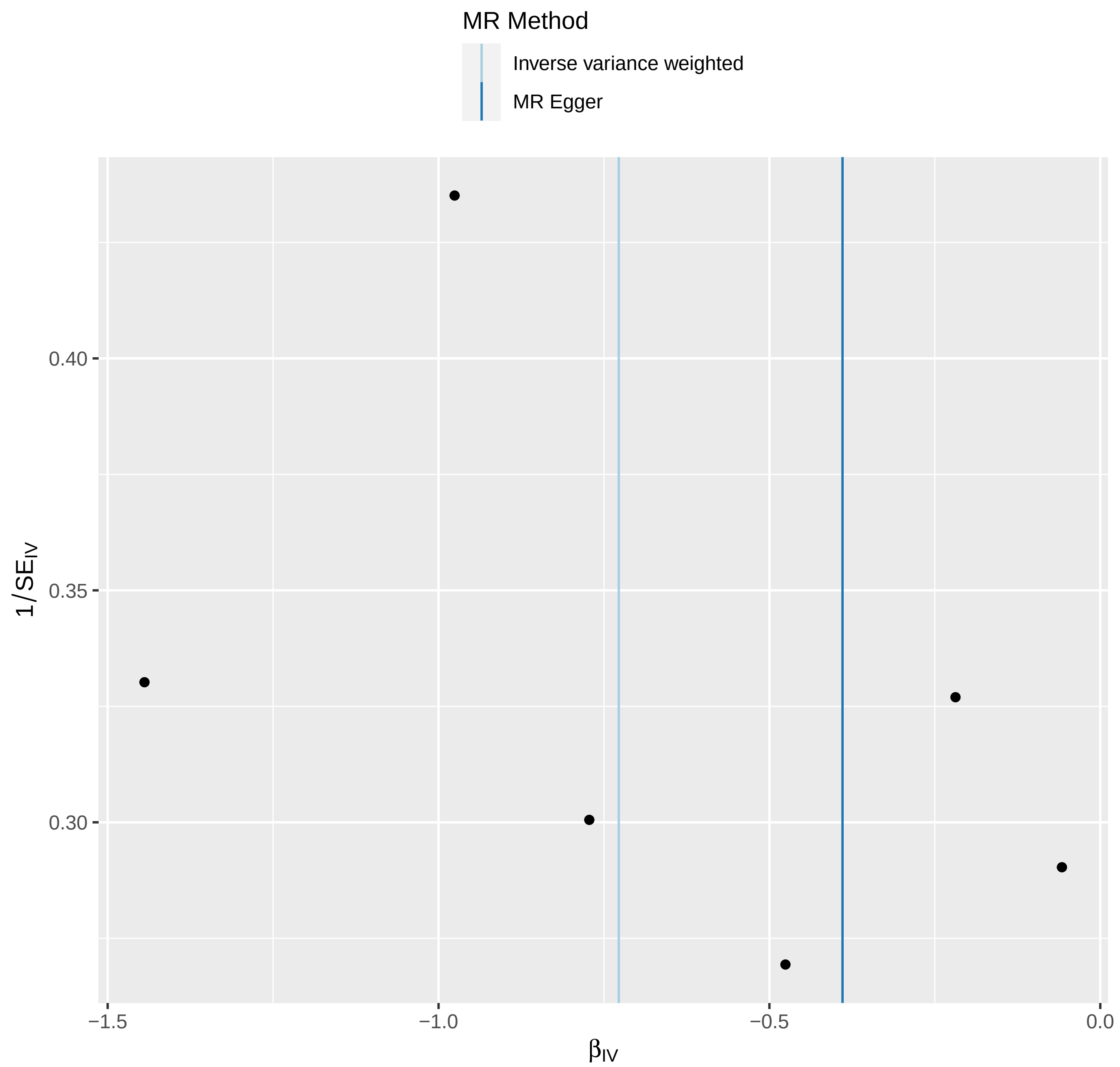

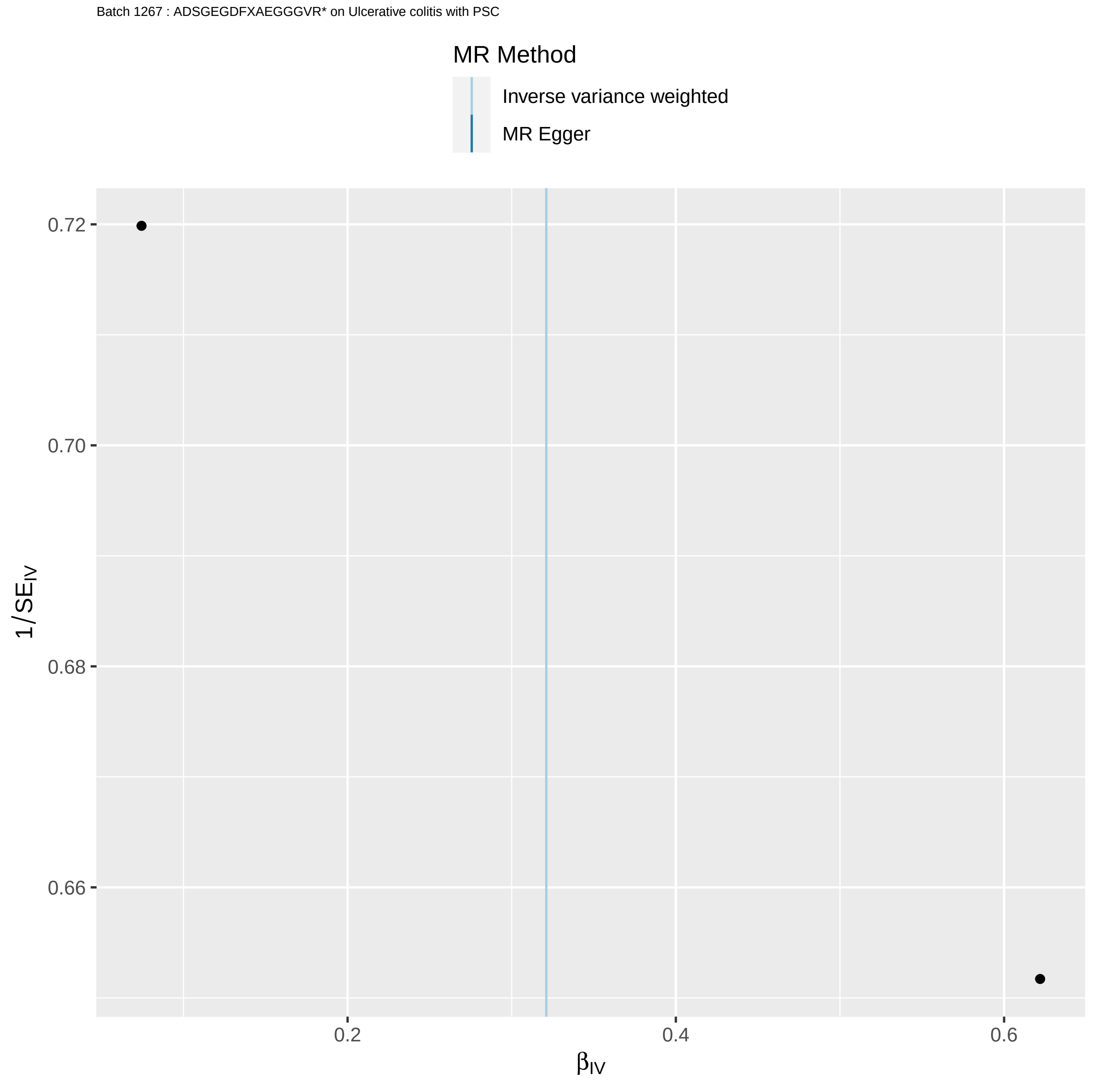
Batch 1267 : ADSGEGDFXAEGGGVR* on Ulcerative colitis with PSC
MR Method
Inverse variance weighted
MR Egger
0.72
0.70
0.68
0.66
1/SE$_{IV}$
0.2
0.4
0.6
β$_{IV}$

Batch 1269 : X−11793−−oxidized bilirubin* on Ulcerative colitis with PSC

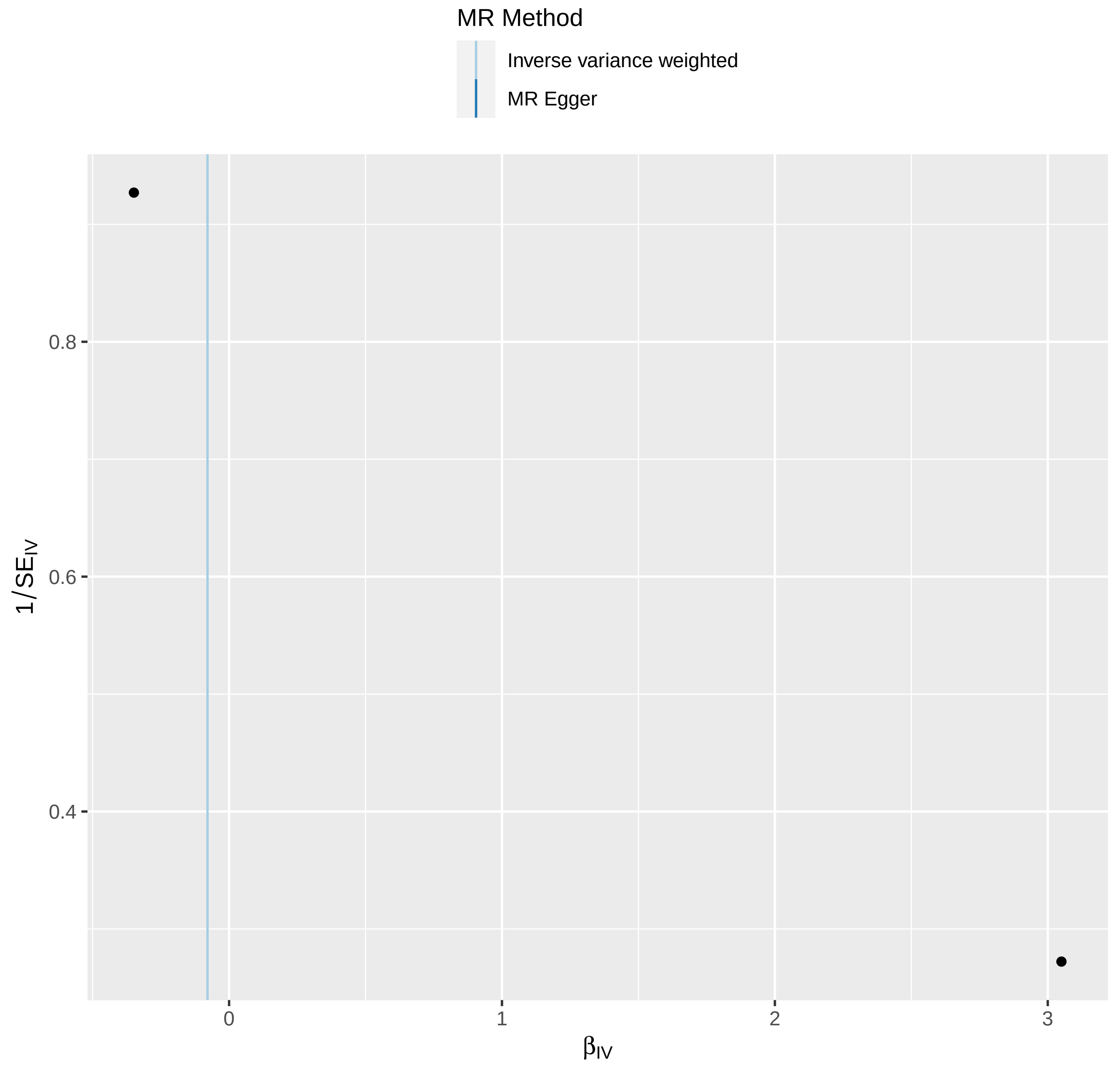

Batch 1270 : 1−arachidonoylglycerophosphocholine* on Ulcerative colitis with PSC

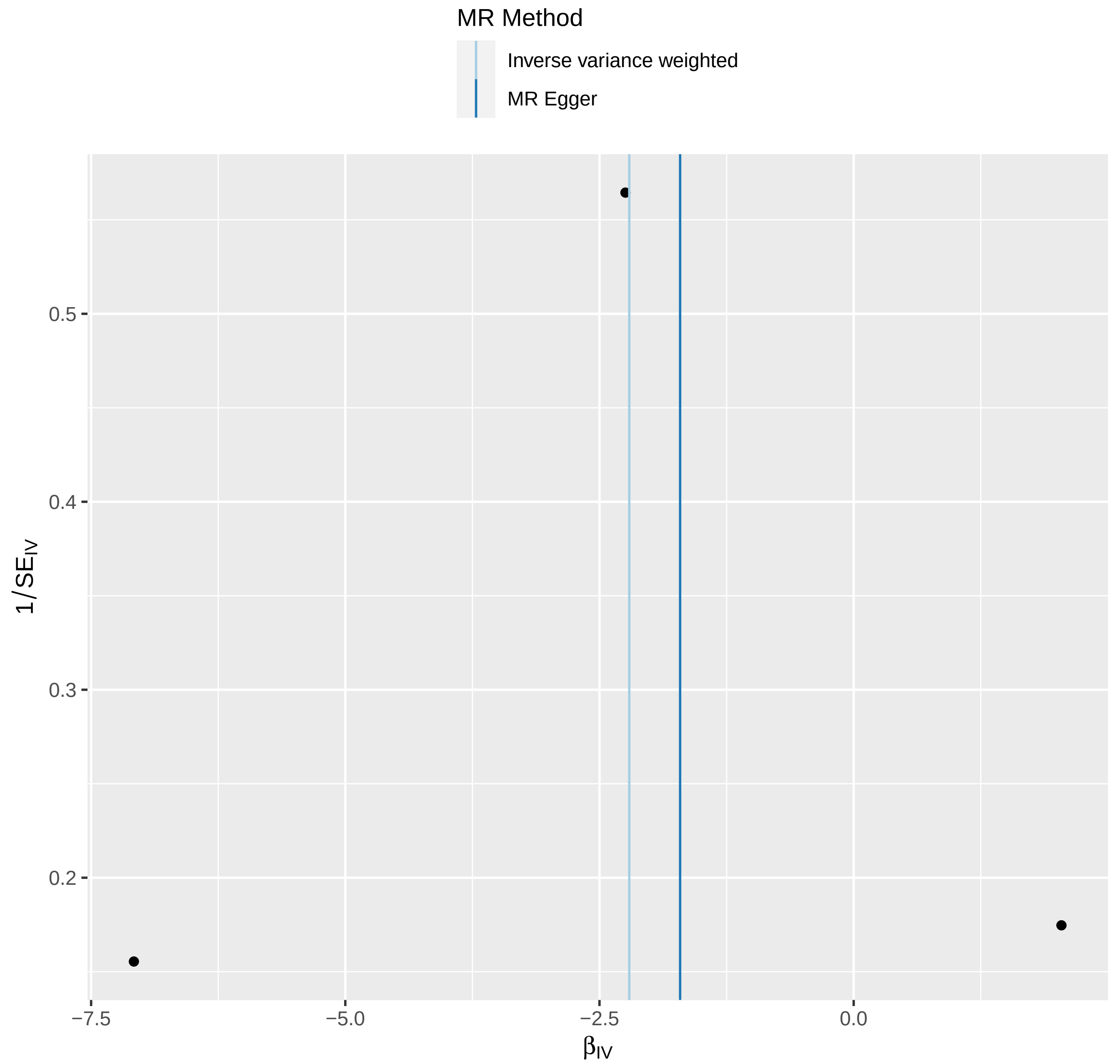

Batch 1272 : Gamma−glutamylmethionine* on Ulcerative colitis with PSC

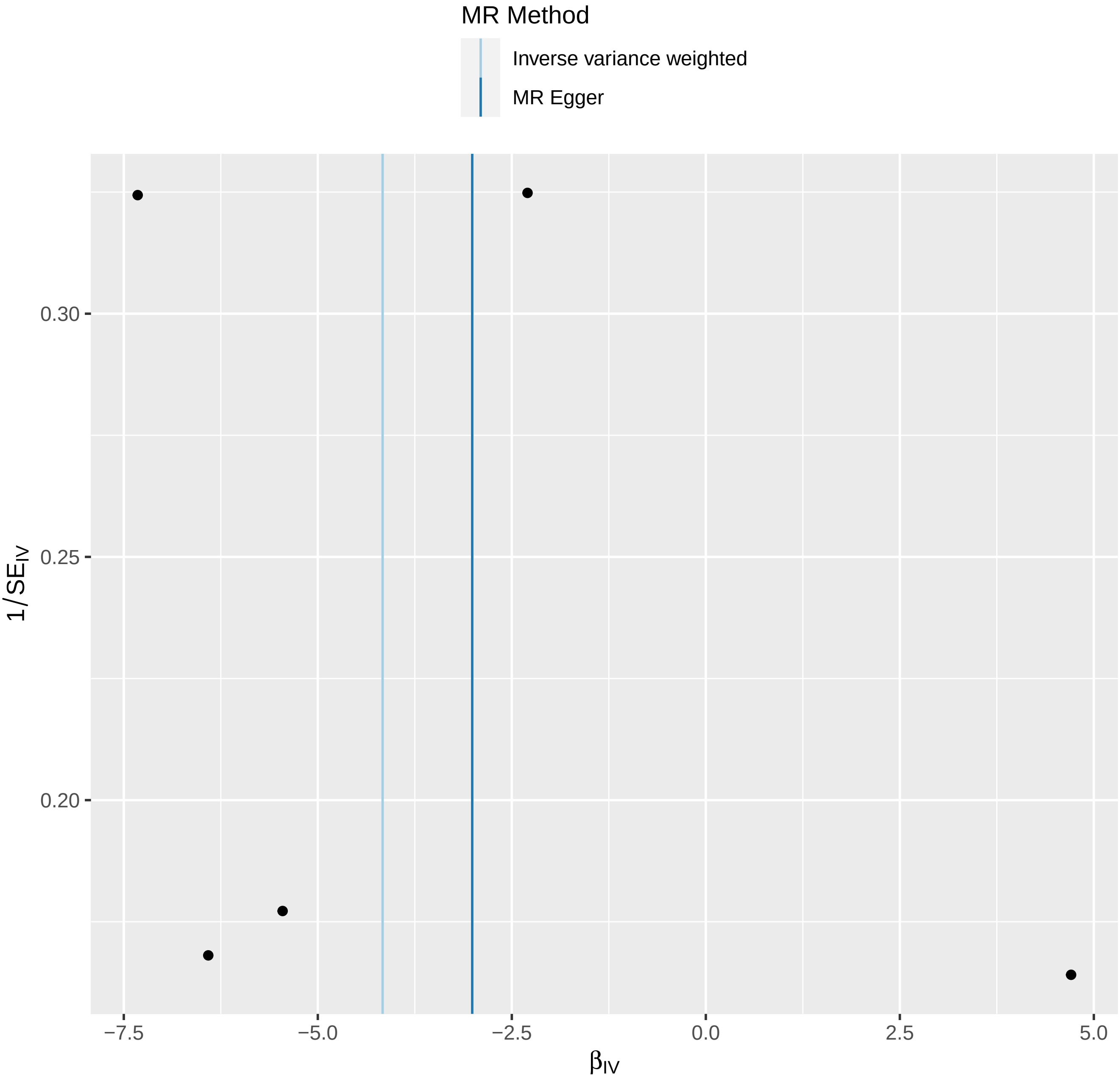

Batch 1273 : Gamma−glutamylthreonine* on Ulcerative colitis with PSC

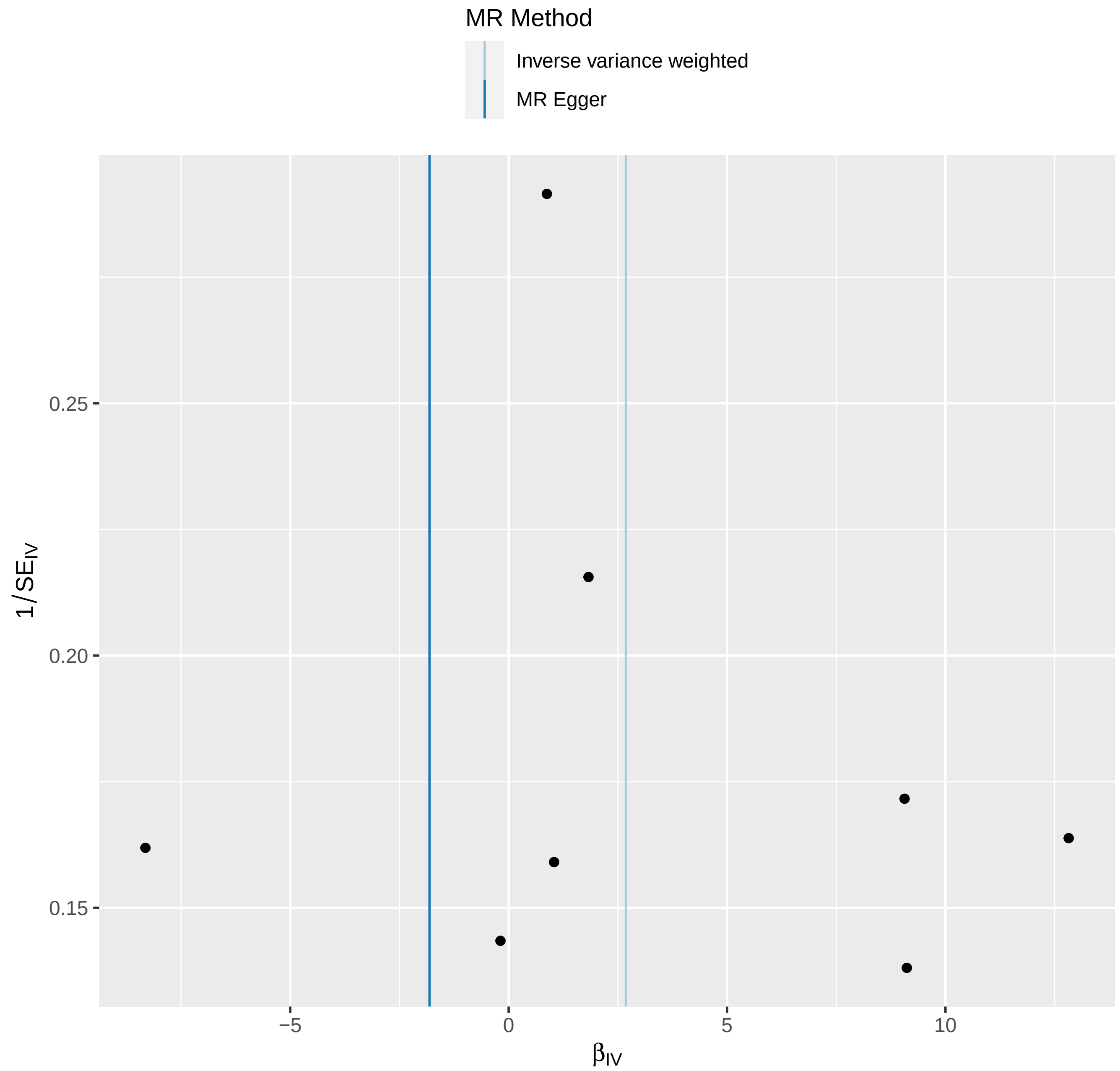

Batch 1274 : Gamma−tocopherol on Ulcerative colitis with PSC

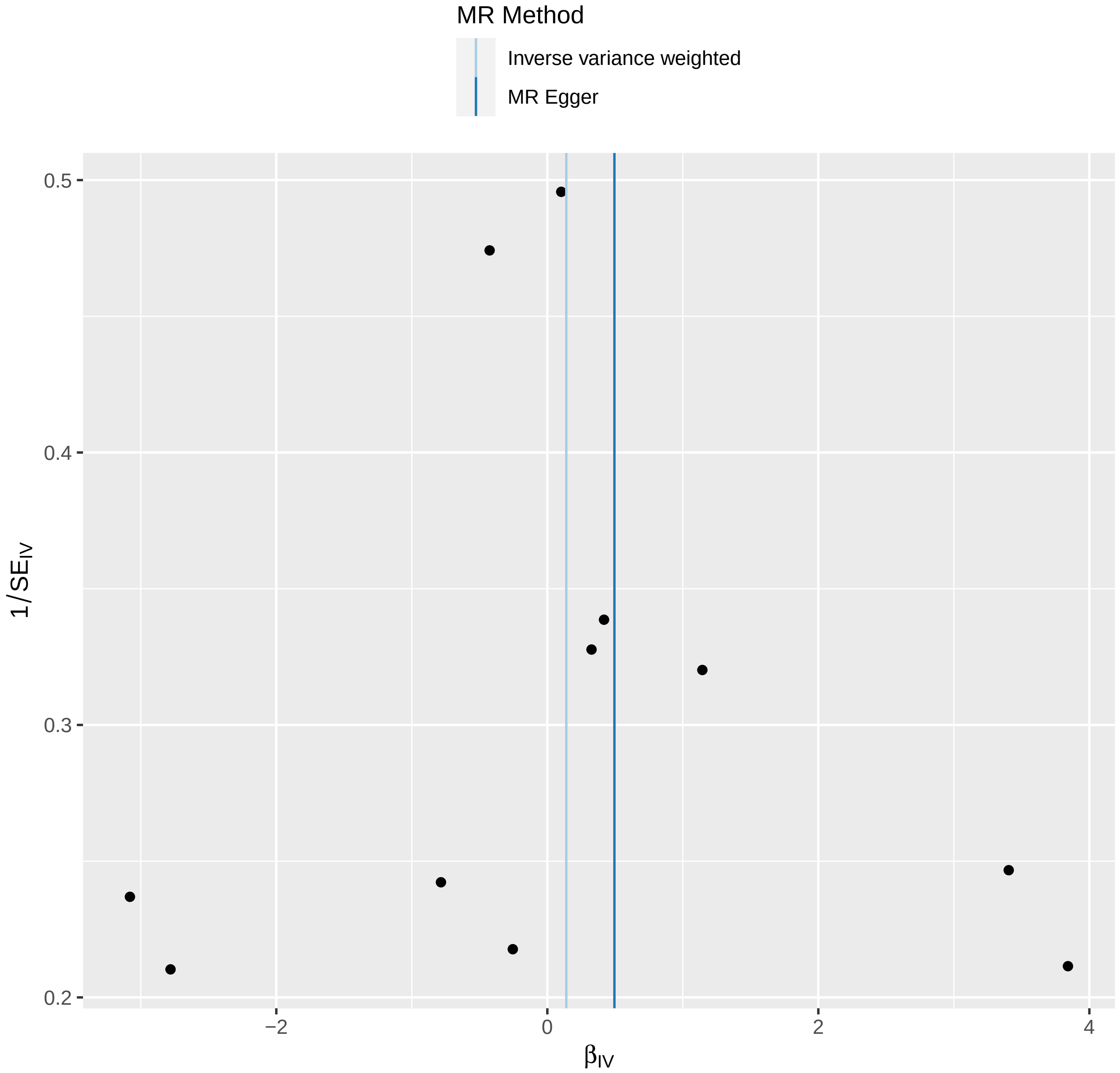

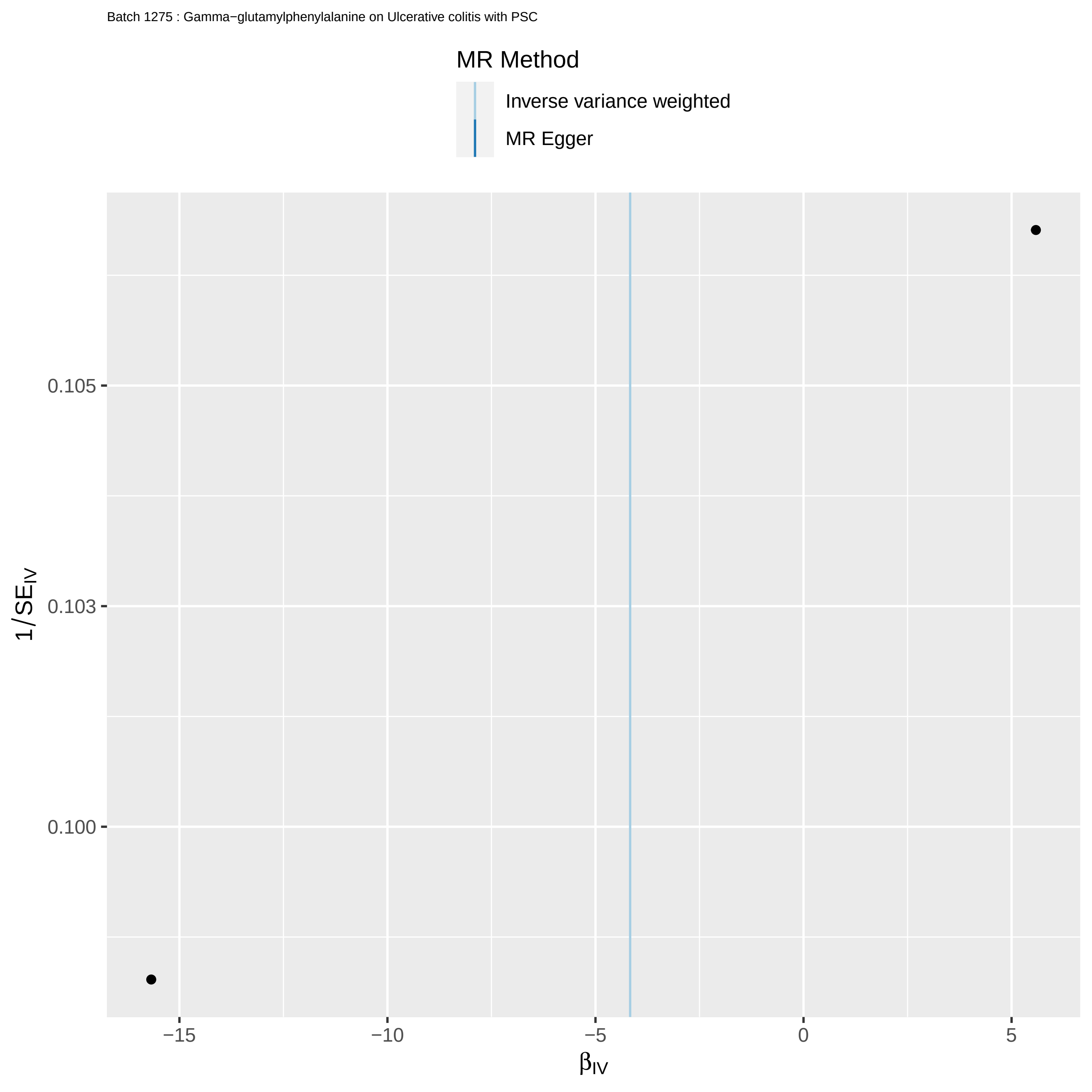
Batch 1275 : Gamma−glutamylphenylalanine on Ulcerative colitis with PSC
MR Method
Inverse variance weighted
MR Egger
0.105
0.103
0.100
−15
−10
−5
0
5
$1/SE_{IV}$
β_{IV}

Batch 1276 : Isobutyrylcarnitine on Ulcerative colitis with PSC

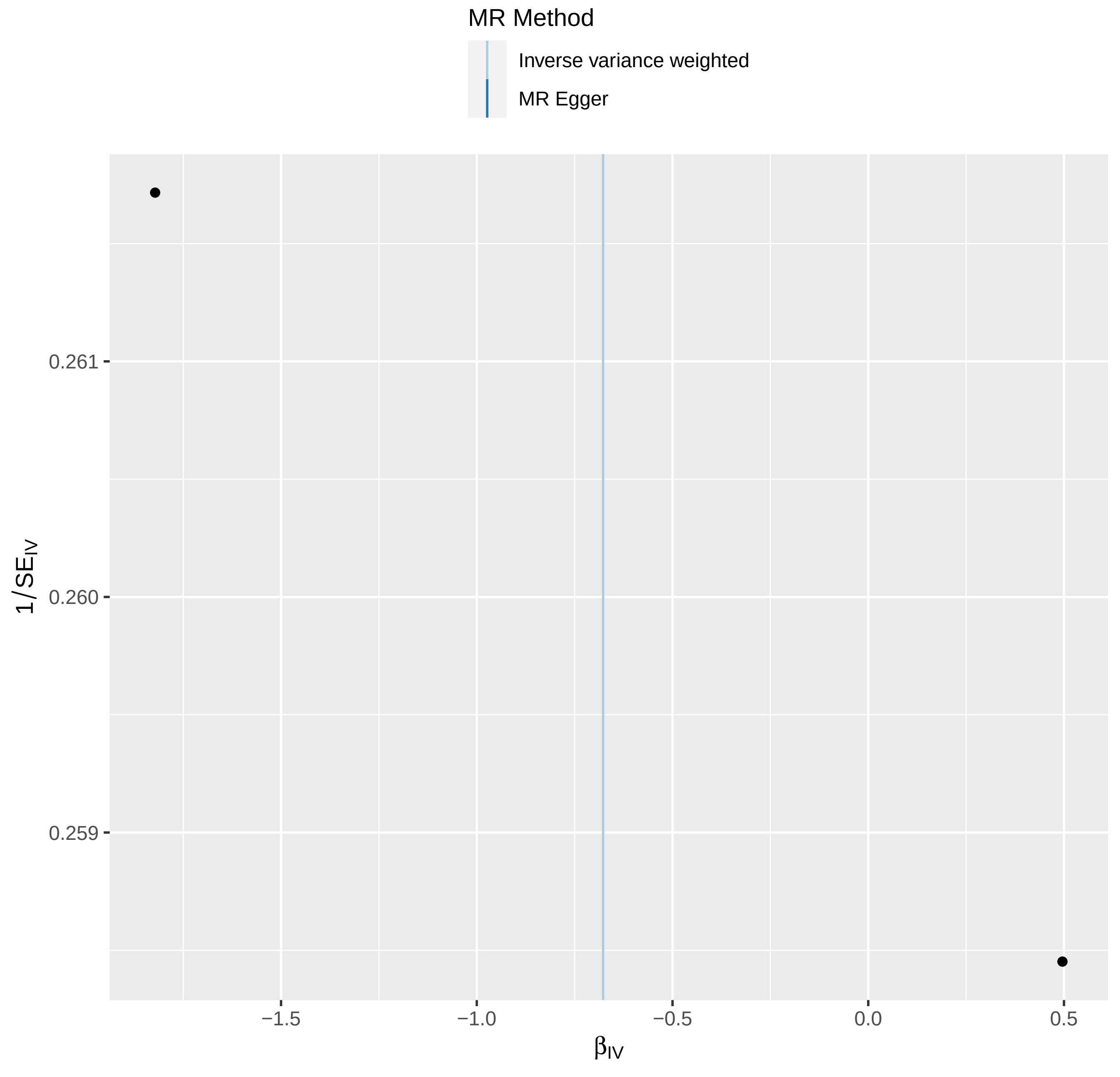

Batch 1277 : Pseudouridine on Ulcerative colitis with PSC

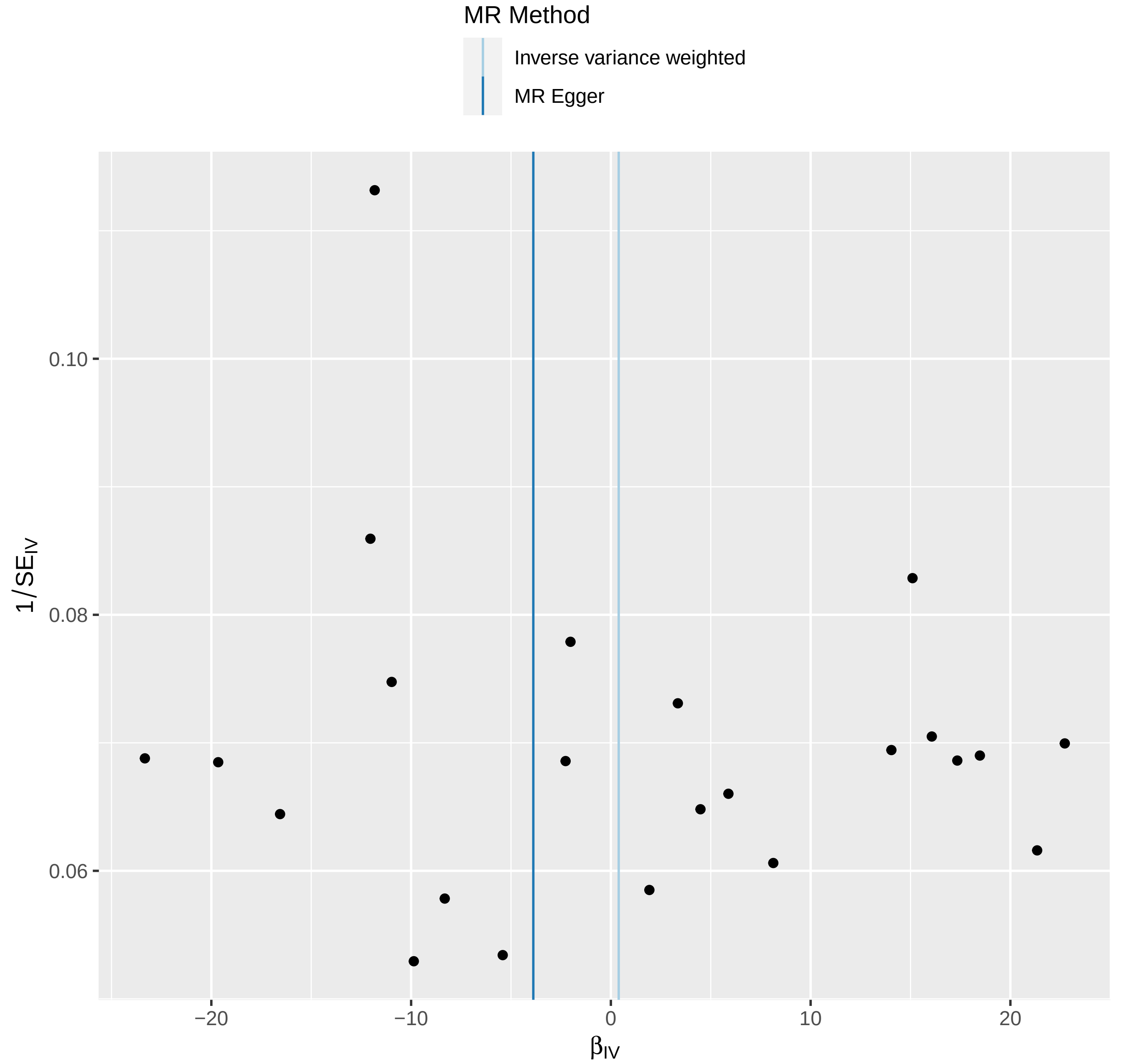

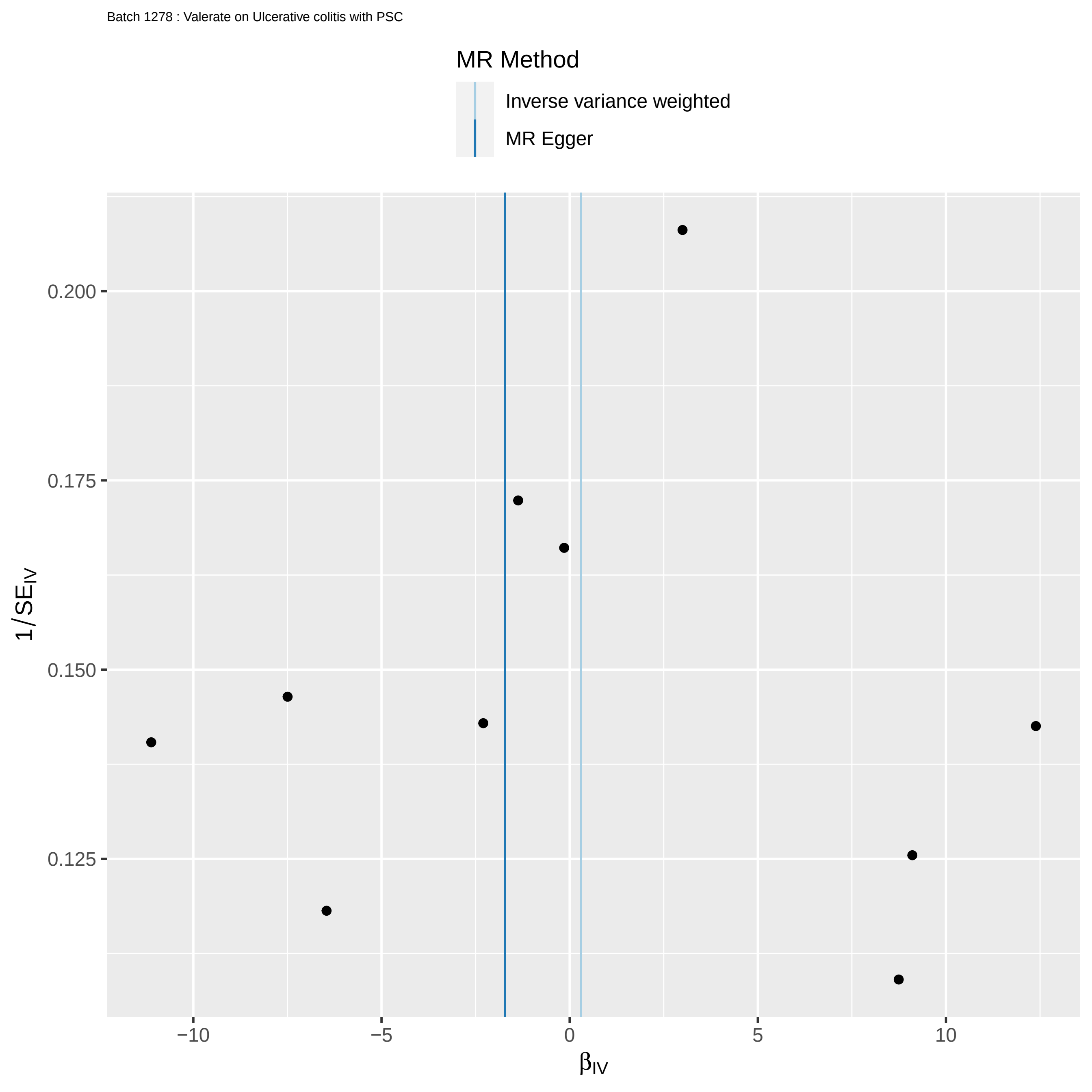

Batch 1278 : Valerate on Ulcerative colitis with PSC
MR Method
Inverse variance weighted
MR Egger
0.200
0.175
0.150
0.125
1/SE_IV
−10
−5
0
5
10
β_IV

Batch 1281 : Erythronate* on Ulcerative colitis with PSC

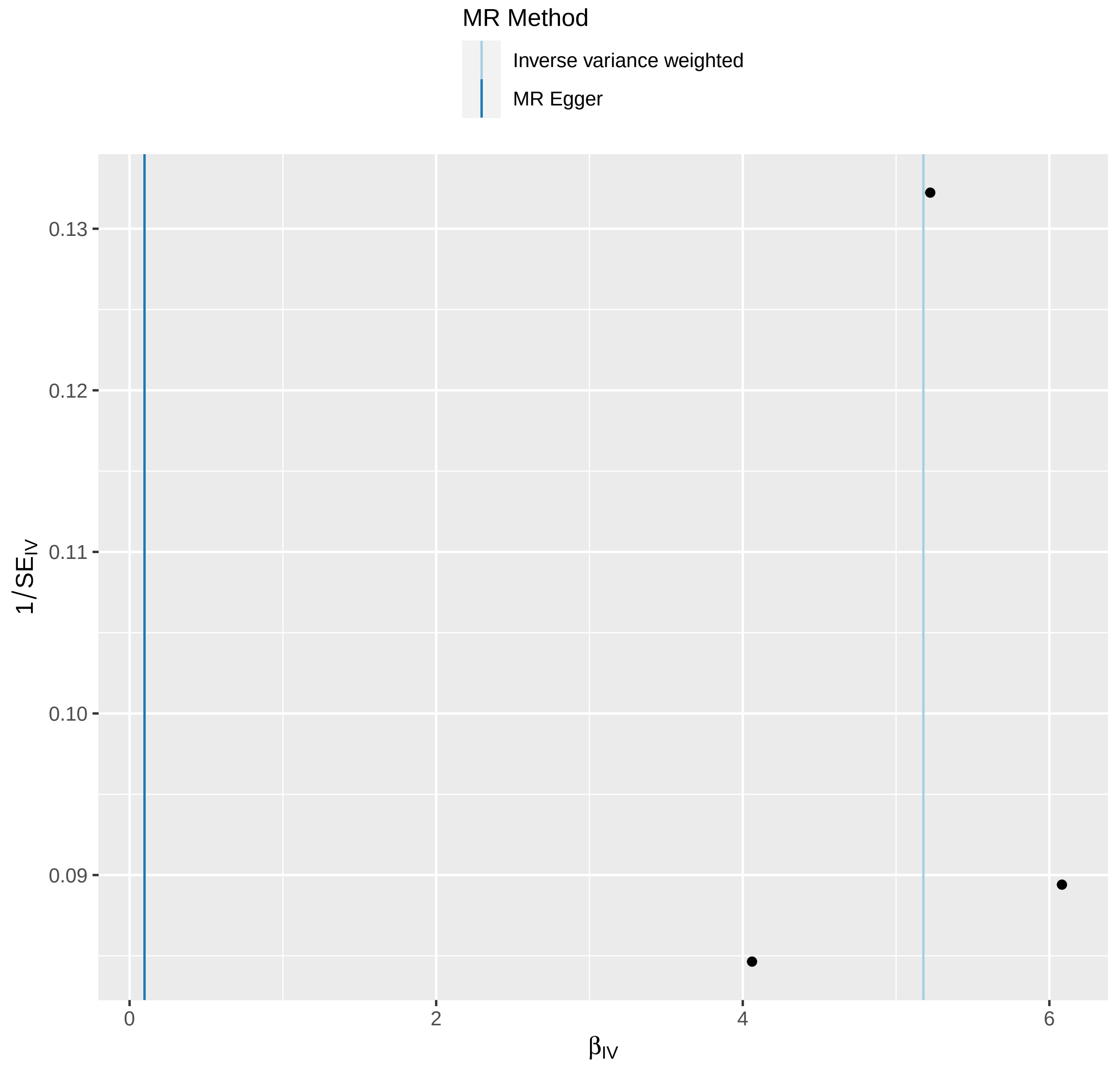

Batch 1282 : Lathosterol on Ulcerative colitis with PSC

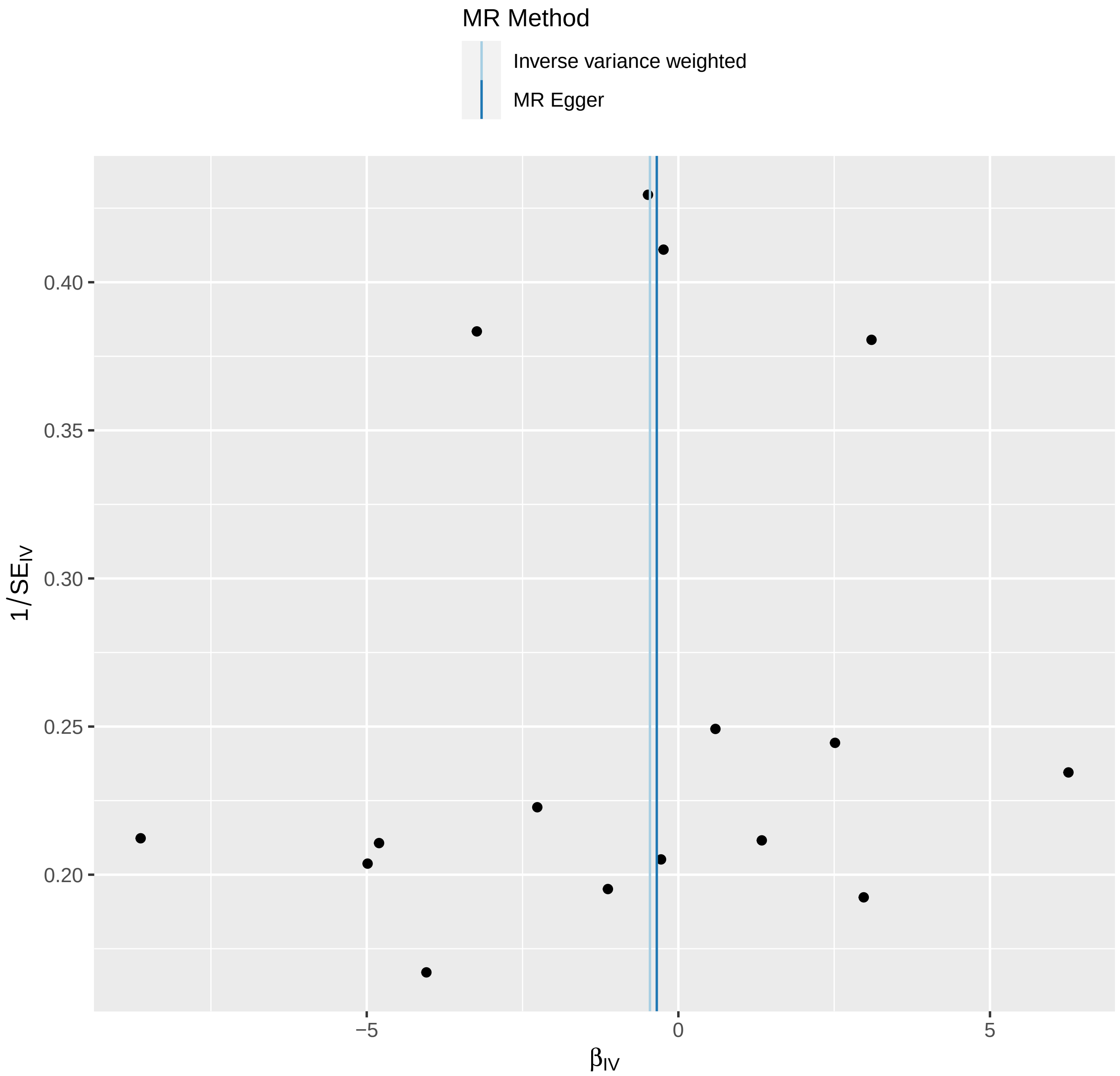

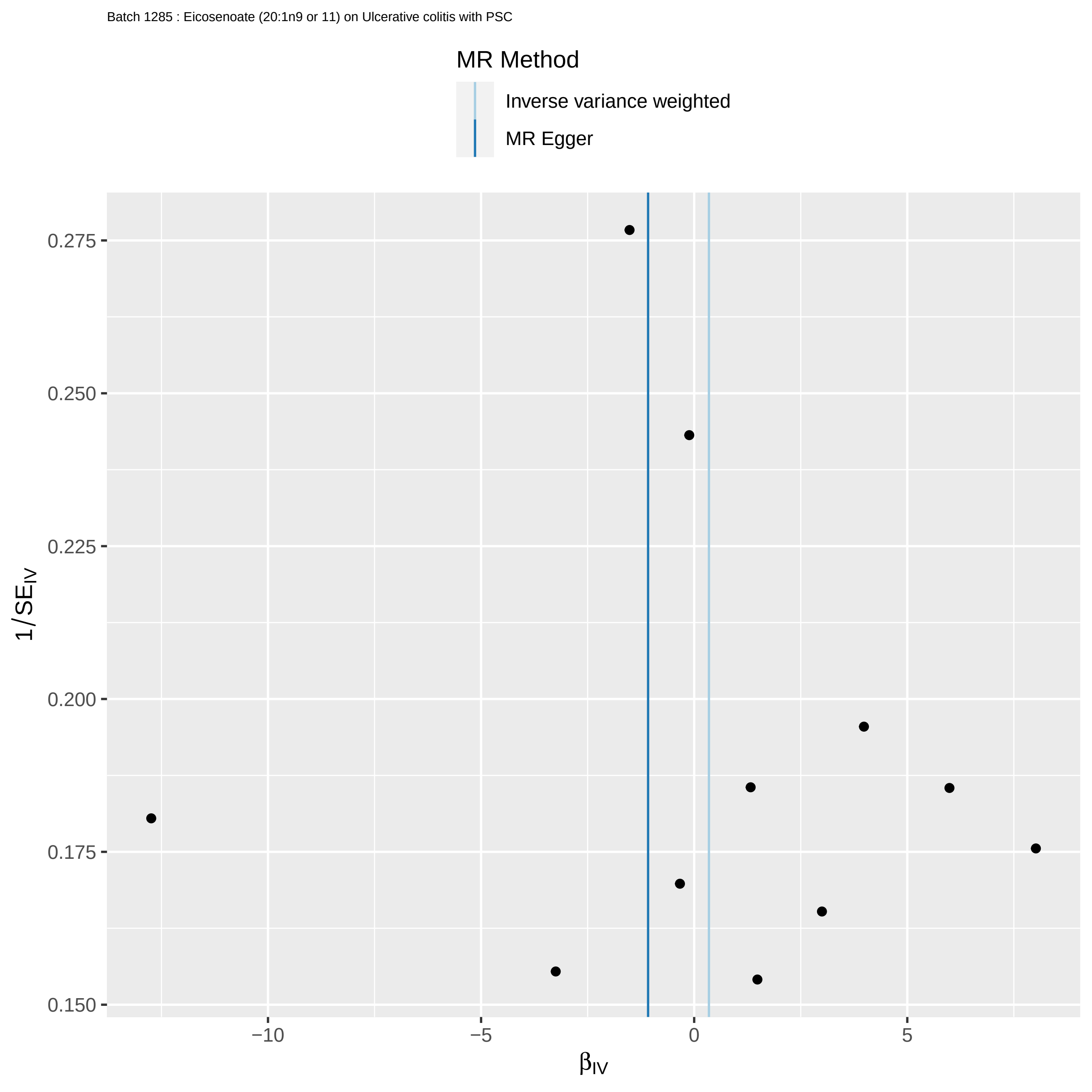
Batch 1285 : Eicosenoate (20:1n9 or 11) on Ulcerative colitis with PSC
MR Method
Inverse variance weighted
MR Egger
0.275
0.250
0.225
0.200
0.175
0.150
1/SE$_{IV}$
−10
−5
0
5
β_{IV}

Batch 1286 : X−12244−−N−acetylcarnosine on Ulcerative colitis with PSC

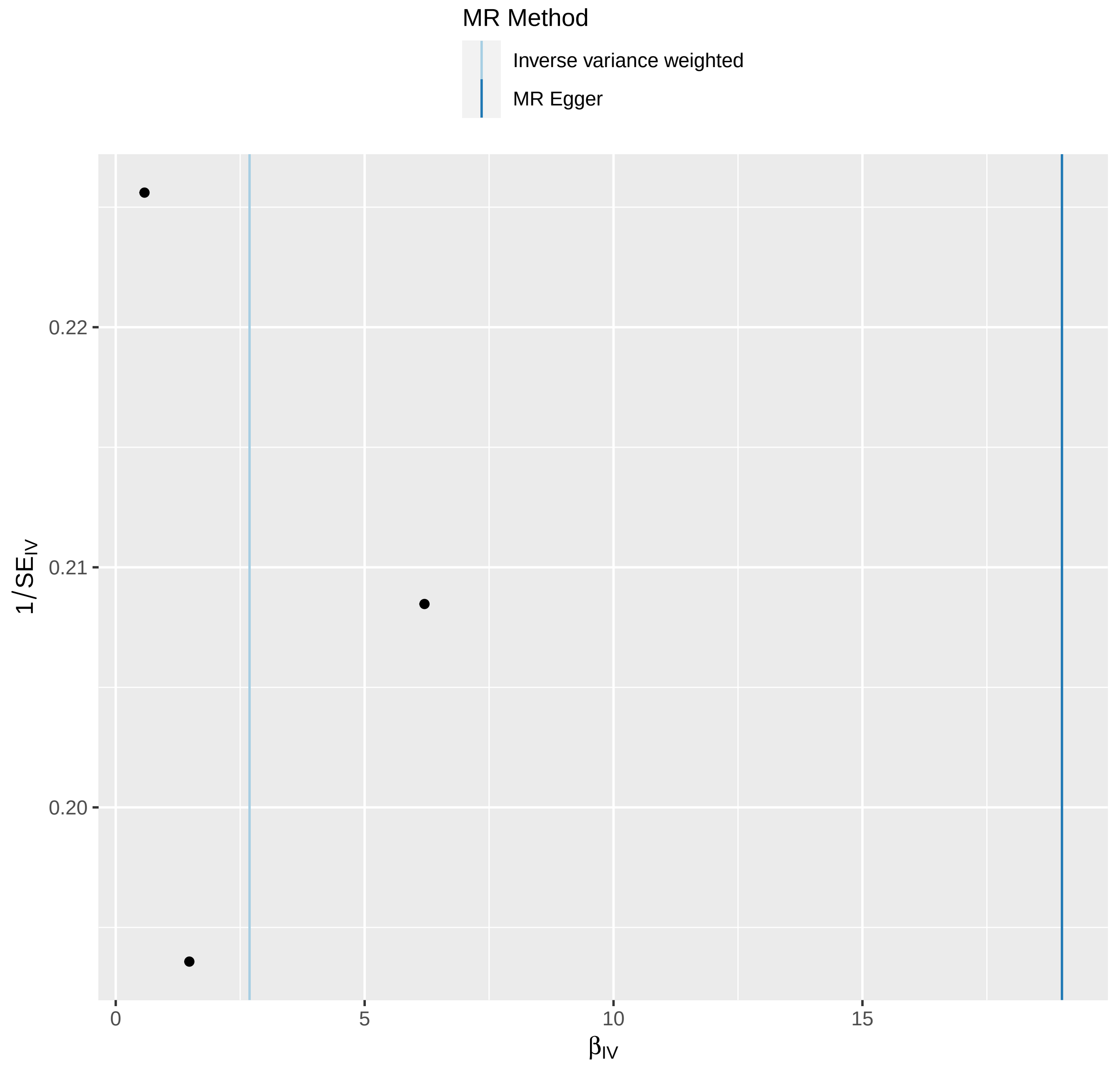

Batch 1288 : 1−eicosatrienoylglycerophosphocholine* on Ulcerative colitis with PSC

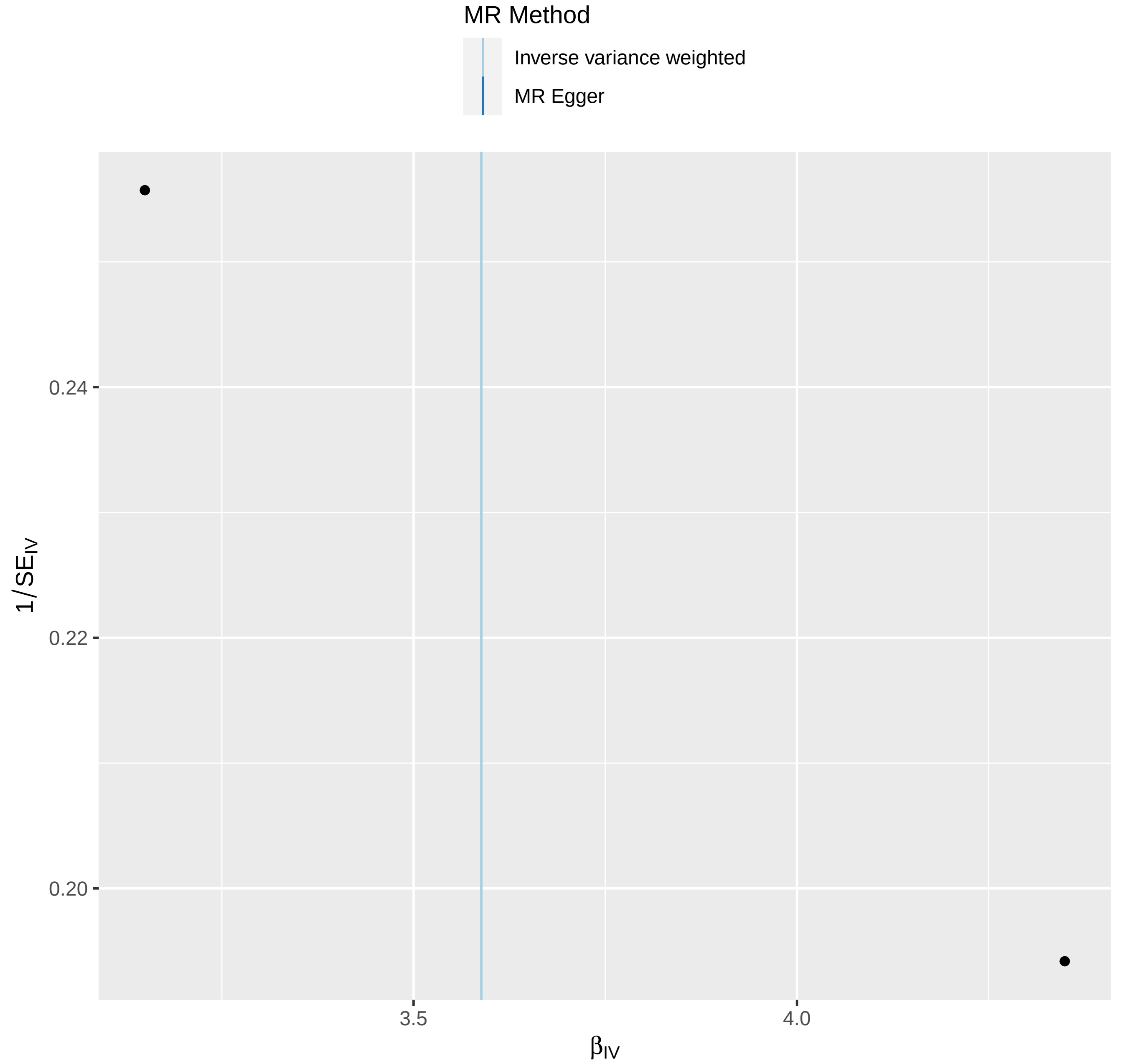

Batch 1289 : 1−docosahexaenoylglycerophosphocholine* on Ulcerative colitis with PSC

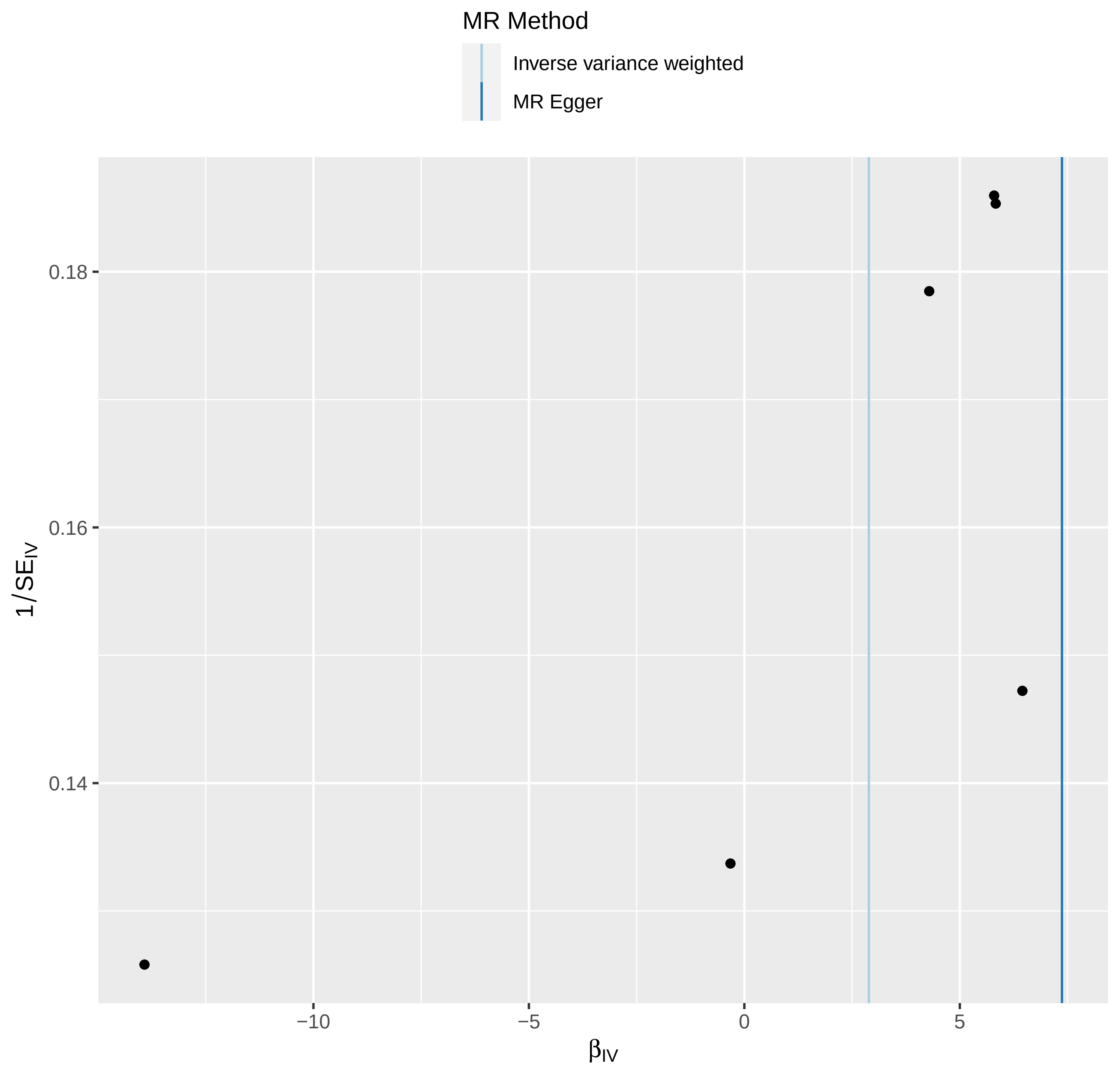

Batch 1293 : Octanoylcarnitine on Ulcerative colitis with PSC

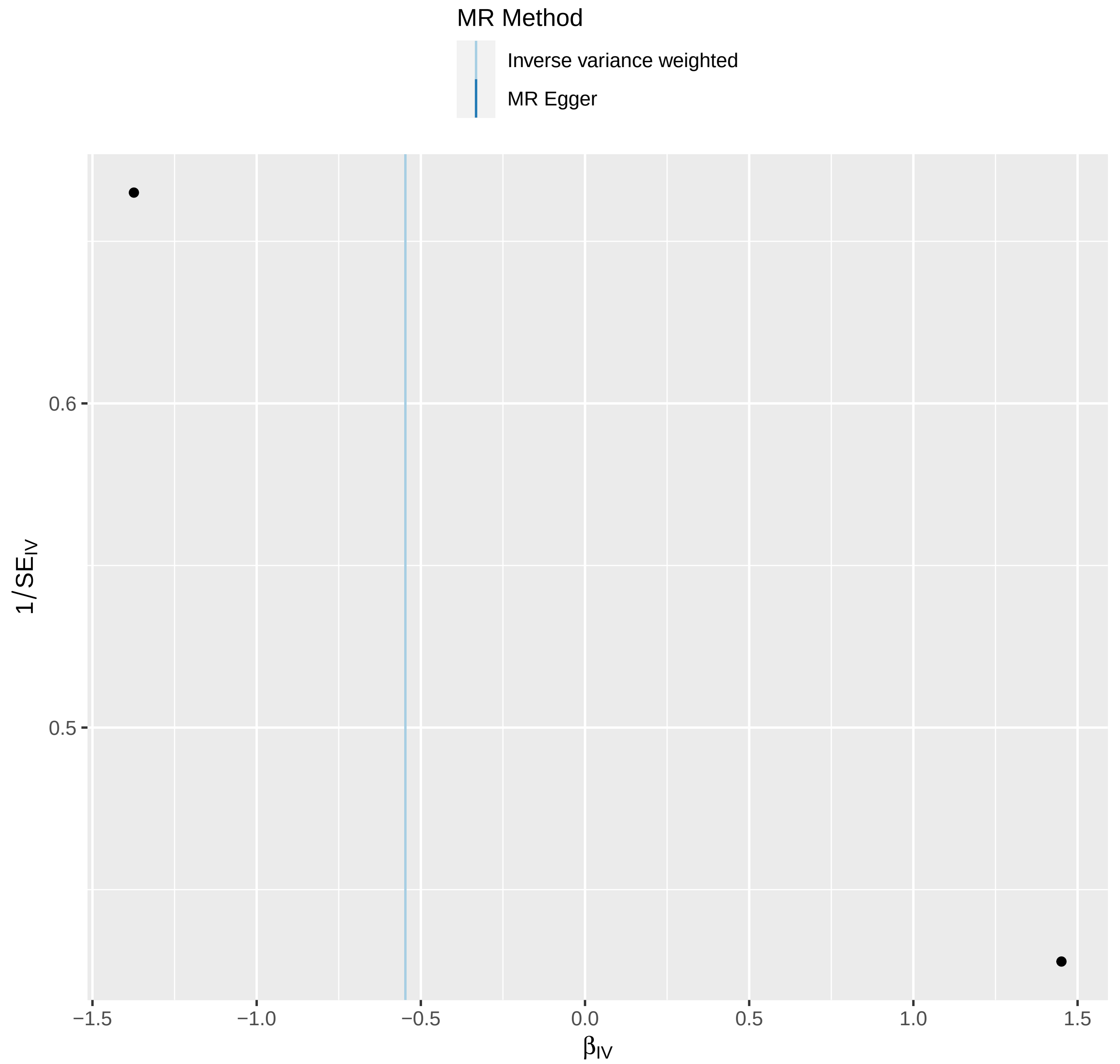

Batch 1294 : Alpha−hydroxyisovalerate on Ulcerative colitis with PSC

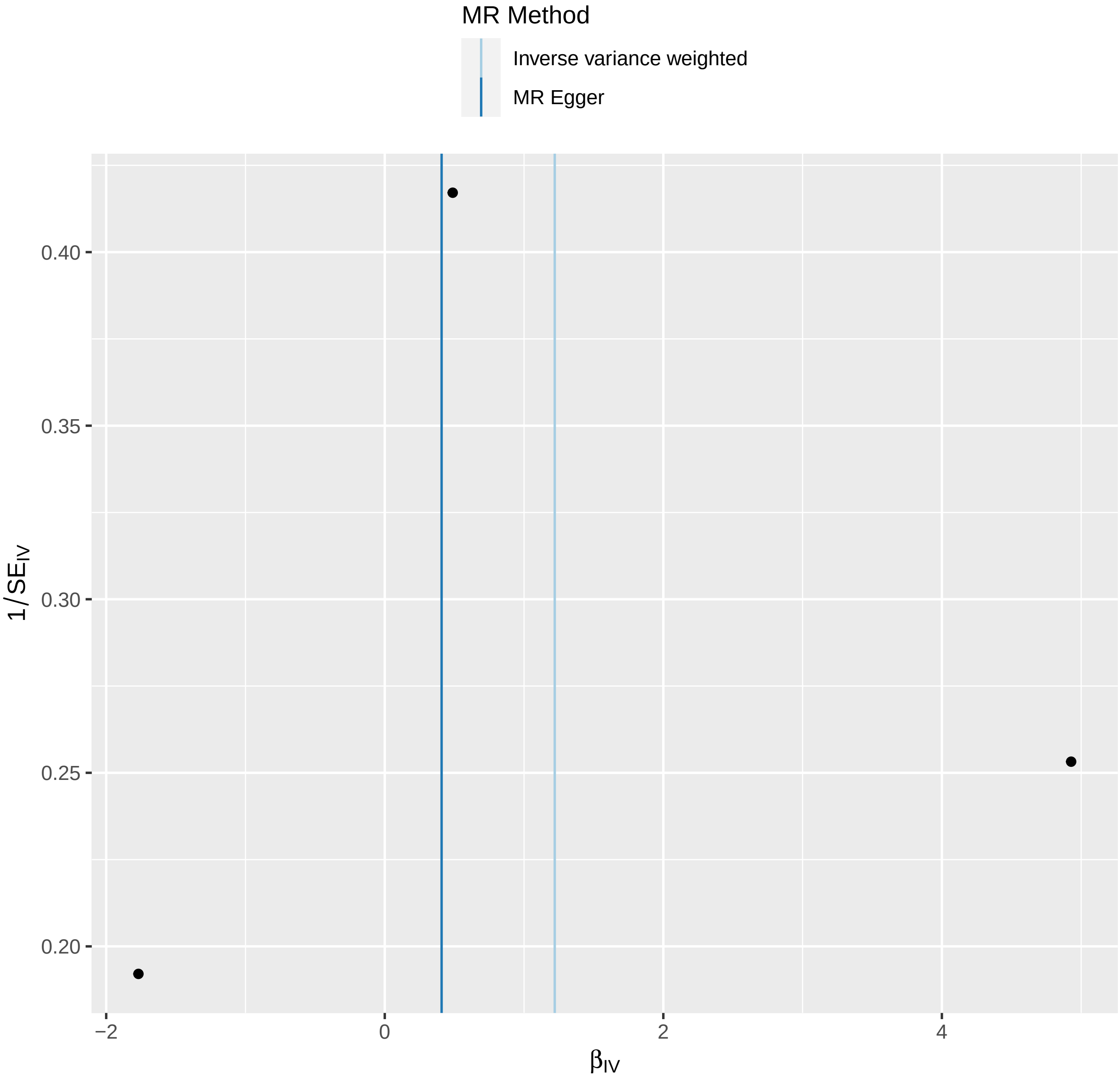

Batch 1295 : N−acetylthreonine on Ulcerative colitis with PSC

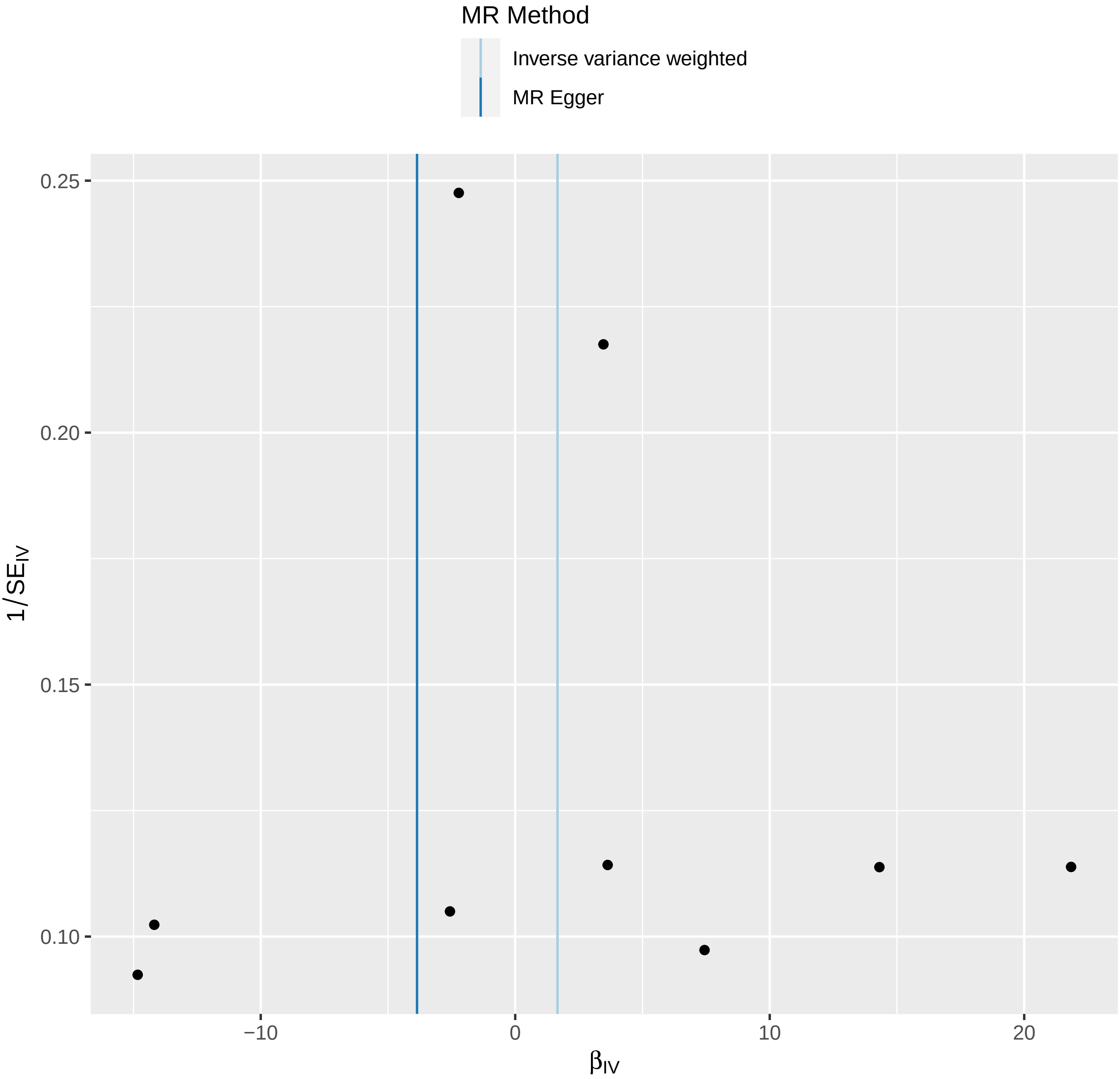

Batch 1296 : Decanoylcarnitine on Ulcerative colitis with PSC

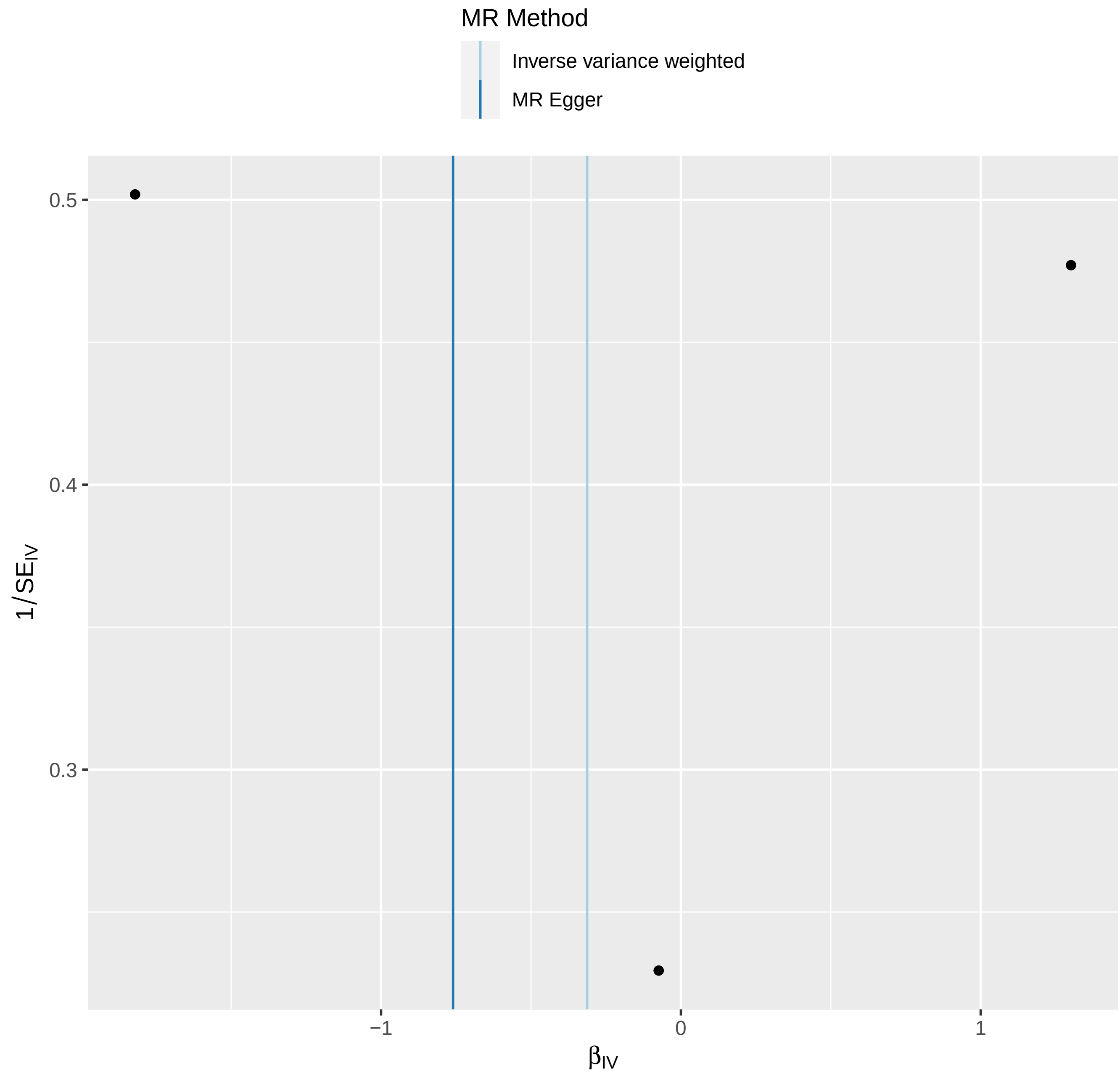

Batch 1297 : 1−palmitoylglycerophosphocholine on Ulcerative colitis with PSC

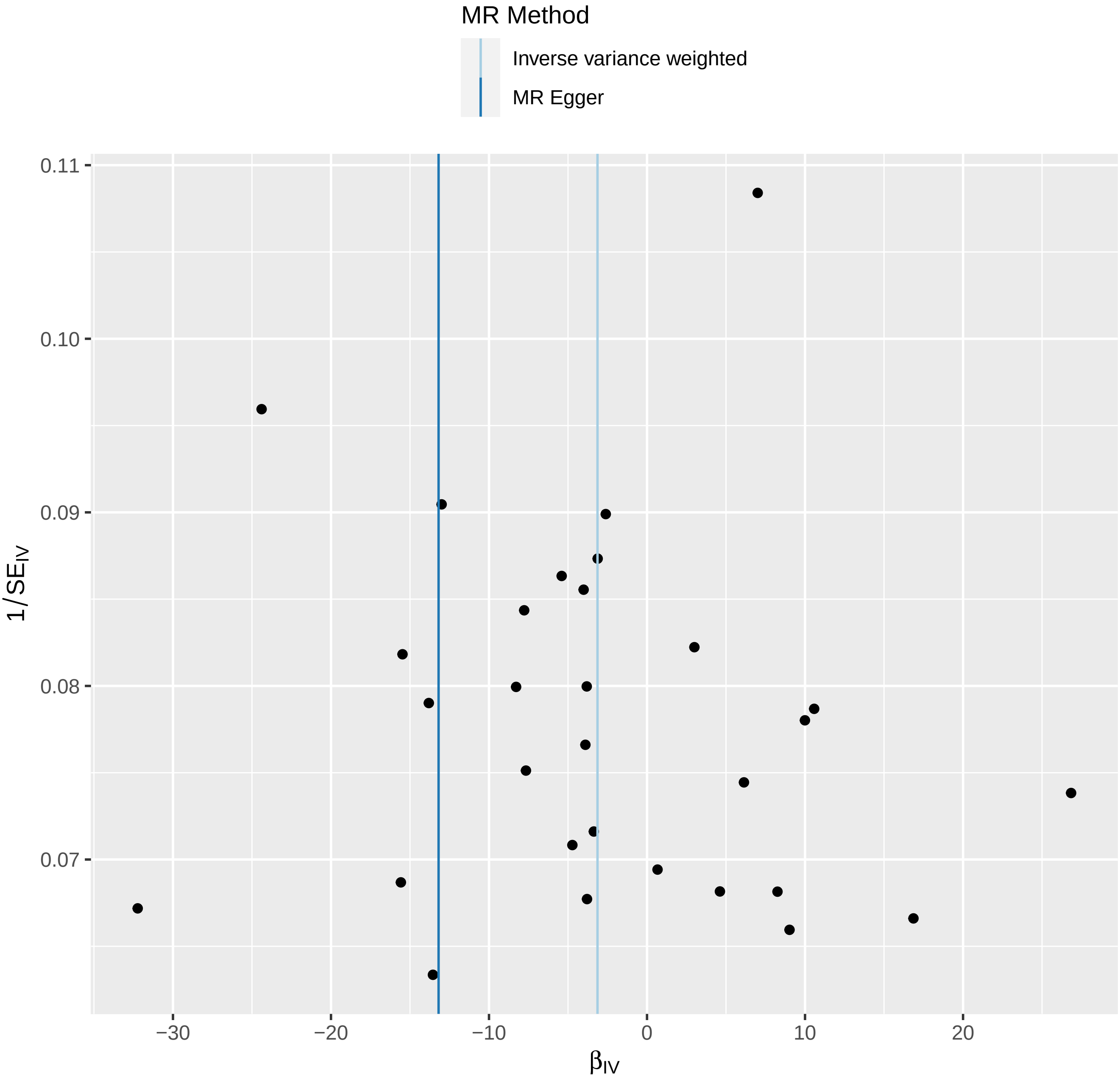

Batch 1298 : 1−heptadecanoylglycerophosphocholine on Ulcerative colitis with PSC

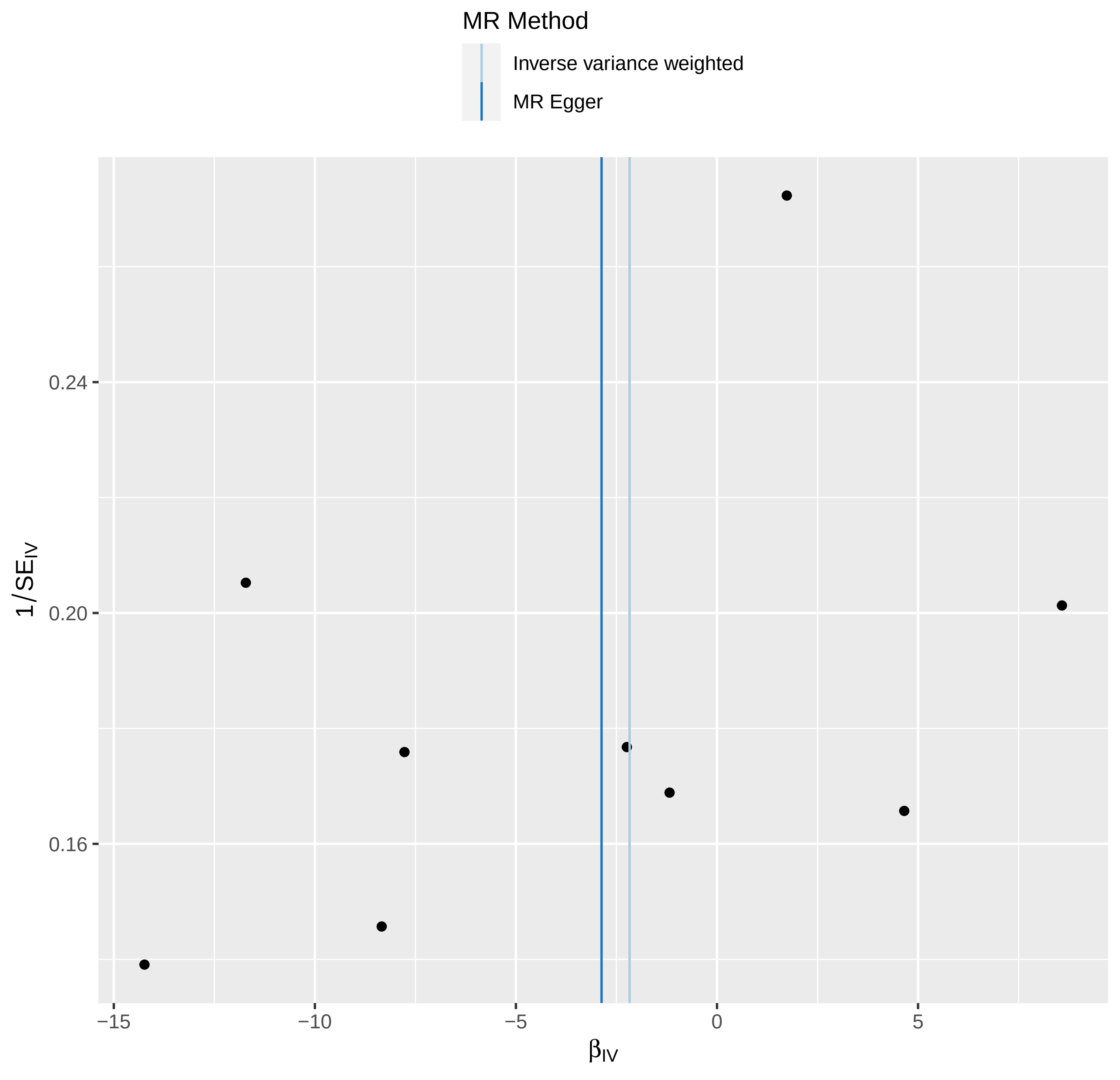

Batch 1299 : 1−oleoylglycerophosphocholine on Ulcerative colitis with PSC

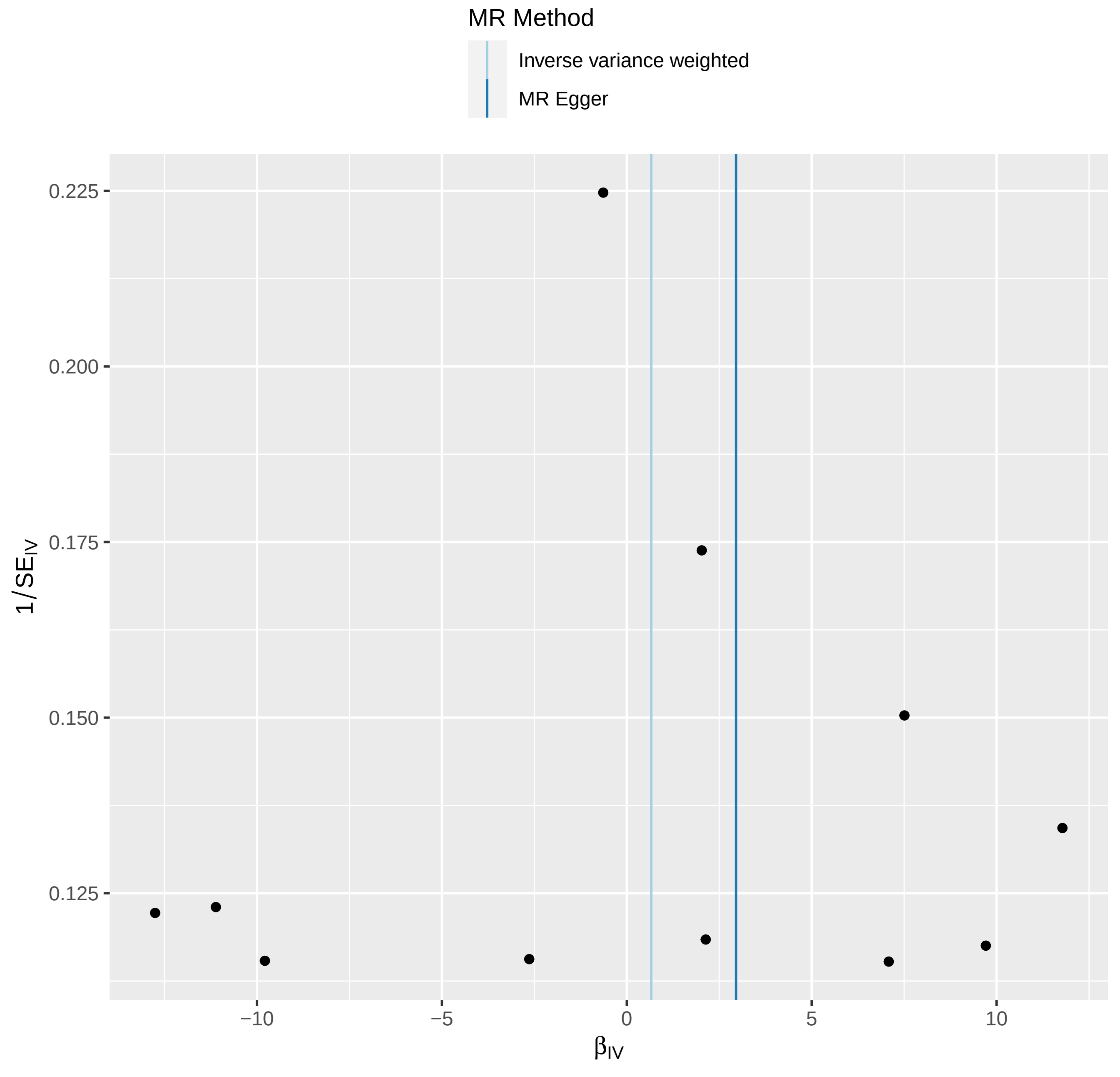

Batch 1300 : 1−stearoylglycerophosphocholine on Ulcerative colitis with PSC

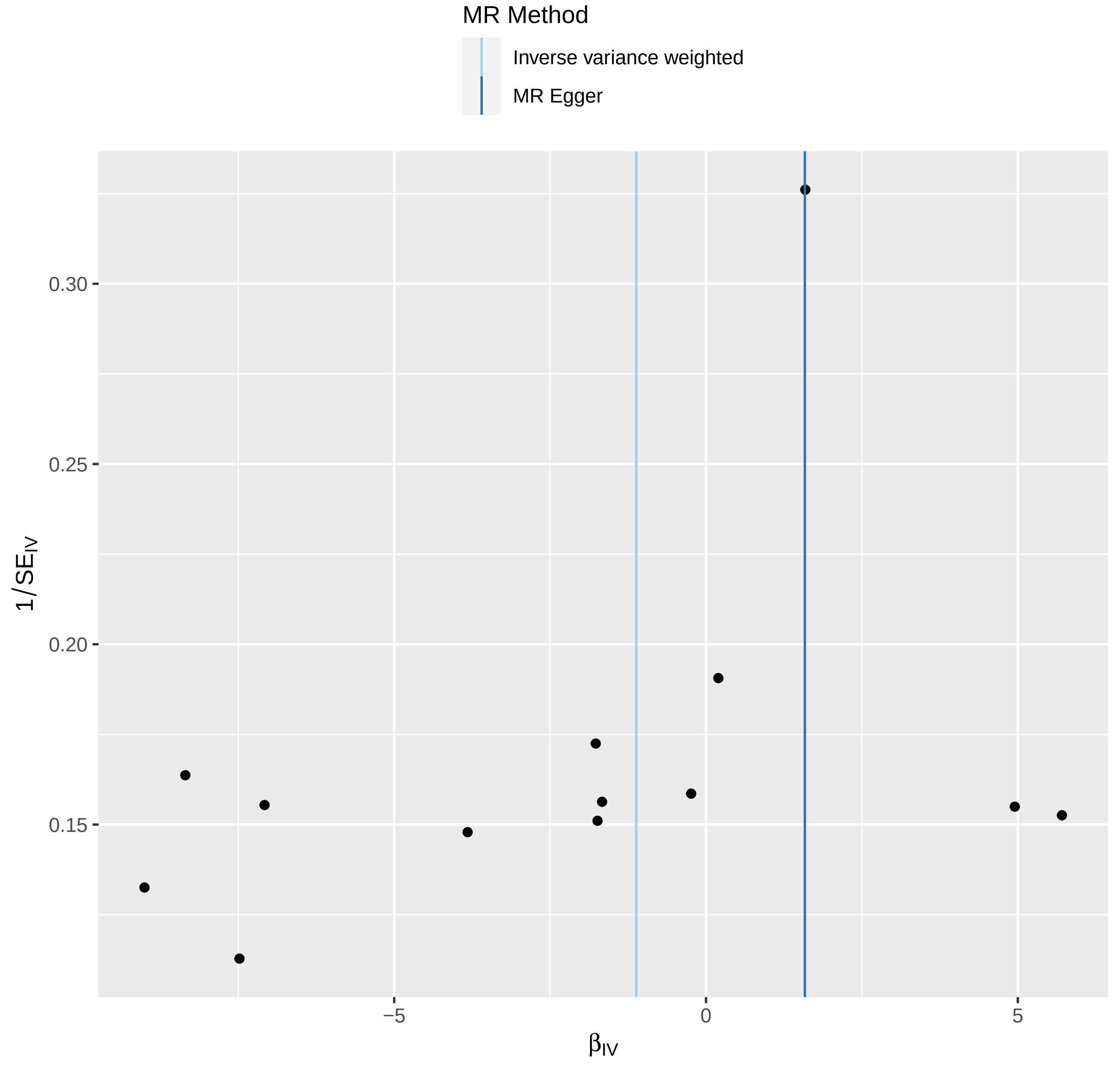

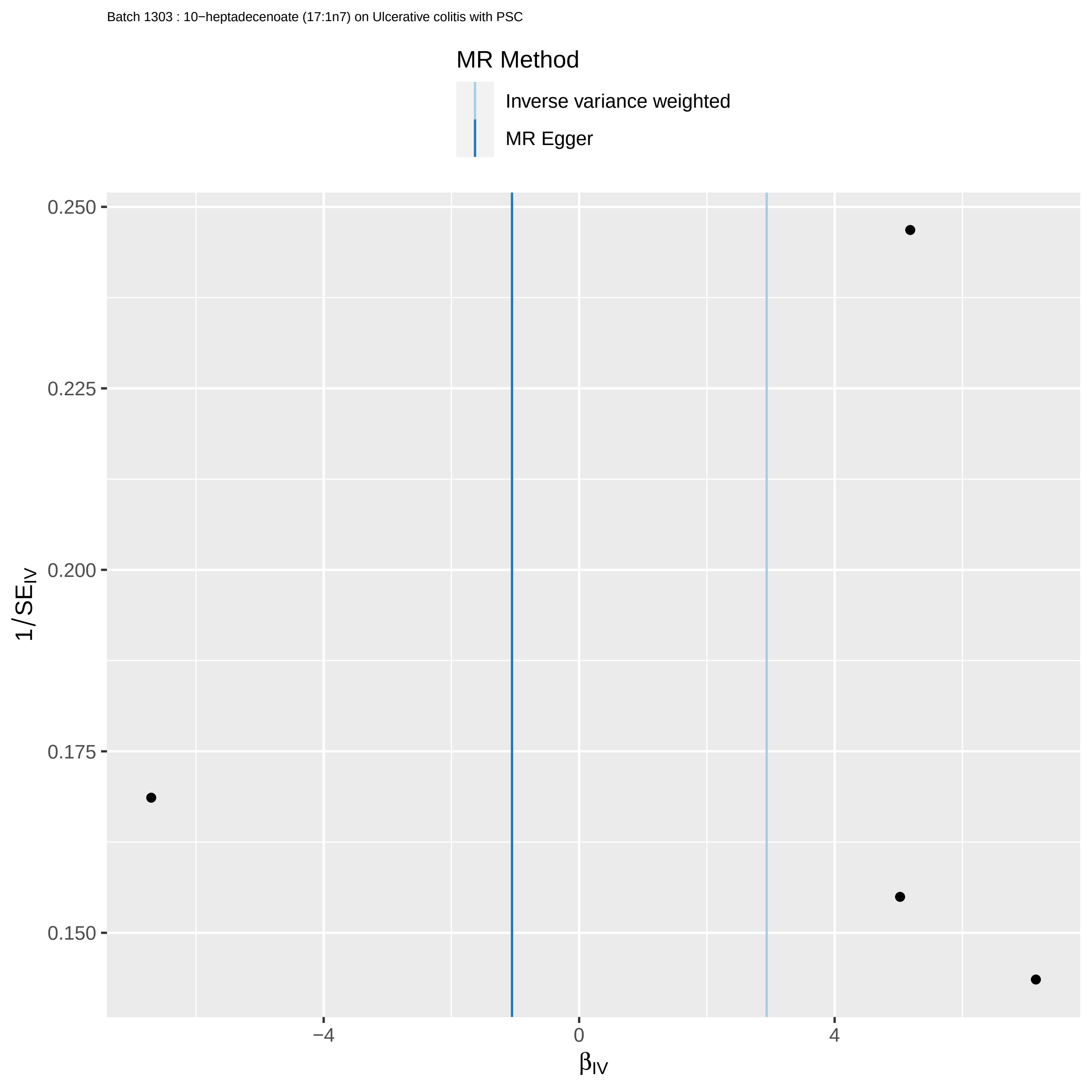
Batch 1303 : 10−heptadecenoate (17:1n7) on Ulcerative colitis with PSC
MR Method
Inverse variance weighted
MR Egger
0.250
0.225
0.200
0.175
0.150
1/SE$_{IV}$
−4
0
4
β_{IV}

Batch 1305 : Epiandrosterone sulfate on Ulcerative colitis with PSC

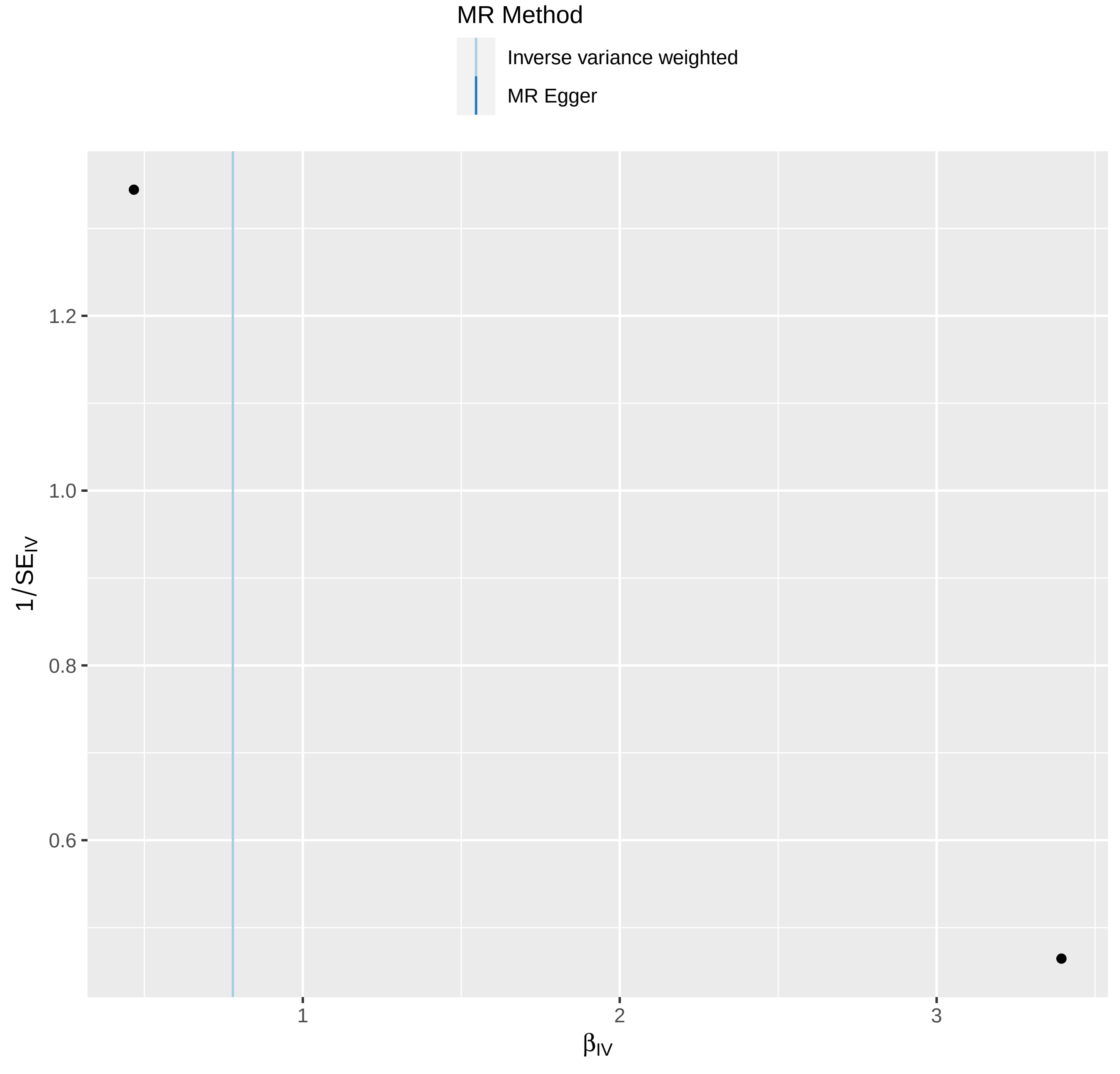

Batch 1306 : Linolenate [alpha or gamma; (18:3n3 or 6)] on Ulcerative colitis with PSC

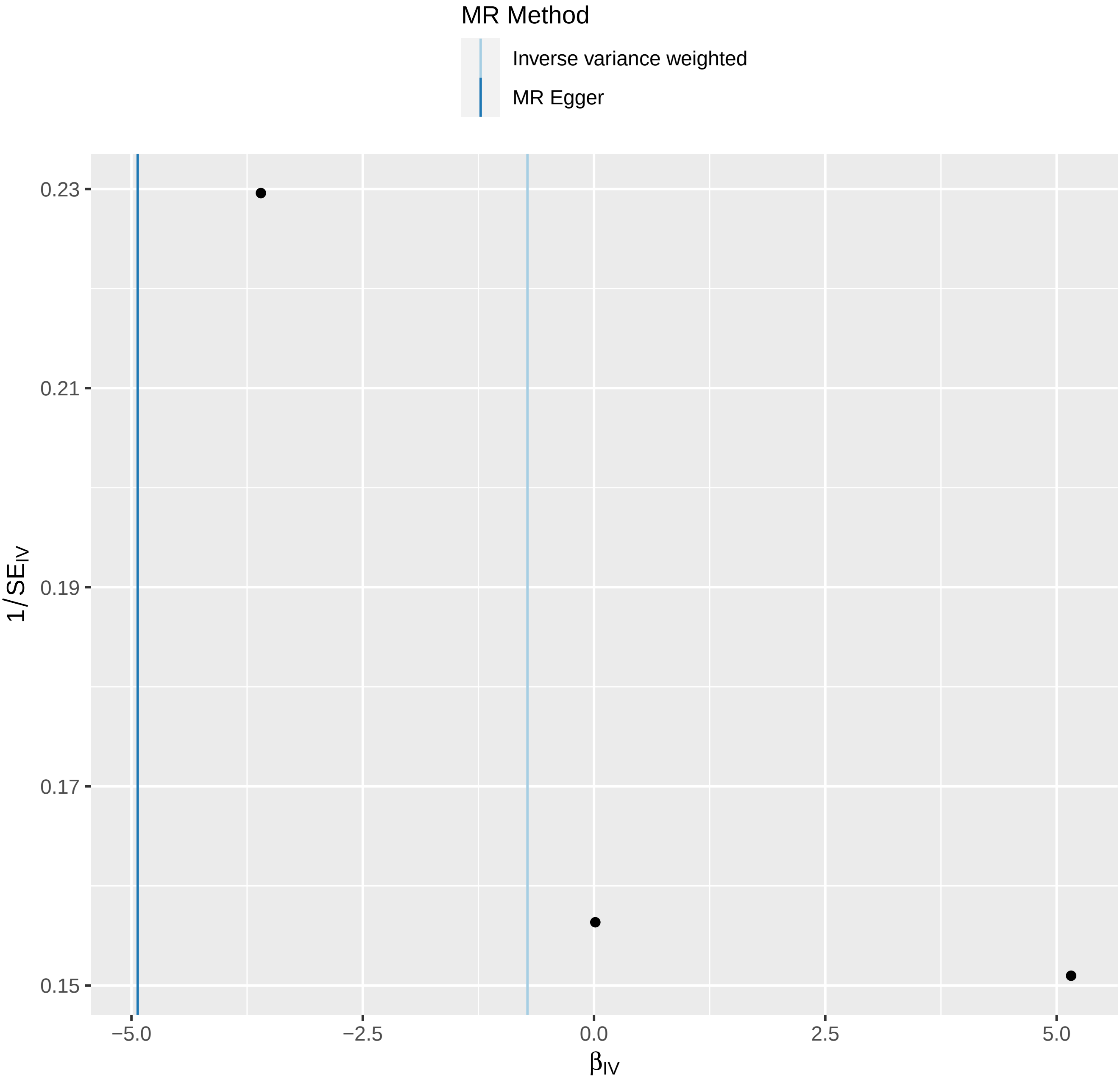

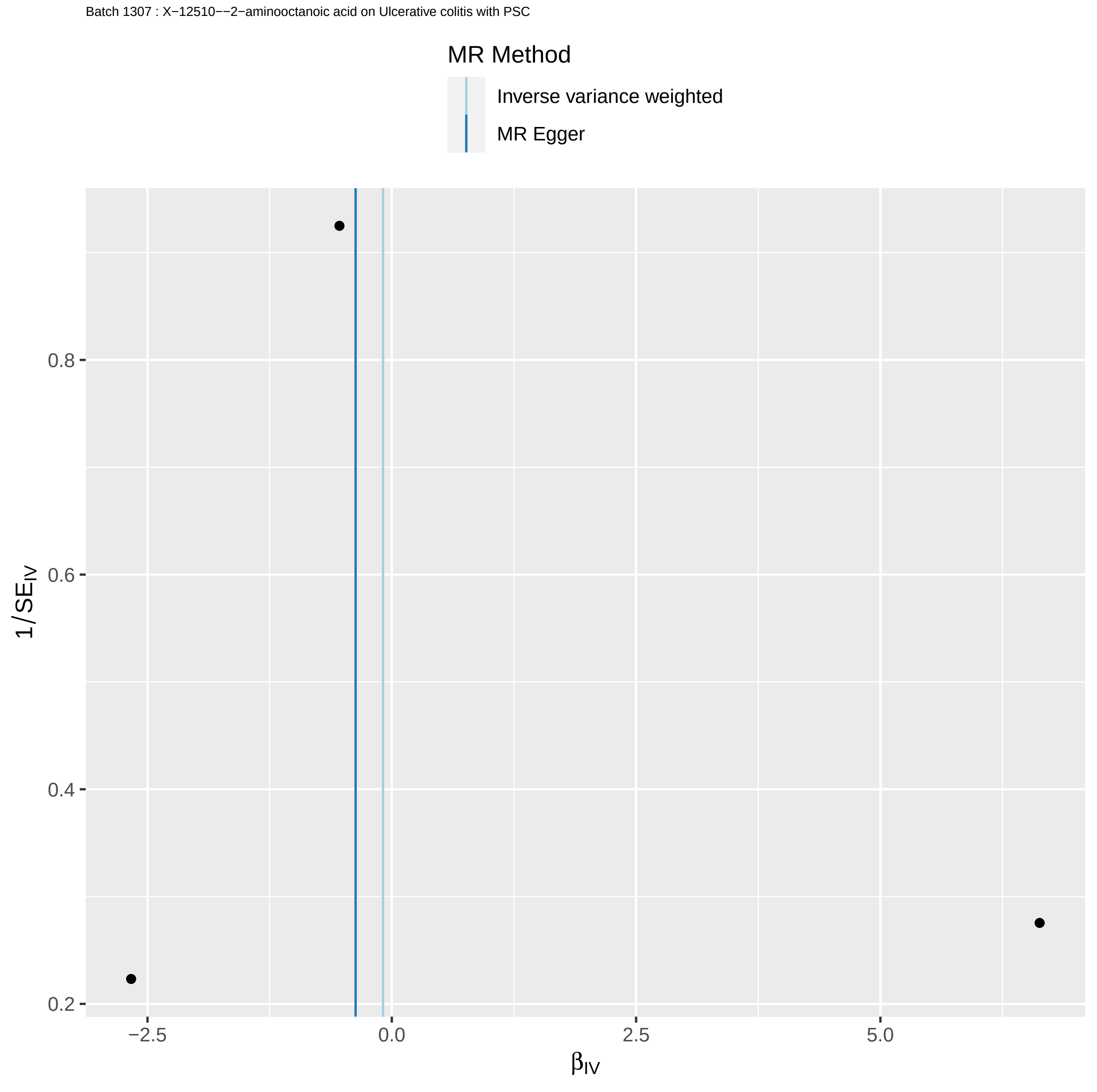
Batch 1307 : X−12510−−2−aminooctanoic acid on Ulcerative colitis with PSC
MR Method
Inverse variance weighted
MR Egger
0.8
0.6
0.4
0.2
1/SE_IV
−2.5
0.0
2.5
5.0
β_IV

Batch 1310 : Asparagine on Ulcerative colitis with PSC

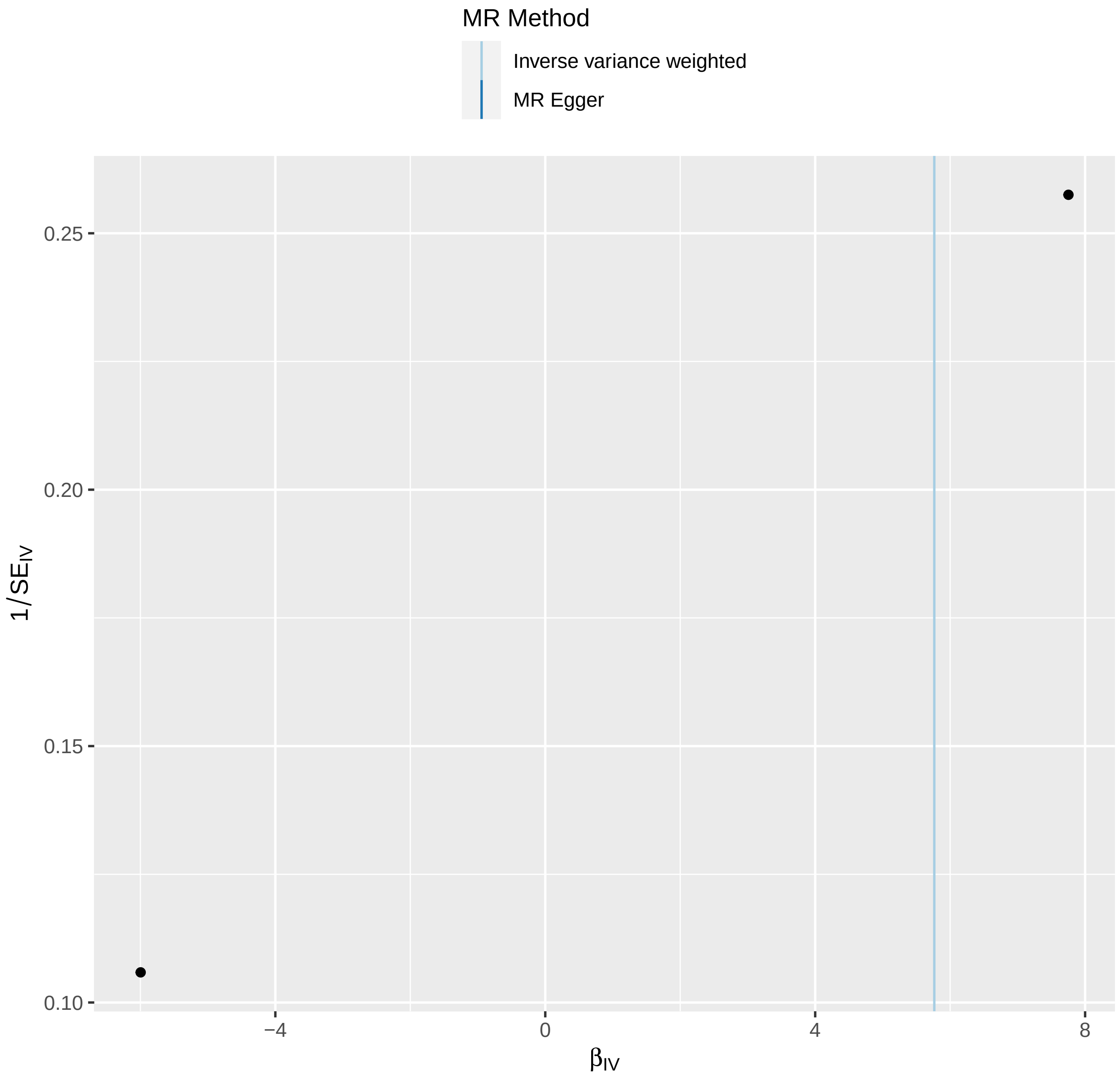

Batch 1311 : Stachydrine on Ulcerative colitis with PSC

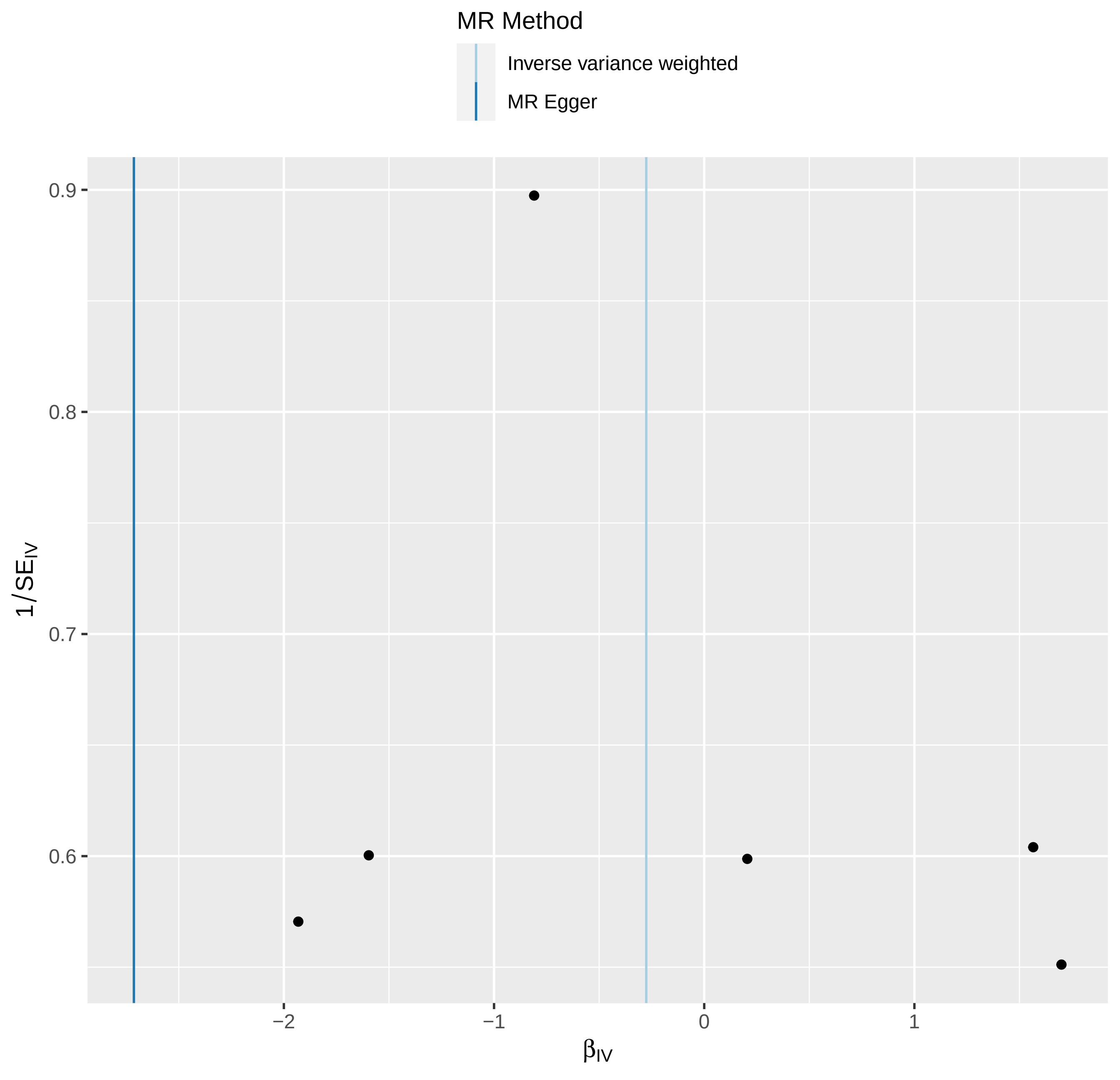

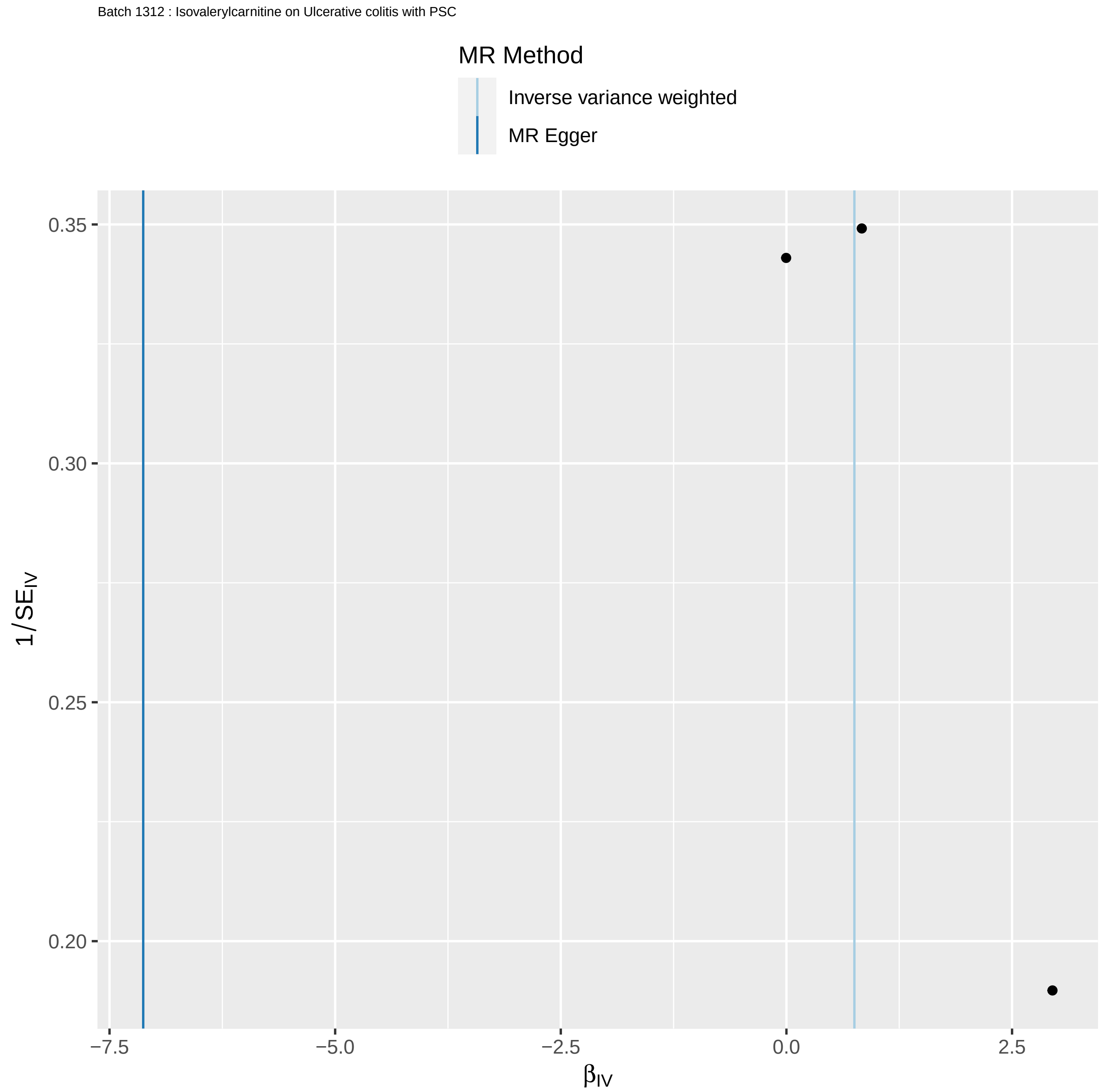
Batch 1312 : Isovalerylcarnitine on Ulcerative colitis with PSC
MR Method
Inverse variance weighted
MR Egger
0.35
0.30
0.25
0.20
1/SE_IV
−7.5
−5.0
−2.5
0.0
2.5
β_IV

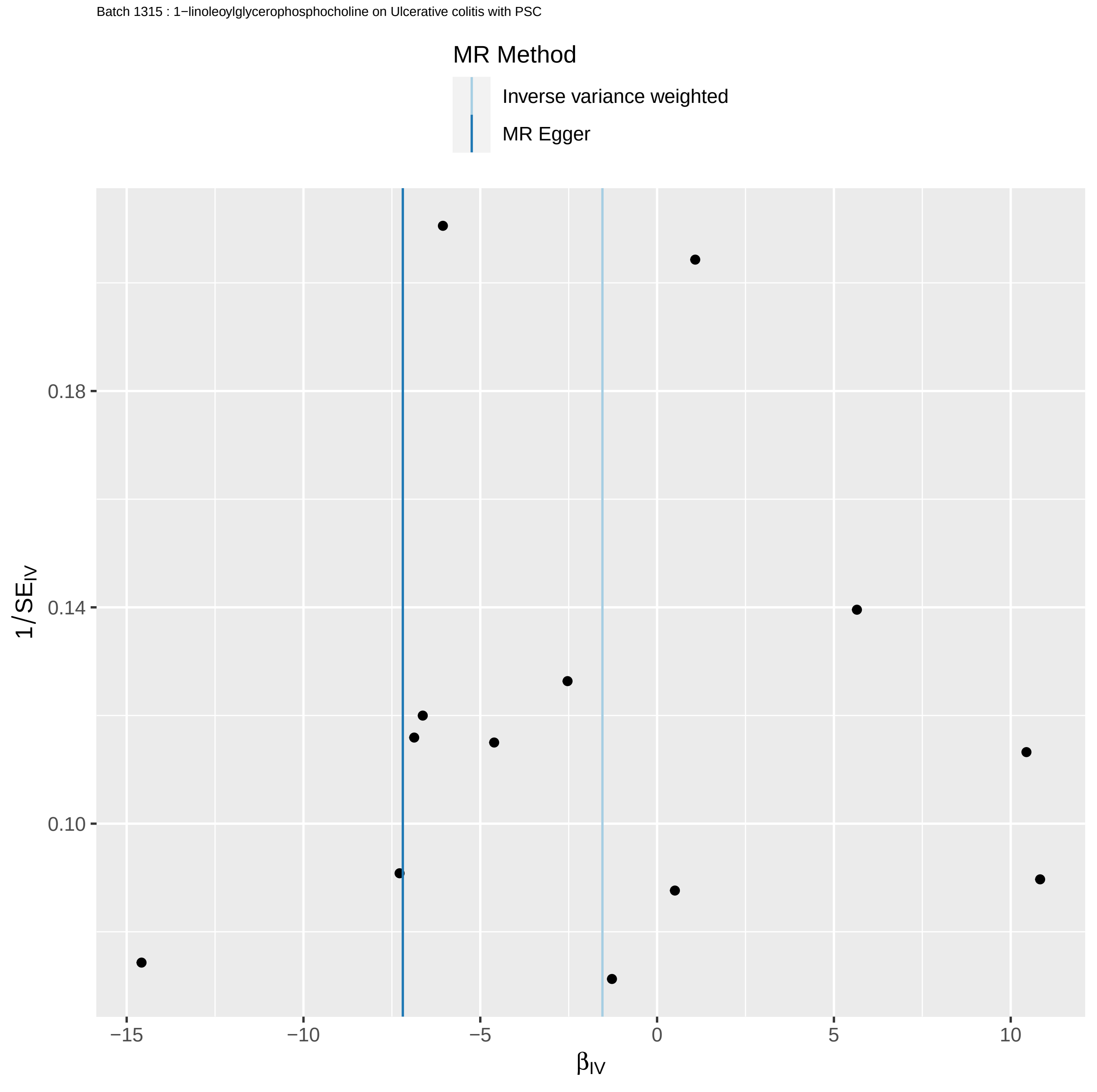
Batch 1315 : 1−linoleoylglycerophosphocholine on Ulcerative colitis with PSC
MR Method
Inverse variance weighted
MR Egger
0.18
0.14
0.10
$1/SE_{IV}$
−15
−10
−5
0
5
10
β_{IV}

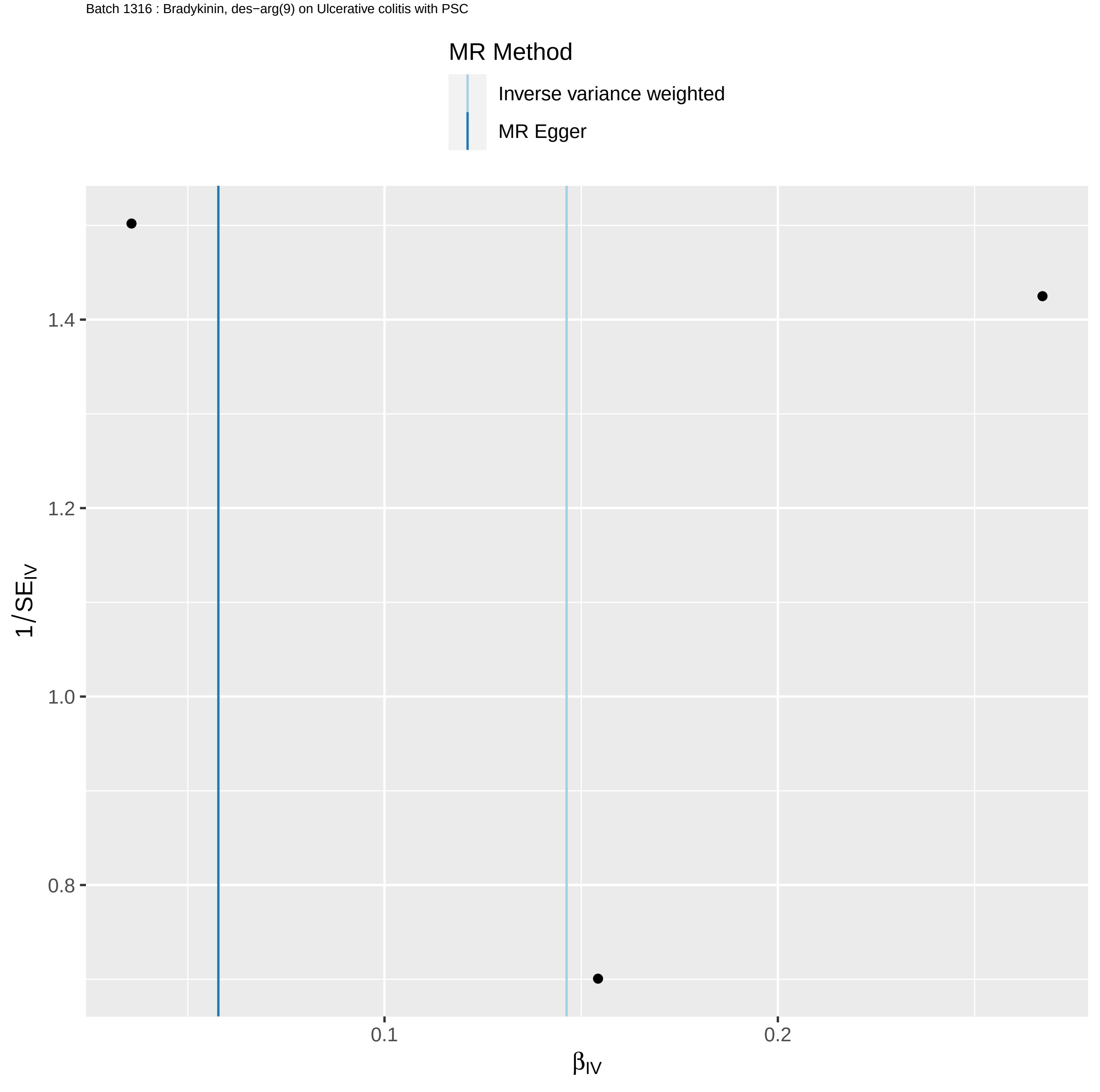

Batch 1316 : Bradykinin, des−arg(9) on Ulcerative colitis with PSC
MR Method
Inverse variance weighted
MR Egger
1.4
1.2
1.0
0.8
$1/SE_{IV}$
0.1
0.2
β_{IV}

Batch 1317 : Gamma−glutamylisoleucine* on Ulcerative colitis with PSC

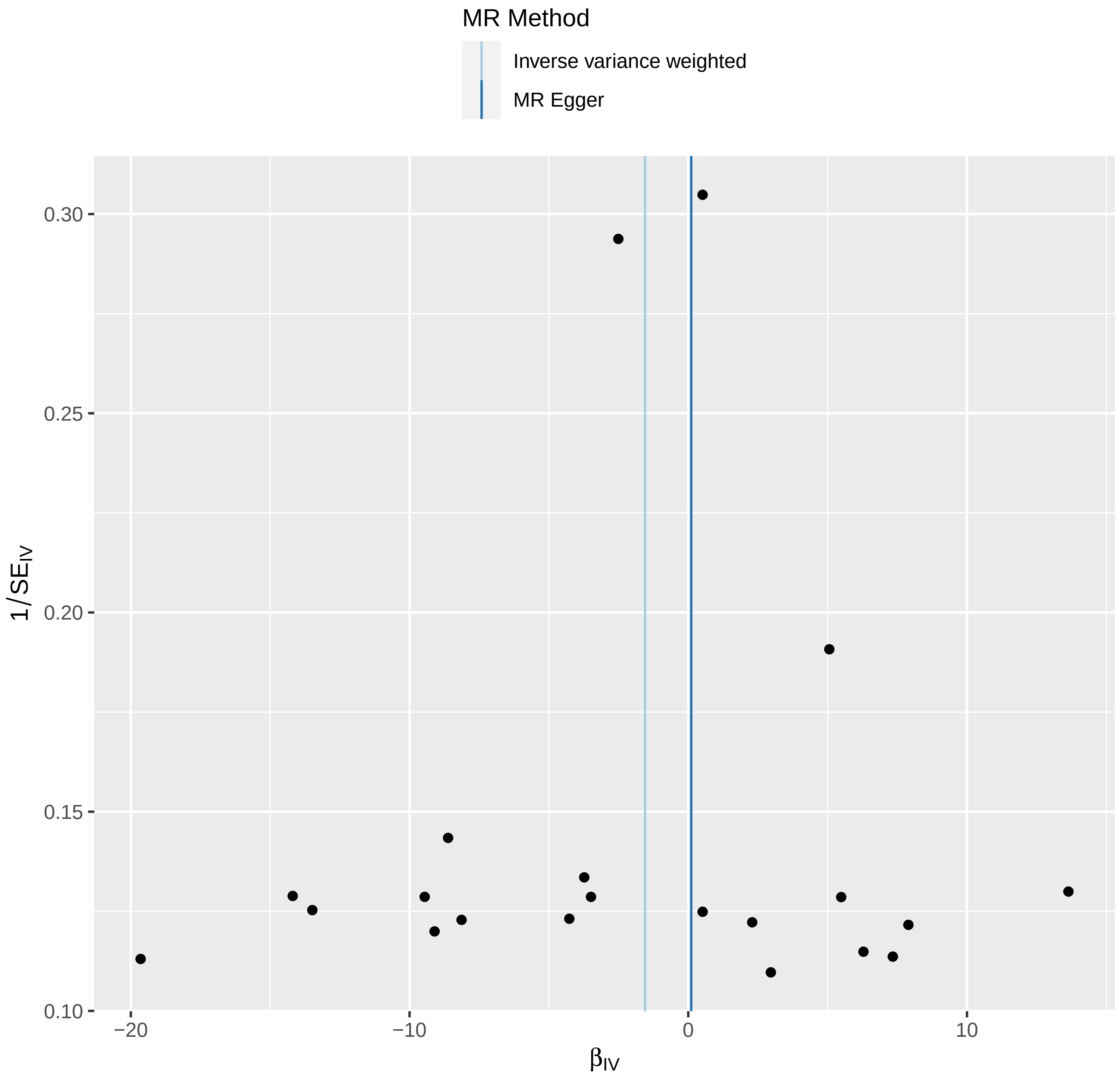

Batch 1320 : Isovalerate on Ulcerative colitis with PSC

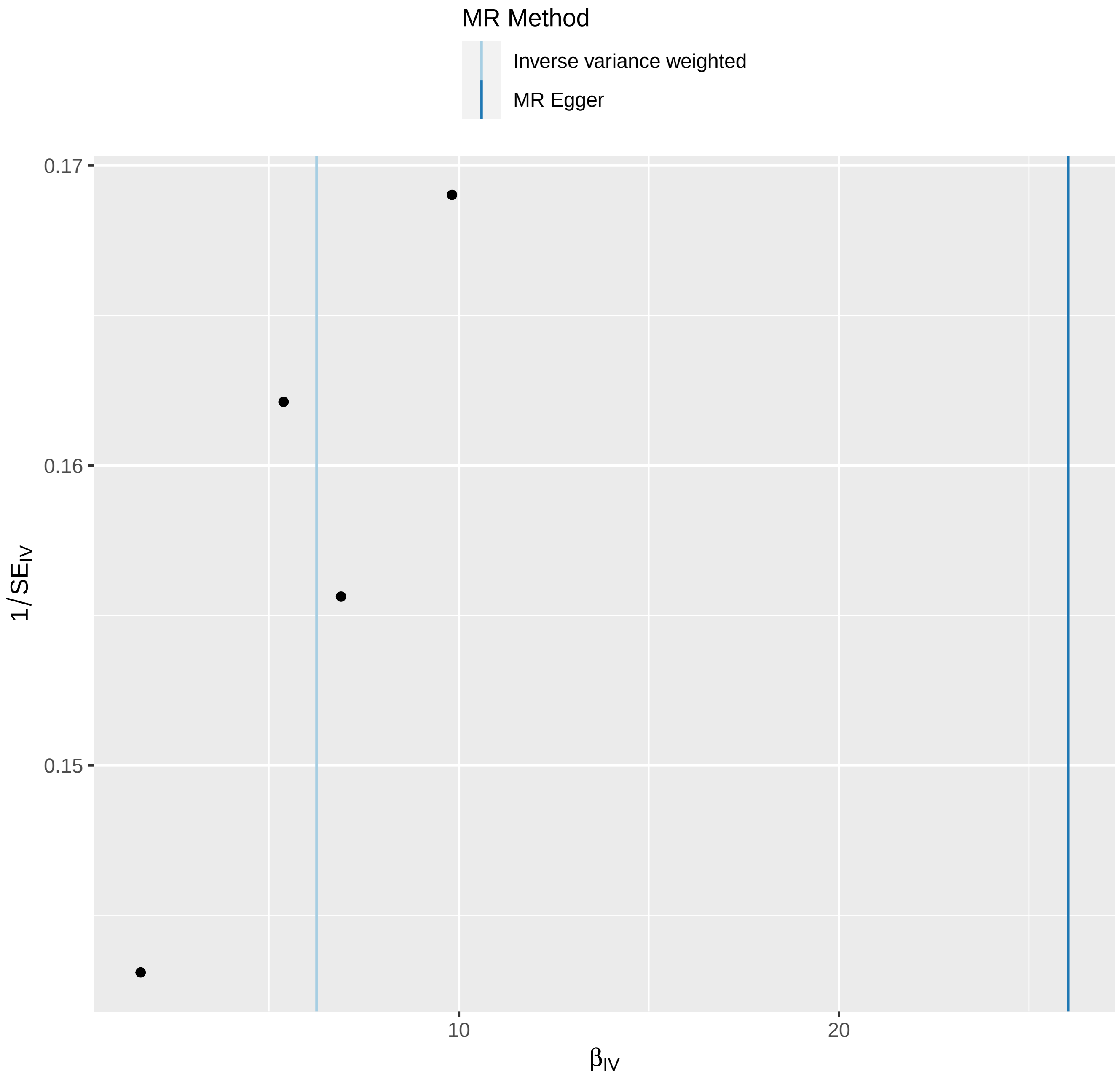

Batch 1321 : X−13183−−stearamide on Ulcerative colitis with PSC

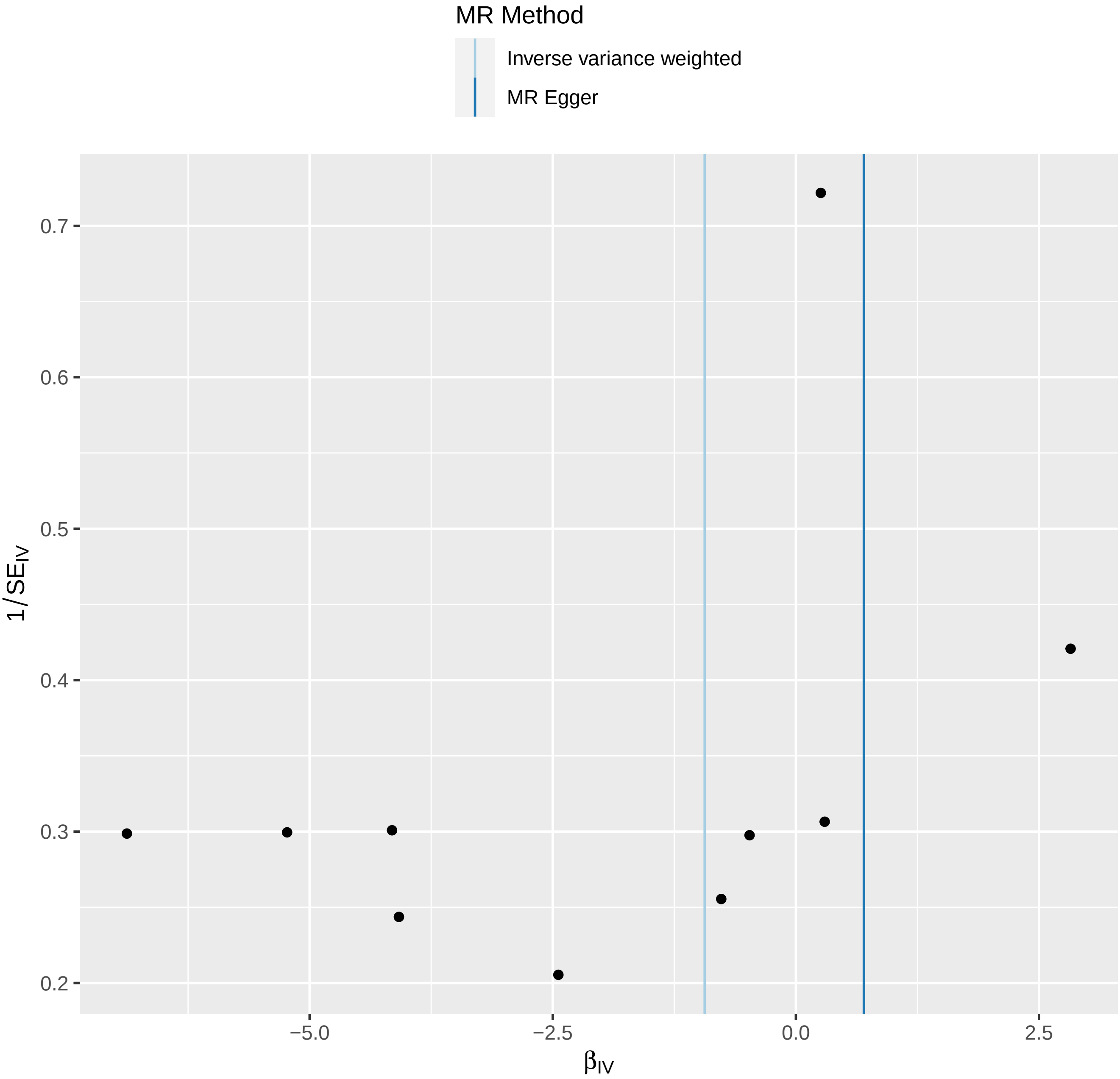

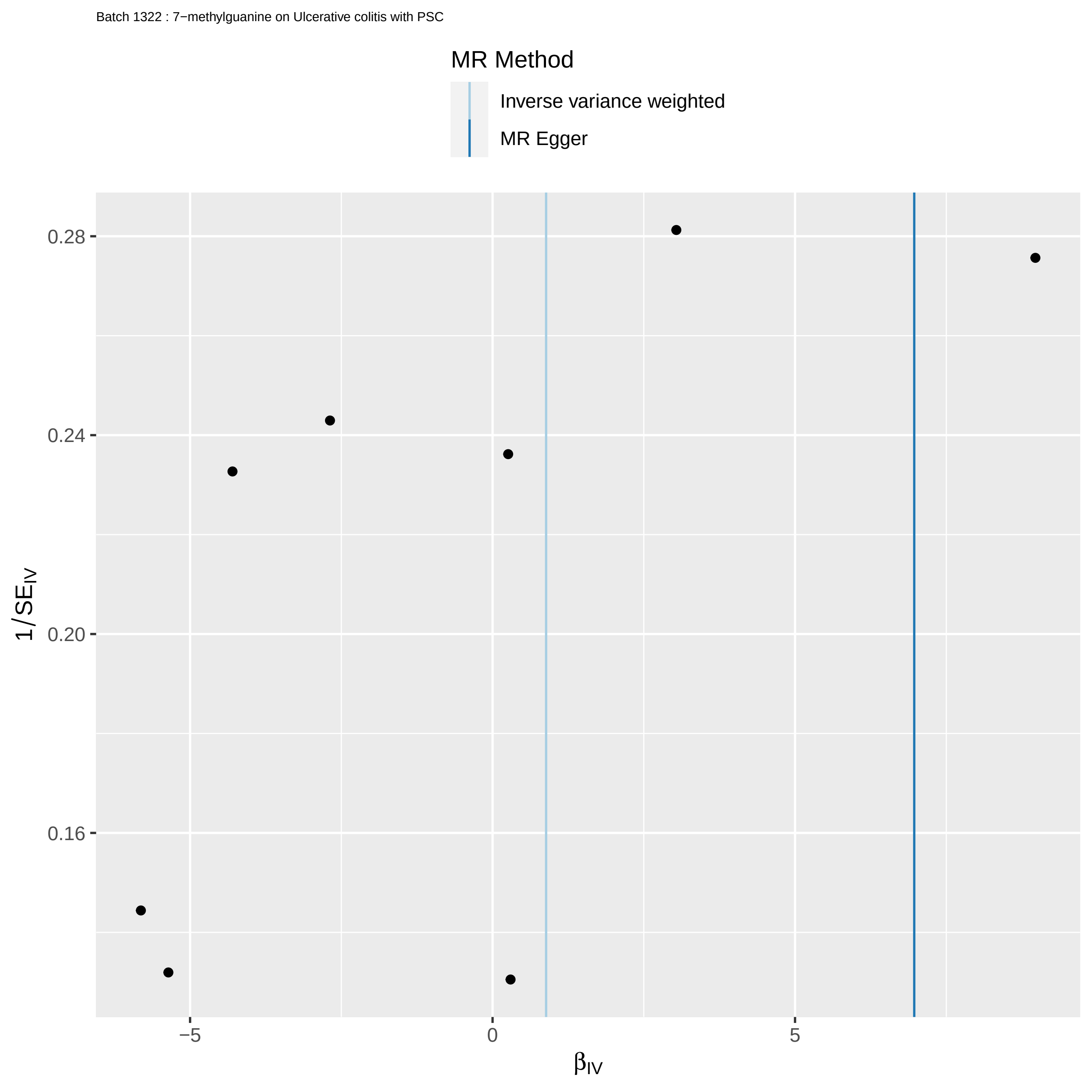

Batch 1322 : 7−methylguanine on Ulcerative colitis with PSC
MR Method
Inverse variance weighted
MR Egger
0.28
0.24
0.20
0.16
1/SE_IV
−5
0
5
β_IV

Batch 1329 : X−13431−−nonanoylcarnitine* on Ulcerative colitis with PSC

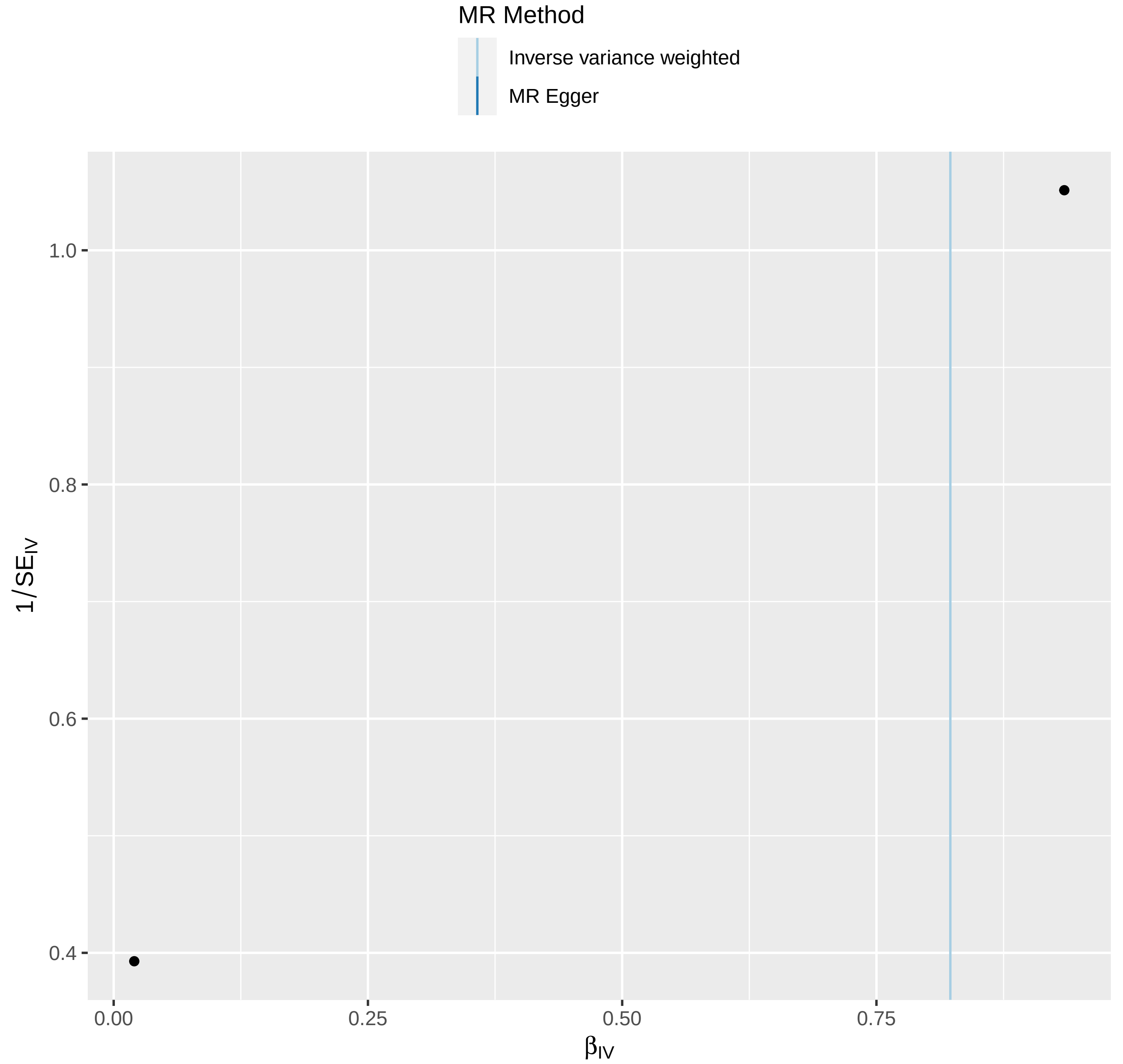

Batch 1330 : 2−palmitoylglycerophosphocholine* on Ulcerative colitis with PSC

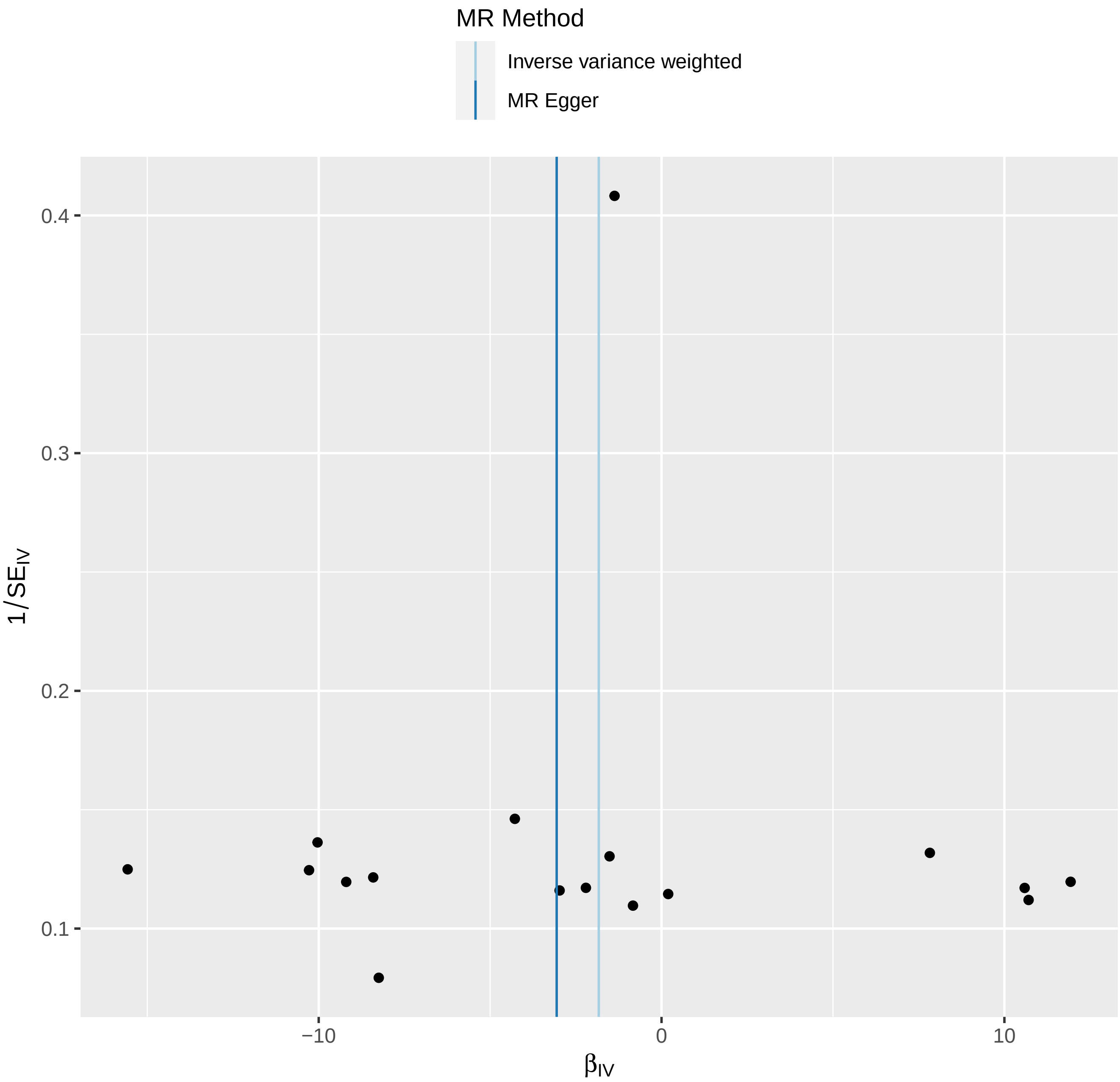

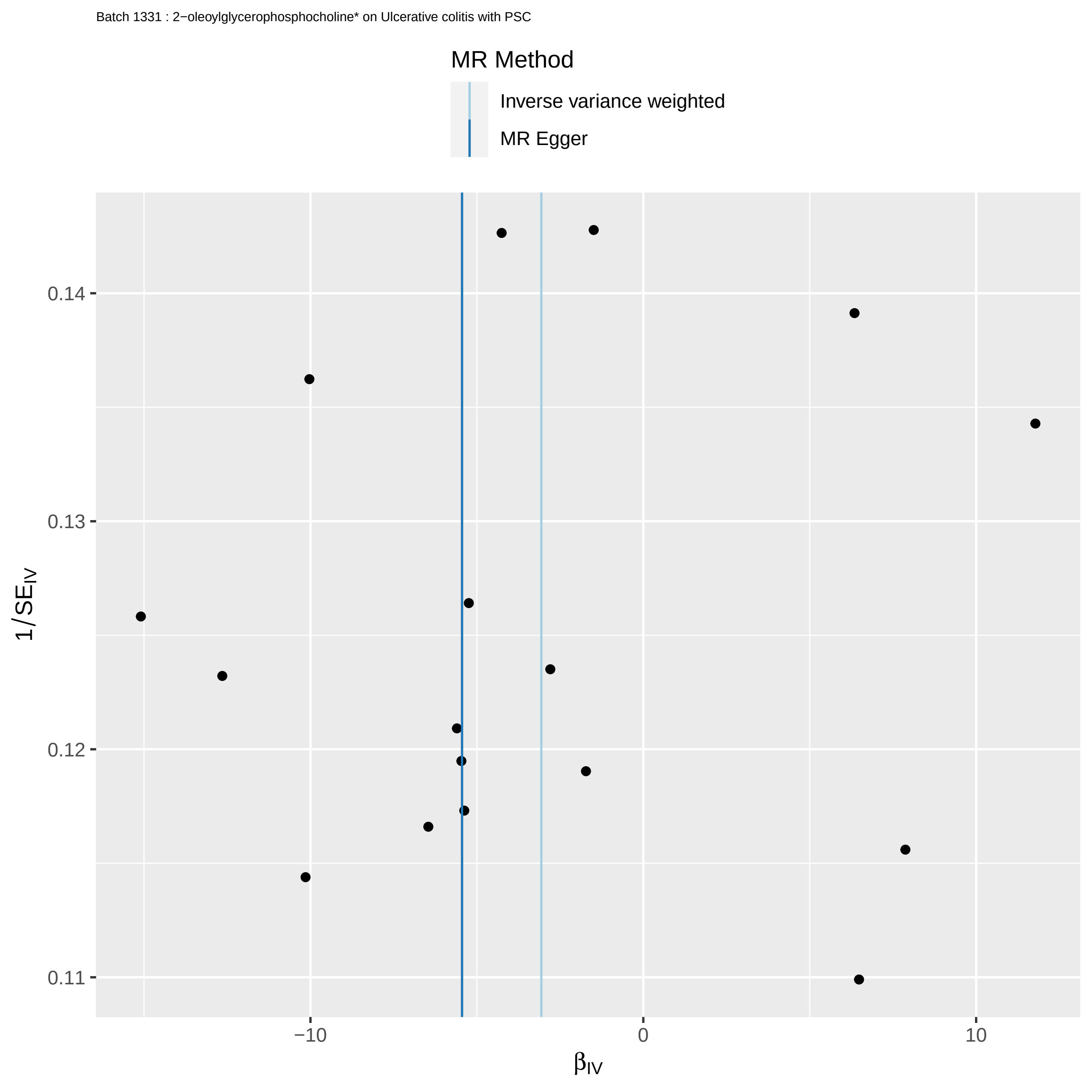

Batch 1331 : 2−oleoylglycerophosphocholine* on Ulcerative colitis with PSC
MR Method
Inverse variance weighted
MR Egger
0.14
0.13
0.12
0.11
1/SE$_{IV}$
−10
0
10
β_{IV}

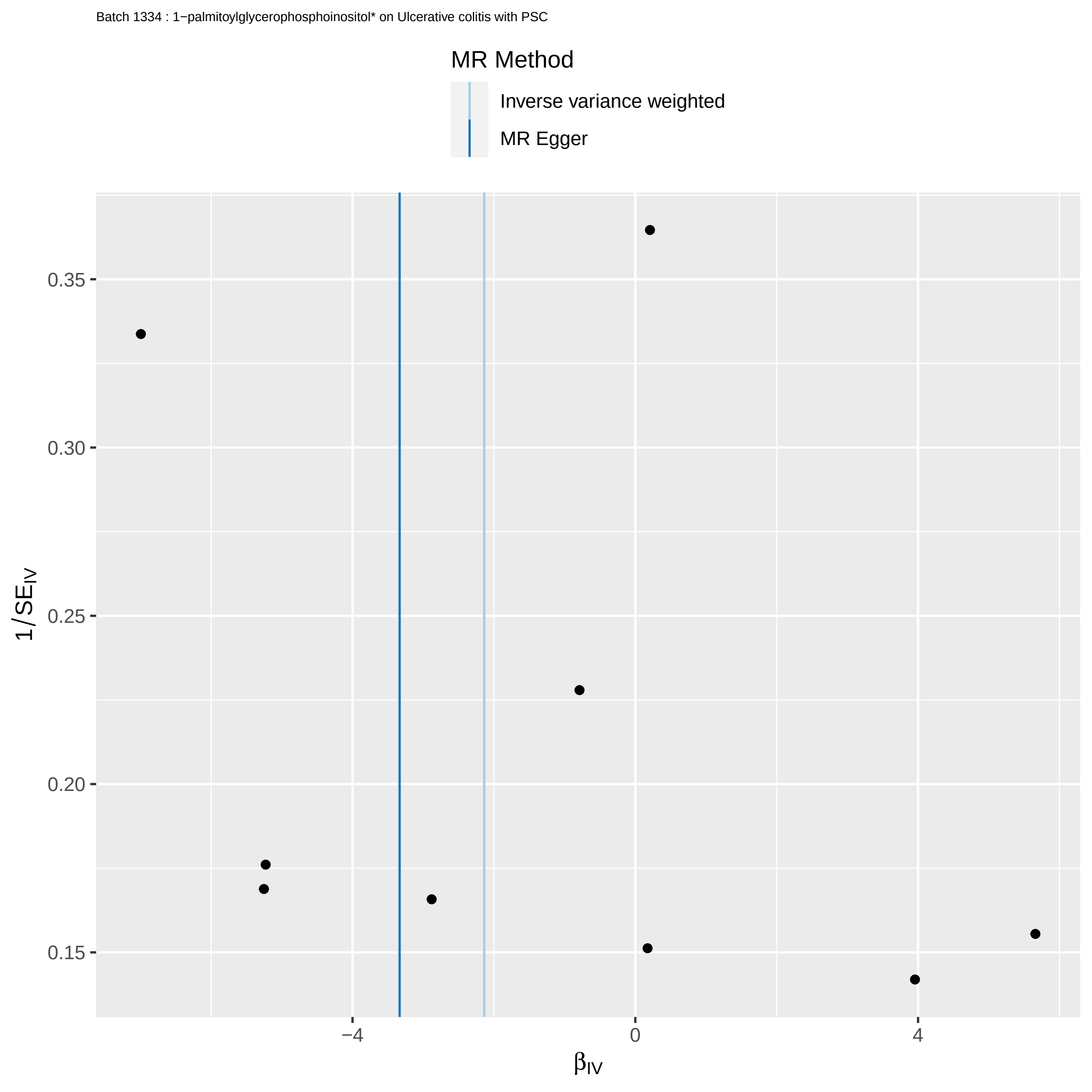
Batch 1334 : 1−palmitoylglycerophosphoinositol* on Ulcerative colitis with PSC
MR Method
Inverse variance weighted
MR Egger
0.35
0.30
0.25
0.20
0.15
$1/SE_{IV}$
−4
0
4
β_{IV}

Batch 1336 : Hydroxyisovaleroyl carnitine on Ulcerative colitis with PSC

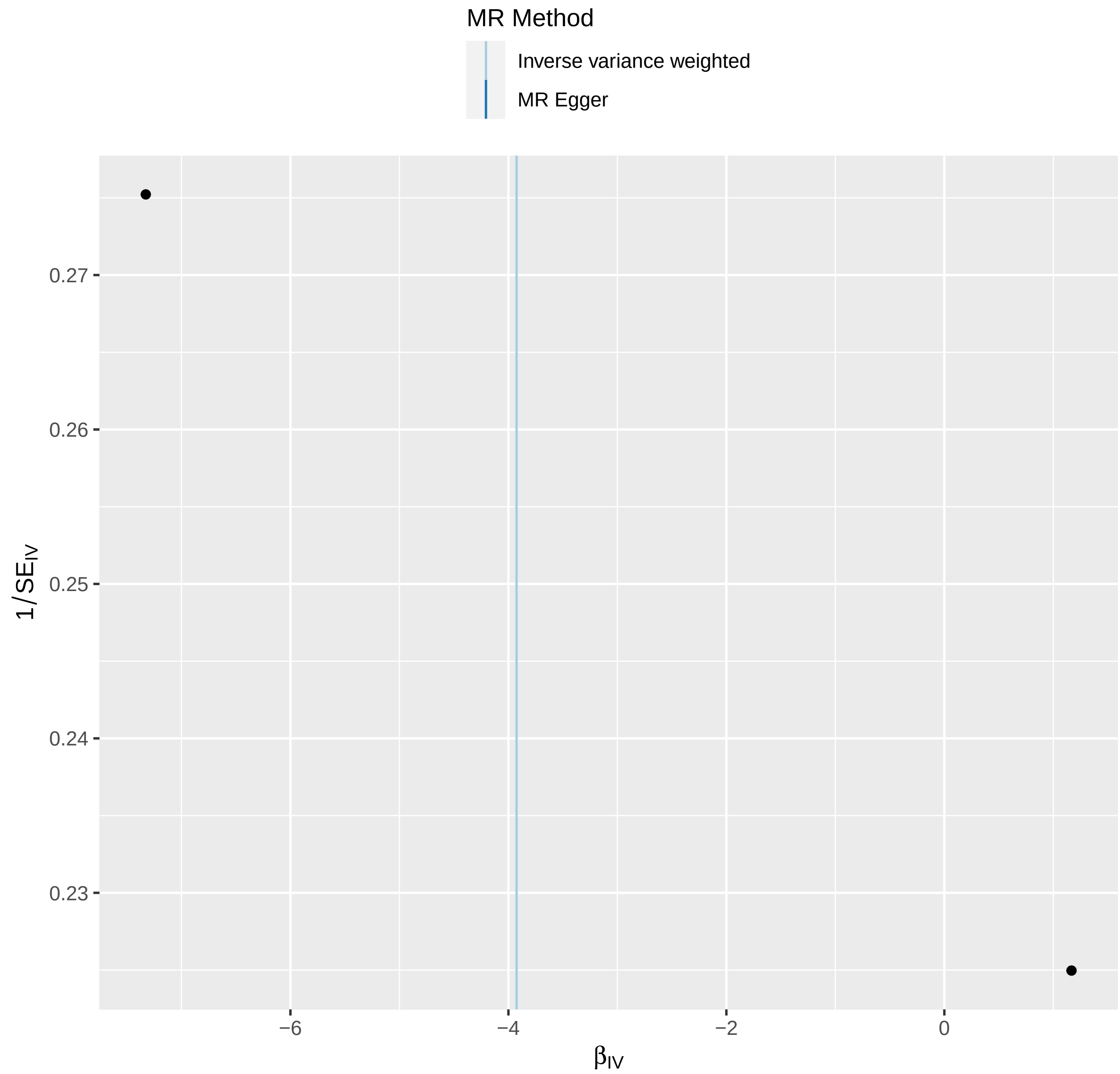

Batch 1337 : Glutaroyl carnitine on Ulcerative colitis with PSC

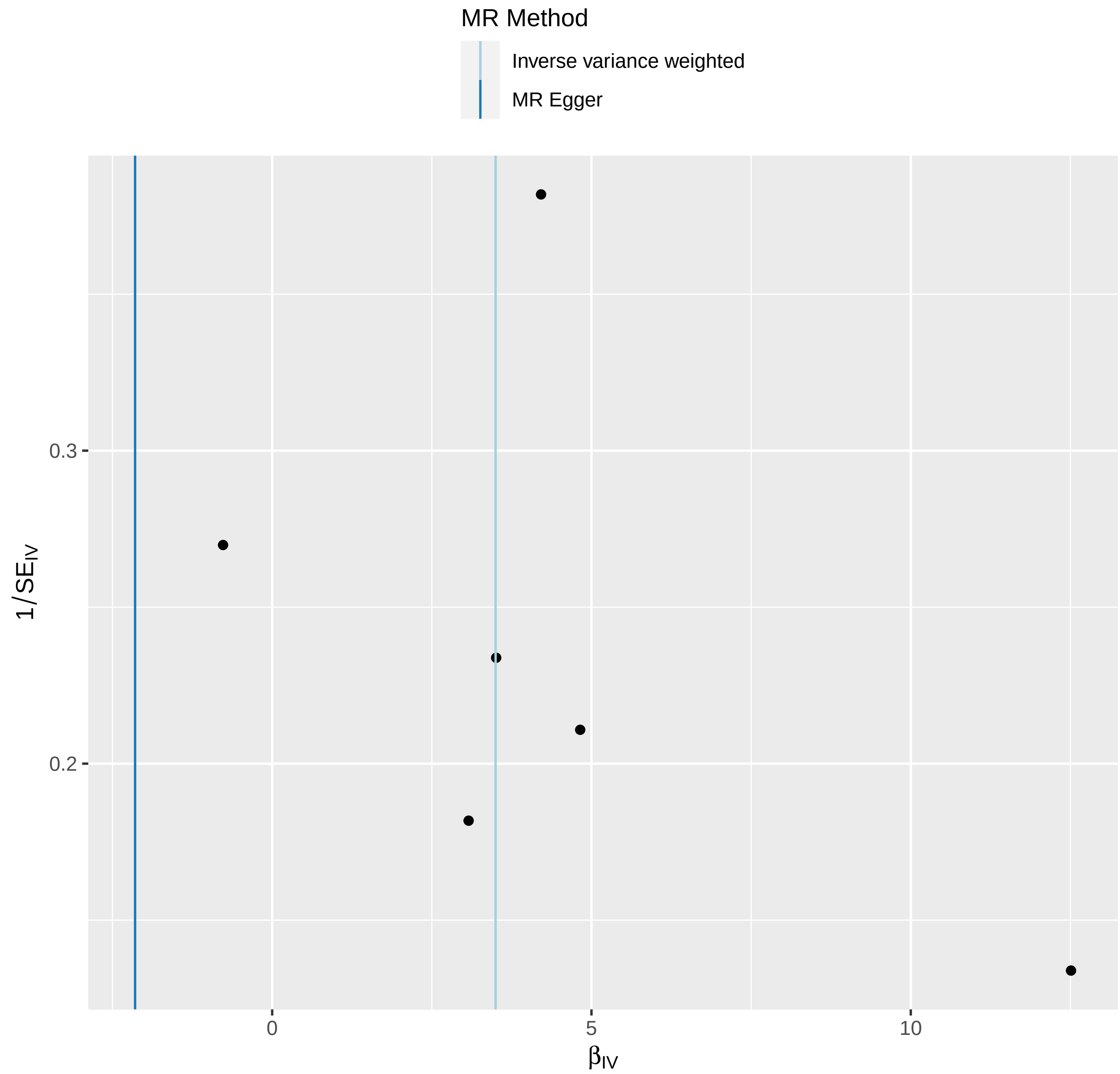

Batch 1339 : 4−hydroxyhippurate on Ulcerative colitis with PSC

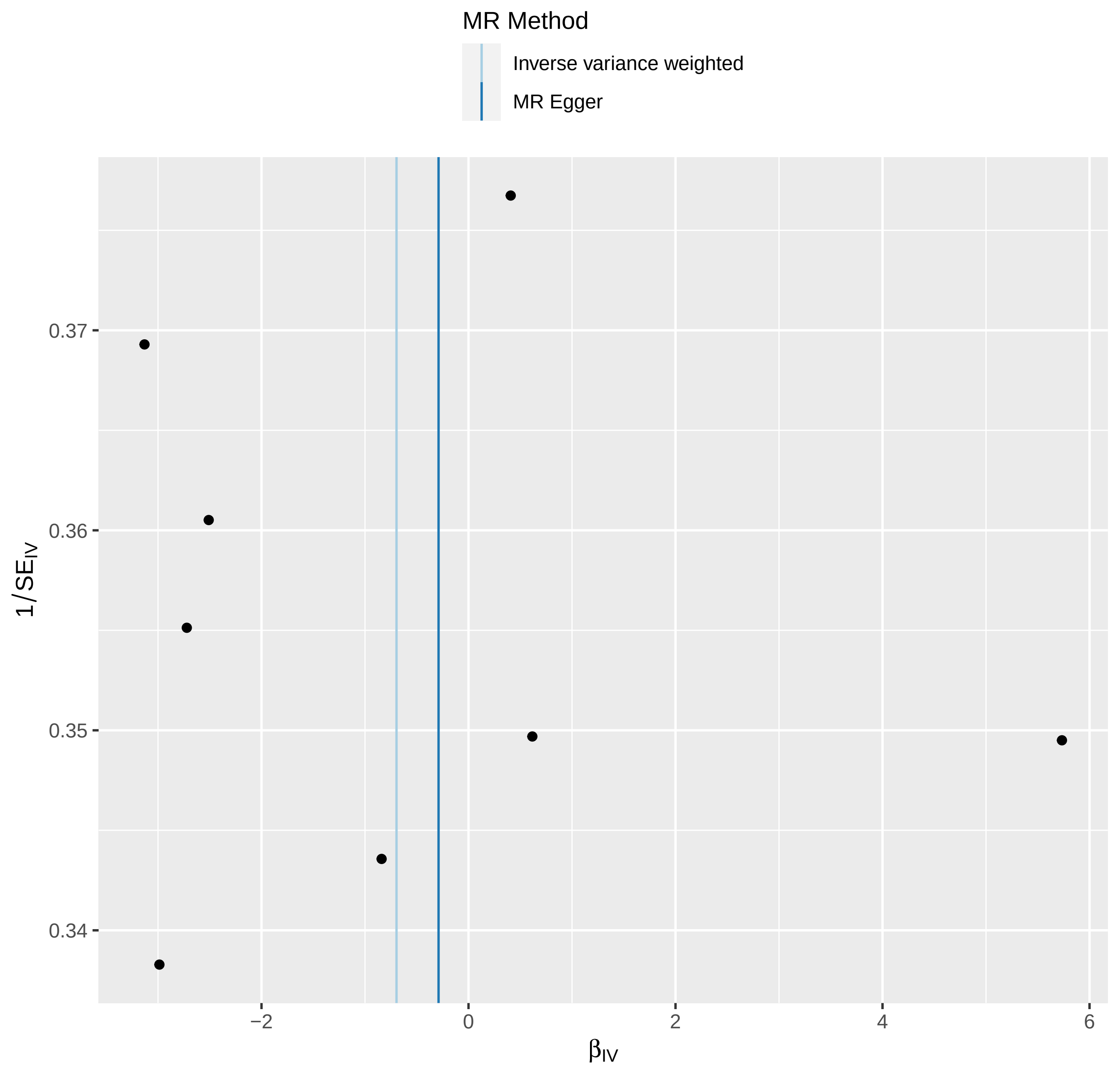

Batch 1340 : 1−myristoylglycerophosphocholine on Ulcerative colitis with PSC

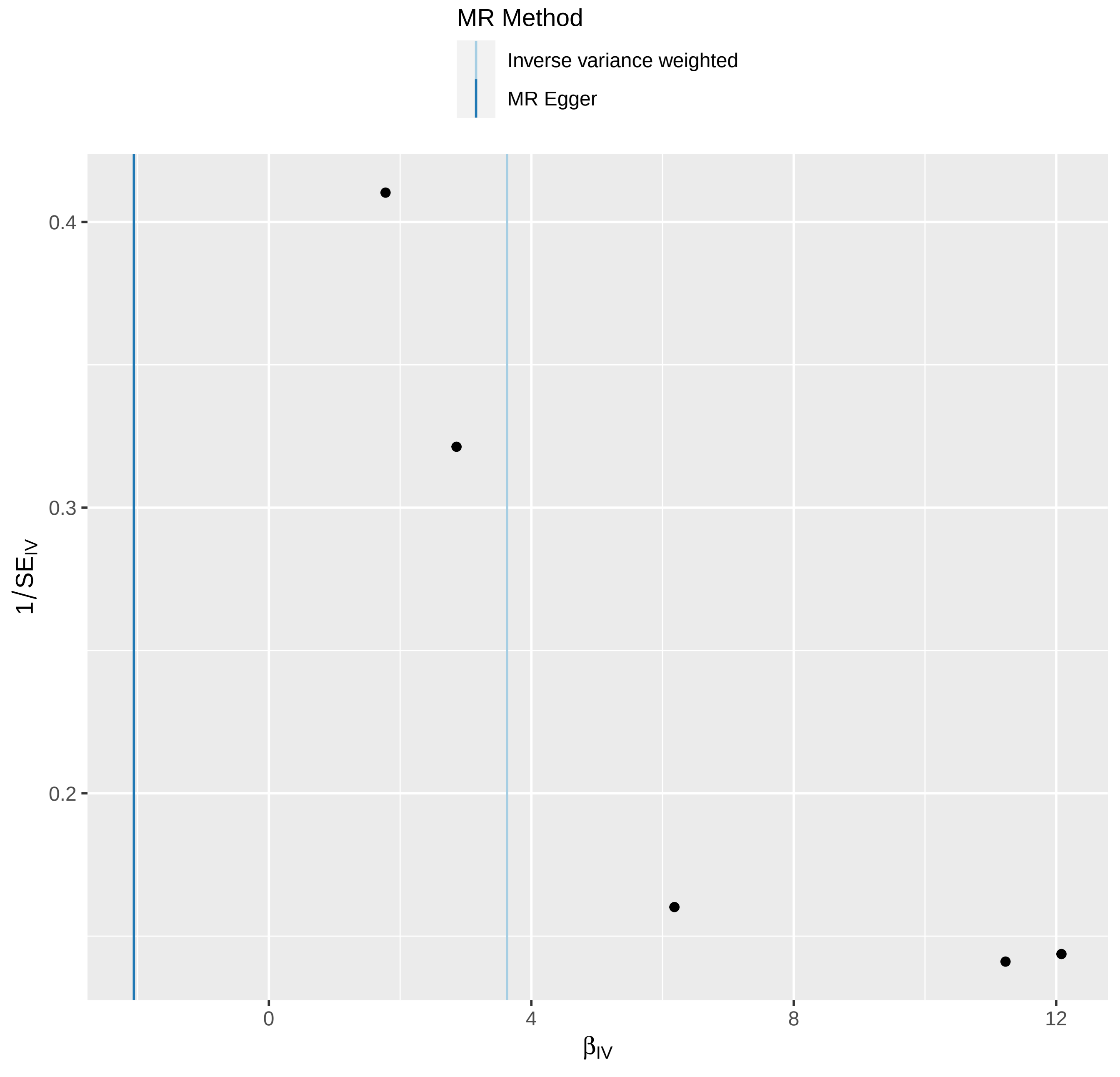

Batch 1343 : 3−(3−hydroxyphenyl)propionate on Ulcerative colitis with PSC

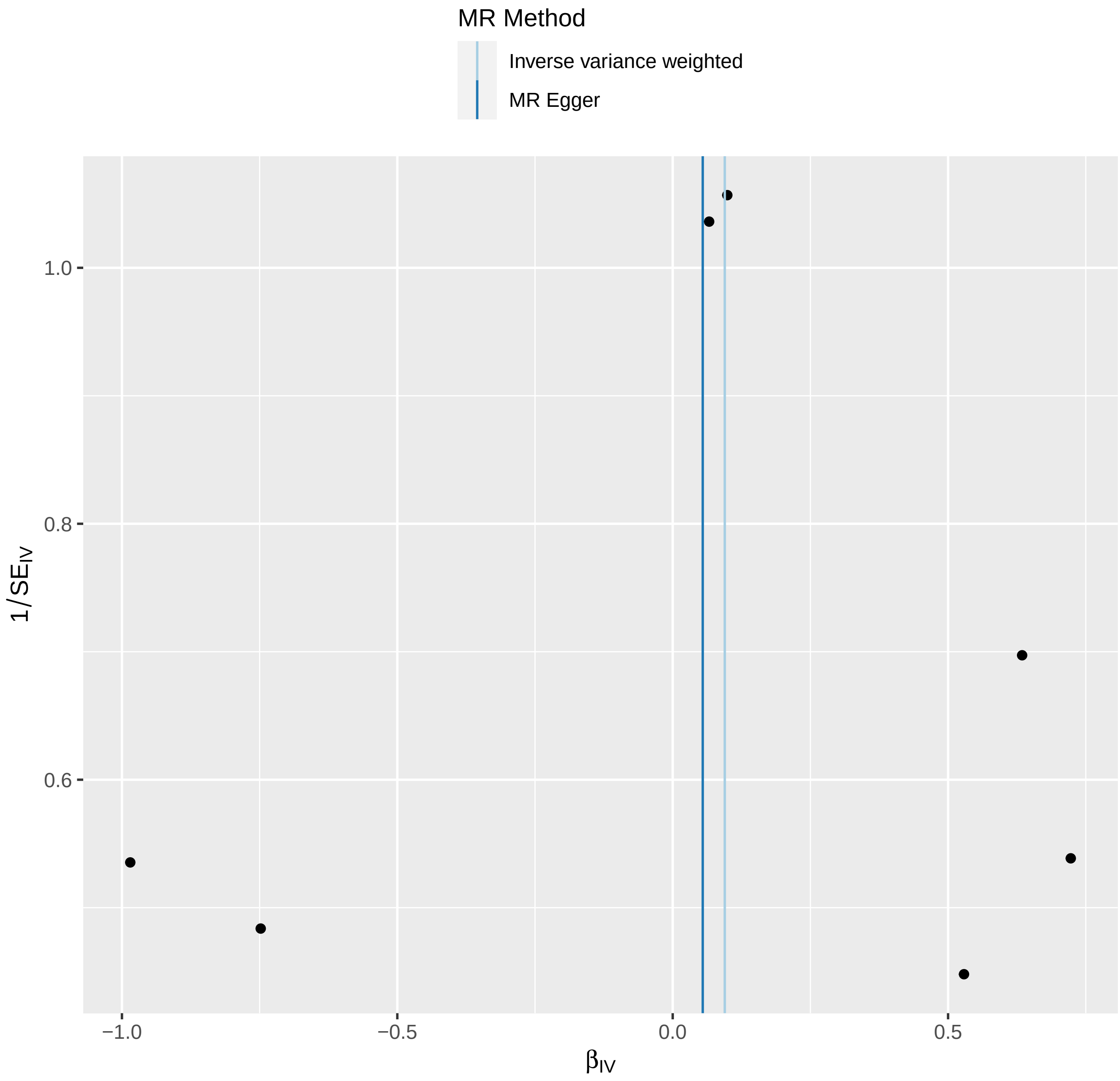

Batch 1347 : Dihomo−linolenate (20:3n3 or n6) on Ulcerative colitis with PSC

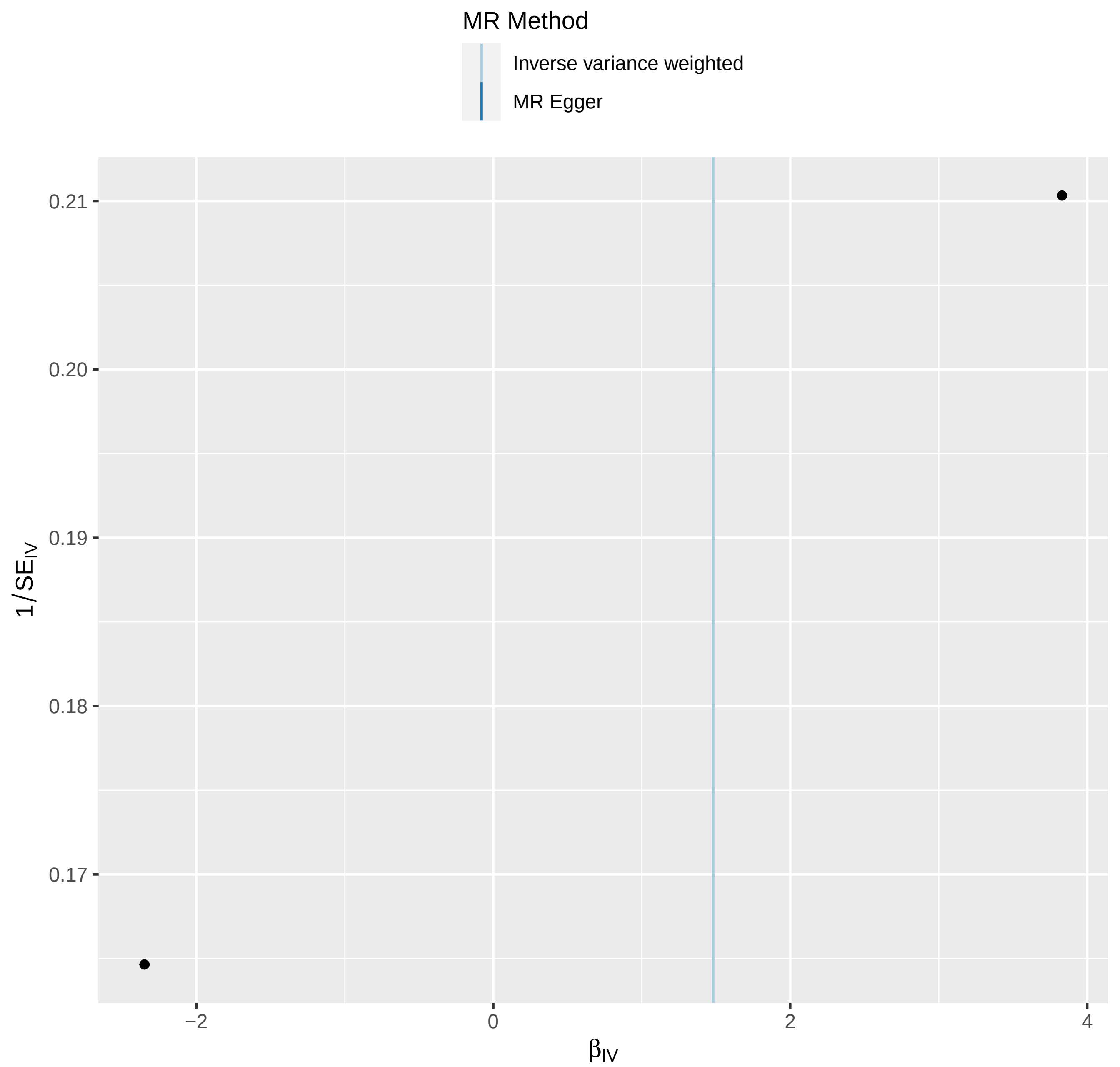

Batch 1348 : Threitol on Ulcerative colitis with PSC

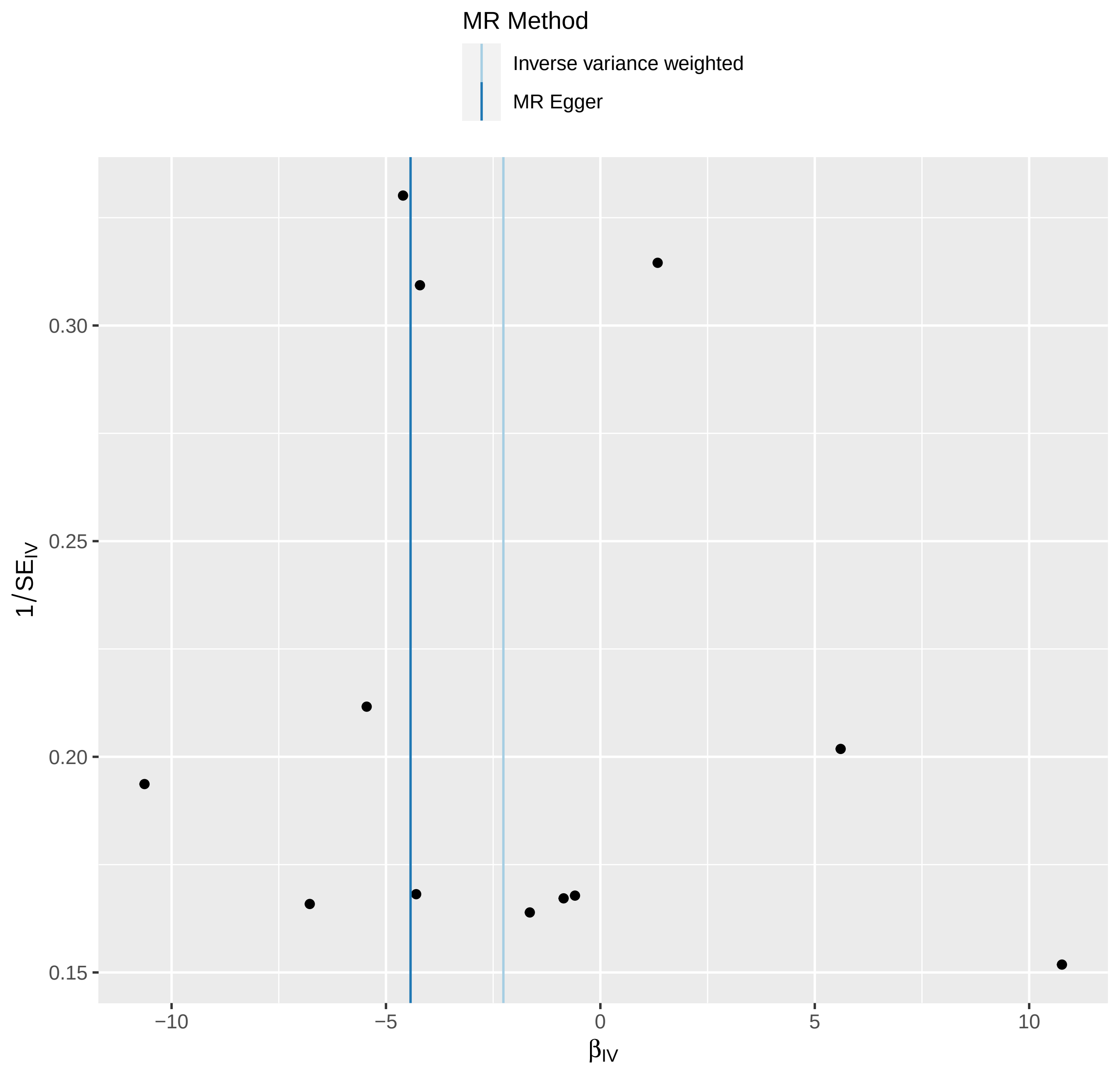

Batch 1349 : P−cresol sulfate on Ulcerative colitis with PSC

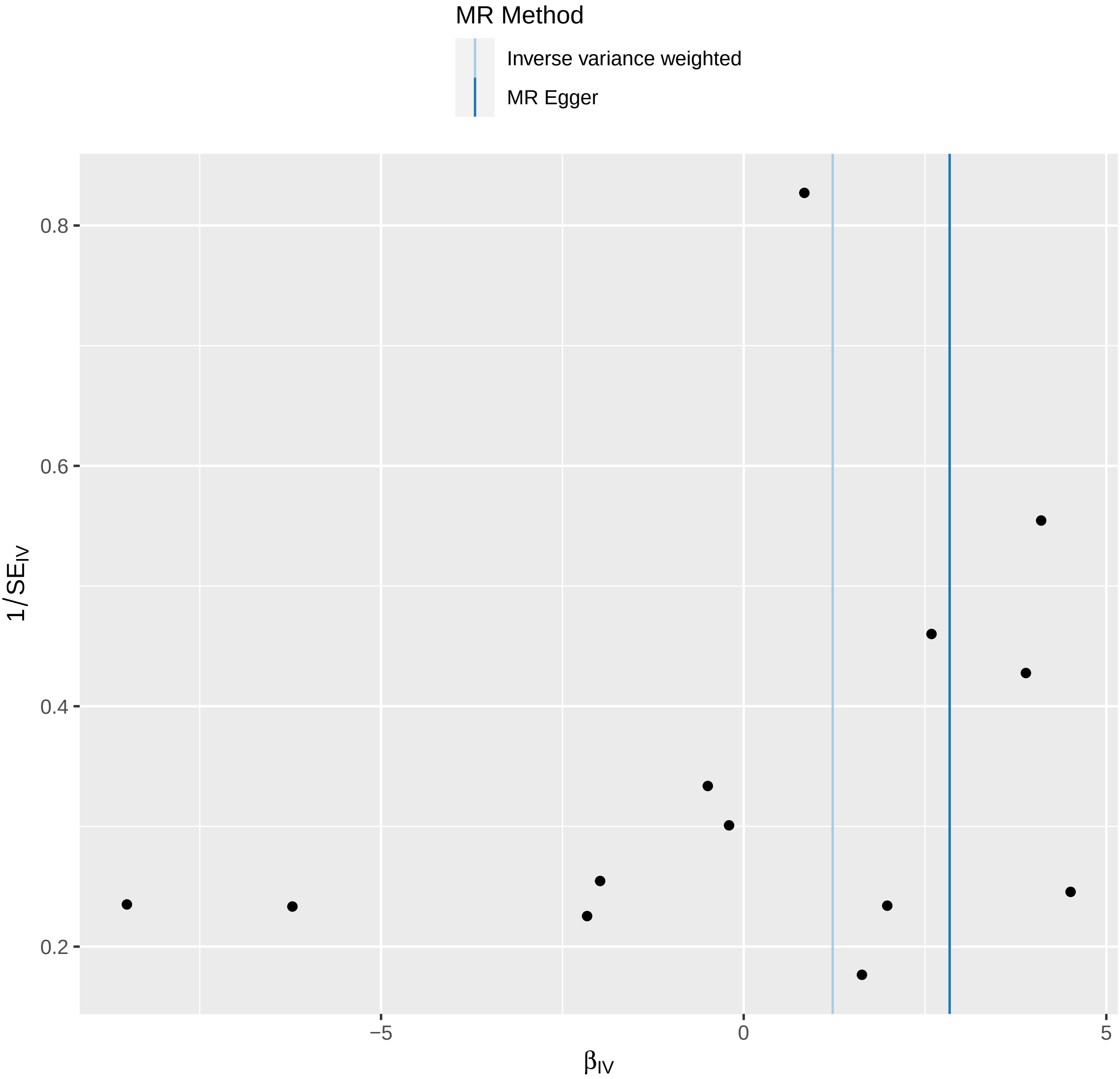

Batch 1352 : X−14208−−phenylalanylserine on Ulcerative colitis with PSC

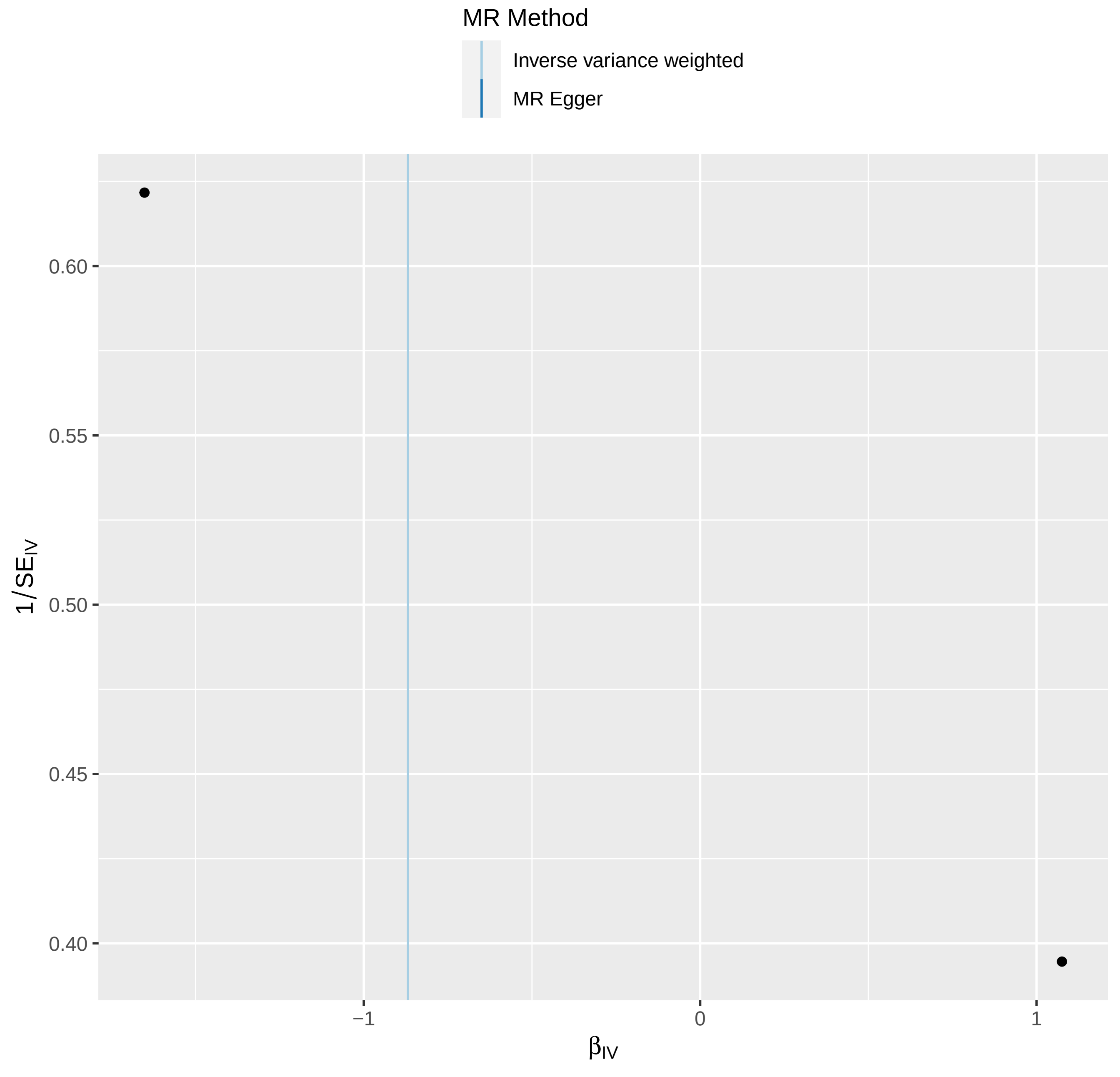

Batch 1354 : X−14450−−phenylalanylleucine on Ulcerative colitis with PSC

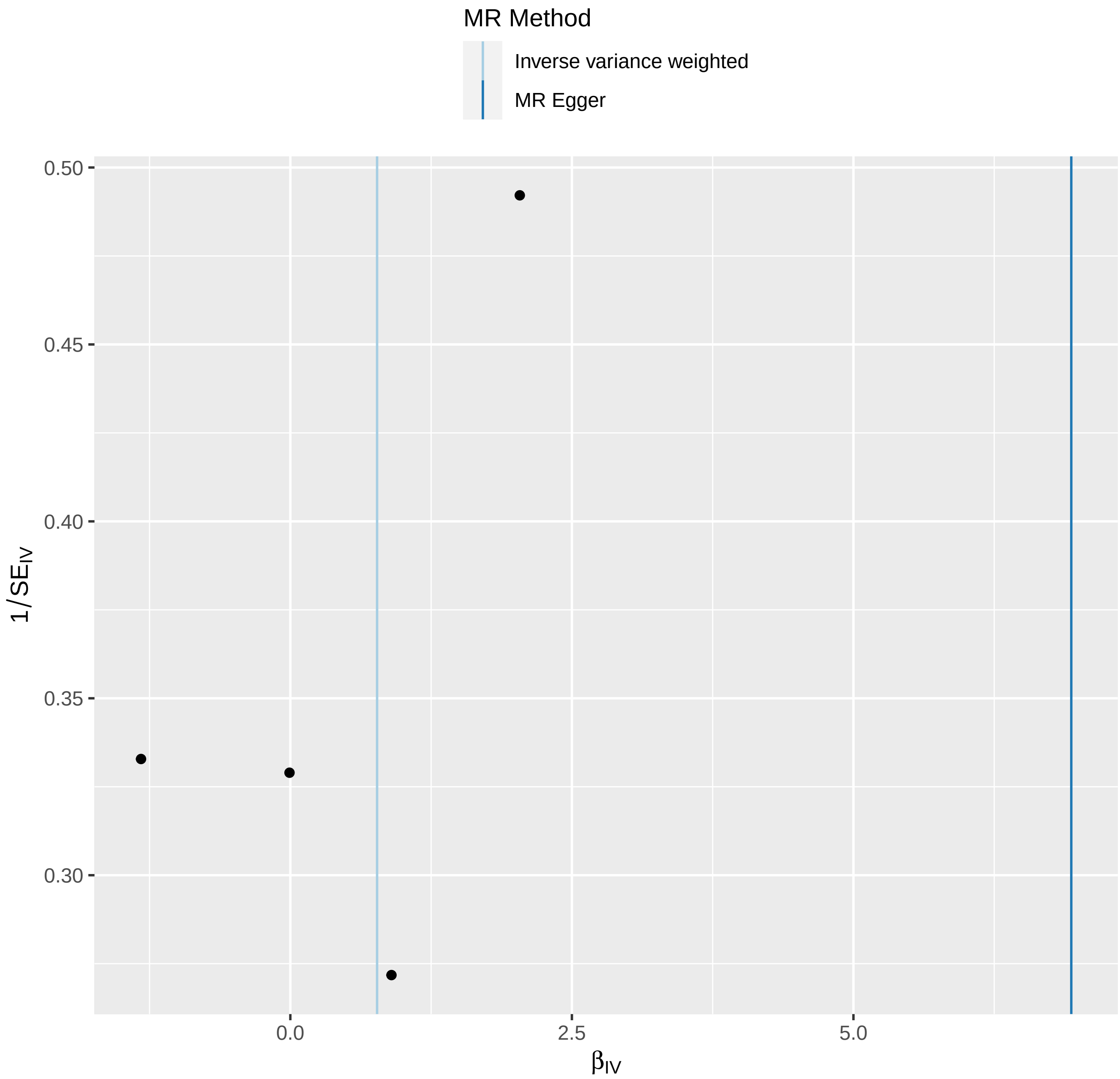

Batch 1355 : Gamma−glutamylglutamate on Ulcerative colitis with PSC

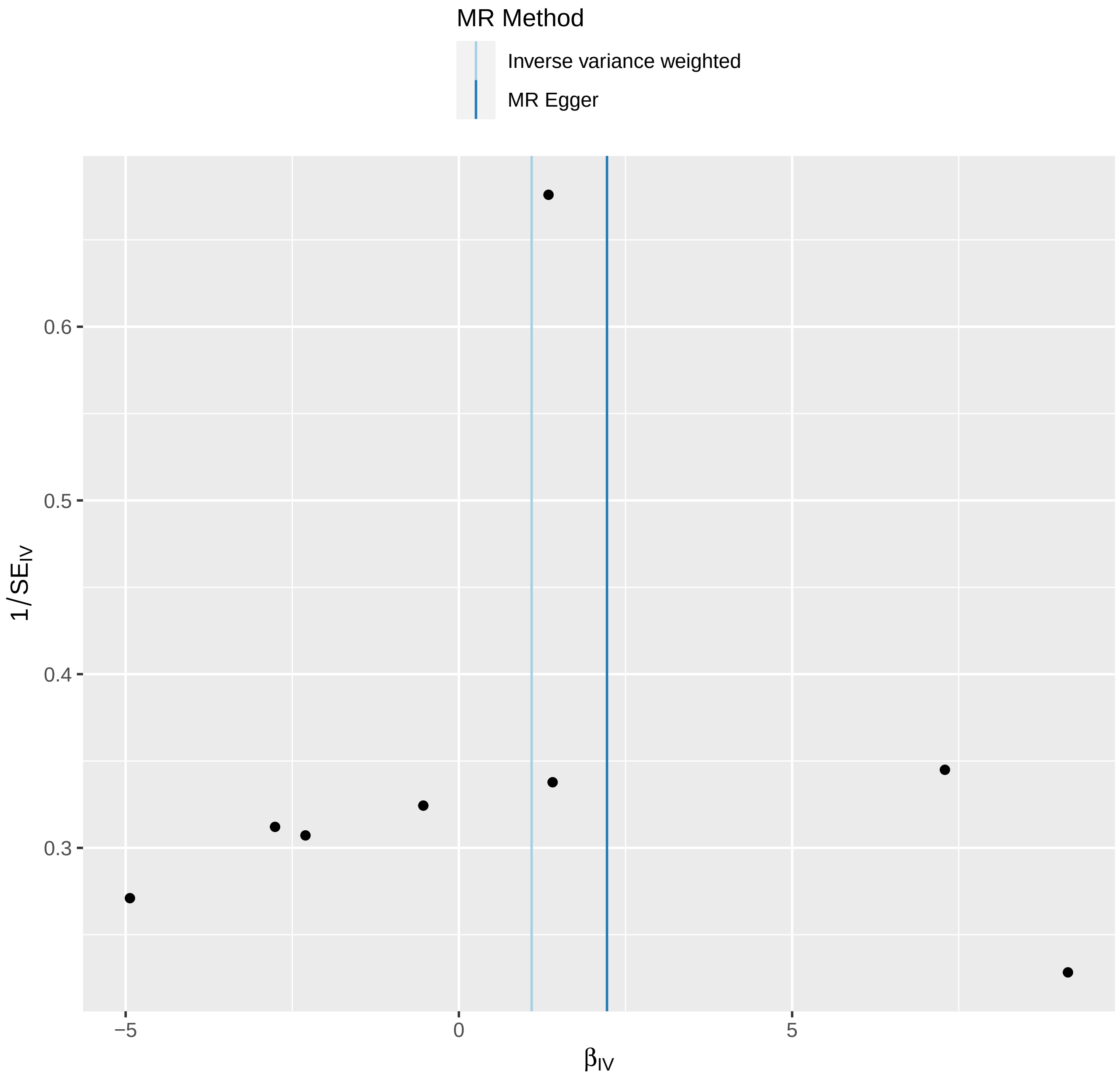

Batch 1356 : Octadecanedioate on Ulcerative colitis with PSC

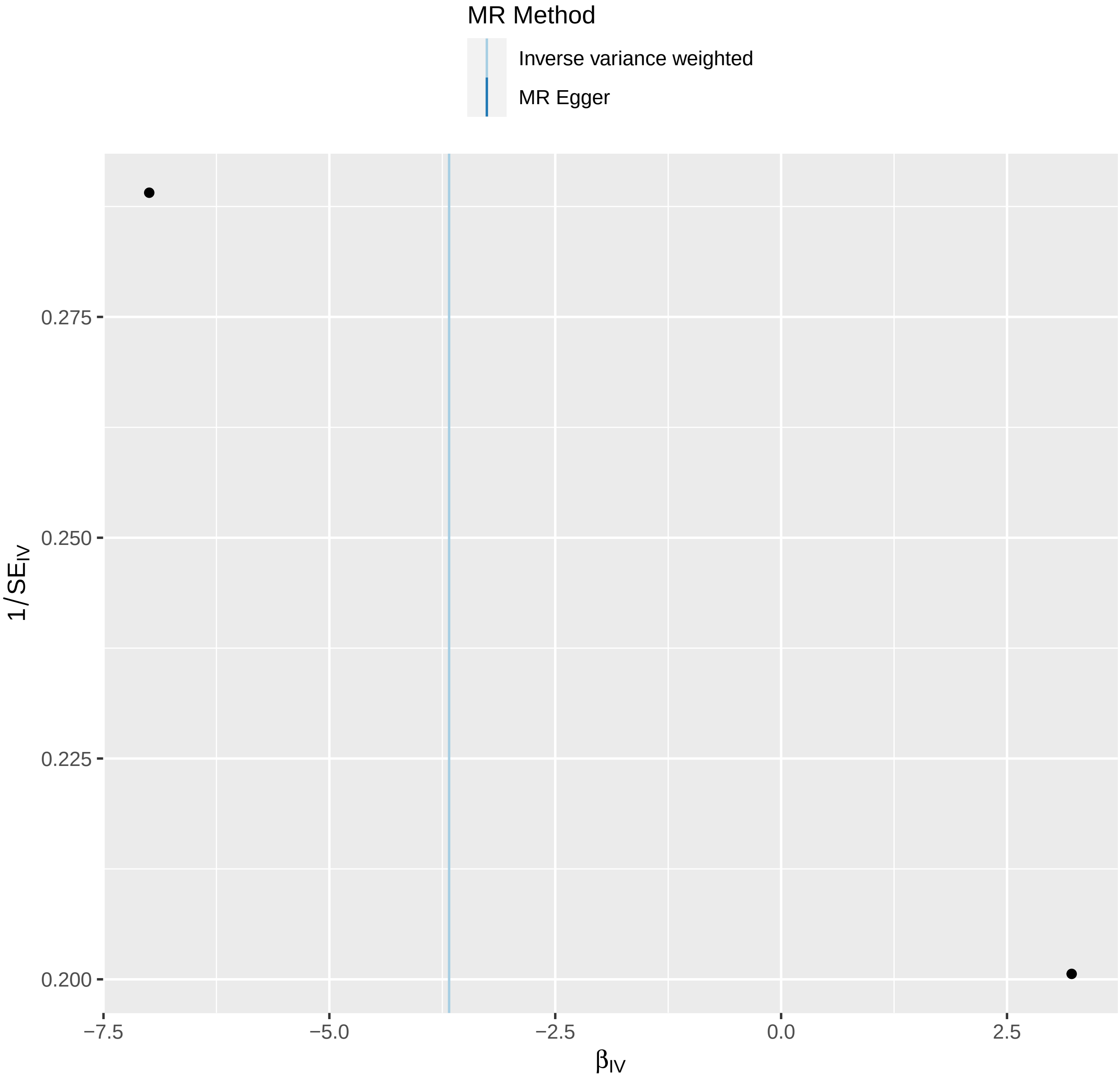

Batch 1357 : Leucylleucine on Ulcerative colitis with PSC

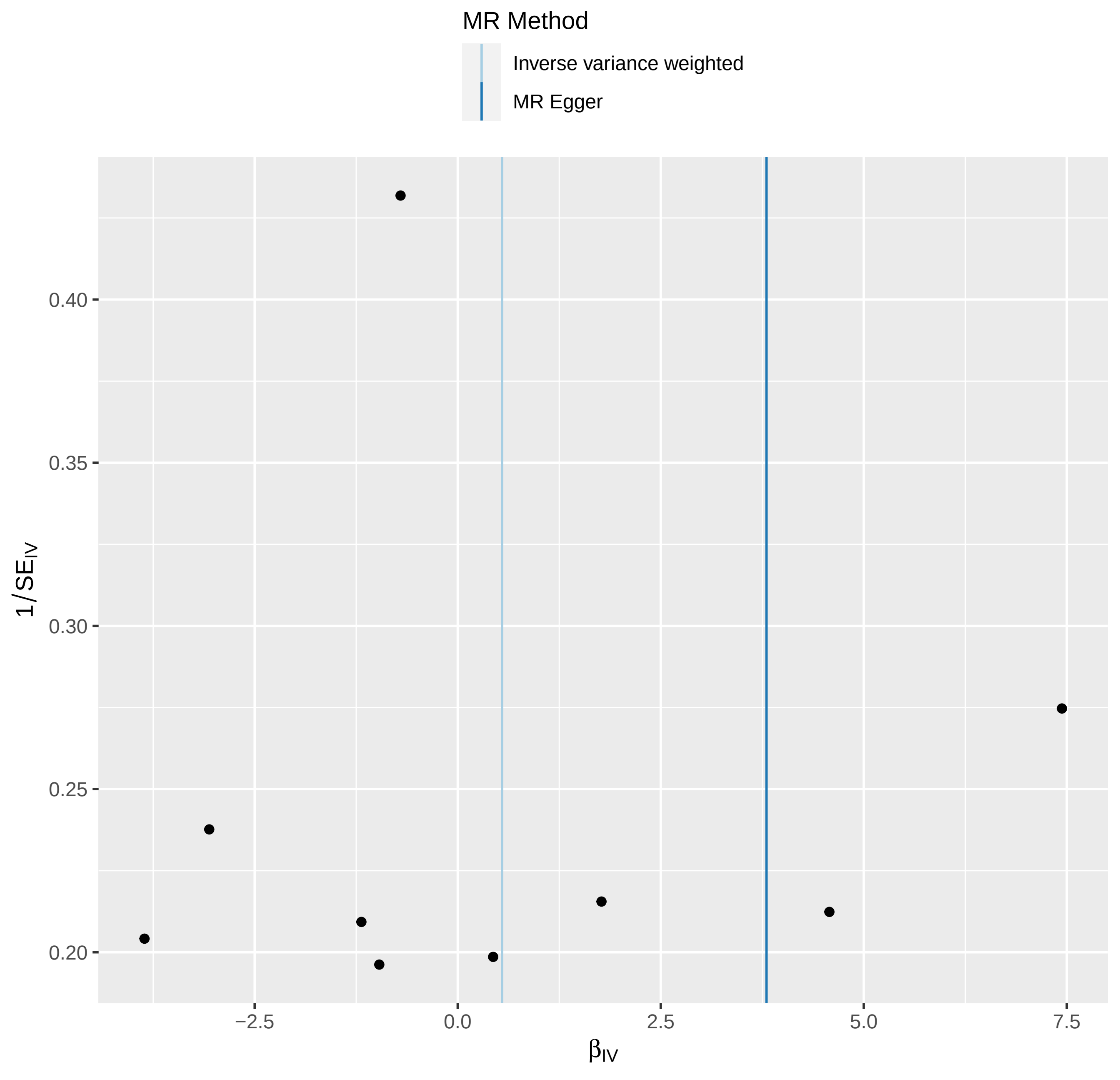

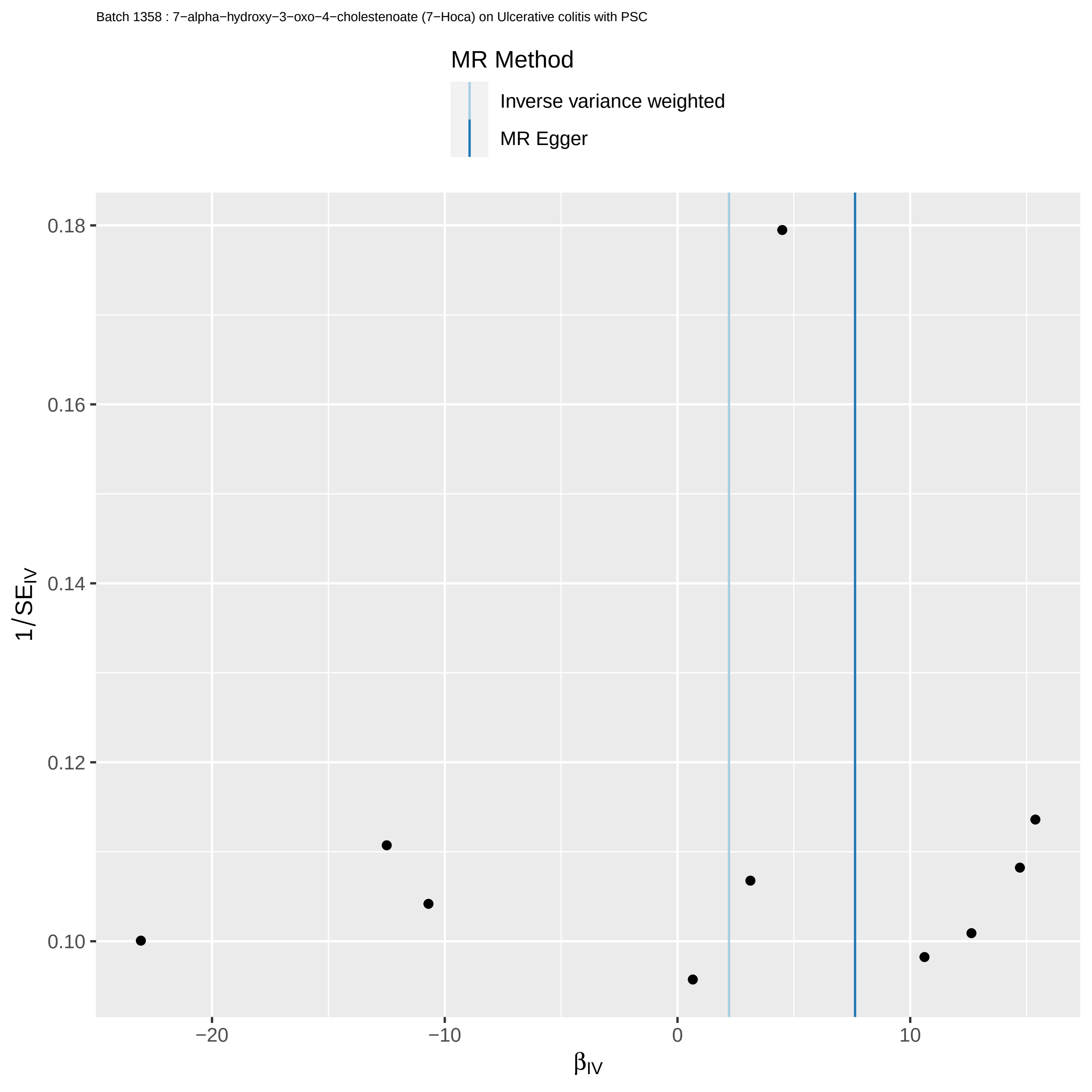

Batch 1358 : 7−alpha−hydroxy−3−oxo−4−cholestenoate (7−Hoca) on Ulcerative colitis with PSC
MR Method
Inverse variance weighted
MR Egger
0.18
0.16
0.14
0.12
0.10
1/SE_IV
−20
−10
0
10
β_IV

Batch 1359 : N−Butyl Oleate on Ulcerative colitis with PSC

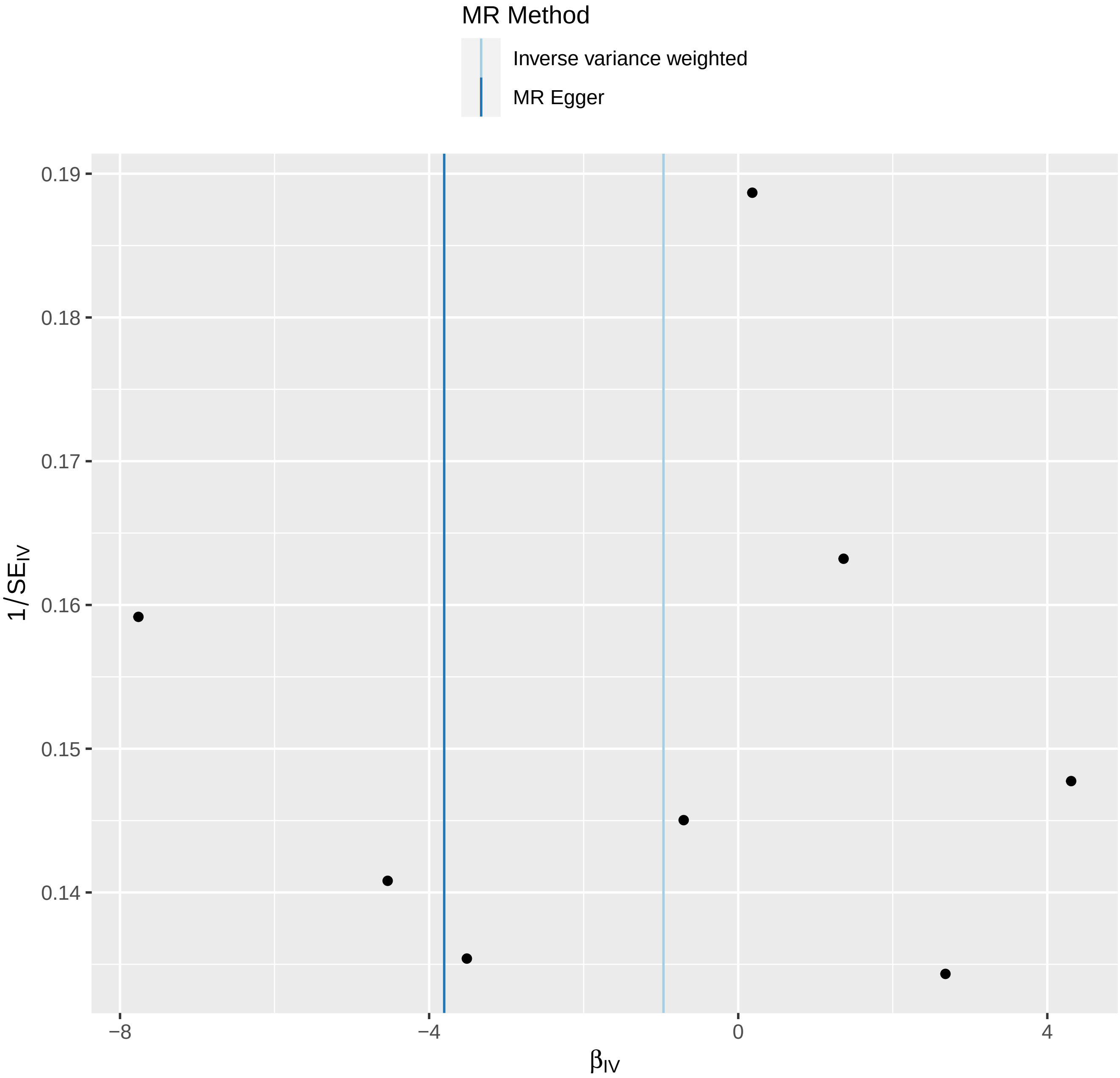

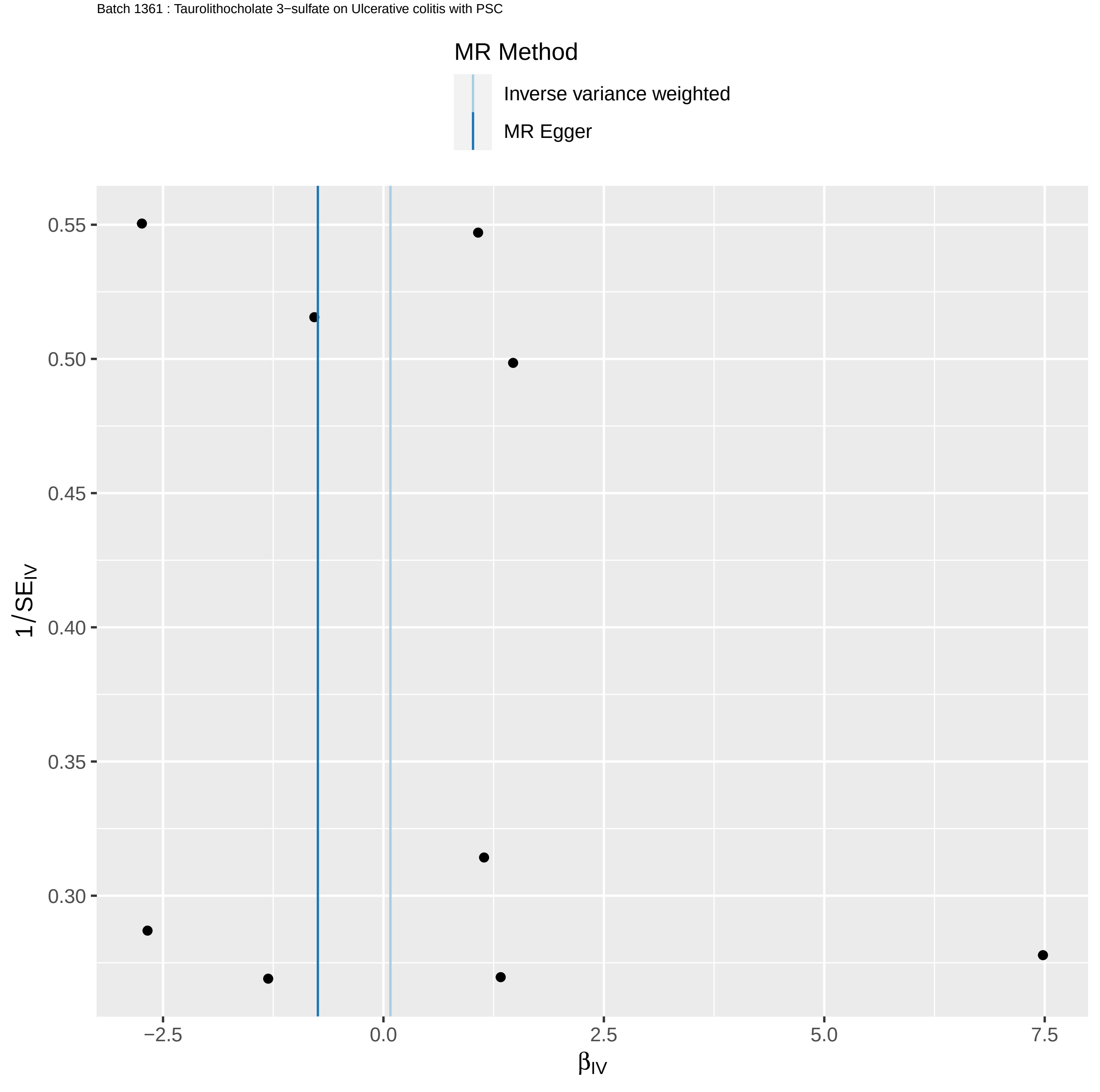

Batch 1361 : Taurolithocholate 3−sulfate on Ulcerative colitis with PSC
MR Method
Inverse variance weighted
MR Egger
0.55
0.50
0.45
0.40
0.35
0.30
1/SE$_{IV}$
−2.5
0.0
2.5
5.0
7.5
β$_{IV}$

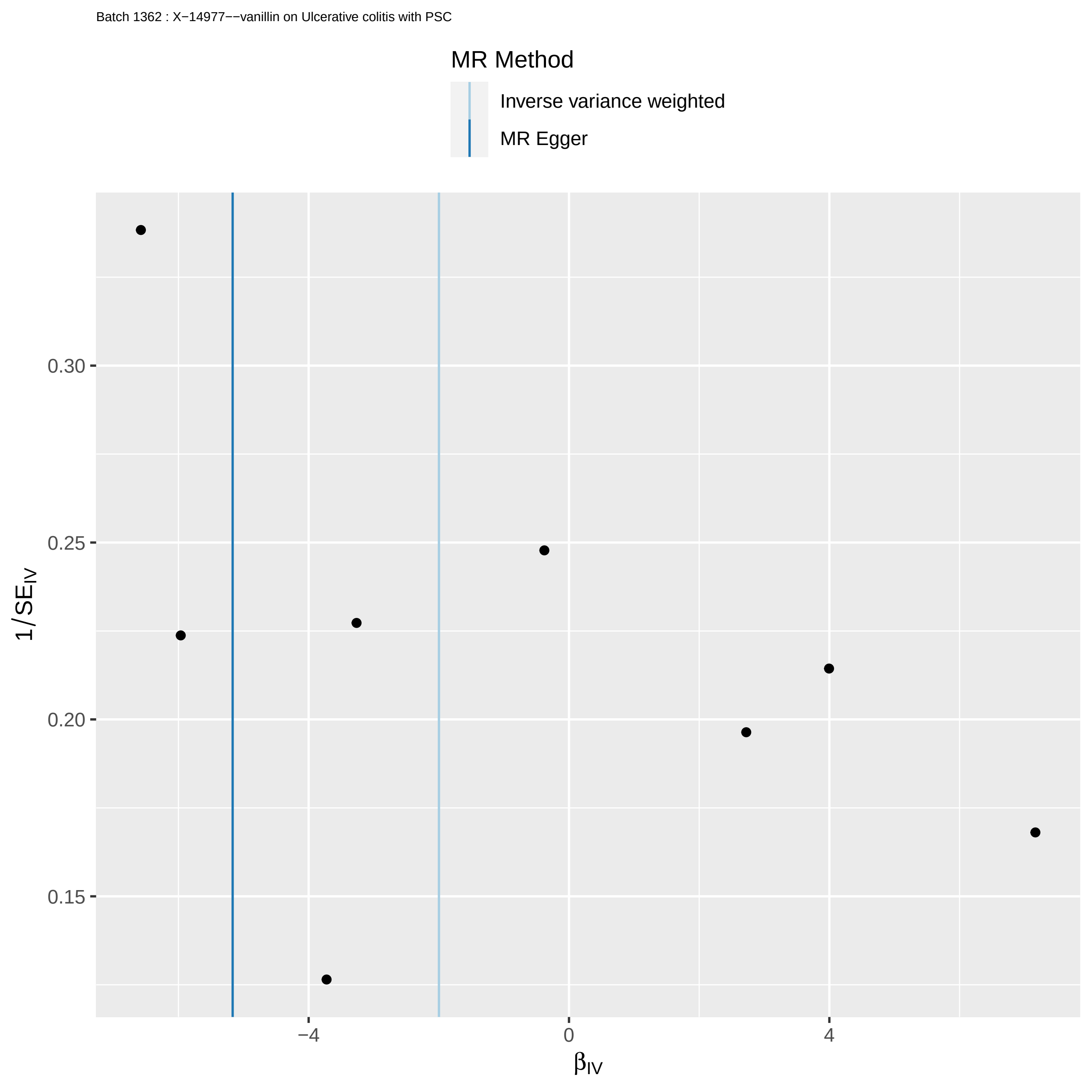
Batch 1362 : X−14977−−vanillin on Ulcerative colitis with PSC
MR Method
Inverse variance weighted
MR Egger
0.30
0.25
0.20
0.15
1/SE$_{IV}$
−4
0
4
β$_{IV}$

Batch 1363 : Succinylcarnitine on Ulcerative colitis with PSC

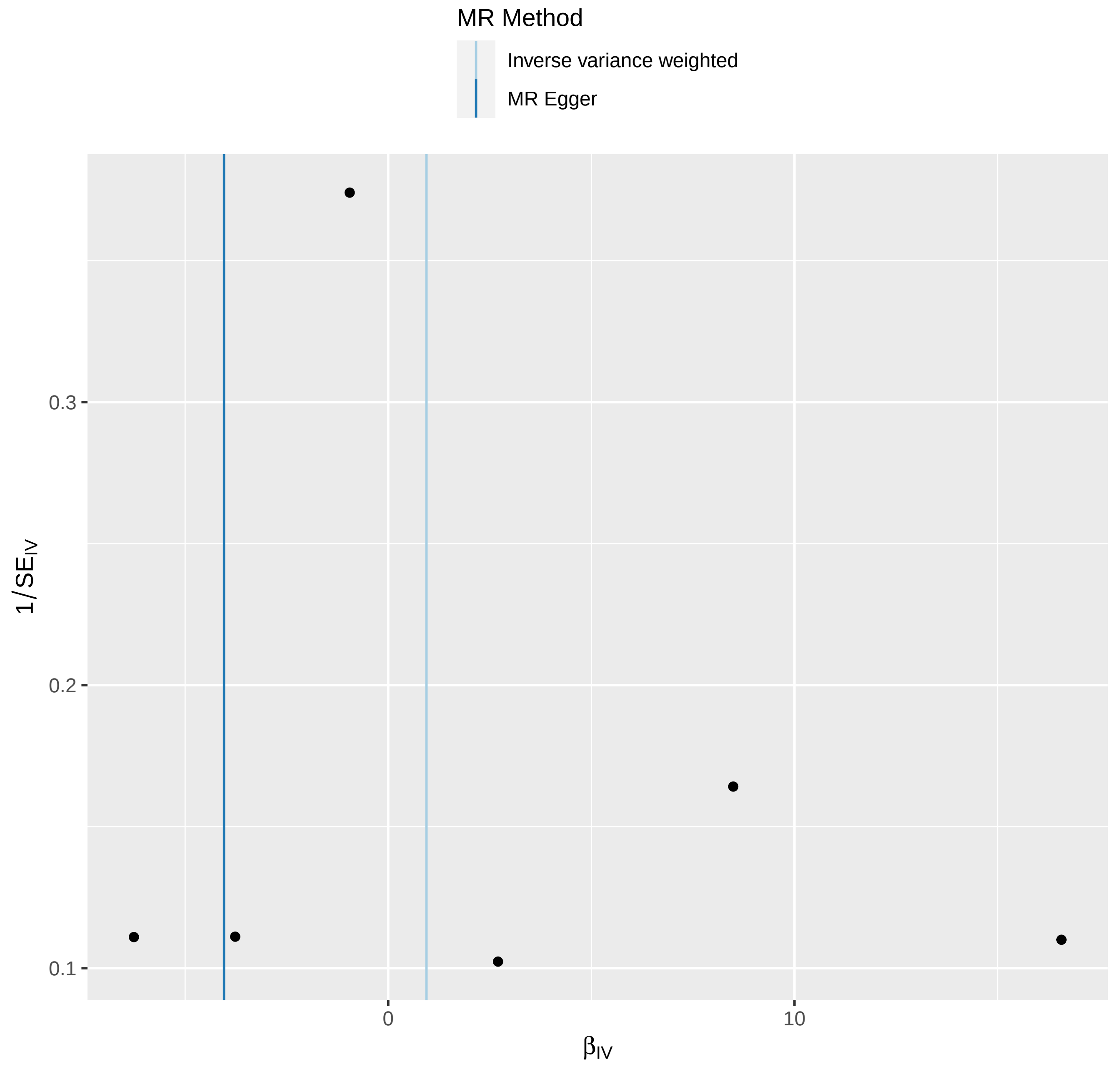

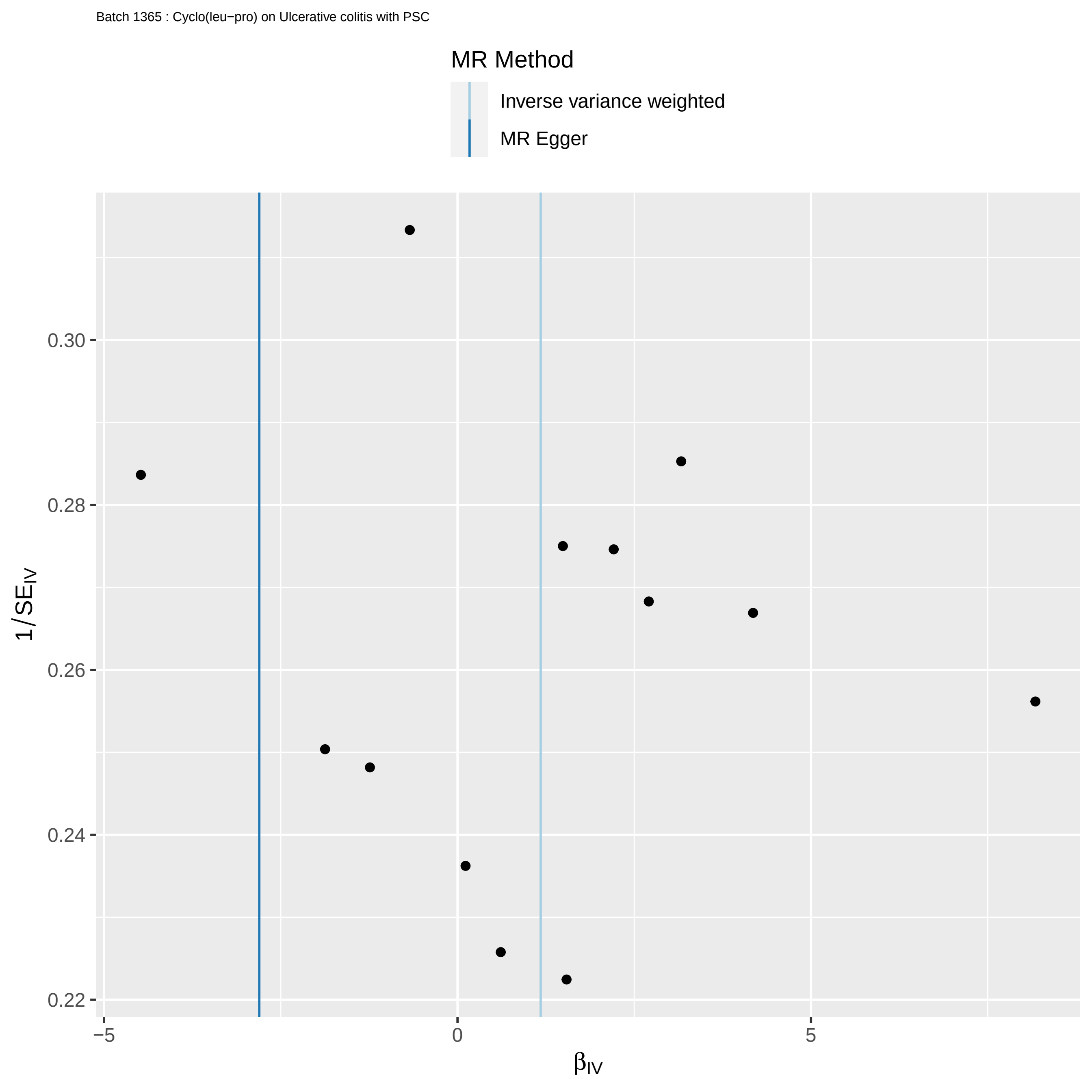
Batch 1365 : Cyclo(leu−pro) on Ulcerative colitis with PSC
MR Method
Inverse variance weighted
MR Egger
0.30
0.28
0.26
0.24
0.22
1/SE_IV
−5
0
5
β_IV

Batch 1366 : Chiro−inositol on Ulcerative colitis with PSC

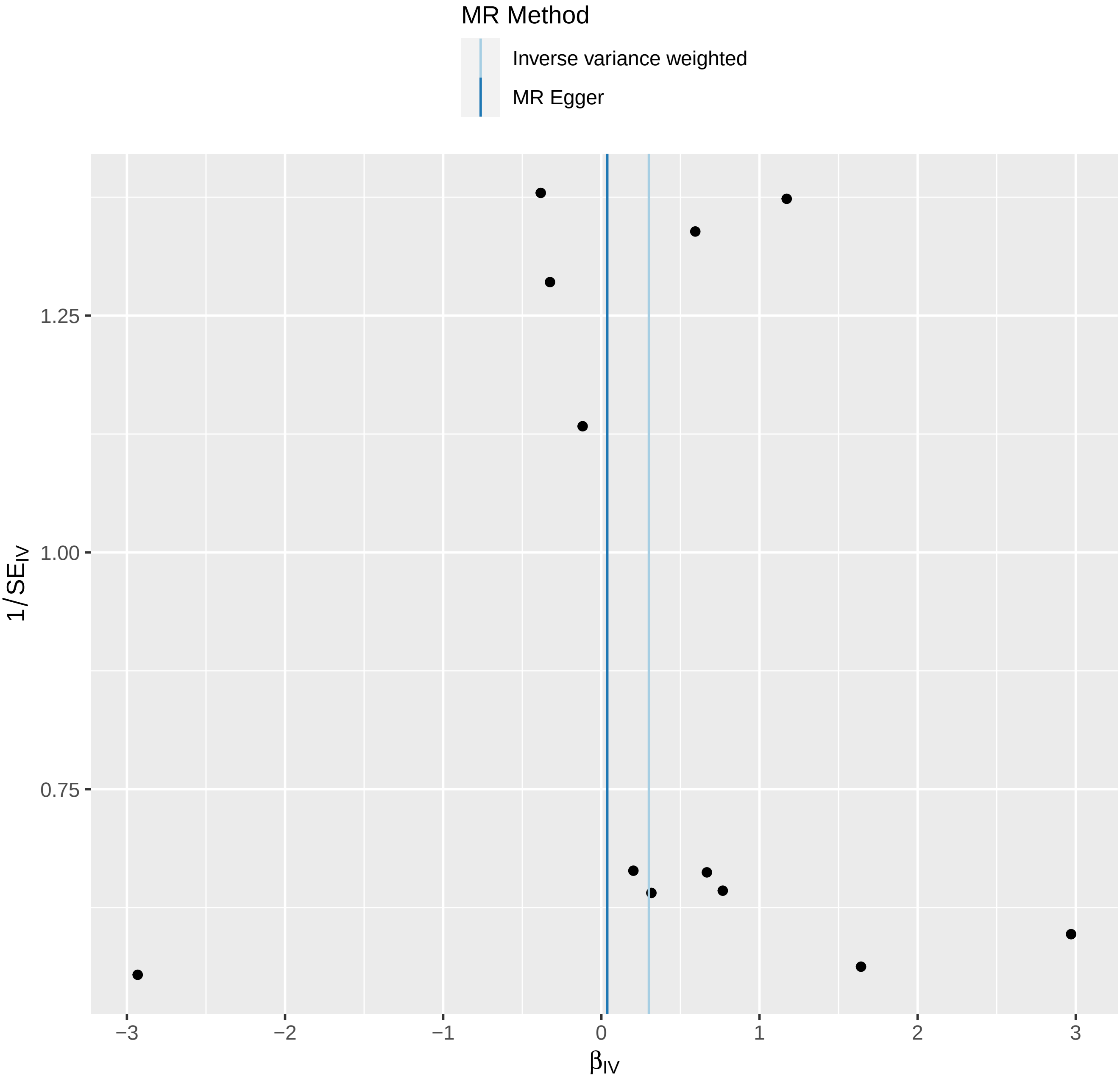

Batch 1370 : 2−hydroxyglutarate on Ulcerative colitis with PSC

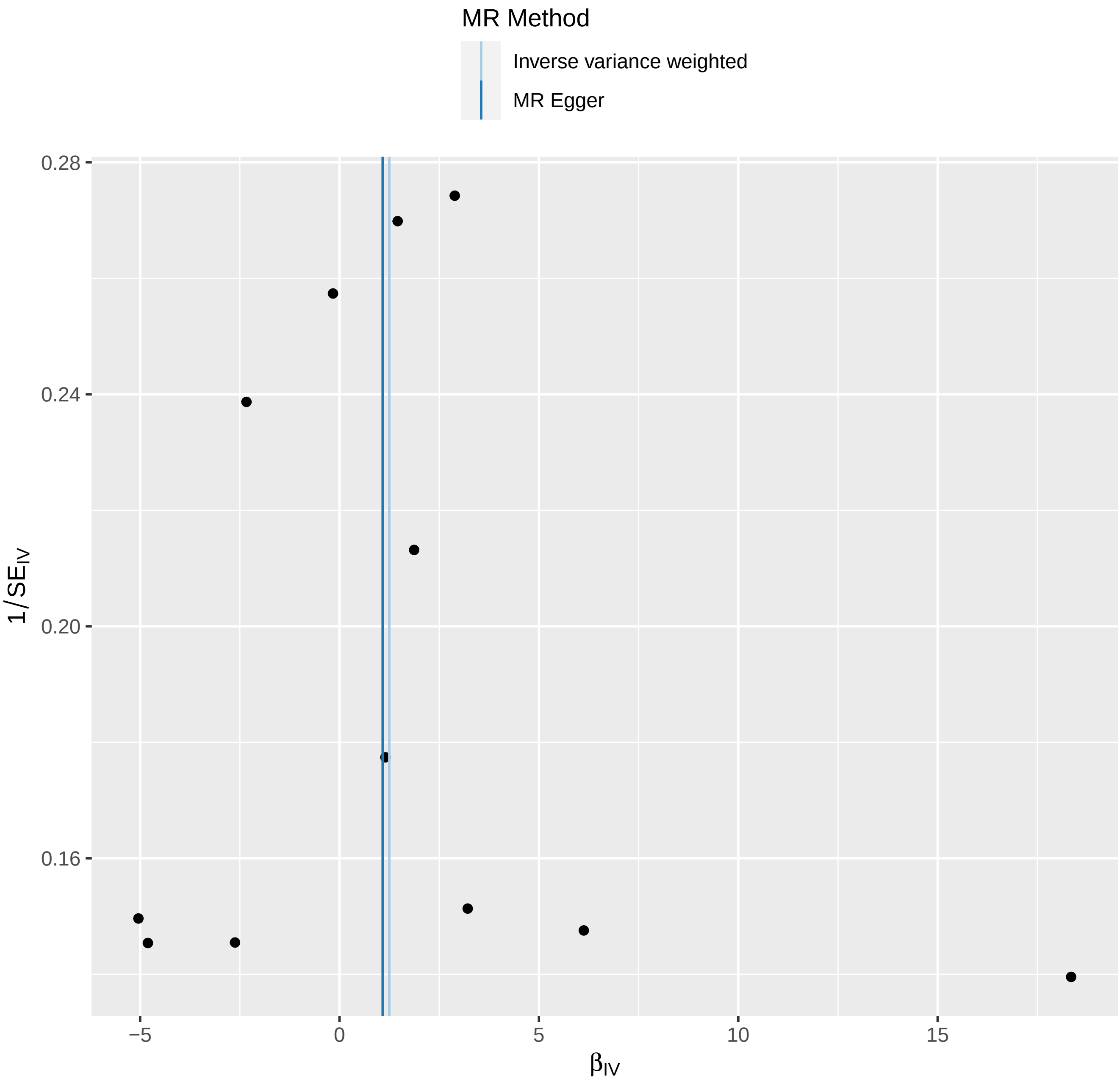

Batch 1371 : Ergothioneine on Ulcerative colitis with PSC

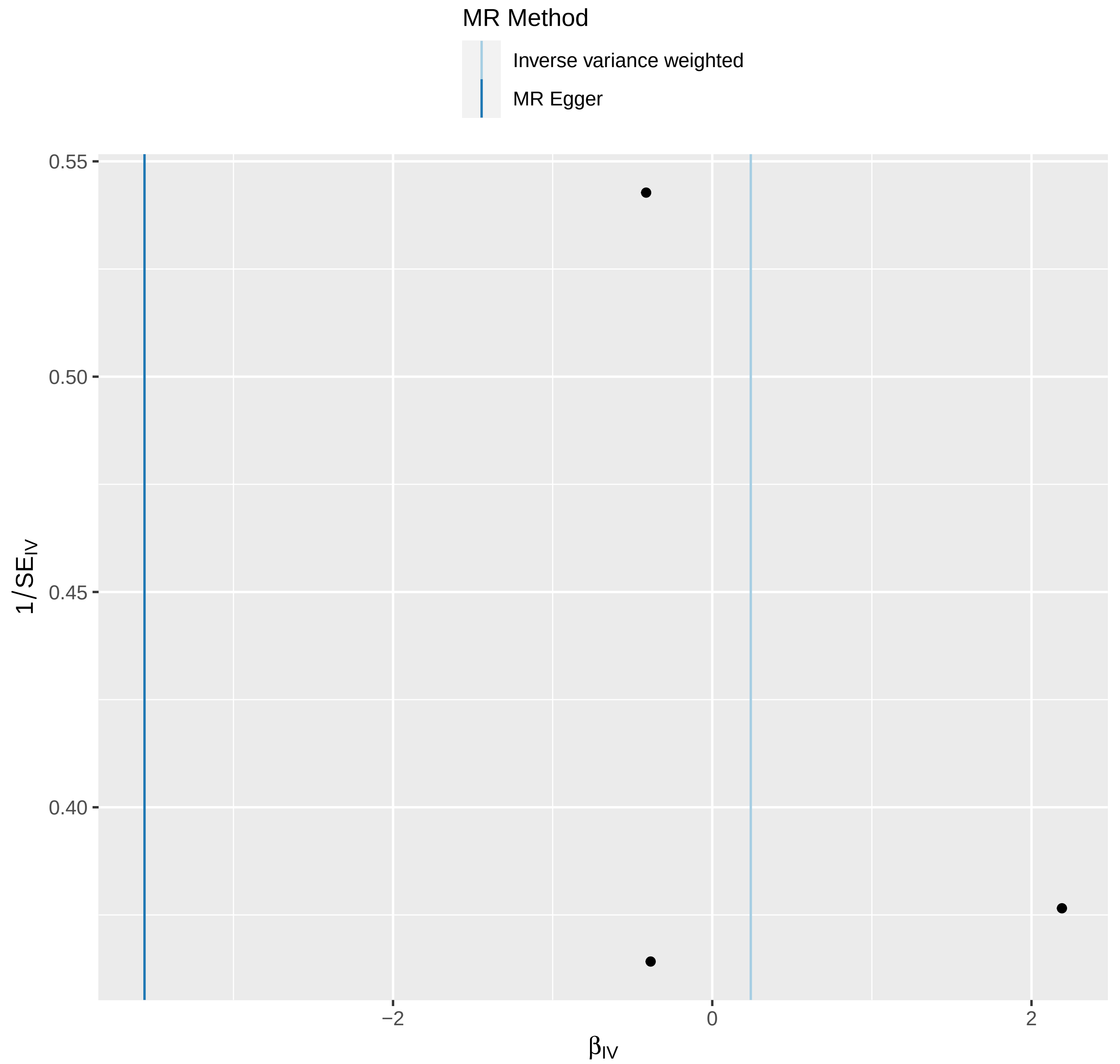

Batch 1373 : Phenylalanylphenylalanine on Ulcerative colitis with PSC

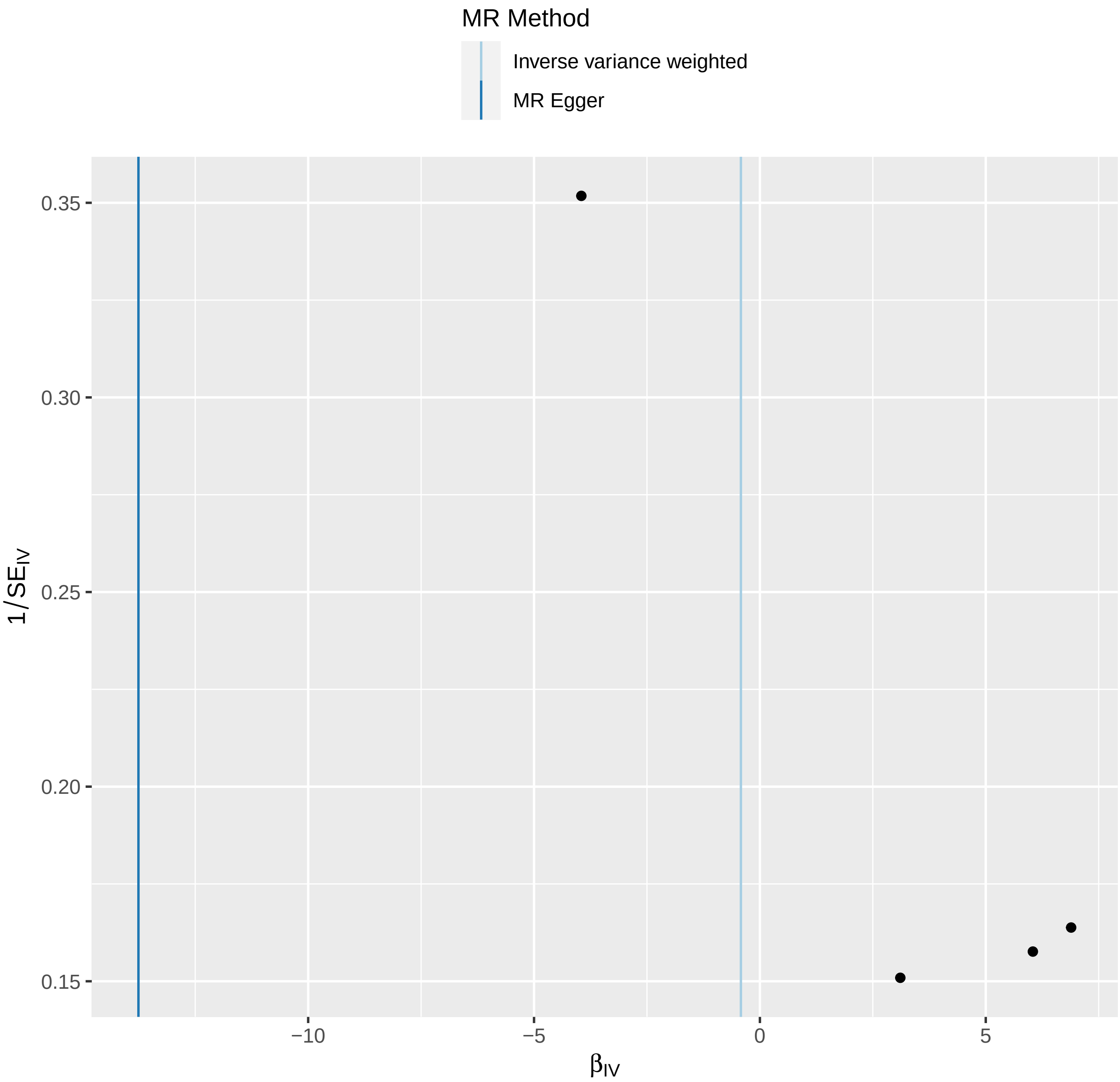

Batch 1374 : Cis−4−decenoyl carnitine on Ulcerative colitis with PSC

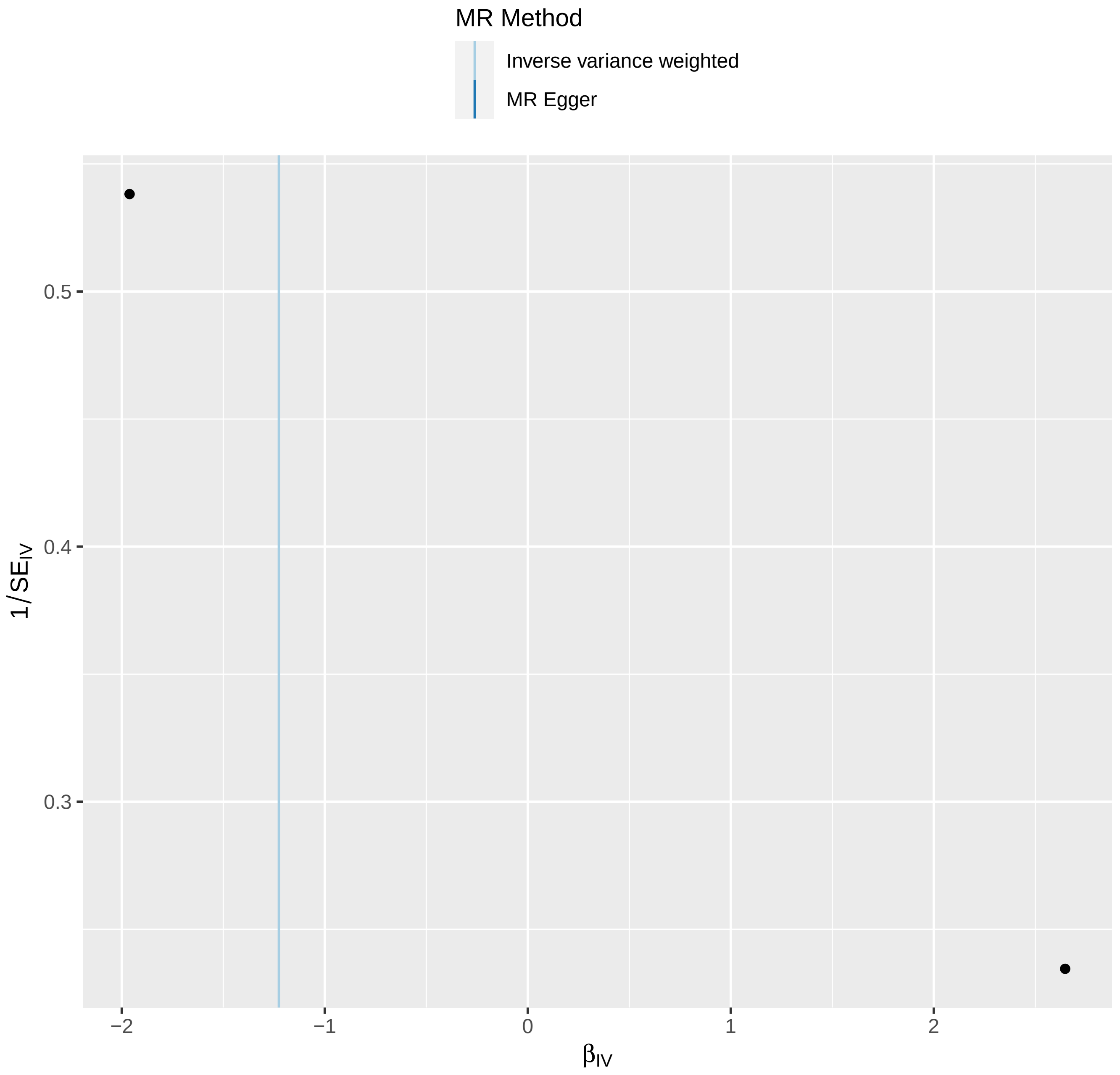

Batch 1375 : 15−methylpalmitate (isobar with 2−methylpalmitate) on Ulcerative colitis with PSC

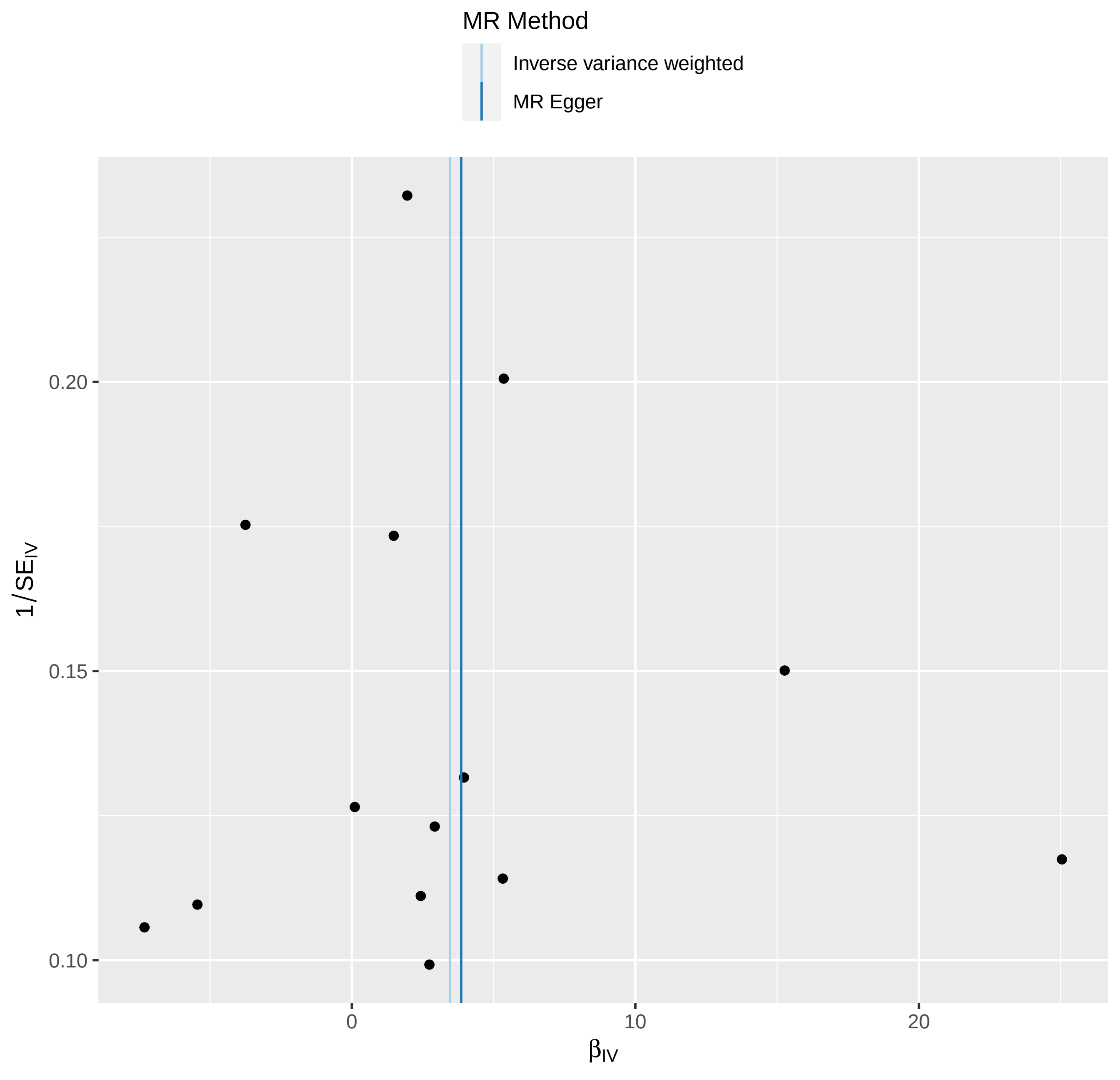

Batch 1377 : Tryptophan on Left−sided ulcerative colitis

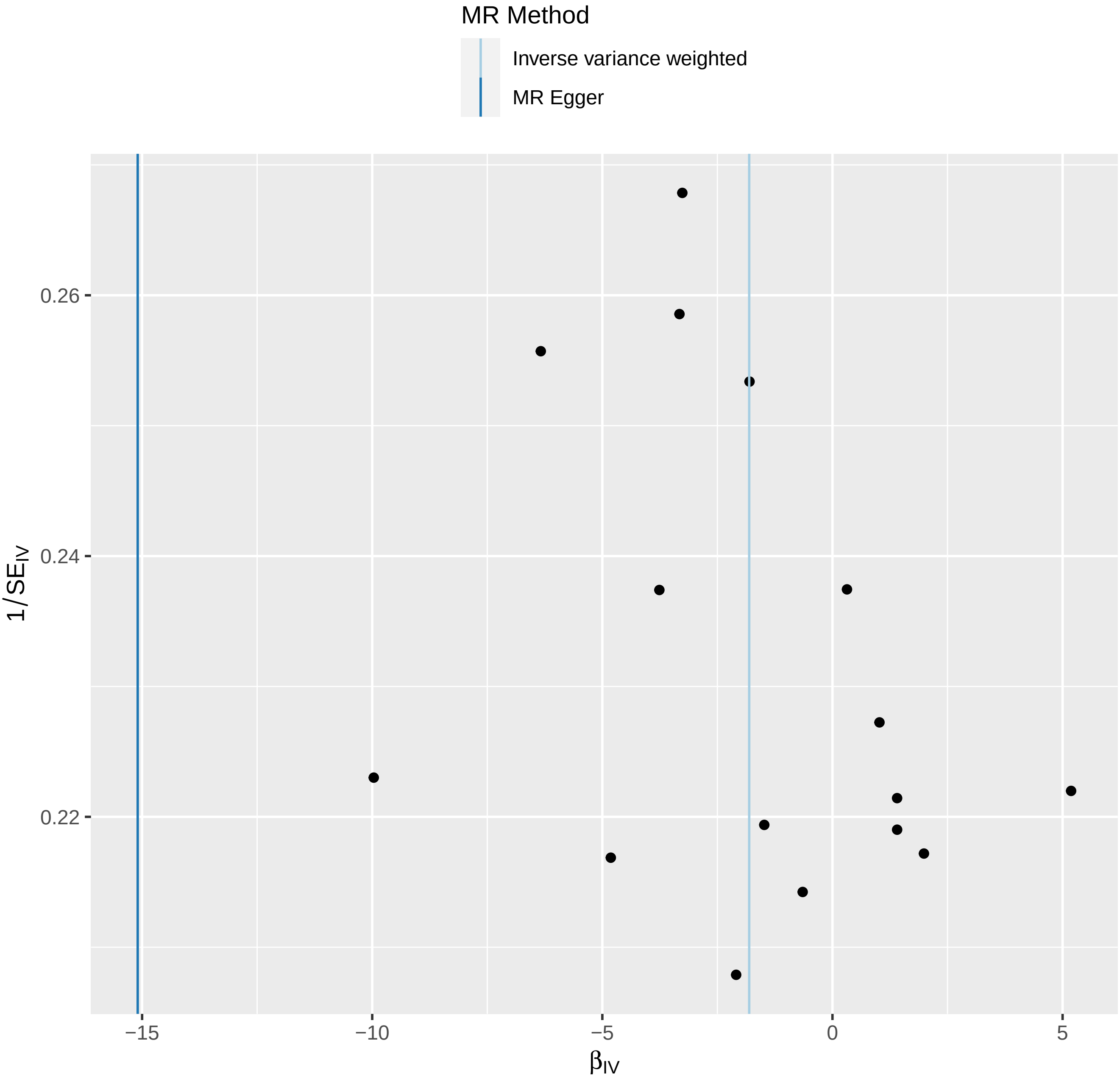

Batch 1379 : Leucine on Left−sided ulcerative colitis

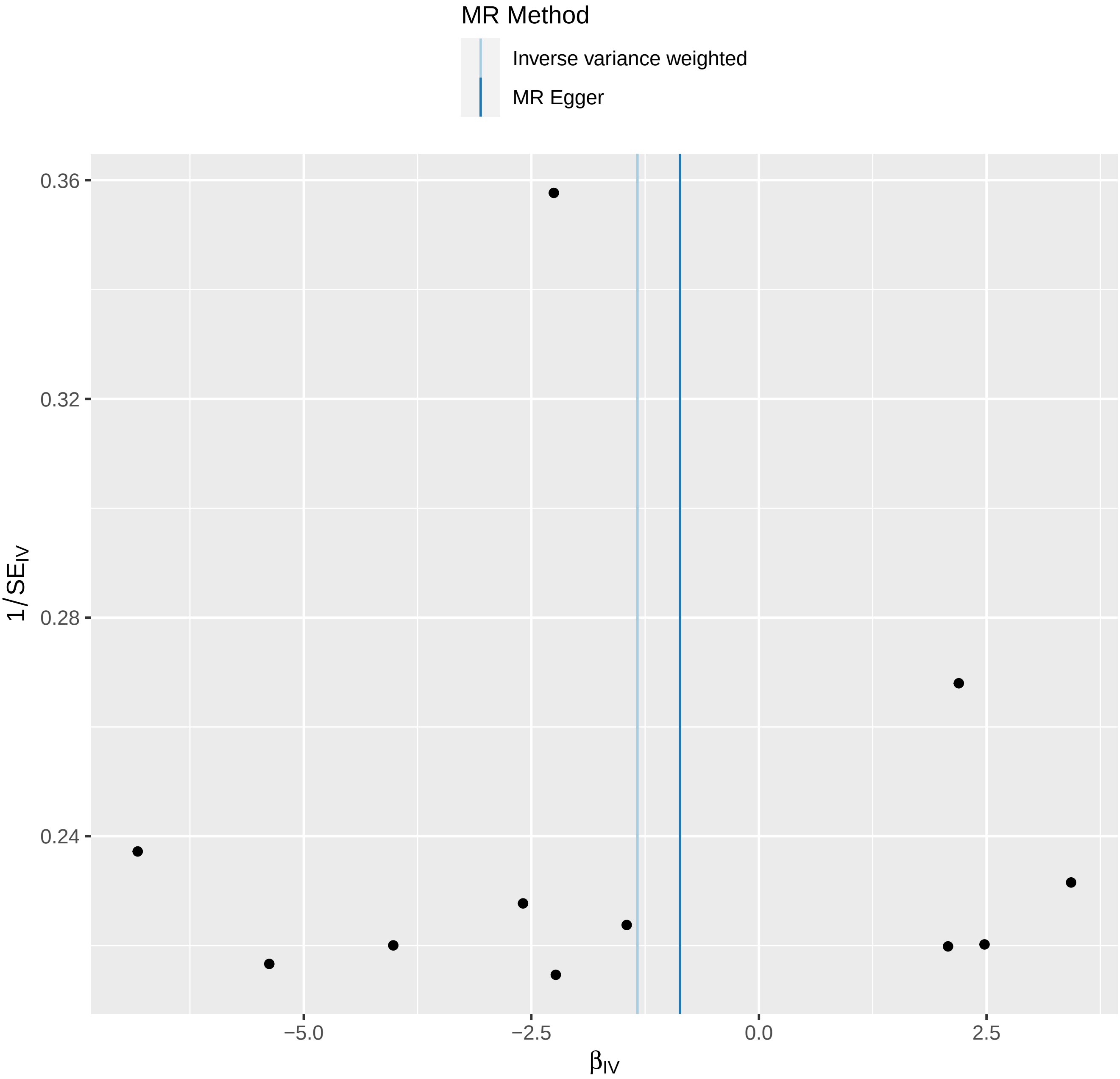

Batch 1383 : Lactate on Left−sided ulcerative colitis

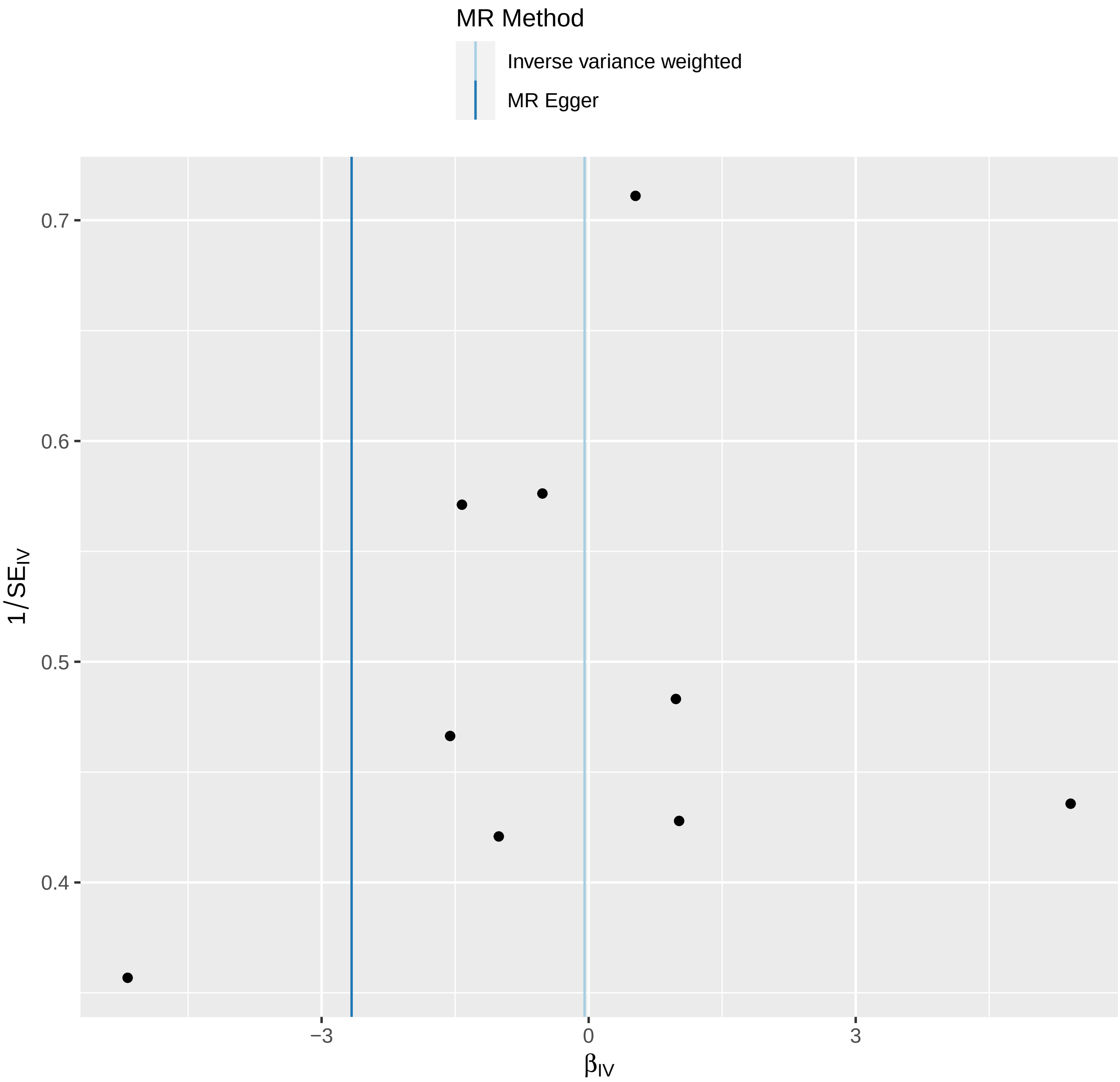

Batch 1384 : 3−hydroxybutyrate (BHBA) on Left−sided ulcerative colitis

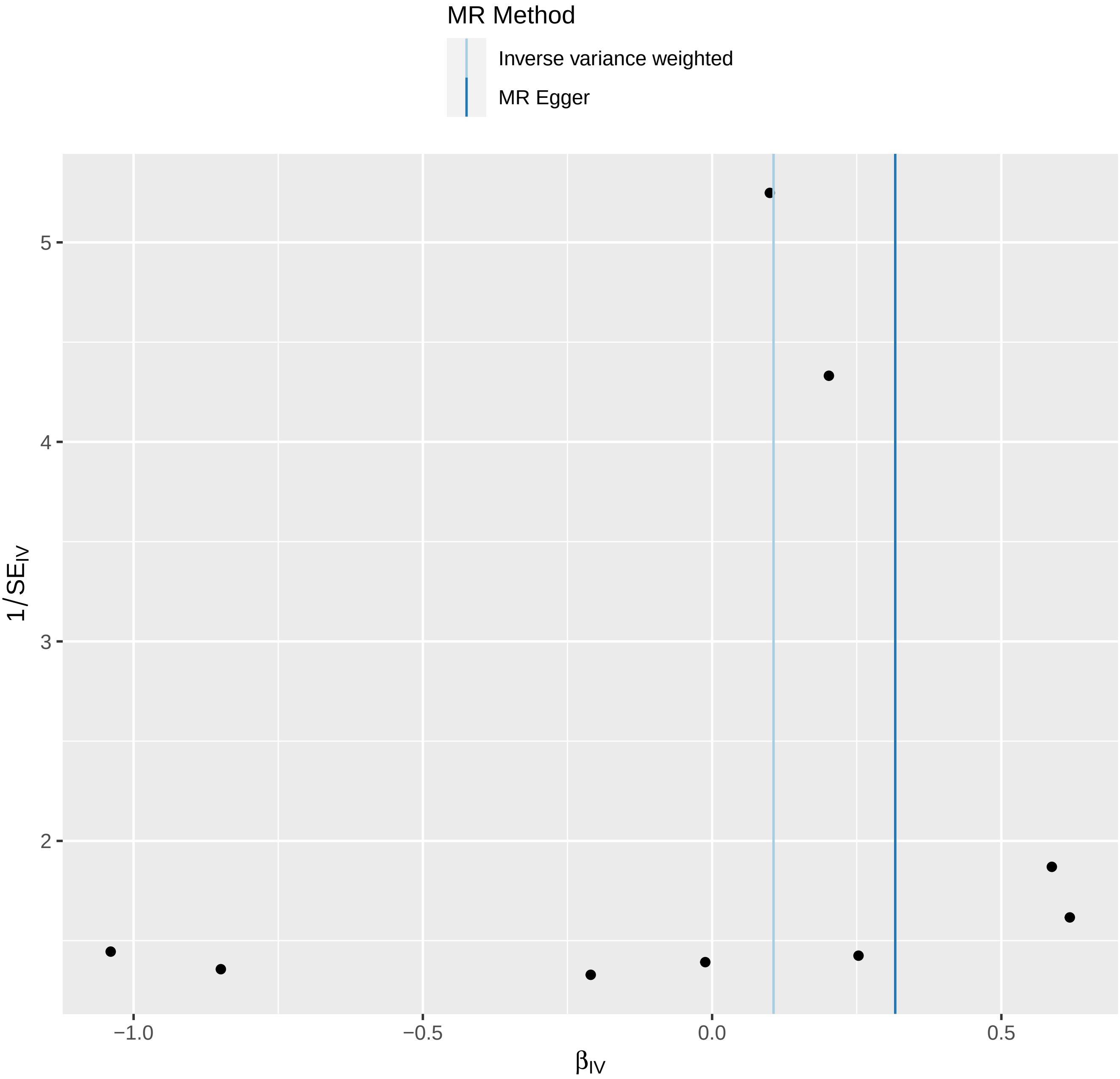

Batch 1385 : Arabinose on Left−sided ulcerative colitis

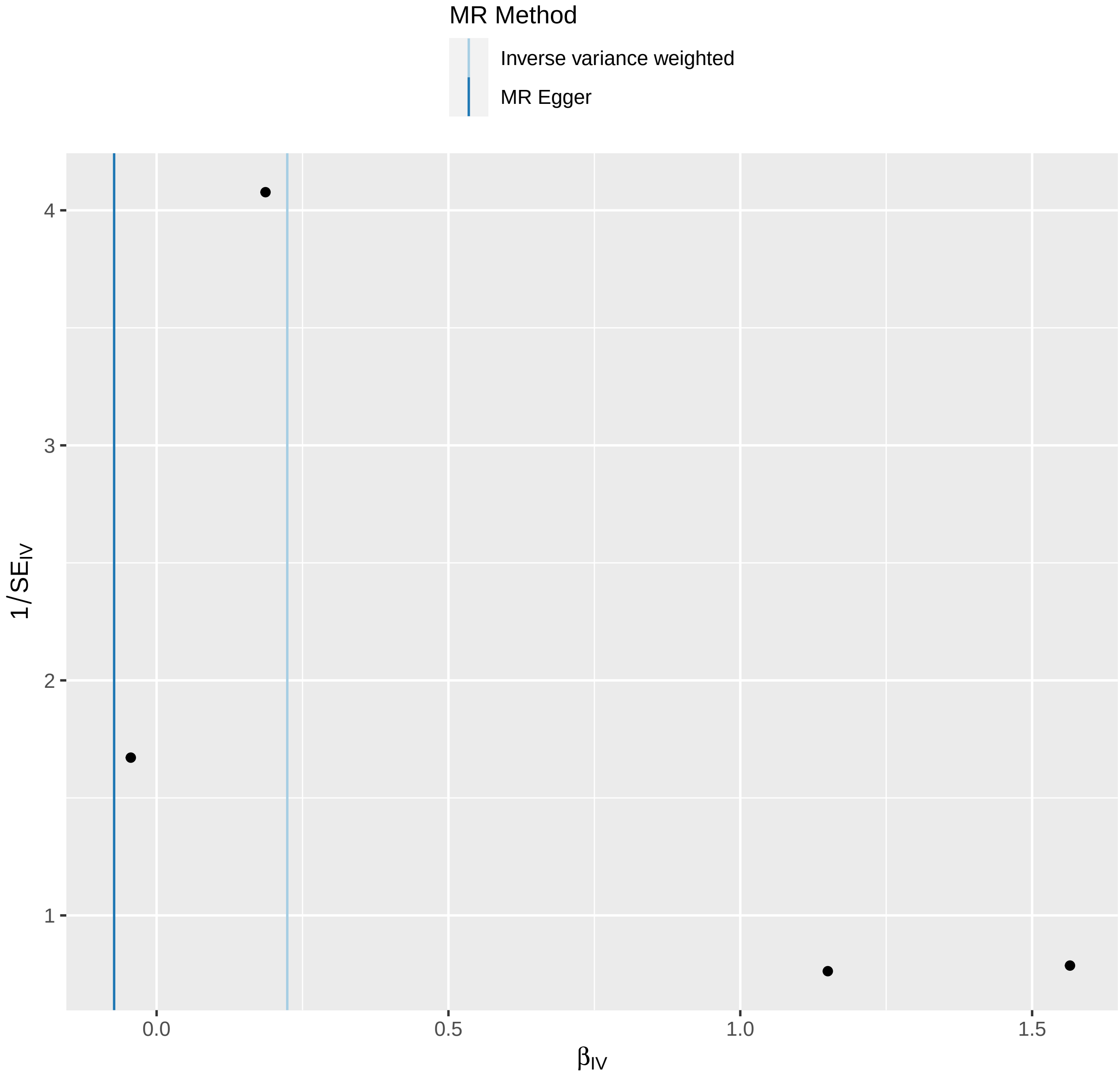

Batch 1386 : Fructose on Left−sided ulcerative colitis

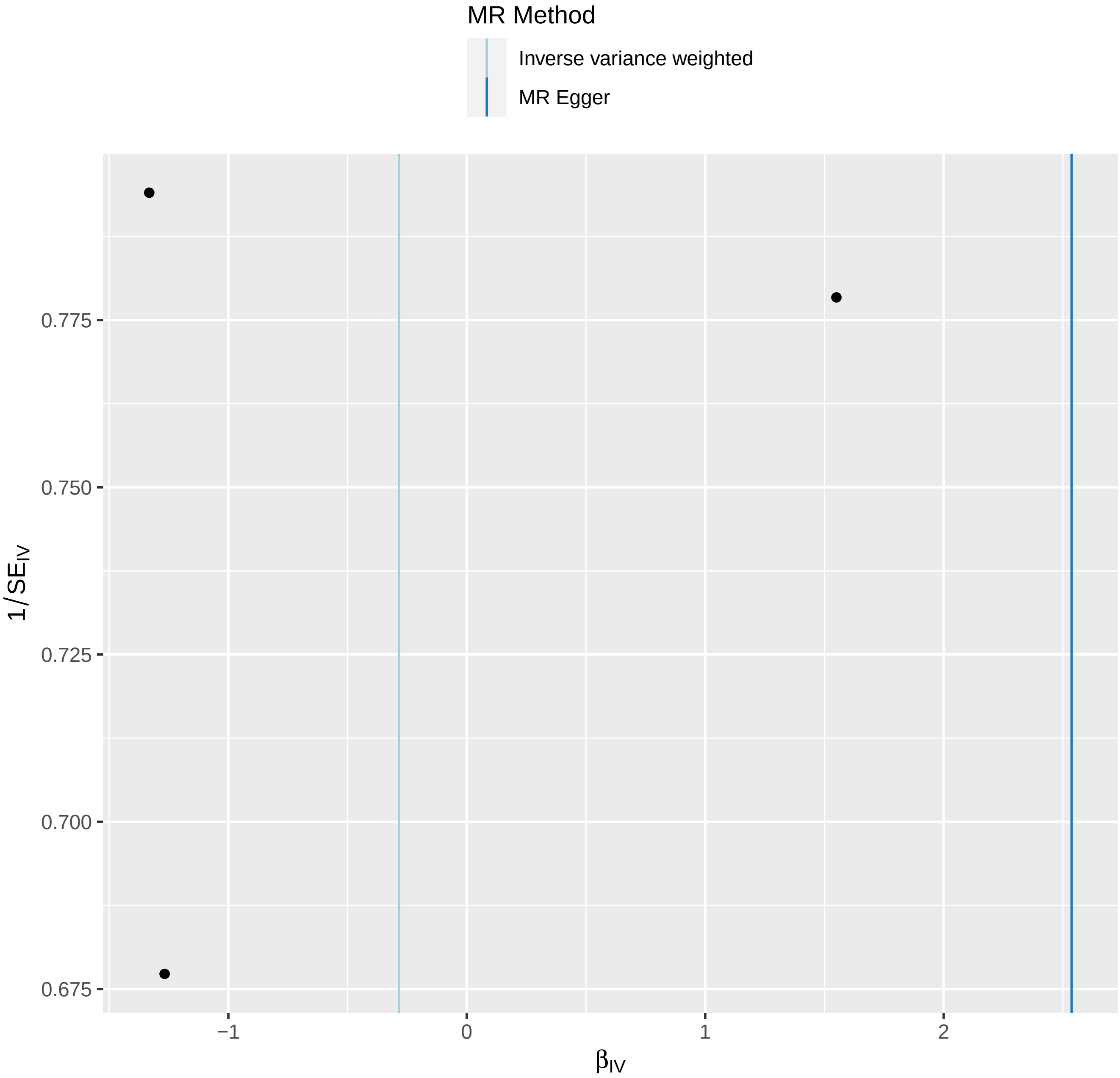

Batch 1388 : Pyruvate on Left−sided ulcerative colitis

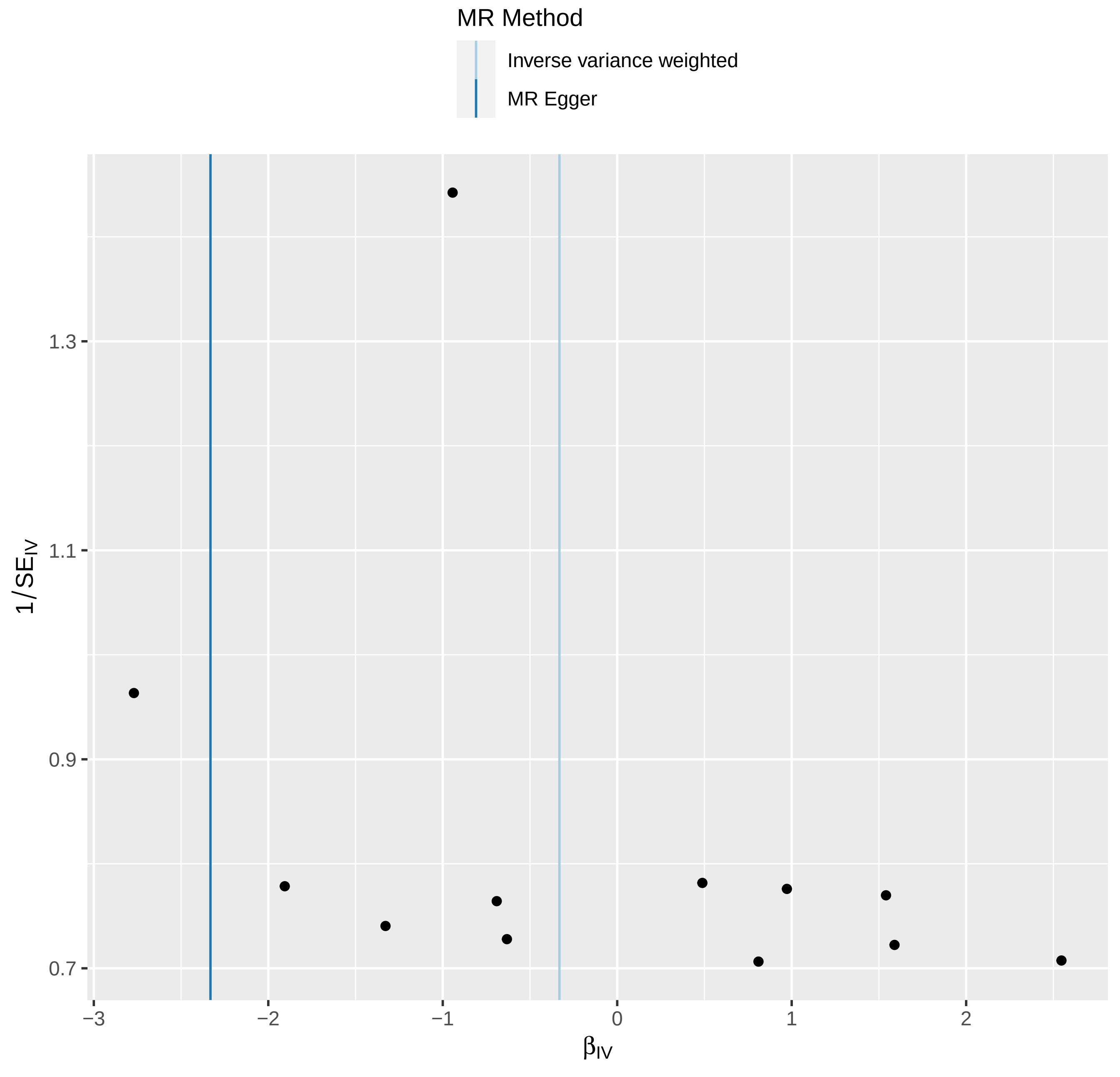

Batch 1389 : Uridine on Left−sided ulcerative colitis

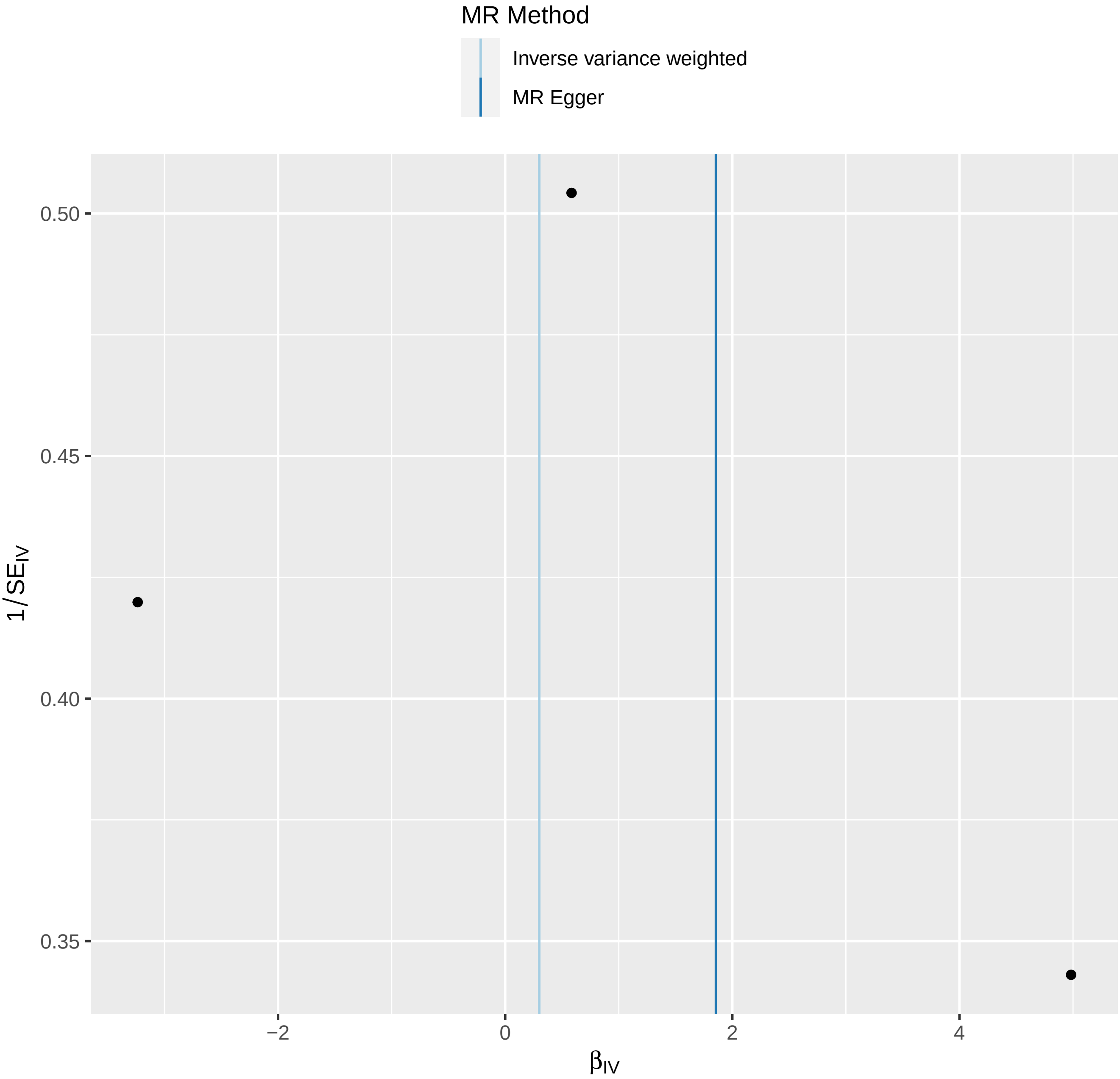

Batch 1390 : Linoleate (18:2n6) on Left−sided ulcerative colitis

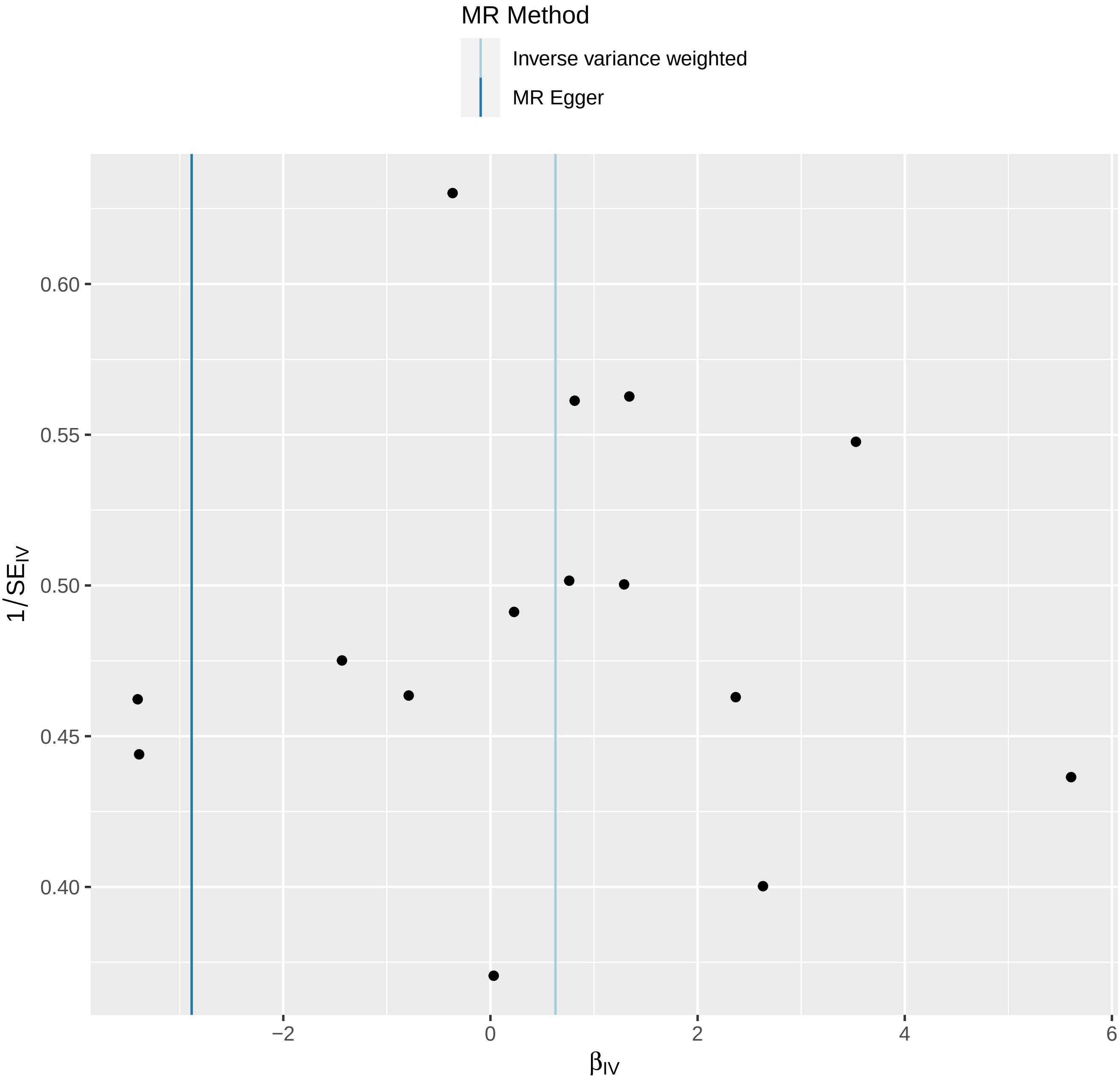

Batch 1391 : Allantoin on Left−sided ulcerative colitis

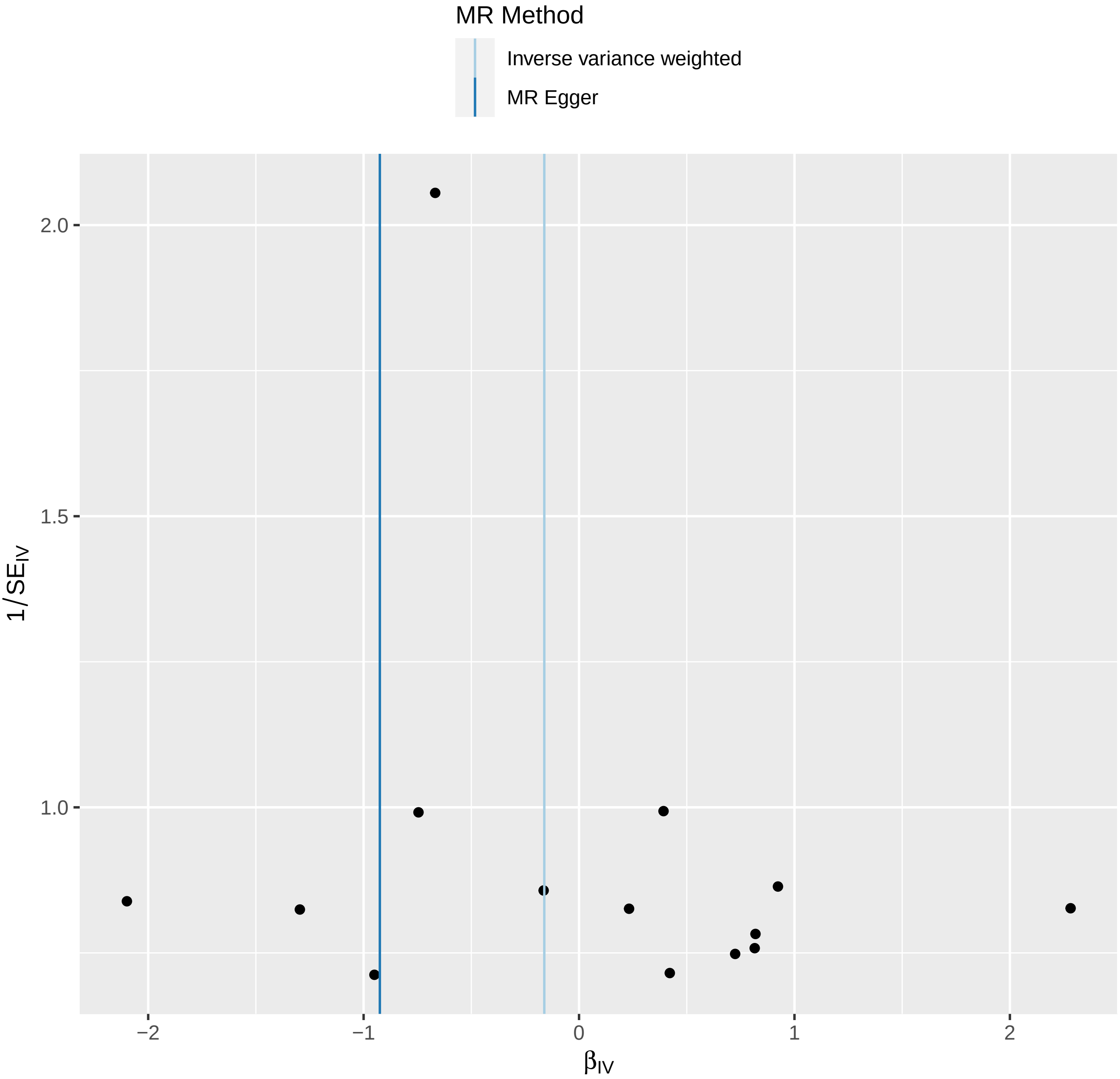

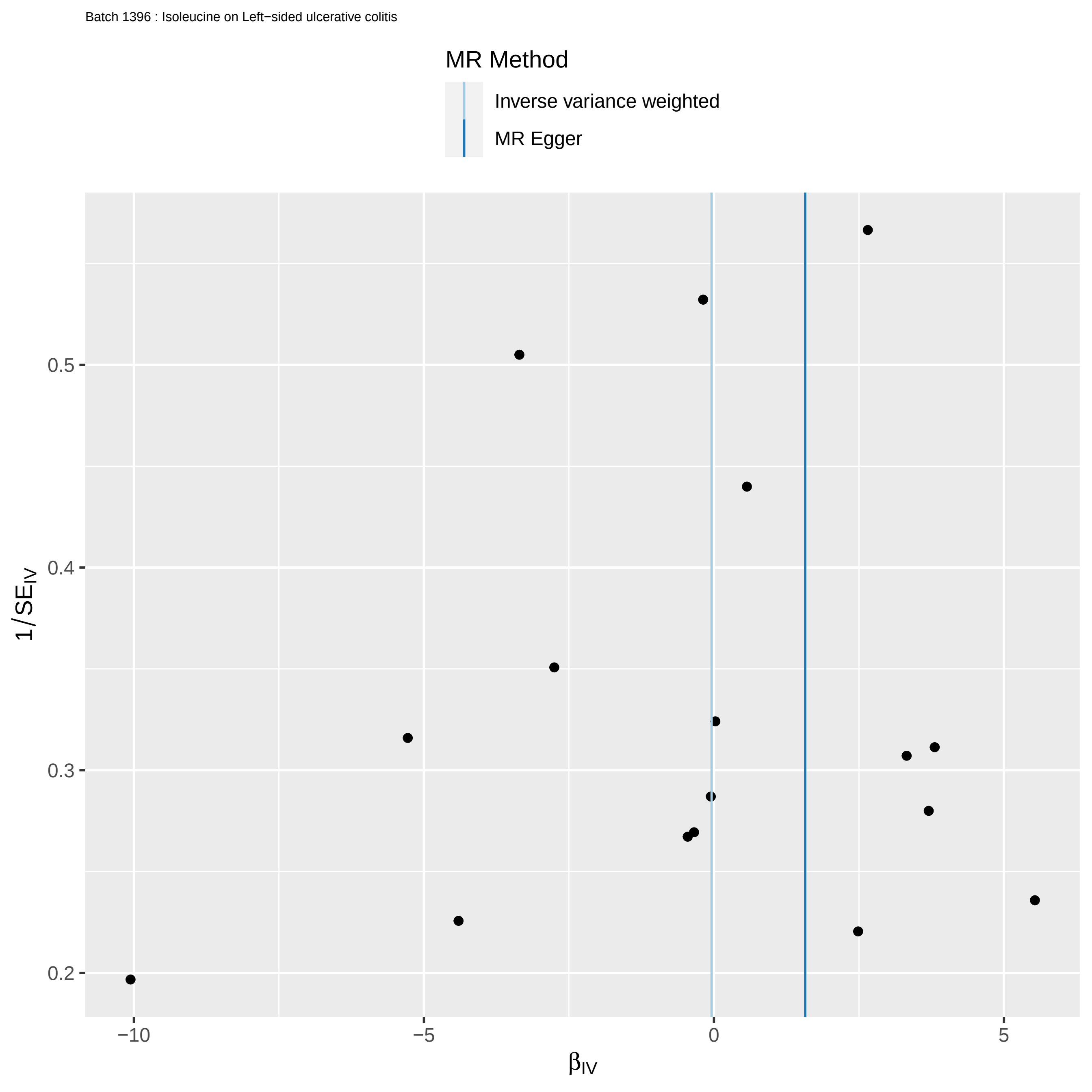
Batch 1396 : Isoleucine on Left−sided ulcerative colitis
MR Method
Inverse variance weighted
MR Egger
0.5
0.4
0.3
0.2
1/SE$_{IV}$
−10
−5
0
5
β_{IV}

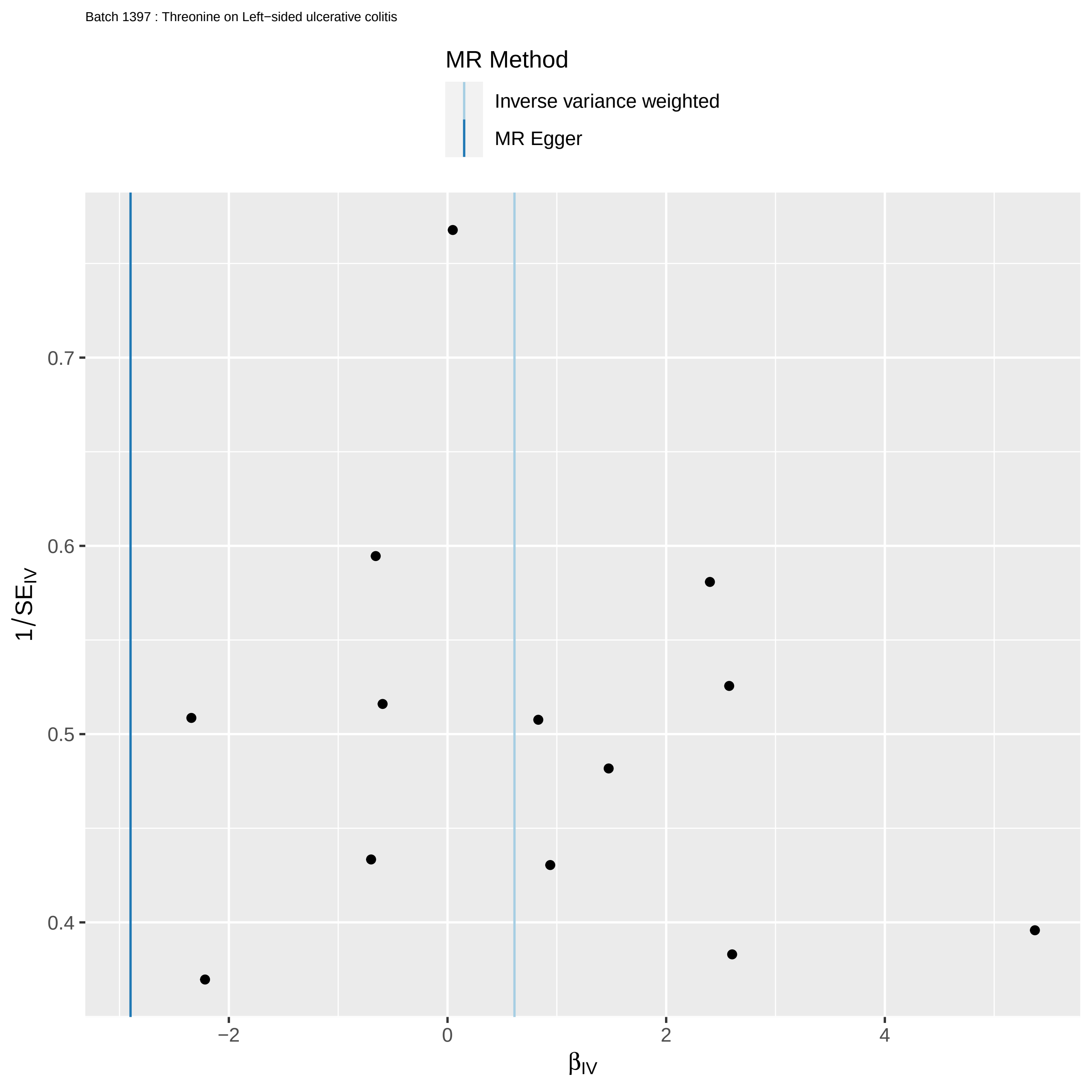

Batch 1397 : Threonine on Left−sided ulcerative colitis
MR Method
Inverse variance weighted
MR Egger
0.7
0.6
0.5
0.4
$1/SE_{IV}$
−2
0
2
4
β_{IV}

Batch 1398 : Tyrosine on Left−sided ulcerative colitis

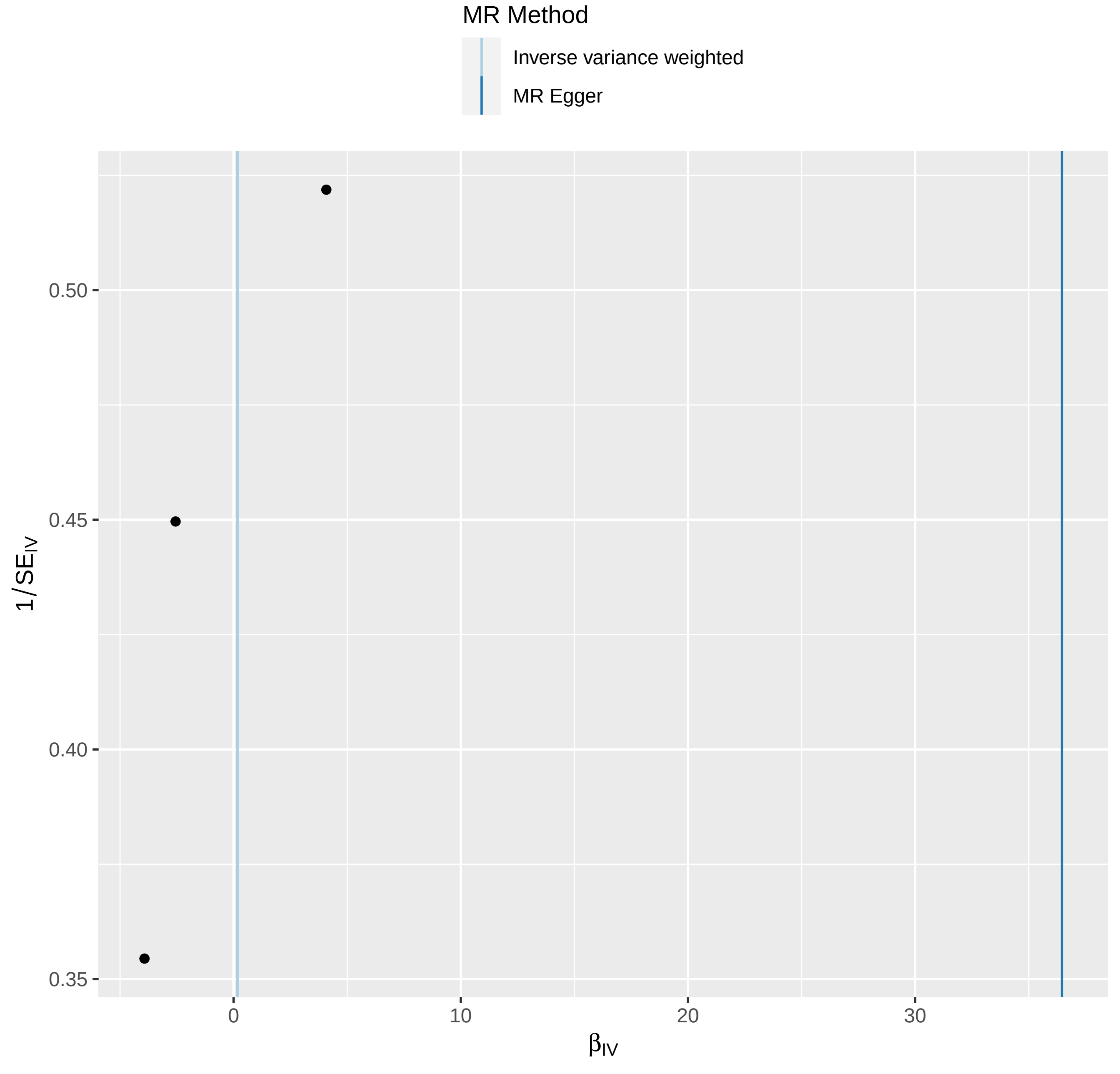

Batch 1404 : Stearate (18:0) on Left−sided ulcerative colitis

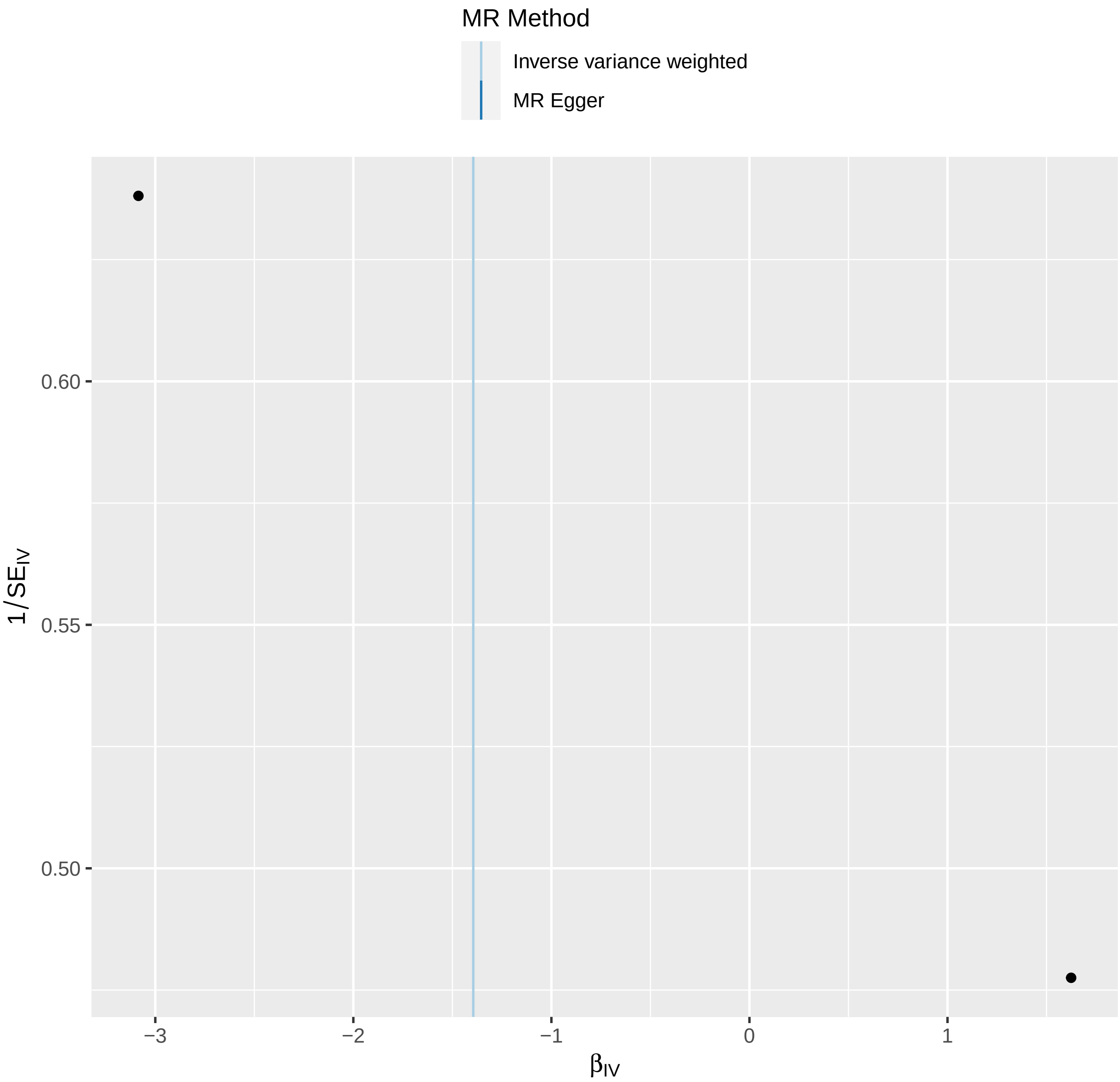

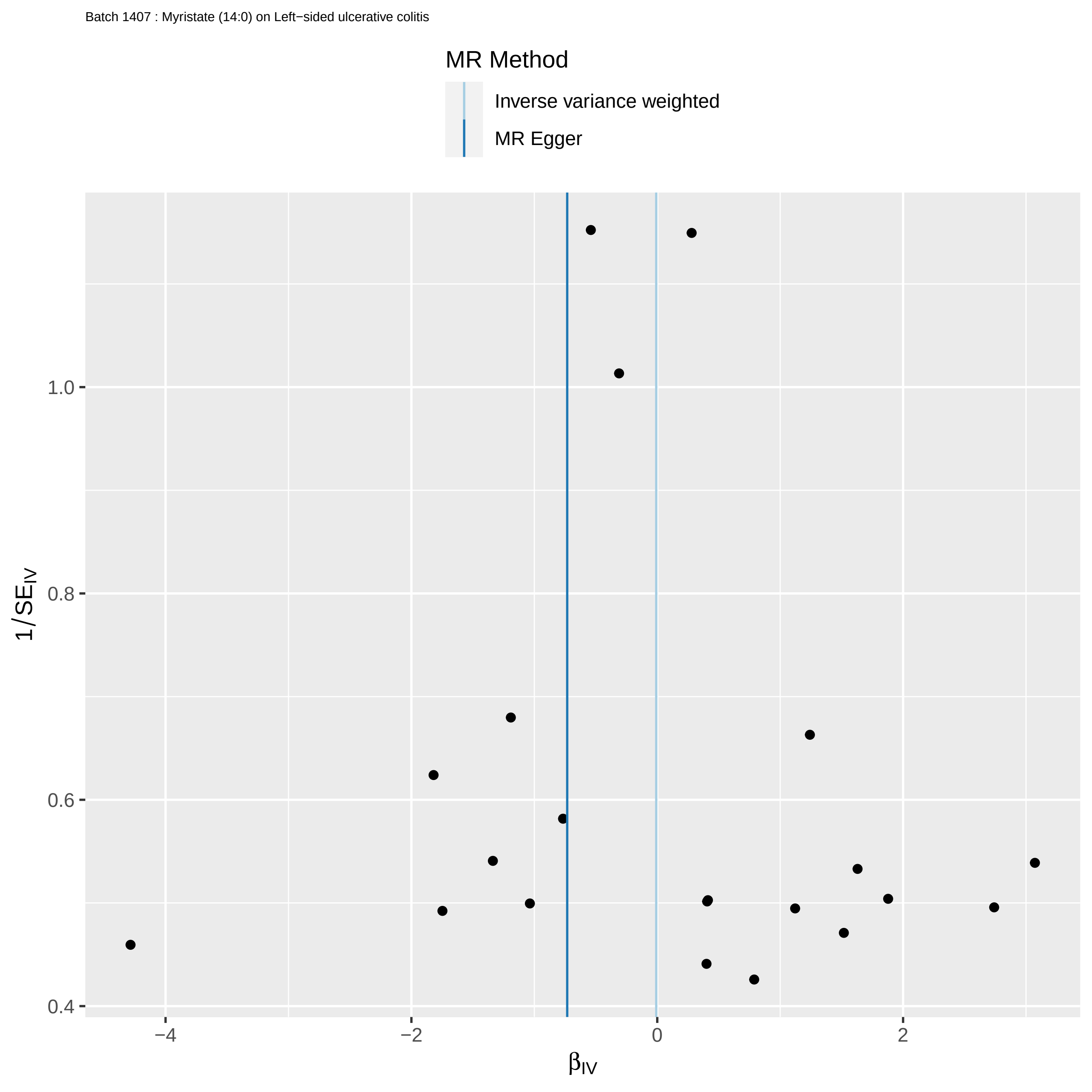
Batch 1407 : Myristate (14:0) on Left−sided ulcerative colitis
MR Method
Inverse variance weighted
MR Egger
1/SE_IV
1.0
0.8
0.6
0.4
−4
−2
0
2
β_IV

Batch 1409 : Ornithine on Left−sided ulcerative colitis

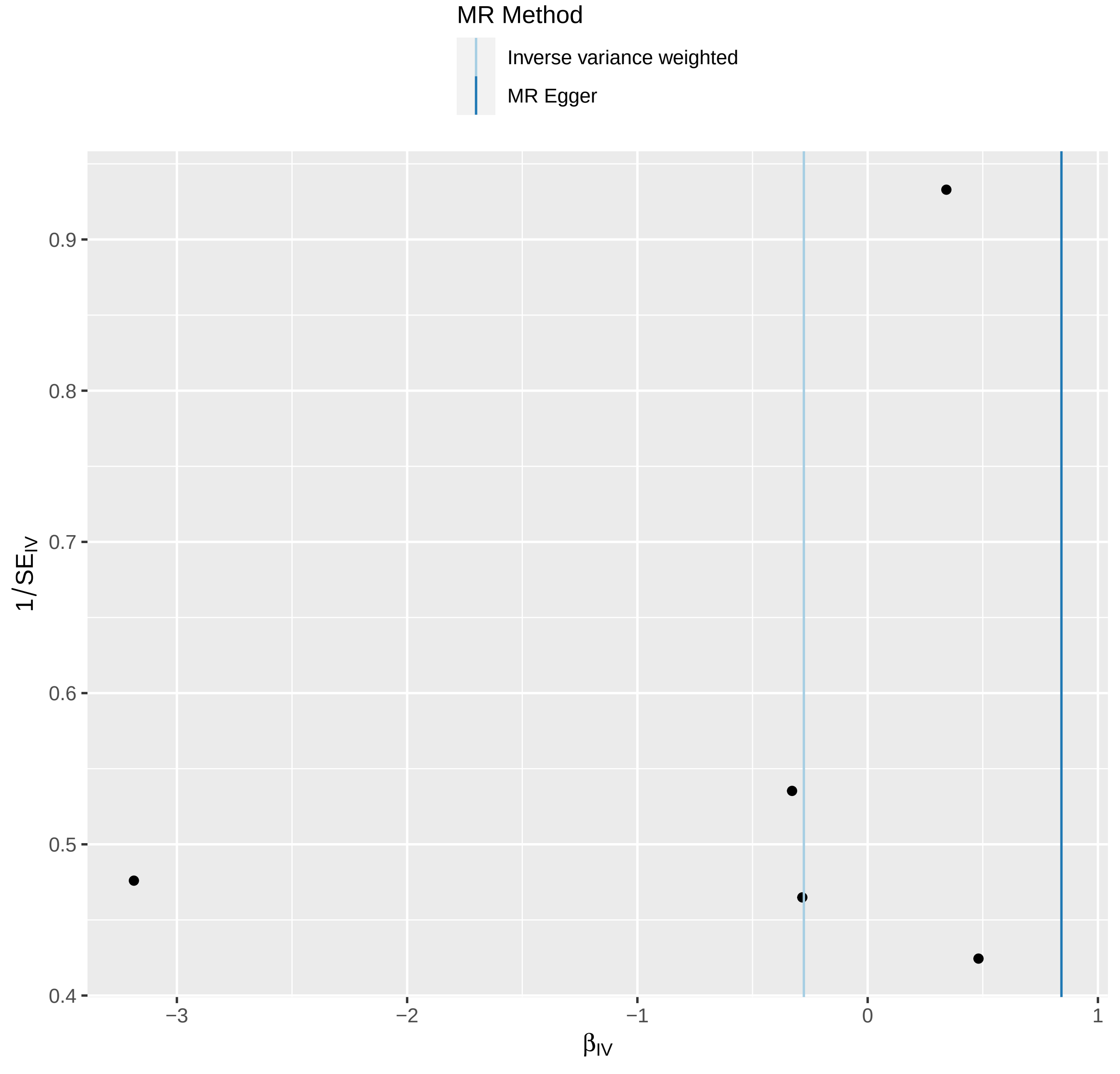

Batch 1412 : 4−acetamidobutanoate on Left−sided ulcerative colitis

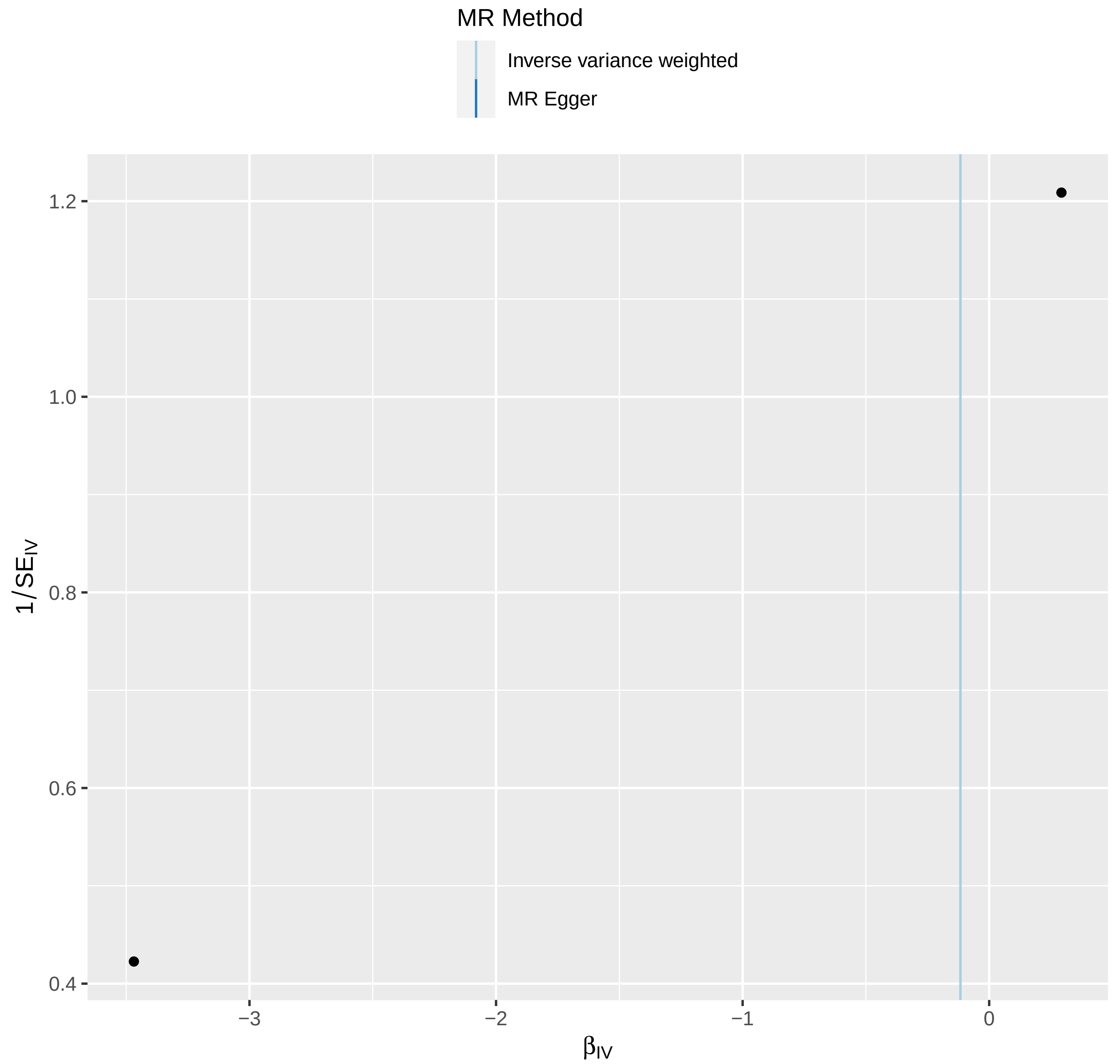

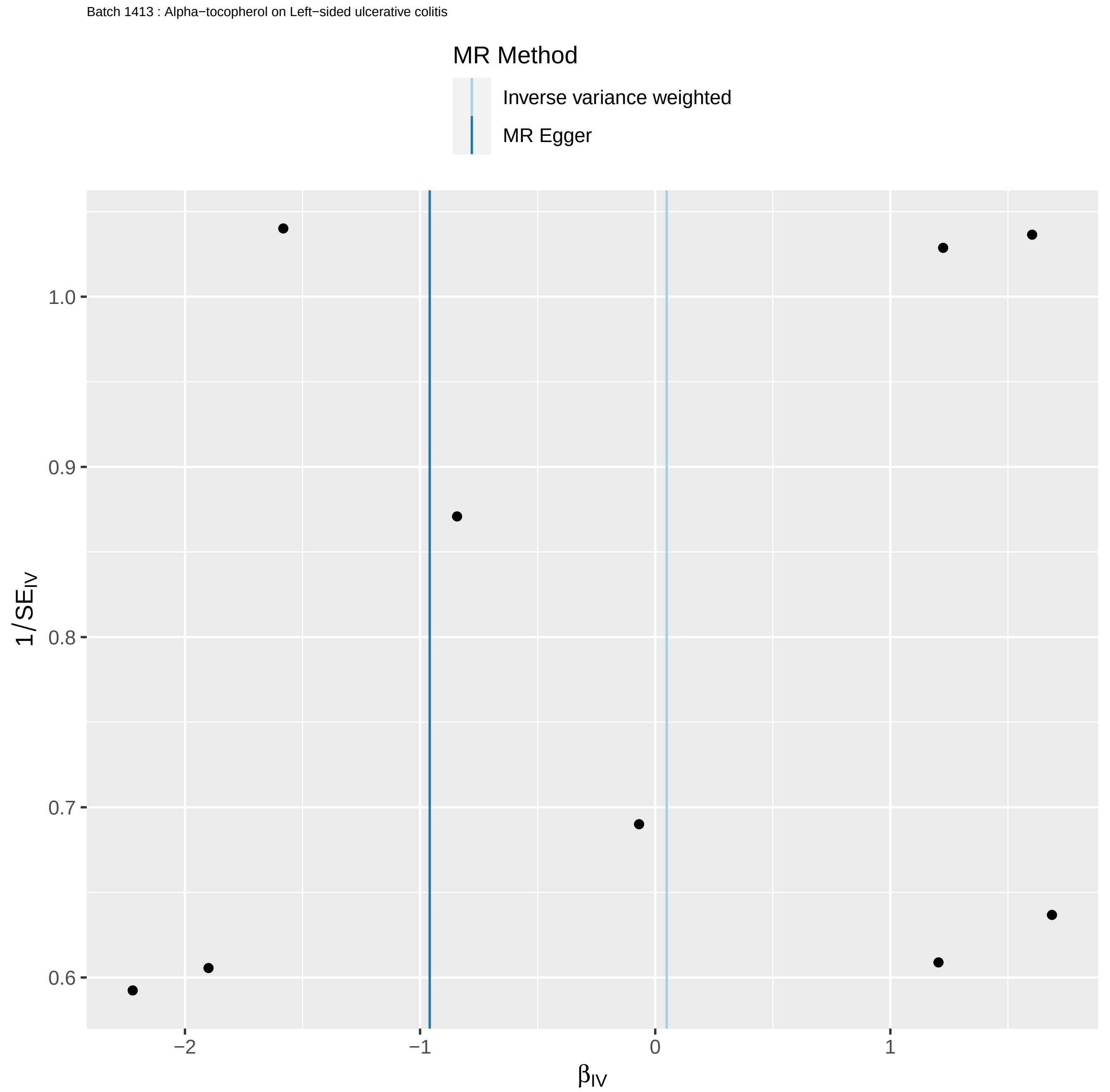
Batch 1413 : Alpha−tocopherol on Left−sided ulcerative colitis
MR Method
Inverse variance weighted
MR Egger
1.0
0.9
0.8
0.7
0.6
$1/SE_{IV}$
−2
−1
0
1
β_{IV}

Batch 1414 : Citrate on Left−sided ulcerative colitis

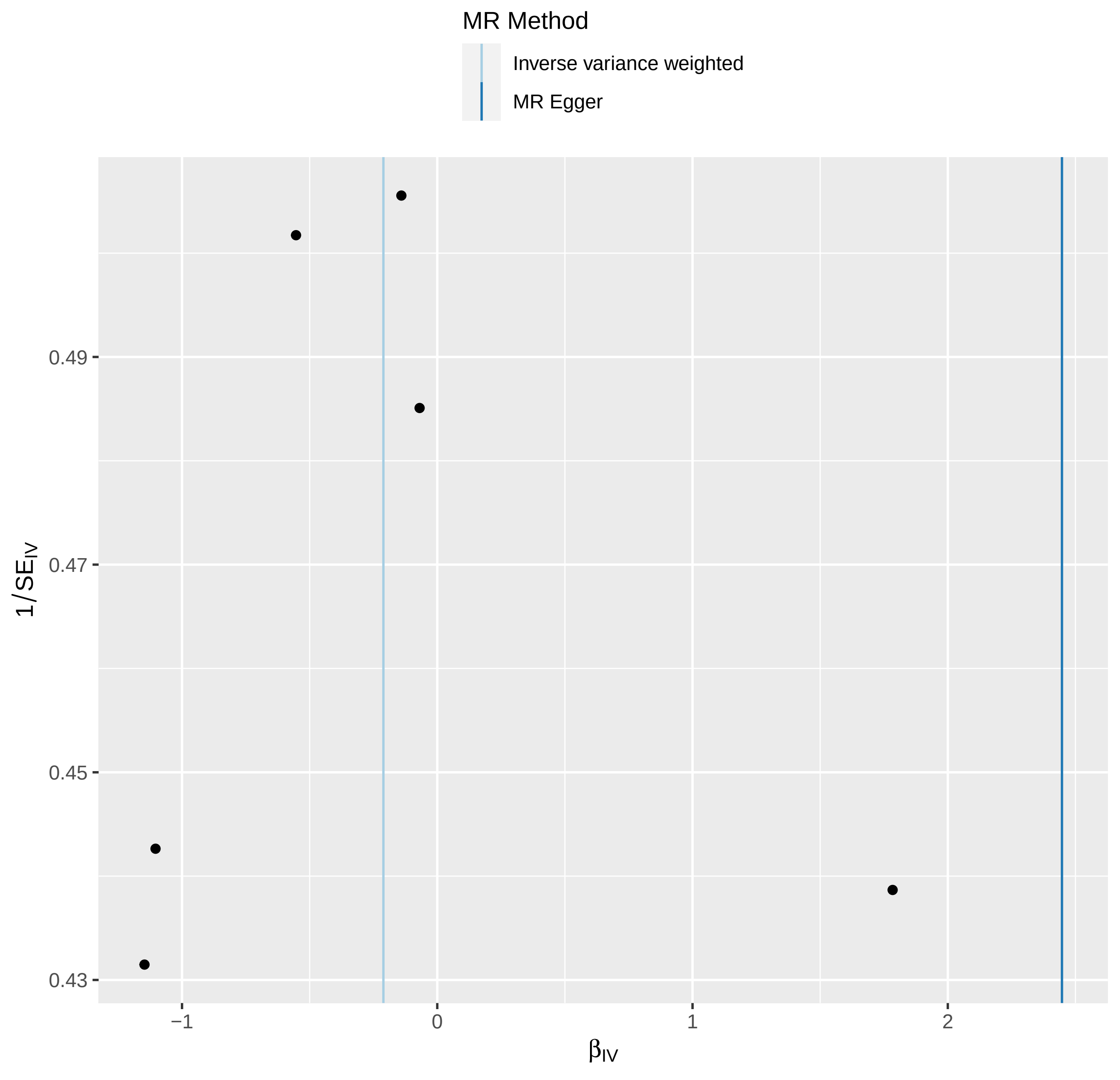

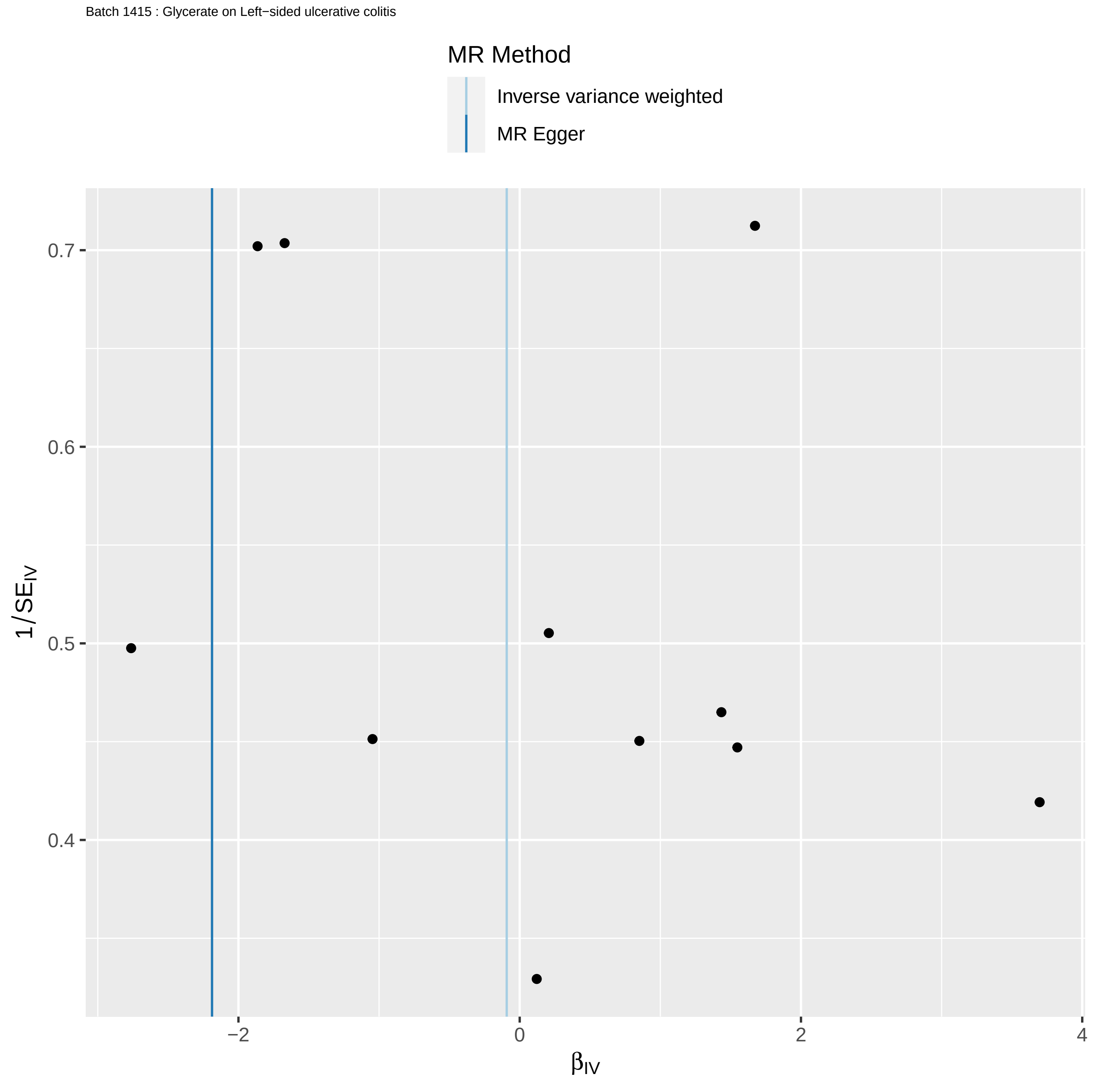

Batch 1415 : Glycerate on Left−sided ulcerative colitis
MR Method
Inverse variance weighted
MR Egger
0.7
0.6
0.5
0.4
1/SE_IV
−2
0
2
4
β_IV

Batch 1416 : Guanosine on Left−sided ulcerative colitis

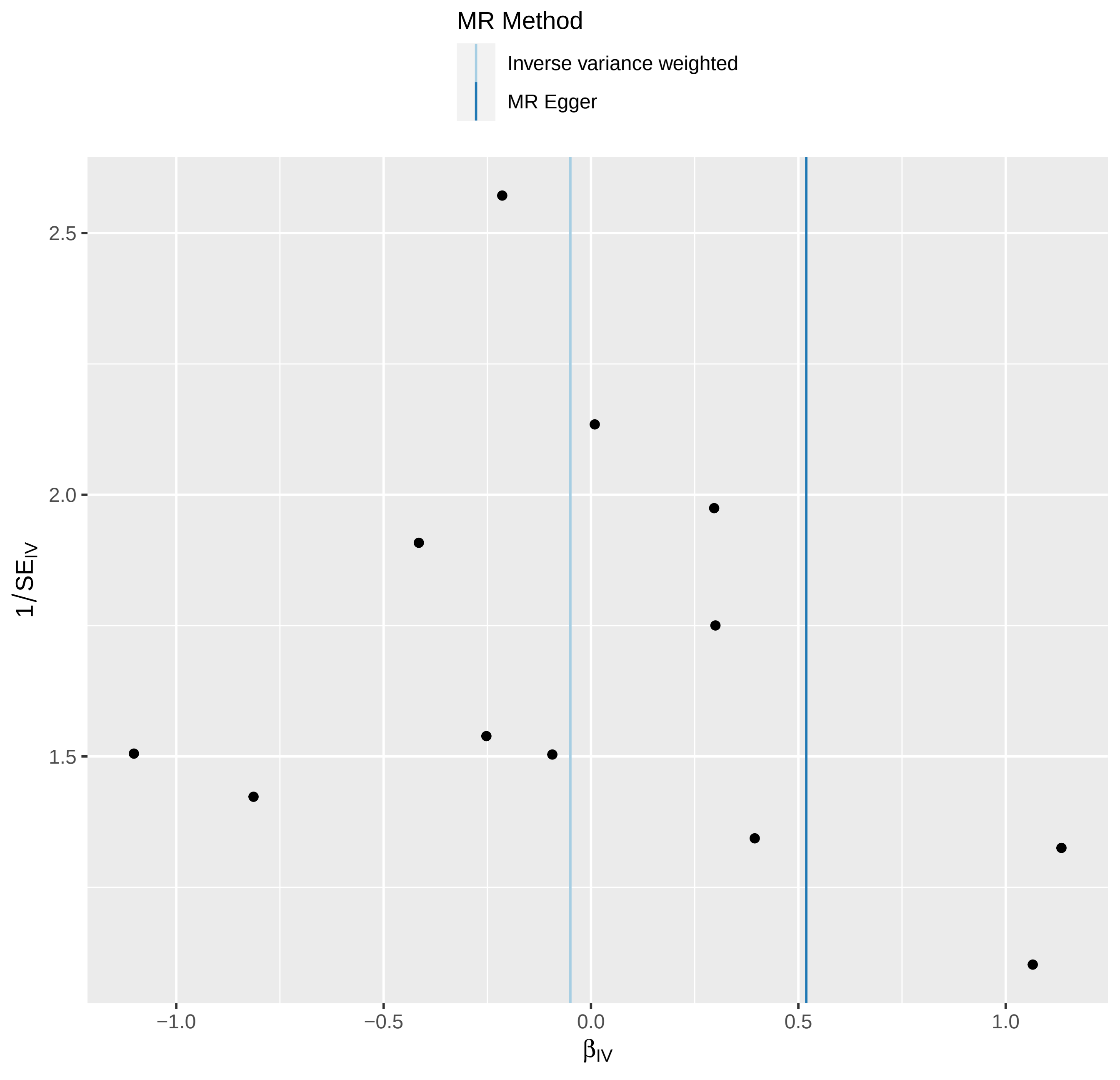

Batch 1419 : Ursodeoxycholate on Left−sided ulcerative colitis

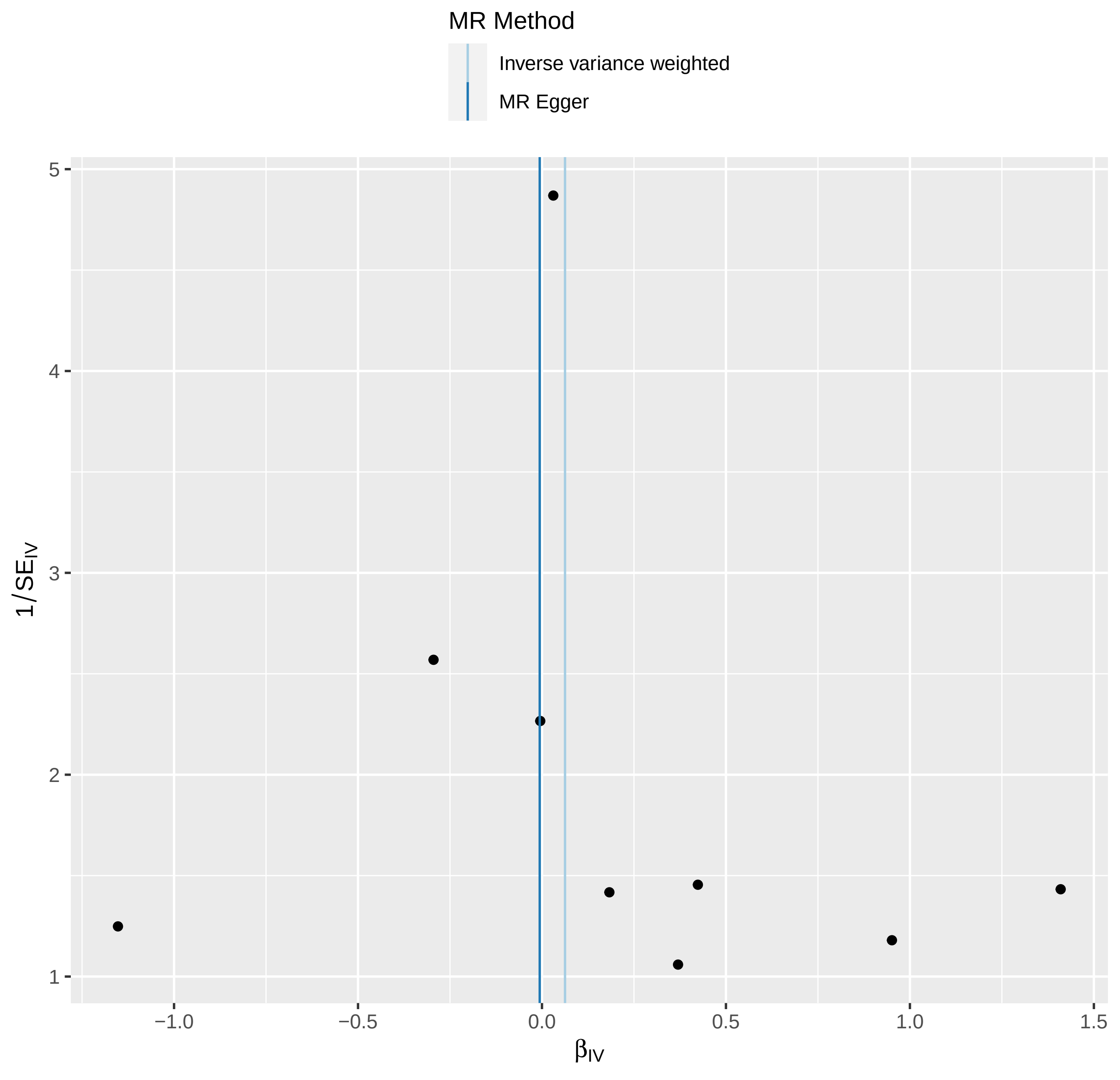

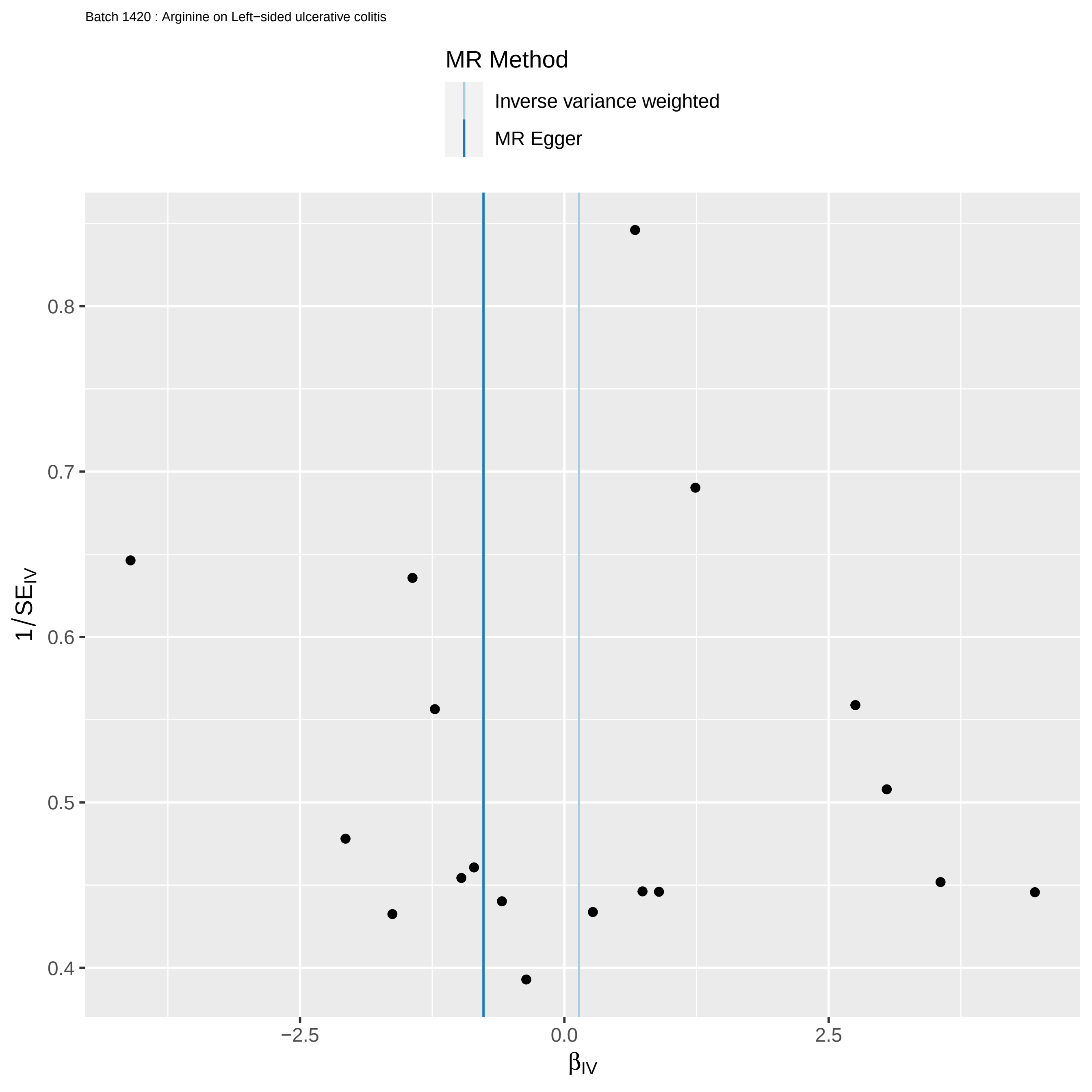

Batch 1420 : Arginine on Left−sided ulcerative colitis
MR Method
Inverse variance weighted
MR Egger
0.8
0.7
0.6
0.5
0.4
1/SE_IV
−2.5
0.0
2.5
β_IV

Batch 1421 : Ascorbate (Vitamin C) on Left−sided ulcerative colitis

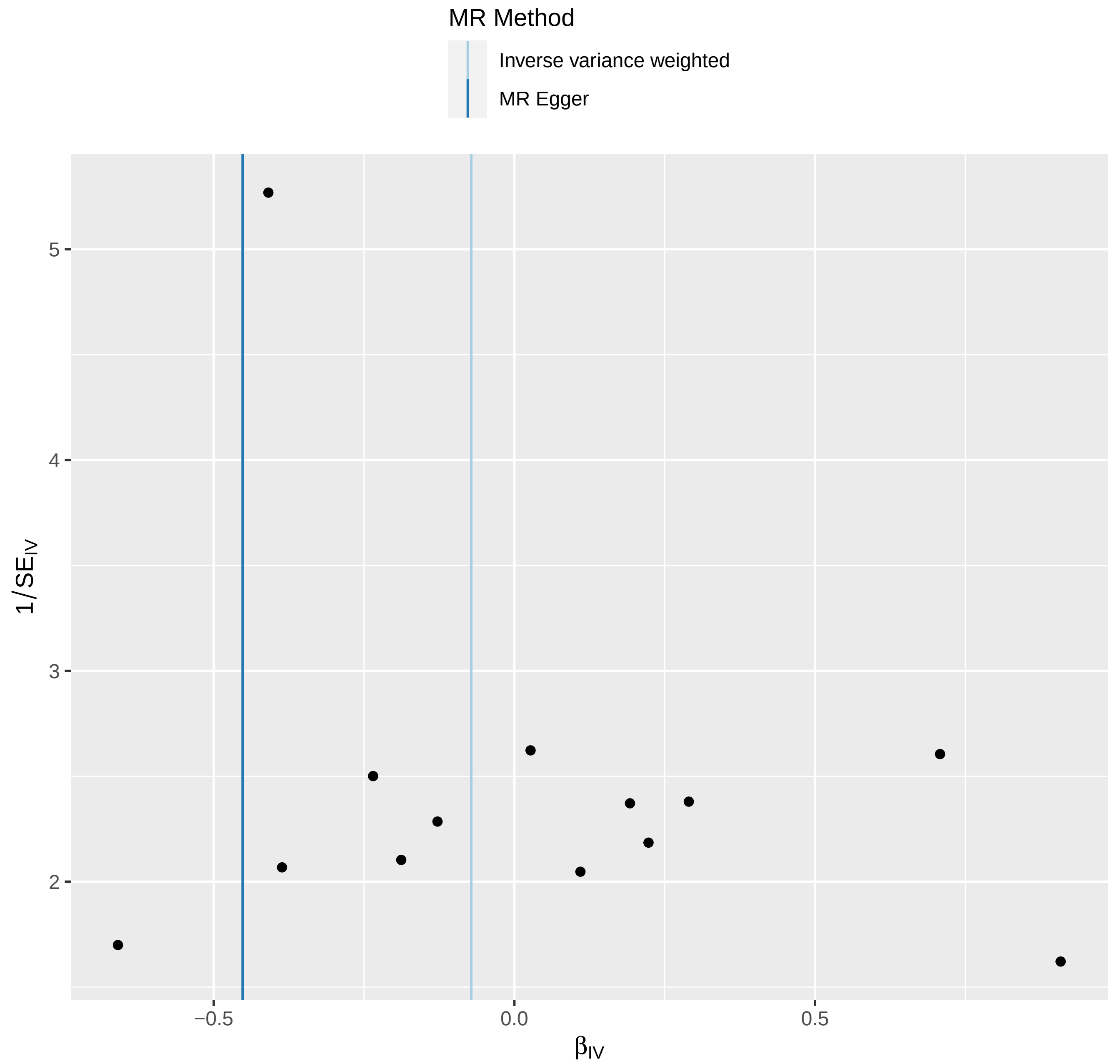

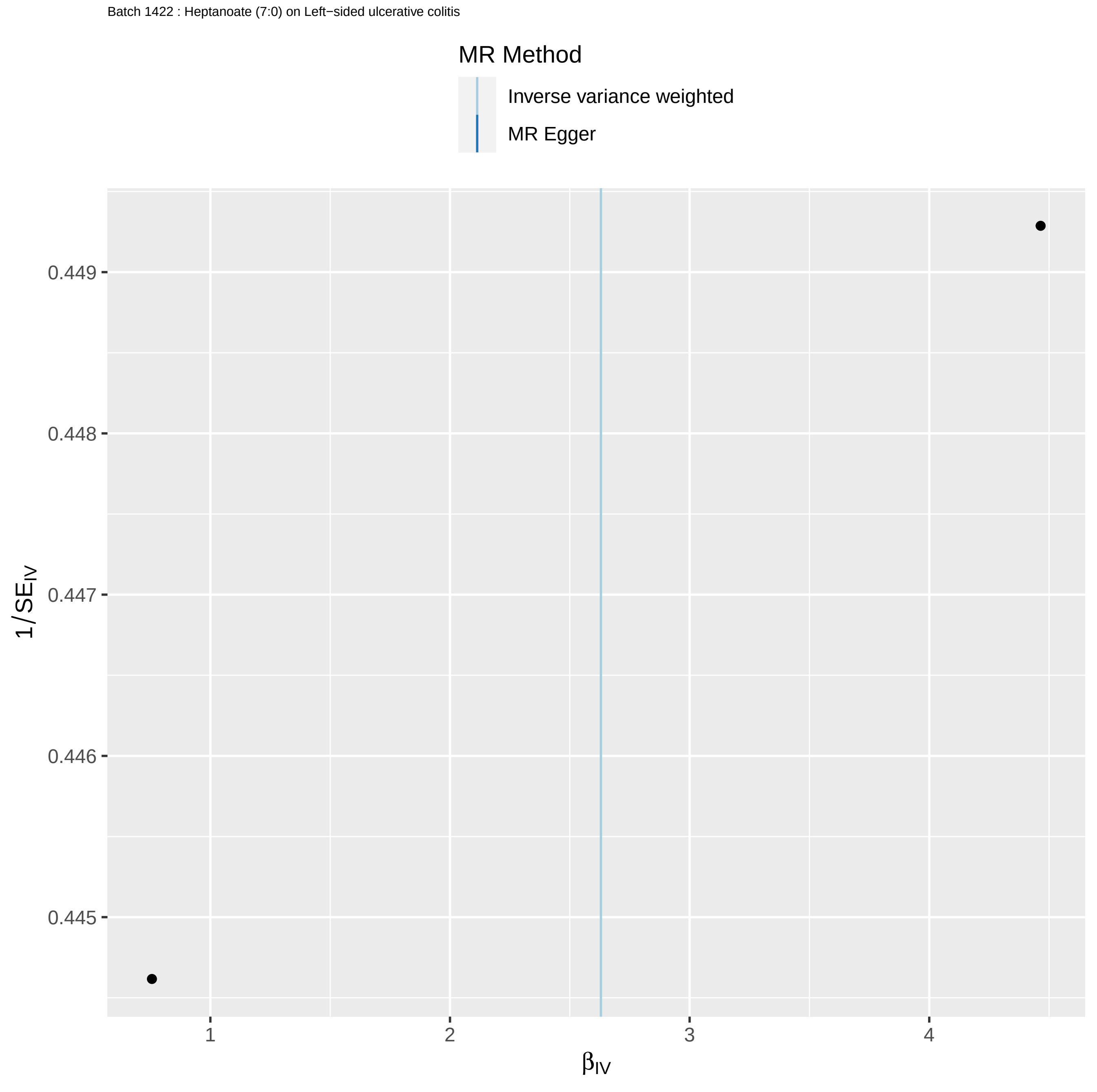

Batch 1422 : Heptanoate (7:0) on Left−sided ulcerative colitis
MR Method
Inverse variance weighted
MR Egger
0.449
0.448
0.447
0.446
0.445
$1/SE_{IV}$
1
2
3
4
β_{IV}

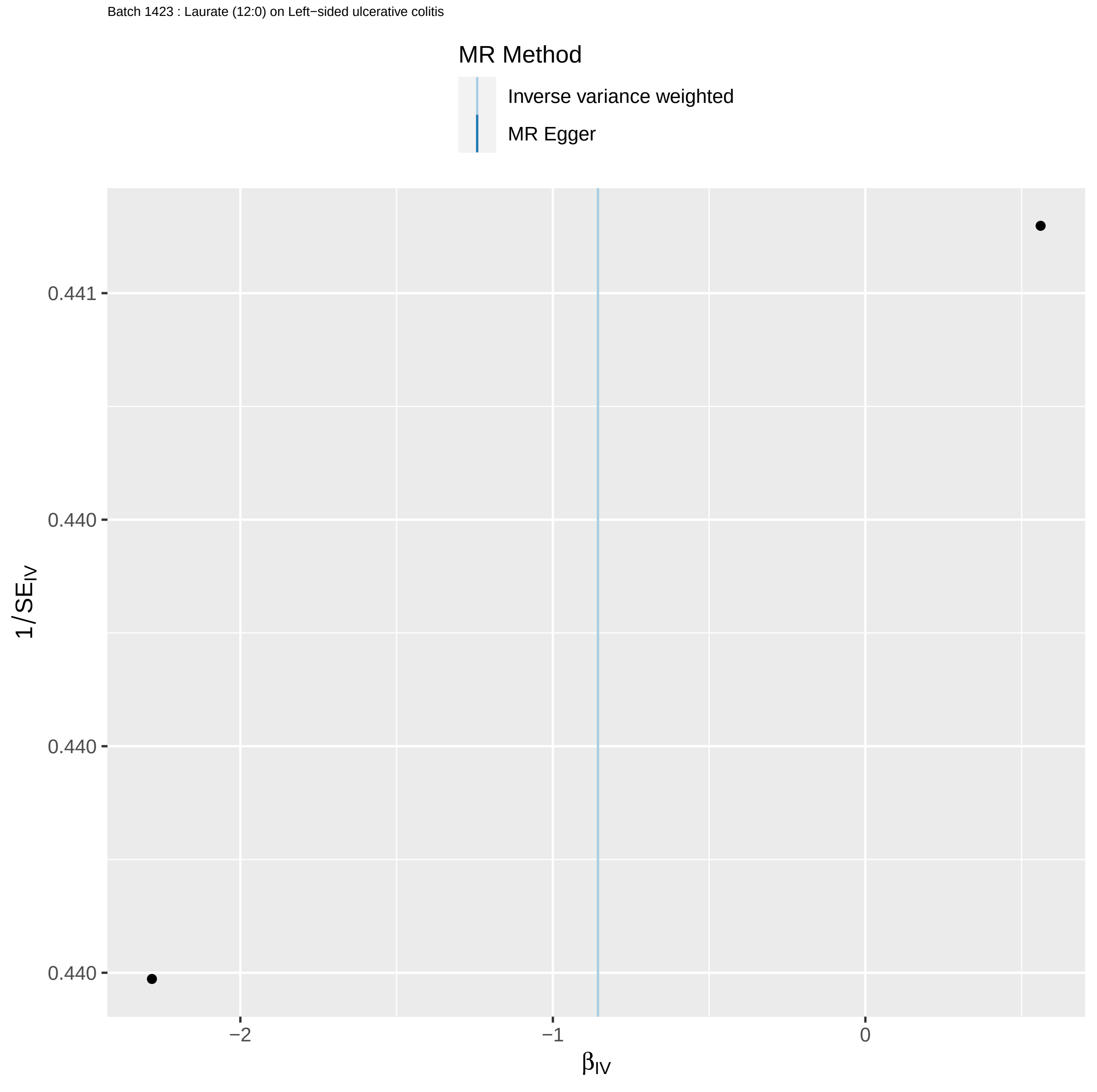
Batch 1423 : Laurate (12:0) on Left−sided ulcerative colitis
MR Method
Inverse variance weighted
MR Egger
0.441
0.440
0.440
0.440
1/SE_IV
−2
−1
0
β_IV

Batch 1425 : Urea on Left−sided ulcerative colitis

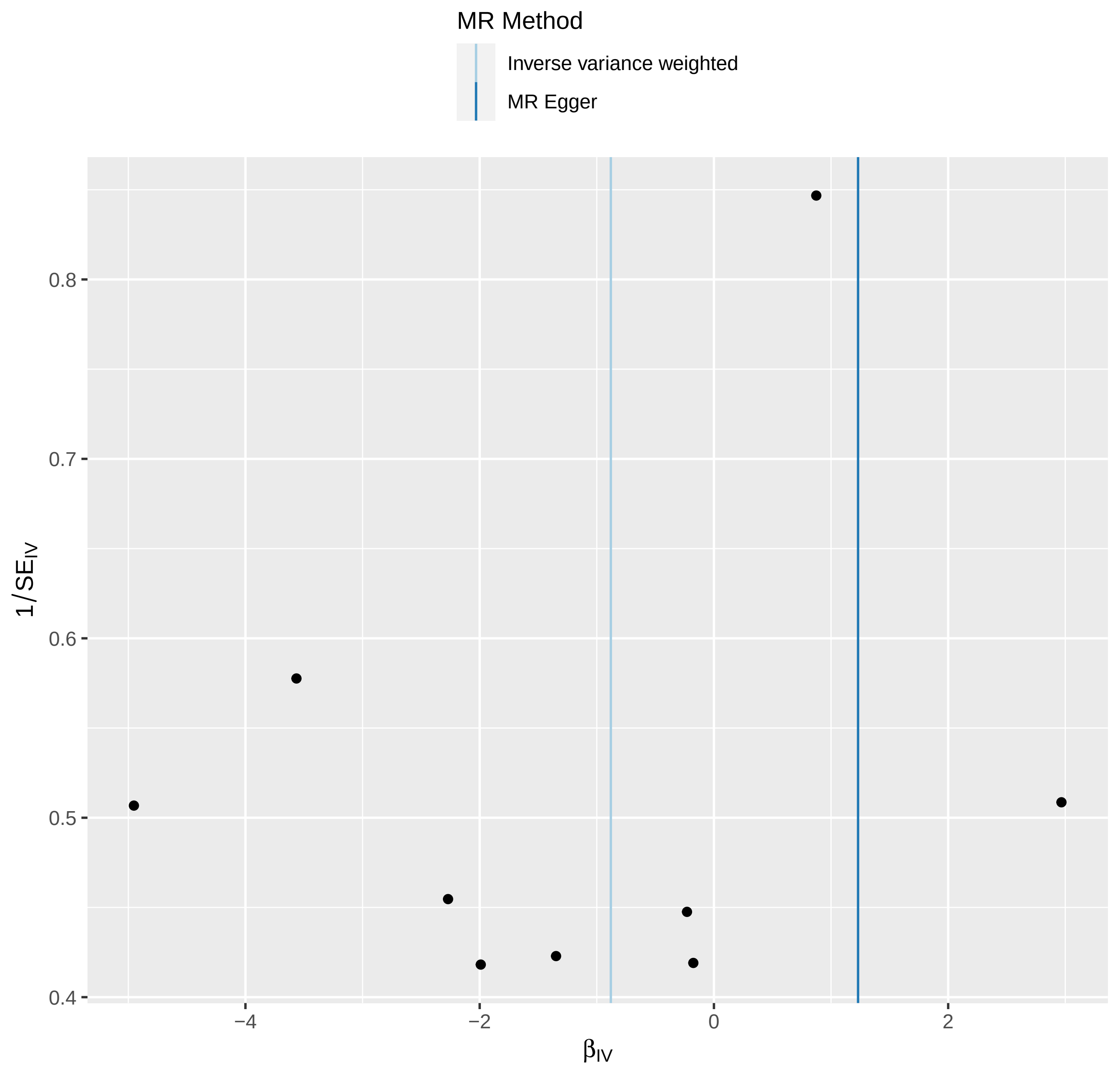

Batch 1426 : Cortisol on Left−sided ulcerative colitis

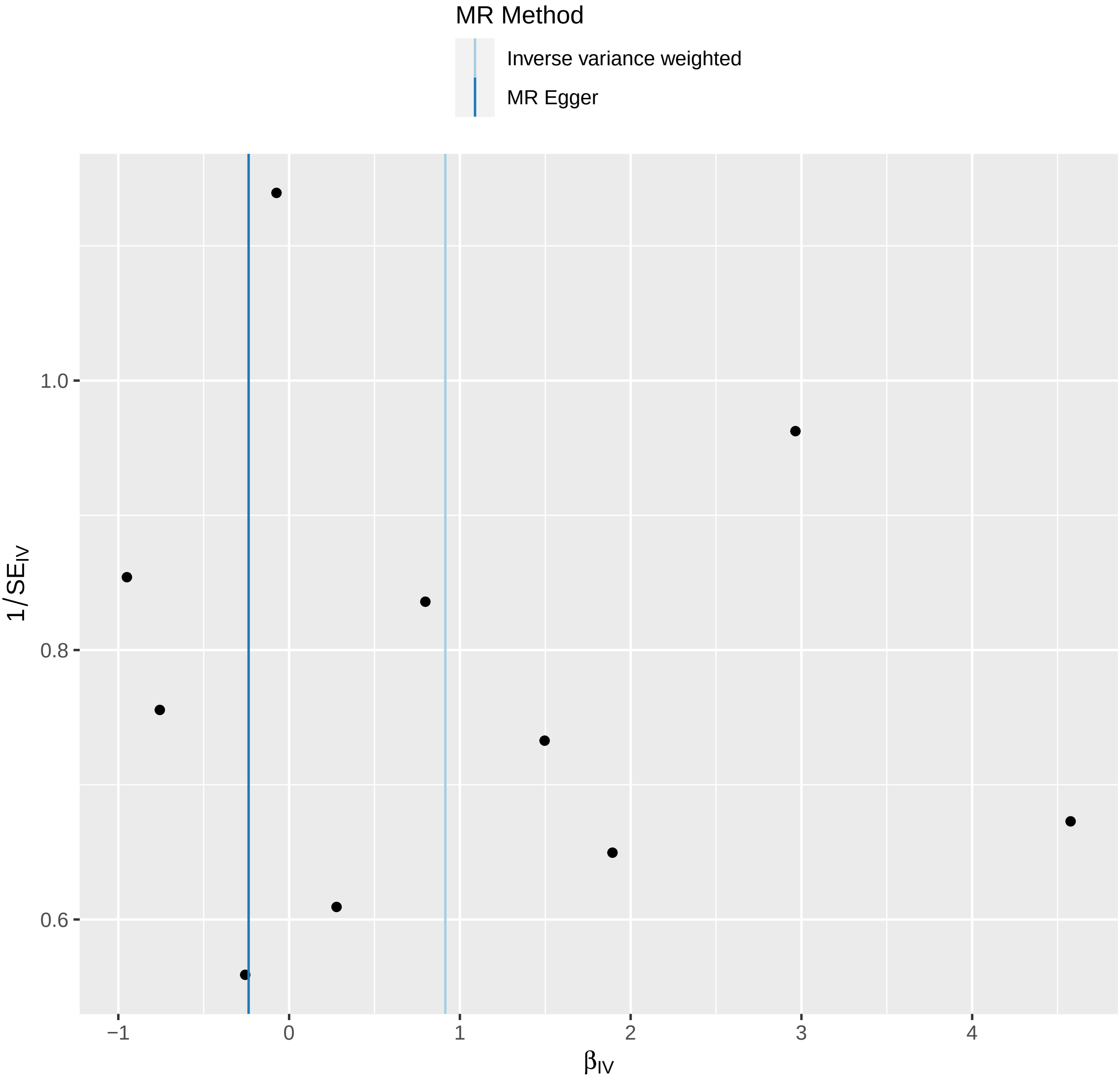

Batch 1429 : Citrulline on Left−sided ulcerative colitis

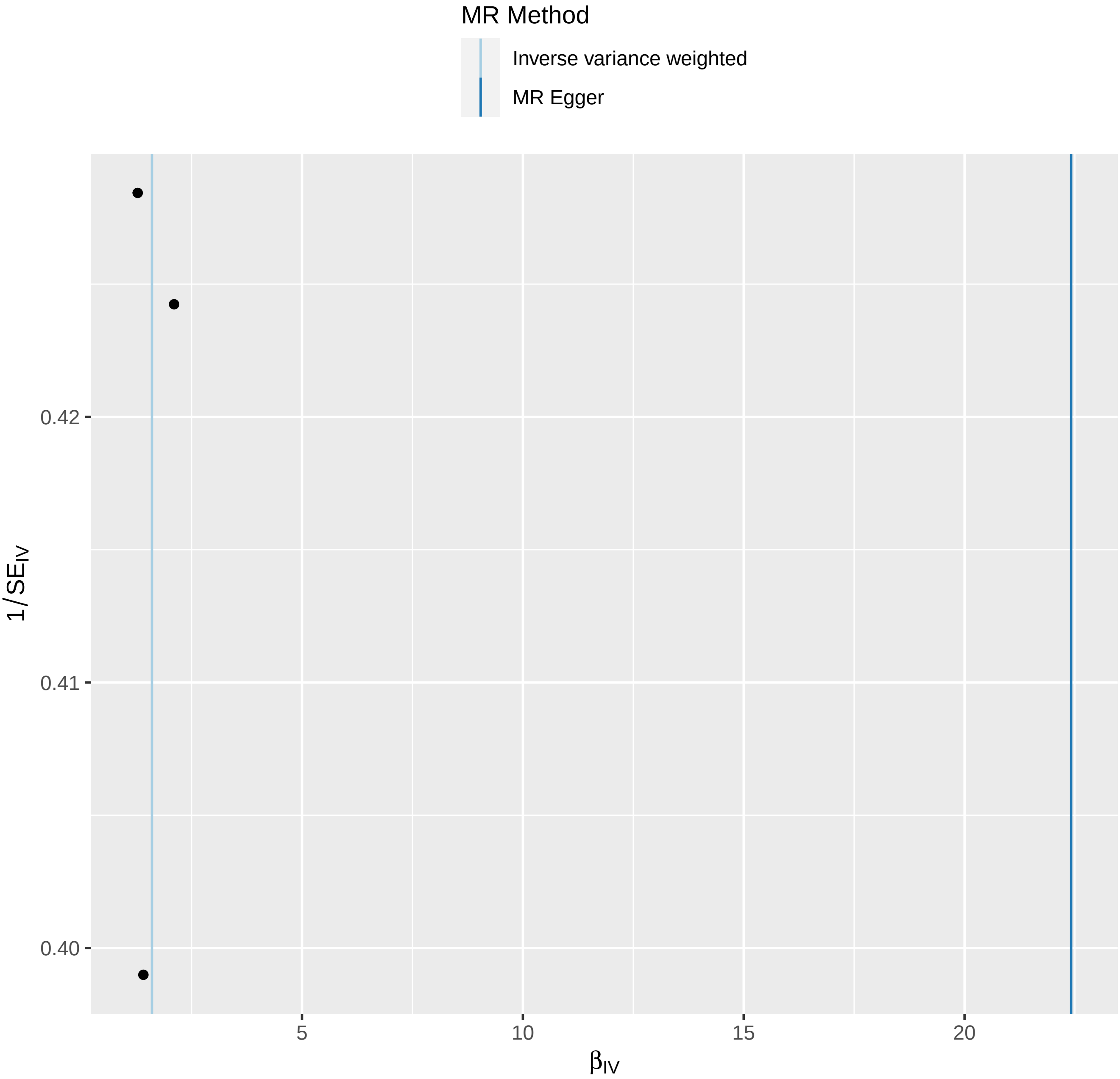

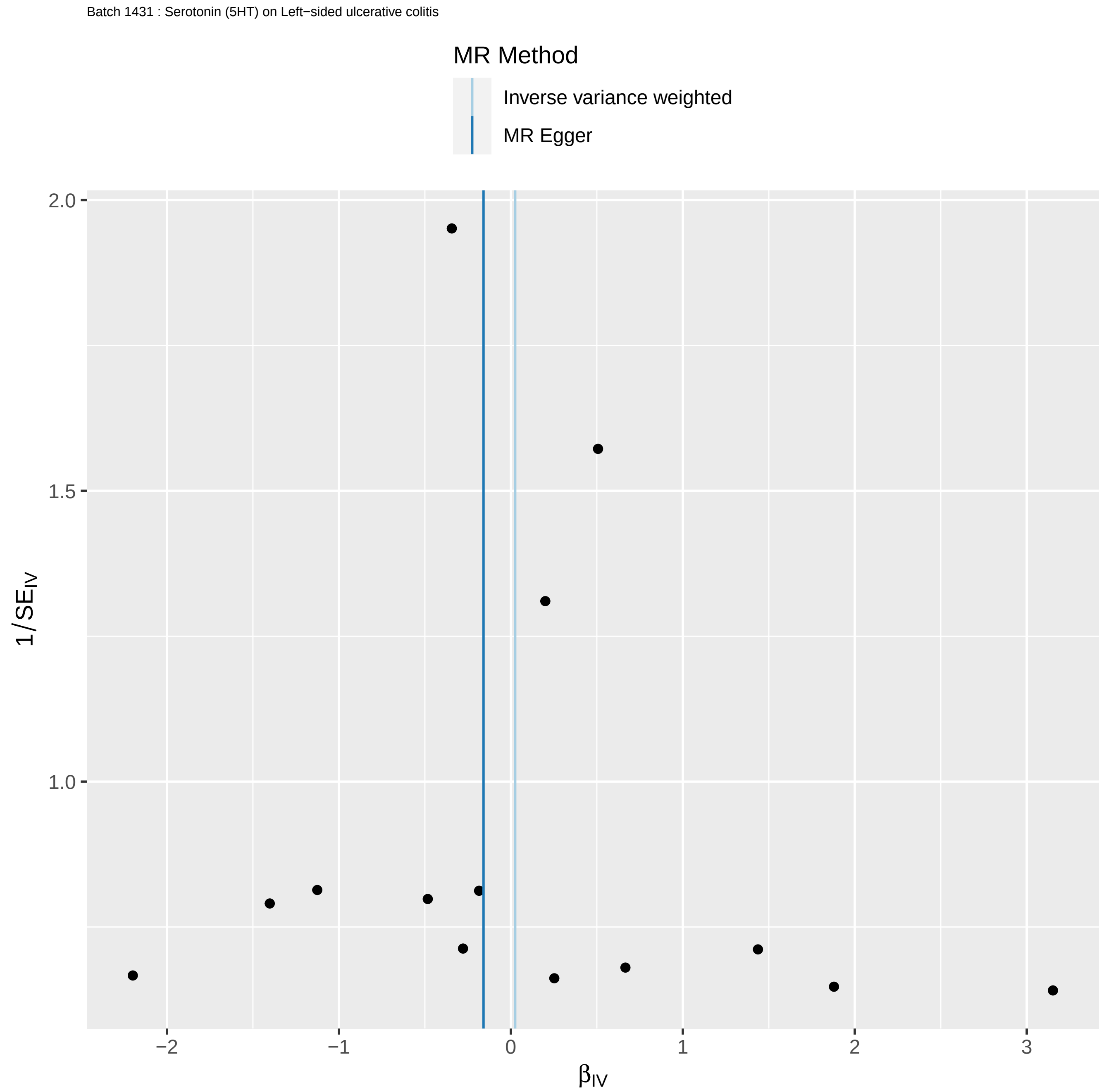

Batch 1431 : Serotonin (5HT) on Left−sided ulcerative colitis
MR Method
Inverse variance weighted
MR Egger
2.0
1.5
1.0
1/SE_IV
−2
−1
0
1
2
3
β_IV

Batch 1432 : Gamma−glutamylglutamine on Left−sided ulcerative colitis

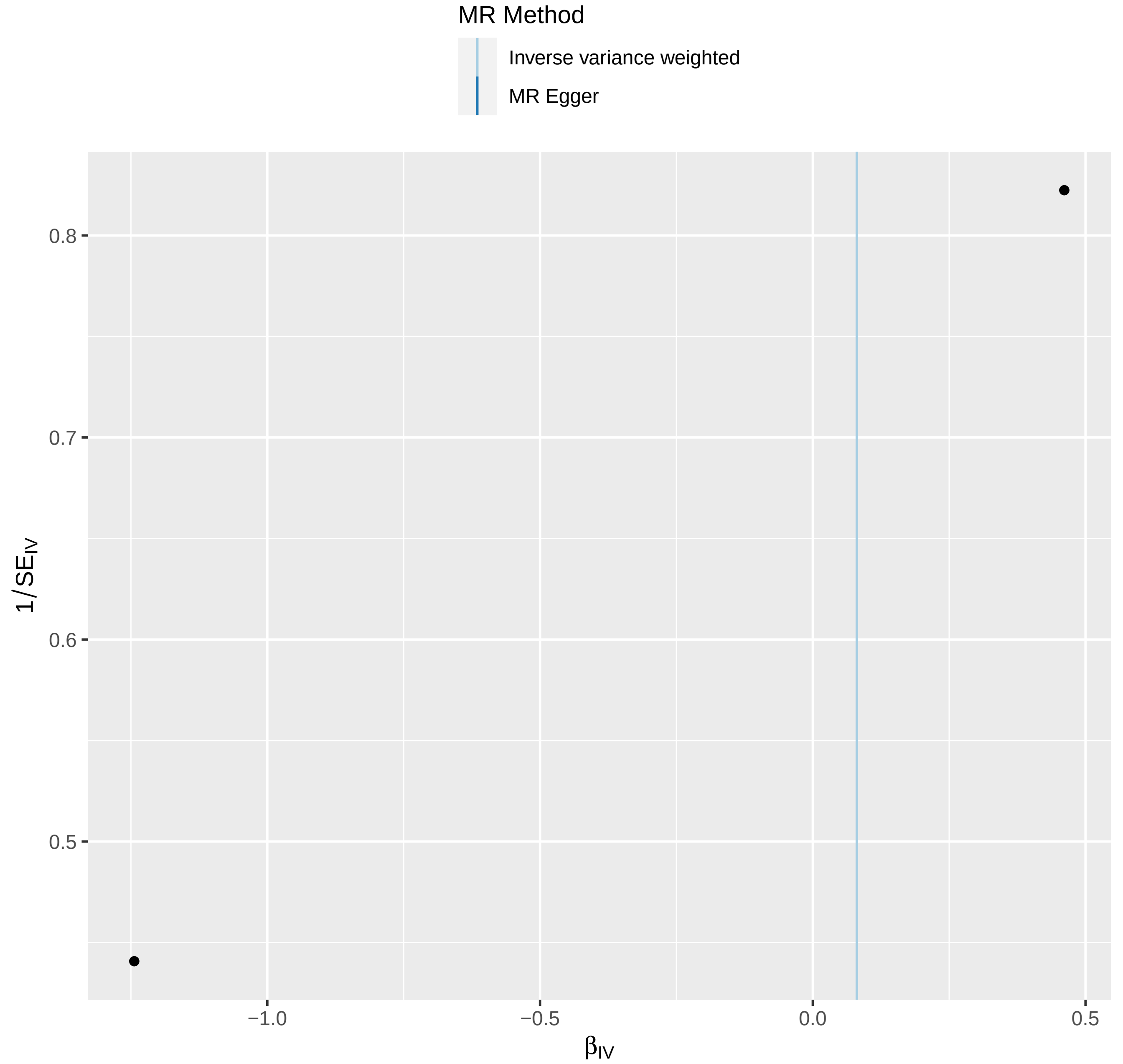

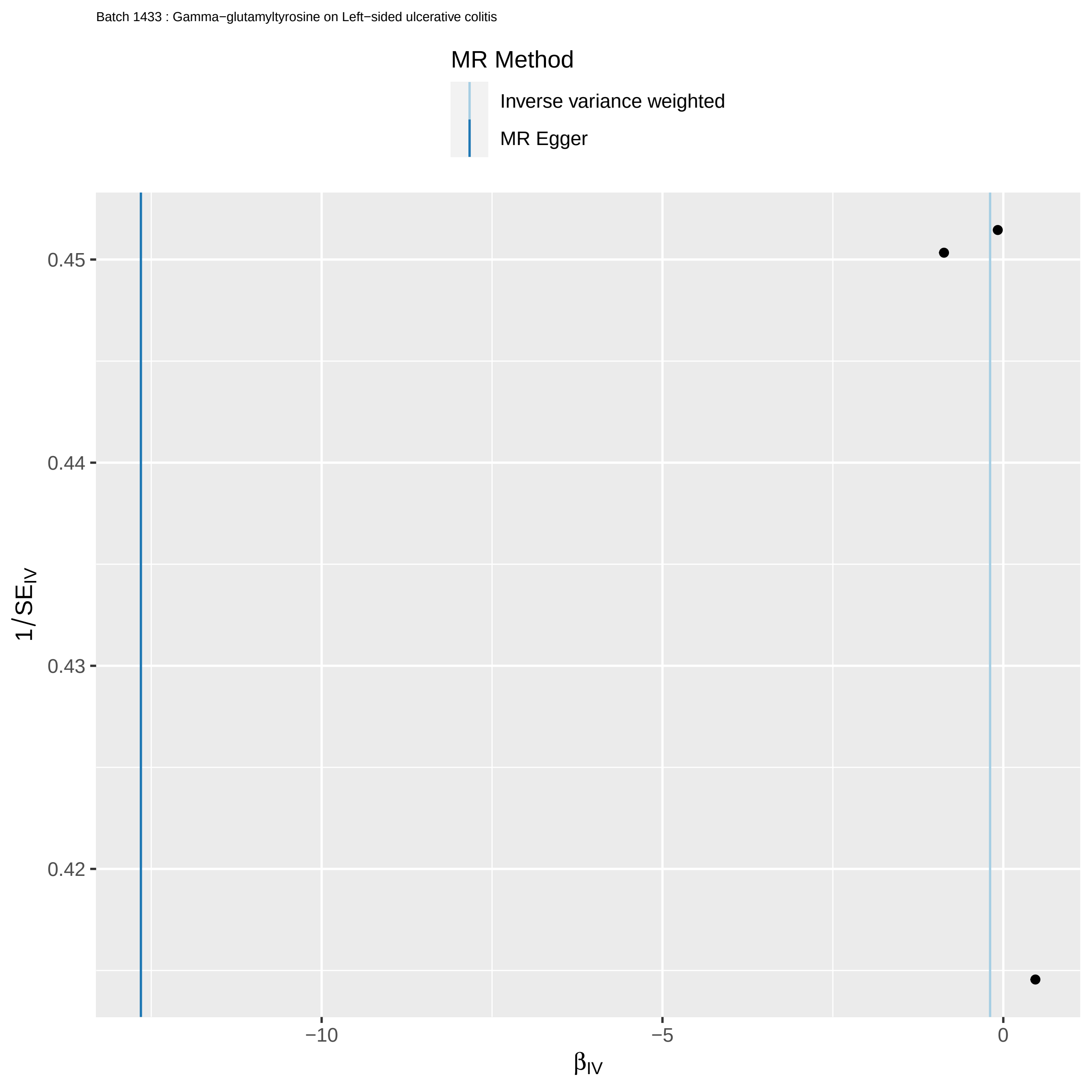
Batch 1433 : Gamma−glutamyltyrosine on Left−sided ulcerative colitis
MR Method
Inverse variance weighted
MR Egger
0.45
0.44
0.43
0.42
1/SE_IV
−10
−5
0
β_IV

Batch 1435 : Betaine on Left−sided ulcerative colitis

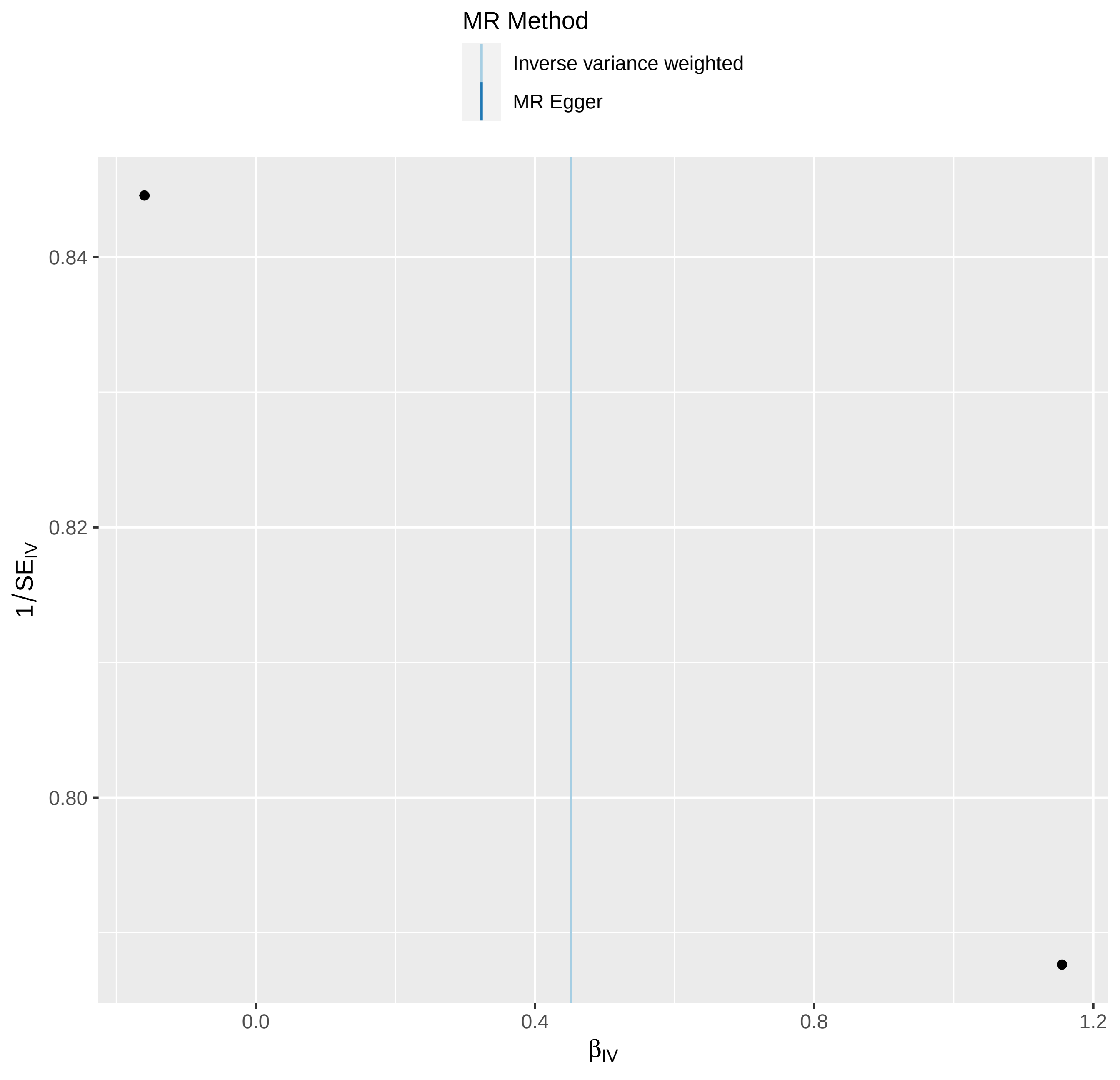

Batch 1436 : Xanthine on Left−sided ulcerative colitis

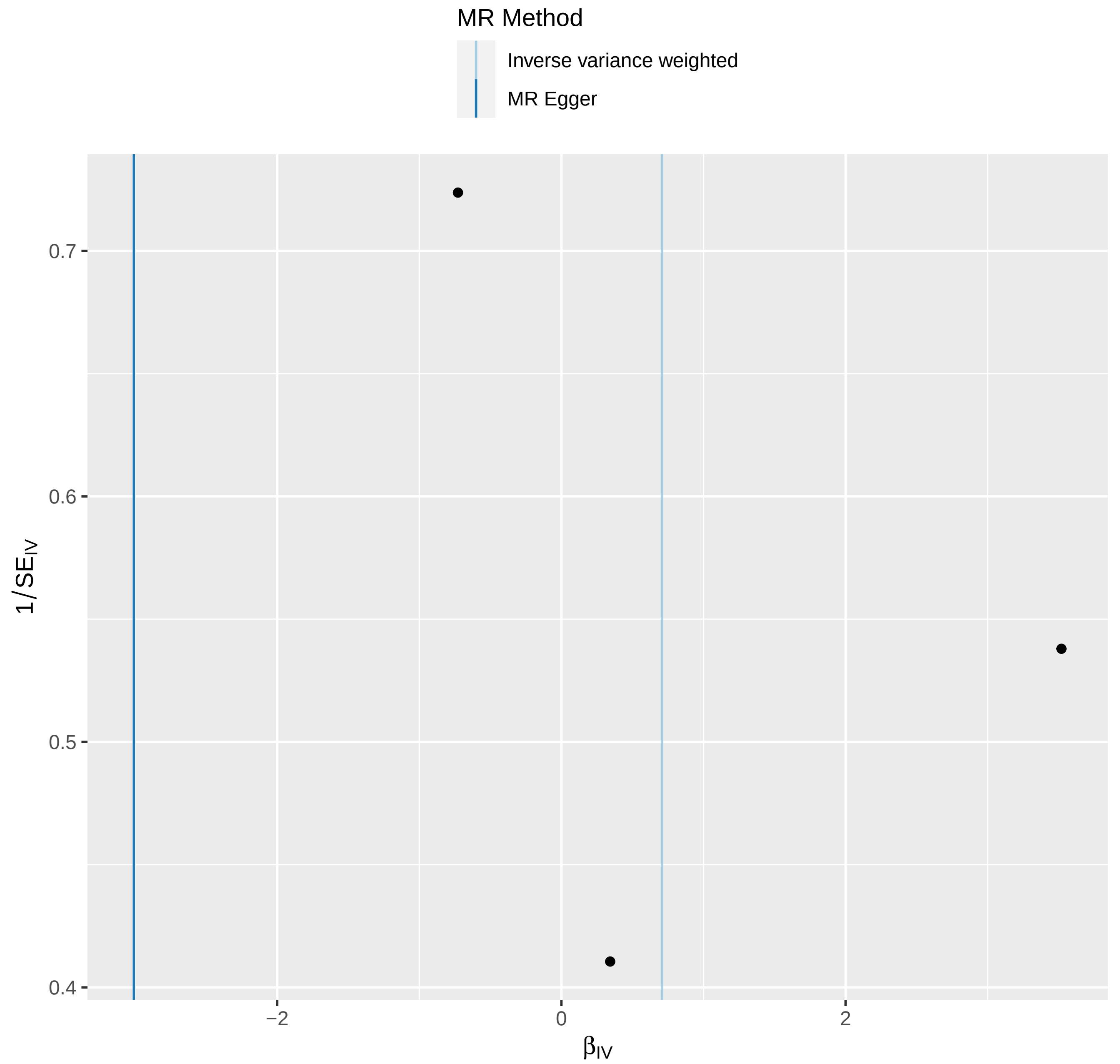

Batch 1437 : Phosphate on Left−sided ulcerative colitis

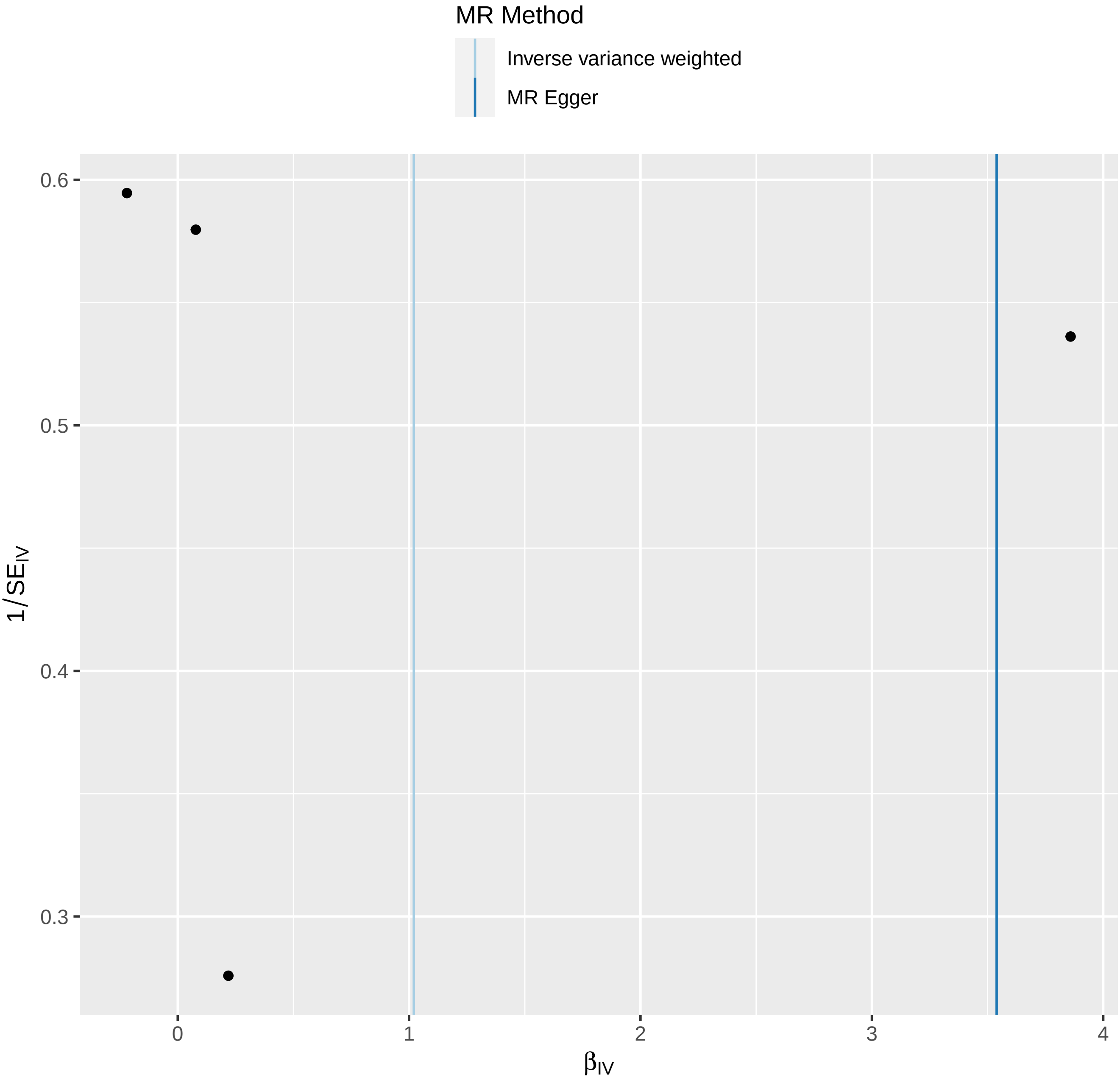

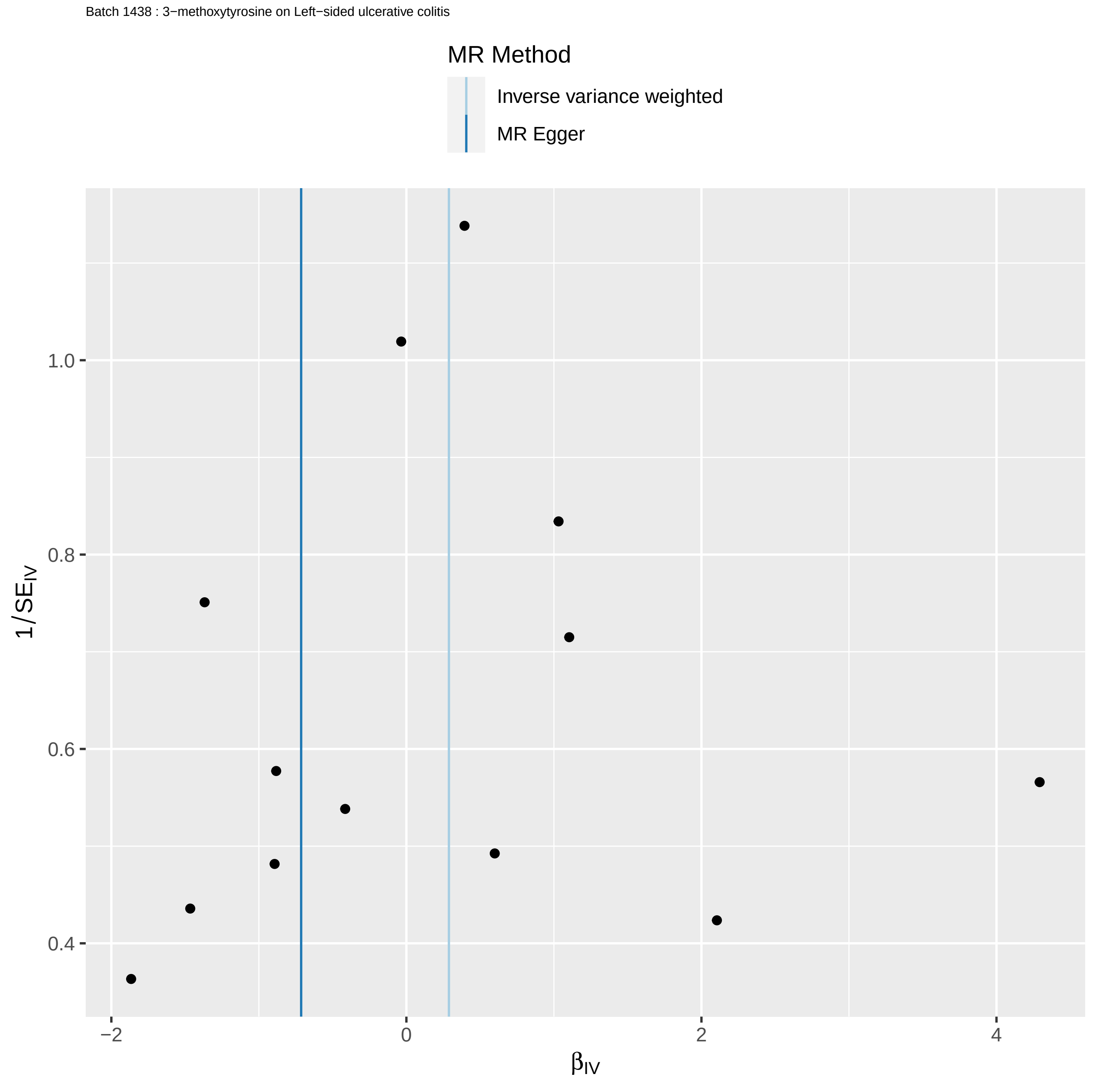

Batch 1438 : 3−methoxytyrosine on Left−sided ulcerative colitis
MR Method
Inverse variance weighted
MR Egger
1.0
0.8
0.6
0.4
1/SE_IV
−2
0
2
4
β_IV

Batch 1440 : Undecanoate (11:0) on Left−sided ulcerative colitis

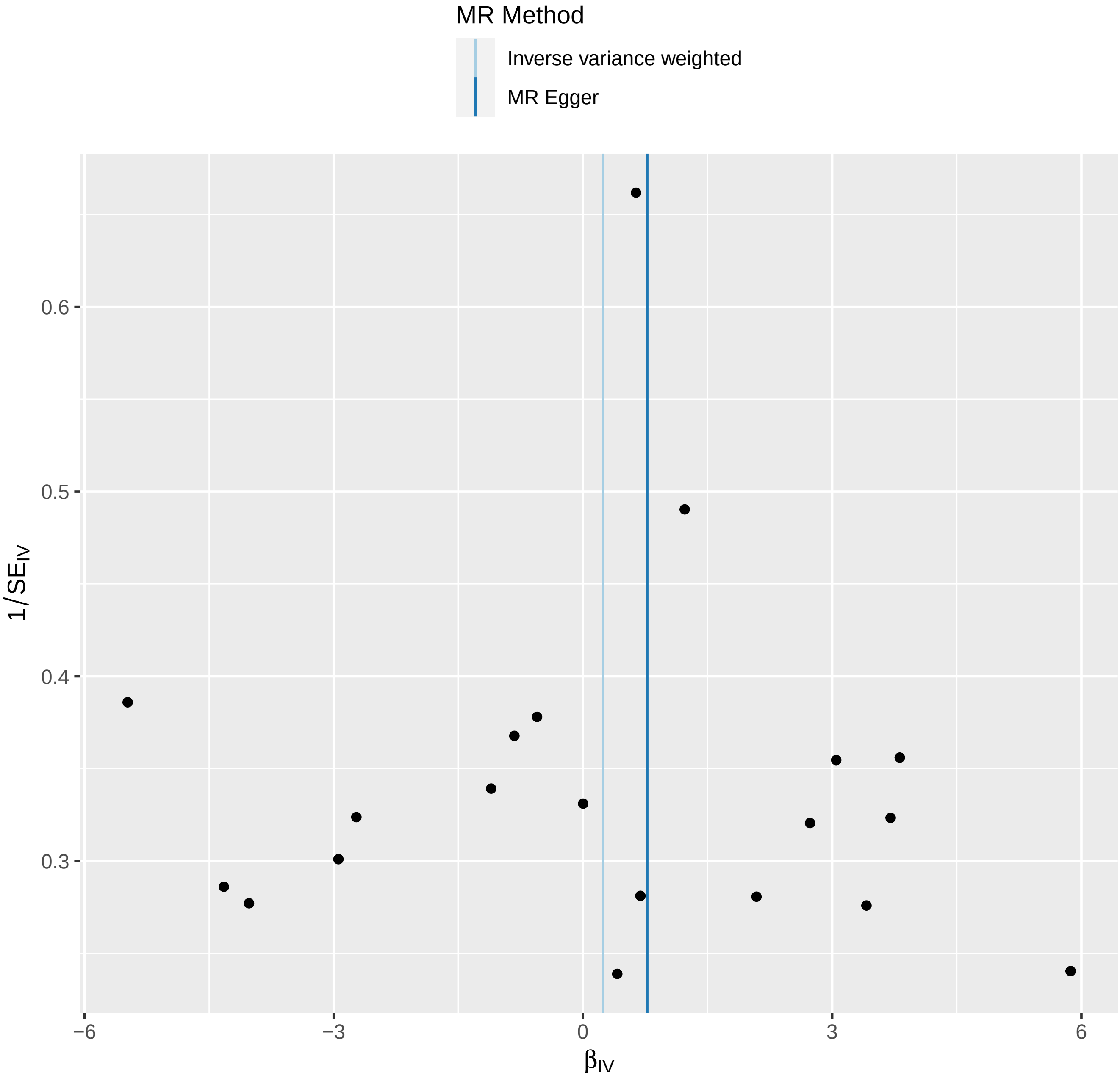

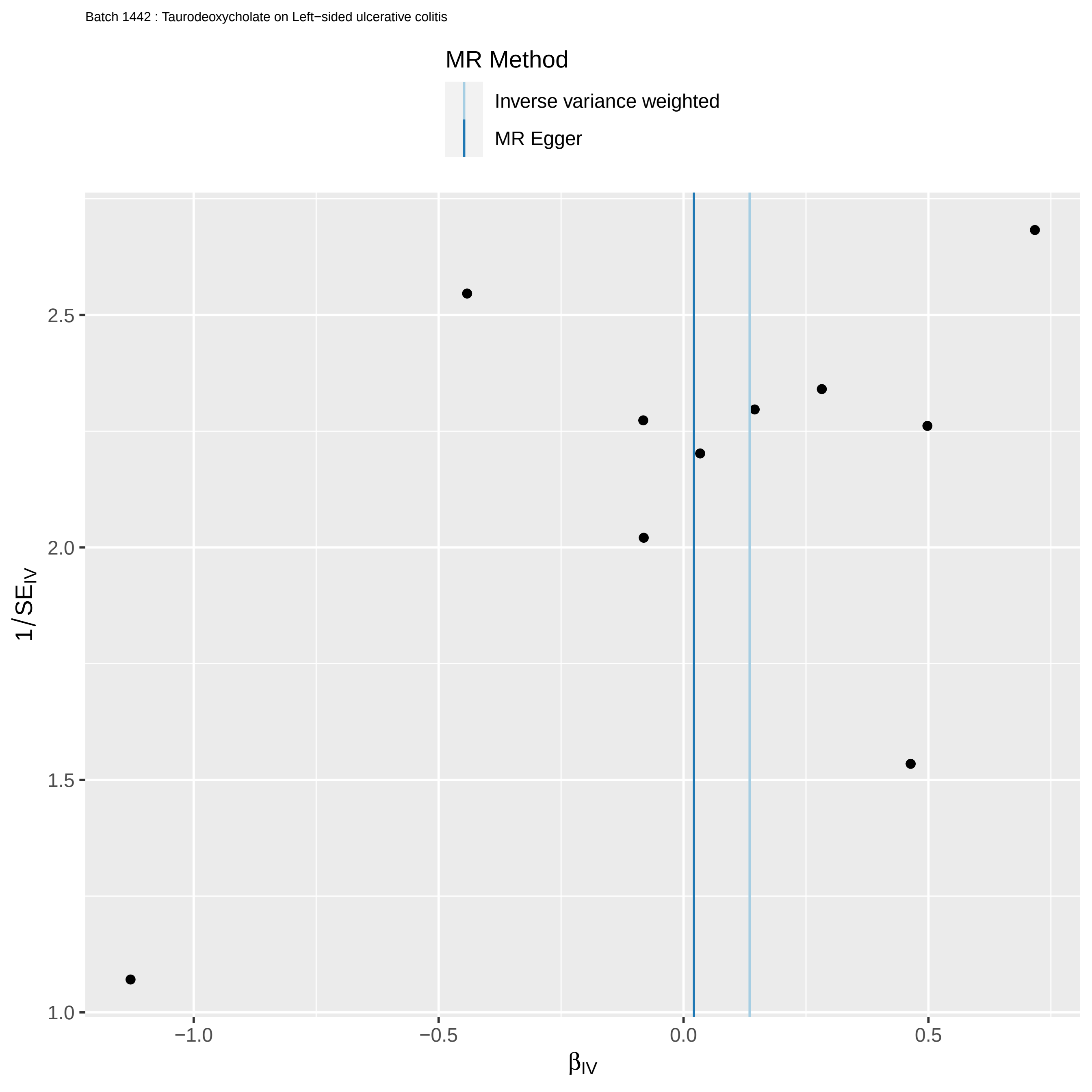

Batch 1442 : Taurodeoxycholate on Left−sided ulcerative colitis
MR Method
Inverse variance weighted
MR Egger
2.5
2.0
1.5
1.0
1/SE$_{IV}$
−1.0
−0.5
0.0
0.5
β_{IV}

Batch 1443 : Glycerol on Left−sided ulcerative colitis

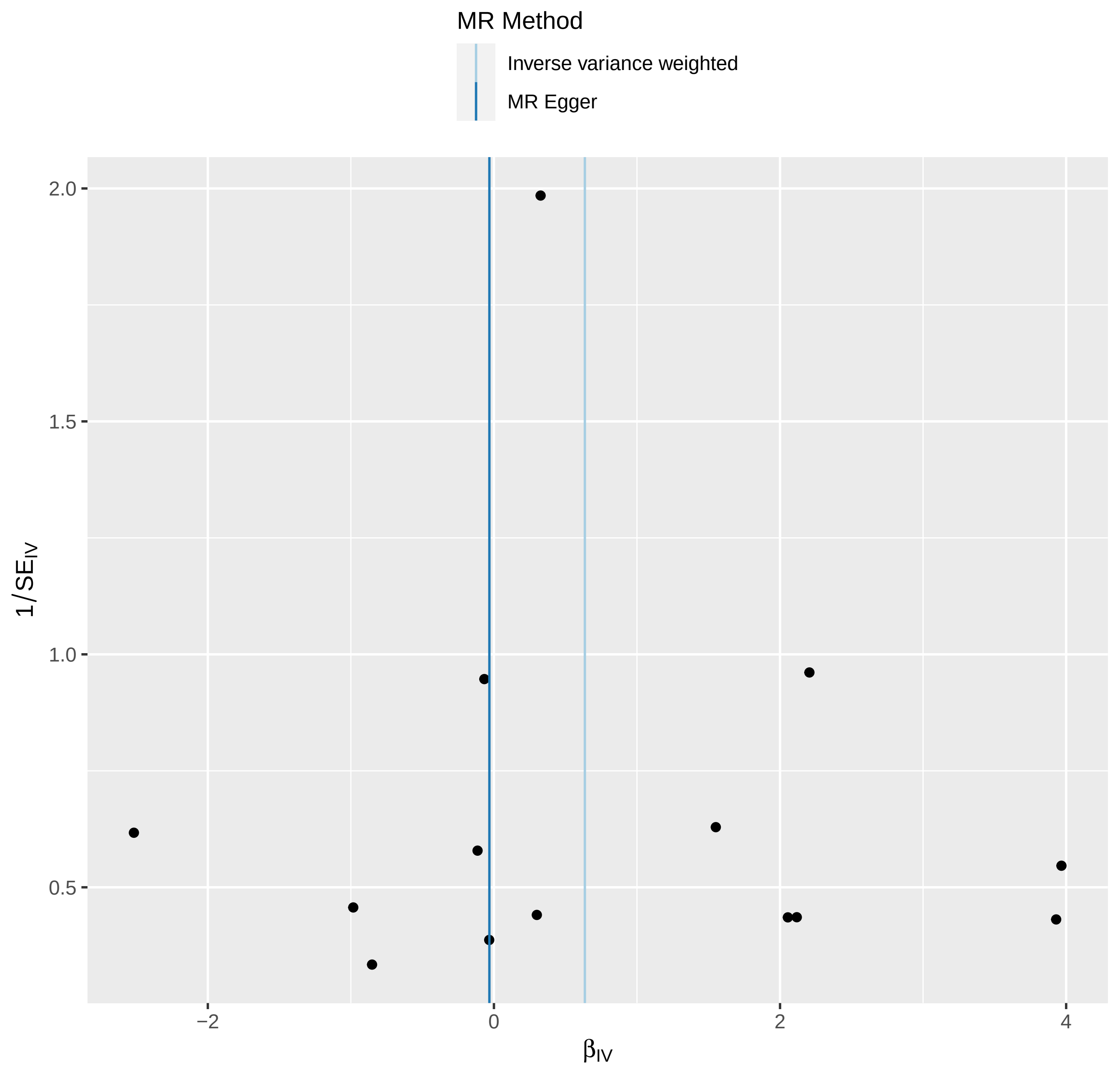

Batch 1444 : Kynurenine on Left−sided ulcerative colitis

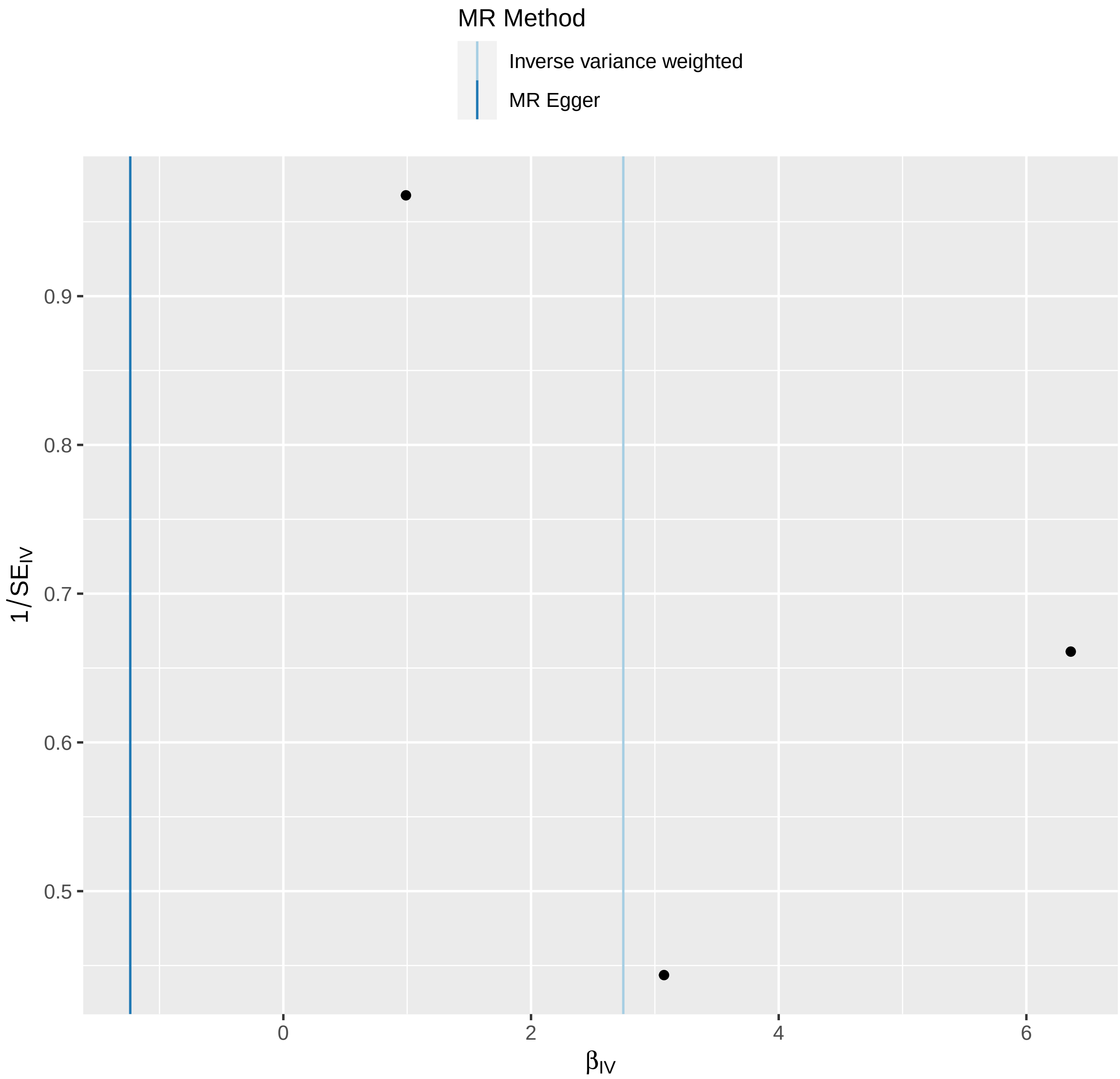

Batch 1445 : Mannitol on Left−sided ulcerative colitis

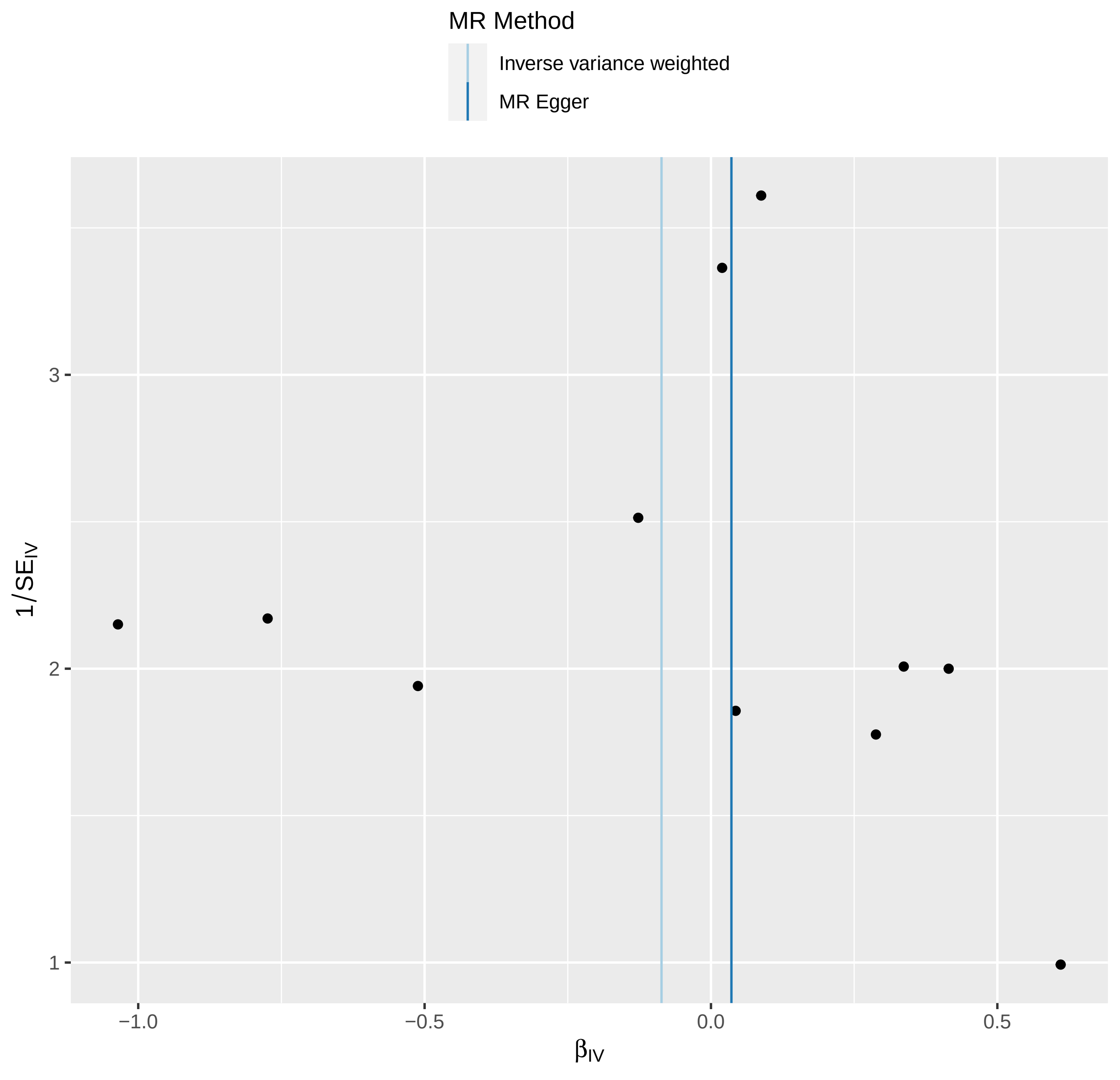

Batch 1446 : Glycerol 3−phosphate (G3P) on Left−sided ulcerative colitis

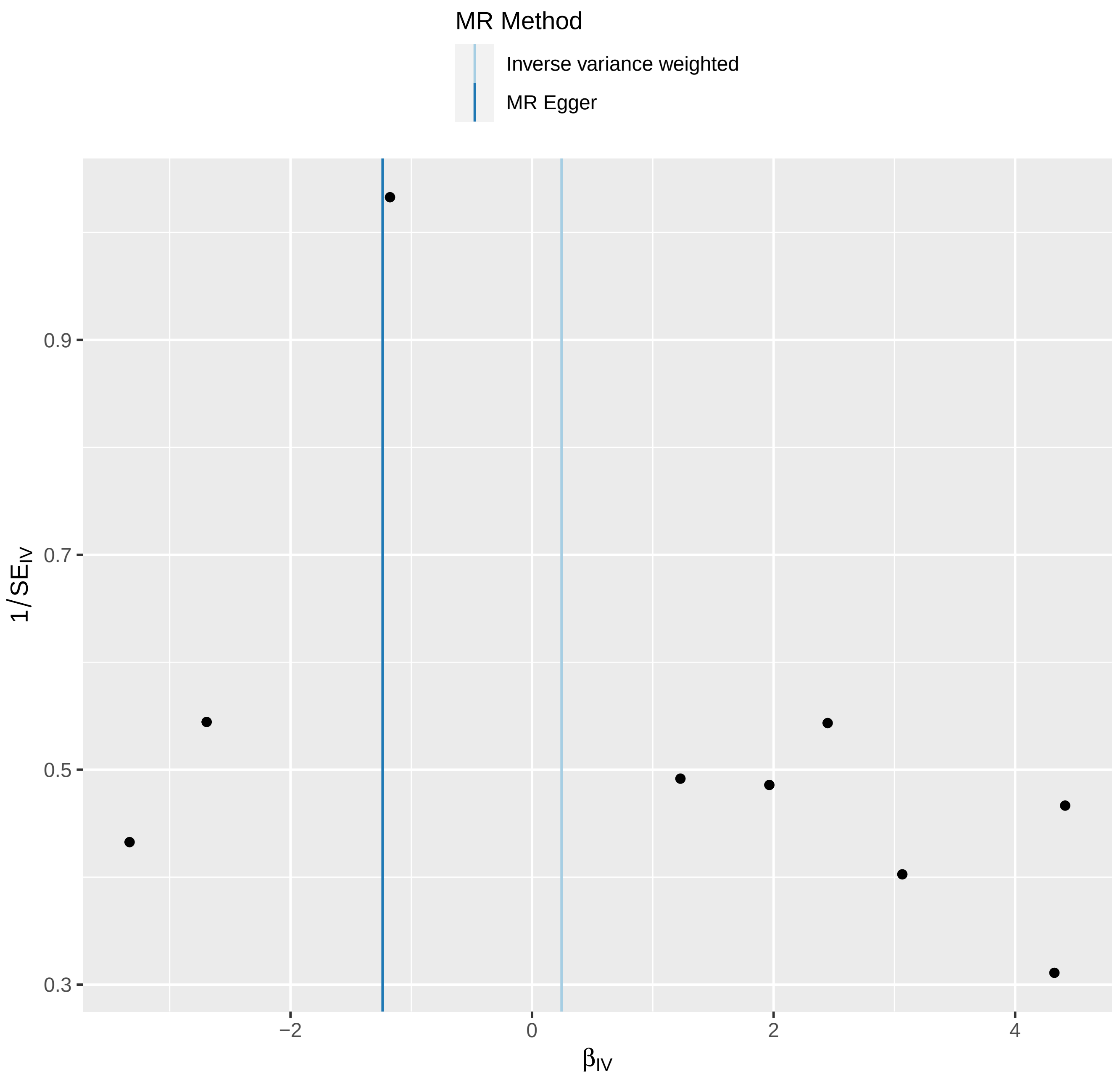

Batch 1448 : Carnitine on Left−sided ulcerative colitis

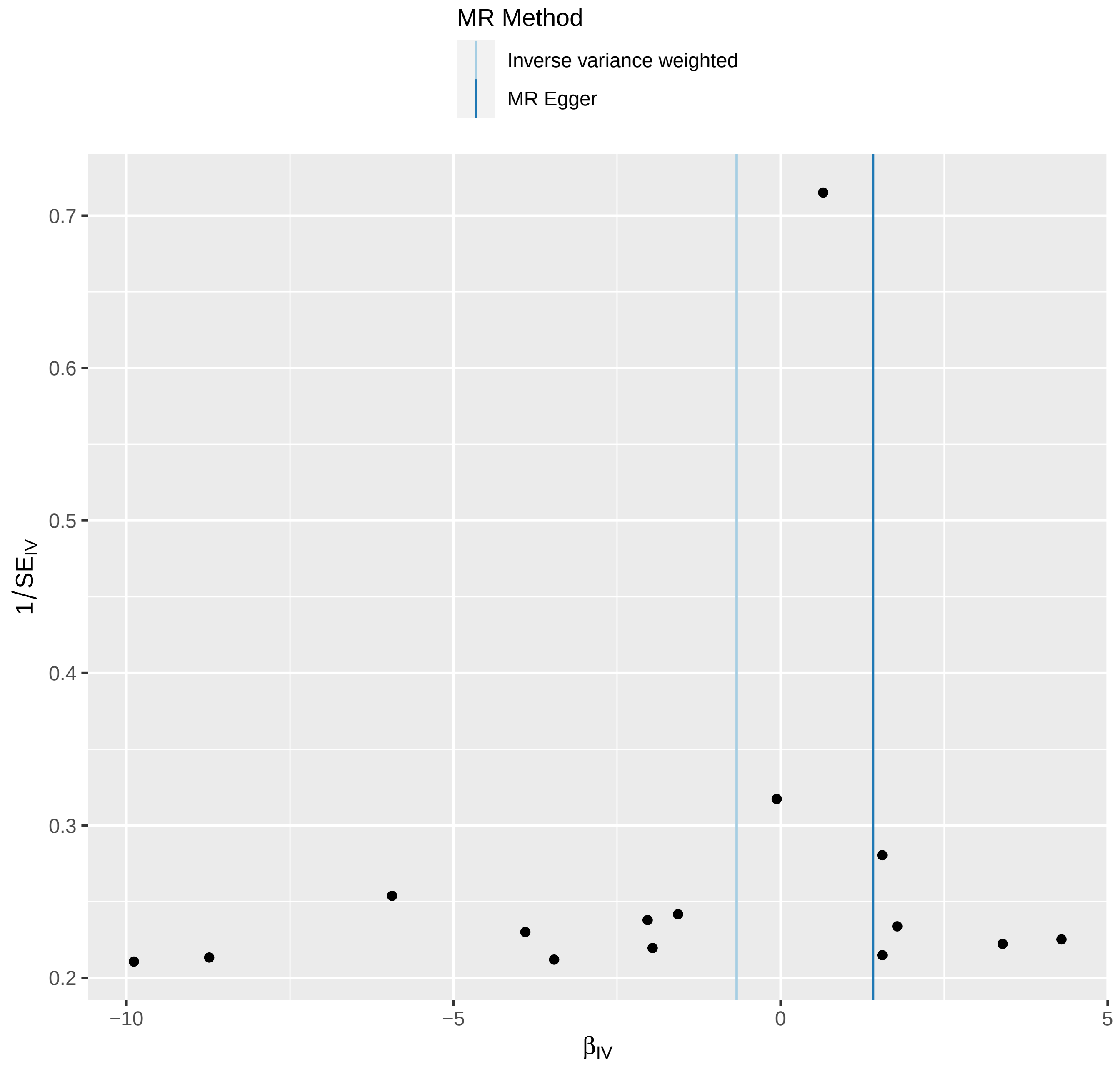

Batch 1449 : Choline on Left−sided ulcerative colitis

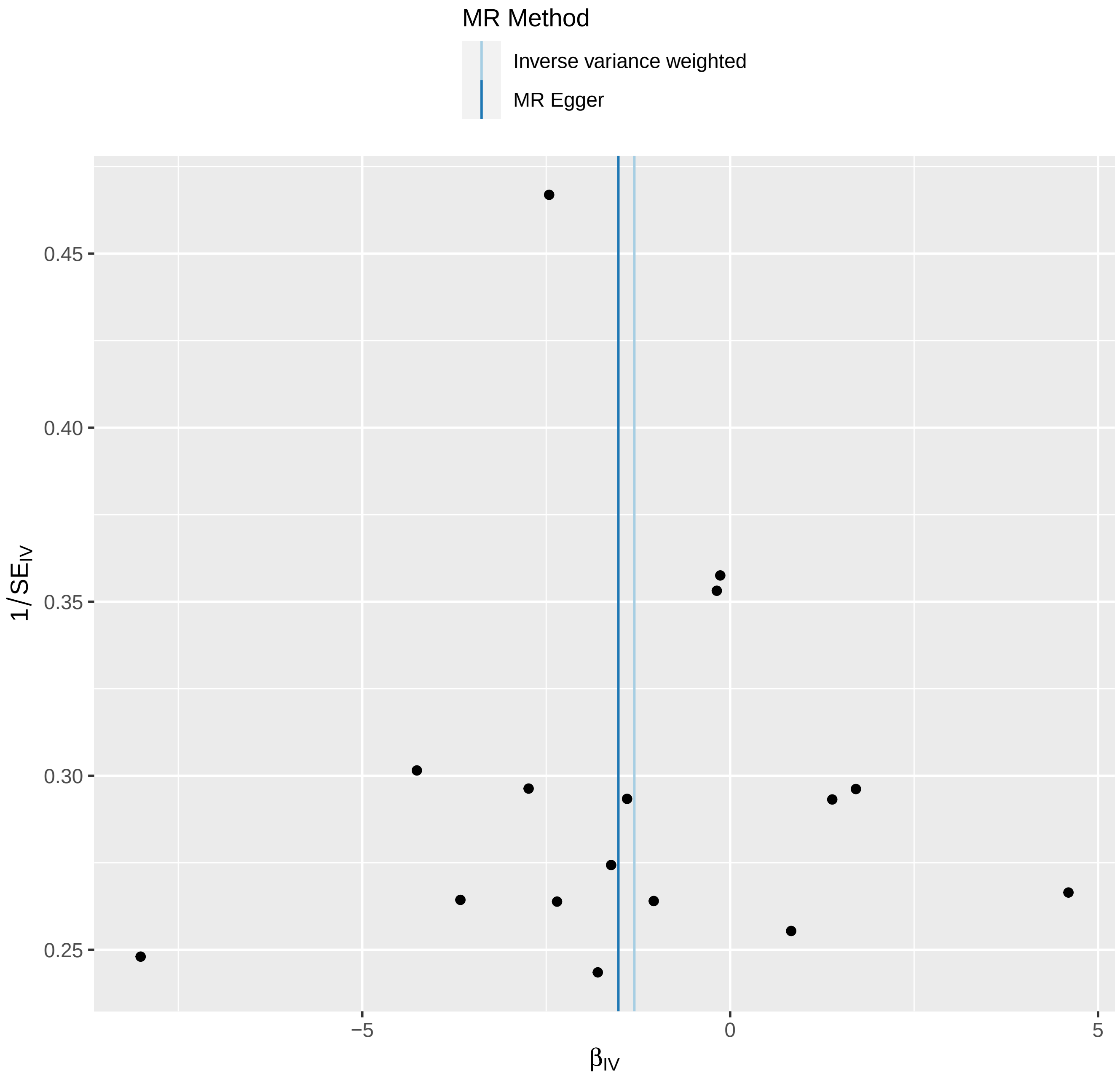

Batch 1452 : 3−methyl−2−oxovalerate on Left−sided ulcerative colitis

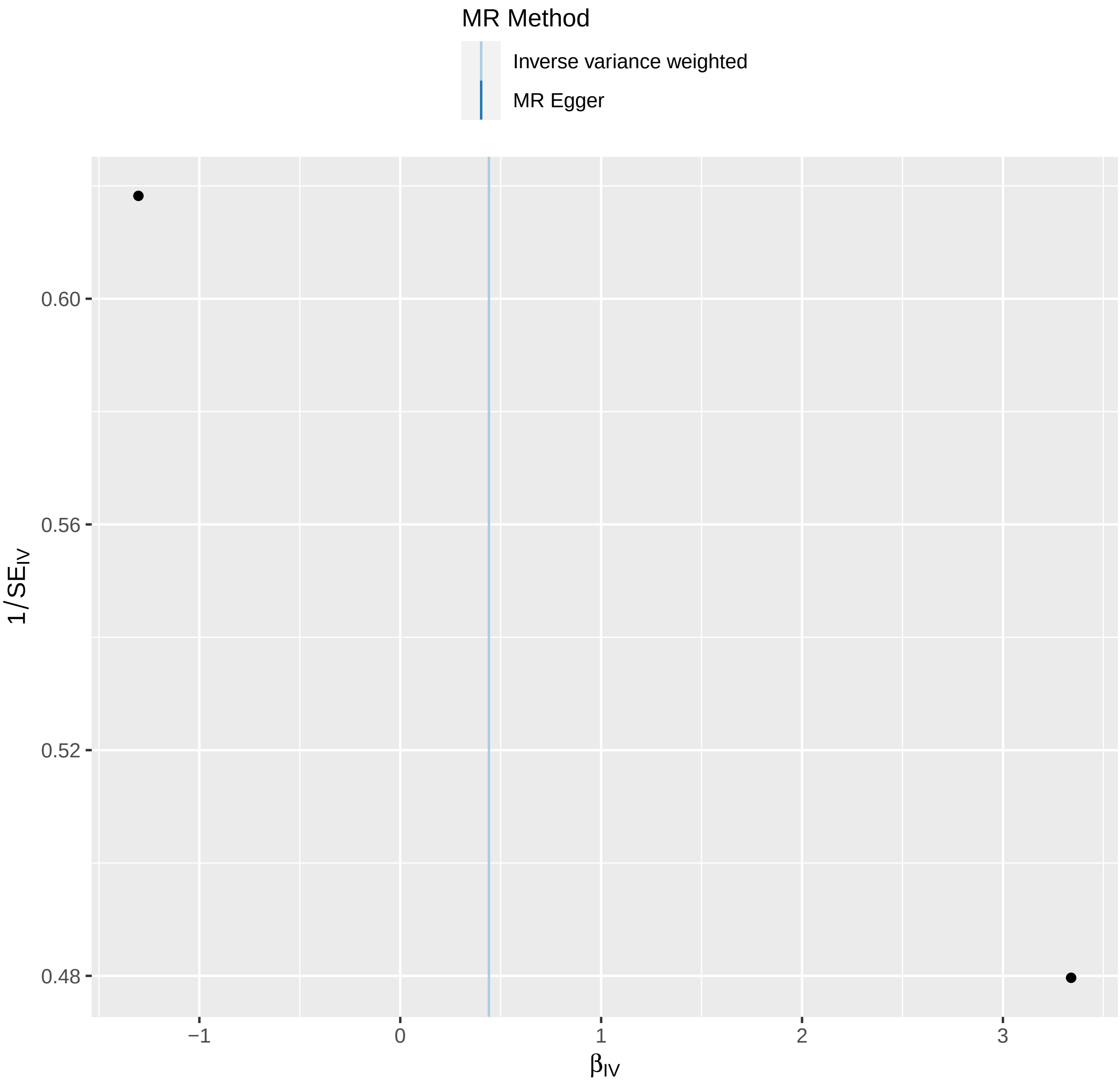

Batch 1453 : 3−methylhistidine on Left−sided ulcerative colitis

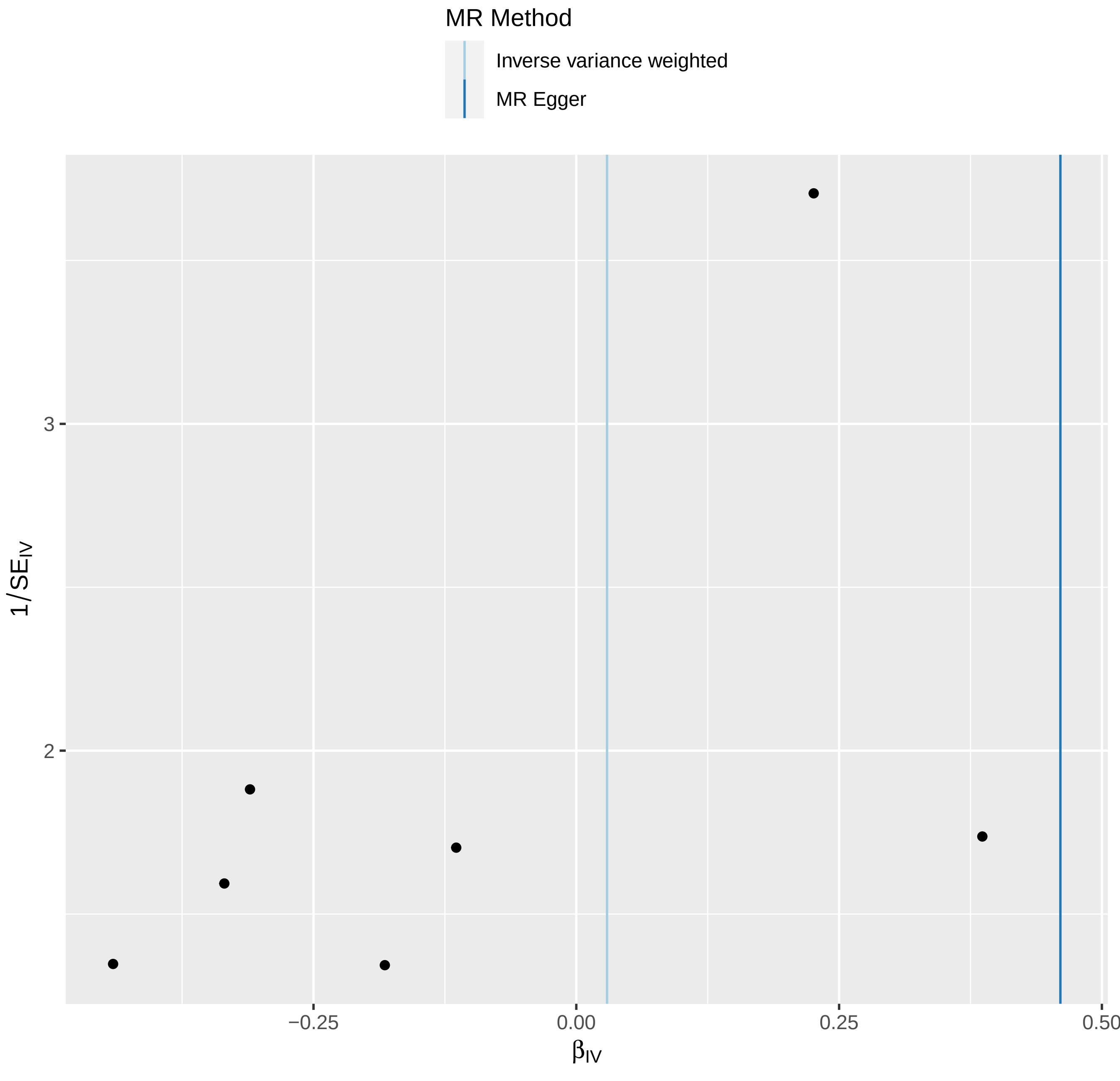

Batch 1455 : Phenylacetate on Left−sided ulcerative colitis

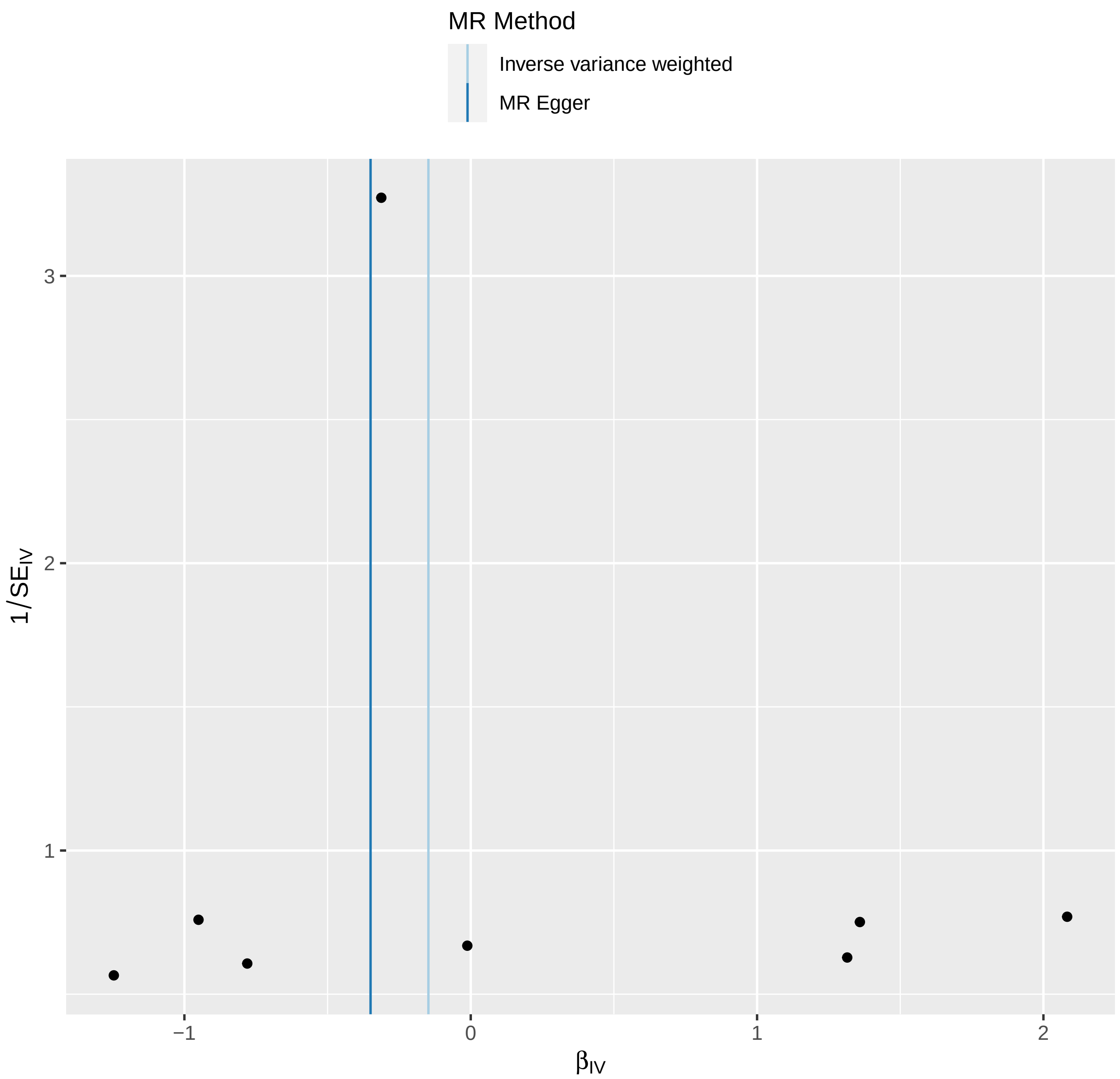

Batch 1456 : Glycerophosphorylcholine (GPC) on Left−sided ulcerative colitis

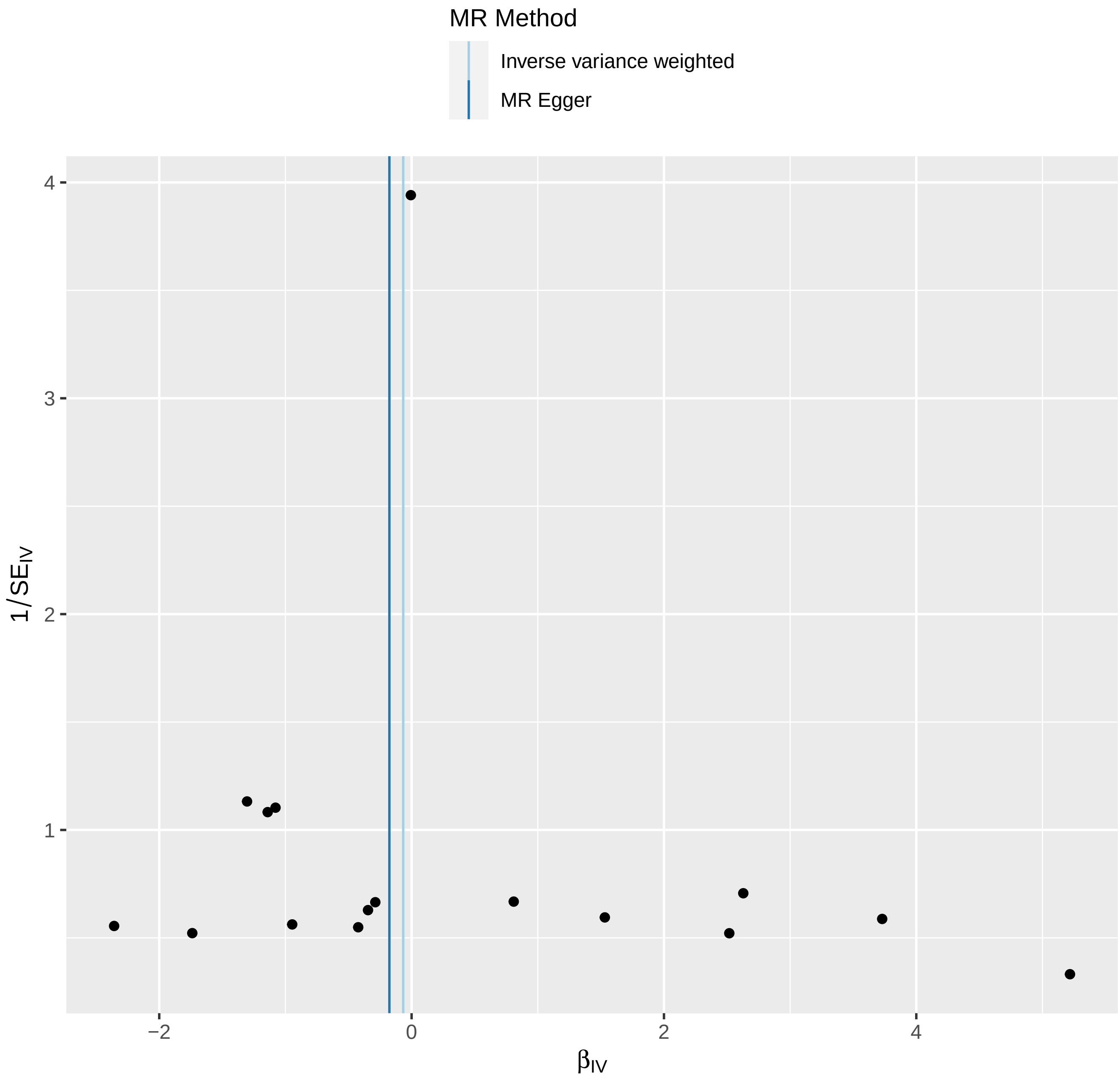

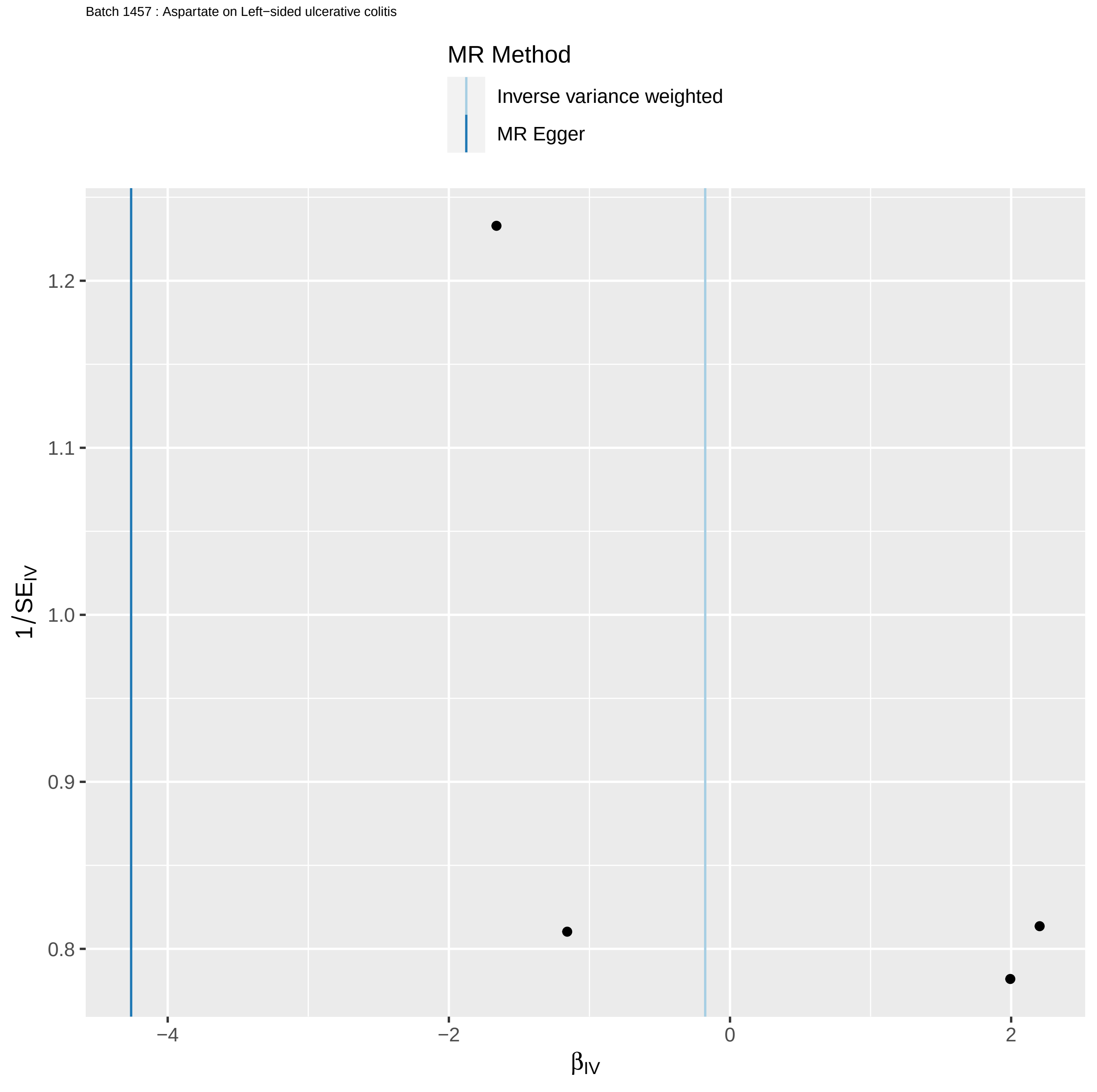
Batch 1457 : Aspartate on Left−sided ulcerative colitis
MR Method
Inverse variance weighted
MR Egger
1.2
1.1
1.0
0.9
0.8
1/SE$_{IV}$
−4
−2
0
2
β$_{IV}$

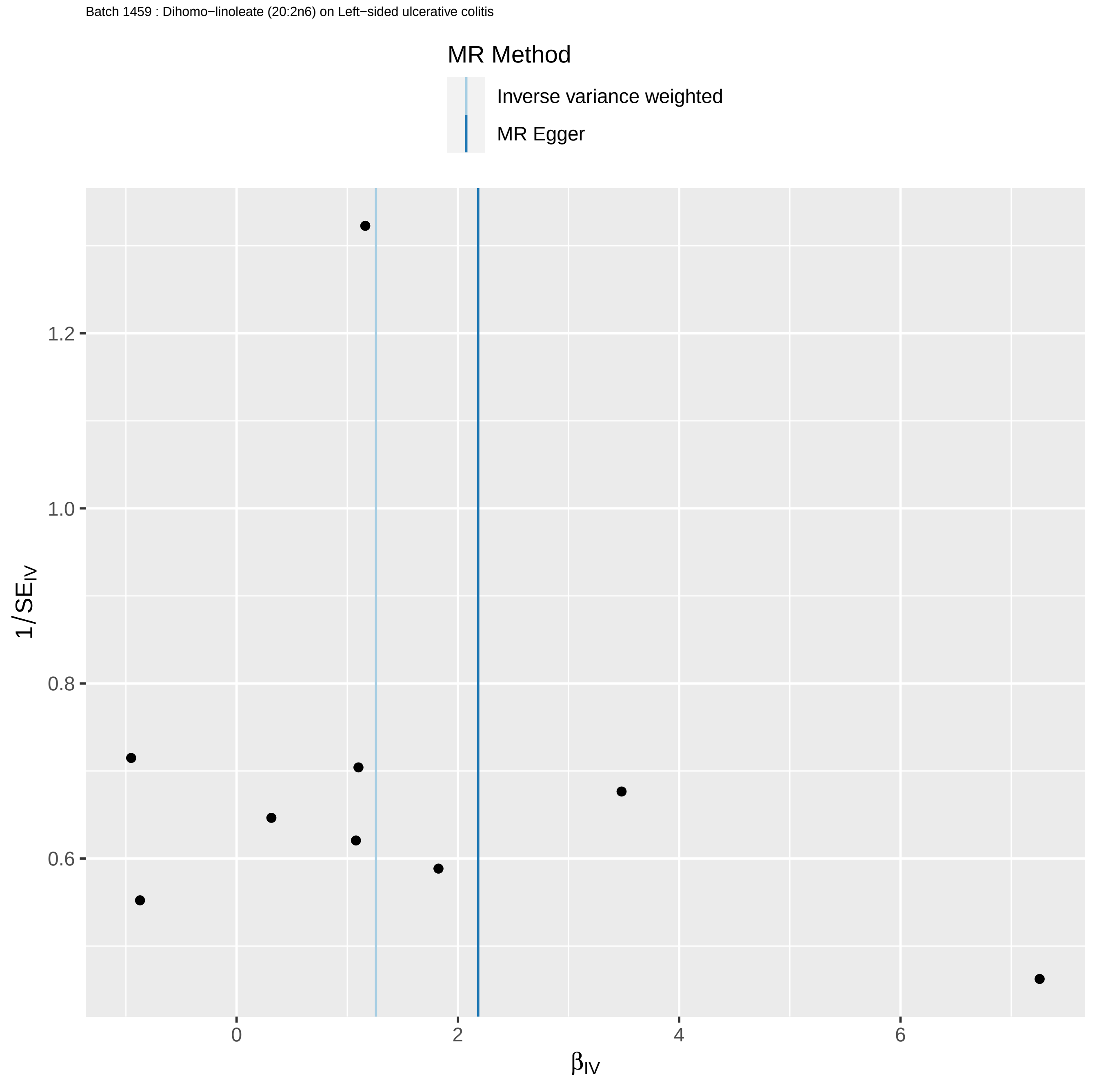
Batch 1459 : Dihomo−linoleate (20:2n6) on Left−sided ulcerative colitis
MR Method
Inverse variance weighted
MR Egger
1/SE_IV
1.2
1.0
0.8
0.6
0
2
4
6
β_IV

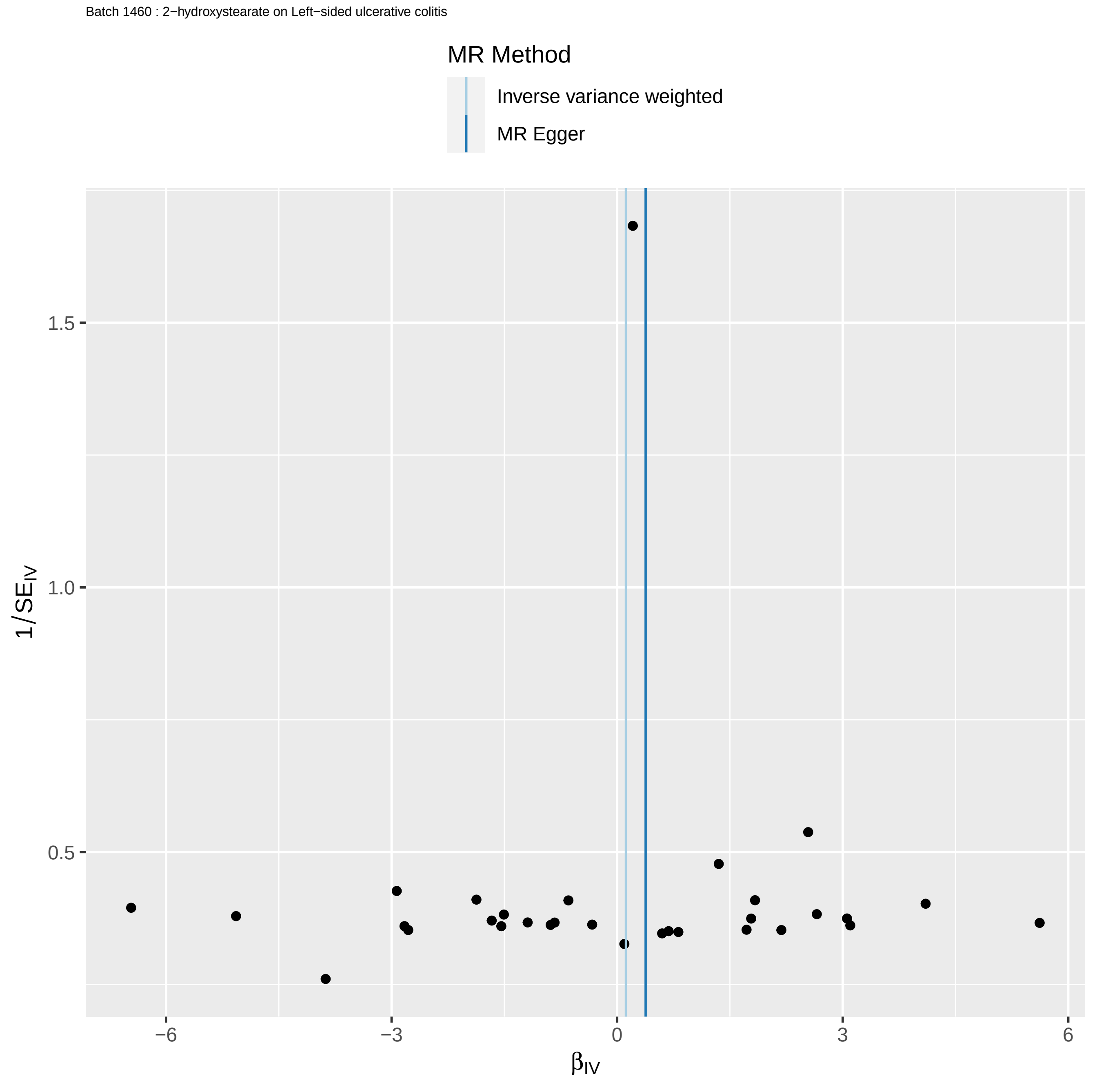

Batch 1460 : 2−hydroxystearate on Left−sided ulcerative colitis
MR Method
Inverse variance weighted
MR Egger
1.5
1.0
0.5
1/SE_IV
−6
−3
0
3
6
β_IV

Batch 1462 : Glycylvaline on Left−sided ulcerative colitis

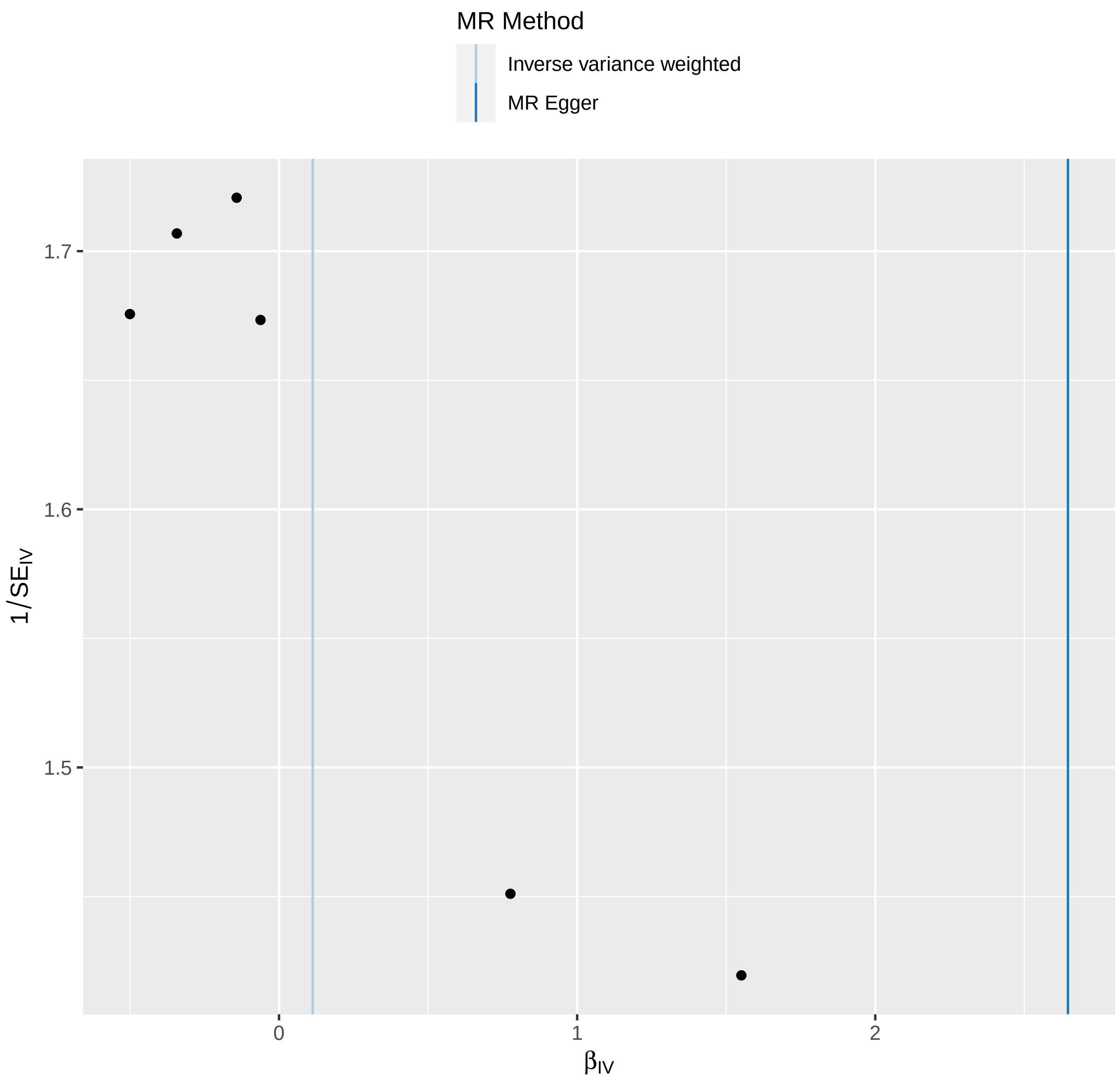

Batch 1463 : Gamma−glutamylleucine on Left−sided ulcerative colitis

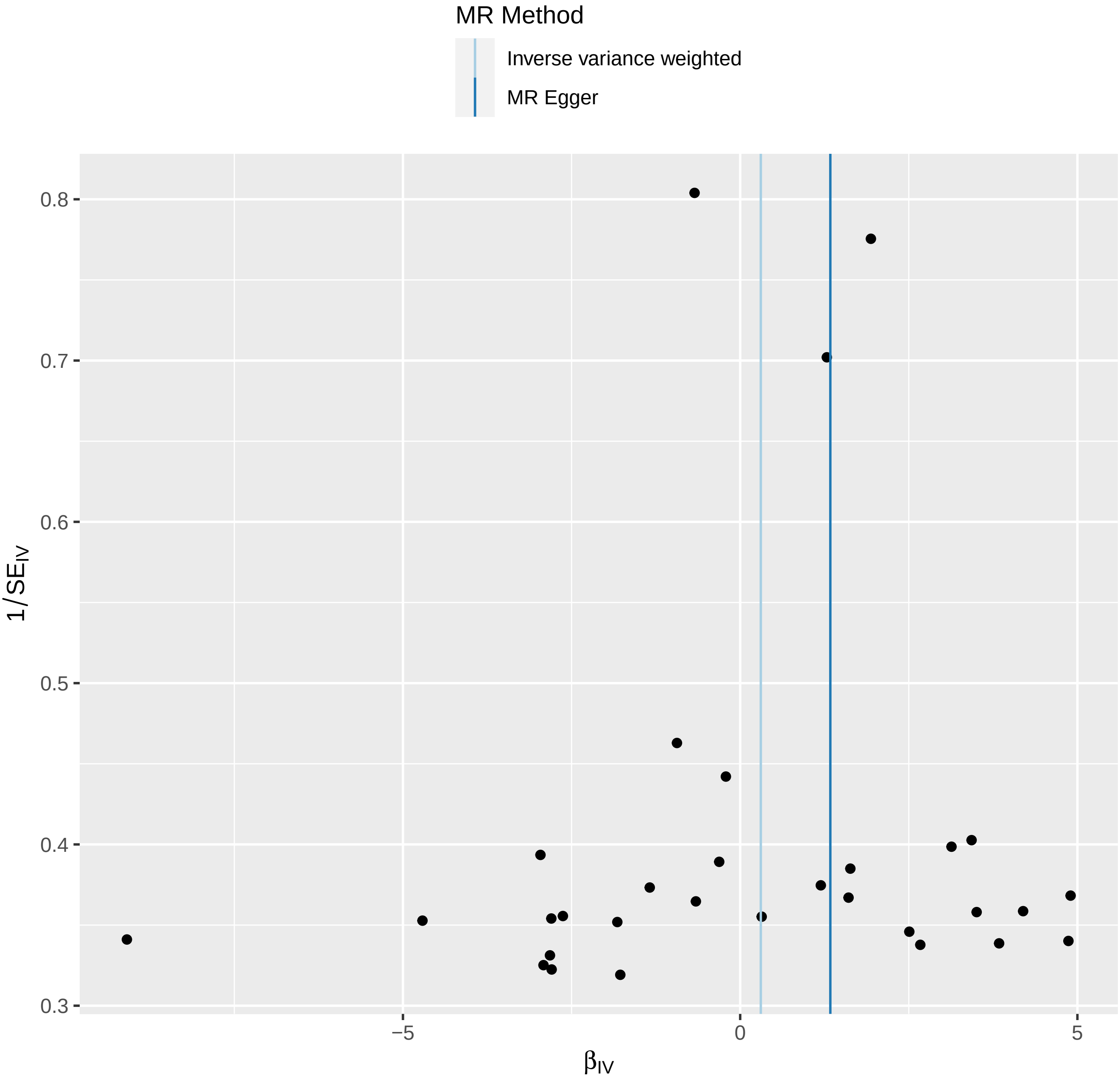

Batch 1466 : Glycocholate on Left−sided ulcerative colitis

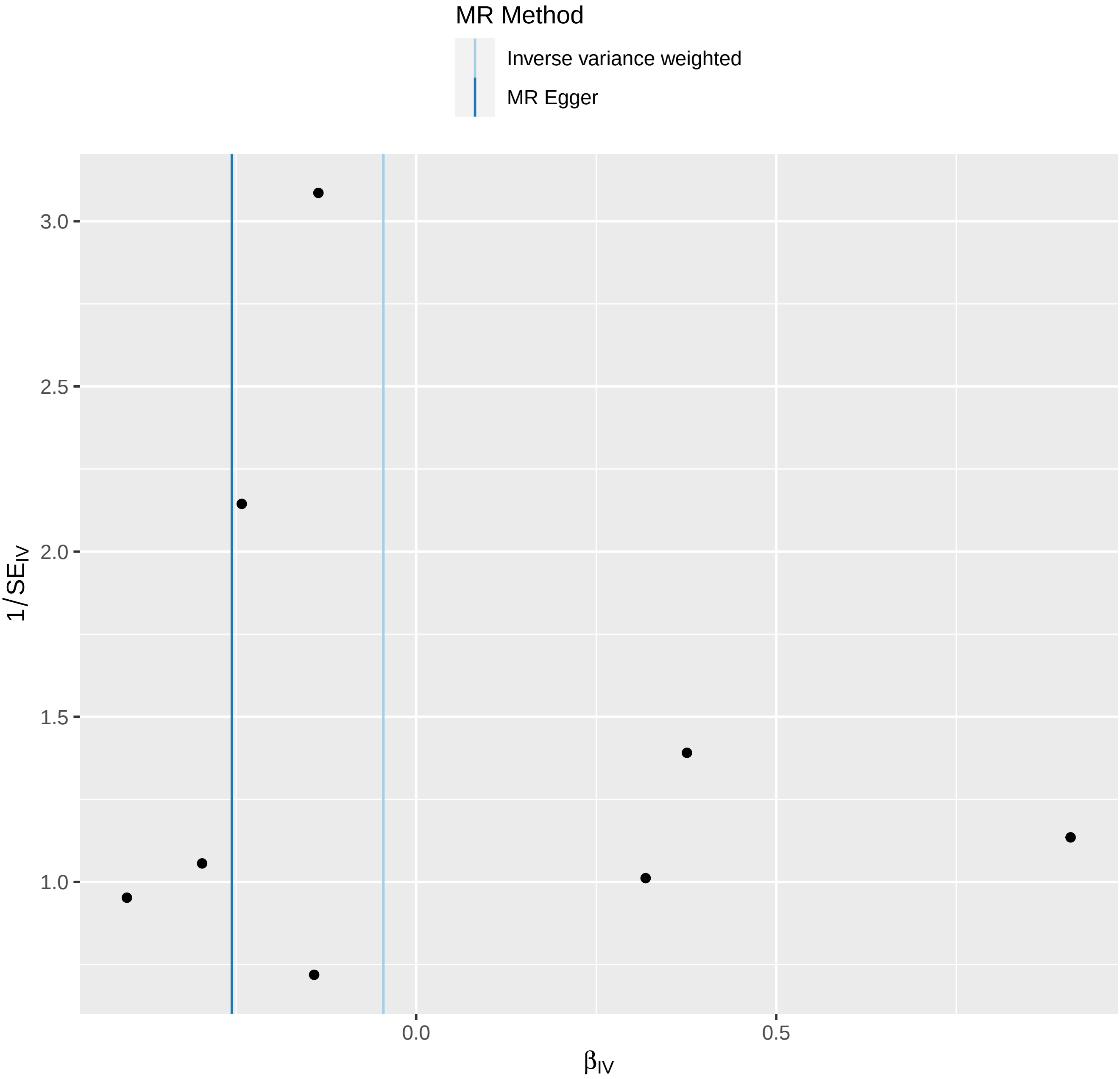

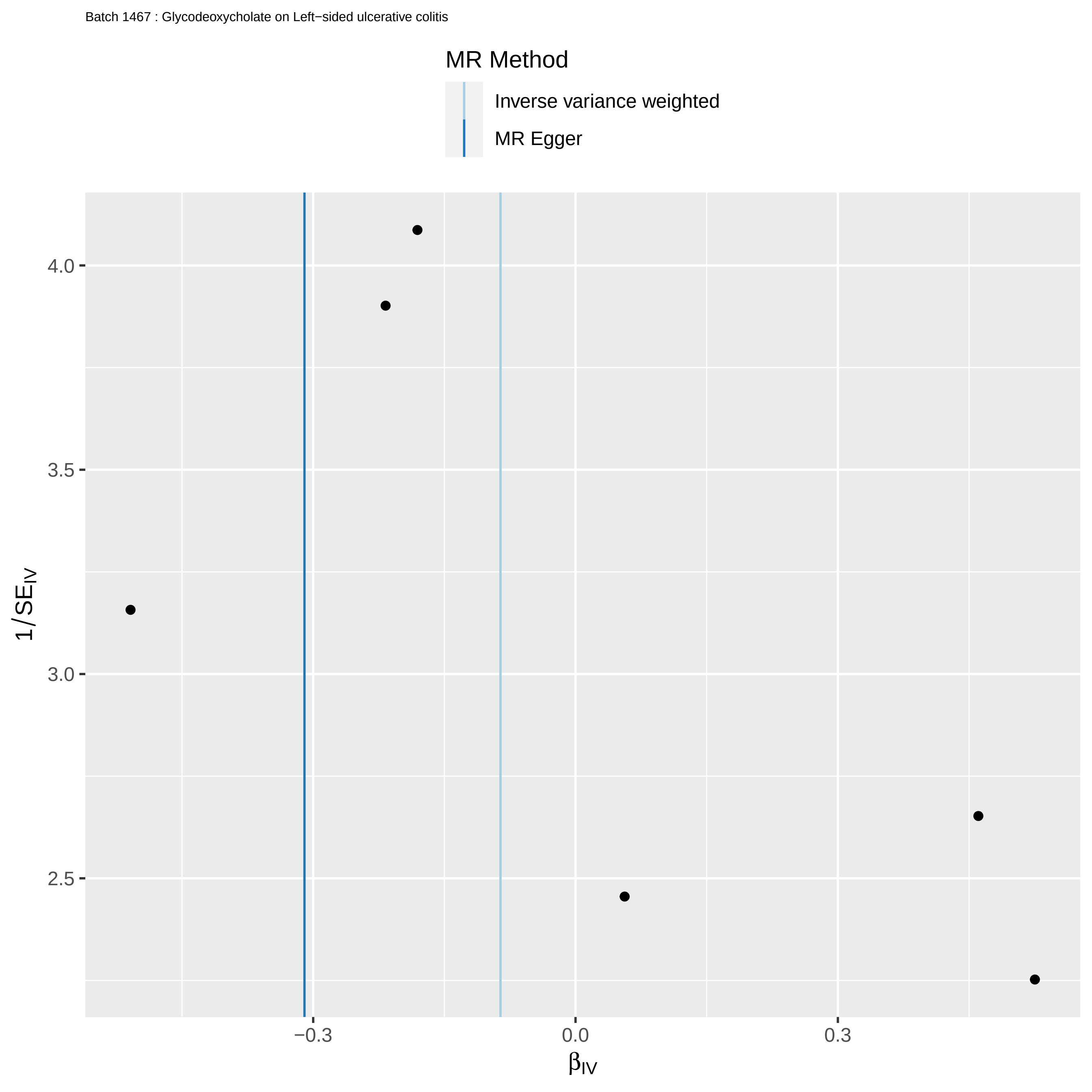

Batch 1467 : Glycodeoxycholate on Left−sided ulcerative colitis
MR Method
Inverse variance weighted
MR Egger
4.0
3.5
3.0
2.5
1/SE_IV
−0.3
0.0
0.3
β_IV

Batch 1468 : Taurochenodeoxycholate on Left−sided ulcerative colitis

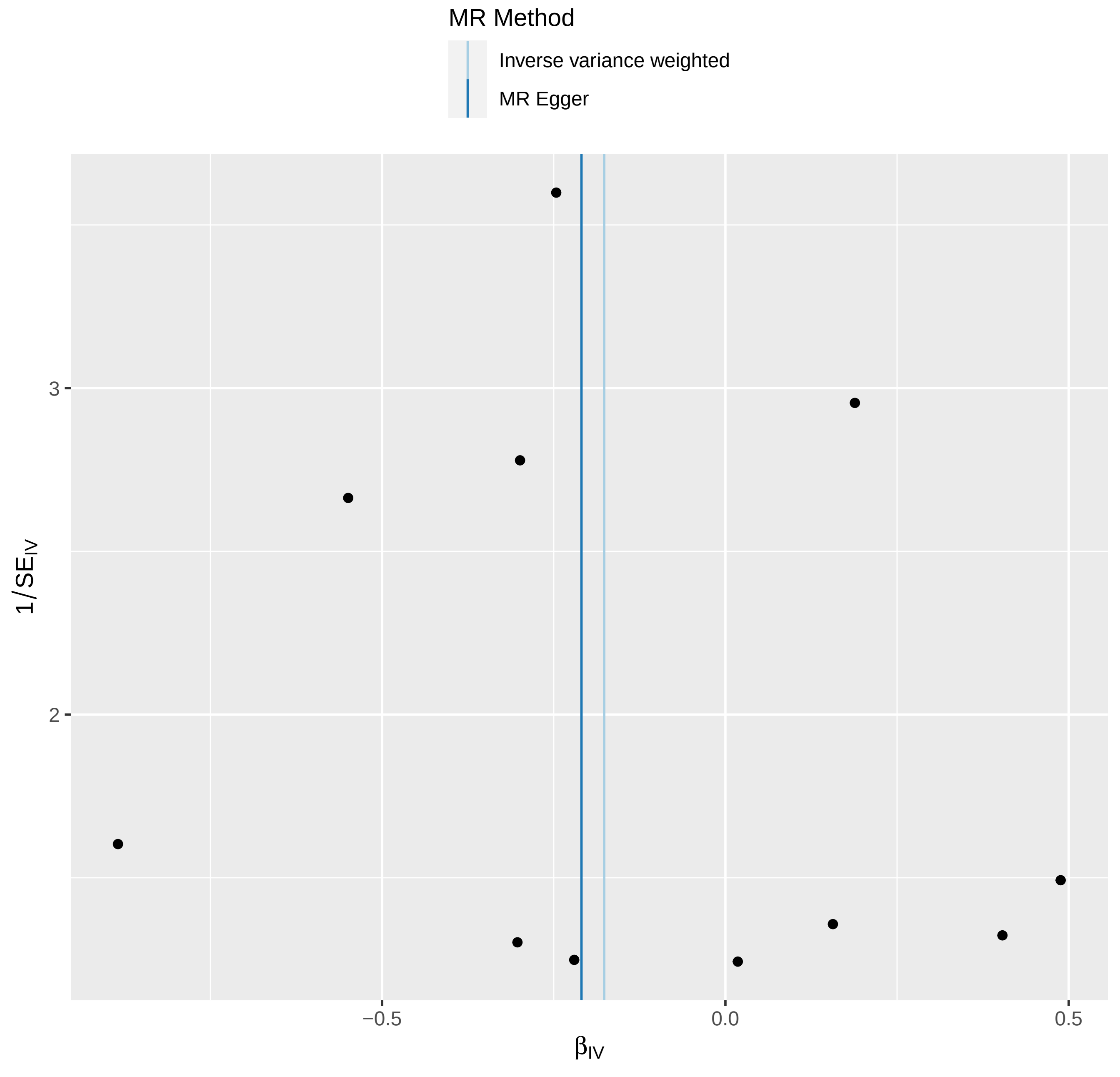

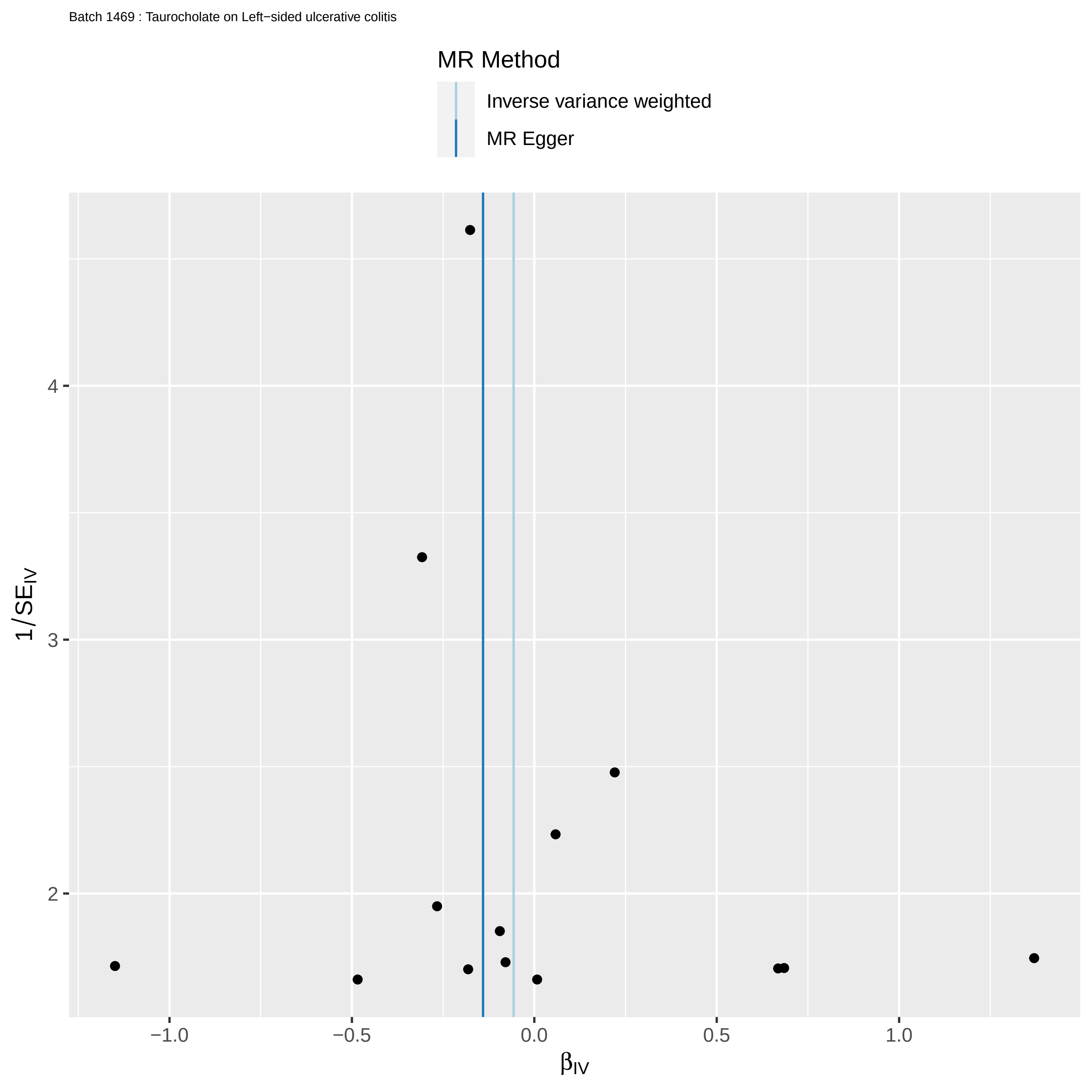

Batch 1469 : Taurocholate on Left−sided ulcerative colitis
MR Method
Inverse variance weighted
MR Egger
1/SE$_{IV}$
4
3
2
−1.0
−0.5
0.0
0.5
1.0
β_{IV}

Batch 1471 : 1−stearoylglycerophosphoinositol on Left−sided ulcerative colitis

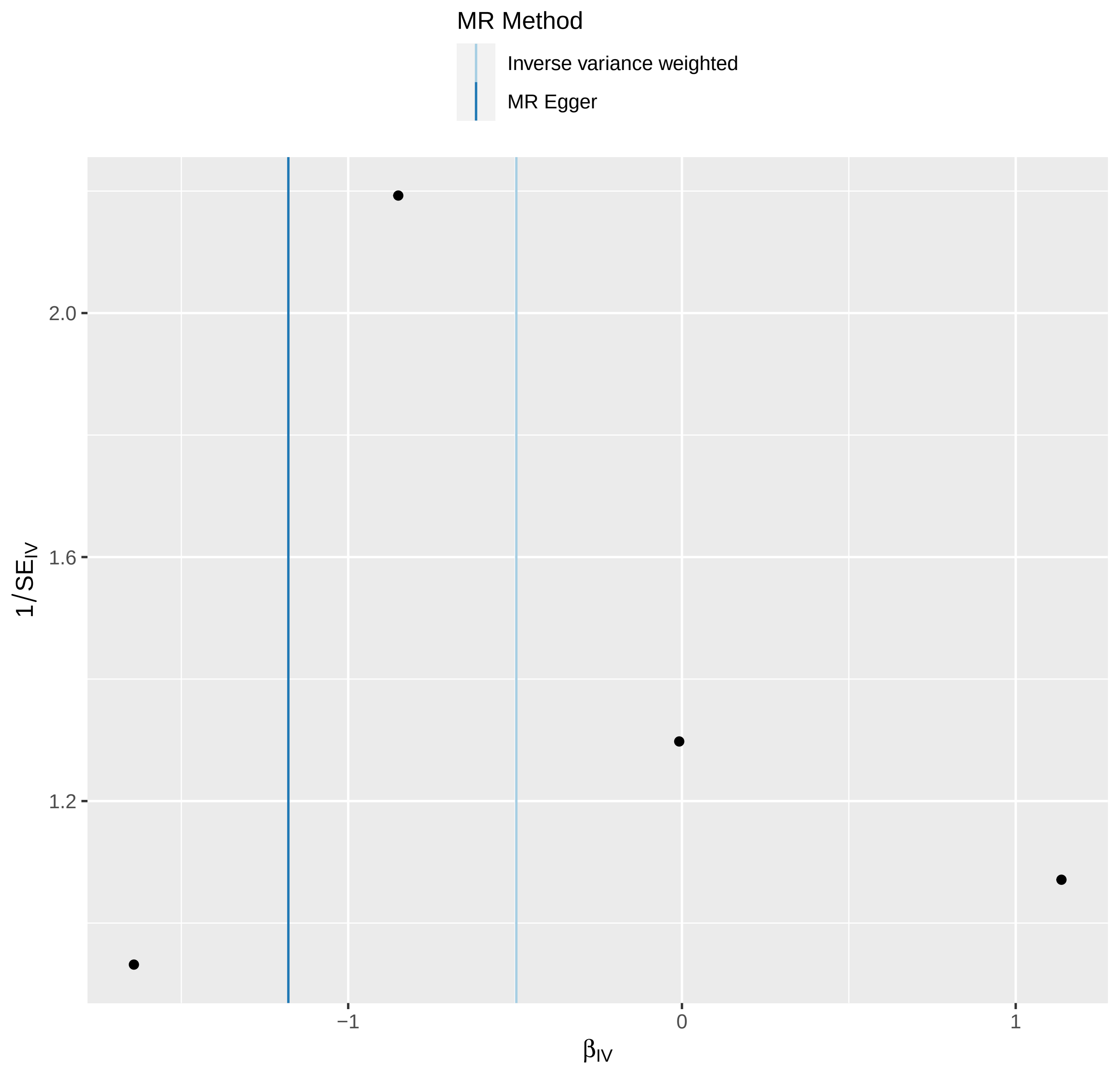

Batch 1472 : Myo−inositol on Left−sided ulcerative colitis

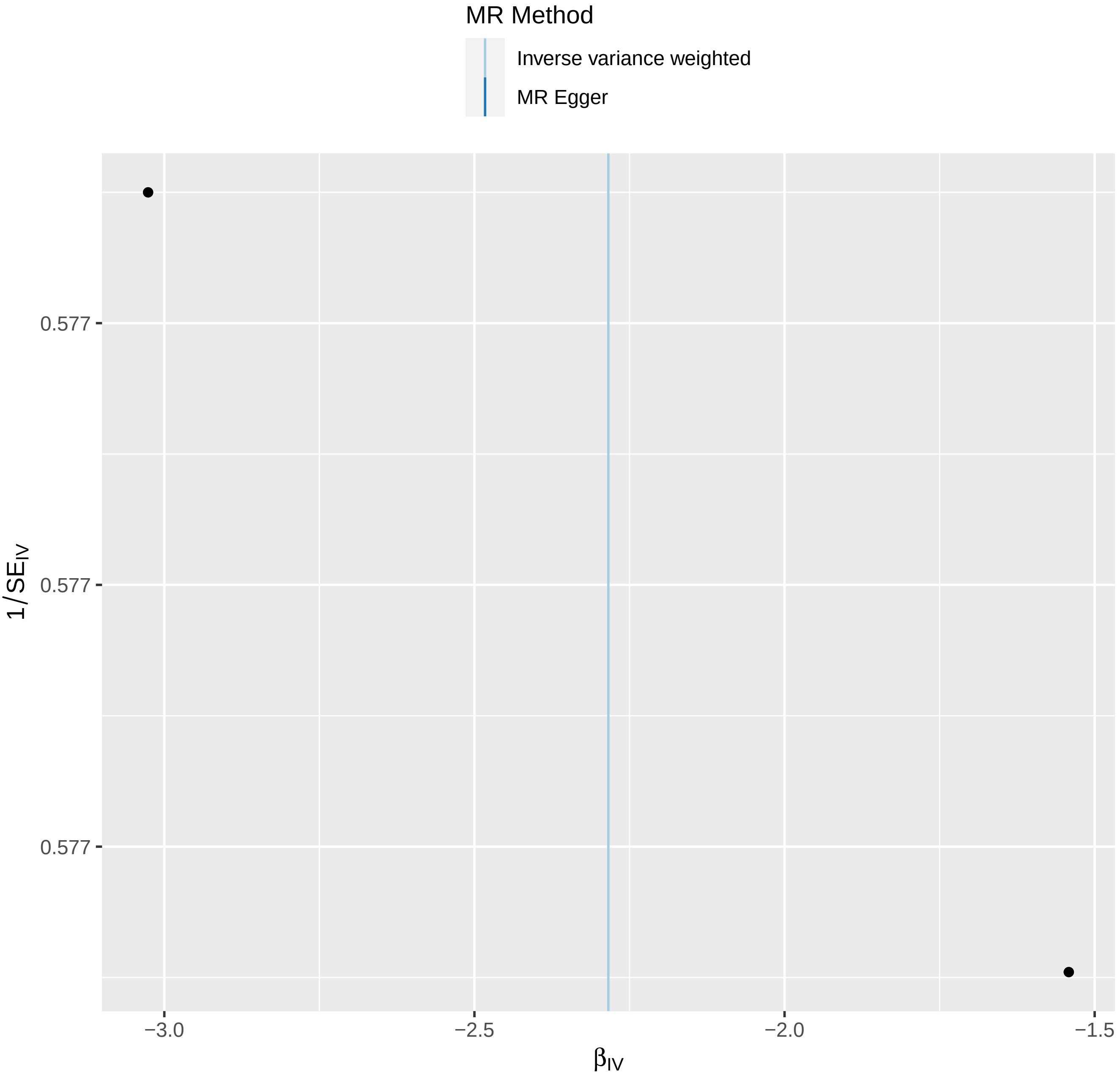

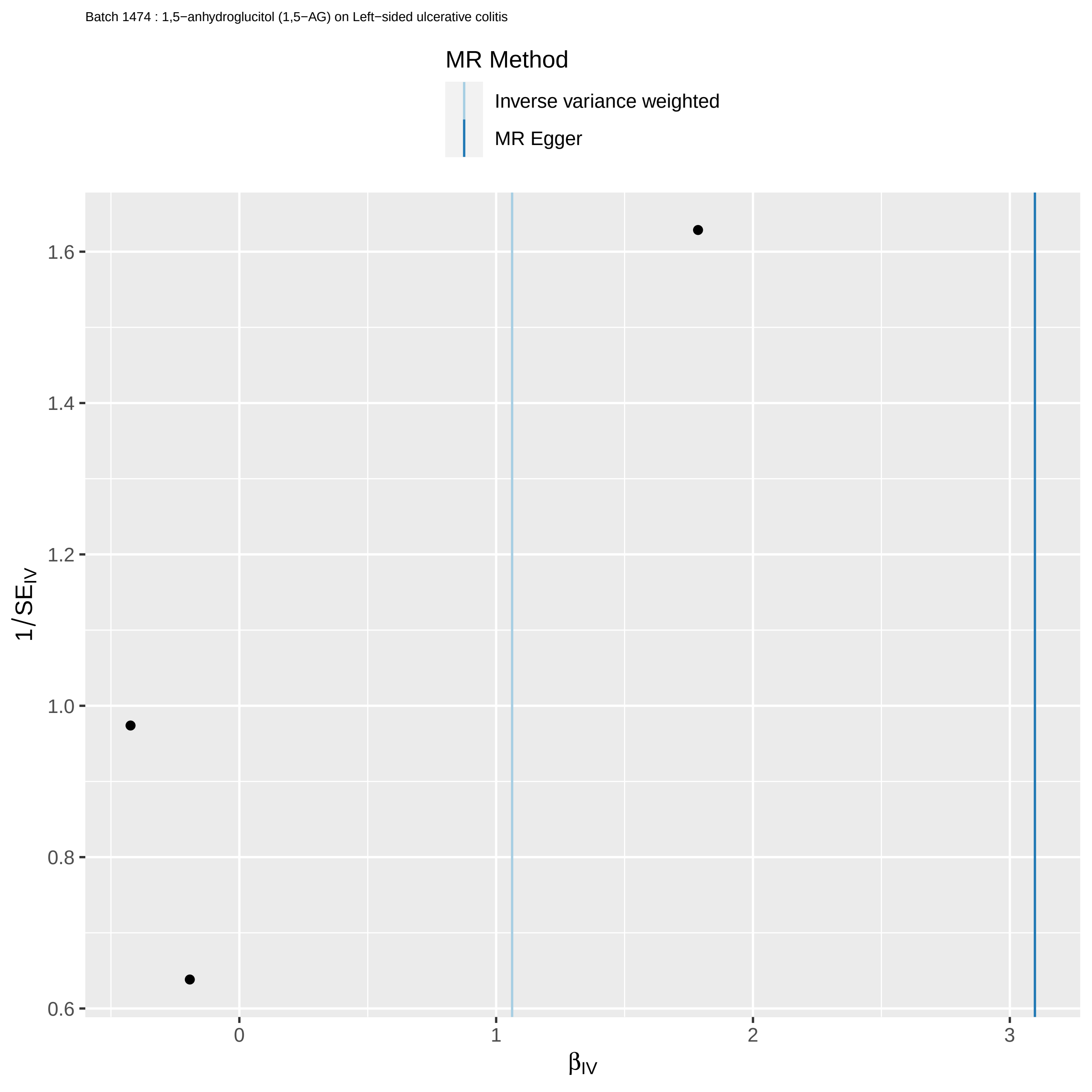

Batch 1474 : 1,5−anhydroglucitol (1,5−AG) on Left−sided ulcerative colitis
MR Method
Inverse variance weighted
MR Egger
1.6
1.4
1.2
1.0
0.8
0.6
1/SE_IV
0
1
2
3
β_IV

Batch 1475 : 2−hydroxybutyrate (AHB) on Left−sided ulcerative colitis

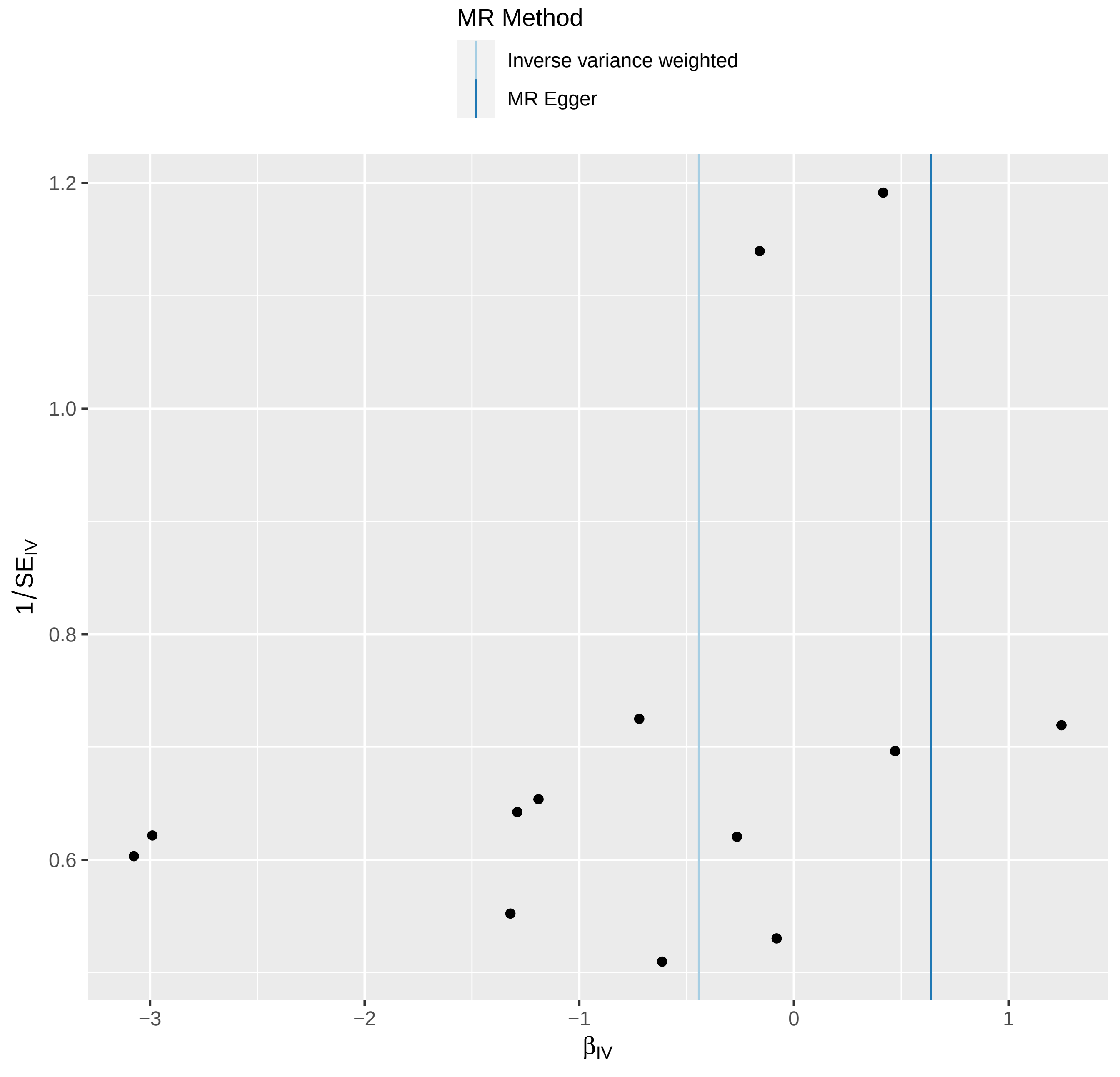

Batch 1478 : 1−palmitoylglycerol (1−monopalmitin) on Left−sided ulcerative colitis

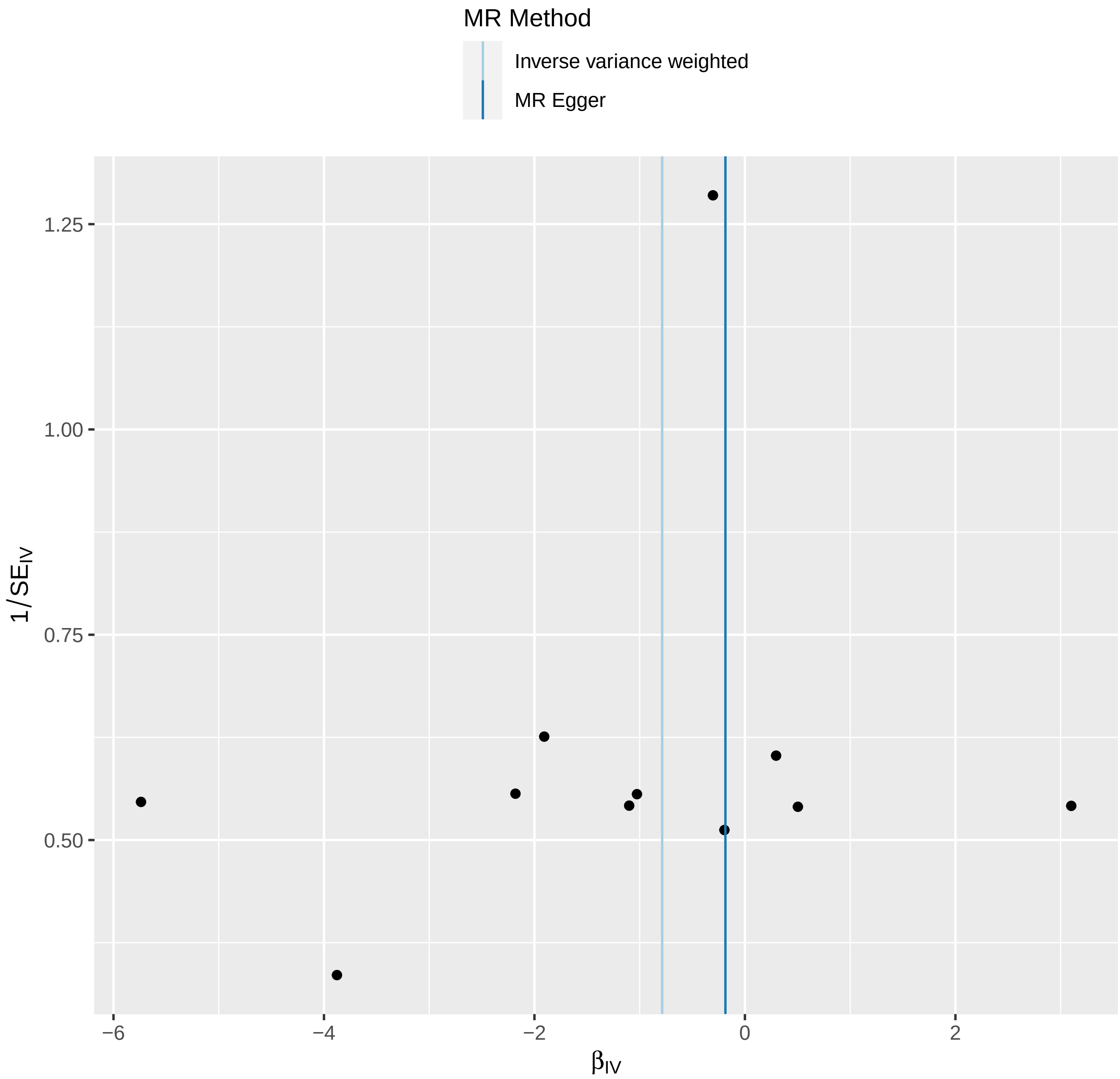

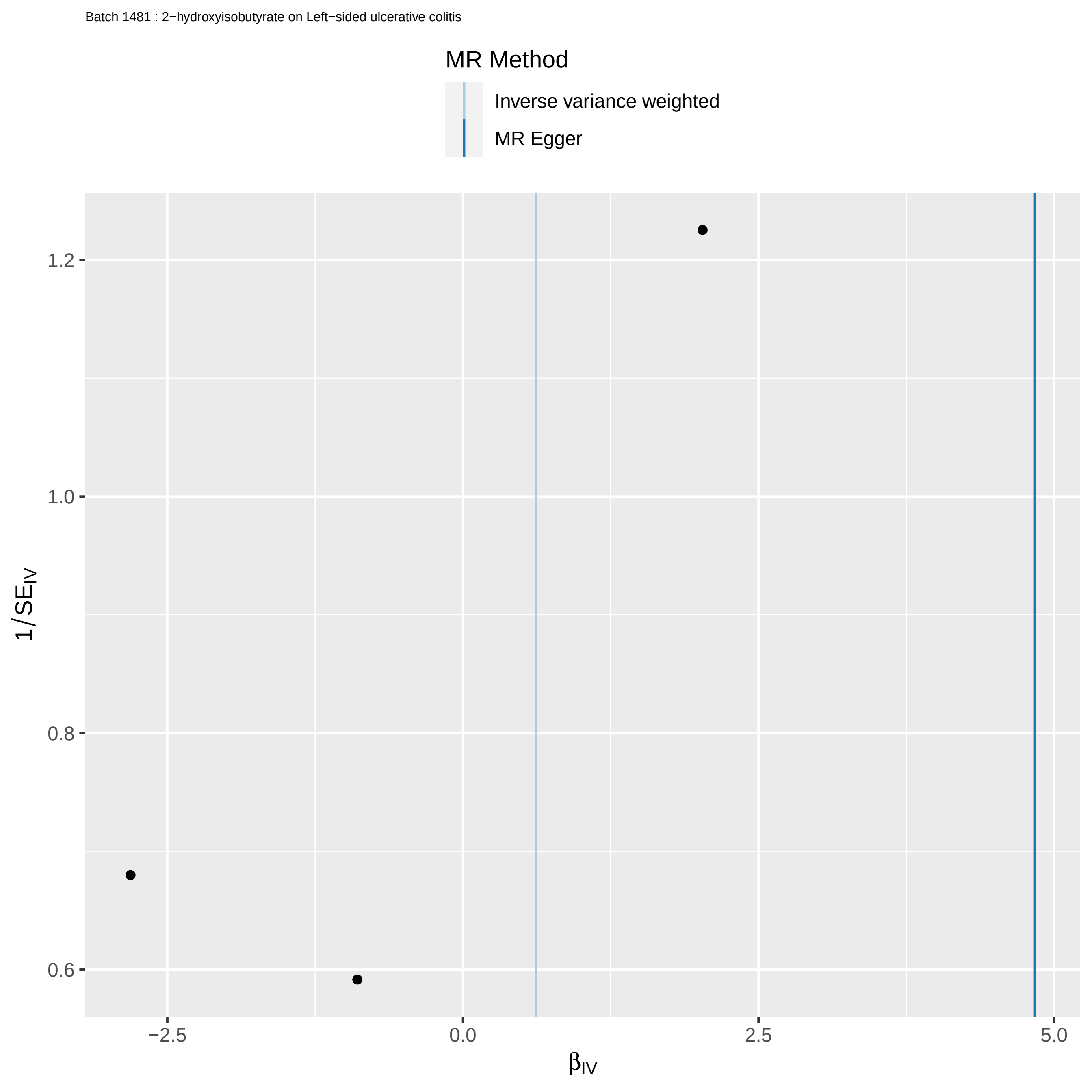

Batch 1481 : 2−hydroxyisobutyrate on Left−sided ulcerative colitis
MR Method
Inverse variance weighted
MR Egger
1.2
1.0
0.8
0.6
1/SE$_{IV}$
−2.5
0.0
2.5
5.0
β$_{IV}$

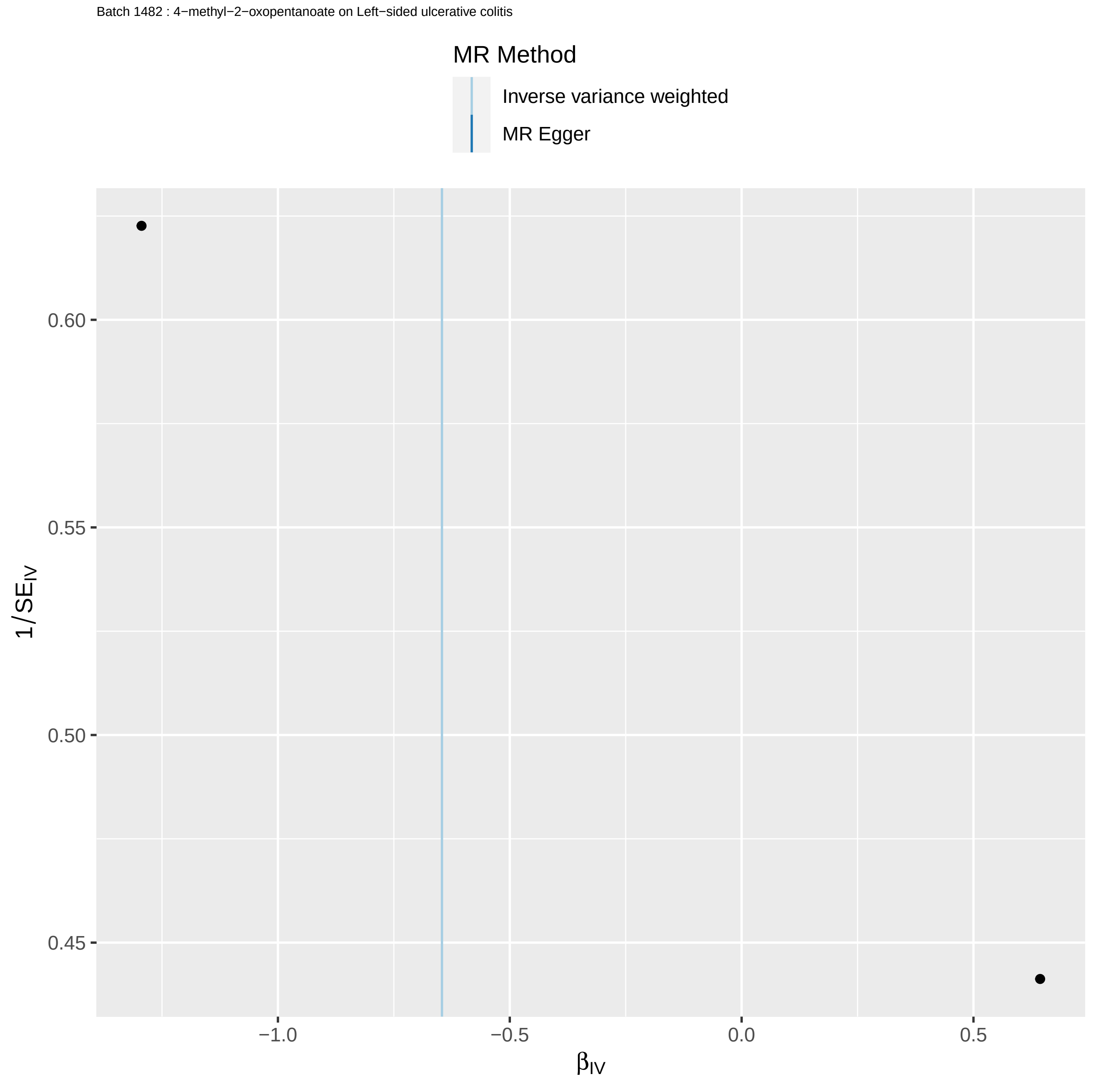

Batch 1482 : 4−methyl−2−oxopentanoate on Left−sided ulcerative colitis
MR Method
Inverse variance weighted
MR Egger
0.60
0.55
0.50
0.45
1/SE_IV
−1.0
−0.5
0.0
0.5
β_IV

Batch 1486 : Levulinate (4−oxovalerate) on Left−sided ulcerative colitis

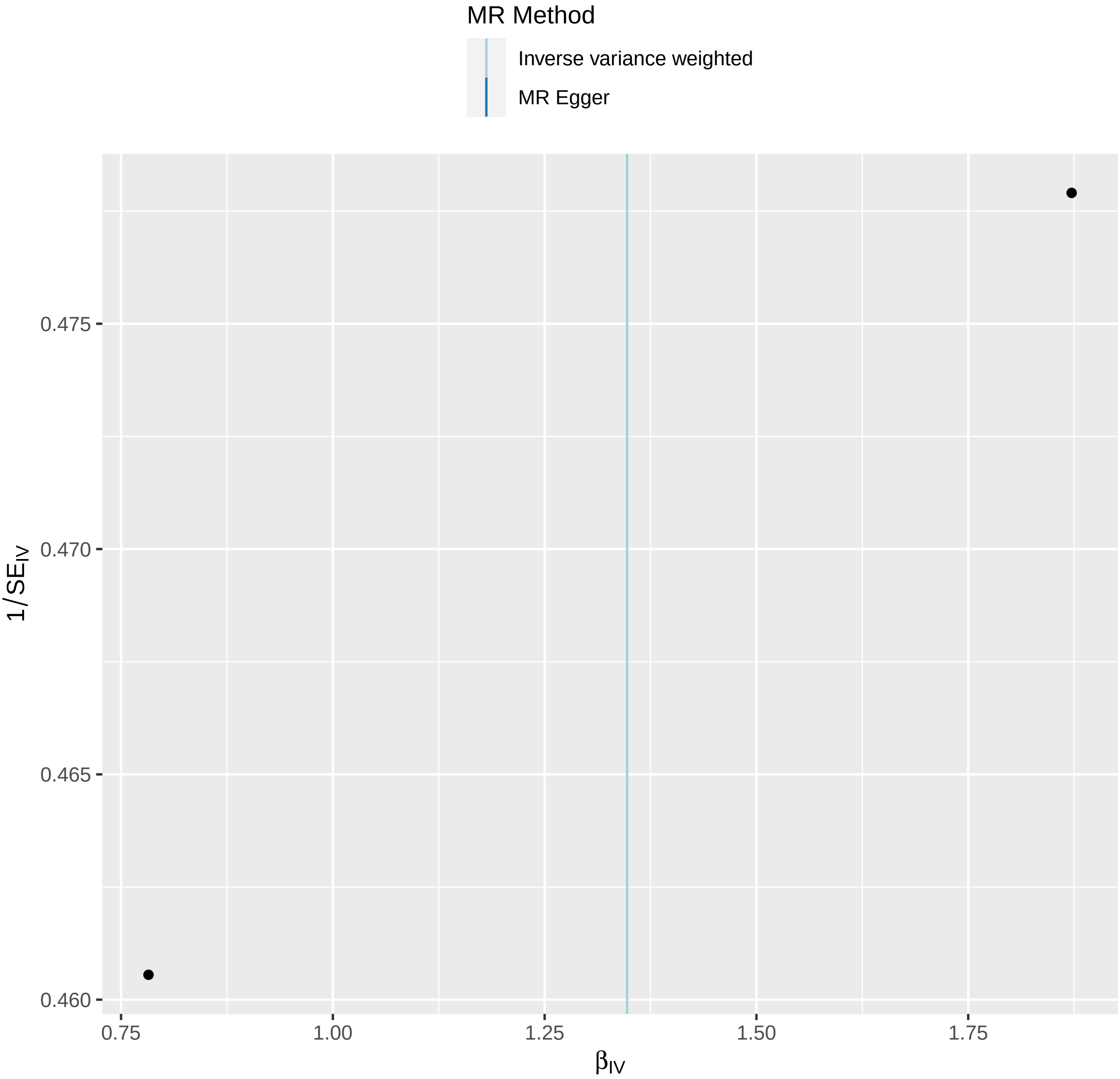

Batch 1488 : Cholate on Left−sided ulcerative colitis

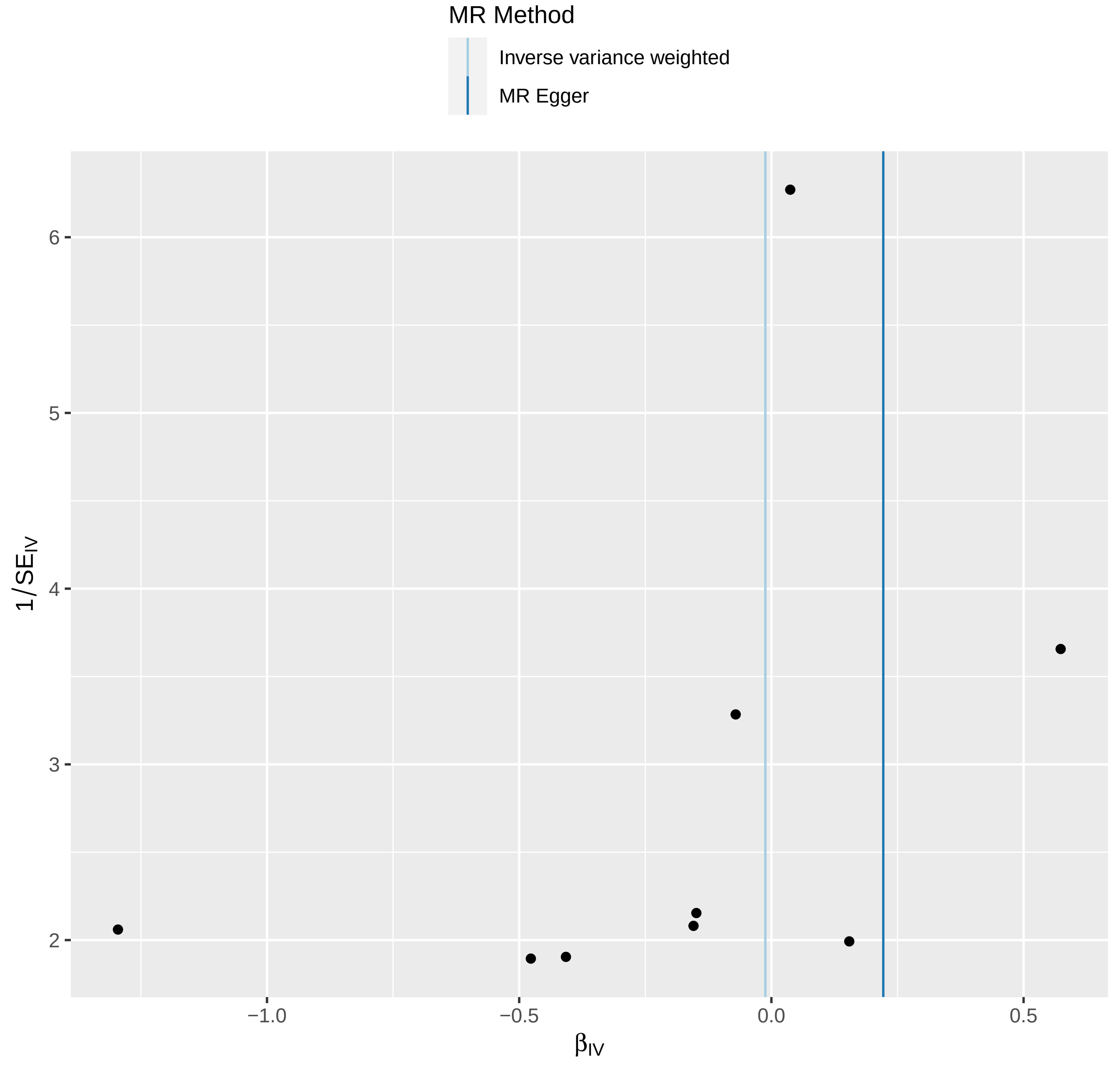

Batch 1491 : Hyodeoxycholate on Left−sided ulcerative colitis

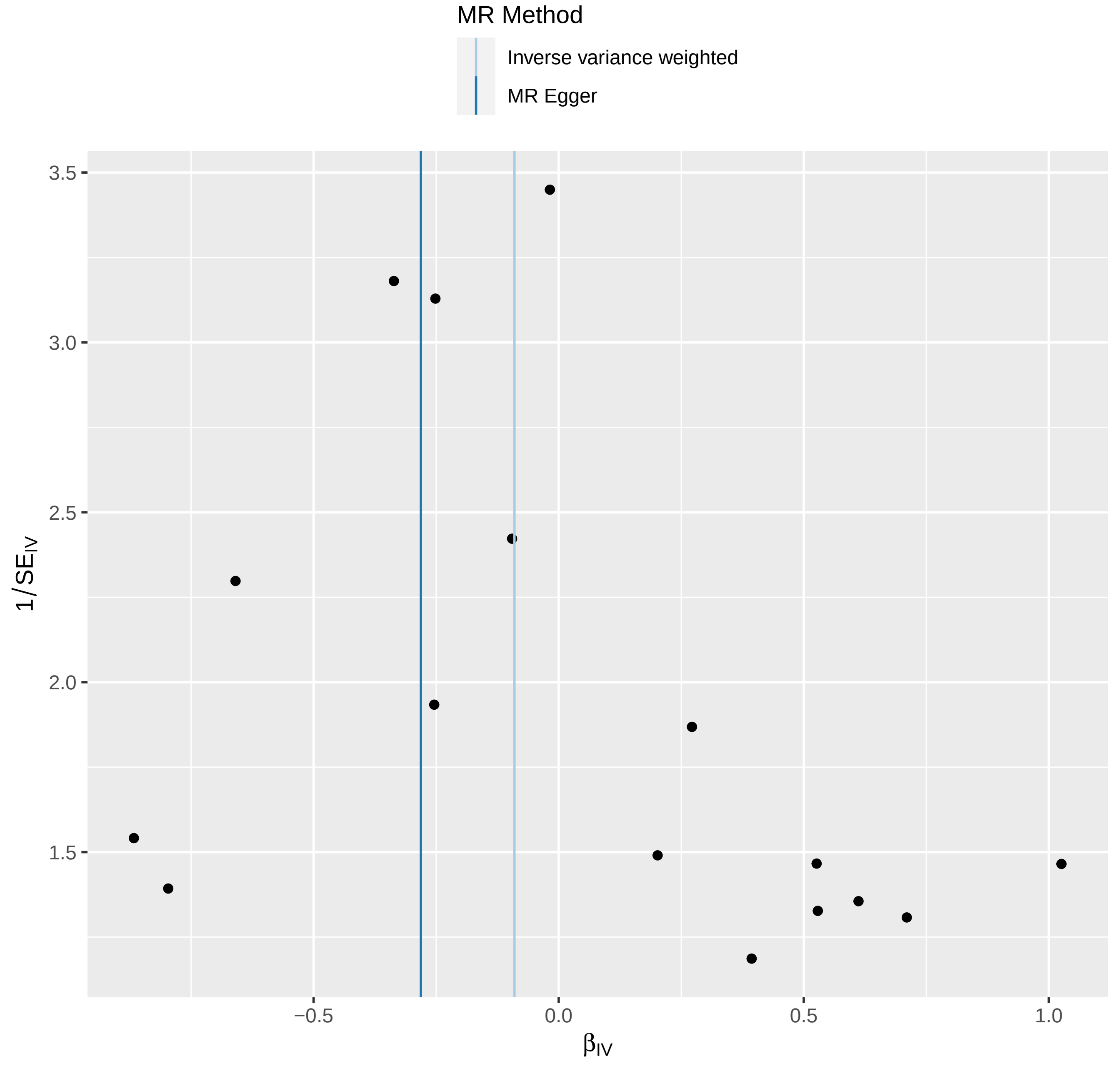

Batch 1492 : 3−indoxyl sulfate on Left−sided ulcerative colitis

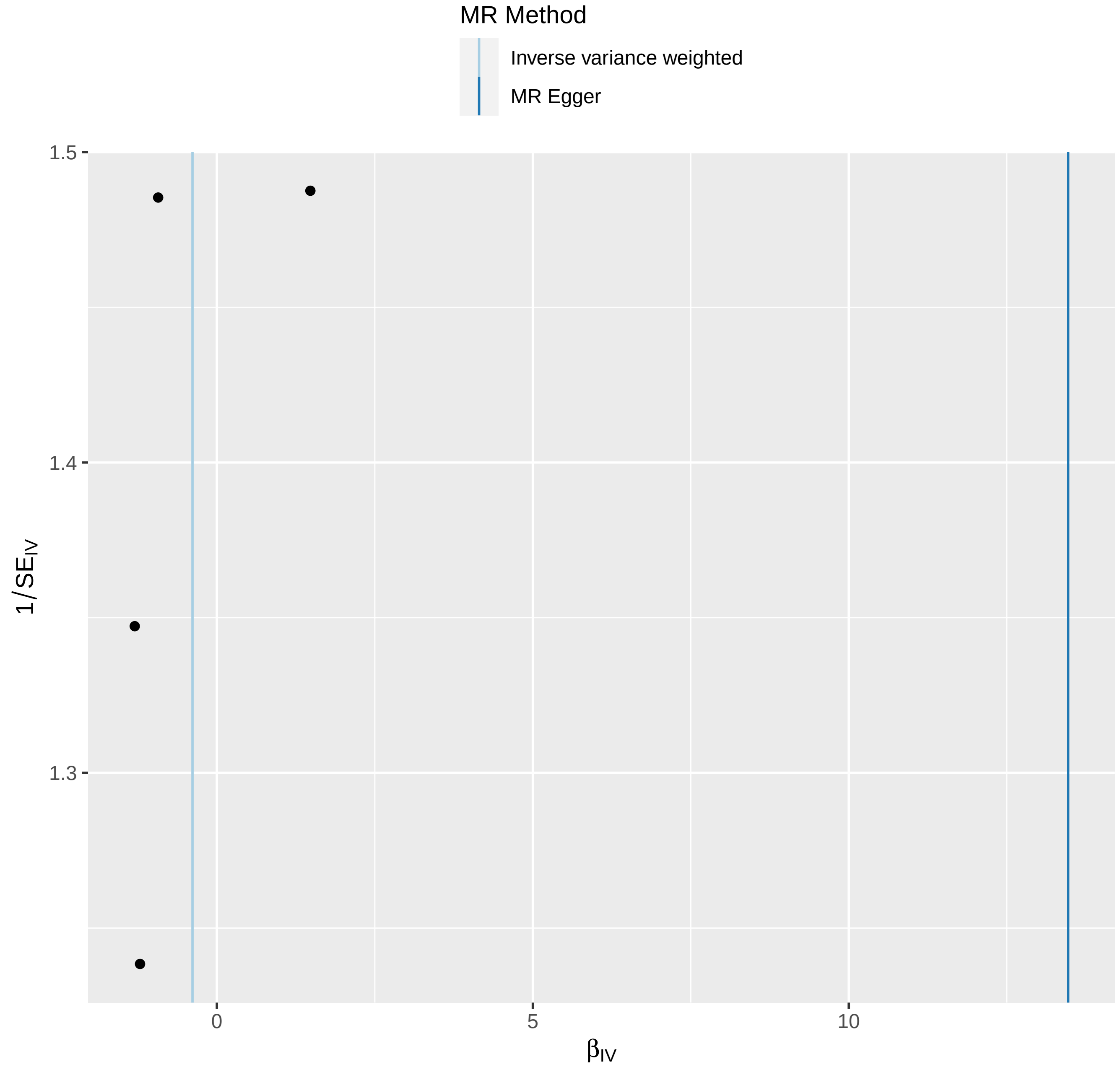

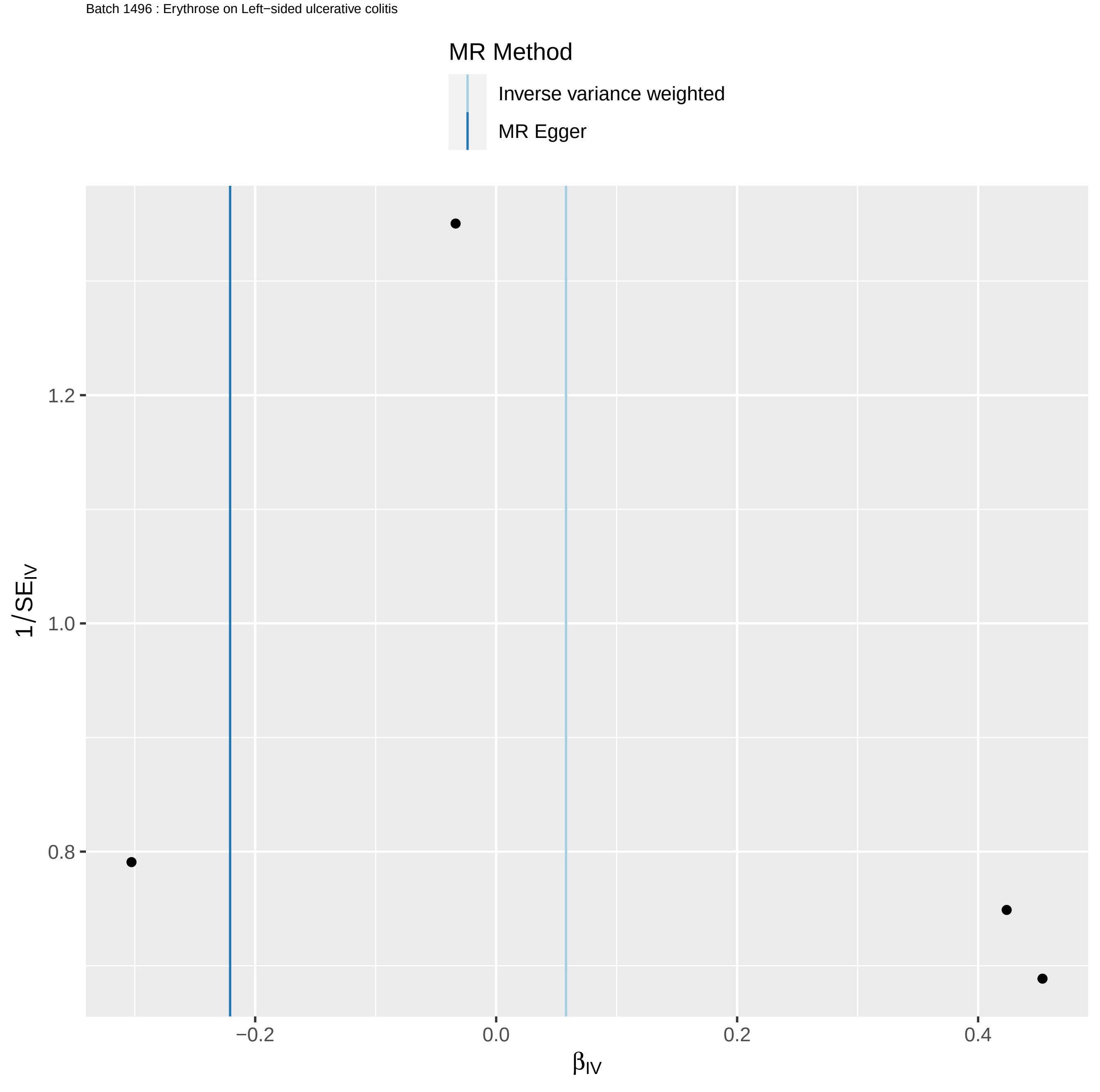

Batch 1496 : Erythrose on Left−sided ulcerative colitis
MR Method
Inverse variance weighted
MR Egger
1.2
1.0
0.8
1/SE$_{IV}$
−0.2
0.0
0.2
0.4
β$_{IV}$

Batch 1497 : Threonate on Left−sided ulcerative colitis

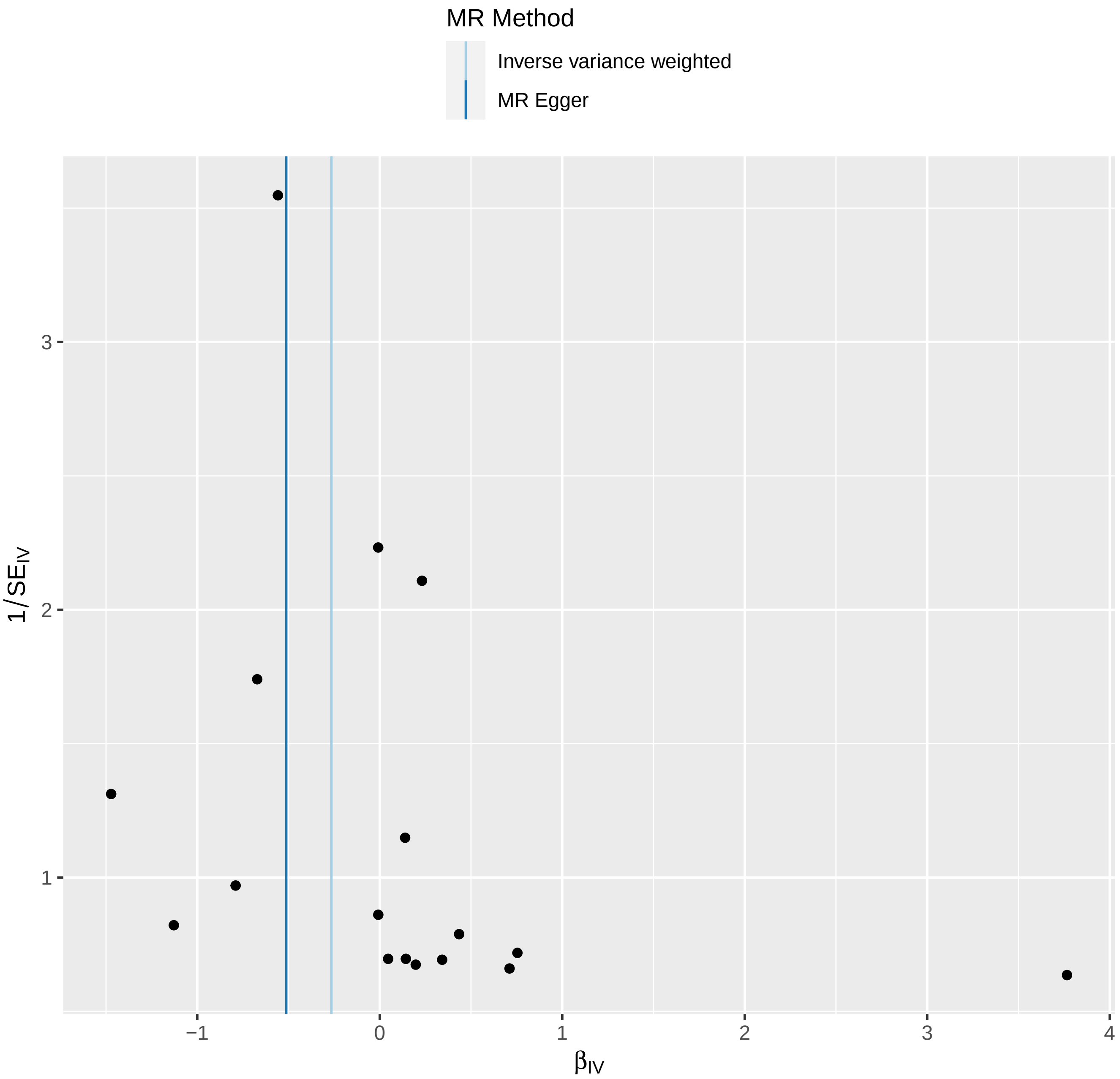

Batch 1498 : Cysteine on Left−sided ulcerative colitis

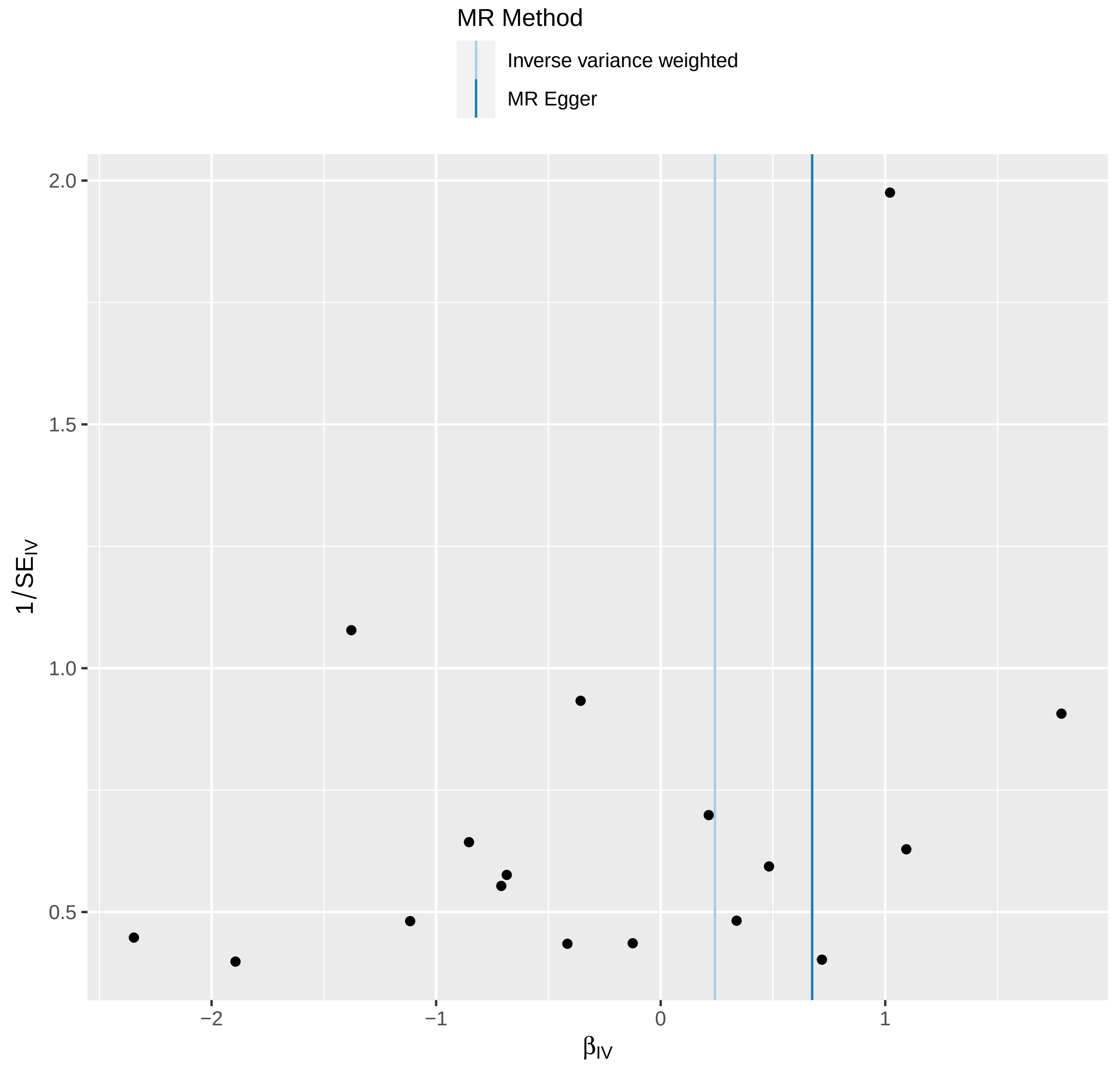

Batch 1499 : Pyroglutamylglycine on Left−sided ulcerative colitis

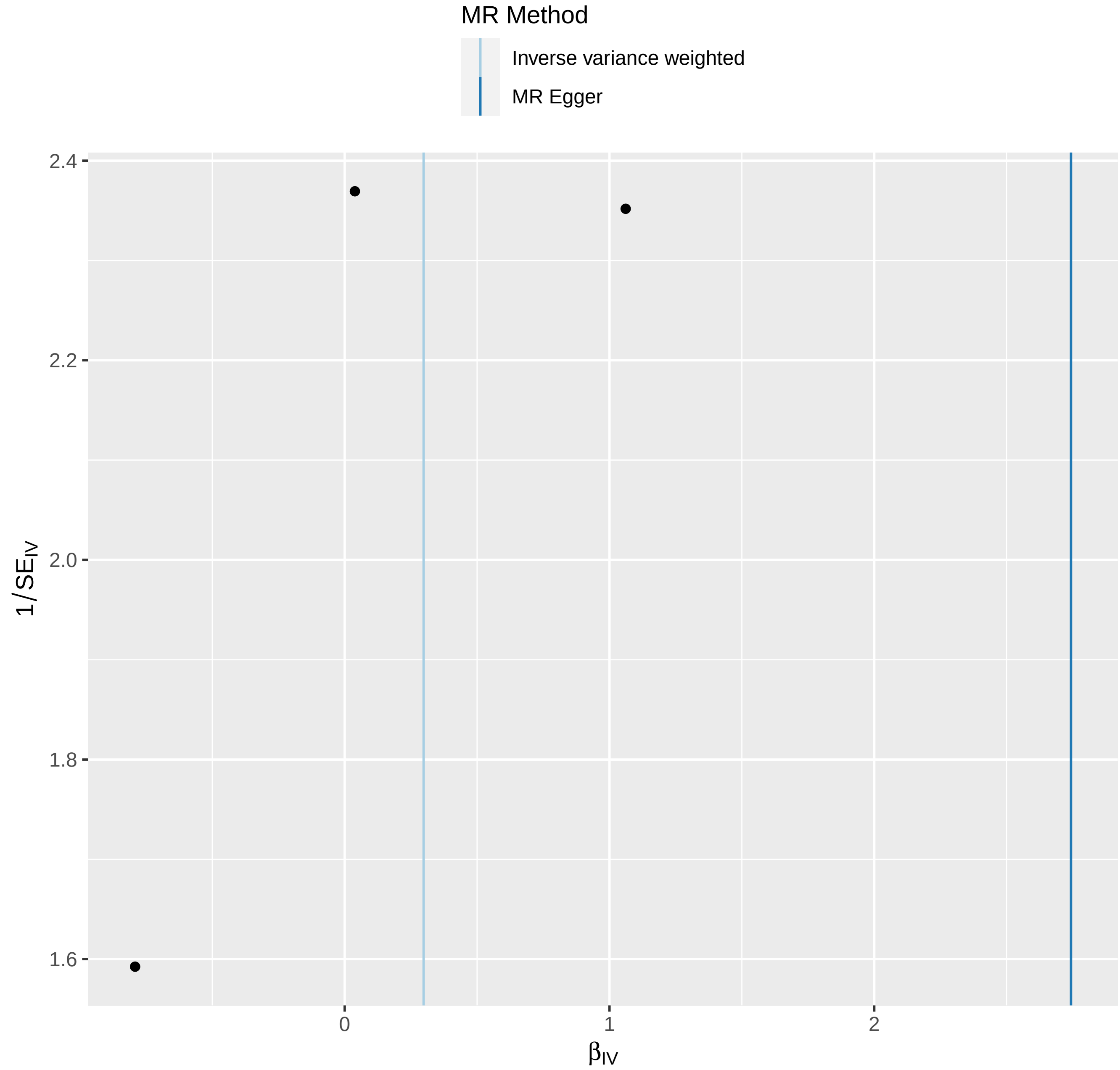

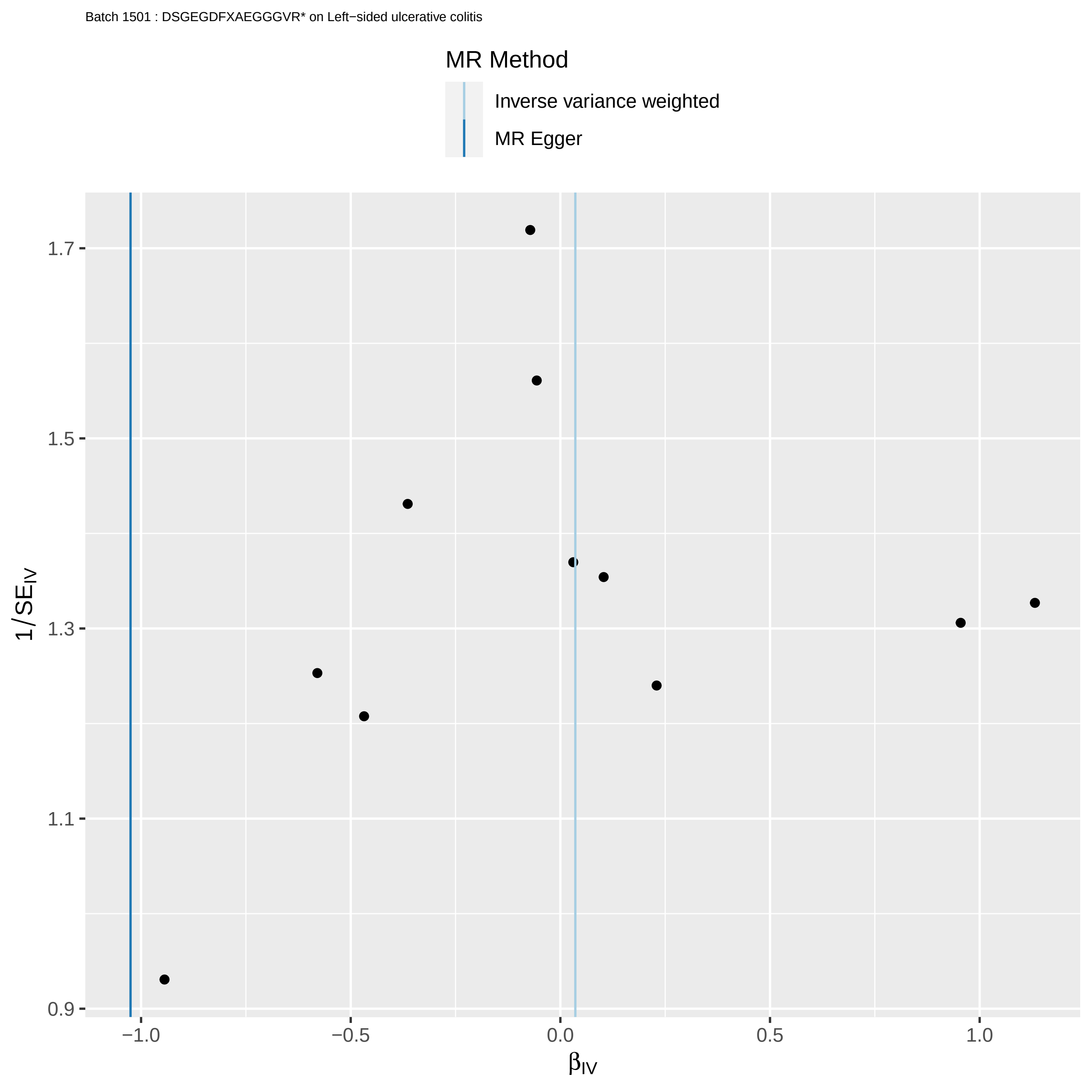

Batch 1501 : DSGEGDFXAEGGGVR* on Left−sided ulcerative colitis
MR Method
Inverse variance weighted
MR Egger
1.7
1.5
1.3
1.1
0.9
−1.0
−0.5
0.0
0.5
1.0
$1/SE_{IV}$
β_{IV}

Batch 1502 : Pyridoxate on Left−sided ulcerative colitis

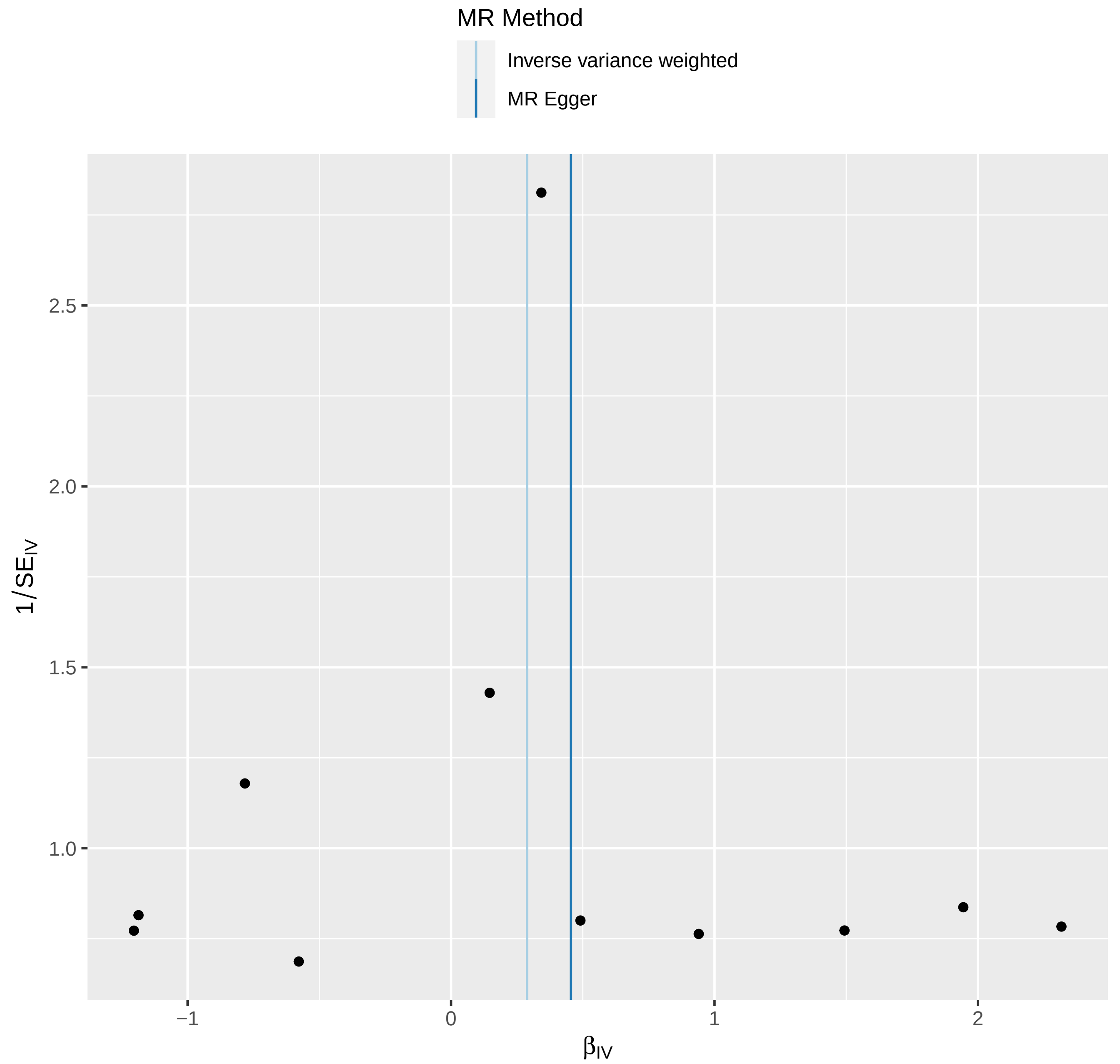

Batch 1503 : Androsterone sulfate on Left−sided ulcerative colitis

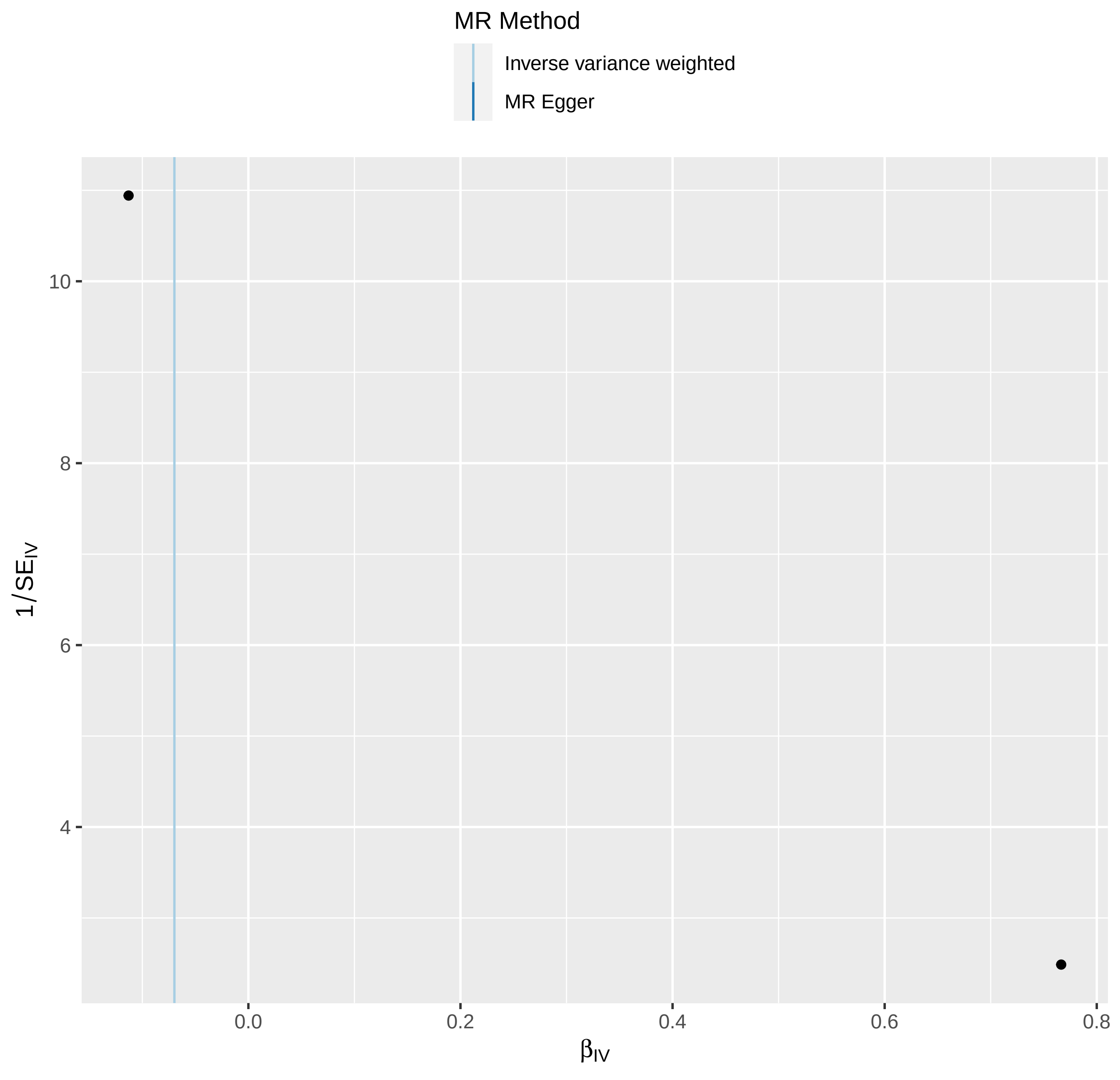

Batch 1507 : Serine on Left−sided ulcerative colitis

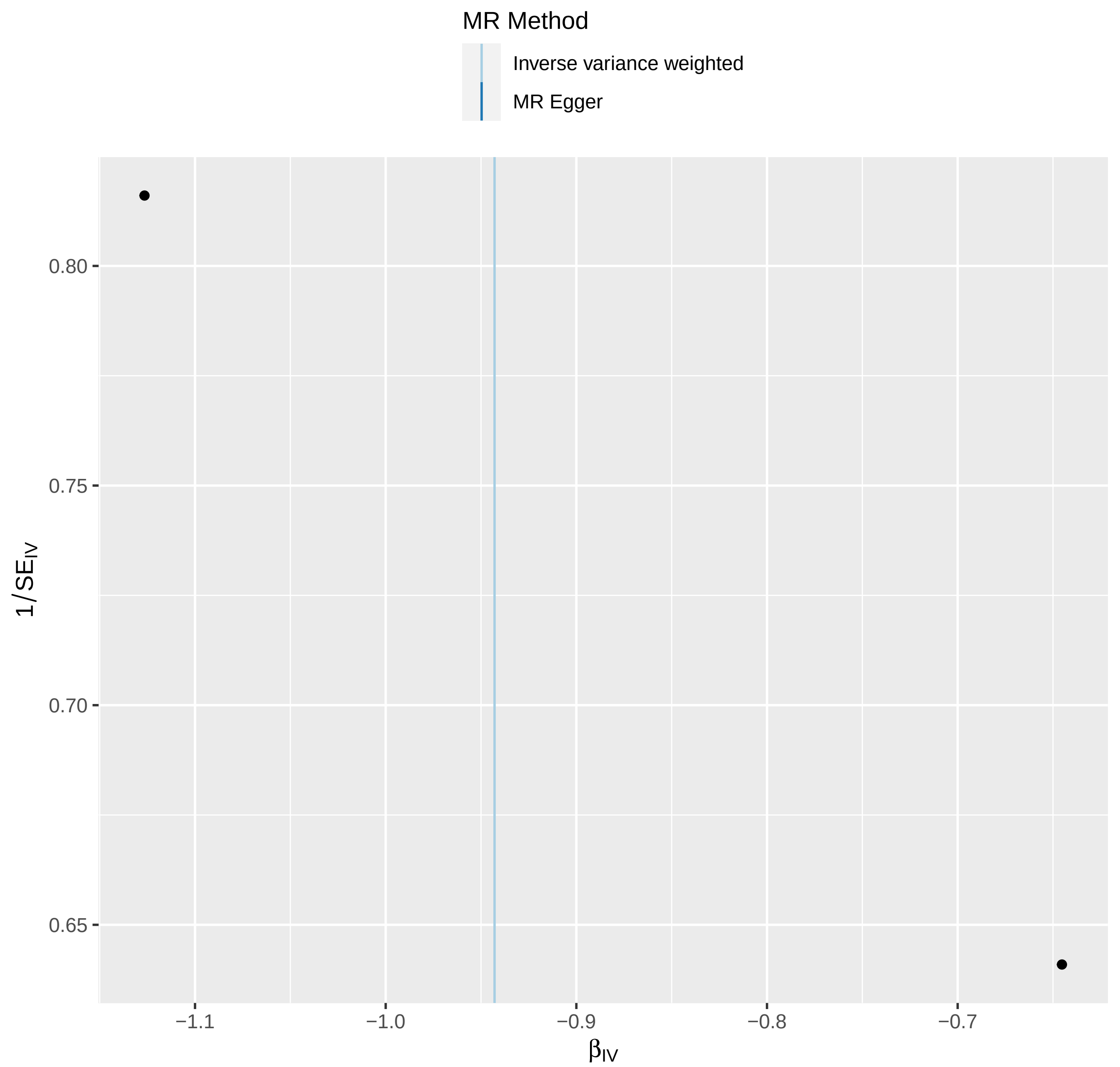

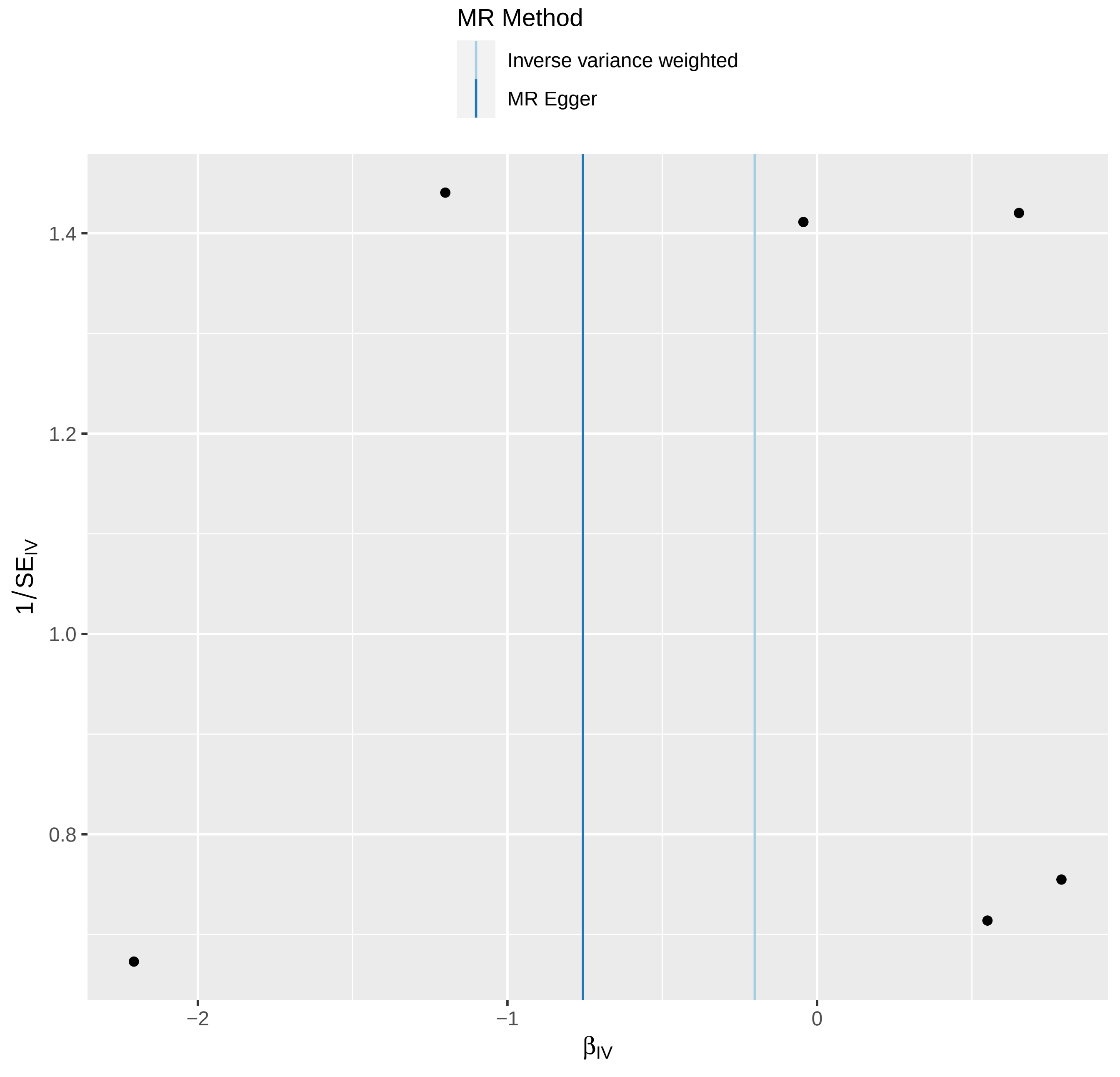
MR Method
Inverse variance weighted
MR Egger
1.4
1.2
1.0
0.8
1/SE_IV
−2
−1
0
β_IV

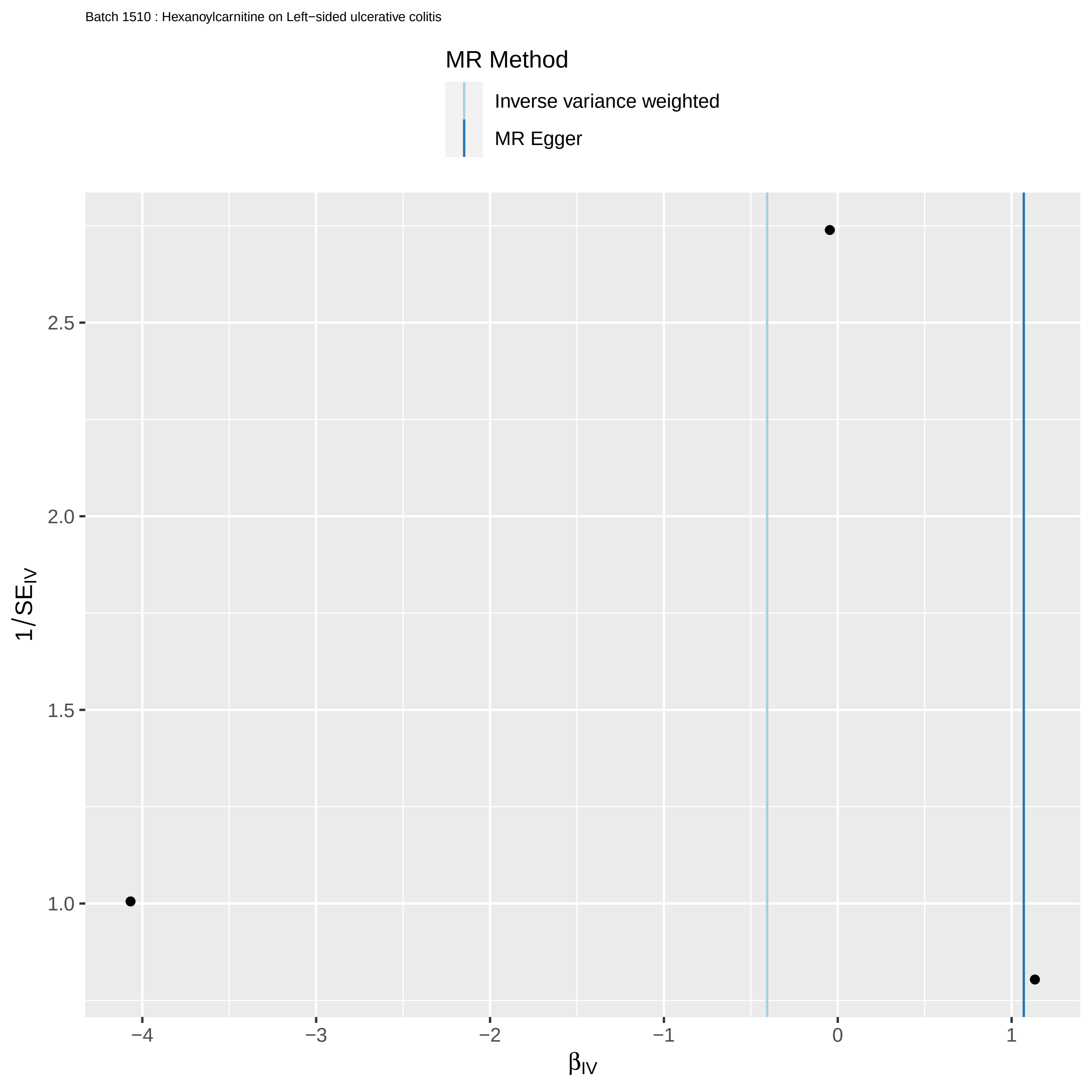
Batch 1510 : Hexanoylcarnitine on Left−sided ulcerative colitis
MR Method
Inverse variance weighted
MR Egger
2.5
2.0
1.5
1.0
1/SE_IV
−4
−3
−2
−1
0
1
β_IV

Batch 1514 : 2−aminobutyrate on Left−sided ulcerative colitis

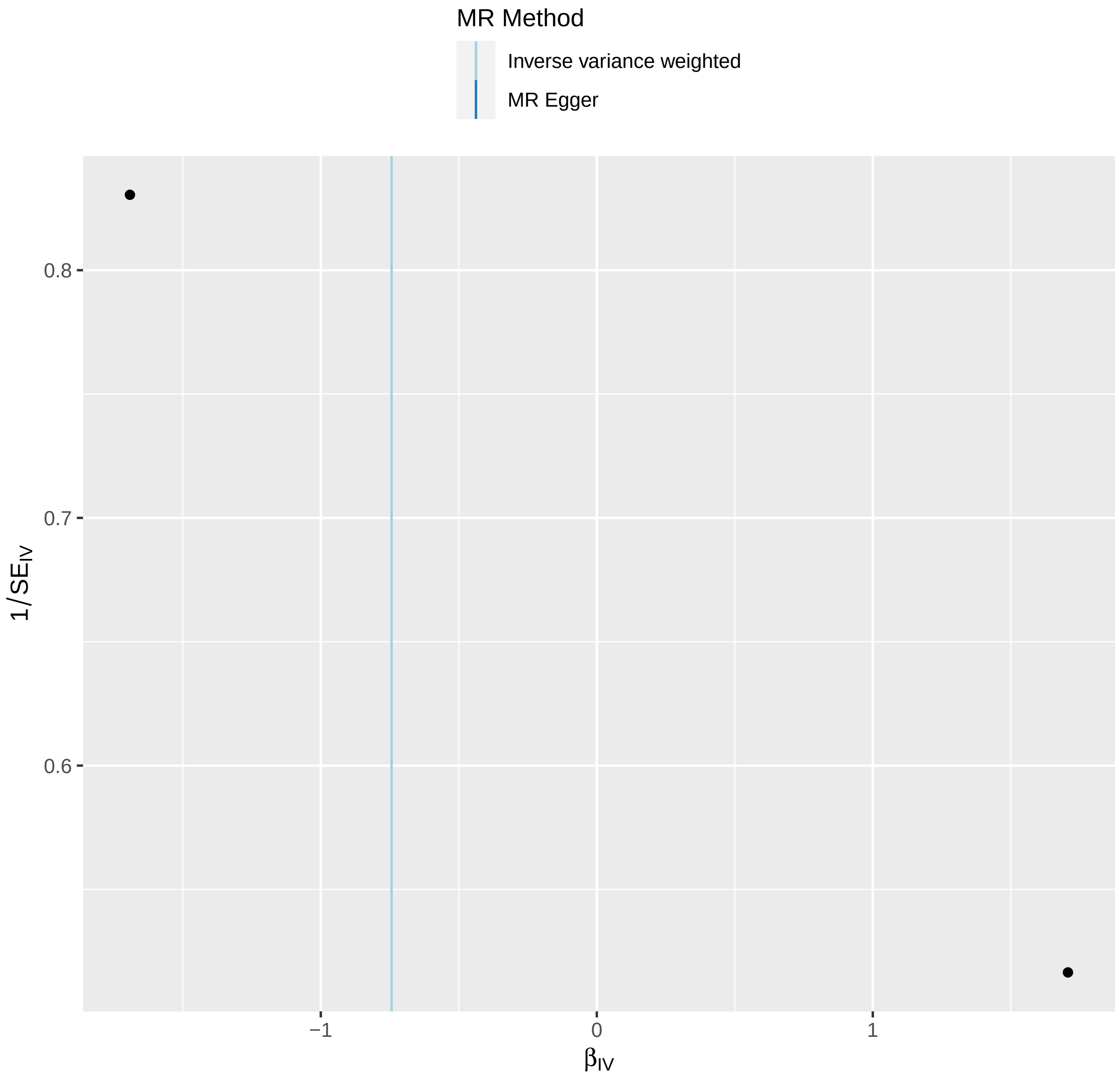

Batch 1519 : Butyrylcarnitine on Left−sided ulcerative colitis

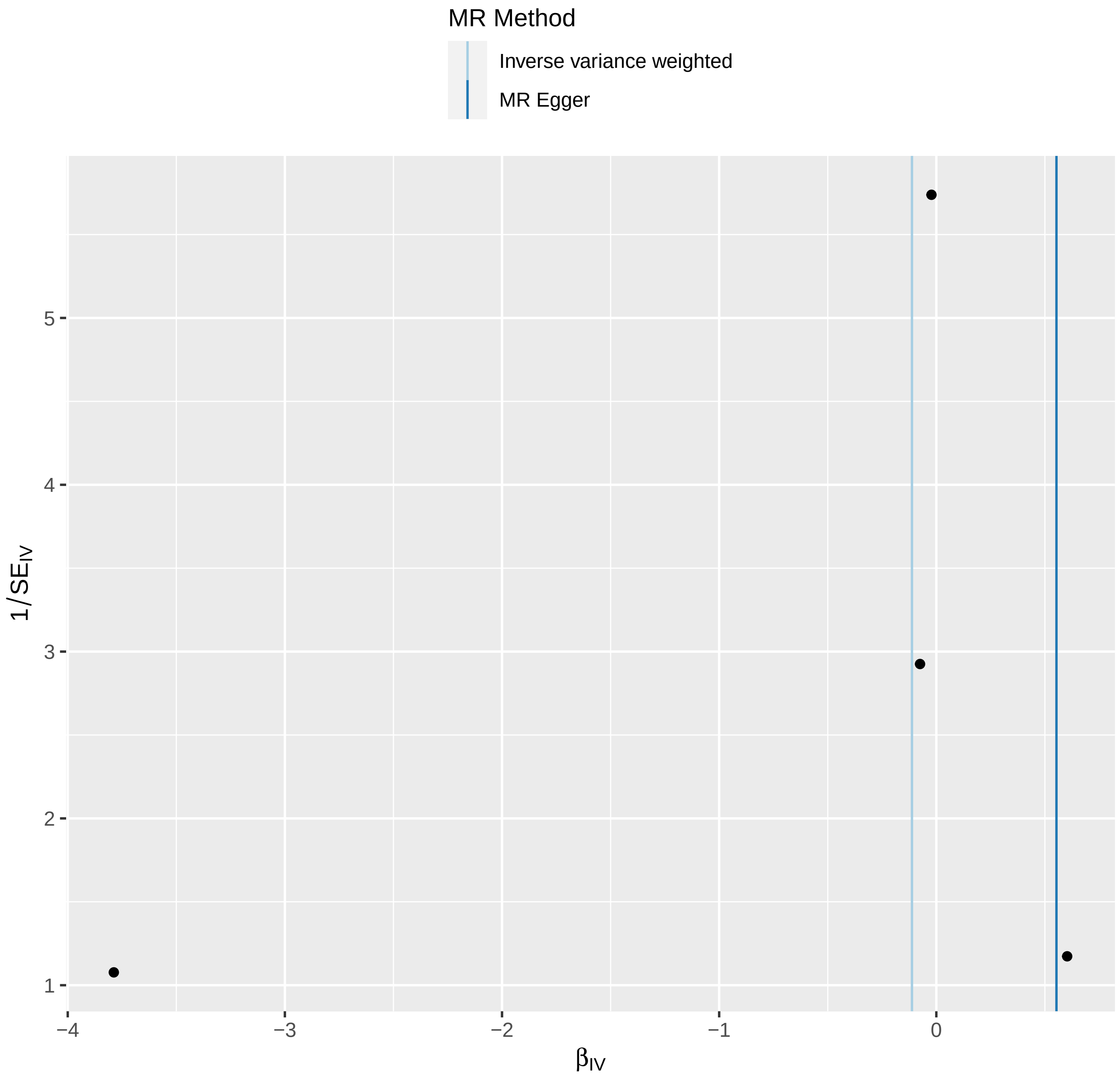

Batch 1522 : Propionylcarnitine on Left−sided ulcerative colitis

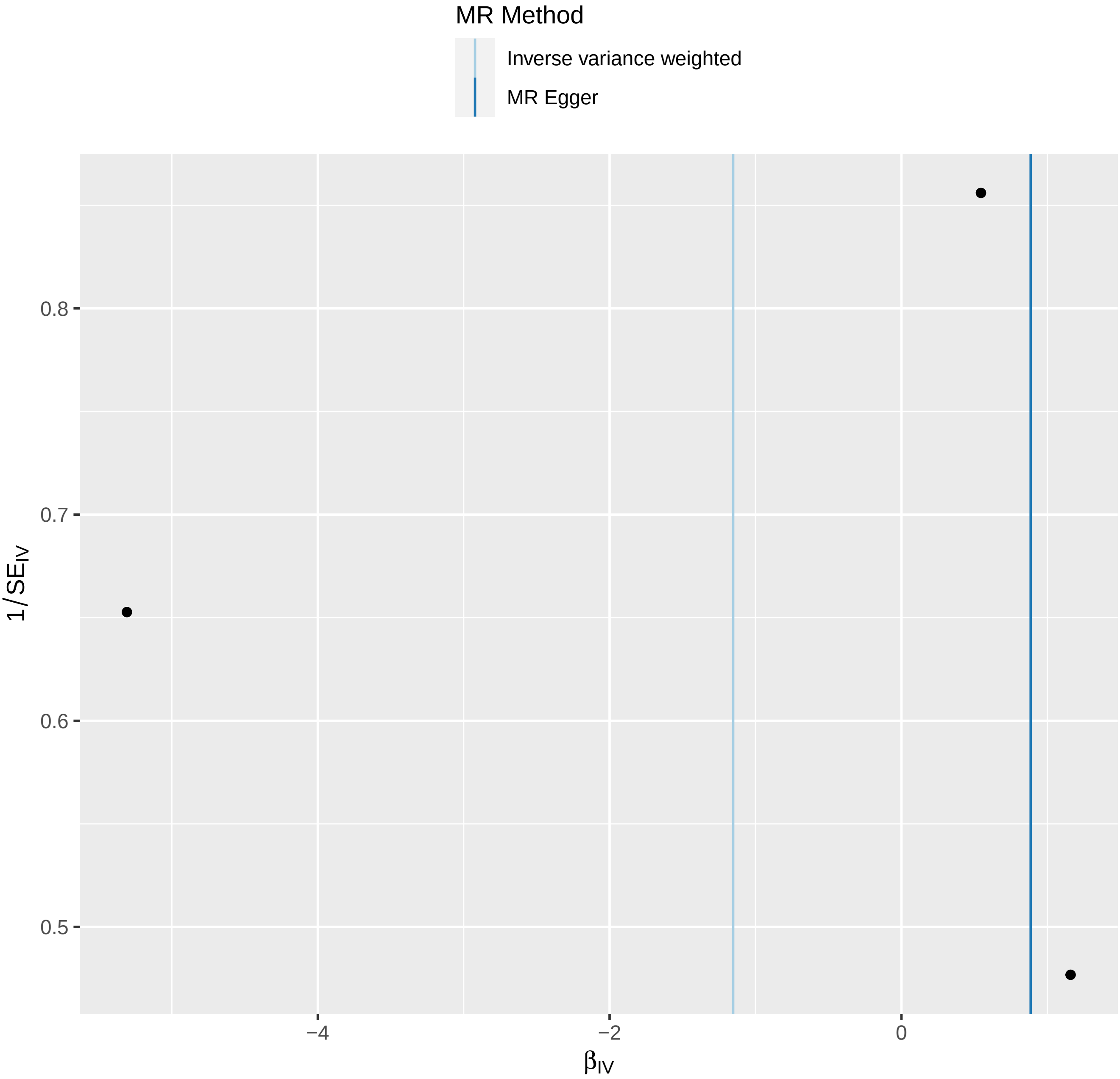

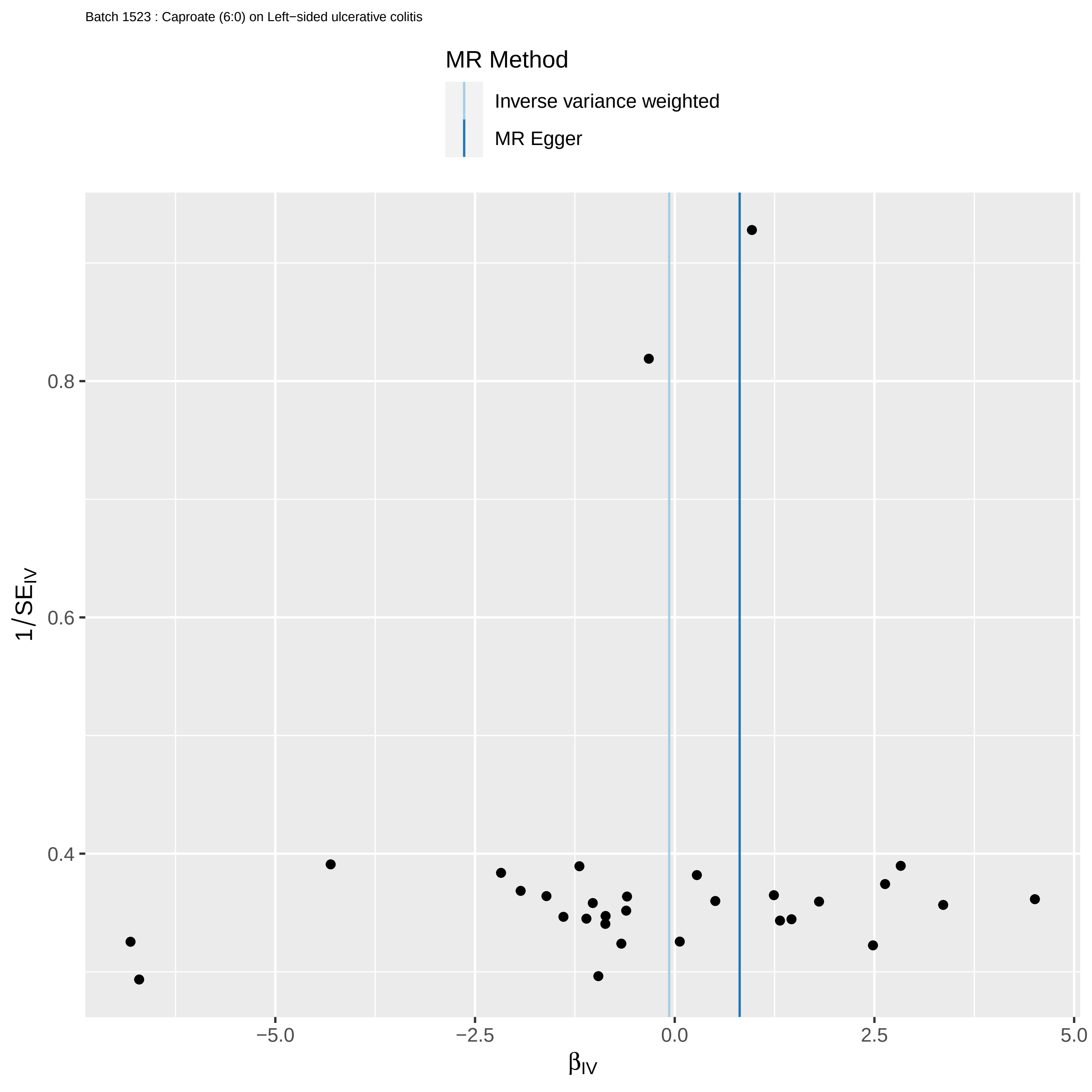

Batch 1523 : Caproate (6:0) on Left−sided ulcerative colitis
MR Method
Inverse variance weighted
MR Egger
0.8
0.6
0.4
1/SE_IV
−5.0
−2.5
0.0
2.5
5.0
β_IV

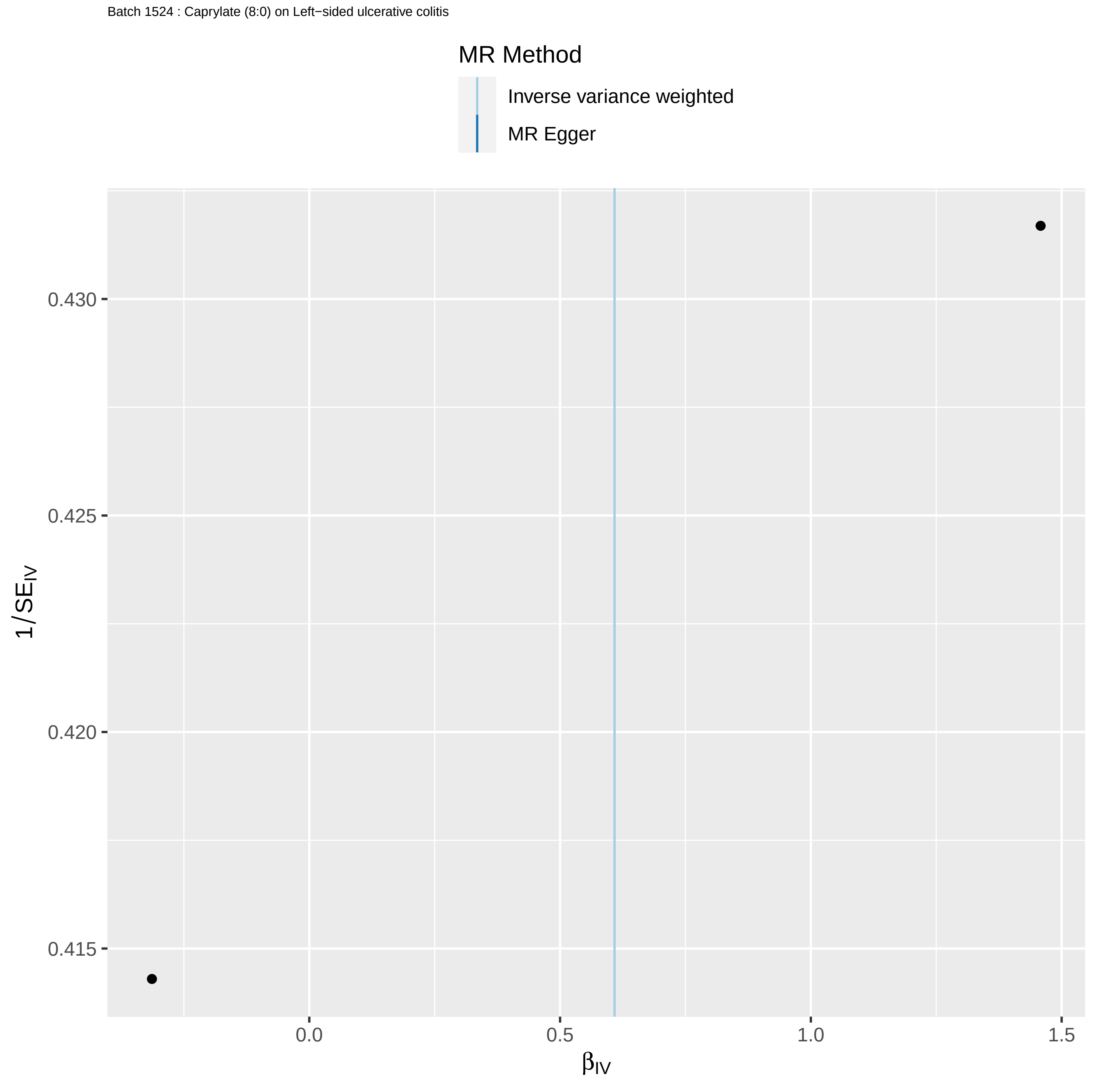

Batch 1524 : Caprylate (8:0) on Left−sided ulcerative colitis
MR Method
Inverse variance weighted
MR Egger
0.430
0.425
0.420
0.415
1/SE_IV
0.0
0.5
1.0
1.5
β_IV

Batch 1525 : 10−undecenoate (11:1n1) on Left−sided ulcerative colitis

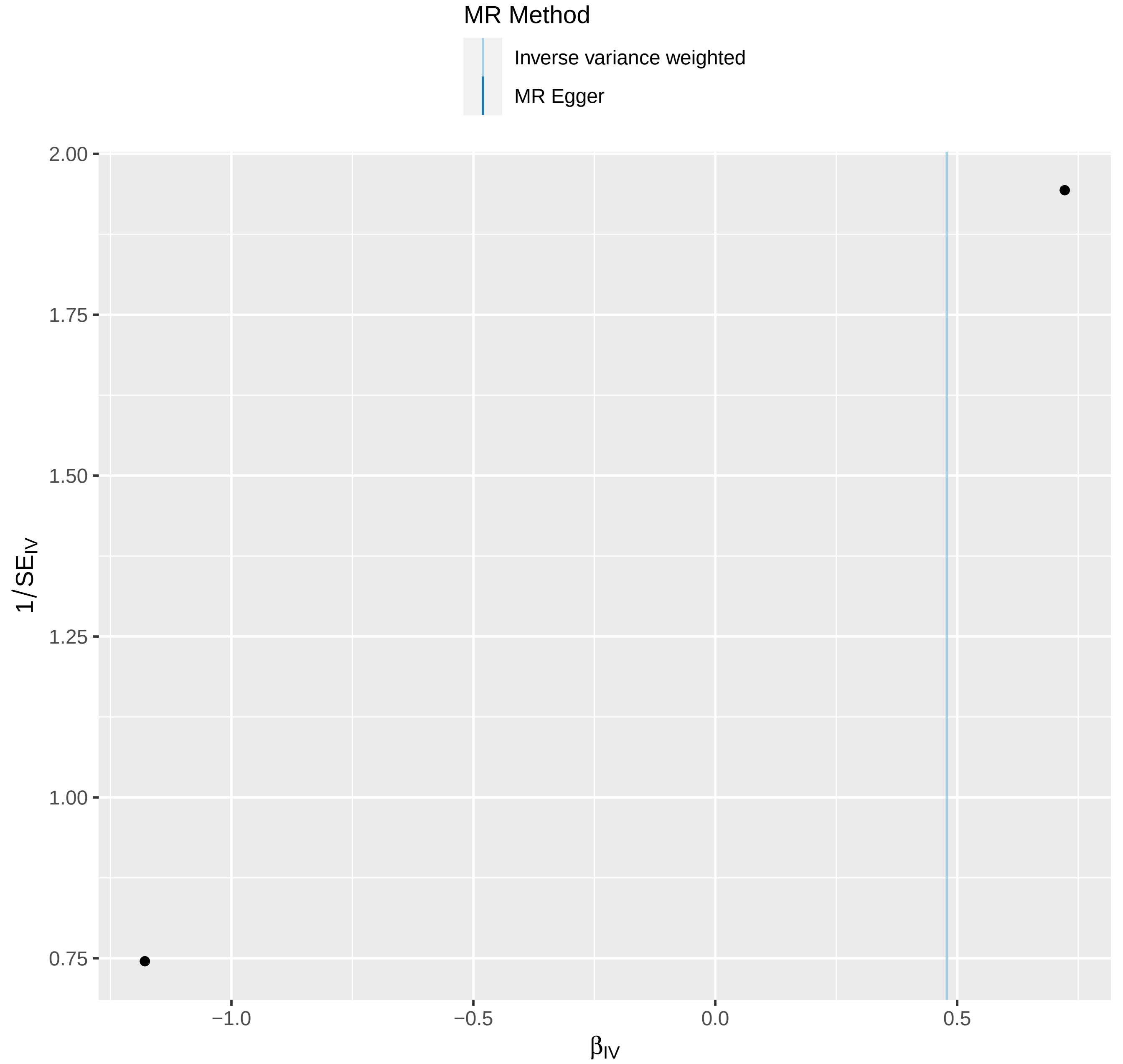

Batch 1527 : Phenol sulfate on Left−sided ulcerative colitis

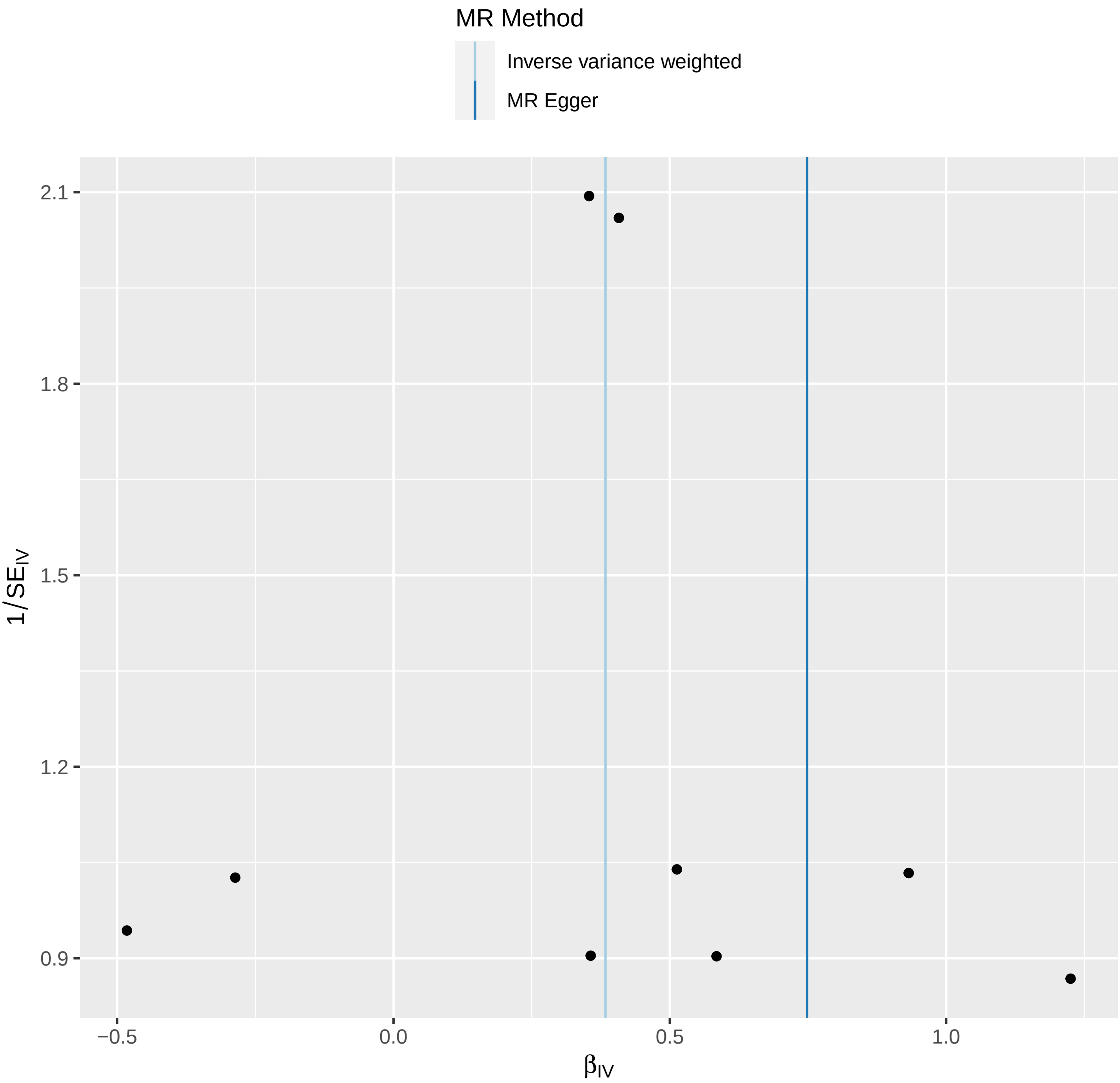

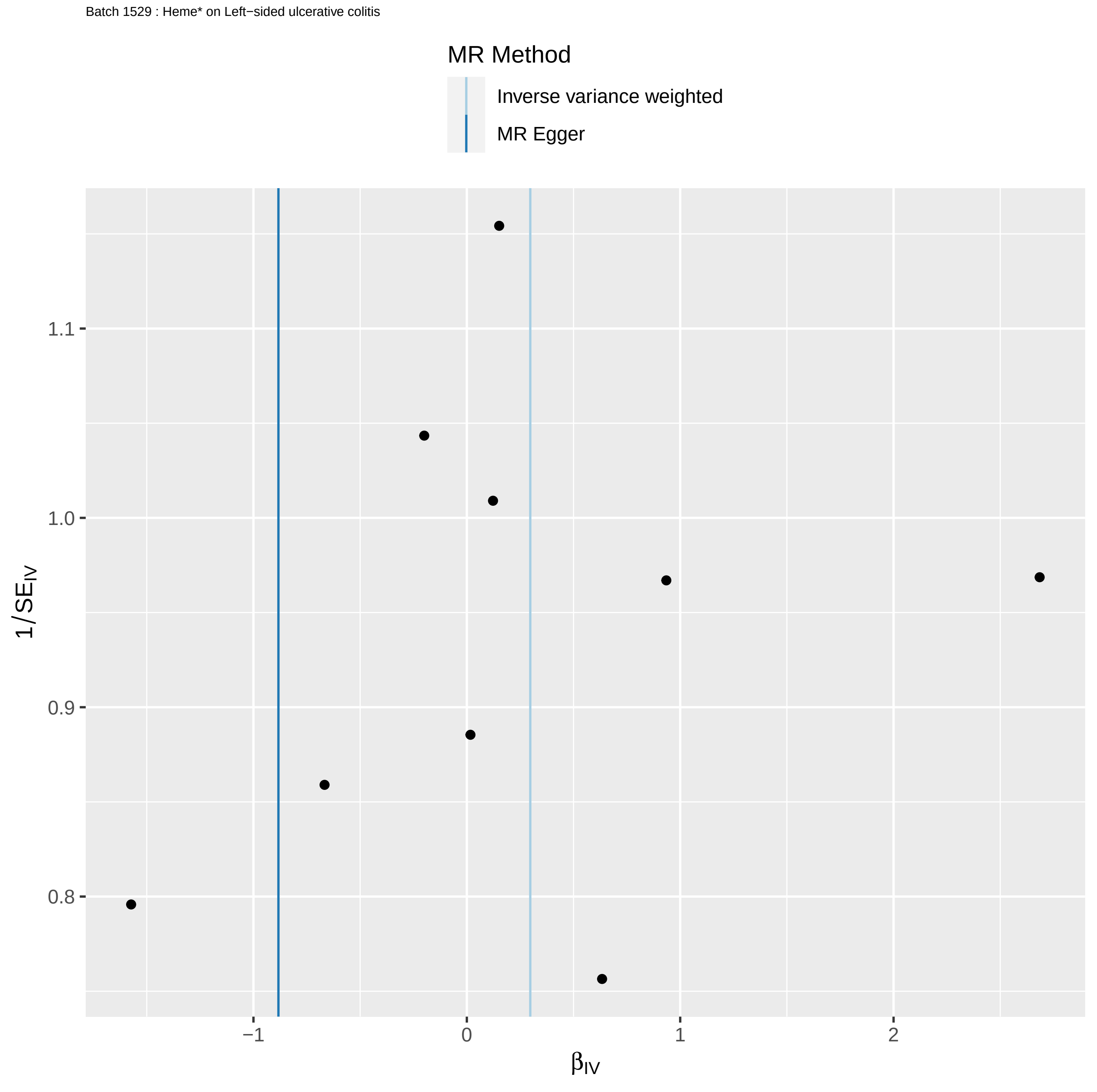
Batch 1529 : Heme* on Left−sided ulcerative colitis
MR Method
Inverse variance weighted
MR Egger
1.1
1.0
0.9
0.8
$1/SE_{IV}$
−1
0
1
2
β_{IV}

Batch 1532 : Pyroglutamine* on Left−sided ulcerative colitis

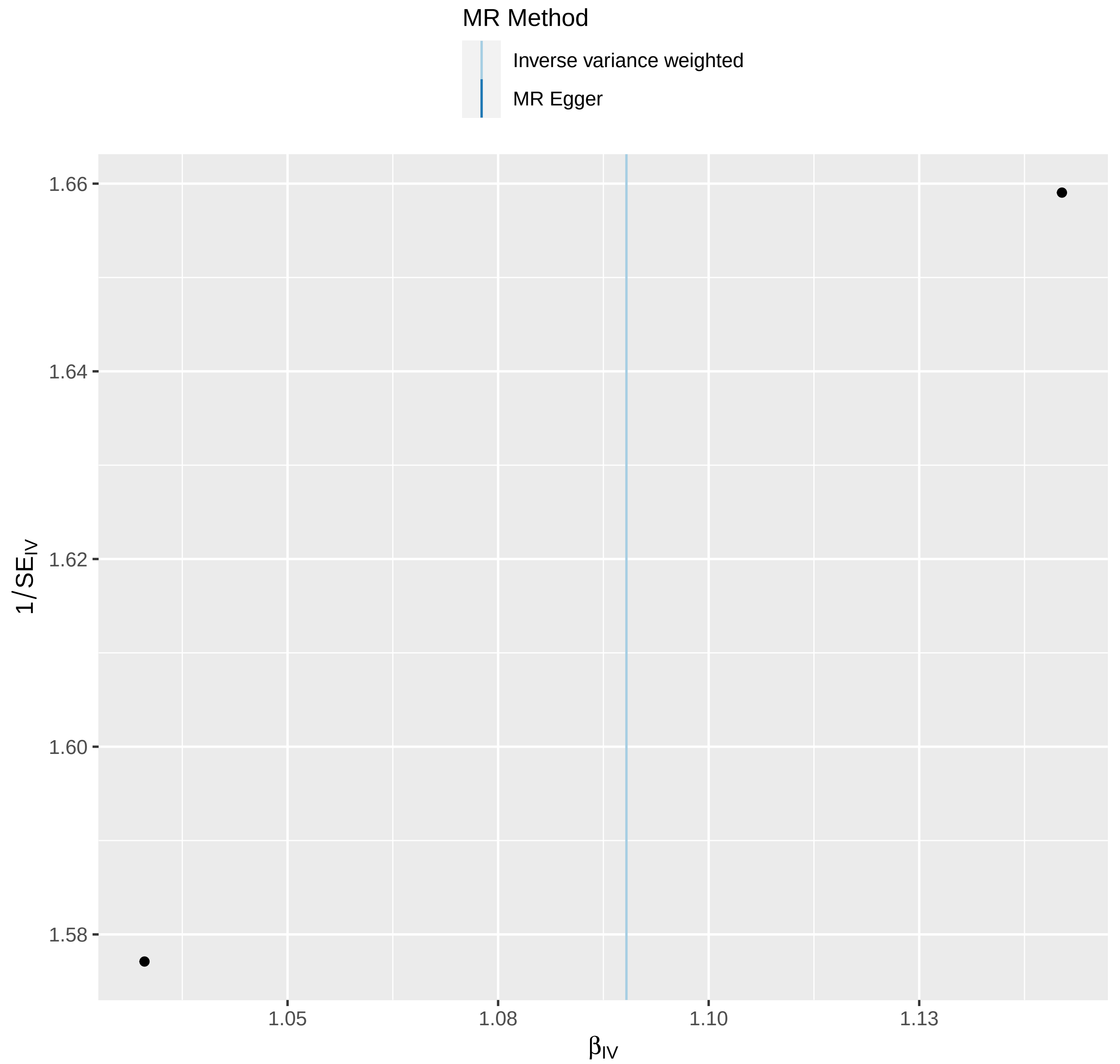

Batch 1535 : X−11422−−xanthine on Left−sided ulcerative colitis

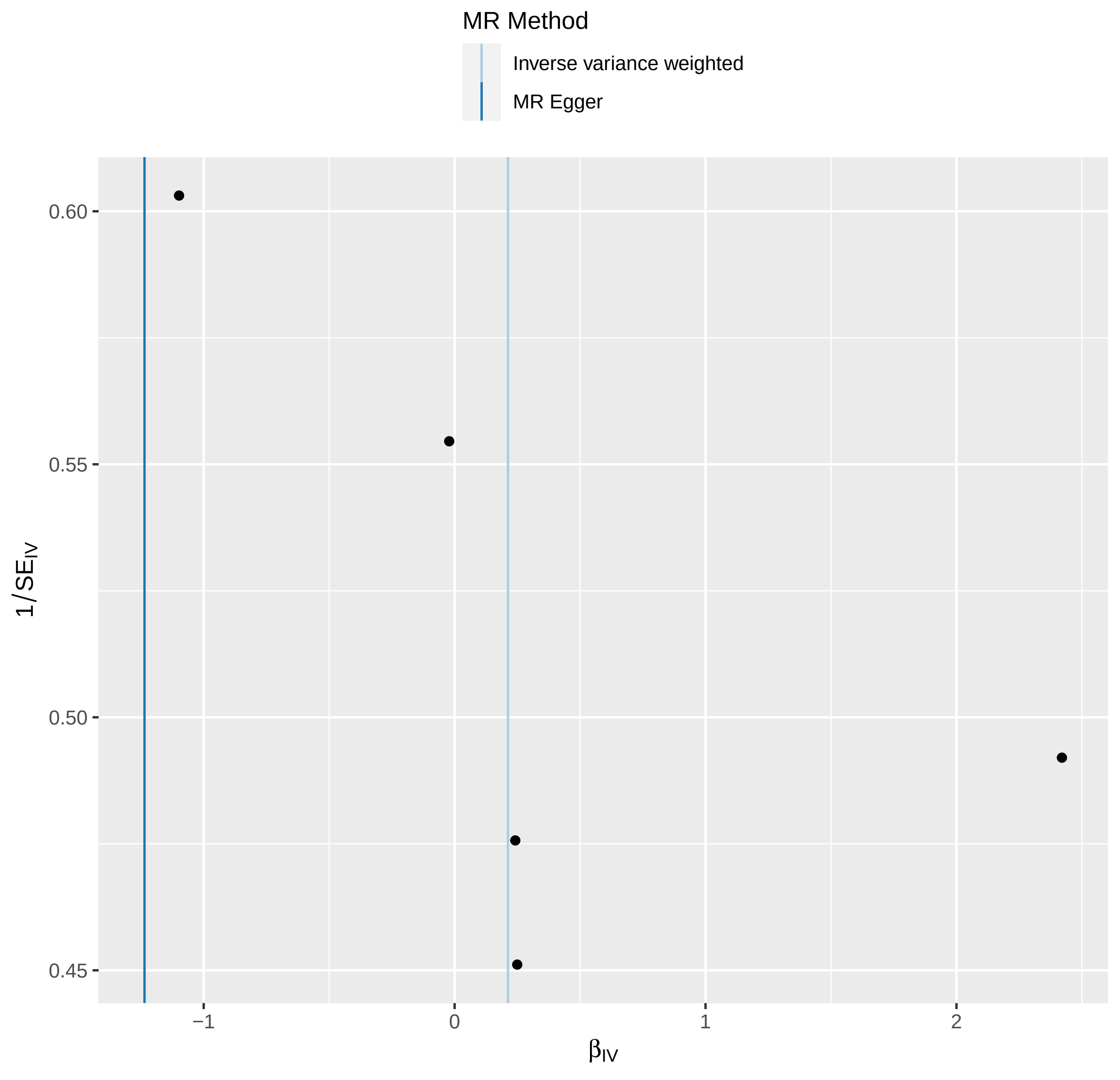

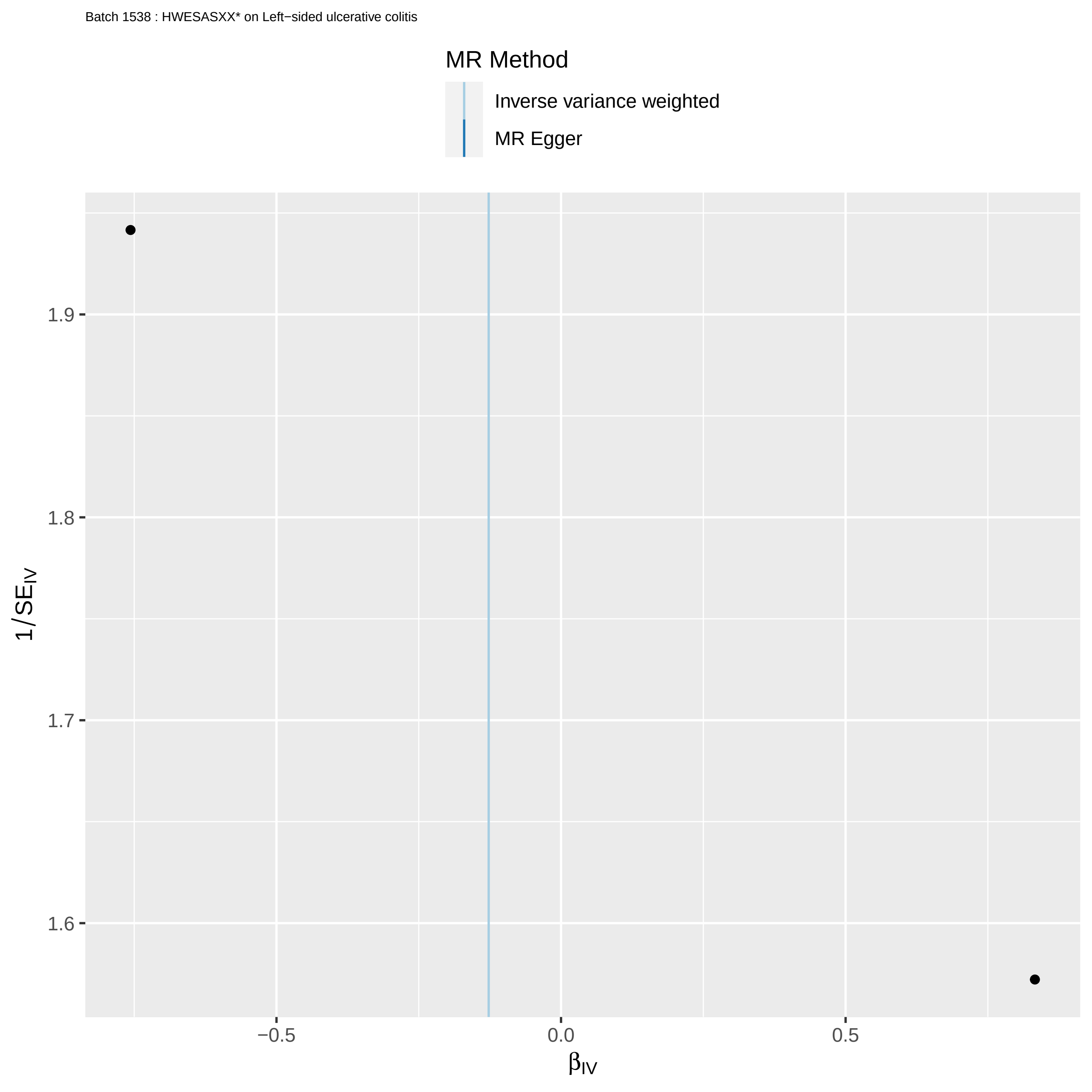

Batch 1538 : HWESASXX* on Left−sided ulcerative colitis
MR Method
Inverse variance weighted
MR Egger
1.9
1.8
1.7
1.6
1/SE_IV
−0.5
0.0
0.5
β_IV

Batch 1539 : X−11593−−O−methylascorbate* on Left−sided ulcerative colitis

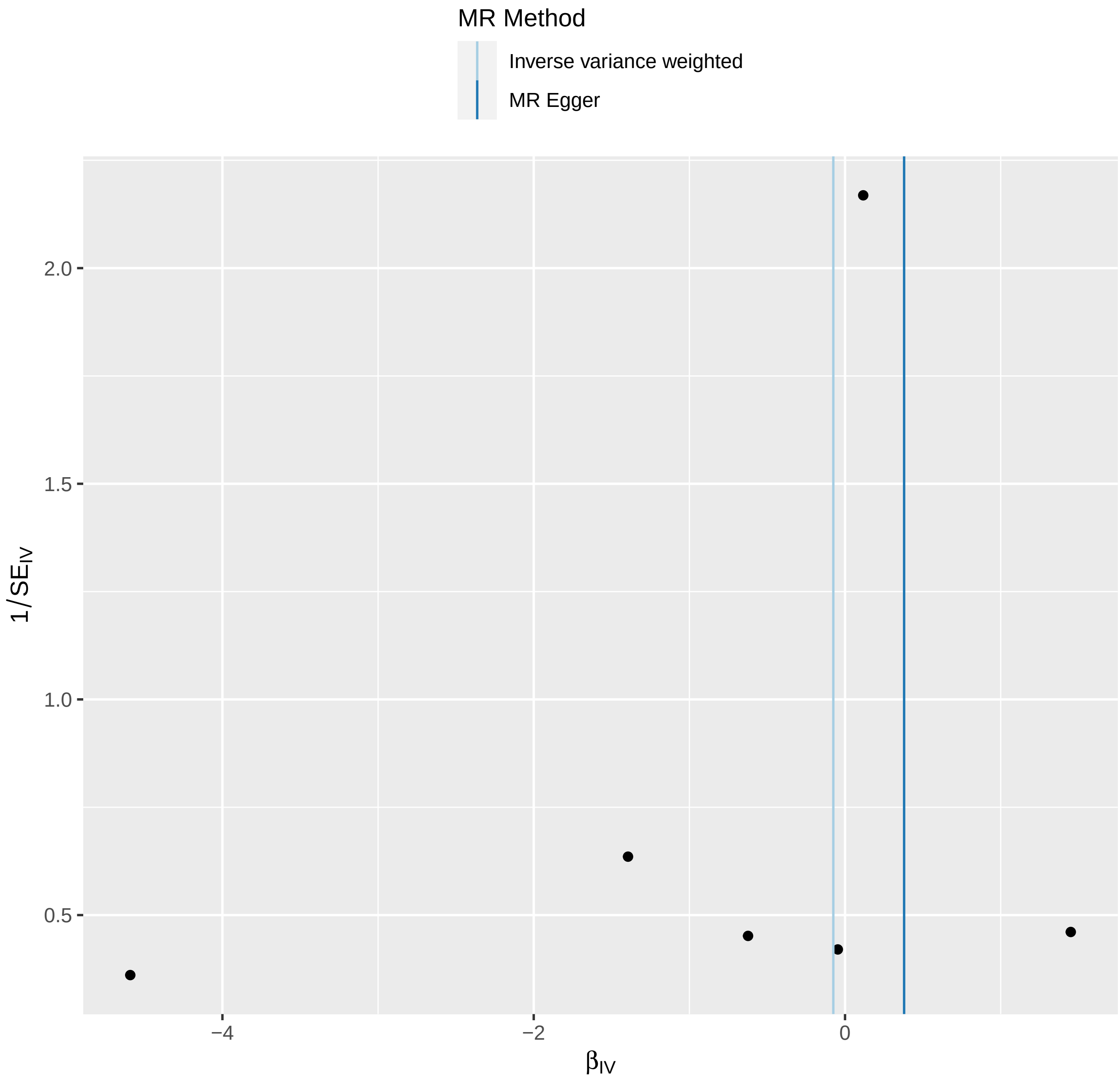

Batch 1541 : Homostachydrine* on Left−sided ulcerative colitis

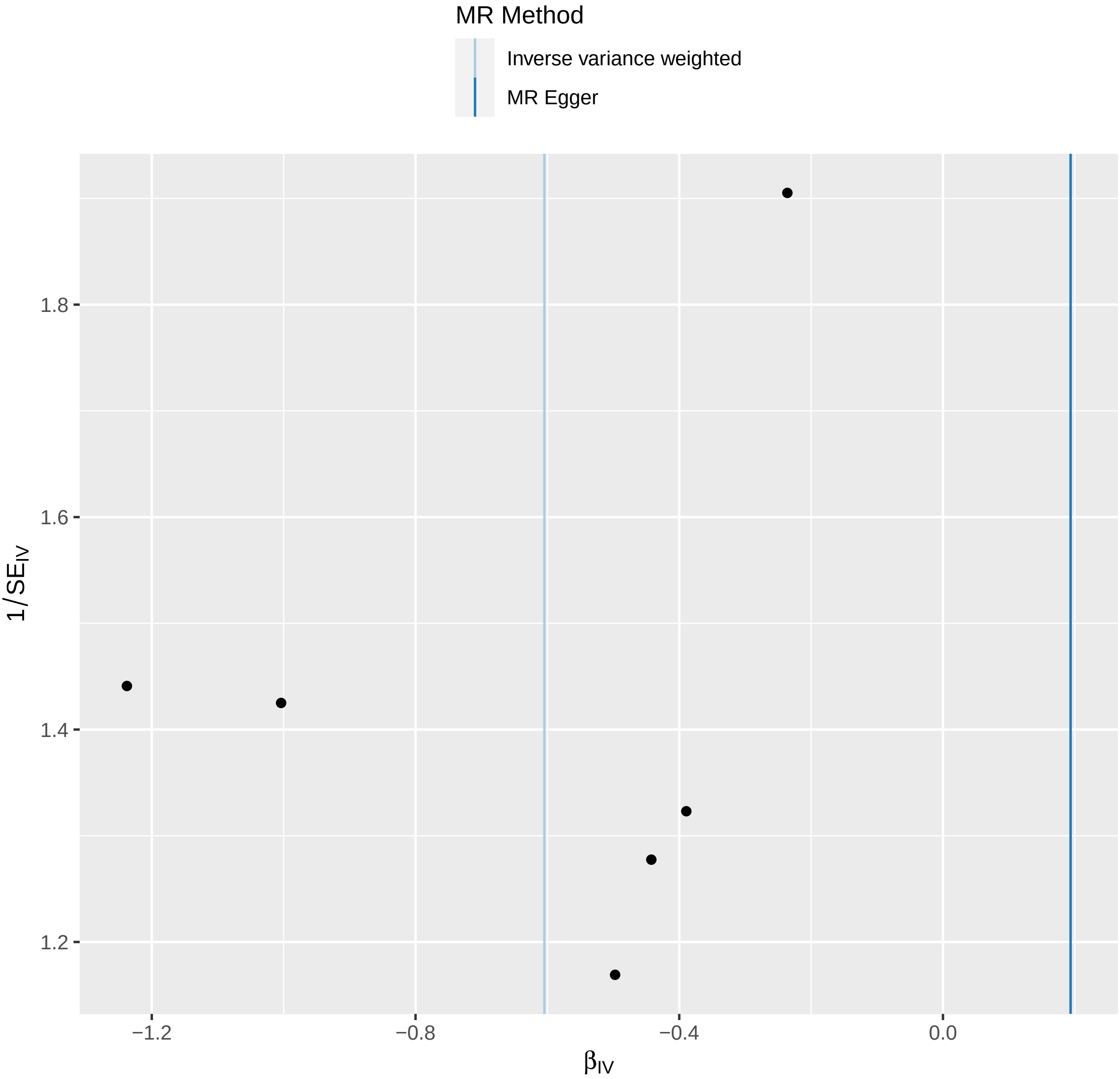

Batch 1542 : ADSGEGDFXAEGGGVR* on Left−sided ulcerative colitis

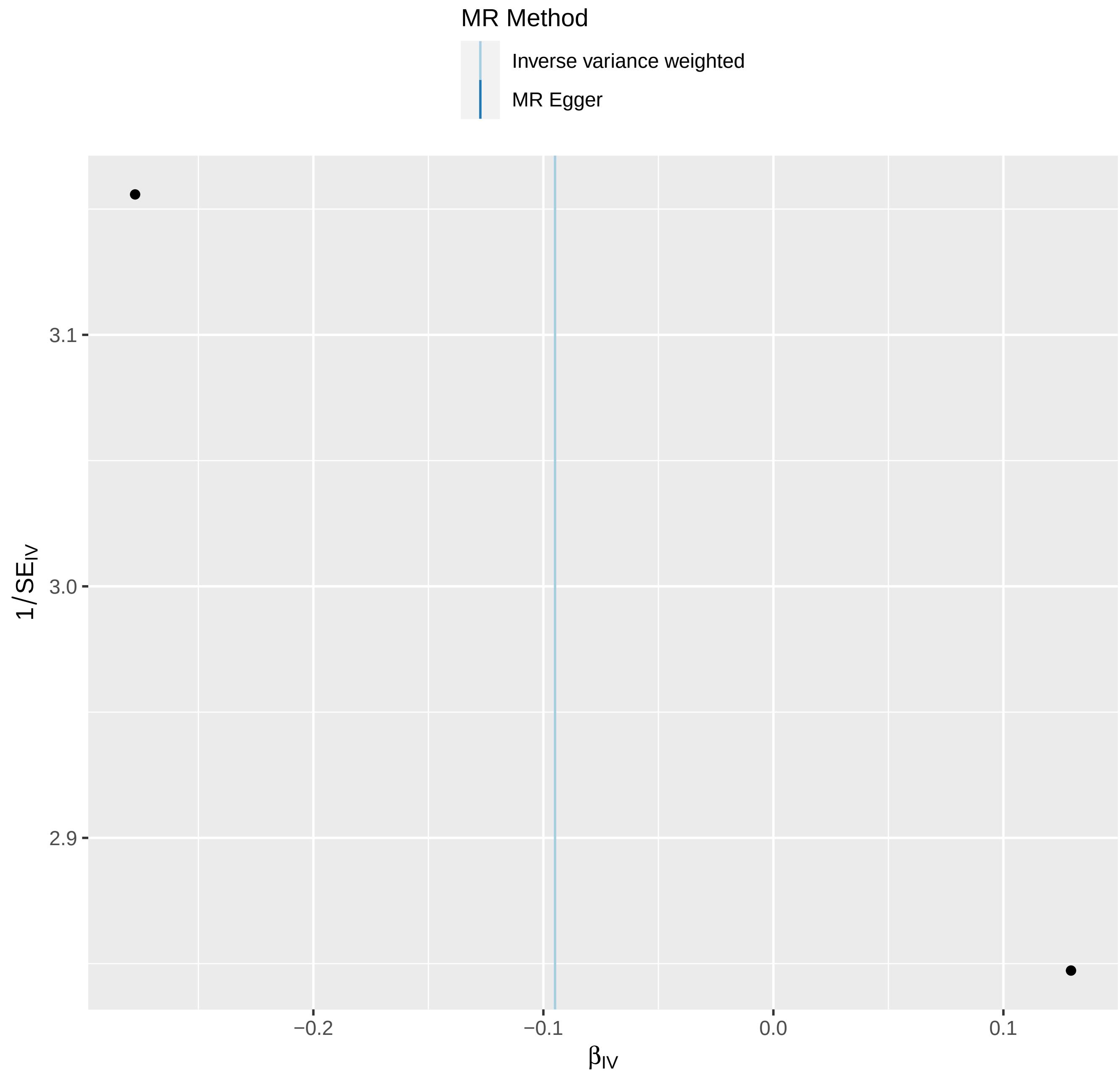

Batch 1544 : X−11793−−oxidized bilirubin* on Left−sided ulcerative colitis

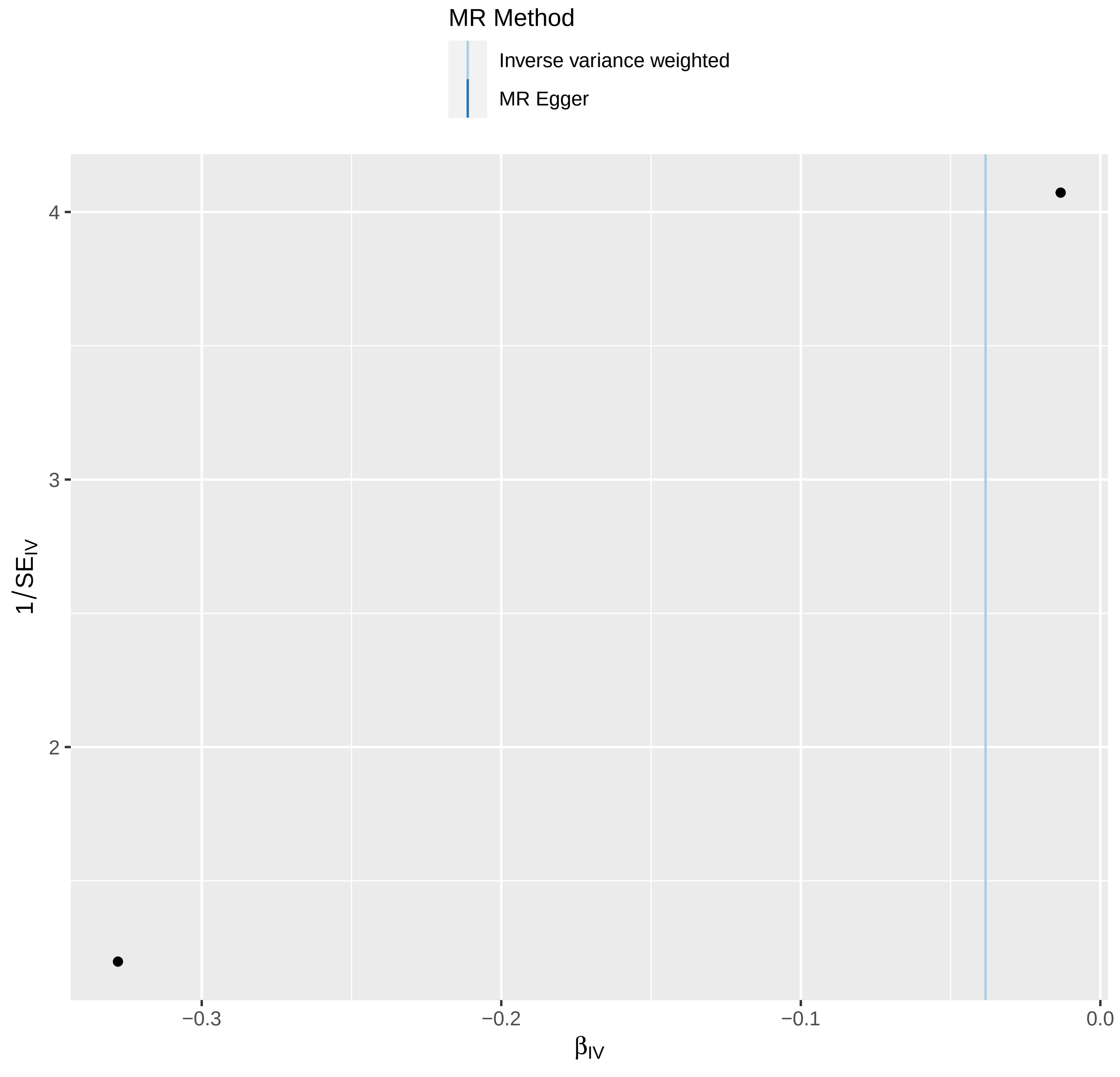

Batch 1545 : 1−arachidonoylglycerophosphocholine* on Left−sided ulcerative colitis

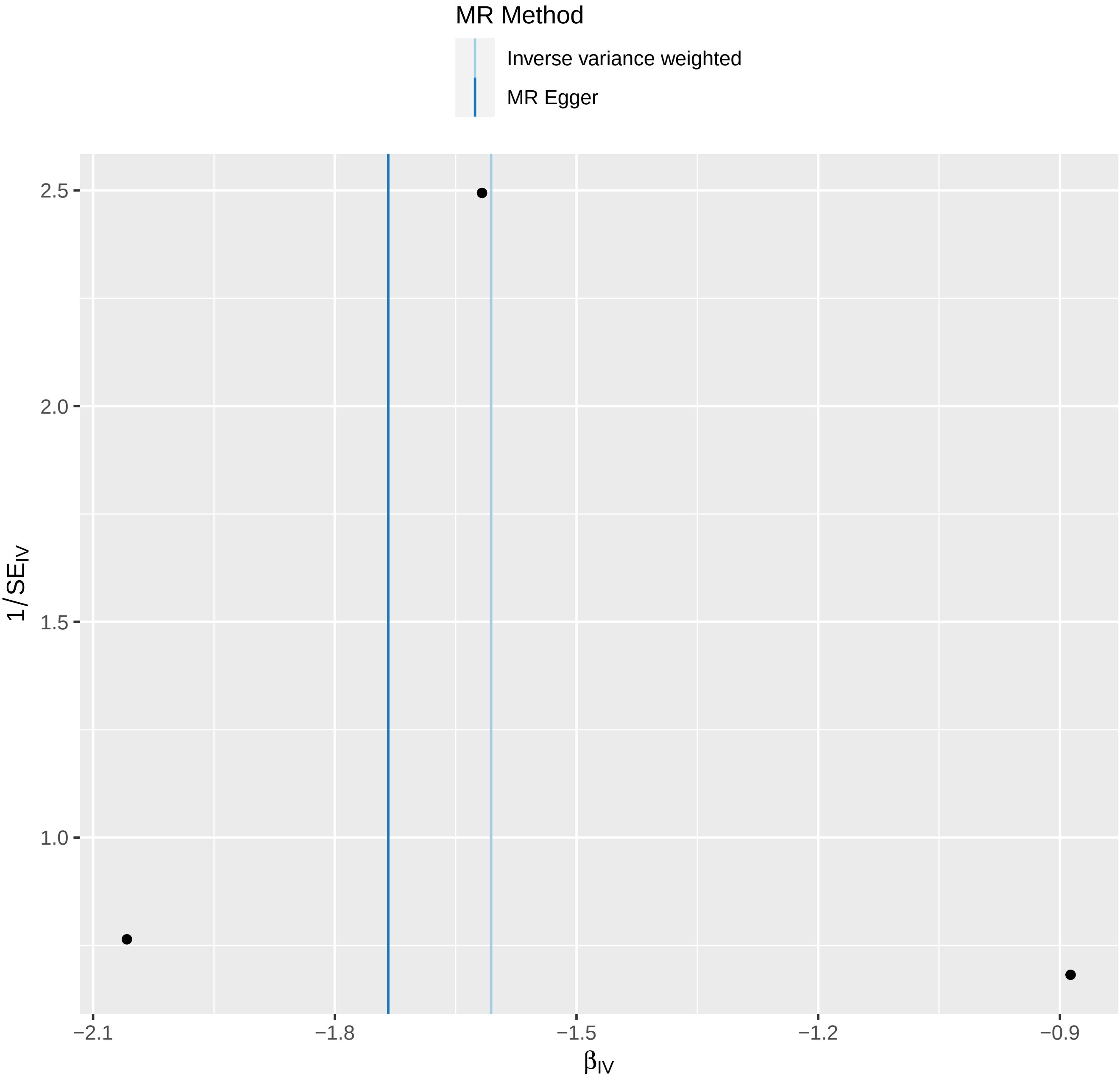

Batch 1547 : Gamma−glutamylmethionine* on Left−sided ulcerative colitis

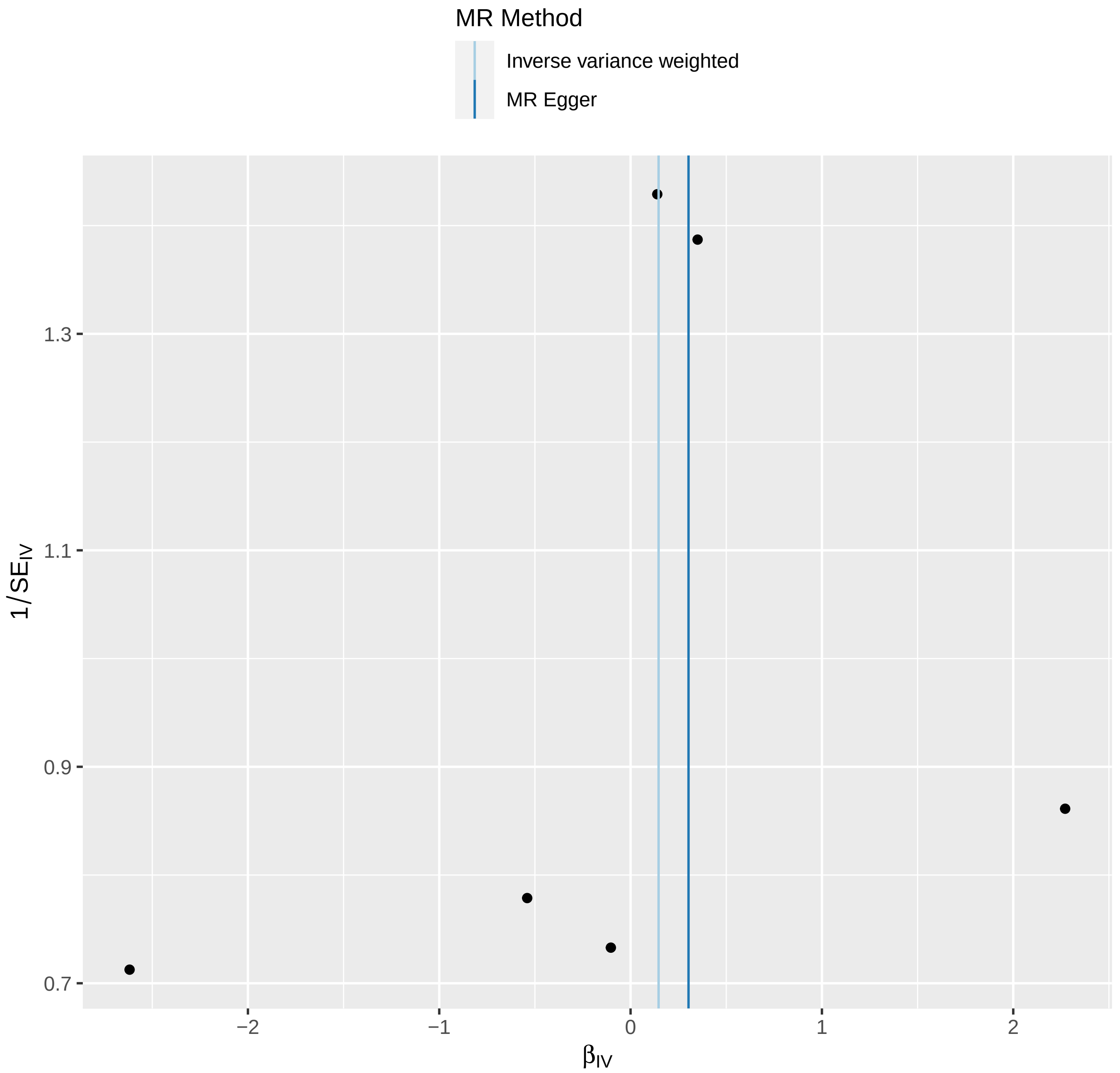

Batch 1548 : Gamma−glutamylthreonine* on Left−sided ulcerative colitis

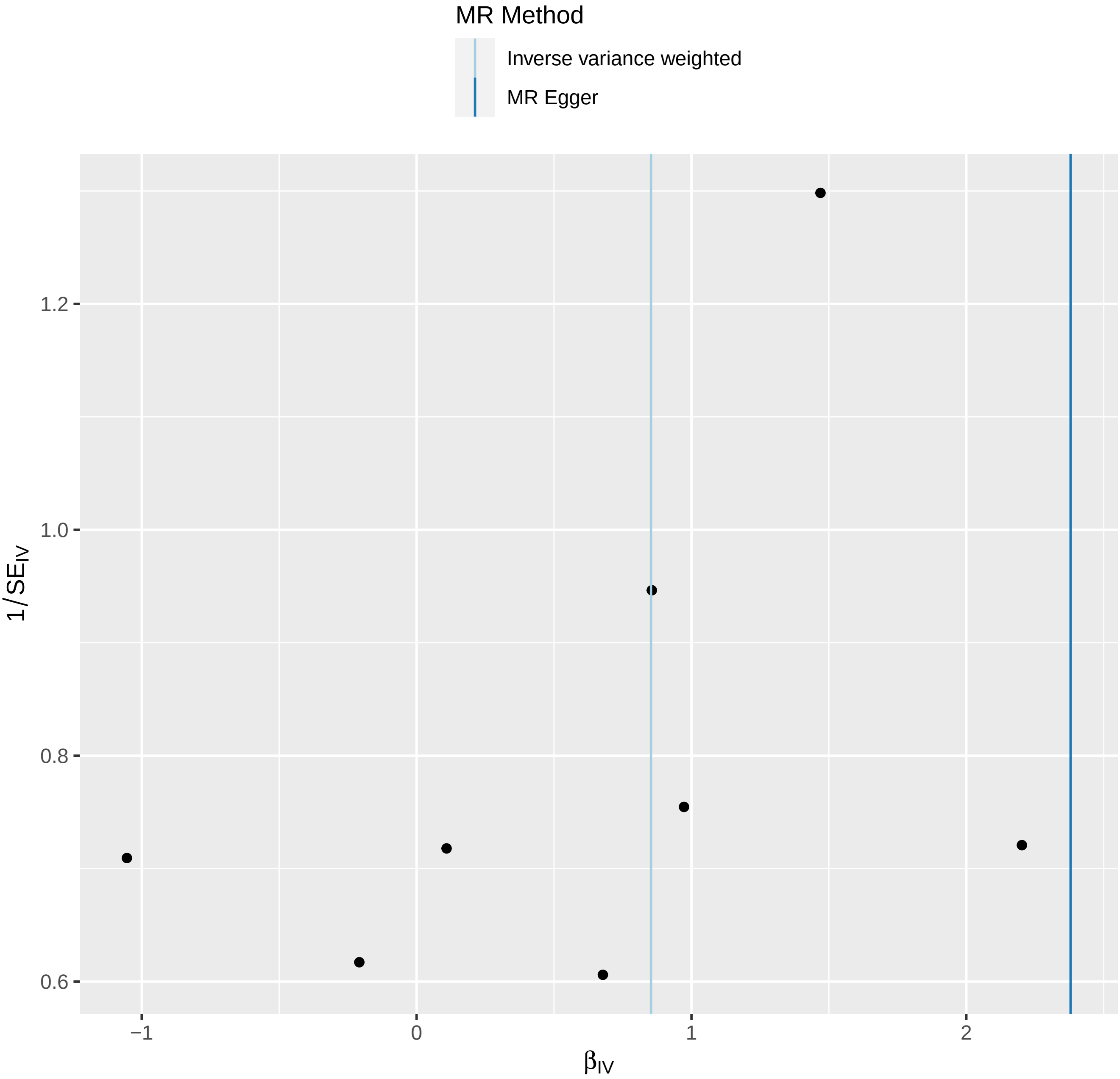

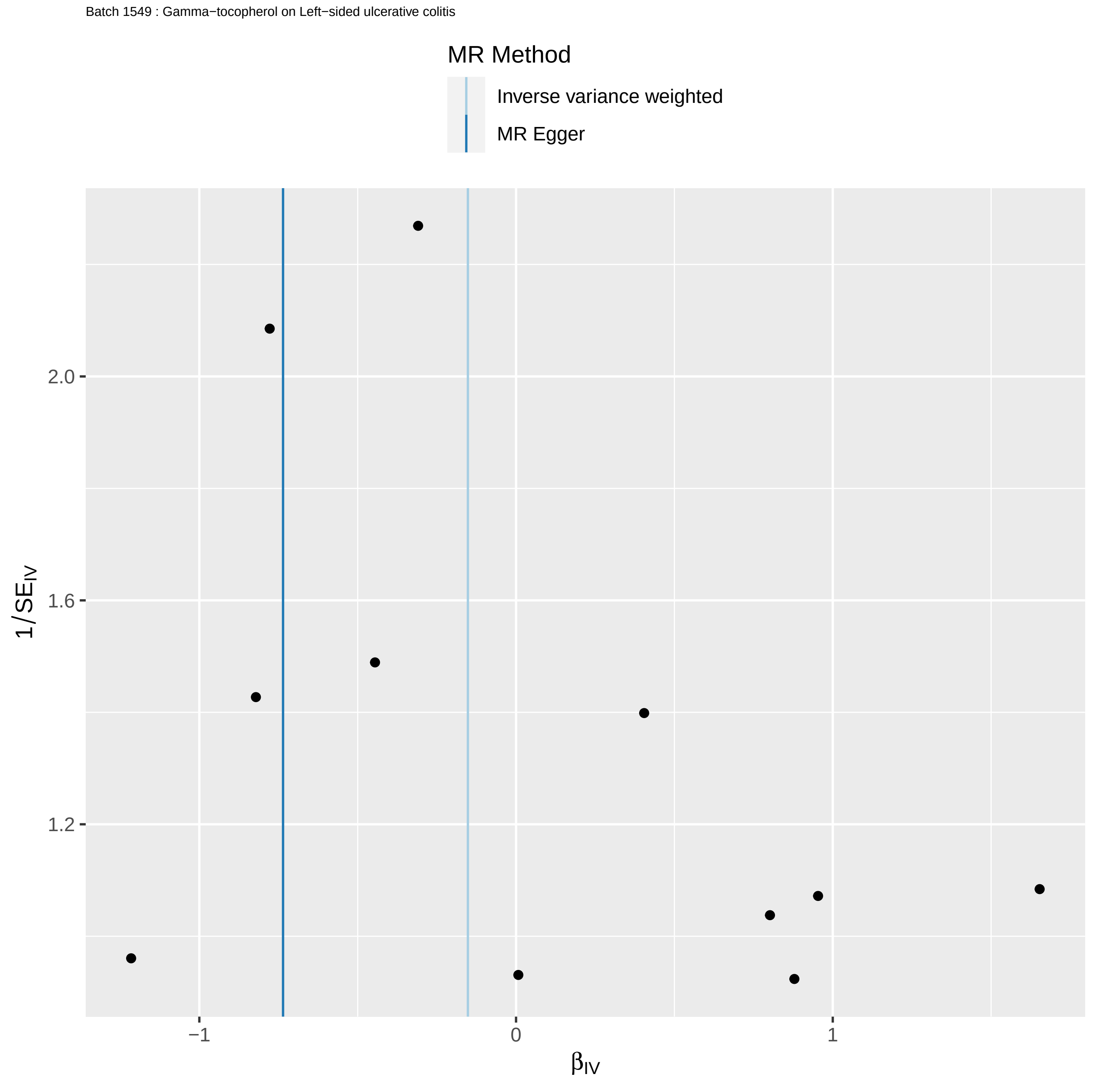

Batch 1549 : Gamma−tocopherol on Left−sided ulcerative colitis
MR Method
Inverse variance weighted
MR Egger
2.0
1.6
1.2
$1/SE_{IV}$
−1
0
1
β_{IV}

Batch 1550 : Gamma−glutamylphenylalanine on Left−sided ulcerative colitis

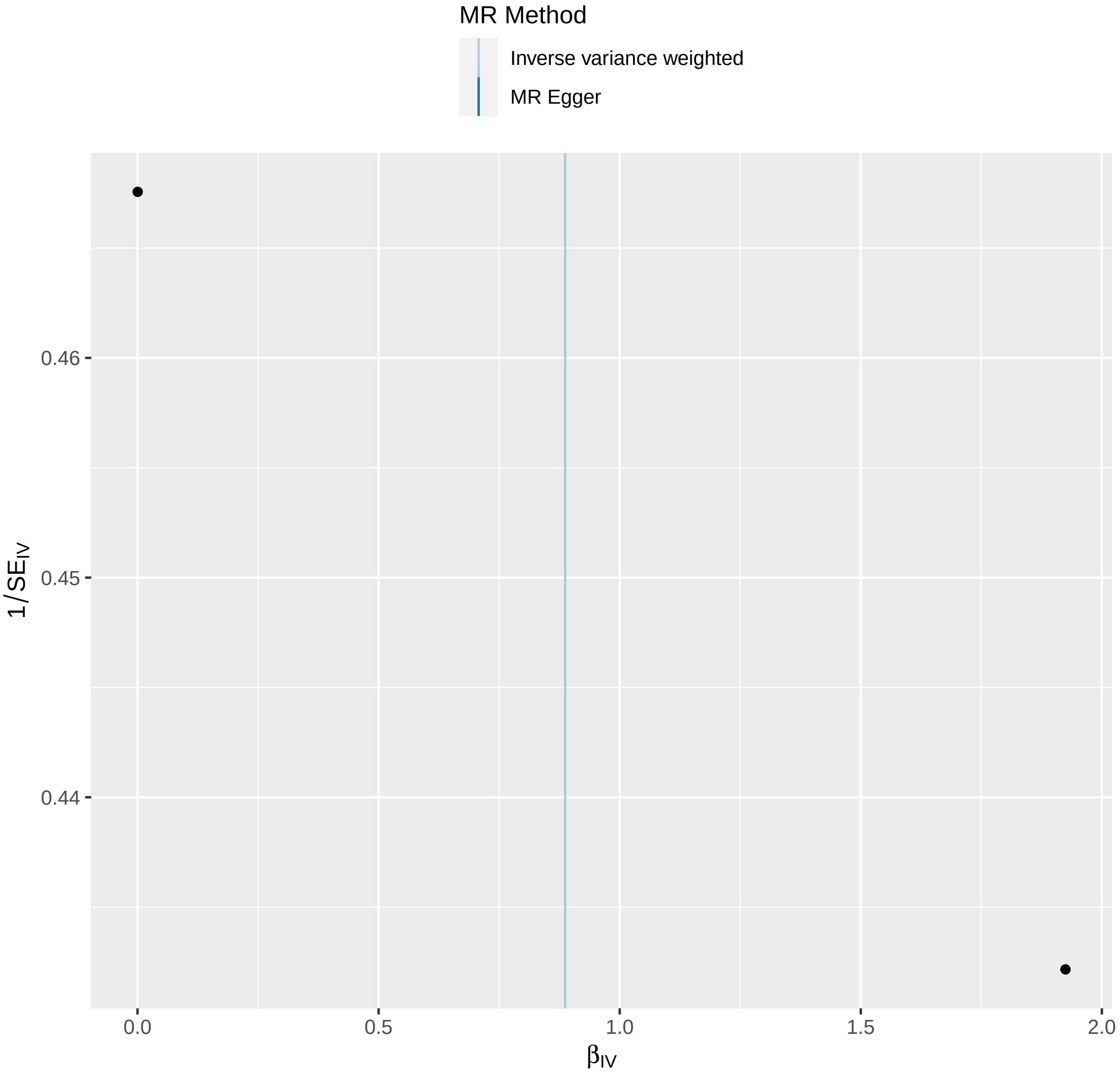

Batch 1551 : Isobutyrylcarnitine on Left−sided ulcerative colitis

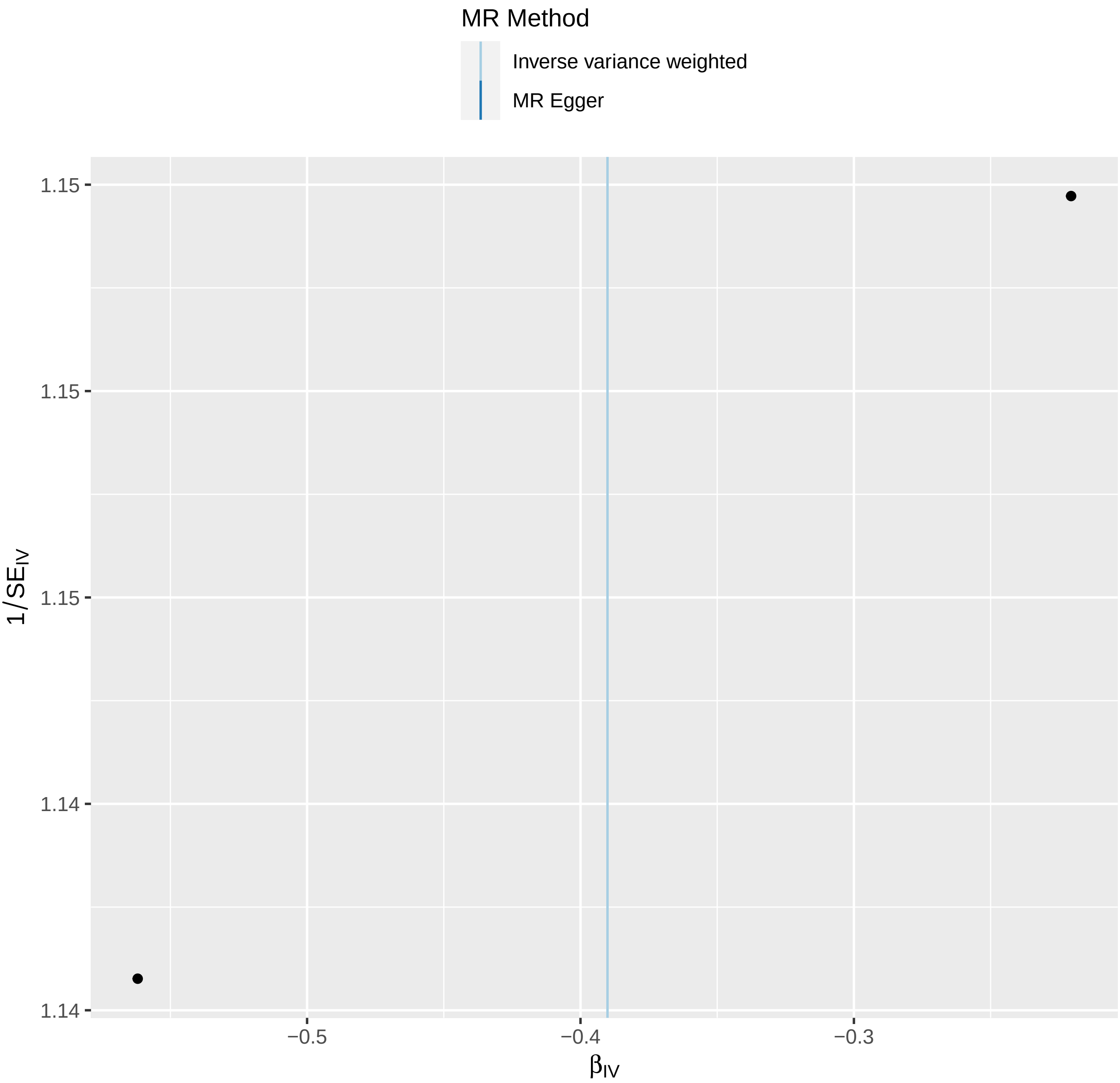

Batch 1552 : Pseudouridine on Left−sided ulcerative colitis

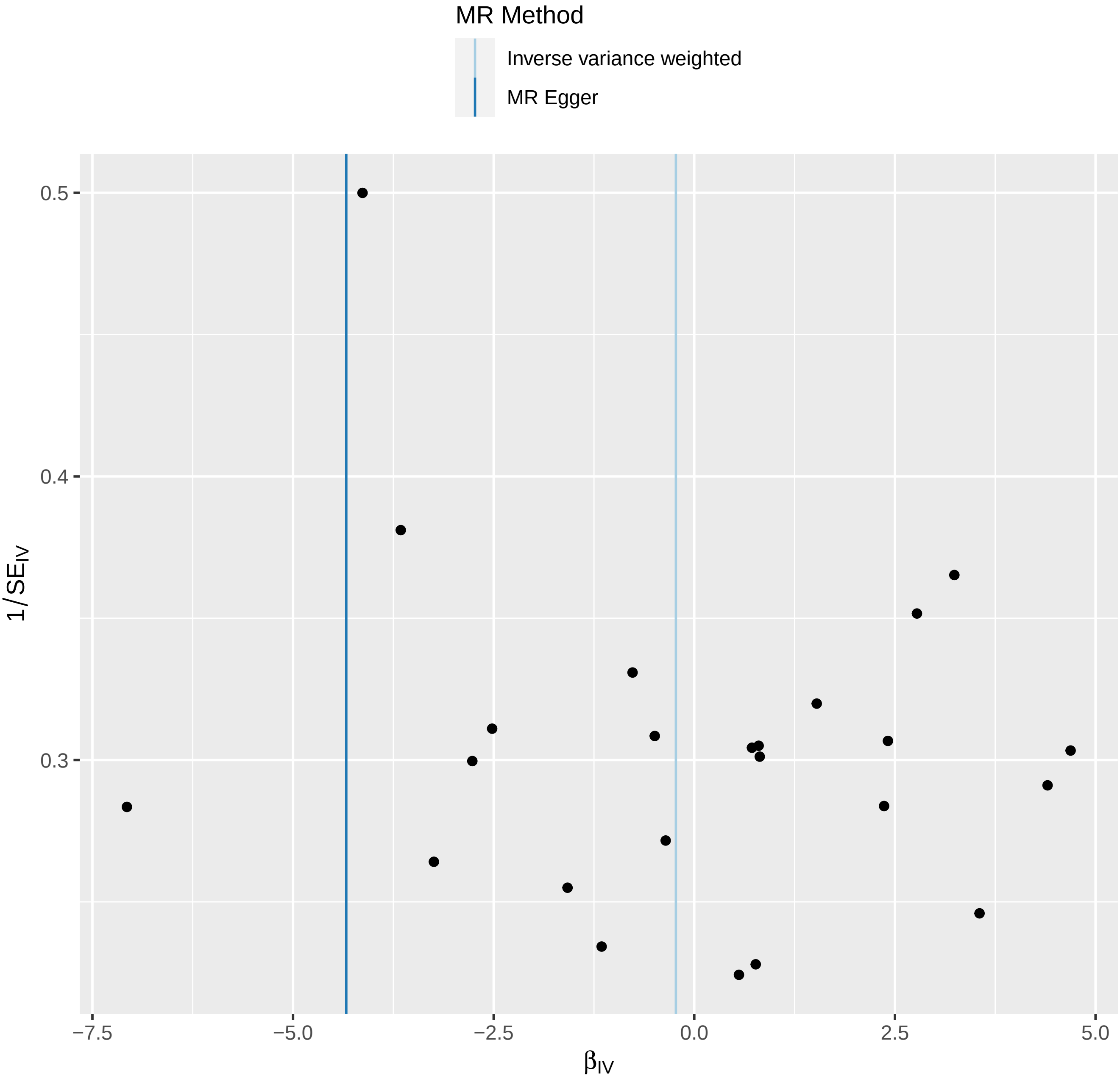

Batch 1553 : Valerate on Left−sided ulcerative colitis

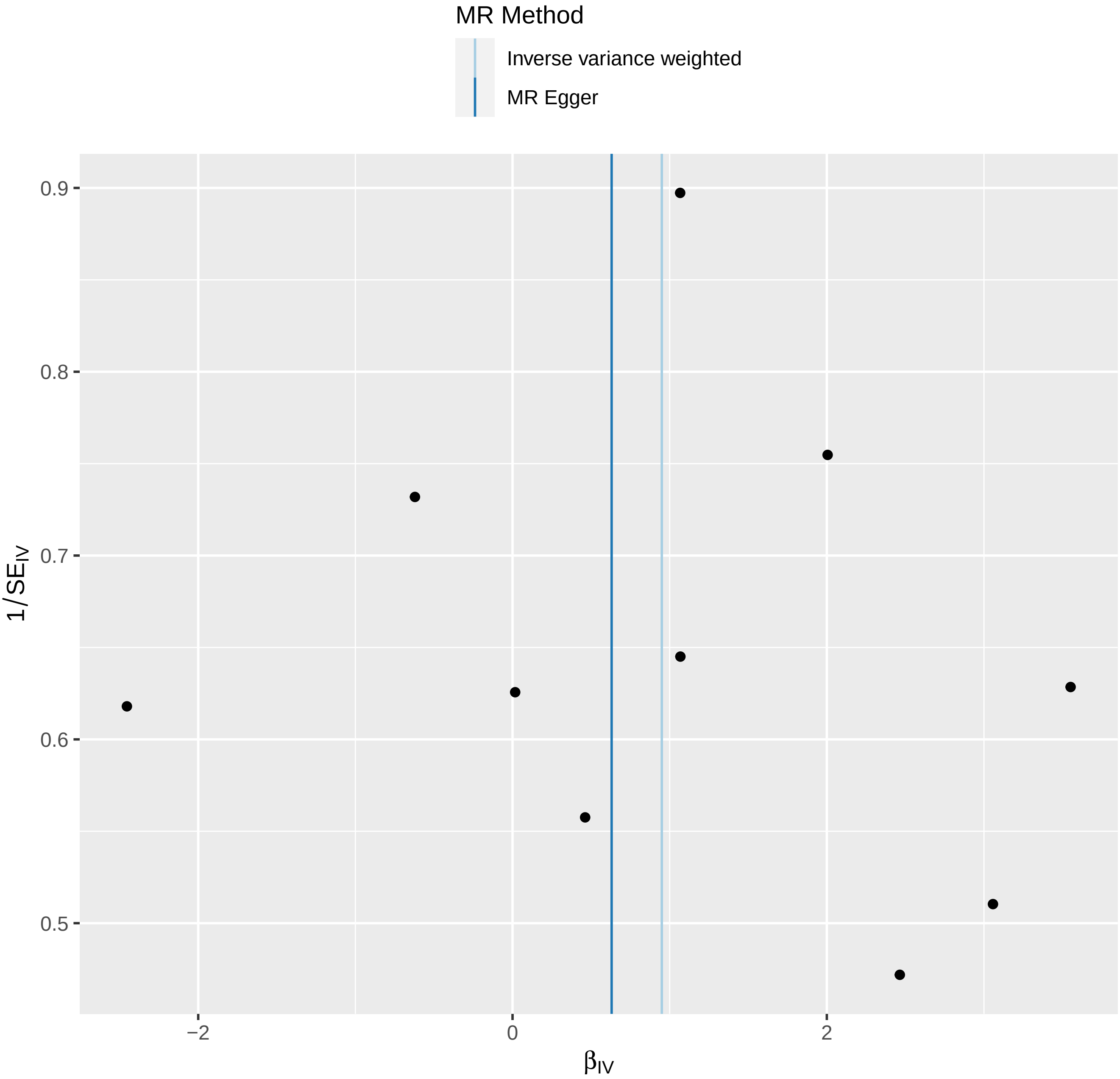

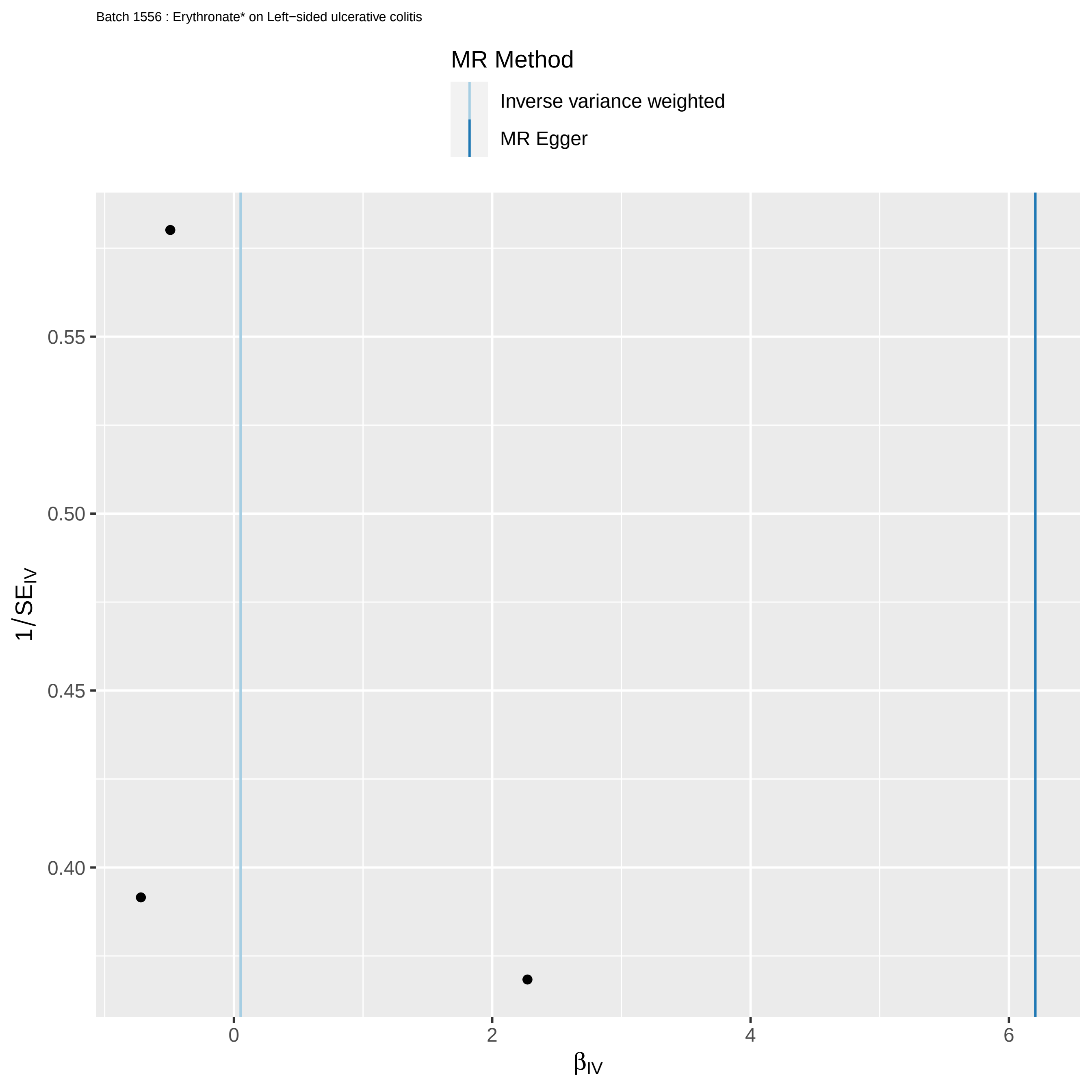

Batch 1556 : Erythronate* on Left−sided ulcerative colitis
MR Method
Inverse variance weighted
MR Egger
0.55
0.50
0.45
0.40
1/SE_IV
0
2
4
6
β_IV

Batch 1557 : Lathosterol on Left−sided ulcerative colitis

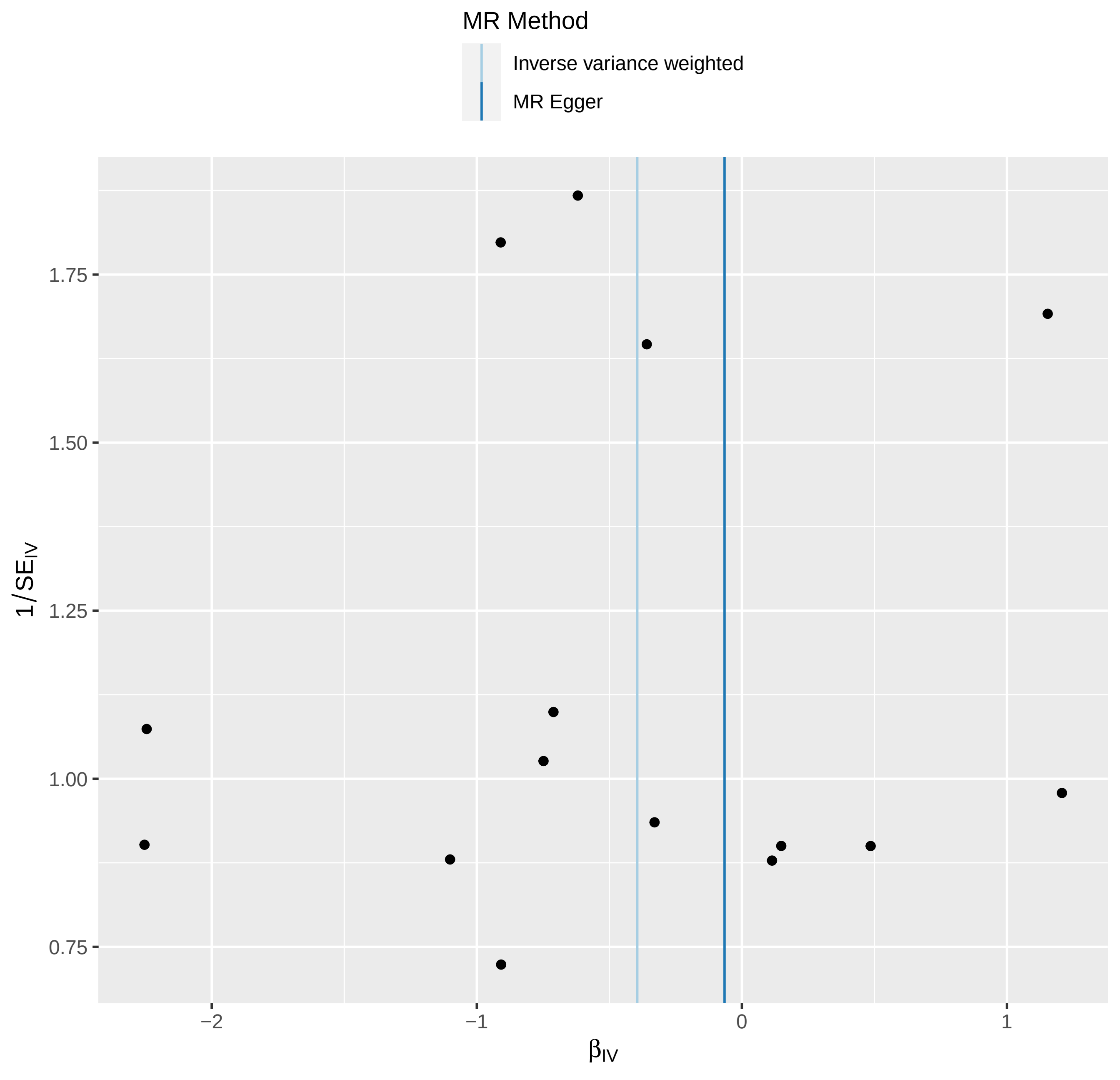

Batch 1560 : Eicosenoate (20:1n9 or 11) on Left−sided ulcerative colitis

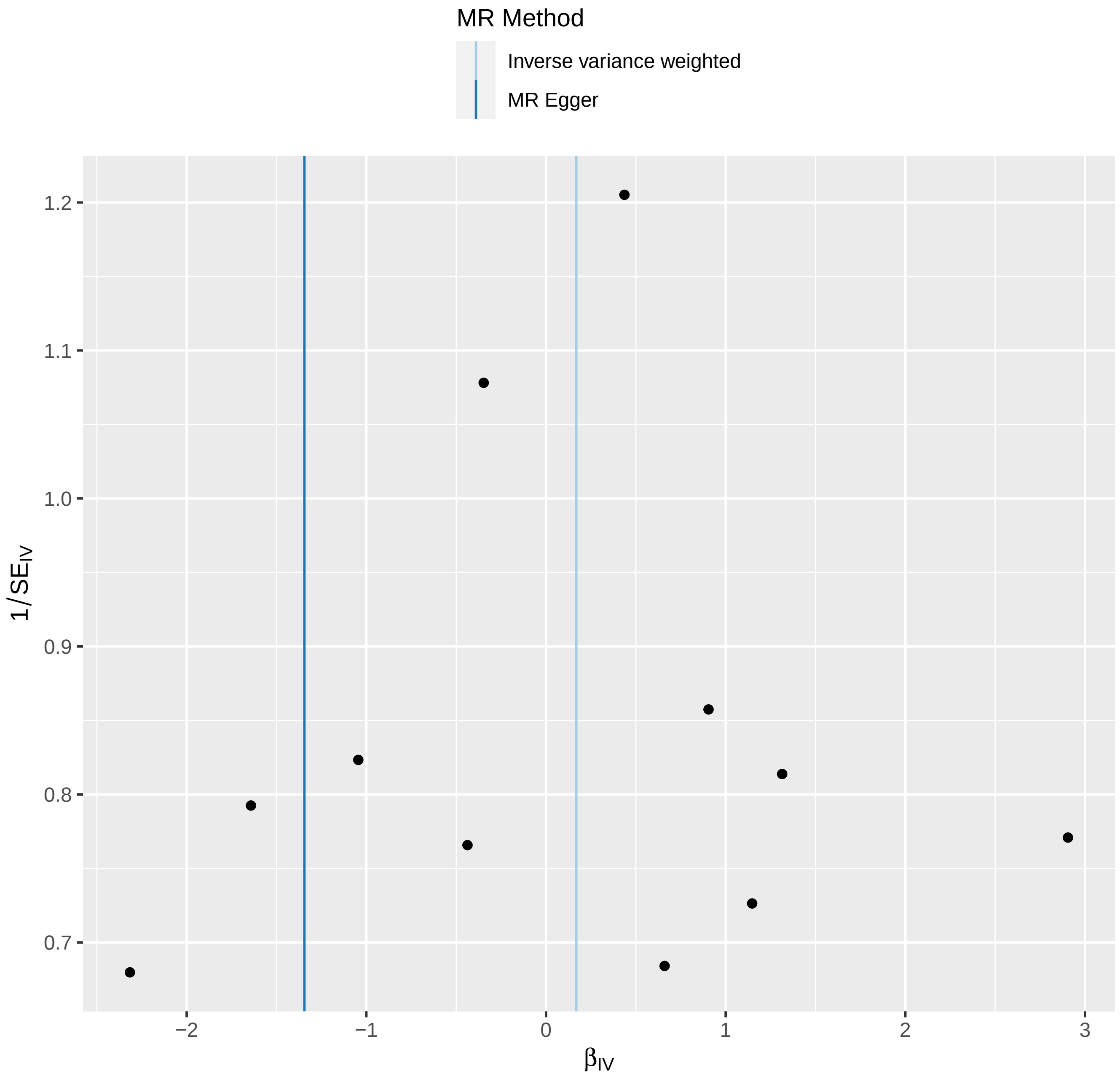

Batch 1561 : X−12244−−N−acetylcarnosine on Left−sided ulcerative colitis

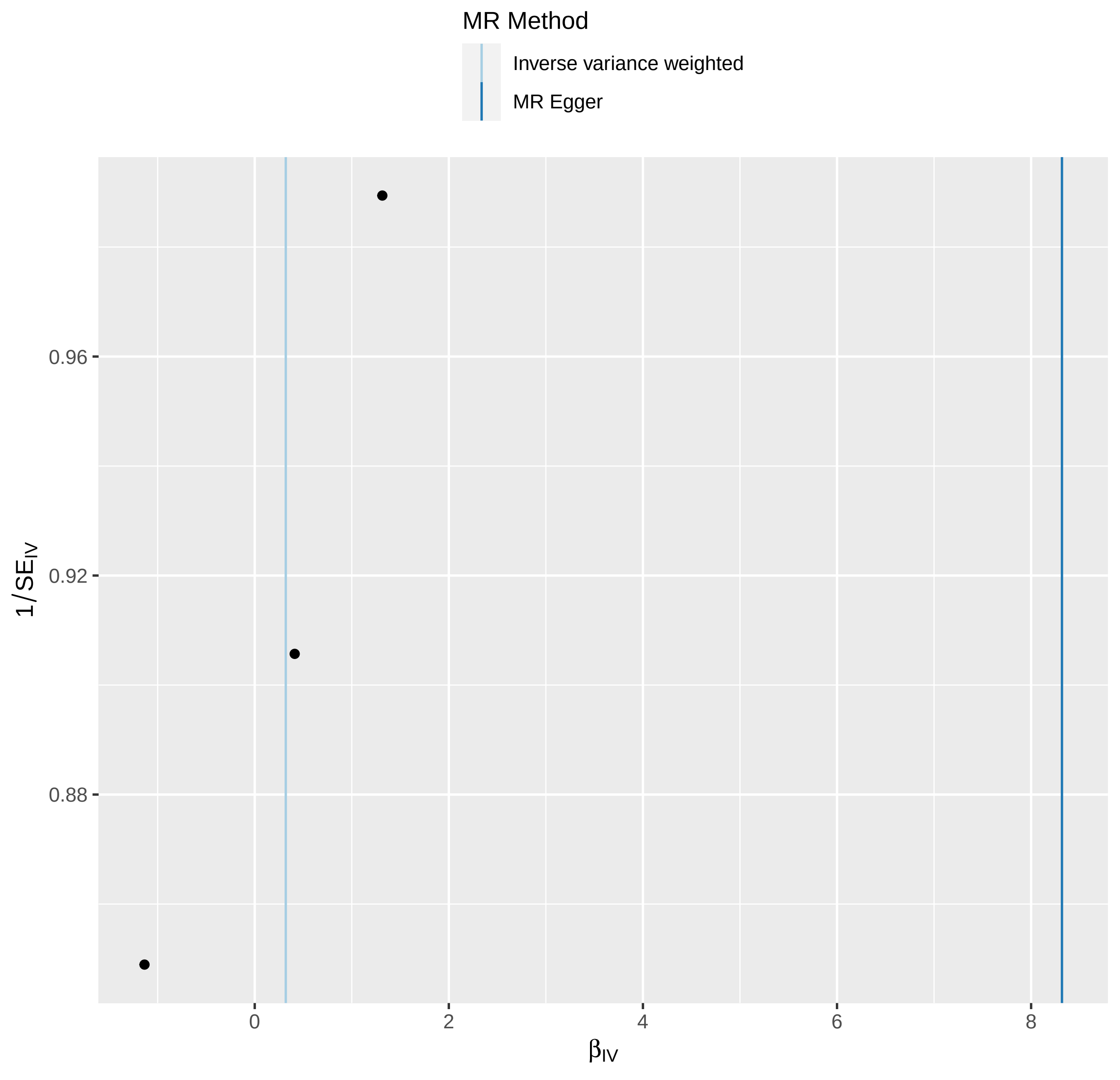

Batch 1563 : 1−eicosatrienoylglycerophosphocholine* on Left−sided ulcerative colitis

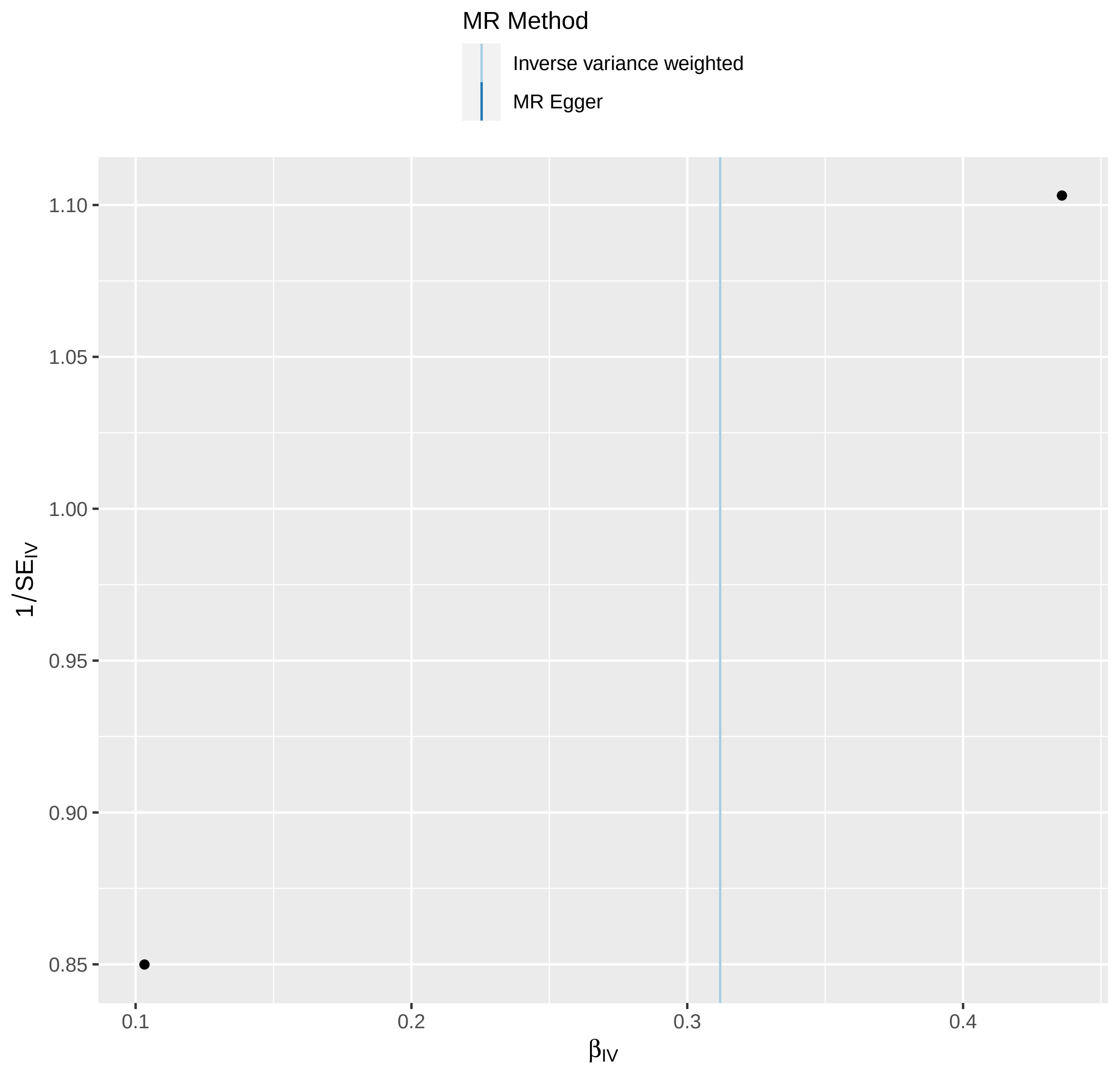

Batch 1564 : 1−docosahexaenoylglycerophosphocholine* on Left−sided ulcerative colitis

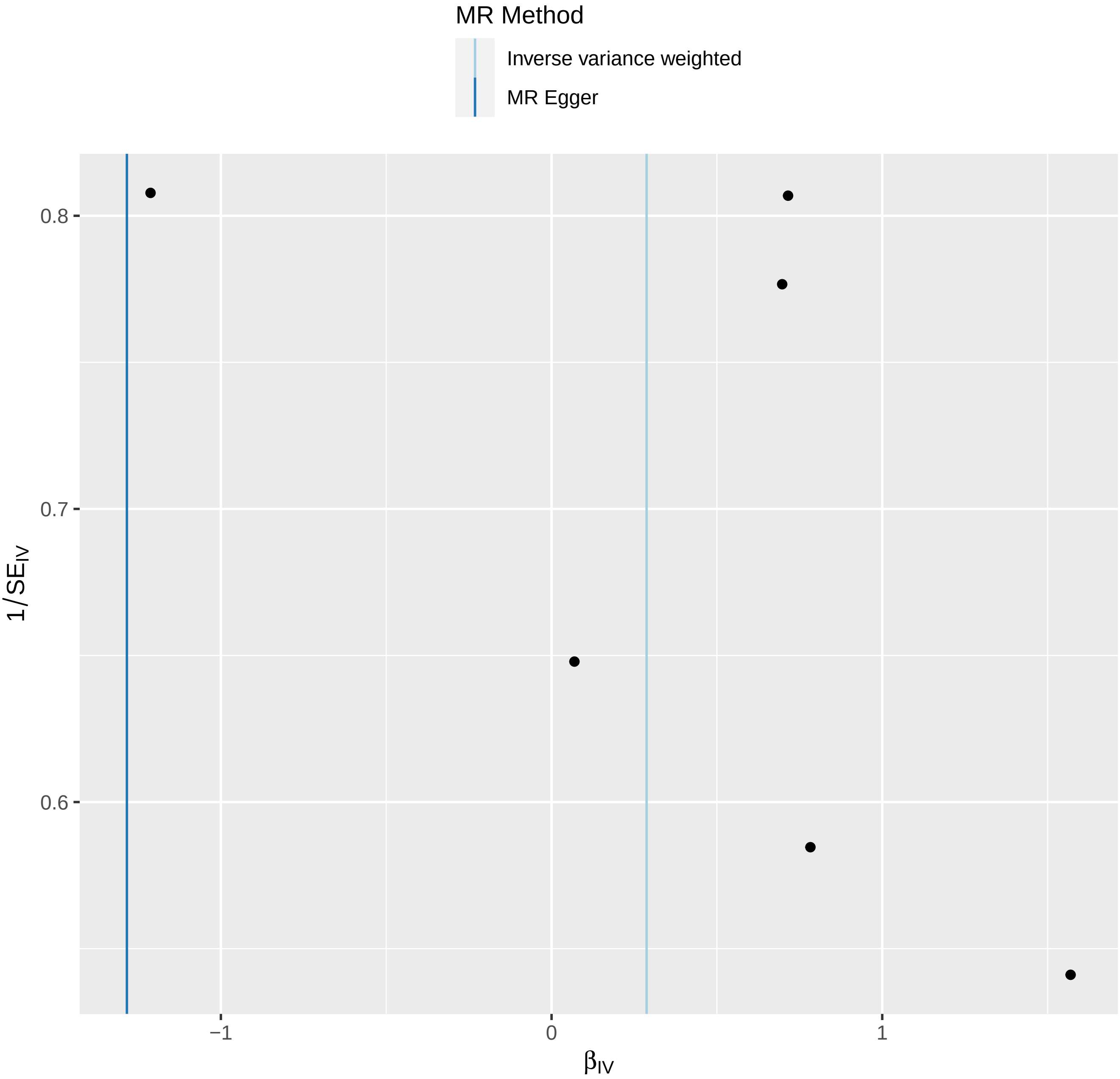

Batch 1568 : Octanoylcarnitine on Left−sided ulcerative colitis

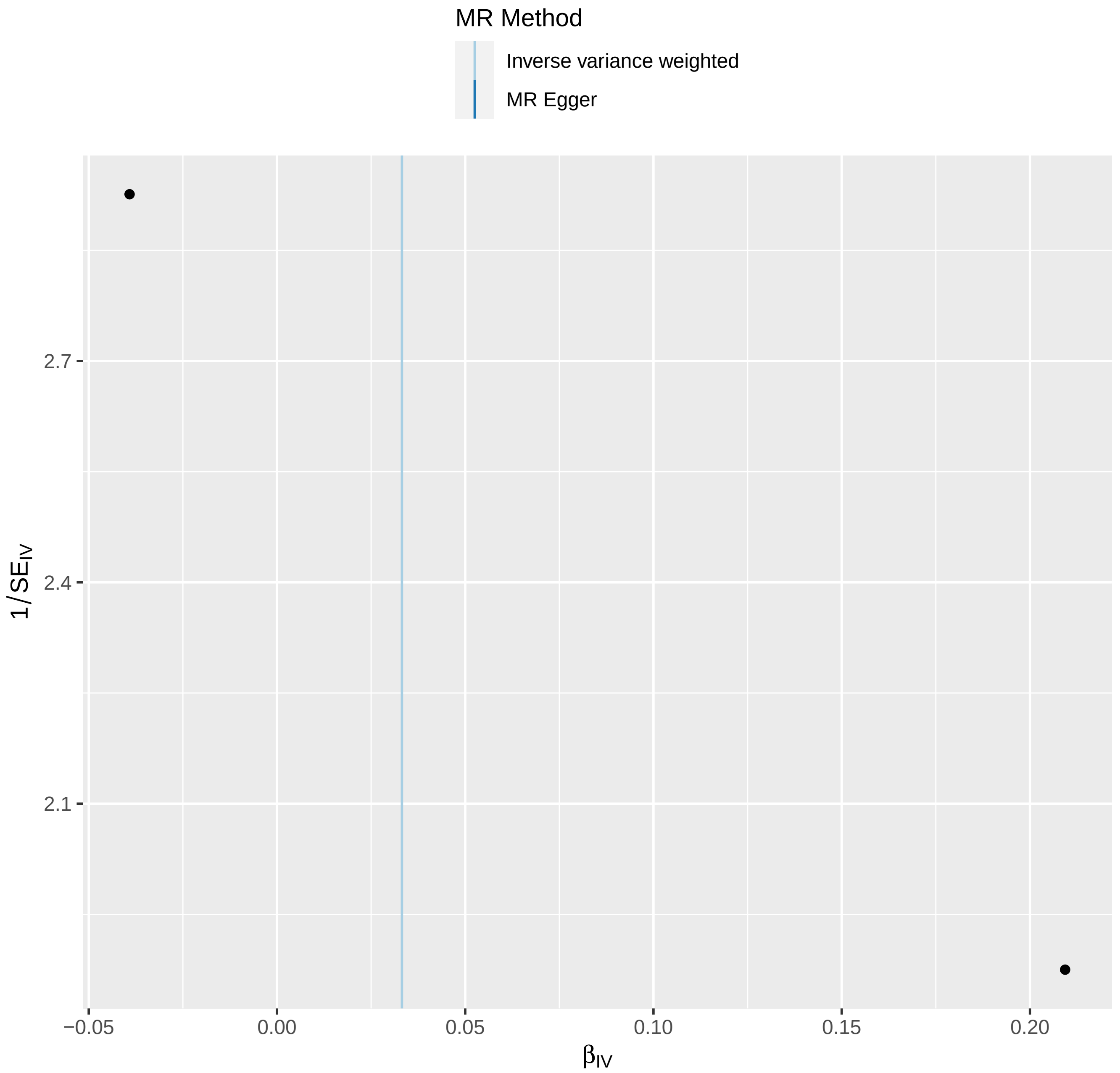

Batch 1569 : Alpha−hydroxyisovalerate on Left−sided ulcerative colitis

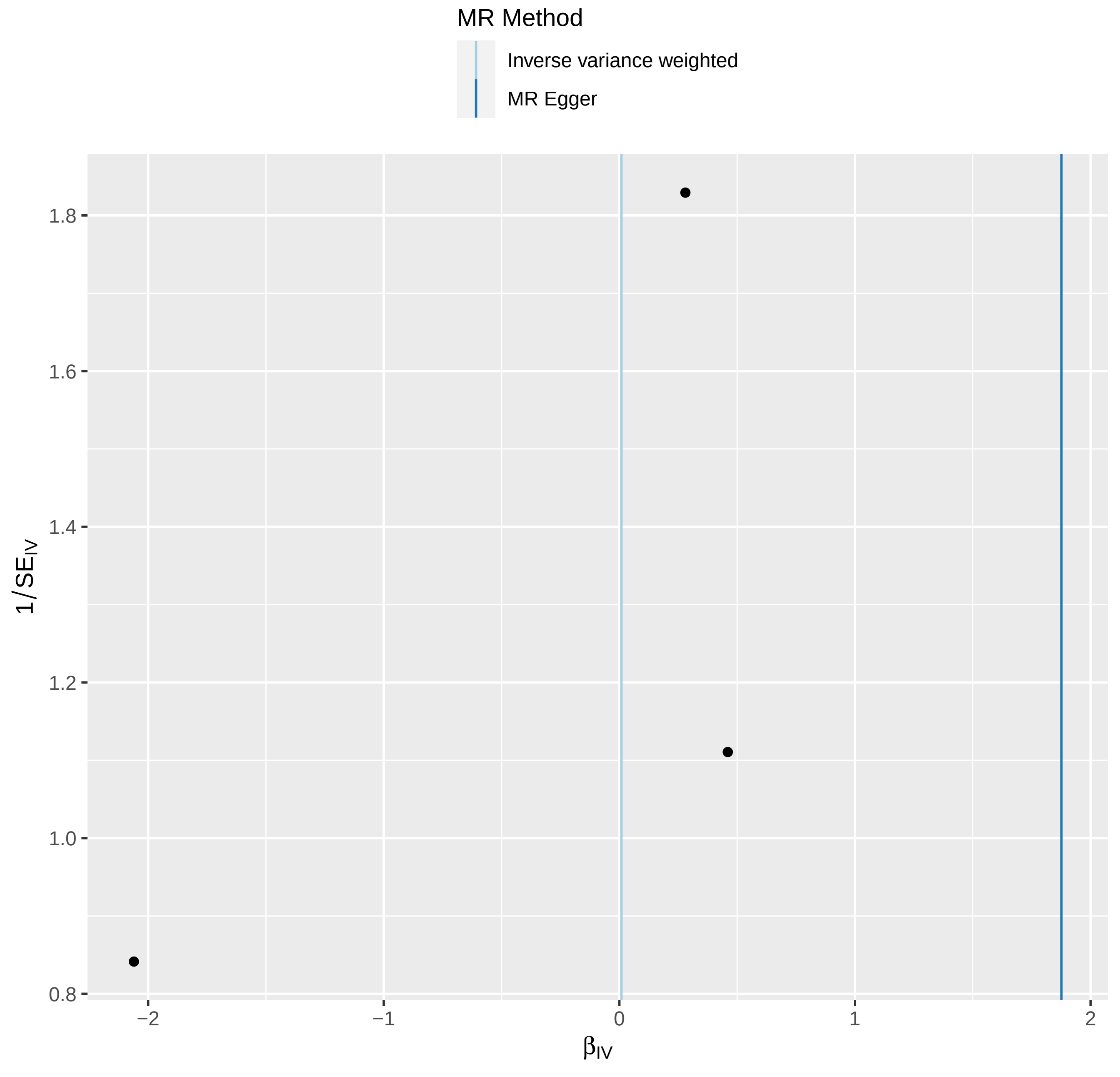

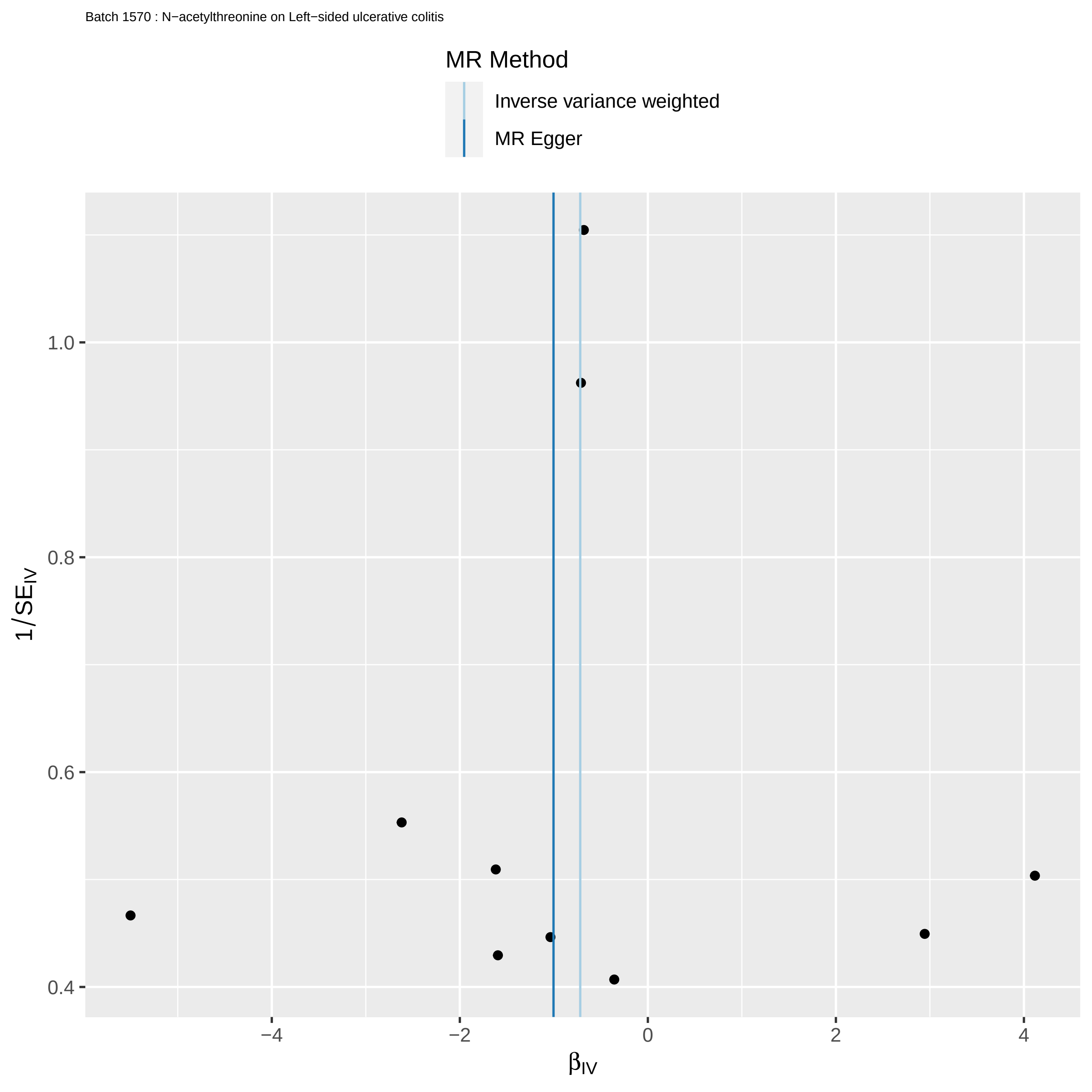
Batch 1570 : N−acetylthreonine on Left−sided ulcerative colitis
MR Method
Inverse variance weighted
MR Egger
1.0
0.8
0.6
0.4
1/SE$_{IV}$
−4
−2
0
2
4
β$_{IV}$

Batch 1571 : Decanoylcarnitine on Left−sided ulcerative colitis

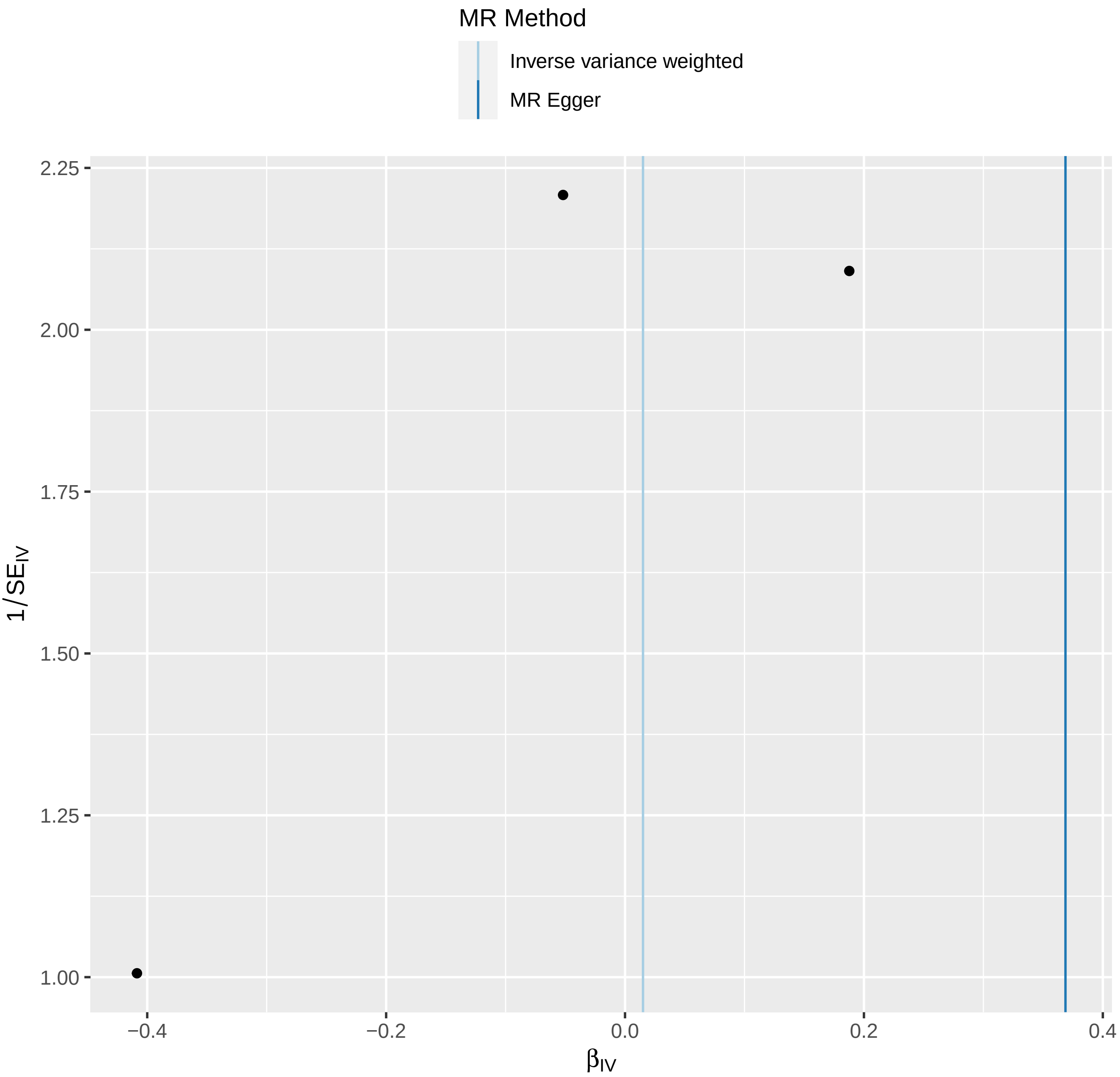

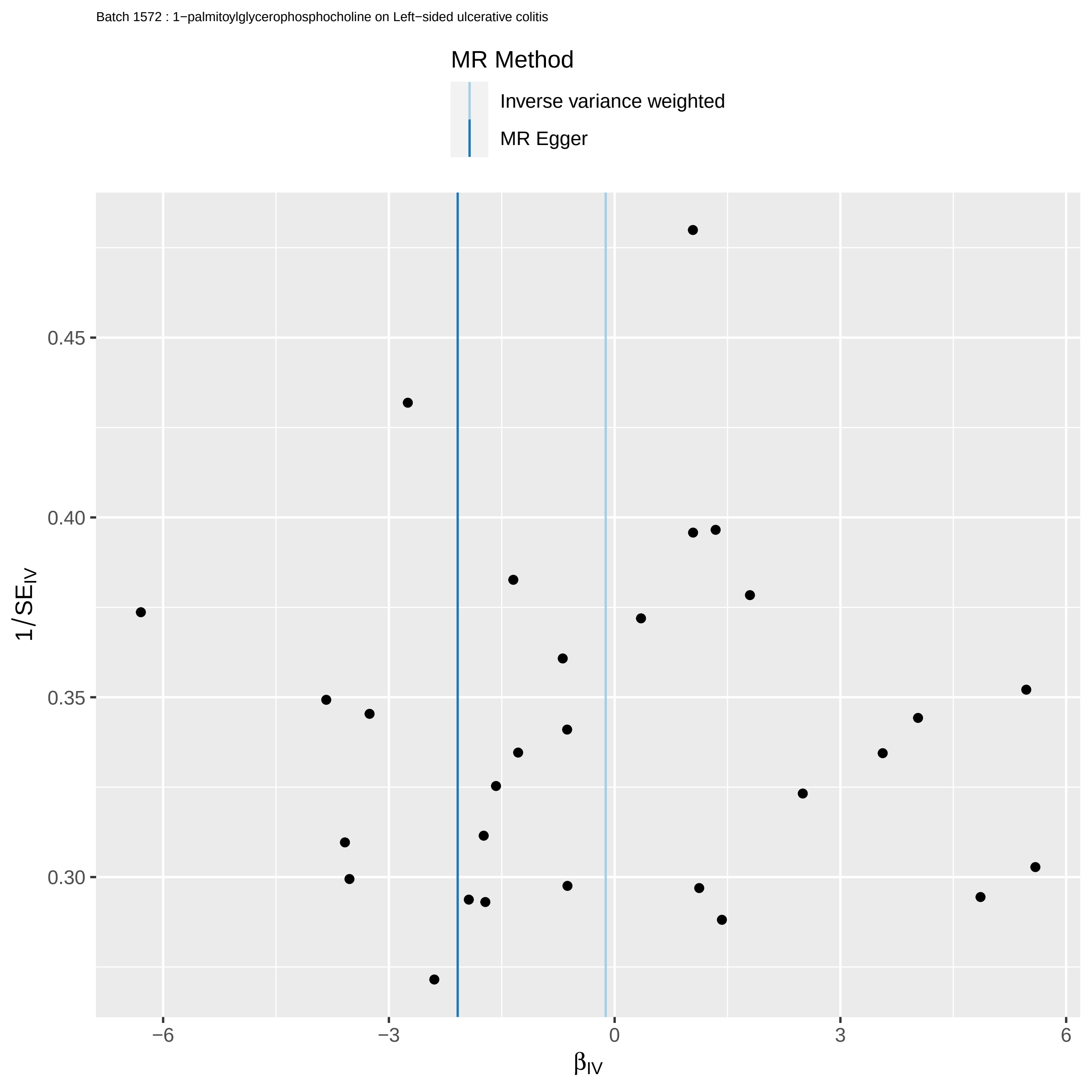

Batch 1572 : 1−palmitoylglycerophosphocholine on Left−sided ulcerative colitis
MR Method
Inverse variance weighted
MR Egger
0.45
0.40
0.35
0.30
1/SE_IV
−6
−3
0
3
6
β_IV

Batch 1573 : 1−heptadecanoylglycerophosphocholine on Left−sided ulcerative colitis

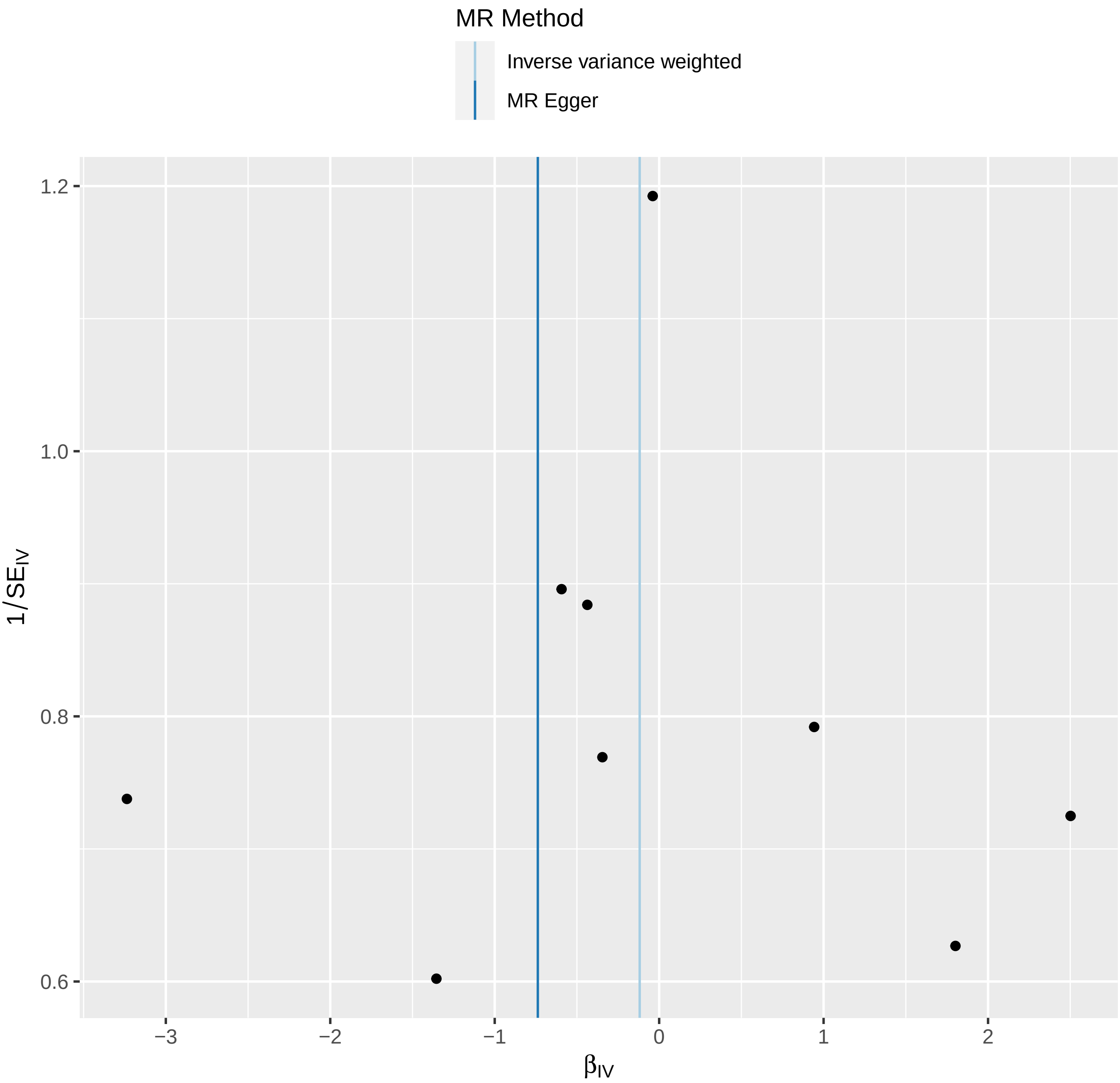

Batch 1574 : 1−oleoylglycerophosphocholine on Left−sided ulcerative colitis

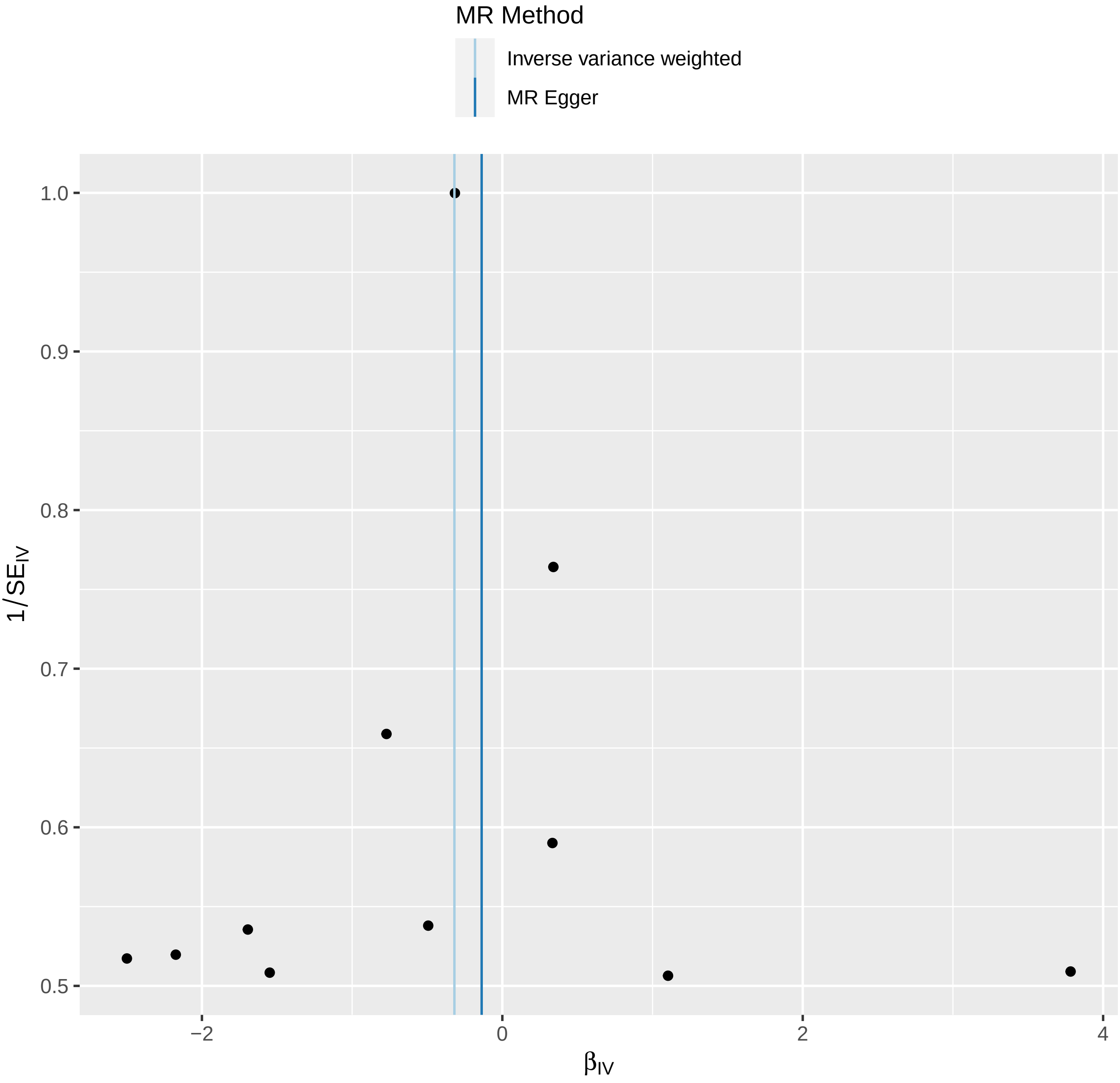

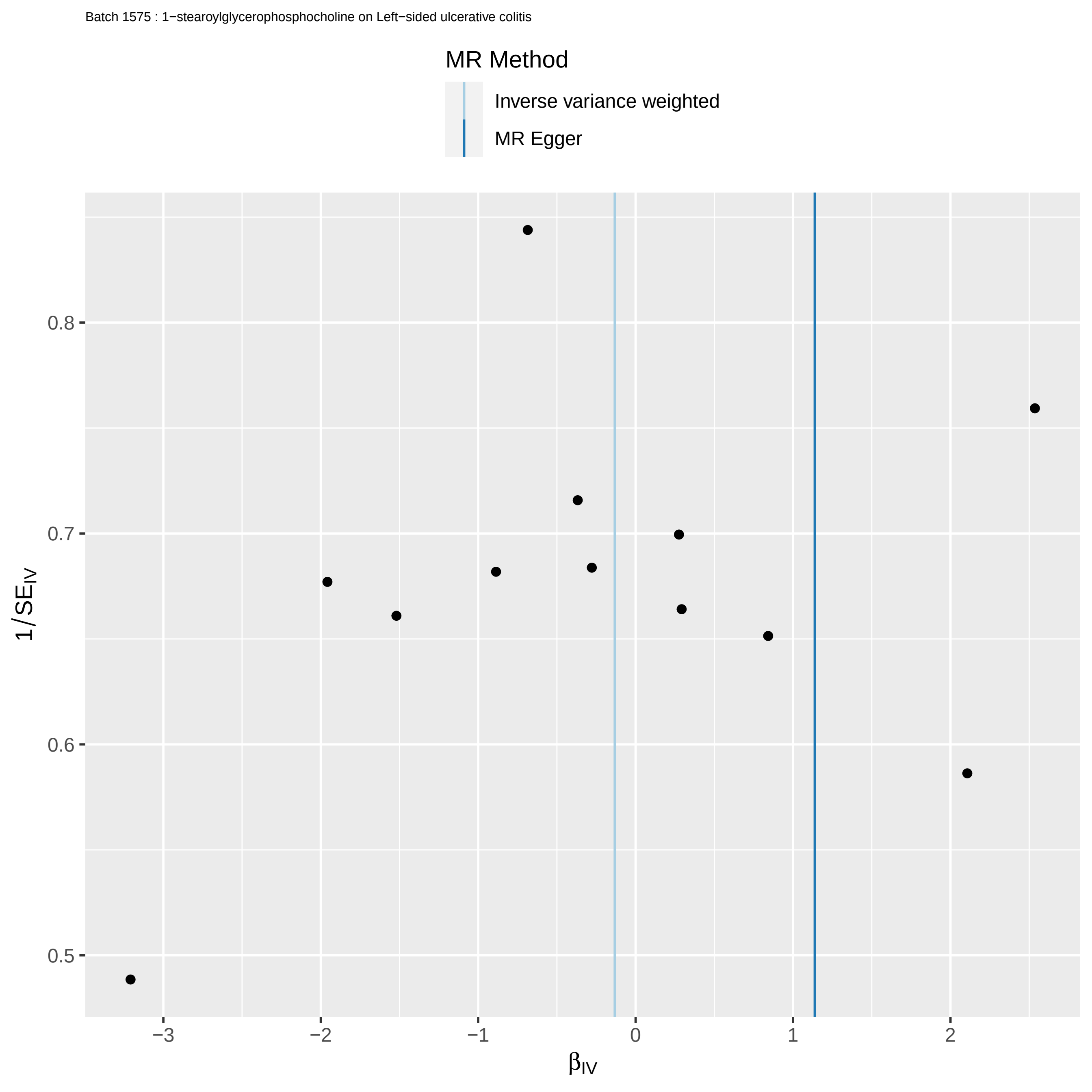
Batch 1575 : 1−stearoylglycerophosphocholine on Left−sided ulcerative colitis
MR Method
Inverse variance weighted
MR Egger
0.8
0.7
0.6
0.5
1/SE_IV
−3
−2
−1
0
1
2
β_IV

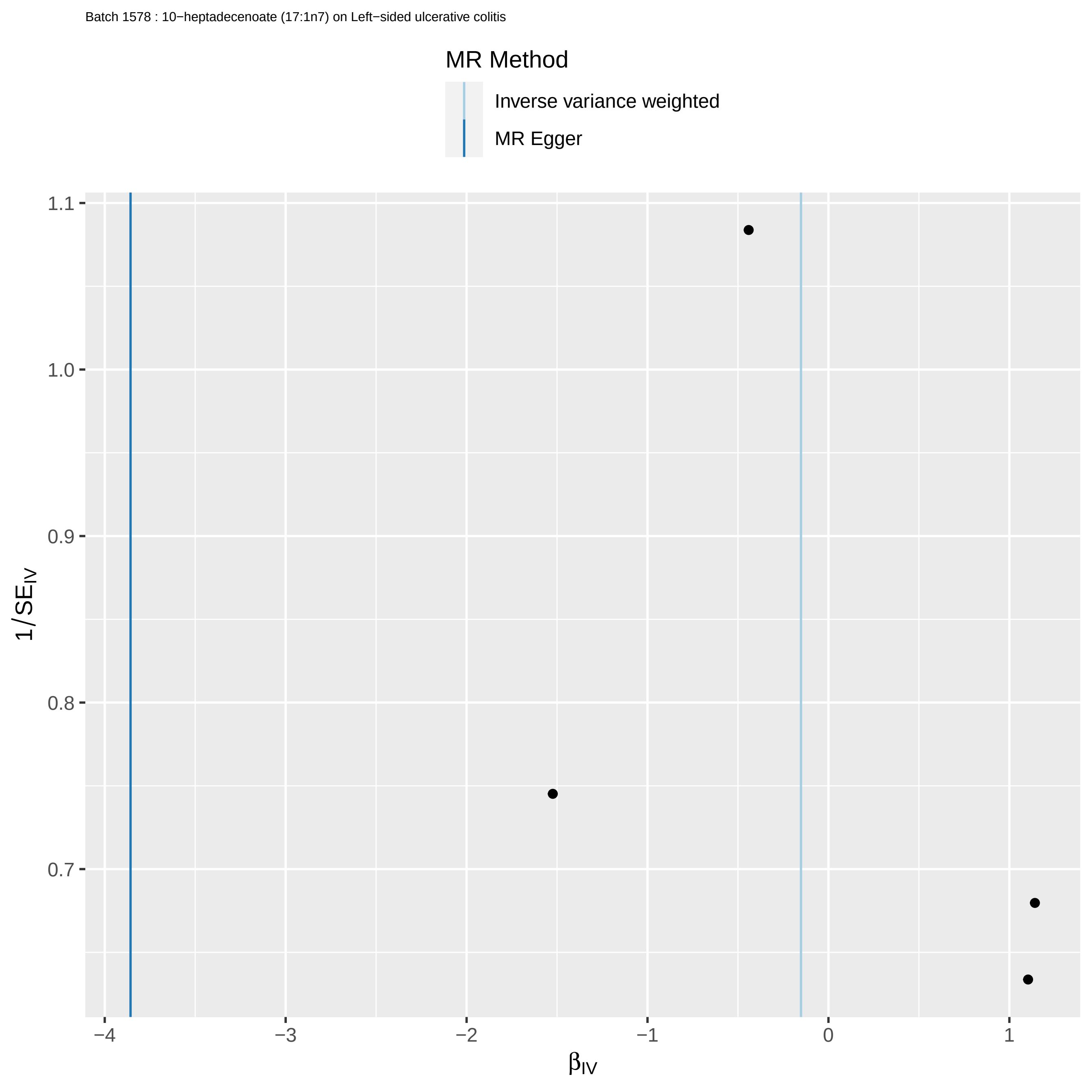
Batch 1578 : 10−heptadecenoate (17:1n7) on Left−sided ulcerative colitis
MR Method
Inverse variance weighted
MR Egger
1.1
1.0
0.9
0.8
0.7
$1/SE_{IV}$
−4
−3
−2
−1
0
1
β_{IV}

Batch 1580 : Epiandrosterone sulfate on Left−sided ulcerative colitis

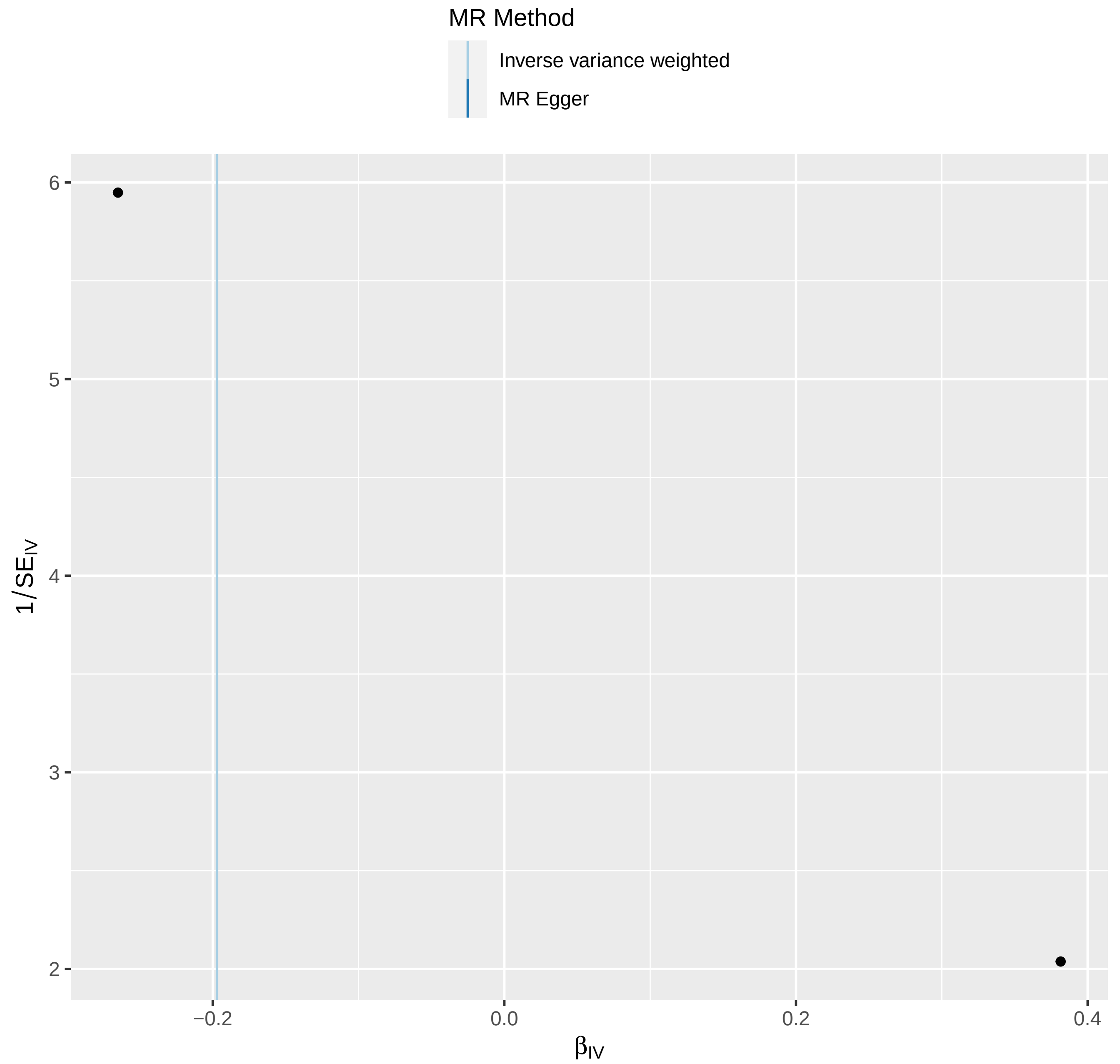

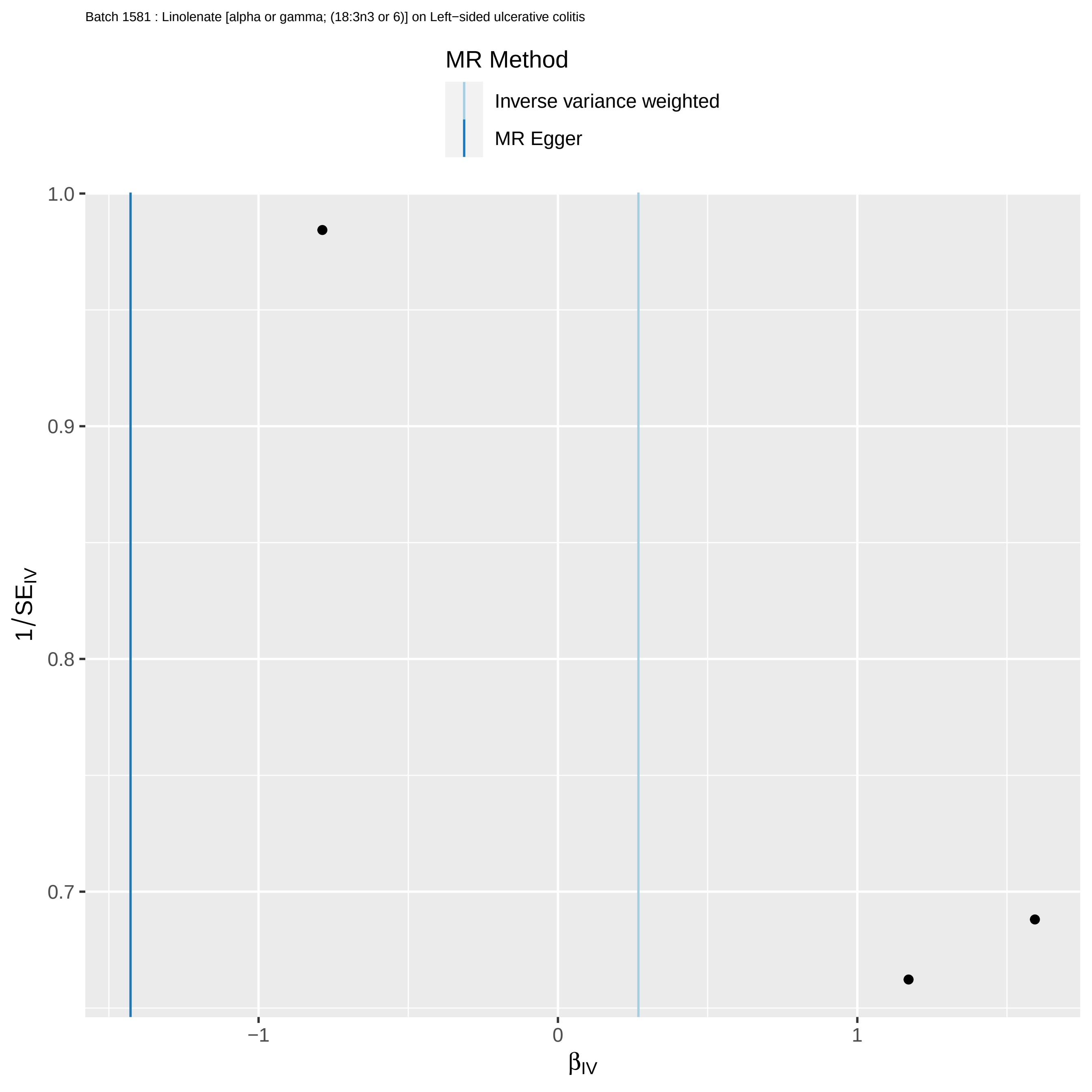
Batch 1581 : Linolenate [alpha or gamma; (18:3n3 or 6)] on Left−sided ulcerative colitis
MR Method
Inverse variance weighted
MR Egger
1.0
0.9
0.8
0.7
$1/SE_{IV}$
−1
0
1
β_{IV}

Batch 1582 : X−12510−−2−aminooctanoic acid on Left−sided ulcerative colitis

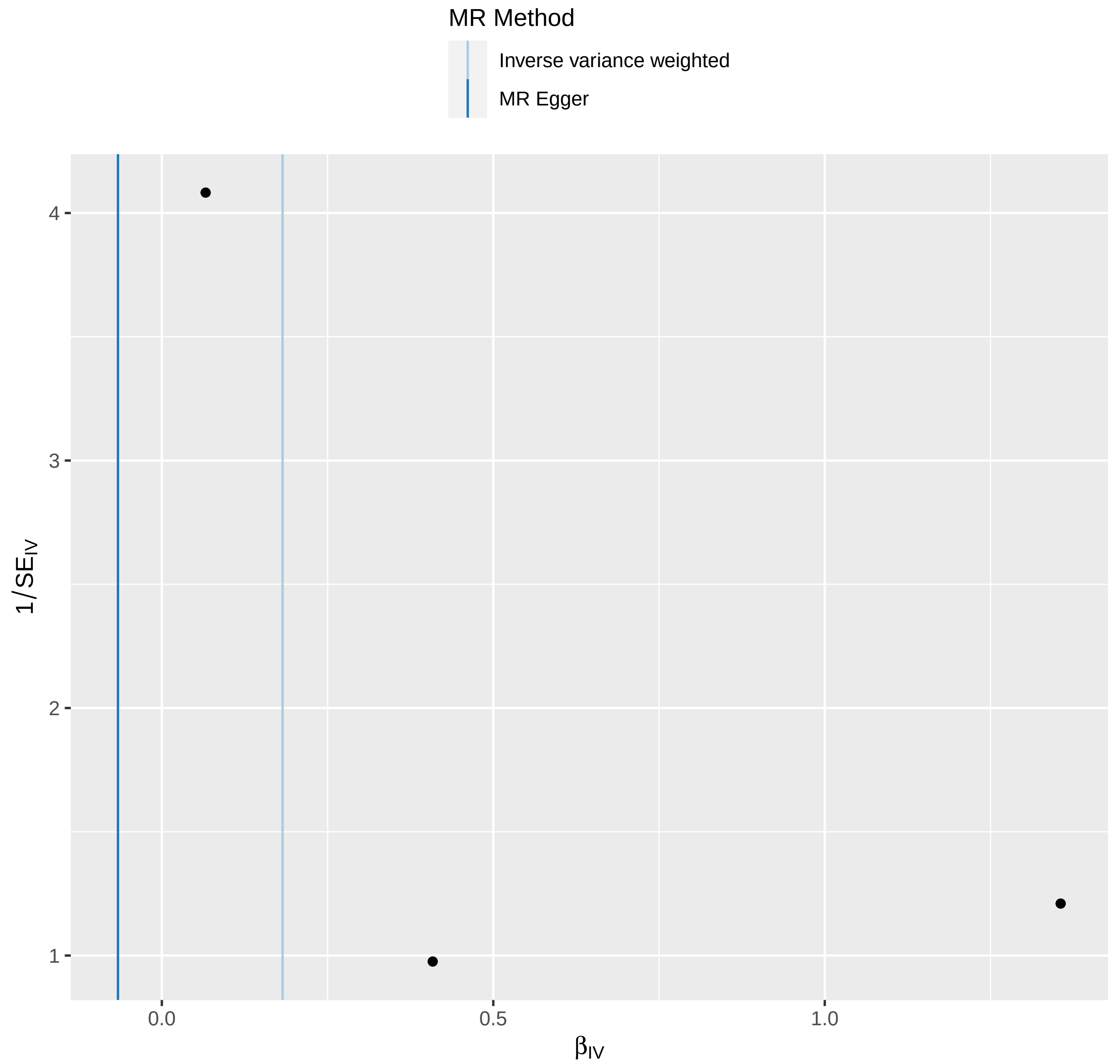

Batch 1585 : Asparagine on Left−sided ulcerative colitis

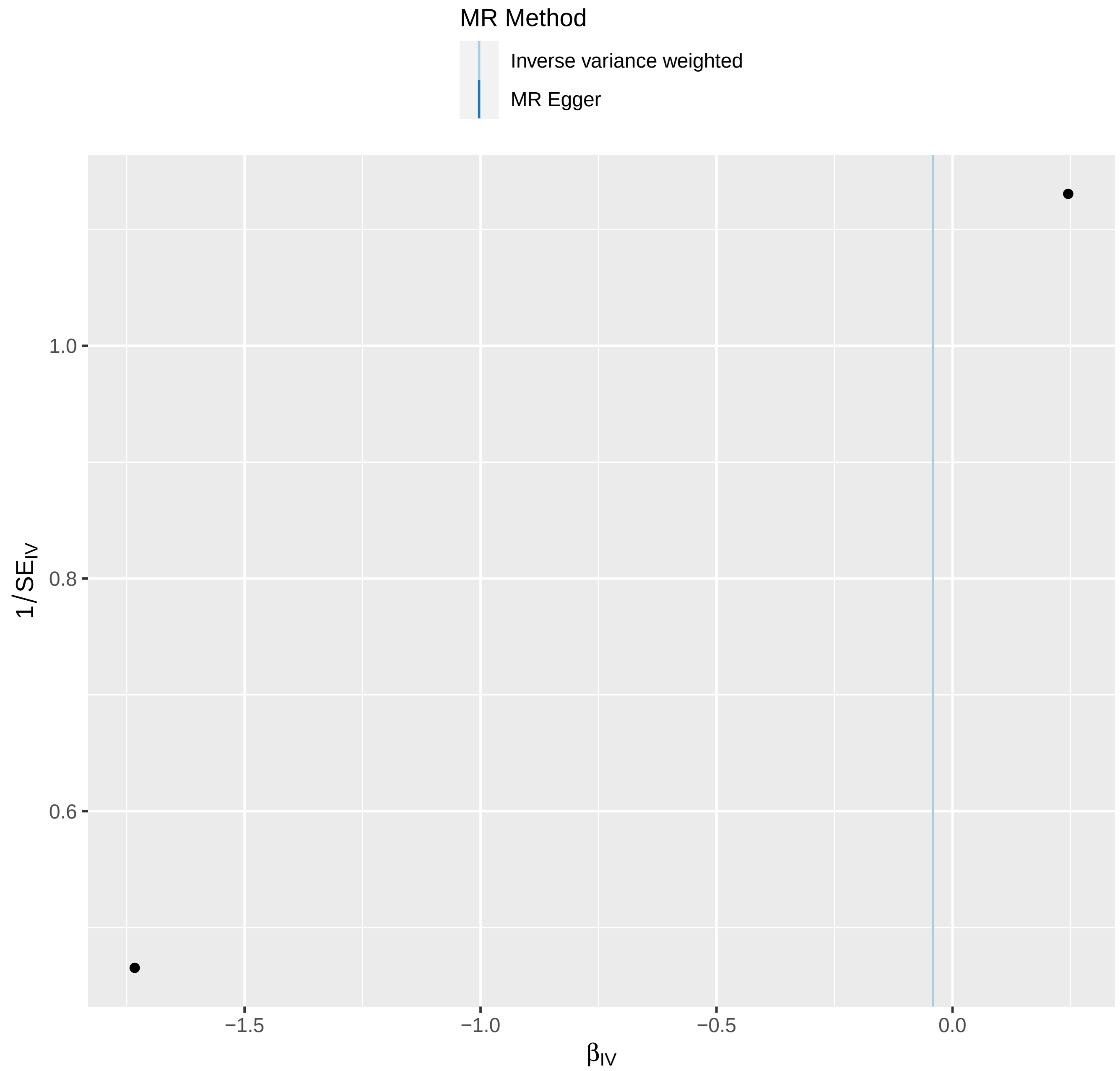

Batch 1586 : Stachydrine on Left−sided ulcerative colitis

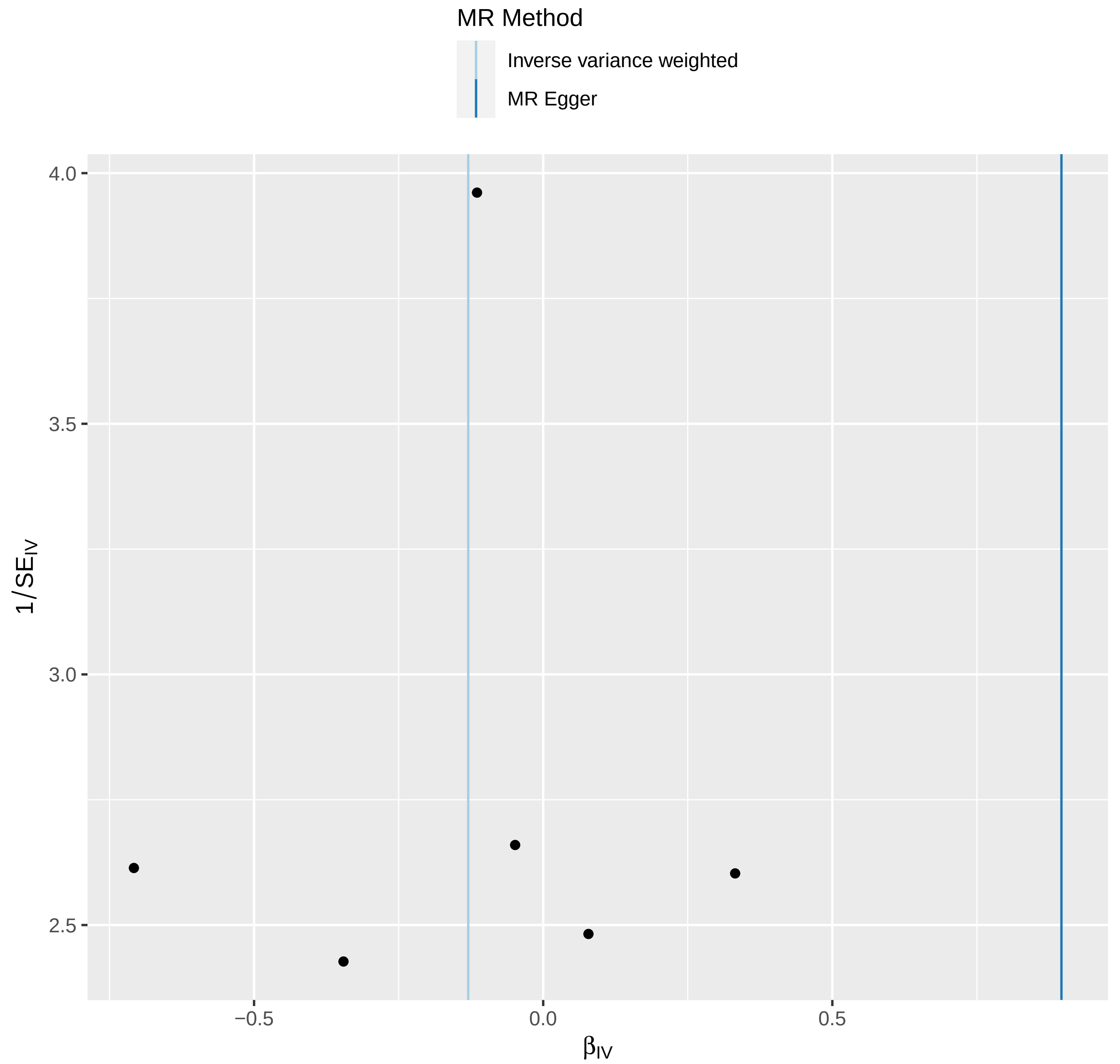

Batch 1587 : Isovalerylcarnitine on Left−sided ulcerative colitis

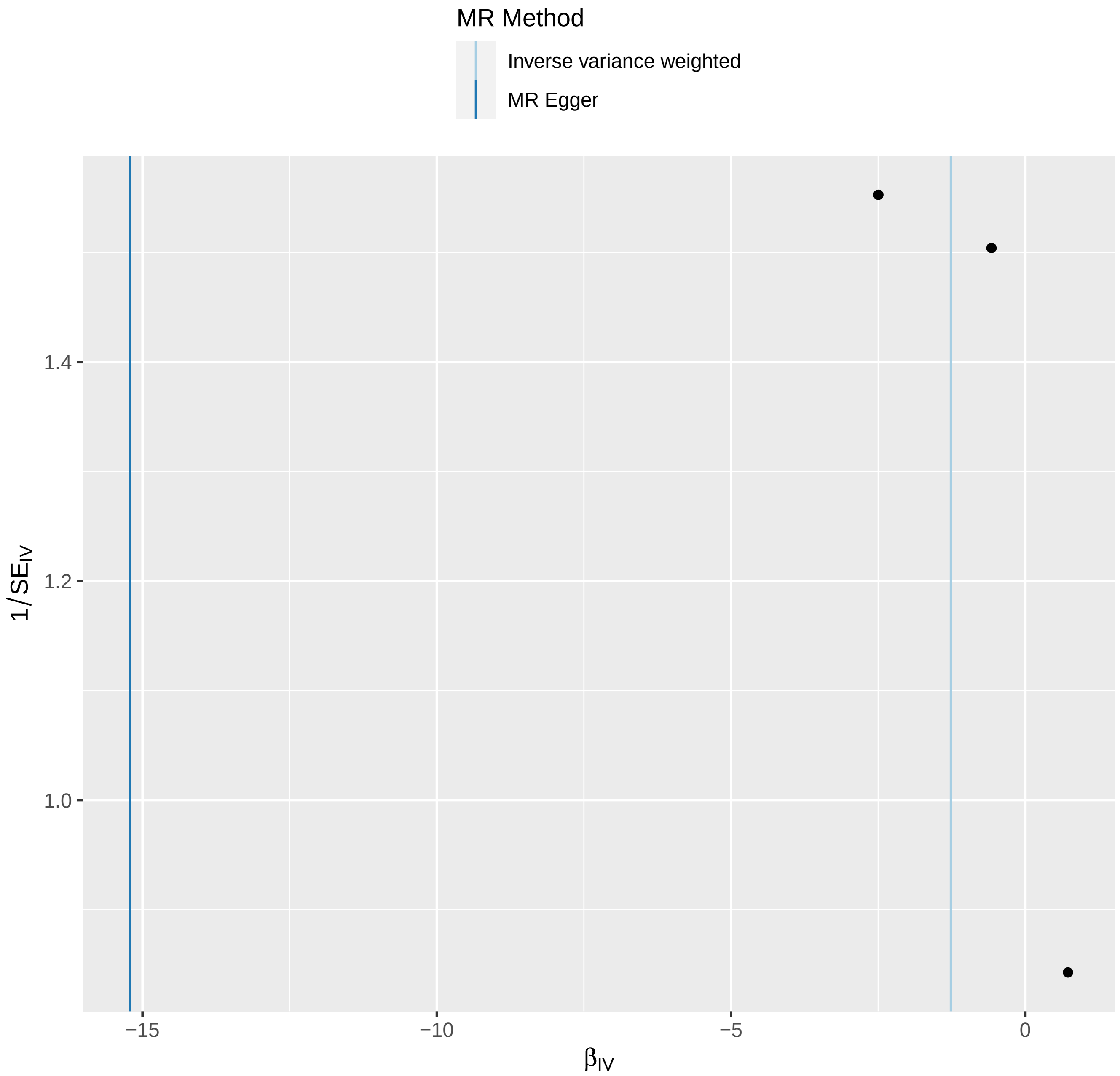

Batch 1590 : 1−linoleoylglycerophosphocholine on Left−sided ulcerative colitis

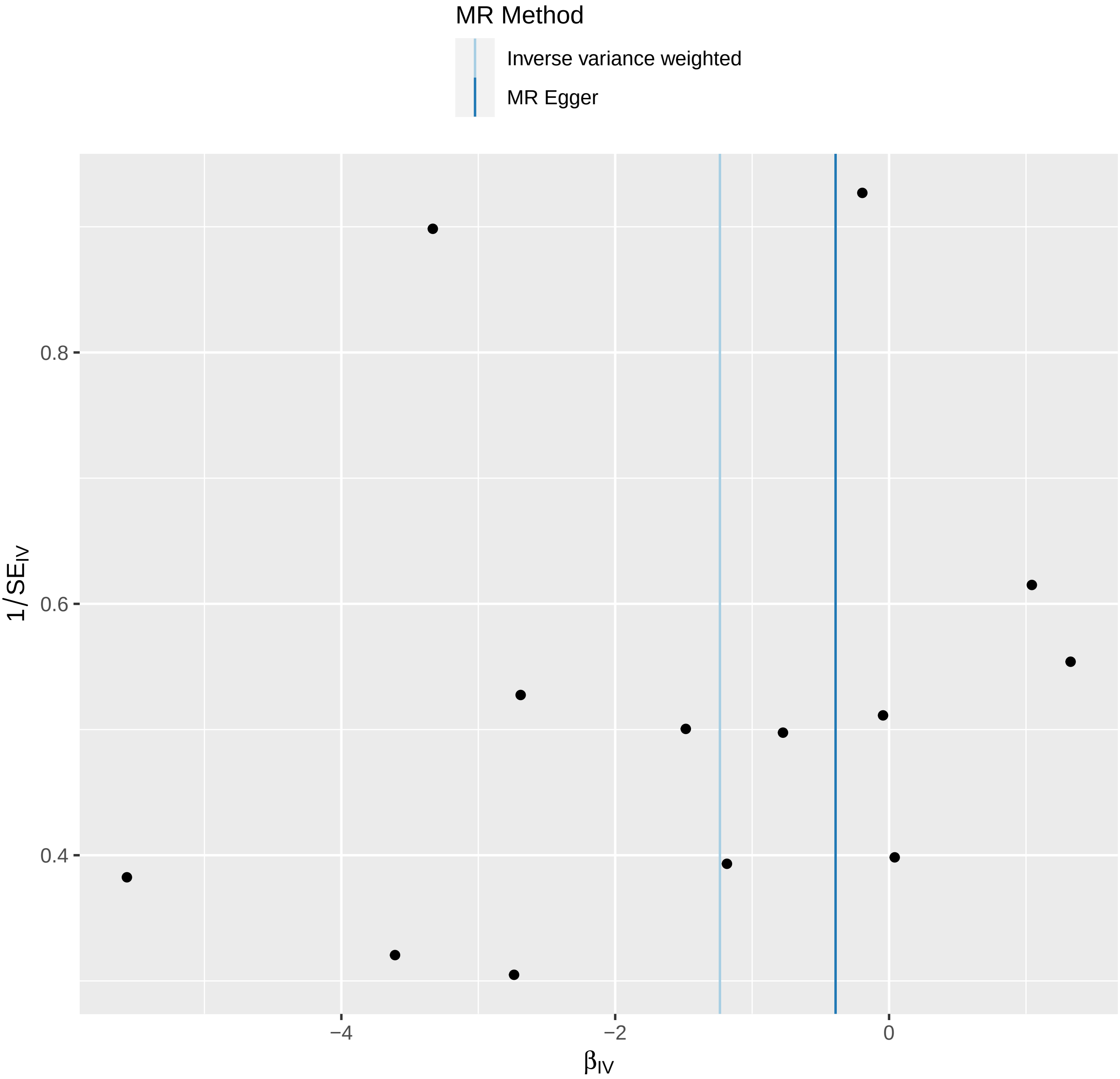

Batch 1591 : Bradykinin, des−arg(9) on Left−sided ulcerative colitis

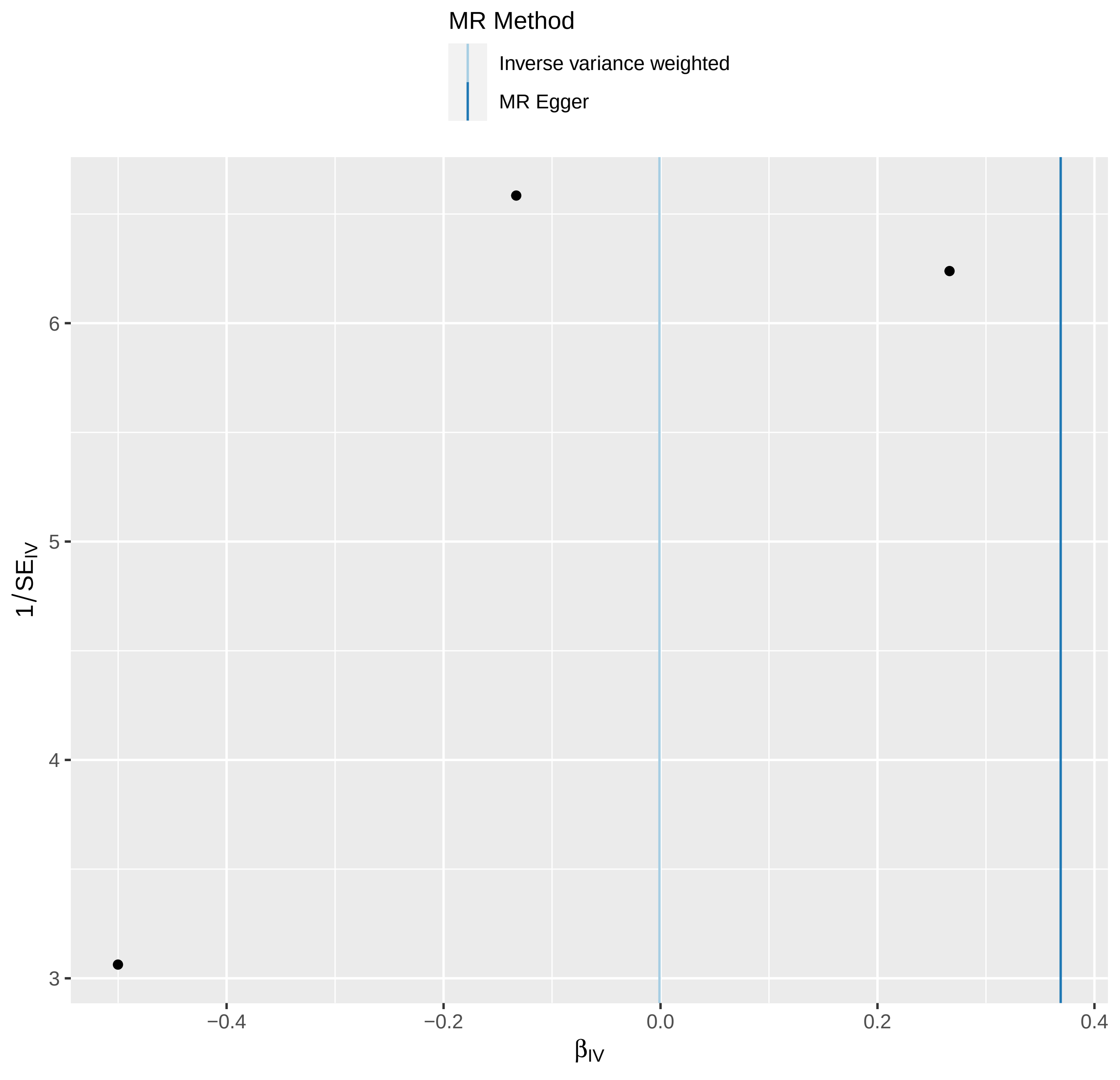

Batch 1592 : Gamma−glutamylisoleucine* on Left−sided ulcerative colitis

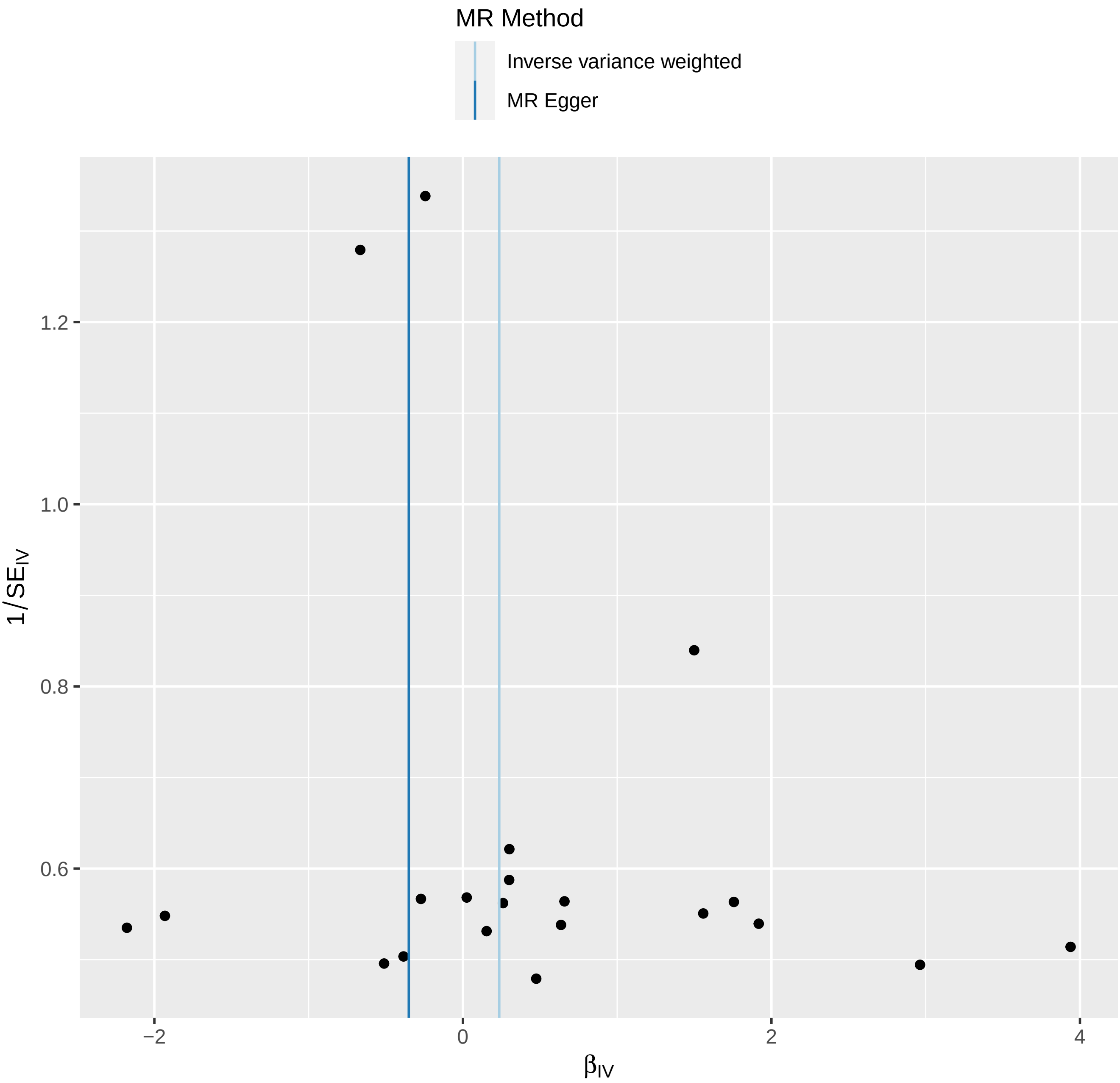

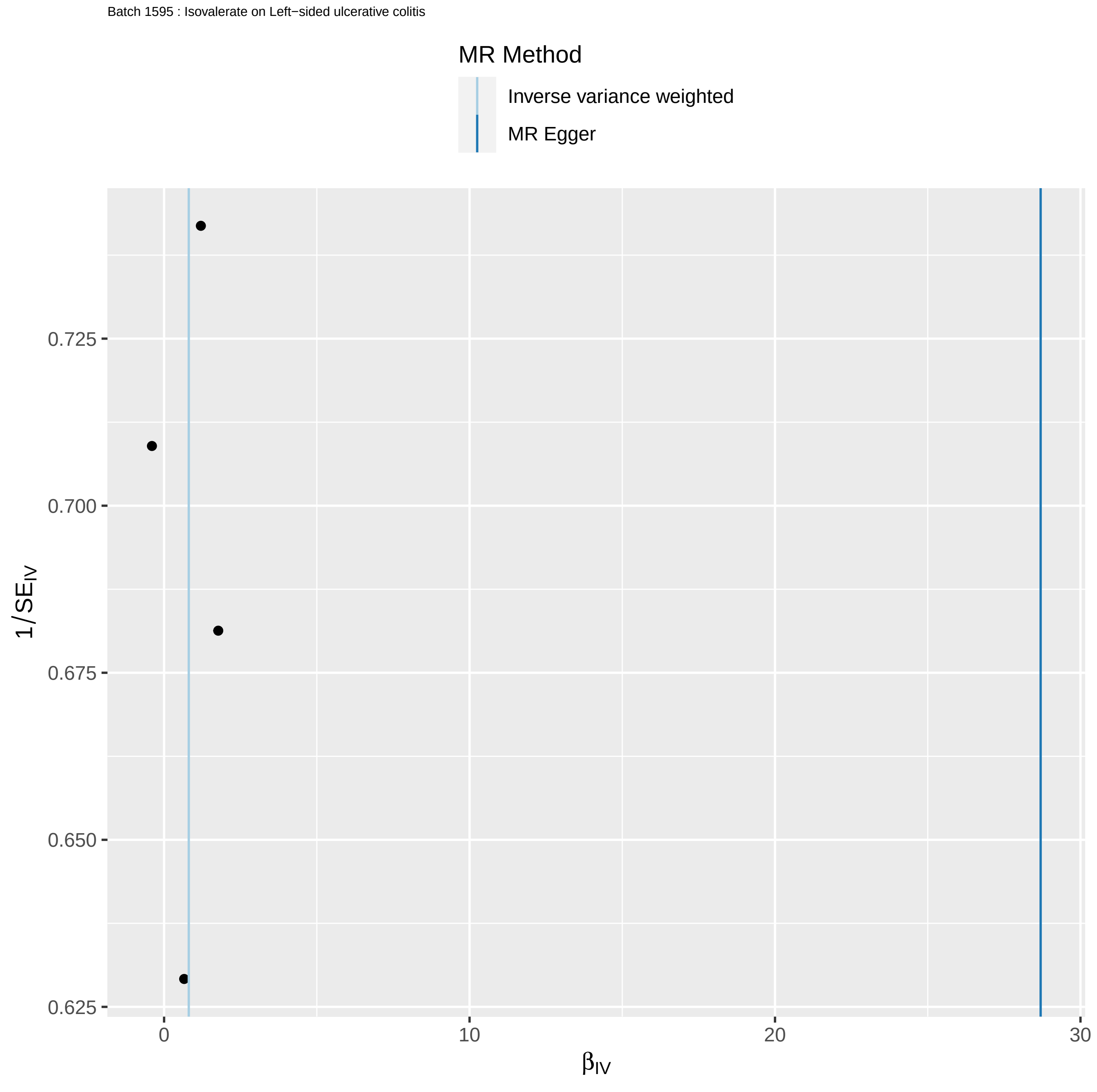
Batch 1595 : Isovalerate on Left−sided ulcerative colitis
MR Method
Inverse variance weighted
MR Egger
0.725
0.700
0.675
0.650
0.625
$1/SE_{IV}$
0
10
20
30
β_{IV}

Batch 1596 : X−13183−−stearamide on Left−sided ulcerative colitis

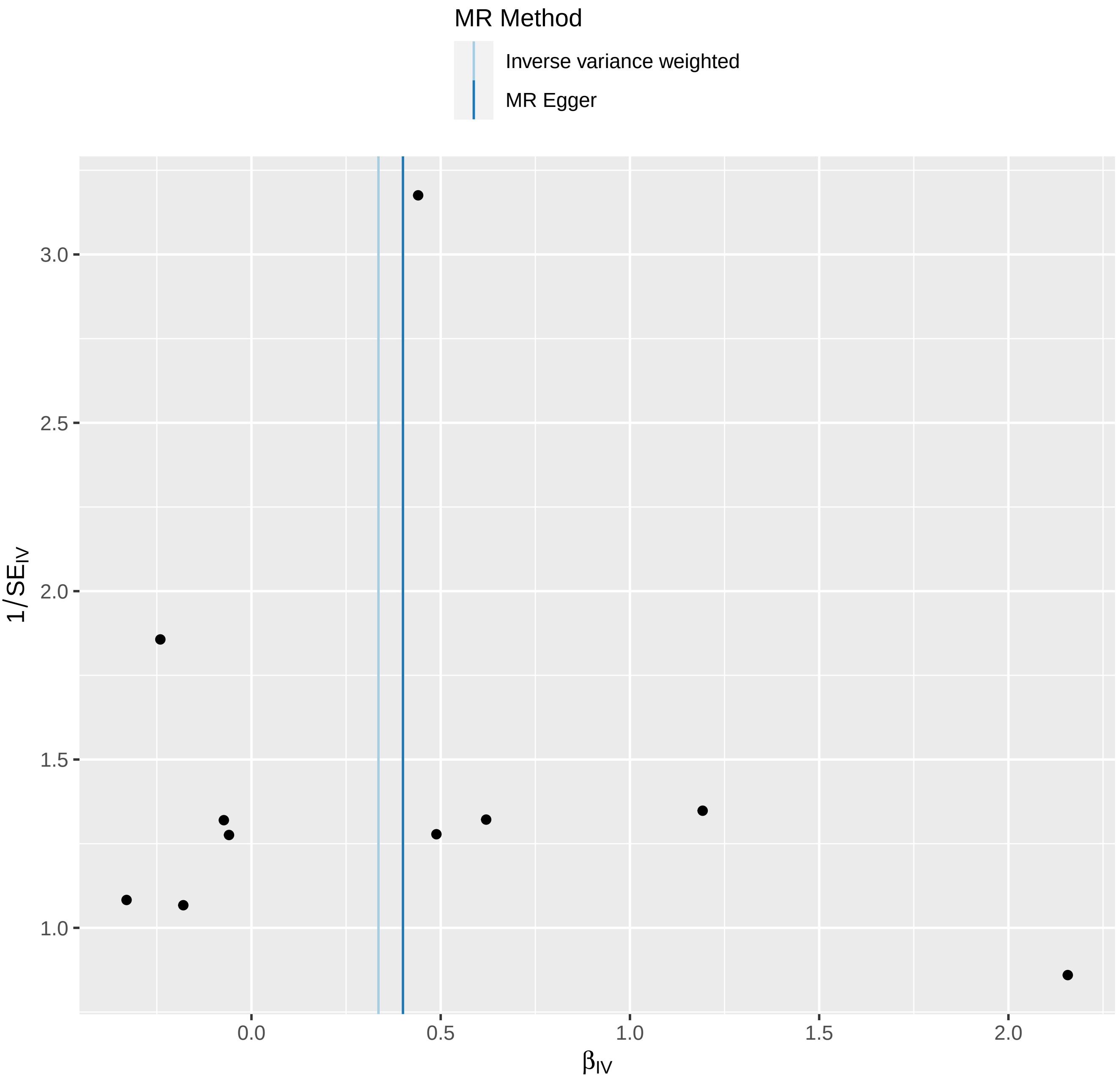

Batch 1597 : 7−methylguanine on Left−sided ulcerative colitis

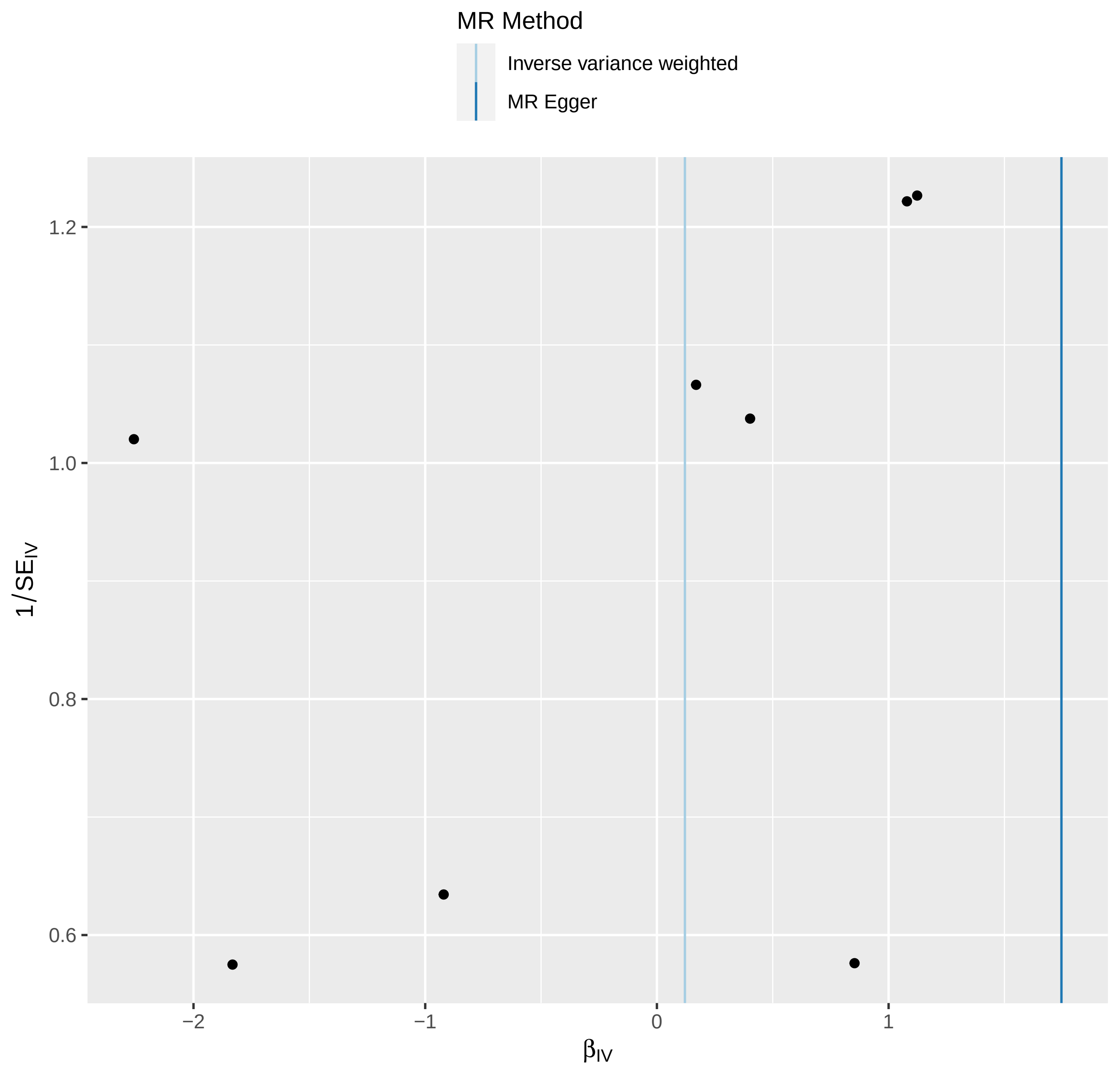

Batch 1604 : X−13431−−nonanoylcarnitine* on Left−sided ulcerative colitis

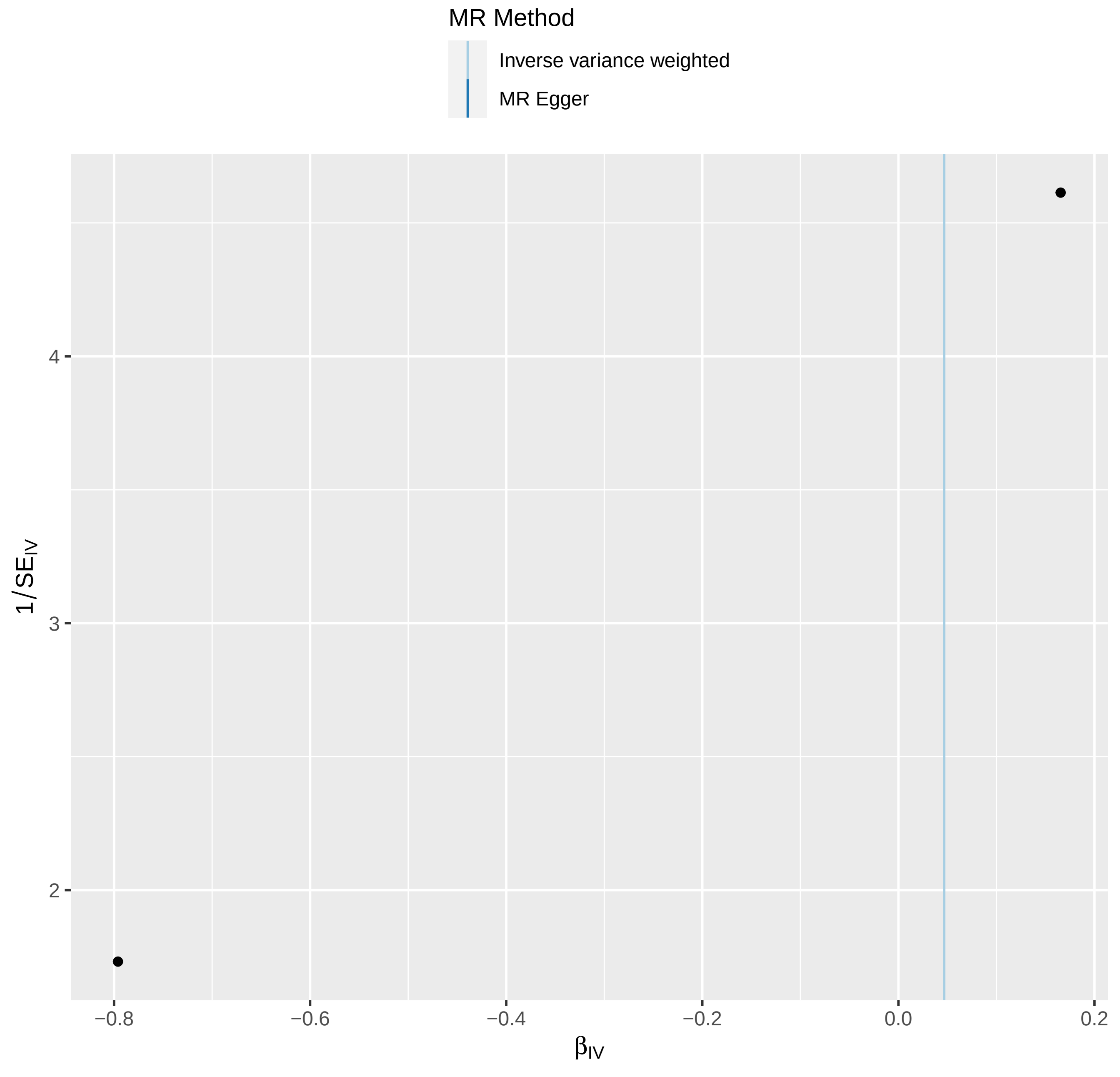

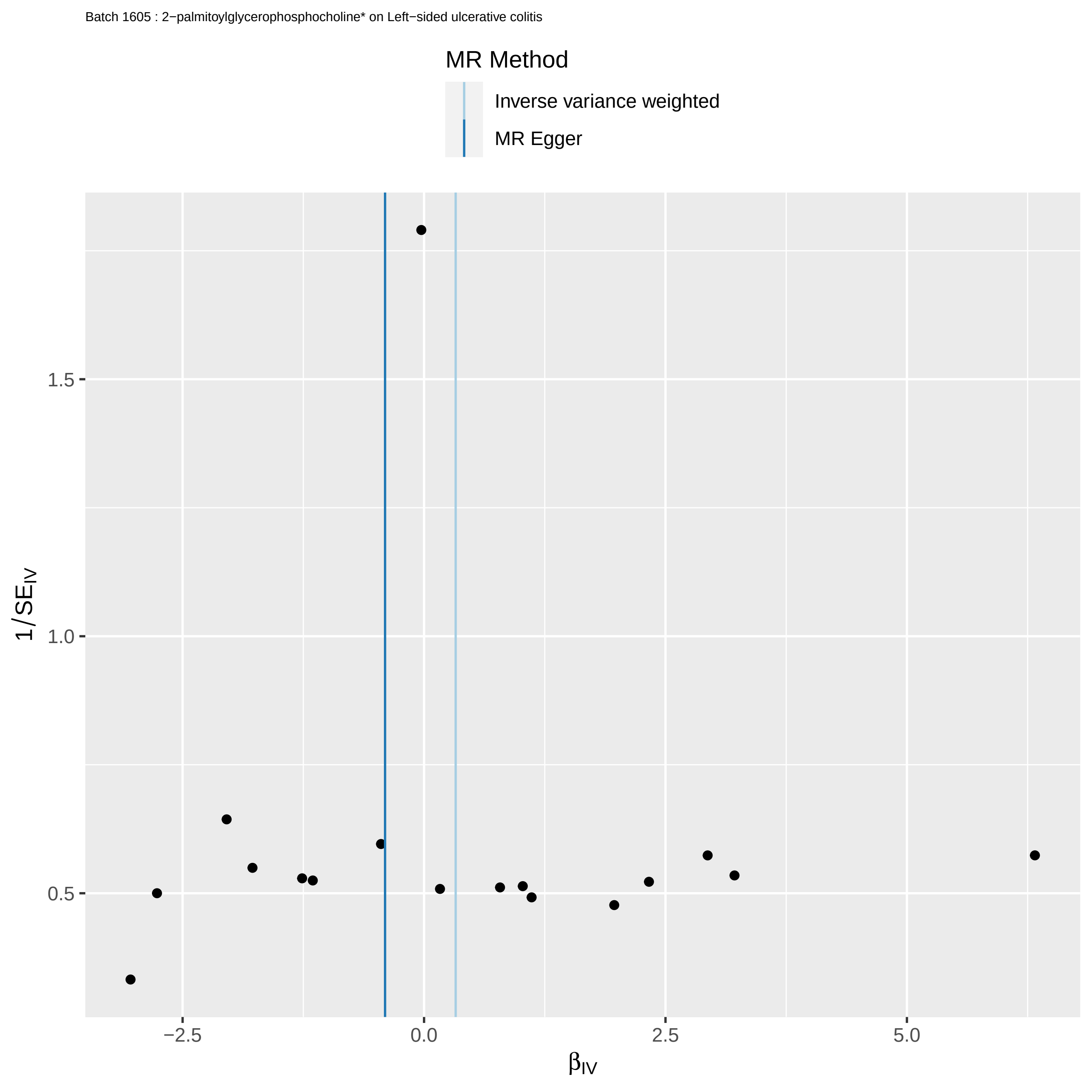

Batch 1605 : 2−palmitoylglycerophosphocholine* on Left−sided ulcerative colitis
MR Method
Inverse variance weighted
MR Egger
1.5
1.0
0.5
1/SE_IV
−2.5
0.0
2.5
5.0
β_IV

Batch 1606 : 2−oleoylglycerophosphocholine* on Left−sided ulcerative colitis

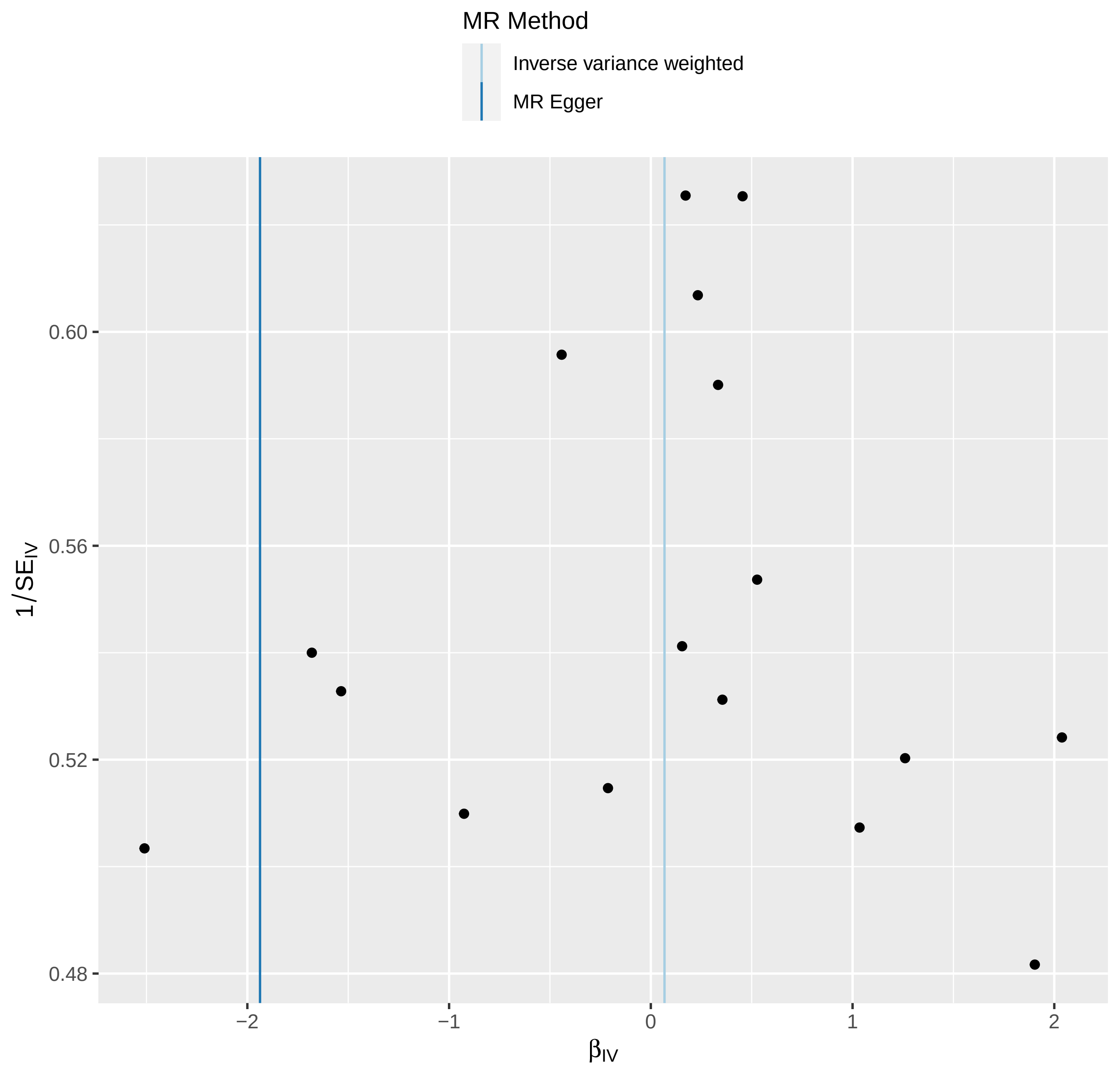

Batch 1609 : 1−palmitoylglycerophosphoinositol* on Left−sided ulcerative colitis

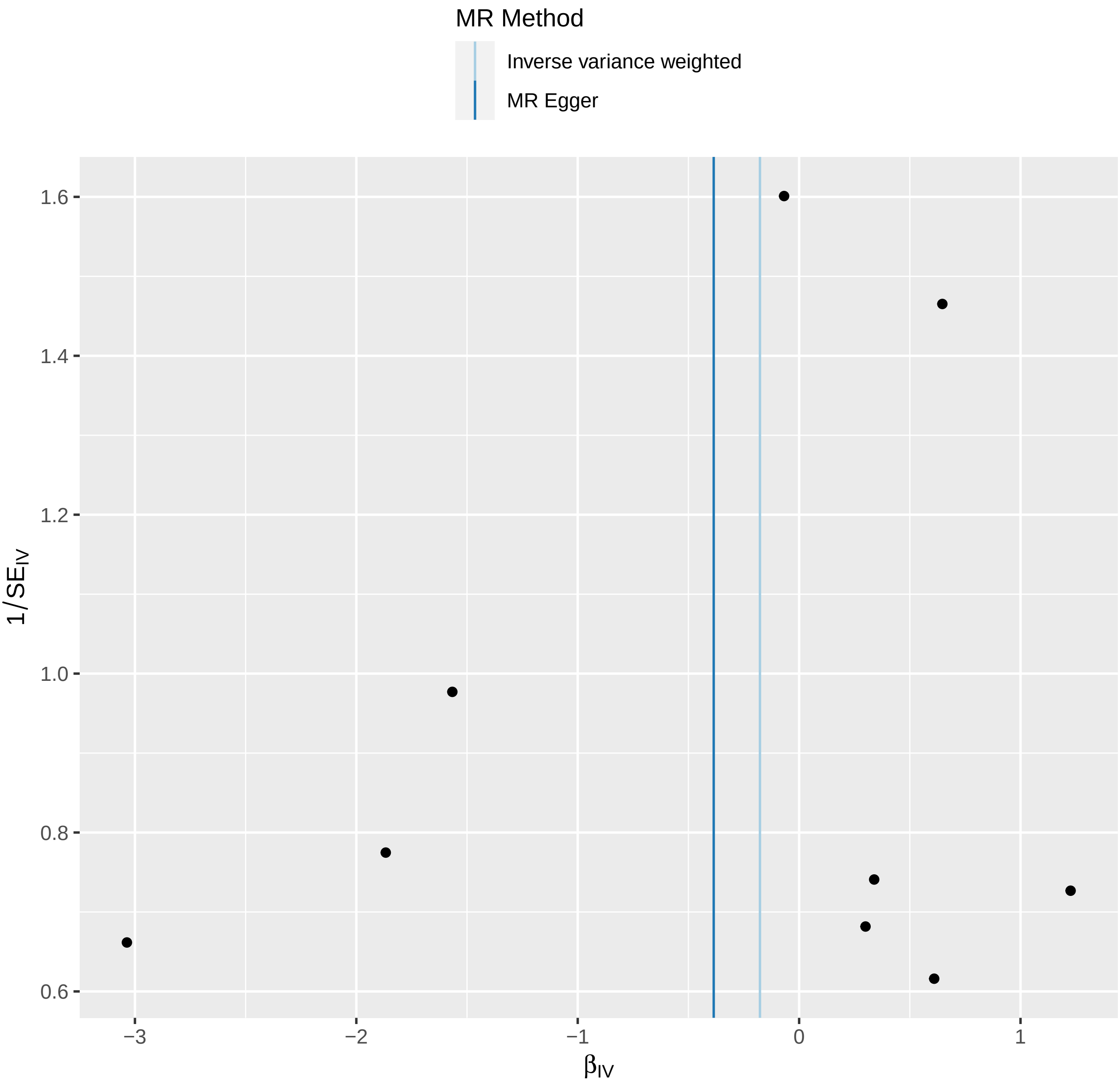

Batch 1612 : Glutaroyl carnitine on Left−sided ulcerative colitis

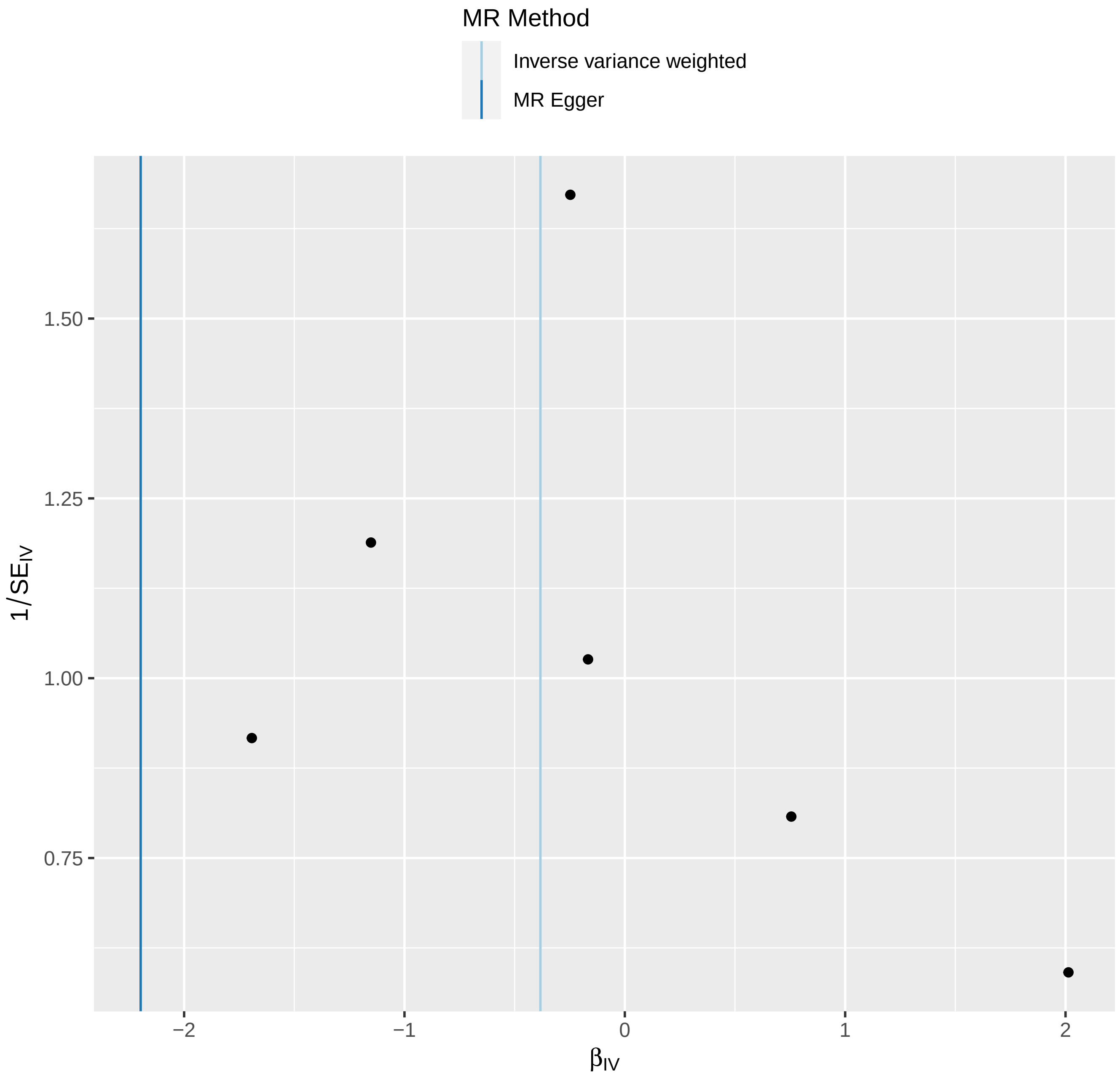

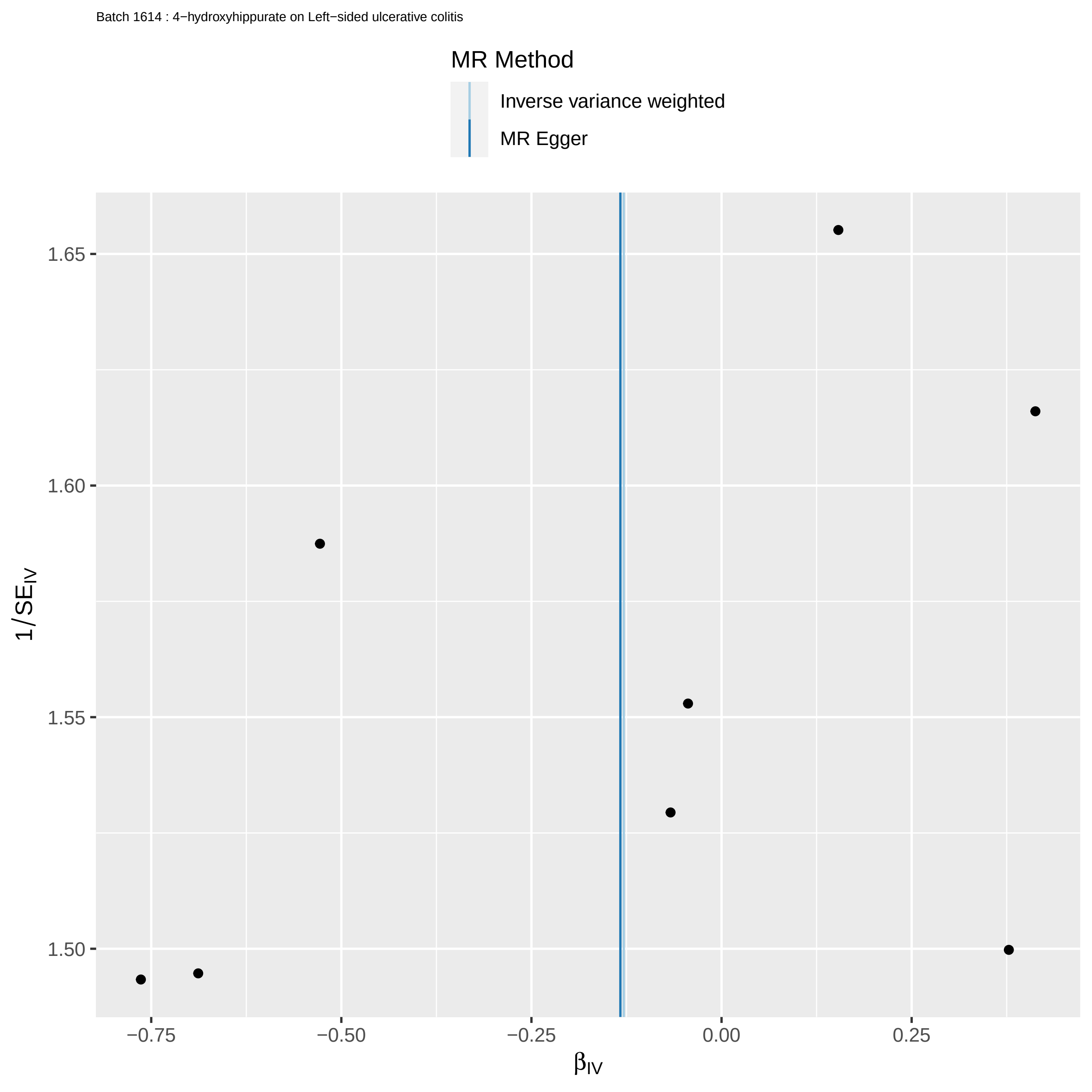

Batch 1614 : 4−hydroxyhippurate on Left−sided ulcerative colitis
MR Method
Inverse variance weighted
MR Egger
1.65
1.60
1.55
1.50
1/SE$_{IV}$
−0.75
−0.50
−0.25
0.00
0.25
β_{IV}

Batch 1615 : 1−myristoylglycerophosphocholine on Left−sided ulcerative colitis

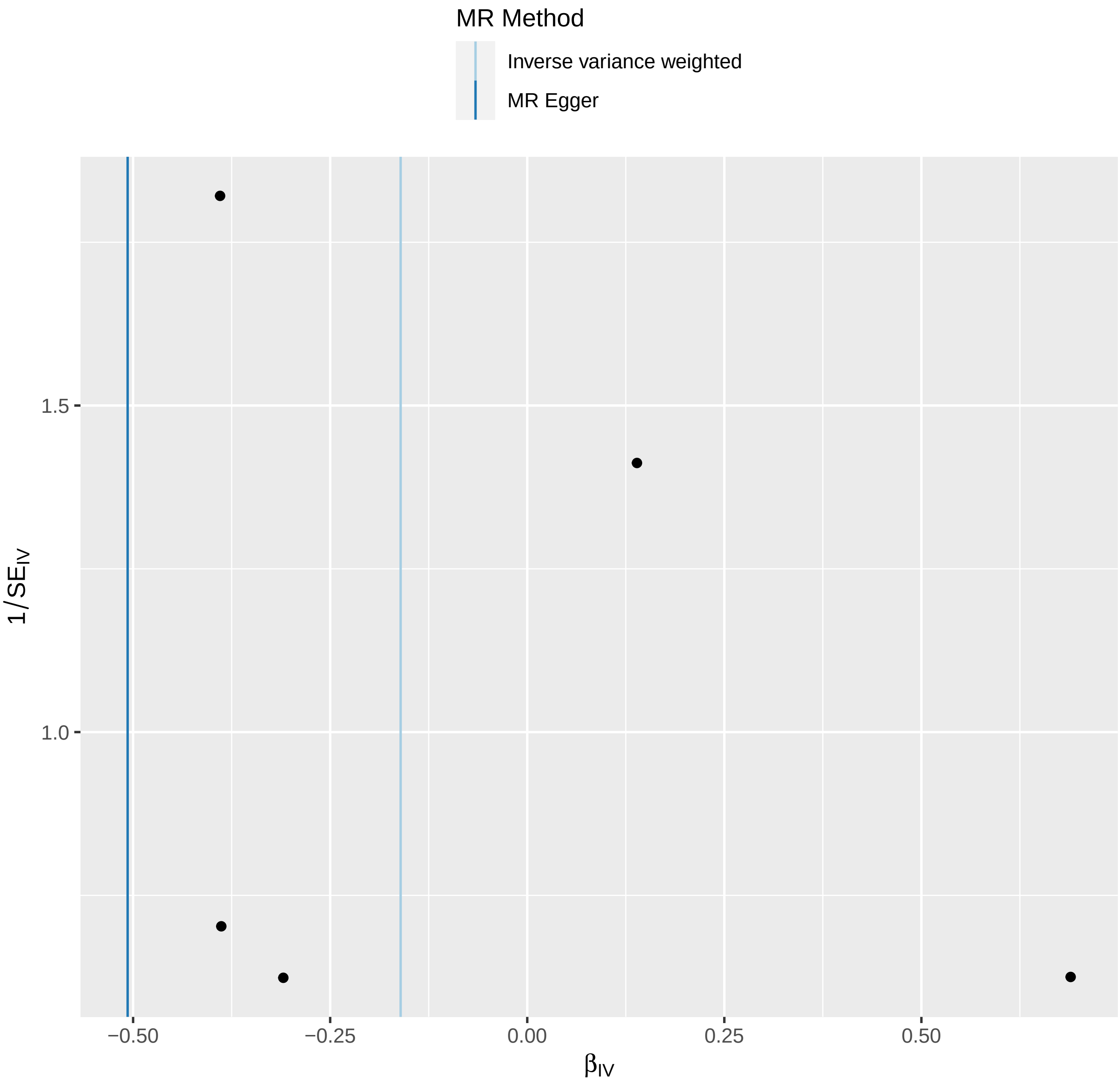

Batch 1618 : 3−(3−hydroxyphenyl)propionate on Left−sided ulcerative colitis

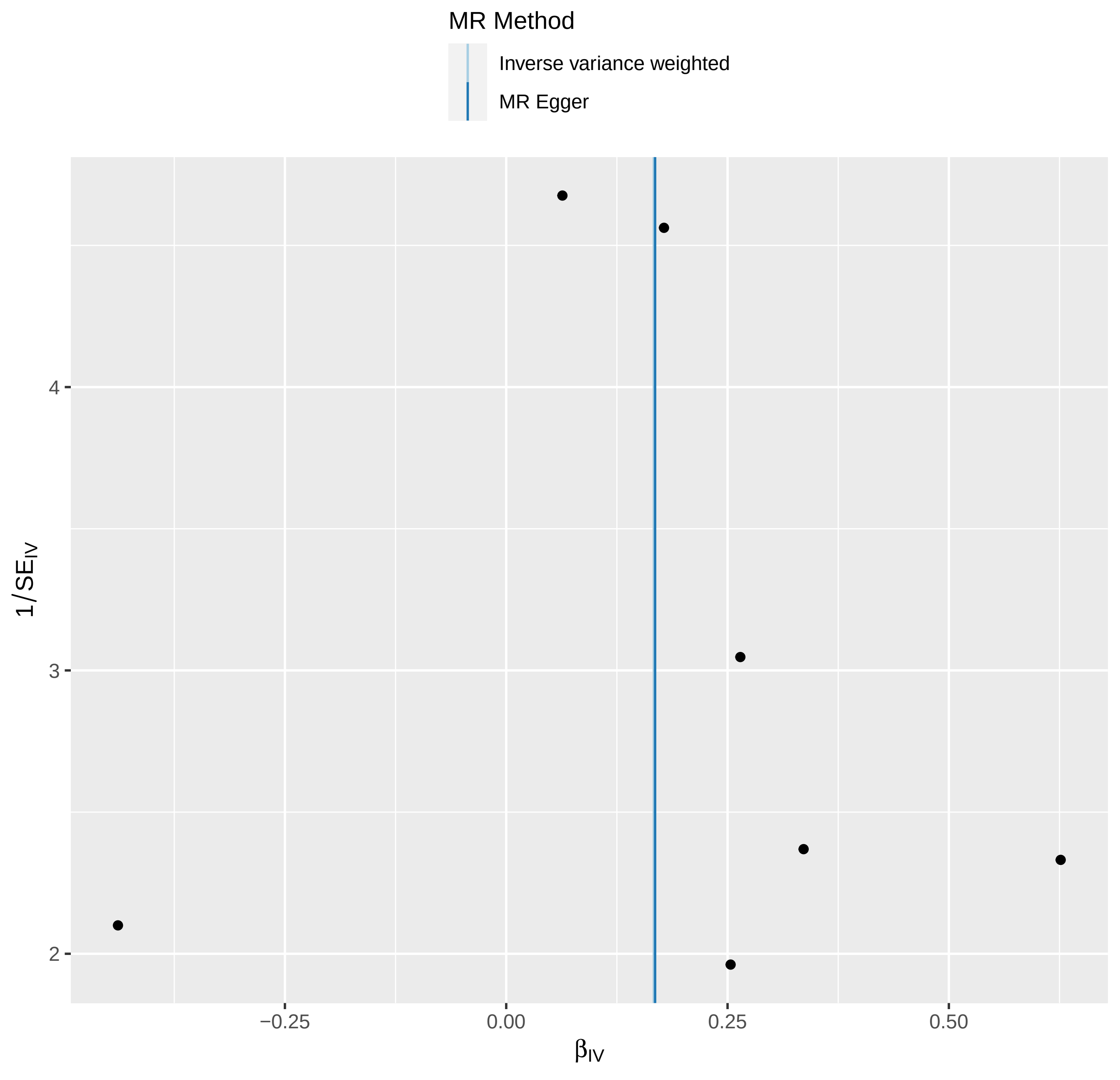

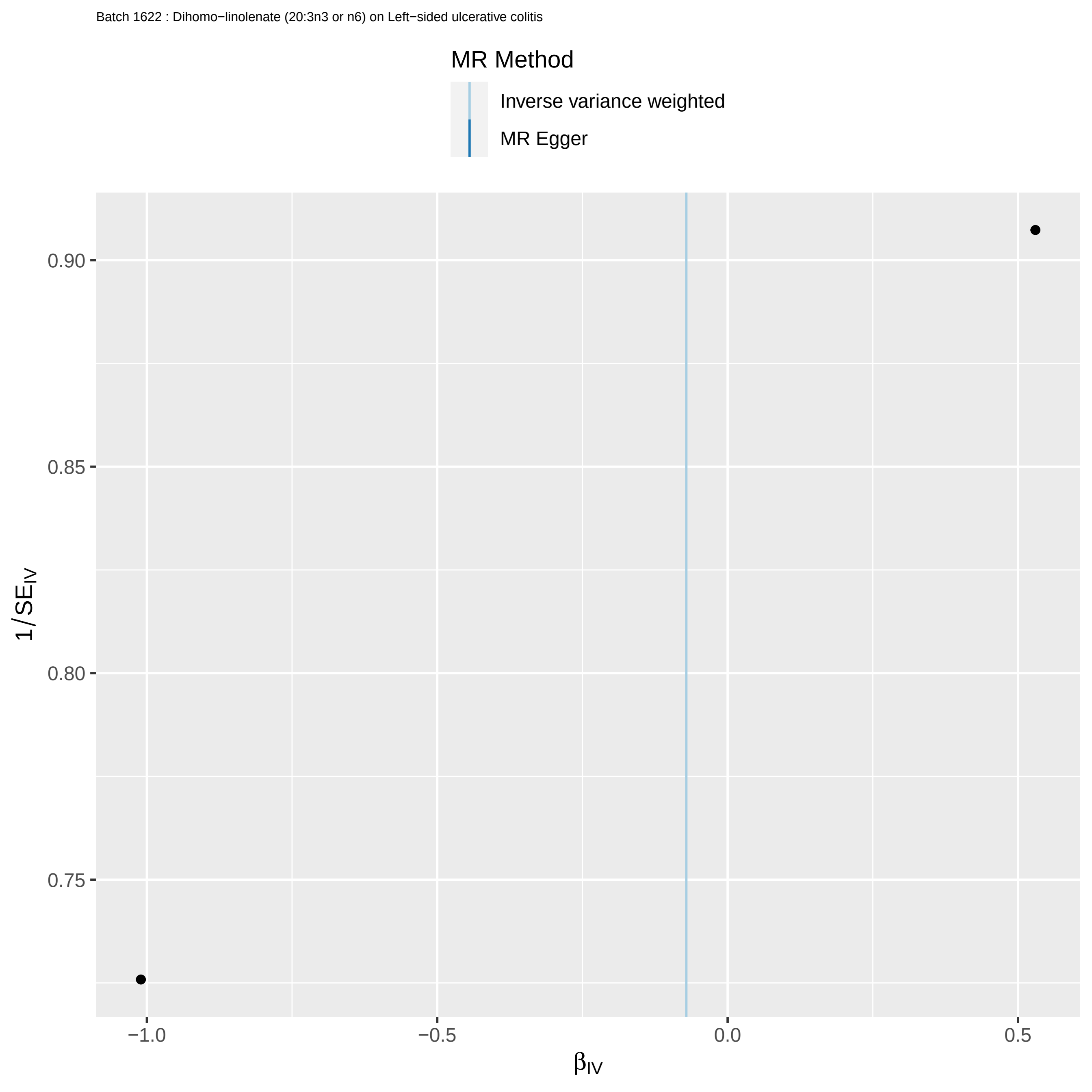

Batch 1622 : Dihomo−linolenate (20:3n3 or n6) on Left−sided ulcerative colitis
MR Method
Inverse variance weighted
MR Egger
0.90
0.85
0.80
0.75
1/SE_IV
−1.0
−0.5
0.0
0.5
β_IV

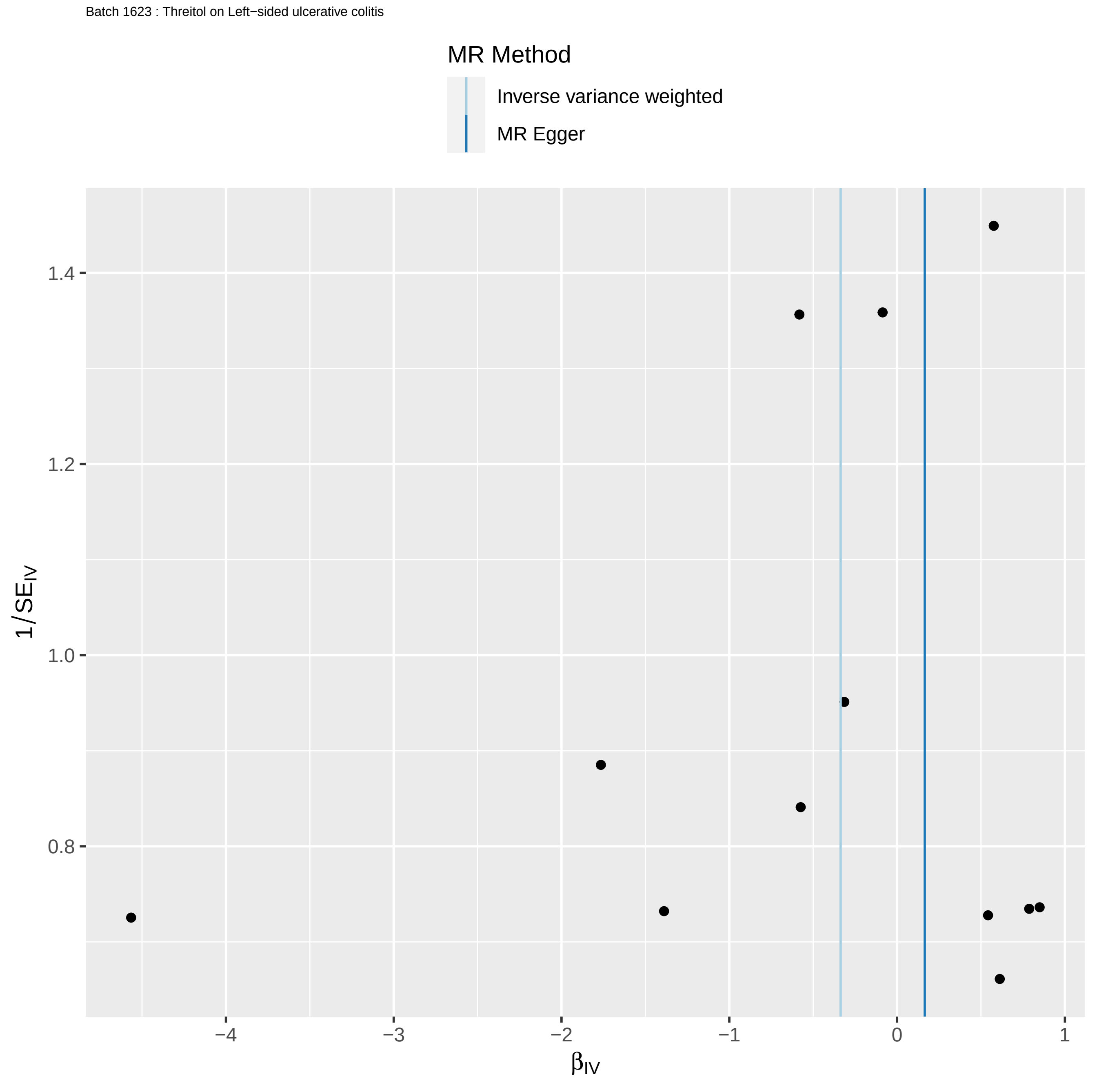

Batch 1623 : Threitol on Left−sided ulcerative colitis
MR Method
Inverse variance weighted
MR Egger
1.4
1.2
1.0
0.8
1/SE$_{IV}$
−4
−3
−2
−1
0
1
β$_{IV}$

Batch 1624 : P−cresol sulfate on Left−sided ulcerative colitis

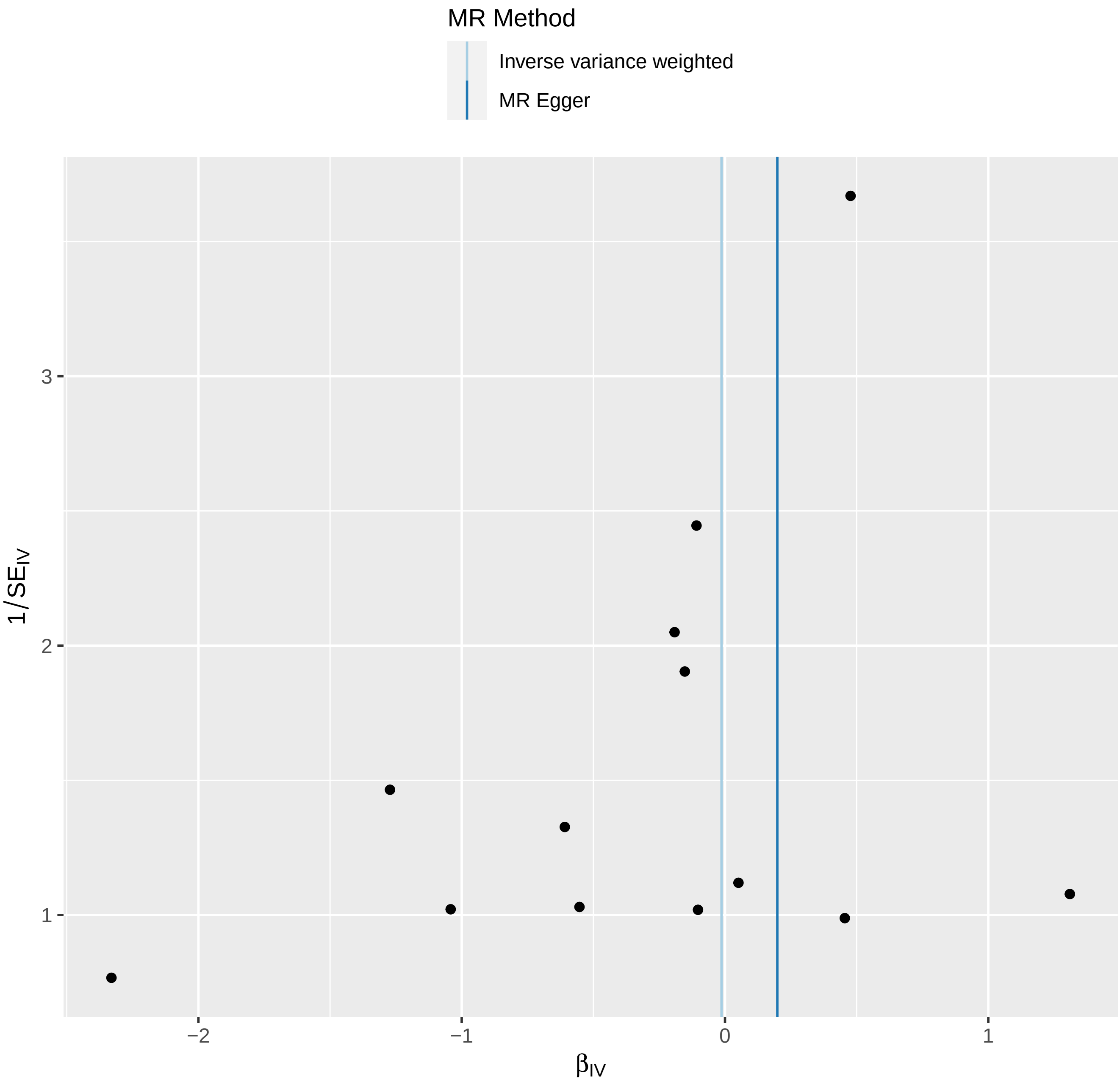

Batch 1629 : X−14450−−phenylalanylleucine on Left−sided ulcerative colitis

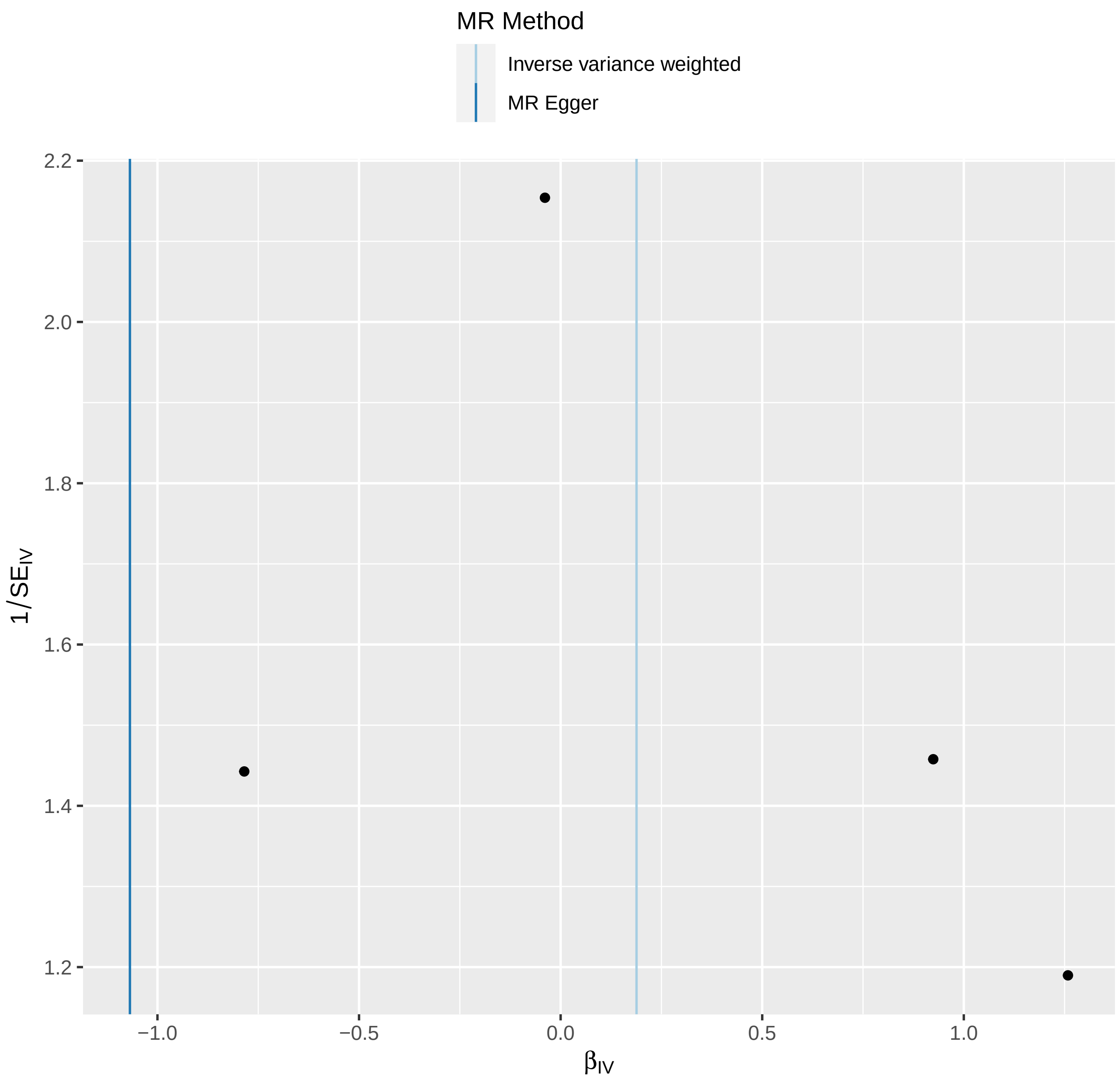

Batch 1630 : Gamma−glutamylglutamate on Left−sided ulcerative colitis

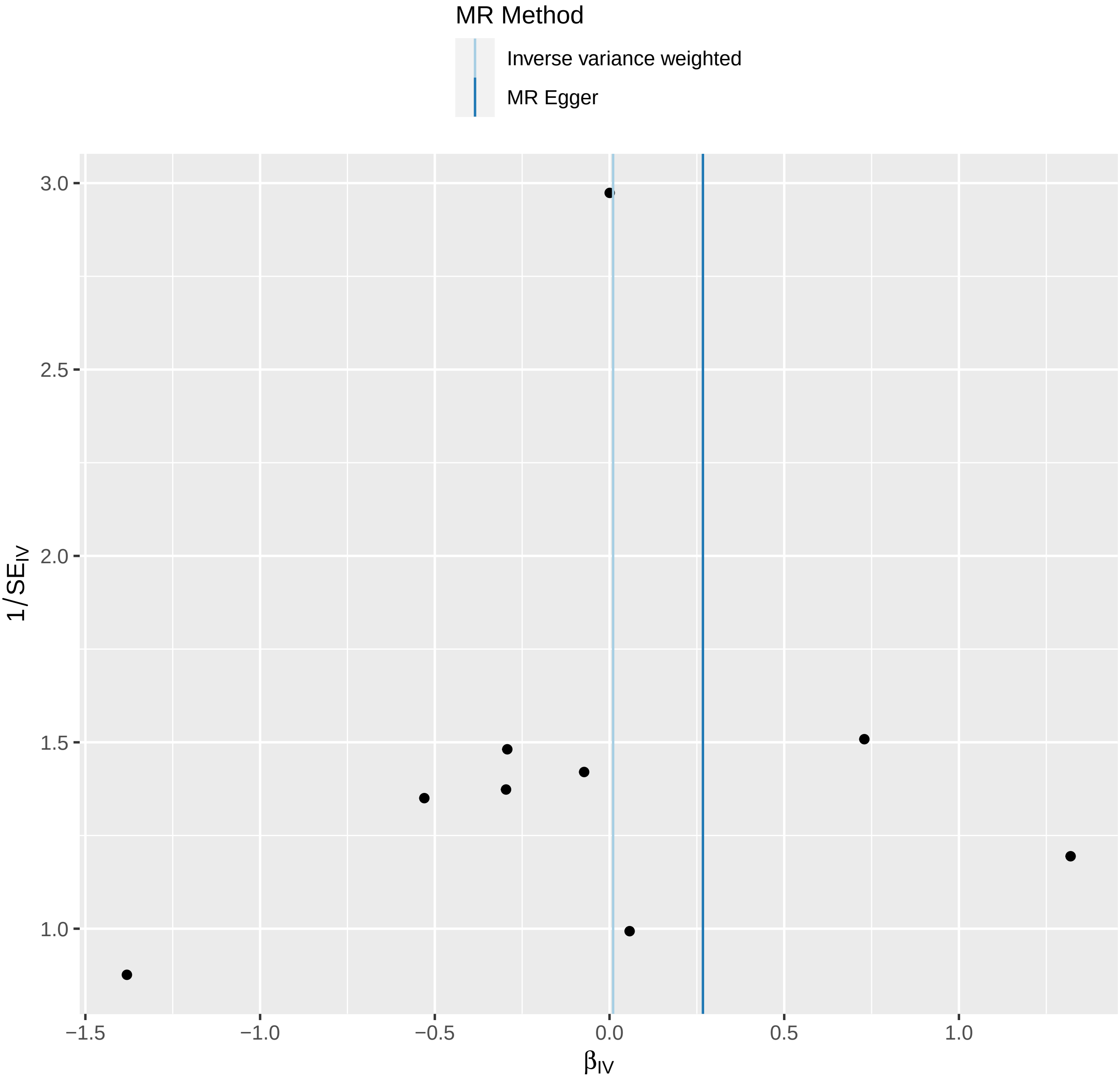

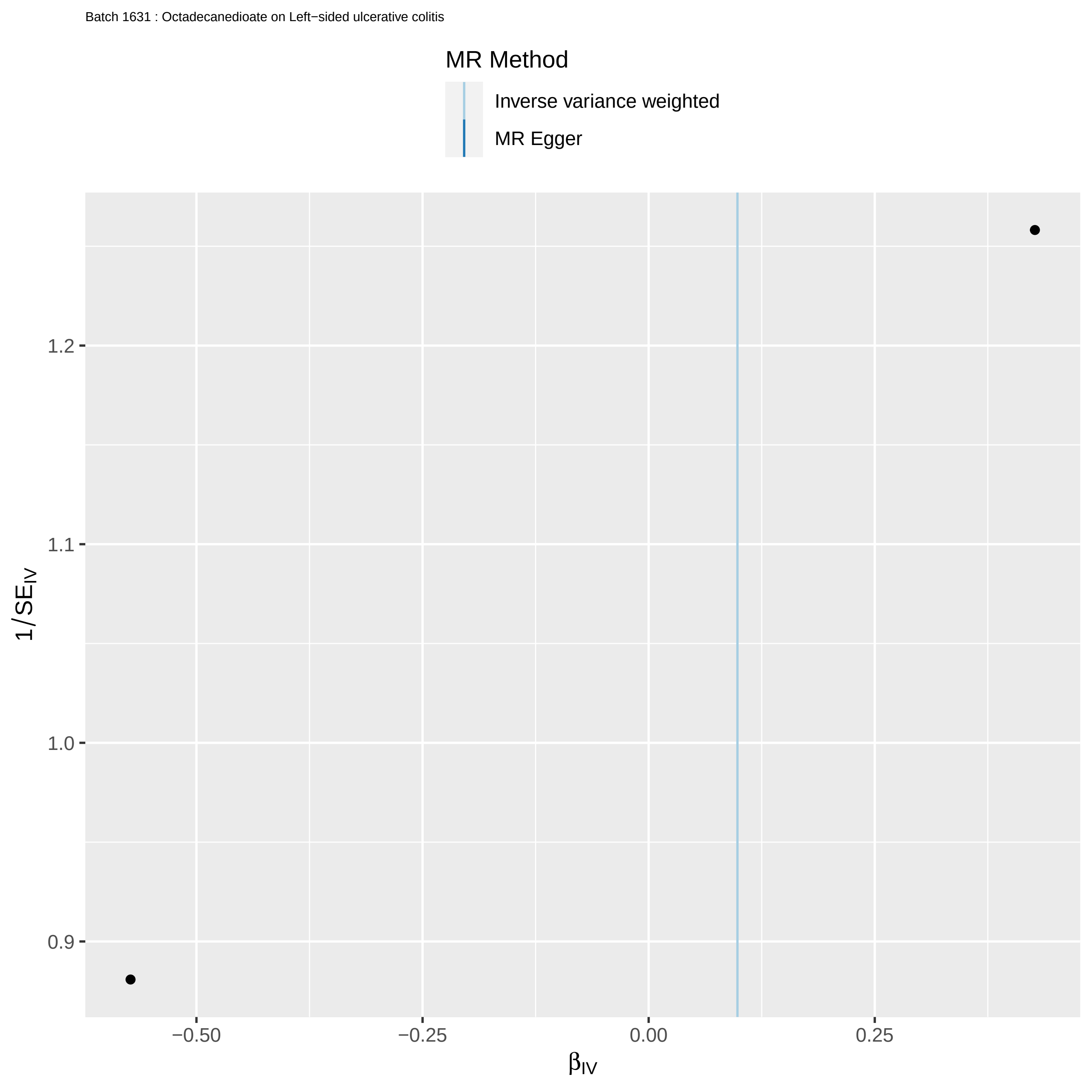
Batch 1631 : Octadecanedioate on Left−sided ulcerative colitis
MR Method
Inverse variance weighted
MR Egger
1.2
1.1
1.0
0.9
1/SE_IV
−0.50
−0.25
0.00
0.25
β_IV

Batch 1632 : Leucylleucine on Left−sided ulcerative colitis

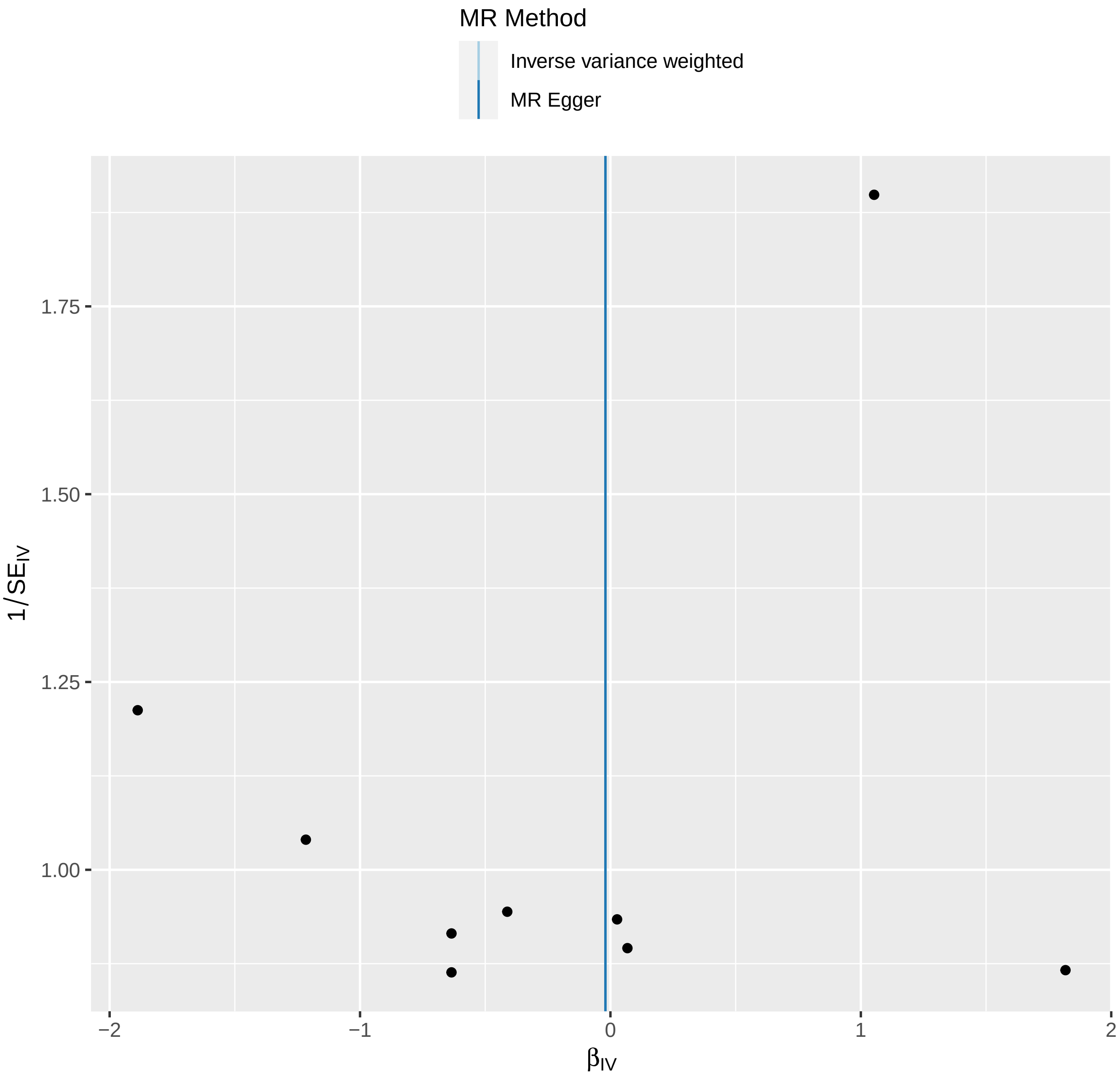

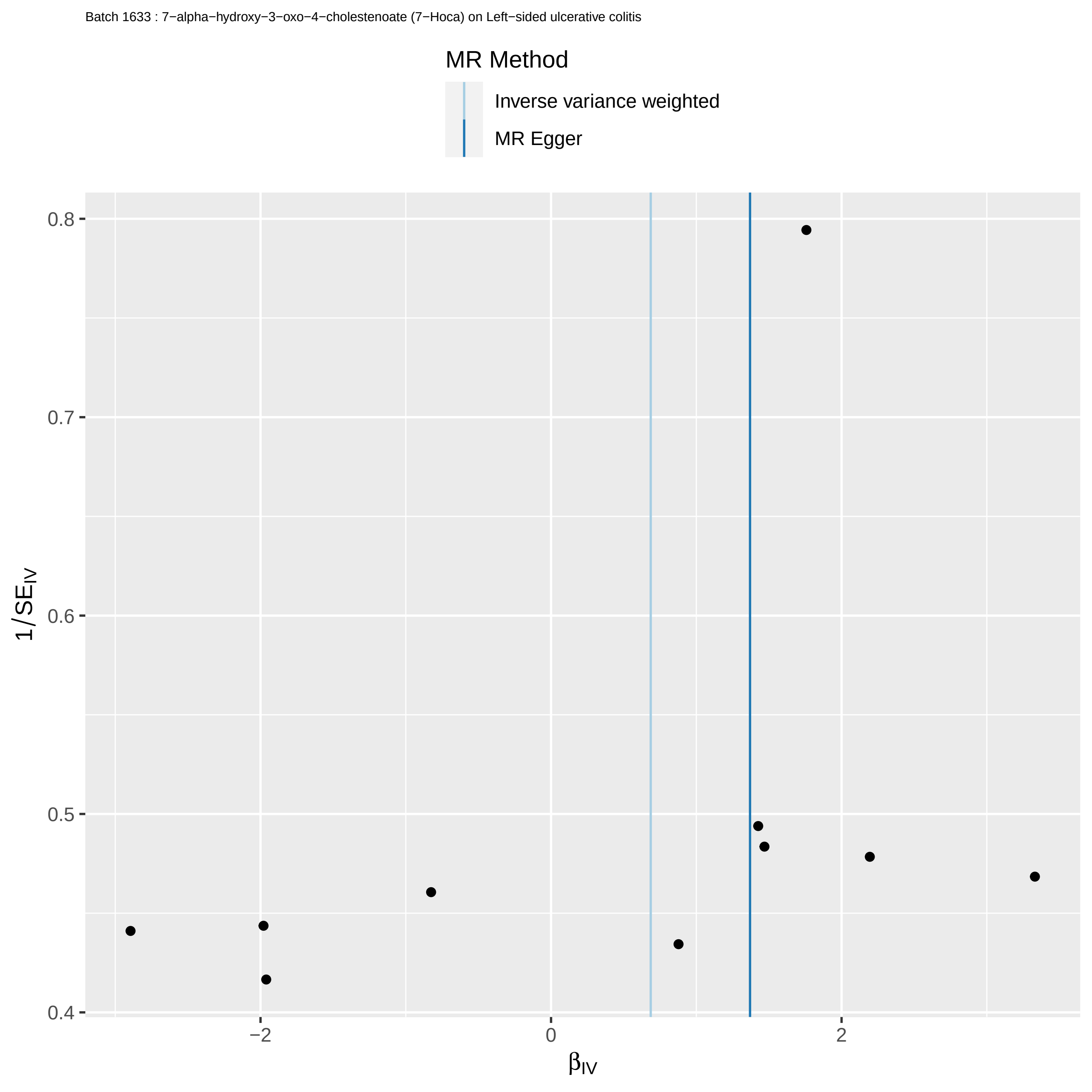

Batch 1633 : 7−alpha−hydroxy−3−oxo−4−cholestenoate (7−Hoca) on Left−sided ulcerative colitis
MR Method
Inverse variance weighted
MR Egger
0.8
0.7
0.6
0.5
0.4
1/SE_IV
−2
0
2
β_IV

Batch 1634 : N−Butyl Oleate on Left−sided ulcerative colitis

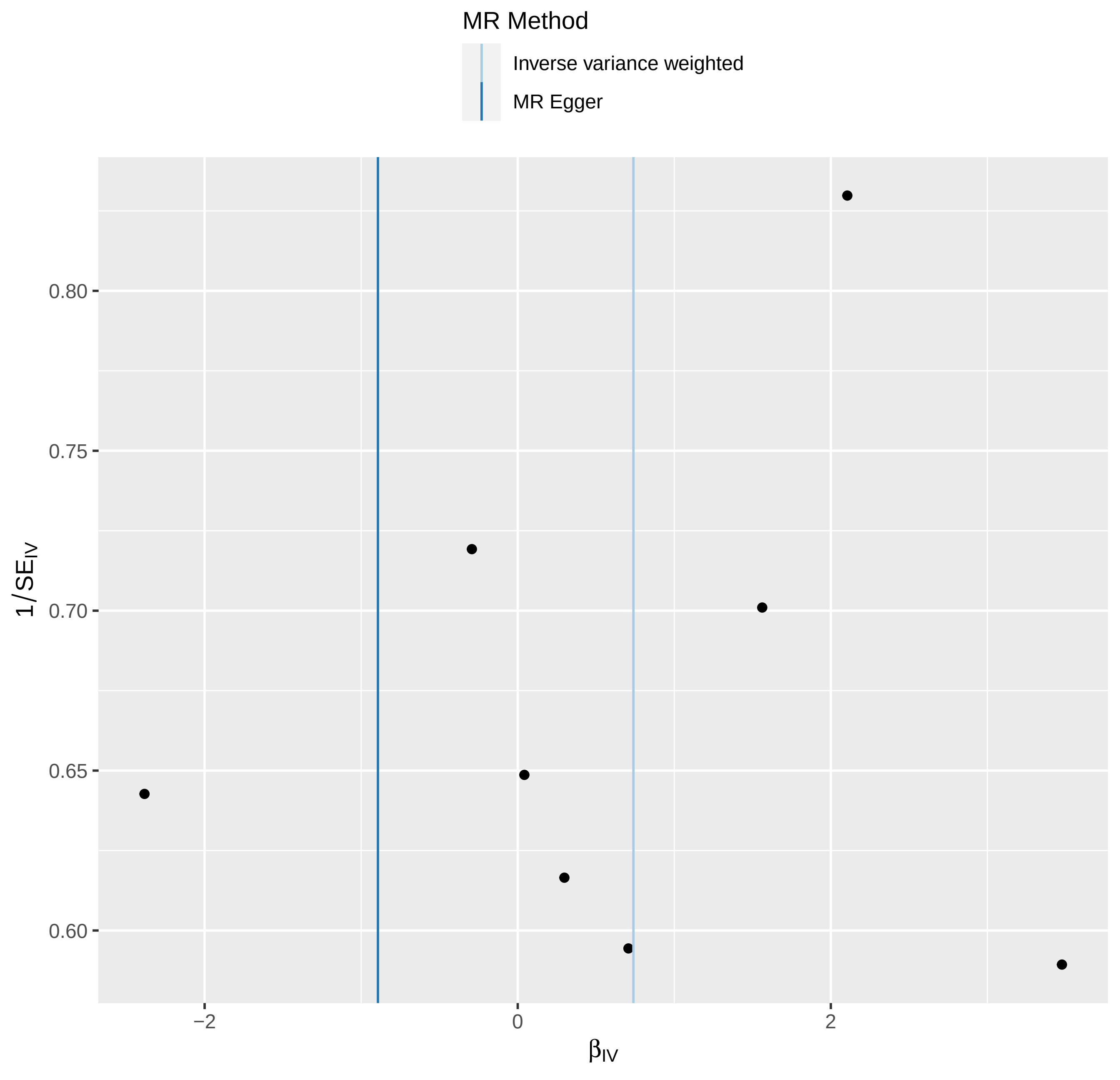

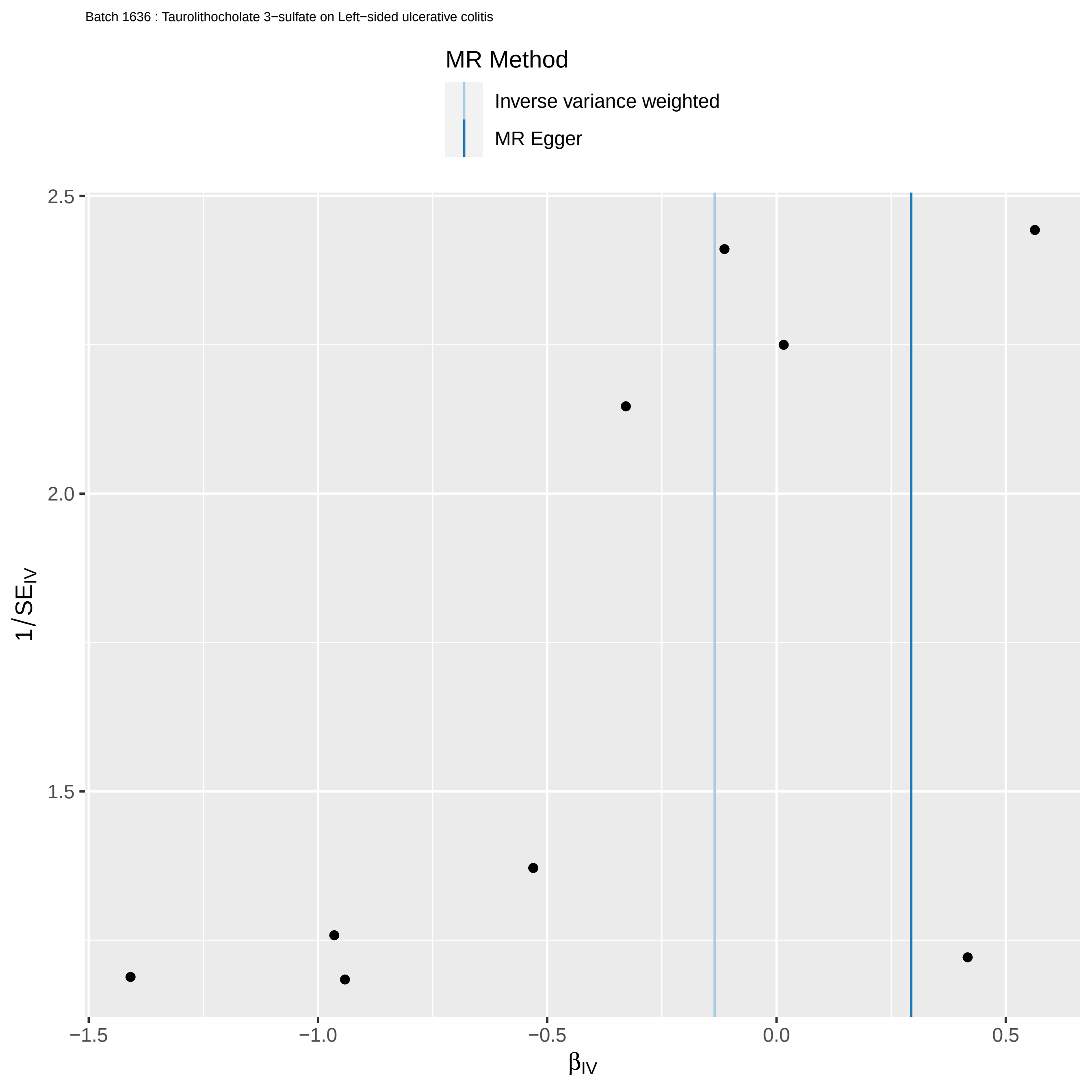
Batch 1636 : Taurolithocholate 3−sulfate on Left−sided ulcerative colitis
MR Method
Inverse variance weighted
MR Egger
2.5
2.0
1.5
1/SE_IV
−1.5
−1.0
−0.5
0.0
0.5
β_IV

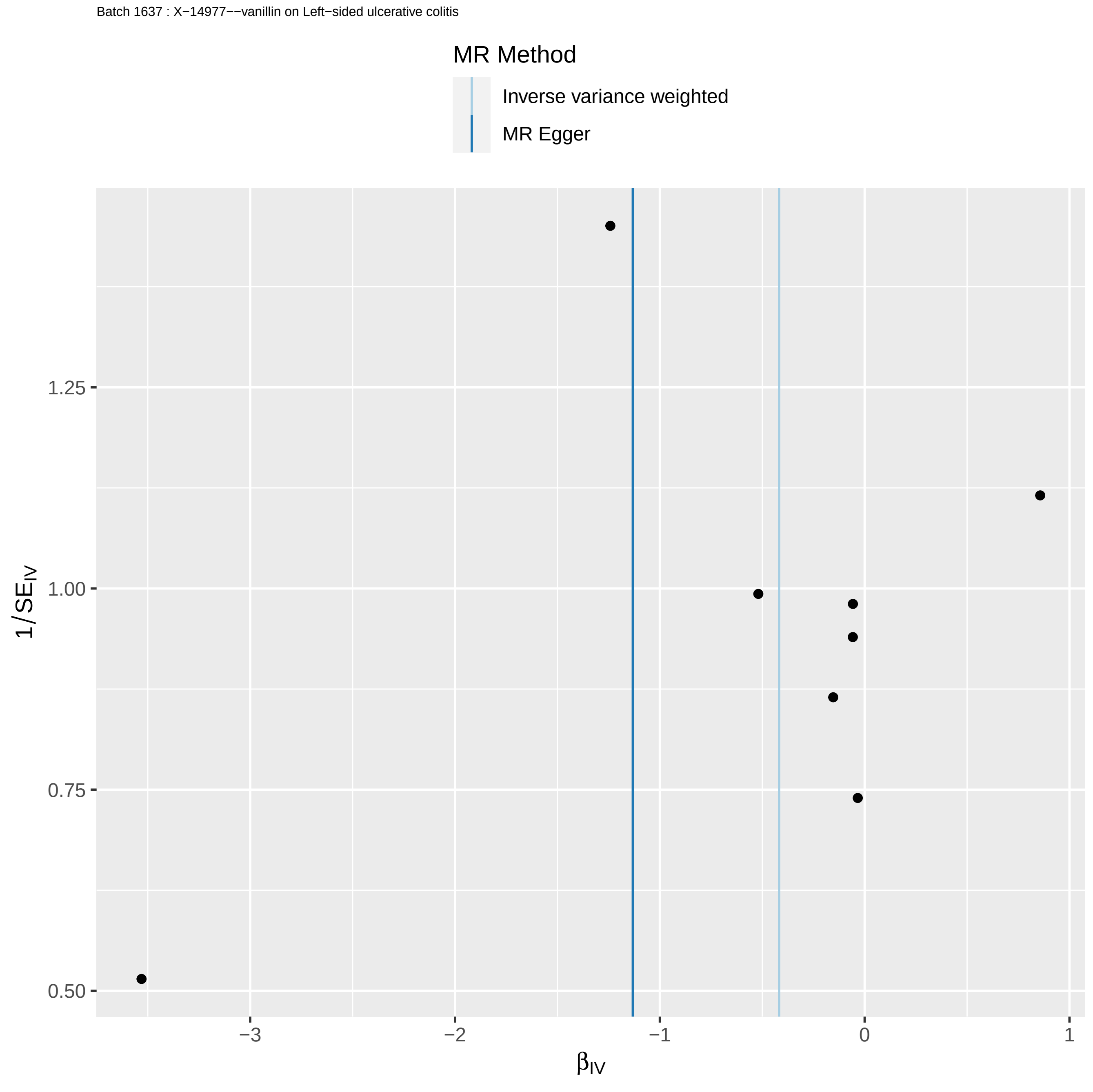
Batch 1637 : X−14977−−vanillin on Left−sided ulcerative colitis
MR Method
Inverse variance weighted
MR Egger
1.25
1.00
0.75
0.50
1/SE$_{IV}$
−3
−2
−1
0
1
β$_{IV}$

Batch 1638 : Succinylcarnitine on Left−sided ulcerative colitis

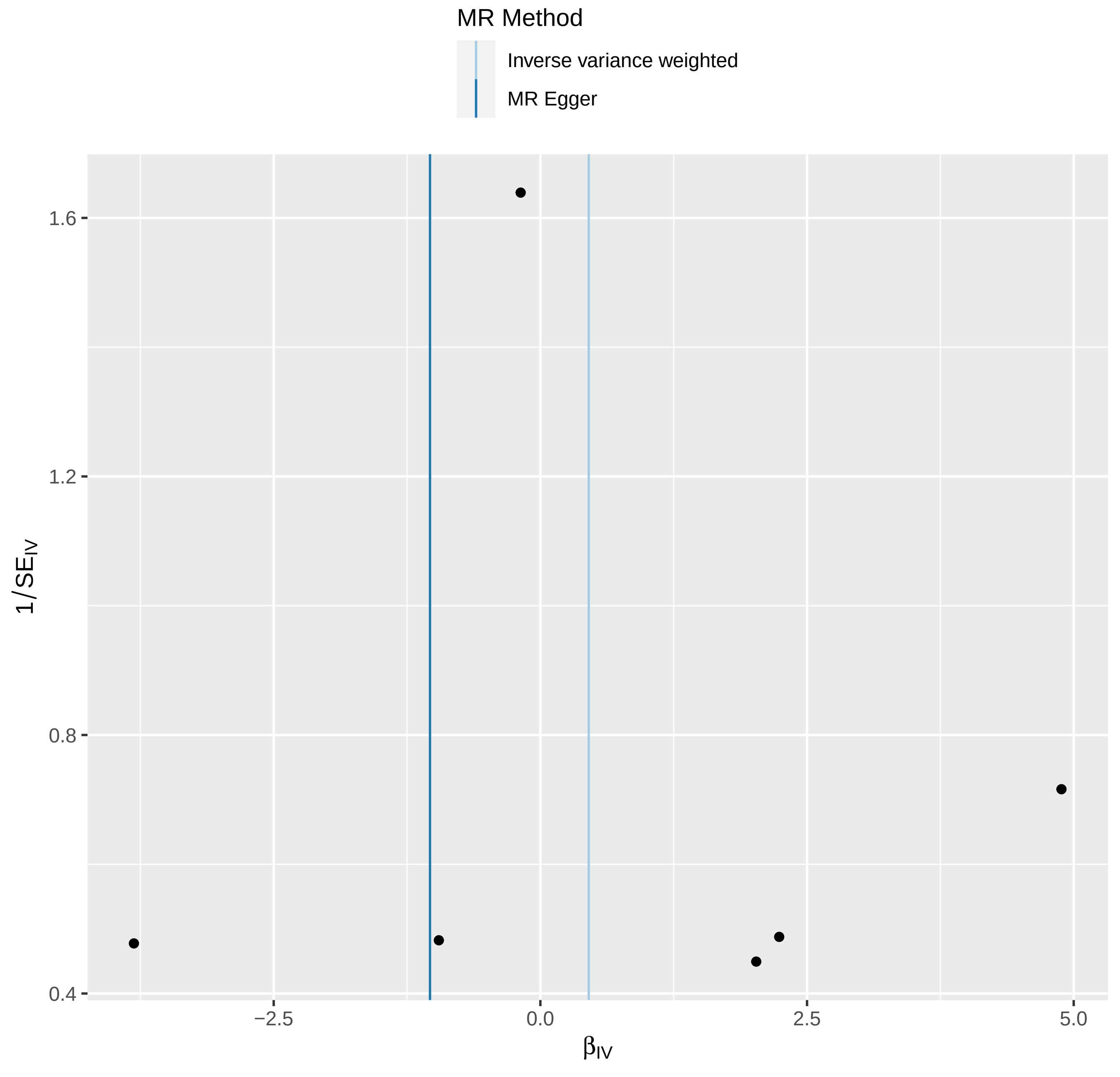

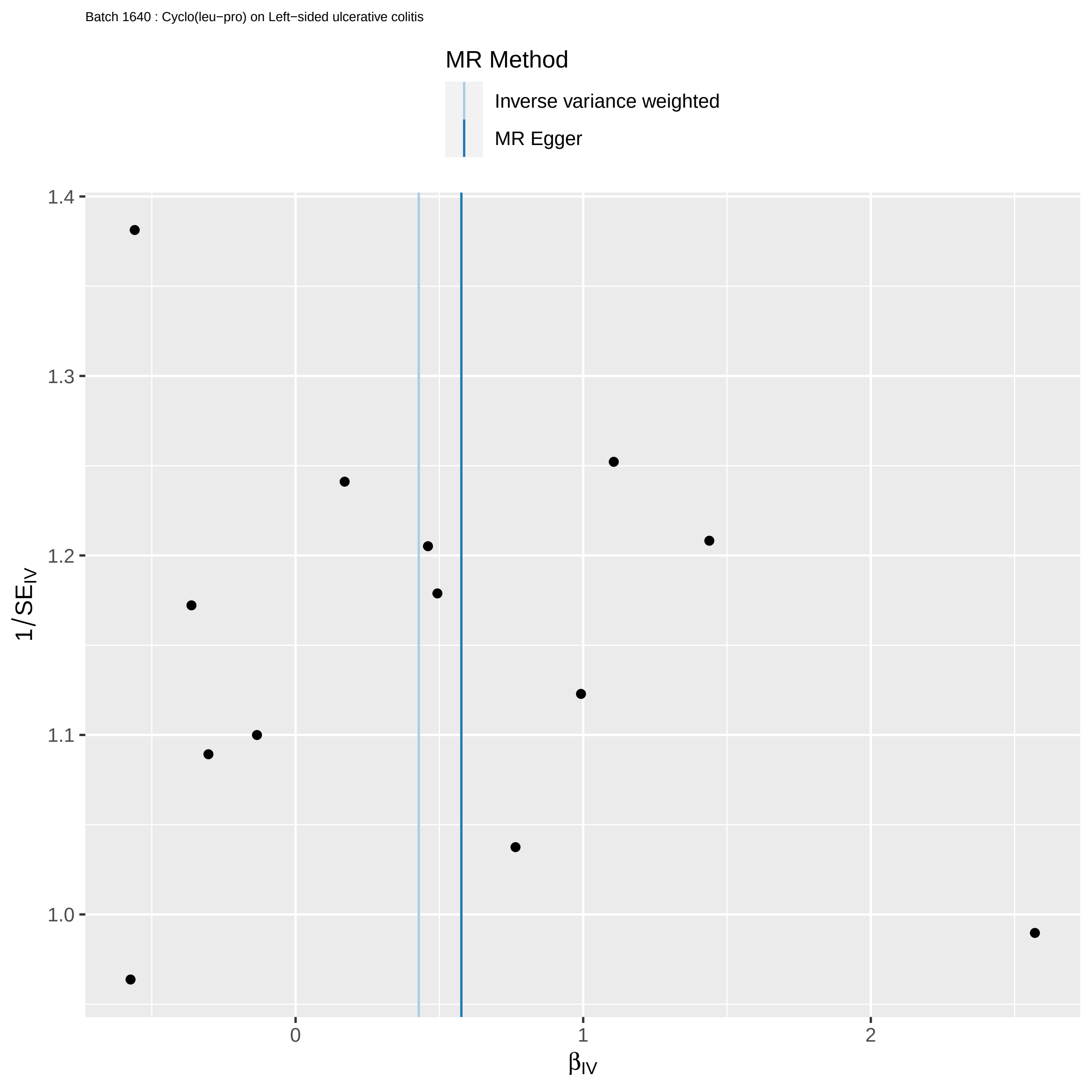

Batch 1640 : Cyclo(leu−pro) on Left−sided ulcerative colitis
MR Method
Inverse variance weighted
MR Egger
1.4
1.3
1.2
1.1
1.0
1/SE_IV
0
1
2
β_IV

Batch 1641 : Chiro−inositol on Left−sided ulcerative colitis

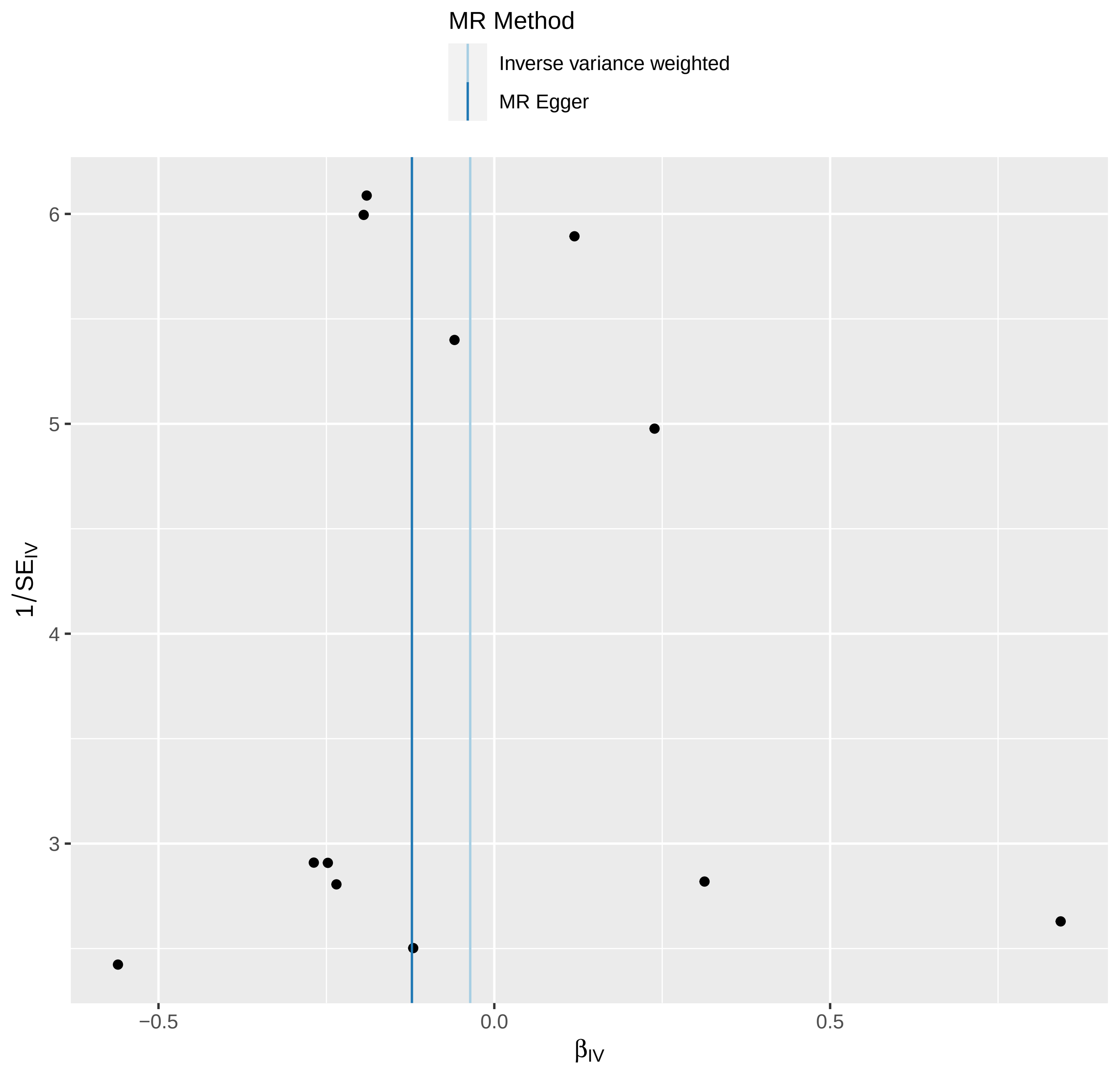

Batch 1645 : 2−hydroxyglutarate on Left−sided ulcerative colitis

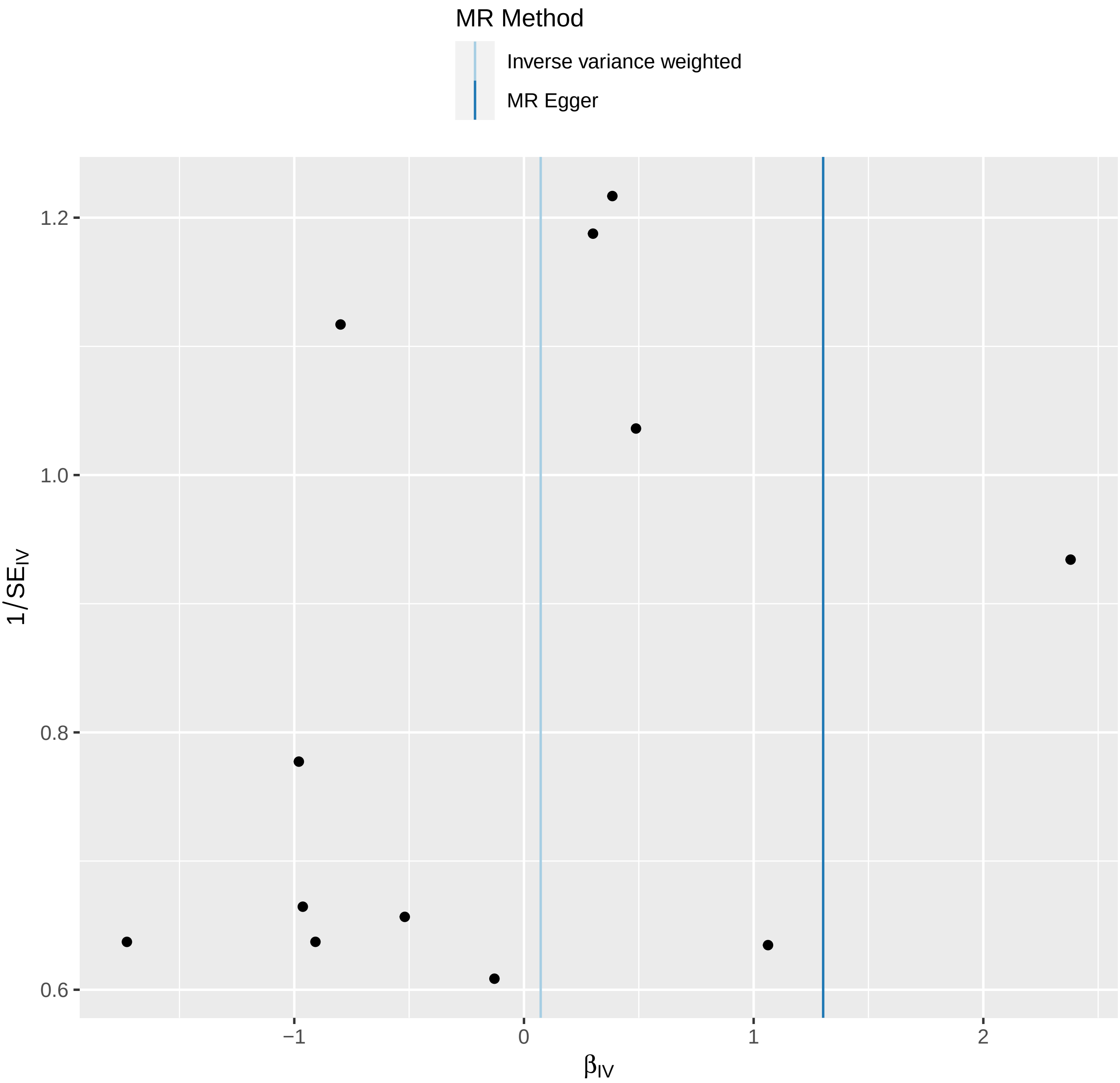

Batch 1646 : Ergothioneine on Left−sided ulcerative colitis

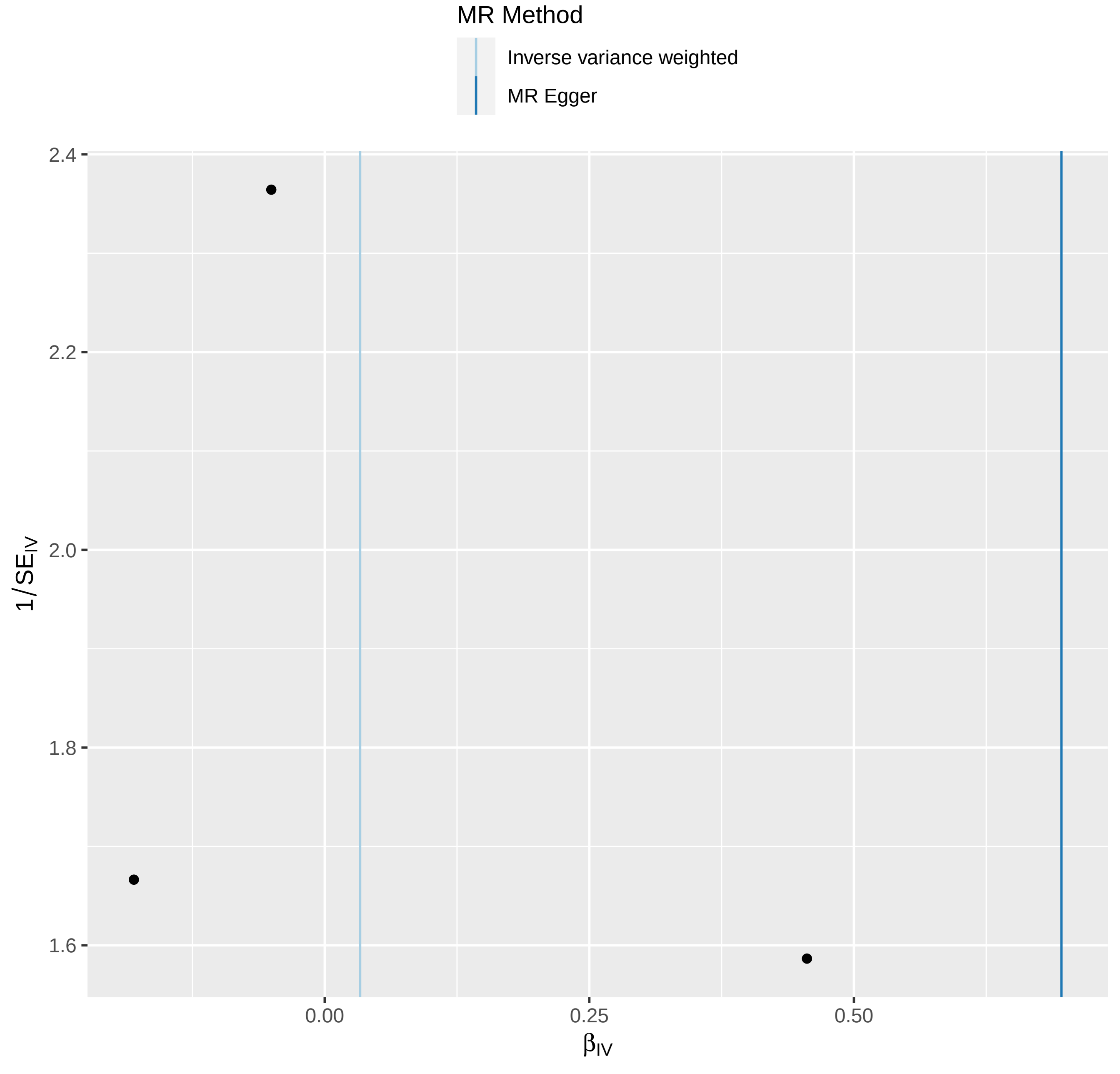

Batch 1648 : Phenylalanylphenylalanine on Left−sided ulcerative colitis

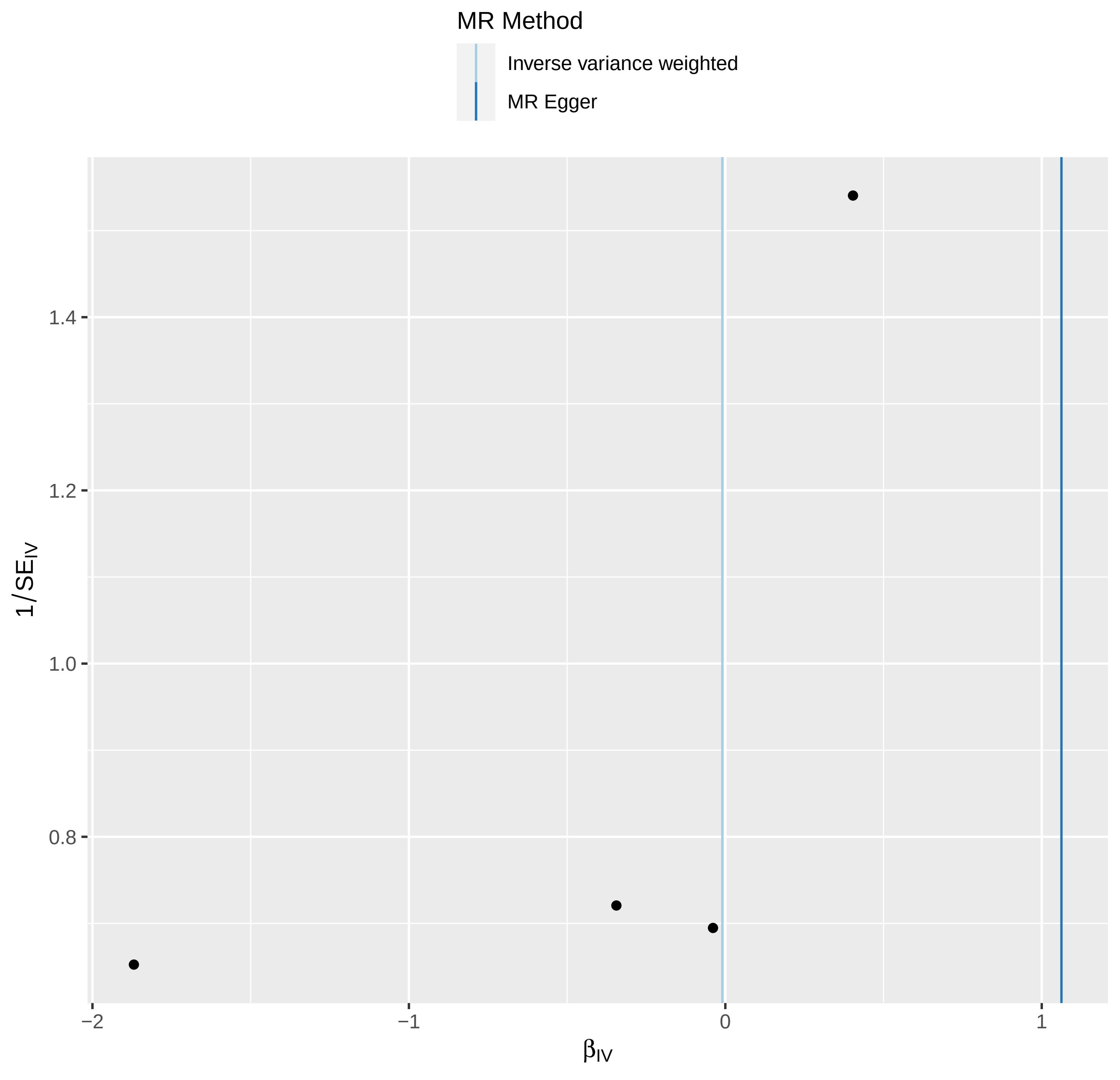

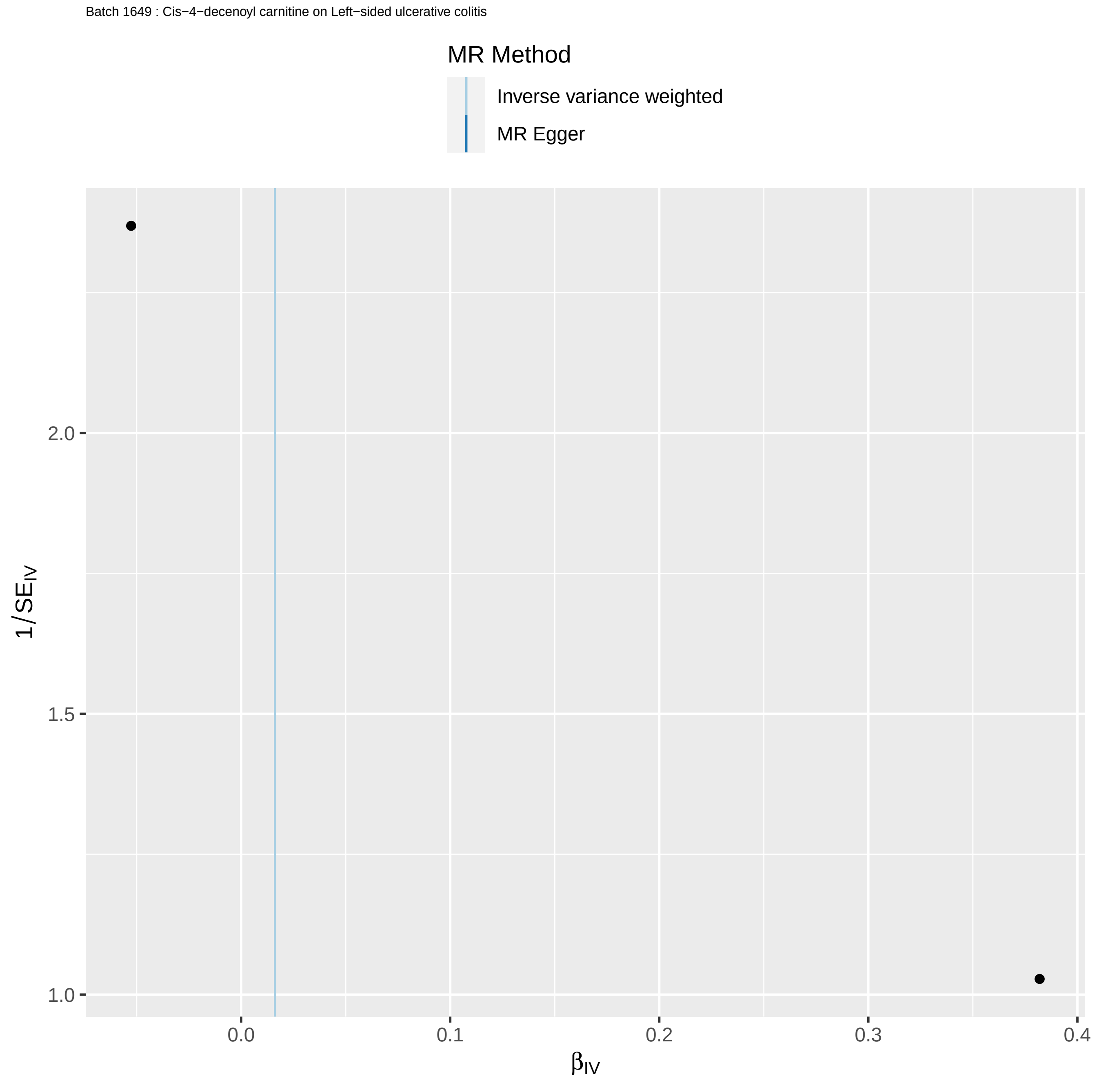
Batch 1649 : Cis−4−decenoyl carnitine on Left−sided ulcerative colitis
MR Method
Inverse variance weighted
MR Egger
2.0
1.5
1.0
$1/SE_{IV}$
0.0
0.1
0.2
0.3
0.4
β_{IV}

Batch 1650 : 15−methylpalmitate (isobar with 2−methylpalmitate) on Left−sided ulcerative colitis

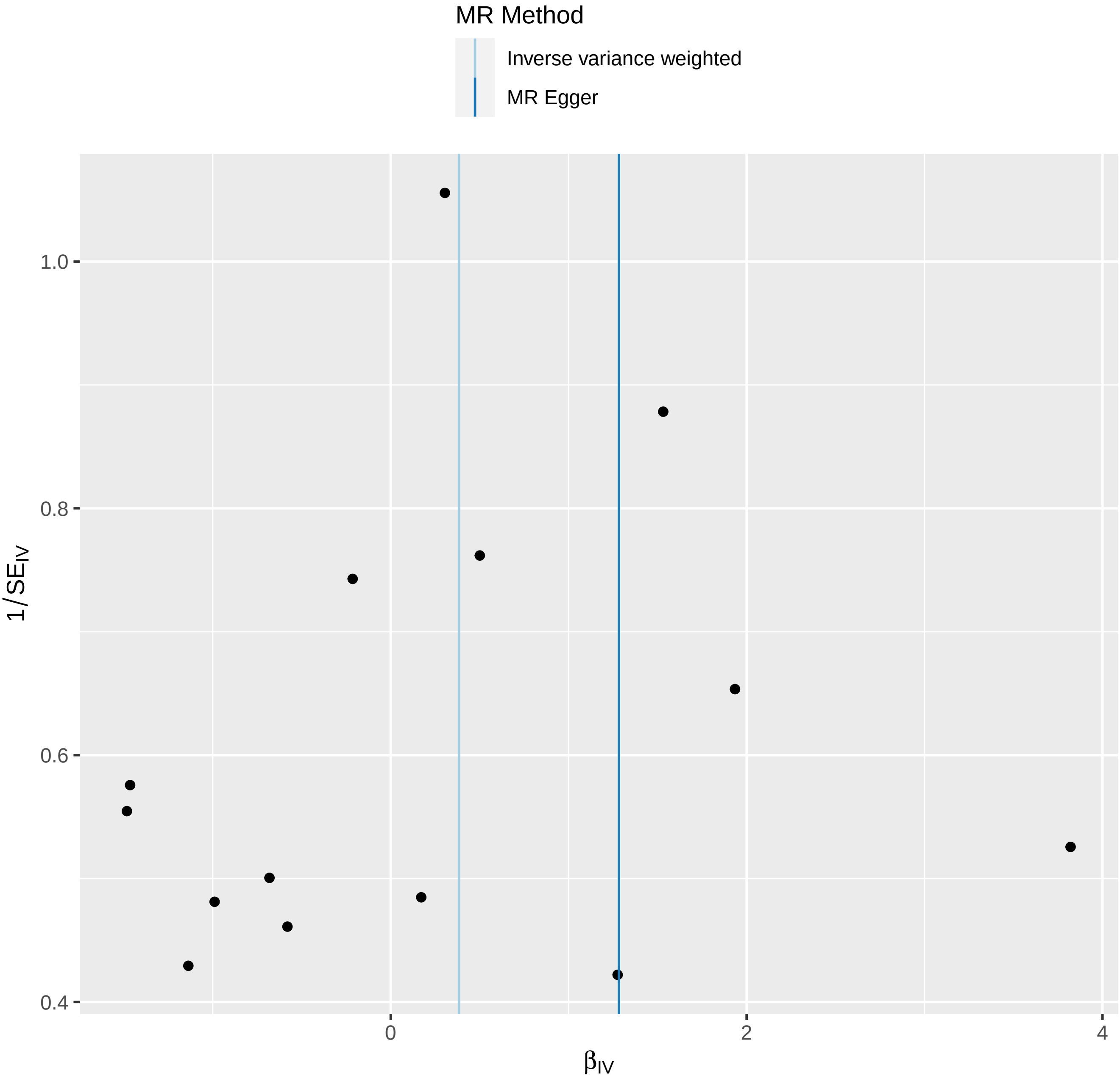

Batch 1652 : Tryptophan on Arthropathy in ulcerative colitis

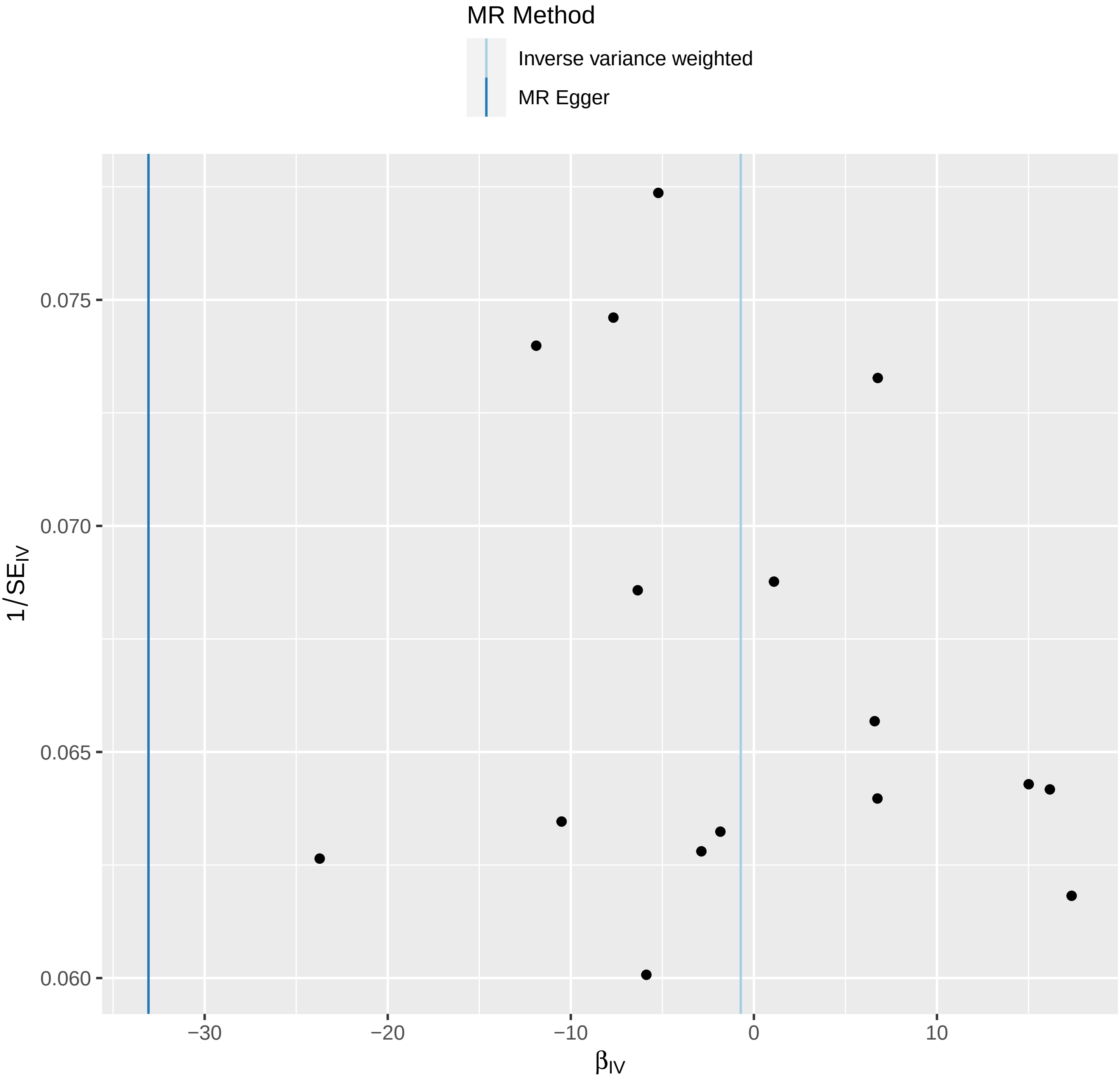

Batch 1654 : Leucine on Arthropathy in ulcerative colitis

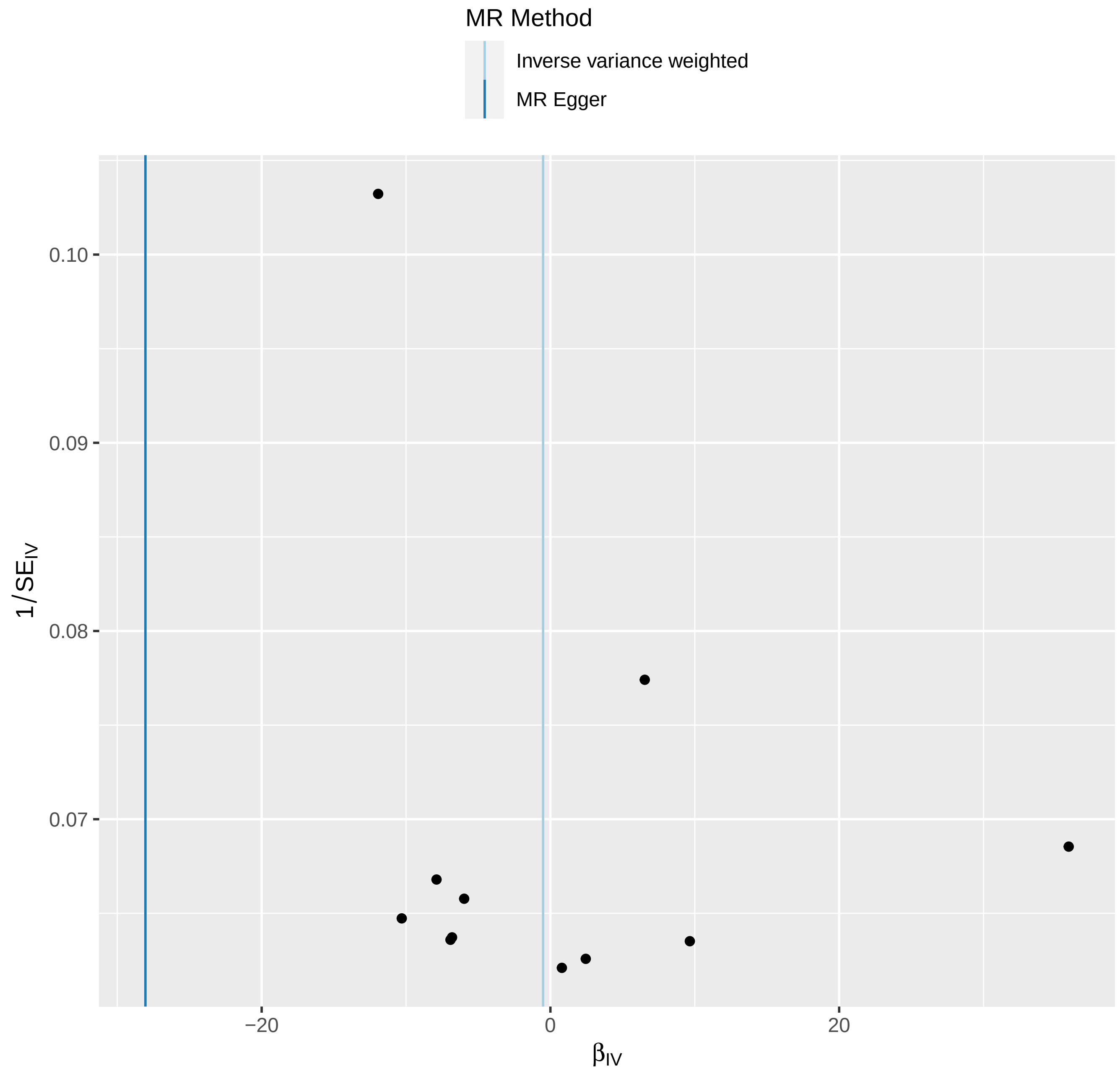

Batch 1658 : Lactate on Arthropathy in ulcerative colitis

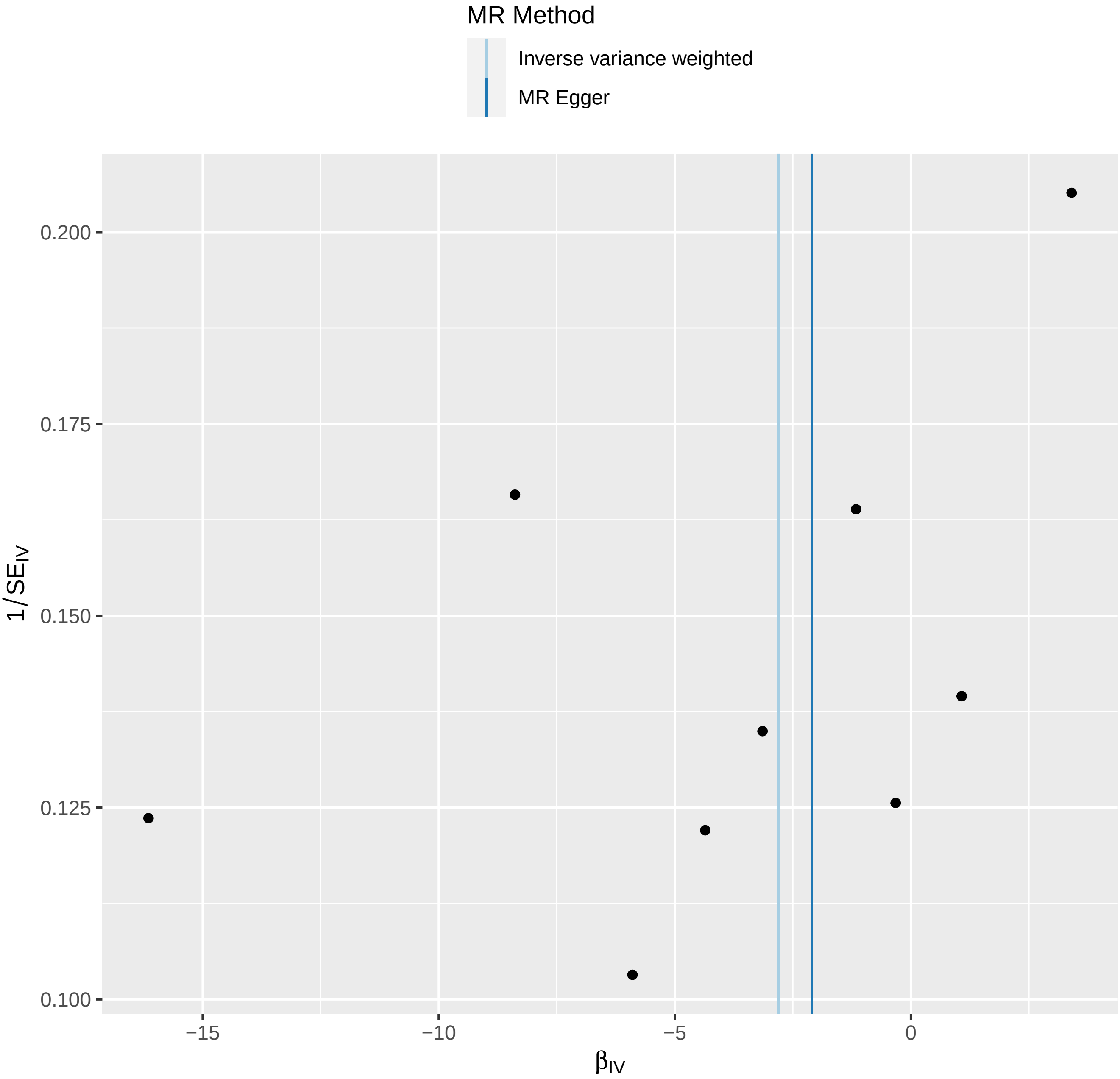

Batch 1659 : 3−hydroxybutyrate (BHBA) on Arthropathy in ulcerative colitis

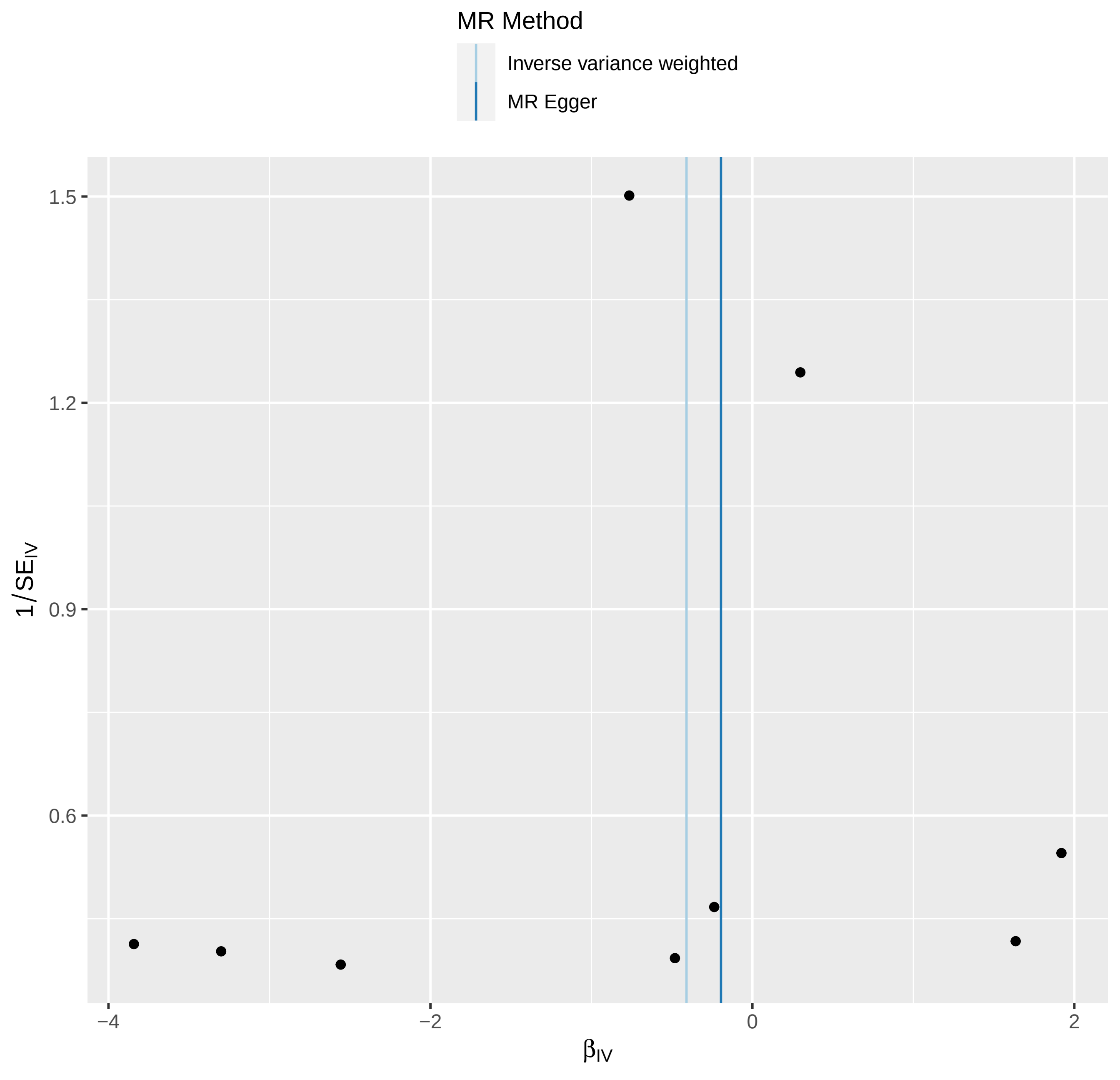

Batch 1660 : Arabinose on Arthropathy in ulcerative colitis

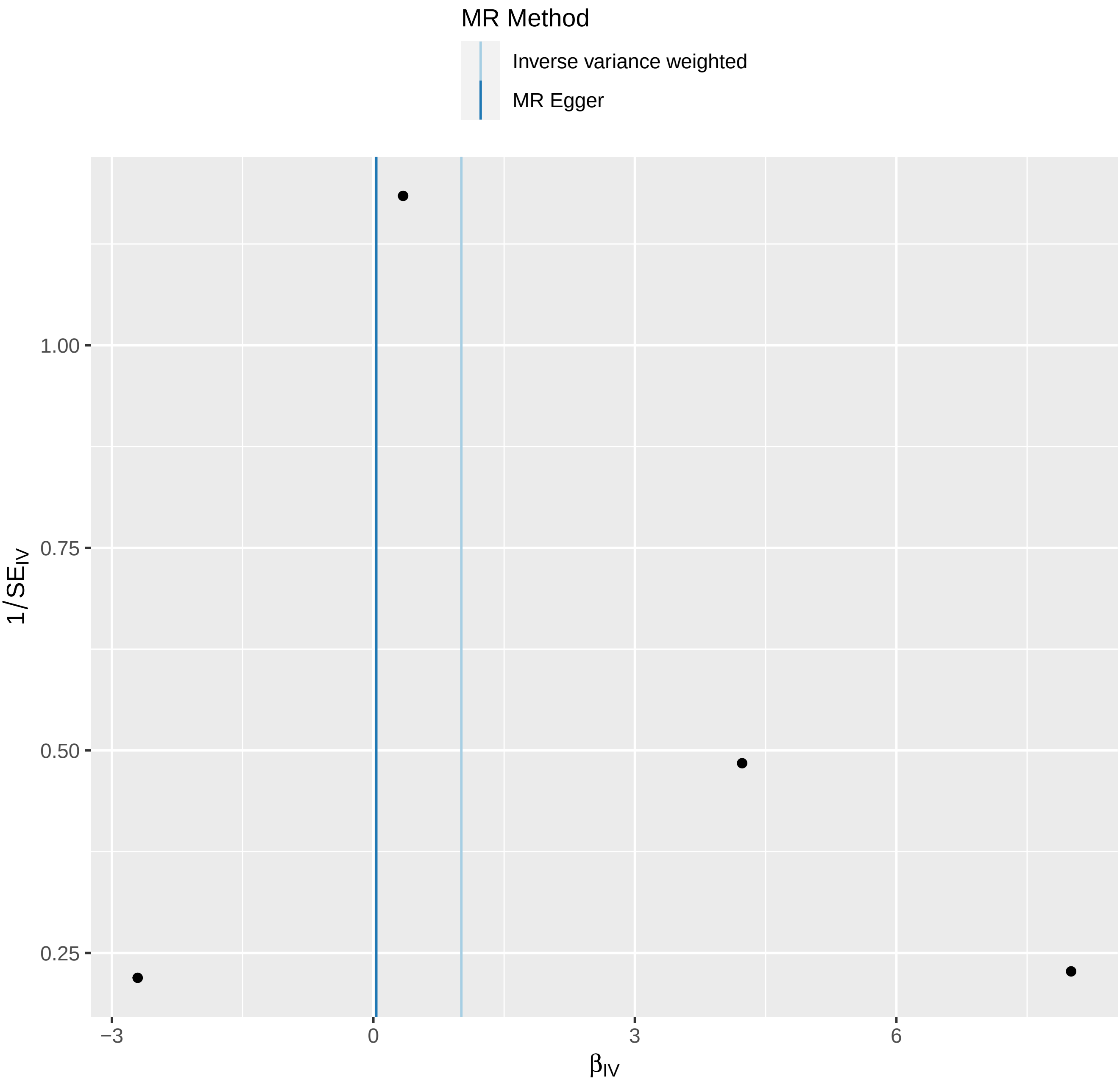

Batch 1661 : Fructose on Arthropathy in ulcerative colitis

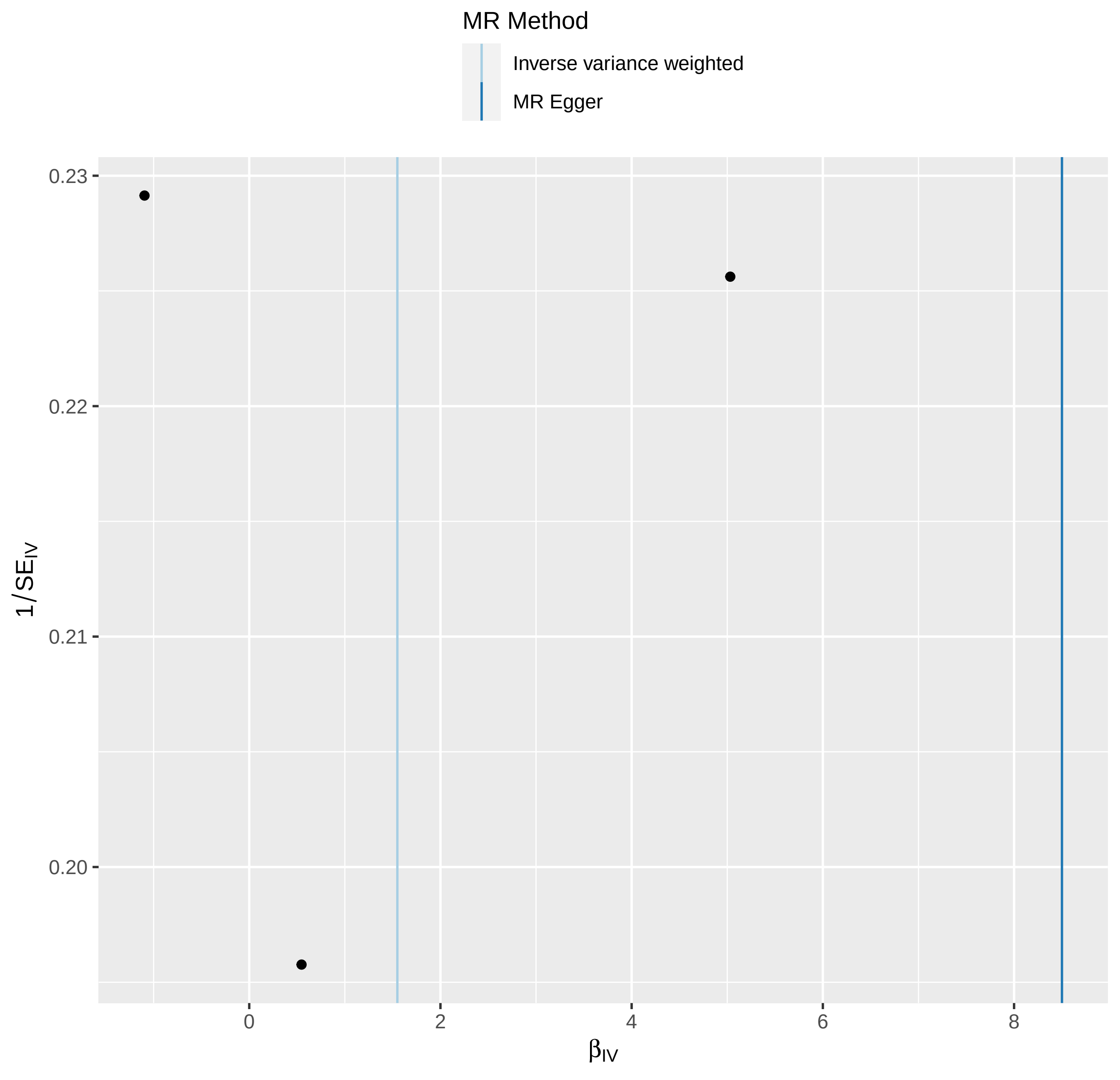

Batch 1663 : Pyruvate on Arthropathy in ulcerative colitis

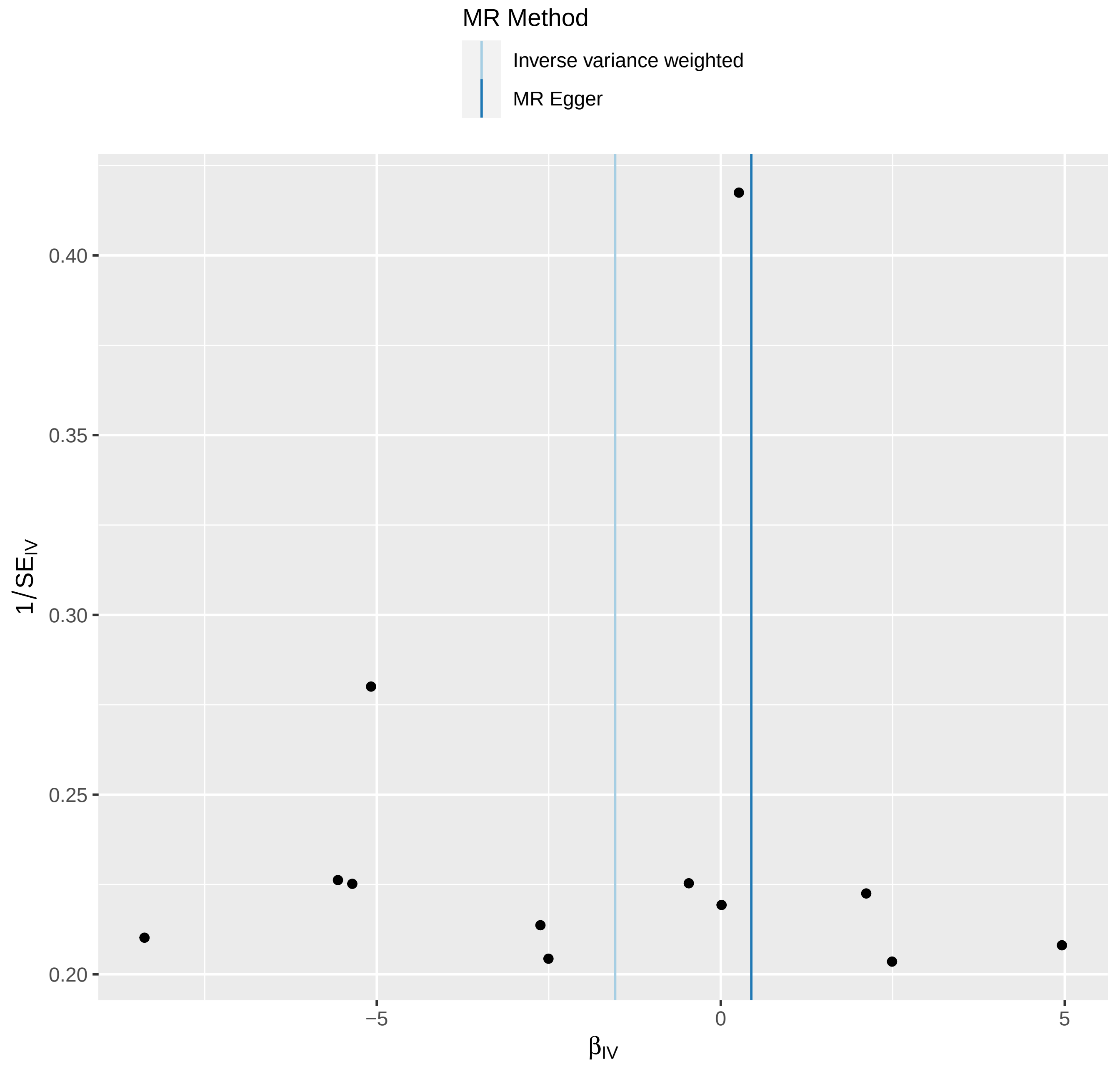

Batch 1664 : Uridine on Arthropathy in ulcerative colitis

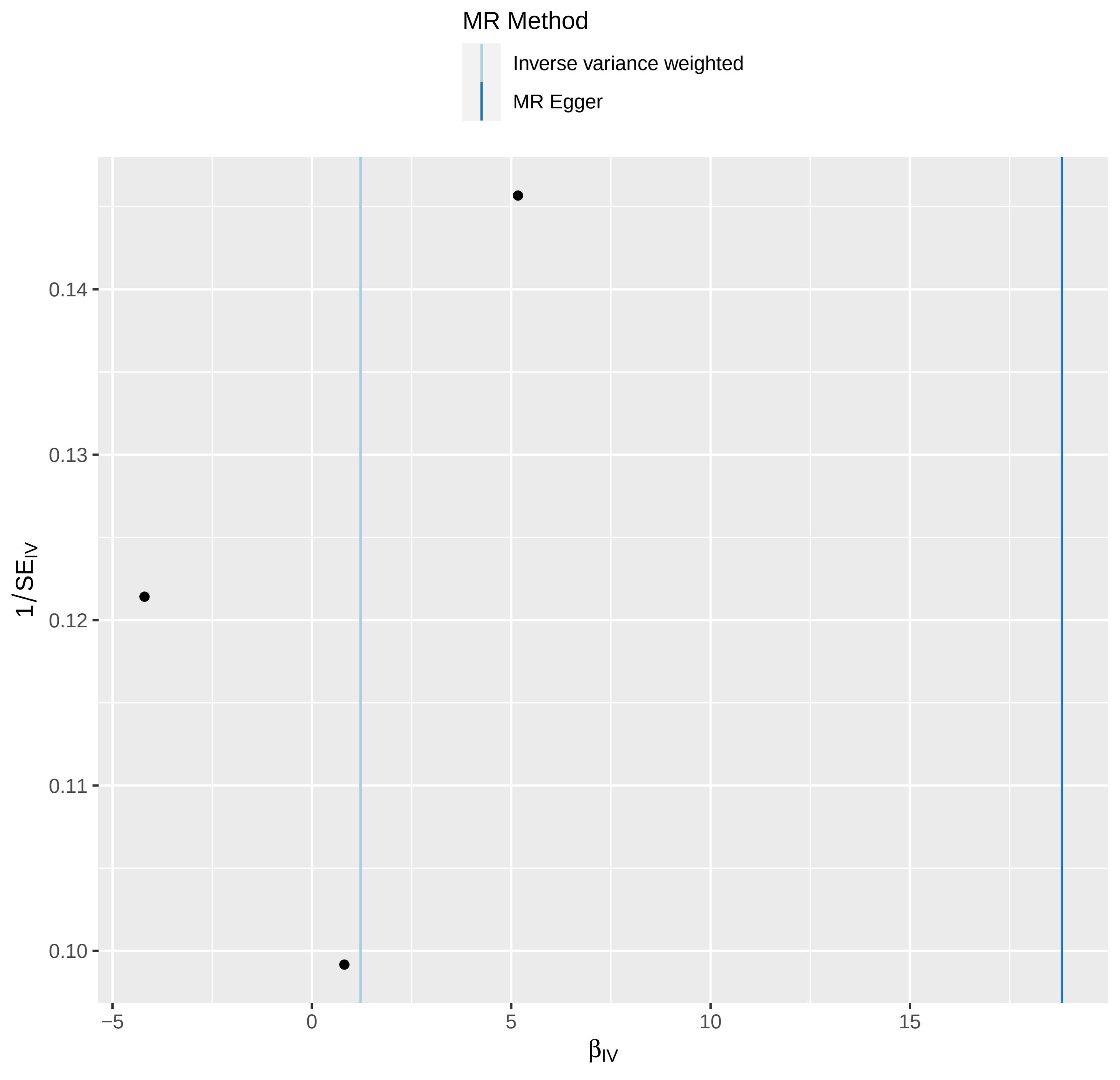

Batch 1665 : Linoleate (18:2n6) on Arthropathy in ulcerative colitis

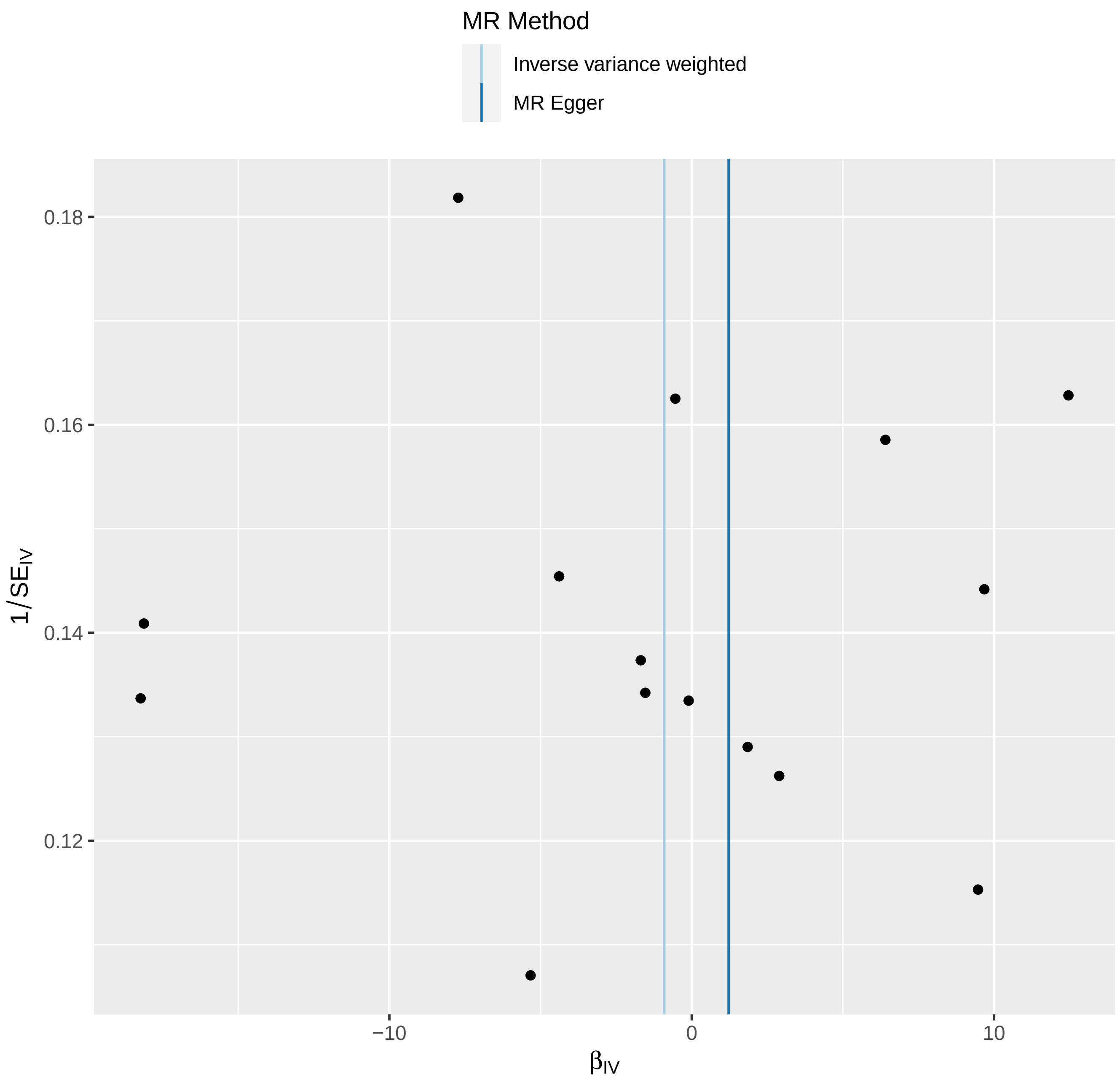

Batch 1666 : Allantoin on Arthropathy in ulcerative colitis

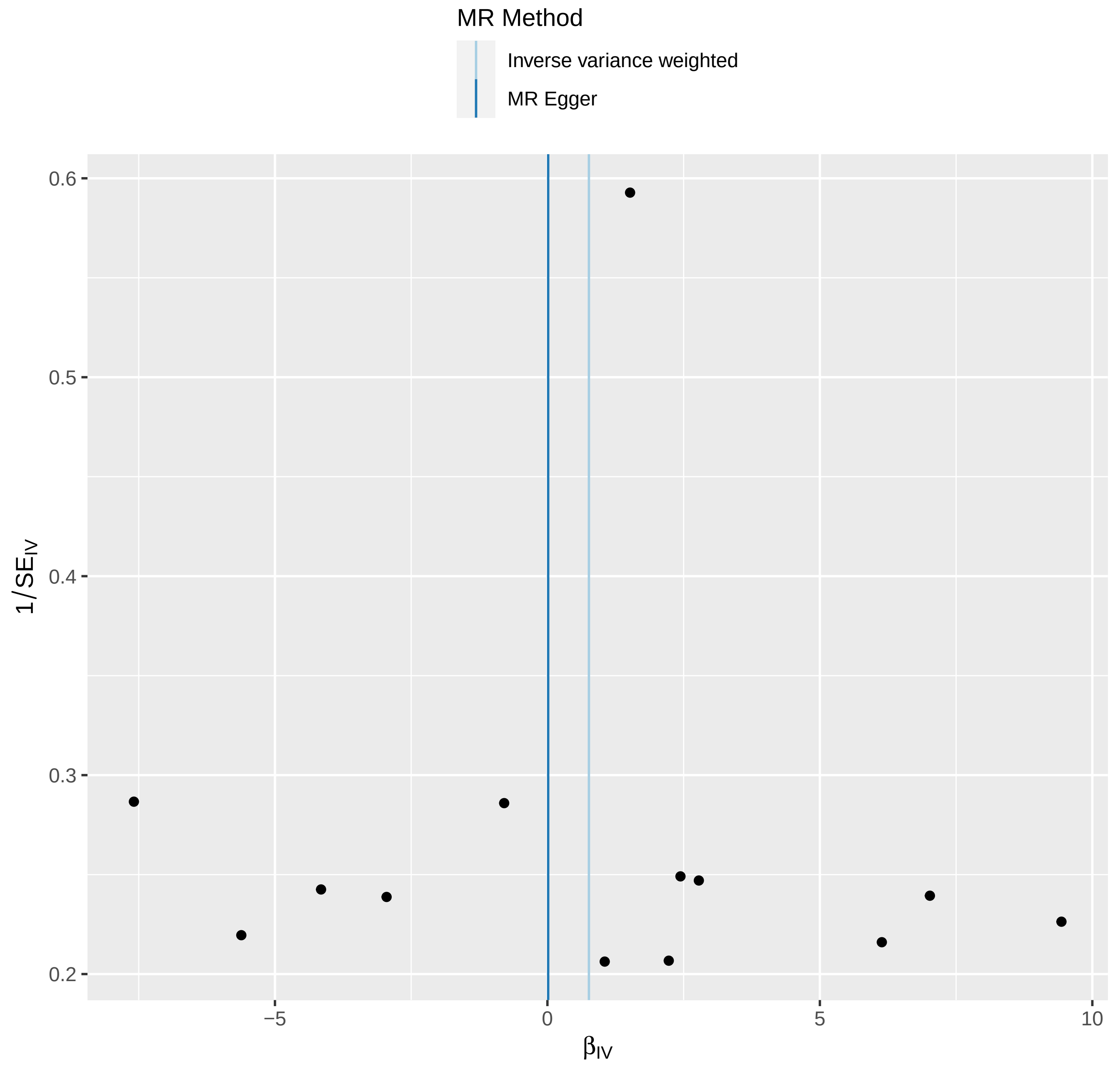

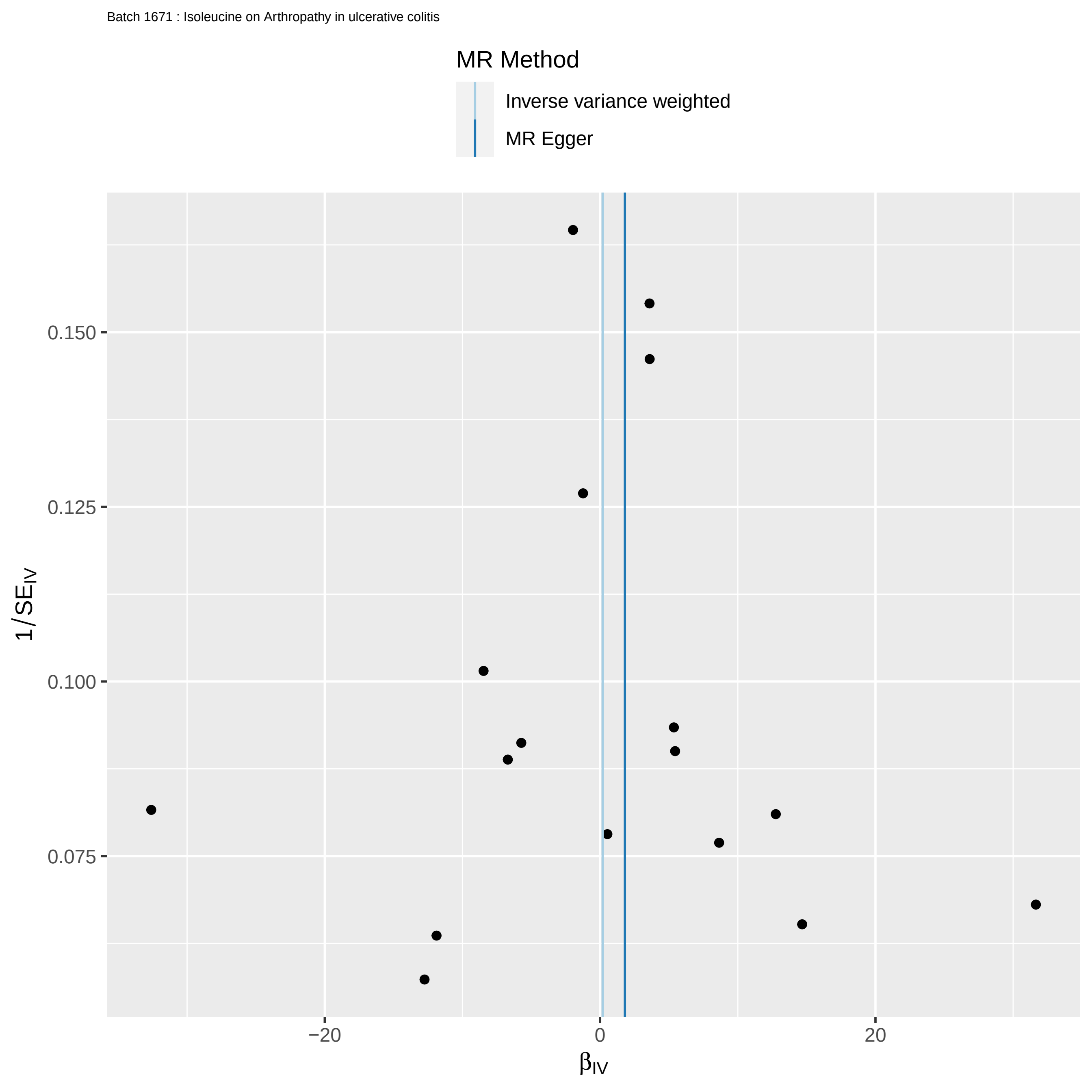

Batch 1671 : Isoleucine on Arthropathy in ulcerative colitis
MR Method
Inverse variance weighted
MR Egger
0.150
0.125
0.100
0.075
1/SE$_{IV}$
−20
0
20
β$_{IV}$

Batch 1672 : Threonine on Arthropathy in ulcerative colitis

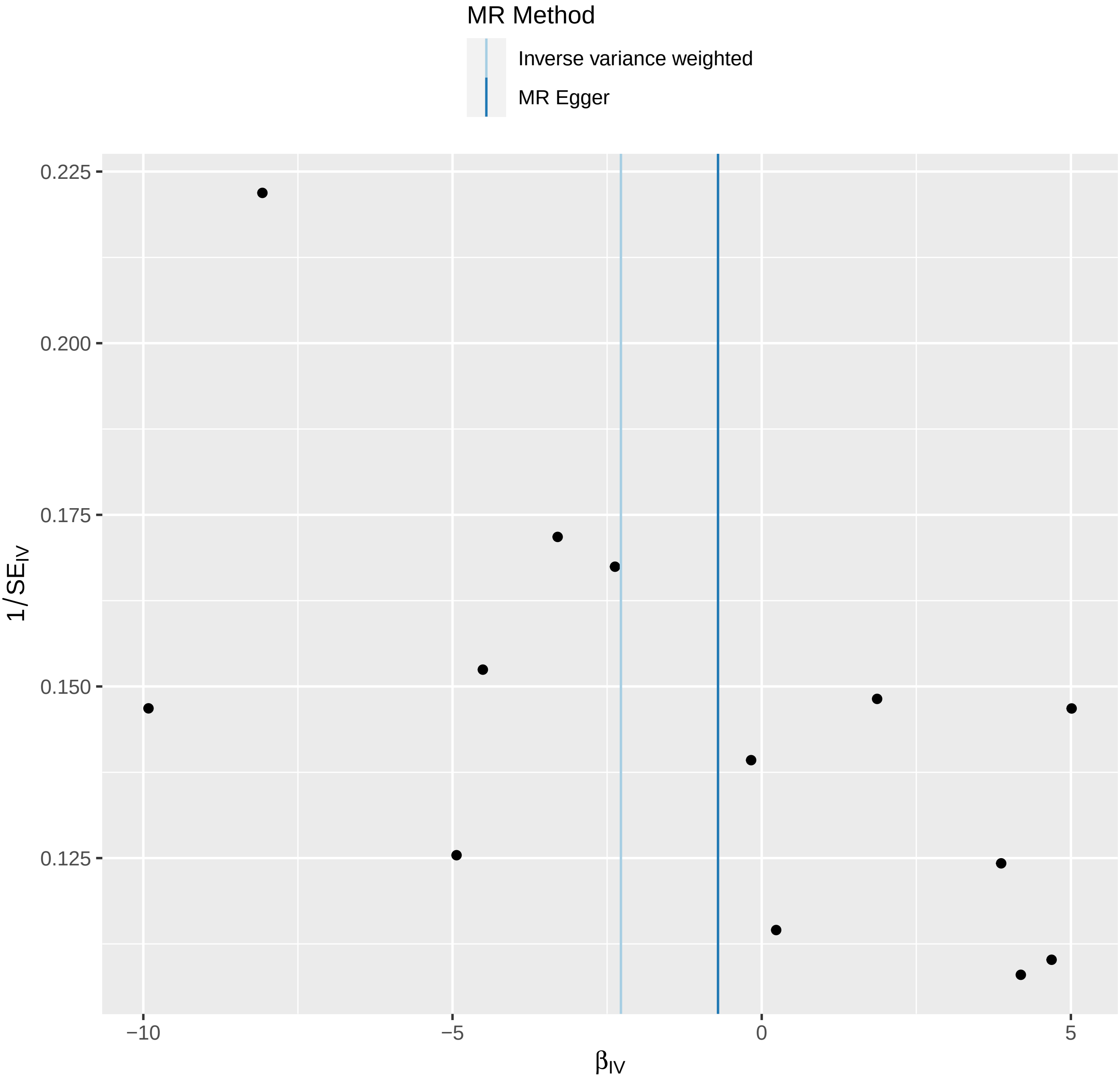

Batch 1673 : Tyrosine on Arthropathy in ulcerative colitis

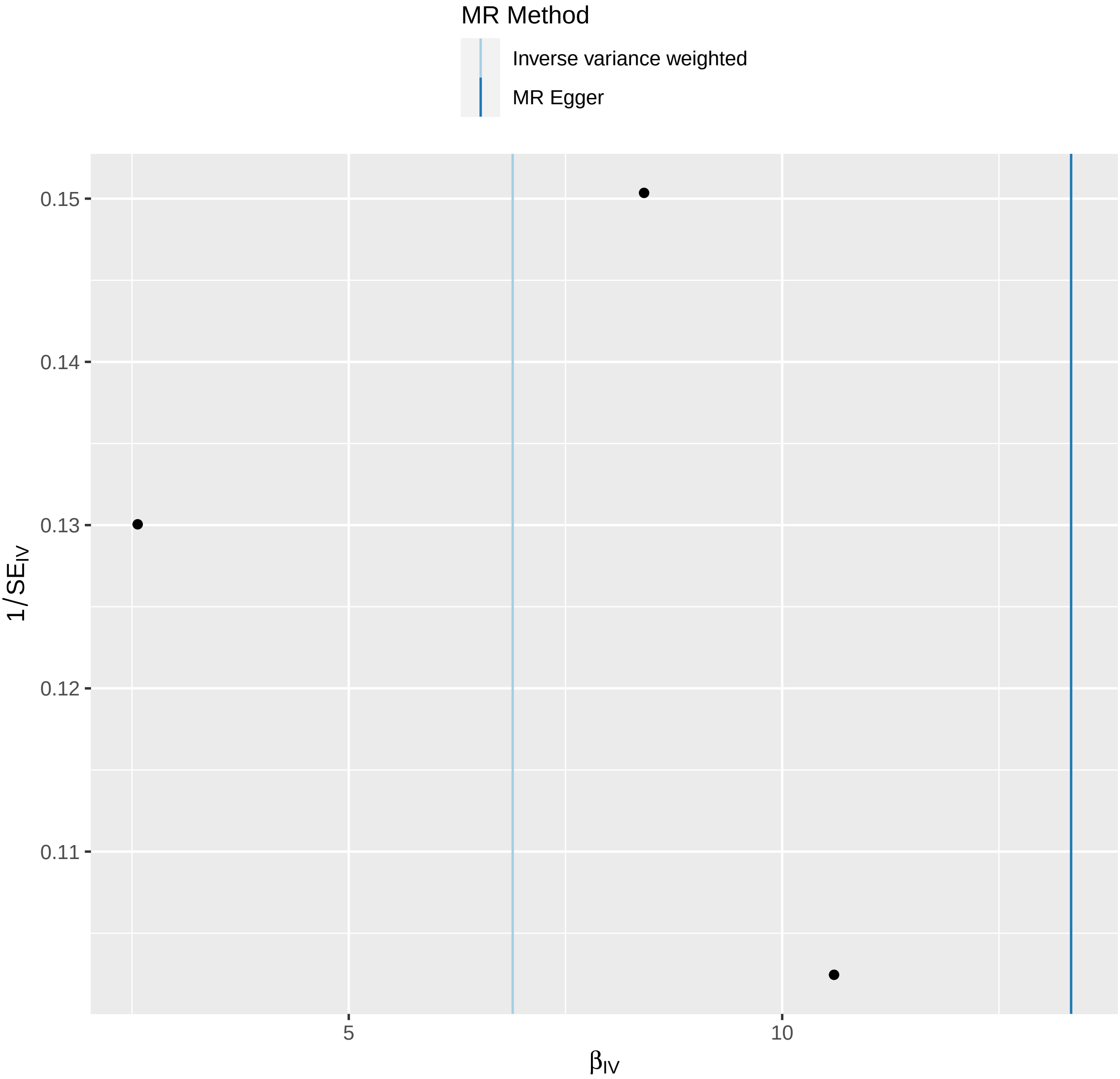

Batch 1679 : Stearate (18:0) on Arthropathy in ulcerative colitis

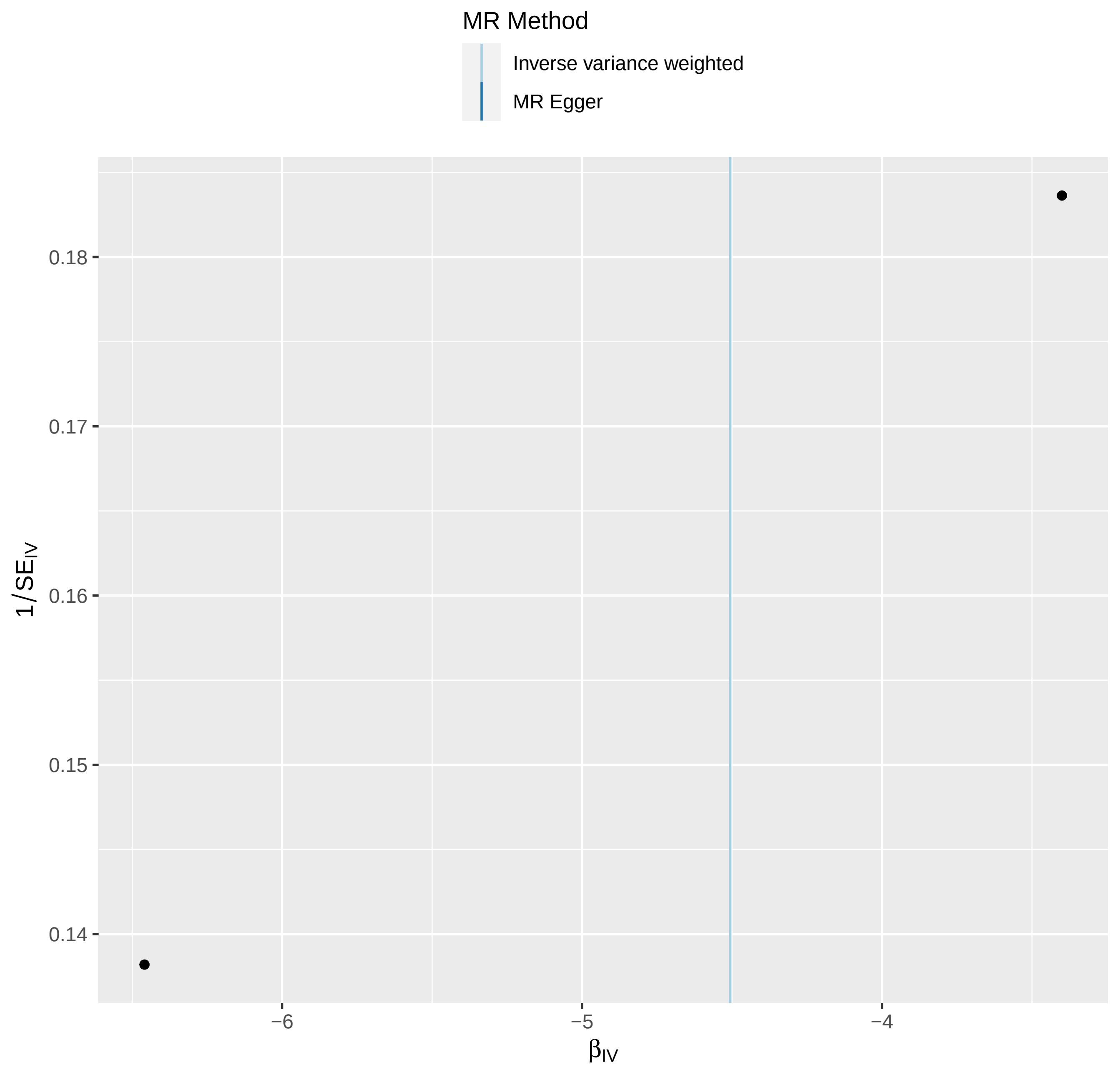

Batch 1682 : Myristate (14:0) on Arthropathy in ulcerative colitis

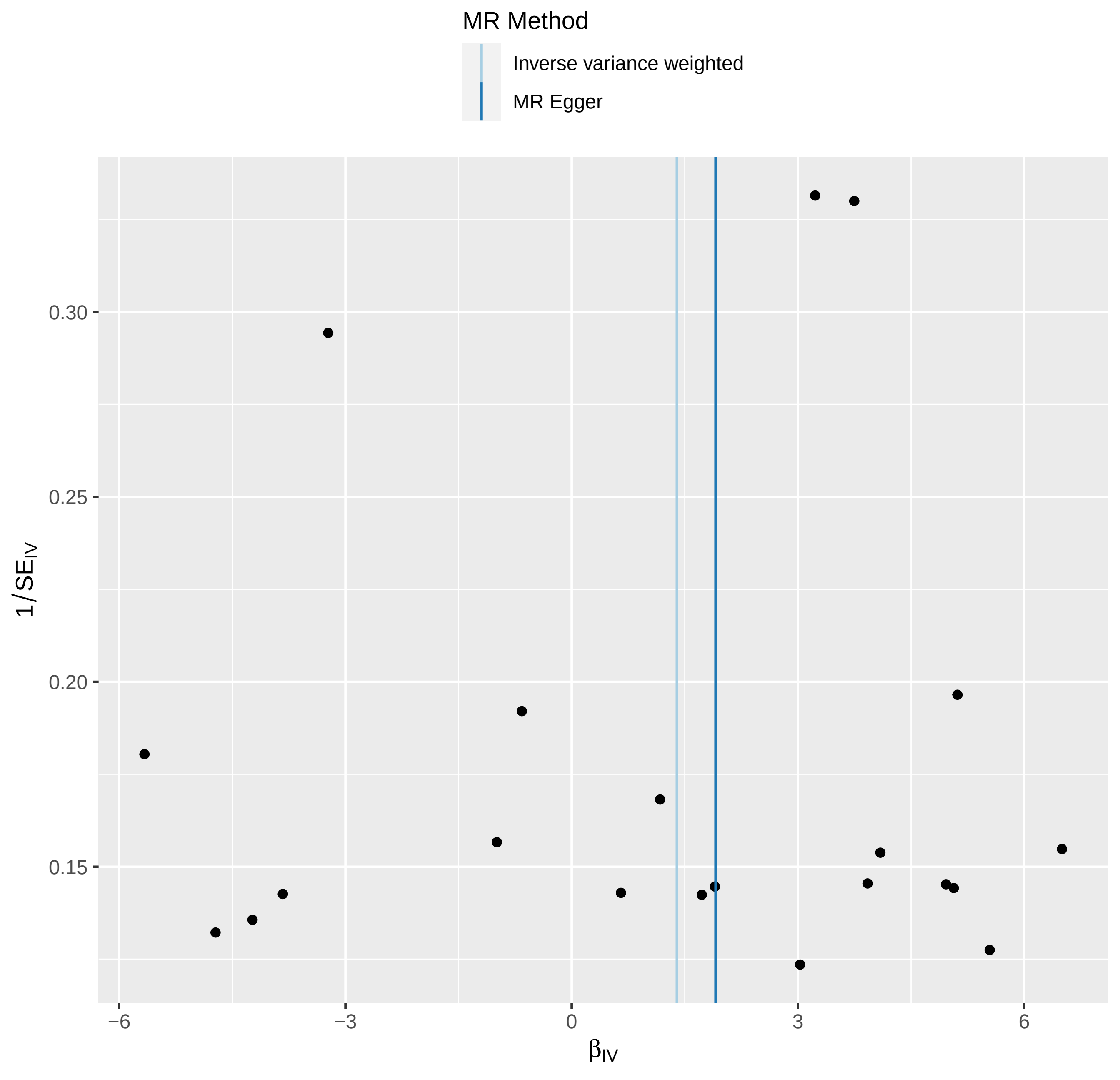

Batch 1684 : Ornithine on Arthropathy in ulcerative colitis

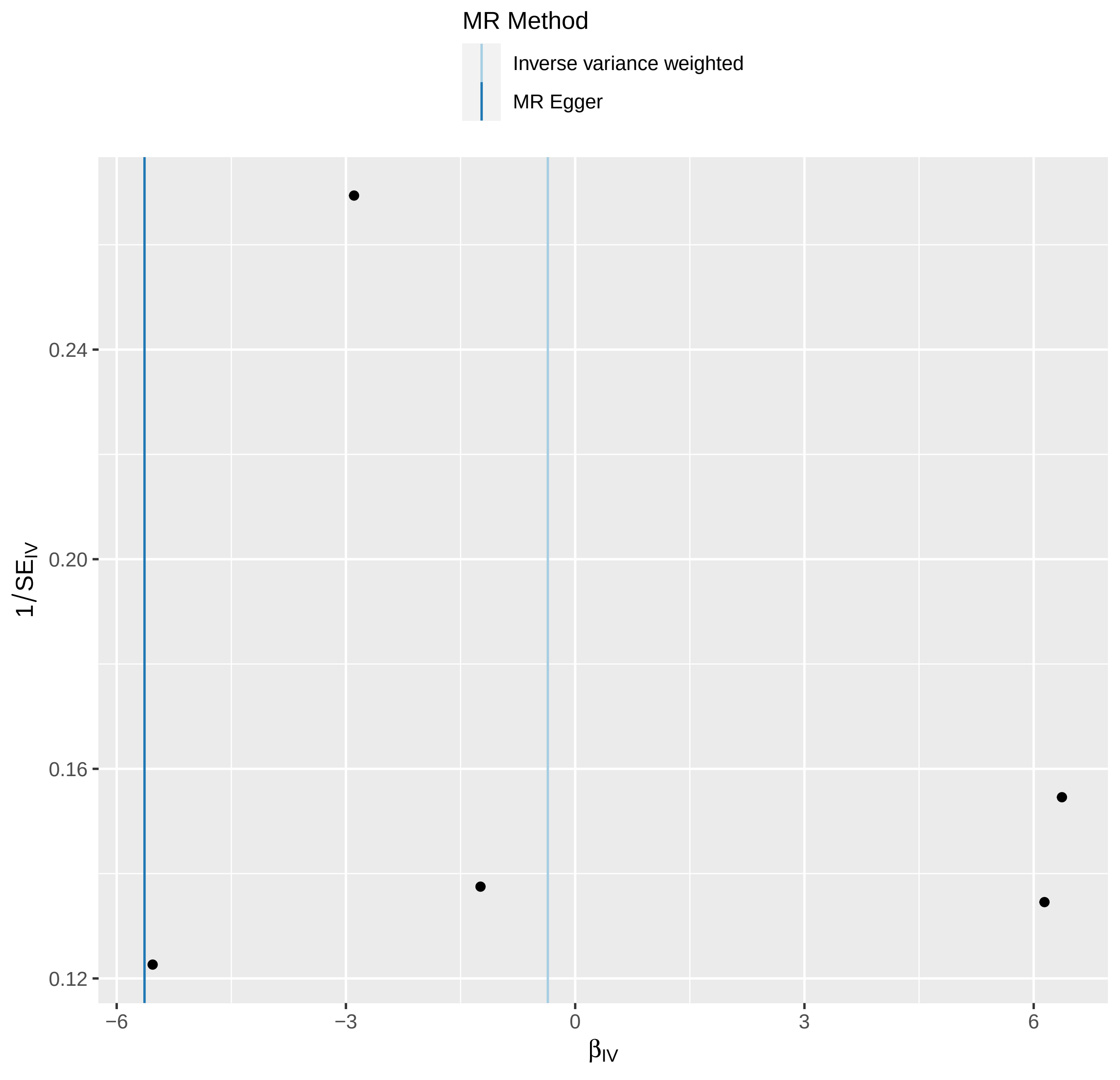

Batch 1688 : Alpha−tocopherol on Arthropathy in ulcerative colitis

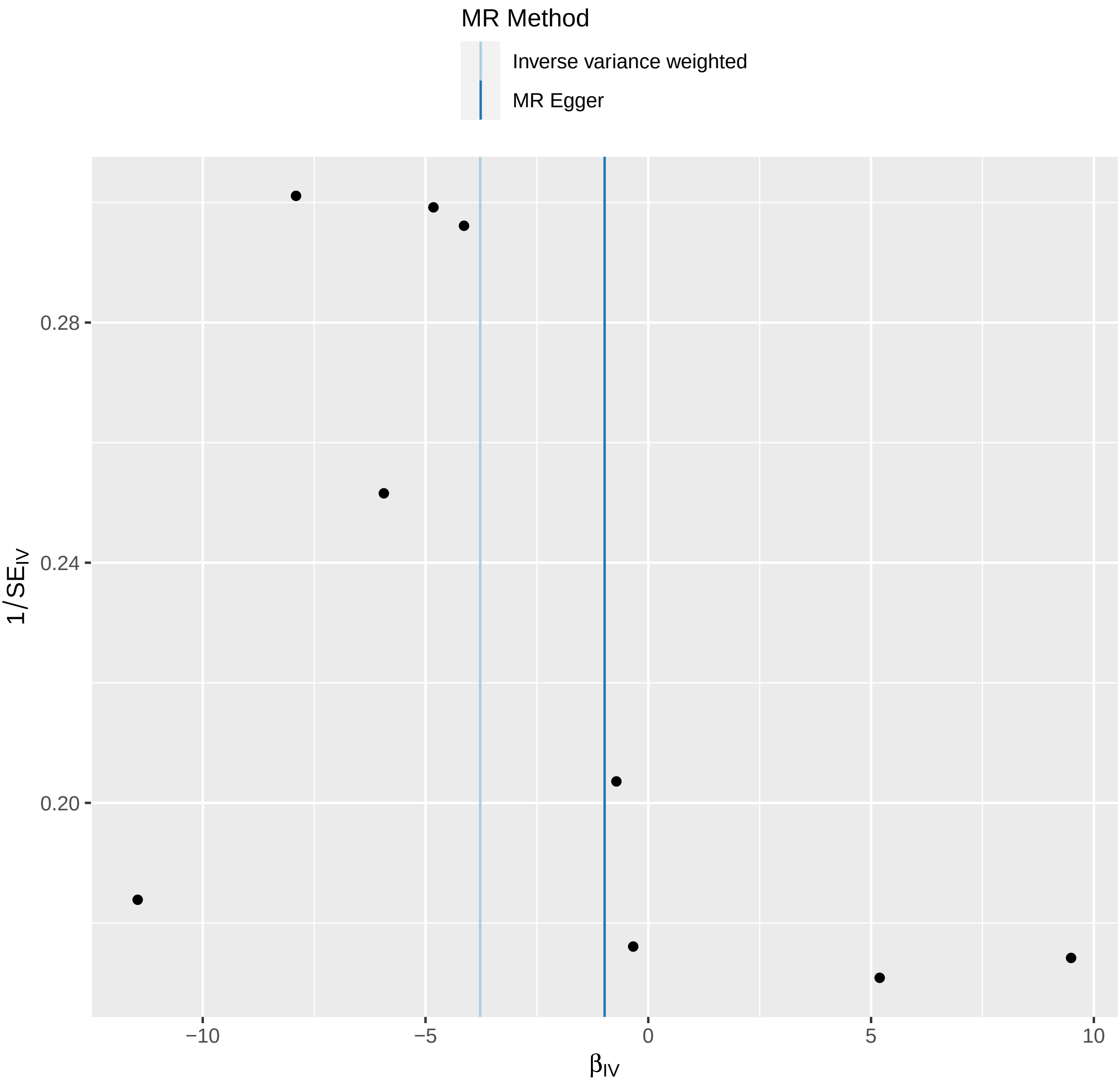

Batch 1689 : Citrate on Arthropathy in ulcerative colitis

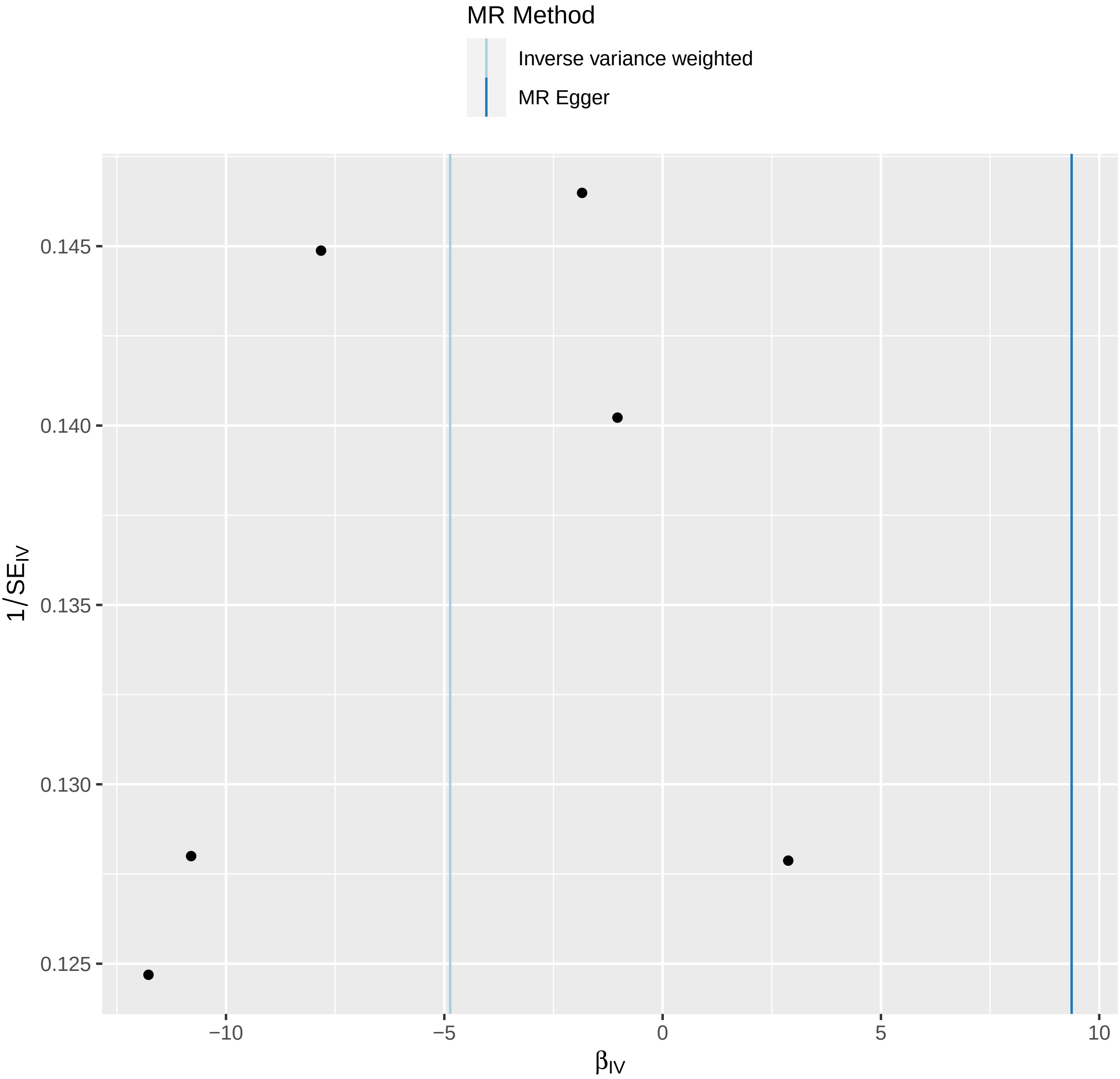

Batch 1690 : Glycerate on Arthropathy in ulcerative colitis

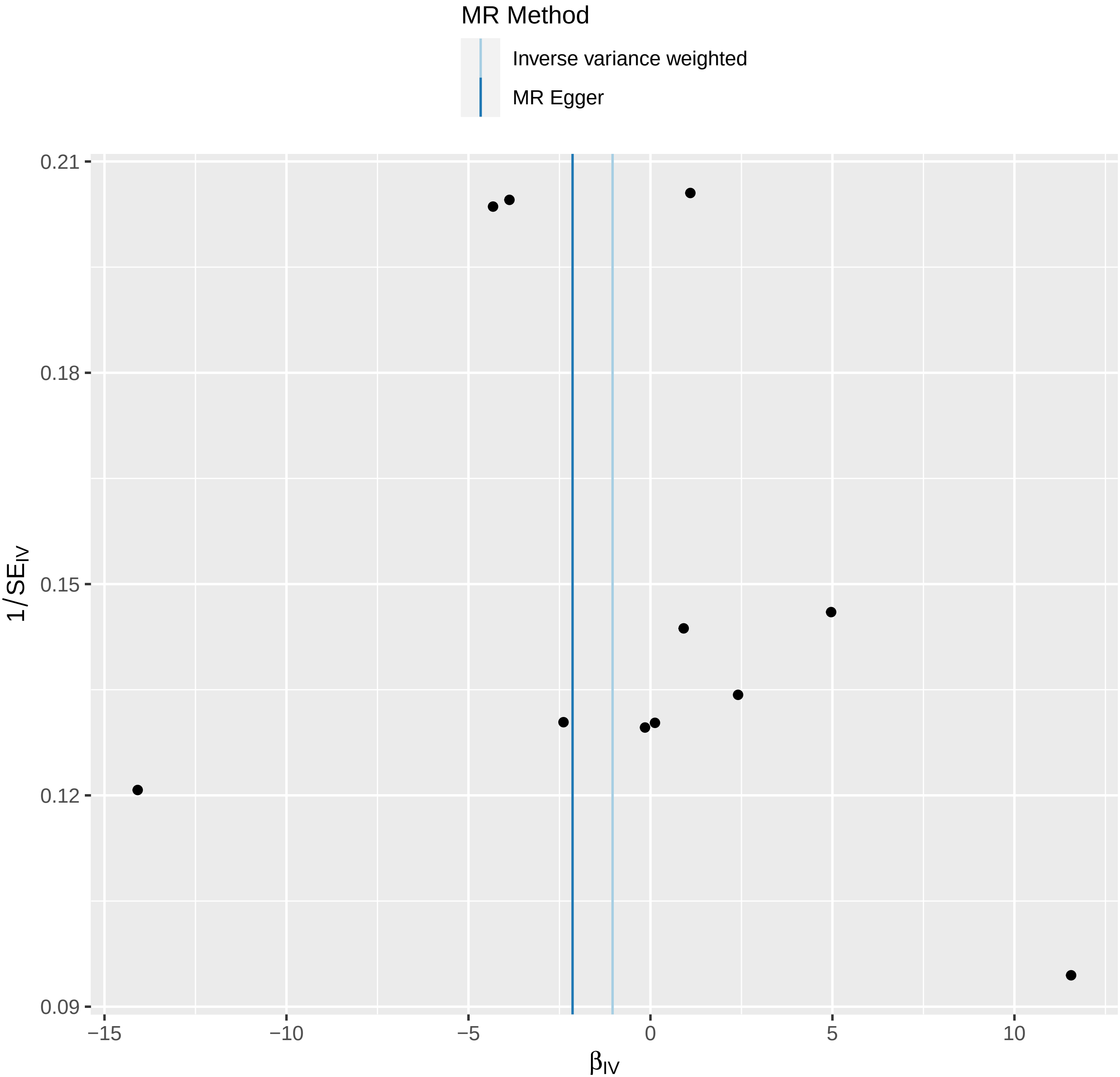

Batch 1691 : Guanosine on Arthropathy in ulcerative colitis

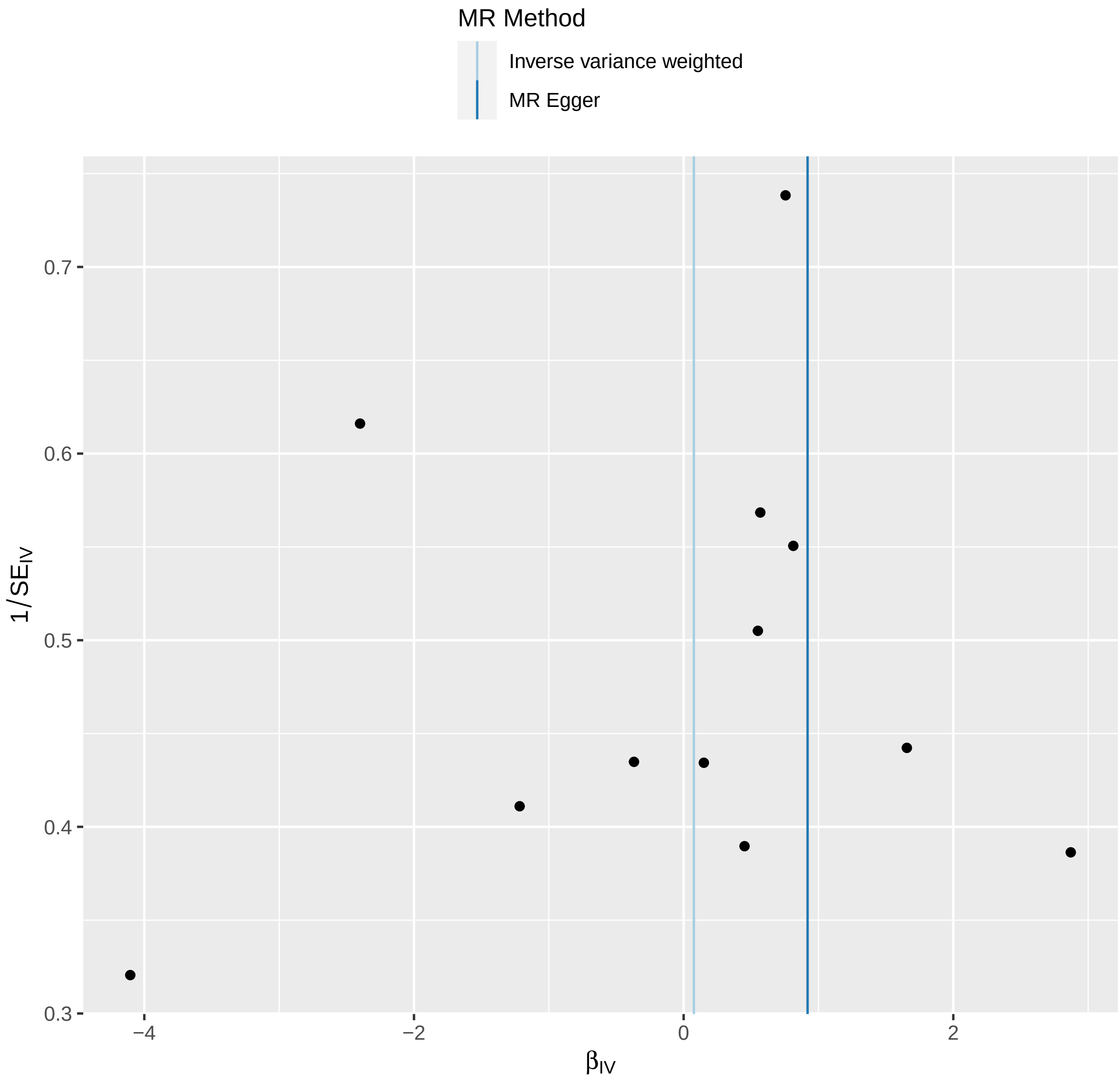

Batch 1694 : Ursodeoxycholate on Arthropathy in ulcerative colitis

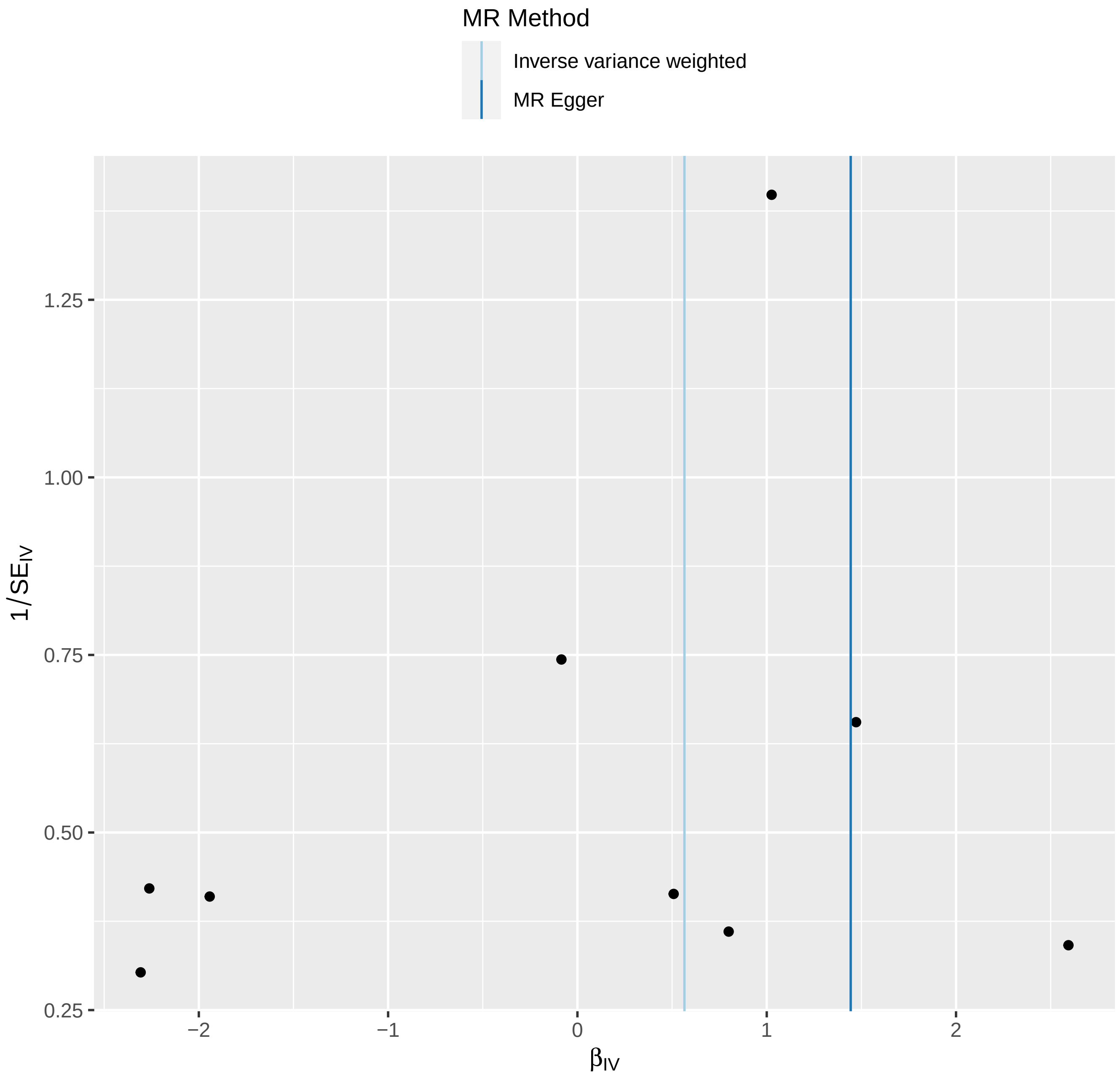

Batch 1695 : Arginine on Arthropathy in ulcerative colitis

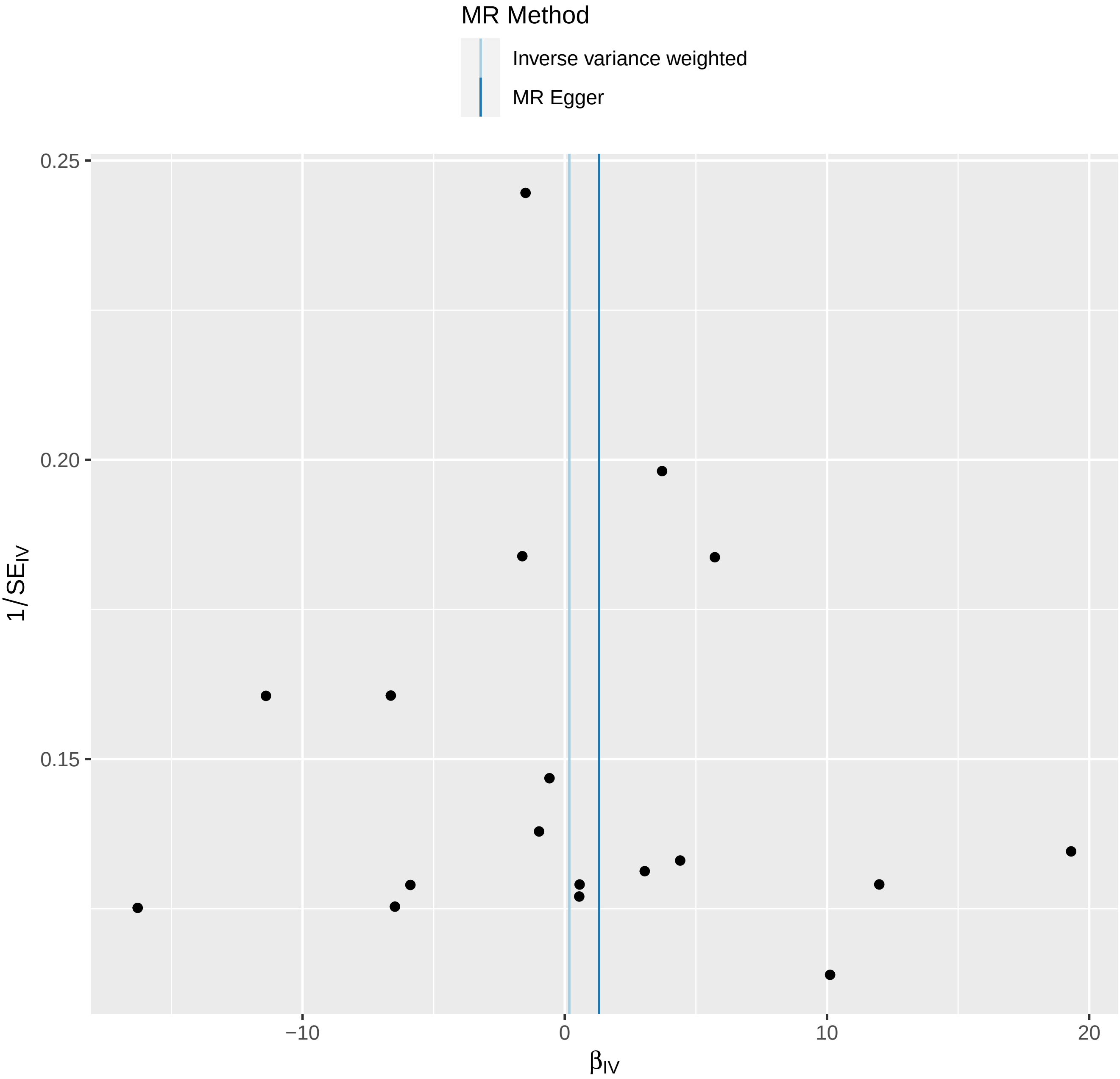

Batch 1696 : Ascorbate (Vitamin C) on Arthropathy in ulcerative colitis

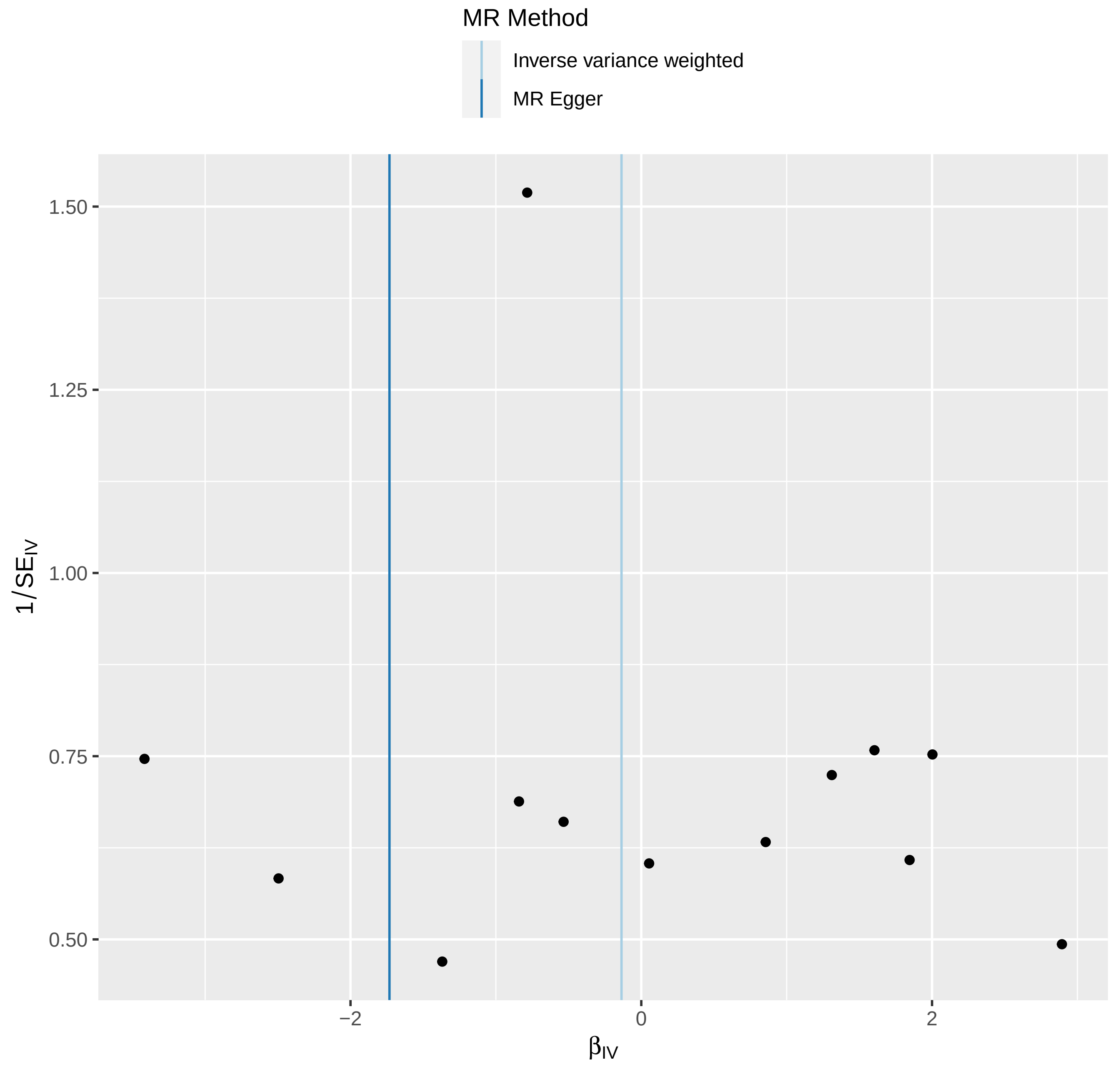

Batch 1697 : Heptanoate (7:0) on Arthropathy in ulcerative colitis

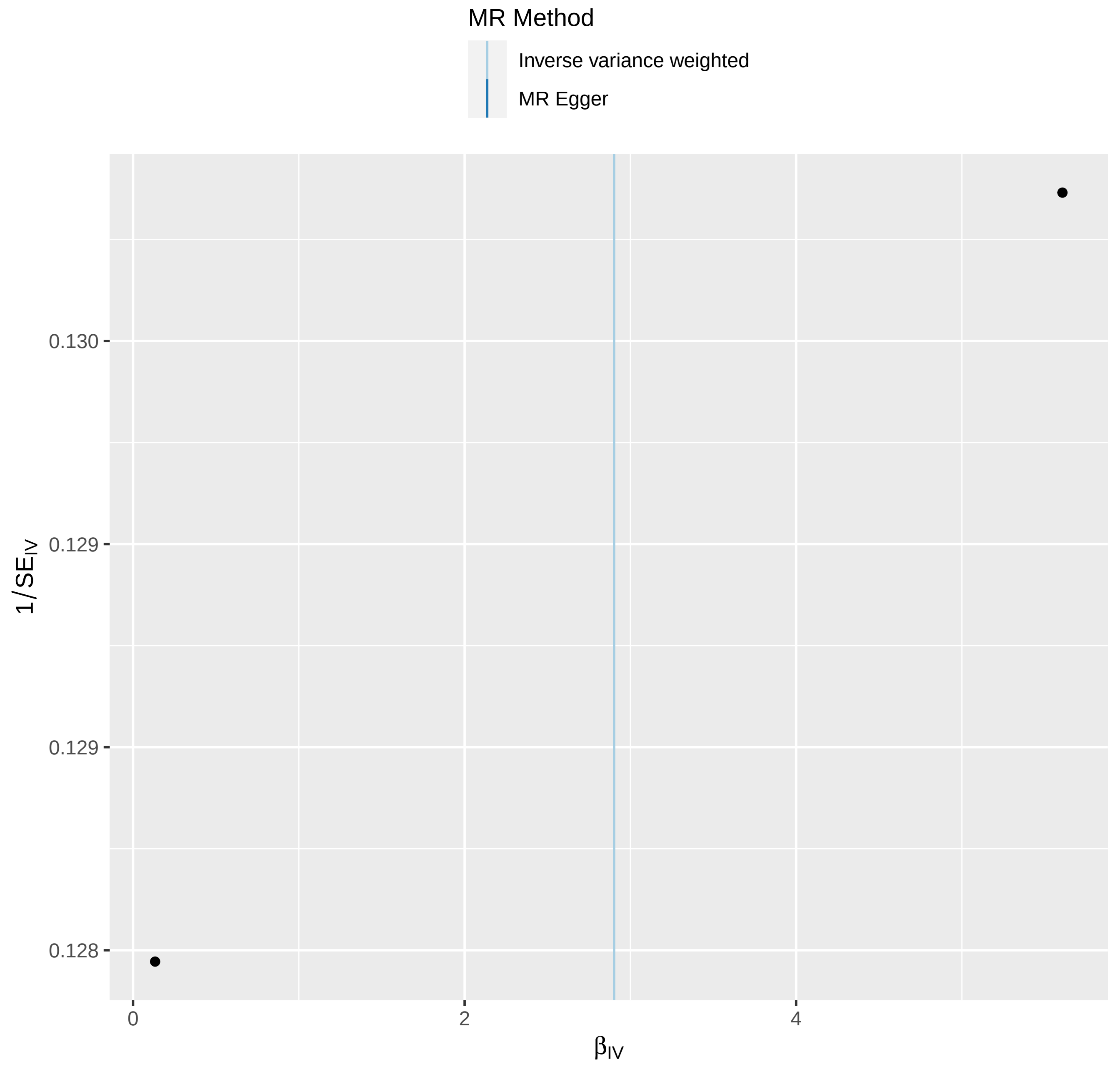

Batch 1698 : Laurate (12:0) on Arthropathy in ulcerative colitis

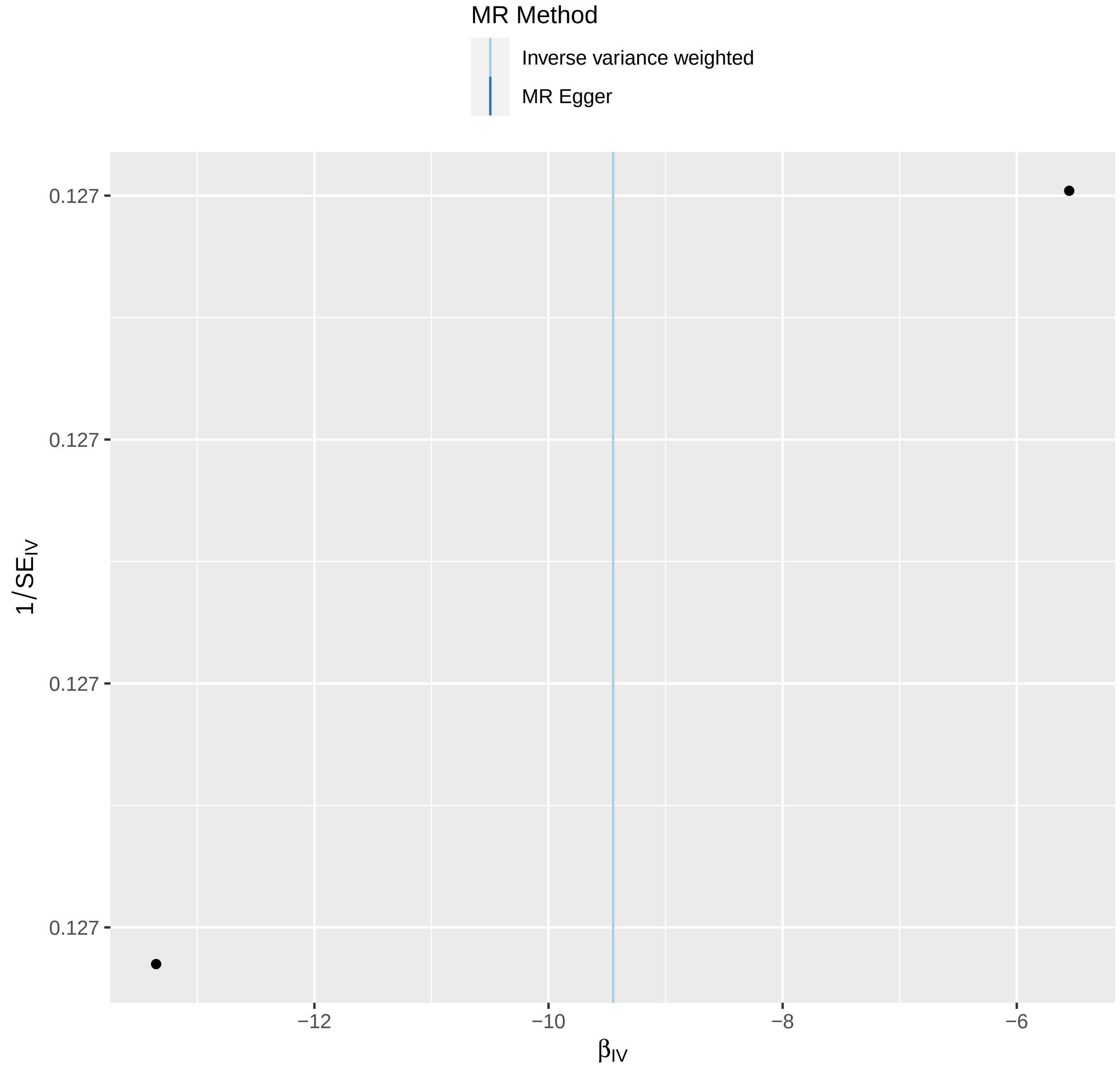

Batch 1700 : Urea on Arthropathy in ulcerative colitis

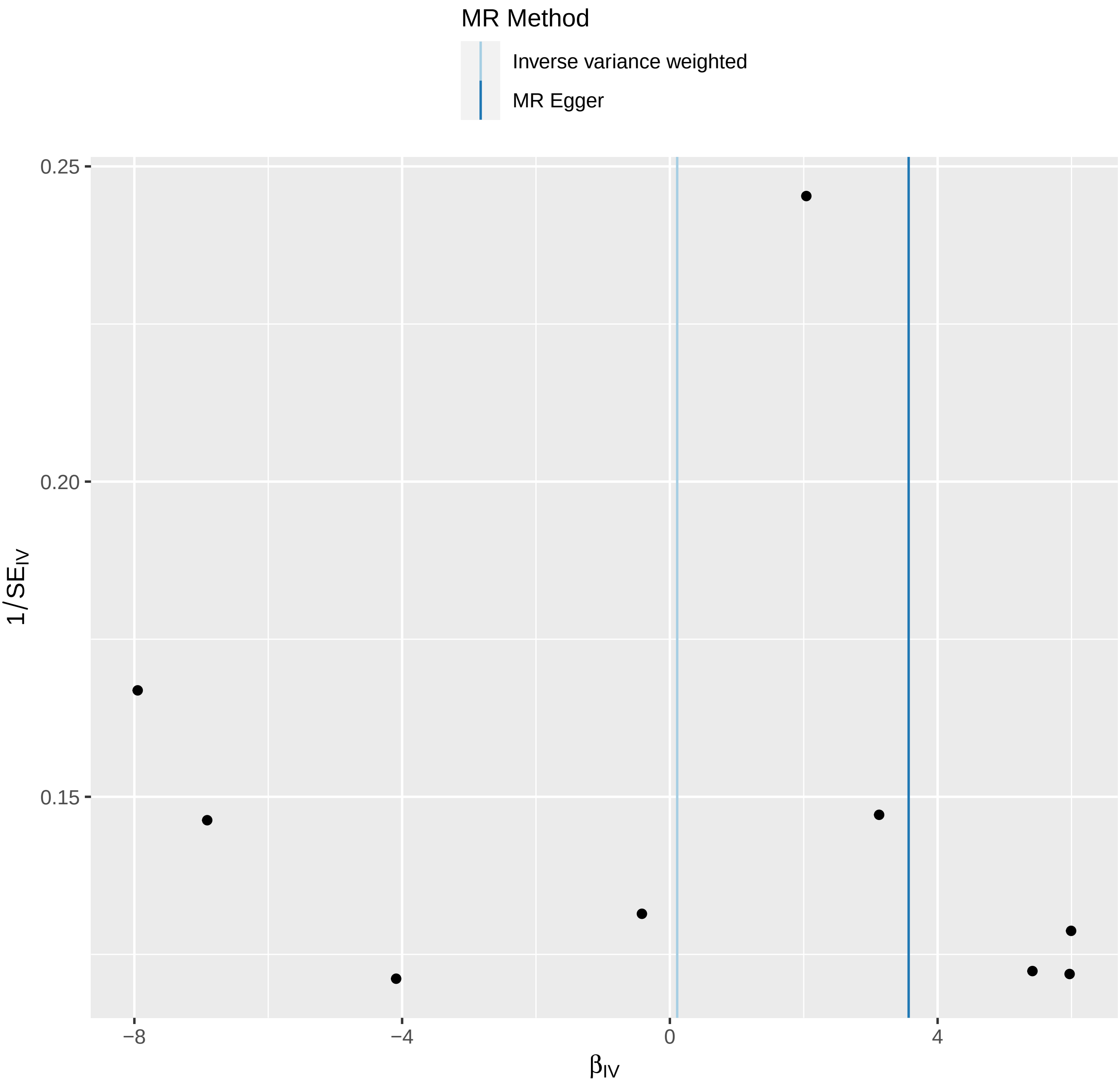

Batch 1701 : Cortisol on Arthropathy in ulcerative colitis

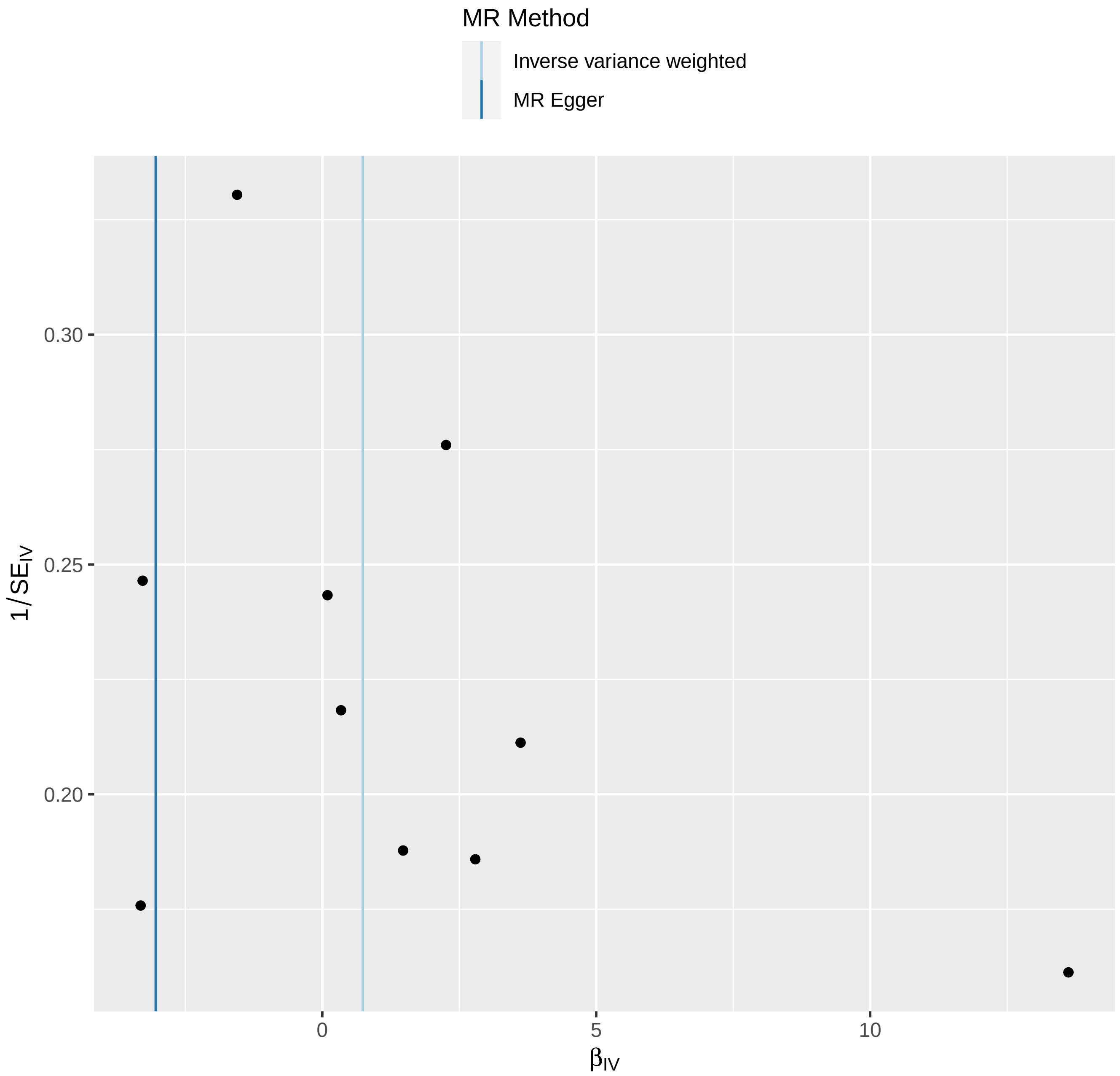

Batch 1704 : Citrulline on Arthropathy in ulcerative colitis

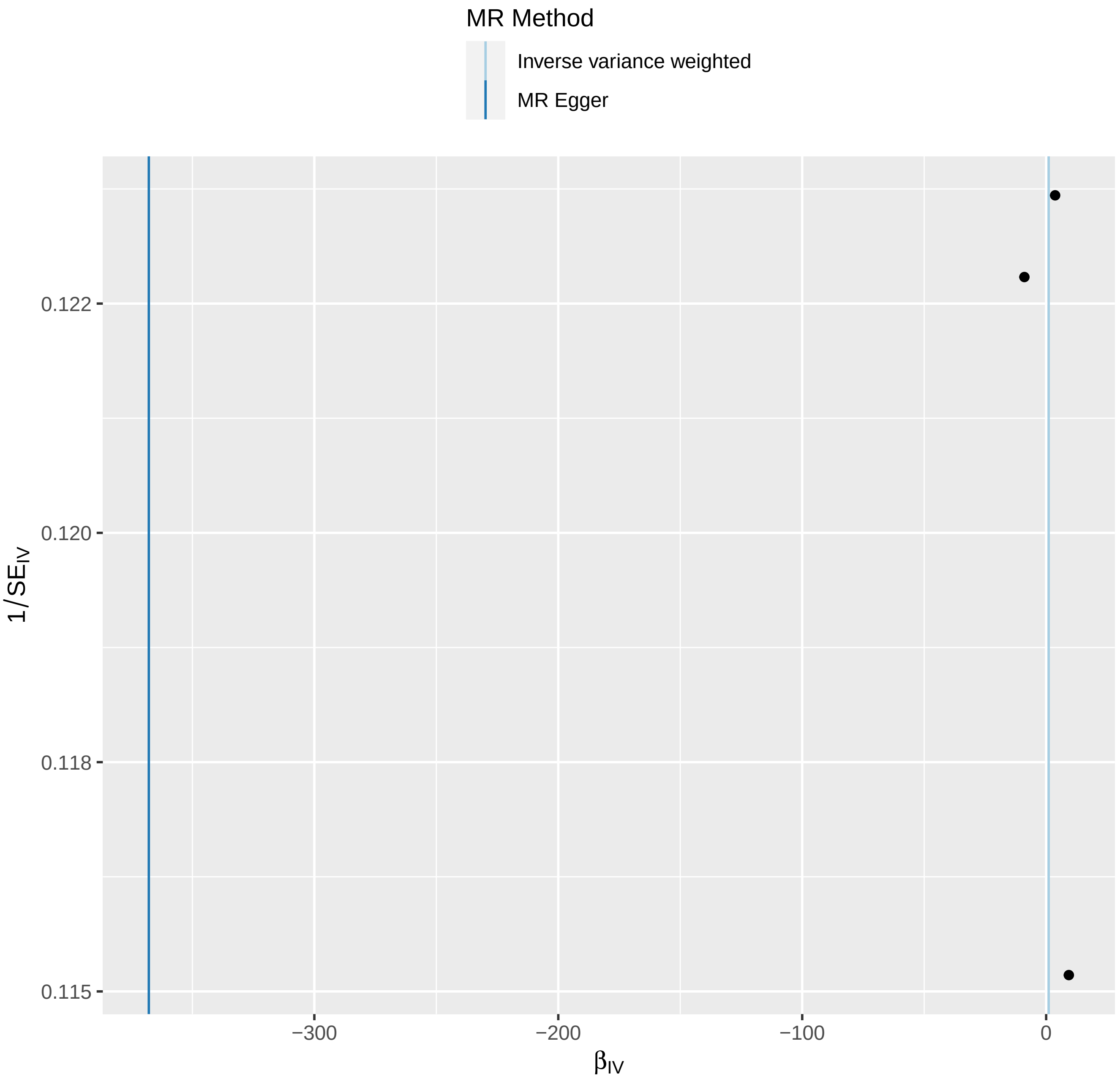

Batch 1706 : Serotonin (5HT) on Arthropathy in ulcerative colitis

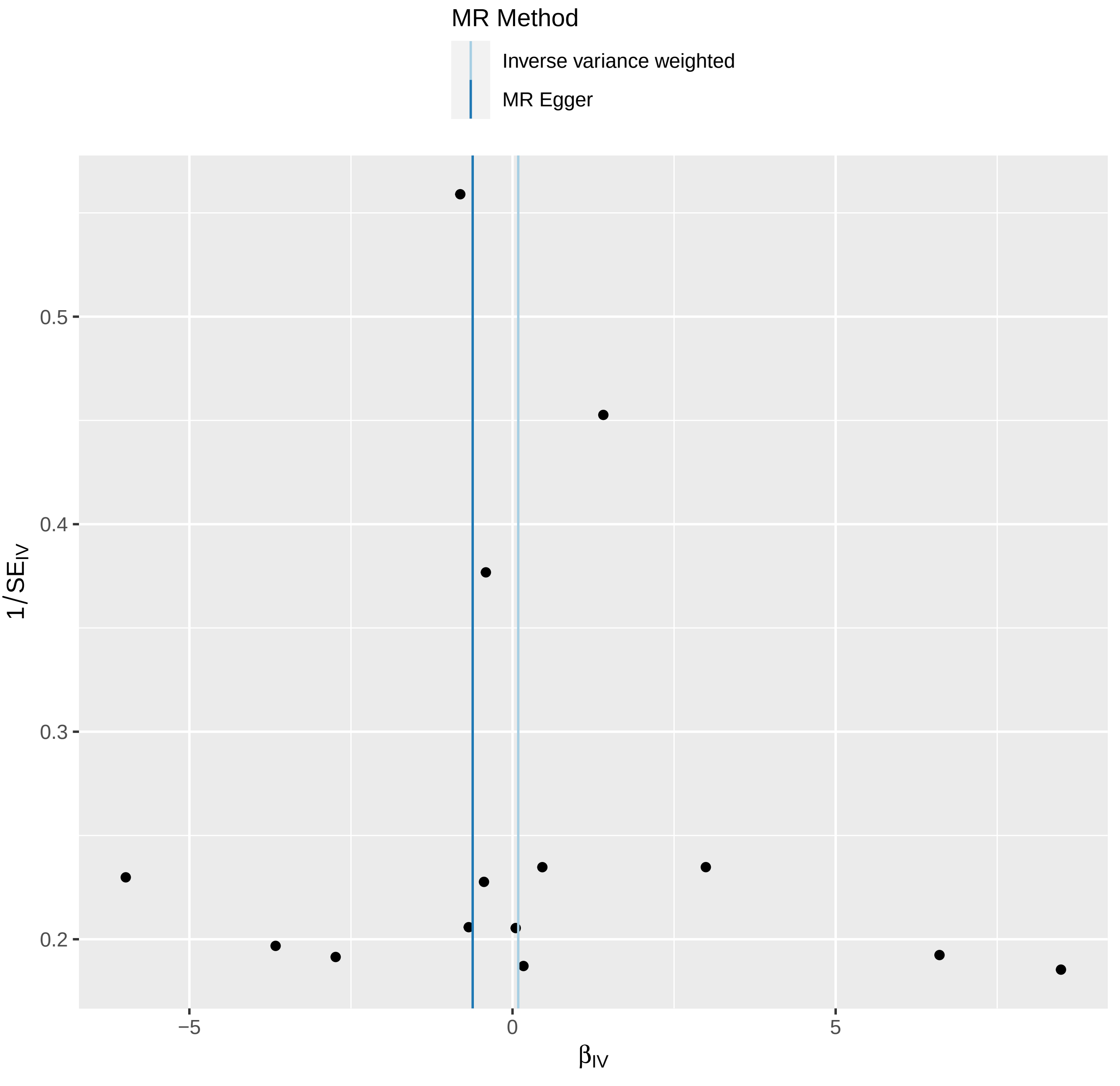

Batch 1707 : Gamma−glutamylglutamine on Arthropathy in ulcerative colitis

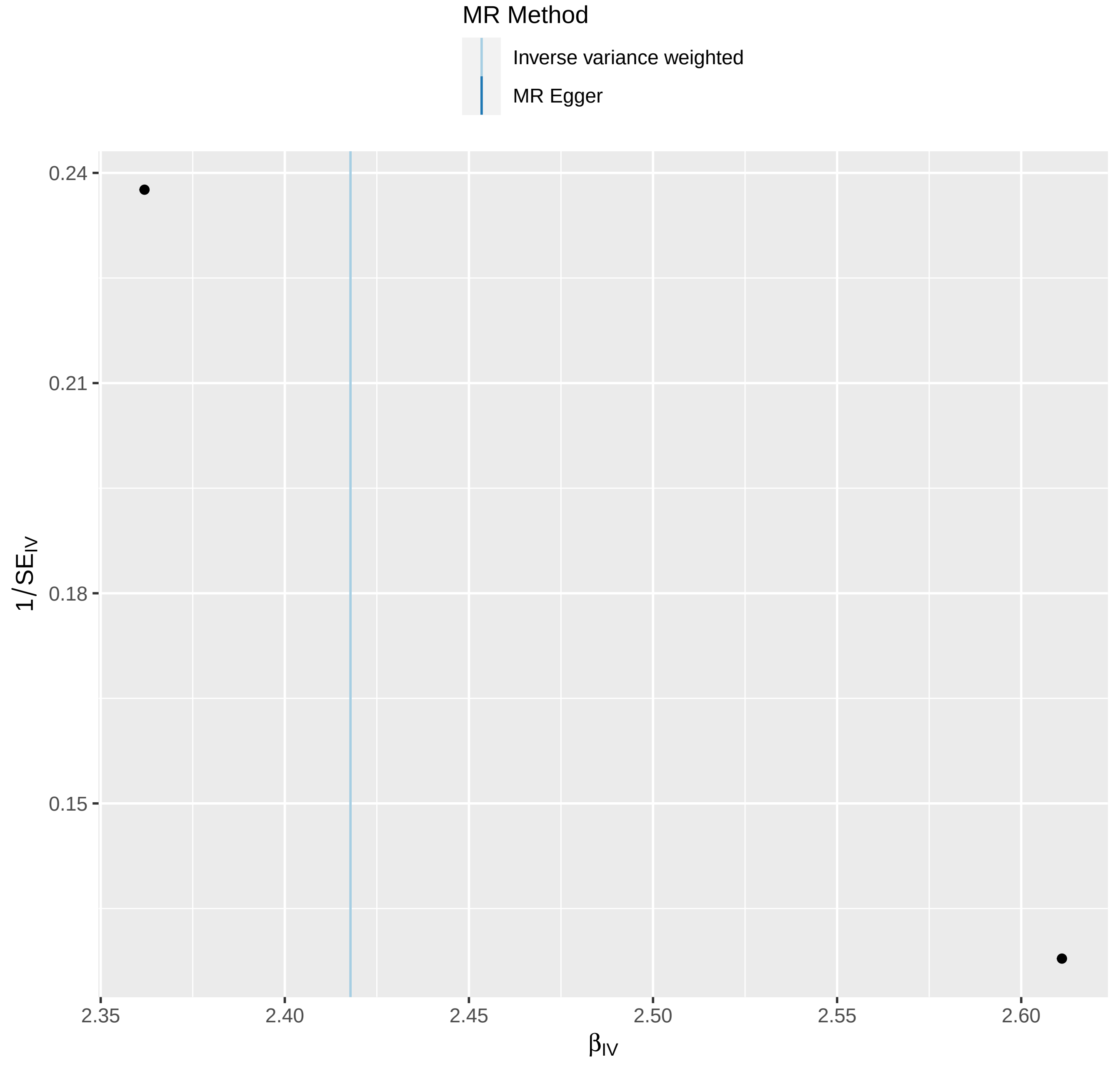

Batch 1708 : Gamma−glutamyltyrosine on Arthropathy in ulcerative colitis

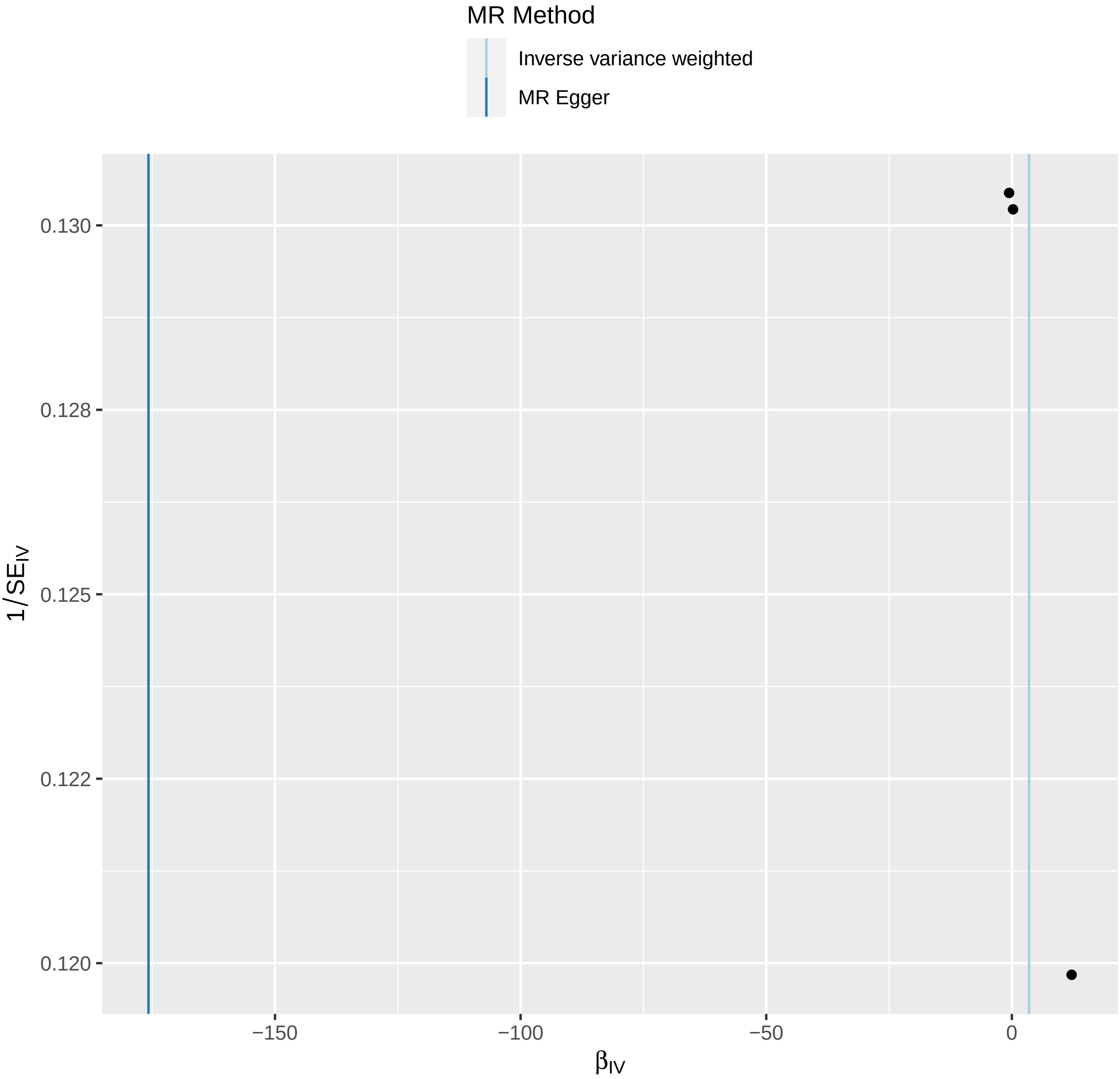

Batch 1710 : Betaine on Arthropathy in ulcerative colitis

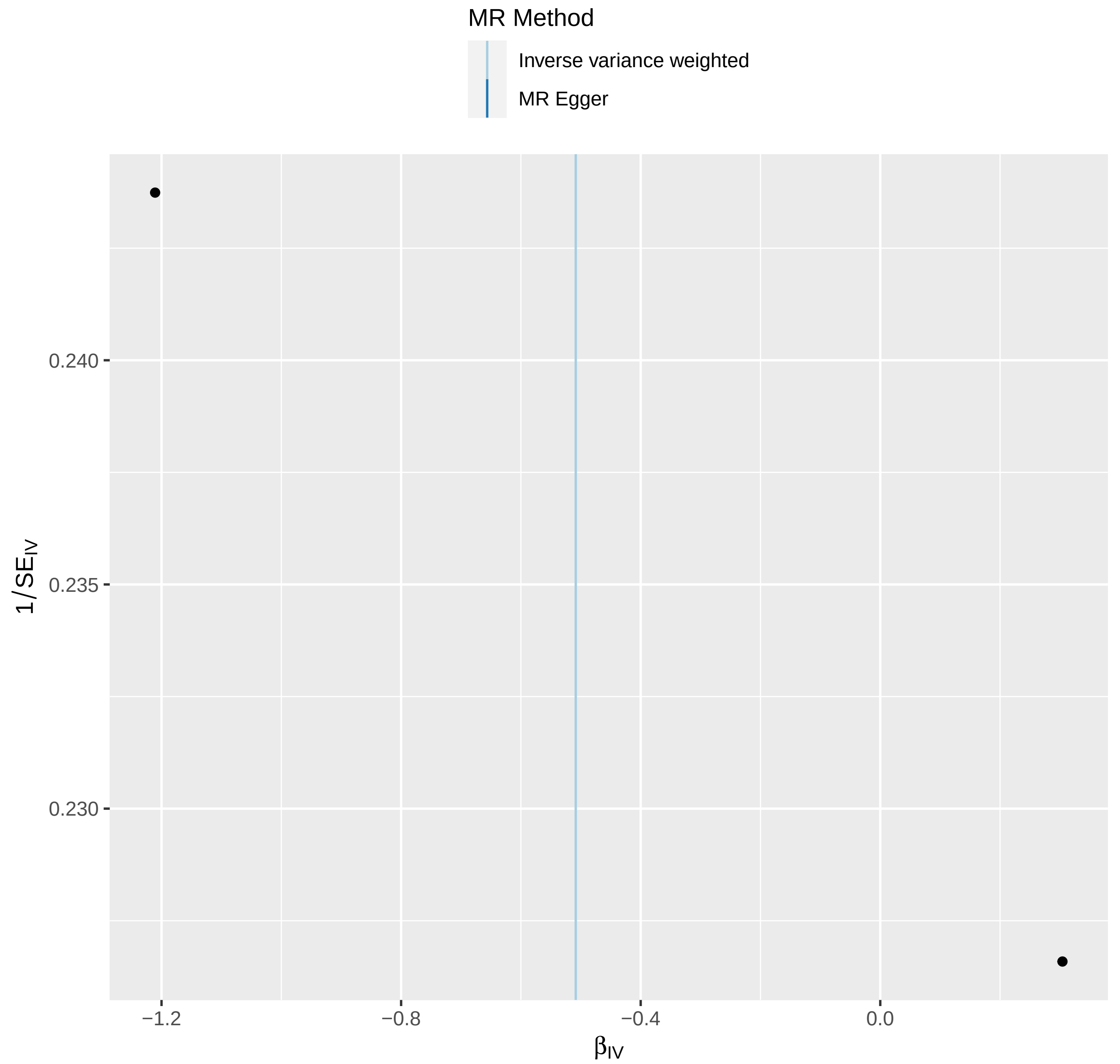

Batch 1711 : Xanthine on Arthropathy in ulcerative colitis

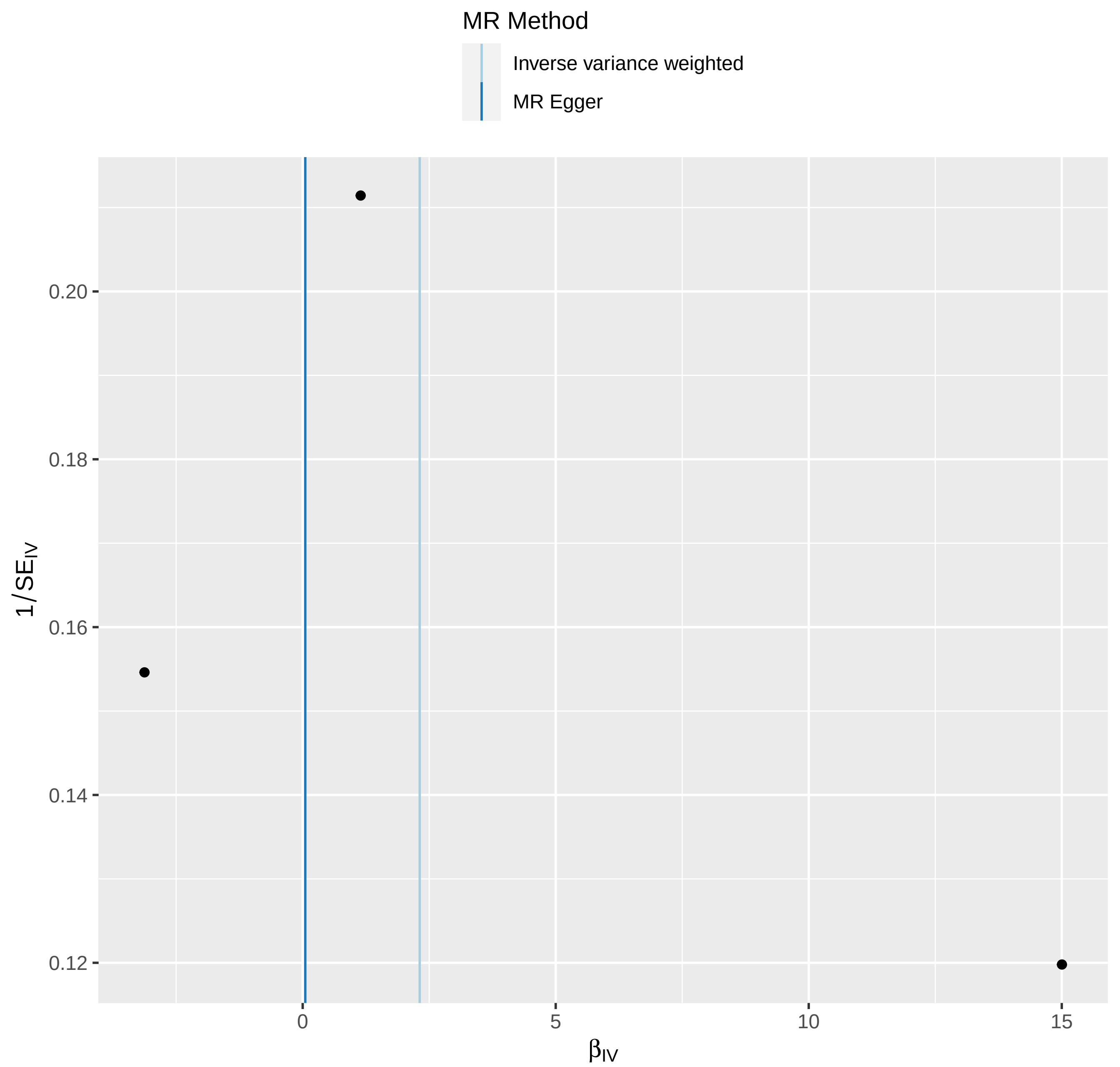

Batch 1712 : Phosphate on Arthropathy in ulcerative colitis

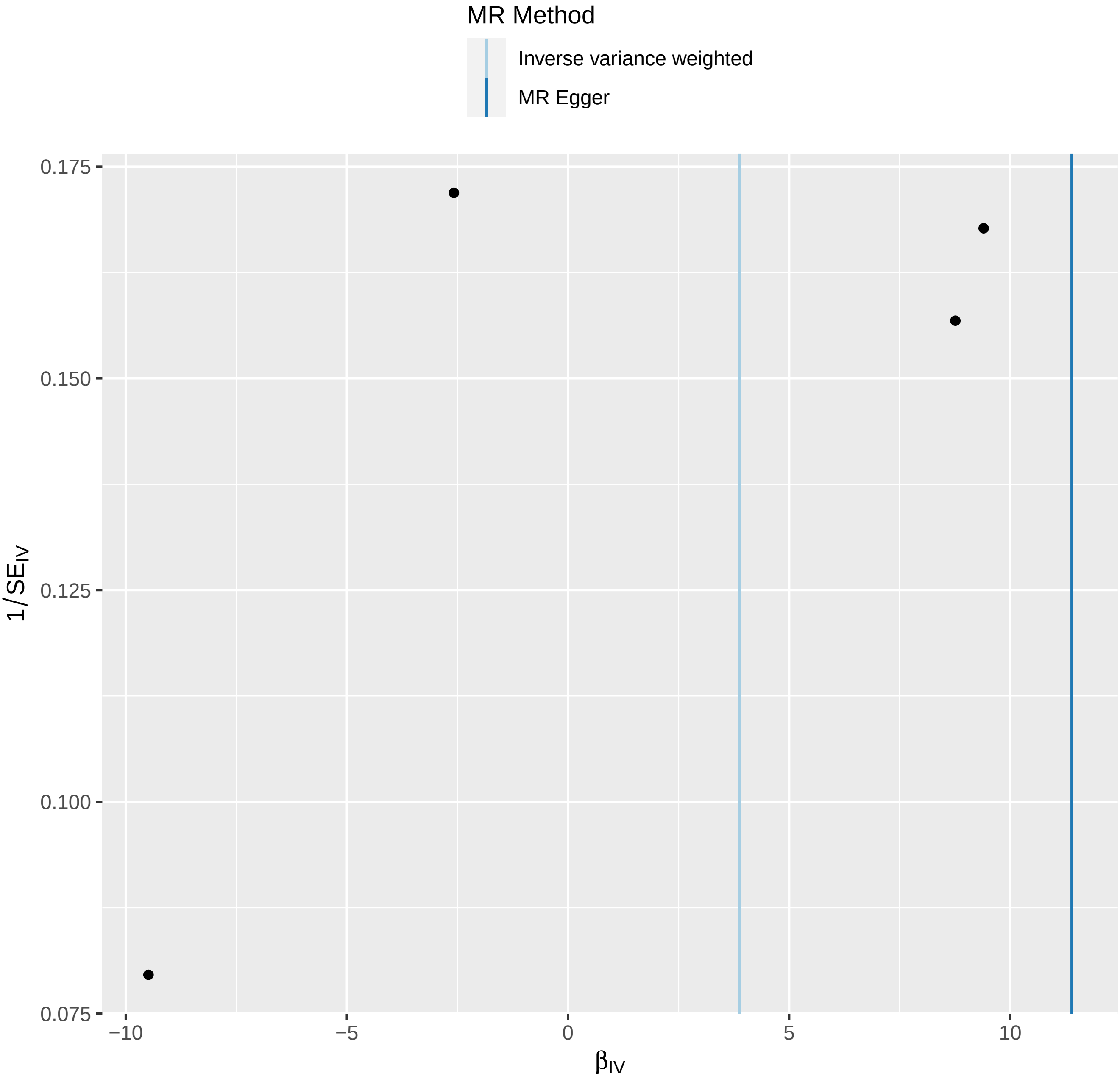

Batch 1713 : 3−methoxytyrosine on Arthropathy in ulcerative colitis

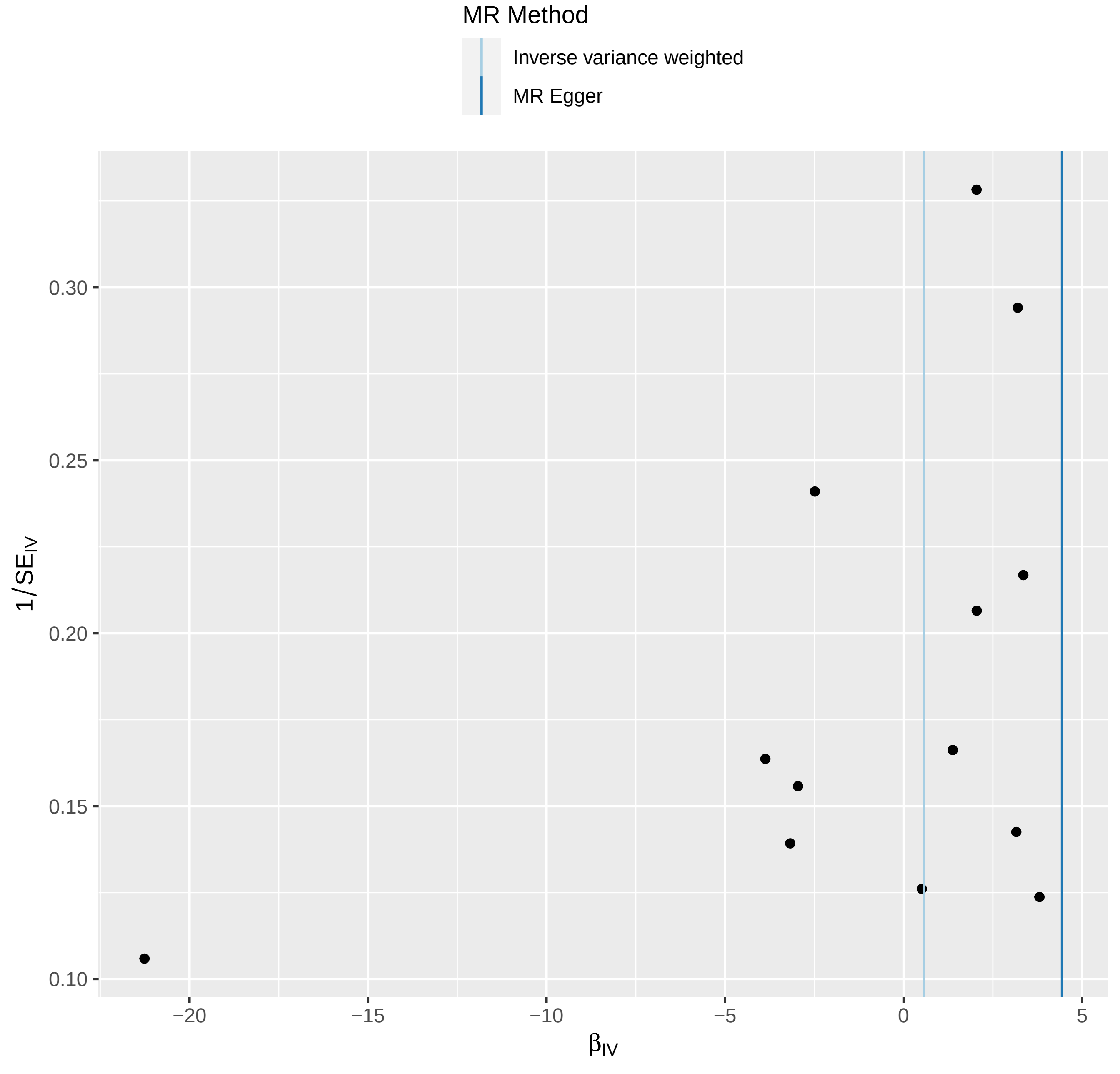

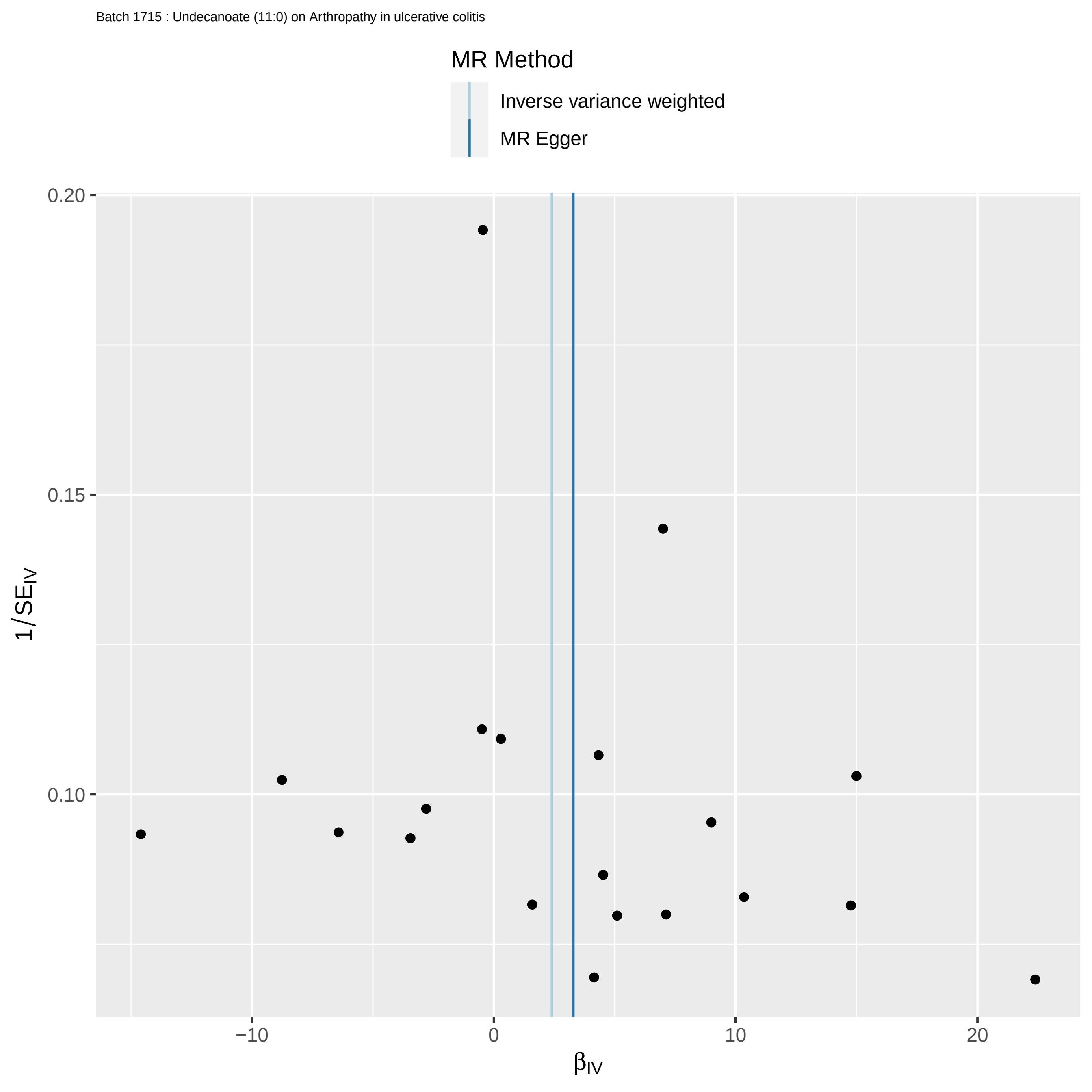

Batch 1715 : Undecanoate (11:0) on Arthropathy in ulcerative colitis
MR Method
Inverse variance weighted
MR Egger
0.20
0.15
0.10
1/SE_IV
−10
0
10
20
β_IV

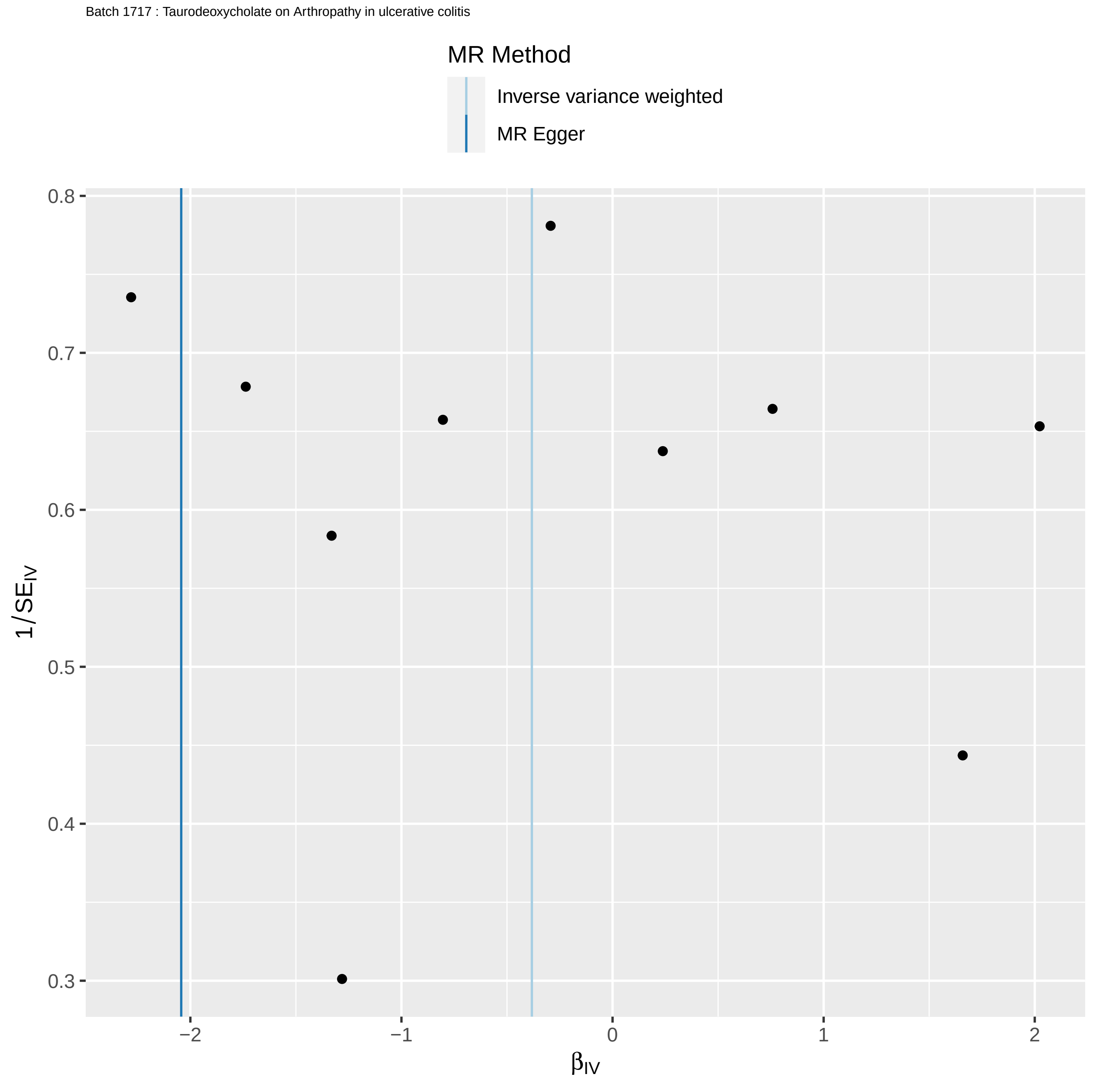
Batch 1717 : Taurodeoxycholate on Arthropathy in ulcerative colitis
MR Method
Inverse variance weighted
MR Egger
0.8
0.7
0.6
0.5
0.4
0.3
1/SE$_{IV}$
−2
−1
0
1
2
β$_{IV}$

Batch 1718 : Glycerol on Arthropathy in ulcerative colitis

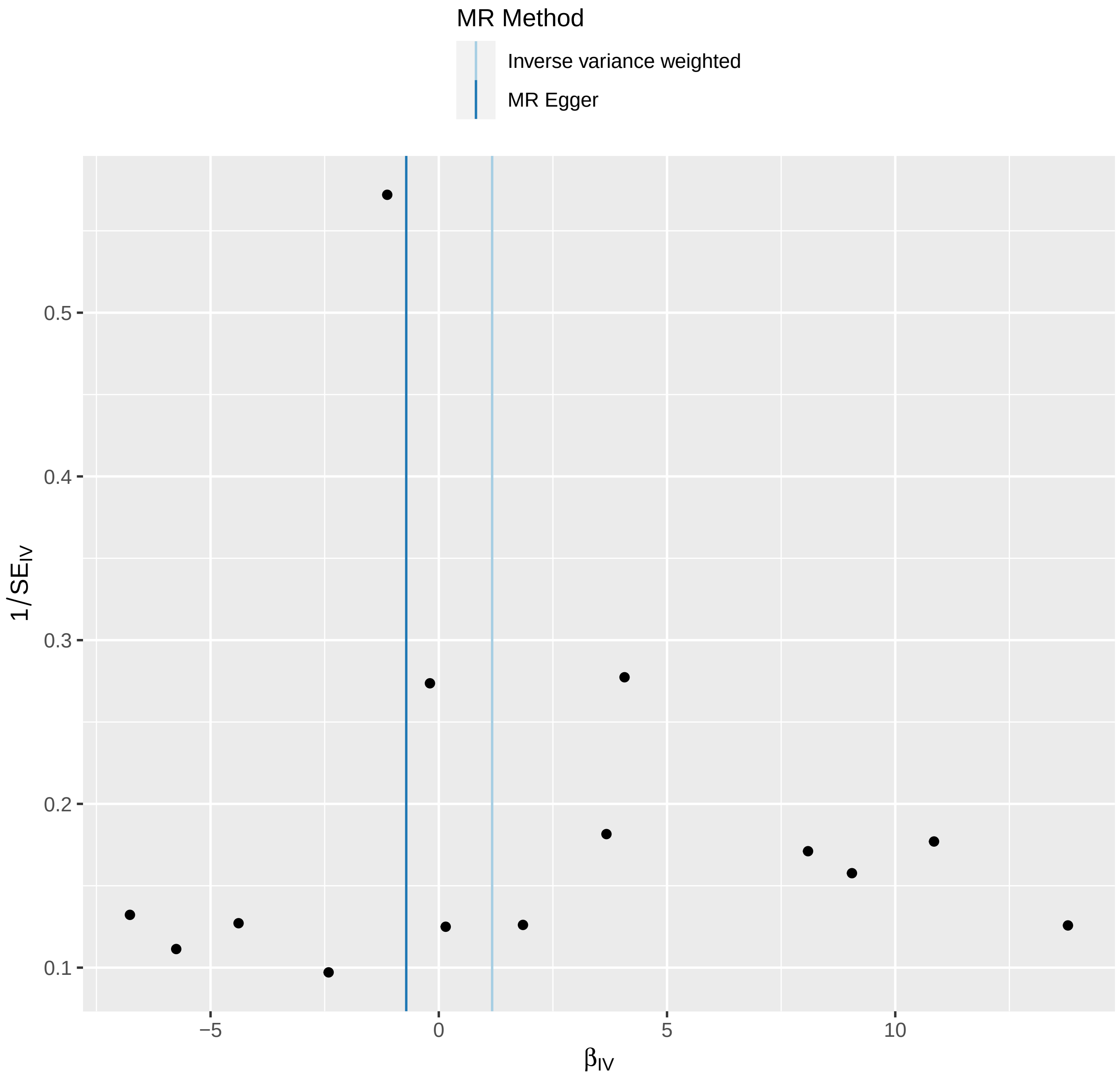

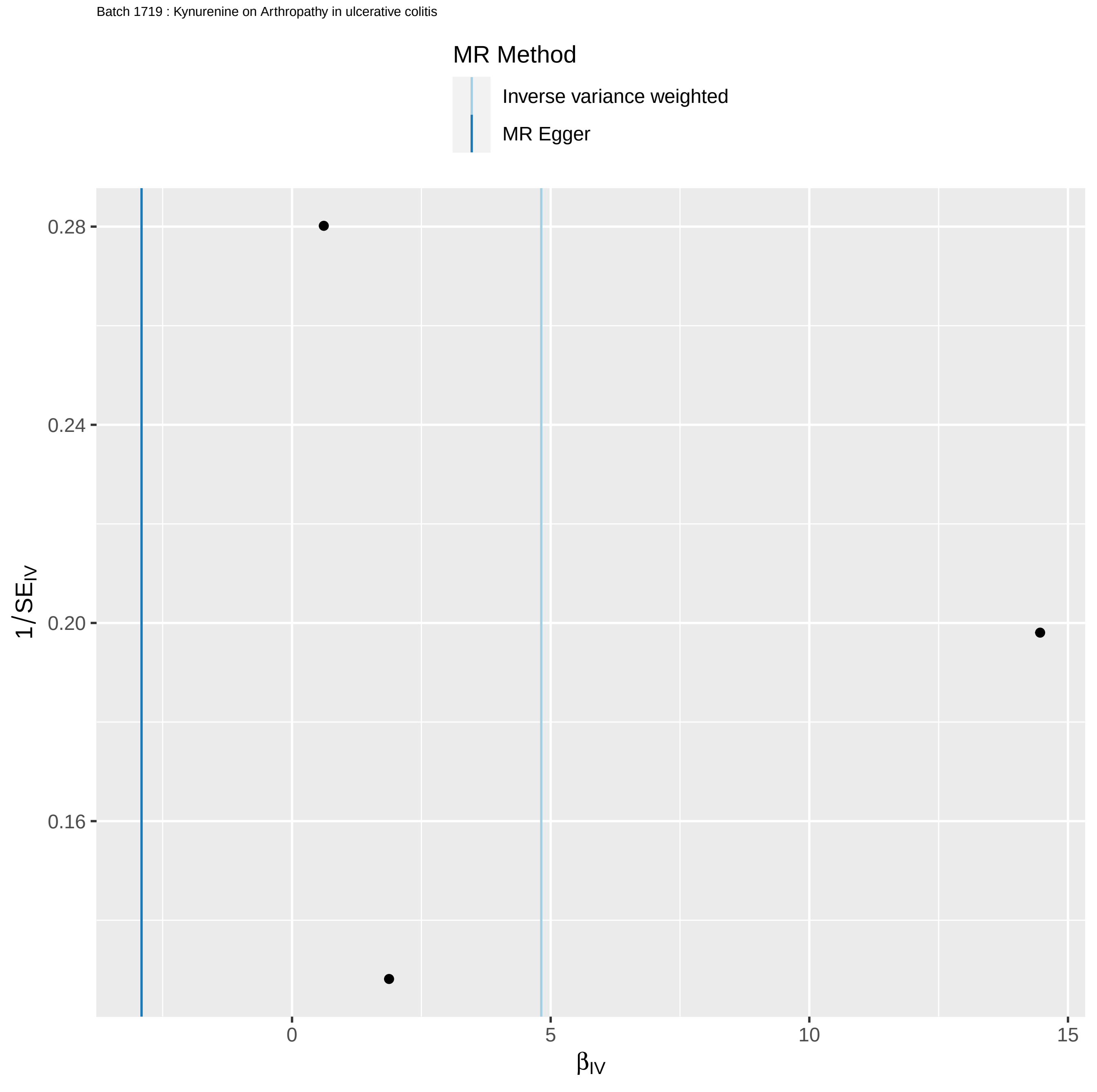

Batch 1719 : Kynurenine on Arthropathy in ulcerative colitis
MR Method
Inverse variance weighted
MR Egger
0.28
0.24
0.20
0.16
$1/SE_{IV}$
0
5
10
15
β_{IV}

Batch 1720 : Mannitol on Arthropathy in ulcerative colitis

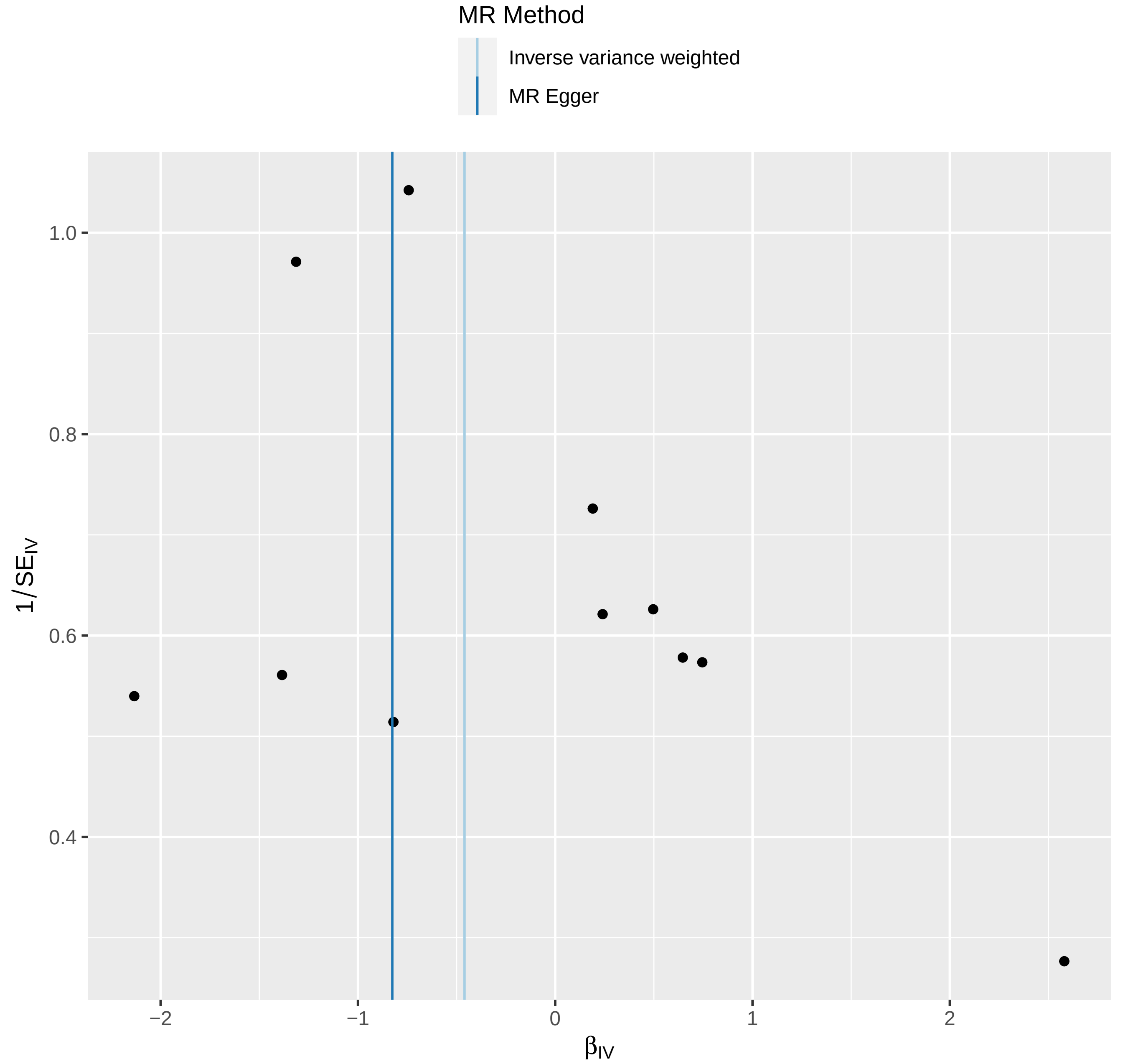

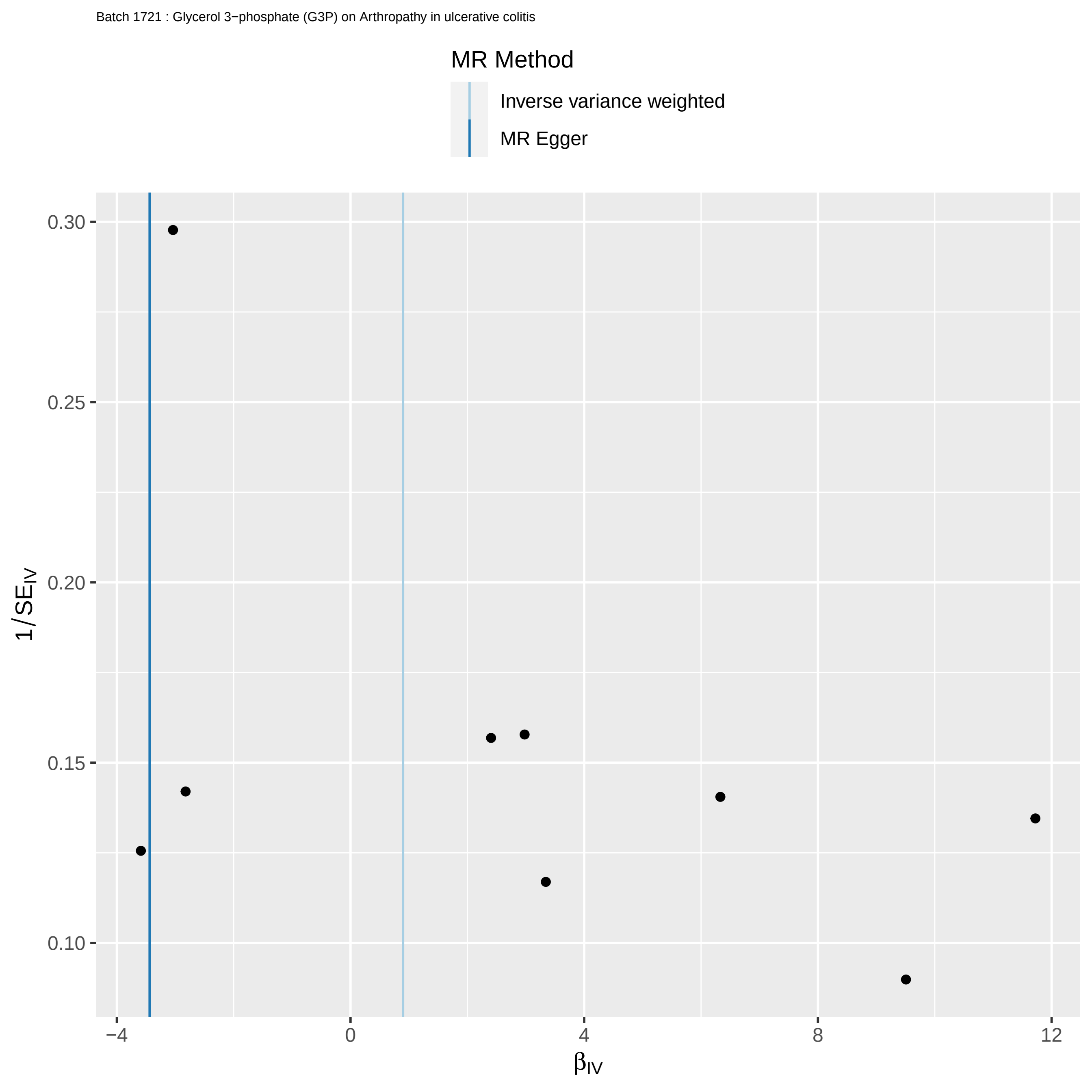
Batch 1721 : Glycerol 3−phosphate (G3P) on Arthropathy in ulcerative colitis
MR Method
Inverse variance weighted
MR Egger
0.30
0.25
0.20
0.15
0.10
$1/SE_{IV}$
−4
0
4
8
12
β_{IV}

Batch 1723 : Carnitine on Arthropathy in ulcerative colitis

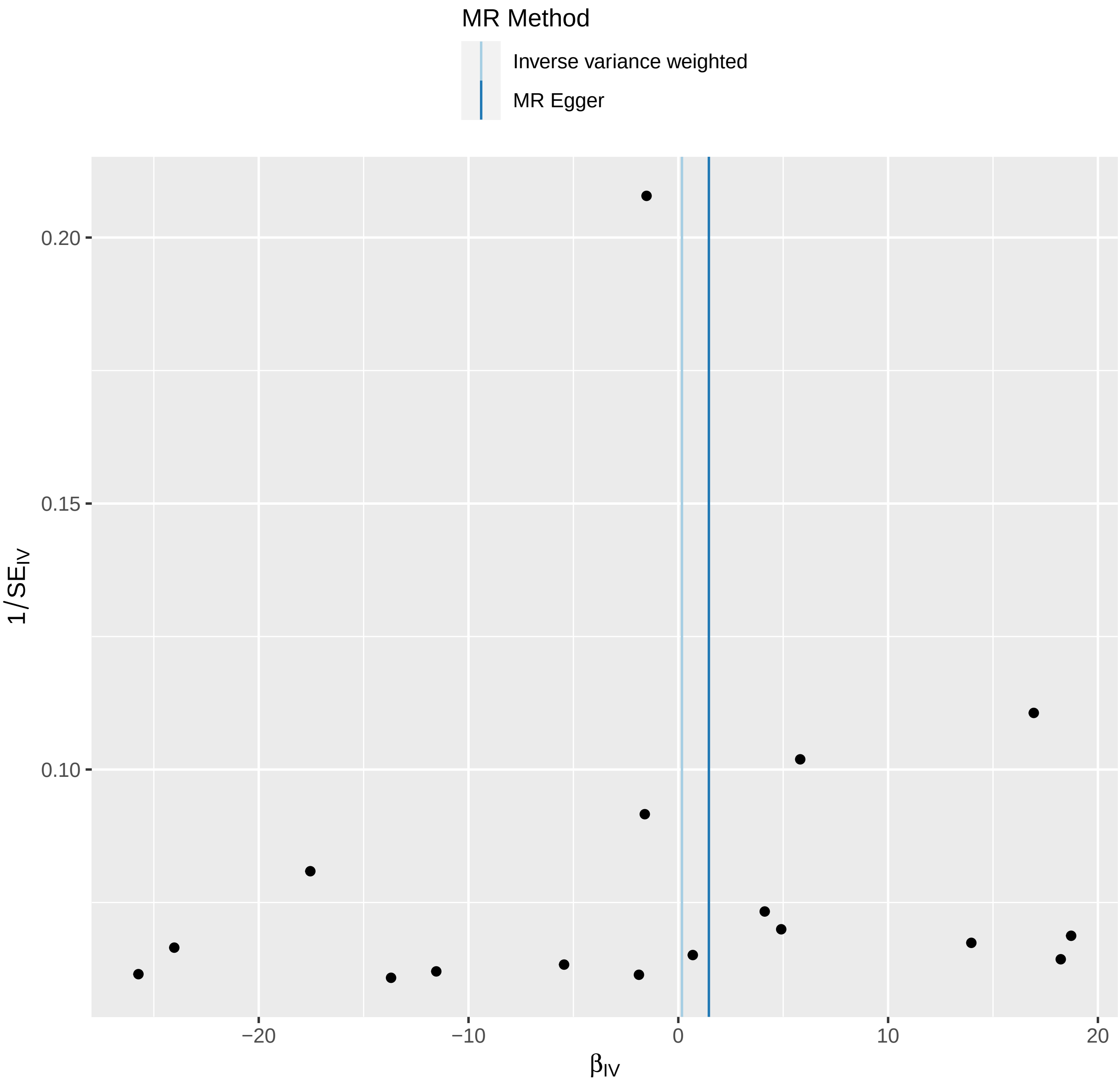

Batch 1724 : Choline on Arthropathy in ulcerative colitis

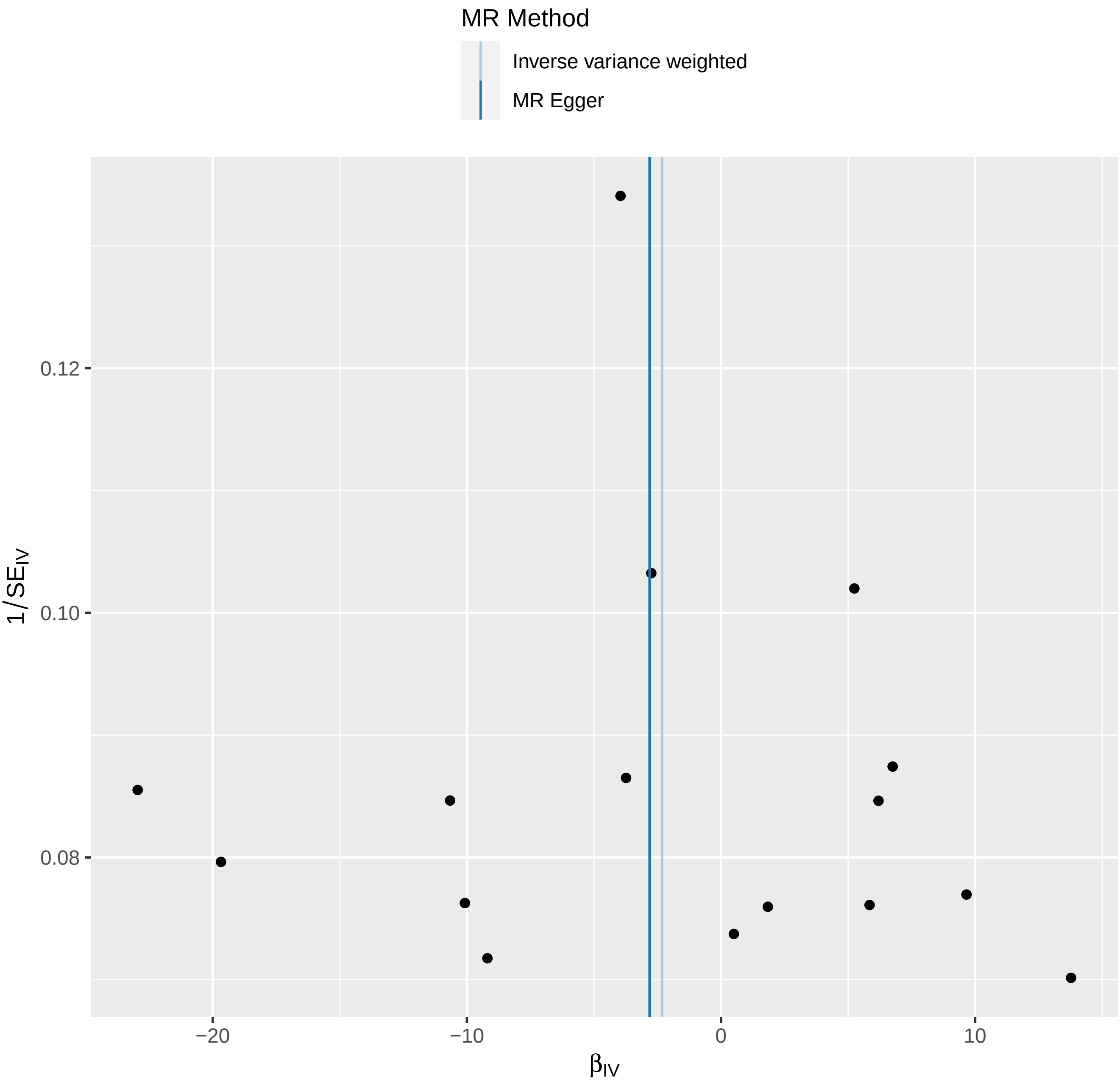

Batch 1727 : 3−methyl−2−oxovalerate on Arthropathy in ulcerative colitis

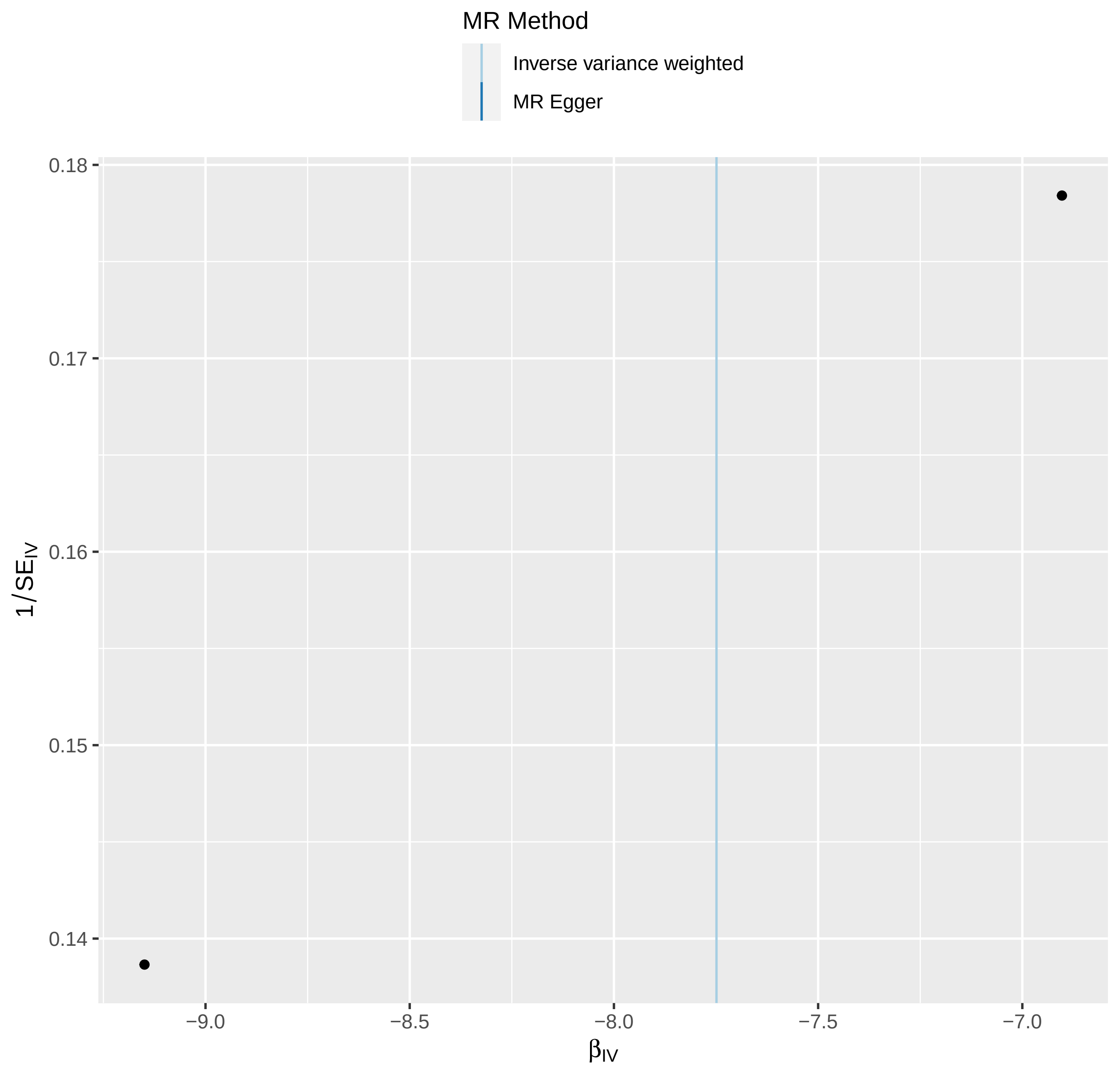

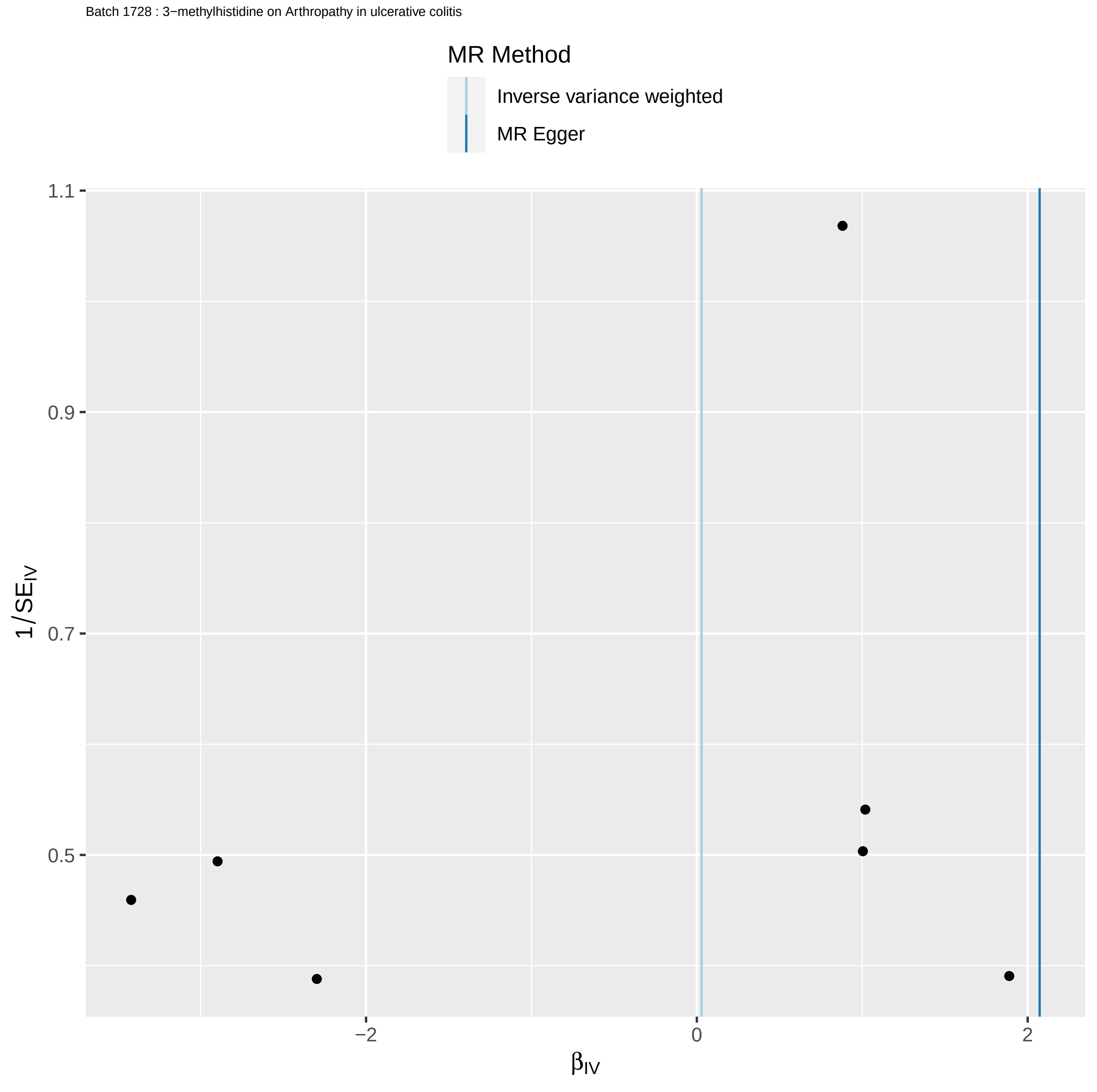
Batch 1728 : 3−methylhistidine on Arthropathy in ulcerative colitis
MR Method
Inverse variance weighted
MR Egger
1.1
0.9
0.7
0.5
1/SE_IV
−2
0
2
β_IV

Batch 1730 : Phenylacetate on Arthropathy in ulcerative colitis

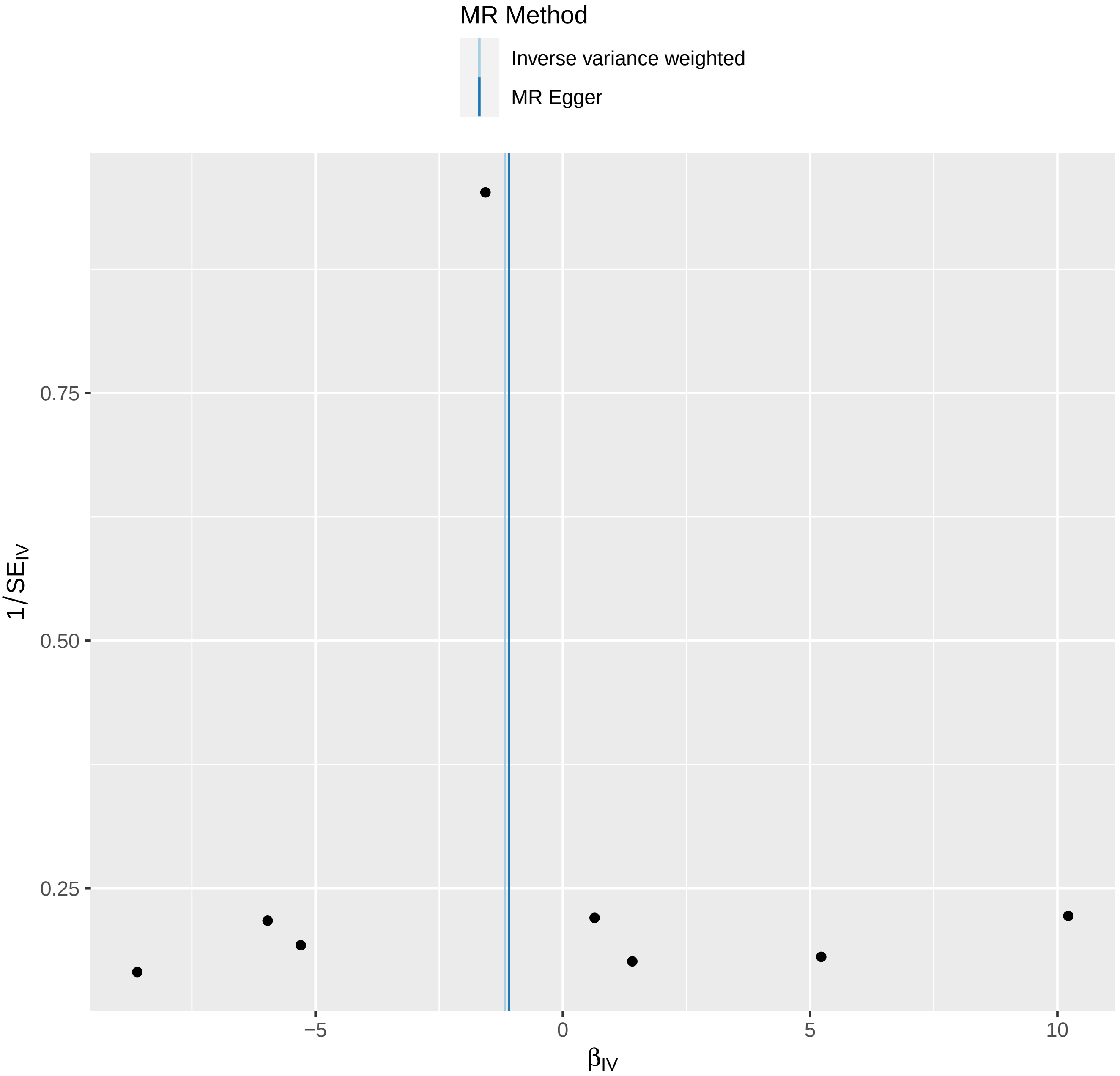

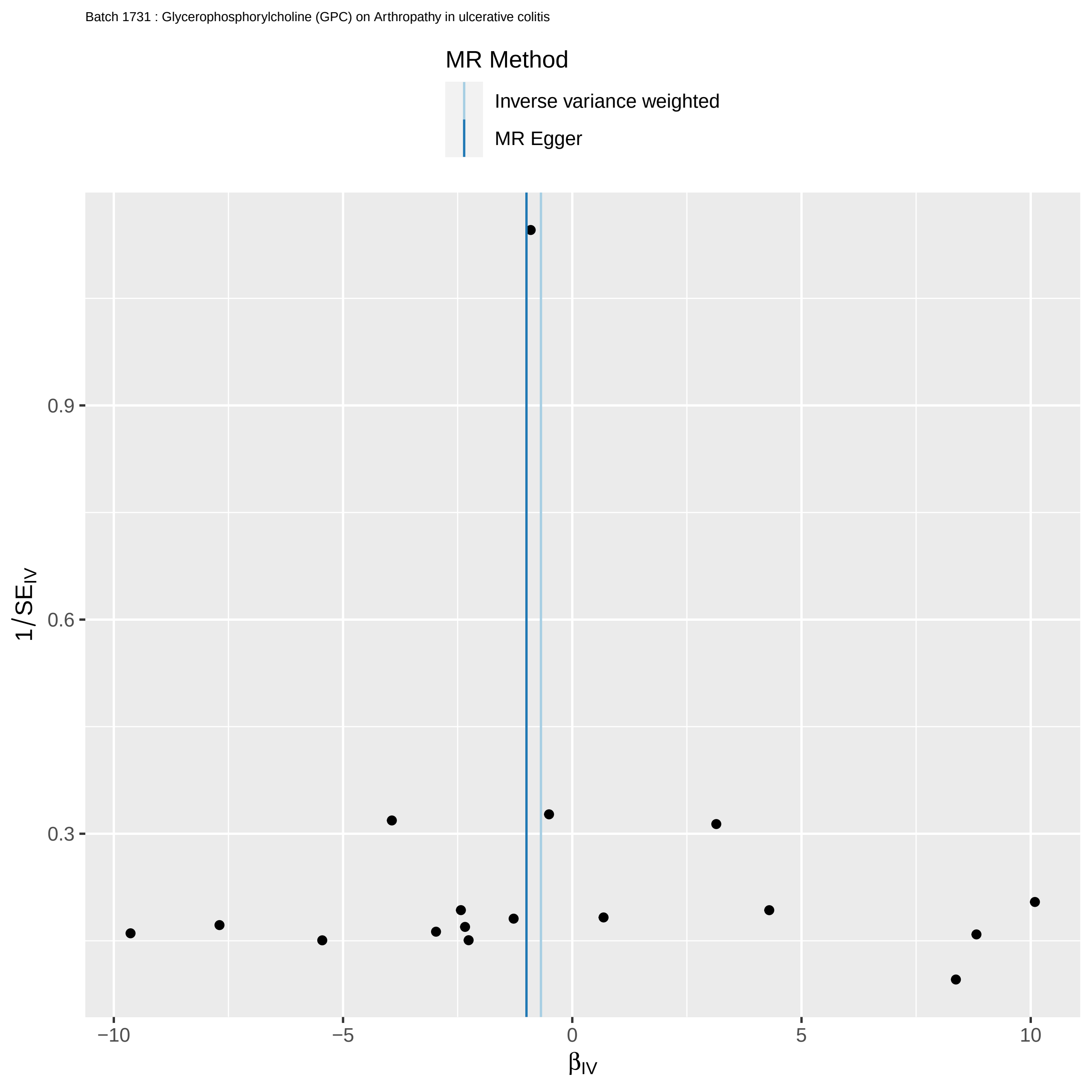

Batch 1731 : Glycerophosphorylcholine (GPC) on Arthropathy in ulcerative colitis
MR Method
Inverse variance weighted
MR Egger
0.9
0.6
0.3
$1/SE_{IV}$
−10
−5
0
5
10
β_{IV}

Batch 1732 : Aspartate on Arthropathy in ulcerative colitis

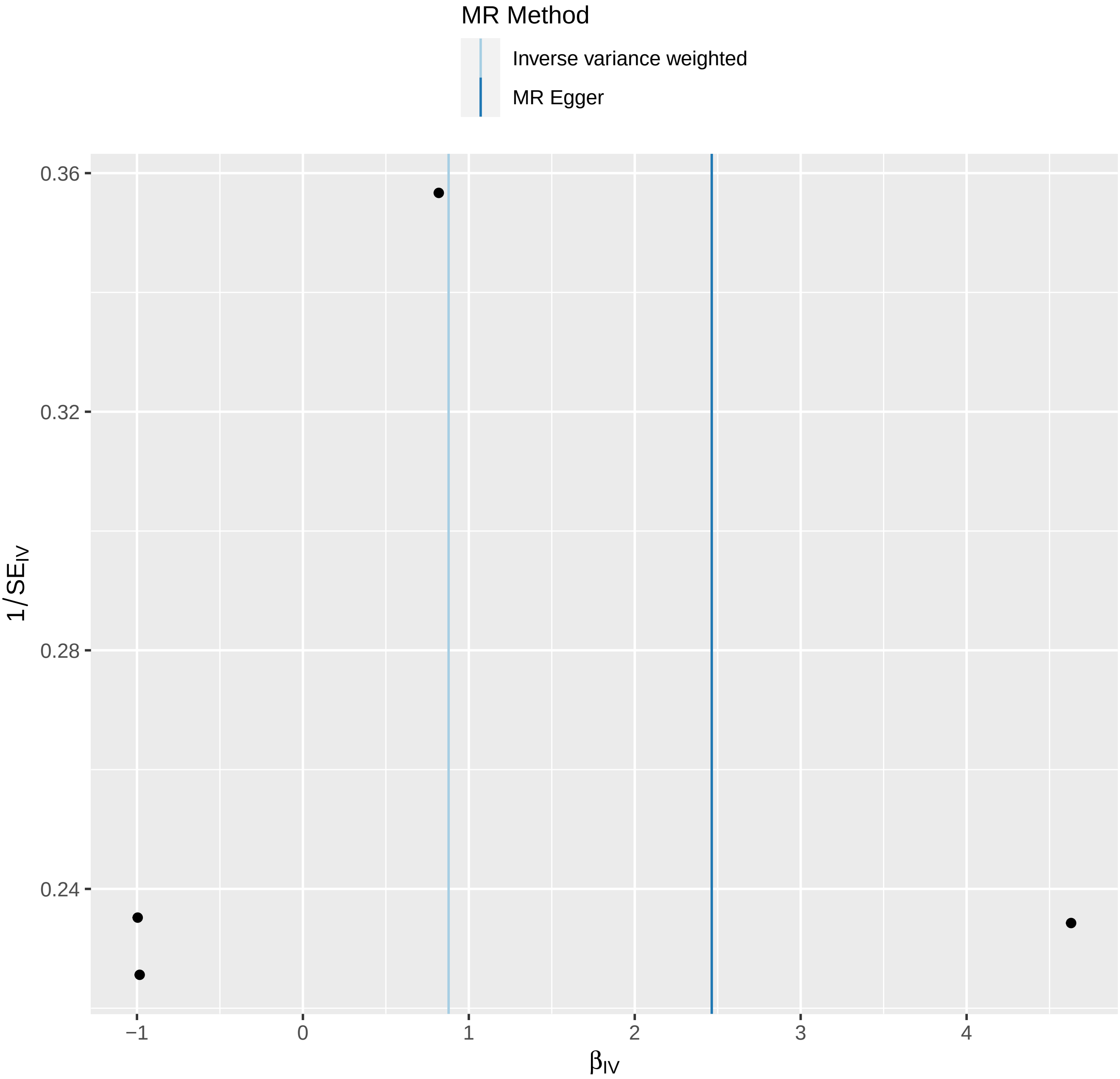

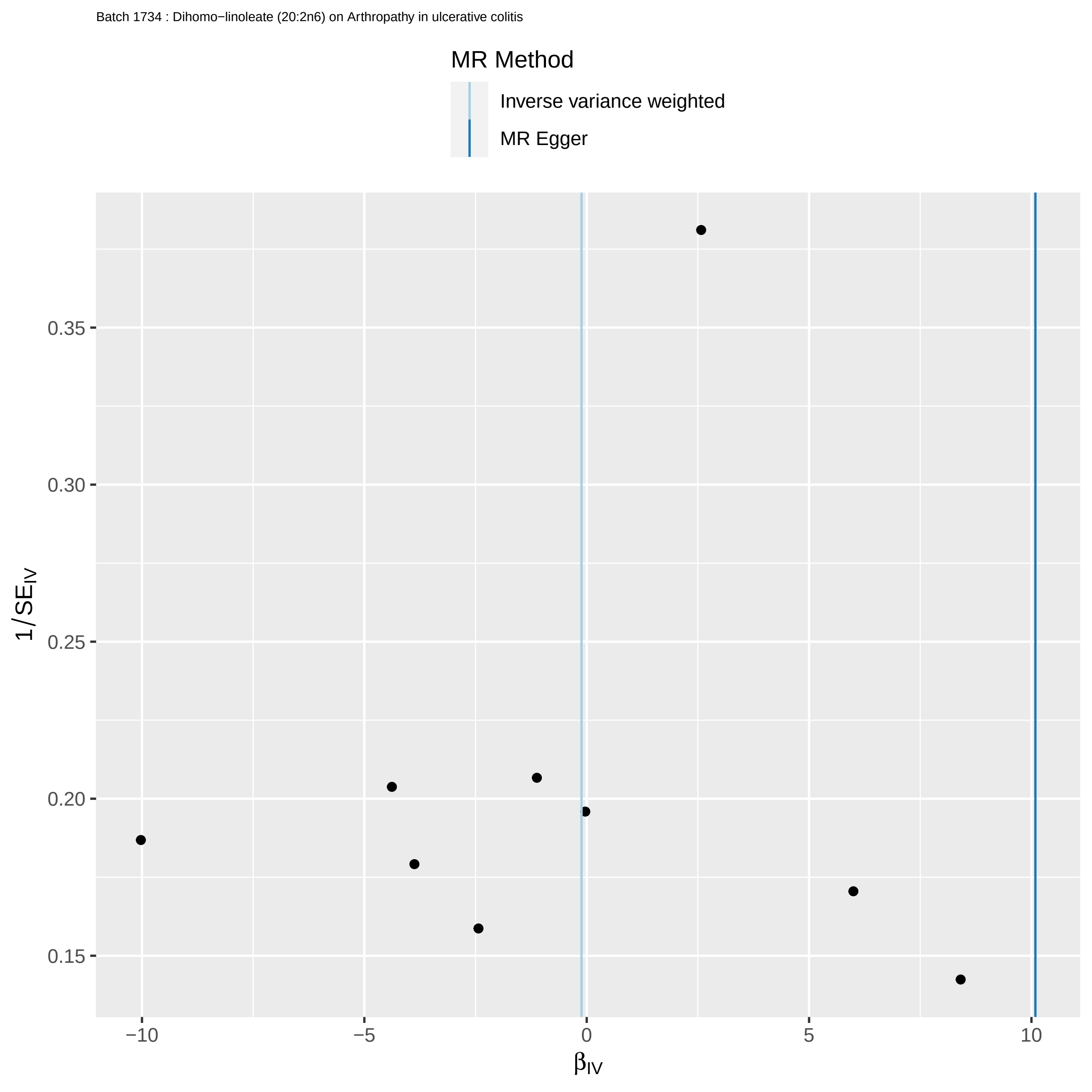
Batch 1734 : Dihomo−linoleate (20:2n6) on Arthropathy in ulcerative colitis
MR Method
Inverse variance weighted
MR Egger
0.35
0.30
0.25
0.20
0.15
$1/SE_{IV}$
−10
−5
0
5
10
β_{IV}

Batch 1735 : 2−hydroxystearate on Arthropathy in ulcerative colitis

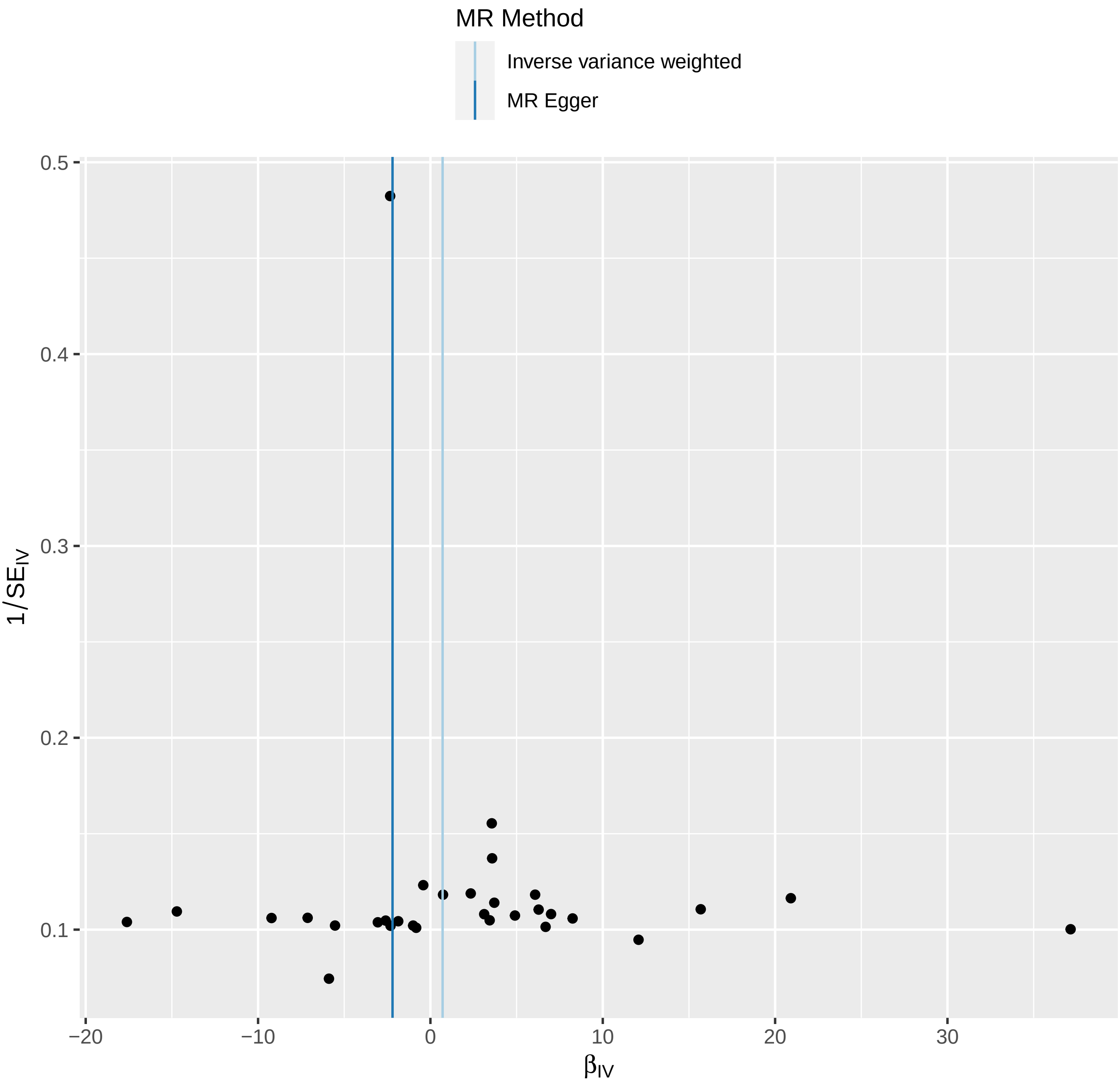

Batch 1737 : Glycylvaline on Arthropathy in ulcerative colitis

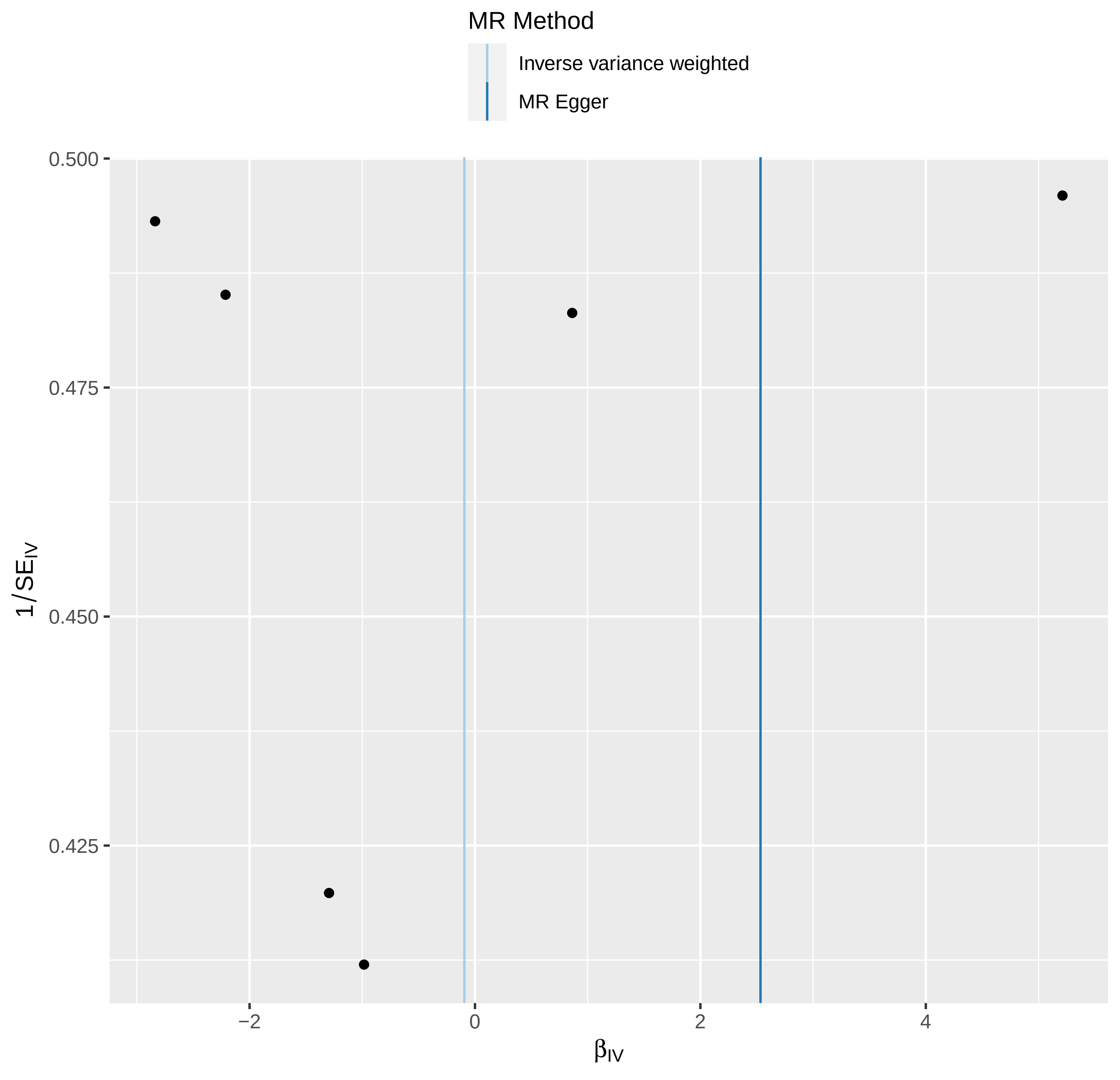

Batch 1738 : Gamma−glutamylleucine on Arthropathy in ulcerative colitis

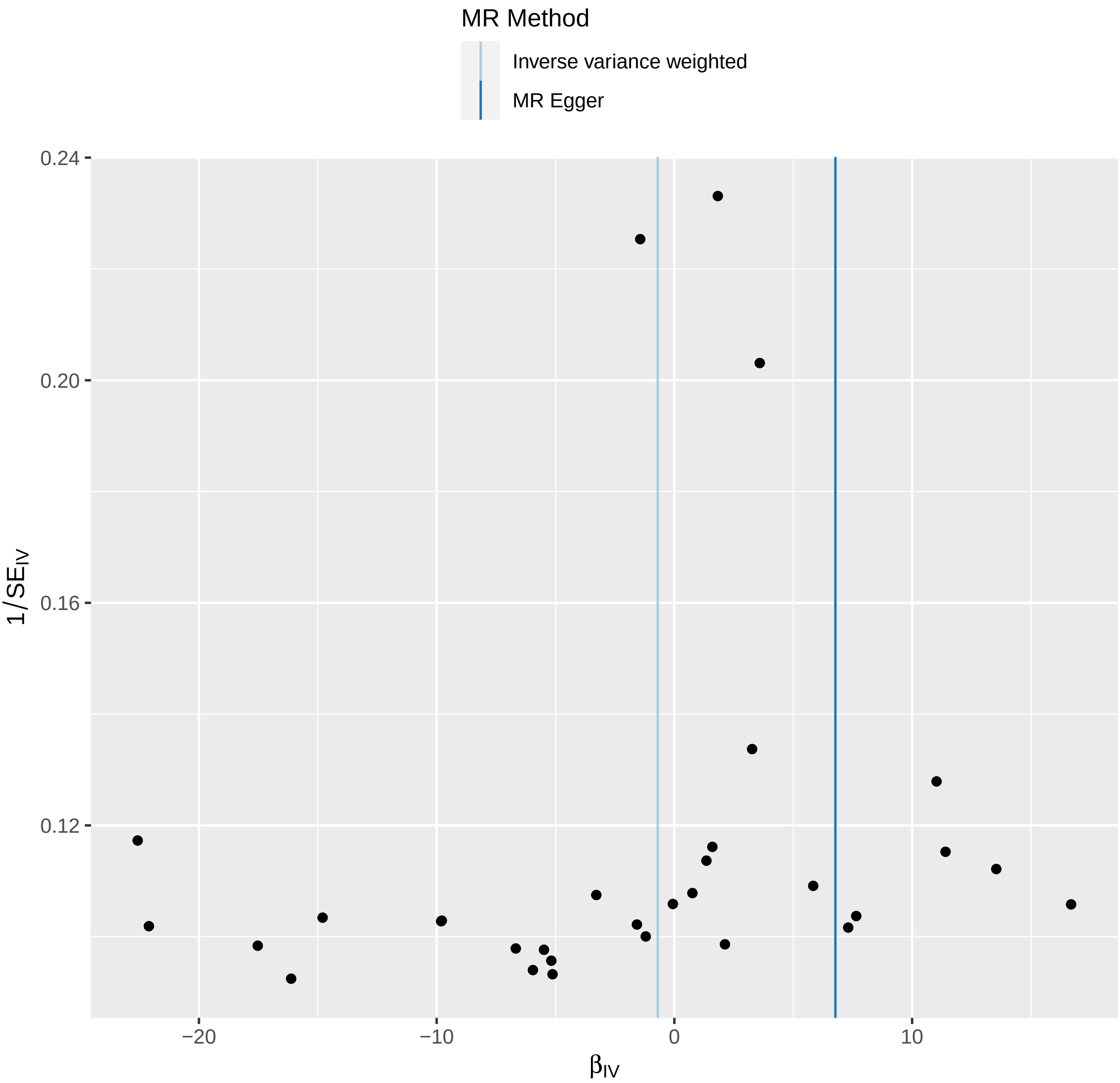

Batch 1741 : Glycocholate on Arthropathy in ulcerative colitis

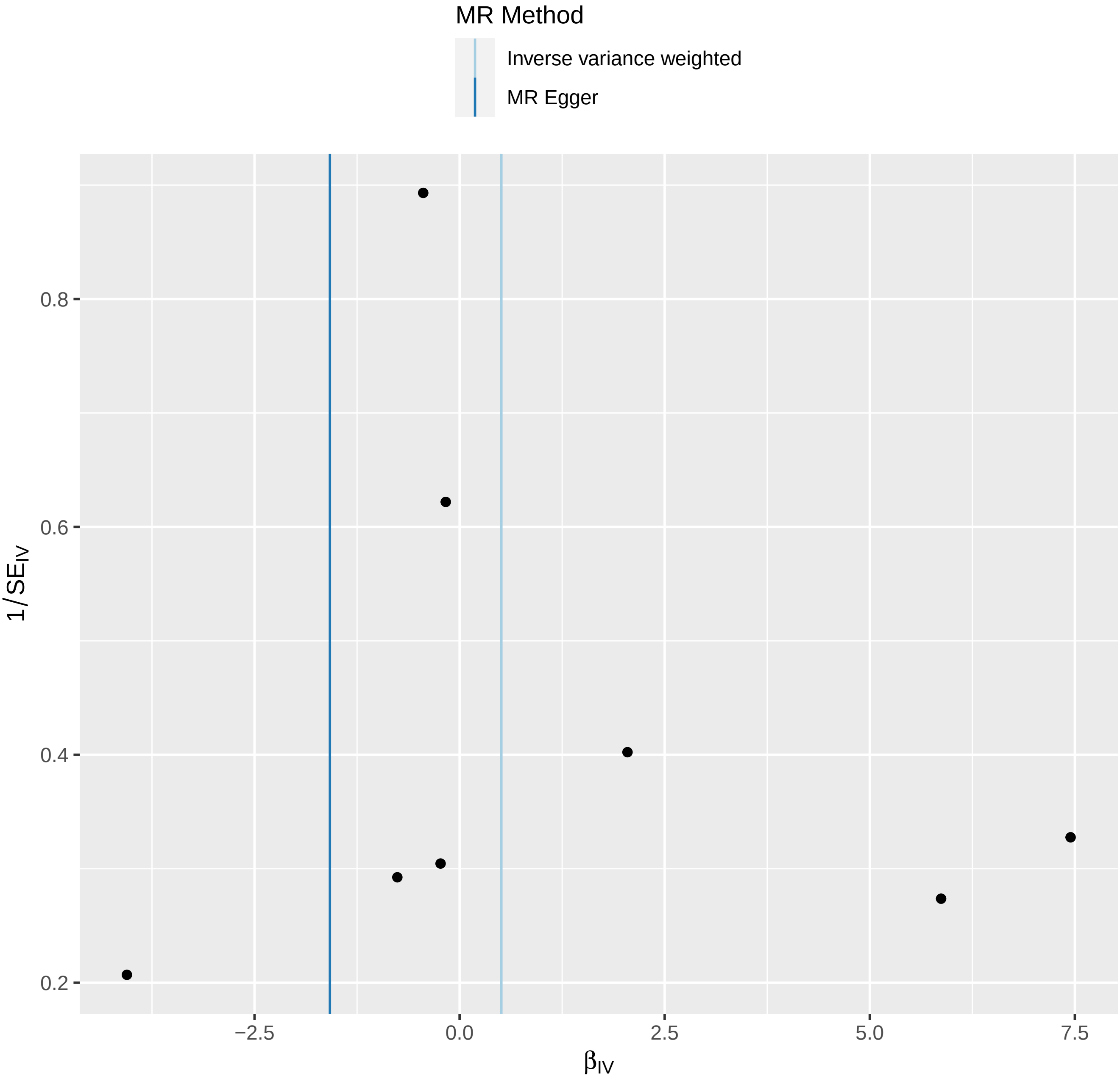

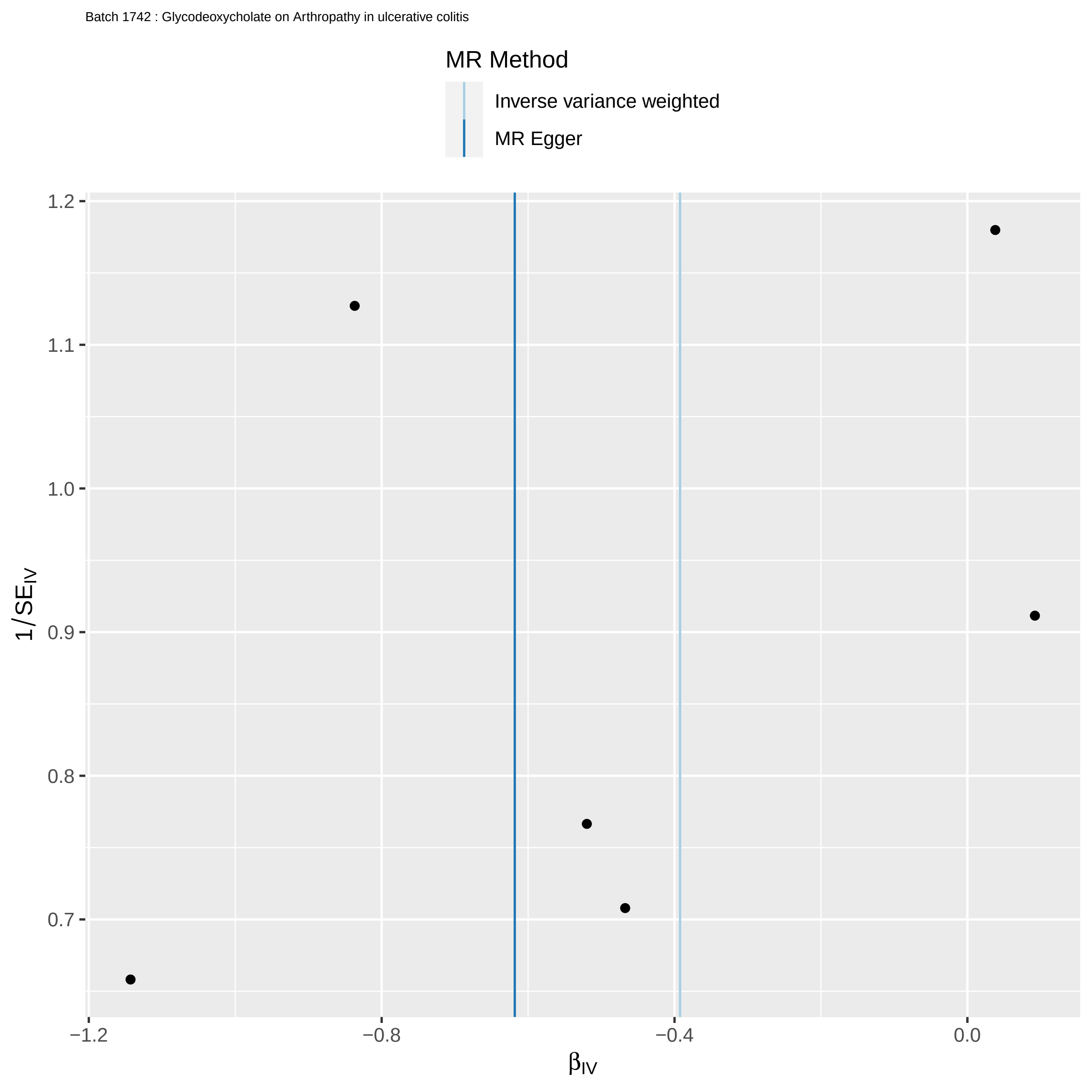

Batch 1742 : Glycodeoxycholate on Arthropathy in ulcerative colitis
MR Method
Inverse variance weighted
MR Egger
1.2
1.1
1.0
0.9
0.8
0.7
$1/SE_{IV}$
−1.2
−0.8
−0.4
0.0
β_{IV}

Batch 1743 : Taurochenodeoxycholate on Arthropathy in ulcerative colitis

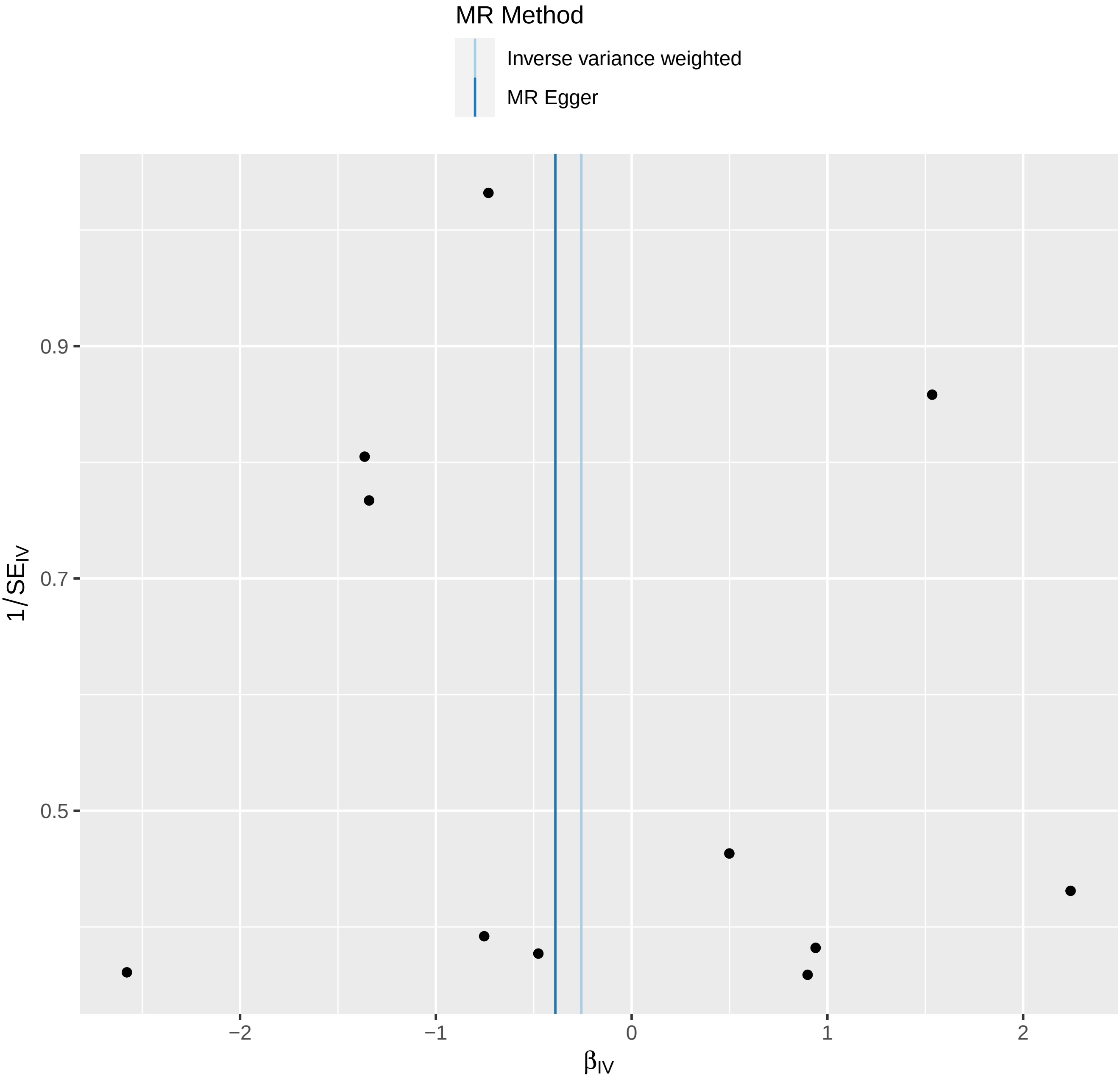

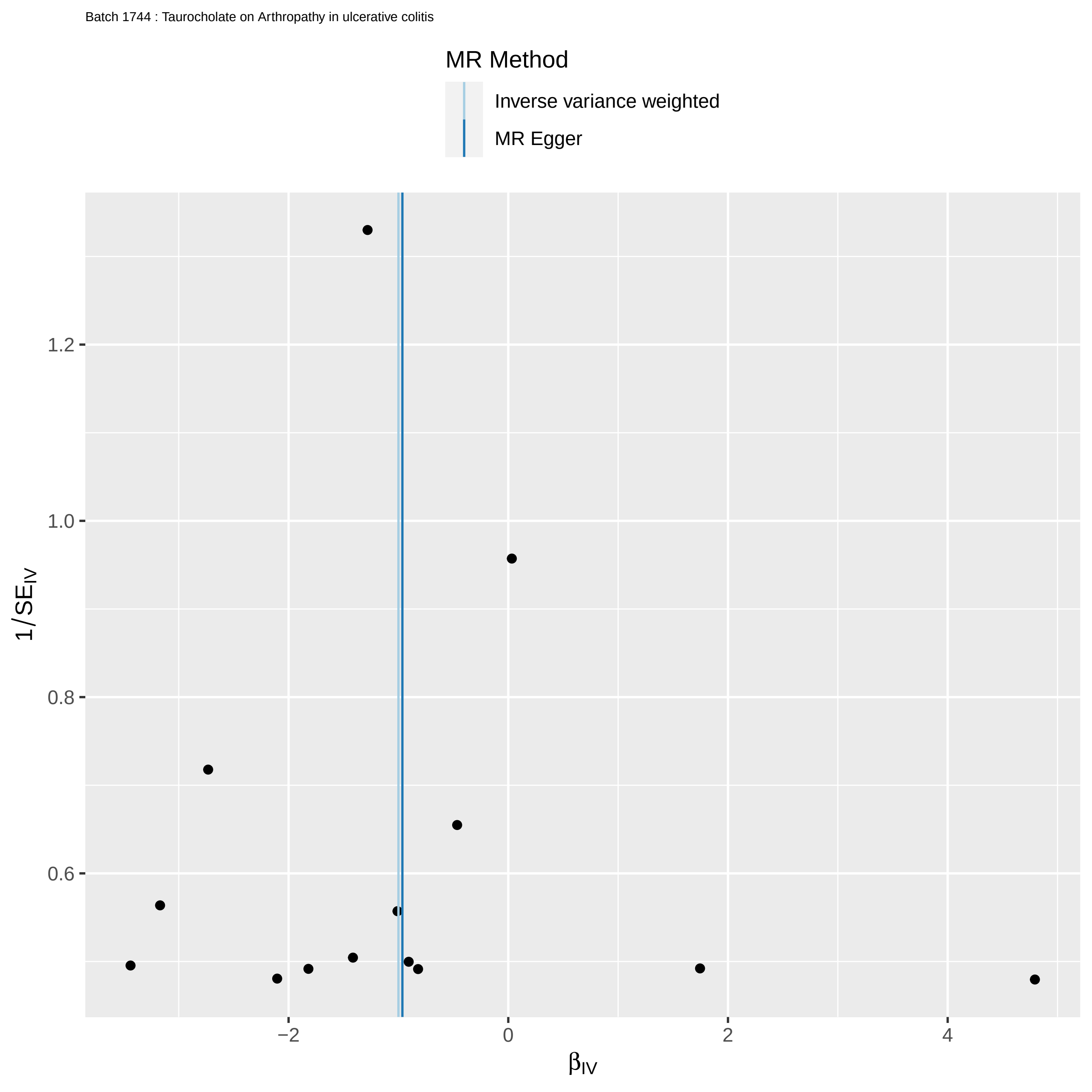
Batch 1744 : Taurocholate on Arthropathy in ulcerative colitis
MR Method
Inverse variance weighted
MR Egger
1.2
1.0
0.8
0.6
1/SE$_{IV}$
−2
0
2
4
β$_{IV}$

Batch 1746 : 1−stearoylglycerophosphoinositol on Arthropathy in ulcerative colitis

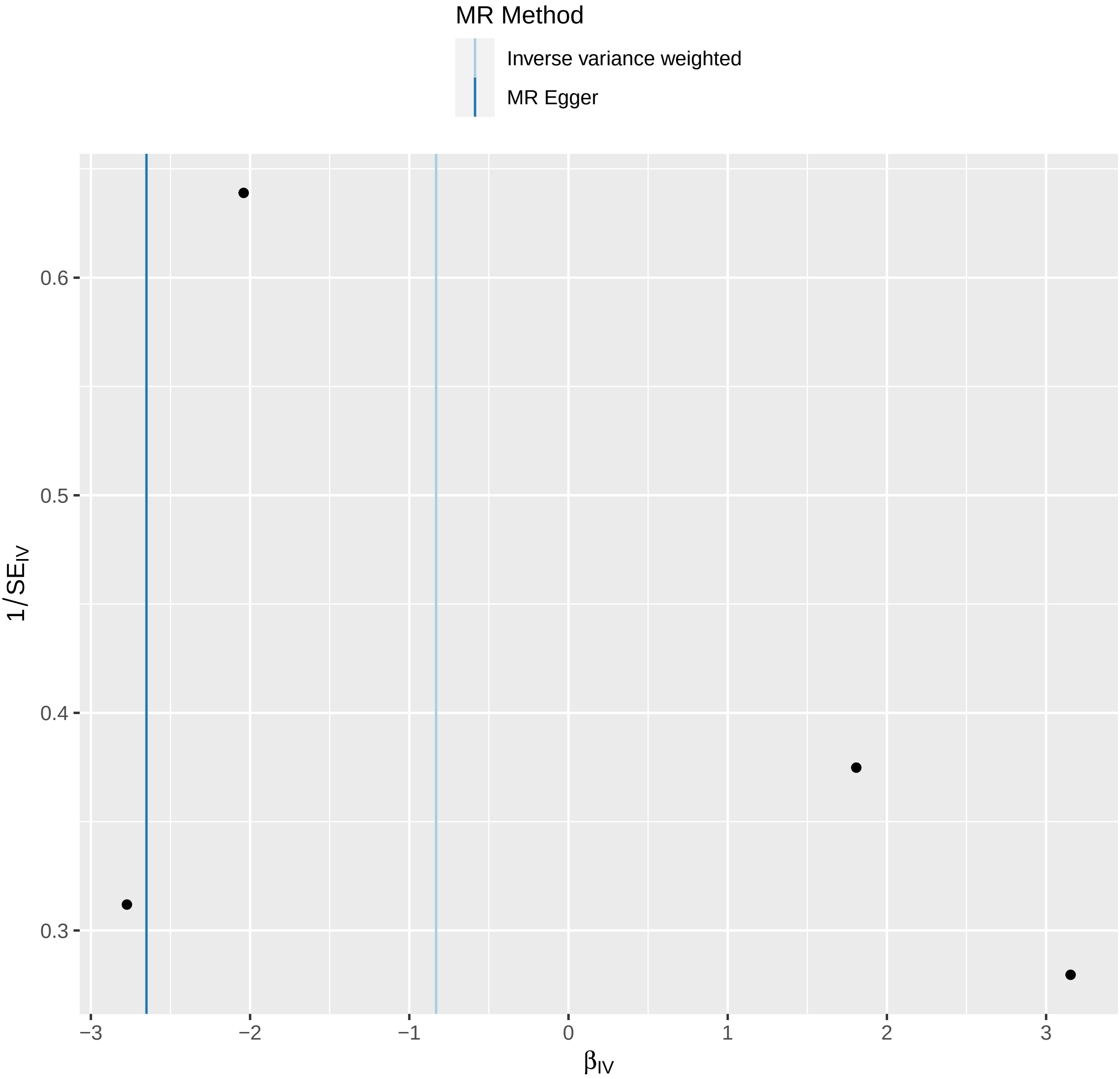

Batch 1747 : Myo−inositol on Arthropathy in ulcerative colitis

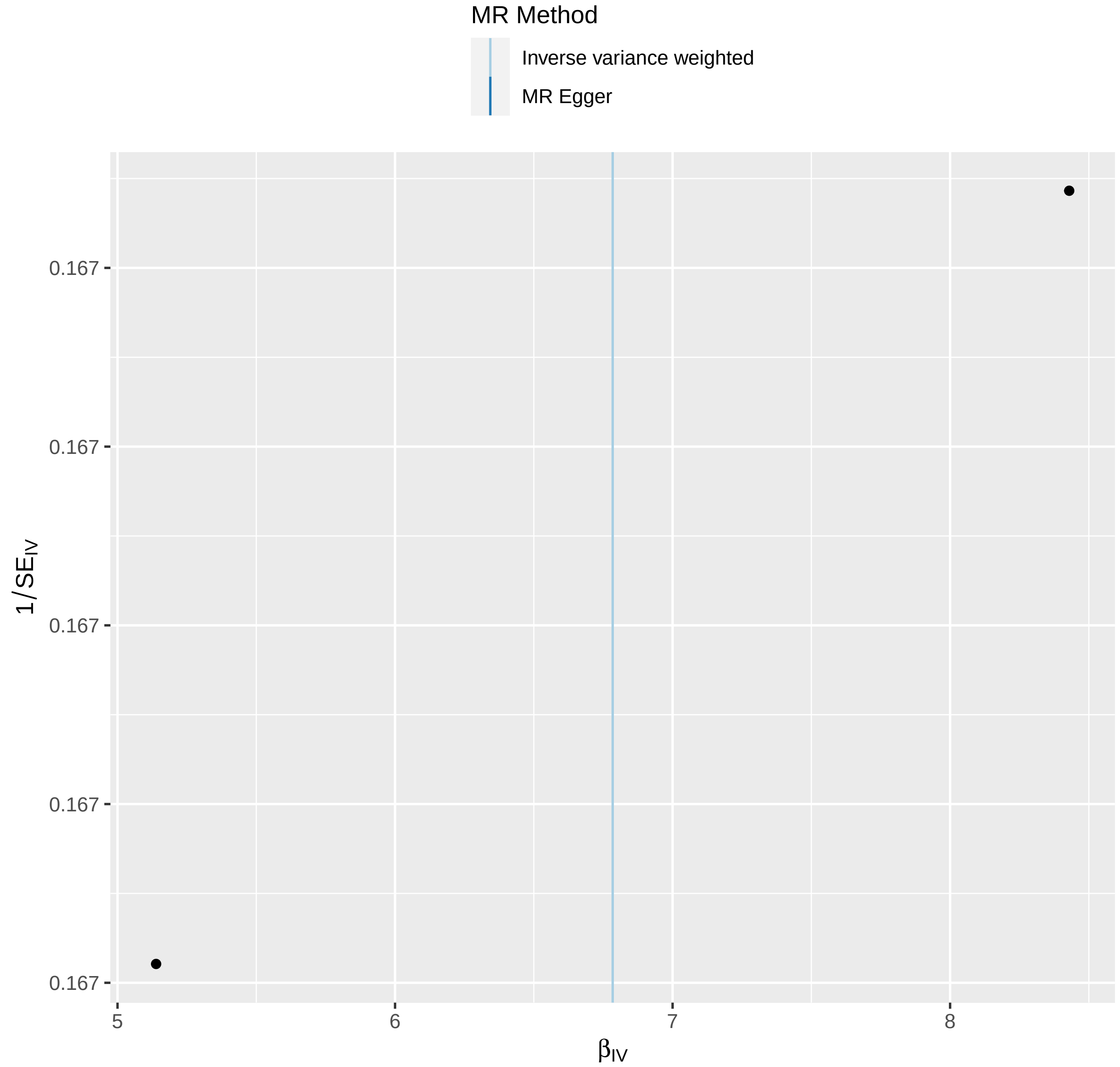

Batch 1749 : 1,5−anhydroglucitol (1,5−AG) on Arthropathy in ulcerative colitis

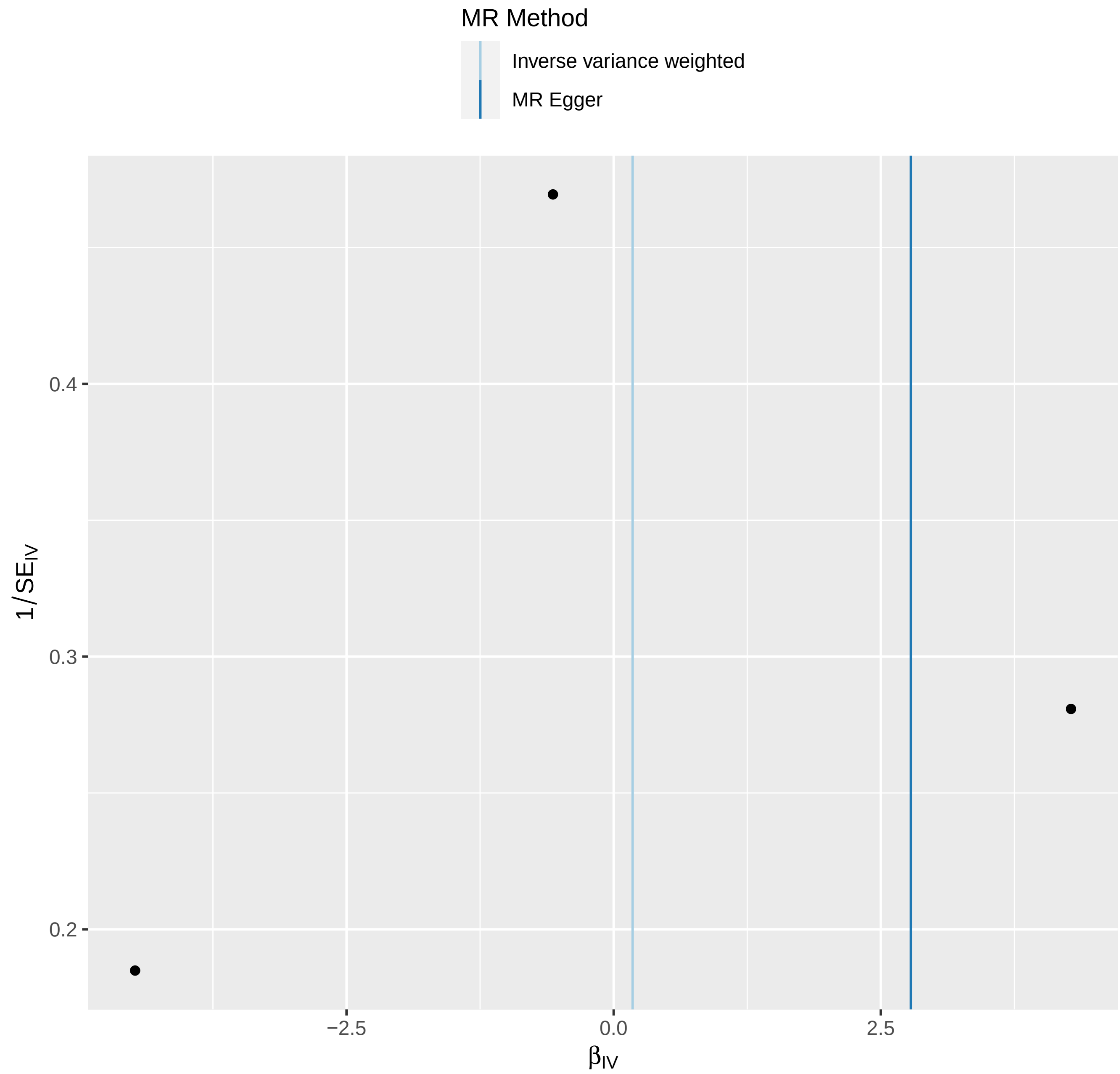

Batch 1750 : 2−hydroxybutyrate (AHB) on Arthropathy in ulcerative colitis

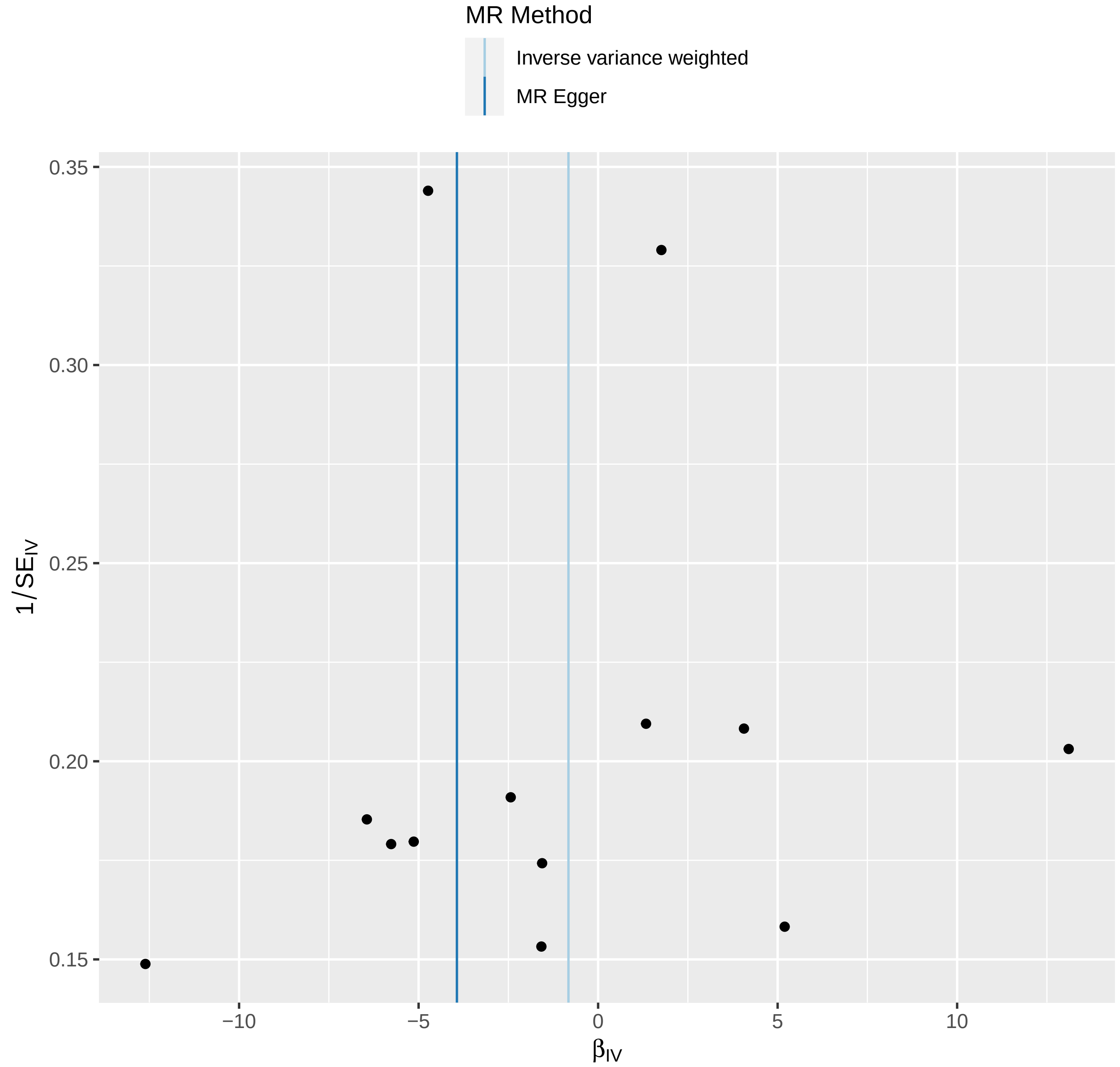

Batch 1753 : 1−palmitoylglycerol (1−monopalmitin) on Arthropathy in ulcerative colitis

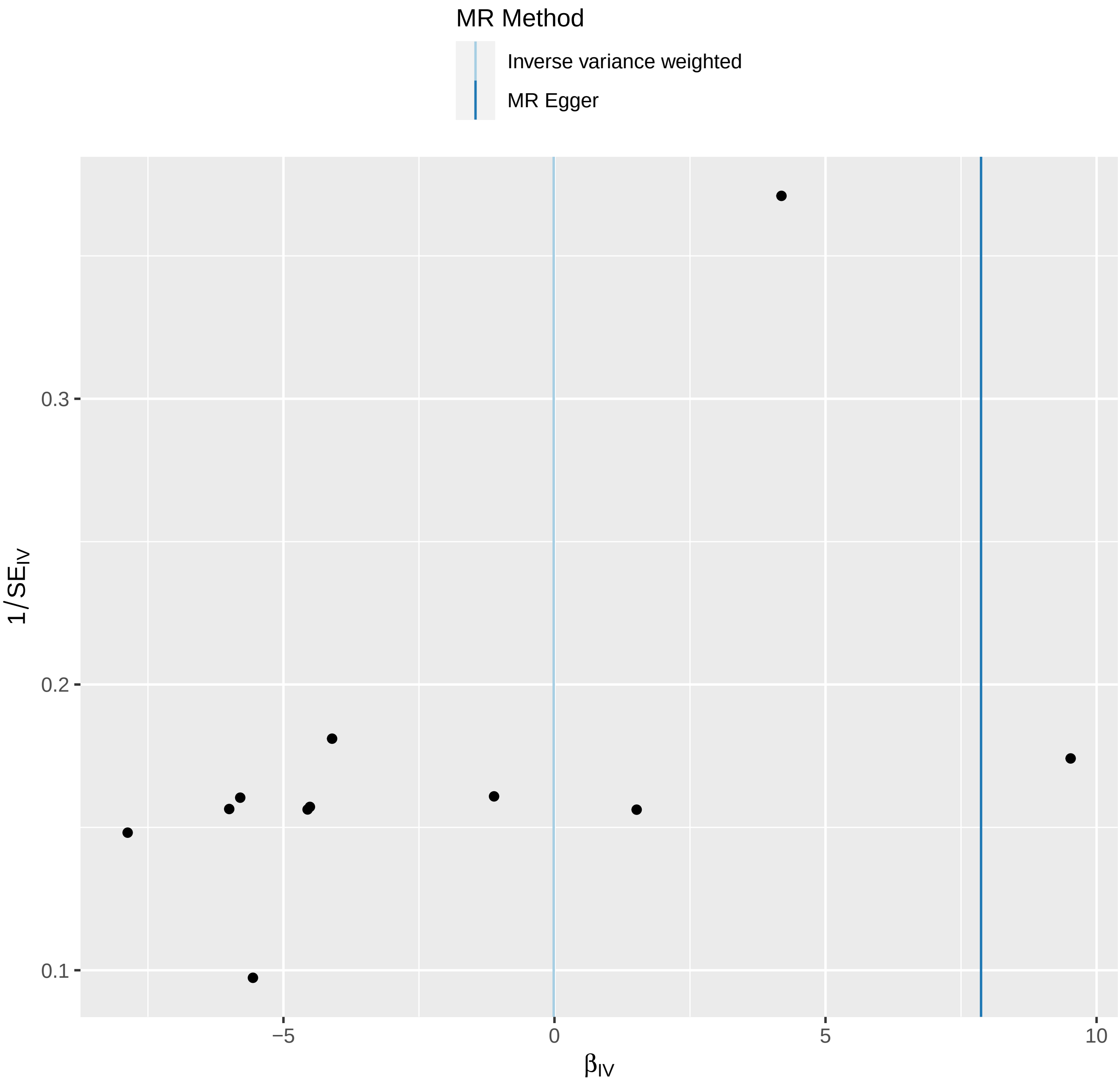

Batch 1756 : 2−hydroxyisobutyrate on Arthropathy in ulcerative colitis

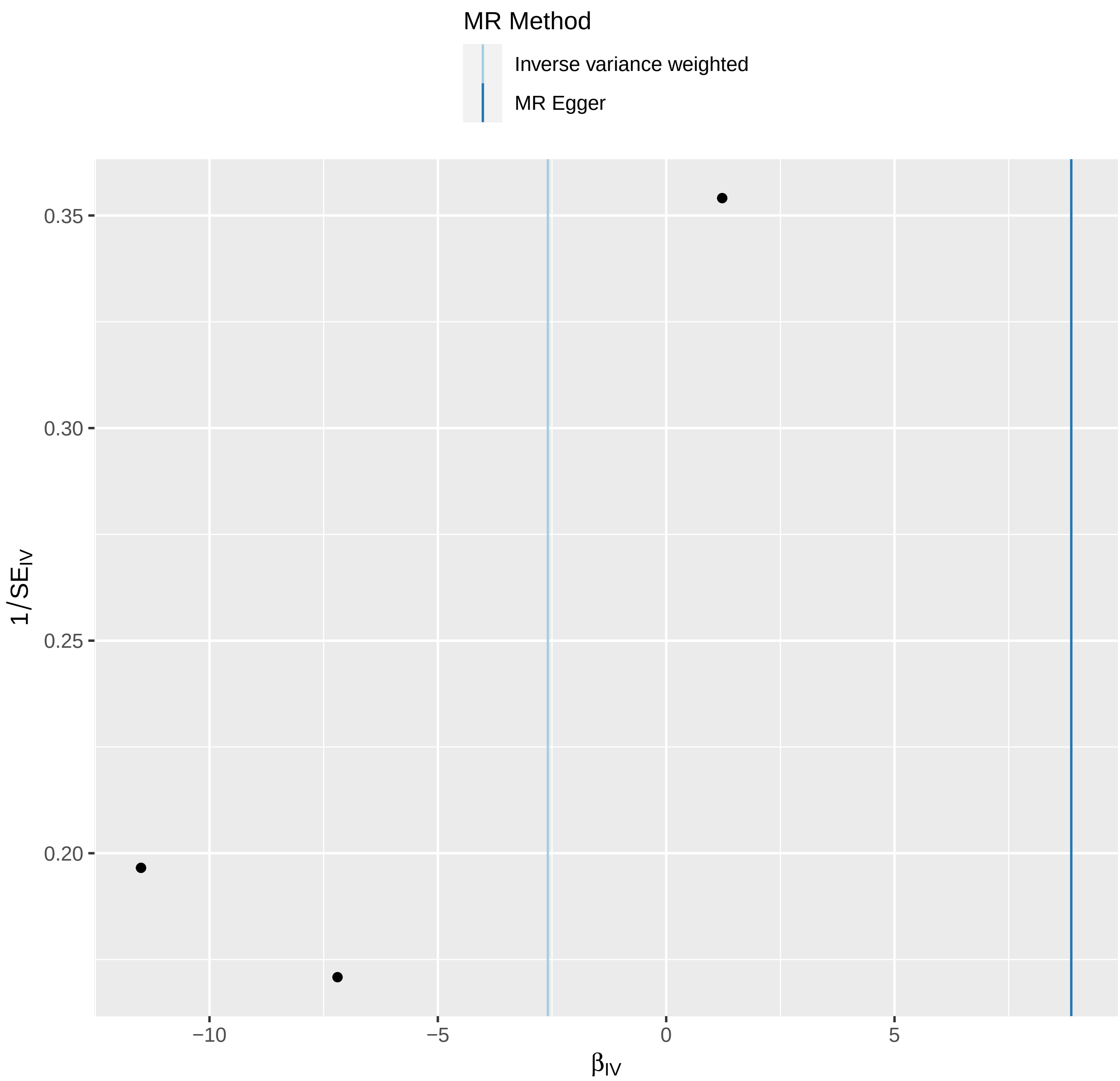

Batch 1757 : 4−methyl−2−oxopentanoate on Arthropathy in ulcerative colitis

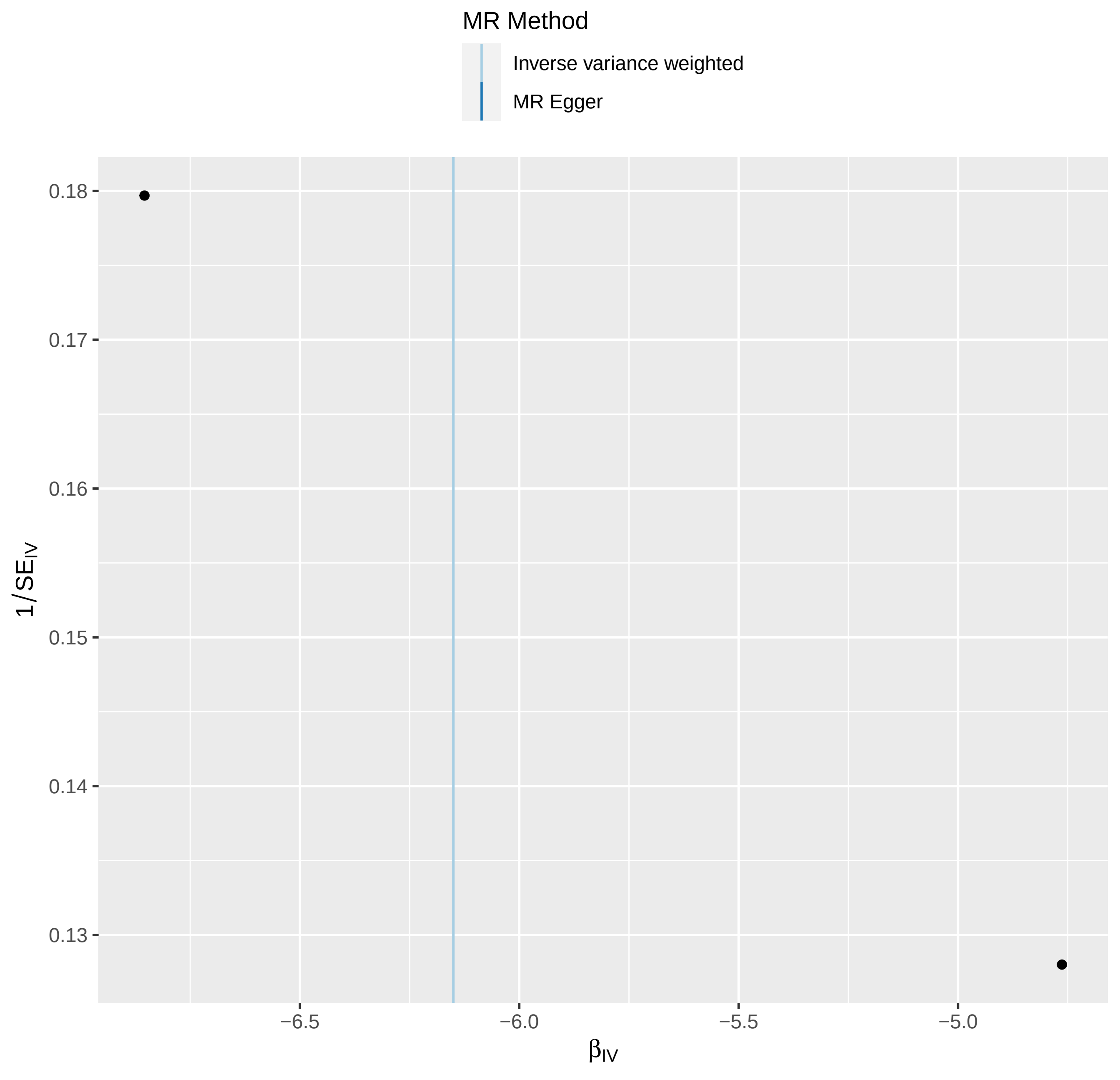

Batch 1761 : Levulinate (4−oxovalerate) on Arthropathy in ulcerative colitis

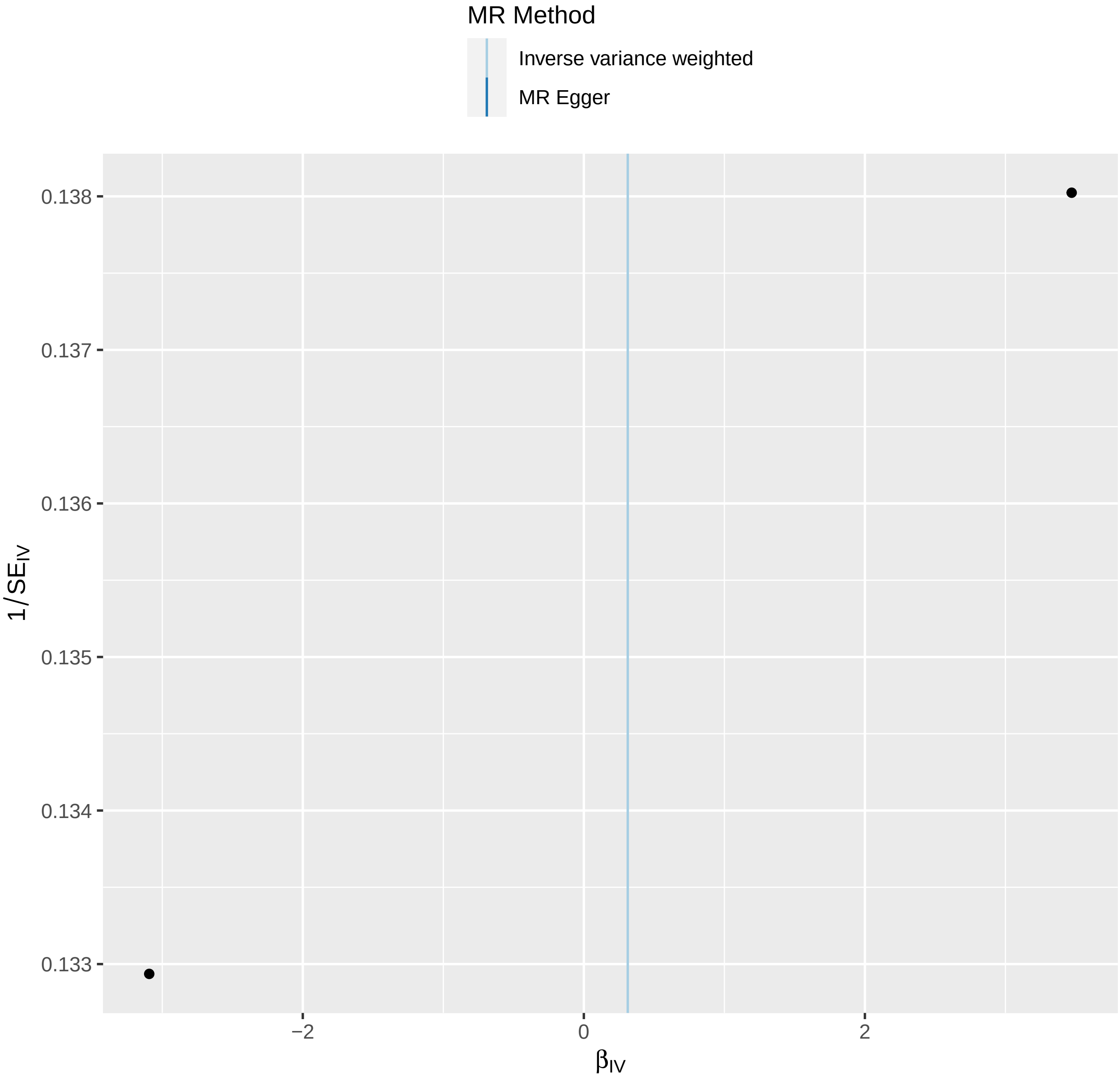

Batch 1763 : Cholate on Arthropathy in ulcerative colitis

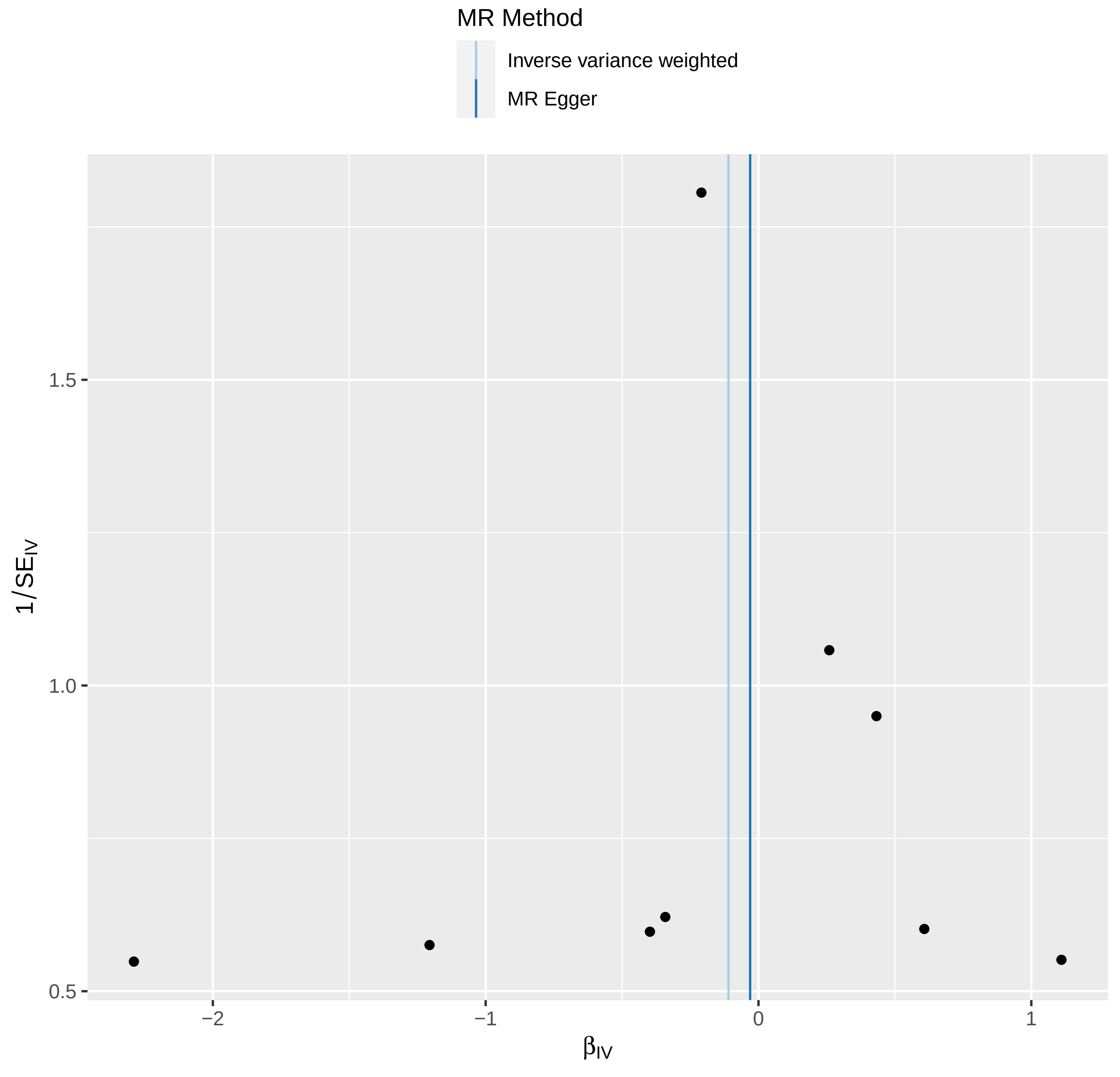

Batch 1766 : Hyodeoxycholate on Arthropathy in ulcerative colitis

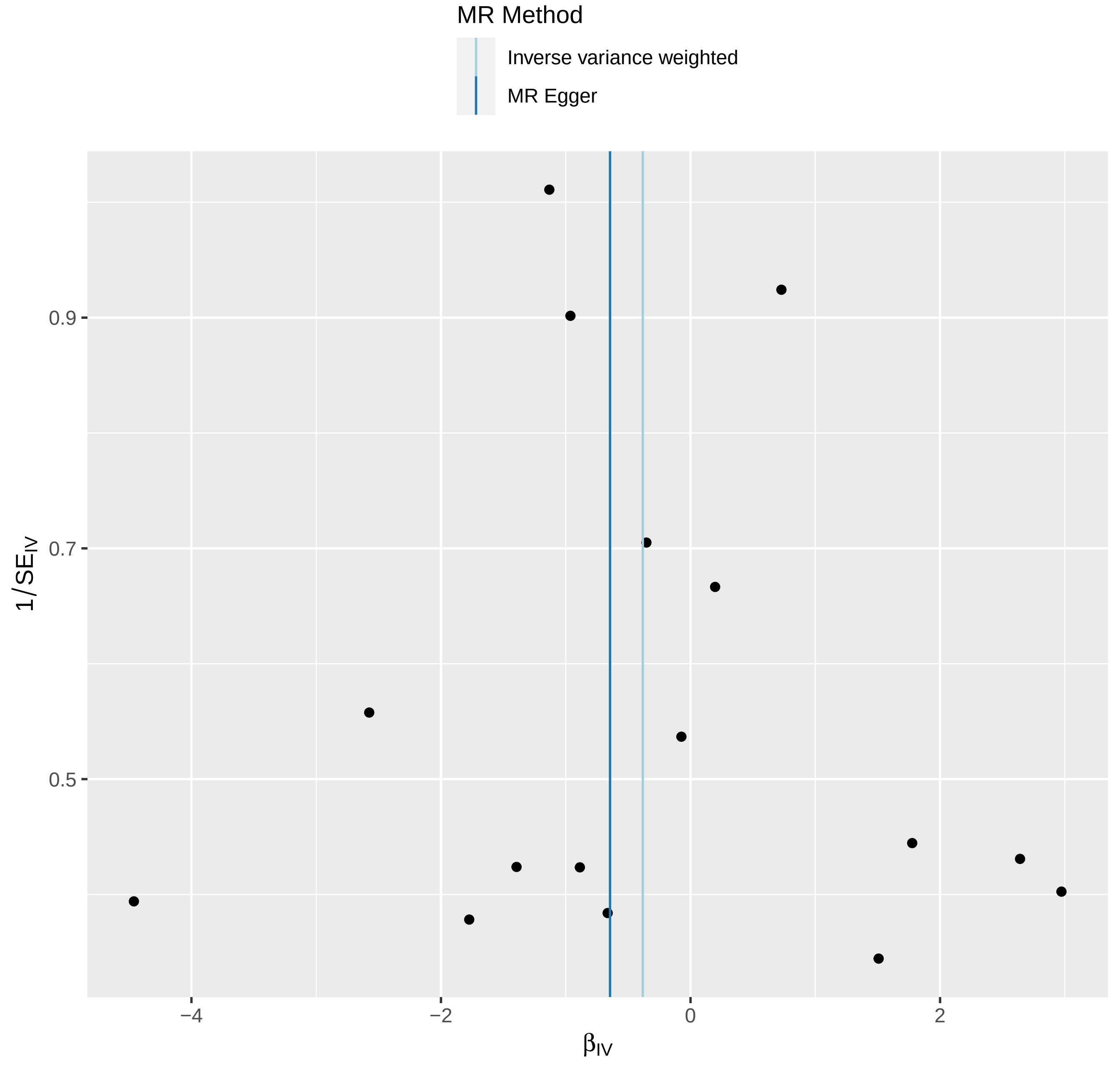

Batch 1767 : 3−indoxyl sulfate on Arthropathy in ulcerative colitis

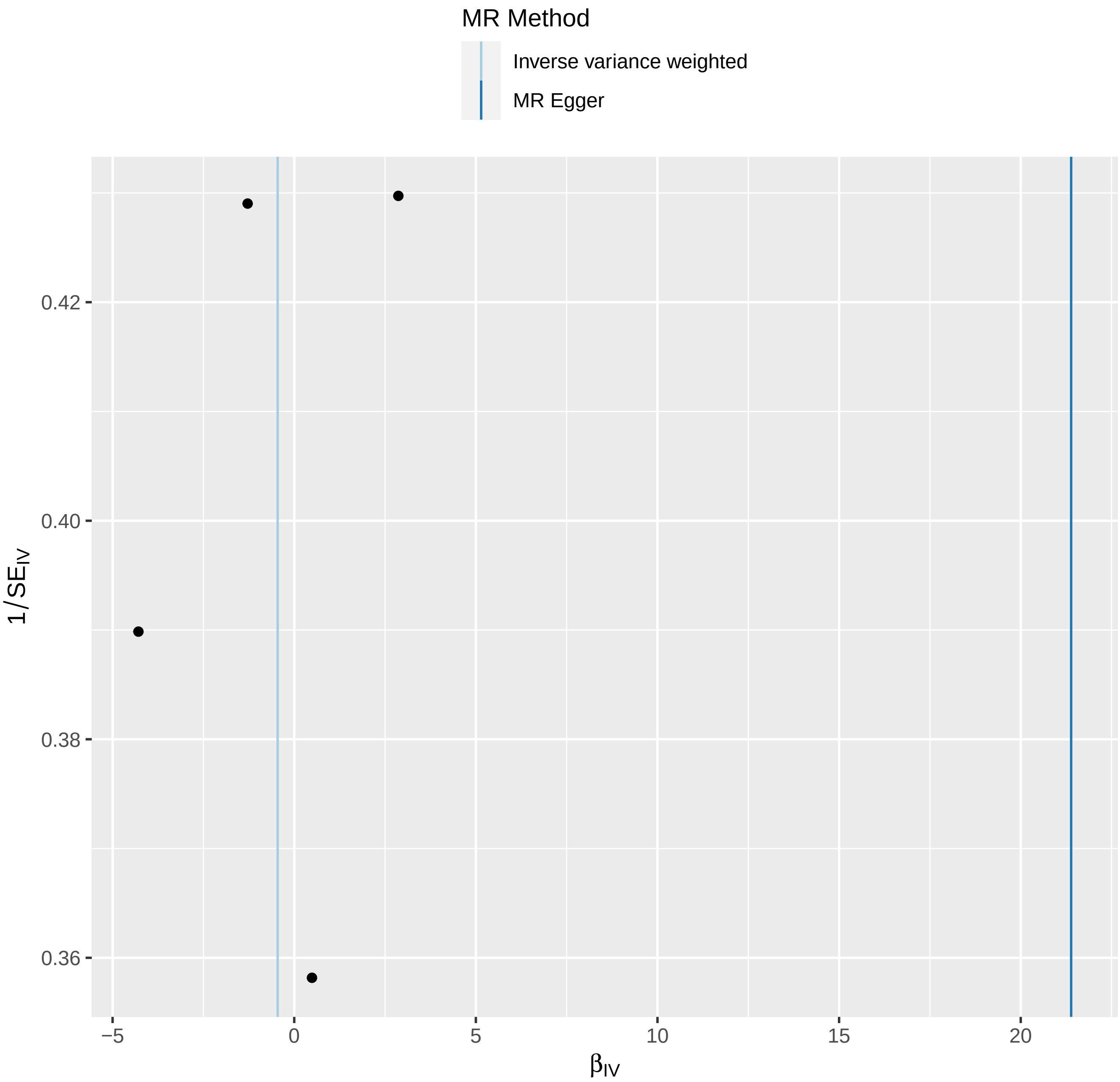

Batch 1771 : Erythrose on Arthropathy in ulcerative colitis

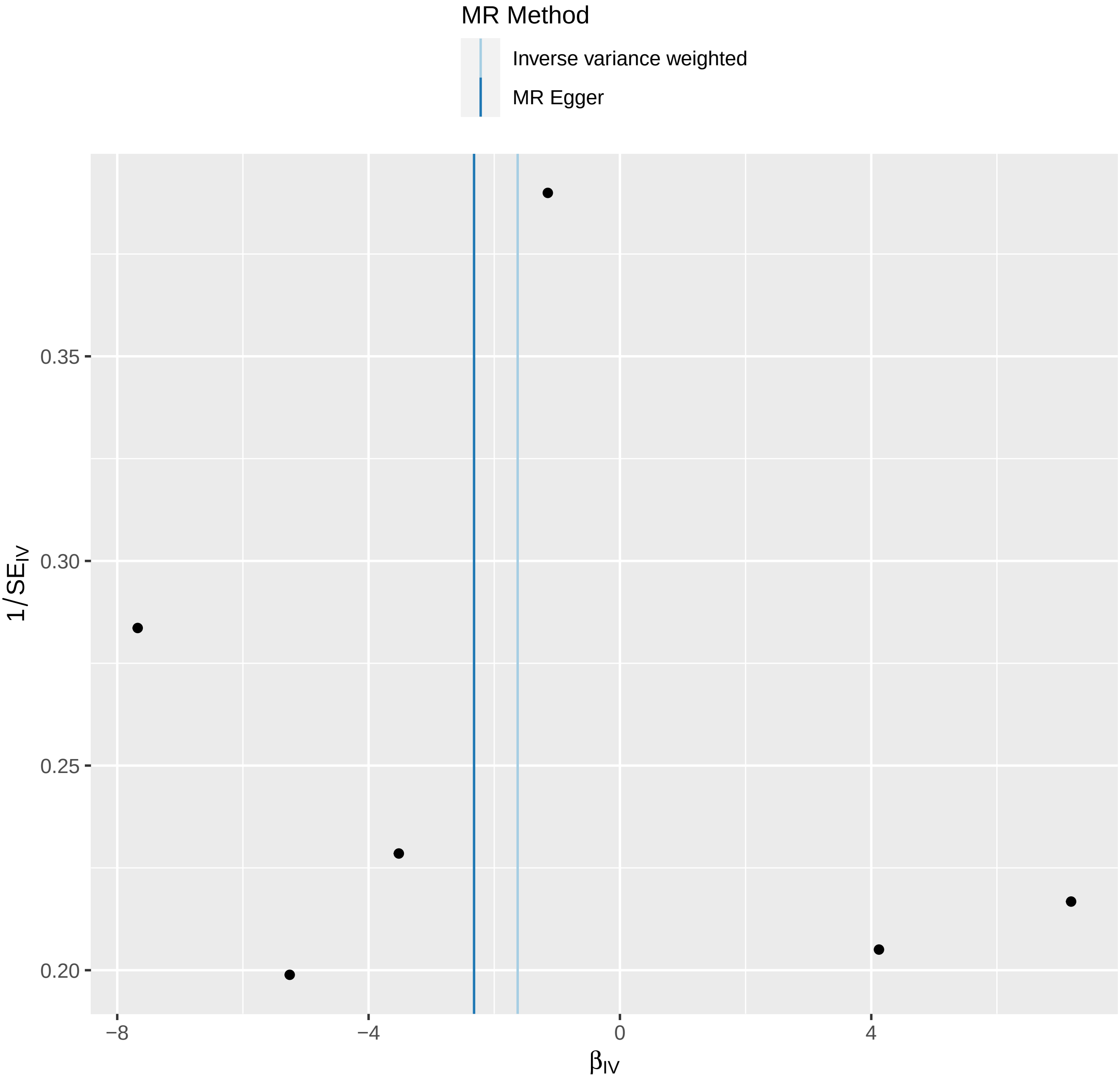

Batch 1772 : Threonate on Arthropathy in ulcerative colitis

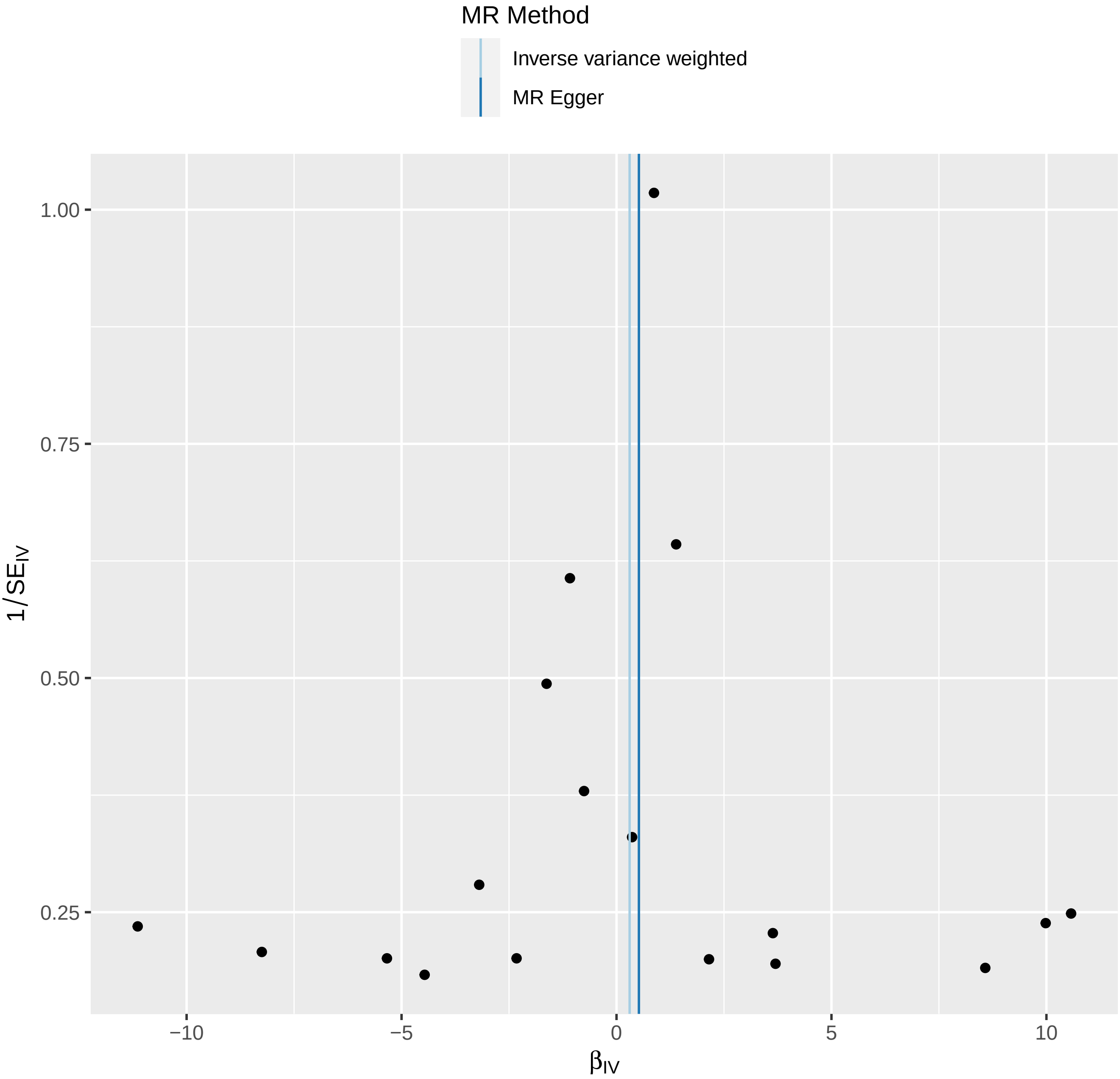

Batch 1773 : Cysteine on Arthropathy in ulcerative colitis

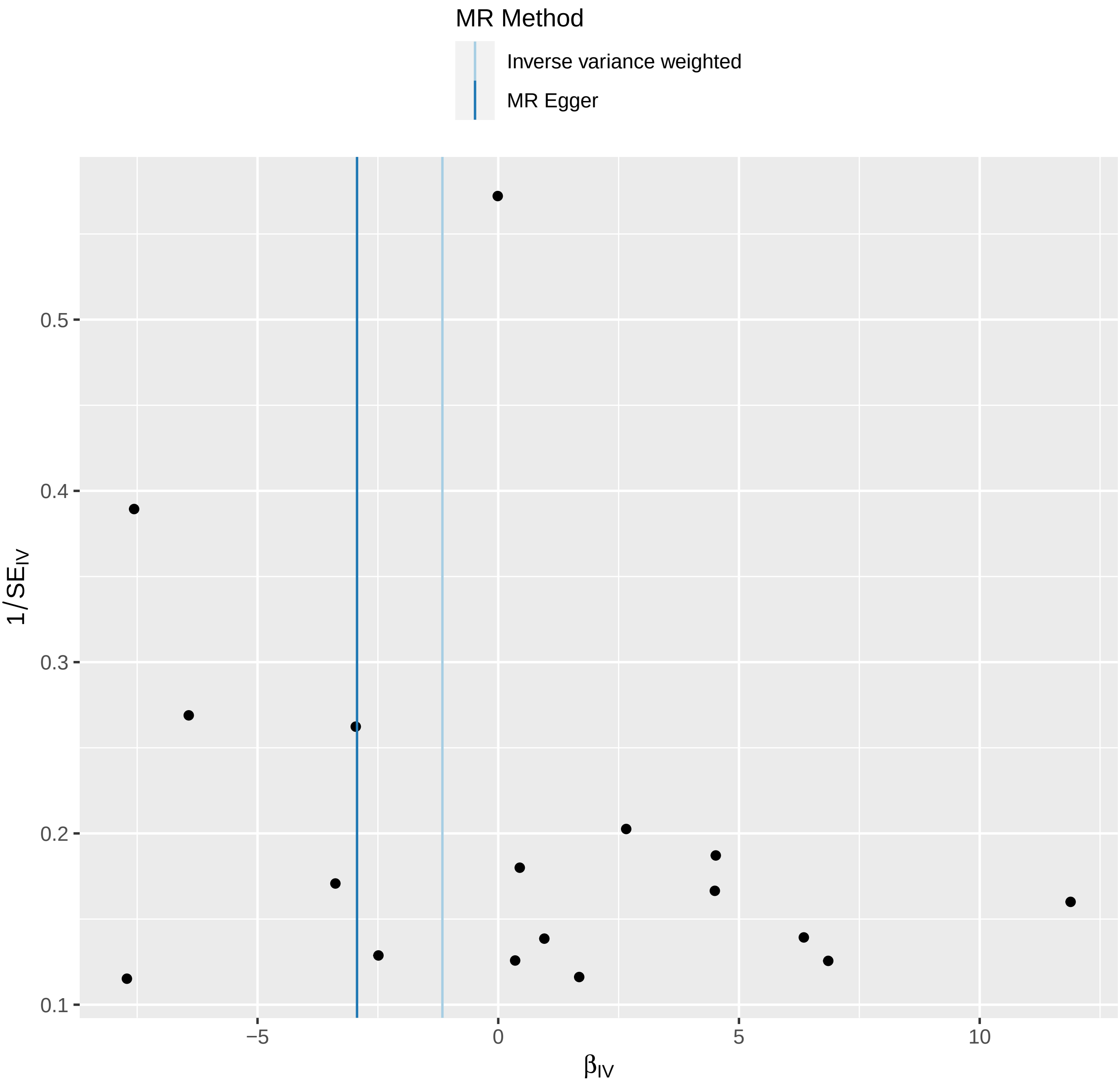

Batch 1774 : Pyroglutamylglycine on Arthropathy in ulcerative colitis

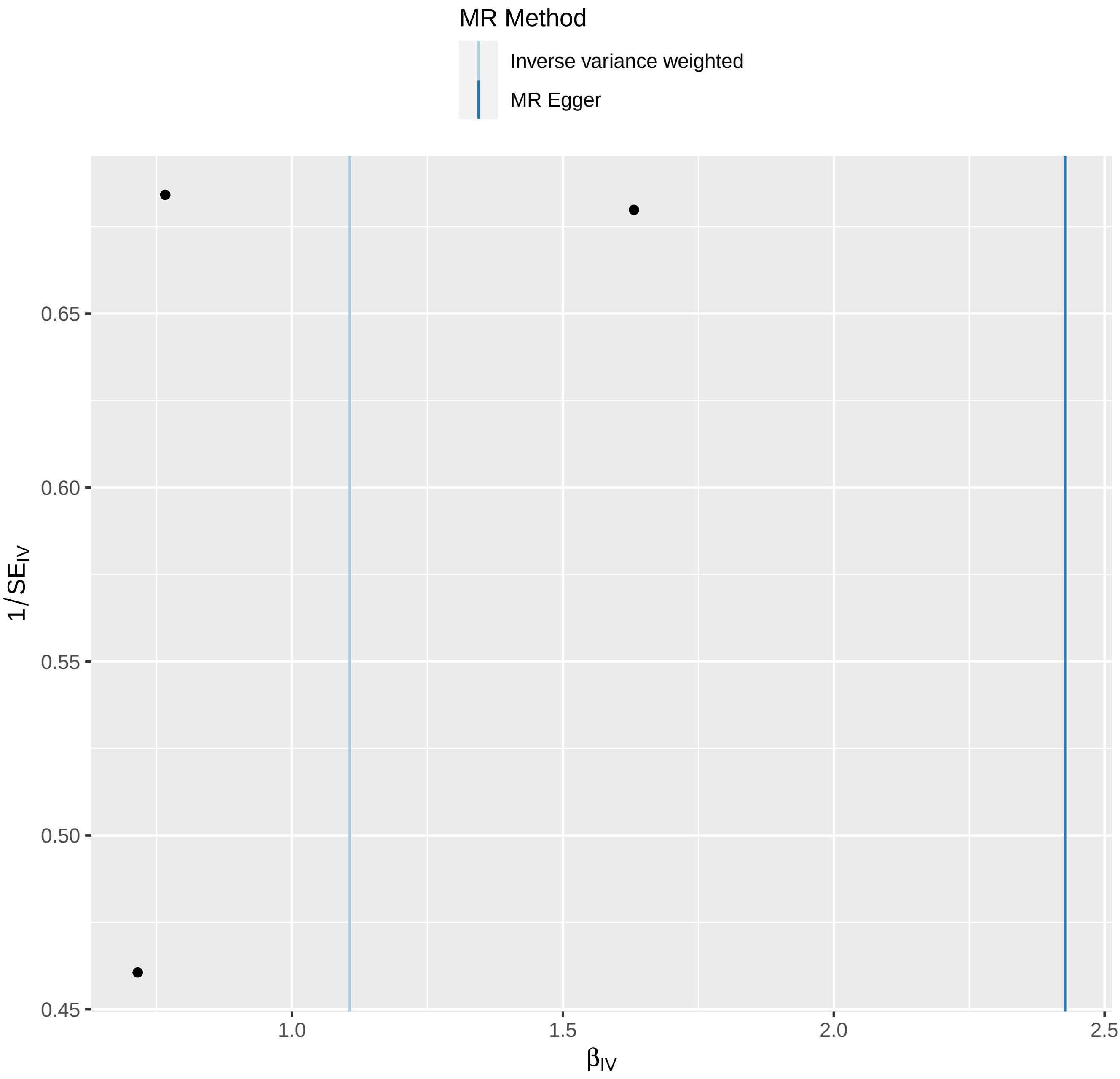

Batch 1776 : DSGEGDFXAEGGGVR* on Arthropathy in ulcerative colitis

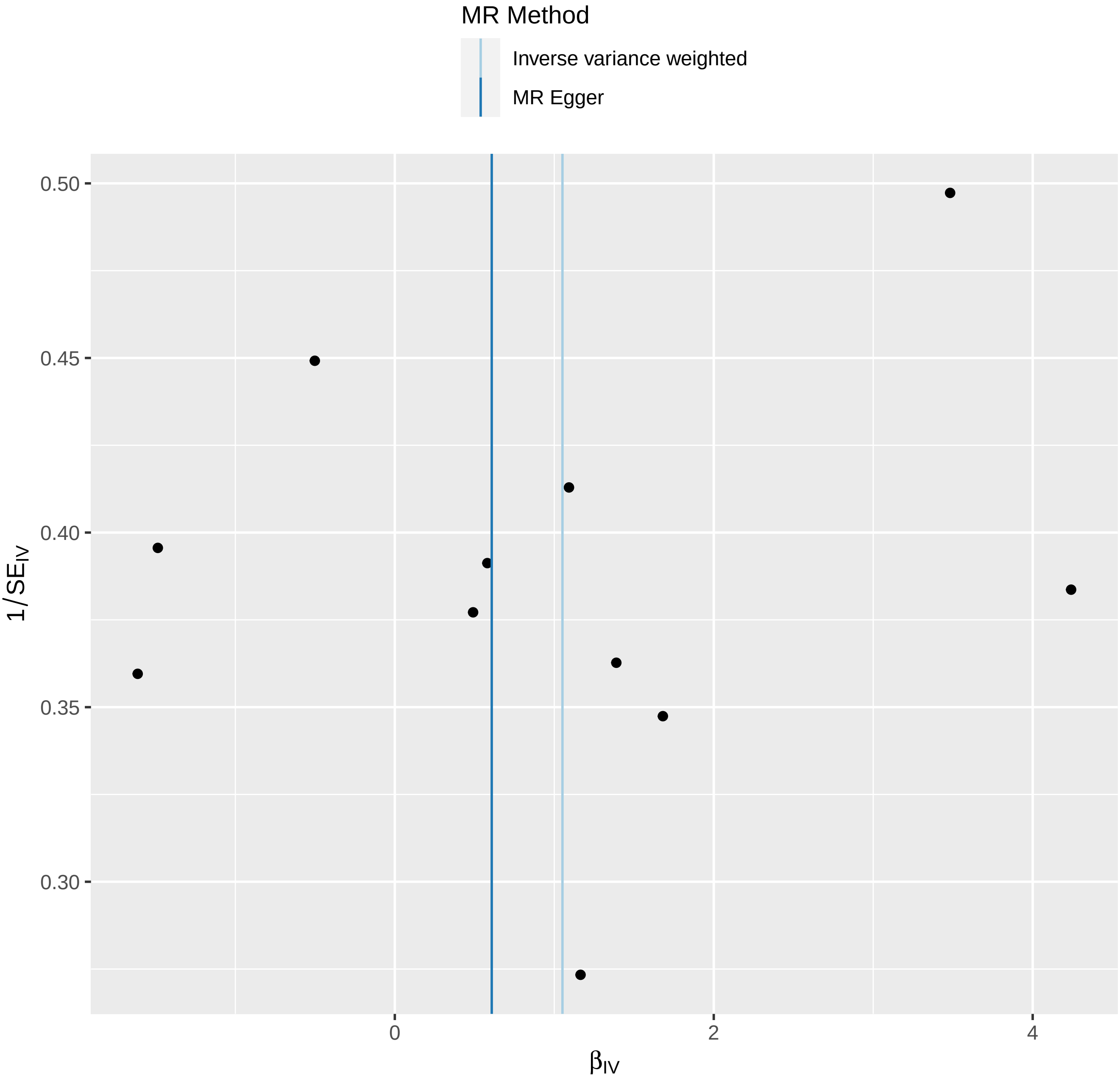

Batch 1777 : Pyridoxate on Arthropathy in ulcerative colitis

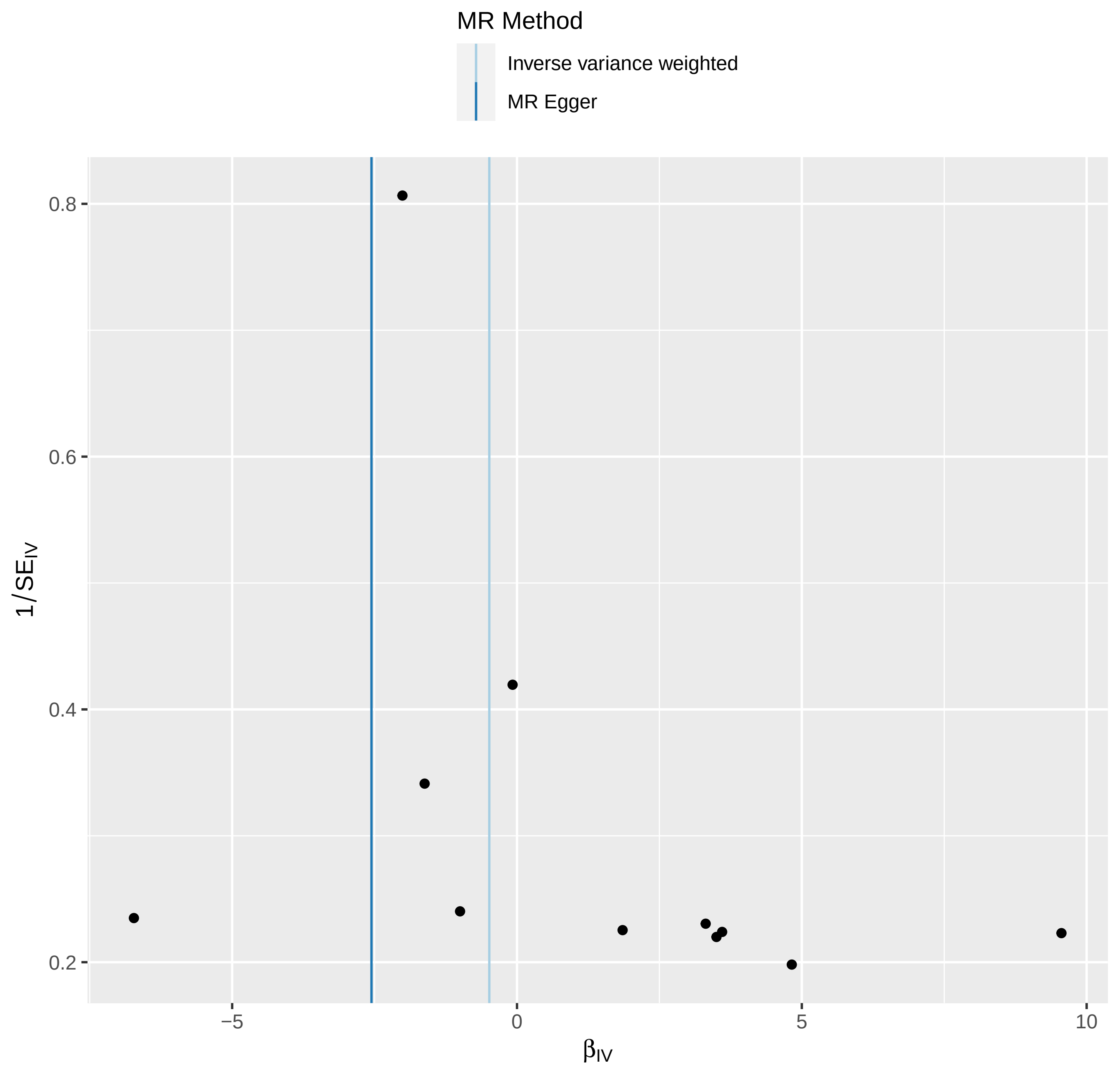

Batch 1778 : Androsterone sulfate on Arthropathy in ulcerative colitis

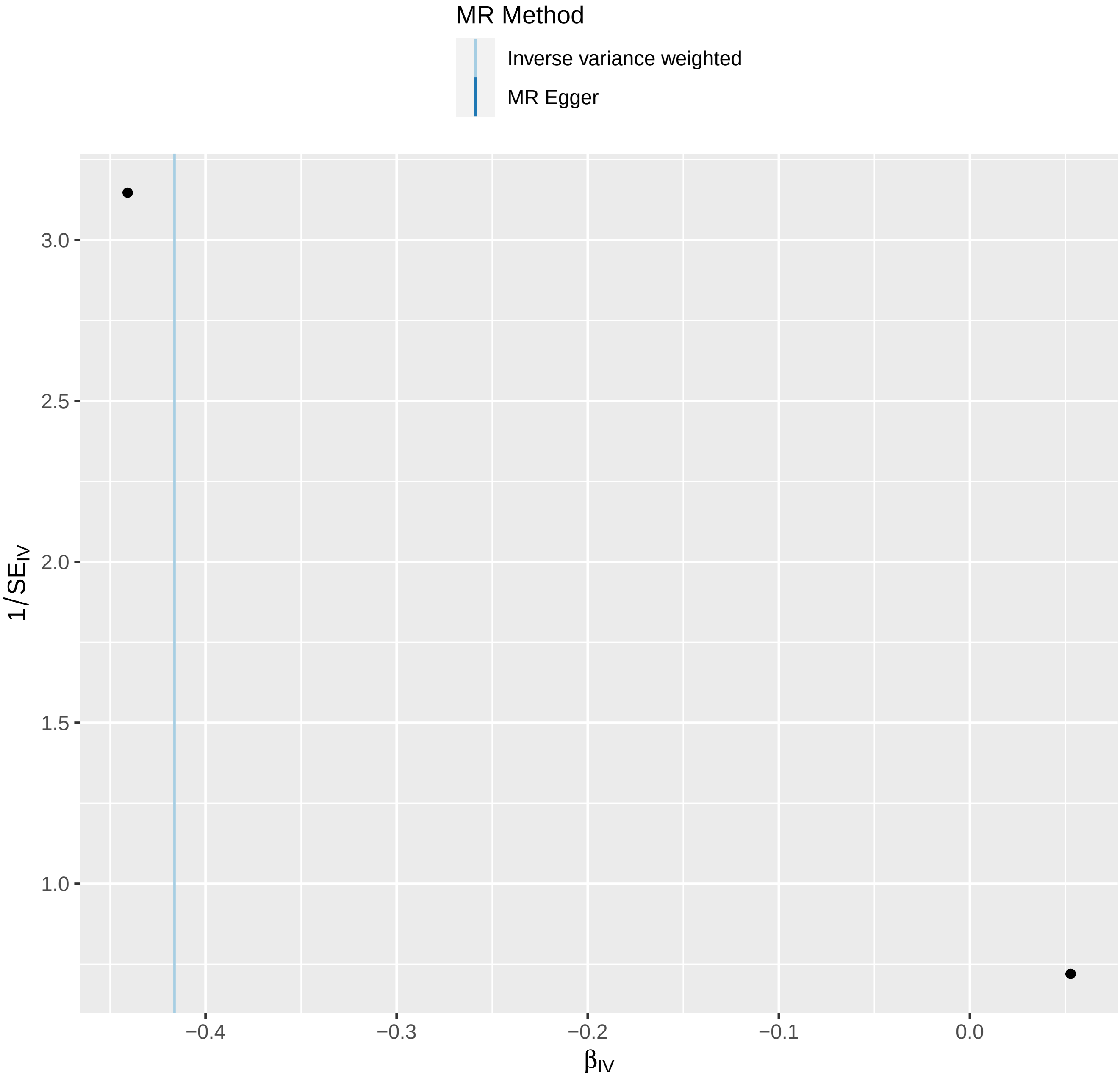

Batch 1781 : Acetylcarnitine on Arthropathy in ulcerative colitis

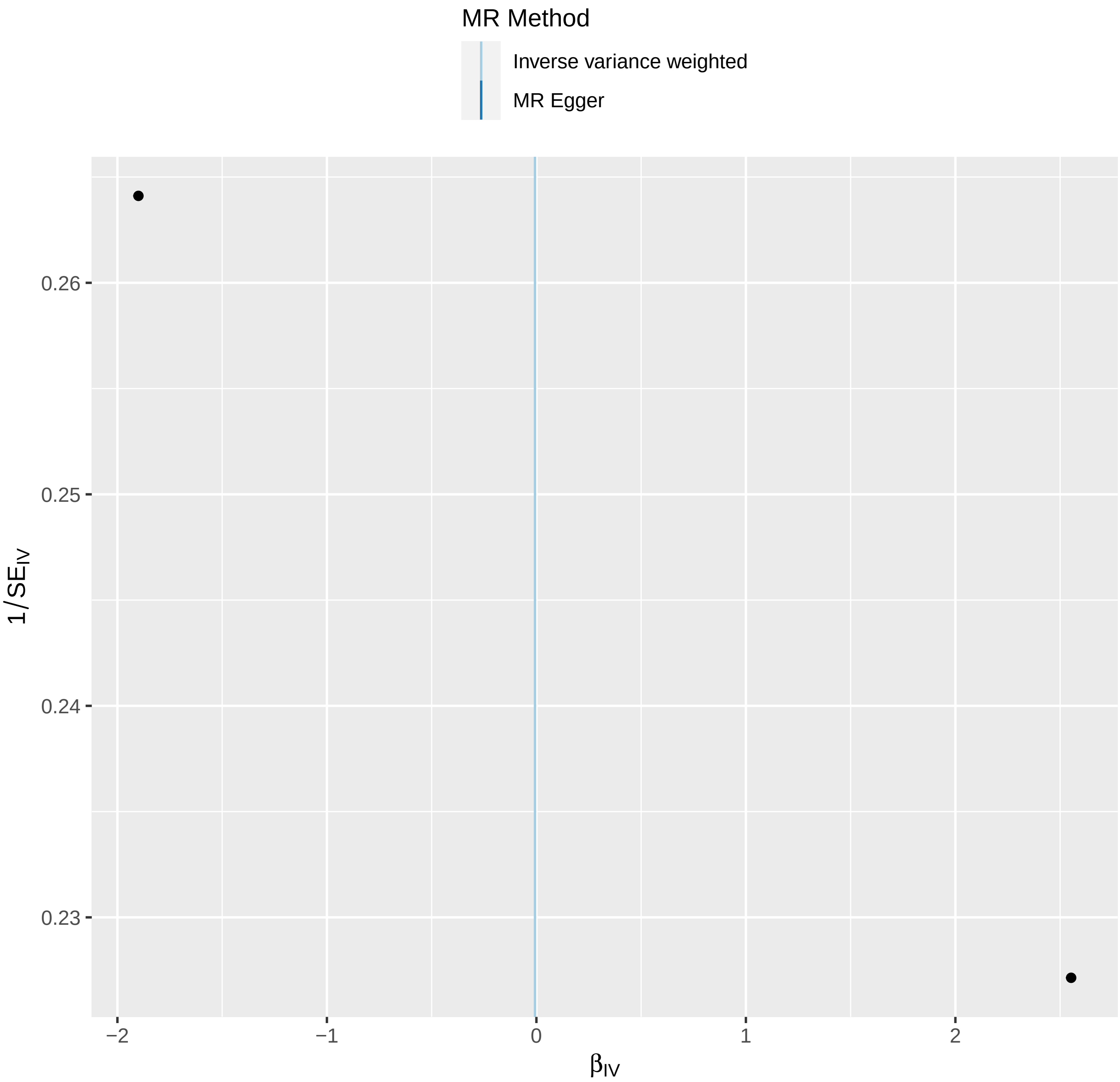

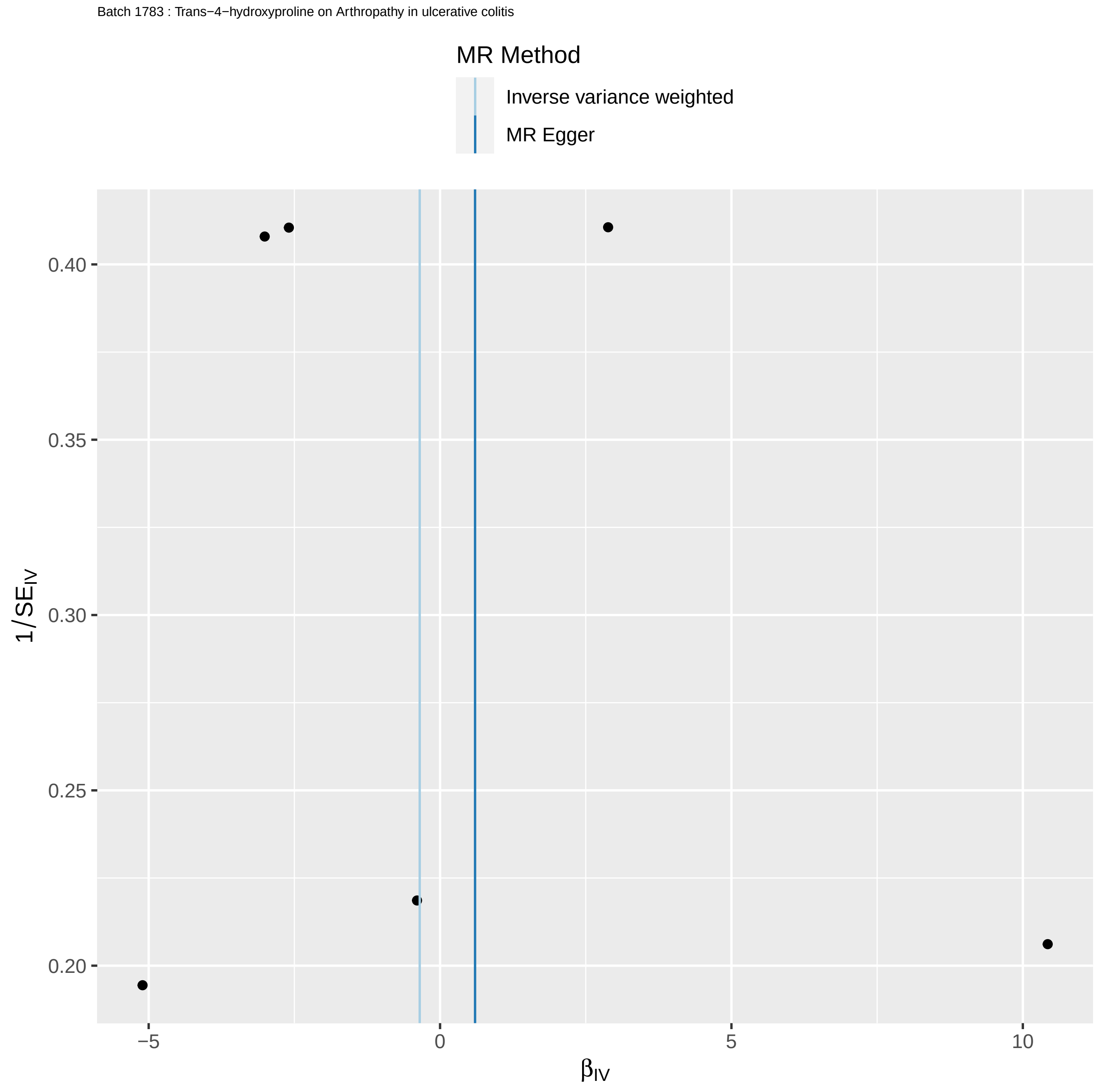
Batch 1783 : Trans−4−hydroxyproline on Arthropathy in ulcerative colitis
MR Method
Inverse variance weighted
MR Egger
0.40
0.35
0.30
0.25
0.20
1/SE_IV
−5
0
5
10
β_IV

Batch 1784 : Glutamate on Arthropathy in ulcerative colitis

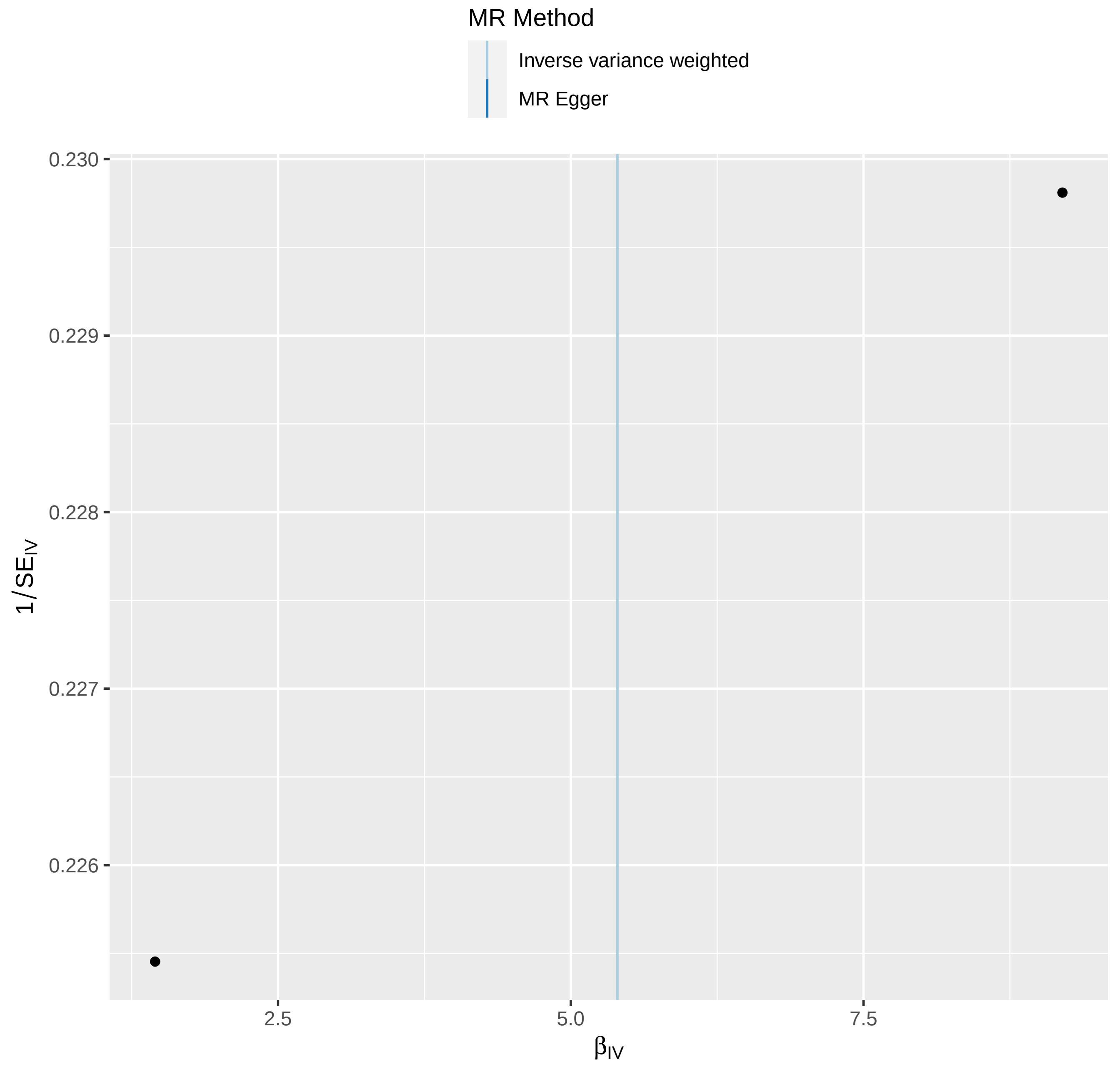

Batch 1785 : Hexanoylcarnitine on Arthropathy in ulcerative colitis

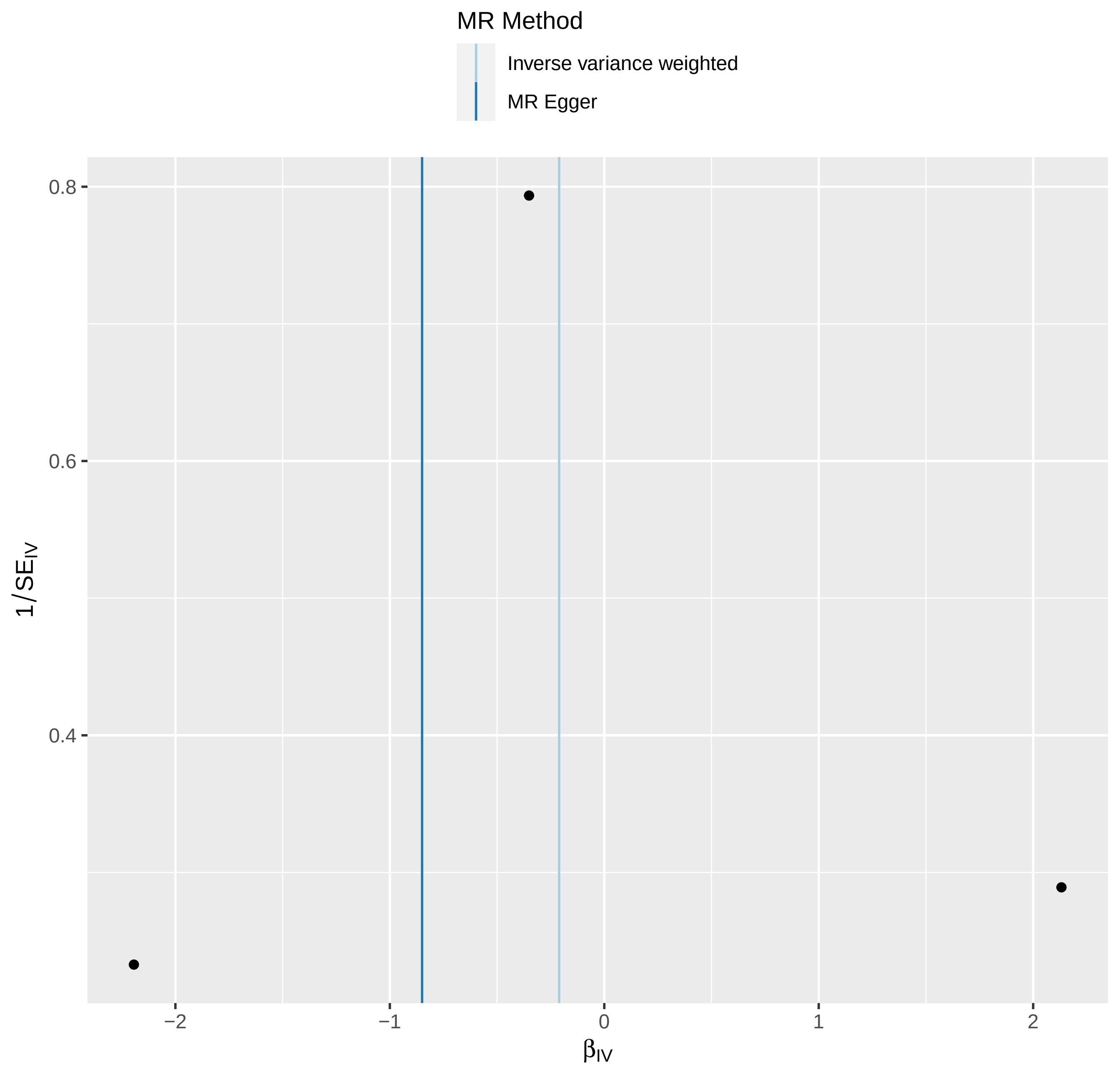

Batch 1789 : 2−aminobutyrate on Arthropathy in ulcerative colitis

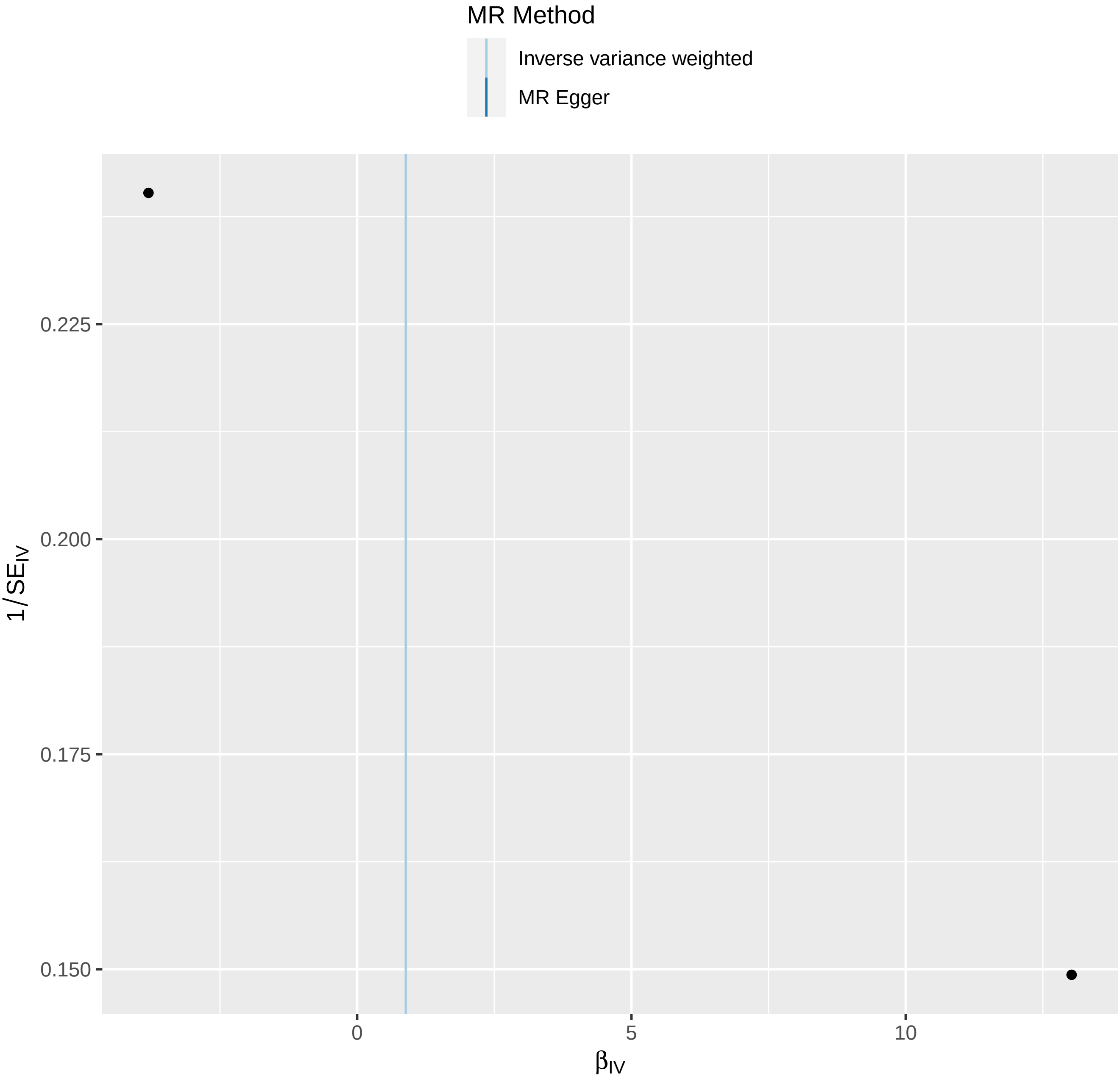

Batch 1794 : Butyrylcarnitine on Arthropathy in ulcerative colitis

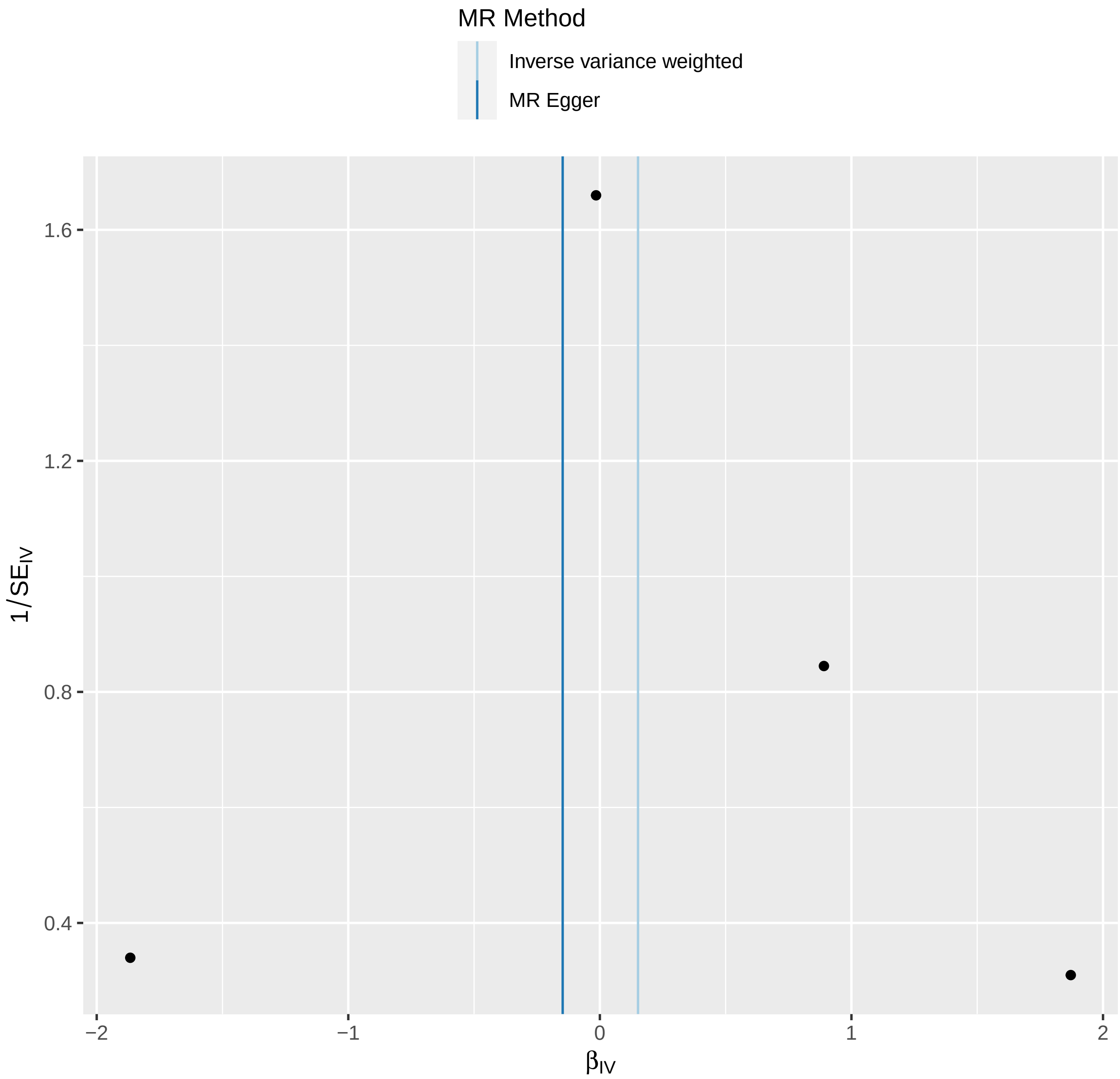

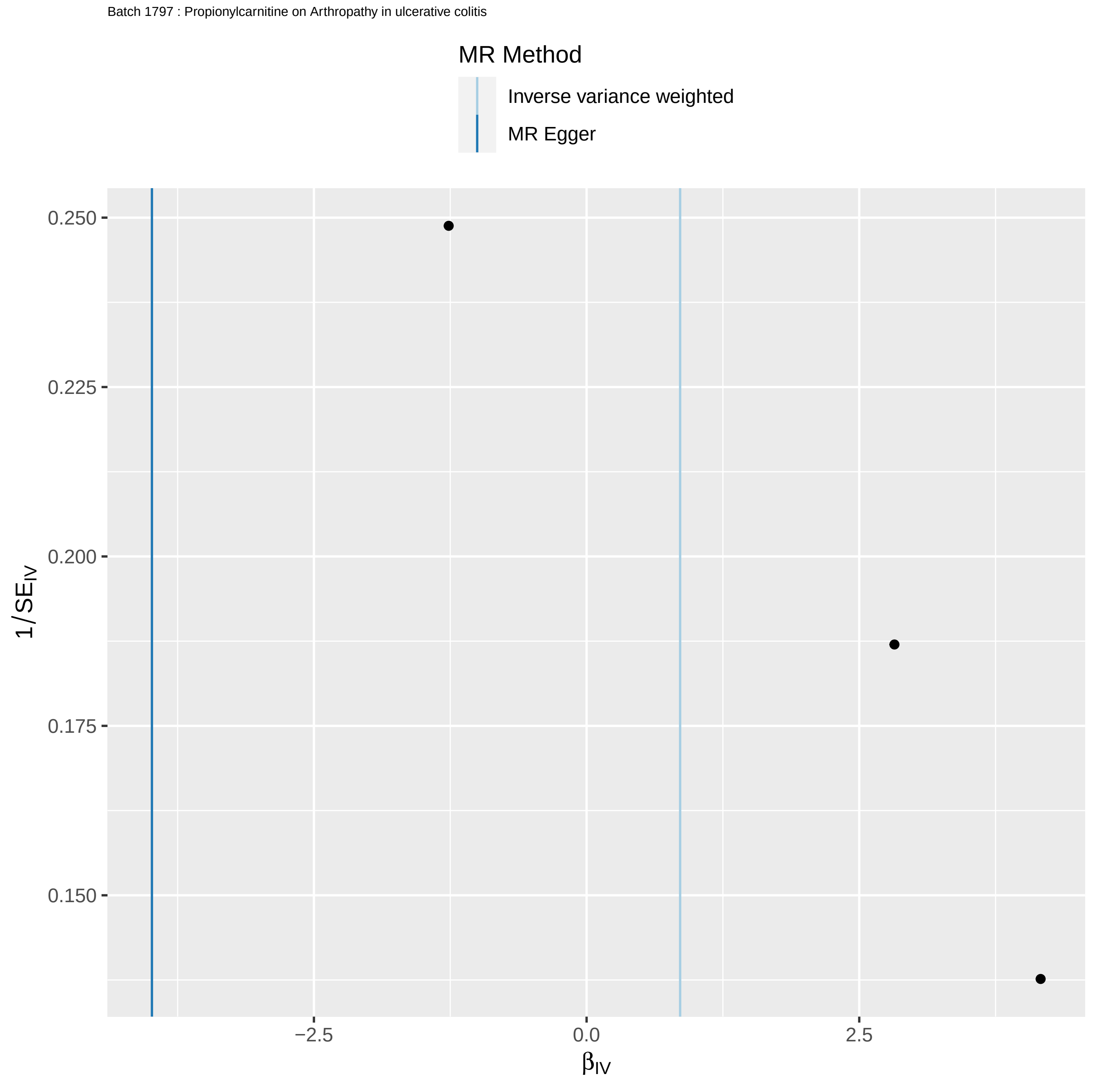

Batch 1797 : Propionylcarnitine on Arthropathy in ulcerative colitis
MR Method
Inverse variance weighted
MR Egger
0.250
0.225
0.200
0.175
0.150
1/SE_IV
−2.5
0.0
2.5
β_IV

Batch 1798 : Caproate (6:0) on Arthropathy in ulcerative colitis

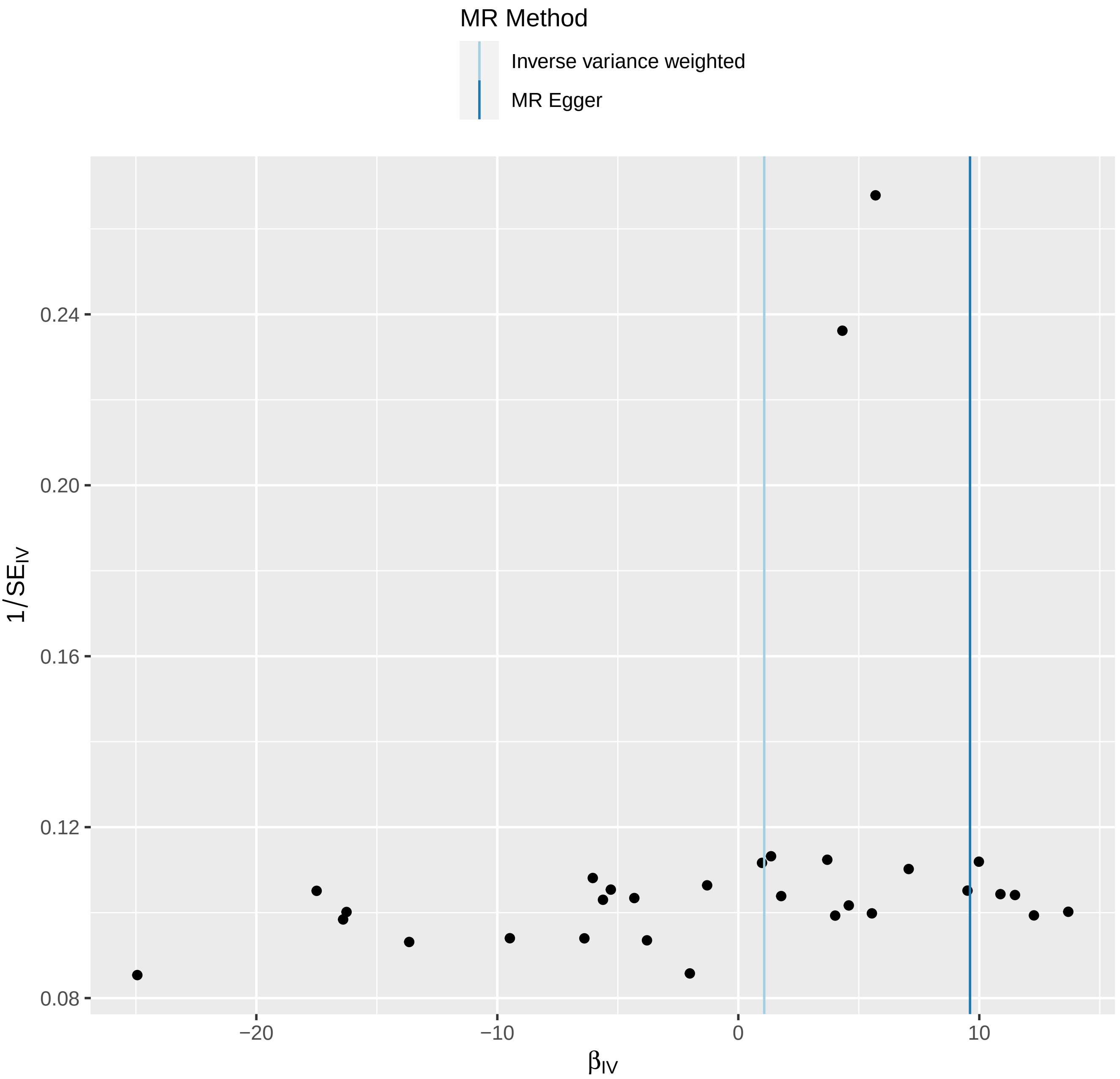

Batch 1799 : Caprylate (8:0) on Arthropathy in ulcerative colitis

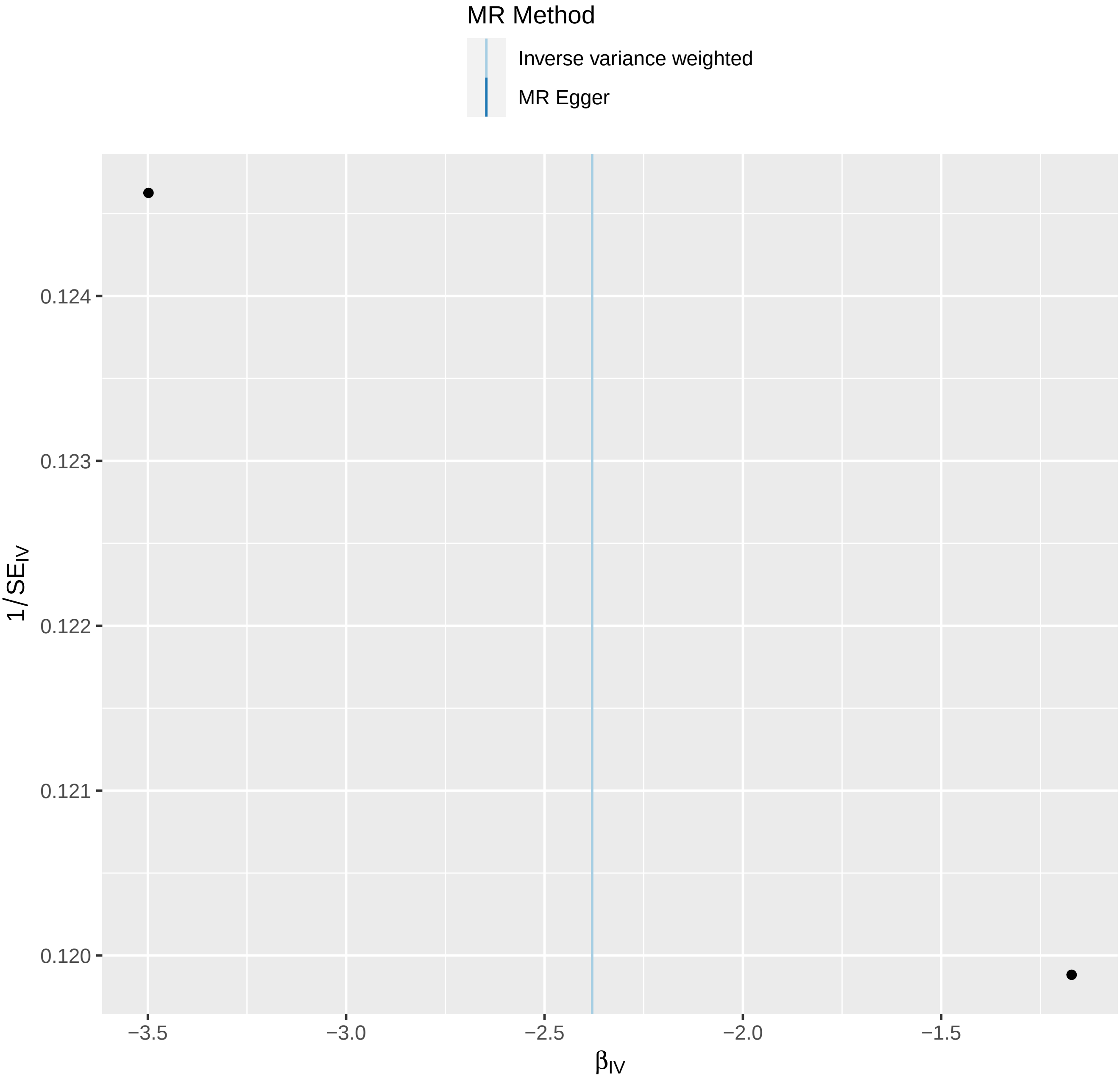

Batch 1800 : 10−undecenoate (11:1n1) on Arthropathy in ulcerative colitis

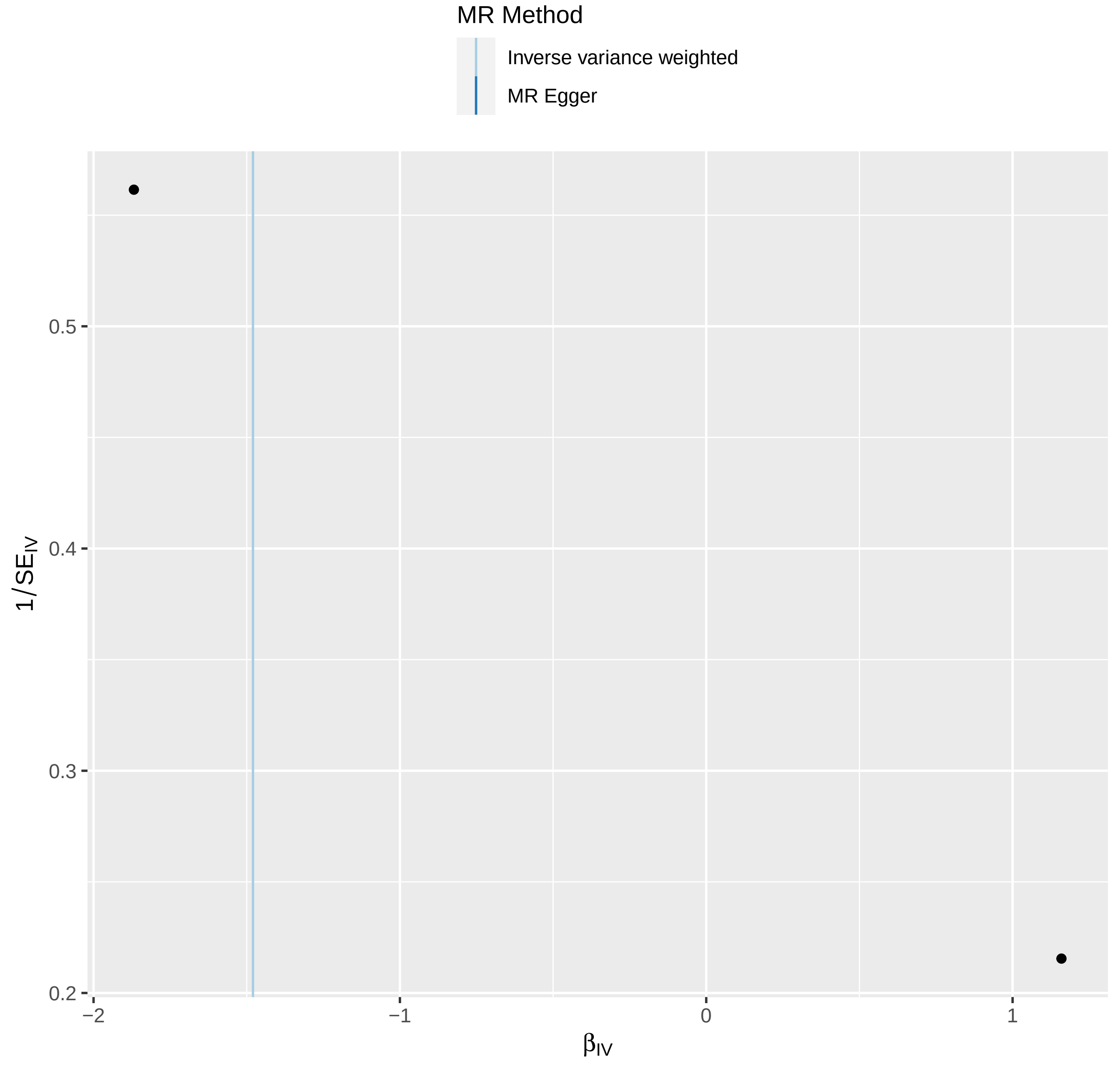

Batch 1802 : Phenol sulfate on Arthropathy in ulcerative colitis

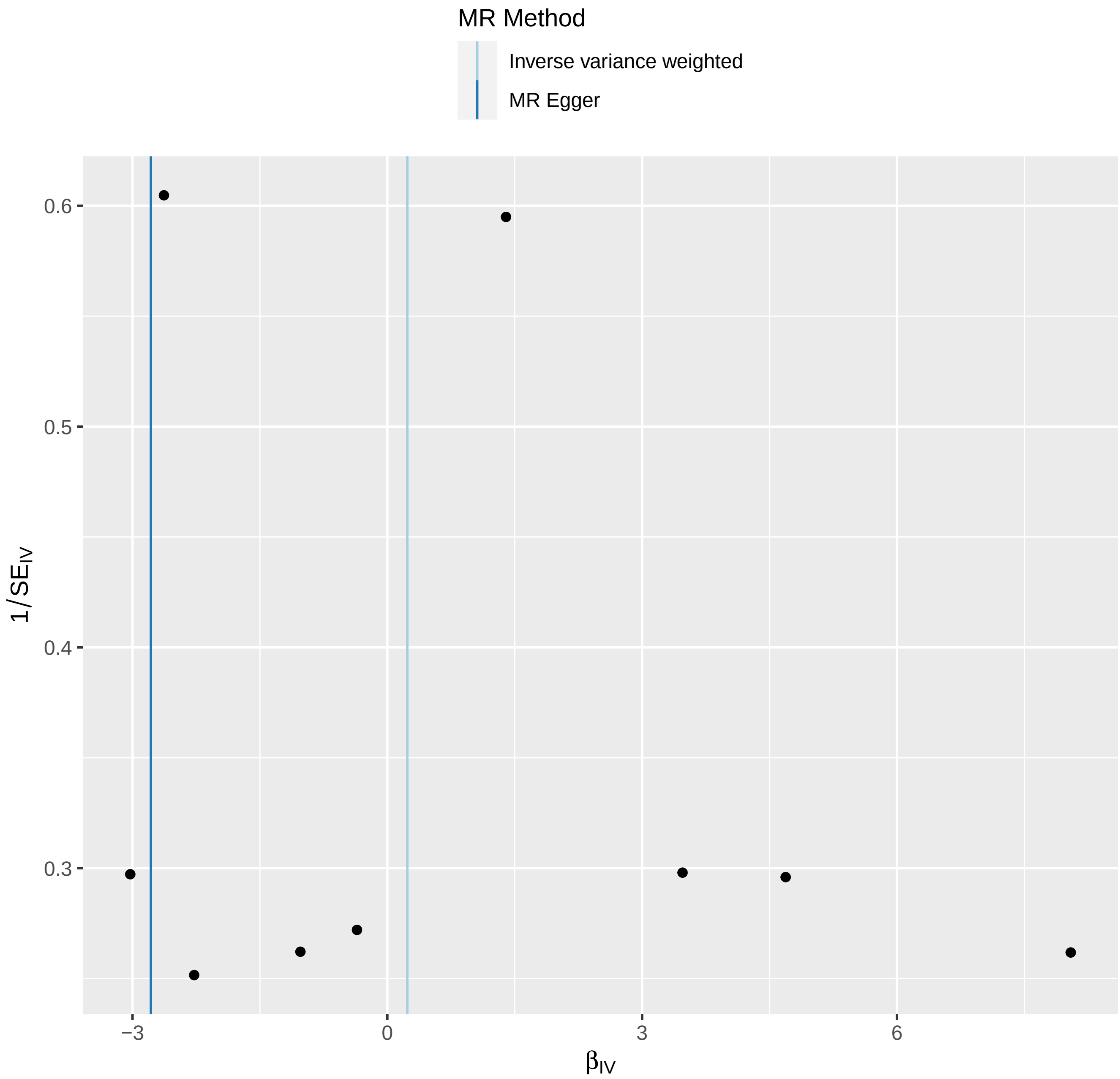

Batch 1804 : Heme* on Arthropathy in ulcerative colitis

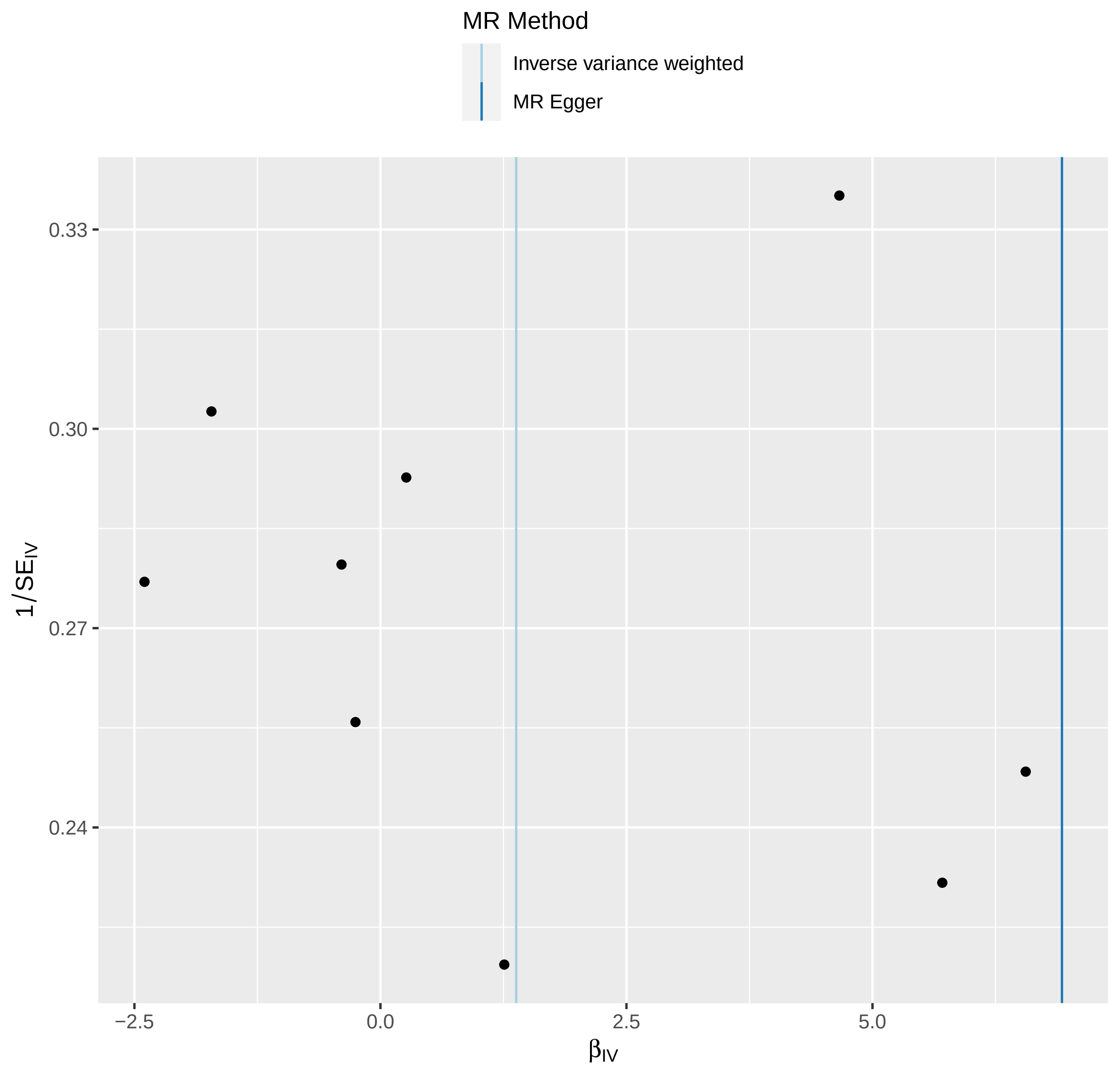

Batch 1806 : 3−dehydrocarnitine* on Arthropathy in ulcerative colitis

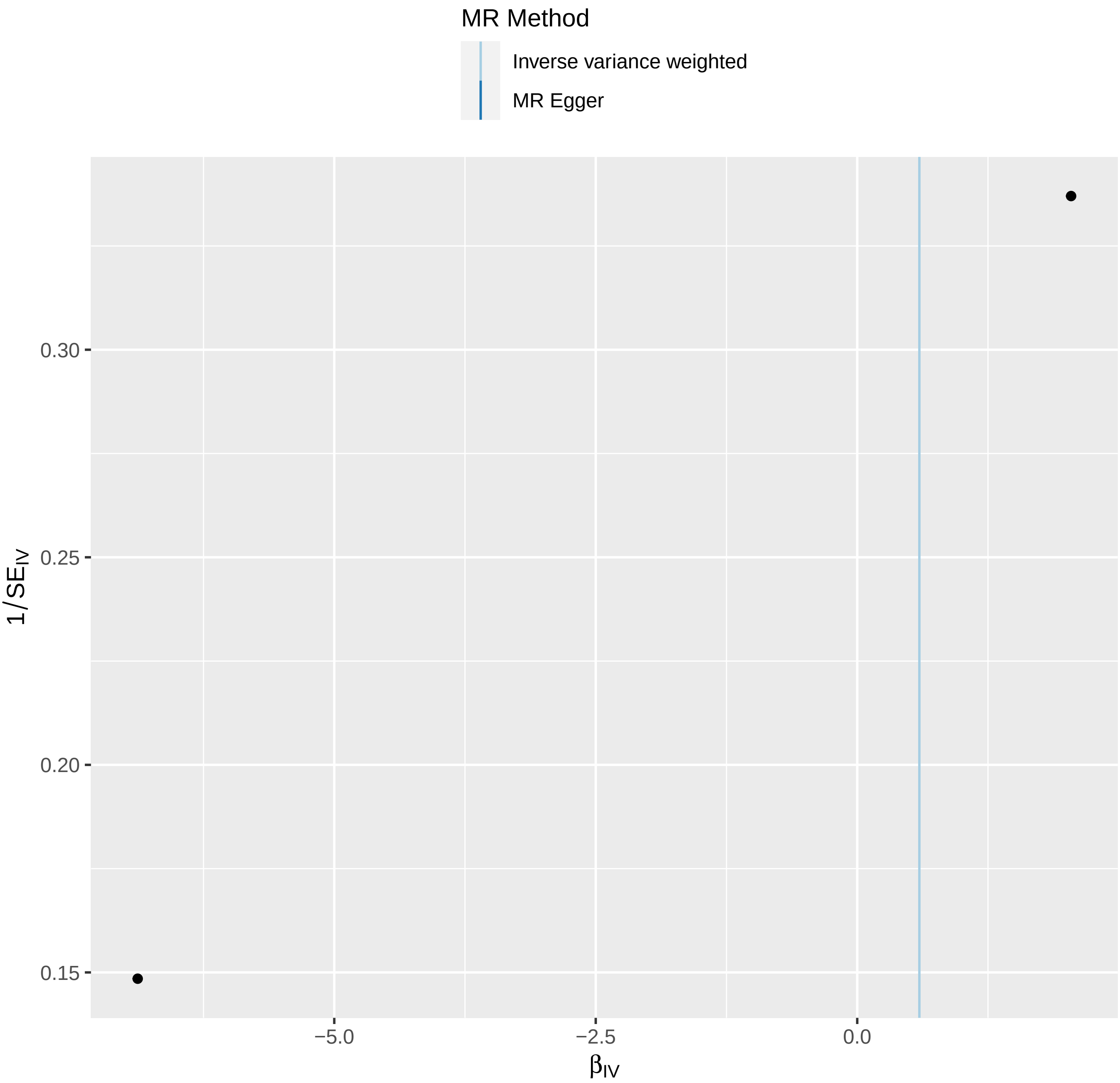

Batch 1807 : Pyroglutamine* on Arthropathy in ulcerative colitis

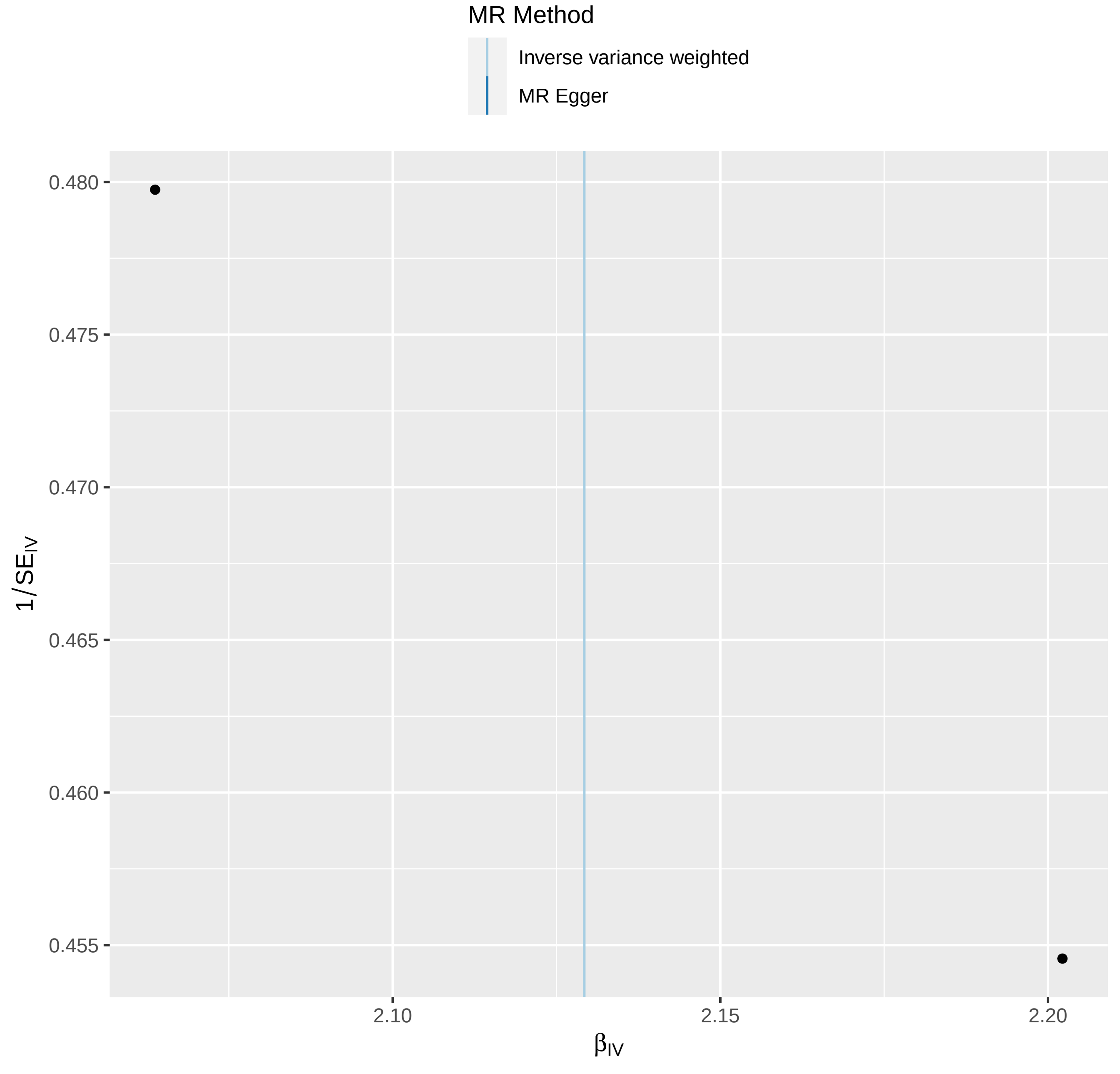

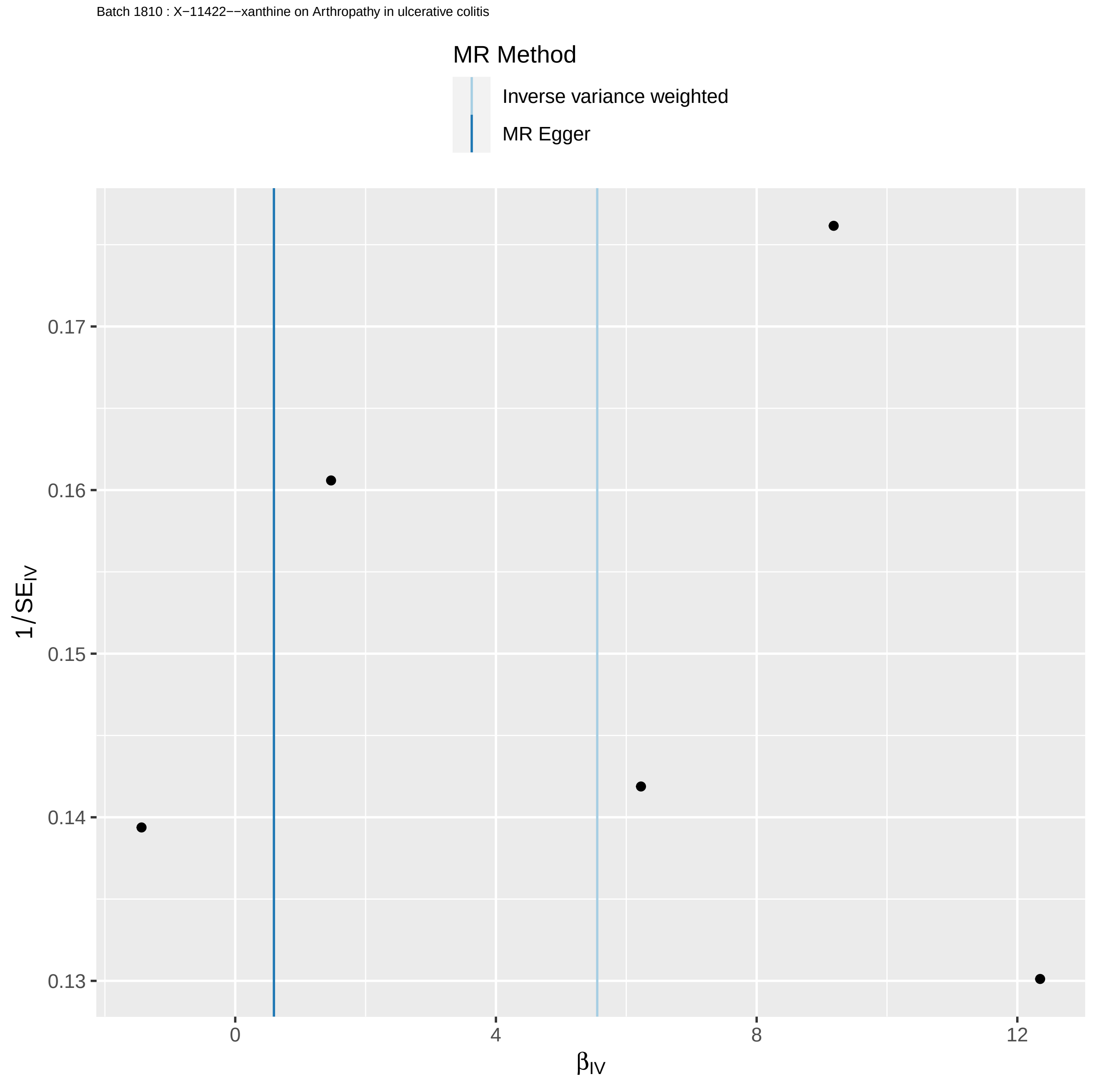

Batch 1810 : X−11422−−xanthine on Arthropathy in ulcerative colitis
MR Method
Inverse variance weighted
MR Egger
0.17
0.16
0.15
0.14
0.13
1/SE_IV
0
4
8
12
β_IV

Batch 1813 : HWESASXX* on Arthropathy in ulcerative colitis

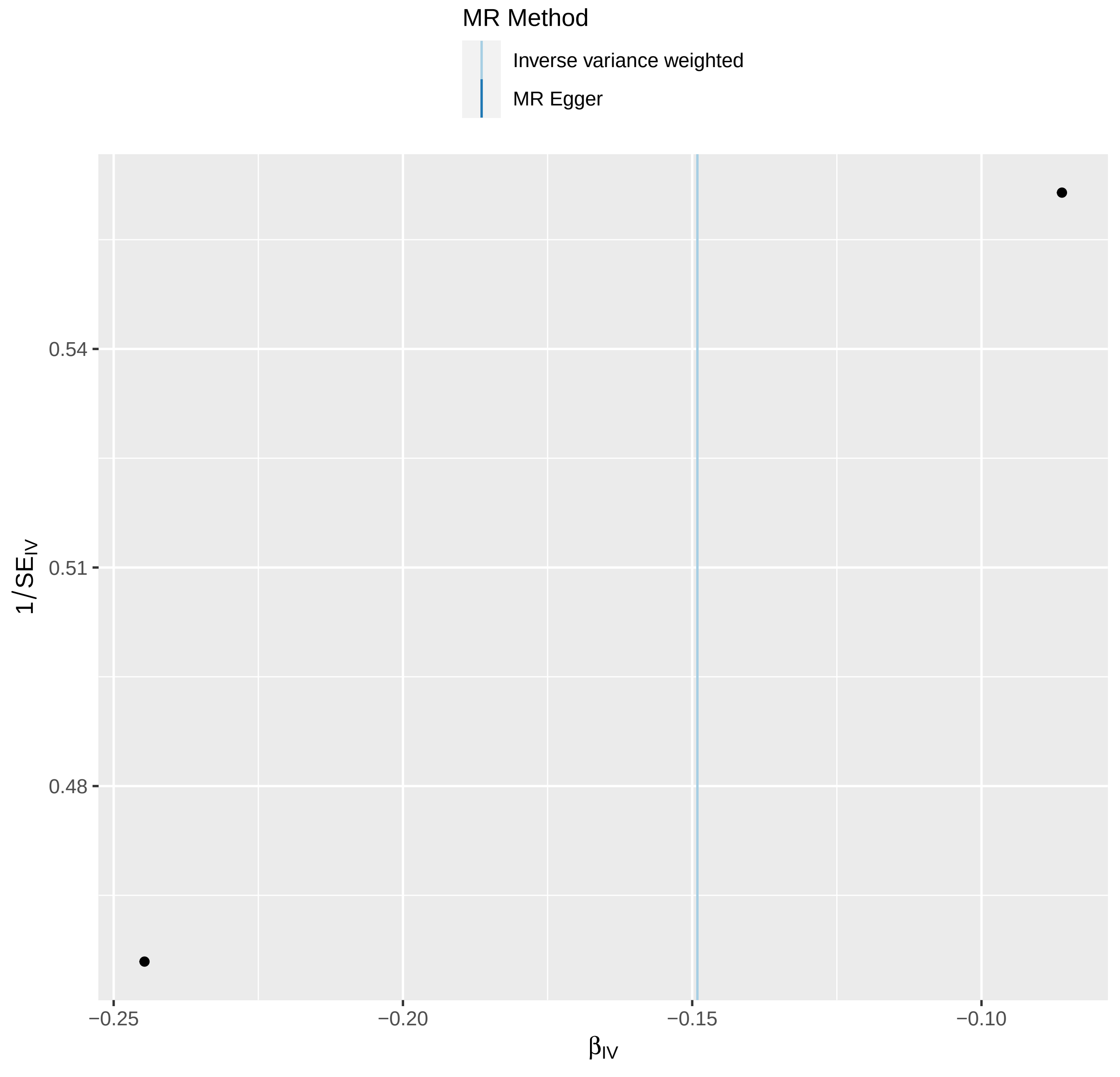

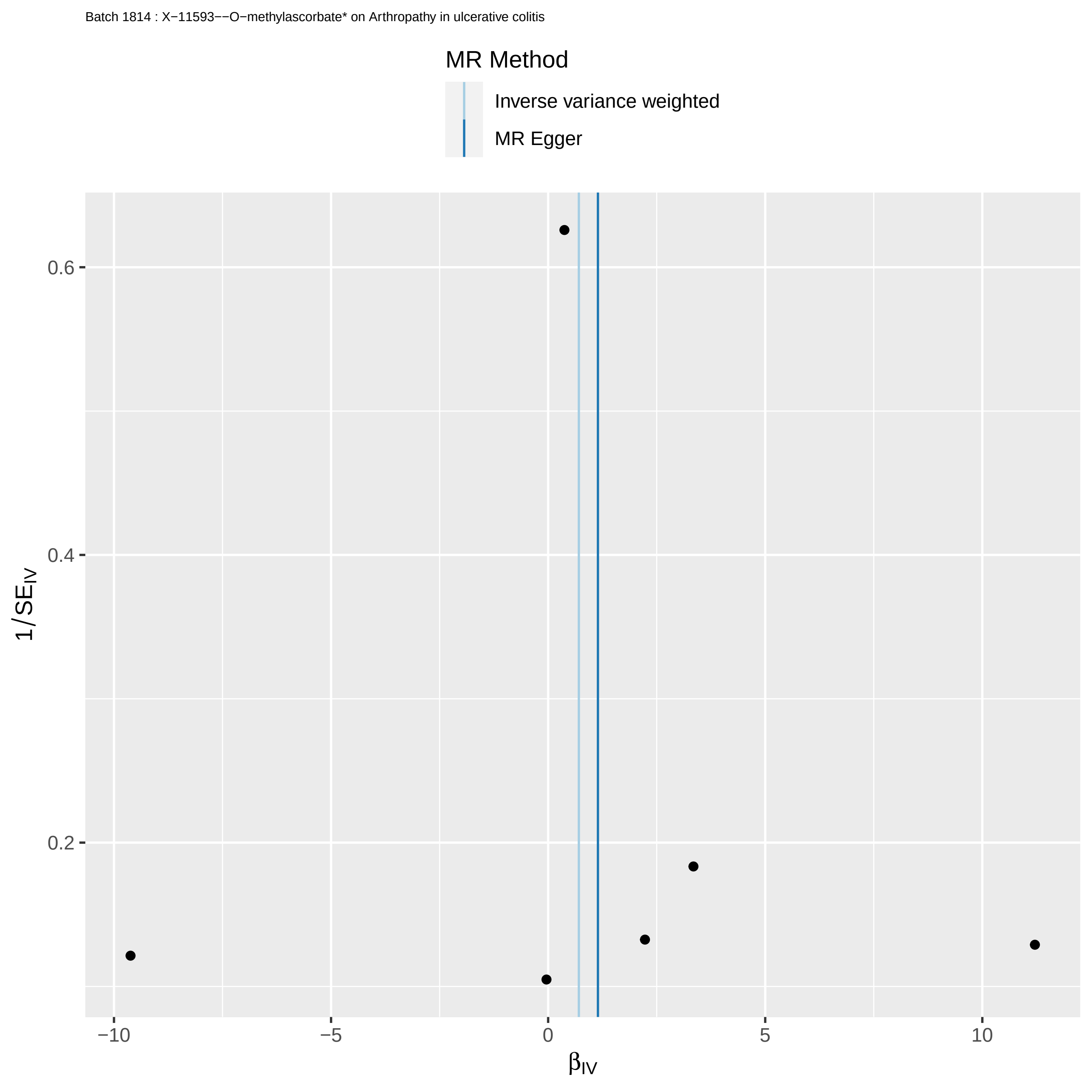
Batch 1814 : X−11593−−O−methylascorbate* on Arthropathy in ulcerative colitis
MR Method
Inverse variance weighted
MR Egger
0.6
0.4
0.2
1/SE_IV
−10
−5
0
5
10
β_IV

Batch 1816 : Homostachydrine* on Arthropathy in ulcerative colitis

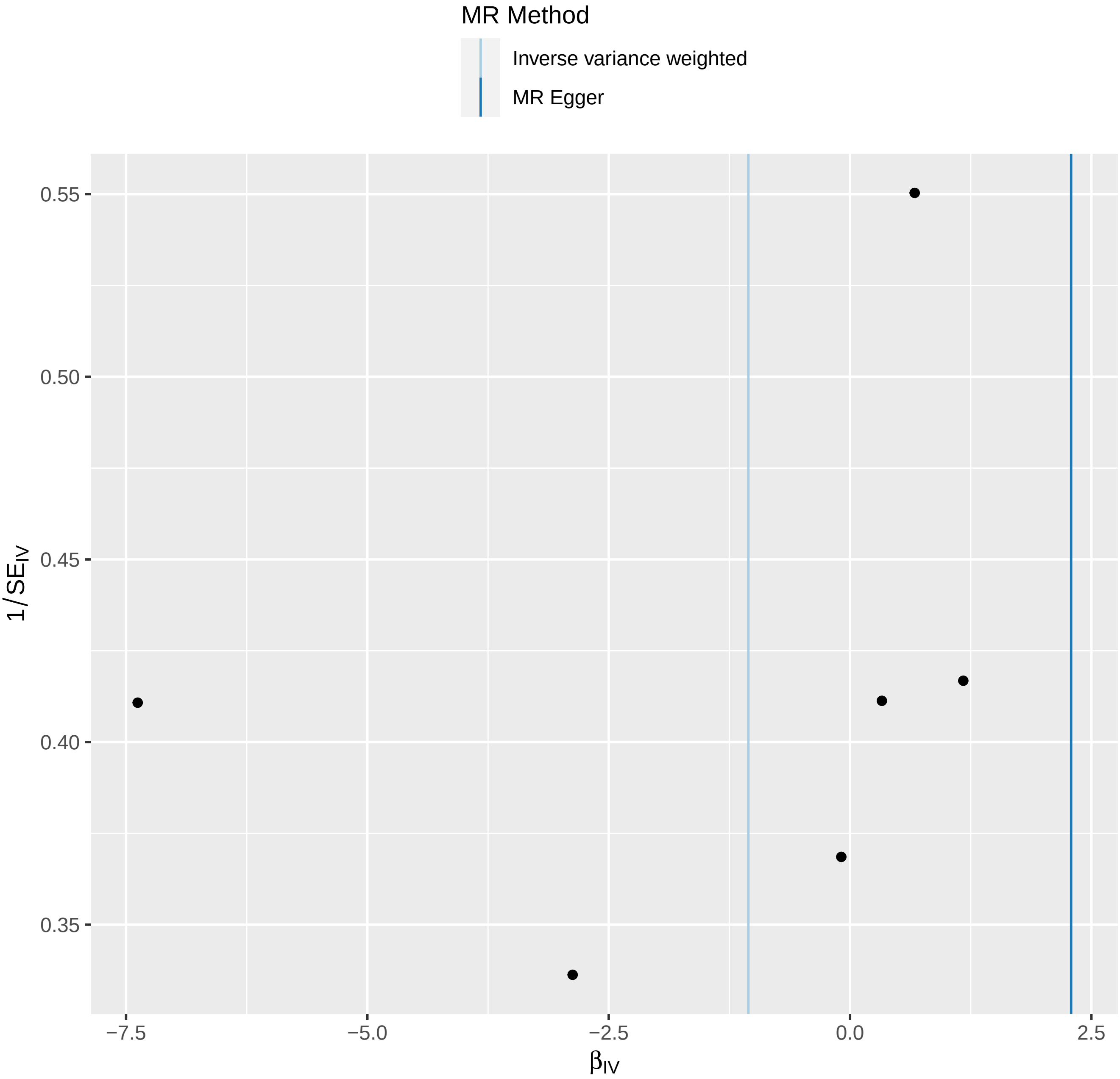

Batch 1817 : ADSGEGDFXAEGGGVR* on Arthropathy in ulcerative colitis

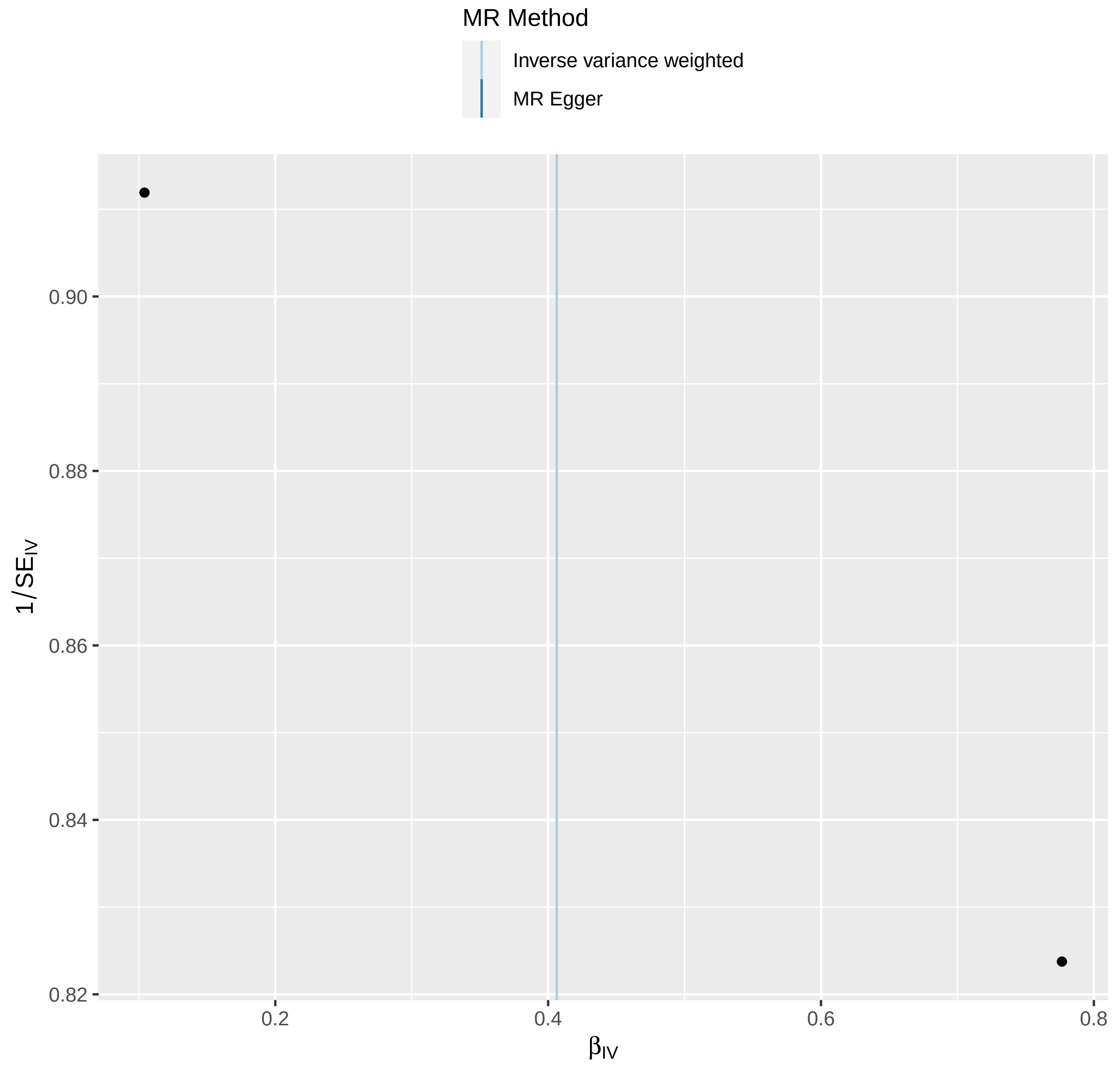

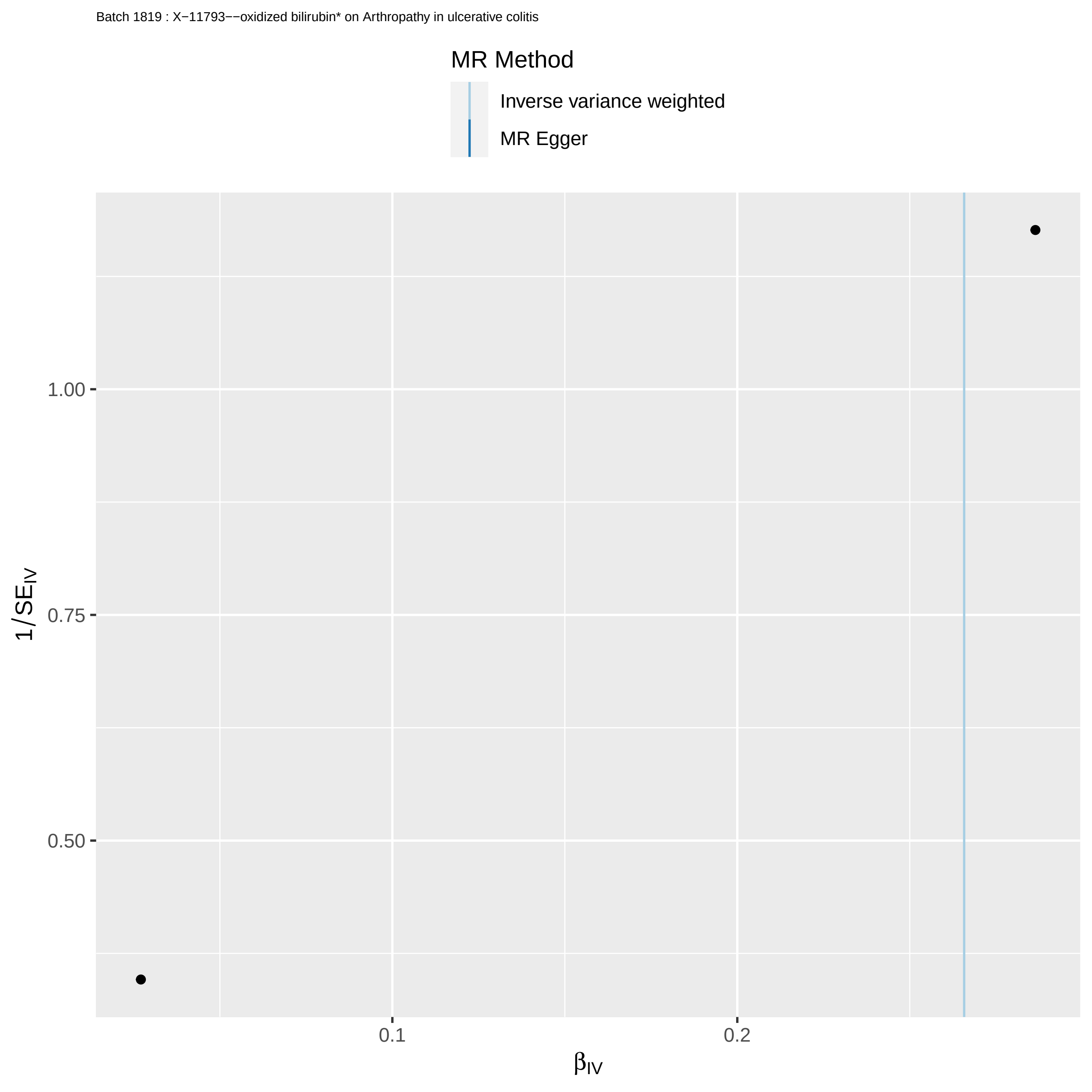

Batch 1819 : X−11793−−oxidized bilirubin* on Arthropathy in ulcerative colitis
MR Method
Inverse variance weighted
MR Egger
1.00
0.75
0.50
1/SE$_{IV}$
0.1
0.2
β$_{IV}$

Batch 1820 : 1−arachidonoylglycerophosphocholine* on Arthropathy in ulcerative colitis

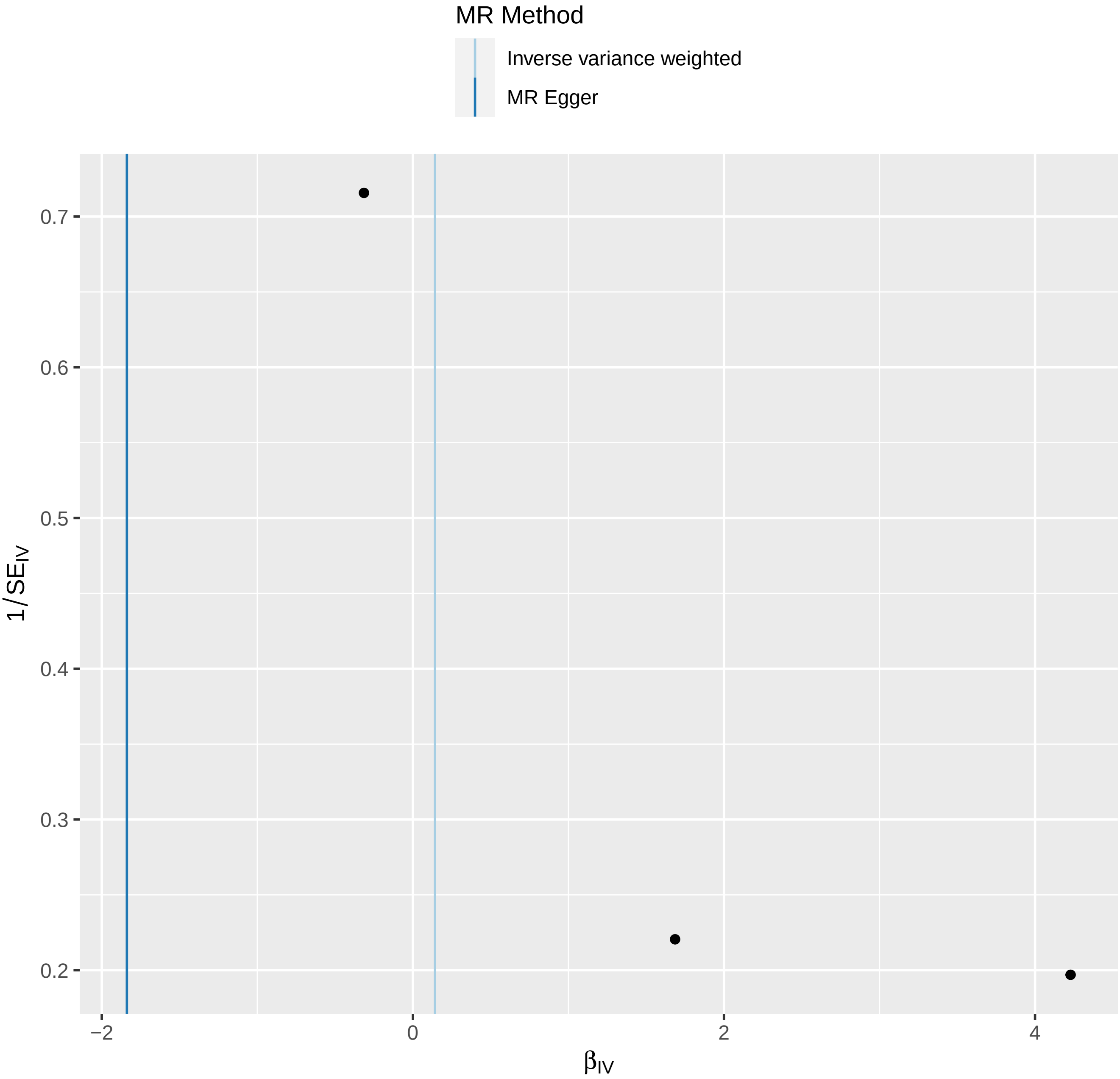

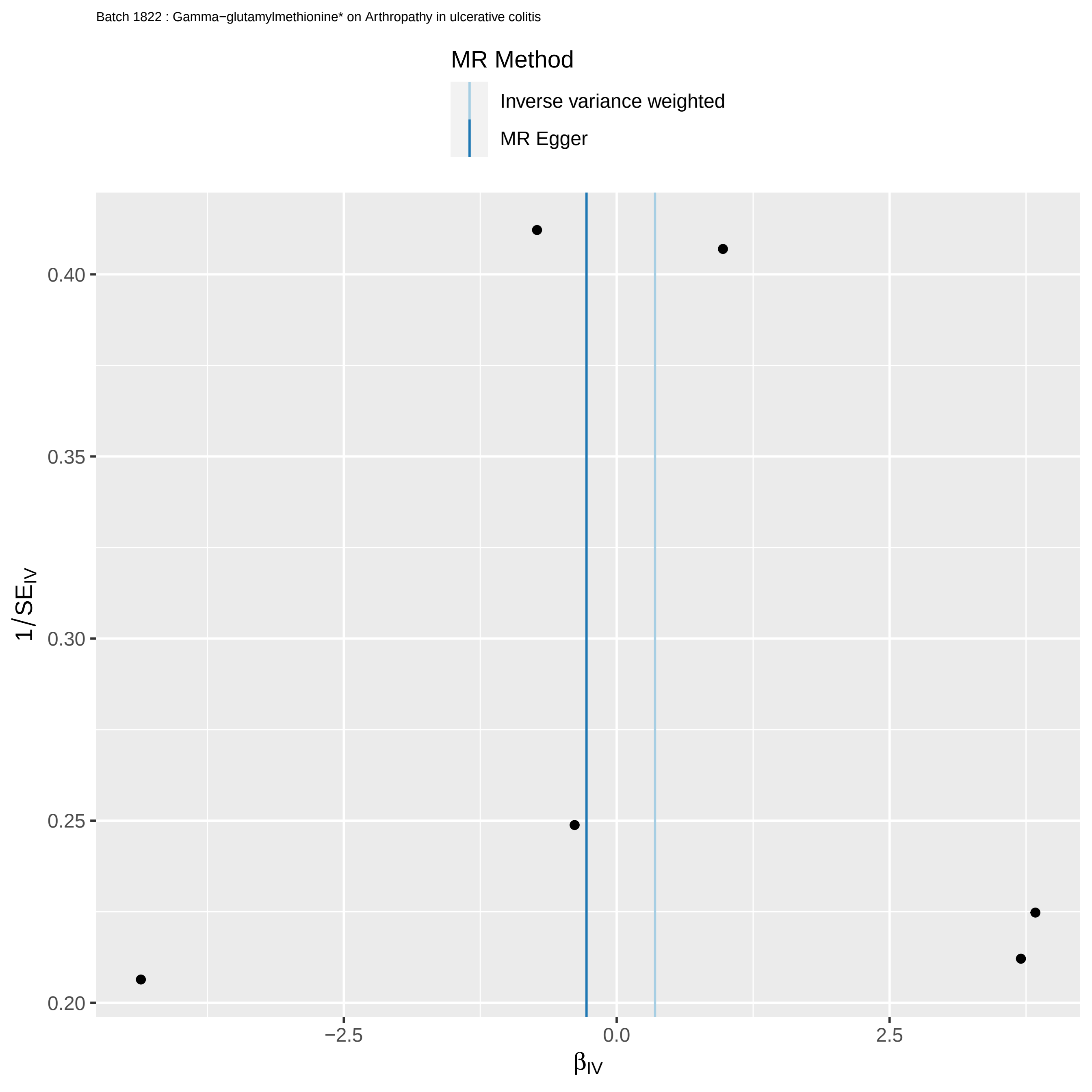
Batch 1822 : Gamma−glutamylmethionine* on Arthropathy in ulcerative colitis
MR Method
Inverse variance weighted
MR Egger
0.40
0.35
0.30
0.25
0.20
1/SE_IV
−2.5
0.0
2.5
β_IV

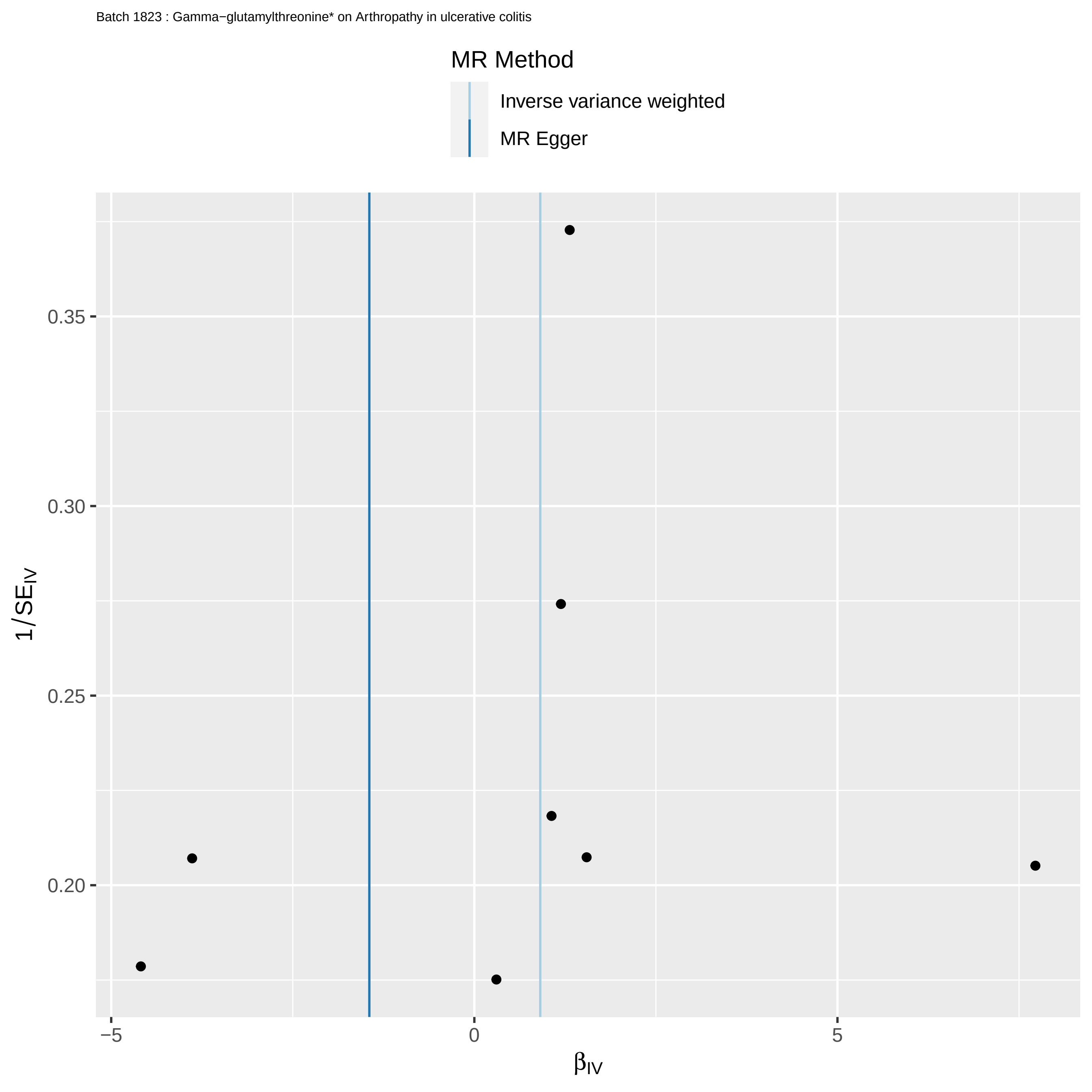
Batch 1823 : Gamma−glutamylthreonine* on Arthropathy in ulcerative colitis
MR Method
Inverse variance weighted
MR Egger
0.35
0.30
0.25
0.20
1/SE$_{IV}$
−5
0
5
β$_{IV}$

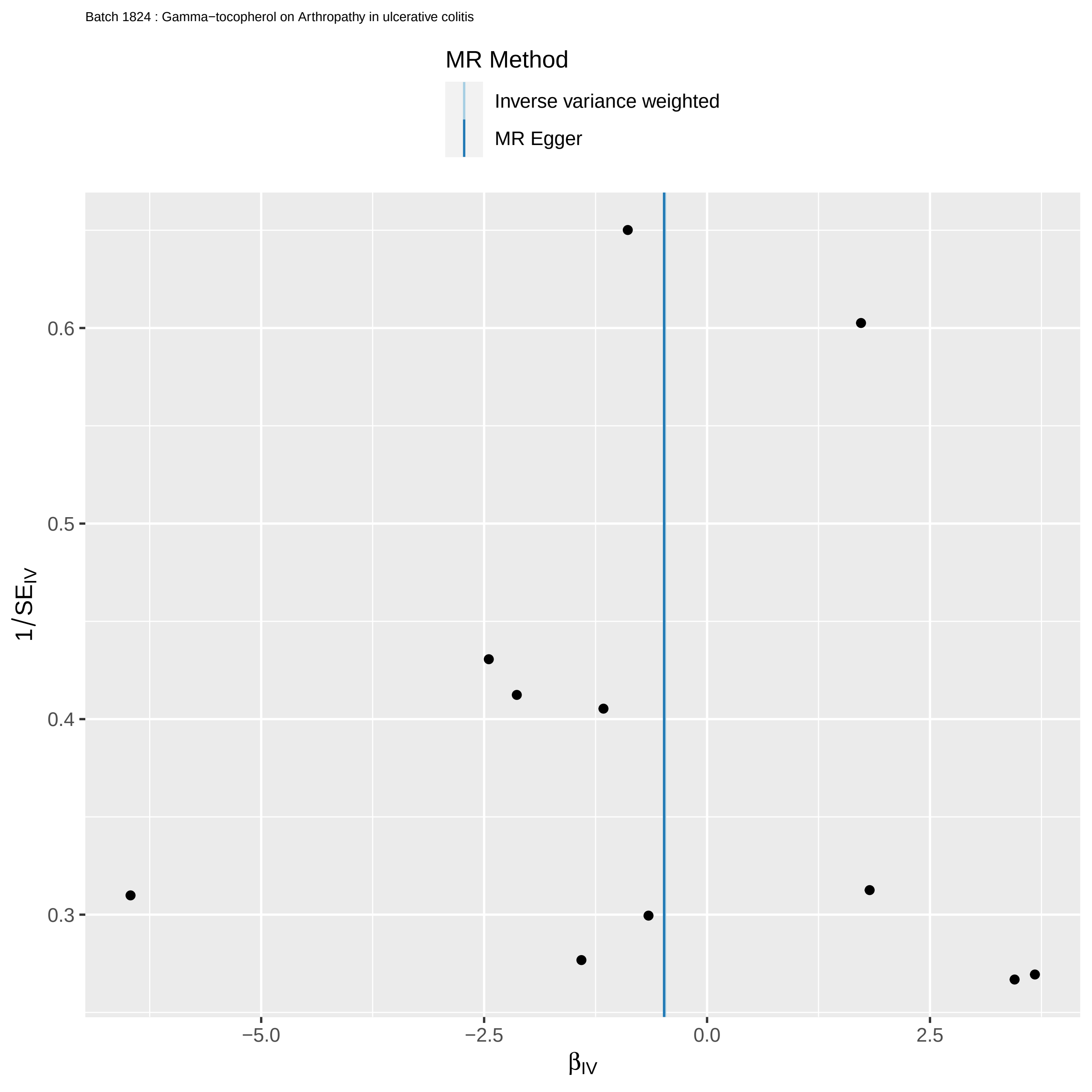
Batch 1824 : Gamma−tocopherol on Arthropathy in ulcerative colitis
MR Method
Inverse variance weighted
MR Egger
0.6
0.5
0.4
0.3
1/SE_IV
−5.0
−2.5
0.0
2.5
β_IV

Batch 1825 : Gamma−glutamylphenylalanine on Arthropathy in ulcerative colitis

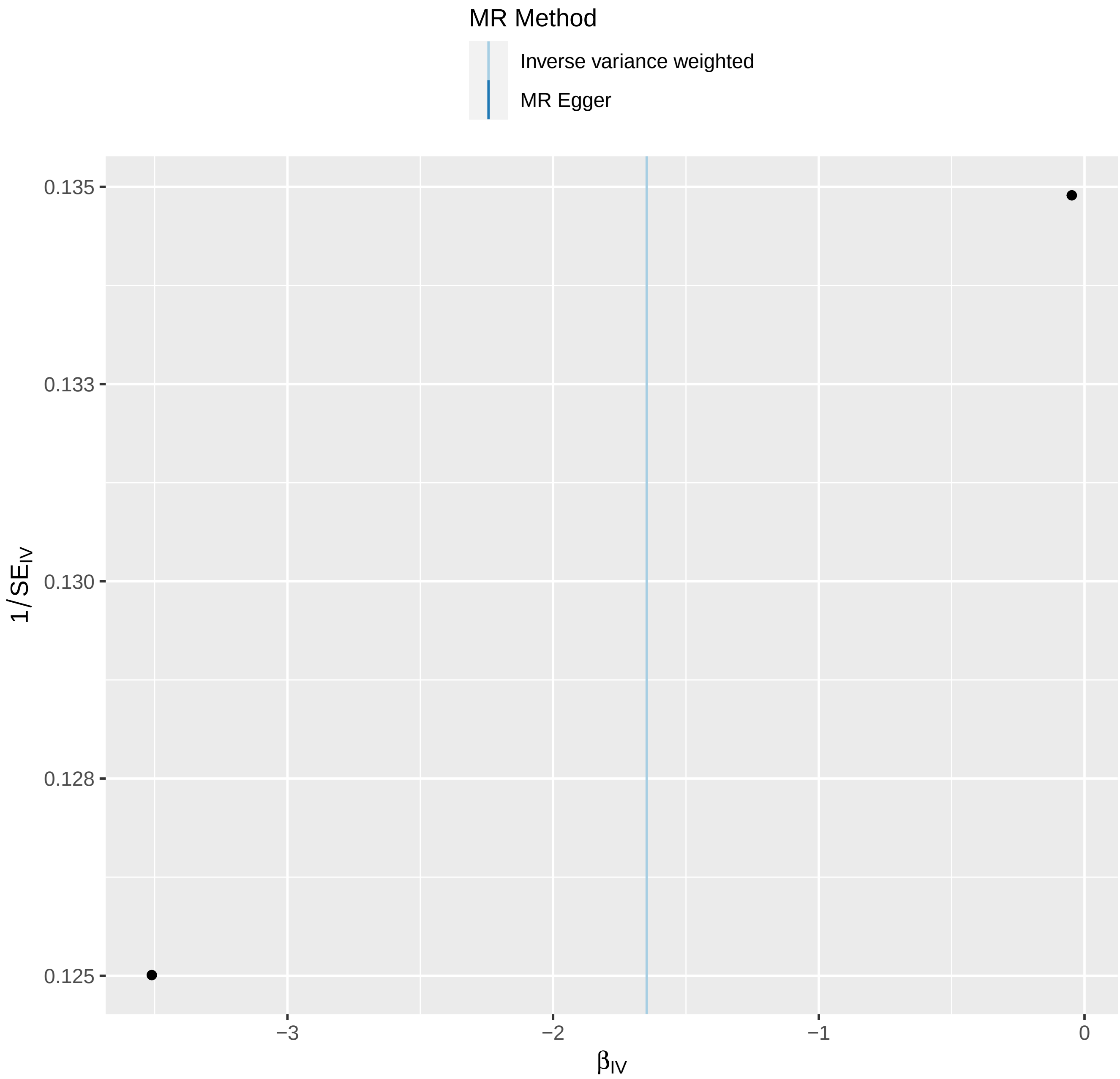

Batch 1827 : Pseudouridine on Arthropathy in ulcerative colitis

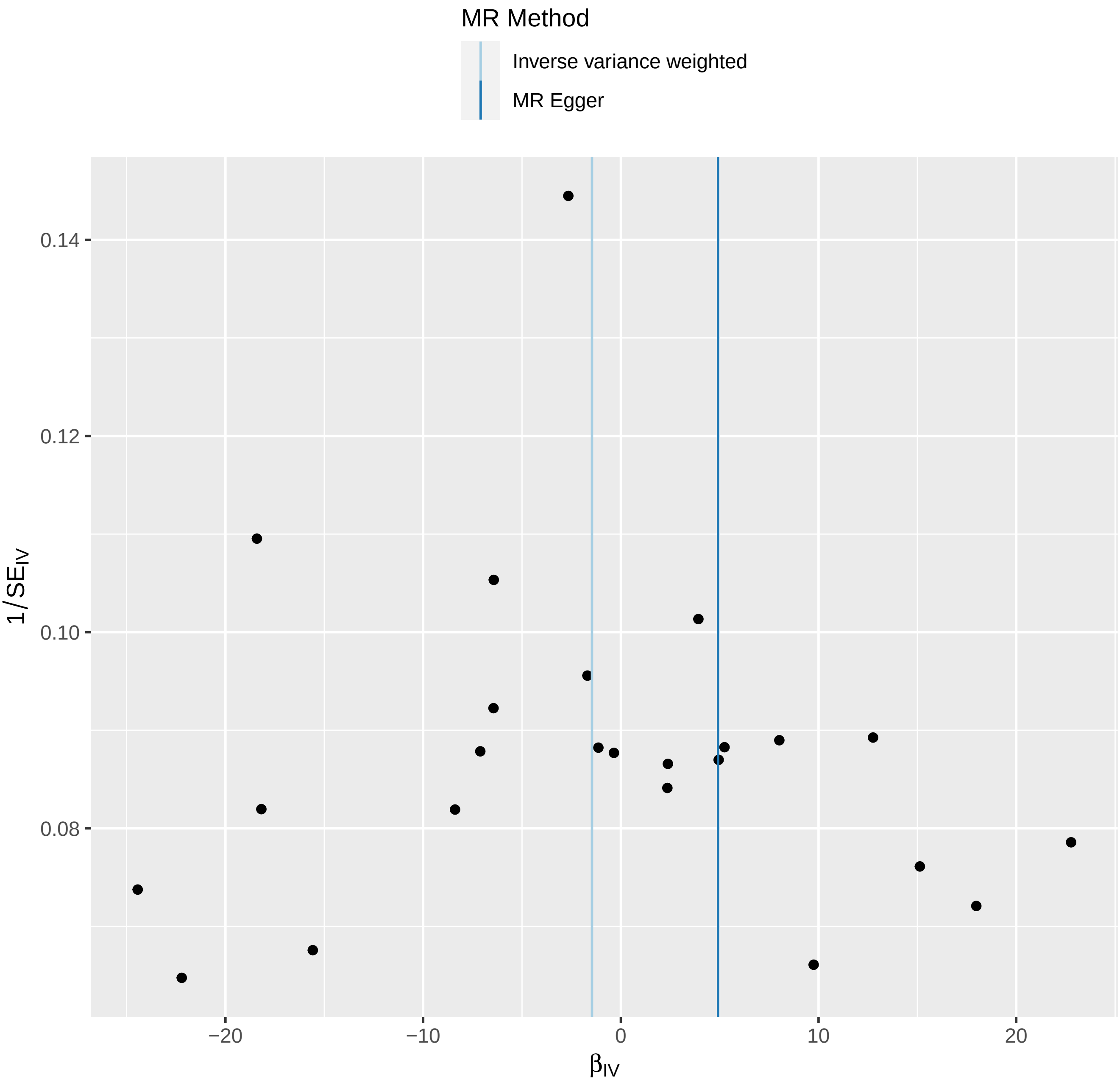

Batch 1828 : Valerate on Arthropathy in ulcerative colitis

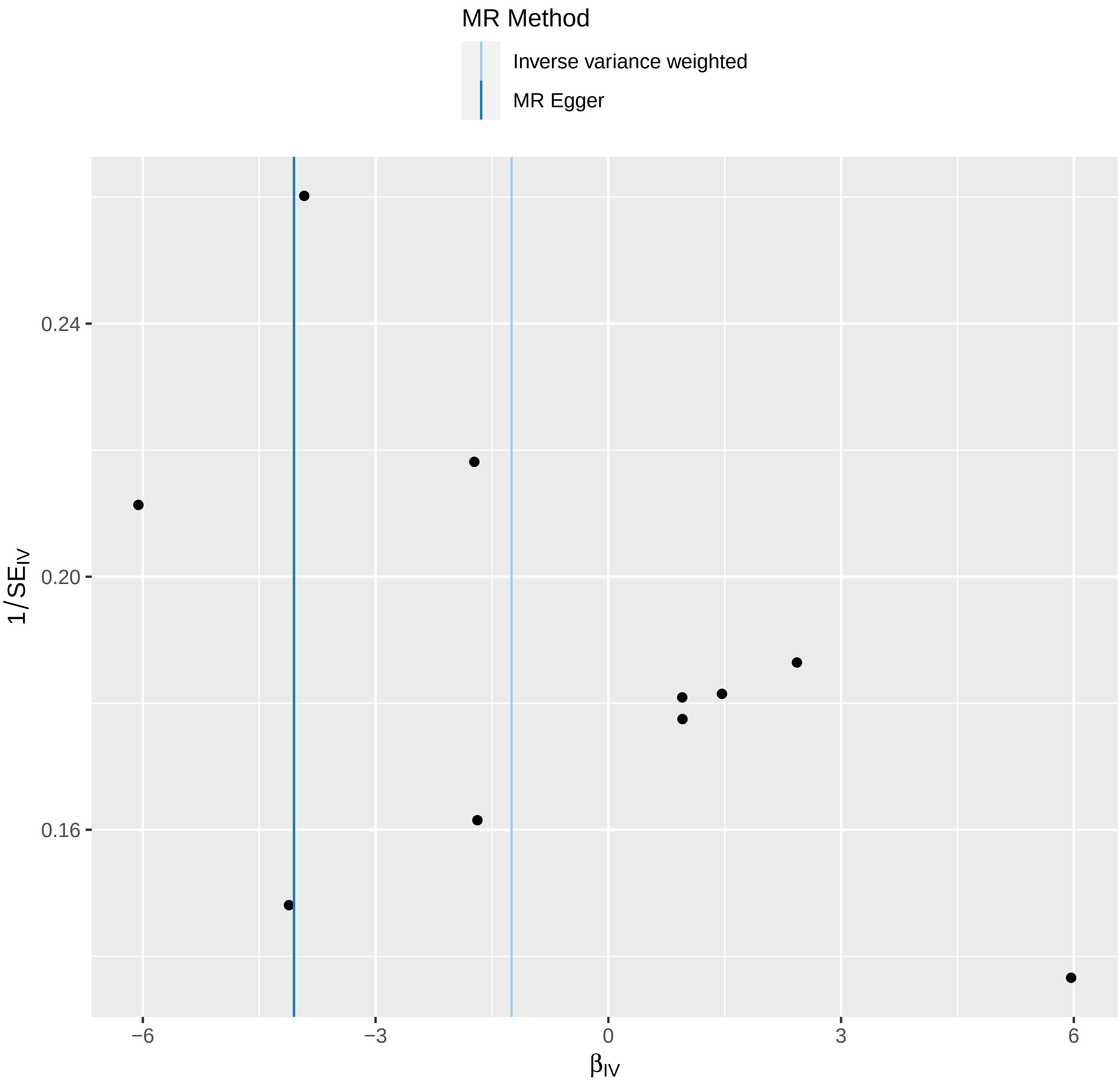

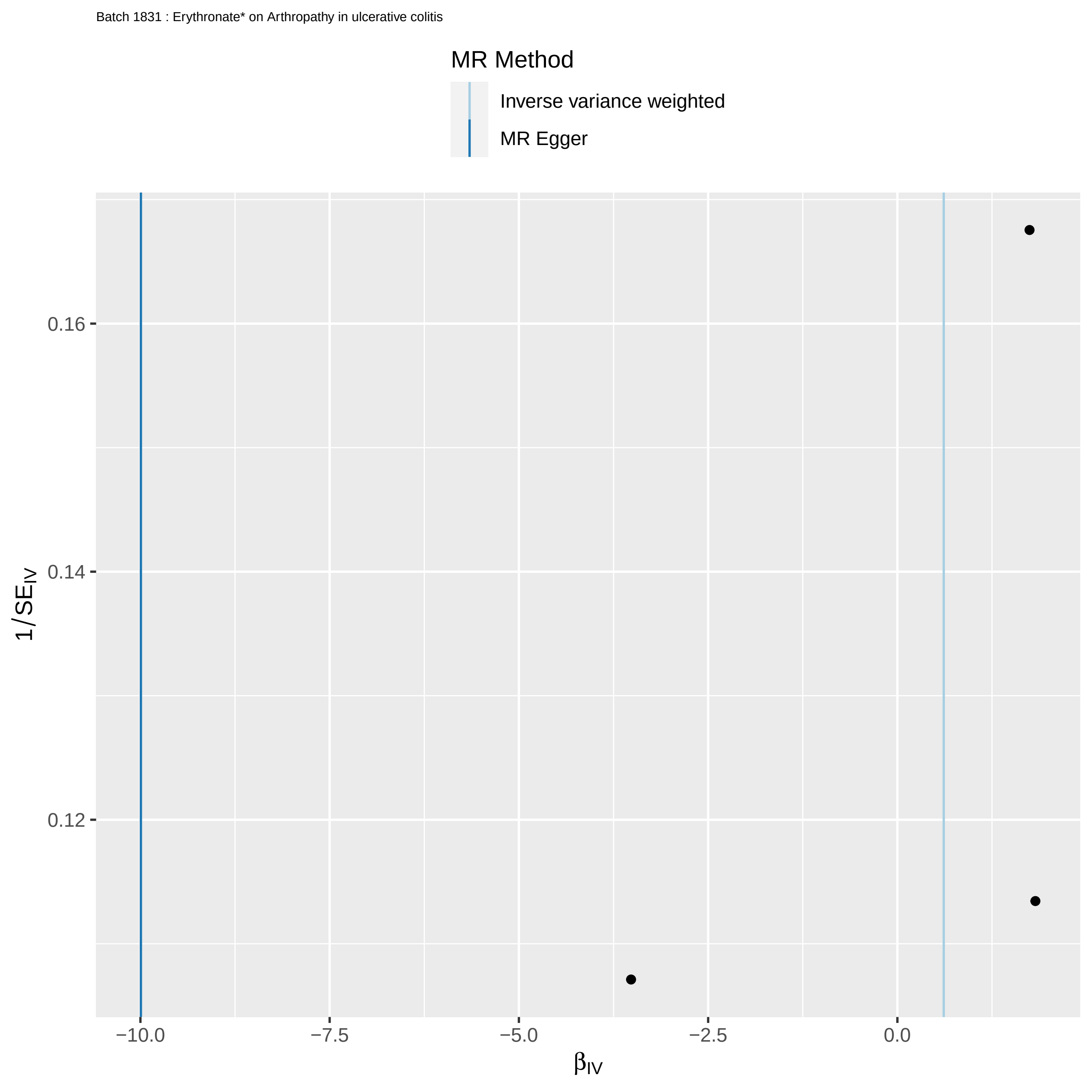
Batch 1831 : Erythronate* on Arthropathy in ulcerative colitis
MR Method
Inverse variance weighted
MR Egger
0.16
0.14
0.12
1/SE_IV
−10.0
−7.5
−5.0
−2.5
0.0
β_IV

Batch 1832 : Lathosterol on Arthropathy in ulcerative colitis

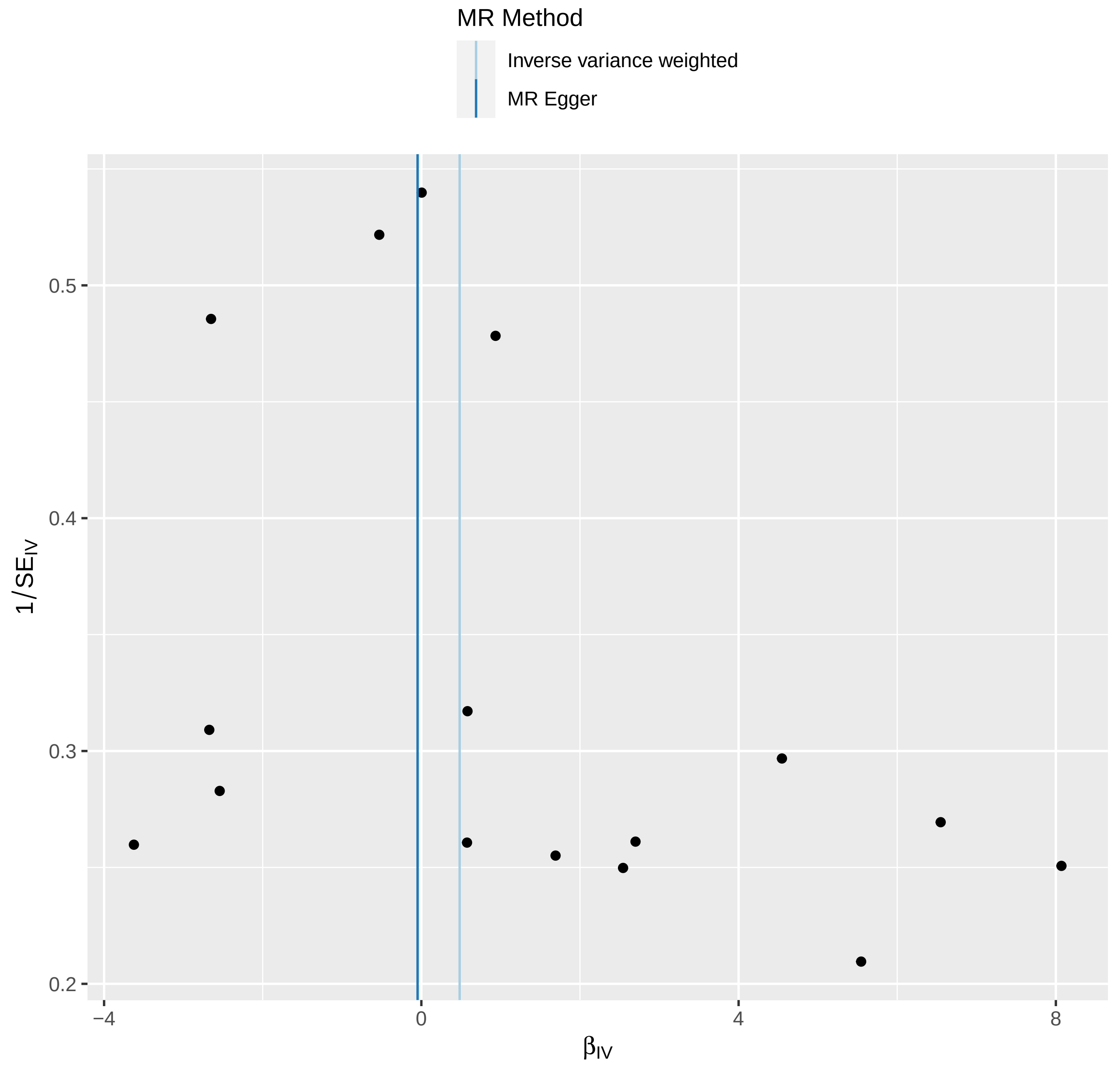

Batch 1835 : Eicosenoate (20:1n9 or 11) on Arthropathy in ulcerative colitis

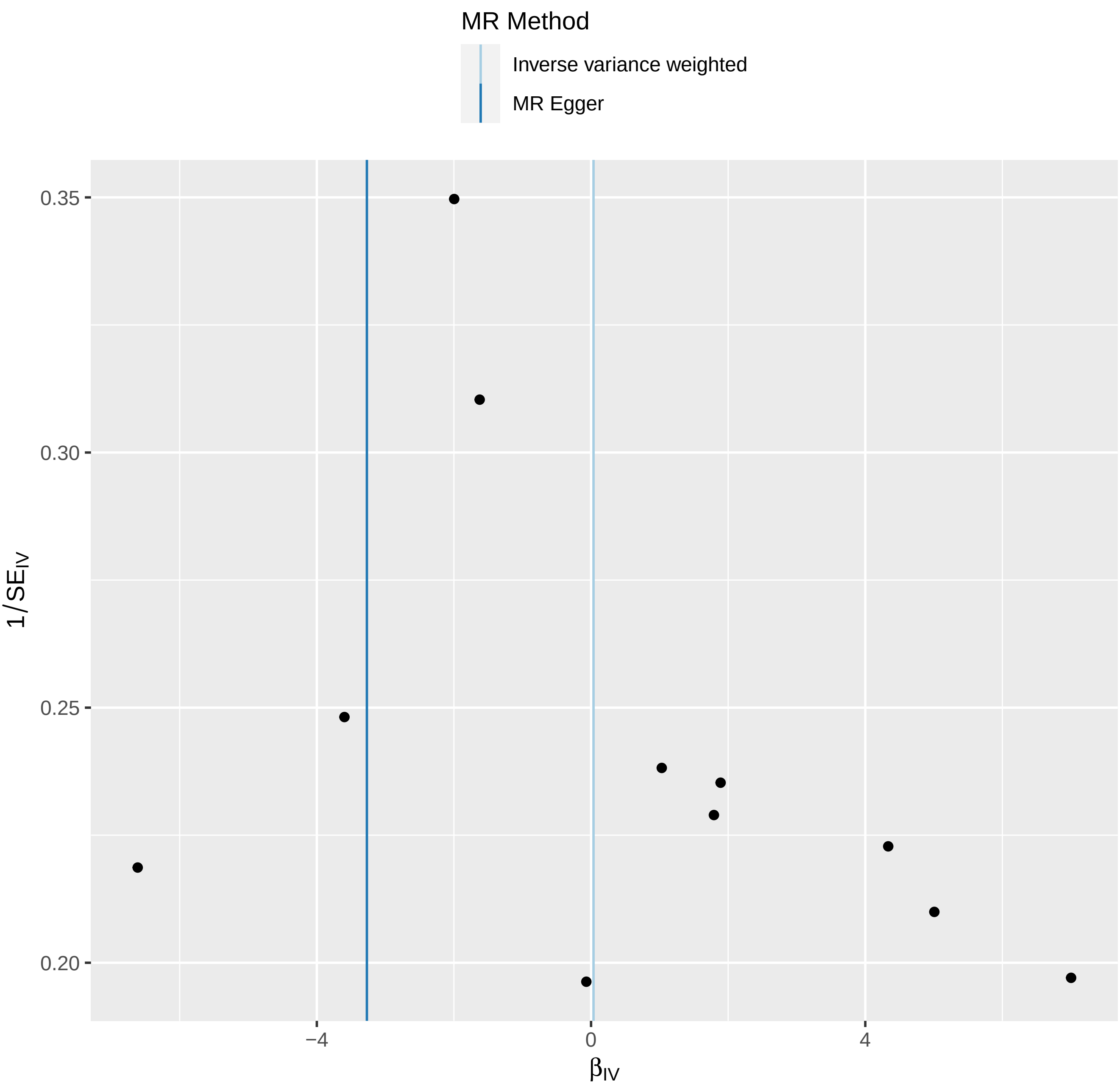

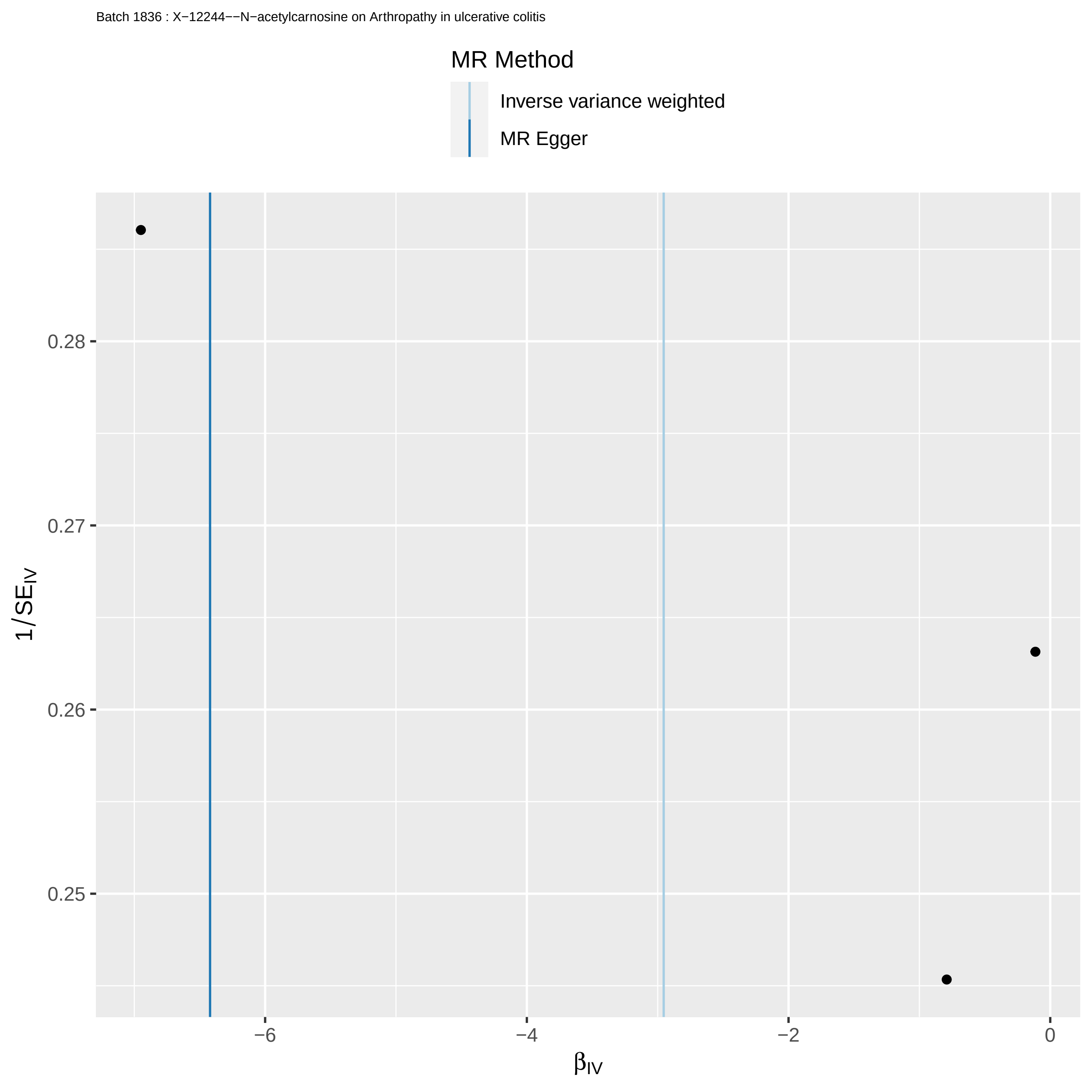
Batch 1836 : X−12244−−N−acetylcarnosine on Arthropathy in ulcerative colitis
MR Method
Inverse variance weighted
MR Egger
0.28
0.27
0.26
0.25
1/SE_IV
−6
−4
−2
0
β_IV

Batch 1838 : 1−eicosatrienoylglycerophosphocholine* on Arthropathy in ulcerative colitis

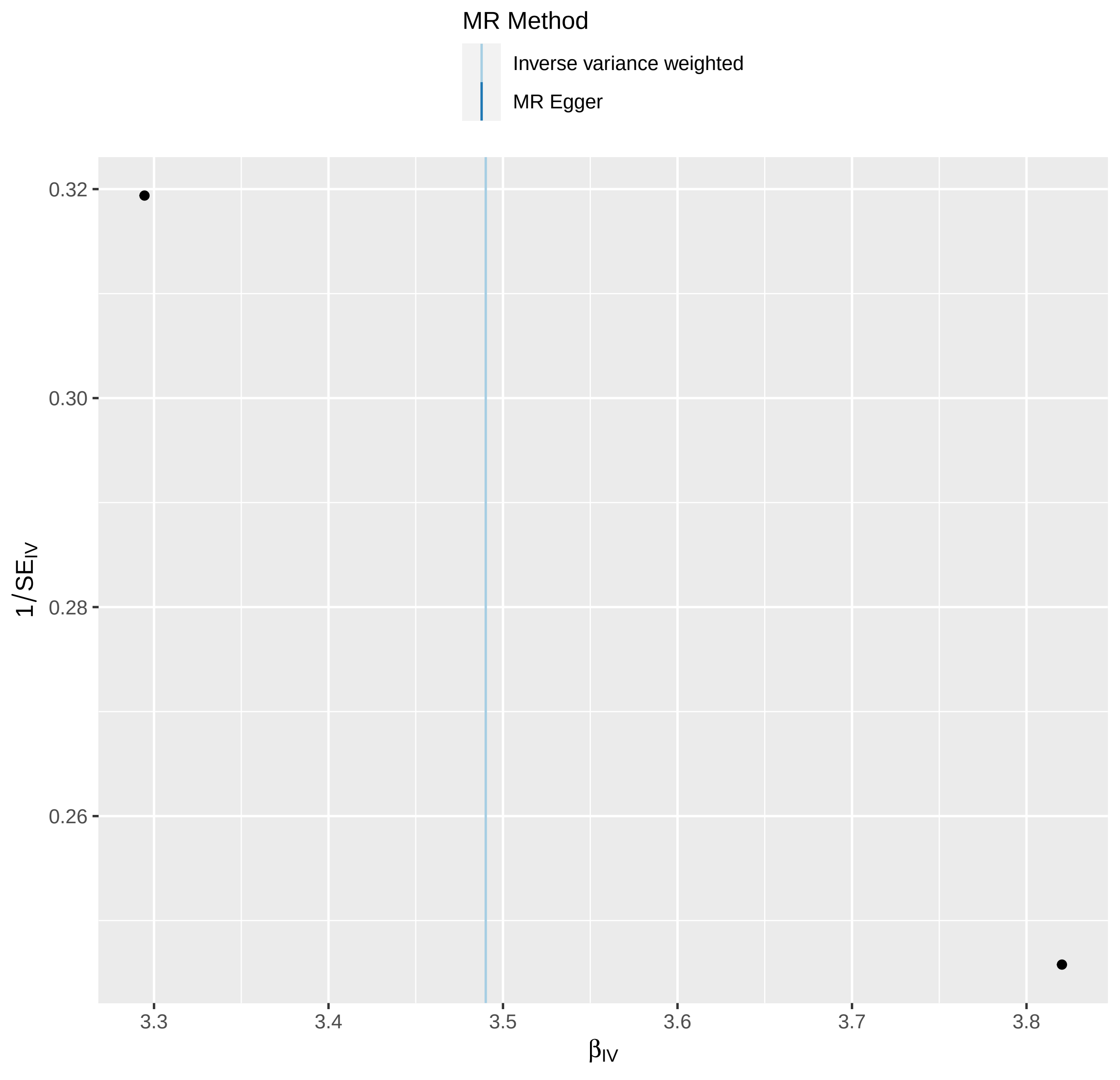

Batch 1839 : 1−docosahexaenoylglycerophosphocholine* on Arthropathy in ulcerative colitis

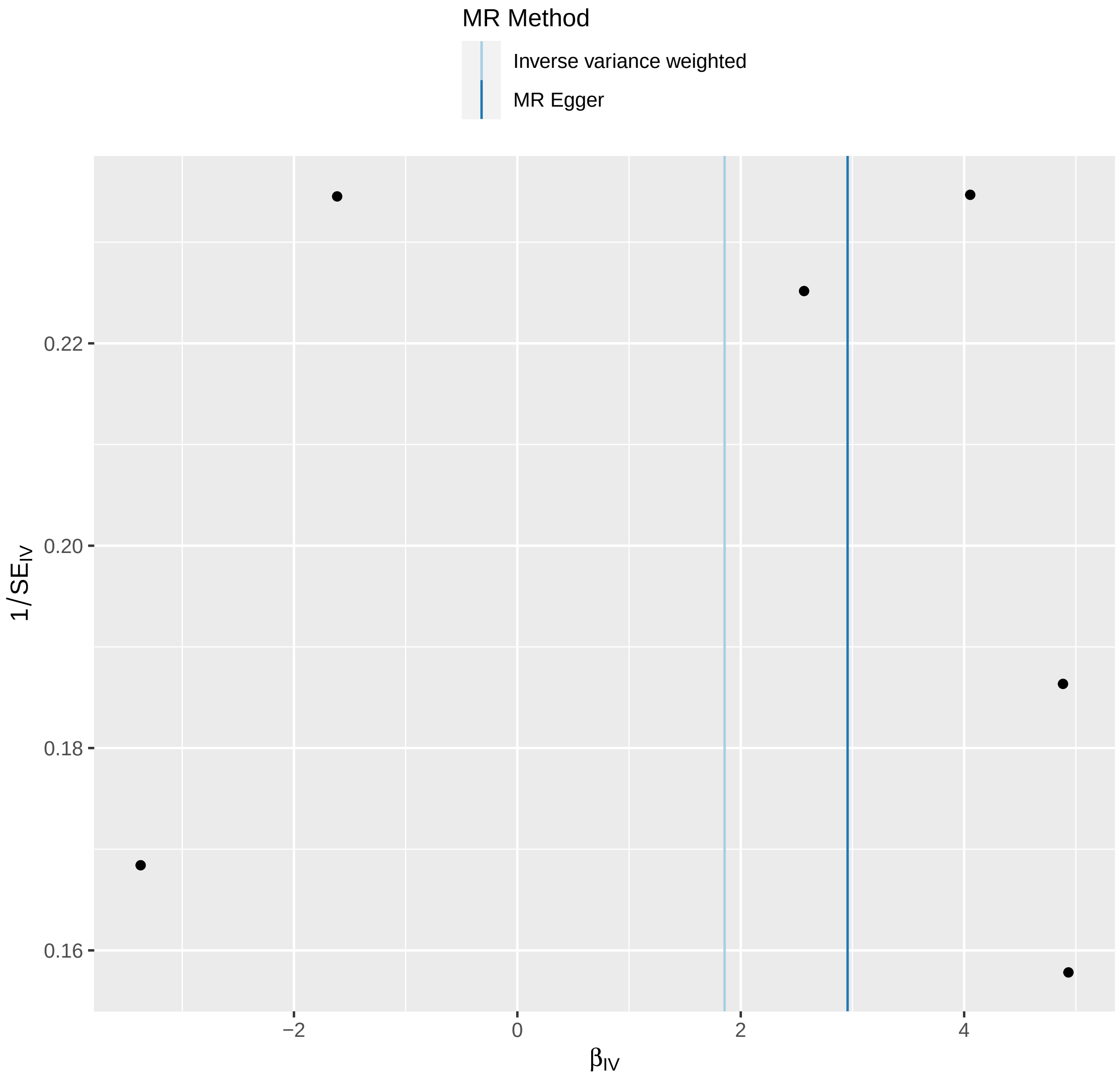

Batch 1843 : Octanoylcarnitine on Arthropathy in ulcerative colitis

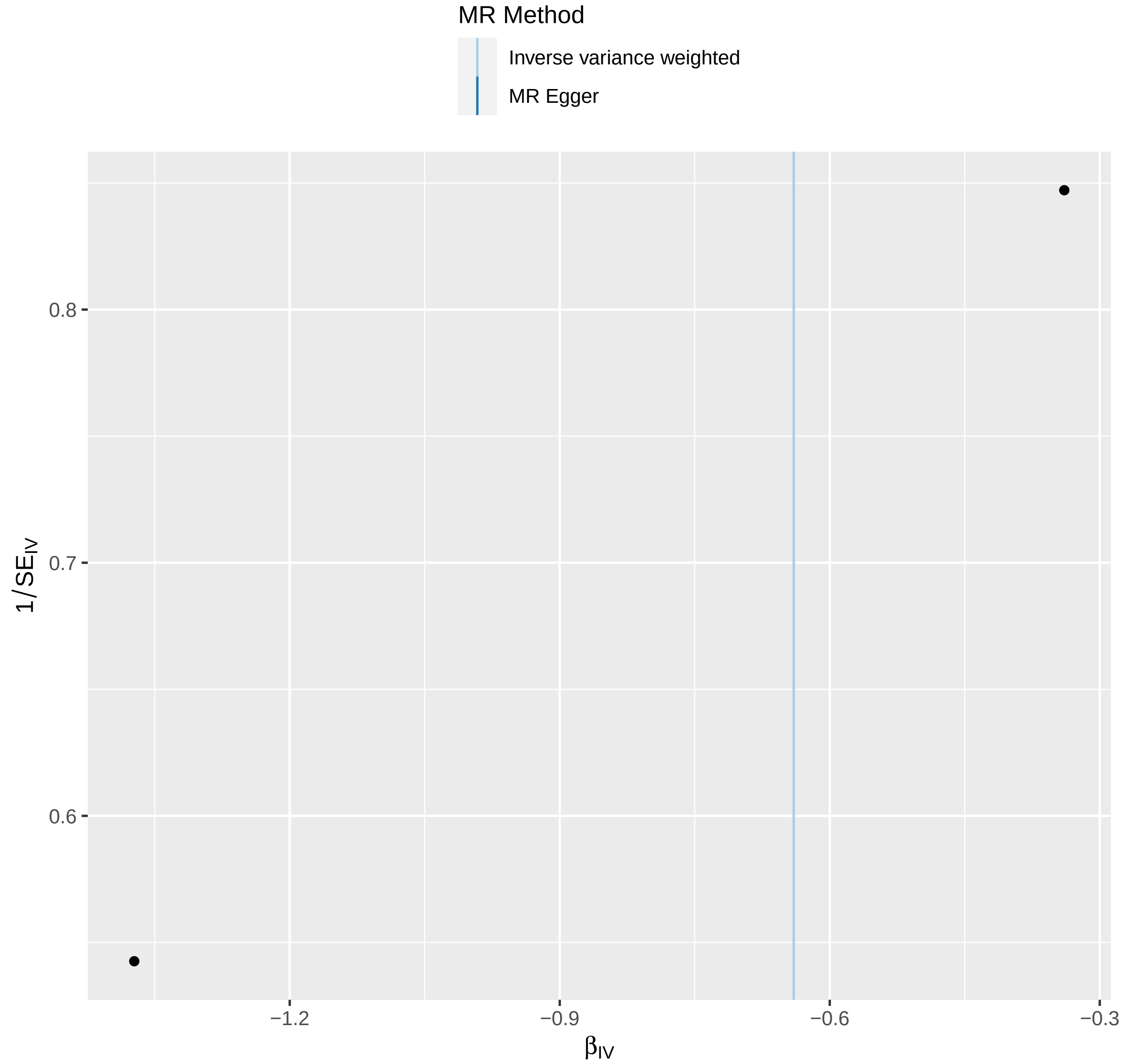

Batch 1844 : Alpha−hydroxyisovalerate on Arthropathy in ulcerative colitis

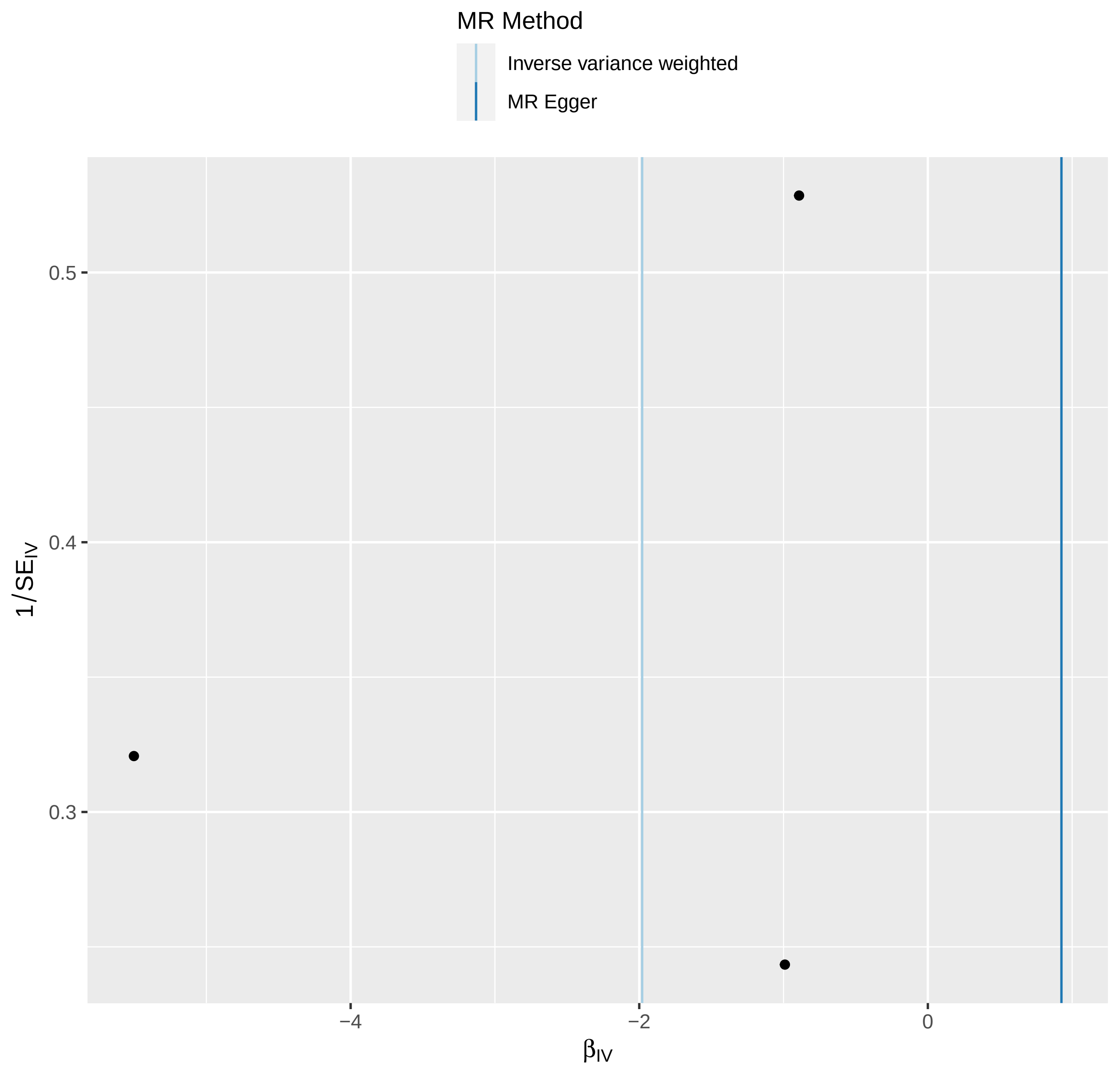

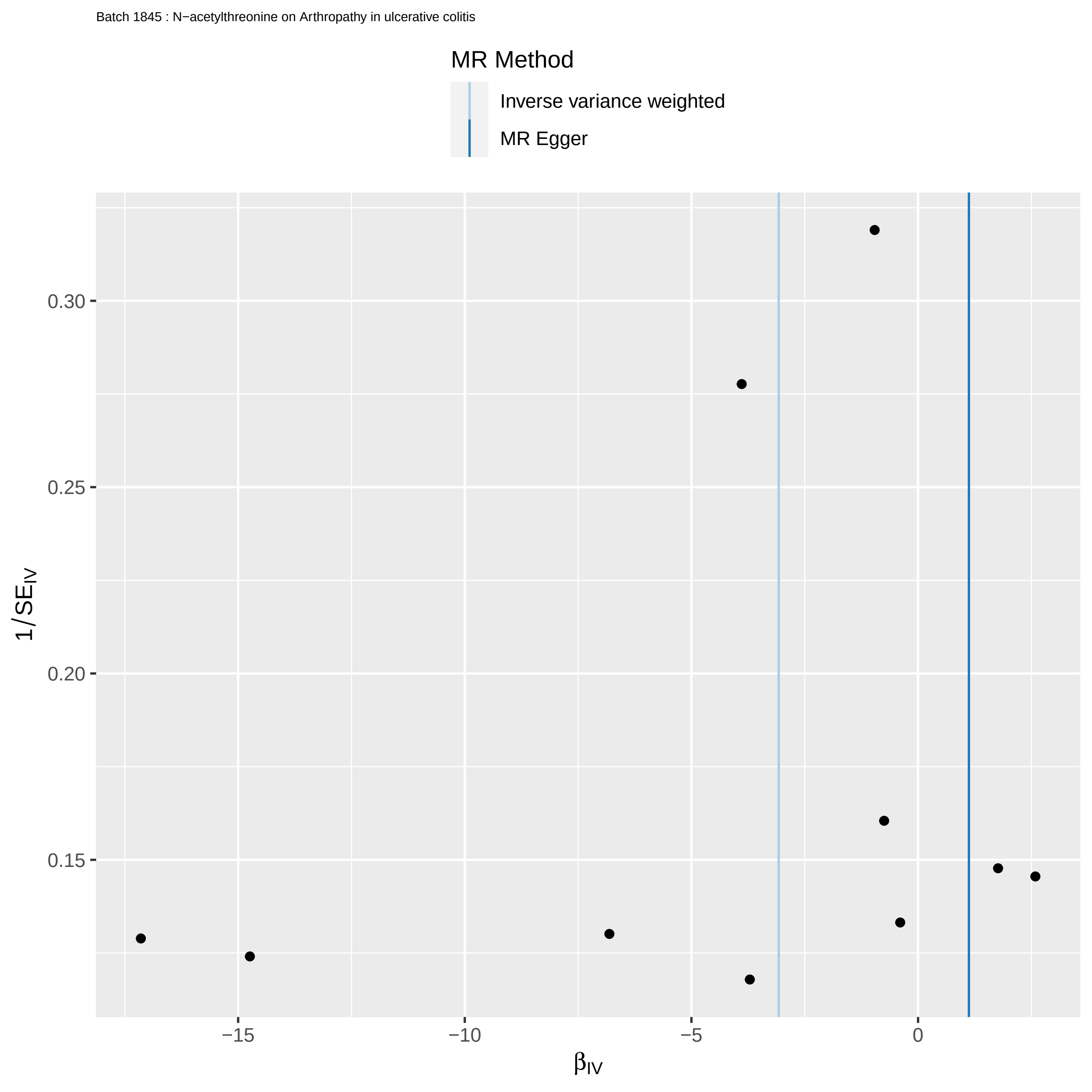
Batch 1845 : N−acetylthreonine on Arthropathy in ulcerative colitis
MR Method
Inverse variance weighted
MR Egger
0.30
0.25
0.20
0.15
1/SE_IV
−15
−10
−5
0
β_IV

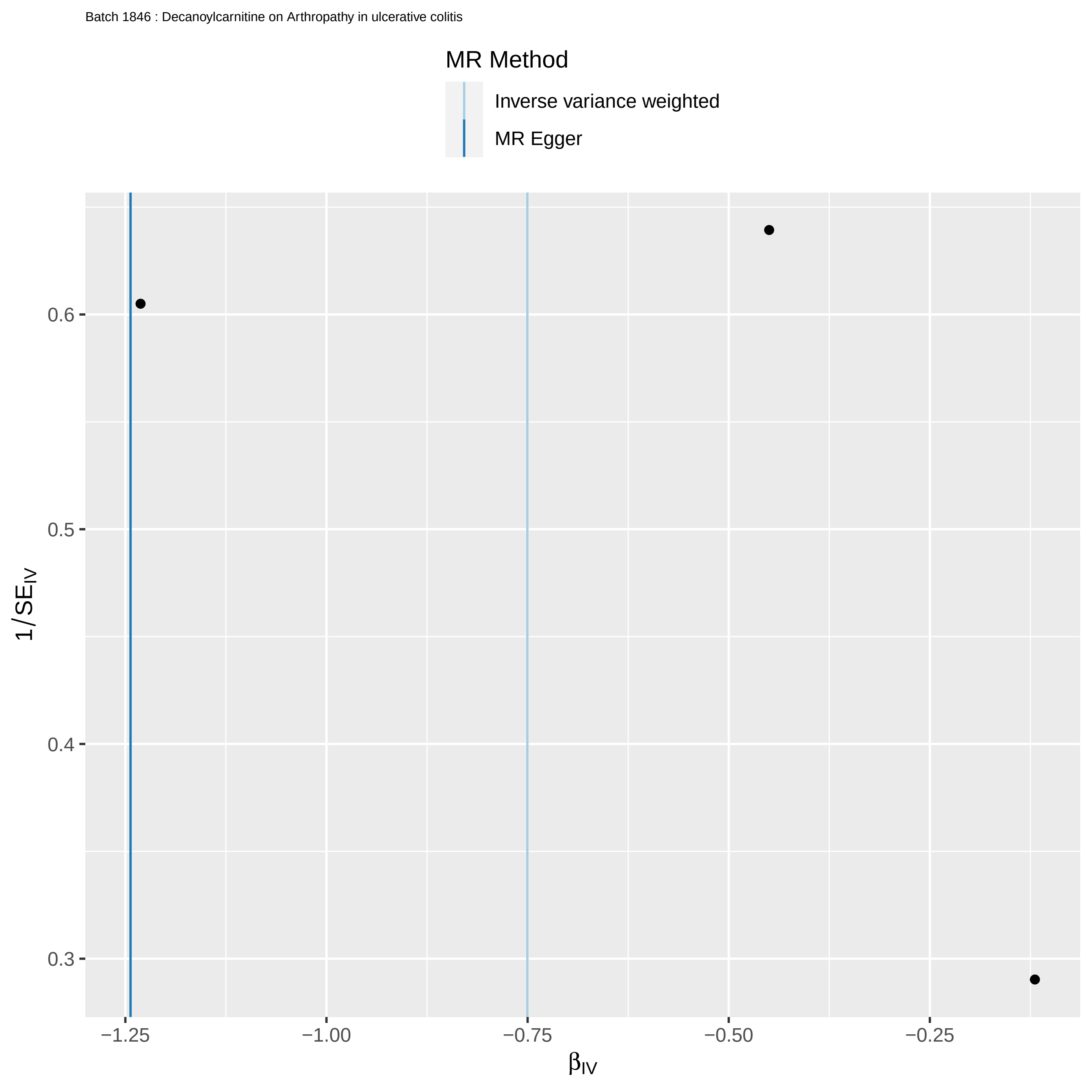

Batch 1846 : Decanoylcarnitine on Arthropathy in ulcerative colitis
MR Method
Inverse variance weighted
MR Egger
0.6
0.5
0.4
0.3
1/SE$_{IV}$
−1.25
−1.00
−0.75
−0.50
−0.25
β$_{IV}$

Batch 1847 : 1−palmitoylglycerophosphocholine on Arthropathy in ulcerative colitis

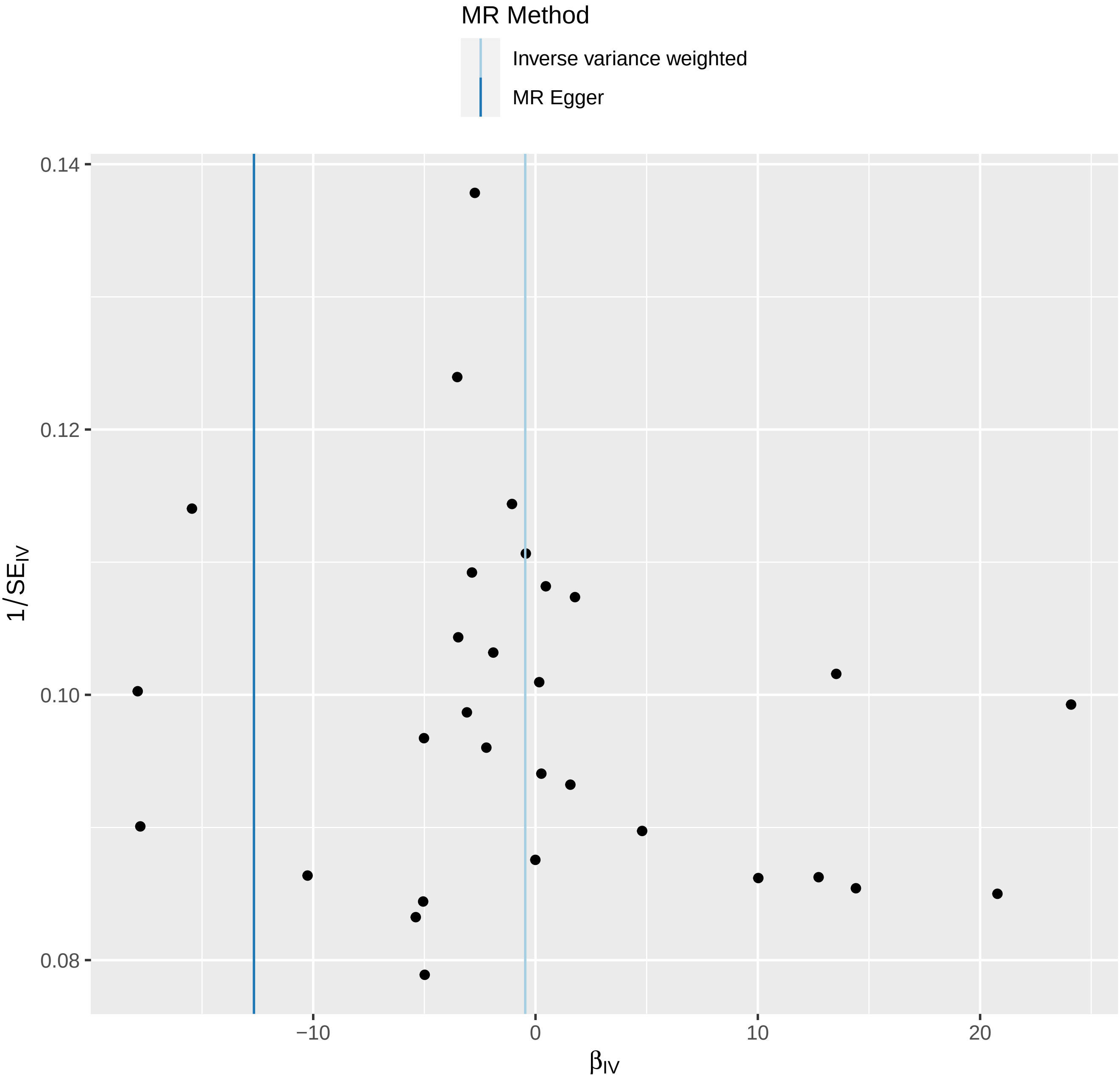

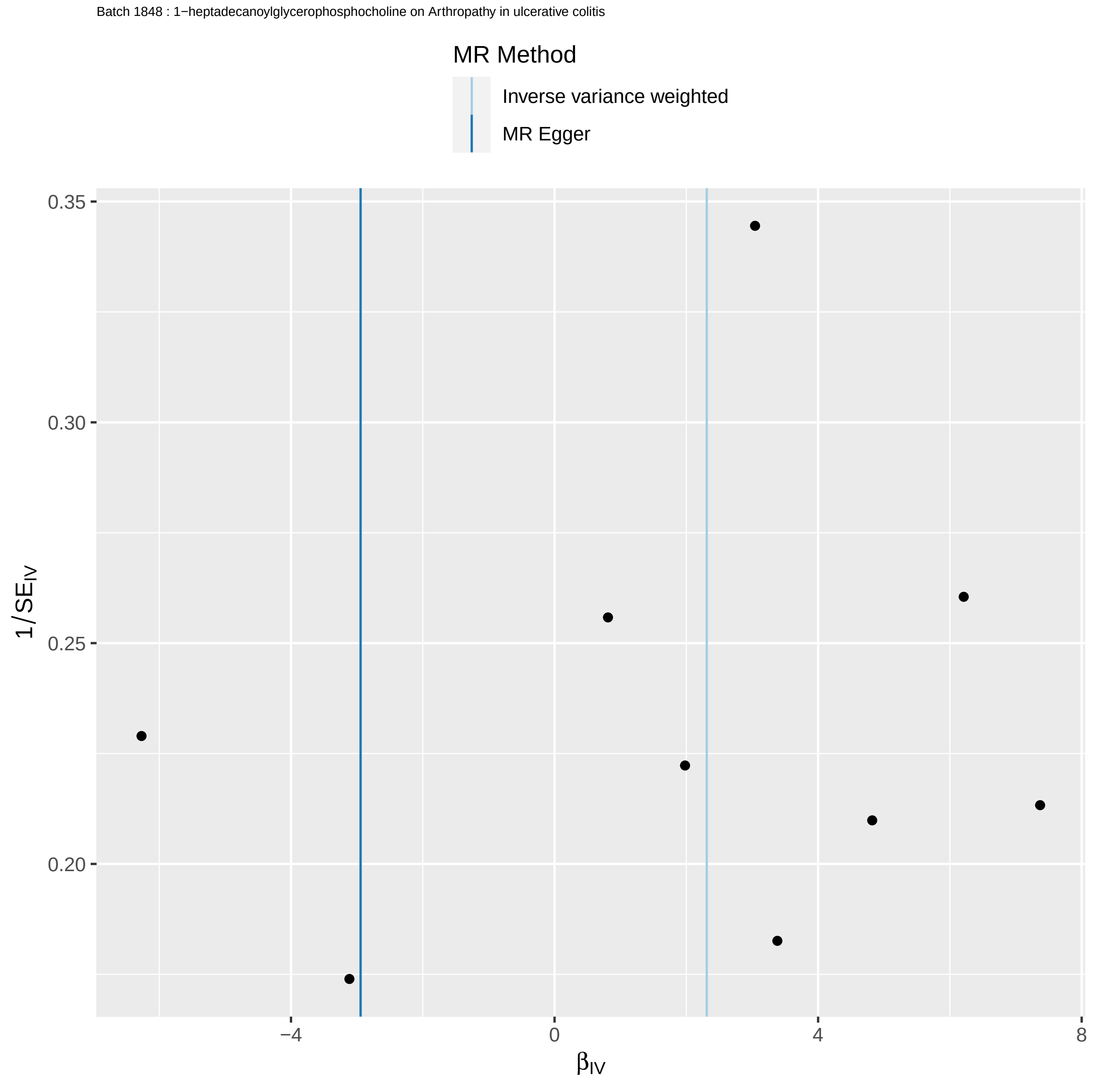

Batch 1848 : 1−heptadecanoylglycerophosphocholine on Arthropathy in ulcerative colitis
MR Method
Inverse variance weighted
MR Egger
0.35
0.30
0.25
0.20
1/SE$_{IV}$
−4
0
4
8
β$_{IV}$

Batch 1849 : 1−oleoylglycerophosphocholine on Arthropathy in ulcerative colitis

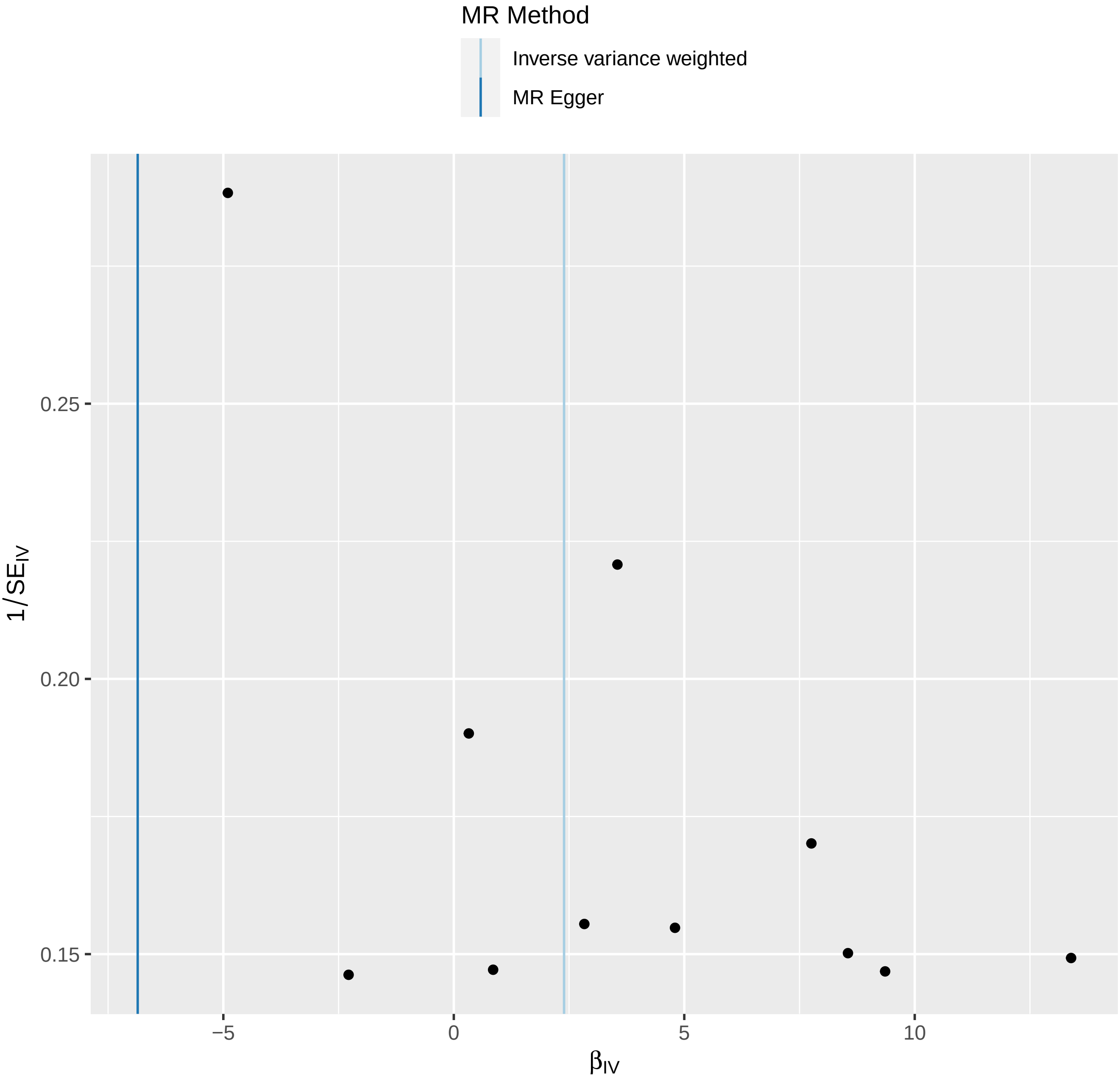

Batch 1850 : 1−stearoylglycerophosphocholine on Arthropathy in ulcerative colitis

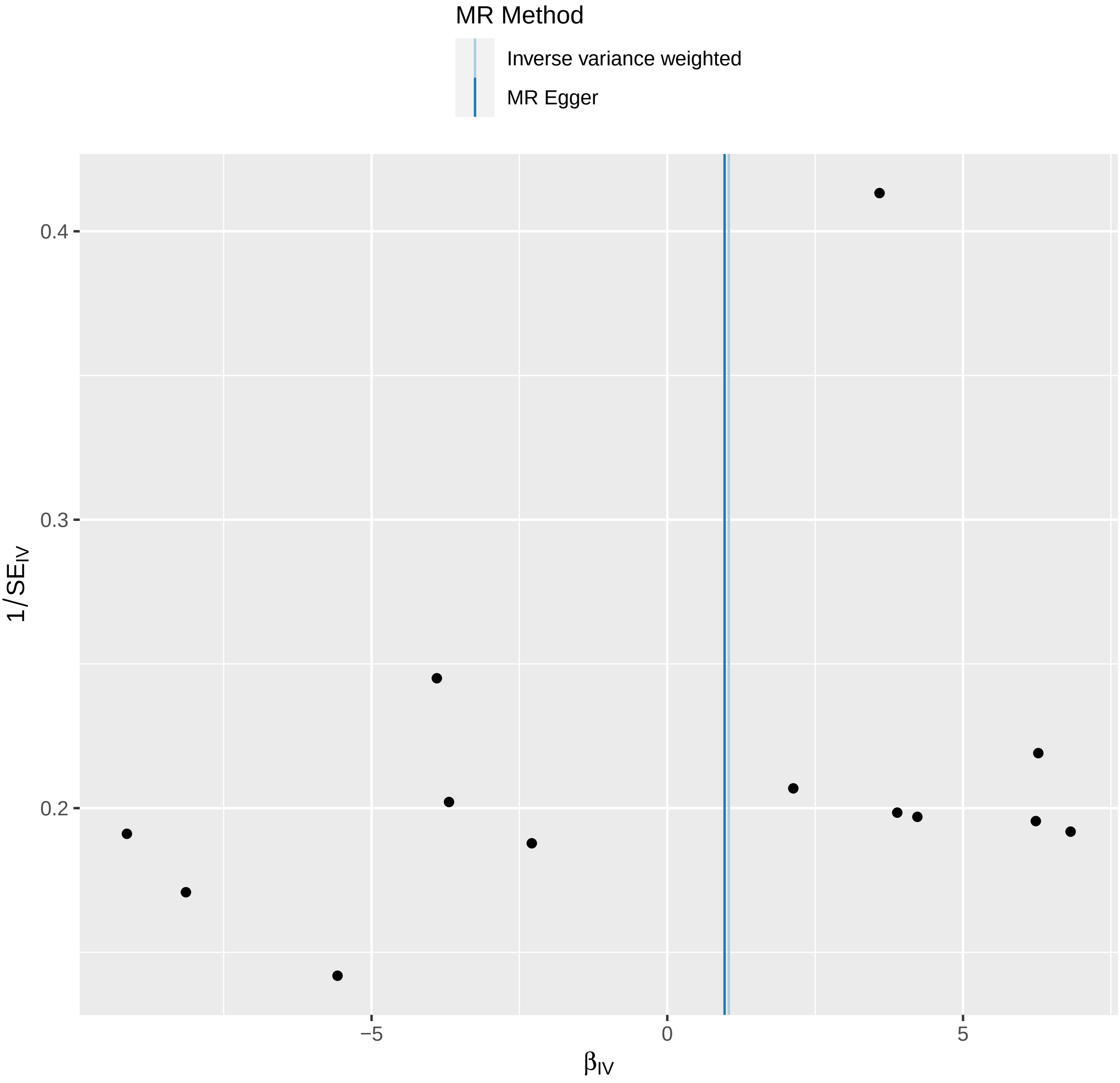

Batch 1853 : 10−heptadecenoate (17:1n7) on Arthropathy in ulcerative colitis

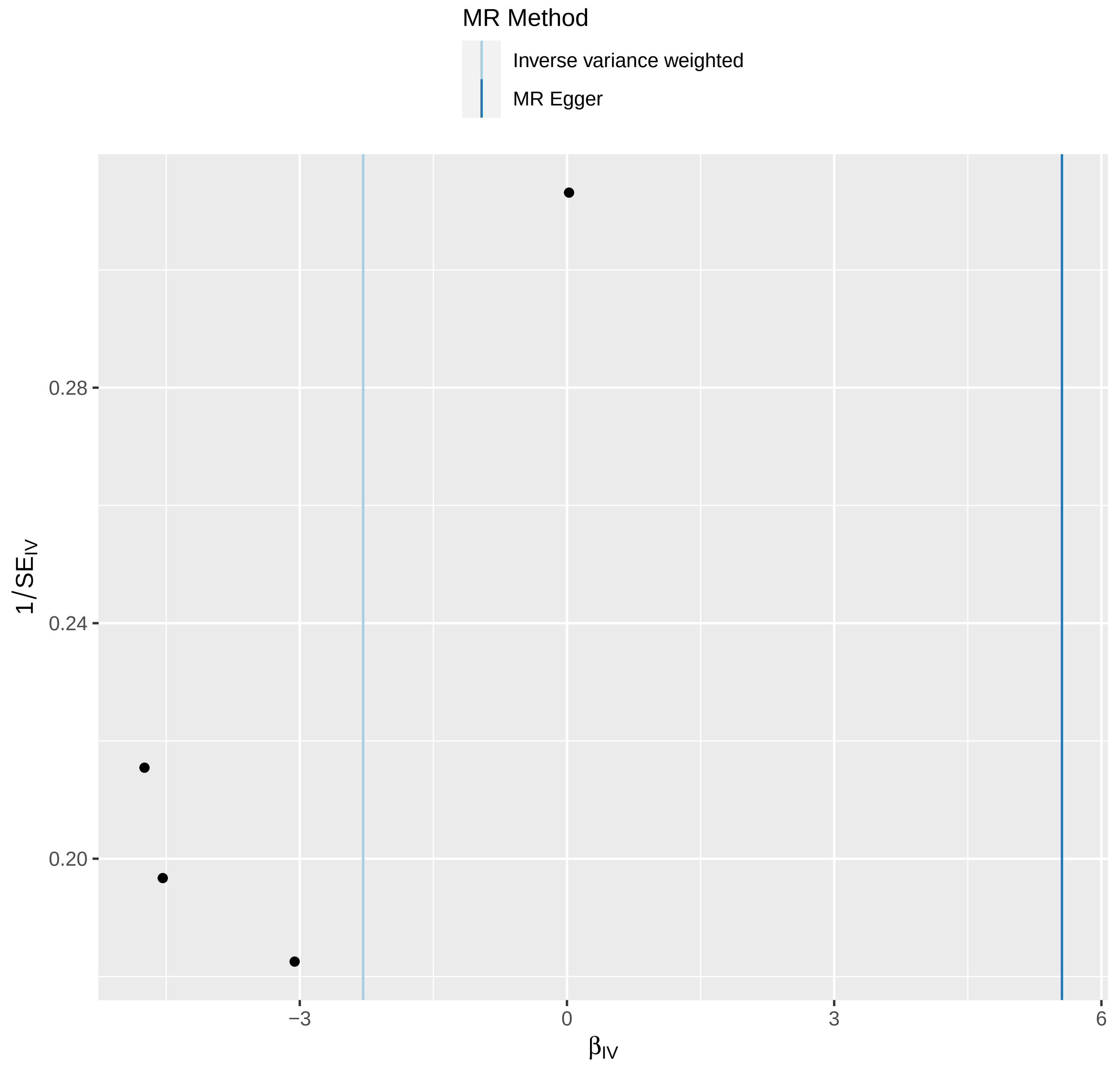

Batch 1856 : Linolenate [alpha or gamma; (18:3n3 or 6)] on Arthropathy in ulcerative colitis

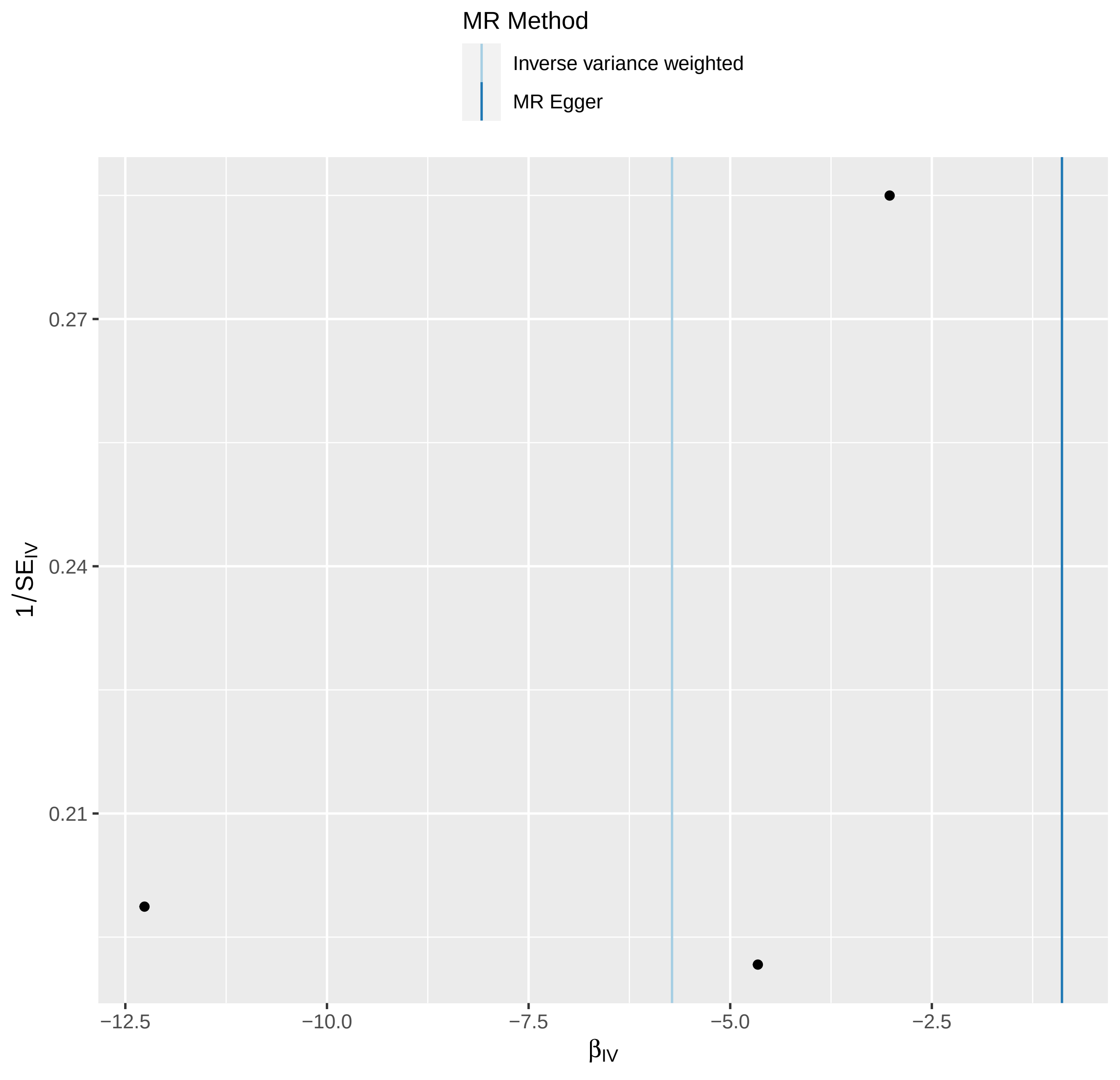

Batch 1857 : X−12510−−2−aminooctanoic acid on Arthropathy in ulcerative colitis

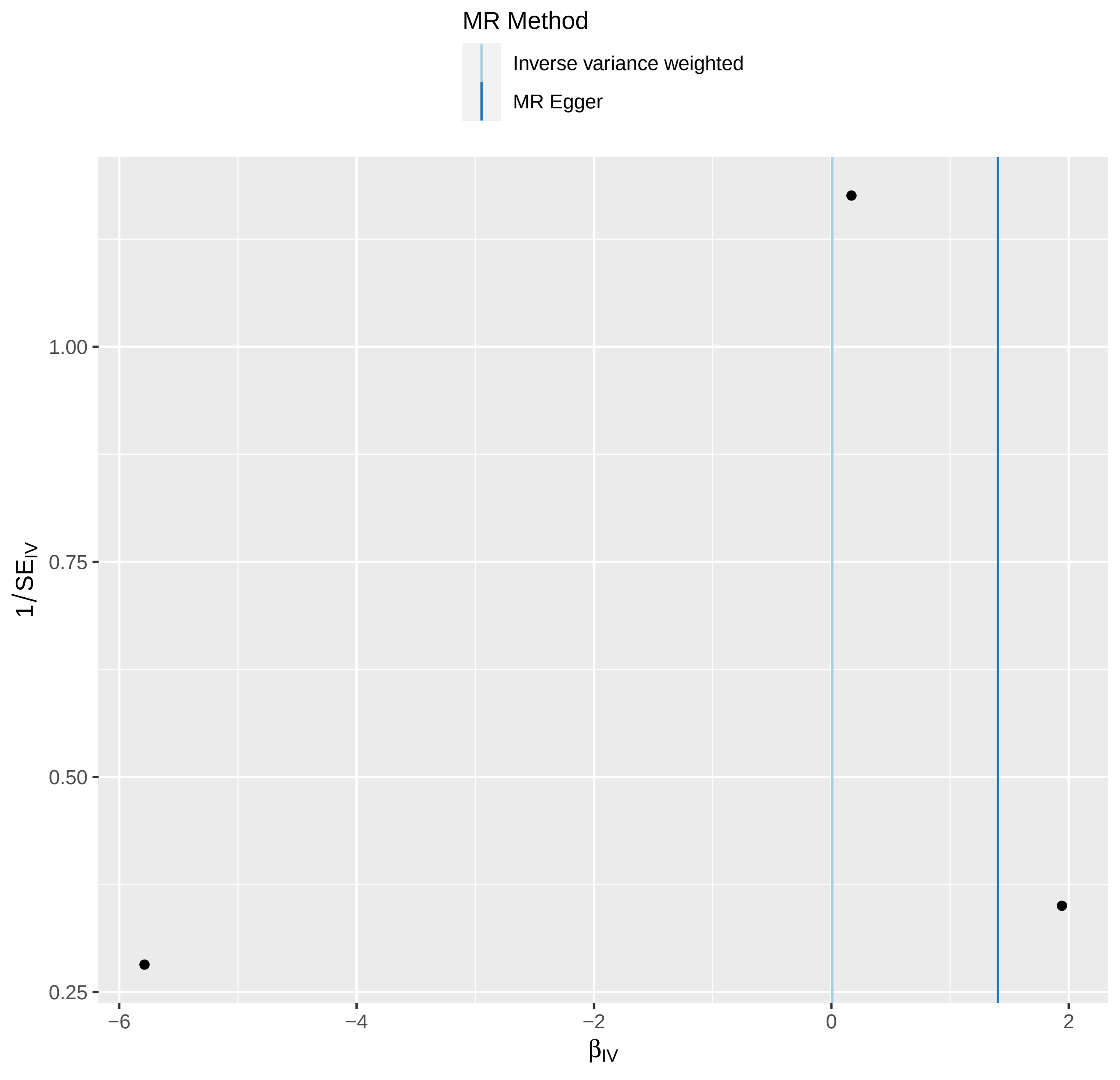

Batch 1860 : Asparagine on Arthropathy in ulcerative colitis

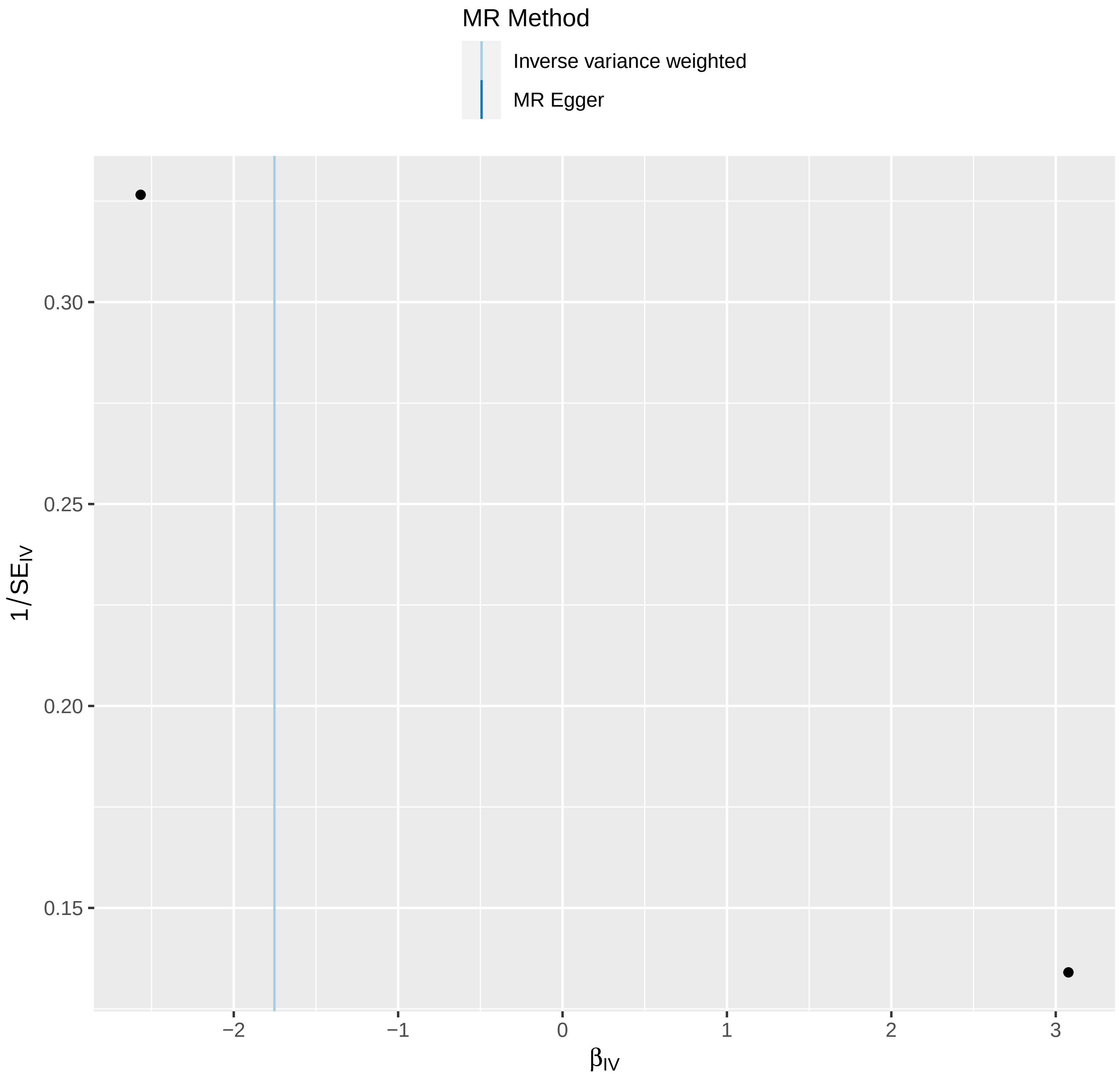

Batch 1861 : Stachydrine on Arthropathy in ulcerative colitis

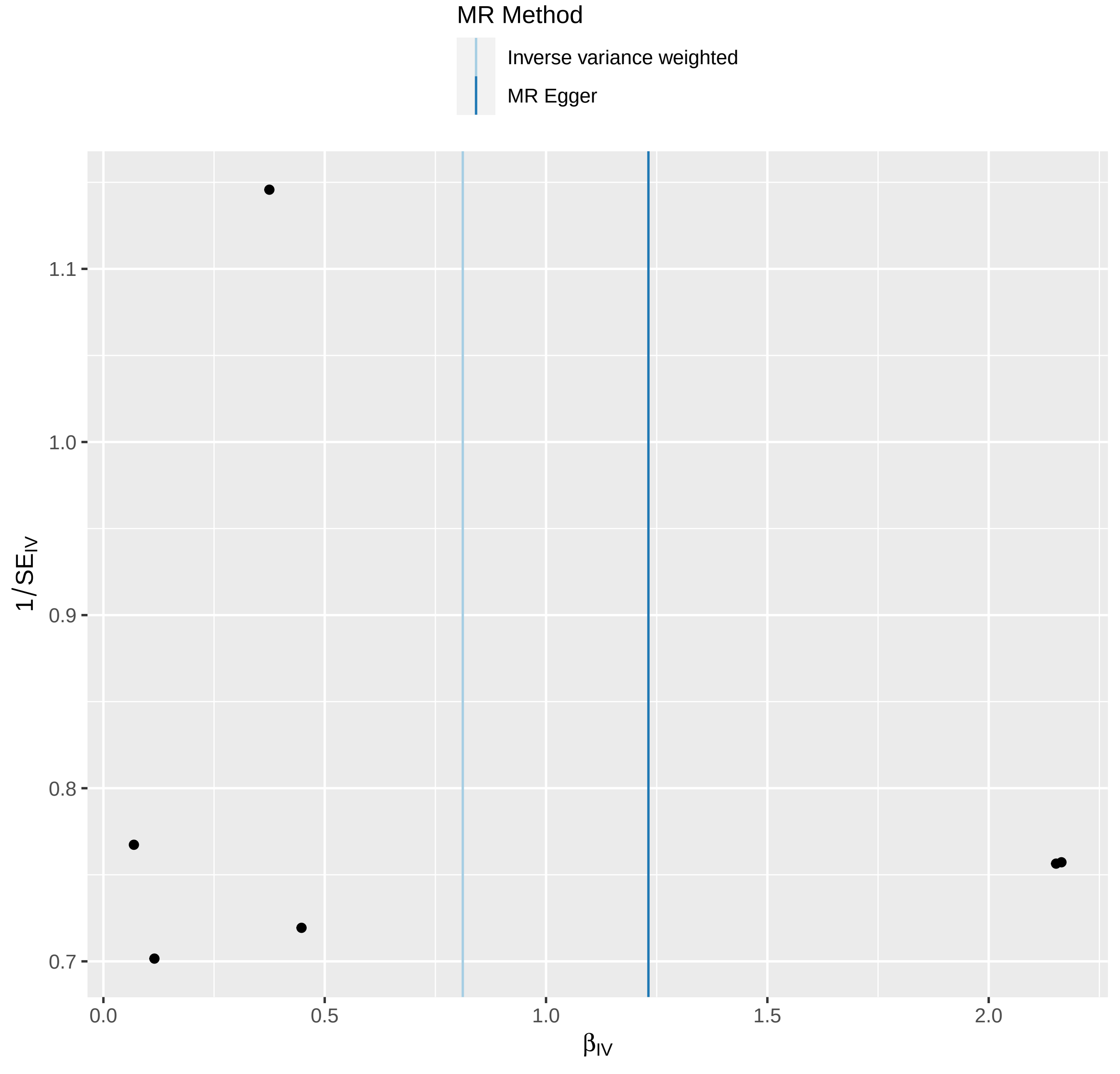

Batch 1862 : Isovalerylcarnitine on Arthropathy in ulcerative colitis

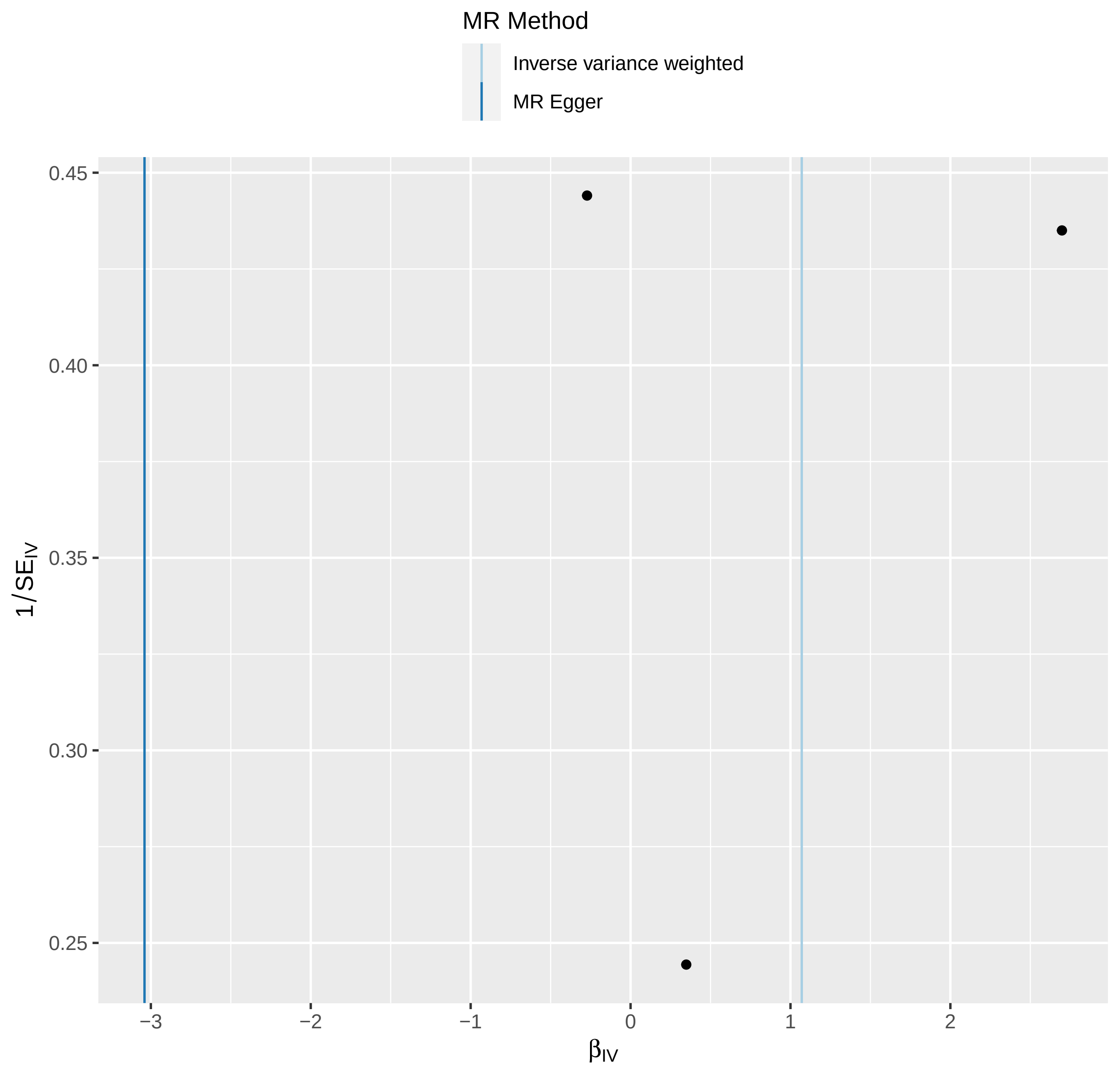

Batch 1865 : 1−linoleoylglycerophosphocholine on Arthropathy in ulcerative colitis

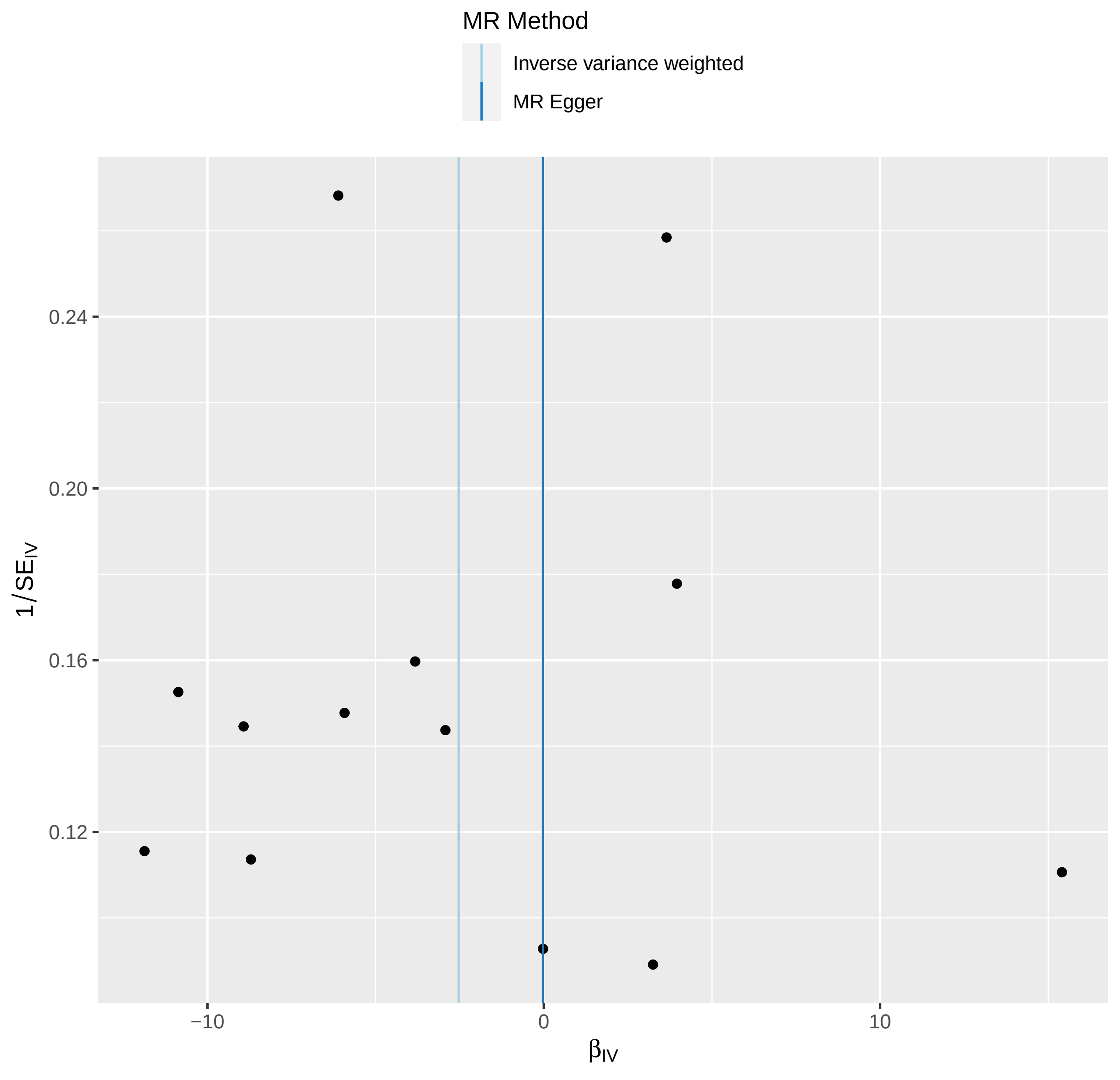

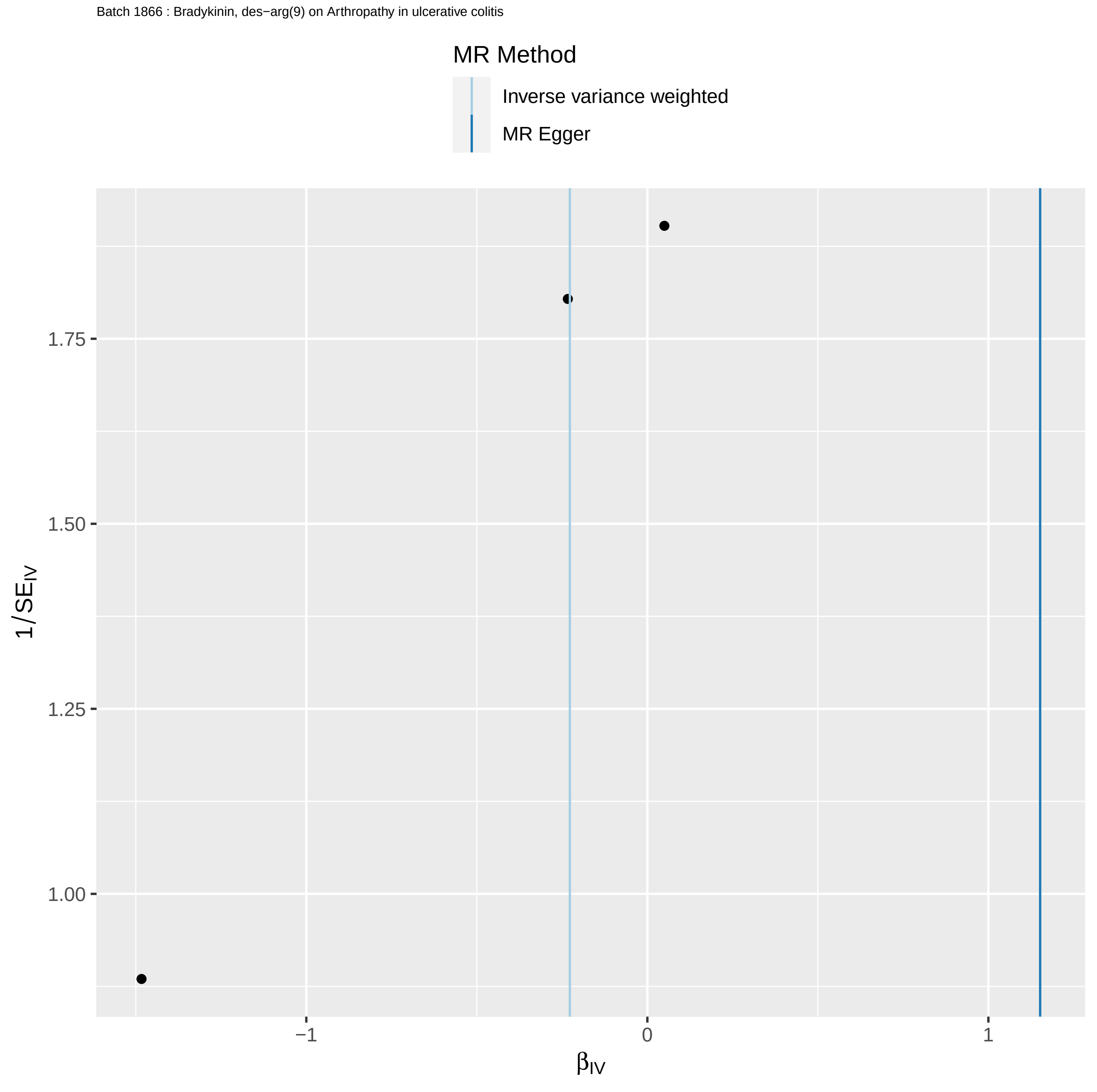
Batch 1866 : Bradykinin, des−arg(9) on Arthropathy in ulcerative colitis
MR Method
Inverse variance weighted
MR Egger
1.75
1.50
1.25
1.00
1/SE_IV
−1
0
1
β_IV

Batch 1867 : Gamma−glutamylisoleucine* on Arthropathy in ulcerative colitis

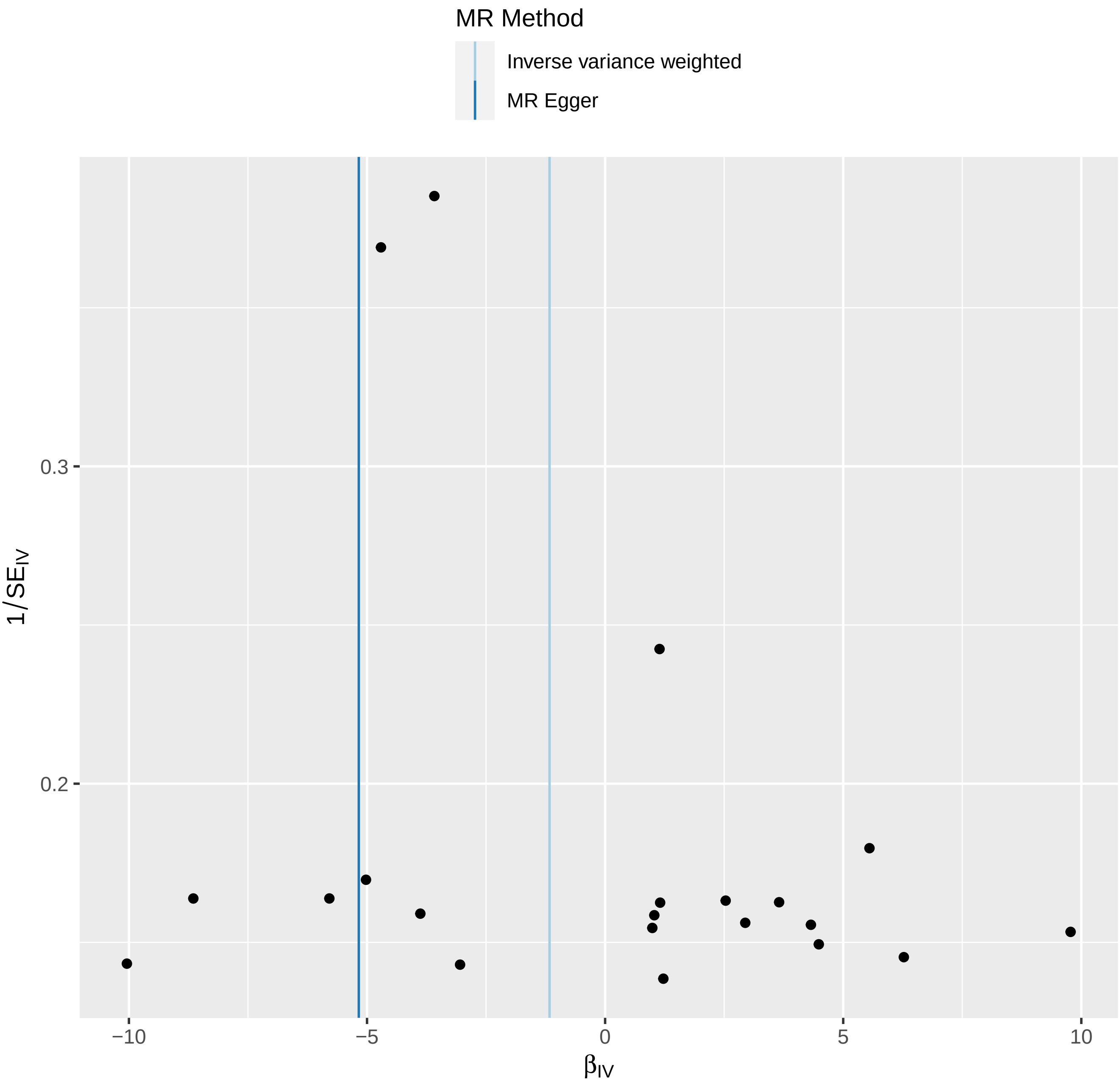

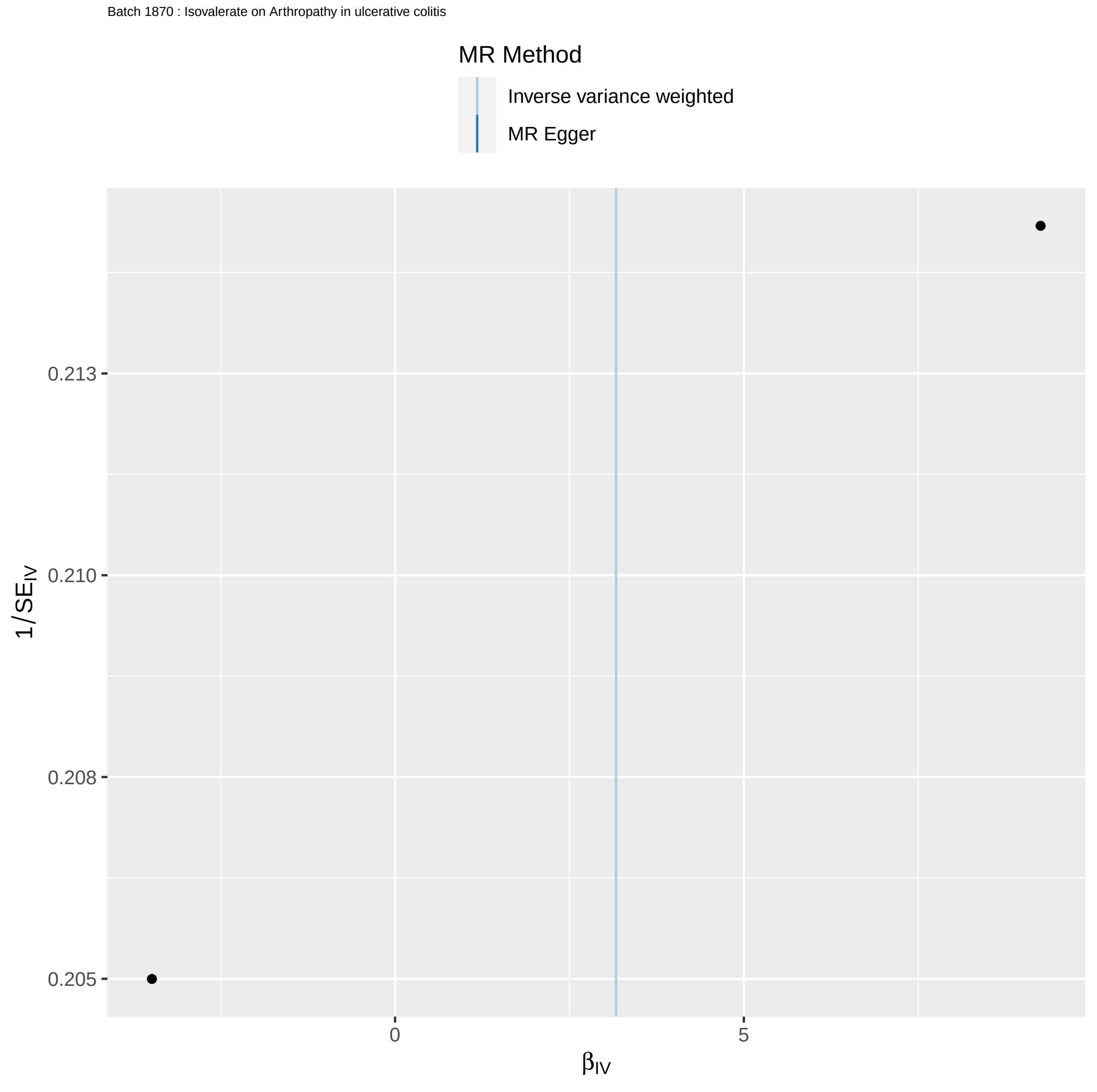
Batch 1870 : Isovalerate on Arthropathy in ulcerative colitis
MR Method
Inverse variance weighted
MR Egger
0.213
0.210
0.208
0.205
1/SE_IV
0
5
β_IV

Batch 1871 : X−13183−−stearamide on Arthropathy in ulcerative colitis

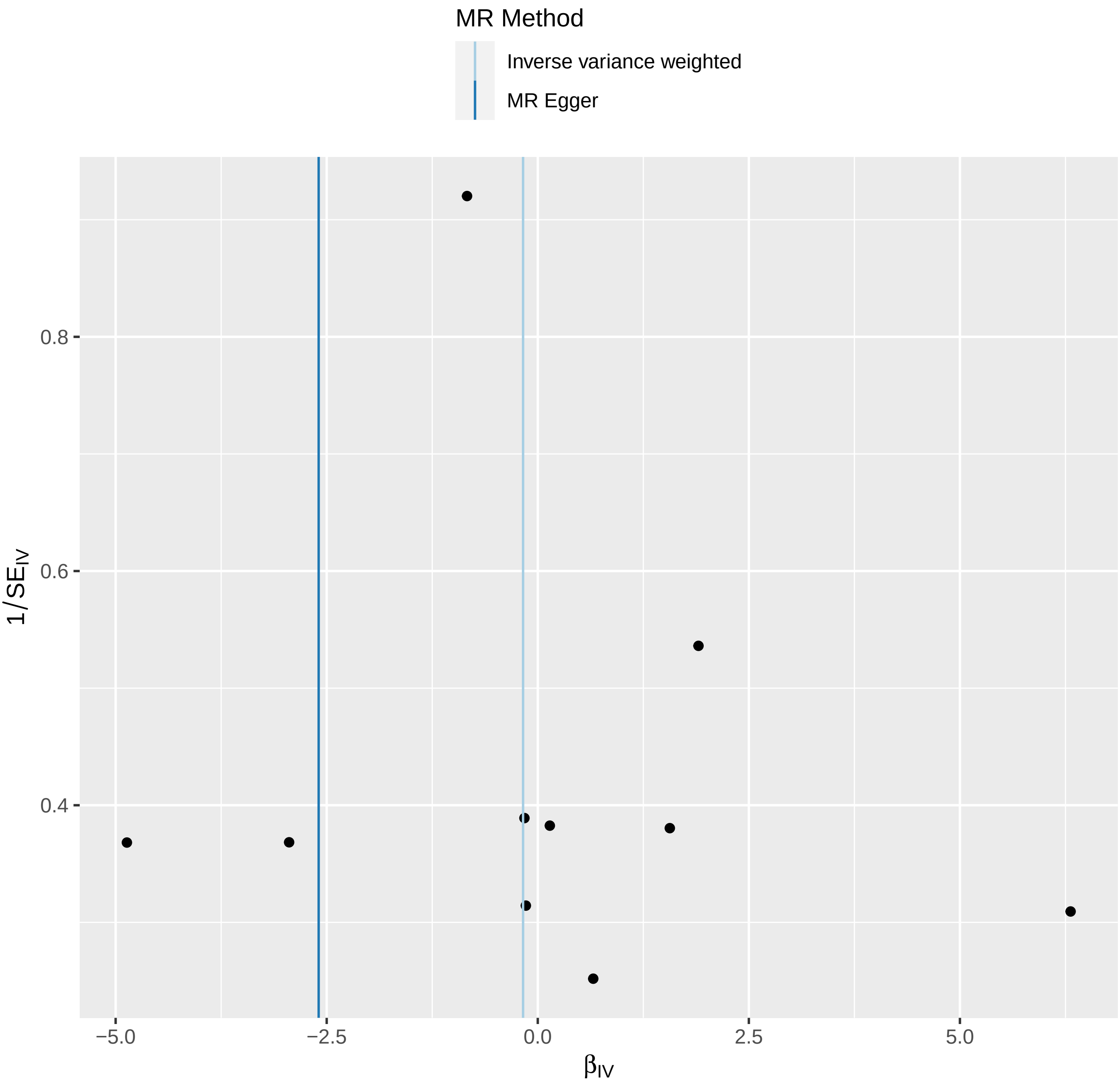

Batch 1872 : 7−methylguanine on Arthropathy in ulcerative colitis

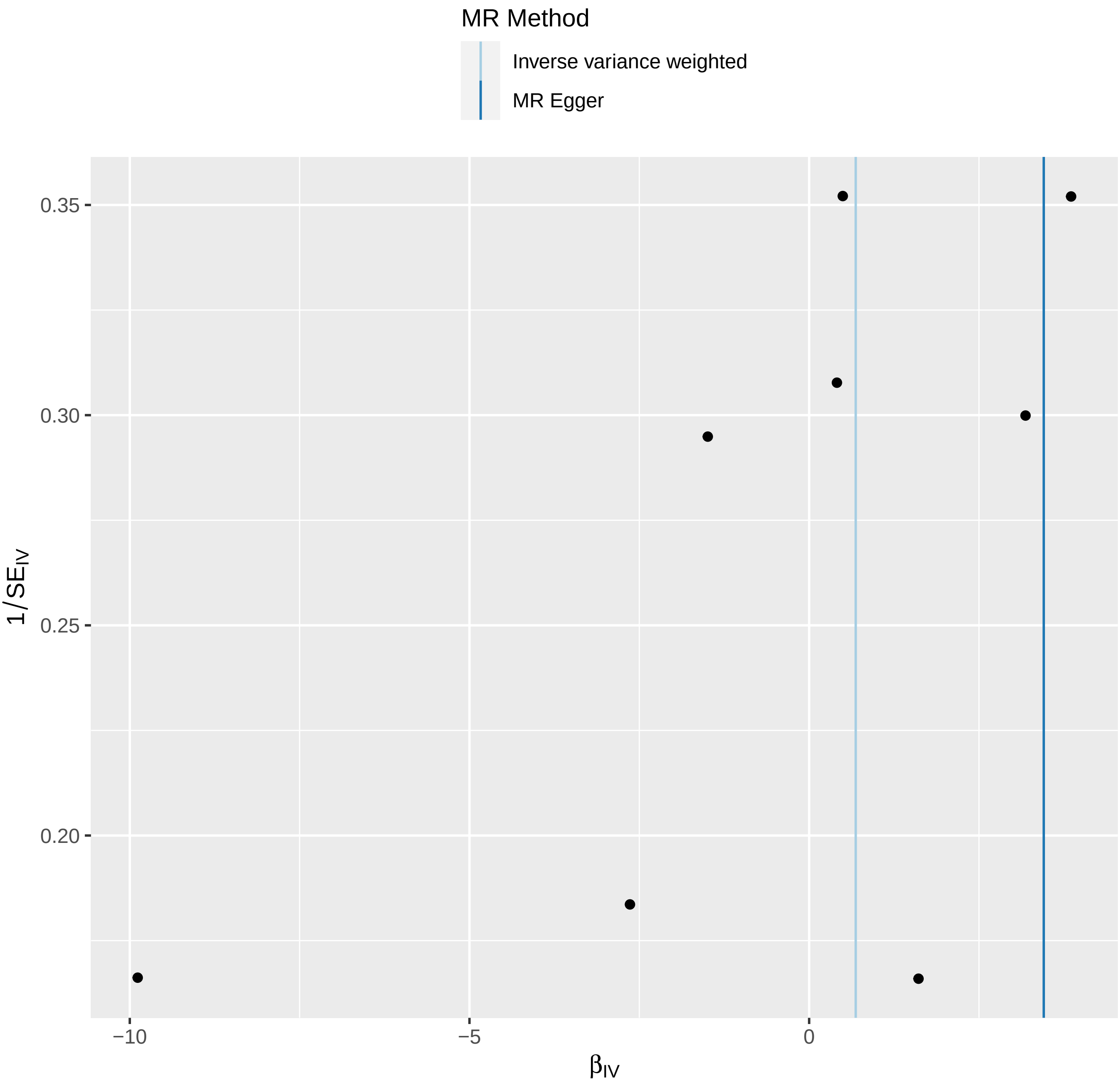

Batch 1879 : X−13431−−nonanoylcarnitine* on Arthropathy in ulcerative colitis

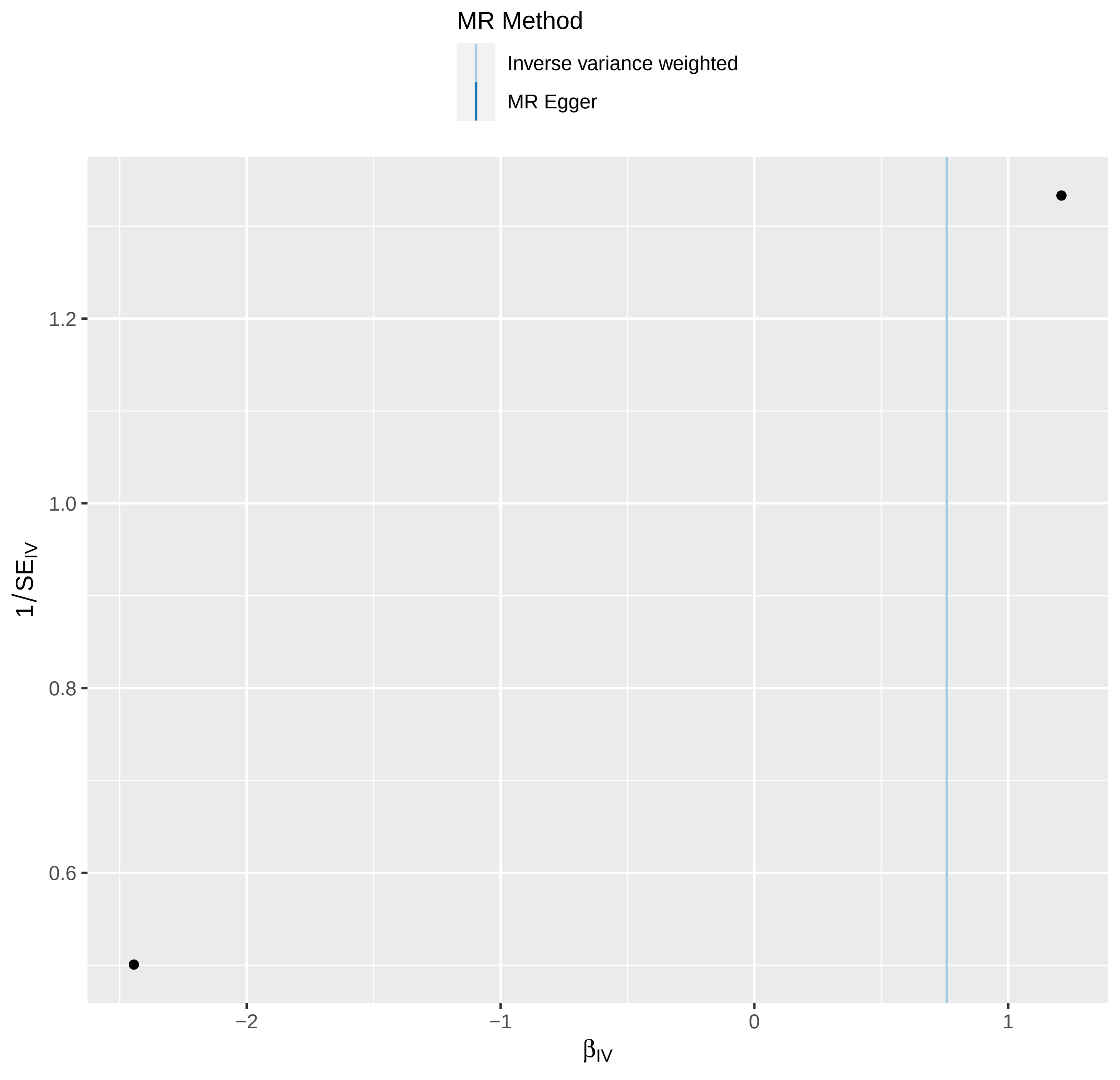

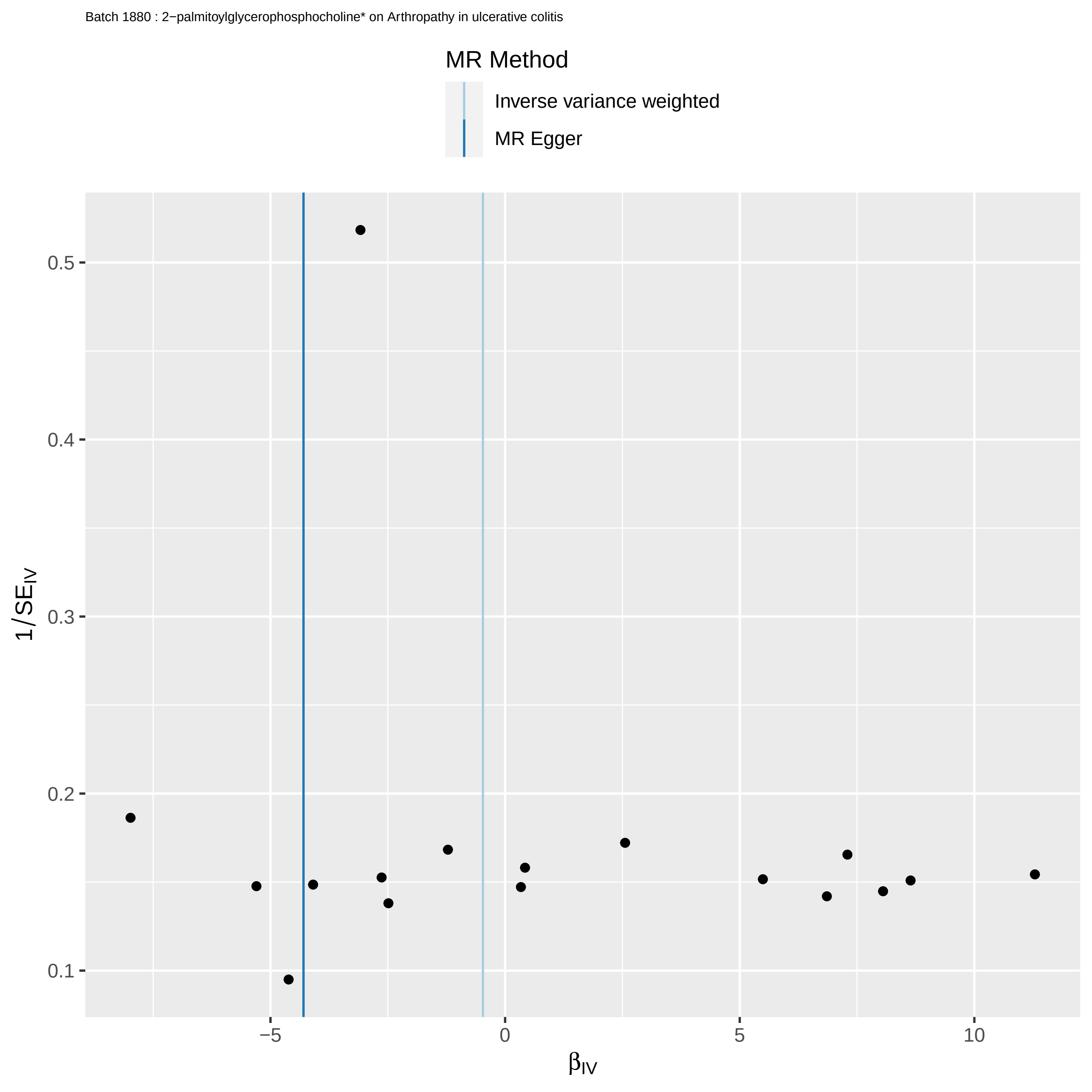
Batch 1880 : 2−palmitoylglycerophosphocholine* on Arthropathy in ulcerative colitis
MR Method
Inverse variance weighted
MR Egger
0.5
0.4
0.3
0.2
0.1
1/SE_IV
−5
0
5
10
β_IV

Batch 1881 : 2−oleoylglycerophosphocholine* on Arthropathy in ulcerative colitis

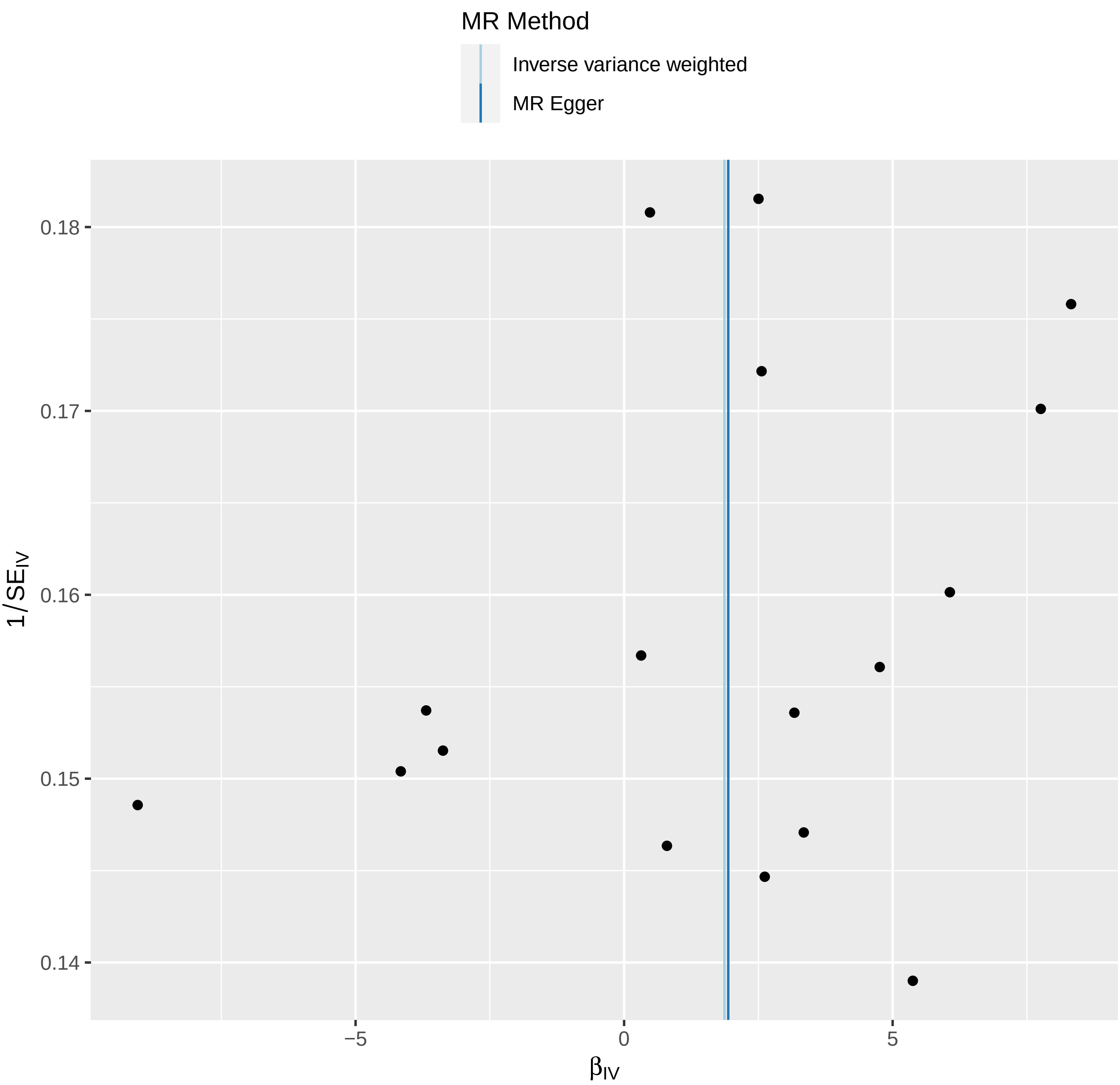

Batch 1884 : 1−palmitoylglycerophosphoinositol* on Arthropathy in ulcerative colitis

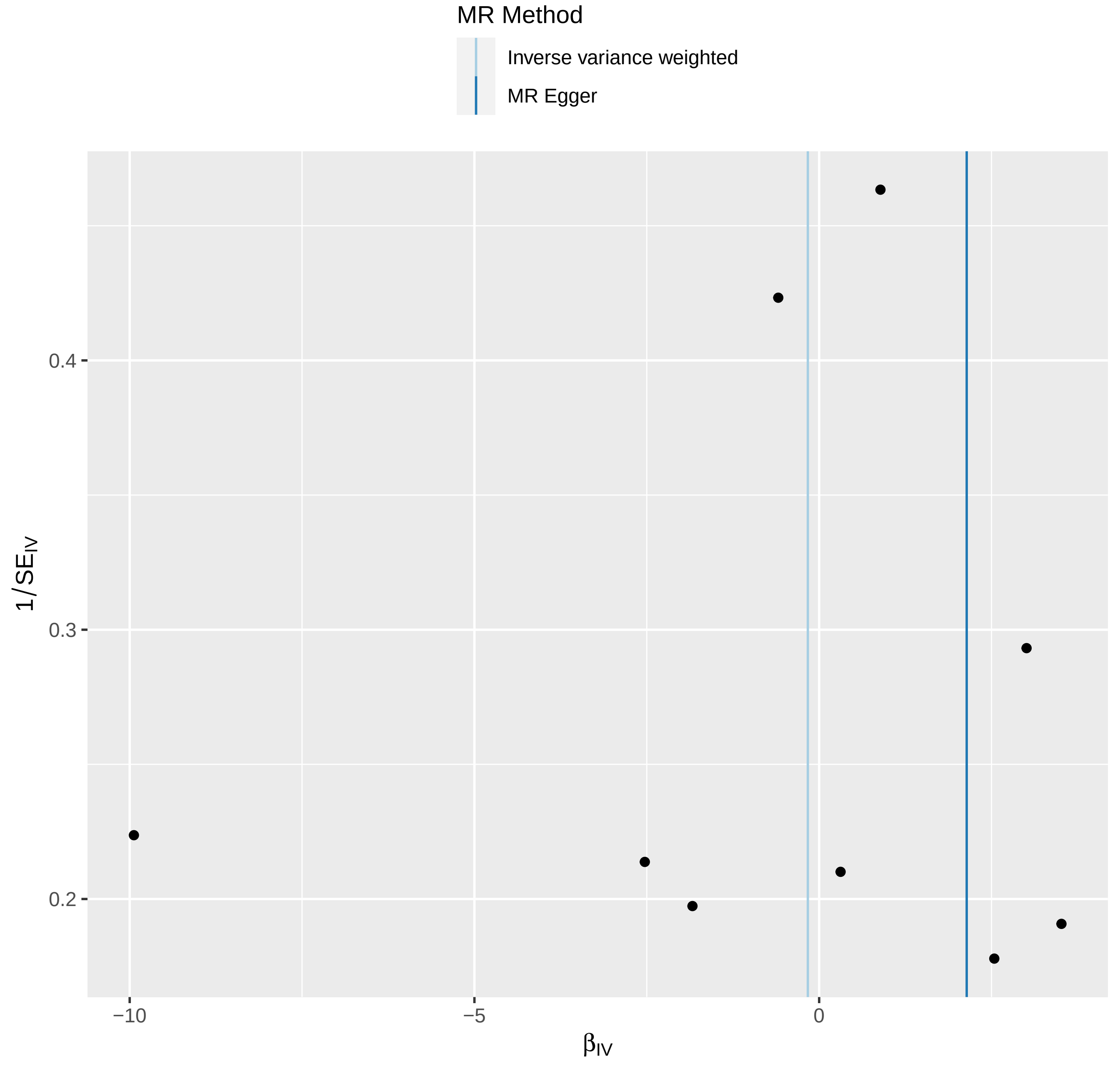

Batch 1886 : Hydroxyisovaleroyl carnitine on Arthropathy in ulcerative colitis

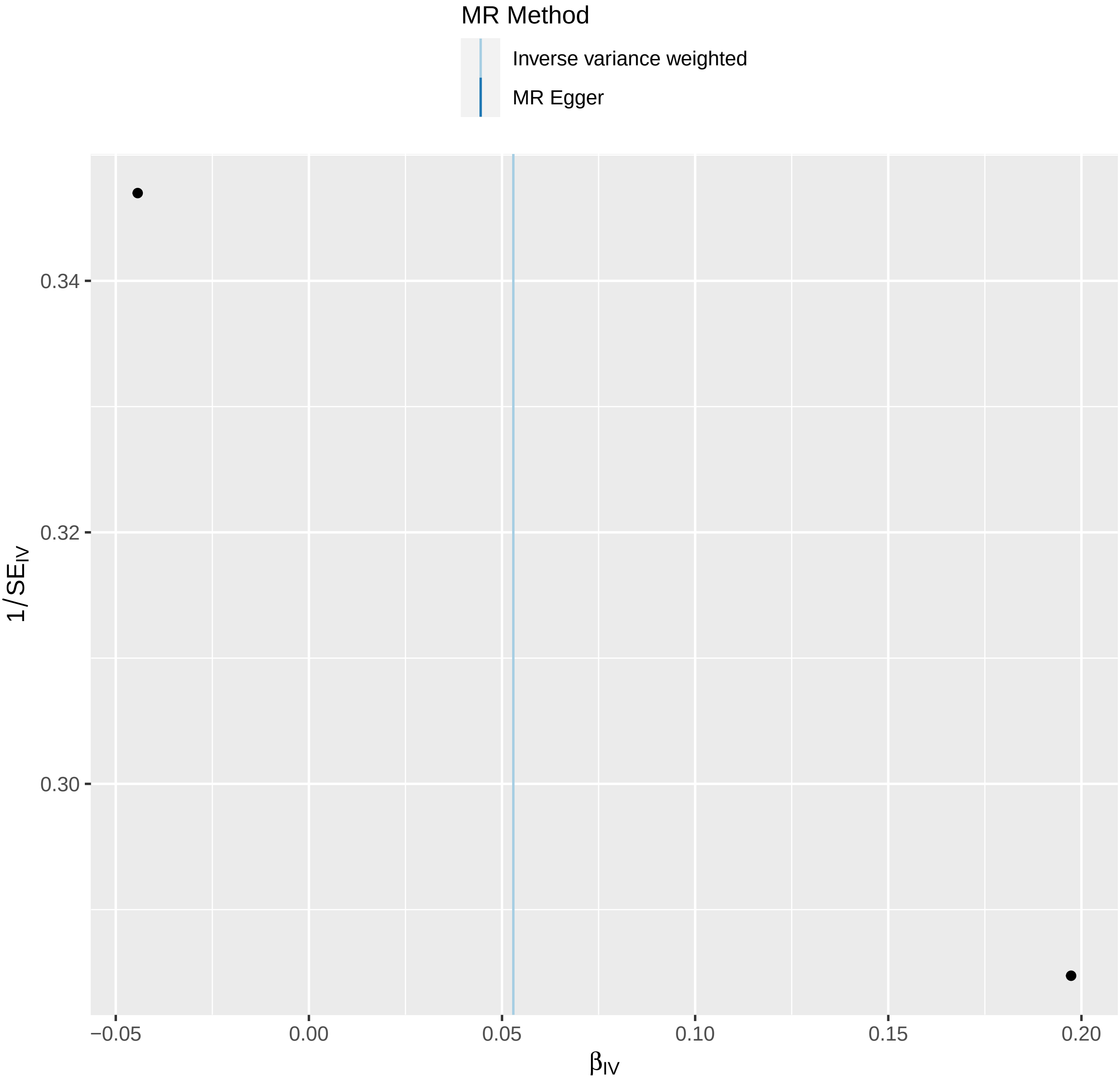

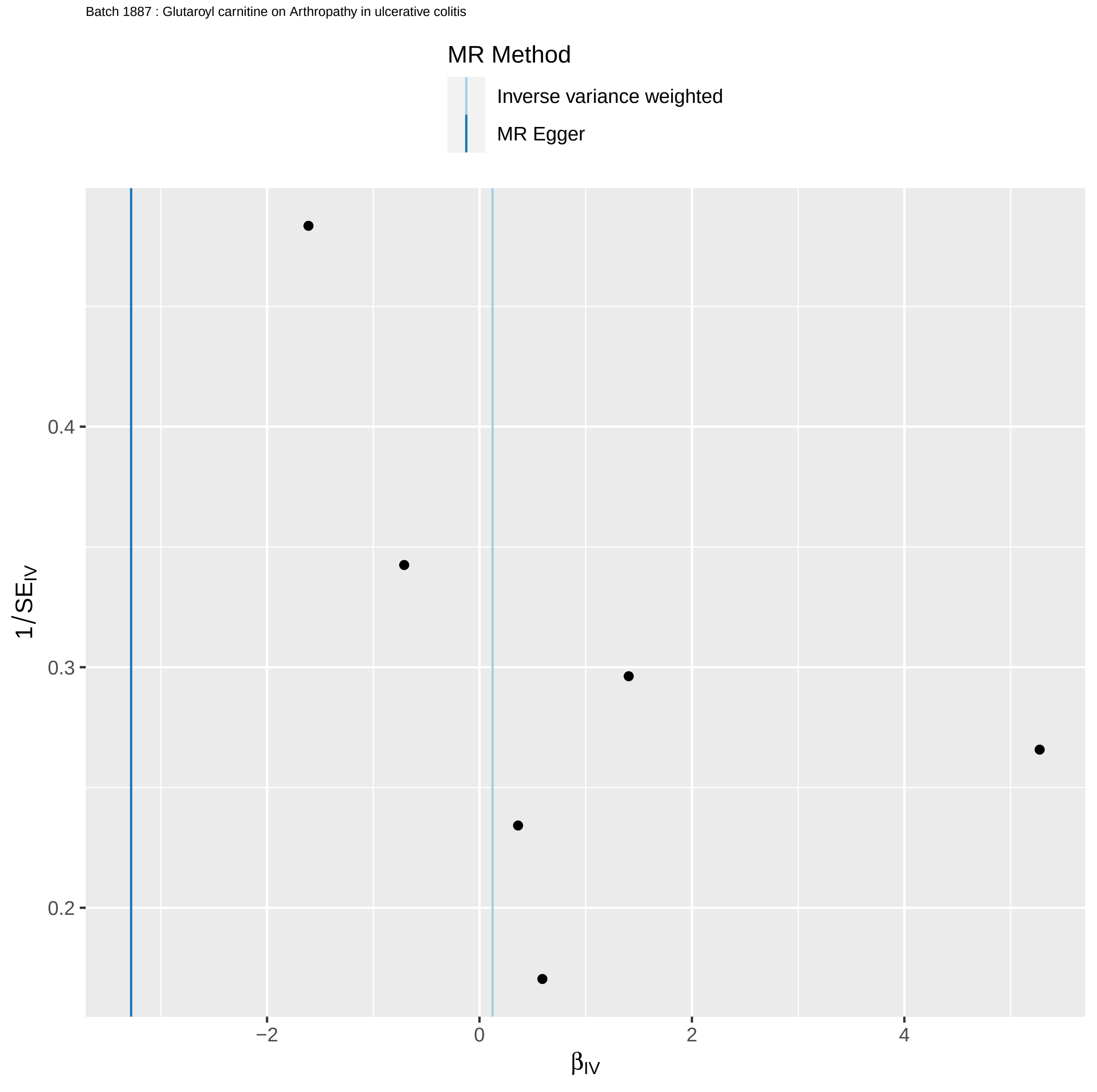
Batch 1887 : Glutaroyl carnitine on Arthropathy in ulcerative colitis
MR Method
Inverse variance weighted
MR Egger
0.4
0.3
0.2
1/SE_IV
−2
0
2
4
β_IV

Batch 1889 : 4−hydroxyhippurate on Arthropathy in ulcerative colitis

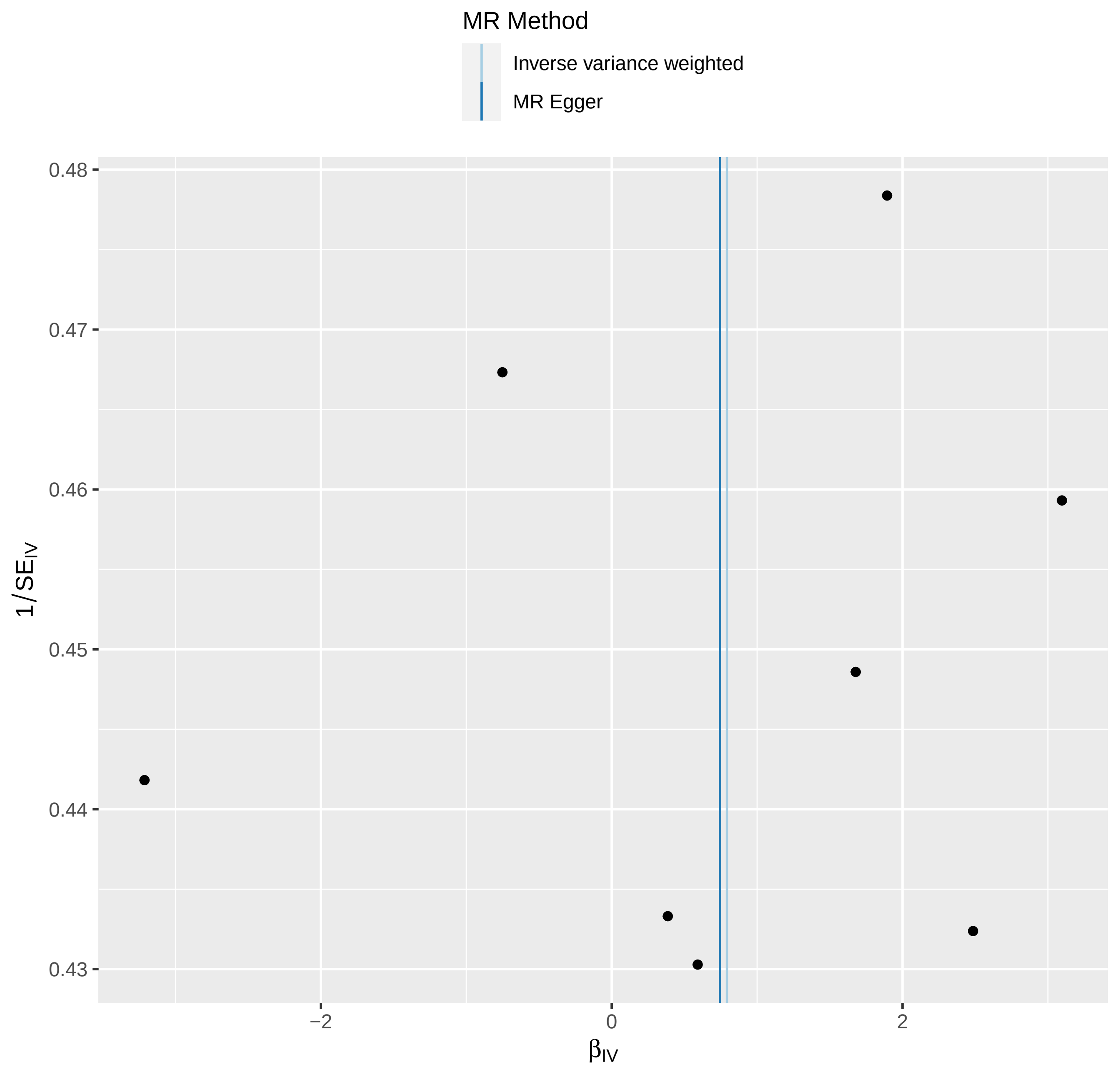

Batch 1890 : 1−myristoylglycerophosphocholine on Arthropathy in ulcerative colitis

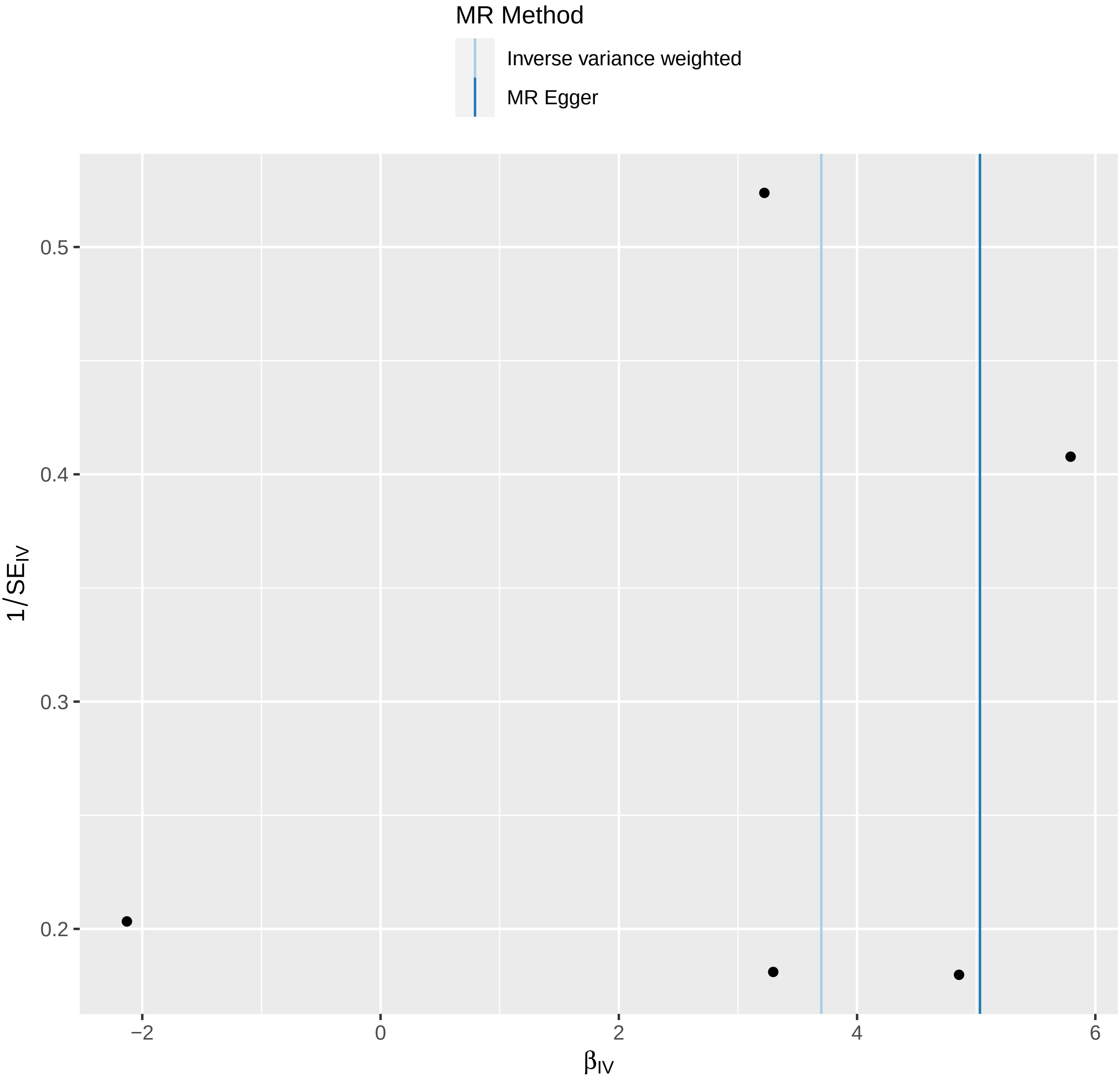

Batch 1893 : 3−(3−hydroxyphenyl)propionate on Arthropathy in ulcerative colitis

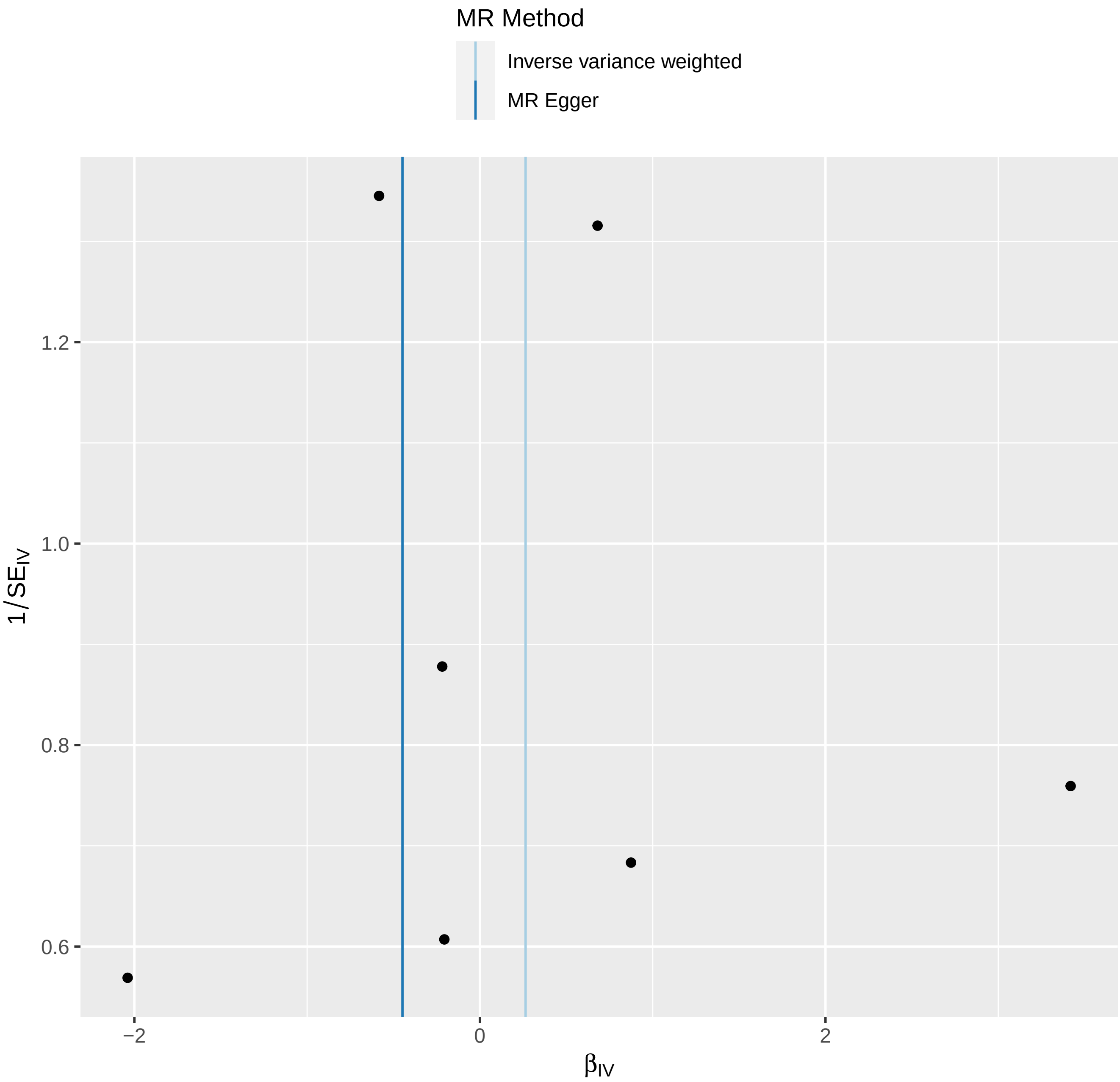

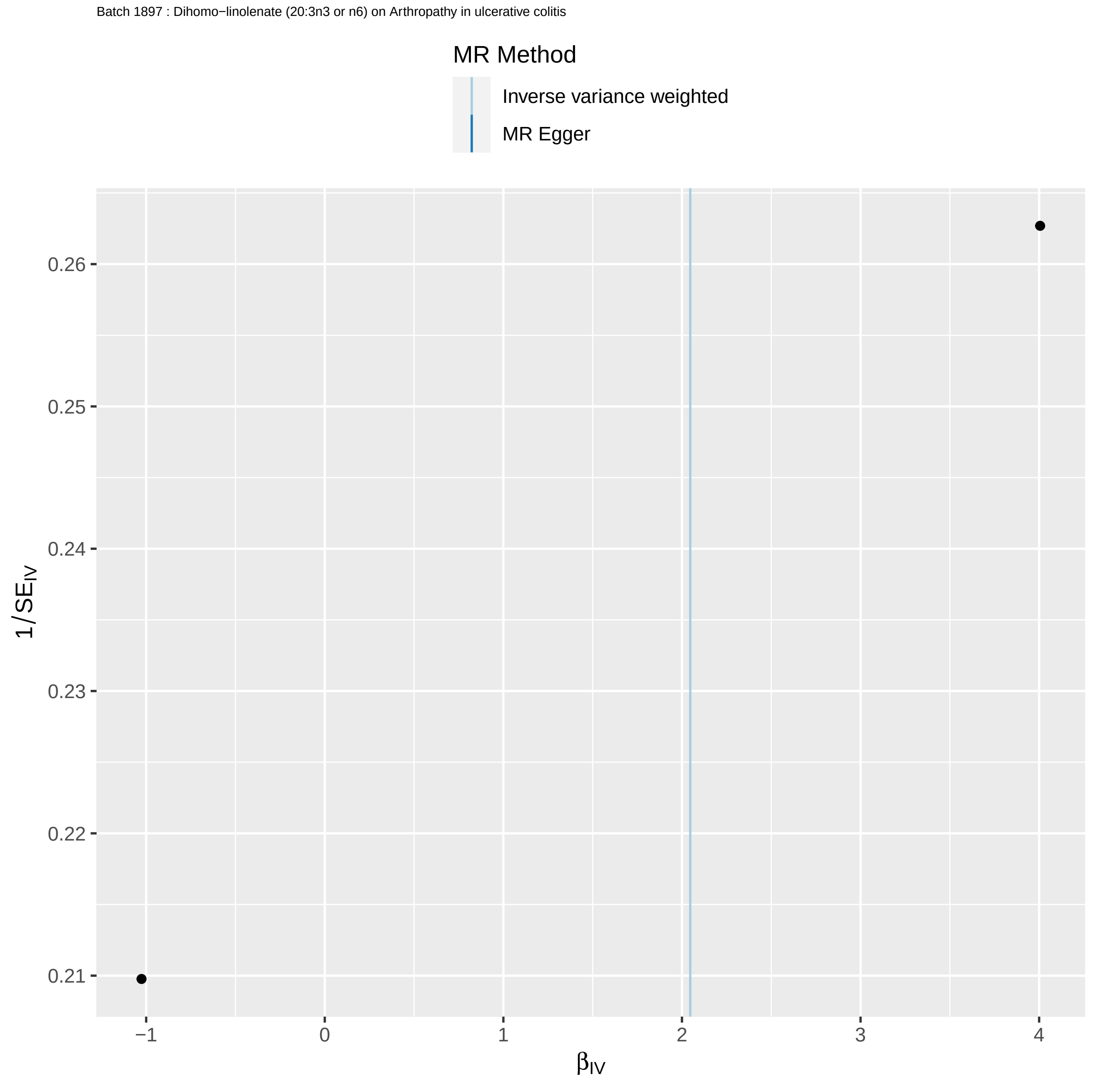
Batch 1897 : Dihomo−linolenate (20:3n3 or n6) on Arthropathy in ulcerative colitis
MR Method
Inverse variance weighted
MR Egger
0.26
0.25
0.24
0.23
0.22
0.21
1/SE_IV
−1
0
1
2
3
4
β_IV

Batch 1898 : Threitol on Arthropathy in ulcerative colitis

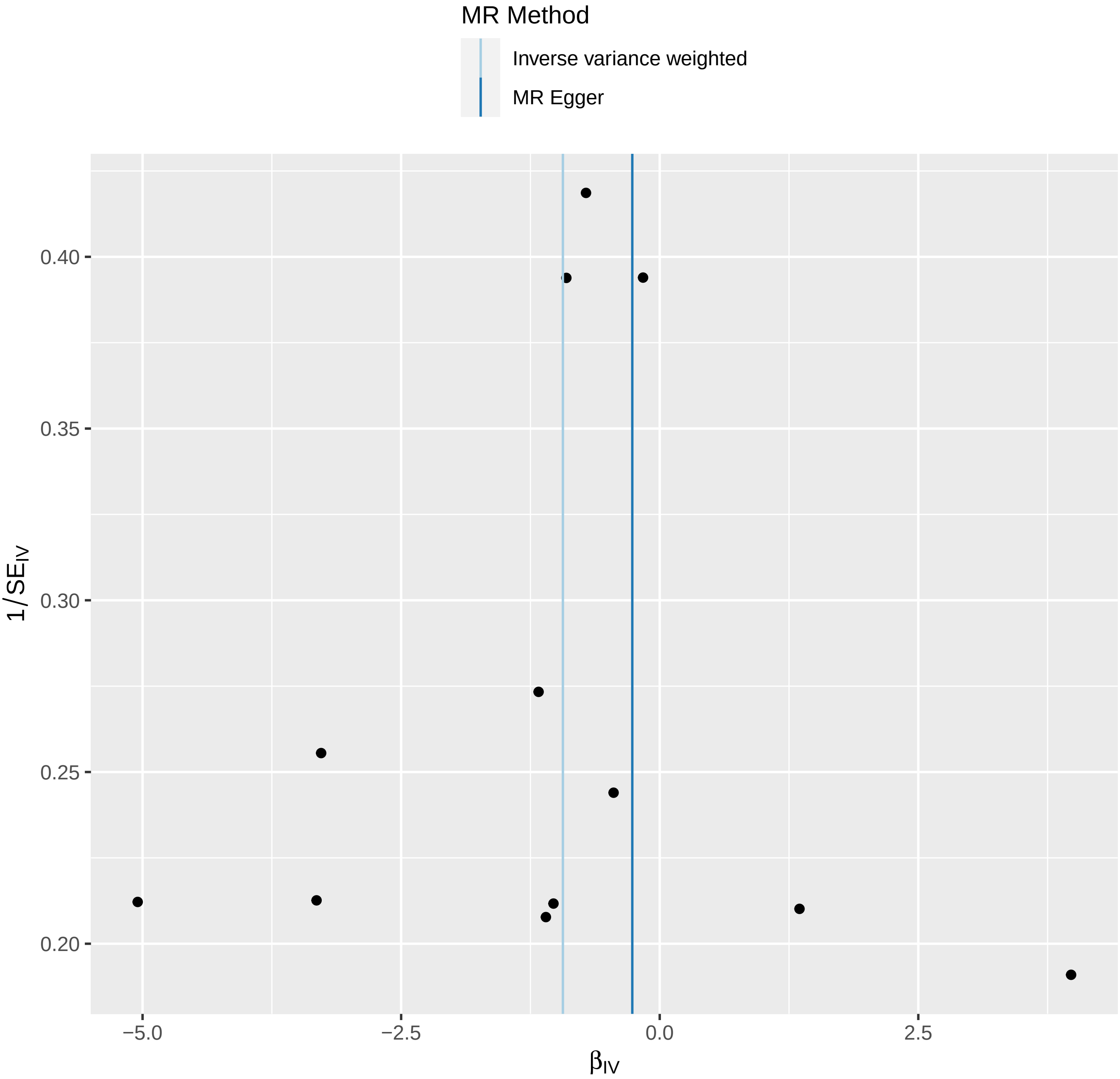

Batch 1899 : P−cresol sulfate on Arthropathy in ulcerative colitis

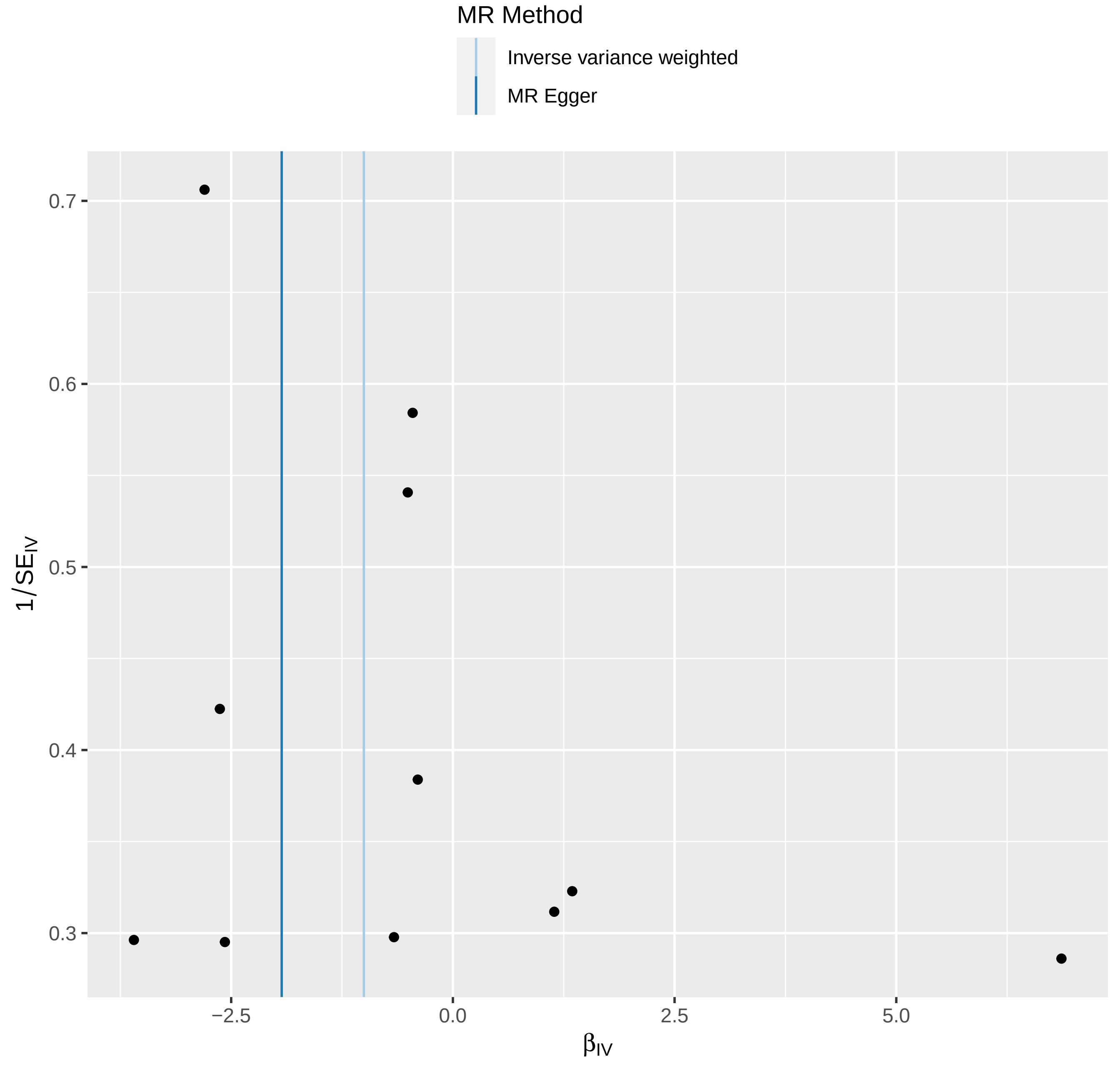

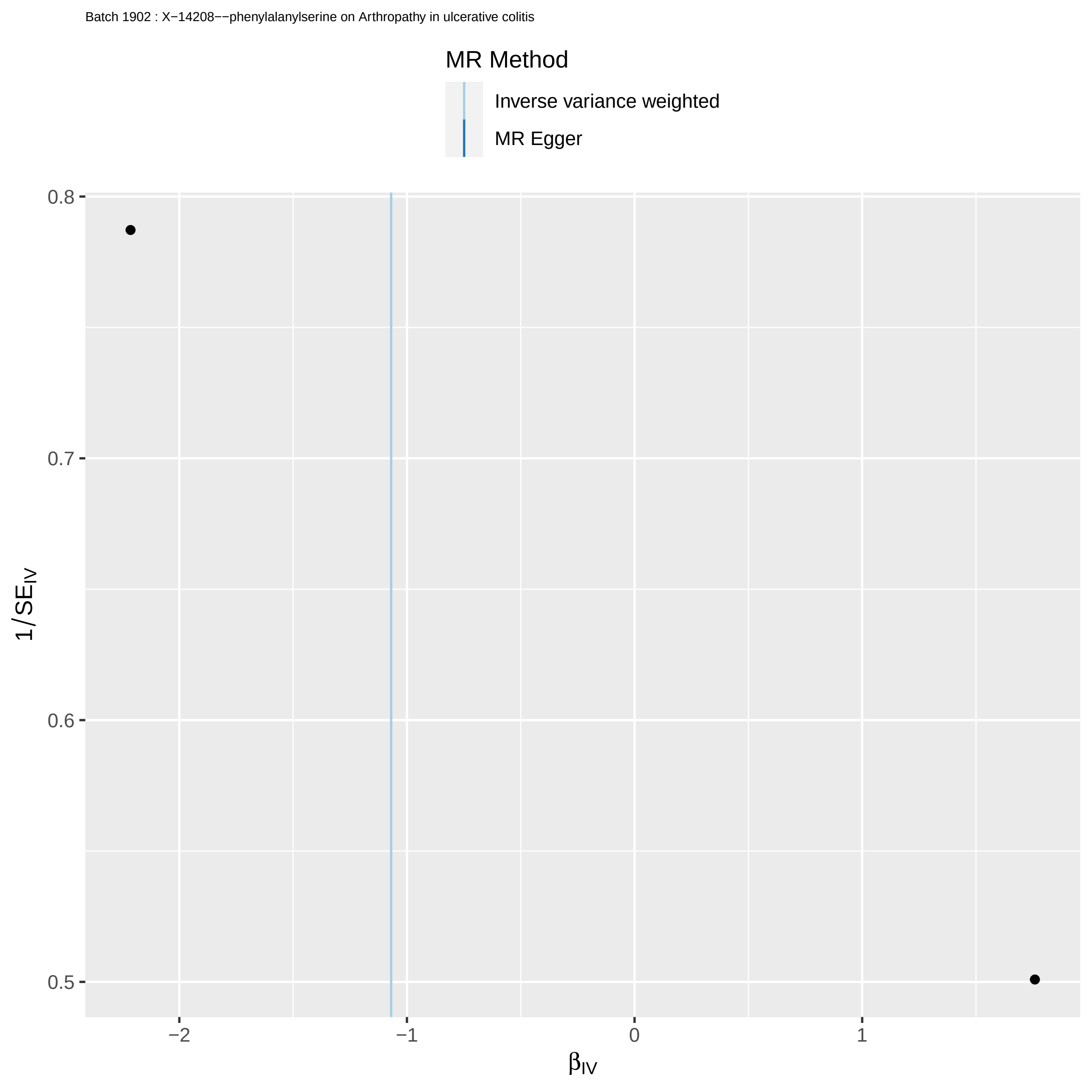
Batch 1902 : X−14208−−phenylalanylserine on Arthropathy in ulcerative colitis
MR Method
Inverse variance weighted
MR Egger
0.8
0.7
0.6
0.5
1/SE_IV
−2
−1
0
1
β_IV

Batch 1904 : X−14450−−phenylalanylleucine on Arthropathy in ulcerative colitis

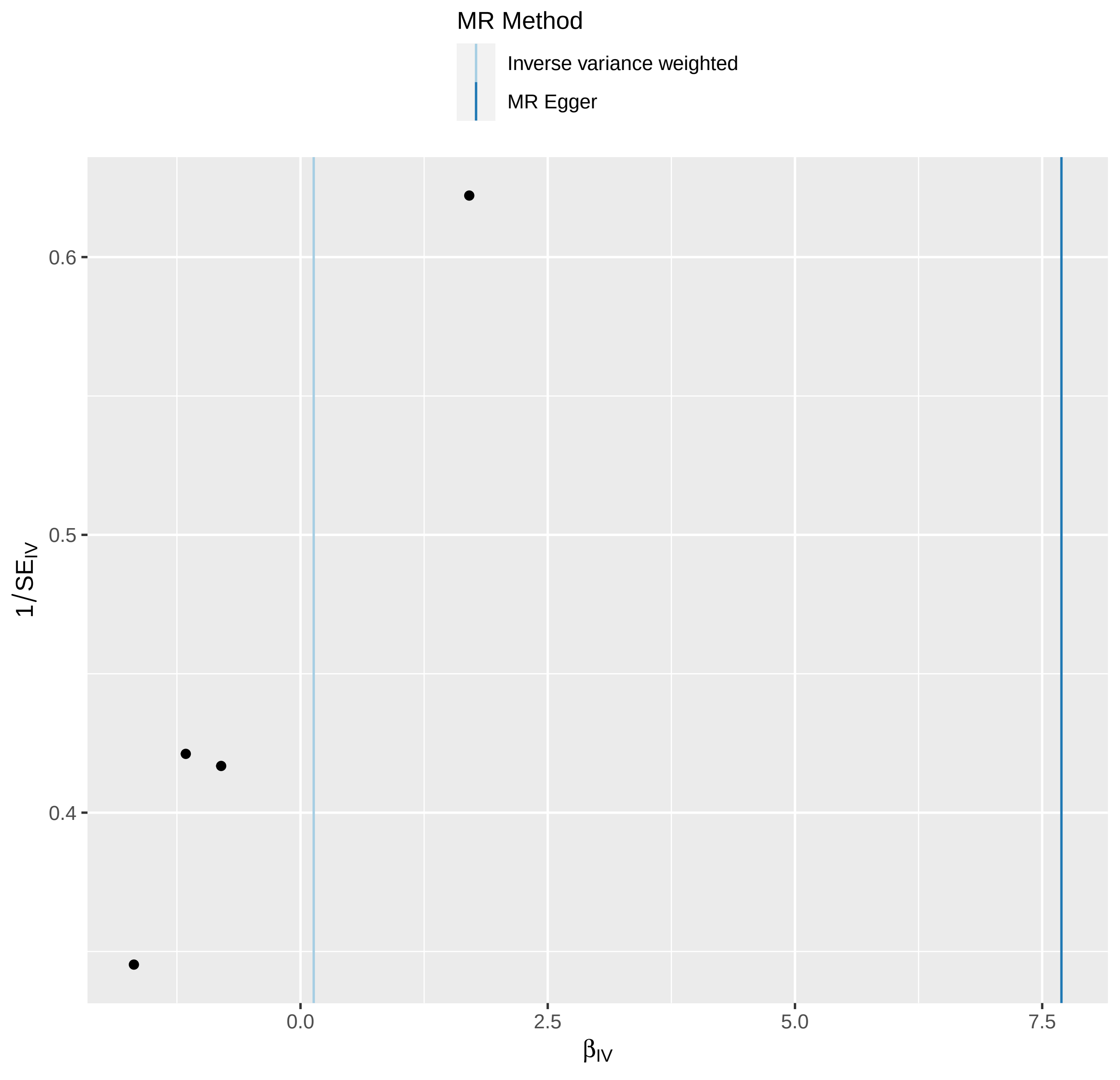

Batch 1905 : Gamma−glutamylglutamate on Arthropathy in ulcerative colitis

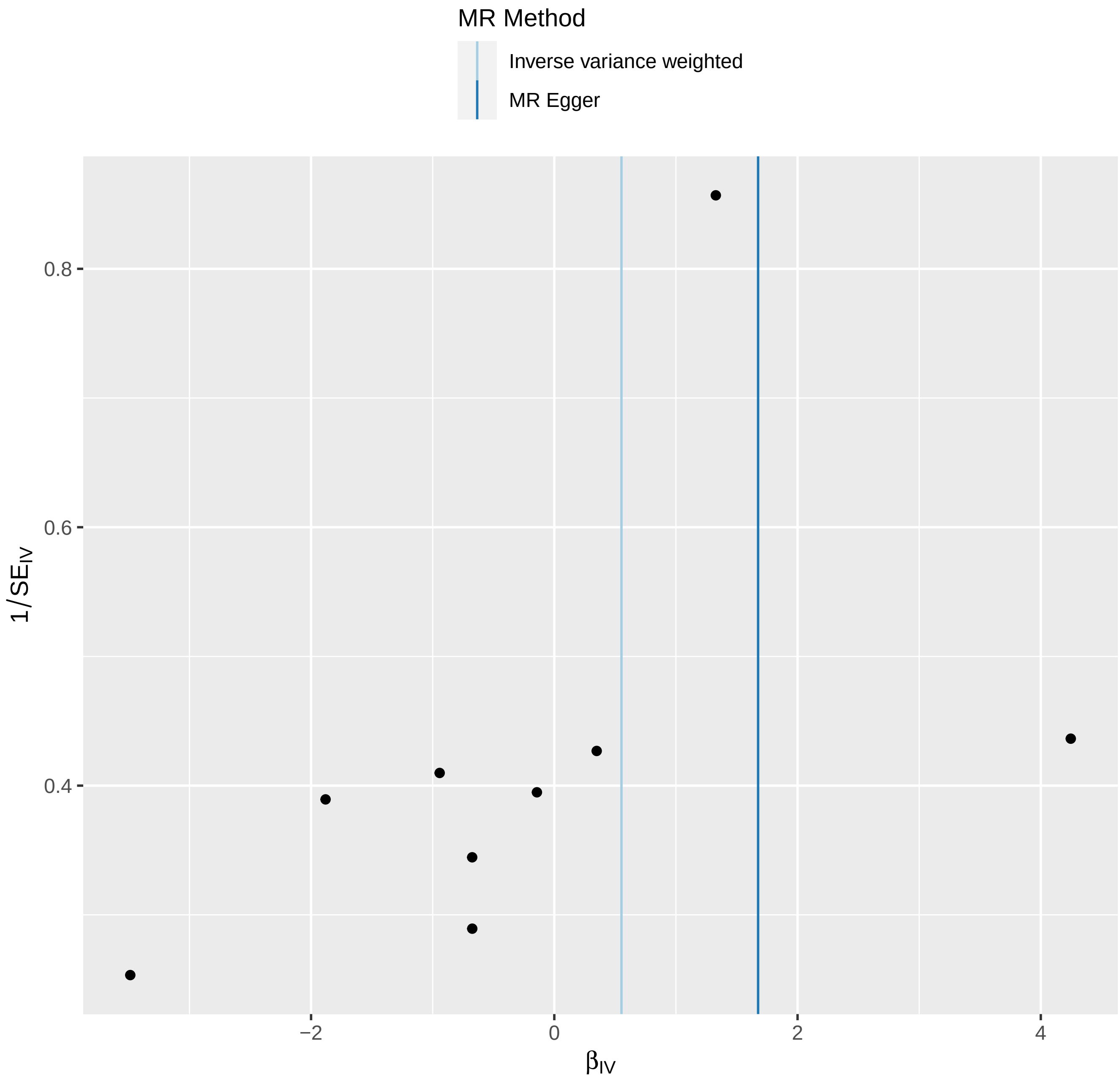

Batch 1906 : Octadecanedioate on Arthropathy in ulcerative colitis

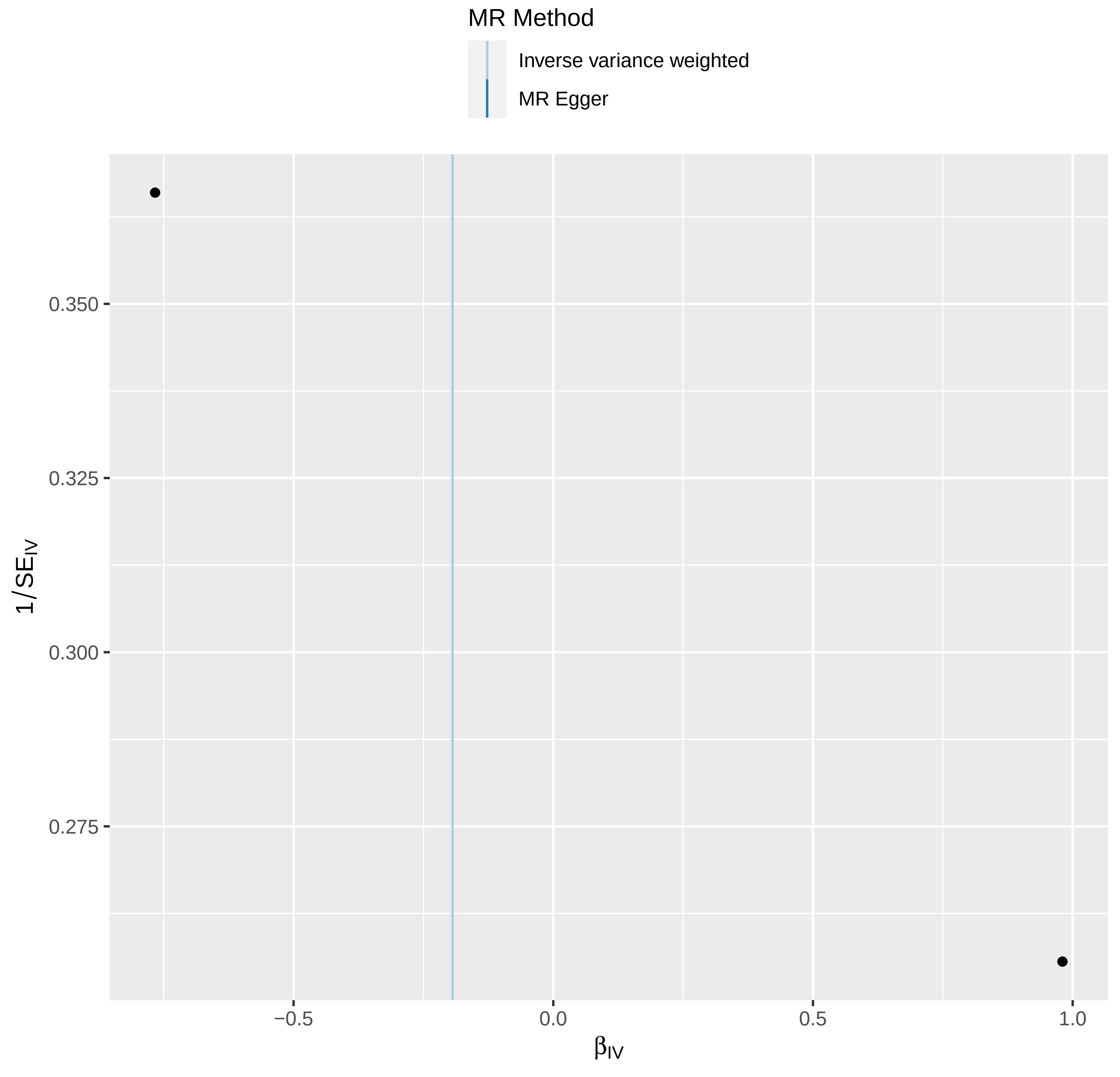

Batch 1907 : Leucylleucine on Arthropathy in ulcerative colitis

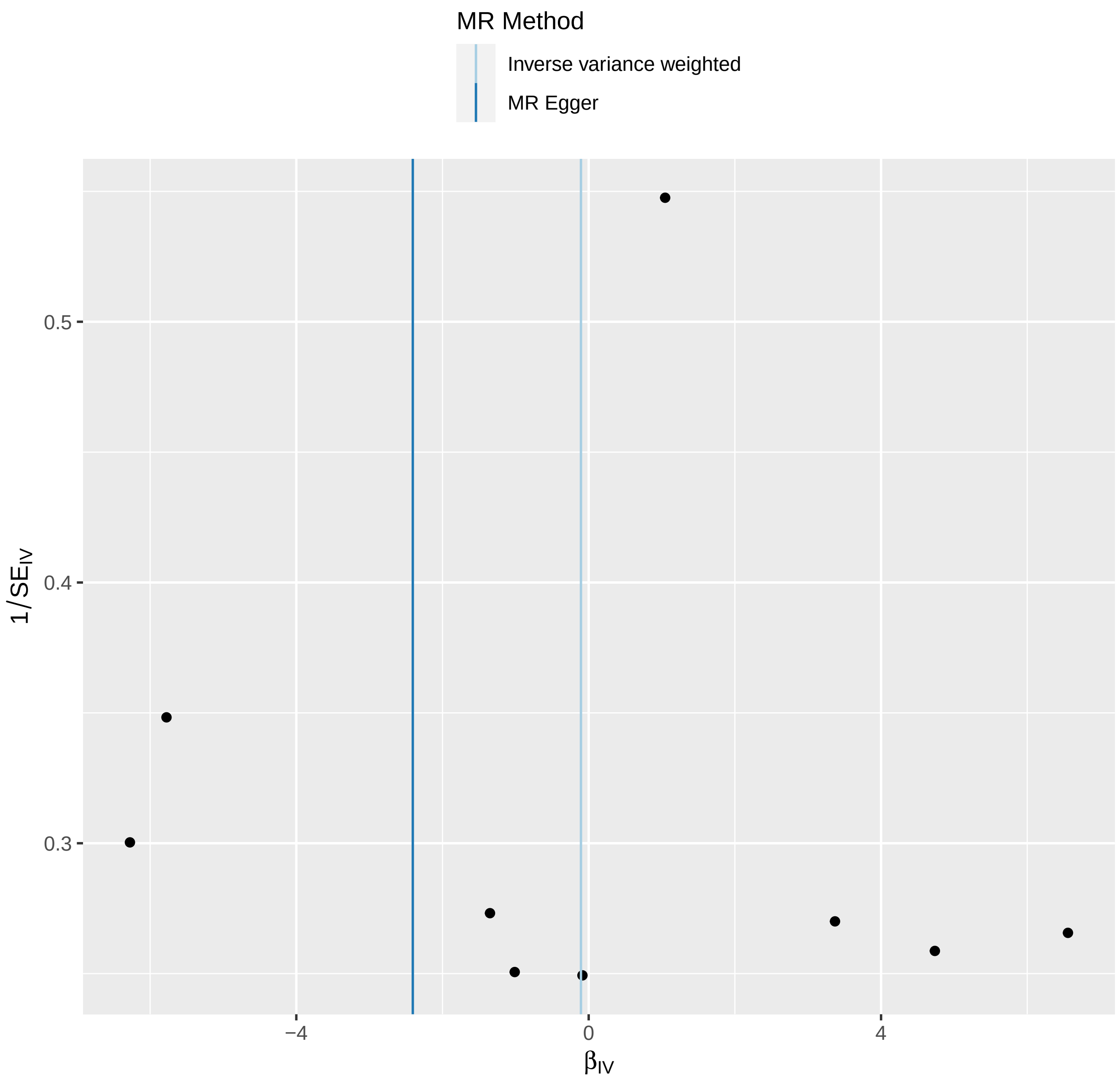

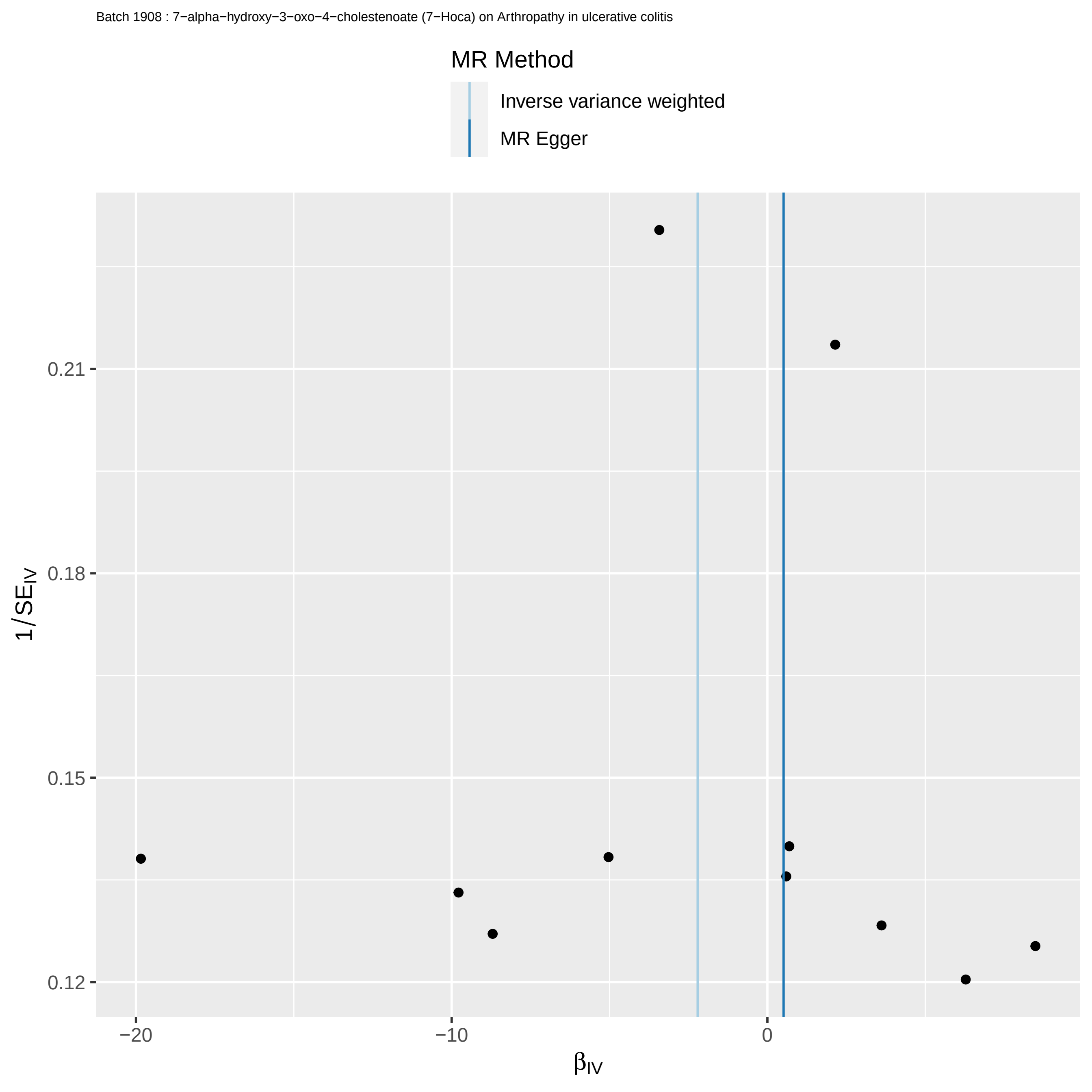
Batch 1908 : 7−alpha−hydroxy−3−oxo−4−cholestenoate (7−Hoca) on Arthropathy in ulcerative colitis
MR Method
Inverse variance weighted
MR Egger
0.21
0.18
0.15
0.12
1/SE$_{IV}$
−20
−10
0
β$_{IV}$

Batch 1909 : N−Butyl Oleate on Arthropathy in ulcerative colitis

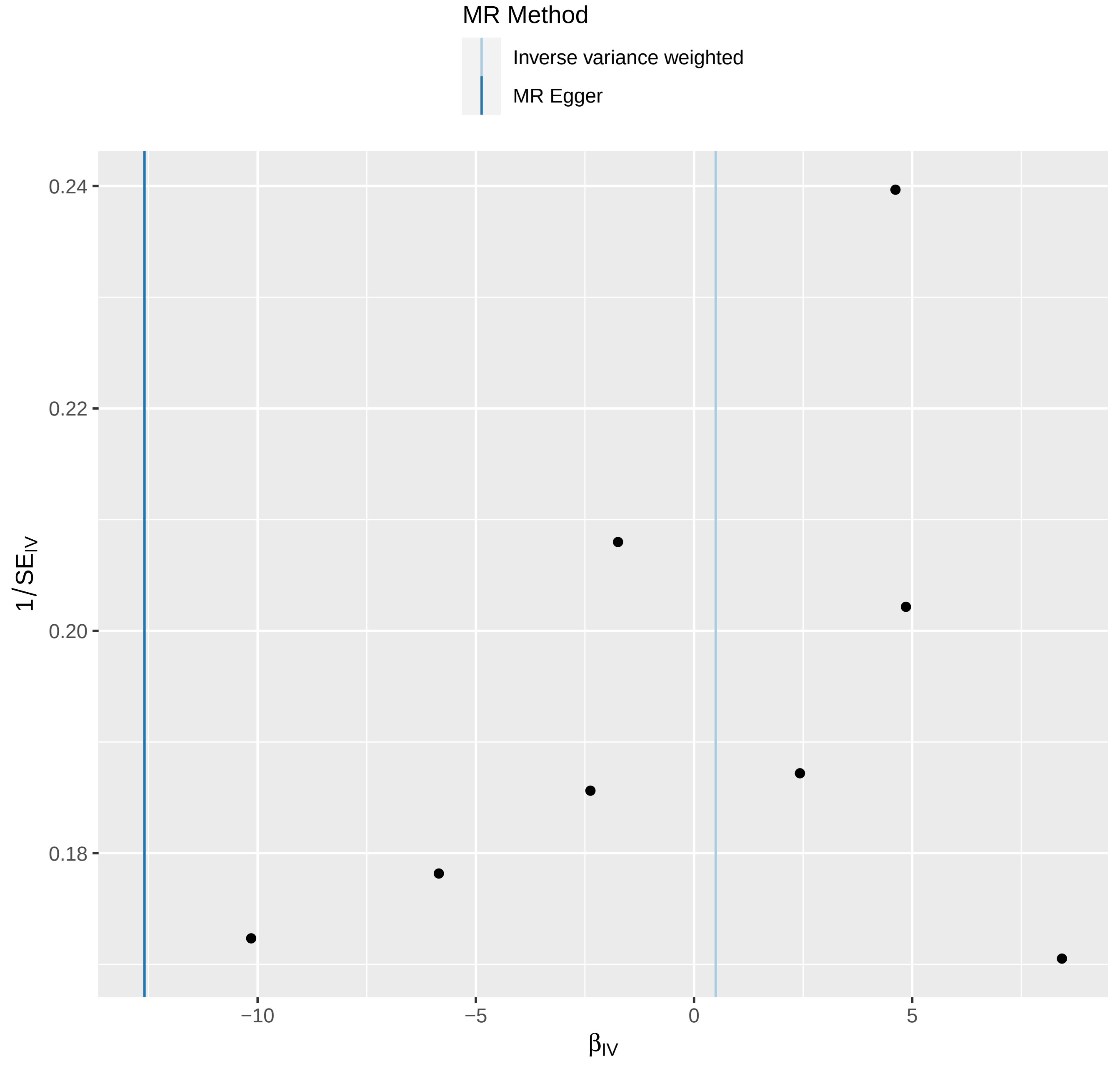

Batch 1911 : Taurolithocholate 3−sulfate on Arthropathy in ulcerative colitis

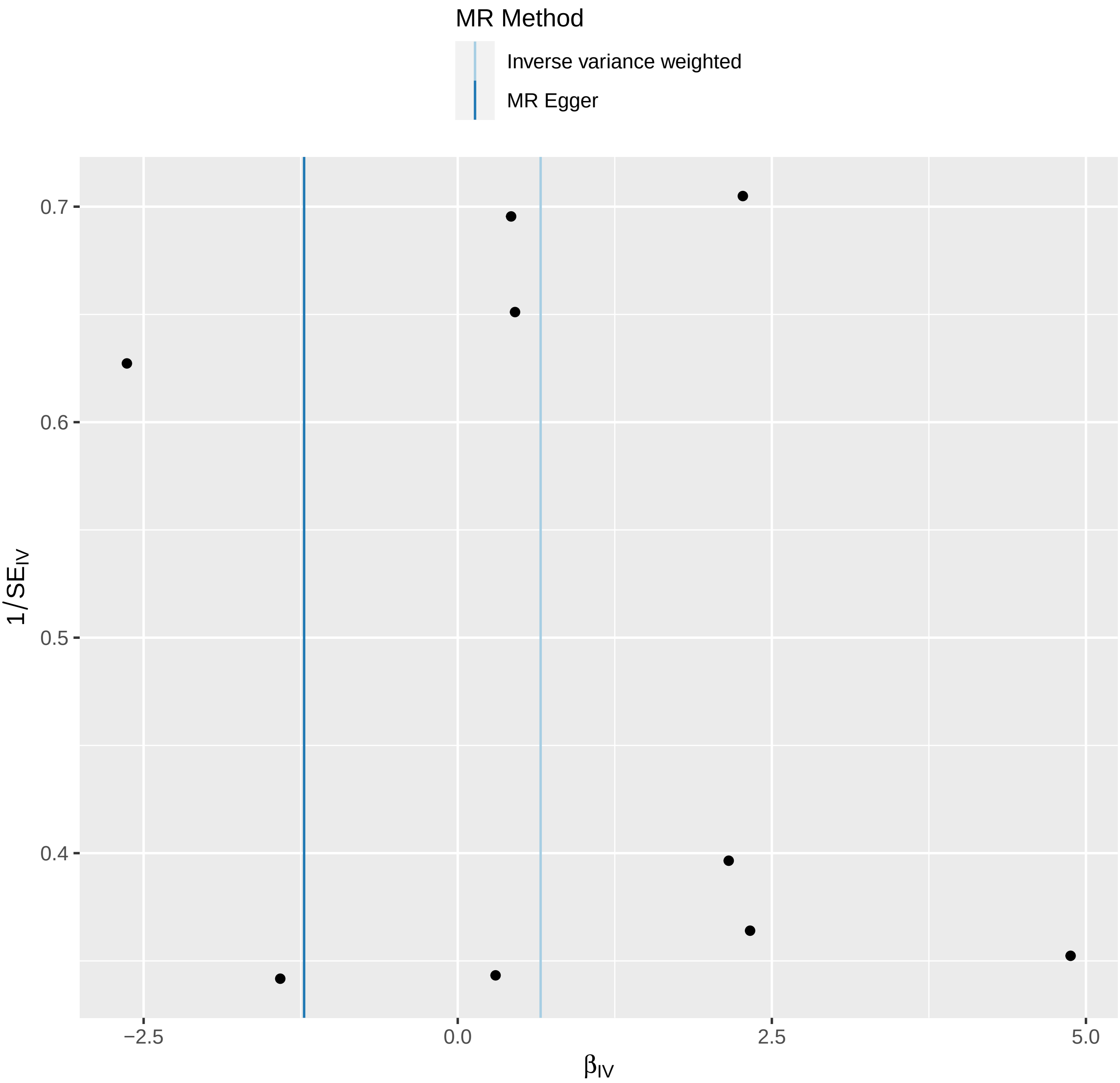

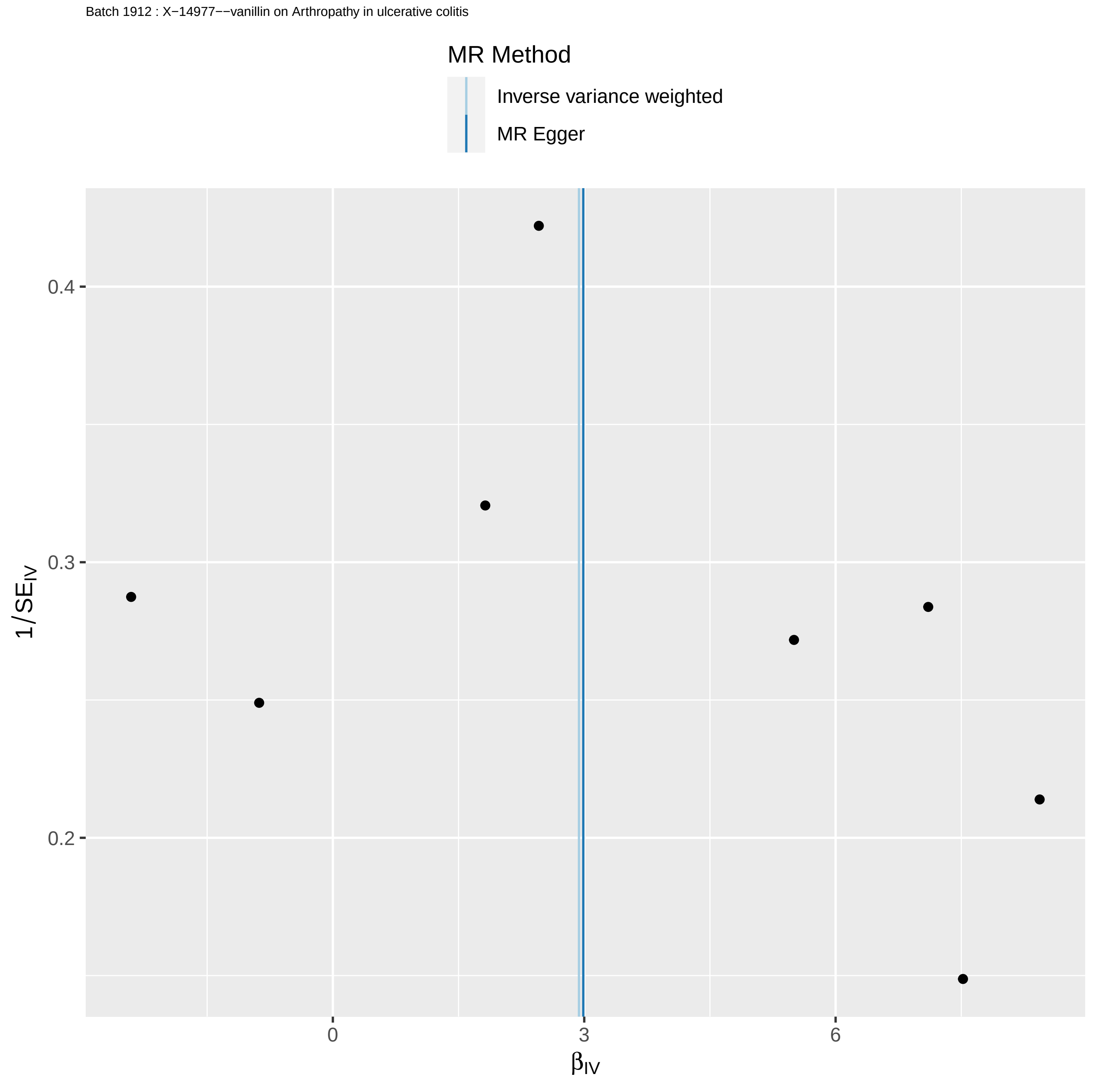
Batch 1912 : X−14977−−vanillin on Arthropathy in ulcerative colitis
MR Method
Inverse variance weighted
MR Egger
0.4
0.3
0.2
1/SE_IV
0
3
6
β_IV

Batch 1913 : Succinylcarnitine on Arthropathy in ulcerative colitis

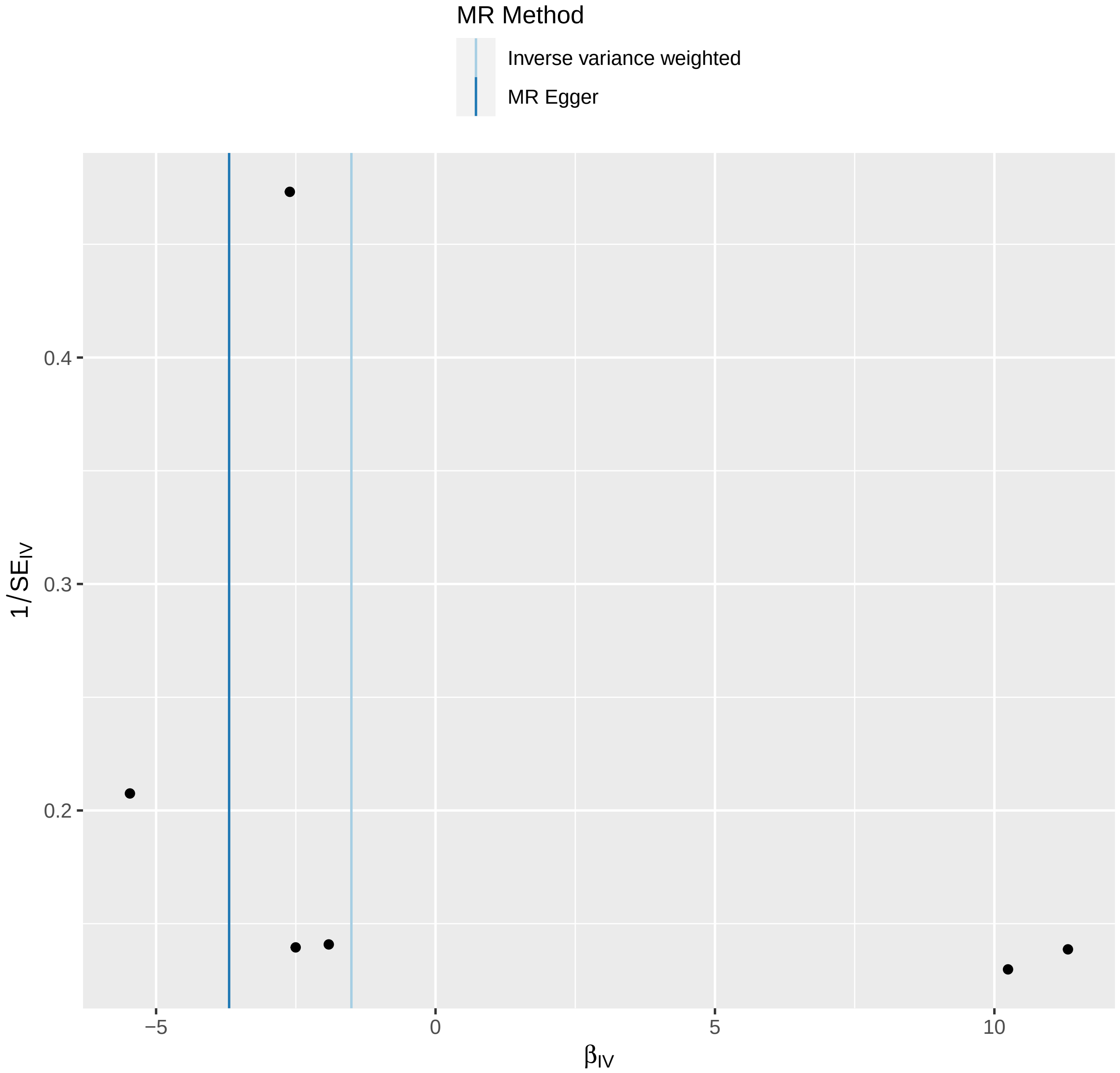

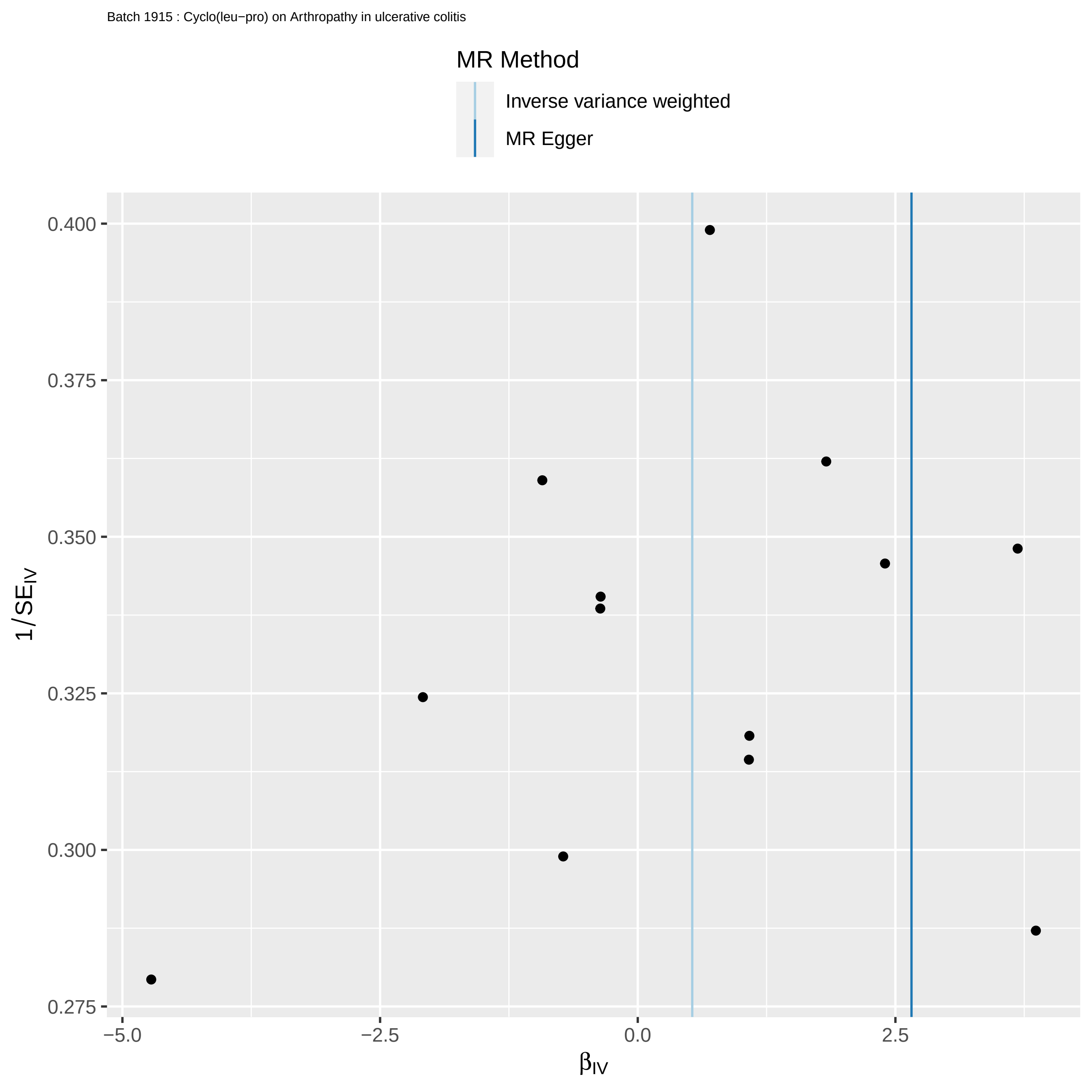
Batch 1915 : Cyclo(leu−pro) on Arthropathy in ulcerative colitis
MR Method
Inverse variance weighted
MR Egger
0.400
0.375
0.350
0.325
0.300
0.275
1/SE_IV
−5.0
−2.5
0.0
2.5
β_IV

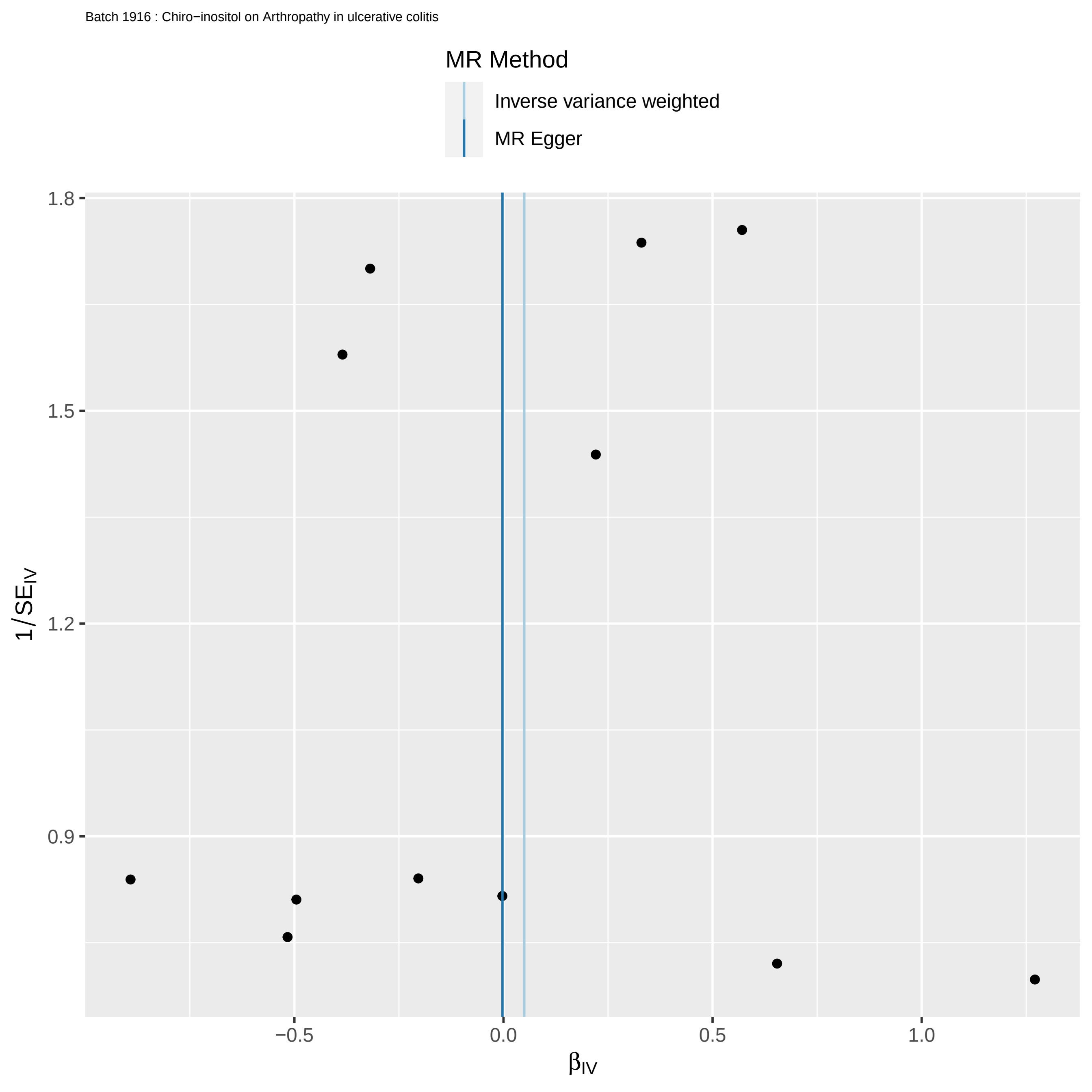
Batch 1916 : Chiro−inositol on Arthropathy in ulcerative colitis
MR Method
Inverse variance weighted
MR Egger
1.8
1.5
1.2
0.9
1/SE$_{IV}$
−0.5
0.0
0.5
1.0
β_{IV}

Batch 1920 : 2−hydroxyglutarate on Arthropathy in ulcerative colitis

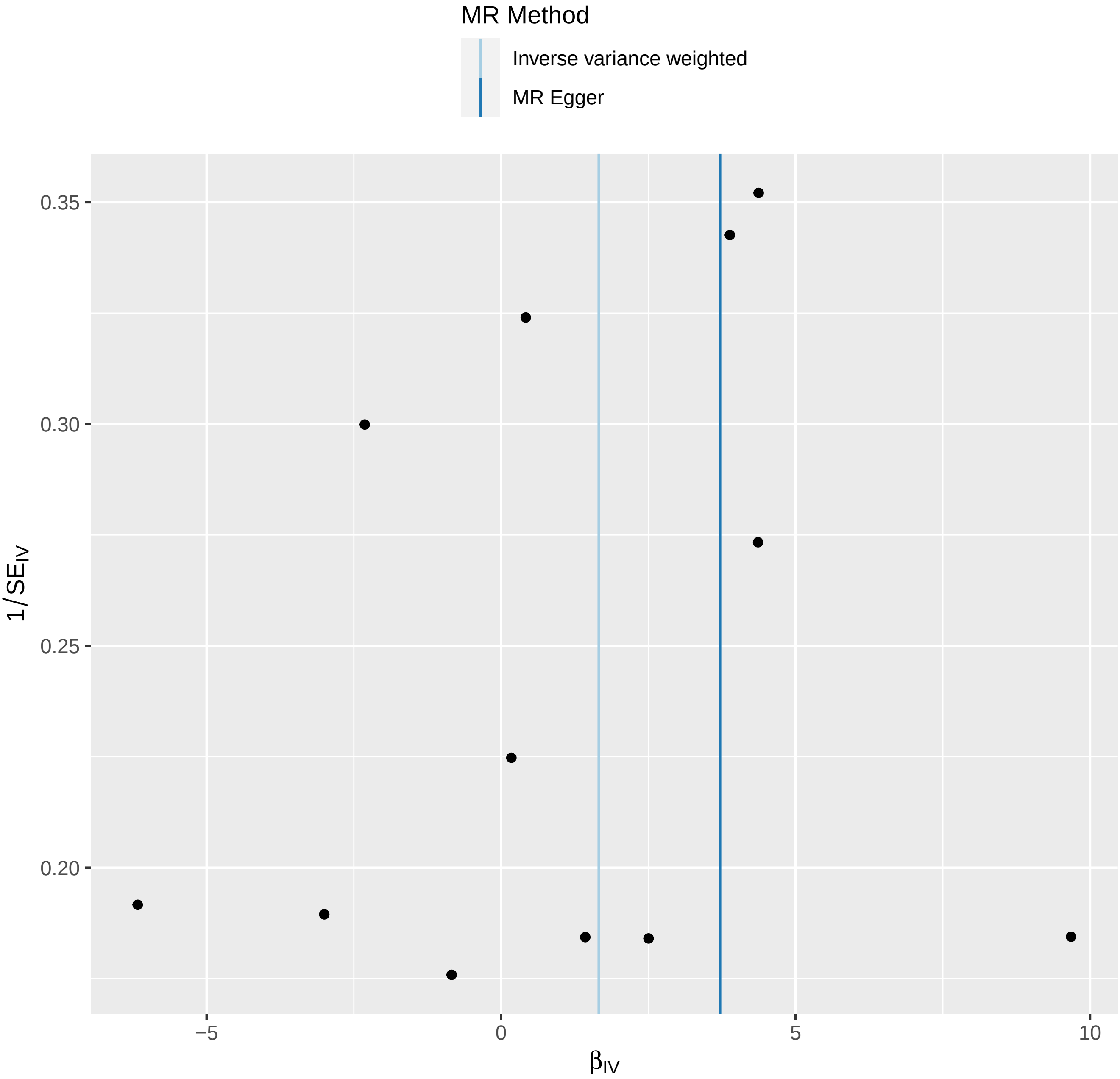

Batch 1921 : Ergothioneine on Arthropathy in ulcerative colitis

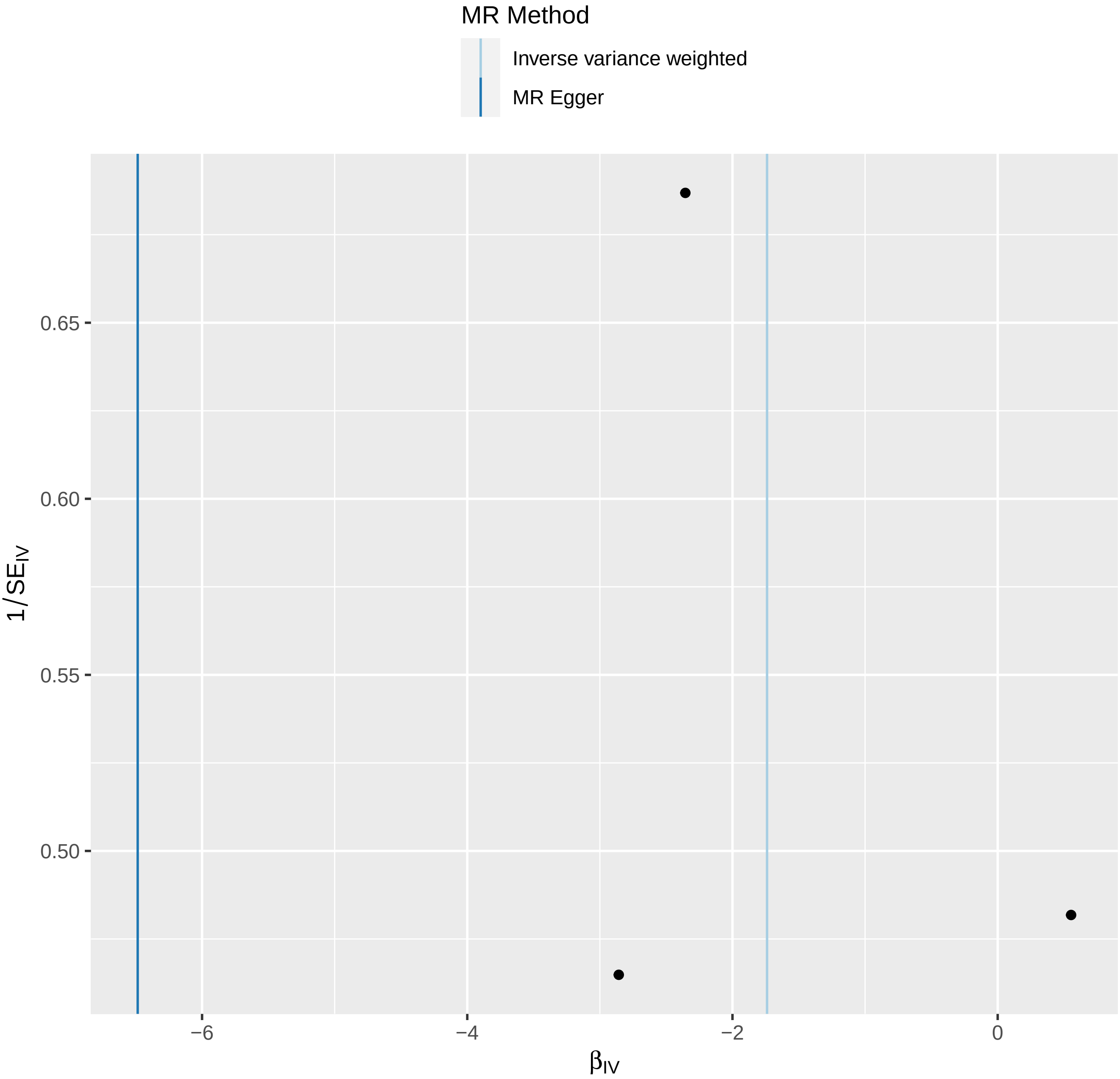

Batch 1923 : Phenylalanylphenylalanine on Arthropathy in ulcerative colitis

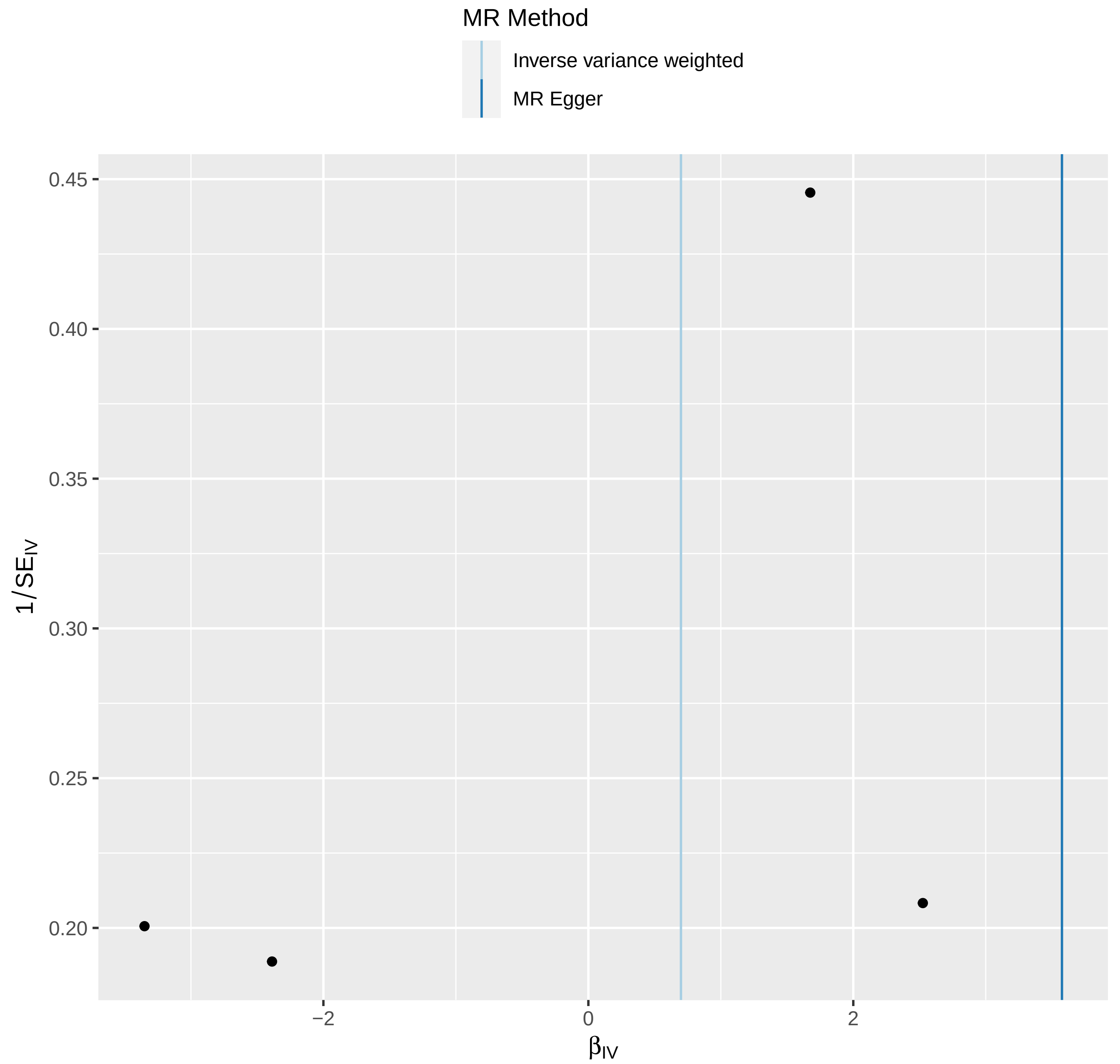

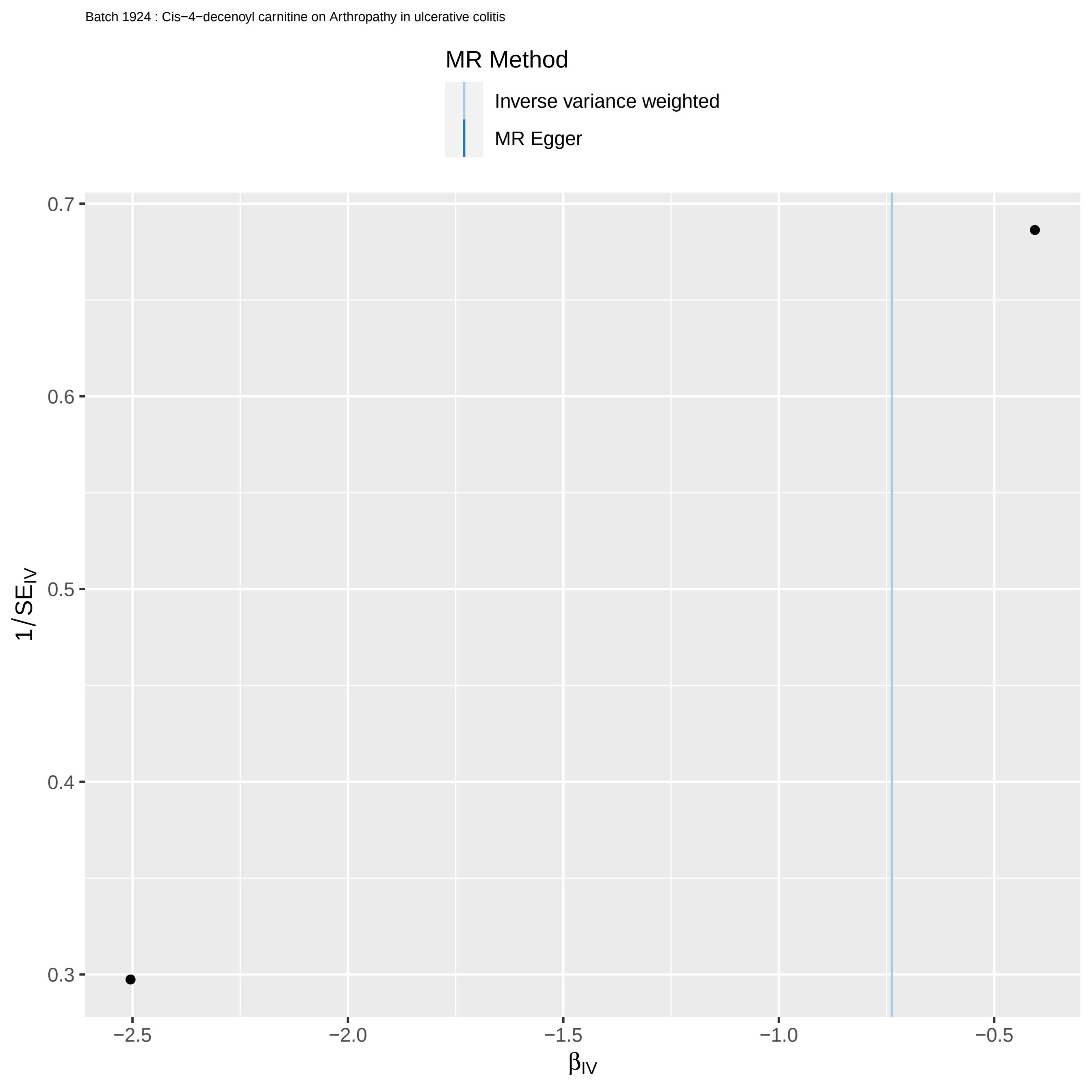

Batch 1924 : Cis−4−decenoyl carnitine on Arthropathy in ulcerative colitis
MR Method
Inverse variance weighted
MR Egger
0.7
0.6
0.5
0.4
0.3
1/SE_IV
−2.5
−2.0
−1.5
−1.0
−0.5
β_IV

Batch 1925 : 15−methylpalmitate (isobar with 2−methylpalmitate) on Arthropathy in ulcerative colitis

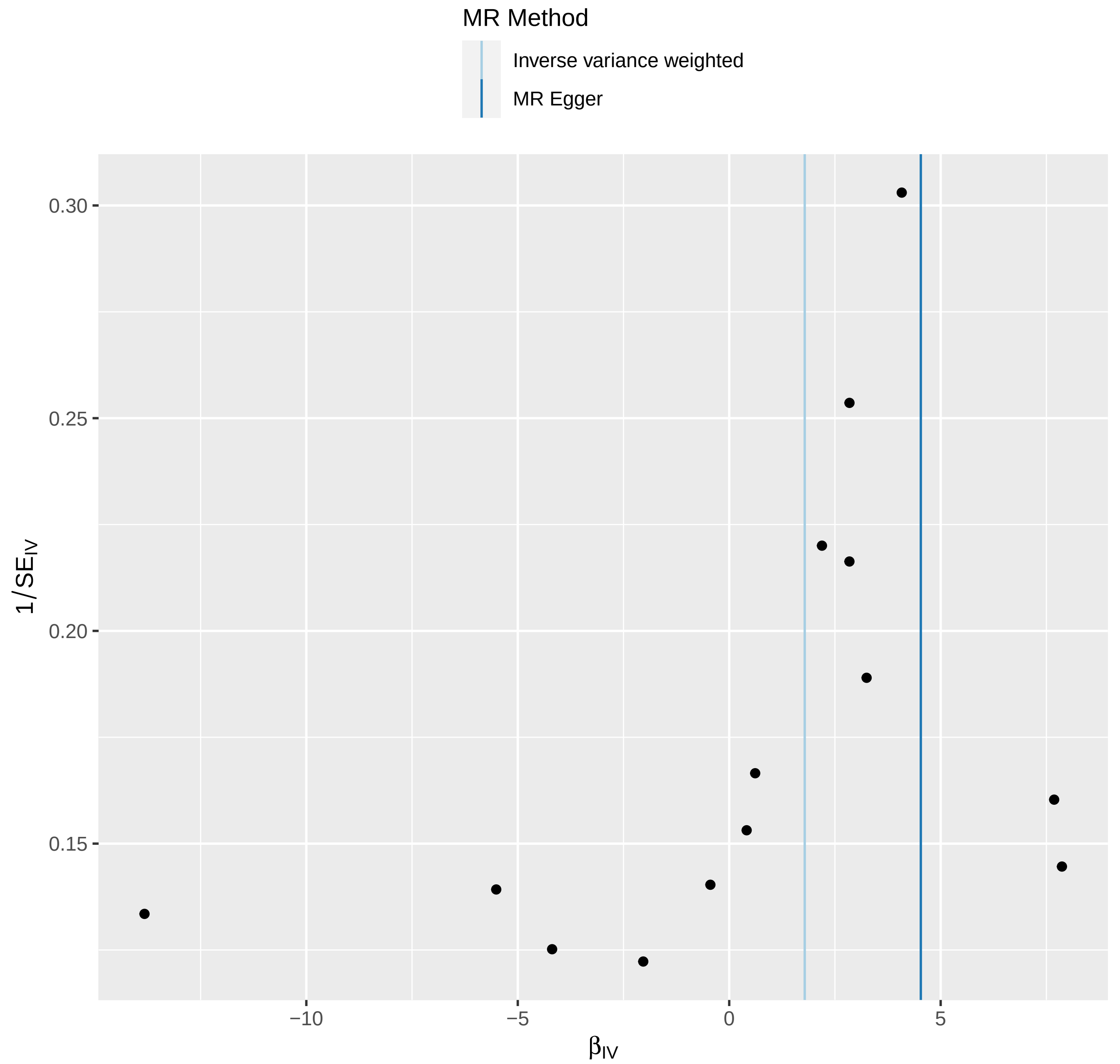

Batch 1927 : Tryptophan on Ulcerative pancolitis

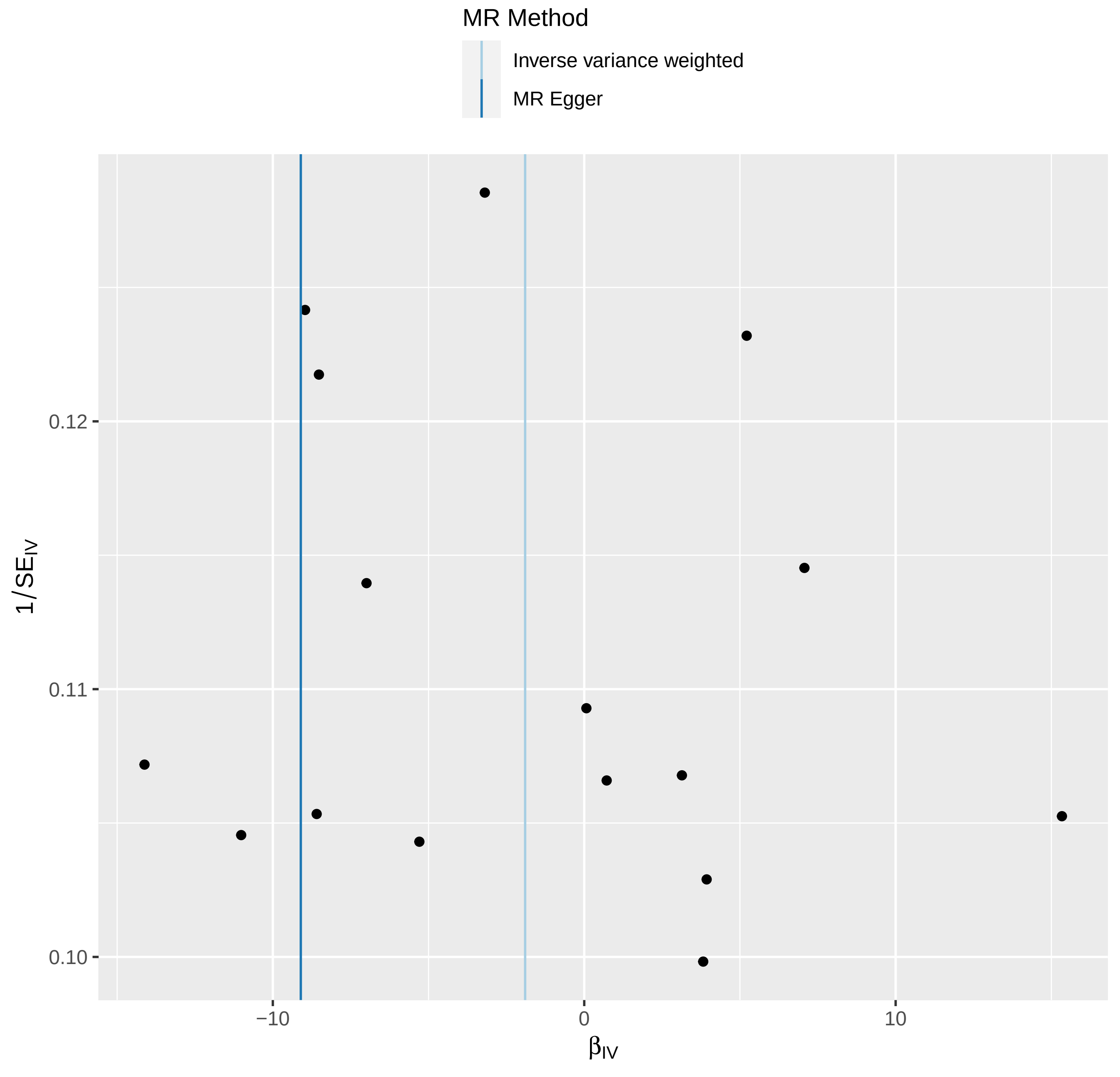

Batch 1929 : Leucine on Ulcerative pancolitis

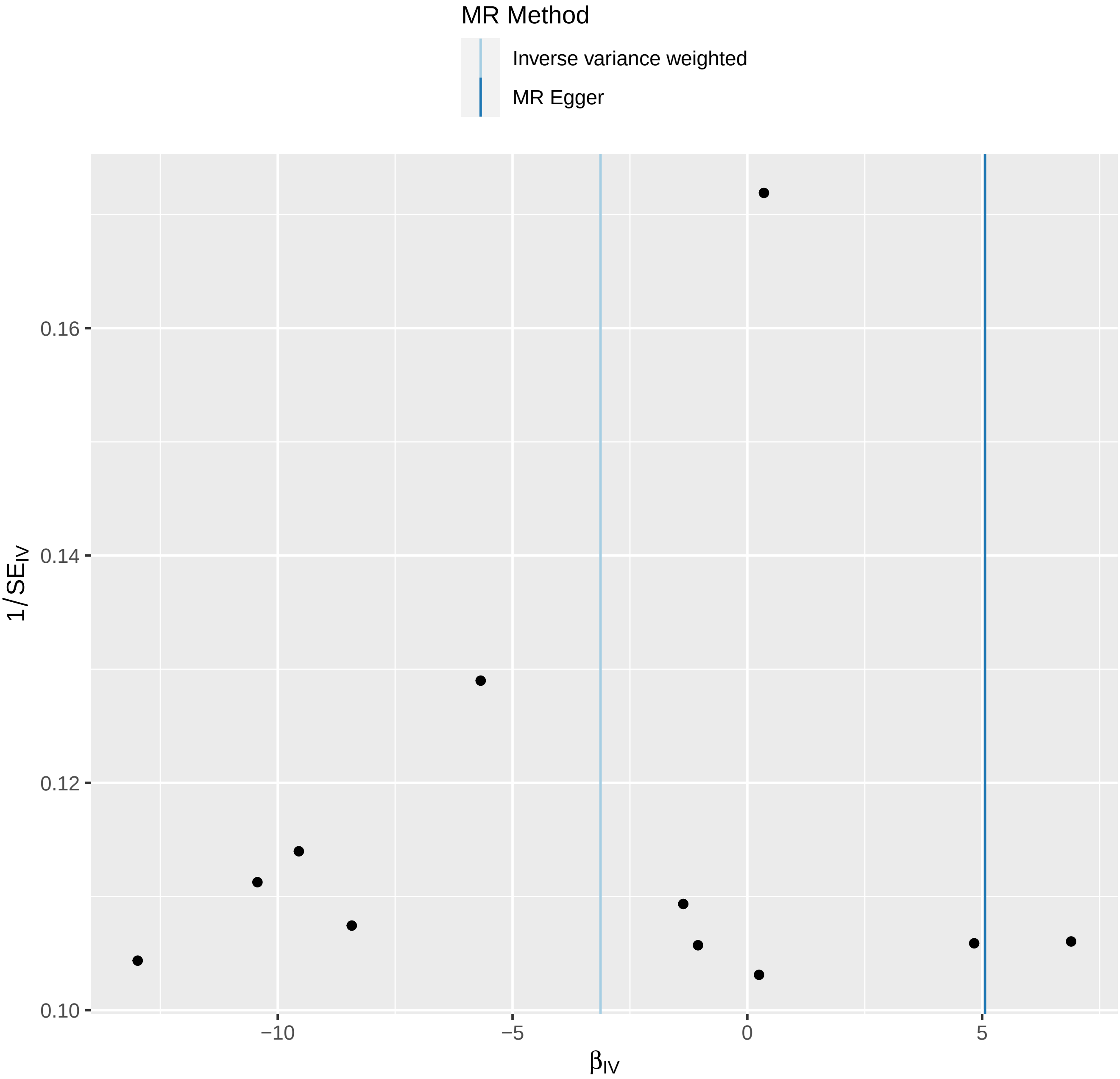

Batch 1933 : Lactate on Ulcerative pancolitis

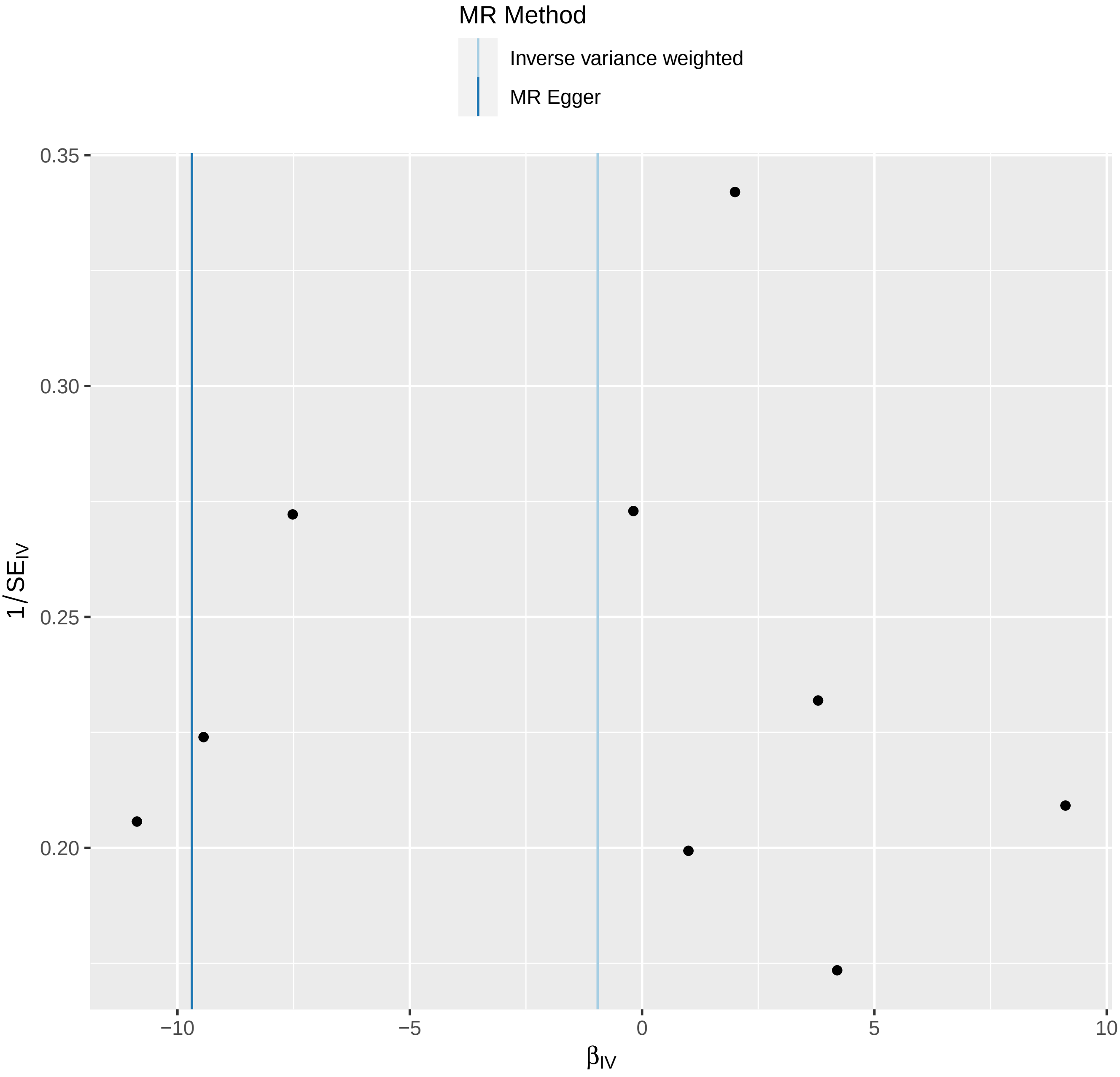

Batch 1934 : 3−hydroxybutyrate (BHBA) on Ulcerative pancolitis

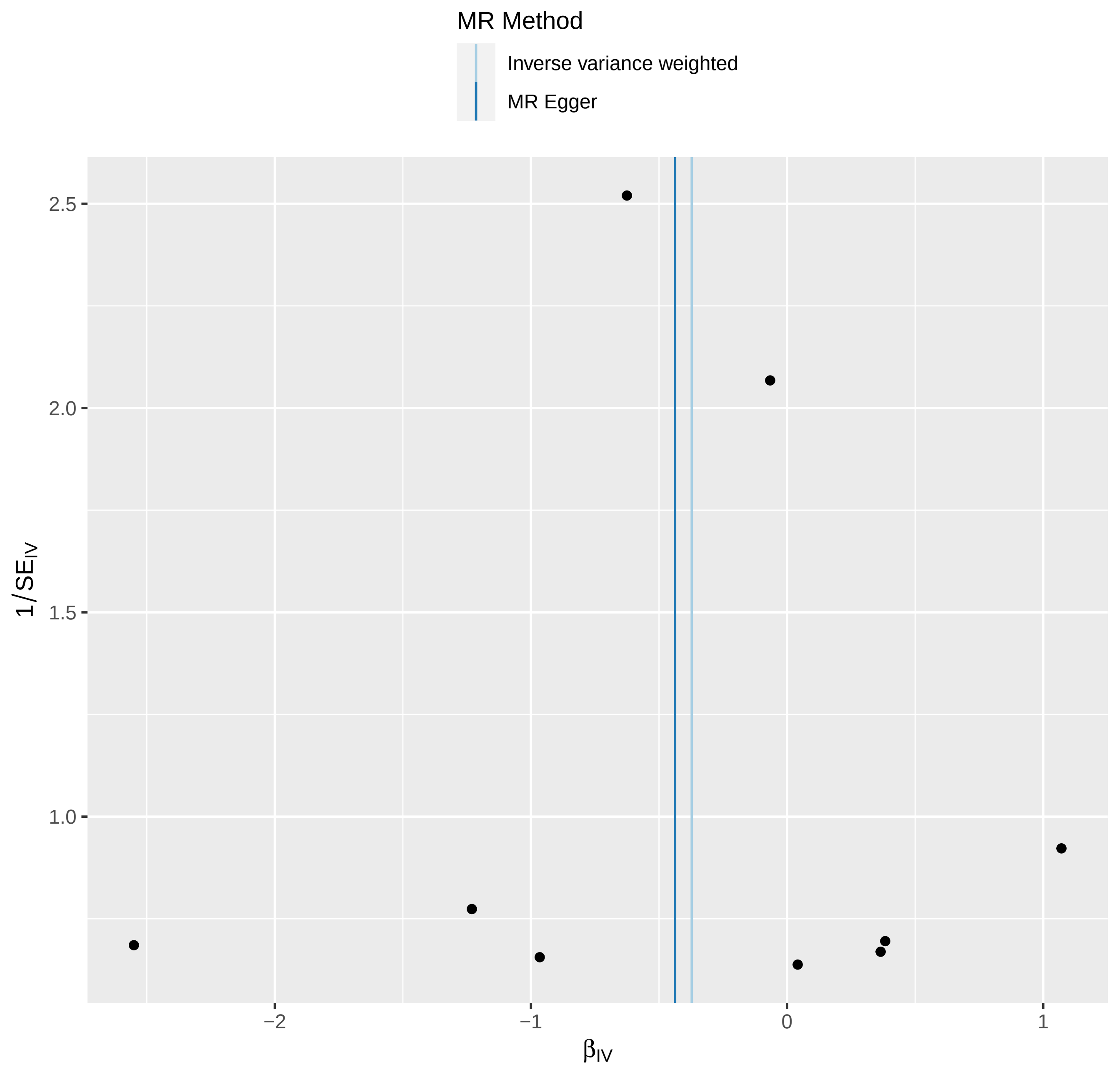

Batch 1935 : Arabinose on Ulcerative pancolitis

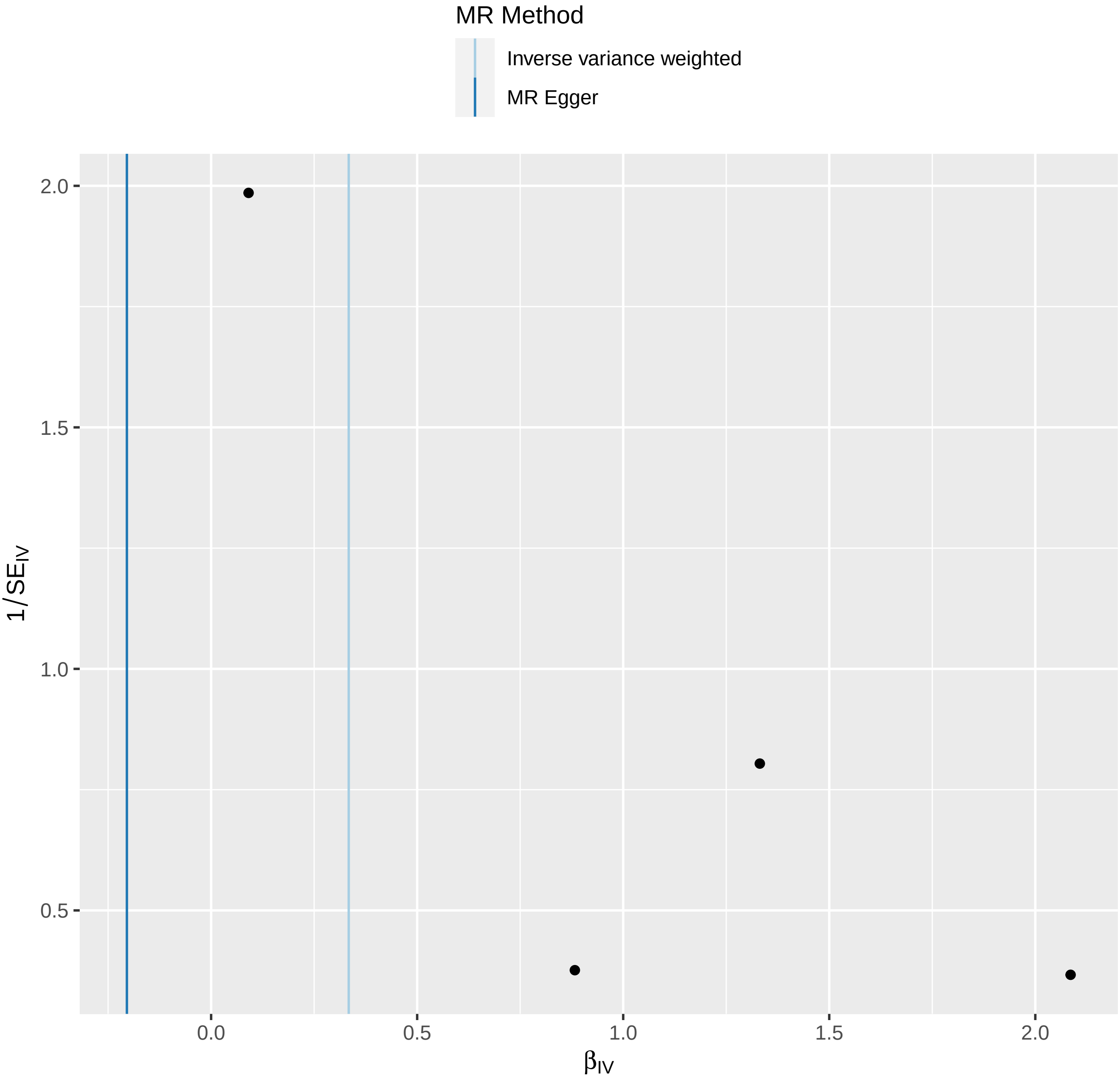

Batch 1936 : Fructose on Ulcerative pancolitis

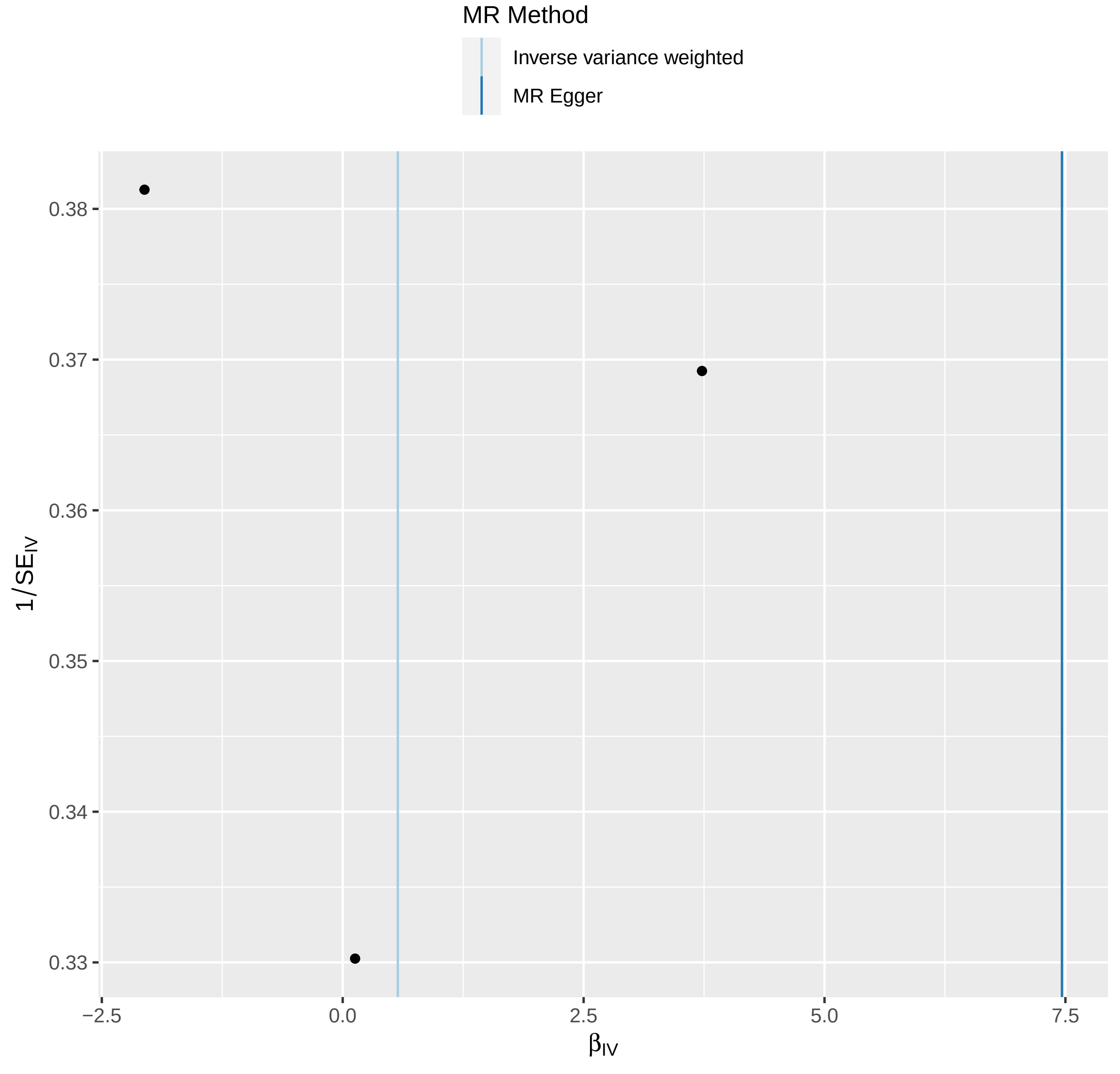

Batch 1938 : Pyruvate on Ulcerative pancolitis

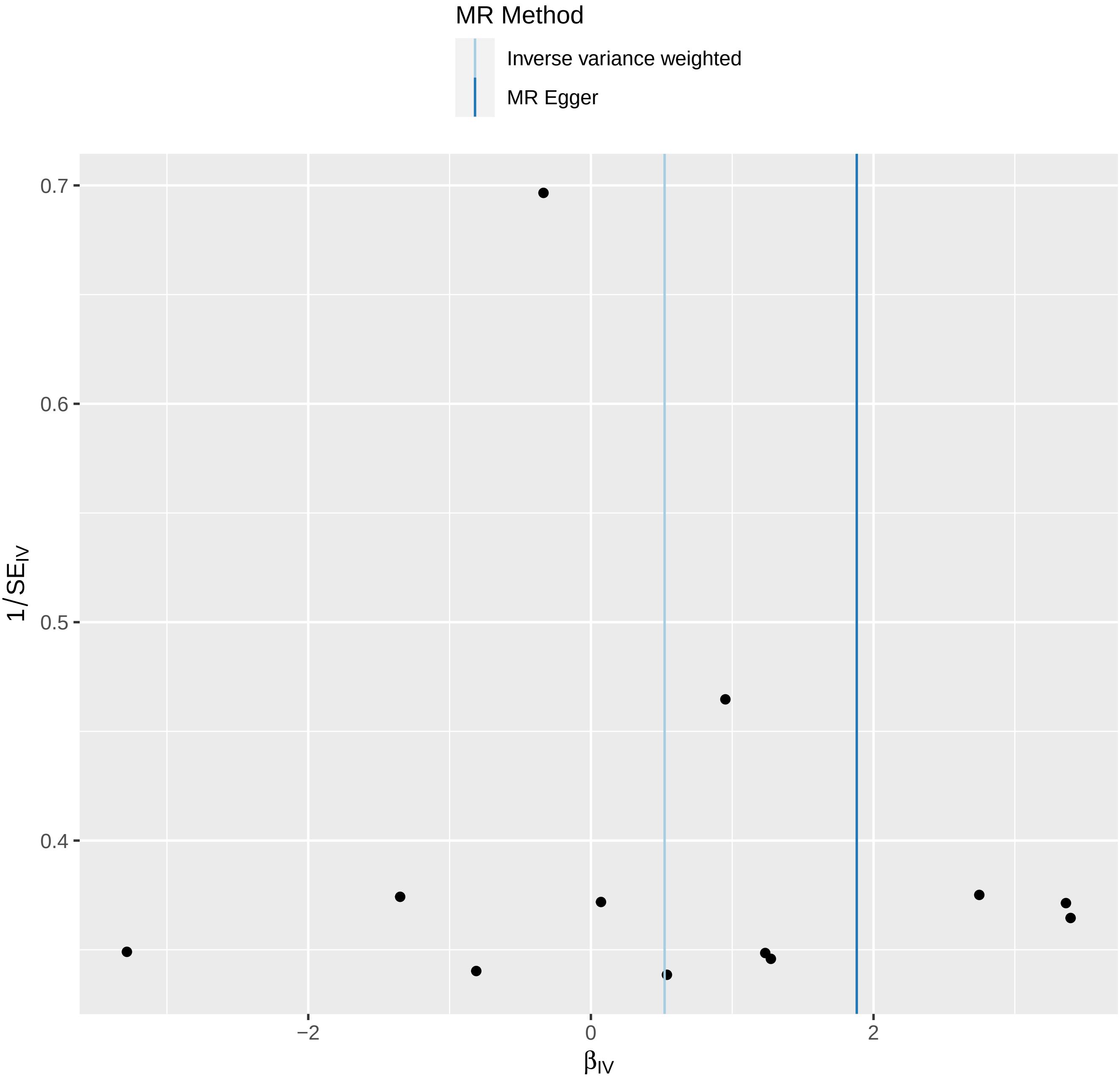

Batch 1939 : Uridine on Ulcerative pancolitis

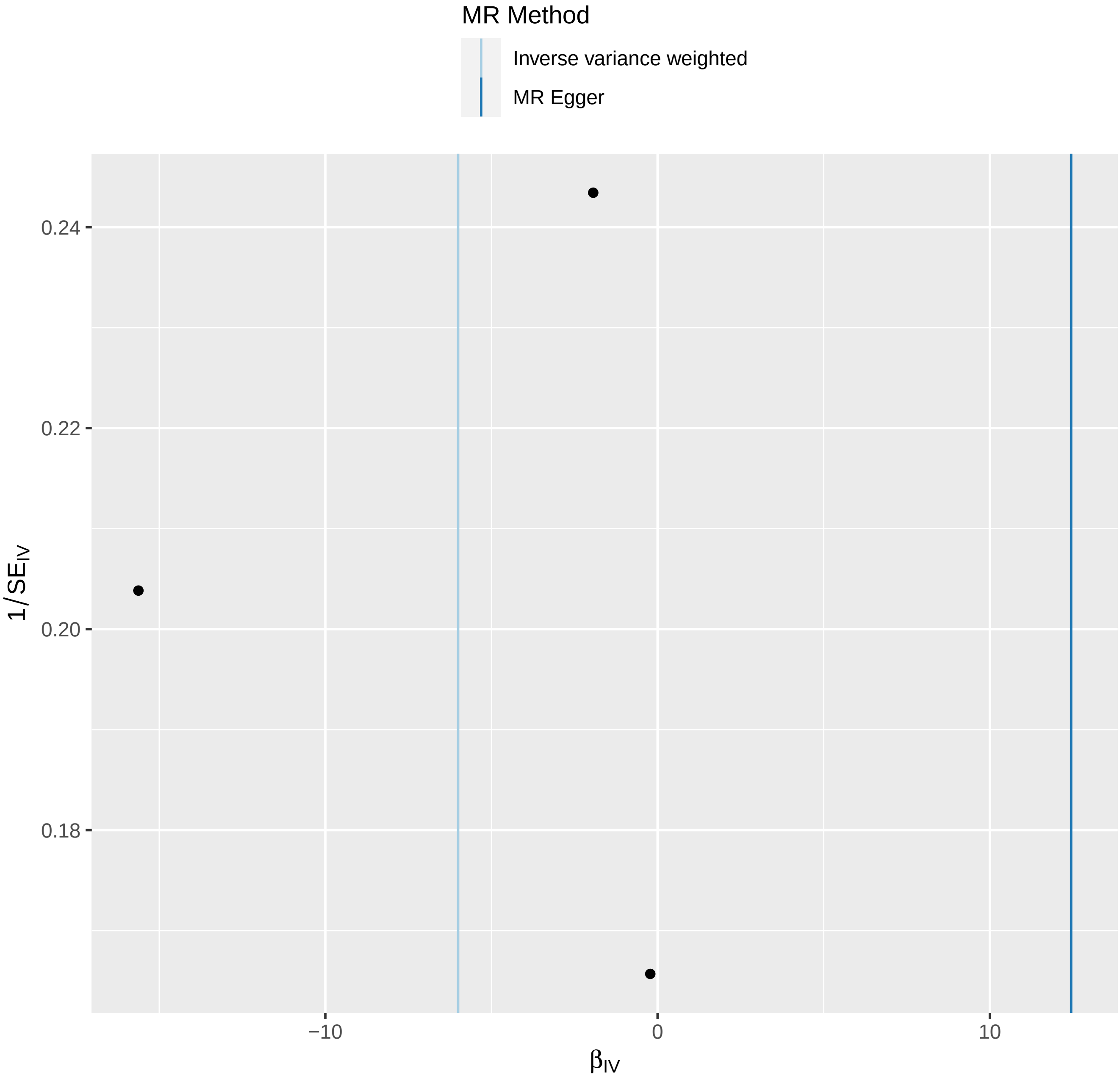

Batch 1940 : Linoleate (18:2n6) on Ulcerative pancolitis

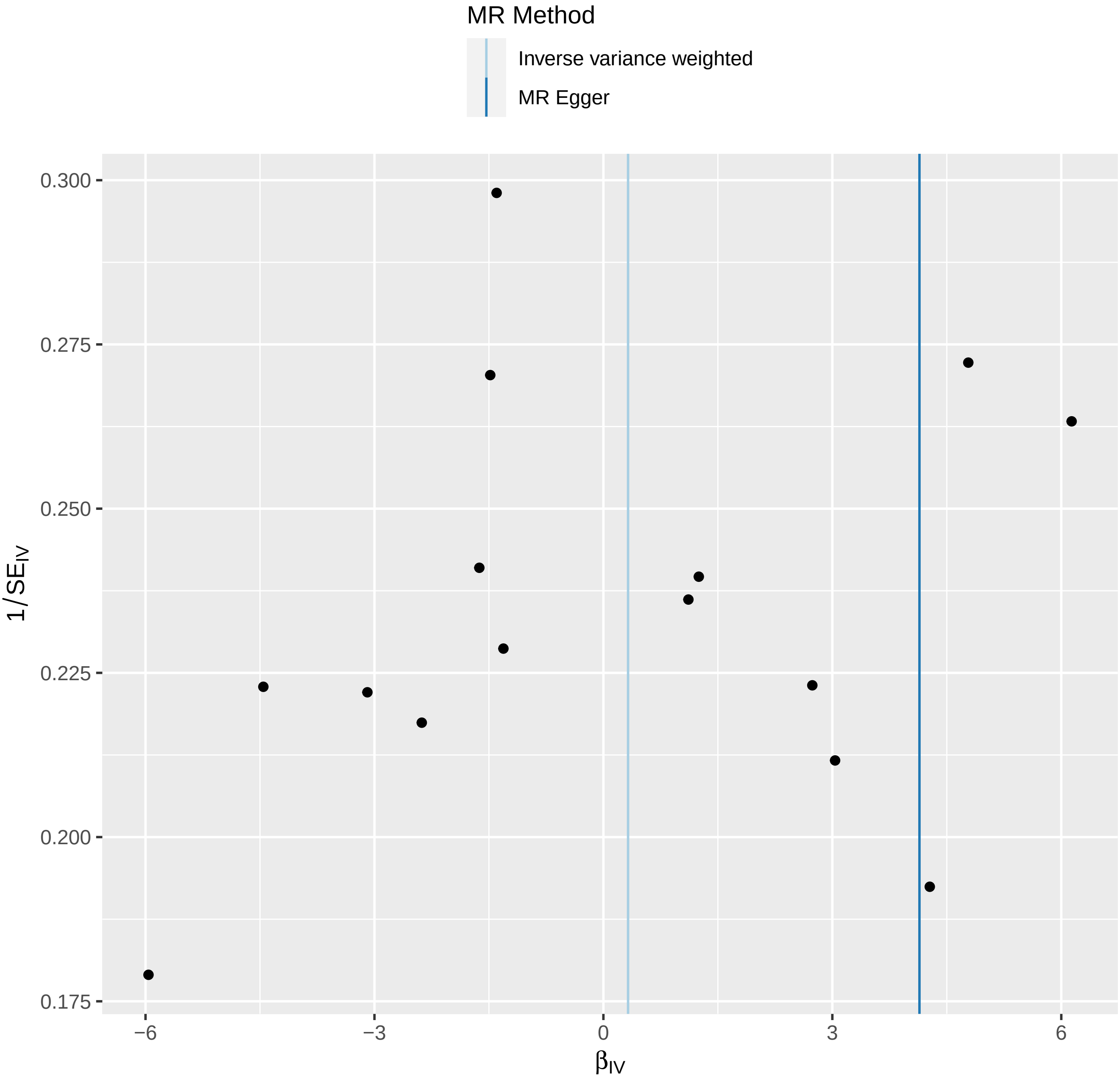

Batch 1941 : Allantoin on Ulcerative pancolitis

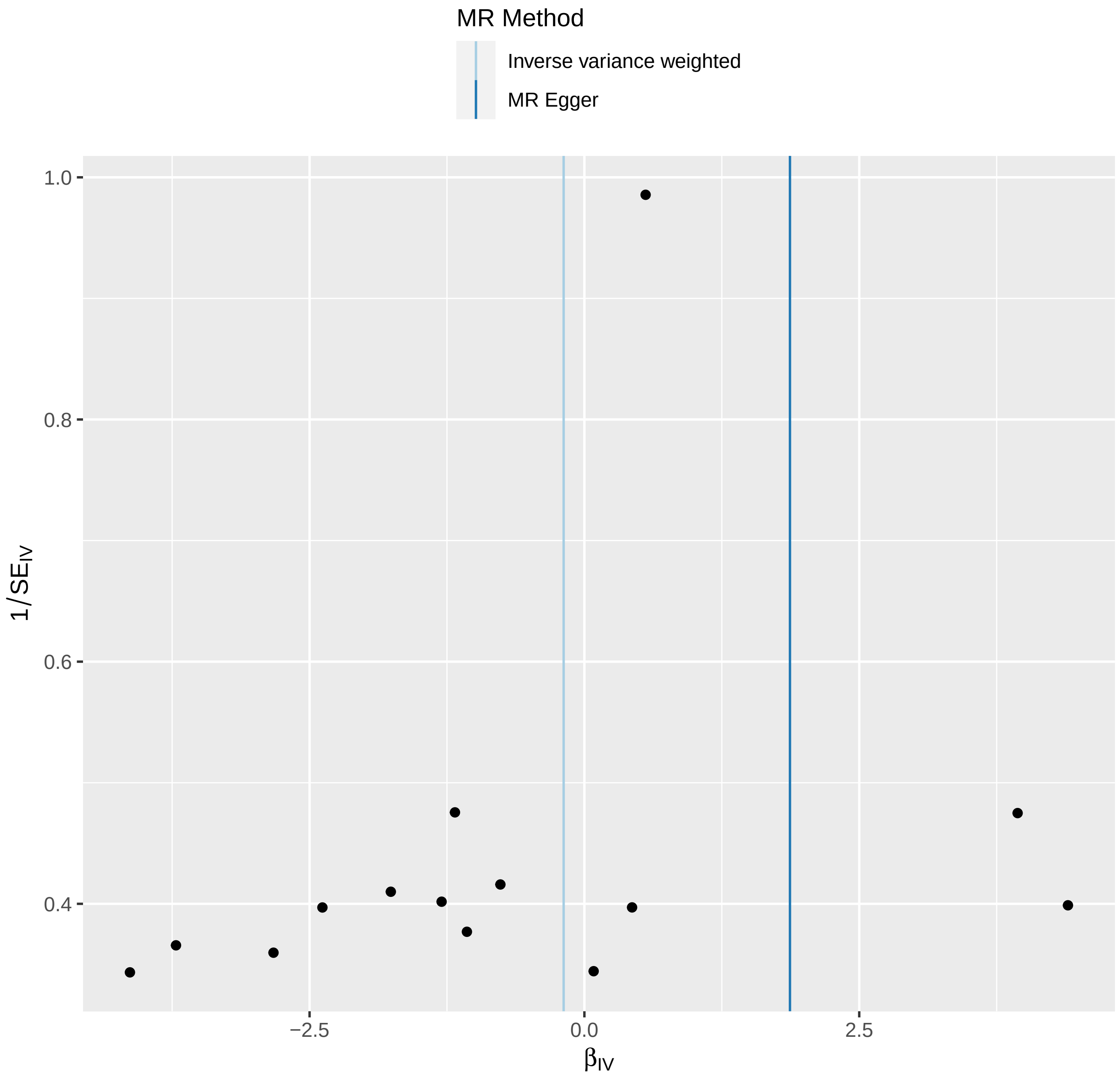

Batch 1946 : Isoleucine on Ulcerative pancolitis

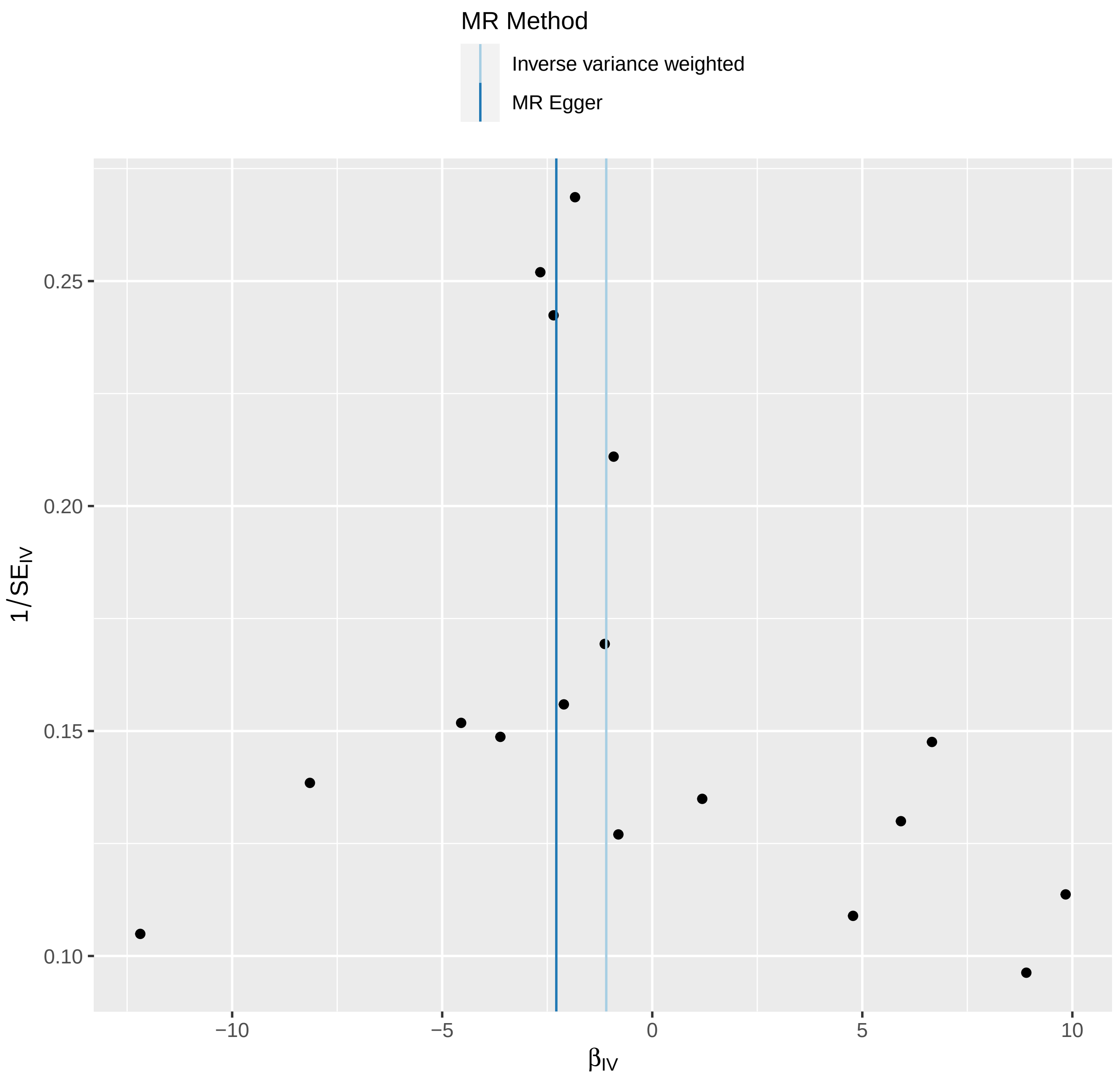

Batch 1947 : Threonine on Ulcerative pancolitis

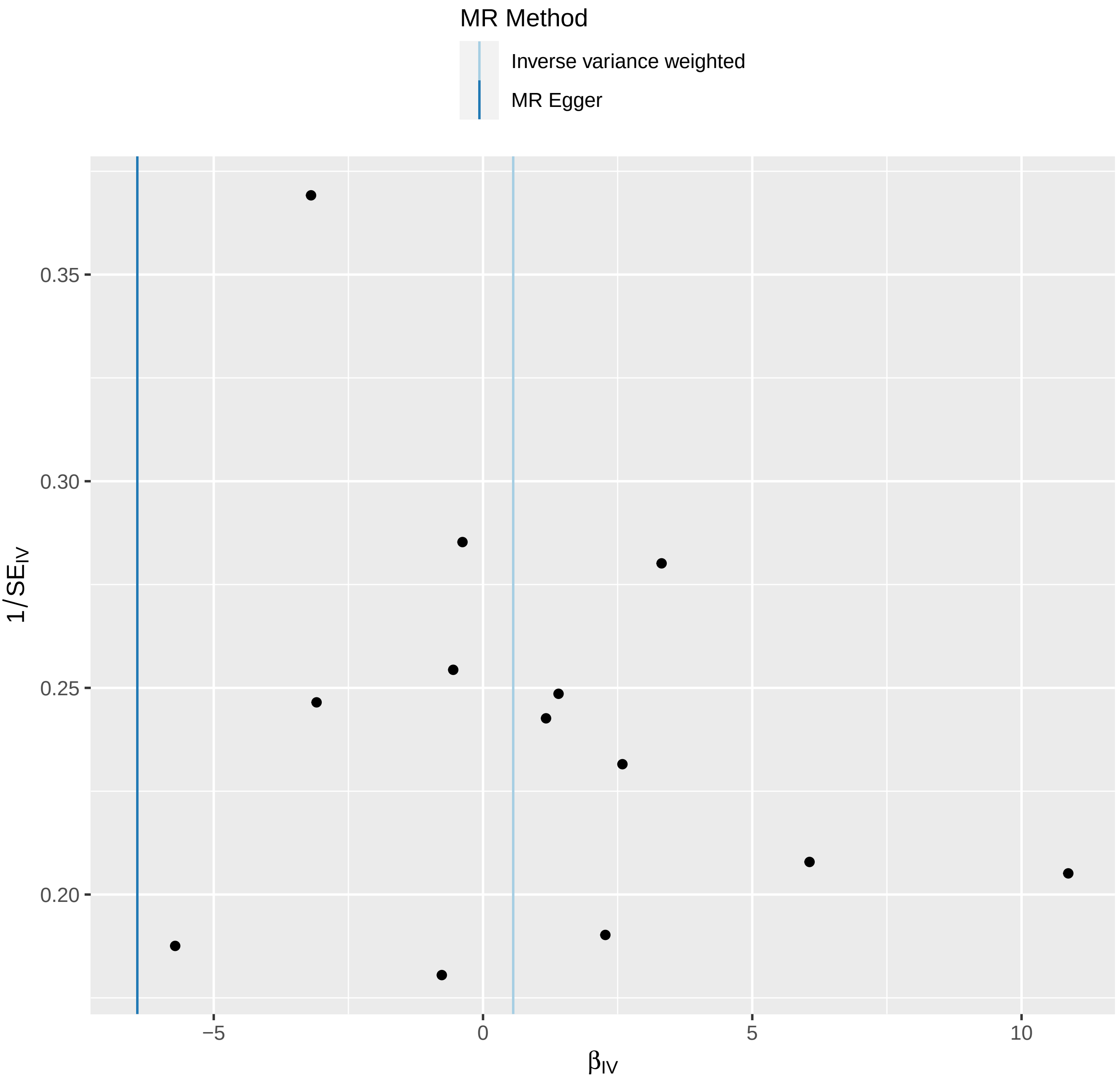

Batch 1948 : Tyrosine on Ulcerative pancolitis

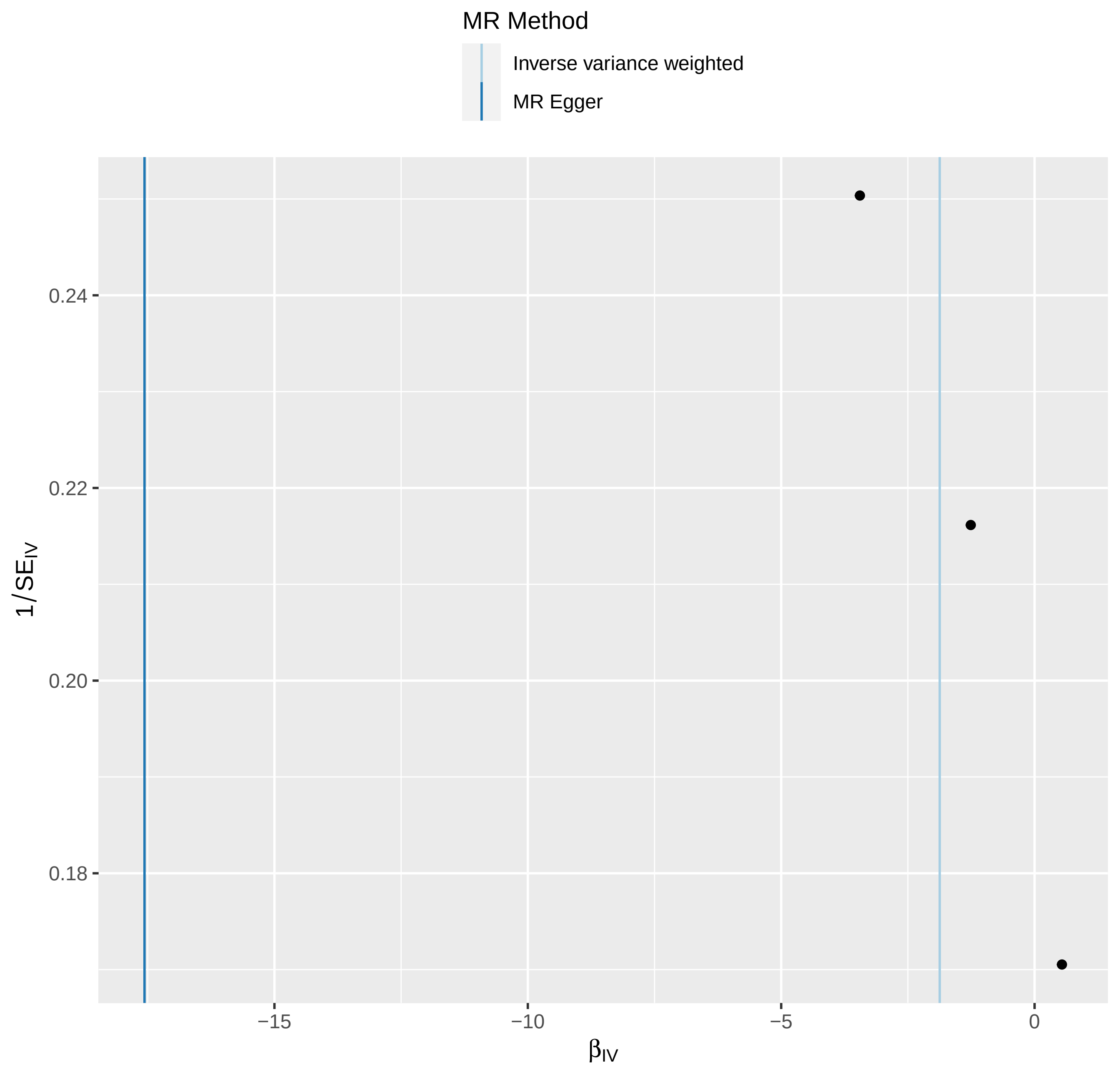

Batch 1954 : Stearate (18:0) on Ulcerative pancolitis

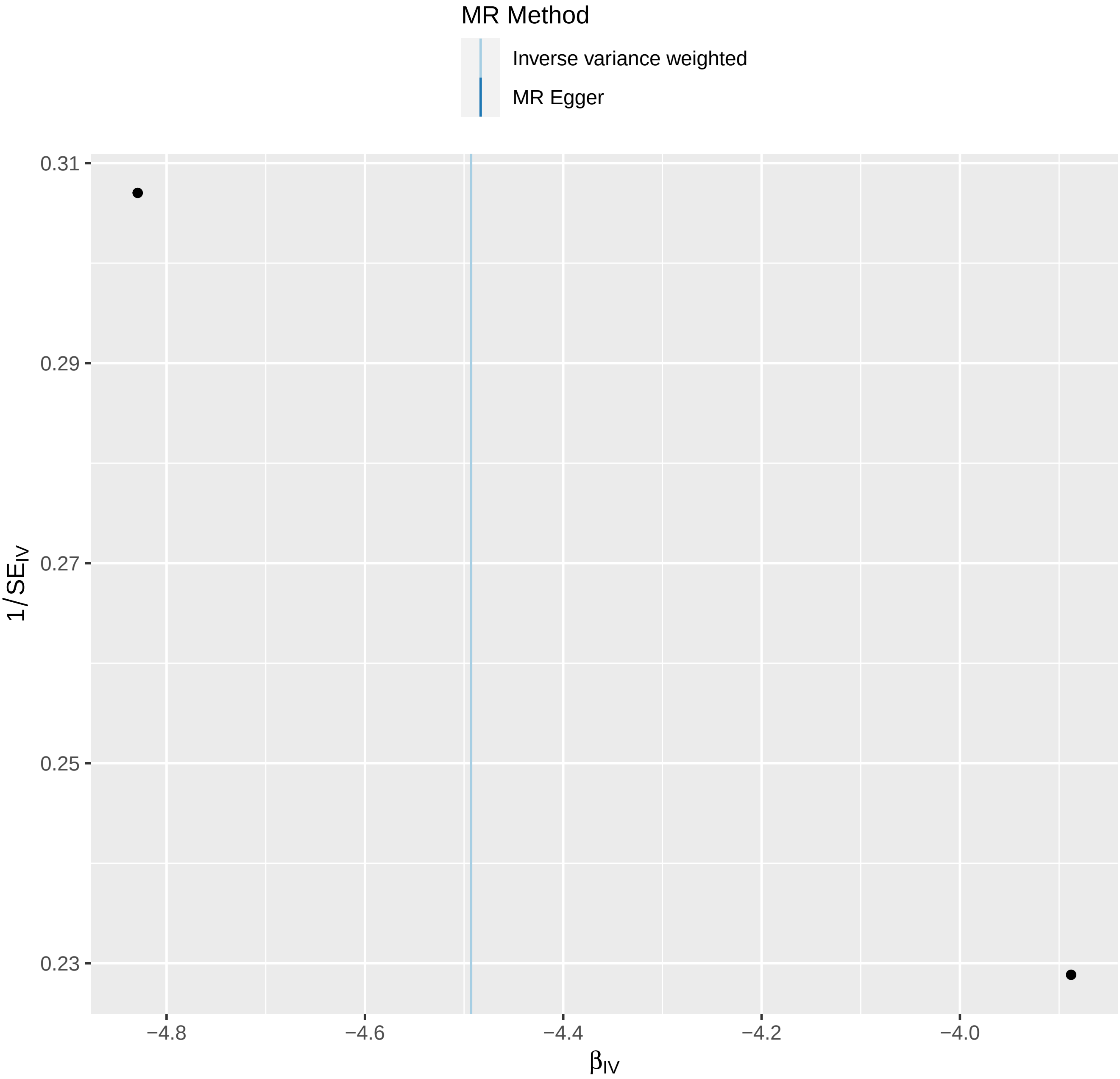

Batch 1957 : Myristate (14:0) on Ulcerative pancolitis

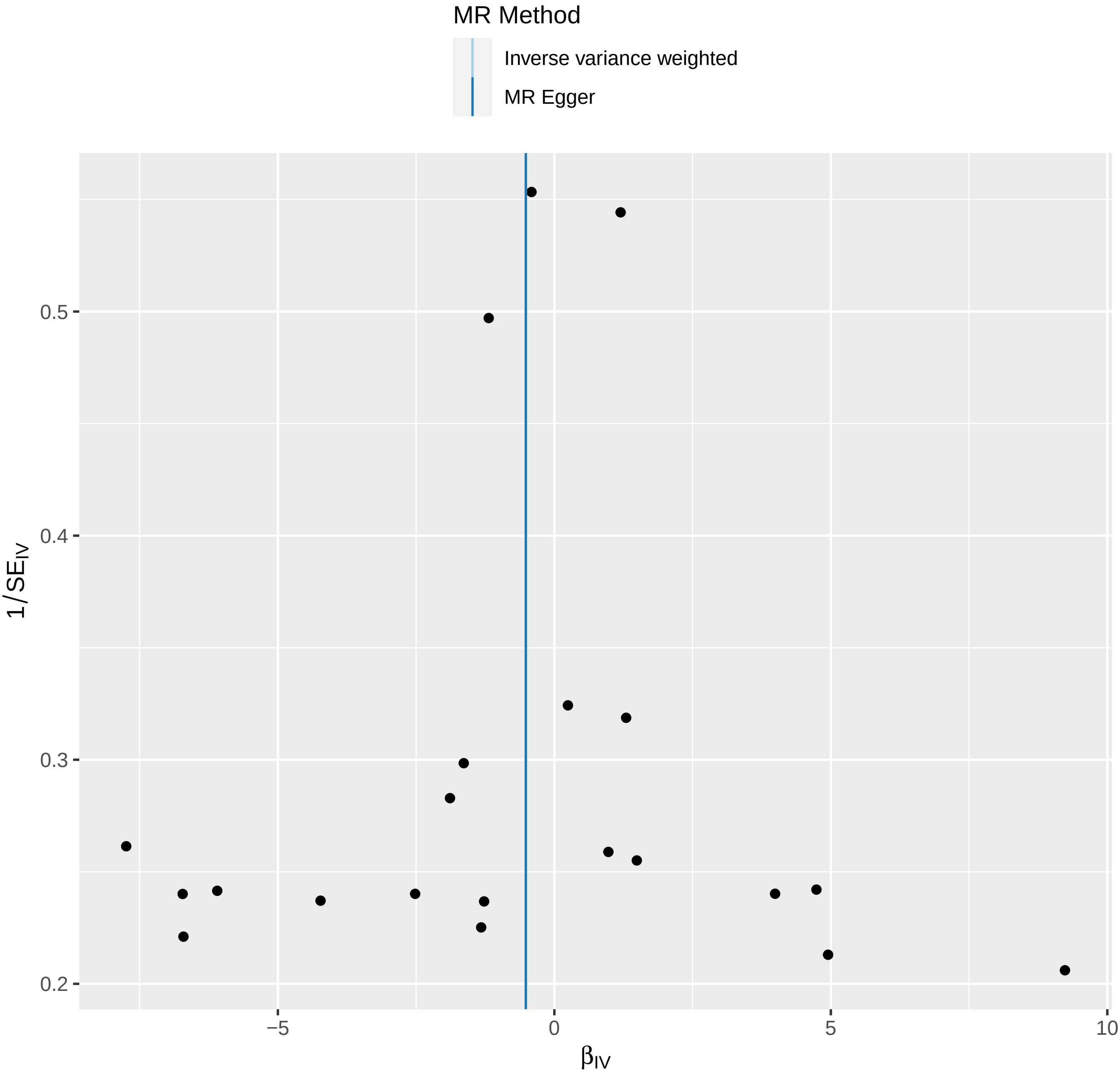

Batch 1959 : Ornithine on Ulcerative pancolitis

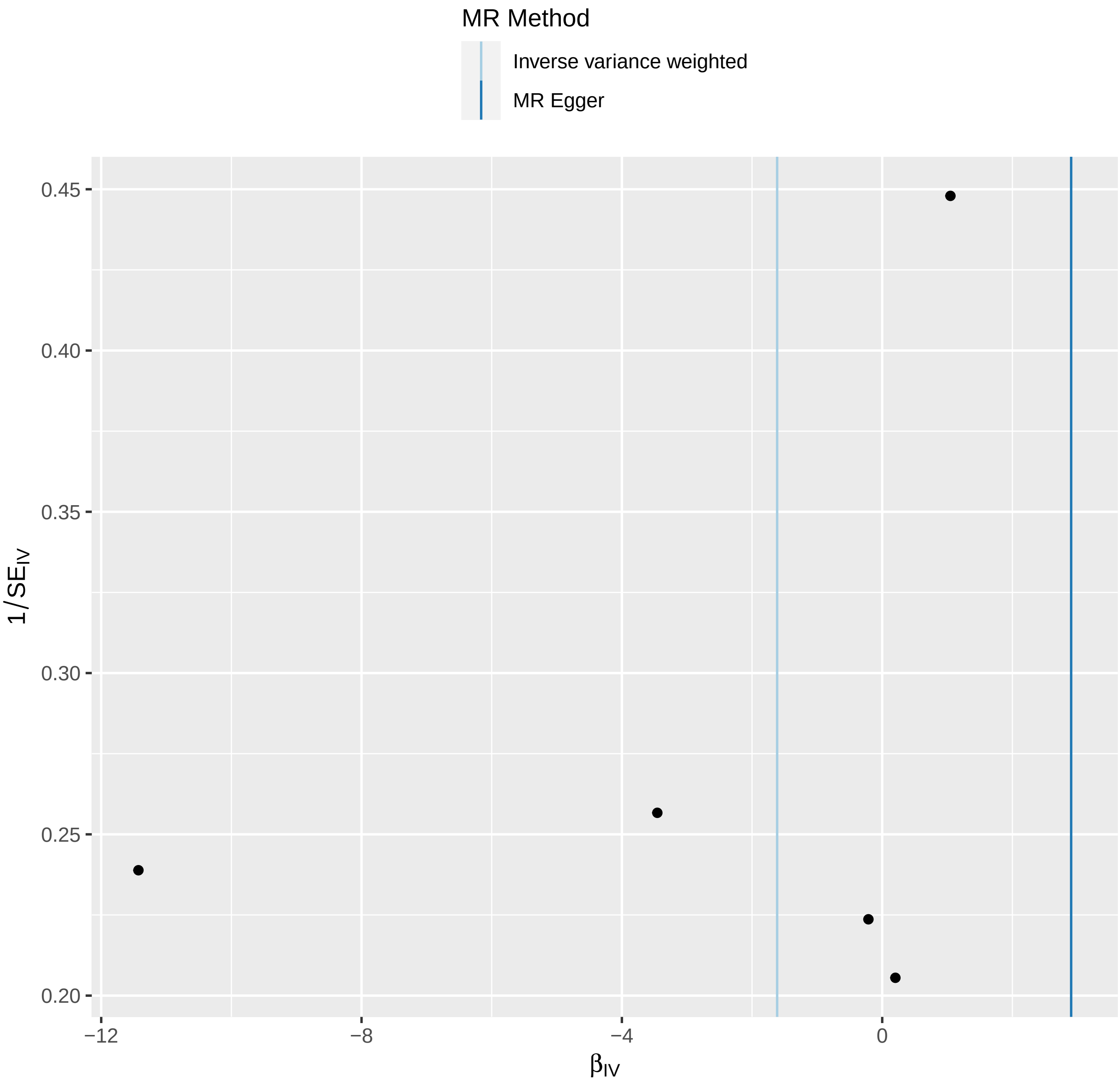

Batch 1962 : 4−acetamidobutanoate on Ulcerative pancolitis

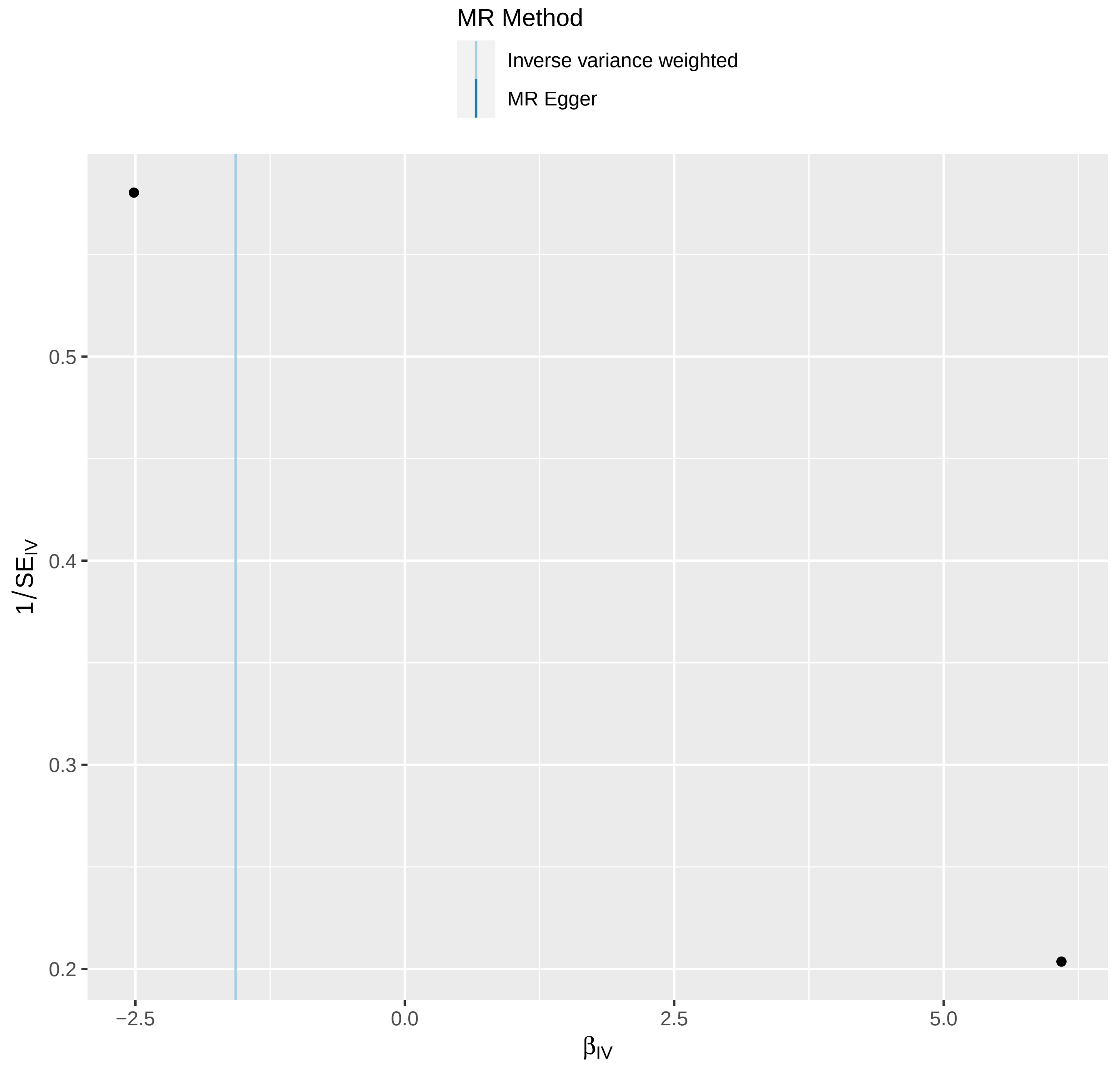

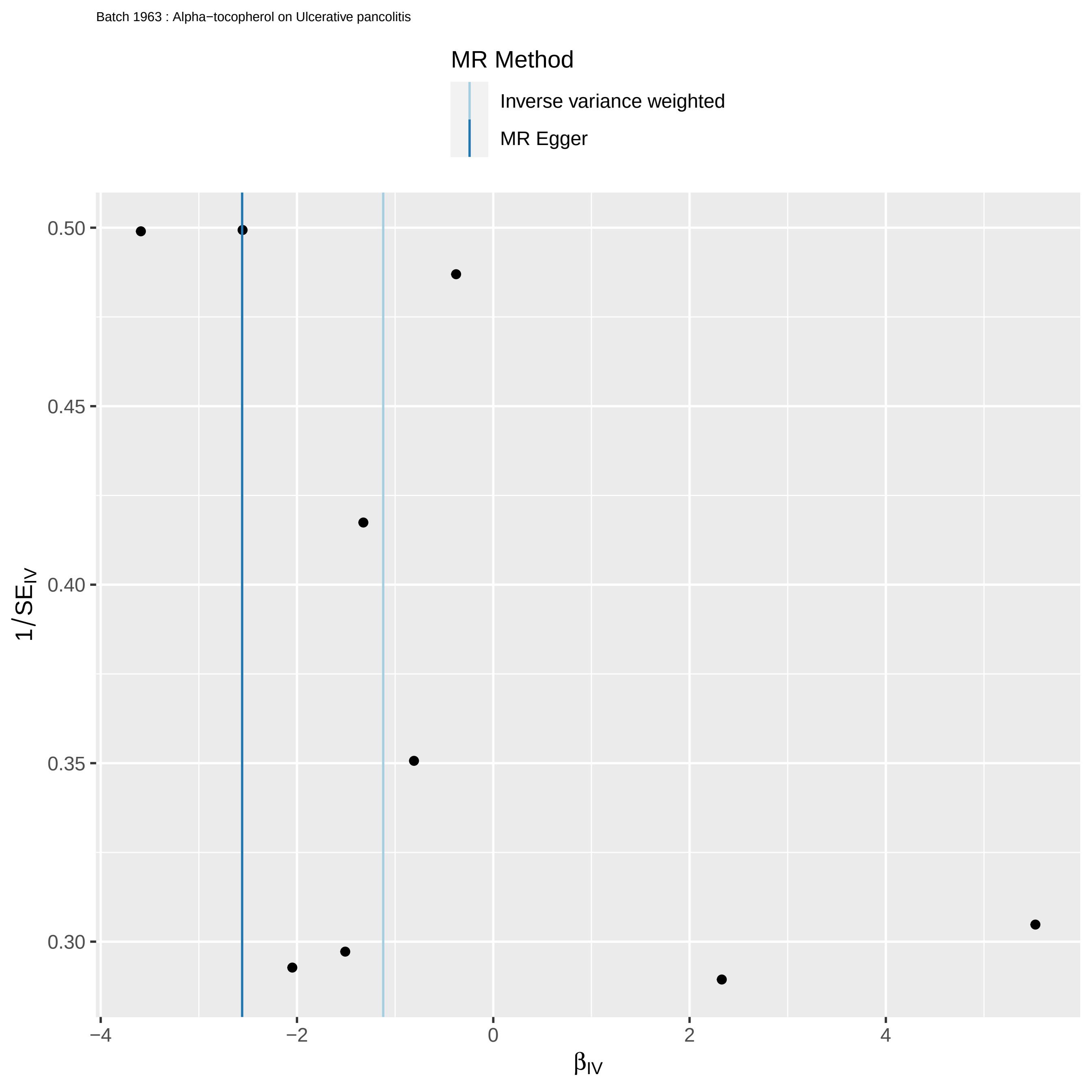

Batch 1963 : Alpha−tocopherol on Ulcerative pancolitis
MR Method
Inverse variance weighted
MR Egger
0.50
0.45
0.40
0.35
0.30
1/SE_IV
−4
−2
0
2
4
β_IV

Batch 1964 : Citrate on Ulcerative pancolitis

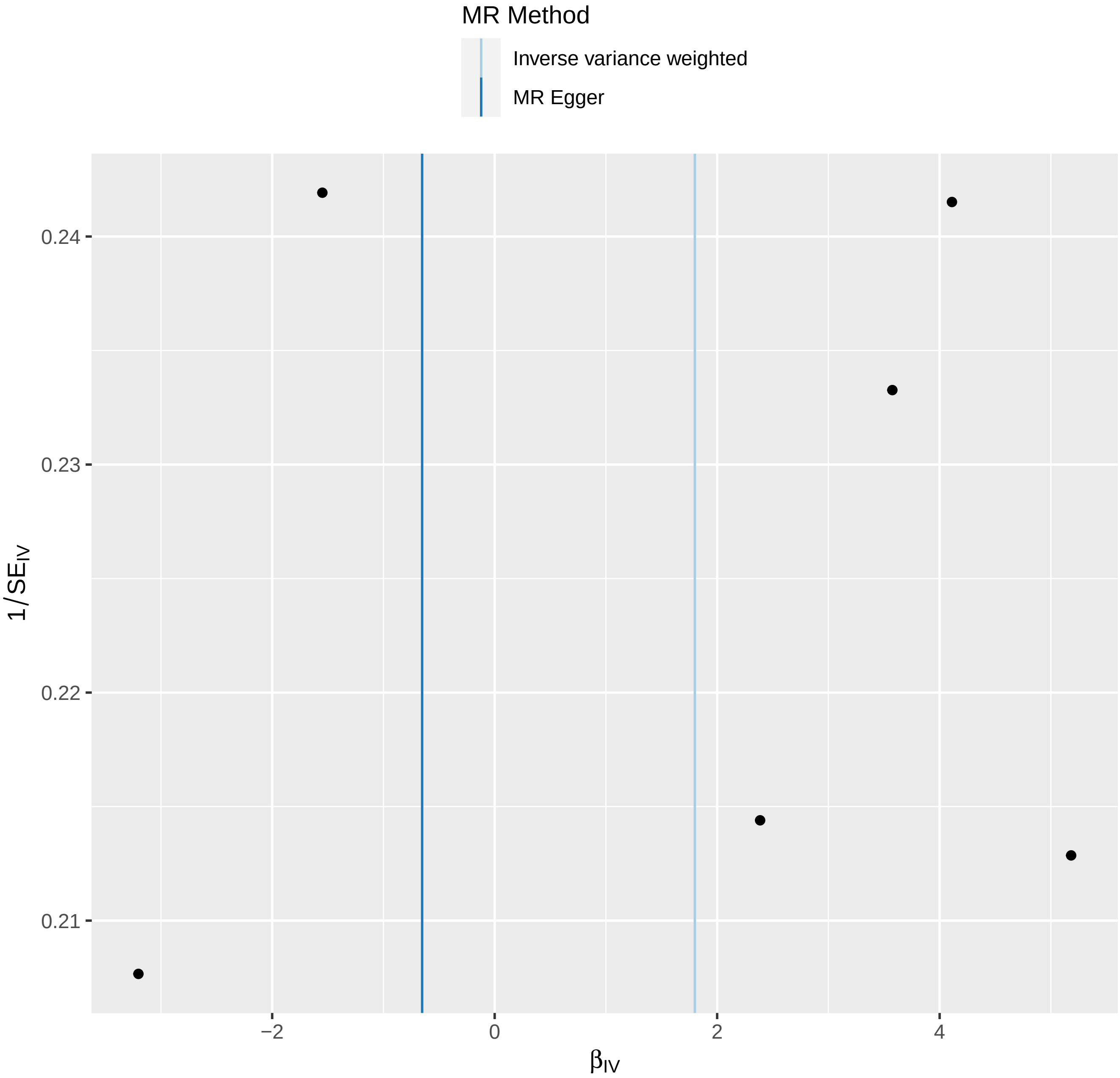

Batch 1965 : Glycerate on Ulcerative pancolitis

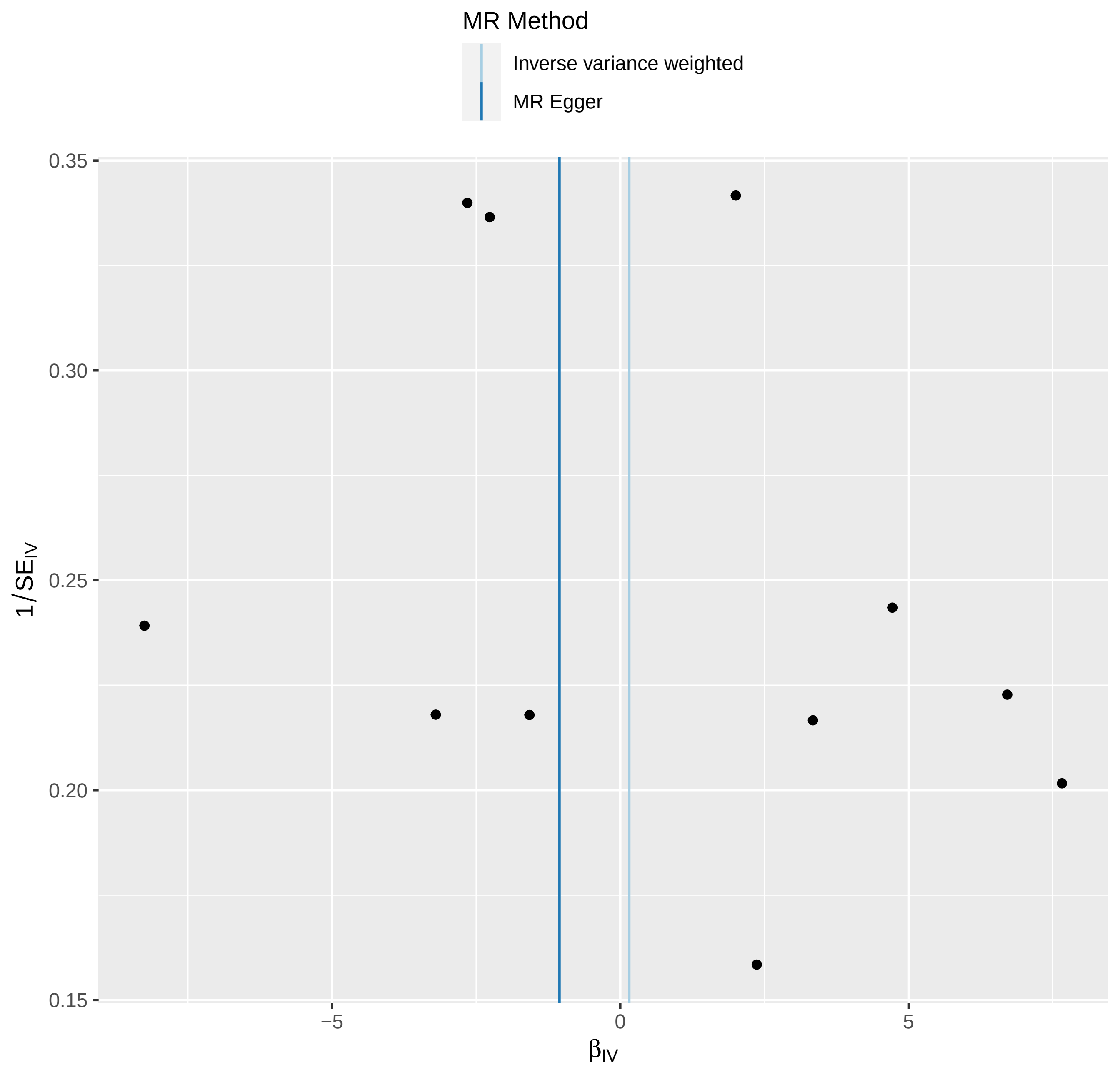

Batch 1966 : Guanosine on Ulcerative pancolitis

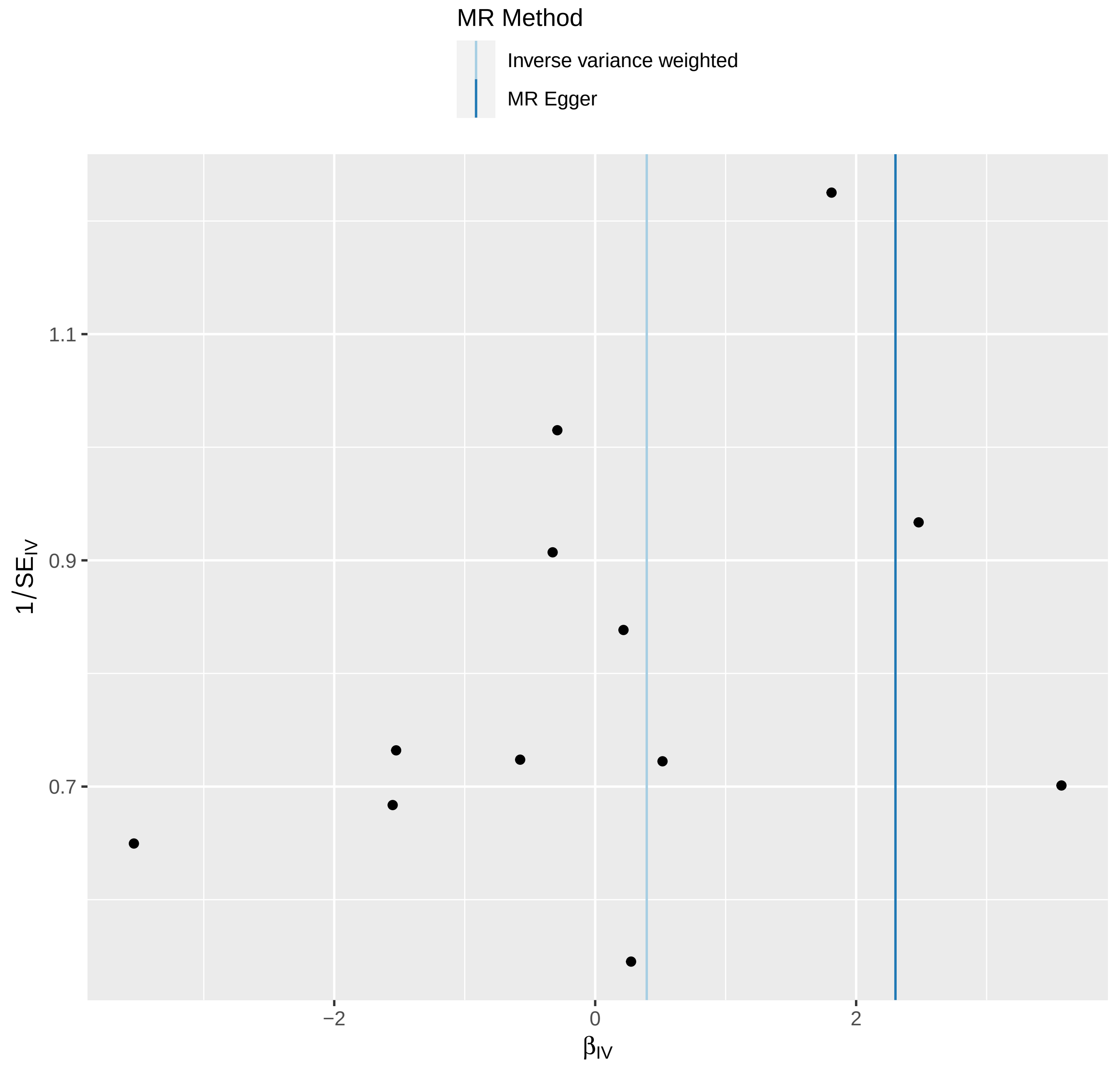

Batch 1969 : Ursodeoxycholate on Ulcerative pancolitis

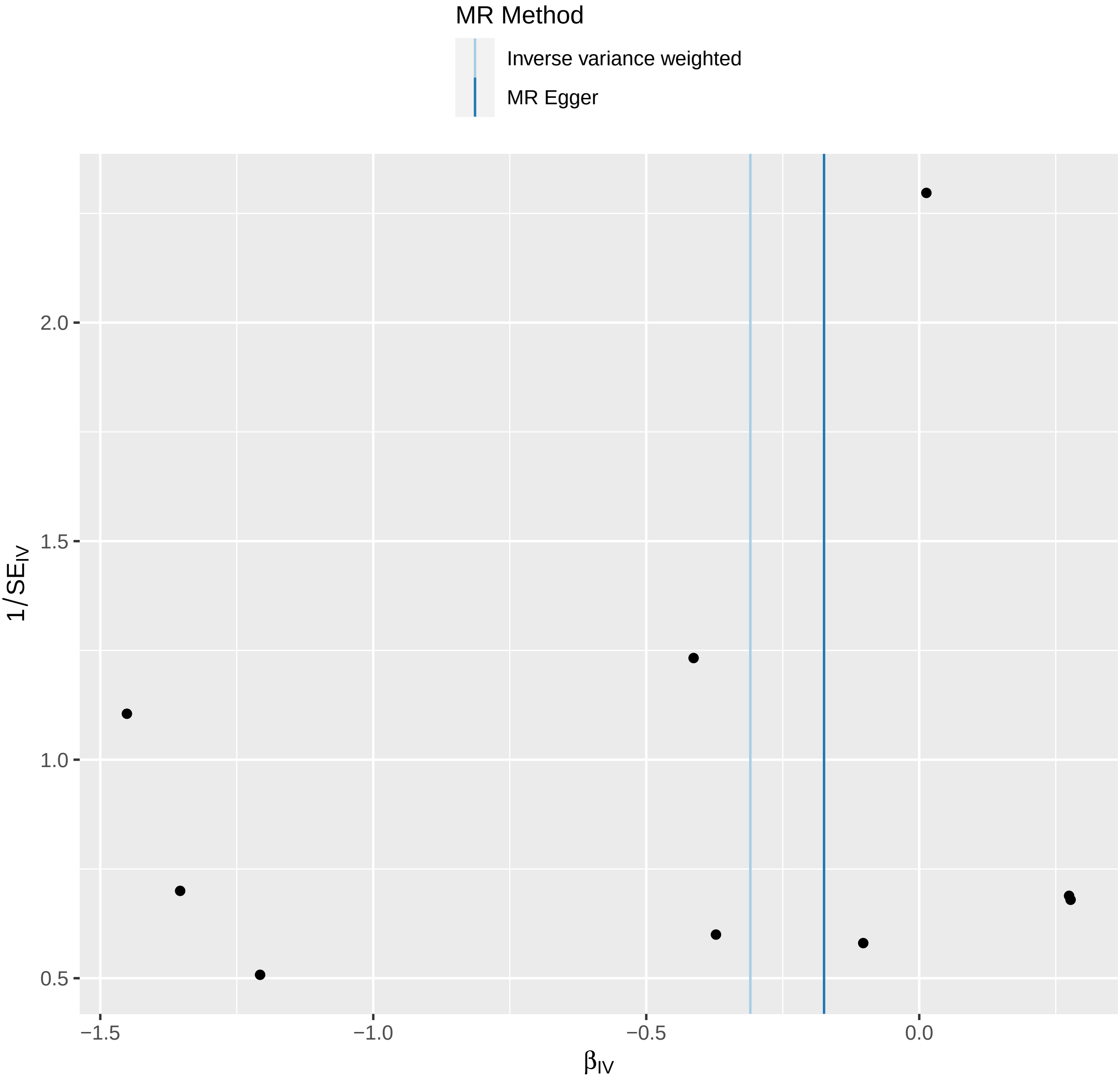

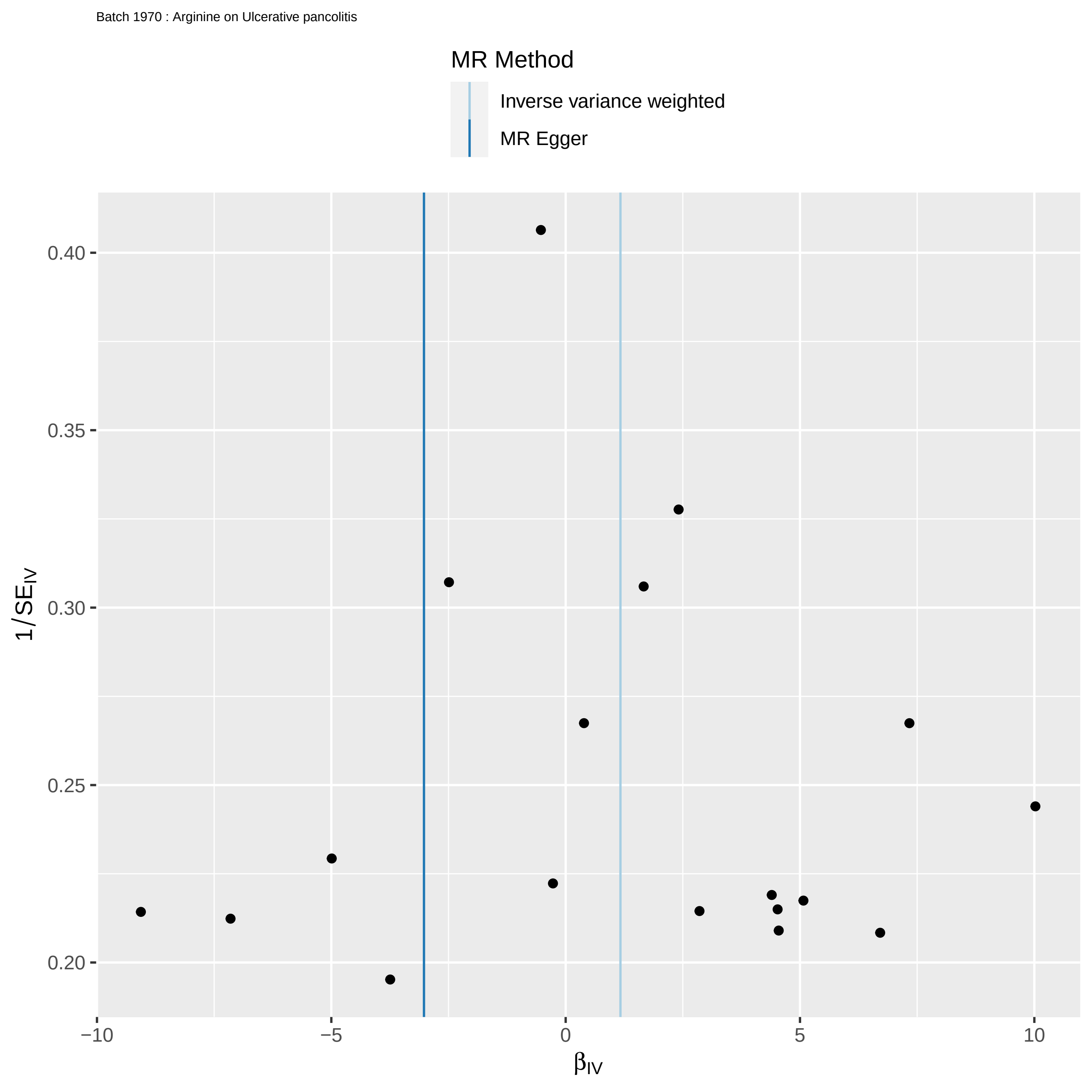
Batch 1970 : Arginine on Ulcerative pancolitis
MR Method
Inverse variance weighted
MR Egger
0.40
0.35
0.30
0.25
0.20
1/SE_IV
−10
−5
0
5
10
β_IV

Batch 1971 : Ascorbate (Vitamin C) on Ulcerative pancolitis

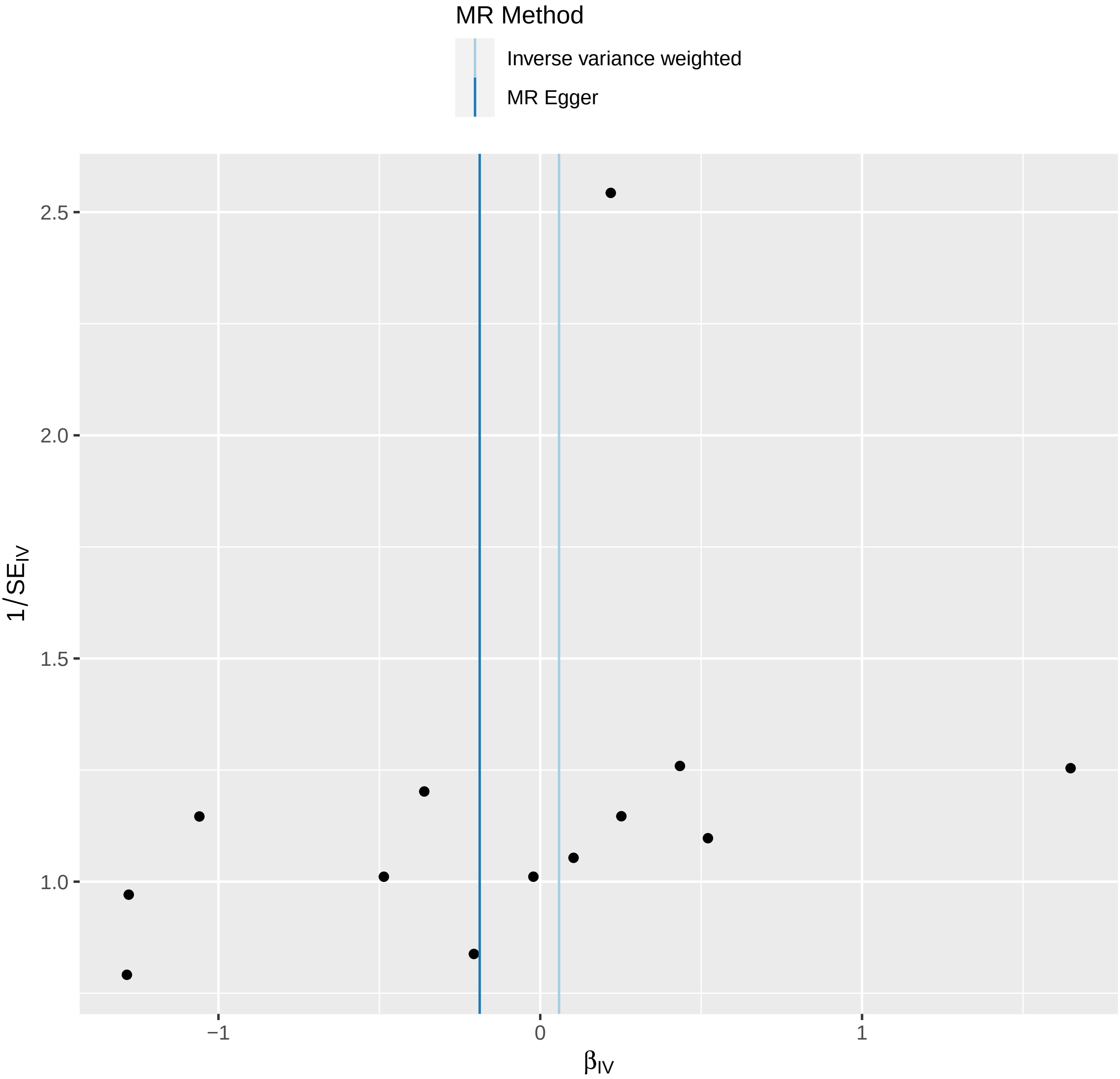

Batch 1972 : Heptanoate (7:0) on Ulcerative pancolitis

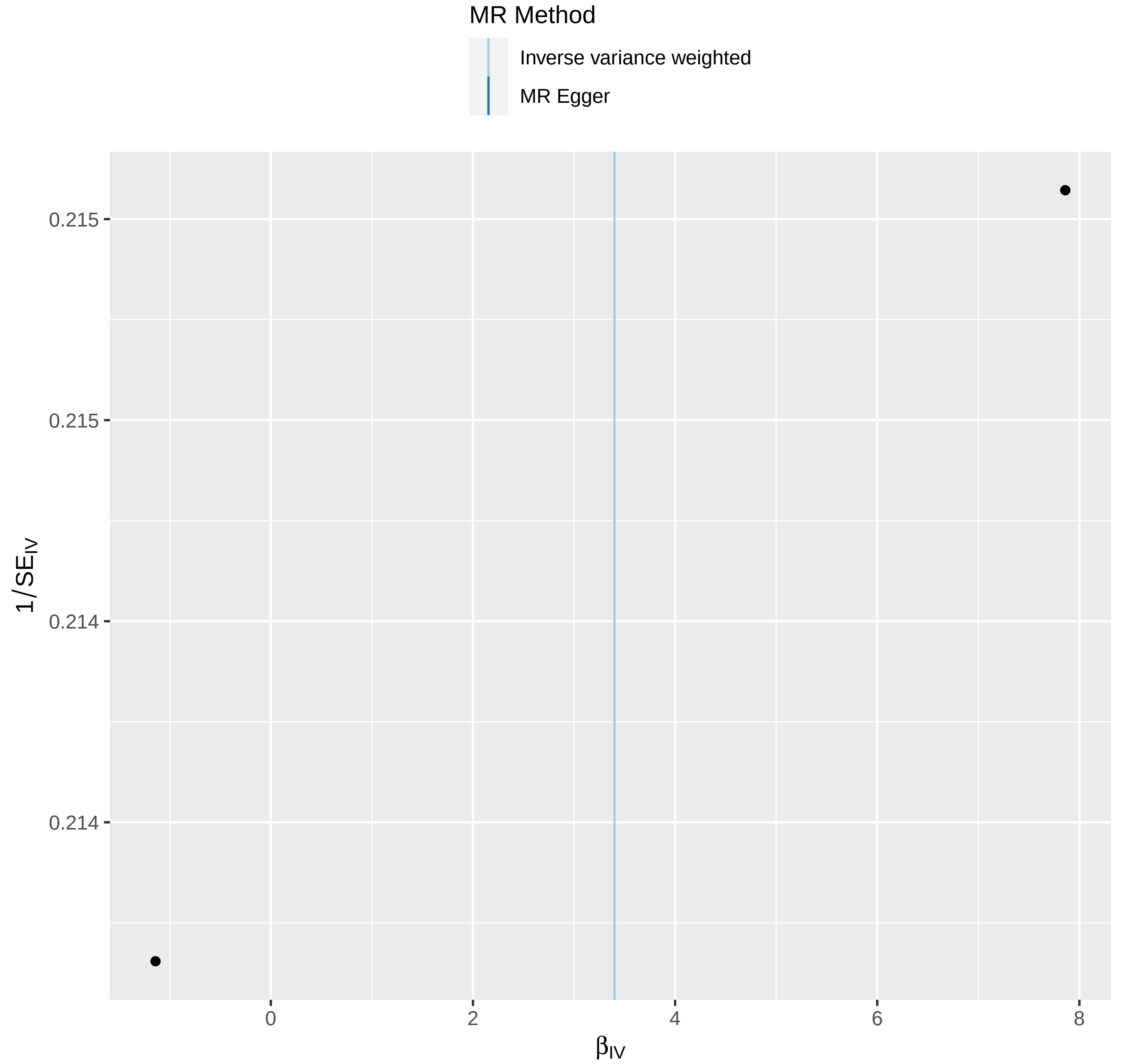

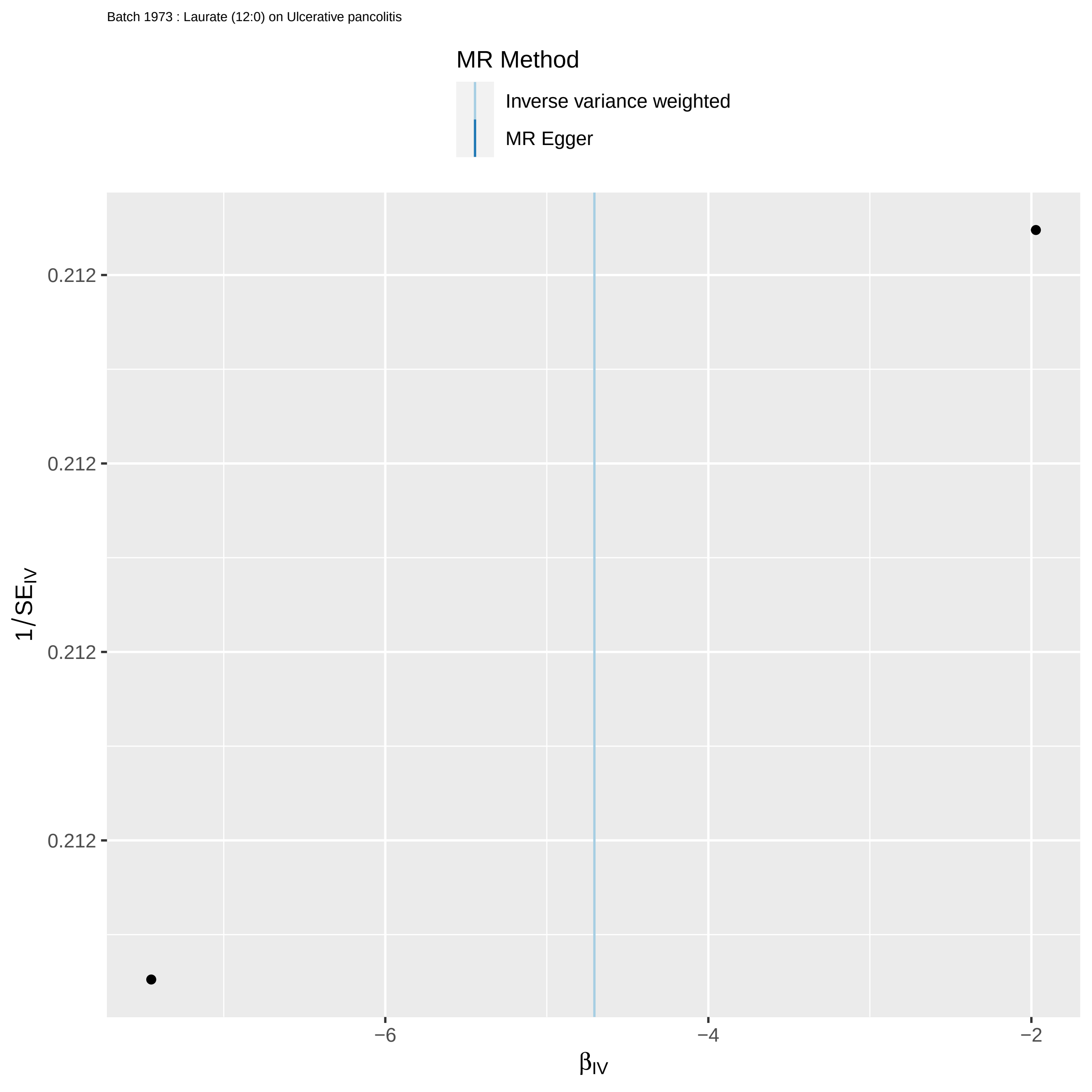

Batch 1973 : Laurate (12:0) on Ulcerative pancolitis
MR Method
Inverse variance weighted
MR Egger
0.212
0.212
0.212
0.212
1/SE_IV
−6
−4
−2
β_IV

Batch 1975 : Urea on Ulcerative pancolitis

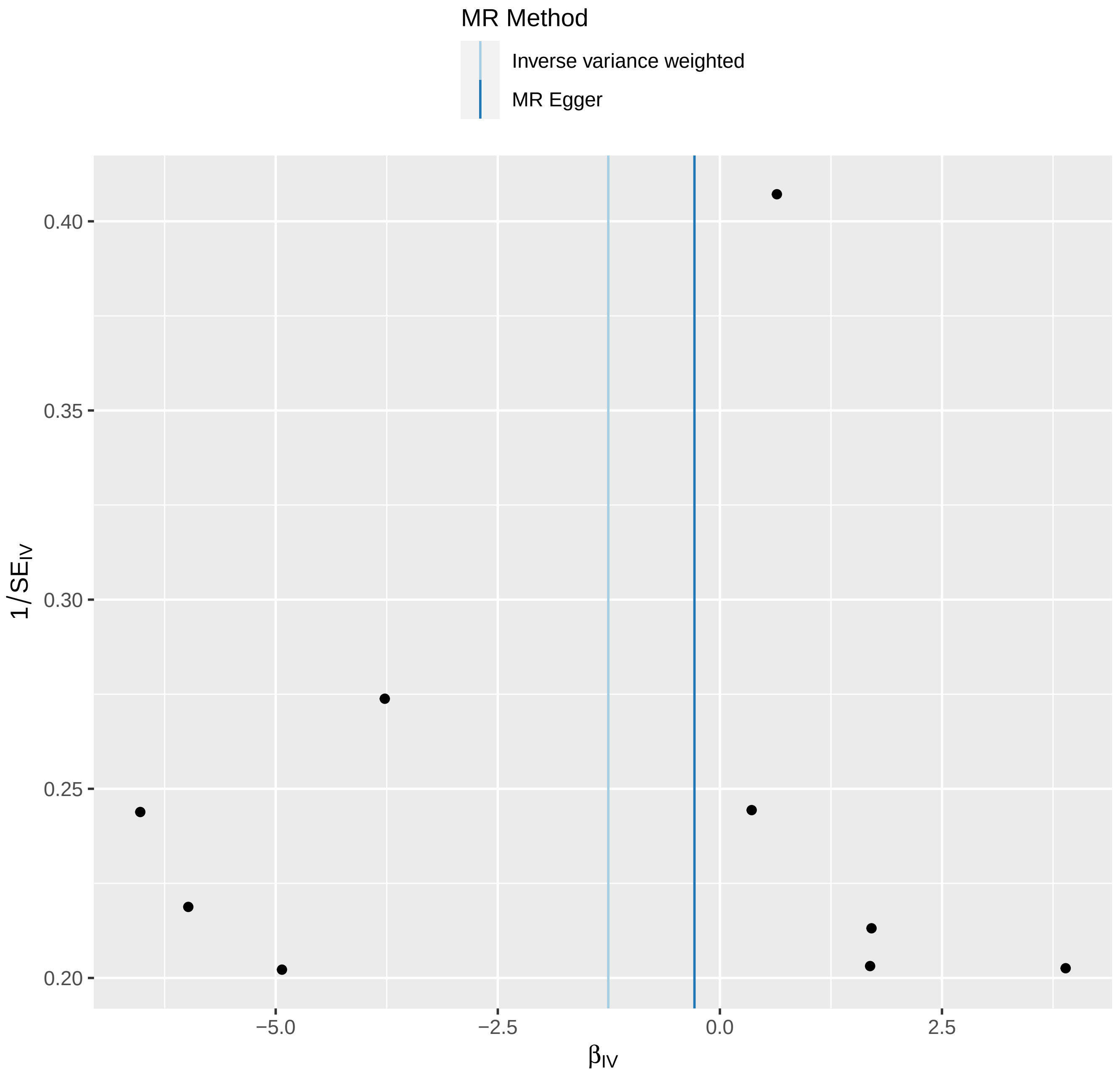

Batch 1976 : Cortisol on Ulcerative pancolitis

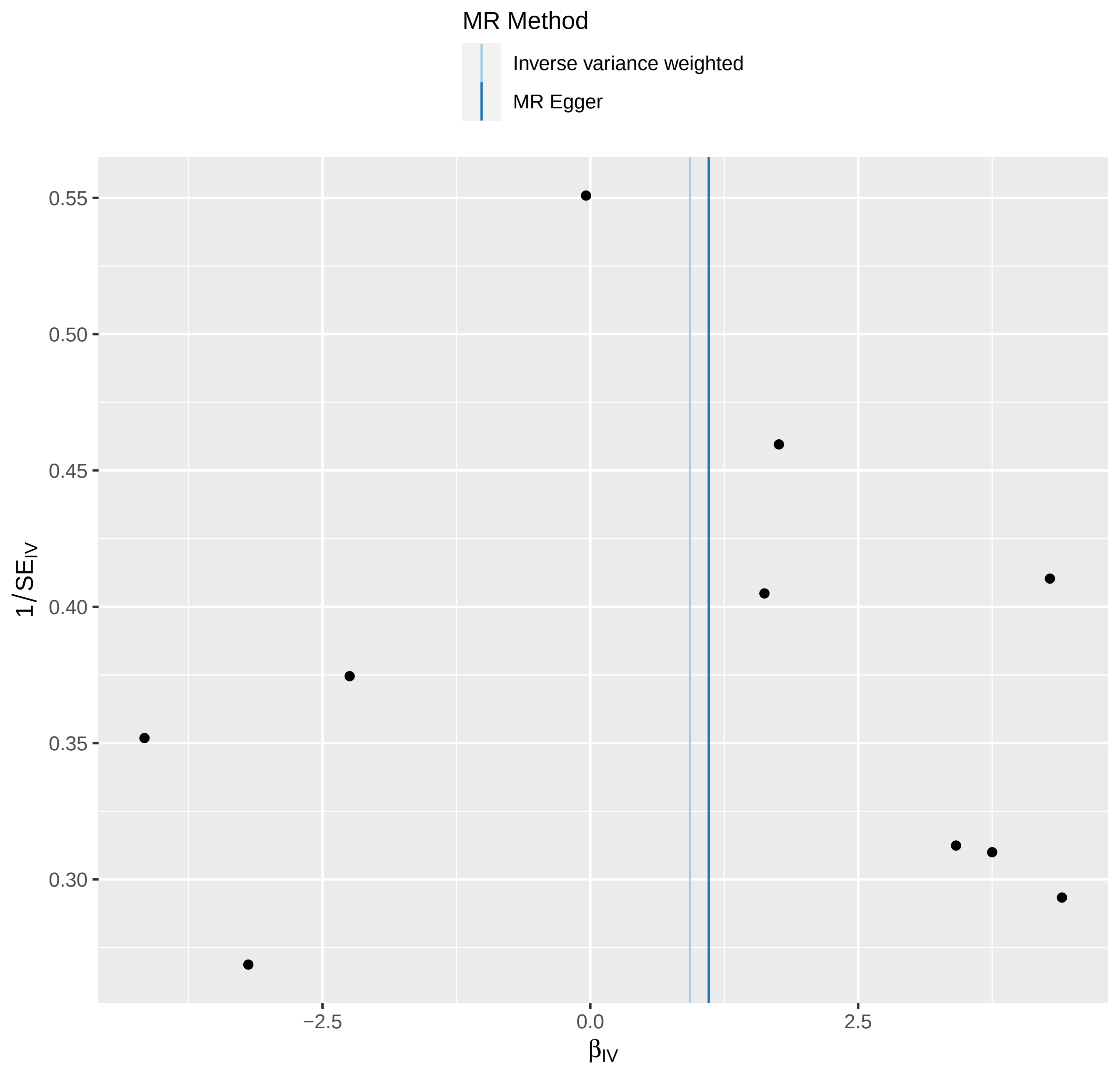

Batch 1979 : Citrulline on Ulcerative pancolitis

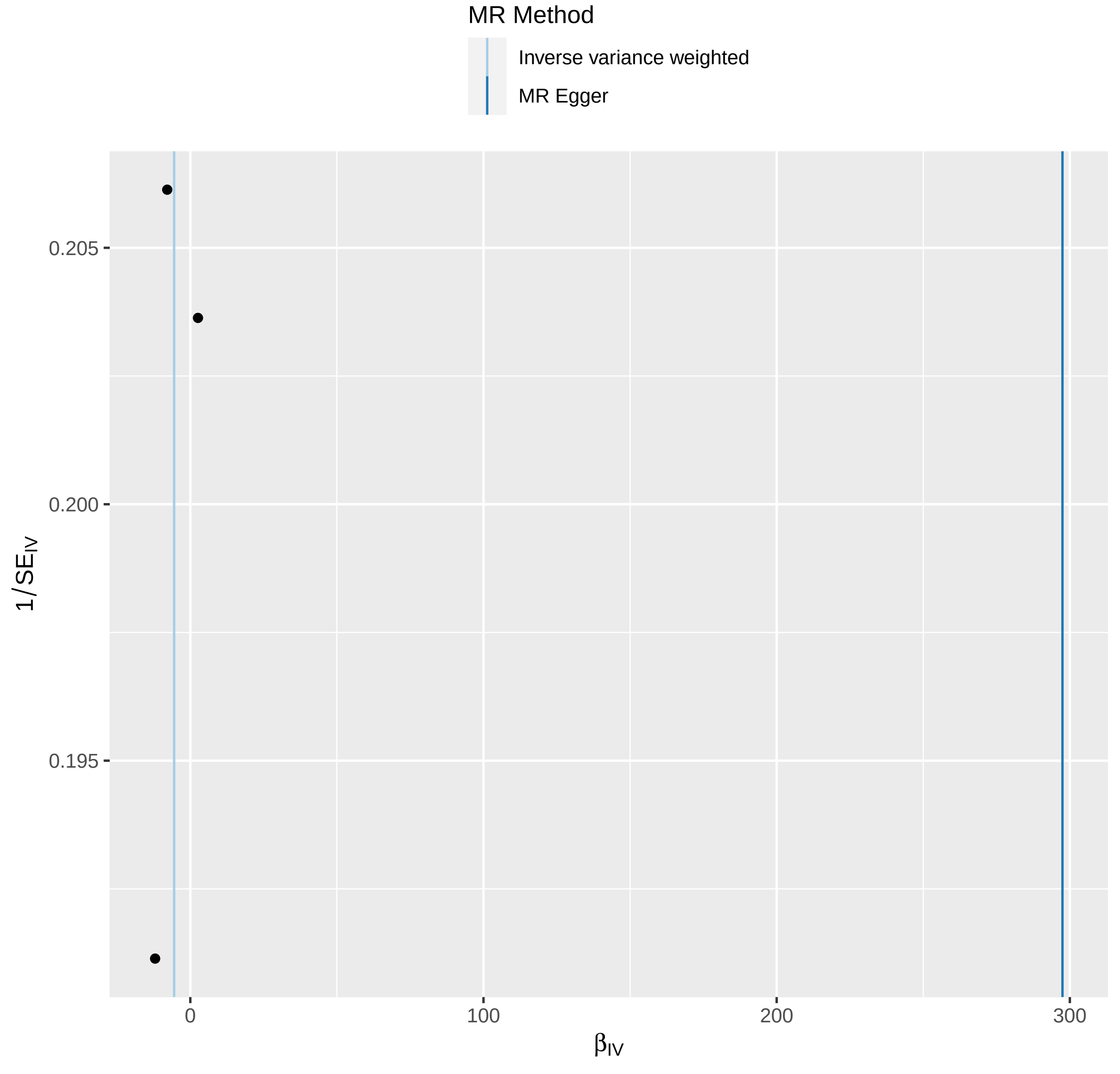

Batch 1981 : Serotonin (5HT) on Ulcerative pancolitis

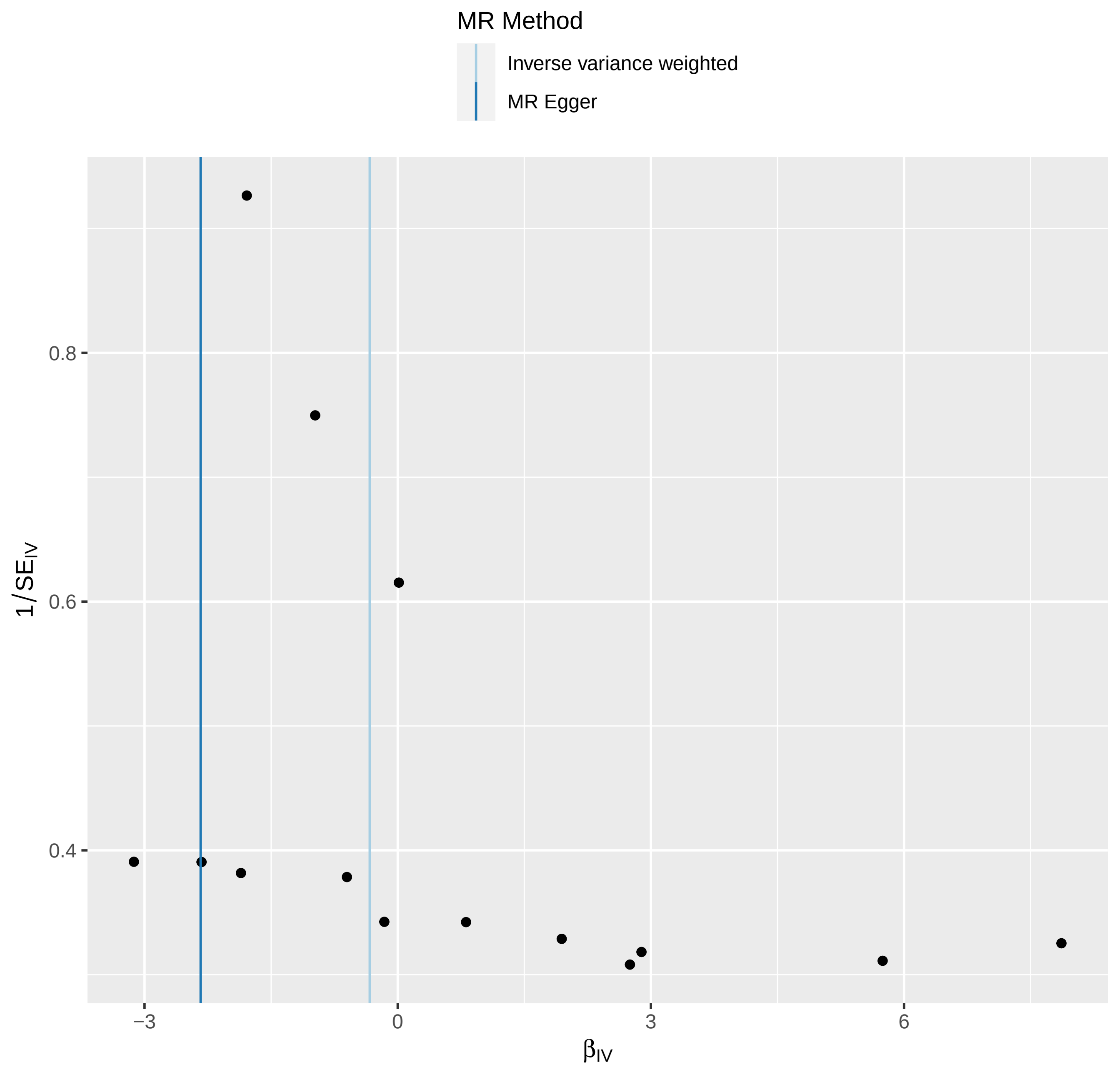

Batch 1982 : Gamma−glutamylglutamine on Ulcerative pancolitis

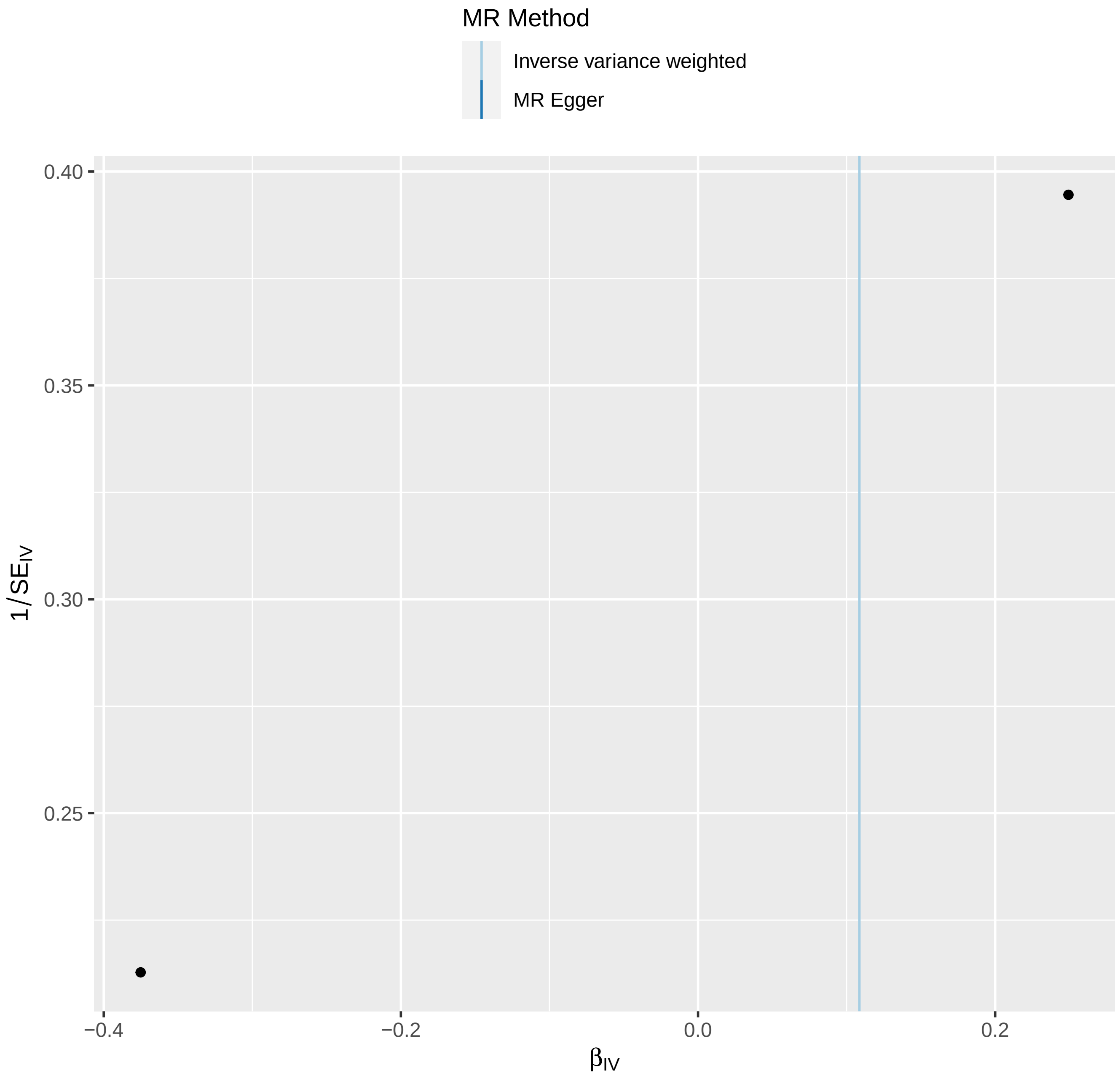

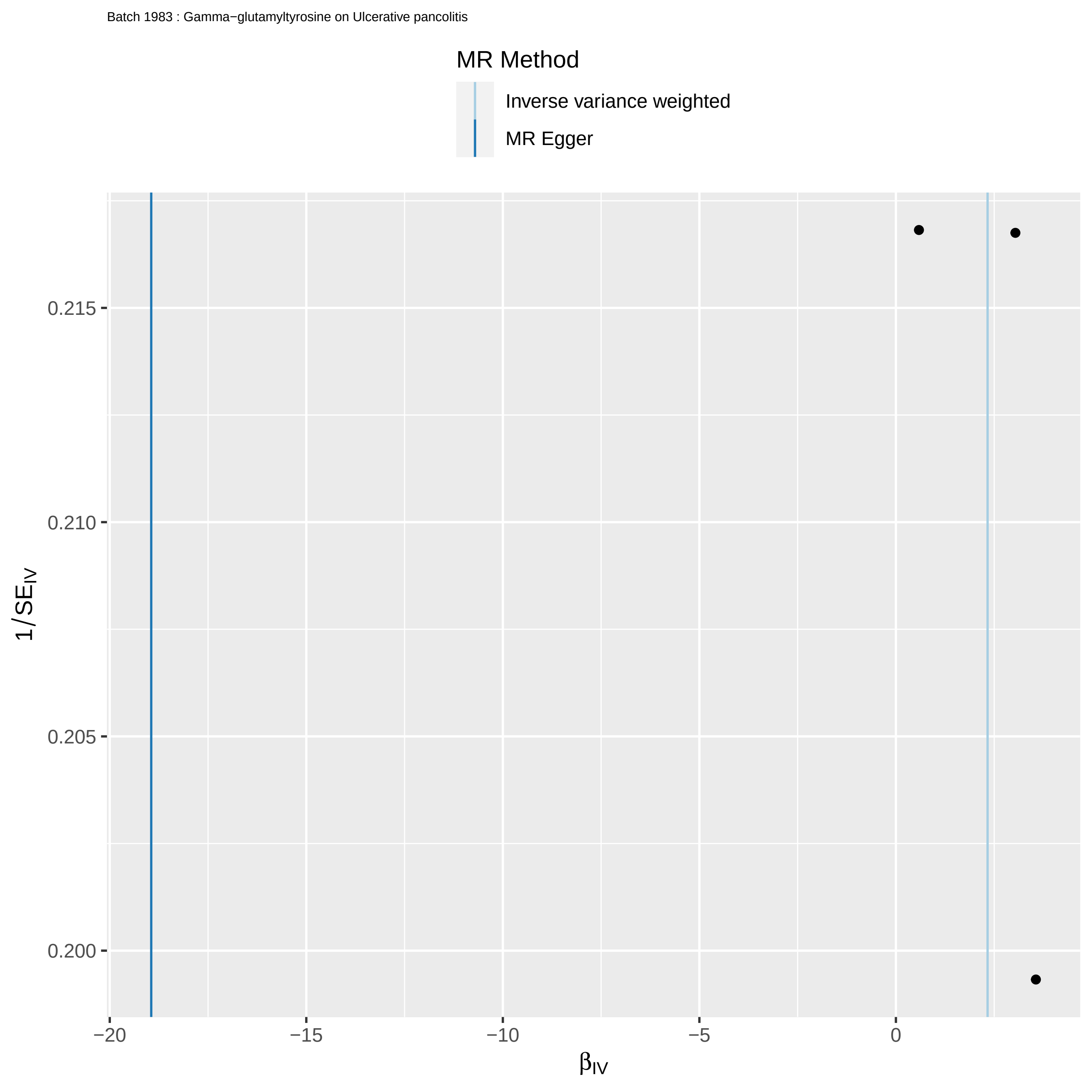

Batch 1983 : Gamma−glutamyltyrosine on Ulcerative pancolitis
MR Method
Inverse variance weighted
MR Egger
0.215
0.210
0.205
0.200
1/SE_IV
−20
−15
−10
−5
0
β_IV

Batch 1985 : Betaine on Ulcerative pancolitis

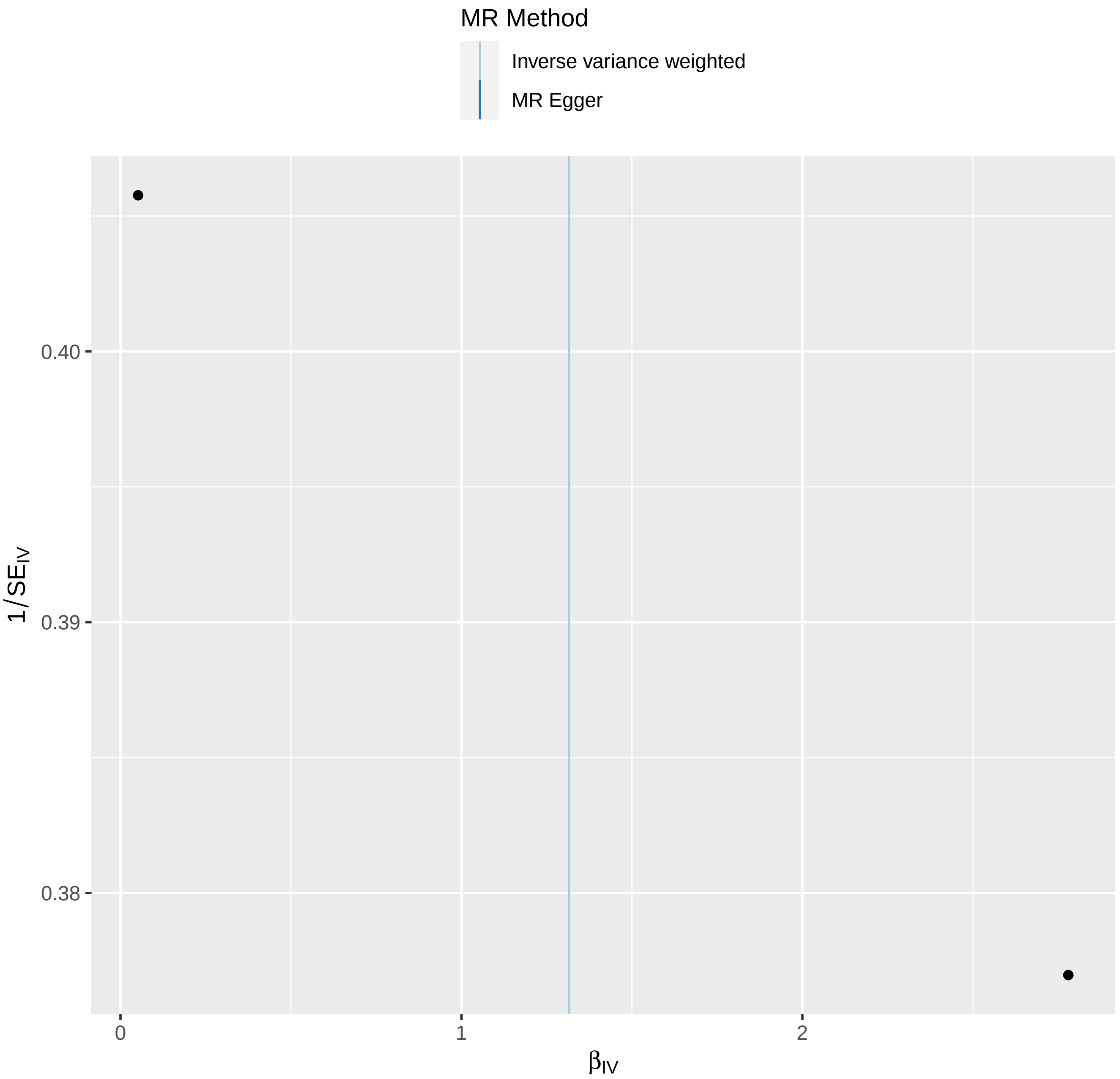

Batch 1986 : Xanthine on Ulcerative pancolitis

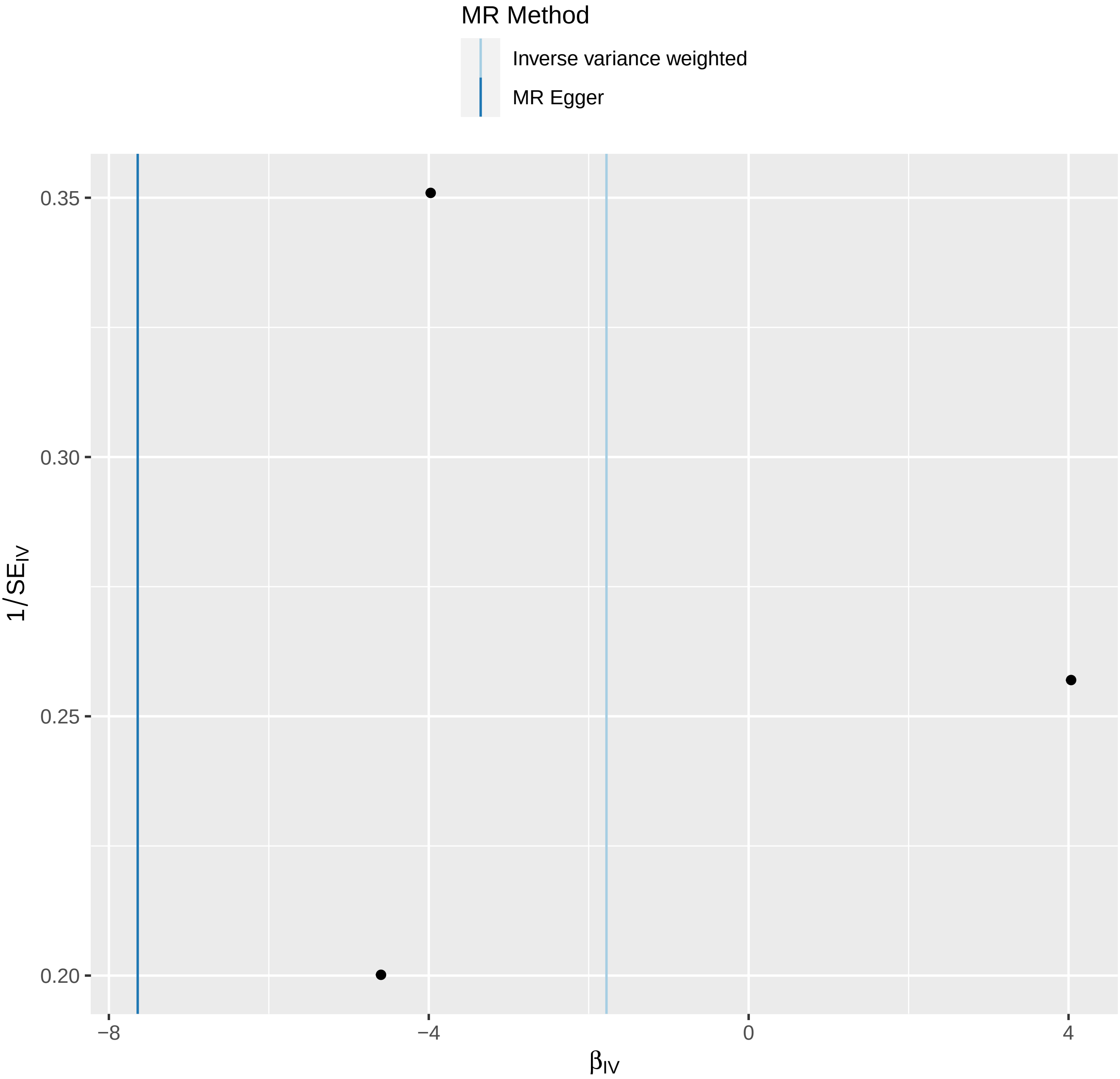

Batch 1987 : Phosphate on Ulcerative pancolitis

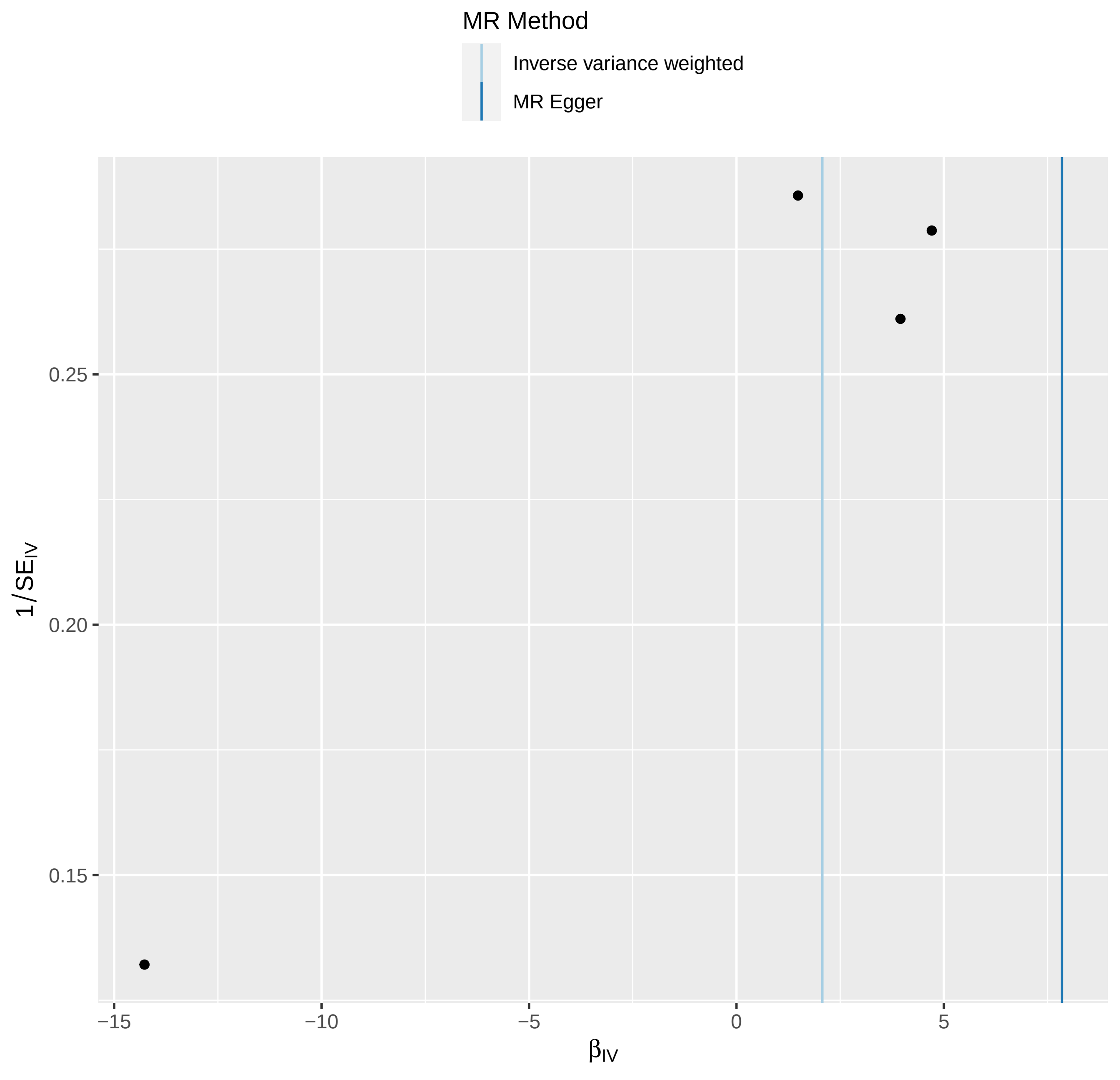

Batch 1988 : 3−methoxytyrosine on Ulcerative pancolitis

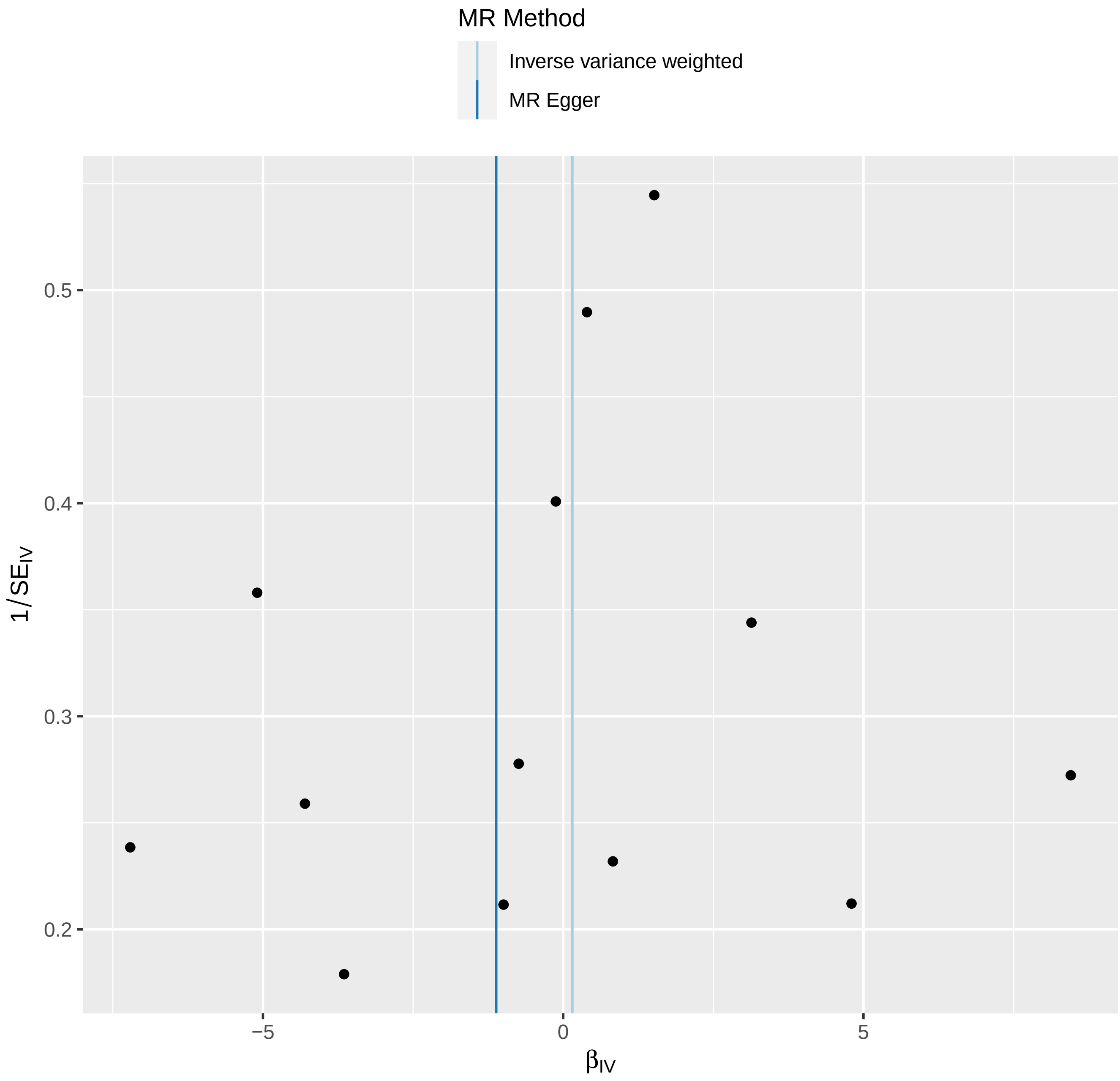

Batch 1990 : Undecanoate (11:0) on Ulcerative pancolitis

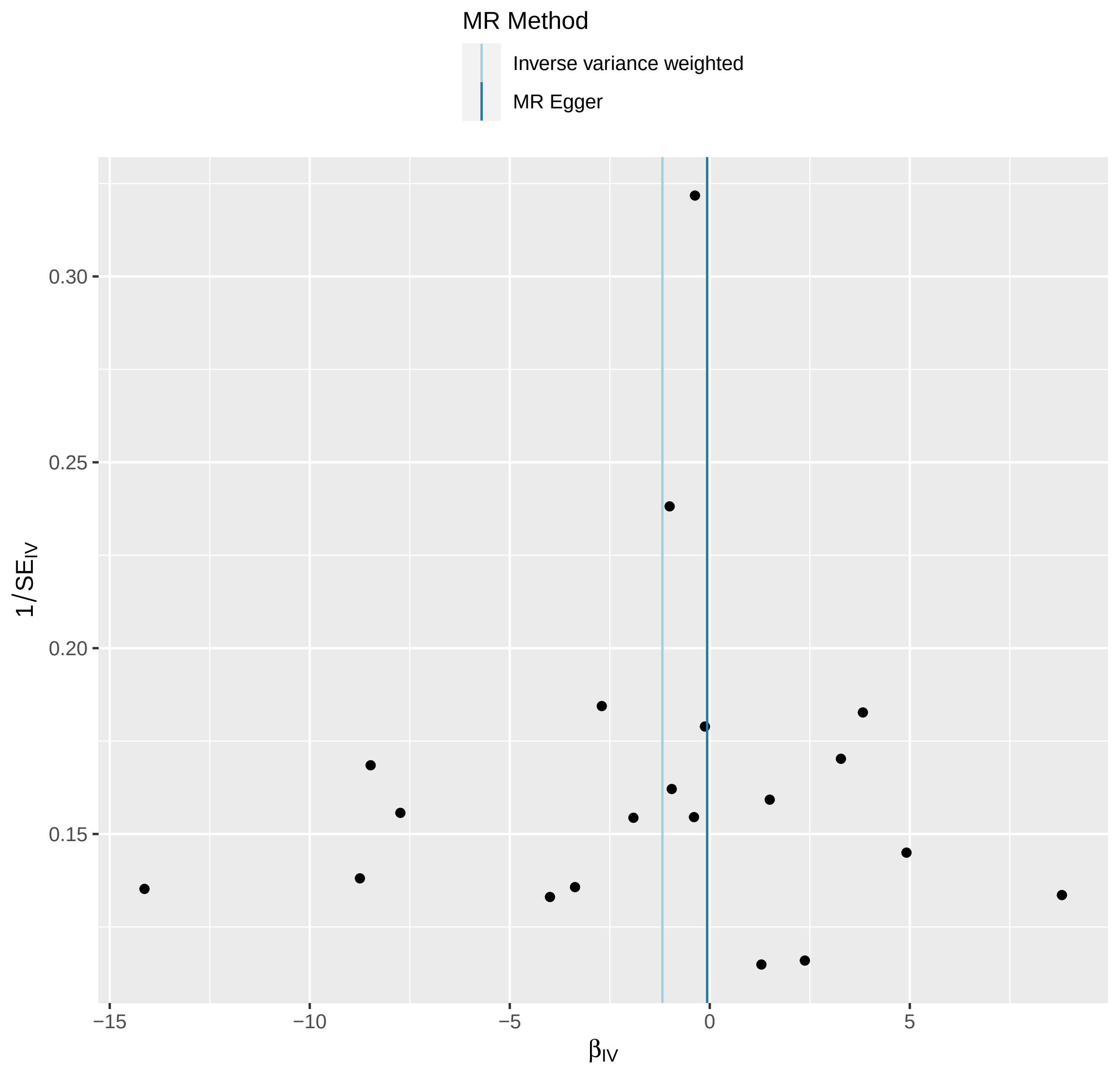

Batch 1992 : Taurodeoxycholate on Ulcerative pancolitis

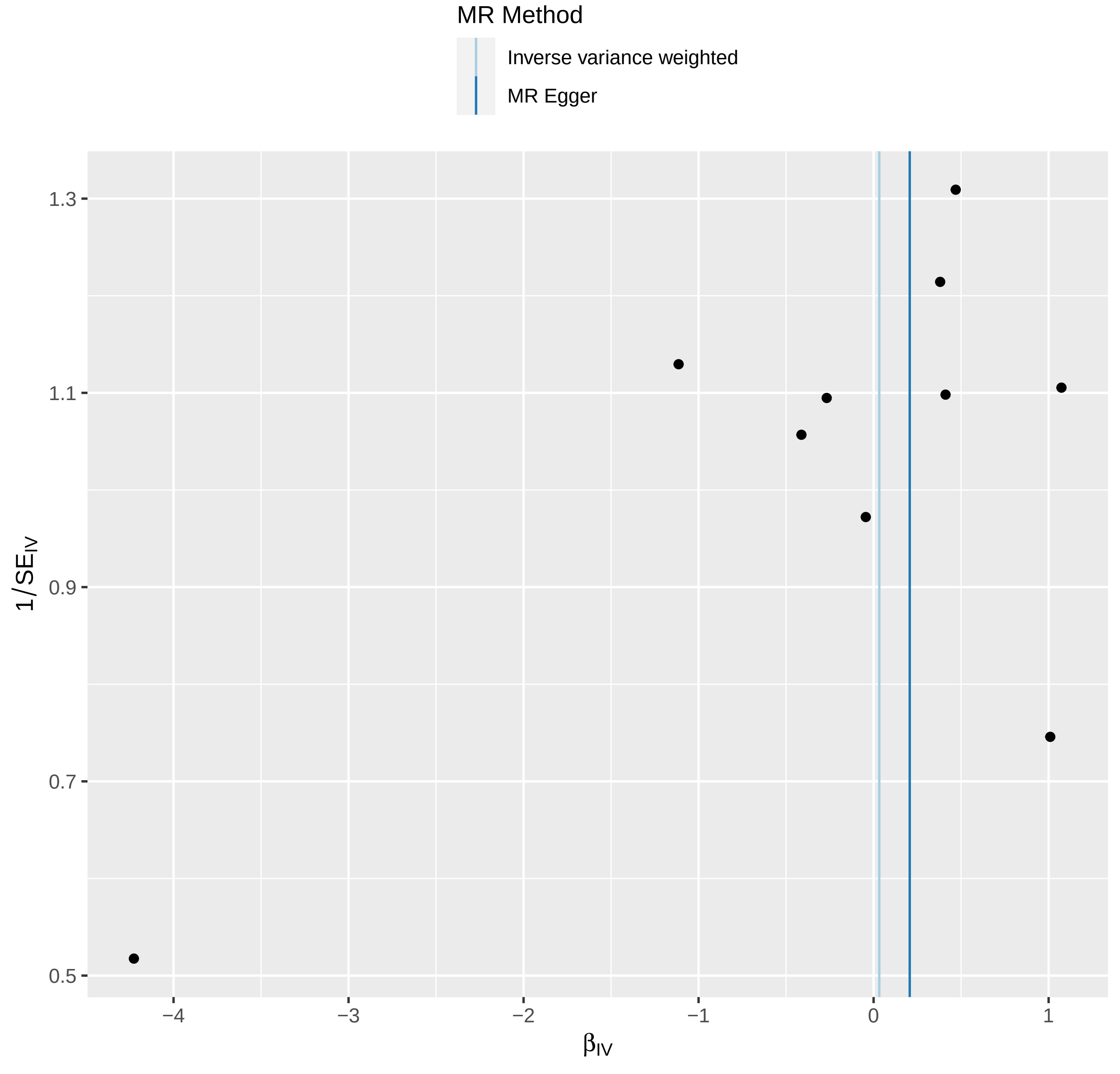

Batch 1993 : Glycerol on Ulcerative pancolitis

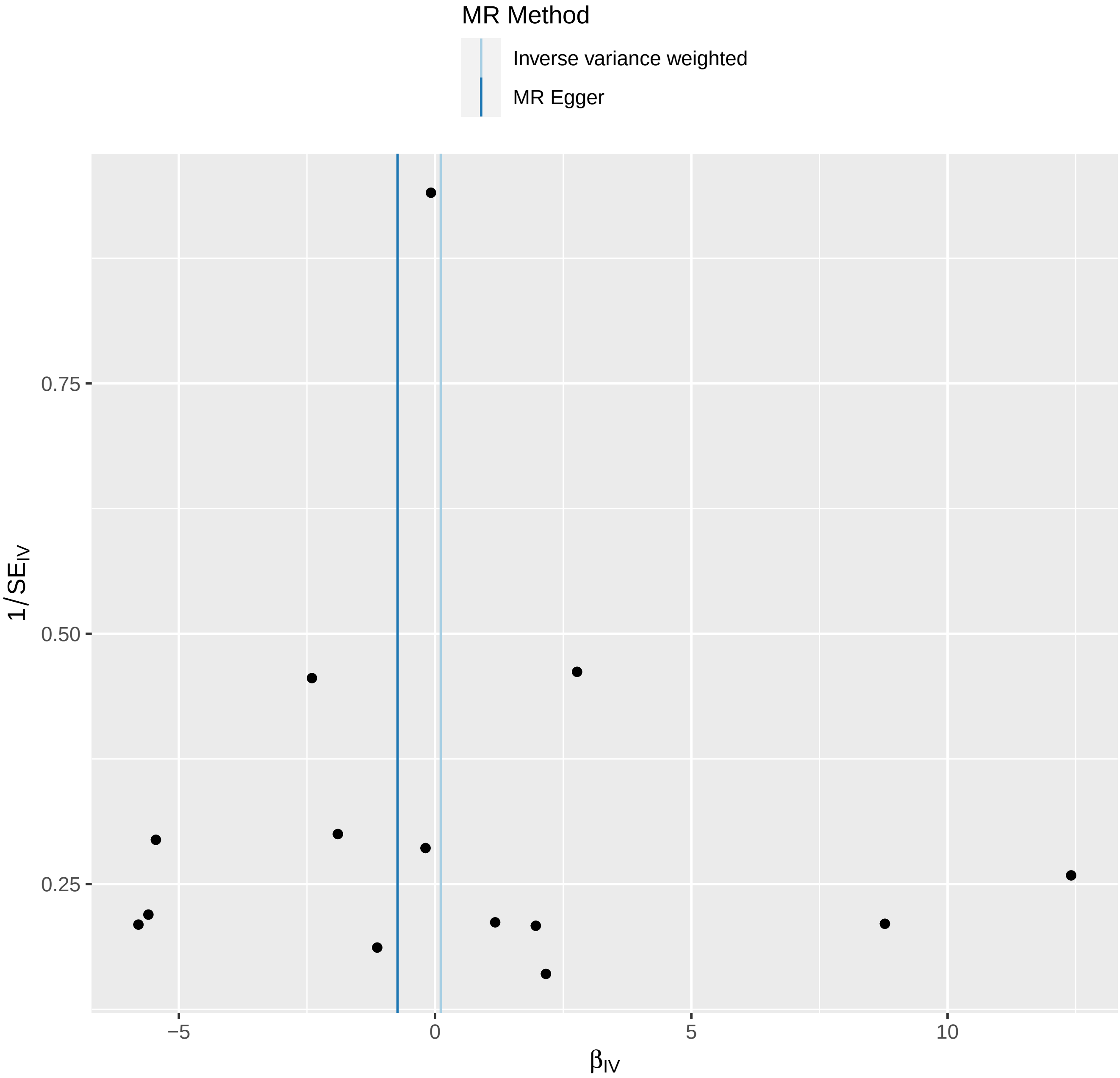

Batch 1994 : Kynurenine on Ulcerative pancolitis

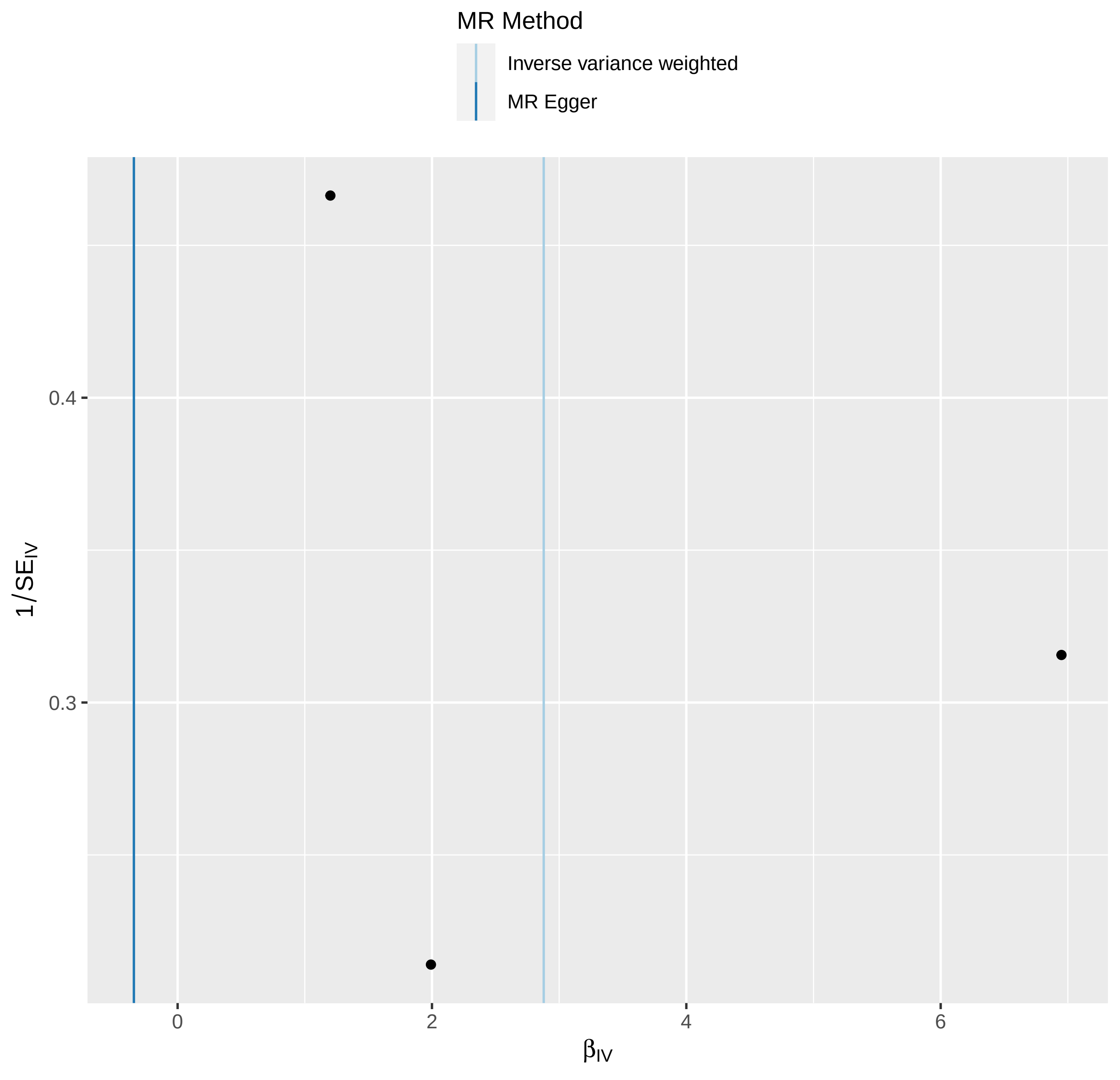

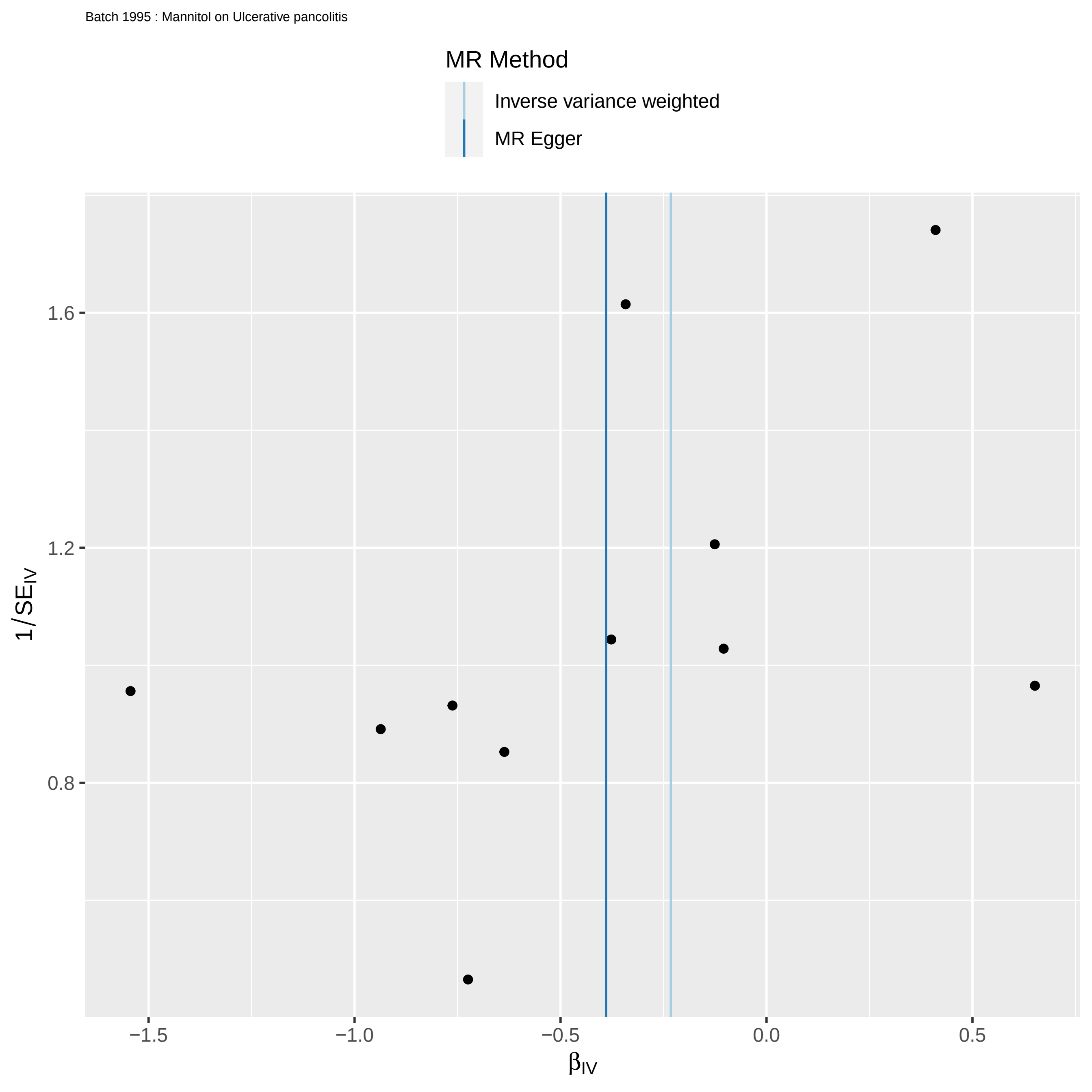
Batch 1995 : Mannitol on Ulcerative pancolitis
MR Method
Inverse variance weighted
MR Egger
1.6
1.2
0.8
1/SE$_{IV}$
−1.5
−1.0
−0.5
0.0
0.5
β$_{IV}$

Batch 1996 : Glycerol 3−phosphate (G3P) on Ulcerative pancolitis

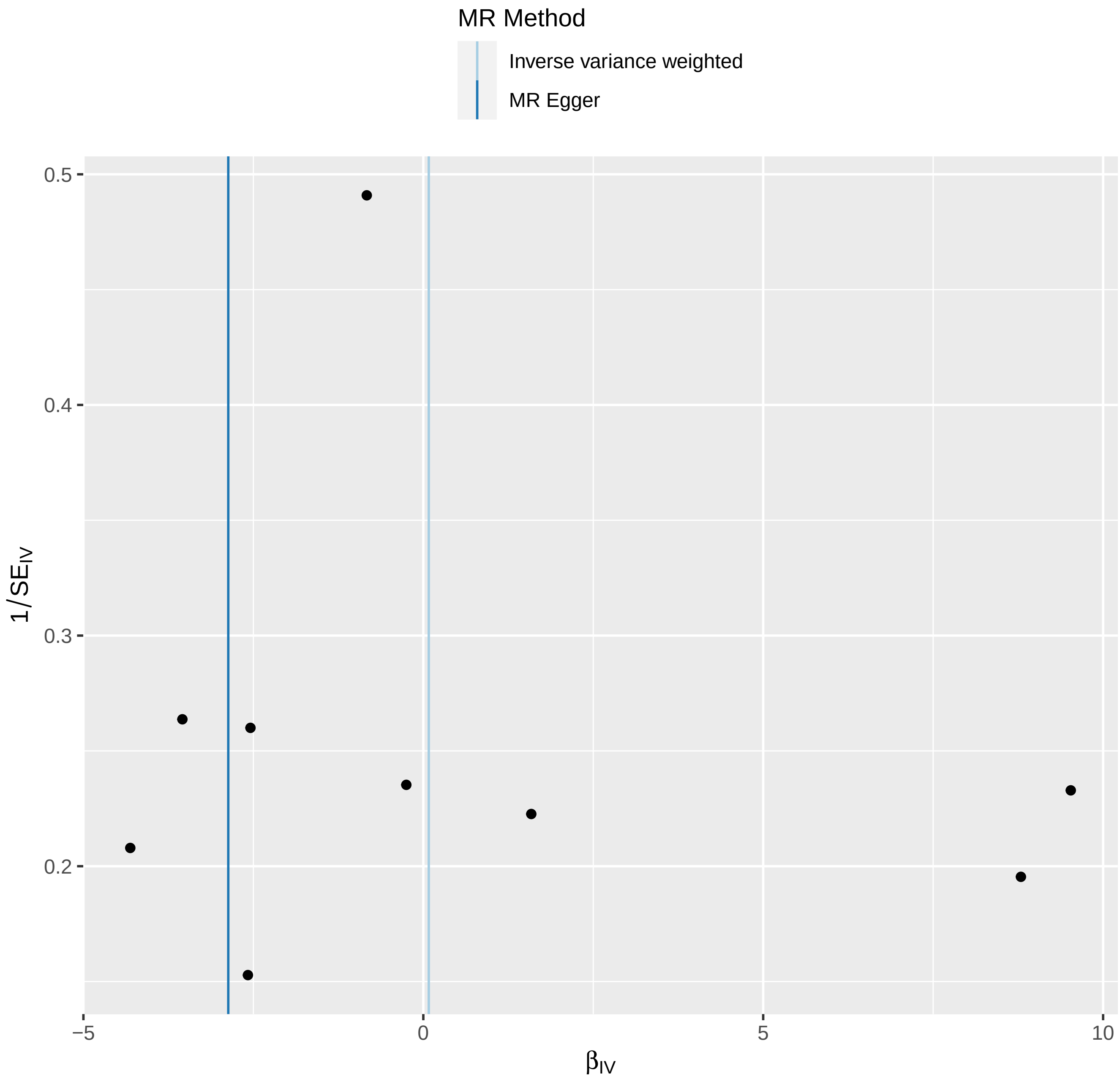

Batch 1998 : Carnitine on Ulcerative pancolitis

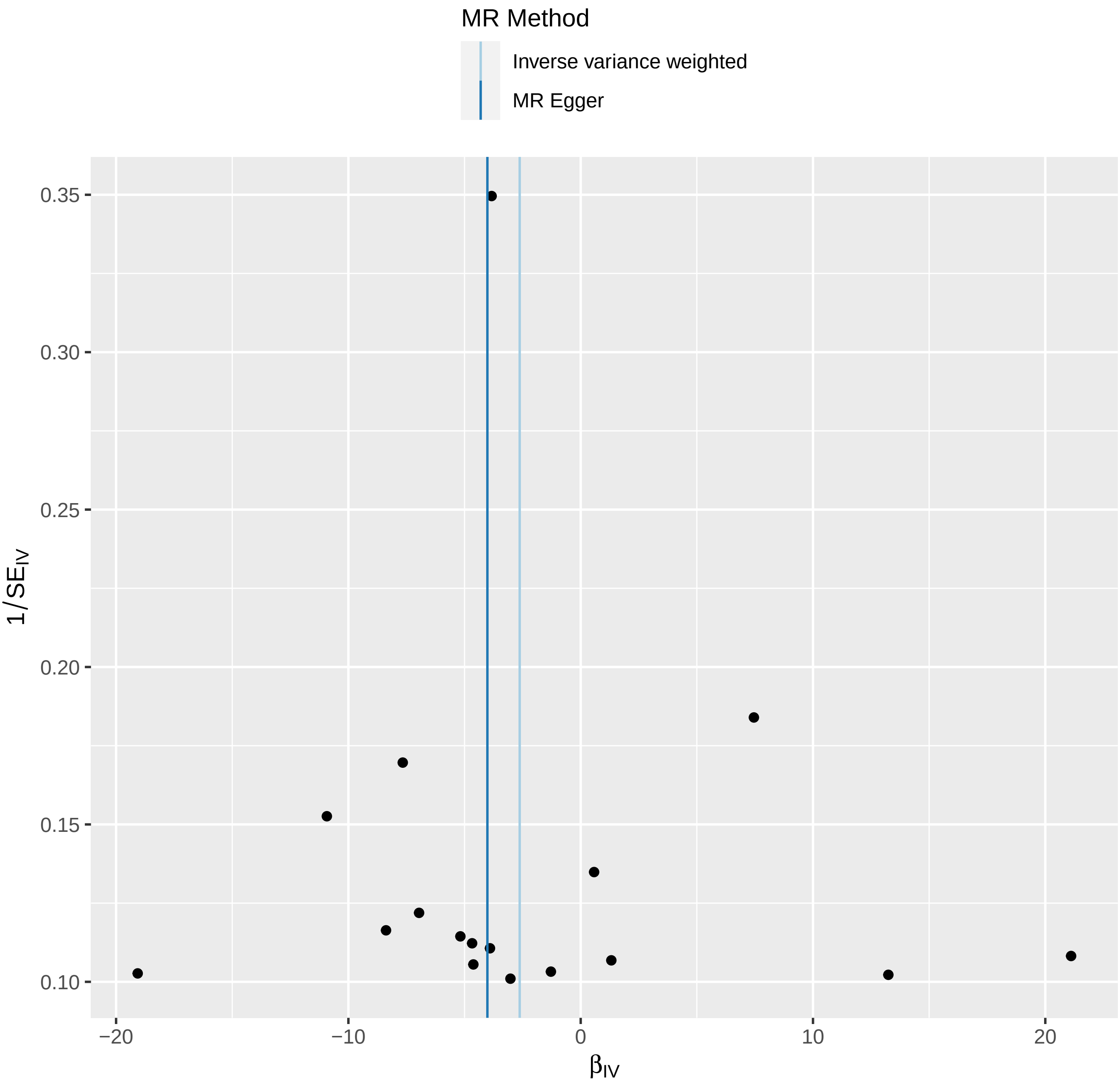

Batch 1999 : Choline on Ulcerative pancolitis

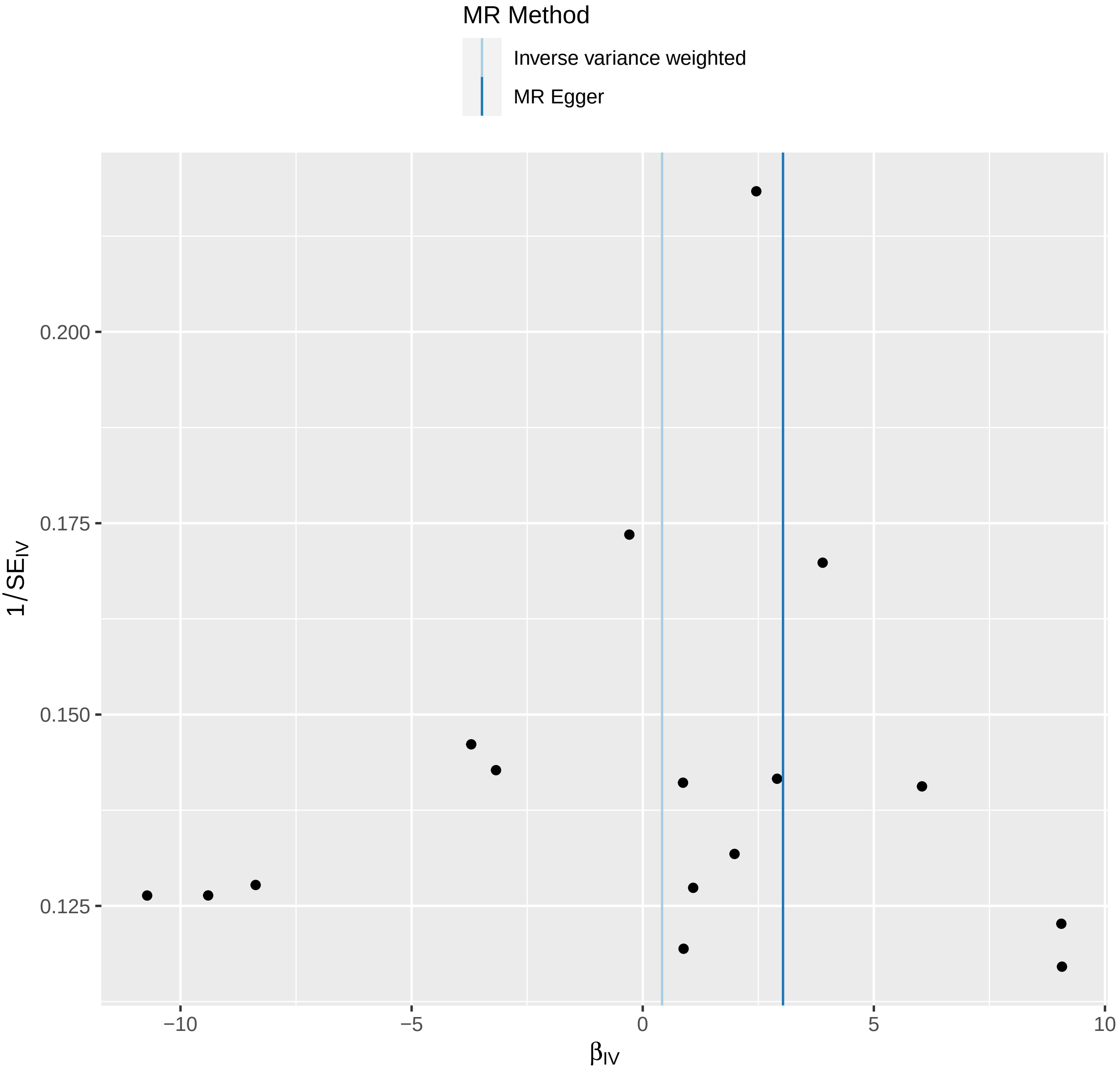

Batch 2002 : 3−methyl−2−oxovalerate on Ulcerative pancolitis

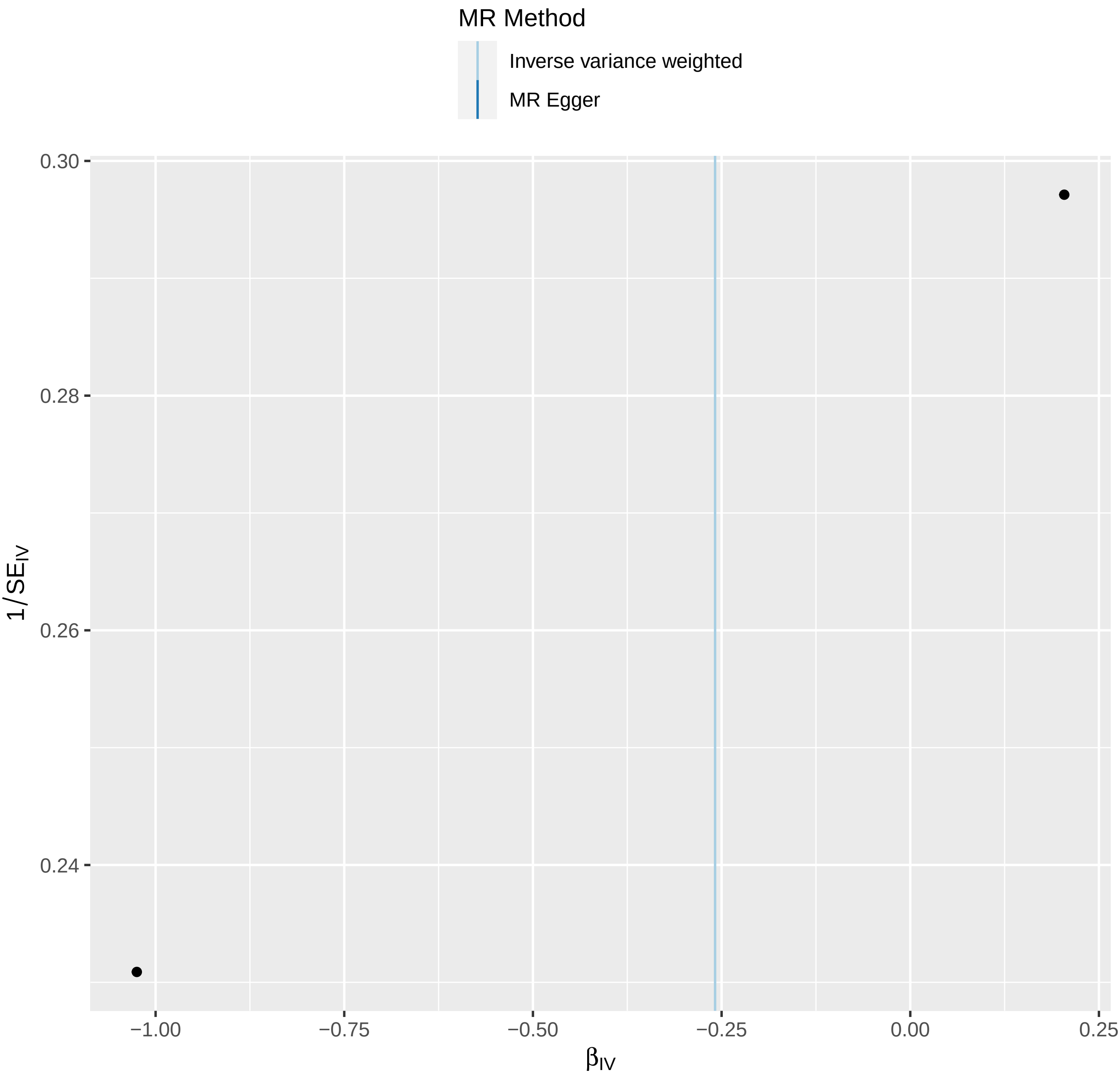

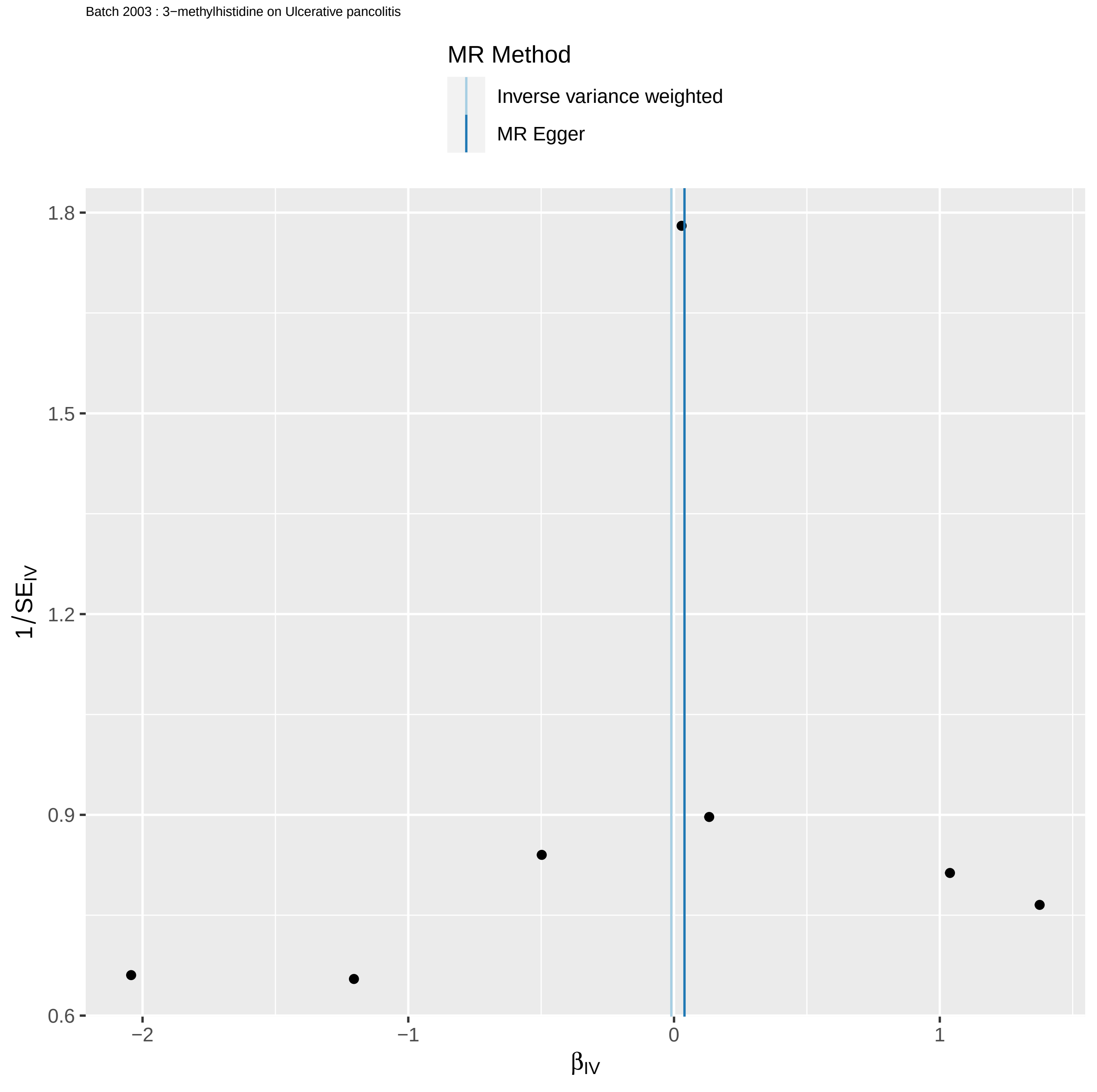
Batch 2003 : 3−methylhistidine on Ulcerative pancolitis
MR Method
Inverse variance weighted
MR Egger
1.8
1.5
1.2
0.9
0.6
1/SE$_{IV}$
−2
−1
0
1
β$_{IV}$

Batch 2005 : Phenylacetate on Ulcerative pancolitis

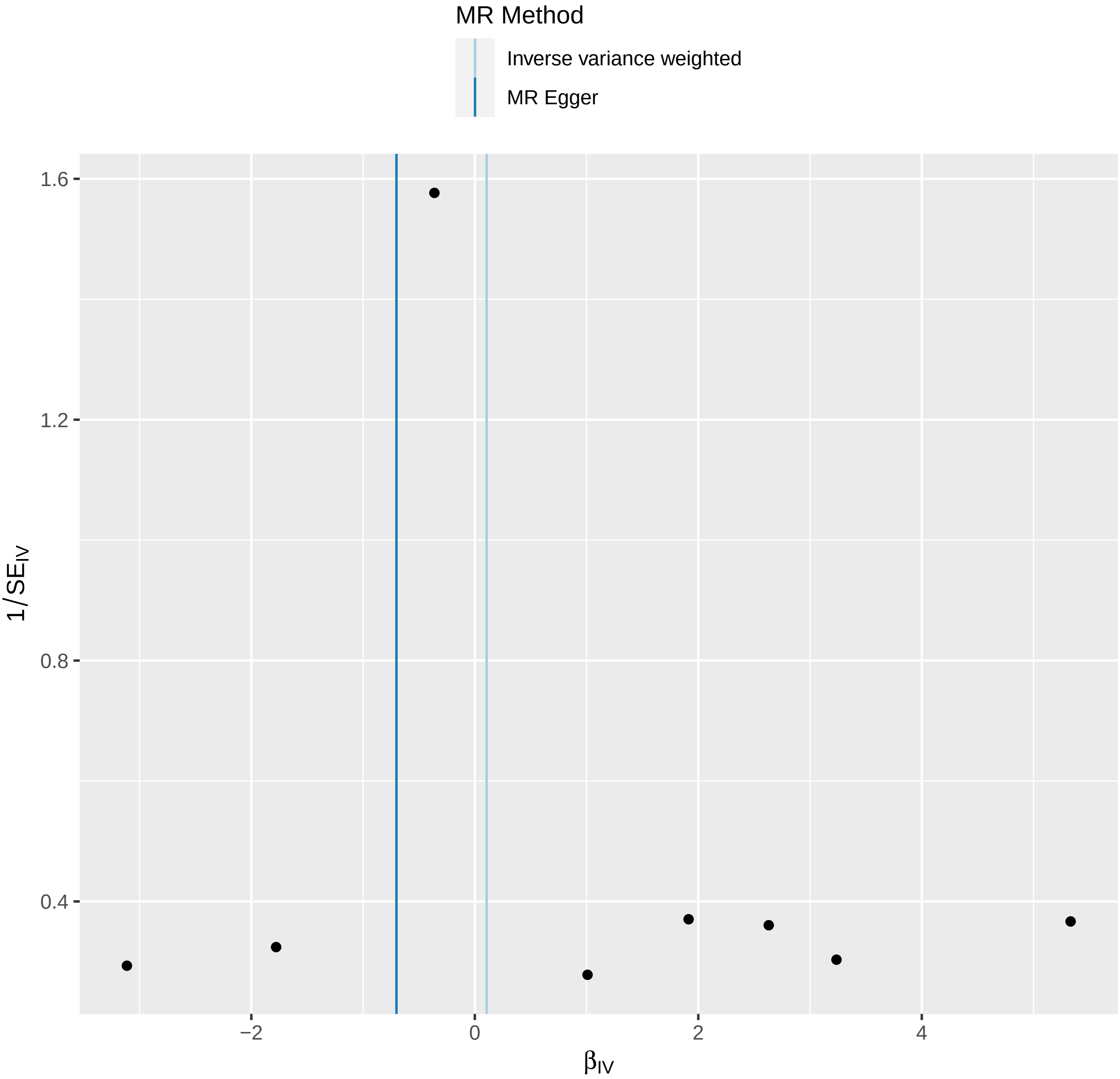

Batch 2006 : Glycerophosphorylcholine (GPC) on Ulcerative pancolitis

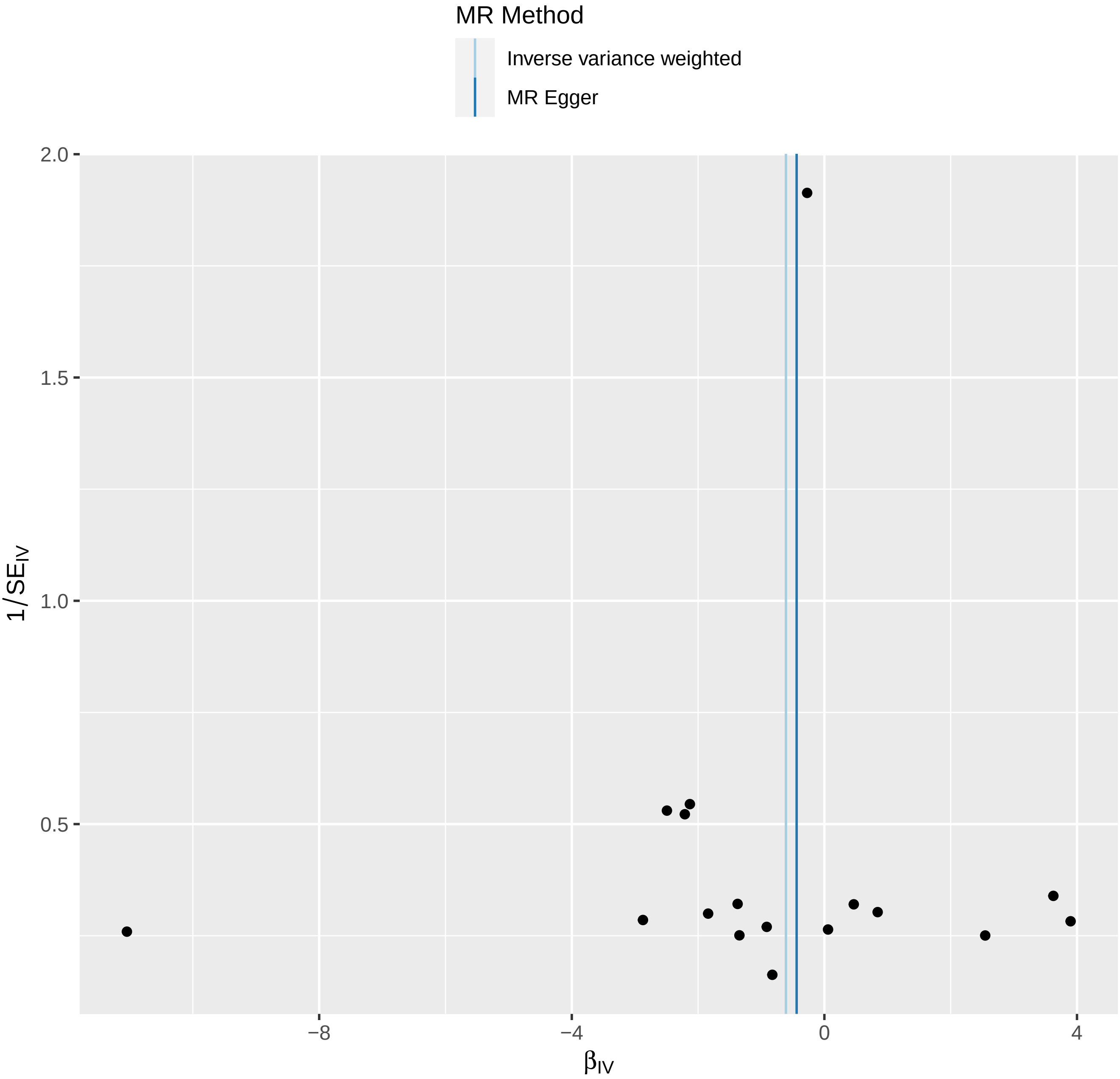

Batch 2007 : Aspartate on Ulcerative pancolitis

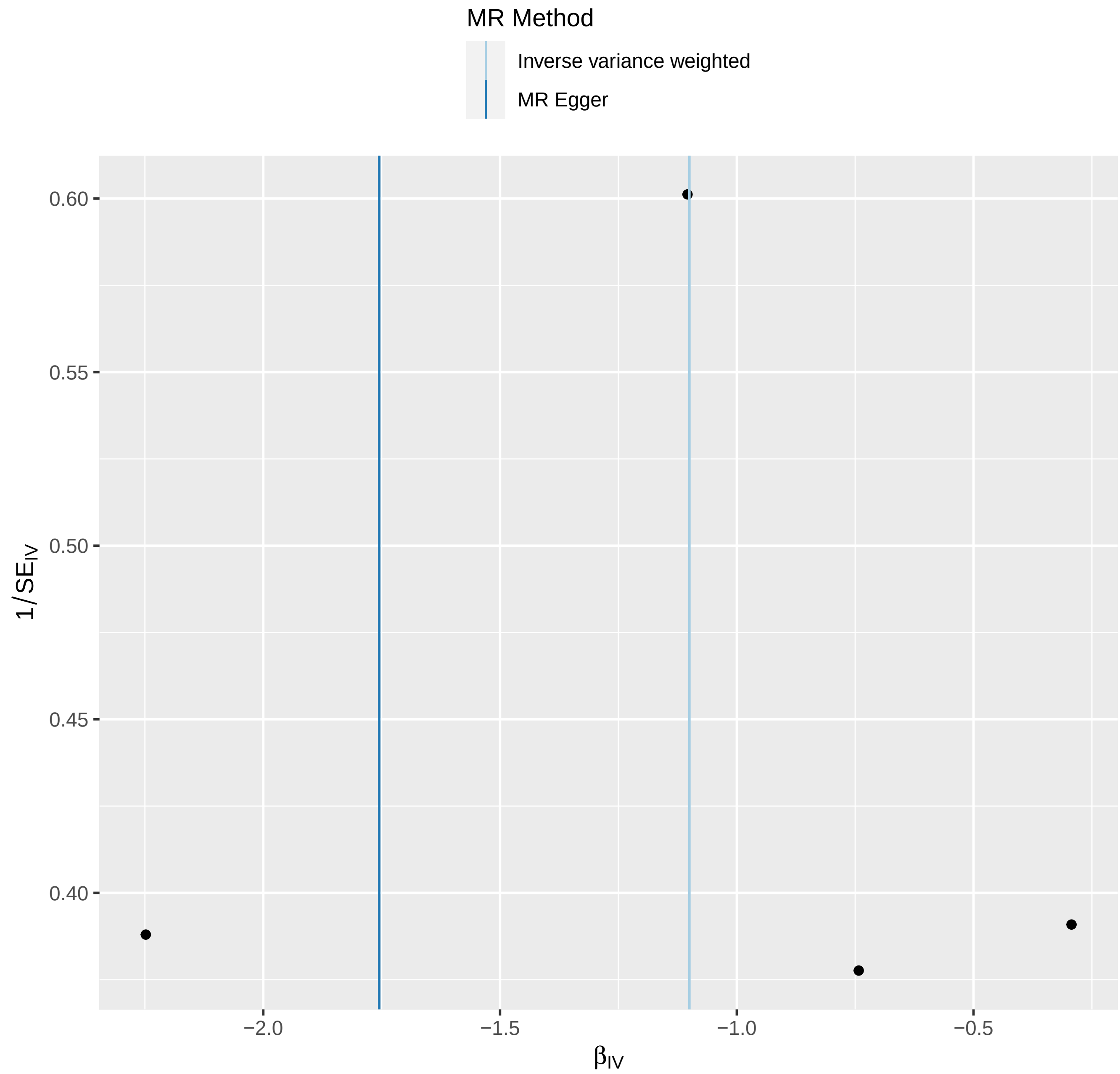

Batch 2009 : Dihomo−linoleate (20:2n6) on Ulcerative pancolitis

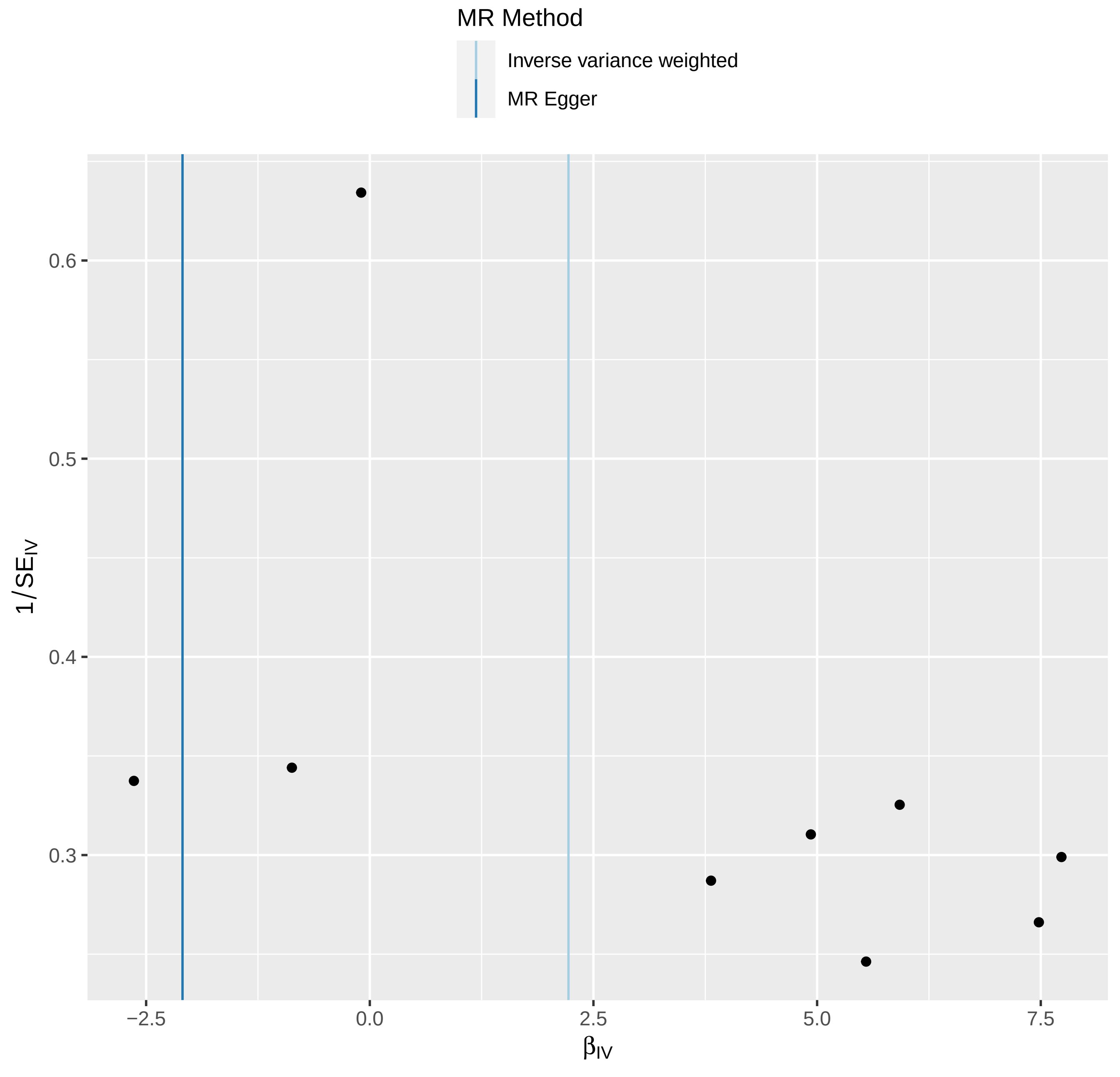

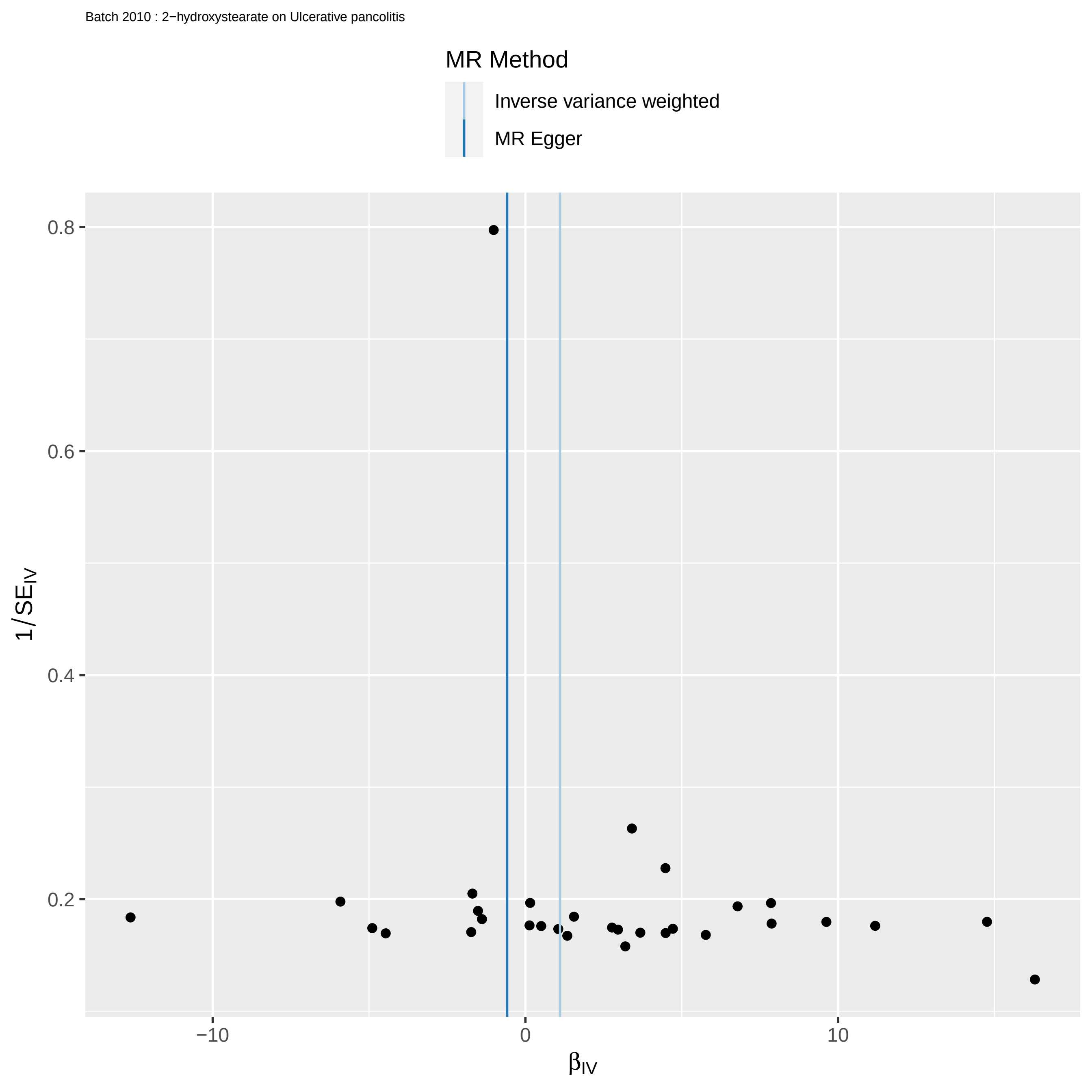
Batch 2010 : 2−hydroxystearate on Ulcerative pancolitis
MR Method
Inverse variance weighted
MR Egger
0.8
0.6
0.4
0.2
1/SE_IV
−10
0
10
β_IV

Batch 2012 : Glycylvaline on Ulcerative pancolitis

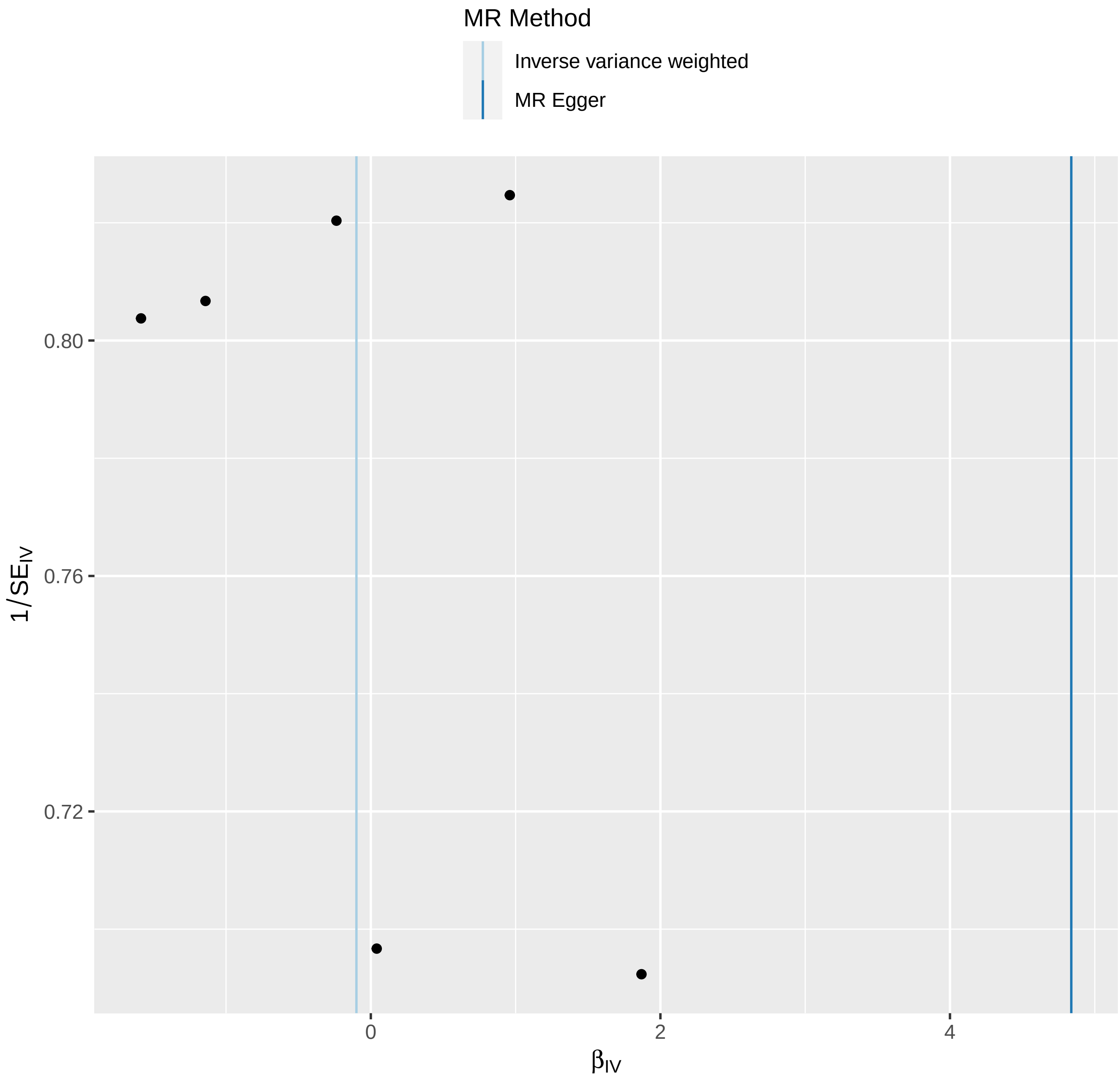

Batch 2013 : Gamma−glutamylleucine on Ulcerative pancolitis

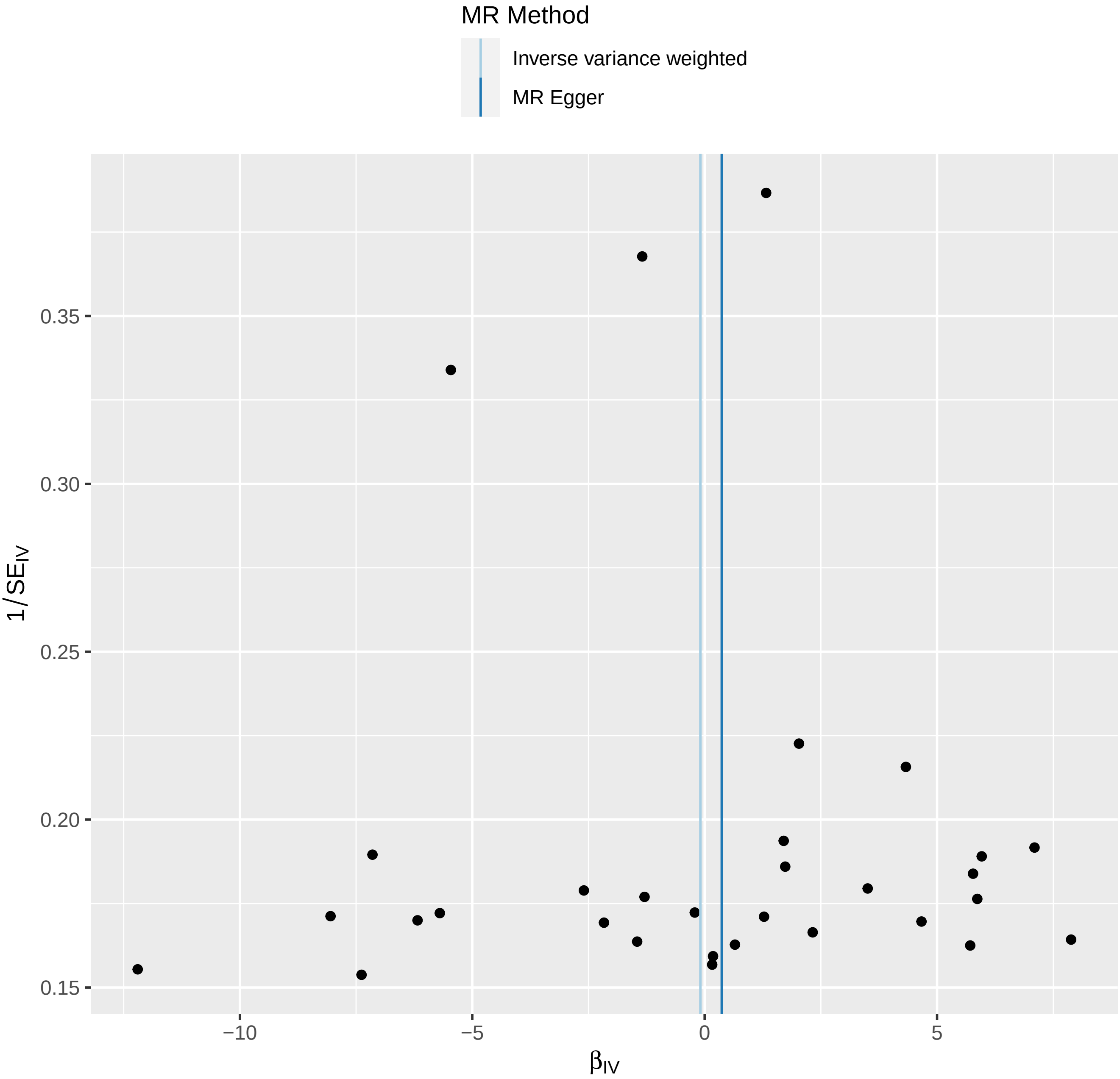

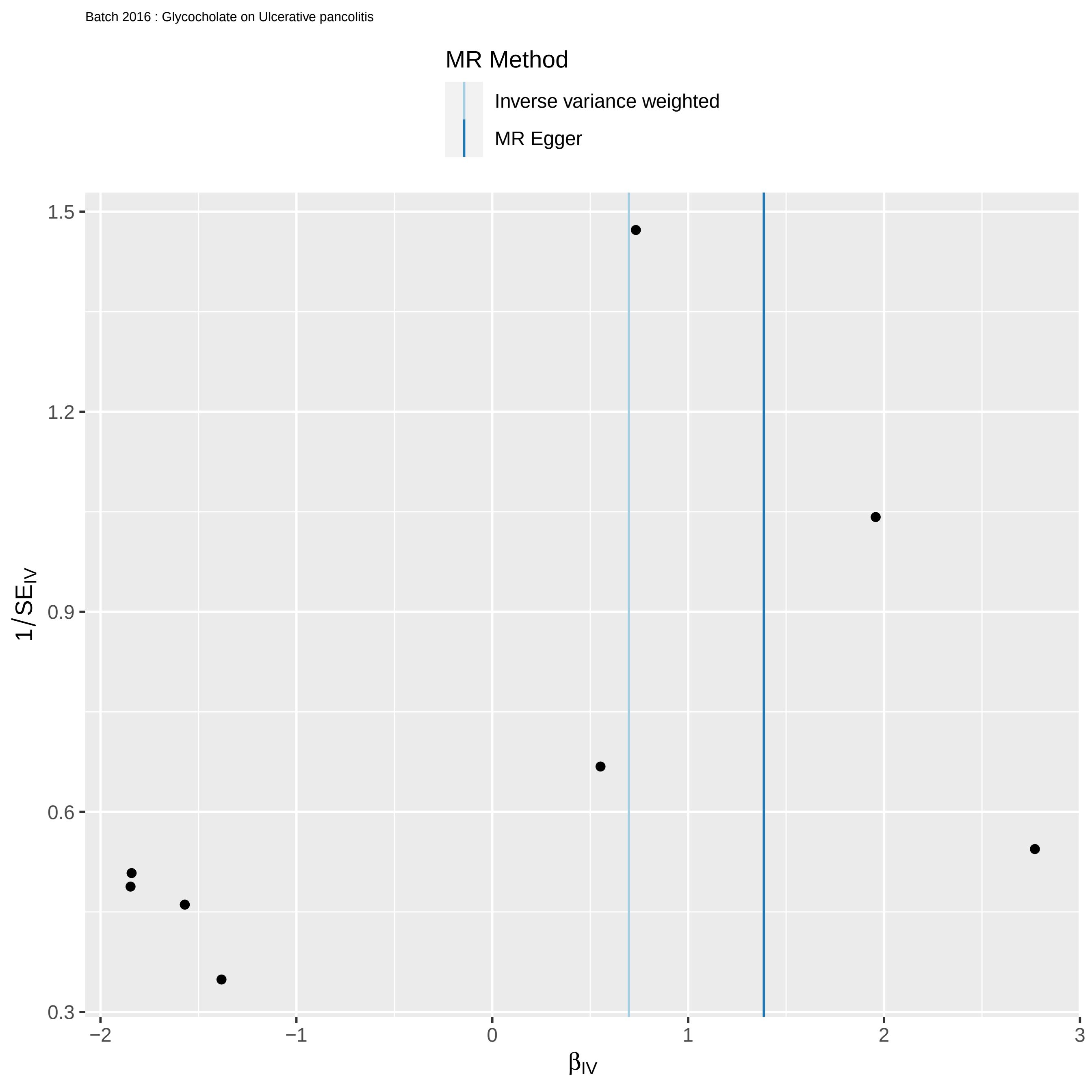

Batch 2016 : Glycocholate on Ulcerative pancolitis
MR Method
Inverse variance weighted
MR Egger
1.5
1.2
0.9
0.6
0.3
1/SE_IV
−2
−1
0
1
2
3
β_IV

Batch 2017 : Glycodeoxycholate on Ulcerative pancolitis

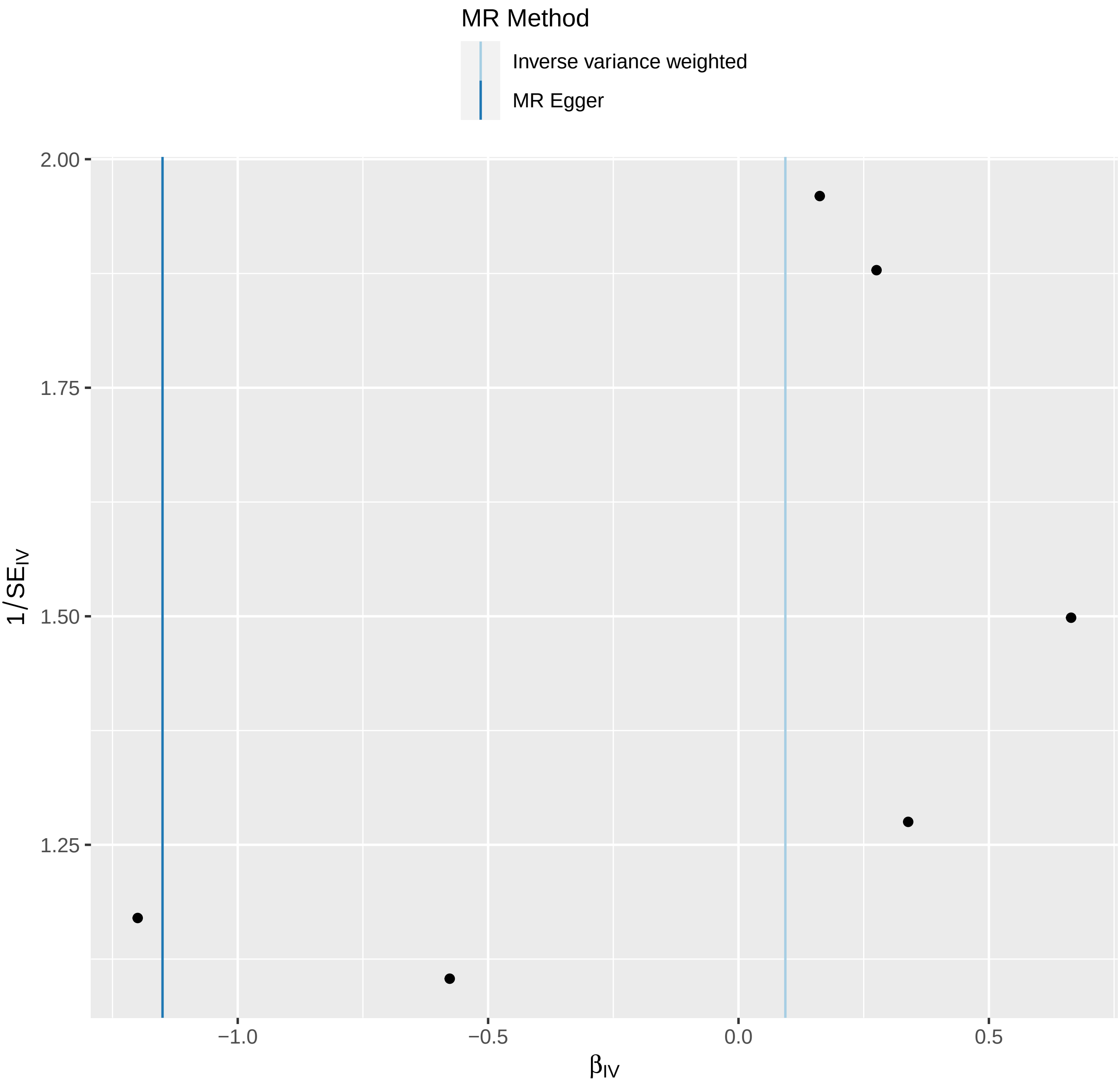

Batch 2018 : Taurochenodeoxycholate on Ulcerative pancolitis

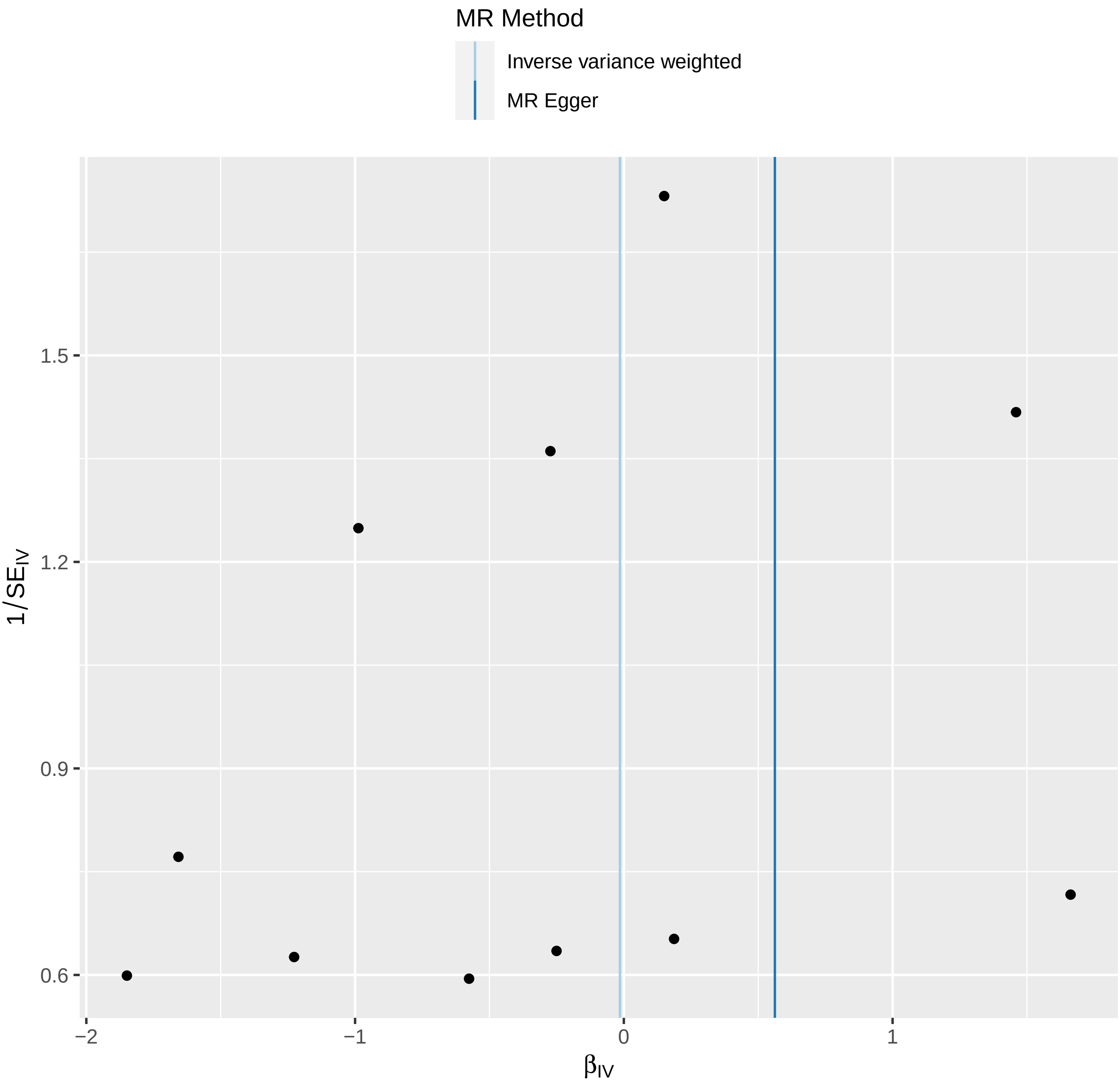

Batch 2019 : Taurocholate on Ulcerative pancolitis

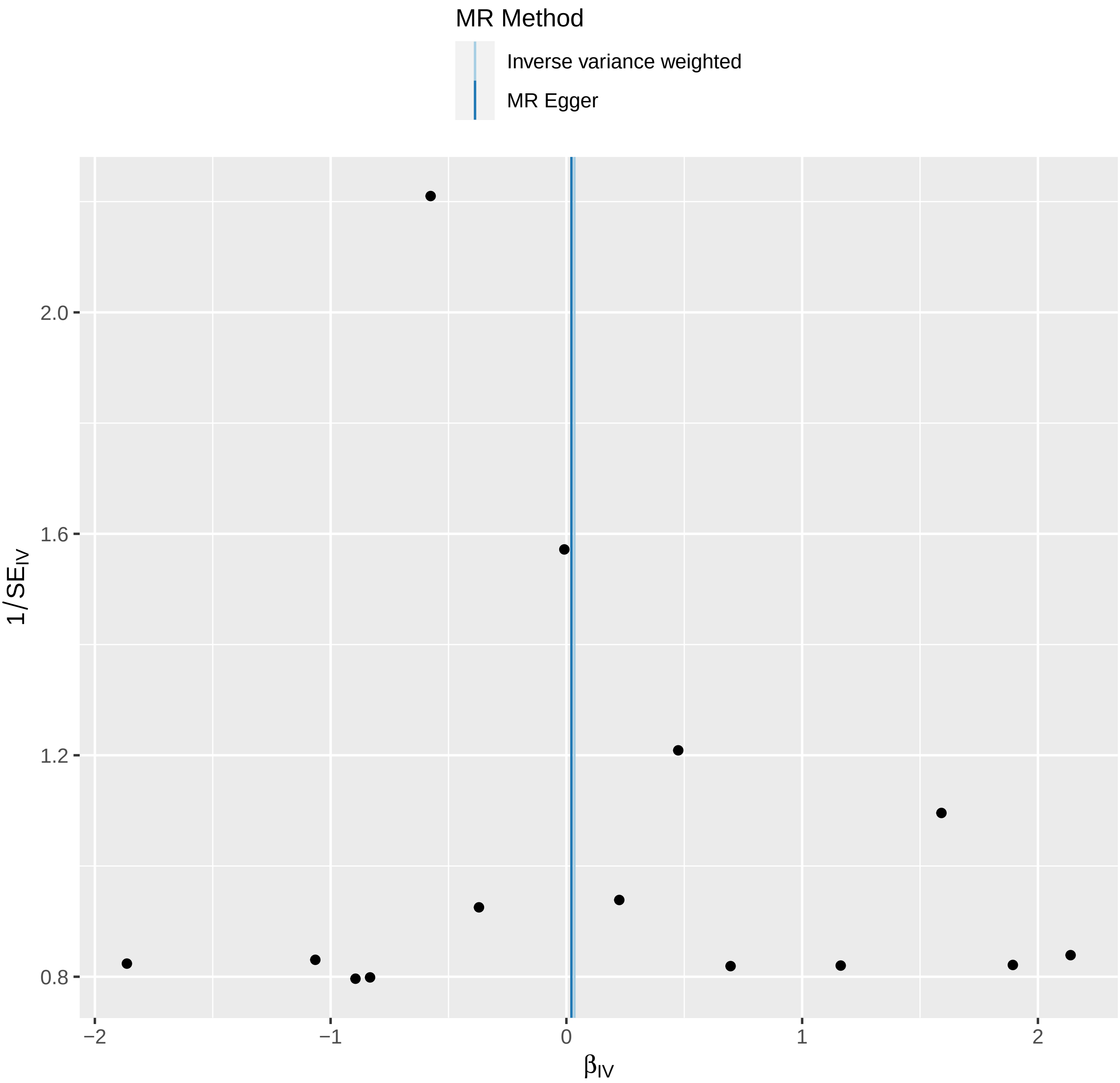

Batch 2021 : 1−stearoylglycerophosphoinositol on Ulcerative pancolitis

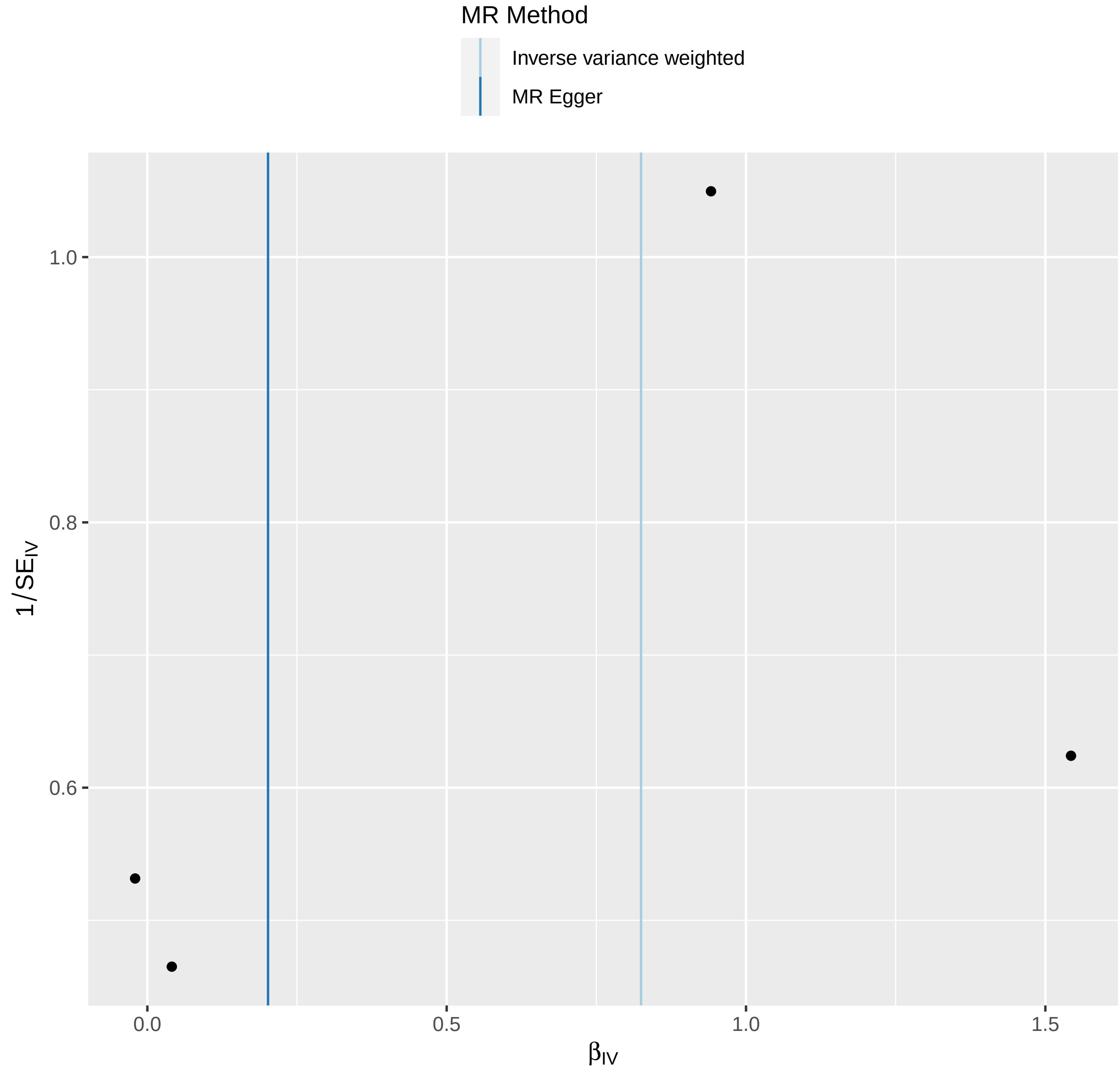

Batch 2022 : Myo−inositol on Ulcerative pancolitis

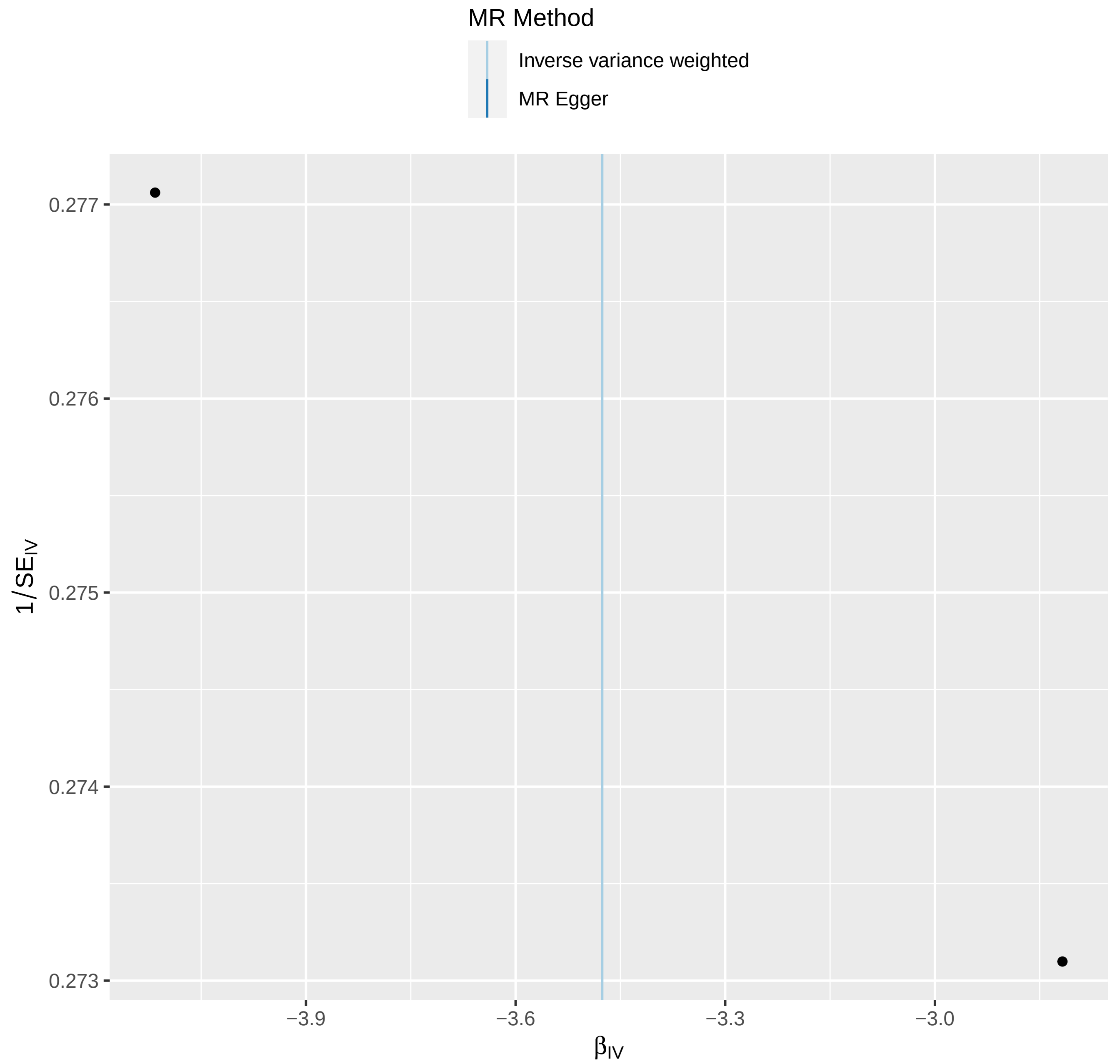

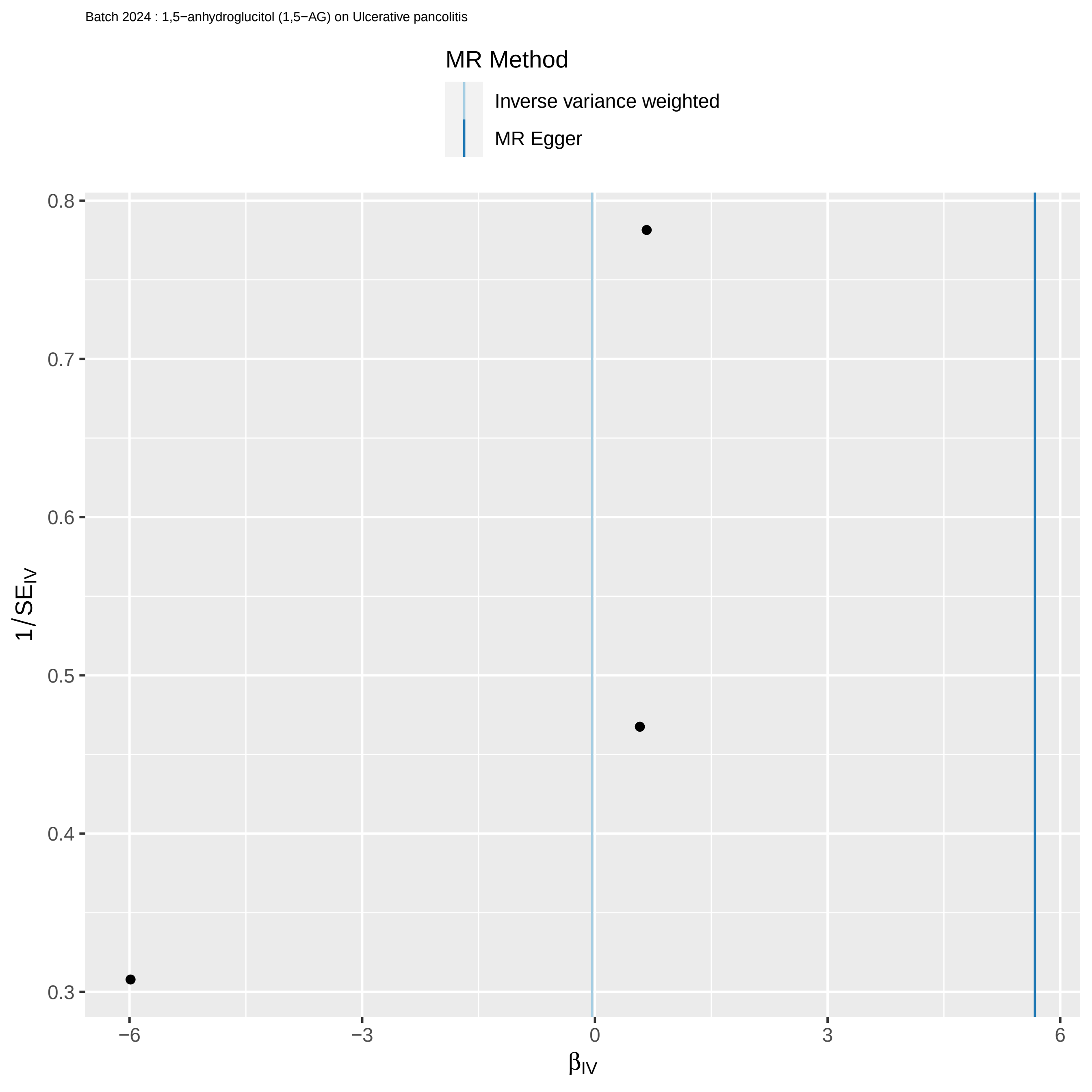

Batch 2024 : 1,5−anhydroglucitol (1,5−AG) on Ulcerative pancolitis
MR Method
Inverse variance weighted
MR Egger
0.8
0.7
0.6
0.5
0.4
0.3
1/SE_IV
−6
−3
0
3
6
β_IV

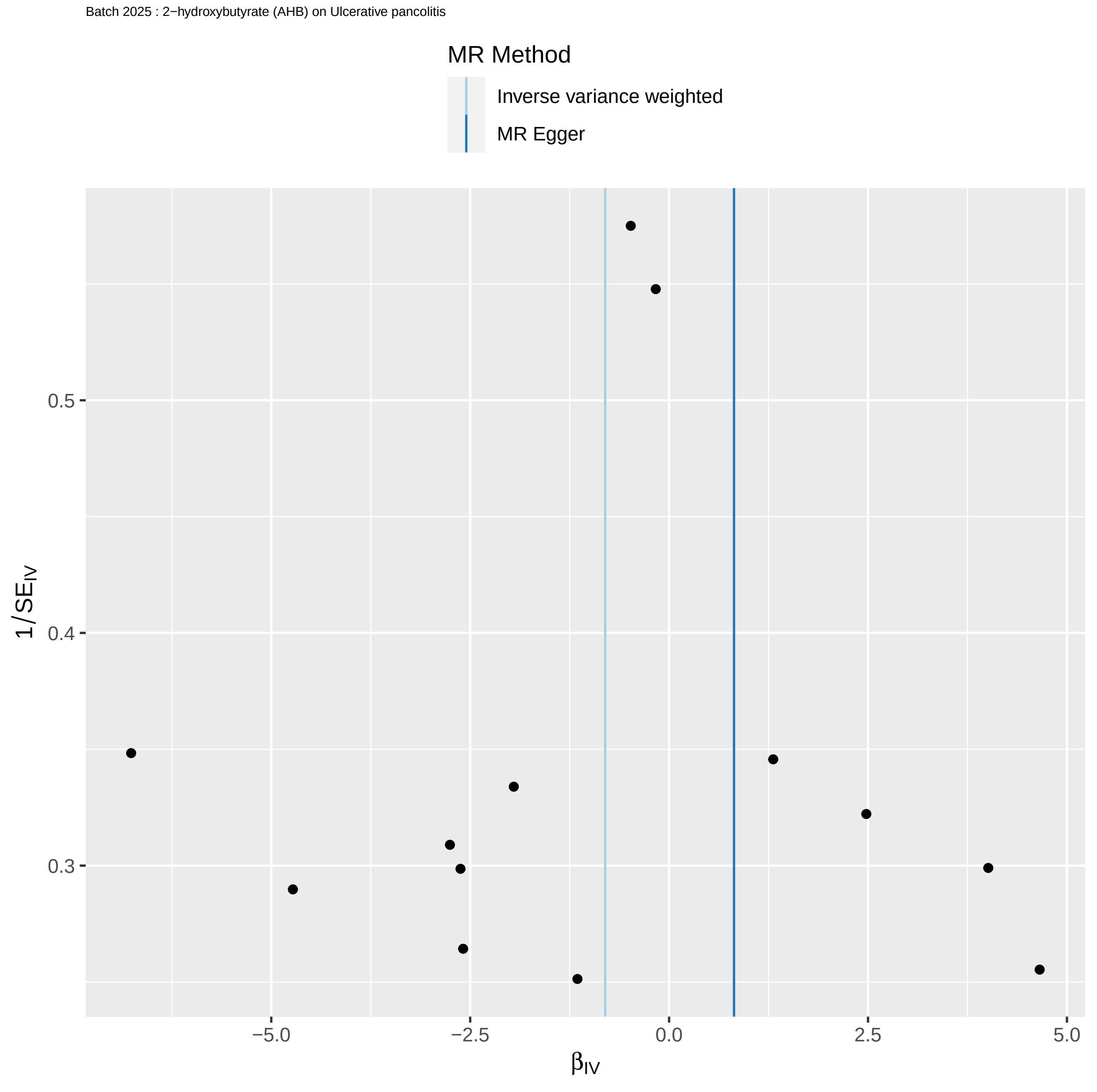
Batch 2025 : 2−hydroxybutyrate (AHB) on Ulcerative pancolitis
MR Method
Inverse variance weighted
MR Egger
0.5
0.4
0.3
1/SE_IV
−5.0
−2.5
0.0
2.5
5.0
β_IV

Batch 2028 : 1−palmitoylglycerol (1−monopalmitin) on Ulcerative pancolitis

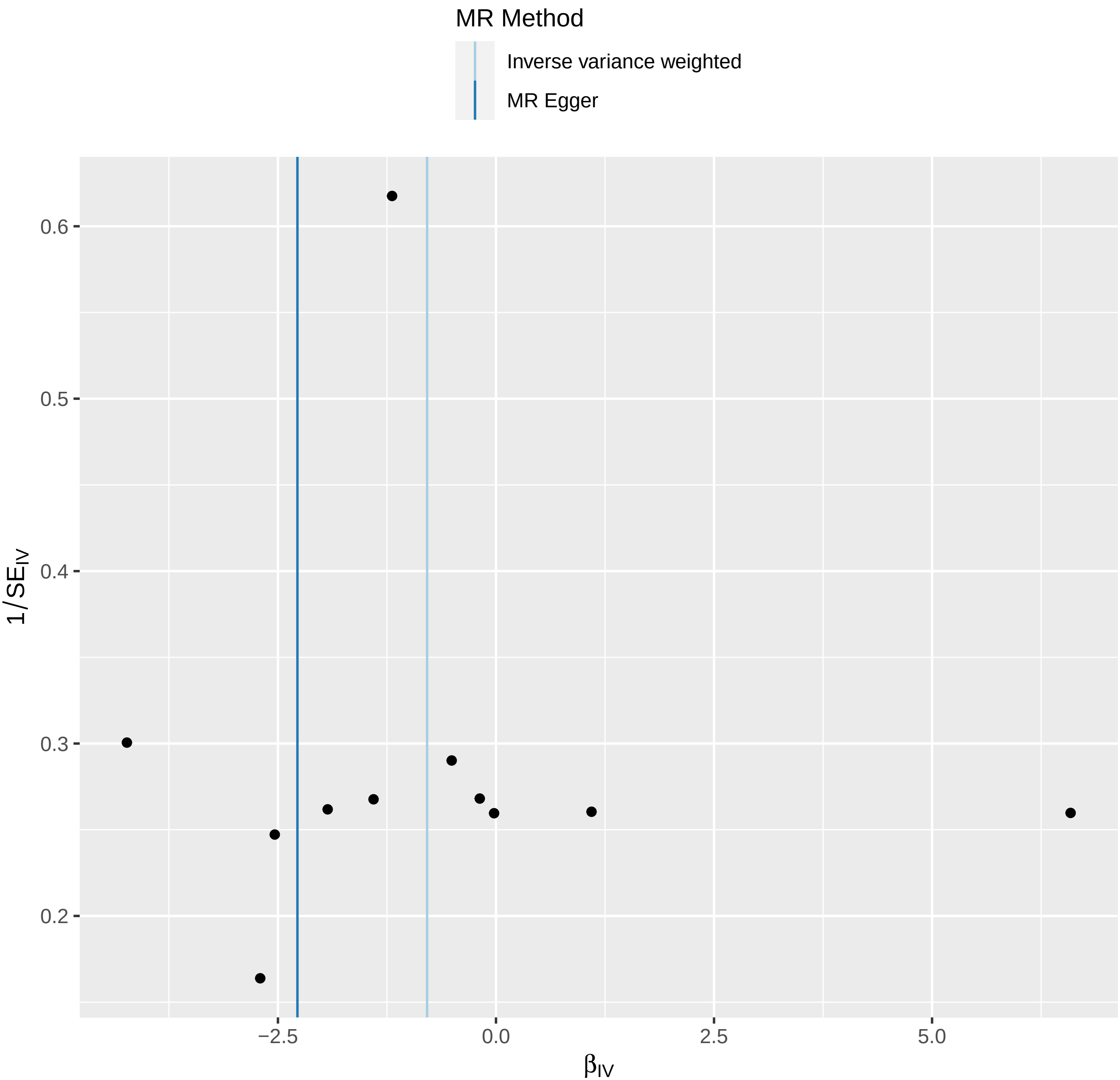

Batch 2031 : 2−hydroxyisobutyrate on Ulcerative pancolitis

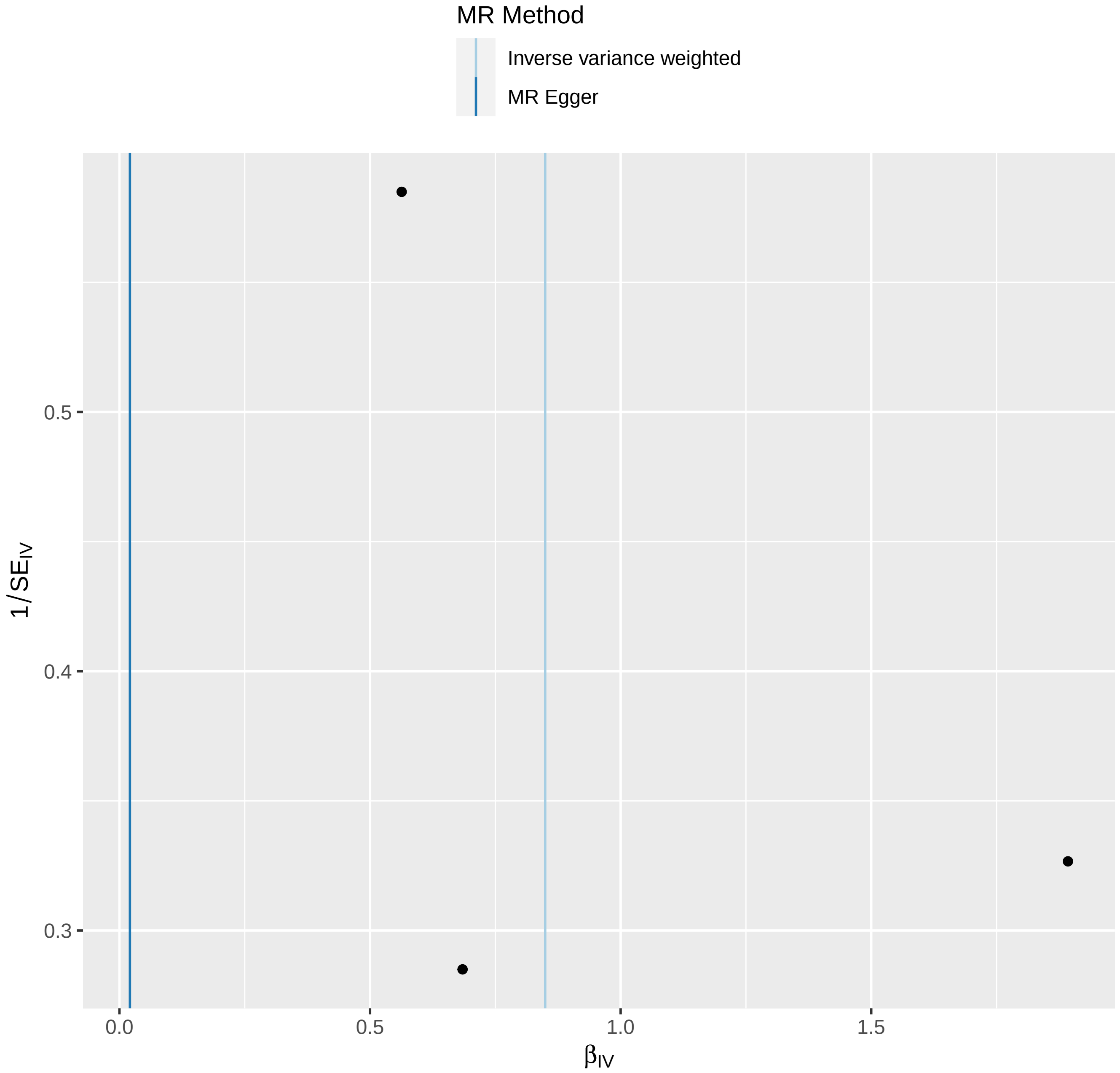

Batch 2032 : 4−methyl−2−oxopentanoate on Ulcerative pancolitis

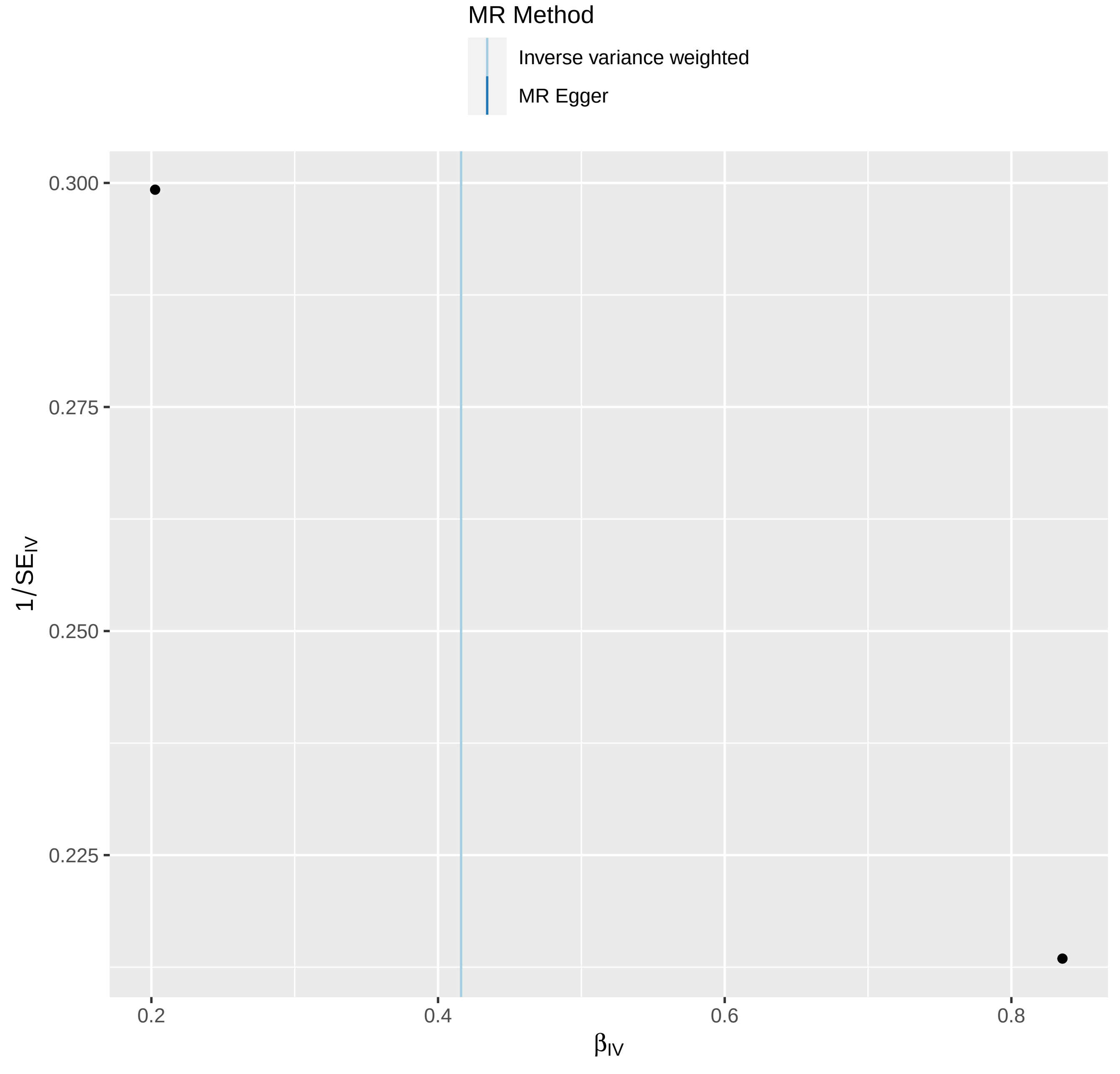

Batch 2036 : Levulinate (4−oxovalerate) on Ulcerative pancolitis

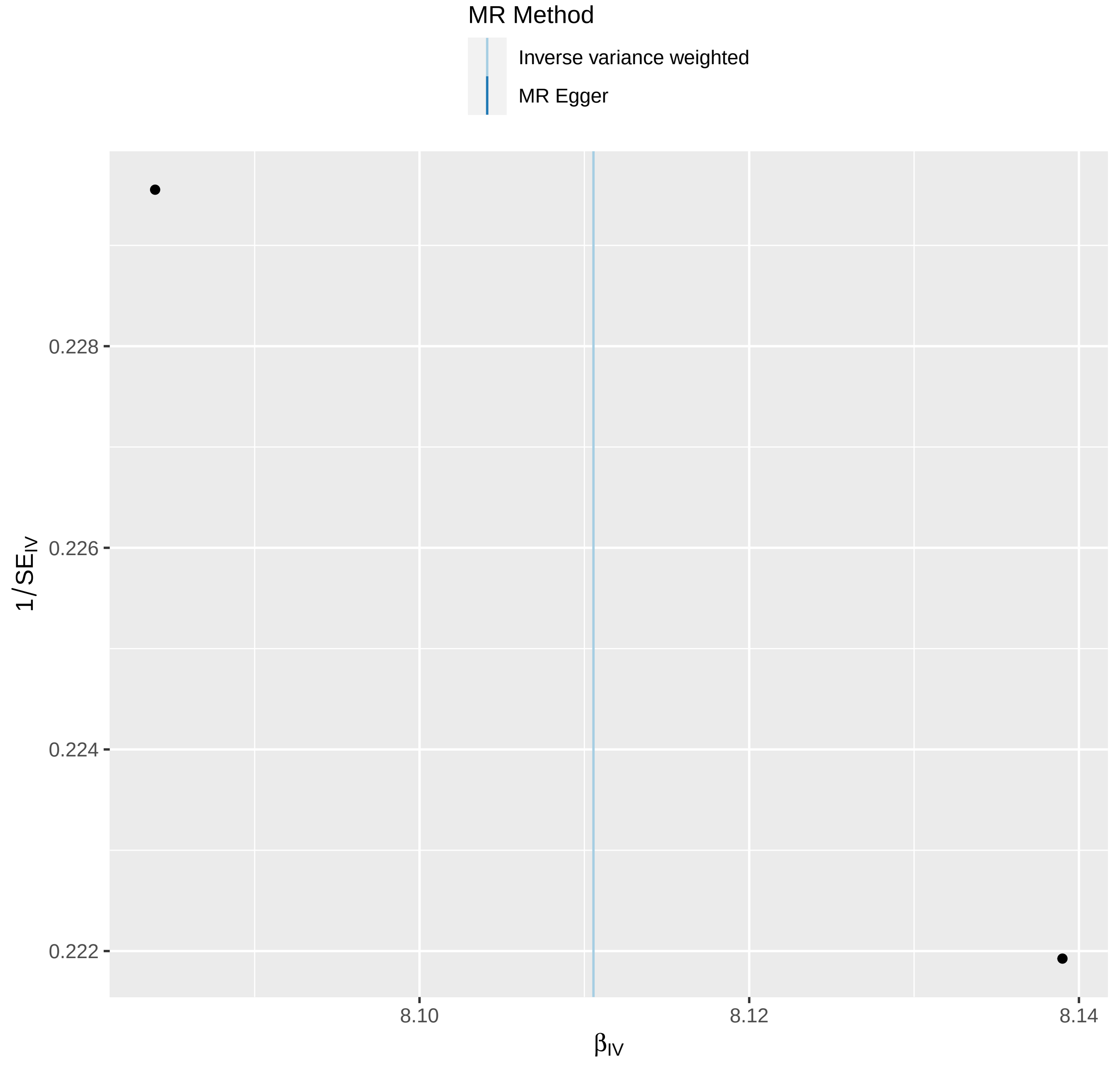

Batch 2038 : Cholate on Ulcerative pancolitis

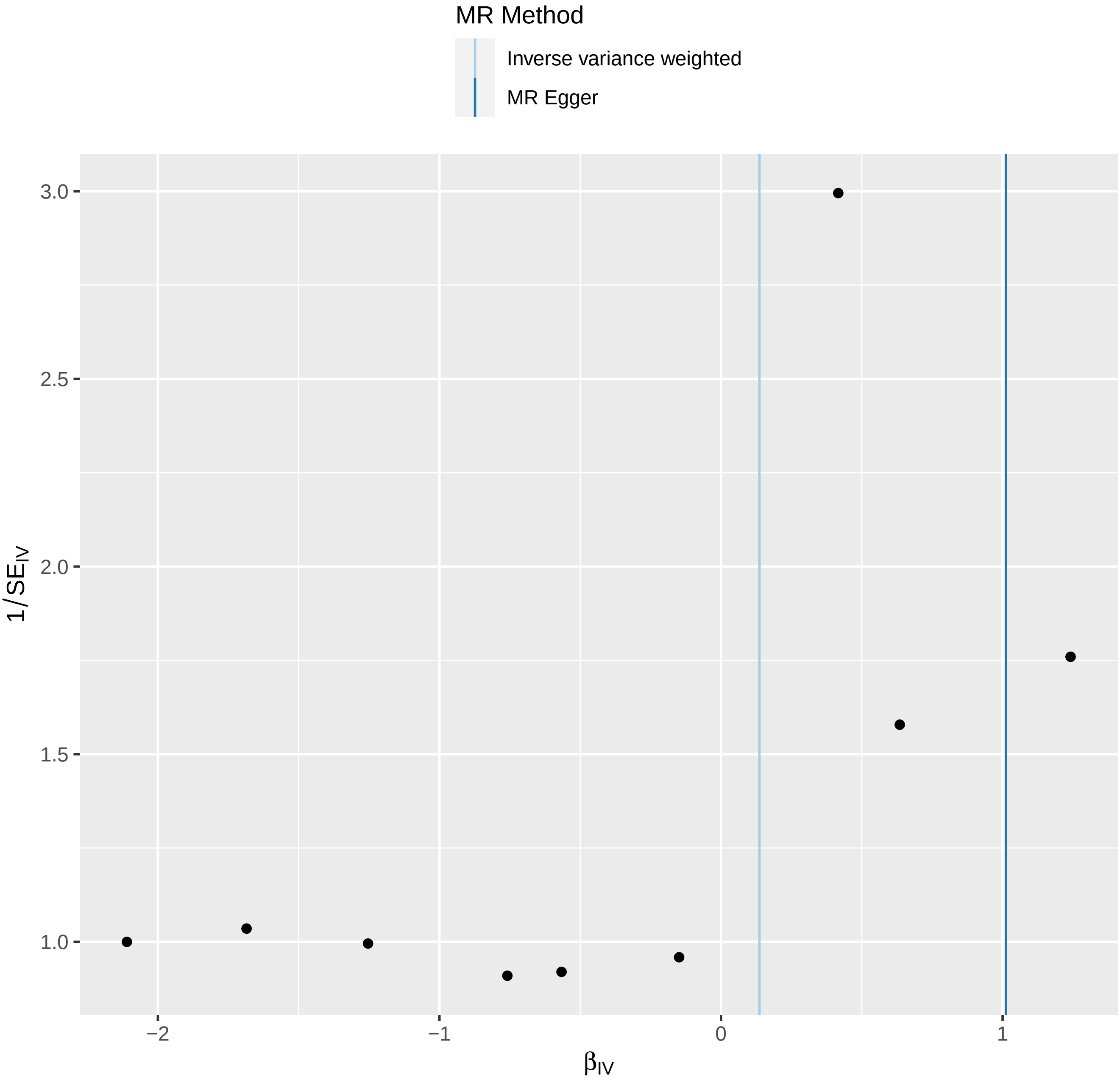

Batch 2041 : Hyodeoxycholate on Ulcerative pancolitis

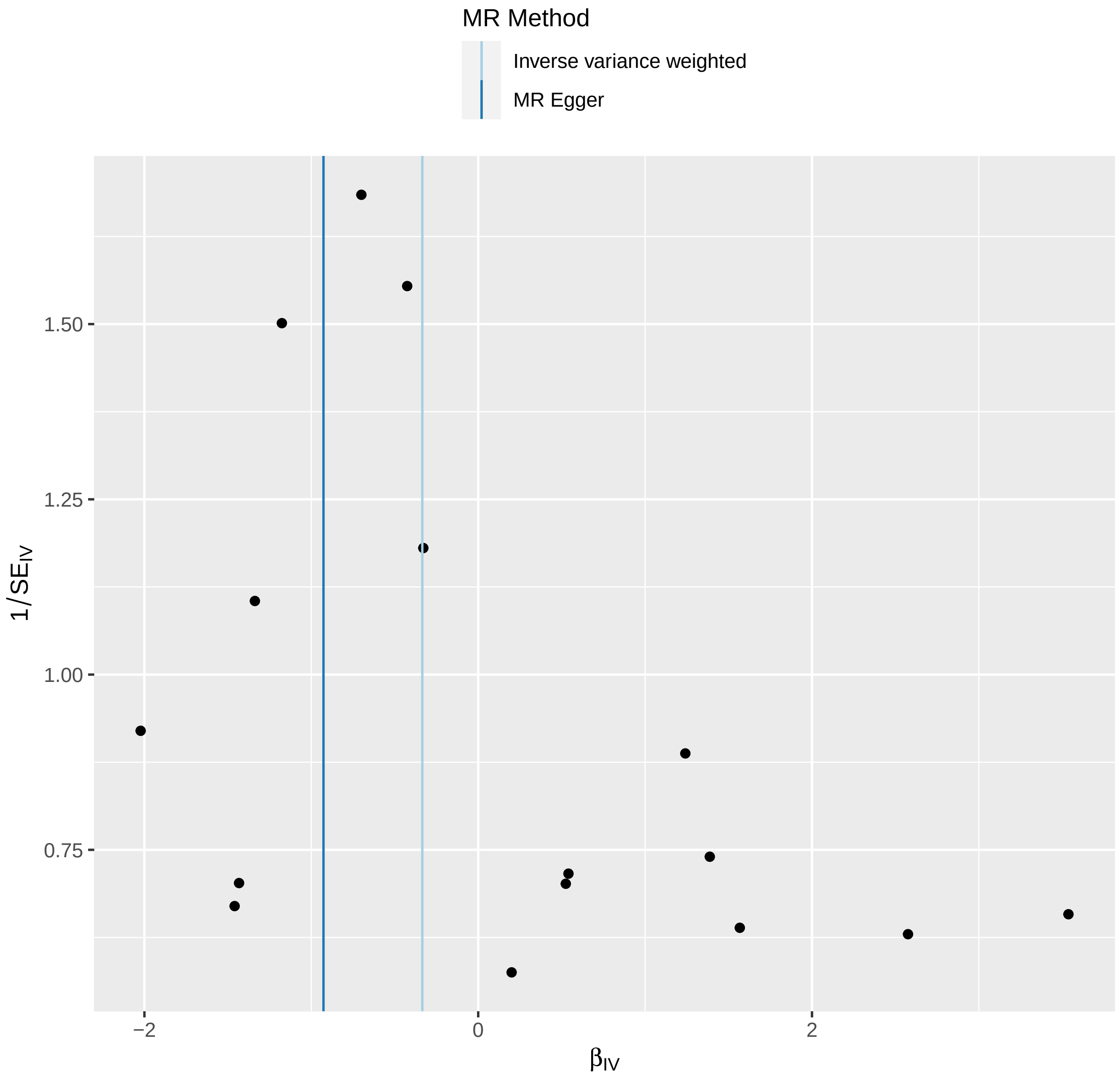

Batch 2042 : 3−indoxyl sulfate on Ulcerative pancolitis

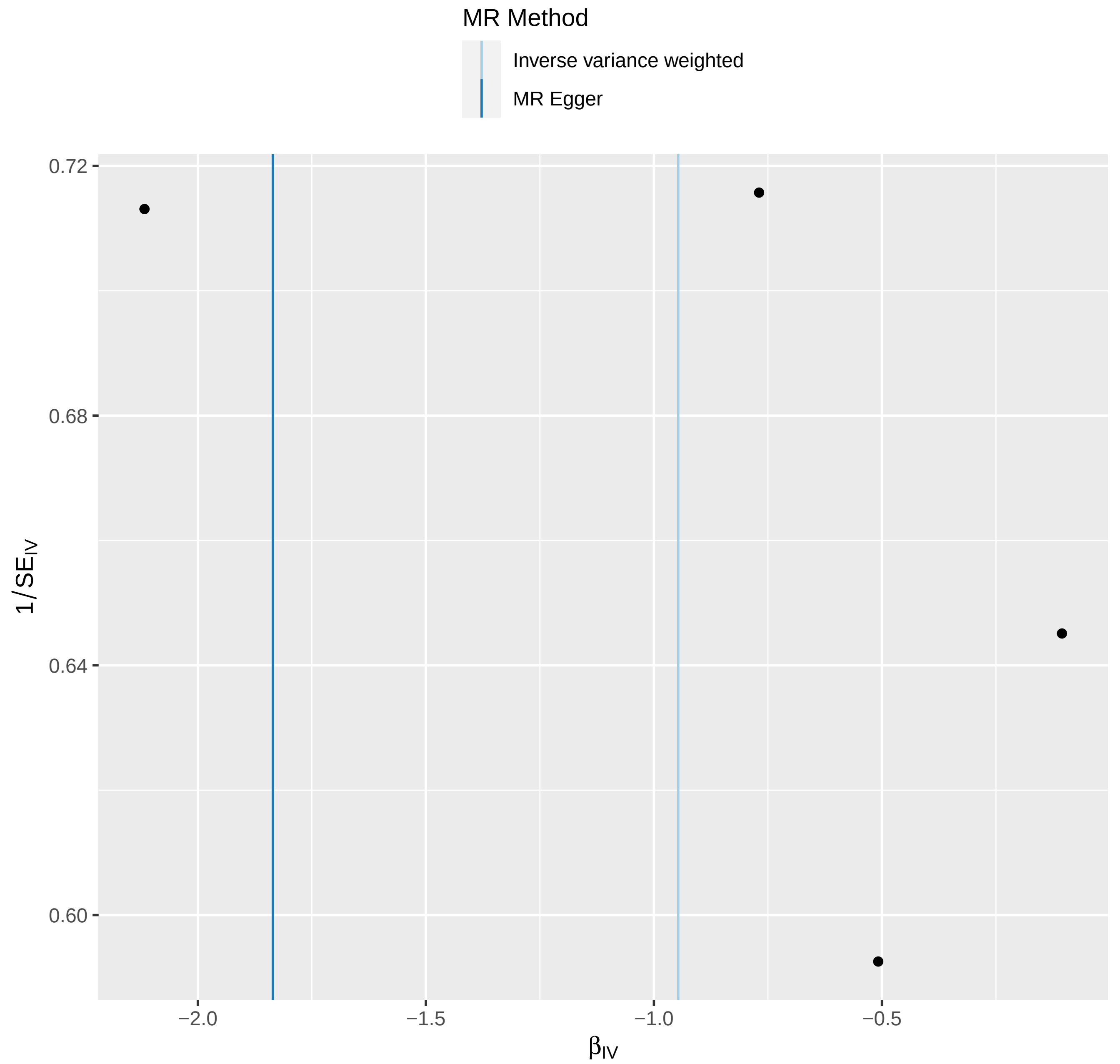

Batch 2046 : Erythrose on Ulcerative pancolitis

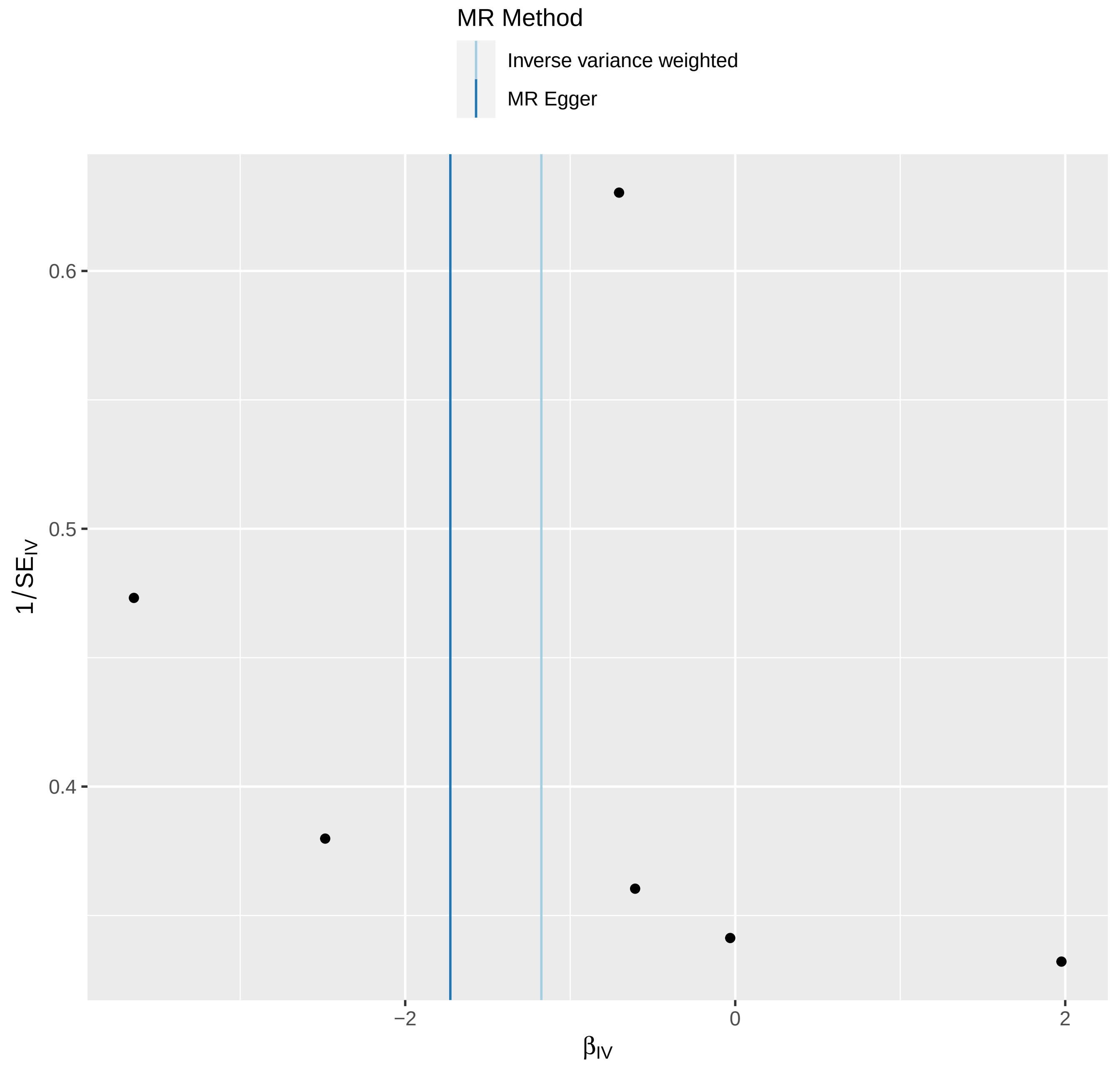

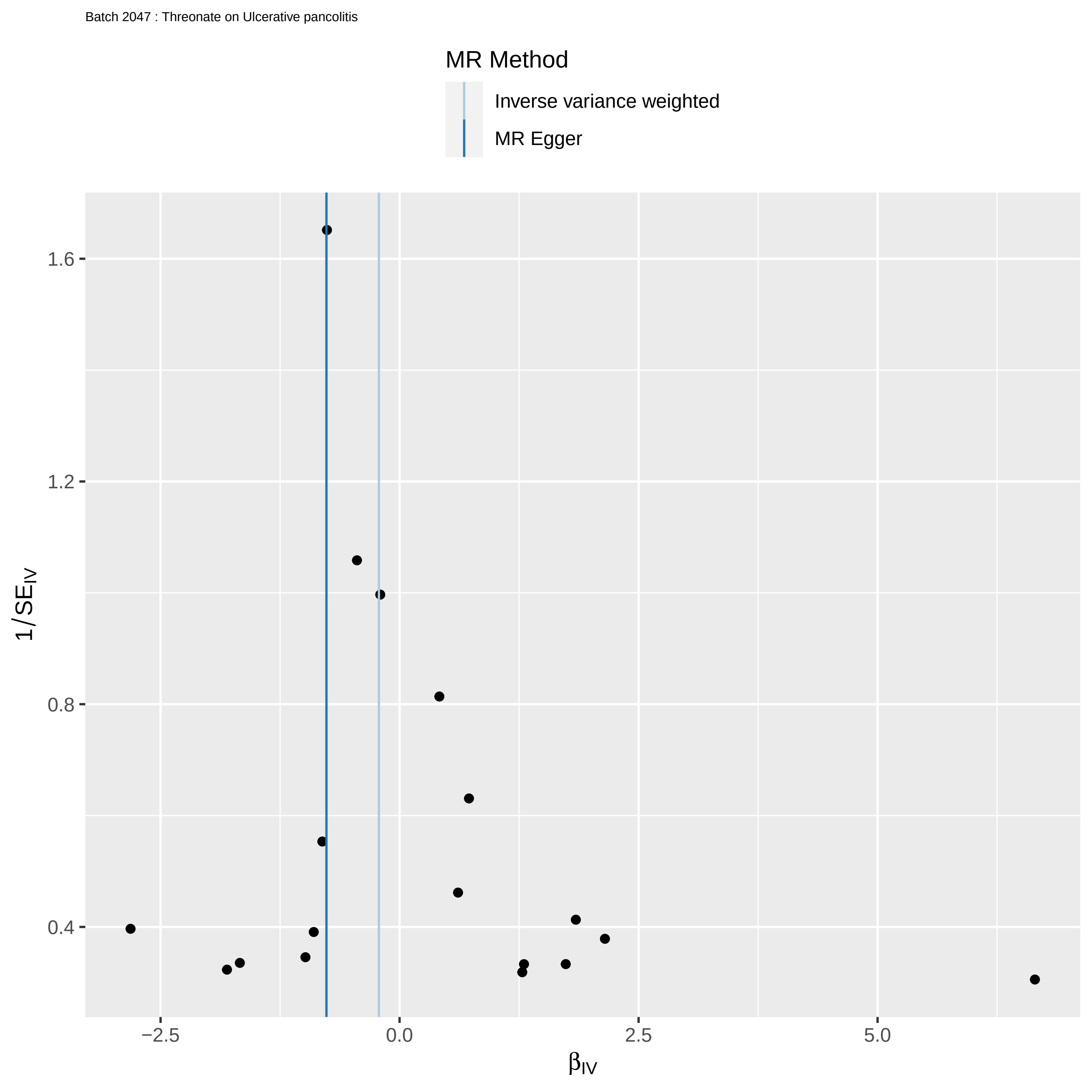
Batch 2047 : Threonate on Ulcerative pancolitis
MR Method
Inverse variance weighted
MR Egger
1.6
1.2
0.8
0.4
1/SE_IV
−2.5
0.0
2.5
5.0
β_IV

Batch 2048 : Cysteine on Ulcerative pancolitis

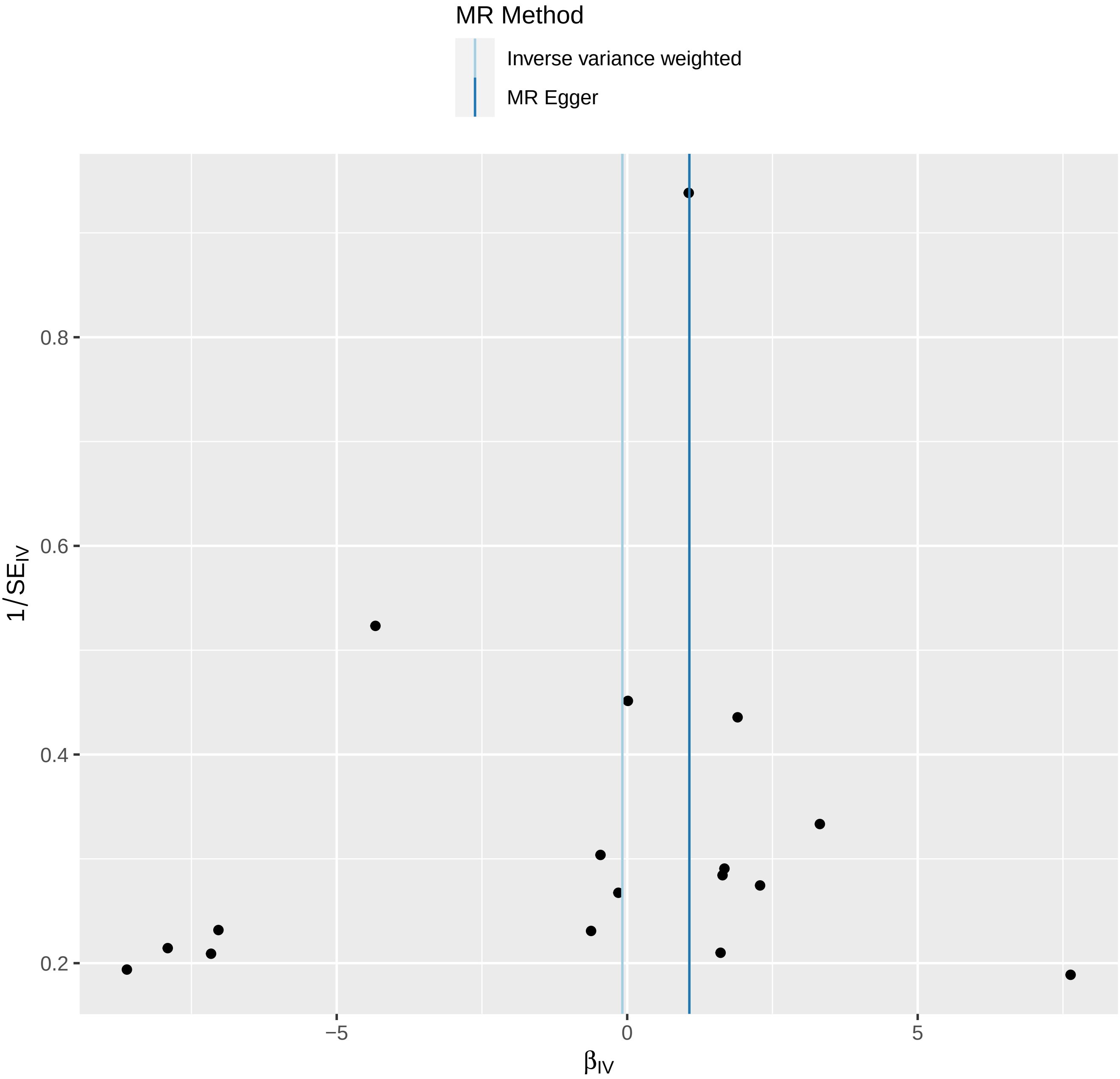

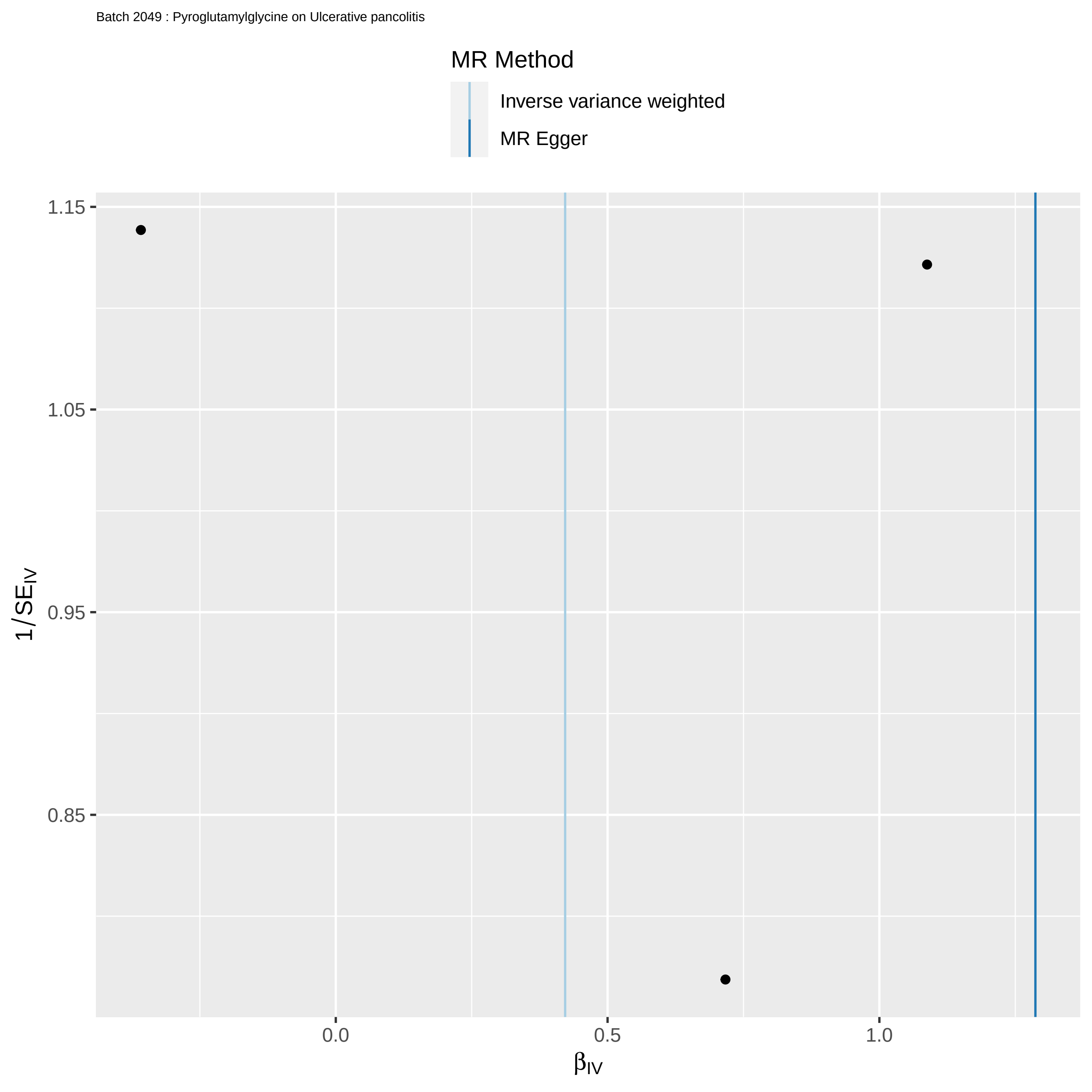

Batch 2049 : Pyroglutamylglycine on Ulcerative pancolitis
MR Method
Inverse variance weighted
MR Egger
1.15
1.05
0.95
0.85
1/SE_IV
0.0
0.5
1.0
β_IV

Batch 2051 : DSGEGDFXAEGGGVR* on Ulcerative pancolitis

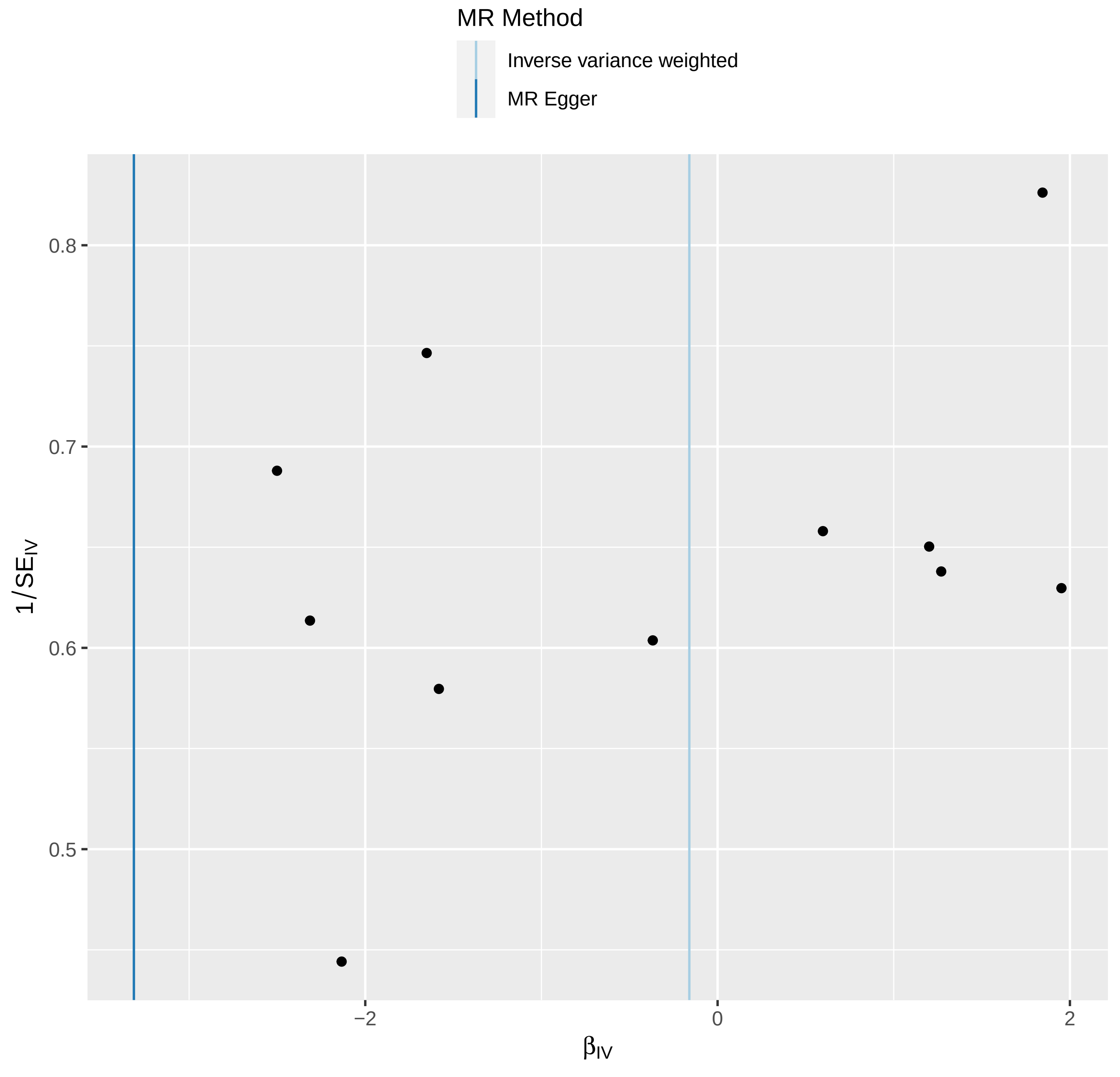

Batch 2052 : Pyridoxate on Ulcerative pancolitis

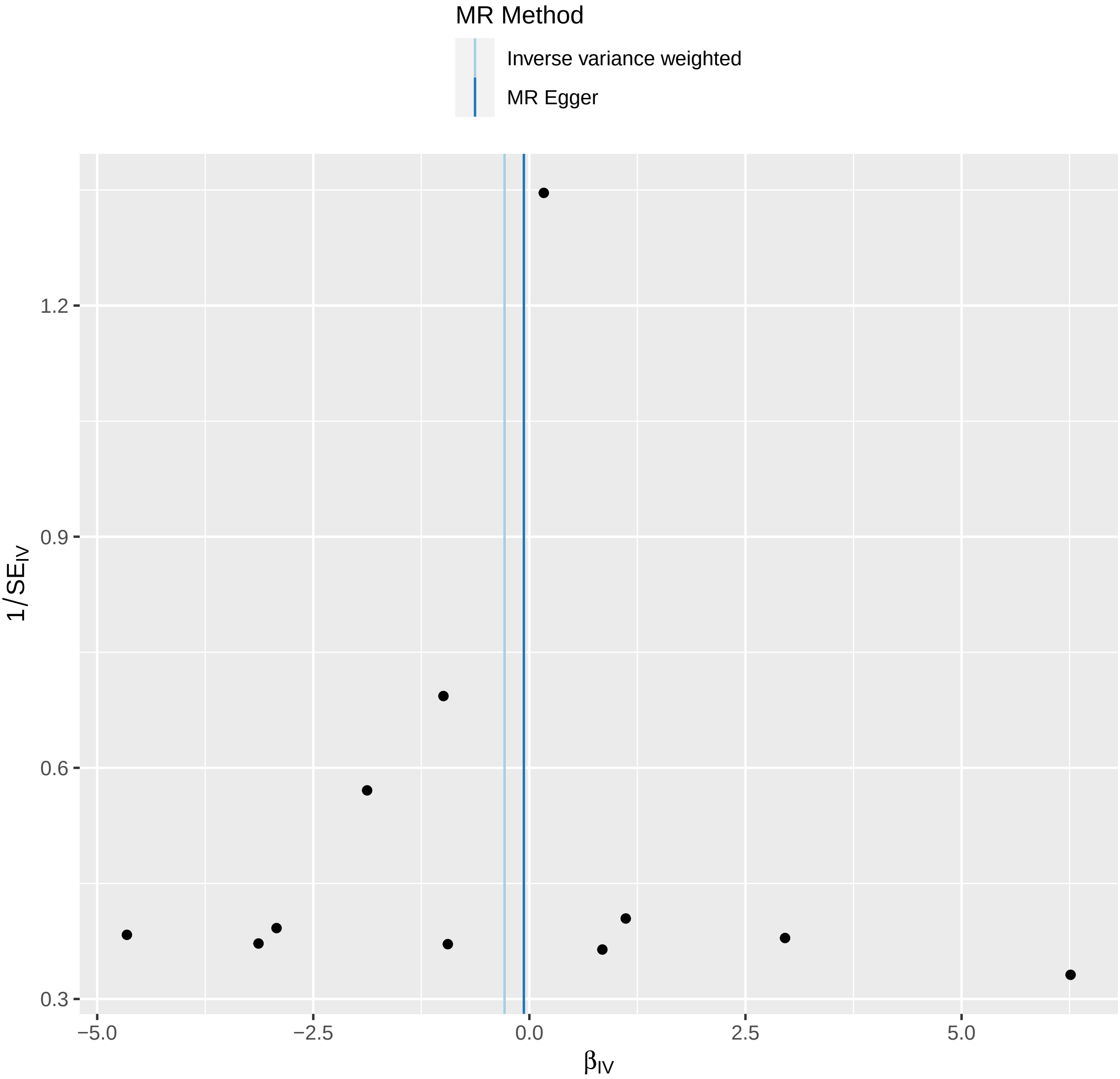

Batch 2053 : Androsterone sulfate on Ulcerative pancolitis

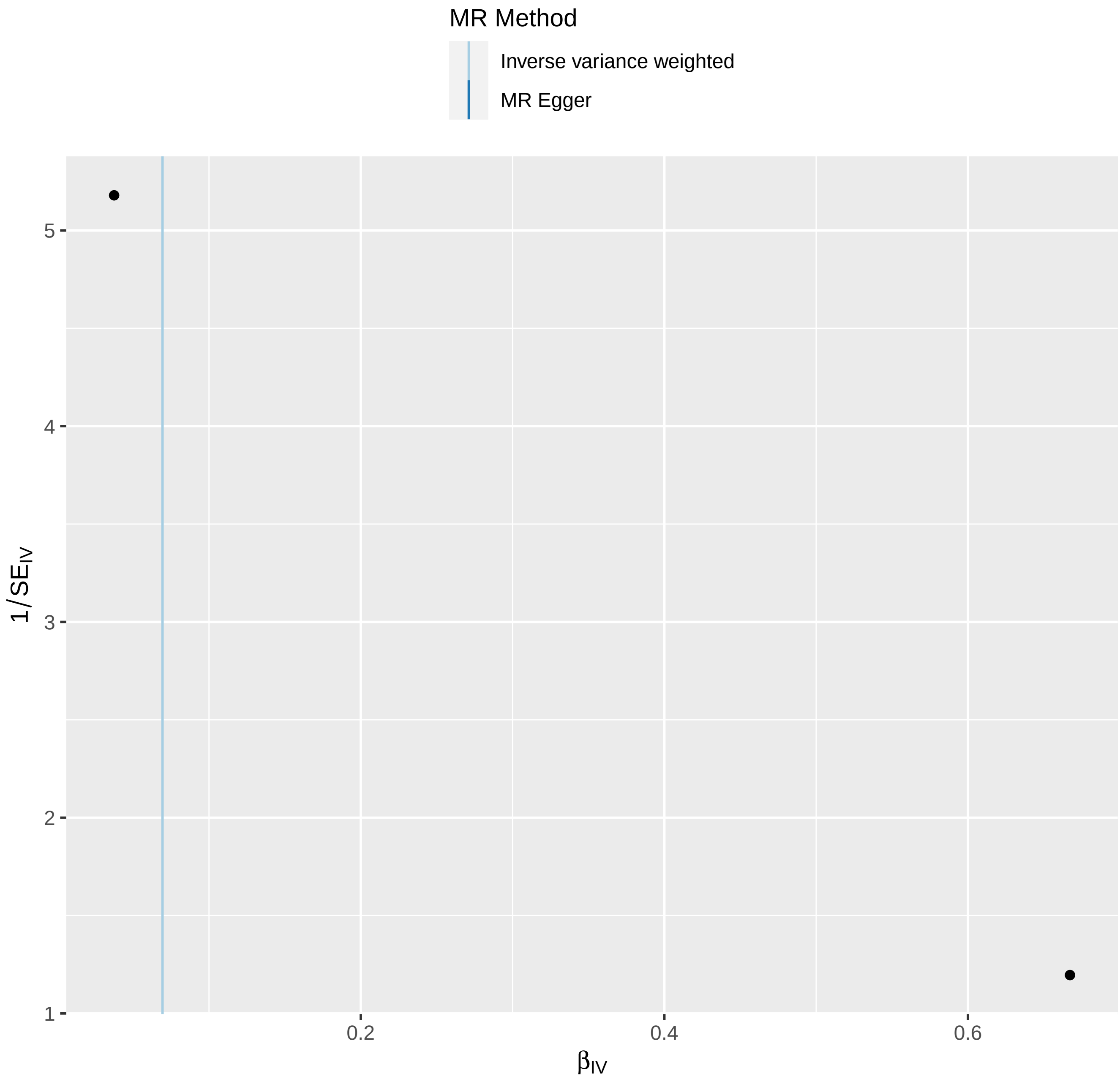

Batch 2056 : Acetylcarnitine on Ulcerative pancolitis

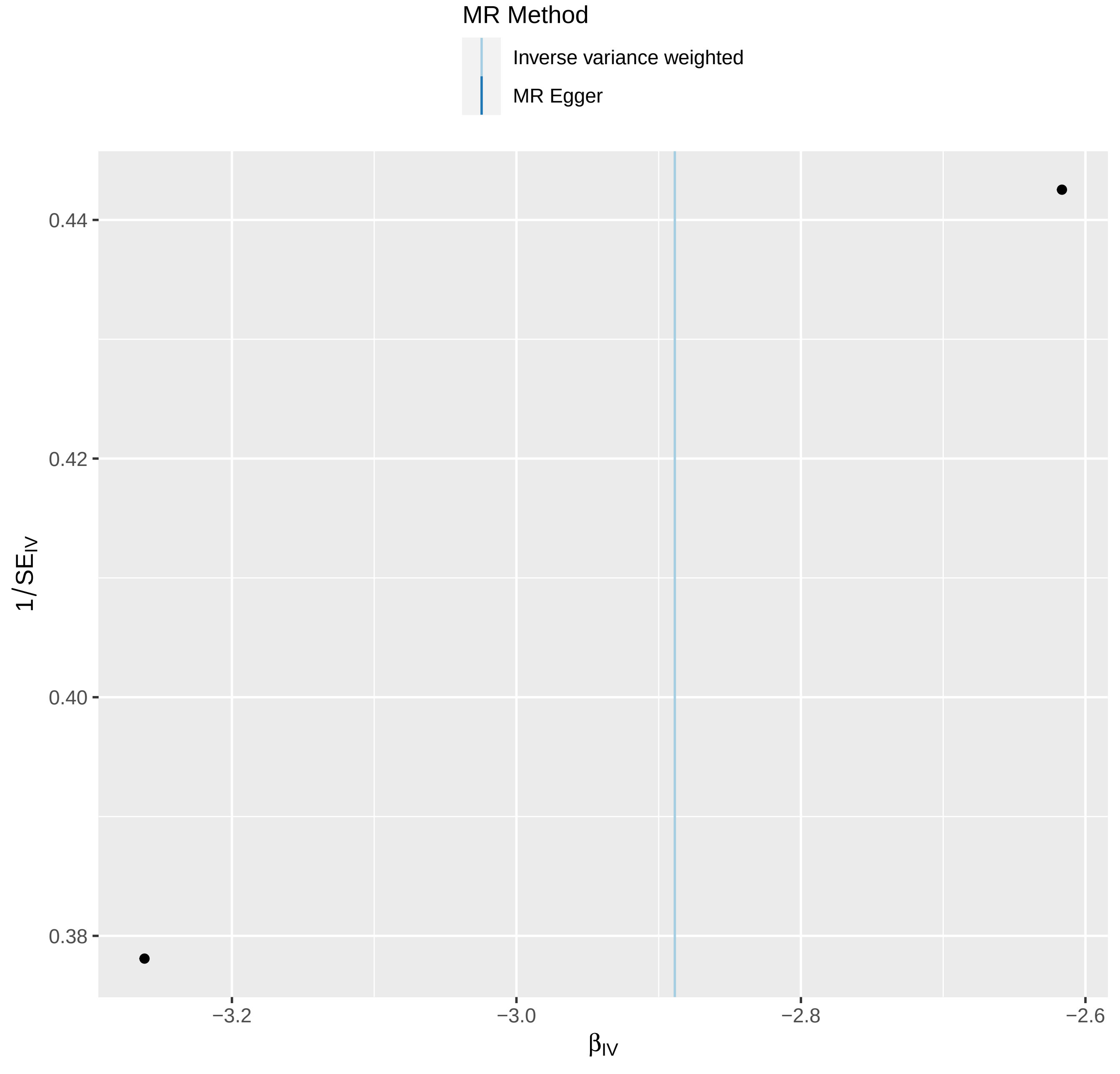

Batch 2057 : Serine on Ulcerative pancolitis

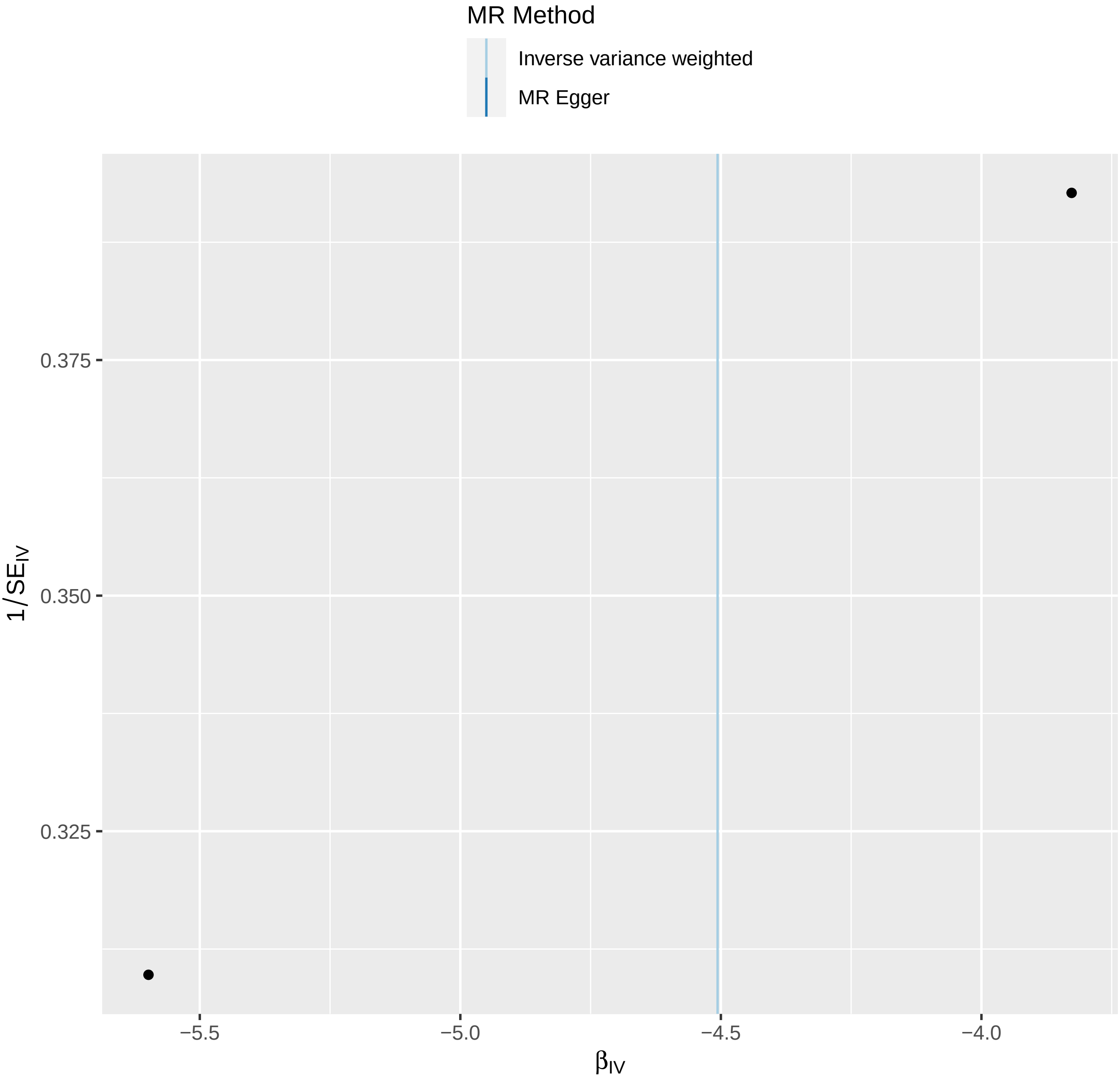

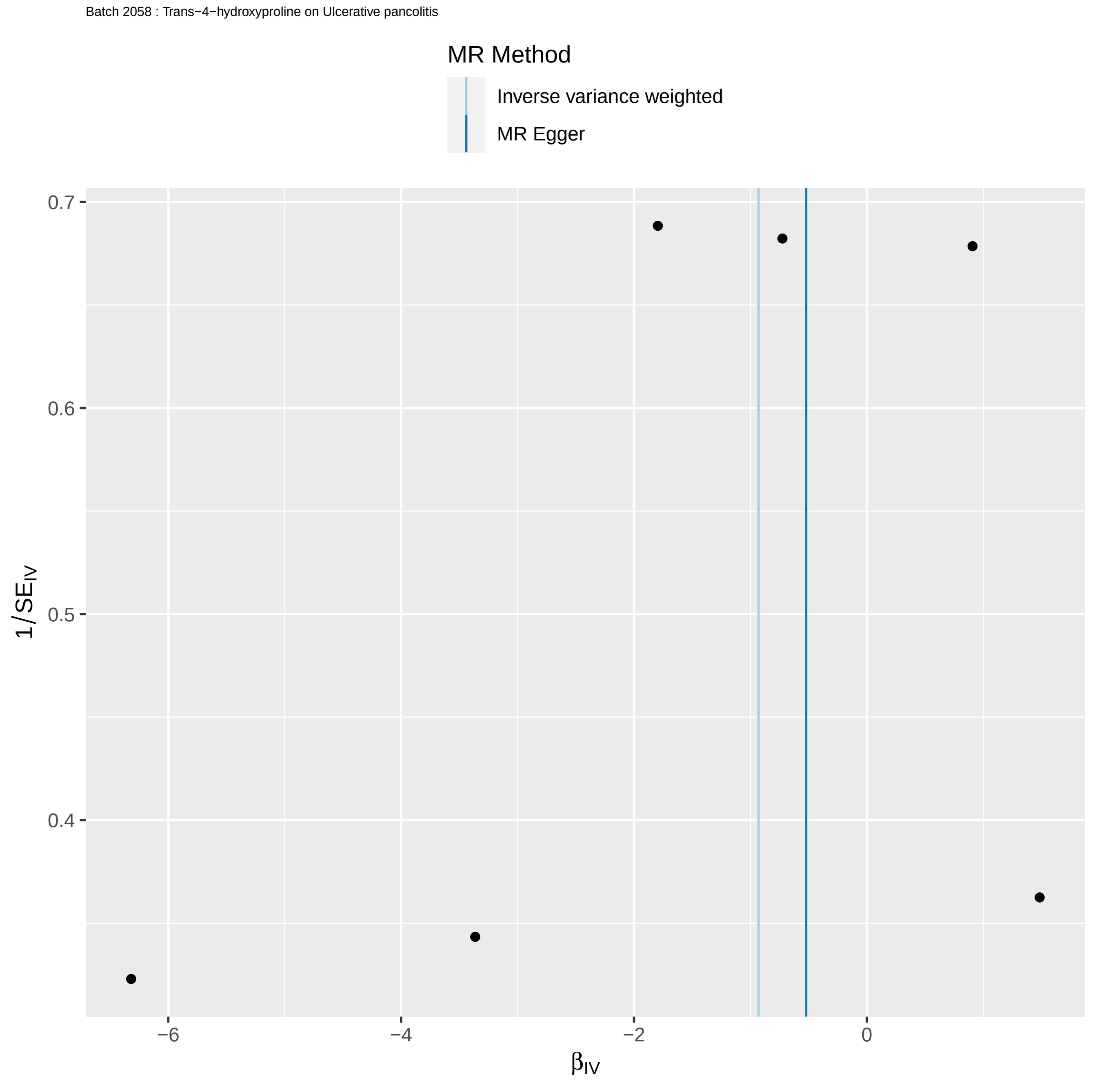
Batch 2058 : Trans−4−hydroxyproline on Ulcerative pancolitis
MR Method
Inverse variance weighted
MR Egger
0.7
0.6
0.5
0.4
1/SE_IV
−6
−4
−2
0
β_IV

Batch 2059 : Glutamate on Ulcerative pancolitis

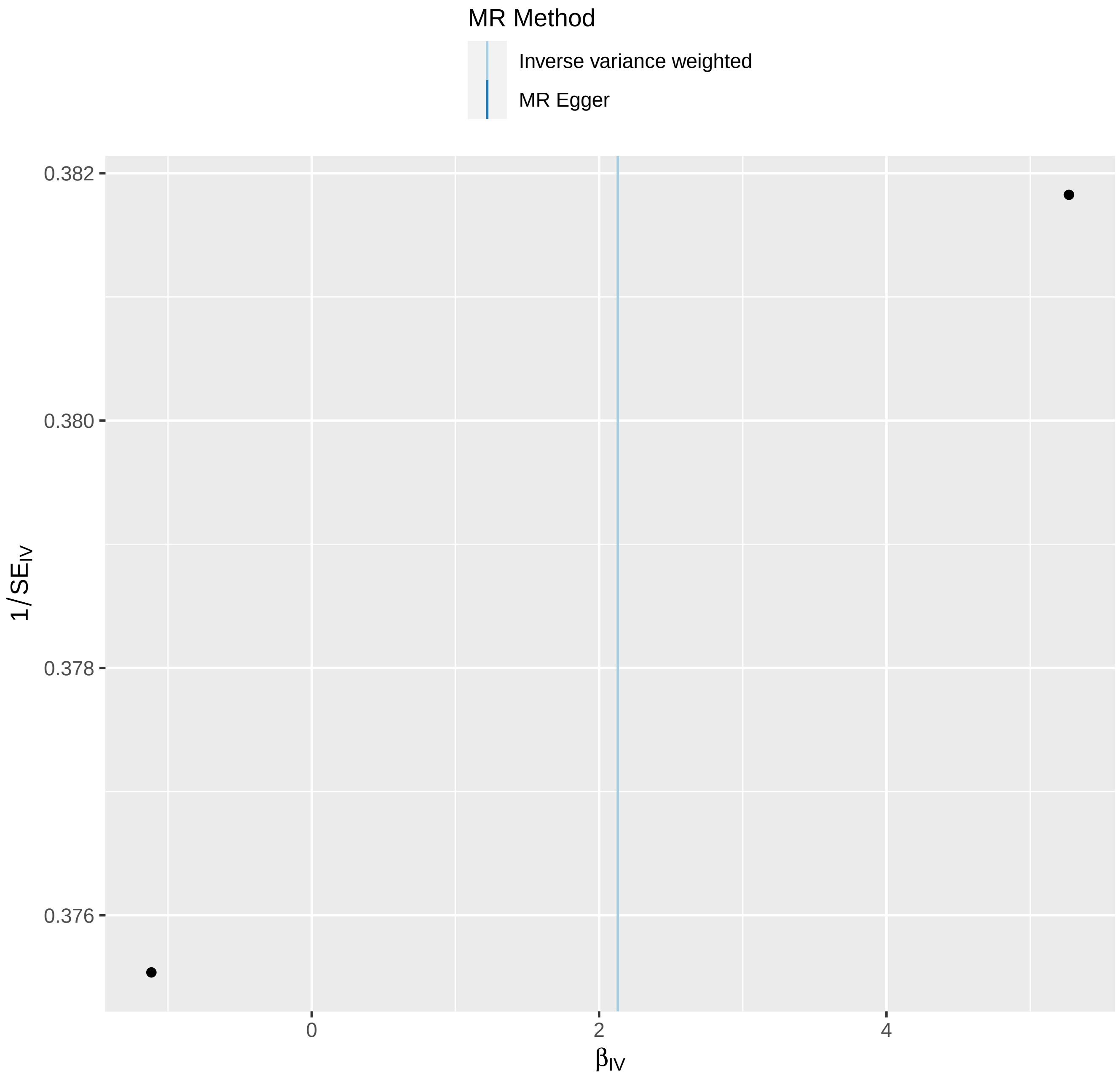

Batch 2060 : Hexanoylcarnitine on Ulcerative pancolitis

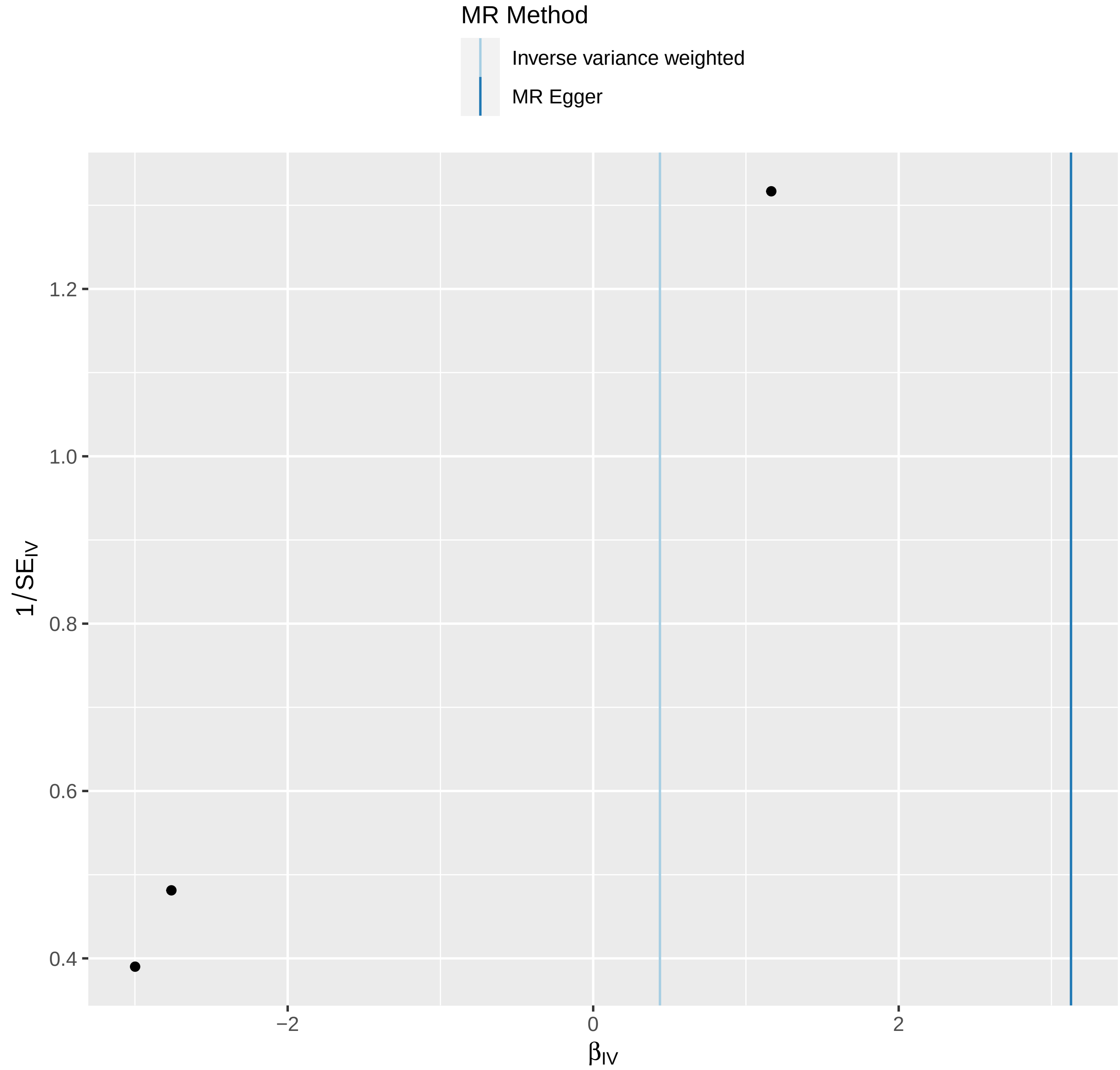

Batch 2064 : 2−aminobutyrate on Ulcerative pancolitis

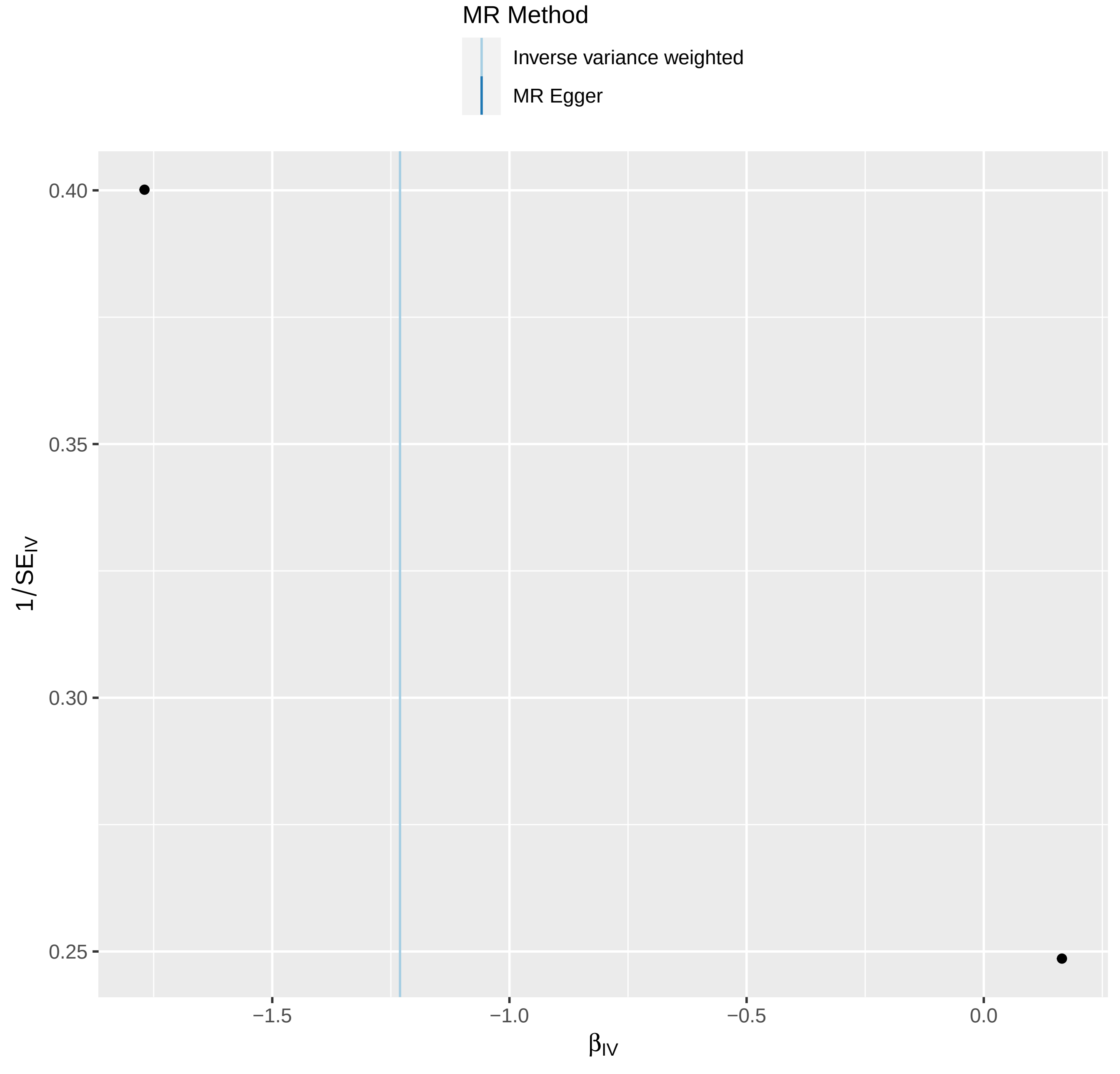

Batch 2069 : Butyrylcarnitine on Ulcerative pancolitis

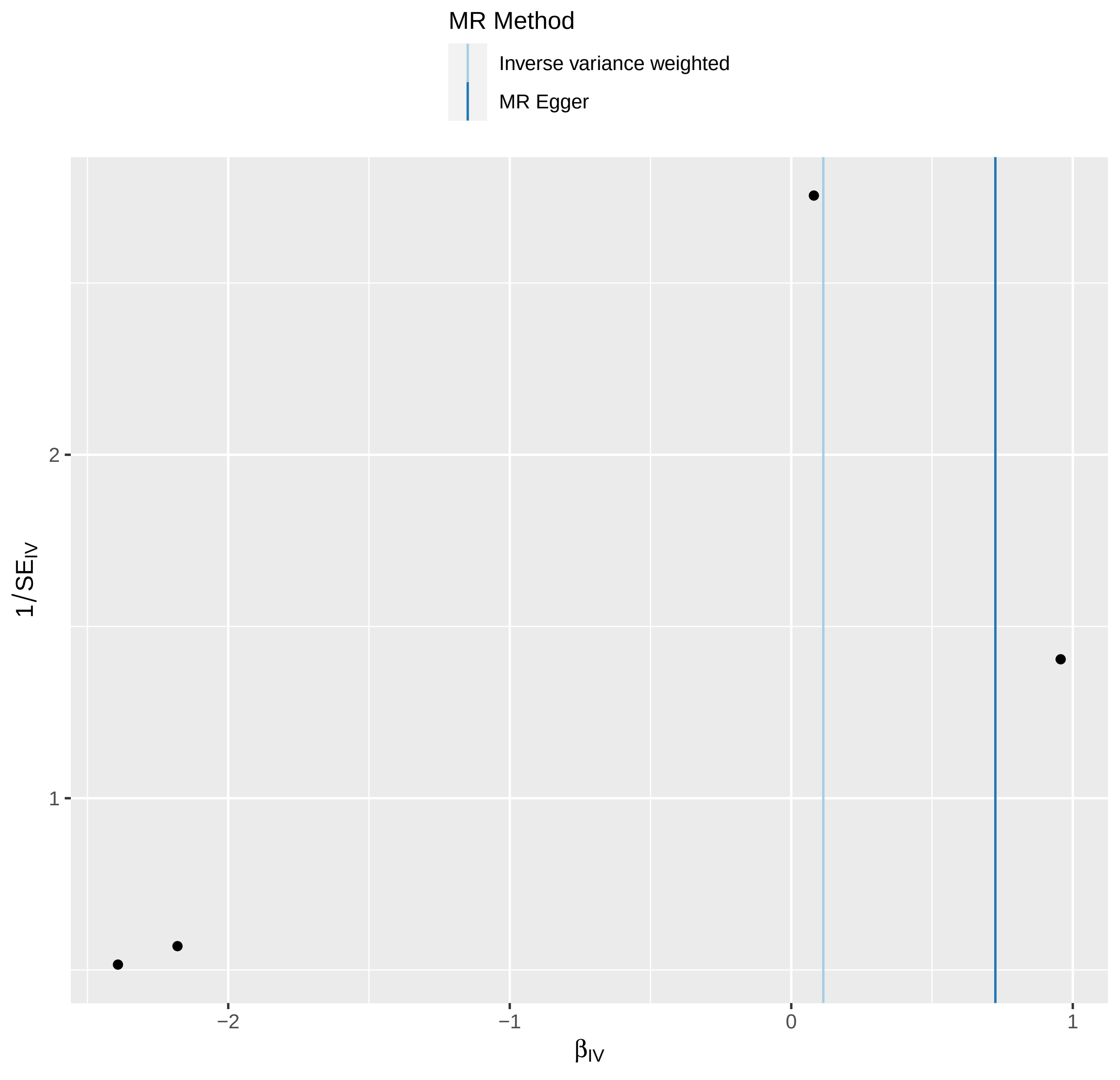

Batch 2072 : Propionylcarnitine on Ulcerative pancolitis

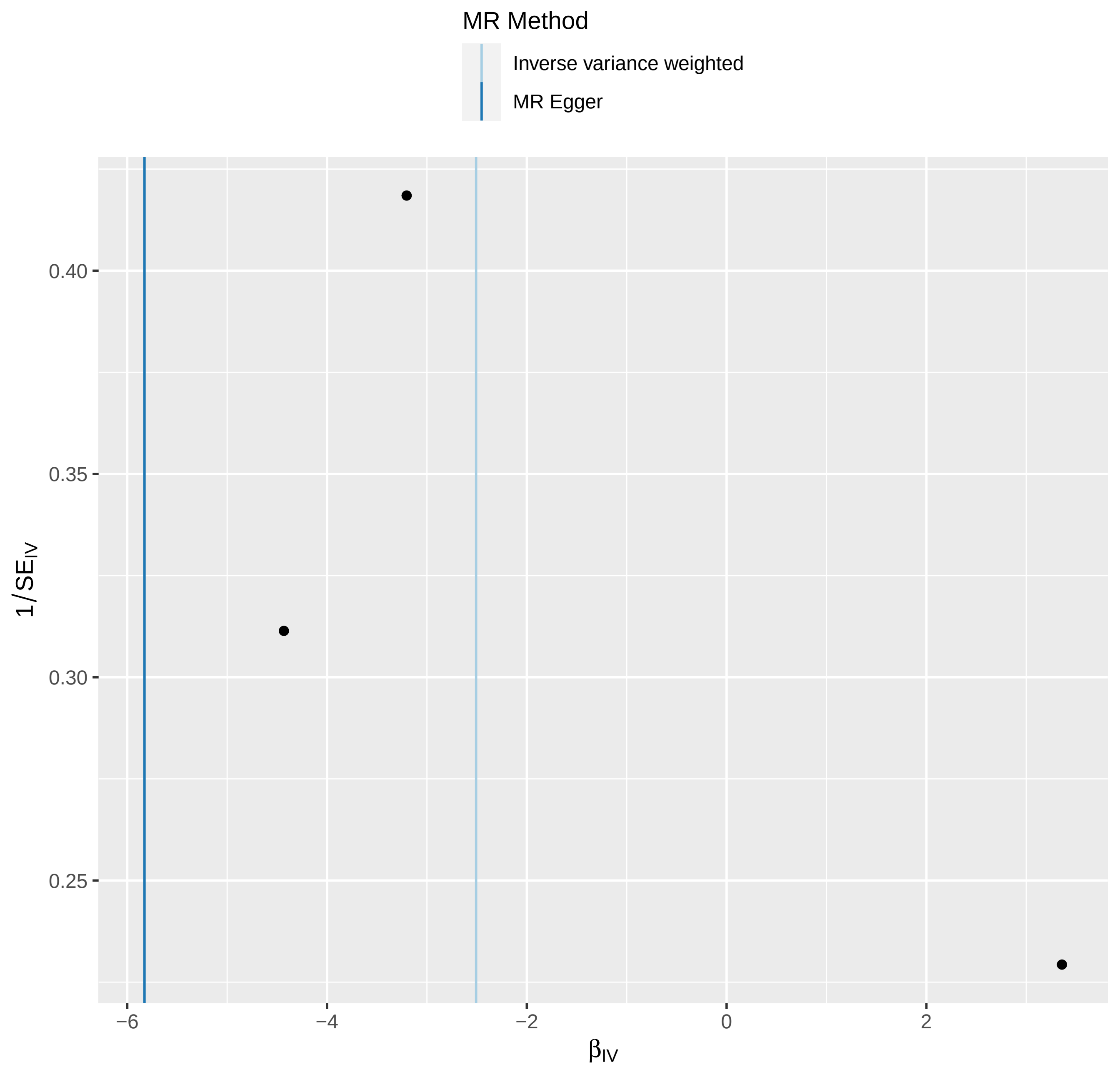

Batch 2073 : Caproate (6:0) on Ulcerative pancolitis

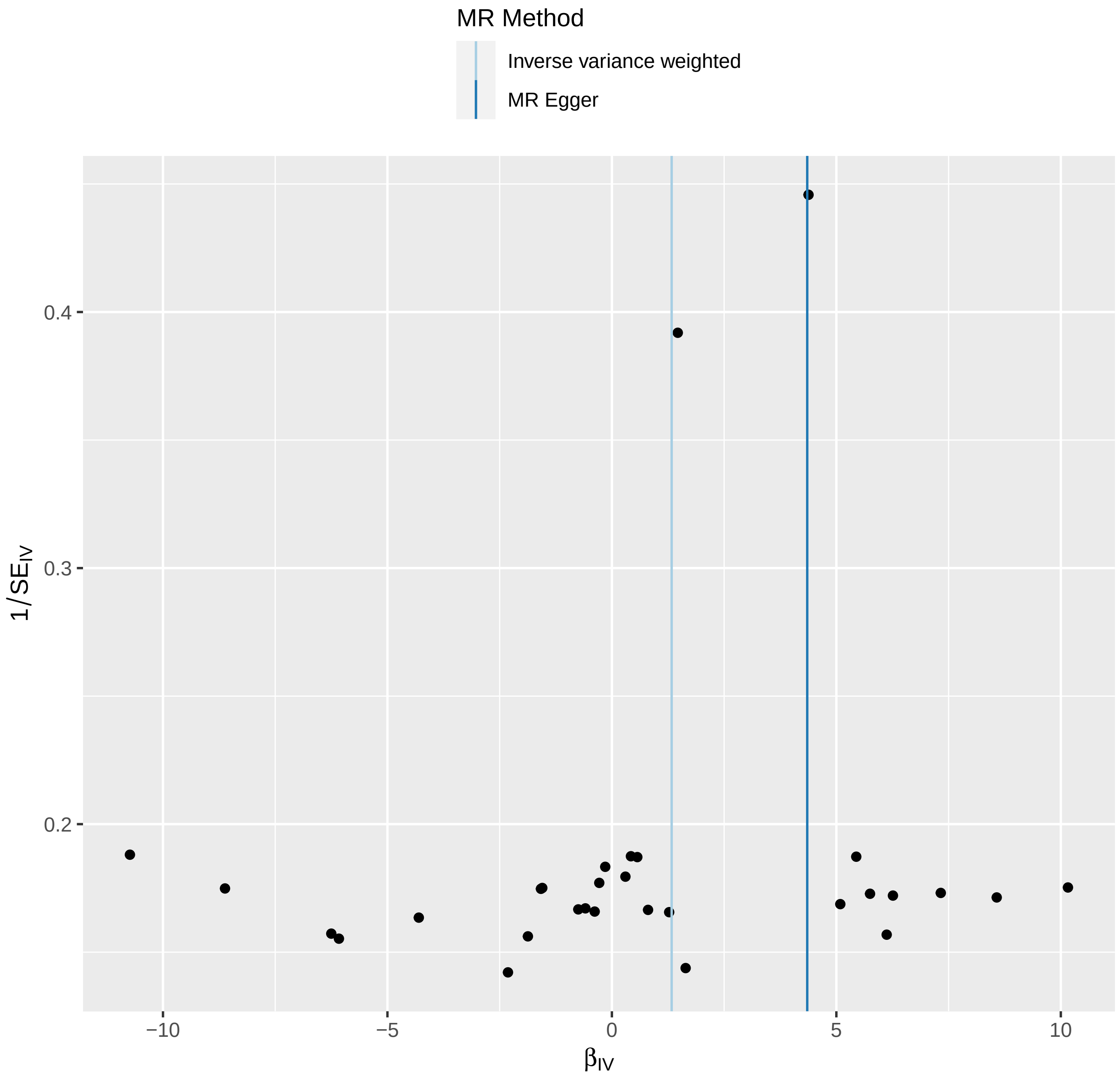

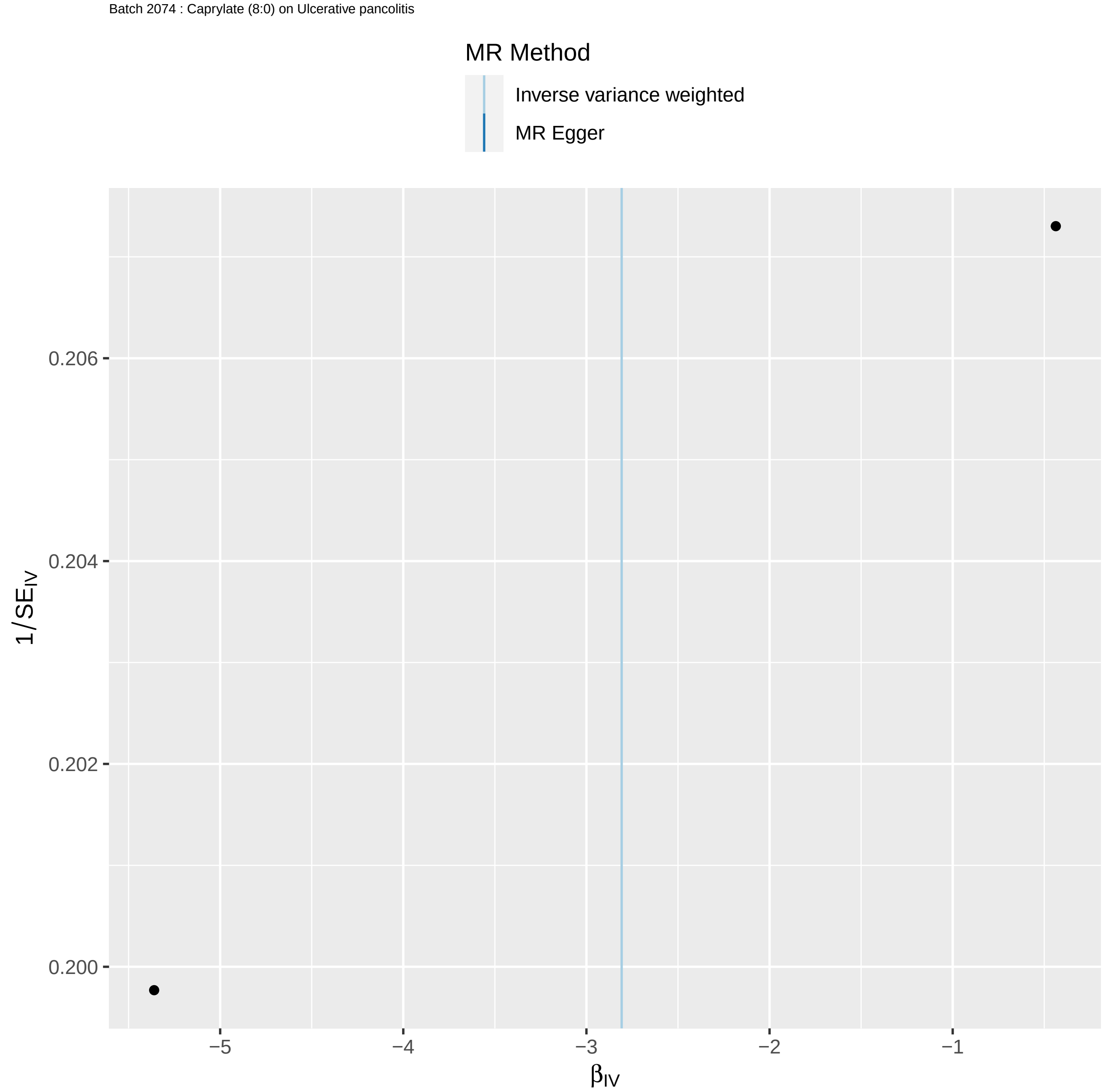
Batch 2074 : Caprylate (8:0) on Ulcerative pancolitis
MR Method
Inverse variance weighted
MR Egger
0.206
0.204
0.202
0.200
1/SE_IV
−5
−4
−3
−2
−1
β_IV

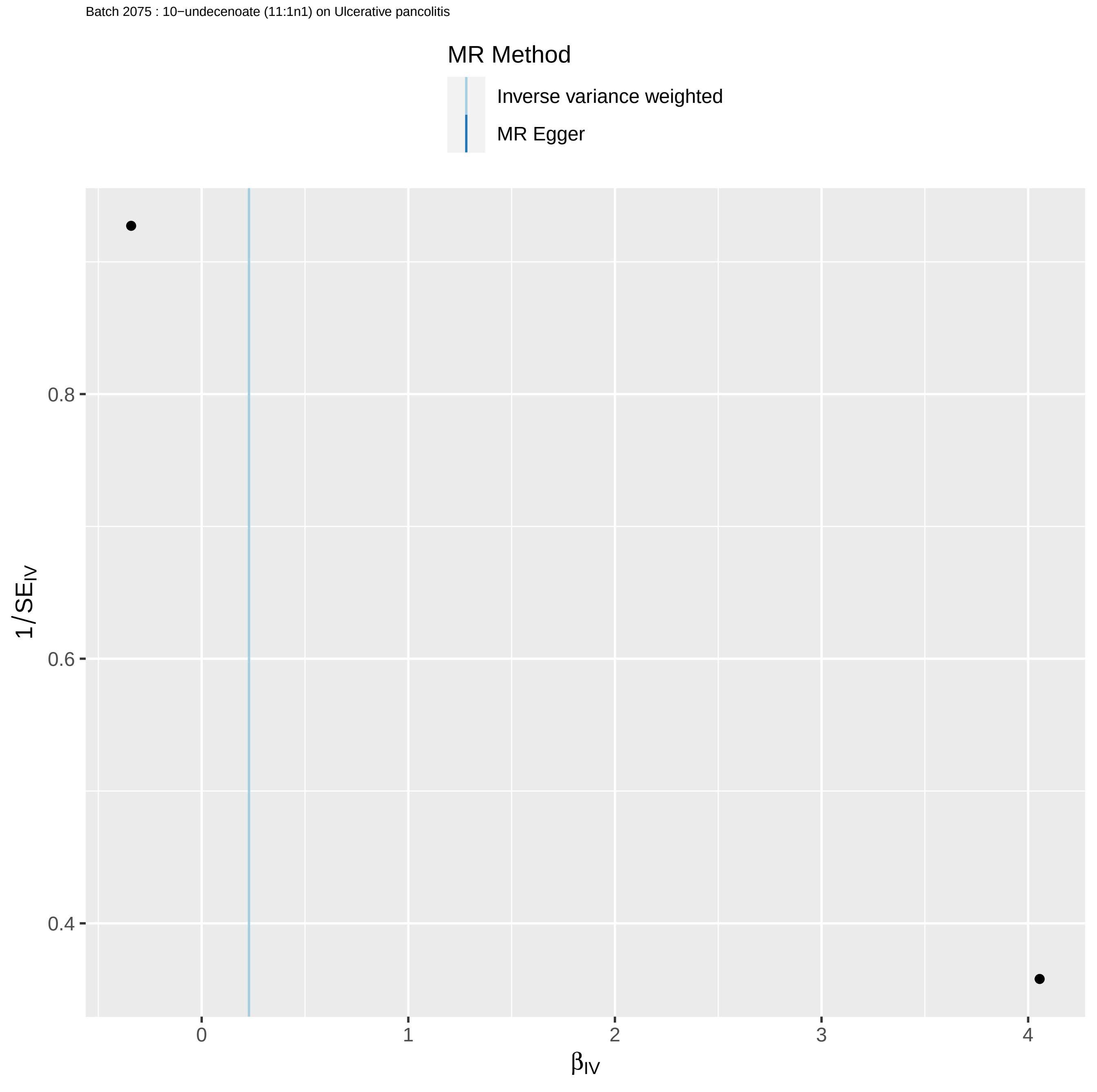

Batch 2075 : 10−undecenoate (11:1n1) on Ulcerative pancolitis
MR Method
Inverse variance weighted
MR Egger
0.8
0.6
0.4
1/SE_IV
0
1
2
3
4
β_IV

Batch 2077 : Phenol sulfate on Ulcerative pancolitis

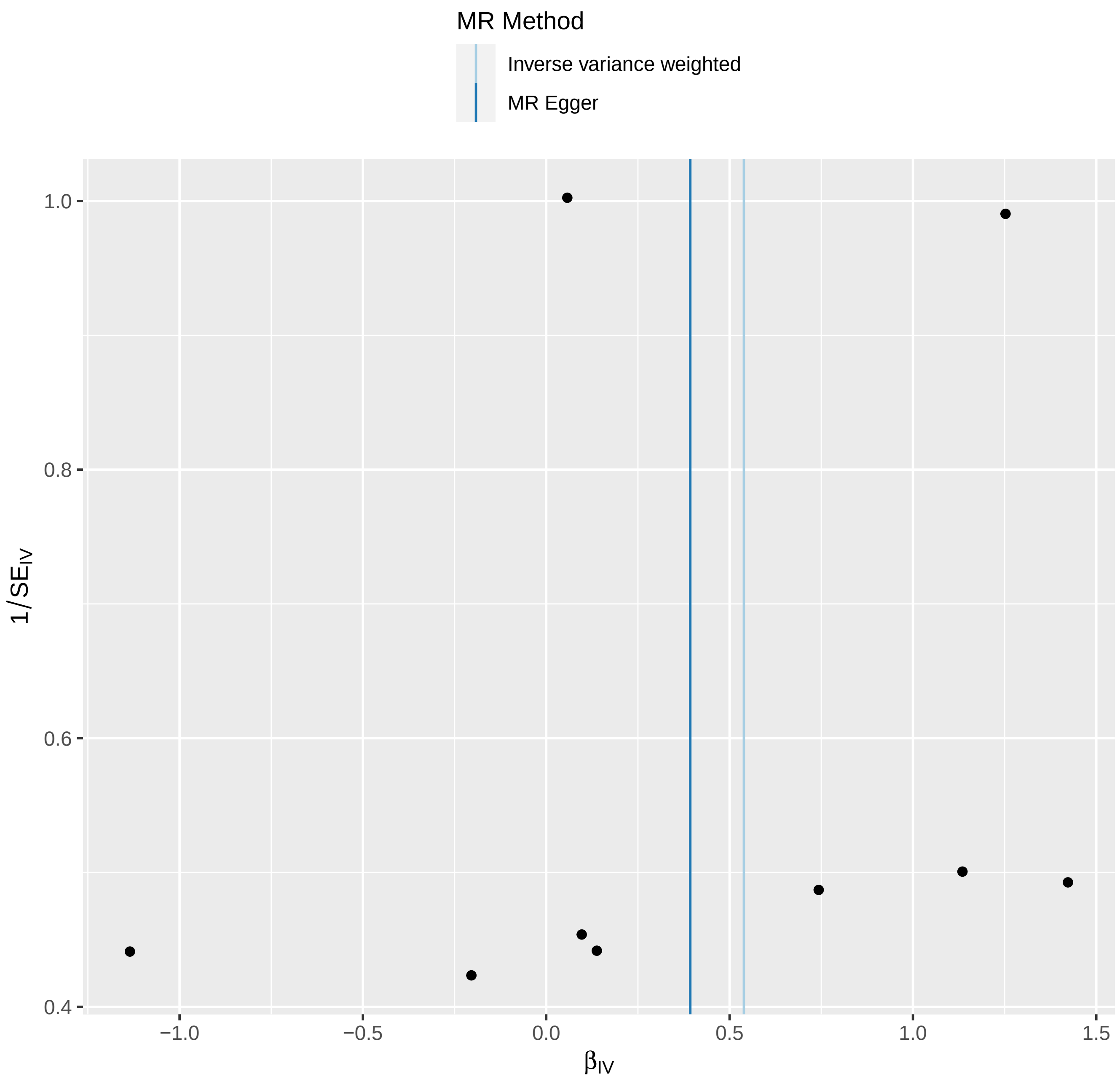

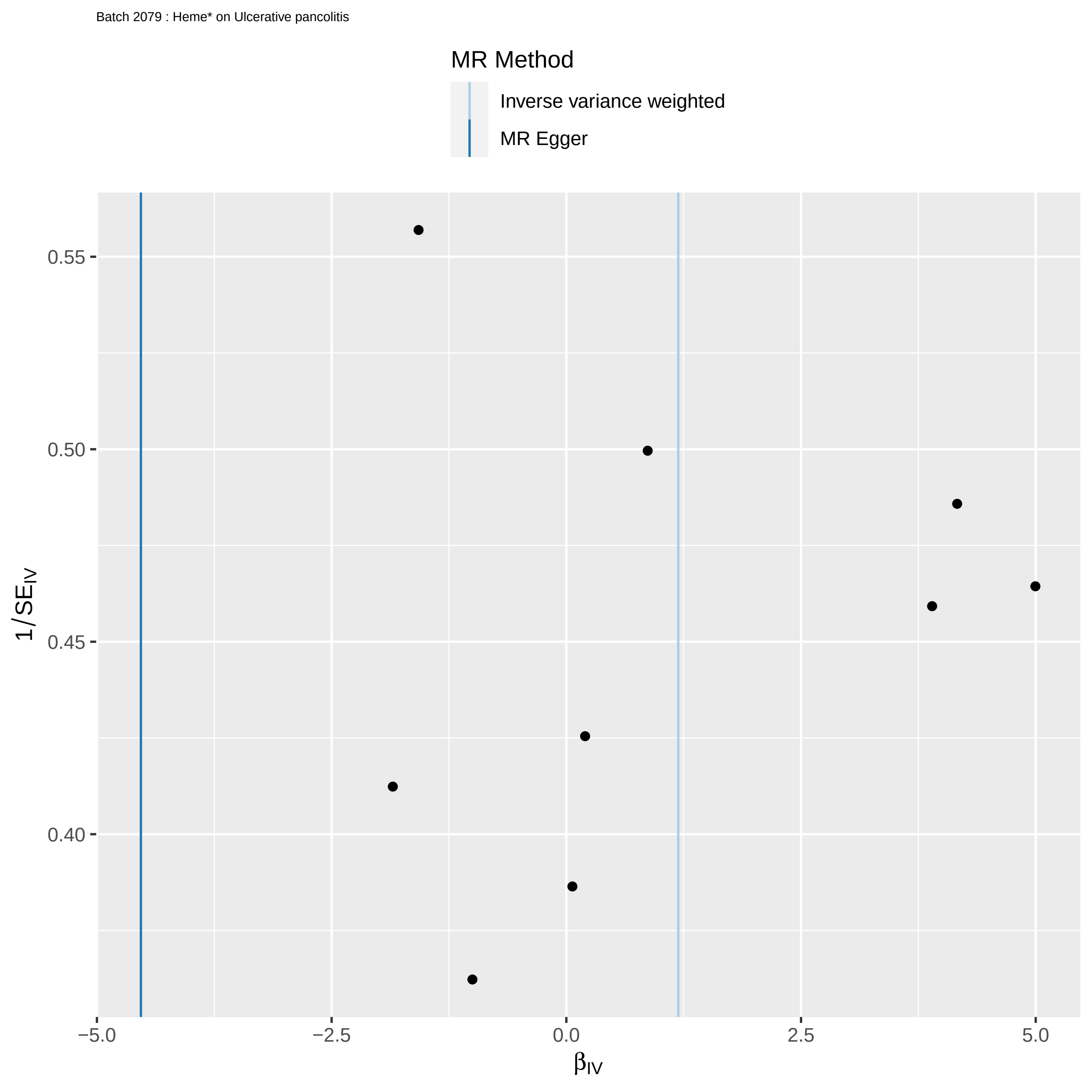
Batch 2079 : Heme* on Ulcerative pancolitis
MR Method
Inverse variance weighted
MR Egger
0.55
0.50
0.45
0.40
1/SE$_{IV}$
−5.0
−2.5
0.0
2.5
5.0
β$_{IV}$

Batch 2081 : 3−dehydrocarnitine* on Ulcerative pancolitis

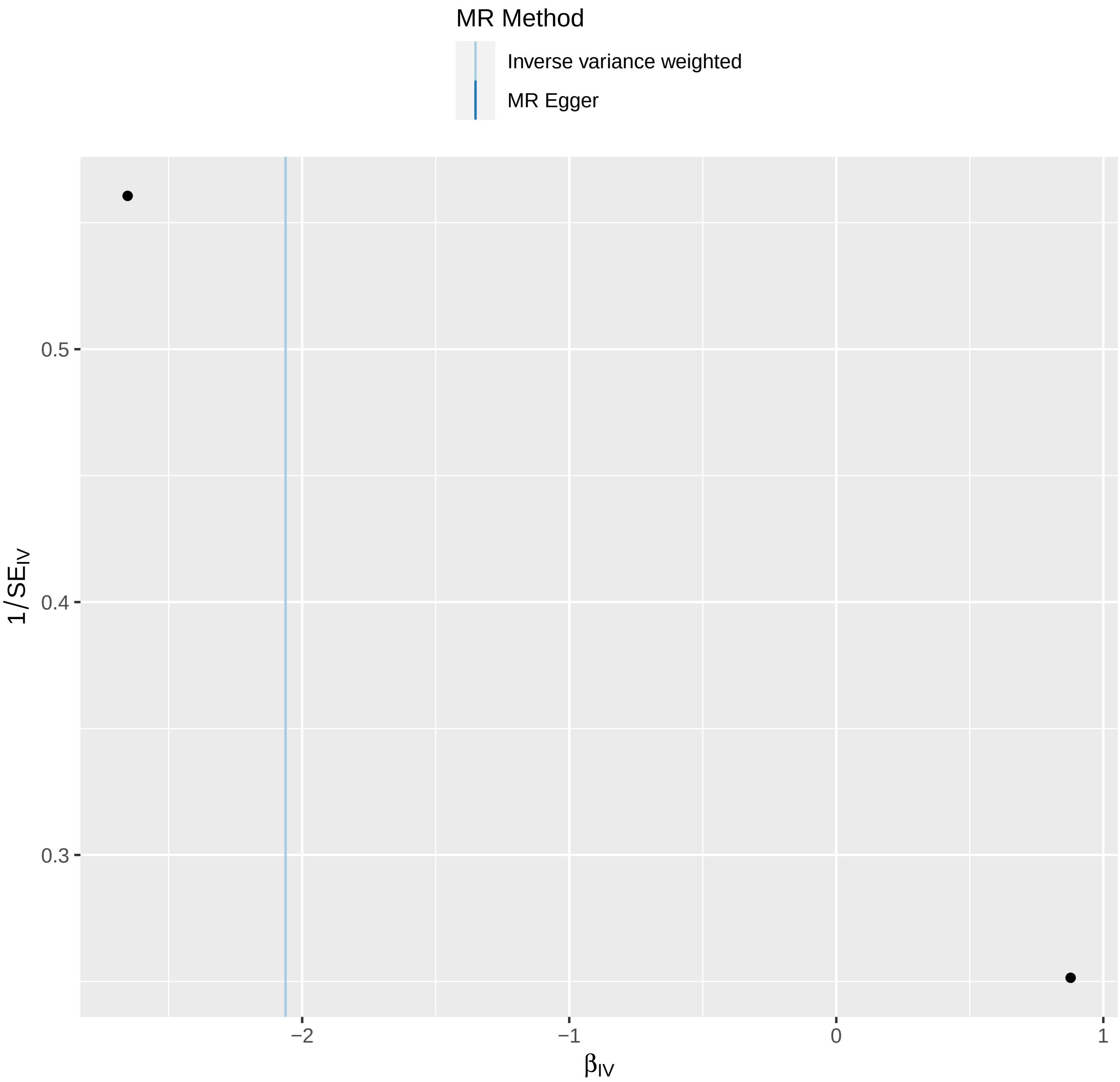

Batch 2082 : Pyroglutamine* on Ulcerative pancolitis

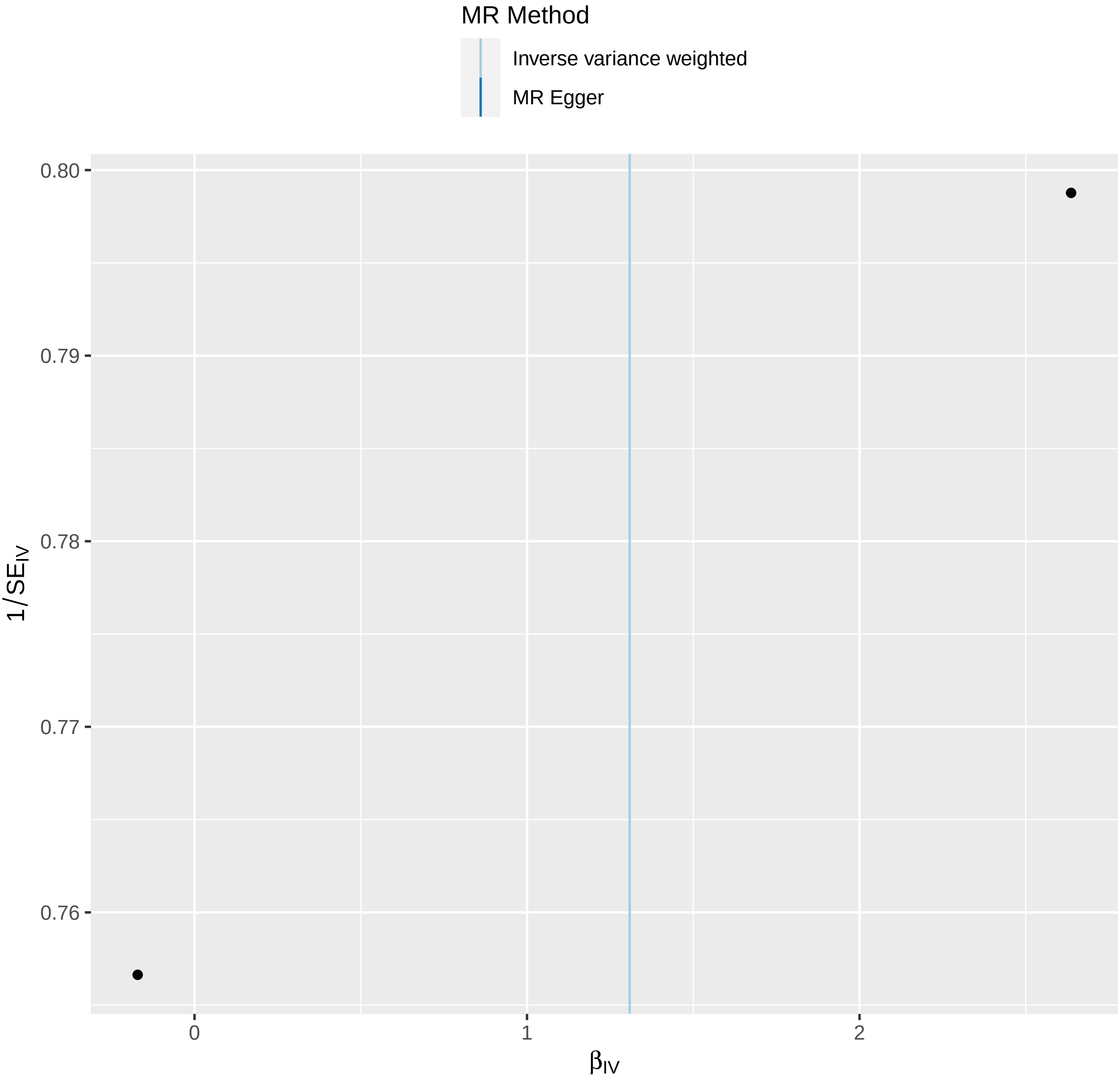

Batch 2085 : X−11422−−xanthine on Ulcerative pancolitis

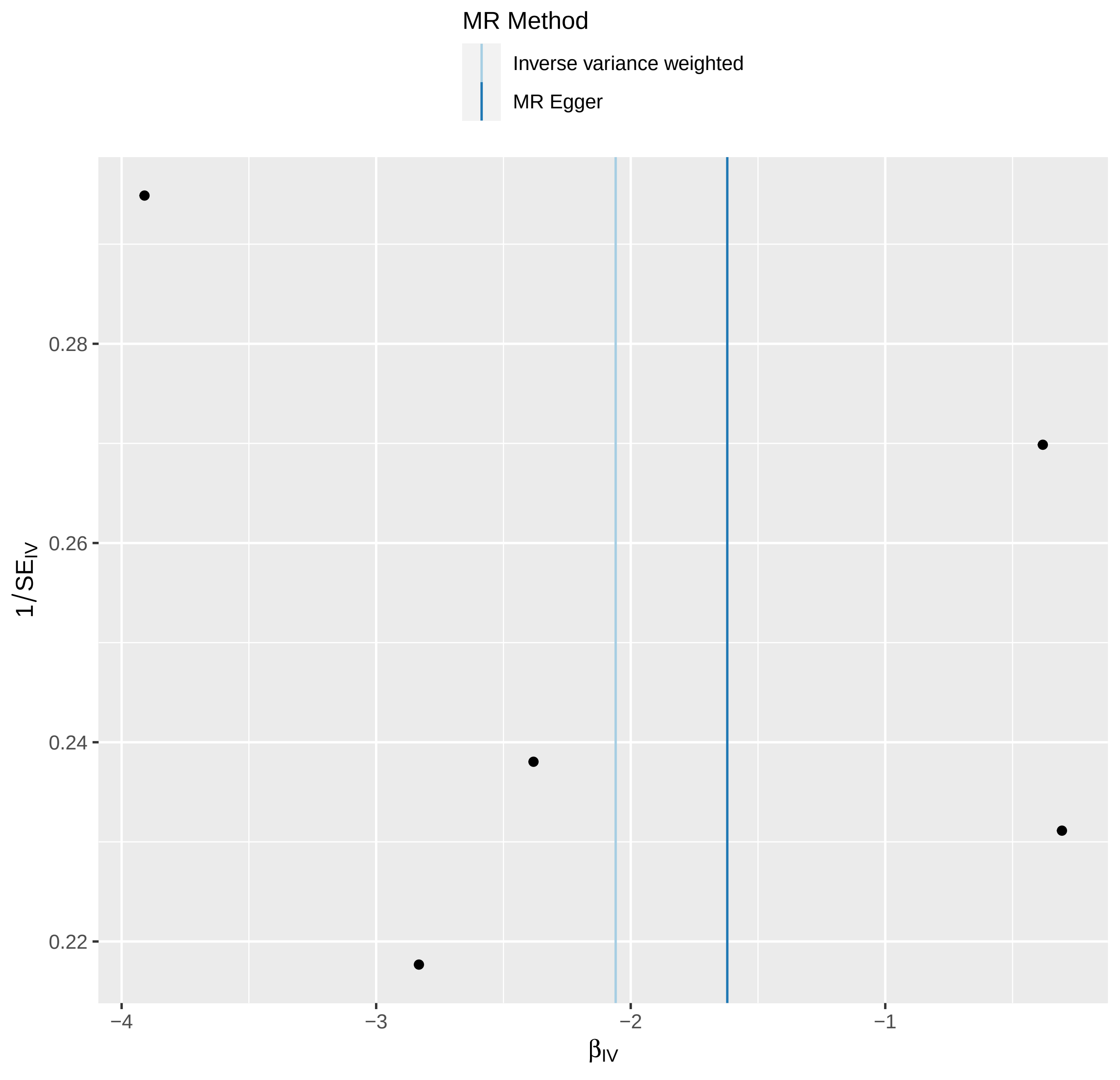

Batch 2088 : HWESASXX* on Ulcerative pancolitis

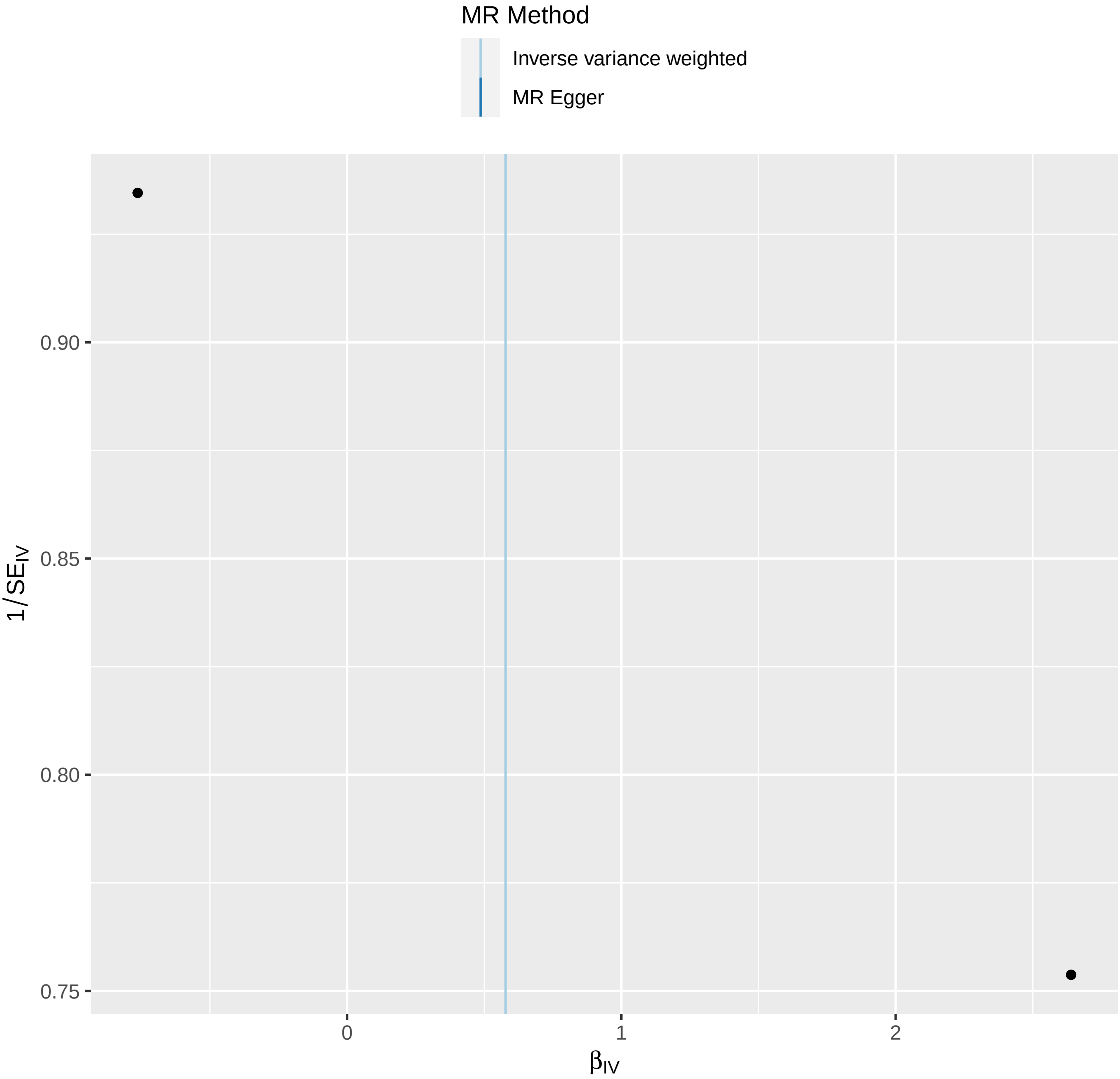

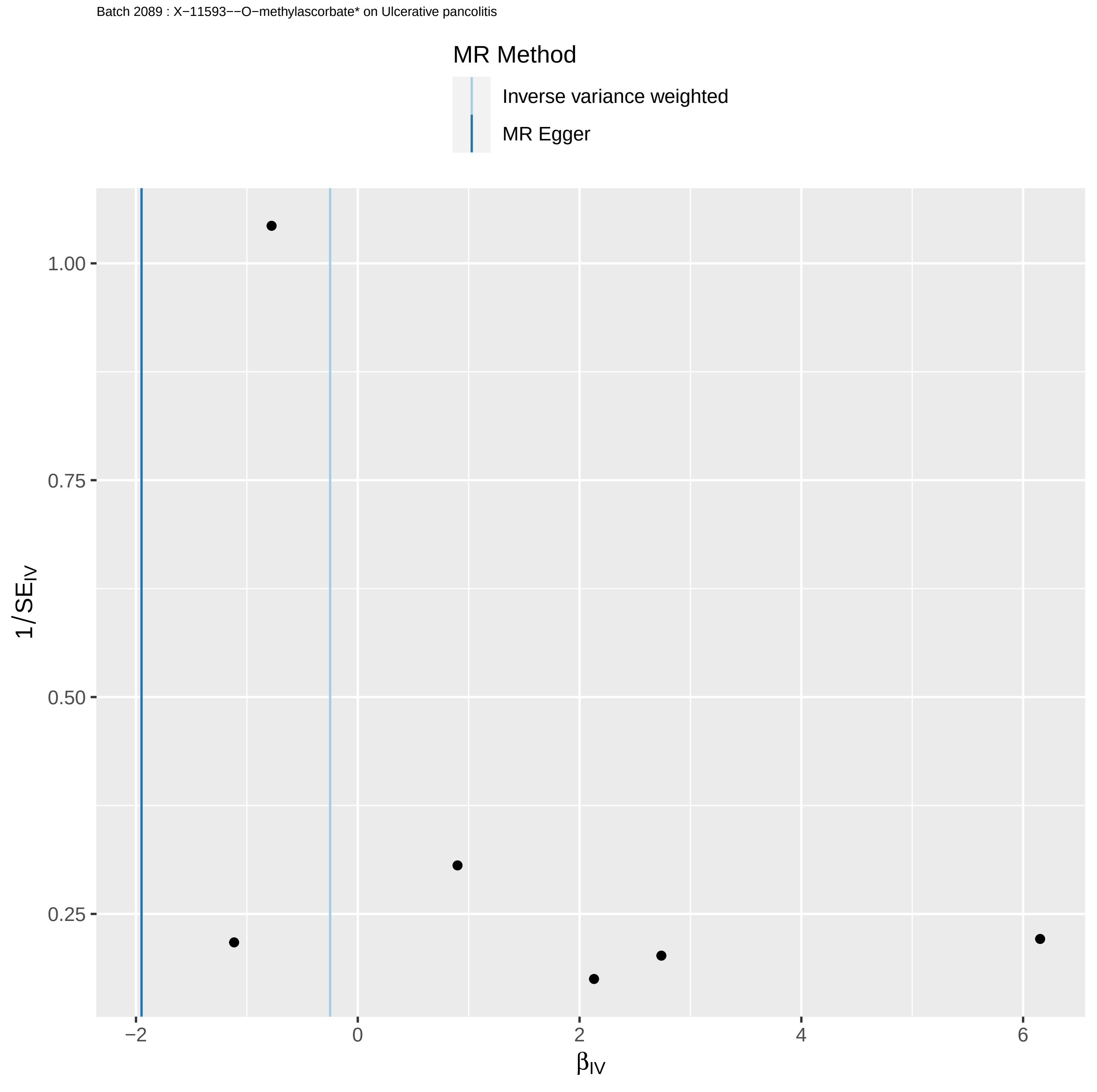

Batch 2089 : X−11593−−O−methylascorbate* on Ulcerative pancolitis
MR Method
Inverse variance weighted
MR Egger
1.00
0.75
0.50
0.25
1/SE_IV
−2
0
2
4
6
β_IV

Batch 2091 : Homostachydrine* on Ulcerative pancolitis

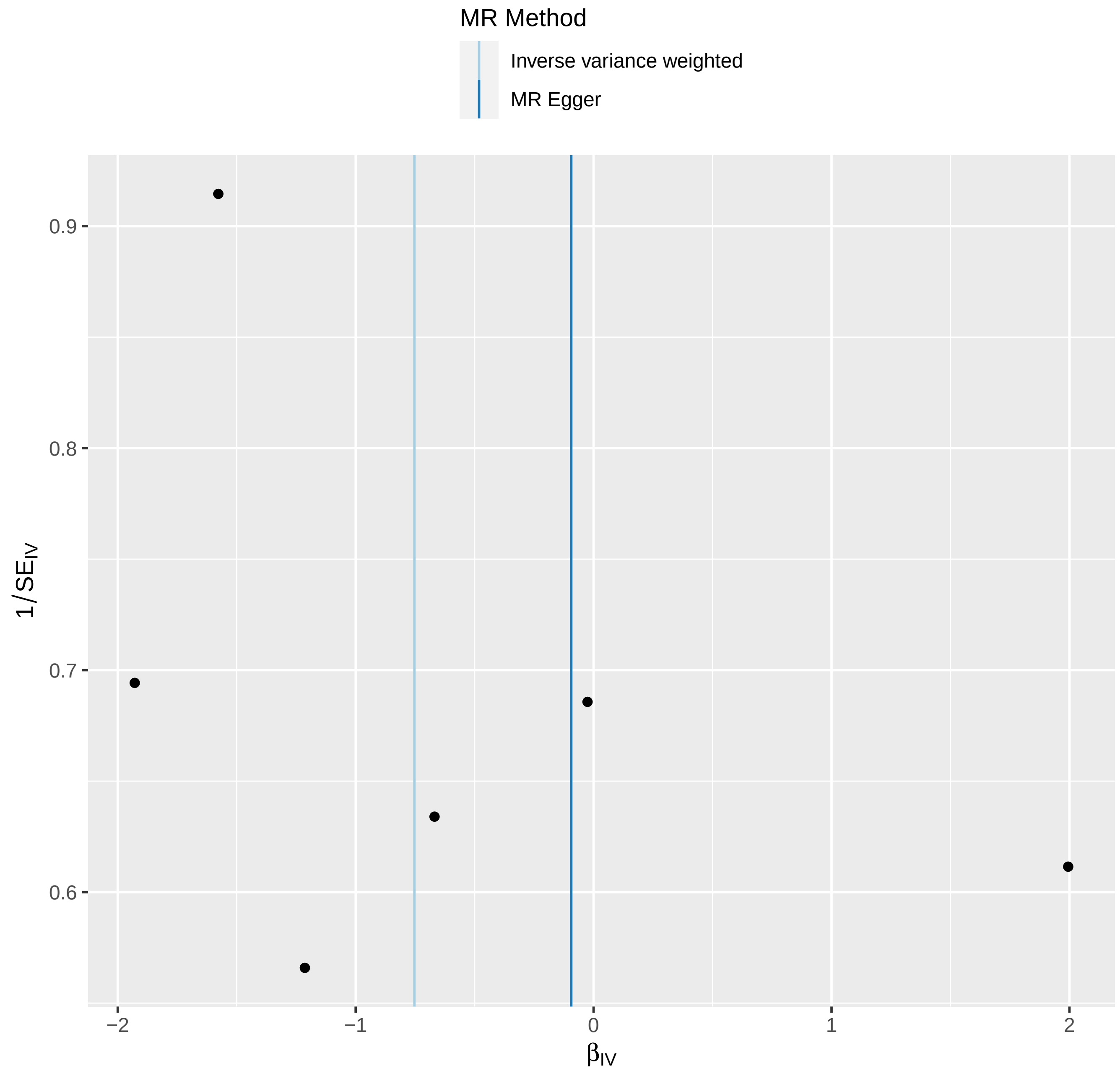

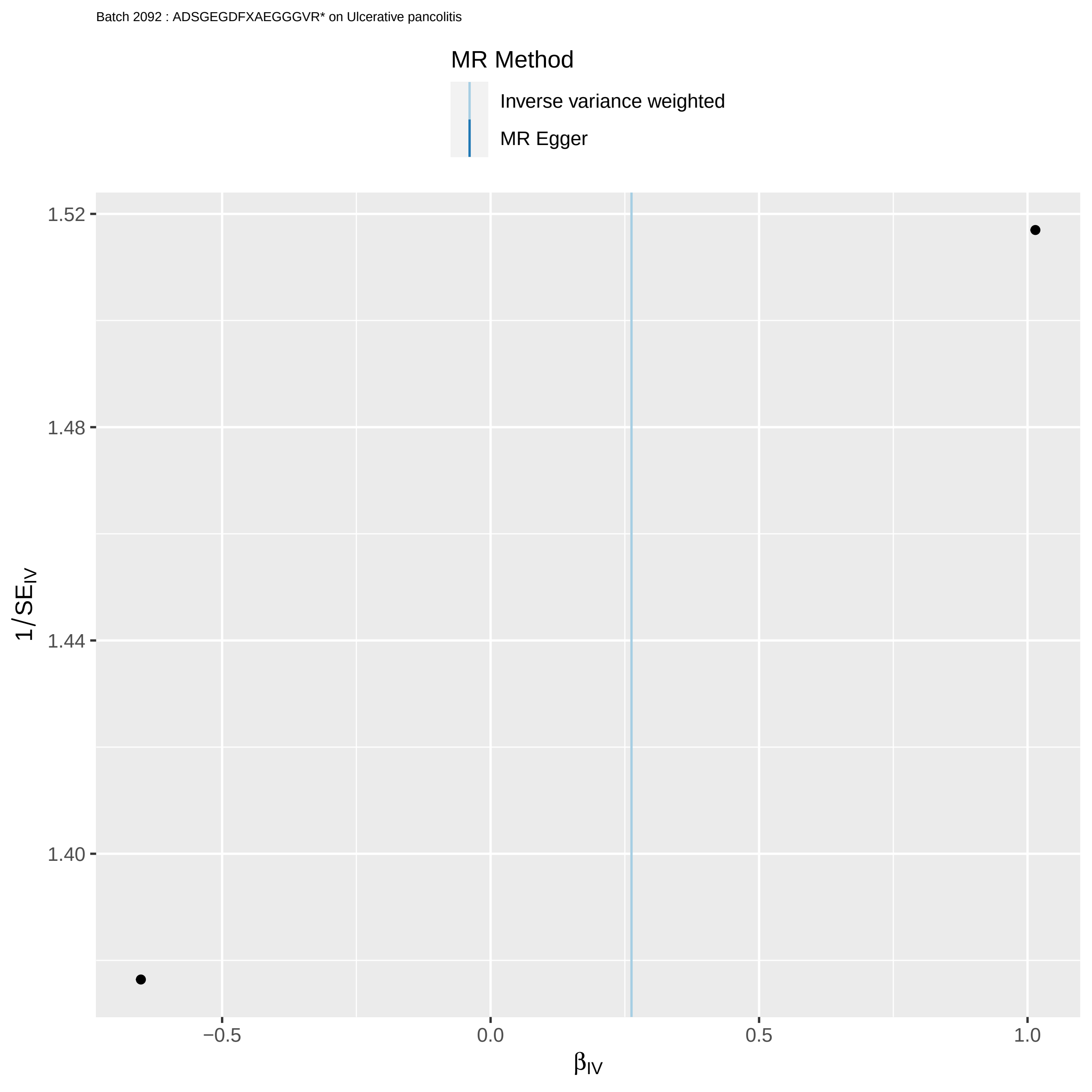
Batch 2092 : ADSGEGDFXAEGGGVR* on Ulcerative pancolitis
MR Method
Inverse variance weighted
MR Egger
1.52
1.48
1.44
1.40
1/SE_IV
−0.5
0.0
0.5
1.0
β_IV

Batch 2094 : X−11793−−oxidized bilirubin* on Ulcerative pancolitis

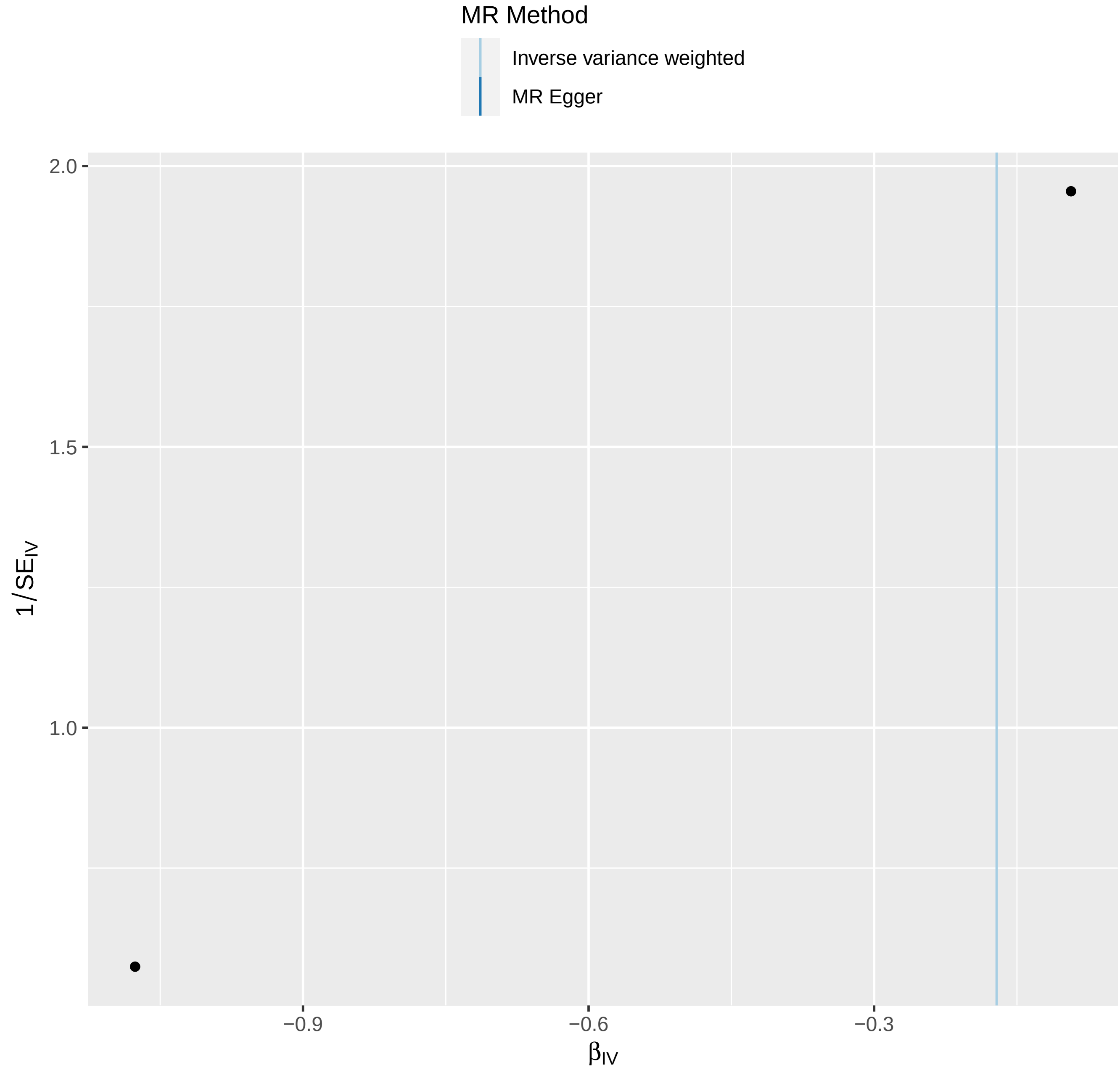

Batch 2095 : 1−arachidonoylglycerophosphocholine* on Ulcerative pancolitis

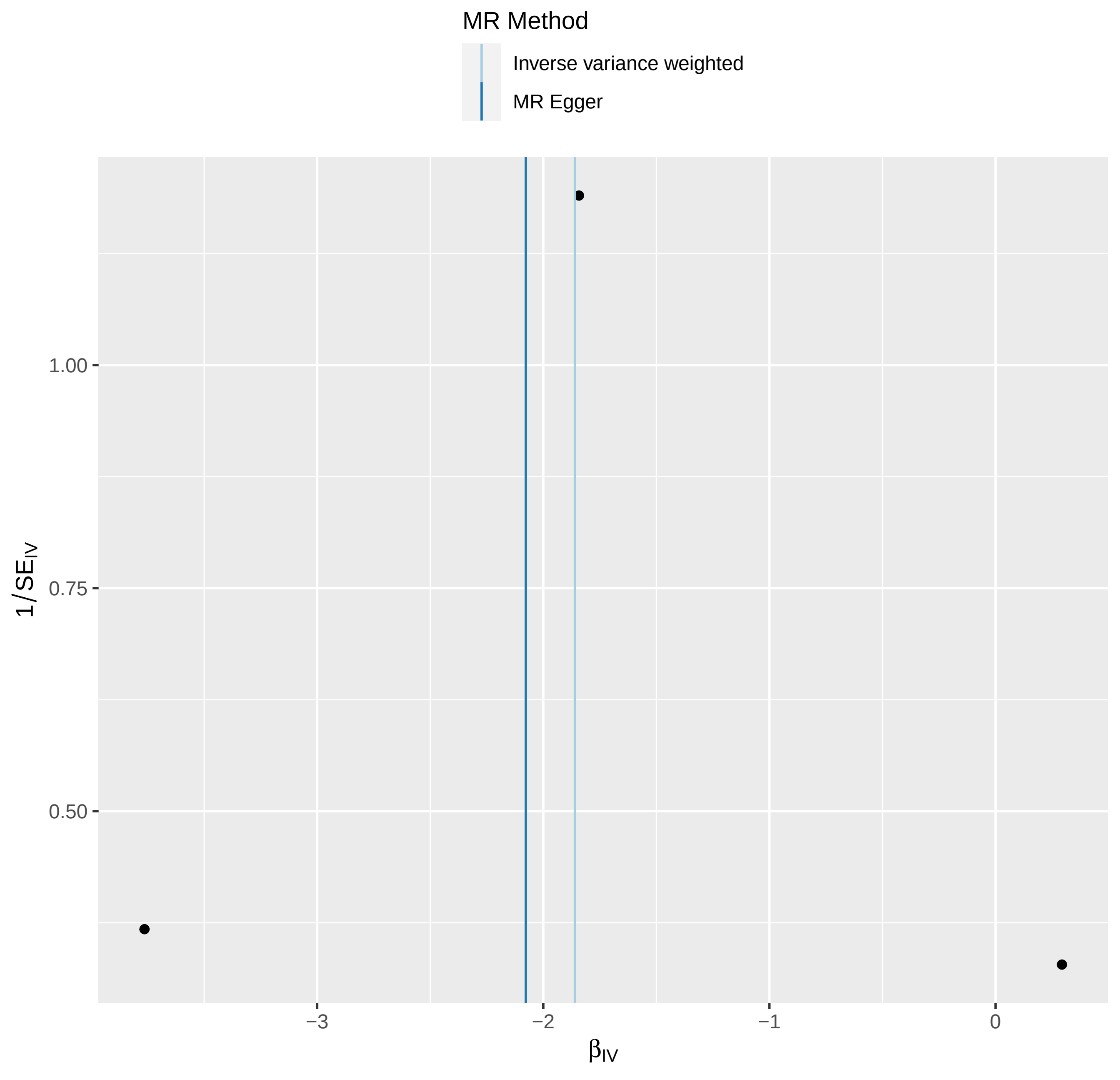

Batch 2097 : Gamma−glutamylmethionine* on Ulcerative pancolitis

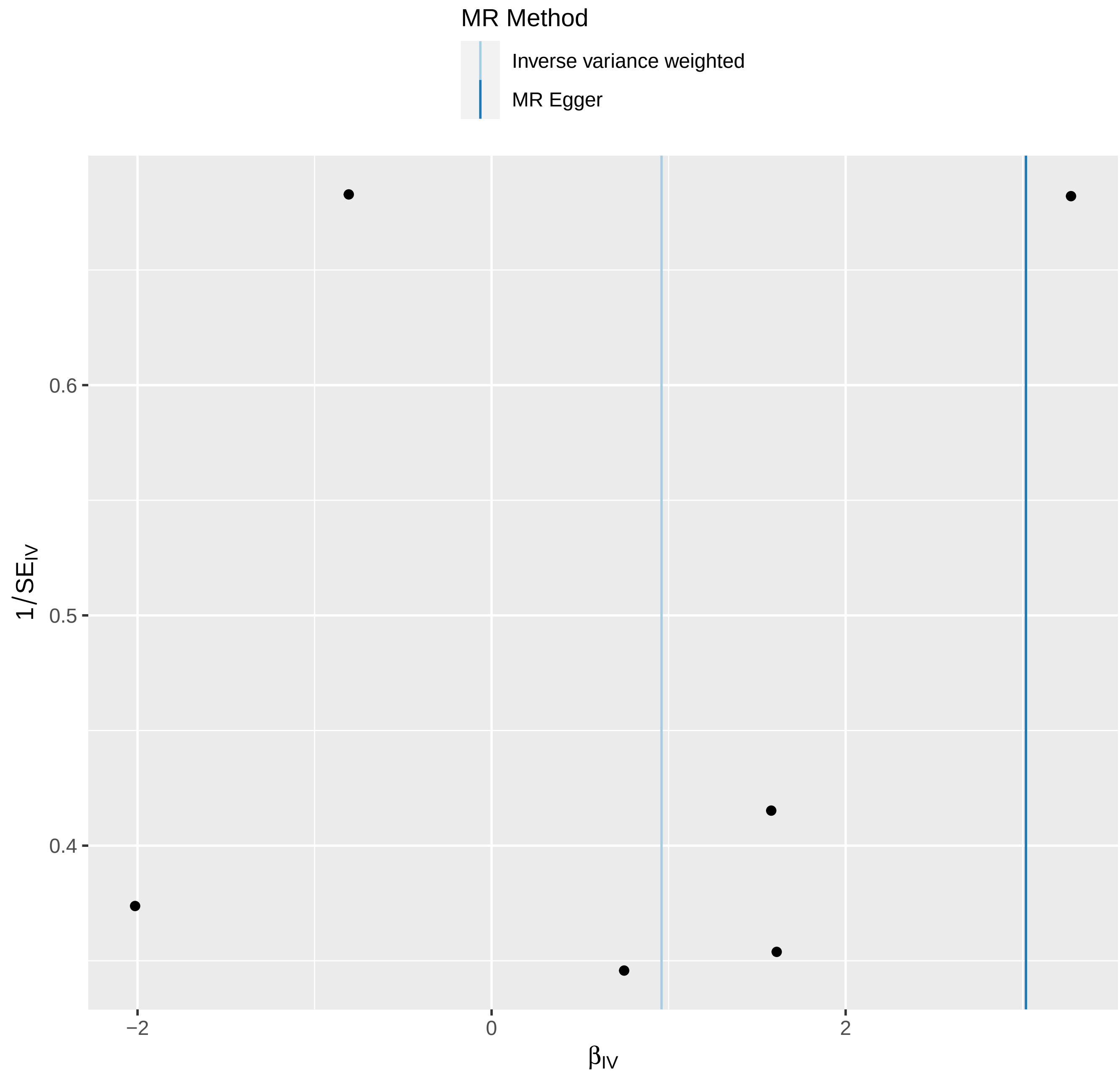

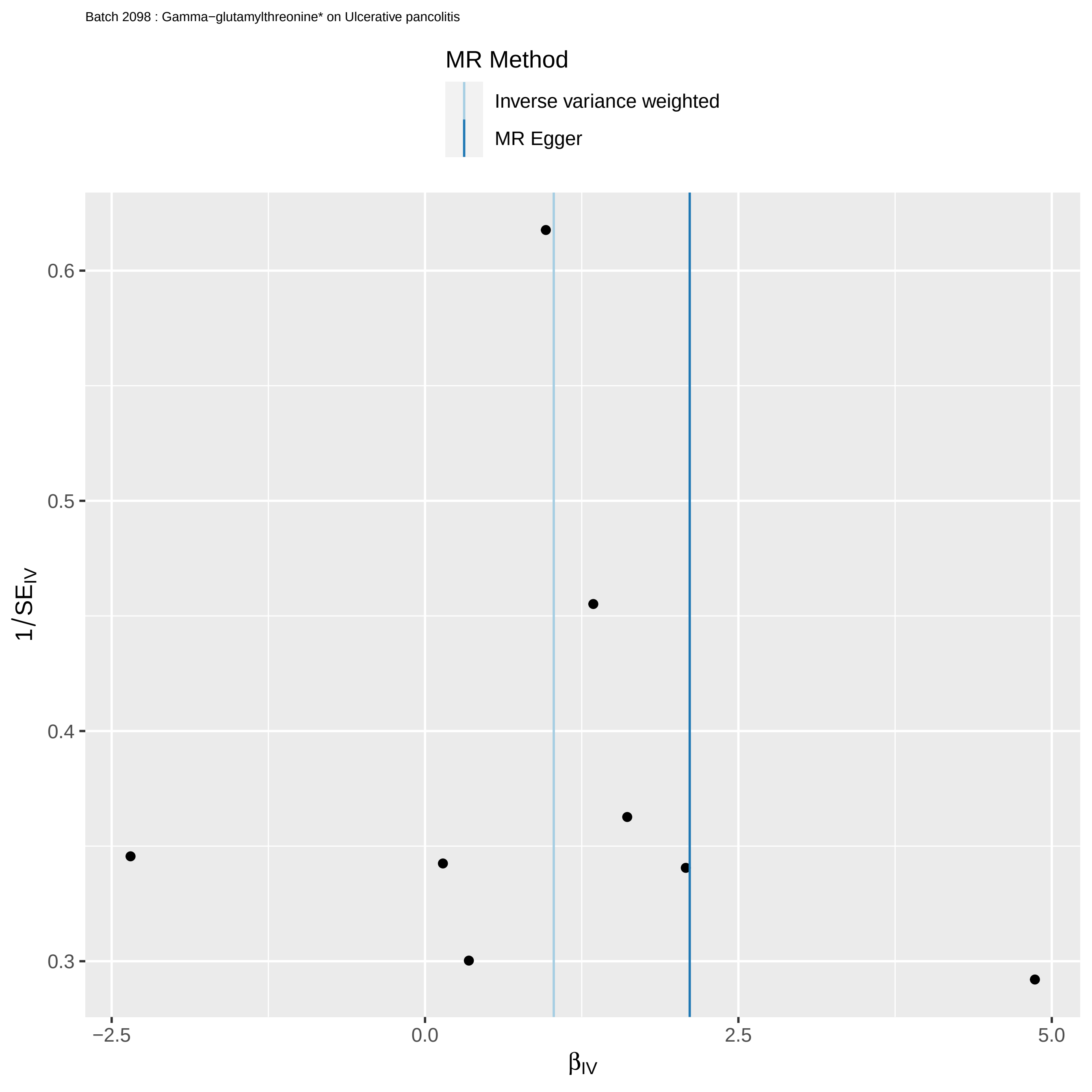

Batch 2098 : Gamma−glutamylthreonine* on Ulcerative pancolitis
MR Method
Inverse variance weighted
MR Egger
0.6
0.5
0.4
0.3
1/SE_IV
−2.5
0.0
2.5
5.0
β_IV

Batch 2099 : Gamma−tocopherol on Ulcerative pancolitis

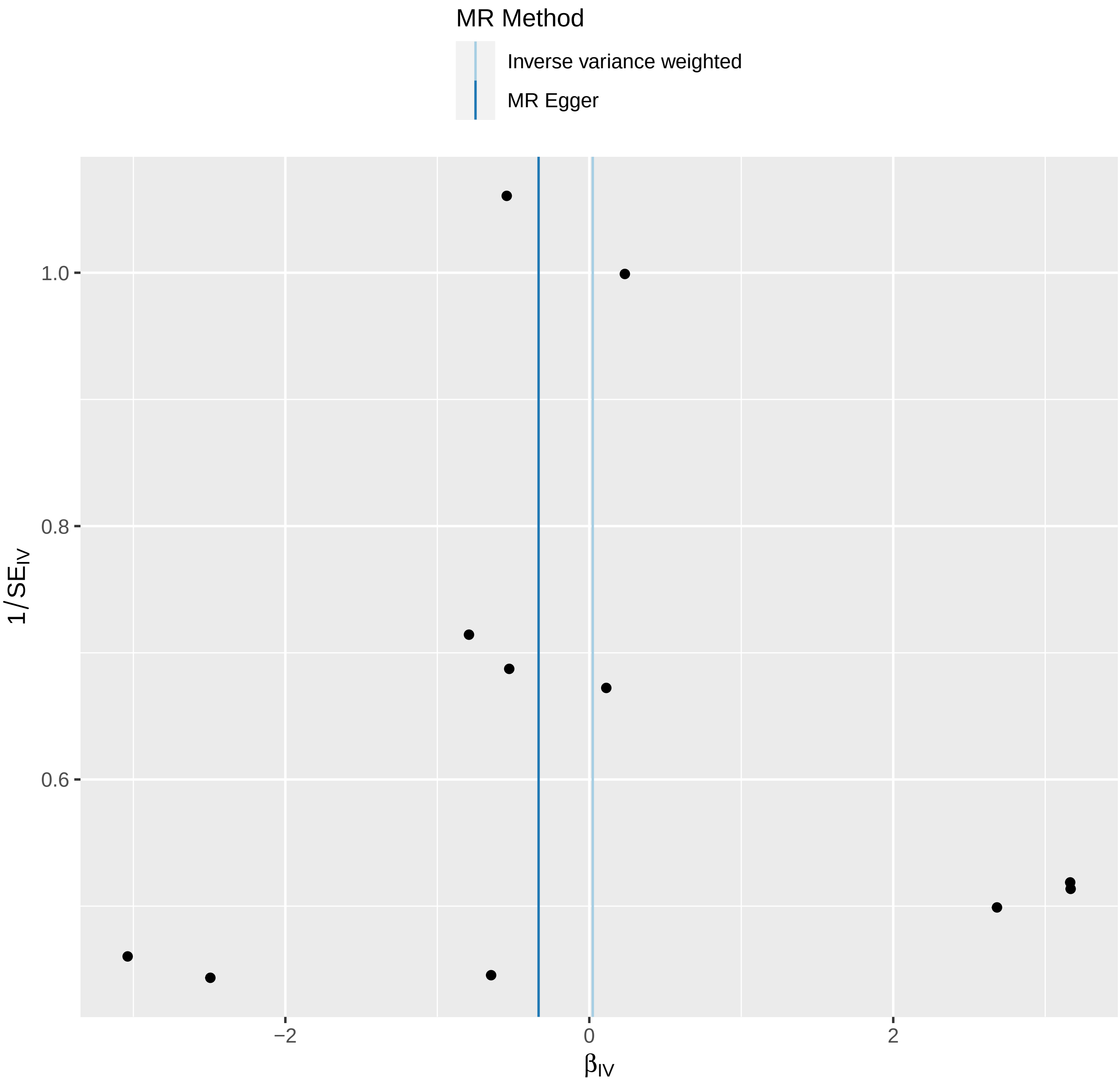

Batch 2100 : Gamma−glutamylphenylalanine on Ulcerative pancolitis

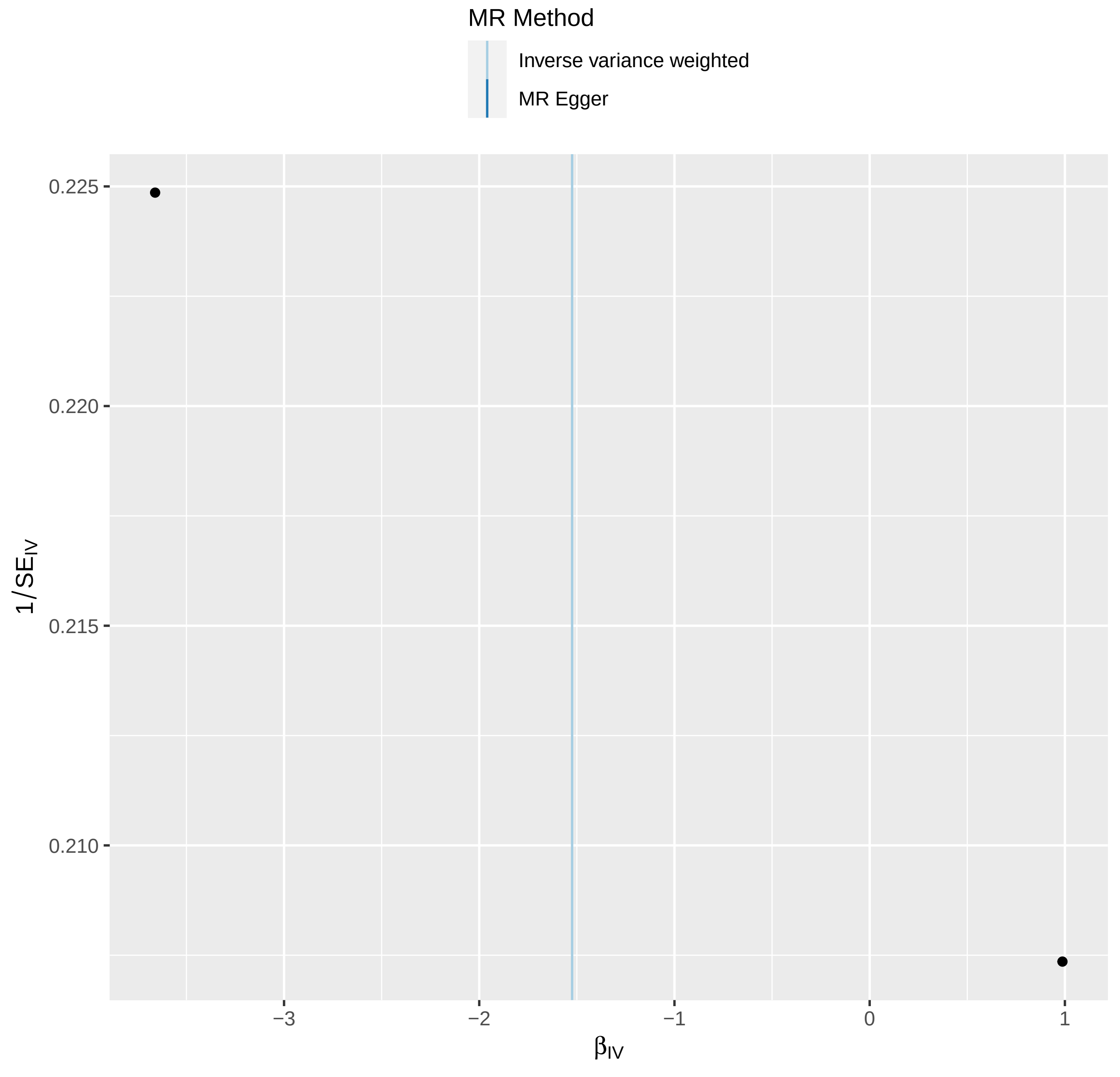

Batch 2101 : Isobutyrylcarnitine on Ulcerative pancolitis

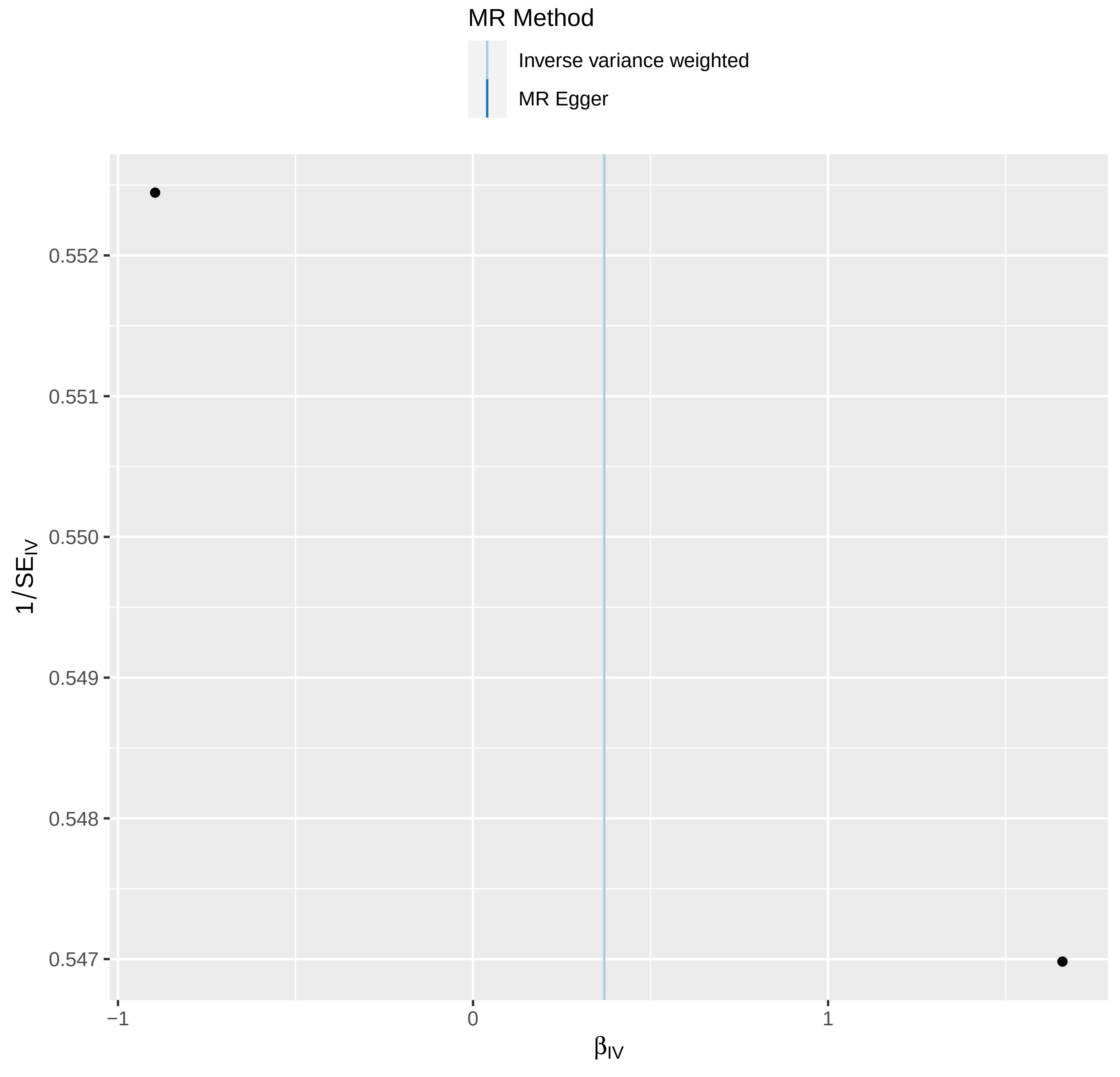

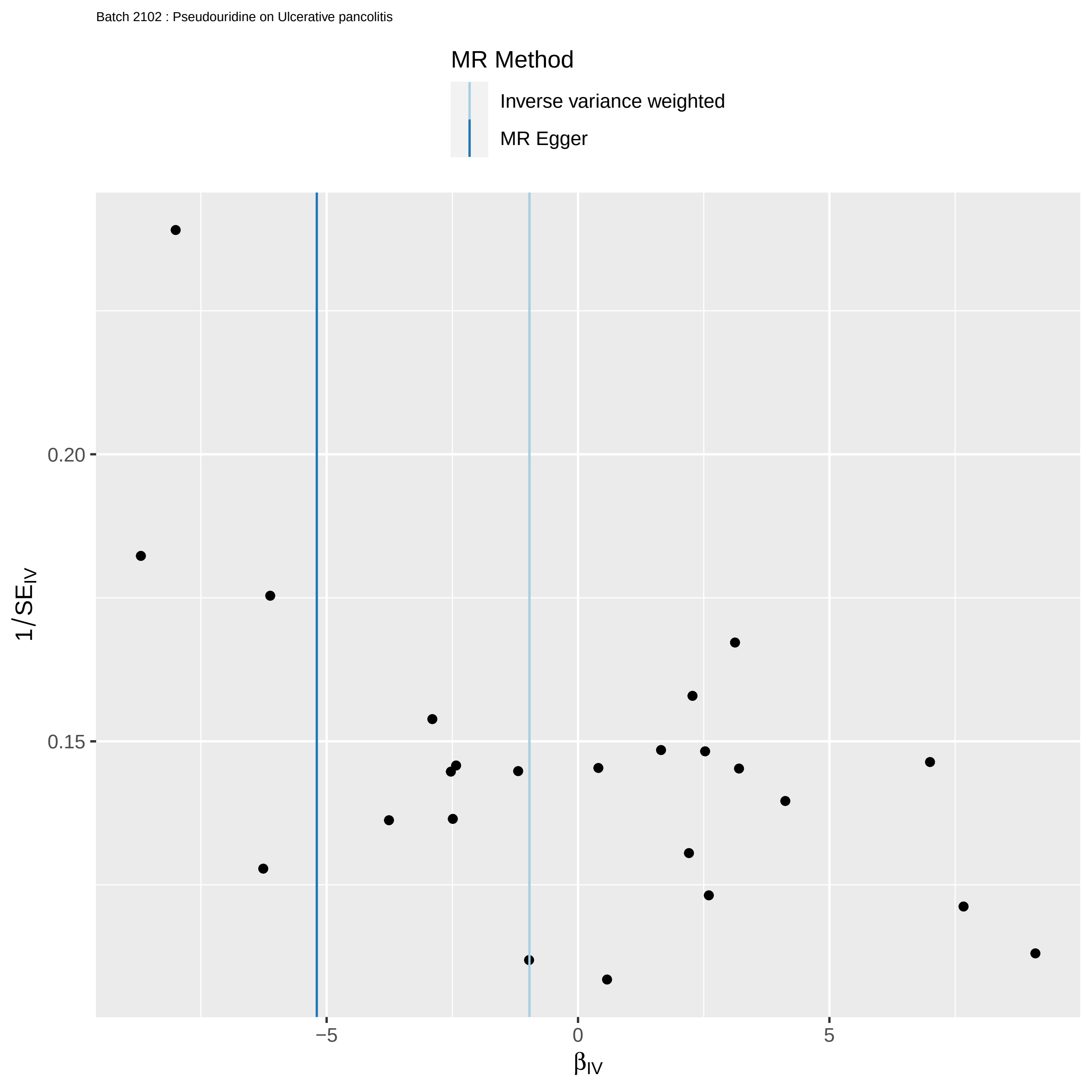

Batch 2102 : Pseudouridine on Ulcerative pancolitis
MR Method
Inverse variance weighted
MR Egger
0.20
0.15
1/SE_IV
−5
0
5
β_IV

Batch 2103 : Valerate on Ulcerative pancolitis

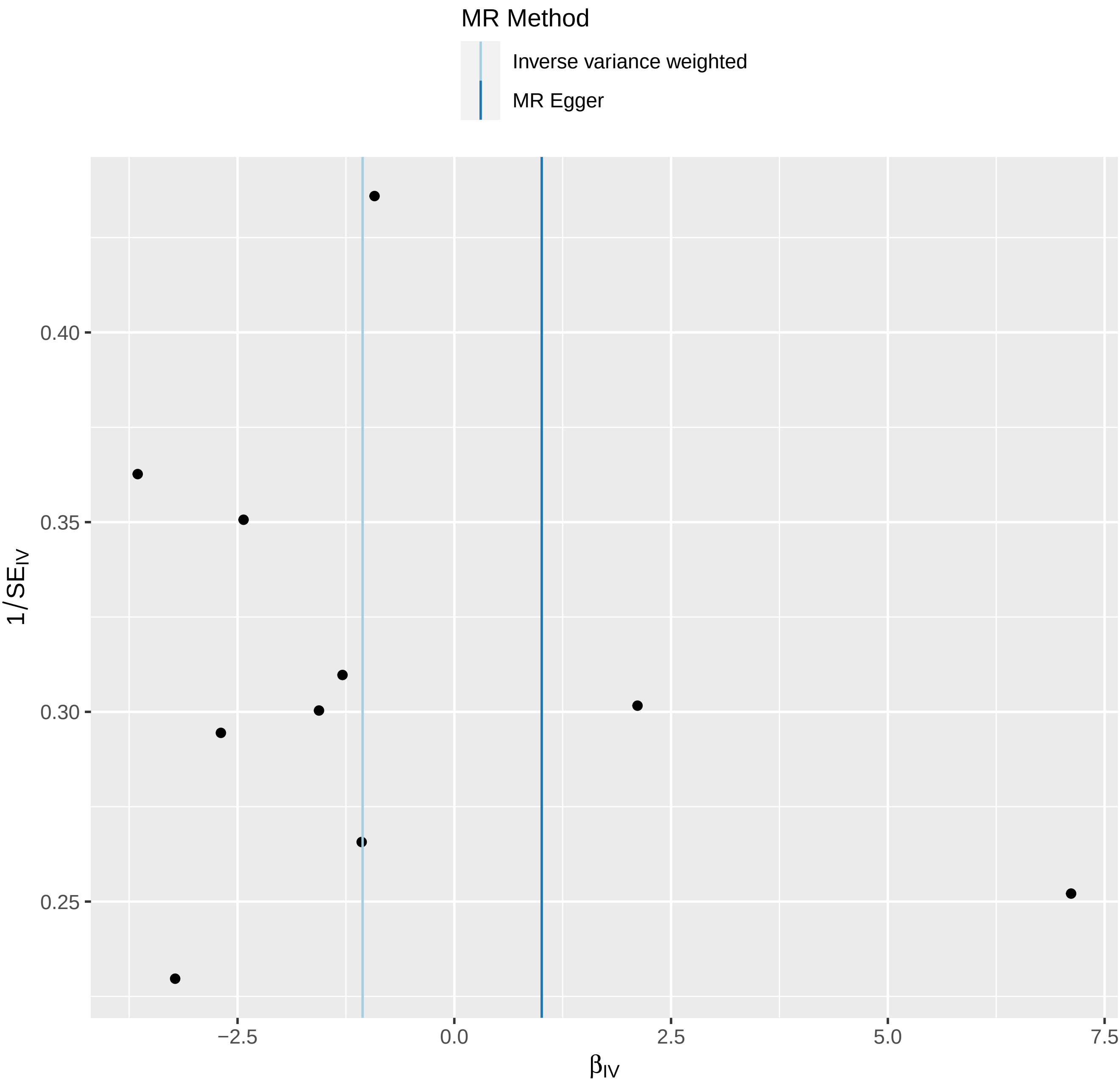

Batch 2106 : Erythronate* on Ulcerative pancolitis

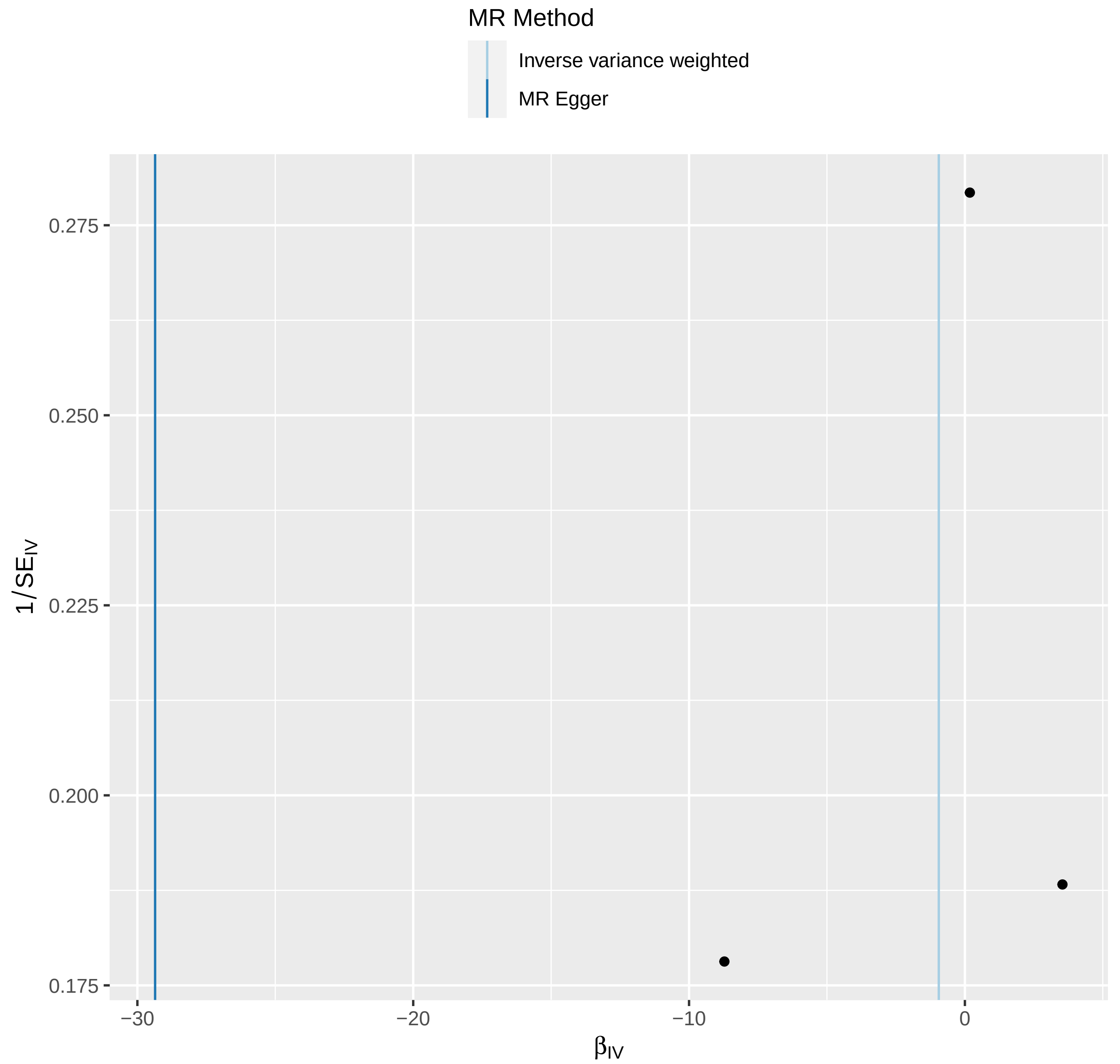

Batch 2107 : Lathosterol on Ulcerative pancolitis

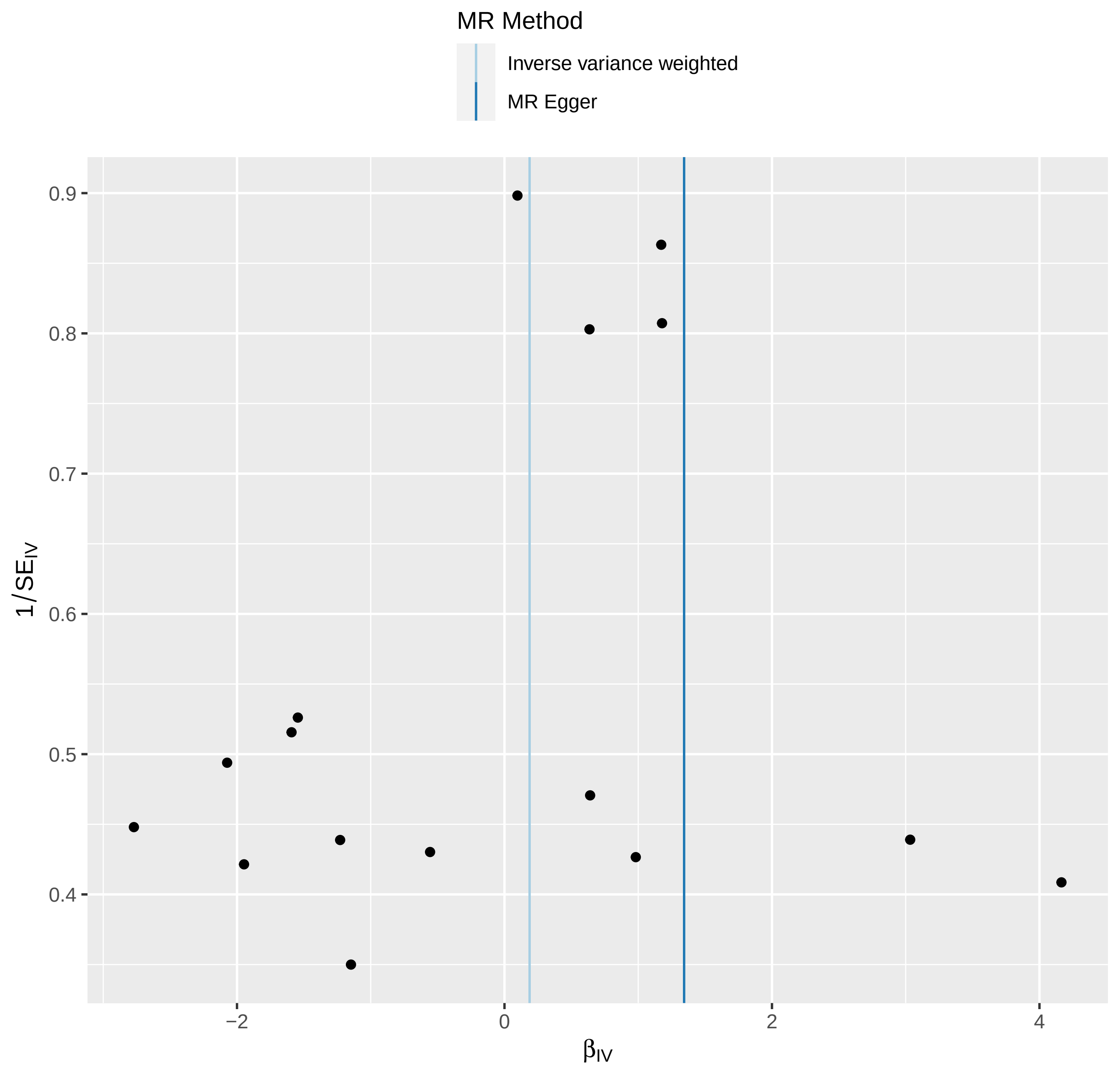

Batch 2110 : Eicosenoate (20:1n9 or 11) on Ulcerative pancolitis

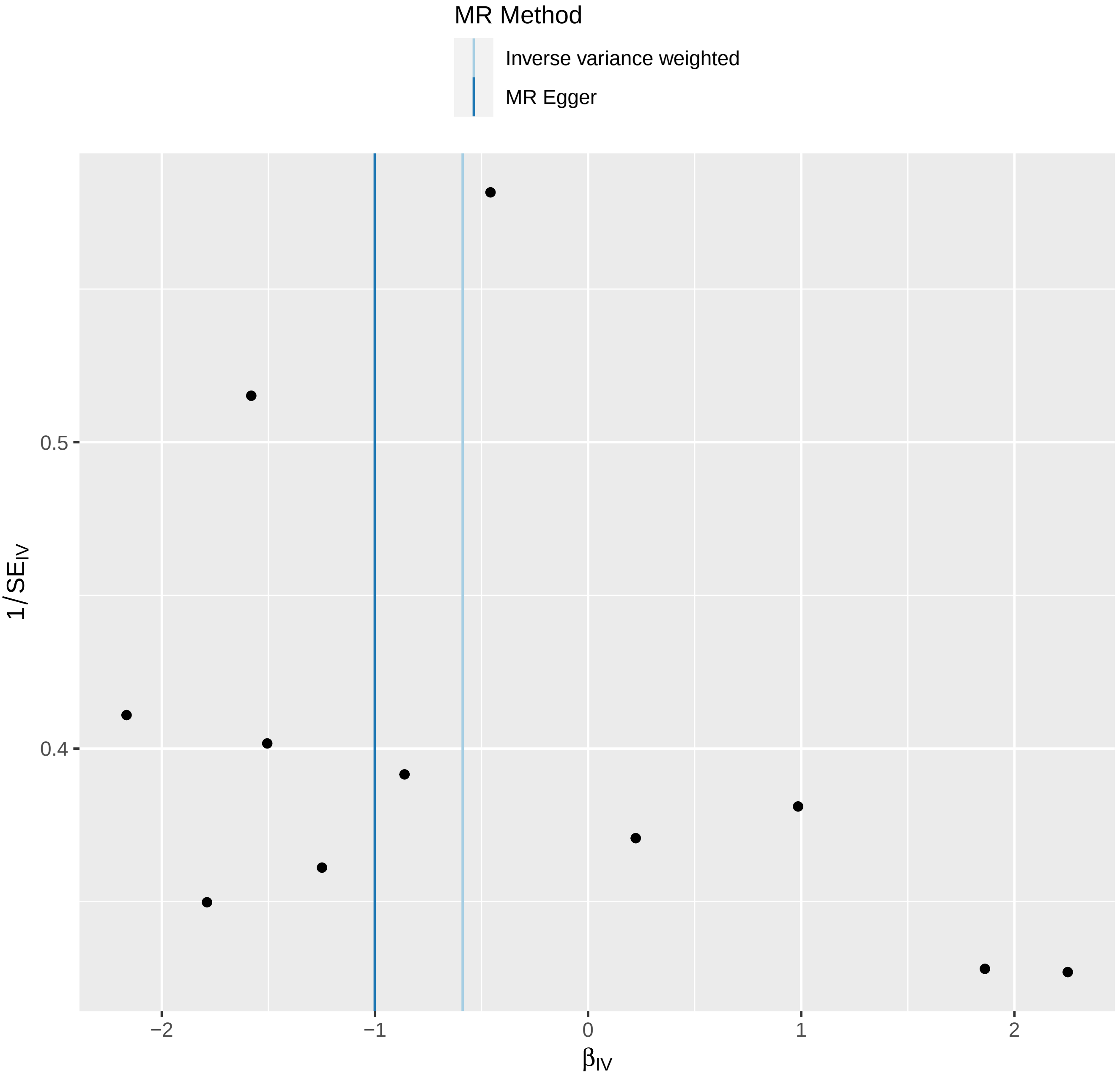

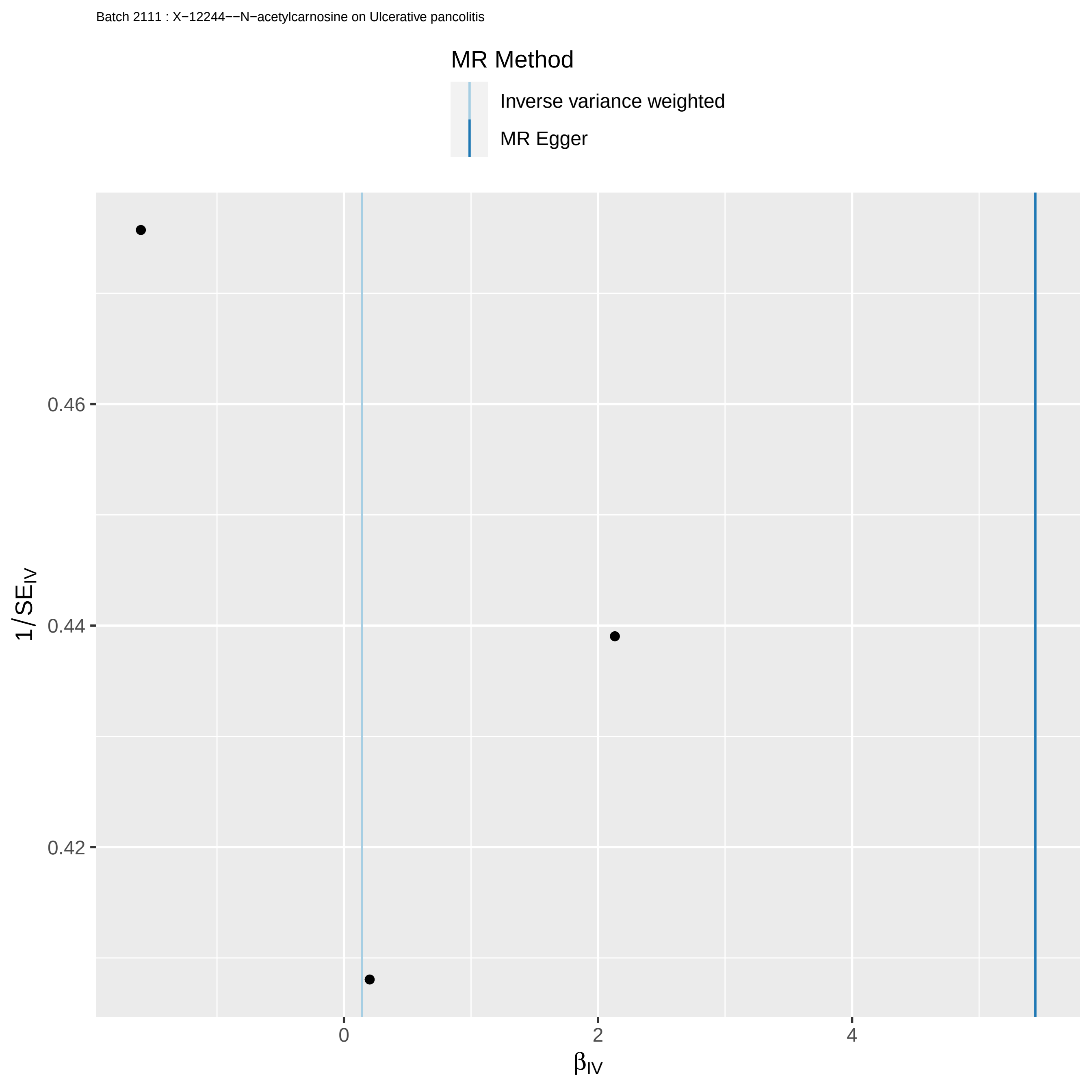
Batch 2111 : X−12244−−N−acetylcarnosine on Ulcerative pancolitis
MR Method
Inverse variance weighted
MR Egger
0.46
0.44
0.42
1/SE_IV
0
2
4
β_IV

Batch 2113 : 1−eicosatrienoylglycerophosphocholine* on Ulcerative pancolitis

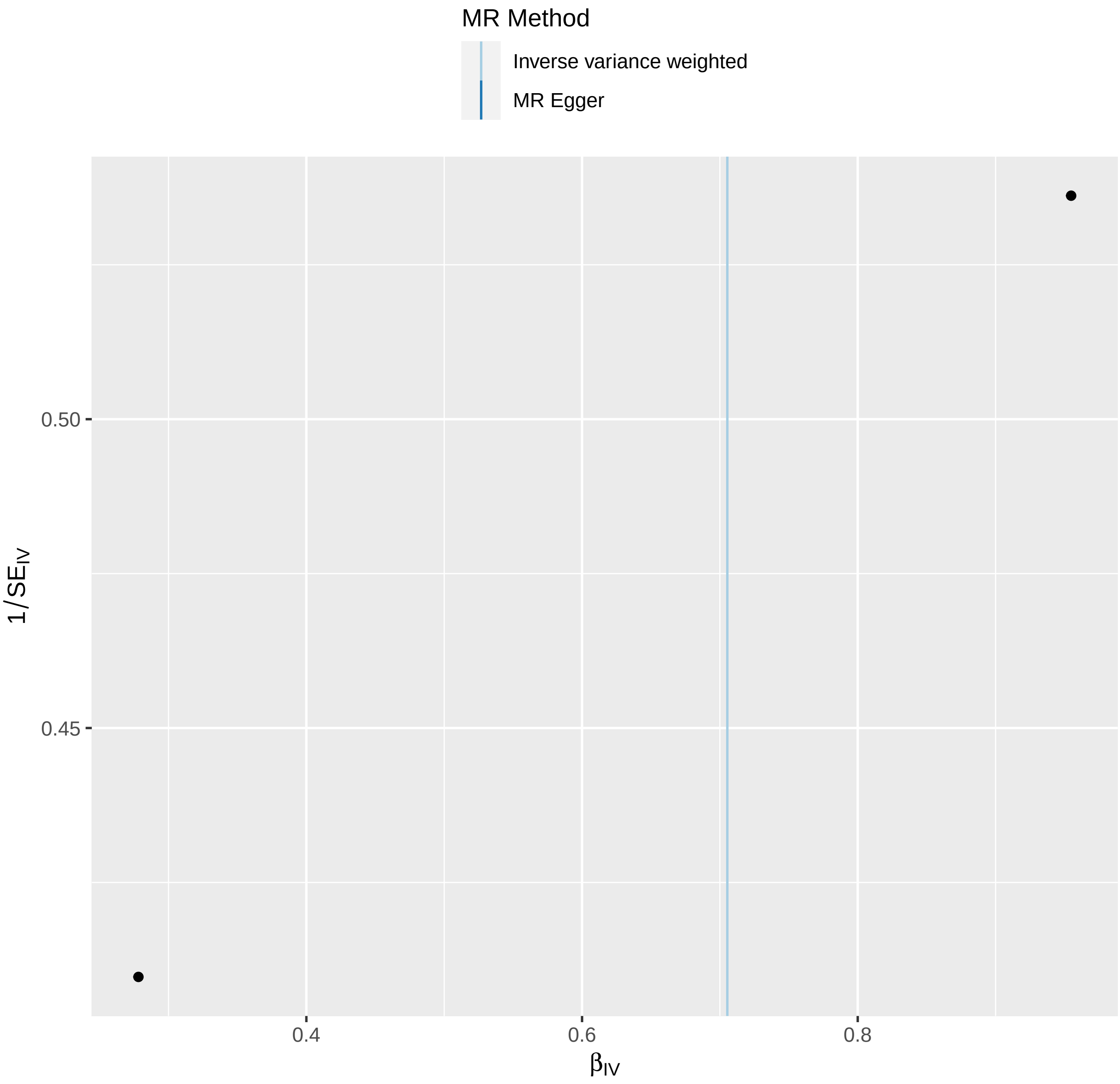

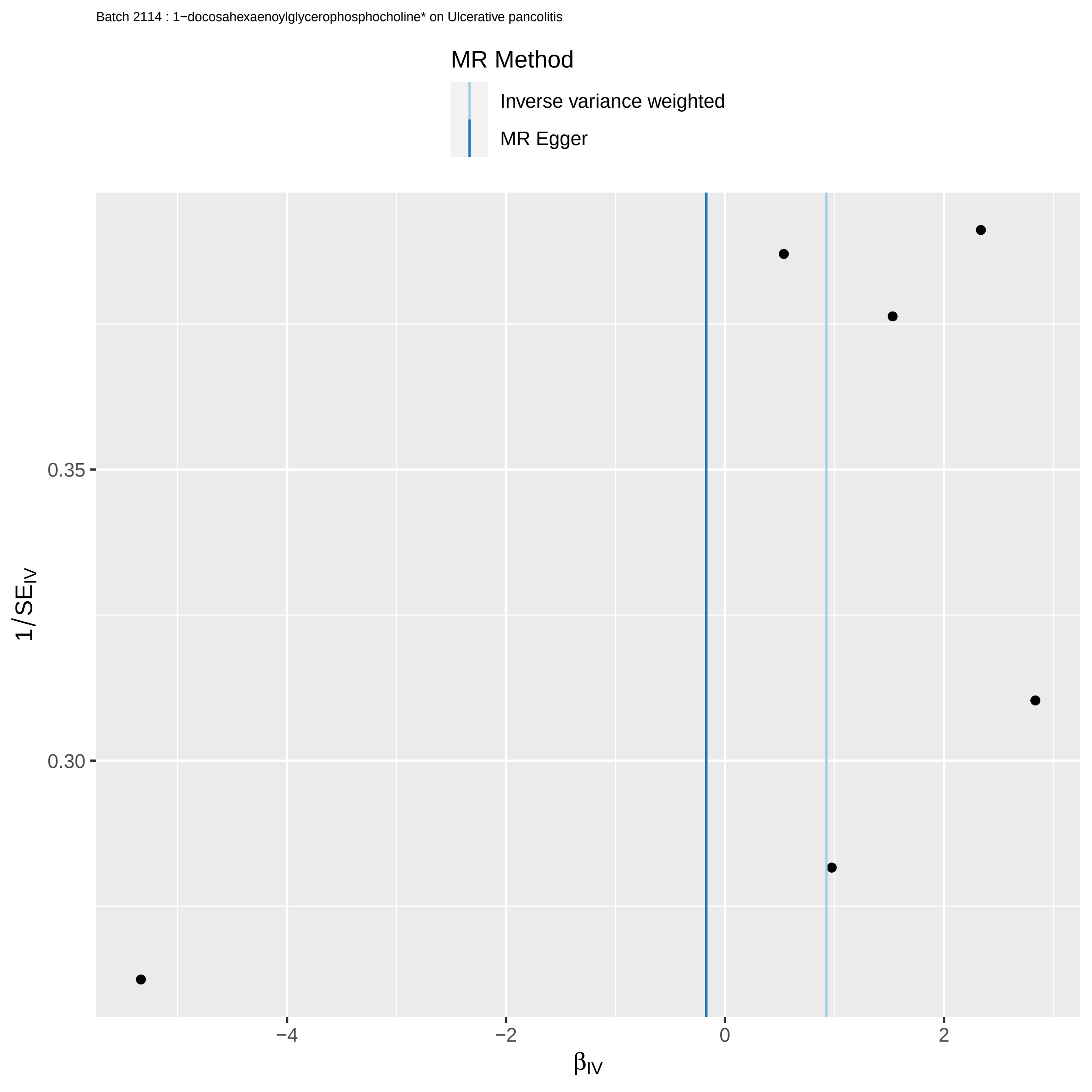
Batch 2114 : 1−docosahexaenoylglycerophosphocholine* on Ulcerative pancolitis
MR Method
Inverse variance weighted
MR Egger
0.35
0.30
1/SE_IV
−4
−2
0
2
β_IV

Batch 2118 : Octanoylcarnitine on Ulcerative pancolitis

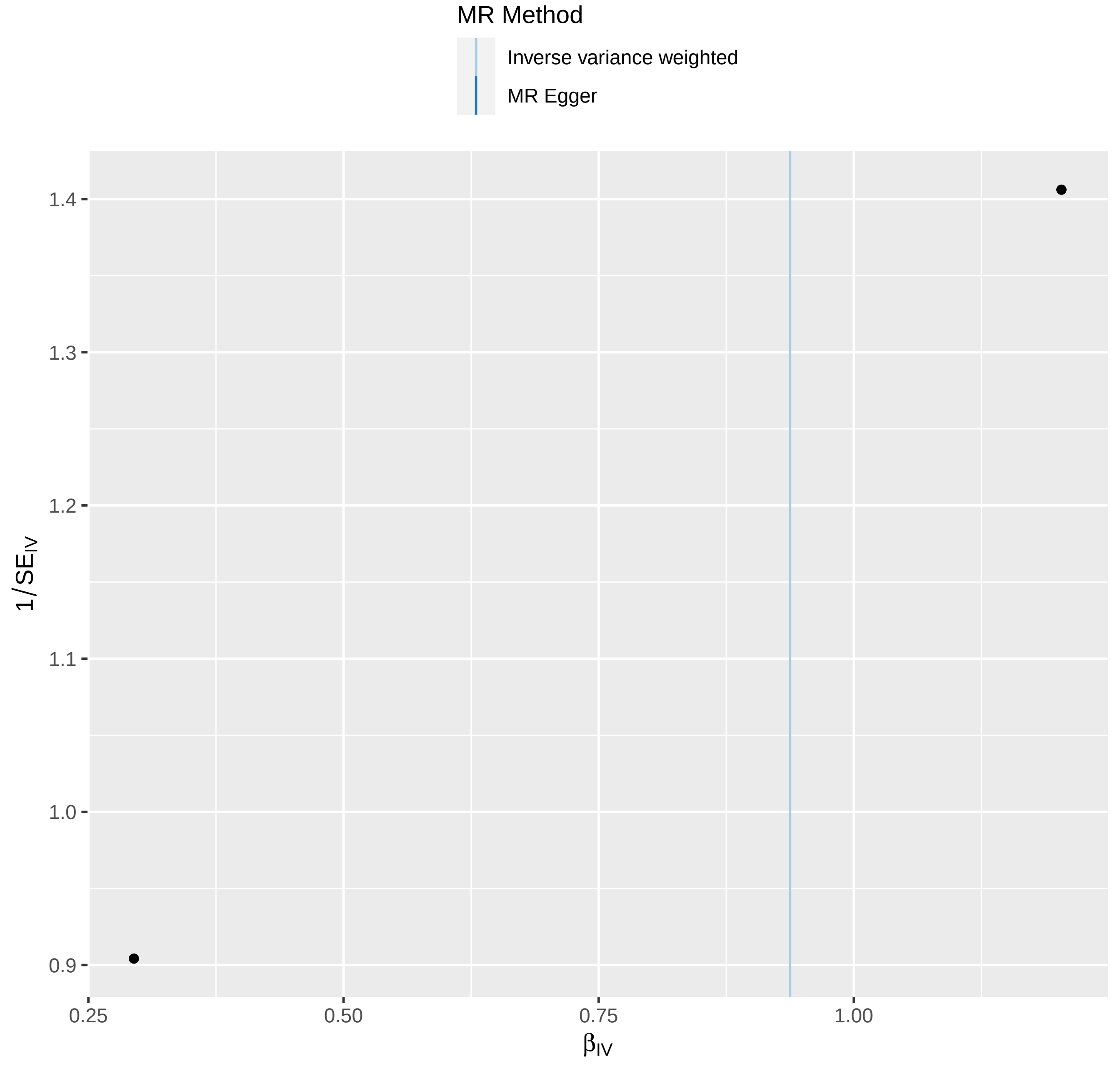

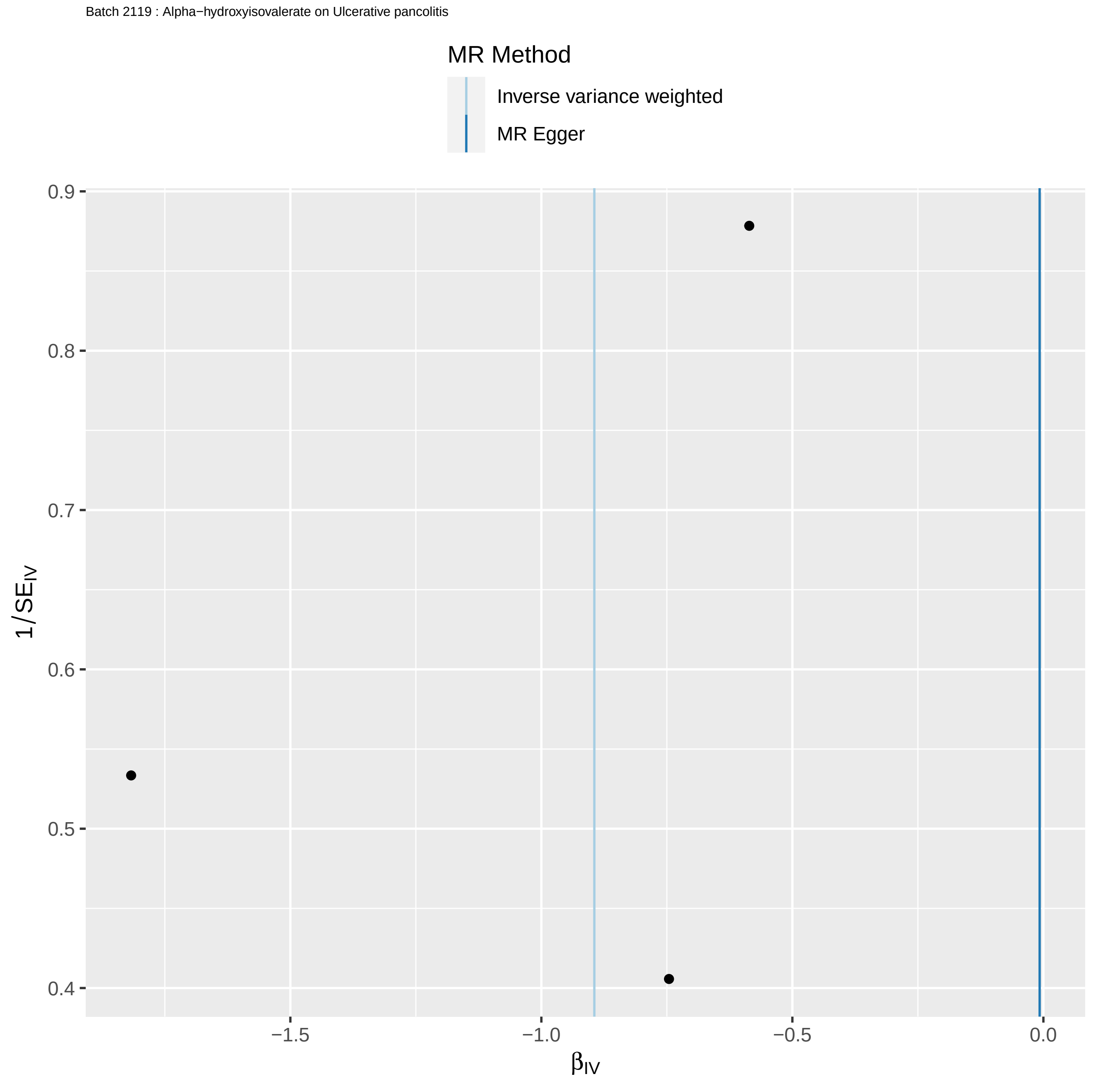

Batch 2119 : Alpha−hydroxyisovalerate on Ulcerative pancolitis
MR Method
Inverse variance weighted
MR Egger
0.9
0.8
0.7
0.6
0.5
0.4
1/SE_IV
−1.5
−1.0
−0.5
0.0
β_IV

Batch 2120 : N−acetylthreonine on Ulcerative pancolitis

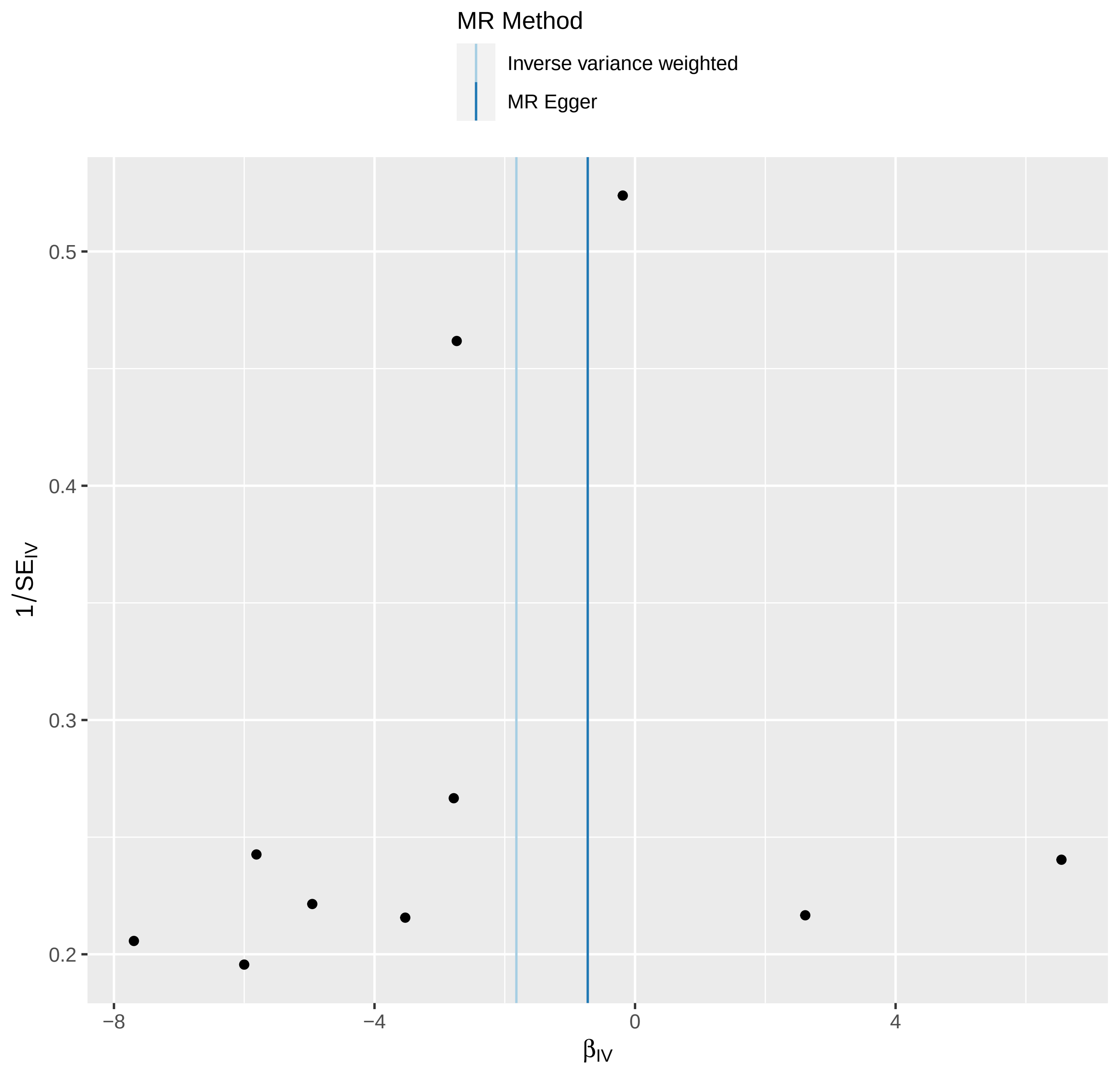

Batch 2121 : Decanoylcarnitine on Ulcerative pancolitis

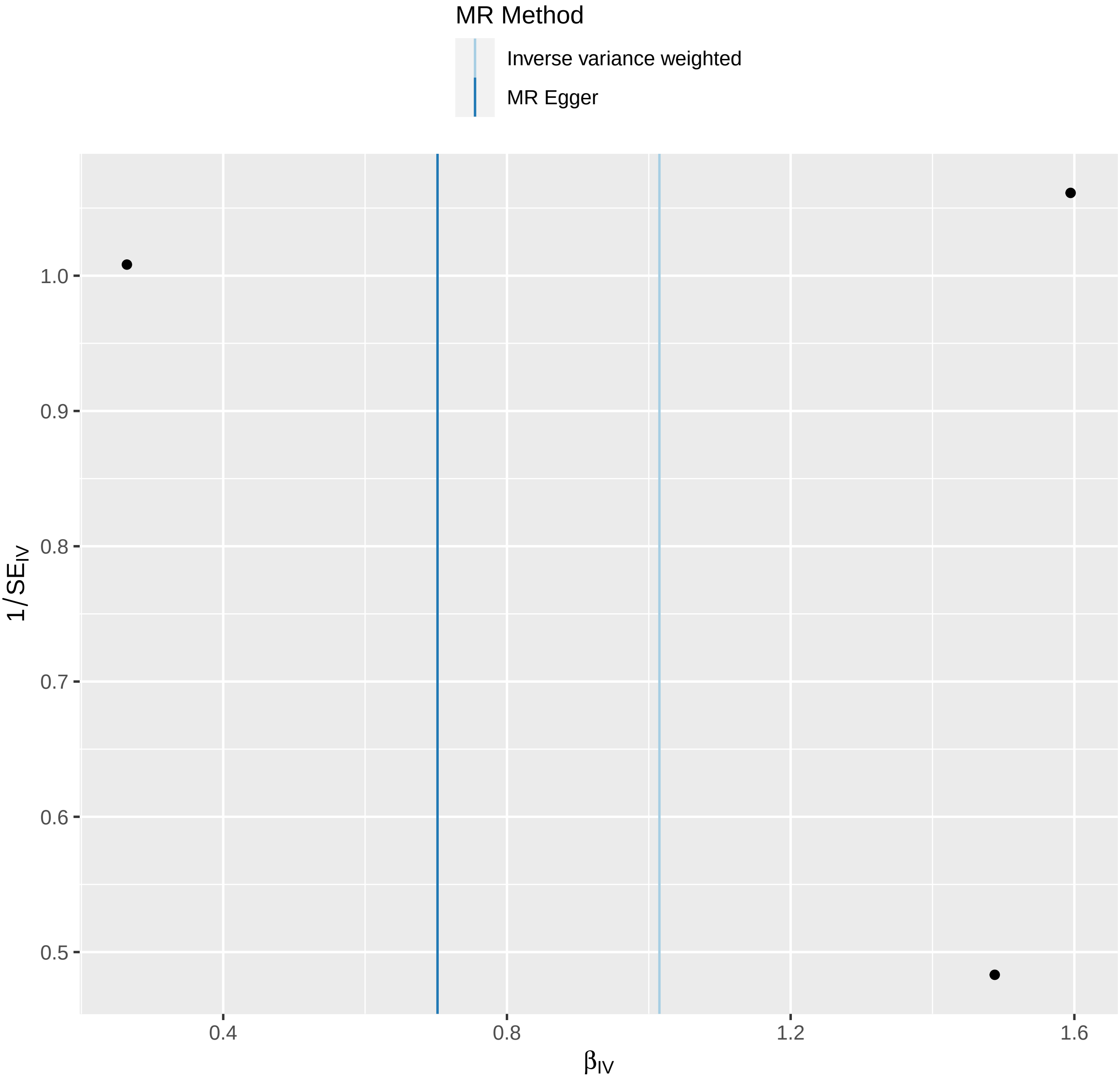

Batch 2122 : 1−palmitoylglycerophosphocholine on Ulcerative pancolitis

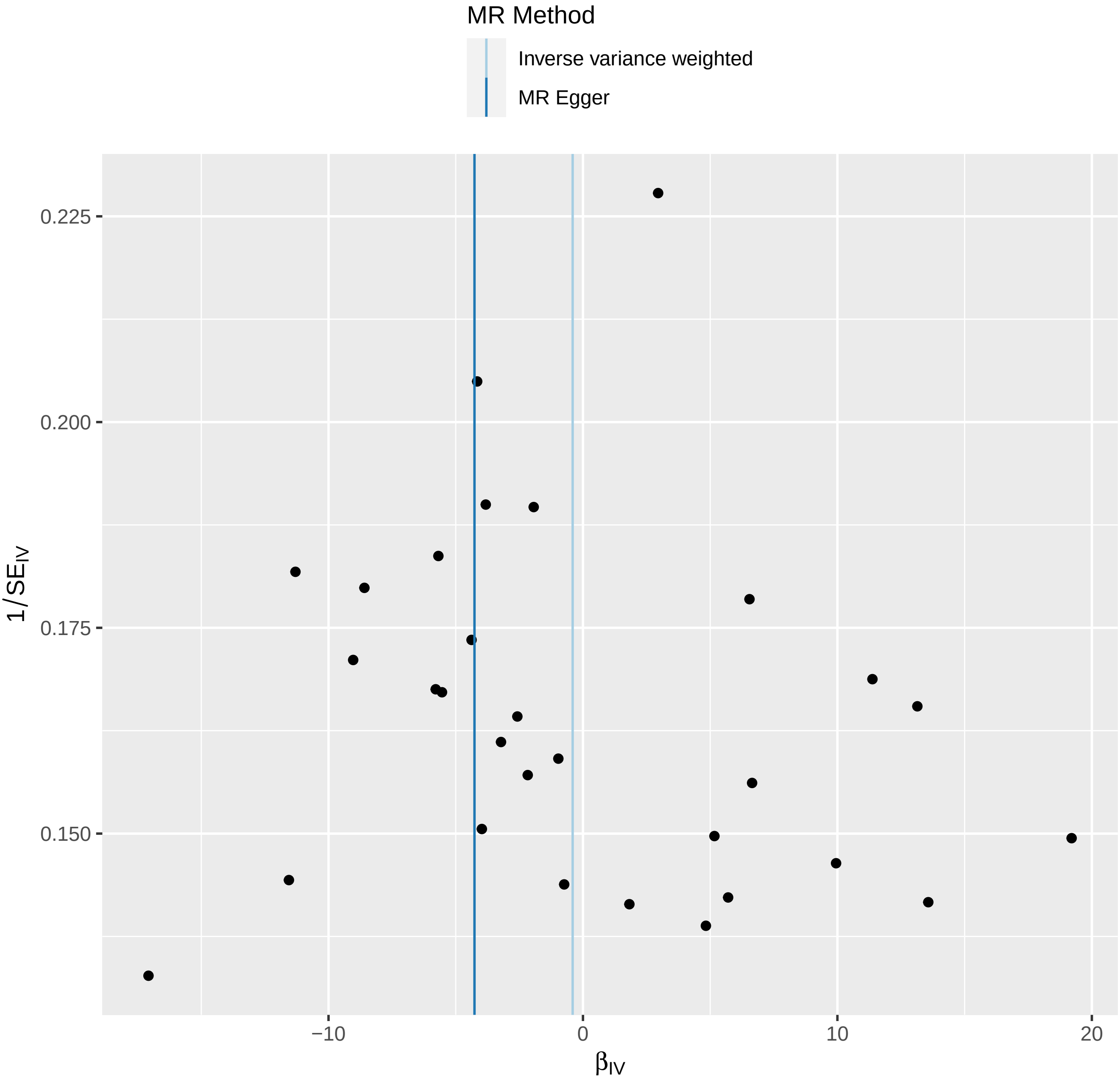

Batch 2123 : 1−heptadecanoylglycerophosphocholine on Ulcerative pancolitis

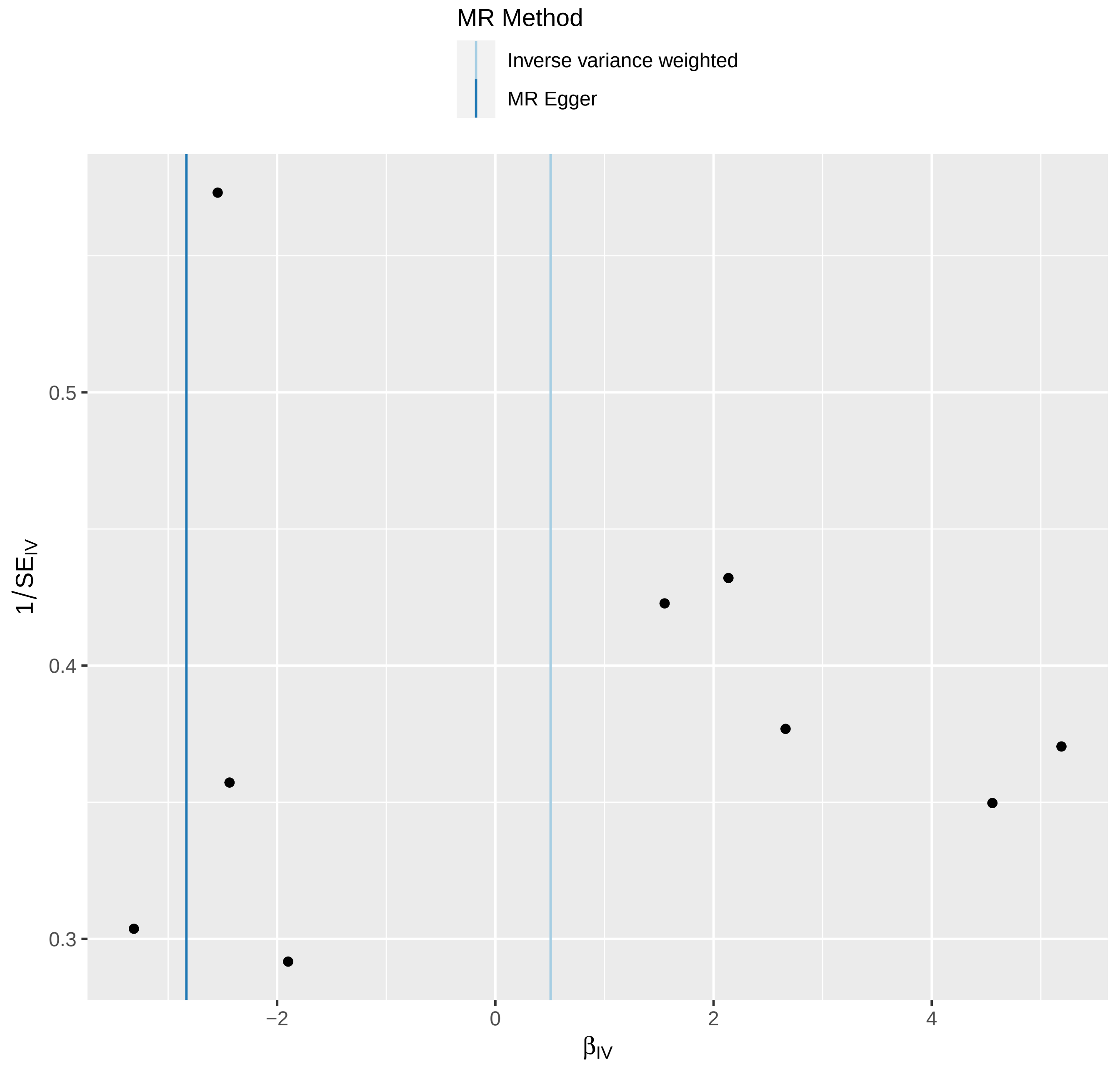

Batch 2124 : 1−oleoylglycerophosphocholine on Ulcerative pancolitis

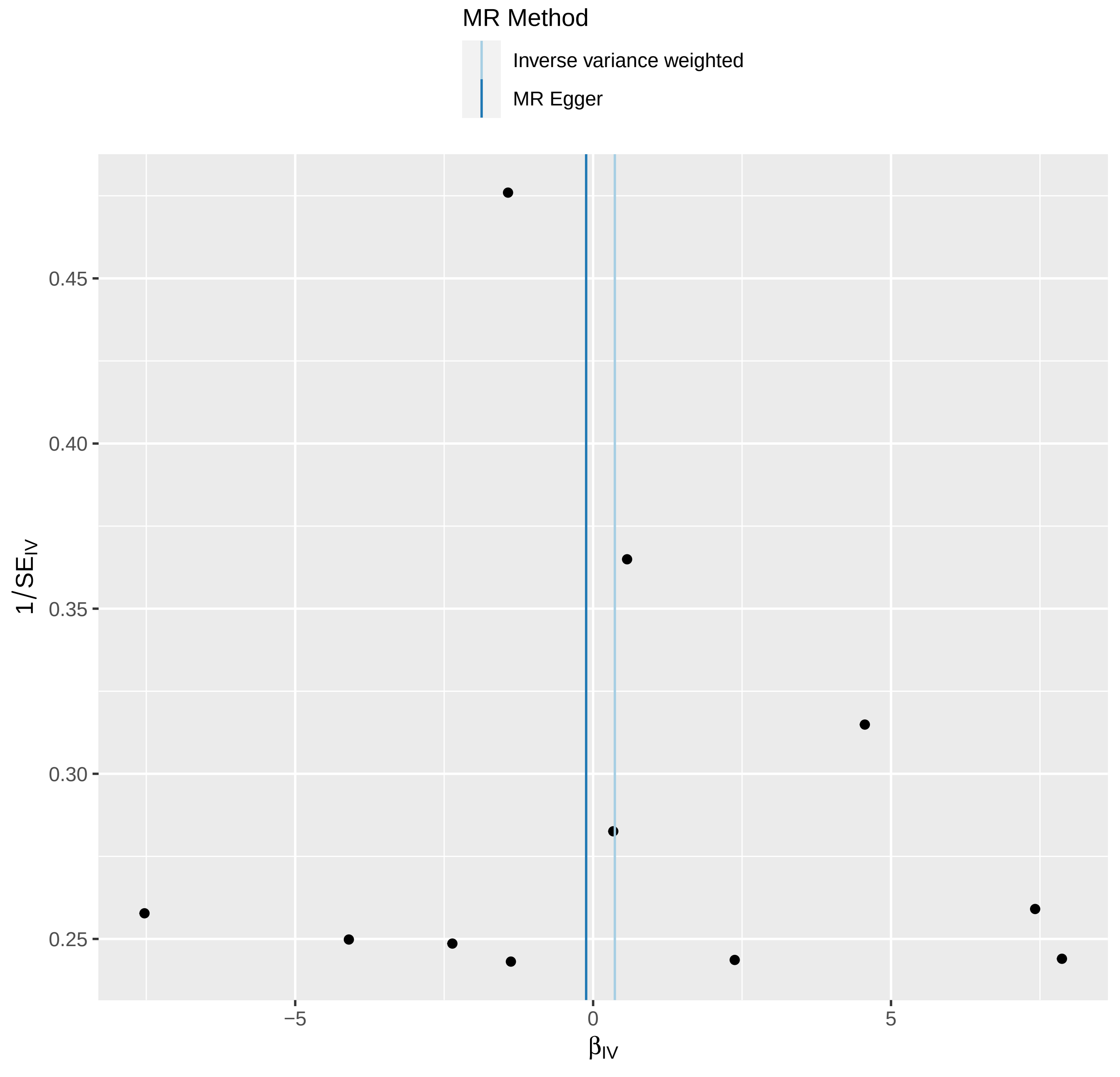

Batch 2125 : 1−stearoylglycerophosphocholine on Ulcerative pancolitis

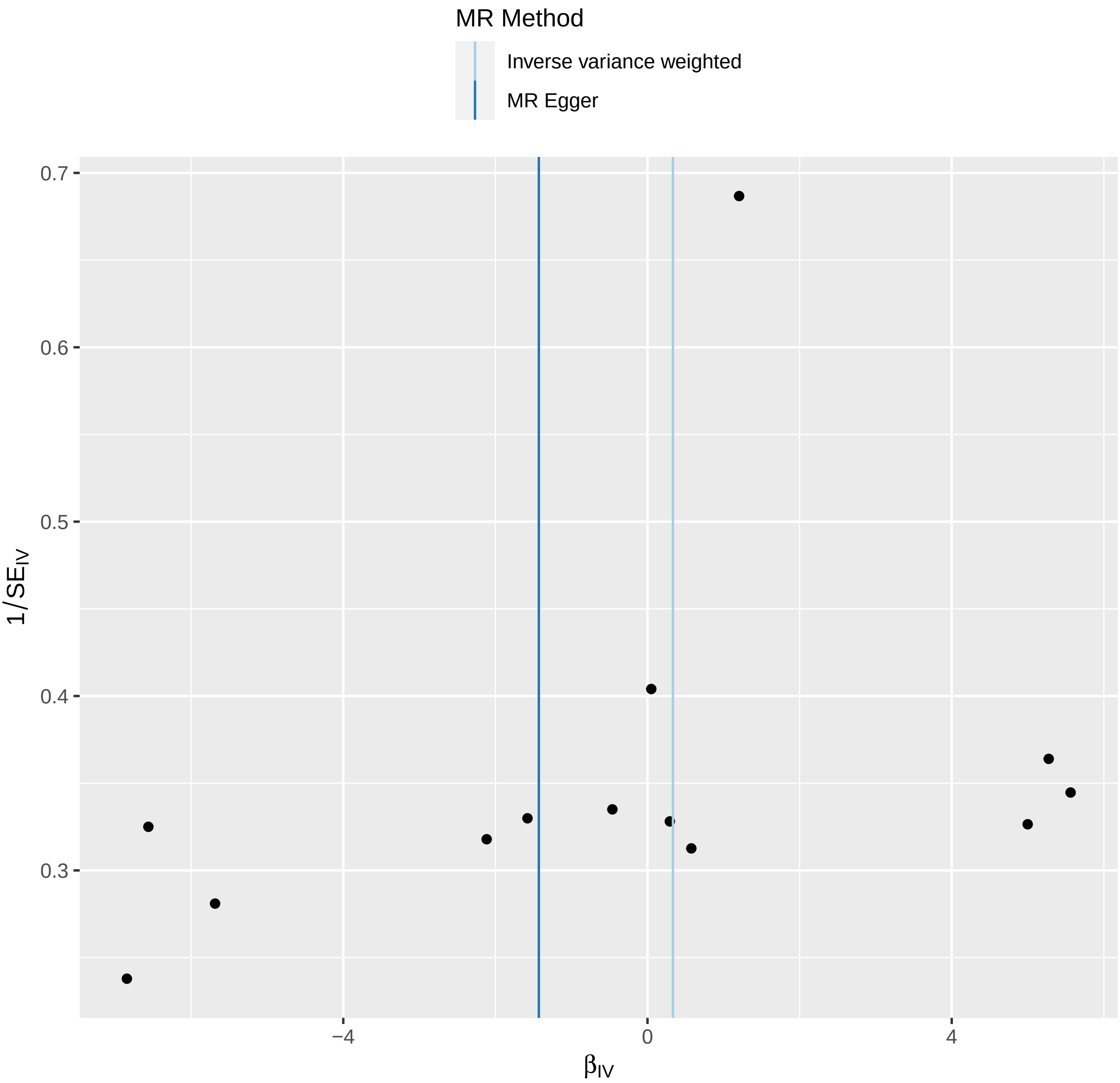

Batch 2128 : 10−heptadecenoate (17:1n7) on Ulcerative pancolitis

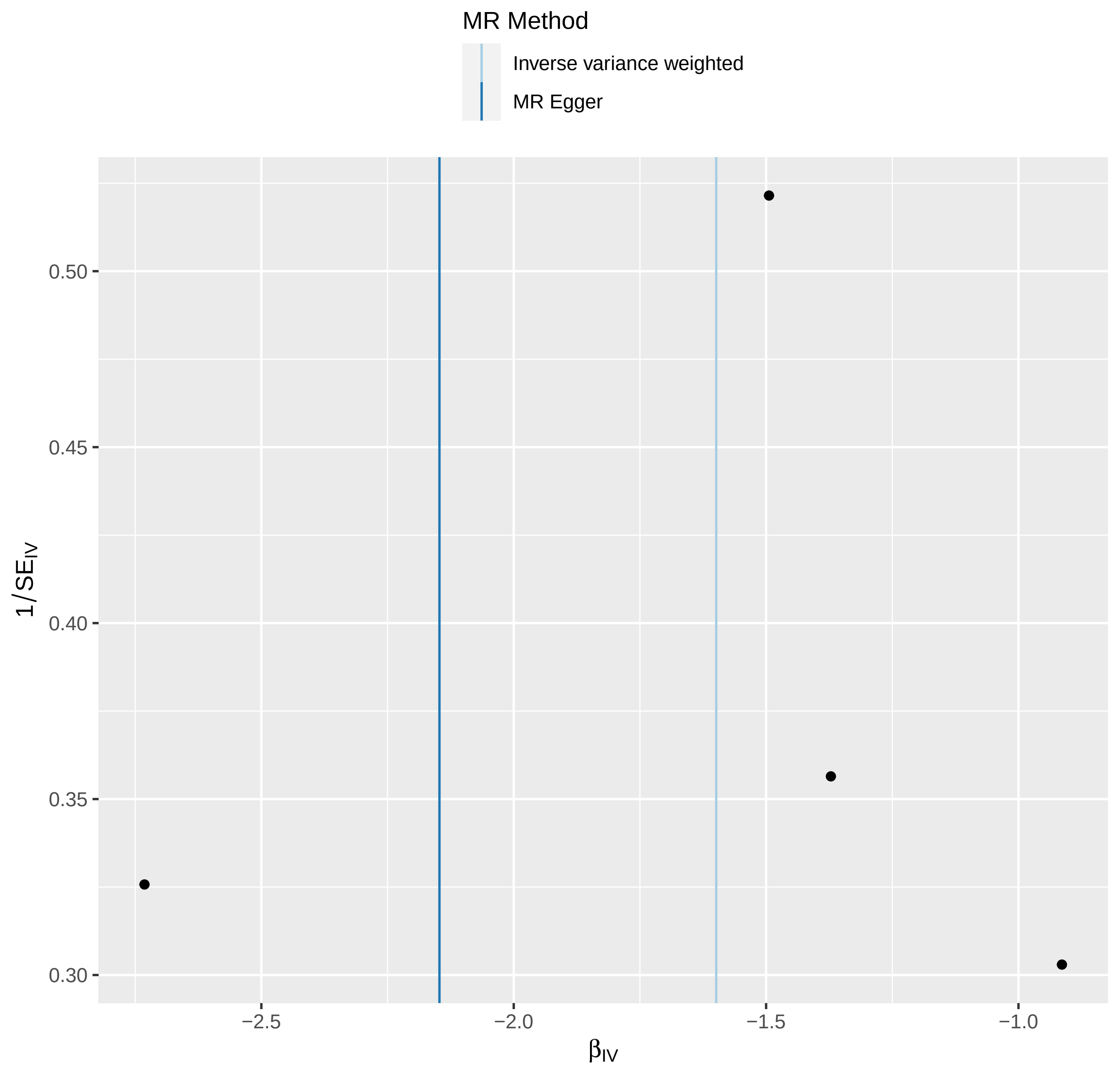

Batch 2130 : Epiandrosterone sulfate on Ulcerative pancolitis

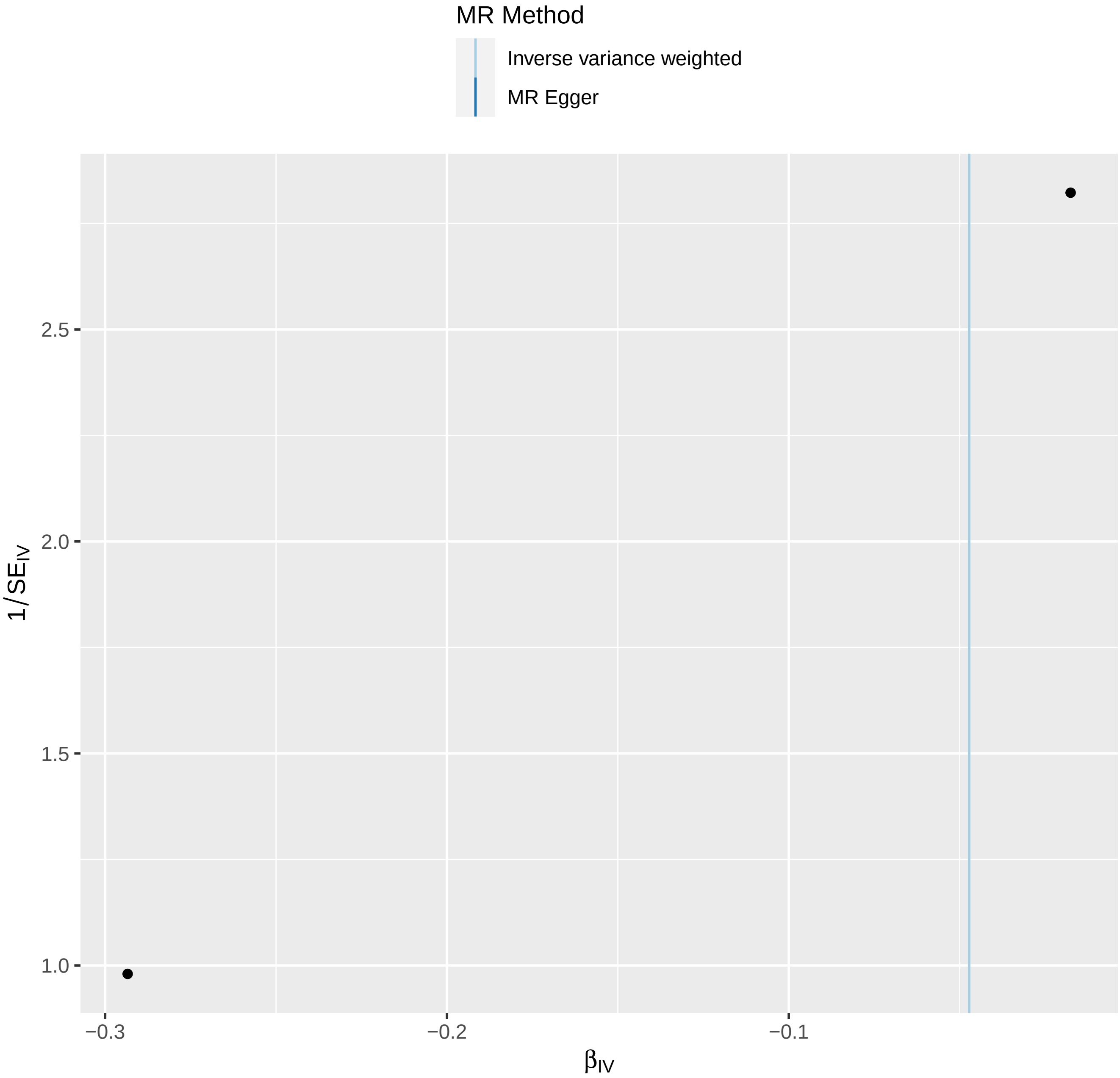

Batch 2131 : Linolenate [alpha or gamma; (18:3n3 or 6)] on Ulcerative pancolitis

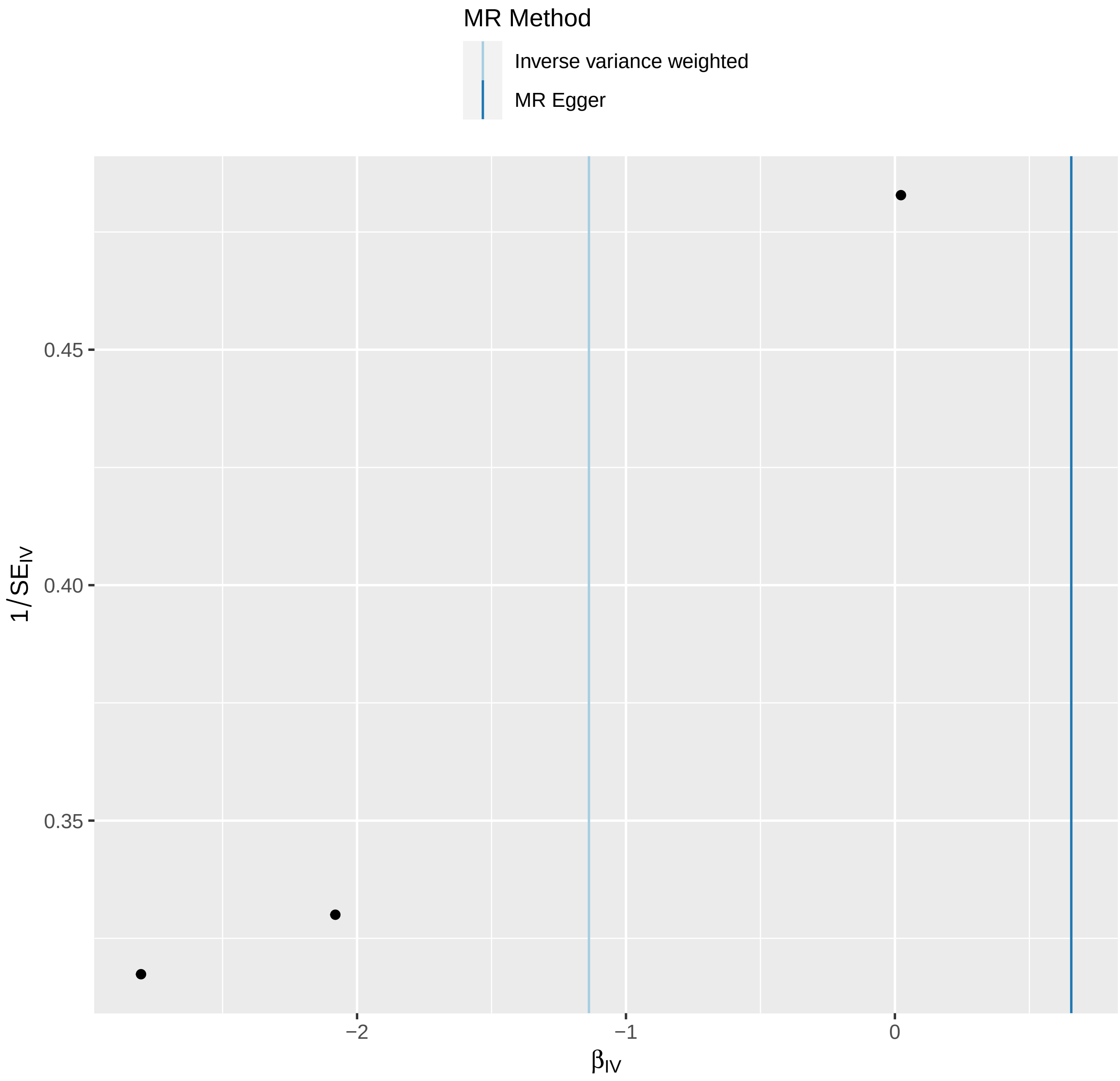

Batch 2132 : X−12510−−2−aminooctanoic acid on Ulcerative pancolitis

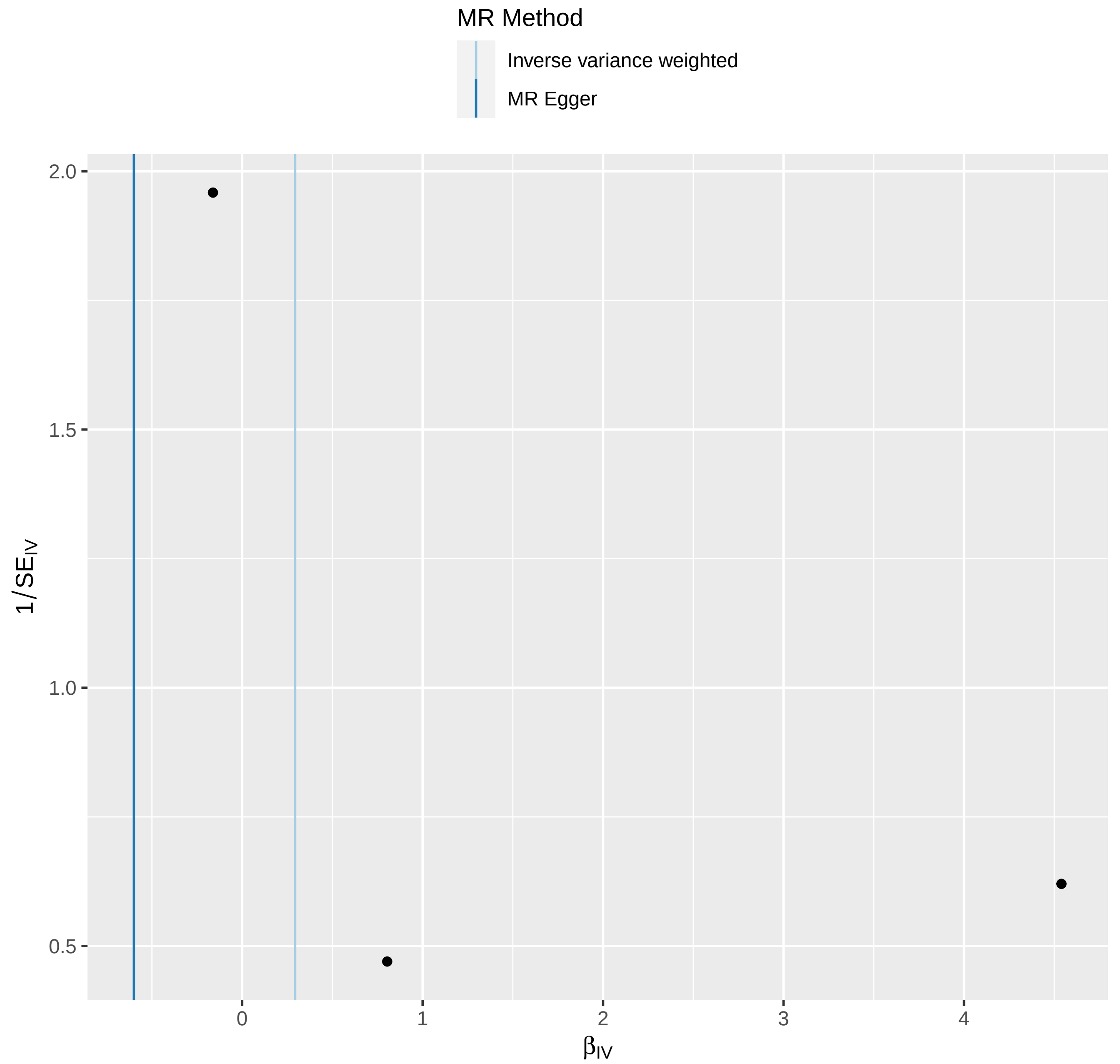

Batch 2135 : Asparagine on Ulcerative pancolitis

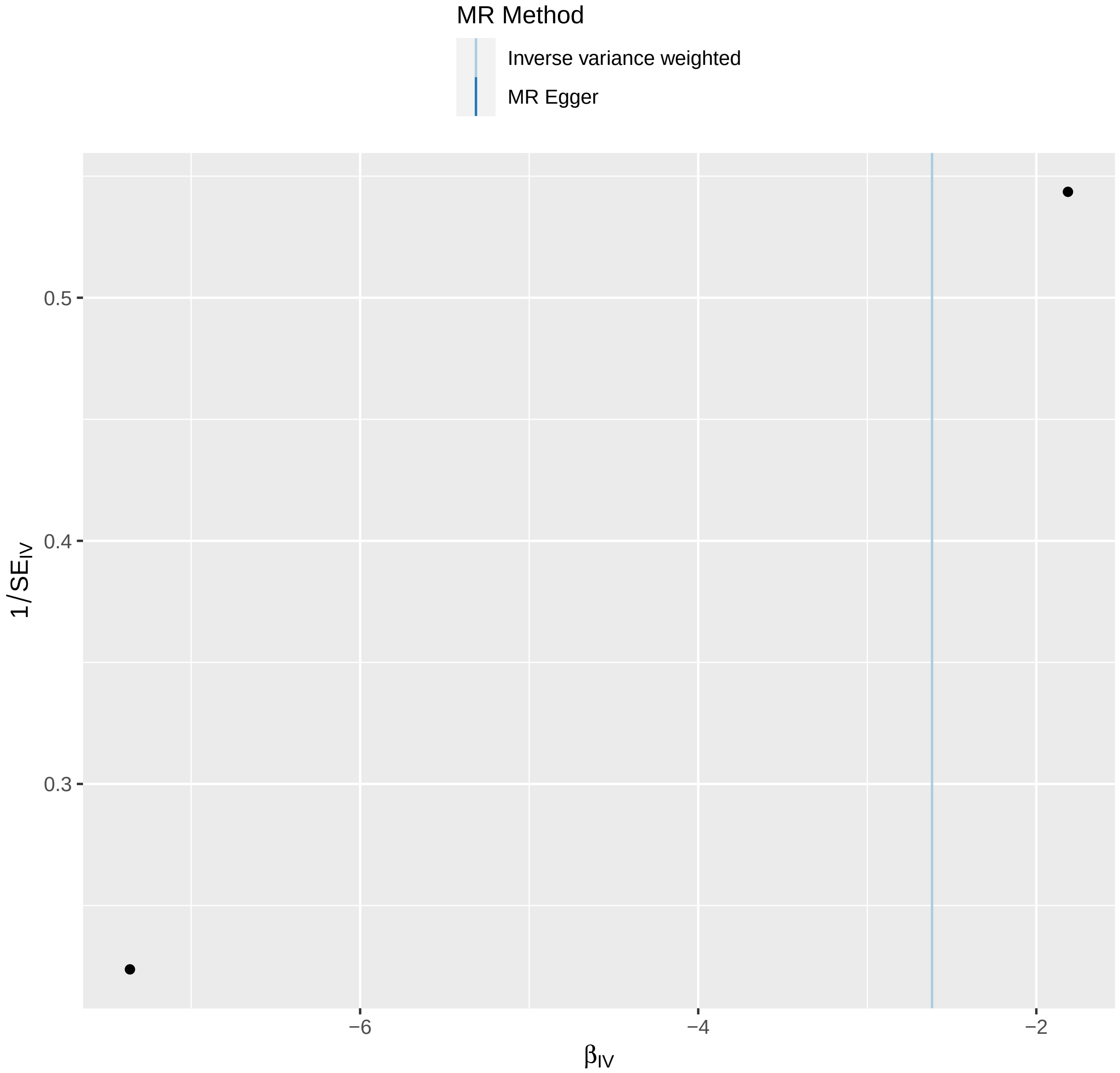

Batch 2136 : Stachydrine on Ulcerative pancolitis

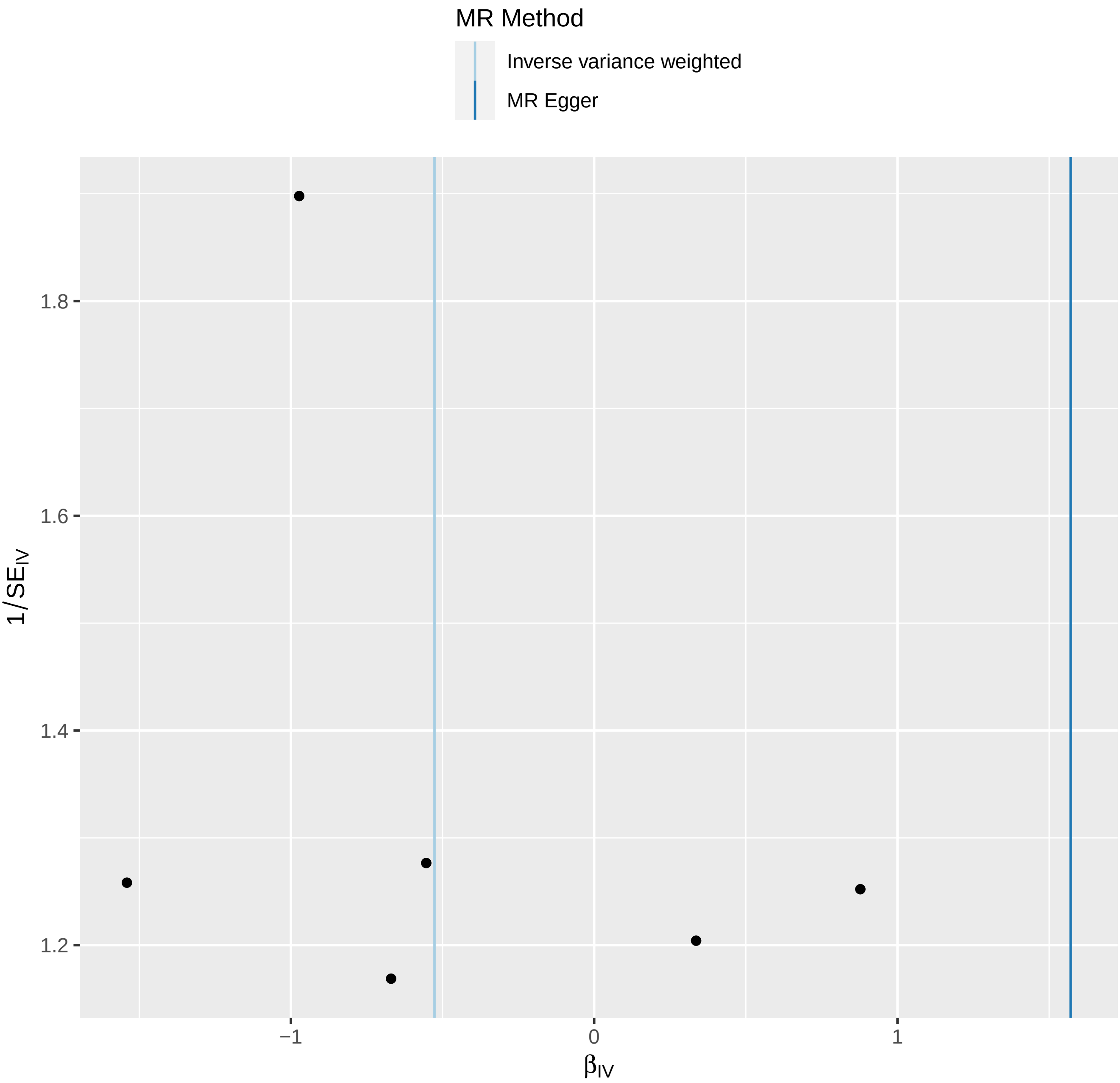

Batch 2137 : Isovalerylcarnitine on Ulcerative pancolitis

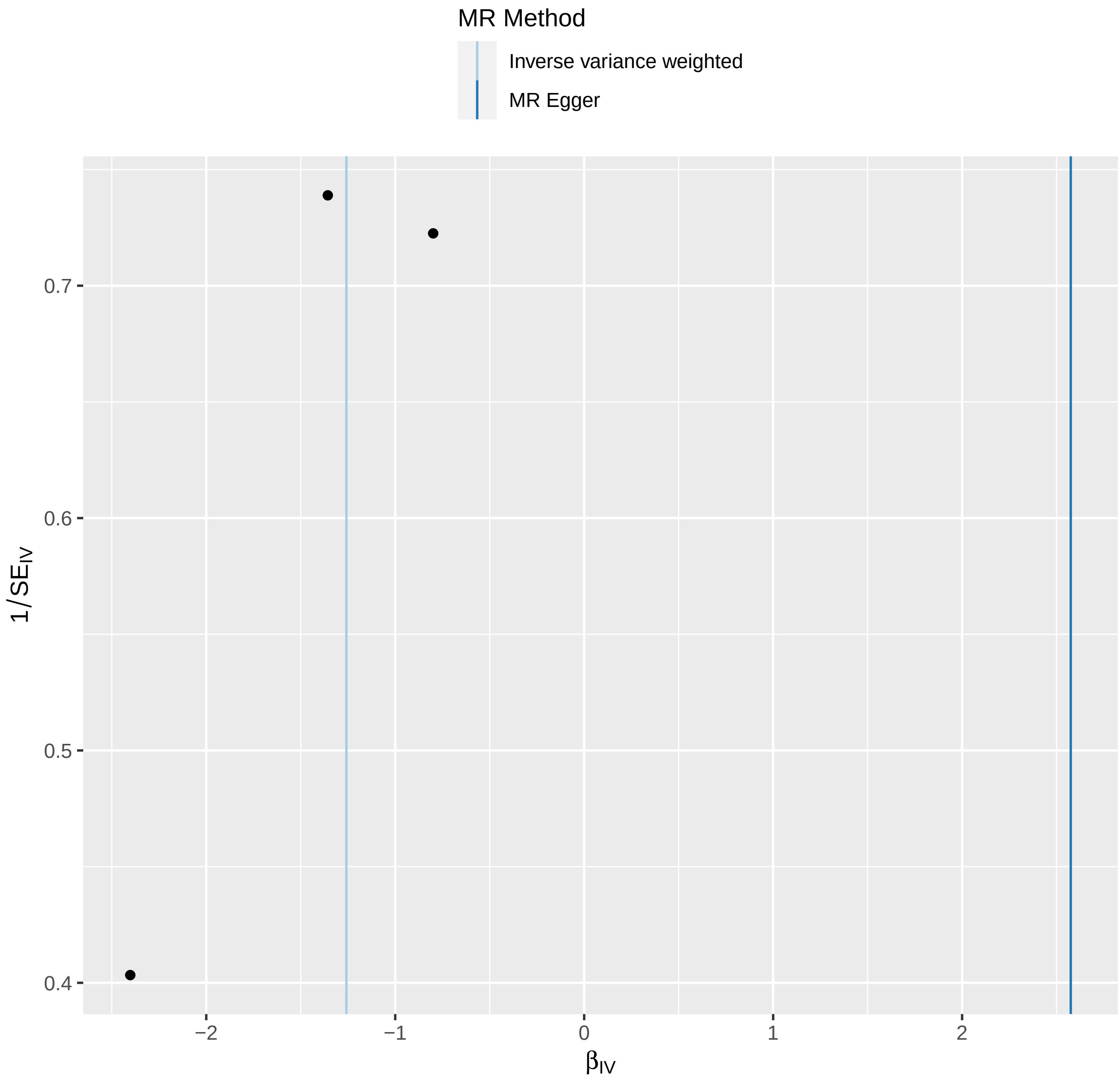

Batch 2140 : 1−linoleoylglycerophosphocholine on Ulcerative pancolitis

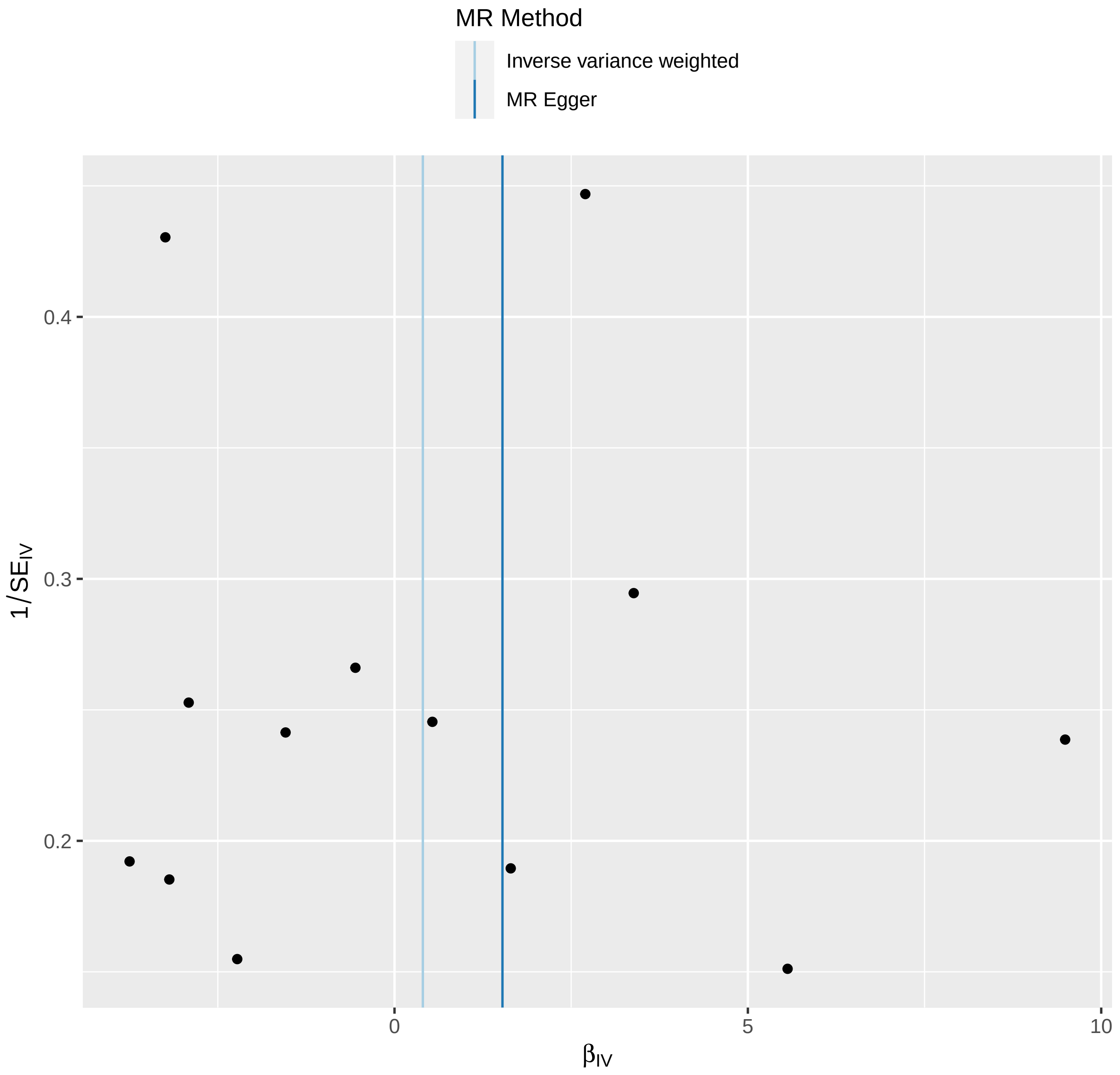

Batch 2141 : Bradykinin, des−arg(9) on Ulcerative pancolitis

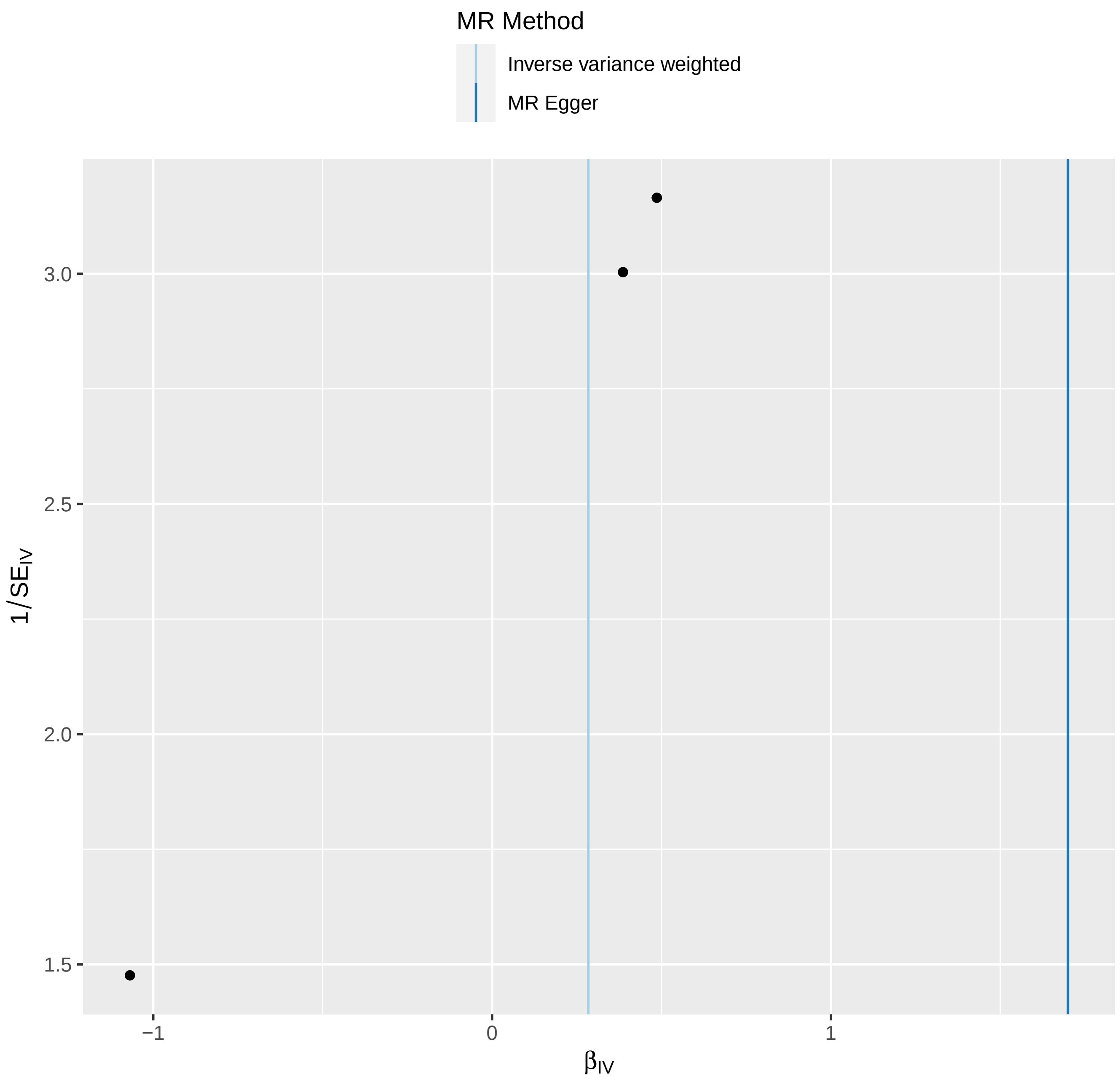

Batch 2142 : Gamma−glutamylisoleucine* on Ulcerative pancolitis

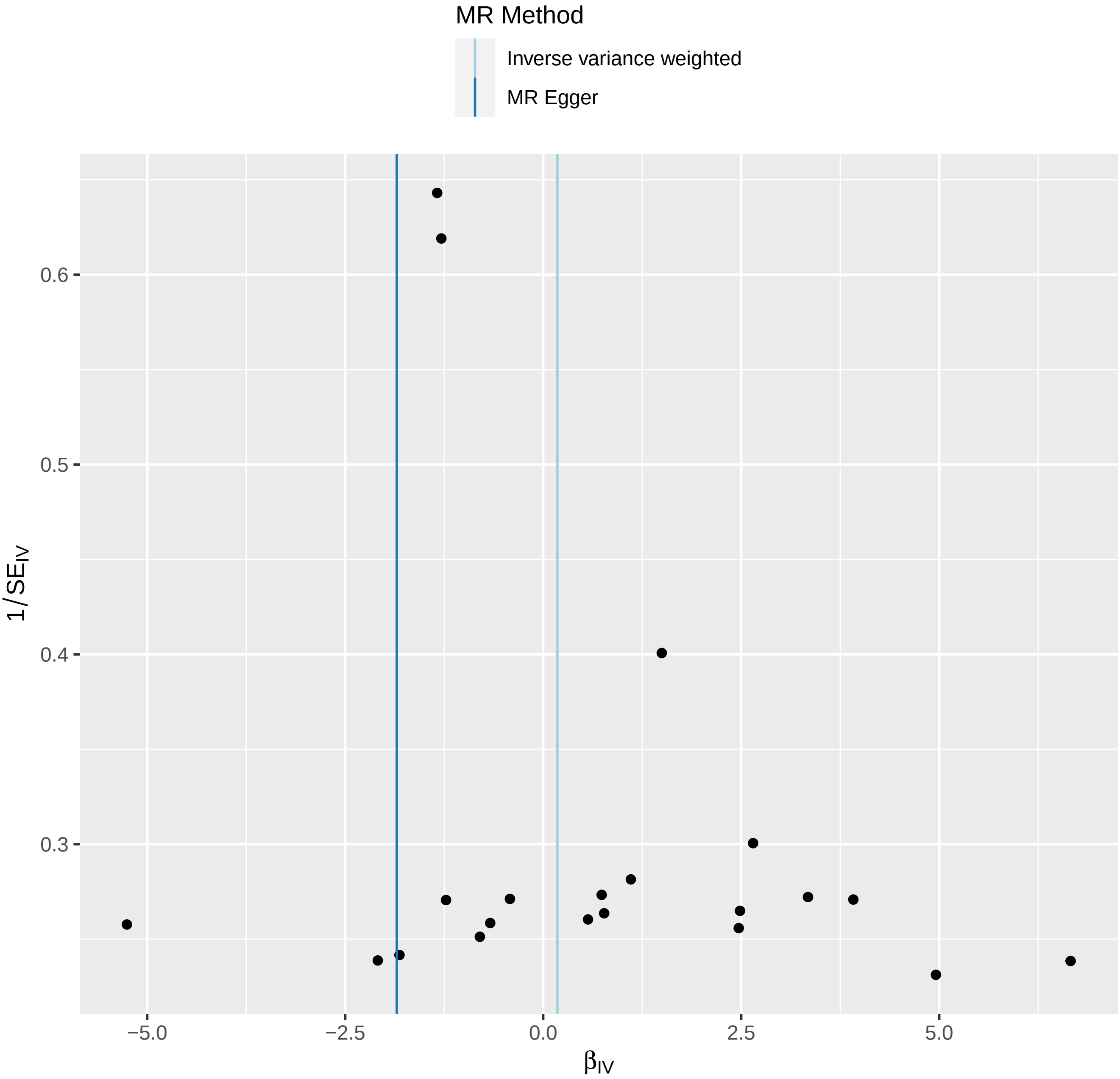

Batch 2145 : Isovalerate on Ulcerative pancolitis

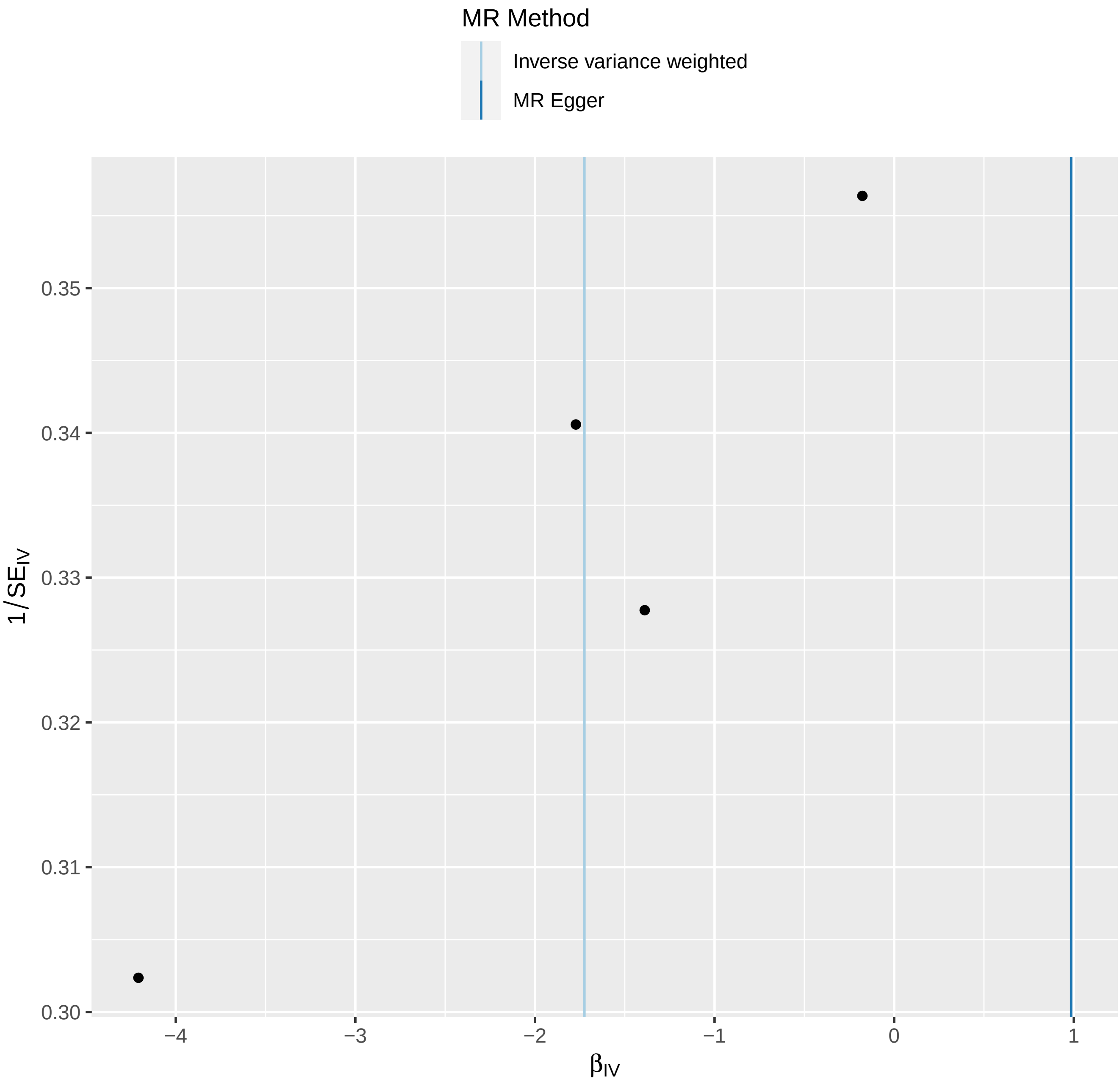

Batch 2146 : X−13183−−stearamide on Ulcerative pancolitis

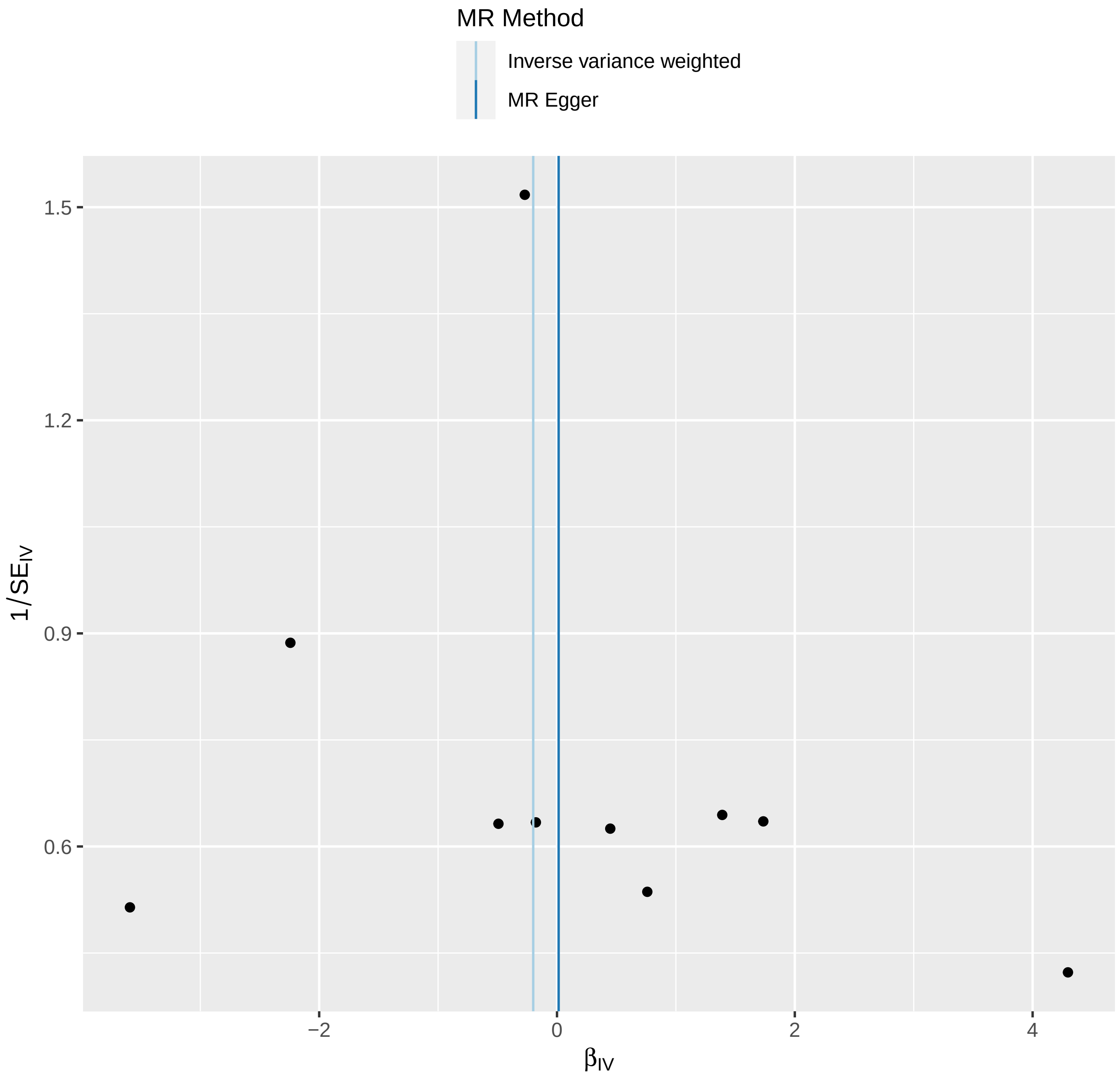

Batch 2147 : 7−methylguanine on Ulcerative pancolitis

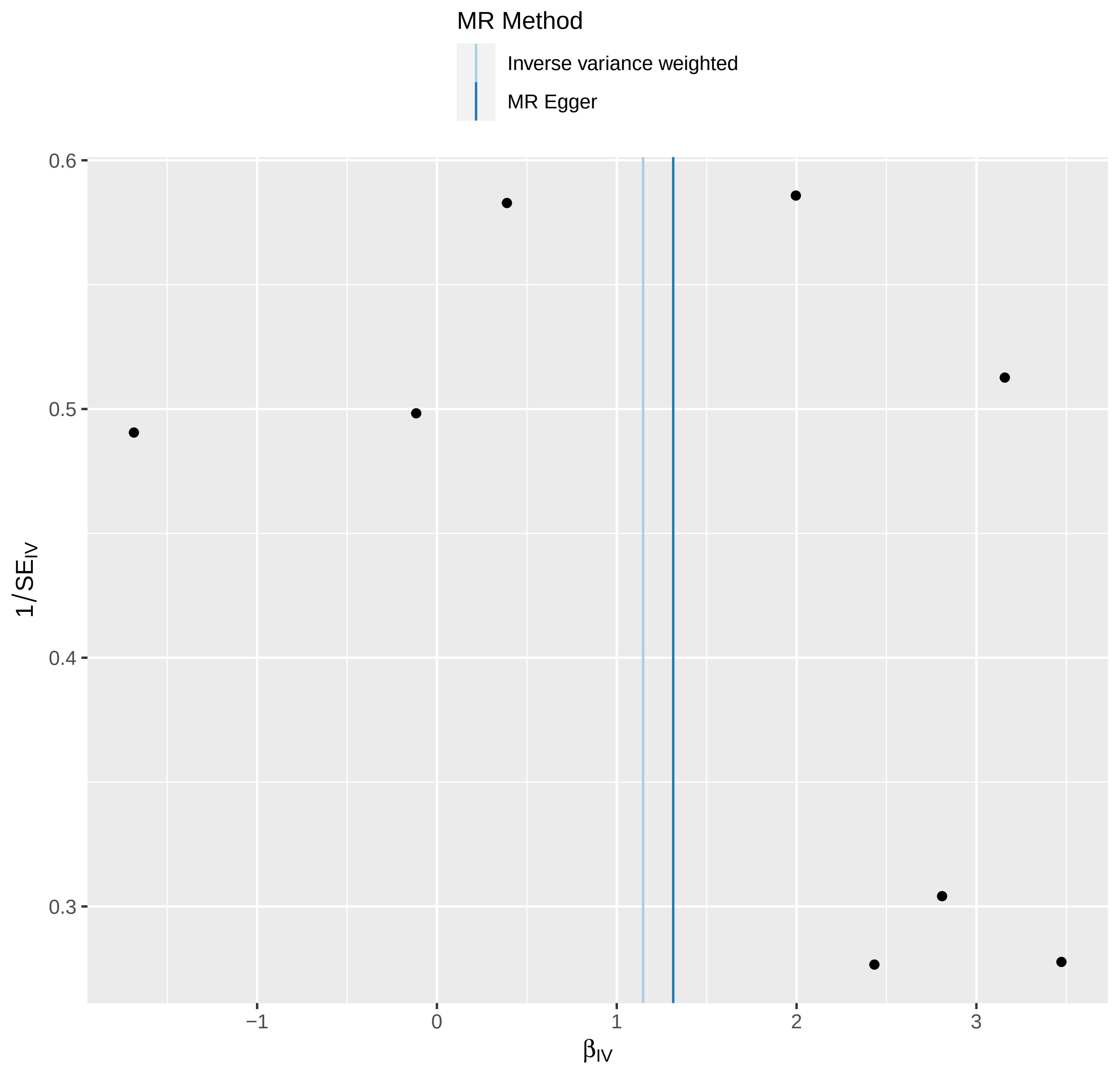

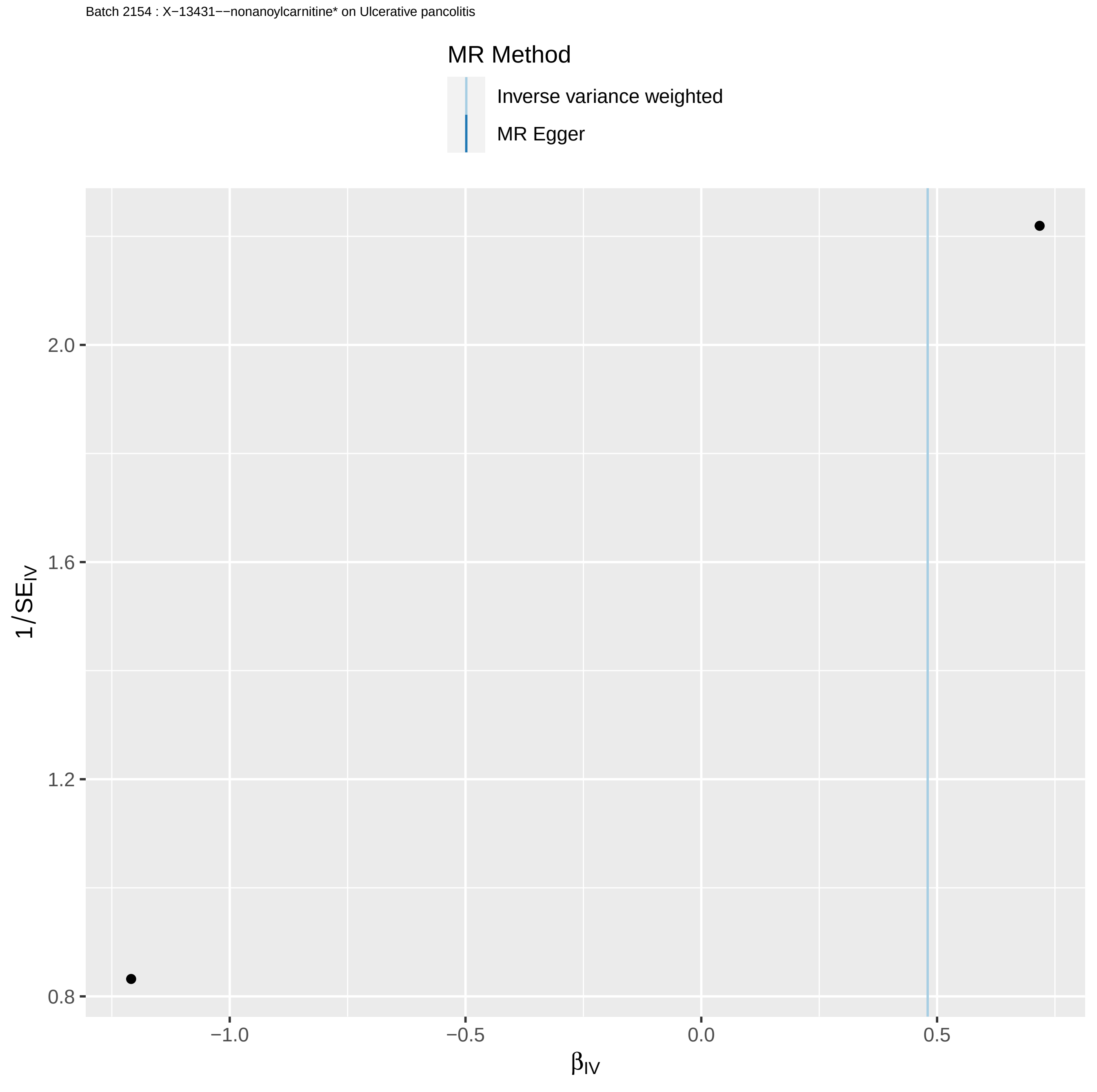

Batch 2154 : X−13431−−nonanoylcarnitine* on Ulcerative pancolitis
MR Method
Inverse variance weighted
MR Egger
2.0
1.6
1.2
0.8
1/SE_IV
−1.0
−0.5
0.0
0.5
β_IV

Batch 2155 : 2−palmitoylglycerophosphocholine* on Ulcerative pancolitis

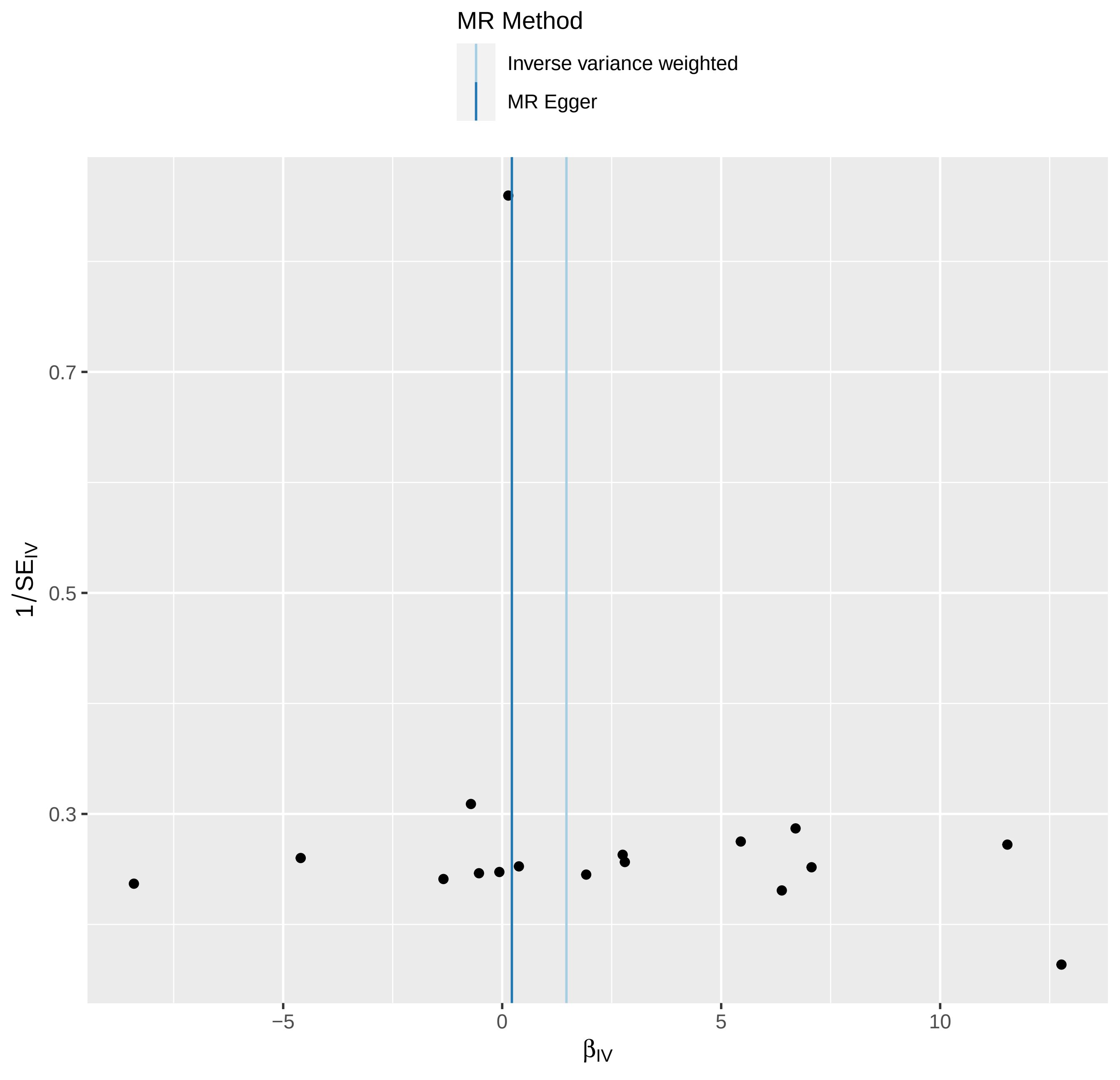

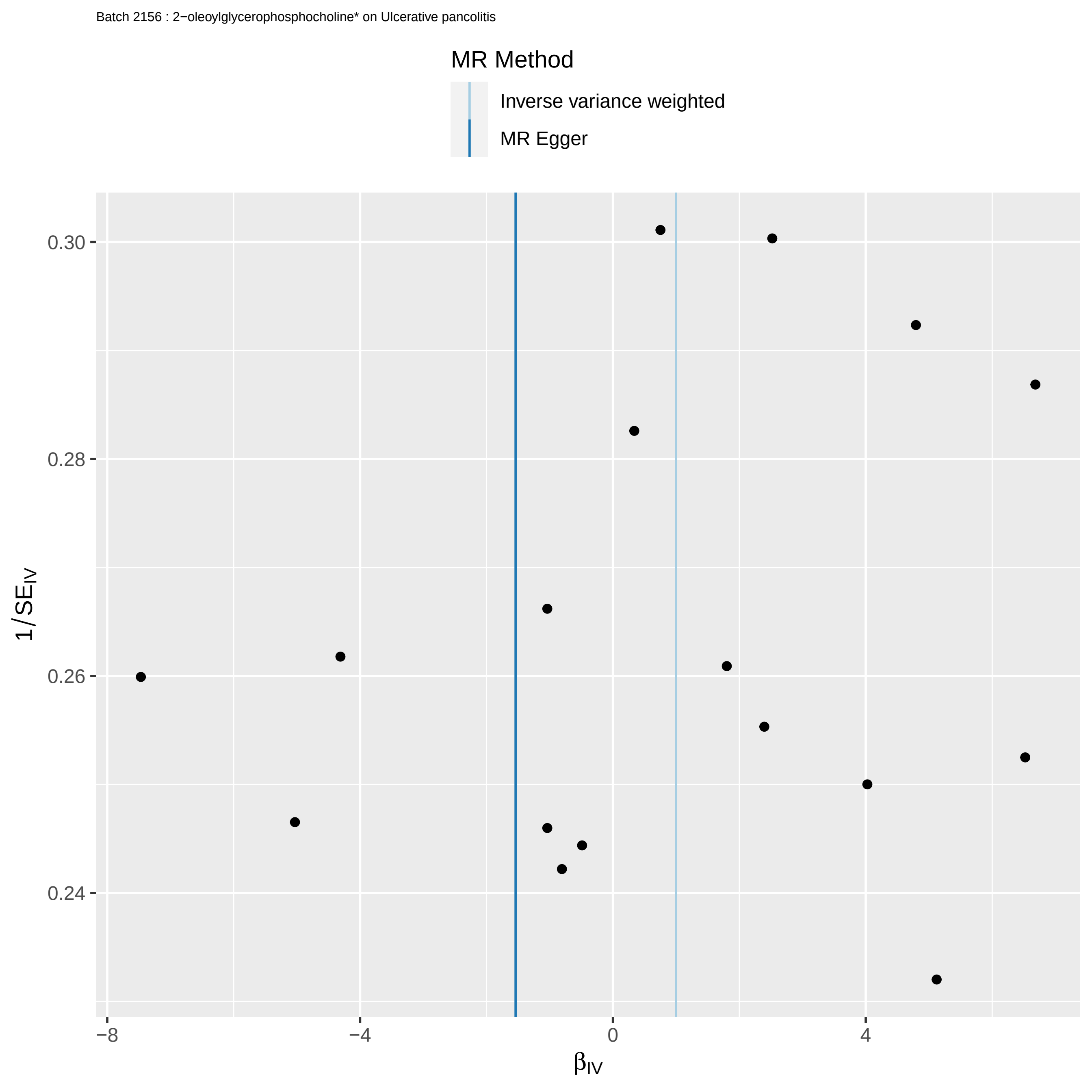
Batch 2156 : 2−oleoylglycerophosphocholine* on Ulcerative pancolitis
MR Method
Inverse variance weighted
MR Egger
1/SE_IV
0.30
0.28
0.26
0.24
−8
−4
0
4
β_IV

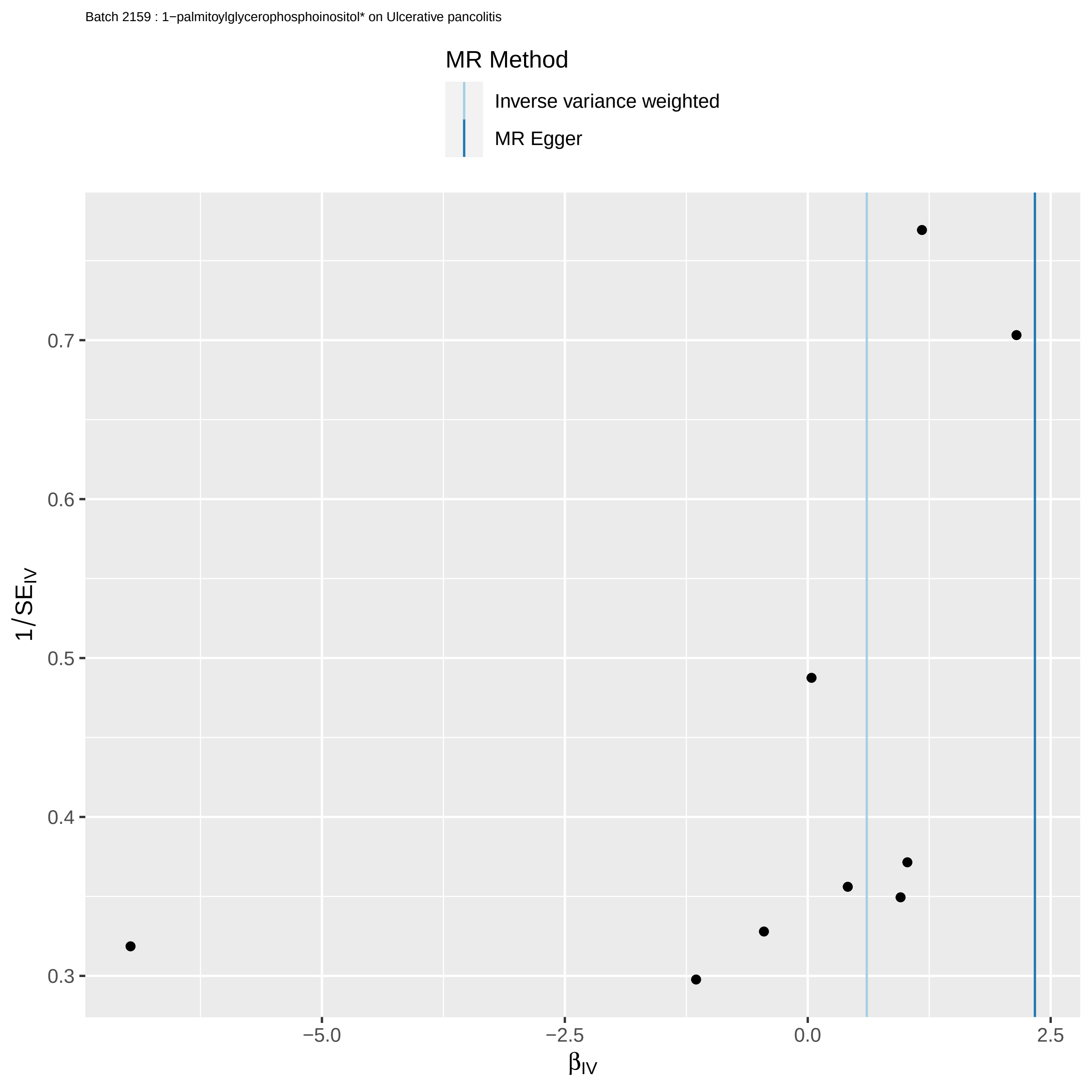

Batch 2159 : 1−palmitoylglycerophosphoinositol* on Ulcerative pancolitis
MR Method
Inverse variance weighted
MR Egger
1/SE_IV
0.7
0.6
0.5
0.4
0.3
−5.0
−2.5
0.0
2.5
β_IV

Batch 2161 : Hydroxyisovaleroyl carnitine on Ulcerative pancolitis

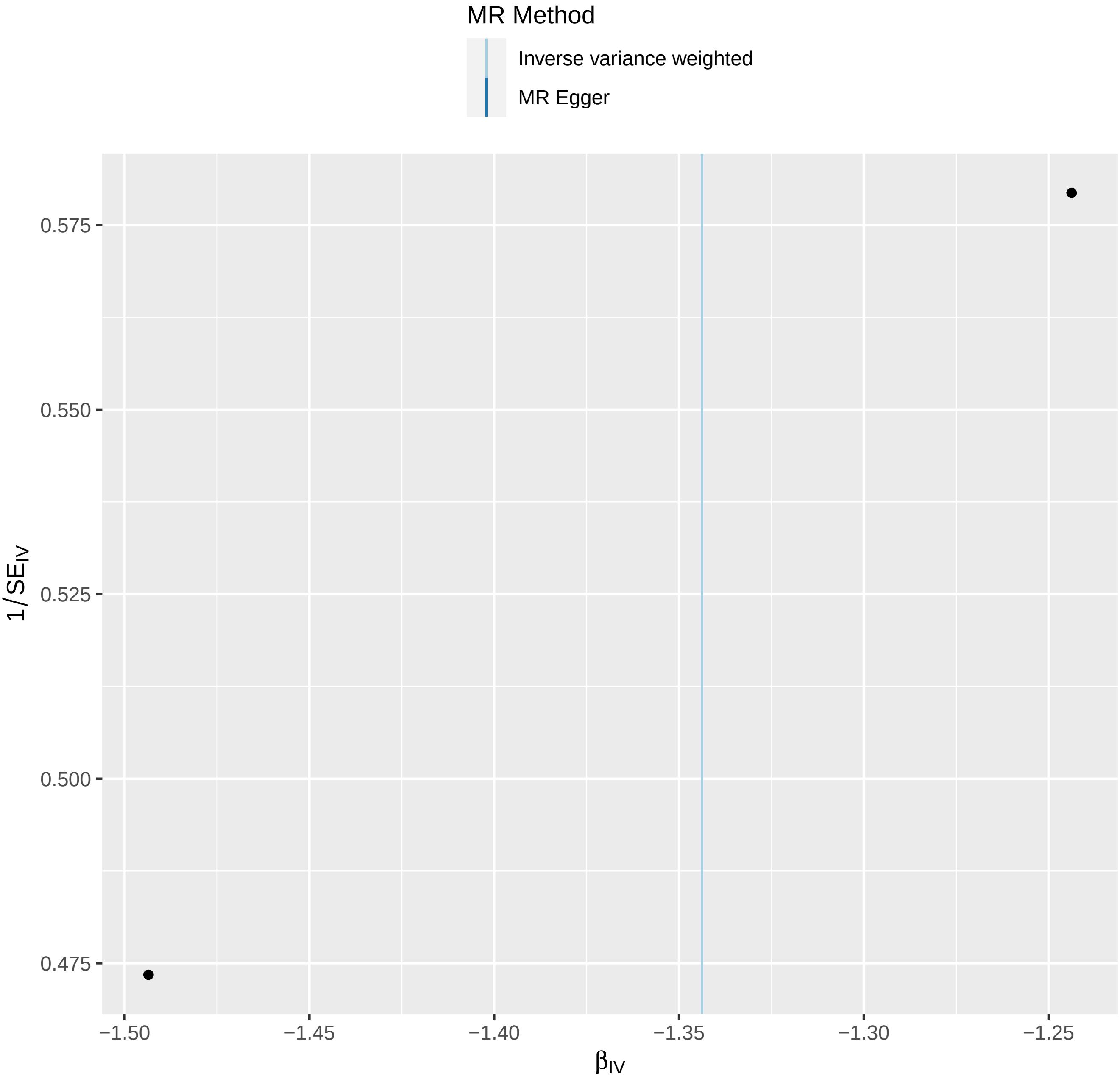

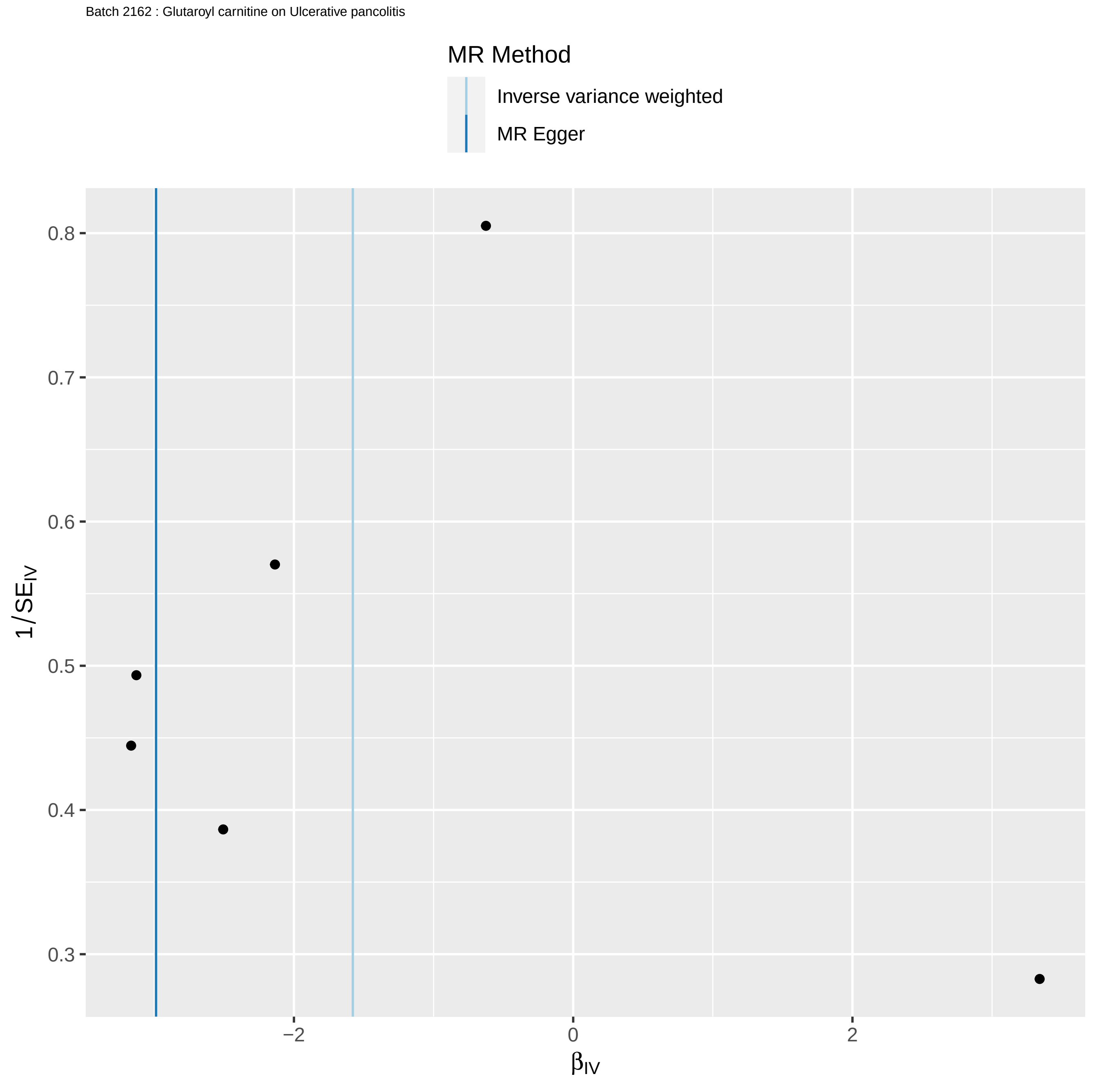

Batch 2162 : Glutaroyl carnitine on Ulcerative pancolitis
MR Method
Inverse variance weighted
MR Egger
0.8
0.7
0.6
0.5
0.4
0.3
1/SE_IV
−2
0
2
β_IV

Batch 2164 : 4−hydroxyhippurate on Ulcerative pancolitis

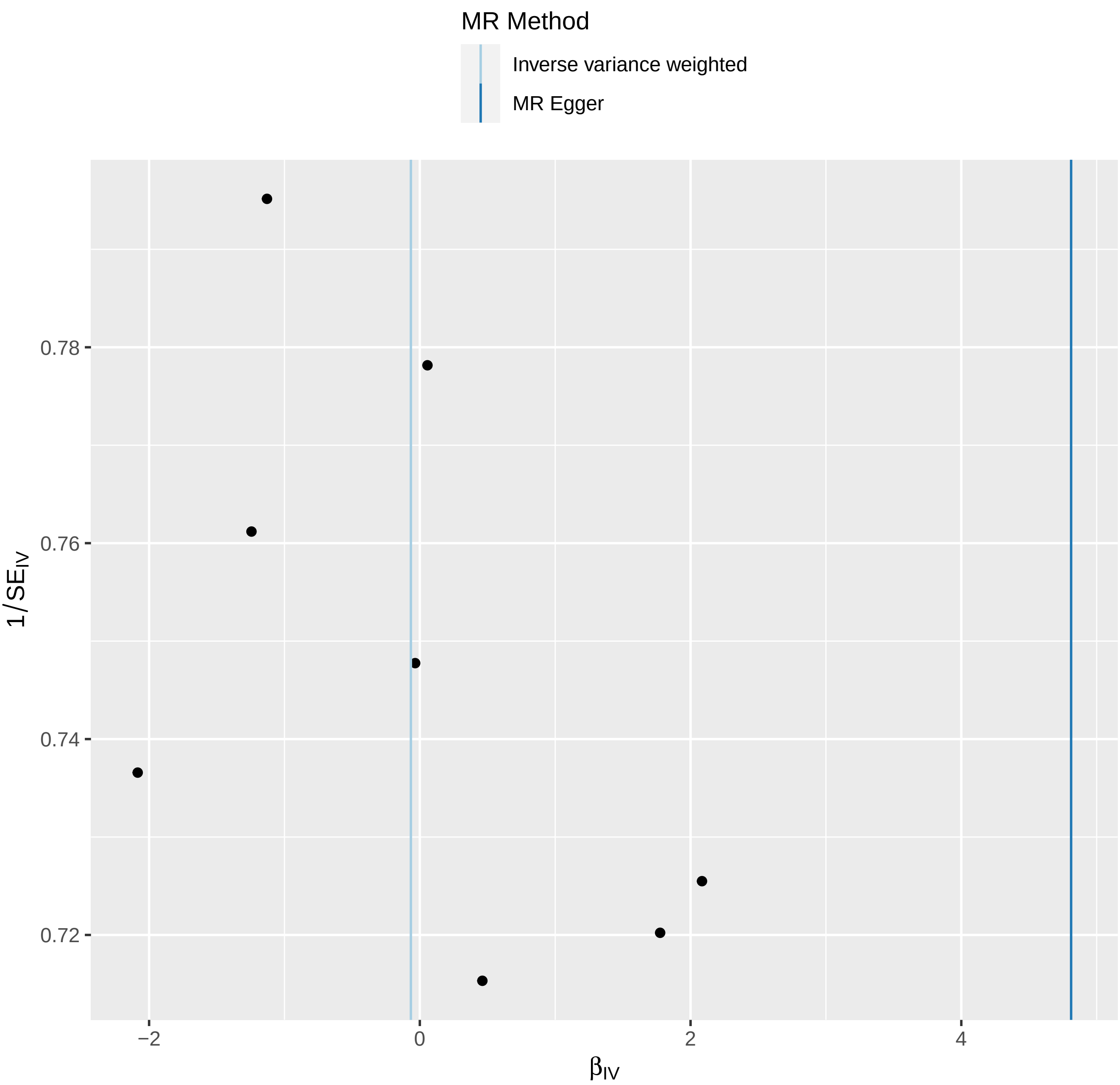

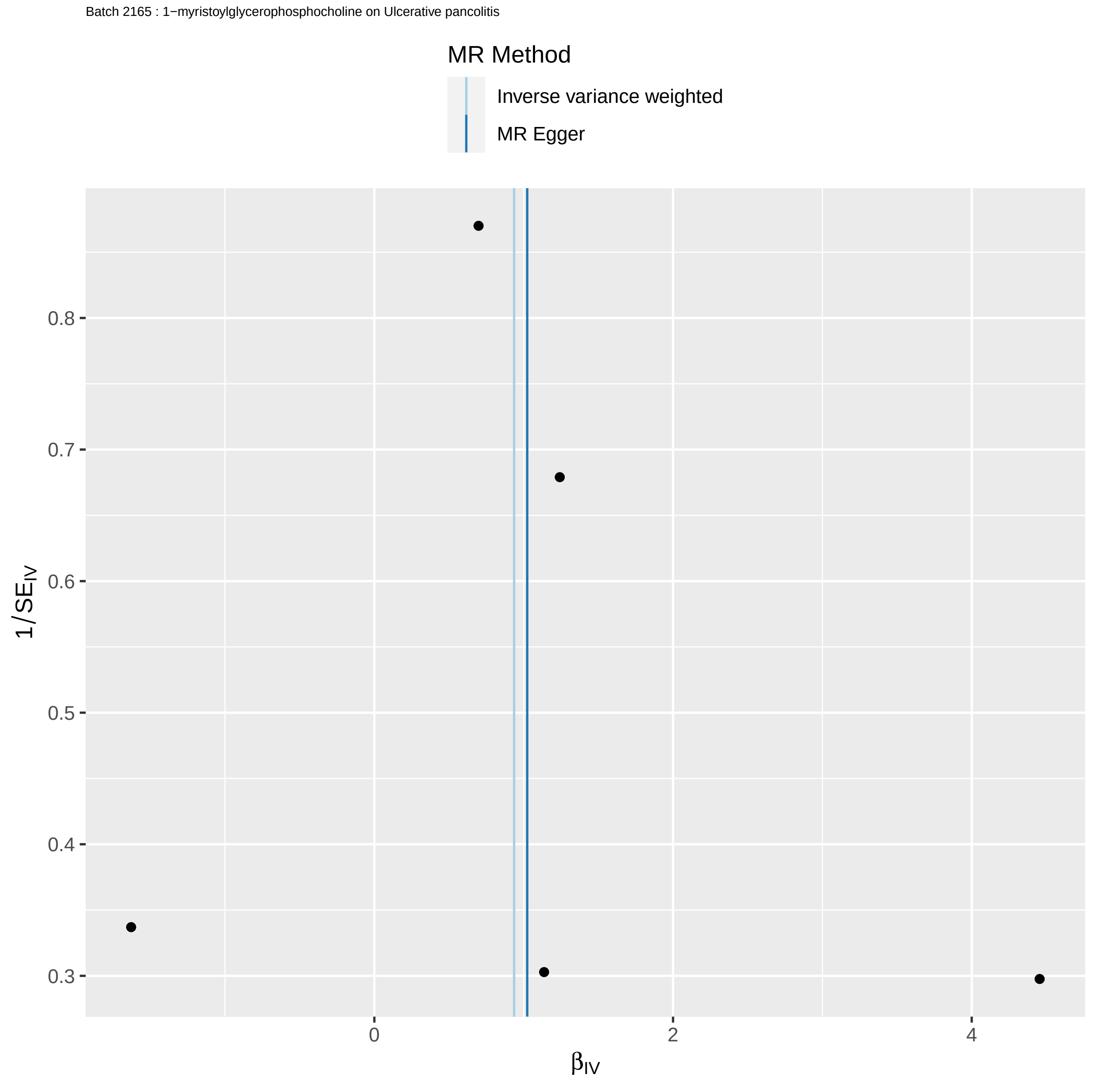
Batch 2165 : 1−myristoylglycerophosphocholine on Ulcerative pancolitis
MR Method
Inverse variance weighted
MR Egger
0.8
0.7
0.6
0.5
0.4
0.3
1/SE_IV
0
2
4
β_IV

Batch 2168 : 3−(3−hydroxyphenyl)propionate on Ulcerative pancolitis

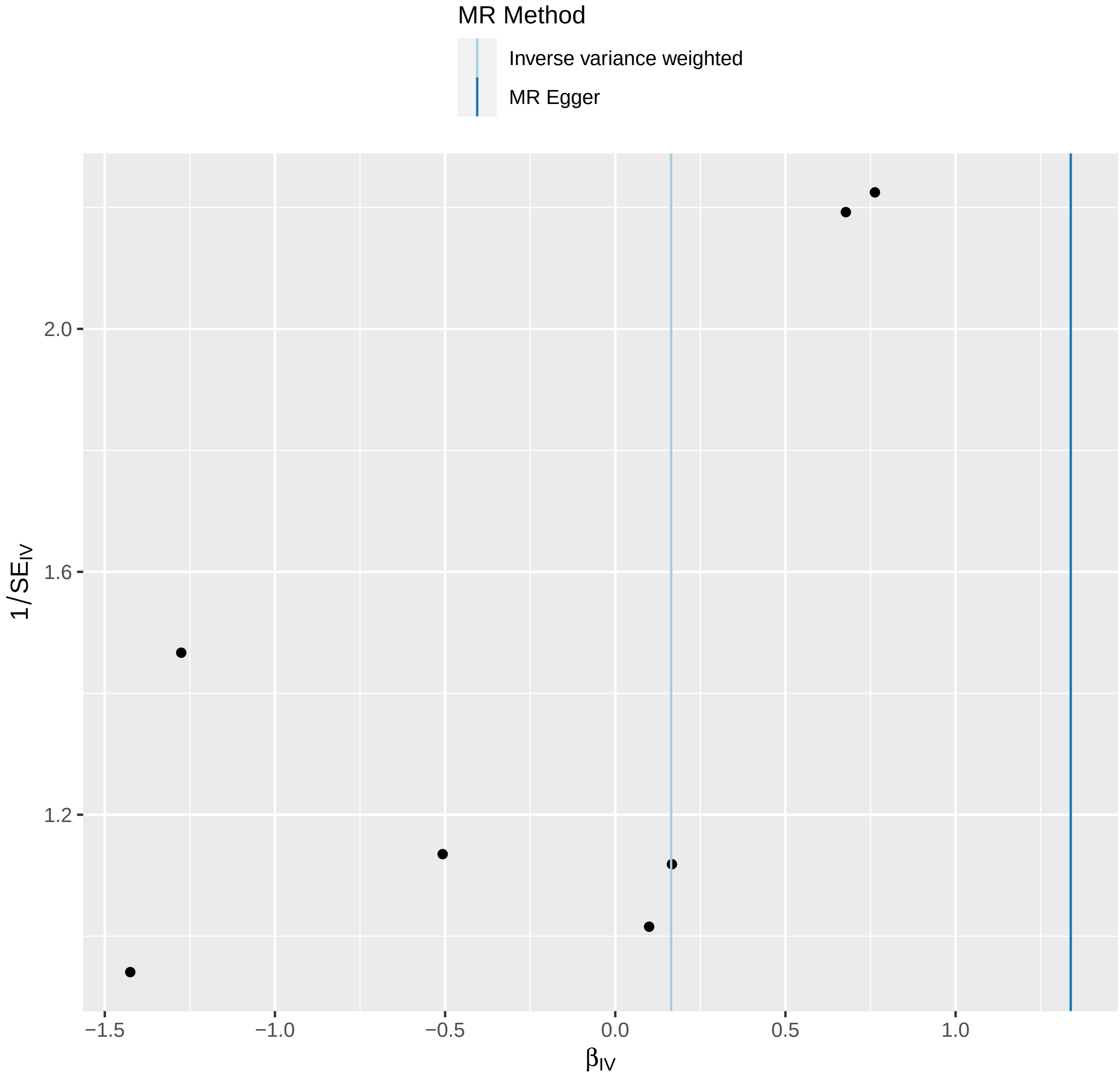

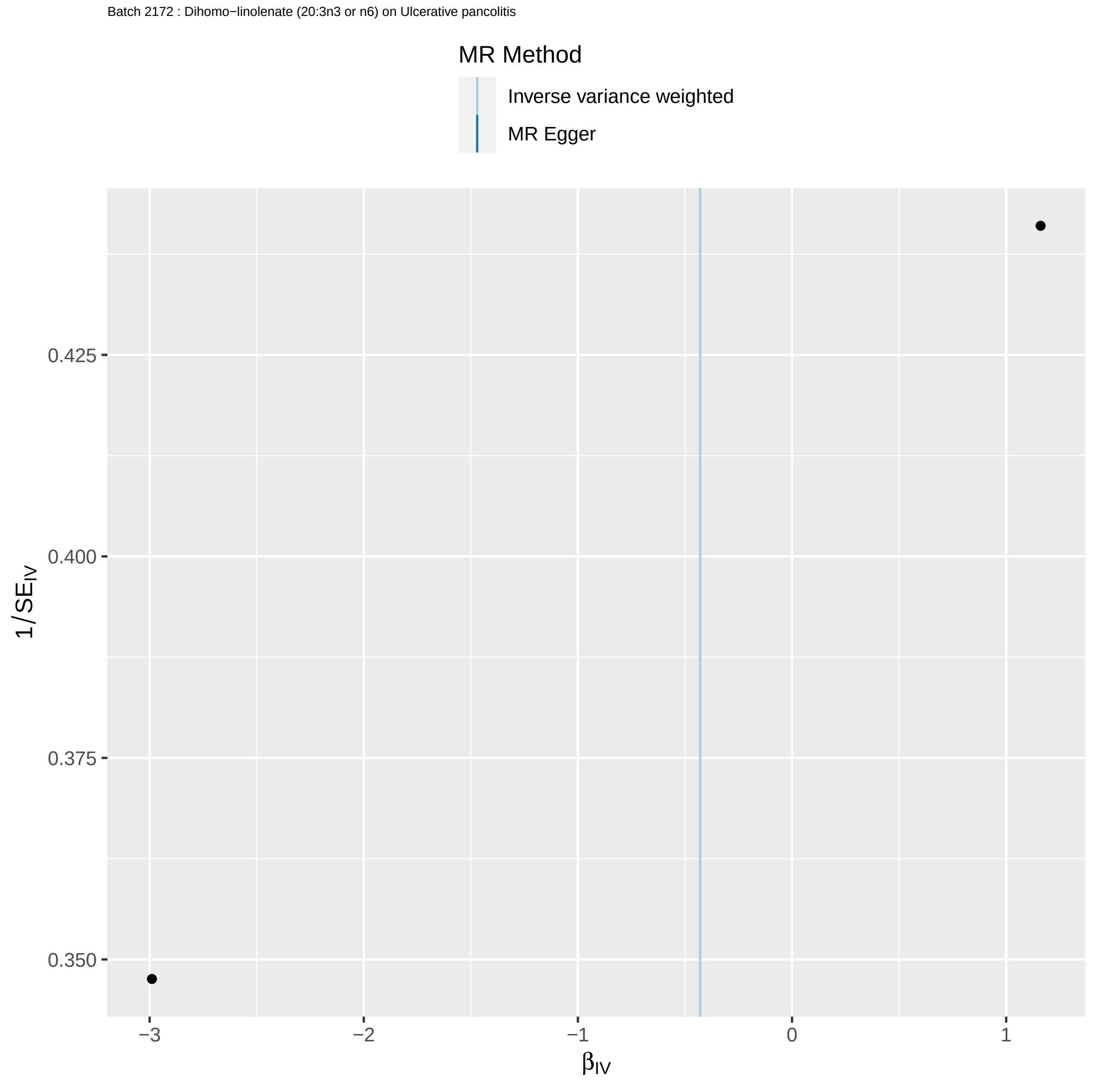
Batch 2172 : Dihomo−linolenate (20:3n3 or n6) on Ulcerative pancolitis
MR Method
Inverse variance weighted
MR Egger
0.425
0.400
0.375
0.350
1/SE$_{IV}$
−3
−2
−1
0
1
β_{IV}

Batch 2173 : Threitol on Ulcerative pancolitis

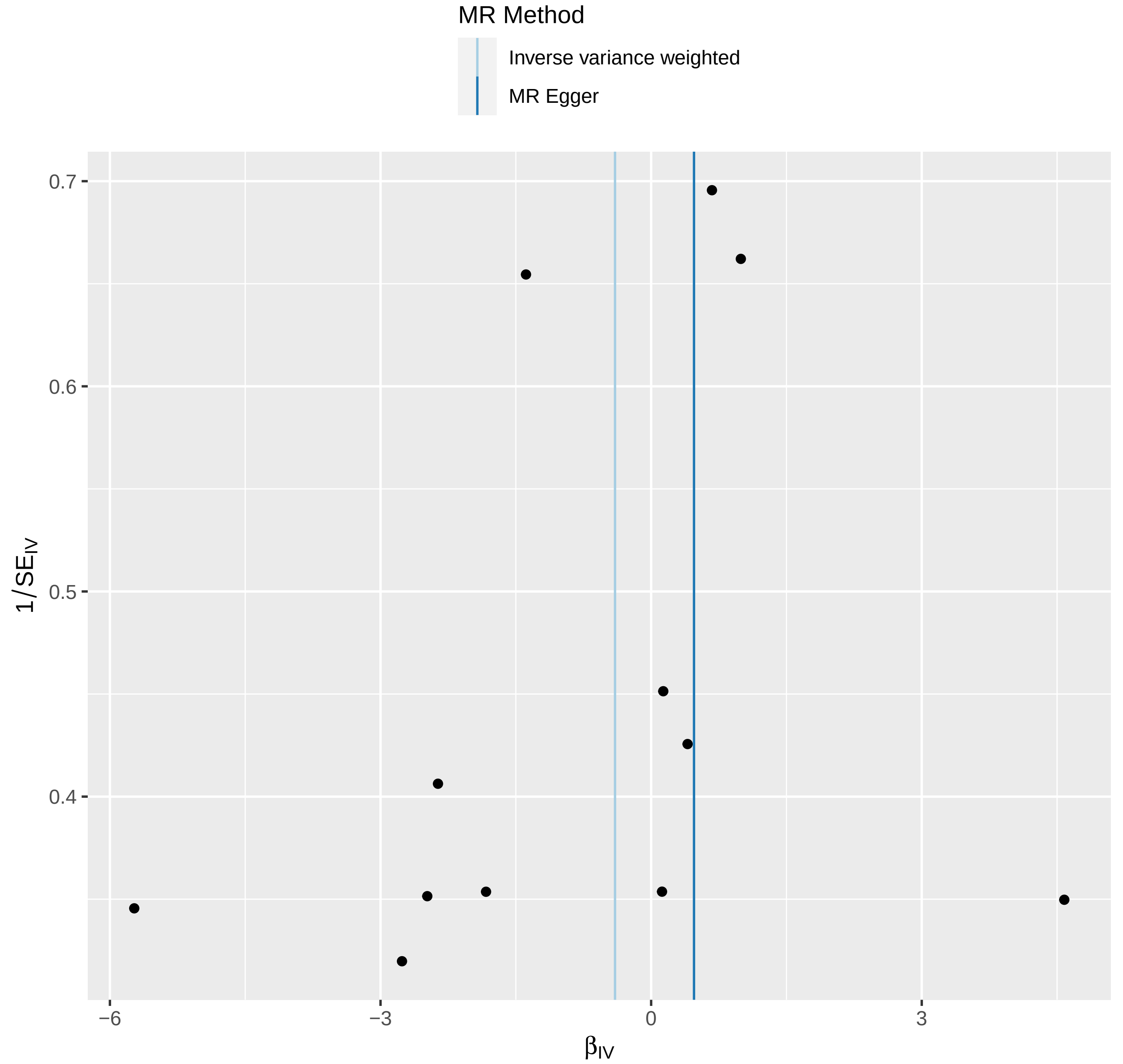

Batch 2174 : P−cresol sulfate on Ulcerative pancolitis

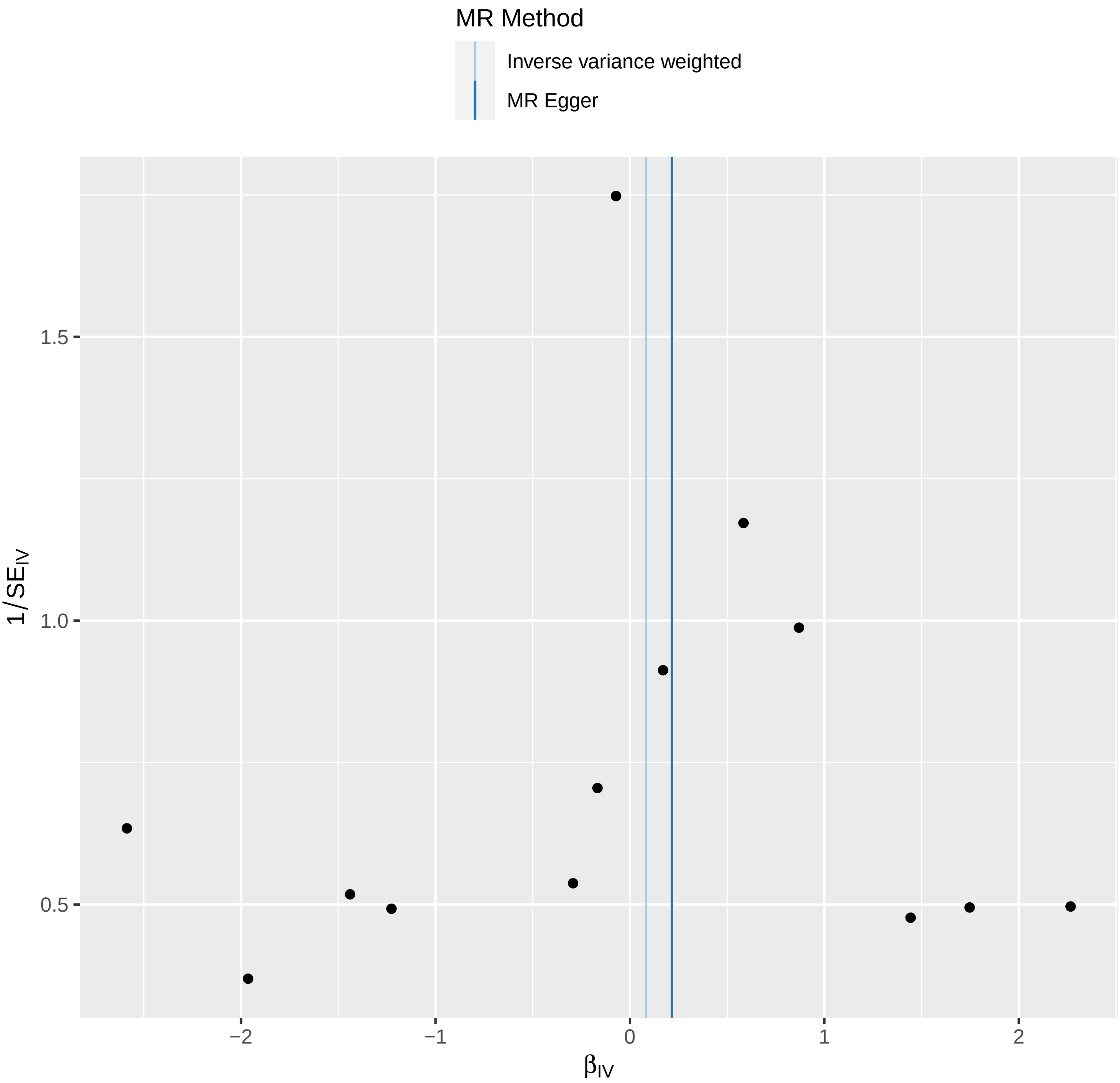

Batch 2177 : X−14208−−phenylalanylserine on Ulcerative pancolitis

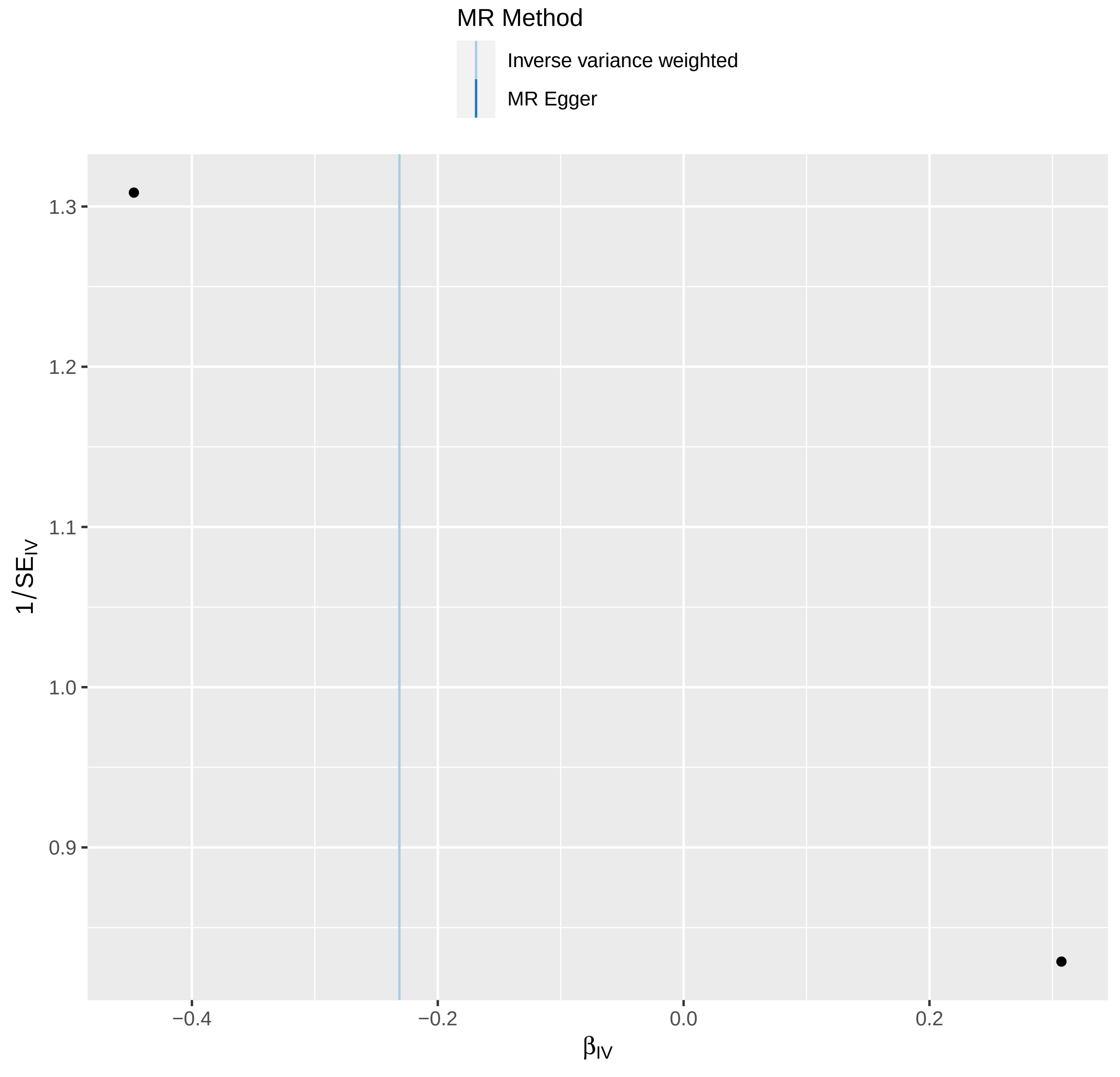

Batch 2179 : X−14450−−phenylalanylleucine on Ulcerative pancolitis

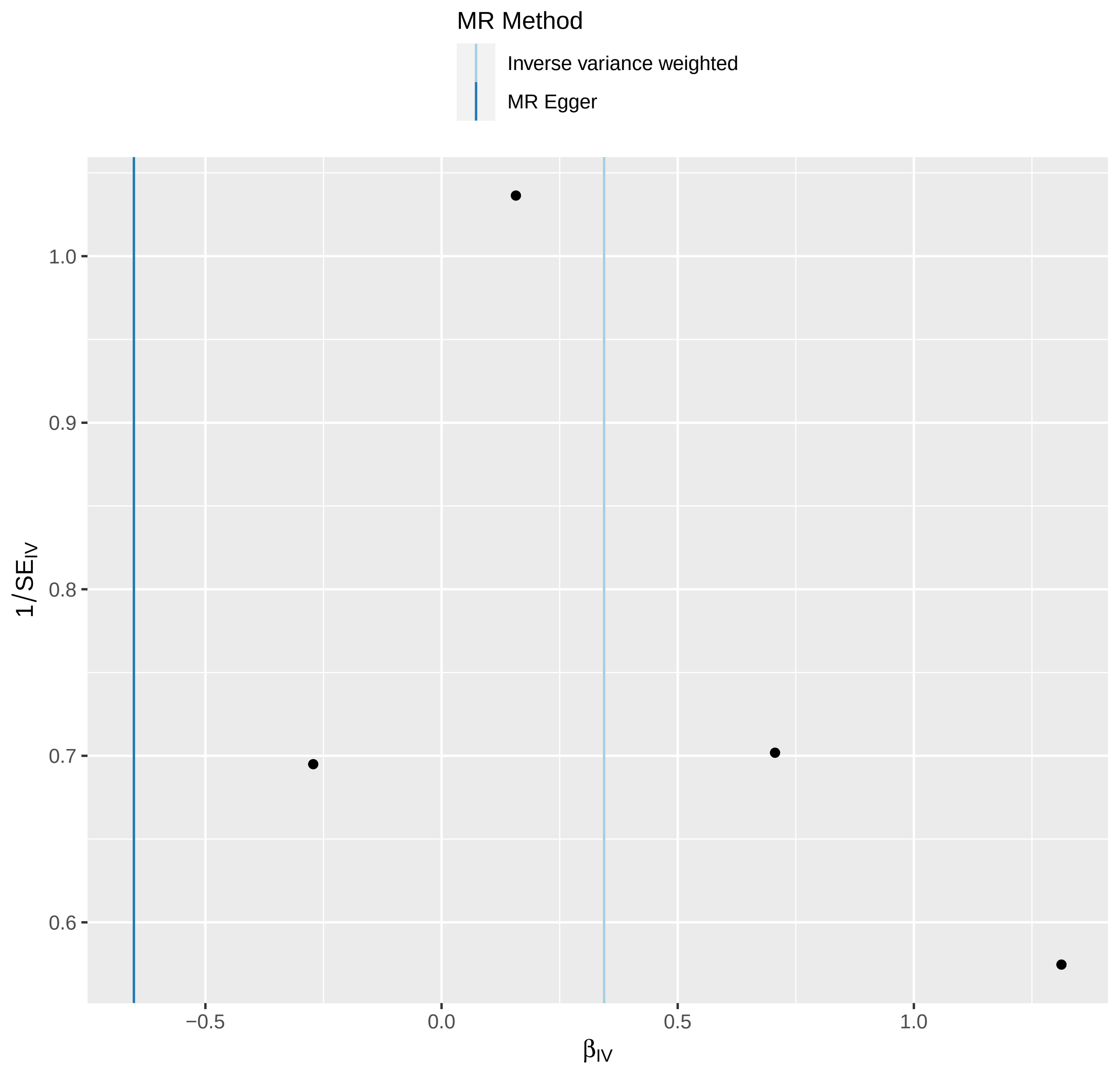

Batch 2180 : Gamma−glutamylglutamate on Ulcerative pancolitis

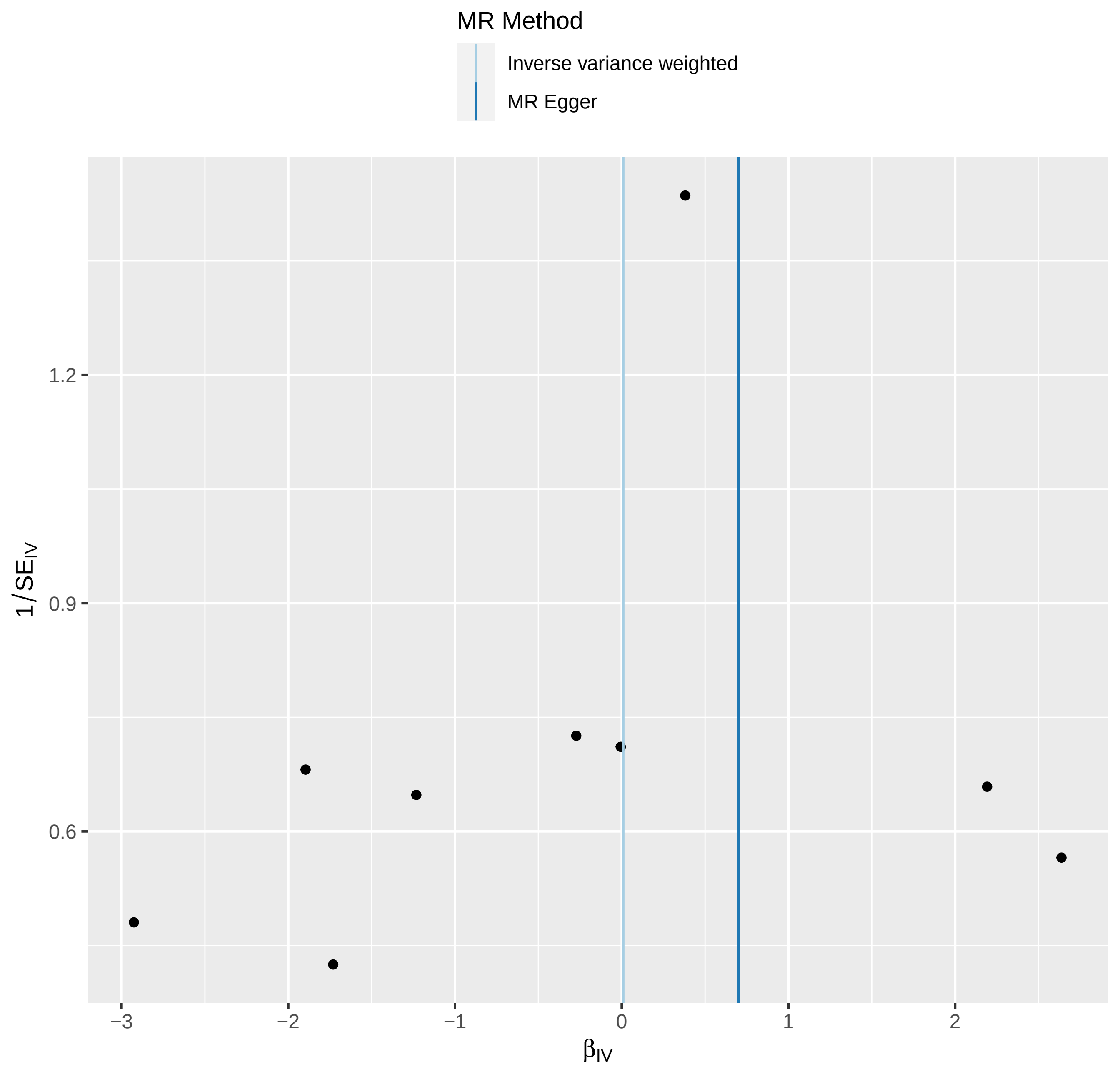

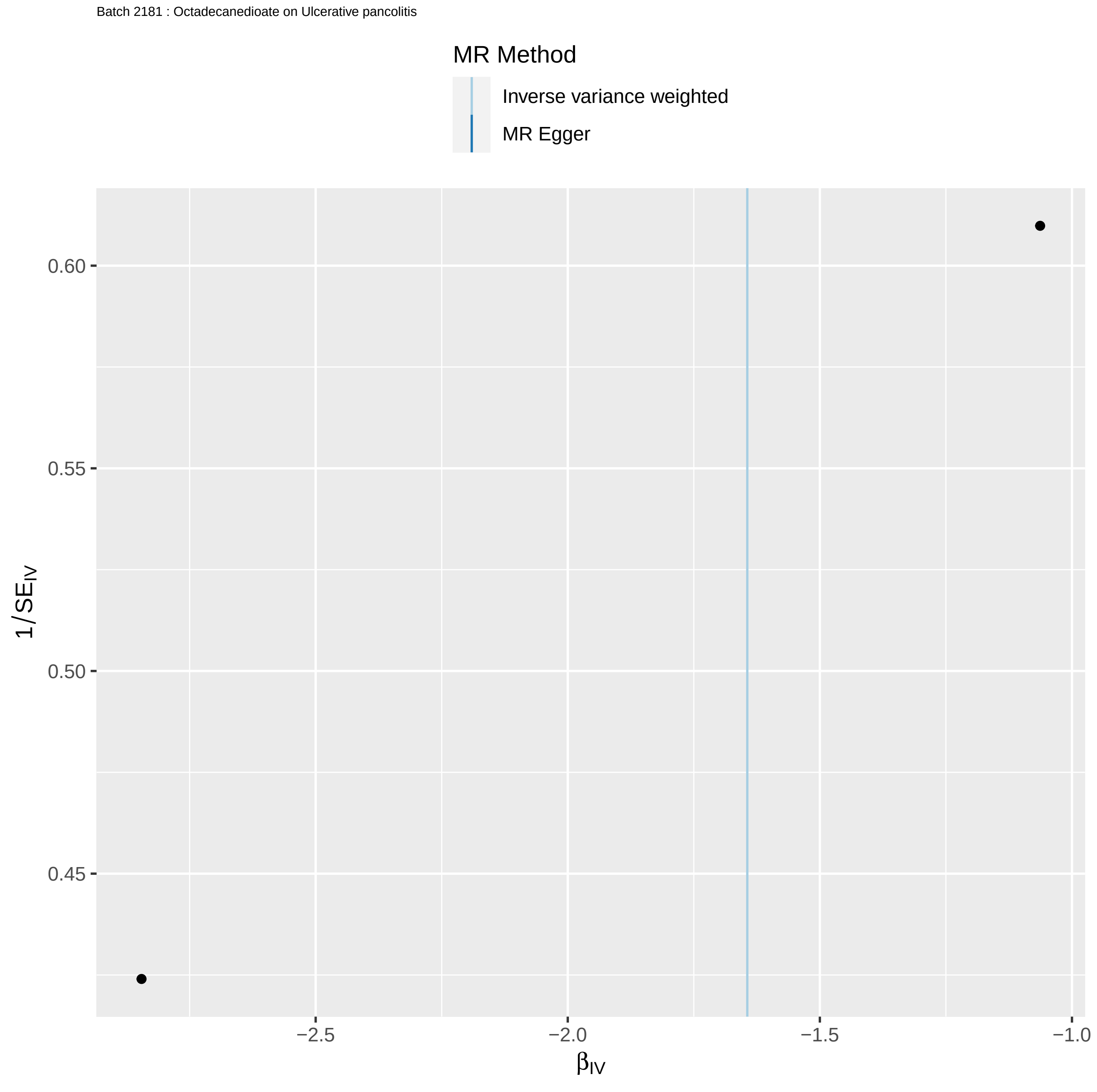
Batch 2181 : Octadecanedioate on Ulcerative pancolitis
MR Method
Inverse variance weighted
MR Egger
0.60
0.55
0.50
0.45
1/SE_IV
−2.5
−2.0
−1.5
−1.0
β_IV

Batch 2182 : Leucylleucine on Ulcerative pancolitis

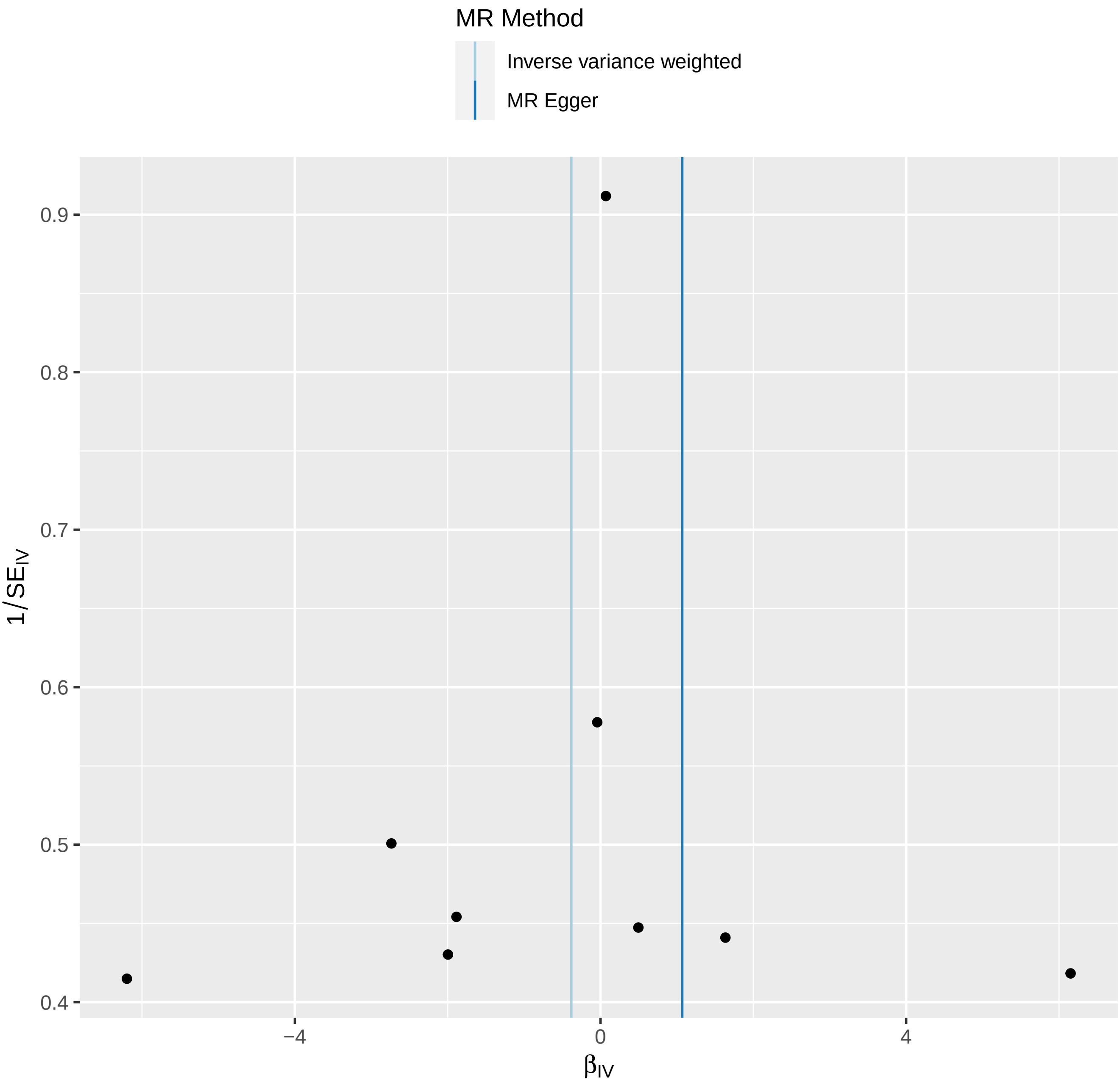

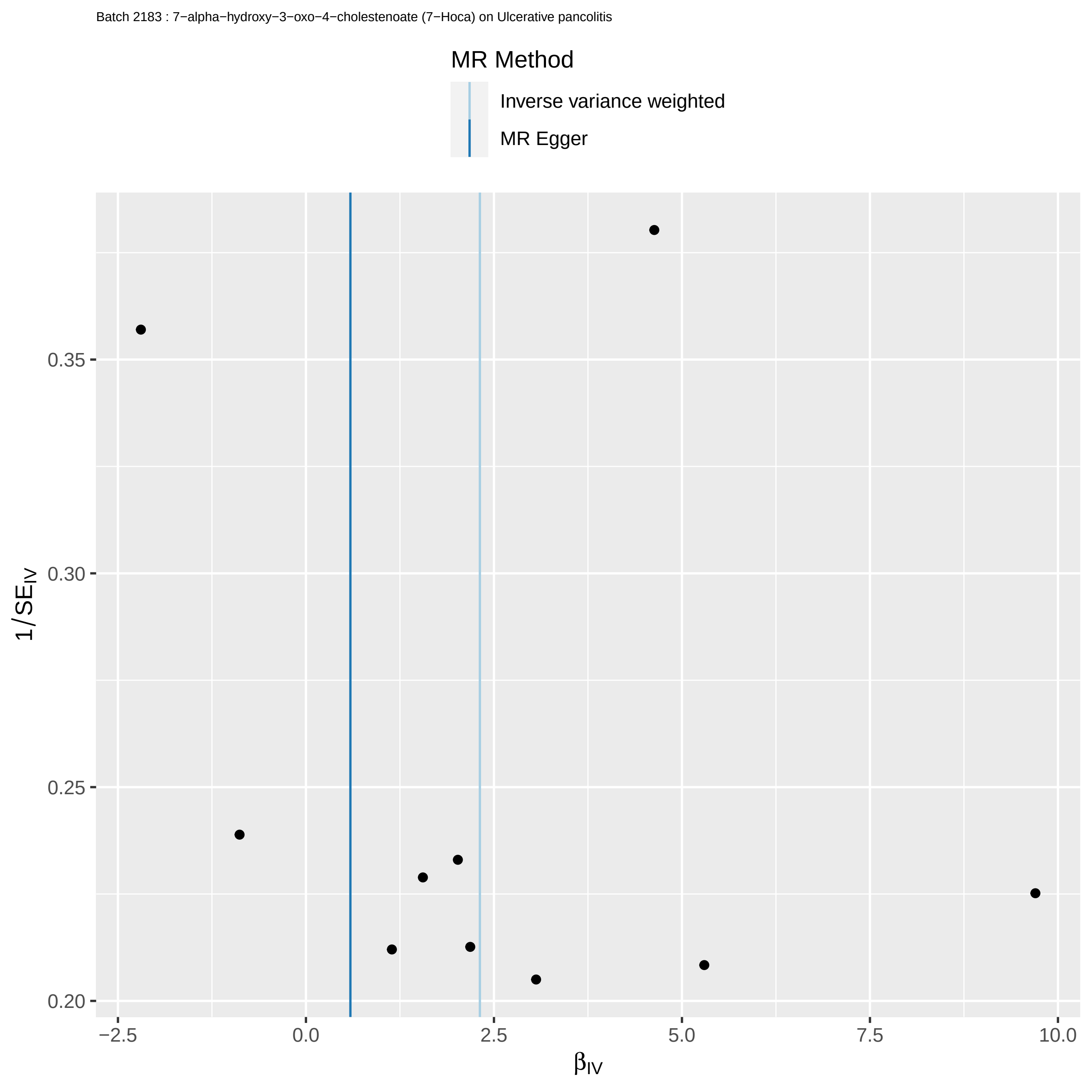
Batch 2183 : 7−alpha−hydroxy−3−oxo−4−cholestenoate (7−Hoca) on Ulcerative pancolitis
MR Method
Inverse variance weighted
MR Egger
0.35
0.30
0.25
0.20
1/SE_IV
−2.5
0.0
2.5
5.0
7.5
10.0
β_IV

Batch 2184 : N−Butyl Oleate on Ulcerative pancolitis

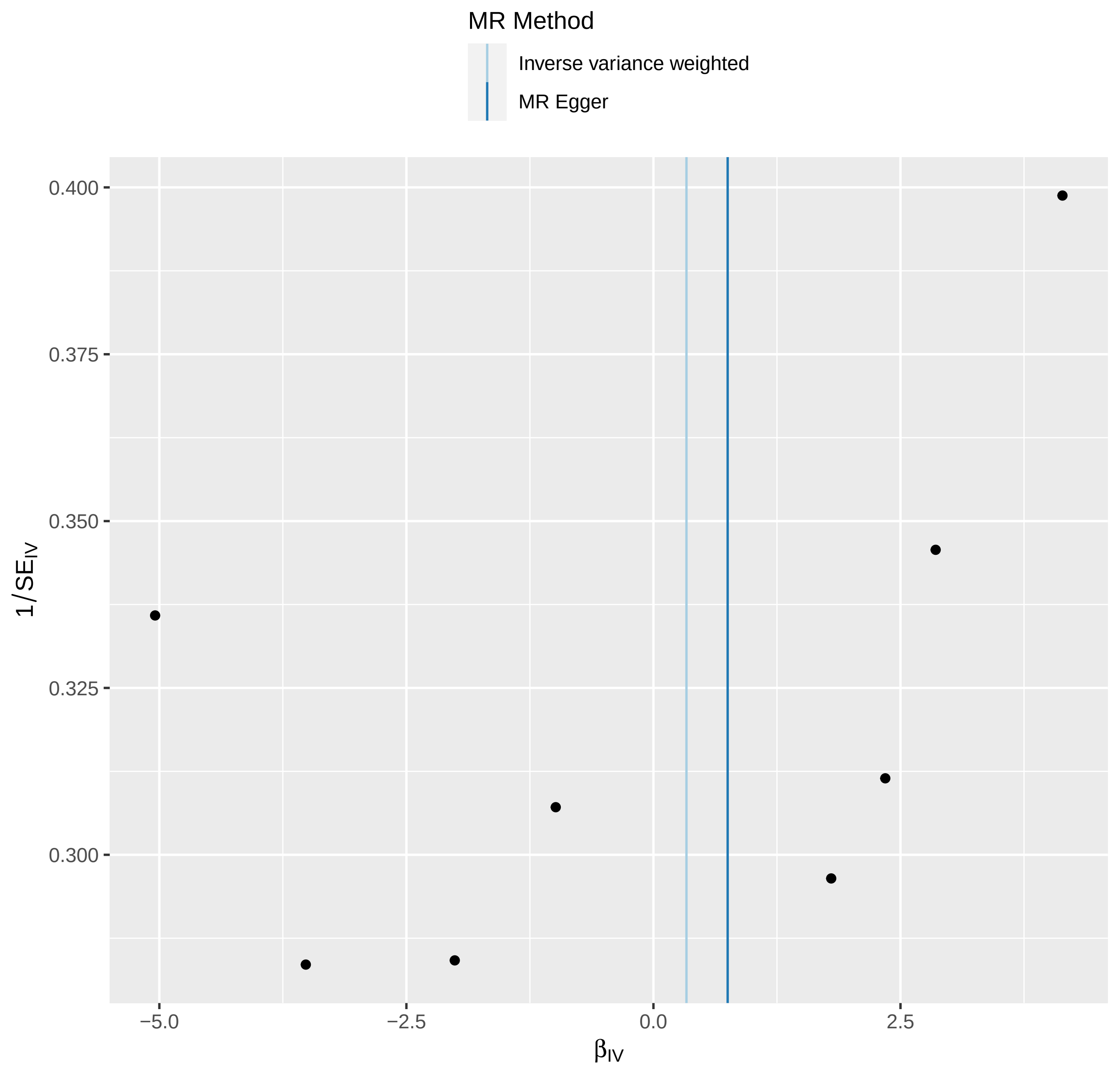

Batch 2186 : Taurolithocholate 3−sulfate on Ulcerative pancolitis

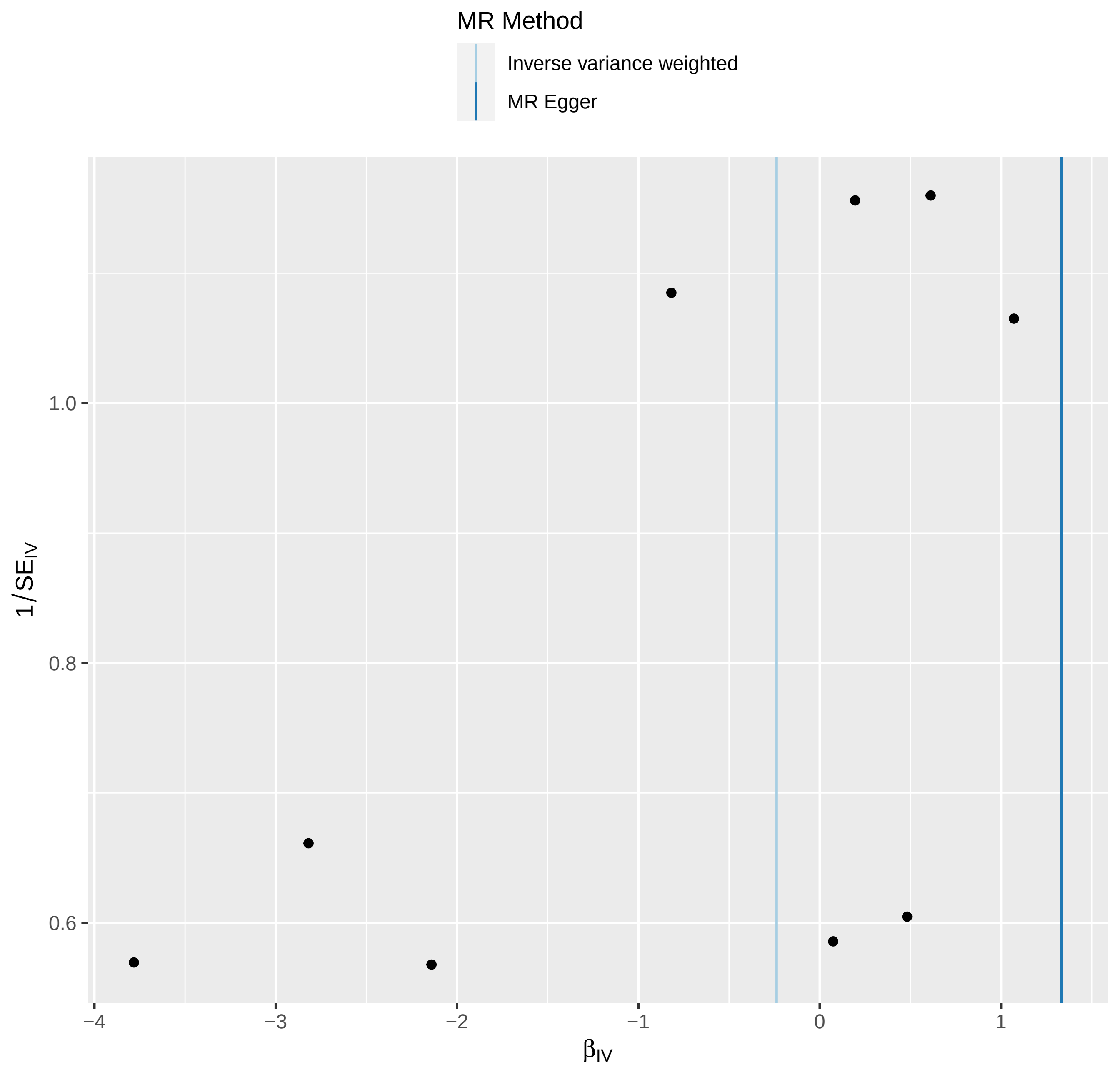

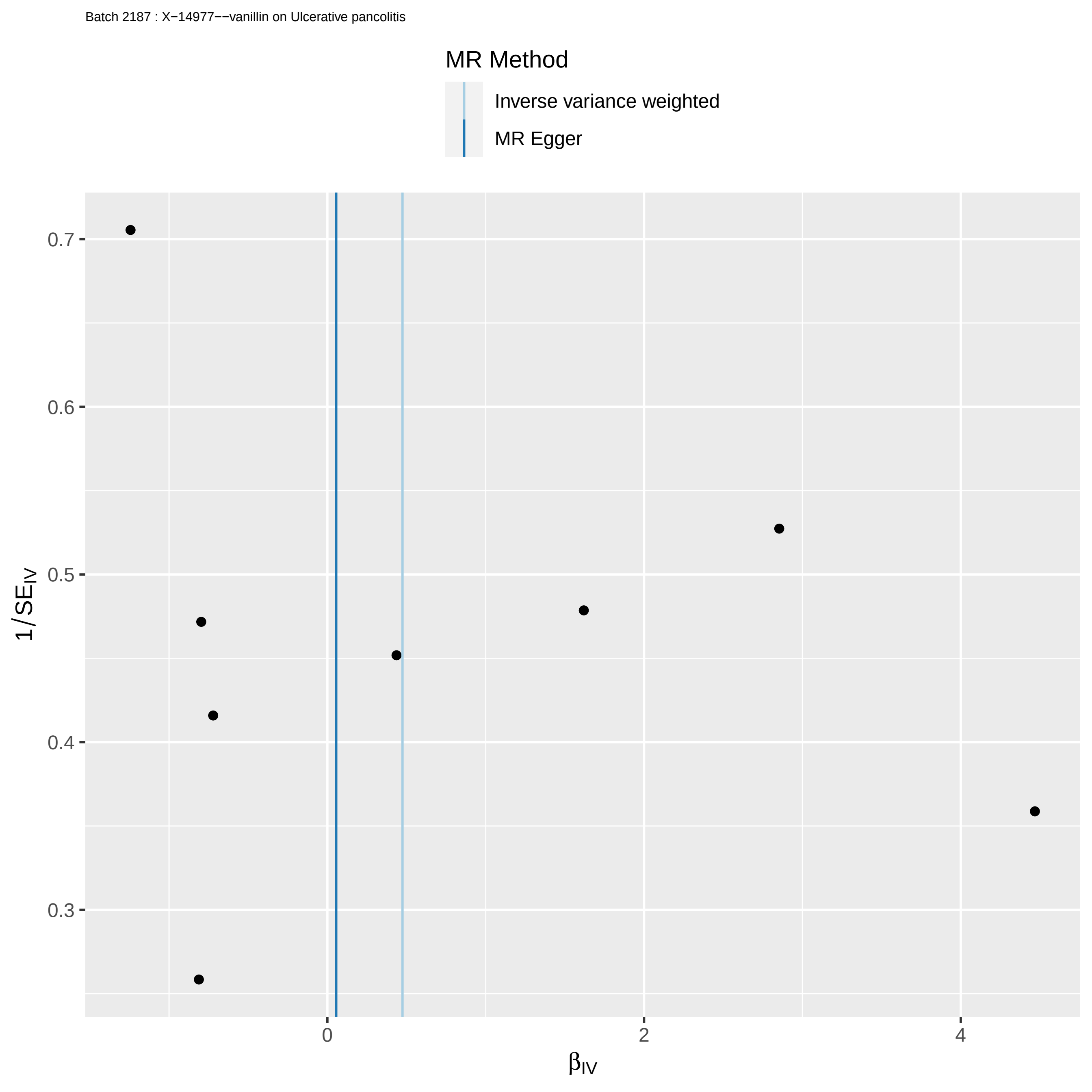
Batch 2187 : X−14977−−vanillin on Ulcerative pancolitis
MR Method
Inverse variance weighted
MR Egger
0.7
0.6
0.5
0.4
0.3
$1/SE_{IV}$
0
2
4
β_{IV}

Batch 2188 : Succinylcarnitine on Ulcerative pancolitis

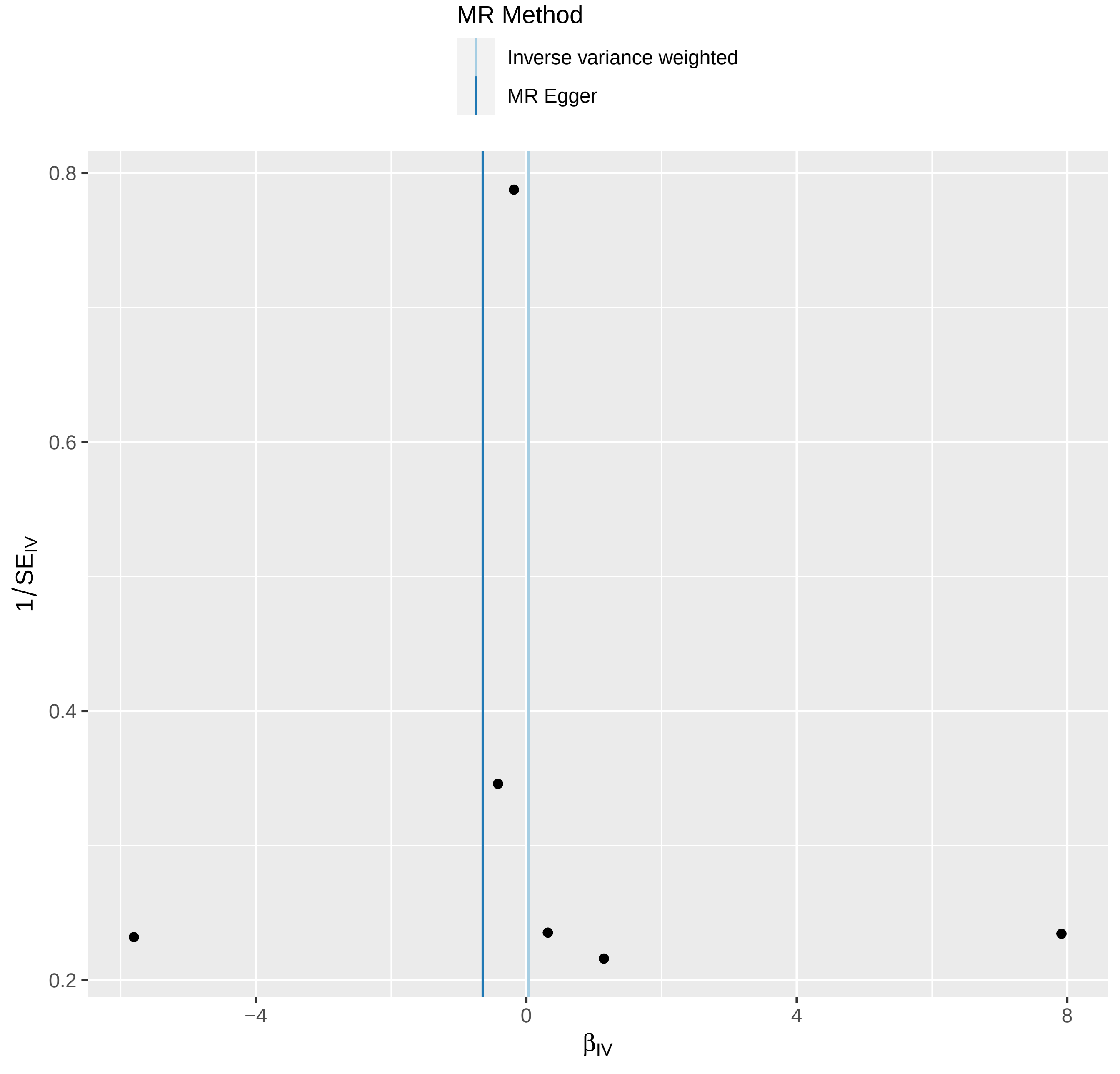

Batch 2190 : Cyclo(leu−pro) on Ulcerative pancolitis

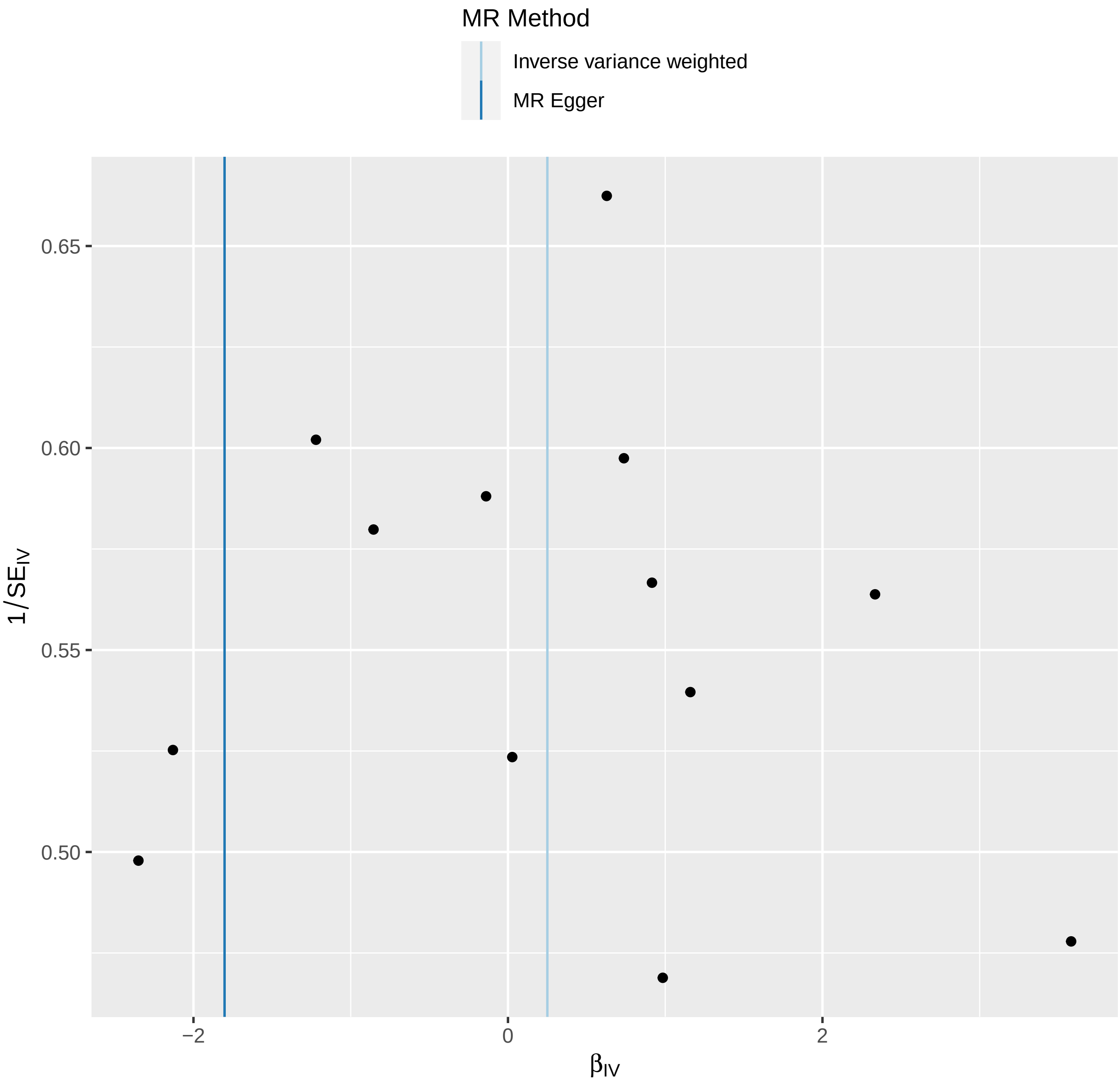

Batch 2191 : Chiro−inositol on Ulcerative pancolitis

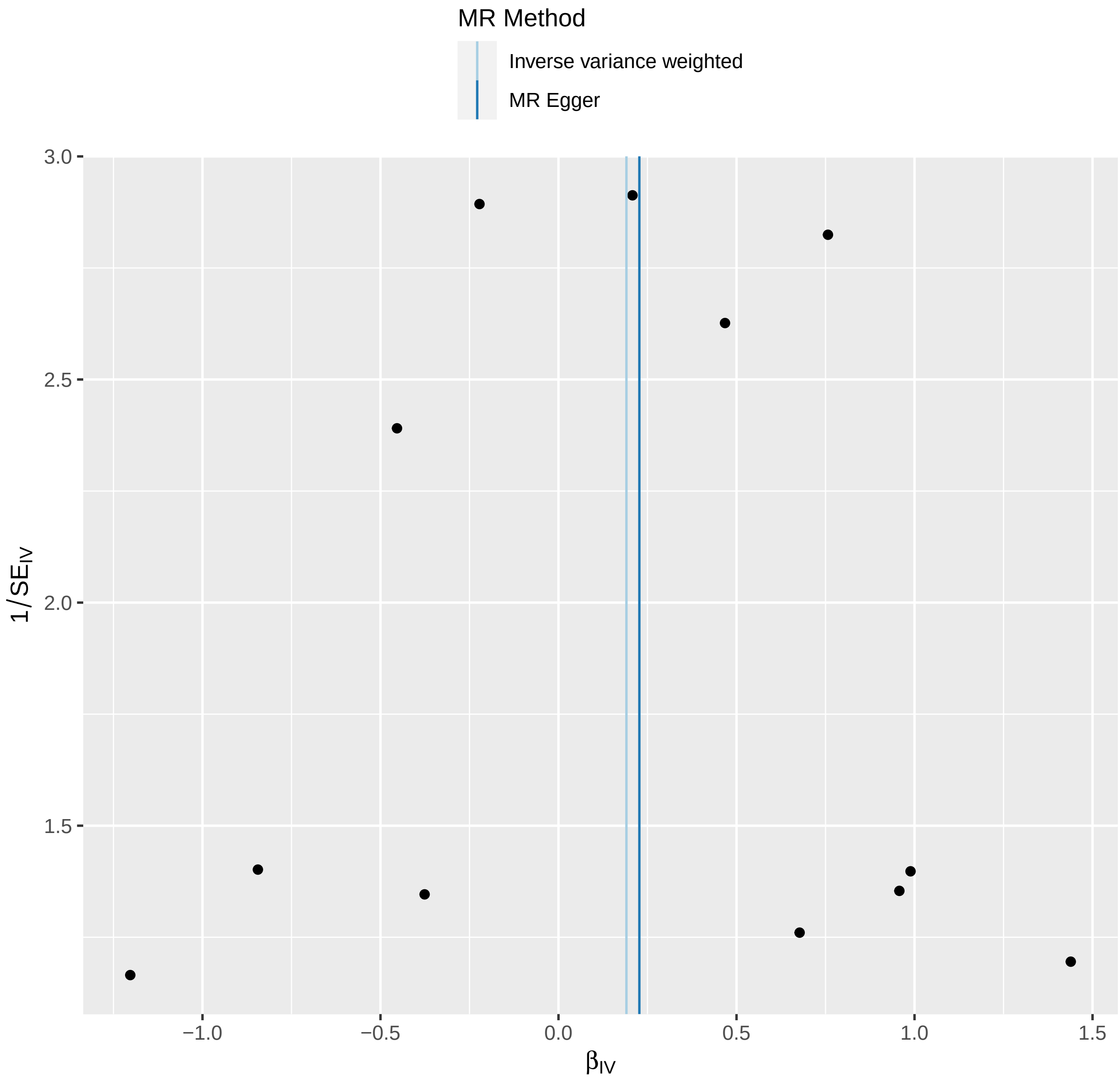

Batch 2195 : 2−hydroxyglutarate on Ulcerative pancolitis

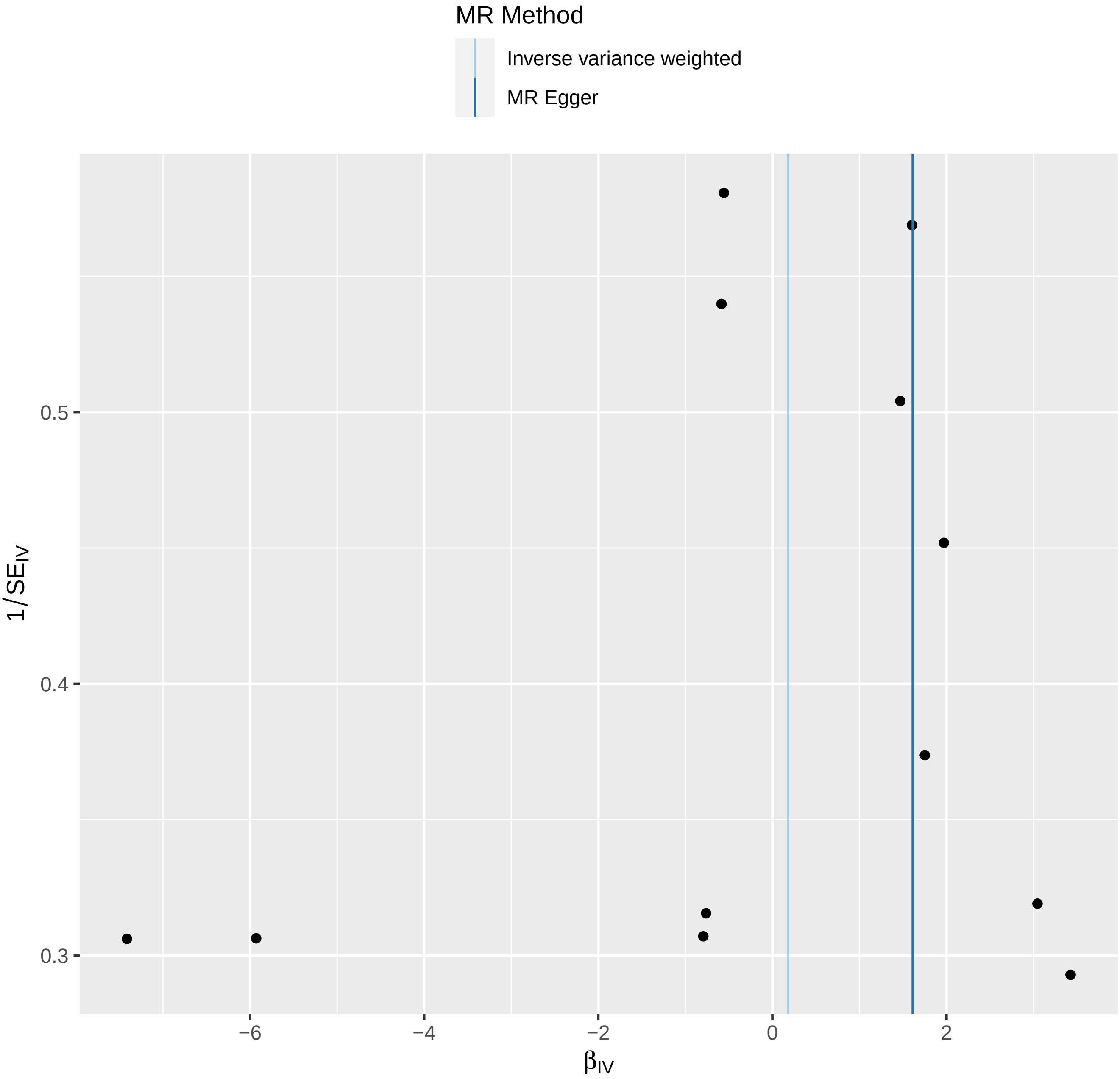

Batch 2196 : Ergothioneine on Ulcerative pancolitis

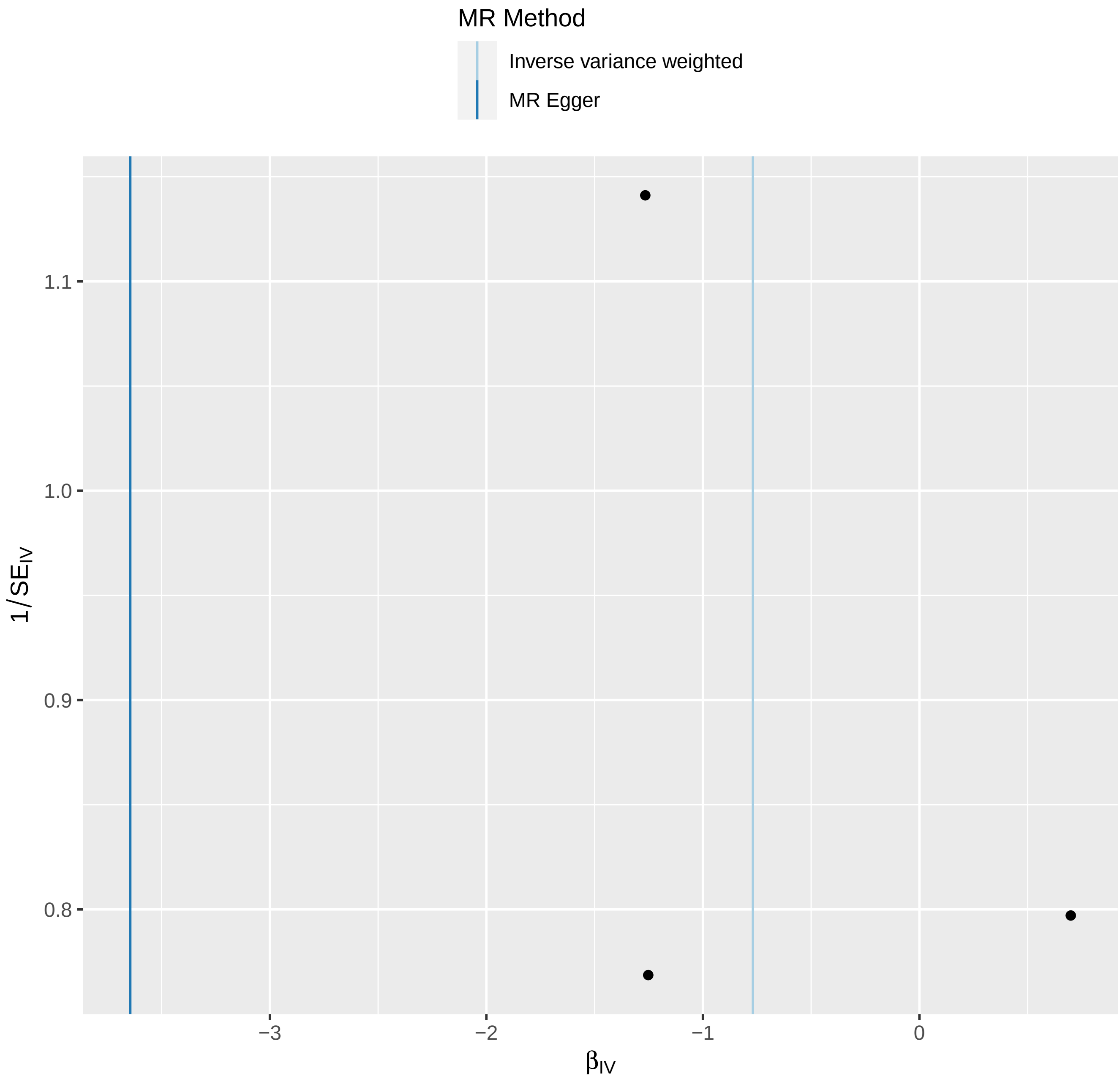

Batch 2198 : Phenylalanylphenylalanine on Ulcerative pancolitis

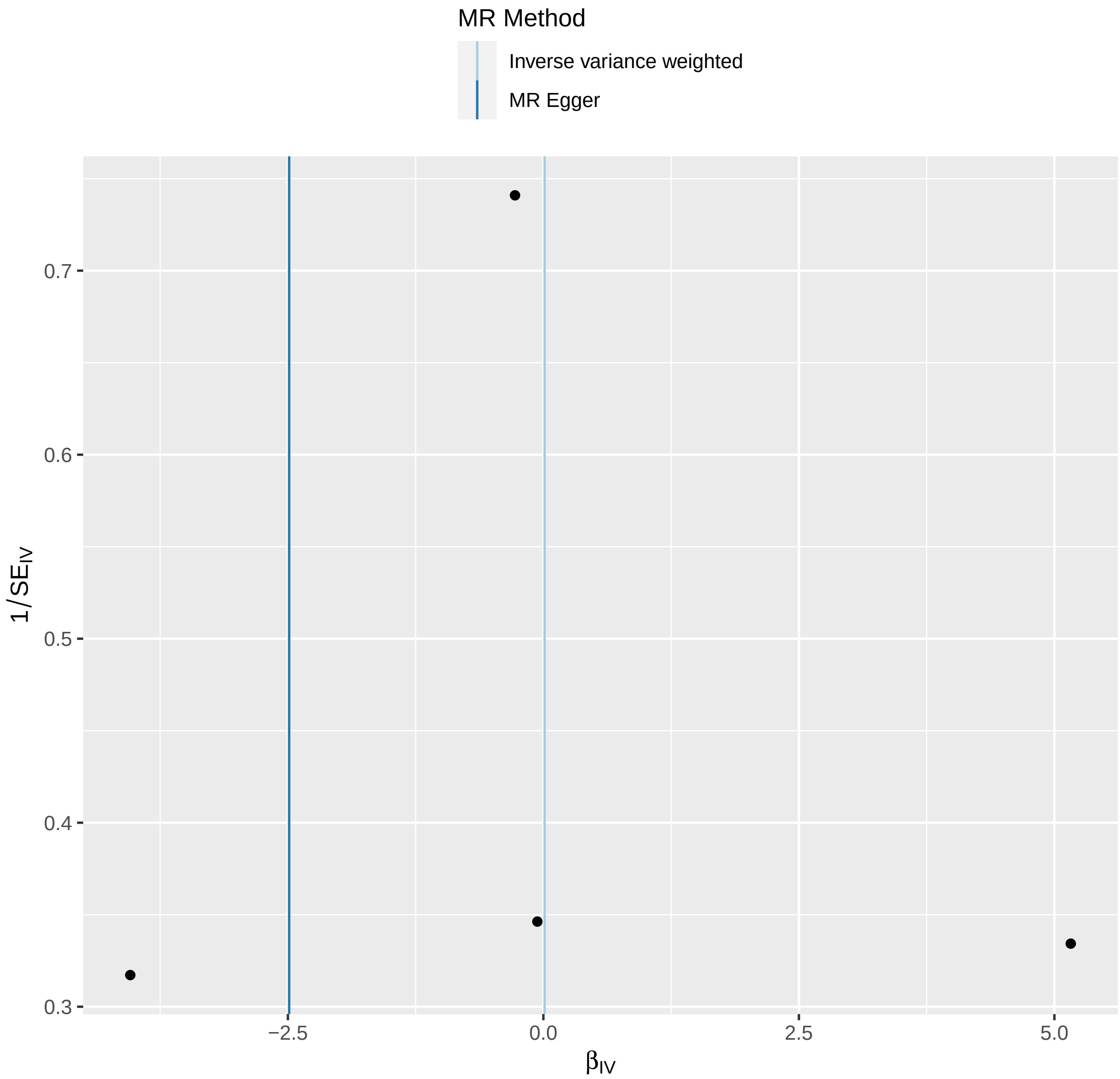

Batch 2199 : Cis−4−decenoyl carnitine on Ulcerative pancolitis

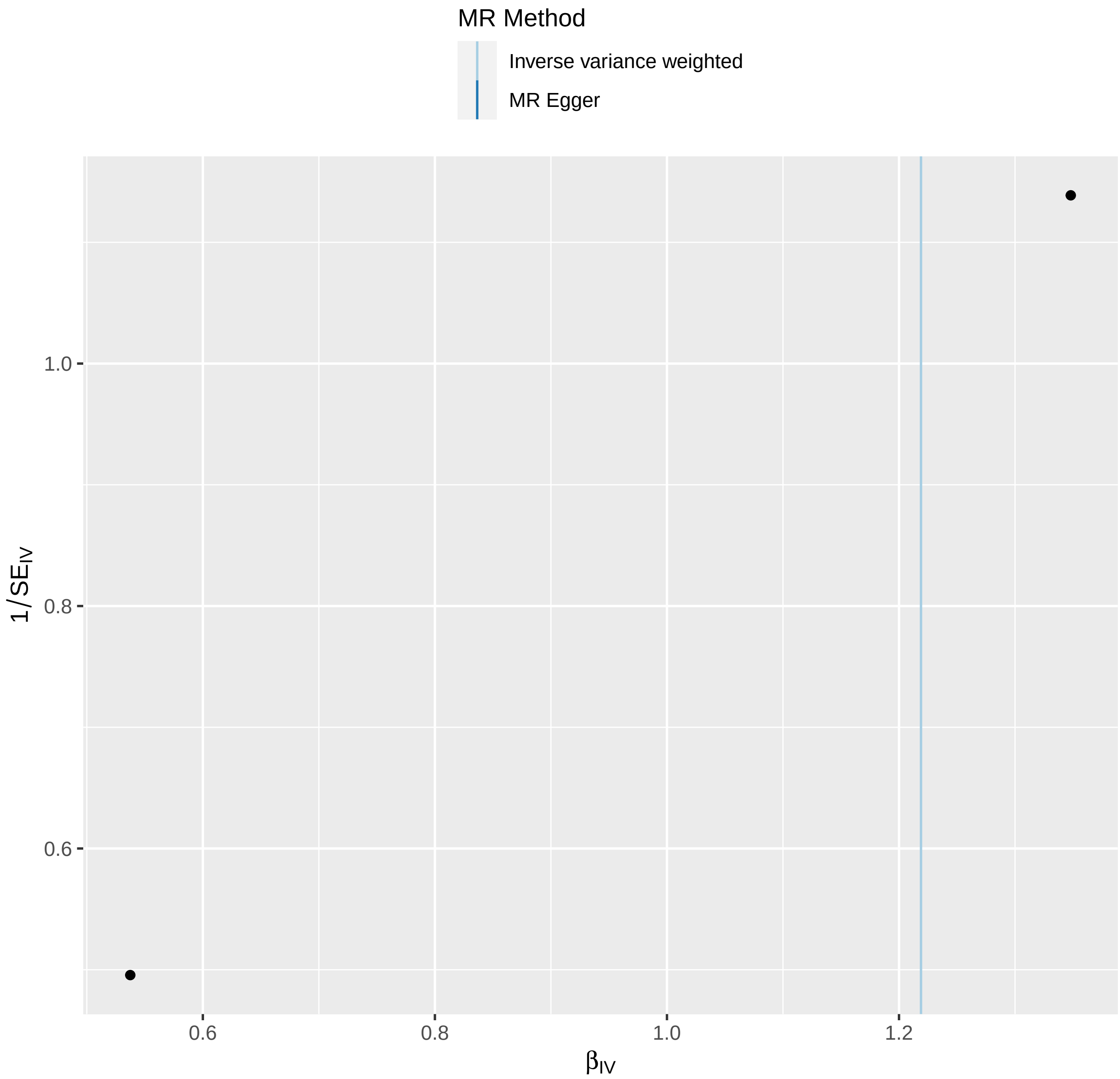

Batch 2200 : 15−methylpalmitate (isobar with 2−methylpalmitate) on Ulcerative pancolitis

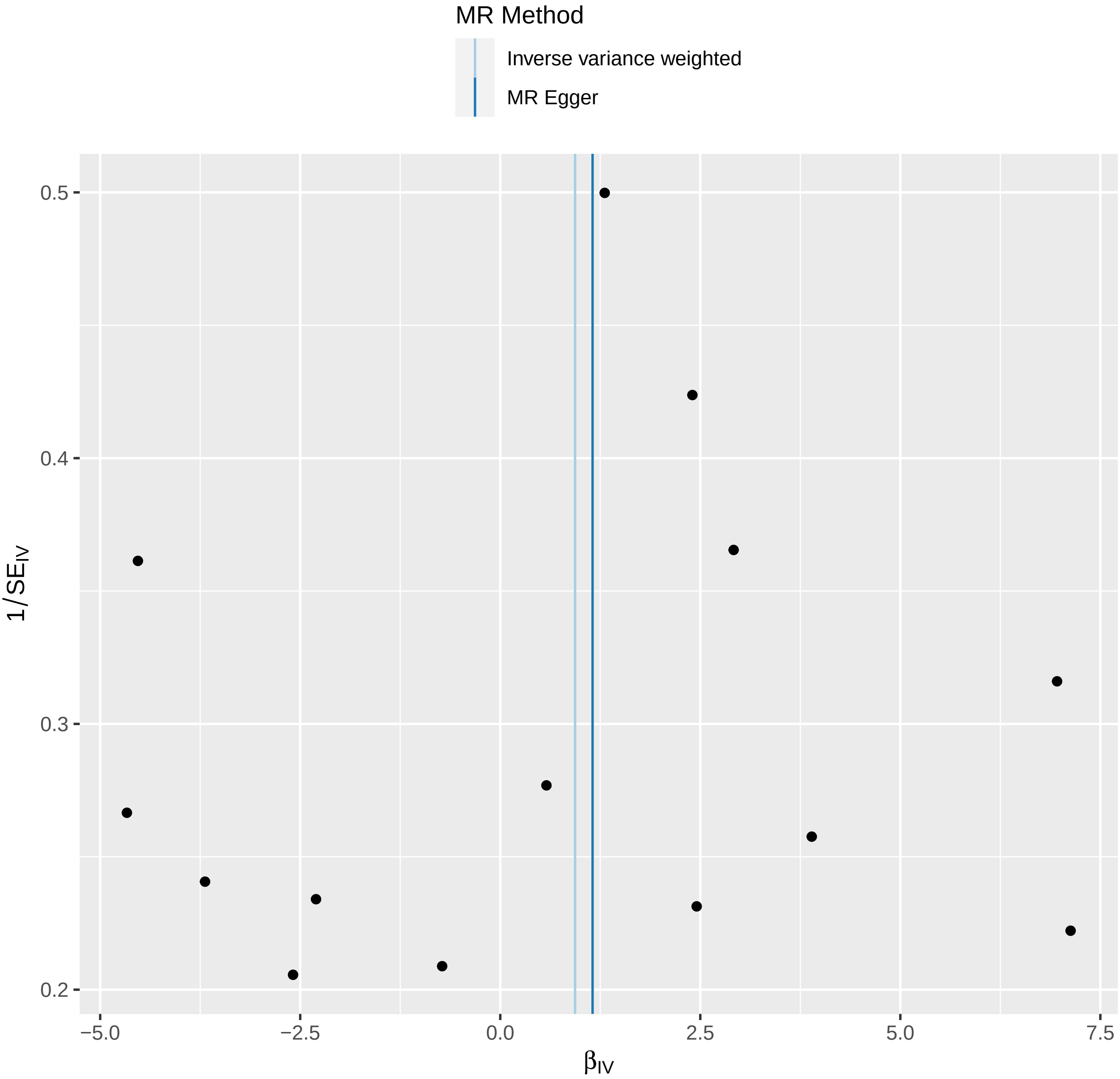

Batch 2202 : Tryptophan on Ulcerative proctitis

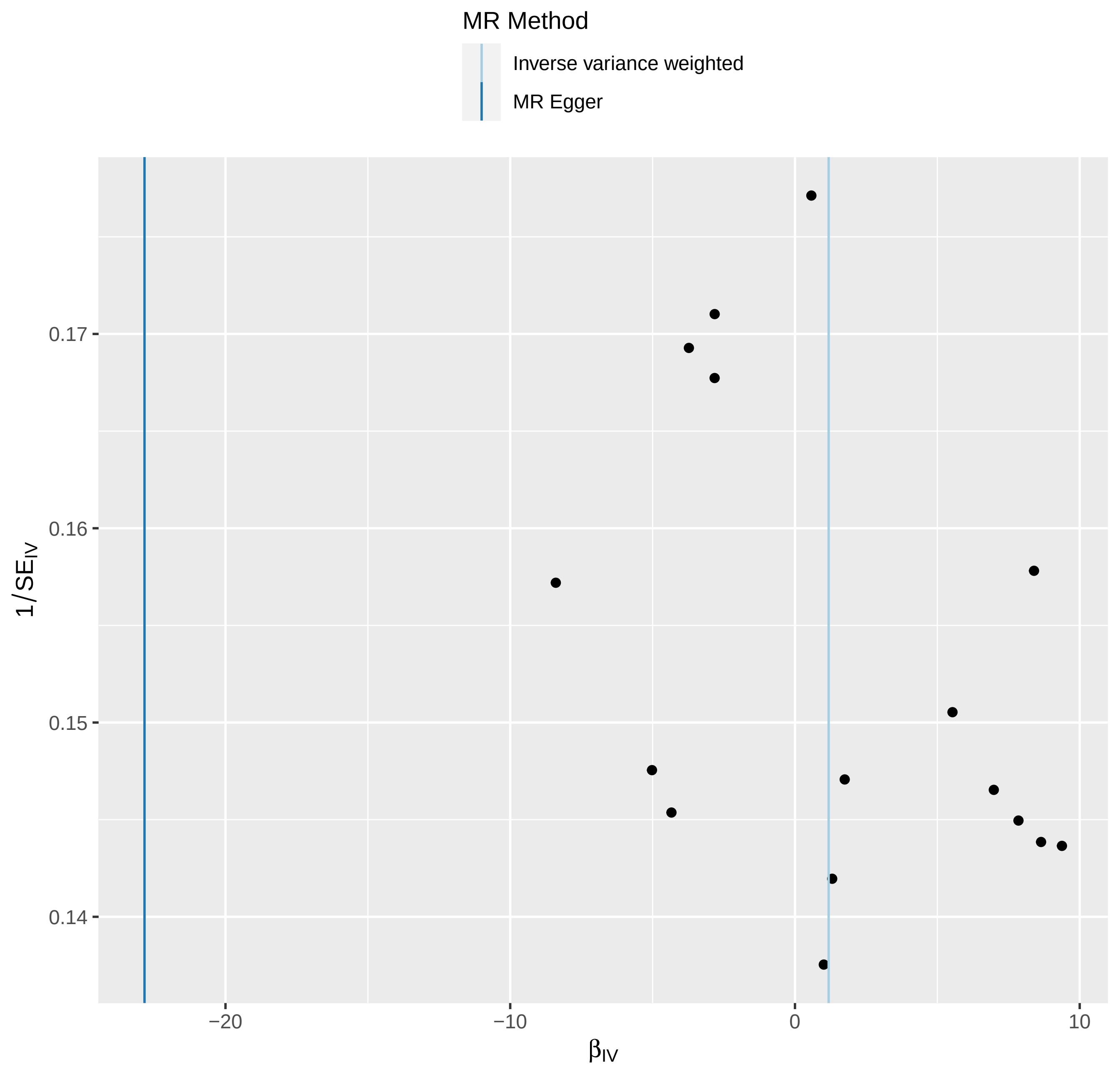

Batch 2204 : Leucine on Ulcerative proctitis

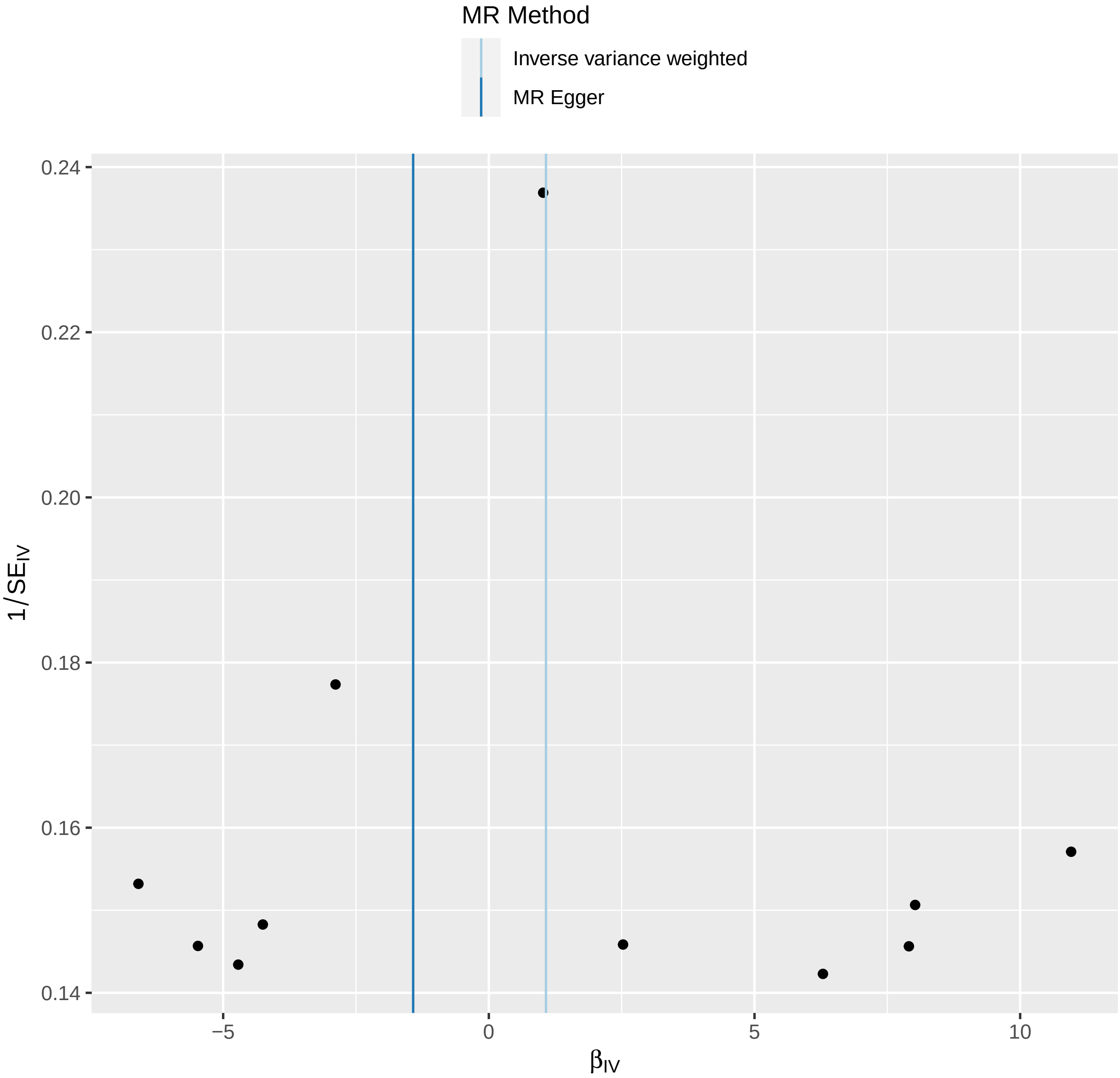

Batch 2208 : Lactate on Ulcerative proctitis

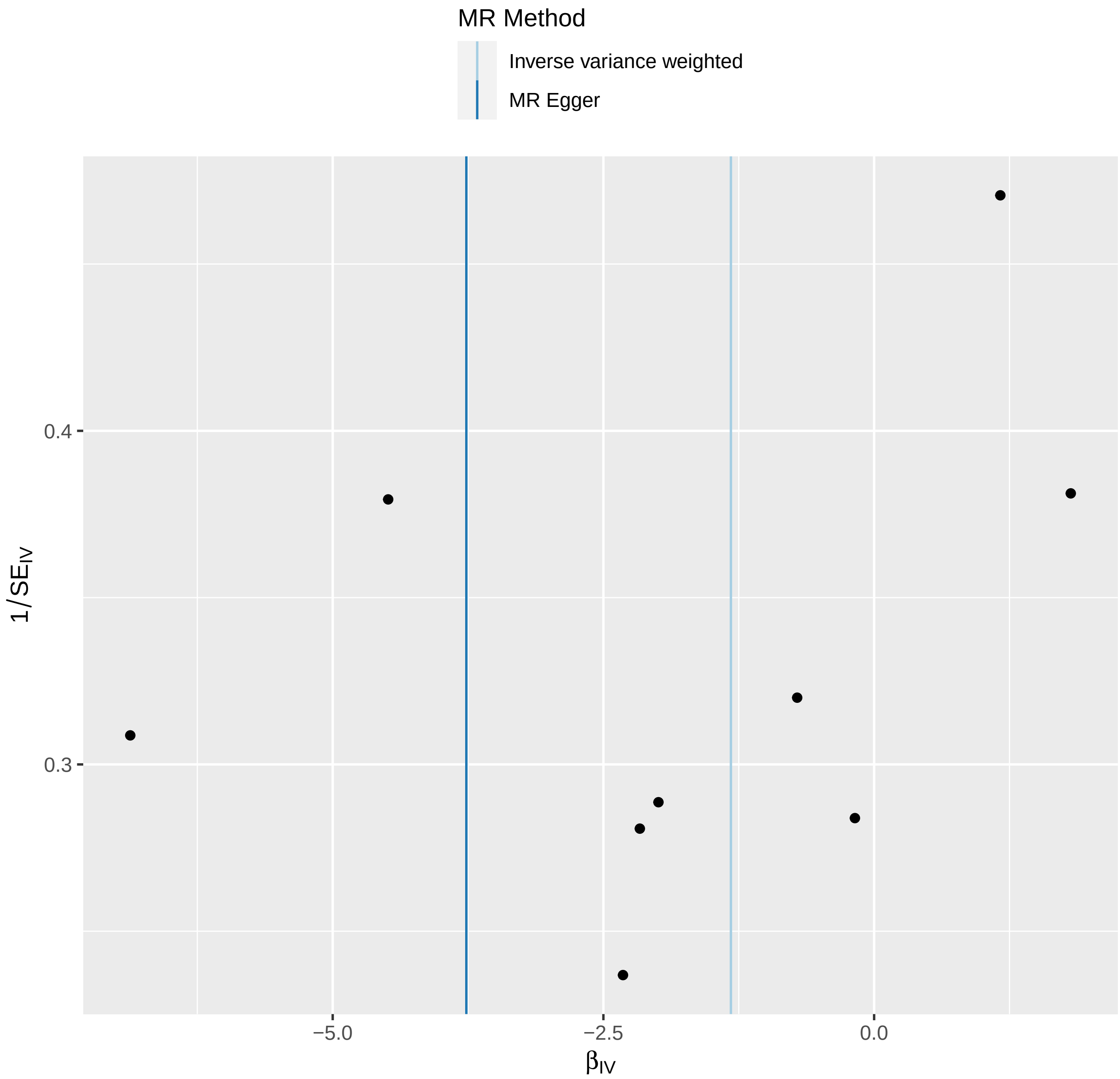

Batch 2209 : 3−hydroxybutyrate (BHBA) on Ulcerative proctitis

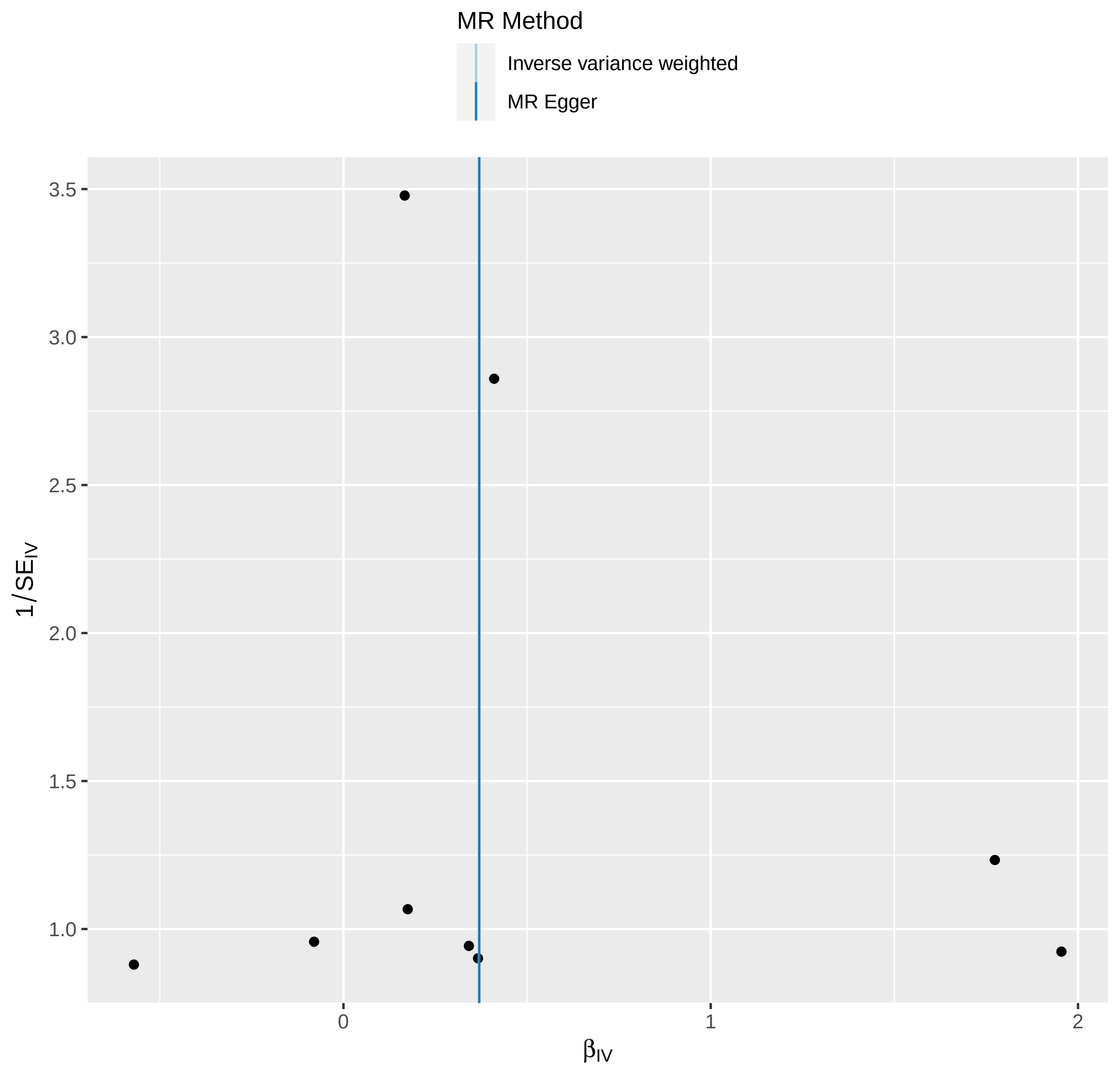

Batch 2210 : Arabinose on Ulcerative proctitis

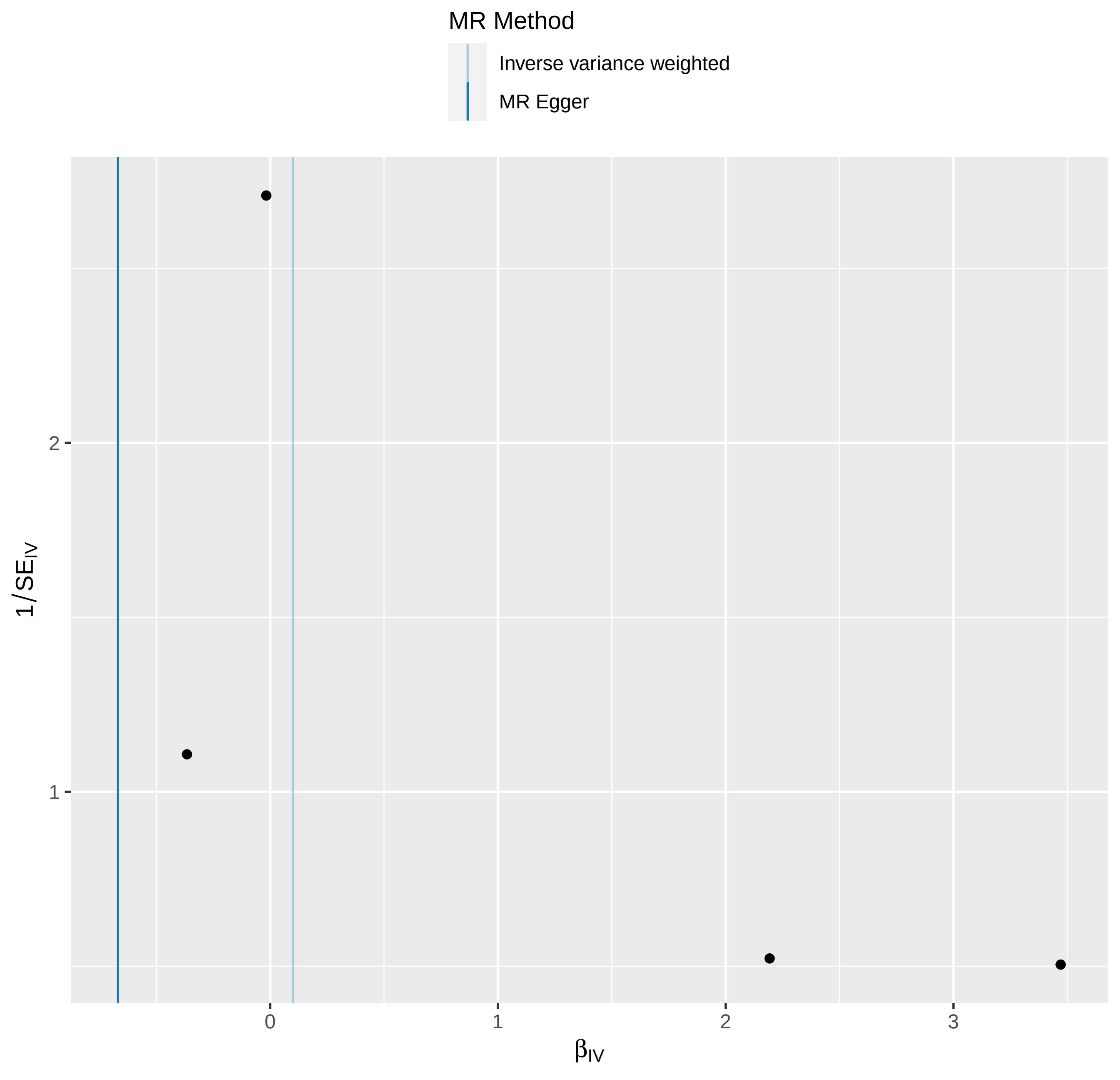

Batch 2211 : Fructose on Ulcerative proctitis

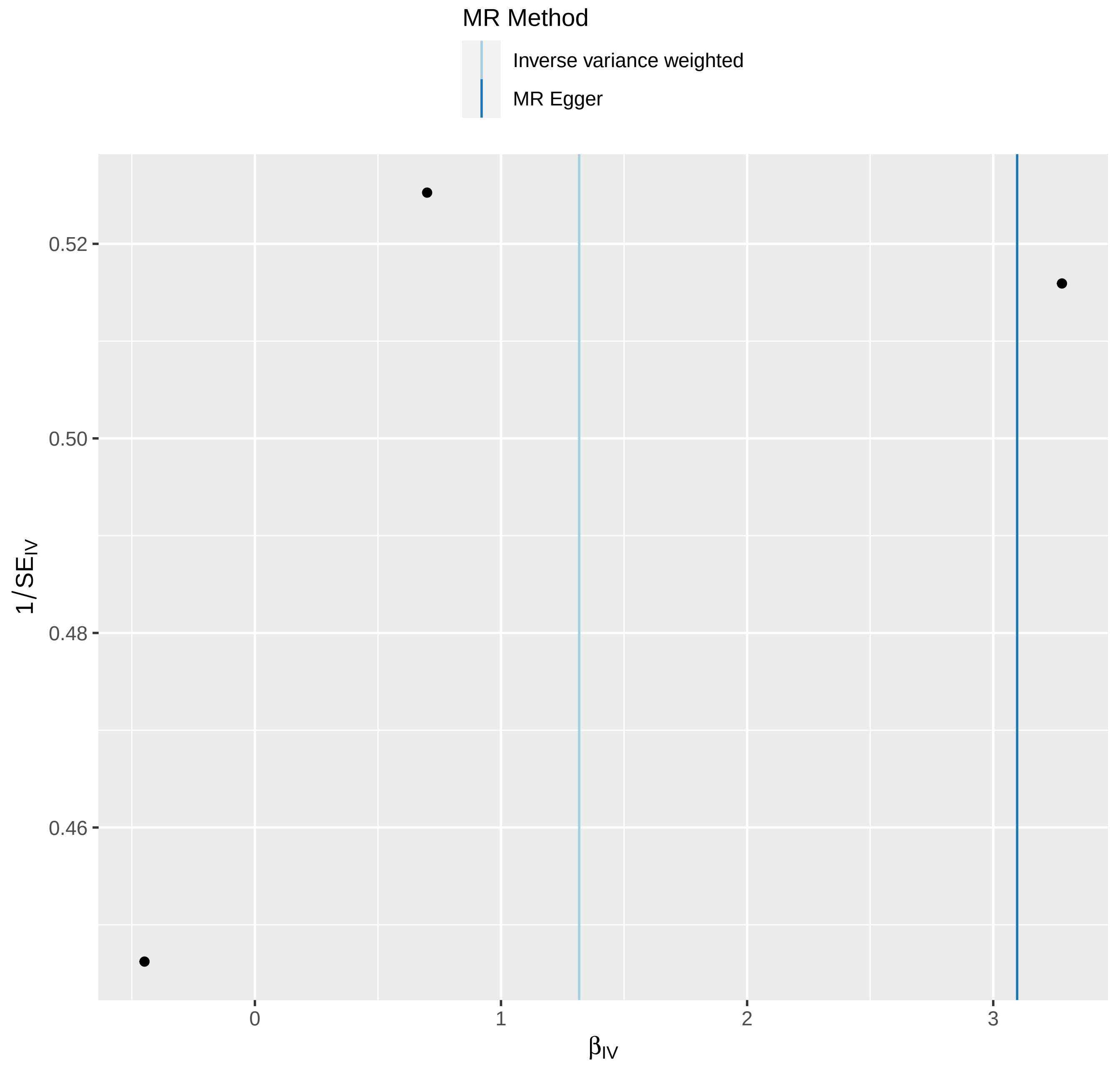

Batch 2213 : Pyruvate on Ulcerative proctitis

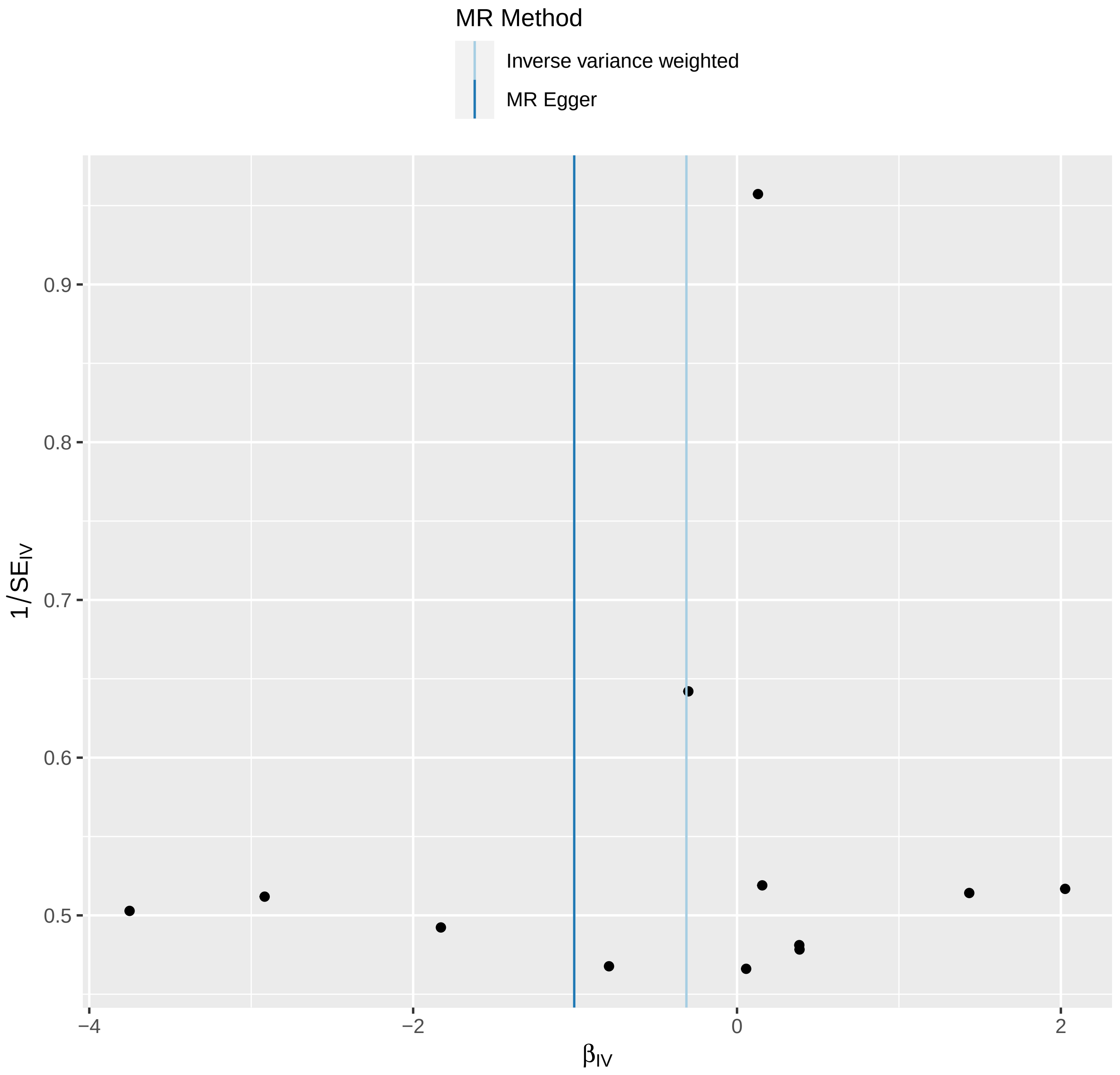

Batch 2214 : Uridine on Ulcerative proctitis

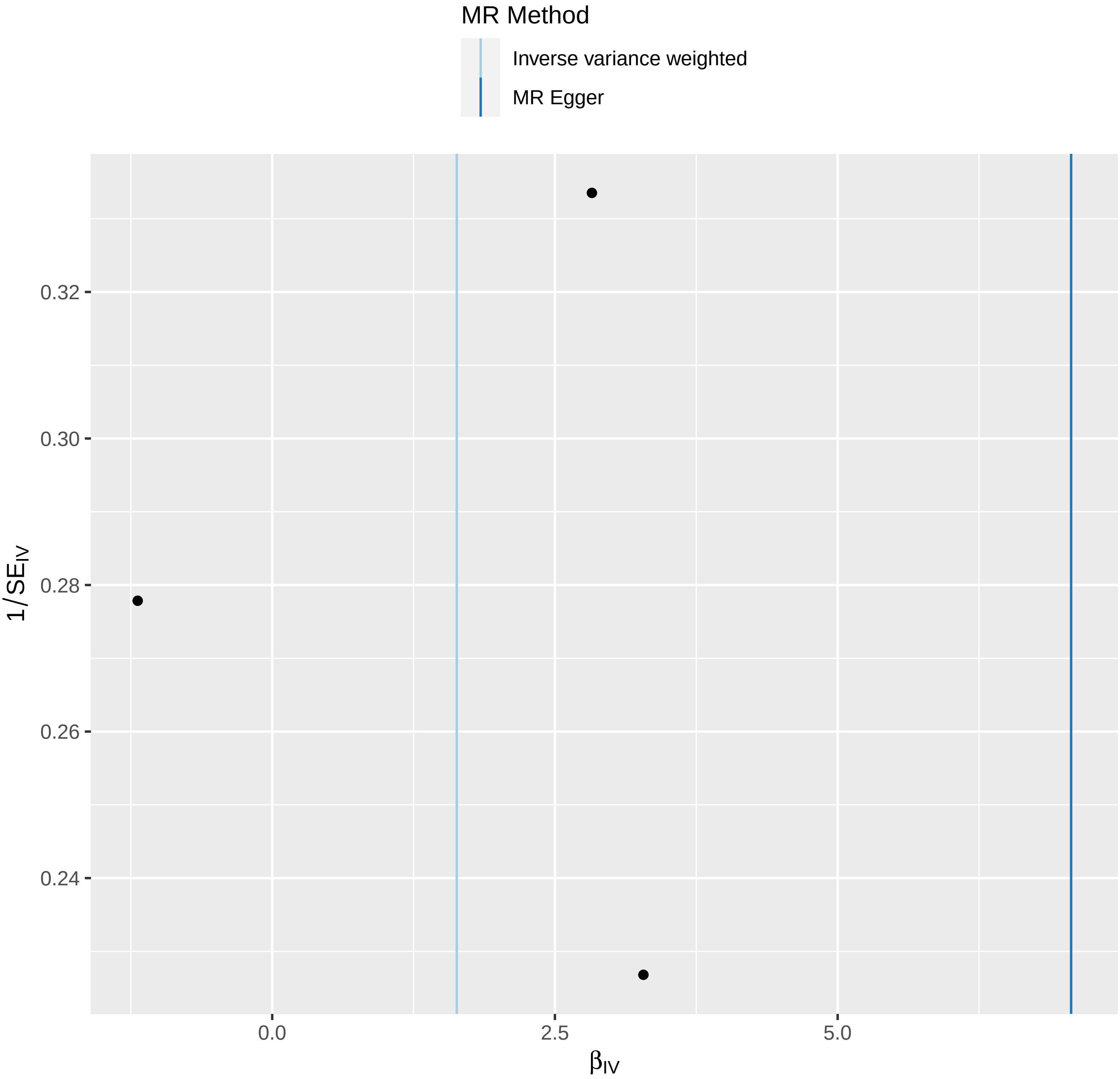

Batch 2215 : Linoleate (18:2n6) on Ulcerative proctitis

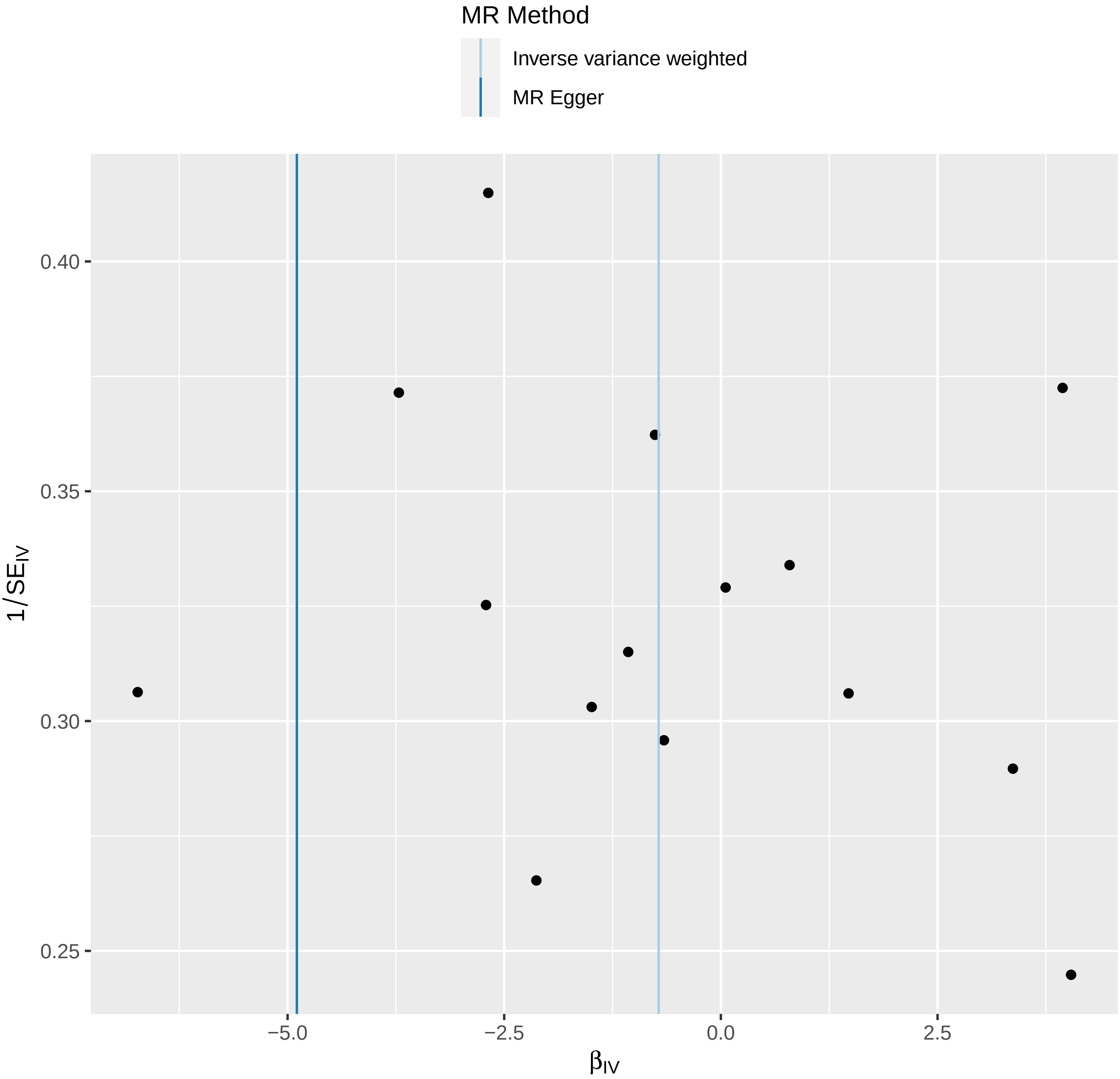

Batch 2216 : Allantoin on Ulcerative proctitis

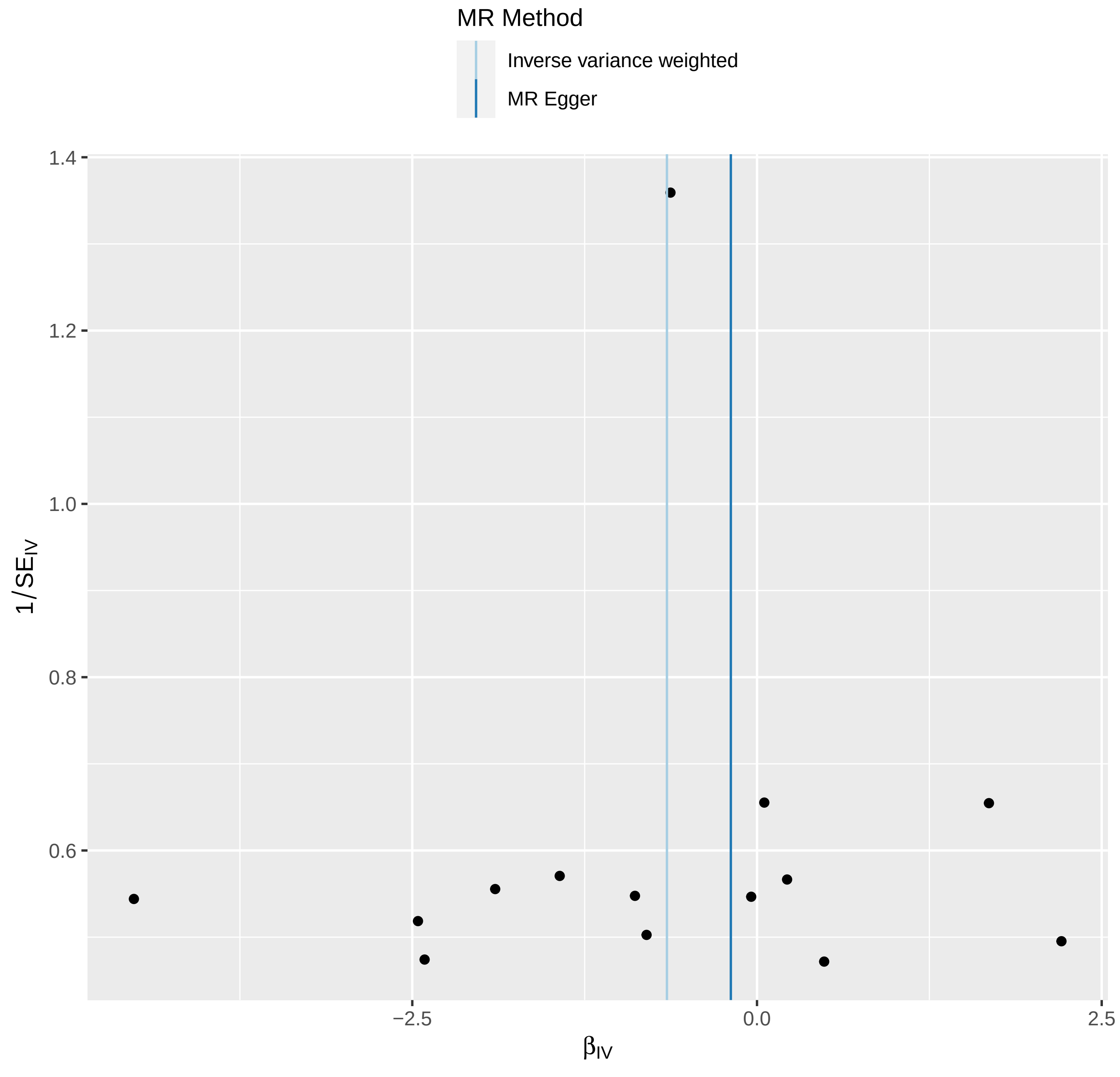

Batch 2221 : Isoleucine on Ulcerative proctitis

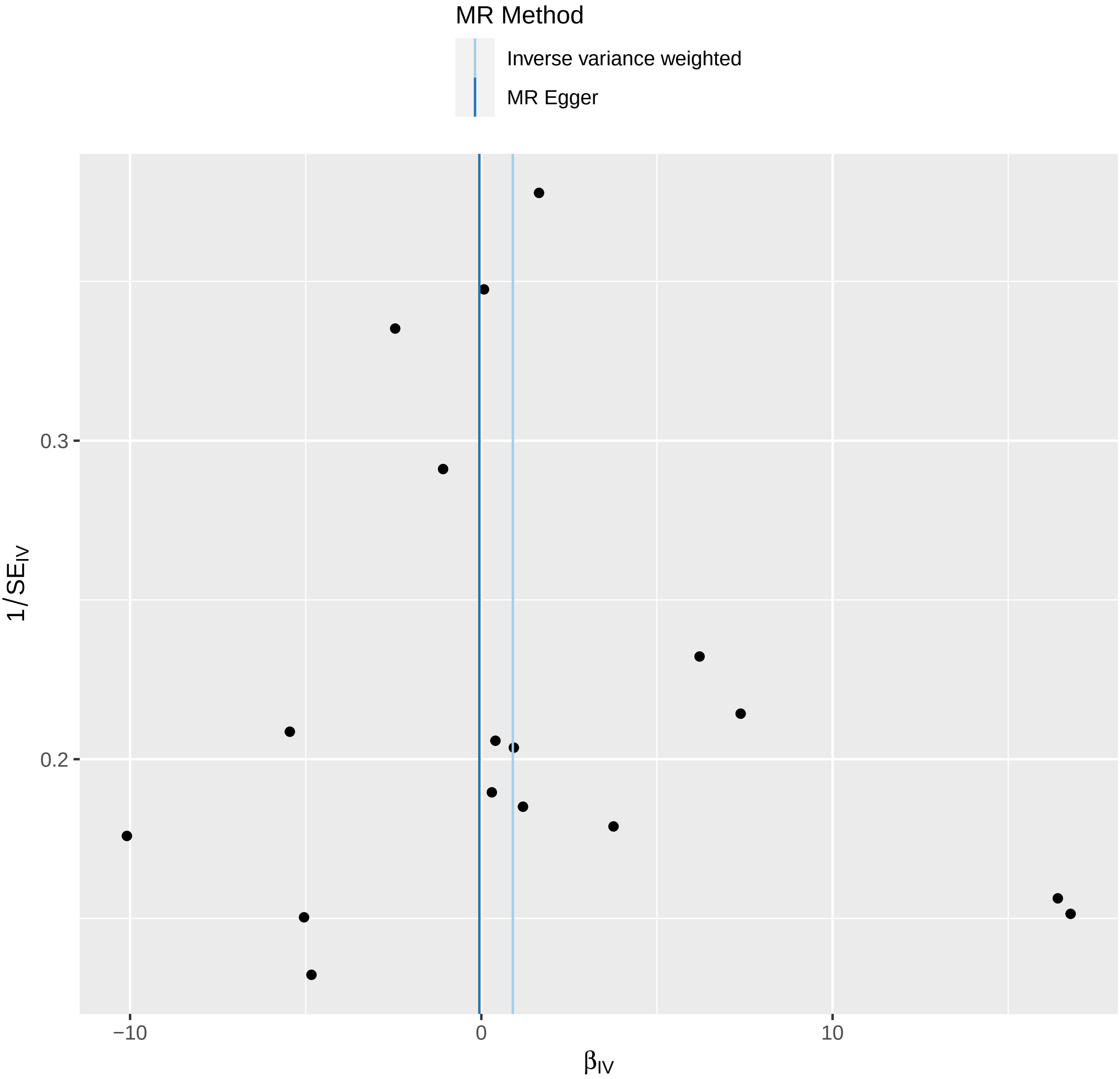

Batch 2222 : Threonine on Ulcerative proctitis

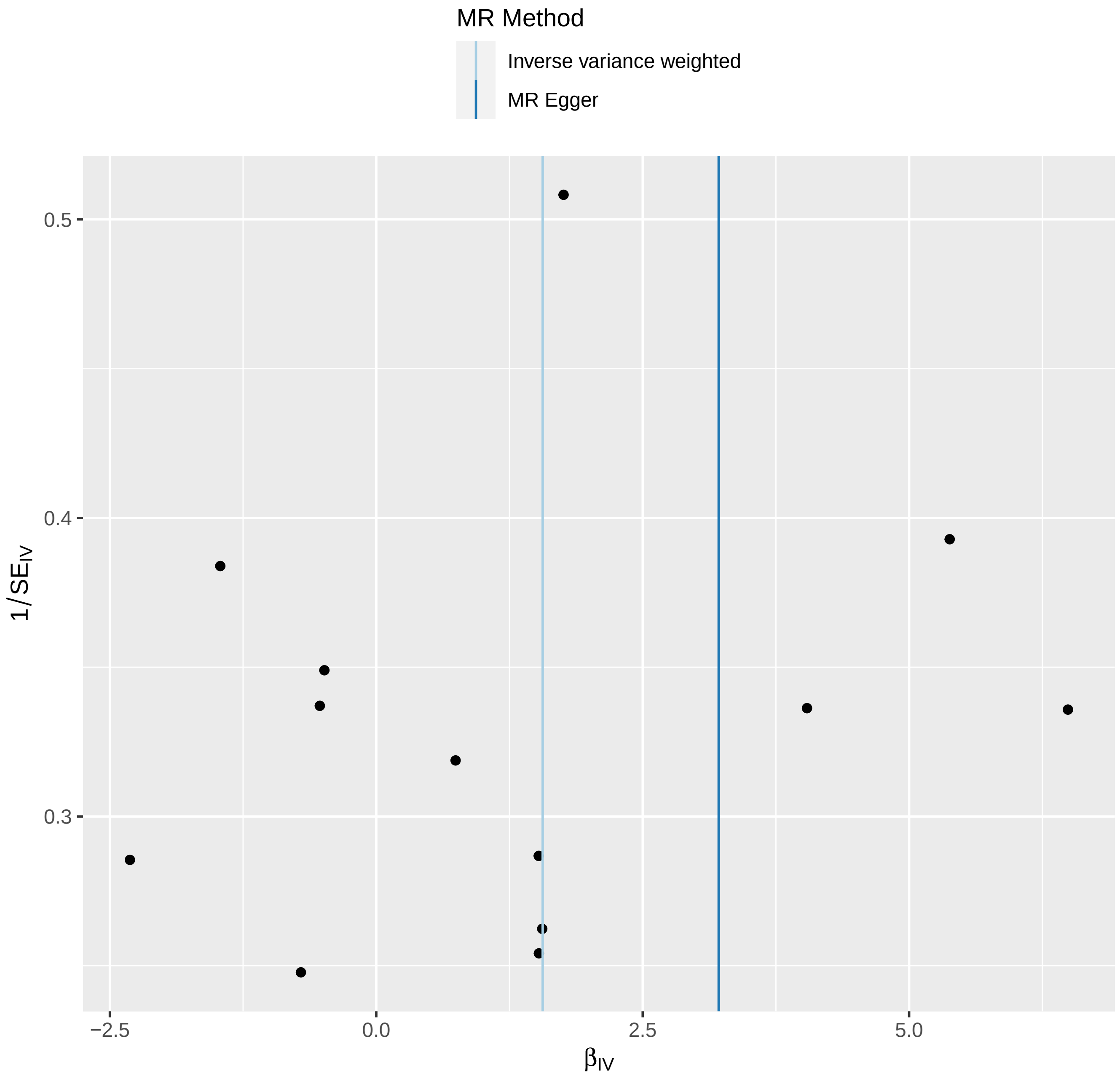

Batch 2223 : Tyrosine on Ulcerative proctitis

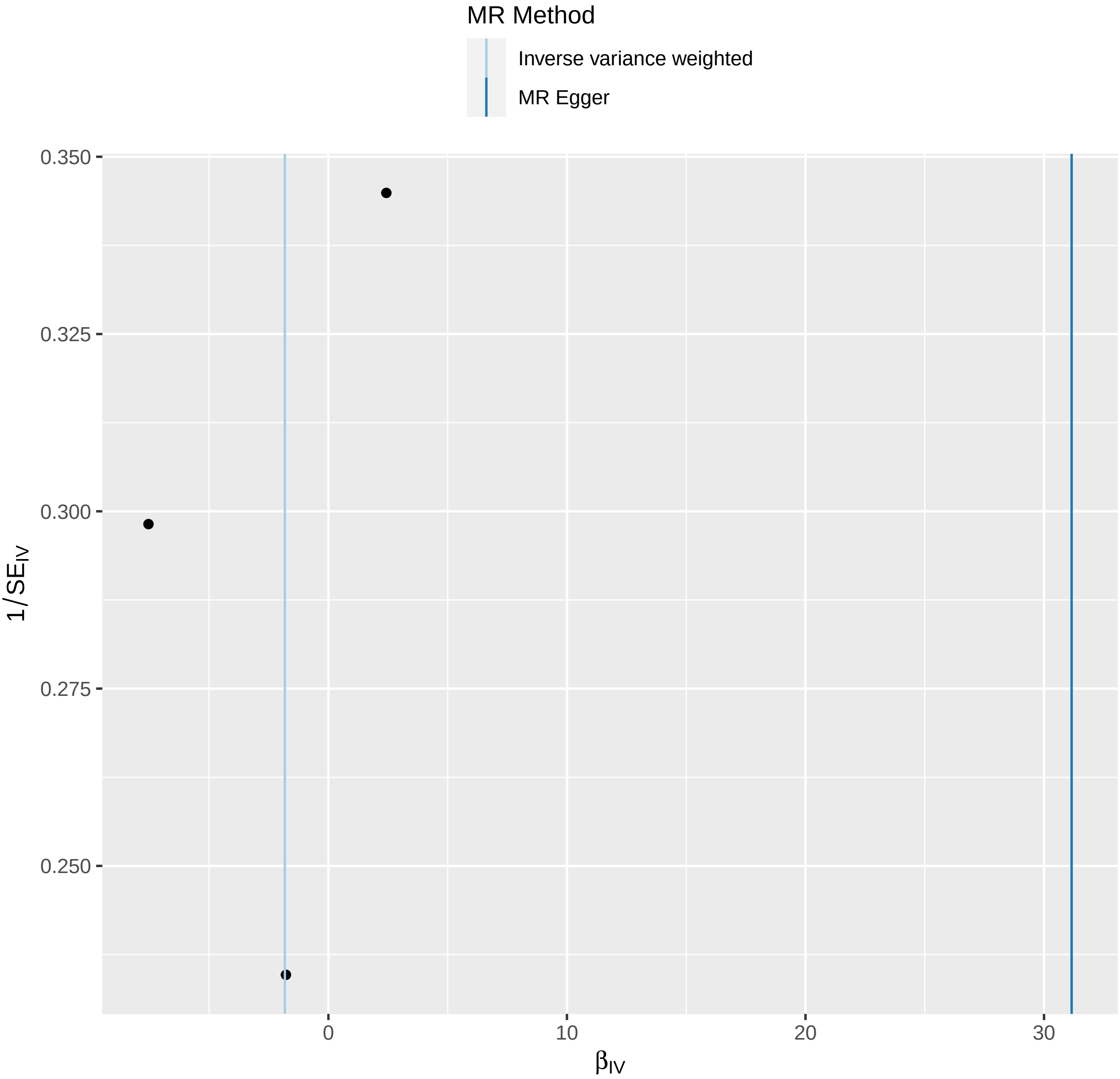

Batch 2229 : Stearate (18:0) on Ulcerative proctitis

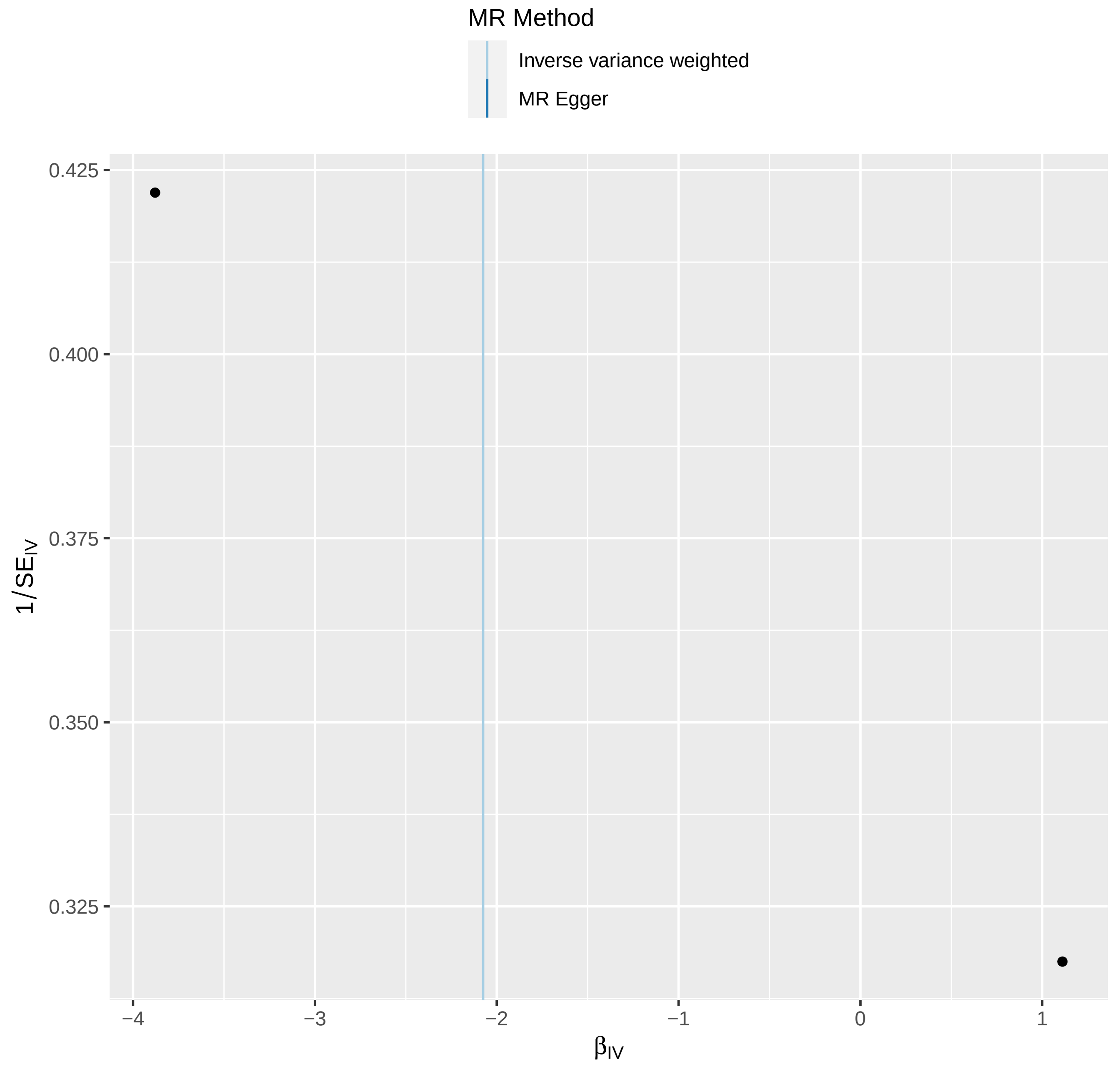

Batch 2232 : Myristate (14:0) on Ulcerative proctitis

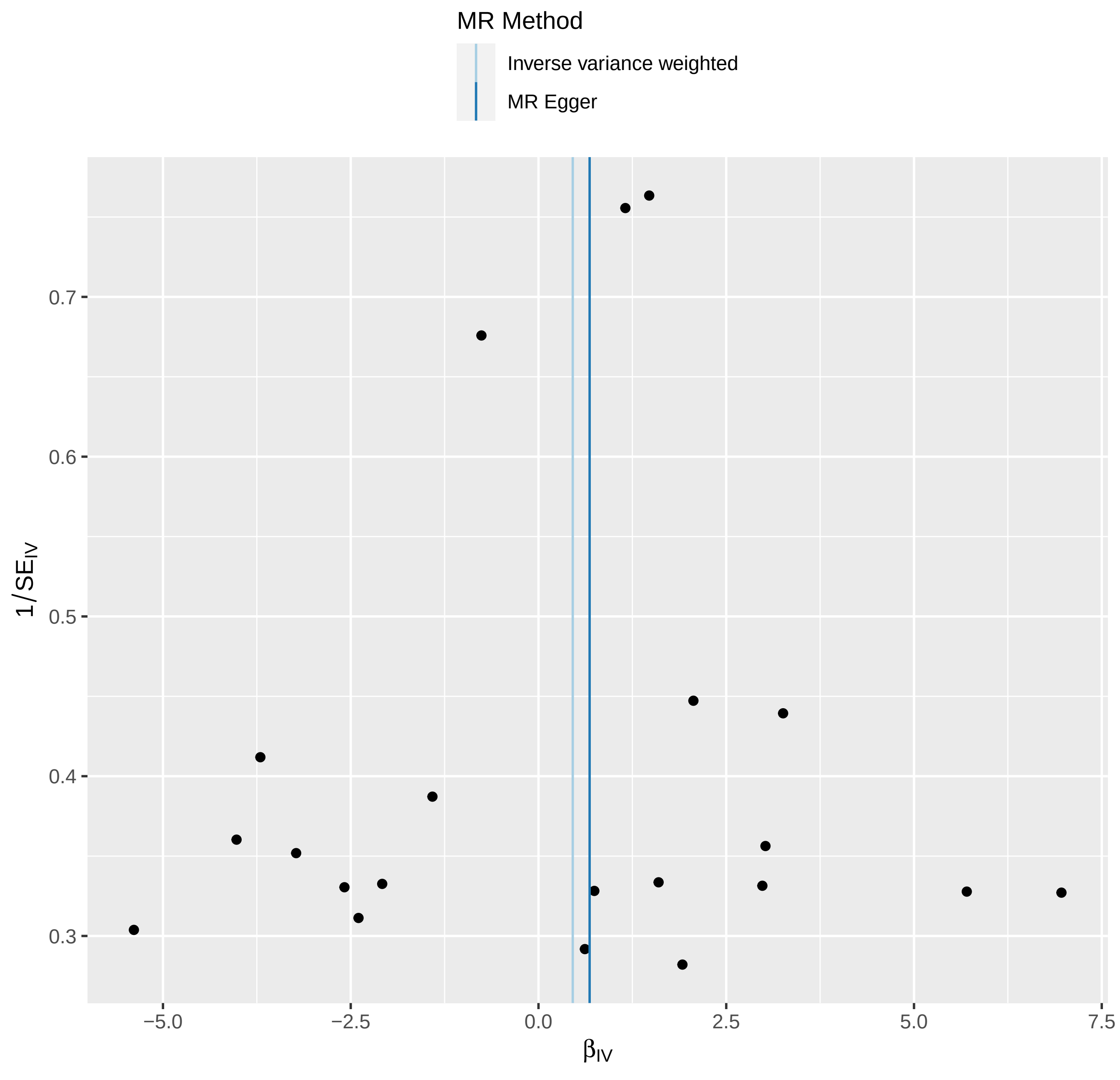

Batch 2234 : Ornithine on Ulcerative proctitis

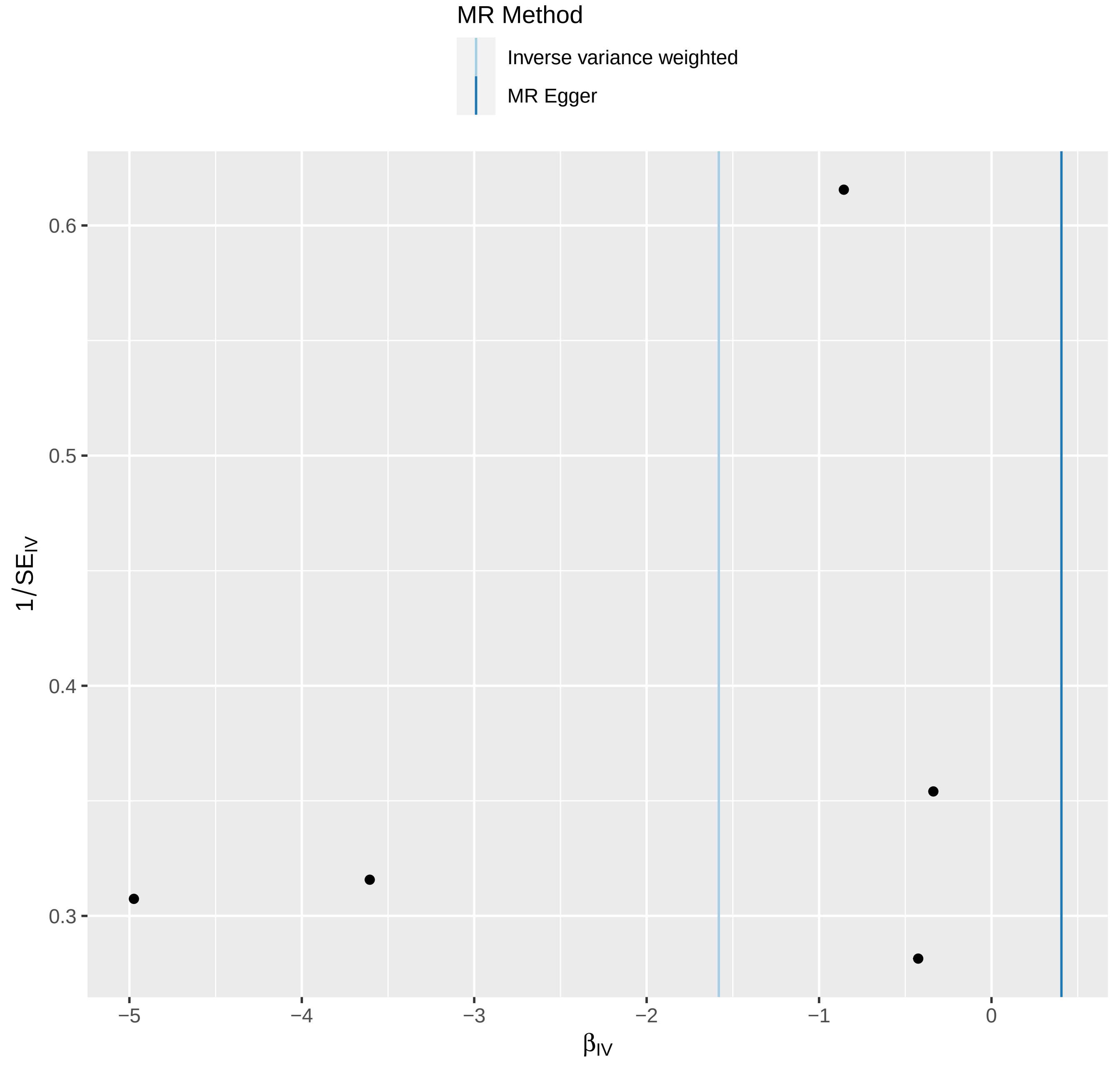

Batch 2237 : 4−acetamidobutanoate on Ulcerative proctitis

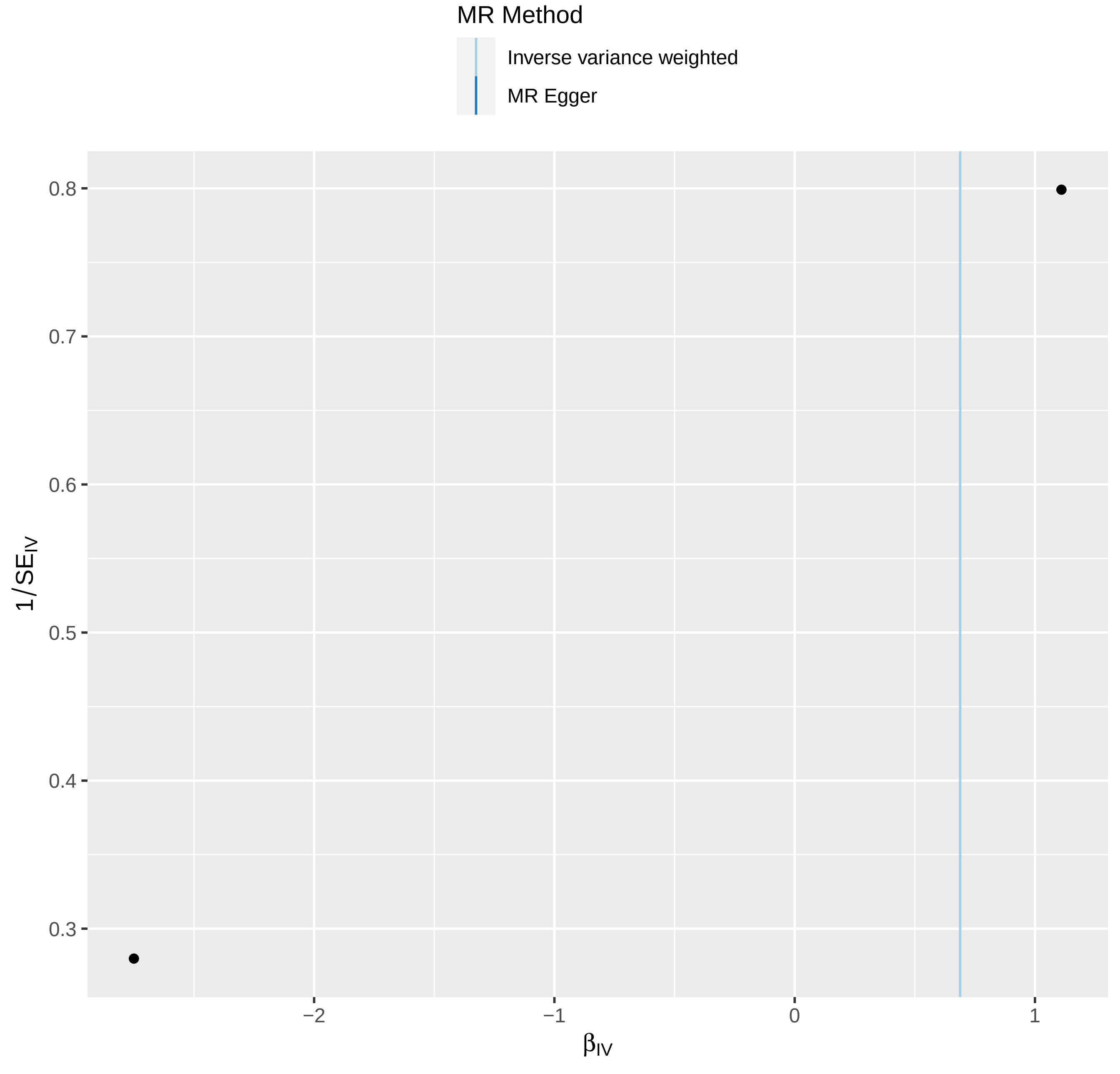

Batch 2238 : Alpha−tocopherol on Ulcerative proctitis

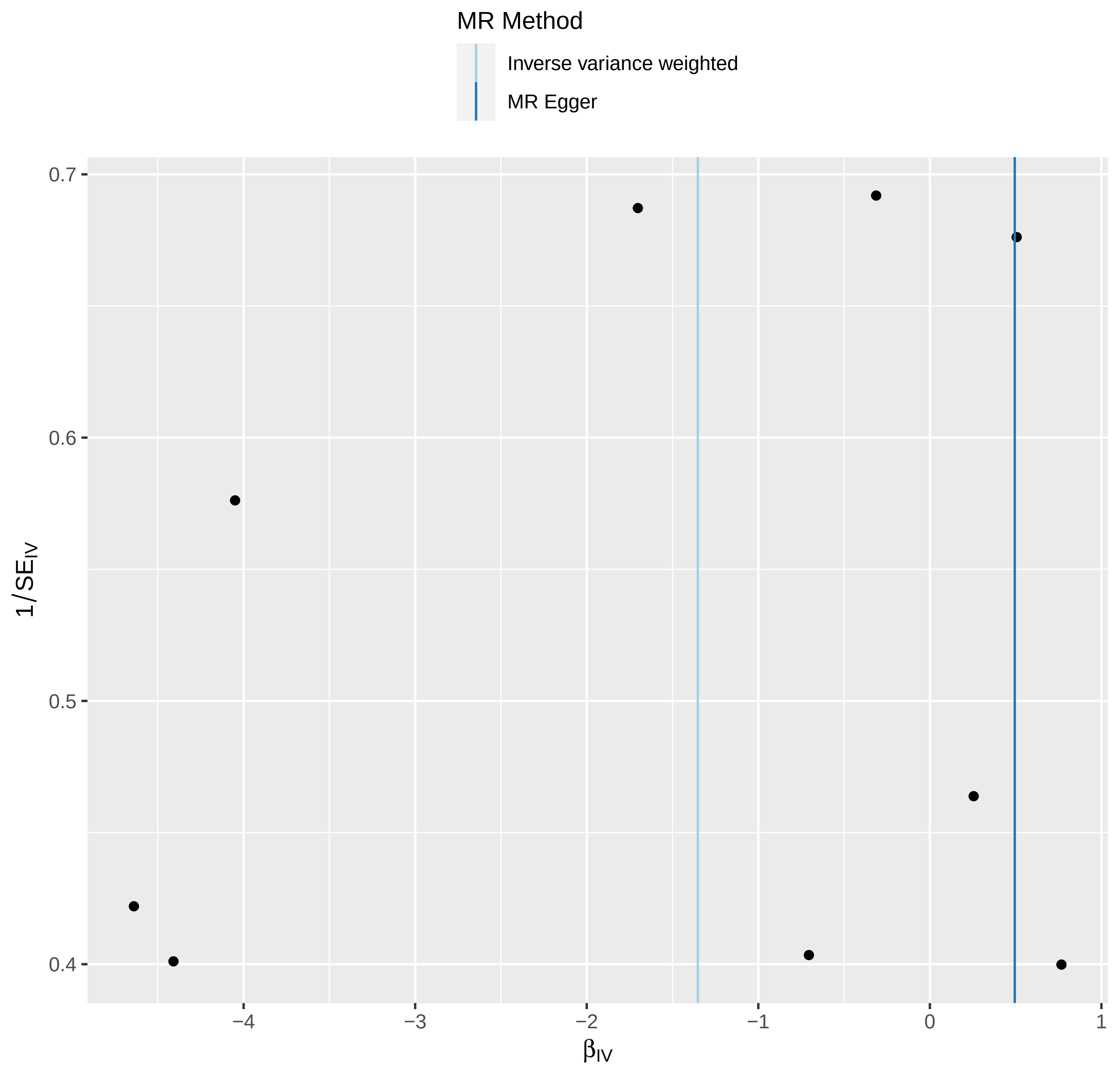

Batch 2239 : Citrate on Ulcerative proctitis

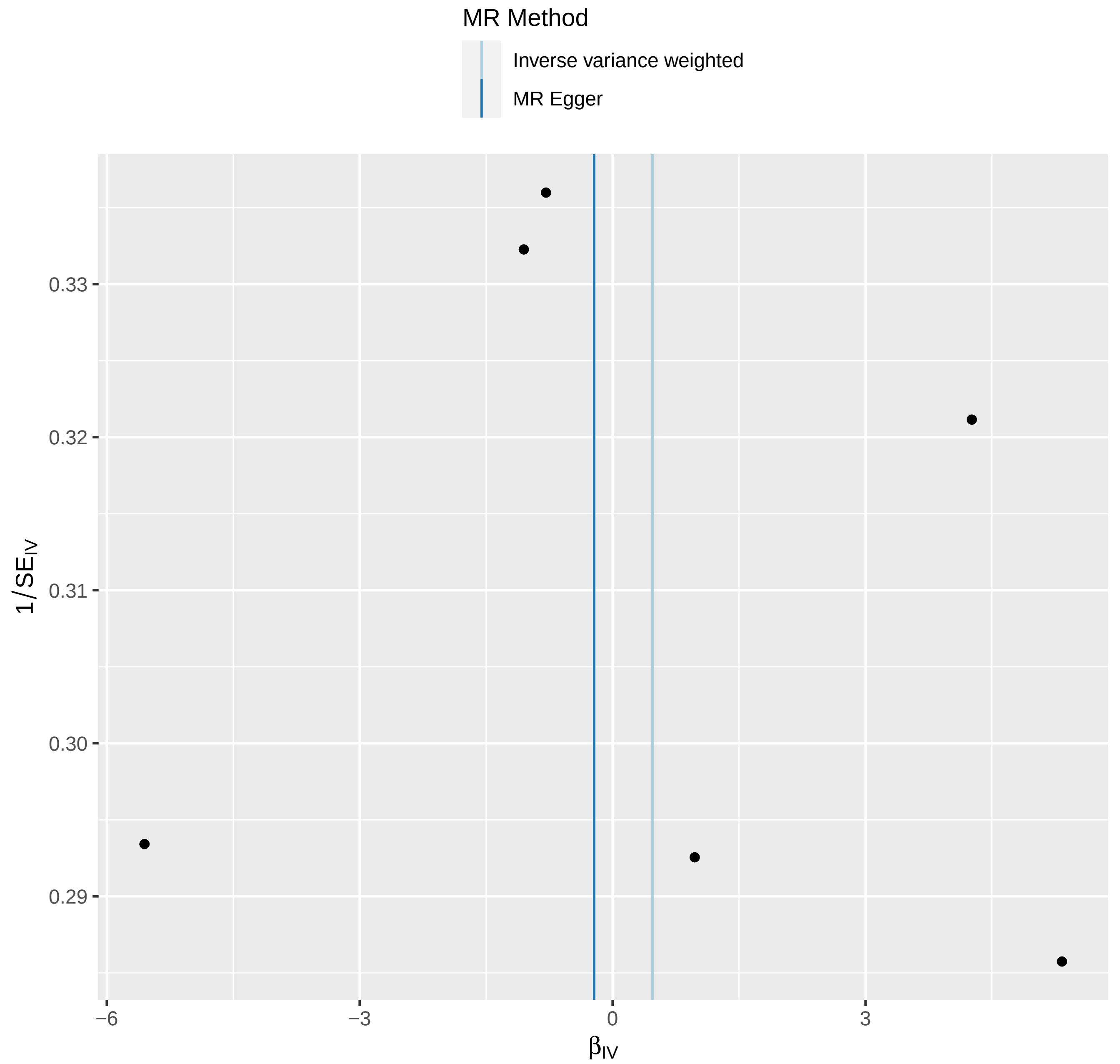

Batch 2240 : Glycerate on Ulcerative proctitis

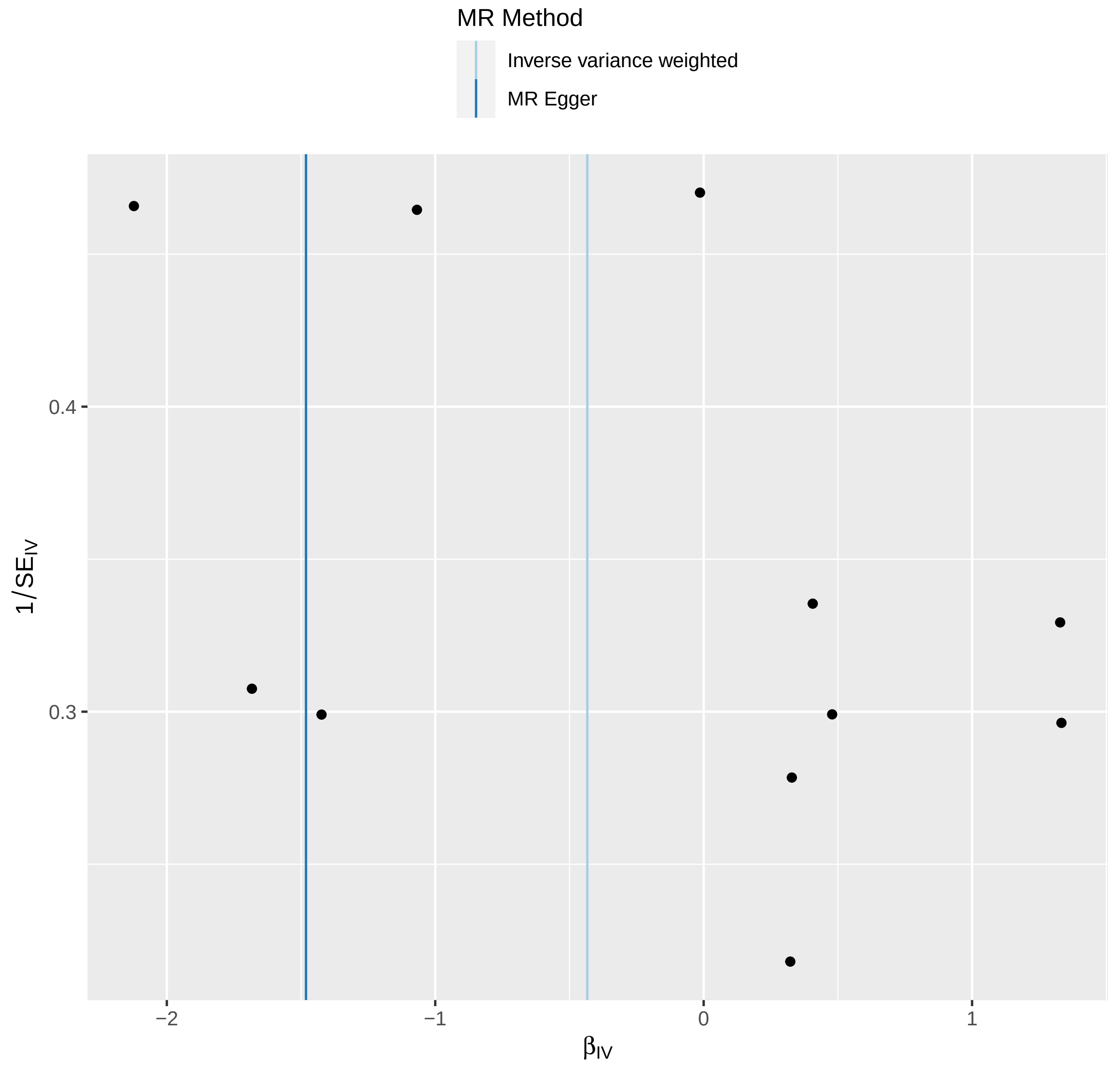

Batch 2241 : Guanosine on Ulcerative proctitis

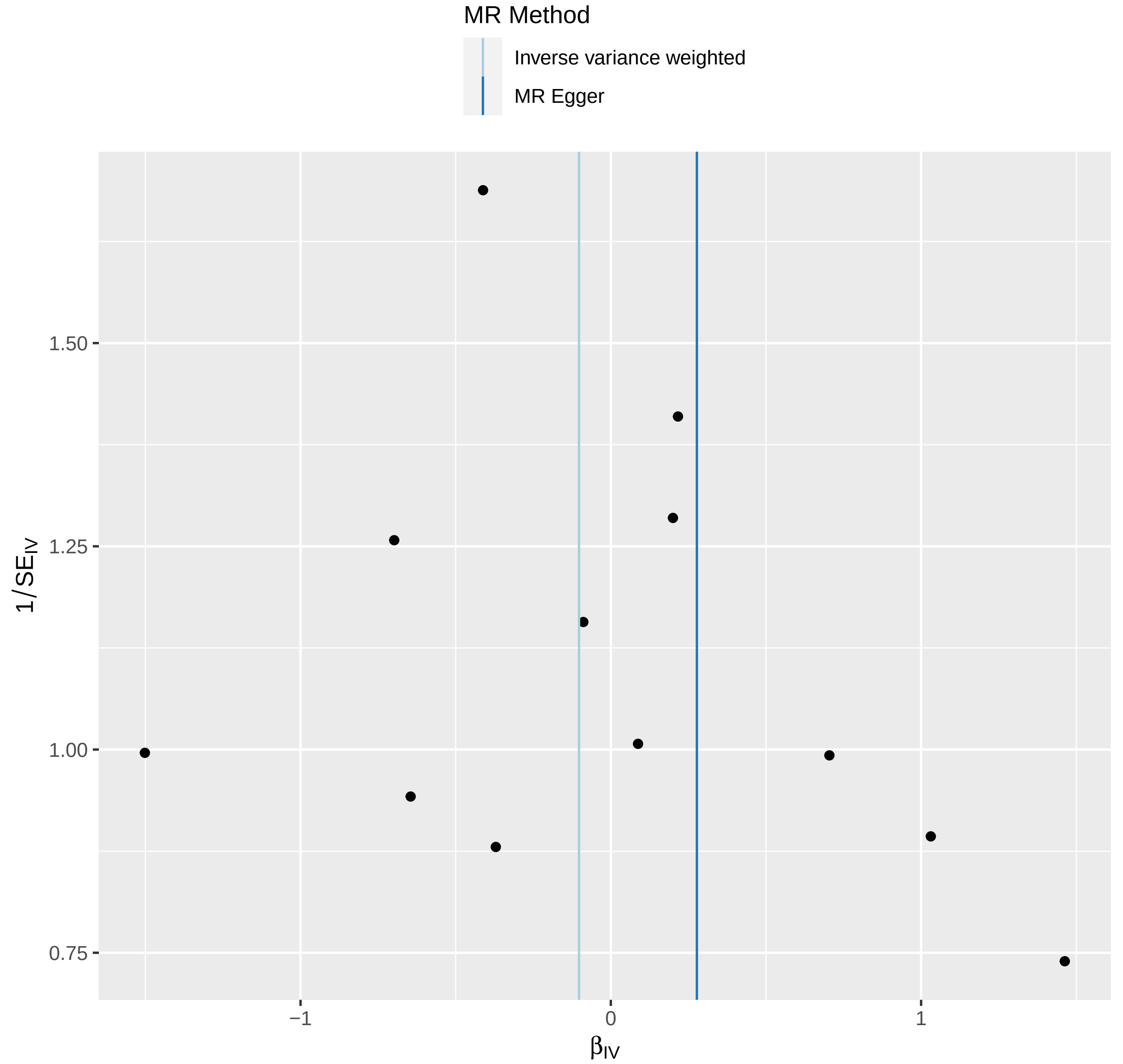

Batch 2244 : Ursodeoxycholate on Ulcerative proctitis

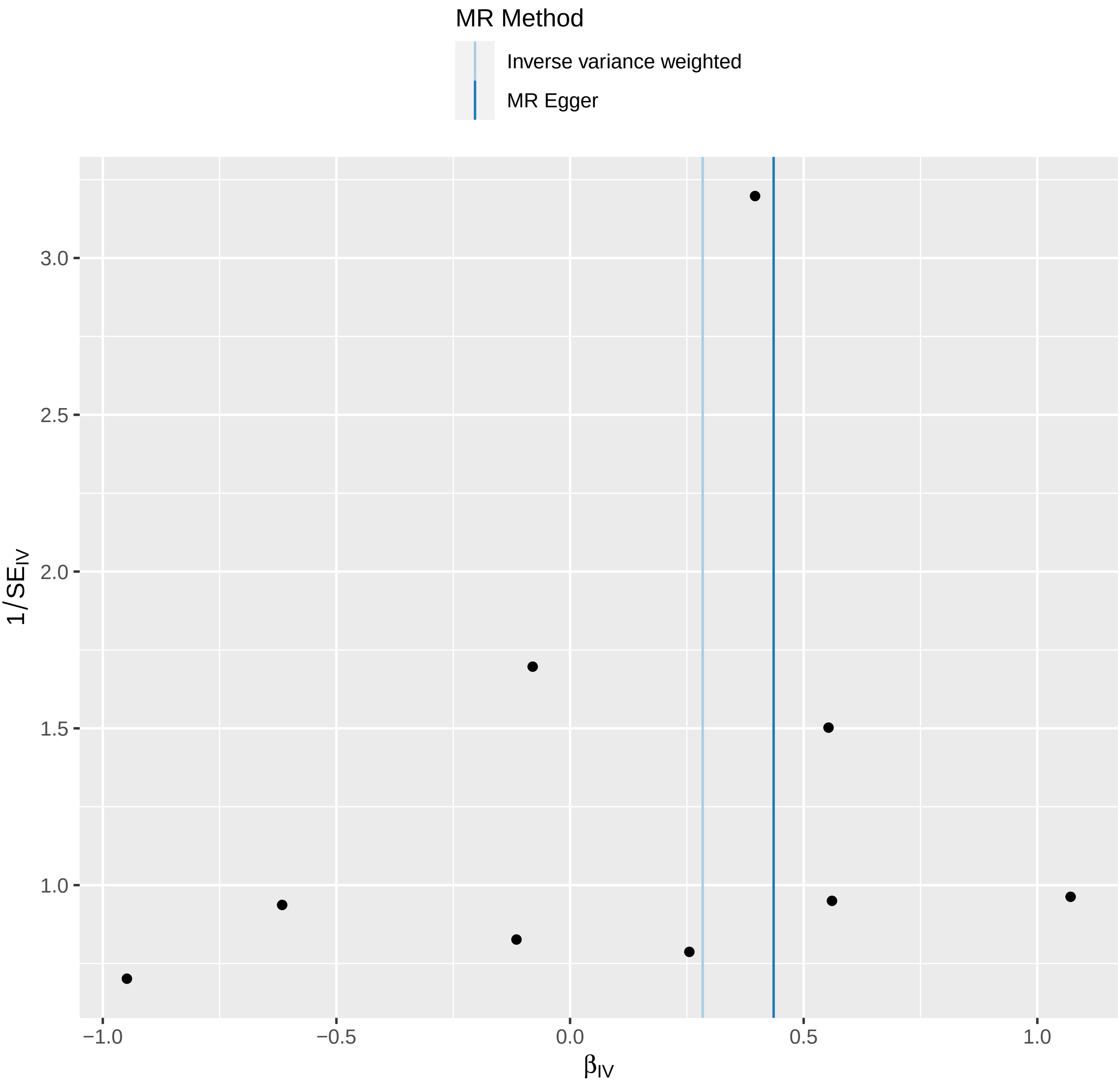

Batch 2245 : Arginine on Ulcerative proctitis

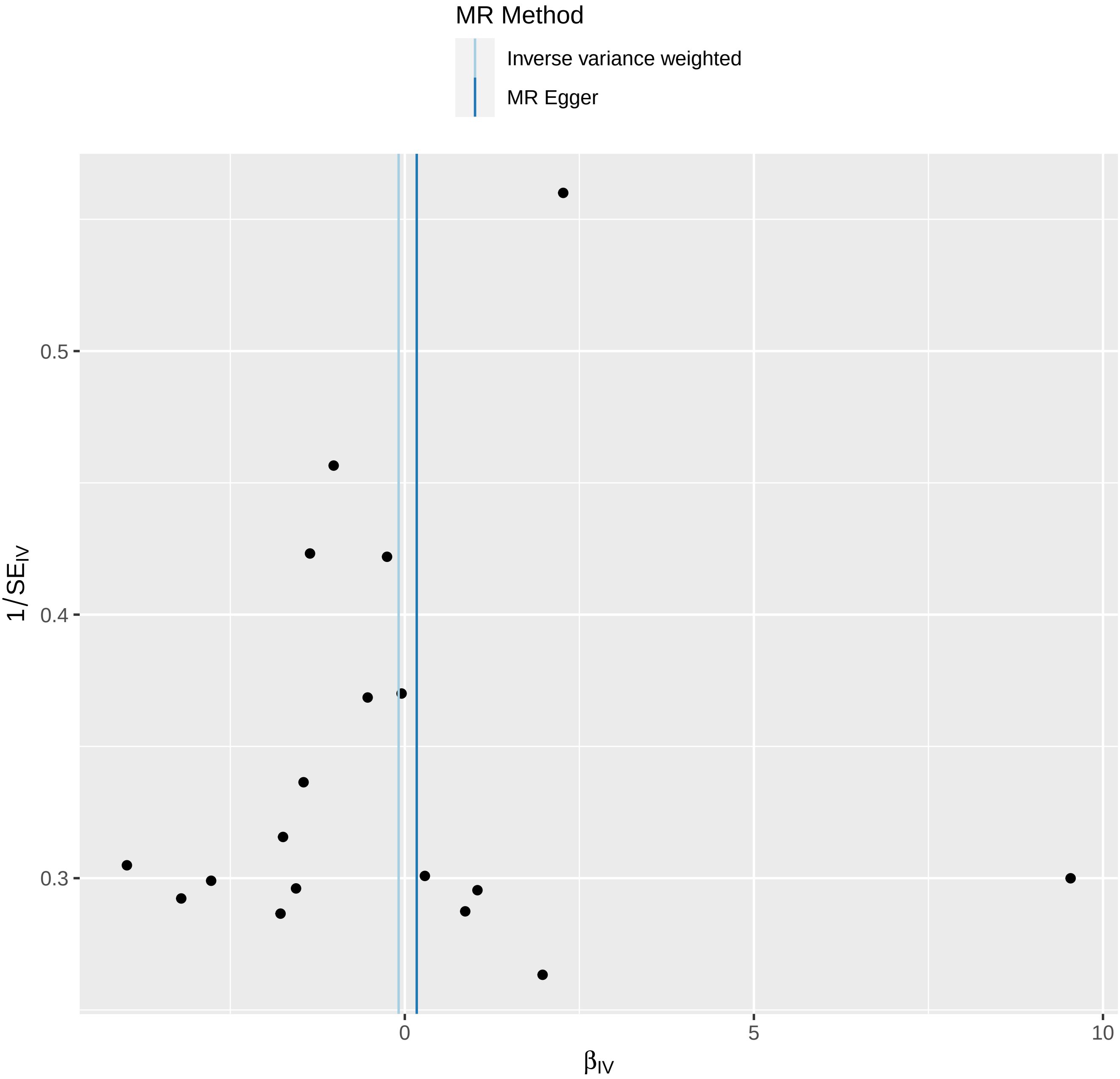

Batch 2246 : Ascorbate (Vitamin C) on Ulcerative proctitis

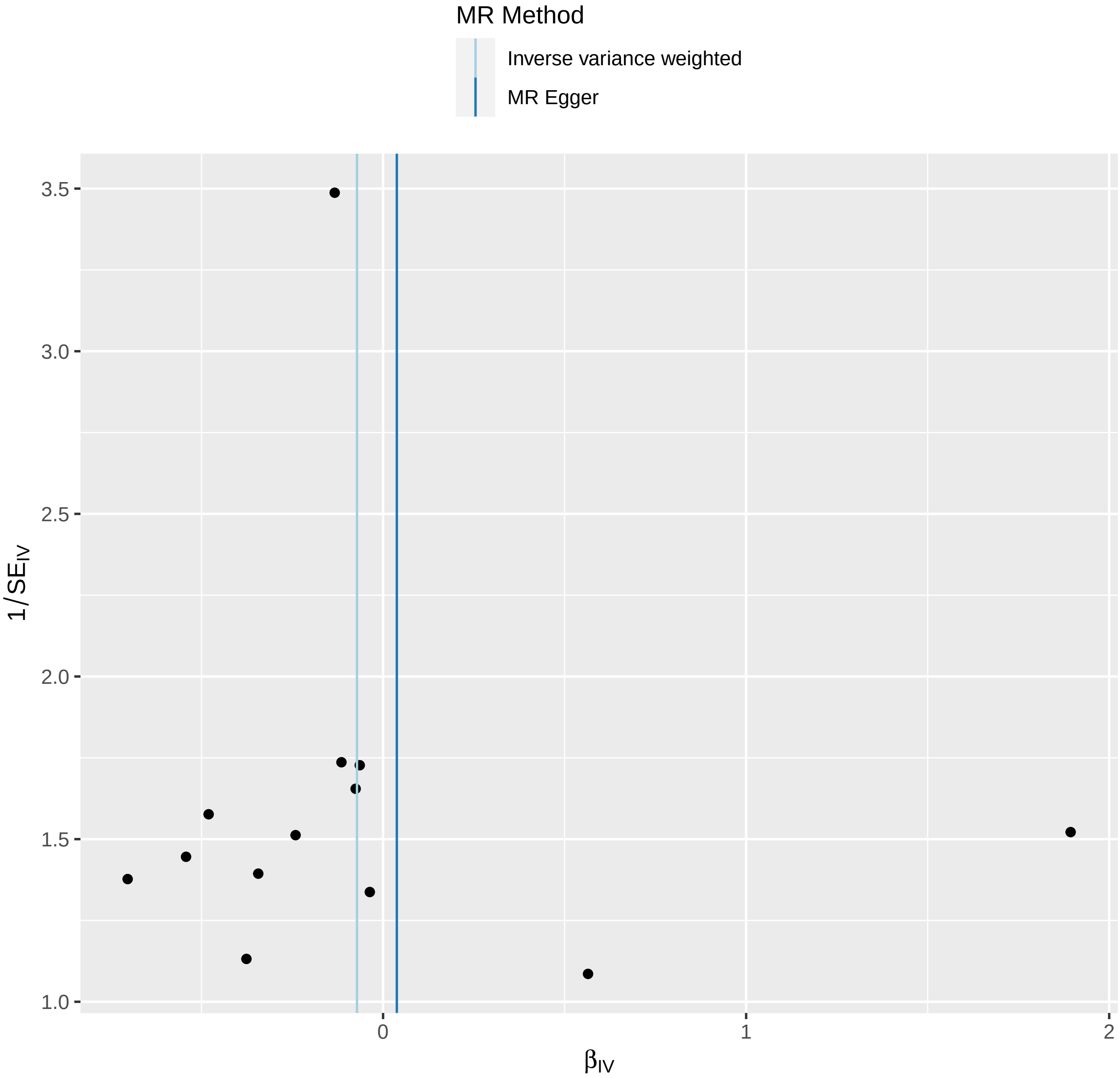

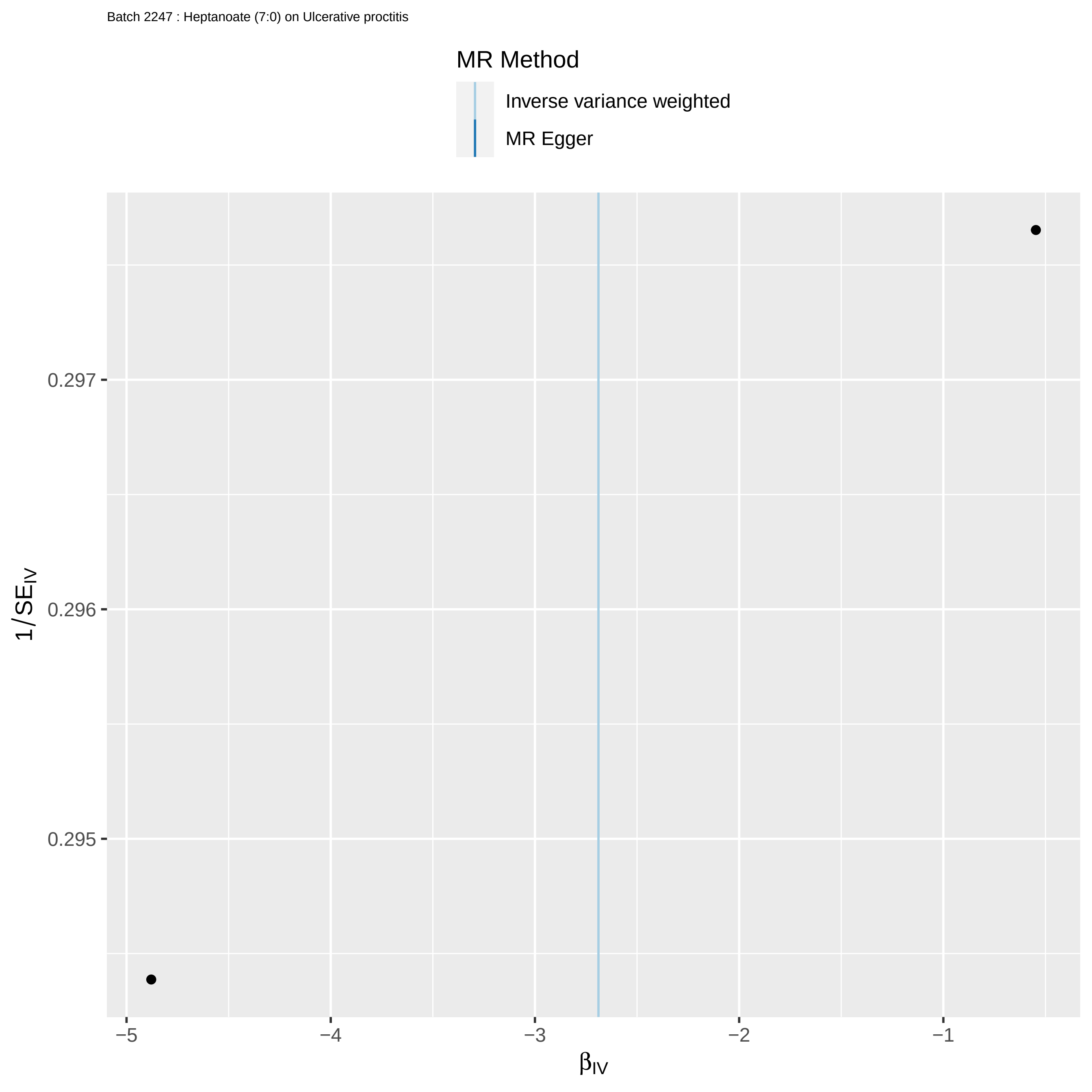

Batch 2247 : Heptanoate (7:0) on Ulcerative proctitis
MR Method
Inverse variance weighted
MR Egger
0.297
0.296
0.295
1/SE$_{IV}$
−5
−4
−3
−2
−1
β$_{IV}$

Batch 2248 : Laurate (12:0) on Ulcerative proctitis

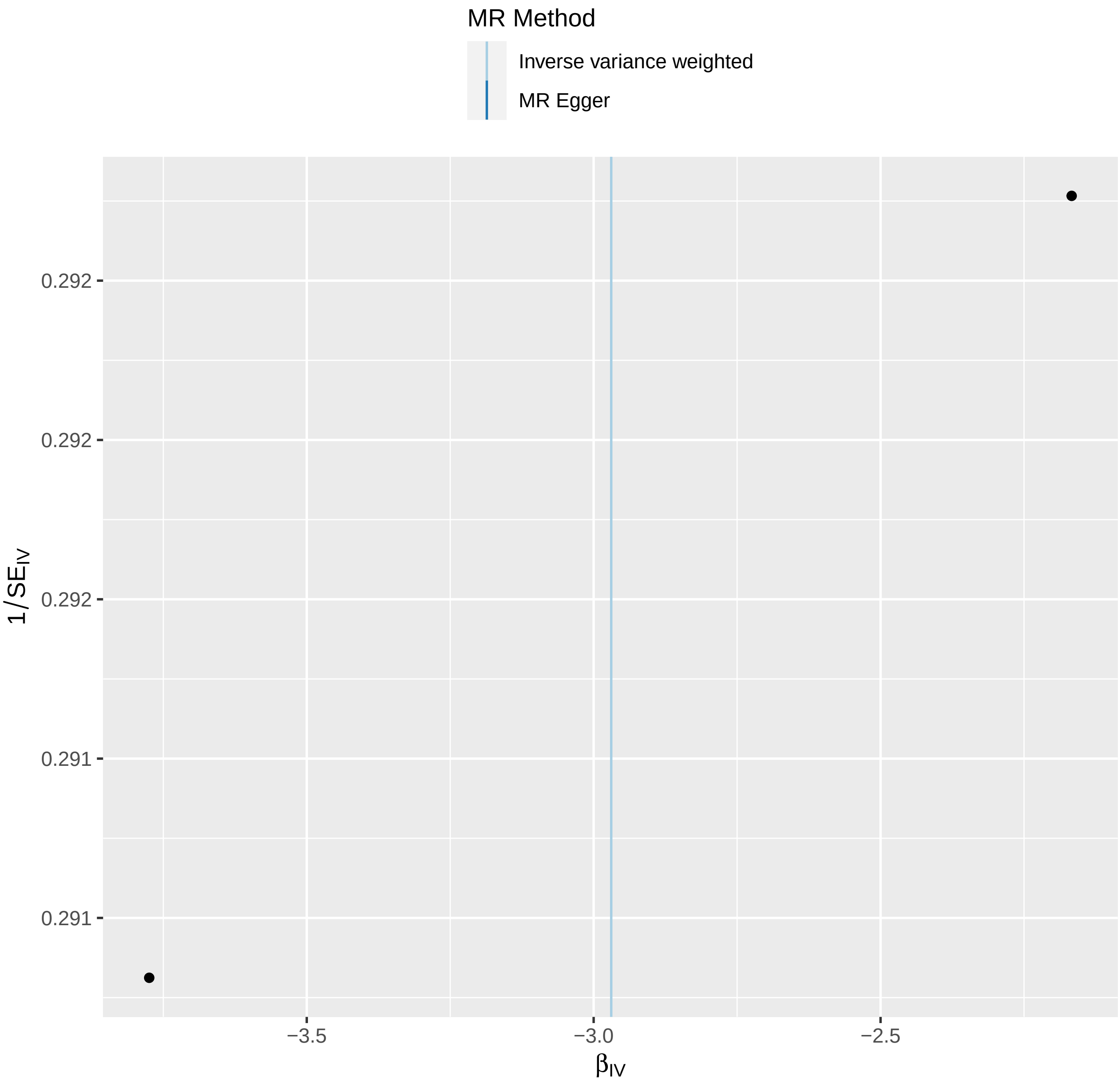

Batch 2250 : Urea on Ulcerative proctitis

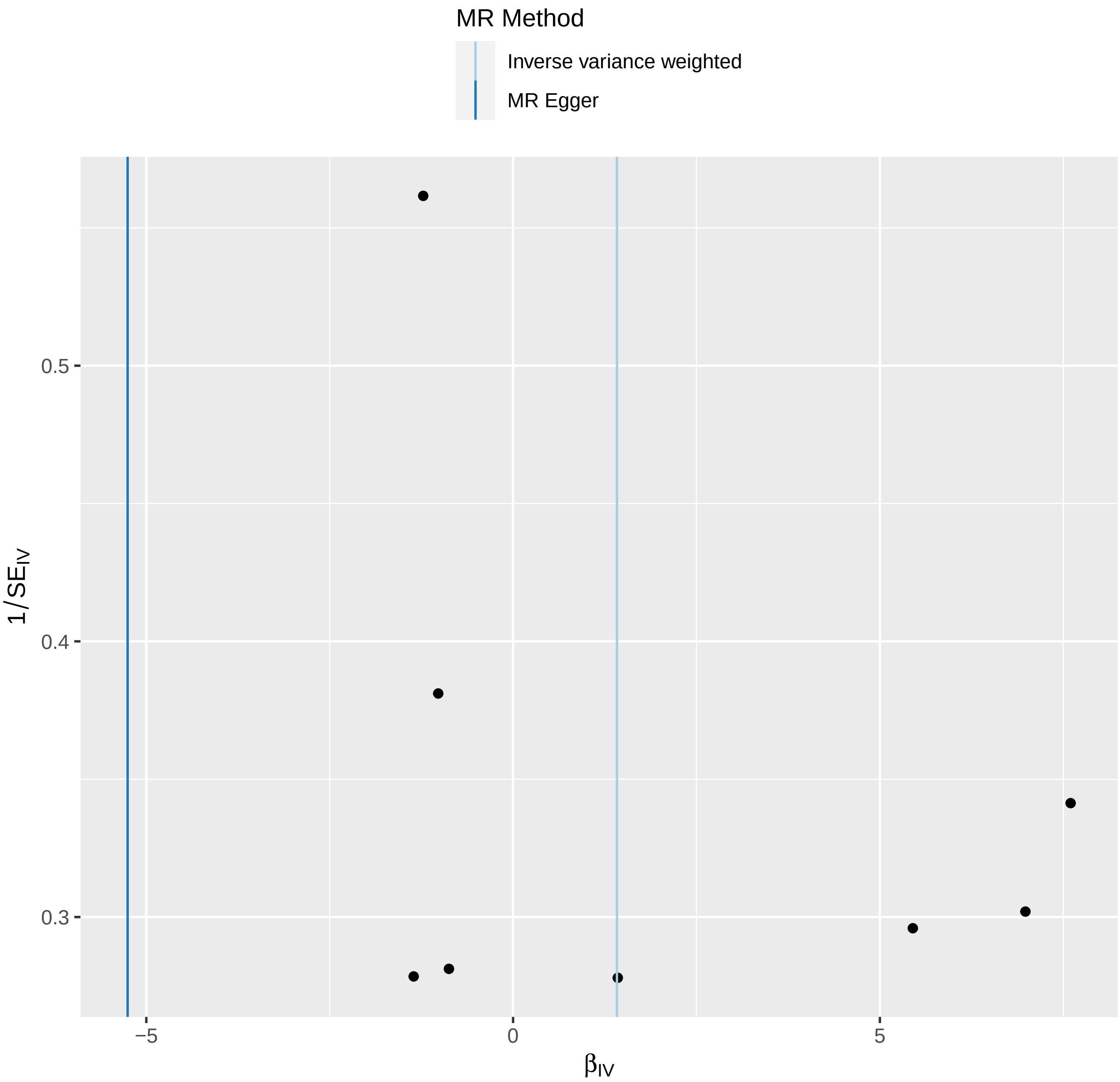

Batch 2251 : Cortisol on Ulcerative proctitis

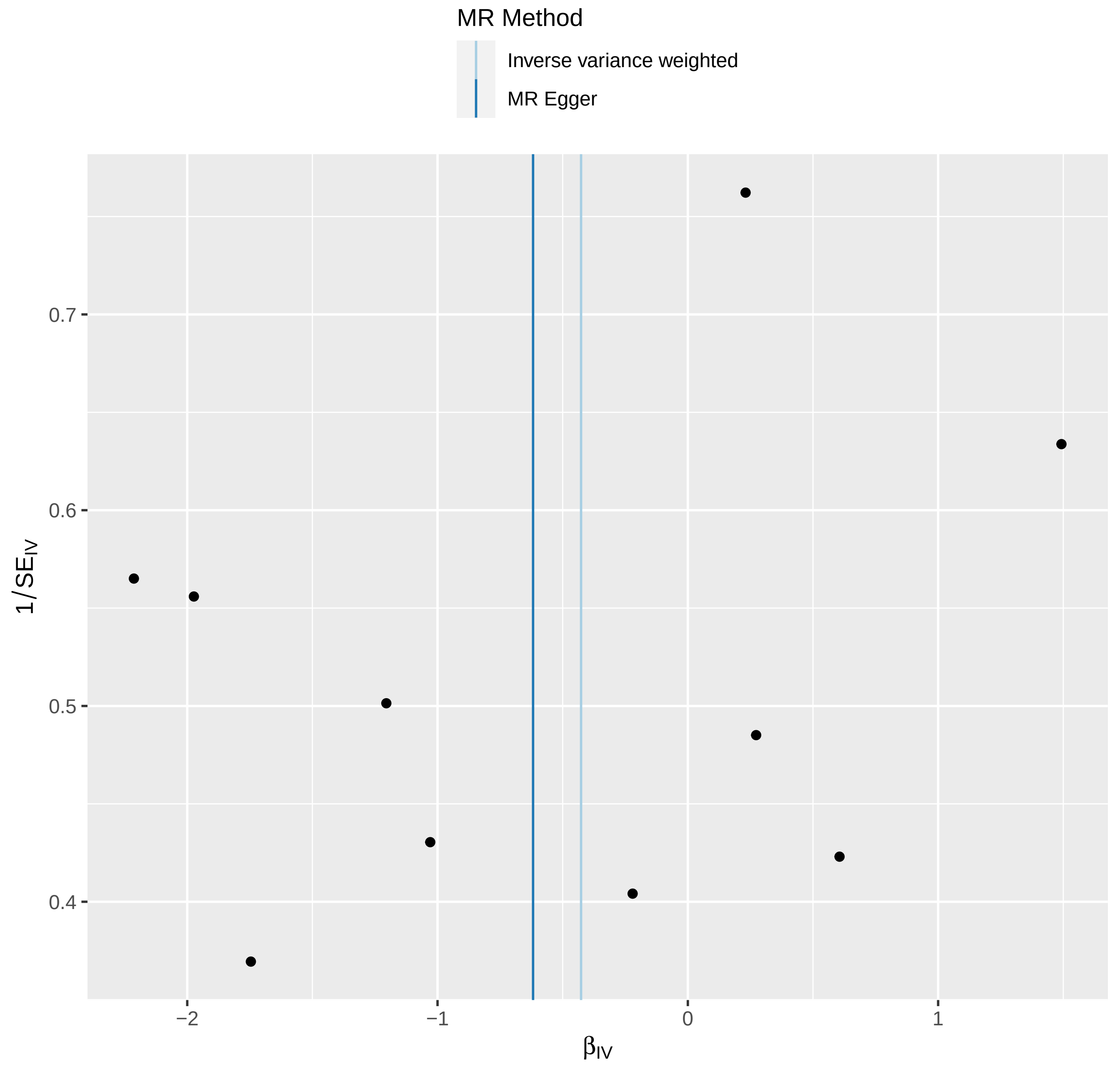

Batch 2254 : Citrulline on Ulcerative proctitis

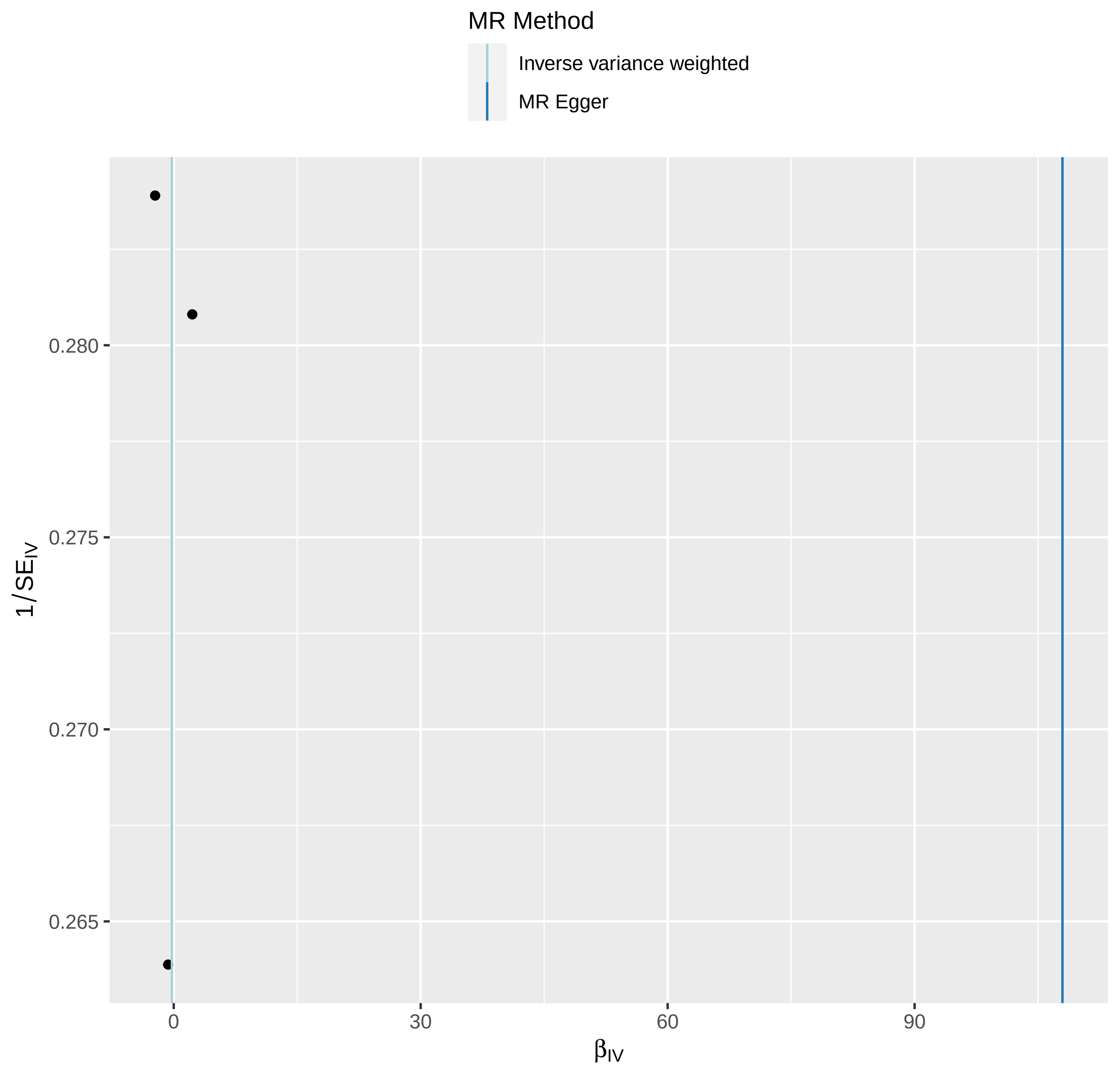

Batch 2256 : Serotonin (5HT) on Ulcerative proctitis

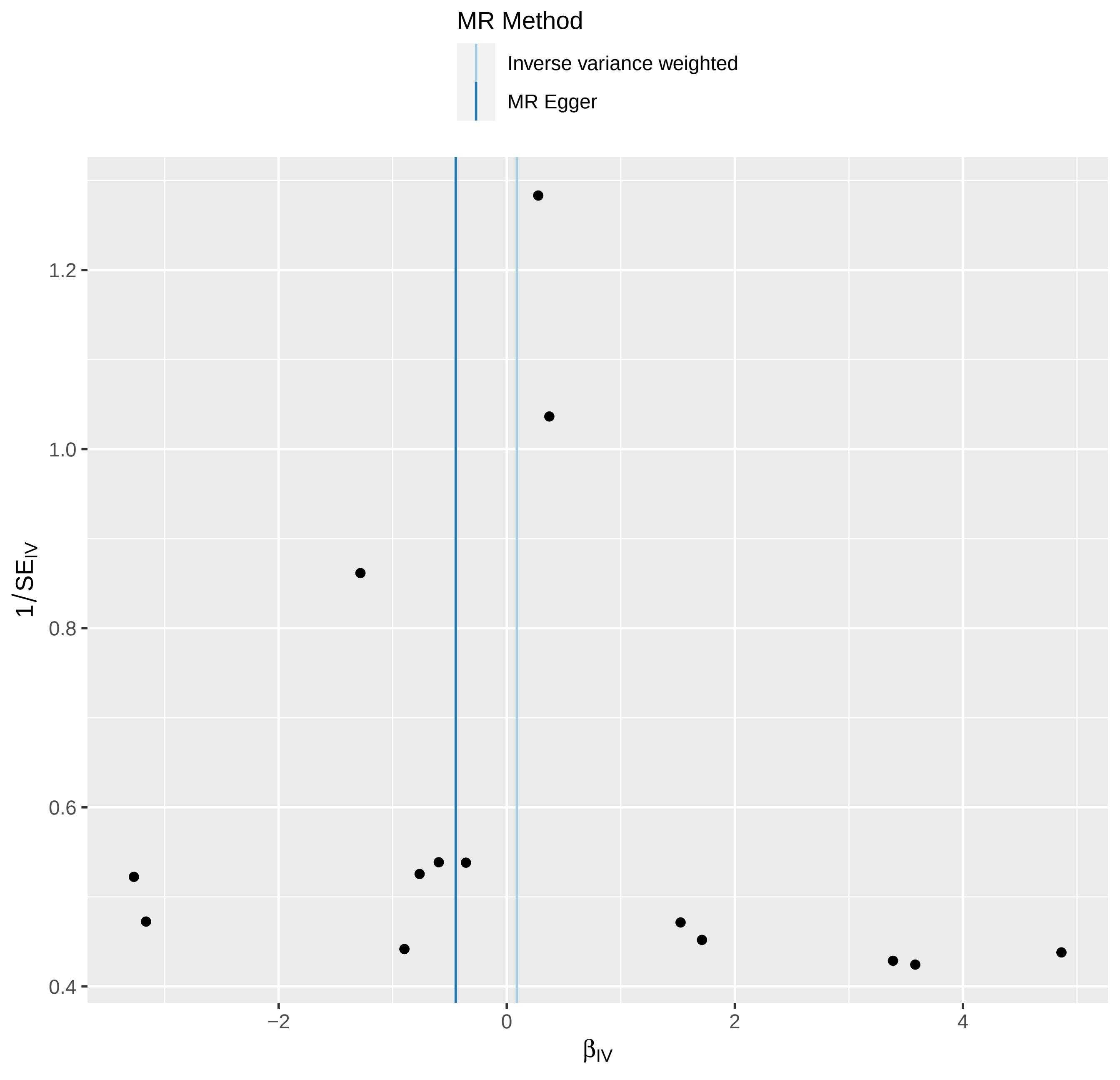

Batch 2257 : Gamma−glutamylglutamine on Ulcerative proctitis

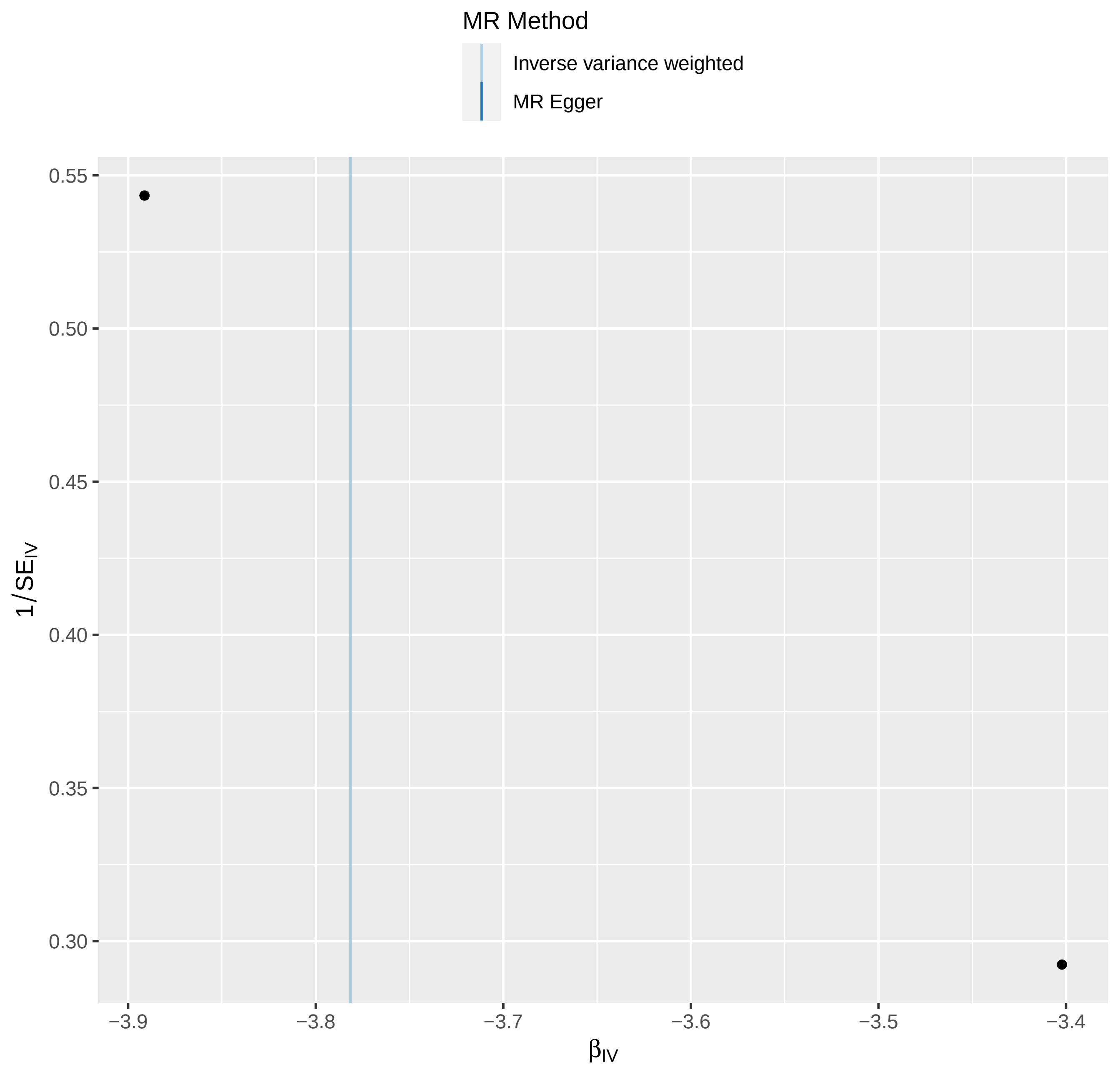

Batch 2258 : Gamma−glutamyltyrosine on Ulcerative proctitis

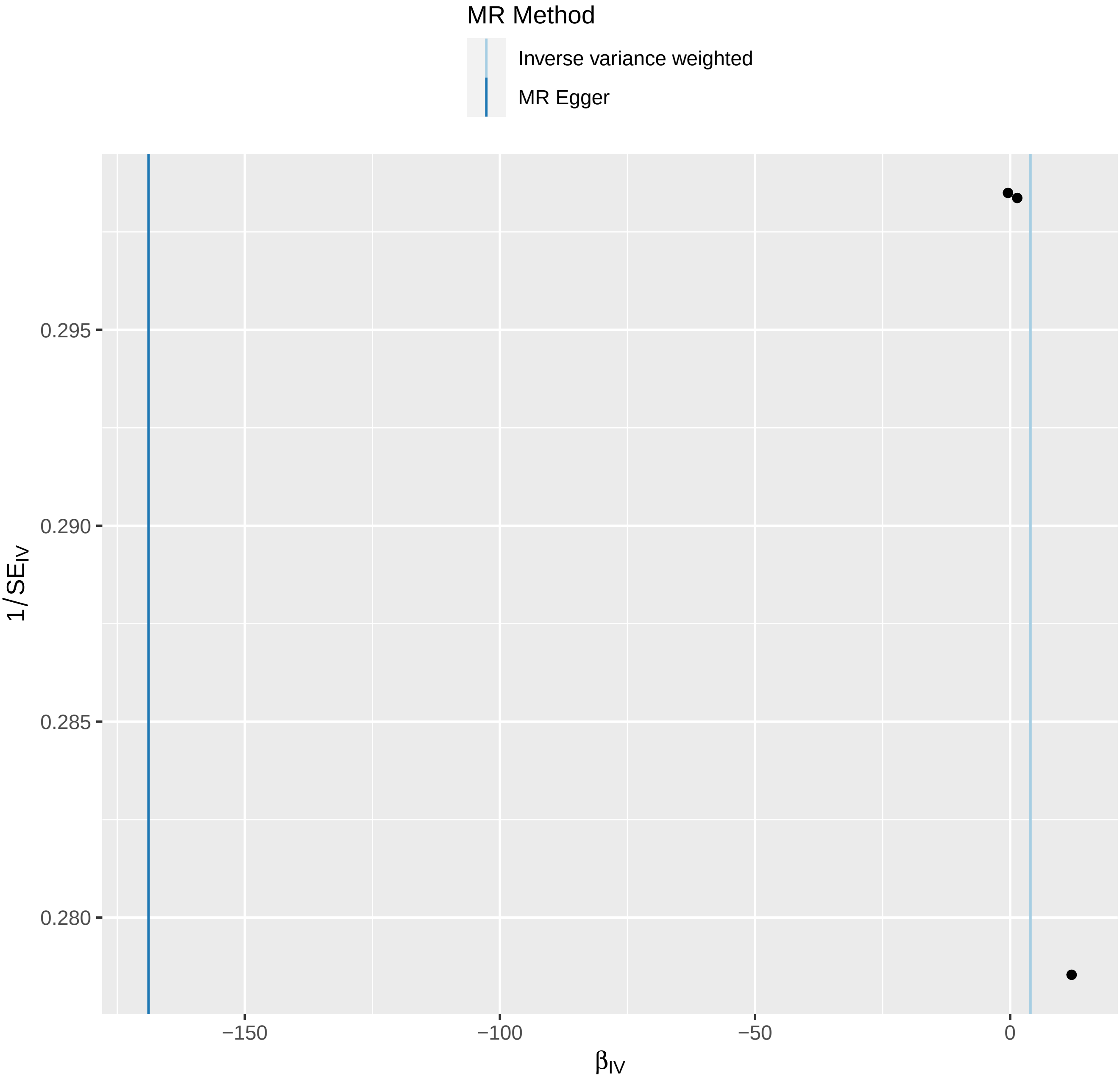

Batch 2260 : Betaine on Ulcerative proctitis

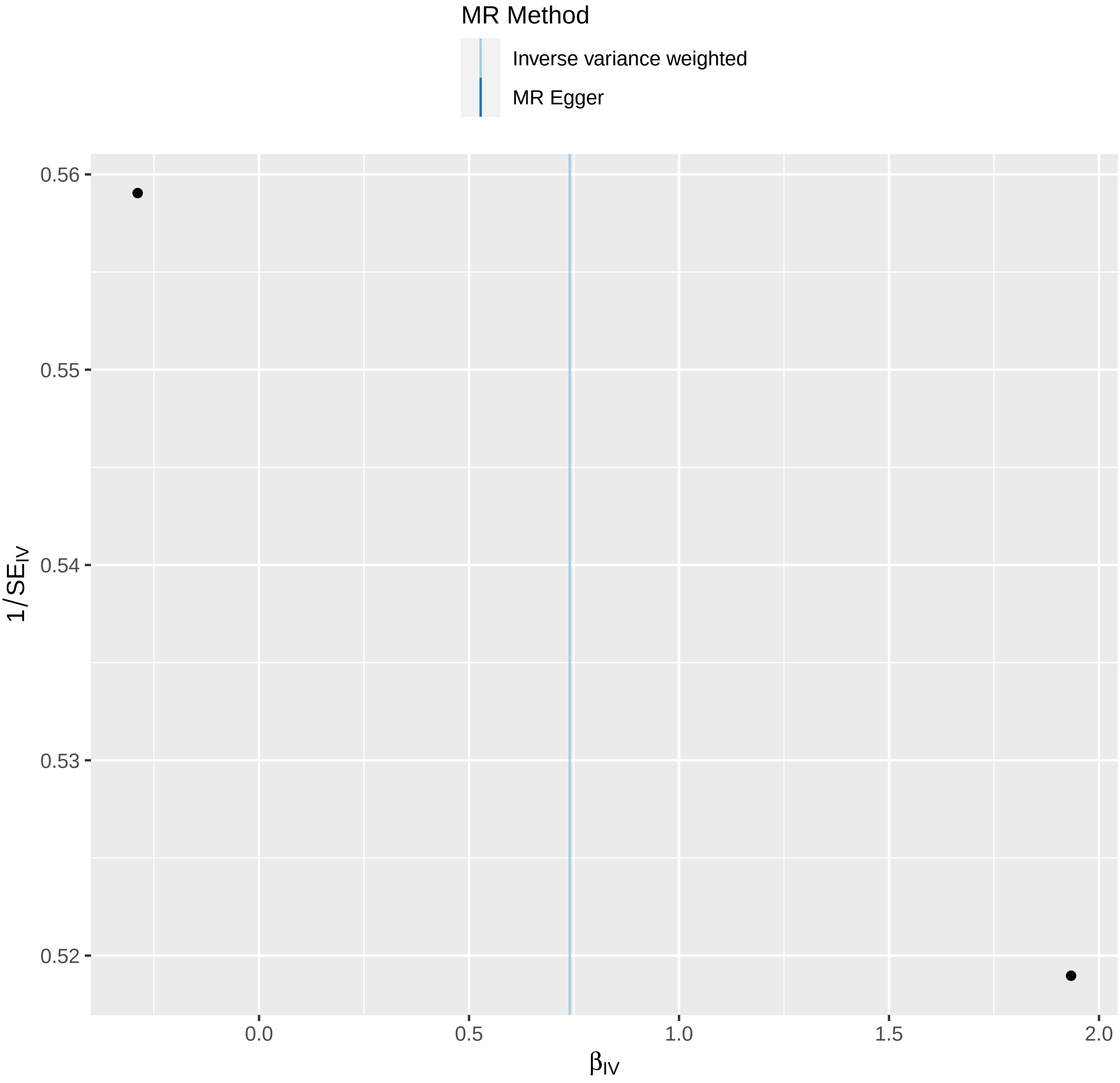

Batch 2261 : Xanthine on Ulcerative proctitis

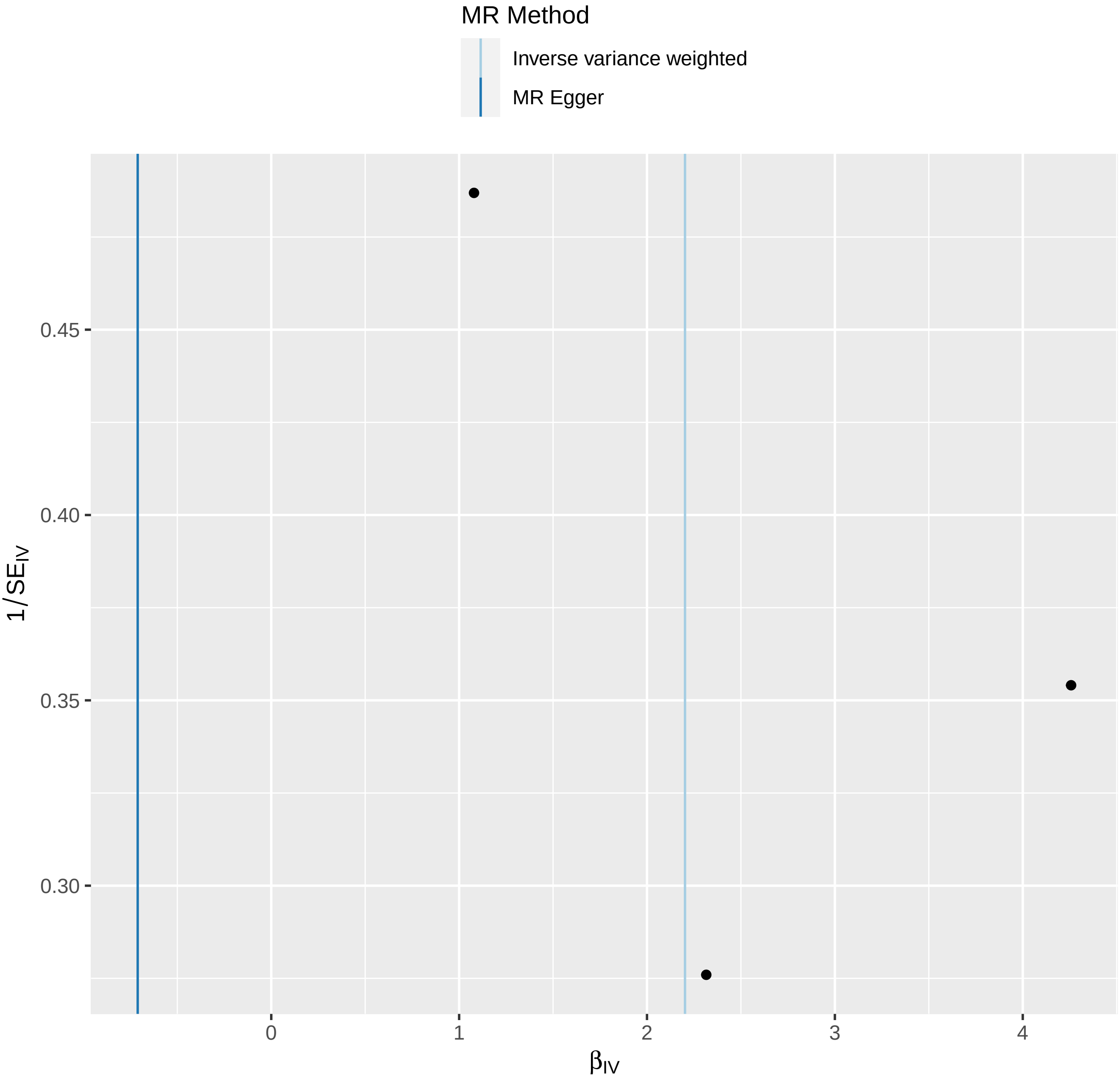

Batch 2262 : Phosphate on Ulcerative proctitis

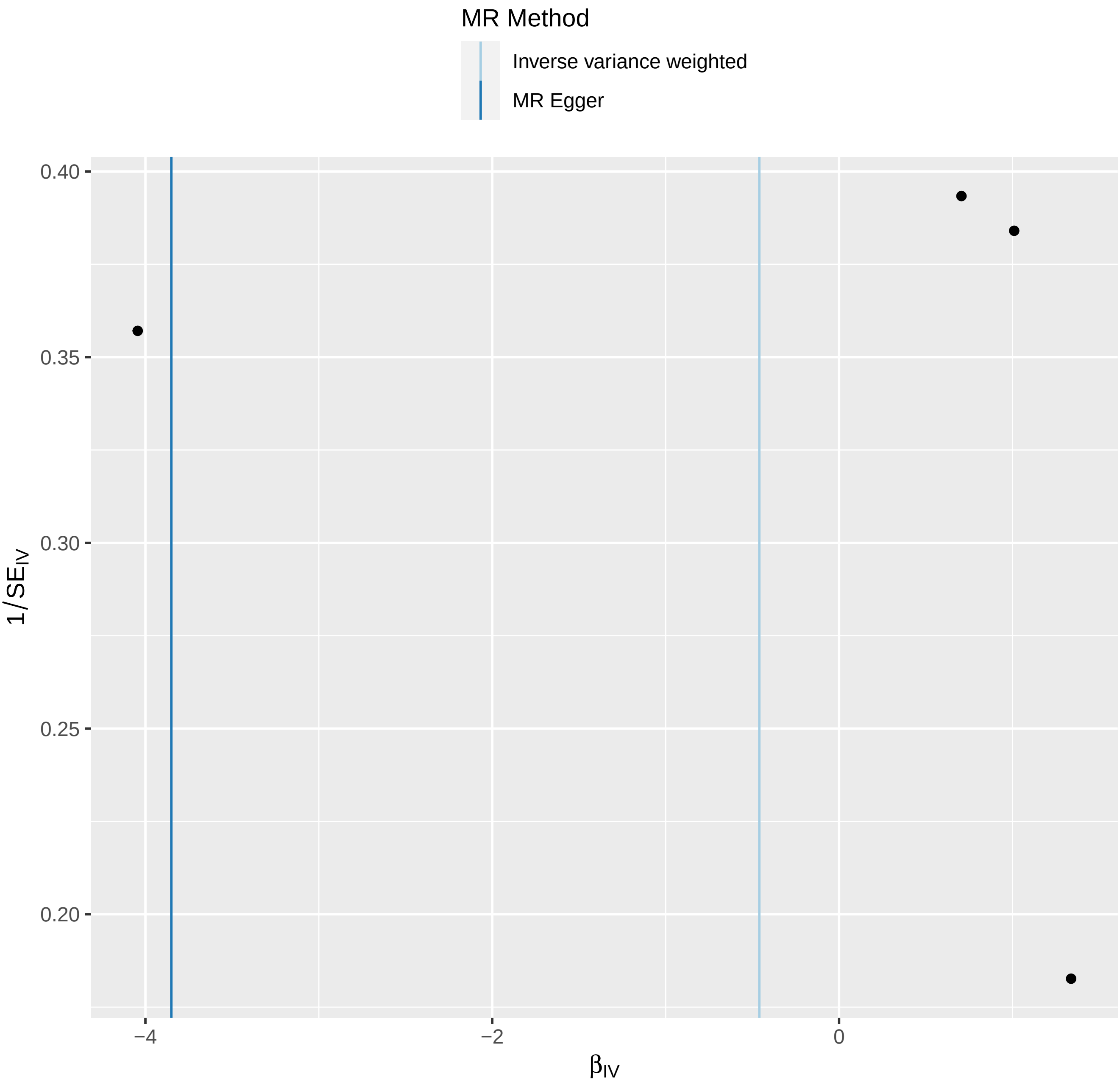

Batch 2263 : 3−methoxytyrosine on Ulcerative proctitis

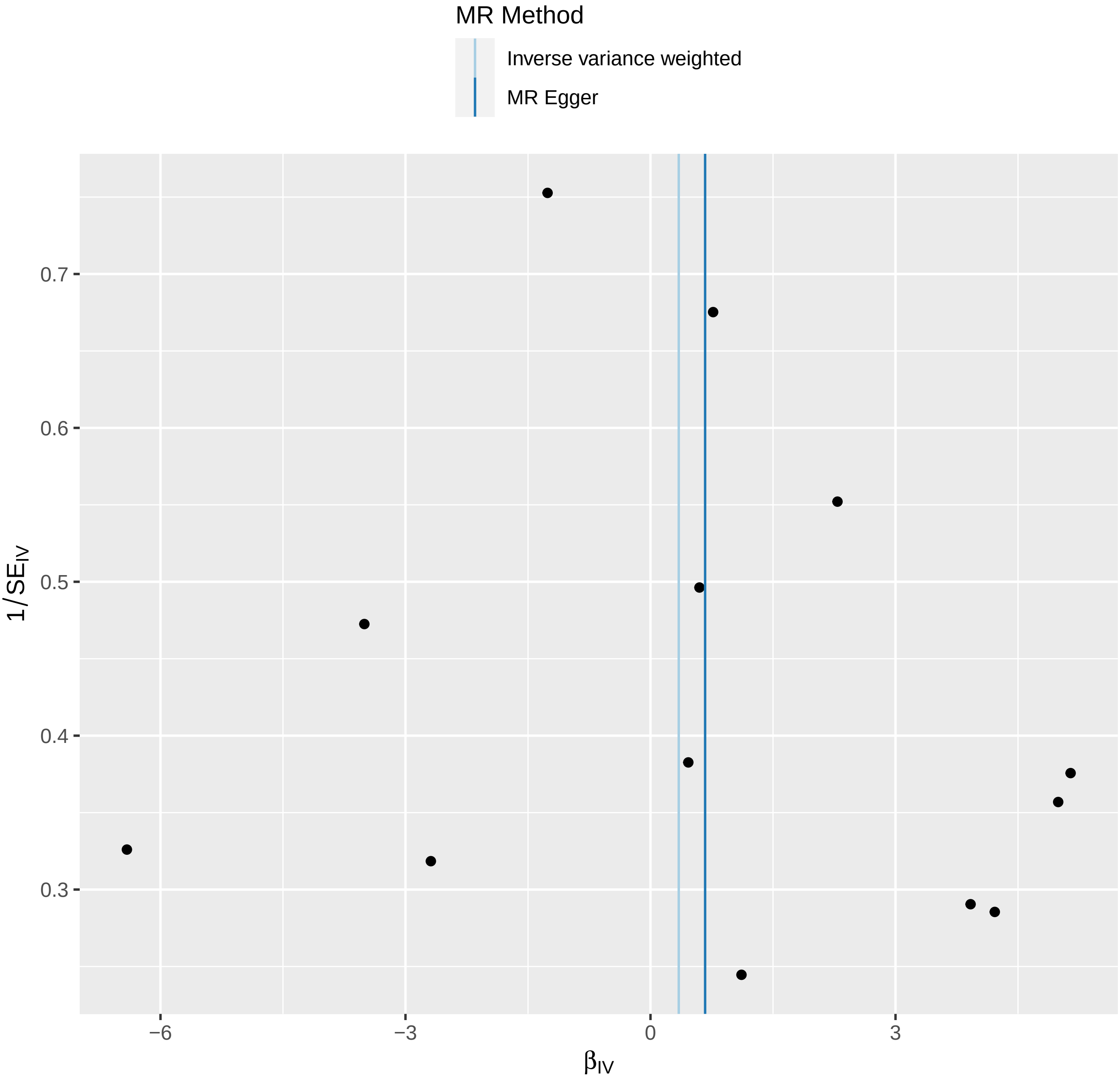

Batch 2265 : Undecanoate (11:0) on Ulcerative proctitis

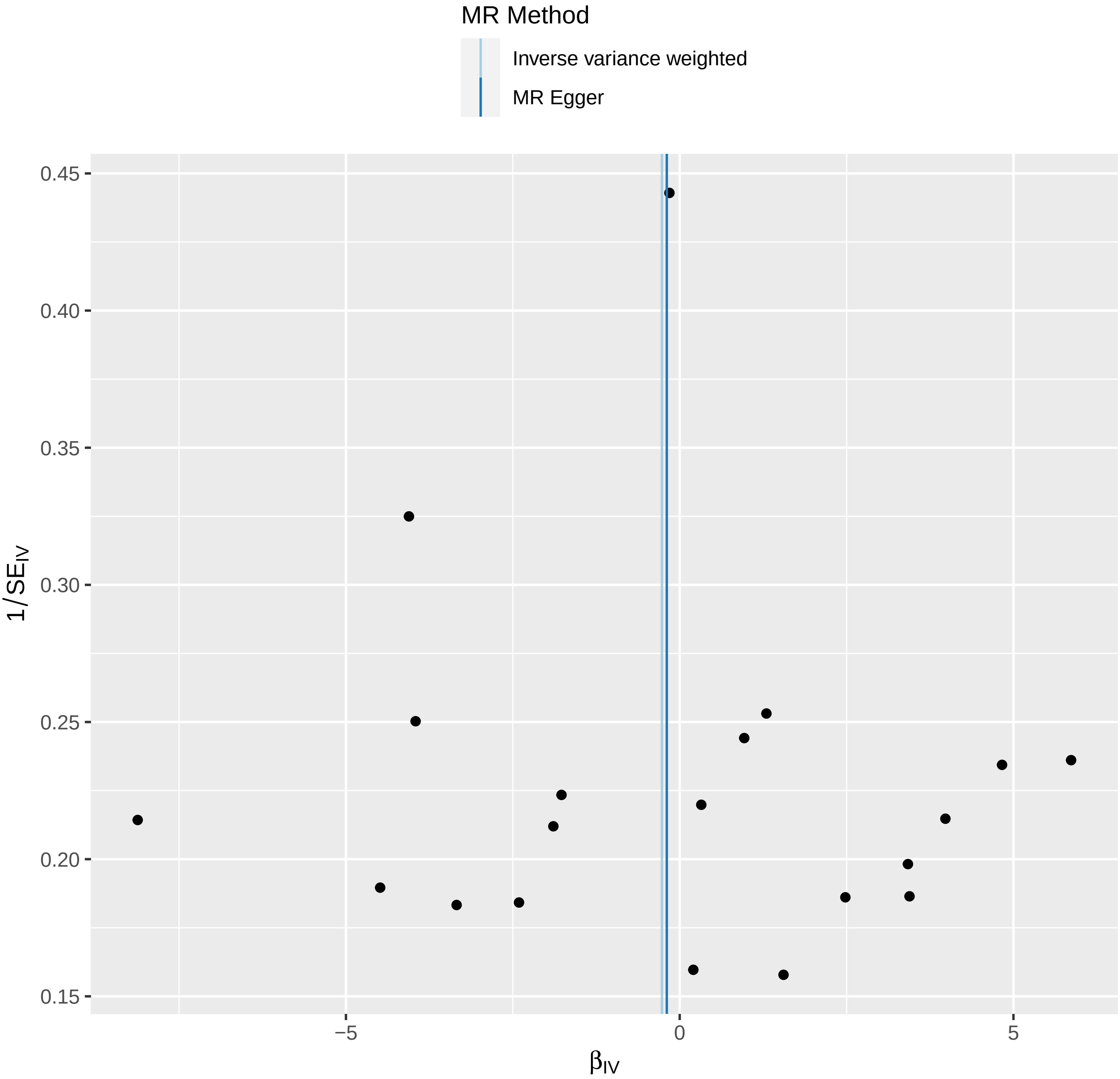

Batch 2267 : Taurodeoxycholate on Ulcerative proctitis

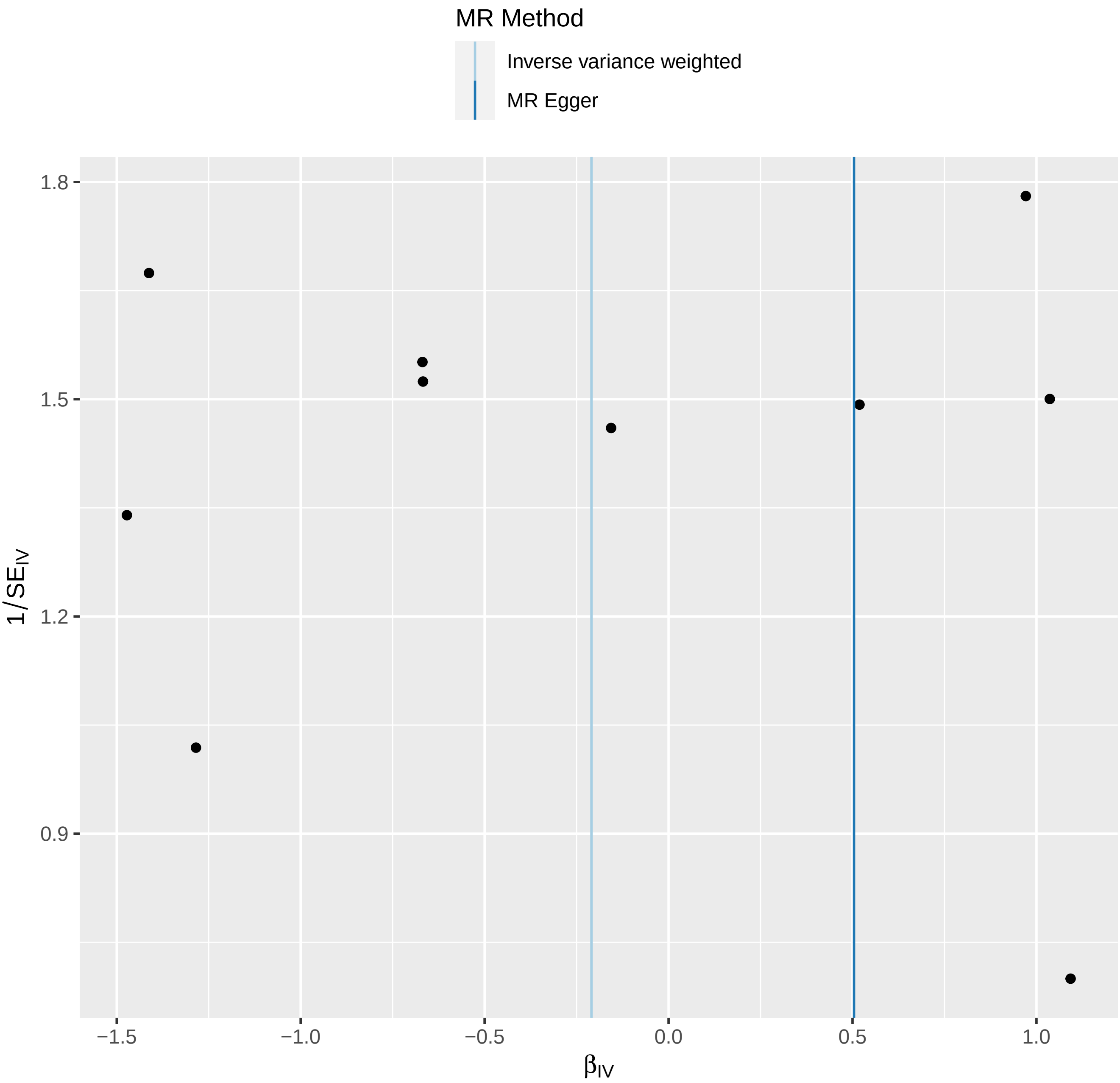

Batch 2268 : Glycerol on Ulcerative proctitis

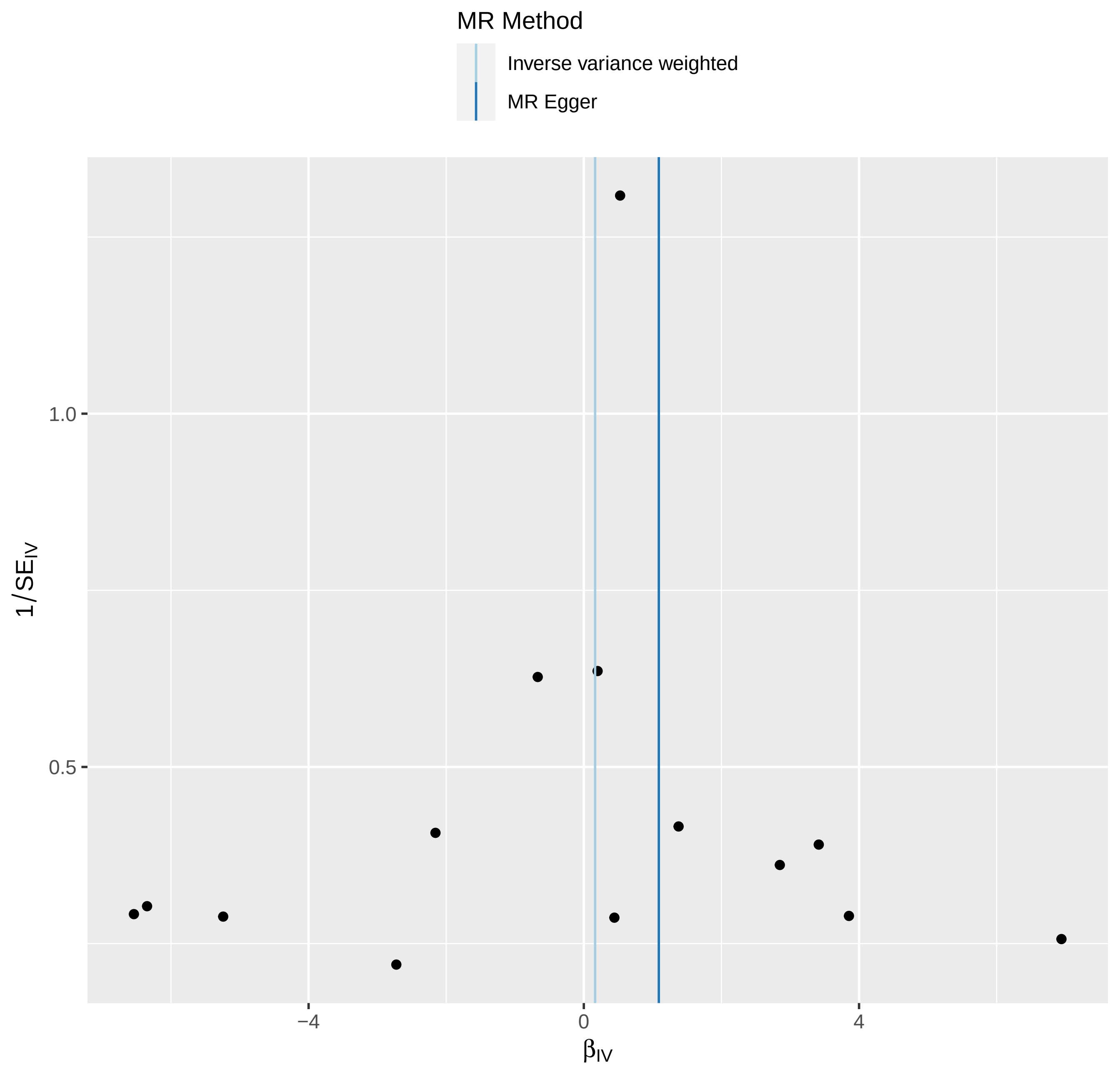

Batch 2269 : Kynurenine on Ulcerative proctitis

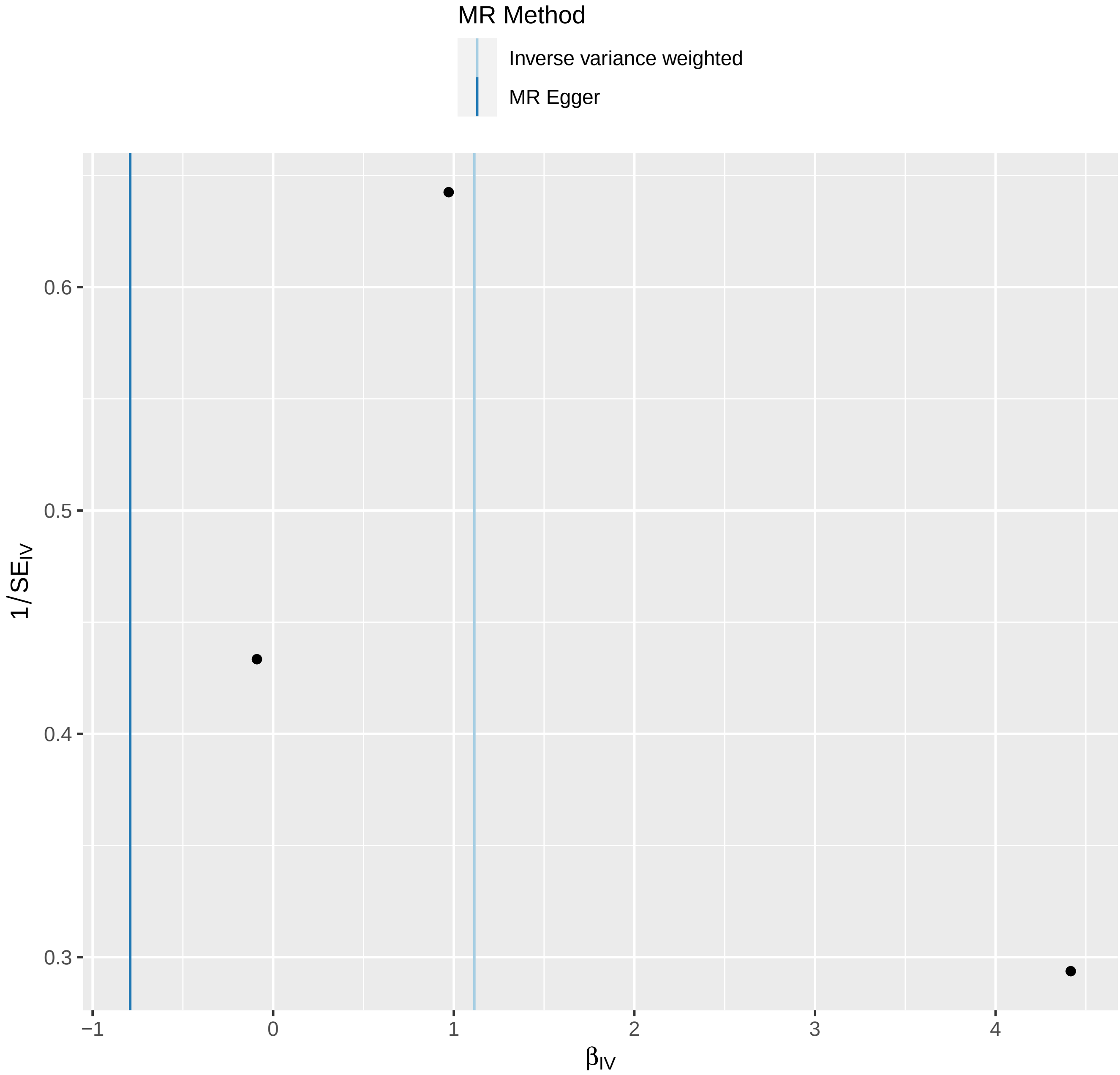

Batch 2270 : Mannitol on Ulcerative proctitis

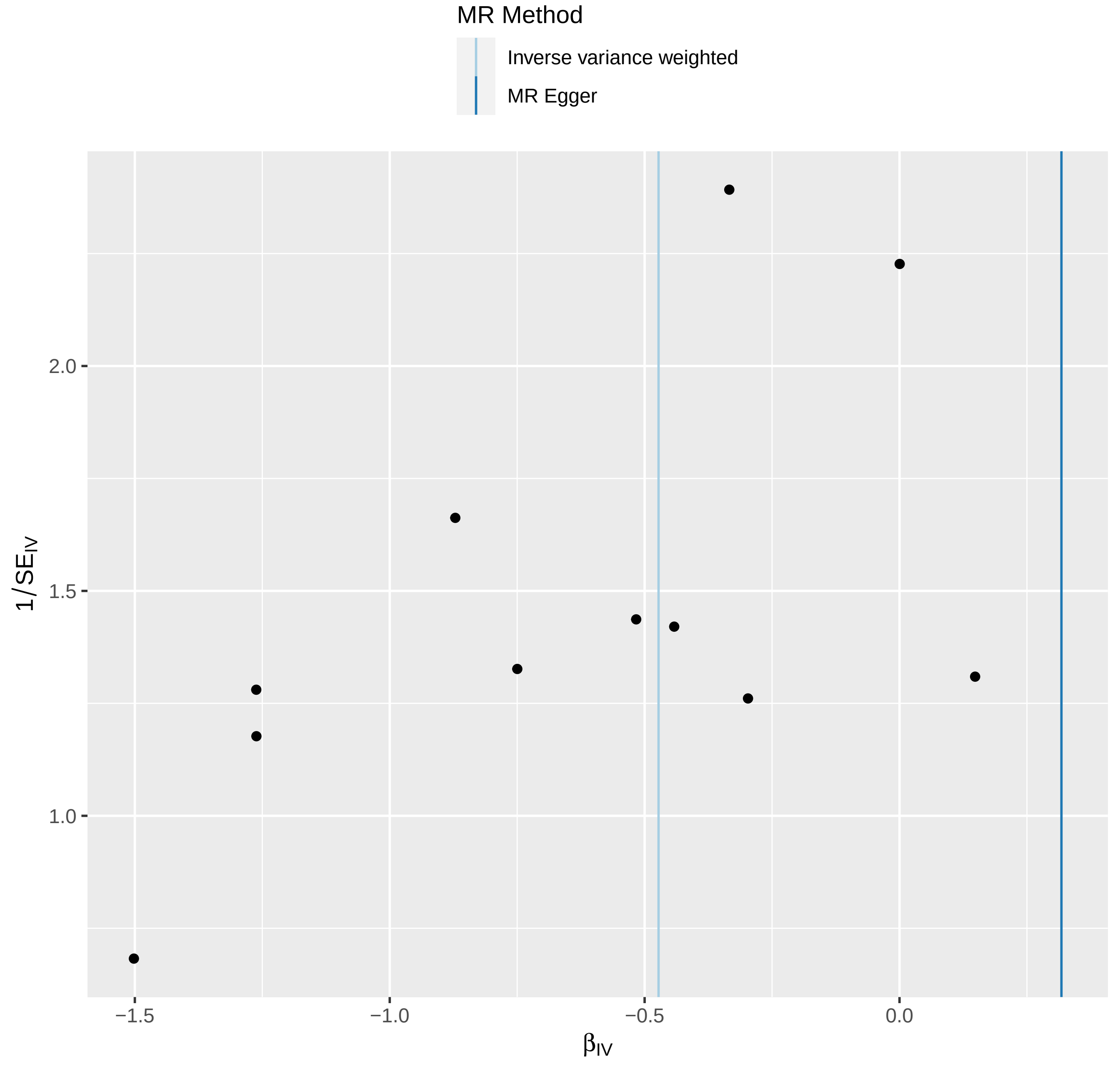

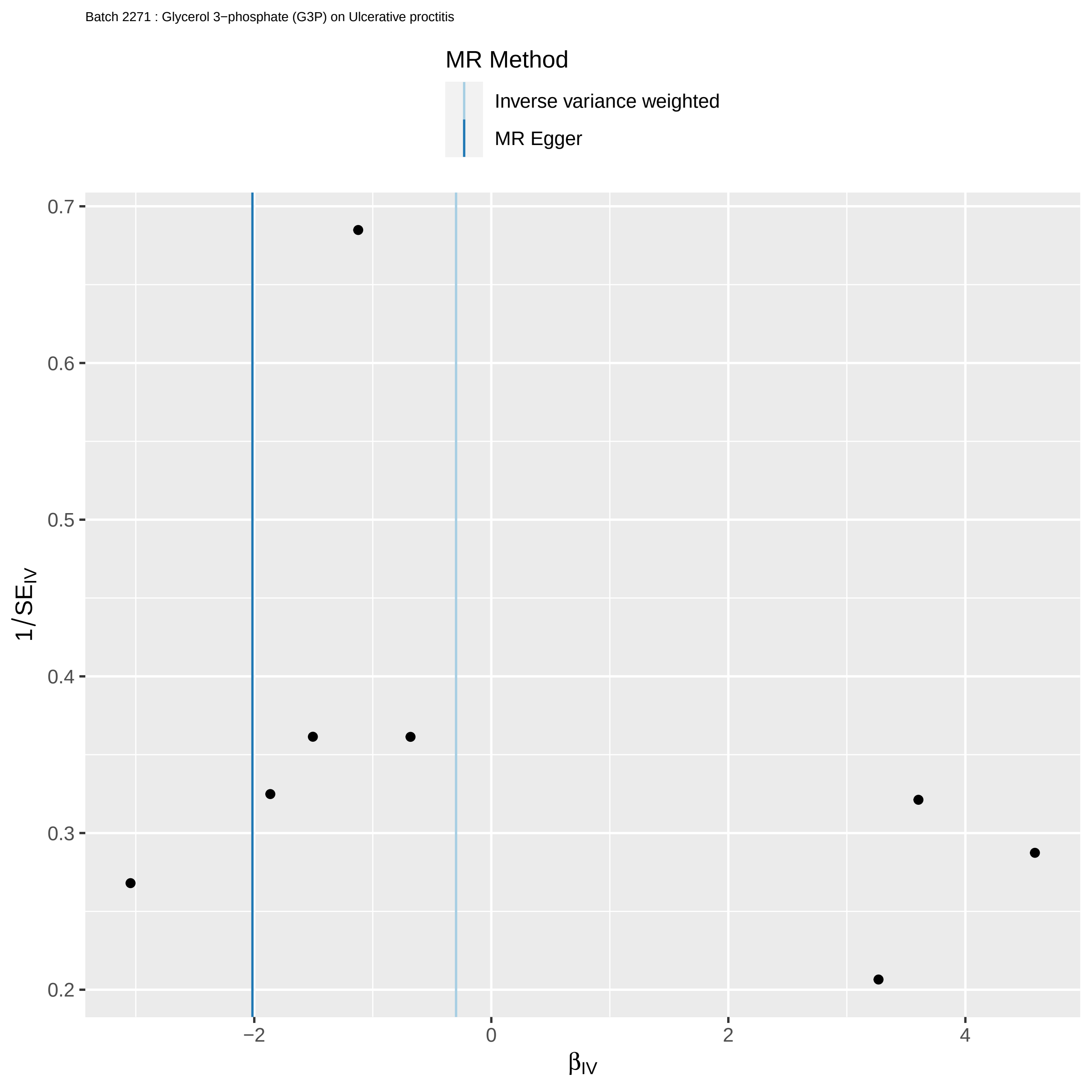
Batch 2271 : Glycerol 3−phosphate (G3P) on Ulcerative proctitis
MR Method
Inverse variance weighted
MR Egger
0.7
0.6
0.5
0.4
0.3
0.2
1/SE$_{IV}$
−2
0
2
4
β$_{IV}$

Batch 2273 : Carnitine on Ulcerative proctitis

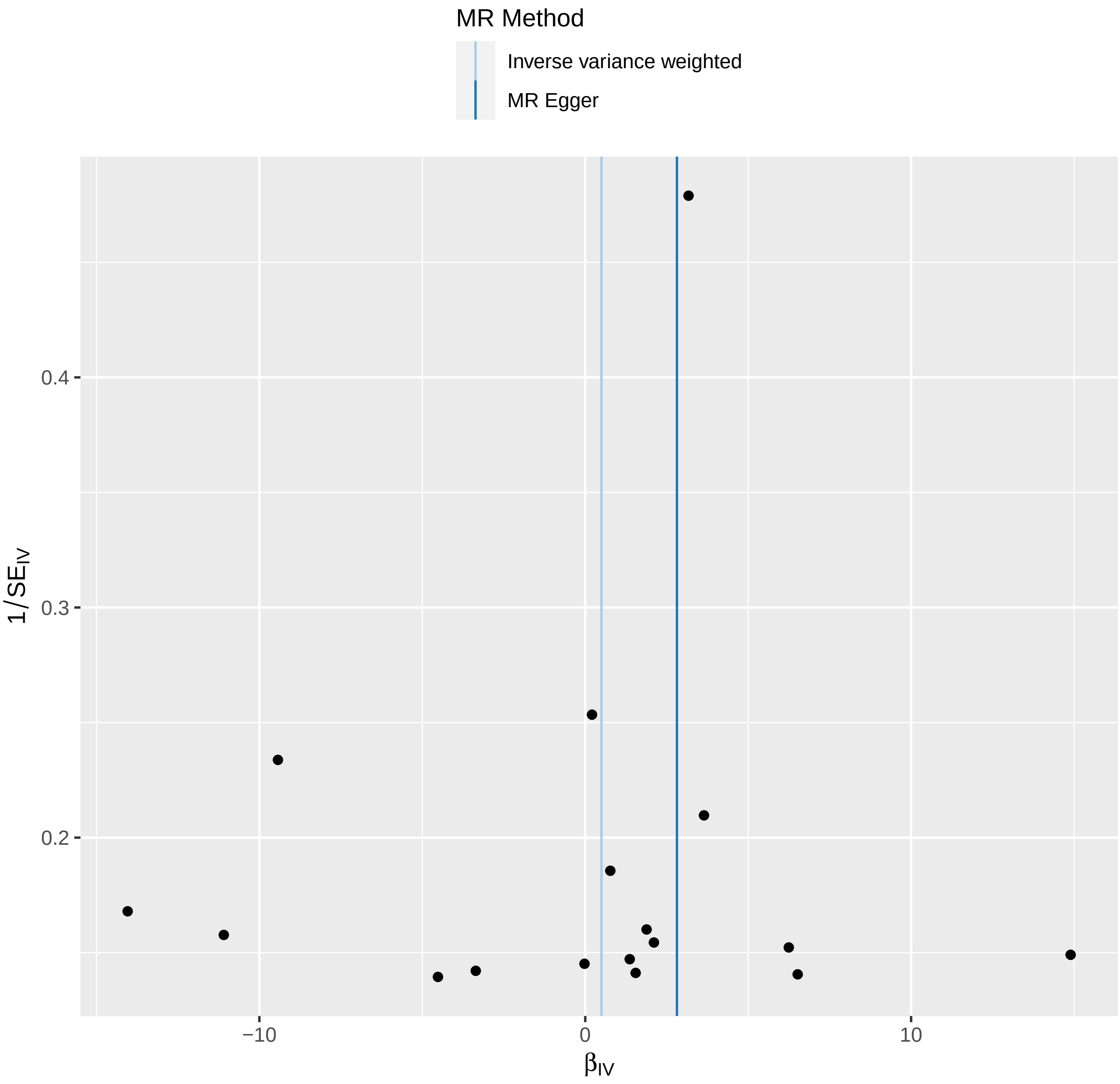

Batch 2274 : Choline on Ulcerative proctitis

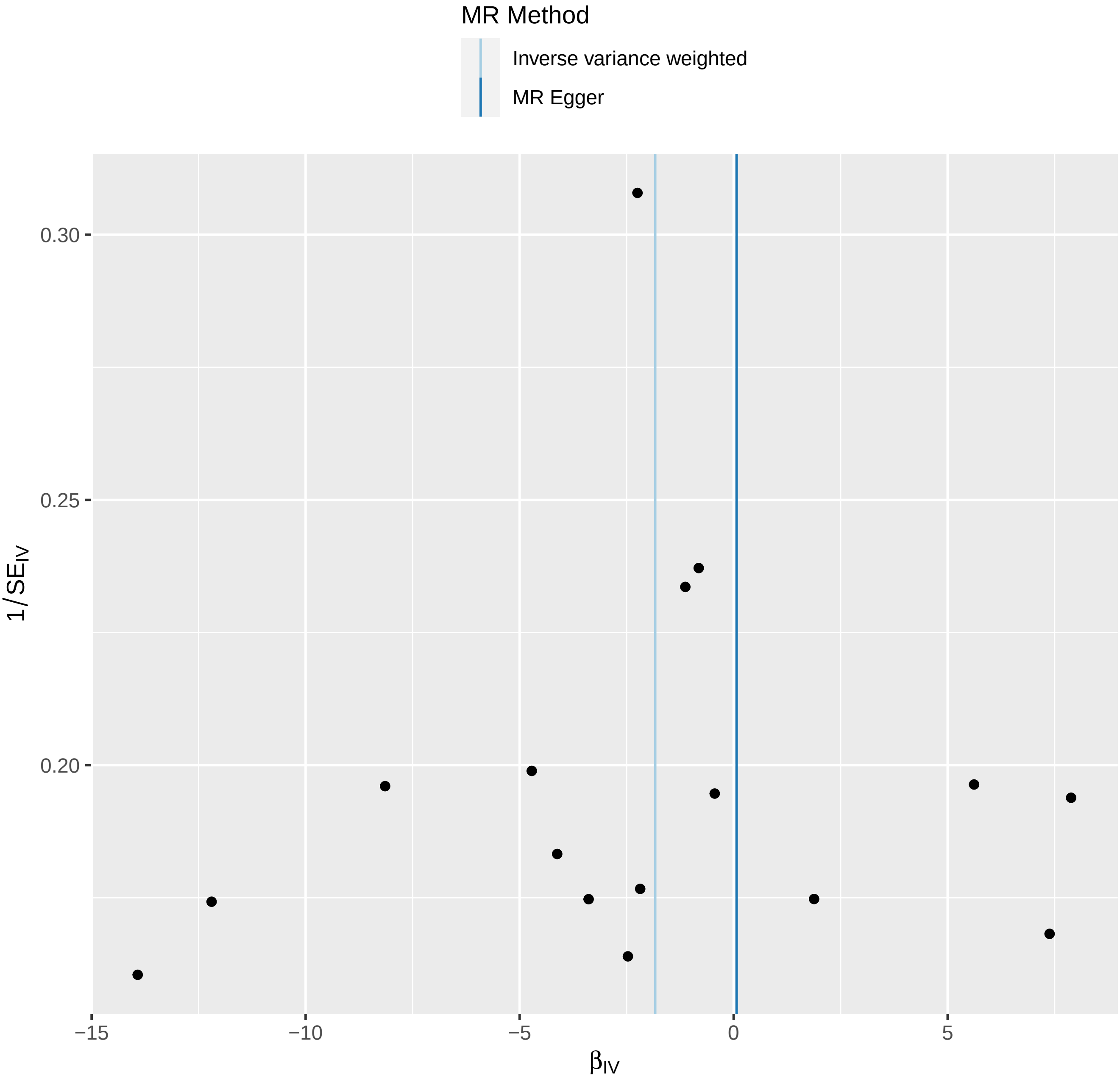

Batch 2277 : 3−methyl−2−oxovalerate on Ulcerative proctitis

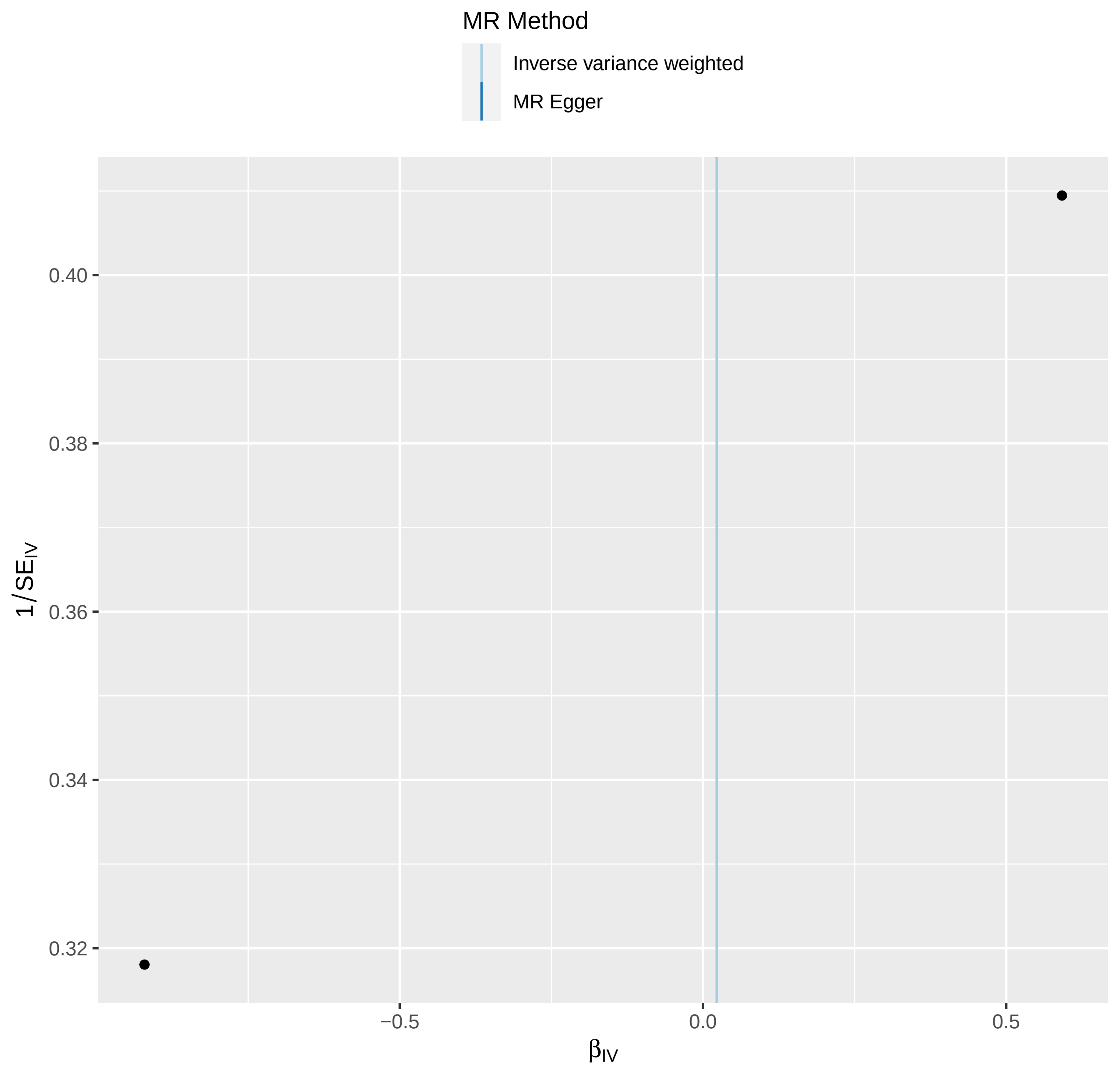

Batch 2278 : 3−methylhistidine on Ulcerative proctitis

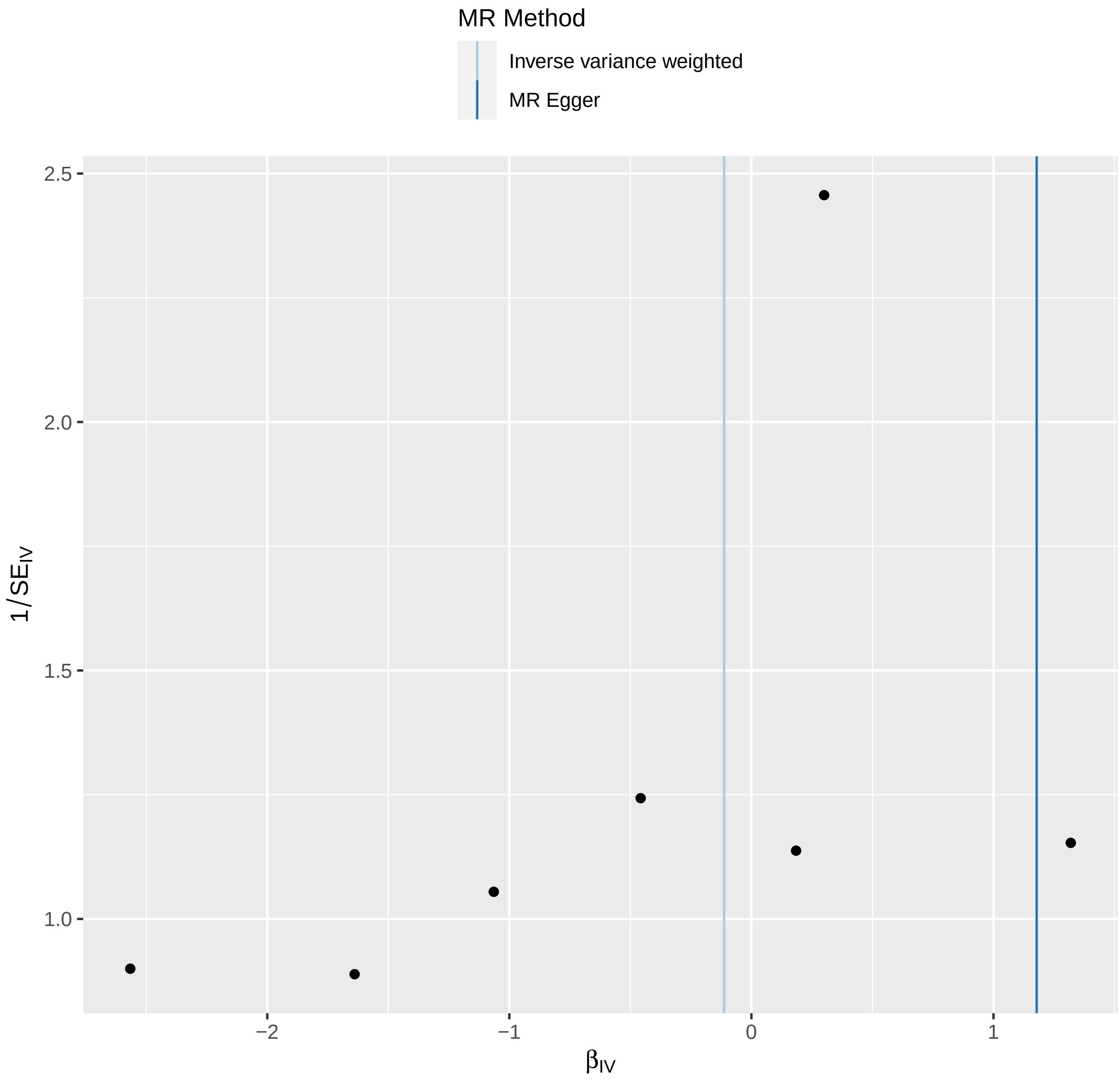

Batch 2280 : Phenylacetate on Ulcerative proctitis

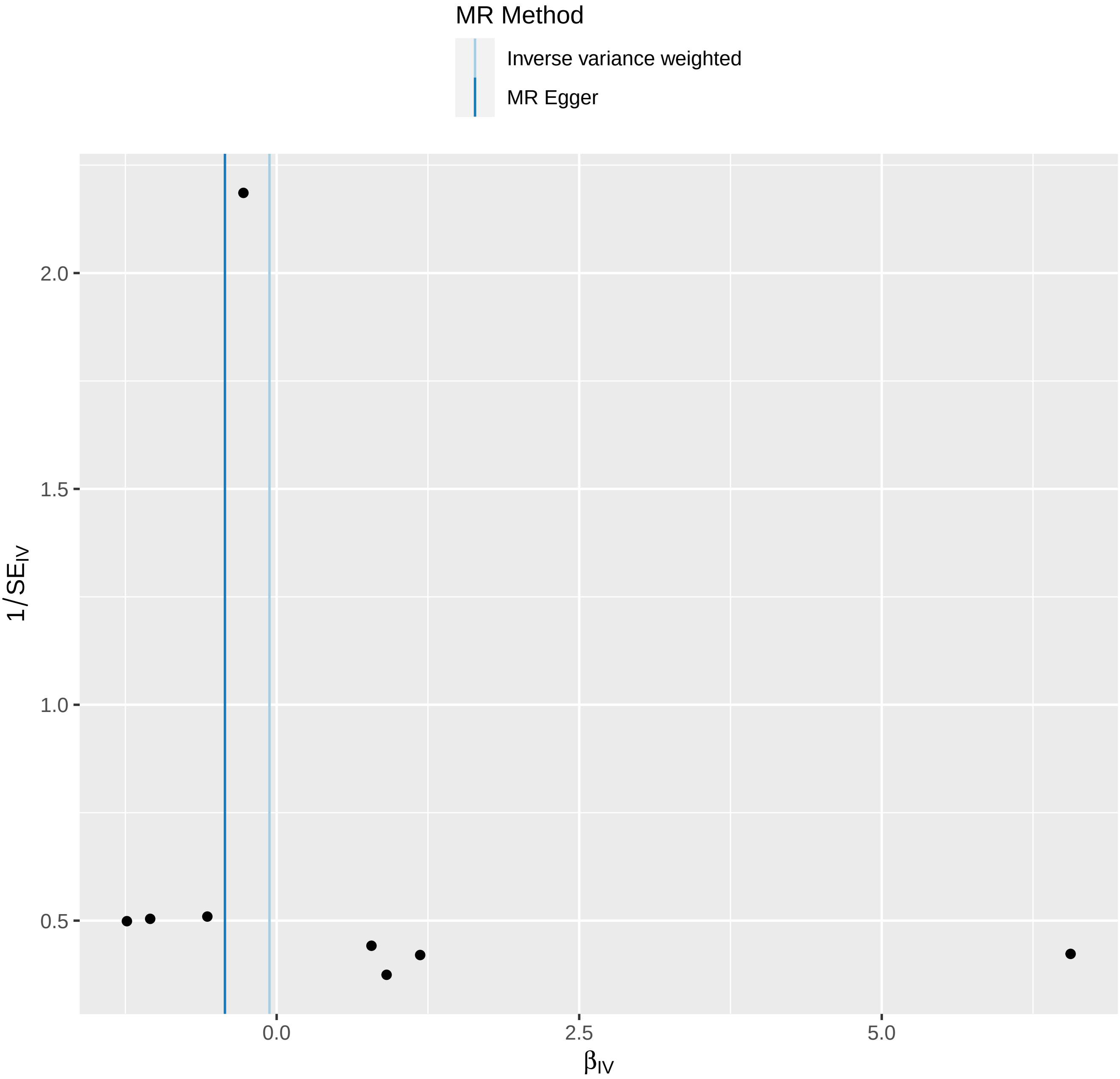

Batch 2281 : Glycerophosphorylcholine (GPC) on Ulcerative proctitis

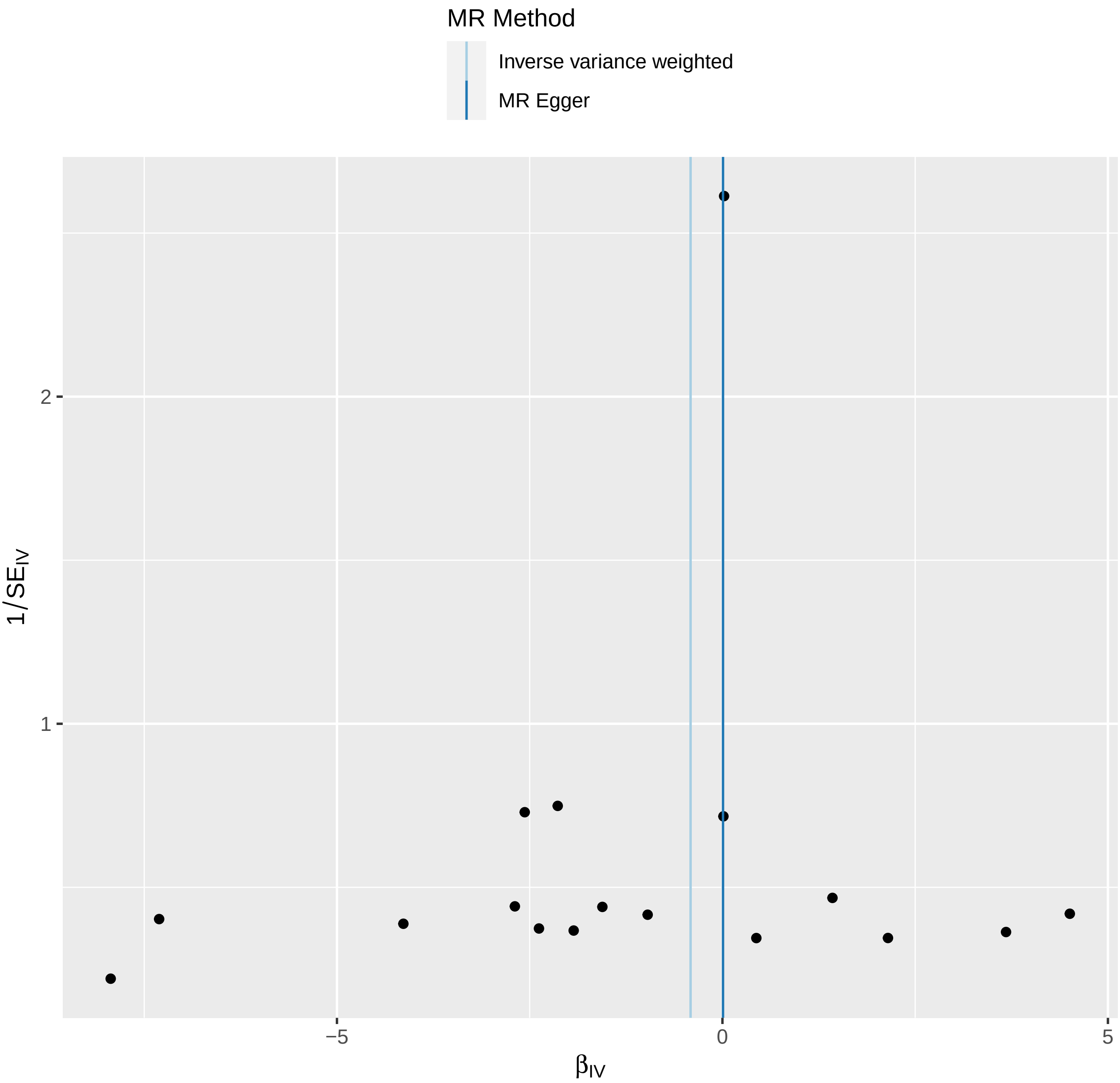

Batch 2282 : Aspartate on Ulcerative proctitis

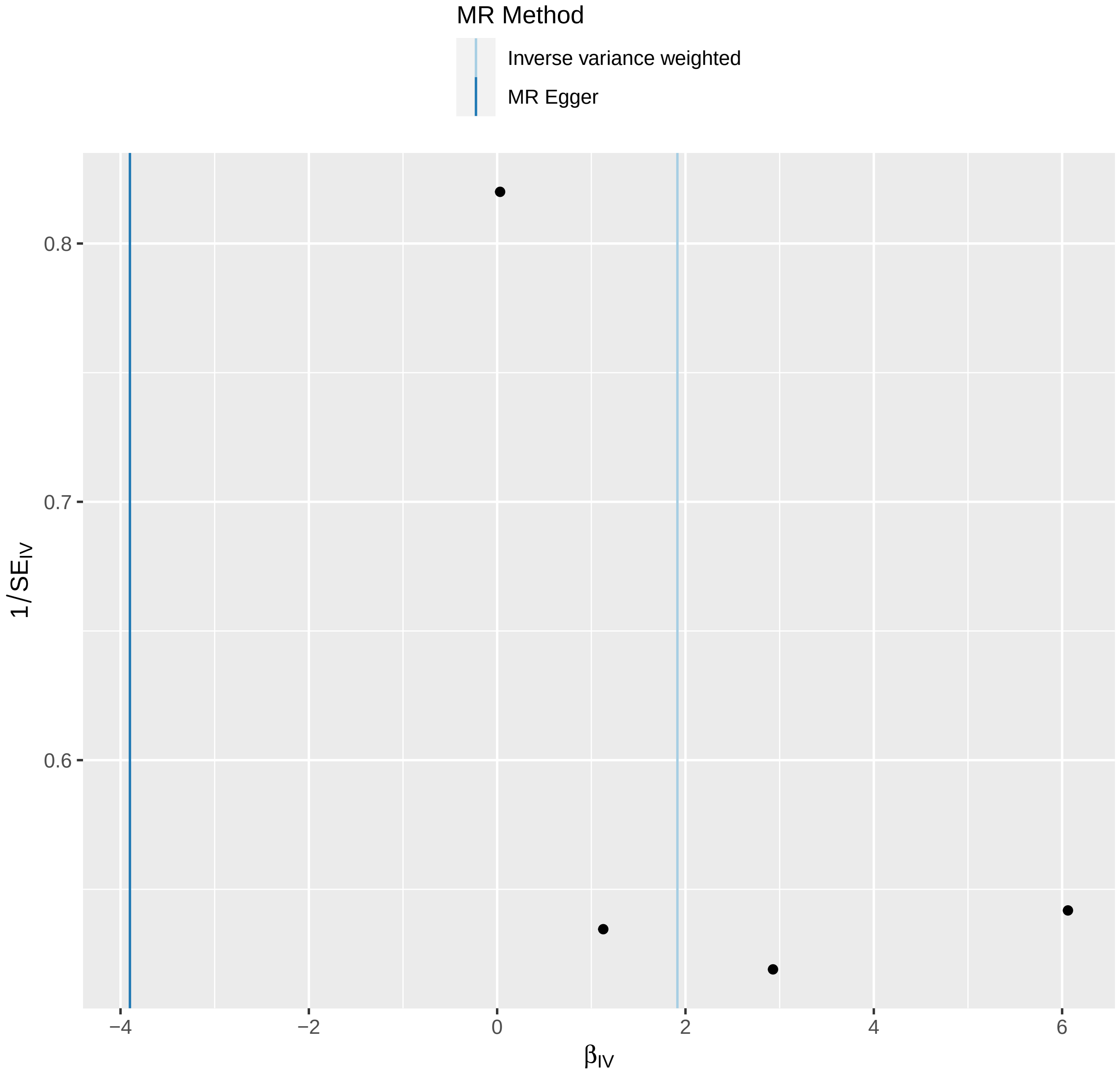

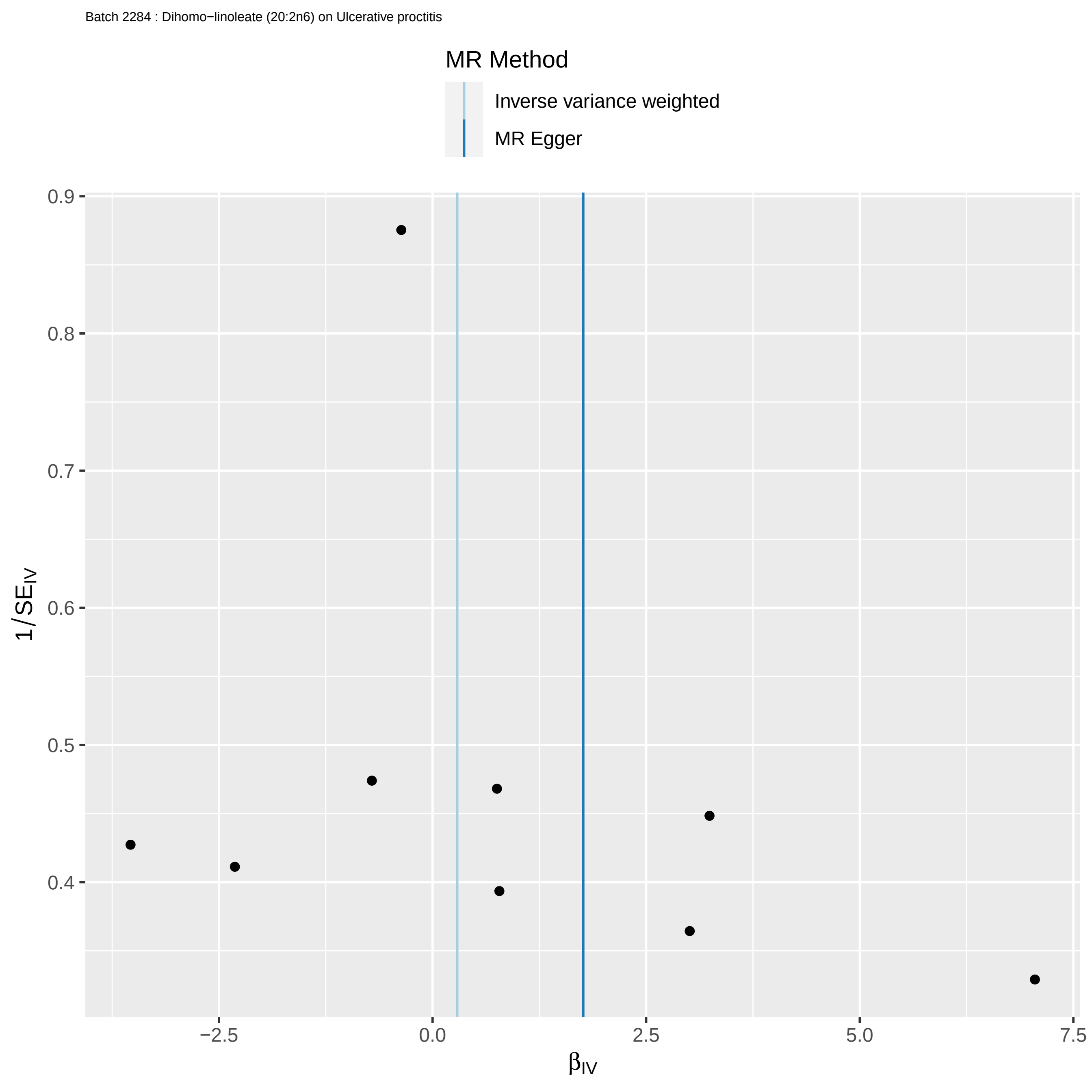

Batch 2284 : Dihomo−linoleate (20:2n6) on Ulcerative proctitis
MR Method
Inverse variance weighted
MR Egger
0.9
0.8
0.7
0.6
0.5
0.4
1/SE_IV
−2.5
0.0
2.5
5.0
7.5
β_IV

Batch 2285 : 2−hydroxystearate on Ulcerative proctitis

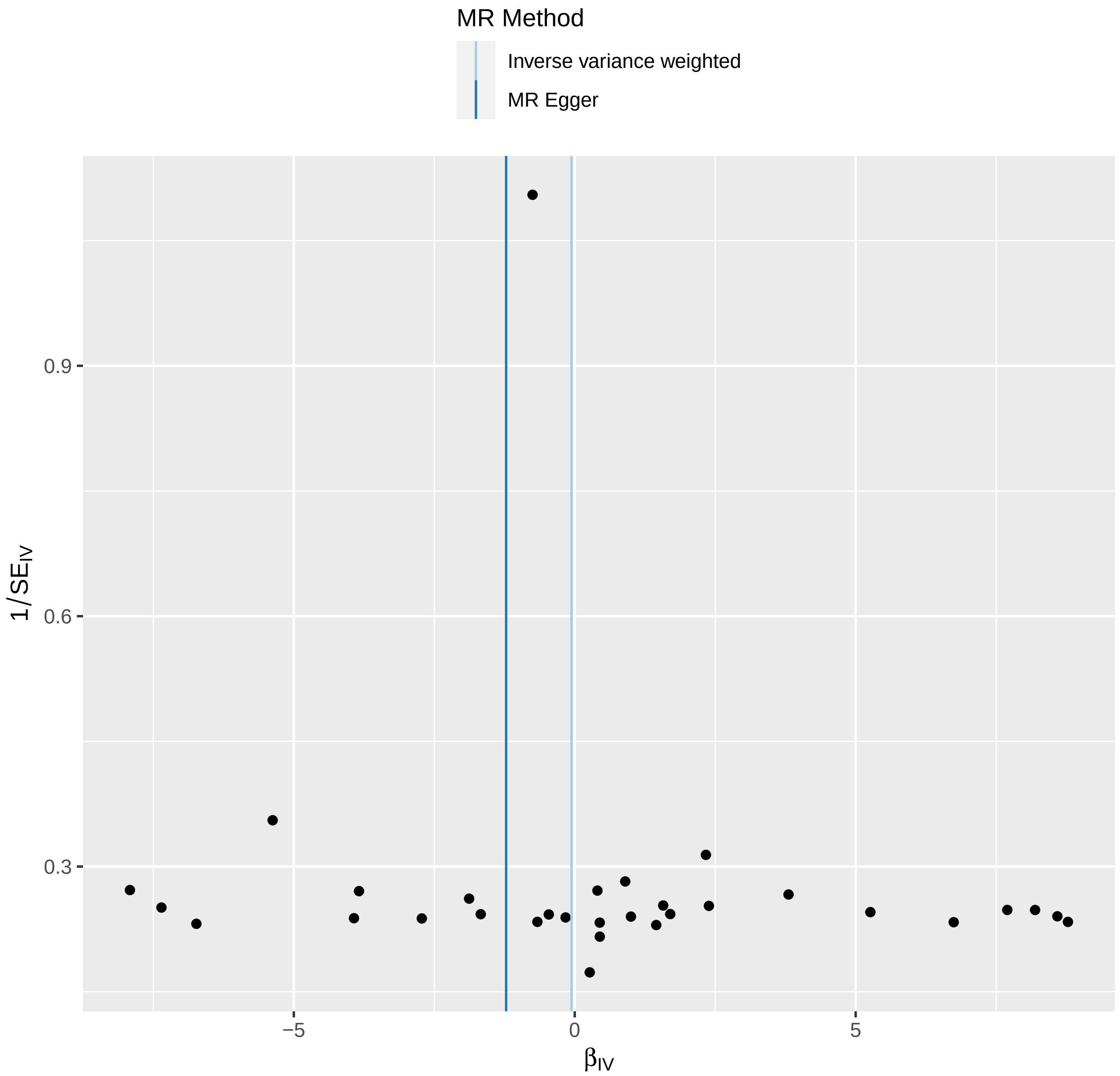

Batch 2287 : Glycylvaline on Ulcerative proctitis

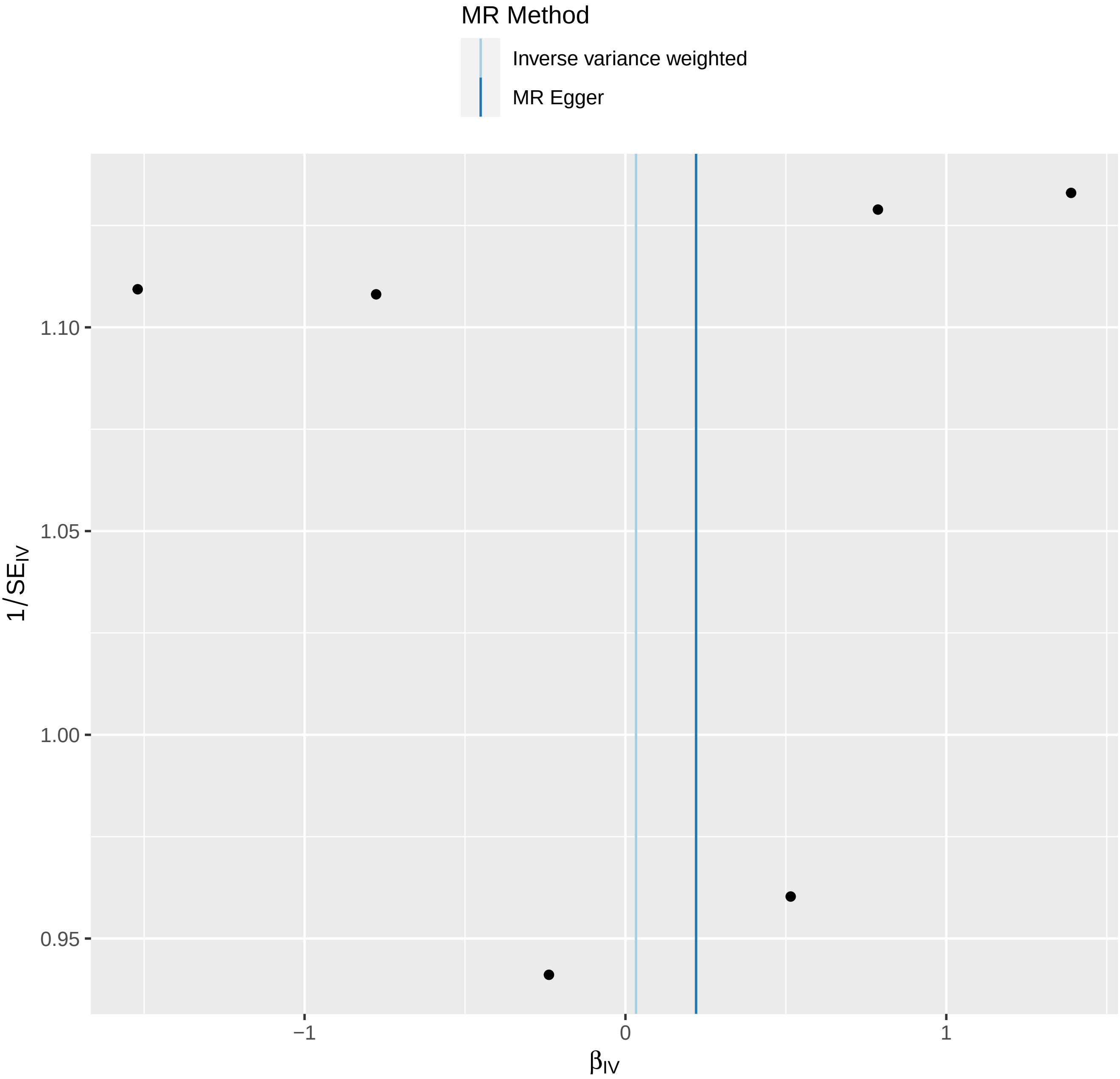

Batch 2288 : Gamma−glutamylleucine on Ulcerative proctitis

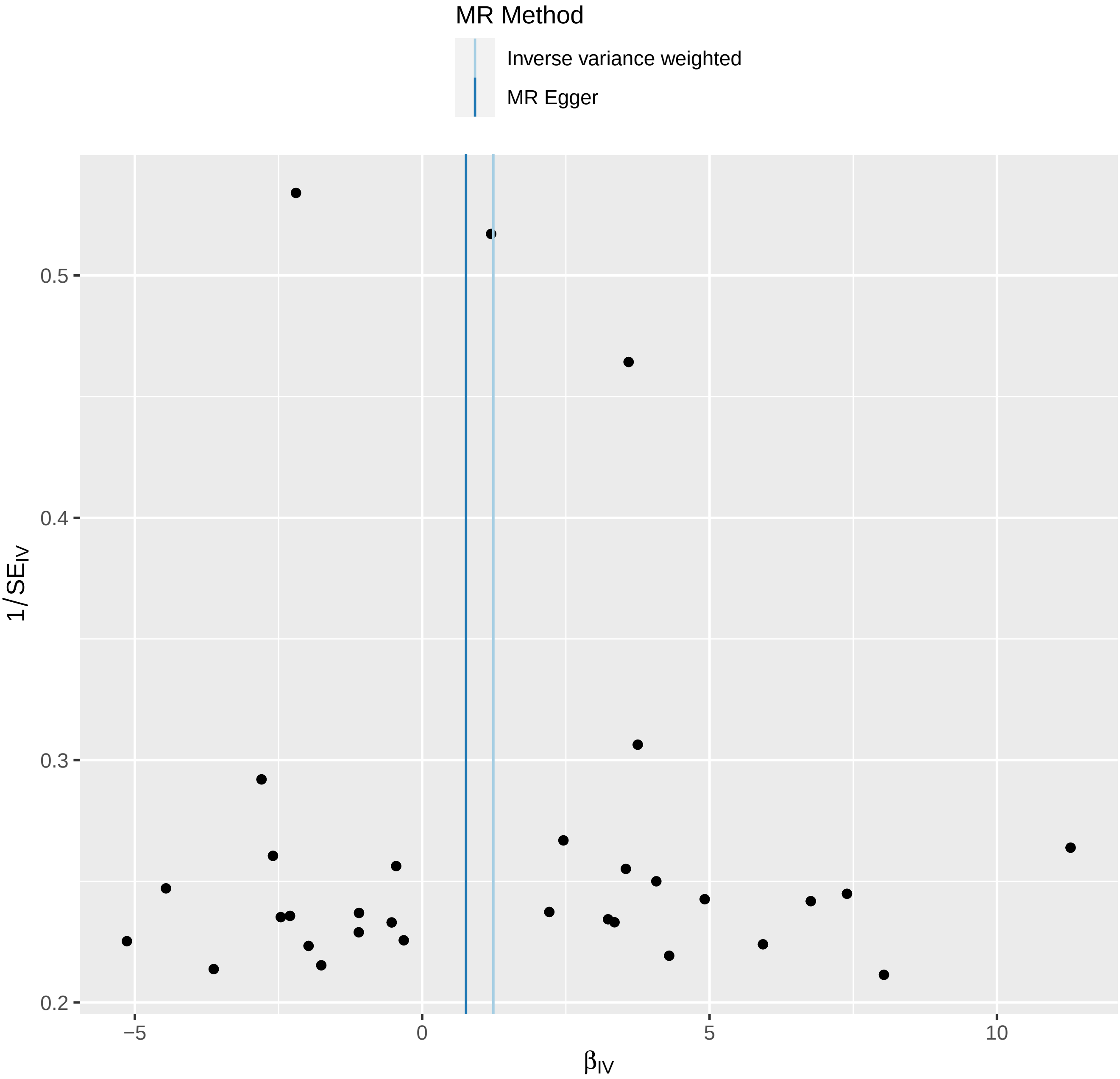

Batch 2291 : Glycocholate on Ulcerative proctitis

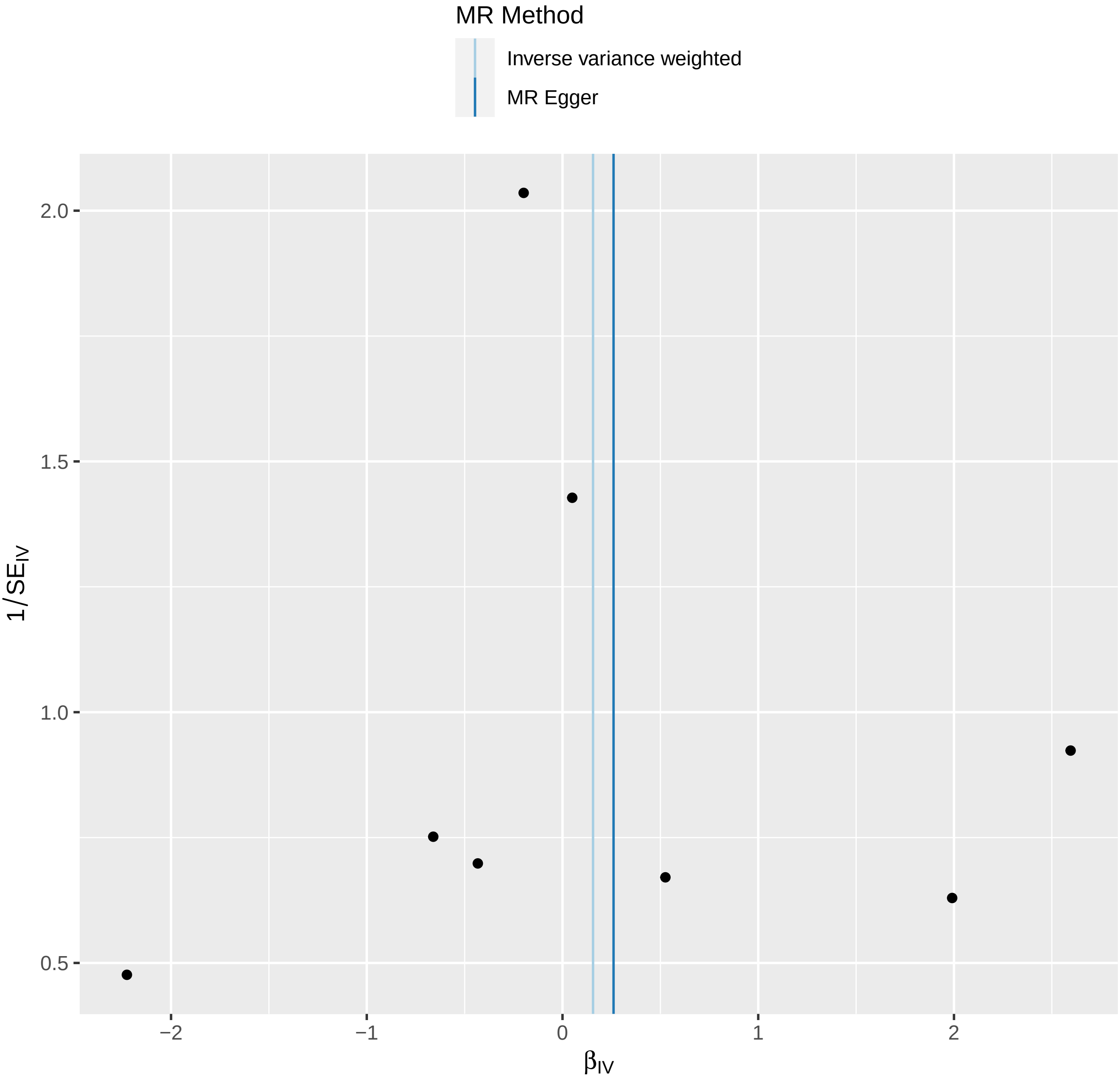

Batch 2292 : Glycodeoxycholate on Ulcerative proctitis

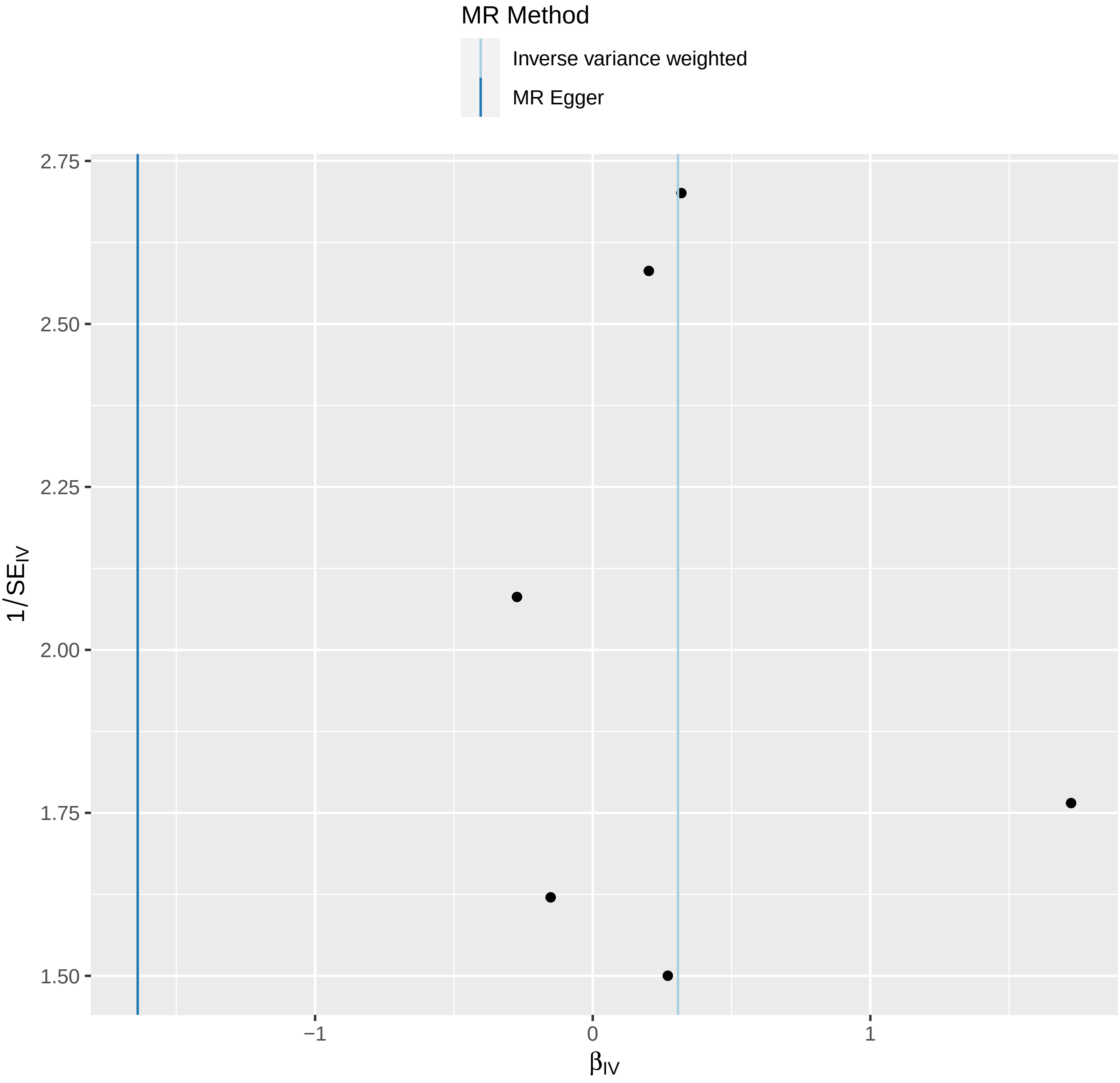

Batch 2293 : Taurochenodeoxycholate on Ulcerative proctitis

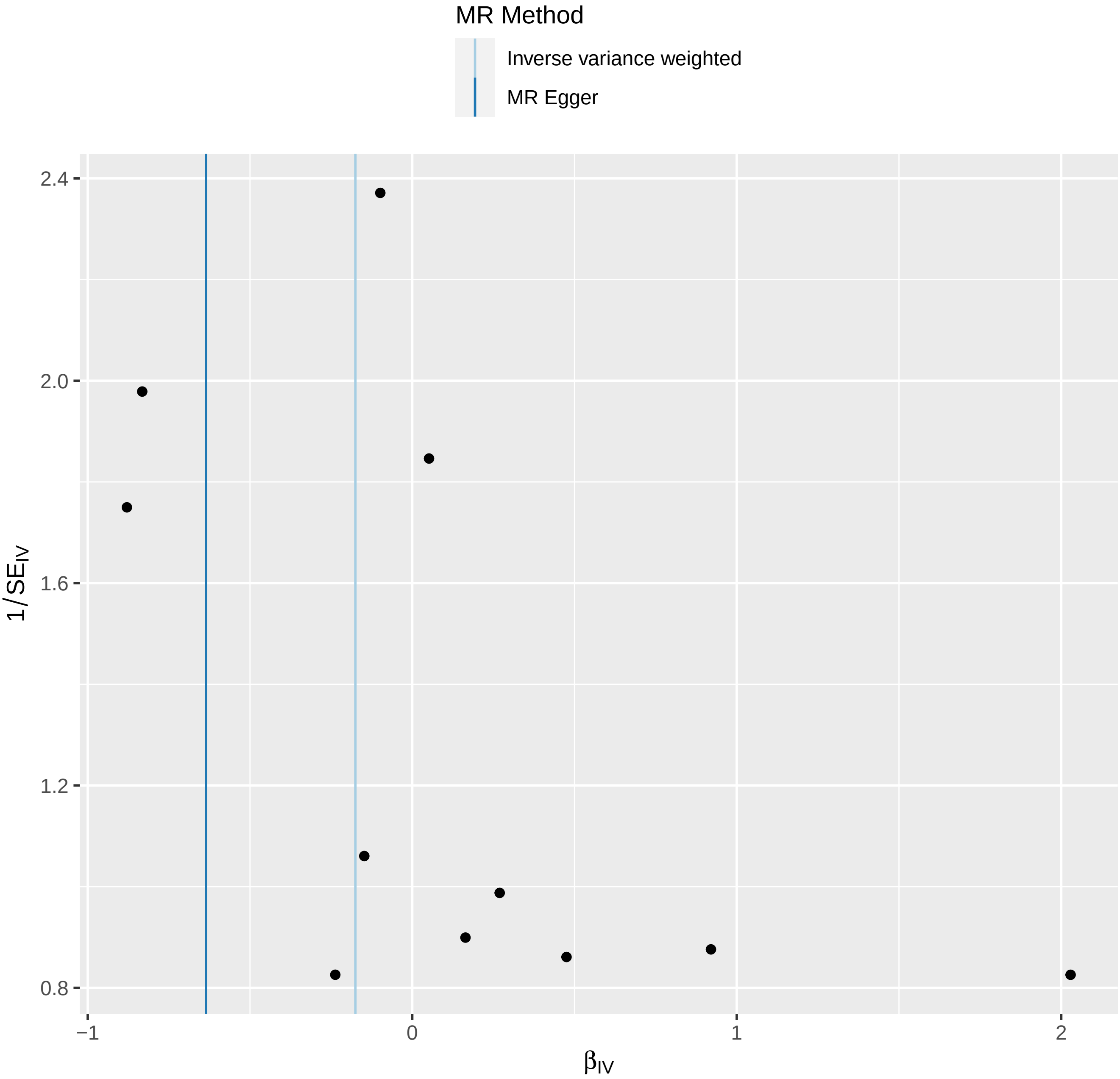

Batch 2294 : Taurocholate on Ulcerative proctitis

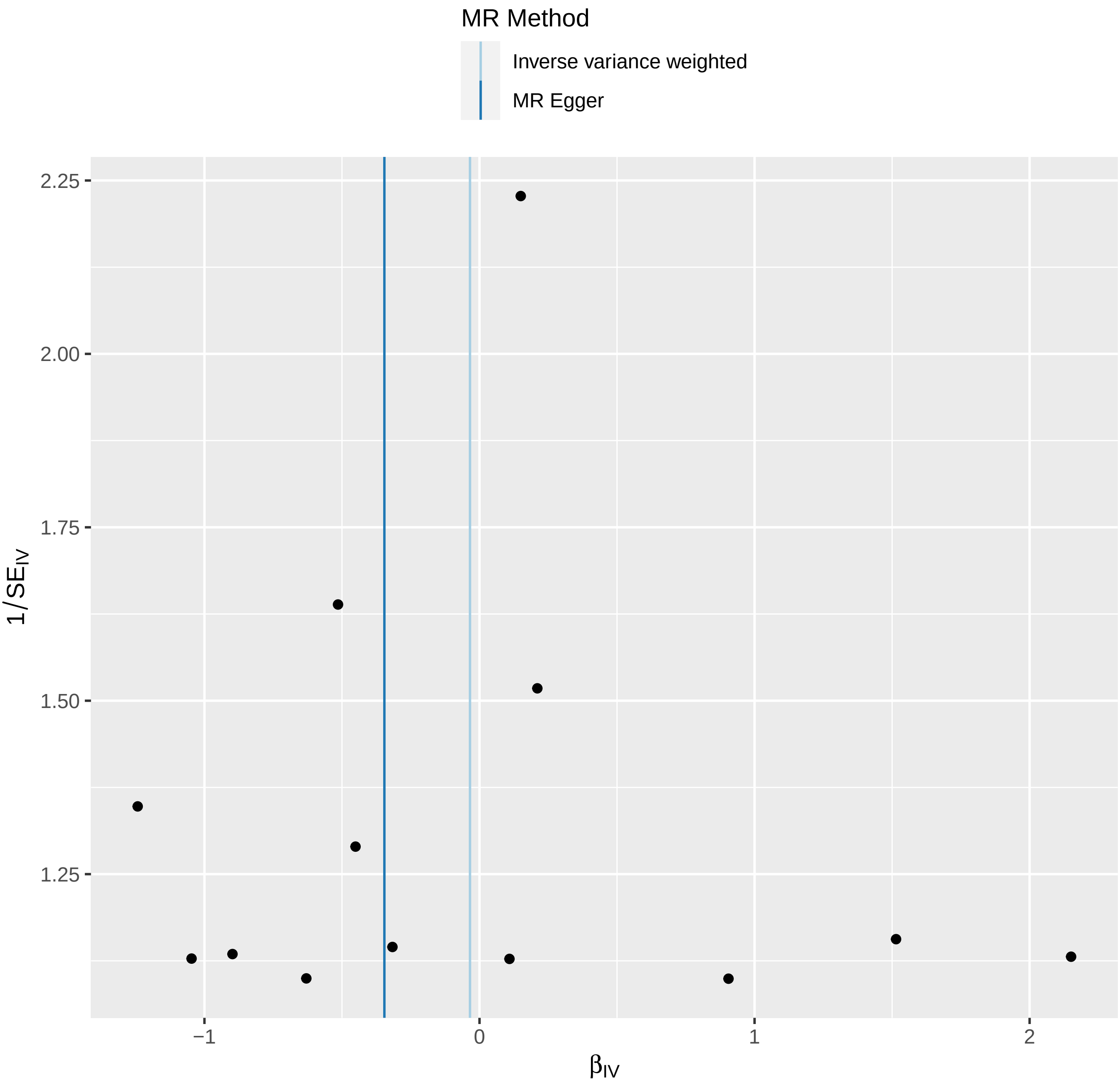

Batch 2296 : 1−stearoylglycerophosphoinositol on Ulcerative proctitis

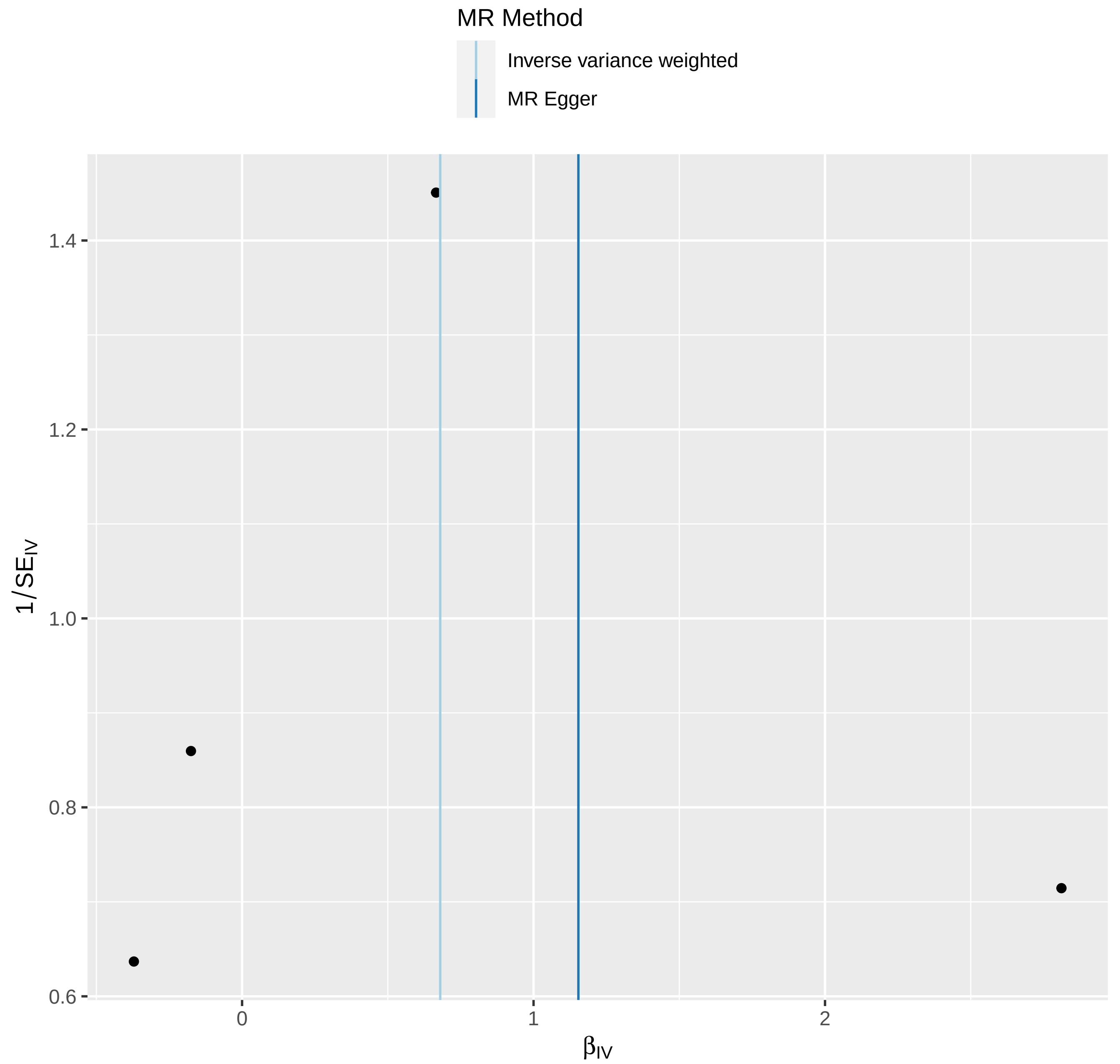

Batch 2297 : Myo−inositol on Ulcerative proctitis

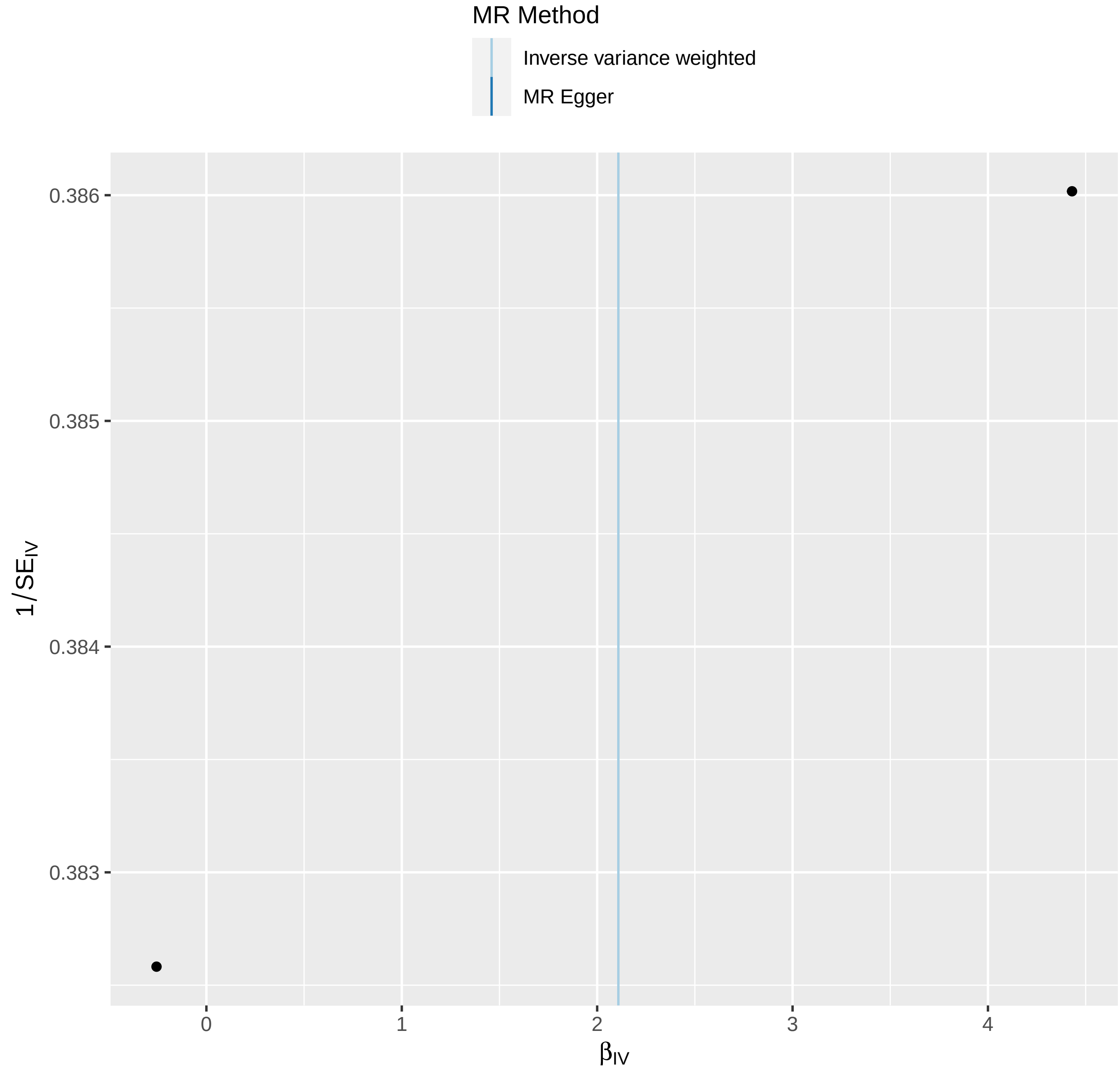

Batch 2299 : 1,5−anhydroglucitol (1,5−AG) on Ulcerative proctitis

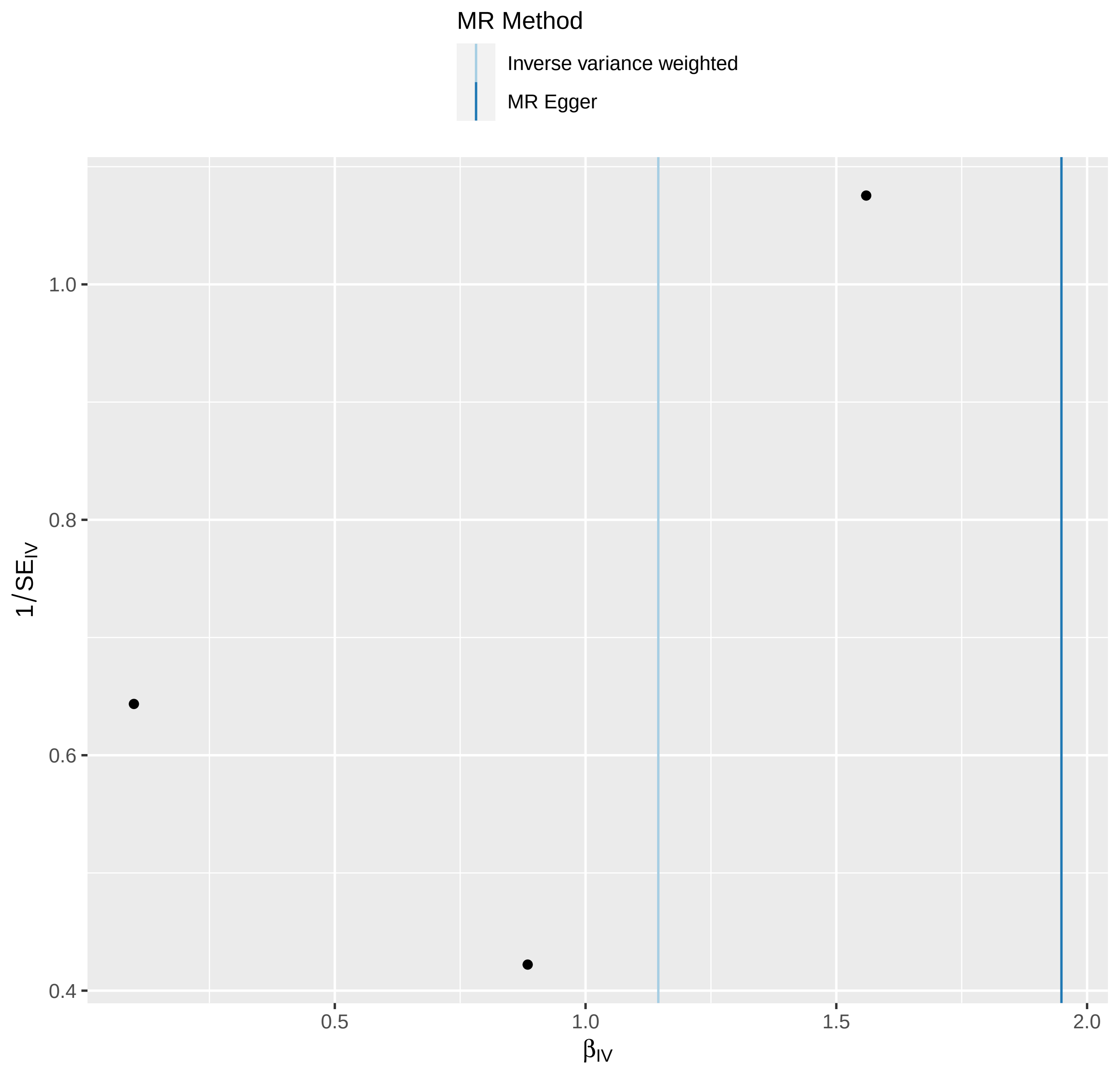

Batch 2300 : 2−hydroxybutyrate (AHB) on Ulcerative proctitis

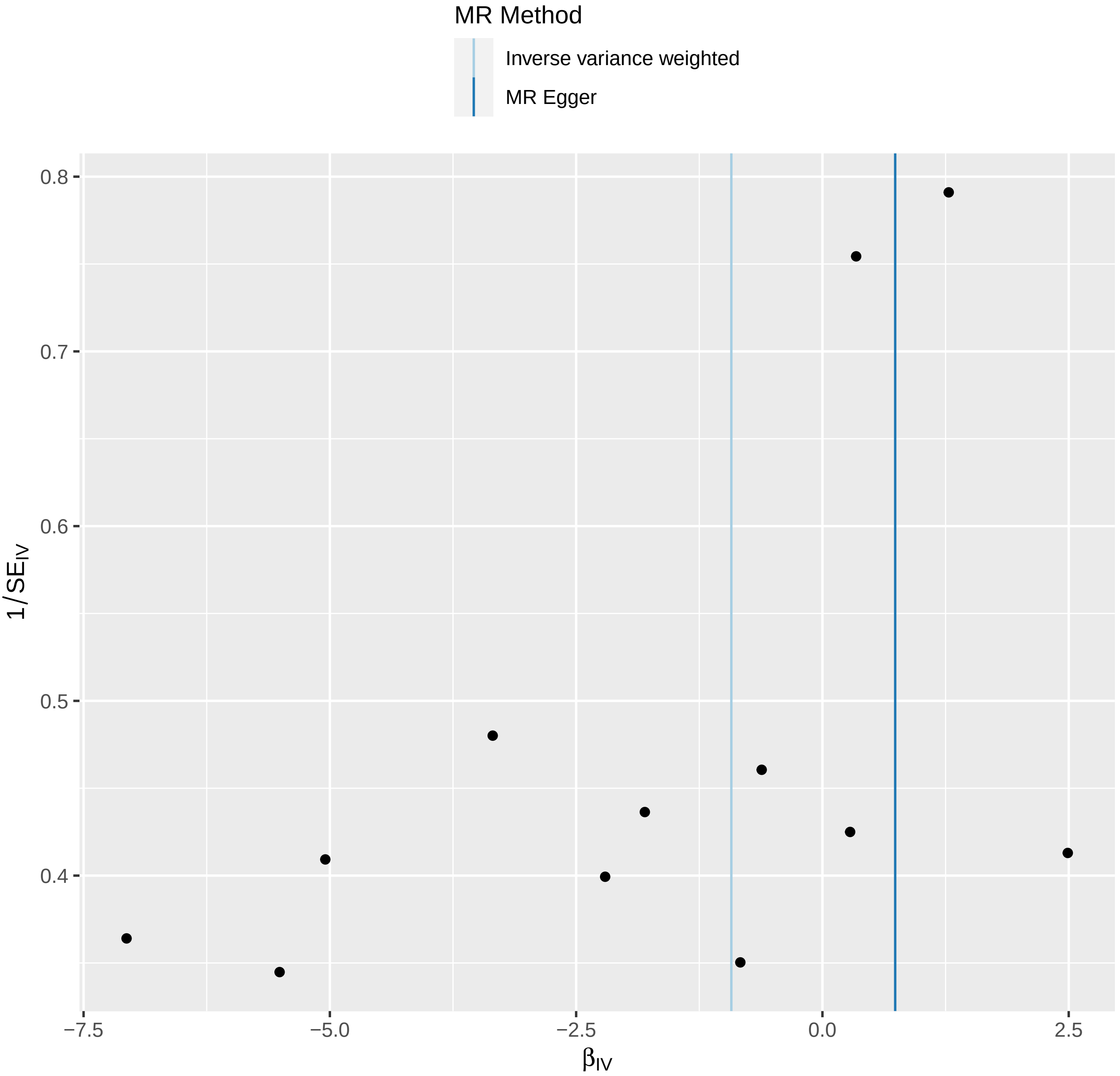

Batch 2303 : 1−palmitoylglycerol (1−monopalmitin) on Ulcerative proctitis

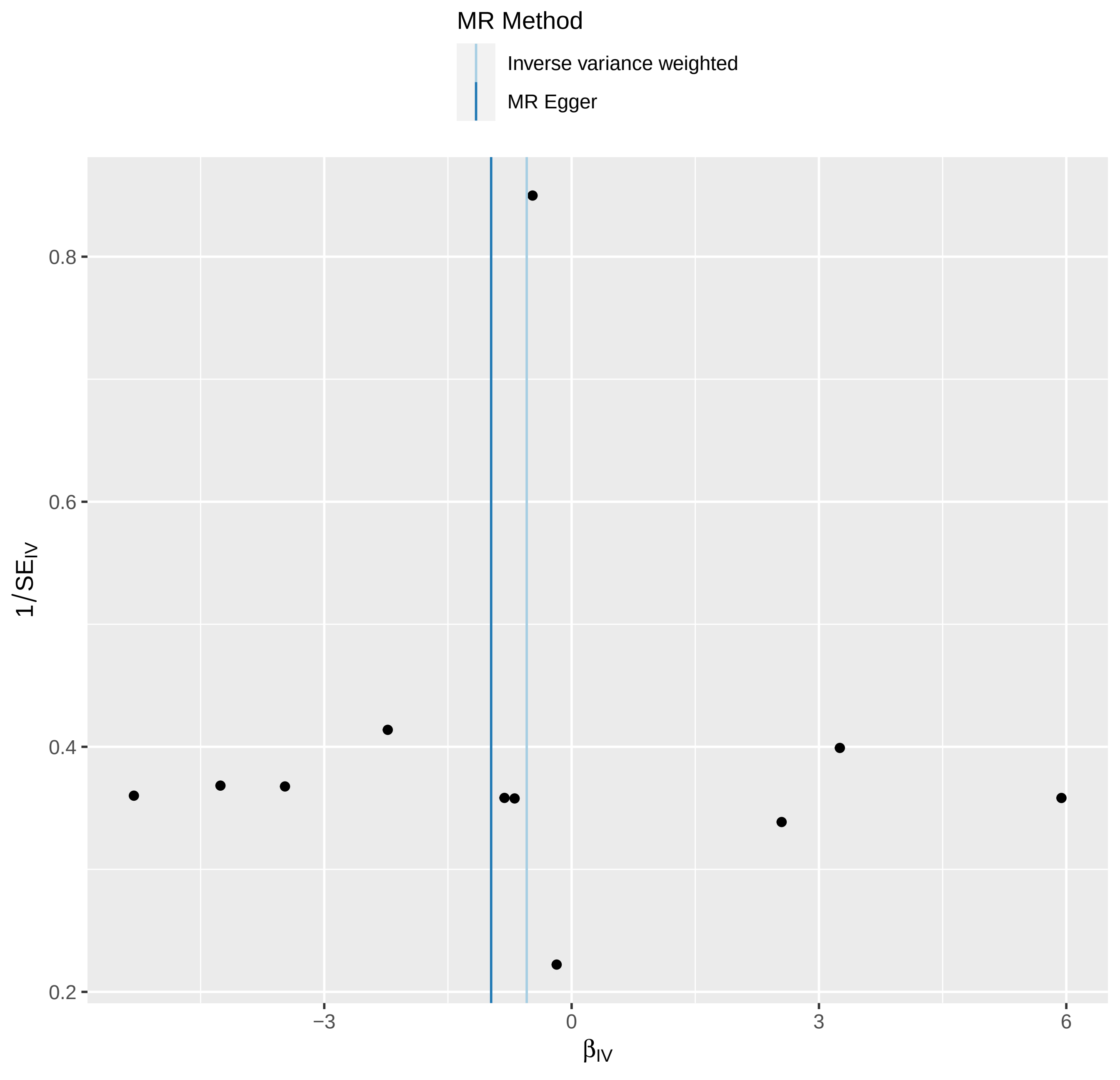

Batch 2306 : 2−hydroxyisobutyrate on Ulcerative proctitis

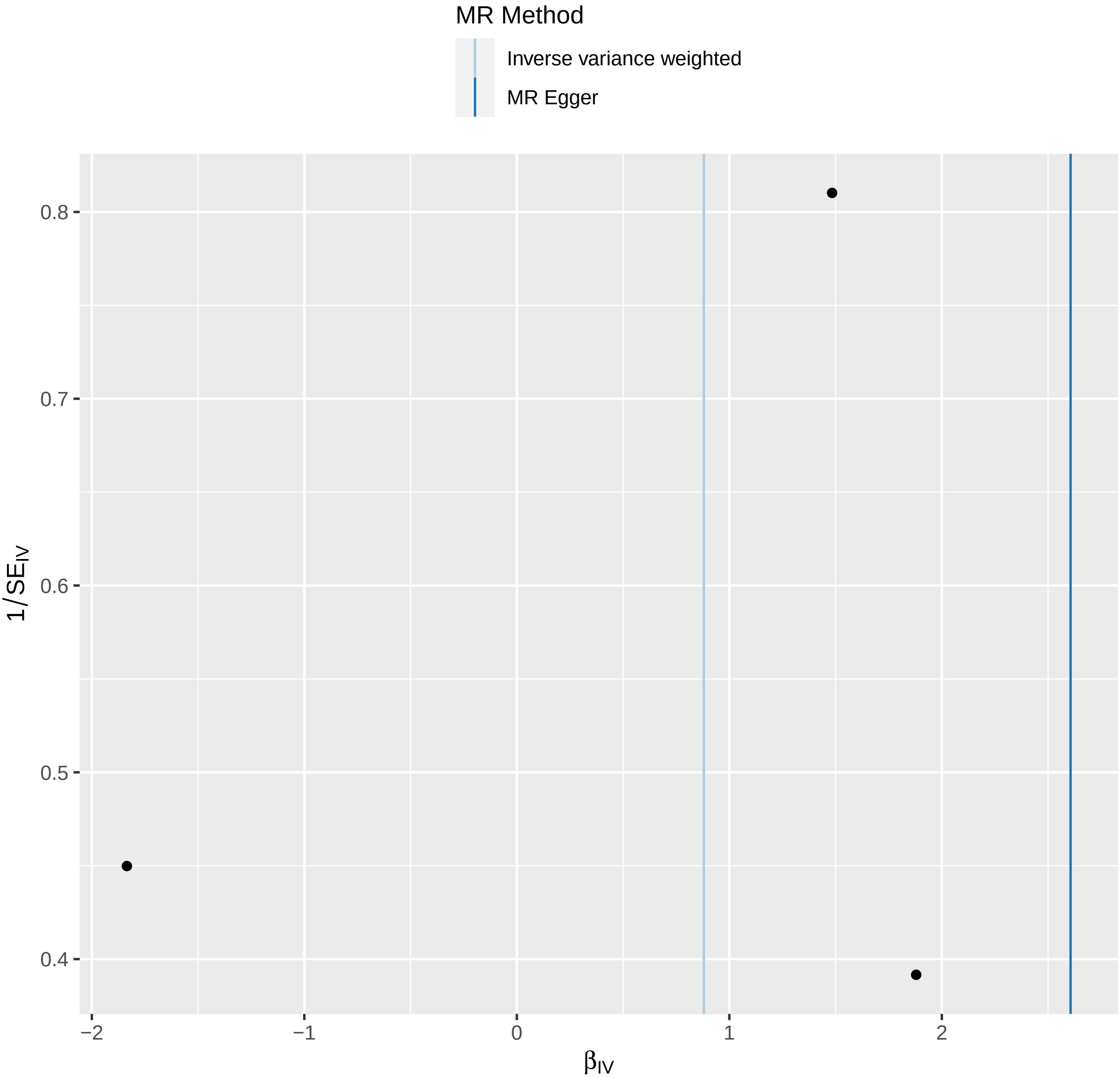

Batch 2307 : 4−methyl−2−oxopentanoate on Ulcerative proctitis

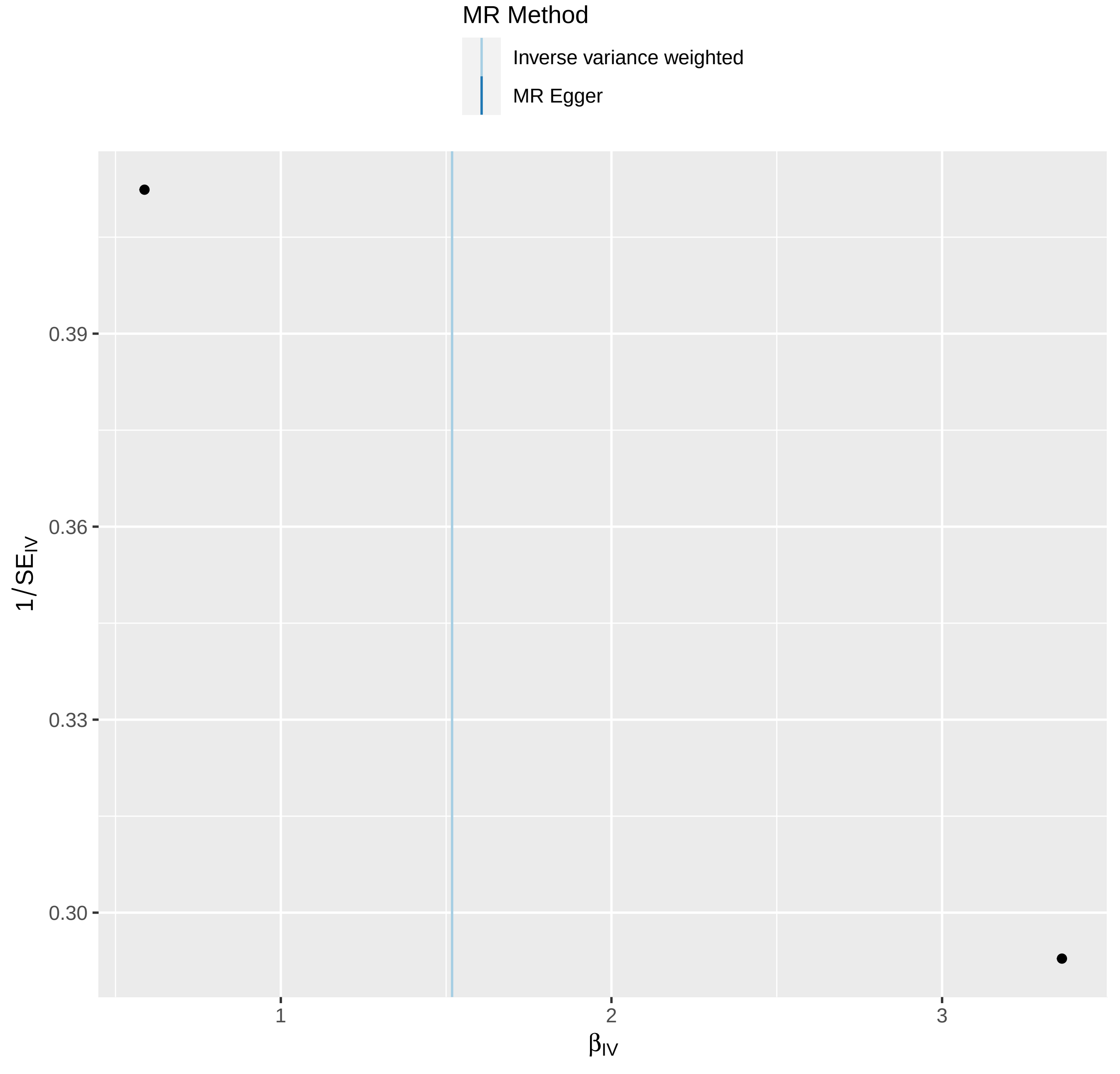

Batch 2311 : Levulinate (4−oxovalerate) on Ulcerative proctitis

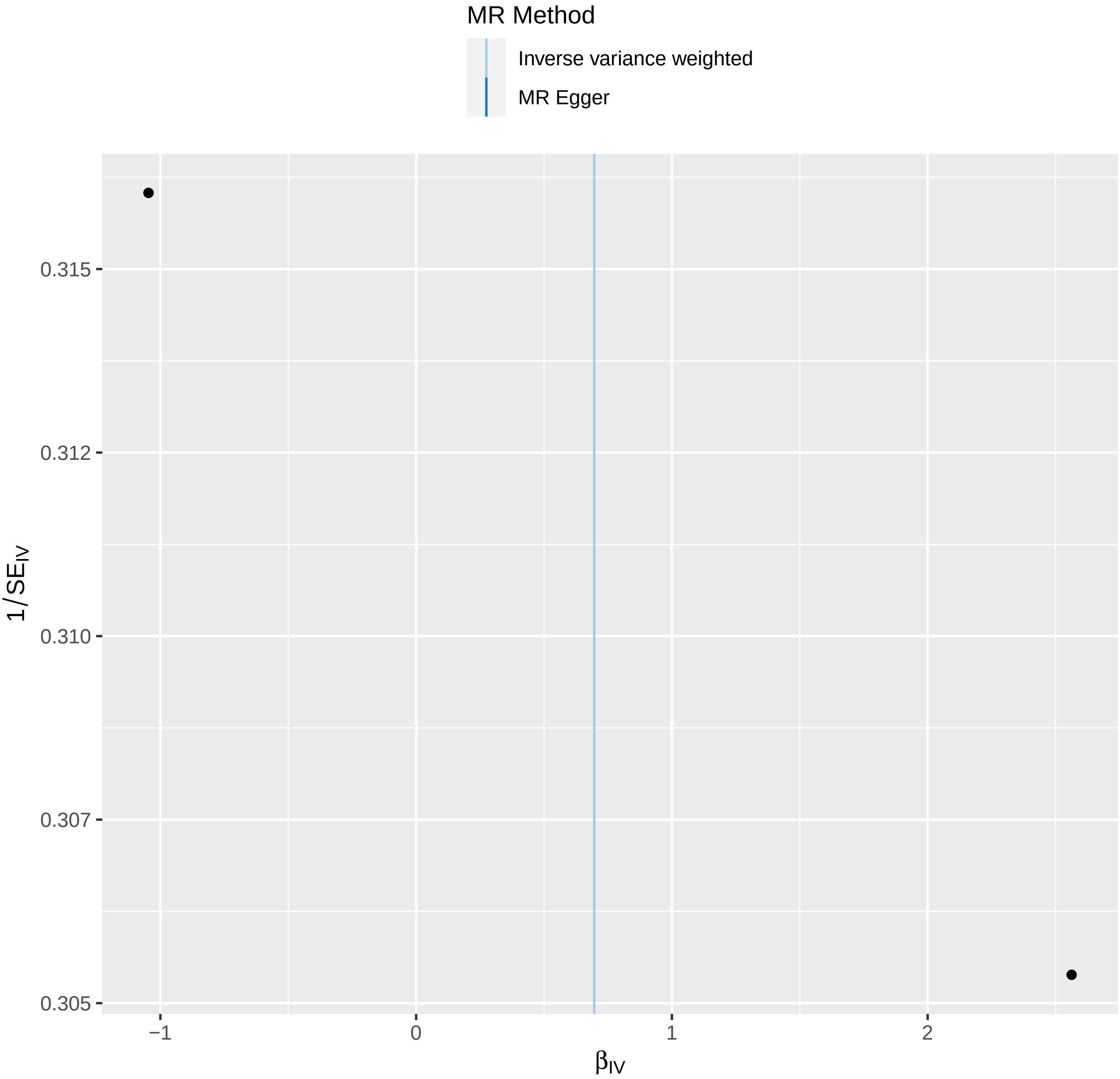

Batch 2313 : Cholate on Ulcerative proctitis

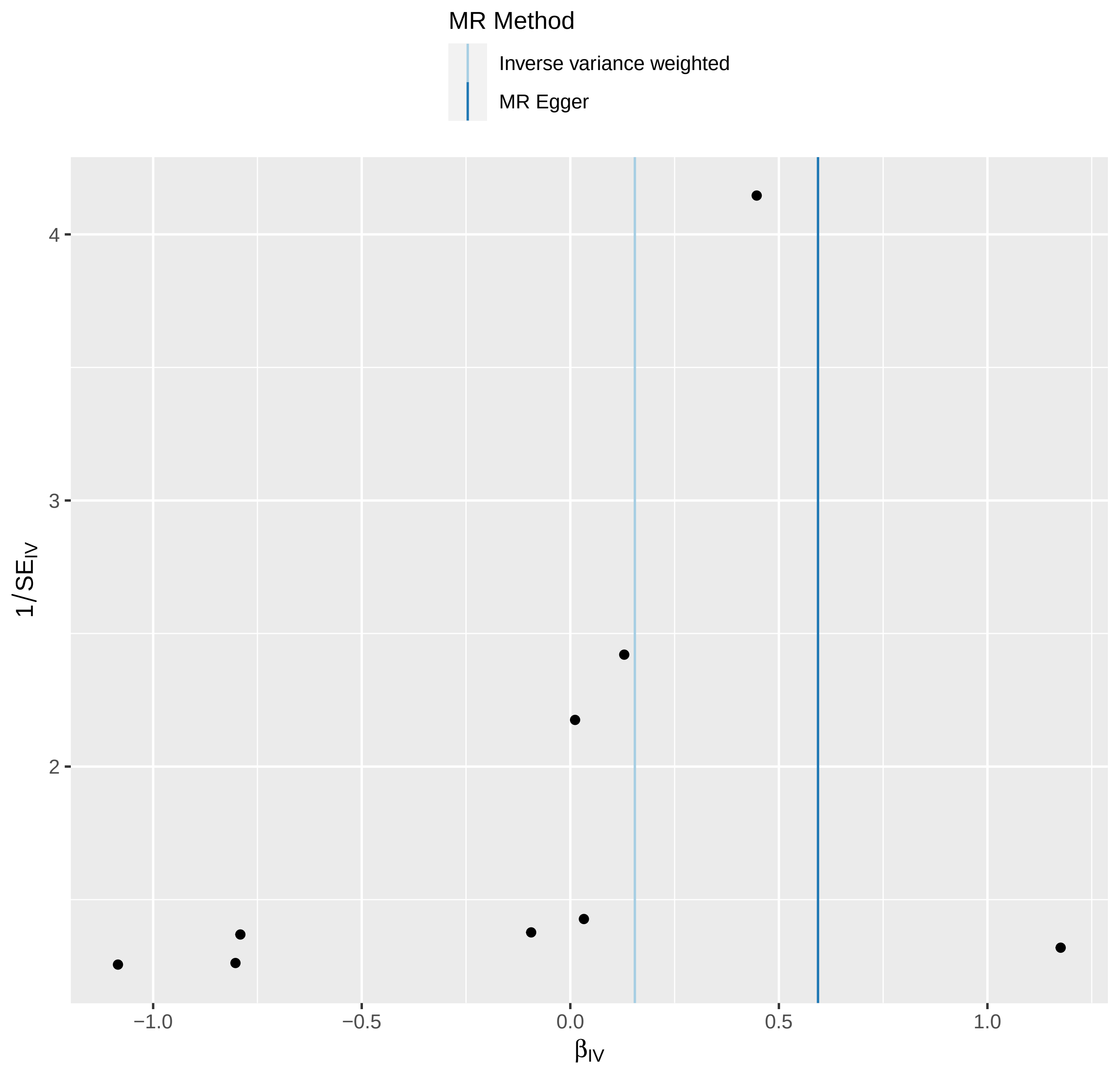

Batch 2316 : Hyodeoxycholate on Ulcerative proctitis

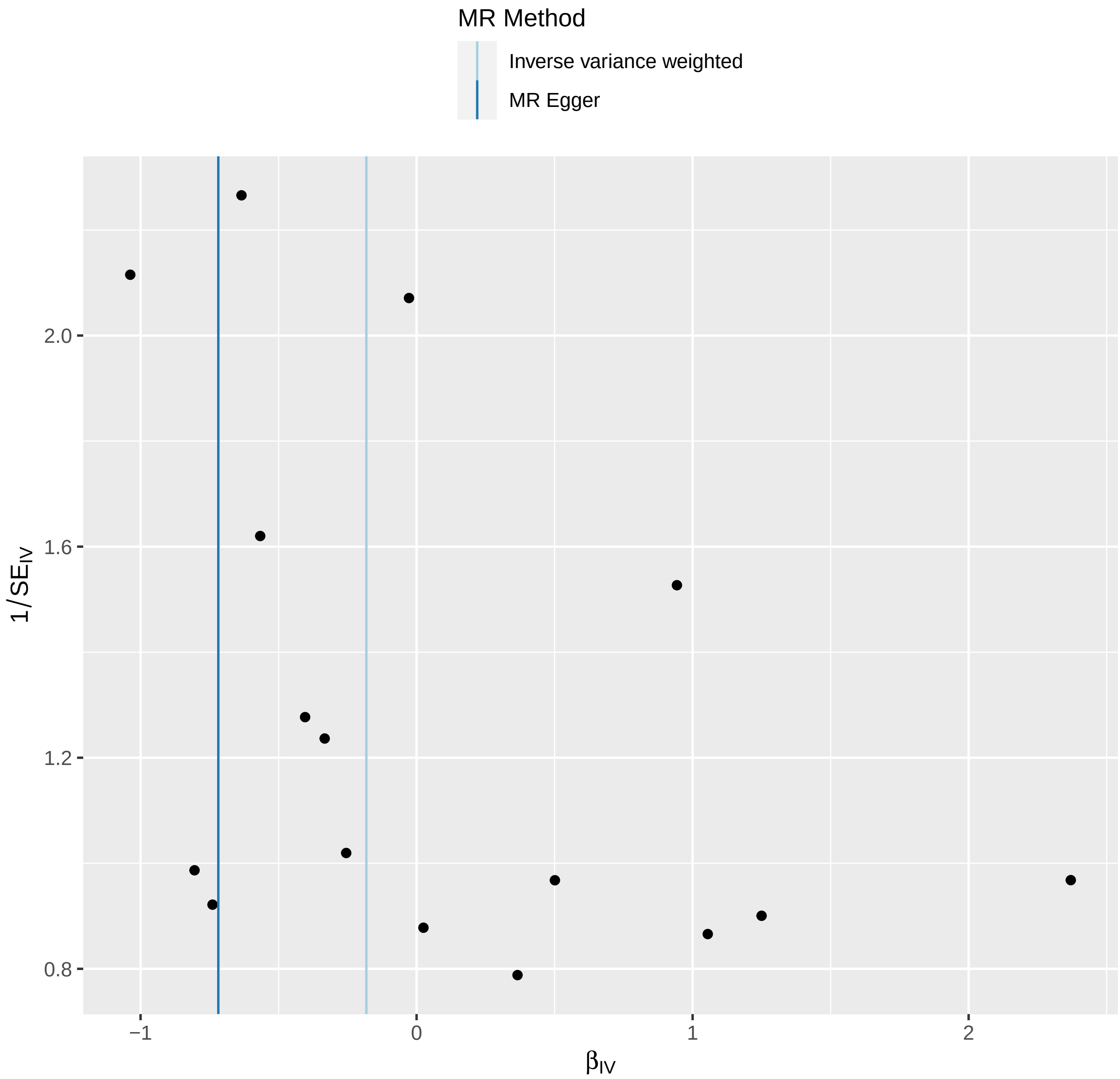

Batch 2317 : 3−indoxyl sulfate on Ulcerative proctitis

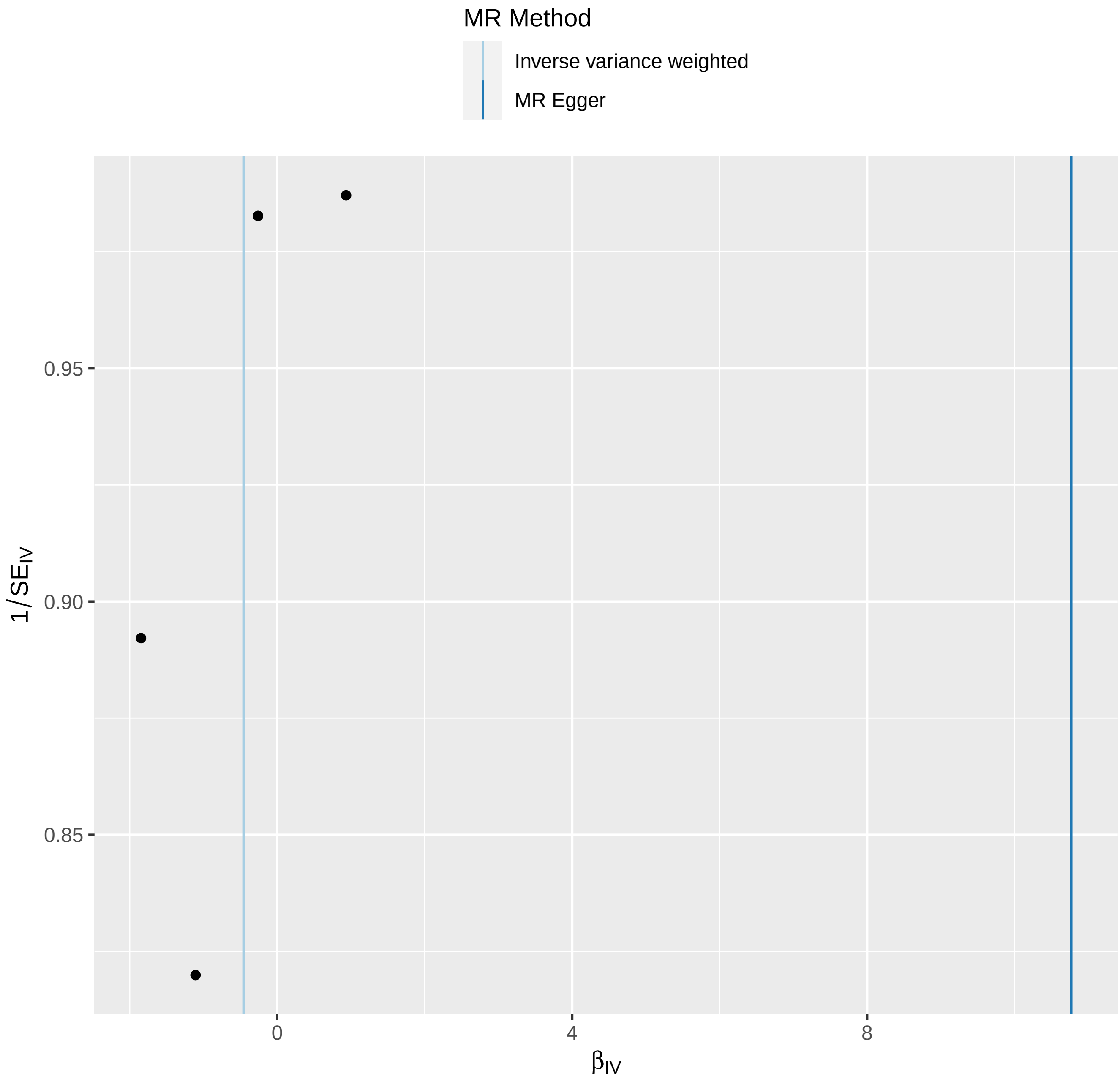

Batch 2321 : Erythrose on Ulcerative proctitis

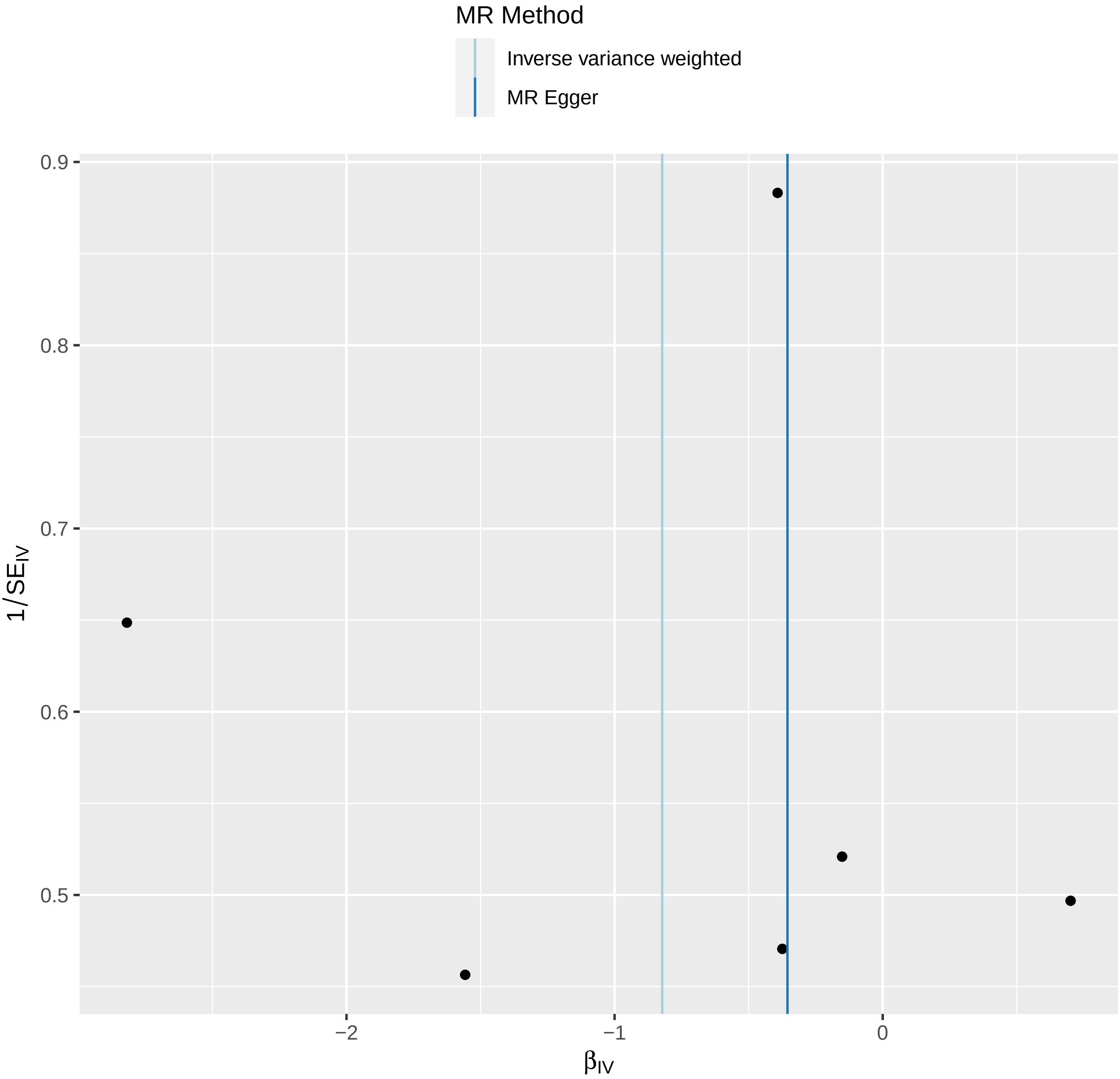

Batch 2322 : Threonate on Ulcerative proctitis

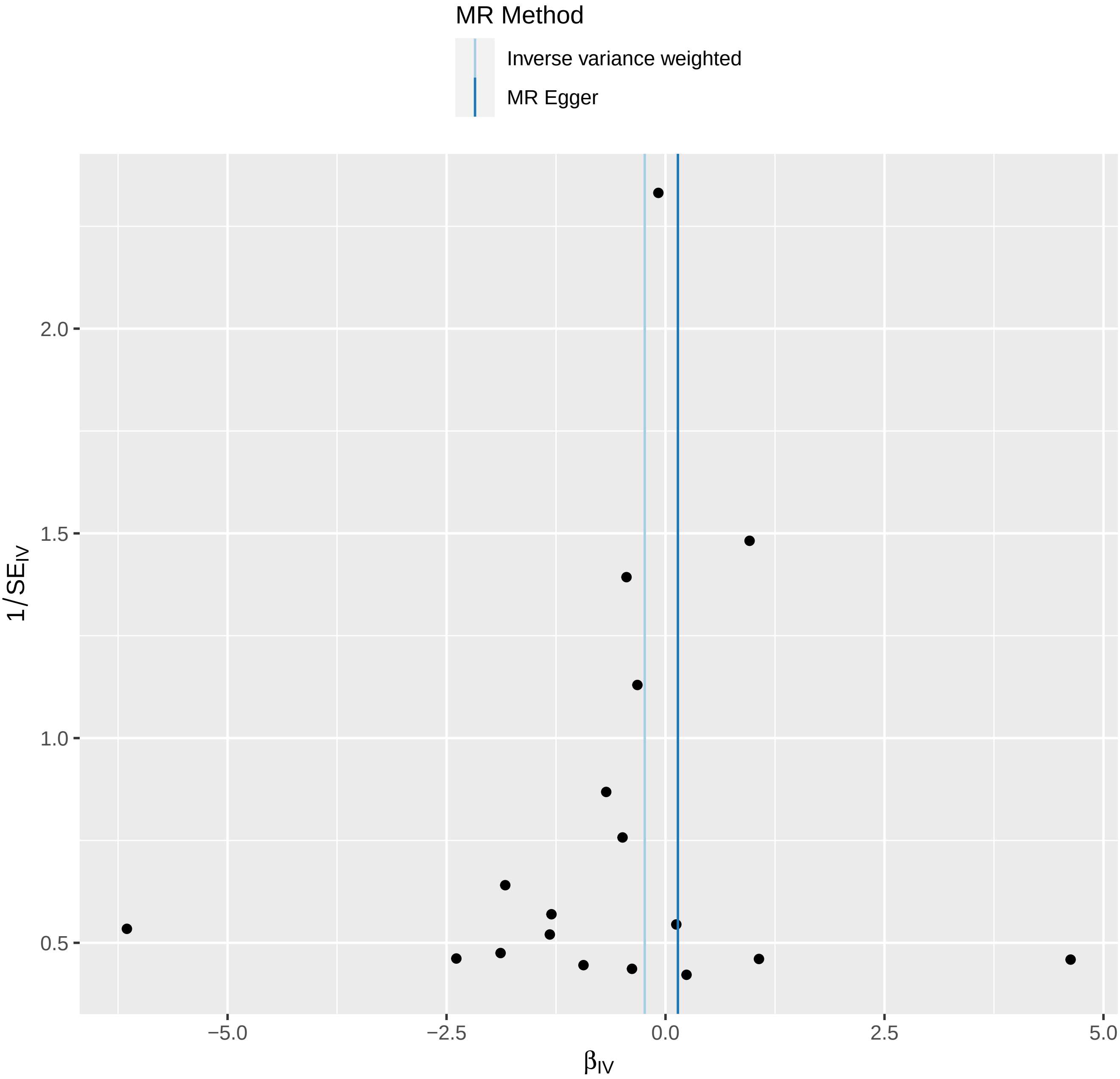

Batch 2323 : Cysteine on Ulcerative proctitis

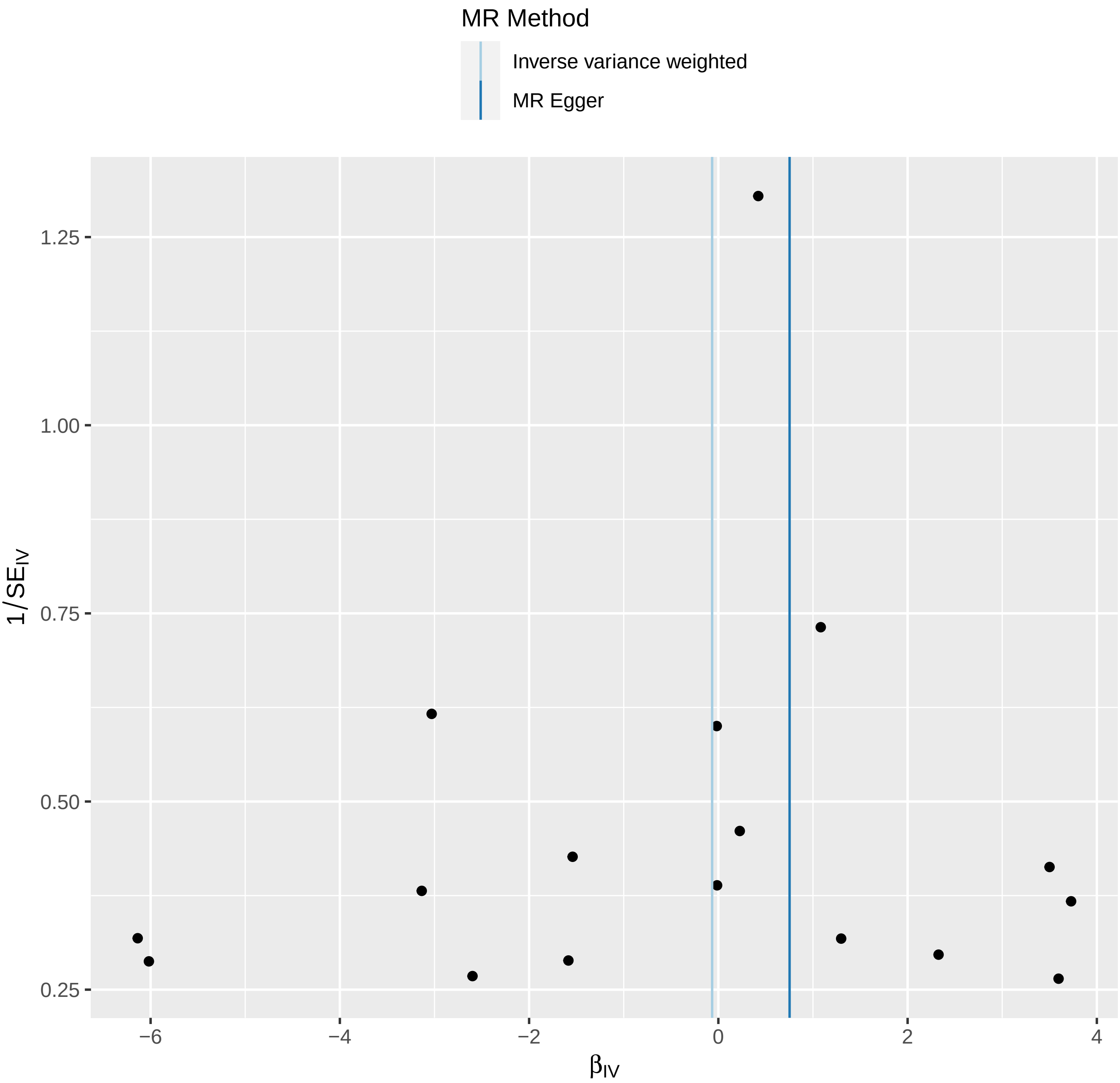

Batch 2324 : Pyroglutamylglycine on Ulcerative proctitis

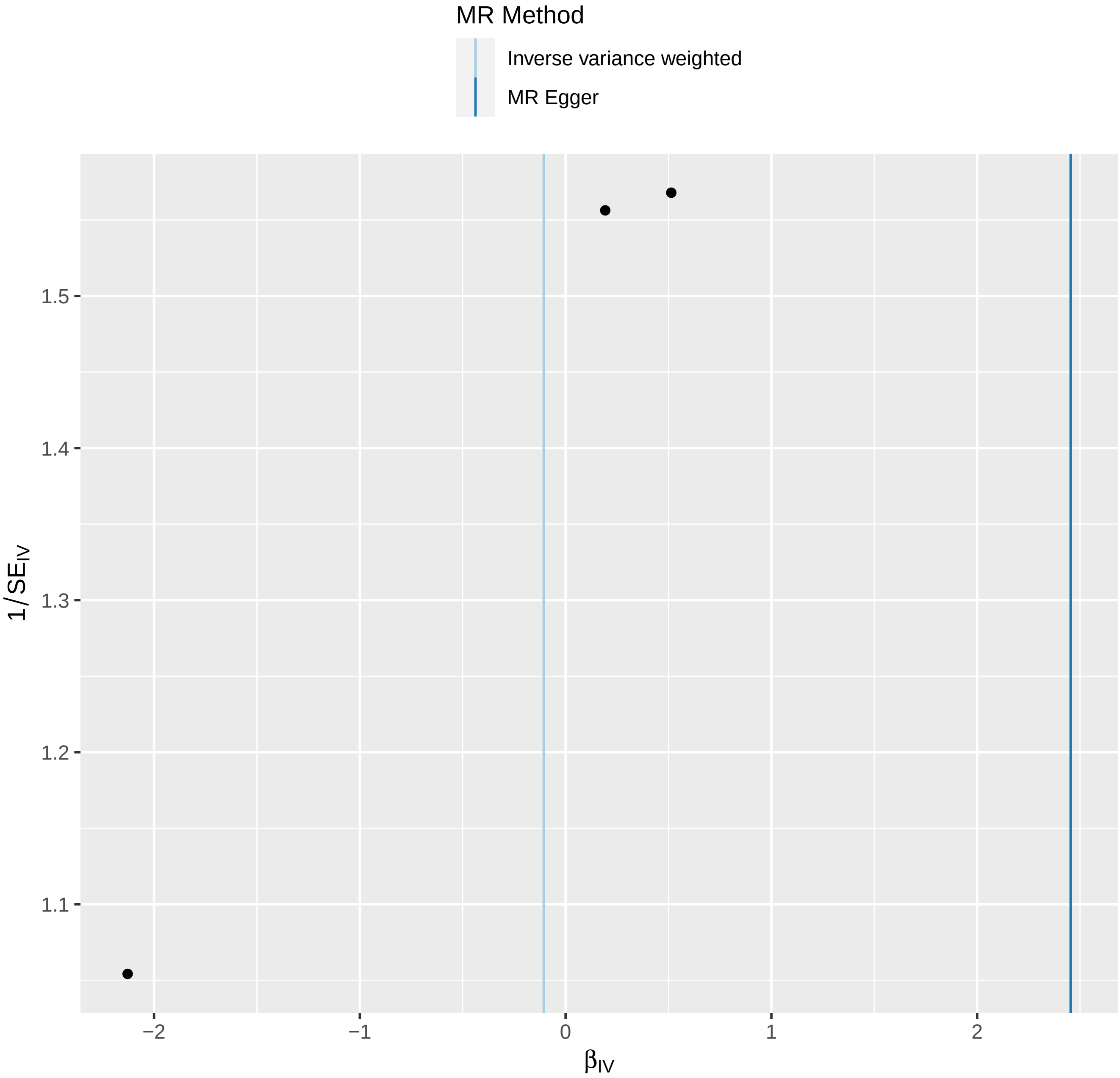

Batch 2326 : DSGEGDFXAEGGGVR* on Ulcerative proctitis

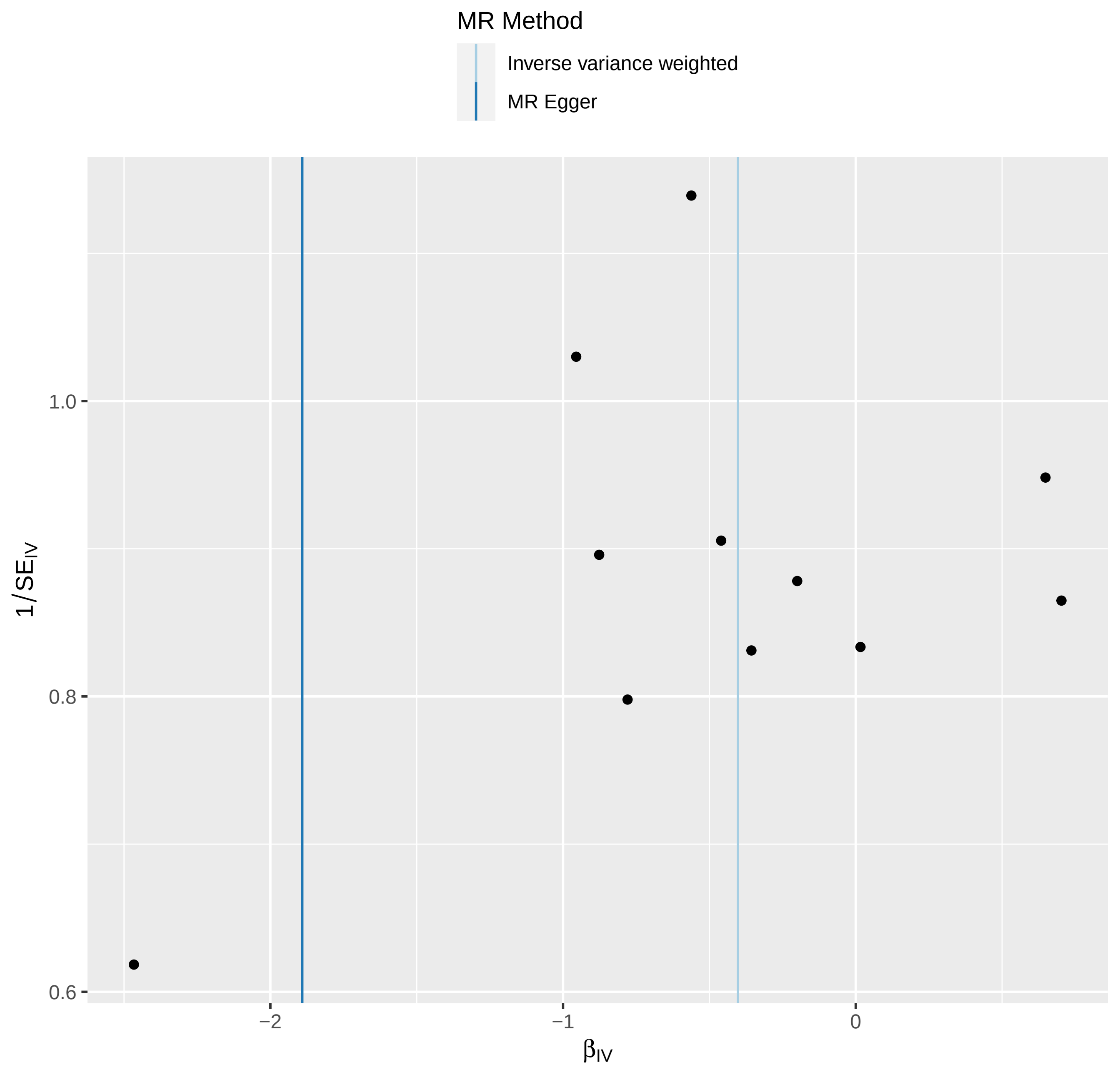

Batch 2327 : Pyridoxate on Ulcerative proctitis

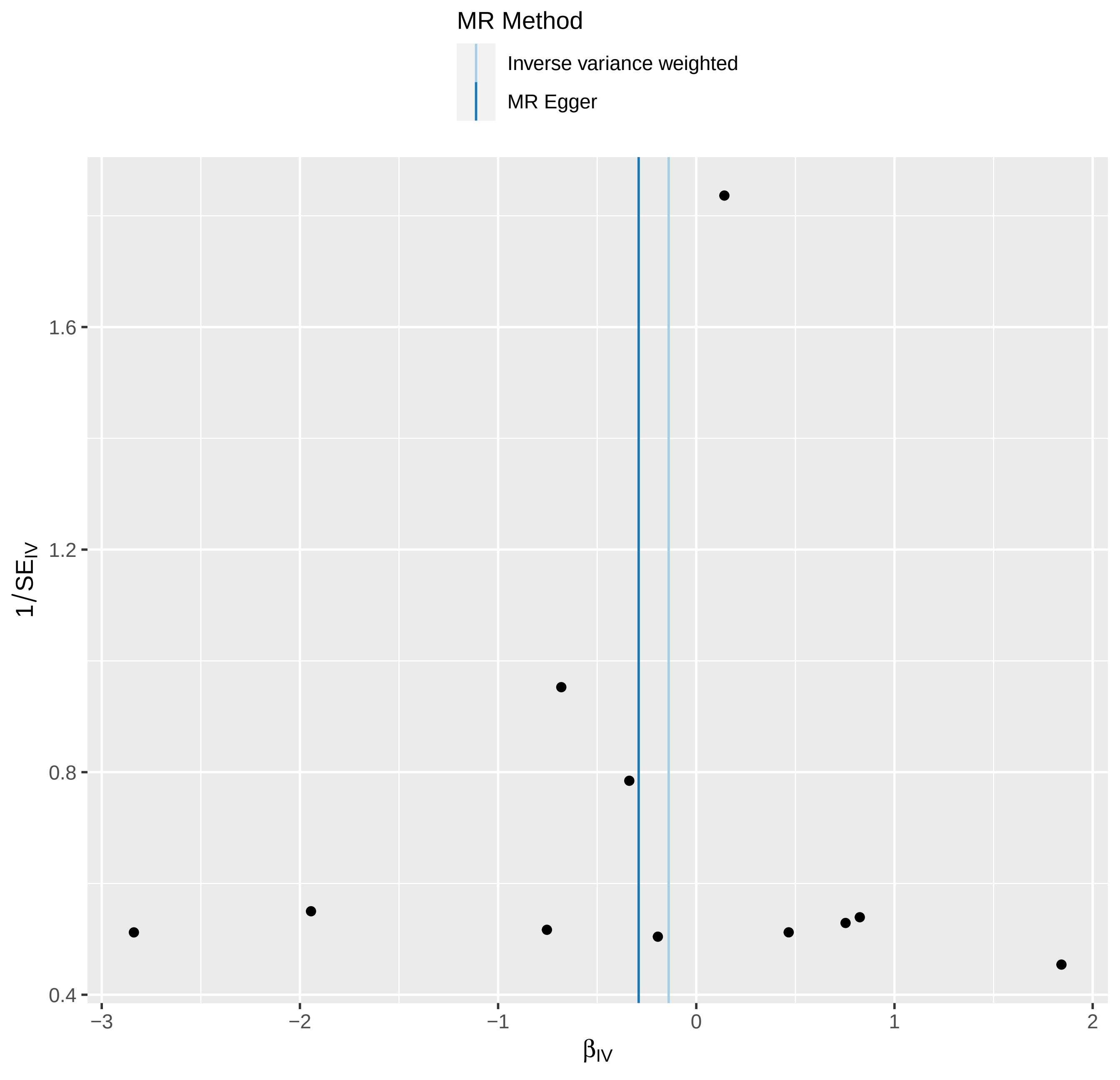

Batch 2328 : Androsterone sulfate on Ulcerative proctitis

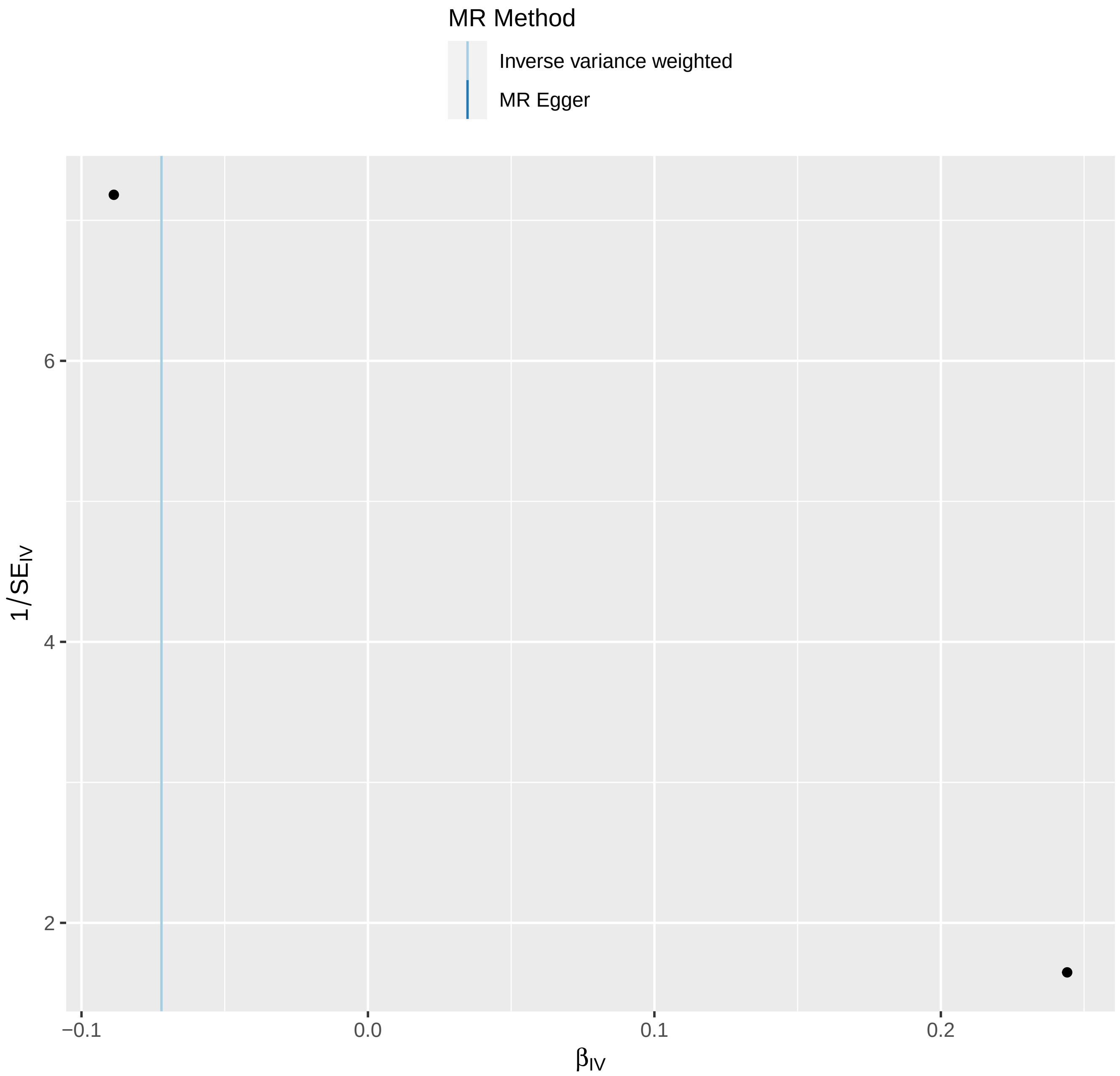

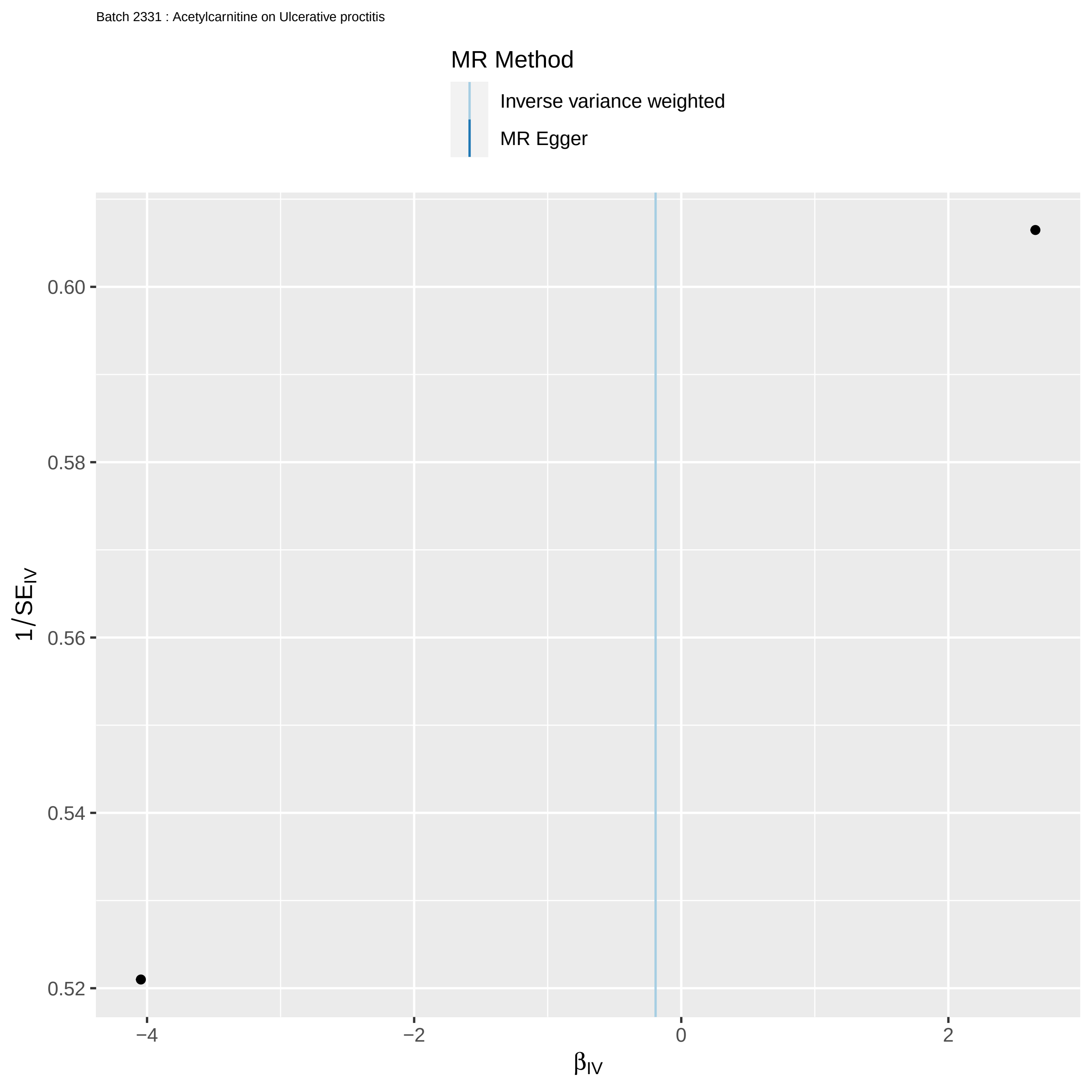

Batch 2331 : Acetylcarnitine on Ulcerative proctitis
MR Method
Inverse variance weighted
MR Egger
0.60
0.58
0.56
0.54
0.52
1/SE_IV
−4
−2
0
2
β_IV

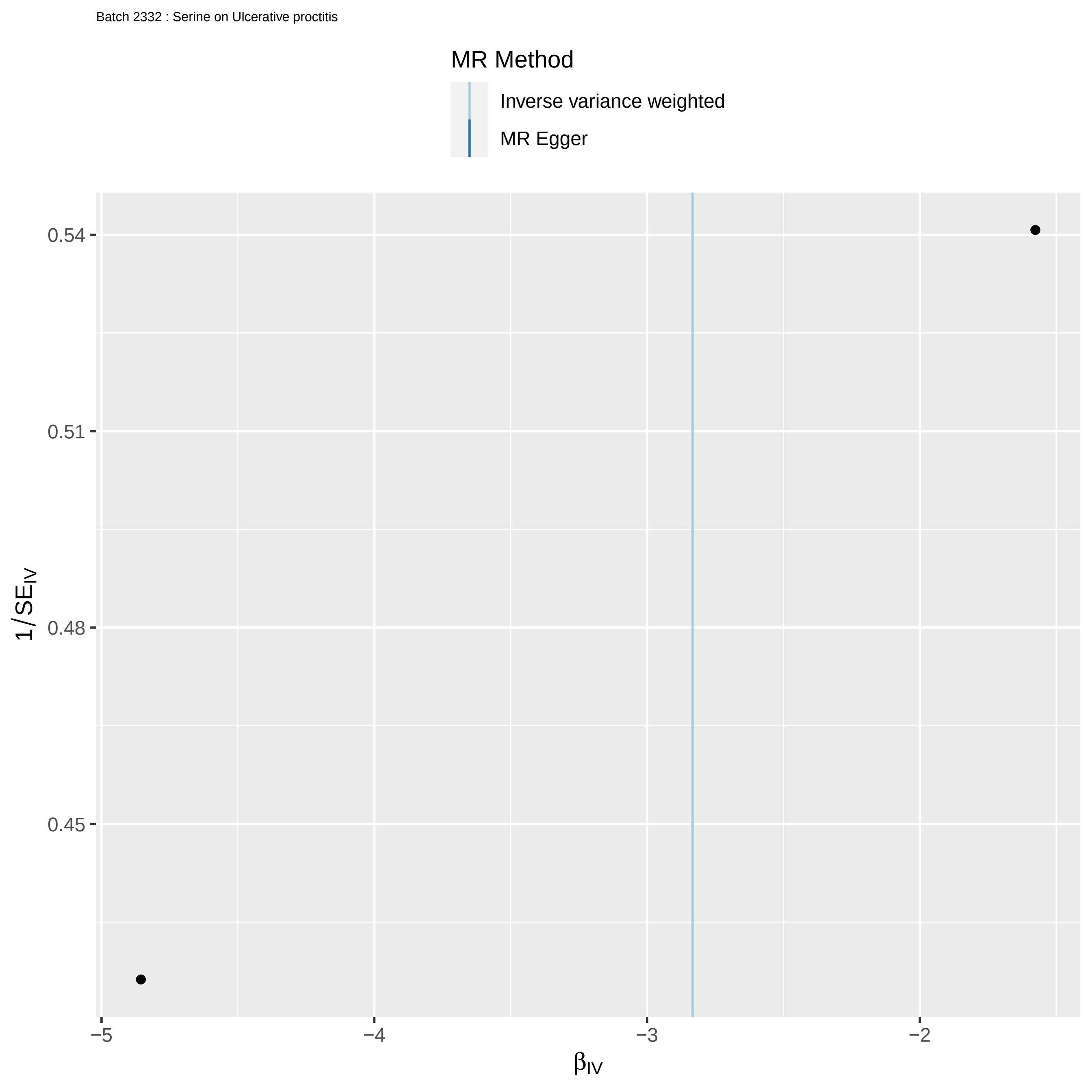

Batch 2332 : Serine on Ulcerative proctitis
MR Method
Inverse variance weighted
MR Egger
0.54
0.51
0.48
0.45
1/SE_IV
−5
−4
−3
−2
β_IV

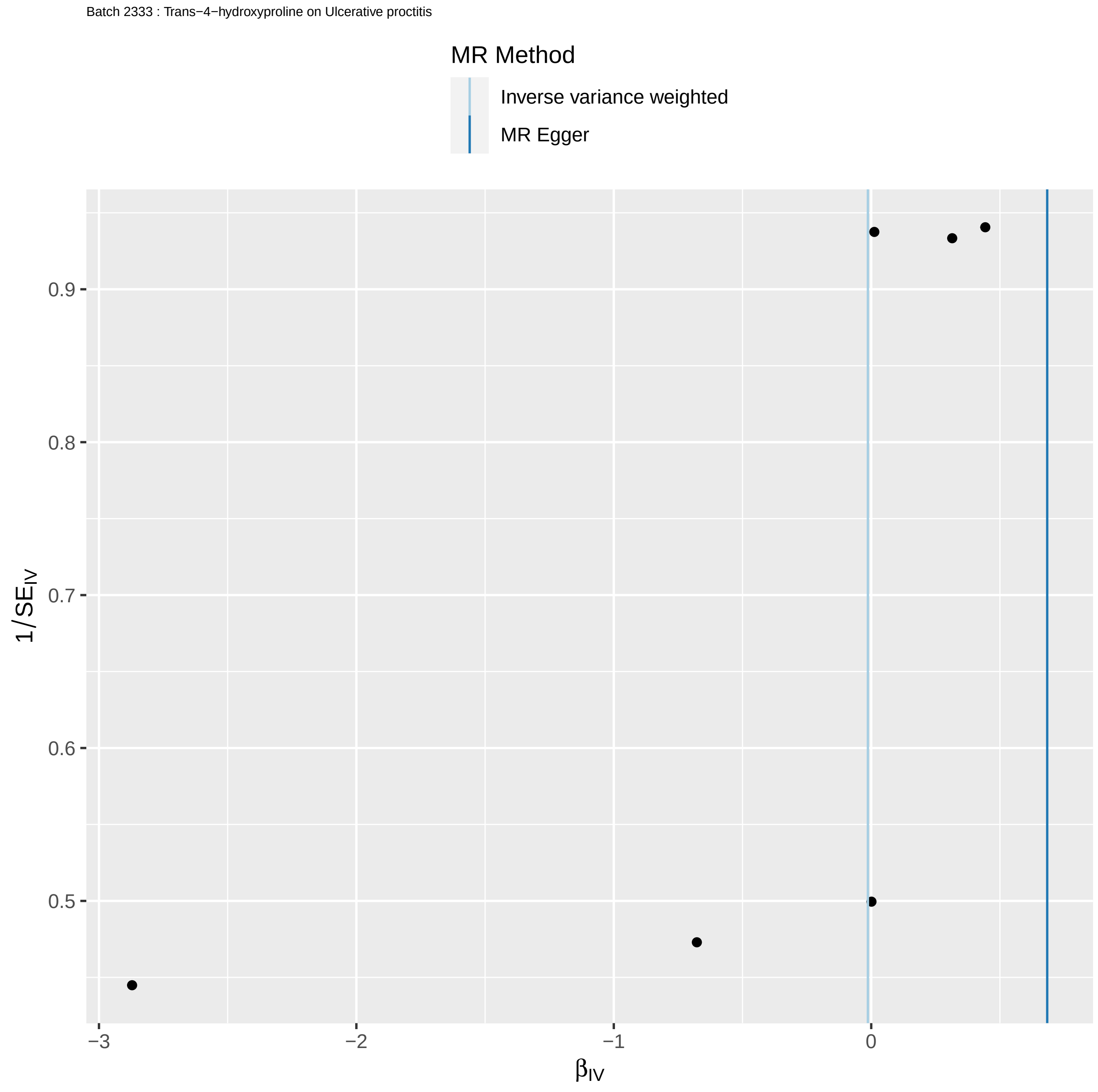
Batch 2333 : Trans−4−hydroxyproline on Ulcerative proctitis
MR Method
Inverse variance weighted
MR Egger
0.9
0.8
0.7
0.6
0.5
1/SE_IV
−3
−2
−1
0
β_IV

Batch 2334 : Glutamate on Ulcerative proctitis

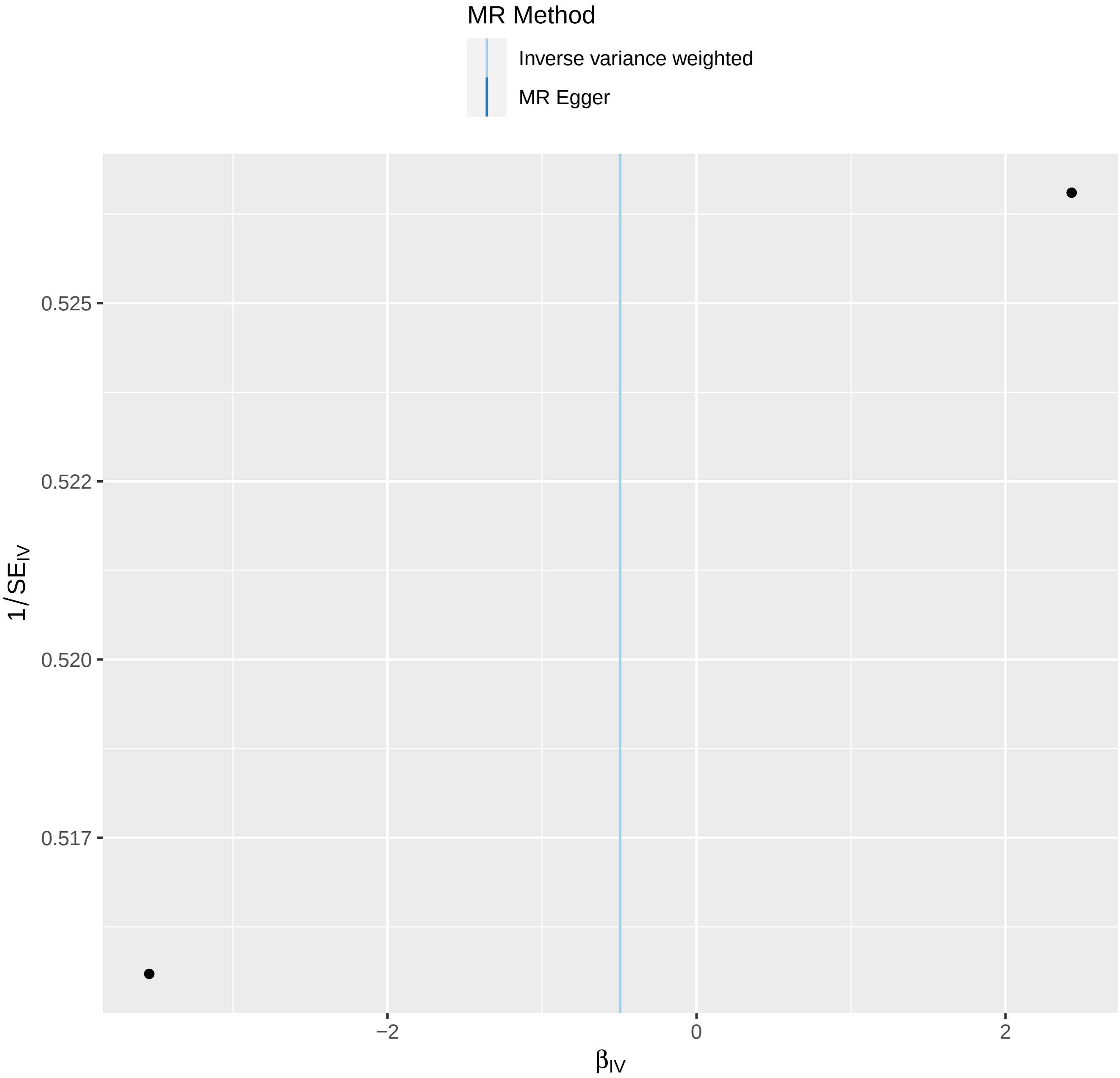

Batch 2335 : Hexanoylcarnitine on Ulcerative proctitis

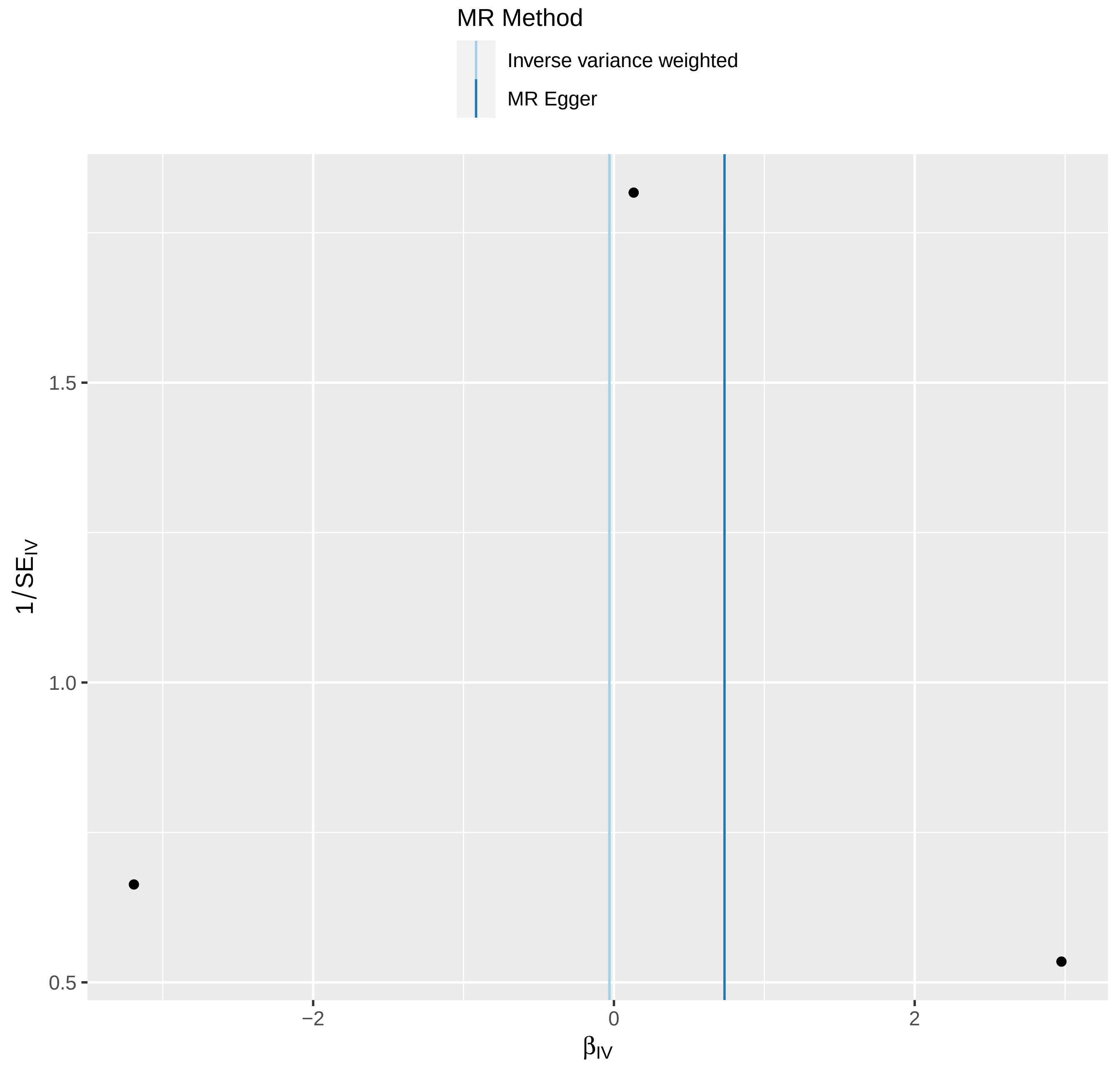

Batch 2339 : 2−aminobutyrate on Ulcerative proctitis

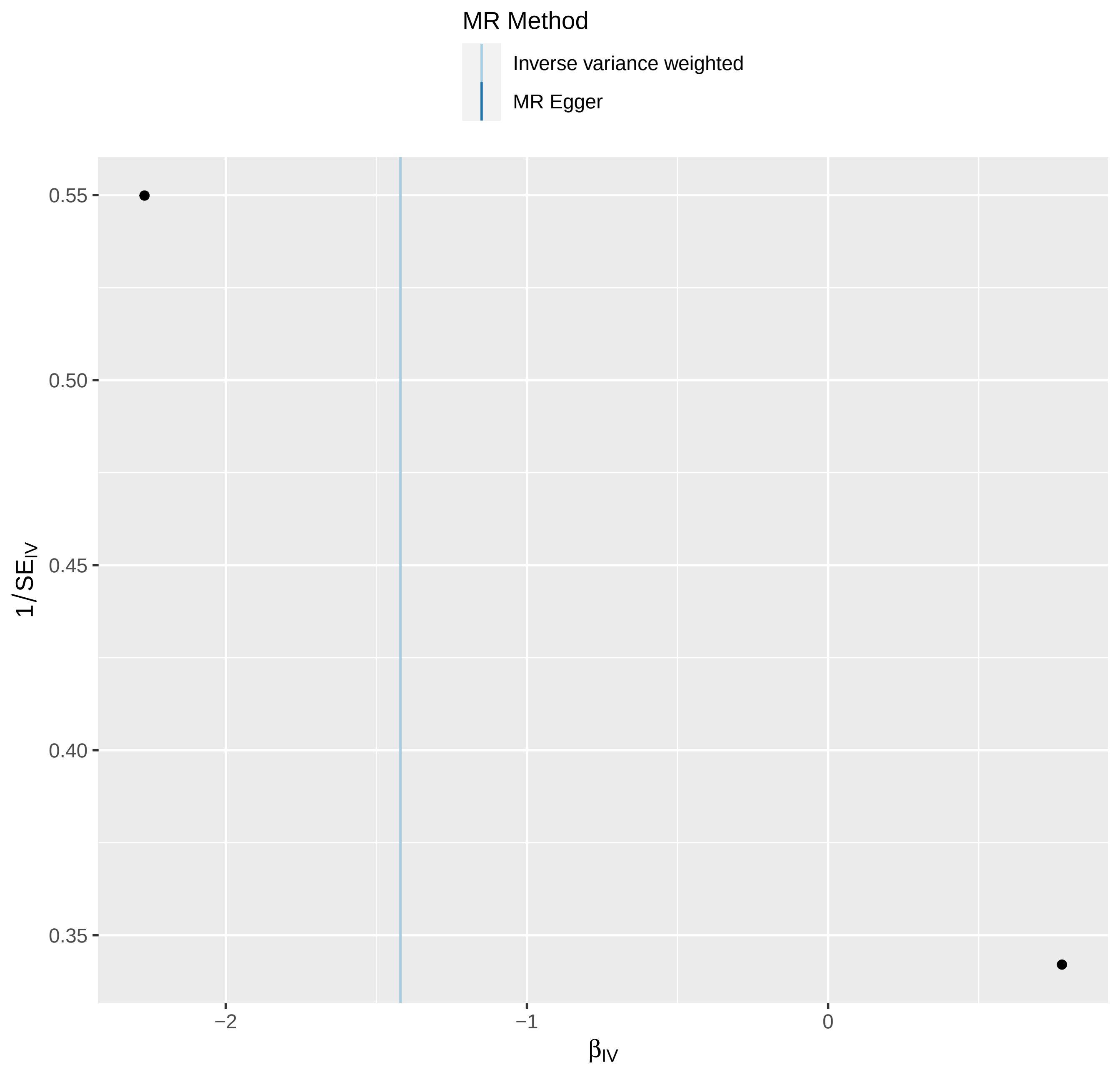

Batch 2344 : Butyrylcarnitine on Ulcerative proctitis

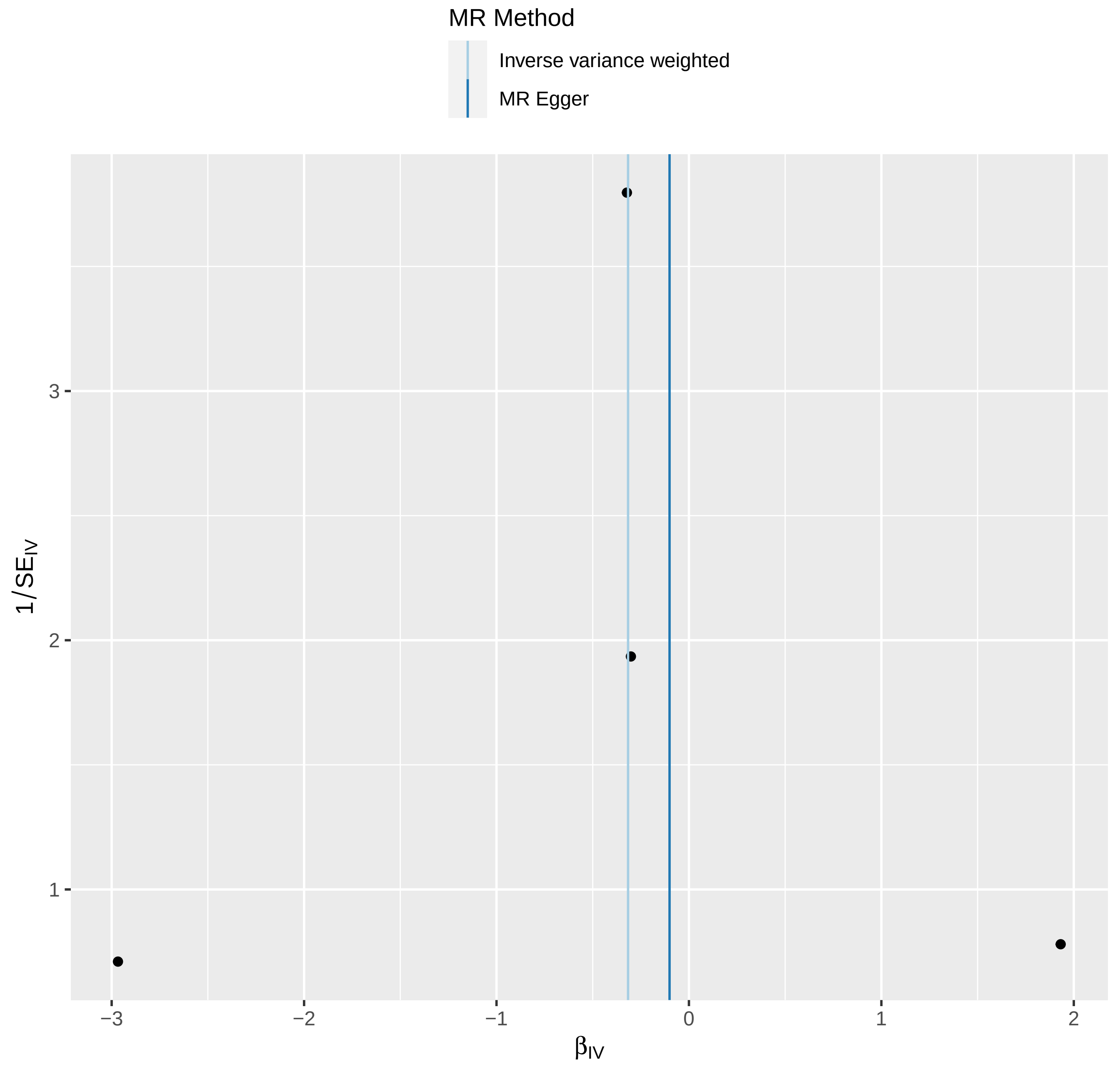

Batch 2347 : Propionylcarnitine on Ulcerative proctitis

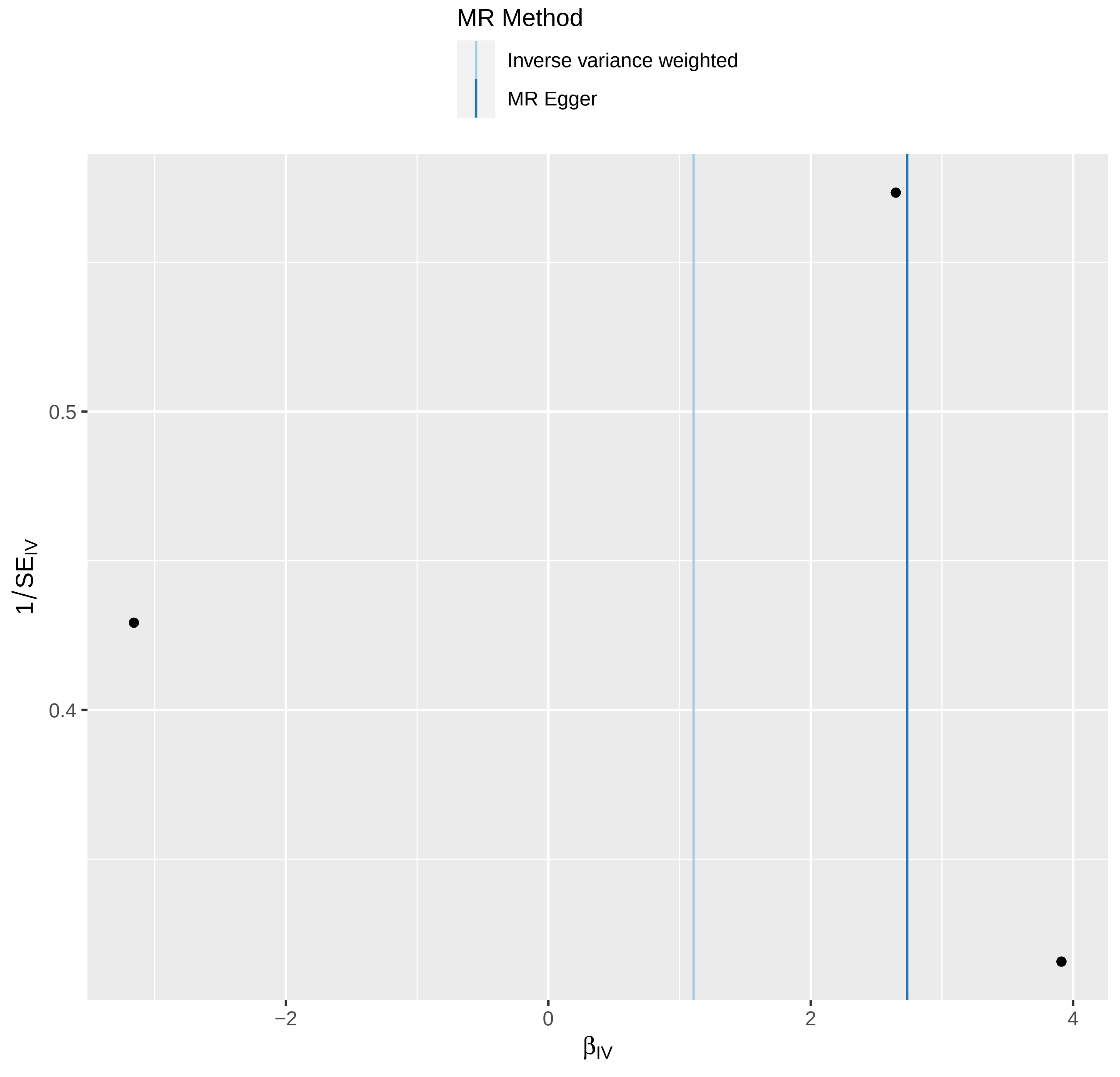

Batch 2348 : Caproate (6:0) on Ulcerative proctitis

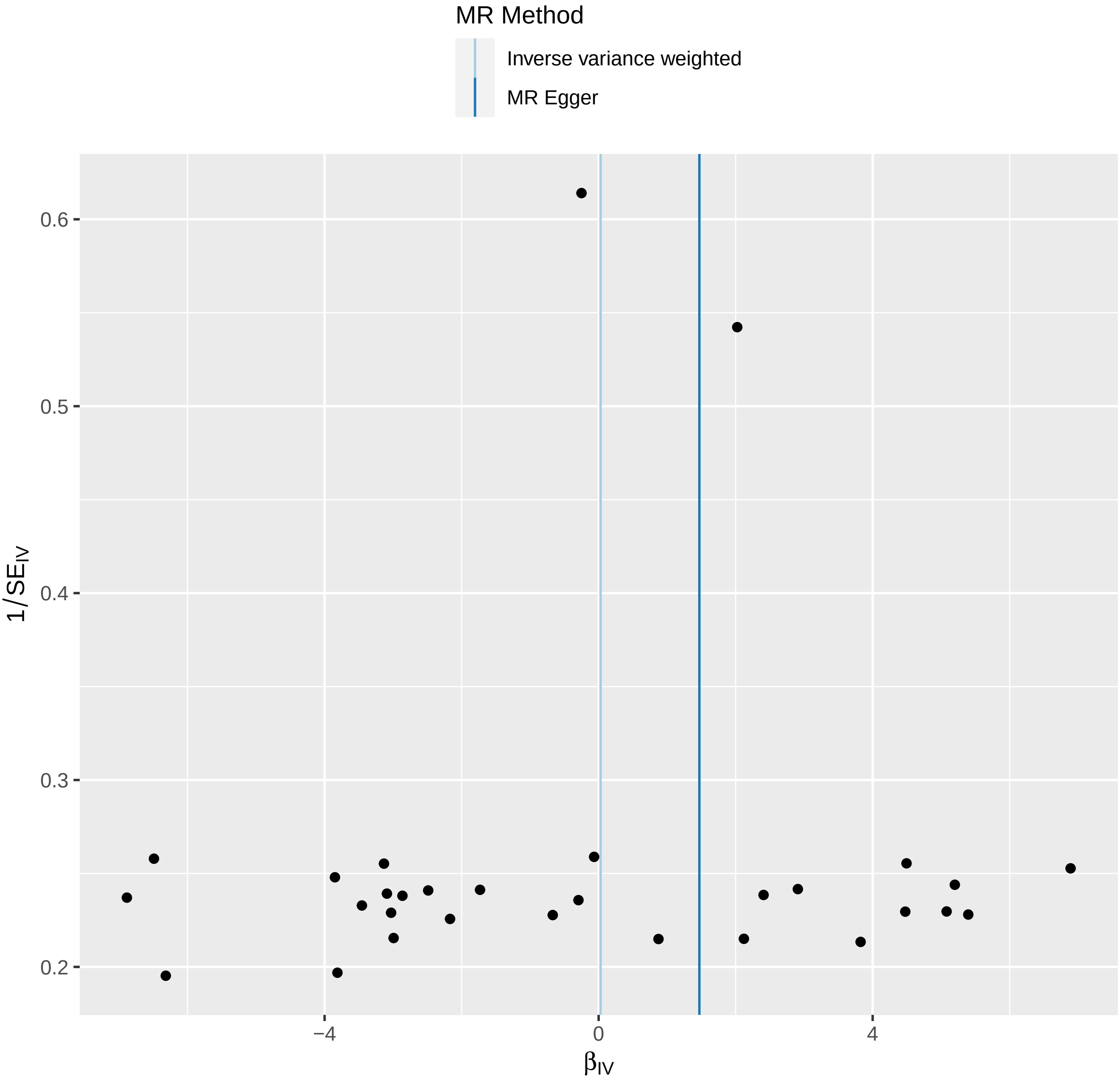

Batch 2349 : Caprylate (8:0) on Ulcerative proctitis

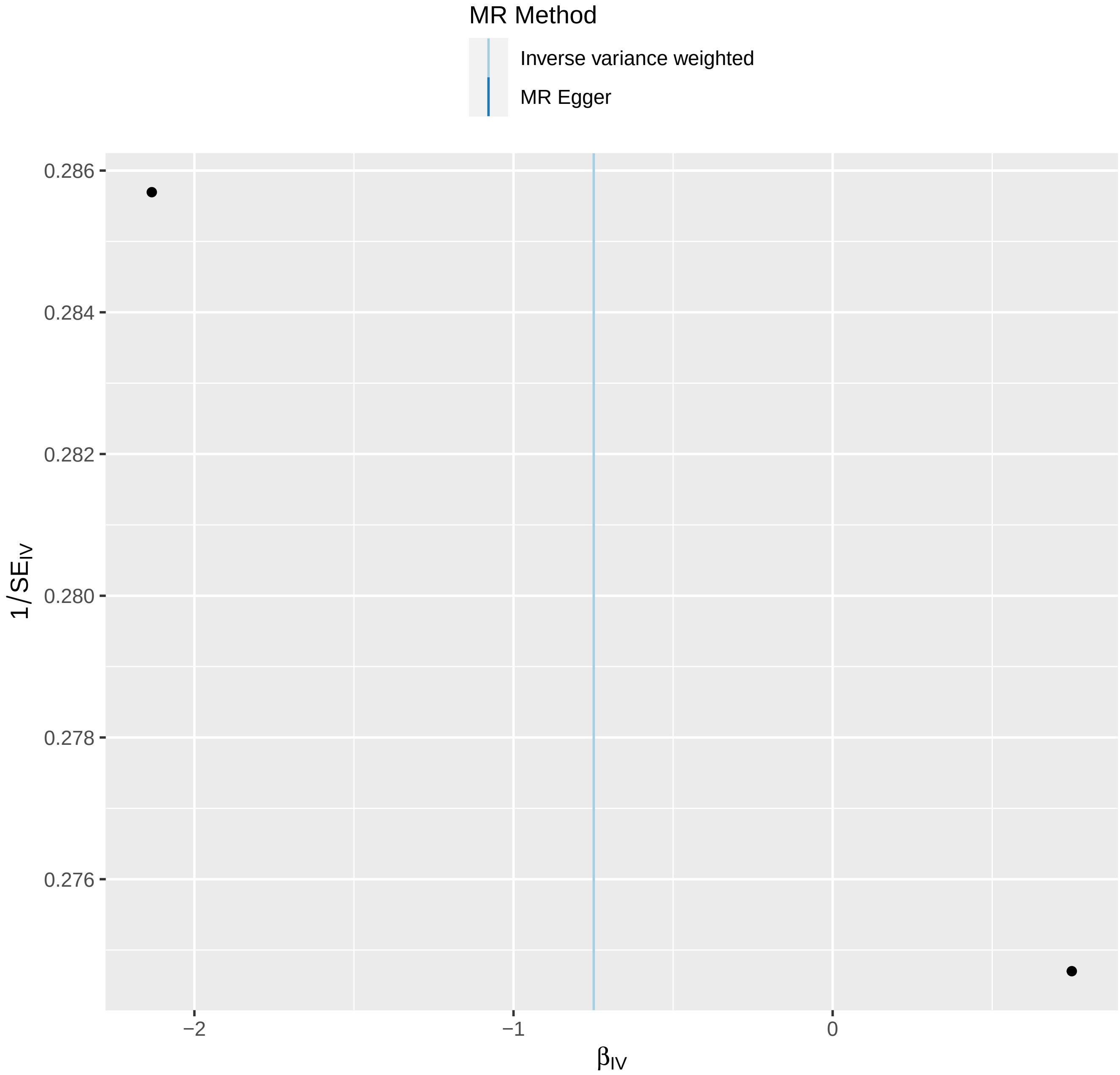

Batch 2350 : 10−undecenoate (11:1n1) on Ulcerative proctitis

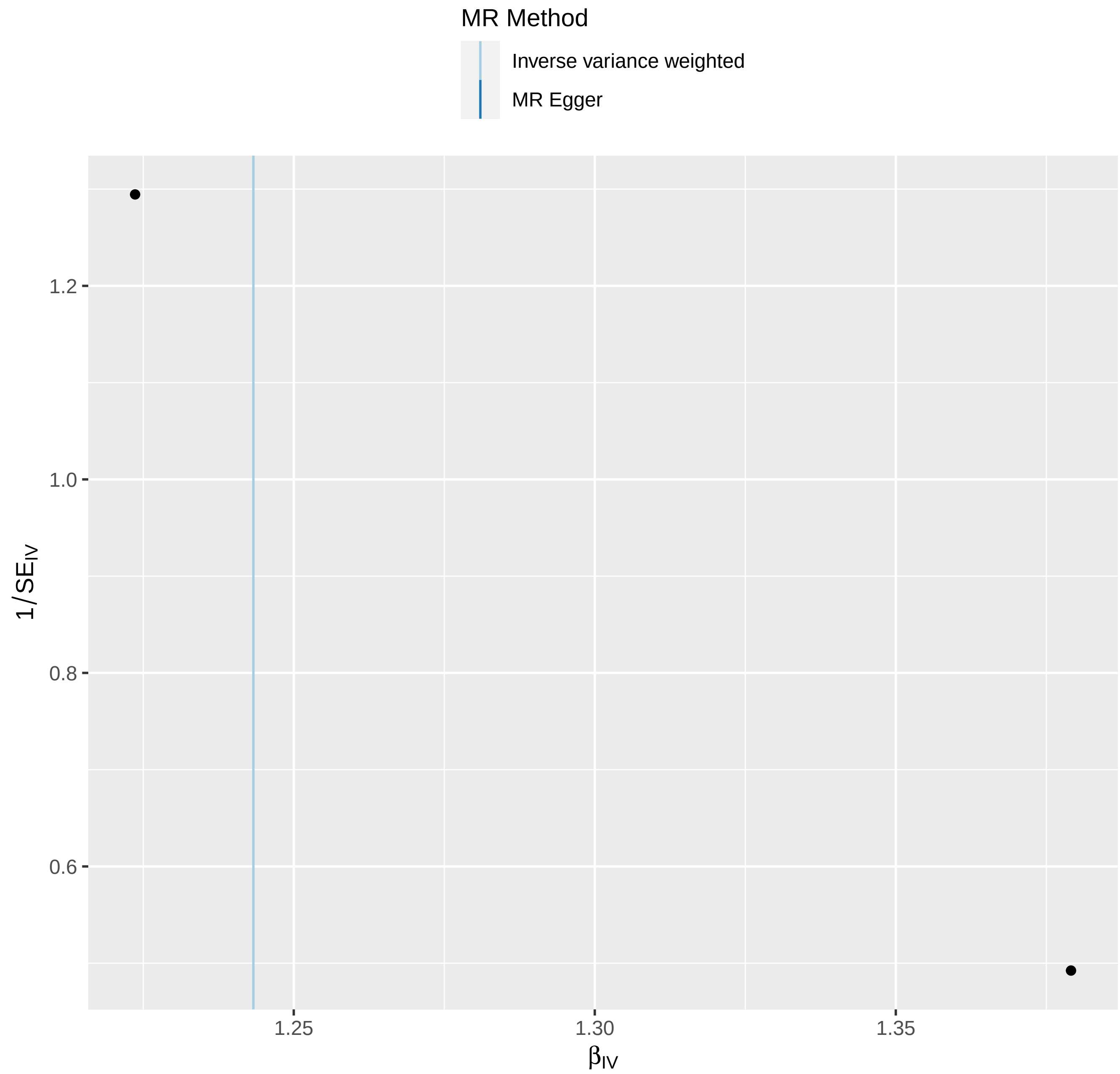

Batch 2352 : Phenol sulfate on Ulcerative proctitis

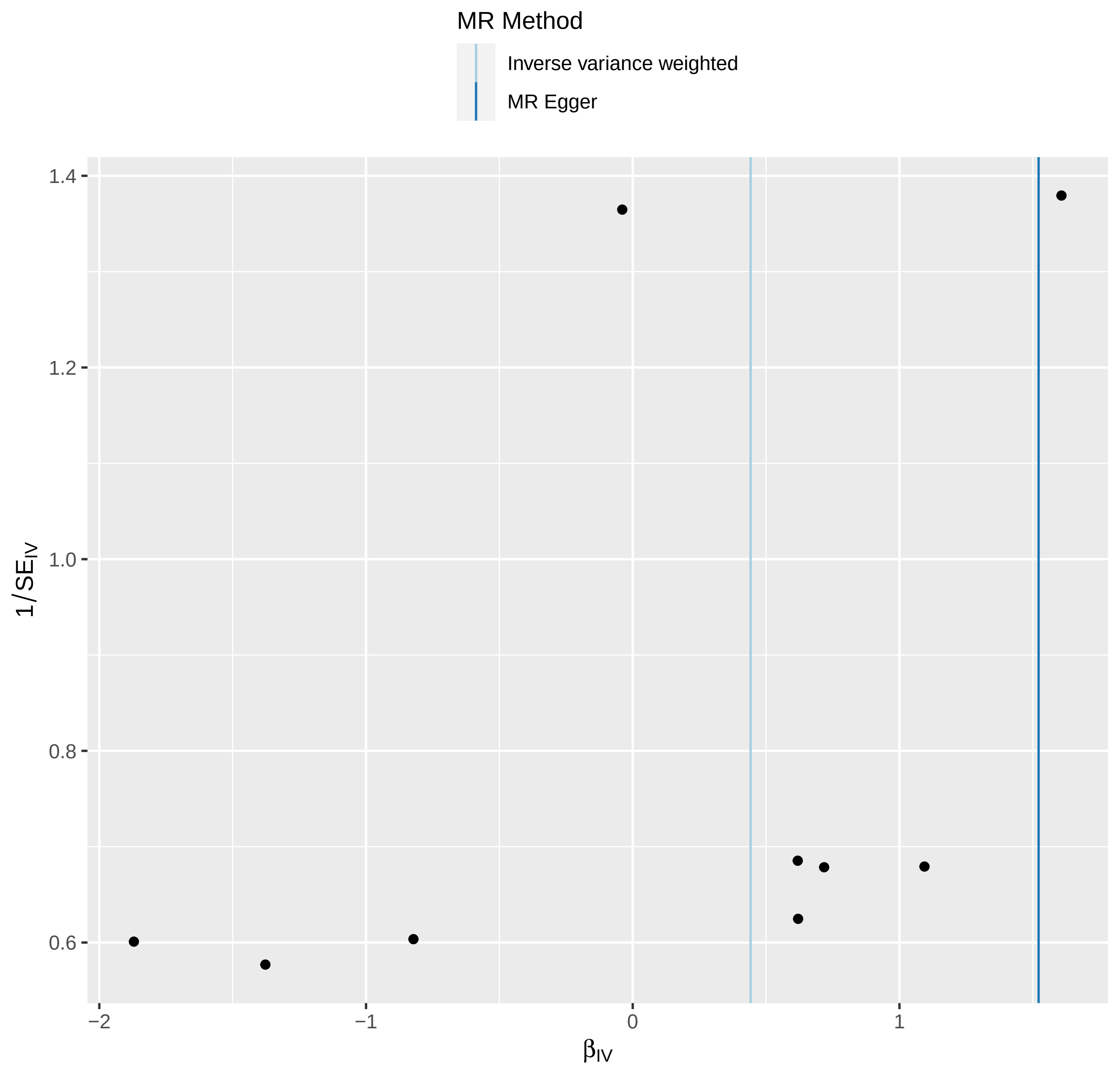

Batch 2354 : Heme* on Ulcerative proctitis

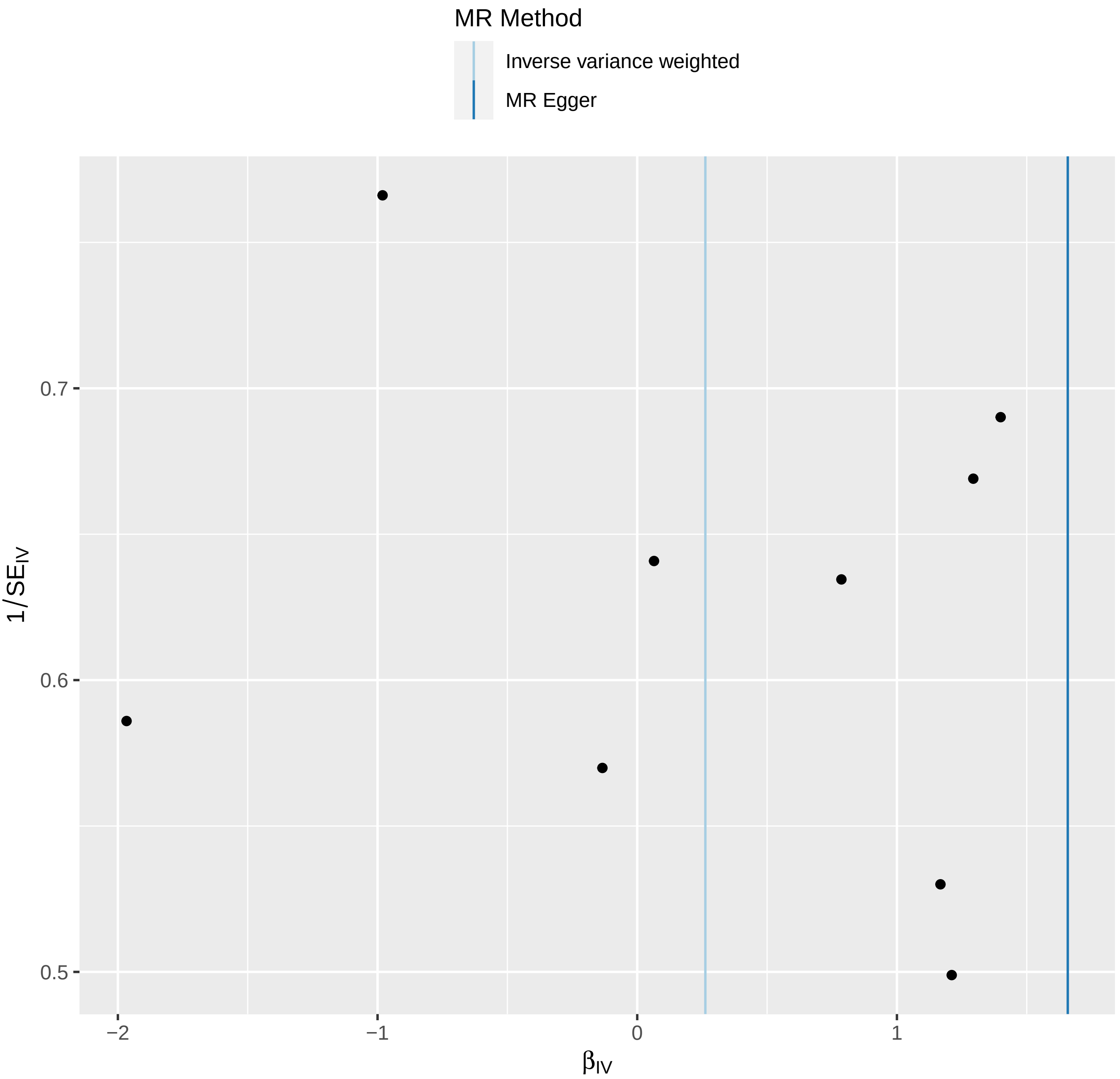

Batch 2356 : 3−dehydrocarnitine* on Ulcerative proctitis

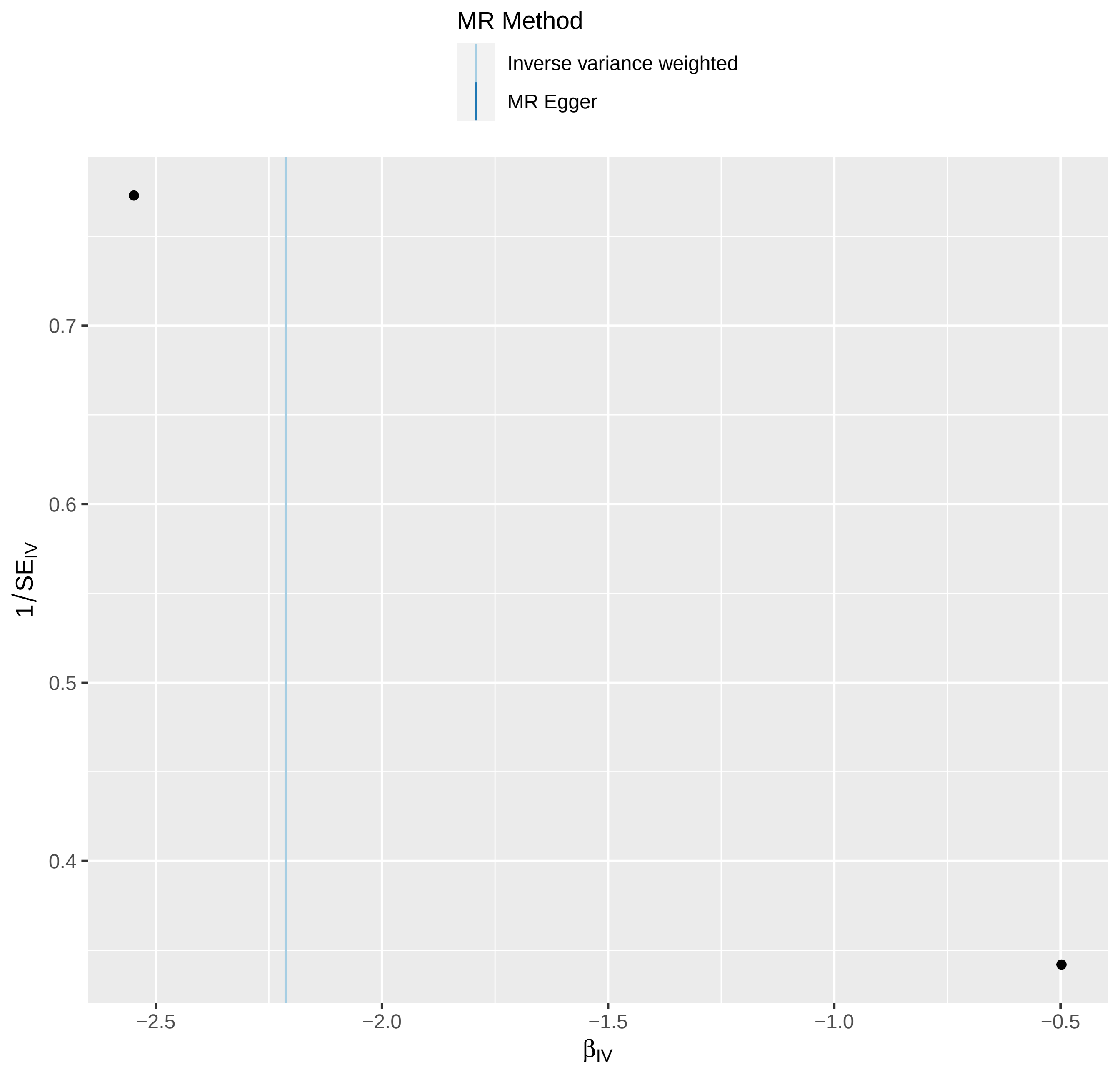

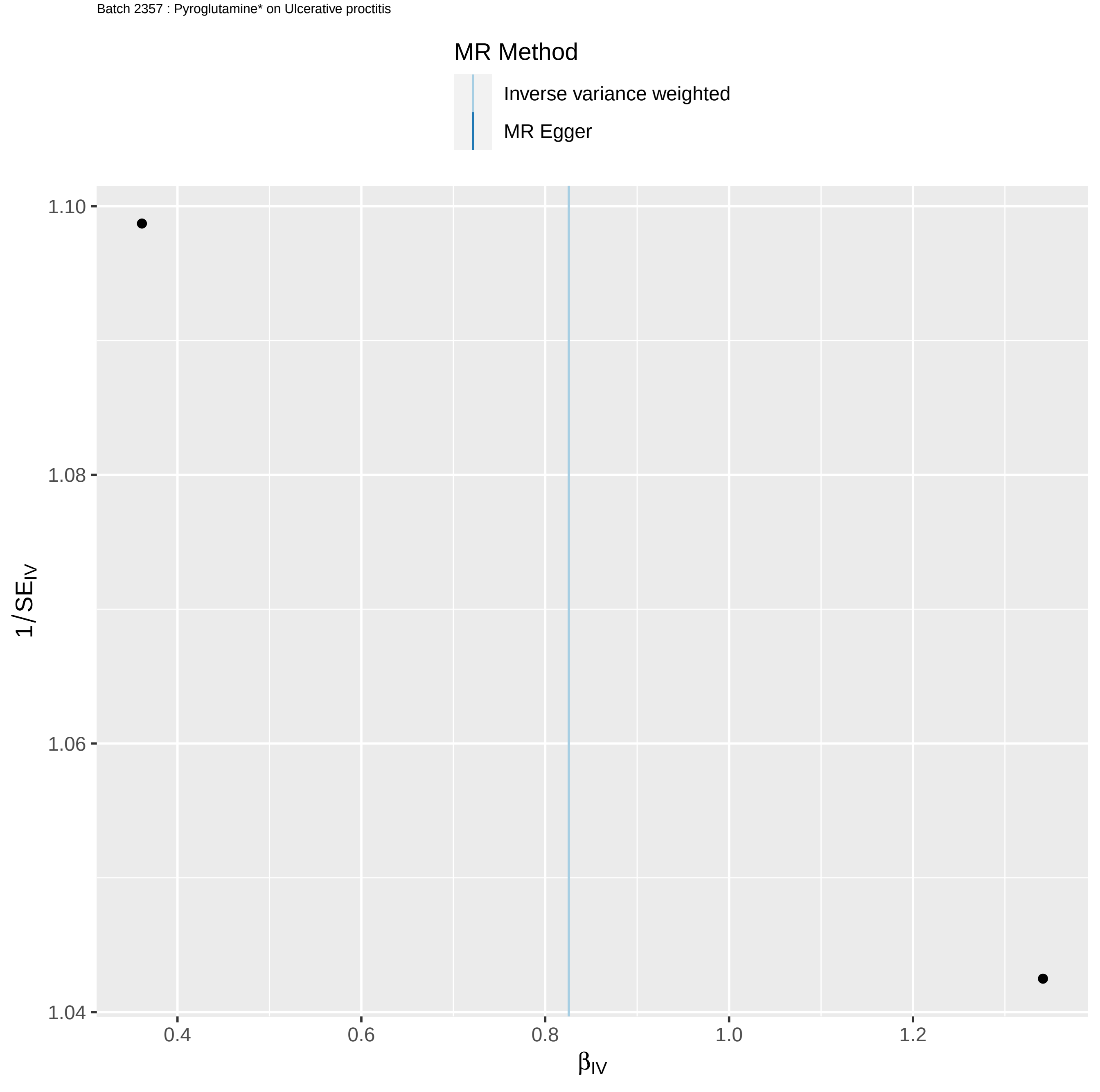
Batch 2357 : Pyroglutamine* on Ulcerative proctitis
MR Method
Inverse variance weighted
MR Egger
1.10
1.08
1.06
1.04
$1/SE_{IV}$
0.4
0.6
0.8
1.0
1.2
β_{IV}

Batch 2360 : X−11422−−xanthine on Ulcerative proctitis

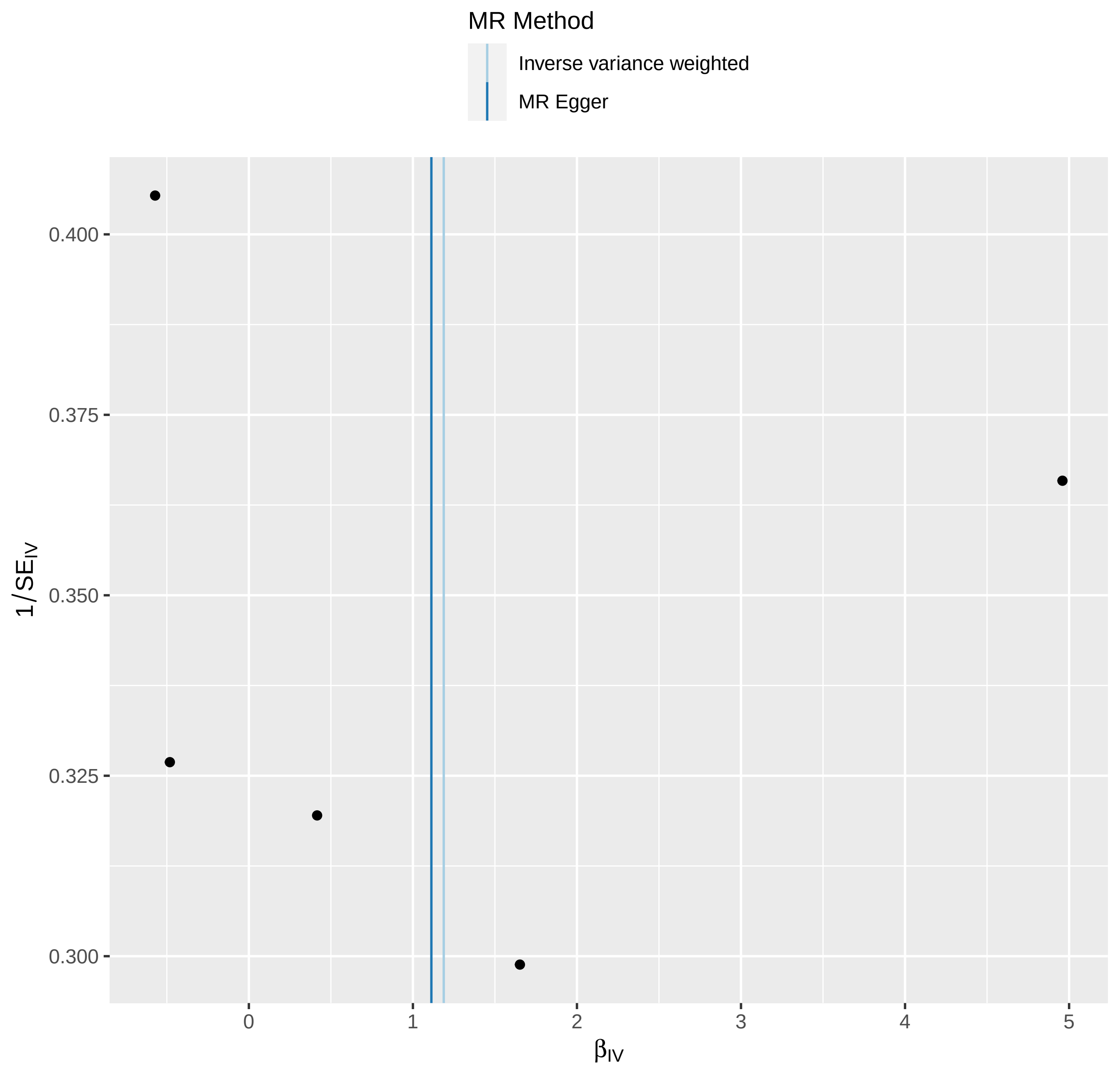

Batch 2363 : HWESASXX* on Ulcerative proctitis

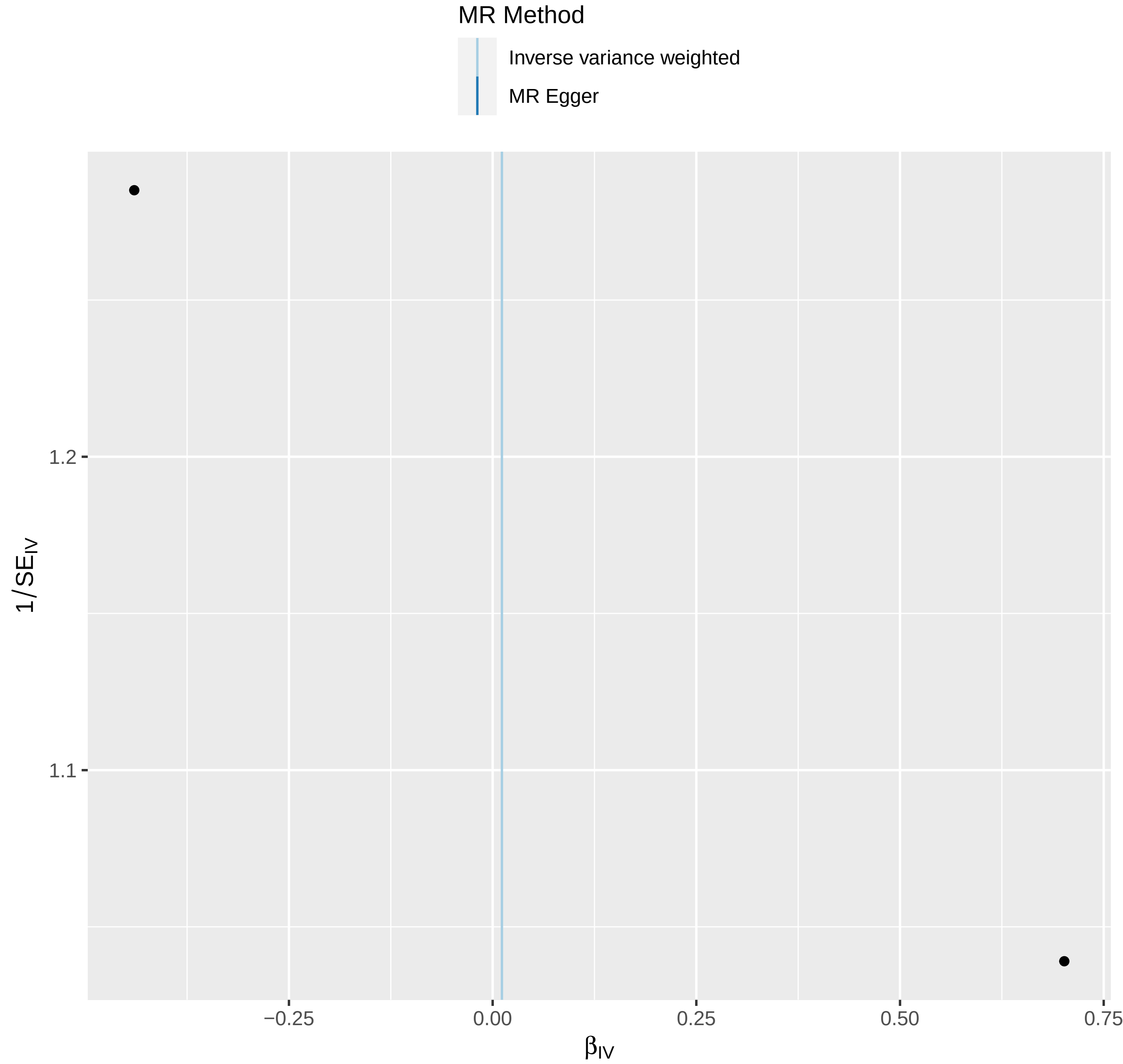

Batch 2364 : X−11593−−O−methylascorbate* on Ulcerative proctitis

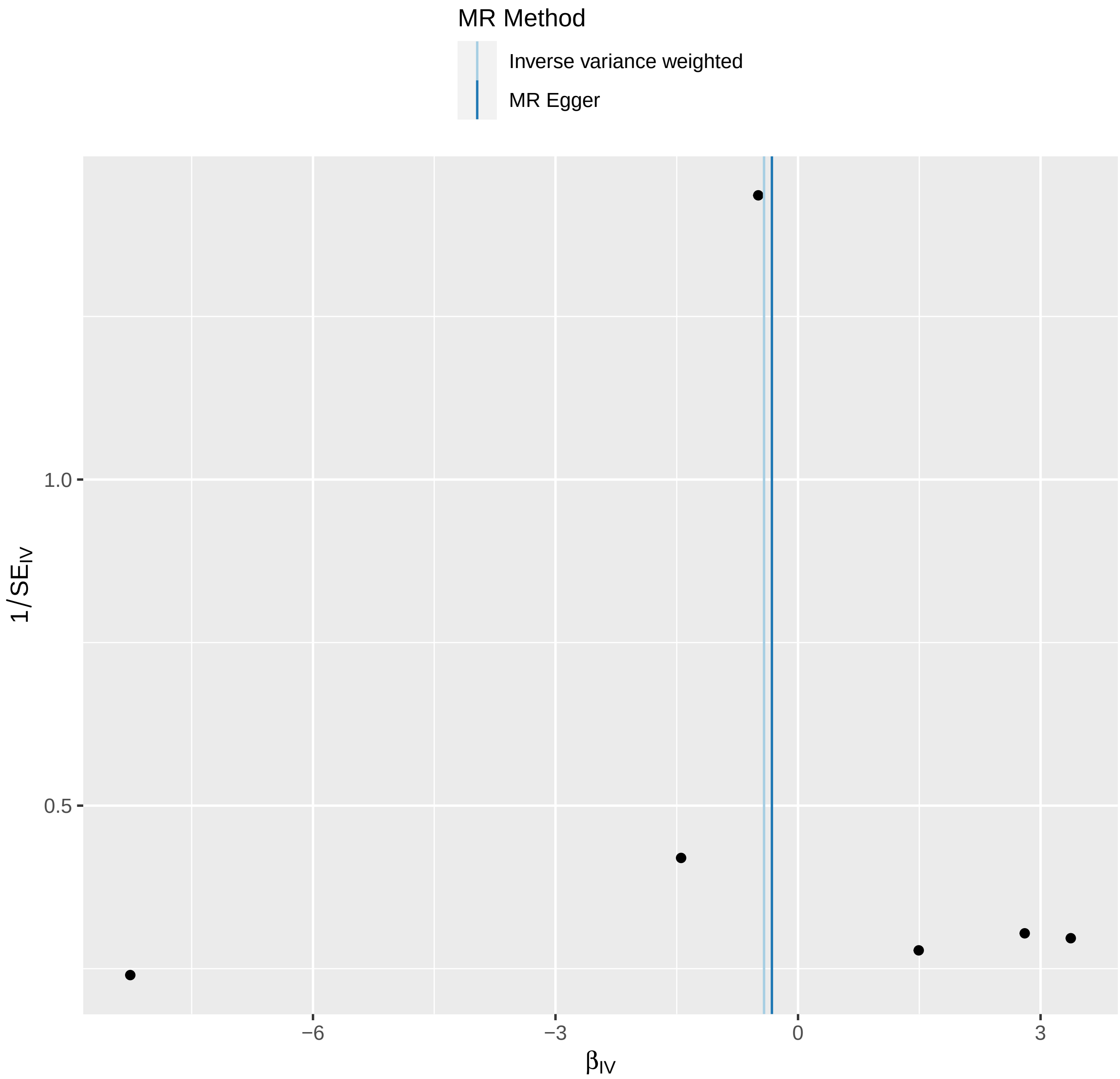

Batch 2366 : Homostachydrine* on Ulcerative proctitis

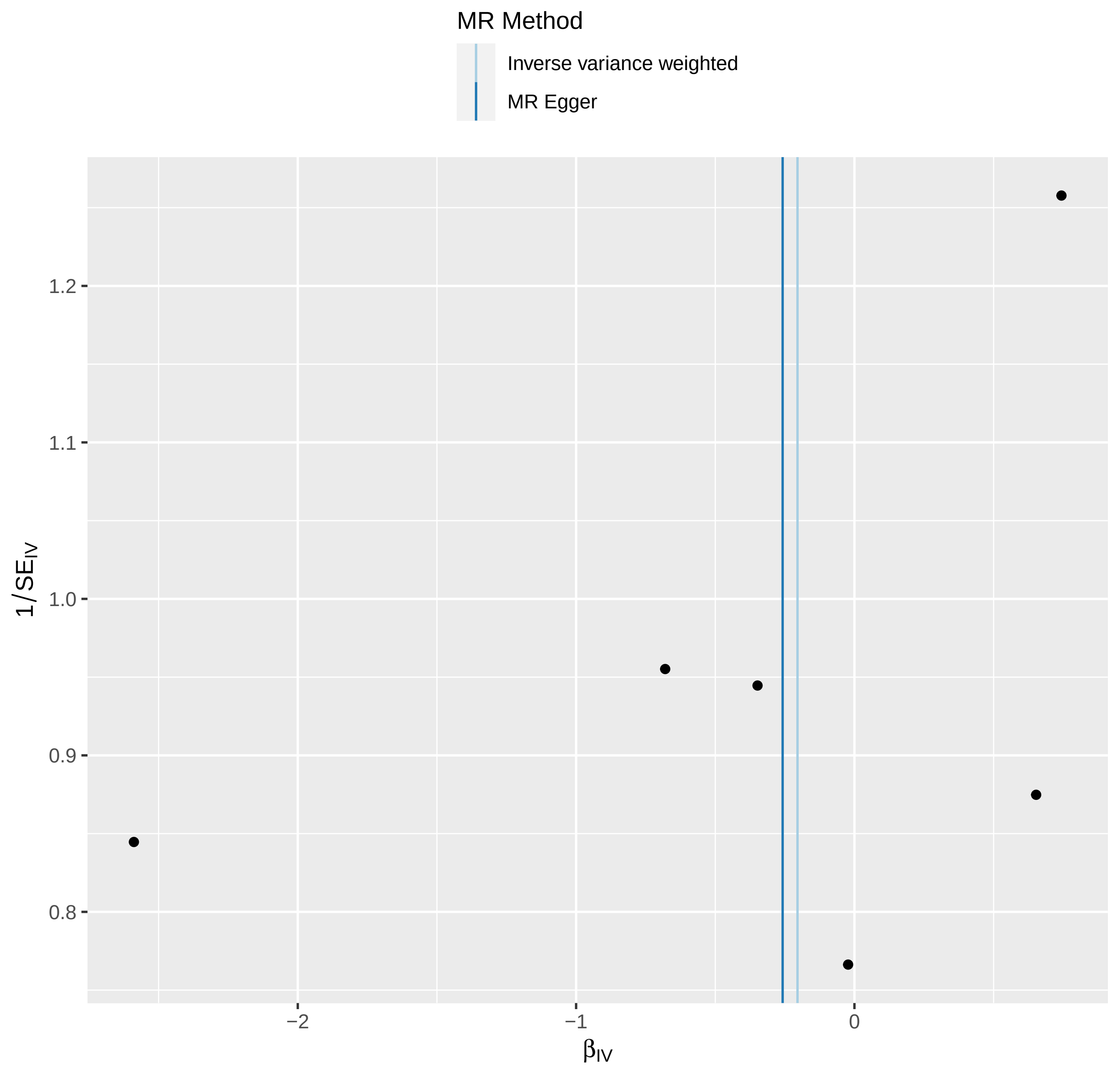

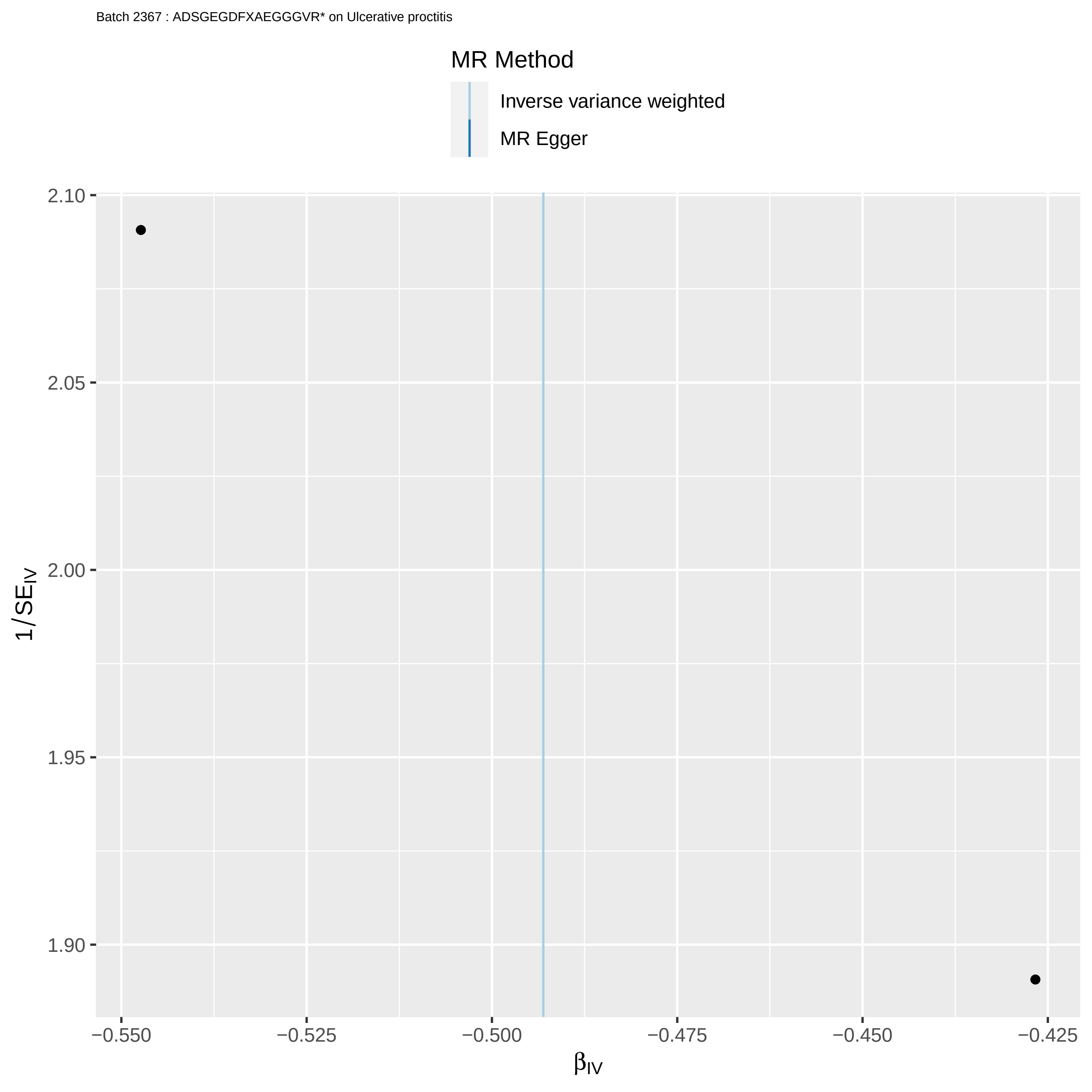
Batch 2367 : ADSGEGDFXAEGGGVR* on Ulcerative proctitis
MR Method
Inverse variance weighted
MR Egger
2.10
2.05
2.00
1.95
1.90
1/SE_IV
−0.550
−0.525
−0.500
−0.475
−0.450
−0.425
β_IV

Batch 2369 : X−11793−−oxidized bilirubin* on Ulcerative proctitis

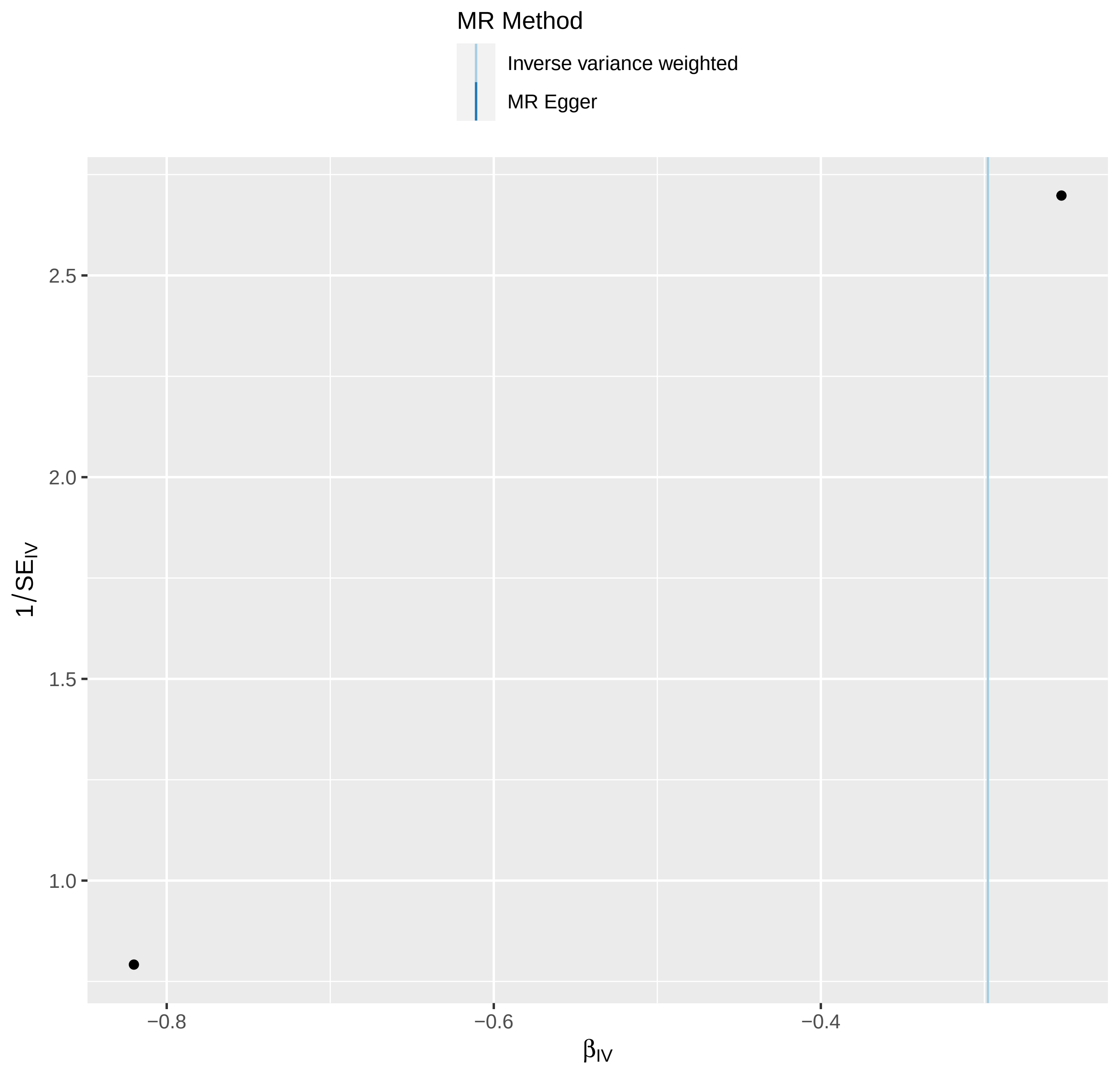

Batch 2370 : 1−arachidonoylglycerophosphocholine* on Ulcerative proctitis

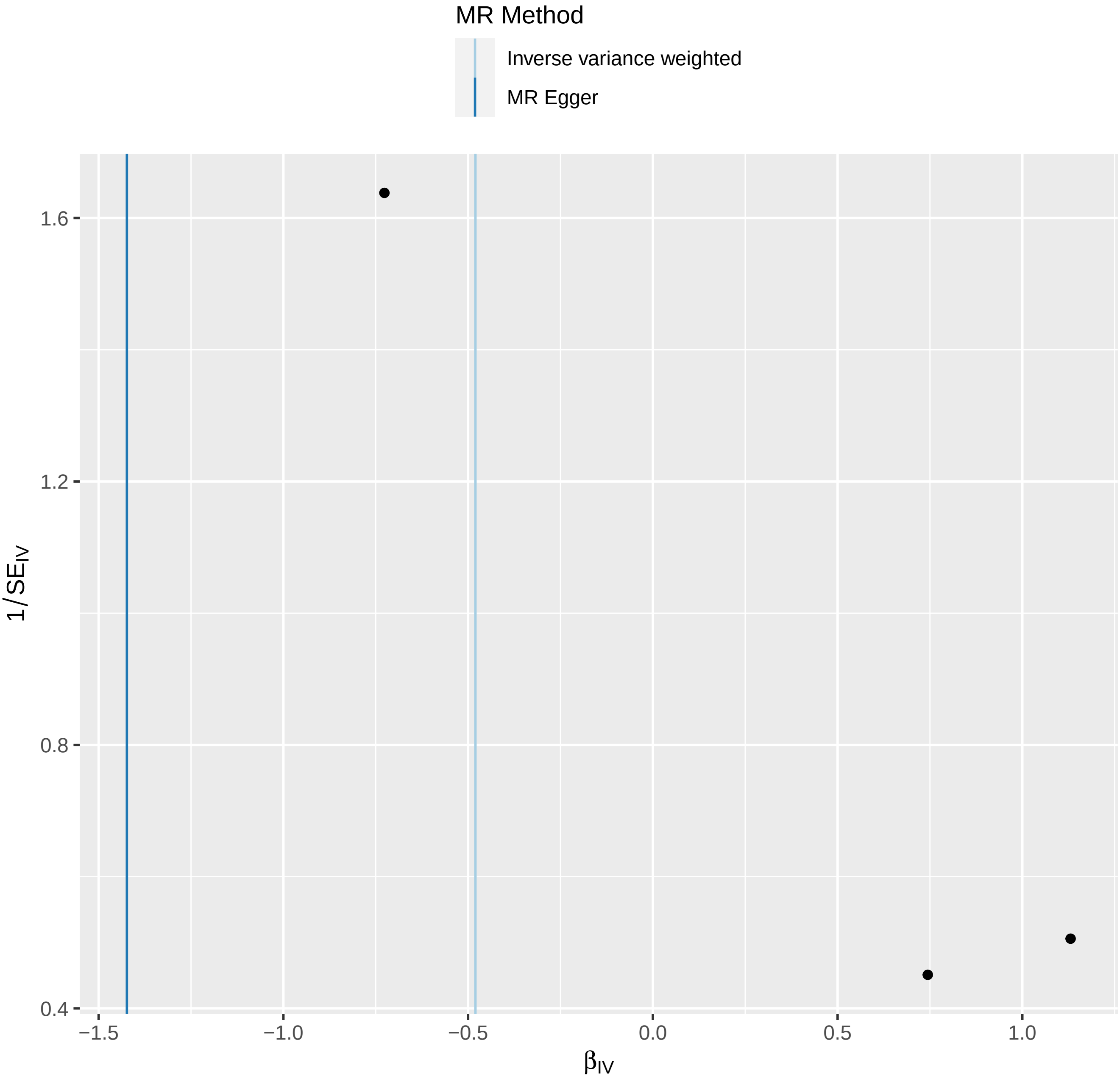

Batch 2372 : Gamma−glutamylmethionine* on Ulcerative proctitis

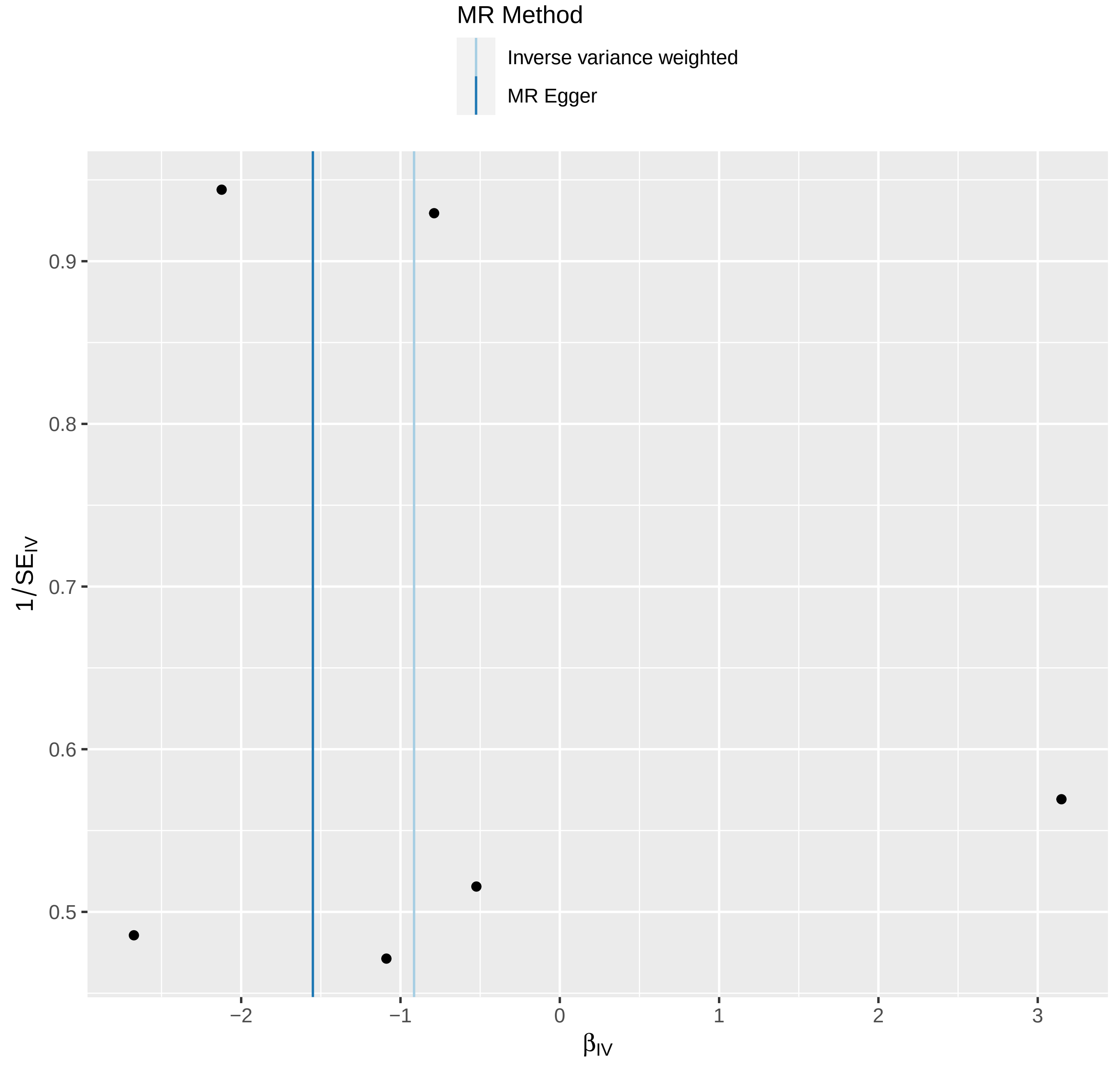

Batch 2373 : Gamma−glutamylthreonine* on Ulcerative proctitis

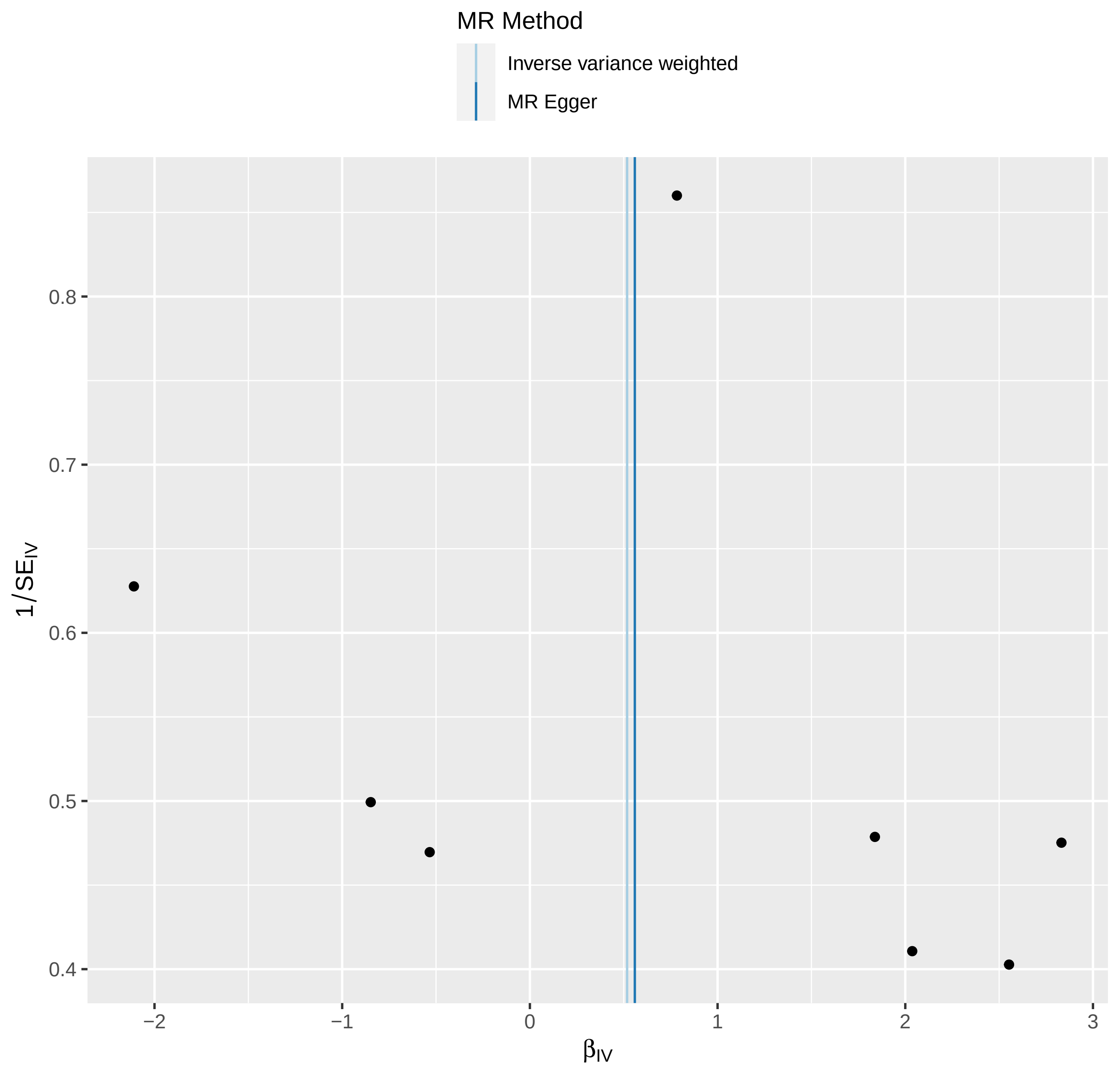

Batch 2374 : Gamma−tocopherol on Ulcerative proctitis

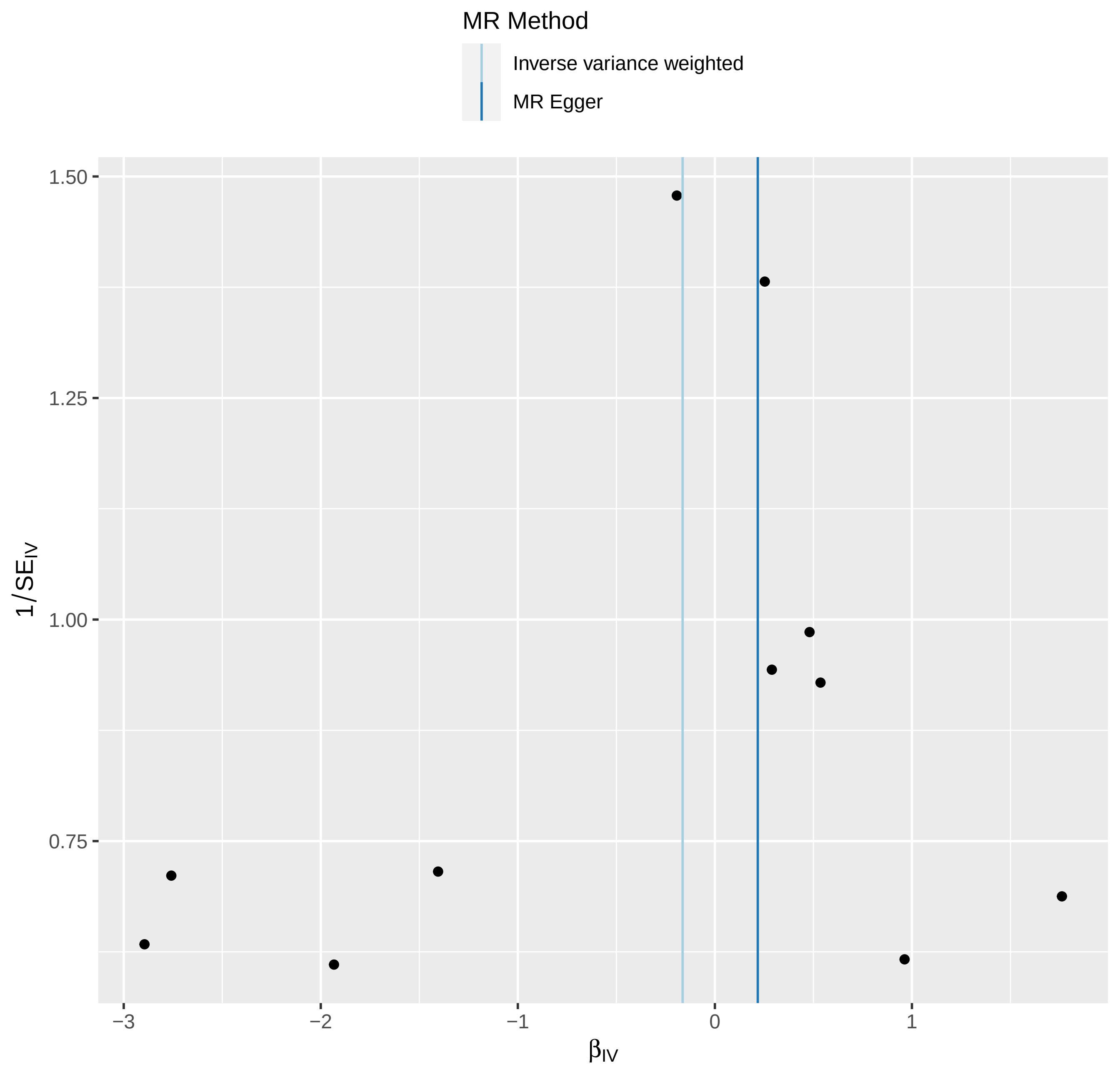

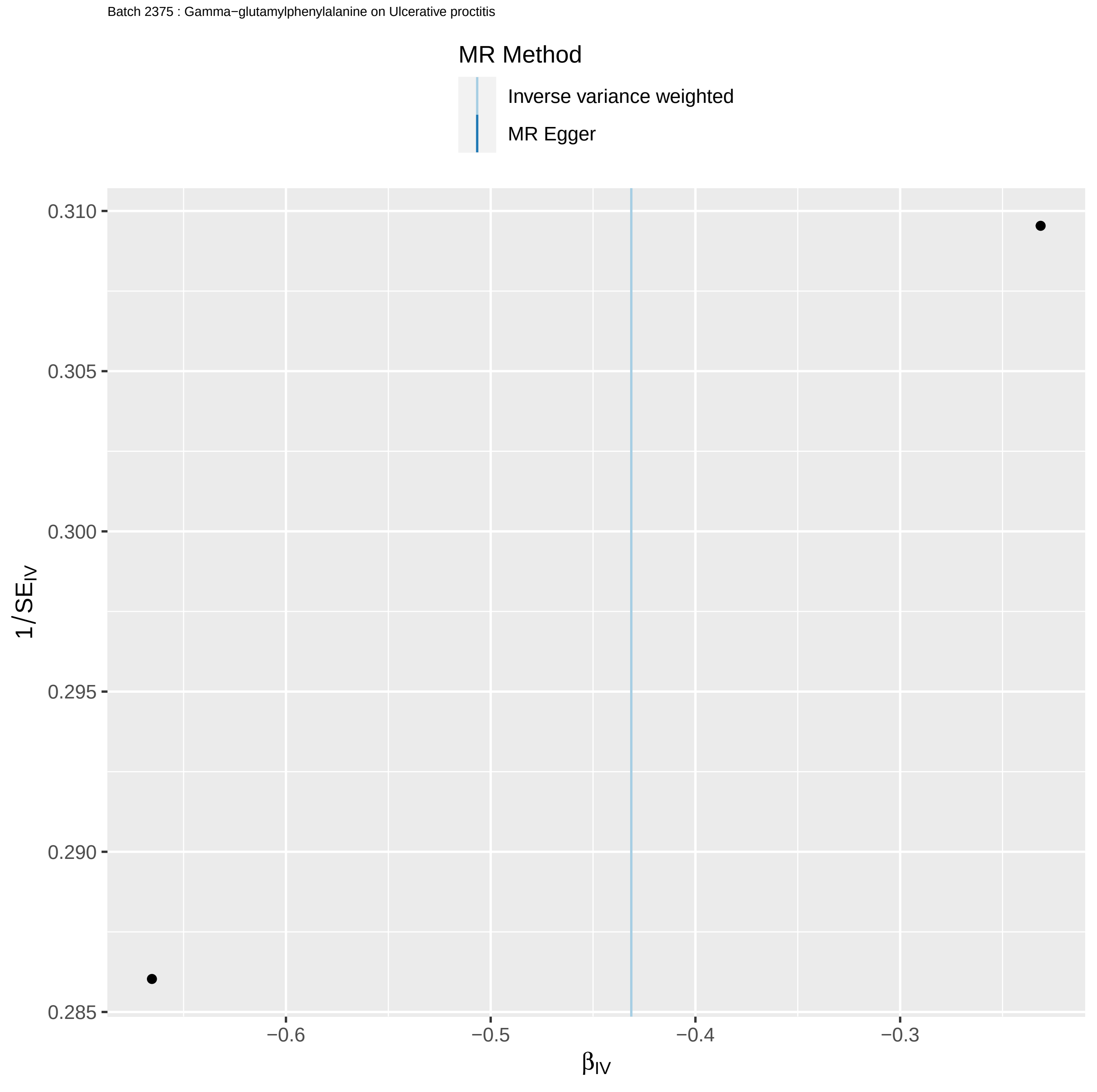
Batch 2375 : Gamma−glutamylphenylalanine on Ulcerative proctitis
MR Method
Inverse variance weighted
MR Egger
0.310
0.305
0.300
0.295
0.290
0.285
1/SE$_{IV}$
−0.6
−0.5
−0.4
−0.3
β$_{IV}$

Batch 2376 : Isobutyrylcarnitine on Ulcerative proctitis

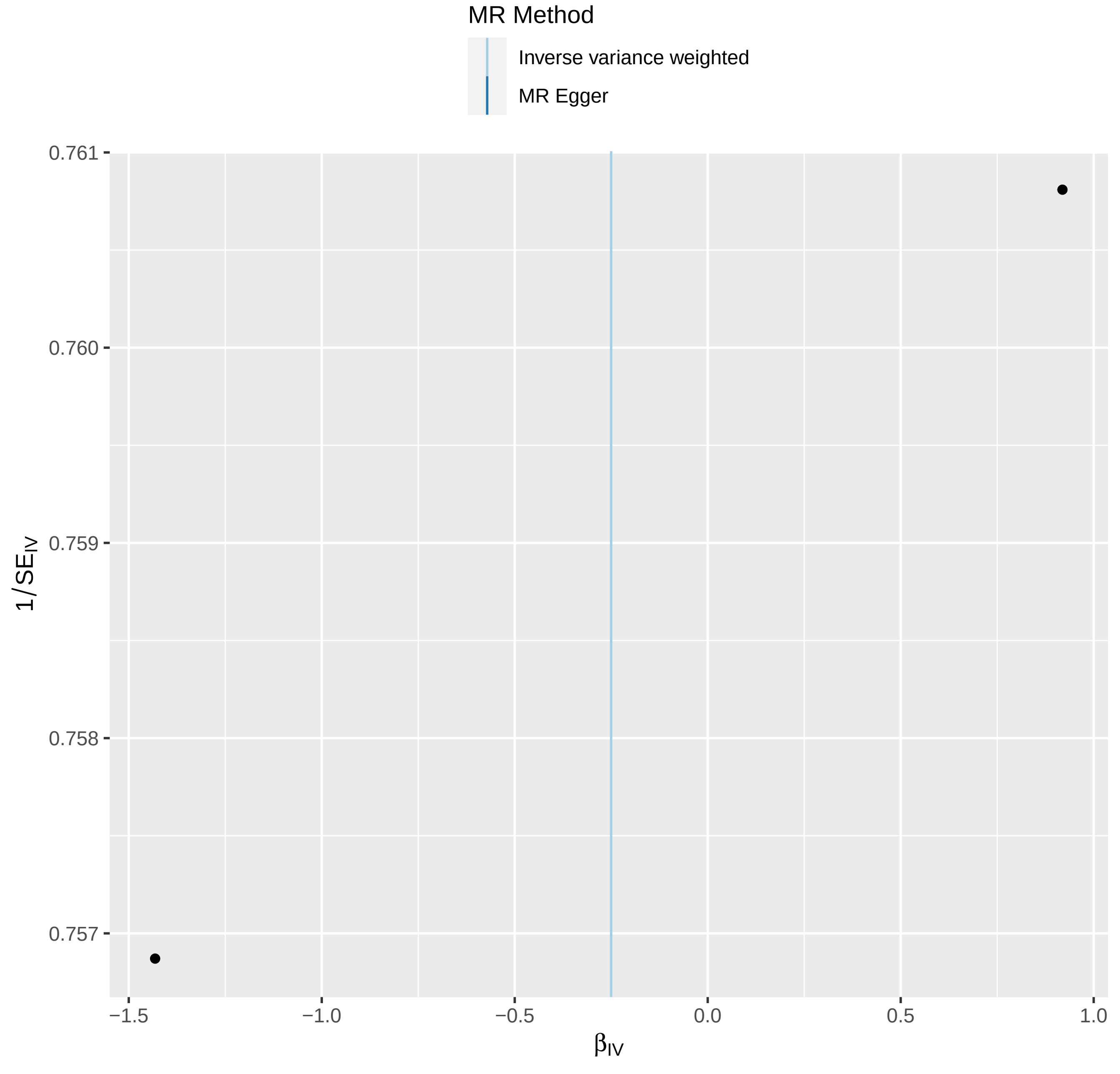

Batch 2377 : Pseudouridine on Ulcerative proctitis

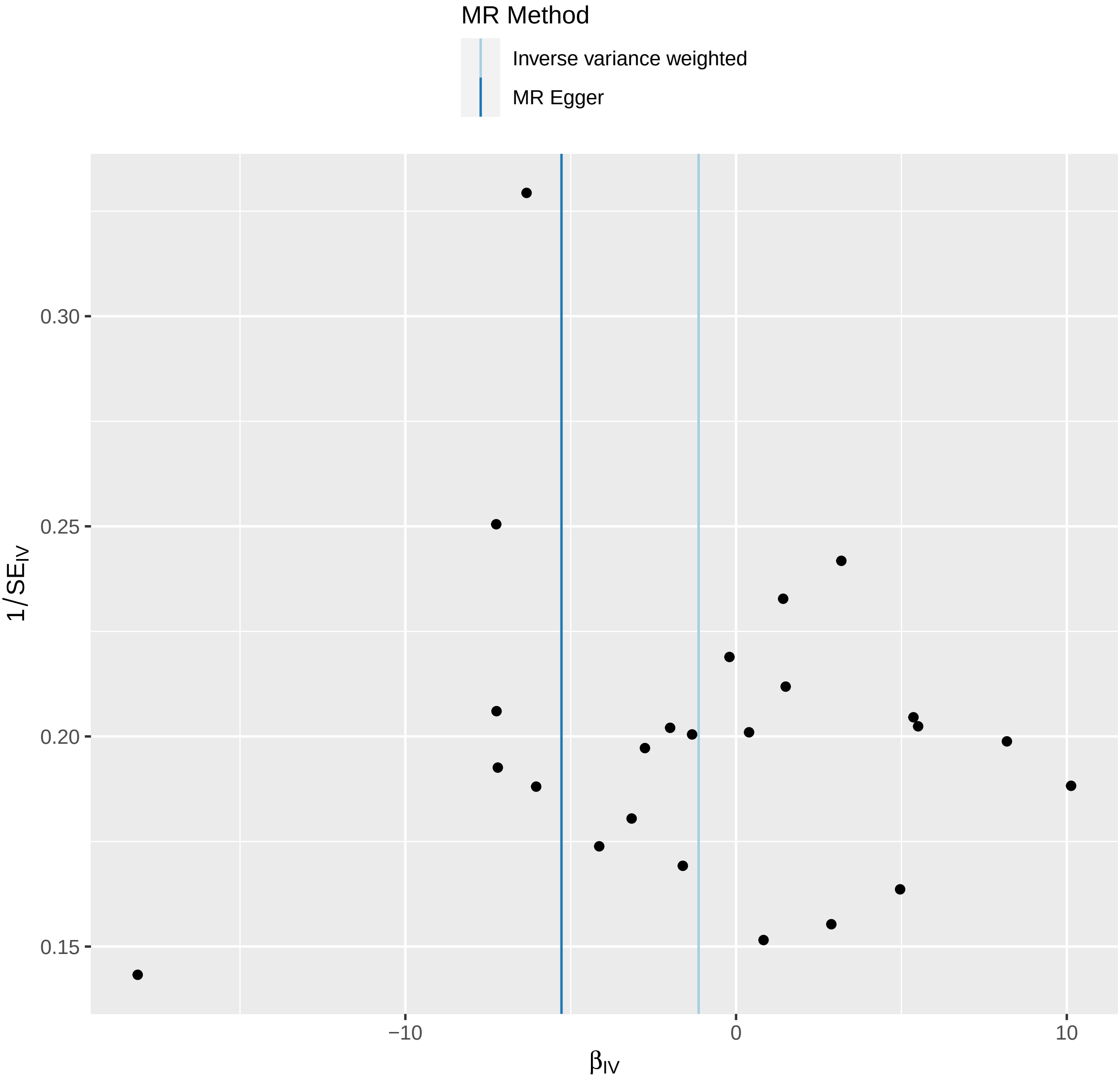

Batch 2378 : Valerate on Ulcerative proctitis

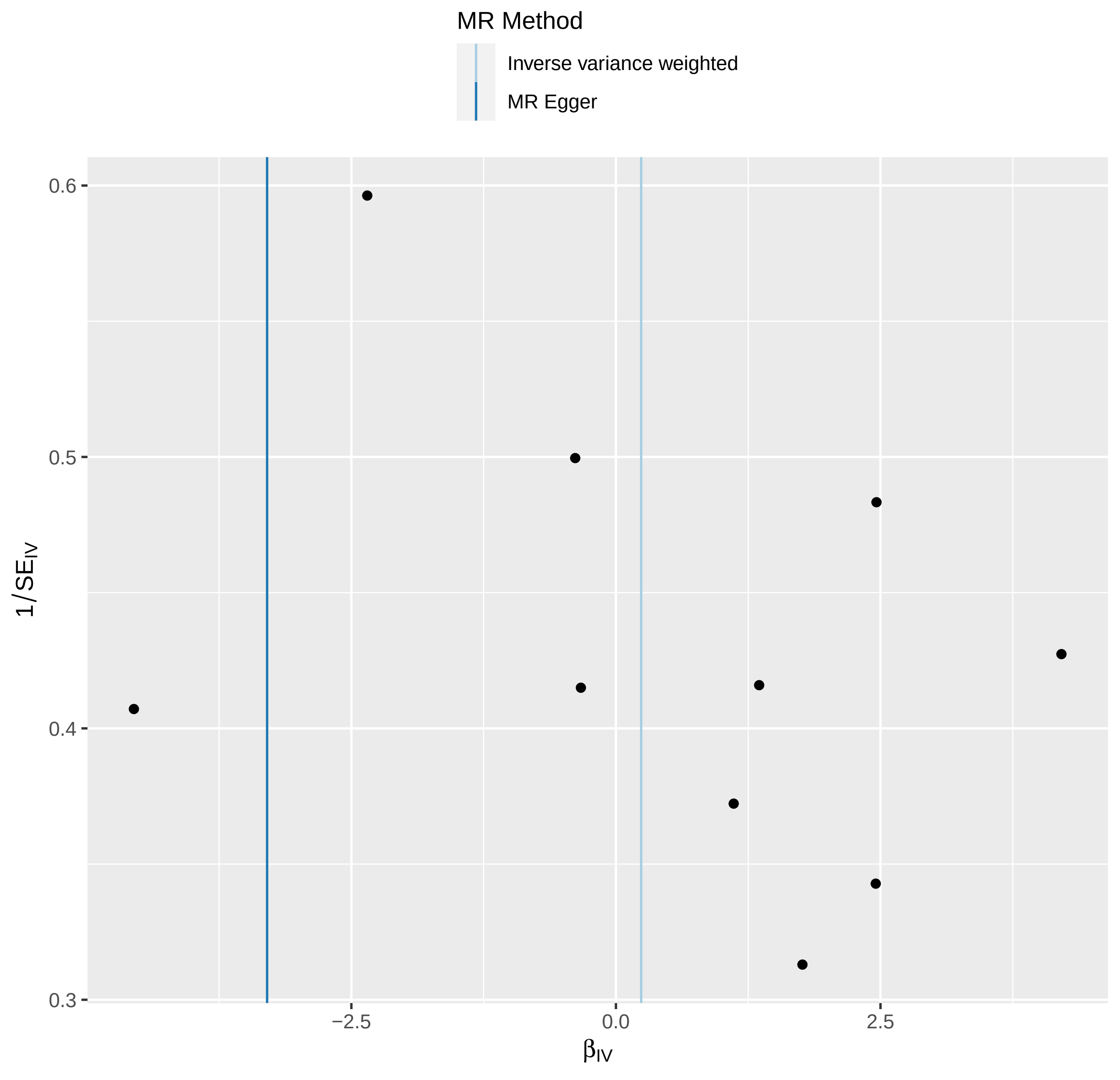

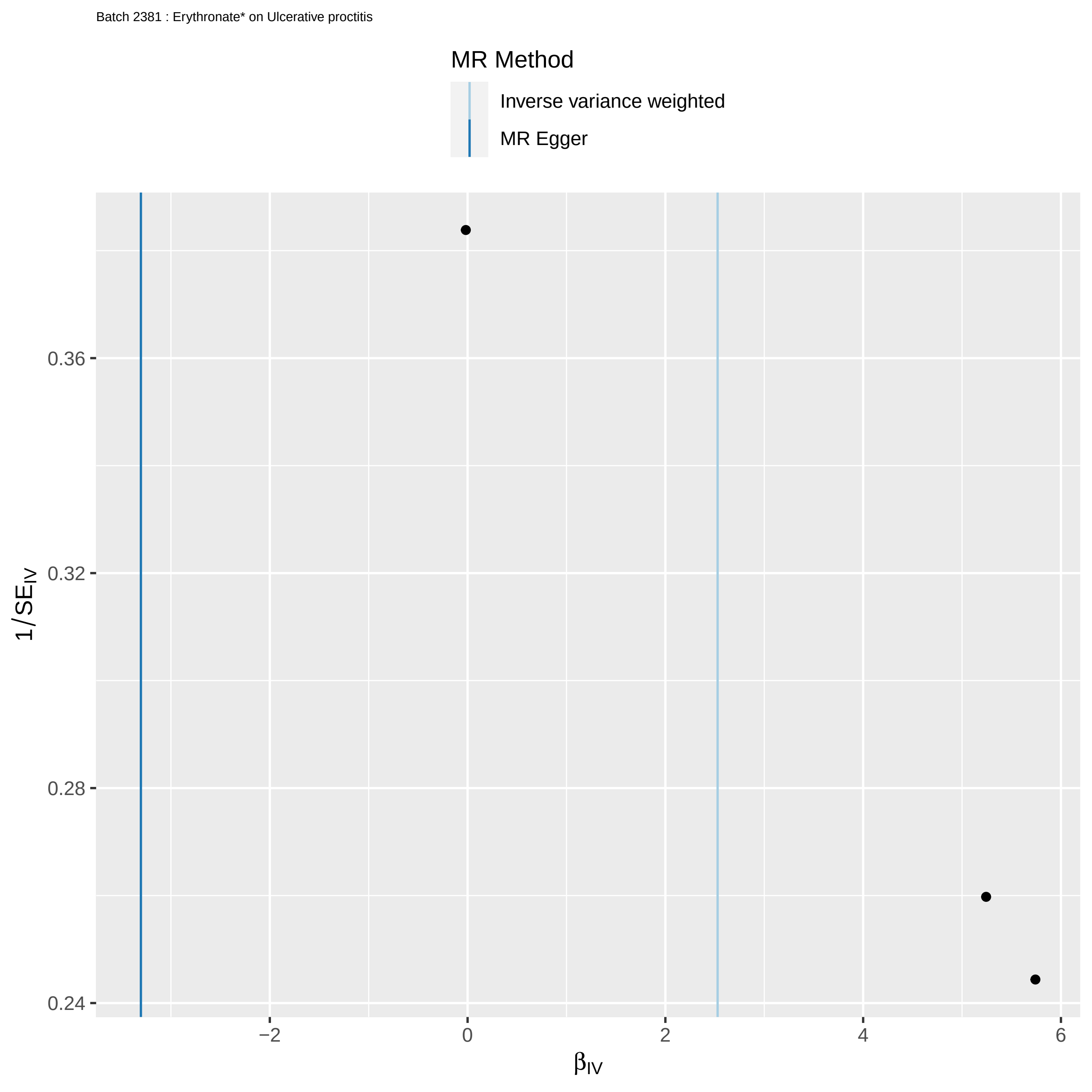

Batch 2381 : Erythronate* on Ulcerative proctitis
MR Method
Inverse variance weighted
MR Egger
0.36
0.32
0.28
0.24
1/SE_IV
−2
0
2
4
6
β_IV

Batch 2382 : Lathosterol on Ulcerative proctitis

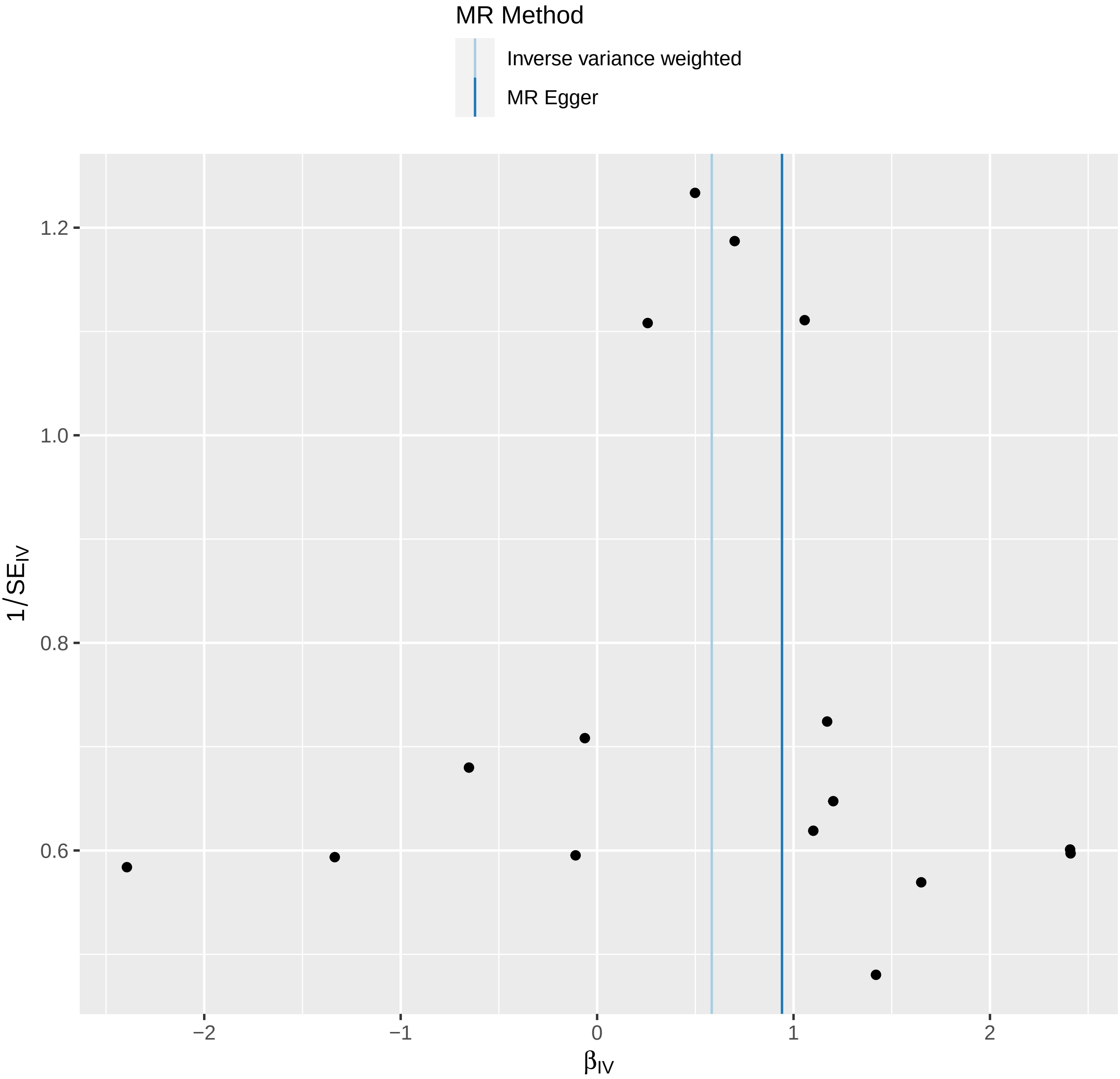

Batch 2385 : Eicosenoate (20:1n9 or 11) on Ulcerative proctitis

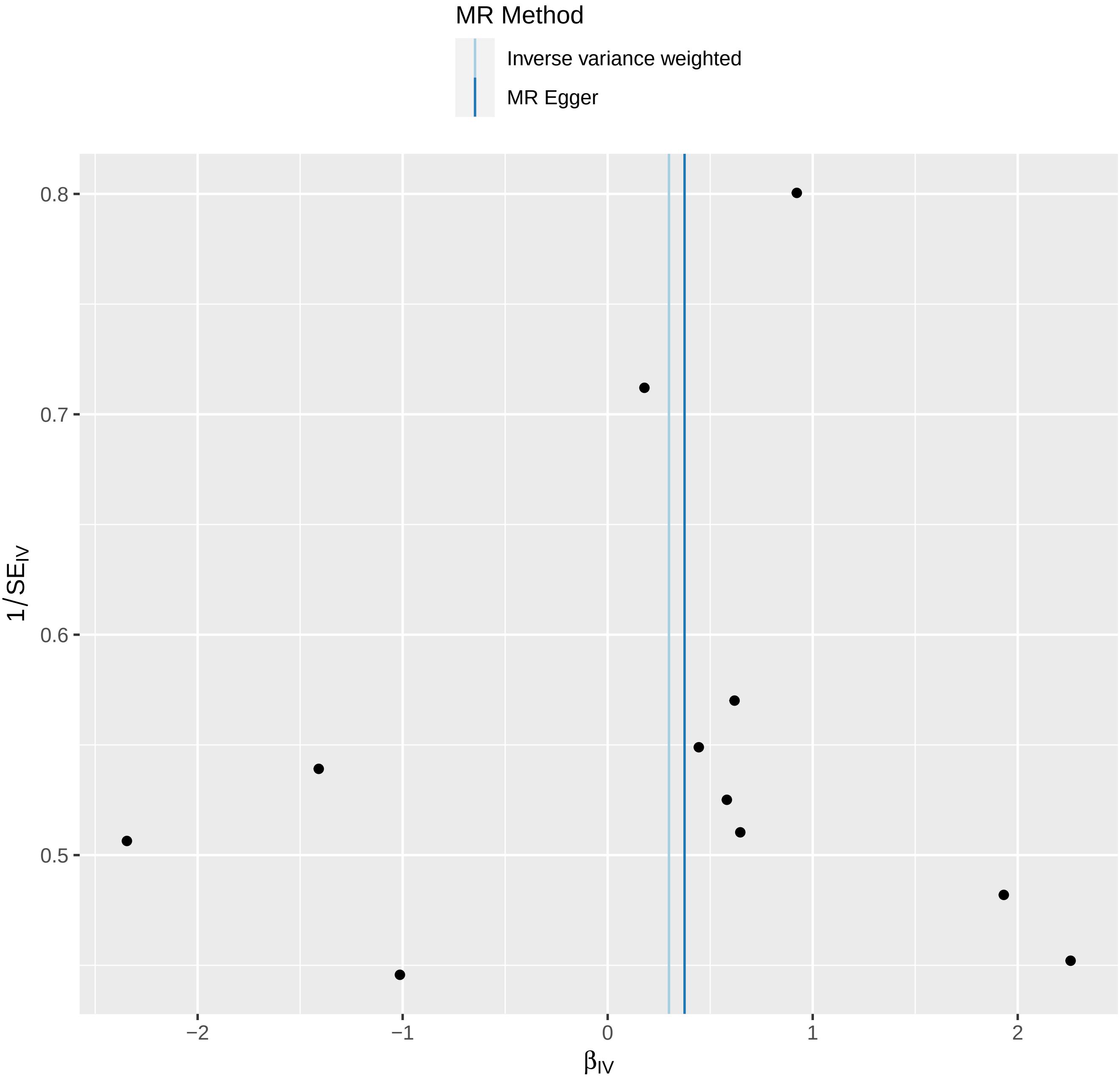

Batch 2386 : X−12244−−N−acetylcarnosine on Ulcerative proctitis

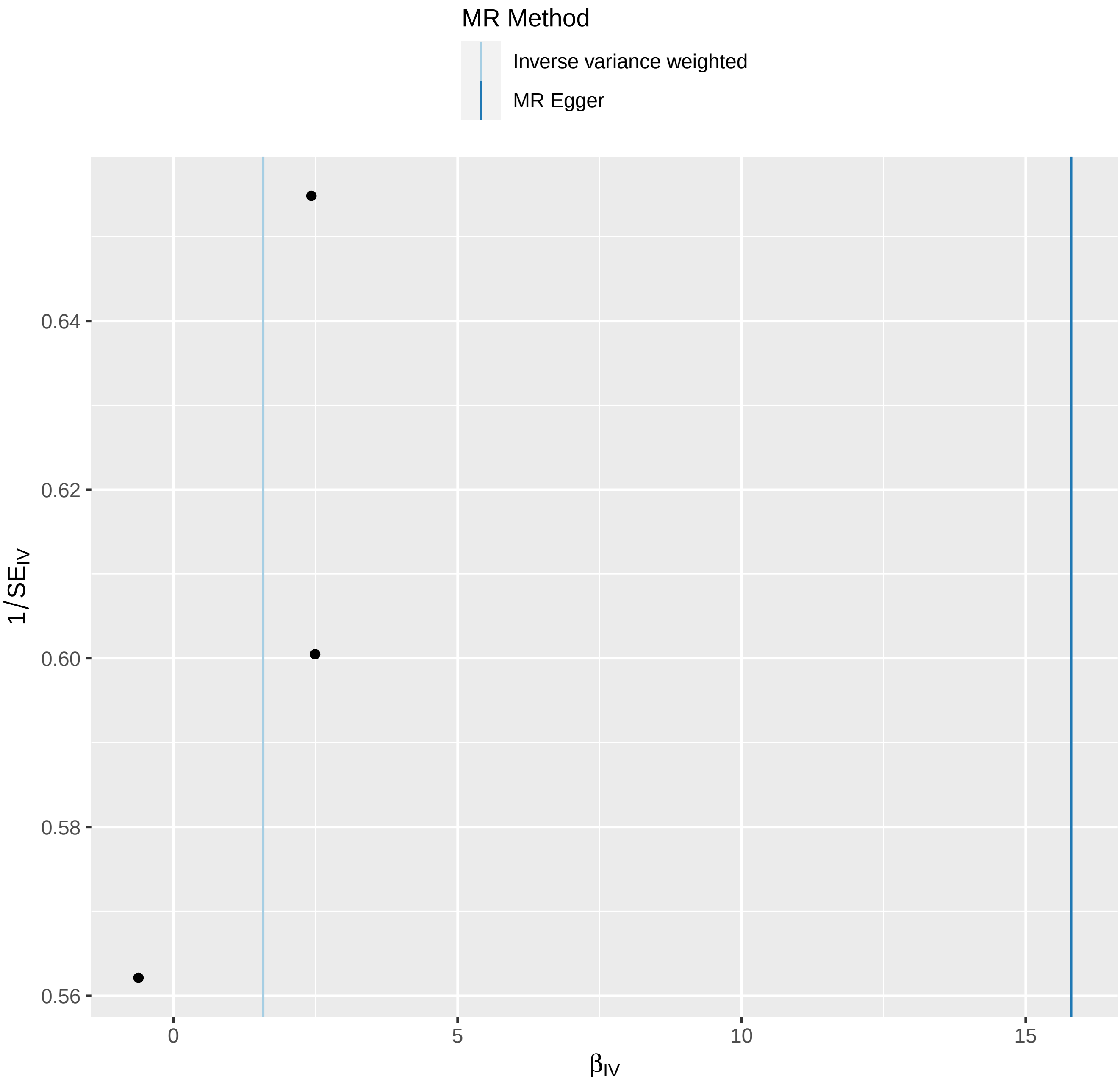

Batch 2388 : 1−eicosatrienoylglycerophosphocholine* on Ulcerative proctitis

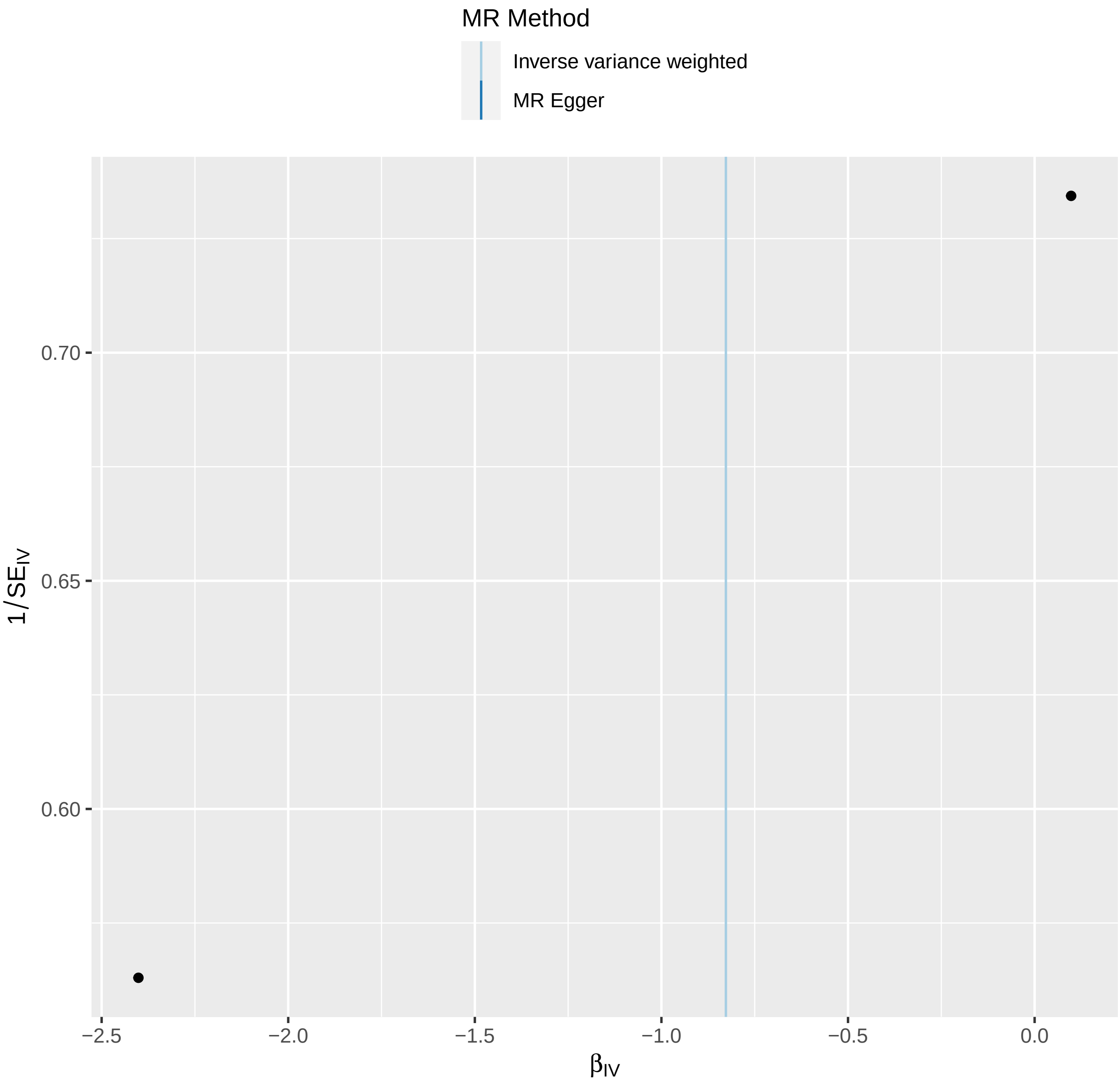

Batch 2389 : 1−docosahexaenoylglycerophosphocholine* on Ulcerative proctitis

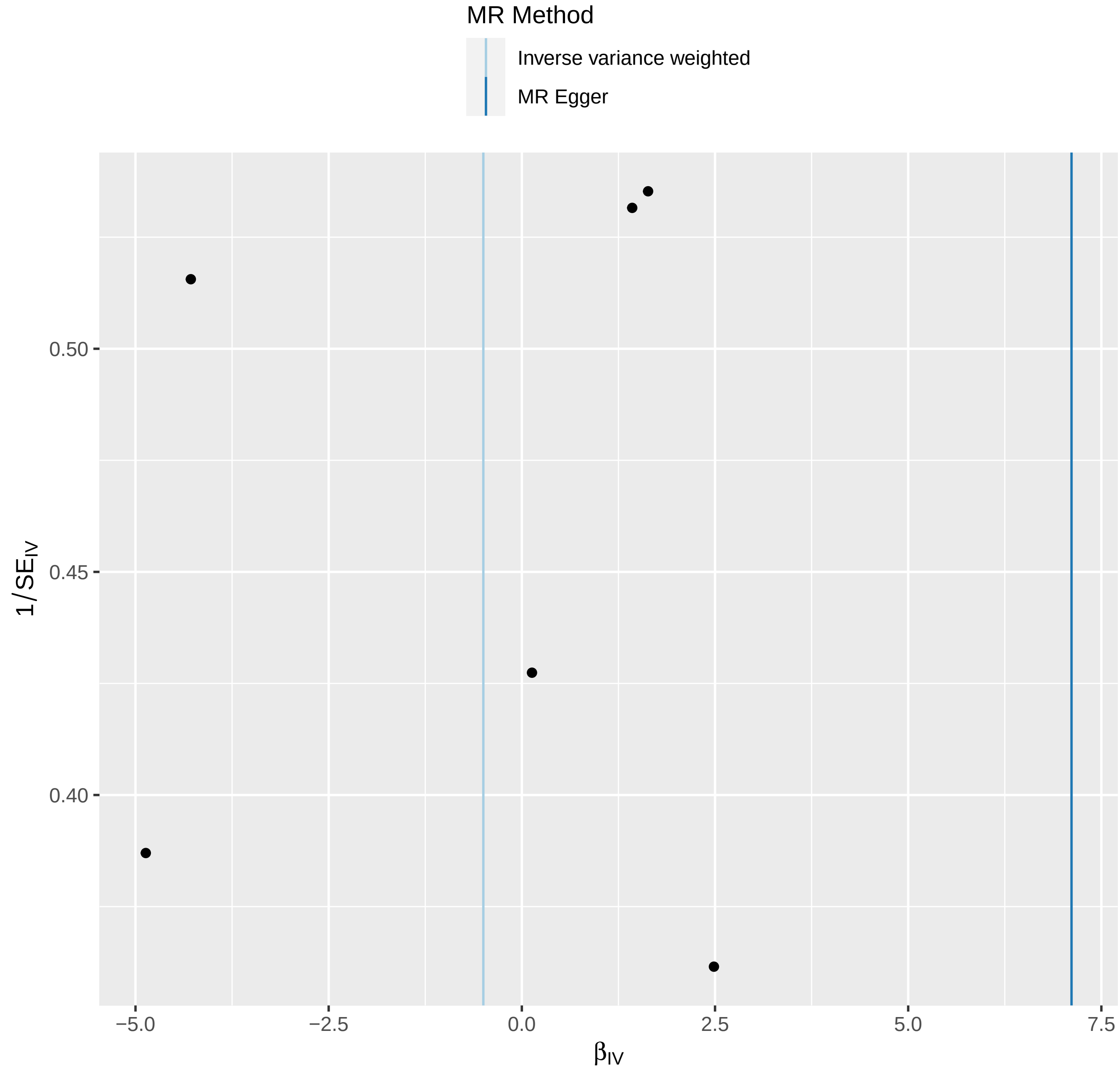

Batch 2393 : Octanoylcarnitine on Ulcerative proctitis

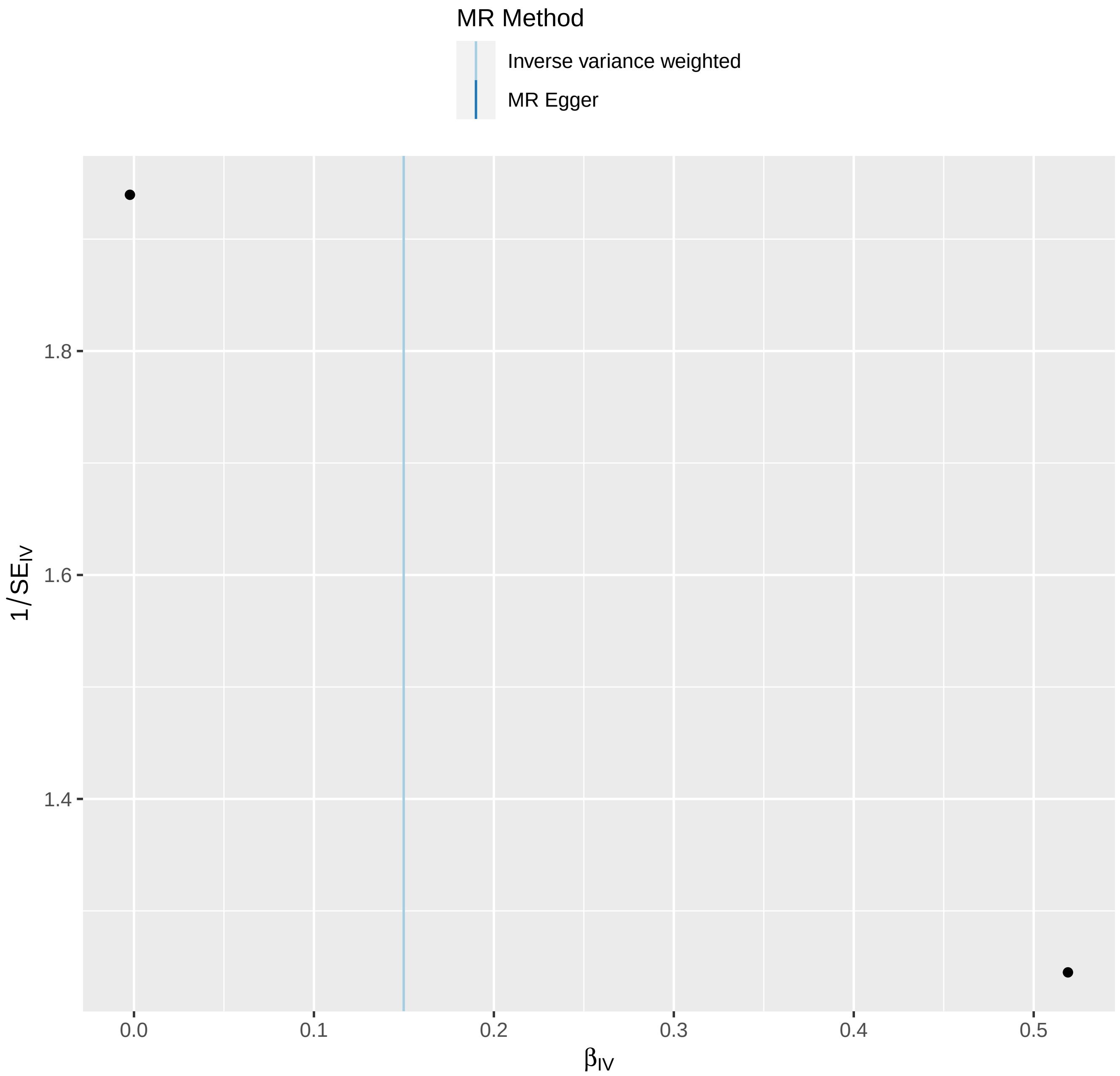

Batch 2394 : Alpha−hydroxyisovalerate on Ulcerative proctitis

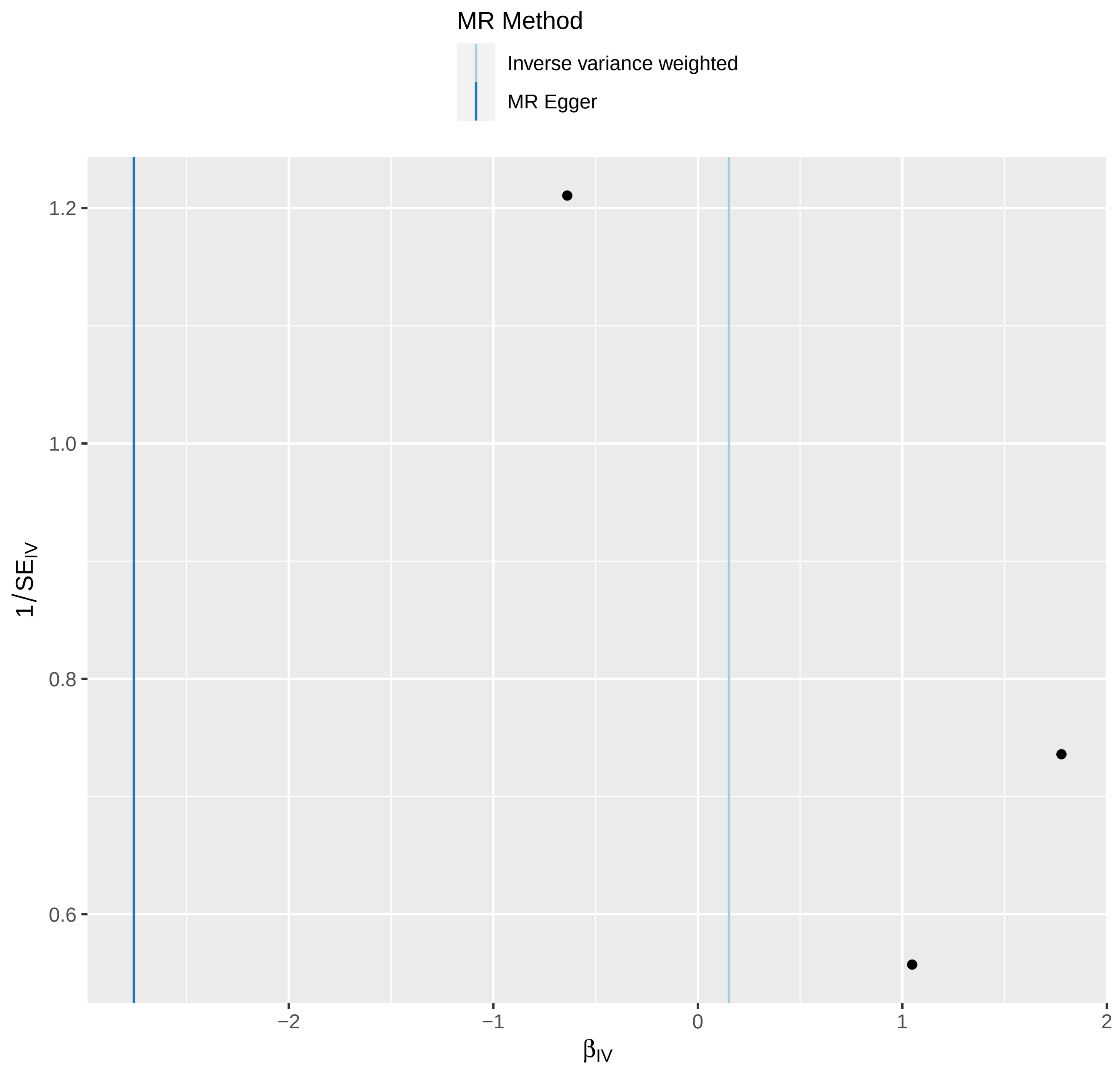

Batch 2395 : N−acetylthreonine on Ulcerative proctitis

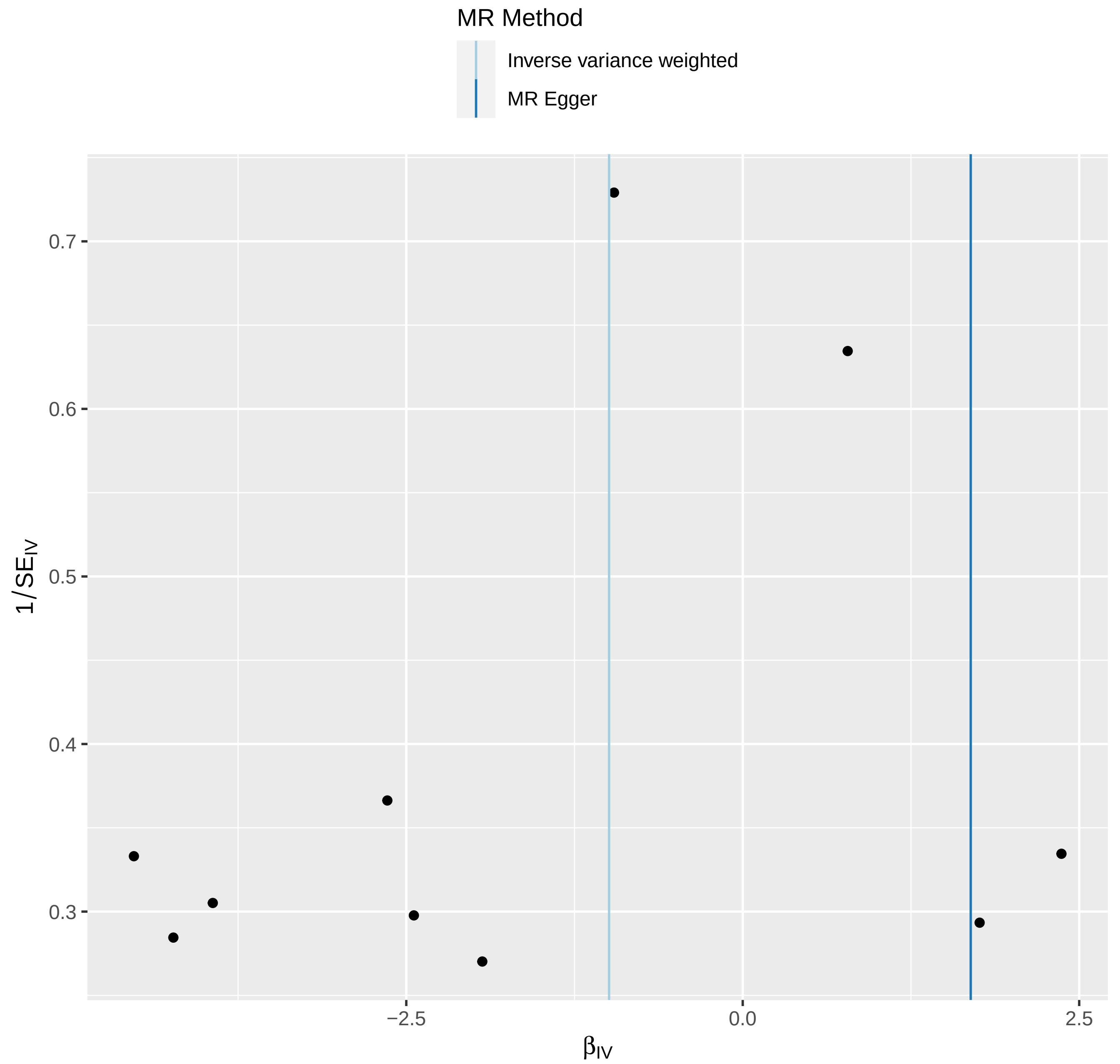

Batch 2396 : Decanoylcarnitine on Ulcerative proctitis

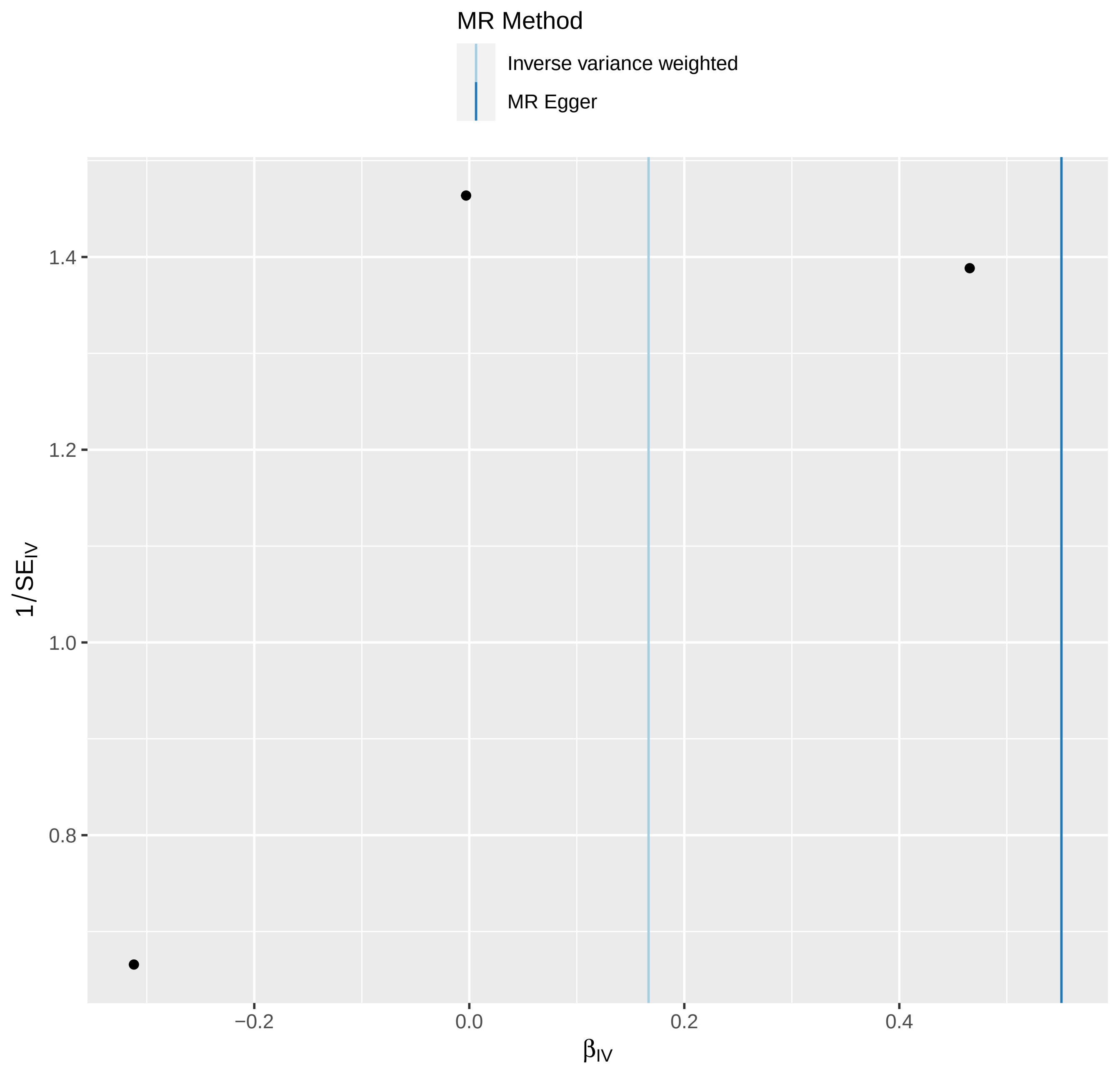

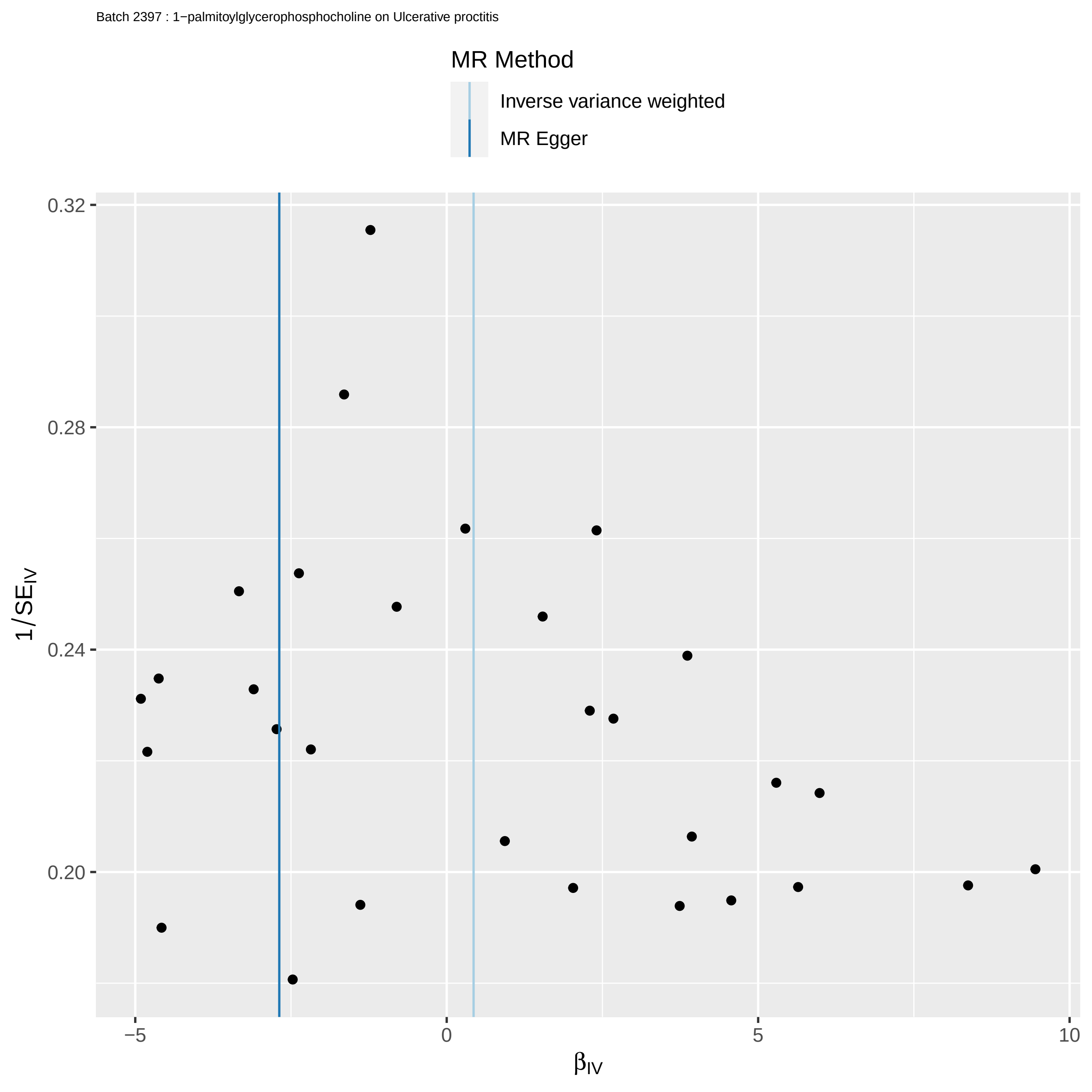

Batch 2397 : 1−palmitoylglycerophosphocholine on Ulcerative proctitis
MR Method
Inverse variance weighted
MR Egger
0.32
0.28
0.24
0.20
1/SE_IV
−5
0
5
10
β_IV

Batch 2398 : 1−heptadecanoylglycerophosphocholine on Ulcerative proctitis

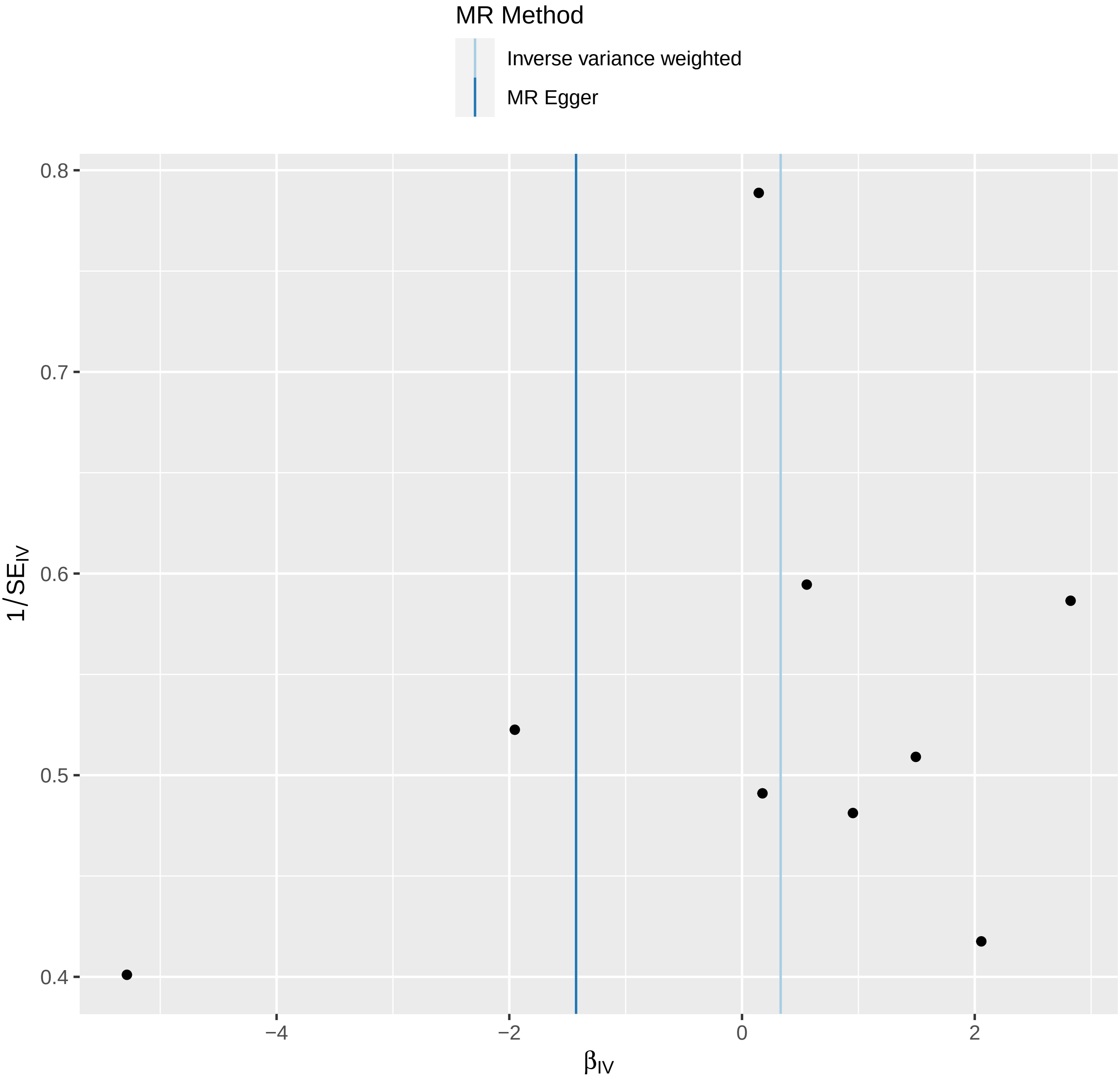

Batch 2399 : 1−oleoylglycerophosphocholine on Ulcerative proctitis

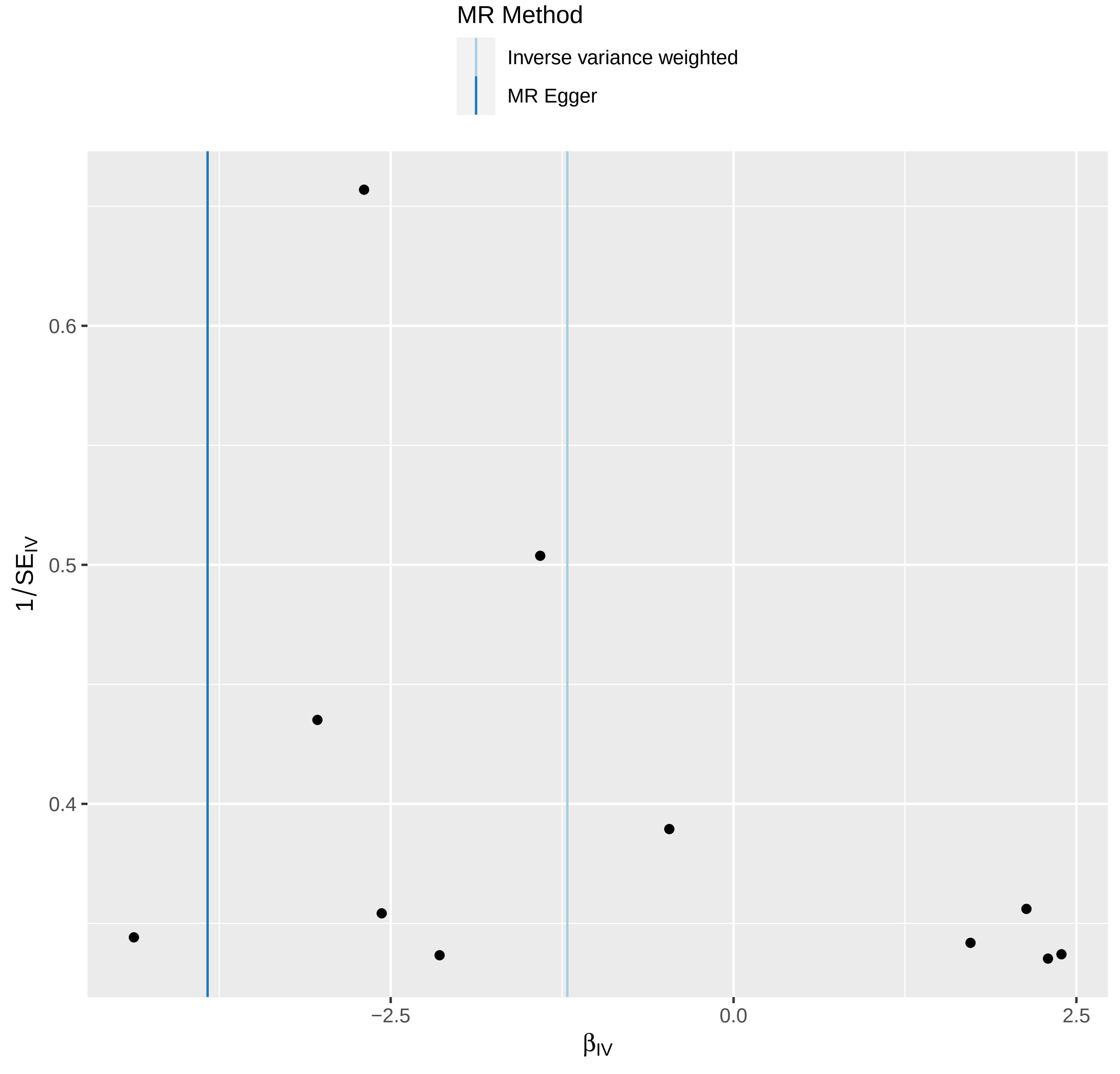

Batch 2400 : 1−stearoylglycerophosphocholine on Ulcerative proctitis

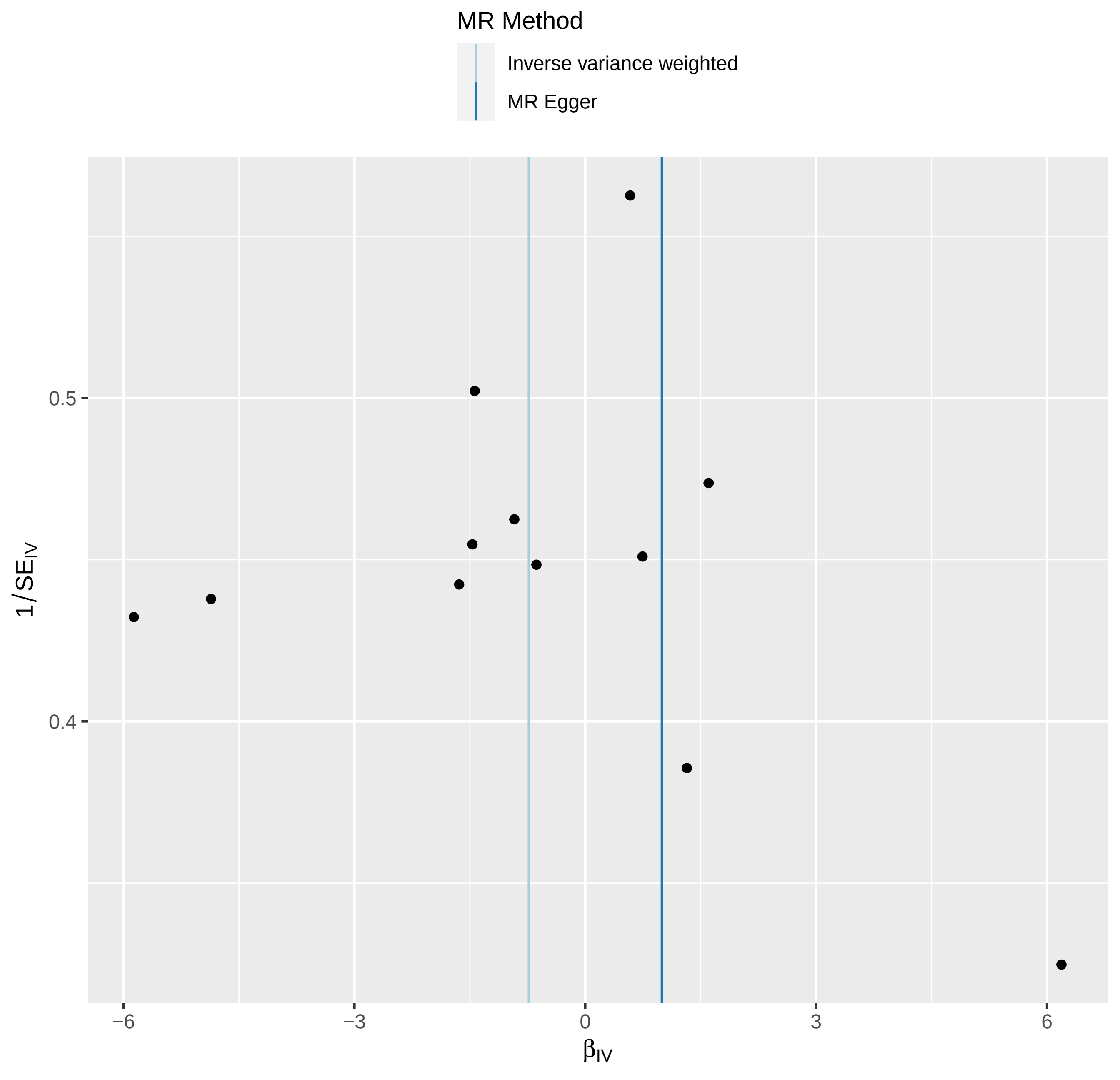

Batch 2403 : 10−heptadecenoate (17:1n7) on Ulcerative proctitis

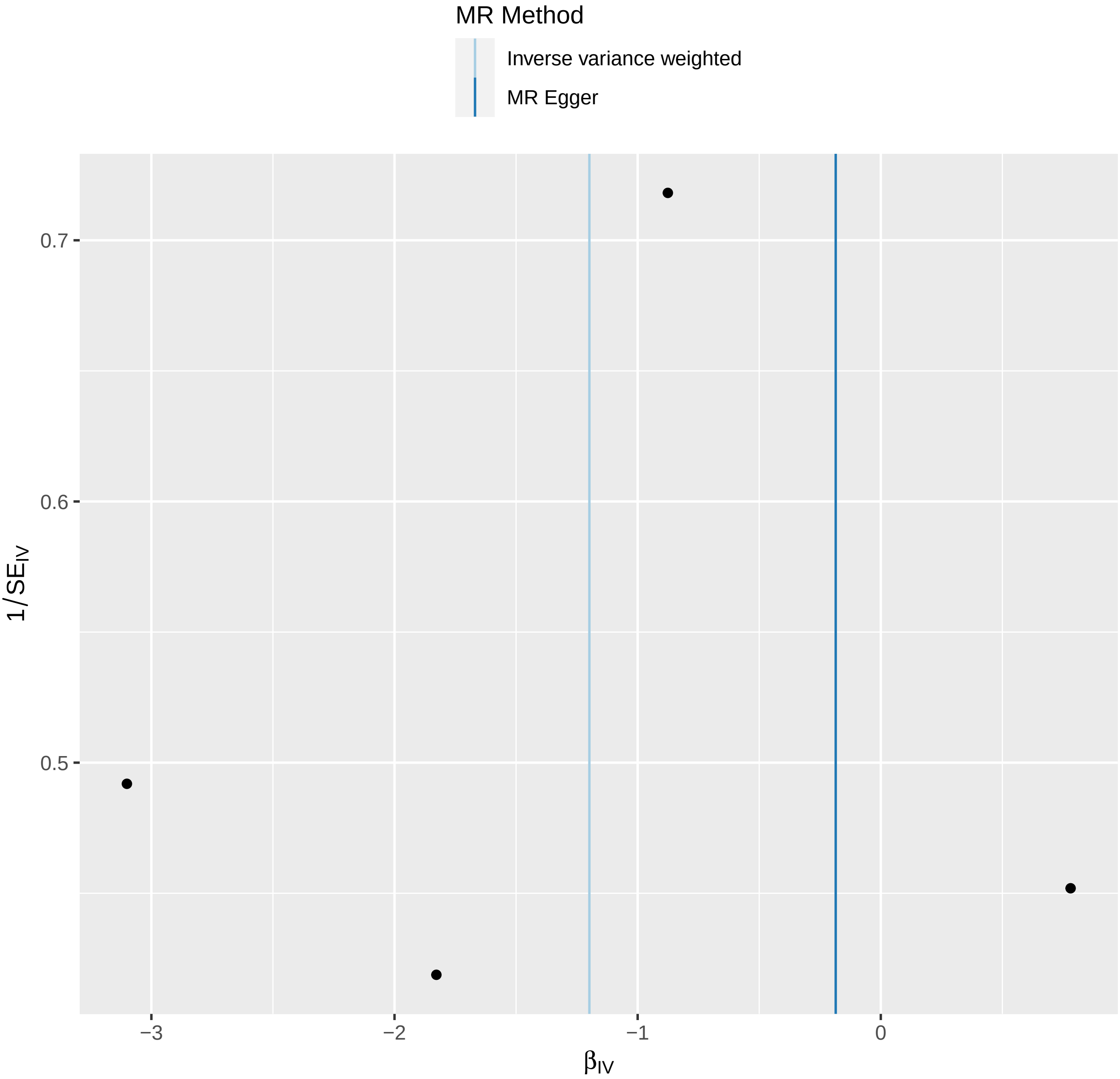

Batch 2405 : Epiandrosterone sulfate on Ulcerative proctitis

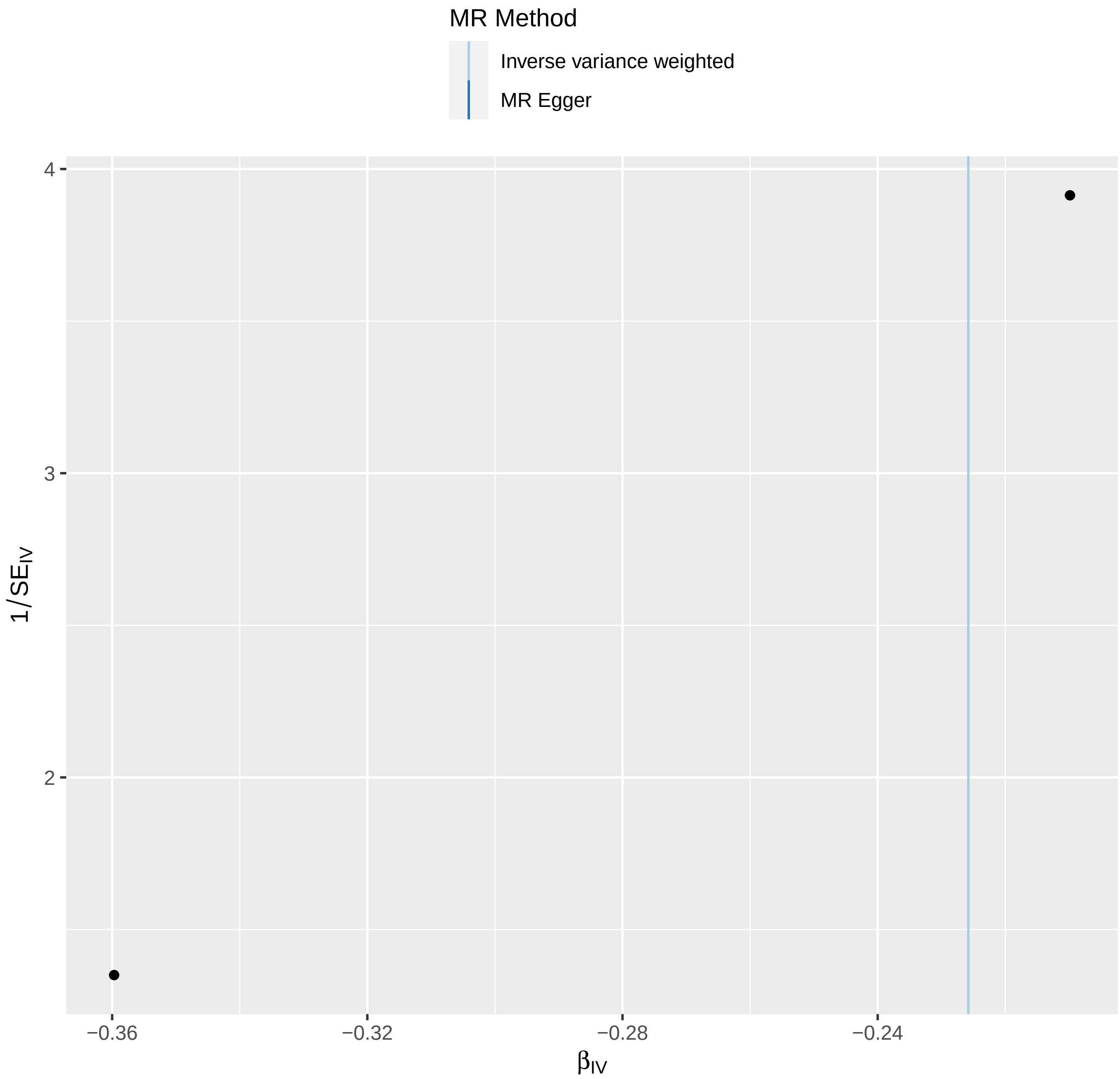

Batch 2406 : Linolenate [alpha or gamma; (18:3n3 or 6)] on Ulcerative proctitis

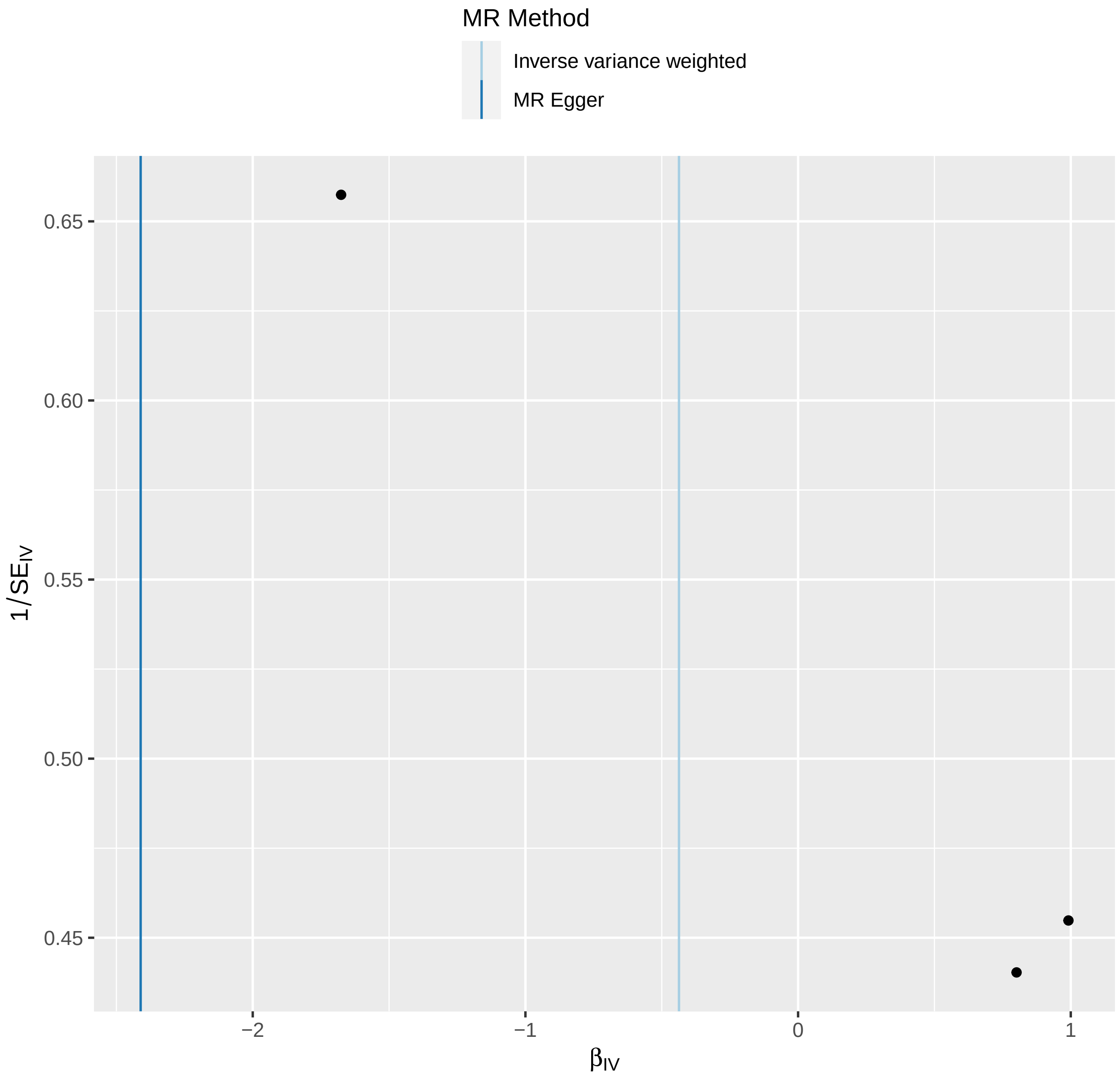

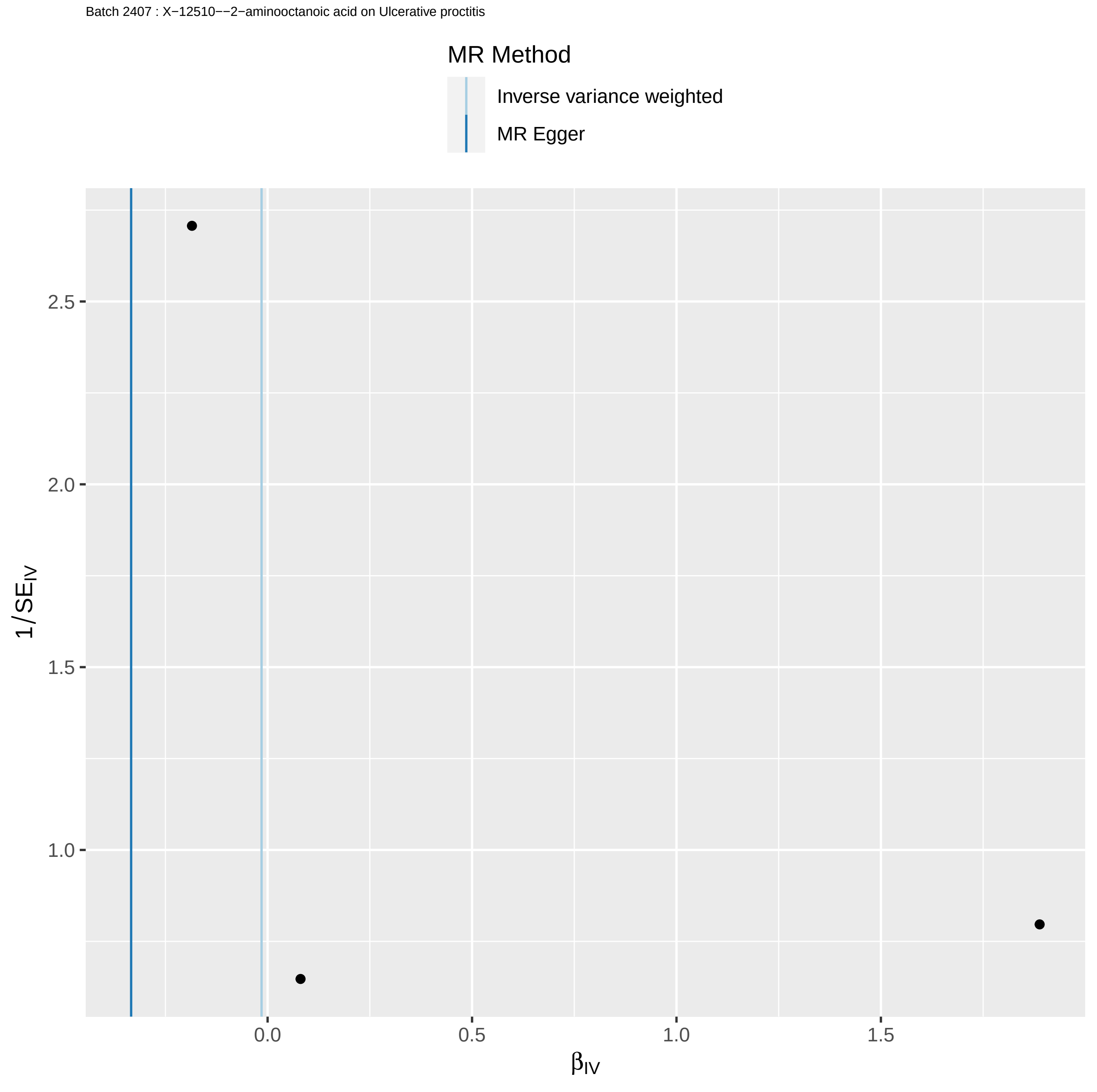

Batch 2407 : X−12510−−2−aminooctanoic acid on Ulcerative proctitis
MR Method
Inverse variance weighted
MR Egger
2.5
2.0
1.5
1.0
1/SE_IV
0.0
0.5
1.0
1.5
β_IV

Batch 2410 : Asparagine on Ulcerative proctitis

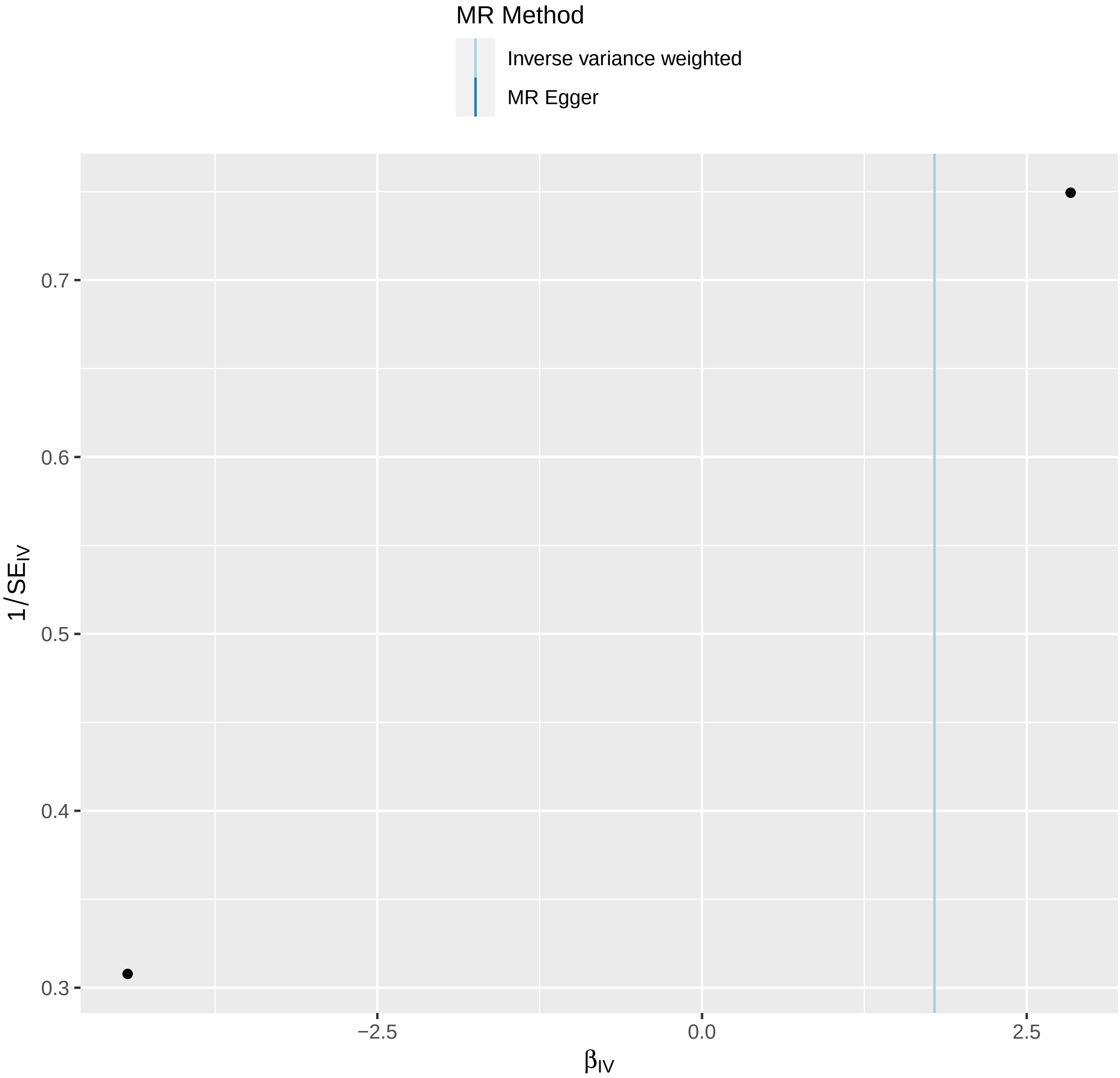

Batch 2411 : Stachydrine on Ulcerative proctitis

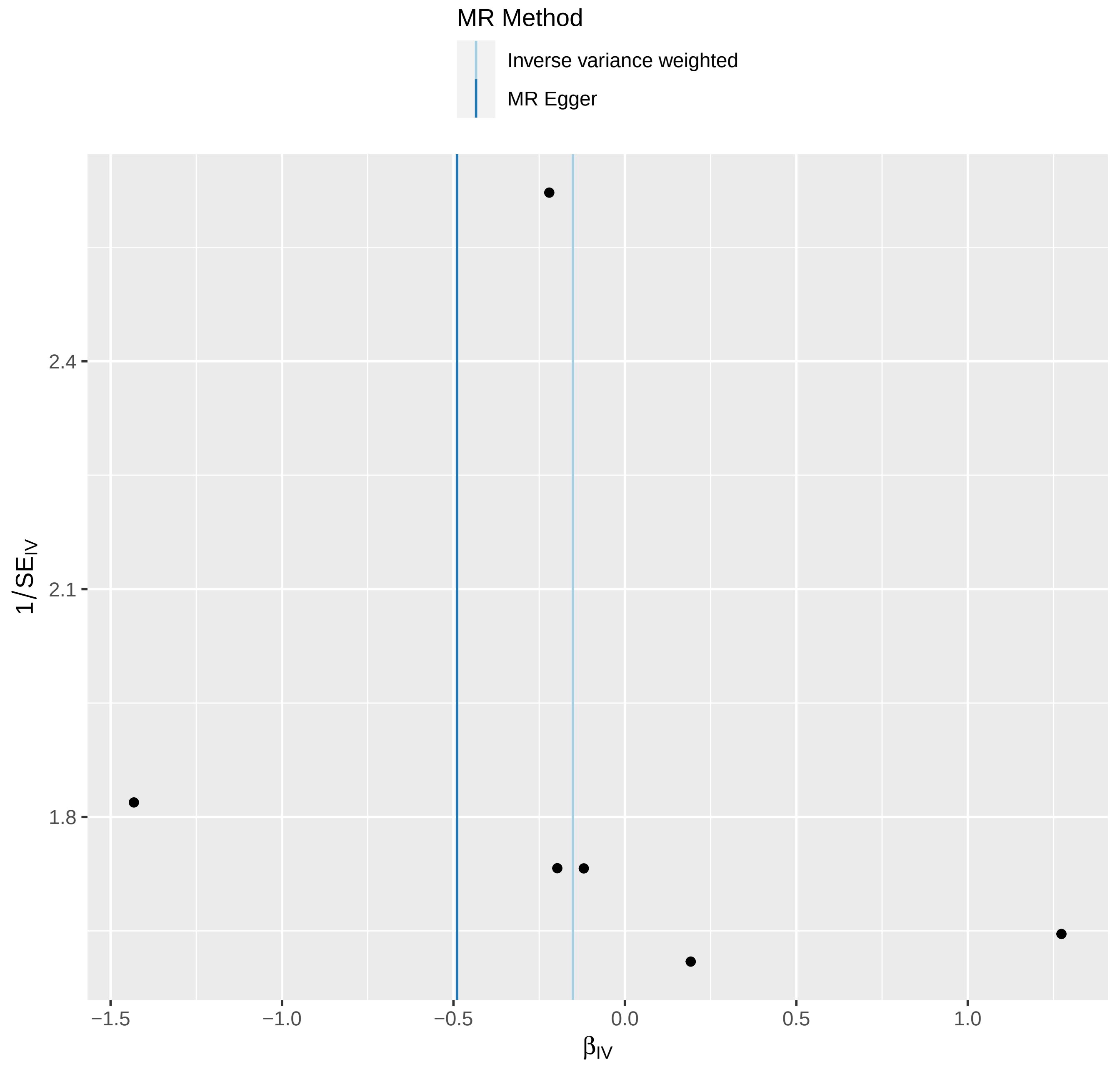

Batch 2412 : Isovalerylcarnitine on Ulcerative proctitis

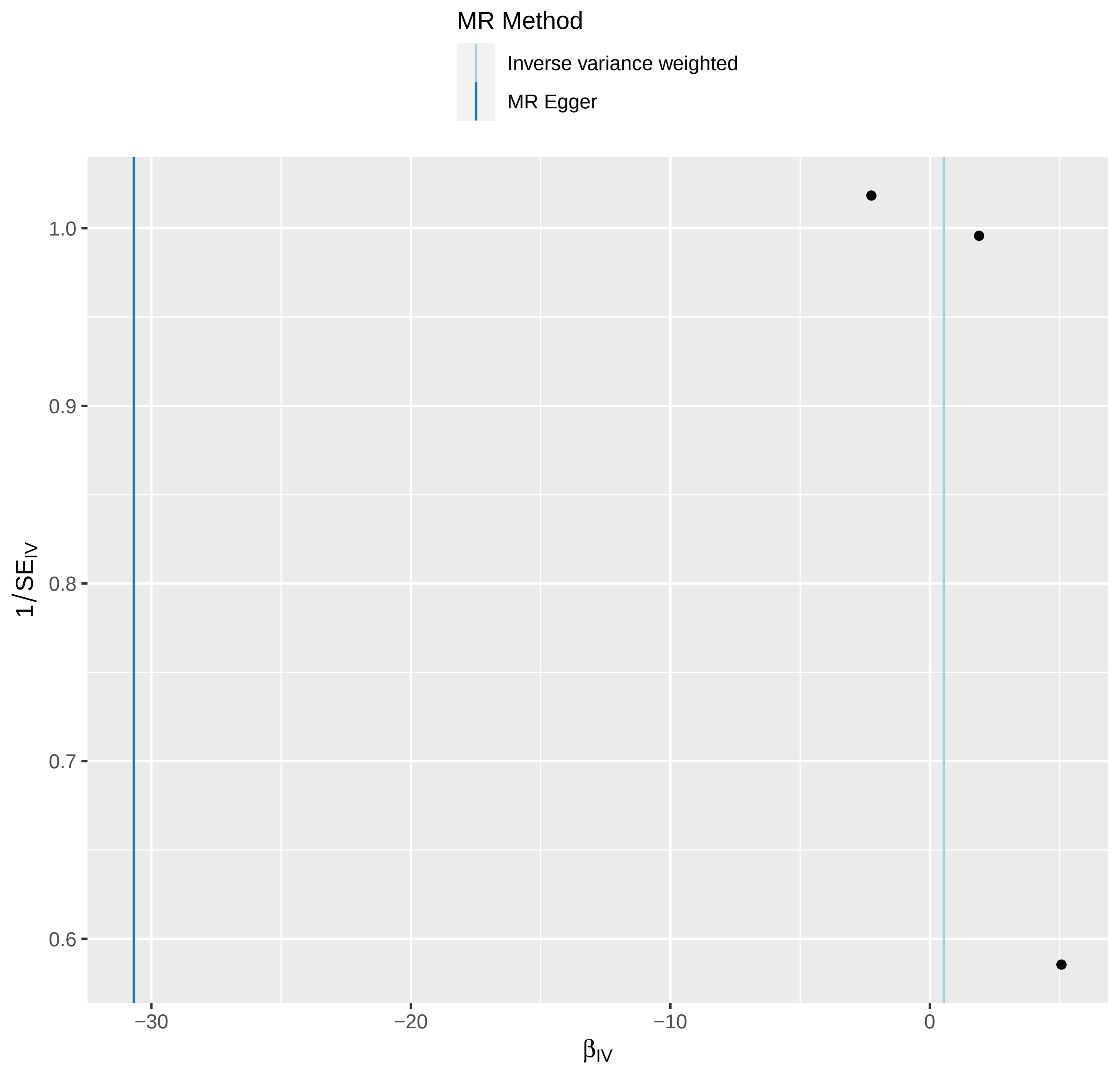

Batch 2415 : 1−linoleoylglycerophosphocholine on Ulcerative proctitis

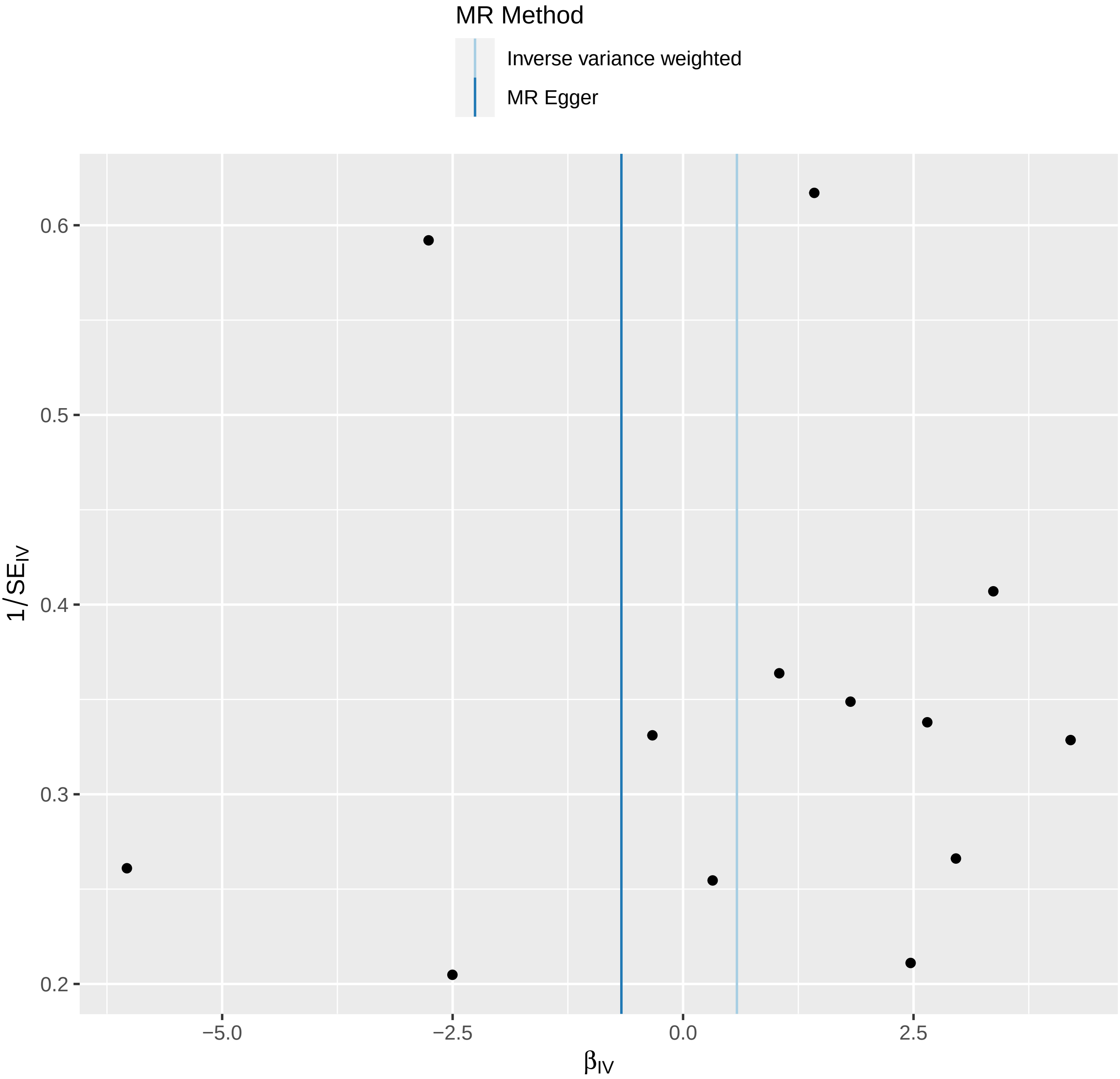

Batch 2416 : Bradykinin, des−arg(9) on Ulcerative proctitis

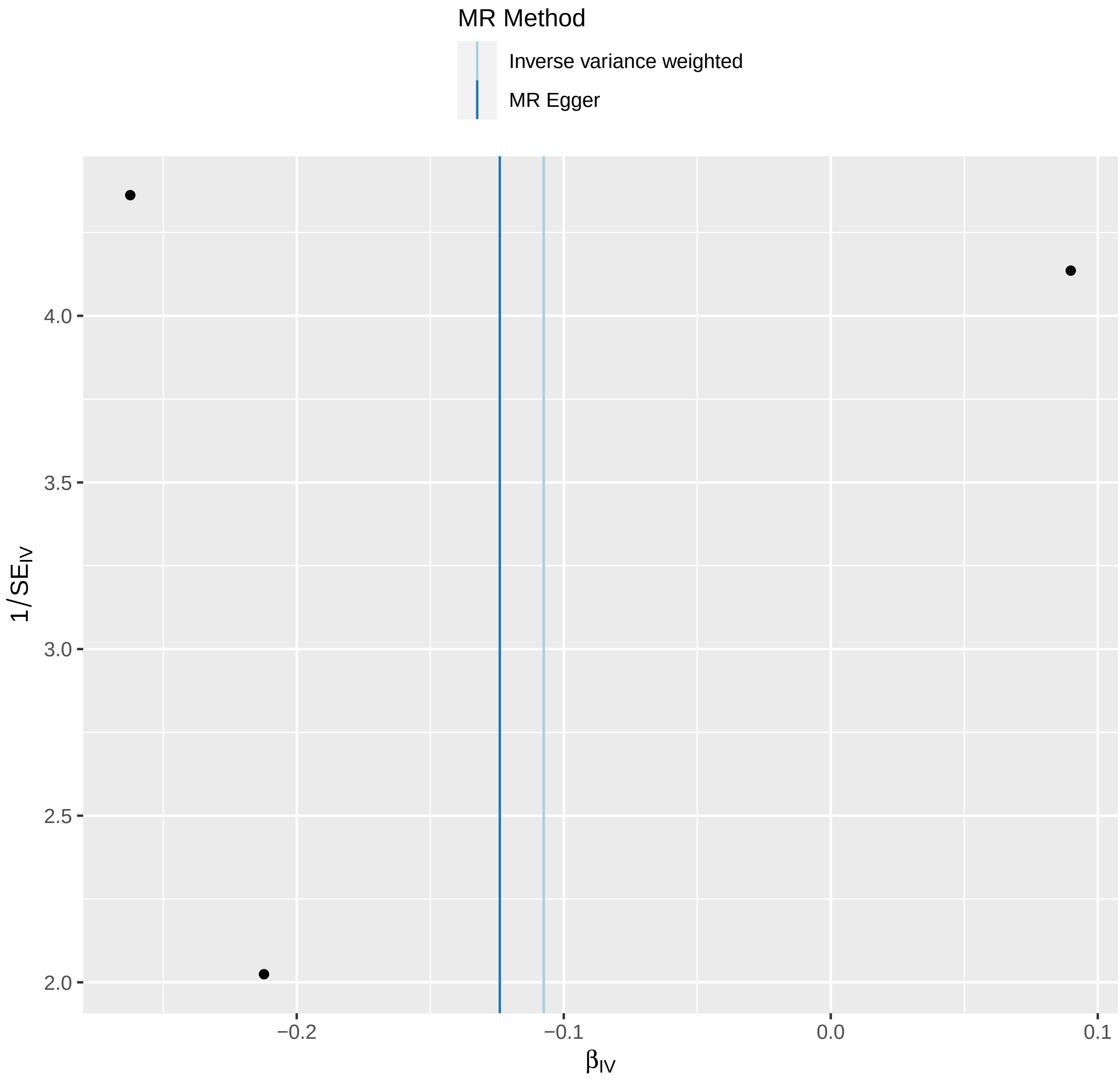

Batch 2417 : Gamma−glutamylisoleucine* on Ulcerative proctitis

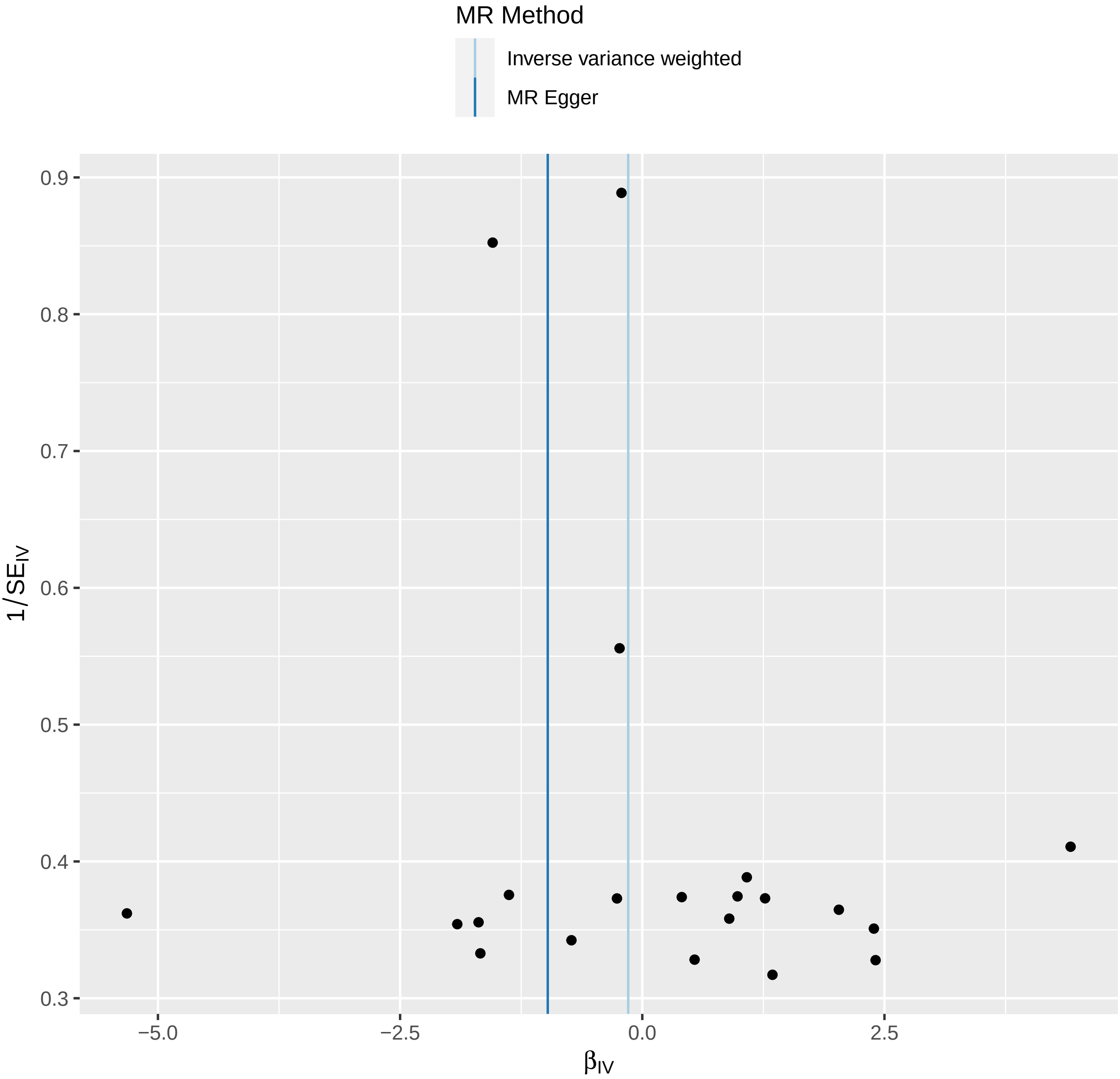

Batch 2420 : Isovalerate on Ulcerative proctitis

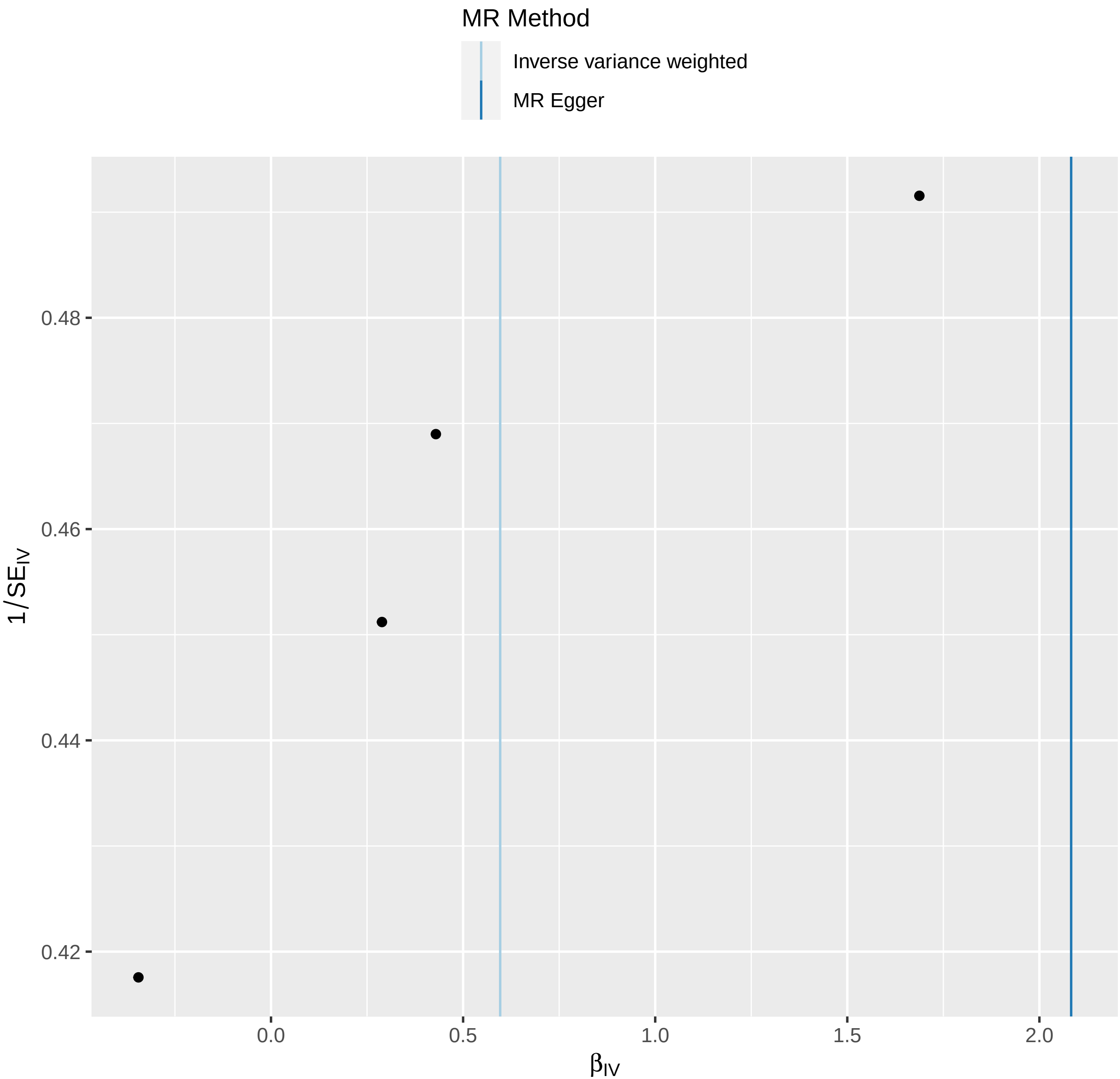

Batch 2421 : X−13183−−stearamide on Ulcerative proctitis

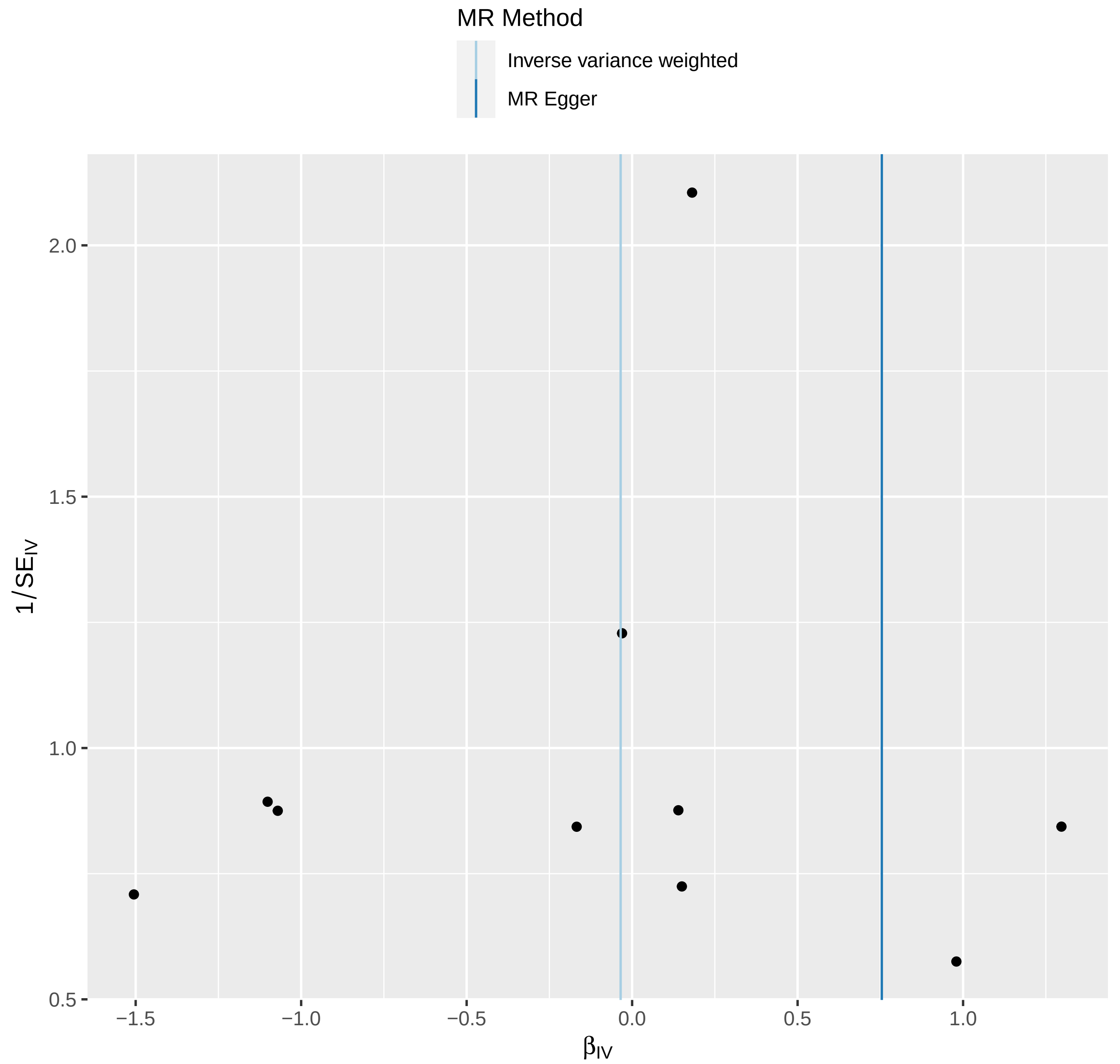

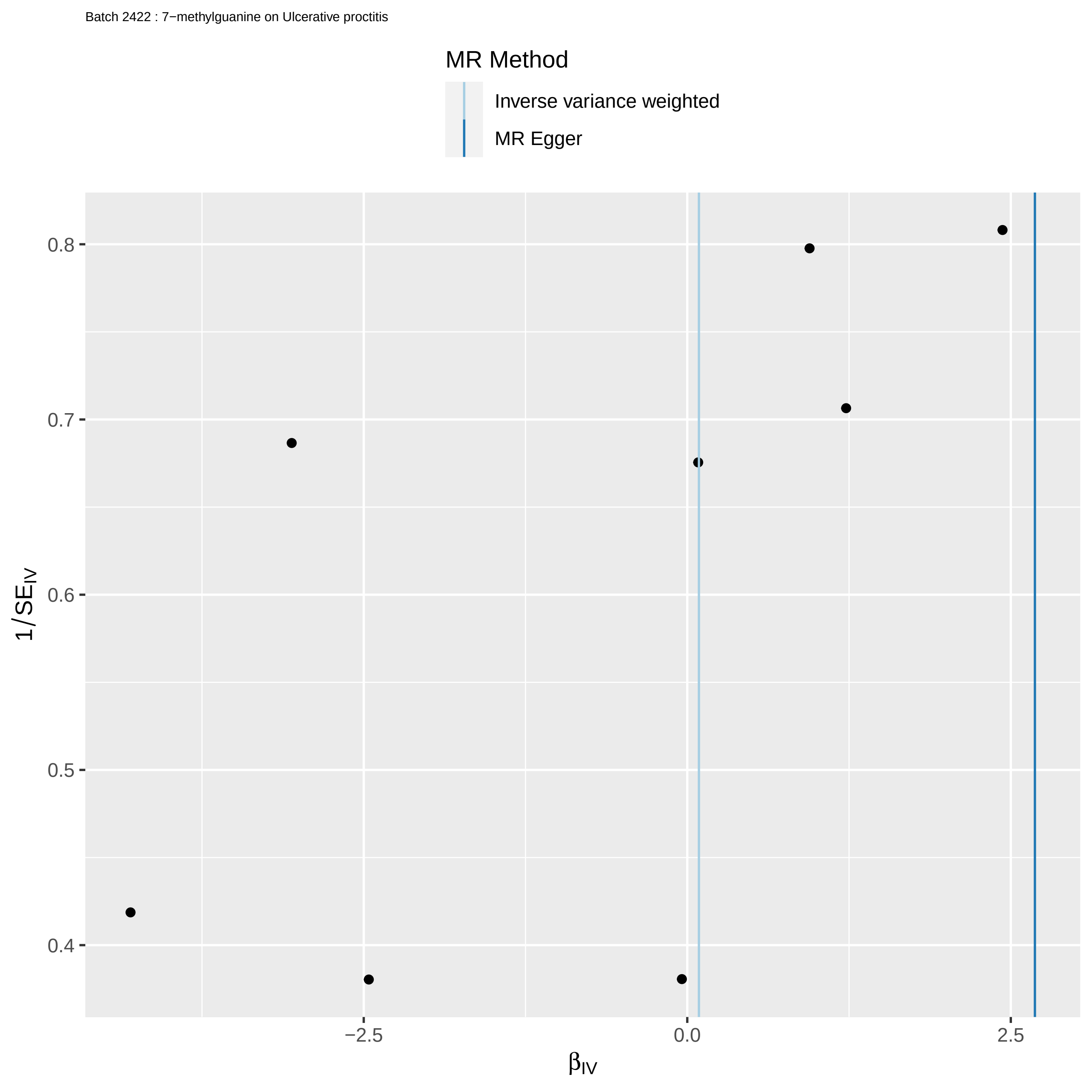
Batch 2422 : 7−methylguanine on Ulcerative proctitis
MR Method
Inverse variance weighted
MR Egger
0.8
0.7
0.6
0.5
0.4
1/SE_IV
−2.5
0.0
2.5
β_IV

Batch 2429 : X−13431−−nonanoylcarnitine* on Ulcerative proctitis

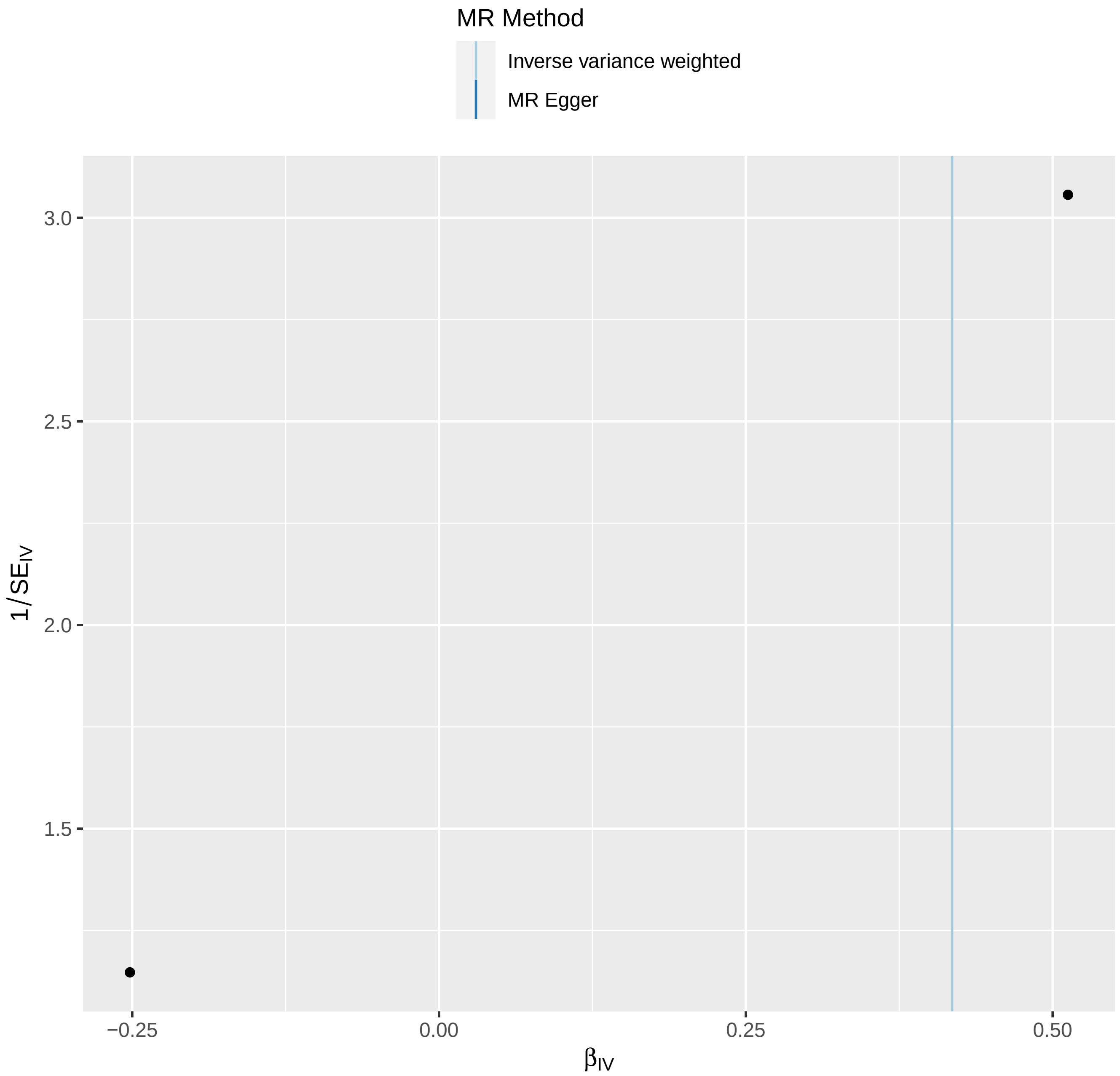

Batch 2430 : 2−palmitoylglycerophosphocholine* on Ulcerative proctitis

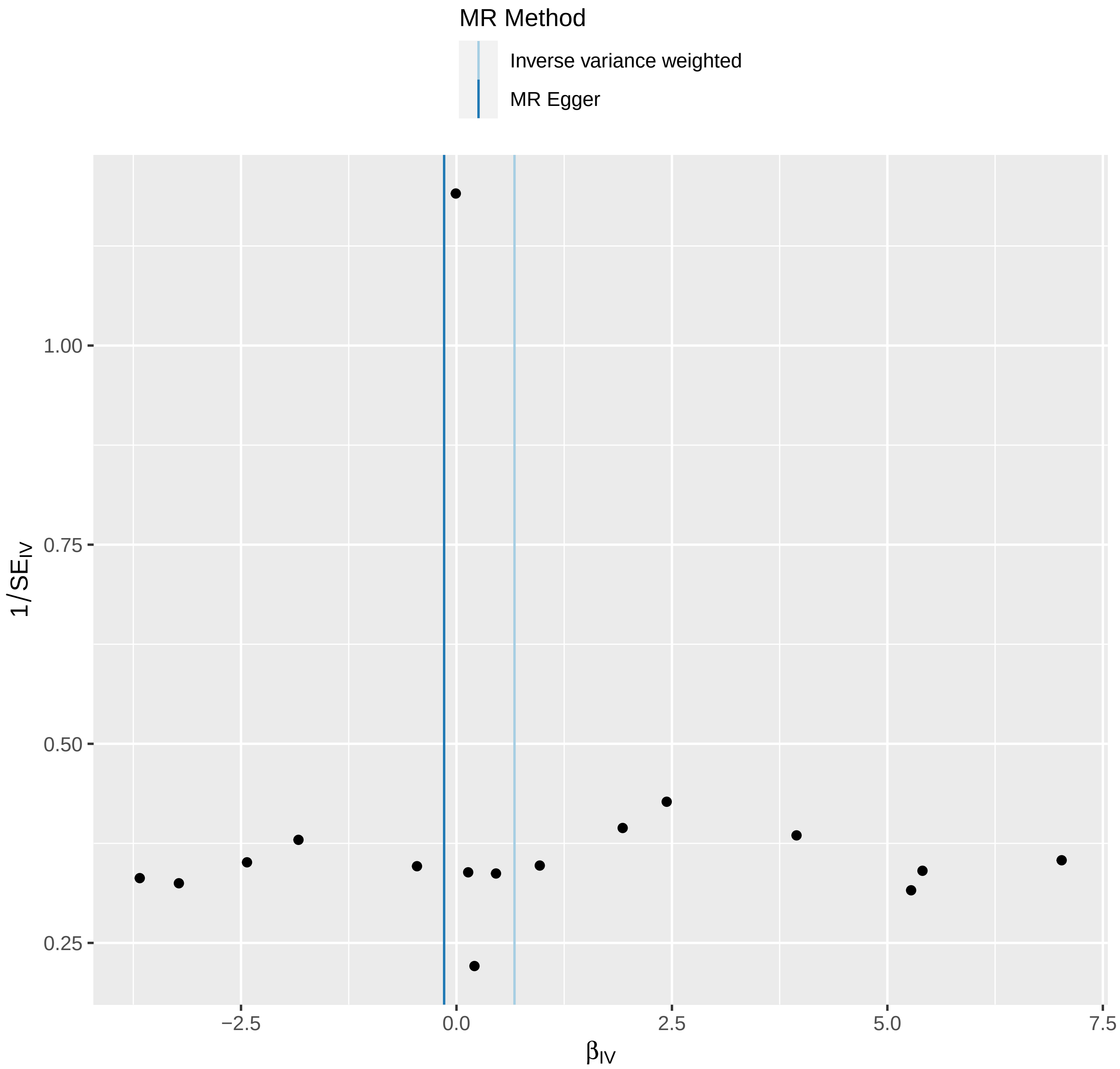

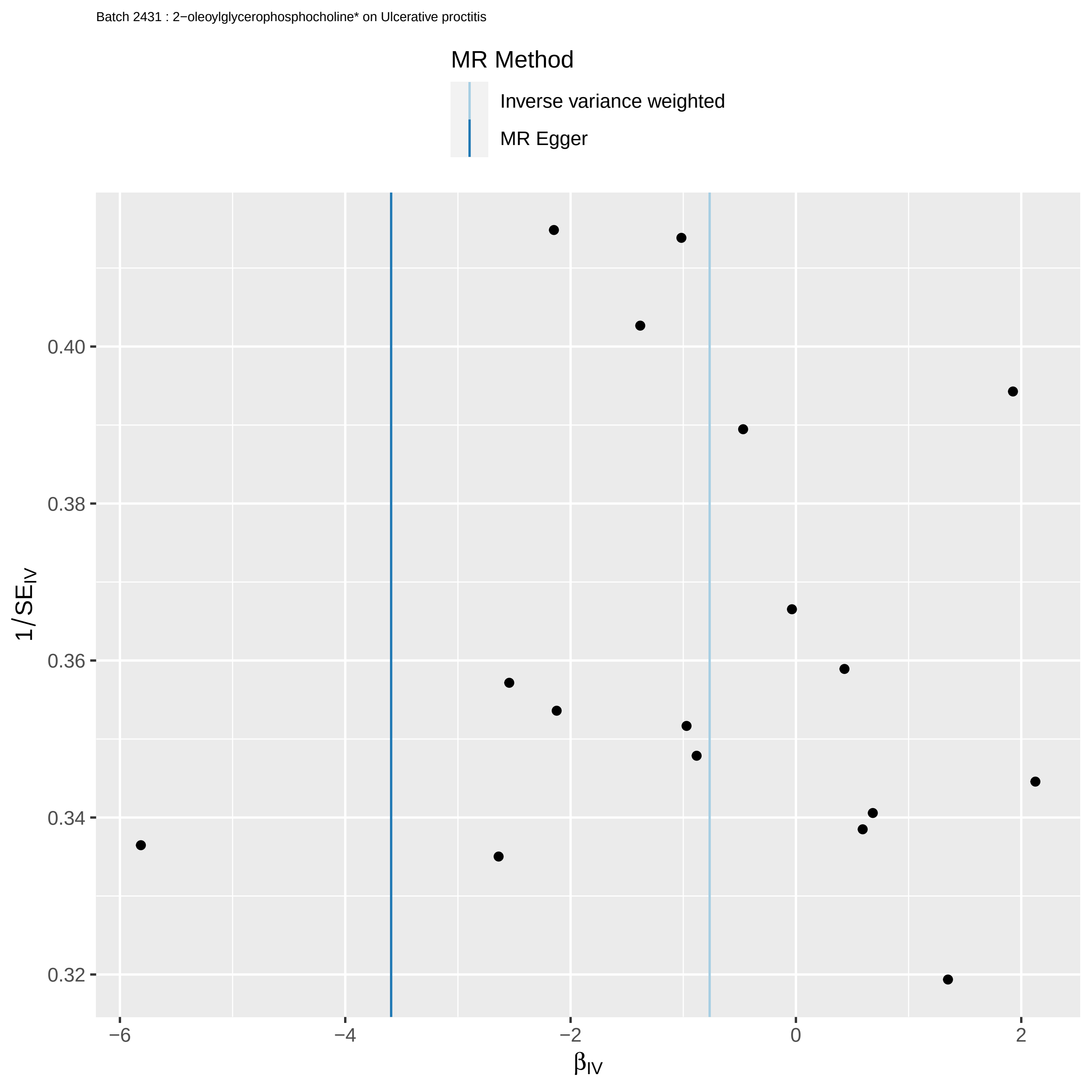

Batch 2431 : 2−oleoylglycerophosphocholine* on Ulcerative proctitis
MR Method
Inverse variance weighted
MR Egger
0.40
0.38
0.36
0.34
0.32
$1/SE_{IV}$
−6
−4
−2
0
2
β_{IV}

Batch 2434 : 1−palmitoylglycerophosphoinositol* on Ulcerative proctitis

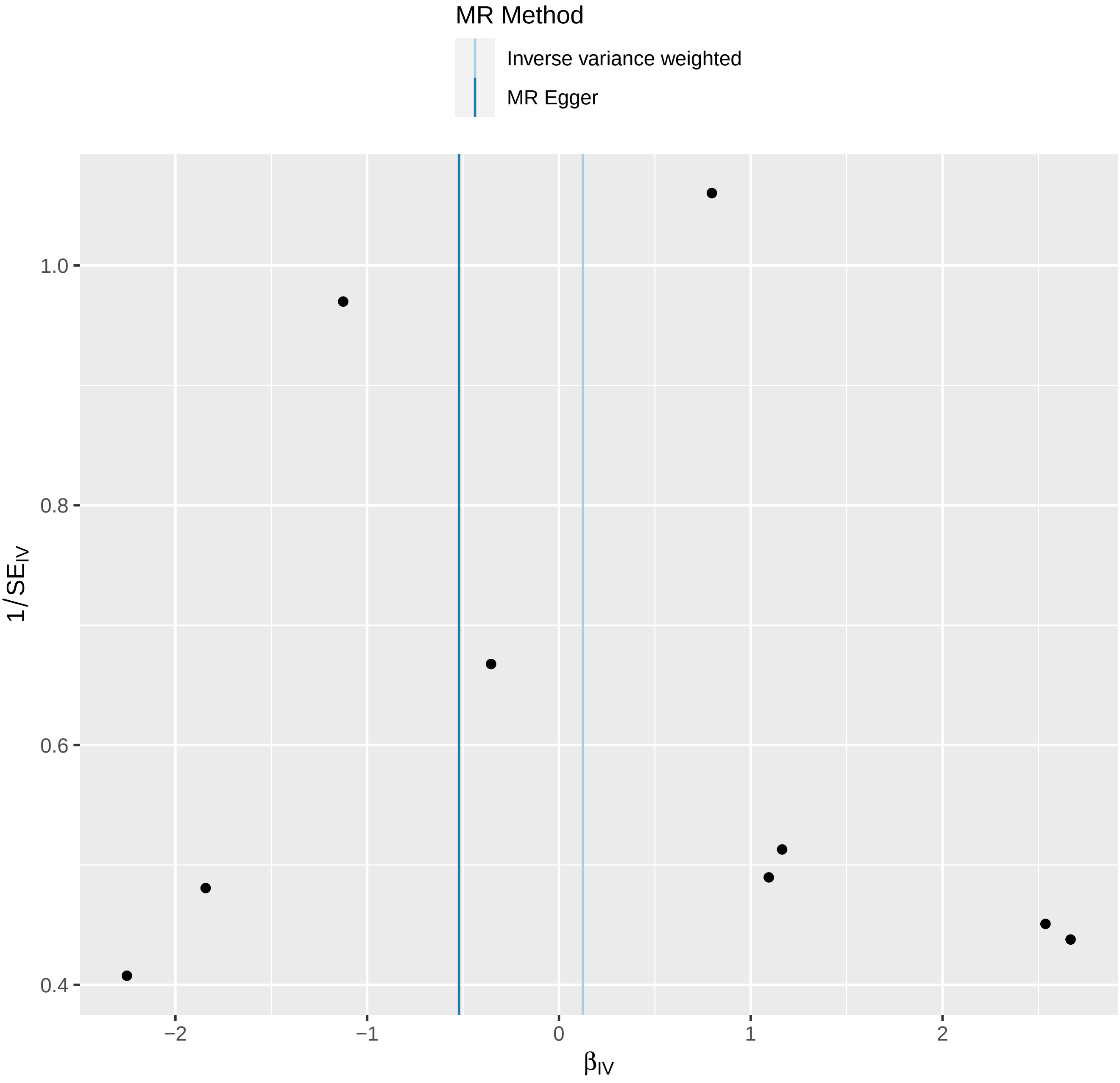

Batch 2437 : Glutaroyl carnitine on Ulcerative proctitis

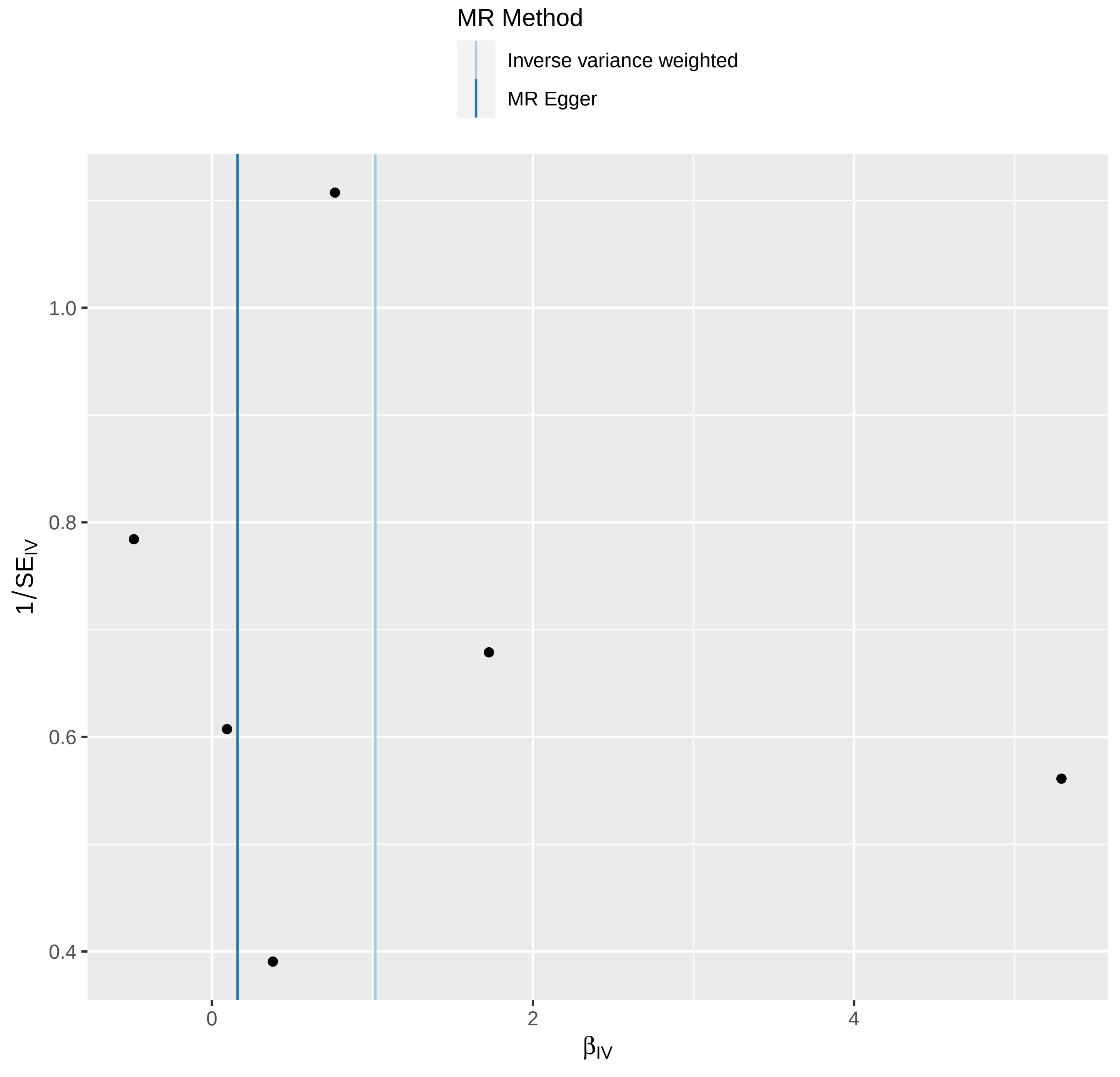

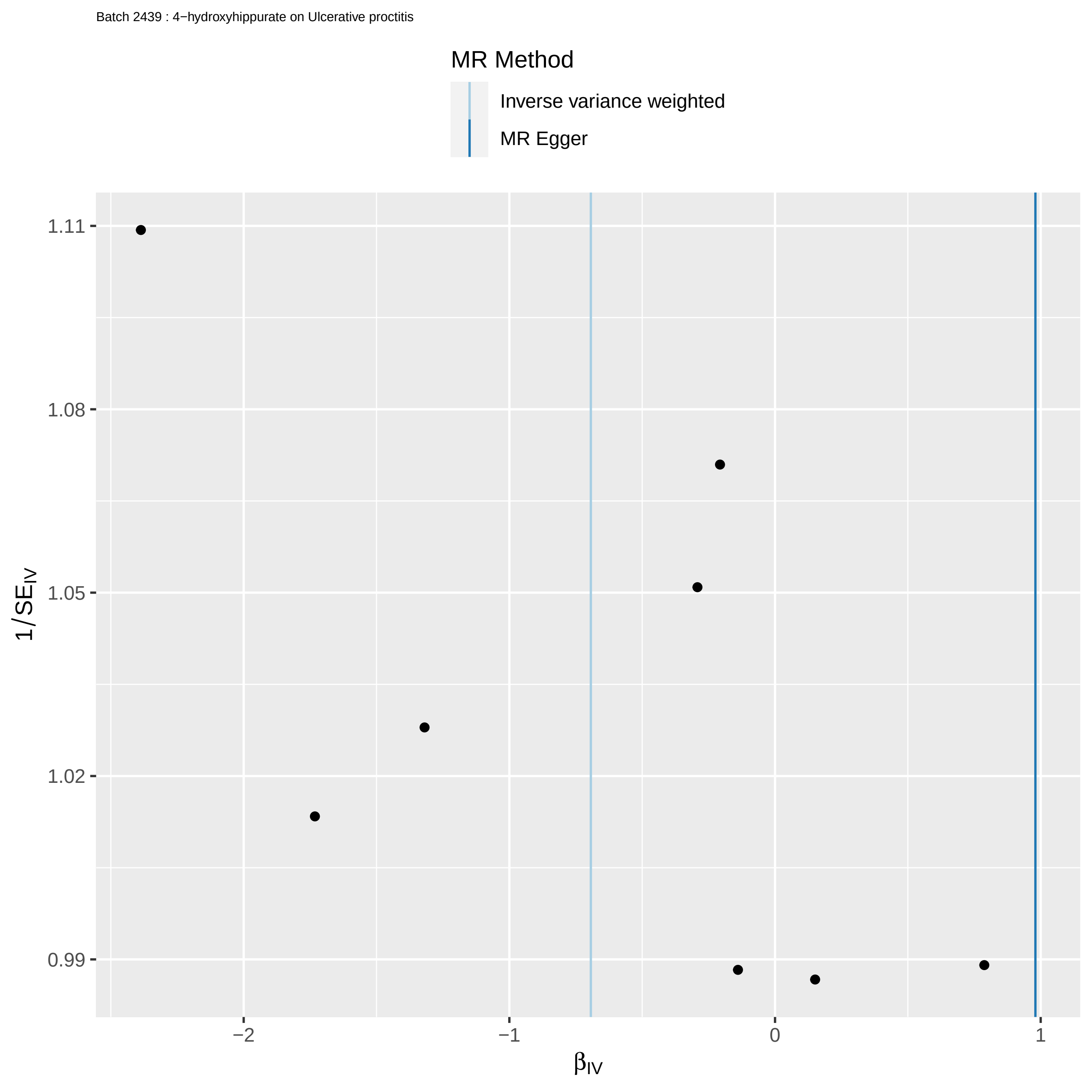
Batch 2439 : 4−hydroxyhippurate on Ulcerative proctitis
MR Method
Inverse variance weighted
MR Egger
1.11
1.08
1.05
1.02
0.99
1/SE_IV
−2
−1
0
1
β_IV

Batch 2440 : 1−myristoylglycerophosphocholine on Ulcerative proctitis

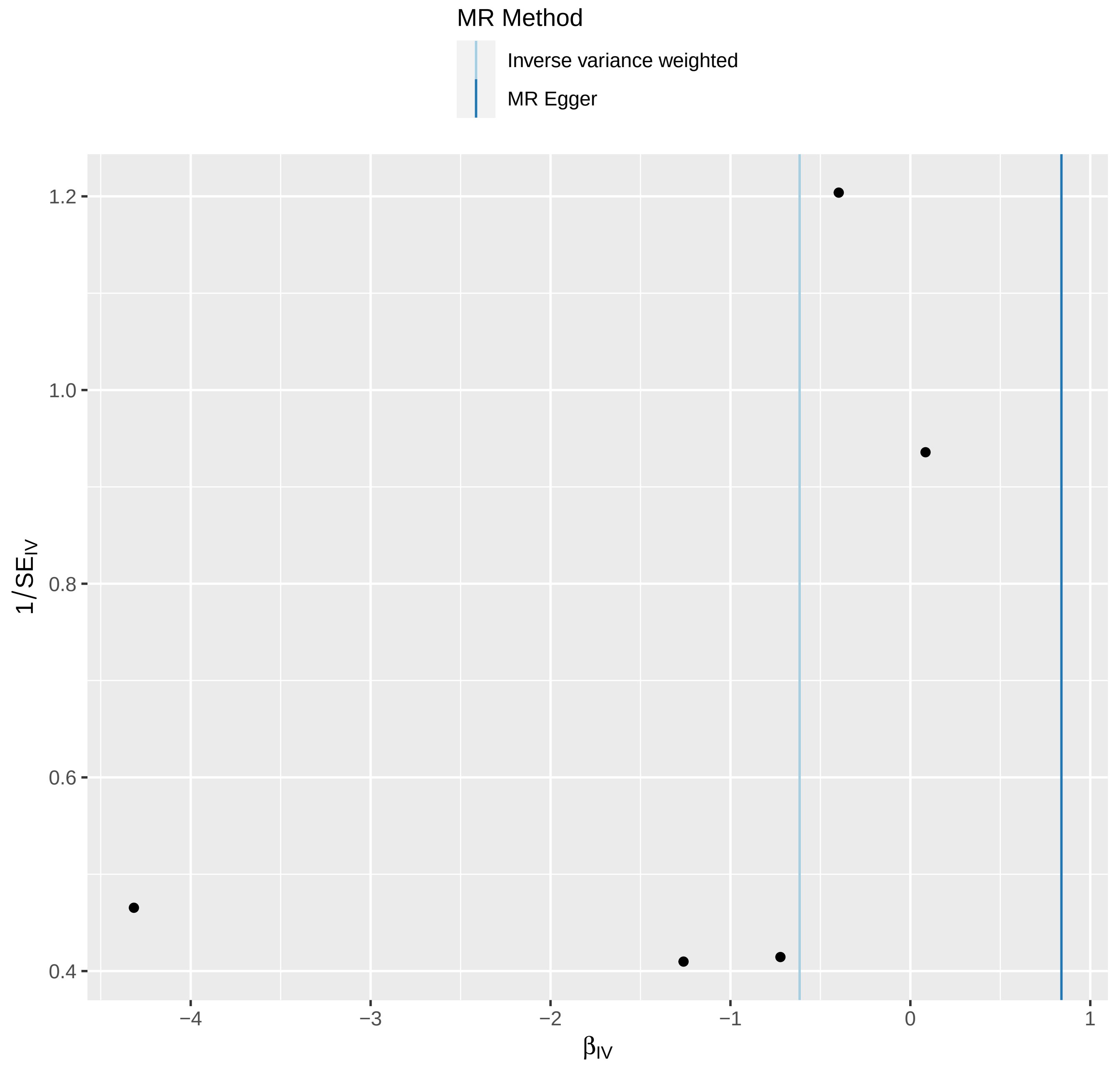

Batch 2443 : 3−(3−hydroxyphenyl)propionate on Ulcerative proctitis

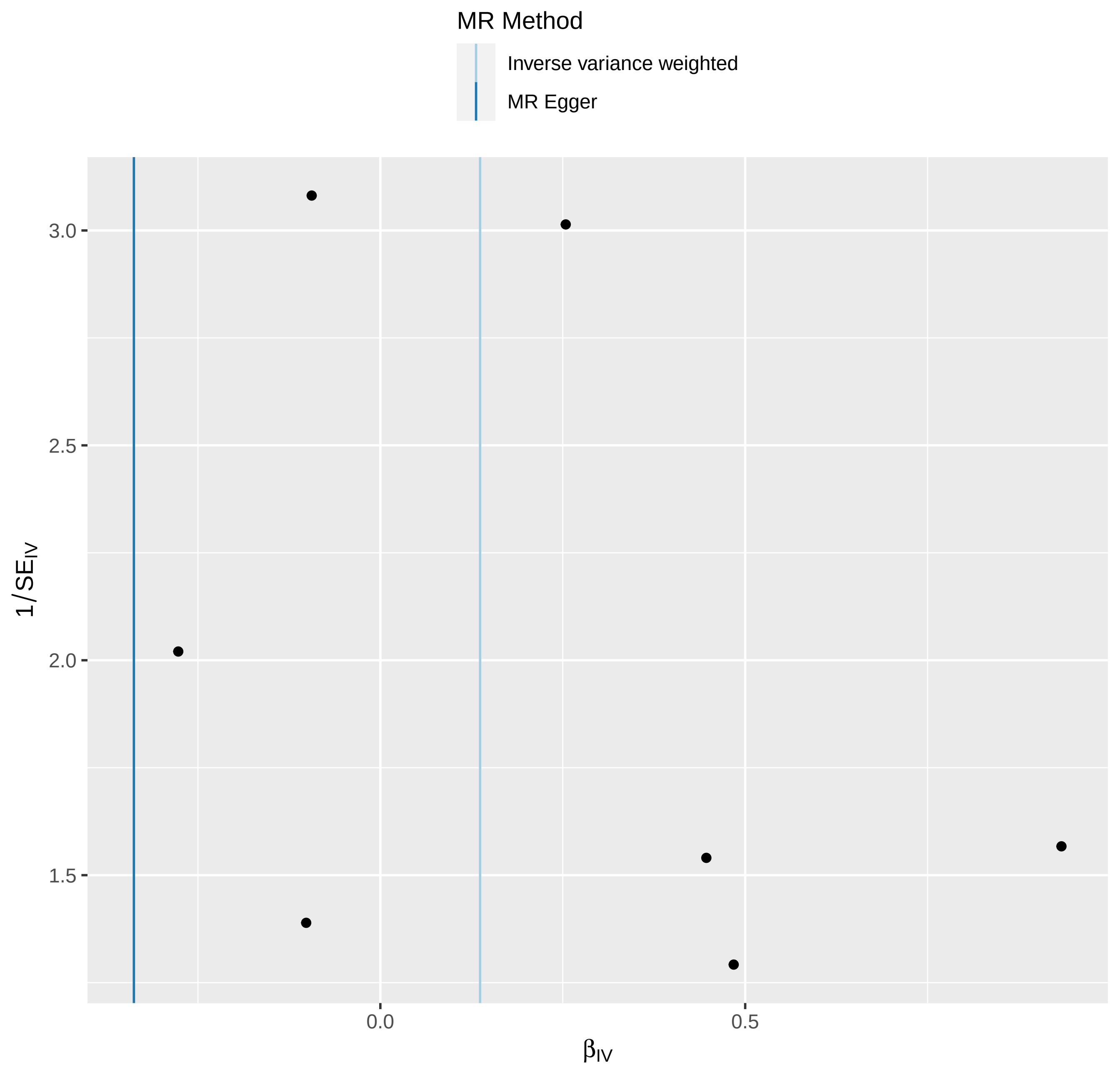

Batch 2447 : Dihomo−linolenate (20:3n3 or n6) on Ulcerative proctitis

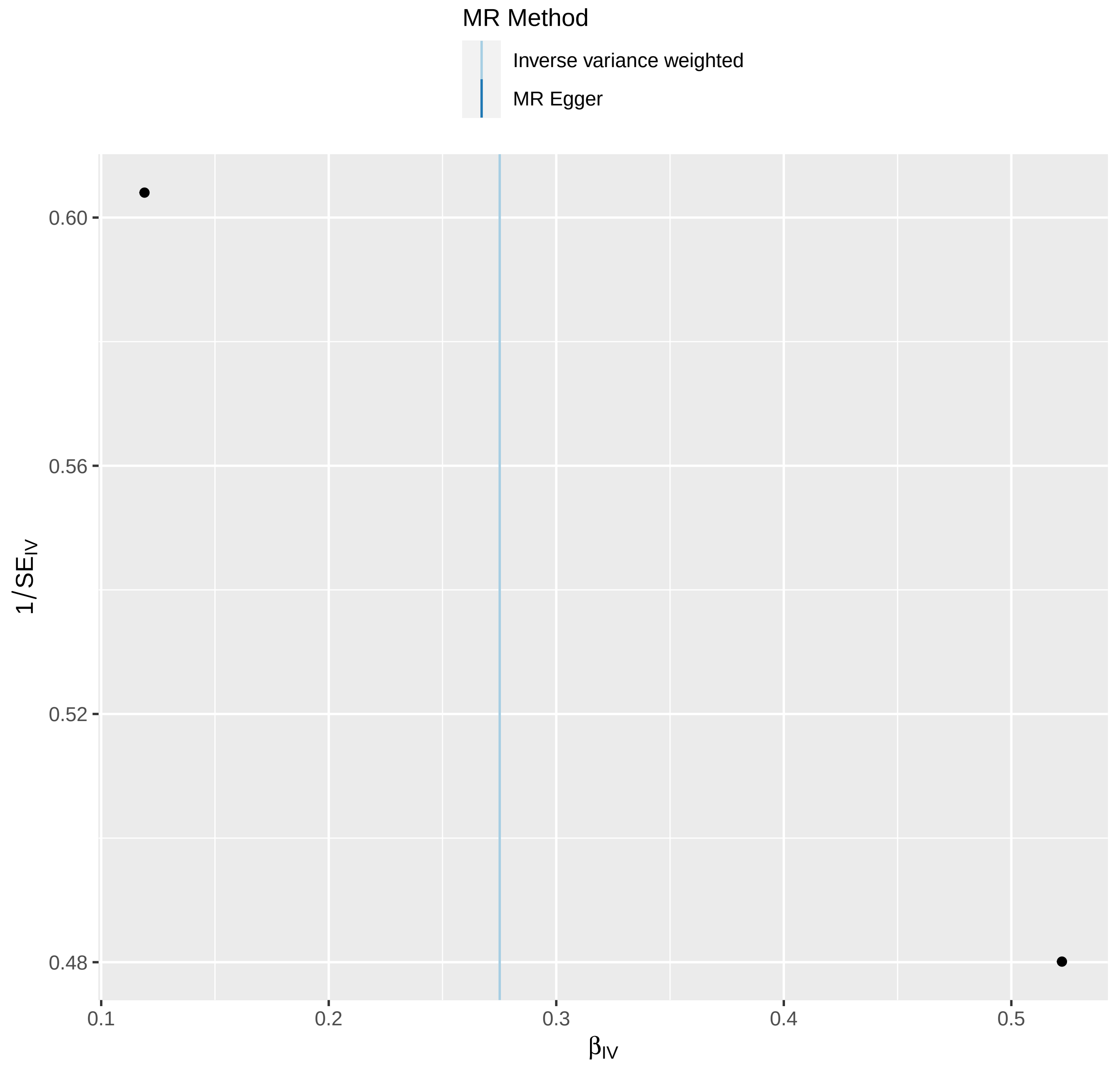

Batch 2448 : Threitol on Ulcerative proctitis

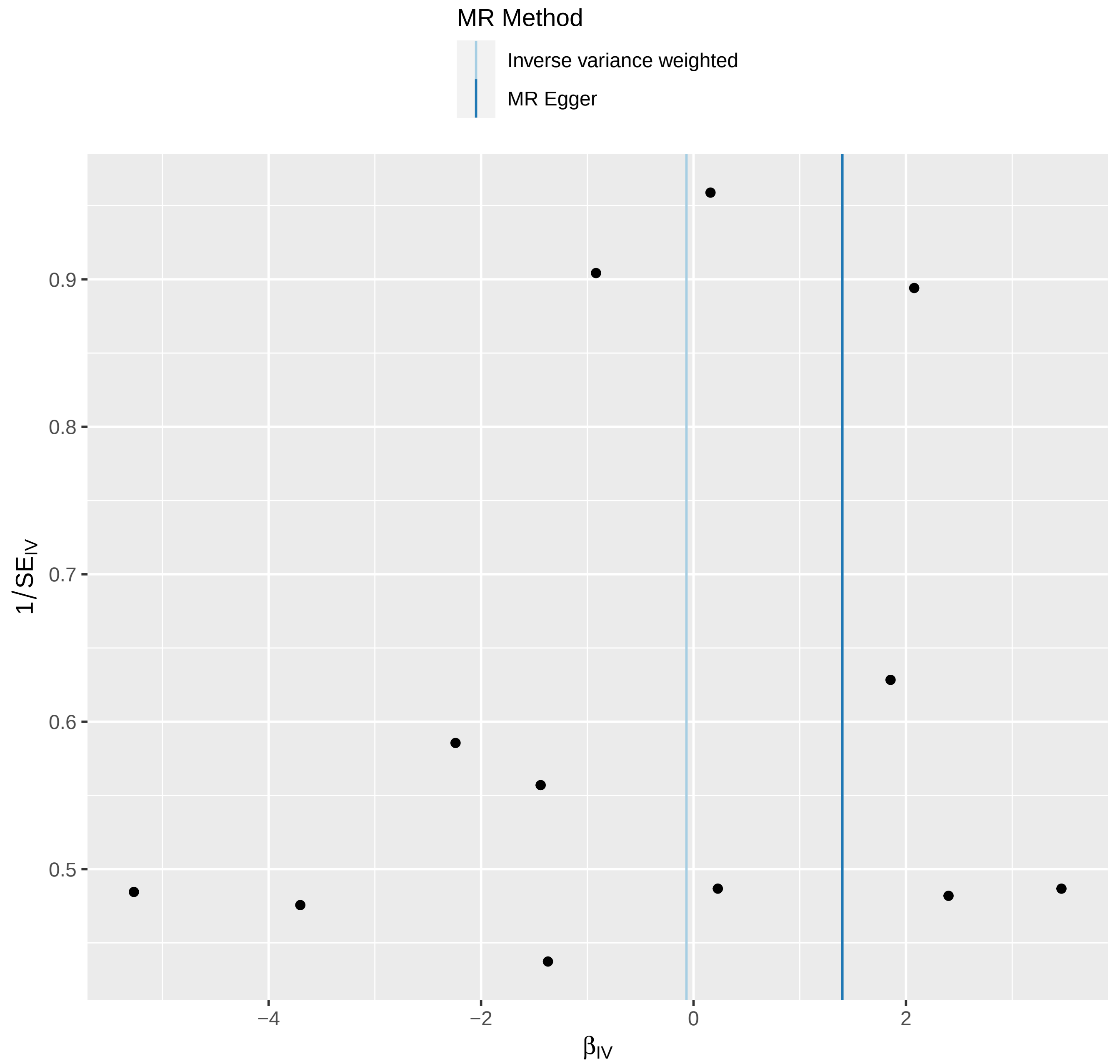

Batch 2449 : P−cresol sulfate on Ulcerative proctitis

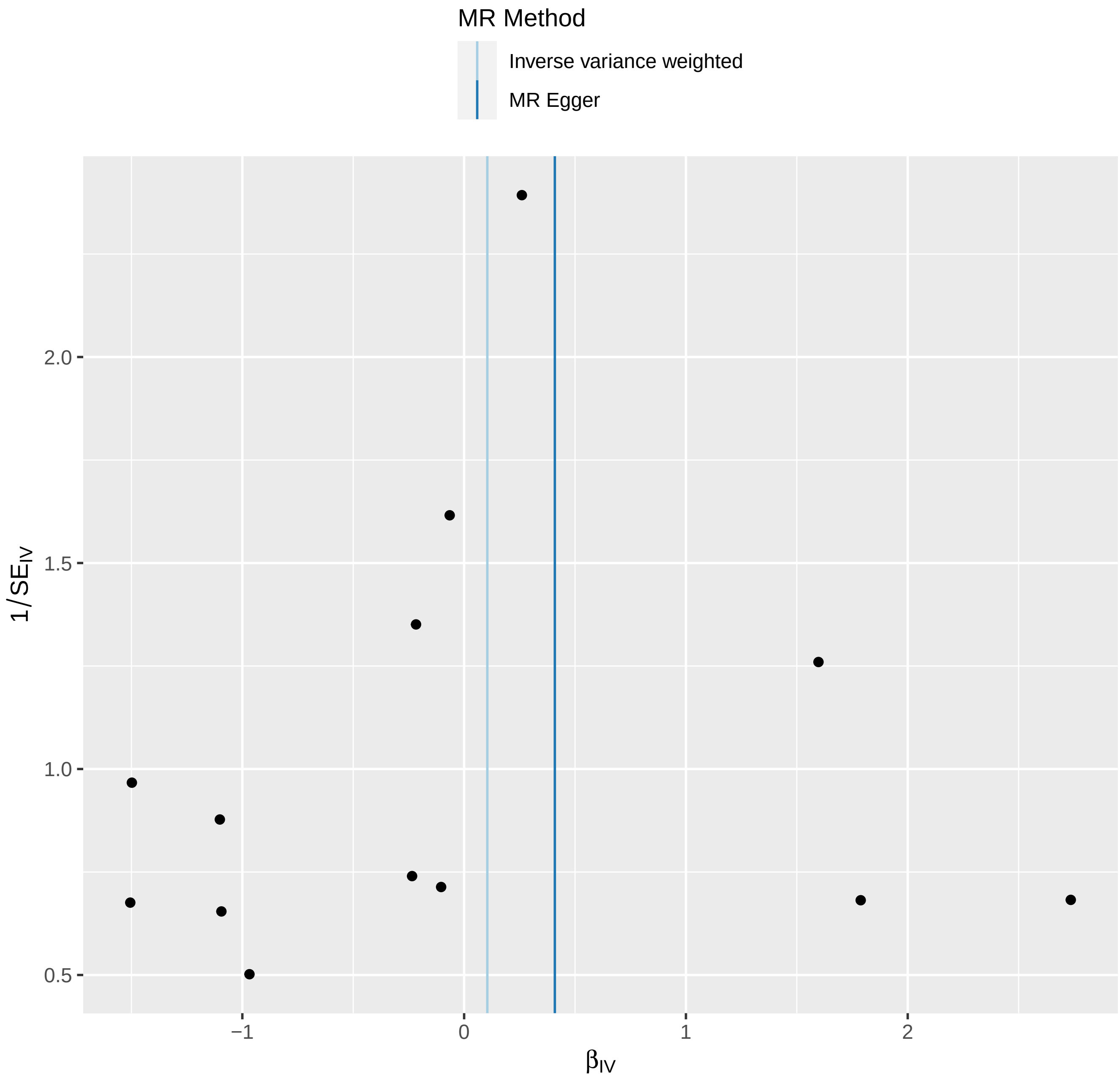

Batch 2452 : X−14208−−phenylalanylserine on Ulcerative proctitis

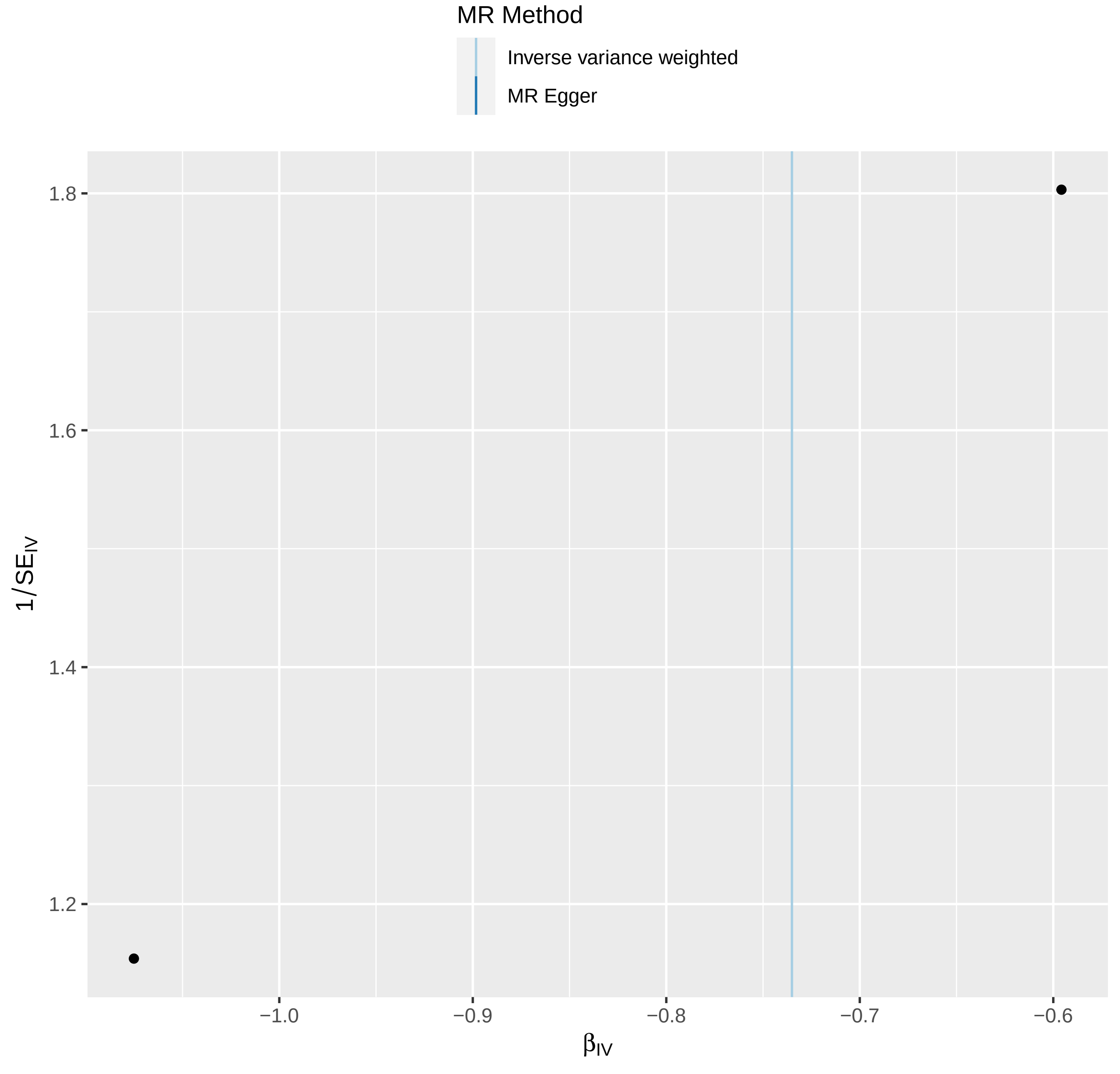

Batch 2454 : X−14450−−phenylalanylleucine on Ulcerative proctitis

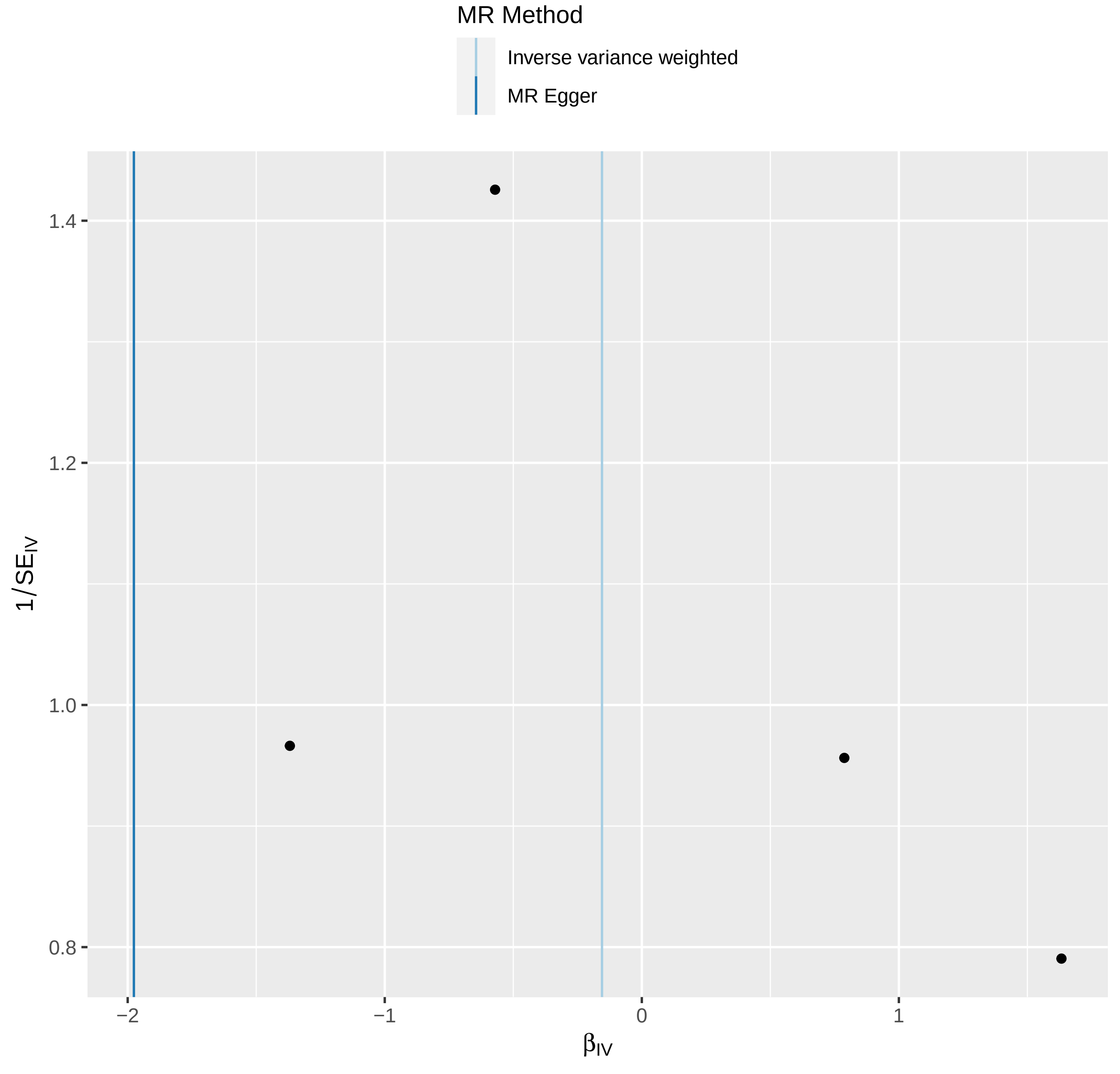

Batch 2455 : Gamma−glutamylglutamate on Ulcerative proctitis

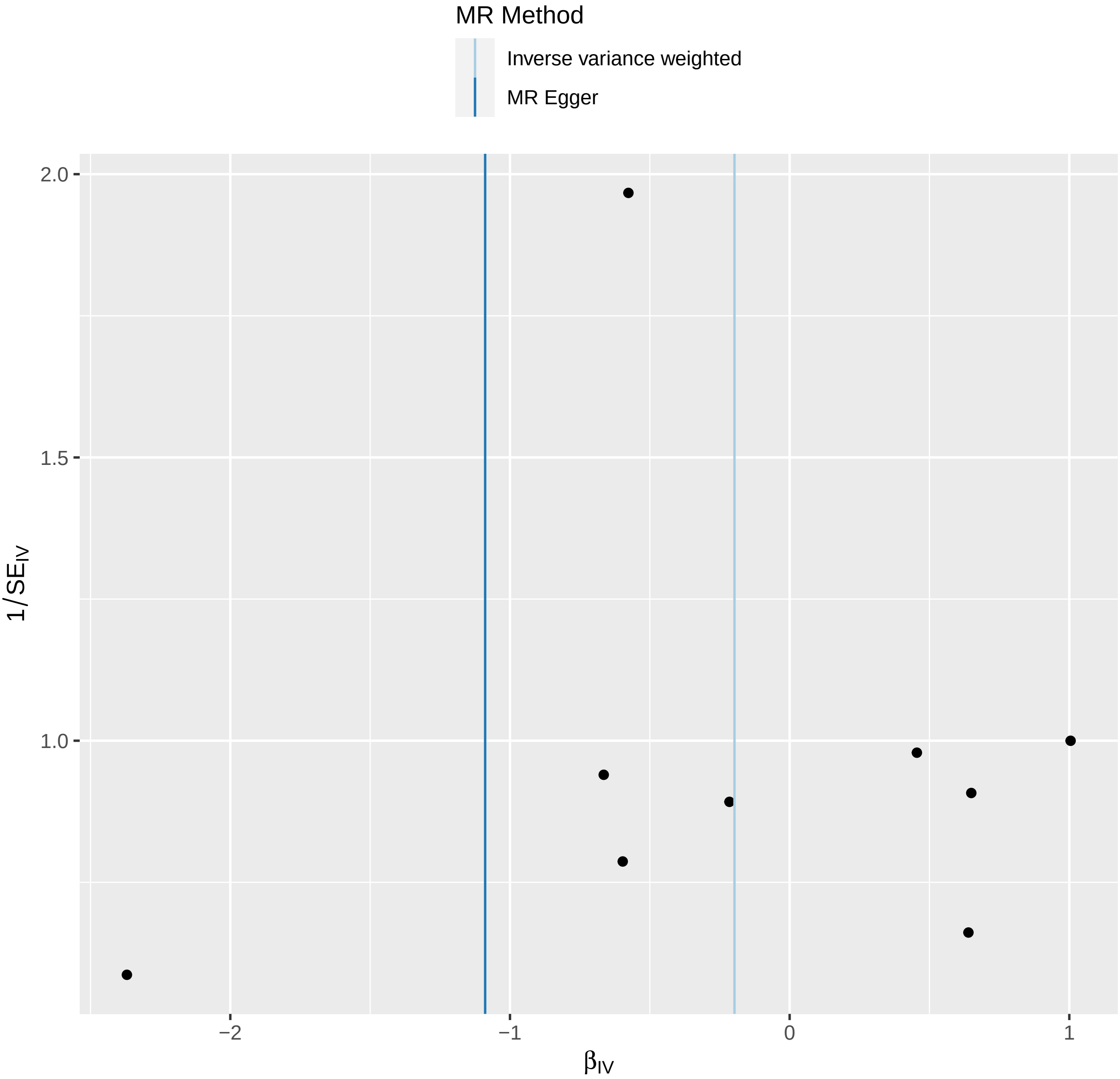

Batch 2456 : Octadecanedioate on Ulcerative proctitis

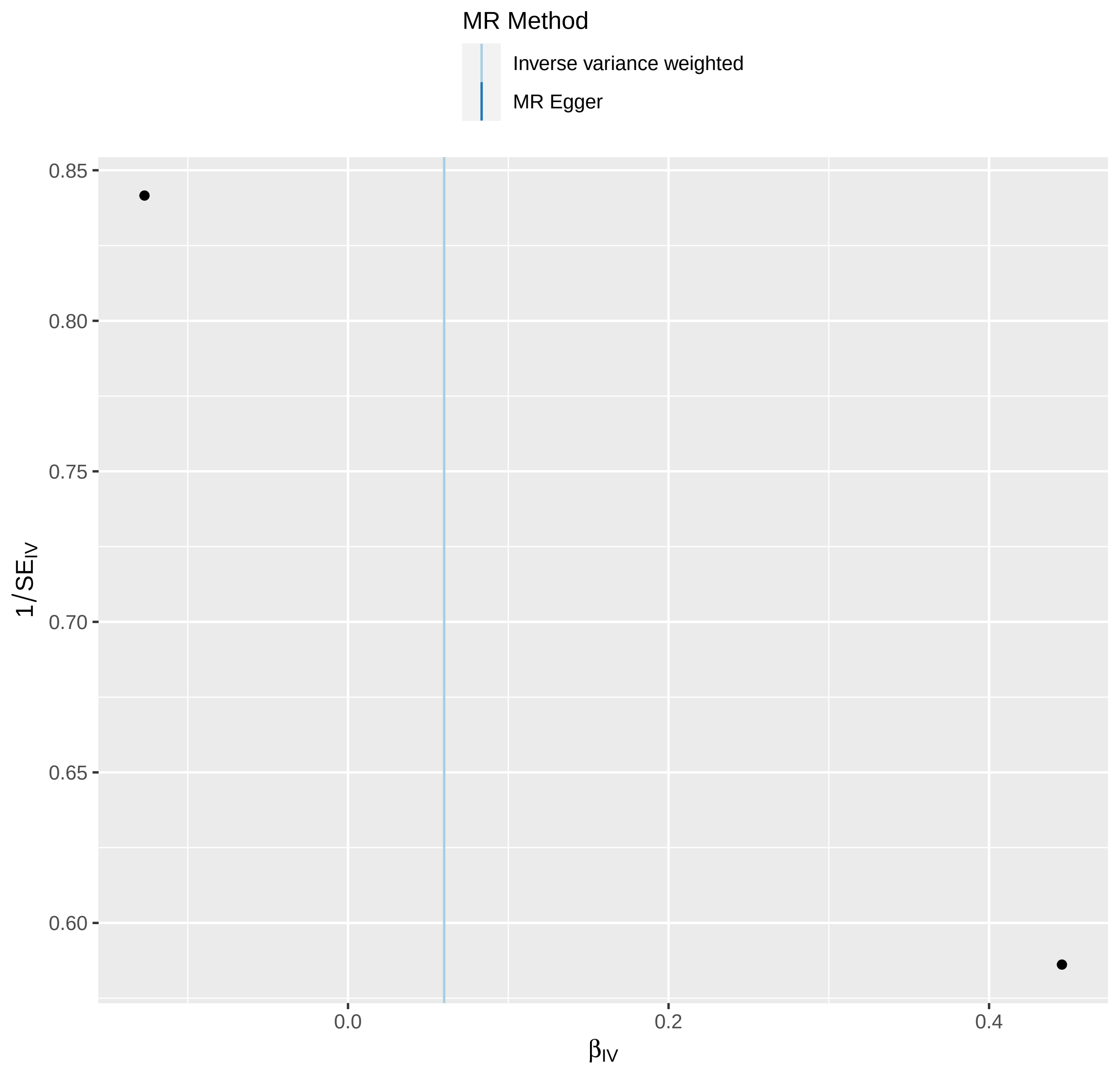

Batch 2457 : Leucylleucine on Ulcerative proctitis

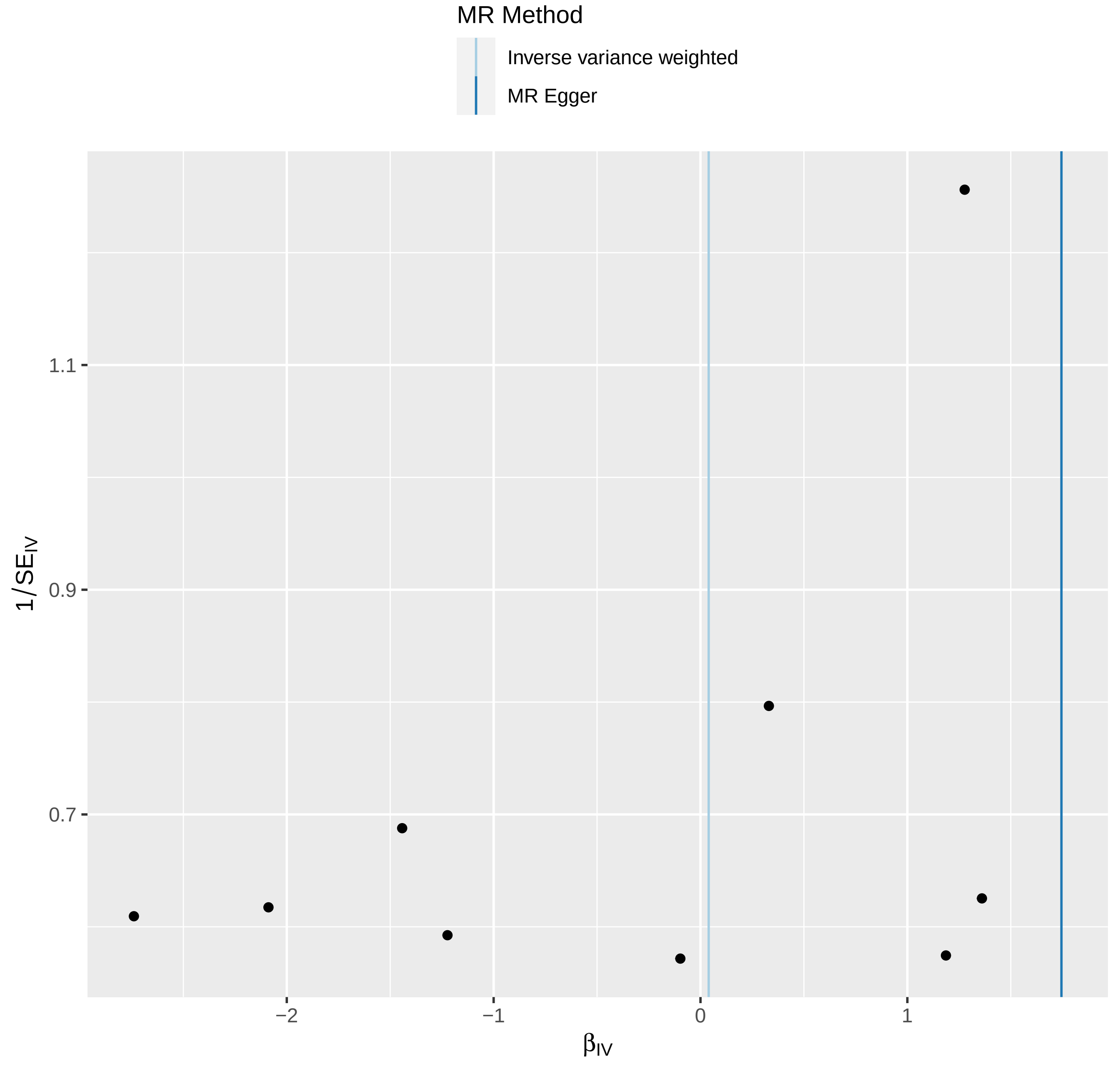

Batch 2458 : 7−alpha−hydroxy−3−oxo−4−cholestenoate (7−Hoca) on Ulcerative proctitis

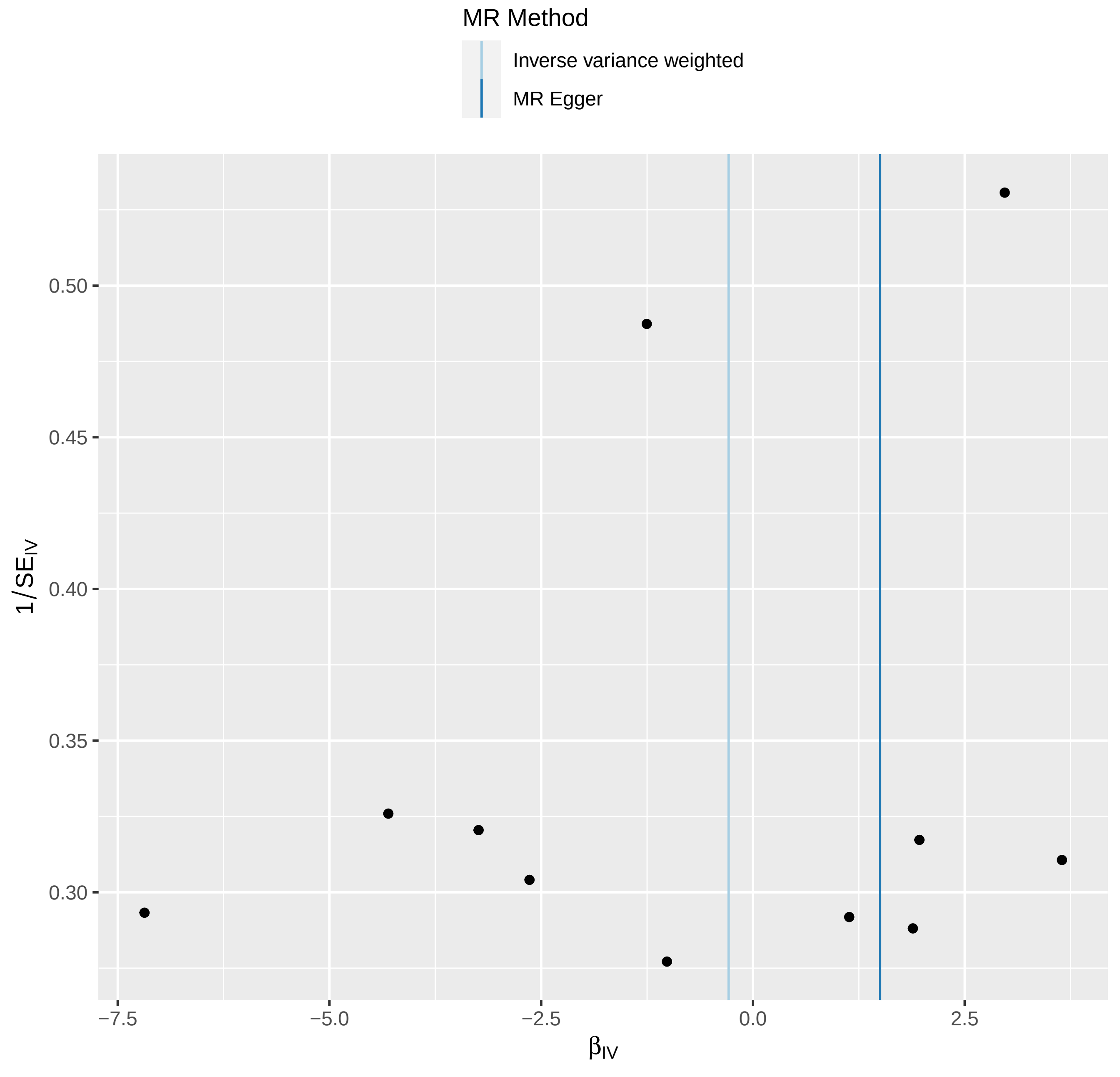

Batch 2459 : N−Butyl Oleate on Ulcerative proctitis

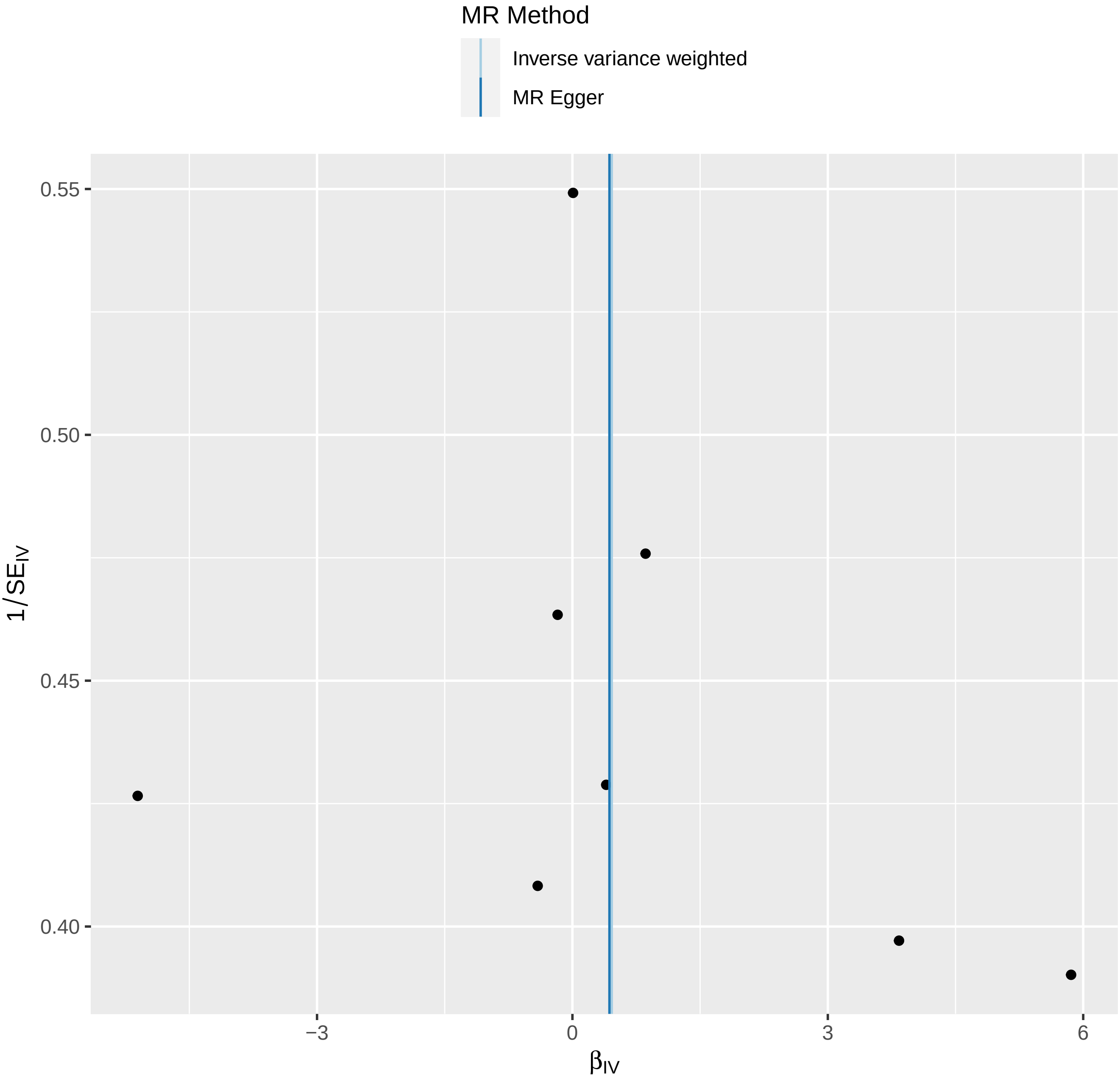

Batch 2461 : Taurolithocholate 3−sulfate on Ulcerative proctitis

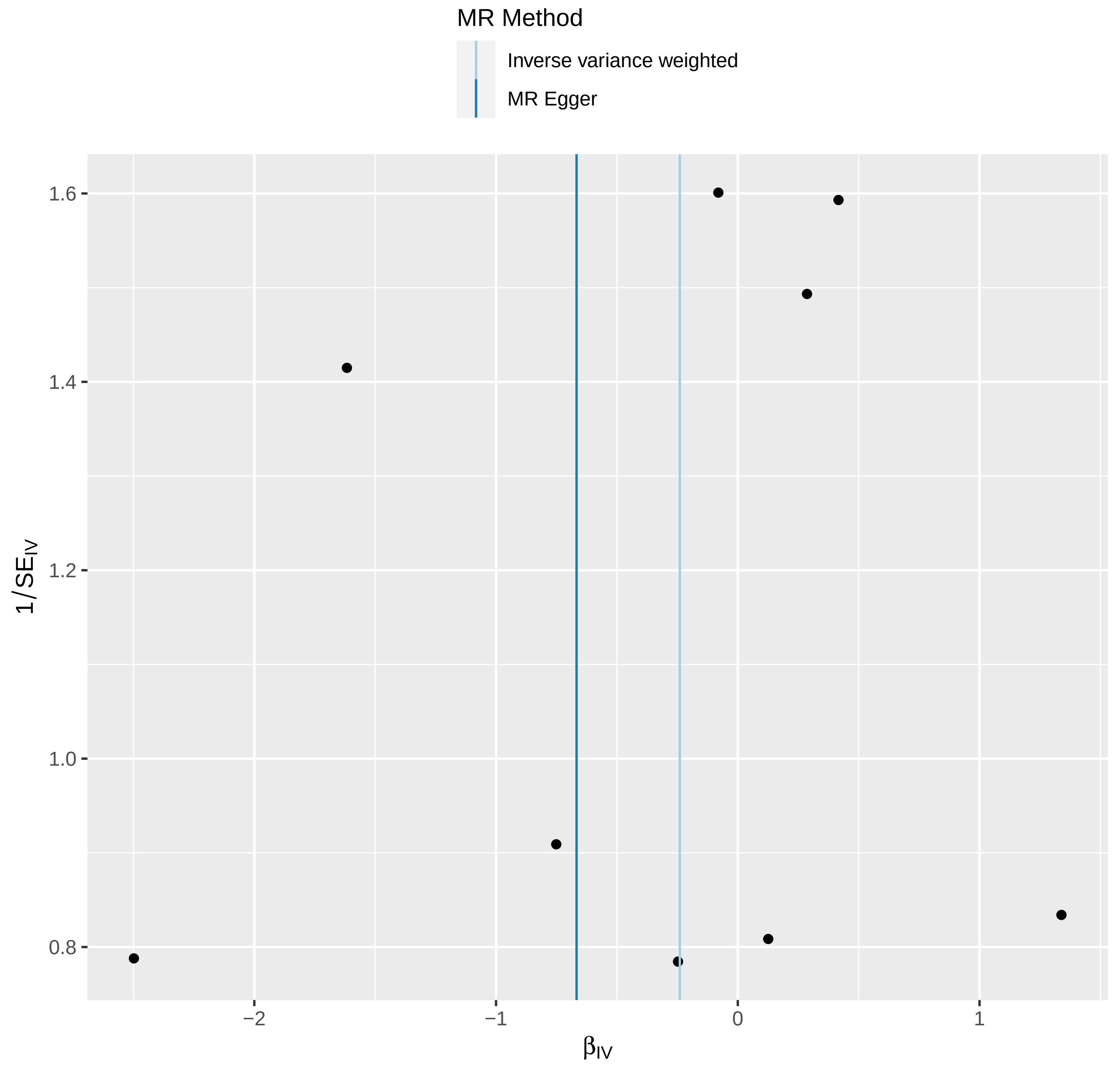

Batch 2462 : X−14977−−vanillin on Ulcerative proctitis

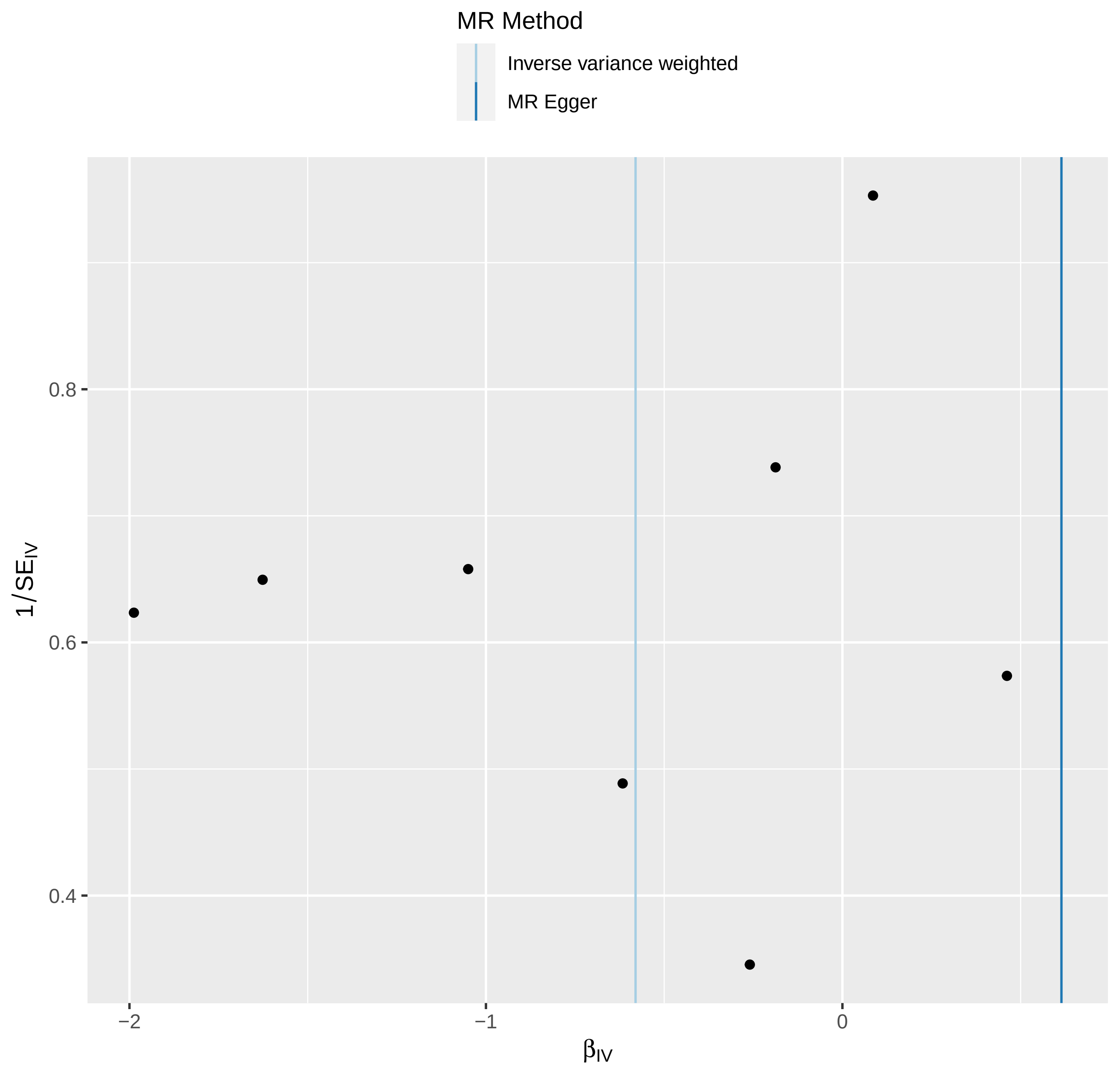

Batch 2463 : Succinylcarnitine on Ulcerative proctitis

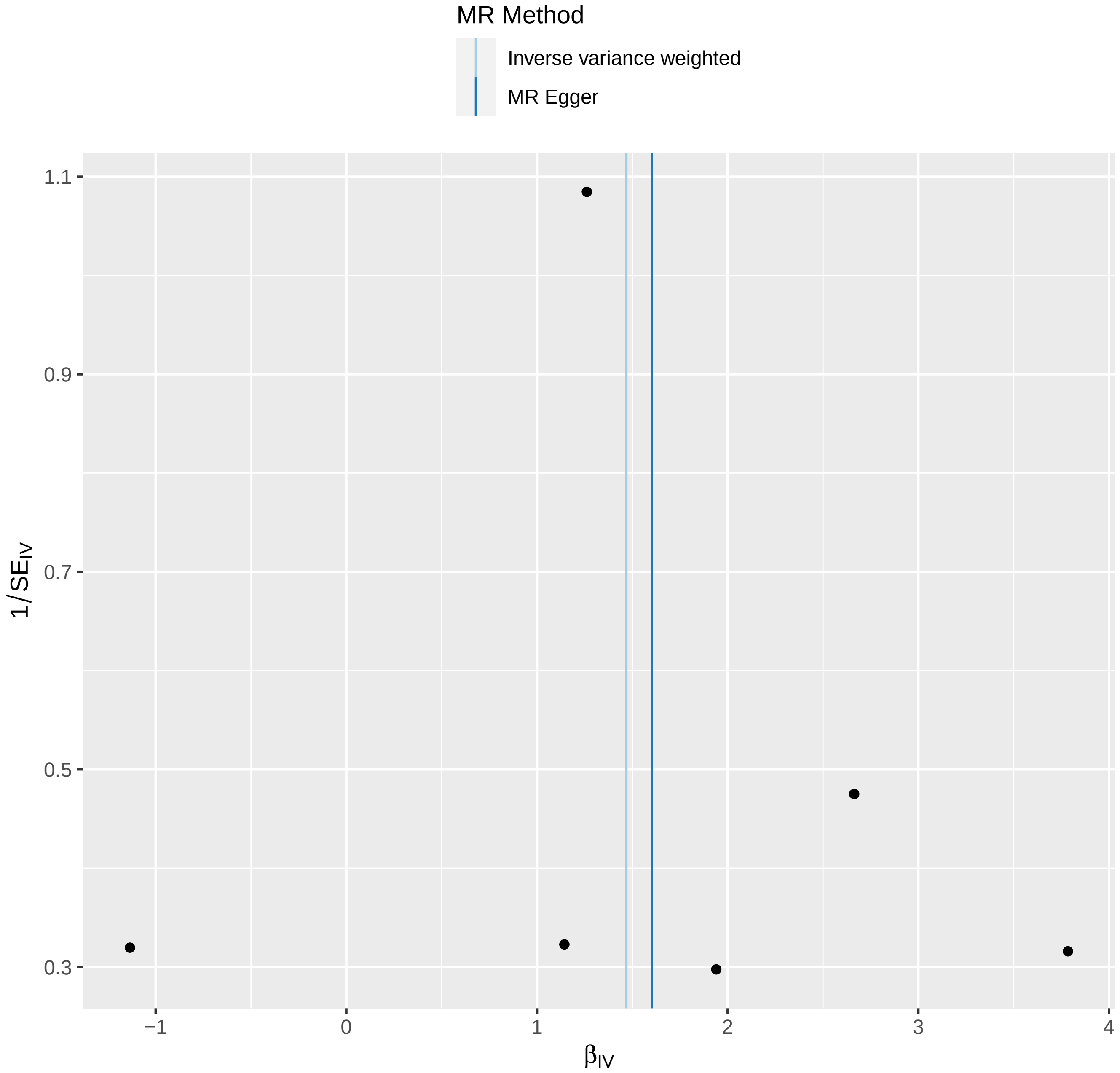

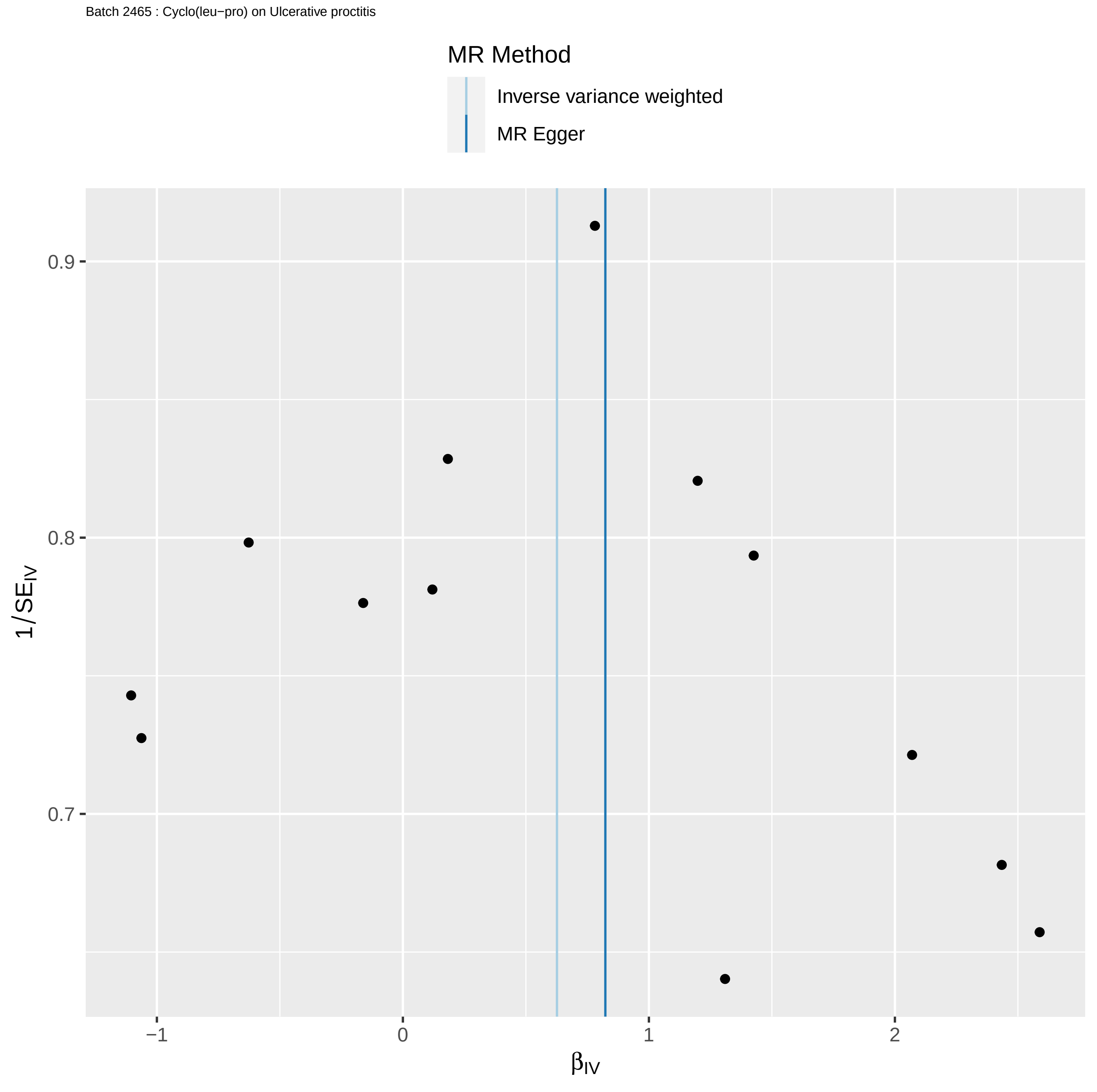

Batch 2465 : Cyclo(leu−pro) on Ulcerative proctitis
MR Method
Inverse variance weighted
MR Egger
0.9
0.8
0.7
1/SE_IV
−1
0
1
2
β_IV

Batch 2466 : Chiro−inositol on Ulcerative proctitis

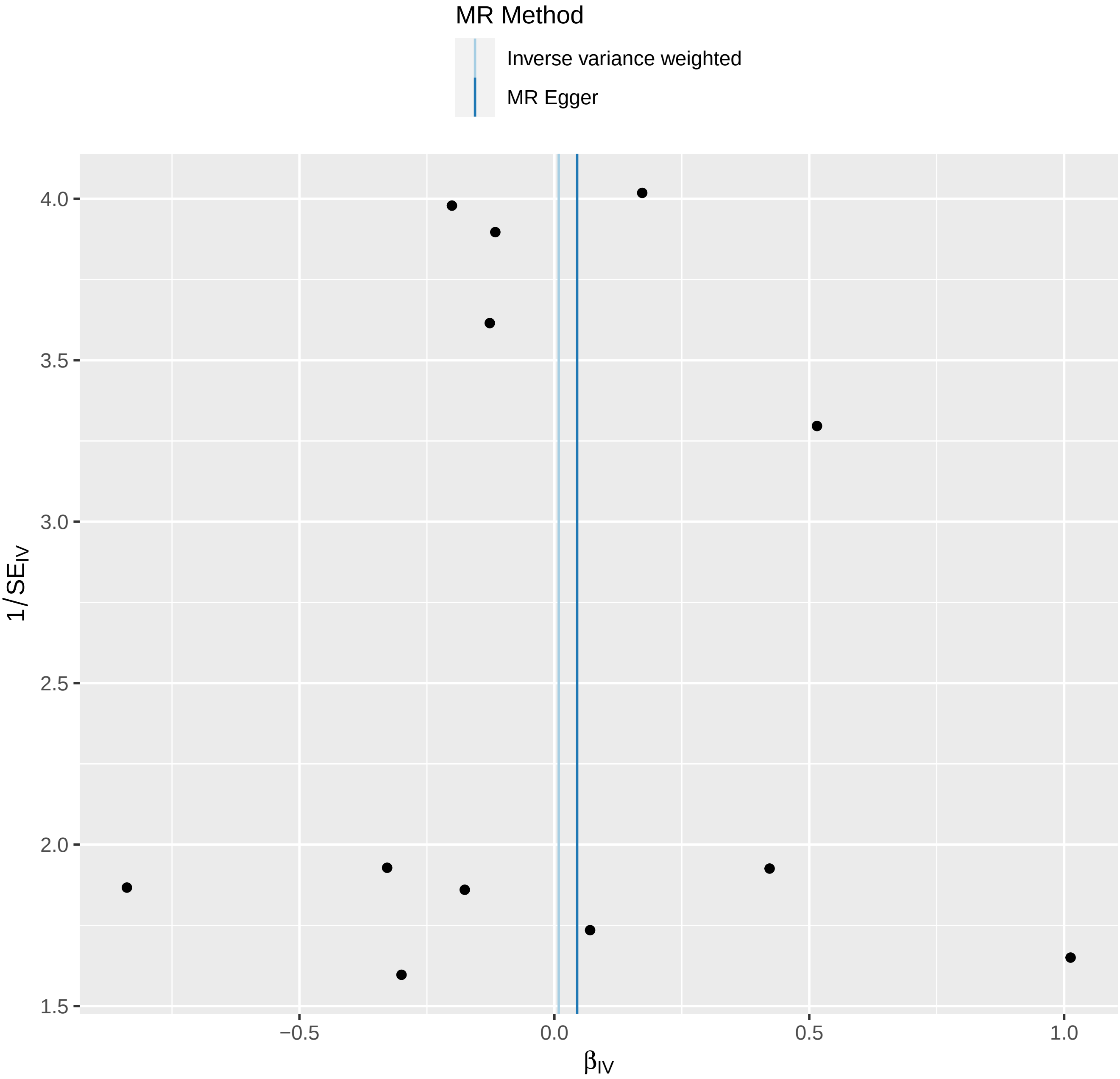

Batch 2470 : 2−hydroxyglutarate on Ulcerative proctitis

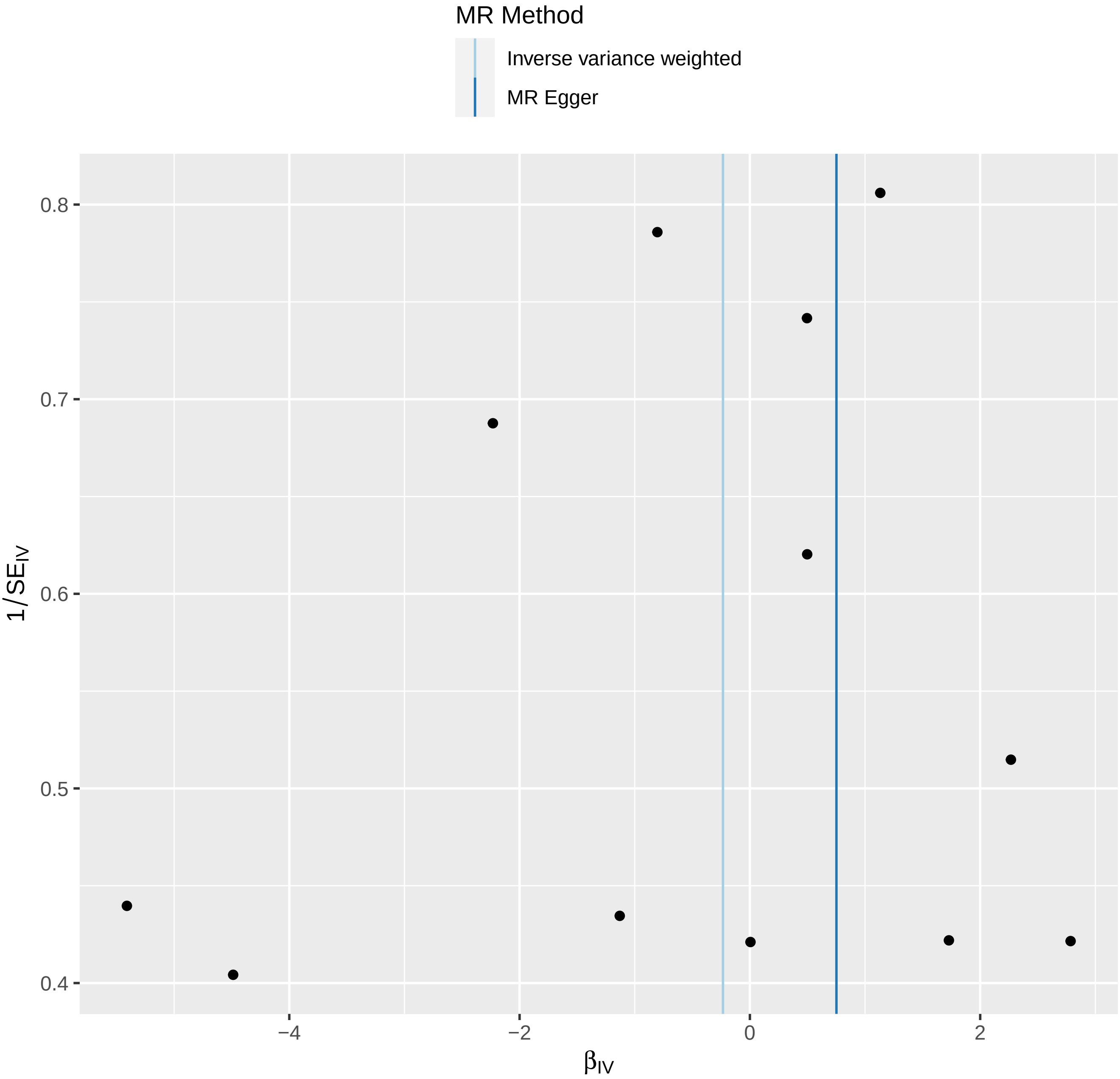

Batch 2471 : Ergothioneine on Ulcerative proctitis

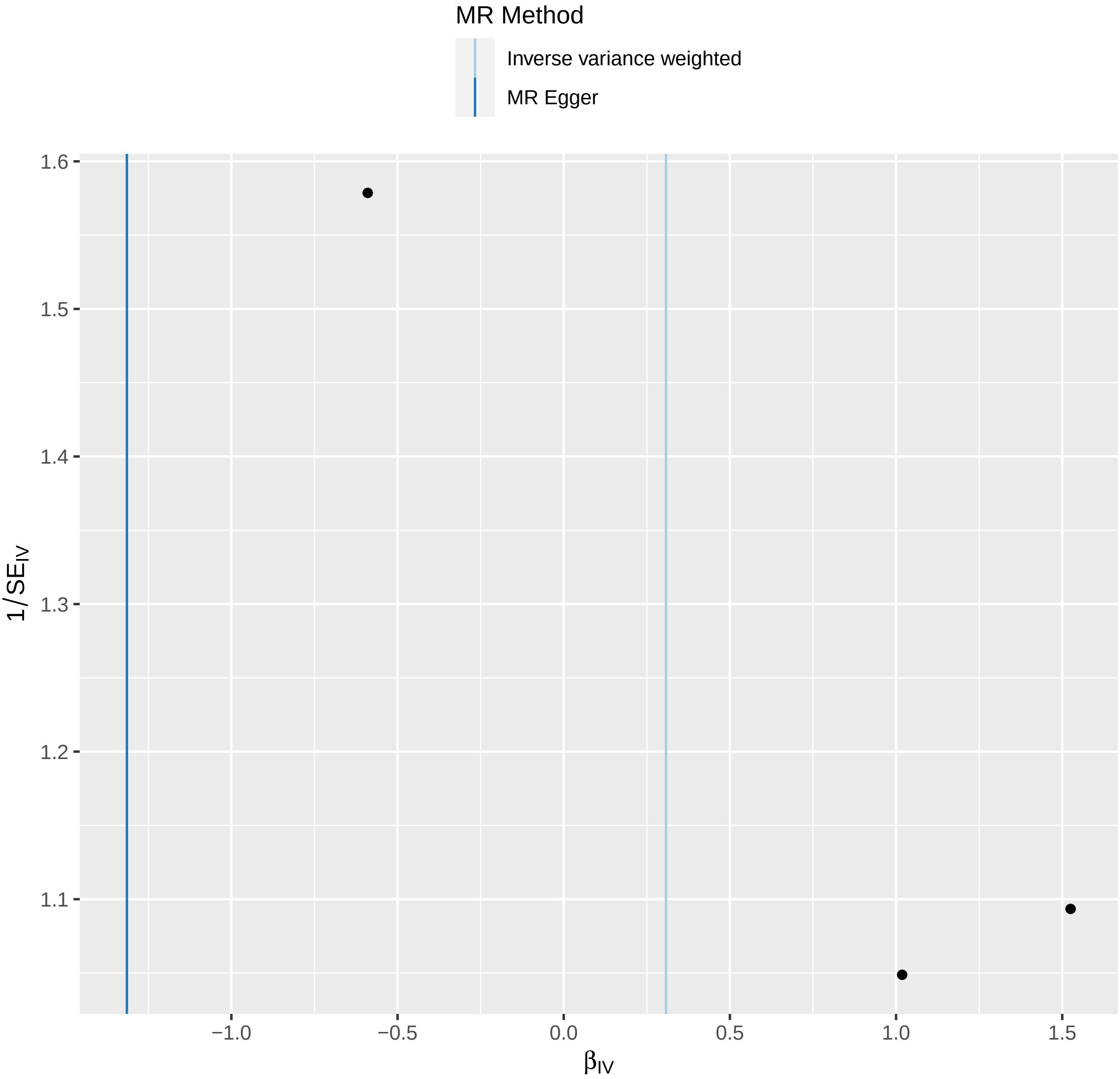

Batch 2473 : Phenylalanylphenylalanine on Ulcerative proctitis

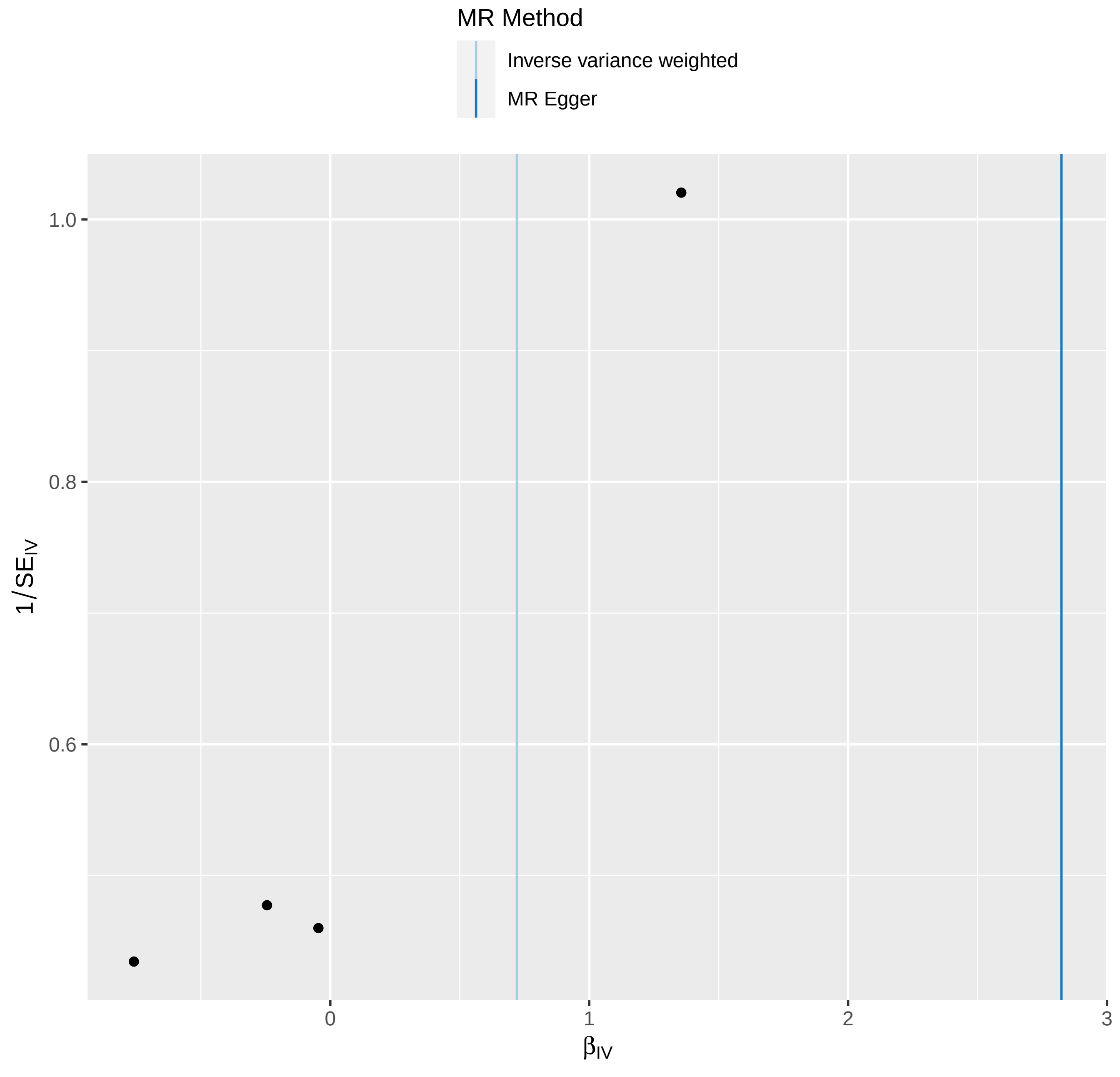

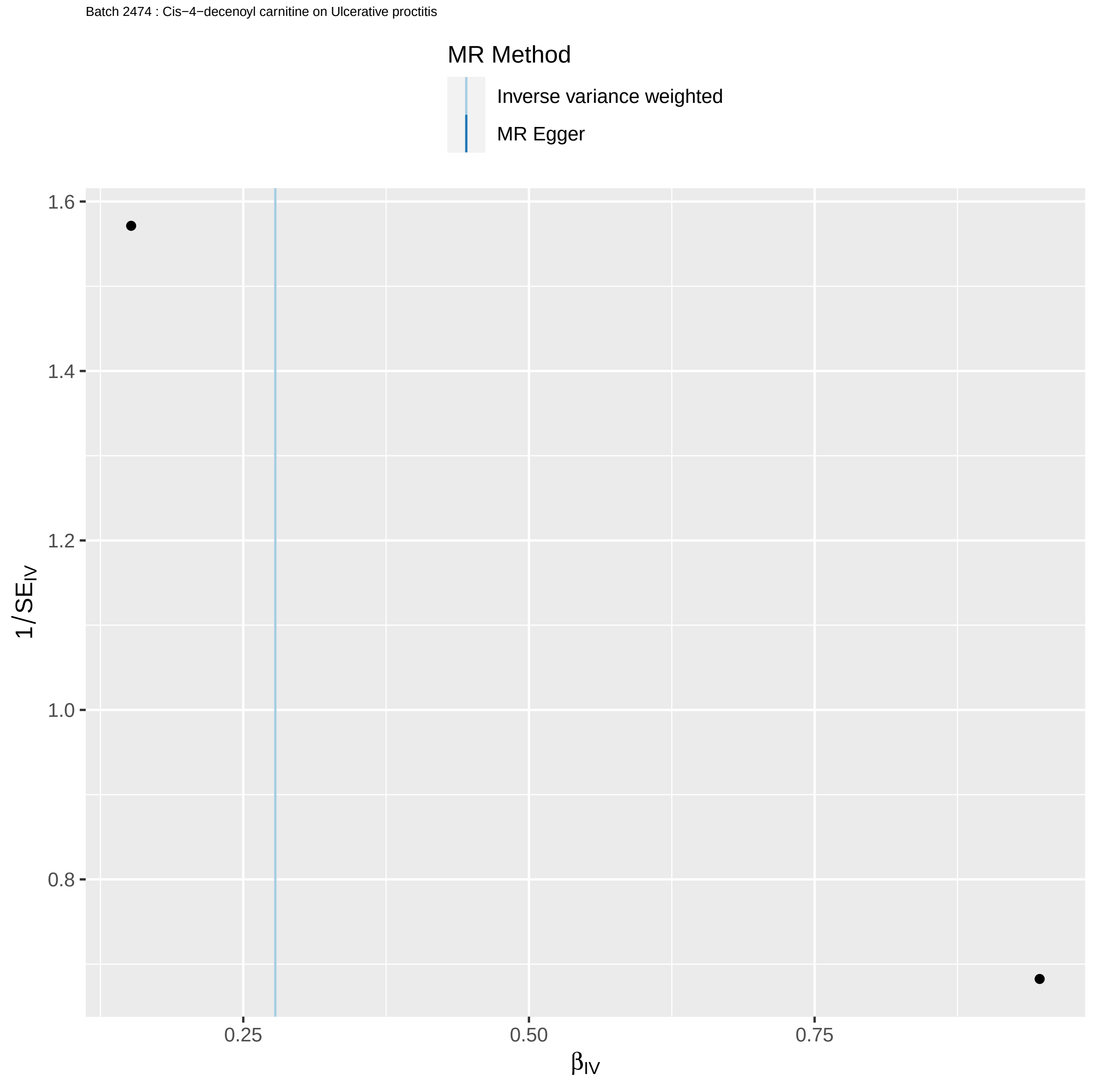

Batch 2474 : Cis−4−decenoyl carnitine on Ulcerative proctitis
MR Method
Inverse variance weighted
MR Egger
1.6
1.4
1.2
1.0
0.8
$1/SE_{IV}$
0.25
0.50
0.75
β_{IV}

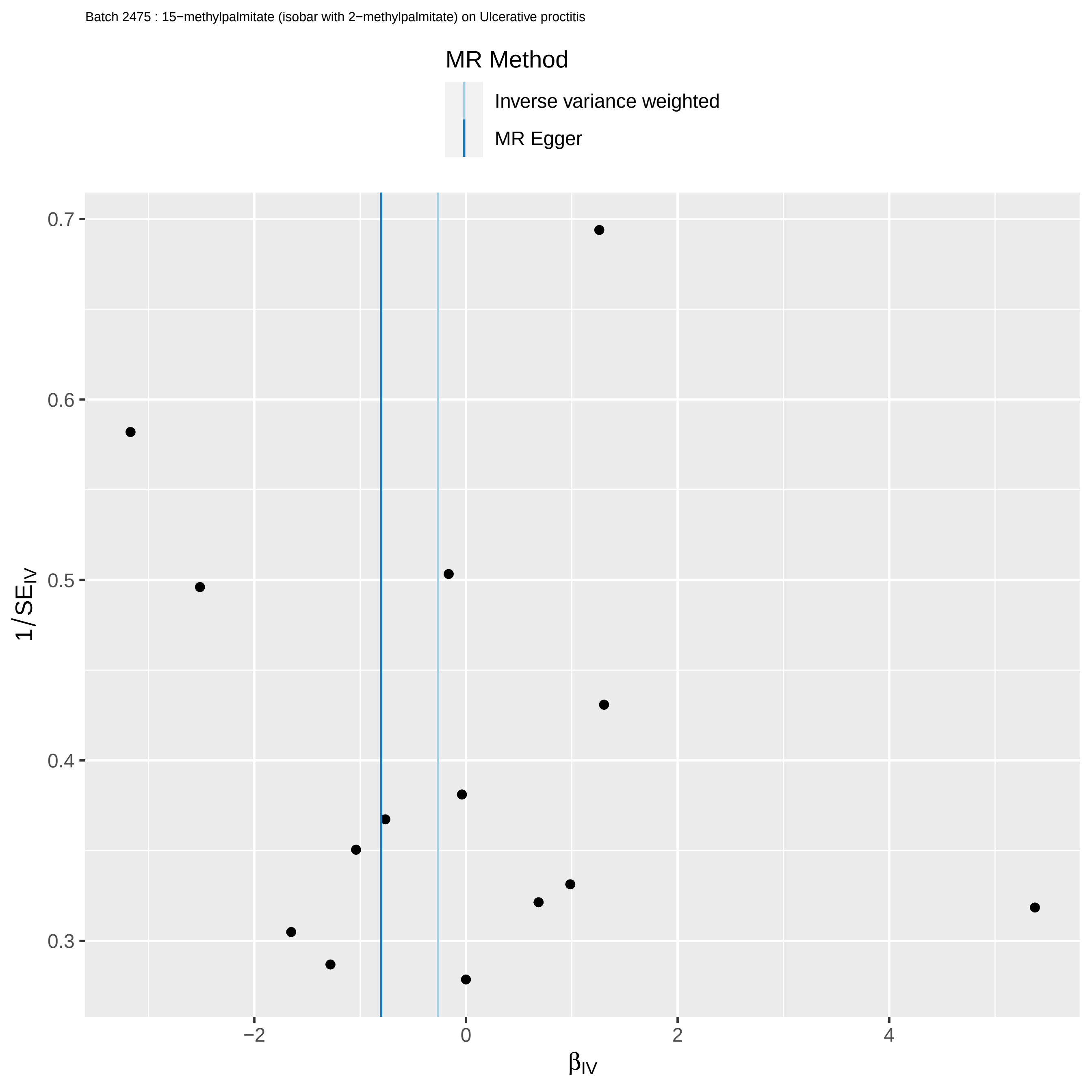

Batch 2475 : 15−methylpalmitate (isobar with 2−methylpalmitate) on Ulcerative proctitis
MR Method
Inverse variance weighted
MR Egger
0.7
0.6
0.5
0.4
0.3
1/SE_IV
−2
0
2
4
β_IV

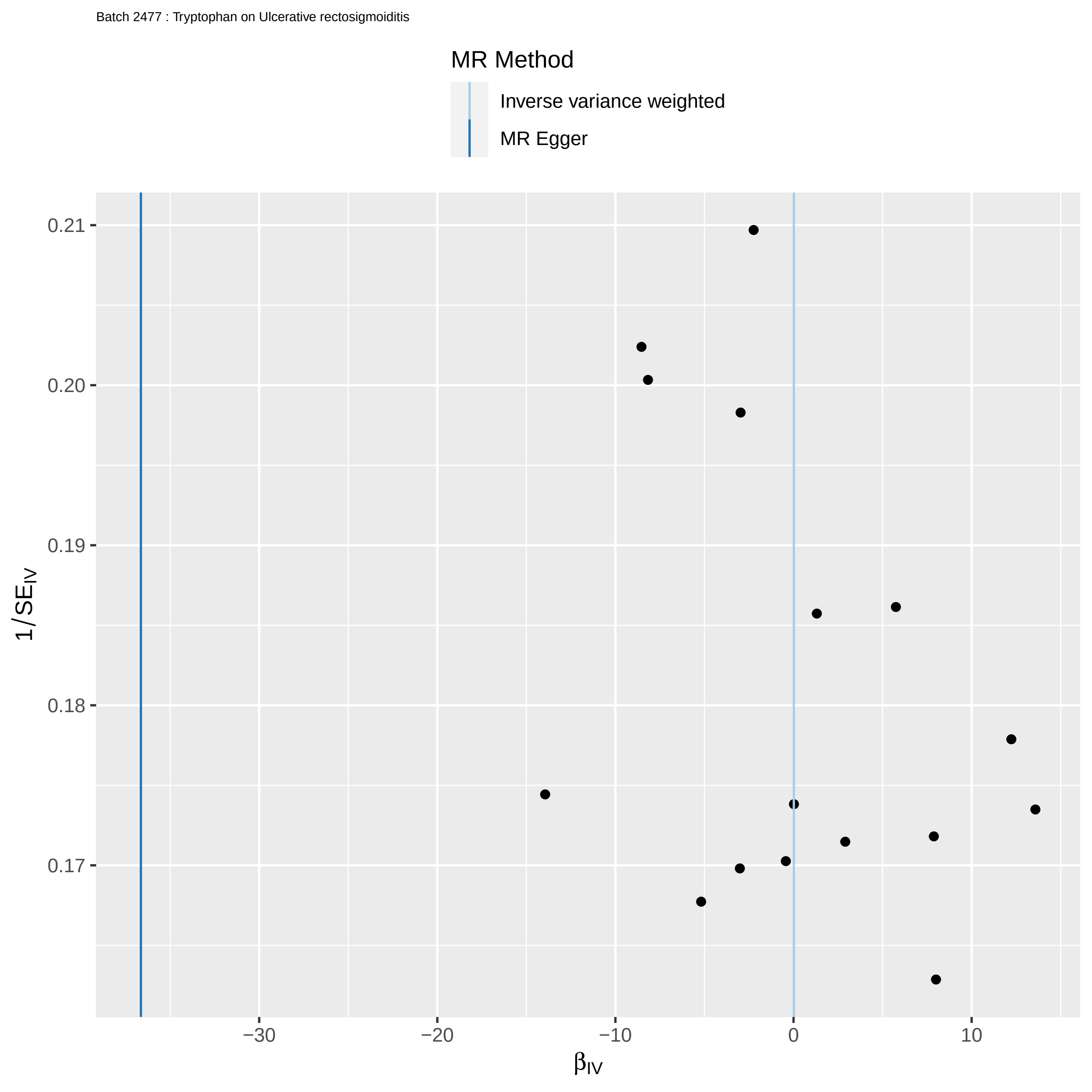
Batch 2477 : Tryptophan on Ulcerative rectosigmoiditis
MR Method
Inverse variance weighted
MR Egger
0.21
0.20
0.19
0.18
0.17
1/SE_IV
−30
−20
−10
0
10
β_IV

Batch 2479 : Leucine on Ulcerative rectosigmoiditis

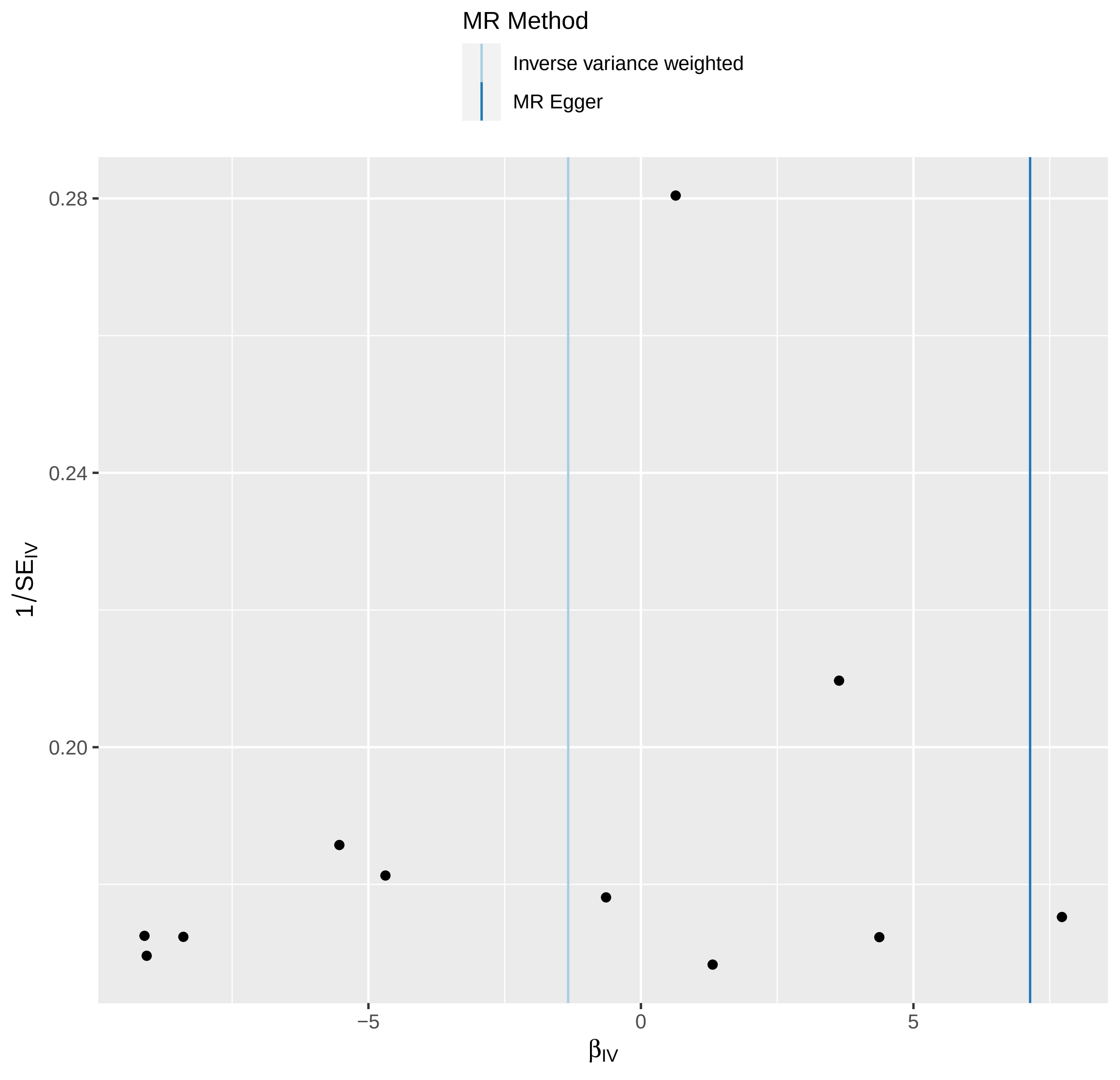

Batch 2483 : Lactate on Ulcerative rectosigmoiditis

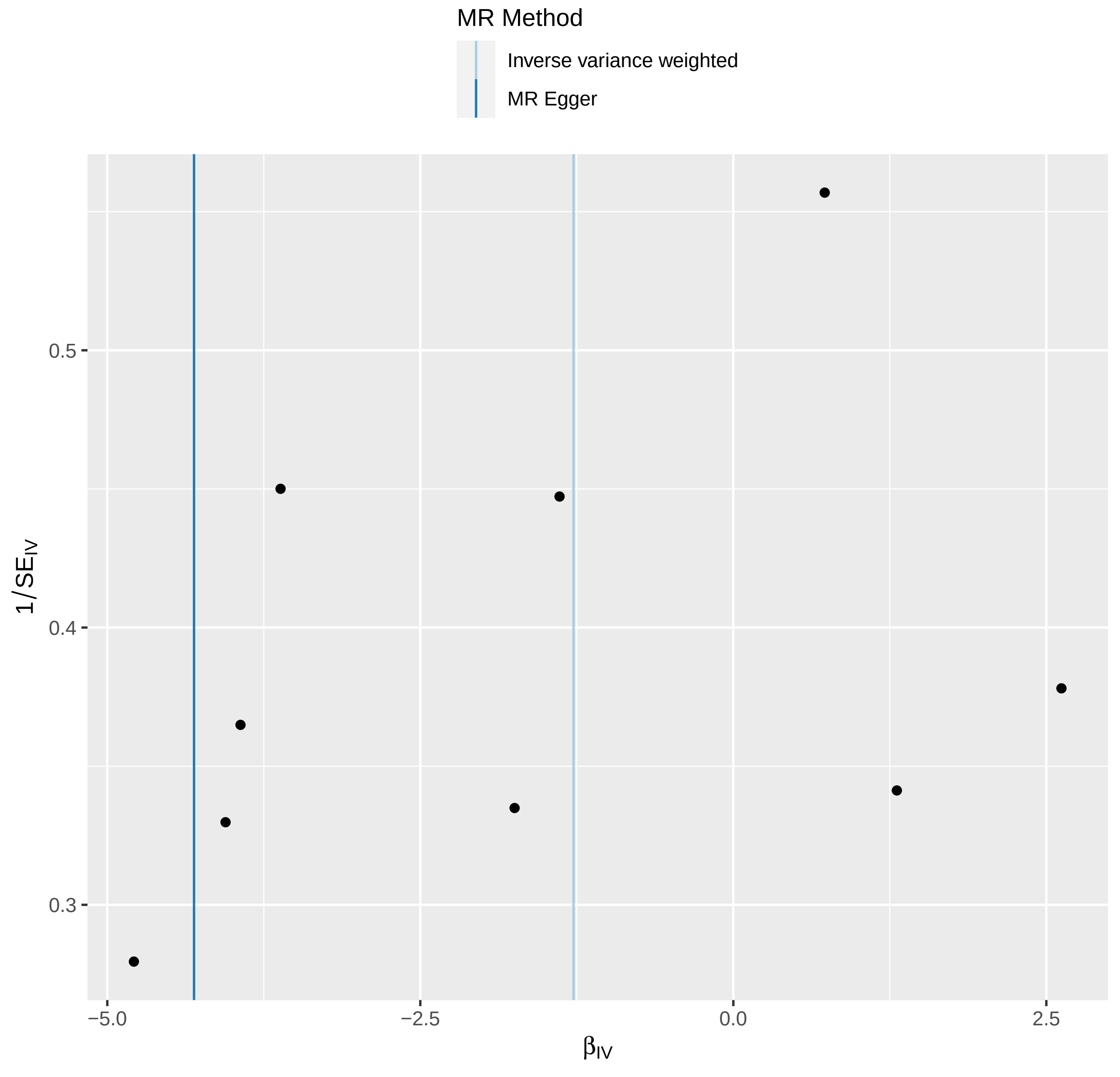

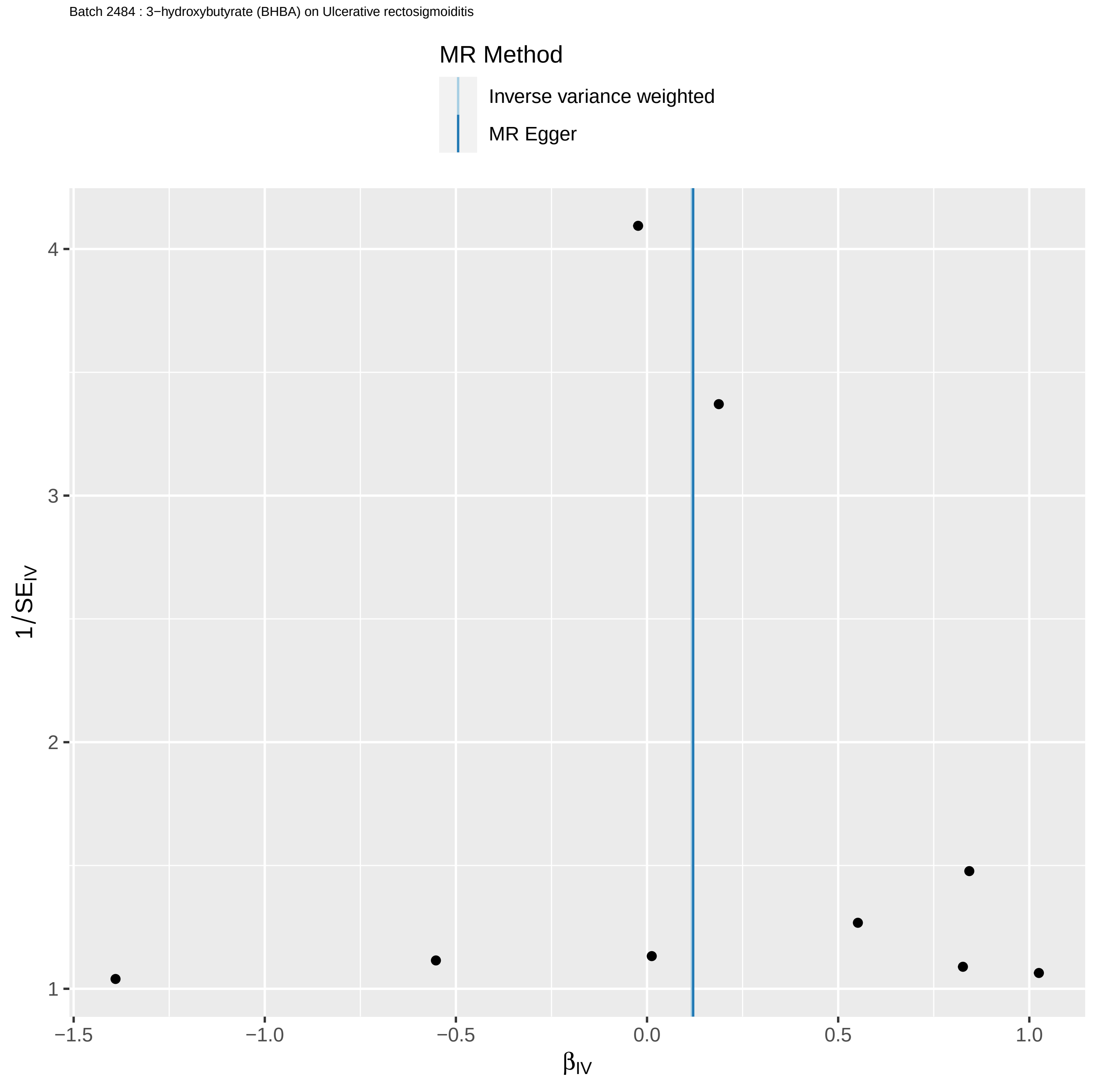

Batch 2484 : 3−hydroxybutyrate (BHBA) on Ulcerative rectosigmoiditis
MR Method
Inverse variance weighted
MR Egger
4
3
2
1
1/SE_IV
−1.5
−1.0
−0.5
0.0
0.5
1.0
β_IV

Batch 2485 : Arabinose on Ulcerative rectosigmoiditis

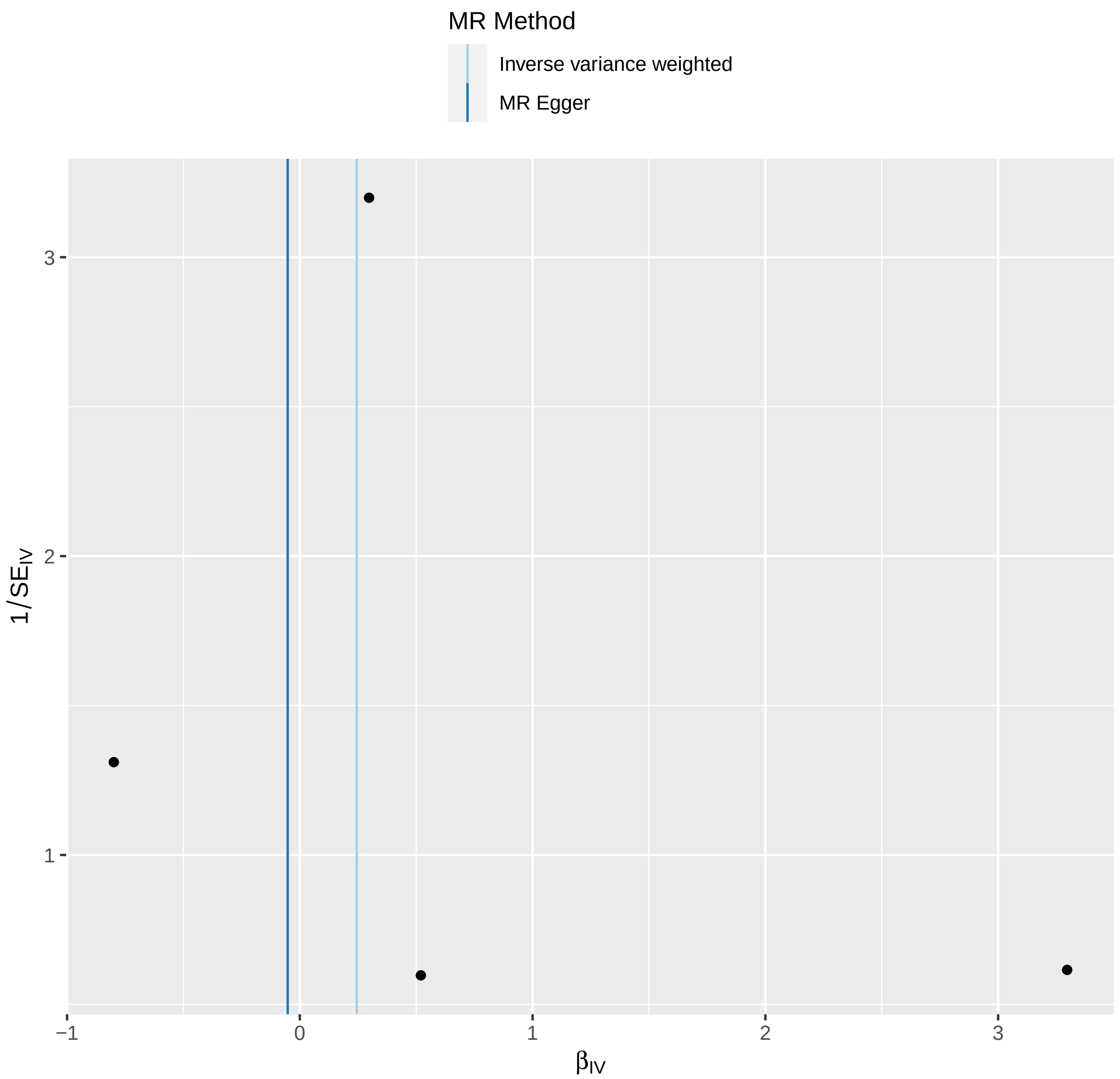

Batch 2486 : Fructose on Ulcerative rectosigmoiditis

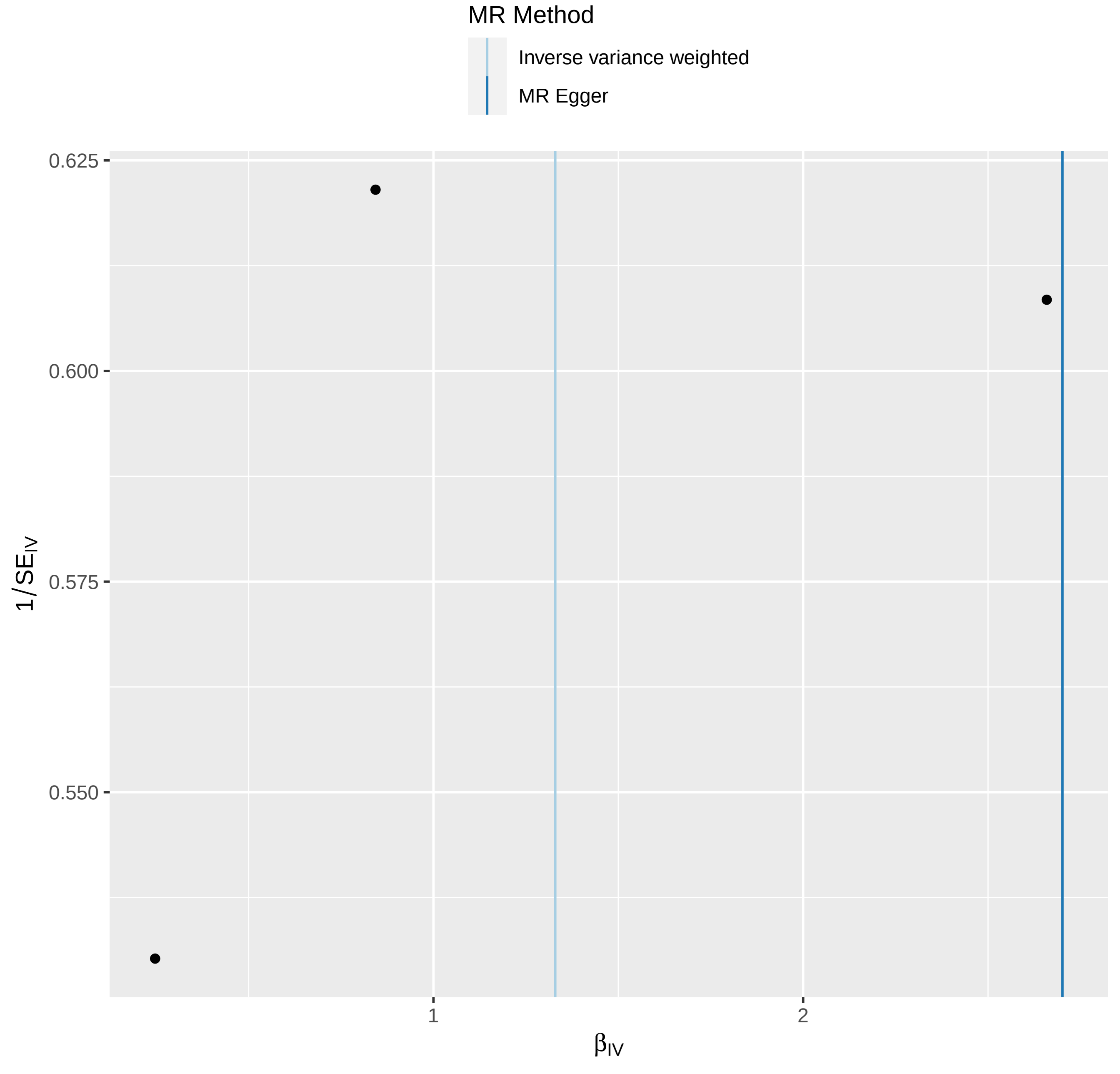

Batch 2488 : Pyruvate on Ulcerative rectosigmoiditis

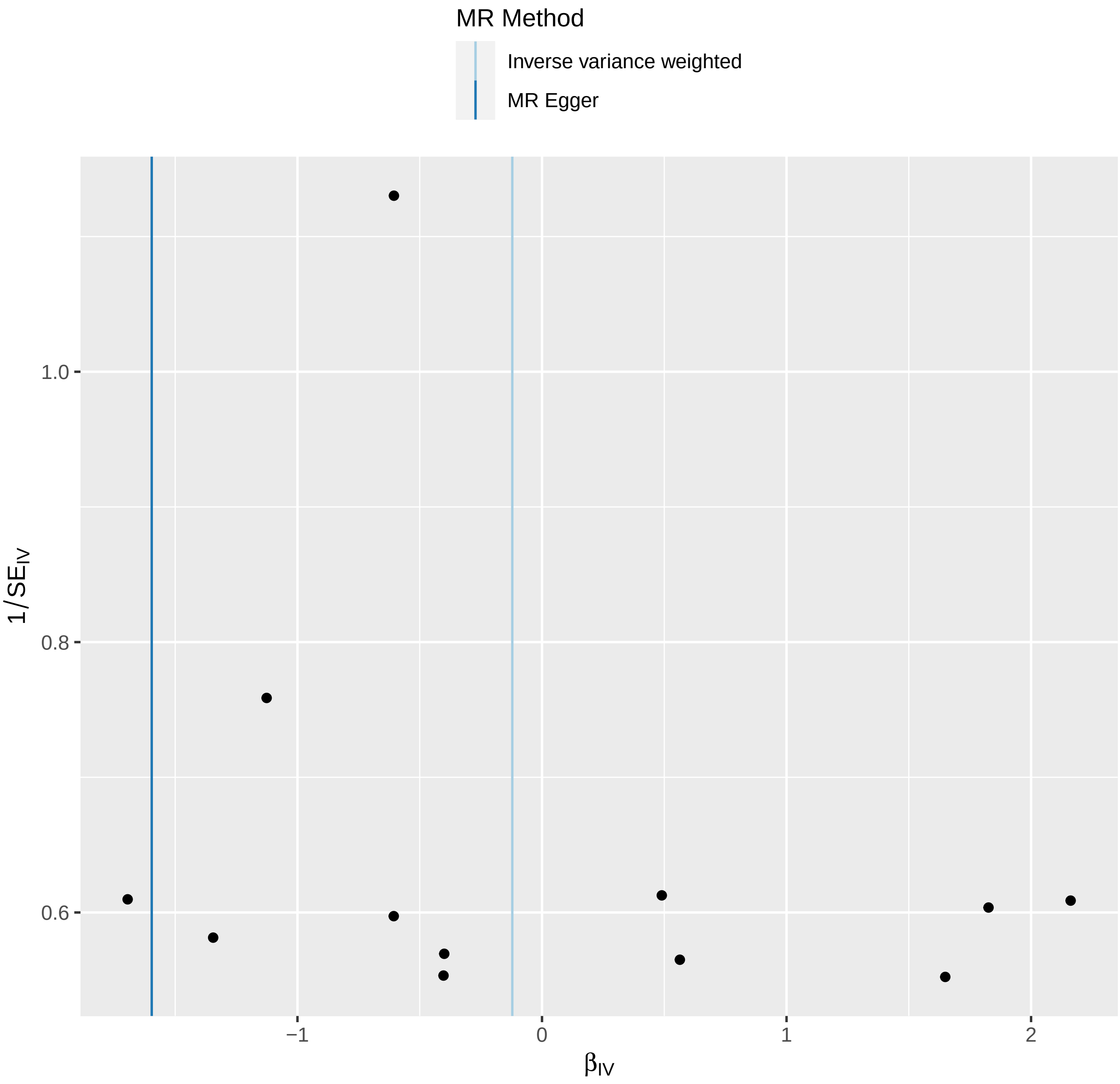

Batch 2489 : Uridine on Ulcerative rectosigmoiditis

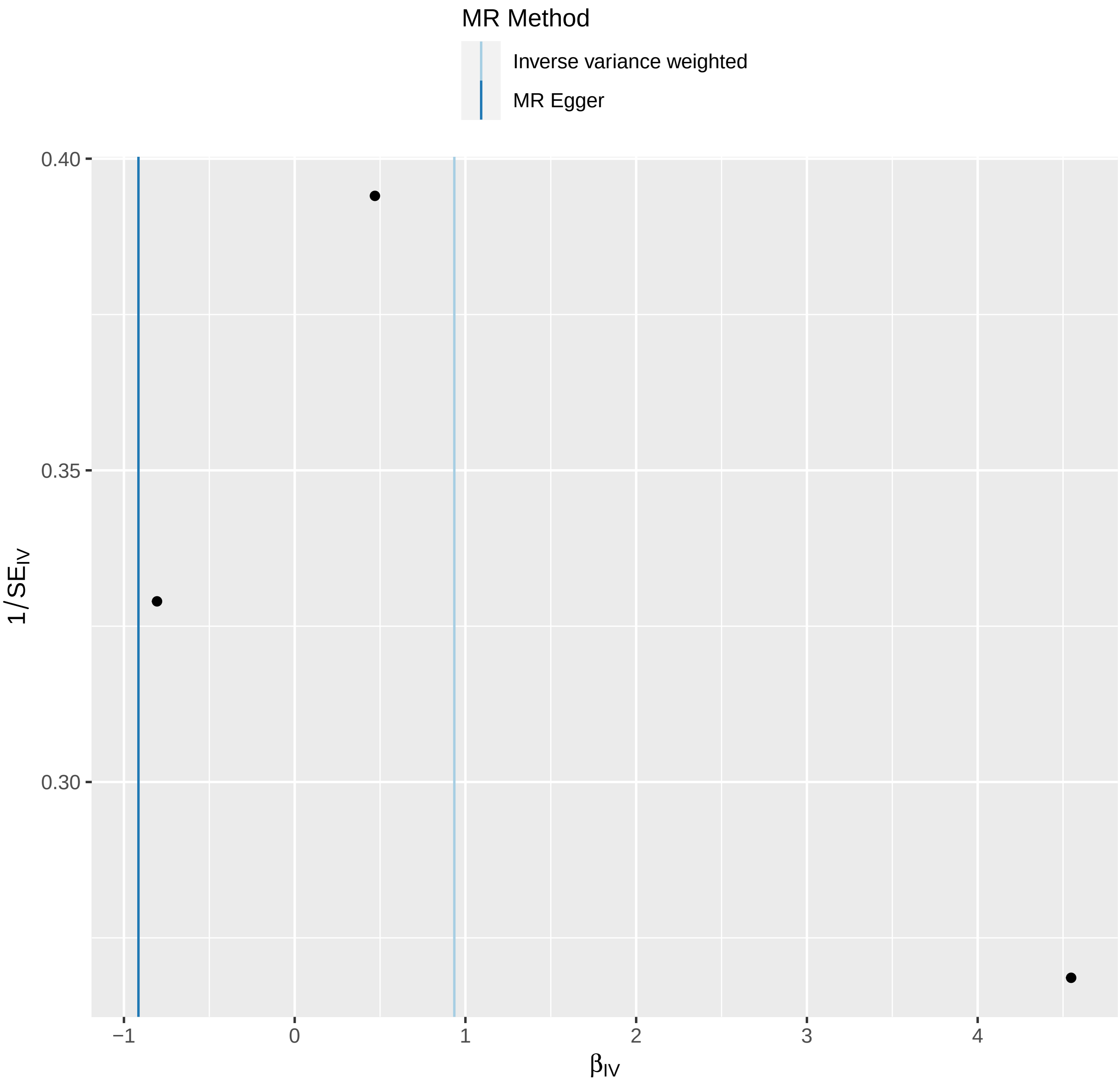

Batch 2490 : Linoleate (18:2n6) on Ulcerative rectosigmoiditis

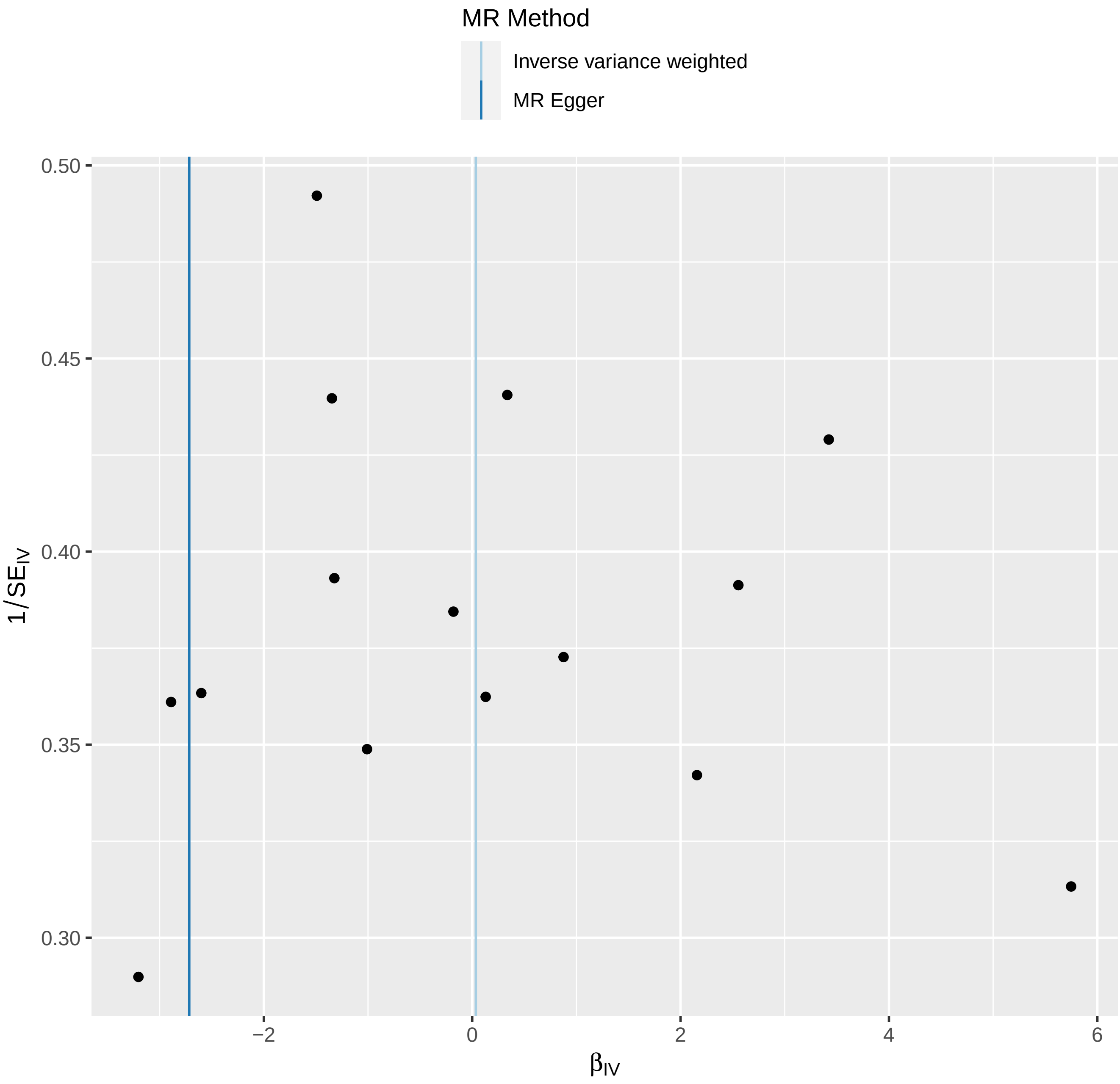

Batch 2491 : Allantoin on Ulcerative rectosigmoiditis

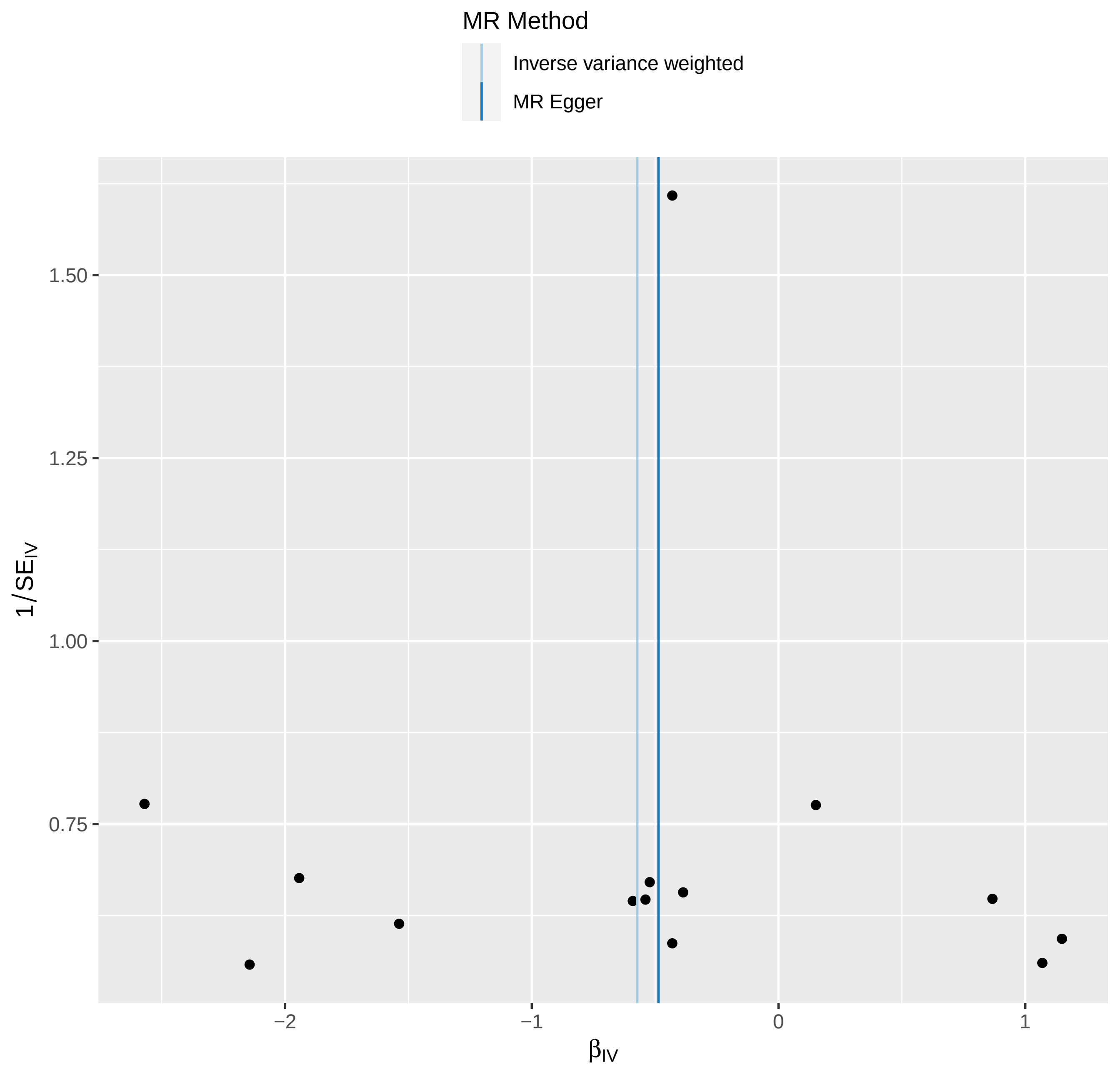

Batch 2496 : Isoleucine on Ulcerative rectosigmoiditis

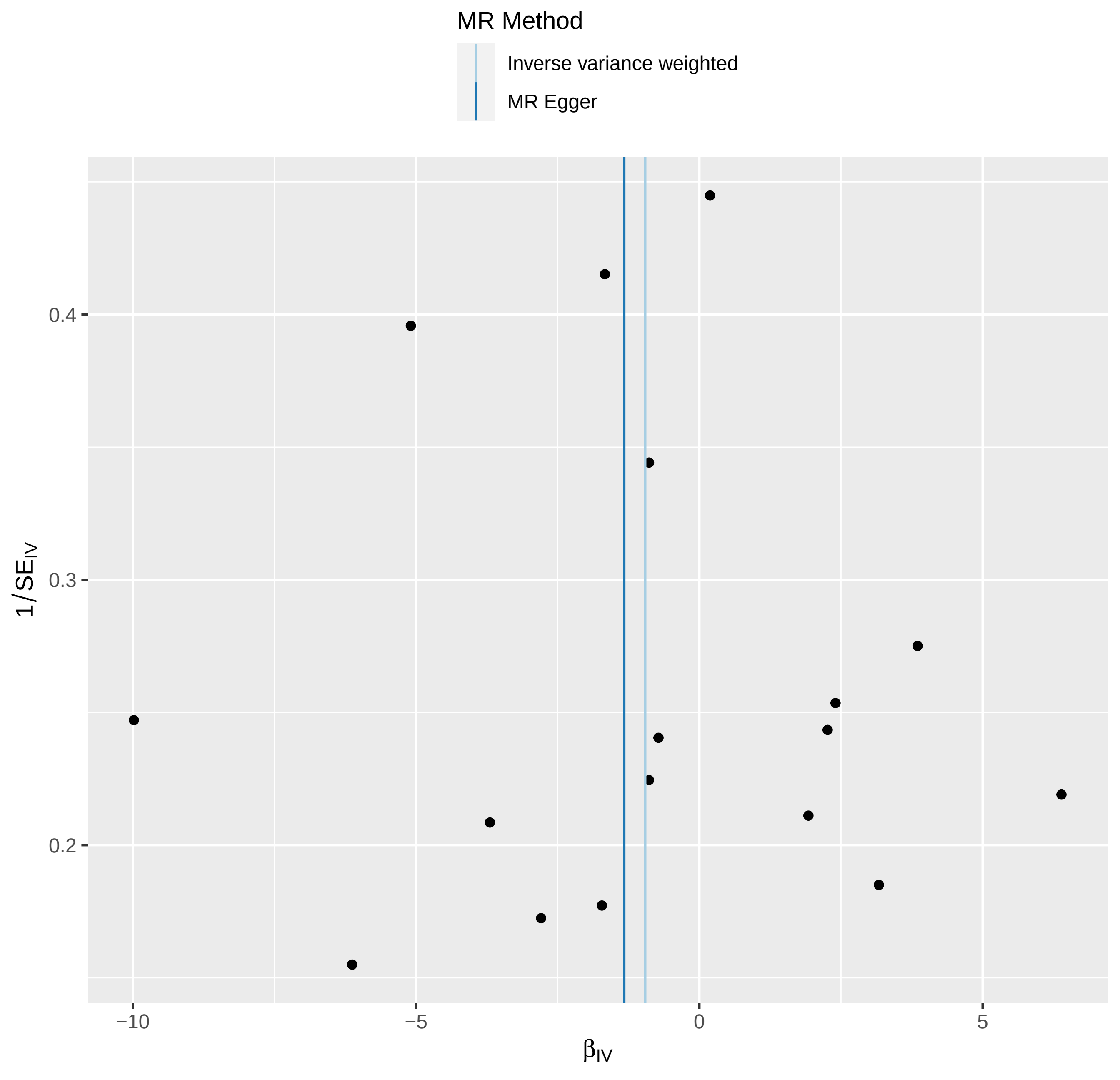

Batch 2497 : Threonine on Ulcerative rectosigmoiditis

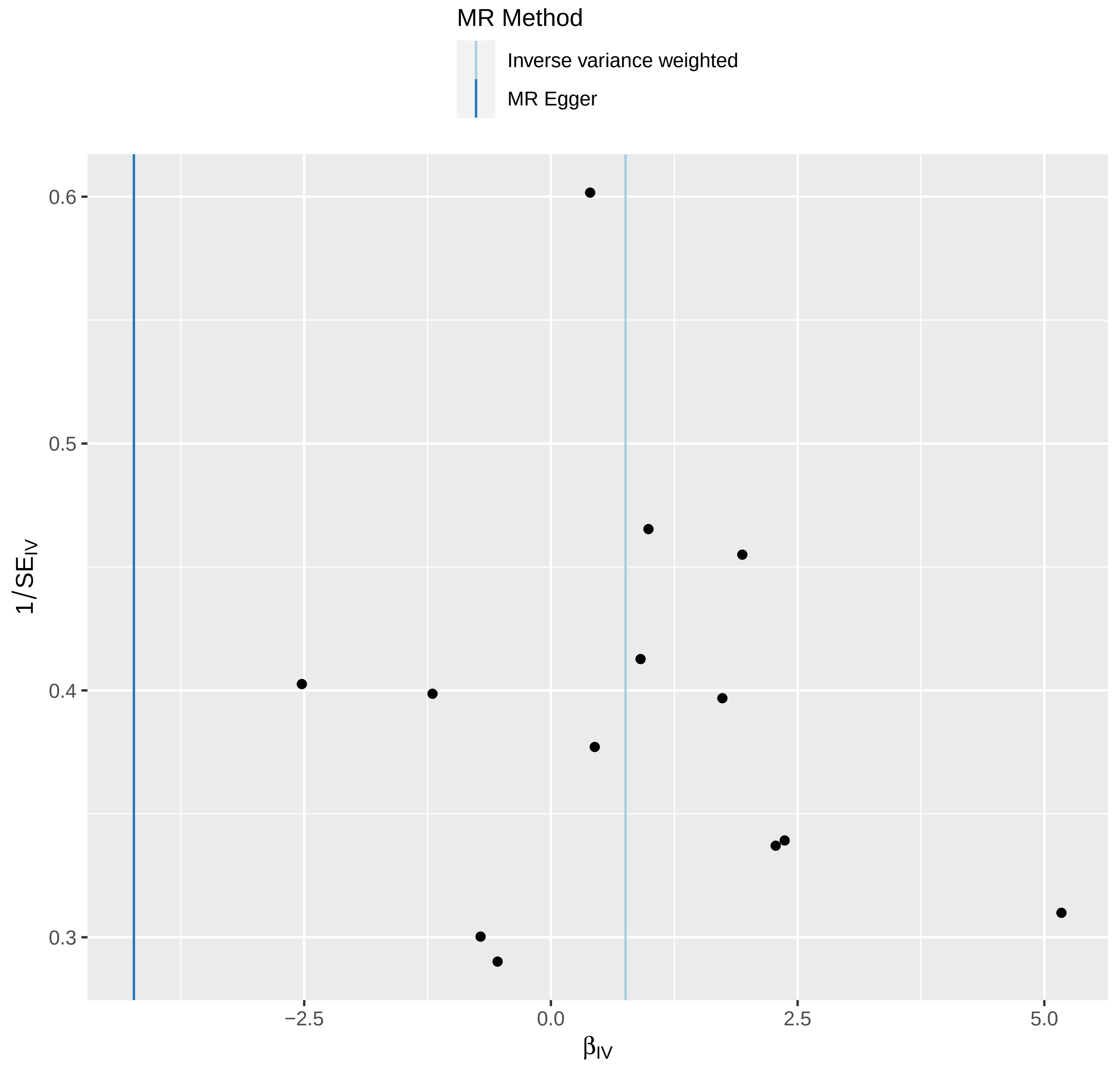

Batch 2498 : Tyrosine on Ulcerative rectosigmoiditis

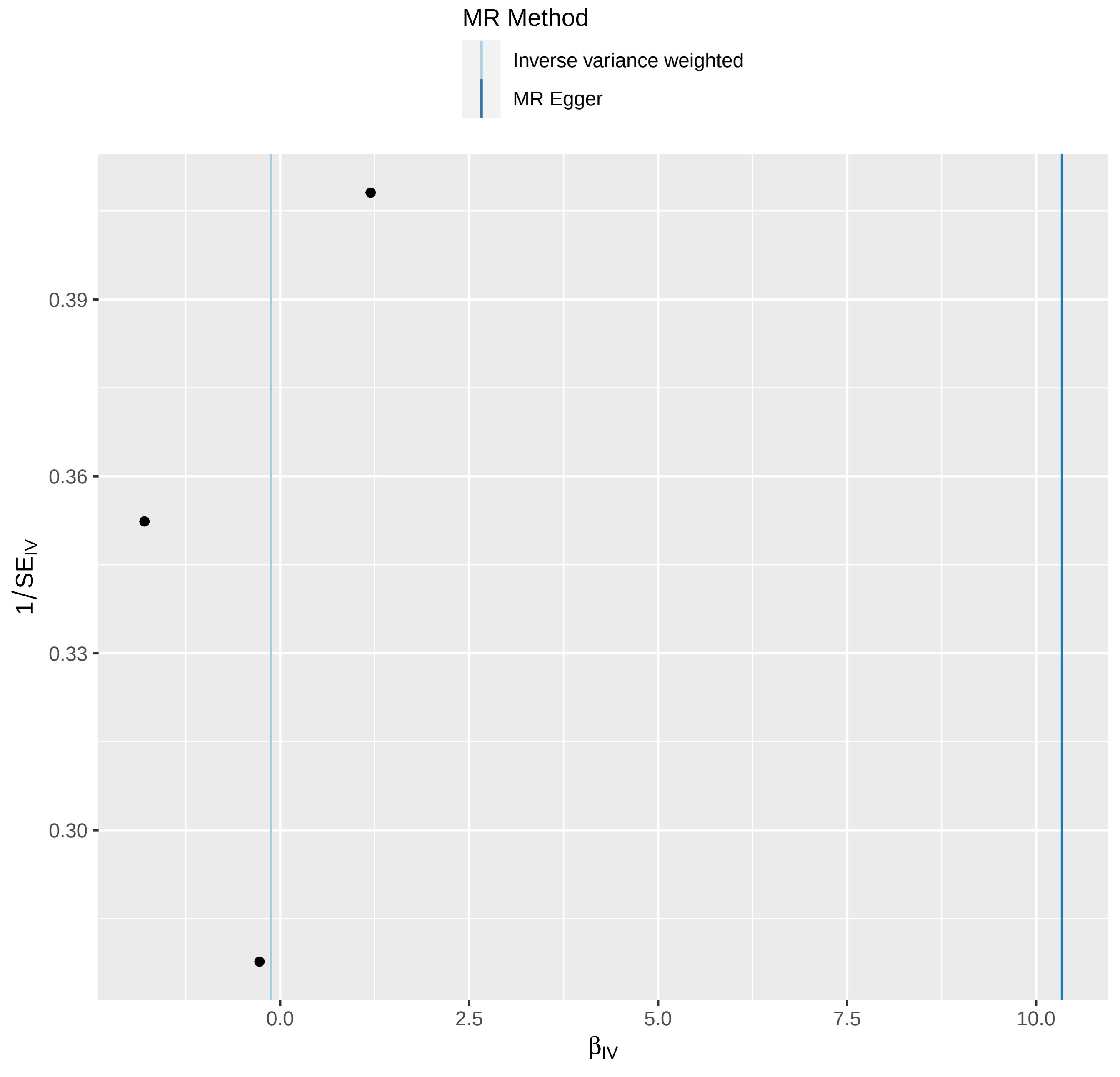

Batch 2504 : Stearate (18:0) on Ulcerative rectosigmoiditis

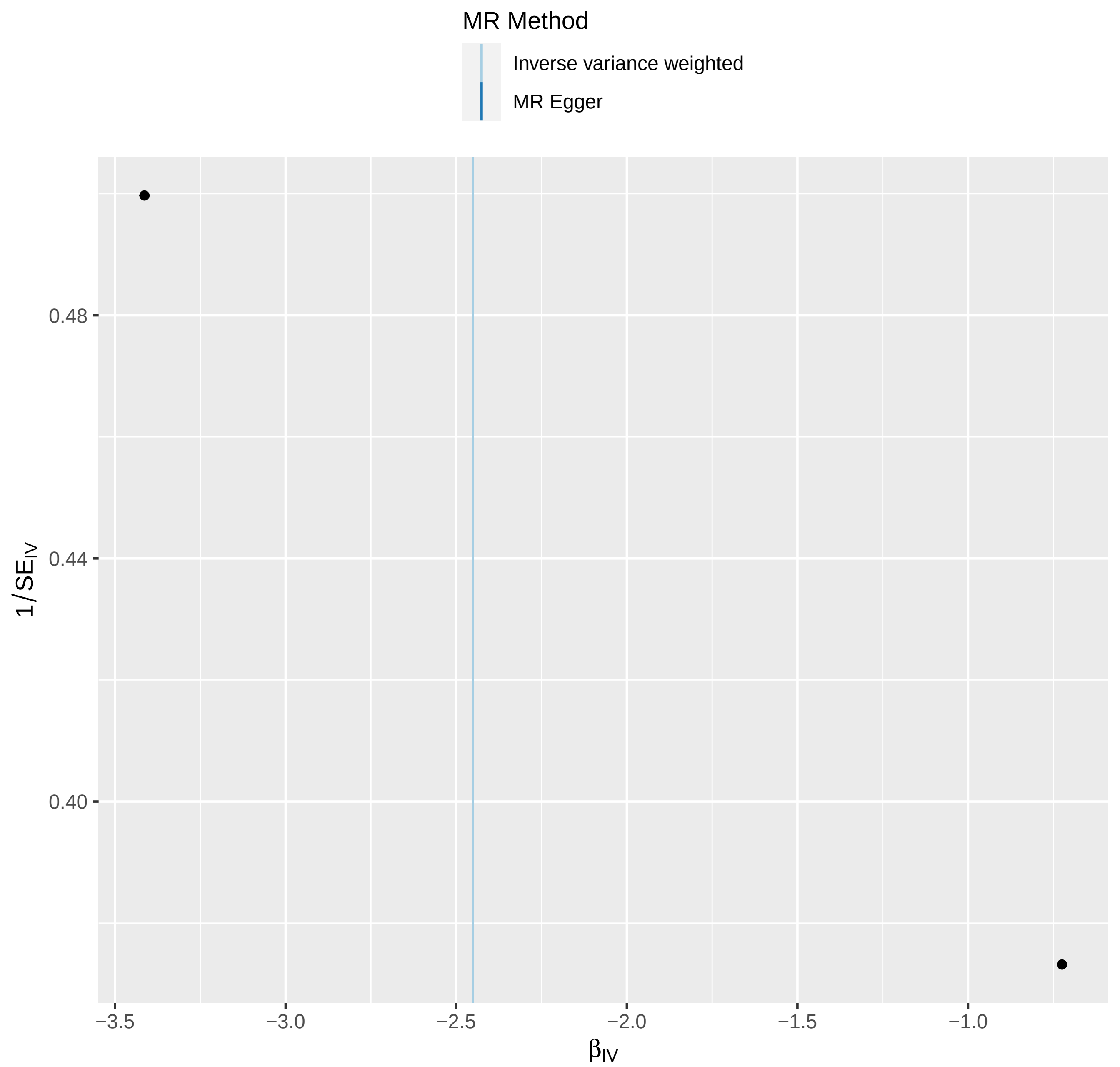

Batch 2507 : Myristate (14:0) on Ulcerative rectosigmoiditis

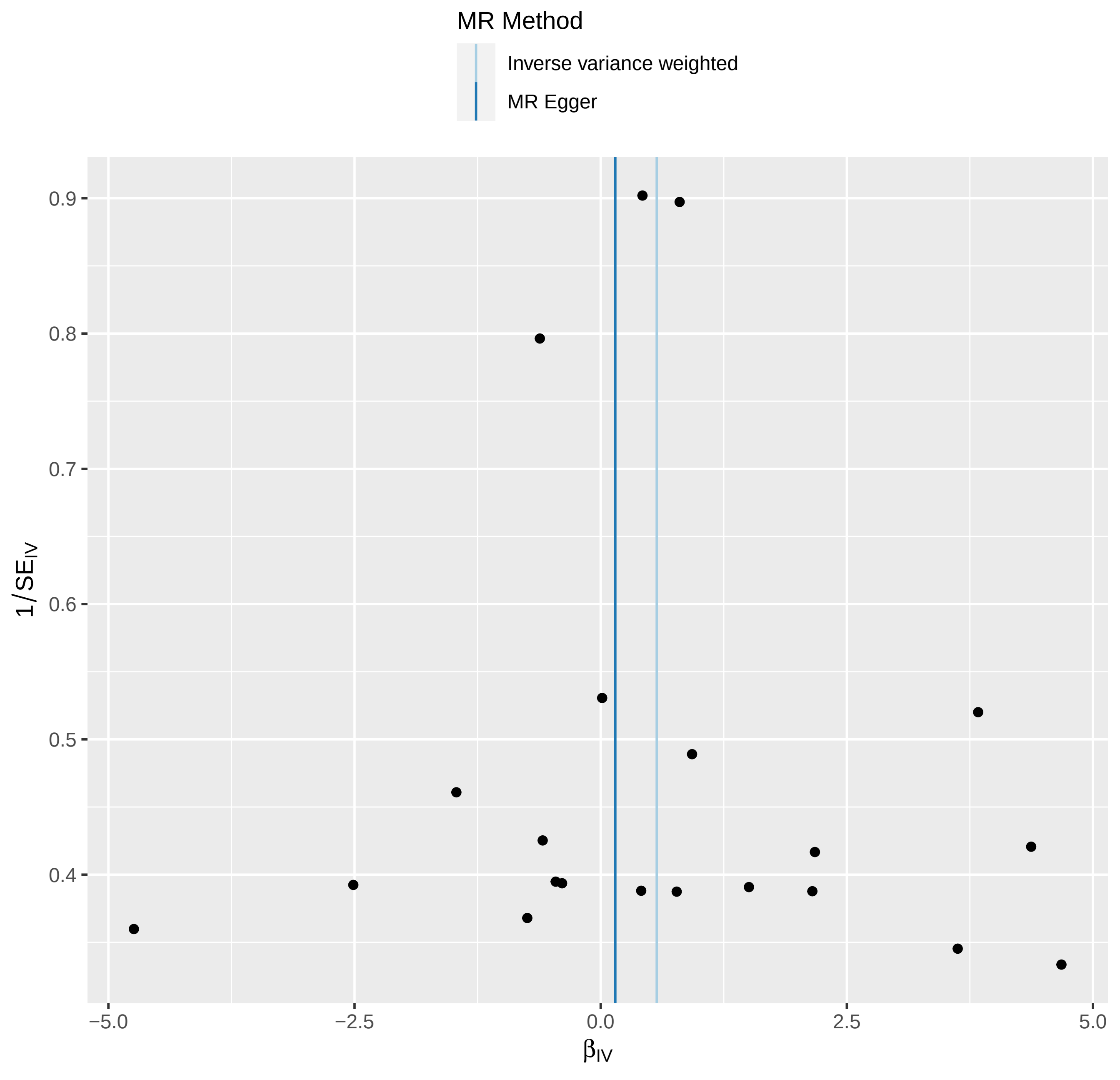

Batch 2509 : Ornithine on Ulcerative rectosigmoiditis

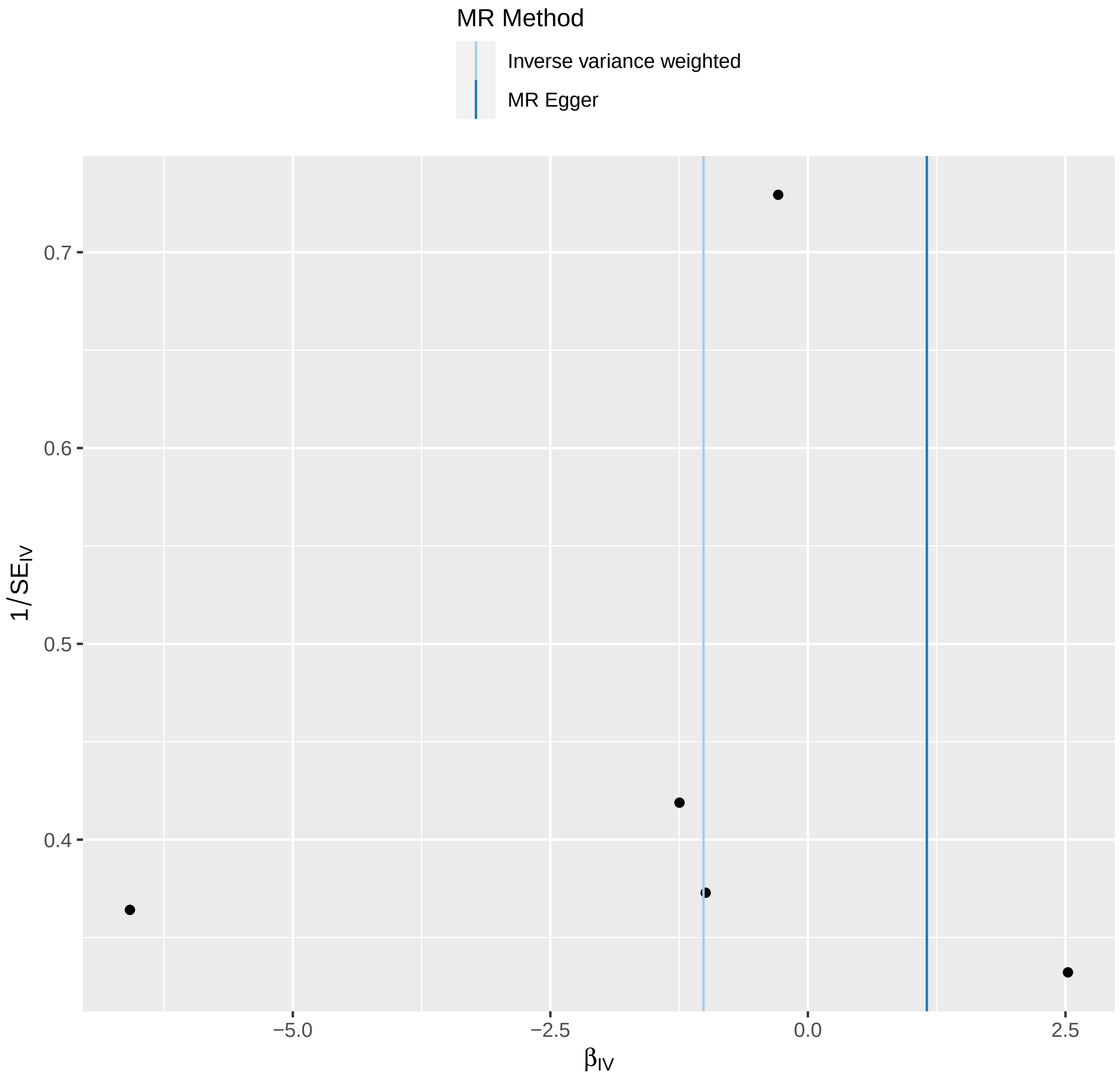

Batch 2512 : 4−acetamidobutanoate on Ulcerative rectosigmoiditis

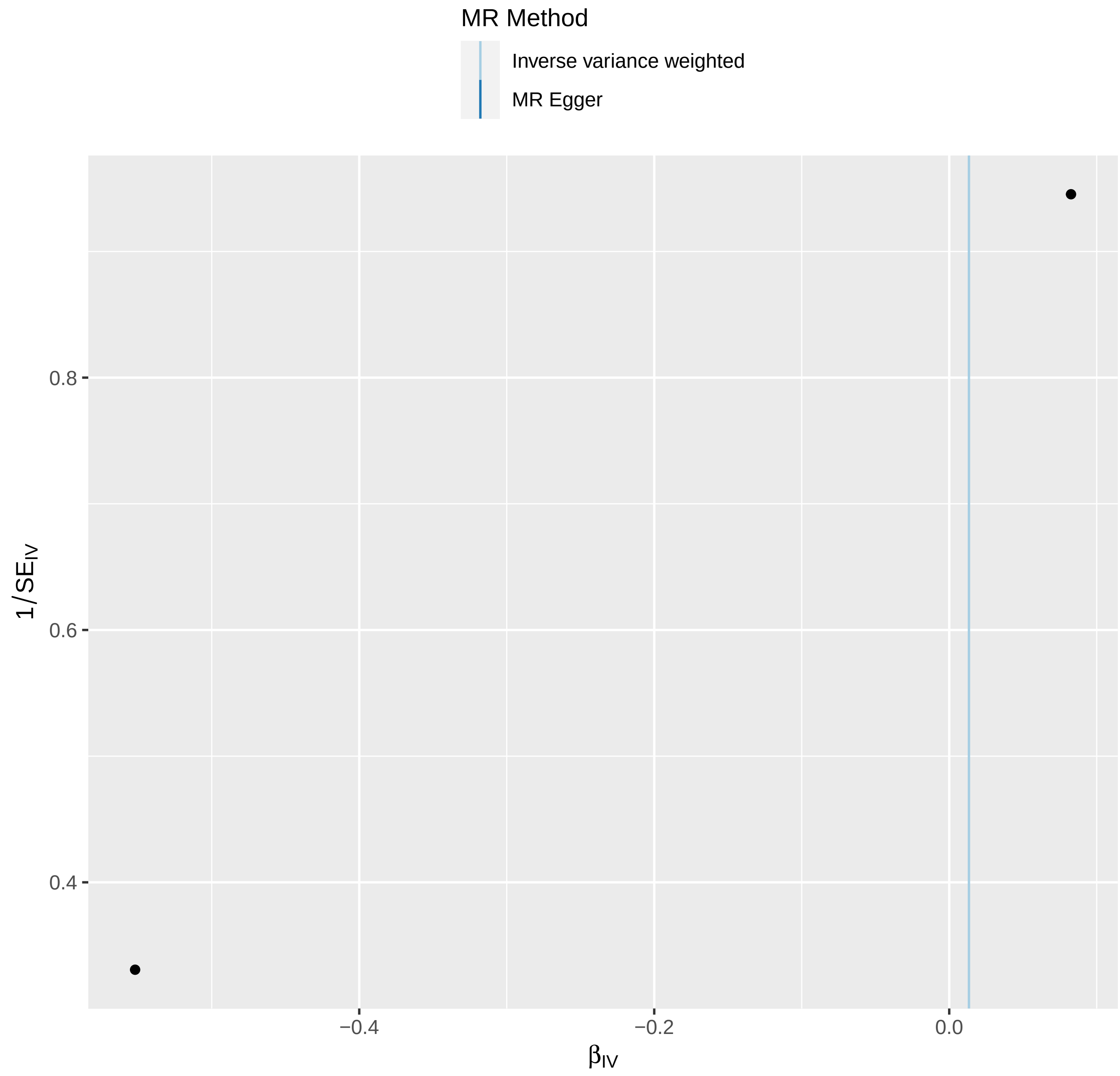

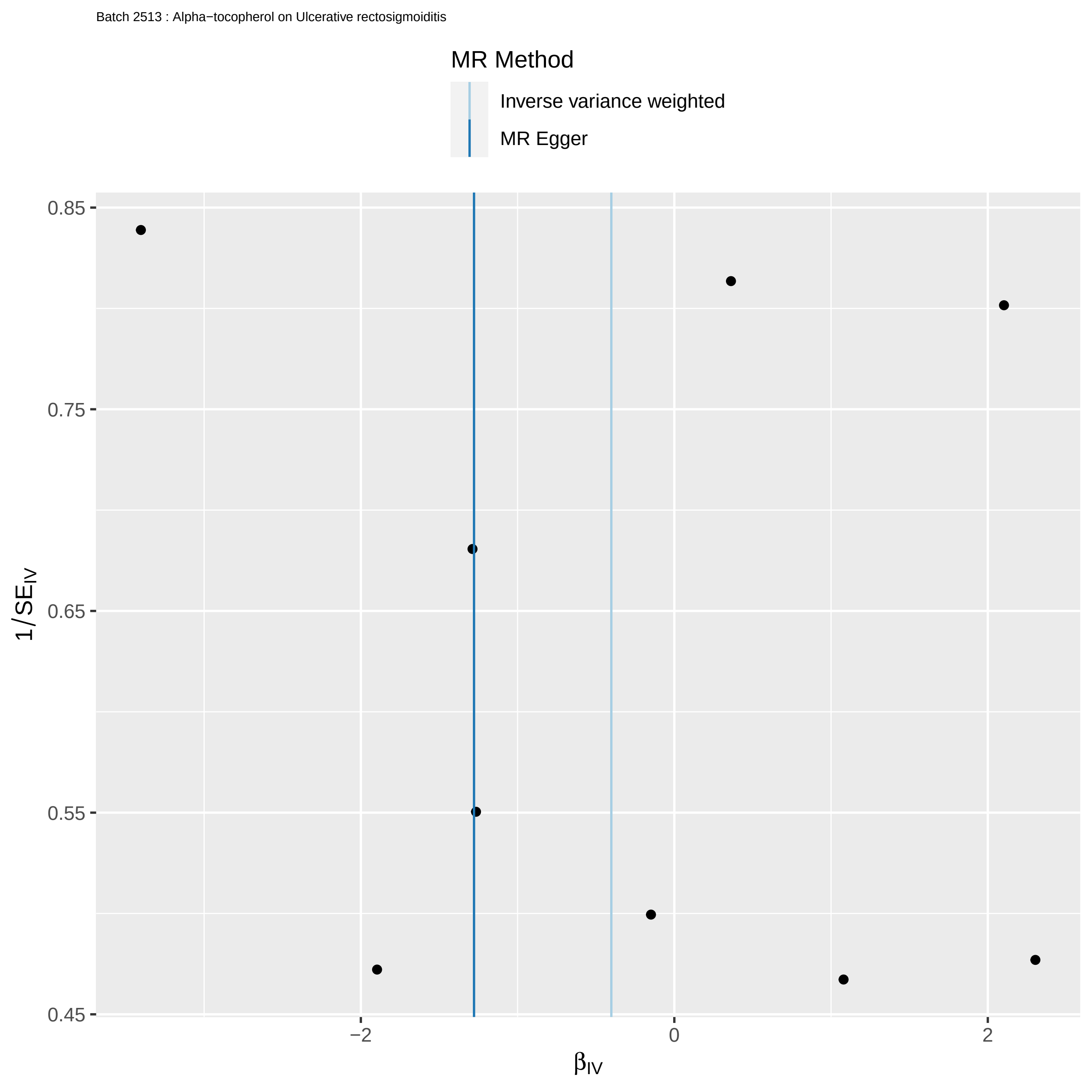

Batch 2513 : Alpha−tocopherol on Ulcerative rectosigmoiditis
MR Method
Inverse variance weighted
MR Egger
0.85
0.75
0.65
0.55
0.45
1/SE_IV
−2
0
2
β_IV

Batch 2514 : Citrate on Ulcerative rectosigmoiditis

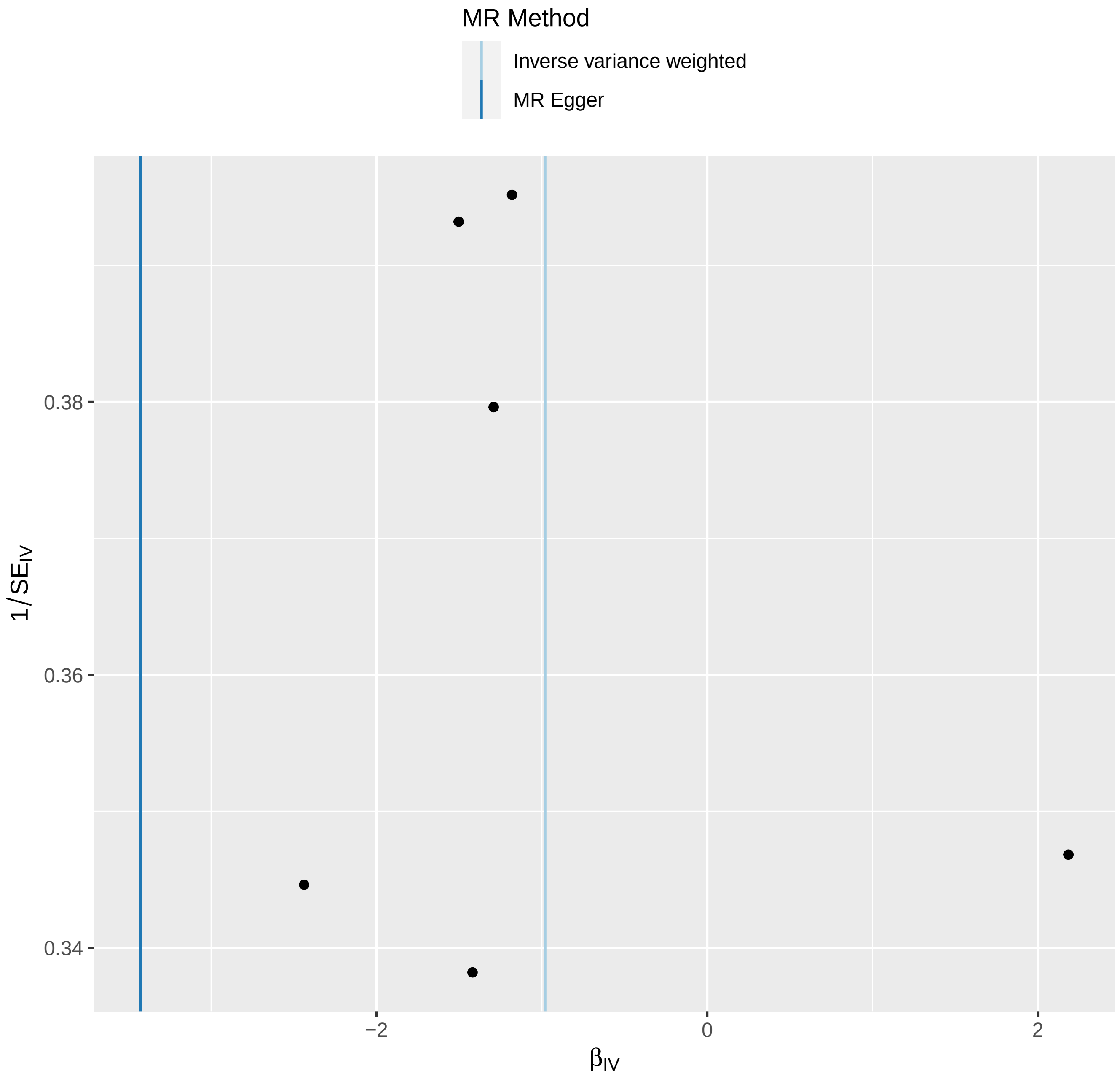

Batch 2515 : Glycerate on Ulcerative rectosigmoiditis

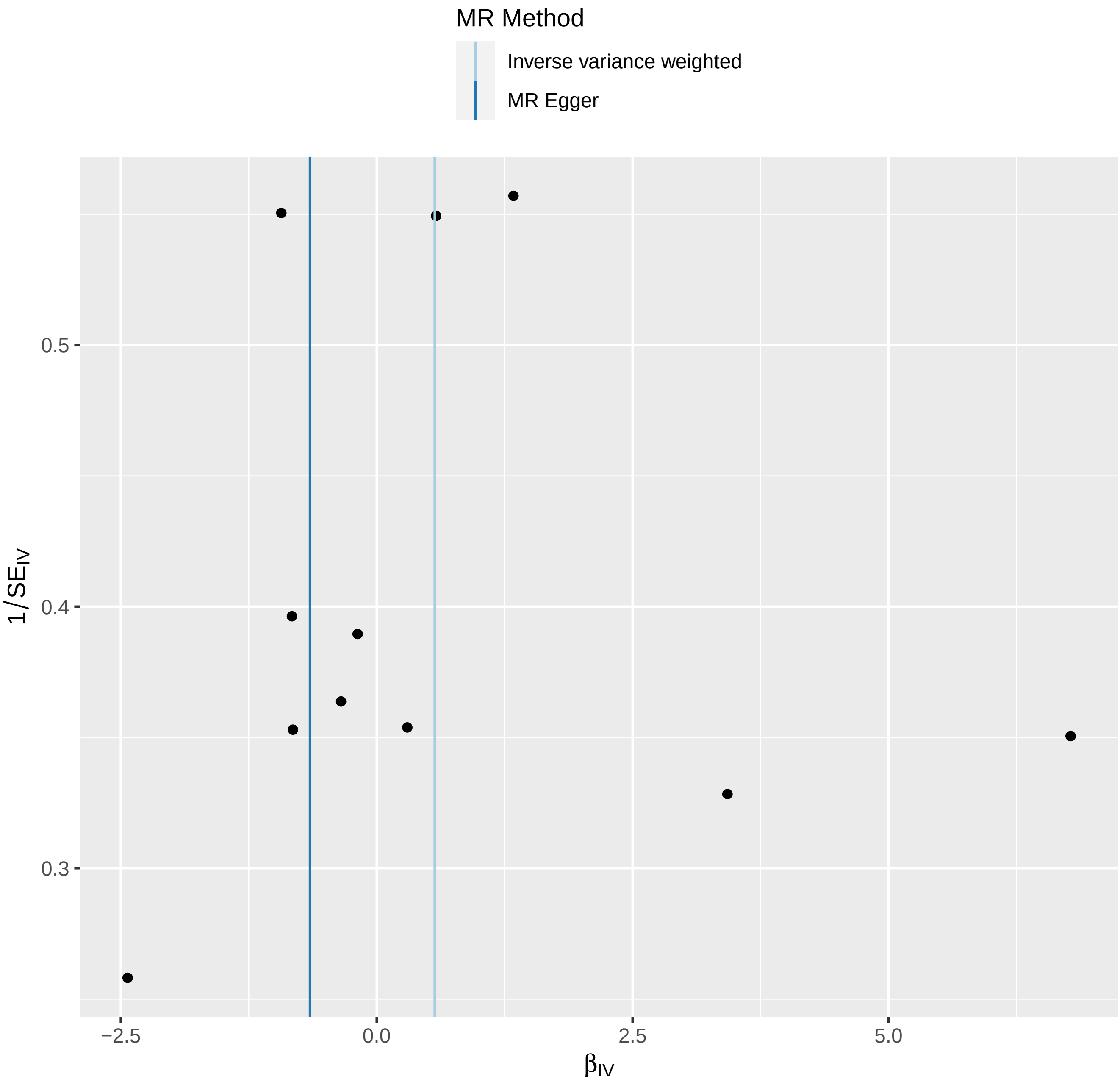

Batch 2516 : Guanosine on Ulcerative rectosigmoiditis

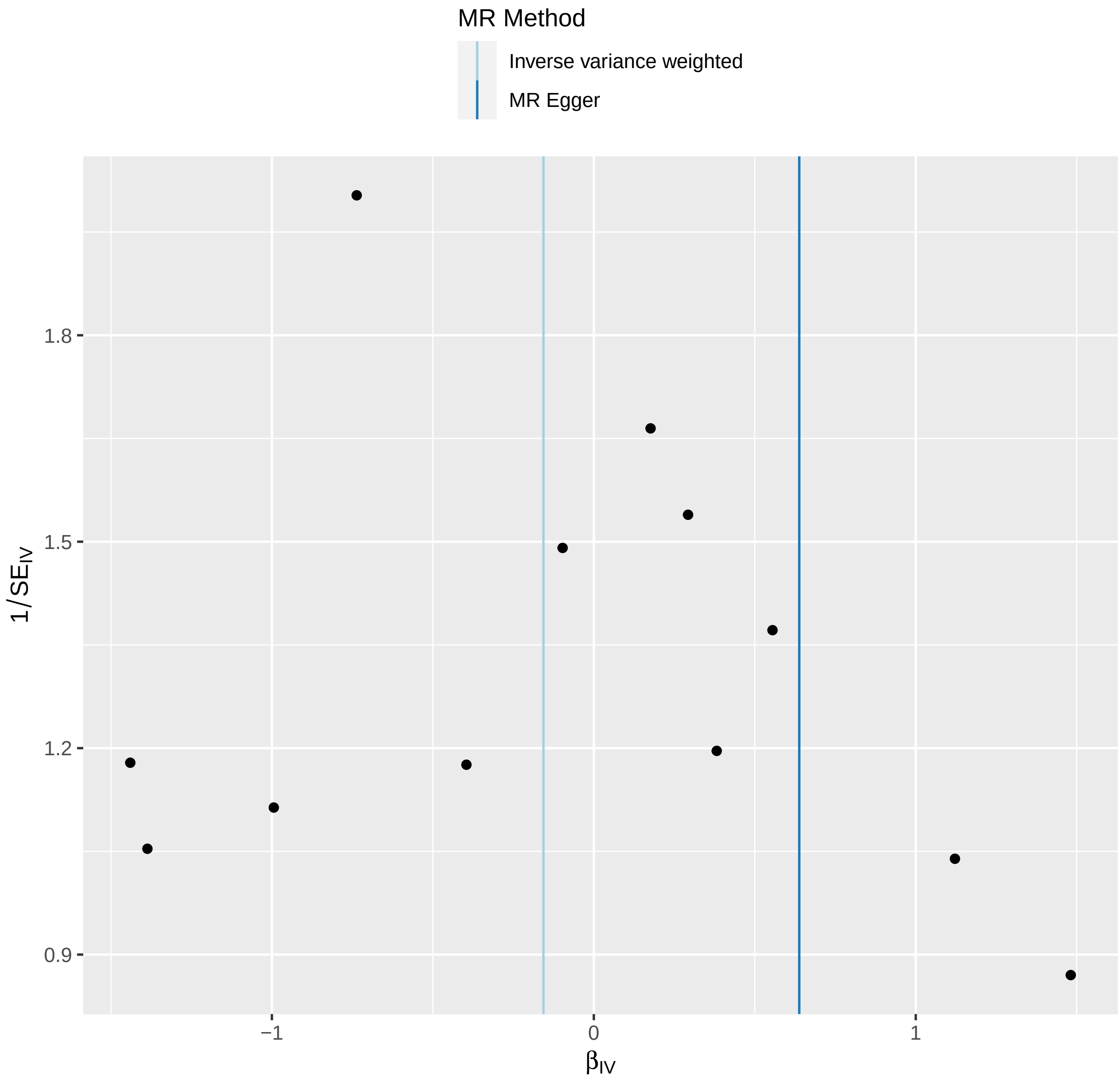

Batch 2519 : Ursodeoxycholate on Ulcerative rectosigmoiditis

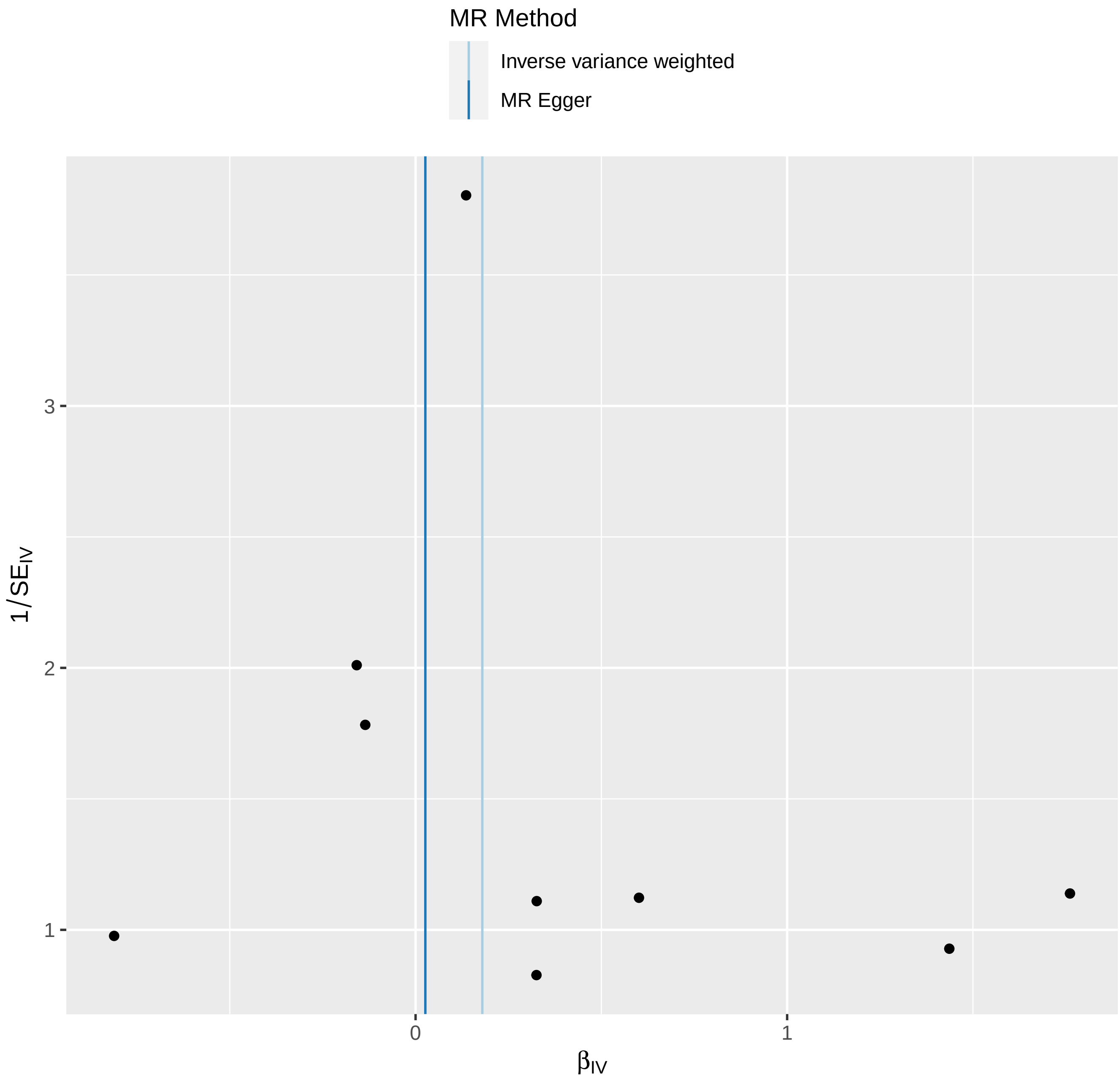

Batch 2520 : Arginine on Ulcerative rectosigmoiditis

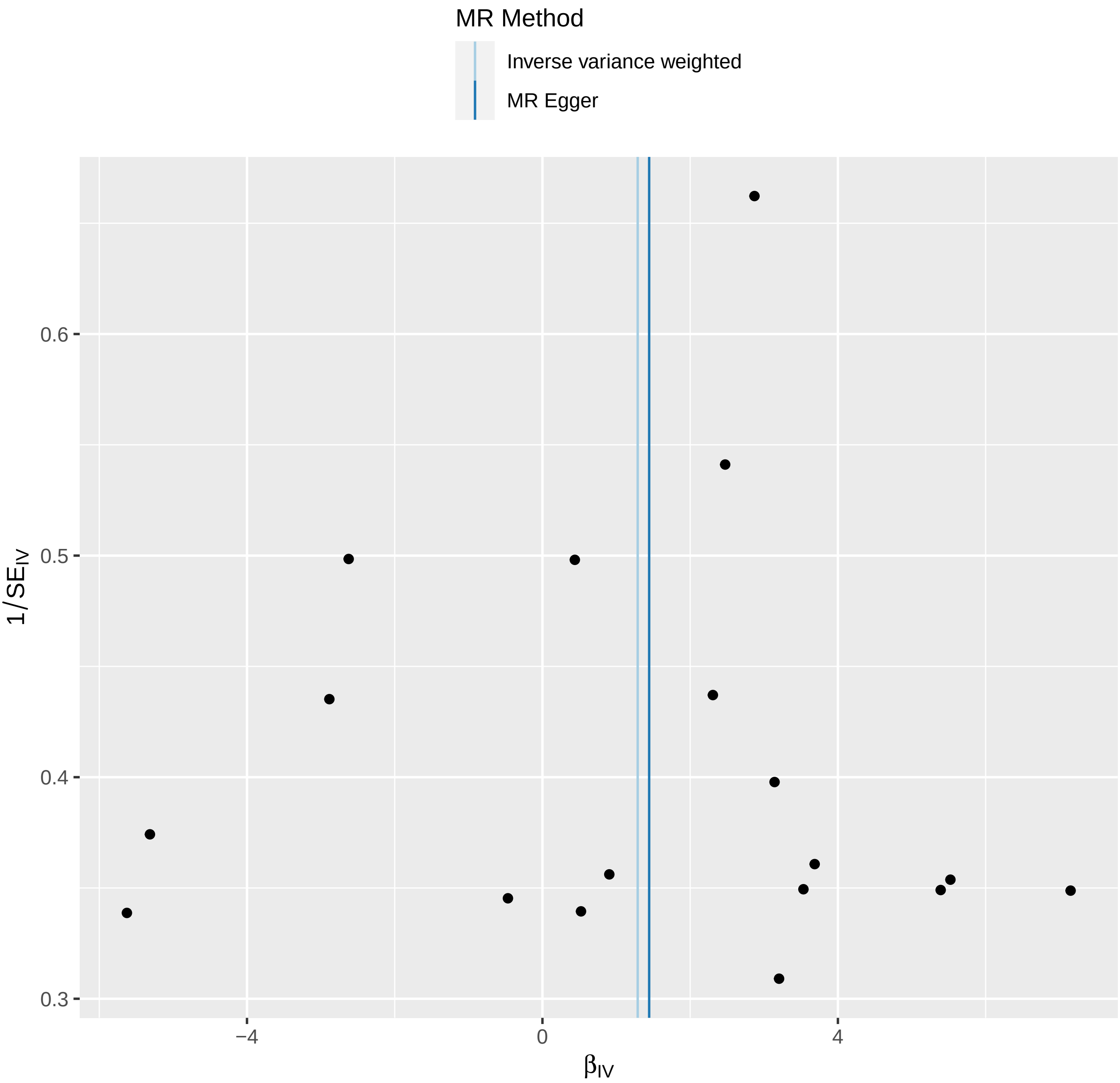

Batch 2521 : Ascorbate (Vitamin C) on Ulcerative rectosigmoiditis

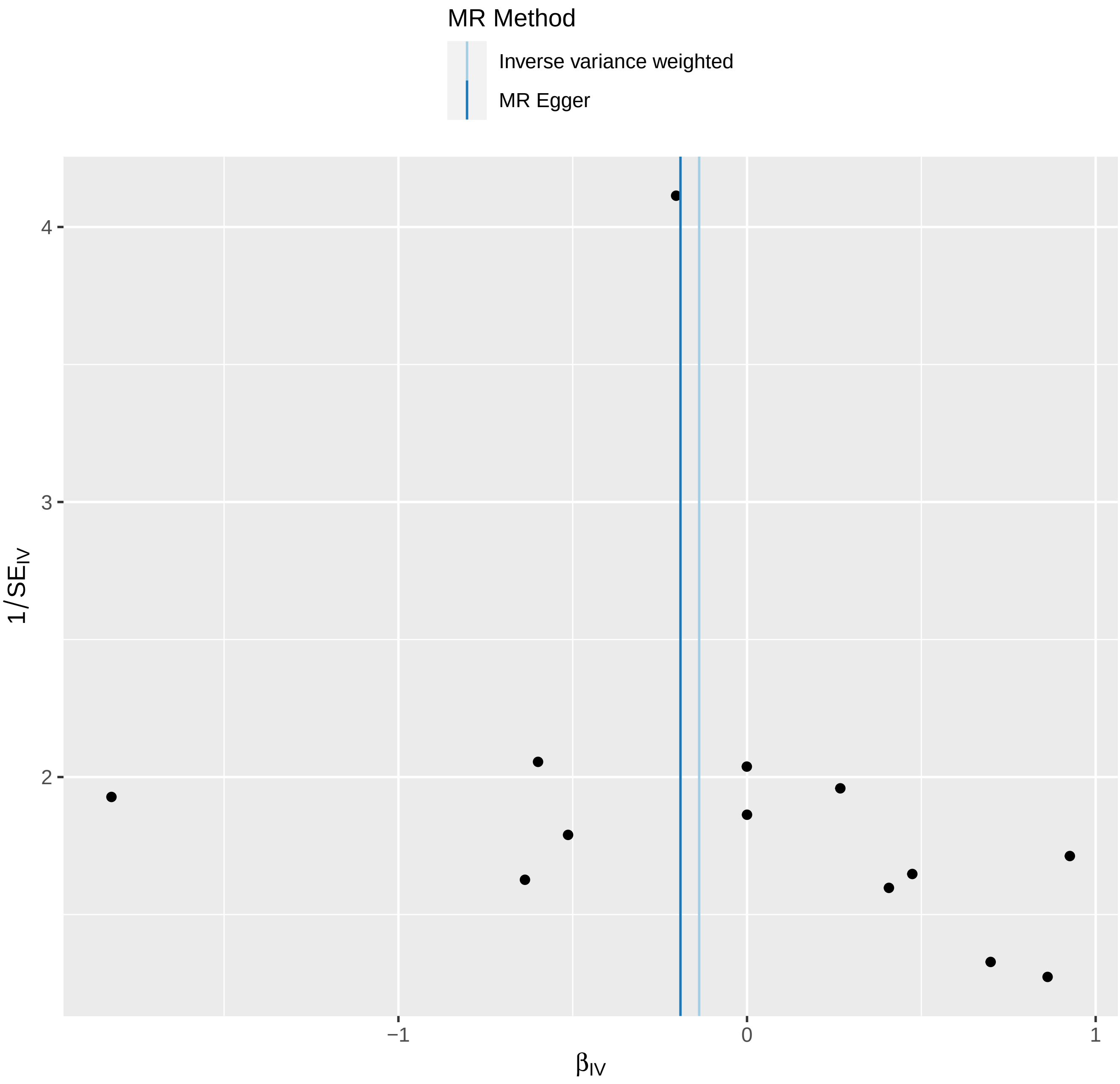

Batch 2522 : Heptanoate (7:0) on Ulcerative rectosigmoiditis

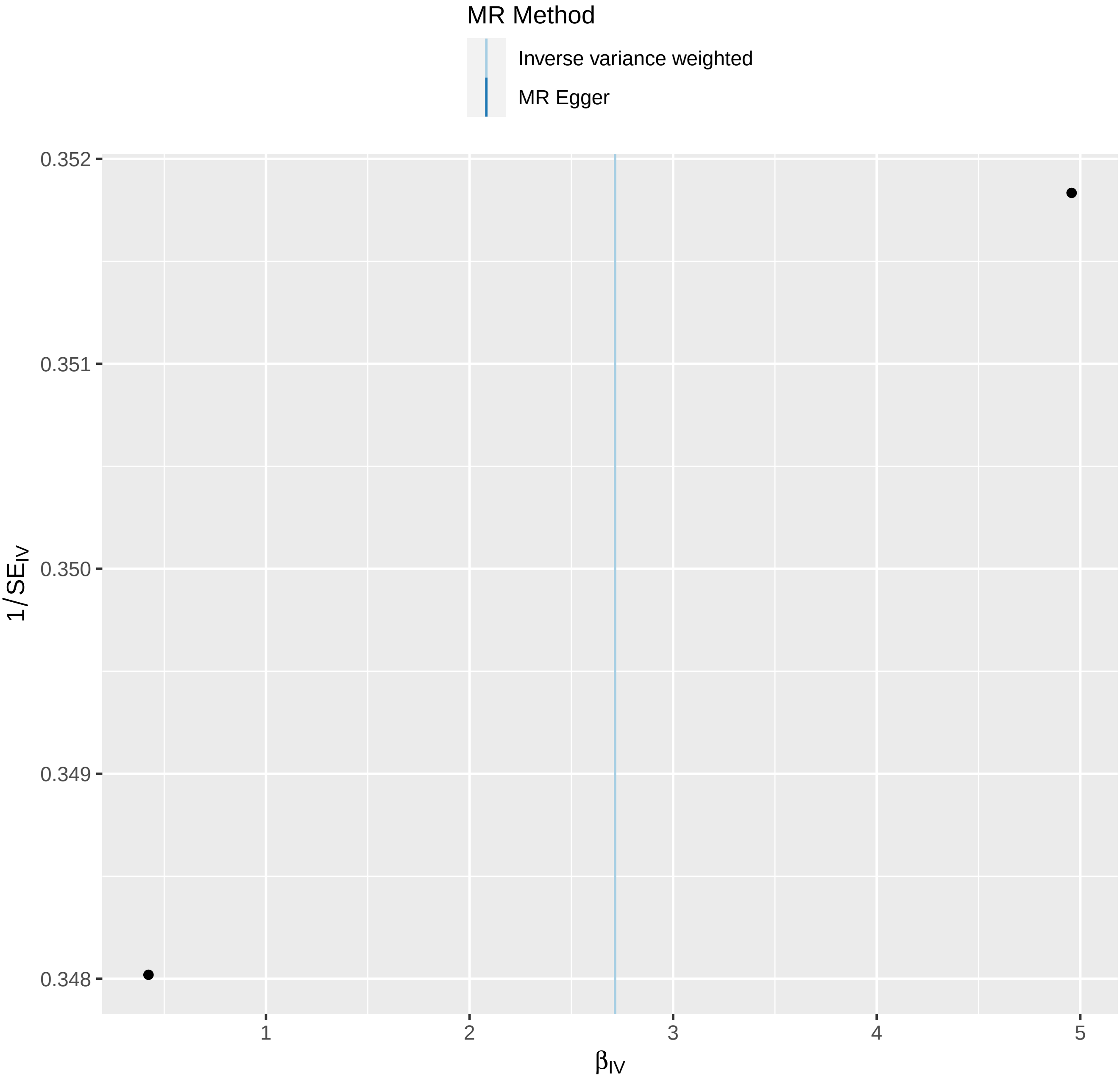

Batch 2523 : Laurate (12:0) on Ulcerative rectosigmoiditis

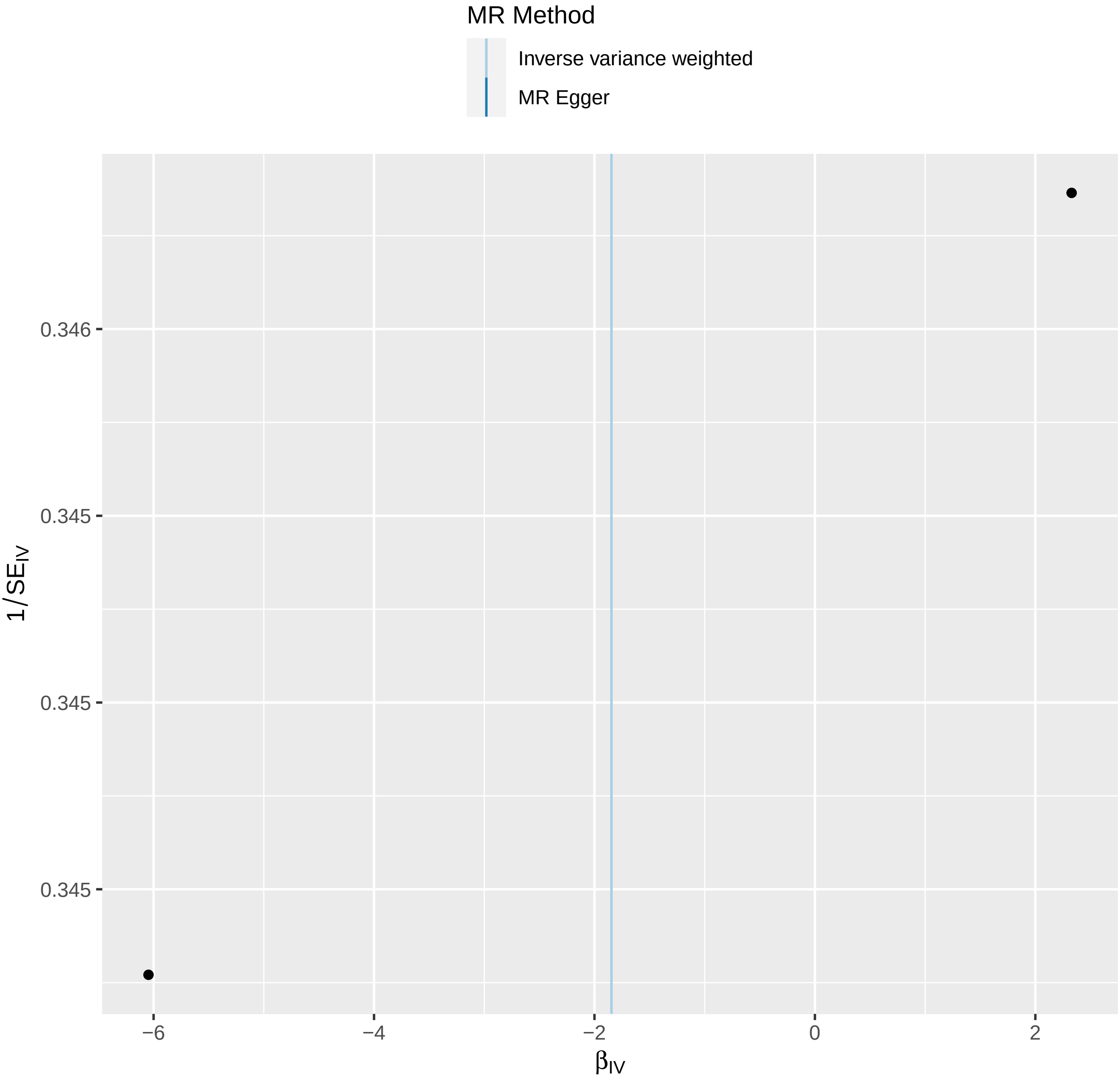

Batch 2525 : Urea on Ulcerative rectosigmoiditis

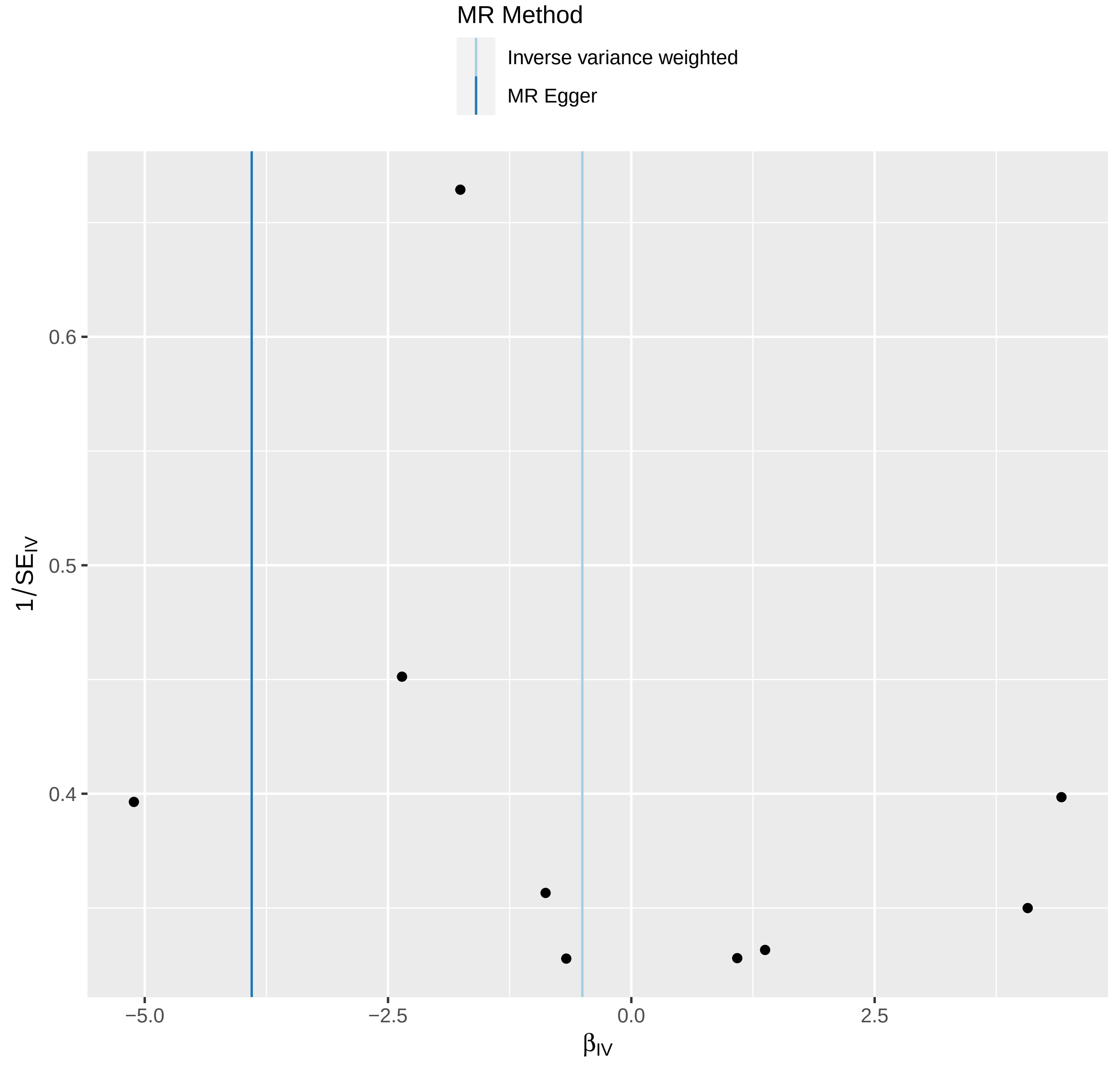

Batch 2526 : Cortisol on Ulcerative rectosigmoiditis

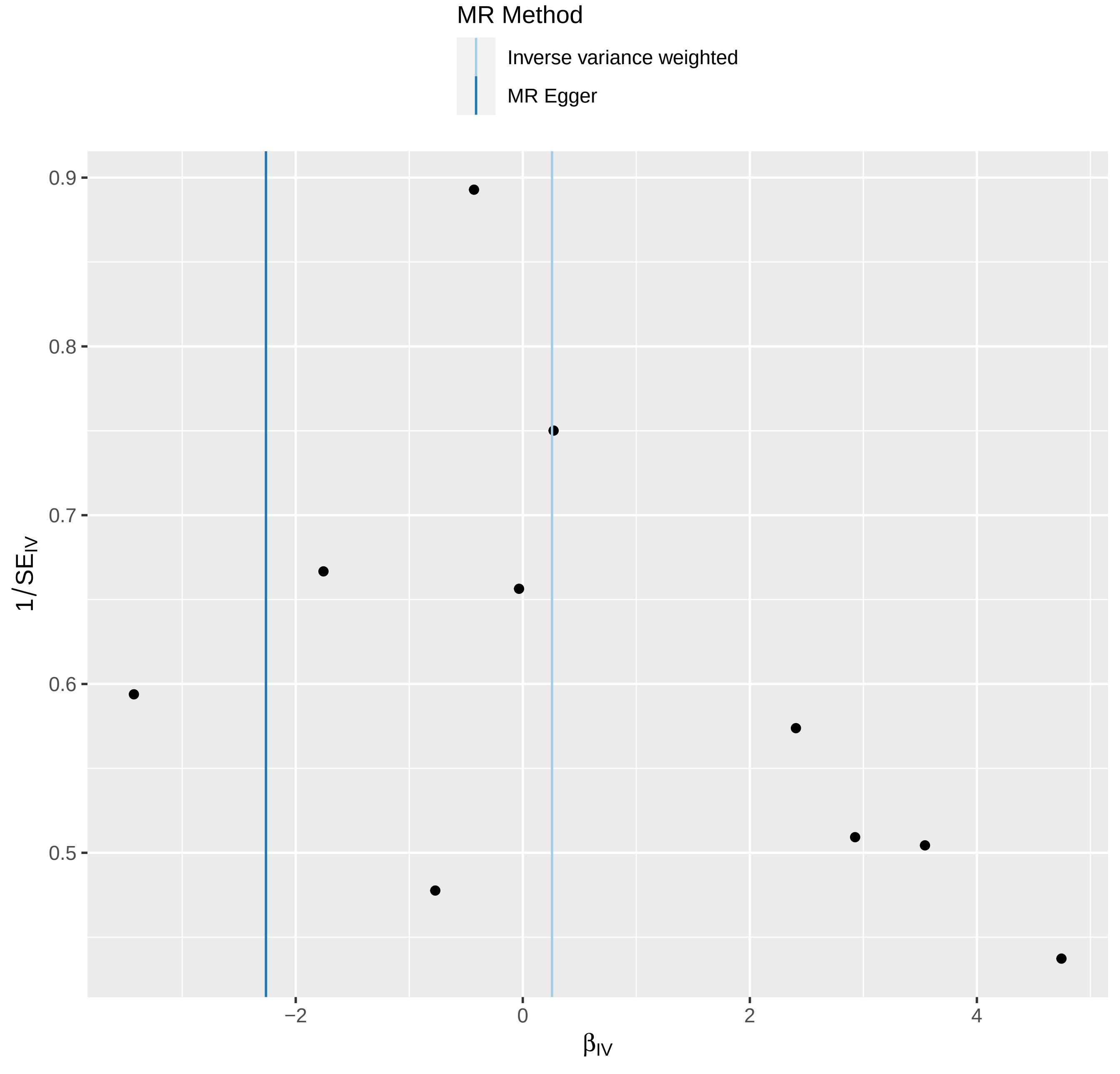

Batch 2529 : Citrulline on Ulcerative rectosigmoiditis

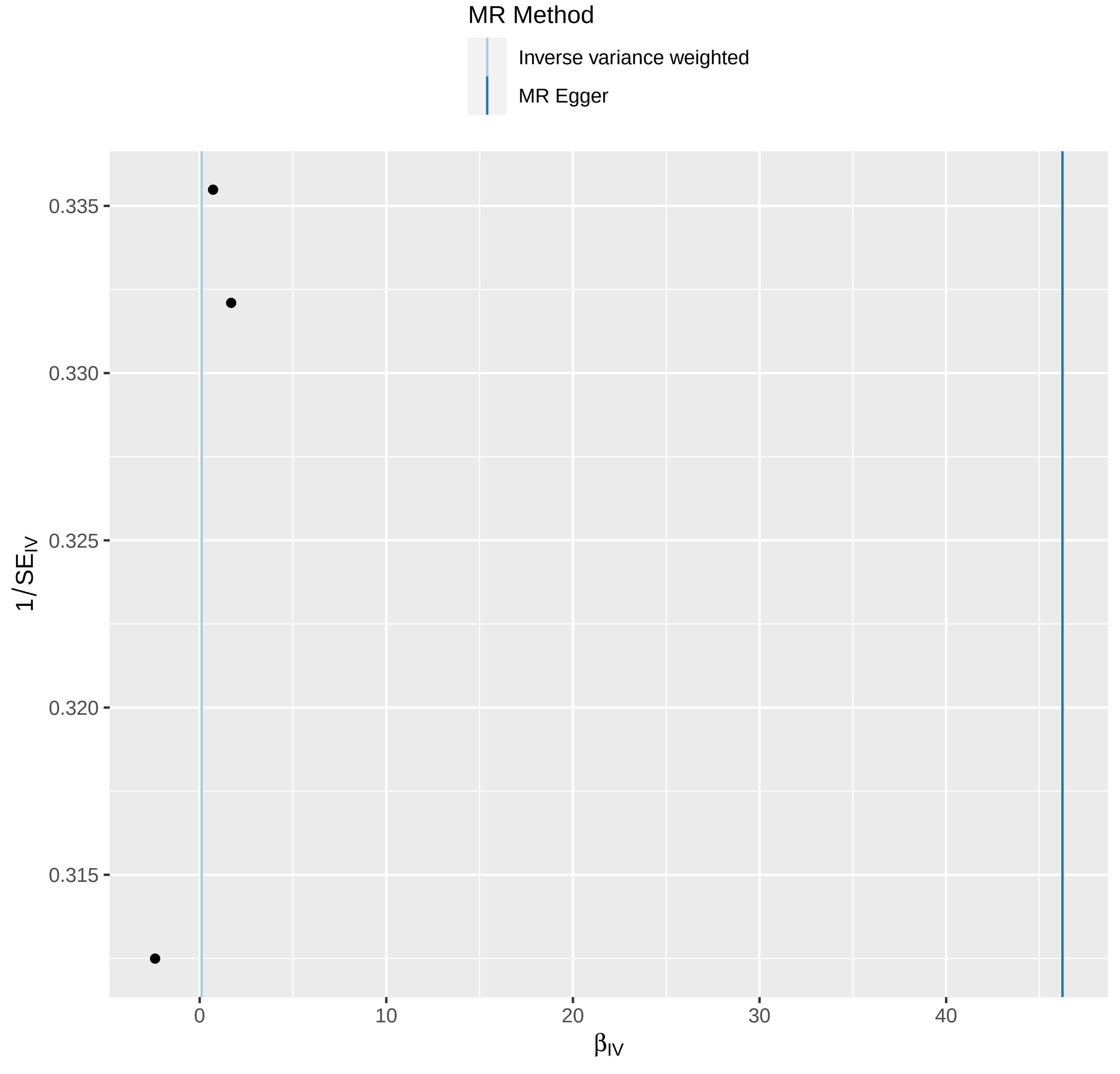

Batch 2531 : Serotonin (5HT) on Ulcerative rectosigmoiditis

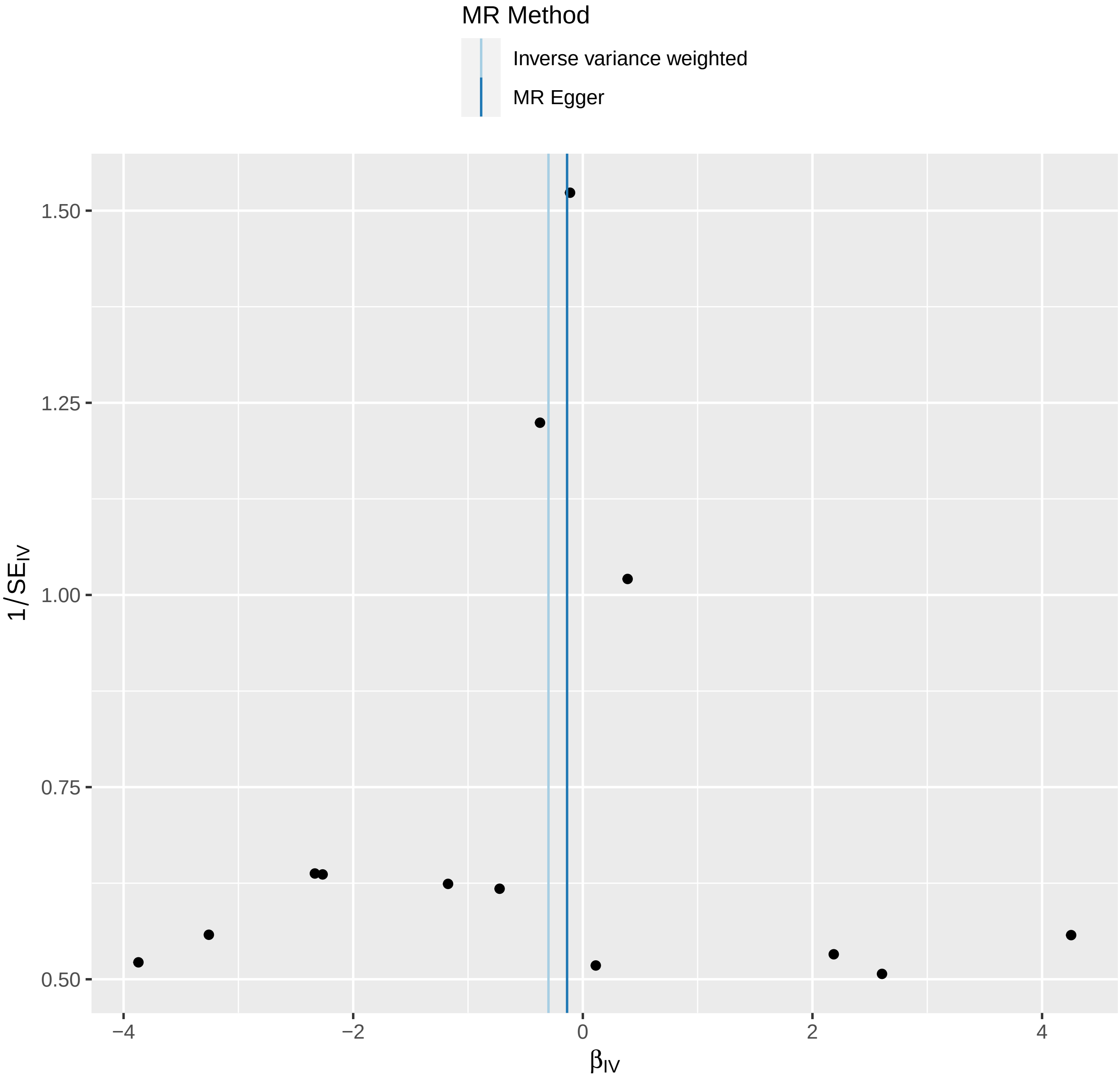

Batch 2532 : Gamma−glutamylglutamine on Ulcerative rectosigmoiditis

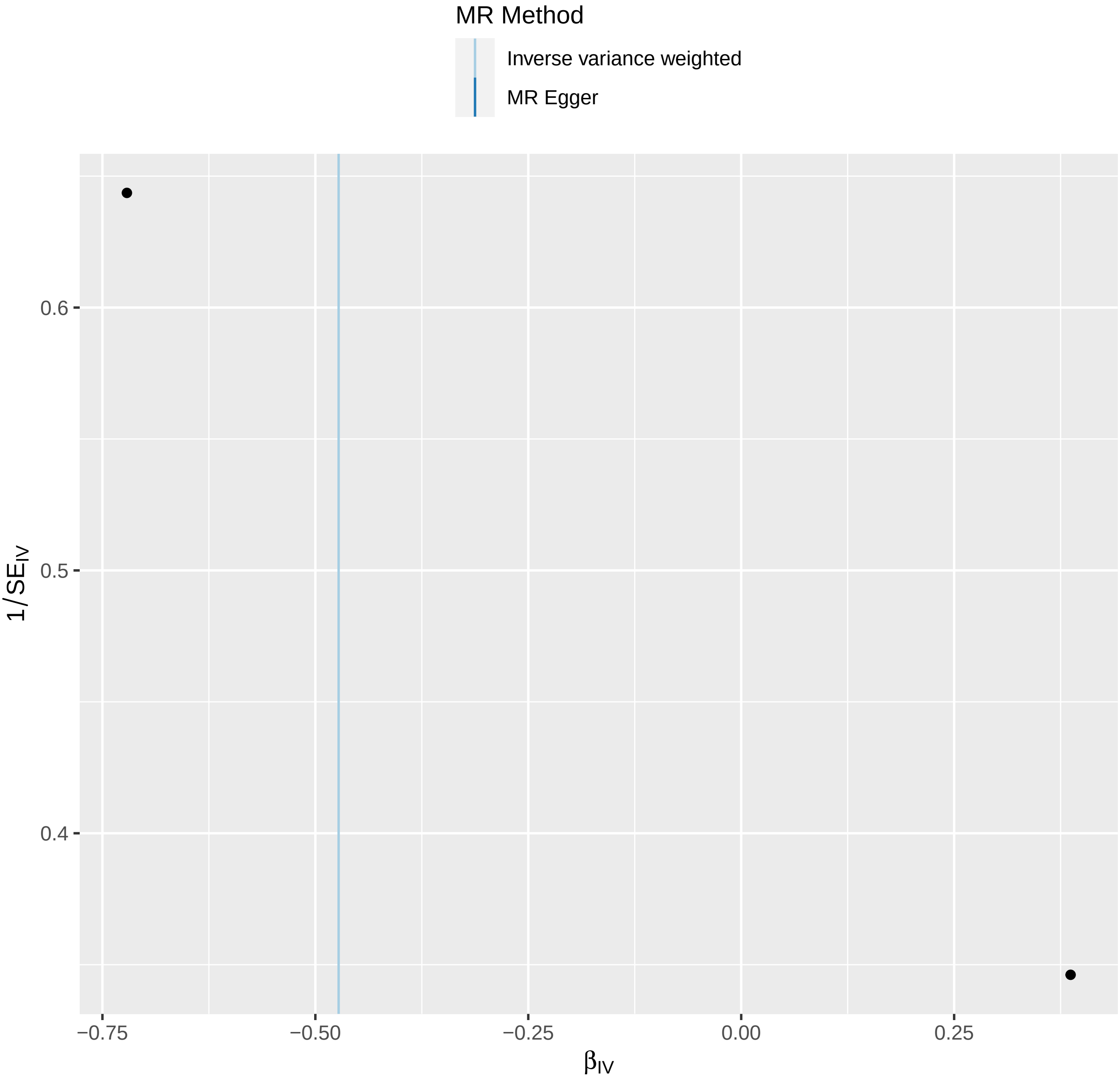

Batch 2533 : Gamma−glutamyltyrosine on Ulcerative rectosigmoiditis

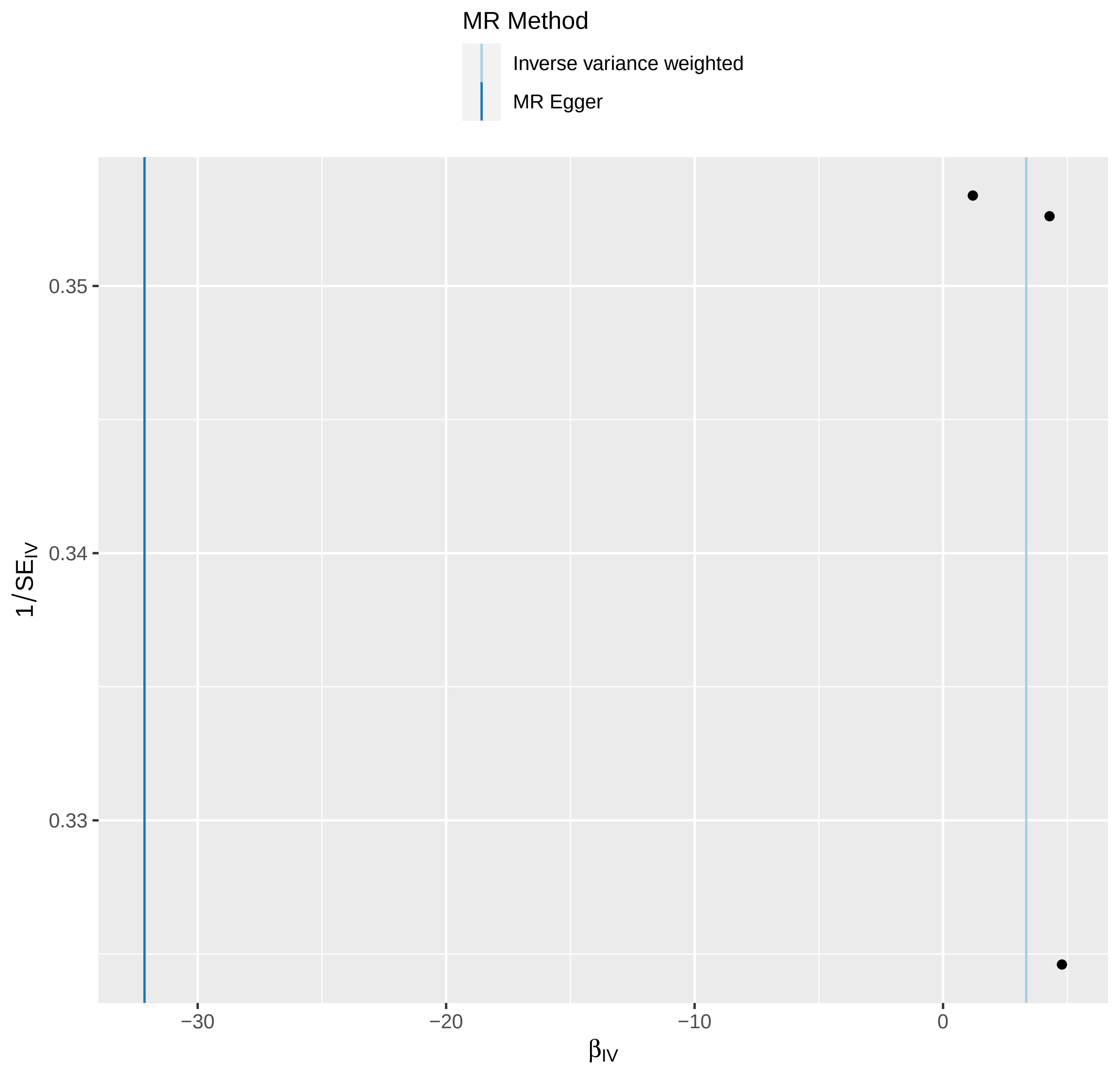

Batch 2535 : Betaine on Ulcerative rectosigmoiditis

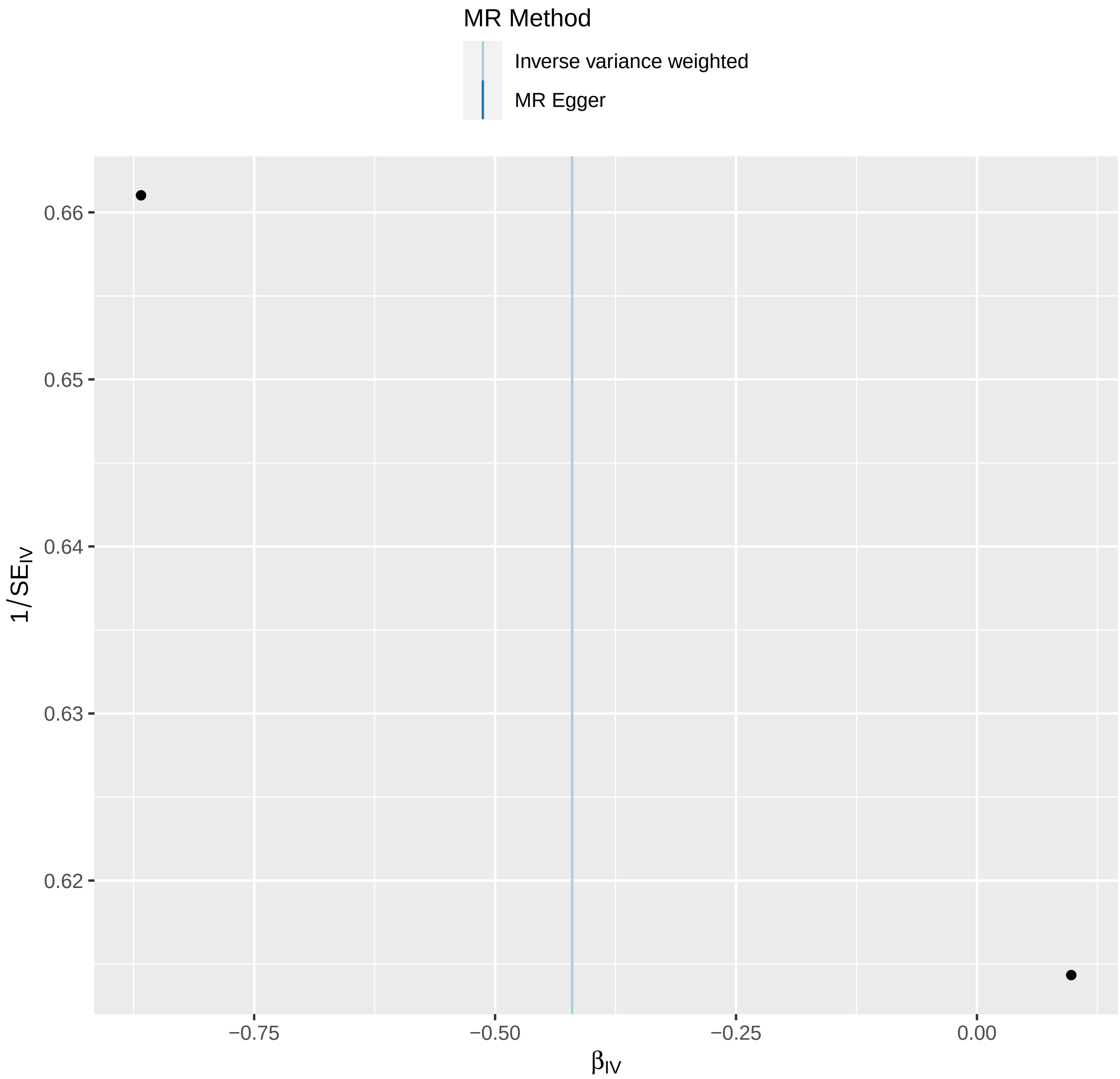

Batch 2536 : Xanthine on Ulcerative rectosigmoiditis

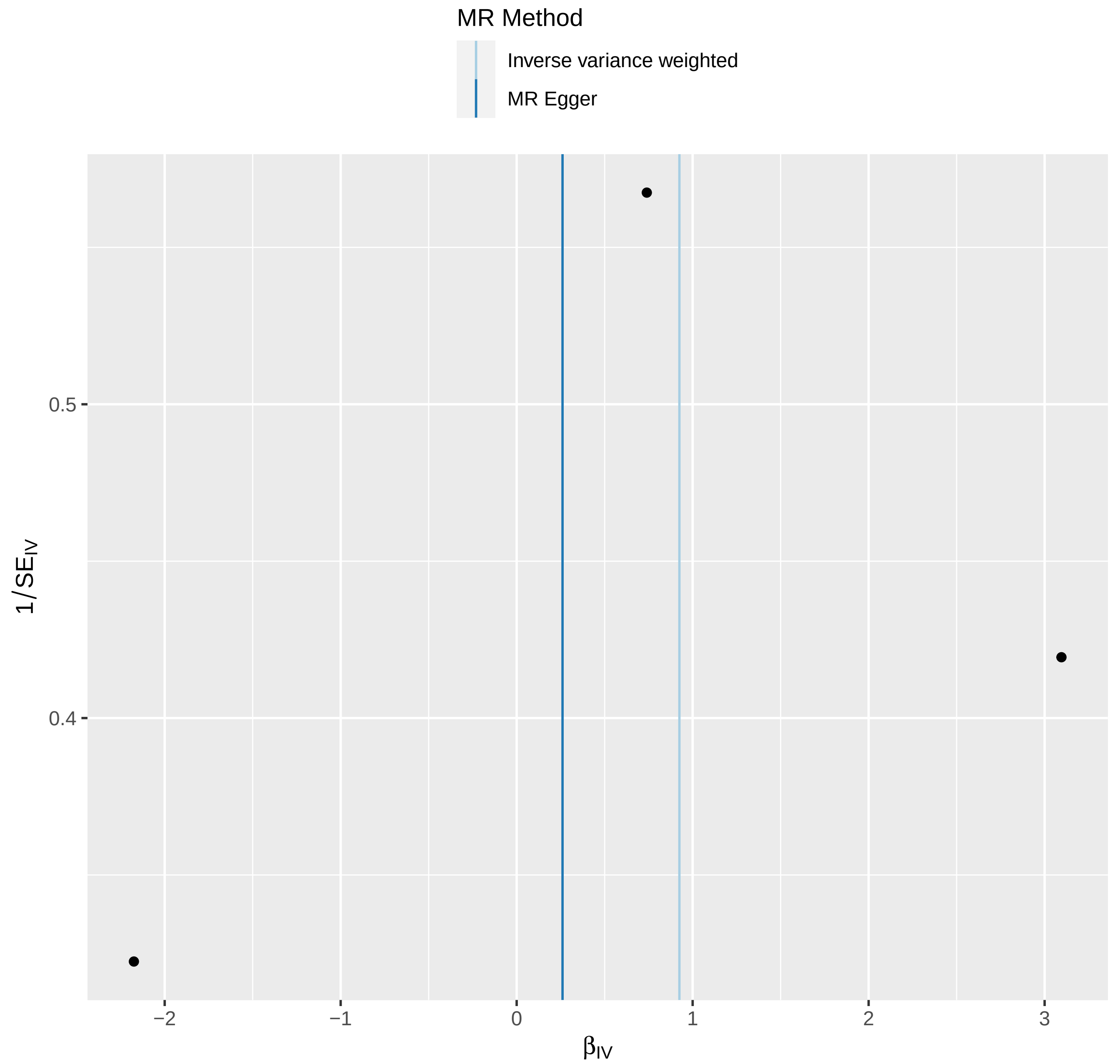

Batch 2537 : Phosphate on Ulcerative rectosigmoiditis

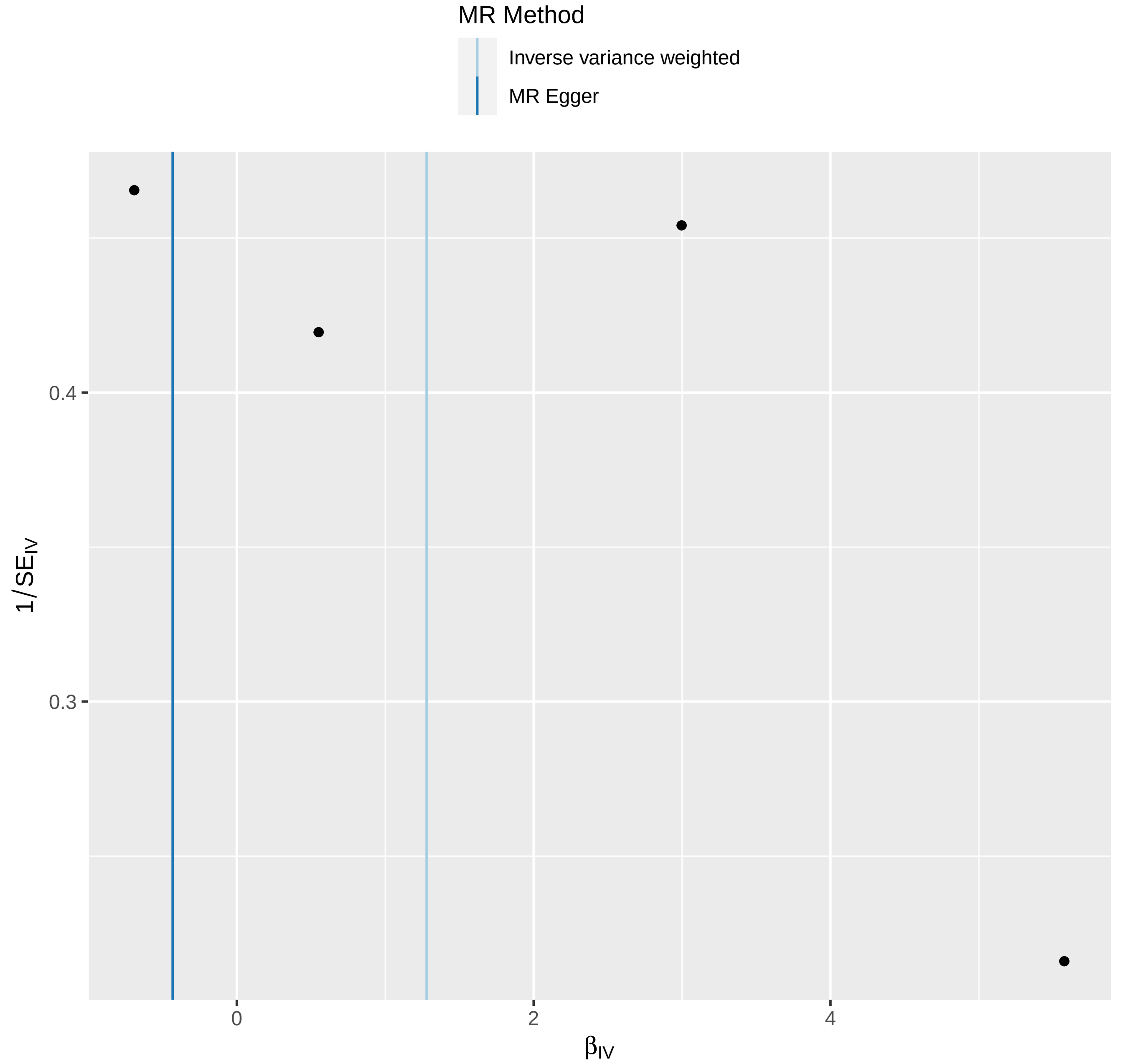

Batch 2538 : 3−methoxytyrosine on Ulcerative rectosigmoiditis

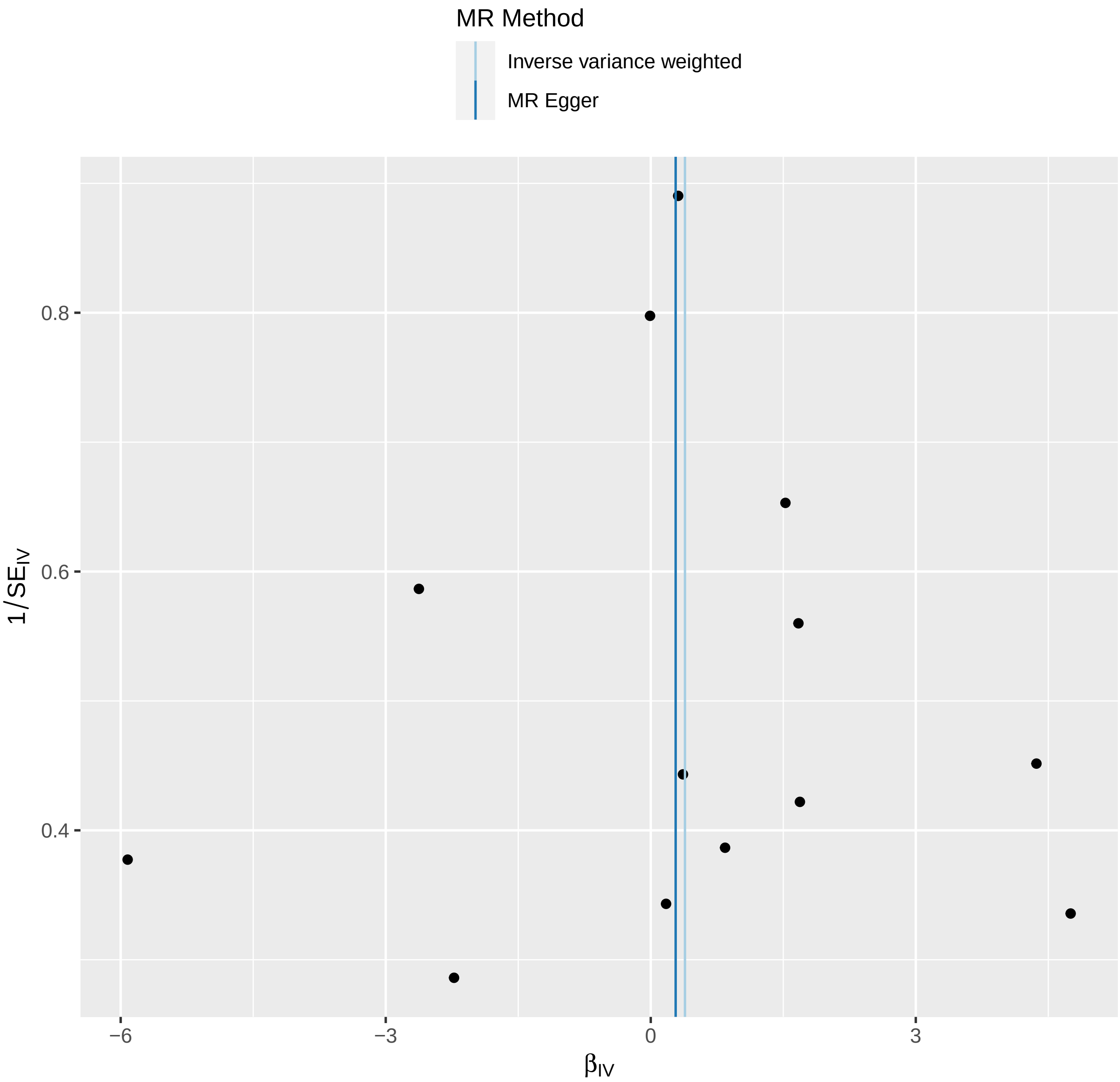

Batch 2540 : Undecanoate (11:0) on Ulcerative rectosigmoiditis

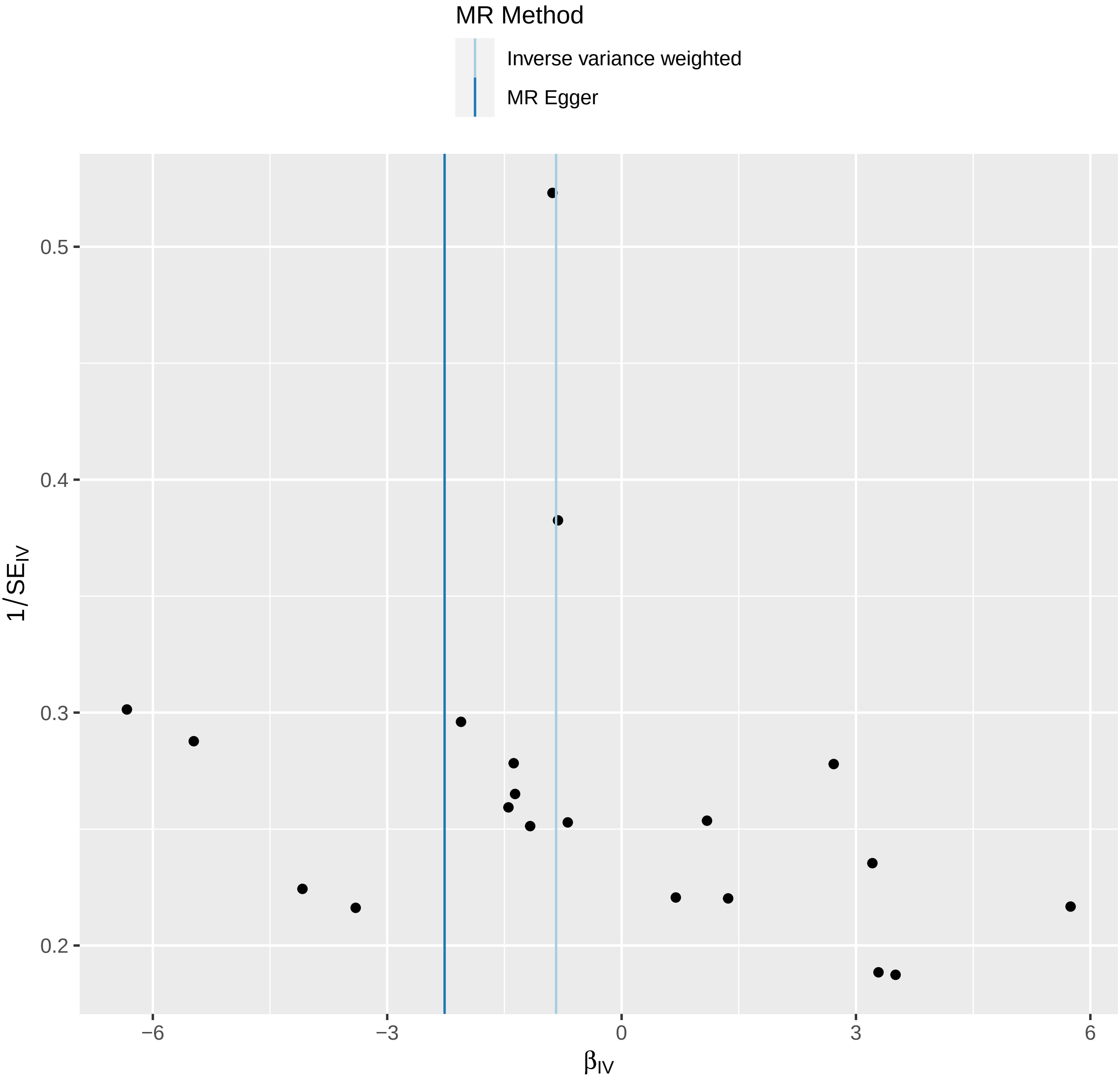

Batch 2542 : Taurodeoxycholate on Ulcerative rectosigmoiditis

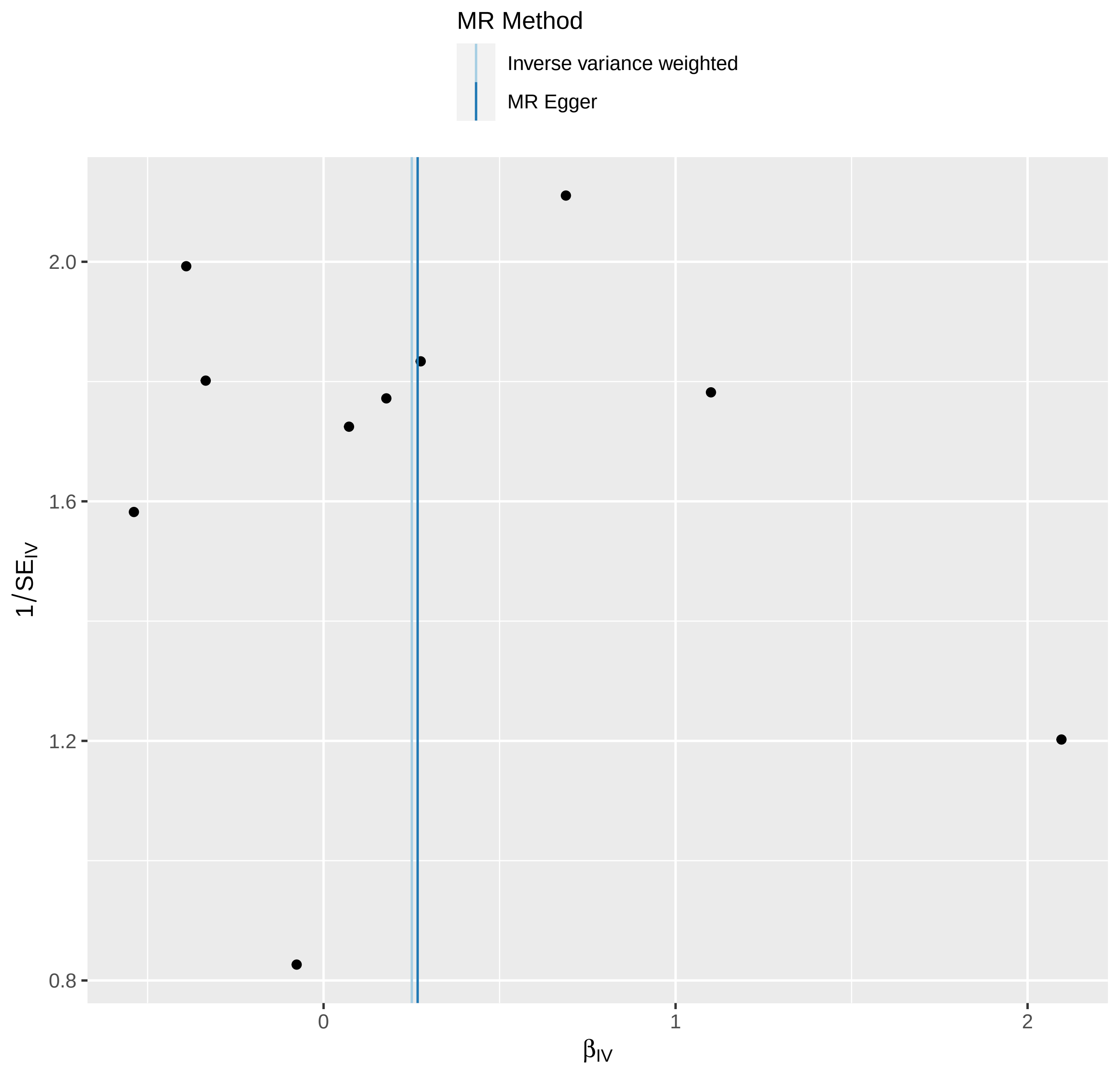

Batch 2543 : Glycerol on Ulcerative rectosigmoiditis

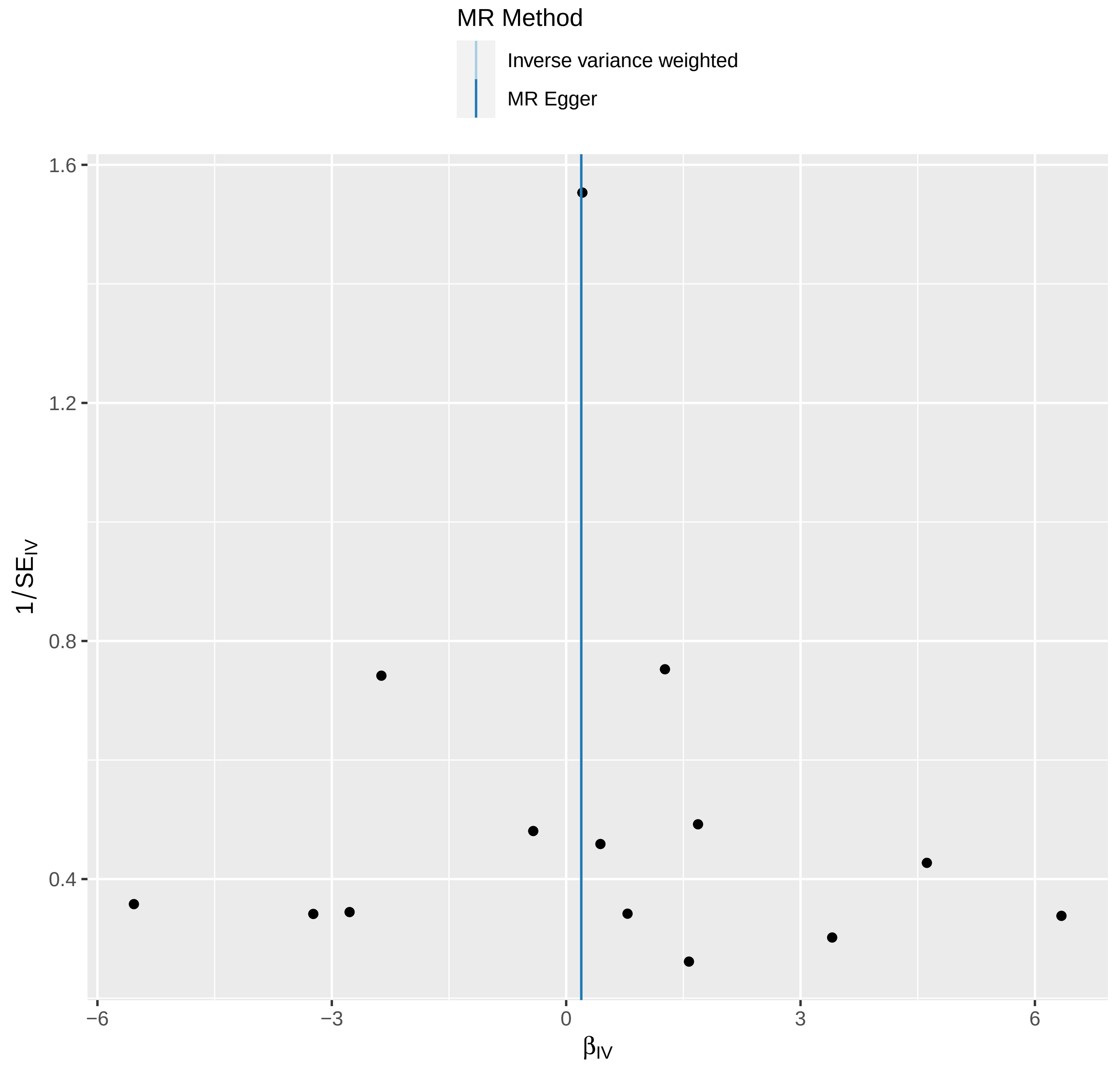

Batch 2544 : Kynurenine on Ulcerative rectosigmoiditis

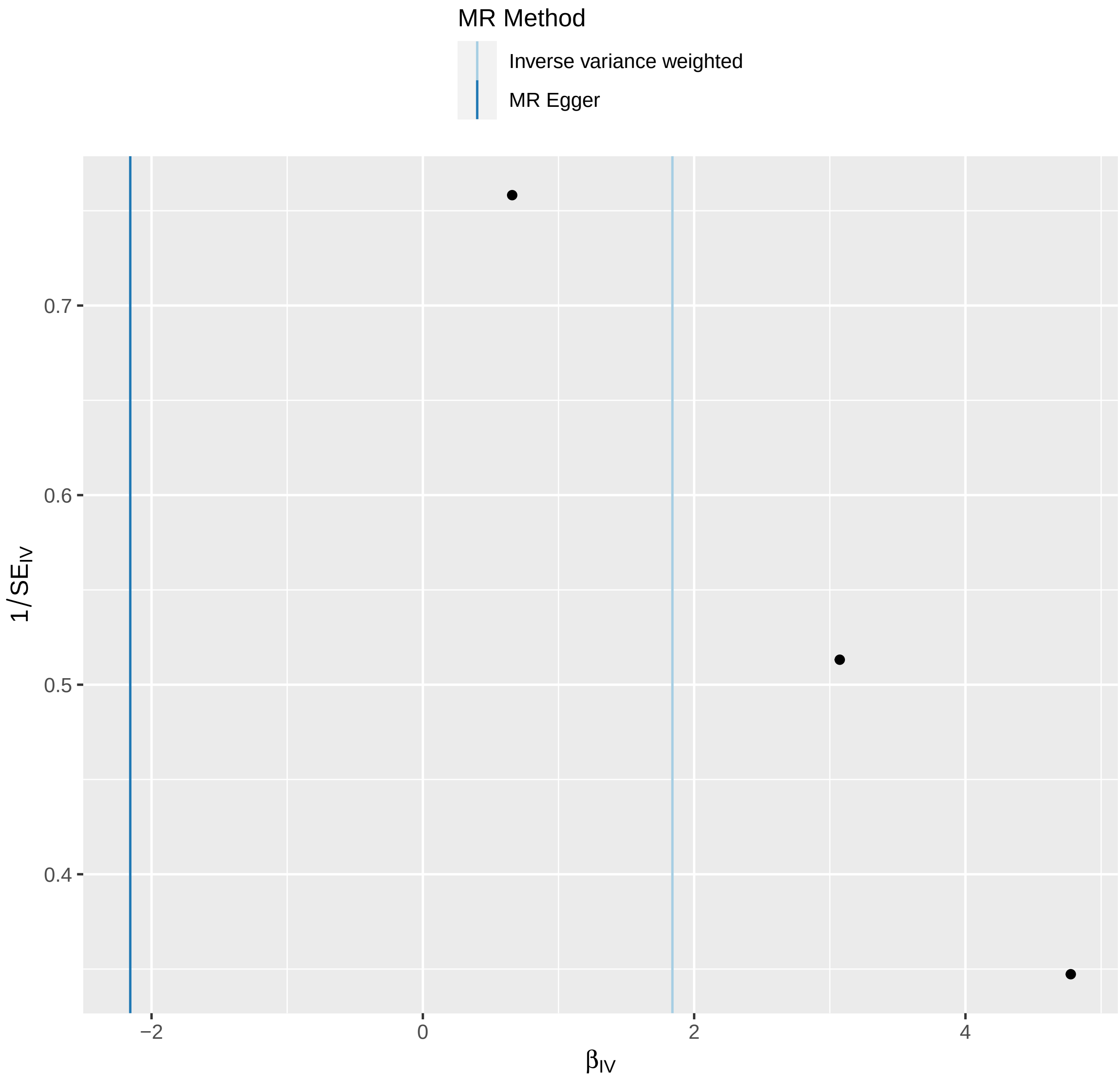

Batch 2545 : Mannitol on Ulcerative rectosigmoiditis

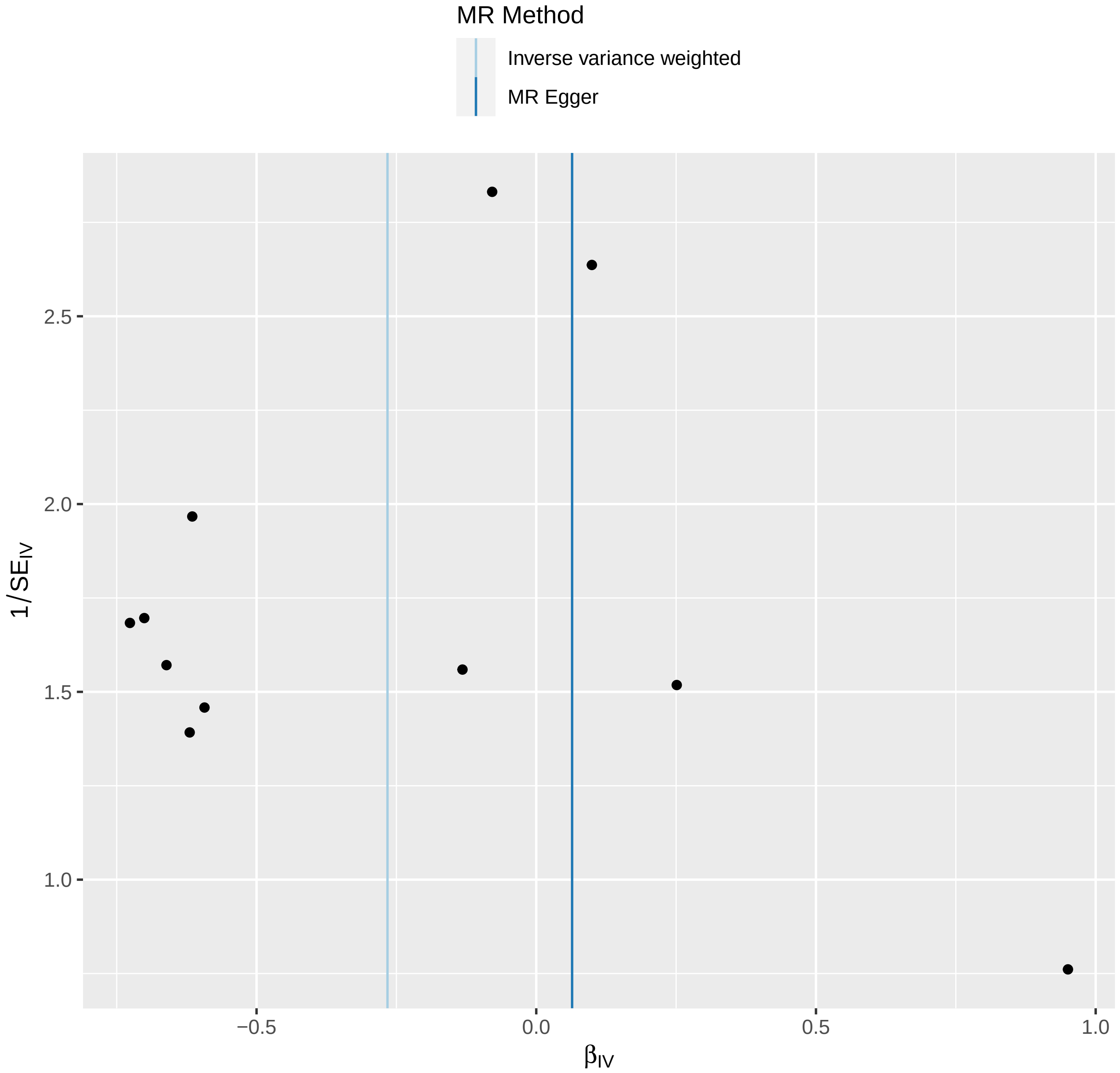

Batch 2546 : Glycerol 3−phosphate (G3P) on Ulcerative rectosigmoiditis

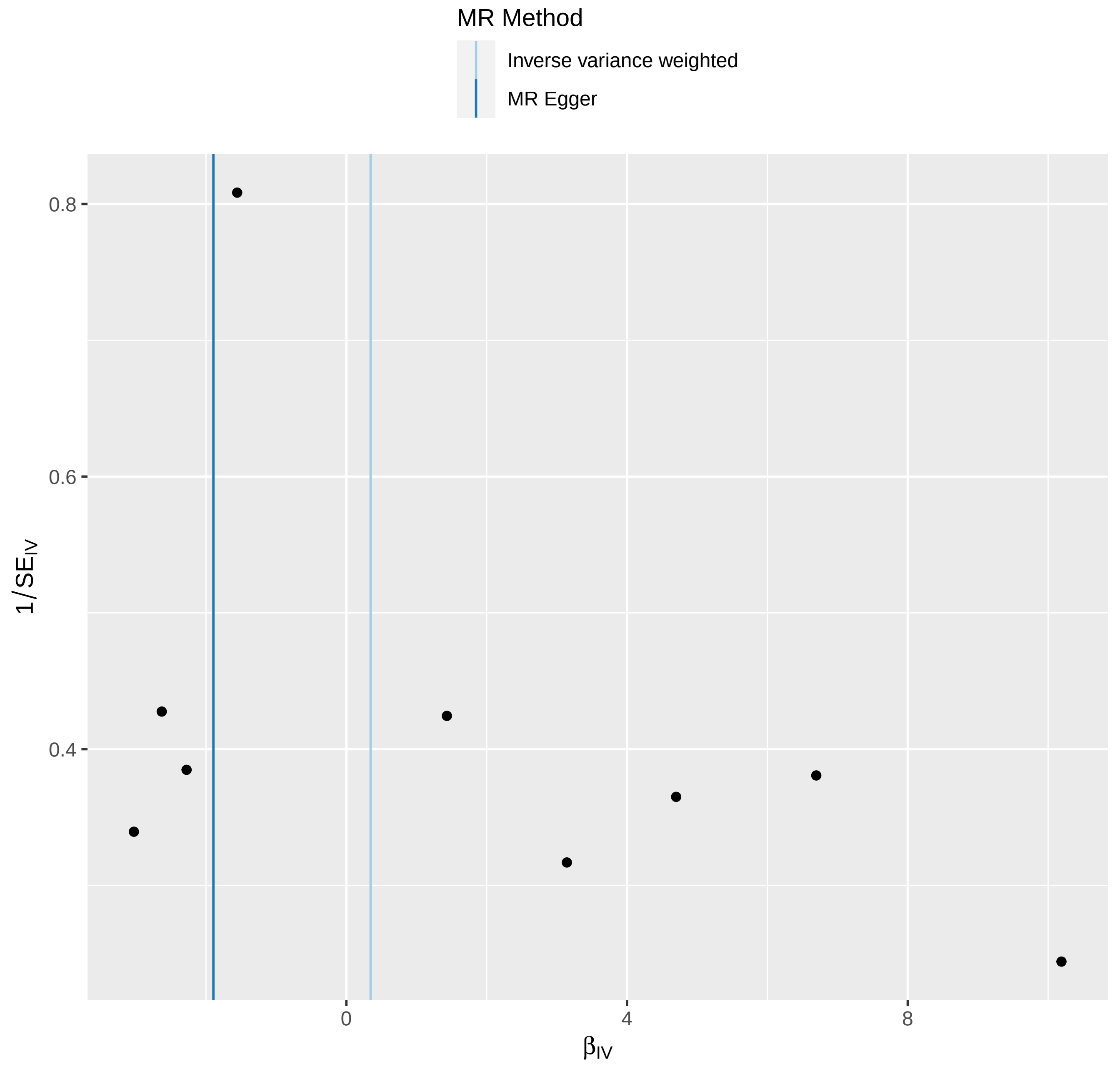

Batch 2548 : Carnitine on Ulcerative rectosigmoiditis

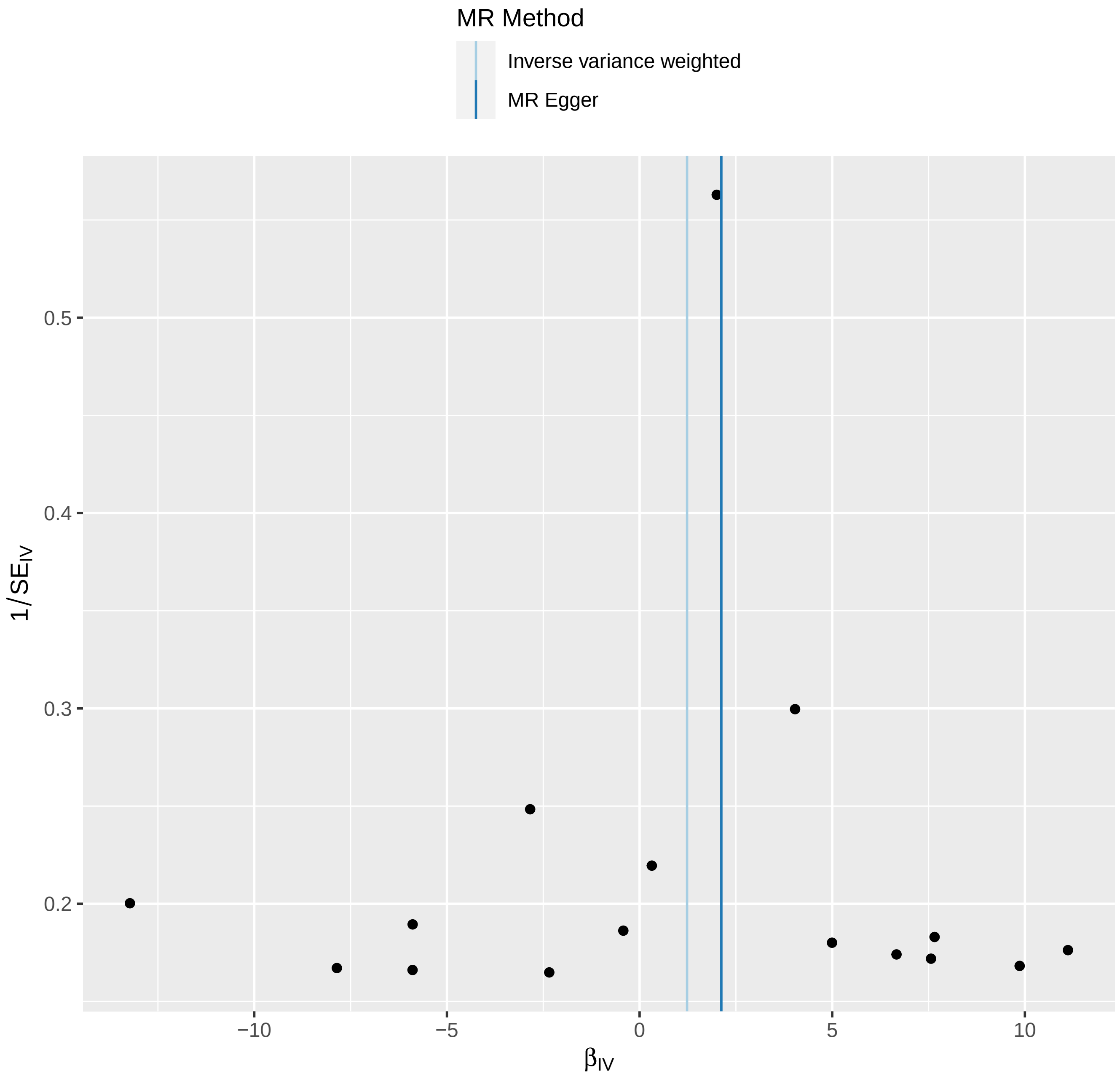

Batch 2549 : Choline on Ulcerative rectosigmoiditis

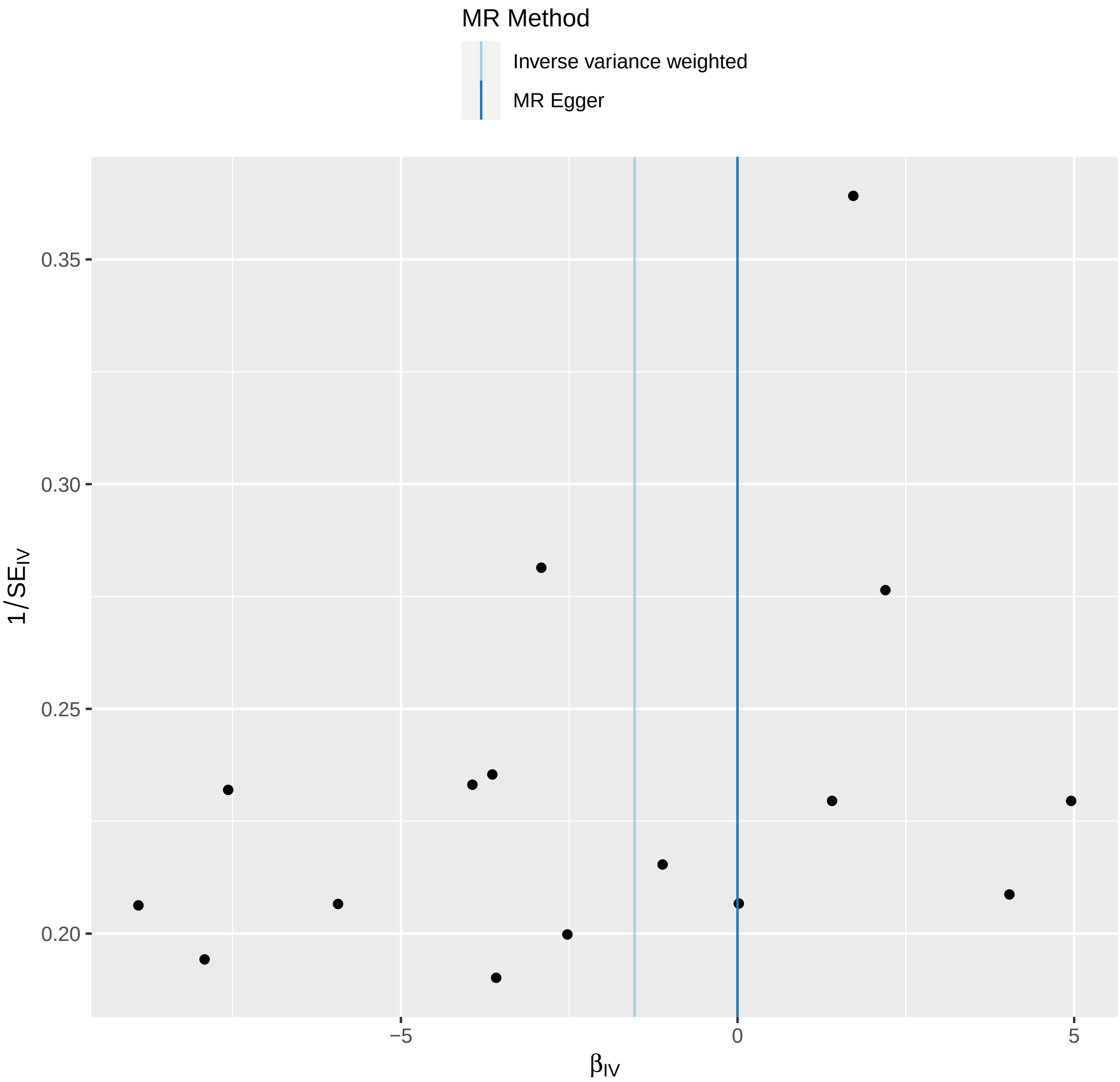

Batch 2552 : 3−methyl−2−oxovalerate on Ulcerative rectosigmoiditis

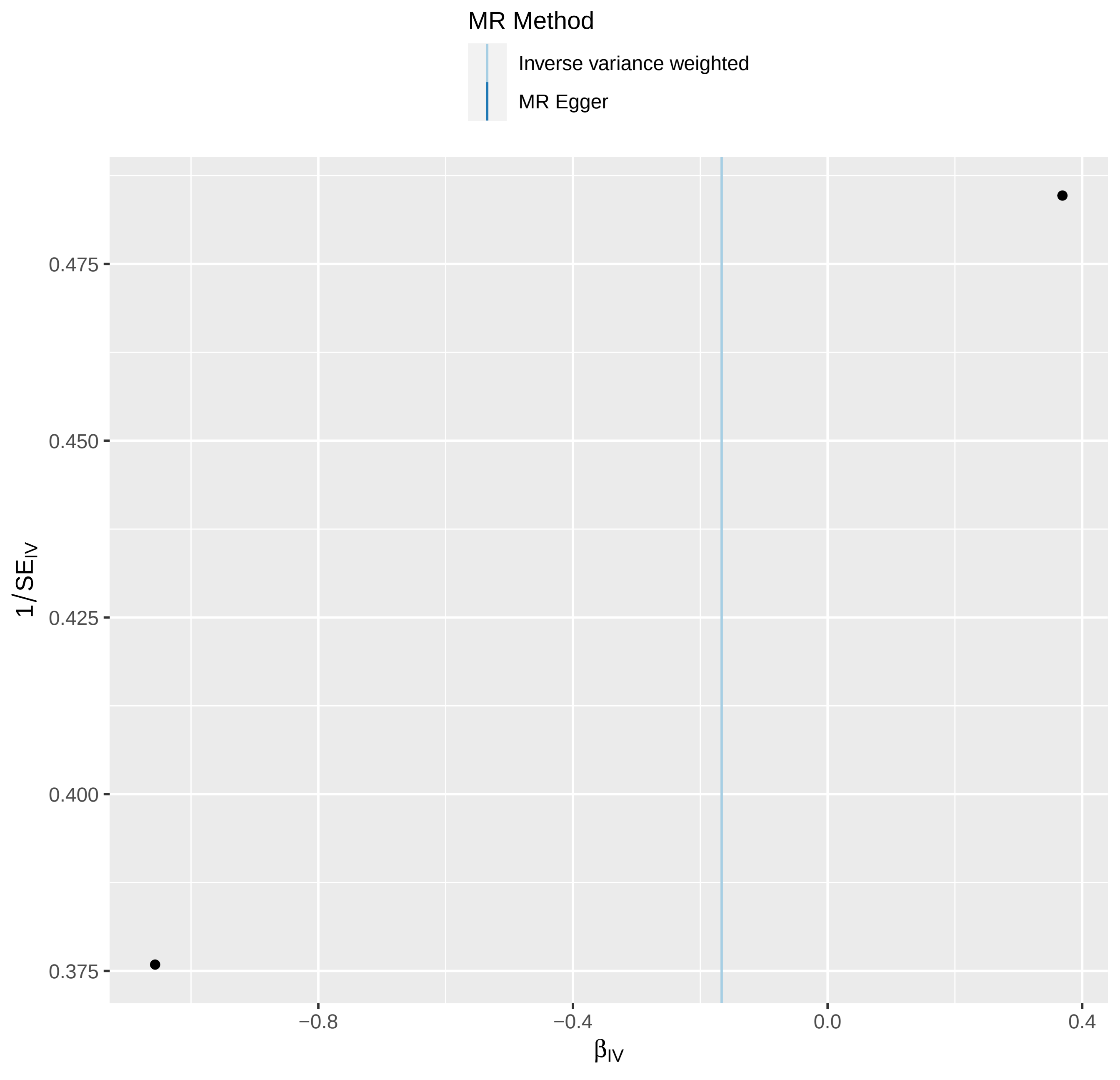

Batch 2553 : 3−methylhistidine on Ulcerative rectosigmoiditis

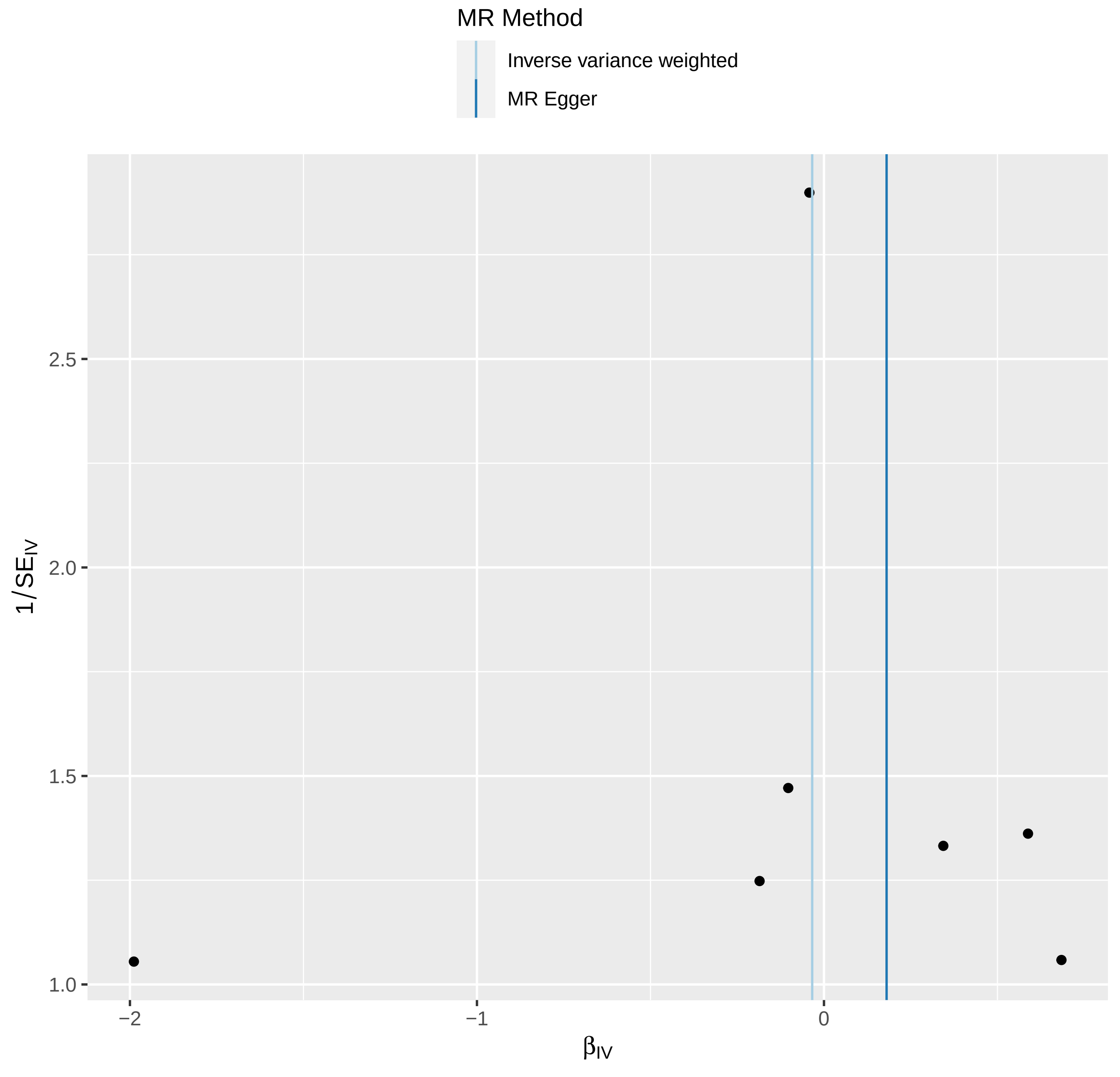

Batch 2555 : Phenylacetate on Ulcerative rectosigmoiditis

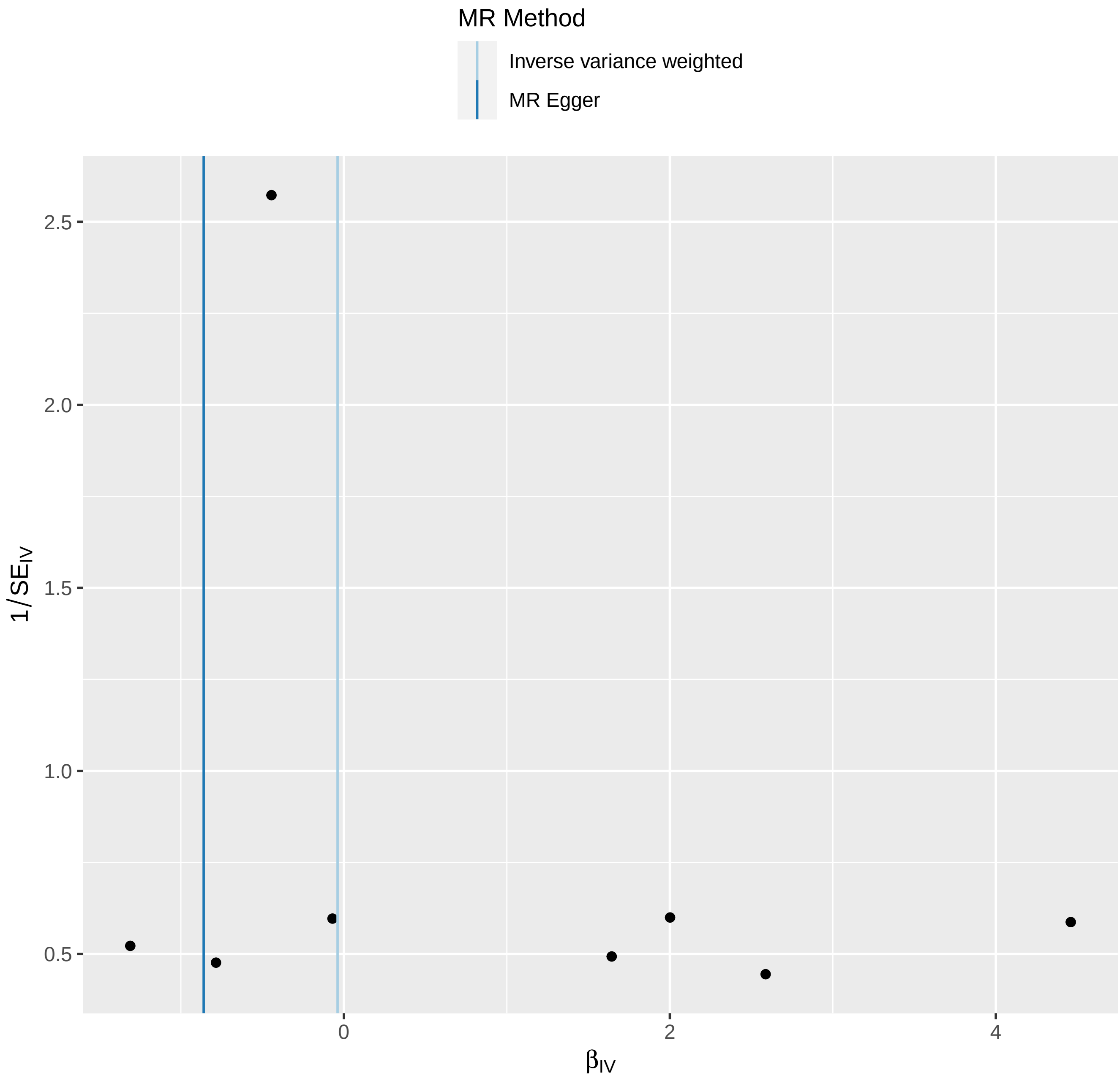

Batch 2556 : Glycerophosphorylcholine (GPC) on Ulcerative rectosigmoiditis

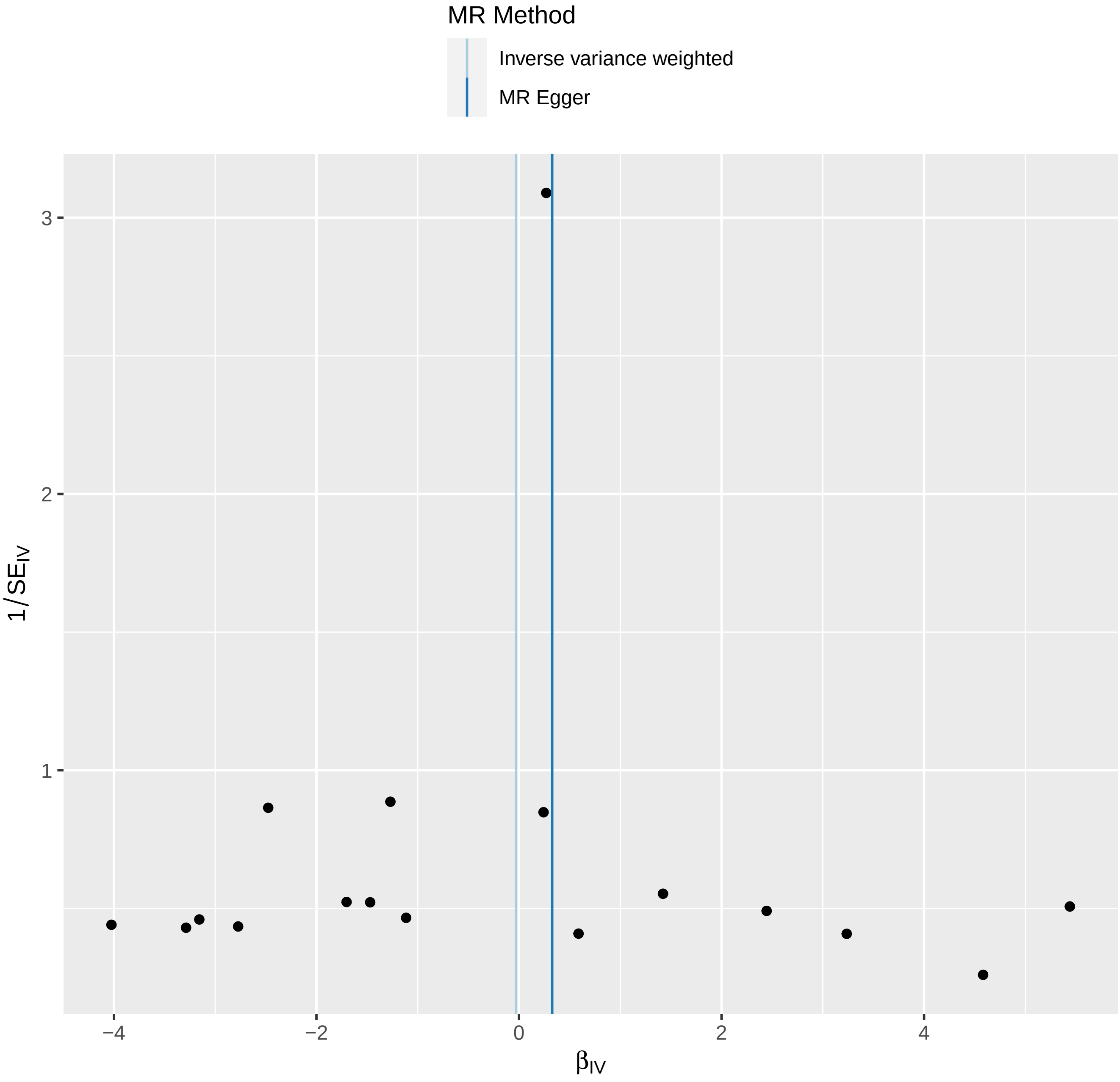

Batch 2557 : Aspartate on Ulcerative rectosigmoiditis

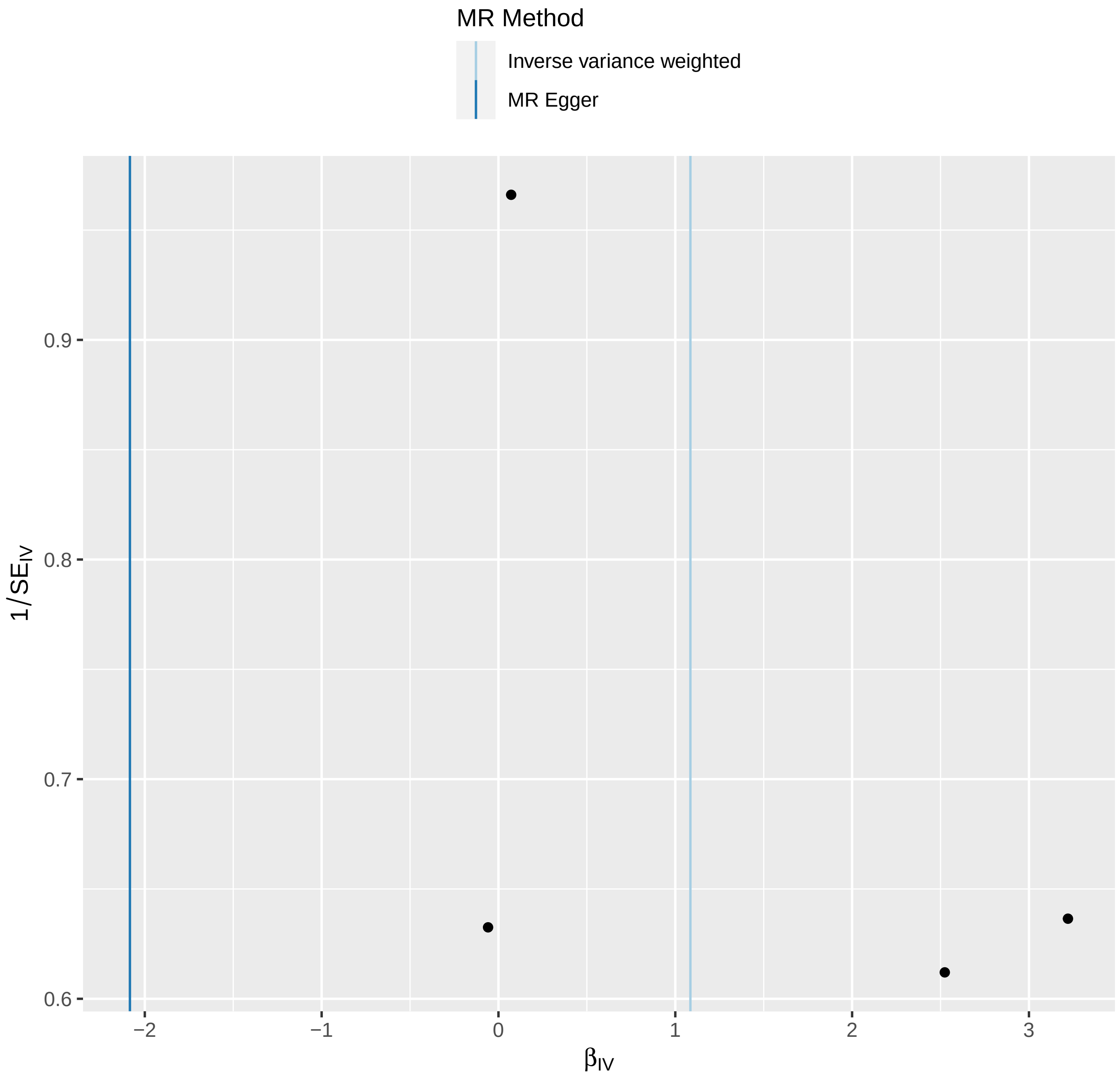

Batch 2559 : Dihomo−linoleate (20:2n6) on Ulcerative rectosigmoiditis

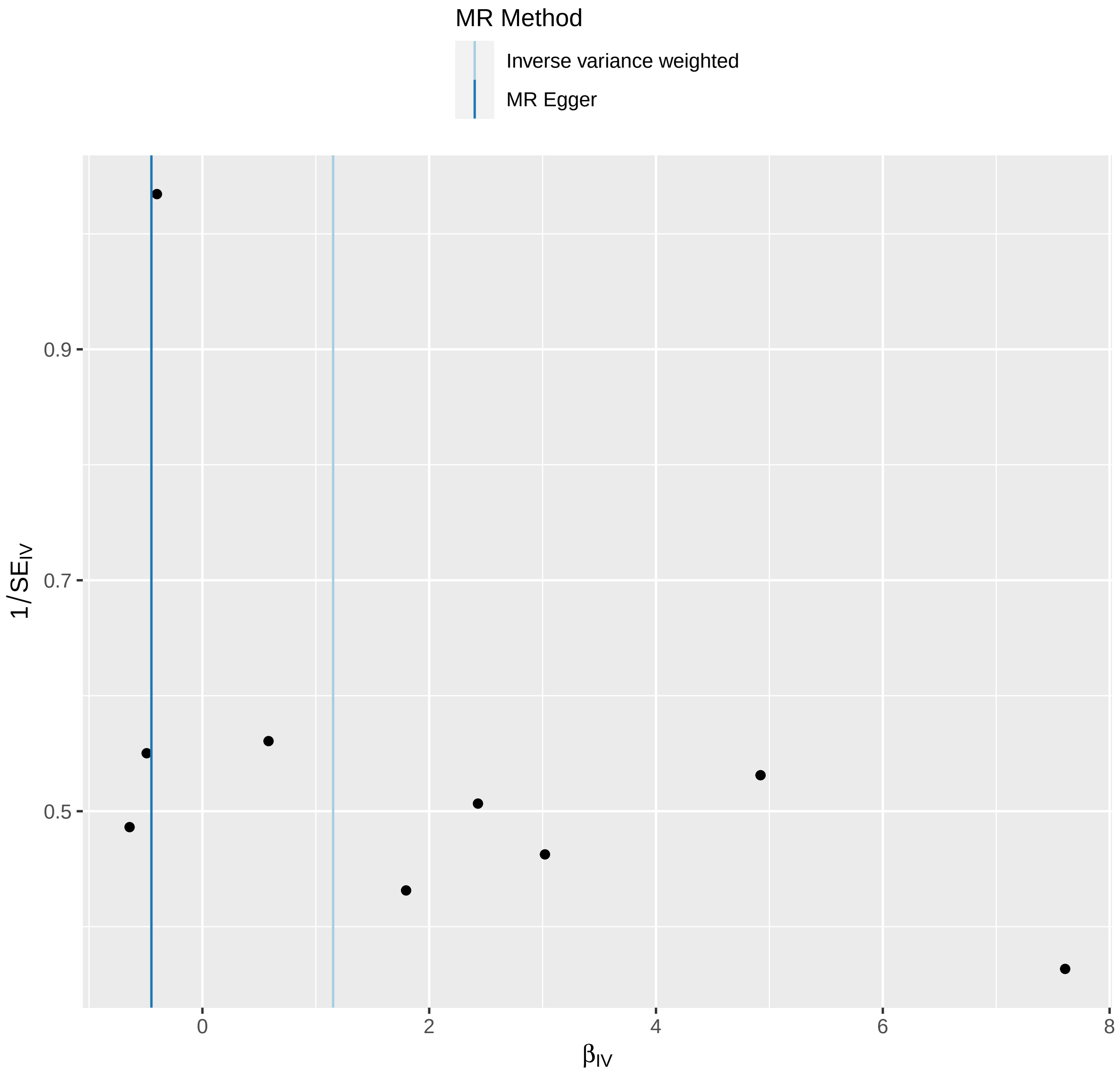

Batch 2560 : 2−hydroxystearate on Ulcerative rectosigmoiditis

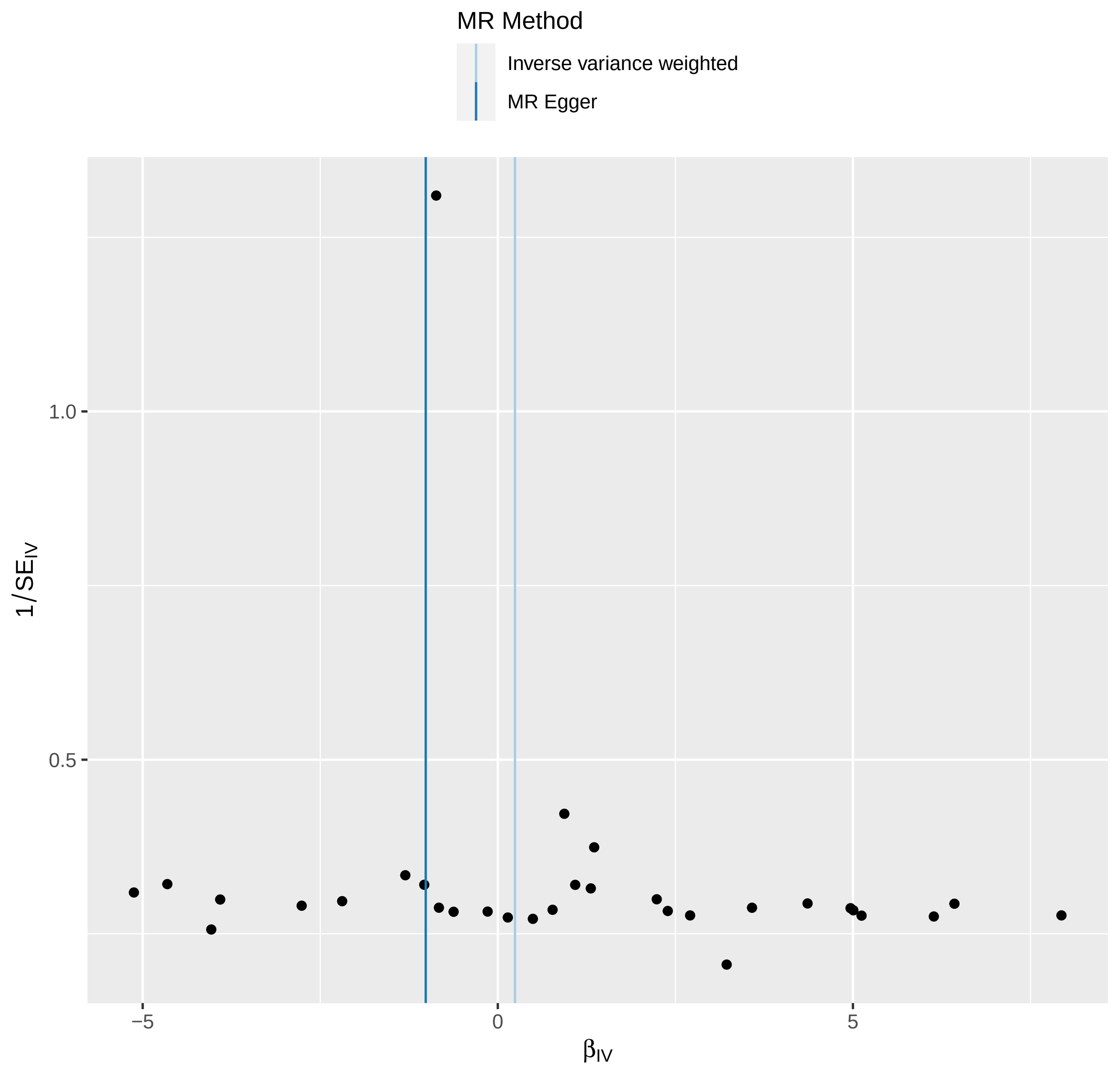

Batch 2562 : Glycylvaline on Ulcerative rectosigmoiditis

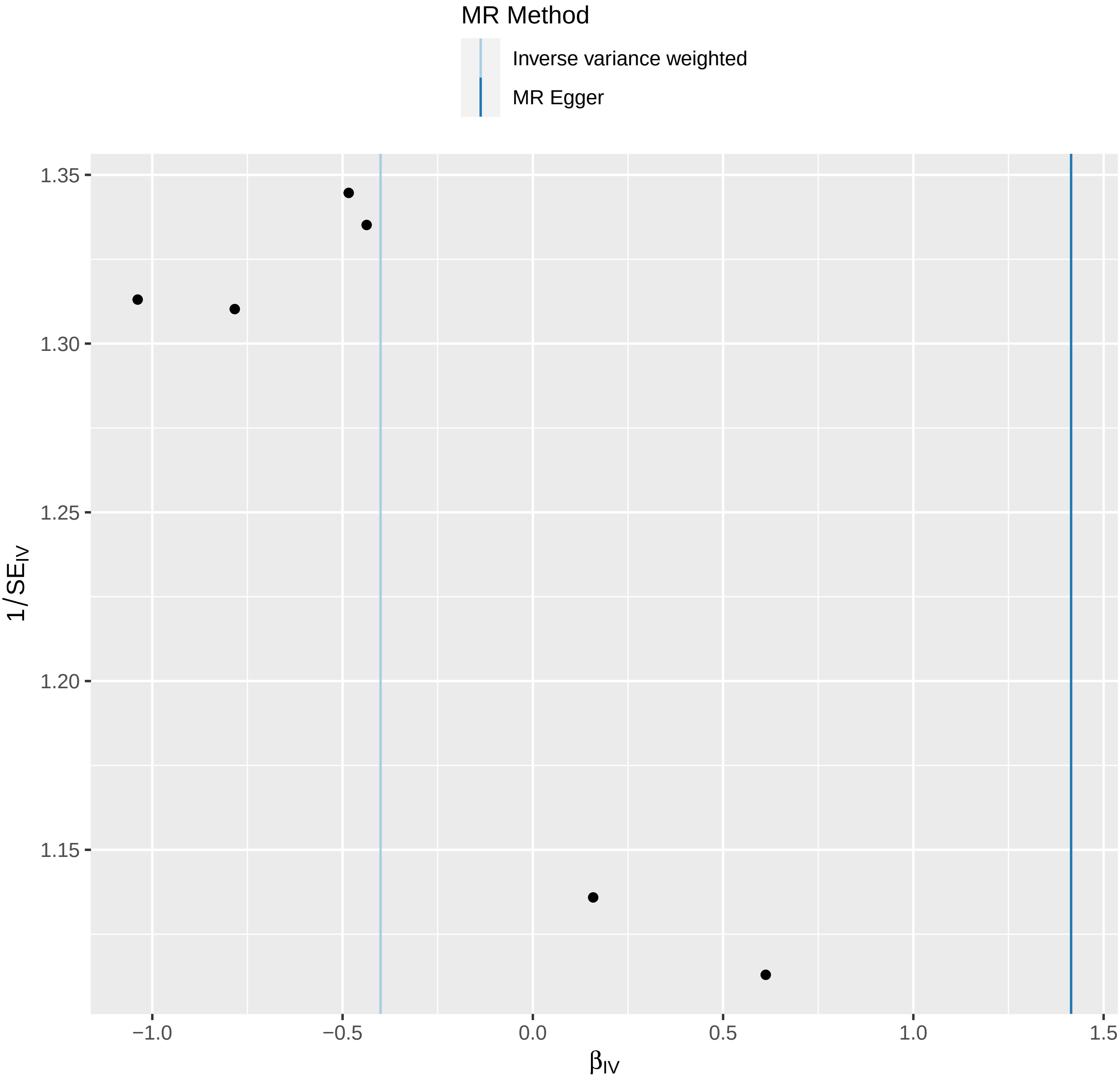

Batch 2563 : Gamma−glutamylleucine on Ulcerative rectosigmoiditis

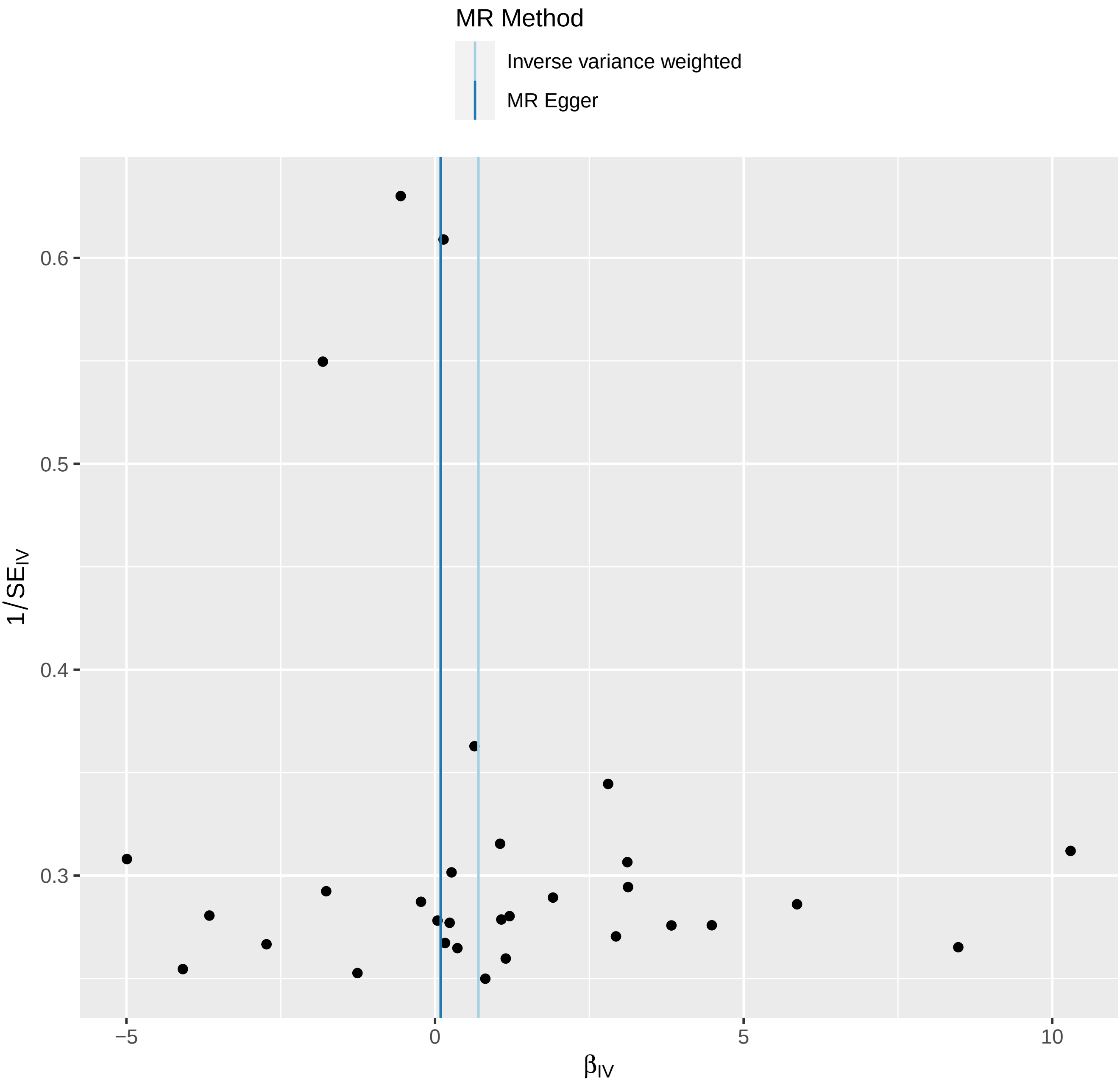

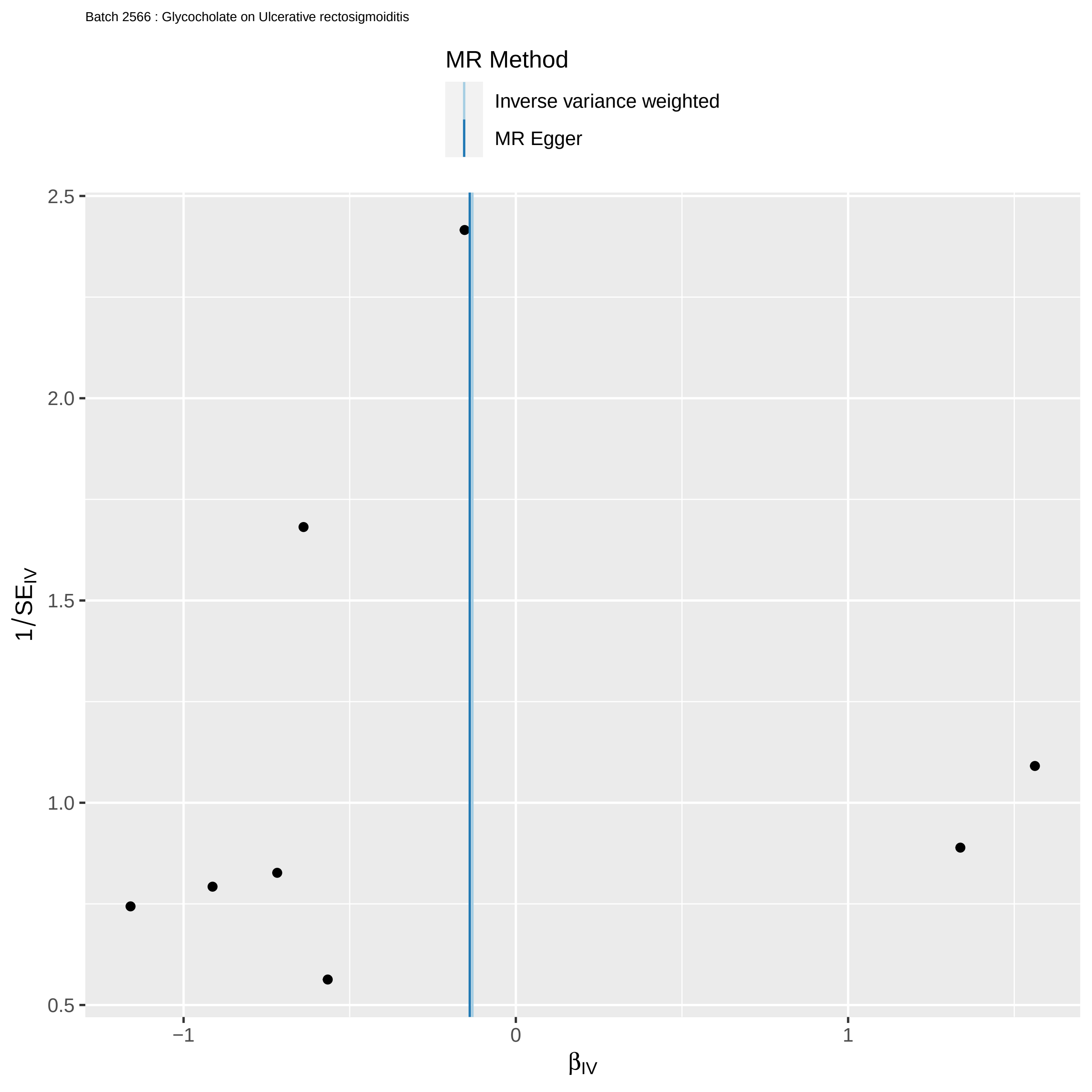

Batch 2566 : Glycocholate on Ulcerative rectosigmoiditis
MR Method
Inverse variance weighted
MR Egger
2.5
2.0
1.5
1.0
0.5
1/SE_IV
−1
0
1
β_IV

Batch 2567 : Glycodeoxycholate on Ulcerative rectosigmoiditis

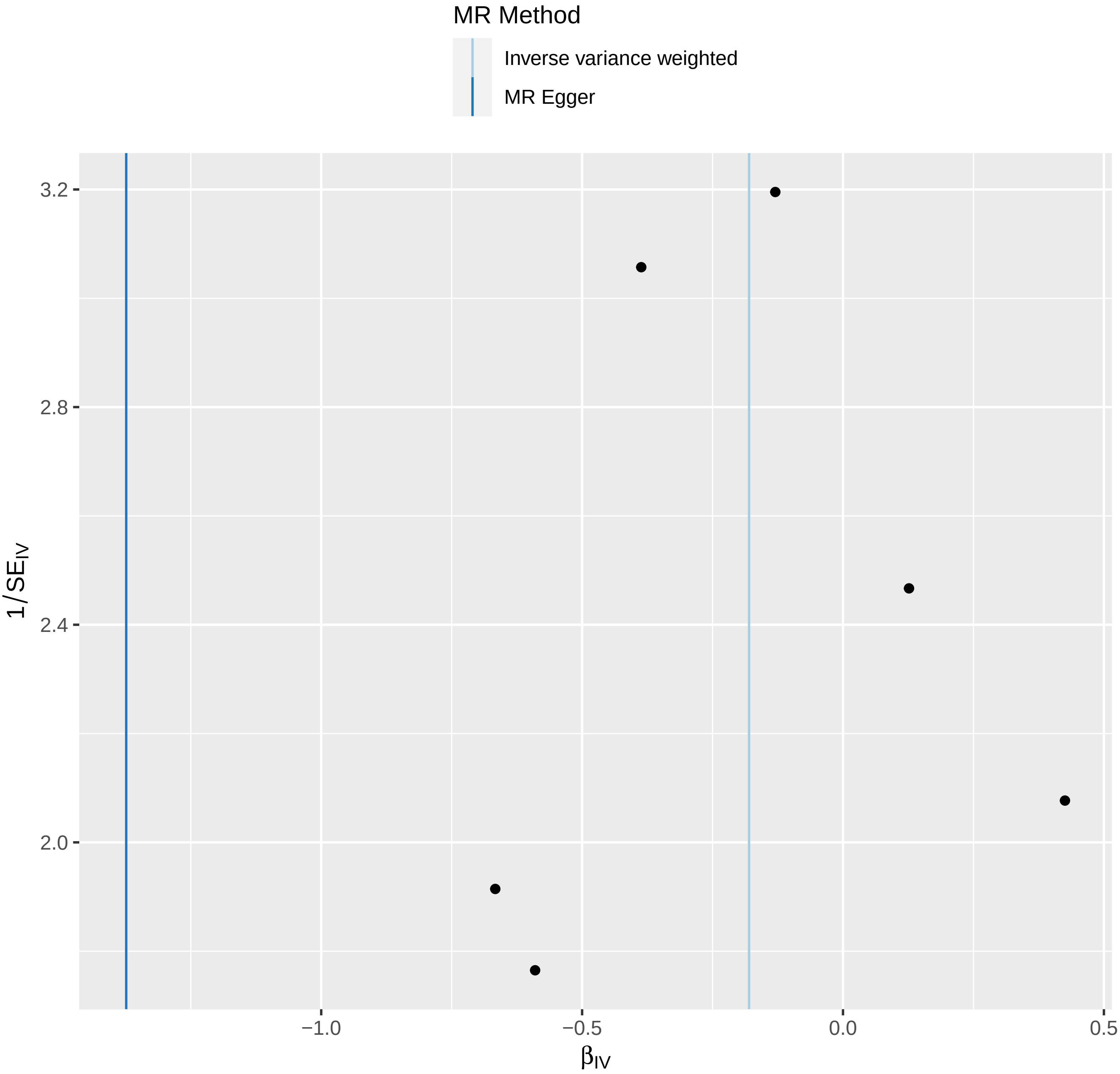

Batch 2568 : Taurochenodeoxycholate on Ulcerative rectosigmoiditis

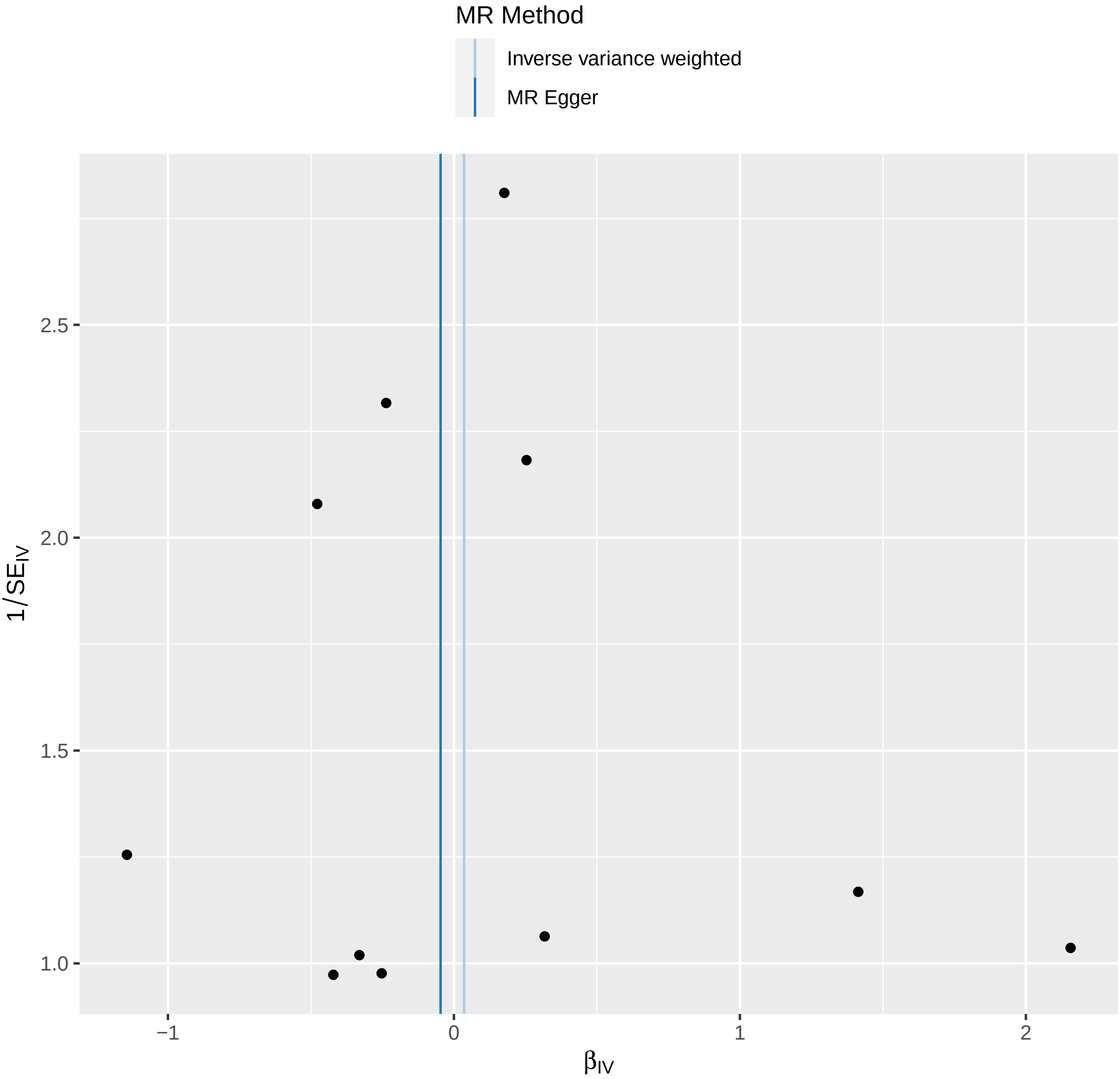

Batch 2569 : Taurocholate on Ulcerative rectosigmoiditis

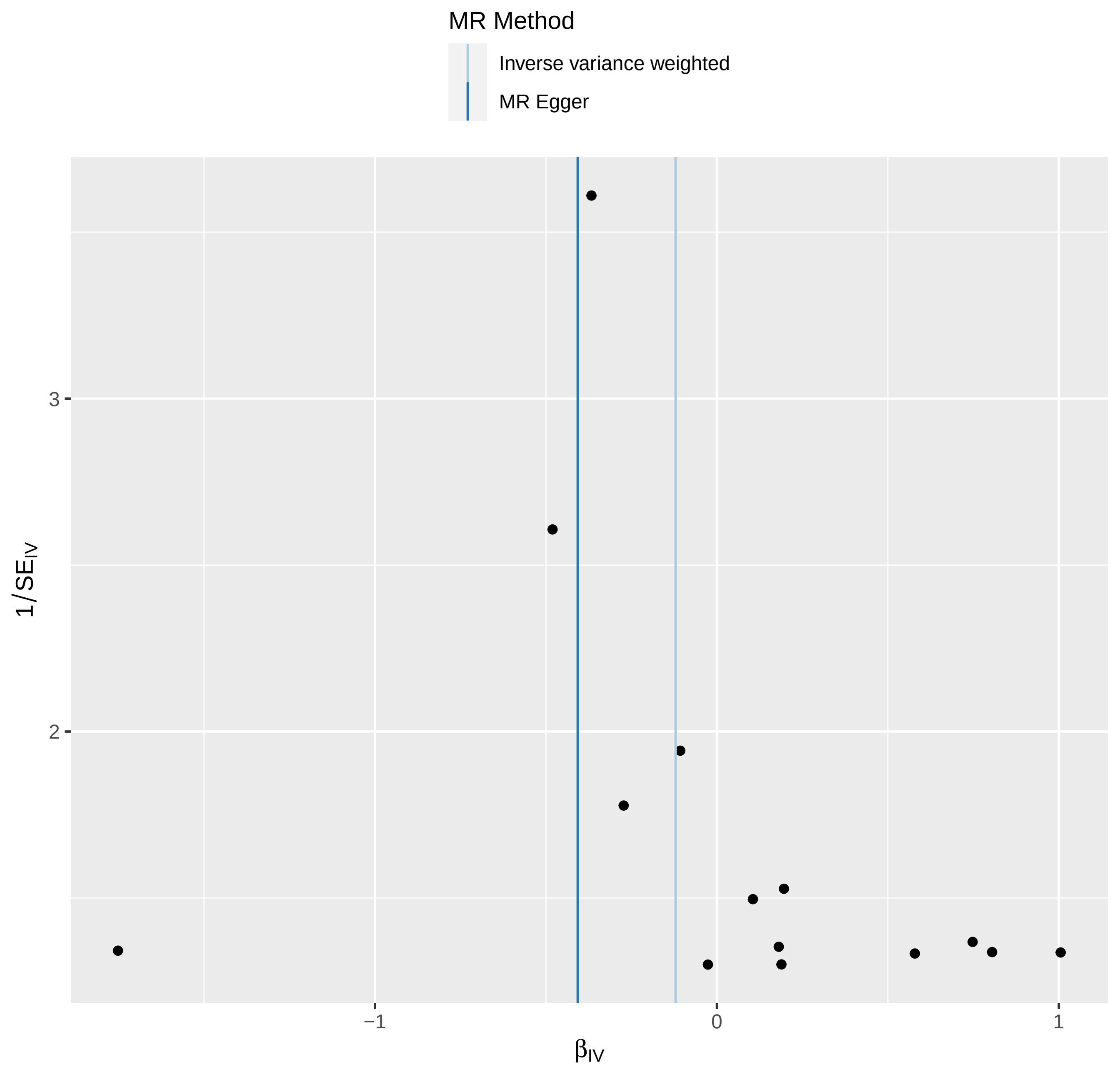

Batch 2571 : 1−stearoylglycerophosphoinositol on Ulcerative rectosigmoiditis

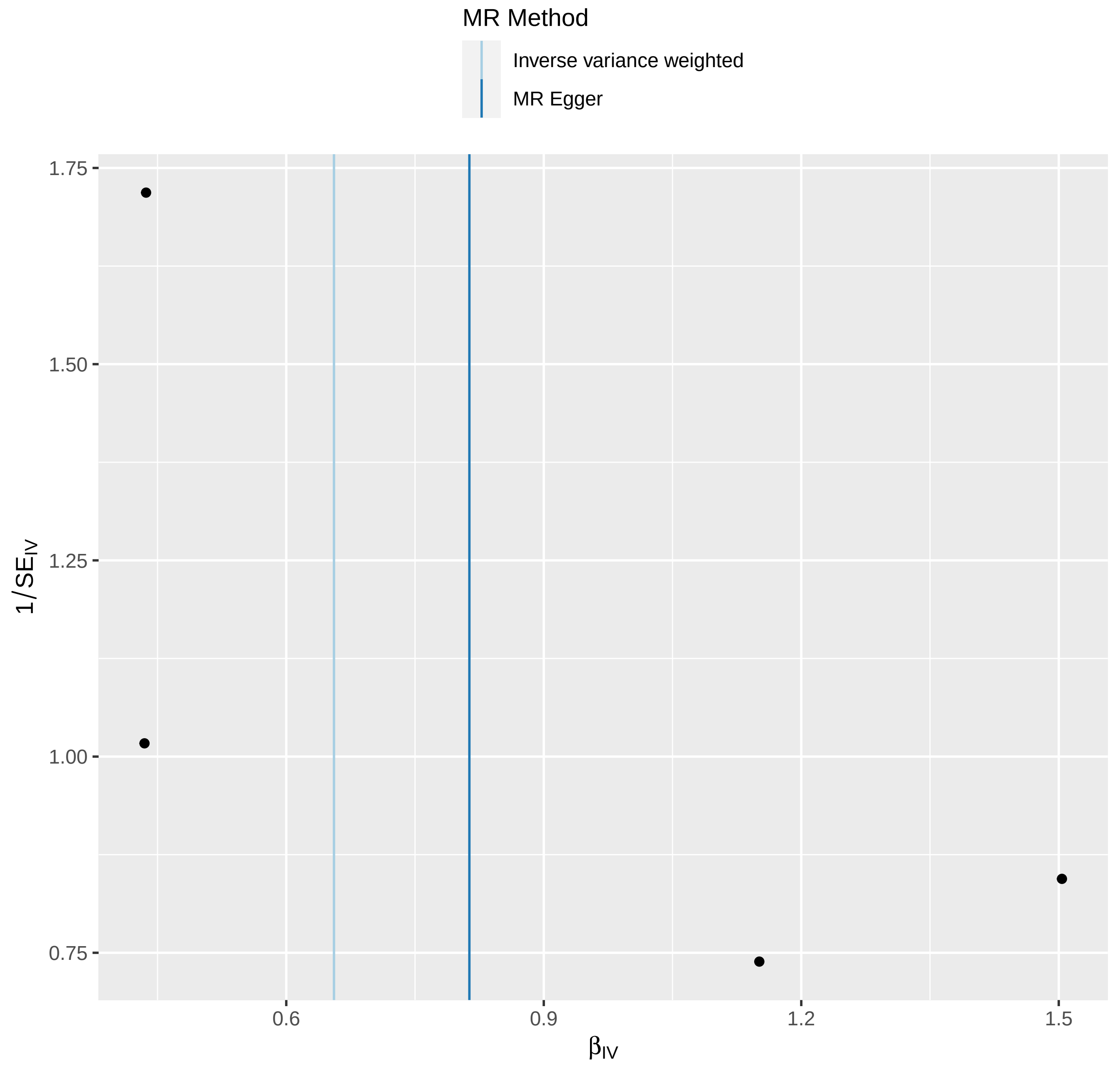

Batch 2574 : 1,5−anhydroglucitol (1,5−AG) on Ulcerative rectosigmoiditis

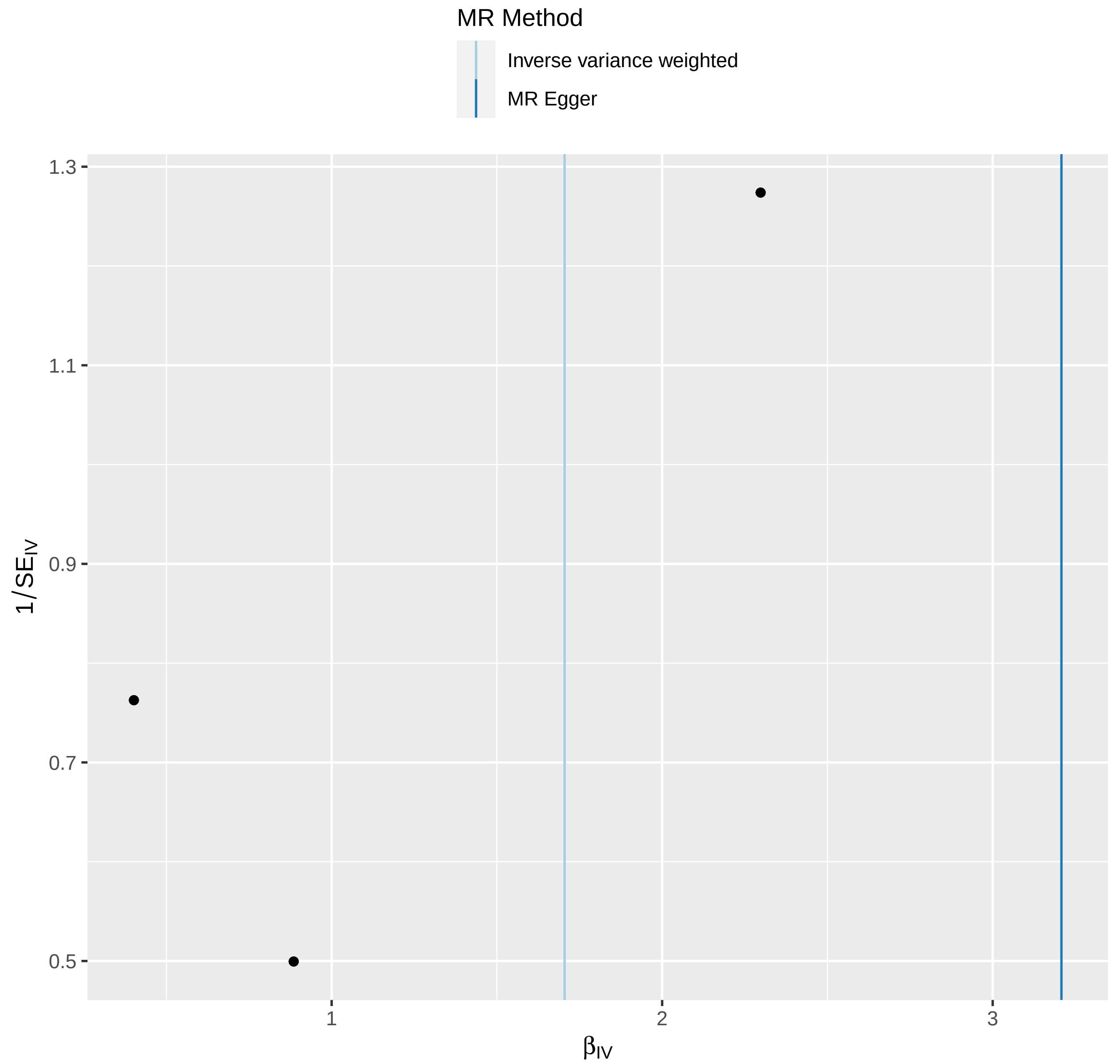

Batch 2575 : 2−hydroxybutyrate (AHB) on Ulcerative rectosigmoiditis

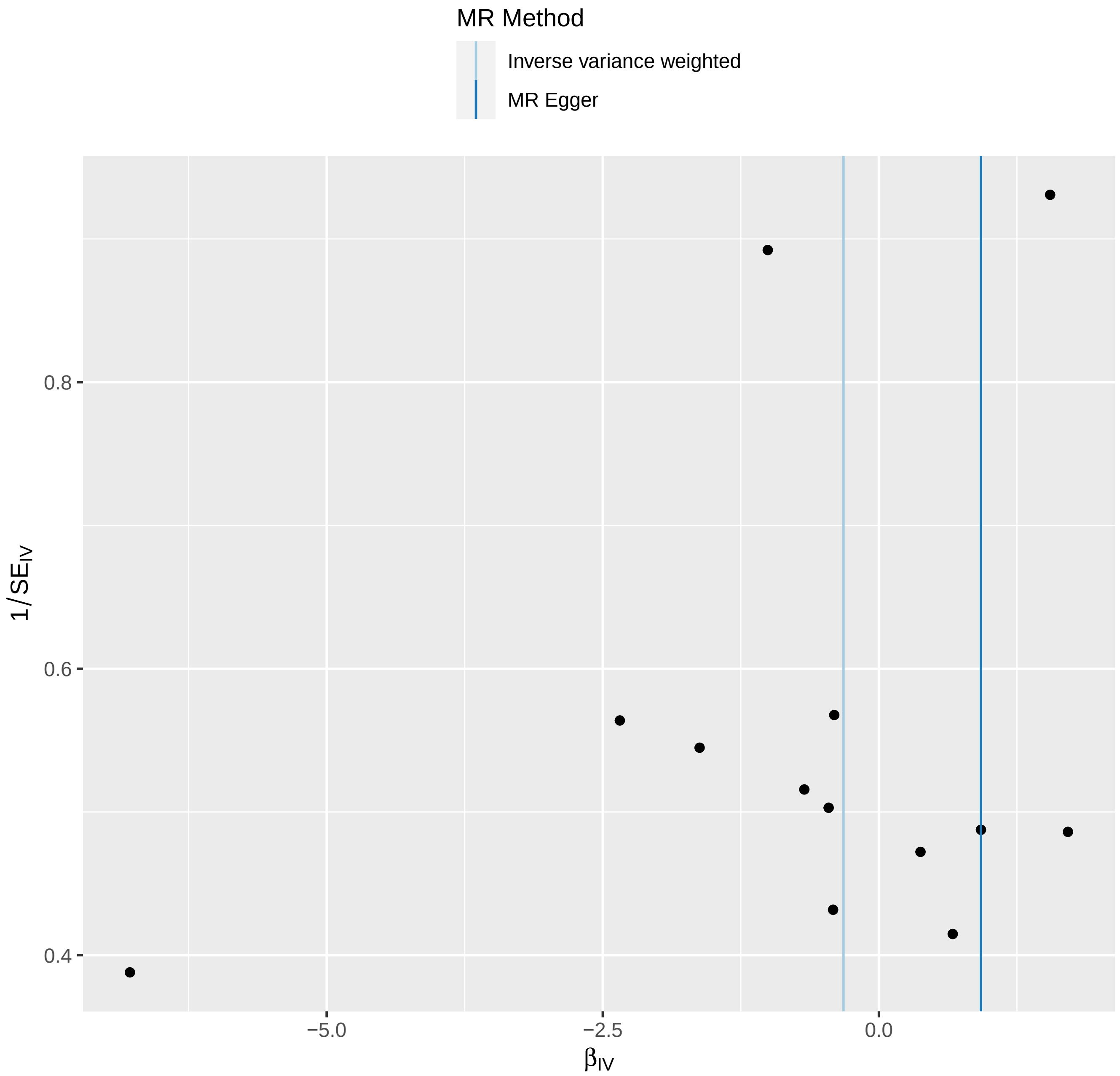

Batch 2578 : 1−palmitoylglycerol (1−monopalmitin) on Ulcerative rectosigmoiditis

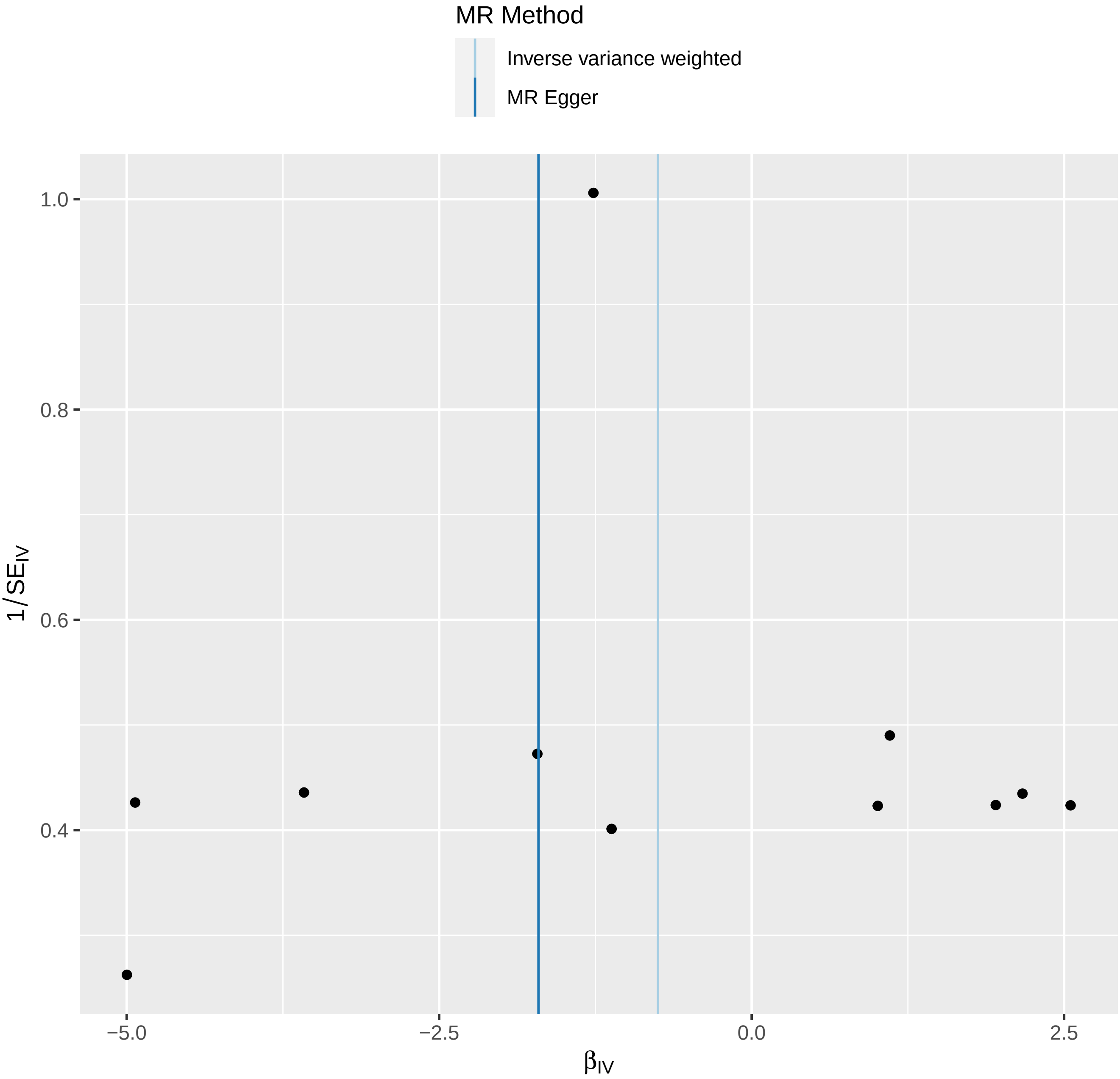

Batch 2581 : 2−hydroxyisobutyrate on Ulcerative rectosigmoiditis

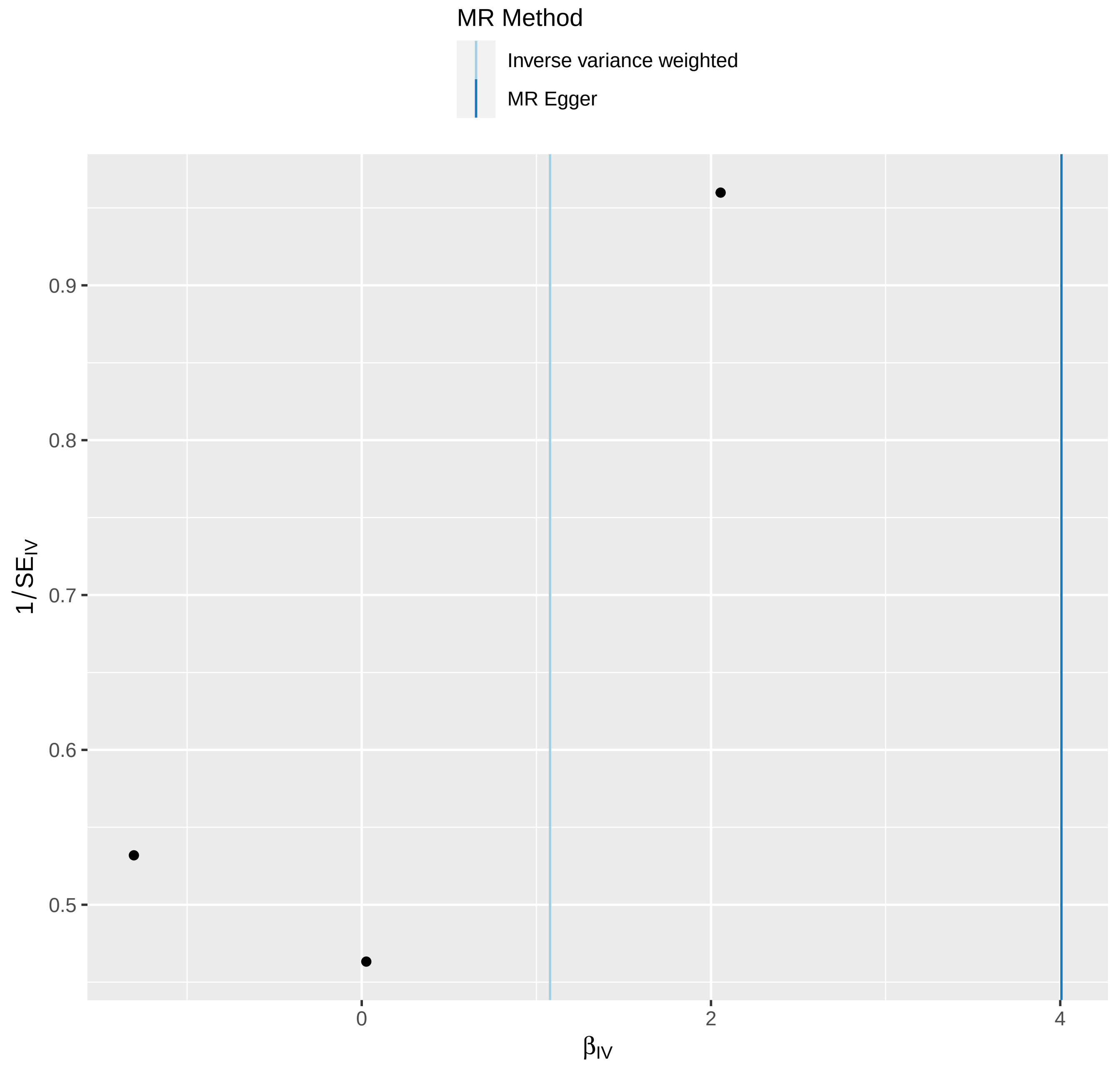

Batch 2582 : 4−methyl−2−oxopentanoate on Ulcerative rectosigmoiditis

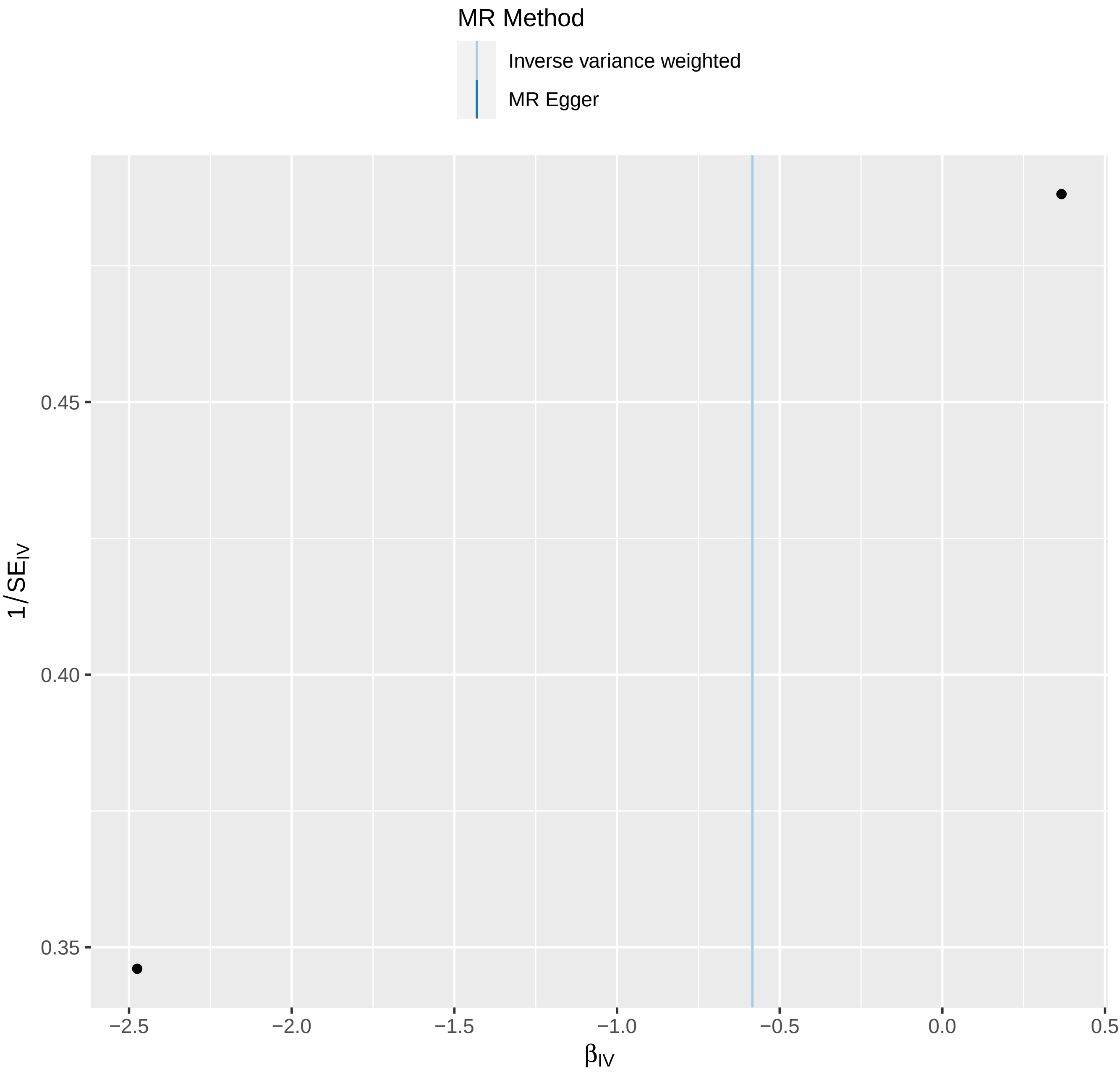

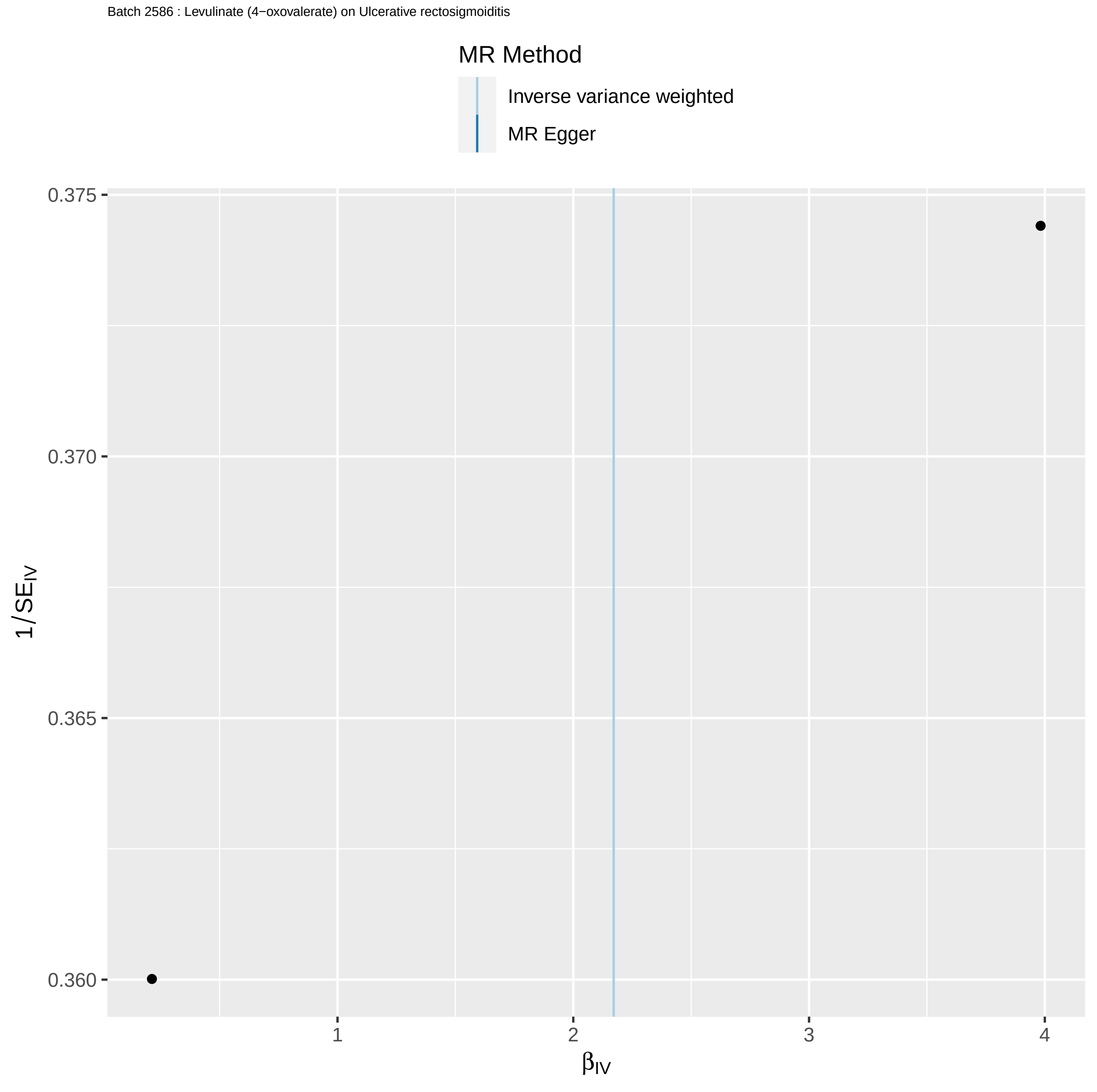

Batch 2586 : Levulinate (4−oxovalerate) on Ulcerative rectosigmoiditis
MR Method
Inverse variance weighted
MR Egger
0.375
0.370
0.365
0.360
1/SE$_{IV}$
1
2
3
4
β$_{IV}$

Batch 2588 : Cholate on Ulcerative rectosigmoiditis

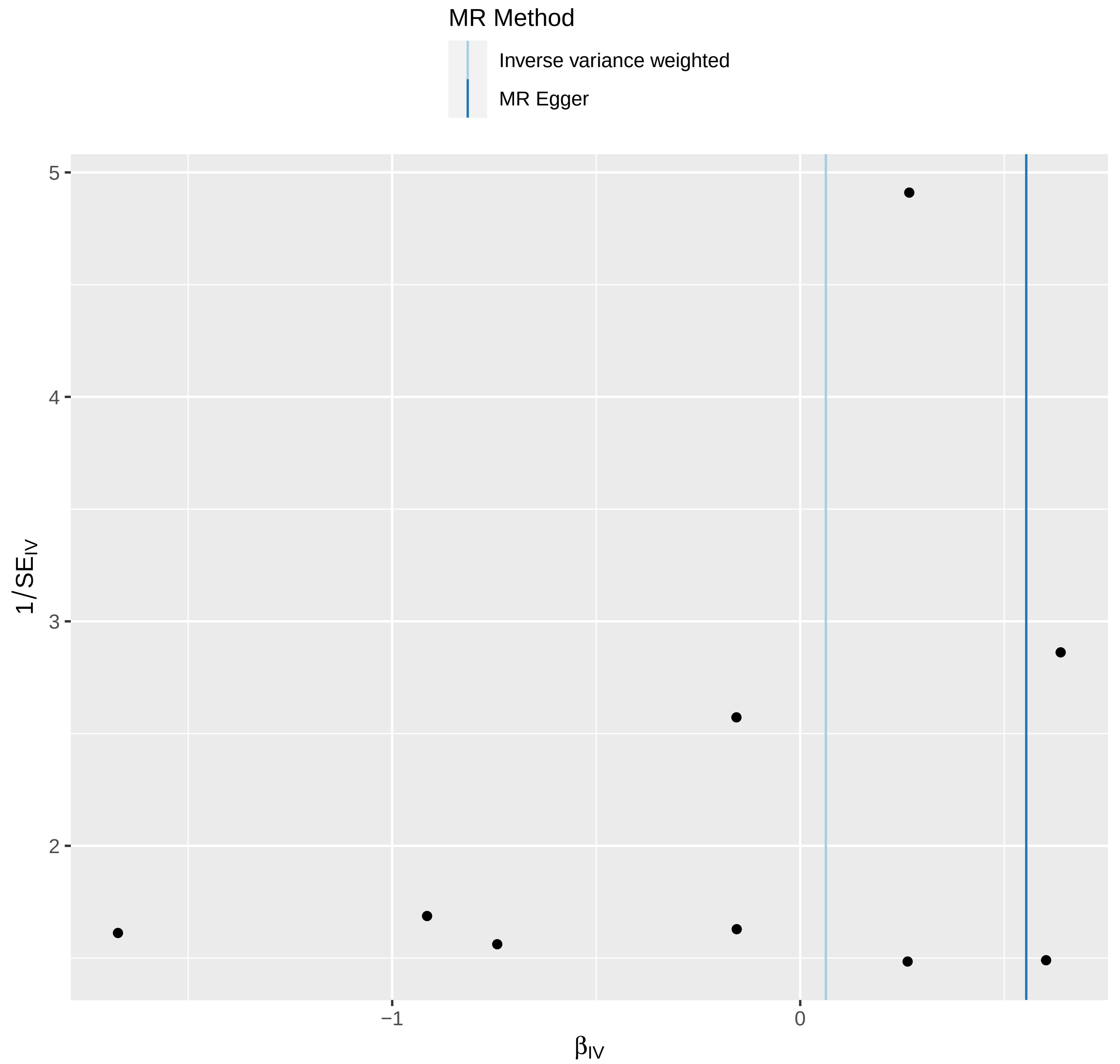

Batch 2591 : Hyodeoxycholate on Ulcerative rectosigmoiditis

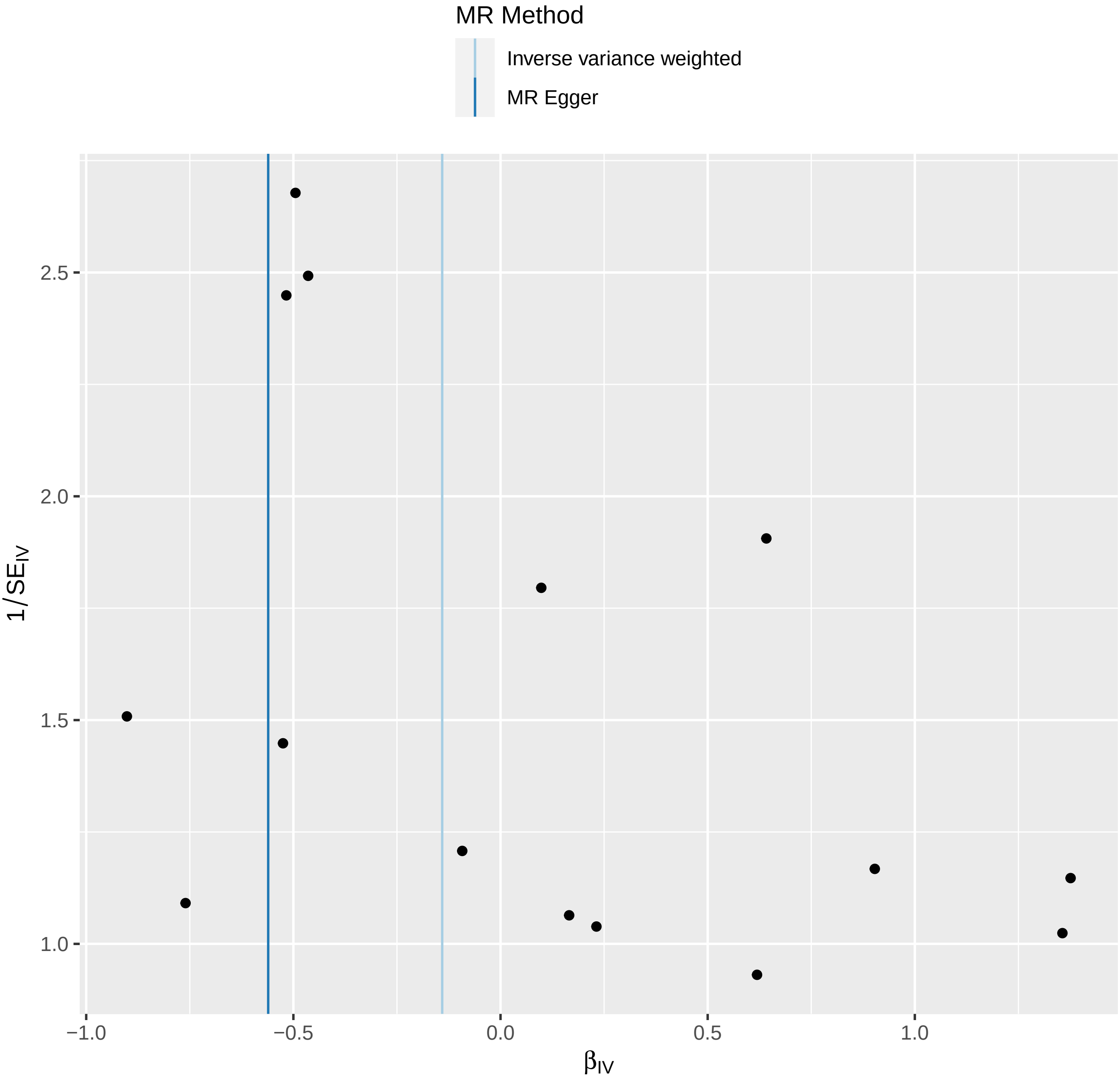

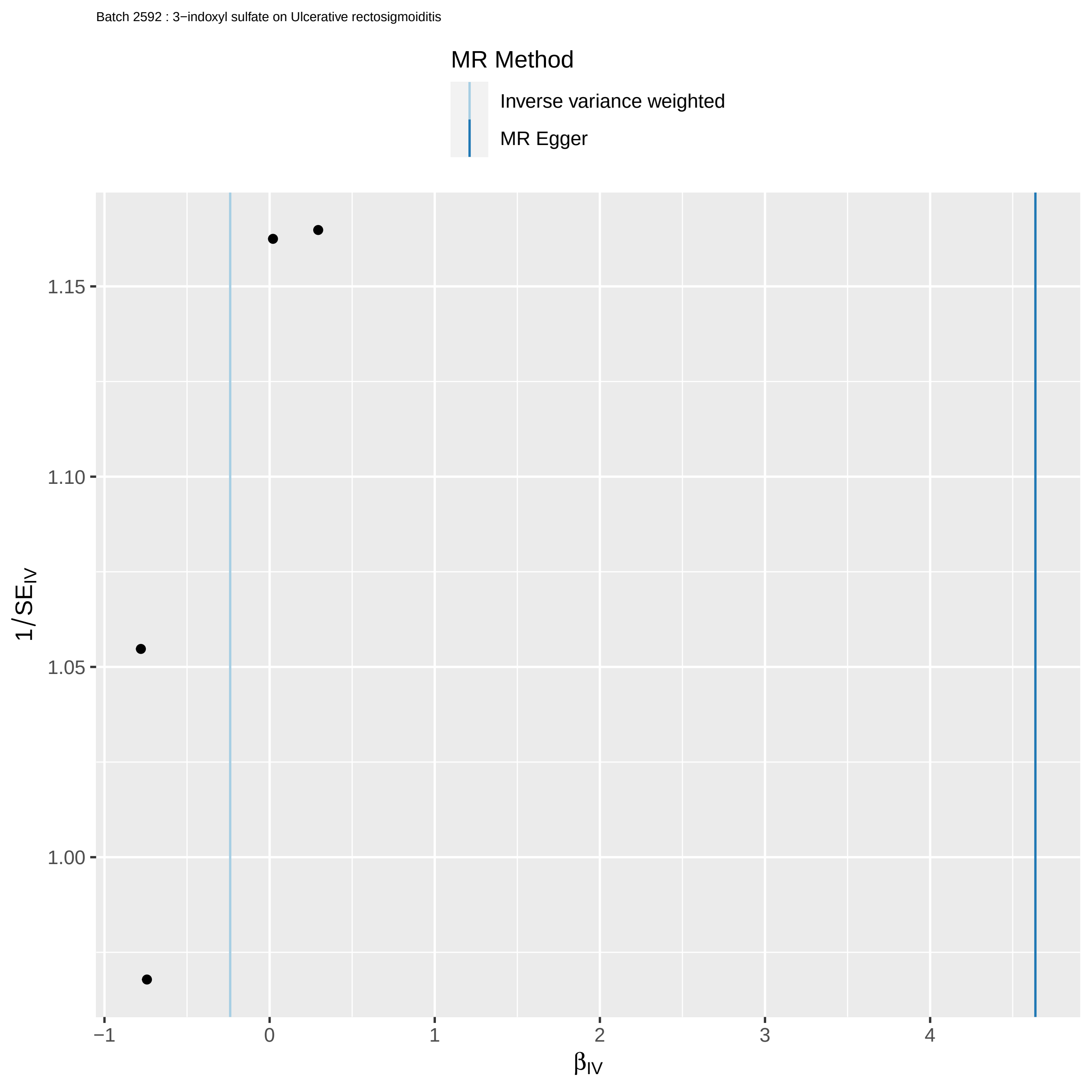
Batch 2592 : 3−indoxyl sulfate on Ulcerative rectosigmoiditis
MR Method
Inverse variance weighted
MR Egger
1.15
1.10
1.05
1.00
$1/SE_{IV}$
−1
0
1
2
3
4
β_{IV}

Batch 2596 : Erythrose on Ulcerative rectosigmoiditis

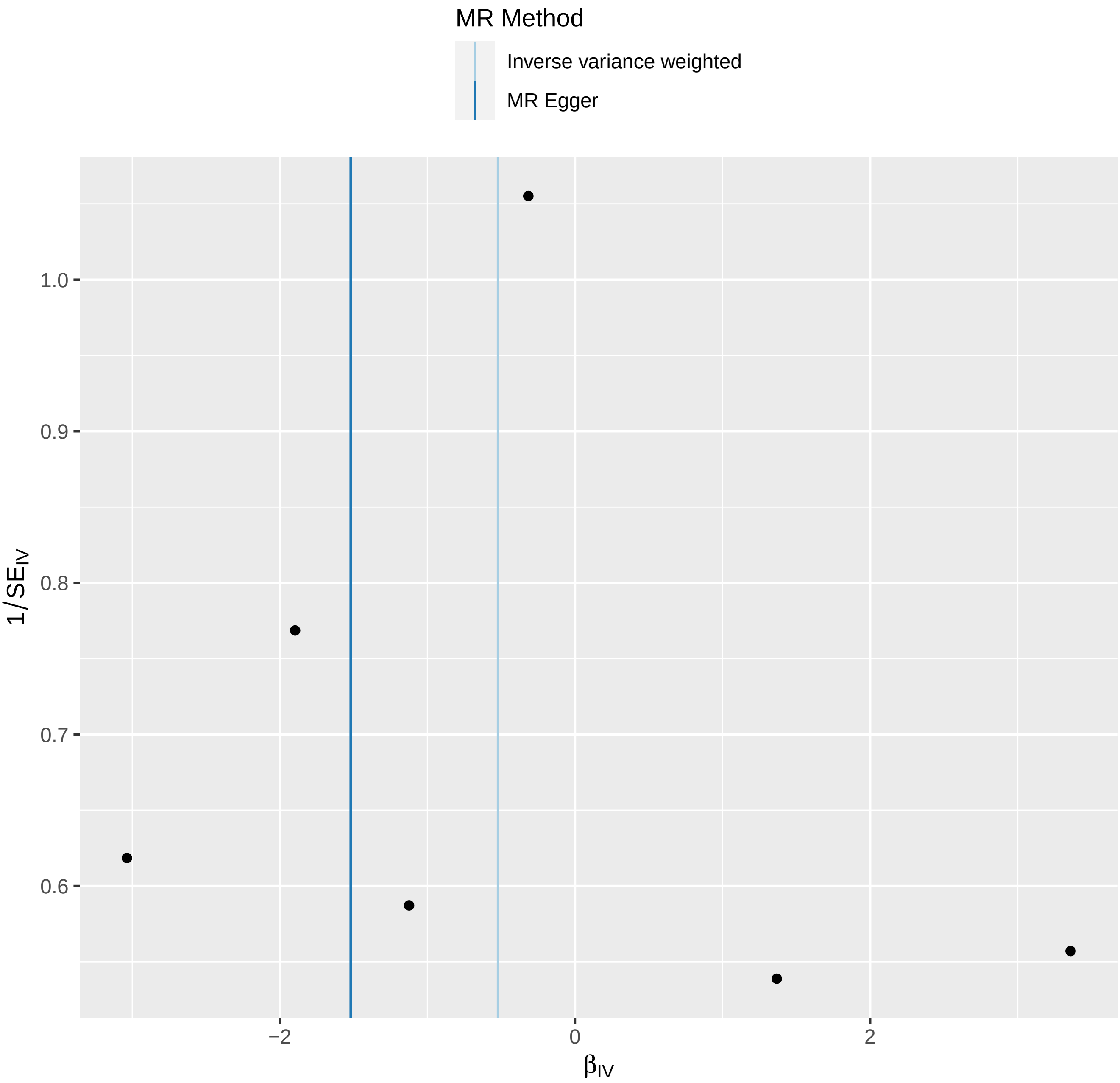

Batch 2597 : Threonate on Ulcerative rectosigmoiditis

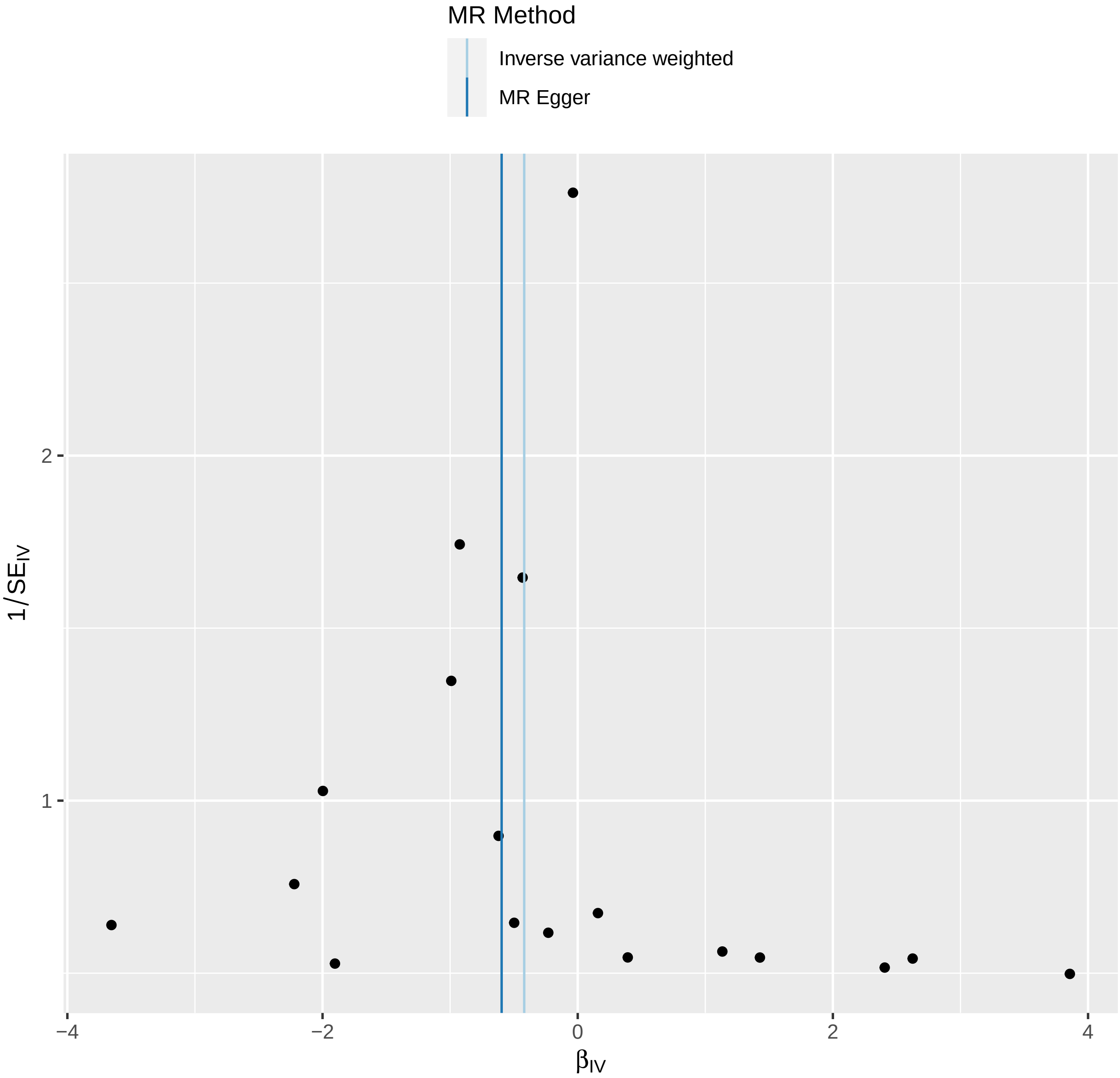

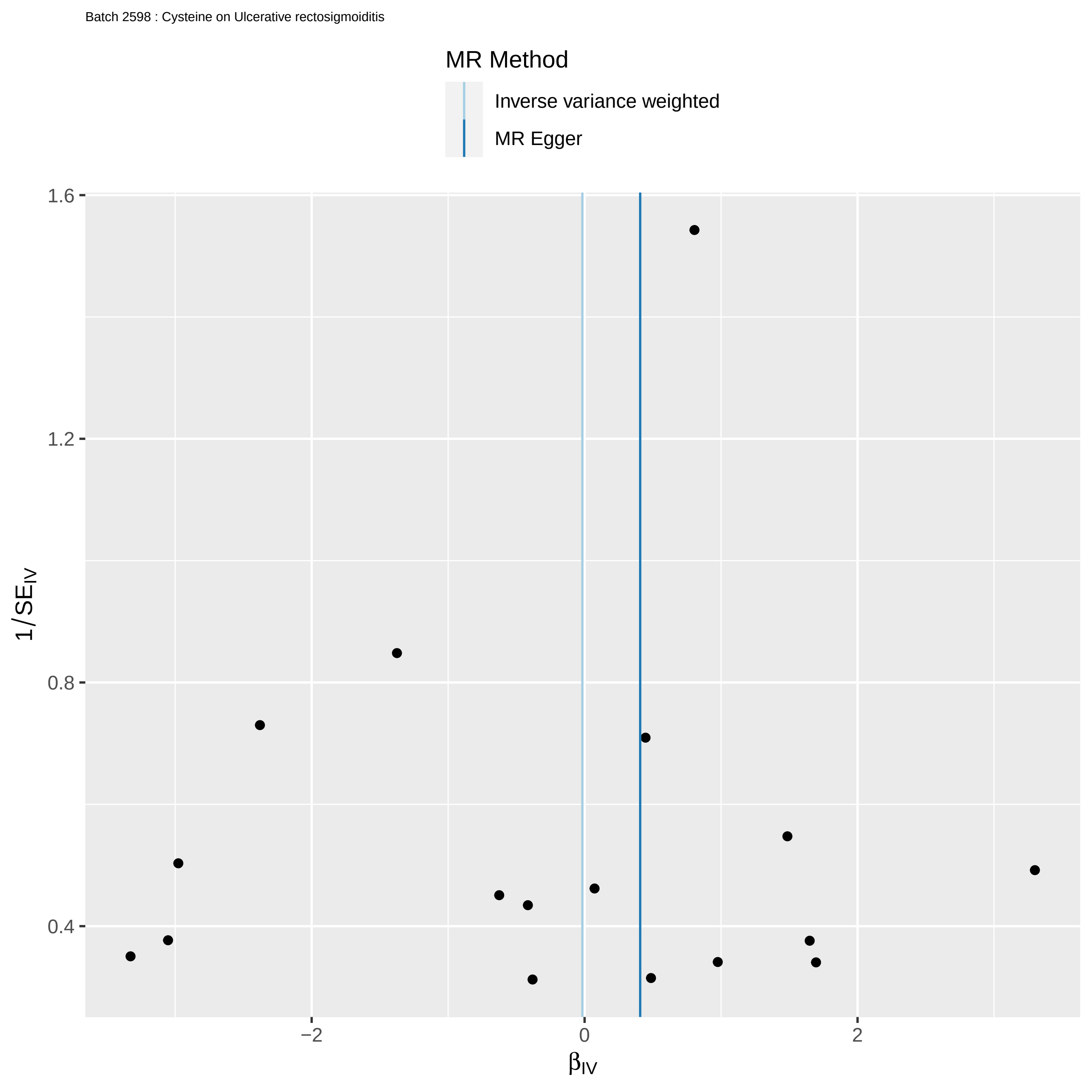
Batch 2598 : Cysteine on Ulcerative rectosigmoiditis
MR Method
Inverse variance weighted
MR Egger
1.6
1.2
0.8
0.4
1/SE_IV
−2
0
2
β_IV

Batch 2599 : Pyroglutamylglycine on Ulcerative rectosigmoiditis

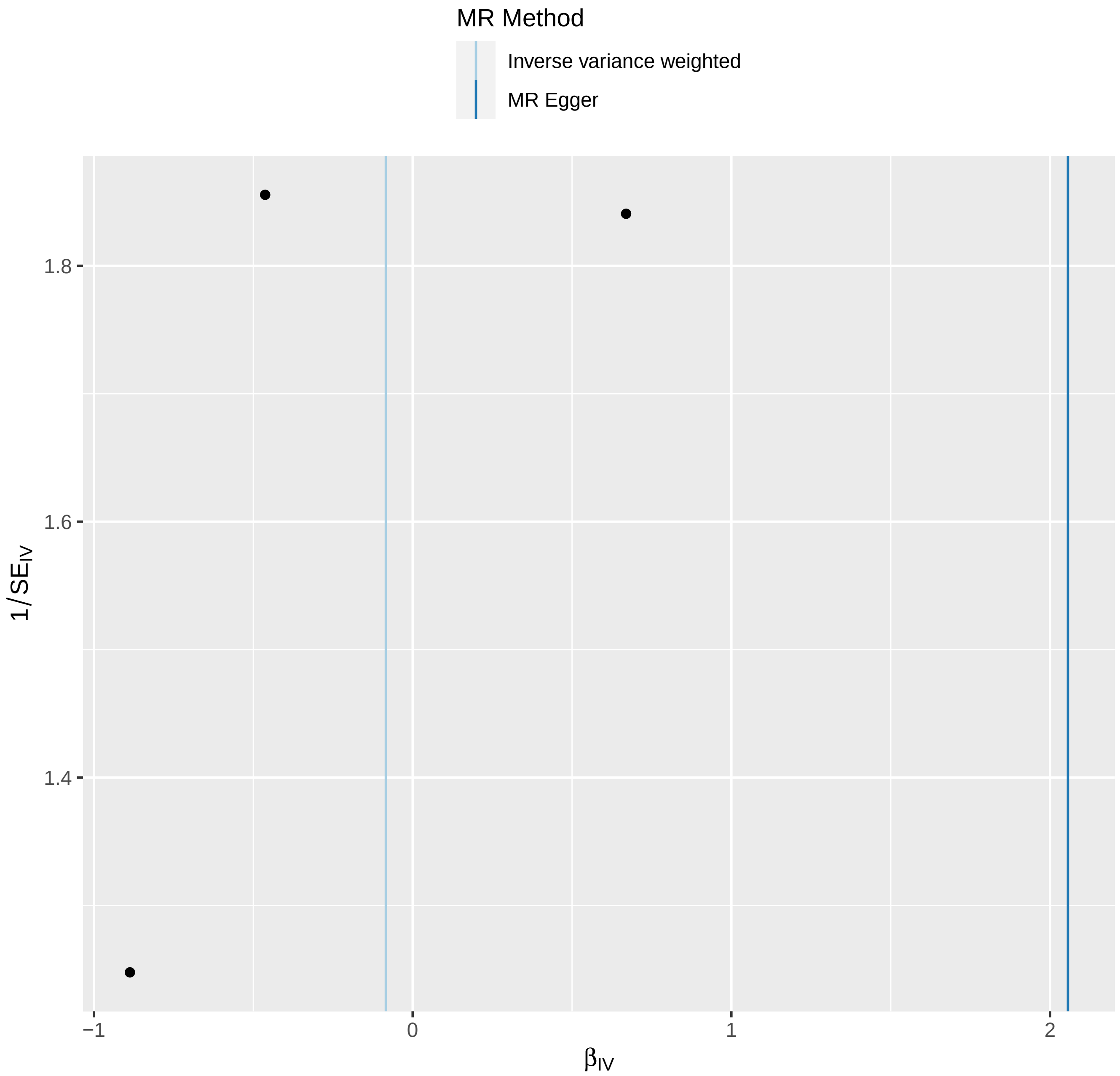

Batch 2601 : DSGEGDFXAEGGGVR* on Ulcerative rectosigmoiditis

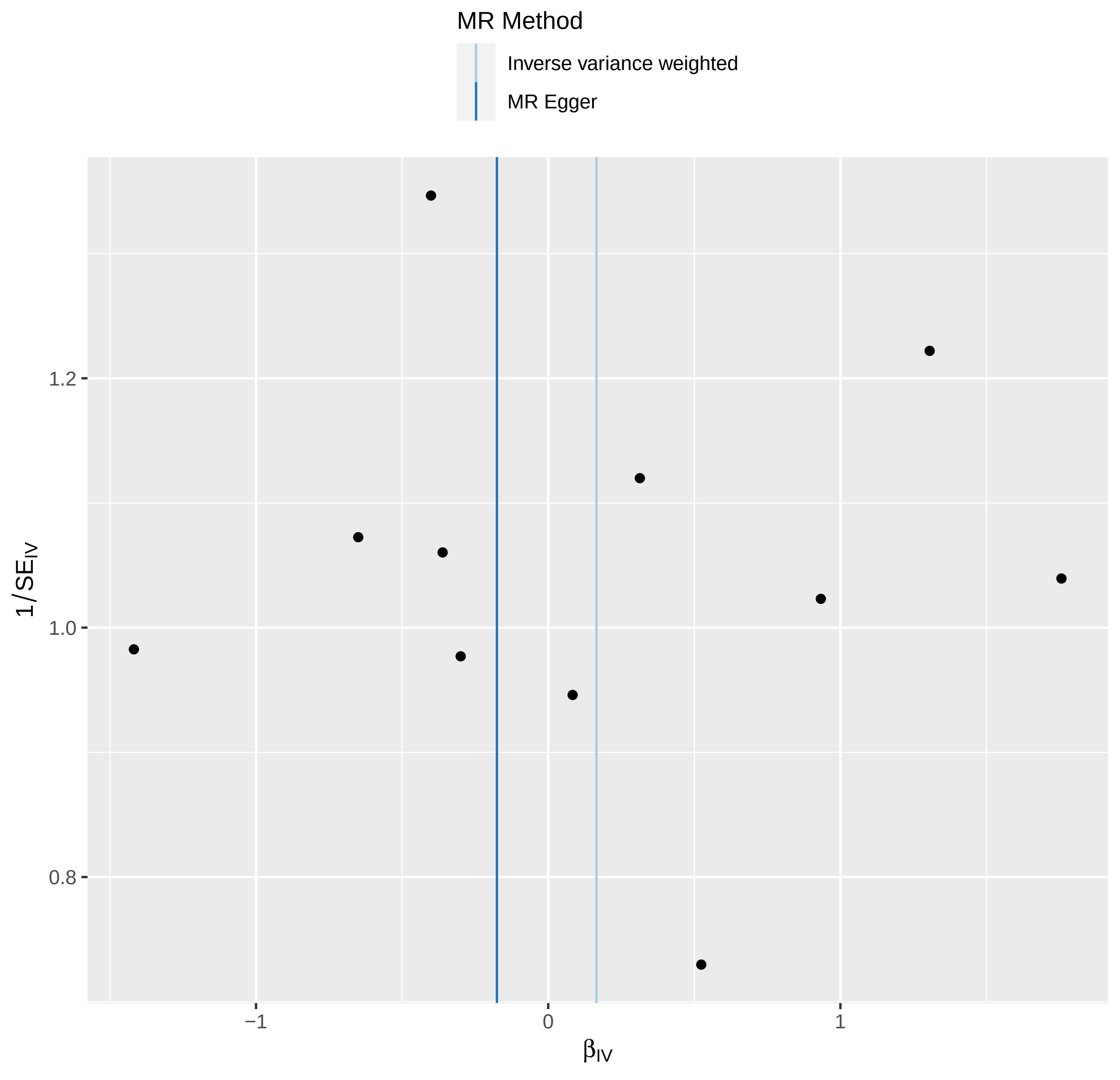

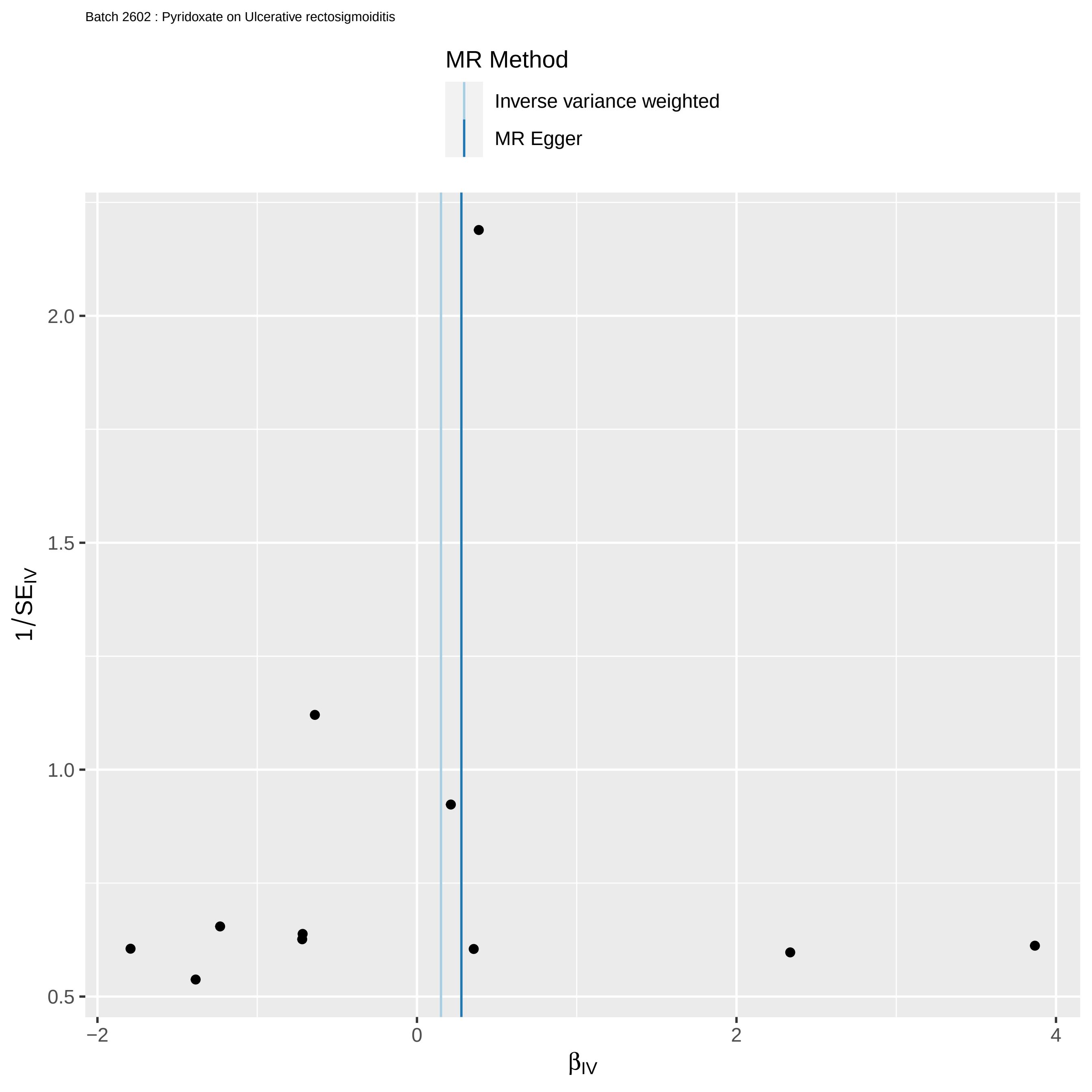
Batch 2602 : Pyridoxate on Ulcerative rectosigmoiditis
MR Method
Inverse variance weighted
MR Egger
2.0
1.5
1.0
0.5
1/SE$_{IV}$
−2
0
2
4
β$_{IV}$

Batch 2603 : Androsterone sulfate on Ulcerative rectosigmoiditis

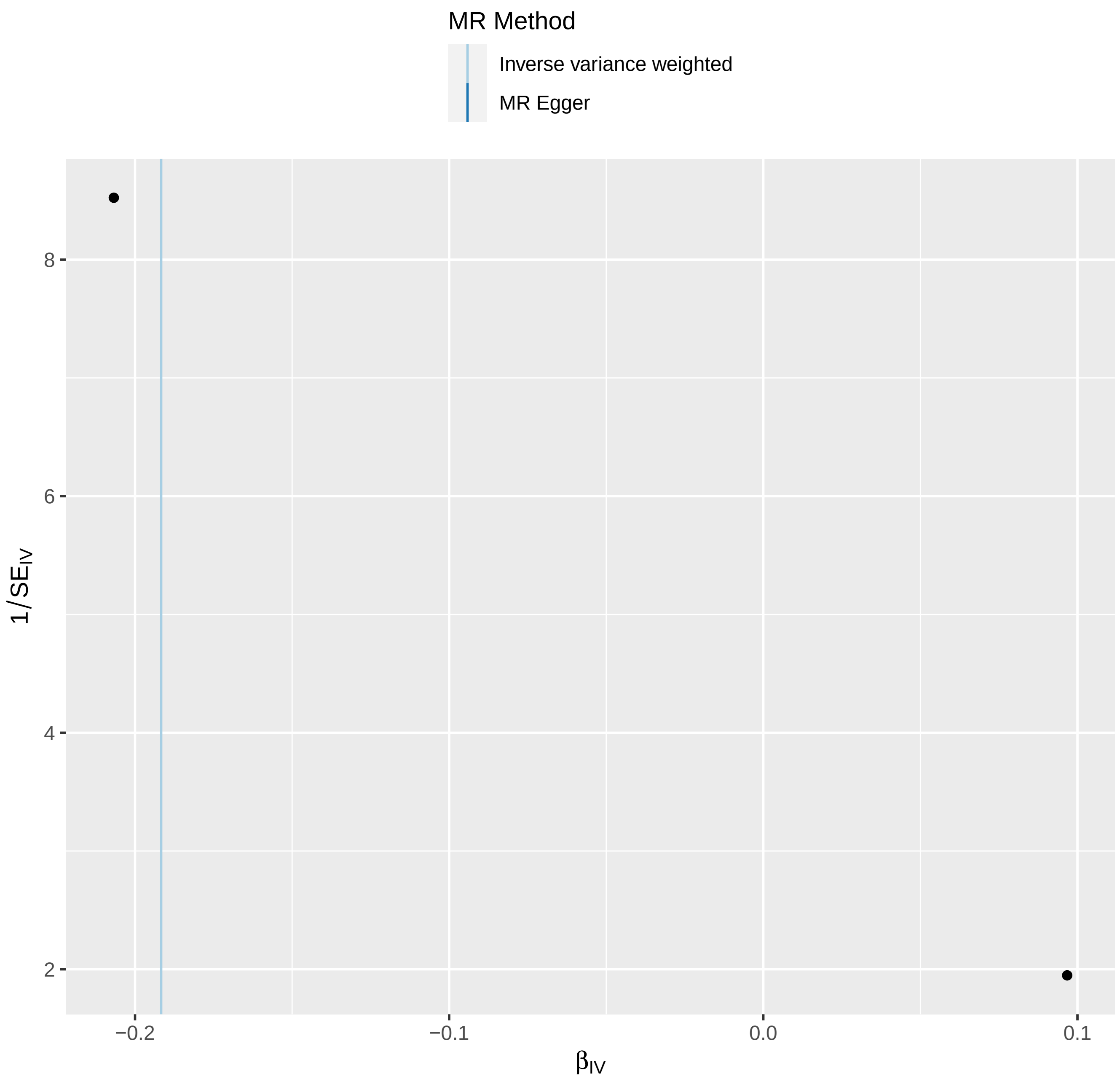

Batch 2607 : Serine on Ulcerative rectosigmoiditis

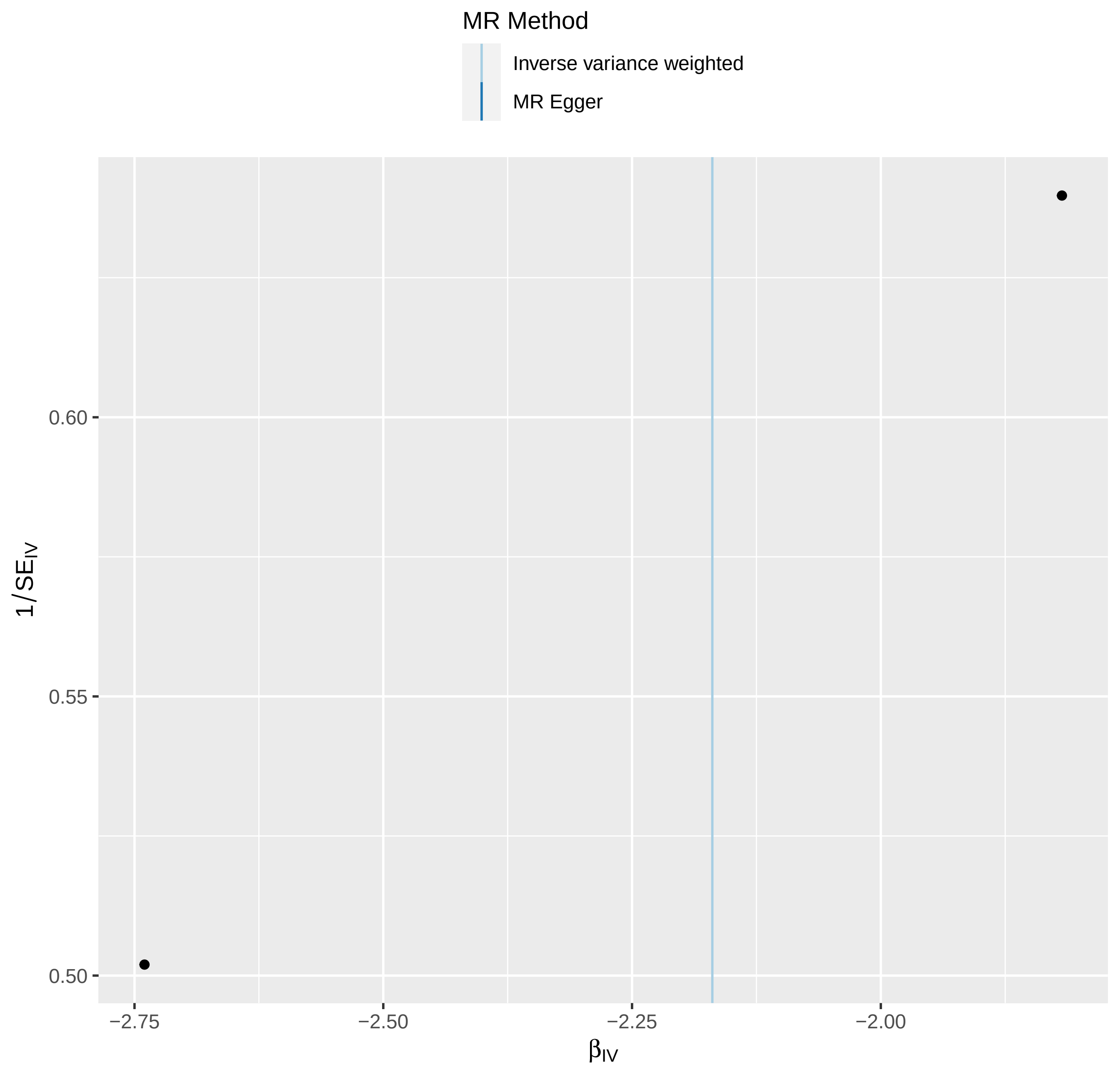

Batch 2608 : Trans−4−hydroxyproline on Ulcerative rectosigmoiditis

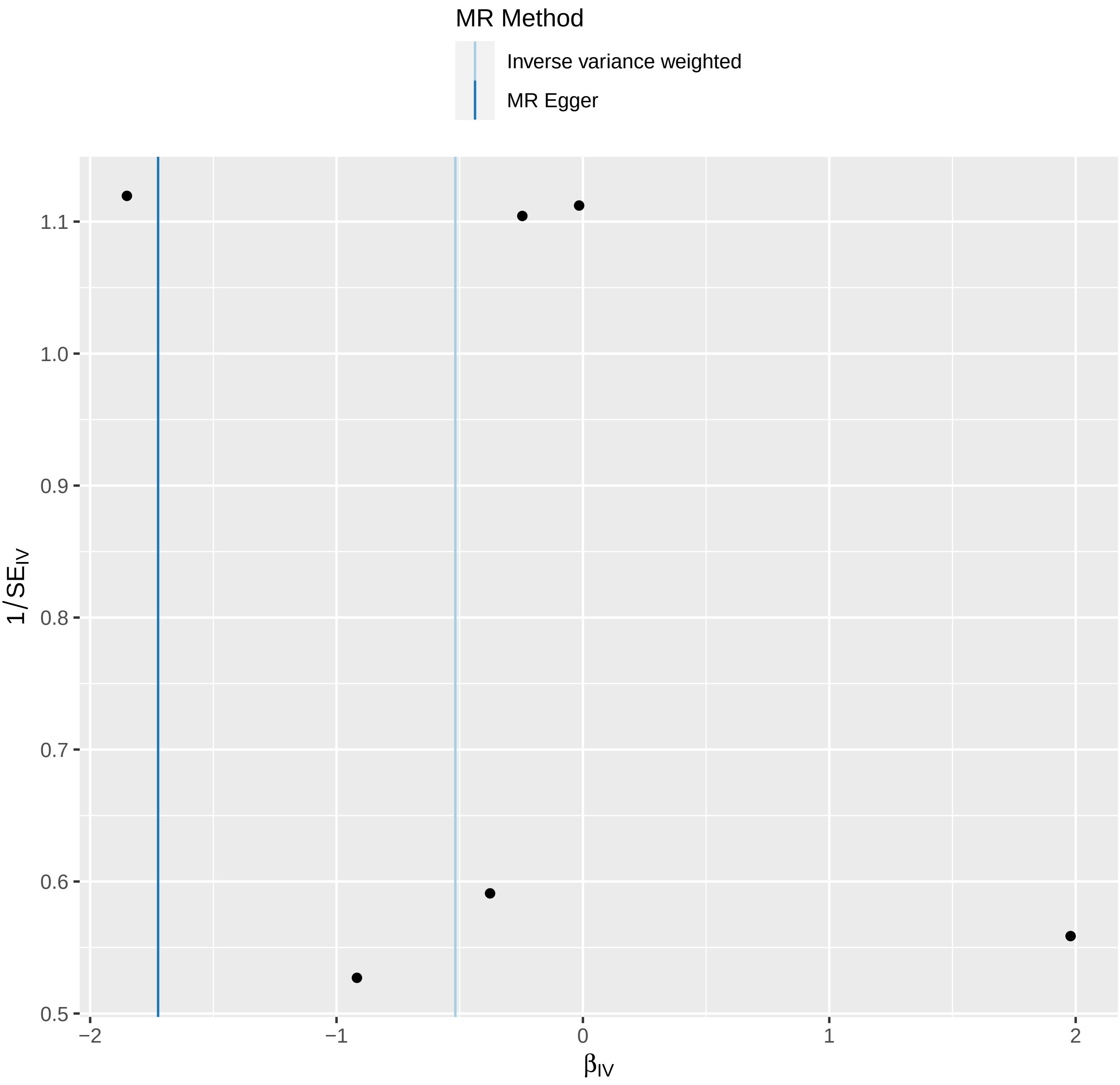

Batch 2610 : Hexanoylcarnitine on Ulcerative rectosigmoiditis

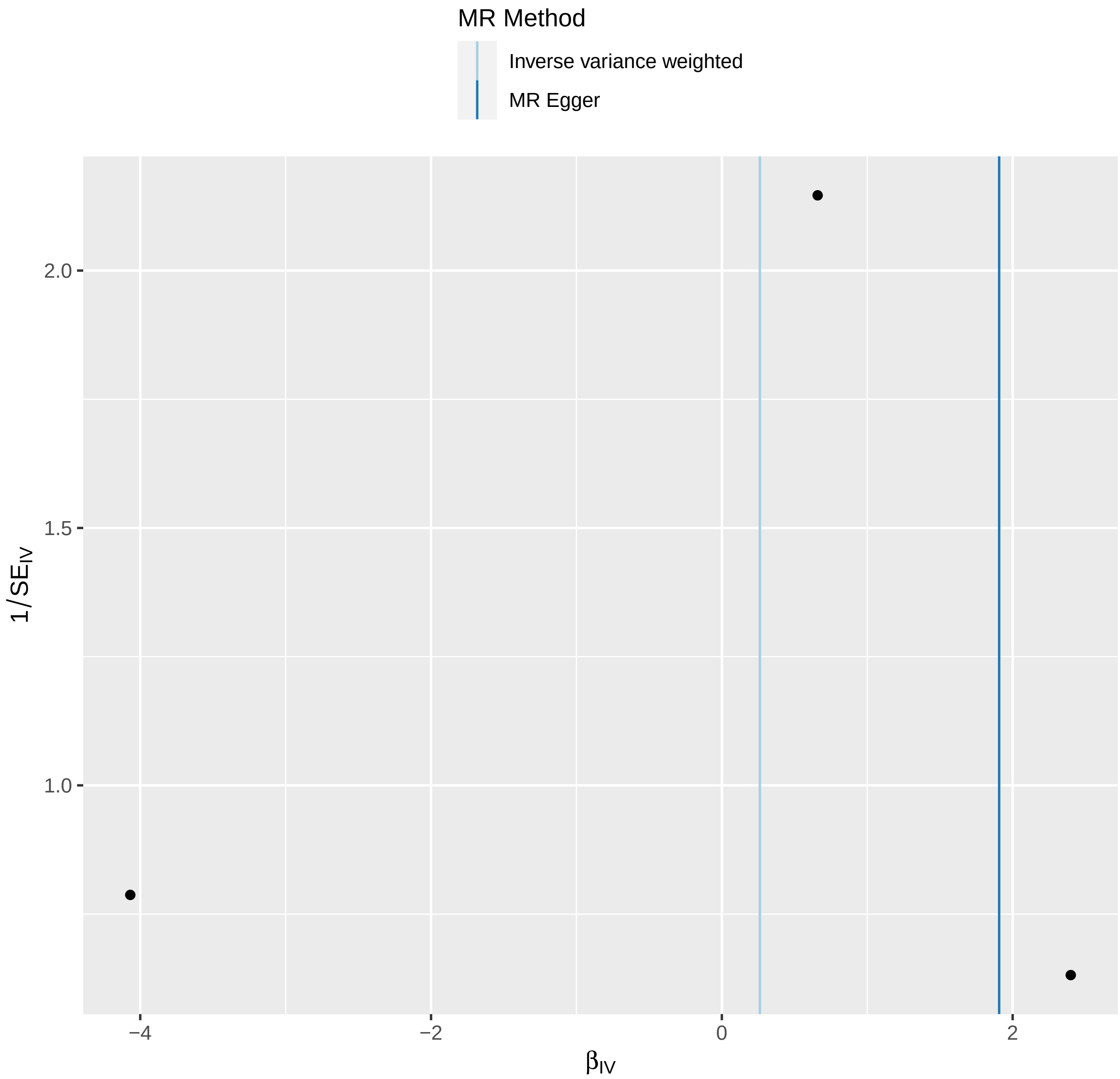

Batch 2619 : Butyrylcarnitine on Ulcerative rectosigmoiditis

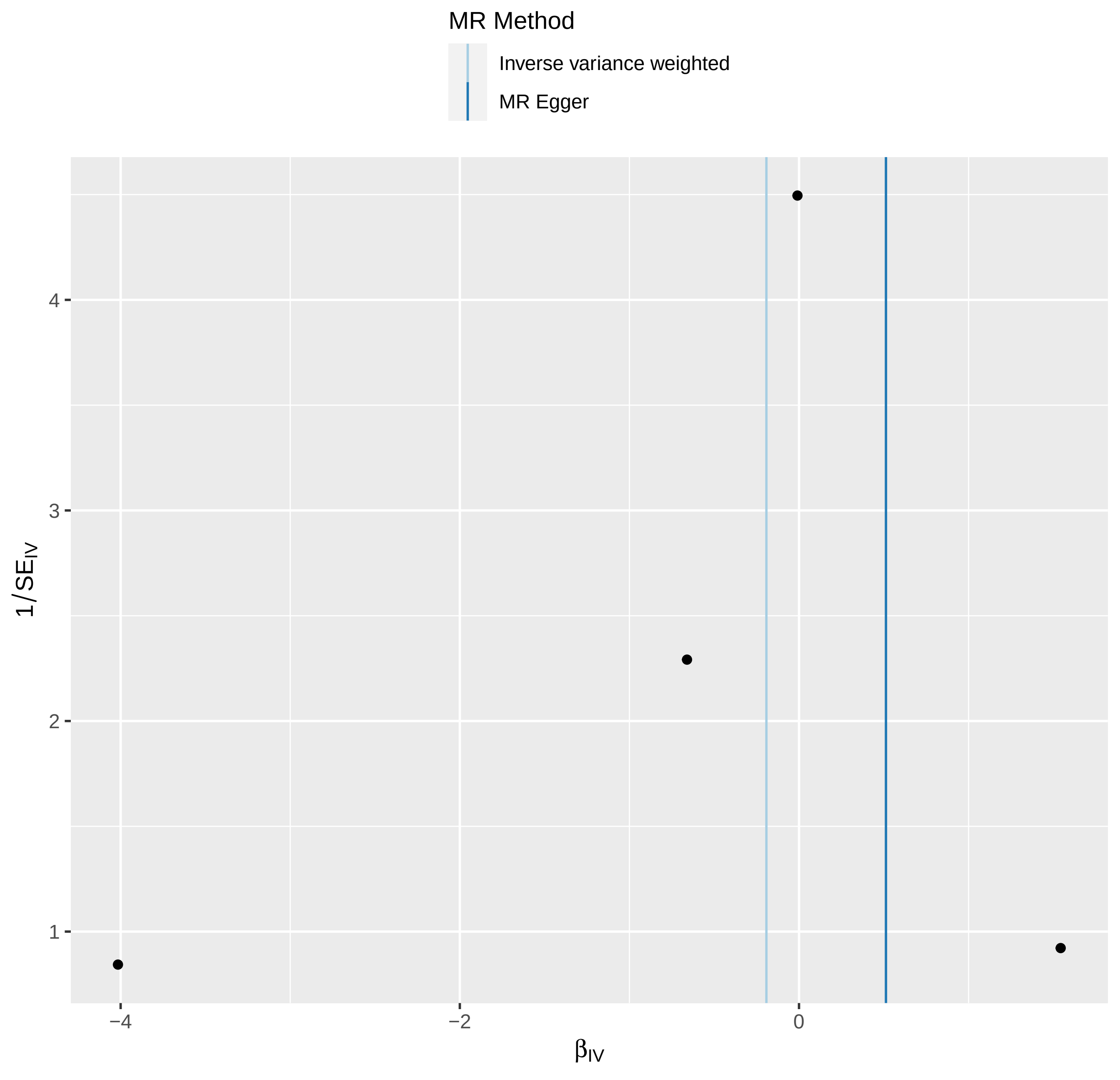

Batch 2622 : Propionylcarnitine on Ulcerative rectosigmoiditis

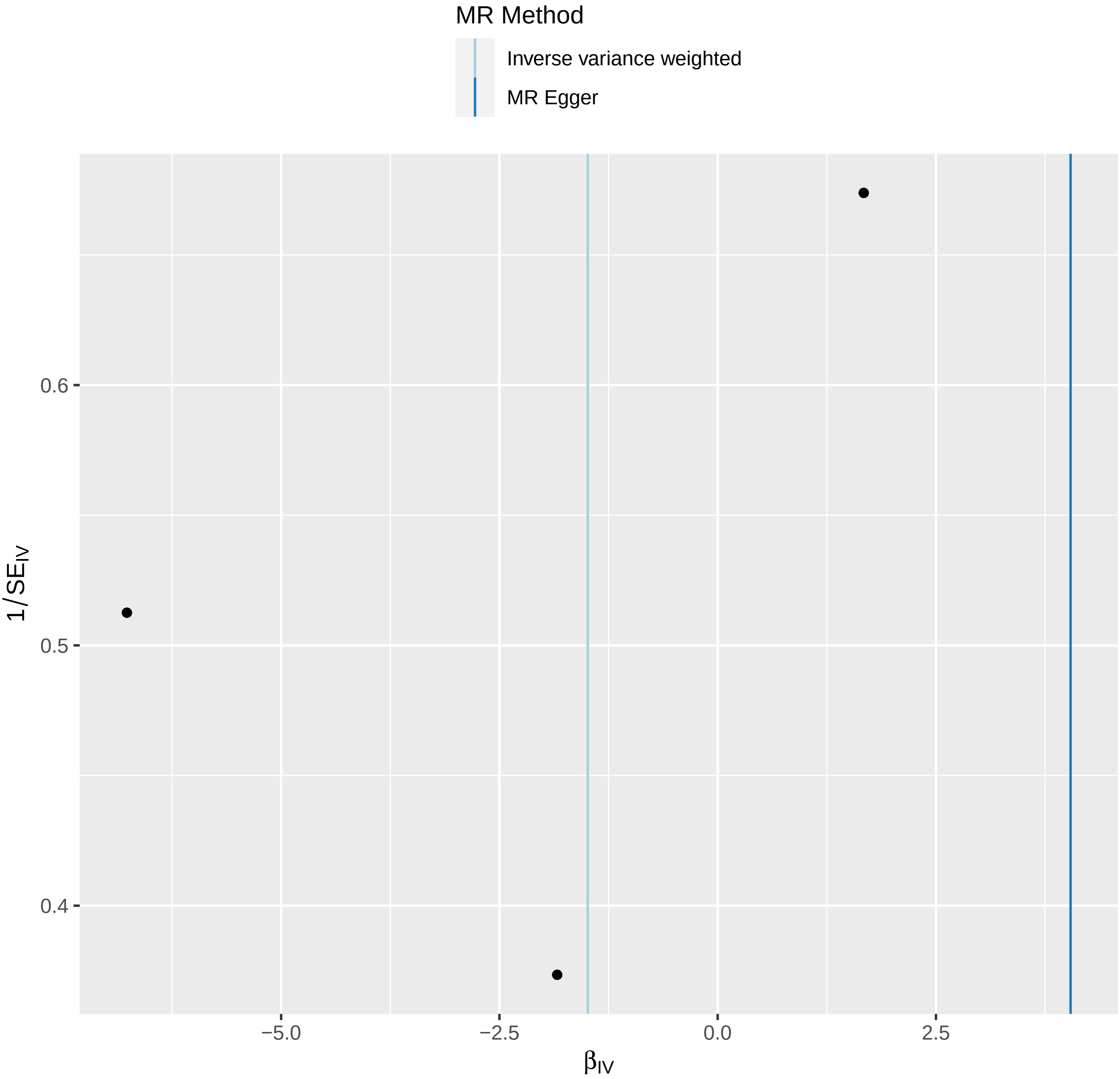

Batch 2623 : Caproate (6:0) on Ulcerative rectosigmoiditis

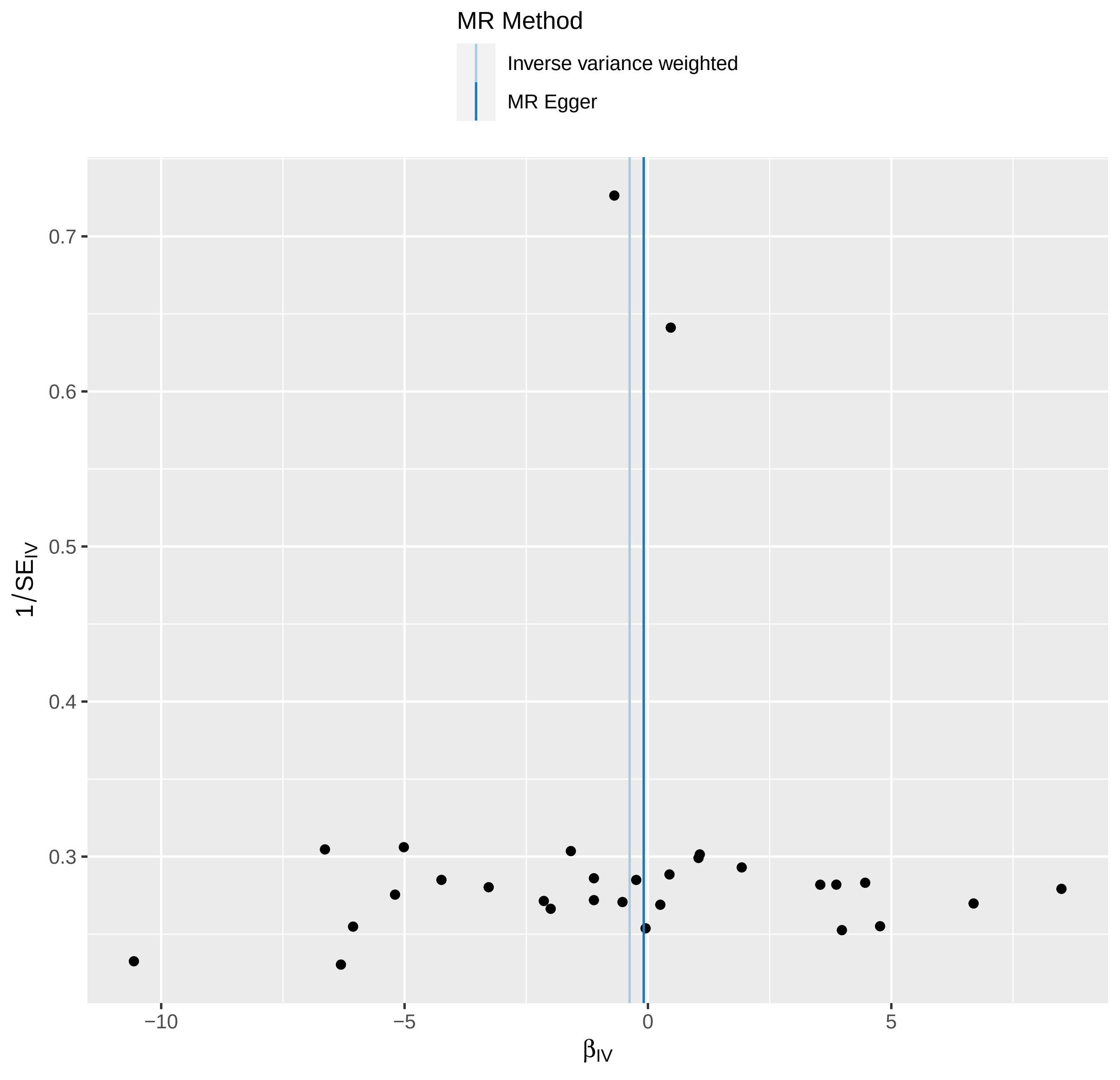

Batch 2624 : Caprylate (8:0) on Ulcerative rectosigmoiditis

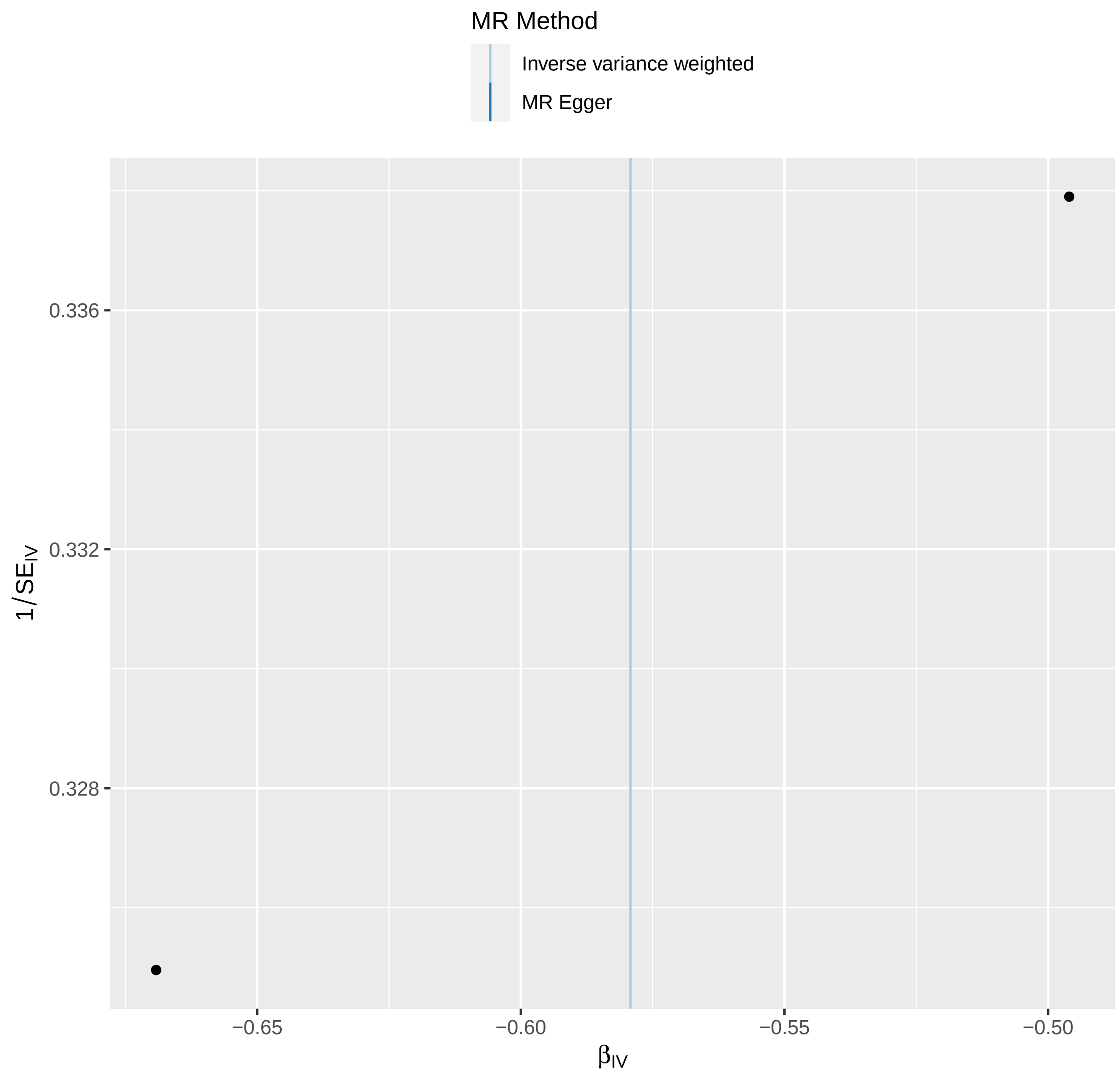

Batch 2625 : 10−undecenoate (11:1n1) on Ulcerative rectosigmoiditis

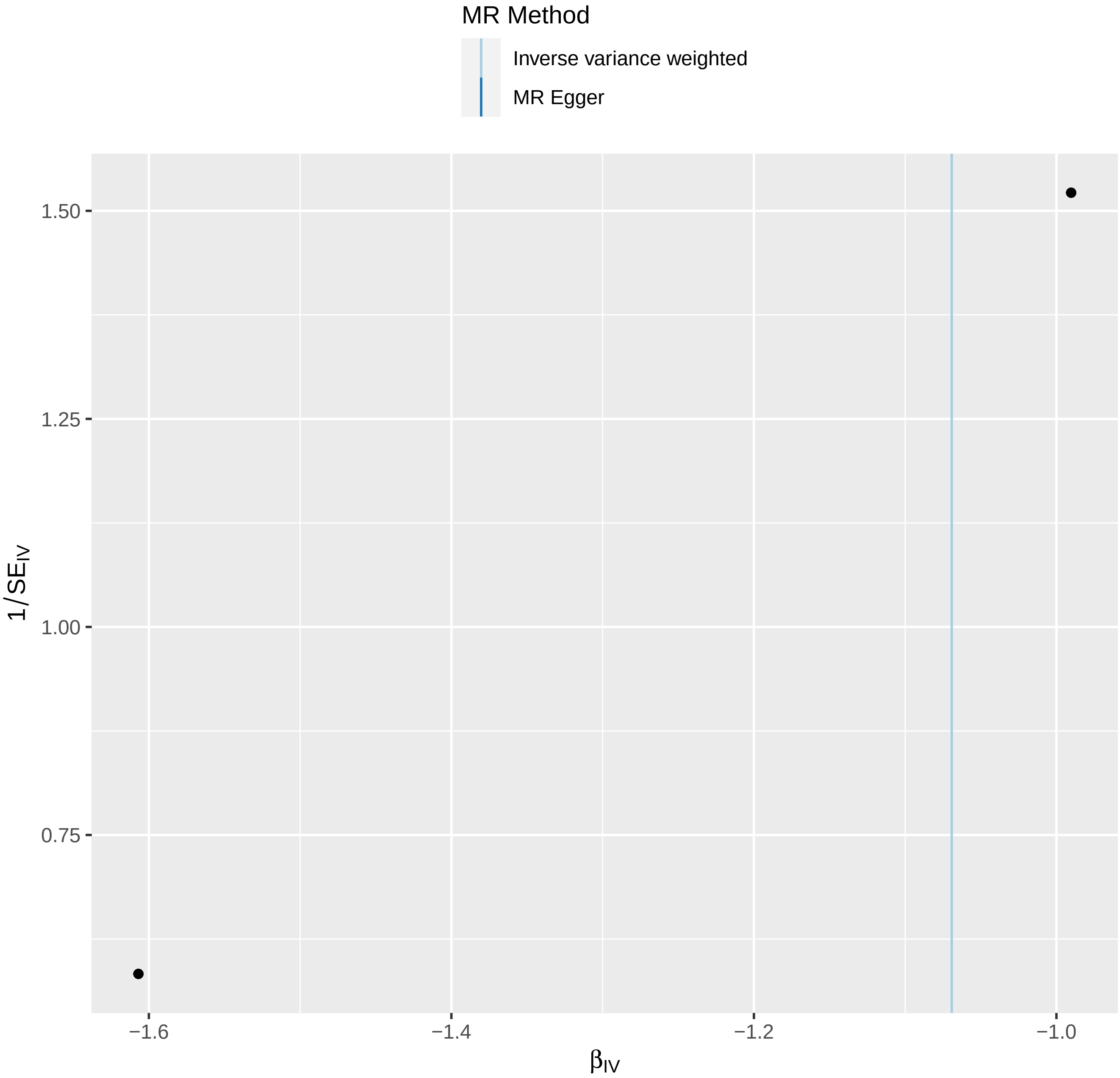

Batch 2627 : Phenol sulfate on Ulcerative rectosigmoiditis

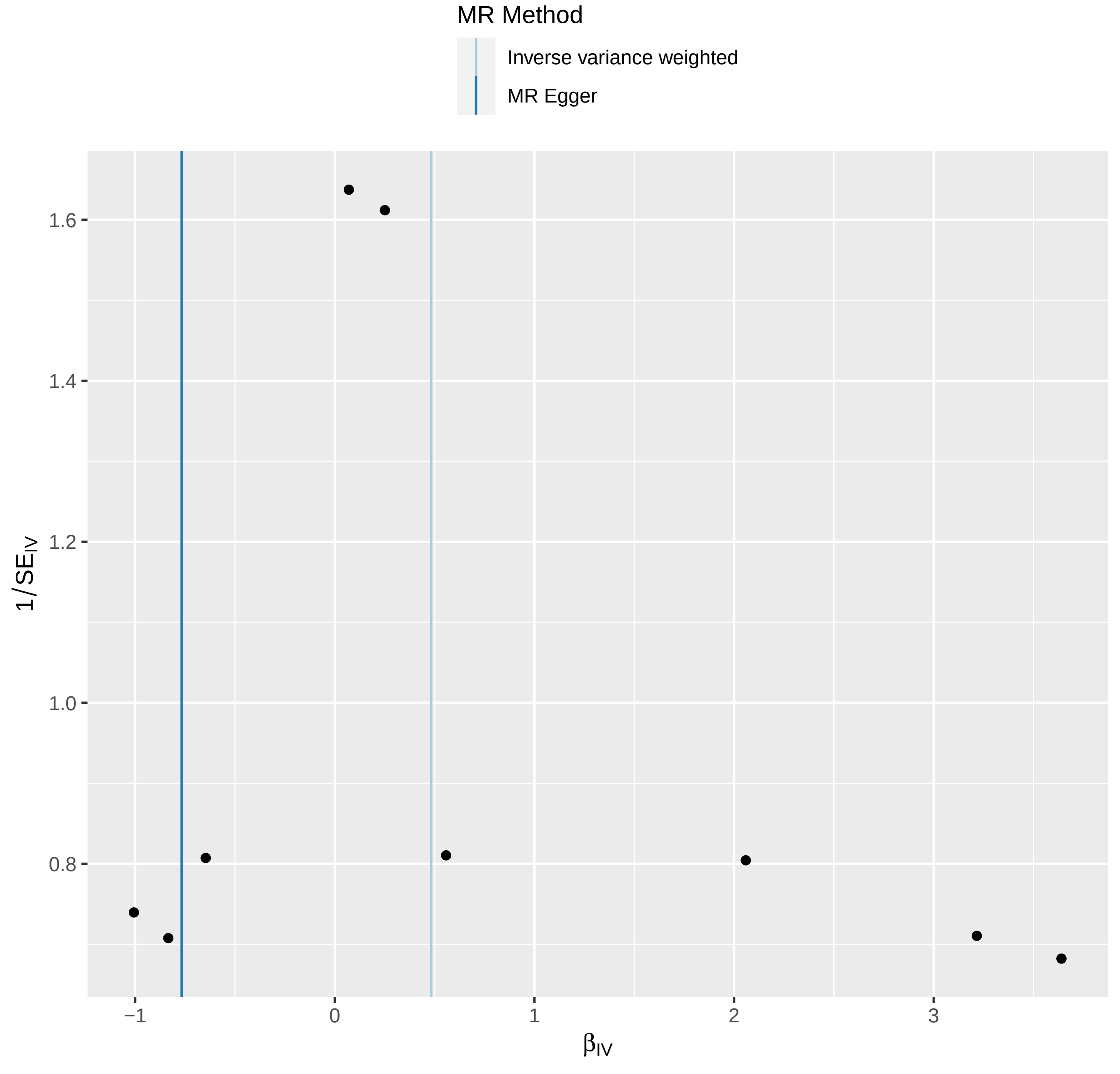

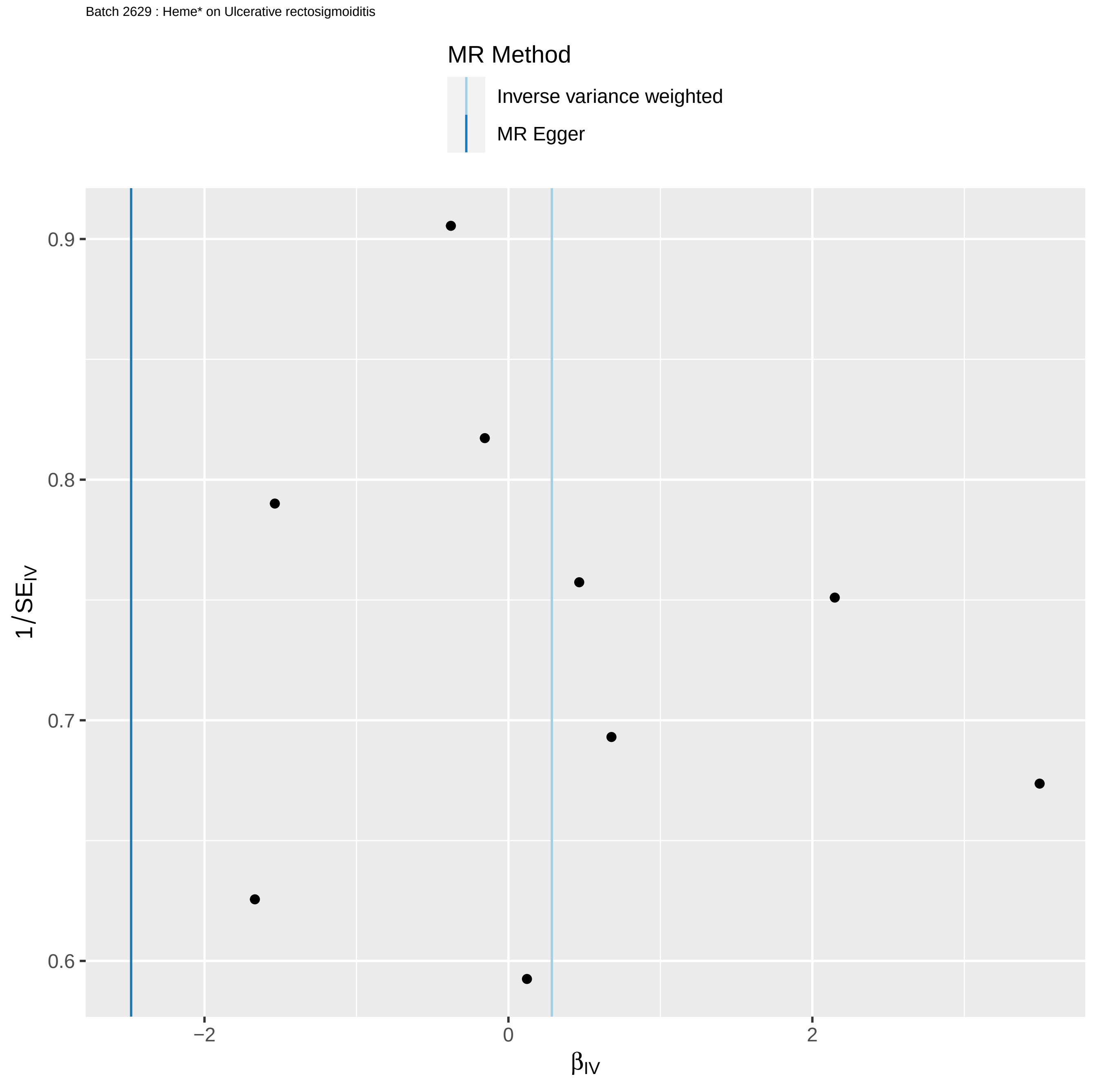
Batch 2629 : Heme* on Ulcerative rectosigmoiditis
MR Method
Inverse variance weighted
MR Egger
0.9
0.8
0.7
0.6
1/SE_IV
−2
0
2
β_IV

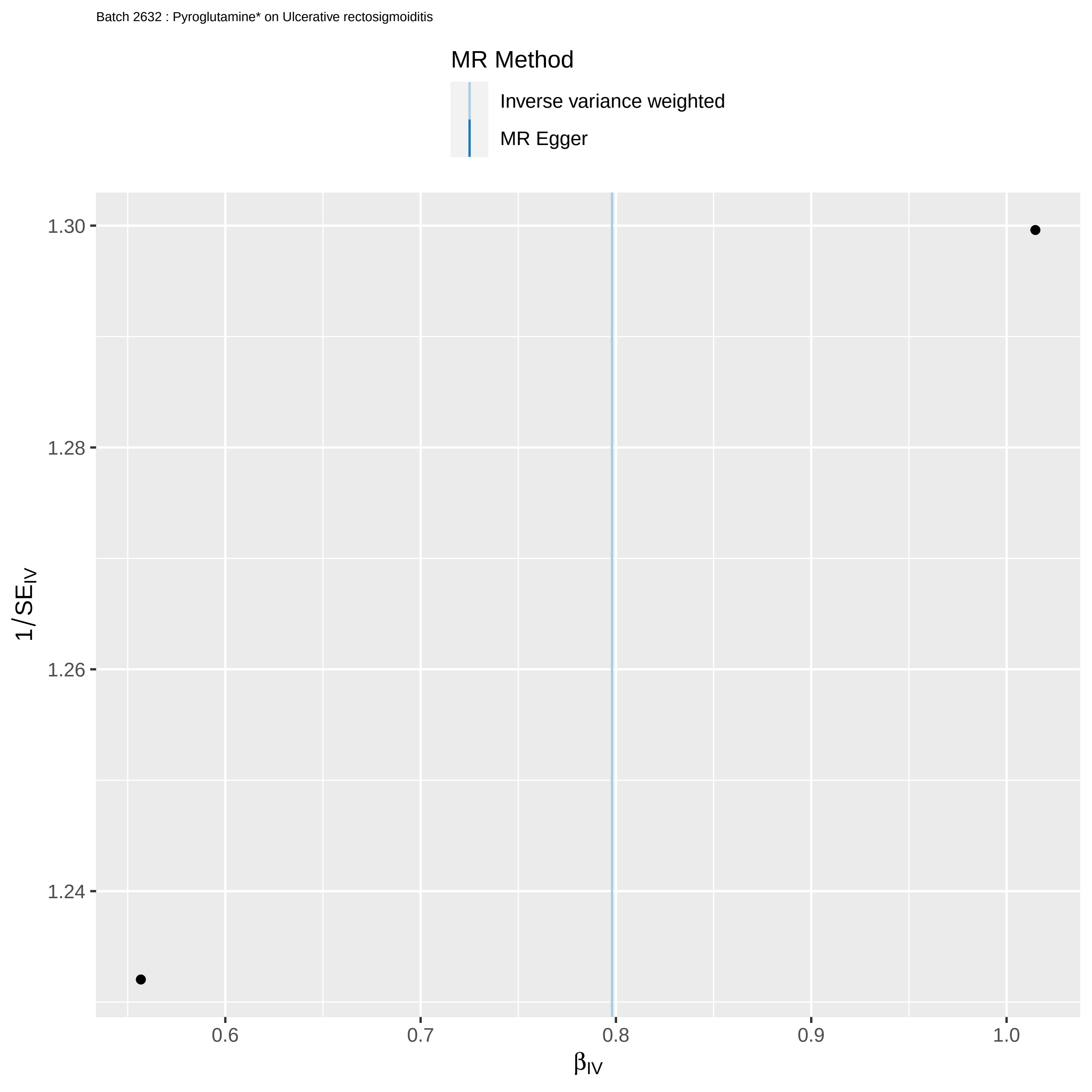

Batch 2632 : Pyroglutamine* on Ulcerative rectosigmoiditis
MR Method
Inverse variance weighted
MR Egger
1.30
1.28
1.26
1.24
1/SE_IV
0.6
0.7
0.8
0.9
1.0
β_IV

Batch 2635 : X−11422−−xanthine on Ulcerative rectosigmoiditis

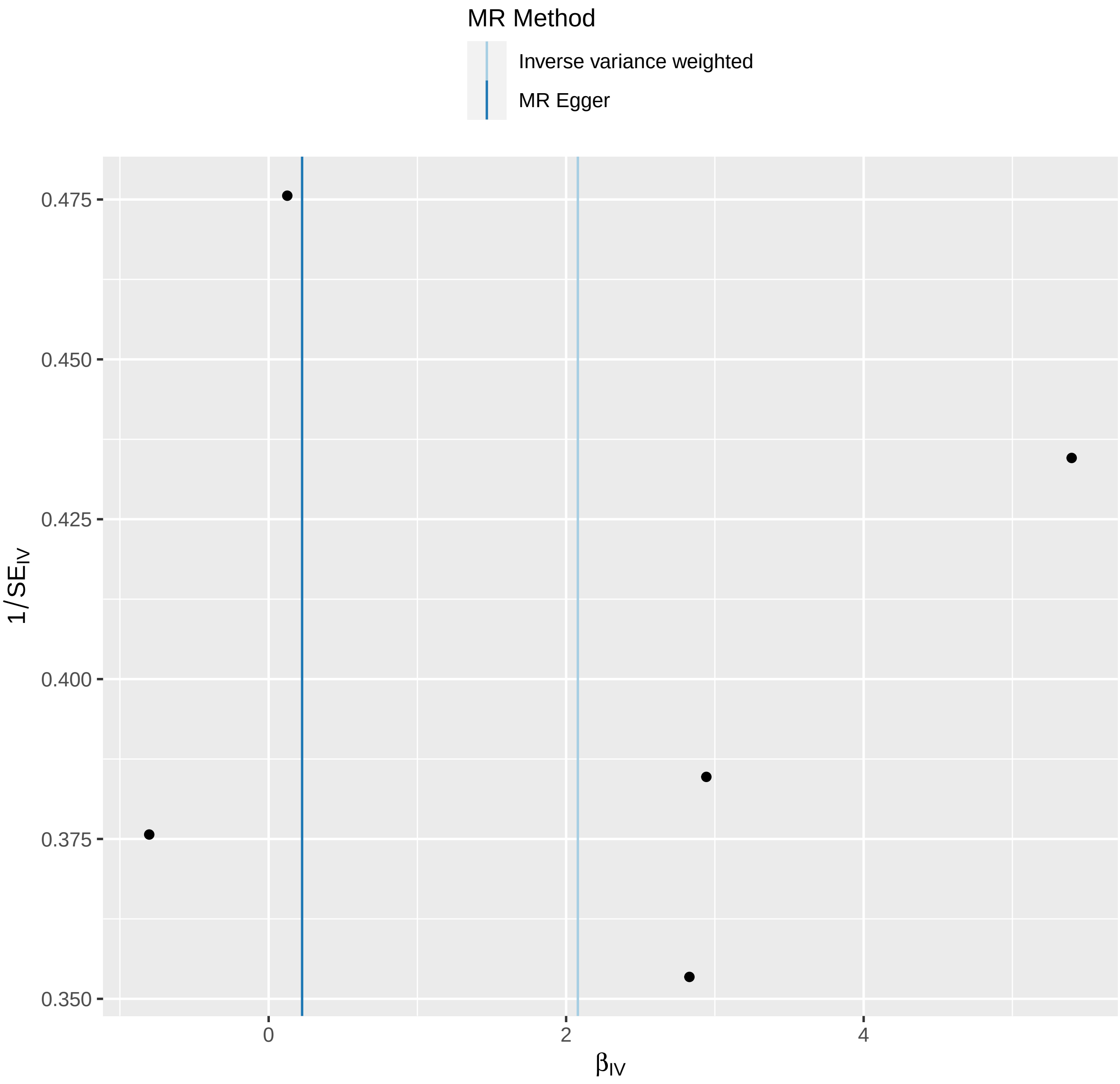

Batch 2638 : HWESASXX* on Ulcerative rectosigmoiditis

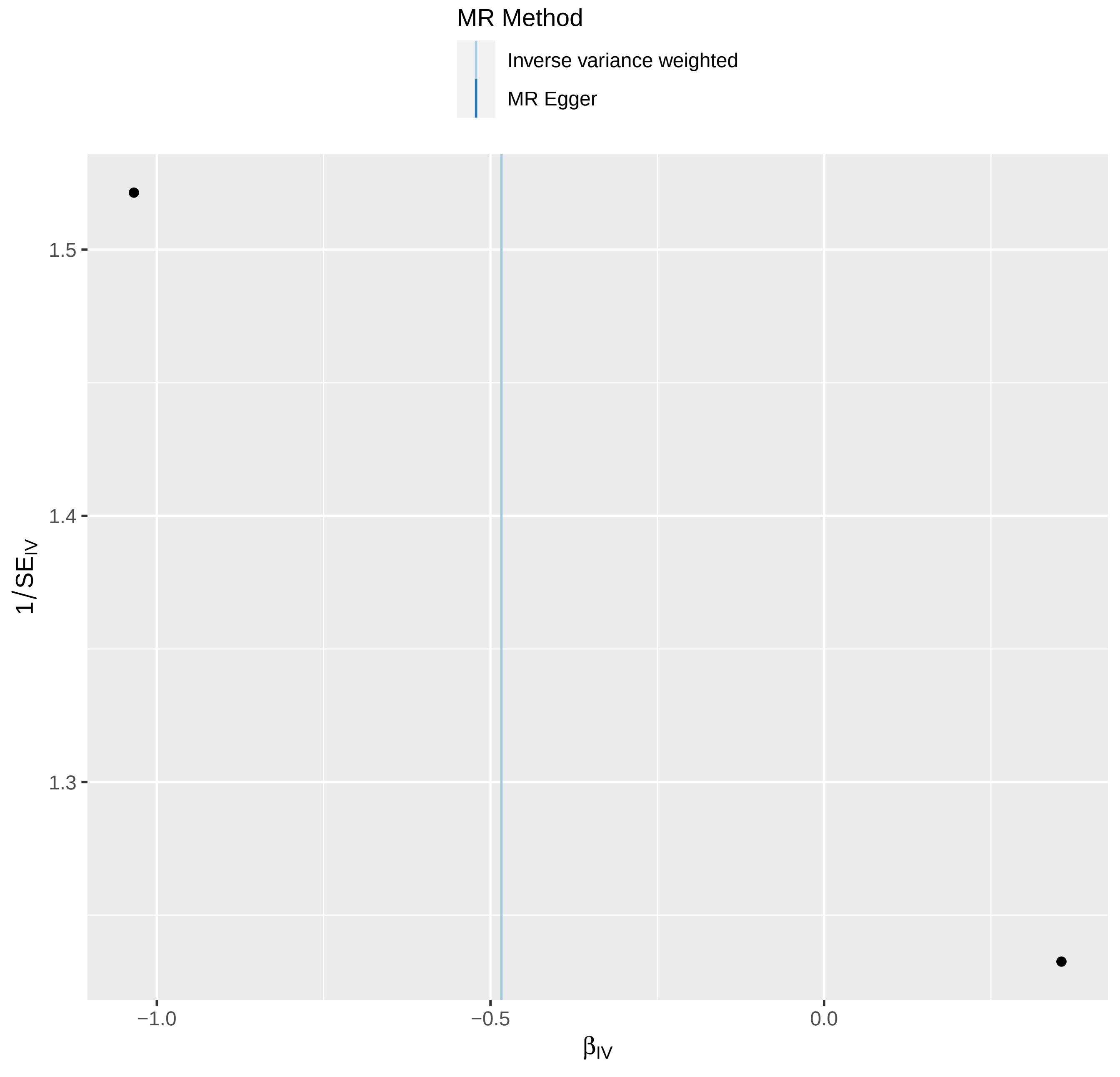

Batch 2639 : X−11593−−O−methylascorbate* on Ulcerative rectosigmoiditis

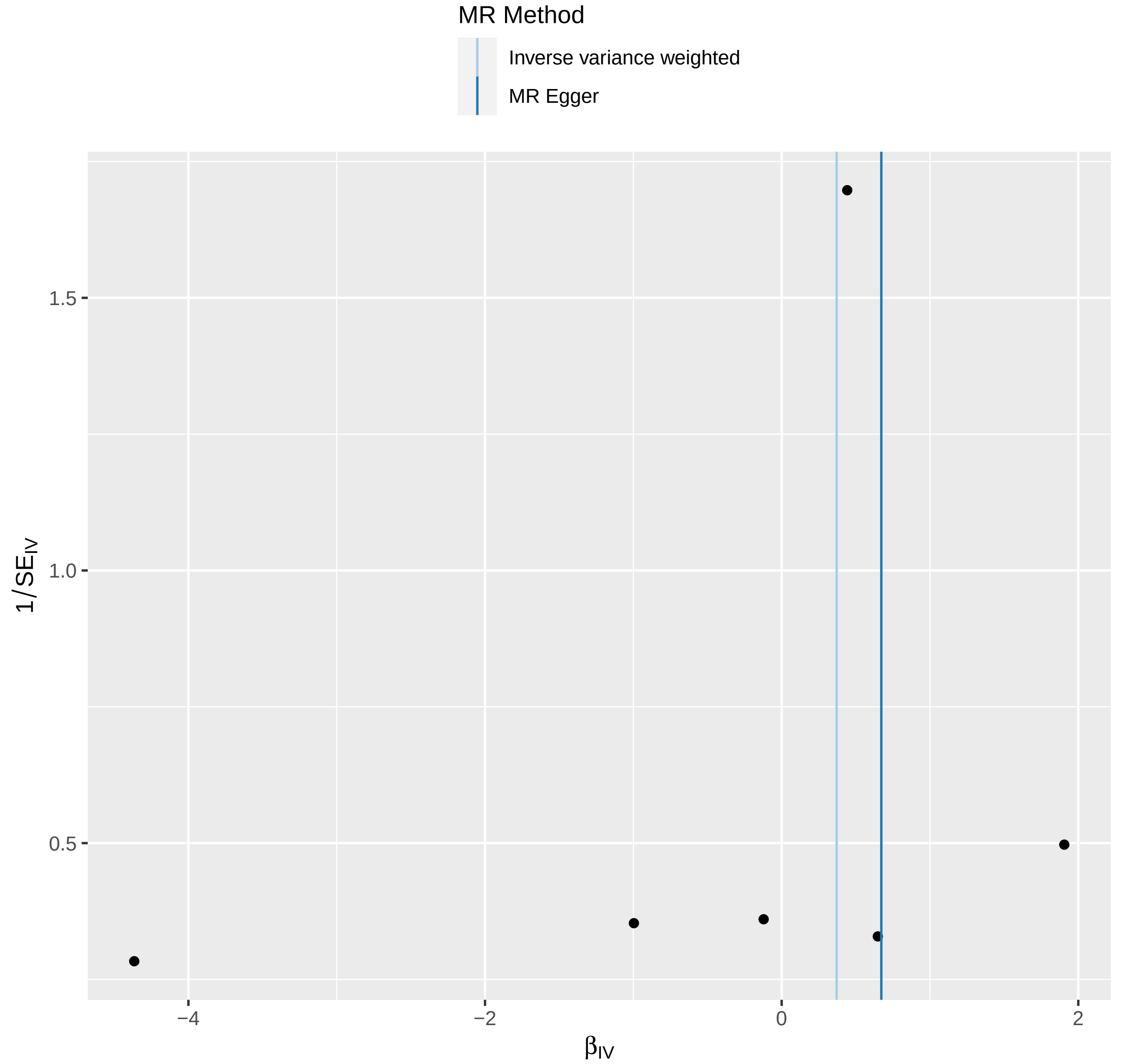

Batch 2641 : Homostachydrine* on Ulcerative rectosigmoiditis

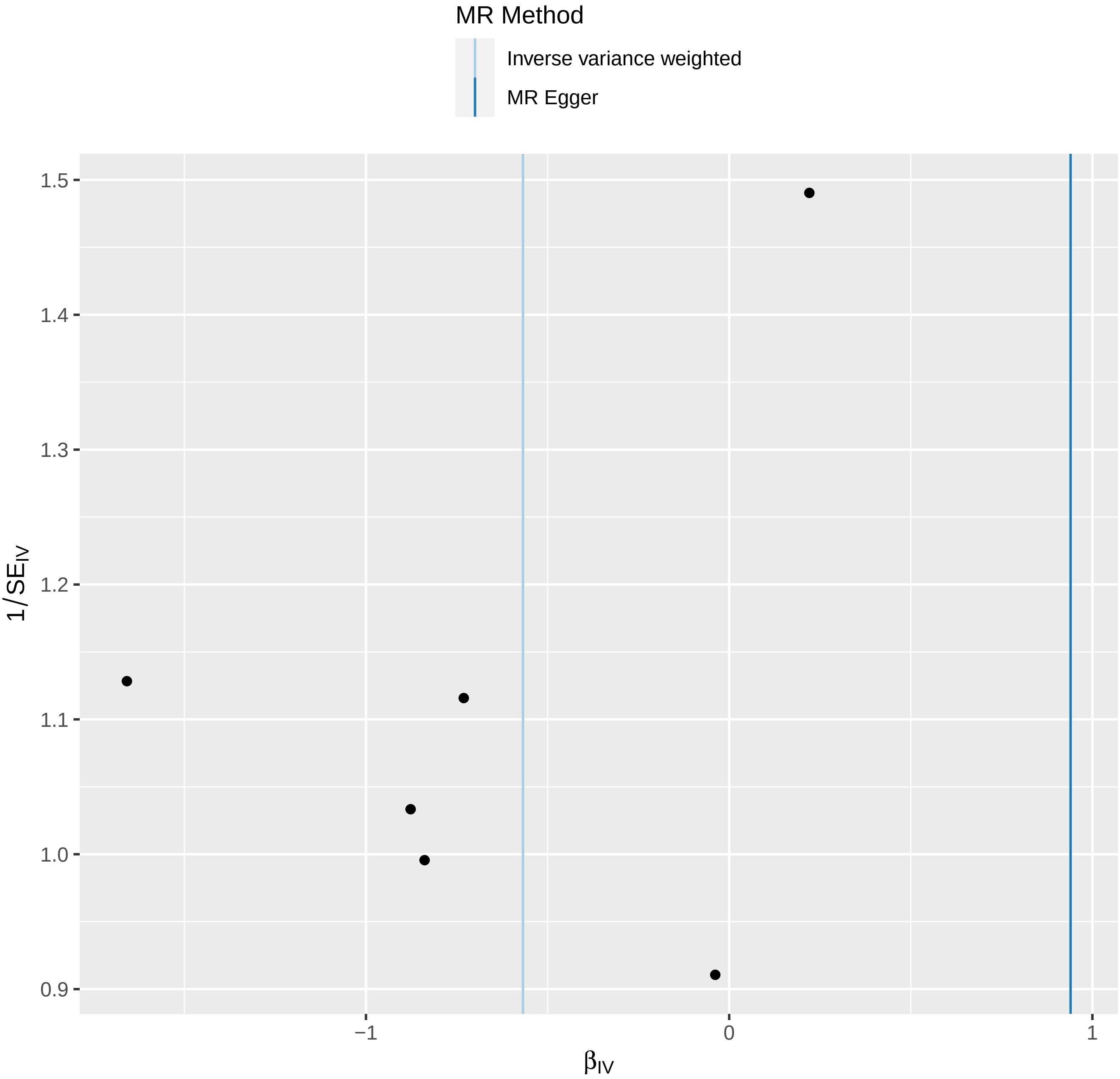

Batch 2642 : ADSGEGDFXAEGGGVR* on Ulcerative rectosigmoiditis

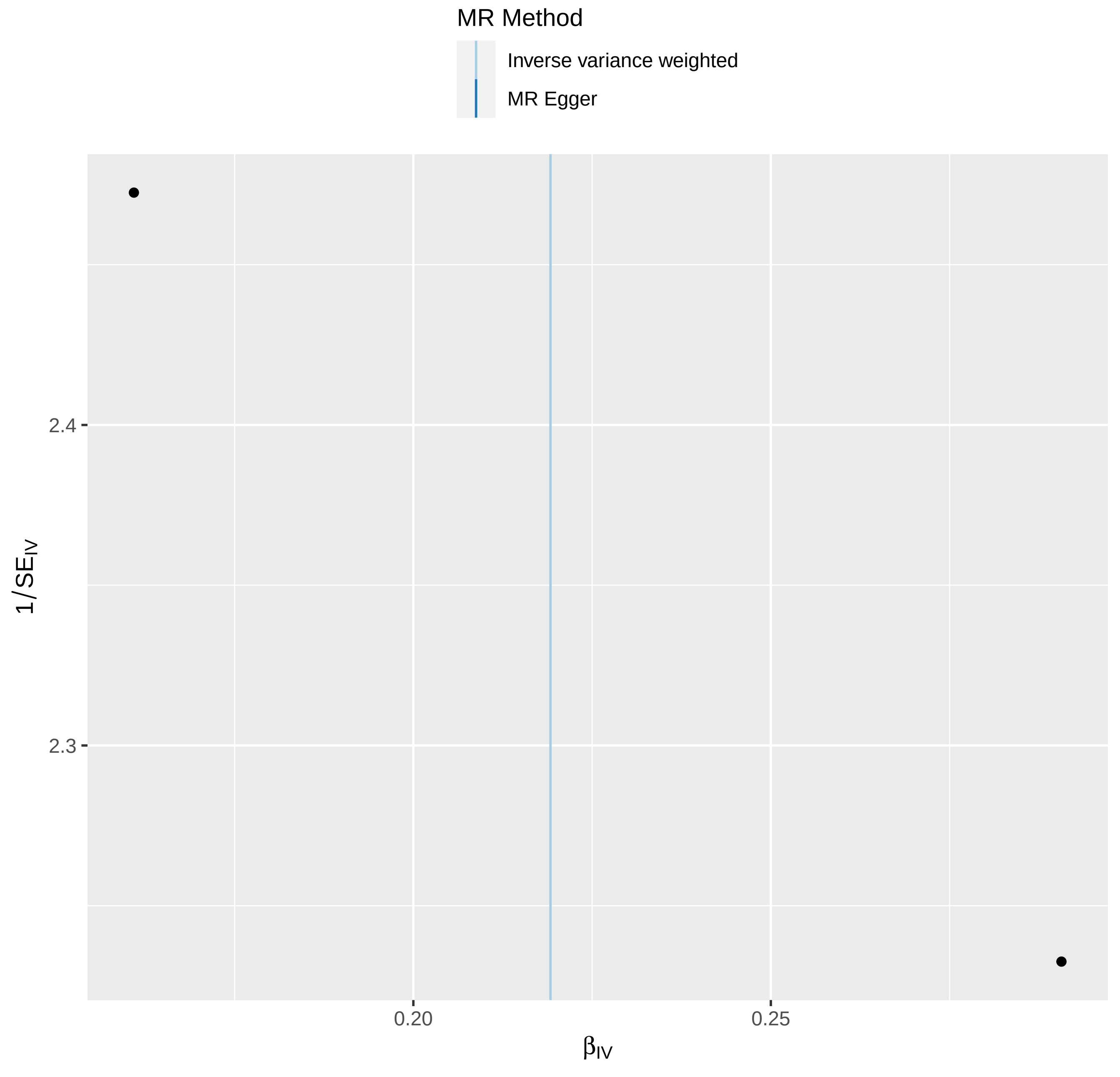

Batch 2644 : X−11793−−oxidized bilirubin* on Ulcerative rectosigmoiditis

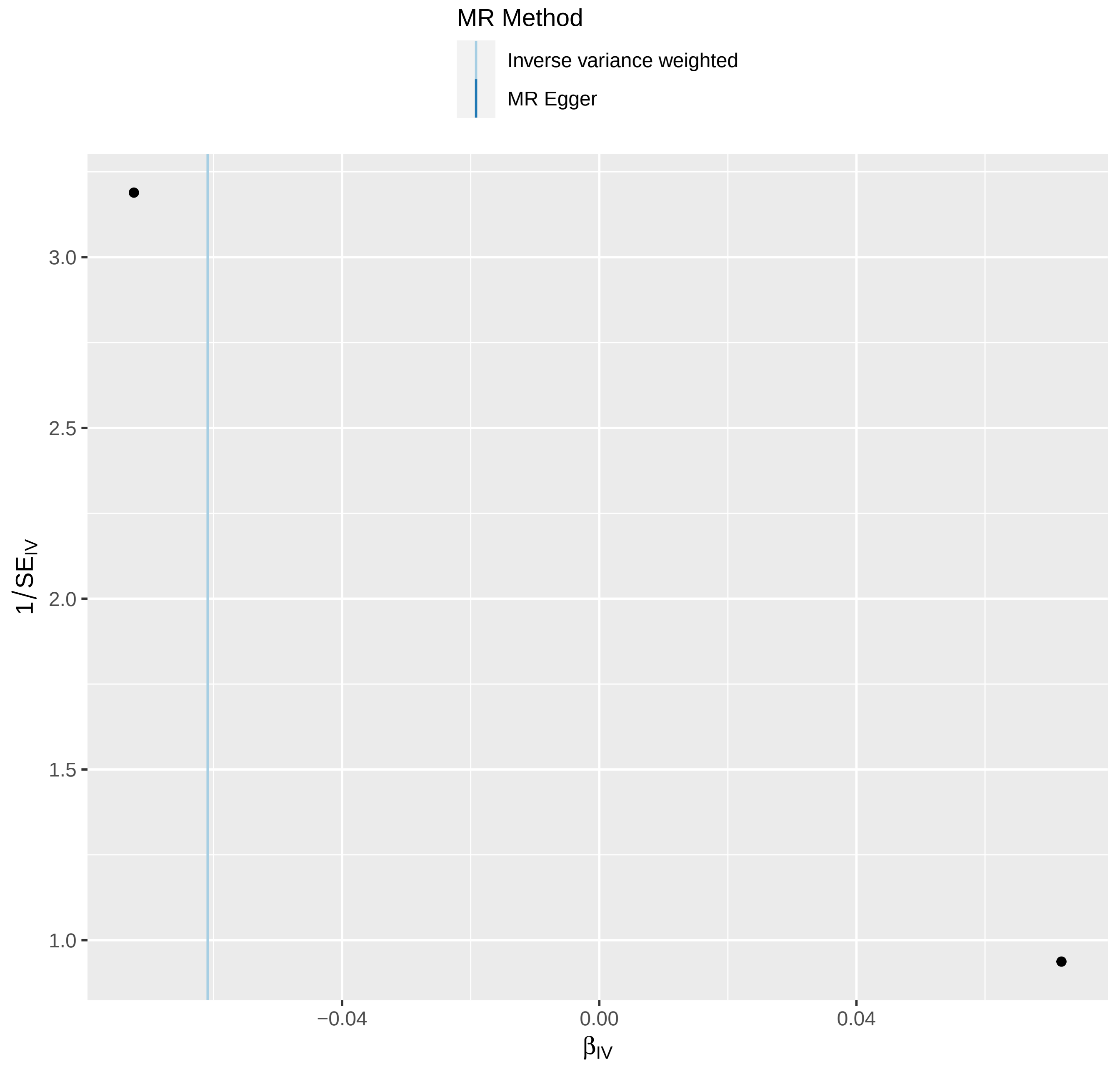

Batch 2645 : 1−arachidonoylglycerophosphocholine* on Ulcerative rectosigmoiditis

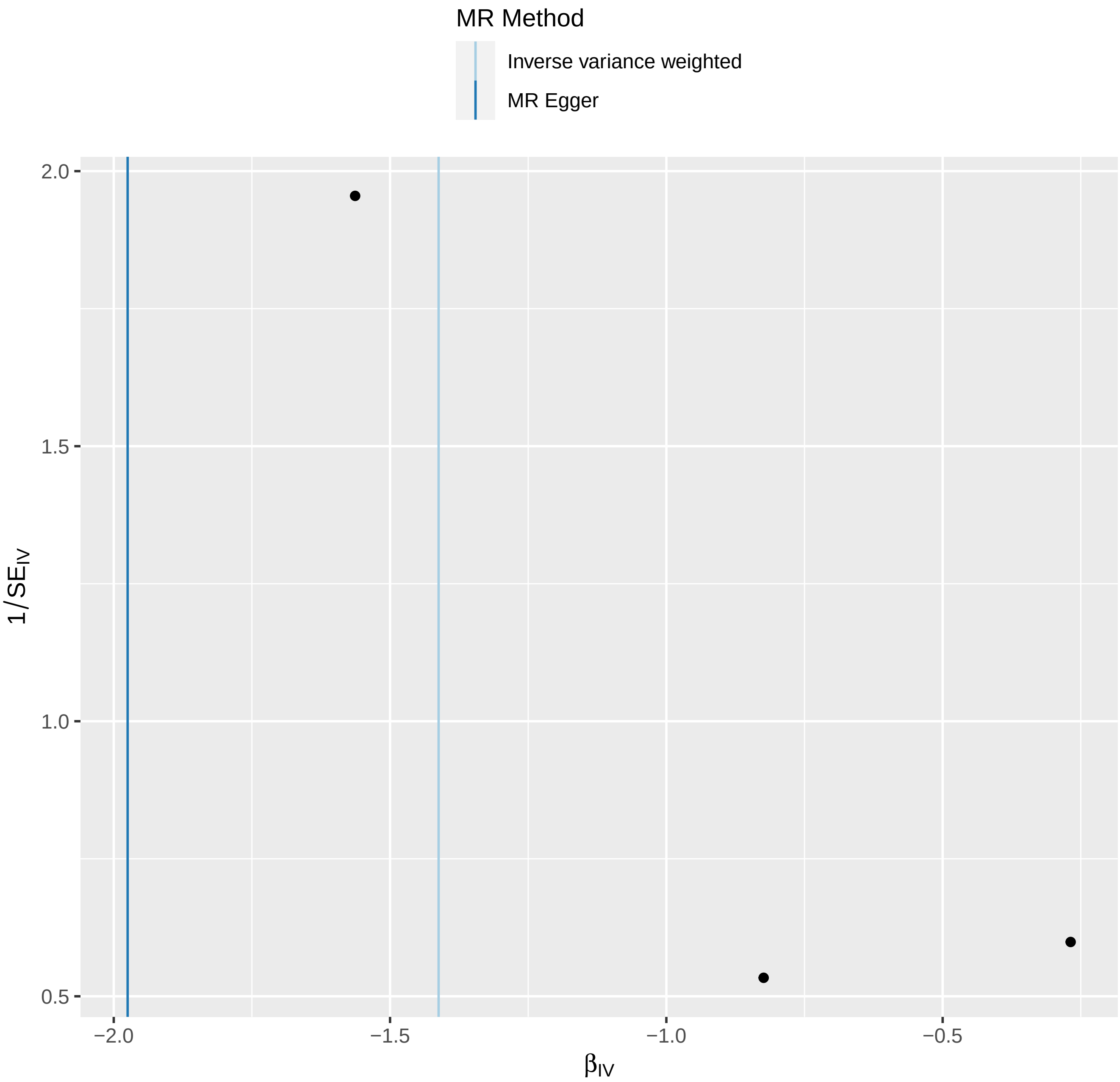

Batch 2647 : Gamma−glutamylmethionine* on Ulcerative rectosigmoiditis

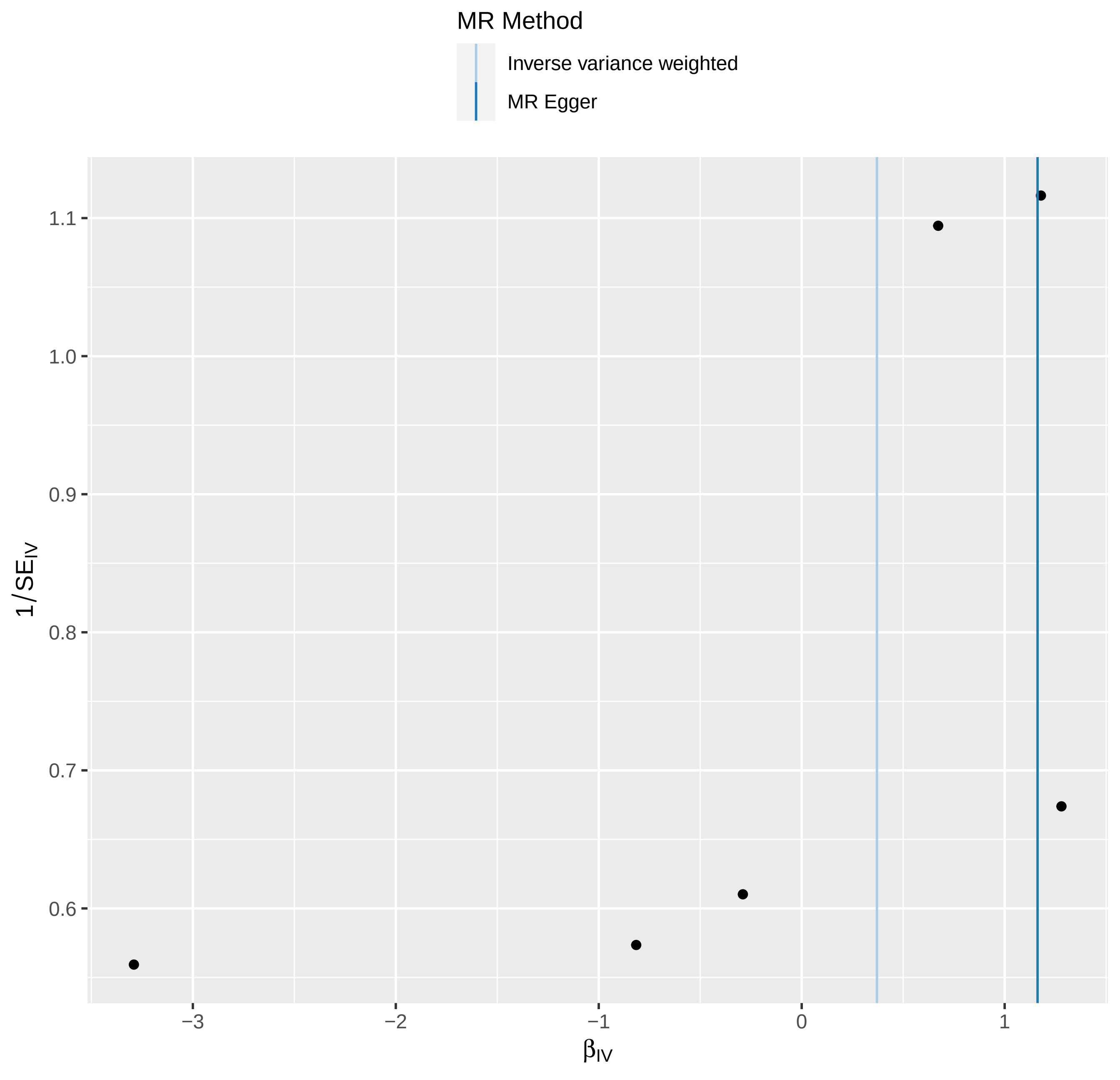

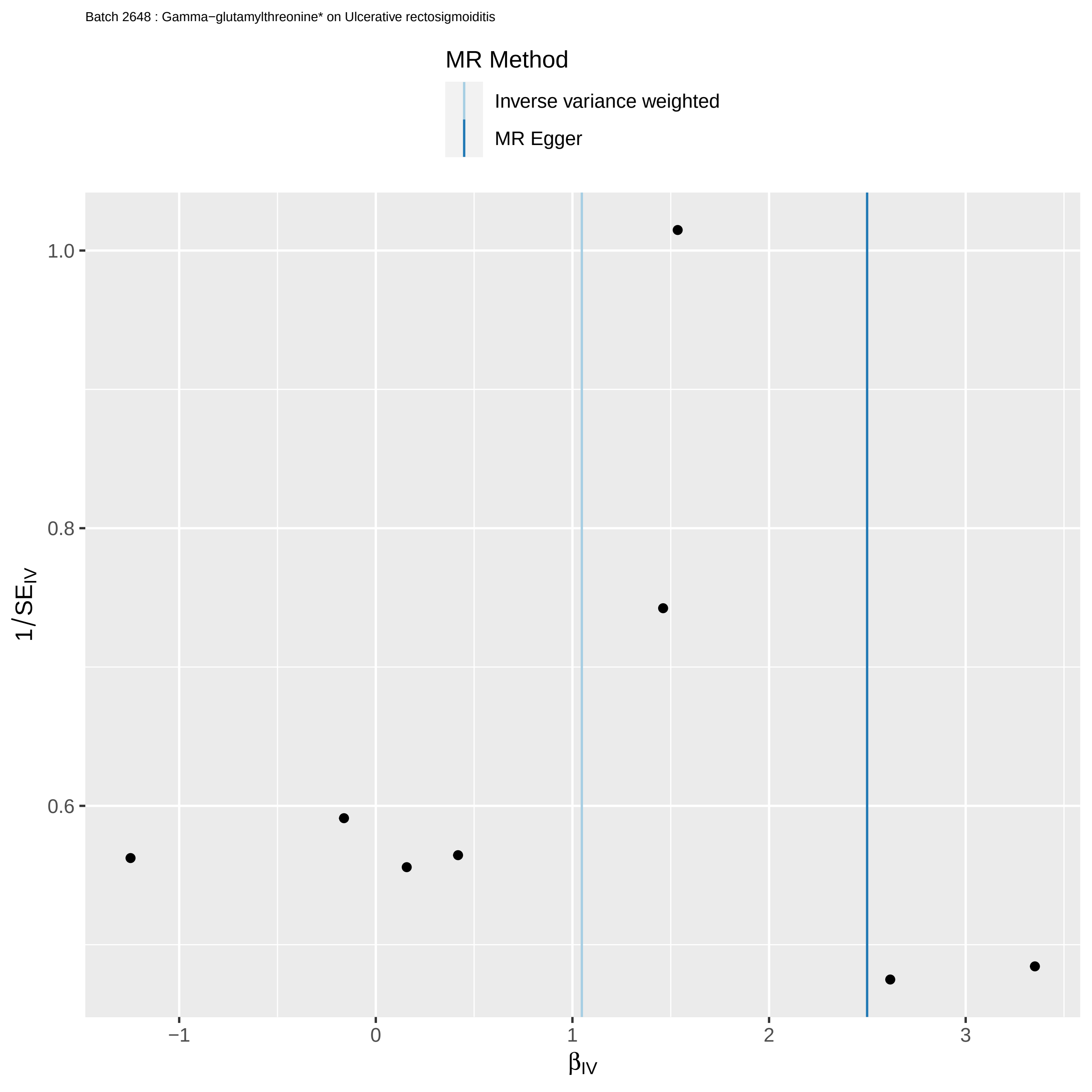
Batch 2648 : Gamma−glutamylthreonine* on Ulcerative rectosigmoiditis
MR Method
Inverse variance weighted
MR Egger
1.0
0.8
0.6
1/SE_IV
−1
0
1
2
3
β_IV

Batch 2649 : Gamma−tocopherol on Ulcerative rectosigmoiditis

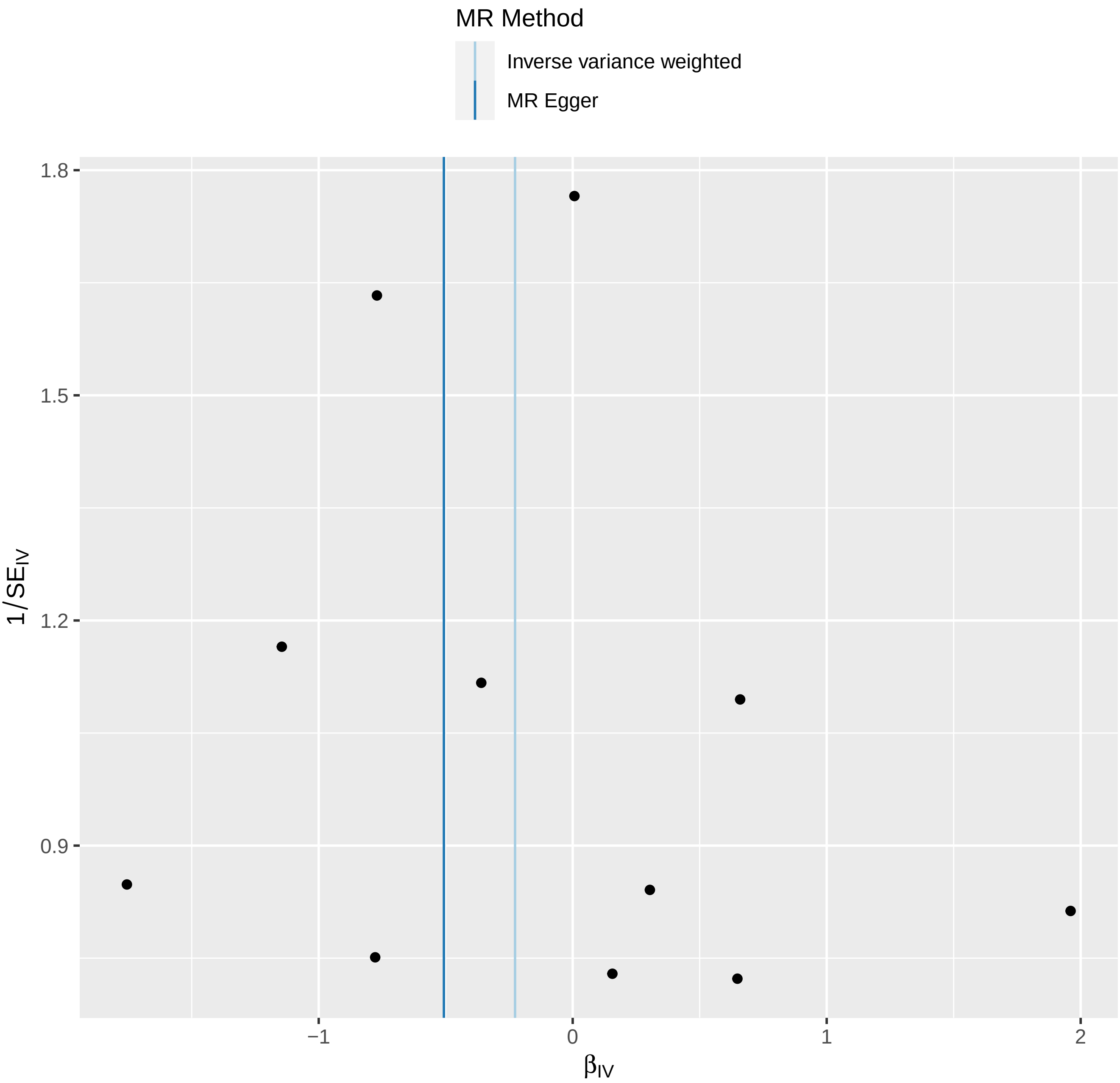

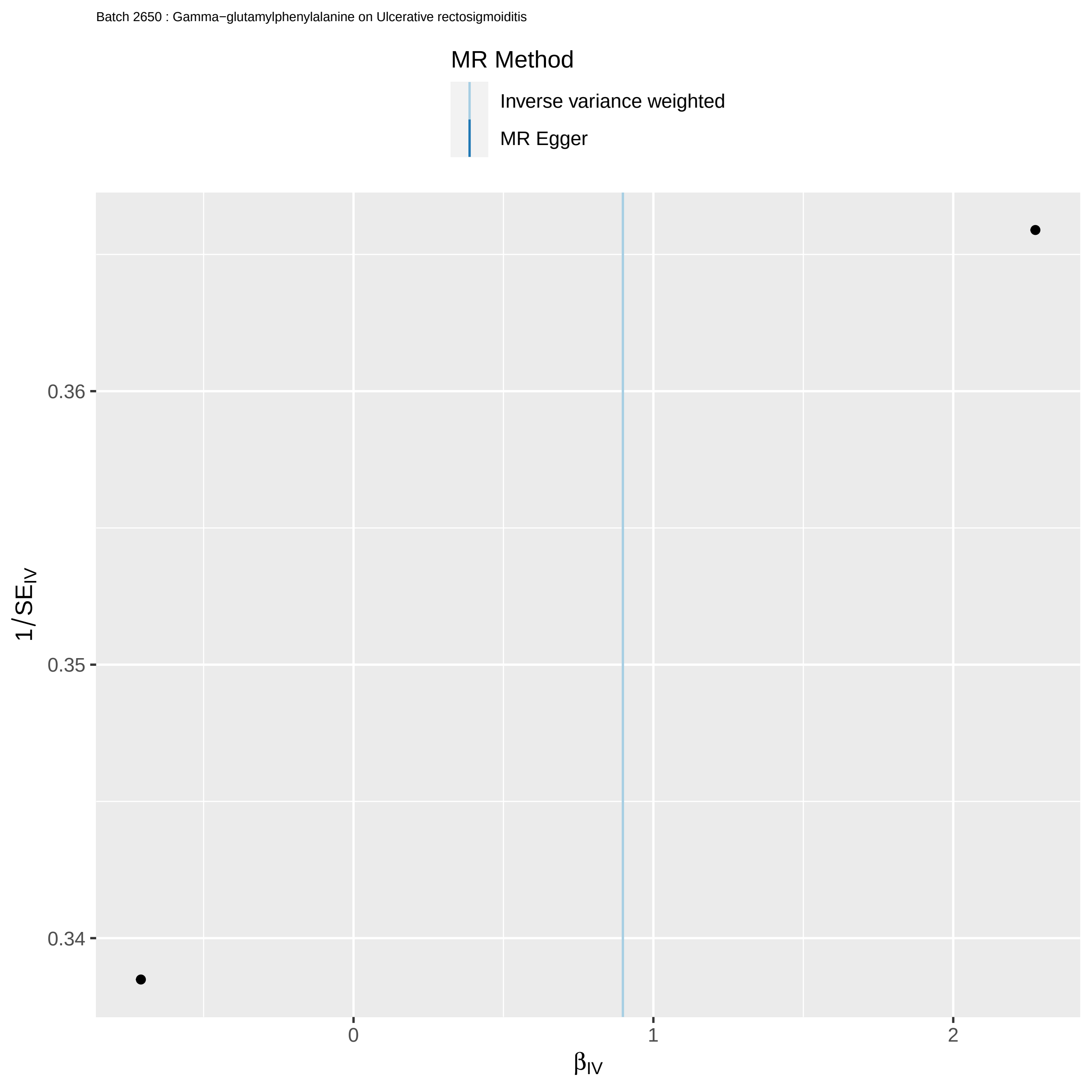
Batch 2650 : Gamma−glutamylphenylalanine on Ulcerative rectosigmoiditis
MR Method
Inverse variance weighted
MR Egger
0.36
0.35
0.34
1/SE_IV
0
1
2
β_IV

Batch 2651 : Isobutyrylcarnitine on Ulcerative rectosigmoiditis

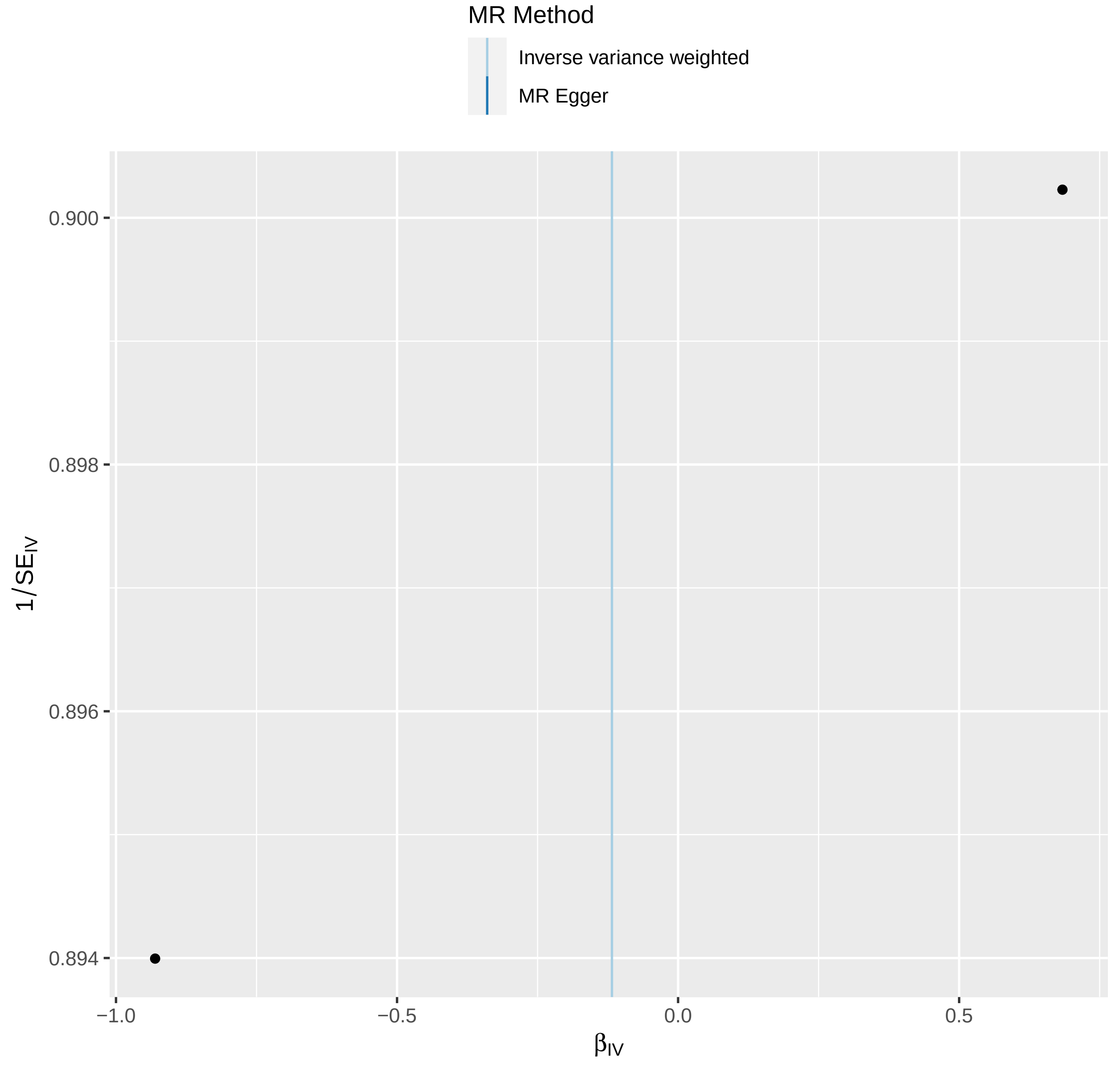

Batch 2652 : Pseudouridine on Ulcerative rectosigmoiditis

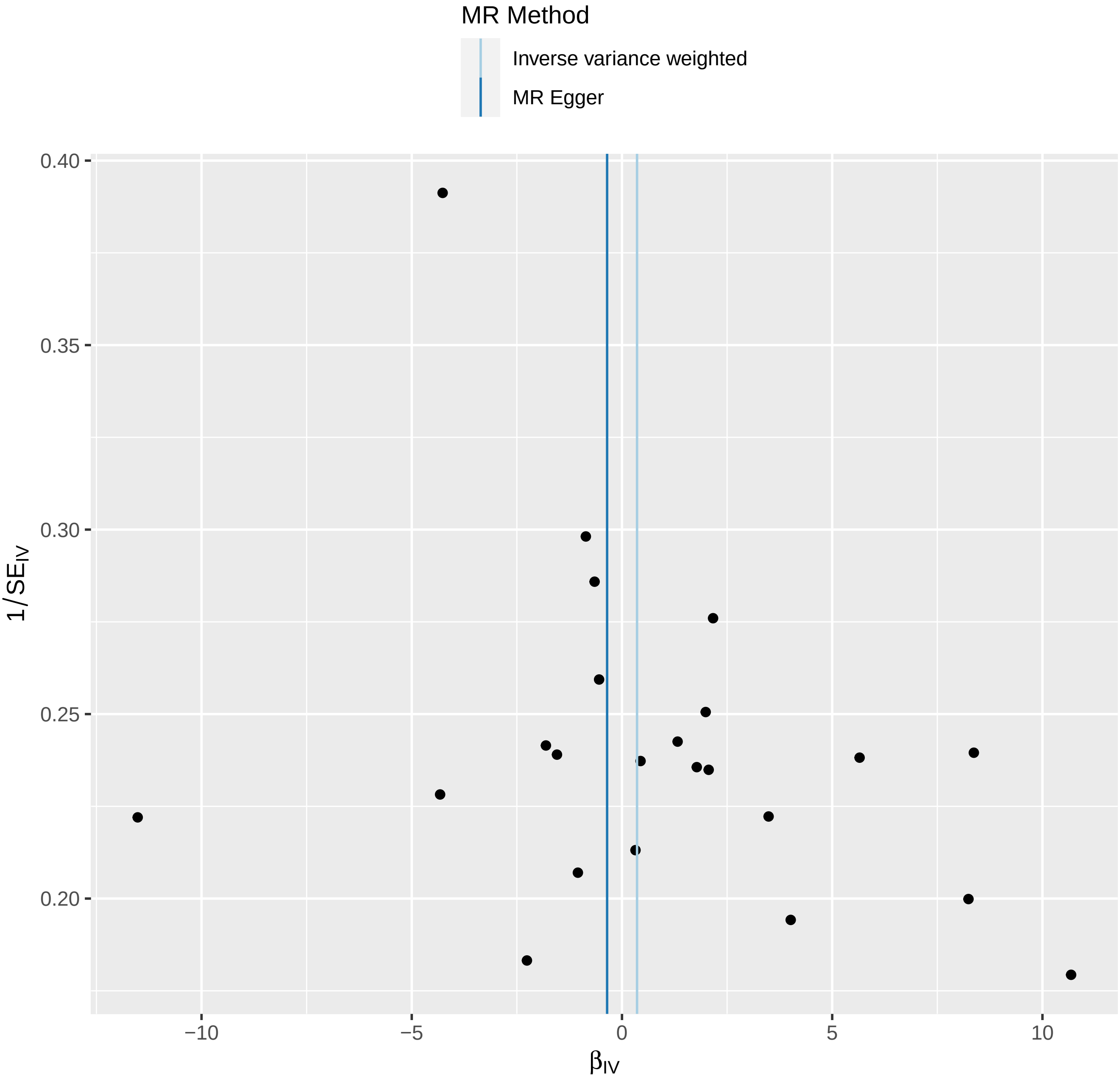

Batch 2653 : Valerate on Ulcerative rectosigmoiditis

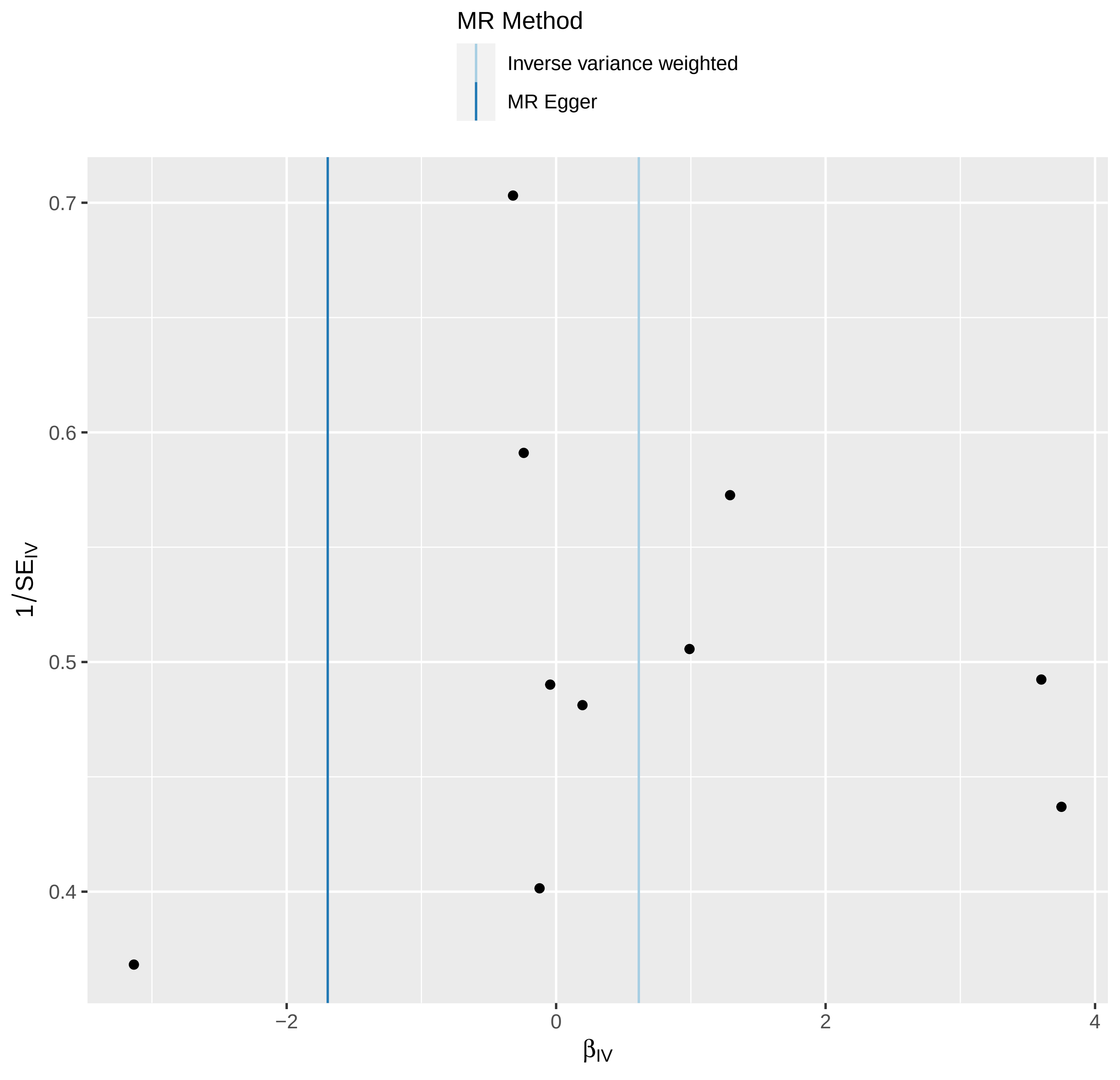

Batch 2656 : Erythronate* on Ulcerative rectosigmoiditis

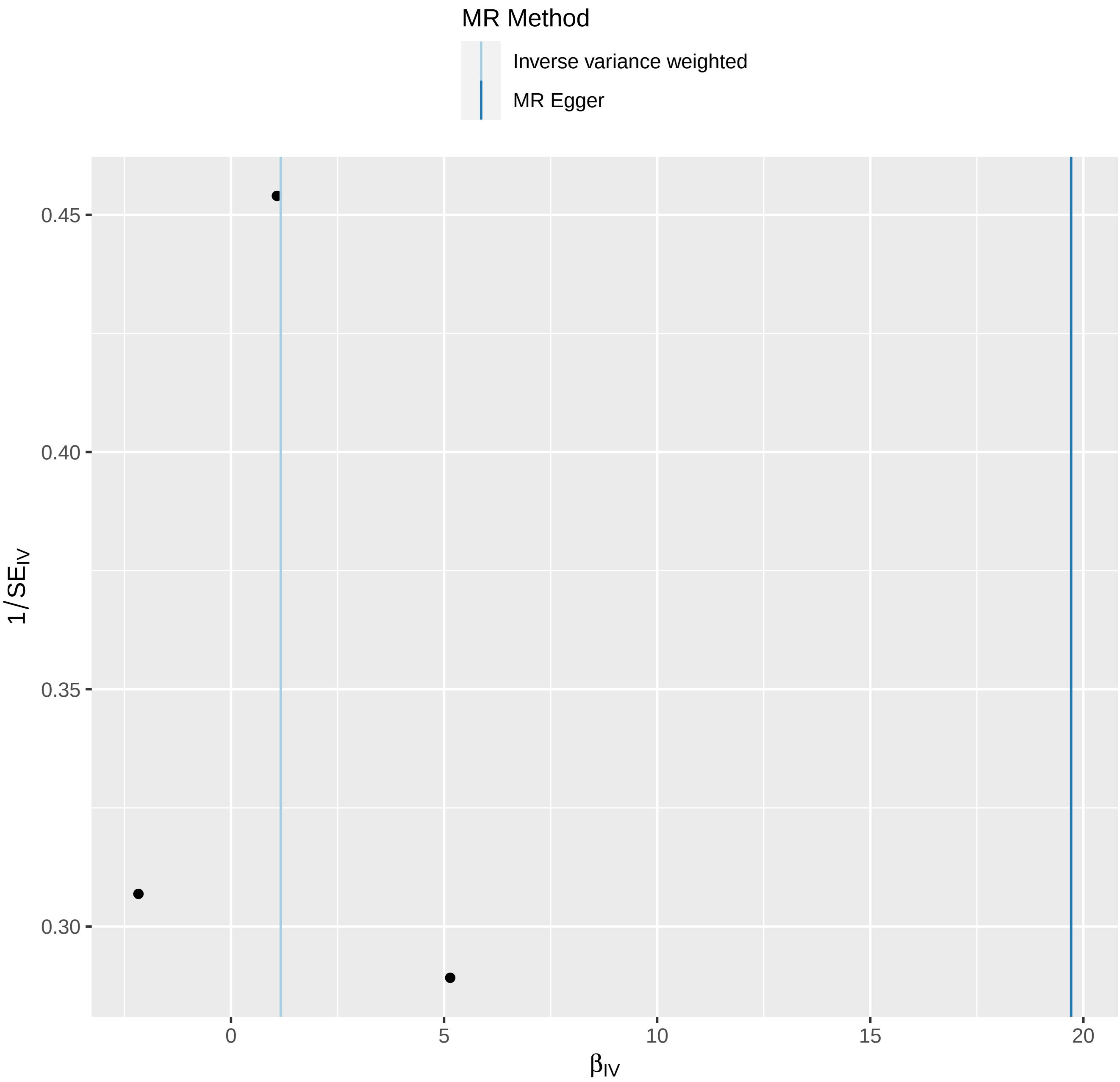

Batch 2657 : Lathosterol on Ulcerative rectosigmoiditis

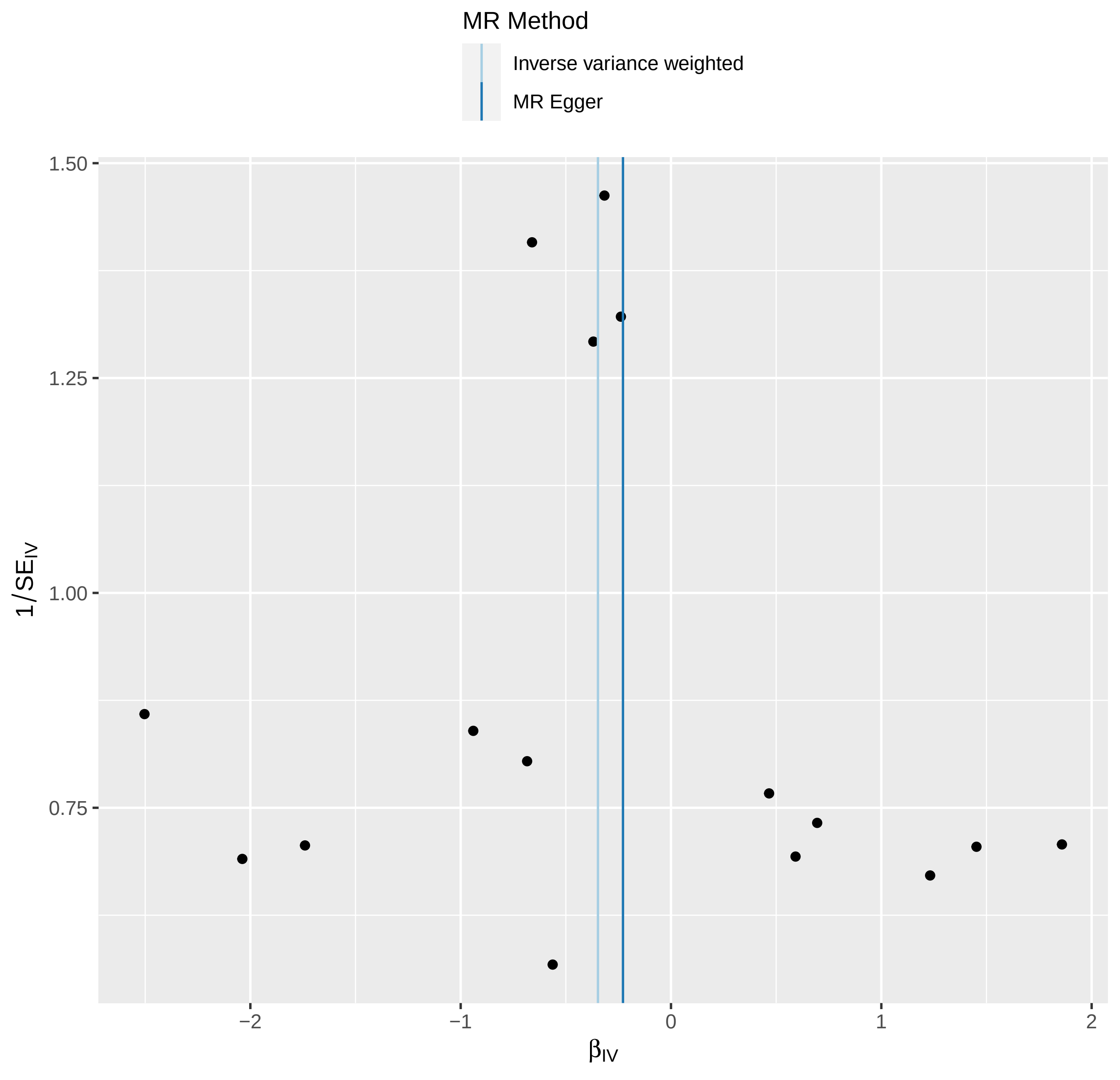

Batch 2660 : Eicosenoate (20:1n9 or 11) on Ulcerative rectosigmoiditis

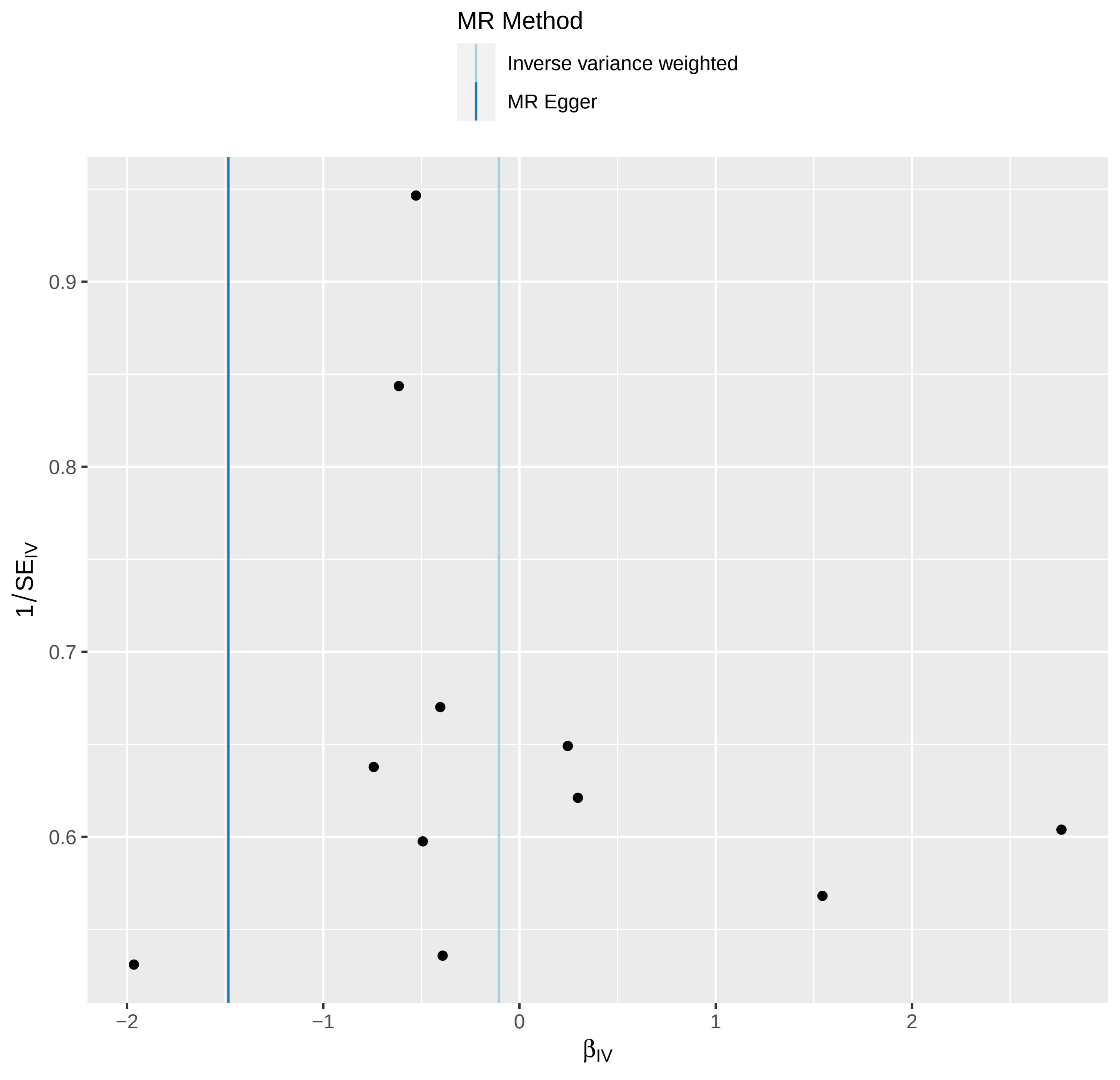

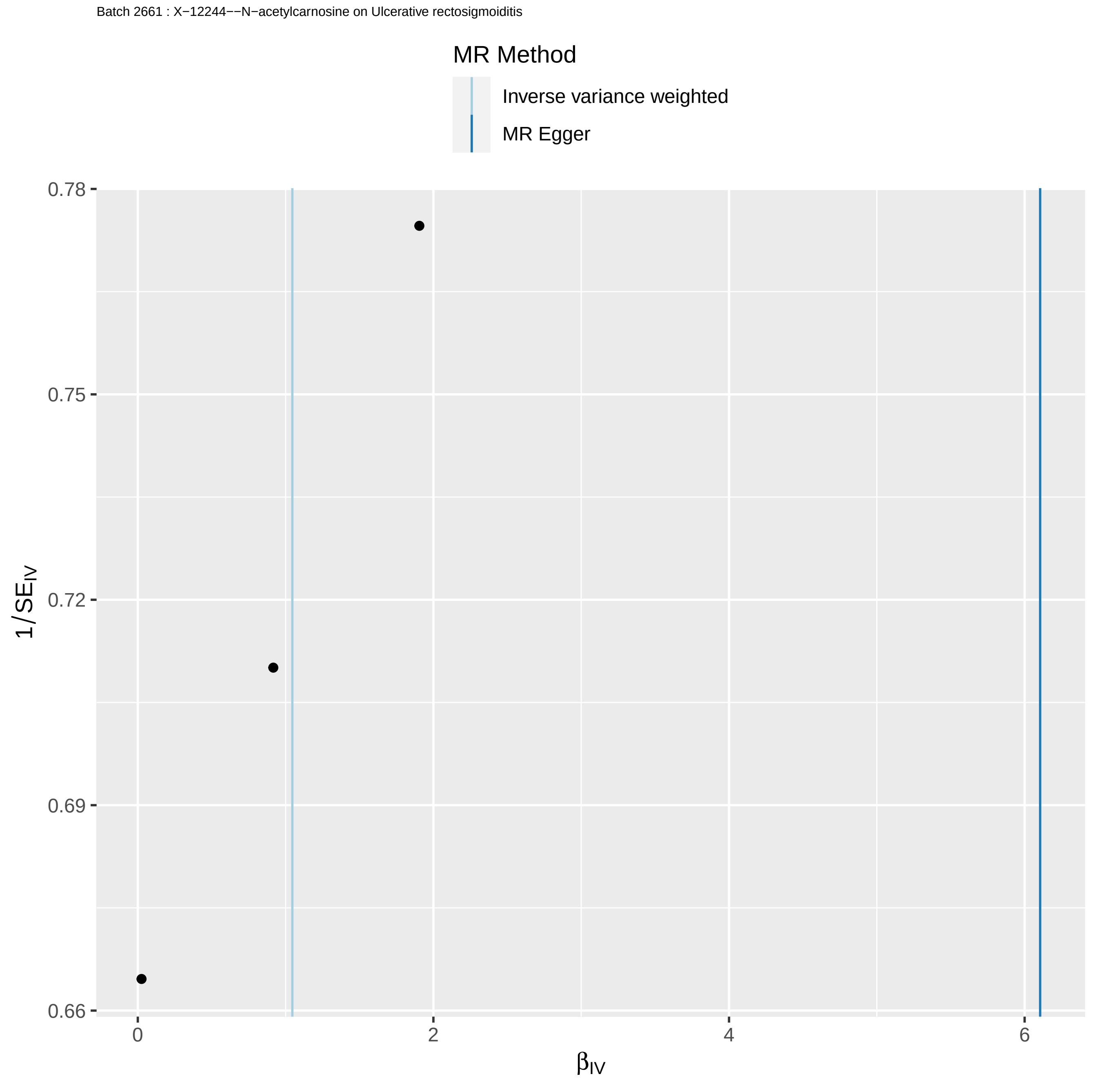
Batch 2661 : X−12244−−N−acetylcarnosine on Ulcerative rectosigmoiditis
MR Method
Inverse variance weighted
MR Egger
0.78
0.75
0.72
0.69
0.66
1/SE_IV
0
2
4
6
β_IV

Batch 2663 : 1−eicosatrienoylglycerophosphocholine* on Ulcerative rectosigmoiditis

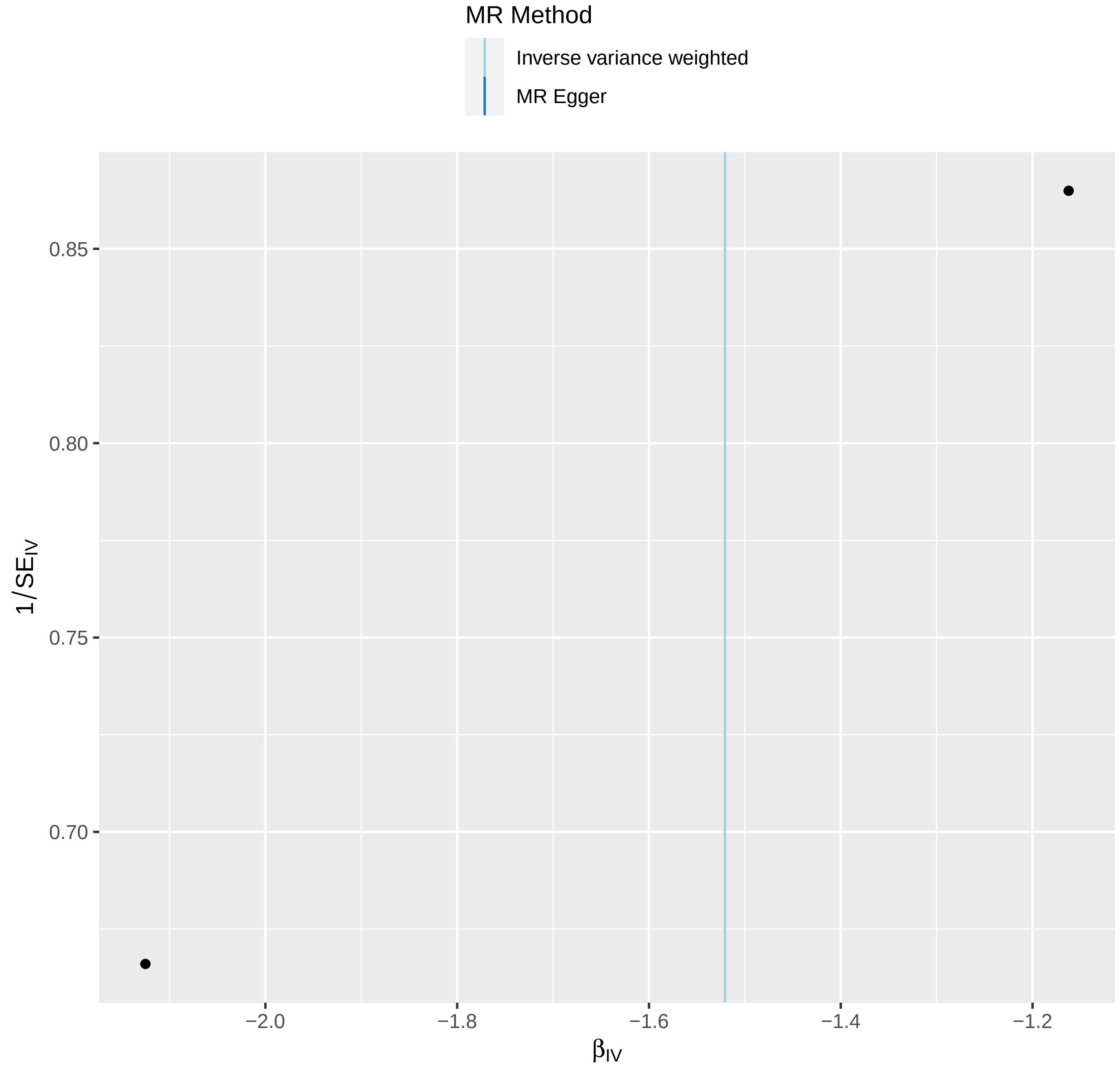

Batch 2664 : 1−docosahexaenoylglycerophosphocholine* on Ulcerative rectosigmoiditis

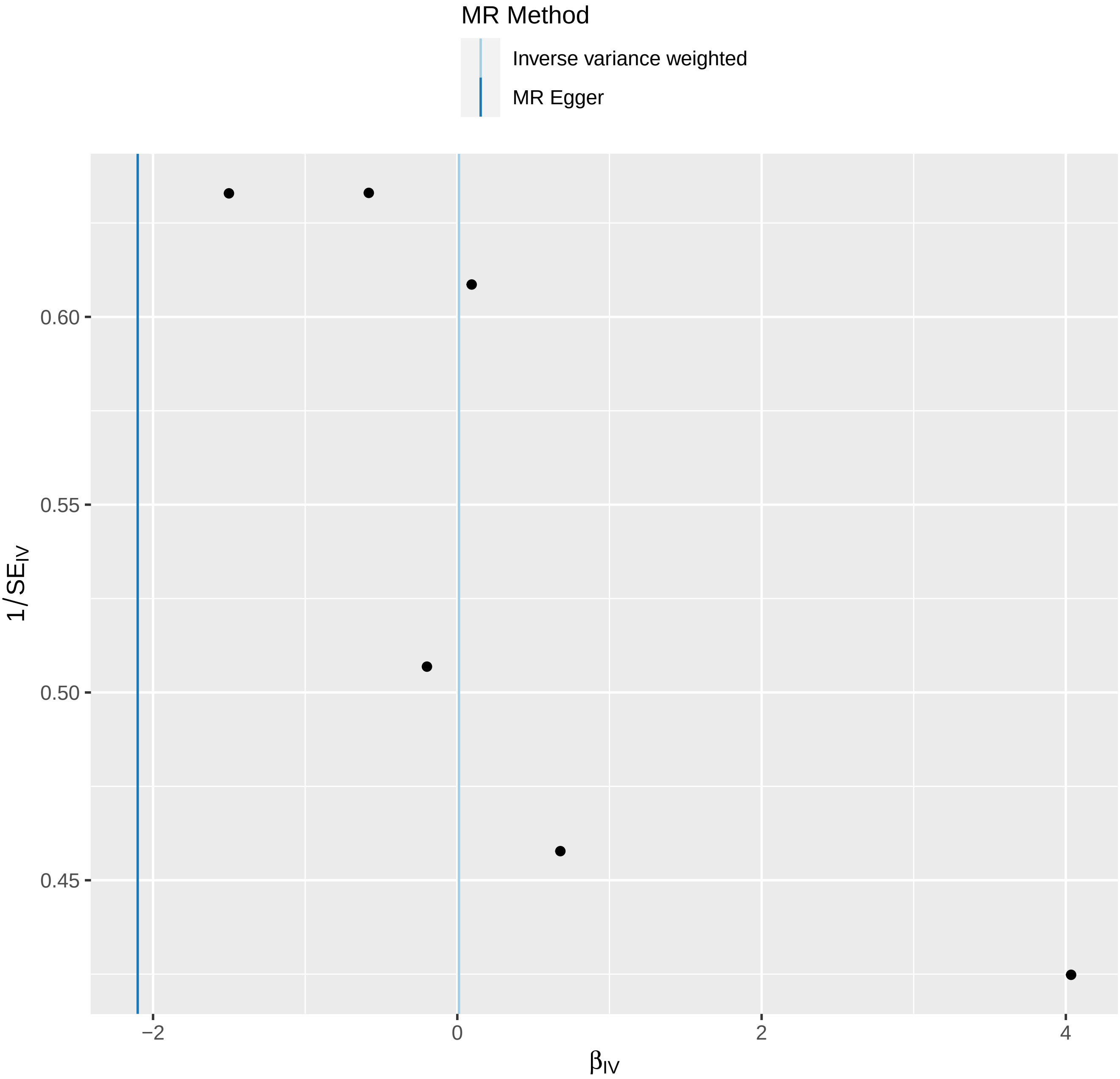

Batch 2668 : Octanoylcarnitine on Ulcerative rectosigmoiditis

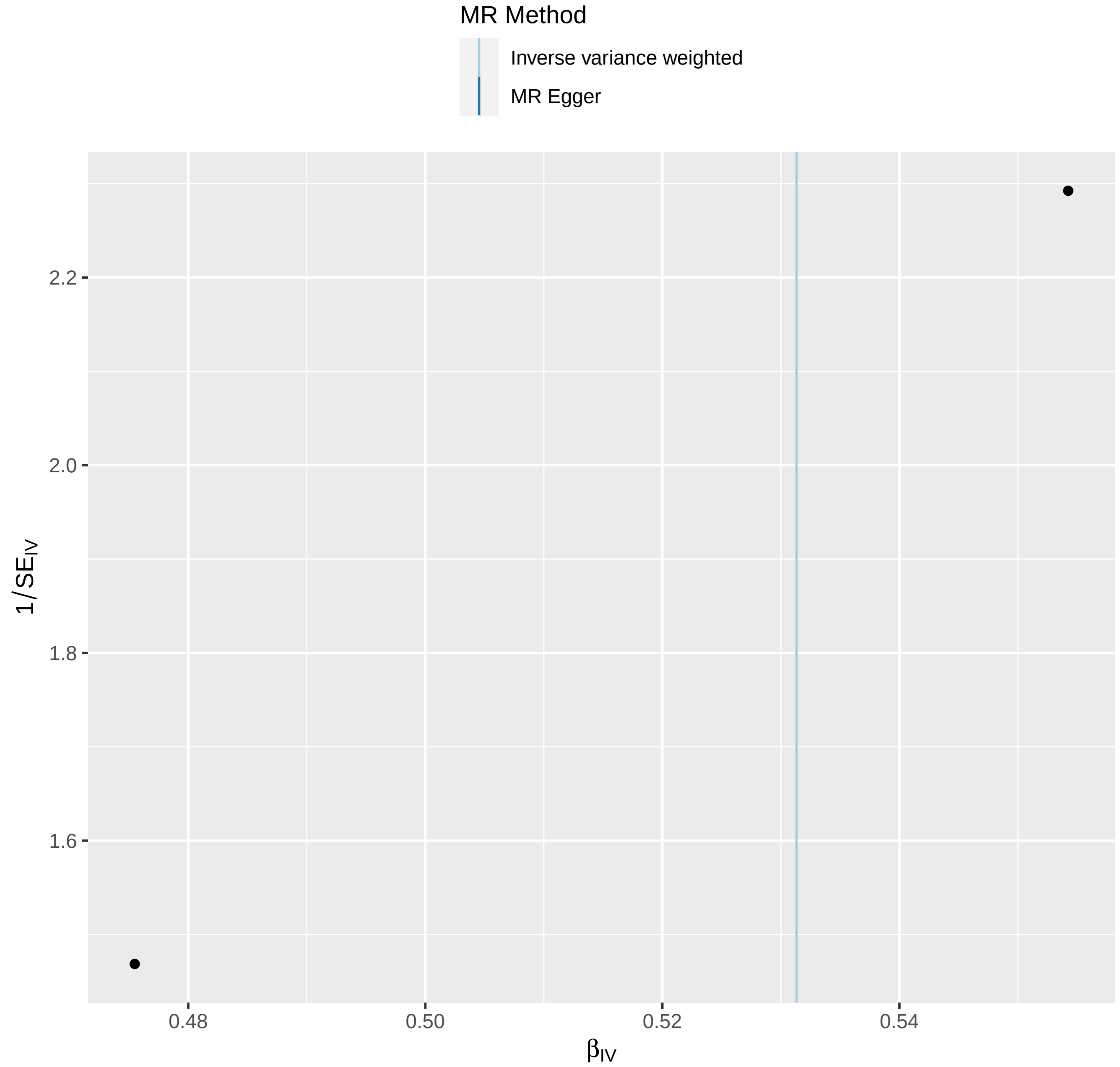

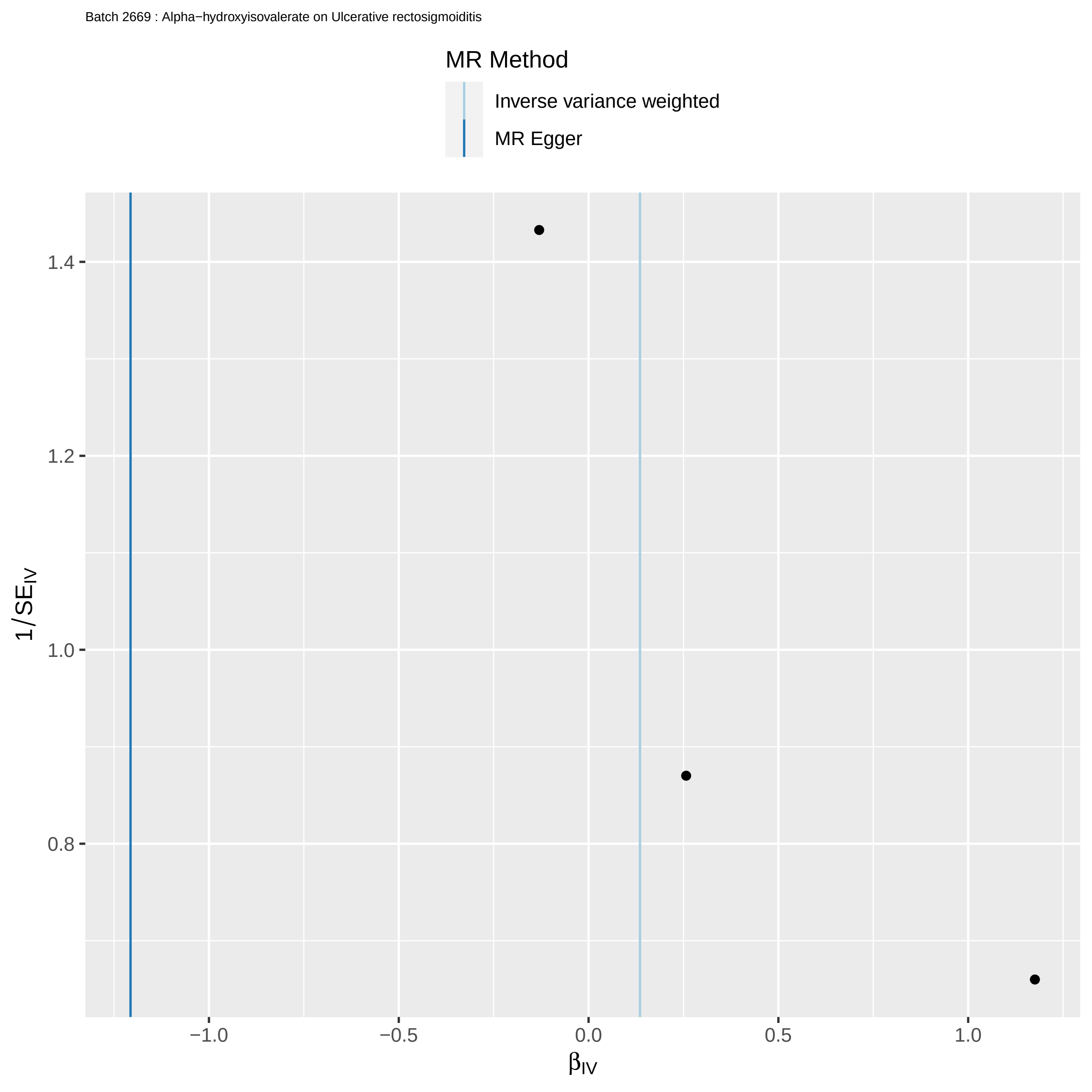

Batch 2669 : Alpha−hydroxyisovalerate on Ulcerative rectosigmoiditis
MR Method
Inverse variance weighted
MR Egger
1.4
1.2
1.0
0.8
1/SE_IV
−1.0
−0.5
0.0
0.5
1.0
β_IV

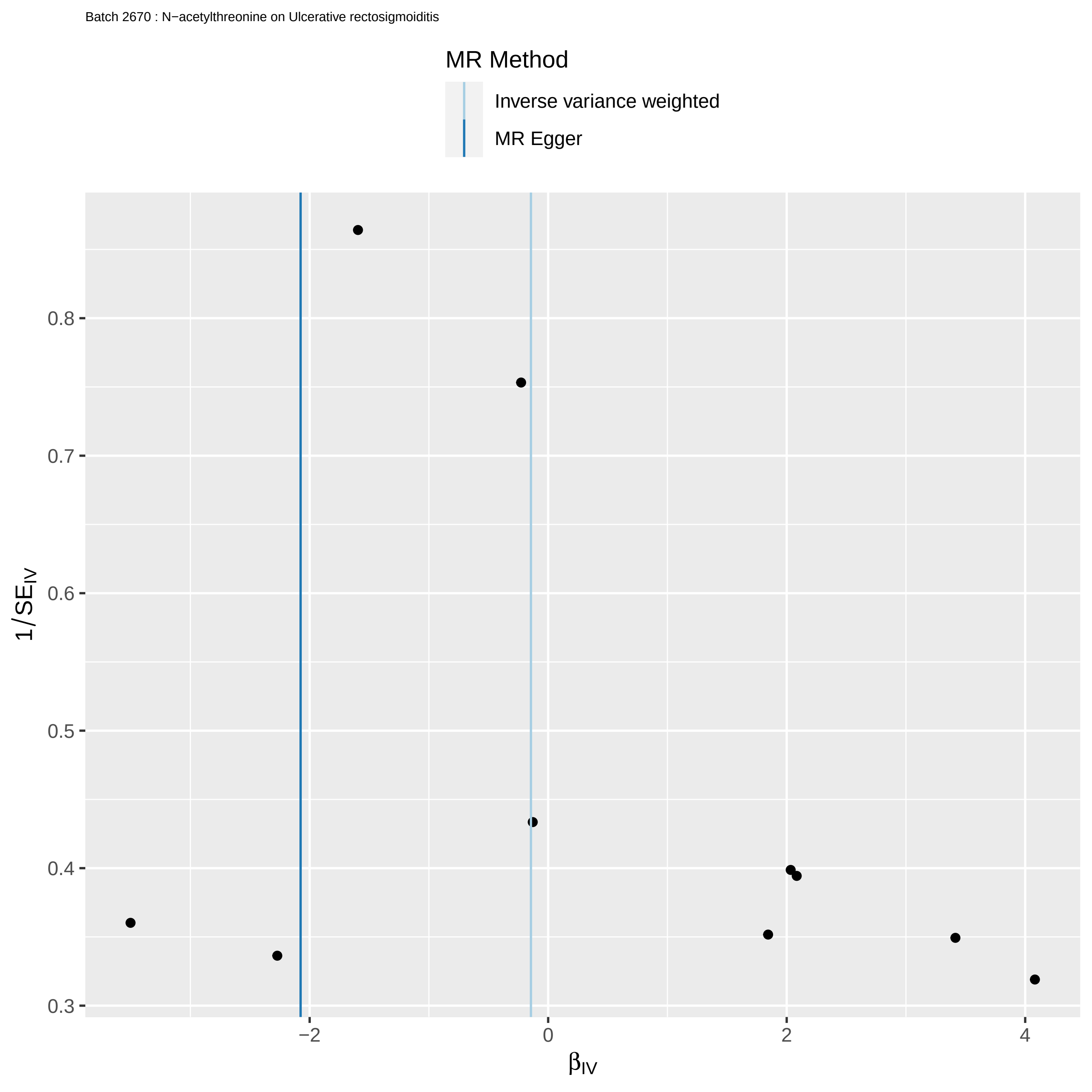

Batch 2670 : N−acetylthreonine on Ulcerative rectosigmoiditis
MR Method
Inverse variance weighted
MR Egger
0.8
0.7
0.6
0.5
0.4
0.3
1/SE_IV
−2
0
2
4
β_IV

Batch 2671 : Decanoylcarnitine on Ulcerative rectosigmoiditis

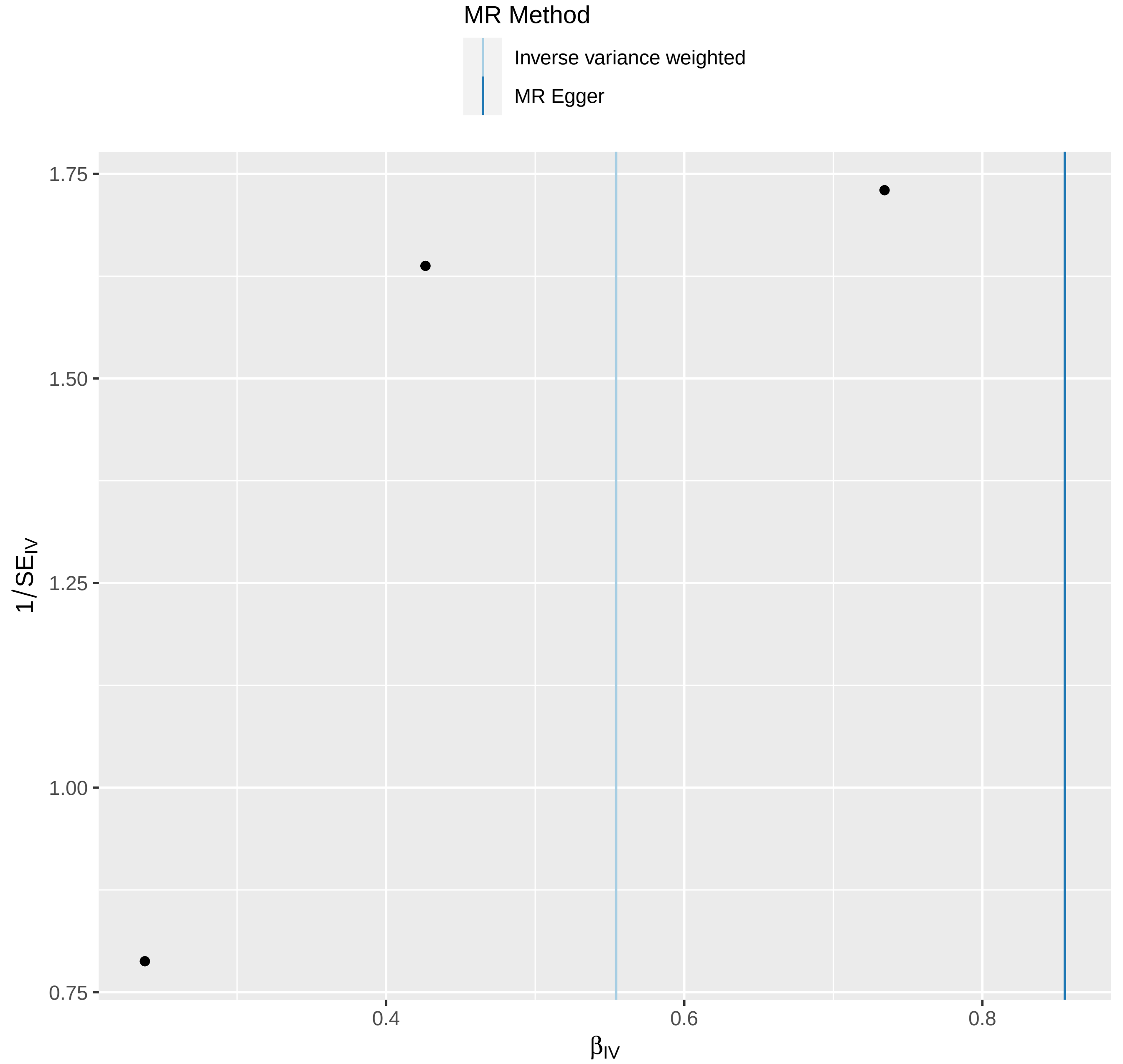

Batch 2672 : 1−palmitoylglycerophosphocholine on Ulcerative rectosigmoiditis

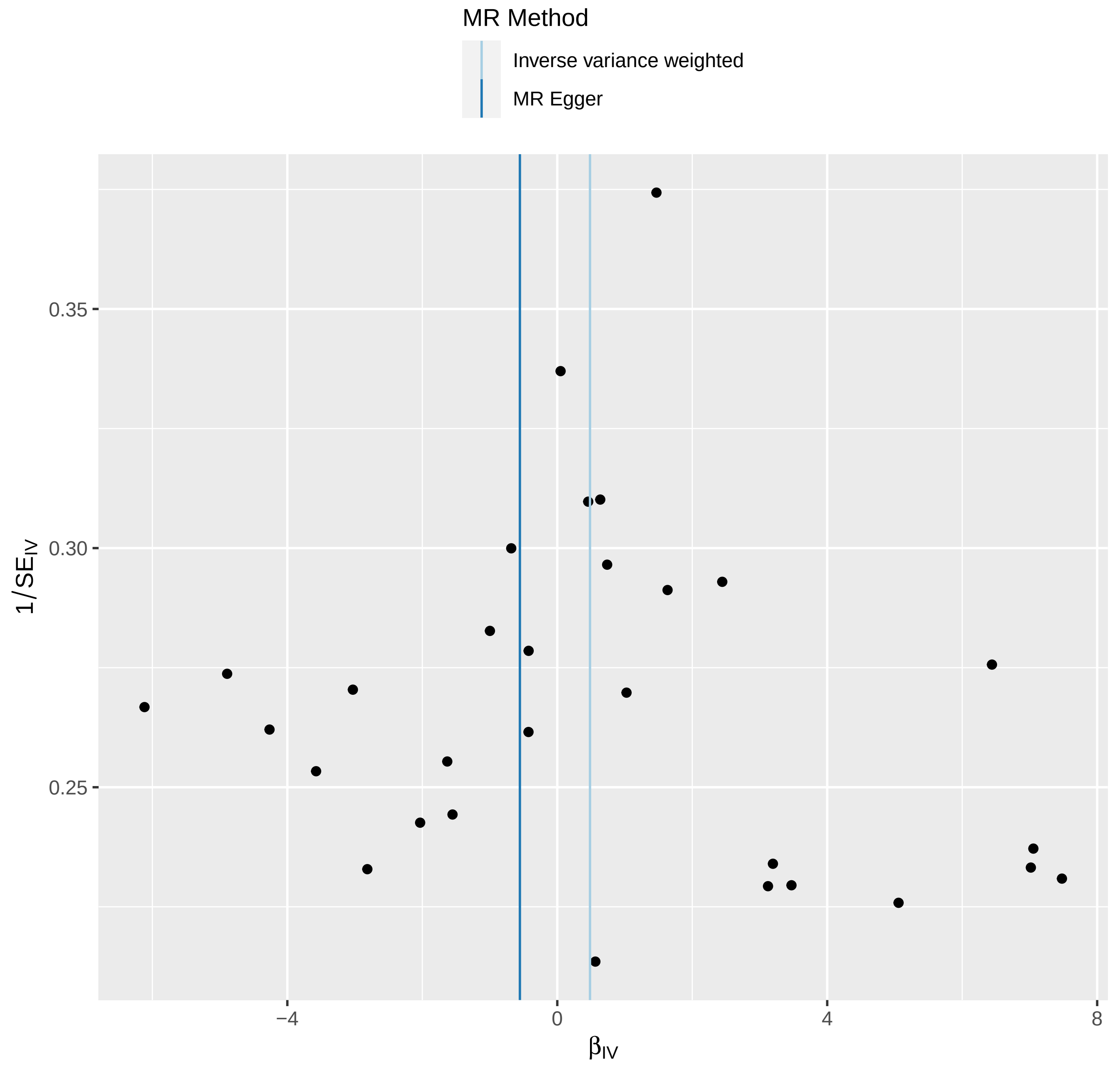

Batch 2673 : 1−heptadecanoylglycerophosphocholine on Ulcerative rectosigmoiditis

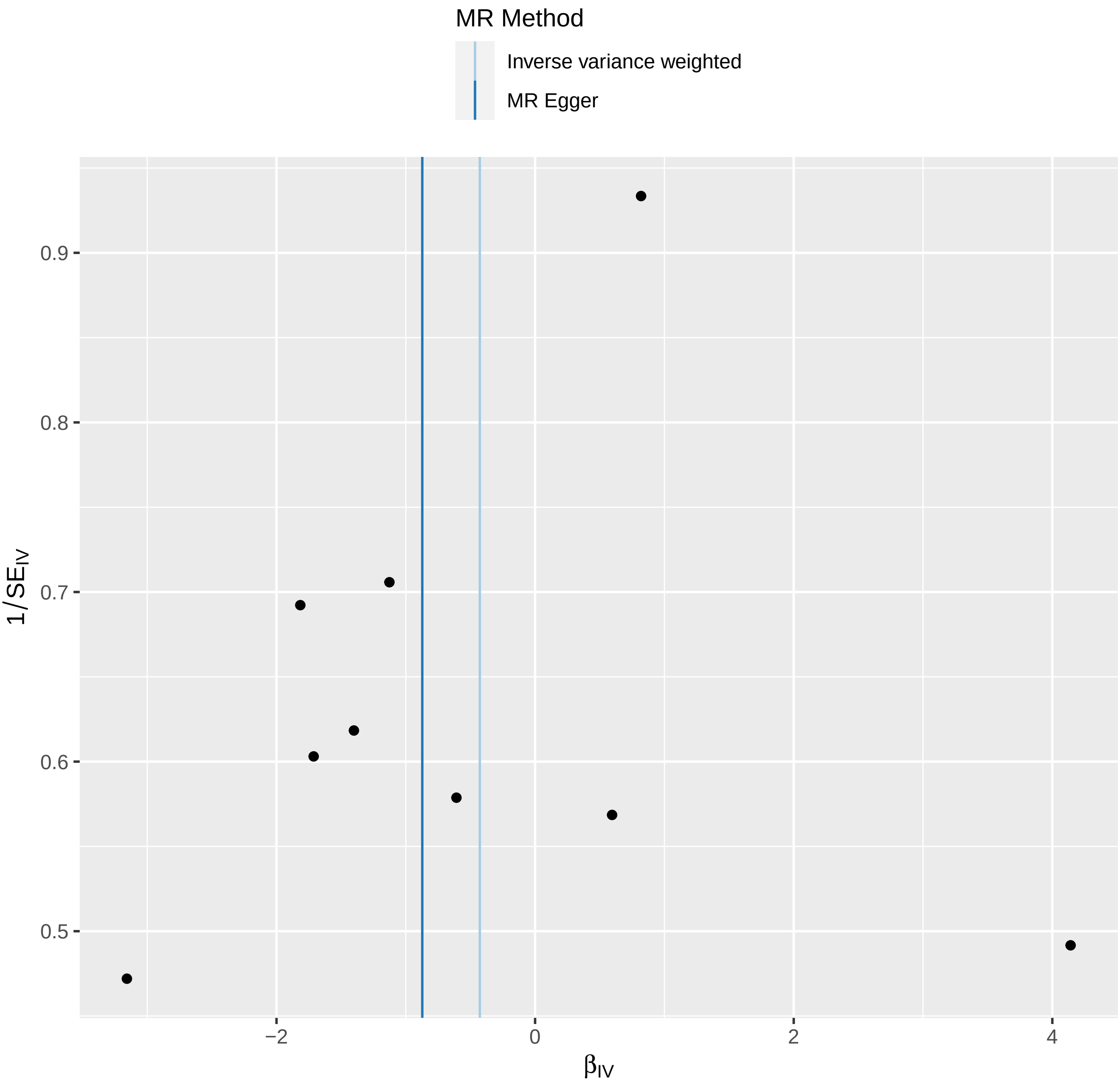

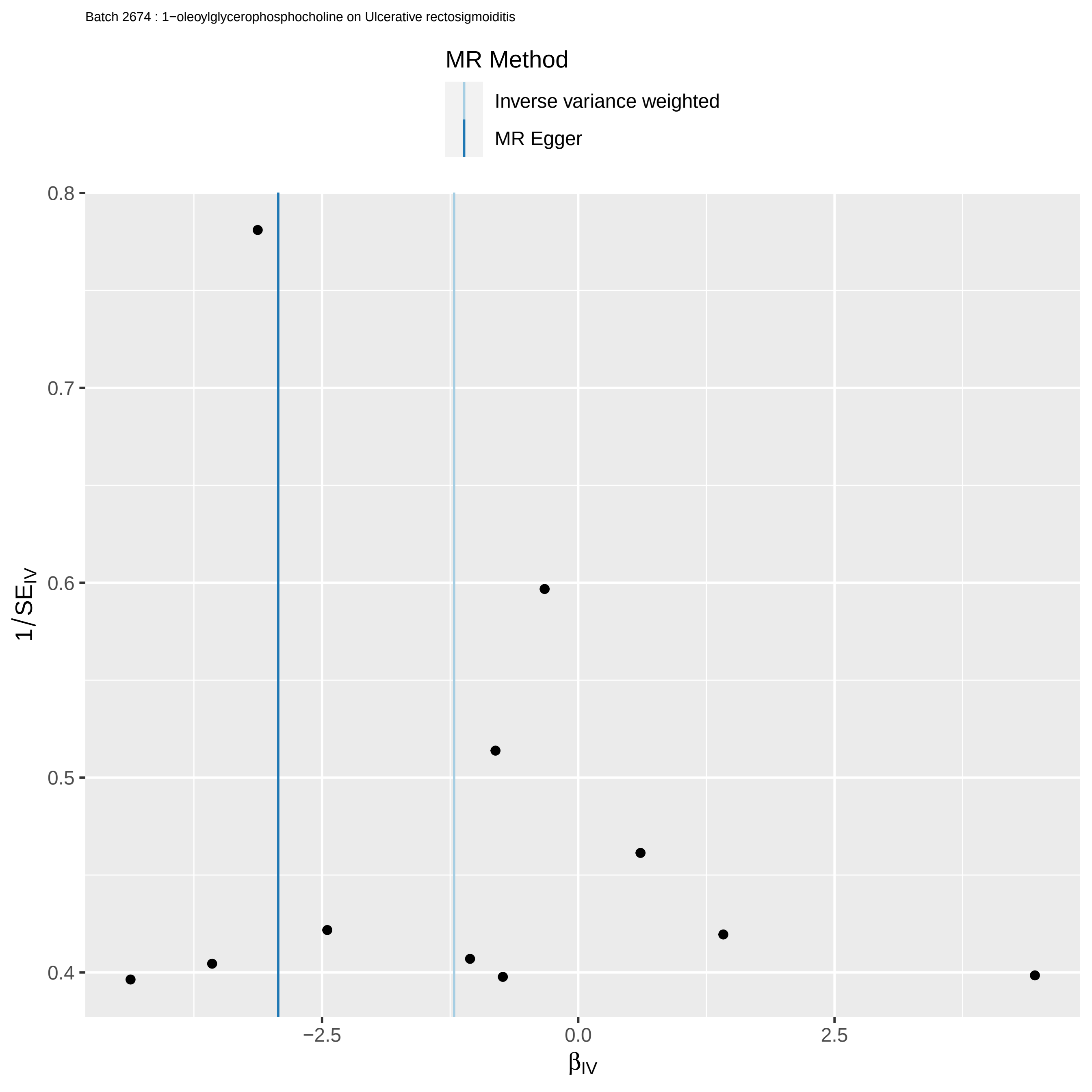
Batch 2674 : 1−oleoylglycerophosphocholine on Ulcerative rectosigmoiditis
MR Method
Inverse variance weighted
MR Egger
0.8
0.7
0.6
0.5
0.4
1/SE_IV
−2.5
0.0
2.5
β_IV

Batch 2675 : 1−stearoylglycerophosphocholine on Ulcerative rectosigmoiditis

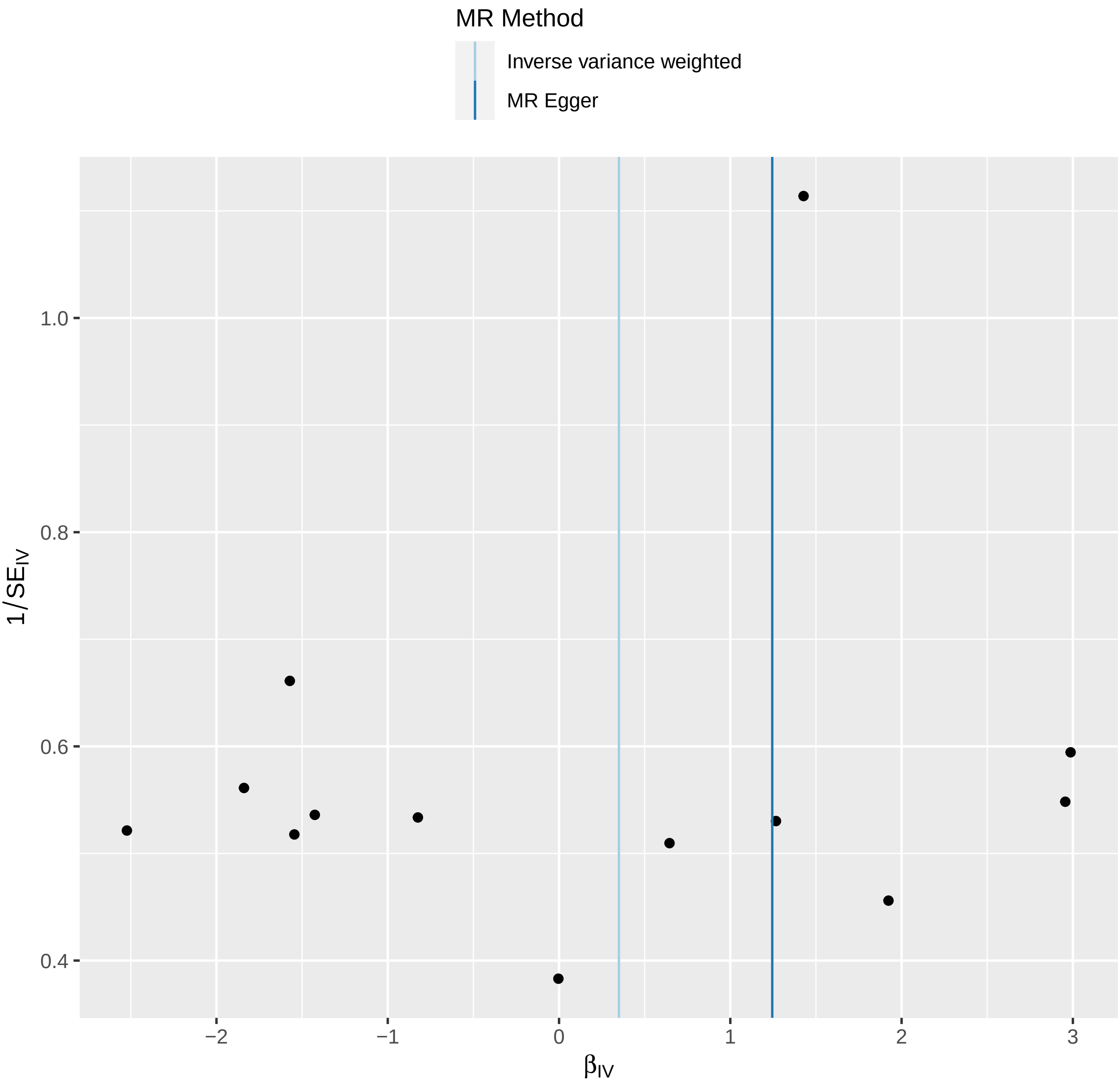

Batch 2678 : 10−heptadecenoate (17:1n7) on Ulcerative rectosigmoiditis

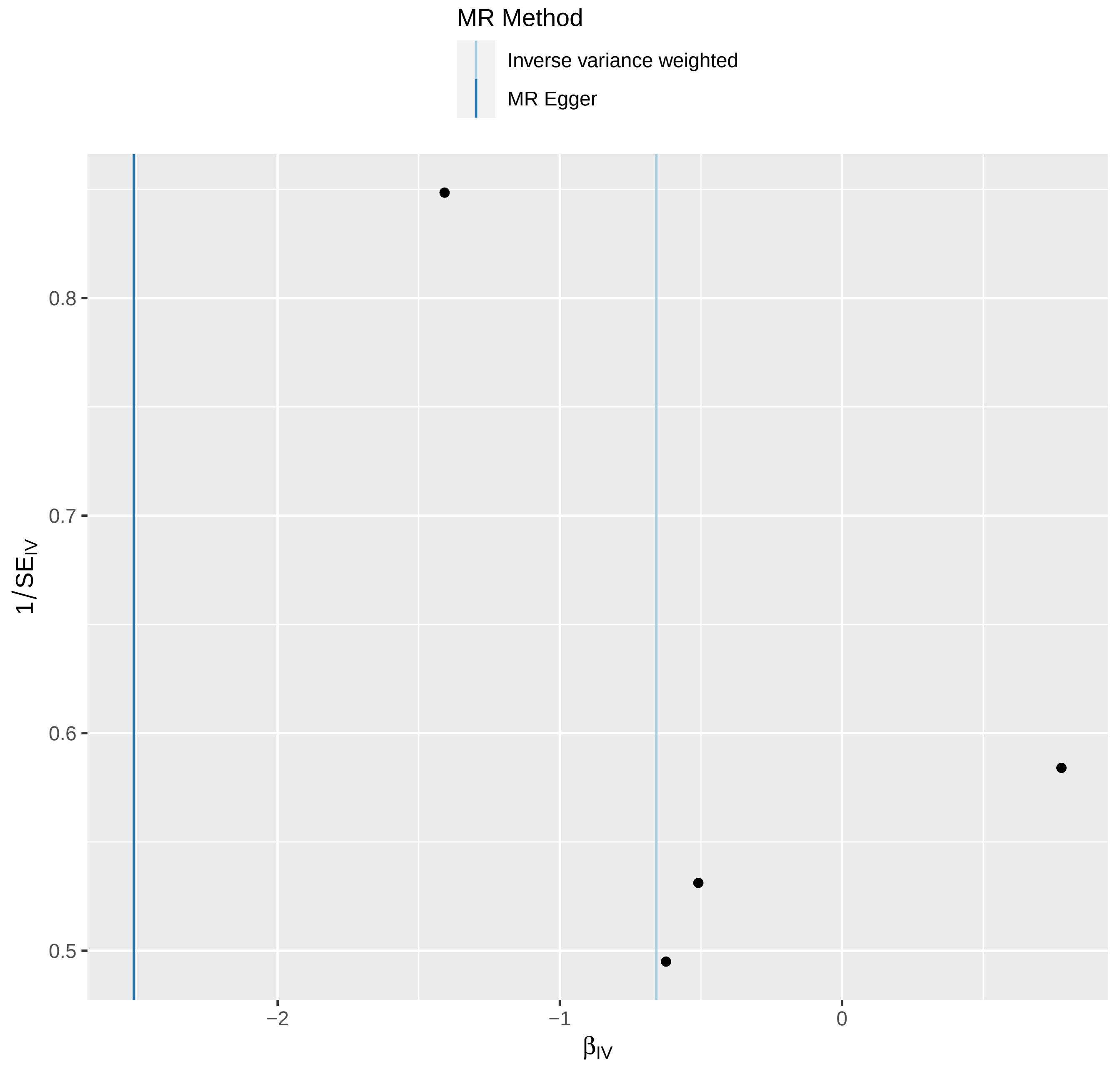

Batch 2680 : Epiandrosterone sulfate on Ulcerative rectosigmoiditis

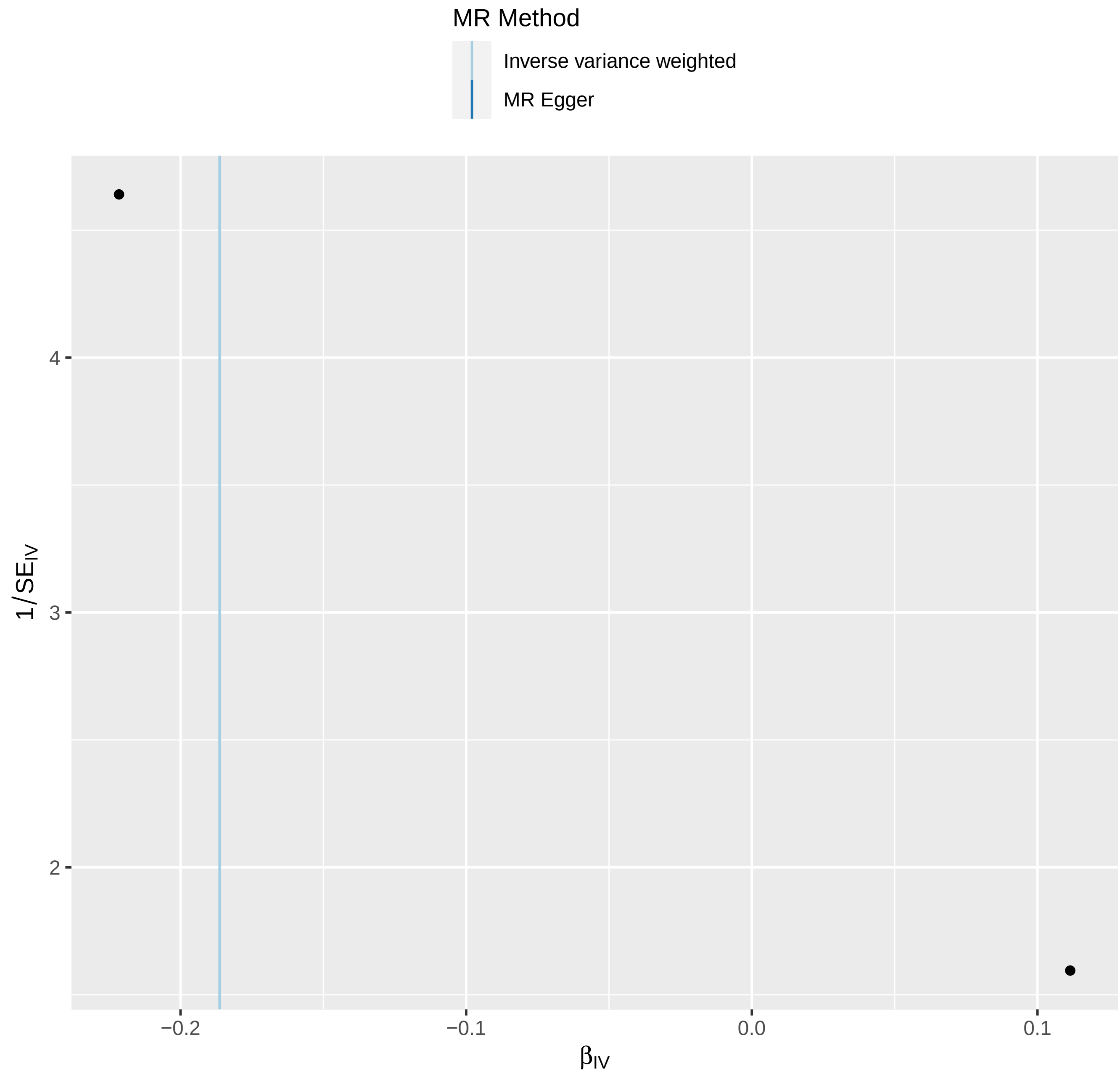

Batch 2681 : Linolenate [alpha or gamma; (18:3n3 or 6)] on Ulcerative rectosigmoiditis

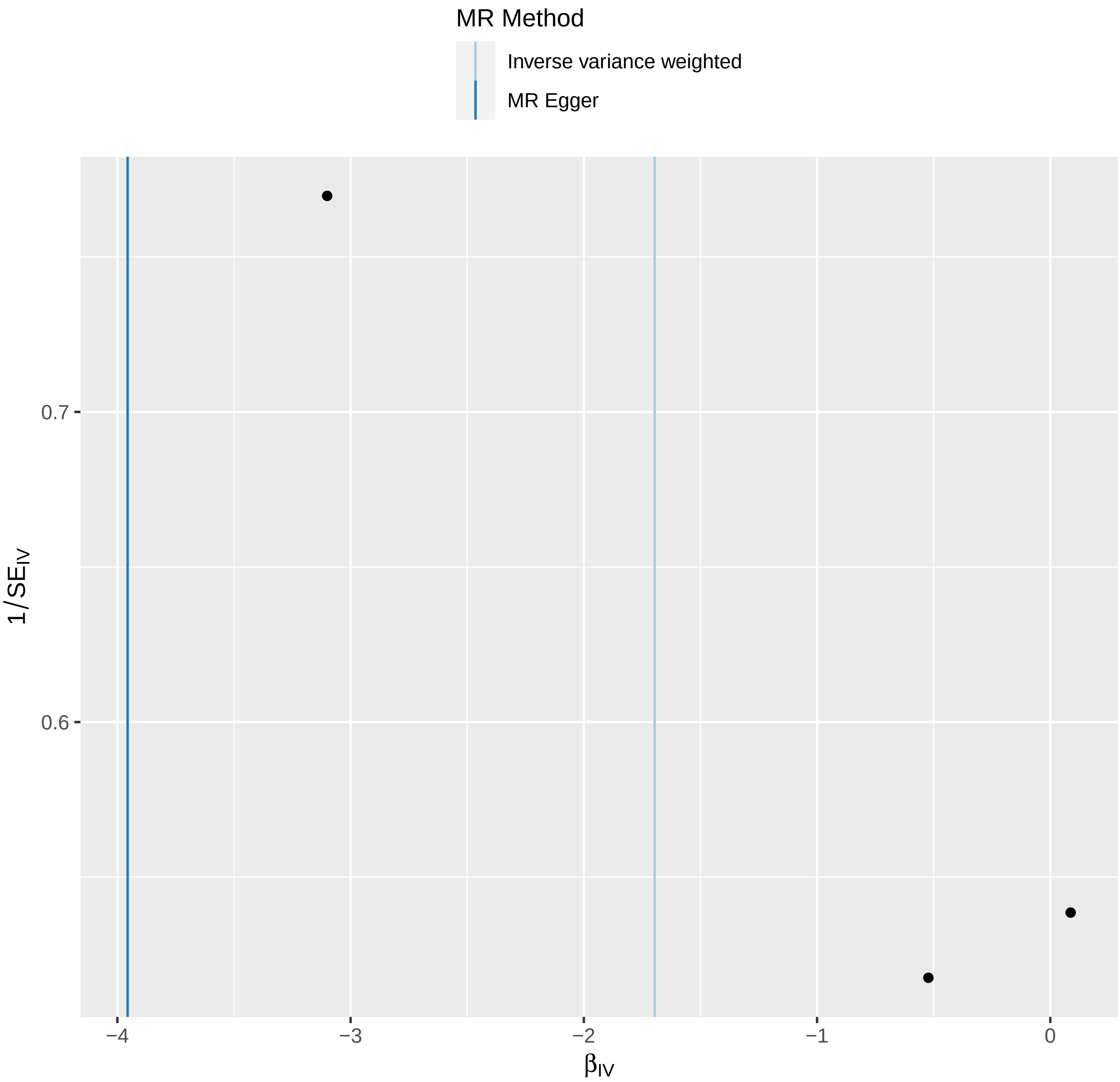

Batch 2682 : X−12510−−2−aminooctanoic acid on Ulcerative rectosigmoiditis

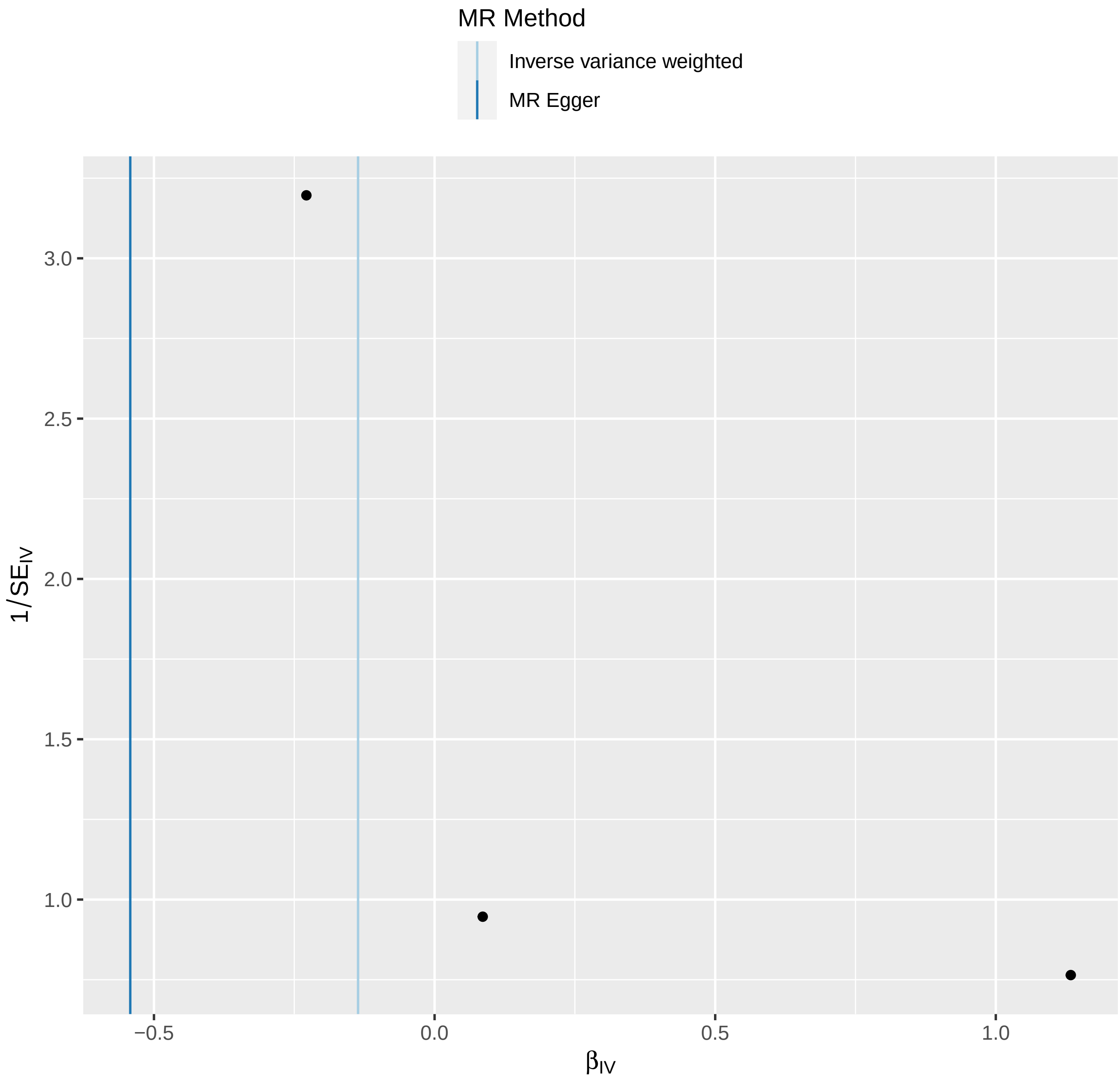

Batch 2685 : Asparagine on Ulcerative rectosigmoiditis

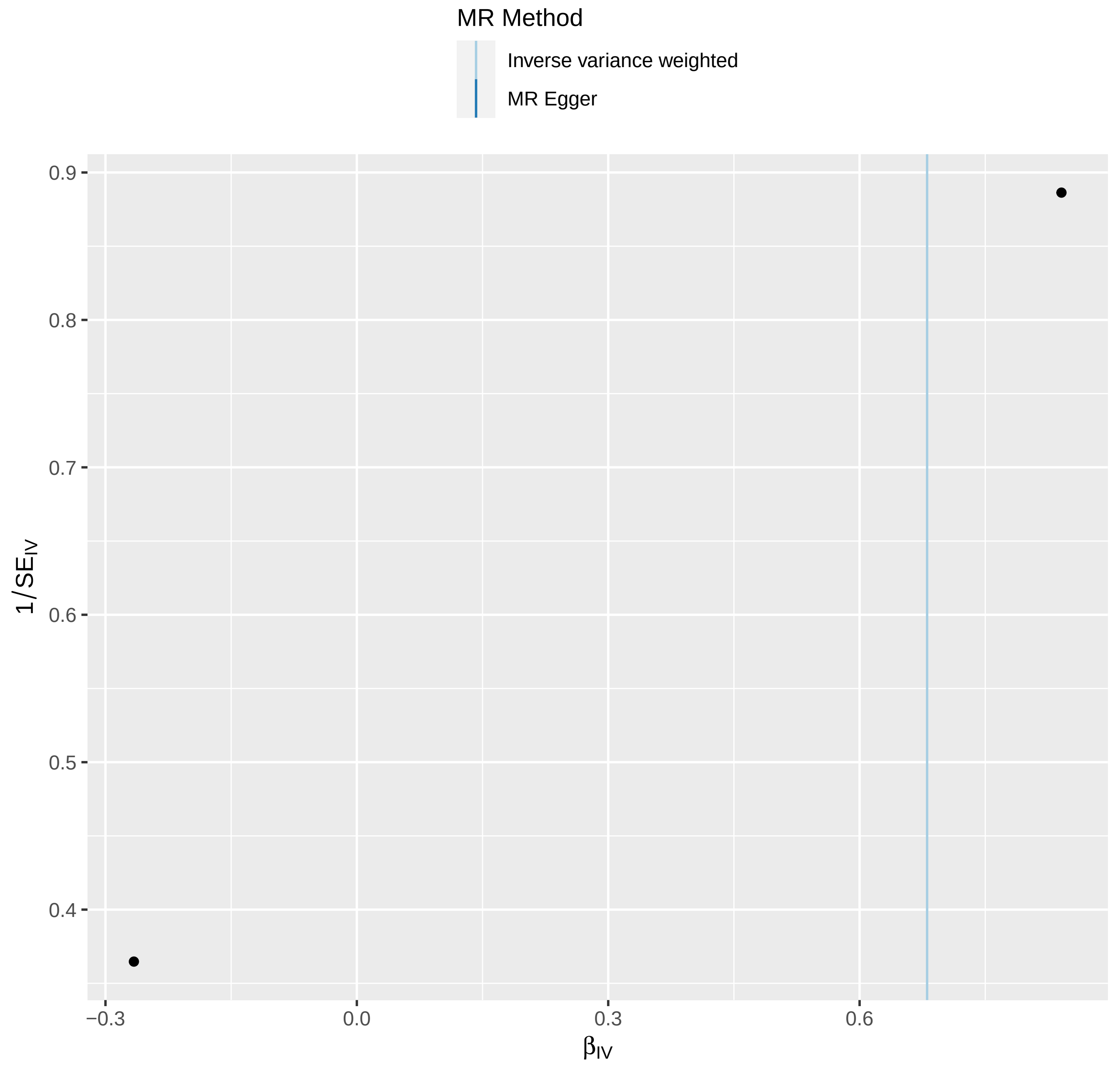

Batch 2686 : Stachydrine on Ulcerative rectosigmoiditis

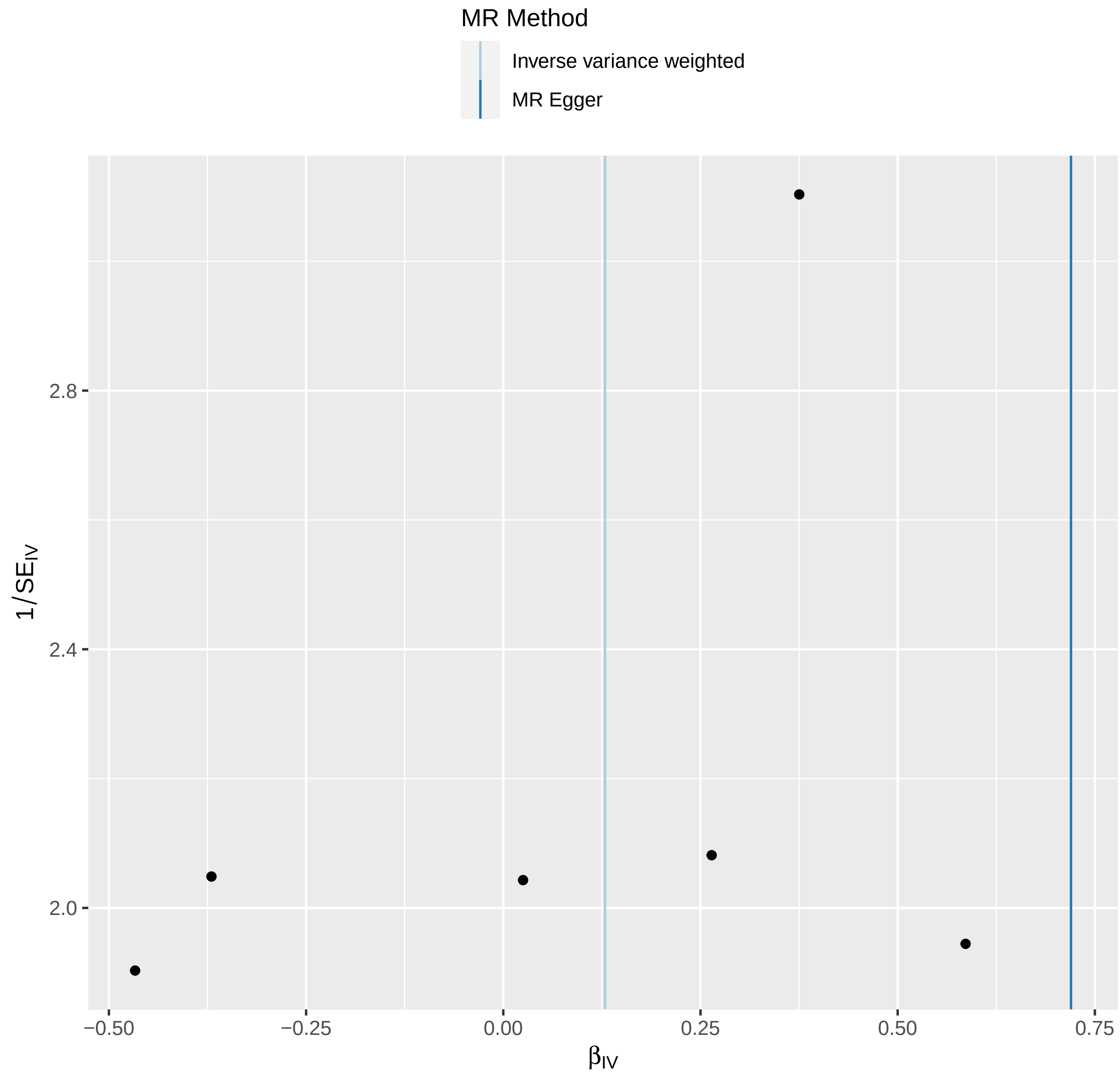

Batch 2687 : Isovalerylcarnitine on Ulcerative rectosigmoiditis

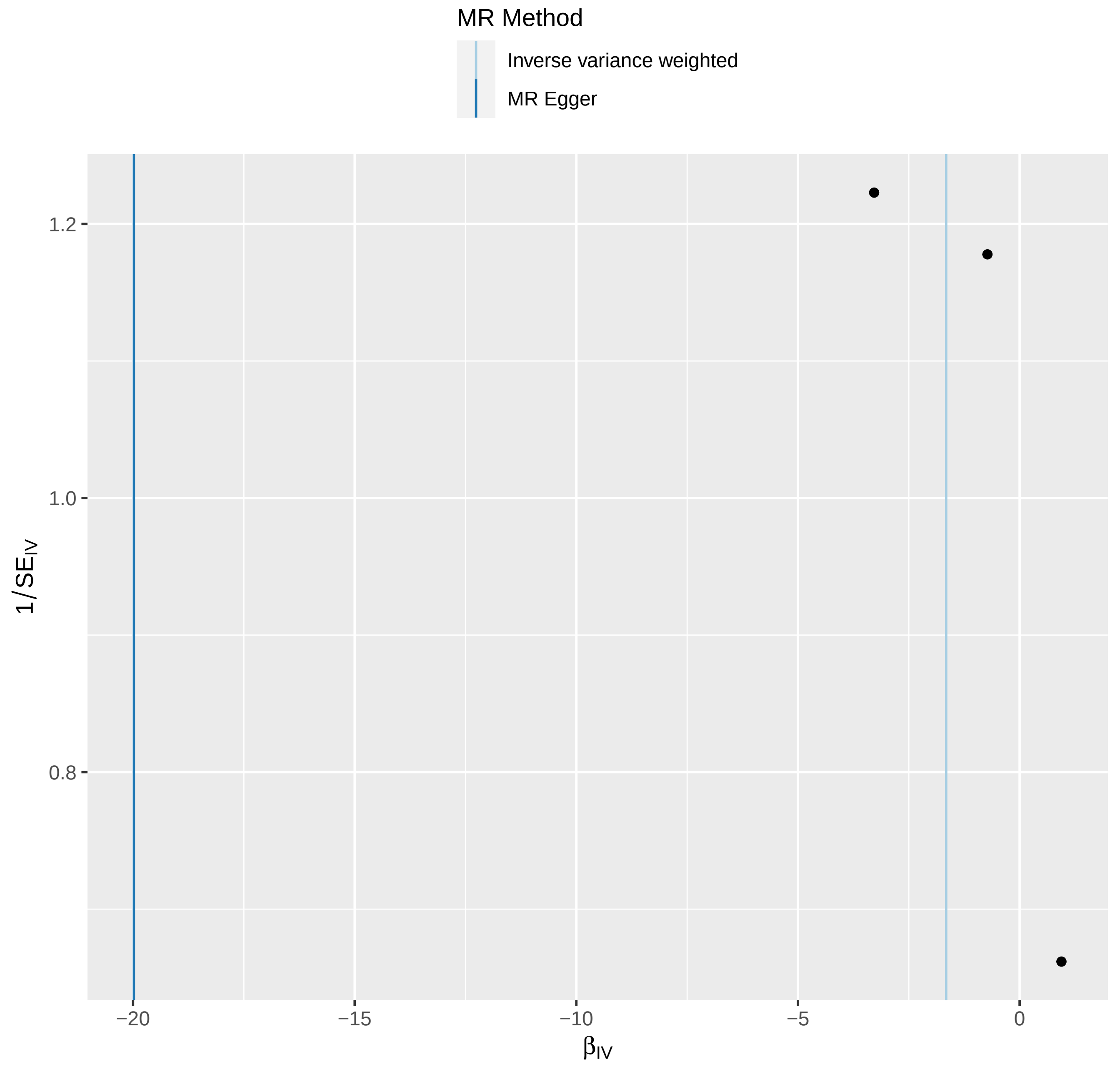

Batch 2690 : 1−linoleoylglycerophosphocholine on Ulcerative rectosigmoiditis

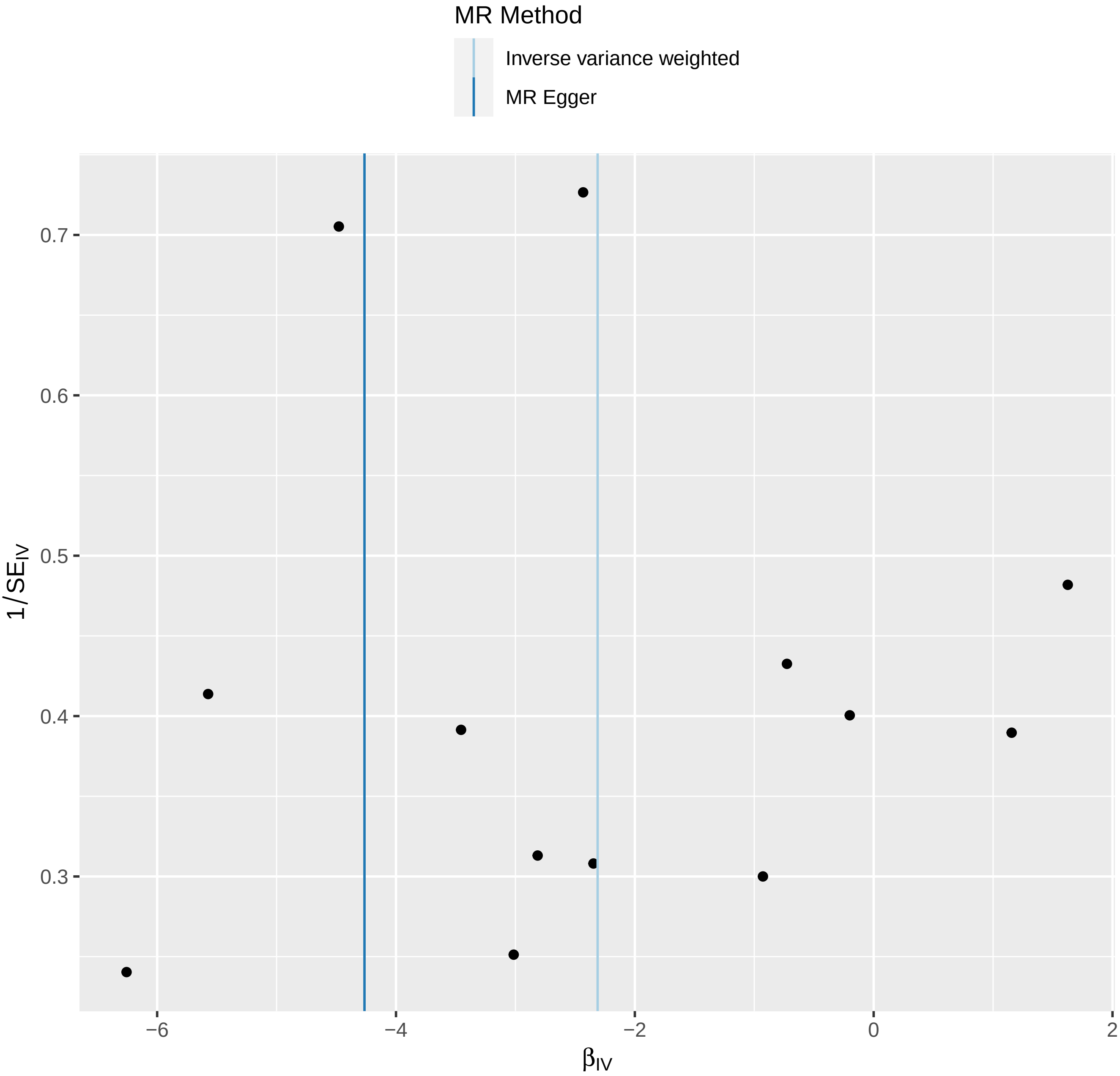

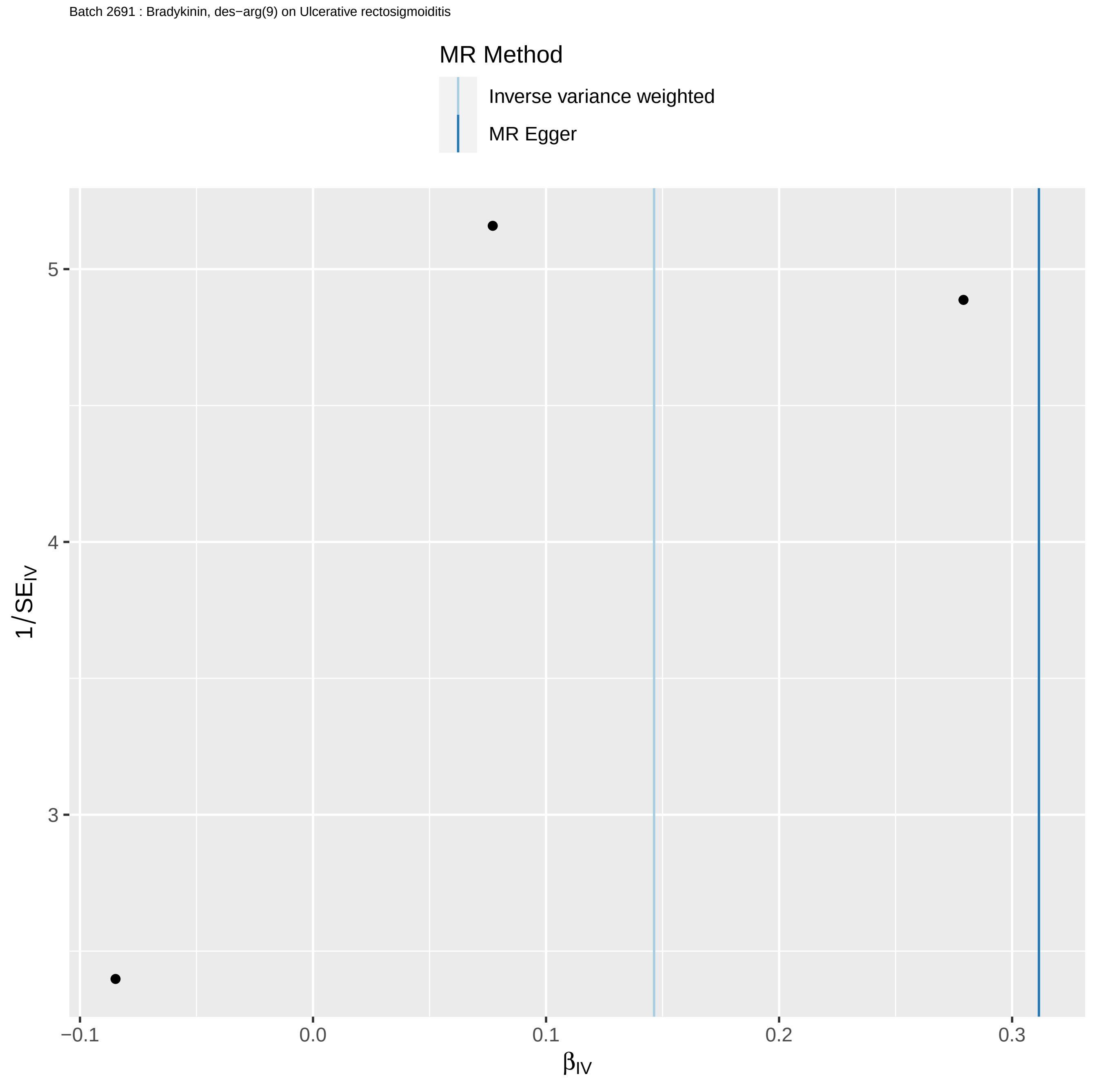
Batch 2691 : Bradykinin, des−arg(9) on Ulcerative rectosigmoiditis
MR Method
Inverse variance weighted
MR Egger
5
4
3
1/SE_IV
−0.1
0.0
0.1
0.2
0.3
β_IV

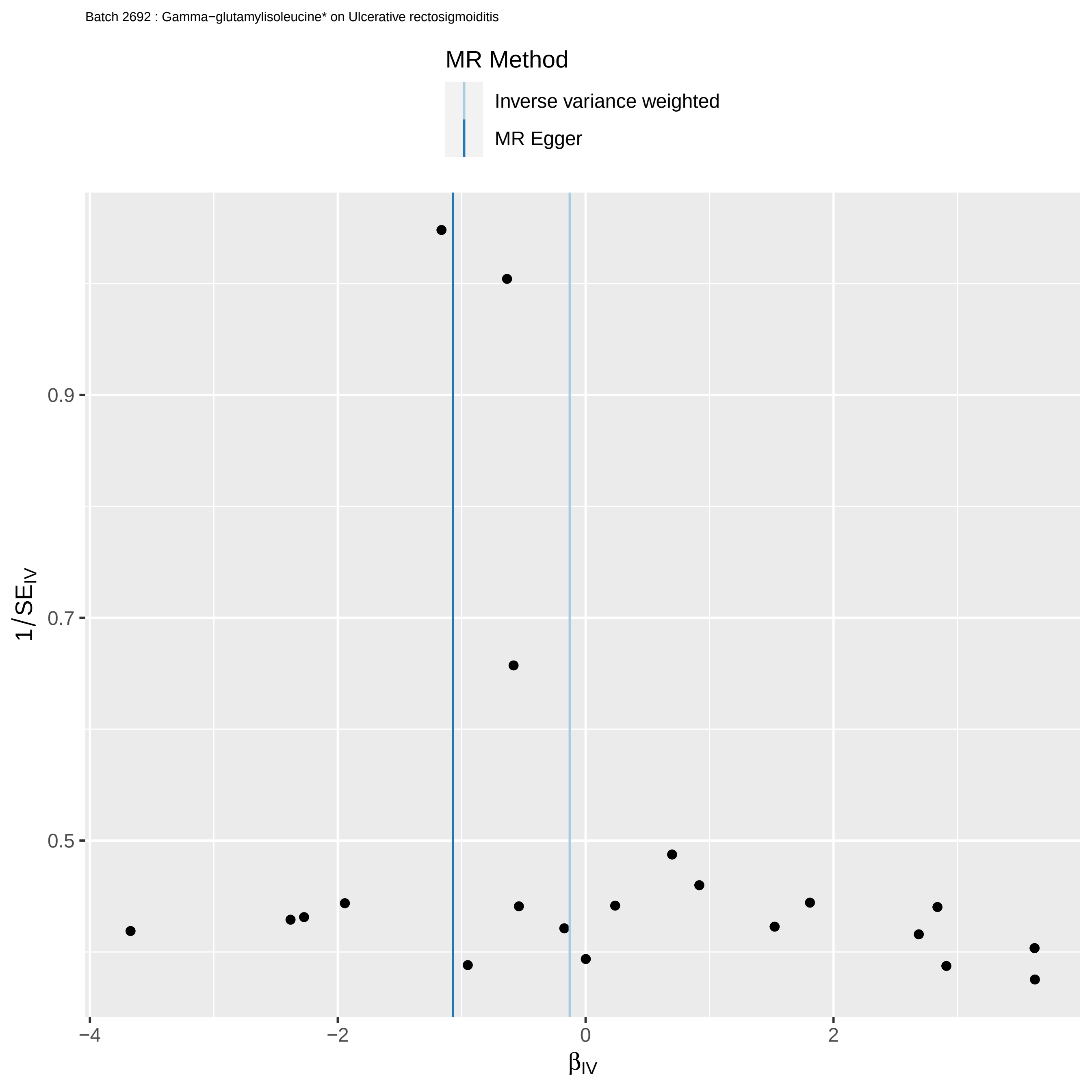
Batch 2692 : Gamma−glutamylisoleucine* on Ulcerative rectosigmoiditis
MR Method
Inverse variance weighted
MR Egger
0.9
0.7
0.5
1/SE$_{IV}$
−4
−2
0
2
β$_{IV}$

Batch 2695 : Isovalerate on Ulcerative rectosigmoiditis

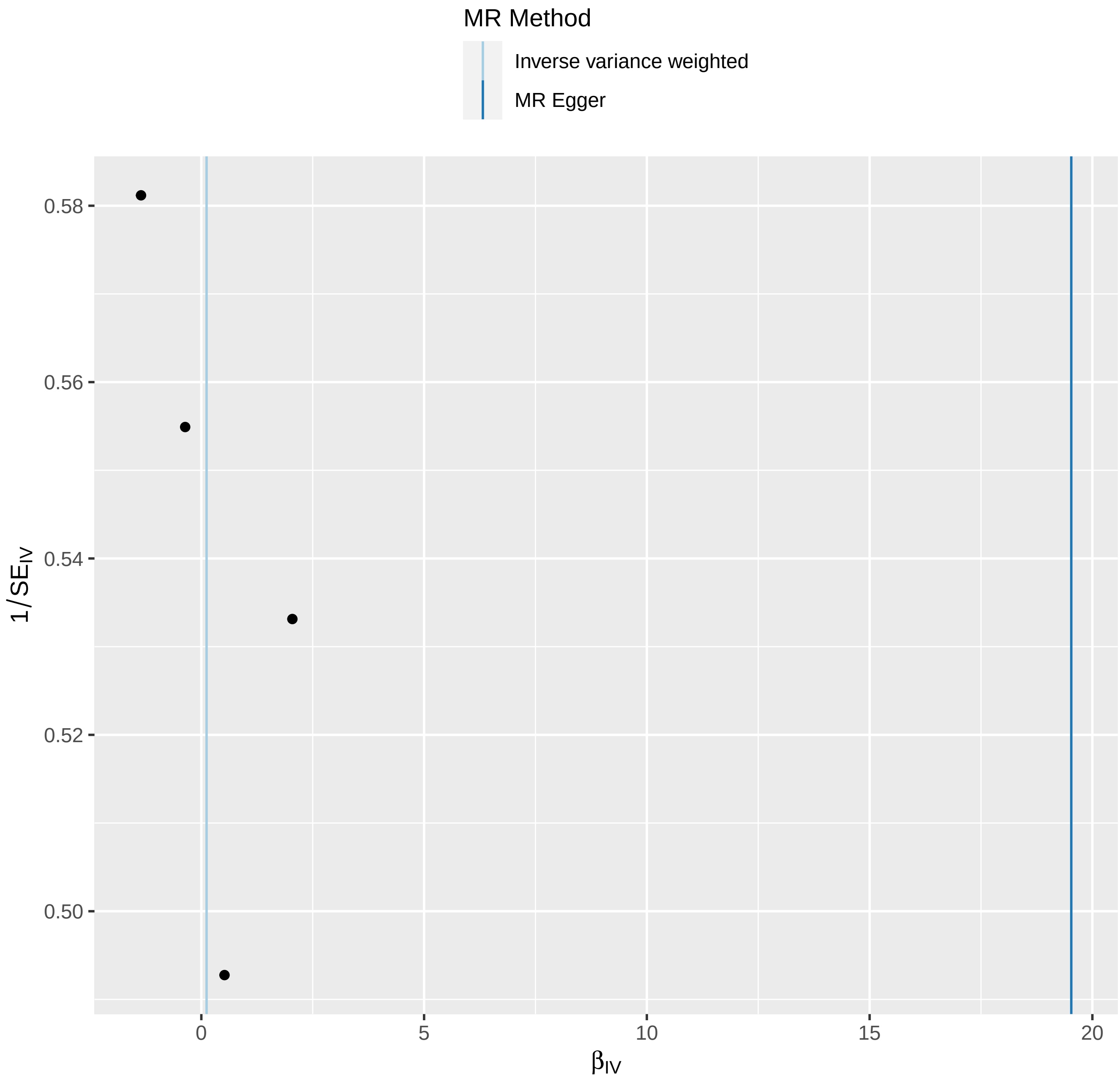

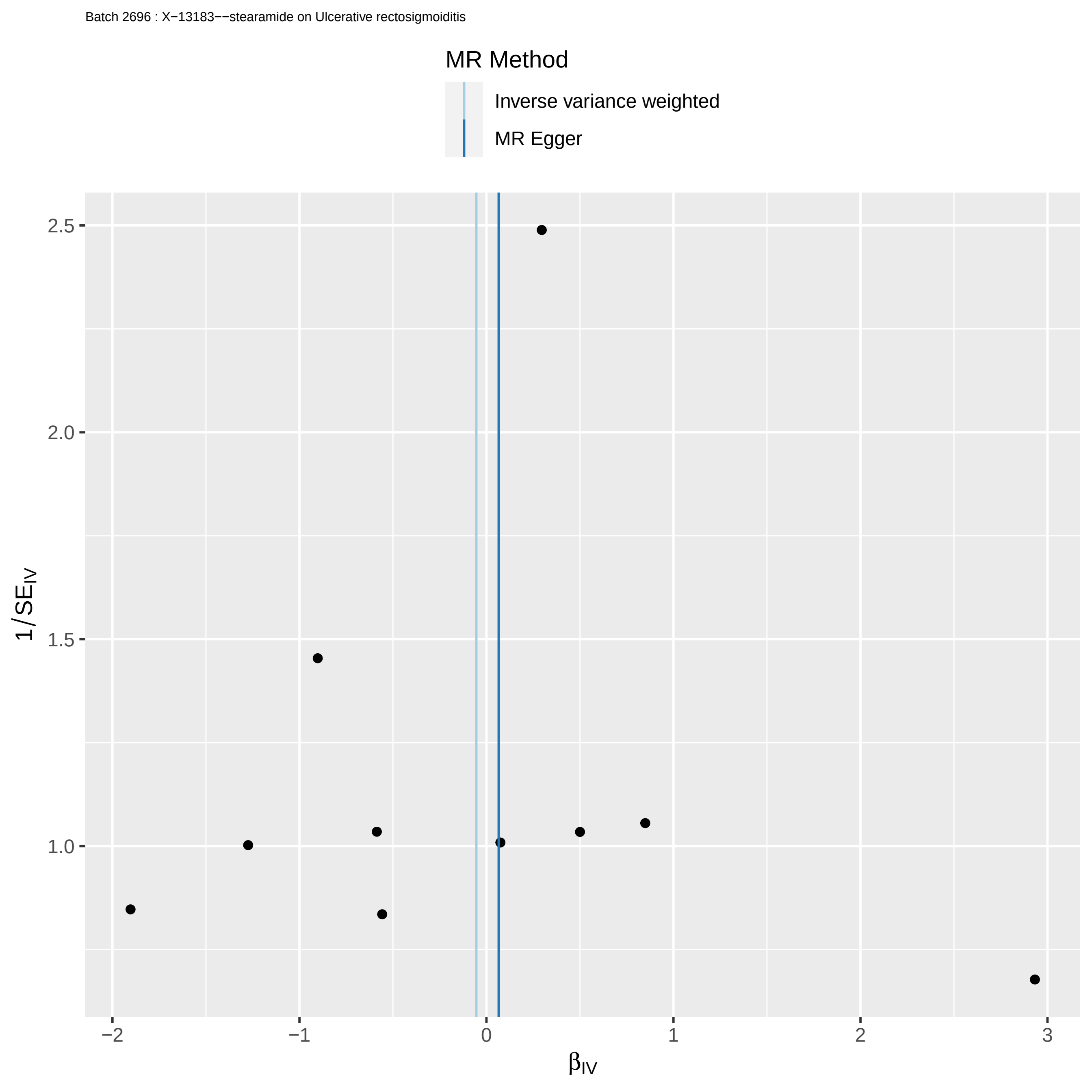
Batch 2696 : X−13183−−stearamide on Ulcerative rectosigmoiditis
MR Method
Inverse variance weighted
MR Egger
2.5
2.0
1.5
1.0
1/SE_IV
−2
−1
0
1
2
3
β_IV

Batch 2697 : 7−methylguanine on Ulcerative rectosigmoiditis

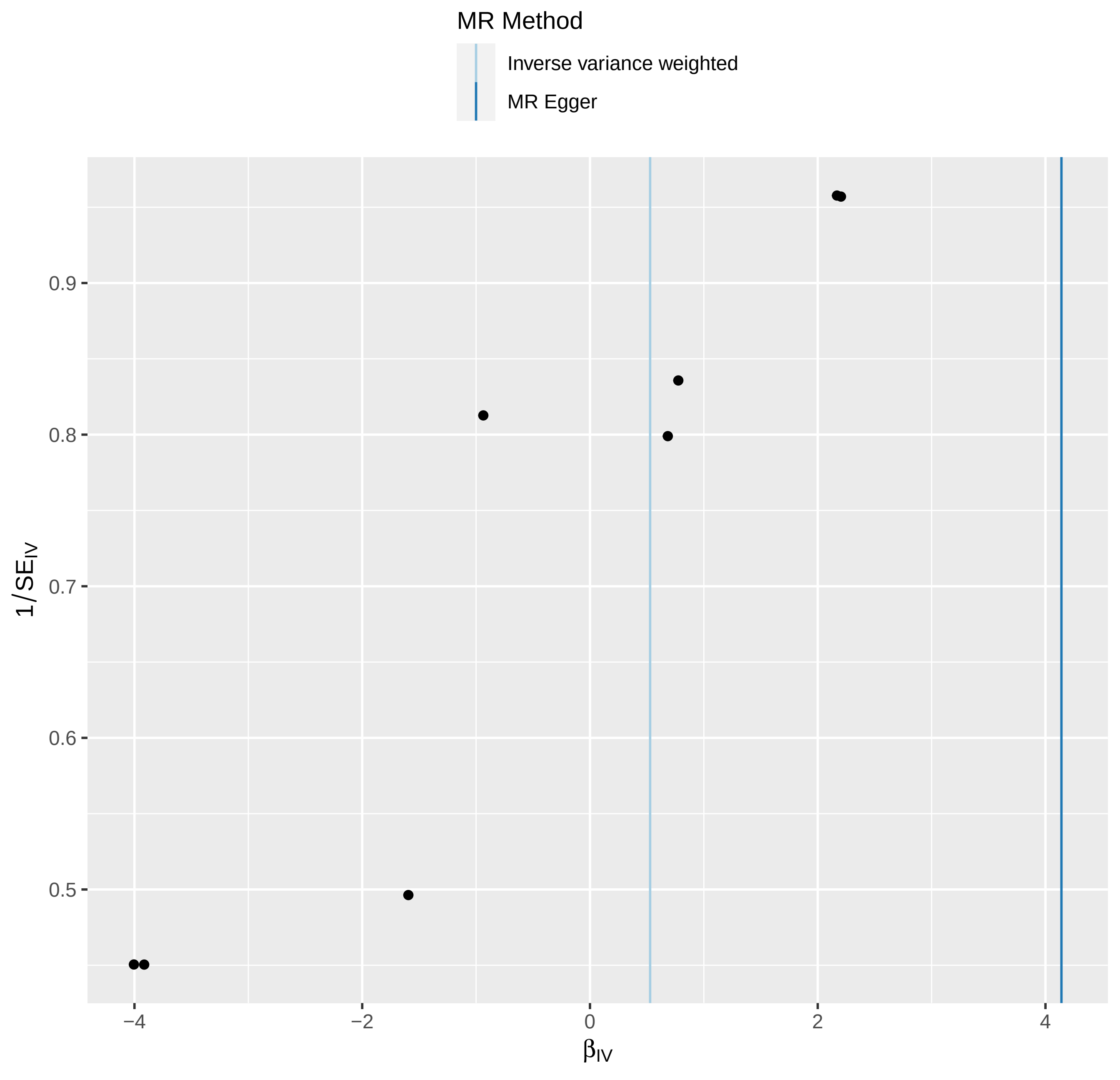

Batch 2704 : X−13431−−nonanoylcarnitine* on Ulcerative rectosigmoiditis

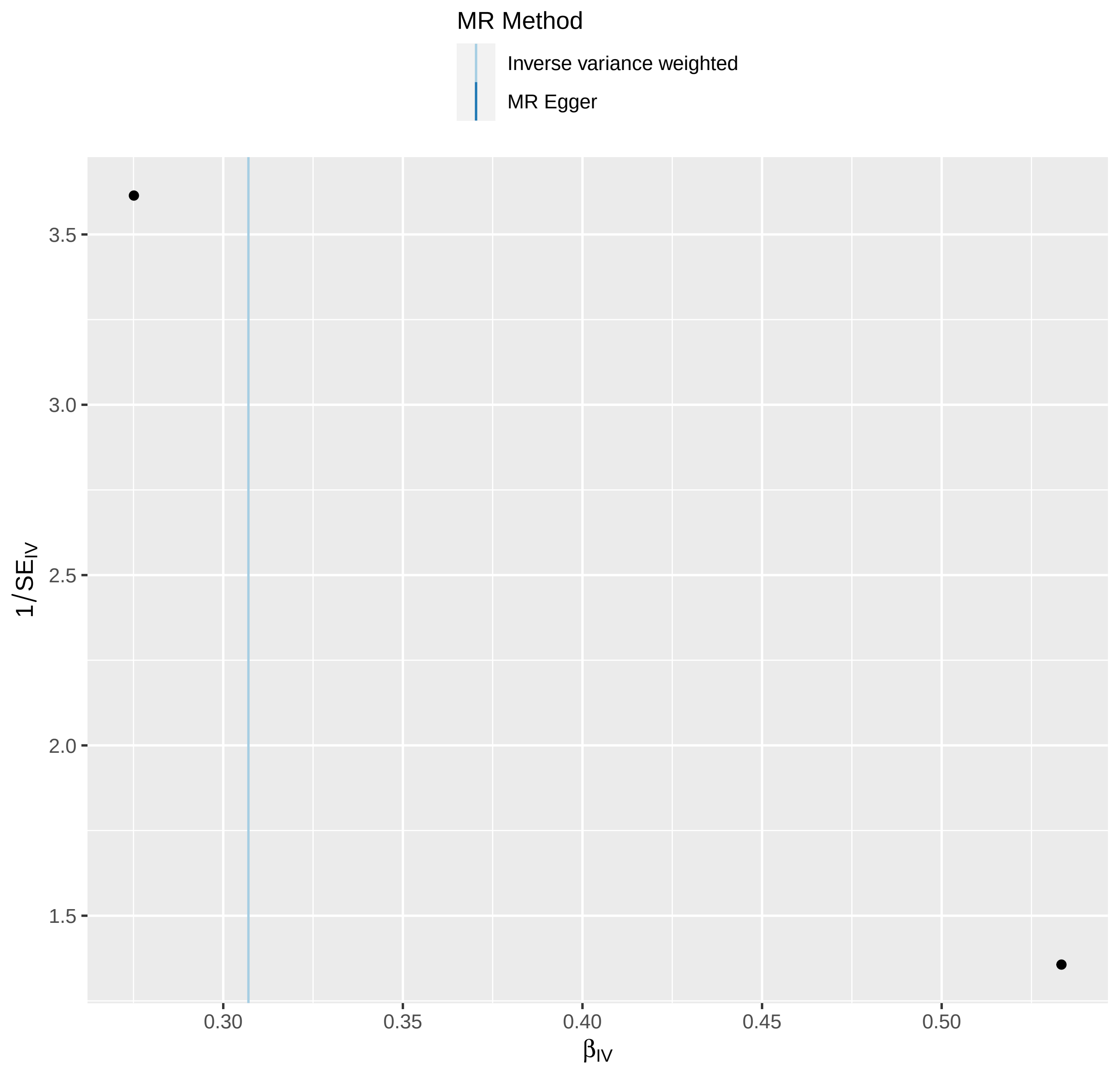

Batch 2705 : 2−palmitoylglycerophosphocholine* on Ulcerative rectosigmoiditis

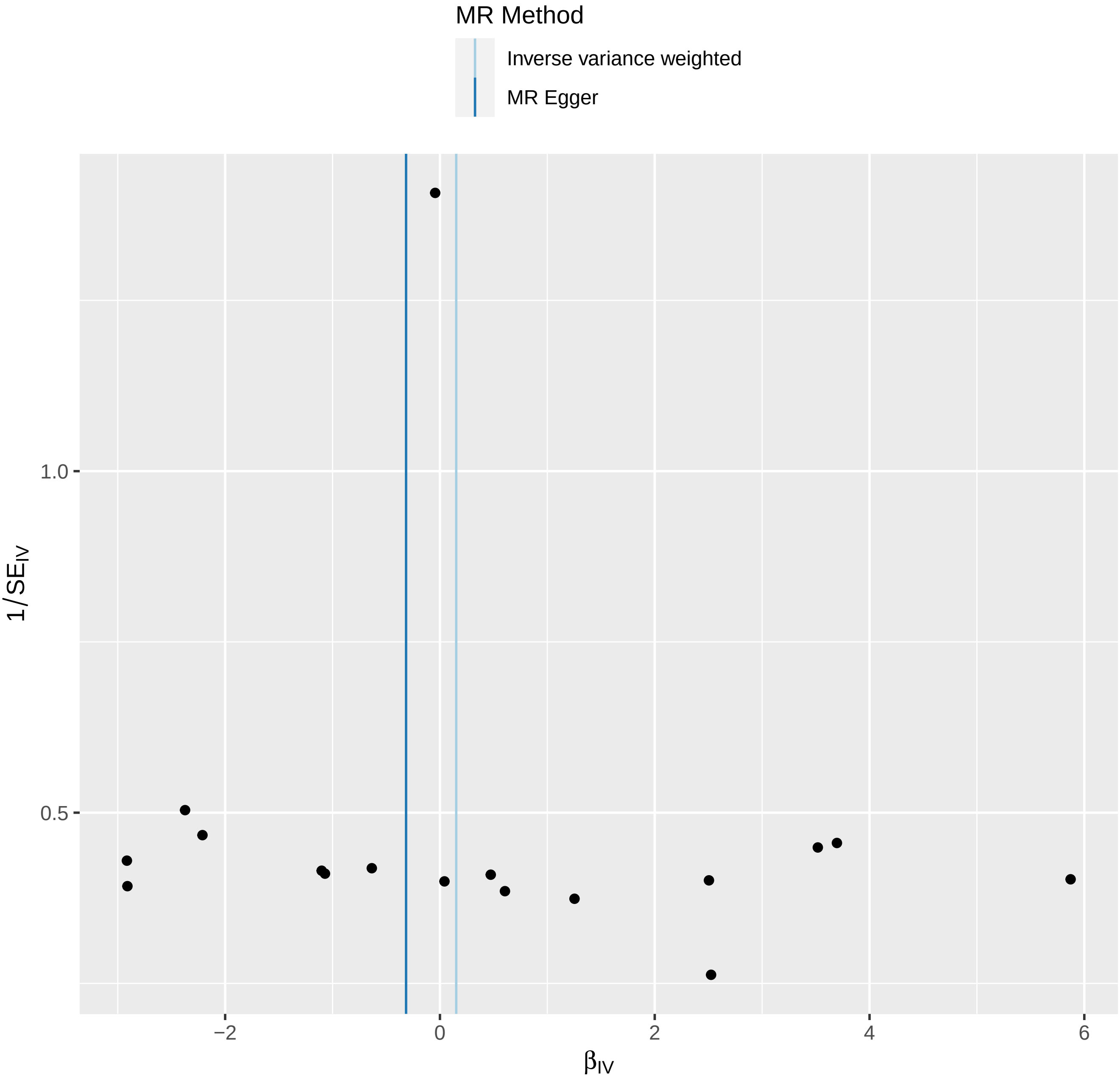

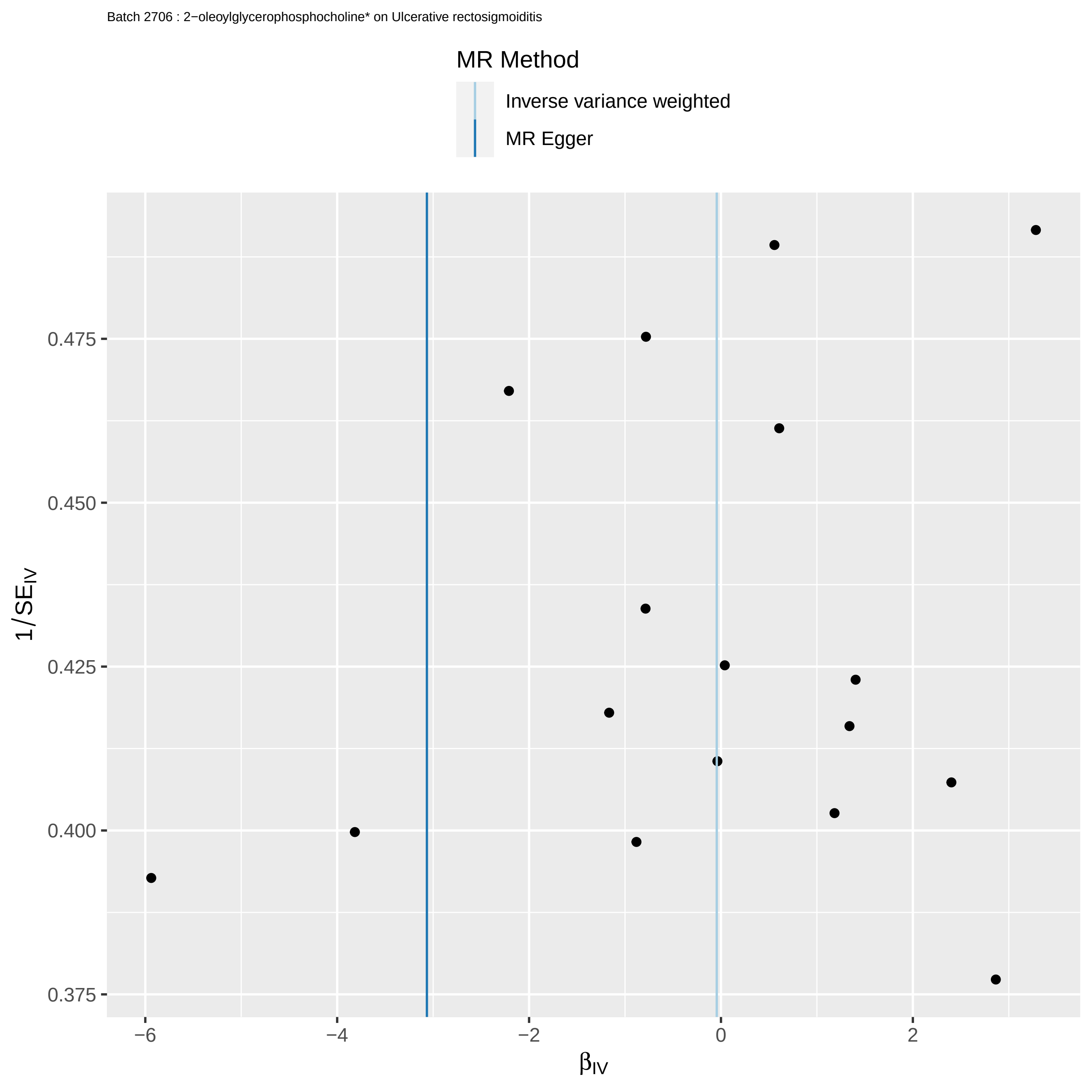

Batch 2706 : 2−oleoylglycerophosphocholine* on Ulcerative rectosigmoiditis
MR Method
Inverse variance weighted
MR Egger
0.475
0.450
0.425
0.400
0.375
1/SE$_{IV}$
−6
−4
−2
0
2
β$_{IV}$

Batch 2709 : 1−palmitoylglycerophosphoinositol* on Ulcerative rectosigmoiditis

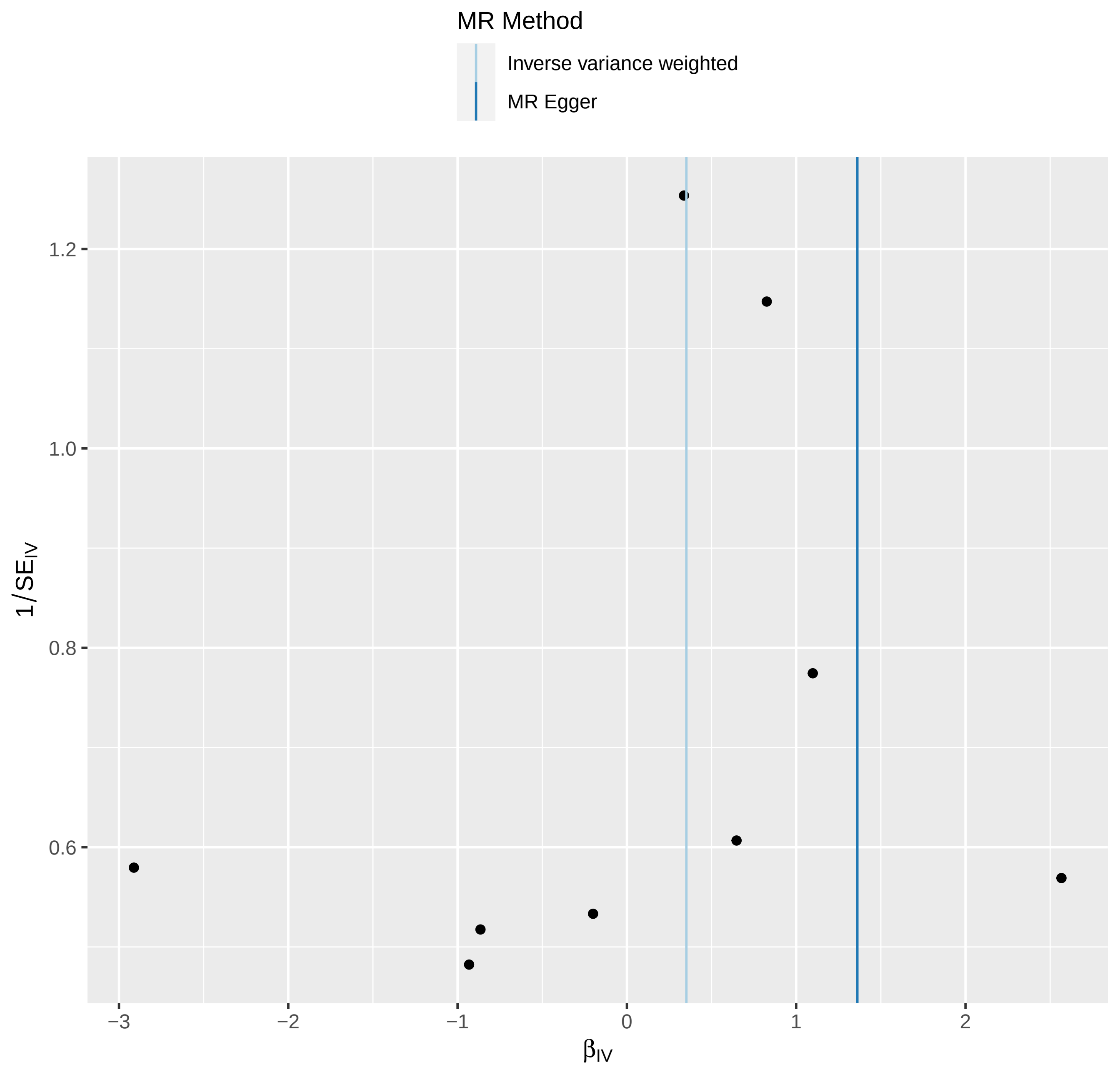

Batch 2712 : Glutaroyl carnitine on Ulcerative rectosigmoiditis

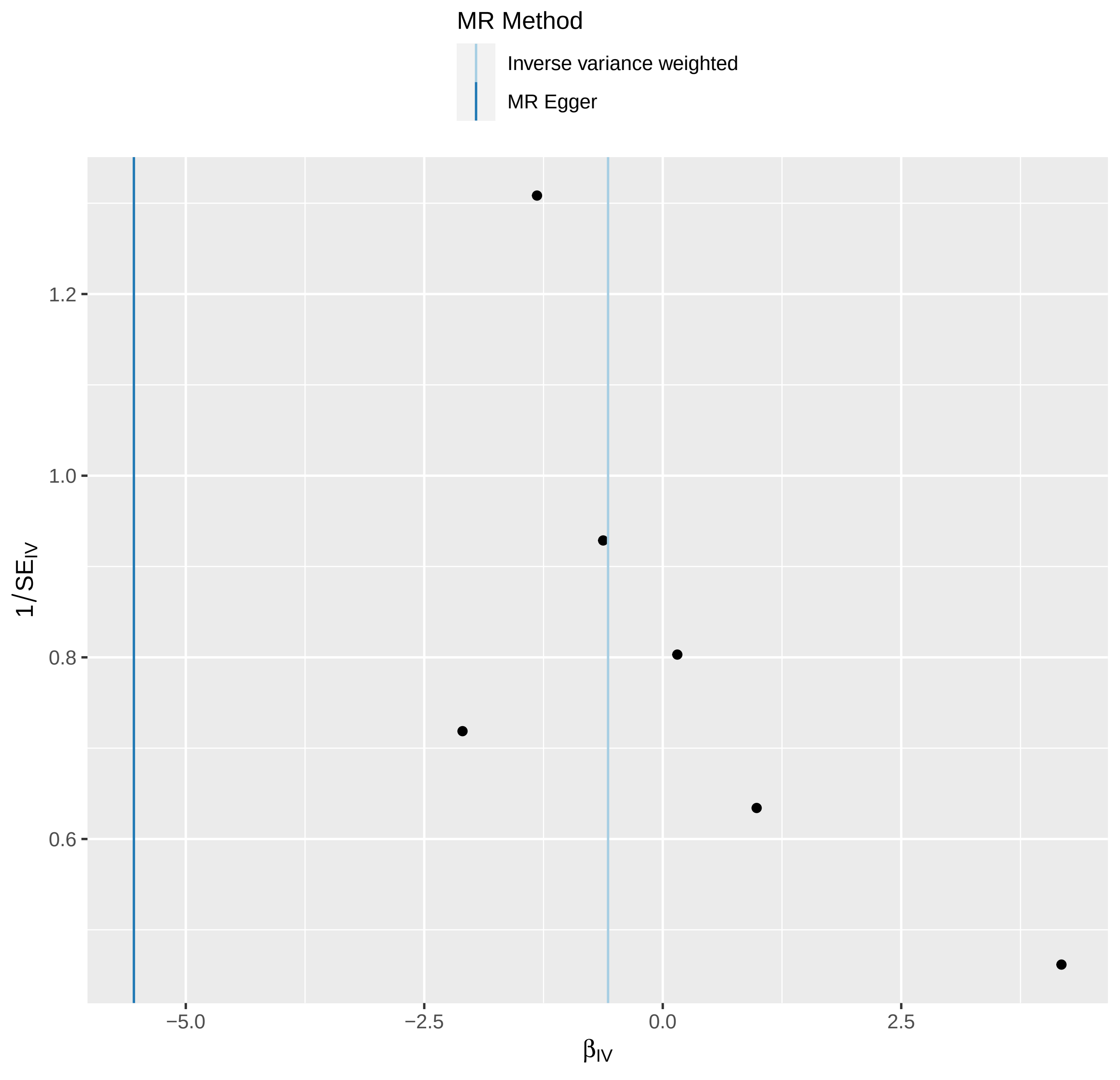

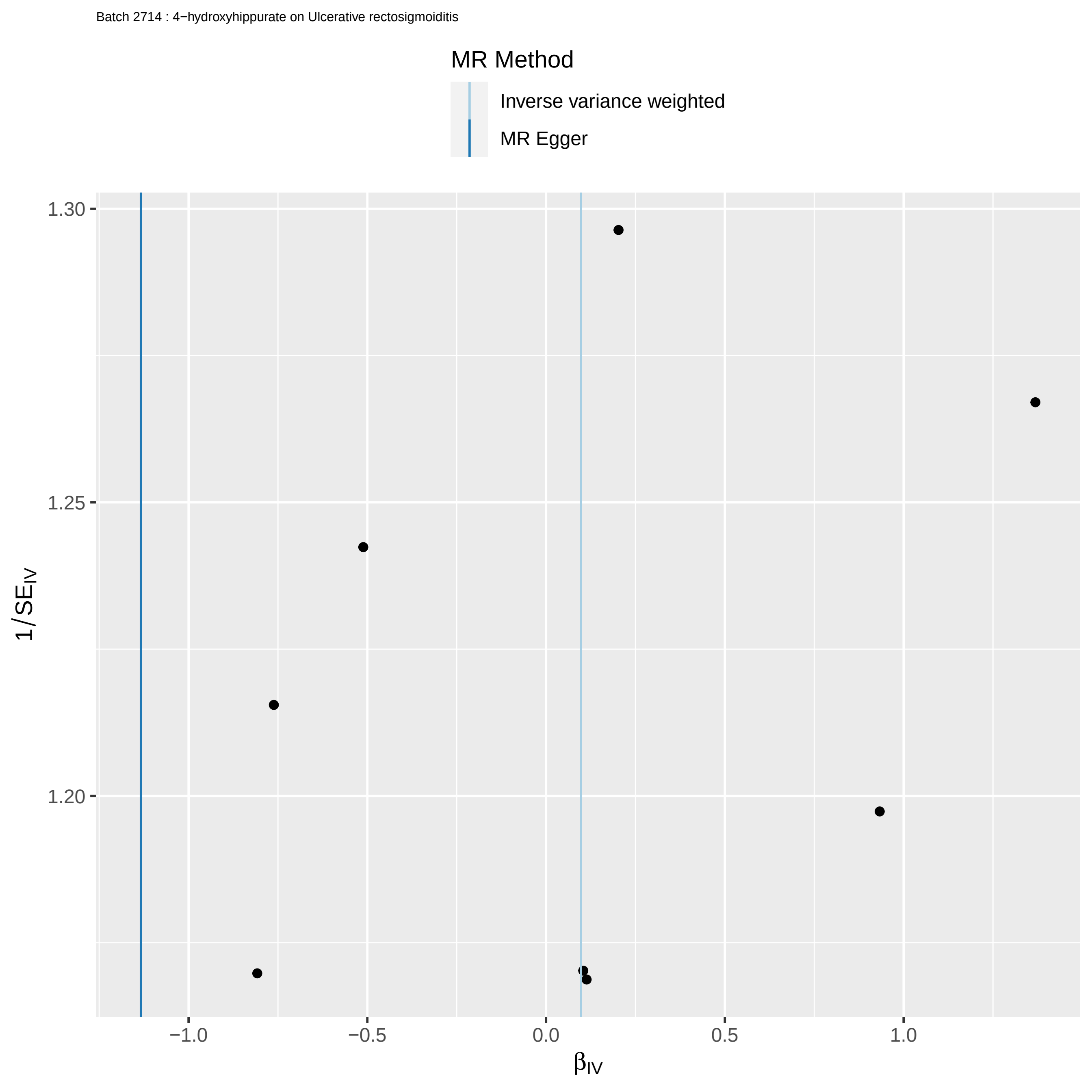
Batch 2714 : 4−hydroxyhippurate on Ulcerative rectosigmoiditis
MR Method
Inverse variance weighted
MR Egger
1.30
1.25
1.20
1/SE_IV
−1.0
−0.5
0.0
0.5
1.0
β_IV

Batch 2715 : 1−myristoylglycerophosphocholine on Ulcerative rectosigmoiditis

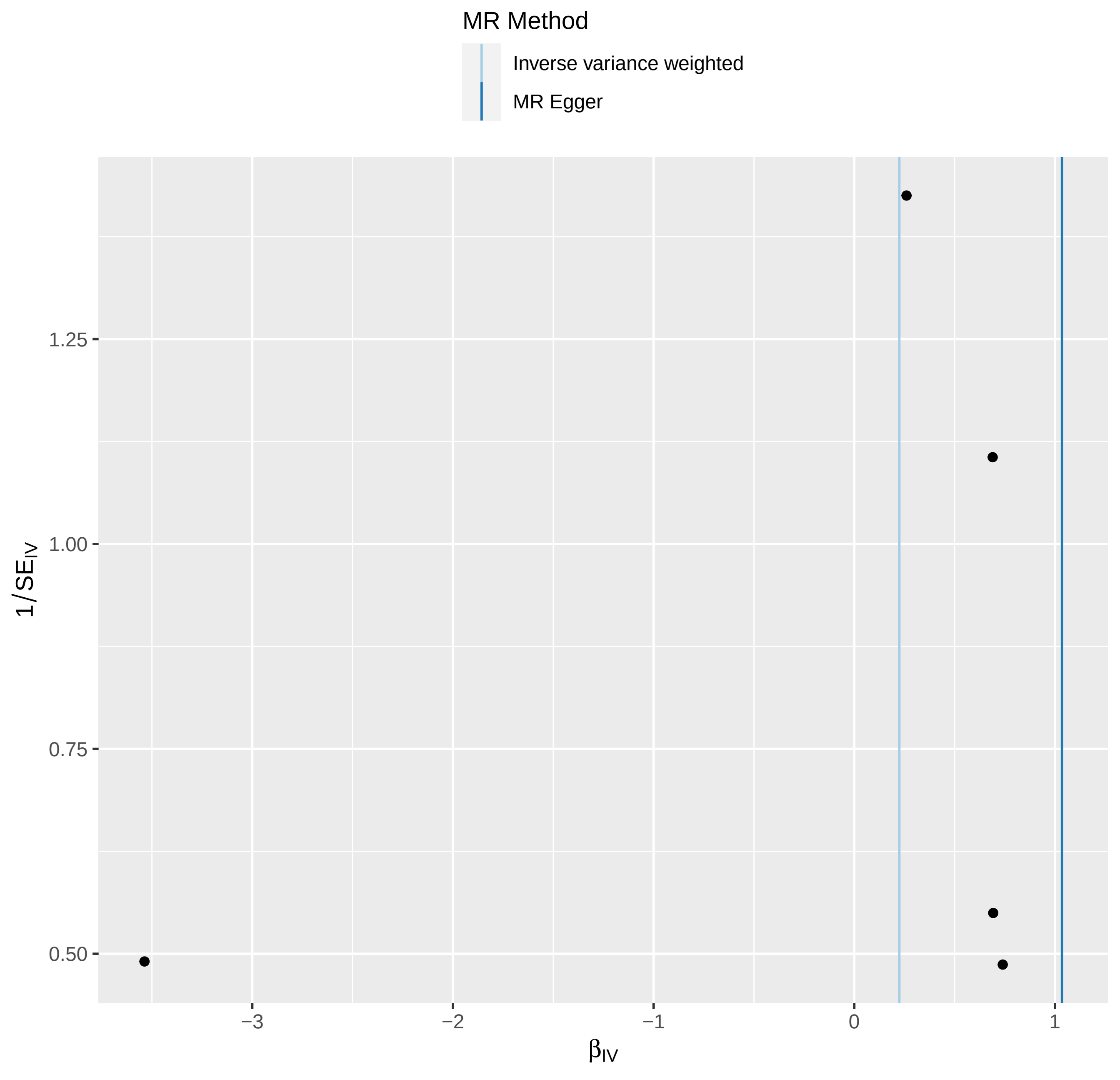

Batch 2718 : 3−(3−hydroxyphenyl)propionate on Ulcerative rectosigmoiditis

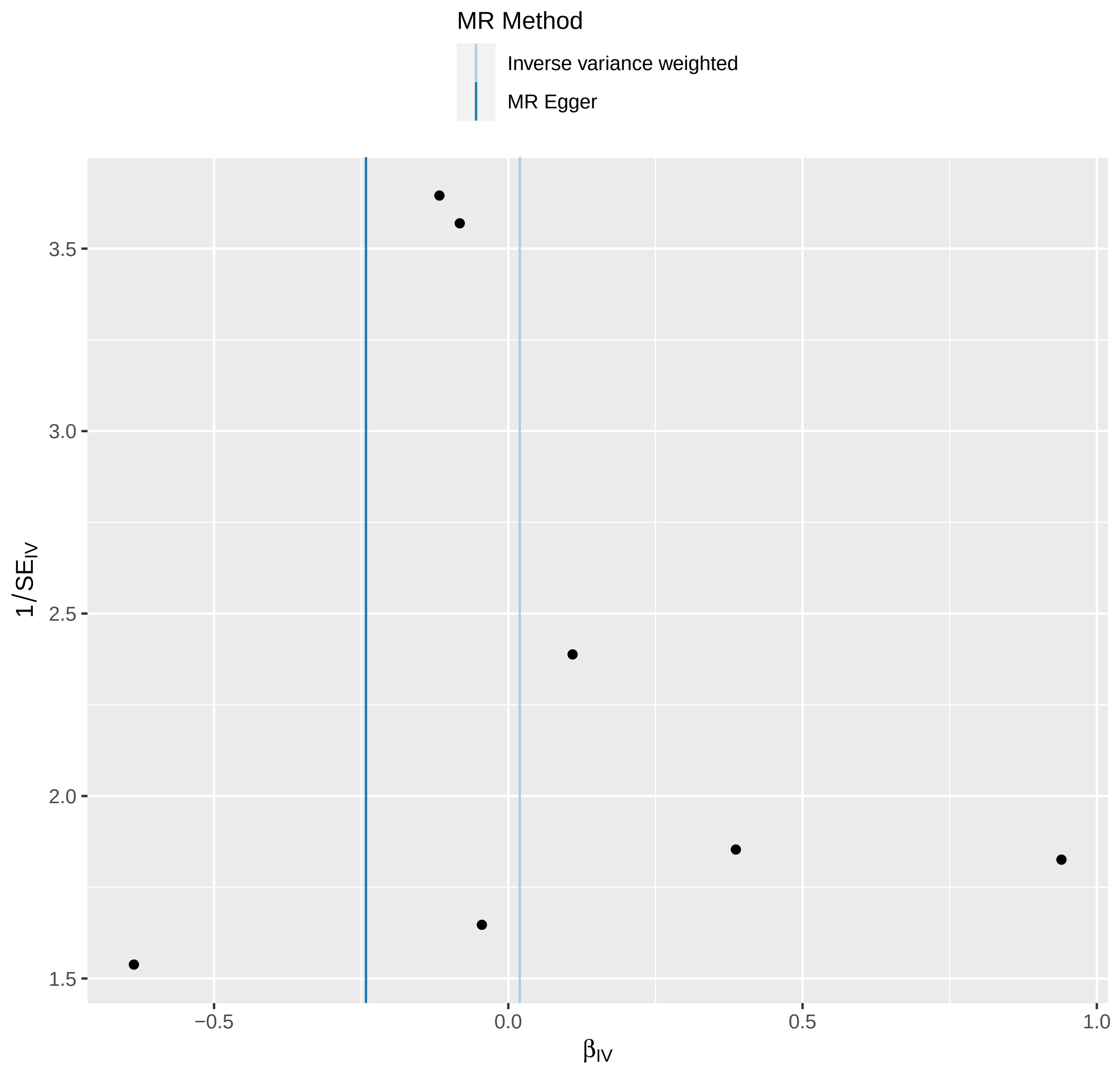

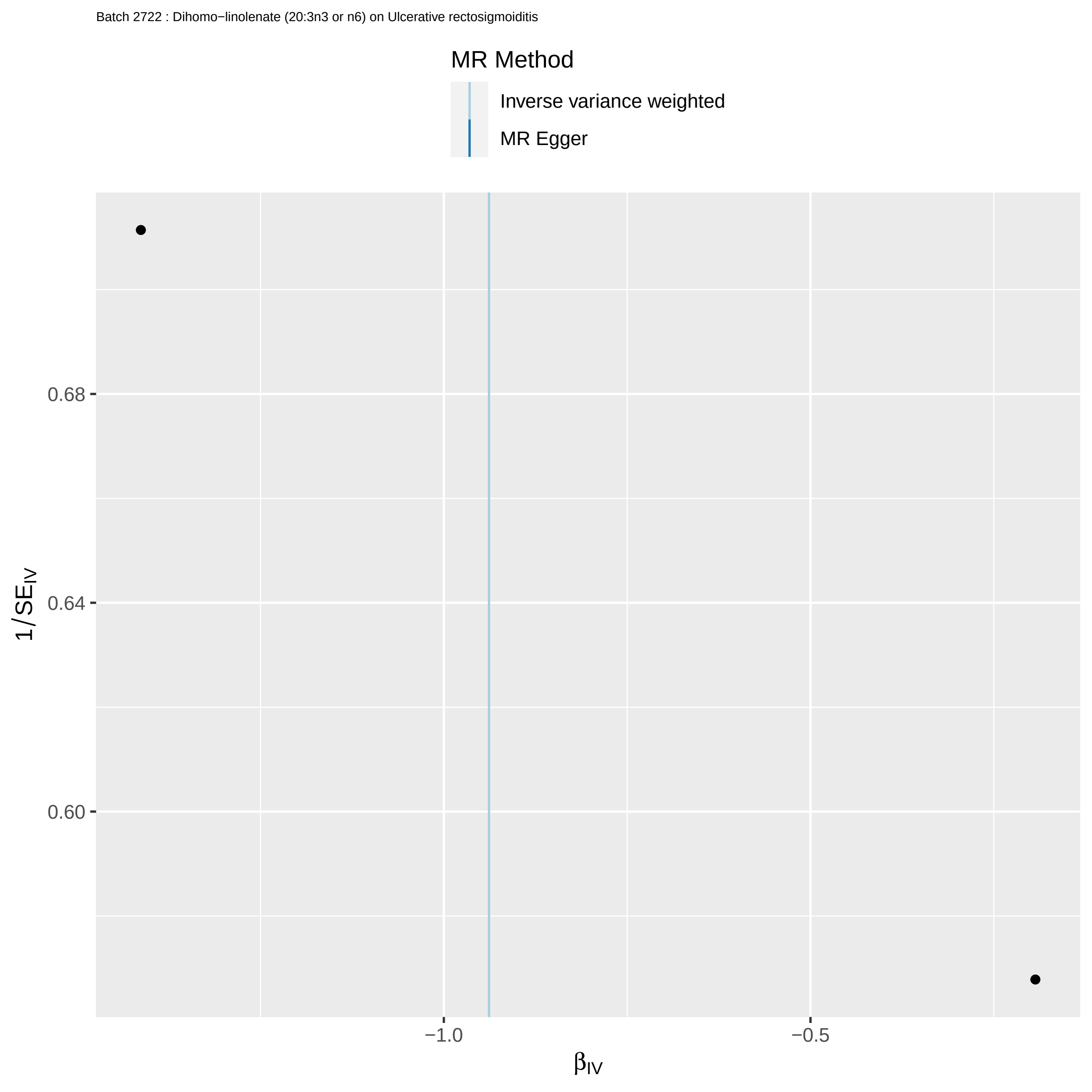
Batch 2722 : Dihomo−linolenate (20:3n3 or n6) on Ulcerative rectosigmoiditis
MR Method
Inverse variance weighted
MR Egger
0.68
0.64
0.60
$1/SE_{IV}$
−1.0
−0.5
β_{IV}

Batch 2723 : Threitol on Ulcerative rectosigmoiditis

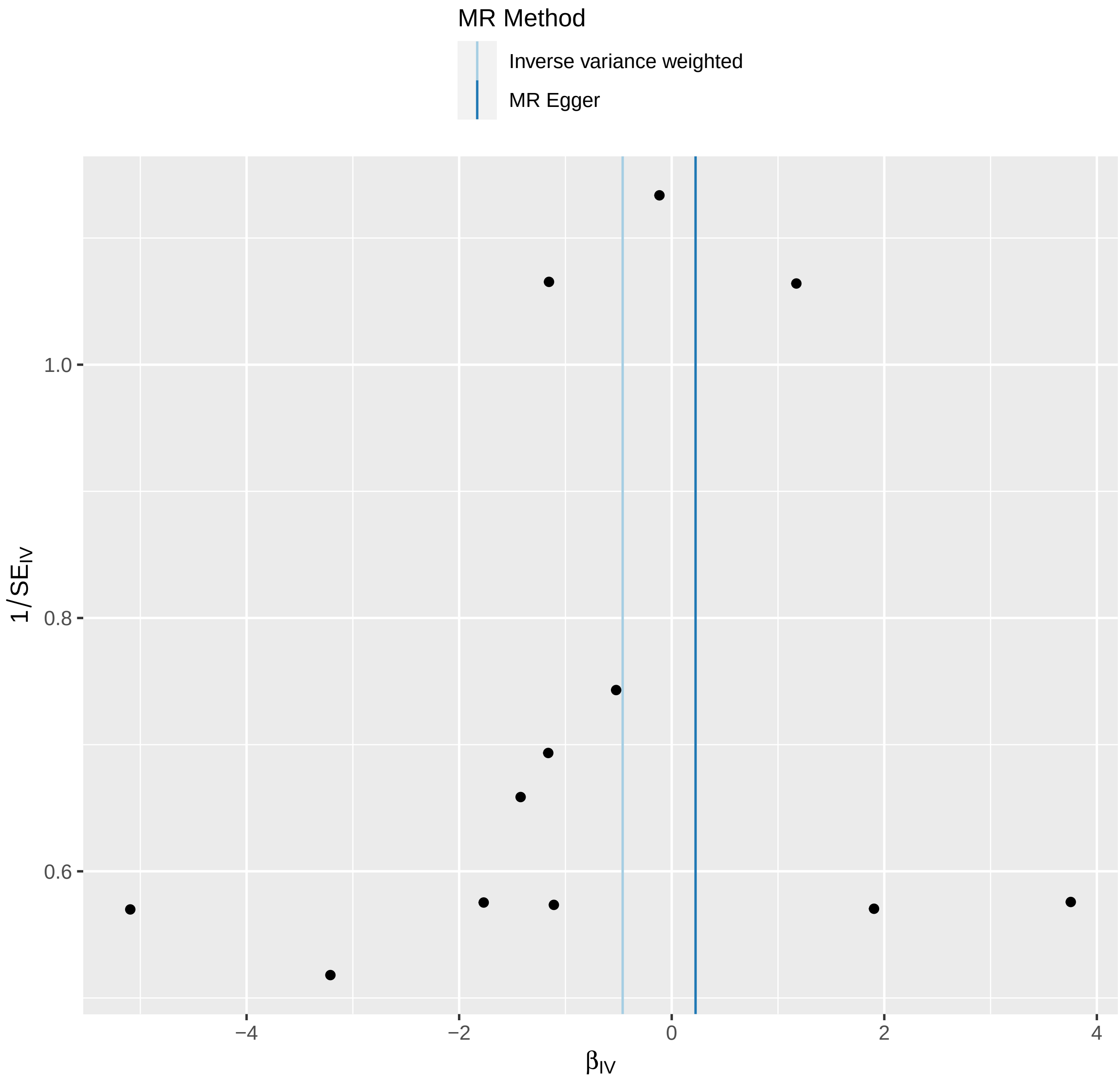

Batch 2724 : P−cresol sulfate on Ulcerative rectosigmoiditis

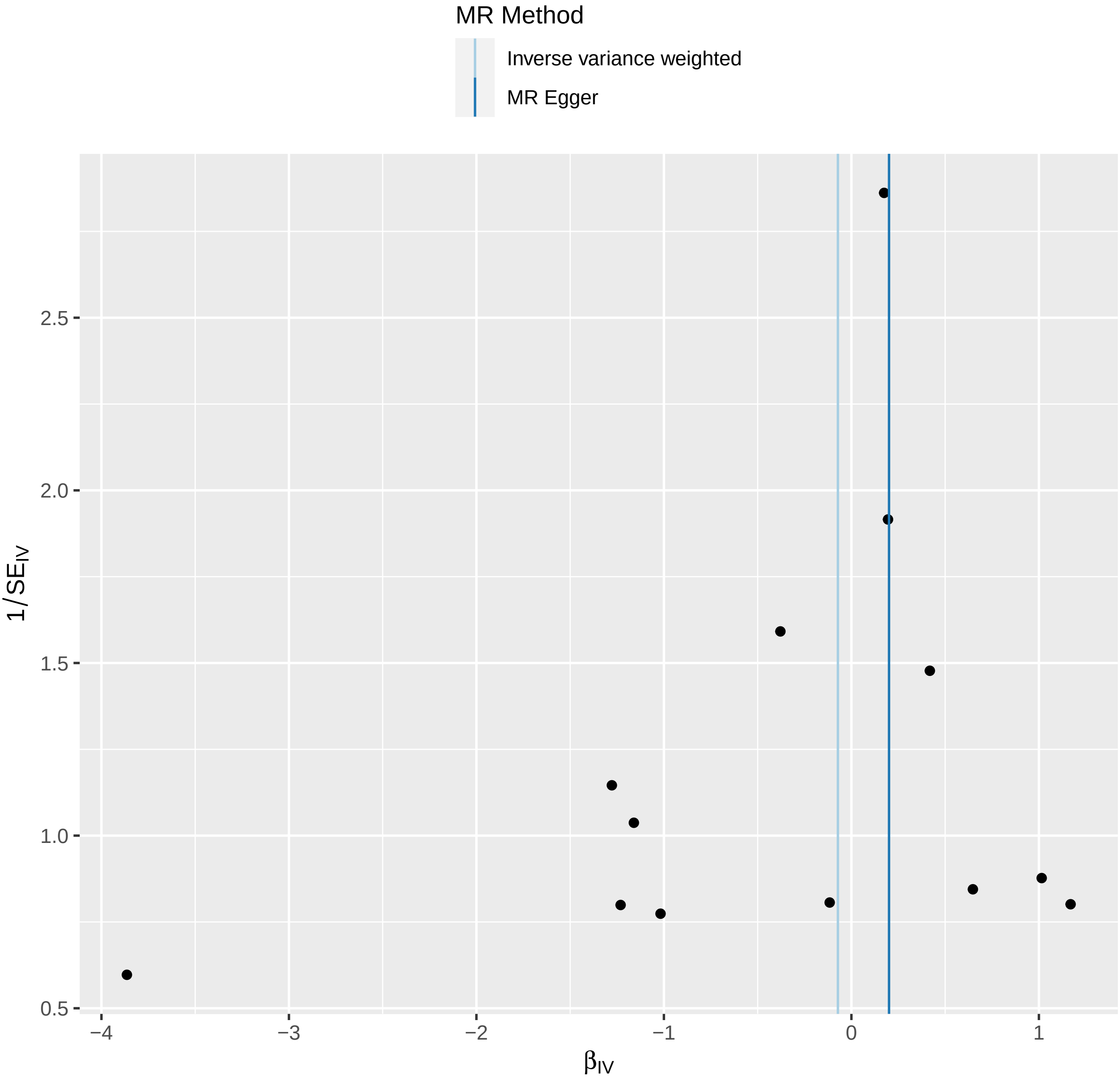

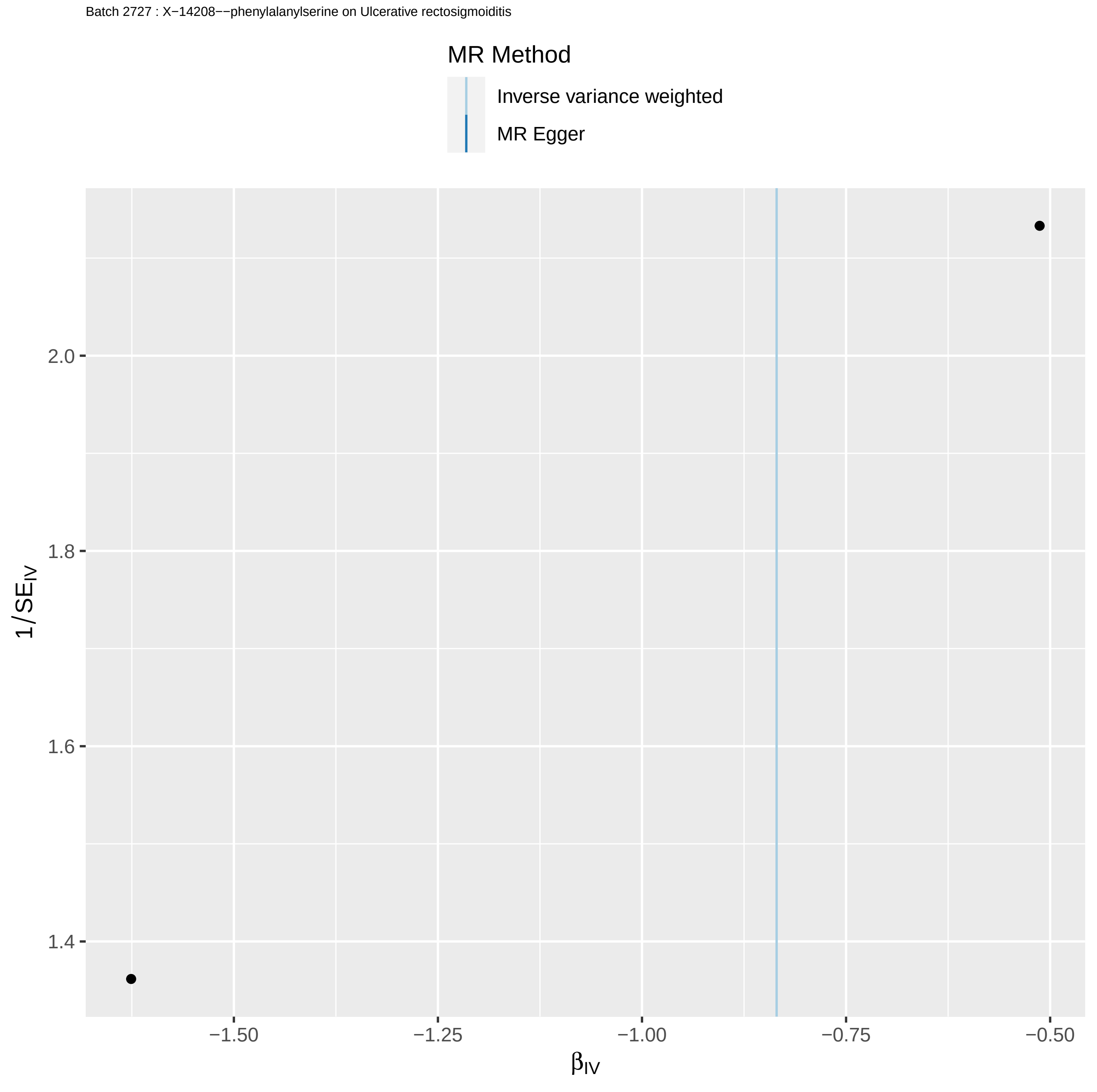

Batch 2727 : X−14208−−phenylalanylserine on Ulcerative rectosigmoiditis
MR Method
Inverse variance weighted
MR Egger
1/SE_IV
2.0
1.8
1.6
1.4
−1.50
−1.25
−1.00
−0.75
−0.50
β_IV

Batch 2729 : X−14450−−phenylalanylleucine on Ulcerative rectosigmoiditis

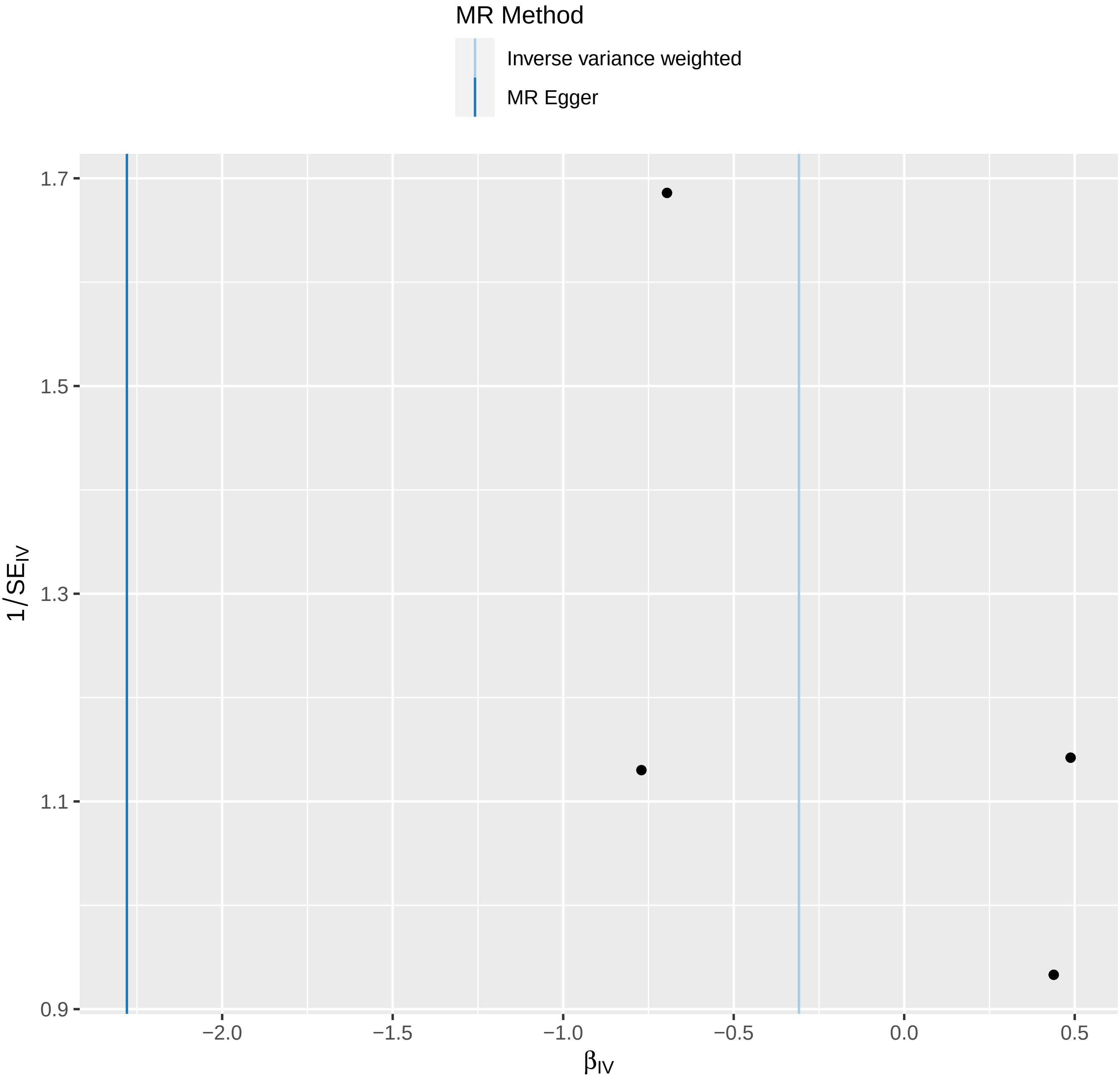

Batch 2730 : Gamma−glutamylglutamate on Ulcerative rectosigmoiditis

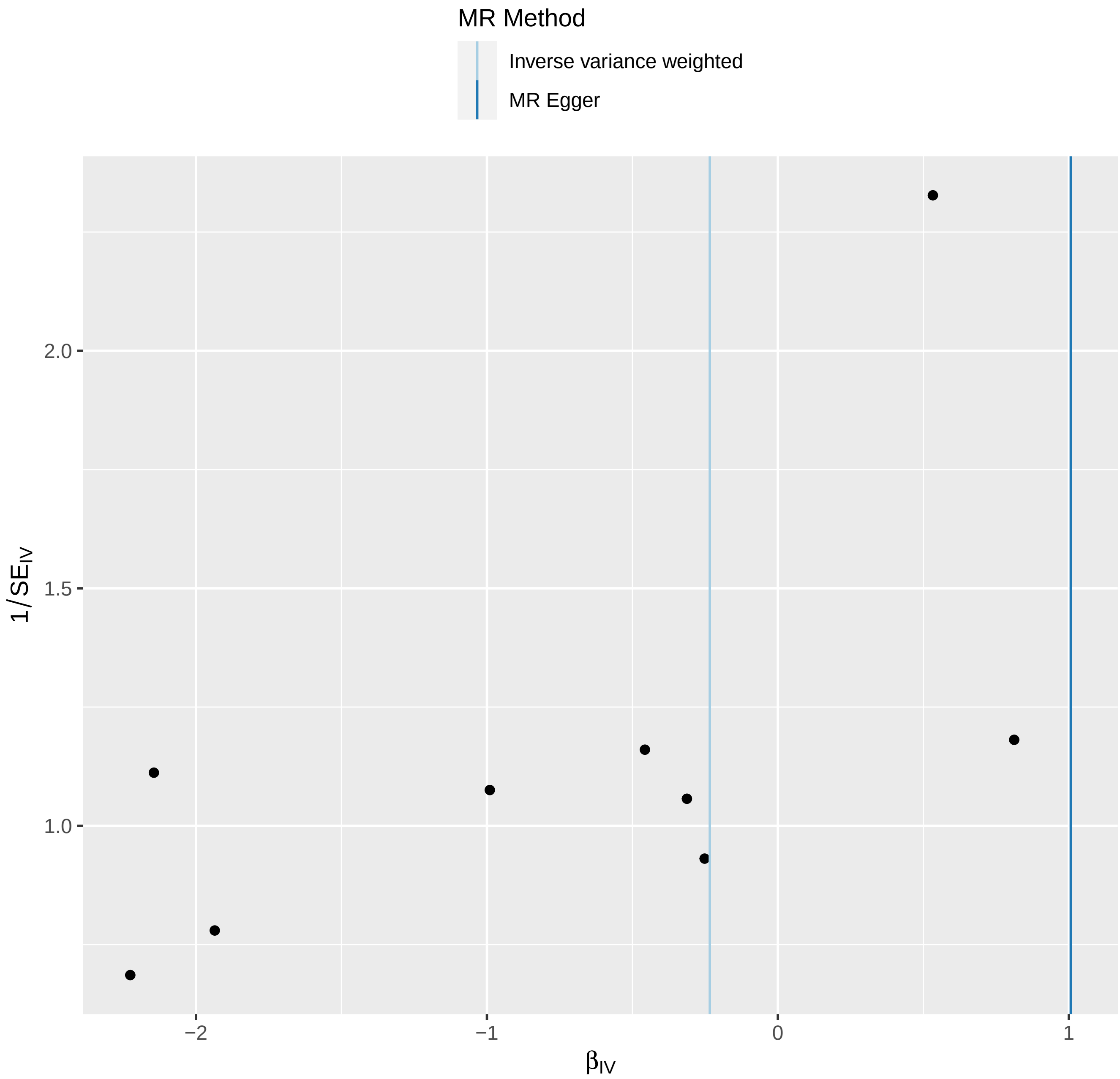

Batch 2731 : Octadecanedioate on Ulcerative rectosigmoiditis

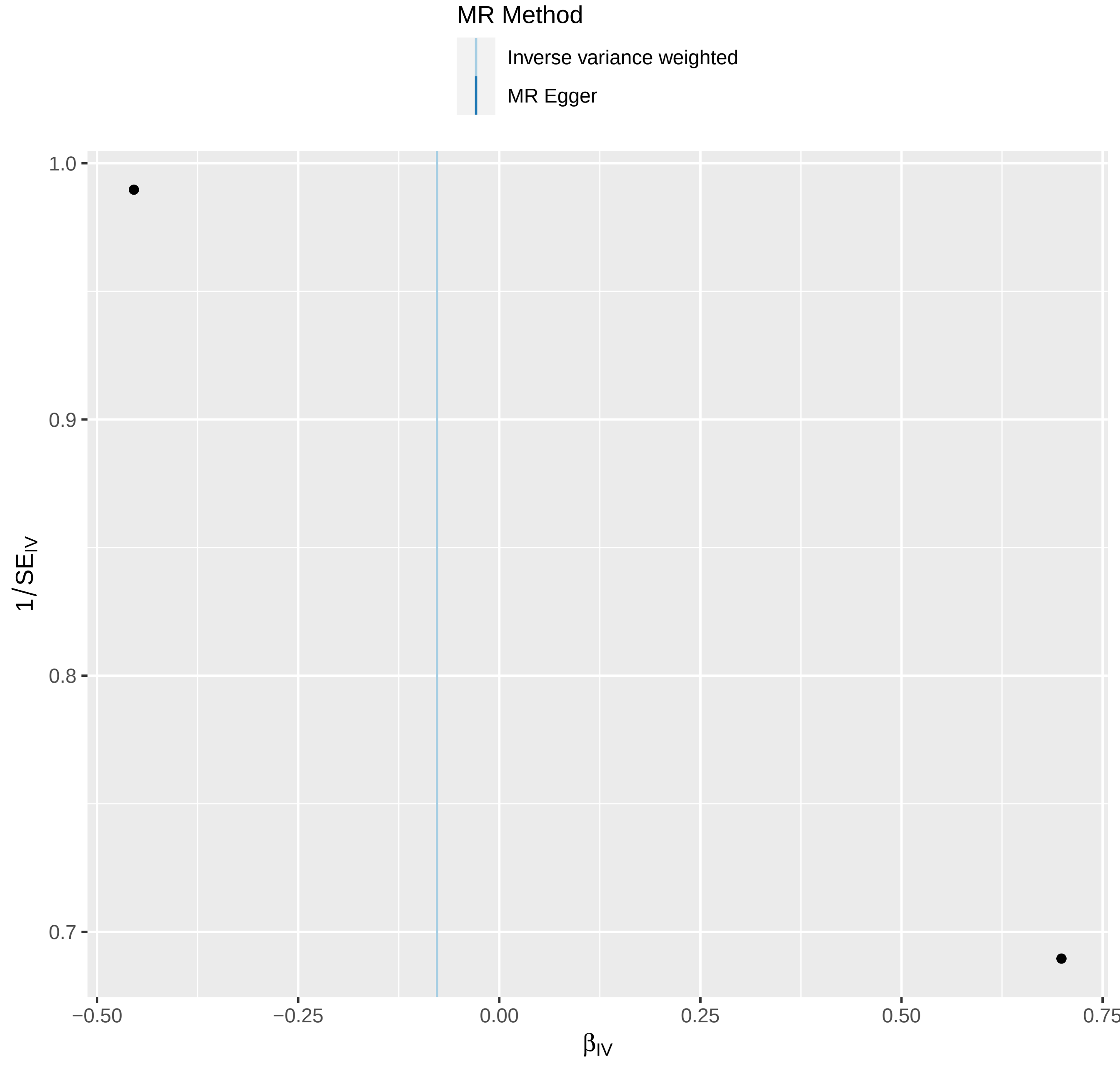

Batch 2732 : Leucylleucine on Ulcerative rectosigmoiditis

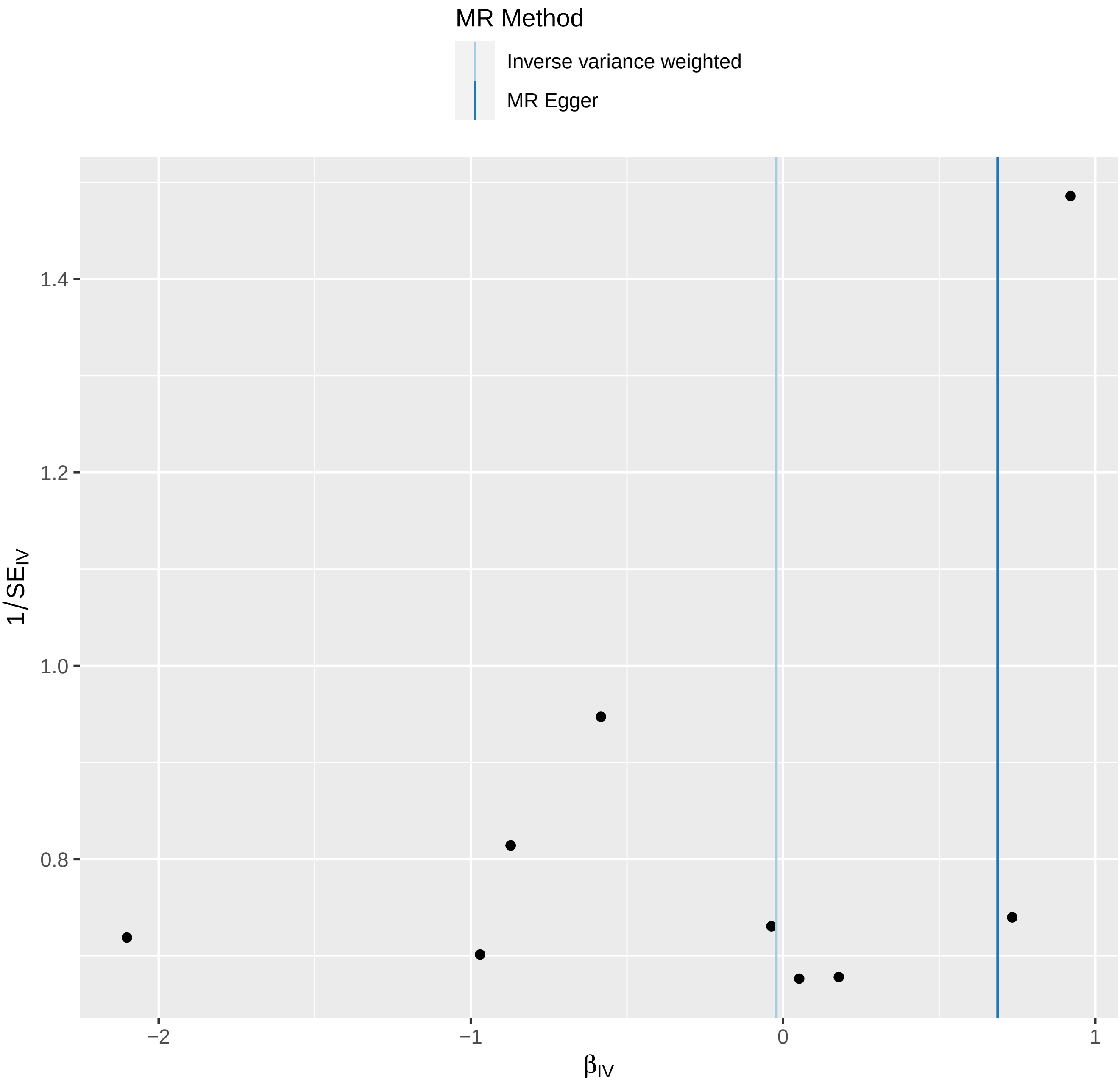

Batch 2733 : 7−alpha−hydroxy−3−oxo−4−cholestenoate (7−Hoca) on Ulcerative rectosigmoiditis

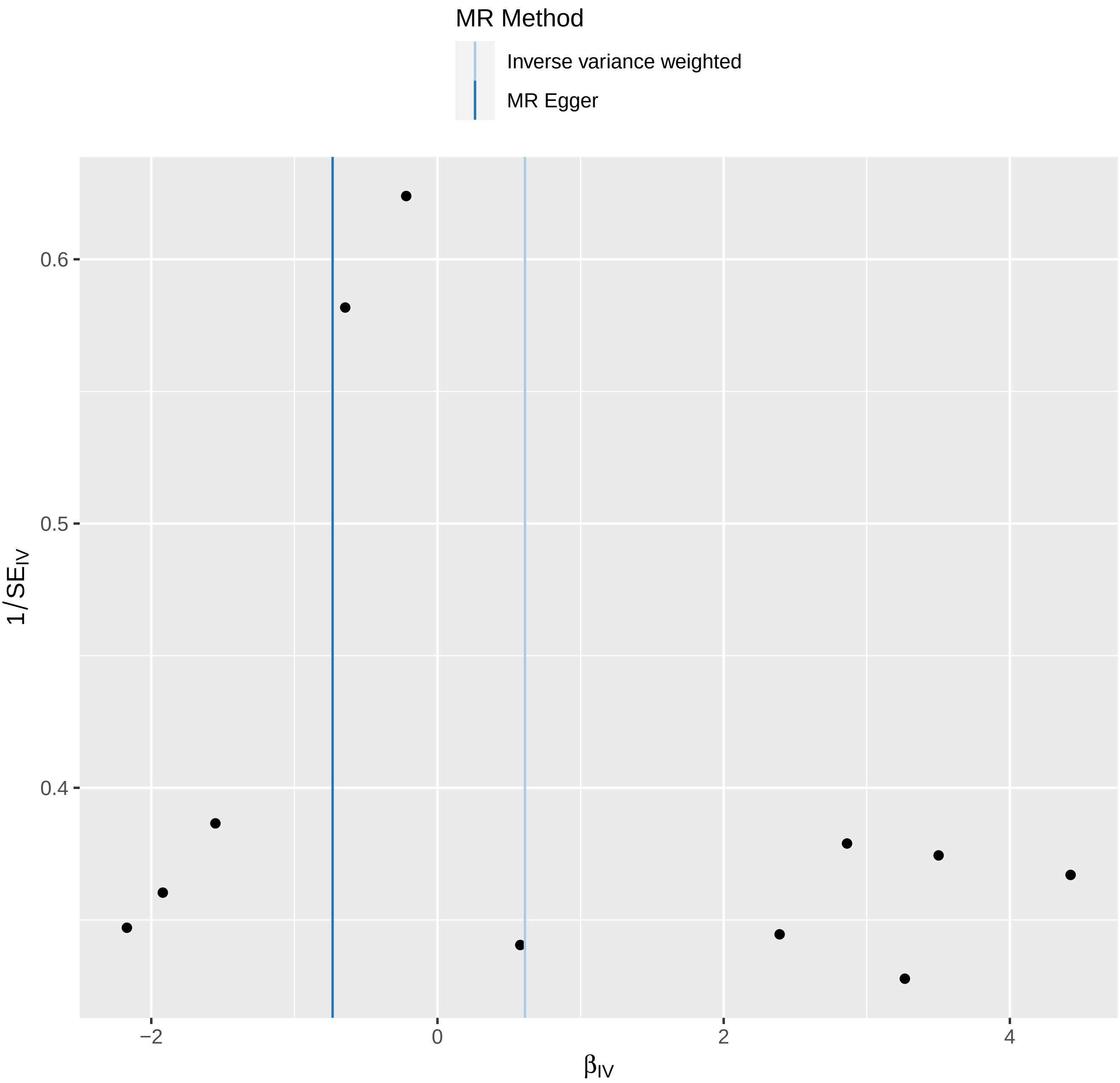

Batch 2734 : N−Butyl Oleate on Ulcerative rectosigmoiditis

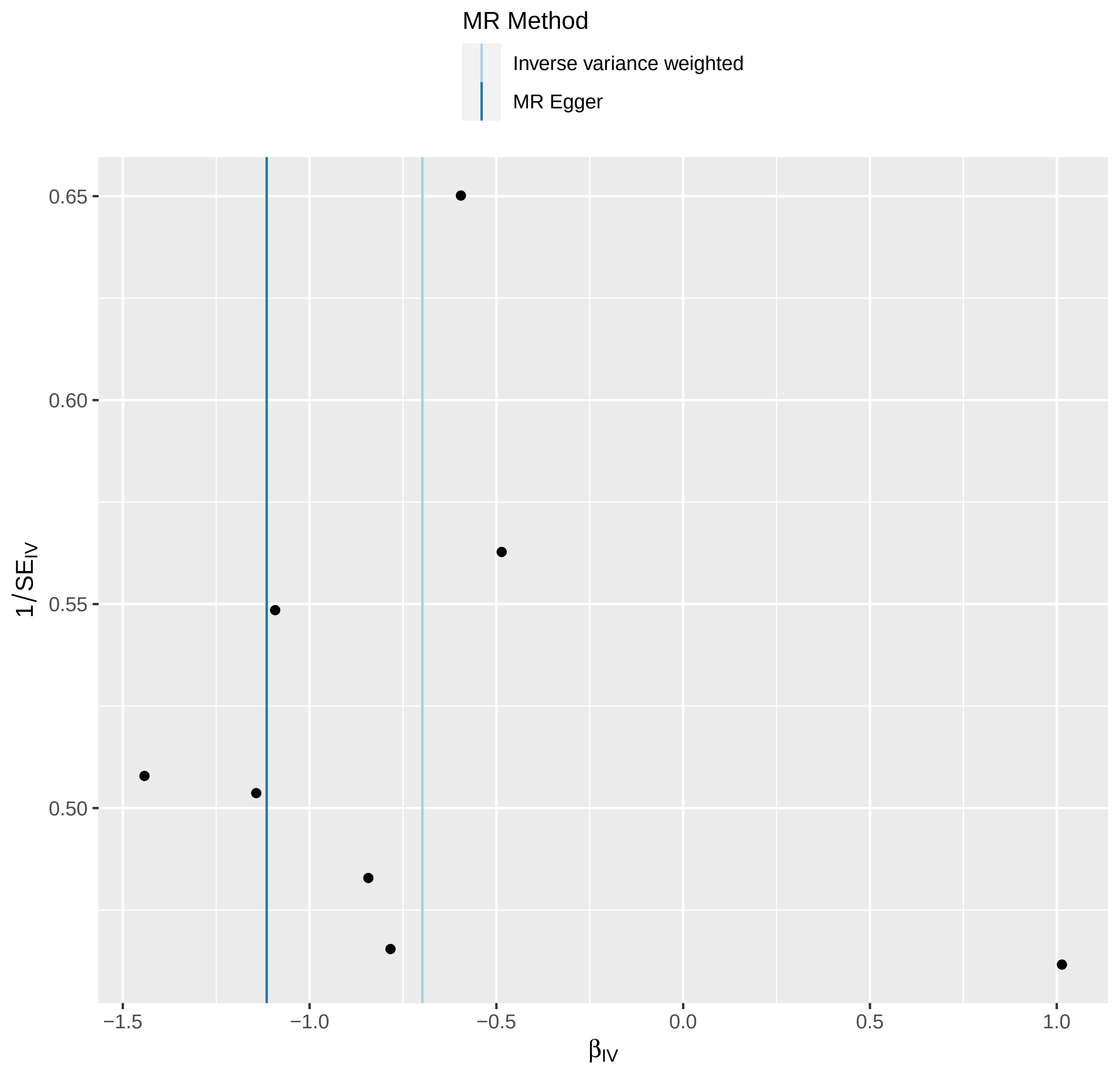

Batch 2736 : Taurolithocholate 3−sulfate on Ulcerative rectosigmoiditis

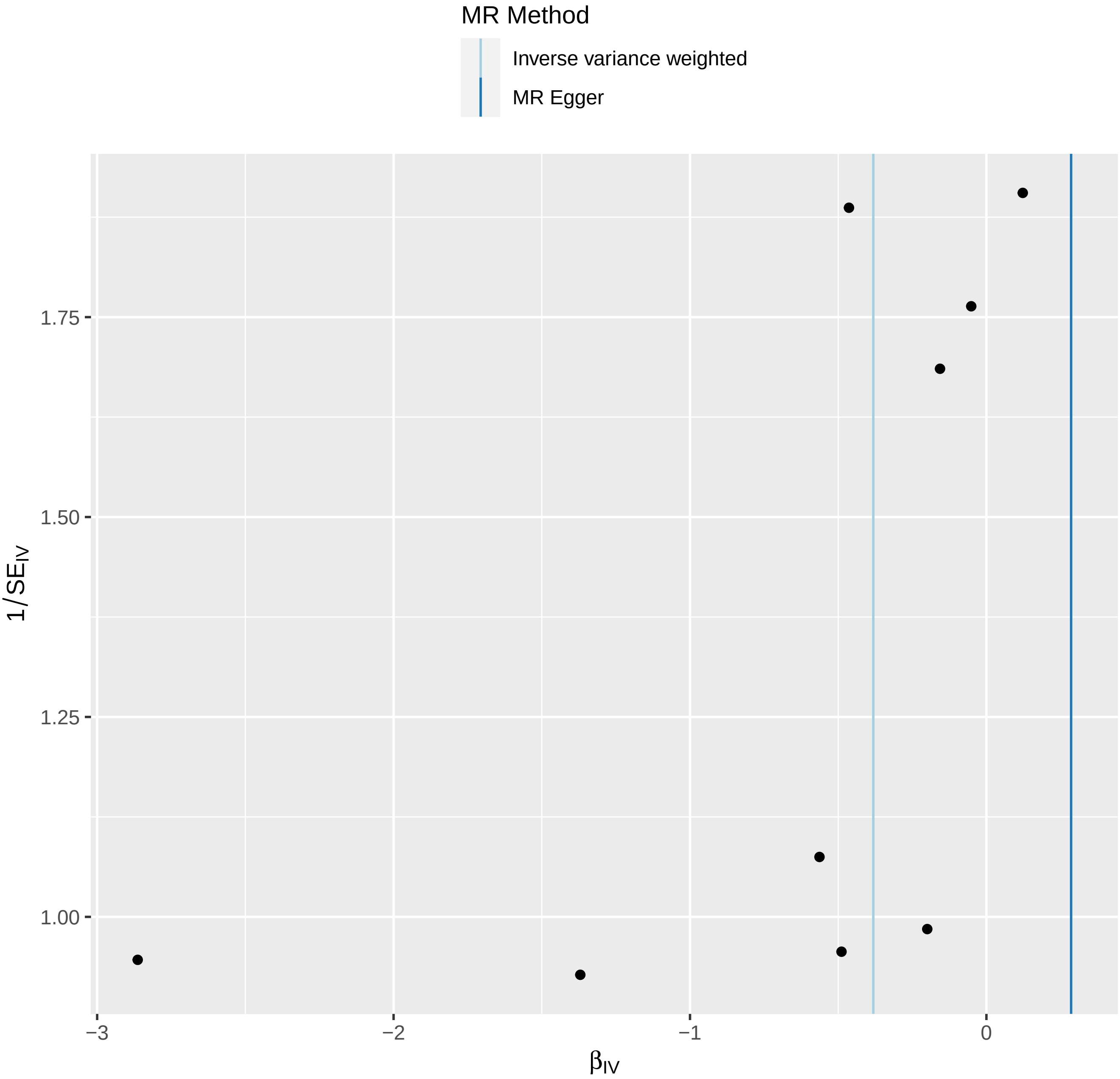

Batch 2737 : X−14977−−vanillin on Ulcerative rectosigmoiditis

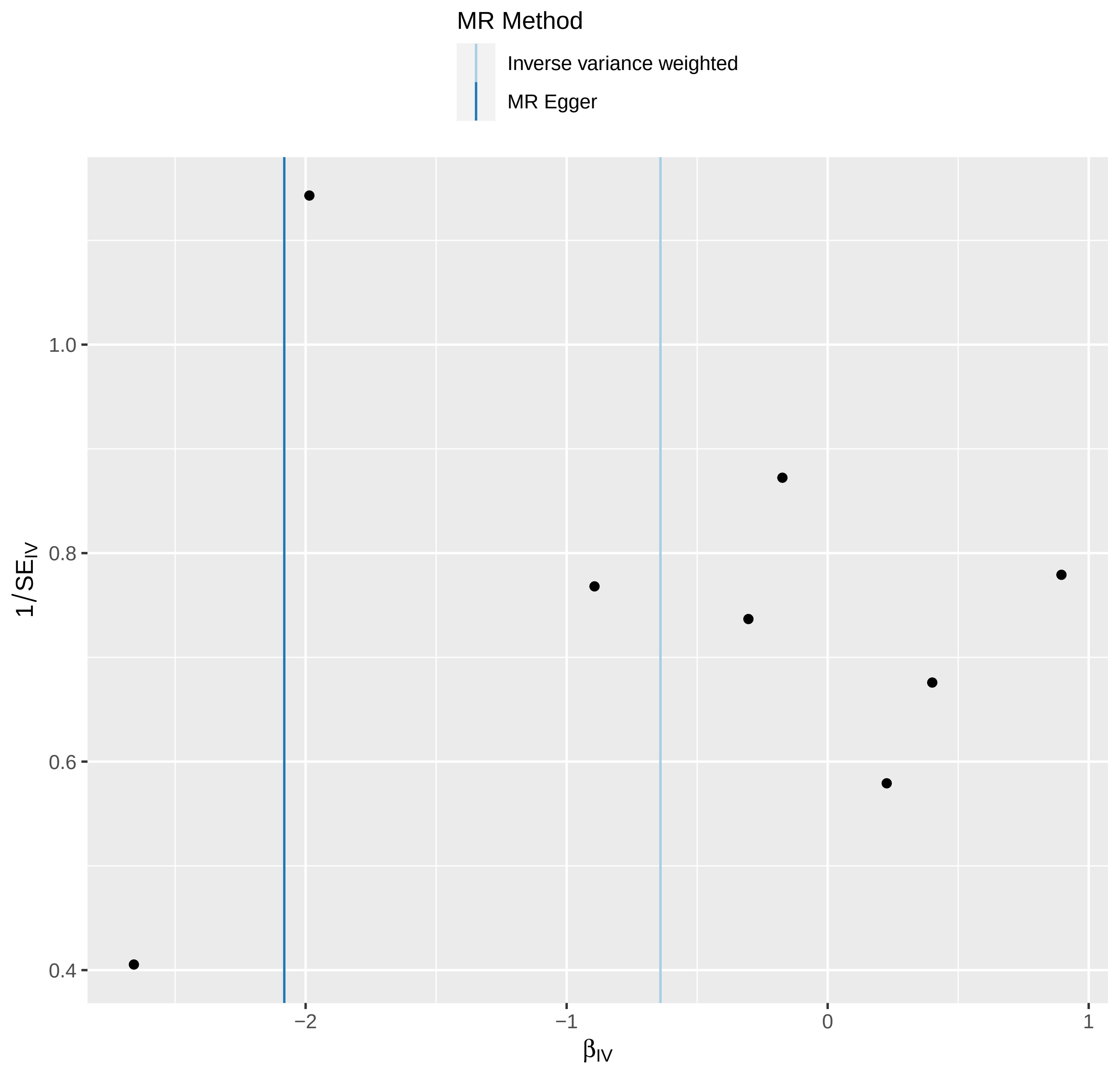

Batch 2738 : Succinylcarnitine on Ulcerative rectosigmoiditis

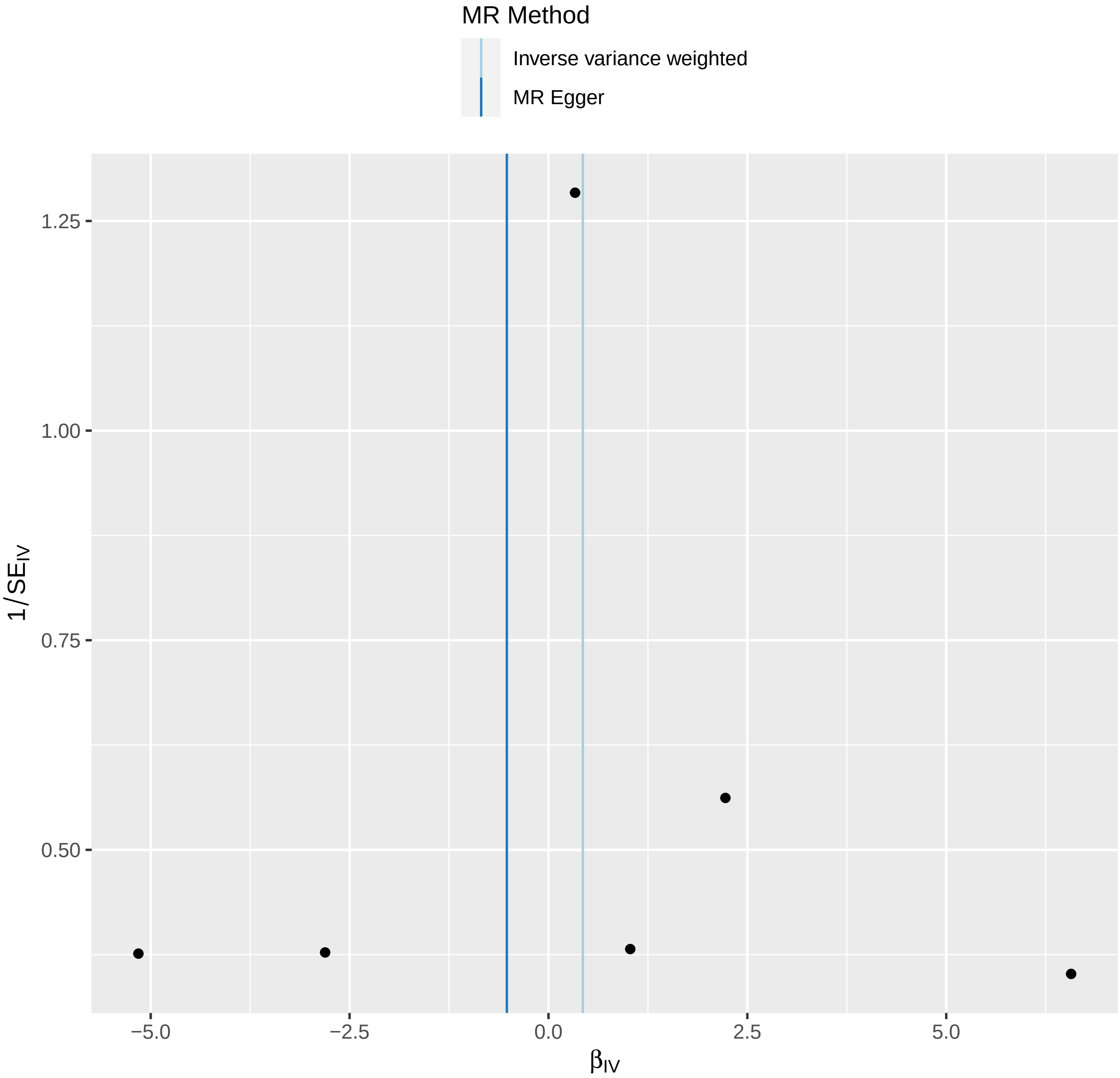

Batch 2740 : Cyclo(leu−pro) on Ulcerative rectosigmoiditis

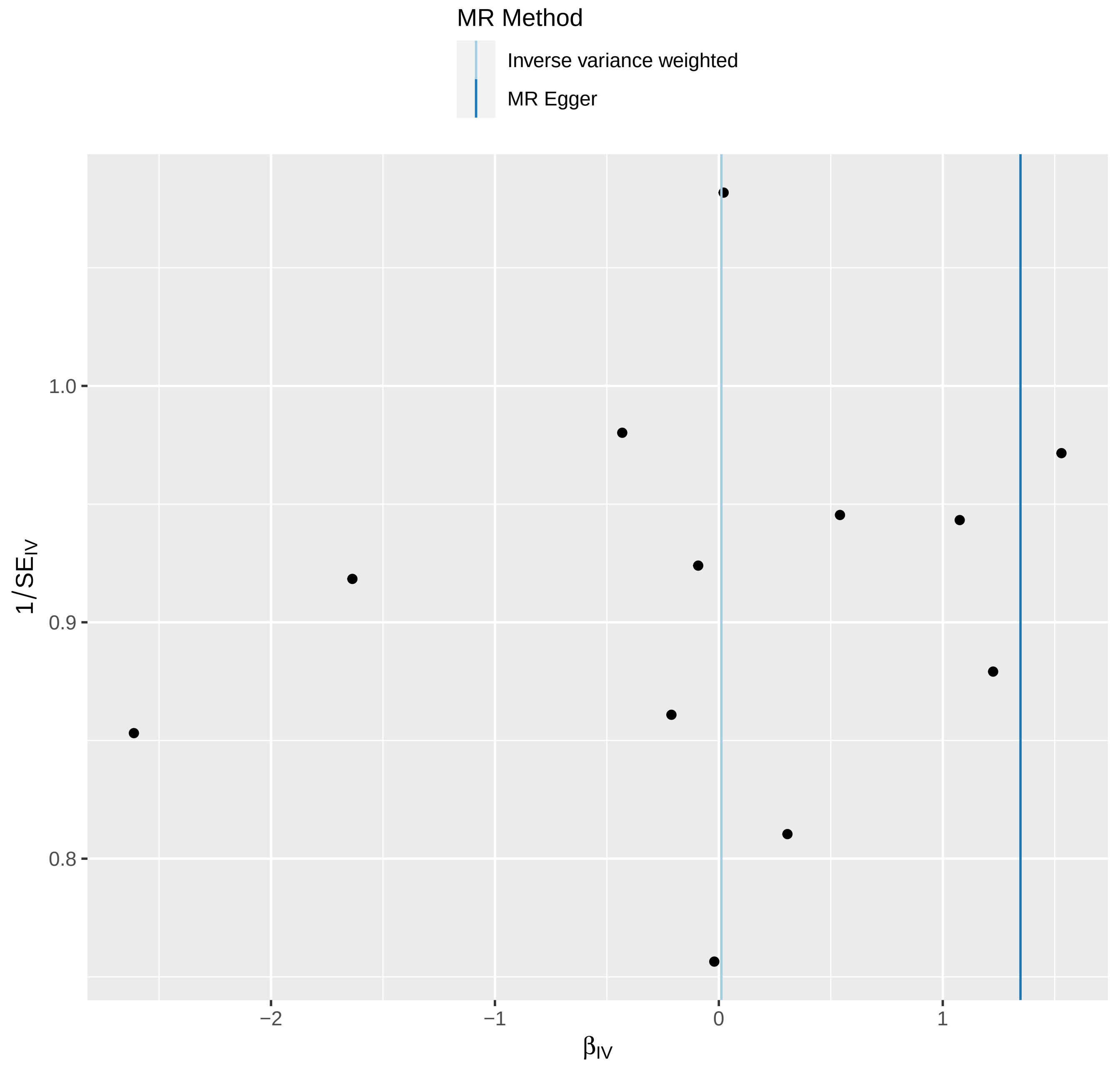

Batch 2741 : Chiro−inositol on Ulcerative rectosigmoiditis

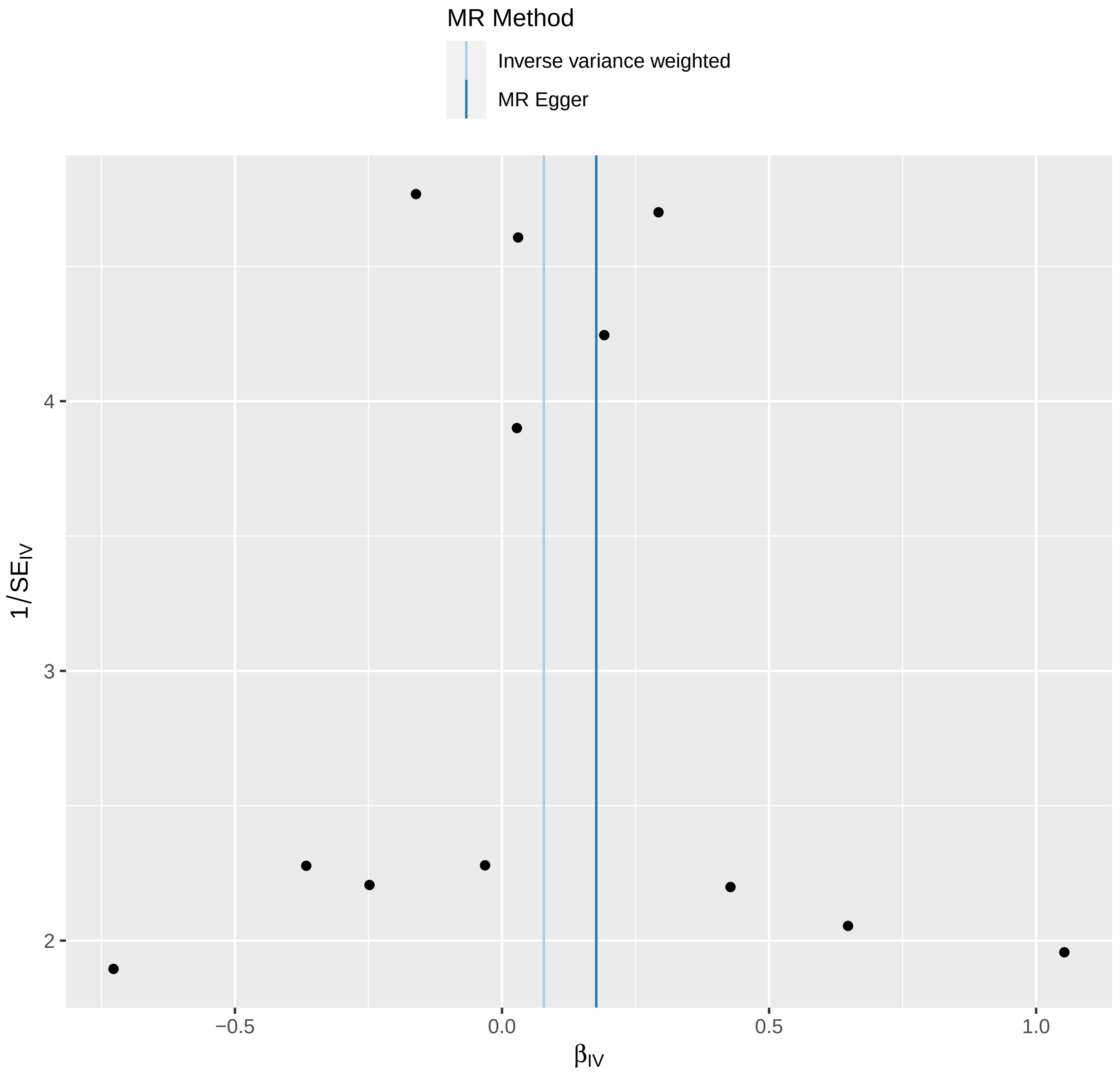

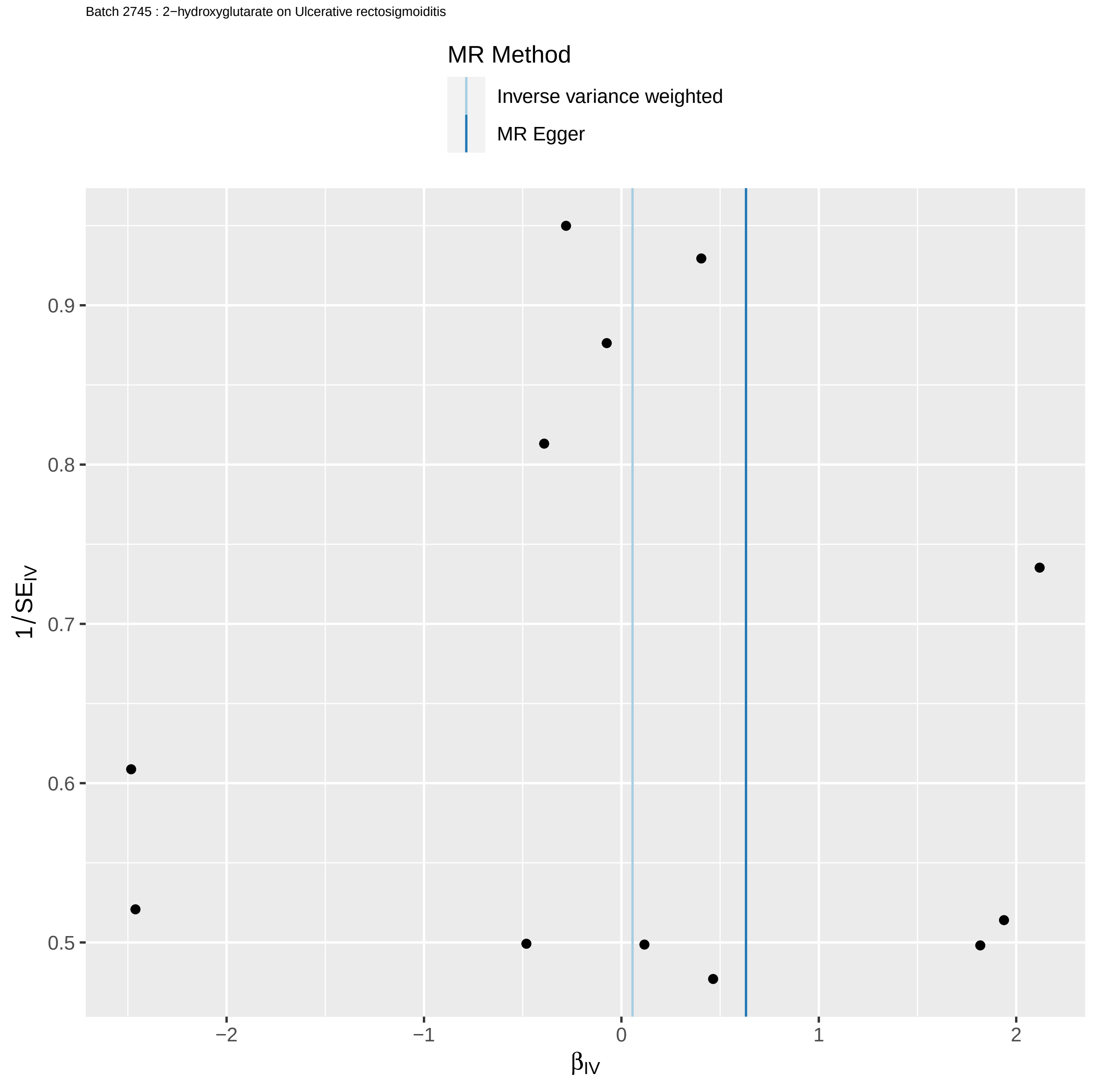
Batch 2745 : 2−hydroxyglutarate on Ulcerative rectosigmoiditis
MR Method
Inverse variance weighted
MR Egger
0.9
0.8
0.7
0.6
0.5
−2
−1
0
1
2
$1/SE_{IV}$
β_{IV}

Batch 2746 : Ergothioneine on Ulcerative rectosigmoiditis

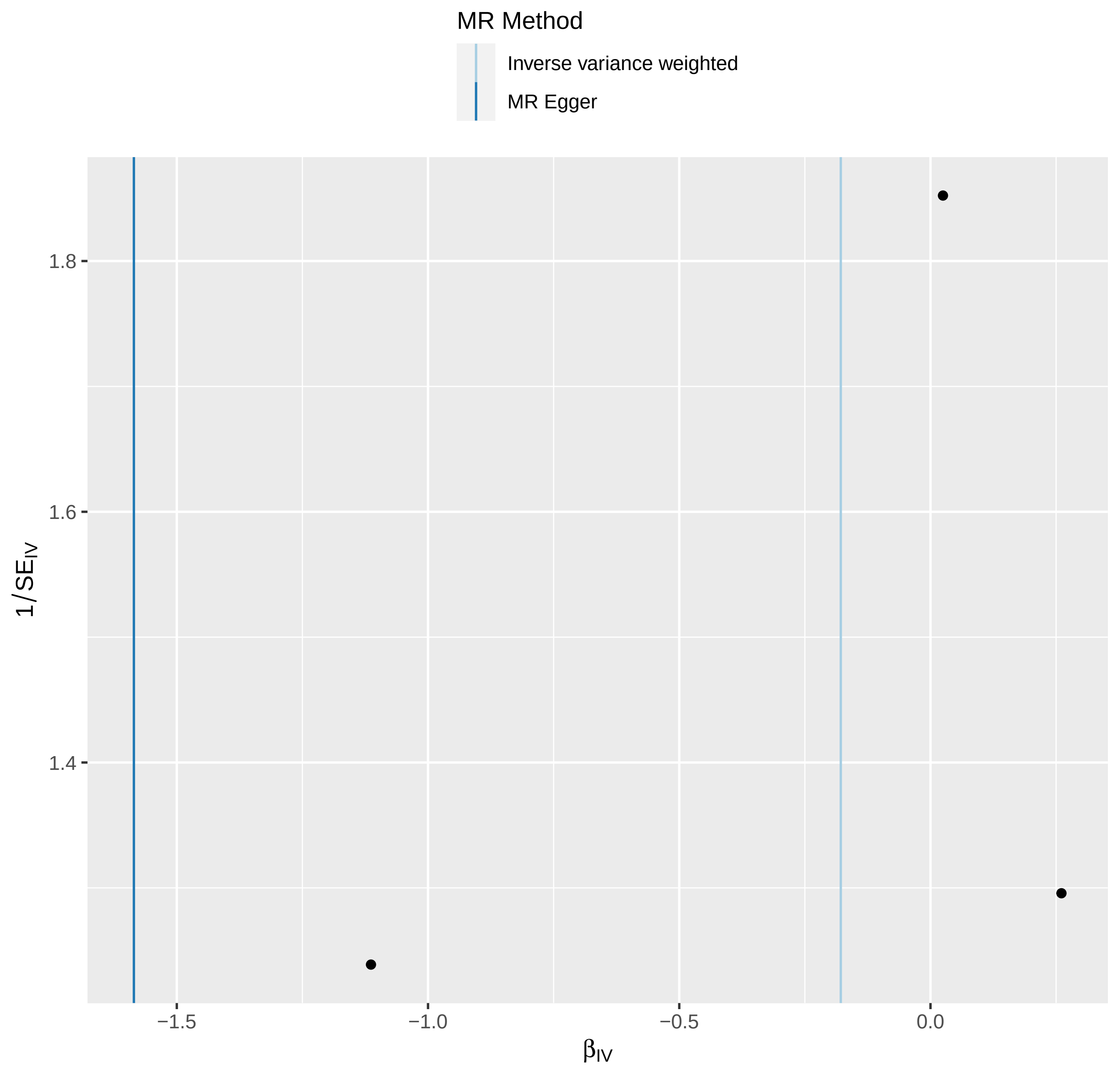

Batch 2748 : Phenylalanylphenylalanine on Ulcerative rectosigmoiditis

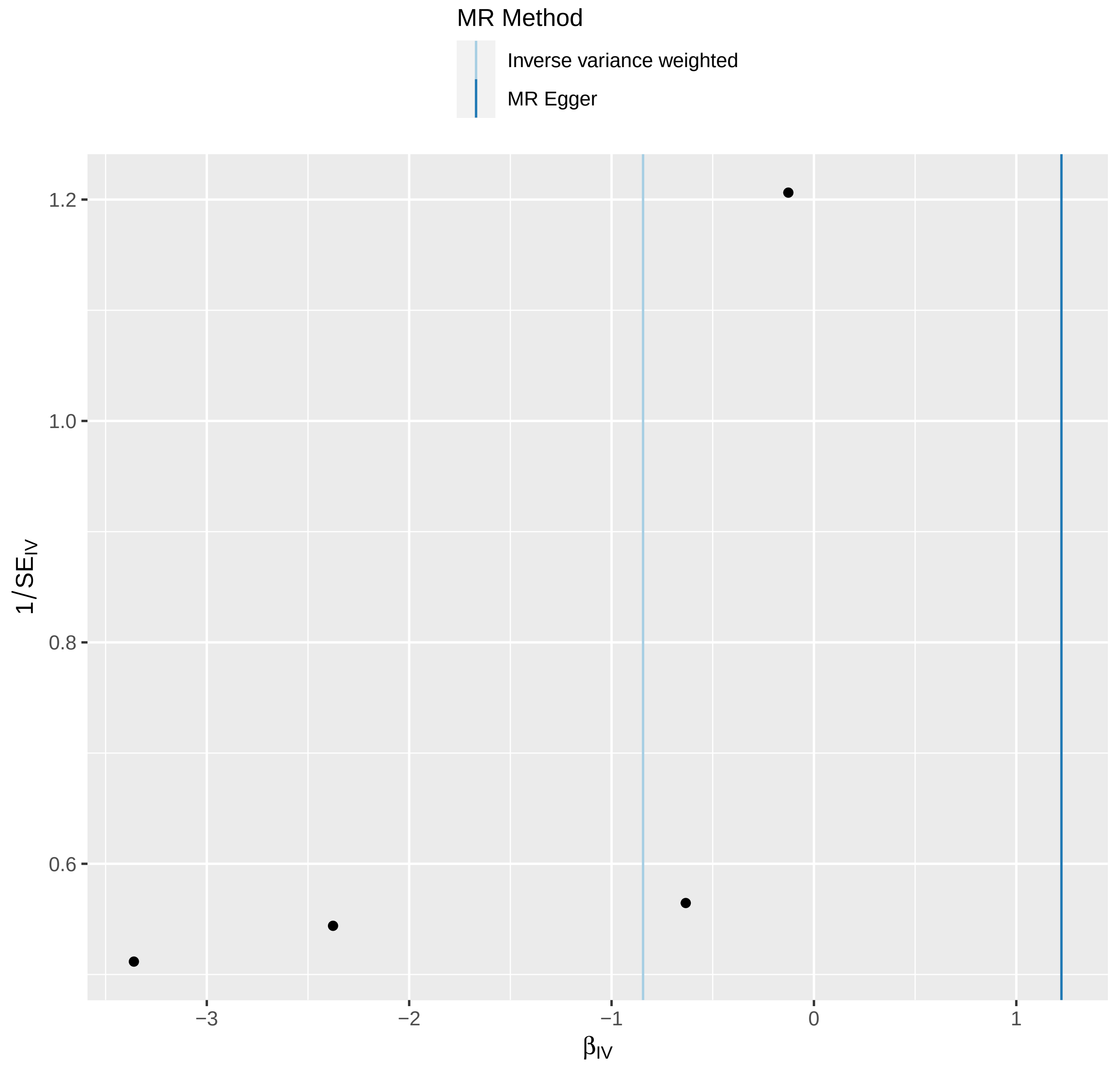

Batch 2749 : Cis−4−decenoyl carnitine on Ulcerative rectosigmoiditis

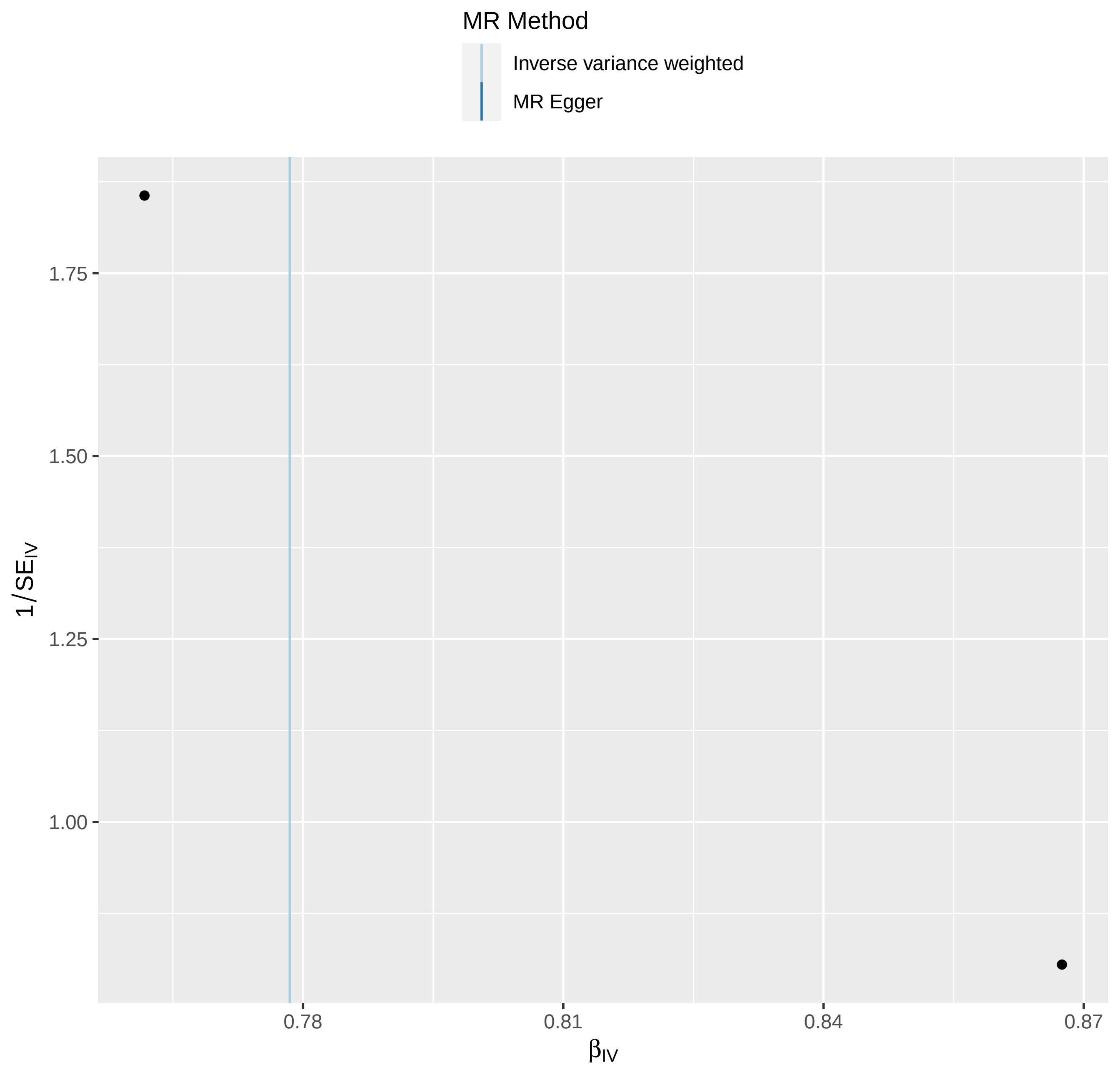

Batch 2750 : 15−methylpalmitate (isobar with 2−methylpalmitate) on Ulcerative rectosigmoiditis

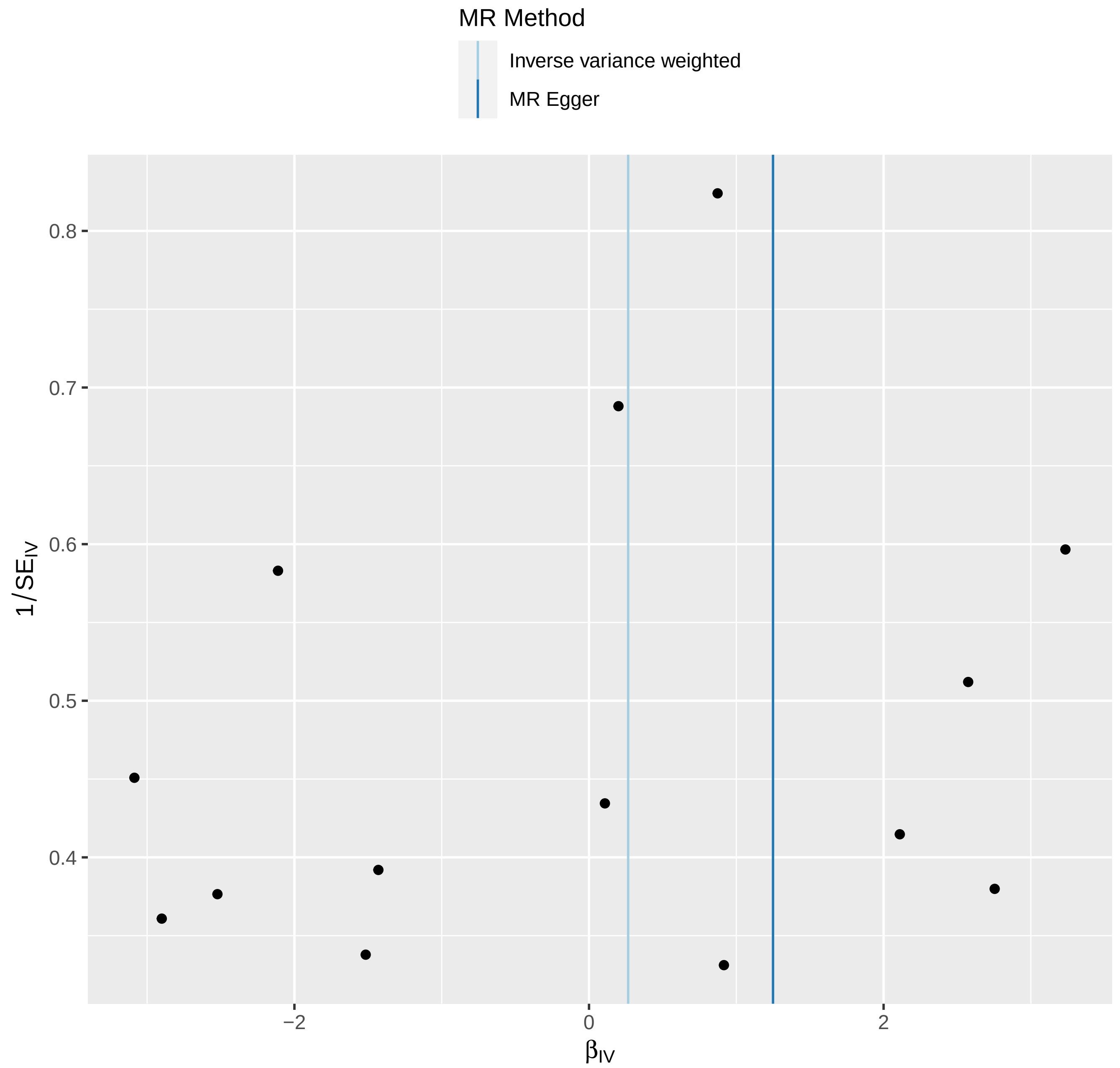

Supplement: Supplementary Figure 4 — Funnel Plots. [file DataSheet_4.pdf]
